# Genome sequencing and analysis of Salmonella enterica subsp. enterica serotype Enteritidis PT4 578: insights into pathogenicity and virulence

**DOI:** 10.1099/acmi.0.000828.v3

**Published:** 2024-11-04

**Authors:** Deisy G. Carneiro, Pedro Marcus P. Vidigal, Túlio Morgan, Maria Cristina D. Vanetti

**Affiliations:** 1 Departamento de Microbiologia, Universidade Federal de Viçosa (UFV), Av. Peter Henry Rolfs, Viçosa, 36570-900, Minas Gerais, Brazil; 2 Núcleo de Análise de Biomoléculas (NuBioMol), Universidade Federal de Viçosa (UFV), Av. Peter Henry Rolfs, Viçosa 36570-900, Minas Gerais, Brazil

**Keywords:** biofilm, foodborne pathogen, genome, rdar morphotype, *Salmonella* enteritidis, virulence determinants

## Abstract

*Salmonella enterica* serotype Enteritidis is a generalist serotype that adapts to different hosts and transmission niches. It has significant epidemiological relevance and is among the most prevalent serotypes distributed in several countries. *Salmonella* Enteritidis causes self-limited gastroenteritis in humans, which can progress to systemic infection in immunocompromised individuals. The *Salmonella* pathogenicity mechanism is multifactorial and complex, including the presence of virulence factors that are encoded by virulence genes. Poultry products are considered significant reservoirs of many *Salmonella* serotypes, and *Salmonella* Enteritidis infections are often related to the consumption of chicken meat and eggs. This study reports the whole-genome sequence of *Salmonella* Enteritidis PT4 strain 578. A total of 165 genes (3.66%) of the 4506 coding sequences (CDS) predicted in its genome are virulence factors associated with cell invasion, intestinal colonization, and intracellular survival. The genome harbours twelve *Salmonella* pathogenicity islands (SPIs), with the SPI-1 and SPI-2 genes encoding type III secretion systems (T3SS) showing high conservation. Six prophage-related sequences were found, with regions of intact prophages corresponding to *Salmon_118970_sal3* and *Gifsy-2*. The genome also contains two CRISPR systems. Comparative genome analysis with *Salmonella* Enteritidis ATCC 13076, *Salmonella* Typhimurium ATCC 13311, and *Salmonella* Typhimurium ATCC 14028 demonstrates that most unshared genes are related to metabolism, membrane, and hypothetical proteins. Finally, the phenotypic characterization evidenced differences among *Salmonella* Enteritidis PT4 578 and the other three serotypes regarding the expression of the red, dry, and rough (rdar) morphotype and biofilm formation. Overall, the genomic characterization and phenotypic properties expand knowledge of the mechanisms of pathogenicity in *Salmonella* Enteritidis PT4 578.

## Data Summary

The *Salmonella* Enteritidis PT4 578 whole-genome shotgun sequencing project was deposited on DDBJ/EMBL/GenBank under accession number PRJNA1081893.

URL: https://www.ncbi.nlm.nih.gov/bioproject/PRJNA1081893/.

BioSample accession number SAMN40191266 (https://www.ncbi.nlm.nih.gov/biosample/SAMN40191266).

Assembly NCBI GenBank accession number GCA_037055345.1 (https://www.ncbi.nlm.nih.gov/datasets/genome/GCA_037055345.1).

NCBI GenBank accession number CP146375.1 (https://www.ncbi.nlm.nih.gov/nuccore/CP146375.1).

All the annotation files are provided as three supplementary files and two supplementary tables.

**Impact Statement**

Genomic sequencing and phenotypic characterization of *Salmonella* Enteritidis PT4 strain 578 provide crucial insights into the virulence mechanisms of this prevalent pathogen. By identifying some of the main virulence factors associated with cellular invasion, intestinal colonization, and intracellular survival, this study reinforces the pathogenic potential of *Salmonella* Enteritidis. Furthermore, the discovery of twelve *Salmonella* pathogenicity islands and the presence of prophage-related sequences shed light on this pathogen’s genetic versatility and adaptability. Comparative genome analysis with other *Salmonella* serotypes highlights distinct metabolic pathways and membrane-associated genes, providing valuable information for understanding serotype-specific pathogenicity. Furthermore, the differences observed in phenotypic characteristics, such as expression of the red, dry, and rough morphotype and biofilm formation, highlight the importance of considering phenotypic variability when studying *Salmonella* pathogenesis. Overall, this comprehensive study increases our understanding of the pathogenic potential of *Salmonella* Enteritidis PT4 578 and its adaptation to different hosts and environments. These findings have significant implications for public health efforts aimed at mitigating *Salmonella* infections, particularly those associated with poultry products, and underscore the importance of continued surveillance and research to combat this widespread foodborne pathogen.

## Introduction

*Salmonella* is one of the major pathogens responsible for millions of foodborne infections worldwide and has significant relevance for public health systems and food production [1,3]. The genus *Salmonella* is a complex group divided into only two species, *Salmonella bongori,* and *Salmonella enterica*, based on differences between *16S rRNA* sequences [4]. The species *S. enterica* can be further subdivided into six subspecies, based on more than 2 659 serotypes based on serotyping [5,6]. Most pathogenic serotypes responsible for causing infections in humans and animals belong to *S. enterica* subsp. *enterica* and are clinically divided into typhoidal and non-typhoidal serotypes (NTS) [7].

*S. enterica* serotype Enteritidis and *S. enterica* serotype Typhimurium are the NTS with the most significant epidemiological relevance globally [8]. Although data on prevalent serotypes in Brazil are scarce, *Salmonella* Enteritidis has been the leading serotype isolated from gastroenteritis cases for decades [9,10]. Most strains belonged to the globally disseminated phage type 4 (PT4) [11]. *Salmonella* Enteritidis is an example of a successful *Salmonella* serotype, with a remarkable ability to adapt to niches and hosts as long as specialization opportunities present themselves [12]. This serotype has been frequently explored in research for understanding behaviours concerning quorum sensing, biofilm, and virulence mechanisms [13,16].

The increasing number of available genomic sequences of *S. enterica* allowed for defining the ‘core’ genome, which is conserved and shared by most of its members, and the ‘accessory’ genomic elements, which are present in some but absent in other *Salmonella* strains [17]. Accessory genomic elements are unnecessary for survival; however, they are a source of diversity, favour adaptability to specific colonization niches, and confer unique virulence attributes [18]. Furthermore, comparative analyses of closely related genomes revealed that these genes were acquired mainly by several horizontal transfer events. Thus, recombination between genomes is a relevant driver of diversity within the *Salmonella* genus [19,20].

In *Salmonella*, many of these horizontally acquired genes linked to infection exist on the chromosome as units of one or a few virulence genes or large cassettes composed of a series of genes and operons called *Salmonella* Pathogenicity Islands (SPIs) [20]. The virulence factors encoded by SPIs, such as type III secretion systems 1 and 2 (T3SS1 and T3SS2, respectively), are responsible for the invasion of the epithelium and induction of the infectious process in the host [21]. The pan-genome of *Salmonella* includes 24 different SPIs, and although not all have undergone experimental validation, most SPIs are in genomes of both species and all subspecies [22]. In addition to SPIs, the main components of the accessory genome of *Salmonella* are prophage sequences, transposable elements, and plasmids [17]. These genes have detectable properties that differentiate them from the rest of the host genome, such as having a different genomic signature and containing mobility genes so they can be integrated into the genome [23].

Thus, the objectives of this work were to perform the sequencing and annotation of the genome of *S. enterica* serotype Enteritidis PT4 578 to expand the understanding of structural features of the core genome and accessory genomic elements. The results help explain the pathogenic characteristics of *Salmonella* Enteritidis PT4 578 and the phenotypic divergences observed between closely related serotypes.

## Methods

### Bacterial strain

This study analysed the *Salmonella* Enteritidis PT4 578 isolated from chicken breasts (GenBank: 16S ribosomal RNA gene MF066708.1) and obtained from the bacterial culture collection of the Oswaldo Cruz Foundation (FIOCRUZ; Rio de Janeiro, Brazil). The PT4 578 strain has clinical importance in Brazil, and it is a model for the study of several subjects, such as virulence, quorum sensing, and biofilm formation [9,13,16]. The culture was maintained in Luria Bertani broth (LB; Himedia, India) (tryptone 1%, yeast extract 0.5%, and NaCl 0.5%) with 20% (v/v) sterile glycerol at −20 °C. The other strains, *Salmonella* Enteritidis ATCC 13076, *Salmonella* Typhimurium ATCC 13311, and *Salmonella* Typhimurium ATCC 14028, were chosen because they are directly related to the Enteritidis PT4 serotype. The maintenance conditions of these strains were the same as those described above.

### Genome sequencing, assembly, and annotation

The total DNA of *Salmonella* Enteritidis PT4 578 was obtained from an overnight culture in 5 ml Tryptic Soy Broth (TSB; Sigma, United States) at 37 °C using Wizard Genomic DNA Purification Kit (Promega, USA) following the instructions provided by the manufacturer. The quality of DNA was assessed using the NanoDrop 2000 (Thermo Fisher Scientific, Delaware, USA) and agarose gel electrophoresis. The double-stranded DNA (dsDNA) was quantified using a Qubit 3.0 fluorometer (Life Technologies, Paisley, UK). The Rapid Sequencing Kit (SQK-RAD004, Oxford Nanopore Technologies, Oxford, UK) produced the genomic library, and the MinION sequencing device equipped with a flow cell (FLO-MIN106 R9.4.1, Oxford Nanopore Technologies, Oxford, UK) sequenced it for approximately 2 h. The MinKNOW software v4.3.4 managed the sequencing run, and the Guppy v5.0.11 performed the real-time base-calling using the high-accuracy mode (parameters: -c dna_r9.4.1_450bps_hac.cfg). The Porechop v0.2.4 (https://github.com/rrwick/Porechop) trimmed the adaptor sequences, and the PycoQC v2.5.2 (https://github.com/tleonardi/pycoQC) assessed the quality of the filtered data. Genomes were *de novo* assembled by Flye v.2.9.0 [24] (parameters: --iterations 5 --plasmids --genome-size 4.8 m –nano-raw). The assembled genomes were corrected using Racon v1.4.3 [25] and Medaka v1.3.3 (Oxford Nanopore Technologies, Oxford, UK). Then, the BUSCO v5.0.0 assessed the genome completeness (parameters: --lineage_dataset enterobacterales_odb10). Finally, the Prokka tool [26] predicted the genes and annotated the assembled genome in FASTA format, generating the standard annotation files, including GFF and GBK. We compared the general characteristics of the *Salmonella* Enteritidis PT4 578 genome with the reference genomes of *Salmonella* Enteritidis P125109 and *Salmonella* Typhimurium LT2.

### Identification of pathogenicity islands (SPIs), virulence factors, and resistance genes

The SPIFinder scanned the genome for SPIs using a threshold of 95% for identity and 60% for minimum length [27]. The virulence factor database (VFDB) [28] allowed the identification of the virulence factors in the genome. The ResFinder 4.1 tool, with thresholds of 90% for identity and 60% for minimum length [29] and the resistance gene identifier from the CARD database (https://card.mcmaster.ca/) assisted in the identification of acquired resistance genes.

### Phage insertion, prediction of plasmid regions, integrative and conjugative elements

The Phage Search Tool (PHAST) [30] scanned the genome for the insertion of prophages. Scores above 90 were known as an intact prophage, scores ranging from 70 to 90 as questionable prophage, and regions scoring below 70 as incomplete prophage [30]. The VFDB was used to identify virulence factors in the prophage regions [28]. PlasmidFinder was used to analyse plasmid regions in the genome [31]. The ICEberg 2.0 identified the integrative and conjugative elements (ICEs) [32].

### Determination of restriction-modification sites (RM) and CRISPR regions

The Restriction-ModificationFinder-1.1 scanned the genome to identify the RM sites [27]. CRISPR regions were analysed using CRISPRCasFinder [33]. Subsequently, the CRISPRTarget [34] processed the file generated by CRISPRCasFinder (result.json) to identify the spacers.

### Comparative genome analysis

The comparative analysis involved the *Salmonella* Enteritidis PT4 578 genome and three other closely related serotypes available in the NCBI GenBank database (https://www.ncbi.nlm.nih.gov/genbank), *Salmonella* Enteritidis ATCC 13076 (accession GCA_001643395.1) and two strains of another NTS, *Salmonella* Typhimurium ATCC 13311 (accession GCA_000743055.1) and *Salmonella* Typhimurium ATCC 14028 (accession GCA_001997115.1). OrthoVenn executed the analysis of orthologous proteins and clustered them with default parameters (*E*-value: 1e-10 and inflation value: 1.5) [35]. In addition, the Roary pipeline 3.11.2 [36] predicted the pan-genome representative of the sequenced *Salmonella* Enteritidis PT4 578 and the three serotypes previously mentioned.

### Phenotypic analysis

#### Swimming and swarming motilities

Swimming and swarming motility analysis was carried out in accordance with published literature with modifications [37]. The four strains were grown for 20 h at 37 °C in a TSB medium. After standardizing the cultures to 10^7^ c.f.u. ml^−1^, an aliquot of 3 µl was plated onto a 0.3% tryptone agar plate (1% tryptone and 0.25% NaCl). For the swarming motility, a volume of 3 µl was inoculated onto a 0.5% LB agar plate supplemented with 0.4% glucose. The plates were incubated at 28 °C for 10 h for swimming motility and 40 h for swarming motility. The evaluation of the halos from the centre to the edge of the swimming and swarming motilities used their diameter measures.

#### Colony morphology

A volume of 5 µl of the standardized inoculum described above was spotted into the LB agar plate, supplemented with 40 mg l^−1^ of Congo red (Sigma-Aldrich, United States) and 20 mg l^−1^ of Coomassie brilliant blue (Sigma-Aldrich, United States) [38]. The colony morphology was judged visually after 72 h incubation under 28 °C as red, dry, and rough (rdar; expression of curli fimbriae and cellulose), pink, dry, and rough (pdar; expression of cellulose), brown, dry, and rough (bdar; expression of fimbriae) and white and smooth (saw; no expression of matrix components). The Canon EOS 450D camera registered the pictures of the colonies.

#### Biofilm formation

In biofilm formation analysis, the measurement was performed in 96-well polystyrene microplates according to the protocol described by Stepanovic *et al.* [39], with modifications. Briefly, the polystyrene microplates were filled with 230 µl of TSB and 20 µl of the standardized inoculum and then incubated for 40 h. After incubation, the total cells' optical density (OD) was determined at 600 nm. After discarding the culture supernatant, the microplate was washed three times with 300 µl of distilled water. The surface-attached cells were then fixed by adding 250 µl of methanol for 15 min. After discarding the methanol, the microplate was air-dried for 20 min and stained with 250 µl of 0.1% (w/v) crystal violet for 30 min. Then, the violet crystal was removed, and the wells were washed three times with water. Next, we air-dried the microplates for 15 min at 40 °C and resolubilized the dye bound to the adherent bacterial cells in 250 µl of 33% glacial acetic acid. The OD of attached cells was determined at 590 nm. The OD was used to classify the isolates, considering the cutoff OD (ODc) of three standard deviations above the mean OD of the negative controls. Thus, we classified the isolates as a non-biofilm producer (OD≤ODc), a weak biofilm producer ODc <OD≤(2×ODc), a moderate biofilm producer (2×ODc) < OD≤(4×ODc), or a strong biofilm producer (4×ODc) < OD.

#### Statistical analysis

GraphPad Prism software executed the statistical analysis to assess the significance of the differences observed in the motilities and biofilm formation between the strains through analysis of variance (ANOVA) and Tukey’s test, considering the *P*<0.05 as the significance threshold.

## Results

### Structure and general information of *Salmonella* Enteritidis PT4 578 genome

**Fig. 1.**
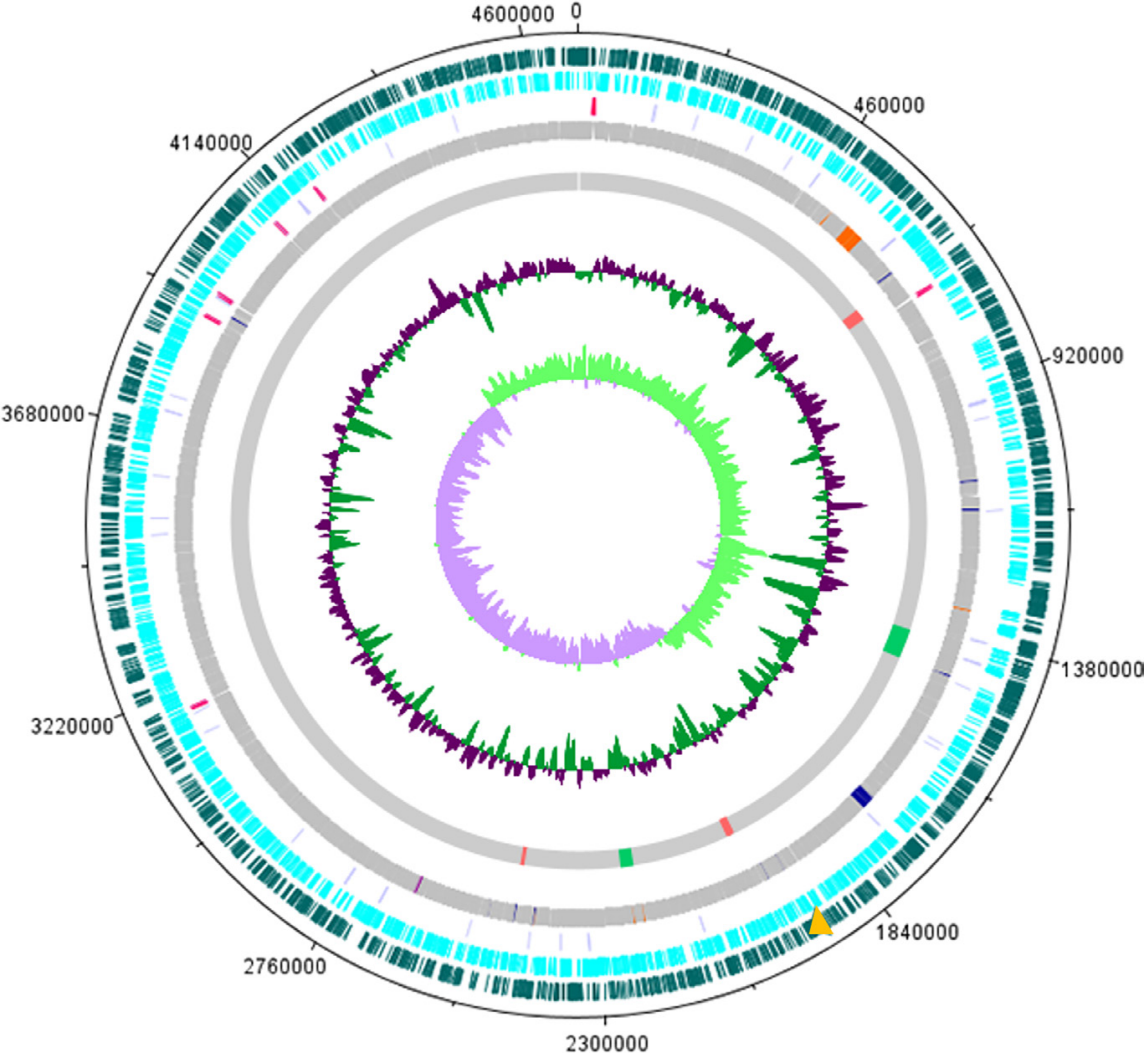
Genome map of the chromosome of *Salmonella* Enteritidis PT4 578. The coding DNA sequences (CDS) on the heavy and light strands are depicted in dark green and light blue, respectively. Light purple marks denote tRNA genes, while pink represents rRNA genes. Resistance genes are in yellow. SPI-1 virulence factors are in orange and SPI-2 in blue. Regions of incomplete prophage are shown in red, while intact prophage regions are in green. The two inner tracks represent G+C content and GC skew. The DNAPlotter [69] generated the circular map.

*Salmonella* Enteritidis PT4 578 presents a chromosome of 4 685 705 bp with an average GC content of 52.20%. It contains 4 615 predicted genes, including 4 506 coding DNA sequences (CDS), seven rRNA genes, and 84 tRNA genes. The general characteristics of this genome are represented in Fig. 1 and summarized in Table 1.

**Table 1.** General genome characteristics of *Salmonella* Enteritidis PT4 578 compared to *Salmonella* Enteritidis P125109 and *Salmonella* Typhimurium LT2

Features	*Salmonella* Enteritidis PT4 578	*Salmonella* Enteritidis P125109	*Salmonella* Typhimurium LT2
Size (bp)	4 685 705	4 685 848	4 857 432
G+C (%)	52.20	52.17	53.0
CDS	4 506	4 318	4 489
rRNA	7	7	22
tRNA	84	84	85
Reference	This work	[46]	[70]

### Pathogenicity islands (SPIs), virulence factors, and antimicrobial resistance genes

**Fig. 2.**
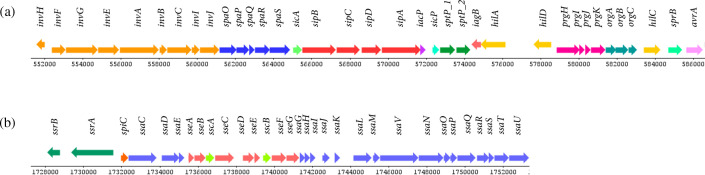
Genetic organization of virulence factors in SPI-1 (**a**) and SPI-2 (**b**) in the *Salmonella* Enteritidis PT4 578 genome. The DNAPlotter [69] generated the linear map.

SPIFinder detected 12 SPIs in the genome of *Salmonella* Enteritidis PT4 578, namely SPI-1, SPI-2, SPI-3, SPI-4, SPI-5, SPI-9, SPI-10, SPI-12, SPI-13, SPI-14, centisome 63 pathogenicity island (C63PI), and centisome 54 pathogenicity island (CS54). Among the predicted CDS, 165 genes (3.66%) encode proteins like the virulence factors of the VFDB (Table S1). Eighty-two genes encode virulence factors associated with the invasion of cells and secretion. Of these, 36 genes are in *Salmonella* Enteritidis PT4 578 SPI-1 (Fig. 2a) and 31 in SPI-2 (Fig. 2b). VFDB results revealed three virulence factors encoding by SPI-5 and SPI-12, both related to T3SS effector. SPI-14 carries one virulence factor, a type IV secretion system effector. *Salmonella* Enteritidis PT4 578 carries the C63PI that encodes four virulence factors related to iron uptake, namely *sitABCD*. Meanwhile, three virulence factors related to intestinal colonization and persistent determinants, *ratB*, *shdA*, and *sinH*, are encoded by CS54. The SPI-3, SPI-4, SPI-9, SPI-10, and SPI-13 did not carry any identified or known virulence factors. In addition to the SPI-encoded secretion apparatus, the *Salmonella* Enteritidis PT4 578 repertoire of virulence factors also include fimbrial and non-fimbrial adherence determinants, macrophage inducible genes, magnesium uptake, stress, and anaerobic adaptation (Table S1). In addition, there are thirteen clusters of fimbriae in the *Salmonella* Enteritidis PT4 578 genome: *csg*, *bcf*, *fim*, *lpf*, *saf*, *sef*, *stb*, *std*, *ste*, *stf*, *sth*, *sti,* and *peg* (Table S1). About the *peg* operon, the genome contains only the *pegA* and *pegD* genes. RESFinder and CARD showed that *Salmonella* Enteritidis PT4 578 contains the cryptic resistance gene, *aac(6′)-Iy*, related to resistance to the aminoglycoside antibiotic class (Fig. 1).

### Mobile elements

The PHAST programme revealed the presence of six prophage regions (Fig. 1), including two intact prophages (regions 2 and 5) and four incomplete prophages. Region 2 contains 62.6 kb, 47.77% of GC content, and 63 ORFs, of which nine encode hypothetical proteins and 54 proteins like bacteriophage proteins. The phage with the highest number of similar proteins was *Salmon_118970_sal3* (GenBank accession NC_031940), with 52 matches in the similarity searches. Region 5 contains 31.1 kb, 47.22% of GC content, and 40 ORFs that encode ten hypothetical proteins and 30 proteins similar to bacteriophage proteins. The phage with the highest number of similar proteins was *Gifsy-2* (accession NC_ 010393), with 16 matches. The similarity searches against the VFDB revealed also identified prophage-encoded virulence factors, such as T3SS effector, *sseK*2 and *nleB*1 in region 2, superoxide dismutase precursor, *sodCI* in region 5, and T3SS effector, *sseI*/*srfH*, *sspH2* and *sspH1* in region 6. However, there aren’t conjugative plasmids in the *Salmonella* Enteritidis PT4 578 genome. In addition, PlasmidFinder did not identify any plasmid regions in the genome. In addition, the genome contains one ICE region of 13 379 bp that encodes thirteen proteins, one integrase, and one relaxase.

### Identification restriction-modification (RM) sites and CRISPR loci

**Fig. 3.**
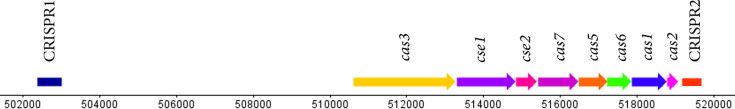
Genetic organization of CRISPR-Cas system in the *Salmonella* Enteritidis PT4 578 genome. The DNAPlotter [69] generated the linear map.

The Restriction-ModificationFinder identified three RM sites in the *Salmonella* Enteritidis PT4 578 genome, comprising types I, II, and III. The type I, *Sty*SBLI, enzymes consist of a hetero-oligomeric protein complex encoded by three closely linked genes, S.Sen1427II (specificity subunit), M.StySBLI (MT), and StySBLI (RE). The recognition CGANNNNNNTRCC motif, in which the residues underlined are the substrates for cleavage. Type II methyltransferase M.Sen158Dcm and M.Sen641III recognize CCWGG and ATGCAT motifs, respectively. In type III, the SenAZII and M.Sen1427I recognize CAGAG and CAGAG motifs, respectively. However, the cleavage site of SenAZII is unknown. The CRISPRCasFinder identified that *Salmonella* Enteritidis PT4 578 contains two CRISPR systems at the evidence of level 4, CRISPR 1 and CRISPR 2 (Fig. 3). Both contain 29 bp, with nine and eight unique spacers, respectively. Three of the seventeen predicted CRISPR systems are protospacers (Table S2), homologs to plasmids, and phage sequences. The Cas cassette, composed of *cas3*, *cse1*, *cse2*, *cas7*, *cas5*, *cas6*, *cas1*, and *cas2*, was found between CRISPR1 and CRISPR2 locus, starting at 510 618 bp and ending at 519 071 bp (Fig. 3).

### Comparison of the genome of *Salmonella* Enteritidis PT4 578 with that of other serotypes

**Fig. 4.**
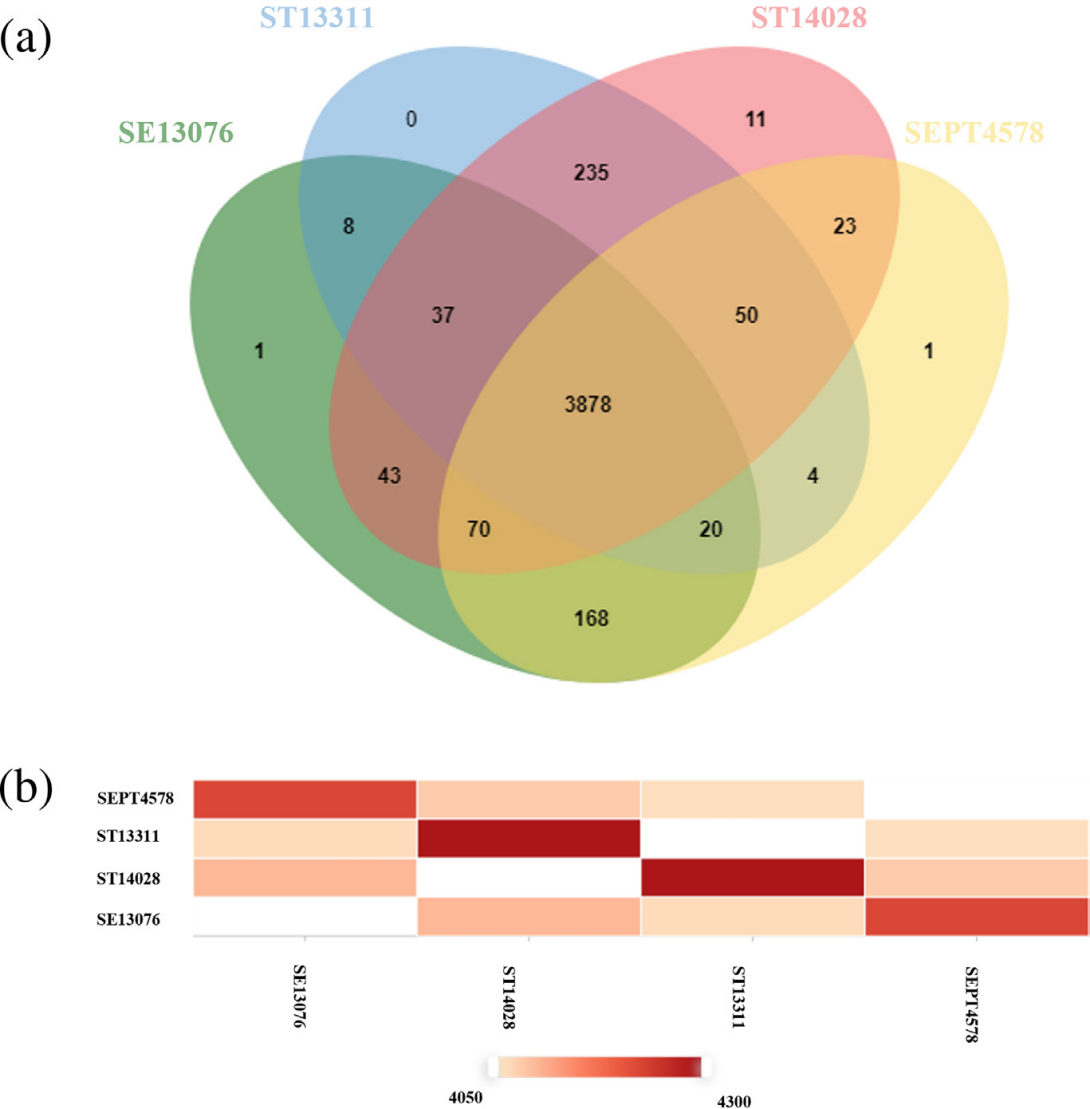
Venn diagram of clusters of orthologous proteins of *Salmonella* Enteritidis PT4 578 and shared with *Salmonella* Enteritidis ATCC 13076, *Salmonella* Typhimurium ATCC 14028, and *Salmonella* Typhimurium ATCC 13311 (**a**). Similarity matrix for pairwise genome comparisons, where the heatmap shows the ortholog cluster between any pair of genomes (**b**).

The comparative analysis of *Salmonella* Enteritidis PT4 578 with three other closely related genomes, one of the same serotype, *Salmonella* Enteritidis ATCC 13076, and two of a different NTS serotype, *Salmonella* Typhimurium ATCC 13311 and *Salmonella* Typhimurium ATCC 14028, showed that they share a core of 3 878 clusters of orthologous proteins (Fig. 4a). In total, *Salmonella* Enteritidis PT4 578 showed 4 214 clusters (Fig. 4a). Of these, only one cluster, which contains two plasma membrane-related proteins, is specific to *Salmonella* Enteritidis PT4 578 (Fig. 4a). The number of clusters shared between strains of the same serotype super passed those shared between strains of different serotypes (Fig. 4b). The pan-genome estimated for these four genomes contains 5 317 genes. Of these, 250 genes were found exclusively in *Salmonella* Enteritidis PT4 578, mainly related to hypothetical proteins, membrane, and metabolism proteins.

### Phenotypes

**Fig. 5.**
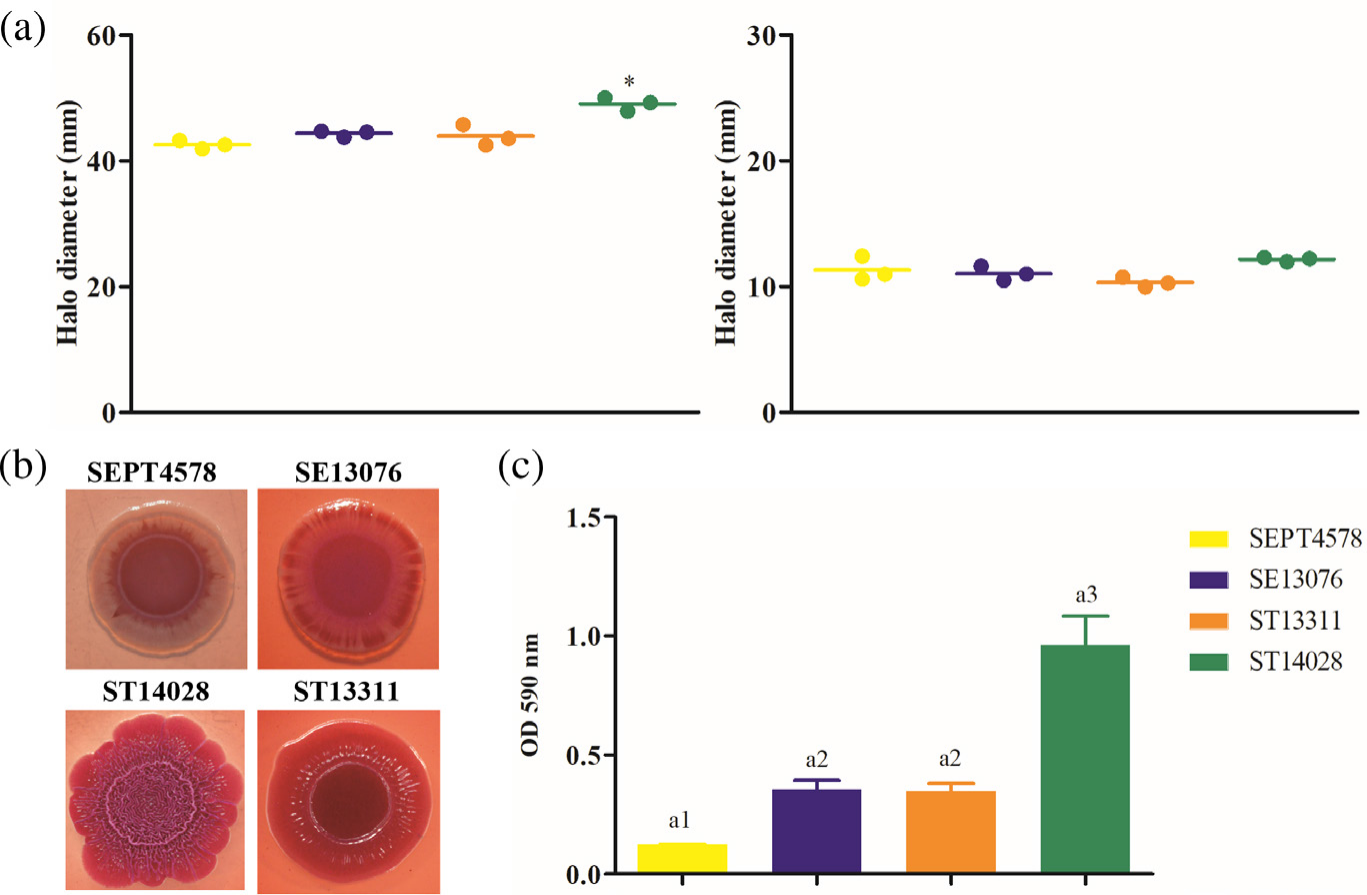
Swimming and swarming motilities of *Salmonella* Enteritidis PT4 578 (SEPT4578), *Salmonella* Enteritidis ATCC 13076 (SE13076), *Salmonella* Typhimurium ATCC 13311 (ST13311), and *Salmonella* Typhimurium ATCC 14028 (ST14028) at 28 °C for 10 and 40 h, respectively (**a**). Colony morphology of SEPT4578, SE13076, ST13311, and ST14028 in Congo red agar (**b**). Adhered cells of SEPT4578, SE13076, ST13311, and ST14028 on polystyrene microplates cultivated at 28 °C for 40 h (**c**). Error bars indicate a standard deviation, and the asterisk (*) and the different numbers mean significant difference (*P*<0.05).

The phenotyping analysis of the four strains assessed their motility, colony morphology, and biofilm formation. All strains showed swimming and swarming motilities at 28 °C after ten hours and 40 h, respectively (Fig. 5a). After ten hours, *Salmonella* Typhimurium ATCC 14028 showed significantly higher swimming motility than the other evaluated strains (Fig. 5a). The colony morphology at 28 °C was highly variable. *Salmonella* Enteritidis PT4 578 did not show a rdar morphotype in Congo red agar, forming white and smooth colonies (saw). *Salmonella* Typhimurium ATCC 14028 was the serotype that showed the typical rdar morphotype. *Salmonella* Typhimurium ATCC 13311 and *Salmonella* Enteritidis ATCC 13076 showed a rdar morphotype with less roughness than *Salmonella* Typhimurium 14 028 (Fig. 5b).

The four strains were grown on polystyrene microplates to assess biofilm formation at 28 °C. The OD 590 nm value was used to classify the strains, according to Stepanovi*c et al*. [39]. *Salmonella* Enteritidis PT4 578 is a non-producing biofilm strain under the condition evaluated. *Salmonella* Typhimurium ATCC 14028 demonstrated strong biofilm formation ability, while *Salmonella* Typhimurium ATCC 13311 and *Salmonella* Enteritidis ATCC 13076 showed a weak biofilm formation (Fig. 5c).

## Discussion

This study analysed the main features of the *Salmonella* Enteritidis PT4 578 genome, a serotype and a phage type of significant clinical relevance and used in several studies of virulence, quorum sensing, and biofilm [14,16,40, 41]. Furthermore, *Salmonella* Enteritidis PT4 is predominant in outbreaks in the western states of the United States and is the most frequently isolated from chicken in Europe and South America [42]. Table 1 shows the general characteristics of this genome and two reference genomes, *Salmonella* Enteritidis P125109 (AM933172.1) and *Salmonella* Typhimurium LT2 (NC_003197.2). Comparatively, the *Salmonella* Enteritidis PT4 578 genome has 143 bp fewer than *Salmonella* Enteritidis P125109 and 171 bp fewer than *Salmonella* Typhimurium LT2. The genomes analysed are according to Lyu *et al.* [43], who analysed genomes of 314 *Salmonella* isolates from retail meat and demonstrated that the average lengths ranged from 4.50 to 5.15 Mbp, with an overall average of 4.78 Mbp. The GC content of the strains is similar. *Salmonella* Enteritidis PT4 578 has more CDS than the reference strains. Minor differences observed in these genomes demonstrate high conservation in the functional capacity.

SPIs are important modulators of *Salmonella* pathogenesis. Of the 24 SPIs described [22,44], the genome of *Salmonella* Enteritidis PT4 578 contains 12 SPIs. SPI-1 and SPI-2 encode T3SS-1 and T3SS-2, which play a crucial role in the invasion, survival, and replication of bacteria in their host cells [45]. The completely conserved structure of SPI-1 and SPI-2 indicates that these systems are functional in *Salmonella* Enteritidis PT4 578 (Fig. 2a). Some effectors translocated were variably distributed, including *sopABDE sspH2*, *sseK1*, *sseK2*, and *srfJ*, encoded outside of SPIs and phage-encoded. In *Salmonella* Enteritidis P125109, 14 SPIs have been described, including SPI-6, SPI-17, SPI-18, and SPI-19 [46] that are absent in the *Salmonella* Enteritidis PT4 578 genome. Suez *et al.* [47] reported the universal presence of SPIs 1–5, 9, 13, and 14, the absence of SPIs 7, 8, and 15, and the variable or mosaic presence of SPIs 6, 10–12 and 16–19 in the genome of all non-typhoidal *Salmonella* isolates. Moreover, an analysis of 45 strains of *Salmonella* Enteritidis demonstrated that six SPIs, including SPI-1, SPI-2, SPI-3, SPI-9, SPI-13, and CS54, are conserved in all isolates while SPI-11 and SPI-12 were absent [48].

*Salmonella* Enteritidis PT4 578 also has a diverse repertoire of fimbriae (Table S1). It shares thirteen fimbrial operons (*csg*, *bcf*, *fim*, *lpf*, *saf*, *sef*, *stb*, *std*, *ste*, *stf*, *sth*, *sti,* and *peg*) with the reference genome *Salmonella* Enteritidis P125019. However, the *pegB* and *pegC* genes of the *peg* operon are absent in the *Salmonella* Enteritidis PT4 578 genome. This operon can influence the cecal colonization of chickens by *Salmonella* Enteritidis [49].

Furthermore, the *Salmonella* Enteritidis PT4 578 genome also contains the *mig-14* gene (macrophage-inducible gene-14). This gene, acquired as a mobile genetic element, encodes a periplasmic protein that inhibits the entry of antimicrobial peptides into the *Salmonella* cytoplasm, making the macrophage a good niche for pathogen replication and survival [50].

The presence of mobile genetic elements is crucial for the pathogenesis of *Salmonella*. Prophage sequences are vehicles for horizontal gene exchange between different bacterial species and are responsible for much of the diversity between strains of the same species [51]. No accident, several SPIs are close to phage-encoding genes or phage-binding sites [20].

*Salmonella* Enteritidis PT4 578 has six prophage regions in its genome, two of which are intact (Fig. 1). The intact regions correspond to *Salmon_118970_sal3* and *Gifsy-2*. These are common regions for the serotype Enteritidis. *Gifsy-2* is a lambda-related prophage that may have a significant role in resistance to oxidative stress in *Salmonella* Enteritidis PT4 578 since it harbours superoxide dismutase *sodCI*. The region assigned to *Salmon_118970_sal3* may play a relevant role in invasion as it contains the genes *sseK2* and *nleB1* and those encoding type III effector proteins. Three other genes that also encode type III effector proteins SseI/SrfH (*Gifsy-2*), SspH2, SspH1(*Gifsy-3*) are in the incomplete region 6. It demonstrates that these three genes also originated from lysogenic conversion events followed by disruptions that resulted in incomplete prophages [52].

Plasmids are also part of the accessory genome of bacteria, contributing to antimicrobial resistance and virulence factors, such as *rck* encoded by *pSLT* in *Salmonella* Typhimurium [53]. However, the genome sequencing of *Salmonella* Enteritidis PT4 578 did not detect plasmid sequences. Conjugative plasmids and ICEs are mobile genetic elements that carry genes that encode the machinery necessary for conjugation [54]. The genome of *Salmonella* Enteritidis PT4 578 contains one integrated region predicted as ICE. Modification of aminoglycosides by *N*-acetyltransferases (AACs) is one of the major mechanisms of resistance to these antibiotics [55]. The AAC(6’)-Iy enzyme is classified in sub-family A of AAC(6’)-I enzymes, and have some structural differences with AAC(6’)-Ie/APH(2’)-Ia, the most important aminoglycoside-modifying enzyme in *Staphylococci* and *Enterococci* [56]. It was demonstrated that AAC(6′)-Iy binds to aminoglycosides, proteins, and peptides [57]. However, aminoglycoside resistance in *Salmonella* is typically secondary to increased gene expression following regulatory mutations [58].

The RM system is a significant component of prokaryotic defence mechanisms against the uptake of foreign DNA [59]. There are four categories of RM systems: type I, II, III, and IV [27]. The *Salmonella* Enteritidis PT4 578 genome contains three types of RM systems: I, II, and III. The CRISPR-Cas system is another significant defence mechanism against foreign DNA, and it can increase phage resistance when associated with the RM system [60]. Two CRISPR loci with eight and nine spacers each, of which 11 are typical protospacers found in plasmids and phages, were detected in *Salmonella* Enteritidis PT4 578 (Table S2). This result demonstrates that these systems efficiently generated immunity against exogenous DNA from bacteriophages and plasmids.

Flagella are the main motility structures of bacteria. Although associated with the planktonic lifestyle, flagella also appear necessary for the early stages of biofilm formation [61]. In the evaluated conditions, all serotypes analysed presented the two types of flagellar motility: swimming and swarming.

The rdar is a widely used manifestation of *Salmonella* multicellular behaviour to evaluate the expression of the adhesive extracellular matrix components cellulose and curli fimbriae [62]. Variations in the expression of cellulose and curli fimbriae result in the development of different morphotypes. *Salmonella* Enteritidis PT4 578 presented the saw morphotype, indicating no expression of these components at 28 °C. On the other hand, *Salmonella* Typhimurium ATCC 14028, *Salmonella* Enteritidis ATCC 13076, and *Salmonella* Typhimurium ATCC 13311 presented the rdar morphotype, indicating the expression of fimbriae and cellulose. In most *Salmonella* strains rdar morphotype is activated at temperatures below 30 °C. The divergent *csg* operon is necessary for the curli fimbriae biosynthesis and includes genes for fimbrial protein subunits (*csgBA*), transcriptional regulation (*csgD*), and curli assembly machinery (*csgC* and *csgEFG*) [63,64]. Cellulose is encoded by the *bcsABZC-bcsEFG* operons, which are under the regulation of *adrA* and *csgD* [65]. The comparative analysis confirmed the conservation of the *csg* and *bsc* operons and *adrA* genes in the *Salmonella* Enteritidis PT4 578 genome and the other three serotypes analysed. It indicates that *Salmonella* Enteritidis PT4 578 has all the genetic requirements to form the rdar morphotype. However, for some reason, it has lost this ability. Some strains may lose the ability to express the rdar morphotype due to regulatory mutations that affect *csgD* expression and *rpoS* mutations in within the cellulose biosynthesis pathway or in unknown upstream rdar morphotype regulators [62].

*Salmonella* strains presenting the rdar morphotype have a great capacity to produce biofilm on abiotic surfaces since the curli fimbriae and cellulose are the main components of the matrix of biofilms [66]. The biofilm formation was consistent with the morphotype results (Fig. 5). Under the evaluated conditions, *Salmonella* Enteritidis PT4 578, which expressed the saw morphotype, did not form biofilm. *Salmonella* Typhimurium ATCC 14028 formed more biofilm than *Salmonella* Typhimurium ATCC 13311, *Salmonella* Enteritidis ATCC 13076, and a rougher morphotype. However, despite not expressing the rdar morphotype and being classified as non-biofilm forming, some studies have shown that *Salmonella* Enteritidis PT4 578 is capable of forming a compact and mature biofilm in the presence of acyl-homoserine lactones, a quorum sensing signal molecule [40,67, 68]. This finding reflects the plastic nature of genomes and demonstrates that specific genes might cease to be essential under certain conditions, become redundant, or even lost throughout evolution.

The results of this study expand the understanding of the diversity and pathogenicity of *Salmonella* Enteritidis PT4 578. An increasing number of available genomes allows expanding the species’ pan-genome and specifying the core genome. Although many virulence genes demonstrate the pathogenic potential of the *Salmonella* Enteritidis PT4 578 strain, many genes may lose functionality or become redundant, resulting in different phenotypes. Likewise, horizontal gene transfer can increase virulence, resistance, or host range.

## Conclusion

This work presents a genomic sequencing and phenotypic characterization of *Salmonella* Enteritidis PT4 strain 578 and provides crucial insights into the virulence mechanisms of this serotype with significant clinical relevance. The whole-genome sequencing revealed 12 SPIs, including highly conserved SPI-1 and SPI-2 regions encoding type III secretion systems (T3SS). Additionally, 3.66% of the coding sequences are virulence factors associated with important host invasion mechanisms. Furthermore, the presence of six prophage regions, two of which are intact, underscores its genomic complexity and adaptability of this strain. The genome also contains three types of restriction-modification sites (RM) systems and two CRISPR loci with protospacers typical of plasmids and phages that highlight its genetic defence mechanisms. Intriguingly, although possessing the genetic requirements for the rdar morphotype, *Salmonella* Enteritidis PT4 578 has lost this ability, as evidenced by its failure to form biofilm under evaluated conditions. The comparative analysis with directly related serotypes shows the adaptability and the potential for varied pathogenic expressions. The expansion of knowledge about the accessory genome of *Salmonella* is essential to contribute to developing detection and control strategies for this pathogen.

## supplementary material

10.1099/acmi.0.000828.v3 Uncited Supplementary Material 1.

10.1099/acmi.0.000828.v3 Uncited Supplementary Material 2.

10.1099/acmi.0.000828.v3 Uncited Supplementary Material 3.

10.1099/acmi.0.000828.v3 Uncited Supplementary Material 4.

## Abbreviations

BDAR: brown, dry, and rough
CDS: coding sequence
CRISPR: clustered regularly interspaced short palindromic repeats
dsDNA: double-stranded DNA
ICEs: integrative and conjugative elements
LB: luria bertani broth
NTS: non-typhoidal serotypes
OD: optical density
ODc: cutoff OD
PDAR: pink, dry, and rough
PHAST: phage search tool
PT4: phage type 4
RDAR: red, dry, and rough
RM: restriction-modification sites
SAW: white and smooth
SPI: *Salmonella* pathogenicity islands
TSB: tryptic soy broth
T3SS1: type III secretion systems 1
T3SS2: type III secretion systems 2
VFDB: virulence factor database
v/v: volume per volume
v/w: volume per weight
