# Supplementary material for: Genome sequencing and analysis of Salmonella enterica subsp. enterica serotype Enteritidis PT4 578: insights into pathogenicity and virulence
Source: Access Microbiol. 2024 Nov 4;6(11):000828.v3. doi: 10.1099/acmi.0.000828.v3 (PMC11649194; doi:10.1099/acmi.0.000828.v3)
Supplement: Uncited Supplementary Material 1. [file acmi-6-00828-s001.pdf]

>EECEBICD_00001 hypothetical protein
MCMCVCVCVYVYHVYMLYVYVYIHVCLMCVCLSLCVCVFFYFVYVVVCVCVINVCVCVCM
CVCVCVCVCVYVCMCVCVCVCVCVYVYVYVCVCVCVCMYVYVYVCVCMCVCVCVCMCVCV
CVCVCVCVYIYICIYMYVYMYICVCVCVCIYVCV
>EECEBICD_00002 hypothetical protein
MCVCVCVCVCVCVCVCVCVCVCVCVCVCVCVCVCVCVCVCVCVCVCMCVYVCVCVCVCVC
VCVCVCVCVCVCVCVCVCVCVCVCVCVSVCVYVCVCMYVCVCVYVCCVCVCMCVCVCVCVCV
CVCVCVCVCVCVCVCVCVCVCVCVCVCVCVCVCVLCMCVCVCVCVCVCVCVCVCVCICMF
LLLCVCVCVYVCVYVCMCVYVYIYVAMSVSSIYMFVYILYIYVYIYICSFVCVCVCVCVC
VCVCVCVCVCVGVCVCVCVCVCVCVCMCMCVCVCVCVCVCVCVCVCVCVCVCVCVCVCVC
VCVYVCVCVCVCVCVYVCVCVCVCVGGGGGGGGGVCVCVCVCVCVCVCVYVCVCVCVCVC
VYVYVCVCVYVCVCVCVCVCVCVCVCVCVCVYVCVYMCVCVCVCMYVCVSVCVCVCVVCV
CVCVCVCVCVCVCVCVCVCVCVCVKKIQN
>EECEBICD_00003 Elongation factor Tu 1
MDGAILVVAATDGPMPQTREHILLGRQVGVPYIIVFLNKCDMVDDEELLELVEMEVRELL
SQYDFPGDDTPIVRGSALKALEGDAEWEAKIIELAGFLDSYIPEPERAIDKPFLLPIEDV
FSISGRGTVVTGRVERGIIKVGEEVEIVGIKETQKSTCTGVEMFRKLLDEGRAGENVGVL
LRGIKREEIERGQVLAKPGTIKPHTKFESEVYILSKDEGGRHTPFFKGYRPQFYFRTTDV
TGTIELPEGVEMVMPGDNIKMVVTLIHPIAMDDGLRFAIREGGRTVGAGVVAKVLG
>EECEBICD_00004 Bacterioferritin-associated ferredoxin
MYVCLCNGISDKKIRQAVRQFHPQSFQQLRKFIPVGNQCGKCIRAAREVMQDELTQMPEF
KEIA
>EECEBICD_00005 Bacterioferritin
MKGDVKIINYLNKLLGNELVAINQYFLHARMFKNWGLTRLNDVEYHESIDEMKHADKYIE
RILFLEGIPNLQDLGKLGIGEDVEEMLRSDLRLELEGAKDLREAIAYADSVHDYVSRDMM
IEILADEEGHIDWLETELDLIAKLGMQNYLQSQIKVTD
>EECEBICD_00006 Type 4 prepilin-like proteins leader peptide-processing enzyme
MFVALPFLIFYASFSLLLGIYDARTGLLPDRFTCPLLWGGLLYHQICLPERLPDALWGAI
AGYGGFALIYWGYRLRYQKEGLGYGDVKYLAALGAWHCWETLPLLVFLAAMLACGGFGVA
LLVRGKSALINPLPFGPWLAVAGFITGWKVFFPDG
>EECEBICD_00007 30S ribosomal protein S10
MQNQRIRIRLKAFDHRLIDQSTAEIVETAKRTGAQVRGPIPLPTRKERFTVLISPHVNKD
ARDQYEIRTHKRLVDIVEPTEKTVDALMRLDLAAGVDVQISLG
>EECEBICD_00008 50S ribosomal protein L3
MIGLVGKKVGMTRIFTEDGVSIPVTVIEVEANRVTQVKDLANDGYRAVQVTTGAKKANRV
TKPEAGHFAKAGVEAGRGLWEFRLAEGEEYTVGQSISVELFADVKKVDVTGTSKGKGFAG
TVKRWNFRTQDATHGNSLSHRVPGSIGQNQTPGKVFKGKKMAGQMGNERVTVQSLDVVRV
DAERNLLLVKGGVPGATGCDLIVKPAVKA
>EECEBICD_00009 50S ribosomal protein L4
MELVLKDAQSALTVSETTFGRDFNEALVHQVVVAYAAGARQGTRAQKTRAEVTGSGKKPW
RQKGTGRARSGSIKSPIWRSGGVTFAARPQDHSQKVNKKMYRGALKSILSELVRQDRLIV
VEKFSVEAPKTKLLAQKLKDMALEDVLIITGELDENLFLAARNLHKVDVRDATGIDPVSL
IAFDKVVMTADAVKQVEEMLA
>EECEBICD_00010 50S ribosomal protein L23
MIREERLLKVLRAPHVSEKASTAMEKTNTIVLKVAKDATKAEIKAAVQKLFEVEVEVVNT
LVVKGKVKRHGQRIGRRSDWKKAYVTLKEGQNLDFVGGAE
>EECEBICD_00011 50S ribosomal protein L2
MAVVKCKPTSPGRRHVVKVVNPELHKGKPFAPLVEKNSKSGGRNNNGRITTRHIGGGHKQ
AYRIVDFKRNKDGIPAVVERLEYDPNRSANIALVLYKDGERRYILAPKGLKAGDQIQSGV
DAAIKAGNTLPMRNIPVGSTVHNVEMKPGKGGQLARSAGTYVQIVARDGAYVTLRLRSGE
MRKVEADCRATLGEVGNAEHMLRVLGKAGAARWRGVRPTVRGTAMNPVDHPHGGGEGRNF
GKHPVTPWGVQTKGKKTRSNKRTDKFIVRRRSK
>EECEBICD_00012 30S ribosomal protein S19

MPRSLKKGPFIDLHLLKKVEKAVESGDKKPLRTWSRRSTIFPNMIGLTIAVHNGRQHVPV
FVSDEMVGHKLGEFAPTRTYRGHAADKKAKKK
>EECEBICD_00013 50S ribosomal protein L22
METIAKHRHARSSAQKVRLVADLIRGKKVSQALDILTYTNKKAAVLVKKVLESAIANAEH
NDGADIDDLKVTKIFVDEGPSMKRIMPRAKGRADRILKRTSHITVVVSDR
>EECEBICD_00014 30S ribosomal protein S3
MGQKVHPNGIRLGIVKPWNSTWFANTKEFADNLDSDFKVRQYLTKELAKASVSRIVIERP
AKSIRVTIHTARPGIVIGKKGEDVEKLRKVVADIAGVPAQINIAEVRKPELDAKLVADSI
TSQLERRVMFRRAMKRAVQNAMRLGAKGIKVEVSGRLGGAEIARTEWYREGRVPLHTLRA
DIDYNTSEAHTTYGVIGVKVWIFKGEILGGMAAVEQPEKPAAQPKKQQRKGRK
>EECEBICD_00015 50S ribosomal protein L16
MLQPKRTKFRKMHKGRNRGLAAGADVSFGSFGLKAVGRGRLTARQIEAARRAMTRAVKRQ
GKIWIRVFPDKPITEKPLAVRMGKGKGNVEYWVALIQPGKVLYEMDGVPEELAREAFKLA
AAKLPIKTTFVTKTVM
>EECEBICD_00016 50S ribosomal protein L29
MKAKELREKSVEELNTELLNLLREQFNLRMQAASGQLQQSHLLKQVRRDVARVKTLLTEK
AGA
>EECEBICD_00017 30S ribosomal protein S17
MTDKIRTLQGRVVSDKMEKSIVVAIERFVKHPIYGKFIKRTTKMHVHDENNECGIGDVVE
IRECRPLSKTKSWTLVRVVEKAVL
>EECEBICD_00018 50S ribosomal protein L14
MIQEQTMLNVADNSGARRVMCIKVLGGSHRRYAGVGDIIKITIKEAIPRGKVKKGDVLKA
VVVRTKKGVRRPDGSVIRFDGNACVILNNNSEQPIGTRIFGPVTRELRNEKFMKIISLAP
EVL
>EECEBICD_00019 50S ribosomal protein L24
MAAKIRRDDEVIVLTGKDKGKRGKVKNVLSSGKVIVEGINLVKKHQKPVPALNQPGGIVE
KEAAIQVSNVAIFNAATGKADRVGFRFEDGKKVRFFKSNSETIK
>EECEBICD_00020 50S ribosomal protein L5
MAKLHDYYKDEVVNKLMTEFNYNSVMQVPRVEKITLNMGVGEAIADKKLLDNAAADLTAI
SGQKPLITKARKSVAGFKIRQGYPIGCKVTLRGERMWEFFERLITIAVPRIRDFRGLSAK
SFDGRGNYSMGVREQIIFPEIDYDKVDRVRGLDITITTTAKSDEEGRALLAAFDFPFRK
>EECEBICD_00021 30S ribosomal protein S14
MAKQSMKAREVKRVALADKYFAKRAELKAIISDVNATDEDRWNAVLKLQTLPRDSSPSRQ
RNRCRQTGRPHAFLRKFGLSRIKVREAAMRGEIPGLKKASW
>EECEBICD_00022 30S ribosomal protein S8
MSMQDPIADMLTRIRNGQAANKAAVTMPSSKLKVAIANVLKEEGFIEDFKVEGDTKPELE
LTLKYFQGKAVVESIQRVSRPGLRIYKRKDELPKVMAGLGIAVVSTSKGVMTDRAARQAG
LGGEIICYVA
>EECEBICD_00023 50S ribosomal protein L6
MSRVAKAPVVVPAGVDVKINGQVITIKGKNGELTRTLNDAVEVKHADNALTFGPRDGYAD
GWAQAGTARALLNSMVIGVTEGFTKKLQLVGVGYRAAVKGNVVNLSLGFSHPVDHQLPAG
ITAECPTQTEIVLKGADKQVIGQVAADLRAYRRPEPYKGKGVRYADEVVRTKEAKKK
>EECEBICD_00024 50S ribosomal protein L18
MDKKSARIRRATRARRKLKELGATRLVVHRTPRHIYAQVIAPNGSEVLVAASTVEKAIAE
QLKYTGNKDAAAAVGKAVAERALEKGIKDVSFDRSGFQYHGRVQALADAAREAGLQF
>EECEBICD_00025 30S ribosomal protein S5
MAHIEKQAGELQEKLIAVNRVSKTVKGGRIFSFTALTVVGDGNGRVGFGYGKAREVPAAI
QKAMEKARRNMINVALNNGTLQHPVKGVHTGSRVFMQPASEGTGIIAGGAMRAVLEVAGV
HNVLAKAYGSTNPINVVRATIDGLENMNSPEMVAAKRGKSVEEILGK
>EECEBICD_00026 50S ribosomal protein L30
MAKTIKITQTRSAIGRLPKHKATLLGLGLRRIGHTVEREDTPAVRGMVNAVSFMVKVEE
>EECEBICD_00027 50S ribosomal protein L15
MRLNTLSPAEGSKKAGKRLGRGIGSGLGKTGGRGHKGQKSRSGGGVRRGFEGGQMPLYRR
LPKFGFTSRKAAITAEVRLSDLAKVEGGVVDLNTLKAANIIGIQIEFAKVILAGEVTTPV

TVRGLRVTKGARAAIEAAGGKIEE
>EECEBICD_00028 Protein translocase subunit SecY
MAKQPGLDFQSAKGGLGELKRRLLFVIGALIVFRIGSFIPIPGIDAAVLAKLLEQQRGTI
IEMFNMFSGGALSRASIFALGIMPYISASIIIQLLTVVHPTLAEIKKEGESGRRKISQYT
RYGTLVLAIFQSIGIATGLPNMPGMQGLVMNPGFAFYFTAVVSLVTGTMFLMWLGEQITE
RGIGNGISIIIFAGIVAGLPPAIAHTIEQARQGDLHFLVLLLVAVLVFAVTFFVVFVERG
QRRIVVNYAKRQQGRRVYAAQSTHLPLKVNMAGVIPAIFASSIILFPATIASWFGGGTGW
NWLTTISLYLQPGQPLYVLLYASAIIFFCFFYTALVFNPRETADNLKKSGAFVPGIRPGE
QTAKYIDKVMTRLTLVGALYITFICLIPEFMRDAMKVPFYFGGTSLLIVVVVIMDFMAQV
QTLMMSSQYESALKKANLKGYGR
>EECEBICD_00029 50S ribosomal protein L36
MKVRASVKKLCRNCKIVKRDGVIRVICSAEPKHKQRQG
>EECEBICD_00030 30S ribosomal protein S13
MARIAGINIPDQKHAVIALTSIYGVGKTRSKAILAAAGIAENVKISELSEEQIDTLRDEV
AKFVVEGDLRREISMSIKRLMDLGCYRGLRHRRGLPVRGQRTKTNARTRKGPRKPIKK
>EECEBICD_00031 hypothetical protein
MRDRSHISNISDAETSGVQSTNSRFAAWTRTFNHNFQILDTVFFHGVSATLCCNLSCKRS
GFARTTETGTTGCCPTQRITLTIGNSHDGVVERSMDMSHAVRDLFSYTFTCANWCLCHYS
ITPIIS
>EECEBICD_00032 30S ribosomal protein S4
MARYLGPKLKLSRREGTDLFLKSGVRAIDTKCKIEQAPGQHGARKPRLSDYGVQLREKQK
VRRIYGVLERQFRNYYKEAARLKGNTGENLLALLEGRLDNVVYRMGFGATRAEARQLVSH
KAIMVNGRVVNIASYQVSPNDVVSIREKAKKQSRVKAALELAEQREKPTWLEVDAGKMEG
TYKRKPERSDLSADINEHLIVELYSK
>EECEBICD_00033 DNA-directed RNA polymerase subunit alpha
MQGSVTEFLKPRLVDIEQVSSTHAKVTLEPLERGFGHTLGNALRRILLSSMPGCAVTEVE
IDGVLHEYSTKEGVQEDILEILLNLKGLAVRVQGKDEVILTLNKSGIGPVTAADITHDGD
VEIVKPQHVICHLTDENASISMRIKVQRGRGYVPASTRIHSEEDERPIGRLLVDACYSPV
ERIAYNVEAARVEQRTDLDKLVIEMETNGTIDPEEAIRRAATILAEQLEAFVDLRDVRQP
EVKEEKPEFDPILLRPVDDLELTVRSANCLKAEAIHYIGDLVQRTEVELLKTPNLGKKSL
TEIKDVLASRGLSLGMRLENWPPASIADE
>EECEBICD_00034 50S ribosomal protein L17
MRHRKSGRQLNRNSSHRQAMFRNMAGSLVRHEIIKTTLPKAKELRRVVEPLITLAKTDSV
ANRRLAFARTRDNEIVAKLFNELGPRFASRAGGYTRILKCGFRAGDNAPMAYIELVDRSE
KTEAAAE
>EECEBICD_00035 hypothetical protein
MWLLDQWAERHIIEAQRKGEFDNLPGRGEPLILDDDSHVPAELRAGYRLLKNAGCLPPEL
EQRRDAIQLLDILNSIREDDPRYHQVSRQLSLLELKLRQAGLSTDFLHGEYAEKLLHKIN
DN
>EECEBICD_00036 HTH-type transcriptional regulator ZntR
MYRIGELAKLADVTPDTIRYYEKQQMMDHEVRTEGGFRLYTETDLQRLKFIRYARQLGFT
LDSIRELLSIRIDPEHHTCQESKSIVQERLQEVEARIAELQTMQRSLQRLNDACCGTAHS
SVYCSILEALEQGASGAKSGC
>EECEBICD_00037 hypothetical protein
MSRYQHKKGQIKDNAIEALLHDPLFRQRVEKNKKGKGSYLRKGKHGNRGNWEASGKKVSN
FFTTGLLLIKNGYFVLSADRGFRPIALPL
>EECEBICD_00038 Large-conductance mechanosensitive channel
MSFIKEFREFAMRGNVVDLAVGVIIGAAFGKIVSSLVADIIMPPLGLLIGGIDFKQFAFT
LREAQGDIPAVVMHYGVFIQNVFDFVIVAFAIFVAIKLINRLNRKKAEEPAAPPAPSKEE
VLLGEIRDLLKEQNNRS
>EECEBICD_00039 Trk system potassium uptake protein TrkA
MKIIILGAGQVGGTLAENLVGENNDITVVDTNGERLRSLQDKFDLRVVQGHGSHPRVLRE
AGADDADMLVAVTSSDETNMVACQVAYSLFNTPNRIARIRSPDYVRDADKLFHSEAVPID
HLIAPEQLVIDNIYRLIEYPGALQVVNFAEGKVSLAVVKAYYGGPLIGNALSTMREHMPH

IDTRVAAIFRHDRPIRPQGSTIVEAGDEVFFIAASQHIRAVMSELQRLEKPYKRIMLVGG
GNIGAGLARRLEKDYSVKLIERDQQRAAELAEKLQNTIVFFGDASDQELLAEEHIDQVDL
FIAVTNDDEANIMSAMLAKRMGAKKVMVLIQRRAYVDLVQGSVIDIAISPQQATISALLS
HVRKADIVGVSSLRRGVAEAIEAVAHGDESTSRVVGRVIDEIKLPPGTIIGAVVRGNDVM
IANDNLRIEQGDHVIMFLTDKKFITDVERLFQPSPFFL
>EECEBICD_00040 Ribosomal RNA small subunit methyltransferase B
MKKQNNLRSLAAQAVELVVEQGQSLSNVLPPLQQKVADKDKALLQELCFGVLRTLSQLEW
LINKLMSRPMTGKQRTVHYLIMVGFYQLLYTRVPPHAALAETVEGAVAIKRPQLKGLING
VLRQFQRQQETLLNEFATSDARFLHPGWLVKRLQNAYPTQWQHIIEANNQRPPMWLRVNR
THHTRDGWLGLLEDAGMKGYPHPDYPDAVRLETPAPVHALPGFAEGWVTVQDASAQGCAV
FLAPQNGEHILDLCAAPGGKTTHILEVAPEADVLAVDIDEQRLSRVYDNLKRLGMKATVK
QGDGRYPAQWCGEQQFDRILLDAPCSATGVIRRHPDIKWLRRDRDIAELAQLQAEILDAV
WPRLKPGGTLVYATCSVLPEENRDQIKTFLQRTPDAALSETGTPDQPGQQNLPGGEEGDG
FFYAKLIKK
>EECEBICD_00041 Methionyl-tRNA formyltransferase
MSDSLRIIFAGTPDFAARHLDALLTSGHNVVGVFTQPDRPAGRGKKLMPSPVKVLAEEKG
LPVFQPVSLRPQENQQLVADLHADVMVVVAYGLILPKAVLDMPRLGCINVHGSLLPRWRG
AAPIQRSLWAGDAETGVTIMQMDVGLDTGDMLYKLACPITAEDTSGSLYNKLAELGPQGL
ITTLKQLADGTAAPEAQNEALVTHAEKLSKEEARIDWSLSAVQLERCIRAFNPWPMSWLE
IDGQPVKVWQASVIEDATQSLPGTILAATKQGIQVATGKGILNLLSLQPAGKKAMSAQDL
LNSRREWFIPGNRLA
>EECEBICD_00042 Peptide deformylase
MSVLQVLHIPDERLRKVAKPVEEVNAEIQRIVDDMFETMYAEEGIGLAATQVDIHQRIIV
IDVSENRDERLVLINPELLEKSGETGIEEGCLSIPEQRALVPRAEKVKIRALDRDGNPFE
LEADGLLAICIQHEMDHLVGKLFIDYLSPLKQQRIRQKVEKLDRLNARA
>EECEBICD_00043 hypothetical protein
MARTEIWLRLMYVGDLYGEAMLNMANSLIRQPQINRTHLQEAGLTARQAERFLQLPAGVL
DETLRWLELPQHHFLCADSEIYPPQLRAIDDYPGAIFIDGDPACLHTCQLAVVGSRSHSW
YGERWGRLLCESLAKSGLTITSGLARGIDGVAHNAAVSMGGKSVAVLGNGLAKIYPRRHA
MLAENLIATGGAVVSEFPLSTPPLPQNFPRRNRIISGLSKGVLVIEAALRSGSLVTARCA
LEQGRDVFALPGPIGSPGSEGTHWLIKQGATLVTTPEDILENLQYGLHWLPTTAENSLYS
LNQDEAALPFPELLANVGDEVTPVDVVAERAGQPVPAVVAQLLELELAGWIAAVPGGYVR
LRRASHVRRTNVFV
>EECEBICD_00044 Protein Smg
MFDVLMYLFETYIHNEAELRVDQDRLERDLTDAGFDREDIYNALLWLEKLADYQDGLAEP
MQLASDPLSMRIYTVEECERLDASCRGFLLFLEQIQVLNLETREMVIERVLALDTAEFDL
EDLKWVILMVLFNIPGCENAYQQMEELLFEVNEGMLH
>EECEBICD_00045 DNA topoisomerase 1
MAKSALFSVRKNEPCPQCGAELVIRSGKHGPFLGCSRYPECDYVRPLKSQADGHIVKILE
GQPCPECGAVLVLRQGRFGMFIGCSQYPQCEHTVVIDKPDETAIACPACQQGHLVQRRSR
YGKIFHSCDRYPECQFVINFTPVAGECPECHYPLLIEKKTAQGVKRFCASKQCGKPVPVE
>EECEBICD_00046 Threonylcarbamoyl-AMP synthase
MNNSLPTGSIAAAVDLLNKENVIAYPTEAVFGVGCDPDSETAVTRLLALKQRPVDKGLIL
IAASFEQLKPYIDDSILTAAQRKAVFDCWPGPVTFVFPAPATTPRWLTGRFDSLAVRVTN
HPLVVALCNAYGKPLVSTSANLSGLPPCRTIEEVRAQFGDDFPVVEGATGGRLNPSEIRD
ALTGELFRQG
>EECEBICD_00047 Shikimate dehydrogenase (NADP(+))
MMETYAVFGNPIAHSKSPFIHQQFAQQLDIVHPYGRVLAPINNFINTLDAFFAAGGKGAN
ITVPFKEEAFARSDELTERASLAGAVNTLKRLEDGRLLGDNTDGIGLLSDLERLNFIRPG
LRILLIGAGGASRGVLLPLLSLDCAVTITNRTASRAEALAKIFAHTGSVHATDMDKLDGC
EFDLIINATSSGIRGEIPAIPASLIHPSLCCYDMFYQKGNTPFLSWCVQQGAKRYADGLG
MLVGQAAHAVLLWHGVLPQVEPVIELLQQELLA
>EECEBICD_00048 hypothetical protein
MNQAIQFPDREEWDTAASAIIFPALVNGMQLTCAIKKDVLAYRFGGETAEQWLAIFREYR

WDLEEEAEALILAQQEDDHGWIWLS
>EECEBICD_00049 Protein YrdA
MSDTLRPYKDLFPEIGQRVMIDTSSVVIGDVRLADDIGIWPLVVIRGDVNYVAIGARTNI
QDGSVLHVTHKSSSNPHGNPLIIGEDVTVGHKVMLHGCTIGNRVLVGMGSIVLDGAIIED
DVMIGAGSLVPQHKRLESGYLYLGSPVKQIRPLSDAERSGLQYSANNYVKWKDDYLSQDN
HIQP
>EECEBICD_00056 hypothetical protein
MKRLIPVALLTTLLAGCAHDSPCVPVYDDQGRLVHTNTCMKGTTQDNWETAGAIAGGAAA
VAGLTMGIIALSK
>EECEBICD_00057 Multidrug export protein AcrF
MANFFIRRPIFAWVLAIILMMAGALAIMQLPVAQYPTIAPPAVSISATYPGADAQTVQDT
VTQVIEQNMNGIDNLMYMSSTSDSAGSVTITLTFQSGTDPDIAQVQVQNKLQLATPLLPQ
EVQQQGISVEKSSSSFLMVAGFVSDNPNTTQDDISDYVASNIKDSISRLNGVGDVQLFGA
QYAMRIWLDANLLNKYQLTPVDVINQLKVQNDQIAAGQLGGTPALPGQQLNASIIAQTRL
KDPEEFGKVTLRVNTDGSVVHLKDVARIELGGENYNVVARINGKPASGLGIKLATGANAL
DTATAIKAKLAELQPFFPQGMKVVYPYDTTPFVKISIHEVVKTLFEAIILVFLVMYLFLQ
NIRATLIPTIAVPVVLLGTFAVLAAFGYSINTLTMFGMVLAIGLLVDDAIVVVENVERVM
MEDNLPPREATEKSMSQIQGALVGIAMVLSAVFIPMAFFGGSTGAIYRQFSITIVSAMAL
SVLVALILTPALCATLLKPVSAEHHEKKSGFFGWFNTRFDHSVNHYTNSVSGIVRNTGRY
LIIYLLIVVGMAVLFLRLPTSFLPEEDQGVFLTMIQLPSGATQERTQKVLDQVTHYYLNN
EKANVESVFTVNGFSFSGQGQNSGMAFVSLKPWEERNGEENSVEAVIARATRAFSQIRDG
LVFPFNMPAIVELGTATGFDFELIDQGGLGHDALTKARNQLLGMVAKHPDLLVRVRPNGL
EDTPQFKLDVDQEKAQALGVSLSDINETISAALGGYYVNDFIDRGRVKKVYVQADAQFRM
LPGDINNLYVRSANGEMVPFSTFSSARWIYGSPRLERYNGMPSMELLGEAAPGRSTGEAM
SLMENLASQLPNGIGYDWTGMSYQERLSGNQAPALYAISLIVVFLCLAALYESWSIPFSV
MLVVPLGVVGALLAASLRGLNNDVYFQVGLLTTIGLSAKNAILIVEFAKDLMEKEGRGLI
EATLEASRMRLRPILMTSLAFILGVMPLVISRGAGSGAQNAVGTGVMGGMLTATLLAIFF
VPVFFVVVKRRFNRHHD
>EECEBICD_00058 Multidrug export protein AcrE
MTKHARFSLLPSFIIFSAALLAGCNDQGDTQAHAGEPQVTVHVVETAPLAVTTELPGRTS
AFRIAEVRPQVSGIVLKRNFTEGSDVEAGQSLYQIDPATYQADYDSAKGELAKSEAAAAI
AHLTVKRYVPLVGTKYISQQEYDQAIADARQADAAVVAAKAAVESARINLAYTKVTSPIS
GRIGKSNVTEGALVTNGQSTELATVQQLDPIYVDVTQSSNDFMRLKQSVEQGNLHKDSAS
STVQLVMENGQVYPIKGTLQFSDVTVDESTGSITLRAVFPNPQHSLLPGMFVRARIDEGV
QPNAILVPQQGVTRTPRGDAMVMVVNDKSQVEARNVVAAQAIGDKWLISEGLKPGDKVIV
SGLQKARPGVQVKATTDAPAAKTAQ
>EECEBICD_00059 HTH-type transcriptional regulator AcrR
MAKKTKADALKTRQHLIETAIAQFALRGVANTTLNDIADAADVTRGAIYWHFENKTQLFN
EVWLQQPPLRELIQDRLTGCWNDNPLQDLREKFIAALQYIAAVPRQQALMQILYHKCEFH
NGMISEQAIREKMGFHHQSLLEVLQRCMDKKLISGSLDLDVILIIILHGSFSGIVKNWLMN
PTSYDLYKQAPALVDNVLKMLSPDGSVRQLMPNEQQAEEA
>EECEBICD_00060 putative signaling protein
MPVSEYNHILVAVSFAVAIFASYTALNMAGRVAGSARSNARIWLMGGGFALGVGIWAMHF
VGMLAMDHAMNMRFDPFLTGLSMLIAIGSSLFALWLVSAEKLRLRRLLPGALVMGLGISA
MHYTGMAALQFASIIVWNSAWVALSIIIALLASCGALWLTFRLRHEGTDVALRRAGAAVL
MGIAIAGMHYAGMKAAHFPQNWPMEHRGVDSNWLAVLVSVVALTILGITLLVSLFDARLQ
ARTALLASSLAQANQELAQLALHDTLTRLPNRVLLEDRLEQAISKANRENTSFALLFMDL
DGFKTVNDAYGHDIGDKLLVAVTHRLNQPLSGQFTLARIGGDEFVLLAEVSAPDEAASLA
SALVHSIDAPFTIDPYELVVTLSVGIALYPLDGKNERELMFNADAAMYHTKHTGRNGYHF
FQPSMNMLAQTQLQLMNDLWLALERQELRLVYQPKFQASAGPIVGFEALLRWYHPKQGVL
NPDQFLPLAEKTGLIVTIGSWVIDEACRQLREWHLQGYDLWSVAVNLSALQFEQPGLVDT
ITRSLARHSIRPDLLILEITETTAMNKPEQSVAILTRLTEMGVKASIDDFGTGYSSLLYL
KRLPACELKIDRAFVHELSEAGDGATIVAAIVALAKALNLQIVAEGVENETQQFLTQLG
CHTLQGFLLGKPRTAEEIARDIRDPANIFTRSIYNINSK

>EECEBICD_00061 hypothetical protein
MIRKYWWLVVFAVSVFLFDALLMQWIELMATEQDKCRNMDSVNPLKLINCTDLE
>EECEBICD_00062 DNA adenine methyltransferase YhdJ
MKAECEPQYFGDESKKIIHGDALTELKKLPSESIDLIFADPPYNIGKDFDGMVESWDEAS
FLAWLYECIDECHRVLKKHGTMYIMNSTENMPYIDLKCRTLFTIKSRIVWSYDSSGVQAK
KYFGSMYEPILMMVKNPKSYTFNRDAILVETTTGAKRALIDYRKNPPQPYNQKKVPGNVW
SFPRVRYLMDEYENHPTQKPSALLKRIILASSNPSDTVLDPFAGSFTTGAVAAASGRKFI
GIELNNEYVKMGLRRLSVTSHYSENELAKVKKRKTQNLSKKQRNVGINALSSEK
>EECEBICD_00063 DNA-binding protein Fis
MFEQRVNSDVLTVSTVNSQDQVTQKPLRDSVKQALKNYFAQLNGQDVNDLYELVLAEVEQ
PLLDMVMQYTRGNQTRAALMMGINRGTLRKKLKKYGMN
>EECEBICD_00064 tRNA-dihydrouridine synthase B
MRIGQYQLRNRLIAAPMAGITDRPFRTLCYEMGAGLTVSEMMSSNPQVWESDKSRLRMVH
VDEPGIRTVQIAGSDPVEMADAARINVESGAQIIDINMGCPAKKVNRKLAGSALLQYPDL
VKSILIGVVNAVDVPVTLKIRTGWAPEHRNCVEIAQLAEDCGIQALTIHGRTRACLFNGE
AEYDSIRAVKQKVSIPIIANGDITNPHKARAVLDYTGADALMIGRAAQGRPWIFREIQHY
LDTGELLPPLPLAEVKRLLCTHVRELHDFYGQAKGYRIARKHVSWYLQEHAPDDQFRRTF
NAIEDASEQLEALEAYFENFA
>EECEBICD_00065 hypothetical protein
MLQNNPDSITKYEGDGCDPLKRQYLLSAGSSCLSRRIVTKKLFQAFI
>EECEBICD_00066 Ribosomal protein L11 methyltransferase
MPWIQLKLNTTGANAEELSDALMEAGAVSITFQDTHDTPVFEPLPGETRLWGDTDVIGLF
DAETDMKDVVAILEQHPLLGAGFAHKIEQLEDKDWEREWMDNFHPMRFGERLWICPSWRD
IPDENAVNVMLDPGLAFGTGTHPTTSLCLQWLDGLDLNGKTVIDFGCGSGILAIAALKLG
AAKAIGIDIDPQAIQASRDNAERNGVSDRLELYLPKDQPEAMKADVVVANILAGPLRELA
PLISVLPVEGGLLGLSGILASQAESVCDAYAELFTLDPVVEKEEWCRITGRKK
>EECEBICD_00067 Sodium/pantothenate symporter
MLFARYQSRLLVWLASLSLLVAFIGAMTVQFIGGARLLETAAGIPYETGLLIFGVSIALY
TAFGGFRASVLNDTLQGLVMLVGTIVLLVGVIHAAGGLSQAVDTLHALDPKLVTPQGADD
ILSPAFMTSFWVLVCFGVIGLPHTAVRCISYKDSKAVHRGIIIGTIVVAILMFGMHLAGA
LGRAVLPDLTVPDLVIPTLMVKVLPPFAAGIFLAAPMAAIMSTINAQLLQSSATIIKDLY
LNLRPDQMQNEIRLKRMSAAITLLLGALLLLAAWKPPEMIIWLNLLAFGGLEAVFLWPLV
LGLYWERANAAGALSAMIVGGVLYALLATFNIQYLGFHPIVPALLLSLLAFLIGNRFGSS
ASQATVLSTDK
>EECEBICD_00068 hypothetical protein
MQLEVILPLVAYLVVVFGVSIYAMRKRTAGTFLNEYFLGSRSMGGIVLAMTLTATYISAS
SFIGGPGAAYKYGLGWVLLAMIQLPAVWLSLGILGKNSLSWRVAIMPSRLTICYSRVTKA
ACWCGWQA
>EECEBICD_00069 putative membrane protein YhdT
MDARFVQAHKEARWALWLTLCYLAAWLVAAYLPGDSPGITGLPHWFEMACLLTPLVFILL
CWAMVKFIYRDISLEDDDAA
>EECEBICD_00070 Biotin carboxylase
MLDKIVIANRGEIALRILRACKELGIKTVAVHSSADRDLKHVLLADETVCIGPAPSVKSY
LNIPAIISAAEITGAVAIHPGYGFLSENANFAEQVERSGFIFIGPKADTIRLMGDKVSAI
TAMKKAGVPTVPGSDGPLGDDMNANRAHAKRIGYPVIIKASGGGGGRGMRVVRSDAELAQ
SISMTKAEAKAAFSNDMVYMEKYLENPRHIEIQVLADGQGNAIYLAERDCSMQRRHQKVV
EEAPAPGITPELRRYIGERCAKACVDIGYRGAGTFEFLFENGEFYFIEMNTRIQVEHPVT
EMITGVDLIKEQLRIAAGQPLSITQDEVVVRGHAVECRINAEDPNTFLPSPGKITRFHAP
GGFGVRWESHIYAGYTVPPYYDSMIGKLICYGENRDVAIARMKNALQELIIDGIKTNIDL
QTRIMNDEHFQHGGTNIHYLEKKLGLQEK
>EECEBICD_00071 Biotin carboxyl carrier protein of acetyl-CoA carboxylase
MDIRKIKKLIELVEESGISELEISEGEESVRISRTTANAGFPVMQQAYAAPMMQQPALSN
AVAPAATPAMEAPAAAEISGHIVRSPMVGTFYRTPSPDAKAFIEVGQKVNVGDTLCIVEA
MKMMNQIEADKAGTVKAILVESGQPVEFDEPLVVIE

>EECEBICD_00072 Protein-methionine-sulfoxide reductase heme-binding subunit MsrQ
MRLTAKQITWLKVCLHLAGFLPLLWLFWAINHGGLSADPVKDIQHFTGRTALKFLLATLL
VSPLARYAKQPLLIRTRRLLGLWCFVWATLHLTSYALLELGIHNLALLGSELISRPYLTL
GIISWLVLLALTLTSTQFAQRKLGKRWQTLHNVVYLVAILAPIHYLWSVKILSPQPVIYA
ALALALLALRYRRFRQWWR
>EECEBICD_00073 Protein-methionine-sulfoxide reductase catalytic subunit MsrP
MKKIRPLTEADVTAESAFFMQRRQVLKALGISAAALSLPSTAQADLFSWFKGNDRPKAPA
GKPLEFSQPAAWRSDLALTPEDKVTGYNNFYEFGLDKADPAANAGSLKTEPWTLKISGEV
AKPFTLDYDDLTHRFPLEERIYRMRCVEAWSMVVPWIGFPLYKLLAQAQPTSHAKYVAFE
TLYAPDDMPGQKDRFIGGGLKYPYVEGLRLDEAMHPLTLMTVGVYGKALPPQNGAPIRLI
VPWKYGFKGIKSIVSIKLTRERPLTTWNLSAPNEYGFYANVNPHVDHPRWSQATERFIGS
GGILDVQRQPTLLFNGYANEVASLYRGLNLRENF
>EECEBICD_00074 putative acrylyl-CoA reductase AcuI
MQALILEQQDGKTLASVQHLEESQLPAGDVTVDVHWSSLNYKDALAITGKGKIIRHFPMI
PGIDFAGTVHASEDPRFHAGQEVLLTGWGVGENHWGGLAERARVKGDWLVALPAGLSSRN
AMIIGTAGFTAMLCVMALEDAGIRPQDGEVVVTGASGGVGSTAVALLHKLGYQVAAVSGR
ESTYGYLKSLGANRILSRDEFAESRPLEKQLWAGAIDTVGDKVLAKVLAQMNYGGCVAAC
GLAGGFALPTTVMPFILRNVRLQGVDSVMTPPARRAEAWARLVKDLPESFYAQAATEITL
ADAPKFADAIINNQVQGRTLVKIK
>EECEBICD_00075 RNase E specificity factor CsrD
MRLTTKFSAFITLLTGLTIFVTLIGCSLSFYNAVQYKYVSRVQATATAIDTHLVTHDIVS
LTPQIDELMIASDIVRVDLLQGERSVYSHSRARGYRPAGTSDMYRELVVPLIKHPGMSLR
LAYQDPMGNYFHSLITTAPLTLAIGFIVLILFLSVRWLQRQLSGQELLEIRSTRILNGER
GANVRGSVYEWPARTSSALDVLLSEIQFAHEQRSRLDTLIRSYAAQDTKTGLGNRLFFDN
QLATLLEDQEKVGVHGVVMMIRLPDFNLLRDSLGGNQAEEQMFMLINLLSTFIMRYPGSL
LARYHRSDFAVLLPHRTLKEAESIAGQLLKAVDALPANKMLDRDDMVHIGICAWRSGQST
EQVIEHAEAAARNAALQGGNSWAIYDDTLPEKGRGNVRWRTLIEQMLNRGGPRLYQKPAV
TREGRVHHRELMCRIYDGKEEVSSAEYMPMVLQFGLSEEYDRLQISRLITLLGYWPDENL
AMQLTVESLIRPRFQRWLRDTLMQCEKSQRNRIIIELAEADVCQHICRLQPILRLVNALG
VRVAVTQAGLTLVSTSWIKALNVELLKLHPSLVRNIEKRTENQLLVQSLVEACAGTPTQV
YATGVRSRGEWQTLTKRGVAGGQGDFFASSQLLDTNVKKYSQRYSV
>EECEBICD_00076 Cell shape-determining protein MreB
MLKKFRGMFSNDLSIDLGTANTLIYVKGQGIVLNEPSVVAIRQDRAGSPKSVAAVGHDAK
QMLGRTPGNIAAIRPMKDGVIADFFVTEKMLQHFIKQVHSNSFMRPSPRVLVCVPVGATQ
VERRAIRESAQGAGAREVFLIEEPMAAAIGAGLPVSEATGSMVVDIGGGTTEVAVISLNG
VVYSSSVRIGGDRFDEAIINYVRRNYGSLIGEATAERIKHEIGSAYPGDEVREIEVRGRN
LAEGVPRGFTLNSNEILEALQEPLTGIVSAVMVALEQCPPELASDISERGMVLTGGGALL
RNLDRLLMEETGIPVVVAEDPLTCVARGGGKALEMIDMHGGDLFSEE
>EECEBICD_00077 Cell shape-determining protein MreC
MKPIFSRGPSLQIRLILAVLVALGVIIADSRLGTFSQIRTYMDTAVSPFYFISNGPRELL
DSVSQTLASRDQLELENRALRQELLLKNSDLLMLGQYKQENARLRELLGSPLRQDEQKMV
TQVISTVNDPYSDQVVIDKGSVNGVYEGQPVISDKGVVGQVVAVAKLTSRVLLICDATHA
LPIQVLRNDIRVIAAGNGCTDDLQLEHLPANTDIRVGDVLVTSGLGGRFPEGYPVAVVSS
VKLDTQRAYTVIQARPTAGLQRLRYLLLLWGADRNGANPMTPEEVHRVANERLMQMMPQV
LPSPDAMGPPAPVPDPATGITQPSAGQTAPVSTQPSPSGATTPPARAPGG
>EECEBICD_00078 Rod shape-determining protein MreD
MVASYRSQGRWVIWLSFLIALLLQIMPWPDDIIVFRPNWVLLILLYWILALPHRVNVGTG
FVMGAILDLISGSTLGVRALSMSIVAYLVALKFQLFRNLALWQQALVVMLLSLAVDIIVF
WAEFLVINVSFRPEVFWSSVVNGVLWPWLFLLMRKVRQQFAVQ
>EECEBICD_00079 dTTP/UTP pyrophosphatase
MTTLYLASGSPRRQELLTQLGFSFEQVVPGIEEQRRAQESAQQYVVRLAREKAQAGVALV
PRDLPVLGADTIVVLNGEVLEKPCDAAHAAEMLRLLSGNTHQVMTAVALADSQQTLDCVV

VTEVTFRTLSAQDITGYVASGEPLDKAGAYGIQGRGGCFVRKINGSYHAVVGLPLVETYE
LLSHFNALRDKRDKHDG
>EECEBICD_00080 Ribonuclease G
MTAELLVNVTPSETRVAYIDGGILQEIHIEREARRGIVGNIYKGRVSRVLPGMQAAFVDI
GLDKAAFLHASDIMPHTECVAGDEQKQFTVRDISELVRQGQDLMVQVVKDPLGTKGARLT
TDITLPSRYLVFMPGASHVGVSQRIESESERERLKKVVAEYCDEQGGFIIRTAAEGVCEE
DLASDAAYLKRVWTKVMERKKRPQTRYQMYGELALAQRVLRDFADAQLDRIRVDSRLTYE
SLLEFTAEYIPEMTSKLEHYSGHQPIFDLYDVENEIQRALERKVELKSGGYLIIDQTEAM
TTVDINTGAFVGHRNLDDTIFNTNIEATQAIARQLRLRNLGGIIIIDFIDMNNEDHRRRV
LHSLEQALSKDRVKTSINGFSPLGLVEMTRKRTRESVEHVLCNECPTCHGRGTVKTVETV
CYEIMREIVRVHHAYDSDRFLVYASPAVAEALKGEESHALAEVEIFVGKQVKVQVEPLYN
QEQFDVVMM
>EECEBICD_00081 hypothetical protein
MRRLPGILLLTGAALIVIAALLVSGLRLALPHLDAWRPAILNKIESVTGVPVAASQLSAS
WQNFGPTLEAHNIHAALKDGGELSIKRVTLALDVWQSLLHMRWQFRDLTFWQLNFRTNTP
LQSSDGKGIETSRLSDLFLRQFDHFDLRDSQISFLTLSGQRAELAIPQLTWLNGKERHRA
EGEVSLSSLTGQHGVMQVRMDLRDDDGLLNNGRVWLQADDIDVKPWLGKWMQDNVALQTA
RFSLEGWMTLSKGEIAGGDVWLKQGGASWLGDNTTHTLSVDNLTAQISREQPGWQFYIPD
TRITLDGKPWPSGALTVAWLPQQDVGGENHTRSDELRIRASNLELAGLEALRPLAAKLSP
VLGEIWQATQPSGKIATLALDIPLQATEKTRFQASWENLAWKQWKLLPGAEHFSGTLAGS
VEDGQMKVAMQQAKMPYETVFRAPLEIENGVATLSWLKNENGFQLDGRDIDVKAKAVHAR
GGFRYLQPTGDEPWLGILAGISTDDGSQAWRYFPENLMGKALVDYLSGAIQGGEADNATL
VYGGNPHLFPYKHNEGQFEVLVPLRNATFAFQPDWPALKNLNIELDFLNDGLWMRSDSVD
LGGVKASKLAATIPDYSKEKLLIDADINGPGKAVGPYFDETPLKDSLGSTLAELQLDGDV
NARLHLDIPLDGEQVTAEGDVSLRNNSLFIKPLNSTLKNLNGKFSFVNGALKSGPLTANW
FNQPLNLDFSTTEGAKAYQVAVNLNGNWQPTRMGVLPPQLNDALSGSVTWNGKVGIDLPY
HADTTYHIELNGDLRNVSSHLPSPLNKPAGEAIPVNIQADGNLKSFALTGSAGSKNHFNS
RWLLNQKLTLDRAIWTTDSRTIPPLPAQQGVELNLPALDGAQWLALFQKGAADNVSSSAE
FPQRVTLRTPALSLGGQQWNNLSVVSAPSLNGTKIEAQGREVNATLLMRNHAPWLANIKY
LYYNPGVAKTHASSPTPTSPLASANTISFRGWPDLQLRCEECWLWGQKYGRIDGDFAIKG
NTLTLANGLIDTGFARLKANGEWVNAPGNERTSLKGSLHGSNLDTAAGFFGISTPIQNAS
FNVDYDLHWRNPPWQPEEATLNGILRTRLGKGEFTDLSSGHAGQLLRLLSFDALLRKLRF
DFRDTFSEGFYFDSIHSTAWIKDGVLHTDDTLVDGLEADIAMKGSVDLVRRRLDMEAVVA
PEISATVGVAAAFAVNPIVGAAVFAASKVLGPLWSKVSILRYRITGPVDAPQINEVLRQP
IKESQQ
>EECEBICD_00082 Metalloprotease TldD
MSLNLVSEQLLAANGLNHQDLFAILGQLAERRLDYGDLYFQSSYHESWVLEDRIIKDGSY
NIDQGVGVRAISGEKTGFAYADQISLLALEQSAQAARTIVRENGEGKVKTLAAVAHQPLY
TTLDPLQSMSREEKLDILRRVDKVAREADKRVQEVNASLTGVYELILVAATDGTLAADVR
PLVRLSVSVQVEEDGKRERGASGGGGRFGYEYFLADLDGEVRADAWAKEAVRMALVNLSA
VAAPAGTLPVVLGAGWPGVLLHEAVGHGLEGDFNRRGTSVFSGQIGEQVASALCTVVDDG
TMMNRRGSVAIDDEGTPGQYNVLIENGVLKGYMQDKLNARLMGAAPTGNGRRESYAHLPM
PRMTNTYMLAGQSTPQEIIESVEYGIYAPNFGGGQVDITSGKFVFSTSEAYLIENGKVTT
PVKGATLIGSGIETMQQISMVGNDLKLDNGVGVCGKEGQSLPVGVGQPTLKVDNLTVGGT
A
>EECEBICD_00083 HTH-type transcriptional regulator DmlR
MERLKRMSVFAKVVEFGSFTAAARQLQMSVSSISQTVAKLEDELQVKLLNRSTRSIGLTE
AGKIYYQGCRRMLHEVQDVHEQLYAFNNTPIGTLRIGCSSTMAQNVLAGLTAKLLKEYPG
LAVNLVTGIPAPDLIADGLDVVIRVGALQDSSLFSRRLGAMPMVVCAAKPYLAQYGVPEK
PADLSSHSWLEYSVRPDNEFELIAPEGISTRLIPQGRFVTNDPMTLVRWLTAGTGIAYVP
LMWVIDEINRGDLEILLPRYQSDPRPVYALYTEKDKLPLKVQVVINALTDYFVDVAHLFQ
GMHGRGKEK
>EECEBICD_00084 Protein AaeX
MSLFPVIVVFGLSFPPIFFELLLSLAIFWLVRRMLVPTGIYDFVWHPALFNTALYCCLFY

LISRLFV
>EECEBICD_00085 p-hydroxybenzoic acid efflux pump subunit AaeA
MKTLTRKLSRTAITLVLVILAFIAIFRAWVYYTESPWTRDARFSADVVAIAPDVAGLITH
VNVHDNQLVKKDQVLFTIDQPRYQKALAEAEADVAYYQVLAQEKRQEAGRRNRLGVQAMS
REEIDQANNVLQTVLHQLAKAQATRDLAKLDLERTVIRAPADGWVTNLNVYAGEFITRGS
TAVALVKKNSFYVQAYMEETKLEGVRPGYRAEITPLGSNRVLKGTVDSVAAGVTNASSTS
DAKGMATIDSNLEWVRLAQRVPVRIRLDEQQGNLWPAGTTATVVITGKQDRDASQDSFFR
KLAHRLREFG
>EECEBICD_00086 p-hydroxybenzoic acid efflux pump subunit AaeB
MGIFSIANQHIRFAVKLACAIVLALFIGFHFQLETPRWAVLTAAIVAAGPAFAAGGEPYS
GAIRYRGMLRIIGTFIGCIAALIIIISMIRAPLLMILVCCVWAGFCTWISSLVRIENSYA
WGLSGYTALIIVITIQTEPLLTPQFALERCSEIVIGIGCAILADLLFSPRSIKQEVDREL
DSLLVAQYQLMQLCIKHGDSEEVDNAWGDLVRRTAALEGMRSNLNMESSRWVRANRRLKA
LNTLSLTLITQSCETYLIQNTRPELITDTFRELFETPVETVQDVHRQLKRMRRVIVWTGE
RETPVTLYSWVGAATRYLLLKRGVISNTKISATEEEILQGEPVVKVESAERHHAMVNFWR
TTLSCILGTLFWLWTGWTSGNGAMVMIAVVTSLAMRLPNPRMVCIDFIYGTLAALPLGLL
YFLVIIPNTQQSMLLLCLSLAVLGFFIGIEVQKRRLGSMGALASTINIIVLDNPMTFHFS
QFLDSALGQIVGCMLAFIVILLVRDKSKDRTGRVLLNQFVSAAVSAMTTNVVRRKENRLP
ALYQQLFLLMNKFPGDLPKFRLALTMIIAHQRLRDAPIPVNEDLSVFHRQLRRTADHVIS
AGSDDKRRRYFGQLLDELDIYQEKLRIWEAPTQVTEPVKRLTGMLHKYQNALTDS
>EECEBICD_00087 putative protein YhcO
MNVYTFDFNDIKNQSDFYREFTQTFGLASEKVSDLDTLWDAVMSDILPLPLEIEFVHLPD
KLRRRYGALILLFDEAEEELEGRLRFNVRH
>EECEBICD_00088 hypothetical protein
MKTKYIIASLGLATLLSFGANAAVHQVNAEQAQNLQPMGTISVSQIGSTPMDMRQEIVAK
AEKAGANSYRIIELKEGDNWHATAELYK
>EECEBICD_00089 hypothetical protein
MKIKTTVATLSVLSVLSFGAFAAEPISAEQAQNREAIGSVSVSAIGSSPMDMNAMLSKKA
DEQGATAYHITEARSGSNWHATAELYK
>EECEBICD_00090 Arginine repressor
MRSSAKQEELVRAFKALLKEEKFSSQGEIVLALQDQGFENINQSKVSRMLTKFGAVRTRN
AKMEMVYCLPAELGVPTTSSPLKNLVLDIDYNDAVVVIHTSPGAAQLIARLLDSLGKAEG
ILGTIAGDDTIFTTPASGFSVRDLYEAILELFEQEL
>EECEBICD_00091 Malate dehydrogenase
MKVAVLGAAGGIGQALALLLKNQLPSGSELSLYDIAPVTPGVAVDLSHIPTAVKIKGFSG
EDATPALEGADVVLISAGVARKPGMDRSDLFNVNAGIVKNLVQQIAKTCPKACVGIITNP
VNTTVAIAAEVLKKAGVYDKNKLFGVTTLDIIRSNTFVAELKGKLPTEVEVPVIGGHSGV
TILPLLSQIPGVSFTEQEAAELTKRIQNAGTEVVEAKAGGGSATLSMGQAAARFGLSLVR
ALQGEKGVVECAYVEGDGQYARFFSQPLLLGKNGVEERKSIGTLSAFEQHSLDAMLDTLK
KDIQLGEDFINK
>EECEBICD_00092 hypothetical protein
MKKIQRTQTRDHITQMLRYEILSGNIKAGEELAQESIAEQLGLSRMPVREALQSLEQEGF
LIRLPNRHMQVAHLEADRVSHIFRVIAAMAAEMFSLIPSEVGDALLIRAQALAVAEDKSC
ELECHAMLISYVNNRYLEKVYQQFLDGYVSYVILHLKKDNQESAQLFAELADVIRQGRRD
EIGQVMQRYFLSLAEIMRQHMKDWESAEA
>EECEBICD_00093 hypothetical protein
MQKPKLGKIKLLSAKEQVAAVLRKAILSRELVEGQEITLEGIARMVGVSSMPVREAFQIL
AADGLIKVRPNKGAVVLGINEQTIREHYEIRALLESEAVAKASRPGTDISRIAQVHYAAE
KALAENNSAEYSDLNQAFHMEIWNVAGNEKMKMLLCNMWNGLSMGHKVTEEEYAVISIQE
HKSILQALELHDETLARQRMREHIIRSMENMLTRYVGDPSA
>EECEBICD_00094 Sodium-dependent dicarboxylate transporter SdcS
MEPITLTLCLLVFAIVMFVWEKVPLAVTSMIVCVALVITGVLNIKQAFAGFIDTNVILFV
AMFIVGGALFETGMANKVGGVITRFAKTEKQLIFTIMVVVGLMSGVLSNTGTAAVLIPVV
IGVAAKSGFSRSRLLMPLVFAAALGGNLSLIGAPGNLIAQSALQNIGGGFGFFEYAKIGL

PMLICGILYFLTIGYRFLPNNATGGEVGSVGEQRDYSHVPQWKQRLSLVVLIATILGMIF
EKKIGVSLAVTGCIGALVLVVTGVLTEKQAYKAIDSQTIFIFGGTLALAKALEMTGAGKL
VADYVIGMLGQNSSPFMLLIAVFALSVVMTNFMSNTATTALLVPVSLSIAAGMGADPRAV
LMATVIGGSCAYATPIGMPANMMVLSAGGYKFVDYAKAGIPLIIVSTIVSLILLPILFPF
HP
>EECEBICD_00095 L(+)-tartrate dehydratase subunit alpha
MSKSEQISHMTDVMAKFVGYTGKVLPDDVTAKLEDLHKKETSKLADVIFTTMIENQRLAK
ELDRPSCQDTGVIQFLVECGTNFPLIGELEALLREAVIKATVDSPLRHNSVETFDEYNTG
KNVGKGTPTVFWEIVPNSDQCSIYTYMAGGGCSLPGKAMVLMPGAGYEGVTRFVLDVMTS
YGLNACPPLLVGVGVATSVETAALLSKKALMRPIGSHNENERAASLEKMLEEGINKIGLG
PQGMSGNTSVMGVNIENTARHPSTIGVAVNVGCWSHRKGHIVFDKDLNYTITSHSGVNF
>EECEBICD_00096 L(+)-tartrate dehydratase subunit beta
MTKKILTTPIKDEDLADIKAGDIIYLNGHIVTCRDVAHRRLIEGGRELPVDVRGGAILHA
GPIVRPIKGEDDKFEMVSVGPTTSMRMEKFEKEFIAQTGVKLIVGKGGMGKGTEEGCAEH
KALHCVFPAGCAVVAAVCVEEIEDAQWRDLGMPETLWLCRVKEFGPLIVSIDTHGNNLFE
QNKIIFNQRKEIVADEICQNVSFIK
>EECEBICD_00097 Oxaloacetate decarboxylase gamma chain
MTNAALLLGEGFTLMFLGMGFVLAFLFLLIFAIRGMSAVITRFFPEPVAAPAPRAVPVVD
DFTRLKPVIAAAIHHHRLNA
>EECEBICD_00098 Oxaloacetate decarboxylase alpha chain
MTIAITDVVLRDAHQSLFATRLRLDDMLPIAAALDDVGYGSLECWGGATFDACIRFLGED
PWLRLRELKKAMPKTPLQMLLRGQNLLGYRHYADDVVERFVERAVKNGMDVFRVFDAMND
PRNMKAALQAVRSHGAHAQGTLSYTTSPAHTLQTWLDLTEQLLETGVDSIAIKDMSGILT
PMAAYELVSEIKKRYDVRLHLHCHATTGMAEMALLKAIEAGVDGVDTAISSMSATYGHPA
TEALVATLAGTEHDTGLDILKLENIAAYFREVRKKYHAFEGQLKGYDSRILVAQVPGGML
ANLESQLKQQNAADKLDQVLAEIPRVREDLGFIPLVTPTSQIVGTQAVLNVLTGERYKTI
AKETAGILKGEYGHTPVPVNAALQARVLEGGAPVTCRPADLLKPELAELEADVRRQAQEK
GIQLAGNAIDDVLTVALFPQIGLKFLENRHNPAAFEPVPQAEAAQPVAKAEKPAASGIYT
VEVEGKAFVVKVSDGGDISQLTAASSAPVQAASPVAPAGAGTPVTAPLAGNIWKVIATEG
QSVAEGDVLLILEAMKMETEIRAAQAGTVRGIAVKSGDAVSVGDTLMTLA
>EECEBICD_00099 Oxaloacetate decarboxylase beta chain
MESLNALLQGMGLMHLGAGQAIMLLVSLLLLWLAIAKKFEPLLLLPIGFGGLLSNIPEAG
MALTALESLLAHHDAGQLAVIAAKLNCAPDVHAIKEALALALPSVQSQMENLAVDMGYTP
GVLALFYKVAIGSGVAPLVIFMGVGAMTDFGPLLANPRTLLLGAAAQFGIFATVLGALTL
NYFGLISFTLPQAAAIGIIGGADGPTAIYLSGKLAPELLGAIAVAAYSYMALVPLIQPPI
MRALTSEKERKIRMVQLRTVSKREKILFPVVLLLLVALLLPDAAPLLGMFCFGNLMRESG
VVERLSDTVQNGLINIVTIFLGLSVGAKLVADKFLQPQTLGILLLGVIAFGIGTAAGVLM
AKLMNLCSKNKINPLIGSAGVSAVPMAARVSNKVGLESDPQNFLLMHAMGPNVAGVIGSA
IAAGVMLKYVLAM
>EECEBICD_00100 2-(5''-triphosphoribosyl)-3'-dephosphocoenzyme-A synthase
MSEMVAFRQGTSMPSRETILRYVVETVNQITELEPALHLLPWSGVNSAIYEQRFAQCYDE
GLCAAQTSAPNVPQGILPSTDWAQGIGLLCFAAGYMSAGERPLTHNQLCDFVKQAAVGLS
PIEGEAASGFSTVRSIALPVFRRLQRDGHASRILLLQTLLHLVAWKSASQYARQQAQRLL
WMGGILGEGGSIACLYWIRRCVKKPSGRKVSPLC
>EECEBICD_00101 Serine endoprotease DegS
MFVKLLRSVAIGLIVGAILLAVMPSLRKINPIAVPQFDSTDETPASYNFAVRRAAPAVVN
VYNRSMNSTAHNQLEIRTLGSGVIMDQRGYIITNKHVINDADQIIVALQDGRVFEALLVG
SDSLTDLAVLKINATGGLPTIPINTKRTPHIGDVVLAIGNPYNLGQTITQGIISATGRIG
LNPTGRQNFLQTDASINHGNSGGALVNSLGELMGINTLSFDKSNDGETPEGLGFAIPFQL
ATKIMDKLIRDGRVIRGYIGIGGREIAPLHAQQGSGMDPIQGIVVNEVTPNGPAALAGIQ
VNDLIISVNNKPAVSALETMDQVAEIRPGSVIPVVVMRDDKQLTFQVTVQEYPASN
>EECEBICD_00102 Periplasmic pH-dependent serine endoprotease DegQ
MKKHTQLLSALALSVGLTLSAPFPALASIPGQVPGQATLPSLAPMLEKVLPAVVSVKVEG
TAAQSQKVPEEFKKFFGEDLPDQPSQPFEGLGSGVIIDAAKGYVLTNNHVINQAQKISIQ

LNDGREFDAKLIGGDDQSDIALLQIQNPSKLTQIAIADSDKLRVGDFAVAVGNPFGLGQT
ATSGIISALGRSGLNLEGLENFIQTDASINRGNSGGALLNLNGELIGINTAILAPGGGSI
GIGFAIPSNMAQTLAQQLIQFGEIKRGLLGIKGTEMTADIAKAFKLNVQRGAFVSEVLPN
SGSAKAGVKSGDVIISLNGKPLNSFAELRSRIATTEPGTKVKLGLLRDGKPLEVEVTLDS
NTSSSASAEMIAPALQGATLSDGQLKDGTKGVKVDSVEKSSPAAQAGLQKDDVIIGVNRD
RISSIAEMRKVMAAKPSIIALQVVRGNENIYLLLR
>EECEBICD_00103 Inner membrane protein YhcB
MTWEYALIGLVVGIIIGAVAMRFGNRKLRQQQALQYELEKNKAELEEYREELVSHFARSA
ELLDTMAHDYRQLYQHMAKSSSSLLPEMSAESNPFRNRLAESEASNDQAPVQMPRDYSEG
ASGLLRSGAKRD
>EECEBICD_00104 Cell division protein ZapE
MQSLSPTSRYLQALNEGTHQPDDVQKEAVDRLETLYQALTAKKSSATPPGGLIARLGKLL
GKNEPDAQIPVRGLYMWGGRGSRQNLAHGSLLPQPAG
>EECEBICD_00105 Cell division protein ZapE
MGRGKTWLMDLFYHSLPGERKLRLHFHRFMLRVHEELTALQGQIDPLDIIADRFKTETDV
LCFDEFFVTDITDAMLLGGLMKALFARGITLVATSNIPPDELYRNGLQRARFLPAIDAIK
QHCDIMNVDAGVDYRLRTLTQAHLWLTPLNDETRRQMDKLWLALAGAAREHAPTLEINHR
SLSTLGVENQTLAVSFATLCVEARSQHDYIALSRLFHTVLLFDVPVMTPLMENEARRFIA
LVDEFYERHVKLVVSAAAPLYEIYQGEQLKFEFQRCLSRLQEMQSAEYLKREHMP
>EECEBICD_00106 50S ribosomal protein L13
MKTFTAKPETVKRDWYVVDATGKTLGRLATELARRLRGKHKAEYTPHVDTGDYIIVLNAD
KVAVTGNKRTDKVYYHHTGHIGGIKQATFEEMIARRPERVIEIAVKGMLPKGPLGRAMFR
KLKVYAGNEHNHAAQQPQVLDI
>EECEBICD_00107 30S ribosomal protein S9
MAENQYYGTGRRKSSAARVFIKPGNGKIVINQRSLEQYFGRETARMVVRQPLELVDMVEK
LDLYITVKGGGISGQAGAIRHGITRALMEYDESLRGELRKAGFVTRDARQVERKKVGLRK
ARRRPQFSKR
>EECEBICD_00108 hypothetical protein
MGFFNSLFTKKESLMLSNKWEFFITTVDDHVTGIRVDIGAIQDEKFDRLIHTGFLRVHYT
NCYENGLPQPDETQRLNRIEDWLDEKGKTFPIWLVGVVTQQGWRDFVFMSEEDLNWEKTL
DKLLAGGPEISFSYRESHNDKGNFYRQFLYPTRYDWNWIHDSRVCRGLQEQGDDLTLPRA
IDYYATLPTEVAARDLAQDIAALPYGITLVSIRMNDPQQGFMASFISTDAPQQWHMTEIT
CQLTDLAEKHGGSFDGWGAPVVQA
>EECEBICD_00109 Stringent starvation protein A
MAVAANKRSVMTLFSGPTDIYSHQVRIVLAEKGVSFEIEHVEKDNPPQDLIDLNPNQSVP
TLVDRELTLWESRIIMEYLDERFPHPPLMPVYPVARGESRLYMHRIEKDWYTLMNVIVNG
SASEADSARKQLREELLAIAPVFGQKPYFLSDEFSLVDCYLAPLLWRLPQLGIEFSGAGA
KELKGYMTRVFERDSFLASLTEAEREMRLGRG
>EECEBICD_00110 Stringent starvation protein B
MDLSQLTPRRPYLLRAFYEWLLDNQLTPHLVVDVMLPGVHVPMEYARDGQIVLNIAPRAV
GNLELSNDEVRFNARFGGVPRQVSVPLAAVLAIYARENGAGTMFEPEAAYDEDVVSLNDD
DNTAGAESETVMSVIDGDKPDHDDDSSPDDEPPPPRGGRPALRVVK
>EECEBICD_00111 HTH-type transcriptional repressor NanR
MNAFDSQAEDSPTSLGRSLRRRPLARKKLSEMVEEELEQMIRRHEFGEGEQLPSERELMA
FFNVGRPSVREALAALKRKGLVQINNGERARVSRPSADTIISELSGMAKDFLTHPGGIAH
FEQLRLFFESSLVRYAAEHATDEQIALLTKALEINSQSLDDNALFIRSDVEFHRVLAEIP
GNPIFMAIHVALLDWLIAARPSVPDRELHEHNNVSYQQHIVIVDAIRQRDPDKADRALQT
HLNSVSATWHALGKKSQKMR
>EECEBICD_00112 N-acetylneuraminate lyase
MAKALQGVMAALLTPFDHQQQLDSESLRRLVRFNIGQGIDGLYVGGSTGEAFVQSLAERE
QVLEIVAEEAKGKITLIAHVGTVSTAESQQLASAAKRYGFDAVSAVTPFYYPFSFEEHCD
HYRAIIDSADGLPMVVYNIPALSGVKLTLDQINTLVTLPGVSALKQTSGDLFQMEQIRRA
HPDLVLYNGYDEIFASGLLAGADGGIGSTYNIMGWRYQGIVQALREGDVAKAQRLQTECN
KVIDLLIKTGVFRGLKTVLHYMDVLSVPLCRKPFAPVDEKYLPALKALAQQLMEEKA

>EECEBICD_00113 Sialic acid transporter NanT
MSTSTQNIPWYRHLNRAQWRAFSAAWLGYLLDGFDFVLIALVLTEVQSEFGLTTVQAASL
ISAAFISRWFGGLLLGAMGDRYGRRLAMVSSIILFSVGTLACGFAPGYTTMFIARLVIGM
GMAGEYGSSATYVIESWPKHLRNKASGFLISGFSVGAVVAAQVYSLVVPVWGWRALFFIG
ILPIIFALWLRKNIPEAEDWKEKHAGKAPVRTMVDILYRGEHRIINILMTFAAAAALWFC
FAGNLQNAAIVAGLGLLCAVIFISFMVQSSGKRWPTGVMLMLVVLFAFLYSWPIQALLPT
YLKTELAYDPHTVANVLFFSGFGAAVGCCVGGFLGDWLGTRKAYVCSLLASQILIIPVFA
IGGTNVWVLGLLLFFQQMLGQGIAGILPKLIGGYFDTDQRAAGLGFTYNVGALGGALAPI
LGALIAQRLDLGTALASLSFSLTFVVILLIGLDMPSRVQRWLRPEALRTHDAIDDKPFSG
AVPLGSGKGAFVKTKS
>EECEBICD_00114 Putative N-acetylmannosamine-6-phosphate 2-epimerase 2
MSLLEQLDKNIAASGGLIVSCQPVPGSPLDKPEIVAAMALAAEQAGAVAVRIEGIDNLRM
TRSLVSVPIIGIIKRDLDDSPVRITPFLDDVDALAQAGAAIIAVDGTARQRPVAVEALLA
RIHHHHLLAMADCSSVDDGLACQRLGADIIGTTMSGYTTPDTPEEPDLPLVKALHDAGCR
VIAEGRYNSPALAAEAIRYGAWAVTVGSAITRLEHICGWYNDALKKAAS
>EECEBICD_00115 N-acetylmannosamine kinase
MTTLAIDIGGTKLAAALIDKNLRISQRRELPTPASKTPDALREALKALVEPLRAEARQVA
IASTGIIQEGMLLALNPHNLGGLLHFPLVQTLETIAGLPTLAVNDAQAAAWAEYHALPDD
IRDMVFITVSTGVGGGVVCDGKLLTGKGGLAGHLGHTLADPHGPVCGCGRVGCVEAIASG
RGMAAAARDDLAGCDAKTLFIRAGEGHQQARHLVSQSAQVIARMIADVKATTDCQCVVIG
GSVGLAEGYLEQVRAFLMQEPAPYHVALSAARYRHDAGLLGAALLAQGDTL
>EECEBICD_00116 putative protein YhcH
MMMGEVQSLPSCGLHPRLLDALTLALAARPQEKAPGRYELQGDNVFMNVMQLTTQMPAEK
KAELHEQYIDIQLLLTGVERIAFGMSGAARQCEEMHVEEDYQLCSQIVDEQTITLQAGMF
AVFMPGEPHKPGCAVGEPDDIKKVVVKVRASLLAA
>EECEBICD_00117 Cytosine deaminase
MQNNNITIRQTRLQGHEGLWQITIENGRFSRIEPQEAASLPQGEVLDAEGGLAIPPFVEP
HIHLDTTQTAGEPSWNQSGTLFEGIERWAERKAMLTHEDVKARAMQTLKWQMANGIQYVR
THVDVSDPTLTALKAMLEVKQEVAPWVDLQIVAFPQEGILSYPNGEALLEEAVRLGVDVI
GAIPHFEFTREYGVESLHKTFALAQKYDRLIDVHCDEIDDEQSRFVETVAALAHRDGMGA
RVTASHTTAMHSYNGAYASRLFRLLKMSGINFVANPLVNIHLQGRFDTYPKRRGVTRVKE
MLEAGINVCFGHDDVFDPWYPLGTANMLQVLHMGLHVCQLMGYGQINDGLNLITTHSAKT
LHLQDYGLSVGNAANLVILPAENGFDAVRRQTPARYSIRHGRVIAETVPSQTTLHLTQPE
AVTFKR
>EECEBICD_00118 Cytosine permease
MSQDNNYSQGPVPLAARKGVIPLTFVMLGLTFFSASMWTGGTLGTGLTYHDFFLAVLFGN
LLLGIYTAFLGYIGAKTGLSTHLLARYSFGVKGSWLPSLLLGGTQVGWFGVGVAMFAIPV
SKATGIDANILIAISGLLMTLTIFFGISALTILSIIAVPAIVILGSYSVWLAVSGVGGLE
HLKTIVPQTPLDFSSALALVVGSFVSAGTLTADFVRFGRHAKSAVLIAMVAFFLGNSLMF
IFGAAGAAAVGQADISDVMIAQGLLLPAIVVLGLNIWTTNDNALYASGLGFANITGLSSR
TLSVVNGIIGTVCALWLYNNFVGWLTFLSSAIPPIGGVIIADYLLNRRRYADFNTVRFIP
VNWIAILSVALGIAAGHYVPGIVPVNAVLGGVFSYILLNPLFNRSLAKSPEVSHAEQ
>EECEBICD_00119 Putative nuclease YhcG
MTNPTLVPQSDEYQQIHDGIIRLVDTARTETVRSINAIMTATYWEIGRRIVEFEQGGEAR
AAYGTQLIERLSVDLSQRYKRGFSTRNLWQIRTFYLCFQHIEIPQTLSAEFSNLIPLAKT
FPLPWSAYVRLLSVKNPDARTFYEKETLRNGWSVRQLDRQIATQFYERTLLSHDKSAMLQ
QPAPAEPNVLPEQAIRDPFILEFLNLKDEYSESDLEDALLSHLMDFMLELGNDFAFVGRQ
RRLRIDDSWFRVDLLFFHRRLRCLLLVDLKVGKFGYADAGQMNMYLNYAKEHWTMPGENP
PVGLVLCAGKGAGEAHYALTGLPNTIMASEYKVQLPDEKLLTDELIRSQTMLETQLTRGG
SLTTEKN
>EECEBICD_00120 Glutamate synthase [NADPH] small chain
MSQNVYQFIDLQRVDPPKKPLKLRKIEFVEIYEPFSEGQAKAQADRCLSCGNPYCEWKCP
VHNYIPNWLKLANEGRIFEAAELSHQTNTLPEVCGRVCPQDRLCEGSCTLHDEFGAVTIG
NIERYINDKAFEMGWRPDMTGVRQTDKRVAIIGAGPAGLACADVLTRNGVKAVVFDRHPE

IGGLLTFGIPAFKLEKEVMTRRREIFTGMGIEFKLNTEVGRDVQLEDLLKDYDAVFLGVG
TYQSMRGGLENEDADGVFDALPFLIANTKQIMGFGETSDEPYVSMEGKRVVVLGGGDTAM
DCVRTSIRQGATHVTCAYRRDEENMPGSRREVKNAREEGVEFQFNVQPLGIEVNANGKVS
GVKMVRTEMGEPDAKGRRRAEIVAGSEHVVPADAVVMAFGFRPHSMEWLAKHSVELDSQG
RIIAPERSDNAFQTSNPKIFAGGDIVRGSDLVVTAIAEGRKAADGIMNYLEV
>EECEBICD_00121 Glutamate synthase [NADPH] large chain
MLYDKSLEKDNCGFGLIAHIEGEPSHKVVRTAIHALARMQHRGAILADGKTGDGCGLLLQ
KPDRFFRIVAEERGWRLAKNYAVGMIFLNKDPELAAASRHIVEEELQQETLSIVGWRDVP
TNEGVLGEIALSSLPRIEQIFVNAPAGWRPRDMERRLFIARRRIEKRLQNDKDFYVCSLS
NLVNIYKGLCMPADLPRFYLDLADLRLESAICLFHQRFSTNTVPRWPLAQPFRYLAHNGE
INTITGNRQWARARTYKFQTPLIPDLQSAAPFVNETGSDSSSLDNMLELLLAGGMDIIRA
MRLLVPPAWQNNPDMDQDLRAFFDFNSMHMEPWDGPAGIVMSDGRFAACNLDRNGLRPAR
YVITKDKLITCASEVGIWDYQPDEVVEKGRVGPGELMVIDTRGGRILHSAETDDDLKSRH
PYKEWMEKNVRRLVPFEELPDEEVGSRELDDDLLASYQKQFNYSAEELDSVIRVLGENGQ
EAVGSMGDDTPFAVLSSQPRIIYDYFRQQFAQVTNPPIDPLREAHVMSLATSIGREMNVF
CEAEGQAHRLSFKSPILLYSDFKQLTTMKEEHYRADRLDITFDVTETTLNATVKALCDKA
EQMVRNGTVLLVLSDRNIGKNRLPVPAPMAVGAVQTRLVEQSLRCDANIIVETGSARDPH
HFAVLLGFGATAIYPYLAYETLGRLIDTQAIAKNYRTVMQNYRNGINKGLYKIMSKMGIS
TIASYRCSKLFEAVGLHDDVVNLCFQGVVSRIGGASFDDFQQDLLNLSKRAWLARKPISP
GGLLKYVHGGEYHAYNPDVVRTLQQAVQSGEYSDYQEYAKLVNERPAATLRDLLAIHPDG
EAVTIDEVEPASELFKRFDTAAMSIGALSPEAHEALAEAMNSLGGNSNSGEGGEDPARYG
TNKVSRIKQVASGRFGVTPAYLVNADVIQIKVAQGAKPGEGGQLPGDKVTPYIAKLRYSV
PGVTLISPPPHHDIYSIEDLAQLIFDLKQVNPKAMISVKLVSEPGVGTIATGVAKAYADL
ITIAGYDGGTGASPLSSVKYAGCPWELGLVETQQALVANGLRHKIRLQVDGGLKTGVDII
KAAILGAESFGFGTGPMVALGCKYLRICHLNNCATGVATQDEKLRKNHYHGLPFKVTNYF
EFIAREVRELMASLGVTRLVDLIGRTDLLKELEGFTAKQQKLALSRLLETAEPHPGKALY
CTENNPPFDNGVLNAQLLQQAKPFVDARQSKTFWFDIRNTDRSVGASLSGYIAQTHGDQG
LASDPIKAHFSGTAGQSFGVWNAGGVELYLTGDANDYVGKGMAGGLIAIRPPVGSAFLSH
KASIIGNTCLYGATGGRLYAAGRAGERFGVRNSGAITVVEGIGDNGCEYMTGGIVCVLGK
TGVNFGAGMTGGFAYVLDEDGEFRKRVNPELVEVLDVDSLAIHEEHLRGLITEHVQHTGS
QRGEEILSRWSSFSTQFALVKPKSSDVKALLGHRSRSAAELRVQAQ
>EECEBICD_00122 hypothetical protein
MQLQKLVNMFGGDLLRRYGQKVHKLTLHGGFSCPNRDGTIGRGGCTFCNVASFADEAQQH
HSIAEQLAHQAHLVNRAKRYLAYFQAYTSTFAEVQVLRSMYQQAVSQASIVGLCVGTRPD
CVPQAVVDLLCEYKDQGYEVWLELGLQTAHDKTLHRINRGHDFACYQRTTRIARERGLKV
CAHLIVGLPGEGQAECLQTMERVVETGVDGIKLHPLHIVKGSTMAKAWEAGRLNGIELDD
YTLTAGEMIRHTPPEVIYHRISASARRPTLLAPLWCENRWTGMVELDKYLNEHGVQGSAL
ARPWIPPVA
>EECEBICD_00123 Aerobic respiration control sensor protein ArcB
MKQIRMLAQYYVDLMMKLGLVRFSMLLALALVVLAIVVQMAVTMVLHGQVESIDVIRSIF
FGLLITPWAVYFLSVVVEQLEESRQRLSRLVQKLEEMRERDLKLNVQLKDNIAQLNQEIA
DREKAEAELQETFEQLKVEIKEREEAQIQLEQQSSFLRSFLDASPDLVFYRNEDKEFSGC
NRAMELLTGKSEKQLVHLKPEDVYSPEAAEKVIETDEKVFRHNVSLTYEQWLDYPDGRKA
CFEIRKVPYYDRVGKRHGLMGFGRDITERKRYQDALERASRDKTTFISTISHELRTPLNG
IVGLSRILLDTDLTAEQEKYLKTIHVSAVTLGNIFNDIIDMDKMERRKVQLDNQPVDFTS
FMADLENLSGLQAQQKGLRFVLEPTLPLPHKVITDGTRLRQILWNLISNAVKFTQQGQVT
VRARYDEGDMLHFEVEDSGIGIPQDEQDKIFAMYYQVKDSHGGKPATGTGIGLAVSRRLA
KNMGGDITVSSLPGKGSTFTLTVHAPAVAEEVEDAFDEDDMPLPALHVLLVEDIELNVIV
ARSVLEKLGNSVDVAMTGKAALEMFAPGEYDLVLLDIQLPDMTGLDIARELTRRHTREDL
PPLVALTANVLKDKKEYLDAGMDDVLSKPLSVPALTAMIKKFWDATDKEESTVTPEESDK
AQALLDIPMLEQYIELVGPKLITDGLAVFEKMMPGYLSVLESNLTARDKKGVVEEGHKIK
GAAGSVGLRHLQQLGQQIQSPDLPAWEDNVAEWIEEMKQEWQHDVAVLKAWVANAEKK
>EECEBICD_00124 Glyoxalase ElbB
MKKIGVVLSGCGVYDGTEIHEAVLTLLAIARSGAQAVCFAPDKPQADVINHLTGEAMAET

RNVLIEAARITRGDIRPLSQAQPEELDALIVPGGFGAAKNLSNFASQGSECRVDSDVVAL
AKAMHQSGKPLGFICIAPAMLPKIFDFPLRLTIGTDIDTAEVLEEMGAEHVPCPVDDIVV
DEDNKVVTTPAYMLAQDIAQAASGIDKLVSRVLVLAE
>EECEBICD_00125 Biosynthetic peptidoglycan transglycosylase
MSKRRIAPLTFLRRLLLRILAALAVFWGGGIALFSVVPVPFSAVMAERQISAWLGGEFGY
VAHSDWVSMADISPWMGLAVIAAEDQKFPEHWGFDVPAIEKALAHNERNESRIRGASTLS
QQTAKNLFLWDGRSWLRKGLEAGLTLGIETVWSKKRILTVYLNIAEFGDGIFGVEAAAQR
YFHKPASRLSVSEAALLAAVLPNPLRYKANAPSGYVRSRQAWIMRQMRQLGGESFMTRNQ
LN
>EECEBICD_00126 putative protein YrbL
MILLSEQTPLGAGRHRKCYTHPDNARRCIKVIYNRDHGGDKEIRRELSYYAHLSRYLTDW
SAIPRYYGTVETDCGTGYVYDMITDFNGAPSITLTEFAAQCRYEEDVAVLRRLLKKLKRY
LLDNHIVTMSLKPQNILCQRISESEVVPVVCDNLGESTFIPLATWSTWCCERKLERVWQR
FIAQPALAVALERDEQPKDKKRLALTSHEA
>EECEBICD_00127 hypothetical protein
MFLCIGKAHEMYKSYERVGLRDKWQAGVTRPVLG
>EECEBICD_00128 Phosphocarrier protein NPr
MTVKQTVEVTNKLGMHARPAMKLFELMQGFDAEVLLRNDEGTEAEANSVIALLMLDSAKG
RQIEIEATGPQEVEALAAVIALFNSGFDED
>EECEBICD_00129 RNase adapter protein RapZ
MVLMIVSGRSGSGKSVALRALEDMGFYCVDNLPVVLLPDLARTLADRQISAAVSIDVRNM
PESPEIFEQAMNNLPGAFSPQLLFLDADRNTLIRRYSDTRRLHPLSSKNLSLESAIDKES
DLLEPLRSRADLIVDTSEMSVHELAEMLRTRLLGKRERELTMVFESFGFKHGIPIDADYV
FDVRFLPNPHWDPKLRPMTGLDKPVAAFLDRHTEVHNFIYQTRSYLELWLPMLETNNRSY
LTVAIGCTGGKHRSVYIAEQLADYFRSRGKNVQSRHRTLEKRKT
>EECEBICD_00130 Nitrogen regulatory protein
MINNDTTLQLSSVLNQECTRSGVHCQSKKRALEIISELAAKQLSLPPQVVFEAILTREKM
GSTGIGNGIAIPHGKLEEDTLRAVGVFVQLETPIAFDAIDNQPVDLLFALLVPADQTKTH
LHTLSLVAKRLADKTICRRLRAALNDEELYQIITDTEGEQNEA
>EECEBICD_00131 Ribosome hibernation promoting factor
MQLNITGHNVEITEALREFVTTKFAKLEQYFERINQVYVVLKVEKVTHISDATLHVNGGE
IHASAEGQDMYAAIDGLIDKLARQLTRHKDKLKQH
>EECEBICD_00132 RNA polymerase sigma-54 factor
MKQGLQLRLSQQLAMTPQLQQAIRLLQLSTLELQQELQQALENNPLLEQTDLHDEIDTQQ
PQDNDPLDTADALEQKEMPEELPLDASWDEIYTAGTPSGPSGDYIDDELPVYQGETTQSL
QDYLMWQVELTPFSDTDRAIATSIVDAVDDTGYLTVSLDEIRESMGDVEVDLDEVEAVLK
RIQRFDPVGVAAKDLRDCLLIQLSQFDKSTPWLEEARLIICDHLDLLANHDFRTLMRVTR
LKEEVLKEAVNLIQSLDPRPGQSIQTGEPEYVIPDVLVRKHNGRWTVELNSDSIPRLQIN
QHYAAMCNSARNDADSQFIRSNLQDAKWLIKSLESRNDTLLRVSRCIVEQQQAFFEQGEE
YMKPMVLADIAQAVEMHESTISRVTTQKYLHSPRGIFELKYFFSSHVNTEGGGEASSTAI
RALVKKLIAAENPAKPLSDSKLTSLLSEQGIMVARRTVAKYRESLSIPPSNQRKQLV
>EECEBICD_00133 Lipopolysaccharide export system ATP-binding protein LptB
MATLTAKNLAKAYKGRRVVEDVSLTVNSGEIVGLLGPNGAGKTTTFYMVVGIVPRDAGNI
IIDDEDISLLPLHARARRGIGYLPQEASIFRRLSVFDNLMAVLQIRDDLTAEQREDRANE
LMEEFHIEHLRDSMGQALSGGERRRVEIARALAANPKFILLDEPFAGVDPISVIDIKRII
EHLRDSGLGVLITDHNVRETLAVCERAYIVSQGHLIAHGTPTEILQDEHVKRVYLGEDFR
L
>EECEBICD_00134 Lipopolysaccharide export system protein LptA
MKFKTNKLSLNLMLAGSLLAASIPAFAVTGDTEQPIHIDSDQQSLDMQGNVVTFTGNVVM
TQGTIKINADKVVVTRPGGEQGKEVIDGYGNPATFYQMQDNGKPVKGHASHMHYELAKDF
VVLTGNAYLEQLDSNITGDKITYLVKEQKMQAFSEKGKRVTTVLVPSQLQDKNKGQTPAQ
KKSN
>EECEBICD_00135 Lipopolysaccharide export system protein LptC
MSKTRRWVIILLSLAILVLIGINLADKDDPAAVMVNSNDPTYKSEHTDTVVYSPEGALSY

RLIAQHVEYFSDQAVSWFTQPVLTTFDKDKVPTWSIKADKAKLTNDRMLYLYGHVEVNAL
VPDAQLRRITTDNAQINLVTQDVTSNDLVTLYGTTFNSSGLKMRGNLRSKNAELIEKVRT
SYEIQNKQTQP
>EECEBICD_00136 3-deoxy-D-manno-octulosonate 8-phosphate phosphatase KdsC
MSKAGASLATCYGPVSTHVMTKAENIRLLILDVDGVLSDGLIYMGNNGEELKAFNVRDGY
GIRCALTSNIEVAIITGRKAKLVEDRCATLGIVHLYQGQSNKLIAFSDLLEKLVIAPENV
AYVGDDLIDWPVMEKVGLSVAVADAHPLLIPRADYVTHIAGGRGAVREVCDLLLLAQGKL
DEAKGQSI
>EECEBICD_00137 Arabinose 5-phosphate isomerase KdsD
MSHLALQPGFDFQQAGKEVLEIEREGLAELDQYINQHFTLACEKMFNCTGKVVVMGMGKS
GHIGRKMAATFASTGTSSFFVHPGEAAHGDLGMVTPQDVVIAISNSGESSEIAALIPVLK
RLHVPLICITGRPESSMARAADVHLCVKVPKEACPLGLAPTSSTTATLVMGDALAVALLK
ARGFTAEDFALSHPGGALGRKLLLRVSDIMHTGDEIPHVNKHATLRDALLEITRKNLGMT
VICDESMKIDGIFTDGDLRRVFDMGGDMRQLGIAEVMTPGGIRVRPGILAVDALNLMQSR
HITSVLVADGDQLLGVLHMHDLLRAGVV
>EECEBICD_00138 Inner membrane protein YrbG
MLLAMALLIIGLLLVAYGADRLVFAASILCRTFGIPPLIIGMTVVSIGTSLPEIIVSVAA
SLHGQLDLAVGAALGSNITNILLILGLAALVRPFTVHSDVLRRELPLMLFVSVVAGSVLH
DGQLSRSDGIFLLLLAVLWLLFIVKIARLAERQGNDSLTREQLAELPREGGLPVAFLWLG
IALVIMPMATRMVIDNATVLANYFAMSELTLGLTVIAVGTSLPELATAIAGVRKGENDIA
VGNLIGANIFNLAIVLGLPALIAPGEINPLAFGRDYSVMLLVSVVFALLCWRHPRQIGRG
AGILLTGGFIVWLAMLYWLSPLLVG
>EECEBICD_00139 Intermembrane phospholipid transport system ATP-binding protein MlaF
MGQSAANLVDMRDVSFSRGERCIFDNISLTVPRGKITAIMGPSGIGKTTLLRLIGGQIPP
DKGEILFDGENVPAMSRSRLYTVRKRMSMLFQSGALFTDMNVFDNVAYPLREHTNLPAPL
LKSVVMMKLEAVGLRGAAKLMPSELSGGMARRAALARAIALEPDLIMFDEPFVGQDPITM
GVLVKLISELNSALGVTCVVVSHDVPEVLSIADHAWIMADKKIVAHGSAQALQENTDPRV
RQFLDGIADGPVPFRYPAGDYHLDLLETGS
>EECEBICD_00140 Intermembrane phospholipid transport system permease protein MlaE
MLLNALAALGHSGIKTVRTFGRAGLMLFNAIIGKPEFRKHAPLLVRQLYNVGVLSMLIII
VSGVFIGMVLGLQGYLVLTTYSAETSLGMLVALSLLRELGPVVAALLFAGRAGSALTAEI
GLMRATEQLSSMEMMAVDPLRRVISPRFWAGVISLPLLTIIFVAVGIWGGSLVGVSWKGI
DAGFFWSAMQNAVDWRMDLVNCLIKSVVFAITVTWIALFNGYDAIPTSAGISRATTRTVV
HASLAVLGLDFVLTALMFGN
>EECEBICD_00141 Intermembrane phospholipid transport system binding protein MlaD
MQTKKNEIWVGVFLLVALLAALFVCLKAANVTSMRTEPTYKVYATFDNIGGLKVRSPVRI
GGVVVGRVEDISLDPKTYLPRVTLDIEERYNHIPDTSSLSIRTSGLLGEQYLALNVGFED
PELGTSILKDGSTIQDTKSAMVLEDMIGQFLYNSKGDDNKNSGDAPAATEGHTEATTPAG
ETK
>EECEBICD_00142 Intermembrane phospholipid transport system binding protein MlaC
MFKRLMMVALLVIAPLSAATAADQSNPYKLMNEAAQKTFDRLKNEQPKIRANPDYLRDVV
DQELLPYVQVKYAGALVLGRYYKEATPAQREAYFAAFREYLKQAYGQALAMYHGQTYQIA
PEQPLGDATIVPIRVTIIDPNGRPPVRLDFQWRKNTQTGNWQAYDMIAEGVSMITTKQNE
WSDLLRTKGIDGLTAQLKSISQQKITLDEKQ
>EECEBICD_00143 Intermembrane phospholipid transport system binding protein MlaB
MTPQLTWTREADTLVLAGELDQDVLAPLWDARVEAMTGVTRIDLSQISRVDTGGLALLAH
LVNQAKKQGNAVSLSGVNDKVYALAQLYNLPEDVLPRM
>EECEBICD_00144 Acid stress protein IbaG
MLGCFHYLTNKEPMENHEIQSVLMNALSLQEVHVSGDGSHFQVIAVGEMFDGMSRVKKQQ

TVYGPLMEYIADNRIHAVSIKAYTPAEWARDRKLNGF
>EECEBICD_00145 UDP-N-acetylglucosamine 1-carboxyvinyltransferase
MDKFRVQGPTTLQGEVTISGAKNAALPILFAALLAEEPVEIQNVPKLKDVDTSMKLLSQL
GAKVERNGSVHIDASQVNVFCAPYDLVKTMRASIWALGPLVARFGQGQVSLPGGCTIGAR
PVDLHITGLEQLGATIKLEEGYVKASVEGRLKGAHIVMDKVSVGATVTIMCAATLAEGTT
IIENAAREPEIVDTANFLVTLGAKIAGQGTDRITIEGVERLGGGVYRVLPDRIETGTFLV
AAAISRGKILCRNAQPDTLDAVLAKLRDAGADIEVGEDWISLDMHGKRPKAVNVRTAPHP
AFPTDMQAQFTLLNLVAEGTGFITETVFENRFMHVPELSRMGARAEIESNTVICHGIETL
SGAQVMATDLRASASLVLAGCIAEGTTIVDRIYHIDRGYERIEDKLRALGANIERVKGE
>EECEBICD_00146 hypothetical protein
MESKLIDWHPADIIAGLRKKGTSMAAESRRNGLSSSTLANALTRPWPKGELIIAKALGTE
PWVIWPSRYHDPRTHEFIDRTRLMRARNKDKQNVE
>EECEBICD_00147 Octaprenyl diphosphate synthase
MNLEKINELTAQDMAGVNATILEQLNSDVQLINQLGYYIISGGGKRIRPMIAVLAARAVG
YQGNAHVTIAALIEFIHTATLLHDDVVDESDMRRGKATANAAFGNAASVLVGDFIYTRAF
QMMTSLGSLKVLEVMSEAVNVIAEGEVLQLMNVNDPDITEENYMRVIYSKTARLFEAAAQ
CSGILAGCTPEQEKGLQDYGRYLGTAFQLIDDLLDYSADGEHLGKNVGDDLNEGKPTLPL
LHAMRHGTPEQSAMIRTAIEQGNGRHLLEPVLEAMTTCGSLEWTRQRAEEEADKAISALQ
ILPDTPWREALIGLAHIAVQRDR
>EECEBICD_00148 50S ribosomal protein L21
MYAVFQSGGKQHRVSEGQTVRLEKLDIATGETIEFAEVLMIANGEEVKIGVPFVDGGVIK
AEVVAHGRGEKVKIVKFRRRKHYRKQQGHRQWFTDVKITGISA
>EECEBICD_00149 50S ribosomal protein L27
MAHKKAGGSTRNGRDSEAKRLGVKRFGGEAVLAGSIIVRQRGTKFHAGTNVGCGRDHTLF
AKADGKVKFEVKGPKNRKYISIVAE
>EECEBICD_00150 putative inner membrane transporter YhbE
MKQQAGIGILLALTTAMCWGALPIAMKQVLEVMEPSTIVFYRFLMASIGLGAILAVKRKL
PPLRIFRKPRWLVLLAIATCGLFGNFILFSSSLQYLSPTASQVIGQLSPVGMMVASVFIL
KEKMRGTQVIGALMLLSGLVMFFNTSLIEIFTRLTDYTWGVIFGVGAAMVWVSYGVAQKV
LLRRLASQQILFLLYTLCTIALLPLAKPMVIAQLSDWQLACLIFCGLNTLVGYGALAEAM
ARWQAAQVSAIITLTPLFTLLFSDLLSMAWPDFFARPMLNLLGYLGAFVVVAGAMYSAIG
HRIWGGLRKHETVVSQPRSGE
>EECEBICD_00151 GTPase Obg
MKFVDEASILVVAGDGGNGCVSFRREKYIPKGGPDGGDGGDGGDVWMEADENLNTLIDYR
FEKSFRAERGQNGASRDCTGKRGKDVTIKVPVGTRVIDQGTGETMGDMTKHGQRLLVAKG
GWHGLGNTRFKSSVNRTPRQKTNGTPGDKRDLLLELMLLADVGMLGMPNAGKSTFIRAVS
AAKPKVADYPFTTLVPSLGVVRMDSEKSFVVADIPGLIEGAAEGAGLGIRFLKHLERCRV
LLHLIDIDPIDGSDPVENARIIIGELEKYSQDLAAKPRWLVFNKIDLMDKTEAEEKAKAI
AEALGWEGKYYLISAASQLGVKDLCWDVMTFIIENPIAQAEEAKQPEKVEFMWDDYHRQQ
LAEVEEDADDDWDDDWDEDDEEGVEFIYKR
>EECEBICD_00152 D-alanyl-D-alanine carboxypeptidase DacB
MRFSRFIIGLTTSIAFSVQAANIDEYIKQLPAGANLALMVQKIGAPAPAIDYHSQQMALP
ASTQKVITALAALIQLGPDFRFTTTLETKGNVDNGILKGDVIARFGGDPTLKRQDIRNMV
ATLKKSGVTQIDGNVLIDTSIFASHDKAPGWPWNDLTQCFSAPPAAAIVDRNCFSVSLYS
AQKPNDLAFIRVASYYPVTMFSQVRTLPRGSADAQYCELDVVPGDLNRYTLTGCLPQRAD
PLPLAFAIQDGASYAGAILKQELKEAGITYRGTLLRQTQVNEPGTIVASKQSAPLHDLLK
IMLKKSDNMIADTVFRMIGHVRFNVPGTWRAGSDAVRQILRQQAGIDIGNTIIADGSGLS
RHNLIAPATMMQVLQYIAQHDNELNFISMLPLAGYDGSLQYRAGLHQAGVDGKVSAKTGS
LQGVYNLAGFITTASGQRMAFVQYLSGYAVPPADQRNRRIPLVRFESRLYKDIYQTN
>EECEBICD_00153 hypothetical protein
MVIKEEIVADLGLRARVGVFLLLTLVWHMPGLA
>EECEBICD_00154 Transcription elongation factor GreA
MQAIPMTLRGAEKLREELDFLKSVRRPEIIAAIAEAREHGDLKENAEYHAAREQQGFCEG
RIKDIEAKLSNAQVIDVTKMPNNGRVIFGATVTVLNLDTDEEQTYRIVGDDEADFKQNLI

SVNSPIARGLIGKEQDDVVVIKTPGGDVEYEVLKVEYL
>EECEBICD_00155 RNA-binding protein YhbY
MTRFQSQRKQKYTMNLSTKQKQHLKGLAHPLKPVVMLGNNGLTEGVLAEIEQALEHHELI
KVKIASEDRETKTLIVDAIVRETGACNVQVIGKTLVLYRPTKERKISLPR
>EECEBICD_00156 Ribosomal RNA large subunit methyltransferase E
MPGLNLMKYSKVTNFFKPGMTVVDLGAAPGGWSQYVVTQIGGKGRIIACDLLPMDPIVGV
DFLQGDFRDELVMKALLERVGDSKVQVVMSDMAPNMSGTPAVDIPRAMYLVELALEMCRD
VLAPGGSFVVKVFQGEGFDEYLREIRSLFTKVKVRKPDSSRARSREVYIVATGRK
>EECEBICD_00157 ATP-dependent zinc metalloprotease FtsH
MSDMAKNLILWLVIAVVLMSVFQSFGPSESNGRKVDYSTFLQEVNQDQVREARINGREIN
VTKKDSNRYTTYIPINDPKLLDNLLTKNVKVVGEPPEEPSLLASIFISWFPMLLLIGVWI
FFMRQMQGGGGKGAMSFGKSKARMLTEDQIKTTFADVAGCDEAKEEVAELVEYLREPSRF
QKLGGKIPKGVLMVGPPGTGKTLLAKAIAGEAKVPFFTISGSDFVEMFVGVGASRVRDMF
EQAKKAAPCIIFIDEIDAVGRQRGAGLGGGHDEREQTLNQMLVEMDGFEGNEGIIVIAAT
NRPDVLDPALLRPGRFDRQVVVGLPDVRGREQILKVHMRRVPLATDIDAAIIARGTPGFS
GADLANLVNEAALFAARGNKRVVSMVEFEKAKDKIMMGAERRSMVMTEAQKESTAYHEAG
HAIIGRLVPEHDPVHKVTIIPRGRALGVTFFLPEGDAISASRQKLESQISTLYGGRLAEE
IIYGVEHVSTGASNDIKVATNLARNMVTQWGFSEKLGPLLYAEEEGEVFLGRSVAKAKHM
SDETARIIDQEVKALIERNYNRARQILTDNMDILHAMKDALMKYETIDAPQIDDLMARRE
VRPPAGWEDPNGTNNSDSNGTPQAPRPVDEPRTPNPGNTMSEQLGDK
>EECEBICD_00158 Dihydropteroate synthase
MKLFAQGATLDLTHPHVMGILNVTPDSFSDGGAHNTLIEAVKHANLMVNAGATIIVMVGE
STRPGAAEVSVEEELDRVIPVLEAIAQRFEVWISVDTSKPEVIREATRAGAHIINDVRSL
SEPGALEAAAETGLPVSLMHMQGNPKTMQEAPKYDDVFAEVNRYFIEQIARCEKAGIAKE
KLLLDPGFGFGKNLSHNYTLLARLGEFHHFNLPLLVGMSRKTMVGQLLNVGPSDRLNGSL
ACAVIAAMQGAQIIRVHDVKETVEAMRVVEATLSAKGNKRYE
>EECEBICD_00159 Phosphoglucosamine mutase
MSNRKYFGTDGIRGRVGNAPITPDFVLKLGWAAGKVLARHGSRKIIIGKDTRISGYMLES
ALEAGLAAAGLSASFTGPMPTPAVAYLTRTFRAEAGIVISASHNPFYDNGIKFFSIDGTK
LPDDVEEAIEAEMEKEITCVDSAELGKASRIVDAAGRYIEFCKGTFPNELSLNGLKVVVD
CANGATYHIAPNVLRELGATVIAIGCEPNGVNINEEVGATDVRALQARVLAEKADLGIAL
DGDGDRVIMVDHEGNKVDGDQIMYIIAREGLRQGQLRGGAVGTLMSNMGLELALKQLGIP
FARAKVGDRYVLEKLQEKGWRIGAENSGHVILLDKTTTGDGIVAGLQVLAAMVRNHMSLH
DLCSGMKMFPQILVNVRYTAGSGDPLENEAVKAVTADVEATLGNRGRVLLRKSGTEPLIR
VMVEGEDEAQVTAFAHRIADAVKAV
>EECEBICD_00160 Protein-export membrane protein SecG
MYEALLVVFLIVAIGLVGLIMLQQGKGADMGASFGAGASATLFGSSGSGNFMTRMTAVLA
TLFFIISLVLGNINSNKTNKGSEWENLSAPAKTEQTQPAAPAQPTSDIPR
>EECEBICD_00162 hypothetical protein
MNSEHFVRLALDILKCSQKELAGKLGVSSTQISKWKKGEHMSDDMEKKFRKITNIGEYSP
LLVEWAGSVSNAEKWDRLMHFIADRVHDRAETGYVTTPLLDEEGFLCEETIDTLEKMGLS
APKSFPVELDINYENTDDEETEDLWDSISNNPHSSIIEKIYNSLNDVYGFYAAYVDELIQ
DEGLDIYSTDAINIMYSLMSLAACKIEIDSATAPNFRQFRYEVEKDYENWLSQLKLLAFR
AGIPLRAELLQMVYDSADDLSVAAEAESLDLNKSRIHPDIYMNEILTGMRIIHQVLPVIM
EKLEITDFELDESALHIGR
>EECEBICD_00163 Argininosuccinate synthase
MTTILKHLPAGQRIGIAFSGGLDTSAALLWMRQKGAVPYAYTANLGQPDEDDYDAIPRRA
MEYGAENARLIDCRKQLVAEGIAAIQCGAFHNTTGGLTYFNTTPLGRAVTGTMLVAAMKE
DGVNIWGDGSTYKGNDIERFYRYGLLTNAELQIYKPWLDTDFIDELGGRHEMSEFMIACG
FDYKMSVEKAYSTDSNMLGATHEAKDLEFLNSSVKIVNPIMGVKFWDESVKIPAEEVTVR
FEQGHPVALNGKTFSDDVEMMLEANRIGGRHGLGMSDQIENRIIEAKSRGIYEAPGMALL
HIAYERLLTGIHNEDTIEQYHSHGRQLGKLLYQGRWFDSQALMLRDGLQRWVASQITGEV
TLELRRGNDYSILNTVSDNLTYKAERLTMEKGESVFSPDDRIGQLTMRNLDITDTREKLF
GYAKAGLLTASSATGLPQVENLENKGK

>EECEBICD_00165 Ribosome maturation factor RimP
MSTLEQKLTEMITAPVEALGYELVGIEFIRGRTSTLRIYIDSEDGINVDDCADVSHQVSA
VLDVEDPISVAYNLEVSSPGLDRPMFTADHYARFQGEEVALVLRMAVQNRRKWQGIIKAV
DGEMITVTVEGKDEVFALSNIQKANLVPHF
>EECEBICD_00166 Transcription termination/antitermination protein NusA
MNKEILAVVEAVSNEKALPREKIFEALESALATATKKKYEQEIDVRVEIDRKSGDFDTFR
RWLIVEEVTMPTKEITLEAARFEDESLNVGDYVEDQIESVTFDRITTQTAKQVIVQKVRE
AERAMVVDQFRDQEGEIVTGVVKKVNRDNISLEIKSEGMAGNAEAVILREDMLPRENFRP
GDRIRGVLYAVRPEARGAQLFVTRSKPEMLIELFRIEVPEIGEEVIEIKAAARDPGSRAK
IAVKTNDKRIDPVGACVGMRGARVQAVSTELGGERIDIVLWDDNPAQFVINAMAPADVAS
IVVDEDKHTMDIAVEAGNLAQAIGRNGQNVRLASQLSGWELNVMTVDDLQAKHQAEAHAA
IEIFTKYLDIDEEFATVLVEEGFSTLEELAYVPMKELLEIDGLDEPTVEALRERAKNALA
TLAQDQEASLGDNKPADDLLNLEGLDRDMAFKLAARGVCTLEDLADQGIDDLADIEGLTD
EKAGELIMAARNICWFGDEA
>EECEBICD_00167 Translation initiation factor IF-2
MTDVTLKALAAERQVSVDRLVQQFADAGIRKSADDSVSAQEKQTLLAHLNREAVSGPDKL
TLQRKTRSTLNIPGTGGKSKSVQIEVRKKRTFVKRDPQEAERLAAEEQAQREAEEQARRE
AEEQAKREAQQKAEREAAEQAKREAAEKAKREAAEKDKVSNQQTDDMTKTAQAEKARREN
EAAELKRKAEEEARRKLEEEARRVAEEARRMAEENKWTATPEPVEDTSDYHVTTSQHARQ
AEDENDREVEGGRGRGRNAKAARPAKKGKHAESKADREEARAAVRGGKGGKRKGSSLQQG
FQKPAQAVNRDVVIGETITVGELANKMAVKGSQVIKAMMKLGAMATINQVIDQETAQLVA
EEMGHKVILRRENELEEAVMSDRDTGAAAEPRAPVVTIMGHVDHGKTSLLDYIRSTKVAS
GEAGGITQHIGAYHVETDNGMITFLDTPGHAAFTSMRARGAQATDIVVLVVAADDGVMPQ
TIEAIQHAKAAGVPVVVAVNKIDKPEADPDRVKNELSQYGILPEEWGGESQFVHVSAKAG
TGIDELLDAILLQAEVLELKAVRKGMASGAVIESFLDKGRGPVATVLVREGTLHKGDIVL
CGFEYGRVRAMRNELGQEVLEAGPSIPVEILGLSGVPAAGDEVTVVRDEKKAREVALYRQ
GKFREVKLARQQKSKLENMFANMTEGEVHEVNIVLKADVQGSVEAISDSLLKLSTDEVKV
KIIGSGVGGITETDATLAAASNAILVGFNVRADASARKVIESESLDLRYYSVIYNLIDEV
KAAMSGMLSPELKQQIIGLAEVRDVFKSPKFGAIAGCMVTEGTIKRHNPIRVLRDNVVIY
EGELESLRRFKDDVNEVRNGMECGIGVKNYNDVRVGDMIEVFEIIEIQRTIA
>EECEBICD_00168 30S ribosome-binding factor
MAKEFGRPQRVAQEMQKEIAIILQREIKDPRLGMMTTVSGVEMSRDLAYAKVFVTFLNDK
DEDAVKAGIKALQEASGFIRSLLGKAMRLRIVPELTFFYDNSLVEGMRMSNLVTNVVKHD
EERRVNPDDSKED
>EECEBICD_00169 tRNA pseudouridine synthase B
MSRPRRRGRDIHGVLLLDKPQGMSSNDVLQKVKRIYNANRAGHTGALDPLATGMLPICLG
EATKFSQYLLDSDKRYRVIARLGQRTDTSDADGQIVQERPVTFSAEQLASALETFRGDIE
QIPSMYSALKYQGKKLYEYARQGIEVPREARPITVYELLFIRHEGNELELEVHCSKGTYI
RTIIDDLGEKLGCGAHVTYLRRLTVSKYPVDRMVTLEHLQTLVAQAEQQGVPAAQLLDPL
LMPMDSPASDYPVVNLPLTSSVYFKNGNPVRTTGAPLKGLVRVTEGEDDKFIGMGEIDDE
GRVAPRRLVVEYPA
>EECEBICD_00170 30S ribosomal protein S15
MSLSTEATAKIVSEFGRDANDTGSTDVQVALLTAQINHLQGHFAEHKKDHHSRRGLLRMV
SQRRKLLDYLKRKDVARYTALIERLGLRR
>EECEBICD_00171 Polyribonucleotide nucleotidyltransferase
MPSKIGKDIILLNPIVRKFQYGQHTVTLETGMMARQATAAVMVSMDDTAVFVTVVGQKKA
KPGQDFFPLTVNYQERTYAAGRIPGSFFRREGRPSEGETLIARLIDRPVRPLFPEGFVNE
VQVIATVVSVNPQVNPDIVAMIGASAALSLSGIPFNGPIGAARVGYINDQYVLNPTQDEL
KESKLDLVVAGTEAAVLMVESEAELLSEDTMLGAVVFGHEQQQVVIQAINDLVKEAGKPR
WDWQPEAVNDALNARVAALAESRLSDAYRITDKQERYAQVDVIKSETIEQLIAEDETLDA
NELGEILHAIEKNVVRSRVLAGEPRIDGREKDMIRGLDVRTGVLPRTHGSALFTRGETQA
LVTATLGTARDAQVLDELMGERTDSFLFHYNFPPYSVGETGMVGSPKRREIGHGRLAKRG
VLAVMPDMDKFPYTVRVVSEITESNGSSSMASVCGASLALMDAGVPIKAAVAGIAMGLVK
EGDNYVVLSDILGDEDHLGDMDFKVAGSRDGISALQMDIKIEGITKEIMQVALNQAKGAR

LHILGVMEQAINAPRGDISEFAPRIHTIKISTDKIKDVIGKGGSVIRALTEETGTTIEIE
DDGTVKIAATDGEKAKYAIRRIEEITAEIEVGRIYNGKVTRIVDFGAFVAIGGGKEGLVH
ISQIADKRVEKVTDYLQMGQEVPVKVLEVDRQGRVRLSIKEATEQSQPAAAPEAPASEQA
E
>EECEBICD_00172 Lipoprotein NlpI
MKPFLRWCFVATALTLAGCSNSAWRKSEVLAVPLQPTLQQEVILARMEQILASRALTDDE
RAQLLYERGVLYDSLGLRALARNDFSQALAIRPDMPEVFNYLGIYLTQAGNFDAAYEAFD
SVLELDPTYNYAHLNRGIALYYGGRDKLAQDDLLAFYQDDPNDPYRSLWLYLVEQKLNEK
QAKEALKARFEKSDKEQWGWNIVEFYLGDISEATLMERLKADATDNTSLAEHLSETNFYL
GKYYLSLGDLDSATALFKLAVANNVHNFVEHRYALLELSLLGQDQDDLAESDQQ
>EECEBICD_00173 ATP-dependent RNA helicase DeaD
MAEFETTFADLGLKAPILEALTDLGYEKPSPIQAECIPHLLGGRDVLGMAQTGSGKTAAF
SLPLLNNLDPELKAPQILVLAPTRELAVQVAEAMTDFSKHMRGVNVVALYGGQRYDVQLR
ALRQGPQIVVGTPGRLLDHLKRGTLDLSKLSGLVLDEADEMLRMGFIEDVETIMAQIPEG
HQTALFSATMPEAIRRITRRFMKEPQEVRIQSSVTTRPDISQSYWTVWGMRKNEALVRFL
EAEDFDAAIIFVRTKNATLEVAEALERSGYNSAALNGDMNQALREQTLERLKDGRLDILI
ATDVAARGLDVERISLVVNYDIPMDSESYVHRIGRTGRAGRAGRALLFVENRERRLLRNI
ERTMKLTIPEVELPNAELLGKRRLEKFAAKVQQQLESSDLDQYRALLAKIQPSAEGEELD
LETLAAALLKMAQGERPLILPPDAPMRPKREFRDRDDRGPRERNDRGPRGDREERPRRER
RDVGDMQLYRIEVGRDDGVEVRHIVGAIANEGDISSRYIGNIKLFASHSTIELPKGMPGE
VLQHFTRTRILNKPMNMQLLGDAVPHAGGERRGGGRSFSGERREGGRNFSGERREGGRGD
GRRFSGERRESRGPRRDDSTGRRRFGGDA
>EECEBICD_00174 Tryptophan-specific transport protein
MATLTTTQTSPSLLGGVVIIGGTIIGAGMFSLPVVMSGAWFFWSMAALVFTWFCMLHSGL
MILEANLNYRIGSSFDTITKDLLGKGWNIVNGISIAFVLYILTYAYISASGSILHHTFAE
MSLNVPARAAGFAFALLVAFVVWLSTKAVSRMTAIVLGAKVITFFLTFGSLLGHVQPATL
FNVAESHASYTPYLLMTLPFCLASFGYHGNVPSLMKYYGKDPRTIVKCLIYGTLLALALY
SVWLLGTMGNIPRPEFIGIAQKGGNIDVLVQALSGVLNSRSLDLLLVVFSNFAVASSFLG
VTLGLFDYLADLFGFDDSAMGRFKTALLTFLPPMIGGLLYPNGFLYAIGYAGLAATIWAA
IVPALLARKSRERFGSPKFRVWGGKPMIALILVFGVGNAVIHILSSFNLLPVYQ
>EECEBICD_00175 hypothetical protein
MSEPASFFLHAHITESNLKKFFHSPATNIKDYDDWLPWFTEEQRLYGDPAKMLNNLATCN
SGESEKNIYAEHINFNKETQIVTMDHIFLSESYEIFMPLMACVRGIEKFITLGENNFALI
YYYWWGSEIAIALEFGANGSRITANPKAENLTIADAFFDEHGEALAEELYNKQGFI
>EECEBICD_00176 hypothetical protein
MGRKGLLAIVLLSLFIAFILKFFWLTPYDEDVYLPVEKPVASSLKIIHPGDQLFIRILKA
EDKLELWASANNKPYKLYKTWTICAWSGGLGPKHKQGDGKSPEGFYATNKGLLNPNSRYH
LAFNIGYPNAYDRANGYTGDFIMVHGNCVSAGCYAMTDAGIEEIYQLVAQALNSGQKRVP
VHIFPFTMNDENMRQAQAWPEYNFWRMLKPGYDYFEKNRRLPTITVENRRYKISPTTLP
>EECEBICD_00177 hypothetical protein
MTDKTIPFSVLDLAPIPEGSSAKEAFTHSLDLARLAEKRGYHRYWLAEHHNMTGIASAAT
SVLIGYLAANTTTLHLGSGGVMLPNHSPLVIAEQFGTLNTLYPGRIDLGLGRAPGSDQPT
MRALRRHMSGDIDNFPRDVAELVDWFDARAPNPHVRPVPGYGEQIPVWLLGSSLYSAQLA
AQLGLPFAFASHFAPDMLFQALHLYRTQFKPSARLEKPYAMVCINIIAADSNRDAEFLFT
SMQQAFVKLRRGETGQLPPPIENMETFWSPSEQYGVQQALSMSLVGDKAKVRHGLESILR
ETQADEIMVNGQIFDHQARLHSFDLAMDVKEELLG
>EECEBICD_00178 hypothetical protein
MKYSLGPVLYYWPKETLEDFYQQAAKSSADVIYLGEAVCSKRRATKVGDWLEMAKSLAAS
GKQVVLSTLALVQASSELSELKRYVDNGDFLLEASDLGVVNLCAERKLPFVAGHALNCYN
AVTLRRLLKEGMVRWCMPVELSRDWLVNLLNQCDELGIRNQFEVEVLSYGHLPLAYSARC
FTARSEDRPKDECETCCIKYPNGRDVLSQENQQVFVLNGIQTMSGYVVYNLGNELTSMQGL
VDIVRLSPLGTETFAMLDAFRANENGGAPLALAAHSDCNGYWRRLAGLELQA
>EECEBICD_00179 putative protease YhbU
MELLCPAGNLPALKAAIENGADAVYIGLKDDTNARHFAGLNFTEKKLQEAVSFVHQHRRK

LHIAINTFAHPDGYARWQRAVDMAAQLGADALILADLAMLEYAAERYPHIERHVSVQASA
TNEEAIRFYHRNFDVHRVVLPRVLSIHQVKQLARVTPVPLEVFAFGSLCIMAEGRCYLSS
YLTGESPNTVGACSPARFVRWQQTPQGLESRLNDVLIDRYQDGENAGYPTLCKGRYLVDG
ERYHALEEPTSLNTLELLPELMAANIASVKIEGRQRSPAYVSQVAKVWRQAIDRCKAAPQ
NFVPQRDWMETLGAMSEGTQTTLGAYHRKWQ
>EECEBICD_00180 Ubiquinone biosynthesis accessory factor UbiJ
MLDKLRSRLVHAGPSLMSVPVKLTPFALKRQVLEQVLSWQFRQALADGELEFLEGRWLSI
HVRDIDLKWYTTVENEKLIVSQQADADVSFSADASDLLMIAARKQDPDTLFFQRRLVIEG
DTELGLYVKNLMDAIELEQMPKALRVMLLQLADFVEAGMKNSPETKQTSVGEPC
>EECEBICD_00181 hypothetical protein
MLIRVEIPIDAPGIDALLRRSFESDAEAKLVHDLREDGFLTLGLVATDDEGQVVGYVAFS
PVDVQGEDLQWVGMAPLAVDEKYRGQGLARQLVYEGLDSLNEFGYAAVVTLGDPALYSRF
GFELAAHYDLHCRWPGTESAFQVHRLAEDALEGVTGLVEYHDHFNRF
>EECEBICD_00182 hypothetical protein
MLMATMTPWYLYLIRTADNALYTGITTDVARRYRQHQTGKGAKALRGKGELTLAFAAQVG
NRSLALRIEYRIKQLTKRQKERLVTEREAFEALLSSLQTPVLKND
>EECEBICD_00183 hypothetical protein
MDTLTAIGRWLAKQHVVTWCVHHEGELWCANAFYLFDAQNVALYLLTDDKTRHAQMSGAC
APVAGTVNGQPKTVARIRGVQFKGEIRRLEGQESDAARKAYLRRFPVARVLPAPVWEIRL
DEIKFTDNTLGFGKKLHWLRDSRAQQA
>EECEBICD_00184 Protein/nucleic acid deglycase 2
MLLPGGHSPDYLRGDSRFVDFTRDFVNSGKPVFAICHGPQLLISADVIRGRKLTAVKPII
IDVKNAGAEFYDQEVVVDKDQLVTSRTPDDLPAFNREALRLLGA
>EECEBICD_00185 hypothetical protein
MSQVLITGATGLVGGHLLRMLINTPQVSAIAAPTRRPLTDIVGVYNPHDPQLTDALAQVT
DPVDIVFCCLGTTRREAGSKAAFIHADYTLVVDTALTGRRLGAQHMLVVSAMGANAHSPF
FYNRVKGEMEEALIAQNWPRLTIARPSMLLGDRTTRRVNETLFAPLFRLLPGNWKSIDAR
DVARAMLAEALAPAQEGVTILTSSQLREKAE
>EECEBICD_00186 Osmotically-inducible protein Y
MKAFSPLAVLISALLLQGCVAAAVVGTAAVGTKAATDPRSVGTQVDDGTLELRVSSALSK
DEQIKKETRINVTAYQGKVLLVGQSPNSELSARAKQIAMGVEGTTEVYNEIRQGQPIGLG
TASNDTWITTKVRSQLLTSDQVKSSNVKVTTENGEVFLLGLVTEREGKAAADIASRVSGV
KRVTTAFTYIK
>EECEBICD_00187 DnaA initiator-associating protein DiaA
MLERIKVCFTESIQTQIAAAEALPDAISRAAMTLVHSLLNGNKILCCGNGTSAANAQHFA
ASMINRFETERPSLPAIALNTDNVVLTAIANDRLHDEVYAKQVRALGHAGDVLLAISTRG
NSRDIVKAVEAAVTRDMTIVALTGYDGGELAGLLGPQDVEIRIPSHHSARIQEMHMLTVN
CLCDLIDNTLFPHQDD
>EECEBICD_00188 hypothetical protein
MAQIPARGDCSRQLTRKQAGDAWEAAARRWLESKGLRFIAANVRERGGEIDLIMRDGKTT
VFVEVRYRRSGLYGGAAASVTRSKQHKLLHTARLWLARQNGSFDTVDCRFDVLAFTGNEI
EWFRDAFNDHS
>EECEBICD_00189 Penicillin-binding protein activator LpoA
MVPSTFSRLNAARALPVVLAALLFAGCGTQAPDQSAAYMQGSAQADSAFYLHQMQQSADD
SKTNWQLLAIHALLKEGKSQQAVDLFNQLPQNLNDAQRREQSLLAVEIKLAQKDVAGAQA
LLDKLKPADFAPNQQARYWQAQIVASQGRPSLTLLRALIAQEPLLAAKDKQKNIDATWQA
LSAMTPDQAKTLVINADENVLQGWLDLQRVWFDNRNDPDMLKAGIADWQKRYPQNPGAKM
LPTQLVNVQRFKPASTSKIALLLPLNGQAAVFGRTIQQGFEAAKNLGTQAVEMQPAAAPD
APVEPGVEETQPQMTNGVASPSQASVSDLTDDAPAQSATPVSAPQTPPATASAPADPSAE
LKIYDTSSQPLDQVLAQVQQDGASIVVGPLLKNNVEALMKSNTPLNVLALNQPETVRSFP
NICYFALSPEDEARDAAHHIYDQGKQSPLLLIPRSALGDRVANAFTQEWQKLGGGIVLQQ
KFGSVAELKMGVNGGAGIALTGSPVAASVPAQPGVTIGGLTIPAPPTDAQITGGGRVDAV
YILATPEEIGFIKPMIAMRNGTQSGATLYASSRSAQGTSGPDFRLEMEGLQYSEIPMLAG
GNMPLMQQALSAVHNDYSLARMYAMGVDAWTLANHFSQMRQVQGFEINGNTGALTASPDC

VINRKLSWLKYQQGEIVPAS
>EECEBICD_00190 Ribosomal RNA small subunit methyltransferase I
MKQNESADNSQGQLFIVPTPIGNLADITQRALEVLQAVDLIAAEDTRHTGLLLQHFGINA
RLFALHDHNEQQKAETLVAKLKEGQNIALVSDAGTPLINDPGYHLVRTCREAGIRVVPLP
GPCAAITALSAAGLPSDRFCYEGFLPAKSKGRRDALKAIETEPRTLIFYESTHRLLDSLE
DIVAVLGESRYVVLARELTKTWETIHGAPVGELLAWVKEDENRRKGEMVLIVEGHKAQED
DLPADALRTLALLQAELPLKKAAALAAEIHGVKKNALYKYALEQQEE
>EECEBICD_00191 Glucitol operon repressor
MNSFERRNKIVDLINTQGSVLVMDLSNTFGISEVTIRADLRLLEEKGLVTRFHGGAAKPG
SHLAEGDNQEVILEDRYQLASDPKKRIAQAAAAMVEEGMTIILDSGSTTLLIAEALARKS
NITVITNSLPAAFTLSENKDLTLVVCGGTVRHKTHSMHGTIAERSLHGISADLMFVGADG
IDATNGITTFNEGYSISGVMAAAAHKVIAVLDATKFNRRGFNQVLPMDKIDCVITDDTIS
KQDKAALAKTGVELMIV
>EECEBICD_00192 Galactitol 1-phosphate 5-dehydrogenase
MKSVVIHAEGDVRVEERPLPQLQAEDDVLVKVVSSGLCGSDIPRIFAQGAHYYPITLGHE
FSGYVESYGTGVTDMQPGDAVACVPLLPCFRCPQCERGYFSLCKQYQFVGSRSEGGNAEY
VVVKRANLFRLPSDMPIEDGAFIEPITVGLHAFHLAQGCEGKNVIIVGAGTIGLLALQCA
RELGARSVTAIDINPQKLELAKALGATHTCNSREMTADDIQTALSDIQFDQLVLETAGTP
QTVSLAIDIAGPRAQLALVGTLHHDLTLTTRTFGLILRKELTLLGSWMNYSAPWPGEEWE
TAARLLAEKRLQLTPLIAHRGDAESFAEAVKALNGAPMQGKILLQLS
>EECEBICD_00193 PTS system galactitol-specific EIIC component
MFSEIMRYILDLGPTVMLPLVIIVFSKLLGMKLGDCFKSGLHIGIGFVGIGLVIGLMLDS
IGPAAKAMAEHFQINLHVIDVGWPGSSPMTWASQIALVAIPVAIGVNVLMLVTRMTRVVN
VDIWNIWHMTFTGAMLHLATGSYWLGILGVVVHAAFVYKLGDWFAKDTRDYFGLEGIAIP
HGSSAYLGPVAVLVDTIIEKIPGLNRIHFSADDVQKRFGPFGEPVTVGFVMGLVIGVLAG
YDAKAVLQLAVKTAAVMLLMPRVIKPIMDGLTPIAKHARKRLQAKFGGQEFLIGLDPALL
LGHTSVVSASLIFIPLTILIAVLVPGNQVLPFGDLATIGFFIAMAVAVHQGNLFRTLISG
VIIMGITLWIATQTIGLHTQLAANAGALKAGGQVASLDQGGSPITWLLIQLFTWQNIVGF
AVIAIIYLAGVLLTWRRARQFVAAEKATALQQSQIAS
>EECEBICD_00194 PTS system galactitol-specific EIIB component
MKRKVIVACGGAVATSTMAAEEIKELCDANHIELDLVQCRVTEIETYMDGADLICTTARV
DRAFGDIPVVHGMPFVSGVGIEALQQKILSILMG
>EECEBICD_00195 PTS system galactitol-specific EIIA component
MSQLFVRTGITFDSSQQALVHIGKEMLAKGVVHDSYPQALVEREASFPTGIALERHAVAI
PHCEAVHAKSPAIYLIRPDKPVMFQQADDDEEIAVSLIIALIVENPAAQLKLLRRLFSEL
QNPSTLDALLSAPDAELAIRFQETILEPEQCVQV
>EECEBICD_00196 D-tagatose-1,6-bisphosphate aldolase subunit GatZ
MKEIISRHKAGEQIGICSVCSAHPLVIESALRFDLNSGNKVLIEATSNQVNQFGGYTGMK
PADFRDFVYGIAQEVGFPRERLILGGDHLGPNCWQNEPADTAMEKSVELIKAYVAAGFSK
IHLDASMSCADDPTPLDPMVVAKRAALLCQAAETTATDEQKRHLTYVIGTEVPVPGGEAS
AINAVHVTREQDAARTLQTHQAAFRALGLDEALNRVIAIVVQPGVEFDHTQIIHYQPQAA
QALSAWIKETPMVYEAHSTDYQTRQAYRALVRDHYAILKVGPALTFALREAIFALAQMEN
ELISPEQRSRVLEVIDEVMLNEPGYWKKYYRPTWSQAMVDIHFSLSDRIRYYWPHPRIRQ
SVEKLIANLNNVTLPLGLISQFMPVQFERLSEGVLTPTPHNLIIDKIQDVLRAYRFGCTP
DVA
>EECEBICD_00197 D-tagatose-1,6-bisphosphate aldolase subunit GatY
MFIISGRTMLKKAQQEGYAVPAFNIHNLETLQVVVETAAELRSPLIVAGTPGTFSYAGVG
NIVAIAAELAKSWNHPLAVHLDHHEKLADIKMKVAAGVRSVMIDGSHFPFADNIALVKSV
VDYCHRYDVSVEAELGRLGGQEDDLIVDGKDALYTHPEQAREFVEKTGIDSLAIAIGTAH
GLYTAEPKLDFERLTEIRQRVDVPLVLHGASGLPTRDITRAISLGICKVNVATELKIAFS
GALKNYLTQHAEASDPRHYMIPAKAAMKEVVRKVIADCGCEGKL
>EECEBICD_00198 Galactarate dehydratase (L-threo-forming)
MANIEIRQESPSAFYIKVHETDNVAIIVNDHGLKAGTRFPDGLELTEHIPQGHKVALTDI
PAHGEIIRYGEVIGYAVRDIPRGSWIDESLVELPKAPPLNTLPLATKVPEPLPPLEGYTF

EGYRNADGSVGTKNLLGITTSVHCVAGVVDYVVKVIERDLLPKYPNVDGVVGLNHLYGCG
VAINAPAAVVPIRTIHNIALNPNFGGEVMVIGLGCEKLQPERLLEGTEDVPAIAVESASI
VRLQDEQHVGFKSMVDDILRVAERHLTKLNQRQRETCPASELVVGMQCGGSDAFSGVTAN
PAVGYASDLLVRCGATVMFSEVTEVRDAIHLLTPRAINEAVGKRLLDEMAWYDNYLDMGK
TDRSANPSPGNKKGGLANVVEKALGSIAKSGKSAIVEVLSPGQRPTKRGLIYAATPASDF
VCGTQQVASGITVQVFTTGRGTPYGLMAVPVIKMATRTELANRWYDLMDINAGTIATGEE
TIEDVGWKLFHFILDVASGRKKTFSDQWGLHNQLAVFNPAPVT
>EECEBICD_00199 5-keto-4-deoxy-D-glucarate aldolase
MNNAIFPNKFKAALAAQQVQIGCWSALASPITTEVLGLAGFDWLVLDGEHAPNDVTTLIP
QLMALKGSASAPVVRVPTNEPVIIKRMLDIGFYNFLIPFVETQEEAARAVASTRYPPEGI
RGVSVSHRANMFGTVPDYFAQSNKNITIIVQIESQLGVDNVDAIAATEGVDGIFVGPSDL
AAALGHLGNASHPDVQQTIQHIFARAKAHGKPCGILAPVEADARRYLEWGATFVAVGSDL
GAFRASTQKLADTFKK
>EECEBICD_00200 2-hydroxy-3-oxopropionate reductase
MTMKVGFIGLGIMGKPMSKNLLKAGYSLVVSDRNPEAIADVIAAGAETASTAKAIAEQCD
VIITMLPNSPHVKEVALGENGIIEGAKPGTVLIDMSSIAPLASREISDALKAKGVEMLDA
PVSGGEPKAIDGTLSVMVGGDKAIFDKYYDLMKAMAGSVVHTGDIGAGNVTKLANQVIVA
LNIAAMSEALTLATKAGVNPDLVYQAIRGGLAGSTVLDAKAPMVMDRNFKPGFRIDLHIK
DLANALDTSHGVGAQLPLTAAVMEMMQALRADGHGNDDHSALACYYEKLAKVEVTR
>EECEBICD_00201 Glycerate 2-kinase
MKIVIAPDSYKESLSAAEVAQAIEKGFREIFPDAQYVSVPVADGGEGTVEAMIAATQGVE
RTAWVTGPLGEKVKACWGMSGDGKTAFIEMAAASGLALVPPEKRNPLITTSRGTGELILQ
ALESGASNIIIGIGGSATNDGGAGMMQALGAKLRDANGADIGYGGGSLHCLSDIDISELD
PRLKLCAIRVACDVSNPLIGDNGASRIFGPQKGATEENIVELDRNLAHYADIIKKSLNVD
VKAAPGAGAAGGMGAALMAFLGAELRSGIEIVTAALNLEEHIHDCTLVVTGEGRIDSQSI
RGKVPIGVANVAKKYHKPVIGIAGSLTHDVGIVHHYGIDAVFSVLTRIVTLEEAFRGAFD
NIYRASRNVAAALAIGMRSAG
>EECEBICD_00202 hypothetical protein
MKLTDKPLSFGYSVVDSHSSCRPVKAKTRRSGFYEEVKLTDKPGSVVDSHSSRPAIAHWL
KQPTRVQYGPYLMNPYLALLRVEFTVPRTVTSRAVRSYRTLSPLPDPTCVGHRRFALCCT
GRRFPPRRYLAPCPMEPGLSSPPPVSPKGRRRSGDCLVSFGAQYRGFARLCHPALRIPIA
RAAATFLDAR
>EECEBICD_00203 HTH-type transcriptional regulator TsaR
MNTLVLPKTQHLVVFQEVIRSGSIGSAAKSLGLTQPAVSKIISDVEAYFGVELIVRKNTG
VTLTEAGQVLLSWSESITREMKNMINEMNSMTCNTVVDVSFGFPSLIGFTFMSDMIHKFK
EVFPKAQVSMYEAQLSSFLPALRDGRLDFAIGTLSNEMQLQDLHVEPLFESEFVLVASKS
RTCTGTITLESLKDEQWVLPQTNMGYYSELLTTLQRNGISIENIVKTDSVVTIYNLVLNA
DFLTVIPCDMTTPFGSNQFITIPIKDTLPVARYAAVWSKNYRIKKAASVLVELAKQYSSY
NGCRRRQLIEIE
>EECEBICD_00204 L-threonine dehydratase catabolic TdcB
MHITYDLPVAIEDILEAKKRLAGKIYKTGMPRSNYFSERCKGEIFLKFENMQRTGSFKIR
GAFNKLSSLTEAEKRKGVVACSAGNHAQGVSLSCAMLGIDGKVVMPKGAPKSKVAATCDY
SAEVVLHGDNFNDTIAKVSEIVETEGRIFIPPYDDPKVIAGQGTIGLEIMEDLYDVDNVI
VPIGGGGLIAGIAIAIKSINPTIKVIGVQAENVHGMAASYYAGEITTHRTTGTLADGCDV
SRPGNLTYEIVRELVDDIVLVSEDEIRNSMIALIQRNKVITEGAGALACAALLSGKLDSH
IQNRKTVSIISGGNIDLSRVSQITGLVDA
>EECEBICD_00205 Threonine/serine transporter TdcC
MSTTDSIVSSQAKQSSWRKSDTTWTLGLFGTAIGAGVLFFPIRAGFGGLIPILLMLVLAY
PIAFYCHRALARLCLSGSNPSGNITETVEEHFGKTGGVVITFLYFFAICPLLWIYGVTIT
NTFMTFWENQLQMPALNRGFVALFLLLLMAFVIWFGKDLMVKVMSYLVWPFIASLVLISL
SLIPYWNSAVIDQVDLSNIALTGHDGILVTVWLGISIMVFSFNFSPIVSSFVVSKREEYE
KEFGREFTERKCSQIISRASMLMVAVVMFFAFSCLFTLSPQNMADAKAQNIPVLSYLANH
FASLSGTKSTFATVLEYGASIIALVAIFKSFFGHYLGTLEGLNGLVLKFGYKGDKTKVSM
GKLNTISMIFIMGSTWVVAYANPNILDLIEAMGAPIIASLLCLLPMYAIRKAPSLAKYRG

RLDNVFVTLIGLLTILNIVYKLF
>EECEBICD_00206 Propionate kinase
MNEFPVVLVINCGSSSIKFSVLDFATCDVLMAGIADGMNTENAFLSINGDKPINLSHSNY
EDALKAIAFELEKRDLTDSVALIGHRIAHGGELFTQSVIITDEIIDNIRRVSPLAPLHNY
ANLSGIDAARHLFPAVRQVAVFDTSFHQTLAPEAYLYGLPWEYFSSLGVRRYGFHGTSHR
YVSRRAYELLDLDEKNSGLIVAHLGNGASICAVRNGQSVDTSMGMTPLEGLMMGTRSGDV
DFGAMAWIAKETGQTLSDLERVVNKESGLLGISGLSSDLRVLEKAWHEGHERARLAIKTF
VHRIARHIAGHAASLHRLDGIIFTGGIGENSVLIRQLVIEHLGVLGLTLDVEMNKQPNSH
GERIISANPSQVICAVIPTNEEKMIALDAIHLGNVEAPVEFA
>EECEBICD_00207 PFL-like enzyme TdcE
MKVNIDTSDMLYAEAWRDFKGTDWKEEINVRDFIQHNYTPYEGDESFLADATPATTALWE
KVMAGIRIENATHAPVDFDTNIATTITAHDAGYIEKELEKIVGLQTDKPLKRALHPFGGV
NMIKSSFHAYGREMDADFEYTFTDLRKTHNQGVFDVYSPDMLRCRKSGVLTGLPDGYGRG
RIIGDYRRVALYGIRYLVRERELQFADLQSNLEQGQNLEATIRLREELAEHRRALLQMQE
MAAKYGYDISRPARNAQEAVQWLYFAYLAAVKSQNGGAMSLGRTASFLDIYIERDFNAGL
LTEQQAQELIDHFIMKIRMVRFLRTPEFDSLFSGDPIWATEVIGGMGLDGRTLVTKNSFR
YLHTLHTMGPAPEPNLTILWSEALPVAFKKYAAQVSIVTSSLQYENDDLMRTDFNSDDYA
IACCVSPMVIGKQMQFFGARANLAKTLLYAINGGVDEKLKIQVGPKTAPLMDEVLDYDAV
MESLDHFMDWLAVQYISALNIIHYMHDKYSYEASLMALHDRDVYRTMACGIAGLSVAADS
LSAIRYAQVKPIRDENGLAIDFAIEGEYPQYGNNDERVDSIACDLVKRFMQKISVLPTYR
NAVPTQSILTITSNVVYGQKTGNTPDGRRAGTPFAPGANPMHGRDRKGAVASLTSVAKLP
FTYAKDGISYTFSIVPAALGKEDAVRKTNLVGLLDGYFHHEAQVEGGQHLNVNVMNREML
LDAIEHPENYPNLTIRVSGYAVRFNALTREQQQDVISRTFTQAM
>EECEBICD_00208 L-serine dehydratase TdcG
MISAFDIFKIGIGPSSSHTVGPMNAGKCFIDRLIDSGDLPRTTRITVDLYGSLSLTGKGH
ATDTAIIMGLAGNTPQDVNIDSIPAFIQEVARSSRLSVAGGAHVVDFPVADSILFHAETL
ARHENGMRITAWNGQAPLLHKTYYSIGGGFIVEEERFGQSHDVEKSVPYDFHSASELLTL
CERQGLSVSGLMMQNELALRSKEQIDAGFARIWQVMATGIERGMNTEGVLPGPLNVPRRA
VALRRLLVSSDNLSRDPMNVIDWINMFALAVSEENAAGGRVVTAPTNGACGIIPAVLAYY
DKFRRPVNANSIARYLLSSGAIGMLYKMNASISGAEVGCQGEVGVACSMAAAGLTELLGG
SPAQVCIAAEIAMEHNLGLTCDPVAGQVQIPCIERNAINAVKAVNAARMALRRTSEPRVS
LDKVIETMYETGKDMNDKYRETSRGGLAIKVVCG
>EECEBICD_00209 putative serine transporter
MESASNTSVILDASAPARRAGMTESEWREAIKFDSTDTGWVIMSIGMAIGAGIVFLPVQV
GLMGLWVFLLSSIIGYPAMYLFQRLFINTLAESPECKDYPSVISGYLGKNWGILLGALYF
VMLVIWMFVYSTAITNDSASYLHTFGVTEGLLSDSPFYGLVLICILVAISSRGEKLLFKI
STGMVLTKLLVVAALGVSMVGMWHLYNIGALPPMAMLIKNAIITLPFTLTSILFIQTLSP
MVISYRSREKSIEVARHKALRAMNIAFGILFVTVFFYAVSFTLAMGHDEAVKAYEQNISA
LAIAAQFISGDGAGWVKVVSVILNIFAVMTAFFGVYLGFREATQGIVMNILRRKMPAEKI
KENLVQRGIMIFAILLAWSAIVLNAPVLSFTSICSPIFGMVGCLIPAWLVYKVPALHKYK
GASLYLIIITGLLLCVSPFLAFS
>EECEBICD_00210 hypothetical protein
MFESKINPLWQSFILAVQEEVKPALGCTEPISLALAAAAAAAELDGTVERIDAWVSPNLM
KNGMGVTVPGTGMVGLPIAAALGALGGDAKAGLEVLKDASAKAVADAKAMLAAGHVAVML
QEPCNDILFSRAKVYSGDSWACVTIVGDHTNIVRIETNKGVVFTQADNAQEEEKNSPLGV
LSHTSLEEILAFVNAVPFDAIRFILDAARLNGALSQEGLRGSWGLHIGSTLAKQCDRGLL
AKDLSTAILIRTSAASDARMGGATLPAMSNSGSGNQGITATVPVMVVAEHVGADDERLAR
ALMLSHLSAIYIHHQLPRLSALCAATTAAMGAAAGMAWLIDGRYDTIAMAISSMIGDVSG
MICDGASNSCAMKVSTSASAAWKAVLMALDDTAVTGNEGIVAHNVEQSIANLCSLACRSM
QQTDKQIIEIMASKAH
>EECEBICD_00211 hypothetical protein
MSKKSAKKRQPVVKPAVQETMSATVPLGYEEMLTELEAIVADAEARLAEEEAAA
>EECEBICD_00212 Pirin-like protein YhaK
MITTRTAKQCGQADYGWLQARYTFSFGHYFDPTLLGYASLRVLNQEVLAPGASFQPRTYP

KVDILNLILDGEAEYRDSDGNHVQAKAGEALLLAAQPGISYSEHNLSKVKPLTRMQLWLD
ACPERENALVQKIPLSTAQQQLLASPDGEQNSLQLRQQVWVHHITLEKGESLNFQLHGPR
AYLQSIHGTFHAMTHNEEREALTCGDGAFIRDEPNITLVADTPLRALLVDLPV
>EECEBICD_00213 HTH-type transcriptional regulator YhaJ
MAKERALTLEALRVMDAIDRRGSFAAAADELGRVPSALSYTMQKLEEELDVVLFDRSGHR
TKFTNVGRMLLERGRVLLEAADKLTTDAEALARGWETHLTLVTEALVPTPAFFPLIDRLA
AKANTQLSLITEVLAGAWERLEQGRADIVIAPDMHFRSSSEINSRKLYSLMNVYVAAPDH
PIHQEPEPLSEVTRVKYRGVAVADTARERPVLTVQLLDKQPRLTVSTIEDKRQALLAGLG
VATMPYSMVEQDIAEGRLRVVSPESTSEIDIIMAWRRDSMGEAKAWCLREIPKLFAGK
>EECEBICD_00214 Inner membrane protein YhaH
MDWYLKVLKNYLGFGGRARRKEYWMFILVNIIFTFVLGLLDAMLGWQRAGGEGVLTTIYG
VLIFLPWWAVQFRRLHDTDRSAWWLLLLLIPIIGWLIIIAFNCQNGTPGDNRFGPDPKRF
S
>EECEBICD_00215 Glutathionyl-hydroquinone reductase YqjG
MFNSAFDGLGAKAGDYYPPALQSKIDELNGWIYDNVNNGVYKAGFATSQQAYDEAVEKVF
TALARLEQILGQHRYLTGDQLTEADIRLWTTLVRFDPVYVTHFKCDKYRISDYLNLYGFL
RDIYQIPGIAETVNLDHIRHHYFRSHKTINPTGIISIGPWQDLLEPHGRDVRFG
>EECEBICD_00216 Inner membrane protein YqjF
MILSSDNNDAPNRVIAHENSSSRRVGPLENKMKKLEDVGVLIARILMPVLFITAGWGKIS
GYAGTQQYMEAMGVPGFLLPLTILLEFGGGLAILLGFLTRTTALFTAGFTLLTALIFHSN
FAEGVNSLMFMKNLTIAGGFLLLALTGPGAFSLDRLLNKKW
>EECEBICD_00217 hypothetical protein
MSSKGEREKRKALLLSQIQQQRLDLSASRRDWLETTGAYDRGWNTVLSLRSWALVGSSVM
AIWTIRHPNMLVRWAKRGLGIWSAWRLVKTTLRQQQLRG
>EECEBICD_00218 Inner membrane protein YqjE
MADSRQAQGPGKSVLGIGQRIVTIIVEMVETRLRLAVVELEEEKANLFQLLLMVGLTMLF
AAFGLMSLMVLVIWAIDPQYRLNAMIATTVVLLVLALIGGIWTLRKARQSTLLRHTRHEL
ANDRQILEDDQS
>EECEBICD_00219 putative protein YqjD
MSKDNTTEHLRAELKSLTDTLEEVLSSSGEKSKEELSKIRSKAERALKESRYRLGETGDV
IAKQTRVAAARADDYVRENPWTGVGIGAAVGLVLGVLLTRR
>EECEBICD_00220 Protein YqjC
MKYRIALAITLFTLSAGSYANSLCQEKEQDIQKEISYAEKHNNQRRIEGLNKALSEVRAN
CTDSKLRAEHQKKIAEQKEEVAERQRDLAEAKAKGDADKIDKRERKLAEAQDELKKLEAR
DY
>EECEBICD_00221 Modulator protein MzrA
MLKPRITARQLIWISAFLLMLTILMMTWSTLRQQESTLAIRAVNQGASMPDGFSVLHHLD
ANGIHFKSITPKNDMLLITFDSPAQSAAAKTVLDQTLPHGYVVAQQDDDNETVQWLSRLR
ESSHRFG
>EECEBICD_00222 Inner membrane protein YqjA
MELLTQLLNALWAQDFETLANPSMIGMLYFVLFMILFLENGLLPAAFLPGDSLLILVGVL
IAKGAMGFPQTILLLTVAASLGCWVSYIQGRWLGNTRTVQNWLSHLPAHYHQRAHHLFHK
HGLSALLIGRFIAFVRTLLPTIAGISGLNNARFQFFNWMSGLLWVLILTSLGYLLGKTPV
FMKYEDQLMSCLMLLPVVLLFFGLAGSLVMLWKKKYGSRG
>EECEBICD_00223 Serine/threonine transporter SstT
MATQRASGLLQRLAQGSLVKQILVGLVLGILLAWISKPAAEAVGLLGTLFVGALKAVAPV
LVLMLVMASIANHQHGQKTNIRPILFLYLLGTFSAALAAVVFSFAFPSTLHLSSSAQDIV
PPSGIVEVLRGLLMSMVSNPIDALLNANYIGILVWAVGLGFALRHGNETTKNLVNDMSNA
VTFMVKLVIRFAPVGIFGLVSSTLATTGFSTLWGYAHLLVVLIGCMLLVALVVNPLLVFW
KIRRNPYPLVFACLRESGVYAFFTRSSAANIPVNMALCEKLNLDRDTYSVSIPLGATINM
AGAAITITVLTLAAVHTLGVPVDLPTALLLSVVASLCACGASGVAGGSLLLIPLACNMFG
IPNDIAMQVVAVGFIIGVLQDSCETALNSSTDVLFTAAACQAEDERLANNALRS
>EECEBICD_00224 Putative membrane-bound redox modulator Alx
MNTVGTPLLWGGFAVVVVIMLSIDLLLQGRRGAHAMSMKQAAGWSILWVTLSLLFNAAFW

WYLAETQGREVADPQALAFLTGYLIEKSLAVDNVFVWLMLFSYFSVPPALQRRVLVYGVL
GAIVLRTIMIFAGTWLITQFEWLLYVFGAFLLFTGVKMALAKEDESGIGEKPMVRWLRGH
LRMTDTIENEHFFVRKNGLLYATPLLLVLIMVEFSDVIFAVDSIPAIFAVTTDPFIVLTS
NLFAILGLRAMYFLLSGVAERFSMLKYGLAVILVFIGIKMLIVDFYHIPIAISLGVVFGI
LTITLVINAWVNHQRDKKLRAQ
>EECEBICD_00225 scyllo-inositol 2-dehydrogenase (NADP(+)) IolU
MIRFAVIGTNWITRQFVDAAHETGKFRLAAVYSRSLEQAQSFANDYPVEHLFTSLEAMAQ
SDAIEAVYIASPNSLHFSQTQRFLQHKKHVMCEKPLASNLAEVDAAIACARDNQRVLFEA
FKTACLPNFLLLRESLPKIGRMHKAFLNYCQYSSRYQRYLNGENPNTFNPAFSNGSIMDI
GYYCLASAIALWGEPRSVQASANLLESGVDAHGVVVMDYGDFSVTLQHSKVSDSVLASEI
QGERGSLVIEKLSECQKVCFAPRGALMQDLTQPQHINTMLYEAGAFAQLIENHAVEHPGL
SLSRATAKWLTEIRRQTGVIFPADDLTHPLTA
>EECEBICD_00226 hypothetical protein
MLSQCARFIRRLCFTRRALTVACFLLVAAGVALFYSNWLIVNASQHLTWNGIQTVPARNV
GLVLGAKPGNRYFTRRINTAAALYHAGKVKWLLVSGDNGKKEYDEPSAMQQALIAKGVPE
AAIFCDYAGFSTLDSVVRARKVFGESRITIISQAFHNQRAIWLAQQYGIDAIGVNAPDLN
KRHGTYTRLREKLARVSAVLDAKILHRQPKYLGAGVTIGADSAHGCPSRQ
>EECEBICD_00227 UTP pyrophosphatase
MTSLTYLQGYPEHLLAQVRALIAEQRLGAVLEKRYPGAHDYATDKALYHYTQELKSQFLR
NAPPINKVMYDSKIHVLKNALGLHTAVSRVQGGKLKAKAEIRVATVFRNAPEPFLRMIVV
HELAHLKEKDHNKAFYQLCCHMEPQYHQLEFDTRLWLTHQALSAQ
>EECEBICD_00228 Ribosomal RNA large subunit methyltransferase G
MSHVDDGFRSLTLKRFPQTDDVNPLLAWEAADEYLLQQLDETEIRGPVLILNDTFGALSC
ALAEHSPYSIGDSYLSELGTRENLRHNGIAESSVTFLDSTADYPQAPGVVLIKVPKTLAL
LEQQLRALRKVVTAQTRIIAGAKARDIHTSTLELFEKVLGPTTTTLAWKKARLINCTFSH
PQLADAPQTLSWKLEDTGWTIHNHANVFSRTGLDIGARFFMQHLPENLDGEIVDLGCGNG
VIGLSLLAKNPQANVVFVDESPMAVDSSRLNVETNLPEAFERCEFMINNALSGVEPFRFN
AVFCNPPFHQKHALTDNIAWEMFHHARRCLKINGELYIVANRHLDYFHKLKKIFGNCATI
ATNNKFVILKAVKQGHRR
>EECEBICD_00229 2,4-dienoyl-CoA reductase
MSYPSLFAPLDLGFTTLRNRVLMGSMHTGLEEHPDGAERLAAFYAERARHGVALIVTGGI
APVPSGVVMTGGAMLNDASQLTPHRVVTDAVHAQGGKIALQILHTGRYSYQPHLVAPSAI
QAPINRFMPHELTHDEILQLIDDFAHCAQLAREAGYDGVEVMGSEGYLINEFLTRRTNHR
DDEWGGDYTSRMRFAVEVVRAVRQRVGNDFIIIYRLSMLDLVENGGTFDETVQLAQAIEA
AGASLINTGIGWHEARIPTIATPVPRGAFSWVTRKLKGHVSVPLIATNRINDPQVAETIL
TRGDADMVSMARPFLADAEFLTKAQSGRADEINTCIGCNQACLDRIFIGKVTSCLVNPRA
CHETHMPIAPAIRKKNLAVVGAGPAGLAFAINAASRGHHVTLFDAQSEIGGQFTIARQIP
GKEEFYETLRYYRRMIDVTGVTLKLSQRVNAEDLQPFDEAILACGIVPRRPPIDGIDHPK
VLTYLEVLRDKAPVGKRVAIIGCGGIGFDTAMYLSQHGESTSQNIAEFCTEWGIDTSLQQ
AGGLRPEGPRLARSPRQIVMLQRKASKPGEGLGKTTGWIHRATLLARGVKMIPAVSYQKI
DDDGLHLLIGGEPQLLEVDHVVICAGQEPRRELADPLRAAGKTVHLIGGCDVAMELDARR
AIAQGIRLALEI
>EECEBICD_00230 Putrescine aminotransferase
MNRLPSSASALACSAHALNLIEKRTLNHEEMKALNREVIDYFKEHVNPGFLEYRKSVTAG
GDYGAVEWQAGSLNTLVDTQGQEFIDCLGGFGIFNVGHRNPVVVSAVQNQLAKQPLHSQE
LLDPLRAMLAKTLAALTPGKLKYSFFCNSGTESVEAALKLAKAYQSPRGKFTFIATSGAF
HGKSLGALSATAKSTFRRPFMPLLPGFRHVPFGNIDAMSMAFSEGKKTGDEIAAVILEPI
QGEGGVILPPQGYLTEVRKLCDEFGALMILDEVQTGMGRTGKMFACEHENVQPDILCLAK
ALGGGVMPIGATIATEEVFSVLFDNPFLHTTTFGGNPLACAAALATINVLLEQNLPAQAE
QKGDTLLDGFRQLAREYPNLVHDARGKGMLIAIEFVDNETGYRFASEMFRQRVLVAGTLN
NAKTIRIEPPLTLTIELCEQVLKSARNALAAMQVSVEEV
>EECEBICD_00231 Aerotaxis receptor
MSSHPYVSQLNTPLDDDTTLMSTTDLESYITHANDTFVQVSGYQLNELLAQPHNLVRHPD
MPKAAFADMWYTLKQGEPWSGIVKNRRKNGDHYWVRANAVPMIREGRVTGYMSIRTRATD

DEIAAVEPLYQALNEGRCSKRIHKGLVVRQGLLGKLPAMPVRWRVRSIMGLMAVMLALAL
FGTDASWQALLLGALAMLAGTALFEWQIVRPIENVATQALKVATGERNSVQHLNRSDELG
LMLRAVGQLGLMCRWLINDVSSQVSSVRNGSERLAKGNNDLNEHTRQTVENVQETVTTMN
QMAESVKLNSETASAADKLSMAASSAATQGGEAMDTVIKTMDDIAHSTQRIGTITTLIND
IAFQTNILALNAAVEAARAGEQGKGFAVVAGEVRHLASRSANAANDIRKLIDASATKVQS
GSEQVHAAGRTMDDIVAQVQNVTLLIARISQSTQEQTDGLSSLTRAVDELNRITQKNAAL
VEESAQVSAMVKHRASRLEDAVTVLH
>EECEBICD_00232 Methyl-accepting chemotaxis protein III
MFLHNIKIRSKLFMAFGLFIVLMVVSSALSLFSLDRANTGMQDIITNDYPTTVKANLLID
NFNDFIIAQQLMLLDEEGRWSQSSQKELSEISQRISALLDELSRENSHDADSQKIINEIR
EARQQYLESRFRILKDIQSNNRQAAIQEMMTRTVQVQKVYKDKVQELIAVQDAQMHEASV
QVKEDFKNNRTLLITLALISIAAGGVMGWYIVRSITRPLDDAVRFAEAIADGDLTRHITT
DYKDETGVLLQALMAMKTRLLDIVQEVQNGSESISTAAAQIVAGNQDLAARTEEQASSVE
ETAASMEQITATVKNTADHTSEATKLSAGAASVVKNNGEMMNQVTQKMRVINDTANRMSD
IINIIDSIAFQTNILALNAAVEAARAGEHGRGFAVVAGEVRQLAQKSASSASEIRNLIED
STSQTQEGMHLVEKASALINGMVDNVEEMDVILREIGQASREQTDGISQINSAIGLIDAA
TQQNSCLVEESVAAAASLNEQALHLKELVNVFRVREEDTQPA
>EECEBICD_00233 Transcriptional regulator YqjI
MQNQHEGCCKNRDHMHDGCCKDREHQHEGCHSAHQHENASCGGEHRHGHGCGRHGQGGGR
RQRFFGHGELRLVILDILTRDASHGYELIKAIENLTGGGYTPSAGVIYPTLDFLQDQQFI
TISDEEGGRKKIAITANGAQWLDENREHLTHIQARLKARCVGMELRKNPQMKRALDNFKA
VLDLRINHSDINDAQIKRIIGVIDRAALEIAELD
>EECEBICD_00234 NADPH-dependent ferric-chelate reductase
MTTSSVRYPQRVRNELRFRELIVLRVERISAGFQRIVLGGEALDGFISLGFDDHTKVFFP
EPGCRFTPPTVTEEGIIWGEGVRPVSRDYTPLYDEARRELALDFFIHDGGVASRWAMEAR
EGDTLTIGGPRGSLVVPEEYACQVYVCDESGMPALRRRLESLSRLPVRPAVTALVSIQDA
AYRDYLAHLTDITVEYVVGGDEQAMQTRLSQLTIPESDYFIWITGEGKTVKRLSQCFEKG
FDPHLVRAAAYWHRK
>EECEBICD_00236 G/U mismatch-specific DNA glycosylase
MVKDILAPGLRVVFCGINPGLSSANTGFPFAHPANRFWKVIHLAGFTDRQLKPEEAEKLL
DFRCGVTKLVDRPTVQATEVKLHELRSGGRNLIEKIEDYQPAALAVLGKQAFEQGFSQRG
IAWGKQKIAIGATMVWVLPNPSGLNRIKTEKLVEAYRELDQALIMRGL
>EECEBICD_00237 RNA polymerase sigma factor RpoD
MPHIDREAPDSRSERQRPKYKYALALNVDSYTVDTNQTNKCGYRLMEQNPQSQLKLLVTR
GKEQGYLTYAEVNDHLPEDIVDSDQIEDIIQMINDMGIQVMEEAPDADDLLLAENTTSTD
EDAEEAAAQVLSSVESEIGRTTDPVRMYMREMGTVELLTREGEIDIAKRIEDGINQVQCS
VAEYPEAITYLLEQYDRVEAEEARLSDLITGFVDPNAEEEMAPTATHVGSELSQEDLDDD
EDEDEEDGDDDAADDDNSIDPELAREKFAELRAQYVVTRDTIKAKGRSHAAAQEEILKLS
EVFKQFRLVPKQFDYLVNSMRVMMDRVRTQERLIMKLCVEQCKMPKKNFITLFTGNETSE
TWFNAAIAMNKPWSEKLHDVAEEVQRCLQKLRQIEEETGLTIEQVKDINRRMSIGEAKAR
RAKKEMVEANLRLVISIAKKYTNRGLQFLDLIQEGNIGLMKAVDKFEYRRGYKFSTYATW
WIRQAITRSIADQARTIRIPVHMIETINKLNRISRQMLQEMGREPTPEELAERMLMPEDK
IRKVLKIAKEPISMETPIGDDEDSHLGDFIEDTTLELPLDSATTESLRAATHDVLAGLTA
REAKVLRMRFGIDMNTDHTLEEVGKQFDVTRERIRQIEAKALRKLRHPSRSEVLRSFLDD
>EECEBICD_00238 DNA primase
MAGRIPRVFINDLLARTDIVDLIDVRVKLKKQGKNYHACCPFHNEKTPSFTVNGEKQFYH
CFGCGAHGNAIDFLMNYDKLEFVETVEELAAMHNLEIPYEAGTGLSQIERHQRQNLYQLM
NGLNDFYQQSLTHPAAKPARDYLQKRGLSAEIIQRFAIGFAPPGWDNALKRFGNNSDNKA
LLLDAGMLVNNEQGSTYDRFRNRVMFPIRDKRGRVIGFGGRVLGNDTPKYLNSPETDIFH
KGRQLYGLYEAQQYSAEPQRLLVVEGYMDVVALAQYDINYAVASLGTSTTADHMHMLFRA
TNNVICCYDGDRAGRDAAWRALETAMPYMTDGRQVRFMFLPDGEDPDTLVRKEGKAAFEA
RMEQAQPLSTFLFNSLLPQVDLSSPDGSTQLAALALPLINQVPGDAHRIQLRQTLGLKLG
IFDDSQLDRLVPKQAESGVSRPAPQLKRTTMRILIGLLVQNPDLAPLVPPLDALDQNKLP
GLGLFKELVKTCLAQPGLTTGQLLELYRGTNDAATLEKLSMWDDIADKAIAEKTFTDSLN

HMFDSLLQLRQEELIARDRTHGLSSEERRELWTLNQELARK
>EECEBICD_00239 30S ribosomal protein S21
MPVIKVRENEPFDVALRRFKRSCEKAGVLAEVRRREFYEKPTTERKRAKASAVKRHAKKL
ARENARRTRLY
>EECEBICD_00240 tRNA N6-adenosine threonylcarbamoyltransferase
MRVLGIETSCDETGIAIYDDKKGLLANQLYSQVKLHADYGGVVPELASRDHVRKTVPLIQ
AALKEAGLTASDIDAVAYTAGPGLVGALLVGATVGRSLAFAWNVPAIPVHHMEGHLLAPM
LEDNPPEFPFVALLVSGGHTQLISVTGIGQYELLGESIDDAAGEAFDKTAKLLGLDYPGG
PMLSKMASQGTAGRFVFPRPMTDRPGLDFSFSGLKTFAANTIRSNGGDEQTRADIARAFE
DAVVDTLMIKCKRALESTGFKRLVMAGGVSANRTLRAKLAEMMQKRRGEVFYARPEFCTD
NGAMIAYAGMVRFKAGVTADLGVTVRPRWPLAELPAA
>EECEBICD_00241 putative glycerol-3-phosphate acyltransferase
MSAIAPGMILFAYLCGSISSAILVCRIAGLPDPRESGSGNPGATNVLRIGGKGAAVAVLI
FDILKGMLPVWGAYALGVTPFWLGLIAIAACLGHIWPVFFGFKGGKGVATAFGAIAPIGW
DLTGVMAGTWLLTVLLSGYSSLGAIVSALIAPFYVWWFKPQFTFPVSMLSCLILLRHHDN
IQRLWRRQETKIWTKLKKKRQKD
>EECEBICD_00242 Dihydroneopterin aldolase
MDIVFIEQLSVITTIGVYDWEQTIEQKLVFDIEMAWDNRKSAKSDDVADCLSYADIADTV
INHVEGGRFALVERVAEEVADLLLSRFNSPWVRIKLSKPGAVARAANVGVIIERGNNLK
>EECEBICD_00243 Undecaprenyl-diphosphatase
MSDMHSLLIAAILGVVEGLTEFLPVSSTGHMIIVGHLLGFEGDTAKTFEVVIQLGSILAV
VVMFWRRLFGLIGIHFGRPLQREGESKGRLTLIHILLGMIPAVVLGLVFHDTIKSLFNPI
NVMYALVVGGLLLIAAECLKPKEPRAPGLDDMTYRQAFMIGCFQCLALWPGFSRSGATIS
GGMLMGVSRYAASEFSFLLAVPMMMGATVLDLYKSWSFLTAADIPMFAVGFVTAFVVALI
AIKTFLQLIKRISFIPFAIYRFVVAAAVYVVFF
>EECEBICD_00244 Multifunctional CCA protein
MKIYLVGGAVRDALLGLPVKDKDWVVVGATPQEMLDAGYQQVGRDFPVFLHPQTHEEYAL
ARTERKSGSGYTGFTCYAAPDVTLEADLQRRDLTINALARDDDGQIIDPYHGRRDLEARL
LRHVSPAFGEDPLRVLRVARFAARYAHLSFRIADETLALMREMTAAGELEHLTPERVWKE
TENALTTRNPQVYFQVLRDCGALRVLFPEIDALFGVPAPAKWHPEIDTGVHTLMTLSMAA
MLSPQLDVRFATLCHDLGKGLTPKNLWPRHHGHGPAGVKLVEQLCQRLRVPNDLRDLAKL
VAEYHDLIHTFPILQPKTIVKLFDAIDAWRKPQRVEQIALTSEADVRGRTGFEASDYPQG
RWLREAWQVAQAVPTKEVVEAGFKGIEIREELTKRRIAAVANWKEKRCPNPAS
>EECEBICD_00245 hypothetical protein
MPKLRLIGLTLLALSATAVSHAEETRYVSDELNTWVRSGPGDNYRLVGTVNAGEEVTLLQ
SDANYGQIKDSSGRTAWIPLKELNTTPSLRTRVPDLENQVKTLTDKLNNIDTTWNQRTAD
MQQKVAQSDSIINGLKEENQKLKNELIVAQKKVSAANLQLDDKQRTIIMQWFMYGGGVLG
IGLLLGLVLPHMIPSRKRKDRWMN
>EECEBICD_00246 Inorganic triphosphatase
MAQEIELKFIVNHDAVDALRNHLHTLGGEHHAPSQLLNIYFETPDNWLRRHDMGLRIRGE
NGRYEMTMKIAGRVTGGLHQRPEYNVALSEPVLDLTQLPAEVWPDGNLPAGLASSVQPLF
STDFYREKWCLDVDGSRIEIALDLGDVKAGEFAEPICELELELLRGDTRAVLKLAKQLLS
QTGLRQGSLSKAARGYHLAQGNAPRENTPTAILRTAAKATVEQGLEVSLDLALSQWQYHE
ELWLRGDESAEEHVLDAMGLVRHALMLFGGIVPRKASAHLRDLLTQAEATMTSAVSAVTA
VYSTQTAMAKLALTEWLVTKAWQPFLDAKAQAKMADSFKRFADIHLSRHAAELKKVFGQP
LGDKYRDQLPRLTRDIDSVLLLAGYYDAMVAQAWLENWQGLRHAILTGQRIEIEHFRNEA
INQQPFWLHSGKR
>EECEBICD_00247 Bifunctional glutamine synthetase
adenylyltransferase/adenylyl-removing enzyme
MTPLSSPLSQYWQTIVERLPEGFTETSLSVQAKSVLTFSDFALDSVIAHPEWLAELESAS
PQADEWRHYAGWLQEALAGVCDDASLMRELRFFRRRIMVRIAWAQTLSLVDDETILQQLS
HLAETLIVGARDWLYAACCREWGTPCNPQGVPQPLLILGMGKLGGGELNFSSDIDLIFAW
PEHGETRGGRRELDNAQFFTRLGQRLIKALDQPTMDGFVYRVDMRLRPFGDSGPLVLSFA
ALEDYYQEQGRDWERYAMVKARLMGDNDDAWSRELRAMLRPFVFRRYIDFSVIQSLRNMK

GMIAREVRRRGLKDNIKLGAGGIREIEFIVQVFQLIRGGREPSLQSRSLLPTLDAIAALH
LLPENDVAQLRVAYLFLRRLENLLQSINDEQTQTLPADDLNRARLAWGMKAENWPQLVGE
LTDHMANVRRVFNELIGDDEADTPQEEERSEPWREVWQDALQEDDSTPVLAHLADEDRRQ
VLTLIADFRKELDKRPIGPRGRQVLDQLMPHLLADVCSREDAAVTLSRITPLLAGIVTRT
TYLELLSEFPGALKHLIMLCAASPMIASQLARYPLLLDELLDPGTLYQPTATDAYRDELR
QYLLRVPEEDEEQQLEALRQFKQAQLLRIAAADIAGTLPVMKVSDHLTWLAEAMIDAVVQ
QAWTQMVARYGQPAHLDERQGRGFAVVGYGKLGGWELGYSSDLDLIFLHDCPMDVMTNGE
REIDGRQFYLRLAQRIMHLFSTRTSSGILYEVDARLRPSGAAGMLVTSADAFADYQQHEA
WTWEHQALVRARVVYGDPQLTSQFDTVRRTIMTTARDGKTLQTEVREMREKMRAHLGNKH
RDRFDIKADEGGITDIEFIAQYLVLRYAHEKPKLTRWSDNVRILELLAQNGIMDEHEAQA
LTVAYTTLRDELHHLALQELPGHVAQTCFSKERALVQASWRKWLVAV
>EECEBICD_00248 Bifunctional protein HldE
MKVNLPAFERAGVMVVGDVMLDRYWYGPTCRISPEAPVPVVKVNTVEERPGGAANVAMNI
ASLGANARLVGLTGIDDAARALSKTLAEVNVKCDFVSVPTHPTITKLRVLSRNQQLIRLD
FEEGFEGVDPQPLHERINQALGSIGALVLSDYAKGALTSVQTMISLARQAGVPVLIDPKG
TDFERYRGATLLTPNLSEFEAVAGKCKSEDELVERGMKLIADYDLSALLVTRSEQGMTLL
QPNKAPLHMPTQAQEVYDVTGAGDTVIGVLAATLAAGNTLEEACYFANAAAGVVVGKLGT
STVSPIELENAVRGRADTGFGVMTEEELRQAVASARKRGEKVVMTNGVFDILHAGHVSYL
ANARKLGDRLIVAVNSDASTKRLKGDSRPVNPLEQRMIVLGALESVDWVVSFEEDTPQRL
IAGILPDLLVKGGDYKPEEIAGSEEVWANGGEVMVLNFEDGCSTTNIIKKIQTESEK
>EECEBICD_00249 Inner membrane protein YqiK
MDDVFGILPSWMFTAIVAVIVLLIIGIIFARLYRRASAEQAFVRTGLGGQKVVMSGGAIV
MPIFHEIIPINMNTLKLEVSRATVDSLITKDRMRVDVVVAFFVRVKPSVEGIATAAQTLG
QRTLSPEDLRMLVEDKFVDALRATAAQMTMHELQDTRENFVQGVQNTVAEDLSKNGLELE
SVSLTNFNQTSKEHFNPNNAFDAEGLTKLTQETERRRRERNEVEQDVEVAVREKNRDALE
RKLEIEQQEAFMTLEQEQQVKTRTAEQNAKIAAFEAERHREAEQTRILAERQIQETEIER
EQAVRSRKVEAEREVRIKEIEQQQVTEIANQTKSIAIAAKSEQQSQAEARANDALADAVR
AQQNVETTRQTAEADRAKQVALIAAAQDAETKAVELTVRAKAEKEAAELQAAAIIELAEA
TRKKGLAEAEAQRALNDAINVLSDEQTSLKFKLALLQSLPAVIEKSVEPMKSIDGIKIIQ
VDGLNRGATAGDVAAGGANGGNLAEQALSAALTYRTQAPLIDSLLNEIGIAGGSLKALTT
PLVSSATDEINREATVKEQ
>EECEBICD_00250 Inner membrane protein YqiJ
MTLFAEYNSPYLFAIAFVFFIGVLEMISLIFGHFLSGALDAHLDHYDALSSGPAGQALHY
LNIGRVPALVVLCLLAGYFGLFGILIQHGGIMLWQAPLSNLLLVPLSIVLSVFAVHYSGK
ILAPWLPRDESSALREEEFIGGMAIITGHAAVAGTPCEGKFTDKFGQIHYLLLEPEKGKE
FKKGDKVLIVCRLSATRYLAERTFYV
>EECEBICD_00251 Surface composition regulator
MNNNNVYSLNNFDFLARSFARMQAEGRPVDIQAVTGNMDEEHRDWFCKRYALYCQQATQA
KRLELEH
>EECEBICD_00252 Ubiquinone biosynthesis accessory factor UbiK
MIDPKKIEQIARQVHESMPKGIREFGEDIEKKIRQTLQSQLTRLDLVSREEFDVQTQVLL
RTREKLALLEQRLSELEARDKPEEVKPAPAIPPVDPQE
>EECEBICD_00253 3,4-dihydroxy-2-butanone 4-phosphate synthase
MNQTLLSSFGTPFERVELALDALREGRGVMVLDDEDRENEGDMIFPAETMTVEQMALTIR
HGSGIVCLCITEDRRKQLDLPMMVENNTSAYGTGFTVTIEAAEGVTTGVSAADRVTTVRA
AIKDGAKPSDLNRPGHVFPLRAQAGGVLTRGGHTEATIDLMTLAGFKPAGVLCELTNDDG
TMARAPECIAFAGQHNMAVVTIEDLVAYRQAHERKAS
>EECEBICD_00254 Protein-disulfide oxidoreductase DsbI
MDFIKGLWRDLRARPVDTLVRWQEQRFLWLLMAIAMGGLIIILAHSFFQIYLYMAPCEQCV
YIRYAMFVMVIGGVIAAINPKNIVLKLIGCIAAFYGSIMGIKFSIKLNGIHHAVHNADPD
SLFGVQGCSTDPTFPFNLPLAEWAPEWFKPTGDCGYDAPIVPDGVTLSSVQQWFVDLYQQ
SEGWYLLPPWHFMNMAQACMLAFGLCLILLLVMSGAWALKLARGK
>EECEBICD_00255 Thiol:disulfide interchange protein DsbL
MSSKWITSLFKSVVLTAALVTPFAASAFTEGTDYMVLEKPIPNADKTLIKVFSYACPFCY

KYDKAVTGPVSDKVADLVTFTPFHLETKGEYGKQASEVFAVLIAKDKAAGISLFDAKSQF
KKAKFAWYAAYHDKKERWSDGKDPAAFIKTGLDAAGMSQADFEALKDPAVQETLEKWKA
AYDVAKIQGVPAYVVNGKYLIYTKNIKSIDSMAELVRELATKK
>EECEBICD_00256 Arylsulfate sulfotransferase AssT
MFDQYRKTILAGAVALTCGLTAASTFAAGFQPAQPAGKLGAVVVDPYGNAPLTALVELDS
HIISDVKVTVHGKGEKGVPVTYTVGKESLETYDGIPIFGLYQKFANNVTVEYKENGKAMK
DDYVVQTSAIVNHYMDNRSISDLQQTKVIKVAPGFEDRLYLVNTHTFTPQGAEFHWHGEK
DKNAGILDAGPAGGALPFDIAPYTFVVDTQGEYRWWLDQDTFYDGHDMNINKRGYLMGIR
ETPRGTFTAVQGQHWYEFDMMGQILADHKLPRGFLDASHESIETVNGTVLLRVGKRDYRK
EDGIHVHTIRDQIIEVDKSGRVVDVWDLTKILDPMRDALLGALDAGAVCVNVDLAHAGQQ
AKLEPDTPYGDALGVGAGRNWAHVNSIAYDAKDDSIILSSRHQGIVKIGRDKQVKWILAP
SKGWNKQLASKLLKPVDDHGKPLTCDENGKCKDTDFDFTYTQHTAWLSSKGTLTVFDNGD
GRGLEQPALPTMKYSRFVEYKIDEKKGTVQQVWEYGKERGYDFYSPITSVVEYQKDRDTM
FGFGGSINLFDVGKPTVGKLNEIDYKTKEVKVEIDVLSDKPNQTHYRALLVHPTQMFK
>EECEBICD_00257 Protein YqiD
MFIAWYWIILIVLVVVGYICHMKRYCRAFRQDRDALLEARTKRFRQTSEGDSVMNEQK
>EECEBICD_00258 Zinc transporter ZupT
MSVPLILTLLAGAATFIGAFLGVLGQKPSNRVLAFSLGFAAGIMLLISLMEMLPAALDTE
GMSPVLGYGMFIIGLLGYFGLDRLLPHAHPQDLVQKRQQPLPGSIKRTAILLTLGISLHN
FPEGIATFVTASSNLELGFGIALAVALHNIPEGLAVAGPVYAATGSKRTAIFWAGISGMA
EILGGVLAWLILGSLVSPIVMAAIMAAVAGIMVALSVDELMPLAKEIDPNNNPSYGVLCG
MSIMGLSLVILQTIGIG
>EECEBICD_00259 4,5-DOPA dioxygenase extradiol
MSLTCMPALFLGHGSPMNVLDDNDYTRAWRRLGEALPRPQAIVVVSAHWYTRGTGVTAME
RPQTLHDFGGFPQALYDTHYPAPGSPALAQRLVELLAPVPVALDKEAWGFDHGSWGVLIK
MYPNADIPMVQLSVDSTKPAAWHFEVGRKLATLRDEGVMLVASGNVVHNLRTVRWHGDNI
PYPWAASFNDFVKANLTWQGPVEQHPLVNYLQHEGGALSNPTPEHFLPLLYVLGAWDGKE
PITIPVDGIEMGSISMLSVQVG
>EECEBICD_00260 Putative acid--amine ligase YgiC
MERVSITERPDWRDKATEYGFNFHTMYGEPYWCEDAYYKLTLAQVEKLEDVTAELHQMCL
KVVERVIASDELMTKFRIPKHTWGFVRQSWQTQQPSLYSRLDLAWDGIGEPKLLENNADT
PTSLYEAAFFQWIWLEDQINAGNLPEGSDQFNSLQEKLIERFAELREQYGFQLLHLTCCR
DTVEDRGTIQYLQDCAAEAEIATEFLYIDDIGLGEKGQFTDLQDQVIANLFKLYPWEFML
REMFSTKLEDAGVRWLEPAWKSIISNKALLPLLWEMFPDHPNLLPAYFAEDEHPPMDKYV
VKPIFSREGANVSIIENGKTIESVEGPYGEEGMIVQQFYPLPKFGDSYTLIGSWLINDQP
AGIGIREDRALITQDLSRFYPHIFVEG
>EECEBICD_00261 hypothetical protein
MKRTKSIHHASFRKSWSARHLTPVALAVTAVFMLAGCEKSDDTVSLYQNADDCSAANPGK
SAECTTAYNNALKEAERTAPKYATREDCVAEFGEGQCQQAPAQAGMAPENQAQAQQSSGS
FWMPLMAGYMMGRLMGGGAGFAQQPLFSSKNPASPAYGKYTDAAGKNYGAAQPGRTMTVP
KTAMAPKPATTTTVTRGGFGESVAKQSTMQRSAAGTSTRSMGG
>EECEBICD_00262 Outer membrane protein TolC
MKKLLPILIGLSLSGFSTLSQAENLMQVYQQARLSNPELRKSAADRDAAFEKINEARSPL
LPQLGLGADYTYSNGYRDANGINSNETSASLQLTQTLFDMSKWRGLTLQEKAAGIQDVTY
QTDQQTLILNTANAYFKVLNAIDVLSYTQAQKEAIYRQLDQTTQRFNVGLVAITDVQNAR
AQYDTVLANEVTARNNLDNAVEELRQVTGNYYPELASLNVEHFKTDKPKAVNALLKEAEN
RNLSLLQARLSQDLAREQIRQAQDGHLPTLNLTASTGISDTSYSGSKTNAAQYDDSNMGQ
NKIGLNFSLPLYQGGMVNSQVKQAQYNFVGASEQLESAHRSVVQTVRSSFNNINASISSI
NAYKQAVVSAQSSLDAMEAGYSVGTRTIVDVLDATTTLYDAKQQLANARYTYLINQLNIK
YALGTLNEQDLLALNSTLGKPIPTSPESVAPETPDQDAAADGYNAHSAAPAVQPTAARAN
SNNGNPFRH
>EECEBICD_00263 ADP-ribose pyrophosphatase
MRKSDNLPVTFTKSDVEIIARETRYRGFFSLDVYRFRHRLFNGEMSGEVKREIFERGHAA
VLLPFDPERDEVVLVEQIRIAAYDTSVTPWLLEMVAGMIEEGETIEAVARREAMEEAGLT

VGRTRPVISYLASPGGTSERSSILVGEVDATTAVGIHGLADENEDIRVHVVSREQAYQWV
EEGKIDNAAAVIALQWLQLHHQELKNEWKK
>EECEBICD_00264 hypothetical protein
MKRYTPDFPEMMRLCETNFSQLRRLLPRNDAPGETVSYQVGNVQYRLTIVESTRYTTLVT
IEQTAPAITYWSLPSLTVRLYHDAMVAEVCSSQQIFRFKARYDYPNKKLHQRDEKHQINQ
FLADWLRFCLAHGAMAIPVY
>EECEBICD_00265 3',5'-cyclic adenosine monophosphate phosphodiesterase CpdA
MESLLTLPLAGEARVRILQITDTHLFAEKHETLLGVNTWESYQAVLEAIRAQQYEYDLIV
ATGDLAQDQSAAAYQHFAEGIASFRAPCVWLPGNHDFQPAMYSALQEAGISPAKRVLIGE
QWQILLLDSQVFGVPHGELSEFQLEWLERKLADAPERQTLLLLHHHPLPAGCSWLDQHSL
RNAGELDSVLANFPRVKYLLCGHIHQELDLDWNGRRLLASPSTCVQFKPHCANFTLDTIA
PGWRTLELQANGVLETEVHRLQDTRFRPDTASEGY
>EECEBICD_00266 hypothetical protein
MSTLLYLHGFNSSPRSAKACQLKNWLAERHPHVEMIVPQLPPYPADAAELLESLVLEHGG
APLGLVGSSLGGYYATWLSQCFMLPAVVVNPAVRPFELLTDYLGQNENPYTGQQYVLESR
HIYDLKVMQIDPLEAPDLIWLLQQTGDEVLDYRQAVAYYASCRQTVTEGGNHAFTGFEDY
FNQIVDFLGLHSC
>EECEBICD_00267 DNA topoisomerase 4 subunit B
MTQTYNADAIEVLTGLEPVRRRPGMYTDTTRPNHLGQEVIDNSVDEALAGHAKRVDVILH
ADQSLEVIDDGRGMPVDIHPEEGVPAVELILCRLHAGGKFSNKNYQFSGGLHGVGISVVN
ALSKRVEVTVRRDGQVYNIAFENGEKVQDLQVVGTCGKRNTGTSVHFWPDESFFDSPRFS
VSRLMHVLKAKAVLCPGVEITFKDEVNNSEQRWCYQDGLNDYLGEAVNGLPTLPEKPFIG
NFNGETEAVDWALLWLPEGGELLTESYVNLIPTMQGGRTSTVCARACSTRCANFANTAIF
CRAASNCRRKISGIAALMCSP
>EECEBICD_00268 DNA topoisomerase 4 subunit B
MQDPQFAGQTKERLSSRQCAAFVSGVVKDAFSLWLNQNVQAAEQLAEMAIASAQRRLRAA
KKVVRKKLTSGPALPGKLADCTAQDLNRTELFLVEGDSAGGSAKQARDREYQAIMPLKGK
ILNTWEVSSDEVLASQEVHDISVAIGIDPDSDDLSQLRYGKICILADADSDGLHIATLLC
ALFVRHFRALVKNGHVYVALPPLYRIDLGKEVYYALTEEEKAGVLEQLKRKKGKPNVQRF
KGLGEMNPMQLRETTLDPNTRRLVQLTISDEDDQRTNAMMDMLLAKKRSEDRRNWLQEKG
DLADLDV
>EECEBICD_00269 putative quinol monooxygenase YgiN
MLTVIAEIRTRPGQHHRQAVLDQFAKIIPTVLKEEGCHGYAPMVDHAAGVSFQTLAPDSI
VMIEQWESVAHLEAHLQTPHMKAYSDAVKDDVLEMNIRILESGV
>EECEBICD_00270 NADPH:quinone oxidoreductase MdaB
MSNILIINGAKKFAHSNGQLNDTLTEVADGLLRDLGHQVKIVRADSDYDIKEEVQNFVWA
DVVIWQMPGWWMGAPWTVKKYMDDVFTEGHGTLYASDGRTRSDAAKKYGSGGLVQGKKYM
LSLTWNAPMEAFTEKDQFFHGVGVDGVYLPFHKANQFLGMDALPTFIANDVIKMPDVPRY
TAEYRKHLSEIFA
>EECEBICD_00271 Sensor protein QseC
MKLTQRLSLRVRLTLIFLILVSITWAISSFVAWRKTTDNVDELFDTQLMLFARRLSTLDL
NELNAPQRMAHTPKKLKHGHIDDDALAFAIFSADGKMLLHDGDNGQDIPYRYRREGFDNG
YLKDDNDLWRFLWLNSADGKYRIVVGQEWDYREDMALAIVAAQLTPWLIALPFMLLILLL
LLHRELRPLKKLAQALRFRSPESETPLDAKGVPSEVRPLVEALNQLFSRIHSMMVRERRF
TSDAAHELRSPLAALKVQTEVAQLSGDDPLSRDKALTQLHAGIDRATRLVDQLLTLSRLD
SLNNLQDVAEISLEELLQSAVMDIYHPAQQANIDVRLQLNAHDVIRTGQPLLLSLLVRNL
LDNAIRYSPQGSVVDVTLHARSFTVRDNGPGVAPEILTHIGERFYRPPGQSVTGSGLGLS
IVRRIATLHGMTVSFGNAAEGGFEAVVSW
>EECEBICD_00272 Transcriptional regulatory protein QseB
MRILLVEDDTLIGDGIKAGLSKMGFSVDWFTEGRPGKEALYSAPYDAVILDLTLPGMDGR
DILREWREKGKQEPVLILTARDALAERVEGLRLGADDYLCKPFALIEVAARLEALVRRAS
GQASSELRHGQVTLNPGNLVATLAGEPLALKPKEFALLELLLRNKGRVLPRKLIEEKLYN
WDDDVSSNAVEVHVHHLRRKLGSEFIRTVHGIGYTLGDA

>EECEBICD_00273 Protein YgiW
MKKLAAIVAVMALCSTPVLAAQQGGFSGPSATQTQGGGFVGPNGSSTTVESAKSLRDDAW
VTLRGNIVERISDDLYVFKDATGTINVDIDHKRWNGLTVTPQDTVEIQGEVDKDWNSVEI
DVKQIRKVNP
>EECEBICD_00274 HTH-type transcriptional activator RhaS
MNDLISAAYSERLRRVCDHIERHLDEPLSLEALSRMAHSSPFHFHRQFTVWSGLPLYRYI
QWLRLRRASWRLAFNPQDKVIDIALDAGFQNPESFTRAFKTAFGQSPCRFRQSPDWLAWH
QRVPKLALQEQHVMDVKIVEFPPTRVAMLTHLGHPDKVNASAAKFIAWRRETGQSPIASS
QTFGIAWHDPQTTPPAQFRFDICGSVRQPIAENDVGVVNSEIPGGRCAVVRHQGSLDSLP
ESVWYLFREWLPASGETPRDFPVFFQYLNFVHEVAEHELLTDIYLPLR
>EECEBICD_00275 DNA topoisomerase 4 subunit A
MSDMAERLALHEFTENAYLNYSMYVIMDRALPFIGDGLKPVQRRIVYAMSELGLNATAKF
KKSARTVGDVLGKYHPHGDSACYEAMVLMAQPFSYRYPLVDGQGNWGAPDDPKSFAAMRY
TESRLSKYAELLLSELGQGTADWVPNFDGTMQEPKMLPARLPNILLNGTTGIAVGMATDI
PPHNLREVAKAAITLIEQPKTTLDQLLDIVQGPDYPTEAEIITPRAEIRKIYENGRGSVR
MRAVWTKEDGAVVITALPHQVSGAKVLEQIAAQMRNKKLPMVDDLRDESDHENPTRLVIV
PRSNRVDMEQVMNHLFATTDLEKSYRINLNMIGLDGRPAVKNLLEILTEWLAFRRDTVRR
RLNYRLEKVLKRLHILEGLLVAFLNIDEVIEIIRNEDEPKPALMSRFGISETQAEAILEL
KLRHLAKLEEMKIRGEQDELEKERDQLQGILASERKMNTLLKKELQADSDAYGDDRRSPL
REREEAKAMSEHDMLPSEPVTIVLSQMGWVRSAKGHDIDAPGLNYKAGDSFKAAVKGKSN
QPVVFIDTTGRSYAIDPITLPSARGQGEPLTGKLTLPPGATVEHMLMEGDDQKLLMASDA
GYGFVCTFNDLVARNRAGKALITLPENAHVMPPLVIEDEHDMLLAITQAGRMLMFPVDSL
PQLSKGKGNKIINIPSAEAAKGDDGLAHLYVLPPQSTLTIHVGKRKIKLRPEELQKVVGE
RGRRGTLMRGLQRIDRIEIDSPHRVSHGDSEE
>EECEBICD_00276 1-acyl-sn-glycerol-3-phosphate acyltransferase
MLYIFRLIVTVIYSILVCVFGSIYCLFSPRNPKHVATFGHMFGRLAPLFGLKVECRKPAD
AENYGNAIYIANHQNNYDMVTAANIVQPPTVTVGKKACFGSRFLGNCTG
>EECEBICD_00277 1-acyl-sn-glycerol-3-phosphate acyltransferase
MIDRNNRAKAHSTIAAVVNHFKKRRISIWMFPEGTRSRGRGLLPFKTGAFHAAIAAGVPI
IPVCVSNTSNKVNLNRLNNGLVIVEMLPPVDVSEYGKDQVRELAAHCRALMEQKIAELDK
EVAEREATGKV
>EECEBICD_00278 Cell division protein FtsP
MSFSRRQFLQASGIALCAGAIPLRANAAGQQQPLPVPPLLESRRGQPLFMTLQRAHWSFT
QGTRAPVWGVNGRYLGPTIRVWKGDDVKLIYSNRLAENVSMTVAGLLVPGPLMGGPARMM
SPNADWAPVLPIRQSAATLWYHANTPNRTAQQVYNGLAGMWLVEDDISKTLPIPNHYGVD
DFPVIIQDKRLDNFGTPEYSEPGSGGFVGDTLLVNGAQSPYVEVSRGWVRLRLLNASNSR
RYQLQMSDGRALHVISGDQGFLPAPVSVKQLSLAPGERREILVDMTNGDEVSITCGEAAS
IVDRIRGFFEPSSILVSTLVLTLRPTGLLPLVTDNLPMRLLPTEIMSGAPVRSRDISLGD
DPGINGQLWDVNRIDITAQQGTWERWTVRADMPQSFHIEGVSFLIRNVNGAMPFPEDRGW
KDTVWVDGQVELLVYYGQPSWPHFPFYFNSQTLEMADRGSIGQMLVNPAS
>EECEBICD_00279 C4-dicarboxylate TRAP transporter large permease protein DctM
MDFEYIYPIIVLFGSFAIMLAVGVPITFAIGLSSLFSILIALPPDAAISVISQKMTVGLD
GFTLLAIPFFVLAGNIMNTGGIARRLVNLAQALVGRLPGSLAHCNILANTLFGAISGSAV
ASAAAVGGIMSPLQEKEGYSPAFSTAINVASAPIGLMIPPSNVLIVYSLASGGTSVAALF
LAGYLPGILTALALMTVAALYAHRHHYPVAERINLRQFLSVFRDSLPSLLLIFIIIGGII
GGVFTPTEASAIAVIYSLALAAIYREINVSKLRDILLDSVVTSSIVLLLVGCSMGMSWAM
TNADVPELINEMITSVSENKWVILLIINLILLLVGTFMDITPAILIFTPIFLPIAQHLGI
DPVHFGIIMVFNLTIGLCTPPVGTILFVGCSIGKISIDKVVKPLLPMFLALFVVMAMICY
FPQLSLLLPTLFAPS
>EECEBICD_00280 hypothetical protein
MRILTNALNKILAGCCCIILAIMVACVSWQVAARFIFNAPSSTLDEFTQILFMWMILLGG
VYTAGLKKHLAIDLLAQKLSRTPALVLDSIIQVIITVFALIFMVYGGDIIVEKAAHVSQM
SPVLKWPMDKVYWVMPISGVILVYYTIVNVIDNYHQRHLR

>EECEBICD_00281 Solute-binding protein
MKNTRSFTTSAVLLAGCLLLAFPALAKTTLKLSHNQDKSHAVHKAMSYLADKAKAYSDGE
LNIRIYPNATLGNERESLELMNSGALQMVKVNAASLESFAPEYSVFSLPFLFRDRDHYYN
VLKSDLGKRILASSESKGFVGLTWYDGGARSFYAGKPITQPDDLAGMKIRVQQSPSAIAM
VKALGGVPTPMAQGELYTALQQGVVDGGENNPVVYADMRHAEVAKFYSRDEHTMVPDVLV
ISTKVLNKLSDKERKALYKAADESMQQMKDVIWPAAEKEAYESMKAMNATVVDIDKSAFK
QRVKPLFDEFRAKDAQSAKDLEYIENM
>EECEBICD_00282 hypothetical protein
MSAISLIQPDRDLFSWPQYWAACFGPAPFLPMSRDEMDQLGWDSCDIILVTGDAYVDHPS
FGMAICGRMLEAQGFRVGIIAQPDWNSKDDFMRLGKPNLFFGVTAGNMDSMINRYTADRK
LRHDDAYTPDNVAGKRPDRATLVYTQRCKEAWKDVPVILGGIEASLRRTAHYDYWSDTVR
RSVLVDSKADMLMFGNGERPLVEVAHRLAMGETIDQIRDVRNTAIMVKEALPGWSGVDST
RLDTPGKIDPIPHPYGEDLPCADNKPVAPKKQEAKAITVQPPRPKPWEKTYILLPSFEKV
KGDKVLYAHASRILHHETNPGCARALMQKHGDRYVWINPPAIPLSTEEMDSVFALPYQRV
PHPAYGNARIPAYEMIRFSINIMRGCFGGCSFCSITEHEGRIIQSRSEDSIINEIEAIRD
TVPGFTGVISDLGGPTANMYMLRCKSPRAEQTCRRLSCVYPDICPHMDTDHTPTINLYRR
ARELKGIKKILIASGVRYDIAVEDPRYIKELASHHVGGYLKIAPEHTEEGPLSKMMKPGM
GSYDRFKELFDLYSKQAGKEQYLIPYFISAHPGTRDEDMVNLALWLKRHRFRLDQVQNFY
PSPLANSTTMYYTGKNPLGKIGYKSEDVVVPKGDRQRRLHKALLRYHDPANWPLIRQALE
AMGKKHLIGGRRECLVPAPTIEEMREARRQNRNTRPALTKHTPVEHQRQGLAANKKRGKG
AGR
>EECEBICD_00283 hypothetical protein
MGMIIIPCFIVIAIIAVVIFIMAIKACSMEKREDRQMTLAGIILMGVIAAIPGWFLYEIF
SHAP
>EECEBICD_00284 hypothetical protein
MTCIFCQIVEGKAPCHKVWEDEHHLAFLSIFPNTDGFTVVIPKKHYPSYAFDLPPQALAD
LMLATQKVAKKLDKAFPDVSRTGMFFEGFGVDHVHSKLSPMHGTGDLTHWKPIESRQNKF
FEQYEGYLSSHDHERADDEKLAALAARIREA
>EECEBICD_00285 Putative malate transporter YflS
MCNCGGKKSQGVKWLPFILILVIAAGLWQLTPPSGLSAPAWHSAIIFVATIASIVAKVLP
IGAVGIIGITVFALAYAAGDKTASGAITTALSELNSSLIWLIVVAFMIARGFIKTGLGRR
IALQMIRLLGKRTLGLAYGLAFADLILSPAMPSNTARCGGVIYPIADSLARSFDSHPEDE
SRSKIGTFLITCIGNVNDVTAALFMTGYTGNLLAVKLAANAGVTLSWGSWFIAALLPCLV
SFLIVPLLVYWLTRPEIKHTPDAPDLARKELAQMGSMTRGEWLMLATVGVLLVLWIFGSS
LGVDATTASFVGLSILLLSGVLTWEDVKSEKGAWDTLIWFAALLMMANQLKKLGFTSWFG
NLIGDSIGSTMHGTSWIIILLLLNAAYFYTHYFFASGNAQIAALYAVFLGVGLHLNIPAA
PMALMLAFTSSLYCSLTQYTHARGPILFGAGYVPTGVWWRTGFIISLFNQAVFLTVGLAW
WKVLGLY
>EECEBICD_00286 2,5-diketo-D-gluconic acid reductase A
MANPTIIRLQDGNVMPQLGLGVWKASNEEVIAAIHKALEVGYRSIDTATAYQNEEGVGKA
LKAASVAREELFITTKLWNDDQKRPREALQESLKKLQLDYLDLYLMHWPVPAIDHYVDAW
KGMIALQKEGLVKSIGVCNFQIHHLQRLIDETGVTPVINQIELHPLMQQRQLHAWNATHK
IQTESWSPLAQGGEGVFDQKVIRELADKYGKTPAQIVIRWHLDCGLVVIPKSVTPSRIAE
NFAVWDFRLDKDELGEIAKLDQGKRLGPDPDQFGG
>EECEBICD_00287 Alcohol dehydrogenase YqhD
MNNFKLHTPTRILFGKGAIVDLRDQIPQDARVLITYGGGSVKKTGVLAQVQEALKGLDVR
EFGGIEPNPSYETLMKAVQLVRDENITFLLAVGGGSVLDGTKFIAAAAQYTDGVDPWHIL
ETGGTEIRSAIPMGSVLTLPATGSESNAGAVISRKTTGDKQAFHSSFVQPVFAVLDPVYT
YTLPPRQVANGVVDAFVHTVEQYVTYPVNGKIQDRFAEGILLTLIEEGPKALQEPENYDV
RANVMWAATQALNGLIGAGVPQDWATHMLGHELTAMHGLDHAQTLAIILPALWNEKRDVK
RAKLLQYAERVWNITDGSDDERIDAAIAATRRFFEQMGVPTRLSDYGLDGSTIPALLAKL
EAHGCTNLGENQDITLDVSRRIYEAAR
>EECEBICD_00288 HTH-type transcriptional activator RhaS
MKKWRVMNREAICLQLADKINHLKNNDKIISERLAGIRLLYGVEPGPRTPVMYQPGIIFL

FSGHKIGYINKRKFRYDANEYLLLTVPLPFECETWATPEVPLAGIRLDIDVLQLQELLMD
IGEDERFQLPMAASGINSATLSDEILCAVERLLDVMERPLDARILGKQIIREILYHVLMG
PRGGALLALVSRQTHFSLISRVLKQIEMKYTENLNVEQLAAEANMSVSAFHHNFKAVTST
SPLQYLKSYRLHKARMMMIHDGMKASAAAMRVGYESASQFSREFKRYFGVTPGEDAARMR
TMQGS
>EECEBICD_00289 Inner membrane protein YghB
MAVIQDIIAALWQHDFAALANPHVVSVVYFVMFATLFLENGLLPASFLPGDSLLLLAGAL
IAQDVMHFLPTIGILTTAASLGCWLSYIQGRWLGNTRTVKGWLAQLPAKYHQRATCMFDR
HGLLALLAGRFLAFVRTLLPTMAGISGLSNRRFQFFNWLSGLLWVTVVTSFGYALSMIPF
VKRHEDQVMTFLMILPVALLVAGLLGTLVVVIKKKYCNA
>EECEBICD_00290 Cystathionine beta-lyase MetC
MTDKQLDTKLVNAGRSKKYTLGSVNSVIQRASSLVFDTVEAKKHATRNRANGELFYGRRG
TLTHFSLQEAMCELEGGAGCALFPCGTAAVANTILAFVEQGDHILMTNTAYEPSQDFCSK
ILGKLGVTTSWFDPLIGADITQHIQPNTKVVFLESPGSITMEVHDIPSIVSAVRRVAPEA
VIMIDNTWAAGVLFKALEFDIDISIQAGTKYLIGHSDAMVGTAVANARCWEQLRENAYLM
GQMLDADTAYMTSRGLRTLGVRLRQHQESSLKIAAWLANHPQVARVNHPALPGSKGHAFW
KRDFTGSSGLFSFVLNKKLTEAELSAYLDNFSLFSMAYSWGGYESLIIANQPEQIAAIRP
AGGVDFTGTLVRVHIGLESVDDLIADLAAGFARIV
>EECEBICD_00291 hypothetical protein
MRKRKEKAVKVRQYVNSNENDYQFDVVLILLCSDFVICVLEIQSG
>EECEBICD_00292 Biopolymer transport protein ExbB
MGNNLMQTDLSVWGMYQHADIVVKCVMIGLILASVVTWAIFFSKSVEFFTQKRRLKREQL
QLADARSLDQASDIAAGFSAKSLSAQLINEAQNELELSQGSEDNEGIKERTGFRLERRVA
AVGRYMGRGNGYLATIGAISPFVGLFGTVWGIMNSFIGIAQTQTTNLAVVAPGIAEALLA
TAIGLVAAIPAVVIYNIFARQIGSYKATLGDVAAQVLLLQSRDLDLNASASAQPVRAAQK
LRVG
>EECEBICD_00293 Biopolymer transport protein ExbD
MAMRLNENLDDNGEMHEINVTPFIDVMLVLLIIFMVAAPLATVDVKVNLPASTSTPQPRP
EKPVYLSVKADNTMFIGNDPVTDDTMIAELTALTEGKKDTTIFFRADKTVEYETLMKVMD
MLHQAGYLKIGLVGEETAKAK
>EECEBICD_00294 putative oxidoreductase YghA
MSHLYDPTTQYYTGEYPKQKQPAPGVQAKMTPVPDCGEKSYVGSGRLKDRKALVTGGDSG
IGRAAAIAYAREGADVAINYLPAEEEDAQQVKALIEECGRKAVLLPGDLSDESFARSLVH
KAREALGGLDILALVAGKQTAIPEIKDLTSEQFQQTFAVNVFALFWITQEAIPLLPKGAS
IITTSSIQAYQPSPHLLDYAATKAAILNYSRGLAKQVAEKGIRVNIVAPGPIWTALQISG
GQTQDKIPQFGQQTPMKRAGQPAELAPVYVYLASQESSYVTAEVHGVCGGEHLG
>EECEBICD_00295 hypothetical protein
MKEVEDNNVYLALDNHKSDEFILKQNLAALIASKNATLEKVTQEVVSIPAALVRLKWQNR
REMYTLQVKEEIYGATINAIIEQHPELRDKIMSRLESDWQHLLARETATLRLTRKLSDGD
YRTRNVTTVARQEK
>EECEBICD_00296 hypothetical protein
MLPEHLVSSHFRLSSPPPEATENSKNLPQGKTKLSDYEPDILICANATGQHRNVLFEERD
RHIKERLYCVAKIETFARLINALQAEGNIDAQTLSKILADKTAMINEKGNAIWLNLITRE
TNMPLFYSLEDKDERS
>EECEBICD_00297 hypothetical protein
MNSIFYSVIILLLLTGAILFLMWEVNKKRPGGKIVNLNQTEPMTKEEGEDHFSVLMNSIT
PVWYWRVNHEYIDFLHATIKRMTMTELNETPGLFDAQRRCSDLNSAVYKYYDNIKKRCLN
GEKVPYSDLDVLNLRQCFREFSLEAYPALVALVWPEYQRPQVNPDEI
>EECEBICD_00298 hypothetical protein
MERFLENVMYASRWLLAPVYFGLSLALIALALKFFQEILHVLPNVFALAEADLILVLLSL
VDMTLVGGLLVMVMFSGYENFVSQLDISAGKEKLNWLGKMDATSLKNKVAASIVAISSIH
LLRVFMDAKNVPDNKLMWYVIIHLTFVLSAFVMGYLDRLTRHNH
>EECEBICD_00299 hypothetical protein
MRLLQNFTIRMVMLTILGLFCLLWSGVGLYSVHALSEVSEGNDIDRHLVRQMTVLSQGND

QYFRFVTRLSRAMDVKIGGGTPDFAPARQSLENMRQKLEEMKALSPGPMNPDISREVLSN
WQALLEKGVVPQMQLVQQGSLTAWSEHASTVTPALSRAFGASAERFSHEAGAMLDNTRVM
VDGKTYTIRILLITAVILGIAILIFTDRYLVAMMVKPLERIRQQFQRIAQGDLSQPIEAL
GRNCVGRLVPLLRAMQDSLREAVSTIRAGSDNIWRGATEISTGNNDLSSRTEEQAAALEE
TAASMEQLTATVKMNAEHARQASQLADAASLTAGKGGELVSDVVETMNGISASSQQIAEI
TTVINSIAFQTNILALNAAVEAARAGEQGRGFAVVAGEVRNLASRSAGAAKEIEALIGES
VRRVAQGAQLVQETGATMDAILRGVTEVTTIMKQIASASEEQSKGISQVGVAITQMDSVT
QQNAALVEQVSAAAAALERQTEDLQRSVQQFRLSASEPQQRVTAKAAPGVQRMASAPAQS
TDEWVSF
>EECEBICD_00300 hypothetical protein
MNNHFGKGLMAGLHAPYAYSAHHAVNFCSEYKRGFVLGFTHRMFEKTGDRQLSAWEAGIL
TRRYGLDKEMVMDFFKENHSGMAVRFFMVGYRLEG
>EECEBICD_00301 Hydrogenase-2 small chain
MTGDNTLITSHGINRRDFMKLCAALAATMGLSSKAAAEMAESVSNPQRPPVIWIGAQECT
GCTESLLRATHPTVENLVLETISLEYHEVLSAAFGHQVEENKHNALEKYKGQYVLVVDGS
IPLKDNGIYCMVAGEPIVDHIRKAADGAAAIIAIGSCSAWGGVAAAGVNPTGAVSLQEVL
PGKTVINIPGCPPNPHNFLATVAHIITYGTPPKLDAKNRPTFAYGRLIHEHCERRPHFDA
GRFAKEFGDEGHRQGWCLYHLGCKGPETWGNCSTLQFCDVGGVWPVAIGHPCYGCNEEGI
GFHKGIHQLAHVENQTPRSEKPDVNMKEGGNISAGAVGLLGGVVGLVAGVSVMAVRELGR
QQKKDNADSRGE
>EECEBICD_00302 Formate dehydrogenase, nitrate-inducible, iron-sulfur subunit
MNRRDFIKAASGGALLLGAAPSVSHAAAENRPPIPGSLGMLYDSTLCVGCQACVTKCQDI
NFPARNPEGEQTWSNNDKLSPYTNNIIQVWRSGTGVNKDQEENGYAYIKKQCMHCVDPNC
VSVCPVSALKKDPKTGIVHYDKDVCTGCRYCMVACPYNVPKYDYNNPFGALHKCELCNQK
GVERLDKGGLPGCVEVCPAGAVIFGTREELMAEAKKRLALKPGSEYHYPRQTLKTDDTYL
HTVPKYYPHLYGEKEGGGTQVLVLTGVPYEDLDLPKLDDLSTGARSEHVQHTLYKGMILP
LAALAGLTVLVRRNSKNDHHDGGDDHES
>EECEBICD_00303 putative Ni/Fe-hydrogenase 2 b-type cytochrome subunit
MSHDPKPLGGKIISKPVIIFGPLIVLCMLLIVKRLVFGLGSVSDLNGGFPWGVWIAFDLL
IGTGFACGGWALAWAVYVFNRGQYHPLVRPALLASLFGYSLGGLSITIDVGRYWNLPYFY
IPGHFNVNSVLFETAVCMTIYIGVMALEFAPALFERLGWKVSLKRLNKVMFFIIALGALL
PTMHQSSMGSLMISAGYKVHPLWQSYEMLPLFSVLTAFIMGFSIVIFEGSLVQAGLKGNG
PDEKNLFVKLTNTISVLLAIFVVLRFGELIYRDKLSYAFAGDFYSAMFWIEVVLMVFPLV
VLRVAKLRNDSRMLYLSALSALLGCATWRLTYSLVAFNPGGGYHYFPTWEELLISIGFVA
IEICAYIVLIRLLPILPPLKQNDHNRHEASKA
>EECEBICD_00304 Hydrogenase-2 large chain
MSQRITIDPVTRIEGHLRIDCEIENGVVSKAWASGTMWRGMEEIVKNRDPRDAWMIVQRI
CGVCTTTHAISSVRAAESALNIDVPVNAQYIRNIILAAHTTHDHIVHFYQLSALDWVDIT
SALKADPEKASAMLDGVSSWHLNSAQEFTKVQNKIKDLVASGQLGIFANGYWGHPAMQLP
PEVNLIAVAHYLQALECQRDANRVVALLGGKSPHIQNLAVGGVANPINLDSIGTLNLERL
MYIKSFIDKLSDFVEQVYKVDTAVIAAYYPEWLERGKGAVNYLSAPEFPTDGKNGSFLFP
GGYITNADLSTYRPITSHSDEFLIKGIQESAKHAWYKDEAPQAPWEGTTIPAYDGWSDDG
KYSWVKSPTFYGKTVEVGPLANMLCKLAAGRESTQTKLNEIIALYQKLTGKTLEMAQLHS
TLGRIIGRTVHCCELQNILQDQYNALIVNIGKGDYTTFVKPNIPATGEFKGVGFLEAPRG
MLSHWMVIKDGIISNYQAVVPSTWNSGPRNFNDDVGPYEQSLVGTPIADPAKPLEVVRTI
HSFDPCMACAVHVVDADGNEVVSVKVL
>EECEBICD_00305 Hydrogenase 2 maturation protease
MRILVLGVGNILLTDEAIGVRIVEALEQRYTLPDFVEILDGGTAGMELLGDMASRDHLII
ADAIVSKKNAPGTIMVLRDDEVPALFTNKISPHQLGLADVLSALRFTGEFPKKLTLVGVI
PQSLEPHIGLTPTVEAMIEPALEQVLAALRESGVEAIPKETAHV
>EECEBICD_00306 Hydrogenase-2 operon protein HybE
MSETFSGFDTAPVARVQAAFEEIAHRSMHDLSFLHPTMPVHVSDFTLFEGQWTGTVITPW
MLSALIFPGPDQIWPGRTIGEKLGLQLPYGTMTFTVGELEGVSQYLACSLMSPLSRSLSP

EEGVRLADDCARMLLSLPVSNPDAPQTSRRALLFGRRSCENA
>EECEBICD_00307 Hydrogenase maturation factor HybF
MHELSLCQSAVEIIQQQAEQHGVARVTGVWLEIGALSCVEERAVRFSFDIACQGTLAQGC
ELHIDYRPAQAWCWDCSQVVEILRHDAQCPRCHGDRLRVDTGDSLKVKSIEVE
>EECEBICD_00308 Hydrogenase maturation factor HybG
MCIGVPGQVLAVGDDIHQLAQVEVCGIKRDVNIALICEGKPADLVGQWVLVHVGFAMSII
DEDEAKATLDALRRMEYDITSA
>EECEBICD_00309 putative protein
MNKFLRQLSLLALLFCWPLMSQAARTFTDQLGRQVTVPDTVDRVVVLQHQTLNLLVQMNA
TDKIVGVMANWKQQLGDGYARLAPELAQKASLGDLTHVDPEKLVALRPQVVFVTNYAPQE
MIDKISRLGIPVVAISLRHDVAGERAKMNPTLADEEQAYNRGLREGITLIGDIVNKPQEA
KALIEAMDKGRKMVSDRLQSVPENERVRAYMANPELTTYGSGKYTGLMMAHAGALNVAAS
SVKGFKQVTMEQVIAWDPQVIFVQNRYPKVVNEILHNPQWQVIDAVKHHRVYLMPDYAKA
WGYPMPEAVGIGELWMAKKLYPEKFHDIDMQKAANDWYQRFYRTDYQGVQ
>EECEBICD_00310 hypothetical protein
MRILAAGSLRVVWPQLMAAFQADAVCDFGPAGLLRERIEAGEACDFFASANLAHPQALLE
SGRALRVAPFTTNRLCLSVRAQAMREGEDWLSLLTRRDLRIGTSTAGCDPSGDYTQQLFS
RMGKEGEAVRKRAVALVGGRQTLPLPAGRLAAEWLINHDYTDIFIGYASYAPRLRQVNSL
RVVDIPEPYNPVAEYGFACLSEQGKTLADFLLSARARLILMQHGFSEAPNMTHSQN
>EECEBICD_00311 Disulfide-bond oxidoreductase YghU
MADNTYQPAKVWTWDKSGGGAFANINRPVSGPTHDKTLPVGKHPLQLYSLGTPNGQKVTI
MLEELLALGVTGAEYDAWLIRIGEGDQFSSGFVDINPNSKIPALRDNSHNPPIRVFESGA
ILVYLADKFGHFLPQDLAKRTETLNWLFWLQGAAPFLGGGFGHFYHYAPVKIEYAINRFT
MEAKRLLDVLDKQLAHHPYVAGEEYTIADMAIWPWFGNVVLGNVYDAAEFLDAGSYQNVQ
RWAKQVAERPAVKRGRIVNRTNGPLNEQLHERHDASDFENNTEDKRQS
>EECEBICD_00312 Bifunctional glutathionylspermidine synthetase/amidase
MSKGTTSQDAPFGTLLGYAPGGVAIYSSDYNSLDPWDDDDAAFRSYIDDEYMGHKWQCVE
FARRFLFLNYGVVFTDVGMAWEIFSLRFLREVVNDNILPLQAFPNGSPRAPEAGALLIWQ
KGGEFNETGHVAIITQLLDNKIRIAEQNVIHTPLPPGQQWTRELEMVVENGCYTLRDTFD
DTTILGWMIQTDDTQYSLSQPDIANQSLAIRGARLPEKGQFDGPWLDERDPLQKAYVQAN
GHVINQDPYQYFTITESAEQELIKATNELHLMYLHATDKVLKDDNLLALFDIPKILWPRL
RLSWQRRRHHMITGRMDFCMDERGLKVYEYNADSASCHTEAGLILEKWAEQGYTGKGHNP
AEGLINELAGAWKHSKARPFVHIMQDDDIEEDYHAQFMQQALHQAGFASKILRGLGELRW
DDAGQLIDGDGRLVNCVWKTWAWETAMEQIREVSETEYAAVPIRTGHPENEVRLIDVLLR
PEVLVFEPLWTVIPGNKAILPILWSLFPHHRYLLDTDFTVNDELVQTGYAVKPIAGRCGS
NIDLVSHQEELLDKTSGKFATQKNIYQQLWCLPKVAGKYIQVCTFTVGGNYGGTCLRGDD
SLVIKKESDIEPLIVIKA
>EECEBICD_00313 hypothetical protein
MFKKVLGLRHWNNNVVKIPPPAESGANASDVVVDTPEPYERILCQKILMGISTIDIIRNA
IIKSCEQLNIEKERINELNEQNDKARSSLKSLVEFITEIGTTSSDIGCRMGDLNTSLTQI
NACIKEIQKIANQTNLIAINSAIEAARVGDAGRGFSVISKEVKNLSEDVKHSSKSVSTLT
SVIKDNTARVSEVLDNQQPVIDNITTNINQIVESIGIVIDKSLSMKSVMQYISTVQFLNI
VKVDHVIWKMEVYKLLLNKDINSKITMHDQCRLGKWYYGFEGQQFSNYYSFRSLEAPHKE
VHTAGHSALNYFAAGDMNAMSQELDRMERSSNEVVNQLEMLAVDLLKETTL
>EECEBICD_00314 Uronate isomerase
MATFMTEDFLLKNDIARTLYHKYAAPMPIYDFHCHLSPQEIADDRRFDNLGQIWLEGDHY
KWRALRSAGVDESLITGKETSDYEKYMAWANTVPKTLGNPLYHWTHLELRRPFGITGTLF
GPDTAESIWTQCNEKLATPAFSARGIMQQMNVRMVGTTDDPIDSLEYHRQIAADDSIDIE
VAPSWRPDKVFKIELDGFVDYLRKLEAAADVSITRFDDLRQALTRRLDHFAACGCRASDH
GIETLRFAPVPDDAQLDAILGKRLAGETLSELEIAQFTTAVLVWLGRQYAARGWVMQLHI
GAIRNNNTRMFRLLGPDTGFDSIGDNNISWALSRLLDSMDVTNELPKTILYCLNPRDNEV
LATMIGNFQGPGIAGKVQFGSGWWFNDQKDGMLRQLEQLSQMGLLSQFVGMLTDSRSFLS
YTRHEYFRRILCNLLGQWAQDGEIPDDEAMLSRMVQDICFNNAQRYFTIK
>EECEBICD_00315 Polyol:NADP oxidoreductase

MEQNIATAQVSVARPNWDKSRLVSRIVHLGCGAFHRAHQALFTHHLLEKSDSDWGICEVN
LMPGNDARLIANLKAQNLLYTVAERGAESTELKIIGSMKEALHPEFDGHAGILAAMARPE
TAIVSLTVTEKGYCTDPASGELDVNNPLIQNDLAHPQQPKSAIGYIVEALNMRREQGLKA
FTVLSCDNVRENGHVAKAAVLGLAKARDAALAAWIADNVTFPCTMVDRIVPAATEETLQL
VADQLGVYDPCAIACEPFRQWVIEDNFVNGRPDWDTVGAQFVADVVPFEMMKLRMLNGSH
SFLAYLGYLGGYDTIADTMTNPAYRRAALALMLDEQAPTLSMPEGTDLEGYANLLIARFT
NPSLKHRTWQIAMDGSQKLPQRLLDPVRLHLQQGDDYRRLTLGVAGWMRYVGGIDEQGKT
IDVVDPLLAQYQAIHQQYQTPEERVRGLLAIESIFGSDLPKNHEFVQAVTDAYQQLLQNG
AKATVEALAK
>EECEBICD_00316 Mannonate dehydratase
MKQTWRWYGPNDPVTLSDVRQAGATGVVTALHHIPNGEIWSIDEIQKRKAIVEEAGLEWS
VVESVPIHEDIKTHTGQYDLWIKNYQQTLRNLAQCGIYTVCYNFMPVLDWTRTDLEYVLP
DGSKALRFDQIEFAAFELHILKRPGAEADYTAEEIAQAERRFATMSEEDKARLTRNIIAG
LPGAEEGYTLDQFRQHLATYKDIDKAKLREHFAYFLKAIIPVADEVGVRMAVHPDDPPRP
ILGLPRIVSTIEDMQWMVETVNSMANGFTMCTGSYGVRADNDLVDMIKQFGPRIYFTHLR
STLREENPKTFHEAAHLHGDVDMYEVVKAIVEEEHRRKAEGSDDLIPMRPDHGHQMLDDL
KKKTNPGYSAIGRLKGLAEVRGVELAIQRAFFSK
>EECEBICD_00317 Hexuronate transporter
MKMTKLRWWIIGLVCVGTIVNYLSRSSLSVAAPAMMKELHFDEQQYSWVVSAFQLCYTIA
QPITGYLMDVIGLKIGFFIFALLWSLINMAHALAGGWISLAFLRGLMGLTEASAIPAGIK
ASAEWFPTKERGIAGGLFNIGTSIGAMLAPPLVVWAMLTFADSGIGTEMAFVITGGIGVL
FAITWFLIYNSPNKHPWITHKELRYIEDGQESYLQDDNKKPAVKEIVKKRNFWALAITRF
LADPAWGTLSFWMPLYLINVMHLPLKEIAMFAWLPFLAADFGCVAGGFLAKFFMEKMHMT
TINARRCSFTIGAVLMISIGFVSITTNPYVAIALMSIGGFAHQTLSTVVITMSADLFKKN
EVATVAGLAGSAAWMGQLSFNLFMGALVAIIGYGPFFIALSLFDIIGAIILWVLIKDPEK
HHPPMTEQPLASHR
>EECEBICD_00318 hypothetical protein
MSRNFTVSACQYIVTEINTFEDFITKVRILLNKSQGADVVIFPELFTIELFTLLKKWQER
PISHLTLIDQFTDAYKQLFQQEAKERGQFIIAGSHLEQTGADRYENVAHIWGPDGEHYAH
SKTHIFPAERGWYTQEGDKMAVFQLPFAKVGFNICYEAEIPECAATLAEQGAELILTPSA
TFTEQGFWRVRHCCHARCIENQIYLVHCCLGGNPGGPLPGCWARSSILSPCDVVWKNPQG
IIAEAHVNQEDVISGEINLDVLYENRLGGAATTFKDRRRKAGIYNIWPSHIK
>EECEBICD_00319 Peptidoglycan deacetylase
MGYLKLPEGKRIAVNLGVDVDAQSLWLGGFNRPSPSFMSRGEFGAQVGVPRLLKLFKENN
IKTTFFIPGHTVDTFPEIIKAIFDAGHEIAHHGYYHENPTLVNRDTERRLMDLAFACYKR
HFGIRPVGYRSPYWDYSENTLDLLEEAGFLYDSSLMARDLVPYHPQRWQVNWETGNIAGP
ASAILEIPVSWYLDDFPALAYTGNQEGMSDTDGVLRRWKDIFTYAYHHQENGLYAMALHP
QIIGHGHHMMMLSRLIEHIKGHDGVWFATCEEIASVWVDDEEDRQKMLRPDVRGVAPVPD
DYGWPGK
>EECEBICD_00320 Protein hcp1
MDAIFLKLDNIKGESQVEGFEDQIEIMSYSHNVAMQVNNDVSLTERTSGRAHVGEMSLTK
FVDLATPVLNEYCCSGRLIRTAVLTLCRNDDGKMRSLIVYTLTNVLISQLSVSGGAGGKP
VETMSLNFTKIEWQITAEKSDGAQQESRTSVWDLAMDQIGK
>EECEBICD_00321 Anti-adapter protein IraD
MMTPTIPVALFDRLLVEGISPHELVRRKLMCLFNSCAVPGGETLPPLLTRGMPEWHEVNV
GDKRVLNWFCRELRAAILRYEPSINMLKVSVKDAHHQTLALSLEAMLQDESEPLRLEIAY
SNGRWR
>EECEBICD_00322 Phenylacetaldehyde dehydrogenase
MHEETFGPVCSFIGYQNEKEALSHINASPFGLAASVWSENMSKALRYAEDIDAGMVWVNM
HTFLDPAVPFGGMKGSGSGREFGSAFIDDYTELKSVMVRY
>EECEBICD_00323 Phenylacetaldehyde dehydrogenase
MSDVTLLAEVTTFLRQRHGQFIAGERQAGNGTNFSVTNPATGKIIADVVSATPAQAEEAM
QSARRAFDVWRKMPTLQRGALLLKLADTLAAHREELAQLESVCSGKTITLSRGLELDQSV
AFLRYFAGWAGKITGETLNVSLPSMGEERYTAFTQRQPIGVVVGIVPWNFSIMIAIWKLA

AALVCGCTIVIKPSEYTPLTLLRVAELAKEAGFPDGVINVVNGAGGEIAQQLIAHPDCAK
VSFTGSVETGEKVRRSATSSGKRVTLELGGKNAALFLNDLSAQAMVNGILEAGYLNQGQI
CAAAERFYLPQEKLDTVMTLLRQRLSEIVPGSPLDEKTVMGPLANQVQLEKVLHLIQRAR
EEGDTIVYGGETLPGGGFFYSRQR
>EECEBICD_00324 Gamma-glutamylputrescine oxidoreductase
MNTKIDALNYYGATKKYPLHFPALQDDIDADVVIIGGGFSGINTALELAEQGITNVVVLE
ARHLGYGGTGRNGGQVMAGIGHDIEAVKKYVGAAGMETLFKISNLGAGIIRERIRKYHIE
ADFVPGYGYLAYNQRQLKTLQQWEKEFKAATPEDDIELYTGKDVQQVIGSDVYCGVLKHM
GGGHVHSLNLLLGSAQAANSLGVKIFEYSPVVEVSYGKTVRVRTAMGSVKAAKLLWACDS
FLNNLEPEIYKKTLVTYSYQVSTEPLSSELVERISPLRGAFSDIRPVINYYRLTKENRLL
FGSATRFLEYTPHDFAAWNRTLLTEVFPYLKDVKIDFAWGGPMACSANLFPQIGTLREHN
NVFYVQGYSGFGVTPSHIVCKILAEGINGGSERYRLMSSIPHATIYGRDSLRLLLVSAGK
LIHQTAGFWKGRN
>EECEBICD_00325 hypothetical protein
MHAFKLNQPVPELQPIGSVSLLGALPTEGDPQVAVAMIYGKPEDVFTCGLFSSTRGGFTM
IYPFTEHATVLEGEVELTVAGQEPIRFSPGDSWFVEQGTEVAWKVLTPRFVKHYLAKVES
HKQG
>EECEBICD_00326 Serine/threonine exchanger SteT
MFKNRLASHLERGVIGFPTTLASSVGLIMASPVILTVTSGFGSGGDTFALAVLLAFIMMQ
AQITTFAEAATLIPTTGSVYDYISCGMGRFFAITGALCAYLIVHIFAGTAETILAGVMAL
VNFESVSEHVAMQHNTWMVGVGMVVVFGLLNAIGVEVFGKVEVFLTFGMWTTLTIFGLCG
ILLSPVTHHSGWFGAPLDVSNLSGLFGYIGMAMFMFVGCELVTPMAPEIKKAHRTIPRAM
ALGLLAVAGCMFLYGAAINRQVNNVVVDAANHIFLLDTPMAIPAFANRVMGHVGQYWLGV
GLLLAGCATINTLMAAVPRIIYGMALDGALPRFLTWLHPRFKTPVIAIAFGVAIPCLHAW
YLNGDIDHIVPLILAAVCAWGVAYLLVTLSVVILRIRRPDLPRAYRAPWFPLPQIISSVG
IIIAIINIAPPGMNSRDVLIPFGIMLGLTAAYALFWTLCVQRVNPFLPLPVEEVVERAMG
NYEKRNNEEMTPDVLRPLV
>EECEBICD_00327 hypothetical protein
MSSVPWFKNALMNMVLRDLSGWRCEKLTEHSAVLHLNAFTQVICHVQQKRLFMASIHSCE
FRVKGTINYPLQGKIRVHQPGWLKRYPVIFTGSKSTAGLINYLNCFPNLQQALSELDYRR
FTLVLHHKEWYCGIELWAASEVVCKMPPLRRYLRLERHQRVLLLSVINMINQAMNQWLQQ
DTDAR
>EECEBICD_00328 HTH-type transcriptional regulator MalT
MSALSTTGHNDGITLHCLQSIAQLIPLSSAVFYRVNAYLKPEAYVLHNISNSTHQQYLEH
FQPLDPLSPSRFGQQVITVATMTPGICARHRHYYHEFMLPNHVCDMIEIFIRRGHRIIAG
ISLMRDIPFSSEERLRAQAVQPLLGLAIHDSLQEDNDLASILTAKEREIVGMVCEGASNK
LIARQLNISLSTVKTHLRNIFAKTEVINRTELVSRTRMSSVQHSQHM
>EECEBICD_00329 Anaerobic sulfatase-maturating enzyme
MLNLNTLRQQQIPVMTEYRAQIPFHILAKPIGPACNLACRYCYYPQGETPVEKMNESTLE
VFICRYIAAQPASAREINFVWQGGEPLLAGIGFYKKVIALQQRYAPDGVTISNSLQTNAT
LLNDAWCRLFRDNNFTIGISLEGSEDLQNHHRPGKRGEASYPAVLRGITLLQHYRVDFNV
LIVVHDDMARHAAAIYDHVVSLGARYLQFQPLMNEGNALQQRYQLSADNWGRFMIDIWRQ
WRKRGDMGRVFVINIEQAWAQYFTHISATCVHSARCGTNLVMEPDGKLYACDHLINSQHY
LGQLANNTLAPAVDSATRLPFGIKKSQRRECQRCSVKIVCQGGCPAHINSAGYNRLCSGY
YSFFTEILAPLRAWPRDLNGLKAWRADVMGRFSG
>EECEBICD_00330 Arylsulfatase
MKKEVTLATLSIIFSGTAHSTQNERPDIIVIIADDMGYSDITPFGGEIPTPNLQAMAENG
VRMSQYYTSPMSAPARAMLLTGNTSQQAGIGGMWWYENTIGKEGYELRLTDRVTTMAERF
KDAGYNTLMAGKWHLGFTPGSTPKDRGFRHSFALMGGGASHFDDAVPLGTVEIFHTYYTR
DNQRISLPSSFYSSEAYASQINRWISETPREQPIFAWLAFTAPHDPLQAPDEWISRFKRQ
YEQGYANVYRQRIARLKKLGFLRDDIPLPGLELDKEWQAMTPEQQKYTAKVMQVYAAMIA
NMDAQIGTVIETLKKTGRDKNTILVFLSDNGVNPAEGFHYESEPDFWKQFDNRYENIGRK
NSFISYGPHWADVSNAPYGRYHKTTSGQGGINTSFMISGPGIIHHGAIDNATMAAYDVAP
TLYEFAGIDASKSLSERPTLPMIGVSFKRYLTGESLHAPRTQYGVELHNQAAWIDGEWKL

RRLVTVFPQAGNAPWELFNLQRDPLETHNLAADYVDKVKILSSAYEAFAKQTMVLYAKGK
LIDYVGIDSKTGRYLAVDPQTLQPVPAPLAIPLDTKSDQ
>EECEBICD_00331 Hca operon transcriptional activator HcaR
MIKACGKAGFEPKIGQLVPQISSVINLVSAEMGVSMVPDSMRQVNVKGVVYRPVADQMPV
AKLALAYRRGDTSPTLRNFILKVTG
>EECEBICD_00332 HTH-type transcriptional regulator BenM
MELRHIRYFLAVAEERHFTRAATKLGIGQPPLSQQIKDLERELGAQLFRRVPHGAELTEA
GKAFYDVVKGMPATATRAVLAAQRVARGESGVLRVGFTASAAFNSVVPGAIRTFKRAYPD
VRLQLEEGNTTQLADELNEGSLDVAFLRPGFTGNERFQLRLLSEEPMVIVLAETHPAAAC
KQIALSILKDEFFYSSHVKSACRFMTQ
>EECEBICD_00333 L-malyl-CoA/beta-methylmalyl-CoA lyase
MPDISHTPTRSWLFTPAIRPERFIKAVESAADISIIDLEDSVTPNDKAQARKIAMQFLSS
RPNSSLKIALRINGMNTHAGIEDLHMLLECRFFPDYIILPKTESAAHLQIVDSLIMMAGS
DTRLIGIIESVVGLNAVESIADATPRLCGLMFGAADMAADIGATPAWEPLALARARIVAA
CAMKGLLAIDAPFFDIGDFSGLKEETLQALSFGFSAKSAIHPAQISVINAAFTPTTAEIN
HARAVLTENAKGVGIVSGMMIDAAVARQARRLLARAGIFS
>EECEBICD_00334 Mesaconyl-CoA hydratase
MNTLLPAYKTISEGRYREISGLYWEDFHPGDVFEHRPGRTVLDADNVWFTLLTLNVQPVH
FDAAYASKTEWKKLLVDSTFTLALLTGMSVRTVSAKVVANLGWDKVQAVHPVFAGDTLYA
ESTVLSKRLSNSRPGQGIVTVRTCGINQNGVEVMRFERTMLVYCCGCSPEEDAAY
>EECEBICD_00335 Butyryl-CoA:acetate CoA-transferase
MMNVNAVYAEKCVTPDEAVTLITSGSHLSMGMFAAEPPALLNALAKRAKRGEINDLRVYC
YETASIAGNTIFRYELSDYIHLYSMFITGIERALIRQGIESSGRKIVNYVPSNFHQATRL
LADDIGIDTFIHTVSPMDKYGYFNFGTGNDYSTRIARTAKKLIVEVNKYMPRVHGEGAAI
HISEIDAIVENHVPLIELPIRTAVAEDIAISQIIASLVPDGACLQMGVGALPELICNALK
EHNDLGVHTEALNPGLVSLIQQGVVTNQRKNIDRGMSVFTFAMGQKDMYDYLNDNPSFFS
RPVDYVNDPGIIAQNENVVSINATLQIDLTGACNSEYLLGHQYSASGGQLDFVRGAYASK
GGRSIITTRSTAANDTISRIVPRLEGPVTTPRTDTHWIVTEFGSVNLKGLSSTERALSII
ELAHPNFRQQLRVDAKKMHLI
>EECEBICD_00336 Virulence protein
MIIDRIDHLVLTVSDISTTIRFYEEVLGFSAVTFKQNRKALIFGAQKINLHQQEMEFEPK
ASRPTPGSADLCFITSTPINDVVSEILQAGIPIVEGPVERTGATGEIMSIYIRDPDGNLI
EISQYV
>EECEBICD_00338 hypothetical protein
MTGDPNILIAKSFLDFFTAIIFACSLGIAVSAICAPMLIIQLTLAACATLILPLTTPAMM
GDFSAVGGLLLVATGLRVCGIKMFPVVNMLPALLLAMPISAAWAMFFA
>EECEBICD_00339 hypothetical protein
MVIGPFINAGAILFGGVIGALLSQRLPERIRVSMTSIFGLCSLGIGILLVMKCANLPVMV
LATLVGALIGEFCLLEKGINGAVAKIQQLFMASGKKPTHDSFIQSYVAIIVLFAPAAPVF
SARCMKG
>EECEBICD_00340 Constitutive ornithine decarboxylase
MKSMNIAASGELIPRLSTHRNVVALDSTDFTDVAAVVITTADSRSGILALLKRTGFHLPV
FMLADEPVSAPVGVTAVIGGNAQEWLELENAASRYEAELLPPFYDTLTQYVDMGNSTFAC
PGHQHGEFFRKHPAGRHFYDFFGENLFRADMCNADVKLGDLLIHEGSAKHAQKFAAKVFN
ADKTYFVLNGTSAANKVVTNALLTRGDLVLFDRNNHKSNHHGALIQAGATPVYLEAARNP
FGFIGGIDAHCFDETYLRDQIRDVMPESADALRPFRLAIIQLGTYDGTIYNARQVVDKIG
HLCDYILFDSAWVGYEQFINMMADTSPLRLELNENDPGIFVTQSVHKQQAGFSQTSQIHK
KDNHIRGQARFCPHKRLNNAFMLHASTSPFYPLFAALDVNAKIHEGESGRRLWAECVALG
IDARKAILARCKLLQPFIPLVVDGKPWQAYPTETIASNRRFFSFEPGAKWHGFEGYADDQ
YFVDPCKLLLTTPGIDADSGQYTEFGIPATILAHYLRENGIVPEKCDLNSILFLLTPAES
EEKLARLVAMLAQFERHIEDDTPLADVLPTVFQKYPVRYRDYTLRELCQEMHNLYVSFDV
KDLQKAMFRKESLPHVAMNPQDANSAFIRGDVELVRISEAGGRIAAEGALPYPPGVLCVV
PGEIWGGAAQRYFLALEEGINLLPGFSPELQGVYSETDADGIQRLYGYVLK
>EECEBICD_00341 Nucleoside permease NupG

MAAEVTTPGAMFFVILLNSLAYMPTLGLINTISYYRLQSAGMDIVTDFPPIRIWGTIGFI
LAMWGVSFSGFELSHMQLYIGATLSVLLVLFTFTLPHIPVANQQKNQSWTSMLGLDAFAL
FKNKRMAIFFIFSMMLGAELQITNMFGNTFLHSFDKDPLFASSFIVQHASVMMSISQISE
TLFILTIPFFLSRYGIKNVMLISIVAWMLRFGLFAYGDPTPFGTVLLVLSMIVYGCAFDF
FNISGSVFVEKEVRPEIRASAQGMFLMMTNGFGCILGGIVSGKVVEYYTQNGITDWQTVW
LIFAGYSLVLAFAFVALFKYKHVRVPASSQPVAH
>EECEBICD_00342 Nucleoside permease NupG
MNLKLQLKILSFLQFCLWGSWLTTLGSYMFVTLKFDGASIGAVYSSLGIAAVFMPTLLGI
VADKWLSAKWVYALCHVVAPSRYSWPRKSLRLGRCSL
>EECEBICD_00343 Membrane-bound lytic murein transglycosylase C
MMKKLLALAVIAPLLISCSSSTKKGETYNEAWVKDTNGFDILMGQFANNIENLWGYKEVL
IAGPKDYVKYTDQFQTRSHINFDDGTITVETIAGTEPTAHLRRAIIKTLLMGDDPTSVDL
YSDVDDIKISKEPFLYGQVLDNTGQPIRWEGRATTFADYLLKTRLKSRSNGLRIIYSVTI
NLVPNHLDKRAHKYIGMVRQASRKYGVDESLILAIMQTESSFNPYAVSHADALGLMQVVQ
HSAGKDVFRSQGKSGTPSRNFLFDPASNIDTGTAYLAMLNNVYLSGIENPTSRRYAVITA
YNGGAGSVLRVFSNDKIQAANMINRMSPGDVYQILTTRHPSAESRRYLYKVNSAQRSYRR
R
>EECEBICD_00344 putative Fe(2+)-trafficking protein
MSRTIFCTYLQRDAEGQDFQLYPGELGKRIYNEISKDAWAQWQHKQTMLINEKKLNMMNA
EHRKLLEQEMVSFLFEGKDVHIEGYTPEDKK
>EECEBICD_00345 Adenine DNA glycosylase
MENTLWTLSEQVTPARGVERFNQAMMDLGAMVCTRSKPKCTLCPLQNGCIAAAHESWSCY
PGKKPKQTLPERTGYFLLLQHNQEIFLAQRPPSGLWGGLYCFPQFASEDELREWLAQRHV
NADNLTQLNAFRHTFSHFHLDIVPMWLPVSSLDACMDEGSALWYNLAQPPSVGLAAPVER
LLQQLRTGAPV
>EECEBICD_00346 Adenine DNA glycosylase
MQASQFSAQVLDWYDKYGRKTLPWQINKTPYKVWLSEVMLQQTQVTTVIPYFERFMARFP
TVTDLANAPLDEVLHLWTGLGYYARARNLHKAAQQVATLHGGEFPQTFAEIAALPGVGRS
TAGAILSLALGKHYPILDGNVKRVLARCYAVSGWPGKKRWRIRCGR
>EECEBICD_00347 tRNA (guanine-N(7)-)-methyltransferase
MKNDVISPEFDENGRPLRRIRSFVRRQGRLTKGQEHALENYWPVMGVEFSEAPVDFATLF
GREAPVTLEIGFGMGASLVAMAKARPEQNFLGIEVHSPGVGACLASAHEEGVENLRVMCH
DAVEVLHKMIPDNSLSMVQLFFPDPWHKARHNKRRIVQVPFAELVLSKLKLGGVFHMATD
WEAYAEHMLEVMSSIDGYKNLSESNDYVPRPESRPVTKFEQRGHRLGHGVWDLMFERVK
>EECEBICD_00348 putative protein
MAKNRSRRLRKKMHIDEFQELGFSVAWRFPEGTSEEQVDKTVDDFINDVIEPNKLAFDGS
GYLAWEGLICMQEIGKCTEEHQAIVRKWLEARNLEEVRTSELFDVWWD
>EECEBICD_00349 hypothetical protein
MMRKTLLAAVLTFTAMAAHADYKCSVTPRDDVILSPQTVQVKGENGNLVITPDGNVMYNG
KQYTLSAAQREQAKDYQAELRSALPWIDEGARSRVEKGRVALDKIIAKEVGESSNMRSRL
TKLDAQLKAQMNRIIEHRTDGLTFHYKAIDQVRADGQQLVNQAMGGILQDSINEMGAKVV
LKGGGNPLQGVMGSLGGLQTAIQNEWKNQEKDFQQFGKDVCSRVVTLEDSRKALVGSLK
>EECEBICD_00350 L-asparaginase 2
MEFFRKTALAALVMGFSGAAFALPNITILATGGTIAGGGDSATKSNYTAGKVGVENLVDA
VPQLKDIAIVKGEQVVNIGSQDMNDEVWLTLAKKINTECDSTDGFVITHGTDTMEETAYF
LDLTVKCNKPVVLVGAMRPSTSMSADGPFNLYNAVVTAADKQSANRGVLVVMNDTVMDGR
DVTKTNTTDVATFKAVNYGPLGYIHNGKIDYQRTPERKHTTSTPFDVSKLTALPKVGIVY
NYANASDLPAKALVDAGYDGIVSAGVGNGNLYKTVFDTLATAAHNGTVVVRSSRVPTGAT
TQDAEVDDAKYGFVASGTLNPQKARVLLQLALTQTKDPKQIQTMFNQY
>EECEBICD_00351 hypothetical protein
MVRQWIAGAALFALISGYSWAEVAQPSDNILKEQFSKQYHGILKLDSITLKNLDSTGNQA
TWSAEGDISSREDMYTGVGMAADYYFVEKTWTKDRPVKFSAMLTSKGTPASGWTVNYYSL
QMAASDQGRAIDDIKTNDKYLIVNSDDFNYRFGNIEASWRAQKASIPGLEEQLSALDKKI
AVAKKEADAYWGKGADGKPLTRAEAFKKTLKERDDYVKANDSSVYAEKYEKEVYQPALDA

CRKQSEPCNEAAIQQKRDLDIHEQRRQVFLKSEELRRKAQNDWITLEKGQYPLNIAVQKL
QMQQSDIRVKIMDINDGYERWKKDTDDLRRKGVIK
>EECEBICD_00352 Heme chaperone HemW
MAKLPPLSLYIHIPWCVQKCPYCDFNSHALKGEVPHDDYVQHLLNDLDADVAWAQGREVK
TIFIGGGTPSLLSGPAMQTLLDGVRARLNLAADAEITMEANPGTVEADRFIDYQRAGVNR
ISIGVQSFSEPKLKRLGRIHGPQEAMRAARLANGLGLRSFNLDLMHGLPDQTLEEALNDL
RQAIALNPPHLSWYQLTIEPNTLFGSRPPVLPDDDALWDIFEQGHQLLTAAGYQQYETSA
YAKPGYQCQHNLNYWRFGDYLGIGCGAHGKVTFPGGRILRTTKTRHPRGYMQGRYLESQR
DVSDDDKPFEFFMNRFRLLERAPRAEFVDYTGLTEAVIRQPIDEAIAQGYLTECEQYWQI
TRHGKLFLNSLLELFLAE
>EECEBICD_00353 dITP/XTP pyrophosphatase
MQKVVLATGNAGKVRELASLLSDFGLDVVAQTELGVDSAEETGLTFIENAILKARHAAKM
TGLPAIADDSGLAVDVLGGAPGIYSARYSGENATDQQNLEKLLHTLRDVPDDKRQARFHC
VLVYLRHAEDPTPIVCHGSWPGVITRQAAGNGGFGYDPIFFVPSEGKTAAELTREEKSAI
SHRGQALKLLLDALRNG
>EECEBICD_00354 hypothetical protein
MSAVTRCEDGLVLRLYIQPKASRDSIVGLHGDEVKVAITAPPVDGQANSHLTKFLGKQFR
VAKSQIVIEKGELGRHKQVKIIHPQQIPPEIAALTE
>EECEBICD_00355 hypothetical protein
MNTLTFLLSTVIELYTMVLLLRVWMQWARCDFYNPFSQFVVKITQPIIGPLRRIIPPMGP
IDSASLLVAFILSVIKAIVLFKVITFQAIIWIAAVLILLKTIGLLIFWVLLVMAIMSWVS
QGRSPIEYVLIQLAEPLLSPIRRILPAMGGIDFSPMVLVLLLYVVNMGIAEVLQATGNML
LPGLWMAL
>EECEBICD_00356 Pyridoxal phosphate homeostasis protein
MKDIAHNLAYIRDKISAAATRCGRSSEEVTLLAVSKTKPASDIAEAIAAGQRAFGENYVQ
EGVEKIRHFQEAKVEGLHWHFIGPLQSNKSRLVAEHFDWCHTIDRLRIASRLSEQRPDNL
PALNVLIQINISDENSKSGIPLAELDELAAAVATLPRLRLRGLMAIPAPESDYVRQFEVA
RQMAVAFAGLKARYPDVDTLSLGMSDDMEAAIAAGSTMVRIGTAIFGARDYTKN
>EECEBICD_00357 putative protein YggR
MNMEEIVTLSVKHNVSDLHLCNAWPARWRKQGRMENAPFTAPDVDRLLLDWLNDAQQYQW
RTHGQLDFAVSLSGTRRLRASAFTHQQGTSLALRLLPERCPDLAEIQTPPIVPALLASEN
GLILVTGATGCGKSTTLAAMVGHLNQHADKHILTLEDPIEYRYTSKRCLIQQREIGQHCA
TFAAGLRAALREDPDVILLGELRDSETIRLALTAAETGHLVLATLHTRGAAQAVERLVDS
FPAQEKEPVRSQLAGSLRAVLSQKLEVDRQDGRVALFELLINTPATGNLIREGKLHQLAH
VIQTGQQQGMMTFAQSAQWRQAQGRL
>EECEBICD_00358 hypothetical protein
MSSQPNQSLIDGIRCLQYLVSSDRAIGCRELARLMDINTTRVNRLLMTMASIGLTMQDEH
RRYLPGPGIHALAAQAIRGSALFSHALPILERHAPKDIVVALGVLWEDQIIYIYHSTPGS
QGSQALAGFRMCPAWQSVTGVALLAAESDEALMQRFTPEQWRNLAPHVAQQRQRGYVLWH
HADGEVSMAQPLGKHAAALAFAGMWRIDEAEAAARLQALKALNQLIAQ
>EECEBICD_00359 Putative pre-16S rRNA nuclease
MSDTLLAFDFGTKSIGVAIGQRITGTARPLPAIKAQDGTPDWMLIERLLKEWQPDEIIVG
LPLNMDGTEQPLTARARKFANRIHGRFGVTVTLHDERLSTVEARSGLFERGGYRALNKGK
VDSASAVIILESYFEQGY
>EECEBICD_00360 hypothetical protein
MNLQHHFLIAMPALQDPIFRRSVVYICEHNQDGAMGIIVNKPLENLQIEGILEKLKITPE
PRDSSIRLDKAVMLGGPLAEDRGFILHTPPSRFASSIRISDNTVITTSRDVLETLGTQQQ
PSDVLVALGYASWDKGQLEQELLDNAWLTAPADLNILFKTPIAERWREAAKLIGIDILTM
PGVAGHA
>EECEBICD_00361 Glutathione synthetase
MIKLGIVMDPIAHINIKKDTSFAMLLEAQRRGYELHYMEMADLYLINGEARARTRTLSVE
QNYDKWYEFGSEQEIKLADLDVILMRKDPPFDTEFIYATYILERAEEEGTLIVNKPQSLR
DCNEKLYTAWFADLTPETLVTRNKAQLKAFWEKHGDIIMKPLDGMGGASIFRVKEGDPNI
GVIAETLTELGNRYCMAQNYLPAIKDGDKRVLVVDGEPVPYCLARIPQGGETRGNLAAGG

RGEPRPLSESDWEIARRVGPTLKAKGLIFVGLDIIGDRLTEINVTSPTCVREIEAEYPIS
ITGMLMDAIEARLAK
>EECEBICD_00362 Ribosomal RNA small subunit methyltransferase E
MRIPRIYHPELLTSGTQISLCEDAANHIGRVLRMGAGQALQLFDGSNQVFDAEIISASKK
SVEVQVMKGEIDDRESPLHIHLGQVMSRGEKMEFTIQKSIELGVSLITPLFSERCGVKLD
NERLNKKRQQWQKIAIAACEQCGRNRVPEIRPPMALEAWCAEQDSGLKLNLHPRAHASIN
TLPLPVERVRLLIGPEGGLSADEIAMTARYQFTDILLGPRVLRTETTALTAITALQVRFG
DLG
>EECEBICD_00363 Endonuclease-1
MYRNFSFAAALLAAAFSGQALADGINNFSQAKAASVKVNADAPGSFYCGCQIRWQGKKGV
VDLESCGYKVRKNENRARRIEWEHVVPAWQFGHQRQCWQDGGRKNCAKDPVYRKMESDMH
NLQPAIGEVNGDRGNFMYSQWNGGEGQYGQCAMKVDFKAKLAEPPARARGAIARTYFYMR
DQYQLKLSRQQTQLFNVWDKQYPVTAWECERDARIAKVQGNHNPYVQRACQARKS
>EECEBICD_00364 Protein SprT
MRRLRENLAQANLKLDRHYPEPKLVYTQRGTSAGTAWLESYEIRLNPVLLLENIDTFIAE
VVPHELAHLLVWKHFGRKAPHGKEWKWMMESVLGVPARRTHQFALQSVRRNTFPYHCQCQ
QHQLTVRRHNRVVRGEAVYRCVHCGEPLVAG
>EECEBICD_00365 Galactose-proton symporter
MPDNKKQGRSNKAMTFFVCFLAALAGLLFGLDIGVIAGALPFITDEFQITAHTQEWVVSS
MMFGAAVGAVGSGWLSFKLGRKKSLMIGAILFVAGSLFSAAAPNVEVLIISRVLLGLAVG
VASYTAPLYLSEIAPEKIRGSMISMYQLMITIGILGAYLSDTAFSYSGAWRWMLGVIIIP
AILLLIGVFFLPDSPRWFAAKRRFHDAERVLLRLRDTSAEAKRELDEIRESLQVKQSGWA
LFKENSNFRRAVFLGILLQVMQQFTGMNVIMYYAPKIFELAGYTNTTEQMWGTVIVGLTN
VLATFIAIGLVDRWGRKPTLTLGFLVMAIGMGILGTMMHIGIHSPSAQYFAIAMLLMFII
GFAMSAGPLIWVLCSEIQPLKGRDFGITCSTATNWIANMIVGATFLTMLNNLGNANTFWV
YAGLNVLFILLTLWLVPETKHVSLEHIERNLMKGRKLREIGAHD
>EECEBICD_00366 S-adenosylmethionine synthase
MEEIIKPILPSEWLNTSTKFFINPTGRFVIGGPMGDCGLTGRKIIVDTYGGMARHGGGAF
SGKDPSKVDRSAAYAARYVAKNIVAAGLADRCEIQVSYAIGVAEPTSIMVETFGTEKVPA
EQLILLVREFFDLRPYGLIQMLDLLHPIYKETAAYGHFGRENFPWEKTDKAQLLRDAAGL
K
>EECEBICD_00367 S-adenosylmethionine synthase
MAKHLFTSESVSEGHPDKIADQISDAVLDAILQQDPKARVACETYVKTGMVLVGGEITTS
AWVDIEEITRNTVREIGYVHSDMGFDANSCAVLSAIGKQSPDINQGVDRADPLEQGAGDQ
GLMFGYATNETDVLMPAPITYAHRLVQRQAEVRKNGTLPWLRPDAKARSLSSMTTAKSSV
STPWFSLRSTQKISTKNRCKKR
>EECEBICD_00368 hypothetical protein
MTEDSTMSLKVADTGSLSVNQYGWINIWTAILGHFFAQFPAFFEGRRNVGQNQAINVSDN
ADIMRIYALLFFCVLGIKRDARLIQKHTTFSSCRHFQPG
>EECEBICD_00369 Biosynthetic arginine decarboxylase
MSDDMSMGSPSSAGEQGVLRSMQEVAMSSQEASKMLRTYNIAWWGNNYYDVNELGHISVC
PDPDVPEARVDLAKLVKAREAQGQRLPALFCFPQILQHRLRSINAAFKRARESYGYNGDY
FLVYPIKVNQHRRVIESLIHSGEPLGLEAGSKAELMAVLAHAGMTRSVIVCNGYKDREYI
RLALIGEKMGHKVYLVIEKMSEIAIVLEEAERLNVVPRLGVRARLASQGSGKWQSSGGEK
SKFGLAATQVLQLVETLRDAGRLDSLQLLHFHLGSQMANIRDIATGVRESARFYVELHKL
GVNIQCFDVGGGLGVDYEGTRSQSDCSVNYGLNEYANNIIWAIGDACEEHGLPHPTVITE
SGRAVTAHHTVLVSNIIGVERNEYTDPTAPAEDAPRALQNLWETWQEMHKPGTRRSLREW
LHDSQMDLHDIHIGYSSGAFSLQERAWAEQLYLSMCHEVQKQLDPQNRAHRPIIDELQER
MADKMYVNFSLFQSMPDAWGIDQLFPVLPLEGLDQVPERRAVLLDITCDSDGAIDHYIDG
DGIATTMPMPEYDPENPPMLGFFMVGAYQEILGNMHNLFGDTEAVDVFVFPDGSVEVELS
DEGDTVADMLQYVQLDPKTLLTHFRDQVKQTDLDDALQQQFLEEFEAGLYGYTYLEDE
>EECEBICD_00370 hypothetical protein
MSKFKFNAVIVGLFLLLGGCSSGMQSNNGSSSDVGTAWGGDVHSSVQSVSVERADREPAE
MVIINYSTQYPSGYDKVYSIRISDLEYAVRDANFNSIPITRRYNASMGQWQYSIPARSGM

NYQLYIRNYSHDTNYEIVATVDGLDVLNGKAGSLNHHGYIVNAGDSLAIKGFRKDKHTEA
AFQFADVADAYAAHSAQGDVRNIGVIGFAAFALQGKATNTLPPCSSQAFPADNNGYAPPP
CRK
>EECEBICD_00371 Pyruvate dehydrogenase complex repressor
MEQILTKRRYFDIGLQIEELLYSGVFKAGERLPAERELSERFQTSRTTIREAIIMLELKG
VVEVKQGAGTYFIDSLEKINQKALLPYSDIGPFELLQARQVIESNITGFAATQIRFNELK
QLKRIIEQQEKQIGGDSDKFEELDRQFHNIIAESTQNRVLMKQSAELWRAVRTENPRWKQ
LNYKYLHKKELRMKWVEDHRSIFLALQKRDAEQARQASWTHLENSKNELVKIFQQDDSLE
DFDDFFFAT
>EECEBICD_00372 Mannitol 2-dehydrogenase
MDTIARQLDAARHQFKTTYSRQGMEANIVHIGFGAFHRGHQAVYNDLTNEITGNRWGIFE
LNLFGDAELVDALNAQQGLFSVVETSASAINSRLVRTITGALHTPKSGIQAAIEKLTEPQ
VKIVSLTITEKGYCTDPRSRTLDLSHPLIKHDLADPEHPRSALGLIVEALRIRRQQGLPP
FSVLSCDNIPENGHLTKTAIVTFADNLDPQLAAWIAKHVTFPGTMVDRIVPAMTPEQFDM
IKDQIGFADPCGIVCEDFRQWVIEDNFVAGRPDWDKAGAMFVSDVLPYEEMKLRMLNGSH
SFLAYNGSLAGYEFIYQCMEDDAFKTAVHHLMTEEQAKSLRPNLAVDVHQYAQLLIERFS
NPNIKHKTGQIAMDGSQKLPQRAVDPYLTLQARGVKGRALATLIAGWLHYVINTLSQGQS
VADPLNDTFNAAIKNKNTRWEQALSLLQINSIFGTLAGDNADFLNDIQQAFNHIELHGVT
ATIHRLSSKG
>EECEBICD_00373 L-galactonate-5-dehydrogenase
MKTLICNNPGNIEYIERDIPHLKDDEVLLKIKAVGICGTDIHAFAGRQPFFAYPRVLGHE
ICGVAEILGKSCSTAKVGQRYSVIPCIPCGACAACREGKTNCCENVSLYGVHQDGGFSEY
LAVREQNLVELSDNLTDSAGALVECFAISAHAVRRADVKPQQNIVVVGAGPIGLAAAAIA
KAKGARVAVADIDAERRRLVAEKVGVATLDPSSDDYIDVLKACFSGELAGIVLDATGNKS
SMSRAVDLILHGGKIVFIGLYIGELVIDDPTFHKKETTLLSSRNATREDFECVIELMAQG
AISETMMKNQEFDFYTFGNQYQKNVVENKKLVKGVIKF
>EECEBICD_00374 putative oxidoreductase YjmC
MSTILVKENELKALAFNKLTQAGLDAQTAQQVADVLVHADITGVHSHGVIRVEHYCTRLN
AGGLNPKATFSIEQISPSVAILDSDDGMGHCALIKATDHAISLARETGLGFVSVKNTSHC
GALSWFIEQATSQGMVAIAMTQTDTCVAPYGGAERFLGTNPIAFGFPVKDSHPMIVDMAT
SAIAFGKILHAKETGKPIGHGLALDKEGHITTDPHKIENLLPFGGHKGSGIALAIDALTG
VLMGANFSNHIVRMYGDYDKMRKLASLVIVIDPQMLGNPLFSSIMSTMVNELRAVKPMPG
VDKVLAPNDPQIAYKEKCLKEGIPVAEGIYQYLIG
>EECEBICD_00375 Toxin-antitoxin biofilm protein TabA
MLYGNIEQLTLLPYVNHIIKKLIIEAVKIAEDQPAGRYELSFPESFLMISEGETHSSLNR
KAELHKKYIDVQILLSGYEEIGYSNKIDTRIKELEHLPDDIIFPECVANEQFVTLNPGDF
ALFYPNQIHRPLCTRGKPAPVKKAIVKIPATAFSESSLPCQTKE
>EECEBICD_00376 Non-heme chloroperoxidase
MNHIFKLAFIFIIINIIFLINASATTMDKDKFVEANGIKIHYVEEGEGPPLLLIHGGGLTA
KSWQGLAKEASRYFRVIMPDSRGHGLTNNPQGTFSYDLMTEDMAAFVKALKLEKPLVMGY
SDGGMVVLKLTSRYPDLARAAIVGGATHRFATTHYMQGMEIFYGKGMP
>EECEBICD_00377 hypothetical protein
MDNAHIADGRRGEKIHIPVLLLDGDRDEFFTVEEVTELYRLLPQAEMTLIPGSGHAIFQT
PGKTPLFYALVLEFLQRQLPKAS
>EECEBICD_00378 Agmatinase
MSTLGHQYDNSLVSNAFGFLRLPMNFQPYDSDADWVITGVPFDMATSGRAGGRHGPAAIR
QVSTNLAWEHHRFPWSFDMRERLNVVDCGDLVYAFGDAREMSEKLQAHAEKLLSAGKRML
SFGGDHFVTLPLLRAHAKHFGKMALVHFDAHTDTYANGCEFDHGTMFYTAPKEGLIDPHH
SVQIGIRTEFDKDNGFTVLDACQVNDRGVDDILAQVKQIVGDMPVYLTFDIDCLDPAFAP
GTGTPVIGGLTSDRAIKLVRGLKDLNIVGMDVVEVAPAYDQSEITALAAATLALEMLYIQ
AAKKGE
>EECEBICD_00379 Metalloprotease LoiP
MKIRALLLALGMATVLTGCQNMDSNGLLSSGAEAFQAYTLSDAQVKALSDQSCQELDSKA
KIAPASSEYAKRLAKIAAALGDNINGQPVNYKVYETKDVNAFAMANGCIRVYSGLMDMMT

DNEVEAVIGHEMGHVALGHVKKGMQVALGTNAVRVAAASAGGVVGSLSQSQLGDLGEKLV
NSQFSQRQESEADDYSYDLLRKRGISPAGLATSFEKLAKLEAGRQSSMFDDHPASAARAQ
HVRDRMSADGIK
>EECEBICD_00380 Transketolase 1
MSSRKELANAIRALSMDAVQKAKSGHPGAPMGMADIAEVLWRDFLNHNPTNPSWADRDRF
VLSNGHGSMLIYSLLHLTGYDLPMSELQNFRQLHSKTPGHPEVGYTAGVETTTGPLGQGI
ANAVGMAIAEKTLAAQFNRPGHDIVDHFTYVFMGDGCMMEGISHEVCSLAGTLKLGKLIA
FYDDNGISIDGHVEGWFTDDTAKRFEAYGWHVIRGIDGHDADAIKRATEEARAVTDKPSL
LMCKTIIGFGSPNKQGTHDSHGAPLGDAEIALTREQLGWKYAPFEIPSEIYAQWDAKEAG
QAKESAWNEKFAAYEKAFPQEAAEFTRRMKGDMPADFDAKANEFIAKLQANPSKIASRKA
SQNAIEAFGPLLPEFLGGSADLAPSNLTLWSGSKAINEDAAGNYIHYGVREFGMTAIANG
IALHGGFLPYTSTFLMFVEYARNAVRMAALMKQRQVMVYTHDSIGLGEDGPTHQPVEQVA
SLRVTPNMSTWRPCDQVESAVAWKYGVERQDGPTALILSRQNLAQQERTEEQLANIARGG
YVLKDCAGQPQIIFIATGSEVELAVAAYEKLTAEGVKARVVSMPSTDAFDKQDAAYRESV
LPKDVSARVAIEAGIADYWFKYVGLNGAIVGMTTFGESAPAELLFEEFGFTVDNVIAKAK
ALL
>EECEBICD_00381 Nickel import ATP-binding protein LarO
MLTLNQVAYRWPDAAADCLHAISLELRDGEWLALTGDNGAGKSTLLRIMAGLLSPTSGSV
TLNGEPIAQLKNRQRAAAIGVLFQEAENQIFHSNVAQEVAFGLKLQRLSAEEISRRTQAA
LRLCQLTDVAEAHPLDLHAAPRRMVAVASLEAMAPPVLLLDEPSRDFDAHWLTVFEHWLA
TCRARGTSVVAISHDAAFTRRHFSRVVRLENGRLSKGE
>EECEBICD_00382 Energy-coupling factor transporter ATP-binding protein EcfA1
MVTLEQFRYLPSDATRPPACFDFHYSTPGIVAIVGDNGSGKSTLAQLMAGWYPDYLPGDI
DGTGLLLGVPIGQLPLVEQSPTIQLVQQSPYLQLSGCTFSVEEEVAFGPENLGLHEAEIL
RRIDEALTLTNCQSLRHRHPGTLSGGETQRVVIASALAMQPRLLVLDEAFSRLTSAATGM
LLERLQQWALERHSLIVLFERNHFPFLTRCQRVWQLRDGALTPLC
>EECEBICD_00383 hypothetical protein
MHPFTSLTLWALAACTTLLLPAQTVLPVYSAAAFLCLLALKSTRRRAKYVAWLMLSLGFG
LWLVHGGWLTEWISGQPCDPQRWIYAVTLWLRLLAIVSTSQLWMQYVPVQRFIRALFASR
LPPGIAYLFAGPLLVVEQLKRQLTIVHEAQRARGVPLDEGWYQRLRAMPALIVPLTQNAL
NDLTIRGAALDMRGFRLHRARTTLWAPKDSMLQRVARYGMVLLILAEAGVWIWLR
>EECEBICD_00384 hypothetical protein
MARRPFSSQSLVLIVIAIAINMIGGQLISMLKLPIFLDSIGTLISAVLLGPFIGMLTGLL
TNLLWGLLTDPIAAAFAPVAMVIGLVAGWLARAGWFRTLPKVIVSGVVITLAVTLVAVPL
RTALFGGVTGSGADLFVAWMHSMGQNLVESVAITVIGANLVDKILTAIIVWVLLRQLPLR
TTRHFPAMSAVR
>EECEBICD_00385 hypothetical protein
MTVSHHNASTARFYALRLLPGQEVFSQLHAFVQQNQLHAAWIAGCTGSLTDVALRYAGQE
ATTSLTGTFEVISLNGTLELTGEHLHLAVSDPYGAMLGGHMMPGCTVRTTLELVIGELPA
LTFSRQPCAISGYDELHISSR
>EECEBICD_00386 D-erythrose-4-phosphate dehydrogenase
MTVRIAINGFGRIGRNVVRALYESGRRAEITVVAINELADAAGMAHLLKYDTSHGRFAWE
VRHEREQLFVGDDVIRILHERTLADLPWRELGVDVVLDCTGVYGNREHGEAHIAAGAKKV
LFSHPGSNDLDATVVFGVNQNELRAEHRIVSNASCTTNCIIPVIKLLDDAYGIESGTVTT
IHSAMNDQQVIDAYHSDLRRTRAASQSIIPVDTKLAAGITRIFPQFNDRFEAIAVRVPTI
NVTAIDLSVTVKKPVKASEVNQLLQKAAQGAFHGIVDYTESPLVSIDFNHDPHSAIVDGT
QTRVSGAHLIKTLVWCDNEWGFANRMLDTTLAMAAVGFRLDASASTKL
>EECEBICD_00387 Phosphoglycerate kinase
MSVIKMTDLDLAGKRVFIRADLNVPVKEGKVTSDARIRASLPTIELALKQGAKVMVTSHL
GRPTEGEYNEEFSLLPVVNYLKDKLSNPVRLVKDYLDGVDVAEGELVVLENVRFNKGEKK
DDEALSKKYAALCDVFVMDAFGTAHRAQASTHGIGKFADVACAGPLLAAELDALGKALKE
PARPMVAIVGGSKVSTKLTVLDSLSKIADQLIVGGGIANTFVAAQGHSVGKSLYEADLVD
EAKRLLTTCDIPVPTDVRVATEFSETAPATLKSVNDVKEDEQILDIGDASAQQLAEILKN

AKTILWNGPVGVFEFPNFRKGTEIVANAIADSEAFSIAGGGDTLAAIDLFGIADKISYIS
TGGGAFLEFVEGKVLPAVAMLEERAKK
>EECEBICD_00388 Fructose-bisphosphate aldolase class 2
MSKIFDFVKPGVITGDDVQKVFQVAKENNFALPAVNCVGTDSINAVLETAAKVKAPVIVQ
FSNGGASFIAGKGVKTDVPQGAAILGAISGAHHVHQMAEHYGVPVILHTDHCAKKLLPWI
DGLLDAGEKHFAATGKPLFSSHMIDLSEESLHENIEICSKYLARMSKIGMTLEIELGCTG
GEEDGVDNSHMDASALYTQPEDVDYAYTELSKISPRFTIAASFGNVHGVYKPGNVVLTPT
ILRDSQDYVSKKHNLPHNSLNFVFHGGSGSTAQEIKDSVSYGVVKMNIDTDTQWATWEGV
LNYYKENEAYLQGQLGNPKGEDQPNKKYYDPRVWLRAGQTSMIARLEKAFKELNAIDVL
>EECEBICD_00389 Small-conductance mechanosensitive channel
MEDLNVVDSINGAGTWLVRNQALLLSYAVNIVAAIAIIIIGLIVARVISNTVNRLMRARH
IDATVADFLSALVRYGVIAFTLIAALGRVGVQTASVIAVLGAAGLAVGLALQGSLSNLAA
GVLLVMFRPFRAGEYVDLGGVAGTVLNVQIFSTTMRAVDGKIIVIPNGKIIAGNIINYSR
EPVRRNEFIIGVAYDSDIDQVKQLLTTIIESDDRILKDREMTVRLNELGASSINFVVRVW
SKSSDLQNVYWDVLERIKREFDAAGISFPYPQMDVNFKRVKDNAAE
>EECEBICD_00390 Arginine exporter protein ArgO
MISYYFQGFALGAAMILPLGPQNAFVMNQGIRRQYHLMIALLCALSDLVLISAGIFGGSA
LLMQSPWLLALVTWGGVAFLLWYGLGALKTAMSSNLELASAEVMKQGRWKIIATMLAVTW
LNPHVYLDTFVVLGSLGGQLAMEPKRWFALGTISASFLWFFGLALLAAWLAPRLRTAKAQ
RIINILVGVVMWLIAFQLAREGVAHMHALFN
>EECEBICD_00391 26 kDa periplasmic immunogenic protein
MKFKVMALAALVGLSAMSAQASELPEGPHIVTSGTASVDAVPDIATLAIEVNVAAKDAAT
AKKQADERVAQYLSFLEQNQIAKKDISAANLRTQPDYDYQNGKSILKGYRAVRTVEVTLR
QLDKLNSLLDGALKAGLNEIRSVSLGVAQPDAYKDKARKAAIDDAIHQAQALAAGFNSKL
GPVYSVRYHVSNYQPSPVVRMMKAADAAPVSAQETYEQPTIQFDDQVDVVFQLEPGTGQT
STTAASTQ
>EECEBICD_00392 HTH-type transcriptional regulator ArgP
MKRPDYRTLQALDAVIRERGFERAAQKLCITQSAVSQRIKQLENMFGQPLLVRTVPPRPT
EQGQKLLALLRQVELLEEEWLGDEQTGSTPLLLSLAVNADSLATWLLPALAPVLADSPIR
LNLQVEDETRTQERLRRGEVVGAVSIQHQALPSCLVDKLGALDYLFVASRPFAERYFPNG
VTRSSLLKAPAVAFDHLDDMHQAFLQQNFDLPPGSVPCHIVNSSEAFVQLARQGTTCCMI
PHLQIEKELESGELINLTPGLLQRRMLYWHRFAPESRMMRKVTDALLEYGHKVLRQD
>EECEBICD_00393 Ribose-5-phosphate isomerase A
MTQDELKKAVGWAALQYVQPGTIVGVGTGSTAAHFIDALGTMKGQIEGAVSSSDASTEKL
KGLGIHVFDLNEVDSLGIYVDGADEINGHMQMIKGGGAALTREKIIASVAEKFICIADAS
KQVDILGKFPLPVEVIPMARSAVARQLVKLGGRPEYRQNVVTDNGNVILDVYGMEILDPI
ALENAINAIPGVVTVGLFANRGADVALIGTPDGVKTIVK
>EECEBICD_00394 D-3-phosphoglycerate dehydrogenase
MAKVSLEKDKIKFLLVEGVHQKALESLRAAGYTNIEYHKGALDAEQLKASIRDAHFIGLR
SRTHLTEEVINAAEKLVAIGCFCIGTNQVDLNAAAKRGIPVFNAPFSNTRSVAELVIGEL
LLLLRGVPEANAKAHRGVWNKLAAGSFEARGKKLGIIGYGHIGTQLGILAESLGMHVYFY
DIENKLPLGNATQVQHLSELLNMSDVVSLHVPENASTKNMMGAKEIALMKPGSLLINAAR
GTVVDIPALCDALATKHLAGAAIDVFPTEPATNSDPFTSPLCEFDNVILTPHIGGSTQEA
QENIGLEVAGKLIKYSDNGSTLSAVNFPEVSLPLHGGRRLMHIHENRPGVLTALNQIFAE
QGVNIAAQYLQTSARMGYVVIDIEADGDVAEKALLAMKAIPGTIRARLLY
>EECEBICD_00395 5-formyltetrahydrofolate cyclo-ligase
MIRQRRRALTPEQQRRFGQQAAARMLSFPPVVMAHTVAVFLSFDGELDTQPLIEQLWRAG
KRVYLPVLHPFSPGNLLFLHYHPQSALVTNRLKIREPRLDVRDVLPLAKLDVLVTPLVAF
DEGGQRLGMGGGFYDRTLQNWQQHKIQPVGYAHDCQLVEKLPVEEWDIPLPAVVTPSKIW
EW
>EECEBICD_00396 Cell division protein ZapA
MSAQPVDIQIFGRSLRVNCPPDQRDALNQAADDLNQRLQDLKVRTRVTNTEQLVFIAALN
ISYELTQEKAKTRDYAASMEQRIRMLQQTIEQALLDQGRITEKTGQNFE
>EECEBICD_00397 hypothetical protein

MSIQNEMPGYNEMNRFLNQQGAGLTPAEMHGLISGMICGGNNDSSWQPLLHDLTNEGLAF
GHELAQALRKMHAATSDALEDDGFLFQLYLPEGDDVSVFDRADALAGWVNHFLLGLGVTQ
PKLDKVTGETGEAIDDLRNIAQLGYDESEDQEELEMSLEEIIEYVRVAALLCHDTFTRQQ
PTAPEVRKPTLH
>EECEBICD_00398 Xaa-Pro aminopeptidase
MTQQEYQRRRQALLAQMQPGSAALIFAAPEATRSADSEYPYRQSSDFWYFTGFNEPEAVL
VLIKSDDTHNHSVLFNRVRDLTAEIWFGRRLGQDAAPEKLGVDRALAFSEINQQLFQLLN
GLDVVYHAQGEYAYADEIVLAALGKLRKGSRQNLTAPATMTDWRPIVHEMRLFKSPEEIA
VMRRAGEISALAHIRAMEKCRPGMFEYQLEGEIHHEFNRHGARYPSYNTIVGSGENGCIL
HYTENESEMRDGDLVLIDAGCEYKGYAGDITRTFPVNGKFTPAQREIYDIVLESLETSLR
LFRPGTSIQQVTGEVVRIMITGLVKLGILQGEVDQLIAENAHRPFFMHGLSHWLGLDVHD
VGVYGPDRSRILEPGMVLTVEPGLYIAPDADVPEAYRGIGVRIEDDIVITETGNENLTAG
VVKKADDIEALMAAARQQ
>EECEBICD_00399 2-octaprenyl-6-methoxyphenol hydroxylase
MSVIIVGGGMAGATLALAISQFSHGTLPVHLIEAKAPEADGHPGFDARAIALAAGTCQQL
ARIGVWQAISDCATAINTVHVSDRGHVGFVTLDAQDYSLAALGHVAELHDIGLRLFALLR
KAPGVTLHCPERVASVSRTQQQVNVTLENGNVIAGSVLVAANGTHSALASACGVDWHQEP
YEQLAVIANVATAIPHQGRAFERFTPNGPLAMLPMSHGRCSLVWCHPLDQRDEVLSWSDA
QFCHALQAAFGWRLGRITQAGKRSVYPLALTTAAKVFTHRTVMVGNAAQTLHPIAGQGFN
LGLRDVMSLAETLIAAQDAGEDIGAWHVLSRYQQRRQVDRDTTIGVTNGLVHLFANRWAP
LVIGRNIGLMAMELLPPARDALAQRTLGWVAR
>EECEBICD_00400 2-octaprenylphenol hydroxylase
MQSVDVAIVGGGMVGLAVACGLQGSGLRVAVLDHVVPQPLANDASPQLRVSAINAASEKL
LTRLGVWSNIVARRASCYHGMEVWDKDSFGRITFDDASMGYSHLGHIVENSVIHYALWQK
AQQAADITLMAPAELQQVAWGRTTPS
>EECEBICD_00401 2-octaprenylphenol hydroxylase
MLTARLVIAADGANSWLRNKADIPLTFWDYRHHALVATIRTEEPHGAVARQVFHGEGILA
FLPLSDPHLCSIVWSLSPQDAERMQQSGDEAFNQALTIAFDNRLGLCRLESERQVFPLTG
RYARQFAAHRLALVGDAAHTIHPLAGQGVNLGFMDAAELVDDLRRLQRQGKDIGQHLYLR
RYERSRKHSAAMMLAGMQGFRDLFAGENPAKKLLRDIGLKLADTLPGVKPQLLRQAMGLN
DLPQWLR
>EECEBICD_00402 Aminomethyltransferase
MAQQTPLYEQHTLCGARMVDFHGWMMPLHYGSQLDEHHAVRTDAGMFDVSHMTIVDLHGS
RTREFLRYLLANDVAKLTKTGKALYSGMLNASGGVIDDLIIYYFTEDFFRLVVNSATREK
DLSWITQHAEPYAIDITVRDDLSLIAVQGPNAQEKAATLFTDEQRHAVEGMKPFFGVQVG
DLFIATTGYTGEAGYEIAMPNEKAADFWRALVEAGVKPCGLGARDTLRLEAGMNLYGQEM
DEGISPLAANMGWTIAWEPADRDFIGREALEMQREKGHEQLVGLVMTEKGVLRNELPVRF
TDAQGNQQEGIITSGTFSPTLGYSIALARVPAGIGETAIVQIRNREMPVKVTKPVFVRNG
KAVA
>EECEBICD_00403 Glycine cleavage system H protein
MSNVPAELKYSKEHEWLRKEADGTYTVGITEHAQELLGDMVFVDLPEVGATVSAGDDCAV
AESVKAASDIYAPVSGEIVAVNDALSDSPELVNSEPYAGGWIFKIKASDESELESLLDAT
AYEALLEDE
>EECEBICD_00404 Glycine dehydrogenase (decarboxylating)
MTQTLSQLENRGAFIERHIGPDAAQQQEMLNAVGAESLNALTGQIVPKDIQLATPPQVGE
AATEYAALAELKAIAGRNKRFTSYIGMGYTAVQLPPVILRNMLENPGWYTAYTPYQPEVS
QGRLEALLNFQQVTLDLTGLDMASASLLDEATAAAEAMAMAKRVSKLKNANRFFVASDVH
PQTLDVVRTRAETFGFDVIVDDAAKALDHQDVFGVLLQQVGSTGEIHDYSALISELKARK
VIVSVAADFMALVLLTAPGKQGADIVFGSAQRFGVPMGYGGPHAAFFAAKDEFKRSMPGR
IIGVSKDAAGNTALRMAMQTREQHIRREKANSNICTSQVLLANIASLYAVYHGPVGLKRI
ANRIHRLTDILAAGLQQKGLKLRHAHYFDTLCVEVADKAAVLARAEAAEINLRSDIHNAV
GITLDETTTRENVAQLFNVLLGGSHGLNIETLDKDVALDSRSIQQSMLRDDAILTHPVFN
RYHSETEMMRYMHSLERKDLALNQAMIPLGSCTMKLNAAAEMIPITWPEFAELHPFCPPE
QAEGYHQMISQLSDWLVKLTGYDAVCMQPNSGAQGEYAGLLAIRHYHESRNEGHRDICLI

PASAHGTNPASAHMAGMQVVVVACDKNGNIDLDDLRAKAEQHAANLSCIMVTYPSTHGVY
EETIREVCEVVHQFGGQVYLDGANMNAQVGITSPGFIGADVSHLNLHKTFCIPHGGGGPG
MGPIGVKAHLAPFVPGHSVVQIEGMLTRQGAVSAAPFGSASILPISWMYIRMMGAEGLKQ
ASQVAILNANYIASRLKDAYPVLYTGRDGRVAHECILDIRPLKEETGISELDIAKRLIDY
GFHAPTMSFPVAGTLMVEPTESEGKAELDRFIDAMLAIRAEIDQVKAGVWPLEDNPLVNA
PHIQSELVAEWAHPYSREVAVFPAGVADKYWPTVKRLDDVYGDRNLFCSCVPISDYQ
>EECEBICD_00405 hypothetical protein
MKRFIFLPLMTYFLLMNAMAANGENNPSNLLGAPVNTAISGGSVLPEGKLLTAVNSSFRD
KDHQIEGHGSPDVYSQIWLLKIRYGLTDRLELSTVGSYINNKRDNLSPEHIEGMGDQSVG
VNYALMSQRRGDPFWVTVGGAVLLPTGQDGDNHLPGNSAWGGRVTLTLTKLFTPNIKGDM
GFVYQGPFERGNQDVKRGNEFQWNTQVRYMFSDLPLDLGLESAYSNNASGTKKLSNGSVI
NNHSGTTEWVVGPSFNIAVDSLKLWFGAGAFFPVMQEAKSPTKMEDVRWEFKIGKTW
>EECEBICD_00406 6-phospho-beta-glucosidase BglA
MRKLTLPKDFLWGGAVAAHQVEGGWNKDGKGPSICDVLTGGAHGVPREITQNVVAGKYYP
NHEAVDFYGHYKEDIRLFAEMGFKCFRTSIAWTRIFPNGDESQPNEAGLKFYDDMFDELL
KYNIEPVITLSHFEMPLHLVQHYGGWTNRKVVDFFVRFAEVVFERYKHKVKYWMTFNEIN
NQRNWRAPLFGYCCSGVVYTEHENPEETMYQVLHHQFVASALAVKAARRINPQMKVGCML
AMVALYPFSCKPEDVMFAQESMRERYVFTDVQLRGYYPSYVLNEWERRGFNIKMEDGDLE
VLREGTCDYLGFSYYMTNAVKAEGGSGDAISGFEGSVPNPYVKASDWGWQIDPVGLRYSL
CELYERYQKPLFIVENGFGAYDKVEEDGSINDDYRIDYLRAHIEEMKKAVTYDGVDLMGY
TPWGCIDCVSFTTGQYSKRYGFIYVNKHDDGTGDMSRSRKKSFNWYKEVIASNGEKL
>EECEBICD_00407 hypothetical protein
MQPNDITFFQRFQNDILAGRKTITIRDASESHFKAGDVLRVGRFEDDGYFCTIEVTGTST
VTLDTLNEKHAQQENMSLDELKRVIAEIYPNQTQFYVIDFKCL
>EECEBICD_00408 hypothetical protein
MVQKPLMTQGYSLAEEIANSISHGIGLVFGIVGLVLLLVQAVEANAGMTAIASYSLYGGS
MILLFLASTLYHAIPHQRAKIWLKKFDHCAIYLLIAGTYTPFLLVGLDSPLARGLMIVIW
SLALFGILFKLTIAHRFKVLSLVTYLAMGWLSLIVVYQLAIKLAIGGVTLLAVGGVVYSL
GVIFYVCKRIPYNHAIWHGFVLGGSVCHFLAIYLYVGQA
>EECEBICD_00409 hypothetical protein
MPVPDGGAVVPYPAYENSTCEPDGVALSGYPAFNVISLFQRVGQRDDMQGVGIVANAEDA
FGF
>EECEBICD_00410 tRNA-modifying protein YgfZ
MAFISFPPRHPSSSARLPLTLIALDDWALSTITGVDSEKYIQGQVTADVSQMTEQQHLLA
AHCDAKGKMWSTLRLFRERDGFAWIERRSVREAQLTELKKYAVFSKVVIAPDDERVLLGV
AGFQARAALANVFSELPNSENQVVRDGASTLLWFEHPAERFLLVTDVATANMLTEKLHGE
AELNNSQQWLALDIEAGIPVIDAANSGQFIPQATNLQALGGISFKKGCYTGQEMVARAKF
RGANKRALWLLAGKASRVPEAGEDLELQMGENWRRTGAILAATQLDDGQLLVQAVMNNDL
EAESVFRVRDDANTLHIVPLPYSLEE
>EECEBICD_00411 FAD assembly factor SdhE
MDIHNKARIHWACRRGMRELDISIMPFFEHEYDSLSDEEKRIFVRLLQSDDPDLFNWLMN
HGKPADAELEQMVRLIQTRNRERGPVAI
>EECEBICD_00412 Inner membrane protein YgfX
MVLWQSDLRVSWRAQWISLLIHGLVAAVILLMPWPLSYTPLWMILLSLVVFDCVRSQRRI
NTCQGEIKLLMDGRLRWQGQDWTLLRPPWLLKSGMVLRLRAESGRHQHLWLAADSMEEAE
WRELRRILLQQPISGQH
>EECEBICD_00413 Flavodoxin 2
MNMGLFYGSSTCYTEMAAEKIRDILGPELVTLHNLKDDAPALMEQYDVLILGIPTWDFGE
IQEDWEAVWEQLDDLNLEGKIVALYGMGDQLGYGEWFLDALGMLHDKLALKGVKFVGYWP
TEGYEFTSNKPVIADGQLFVGLALDETNQYDLSDERIQTWCEQILGEMAEHYA
>EECEBICD_00414 Tyrosine recombinase XerD
MEQDLARIEQFLDALWLERNLAENTLSAYRRDLSMVVAWLHHRGKTLATAQADDLQTLLA
ERVEGGYKATSSARLLSAMRRFFQHLYREKYREDDPSAQLASPKLPQRLPKDLSEAQVER
LLQAPLIDQPLELRDKAMLEVLYATGLRVSELVGLTMSDISLRQGVVRVIGKGNKERLVP

LGEEAVYWLETYLEHGRPWLLNGVSIDVLFPSQRAQQMTRQTFWHRIKHYAVLAGIDSEK
LSPHVLRHAFATHLLNHGADLRVVQMLLGHSDLSTTQIYTHVATERLRQLHQQHHPRA
>EECEBICD_00415 Thiol:disulfide interchange protein DsbC
MKKRFMMFTLLAAAFSGVAHADDAAIRQSLAKLGVQSTEIQASPVAGMKTVLTHSGVLYV
TDDGKHIIQGPMYDVSGAHPVNVTNKLLMSQLNALEKEMIVYKAPDEKHVITVFTDITCG
YCHKLHEEMKDYNALGITVRYLAFPRQGLESQAEQDMKSIWCAKDKNKAFDDAMAGKGVK
PASCDVNIADHYALGVQLGVSGTPAIVLSNGYVVPGYQGPKEMKAFLDEHQKQTSGK
>EECEBICD_00416 Single-stranded-DNA-specific exonuclease RecJ
MKGMLPWQQLSGIDNAVEILYNAFREGIRIIVVGDFDADGATSTALSVLGMRALGCDNIS
YLVPNRFEDGYGLSPEVVDQAKARGAQLIVTVDNGISSHAGVAHAKTLGIPVIVTDHHLP
GDTLPEAEAIINPNLRDCEFPSKSLAGVGVAFYLMLALRTFLRDKGWFDERNIAPPNLAE
LLDLVALGTVADVVPLDANNRILTWQGLSRIRAGKCRPGIKALLEISNRDPQQLAASDLG
FALGPRLNAAGRLDDMSVGVALLLCDNLGEARVLASELDALNQTRKEIEQGMQAEALILC
EKLERSSETLPGGLAMYHPEWHQGVVGILASRIKERFHRPVIAFAPAGDGTLKGSGRSIQ
GLHMRDALERLDTLYPDLMIKFGGHAMAAGLSLEEHKFEQFQQRFGELVTEWLDPALLQG
EVISDGPLSAAEMSMEVAQLLRDAGPWGQMFPEPLFDGRFRLLQQRLVGERHLKVMVEPV
GGGPLLDGIAFNIDTTCWPDNGVREVELAYKLDINEFRGNRSLQIIIDDIWPL
>EECEBICD_00417 Peptide chain release factor RF2
MTQGLDDVSGLLELAVEADDEETFNEAVAELNTLEEKLAQLEFRRMFSGEYDSADCYLDI
QAGSGGTEAQDWASMLLRMYLRWAEARGFKTEIIEESEGEVAGIKSATIKISGEYAYGWL
RTETGVHRLVRKSPFDSGGRRHTSFSSAFVYPEVDDDIDIDINPADLRIDVYRASGAGGQ
HVNRTESAVRITHIPTGIVTQCQNDRSQHKNKDQAMKQMKAKLYELEMQKKNAEKQAMED
TKSDIGWGSQIRSYVLDDSRIKDLRTGVETRNTQAVLDGSLDQFIEASLKAGL
>EECEBICD_00418 Lysine--tRNA ligase
MSEQNAQGADEVVDLNNEMKARREKLAALREQGIPFPNDFRRDRTSDQLHAEFDAKEAEE
LEALNIEVSVAGRMMTRRIMGKASFVTLQDVGGRIQLYVARDDLPEGVYNEQFKKWDLGD
ILGAKGKLFKTKTGELSIHCTELRLLTKALRPLPDKFHGLQDQEARYRQRYLDLISNDES
RNTFKTRSKILAGIRQFMVARGFMEVETPMMQVIPGGASARPFITHHNALDLDMYLRIAP
ELYLKRLVVGGFERVFEINRNFRNEGISVRHNPEFTMMELYMAYADYKDLIELTESLFRT
LAQDVLGTTQVPYGDEVFDFGKPFEKLTMREAIKKYRPETDMADLDNFDSAKAIAESIGI
HVEKSWGLGRIVTEIFDEVAEAHLIQPTFITEYPAEVSPLARRNDVNPEITDRFEFFIGG
REIGNGFSELNDAEDQAQRFLDQVNAKAAGDDEAMFYDEDYVTALEHGLPPTAGLGIGID
RMVMLFTNSHTIRDVILFPAMRPVK
>EECEBICD_00419 Isopentenyl-diphosphate Delta-isomerase
MTEEHVVLLDEQDKPSGTLEKYAAHTLNTPLHLAFSCWLFNEDGQLLVTRRSLSKKAWPG
VWTNSVCGHPQQGETTEEAIIRRCRFELGVEITDLTPVYPHFSYRATDPNGIVENEVCPV
FAARATSVLQVNSEEVMDYQWSEFKSVWKSLLATPWAFSPWMVMQASDEQARERLLNYCQ
R
>EECEBICD_00420 Murein hydrolase activator NlpD
MVLSAGRLNKNSLGIAVLLCTGLLLAGCSSNSGSGTYSGSVYTVKRGDTLYRISRATGTS
VKELARLNGISPPYTIEVGQRIKVRGSAKSSSSTRKTSNKTATKTAAVRPSSSVPKSSWP
PVGQRCWVWPANGKVILPYSTAEGGNKGIDIAGARGTPVYAAGAGKVVYVGNQLRGYGNL
IMIKHNEDYITAYAHNDTMLVNNGQSVKAGQKIATMGSTDAASVRLHFQIRYRATAIDPL
RYLPPQGSKPKC
>EECEBICD_00422 hypothetical protein
MKVNTWTILLMSAHLTACAVPGTEKYQTSMDSVTAEKISRIIQSDVIPYKGENHGEVISR
VSSAFLGTPYQADTLIGGPGIPEVLVANFNGVDCFTLADYVEALARSDNQKSFLHNLART
RYAAGKVAYLSRRHFFSDWFAATPRNARDVTPDISPDYVVVDKQLNHKPDGGEYIPGLGI
HPRKINYIPGRAINQQVMNHLKNGDYIGVYSPLDGLDVSHVGIVVRHDEQVWFRNASSLA
ANRKVVDTPFMEYMHSRPGIVVLRAE
>EECEBICD_00423 hypothetical protein
MQTMHKKHMCRWLLPGLLGLALCAPVPHTYAAIIEAGFYPEGTDLQLVLKIIETARQEIR
LMDYSFTSWEVVRFEHSVSTKFSMSK
>EECEBICD_00424 Antitoxin VapB

MHTTLFFSNRTQAVRLPKSISFPEDVKHVEIIAVGRSRIITPVGESWDSWFDGEGASTDF
MSTREQPAVQEREGF
>EECEBICD_00425 tRNA(fMet)-specific endonuclease VapC
MLKFMLDTNTCIFTIKNKPEHIRERFNLNTSRMCISSITLMELIYGAEKSLAPERNLAVV
EGFISRLEVLDYDTQAAIHTGQIRAELARKGTPVGPYDQMIAGHAGSRGLVVVTNNLREF
ERIPGIRIEDWC
>EECEBICD_00426 hypothetical protein
MPLLPDNCRPPPLHSRFLQLKSQTLIYQADMPSFPLYILHTSSCLCVGYGLLGPSMGLAL
TGRRTRRSNLLPAELSLTPVTELSMLPGTHSLATFKQLELFWVYISELLFLSVFIFQFIY
Y
>EECEBICD_00427 Attachment invasion locus protein
MNKMLLAGSAGIVLLSAAASPVWADDNASTFSLGYAQSHTNHAGTLRGVRLANNYEMSPD
WGLTTSFAWLNGSQRYSDESSNGRVTTRYYSLLAGPPGKKKNHLSLLSKGGPVLLHQRDH
GINESDSKVGYGYSAGVAYTPVSSVAITLGYEGADFDATHNSGSLNSNGFNLGVGYRF
>EECEBICD_00428 hypothetical protein
MKSHIMPGVALMVILFSGNIMAATNMTDNVTLNNDKISGQAWQAMRDIGMSRFELFNGRT
QKAEQLAAQAEKLLNDDSTDWNLYVKSDKKAPVEGDHYIRINSSITVAEDYLPAGQKNDA
INKANQKMKEGDKKGTIEALKLAGVSVIENQELIPLQQTRKDVTTALSLMNEGKYYQAGL
LLKSAQDGIVVDSQSVQESPTHSVQHDAAH
>EECEBICD_00429 putative fimbrial-like protein YbgD
MRNKIILAMAAAGMMYGASVYAVDPPTAGPFGSGKITFTGTITNSPCDIAPGDDAITVPF
GQISYRKLNTADATTESKPFTIHLQNCAFDPNTPDTAGSAGKMSKVTVSFSGAANTSKKA
YTTSGIAQHVGVQLLKSDNATIIDPNTPMPDGDAQQLQAGNNELNFFARLIALDNGVTPG
DFNASVTYTLKYL
>EECEBICD_00430 Outer membrane usher protein PapC
MKITRLAILITLTFSVLKSQATEFNASLLDSGNLSNVDLTAFSREGYVAPGNYILDIWLN
DQPVREQYPVRVVPVAGRDAAVICVTTDMVAMLGLKDKIIHGLKPVTGIPDGQCLELRSA
DSQVRYSAENQRLTFIIPQAWMRYQDPDWVPPSRWSDGVTAGLLDYSLMVNRYMPQQGET
STSYSLYGTAGFNLGAWRLRSDYQYSRFDSGQGASQSDFYLPQTYLFRALPALRSKLTLG
QTYLSSAIFDSFRFAGLTLASDERMLPPSLQGYAPKISGIANSNAQVTVSQNGRILYQTR
VSPGPFELPDLSQNISGNLDVSVRESDGSVRTWQVNTASVPFMARQGQVRYKVAAGRPLY
GGTHNNSTVSPDFLLGEATWGAFNNTSLYGGLIASTGDYQSAALGIGQNMGLLGALSADV
TRSDARLPHGQKQSGYSYRINYAKTFDKTGSTLAFVGYRFSDRHFLSMPEYLQRRTTDGG
DAWHEKQSYTVTYSQSVPVLNMSAALSVSRLNYWNAQSNNNYMLSLNKVFSLGDLQGLSA
SVSFARNQYTGGGSQNQVYATISIPWGDSRQVSYSVQKDNRGGLQQTVNYSDFHNPDTTW
NISAGHNRYDTGSSFSGSVQSRLPWGQAAADATLQPGQYRSLGLSWYGSVTATAHGAAFS
QSMAGNEPRMMIDTGDVAGVPVNGNSGVTNRFGVGVVSAGSSYRRSDISVDVAALPEDVD
VSSSVISQVLTEGAVGYRKIDASQGEQVLGHIRLADGASPPFGALVVSGKTGRTAGMVGD
GGLAYLTGLSGEDRRTLNVSWDGRVQCRLTLPETVTLSRGPLLLPCR
>EECEBICD_00431 Chaperone protein PapD
MKNSRRSRVILLALAAAWSQYSPAAVNVDRTRIIMDAPQKTVAITLNNDDKTTPFLAQSW
VTDADGVRTDALMALPPLQRIDAGQKSQVRITQVRGLTDKLPQDRETLFWFNVRGVPPKP
EDDNVLQLAMQSQLKLFYRPKAIIRSSSDQPERKLTAERNAGHLTLRNPTPYYITVAWLG
ADRSHRLSGFREGVMVPPLGNLPLKAVLPAETRTLWVGYIDDYGGLQMNRYTCDALNCAF
KDAGATS
>EECEBICD_00432 hypothetical protein
MTEWIFNLKTKLTVLVMMLCSLCVTKVYAVELGINECAVTSGQNINLRSINLTTDDFKPG
TDSVIYTINQDAVFKCYMGYDTQFPQLVFNQGYFSKFTKTLDAMGLGFRMSIKETGNASS
AVSFSWDEIKSTQSGNELRKEFGTKLPVGTTERKVRITLDFLYTKAYSESSAVTAFTGIS
NVLNIVPFSYSLRQNGFILSGFNVRILRNGLGKVDIVPLQVNFGHIYTTYEPSQTRQANF
TVIATQVLRPAMGQEFTIPLAITFGKGALTQDTGQTLNLVSLDGPNKGQPNGLRLSIKDD
KGKEITFDKQEVLGDITITGAVTGNVSKVYTAVITPTPGGSVKTGTFSAAIPVTVTYN
>EECEBICD_00433 hypothetical protein
MCPDNTHAKKQYLTPGNDIHYPGQTNHDACFIPESVRQYADEPLYIIVAHWCLLQQNWVQ

RNQIAEAFHITARRASYLIAYLRSKTSRVVSICRHQTLPNKARRYEIYVIRVLDSPTPST
RREKAGPPLVSKRRVGNGDRSMANELWNRLCSNRNAGKILKKKEDEDDGT
>EECEBICD_00434 hypothetical protein
MTEHNRMPARQIIVYGDCWPVTIAVAHLVRRFLPGCNCETAYSLPVLLQQLRRKPEAILI
LCLRPREHLFLFYSLRQILPDYPVMIISDELFFSDRVVLKVYGGIPALLEQELAEILIRW
RRDEQWAGGARLRRTGGLDAFLLSPDPVTGFLEVPPIFNNPKRLMNYMDQLMHREILACG
VSLAQLRLLQEVYRGRGRLSALCGRLNTQEKQIWQDKYRLLVKLGMRNRLRELLFGTRFC
KSLQRTPFIAPQ
>EECEBICD_00435 Nickel/cobalt efflux system RcnA
MGEFSTLLQQGNGWFFIPSAILLGILHGLEPGHSKTMMAAFIIAIKGTVKQAVMLGLAAT
LSHTAIVWLIALGGMYLSRAFTAQSVEPWLQLISAIIILSTACWMFWRTWRGEQQWLAGN
HHHDHDHDHDHDHDHDHDHDHDHHGHIHPEGATSKAYQDAHERAHAADIQRRFDGQTVTN
GQILLFGLTGGLIPCPAAITVLLICIQLKAFTLGATMVLSFSLGLALTLVTVGVGAAISV
QQAAKRWSGFSTLARRAPYFSSILIGLVGVYMGIHGYTGIMQ
>EECEBICD_00436 Transcriptional repressor RcnR
MSHTIRDKQKLKARTSKIQGQVIALKKMLDEPHECAAVLQQIAAIRGAVNGLMREVIKGH
LTEHIVHQSDEARREEDLDVILKVLDSYIK
>EECEBICD_00437 Inner membrane transport protein YqeG
MSKIWSKDETLWSFALYGTAVGAGTLFLPIQLGSAGAIVLFITALVAWPLTYWPHKALCQ
FILSSKTSAGEGITGAVTHYYGKKIGSIITTLYFIAFFVVVLIYAVAITNSLTEQLAKHI
QIDIRIRMAVSFGVVLILNMIFLMGRHATLRVMGFLVFPLIAYFLFLSLYLTGSWQPSLL
TGQMSLDSHTLHQVWISIPVMVFAFSHTPIISTFAIDRRENFGDQAMDKCKKIMKVAYLI
ICLSVLFFVFSCLLSIPPSYIEDARNEGVTILSALSMMPNAPAWLSISGIIVAVVAMSKS
FLGTYFGVIEGATEMVRTTLQQVGVKKSRAFNRALSIMLVSGITFIICCINPNAISMIYA
ISGPLIAMILFIMPTLSTYLIPALNPYRSVGNFITLVVGLLCVSVMFFG
>EECEBICD_00438 hypothetical protein
MQHDAIQRRSLPERIFHAVCFEGIATAILAPTTAWLMQRSVLEMGGLTILLATTAMIWNI
IYNALFDRLWPAHQVRRTAKVRALHALGFESGFIVIGVSIVAWVLNVSLLQAFTLEIGFF
LFFLPYTMLYNWAYDVLRQRIVTRRQQRVSA
>EECEBICD_00439 HTH-type transcriptional activator AllS
MRYSPEALMAFVETVSCGSFSAAARRLRKSQSTISTAIAHLEADLGFSLFDRSSRQPVLT
EEGKRVLSYVQAILSASDRLDEVALTLSGETEARLTFVLSDTLHPDSLEELMAQFDRQFP
HTEFECLIGENDDVIDLLQKGRAQIGLIEARDDYPTDIGATRLPVQSWMGVYVATTHPLA
SQTNLQWEQLHTWRELRLNTYLESNANITRGPAWSAPNYLLLLSMAVQGFGWCVLPCALV
EEFAAEKPLAQLEVPGWPRAIAIDLLWNKKTPPGTAGNWLRYHLQQK
>EECEBICD_00440 Acetyl-CoA acetyltransferase
MKEVVIVGALRTPIGCFQGTLARHSAVELGSMVVKALIERTGVDANAIDEVILGQVLTAG
AGQNPARQSAIKGGLPTTVSAITINDVCGSGLKALHLATQAIQCGEADIVIAGGQENMSR
APHVLNDSRTGALPDADNLVDSLVHDGLWDAFNDYHIGVTAENLAREYGISRELQDAYAL
SSQQKARAAIDTGRFKDEIVPIVTQRNGQTAIVDTDEQPRADASAEGLALLHPAFDSLGS
VTAGNASSINDGAAAVMMMSEAKAQALGLPVLARIRAFASVGVDPALMGIAPVYATRRCL
ERAGWQLTEVDLIEANEAFAAQALSVGKMLEWDERRVNVNGGAIALGHPIGASGCRILVS
LVHEMVKRDARKGLATLCIGGGQGVALTIERD
>EECEBICD_00441 4-deoxy-L-threo-5-hexosulose-uronate ketol-isomerase
MMPLNKSKQCFEFYAIAIDLSLEGKVDVRQSIHSEHAKTLDTQALRREFLIENIFVADEY
TMVYSHIDRIIVGGIMPVSHSVEIGGEVGKQLGVSRLLDRRELGVINIGGAGAIIVDGQR
HDIGHRDALYIGKGAKELVFVSNEASRPAKFYYNCAPAHTAYPTKKVSPADVAPVTLGDN
LTSNRRTINKYFVPDVLETCQLSMGLTELAPGNLWNTMPCHTHERRMEVYLYFNMEEDSC
VFHMMGQPQETRHIVMRNEQAVISPSWSIHSGVGTKAYTFIWGMVGENQVFDDMDHVAVQ
DLRQSRPVITPVRDRHDKNKD
>EECEBICD_00442 2-dehydro-3-deoxy-D-gluconate 5-dehydrogenase
MILNAFSLEGKVAVVTGCDTGLGQGMALGLAEAGCDIVGINIVEPTETIARVTALGRRFL
SLTADLRQIDGIPALLERAVAEYGHIDILVNNAGLIRREDAIDFTEKDWDDVMNLNIKSV
FFMSQAAAKHFIAQGNGGKIINIASMLSFQGGIRVPSYTASKSGVMGITRLMANEWAKHN

INVNAIAPGYMSTNNTQQLRADAQRSAEILDRIPAGRWGLPGDLMGPVVFLASSAADYVN
GYTIAVDGGWLAR
>EECEBICD_00443 Arabinose-proton symporter
MVSINHDSALTPRSLRDTRRMNMFVSVSAAVAGLLFGLDIGVIAGALPFITDHFVLTSRL
QEWVVSSMMLGAAIGALFNGWLSFRLGRKYSLMAGAILFVLGSLGSAFASSVEVLIGARV
ILGVAVGIASYTAPLYLSEMASENVRGKMISMYQLMVTLGIVLAFLSDTAFSYSGNWRAM
LGVLALPAVLLIILVVFLPNSPRWLAQKGRHIEAEEVLRMLRDTSEKARDELNEIRESLK
LKQGGWALFKANRNVRRAVFLGMLLQAMQQFTGMNIIMYYAPRIFKMAGFTTTEQQMIAT
LVVGLTFMFATFIAVFTVDKAGRKPALKIGFSVMALGTLVLGYCLMQFDNGTASSGLSWL
SVGMTMMCIAGYAMSAAPVVWILCSEIQPLKCRDFGITCSTTTNWVSNMIIGATFLTLLD
SIGAAGTFWLYTALNIAFIGITFWLIPETKNVTLEHIERKLMAGEKLRNIGV
>EECEBICD_00444 L-aspartate/glutamate-specific racemase
MKTIGLLGGMSWESTIPYYRLINEGIKQQLGGLHSASLLLHSVDFHDIEVCQRRGEWDKA
GDILAQAAQGLQQAGAEGIVLCTNTMHKIAHVIESRCSLPFLHIADATGRAIARQGLHRV
ALLGTRYTMEQDFYRGRLEQQFAIETIVPEADDRARINQVIFDELCQGTFTDASRHYYLQ
VIEQLAAQGAQGVIFGCTEIGLLVPQTQSALPVFDTTAIHAADAVSFMLSSSAPE
>EECEBICD_00445 Octopine catabolism/uptake operon regulatory protein OccR
MAAVNLRHIEIFHAVMTAGNLTEAARLLHTSQPTVSRELARFEKVLGLTLFERTRGRLHP
TVQGLRLFEEVQRSWYGLDRIVSAAESLREFRQGELSIACLPVFSQSFLPPLLPPFLARY
PDVSLNIVPQESPLLEEWLSAQRHDLGLTETLHAPAGTTRTELLTLNEVCVLPCDHPLAA
KTVLTPDDFQGENFISLSRLDSYRQLLDTLFAEHQVKRRMVVETHSAASVCAMVRAGAGV
SIVNPLTALGYAASGVTVRRFSIDVPFTVSLIRPLHRPASALVDAFSKHLQTHLSRLVEP
LEVILGPMTKA
>EECEBICD_00446 Diaminopimelate decarboxylase
MSLPHYYAETDLNAENLLRLPAEFGCPVWVYDAHIIRRQIAALQQFDVVRFAQKACSNIH
ILRLMRAQGVKVDSVSLGEIERALAAGYDPQTCPDDIVFTADVIDAATLARVSELHIPVN
AGSVDMLTQLGQVSPGHRVWLRVNPGFGHGHSQKTNTGGENSKHGIWHSDLPAALAVMQK
YRLKLVGIHMHIGSGVDYGHLEQVCGAMVRQVLECGQDVEAISAGGGLSIPYREGEEPVD
TRHYYGLWNAAREQIARHLGHAVKLEIEPGRFLVAQSGVLVTQVRSVKQMGSRHFVLVDA
GFNDLMRPAMYGSYHRISALAADGRALENGPWVETVVAGPLCESGDVFTQQEGGMVETRA
LPAVIPGDYLVLHDTGAYGASMSSNYNSRPLLPEVLFDNGQARLIRRRQTIEELLALEML
>EECEBICD_00447 HTH-type transcriptional regulator AscG
MATMLDVSRHAGVSKATVSRVLNGTGQVKESTRQKVFTAMQALGYRPNLLARSLANRTSN
SIGLVVSTFDGFYFGSLLRRASRQAEFHNKQLIVTDGHDTPEREQKAVQMLADRQCDAII
LYTRYMDEPAILSLIDATEMPLVIINRNVTQARDRAIFFEQETAAFQAVEYLITQGHRDI
ACITLPVHTPTGTSRVAGYRKALEKYGIPWQPAKVKYGDYTLTRGYDACRELLEEGVTFS
ALFACNDDTALGAAKALRQAGLRIPQDVSLFGFDDAPGATWLEPGLSTVYLPIEDMIATA
IDQAVRLANSEPVAPIPPFTGTLILRESVAAGPFFQRPA
>EECEBICD_00448 HTH-type transcriptional regulator GalR
MATIKDVARLAGVSVATVSRVINDSPKASEASRLAVTSAMESLSYHPNANARALAQQATE
TLGLVVGDVSDPFFGAMVKAVEQVAYHTGNFLLIGNGYHNEQKERQAIEQLIRHRCAALV
VHAKMIPDADLASLMKQIPGMVLINRILPGLEHRCVALDDRYGAWLATRHLIQQGHTRIG
YICSNHTISDAEDRLRGYYDALAESHIPANDRLVTFGEPDESGGEQAMTELLGRGRHFTA
VACYNDSMAAGAMGVLNDNGVGVPGEVSLIGFDDVLVSRYVRPRLTTIRYPIVTMATQAA
ELALALAGKCPTPEVTHVFSPTLVRRHSVSTPTDTGHLSTTD
>EECEBICD_00449 Bifunctional protein Aas
MLFGFFRNLFRVLYRVRVTGDVRALQGNRVLITPNHVSFIDGMLLALFLPVRPVFAVYTS
ISQQWYMRWLTPLIDFVLLDPTKPMSIKHLVRLVEQGRPVVIFPEGRISVTGSLMKIYDG
AGFVAAKSGATVIPLRIDGAELTPFSRLKGLVKRRLFPRIQLHILPPTQIPMPEAPRARD
RRKIAGEMLHQIMMEARMAVRPRETLYESLLAAQYRYGAGKNCIEDINFTPDTYRKLLTK
TLFVGRILEKYSVEGEKIGLMLPNAAISAAVIFGAVSRRRIPAMMNYTAGVKGLTSAITA
AEIKTIFTSRQFLDKGKLWHLPEQLTQVRWVYLEDLKADVTPADKLWIFAHLLAPRLAQV
KQQPEDAAIILFTSGSEGHPKGVVHSHKSILANVEQIKTIADFTANDRFMSALPLFHSFG
LTVGLFTPLLTGAEVFLYPSPLHYRIVPELVYDRNCTVLFGTSTFLGNYARFANPYDFYR

LRYVVAGAEKLQESTKQLWQDKFGLRILEGYGVTECAPVVSINVPMAAKPGTVGRILPGM
DARLLAVPGIENGGRLQLKGPNIMNGYLRVEKPGVLEVPSAENARGETERGWYDTGDIVR
FDENGFVQIQGRAKRFAKIAGEMVSLEMVEQLALGVSADKMHATAIKSDASKGEALVLFT
TDSELTREKLQHYAREHGIPELAVPRDIRYLKQLPLLGSGKPDFVTLKSWVDAPEQHHE
>EECEBICD_00450 Lysophospholipid transporter LplT
MSESVRTNTSIWSKGMLSVIVAQFLSAFGDNALLFATLALLKAQFYPDWSQPVLQMVFVG
AYILFAPFVGQMADSFAKGRVMMVANGLKLAGAADICLGVNPFVGYTLVGIGAAAYSPAK
YGILGELTTGDKLVKANGLMEASTIAAILLGSVAGGVLADWHVIAALVACALAYAGAVAA
NLFIPKLVAARPGQSWRLSAMTRSFFSACVVLWRNGETRFSLVGTGLFWGAGVTLRFLLV
LWVPVALGITDNATPTYLNAMVAVGIVVGAGAAAKLVTLETVSRCMPAGILIGVVVAIFS
LQHALLPAYALLLLIGMLGGFFVVPLNALLQERGKKSVGAGNAIAVQNLGENSAMLLMLG
LYSLAVLVGVPAVAIGIGFGVLFALAIAALWIWQRRQASY
>EECEBICD_00451 Protein tas
MHYHRIPHSSLEVSTLGLGTMTFGEQNSEADAHAQLDYAIANGINLIDAAEMYPVPPRPE
TQGLTESYIGNWLAKRGNREKLIIASKVSGPARNNDQGIRPHQALDRKNIREALHDSLTR
LQTDYLDLYQVHWPQRPTNCFGKLGYNWTDSTPVVSLLETLDALSEFQRAGKIRYIGVSN
ETAFGVMRYLHLAEKHDLPRIVTIQNPYSLLNRSYEVGLAEVSQYEGVELLAYSCLAFGT
LTGKYLNGAKPAGARNTLFSRFTRYSGEQAQKAVAAYVDIAKRHNLDPAQMALAFVRRQP
FVASTLLGATTMAQLKTNVESLHLTLSEEVLAEIEAAHQVYTYPAP
>EECEBICD_00452 putative lipoprotein YgdR
MKKWAVVISAVGLAFAVSGCSSDYVMATKDGRMILTDGKPQVDDDTGLVSYTDAQGNEMQ
INRDEVSQIIER
>EECEBICD_00453 hypothetical protein
MLFAWITDPNAWLALGTLTLLEIVLGIDNIIFLSLVVAKLPTAQRNHARRLGLAAAMVMR
LALLASIAWVTRLTNPLFELFGEAISARDLILLLGGLFLIWKASKEIHESIEGEEEGLKT
RVSSFLGAIVQIMLLDIIFSLDSVITAVGLSDHLFIMMAAVVIAVGVMMFAARPIGEFVD
RHPSVKMLALSFLILVGFTLILESFDVHVPKGYIYFAMFFSIAVESLNLLRNKKNPL
>EECEBICD_00454 DNA mismatch repair protein MutH
MSALCPLLTPPASEALLLAQARQLSGYTLGELAAMAGITTPKDLKRDKGWIGVLLEIWLG
ASAGSKPEQDFAALGVELKTIPVDSLGRPLETTFVCVAPLTGNSGVTWETSHVRHKLKRV
LWVPVEGDRSIPLAERRVGSPLLWSPSEEEDRQLRLDWEELMDMIVLGQVERITARHGEV
LQLRPKAANARALTEAIGARGEPILTLPRGFYLKKFYAGAAGSPFFIAESVTSITRKCAS
AMATRRTDAR
>EECEBICD_00455 RNA pyrophosphohydrolase
MIDDDGYRPNVGIVICNRQGQVMWARRFGQHSWQFPQGGINPGESAEQAMYRELFEEVGL
SRKDVRILASTRNWLRYKLPKRLVRWDTKPVCIGQKQKWFLLQLMSADAEINMQTSSTPE
FDGWRWVSYWYPVRQVVSFKRDVYRRVMKEFASVVMALQDNPPKLQSAPAYRRKRG
>EECEBICD_00456 Phosphoenolpyruvate-dependent phosphotransferase system
MLTRLREIVEKVASAPRLNEALNILVTDICLAMDTEVCSVYLADHDRRCYYLMATRGLKK
PRGRTVALAFDEGIVGLVGRLAEPINLADAQKHPSFKYIPSVKEERFRAFLGVPIIQRRQ
LLGVLVVQQRELRQYDESEESFLVTLATQMAAILSQSQVTALFGQYRQTRIRALPAAPGV
AIATGWQDATMPLMEQVYEASTLDTSLERERLTGALEEAANEFRRYSKRFAAGAQKETAA
IFDLYSHLLSDARLRRELFAEVDKGAVAEWAVKKIIEKFAEQFAALTDNYLKERAGDLRT
LGQRLLFHLDDSVQGPNAWPERFILVADELSATTLAELPQDRLAGVVVRDGAANSHAAIM
VRALGIPTVMGADIQPSVLHRRTLVVDGYRGELLVDPEPVLIQEYQRLISEEIELSRLAE
DDVNLPAQLKSGERIKVMLNAGLSPEHEEKLGSRIDGIGLYRTEIPFMLQSGFPSEEEQV
AQYQGMLQMFNDKPVTLRTLDVGADKQLPYMPISEENPCLGWRGIRITLDQPEIFLIQVR
AMLHANAATGNLSILLPMVTSIDEVDEARRLIERAGREVEEMIGYAIPKPRIGIMLEVPS
MVFMLPHLANRIDFISVGTNDLTQYILAVDRNNTRVASIYDSLHPAMLRALSMIAQEAEK
NGLDLRLCGEMAGDPMCVAILIGLGYRHLSMNGRSVARVKYLLRHIDFEDAQTLARRSLE
AQMATEVRHQVAAFMERRGMGGLIRGGL
>EECEBICD_00457 Phosphatidylglycerol--prolipoprotein diacylglyceryl
transferase
MTSSYLHFPDFDPVIFSIGPVALHWYGLMYLVGFVFAMWLAVRRANRPGSGWTKNEVENL

LYAGFLGVFLGGRIGYVLFYNFPLFLDNPLYLFRVWDGGMSFHGGLIGVILVMIIFARRT
KRSFFQVSDFIAPLIPFGLGAGRLGNFINGELWGRVDPDFRFAMLFPGSRAEDIALLPSH
PQWQPIFDTYGVLPRHPSQLYELALEGVVLFIILNLFIRKPRPMGAVSGLFLIGYGAFRI
IVEFFRQPDAQFTGAWVQYISMGQILSIPMIIAGAIMMVWAYRRRPQQHVS
>EECEBICD_00458 Thymidylate synthase
MKQYLELMQKVLDEGTQKNDRTGTGTLSIFGHQMRFNLQEGFPLVTTKRCHLRSIIHELL
WFLQGDTNIAYLHENNVTIWDEWADENGDLGPVYGKQWRAWPTPDGRHIDQIATVLSQLK
NDPDSRRIIVSAWNVGELDKMALAPCHAFFQFYVADGKLSCQLYQRSCDVFLGLPFNIAS
YALLVHMMAQQCDLDVGDFVWTGGDTHLYSNHMEQTHLQLSREPRALPKLVIKRKPDSLF
DYRFDDFEIEGYDPHPGIKAPVAI
>EECEBICD_00459 hypothetical protein
MKKQQGYTLIETVVAMSLIIILSATGLYGWQRWQQQQRLWQTACQVRDYLLQLREDAHWH
NRDHILRVVSEGGSWCFVSSVATQTSCTPSSPFVFTPDWRDVVLADITPSLAFFGLRNTA
WAGHIVLKNAAGEWRLVVSSWGRIRLCERNEAATCQ
>EECEBICD_00460 hypothetical protein
MSITTRGFSLLEVVLAMAIGSILLLGSARFLPALQREIWHNTRQLSLEDEIWQRTYTVAK
HLQRAGYCHGHCIGEGLHLAEQGQCVIVQWDGNNNGVWDTIPAKEADQTGFRVKDNVLET
LRGATSCQGKGWEKMTDPNTVLITAFTVERRDITGFSPVLMLHLRGASKAEPQTVIDAQY
SVTGFNL
>EECEBICD_00461 hypothetical protein
MNRERGASSLILALLILILGSLLLQGVNQQQASYATRVTTQSLAIQRQALVQSALEWGRG
QLWSGVTEMECRRYSSSGARVCLRRLPGDEVVMAAQDDSMTLWRLGNVIQGSIVFSPHGW
SDFCPLKEVALCRIP
>EECEBICD_00462 hypothetical protein
MSHPLNLQRGFSLPEVLVAMVLMVMIVTALSGYQRVLMHSFALRHQYLQIWRQAWQQTAL
YPFSPAEGWKANRMQTTQSGCVSISVTMVSPSGRQGQMTRLHCPNR
>EECEBICD_00463 RecBCD enzyme subunit RecC
MLRVYHSNRLDVLEALMEFIVERERLDDPFEPEMILVQSTGMAQWLQMTLSQKFGIAANI
AFPLPASFIWEMFVRVLPDIPKESAFSKQSMSWKLMTLLPQLLDKDEFVLLRHYLTDDTD
KRKLFQLSARAADLFDQYLVYRPDWLTQWEAGKTVEGLGEAQNWQAPLWKALVEYTAALG
QPRWHRANLYQRFIQTLESATACPPGLPSRVFICGISALPPVYLRALQALGKHIEIHLLF
TNPCRYYWGDIKDPAWLAKLMARQRRHSFEDRHLPLFRENQNPEALFNSDGEQDIGNPLL
ASWGKLGRDYIYLLSELENSQELDAFVDITPDNLLHRIQADILELESHAVAGVNLEEYSR
SDNKRLLDPEDNSLSFHVCHSPQREVEILHDRLLAMLEADPTLTPRDIIVMVADIDSYSP
FIQAVFGSAPTERYLPYAISDRRARQSHPVLQAFISLLSLPDSRFVSEDVLALLDVPVLA
ARFTINEEGLRYLRLWVNESGIRWGIDDDNVRELELPATGQHTWQFGLTRMLLGYAMESA
QGEWQSVLPYDESSGLIAELVGHLASLLMQLNIWRRGLAQERPLEEWLPVCRDMLNDFFL
PDADTEAAMTLIEQQWQAIIAEGVAAEYGDAVPISLLRDELAQRLDQERISQRFLAGPIN
ICTLMPMRSIPFRVVCLLGMNDGVYPRQLAPLGFDLMSQKPMRGDRSRRDDDRYLFLEAL
ISAQQTLYISYIGRSIQDNSERFPSVLVQELVDYIGQSHYLPGDETLTCDESEARVKAHI
TRLHTRMPFDAQNYQPGEQQSYAREWLPAASQSGKAHSDFVQPLPFTMPETLTLESLQRF
WAHPVRAFFQMRLQVNFRSEESEIPDAEPFELEGLTRYQLNQQLLNTLVEEDDAERLFRR
FRAAGELPYGAFGEIFWDAQCQEMQQLASRVIACRKPSQSLEVDLLCNGVQLTGWLPQVQ
EDGLLRWRPALISVAQGVQLWLEHLVYCASGGSGESRLFLRKEGEWRFPPLDKTQAMAYL
AQLIEGYREGMSSPLLVLPESGGAWIKACYDAENDVMLDDDDTLQKARTKFLQAYEGNMM
VSGEGEDIWYQRLWRQLESETLQAIIAQSRHYLLPLFRFNQSR
>EECEBICD_00464 Protease 3
MPRSTWFKALLLLVALWGPAVQADIGWQPLQETIRKSDKDTRQYQAIRLDNDMVVLLVSD
PQAVKSLSALVVPVGSLEDPEAHQGLAHYLEHMCLMGSKKYPQADSLAEYLKRHGGSHNA
STAPYRTAFYLEVENDALPGAVDRLADAIAAPLLNKKYAERERNAVNAELTMARTRDGMR
MAQVSAETINPAHPGSHFSGGNLETLSDKPGNPVQQALIAFHEKYYSSNLMKAVIYSNKP
LPELASIAAATYGRVPNKQIKKPEITVPVITEAQKGIIIHYVPALPRKVLRVEFRIDNNS
AQFRSKTDELVSYLIGNRSPGTLSDWLQKQGLVEGISADSDPIVNGNSGVFAISATLTDK
GLANRDEVVAAIFSYLNTLREKGIDKRYFDELAHVLDLDFRYPSITRDMDYVEWLADTMI

RVPVAHTLDAANIADRYDPAAIKNRLAMMTPQNARIWYISPQEPHNKIAYFVDAPYQVDK
ISEQTFKNWQQKAQGIALSLPELNPYIPDDFSLVKNDKNYVRPELIVDKADLRVVYAPSR
YFASEPKADVSVVLRNPQAMDSARNQVLFALNDYLAGMALDQLSNQAAVGGISFSTNANN
GLMVTANGYTQRLPQLFLALLEGYFSYDATEEQLAQAKSWYTQMMDSAEKGKAYEQAIMP
VQMISQVPYFSRDERRALLPSITLKEVMAYRNALKTGARPEFLVIGNMSEAQATSLAQDV
QKQLAANGSAWCRNKDVVVEKKQSVIFEKAGSSTDSALAAVFVPVGYDEYVSAAYSAMLG
QIVQPWFYNQLRTEEQLGYAVFAFPMSVGRQWGMGFLLQSNDKQPSYLWQRYQAFFPDAE
AKLRAMKPEEFAQIQQAIITQMRQAPQTLGEEASRLSKDFDRGNMRFDSRDKIIAQIKLL
TPQKLADFFHQAVVEPQGMAILSQIAGSQNGKAEYVHPTGWKVWDNVSALQQTLPLMSEK
NE
>EECEBICD_00465 RecBCD enzyme subunit RecB
MNDVAETLDPLRLPLTGERLIEASAGTGKTFTIAALYLRLLLGLGGSTAFPRPLTVEELL
VVTFTEAATEELRGRIRSNIHELRIACLRESTDNPLYARLLEEISDKKQAAQWLLLAERQ
MDEAAVFTIHGFCQRMLSLNAFESGMLFEQQLIEDESLLRYQACADFWRRHCYPLPRDIA
QVVFDVWKGPKALLKDIDRYLQGEAPVIKAPPSQEETLASRHEQILARINQVKQQWCEAV
SELDALIESSGIDRRKFNRGNQAKWIEKITAWAQEETKNYQLPEALGKFSQRFLAERTKA
GGVTPQHPLFVAIDNLLGEPLSIKDLVLTRALSEIRETVAQEKRRRGELGFDDMLSRLDT
ALRSESGEALAAAIRTRFPVAMIDEFQDTDPQQYRIFRRIWRHQPDTALLLIGDPKQAIY
AFRGADIFTYMKARSEVSAHYTLDTNWRSAPGMVNSVNKLFSQMNDAFMFRDIPFSPVKF
APRNQSLQFKVNDAPQPAMTLWLMEGESCGSGDYQSYMAQVCATQIRDWLRAGQTGDALL
TNGDSSRPVRASDISVLVRSRREAALIRDALTLLAIPSVYLSNRDSVFETLEAQEMLWVL
QAVMAPERENTLRSALATSMMGLTALDIETLNNDENAWDAVVEEFDGYRQIWHKRGVMPM
LRALMSARNIAENLLATAGGERRLTDILHISELLQEAGSQLESEHALVRWLAQHILEPDS
NASSQQLRLESDKHLVQIVTIHKSKGLEYPLVWLPFITHFRVQDQAFYHDRHSYEAVLDL
SHAEESIALAEAERLAEDLRLLYVALTRAVWHCSLGVAPLVRRRSDKKGETDVHQSALGR
LLQKGEPMDAAGLRACIEALCDEDIVCRTPGNTDNDRWQIAAASHAELSARTLQRLLYDS
WRVTSYSGLQQRGHSVAQDLIPRLDIDAAGVGEAAEAPAMTPHHFPRGASPGTFLHNLFE
ELDFTQPINPQWVQEKLELSGFETRWEPVLTRWLDTVLHVPLNETGVSLSVLTEREKQVE
MEFYLPIAQPLTAGELDALIRRYDPLSAGCPALDFMQVRGMLKGFIDLVFRYEGRYYLLD
YKSNWLGEDSAAYTQAAMATAMQAHRYDLQYQLYTLALHRYLRHRMANYDYERHFGGVIY
LFLRGVDSERPQQGIFTTRPAAALINQLDDMFAGEMSEEAQ
>EECEBICD_00466 RecBCD enzyme subunit RecD
MTIQERLLEAVEQKLLRPIDAQFALTVAGNDDPAVTLAAALLSHDAGEGHVCLPLSRLTL
TEEAHPLLVACISETATPIDWKKRLLASAAVSCGDSPAPLILCGERLYLNRMWCNERTVA
RFFNEVNQAIAVDEDQLSRILDALFPPTDEVNWQKVAAAVALTRRISVISGGPGTGKTTT
VAKLLAALIQMADGERCRIRLAAPTGKAAARLTESLGAALRQLPLTDAQKKRIPEDASTL
HRLLGAQPGSQRLRHHAGNPLHLDVLVVDEASMIDLPMMSRLIDALPPHGRVIFLGDRDQ
LASVEAGAVLGDICAYVNAGFTAERARQLSRLTGSAIPAGAGTQAASLRDSLCLLQKSYR
FGSDSGIGKLAAAINCGDRSAIQAVFQQGFSDIEKRTLQSSDDYAGMLDEALAGYGRYLR
LLHEKATPEAILQAFNEYQLLCALREGPFGVRGLNDRIEQAMVQQRKIQRHPHSRWYEGR
PVMIARNDSALGLFNGDIGIALDRGQGLRVWFVMPDGTIKSVQPSRLPEHDTTWAMTVHK
SQGSEFDHAALILPSQRSPVVTRELVYTAVTRARRRLSLYADERILAGAIVTRTERRSGL
ATLFDEVSRTG
>EECEBICD_00467 Amino-acid acetyltransferase
MRGAVKACRSGVRRCHLISYQEDGSLLQELFSRDGIGTQIVMESAEQIRRATINDIGGIL
ELIRPLEQQGILVRRSREQLEMEIDKFTIIQRDNMTIACAALYPFVEEKIGEMACVAVHP
DYRSSSRGEVLLERVAAQARQMGLRKLFVLTTRSIHWFQERGFTPVDIELLPESKKKMYN
YQRRSKVLMADLG
>EECEBICD_00468 Amino-acid acetyltransferase
MIKERKTELVEGFRHSVPYINTHRGKTFVIMLGGEAIEHDNFSSIVSDIGLLHSLGIRLV
VVYGARPQIDANLAAHHHEPIYHKNTRVTDAKALELVKQAAGLLQLDITARLSMSLNNTP
LQGAHINVVSGNFTIAQPLGVDDGVDYCHSGRIRRIDEDAINRQLDNGAIVLMGPVAVSV
TGESFNLTSEEIATQLAVKLKAEKMIGFCSSQGVTNSEGGIISELFPNEAQARVEELEAQ
GDYNSGTVRFCAAR

>EECEBICD_00469 N-acetylmuramoyl-L-alanine amidase AmiC
MSGANSAISRRRLLQGAGAMWLLSVSQVGLAAVSQVVAVRIWPASSYTRVTVESNRLLKY
KQFALSNPDRVVVDIEDVNLNSVLKGIGAQIRSDDPYIKSARVGQFDPKTVRMVFELKQN
VKPQLFALAPVAGFKERLVMDLYPANAQDMQDPLLALLEDYNKGDLDKQVPPSQSGPQPG
KAGRDRPIVIMLDPGHGGEDPGAIGKYKTREKDVVLQIARRLRALIEKEGNMKVYMTRNE
DIFIPLKVRVAKAQKQRADLFVSIHADAFTSRQPSGSSVFALSTKGATSTAAKYLAQTQN
ASDLIGGVSKSGDRYVDHTMFDMVQSLTIADSLKFGKAVLKQLGKINDLHKNKVEQAGFA
VLKAPDIPSILVETAFISNIEEERKLKTATFQQQVAESILAGIKAYFADGATLARRS
>EECEBICD_00472 Membrane-bound lytic murein transglycosylase A
MKGRWAKYVATGVMLAMLAACSSKPTDRGQQYKDGKFTQPFSLVNQPDAVGAPINAGDFA
EQVNQIRSASPRLYTNQSNVYNAVQNWLRSGGDTRTMRQFGIDAWQMEGTDNYGNVQFTG
YYTPVVQARHTRQGAFQYPIYSMPPKRGRLPSRAQIYAGALSDKYILAWSNSLMDNFIMD
VQGSGYIDFGDGSPLNFFSYAGKNGWPYRSIGKVLIDRGEVKKEDMSMQAIREWGEKHSE
AEVRELLEQNPSFVFFKPQSFAPVKGASAVPLIGRASVASDRSIIPPGTTLLAEVPLLDN
NGKFSGQYELRLMVALDVGGAIKGQHFDIYQGIGPDAGHRAGWYNHYGRVWVLKSAPGAG
NVFSG
>EECEBICD_00473 tRNA threonylcarbamoyladenosine dehydratase
MSVVISDAWRQRFGGTARLYGEKALQRFAEAHICVVGIGGVGSWAAEALARTGIGAITLI
DMDDVCVTNTNRQIHALRDNVGLAKAEVMAERIRQINPECRVTVIDDFITPDNVAGYMNA
GFTYVIDAIDSVRPKAALIAYCRRYKVPLVTTGGAGGQIDPTQIQVADLAKTIQDPLAAK
LRERLKHNFGVVKNSKGKLGVDCVFSTEALVYPQADGSVCAMKATAEGPKRMDCASGFGA
ATMVTATFGFVAVSHALKKIMAKAARQE
>EECEBICD_00474 Protein RarD
MEKYLRNGTMFVVLAFILWGLTPLYYQYLSGGNLAQILIYRVFWSIPLLLAVRFLFRQRT
RFHDVWKDKKSFFFCMIAGLLMIVSWSSFIYALTHHLVLDASLGYFINPLFVIALGCIFL
KEKLSLFQAIAVFSGVCGLTFQIIMLRHFPALALTMGLSFALYGLARKFIHYDVMTSITI
ETLWALPVSLLIFLFSDTGPIISANTPFFLYVMTAPVTIIPLVLFAIALNHTSLIVTGLA
QYIEPSLQFLLAIMIFGEHINYAELLCFCAVWFGLFLCISENLYSHYLRARLKPVFGRVQ
RFFR
>EECEBICD_00475 Sulfur acceptor protein CsdE
MTNPLYTGHPFGTTVTEETLRAIFLPLTQWEDKYRQLILLGKQLPALPDECKAQAKEIAG
CENRVWLGFTQSDNGTMHFFGDSEGRIARGLLAVLLTAVEGKNAAELQARSPLALFDELG
LRAQLSASRSQGLNALSEAIVAATHKG
>EECEBICD_00476 Cysteine desulfurase CsdA
MNAFNPTQFRAQFPALADAGVYLDSAATALKPQAVIDATHQFYCLSAGNVHRSQFAQAQR
LTAQYEAARAKAARLLNAPDEKSIVWTRGTTEAINMVAQCYARPRLRPGDEIIVSVAEHH
ANLVPWLMVAQQTGAQVIKLPLNDRRLPDVERLPELITSRSRILALGQMSNVTGGCPDLA
SAISTAHAAGMVVMVDGAQGAVHFPADVQQLDIDFYAFSAHKLYGPTGIGVLYGKPELLE
AMSPWLGGGKMIRDVSFEGFTTQSAPWKLEAGTPNVAGVIGLSAALEWLSDIDIEQAENW
SRGLATLAEDALAKRPGFRSFRCQDSSLLAFDFVGVHHGDMVTLLAEYGIALRAGQHCAQ
PLLAELGVTGTLRASFAPYNTQHDVDALVNAVDRALELLVD
>EECEBICD_00477 putative lipoprotein YgdR
MKKTAAIISACMLTFALSACSGPNYVMHTNDGRSIVTDGKPQTDNDTGMISYKDANGNKQ
QINRTDVKEMVALEN
>EECEBICD_00478 hypothetical protein
MSQIKKTNRLHIVTYLLNSFDIKSRGHVKTITSFKCVTRF
>EECEBICD_00479 Glycine cleavage system transcriptional activator
MSKRLPPLNALRVFDAAARHLSFTRAAEELFVTQAAVSHQIKSLEDFLGLKLFRRRNRSL
LLTEEGQSYFLDIKEIFSQLTEATRKLQARSAKGALTVSLPPSFAIQWLVPRLSSFNSAY
PGIDVRIQAVDRQEDKLADDVDVAIFYGRGNWPGLRVEKLYAEYLLPVCSPLLLTGEKPL
KTPEDLAKHTLLHDASRRDWQAYTRQLGLNHINVQQGPIFSHSAMVLQAAIHGQGIALAN
NVMAQSEIEAGRLVCPFNDVLVSKNAFYLVCHDSQAELGKIAAFRQWILAKAATEQEKFR
FRYEQ
>EECEBICD_00480 hypothetical protein

MTSRFMLIVAAISGFIYVALGAFGAHVLSKTLGVVEMGWIQTGLQYQAFHTLAIFGLAVA
MQRRISIWFYWSSVFLALGTVLFSGSLYCLALSHLRLWAYITPVGGVSFLVGWALLLIGA
IRMKRKGASHE
>EECEBICD_00481 Ribosomal RNA large subunit methyltransferase M
MNKVVLLCRPGFEKECAAEITDKAGKREIFGFARVKENAGYVIYECYQPEDGEKLISELP
FSSLIFARQWFVVGELLQHLPPEDRITPIVGMLQGVVEKGGELRVEVADTNESKELMKFC
RKFTVPLRAALRDAGVLTNYETPKRPVVHVFFIAPGCCYTGYSFAHNNSPFYMGIPRLKF
PSDAPSRSTLKLEEALHVFIPEDEWDERLANGMYAVDLGACPGGWTYQLVKRNMWVYSVD
NGPMAQSLMDTGQVTWLREDGFRYRPNRNNISWMVCDMVEKPAKVTALMAQWLVNGWCRE
TIFNLKLPMKKRYEEVSHNLAYLQAQLDEHGVNAQIQARQLYHDREEVTVHVRRLWAAVG
GRRDER
>EECEBICD_00482 putative HTH-type transcriptional regulator YdjF
MKAARQQAIVDLLINHKSLTTEALATRLNVSKETIRRDLSELQTQGKVLRNHGRAKYIHR
ENQDSGDPFHIRLKSHYAHKADIAREALAWIEEGMTIALDASSTCWYLARQLPDIPIQVF
TNSHPICQELGKRERITLISSGGQLERKYGCYVNPSLISQLKSLDIDLFIFSCEGIDGGG
DLWDSNAINADFKSILLRRASQALLLIDKSKFNRSGEARIGHLDDVTHIVSDAPQS
>EECEBICD_00483 L-fucose mutarotase
MLKTISPLISPTLLKVLAEMGHGDEIIFSDAHFPAHSLGPQVIRADGLSVSDLLRAIIPL
FELDSYAPPLVMMAAVEGDTLDPSVEARYRDALSLEAPCPDIVRIDRYAFYERAQKAFAI
VITGECAKYGNILLKKGVTP
>EECEBICD_00484 L-fuculokinase
MKQDVILVLDCGATNVRAIAVDRQGKIVARASTANASDIAAENSAWHQWSLDAILQRFAD
CCRSLSSALSECVVRGITVTTFGVDGALVDAQGKLLYPVISWKCPRTAAVMETIERFISP
RQLQTLSGVGAFSFNTLYKLVWLKENHPRLLEQAHCWLFISSLINHRLTGEFTTDITMAG
TSQLLDIHQRDFSPEILQATGLARRLFPRIVEAGAQIGTLQTDAARLLGLPAGVPVISAG
HDTQFALFGAGAQQGEPVLSSGTWEILMVRSGQVDTSLLSQYPGSTCELDSQSGLYNPGM
QWLASGVLEWVRKLLWTPETPWQTLIDEARAISAGAEGVRMQCDLLACQNAGWQGVTLNT
TRGHFYRAALEGLTAQLQRNLRTLEKIGHFNATELLLVGGGSRNALWNQIKANQLDIPIK
VLDDAETTVAGAAMFGWYGVGEFSSPEQARAQVNYQYRYFWPQTEPEIIEGV
>EECEBICD_00485 L-fucose isomerase
MKKISLPKIGIRPVIDGRRMGVRESLEEQTMNMAKATAALITEKMRHACGAQVECVIADT
CIAGMAESAACEEKFSSQNVGVTITVTPCWCYGSETIDMDPMRPKAIWGFNGTERPGAVY
LAAALAAHSQKGIPAFSIYGHDVQDADDTSIPADVEEKLLRFARAGLAVASMKGKSYLSV
GGVSMGIAGSIVDHNFFESWLGMKVQAVDMTELRRRIDQKIYDEAELEMALAWADKNFRY
GEDQNASQYKRNEAQNRAVLKESLLMAMCIRDMMQGNKTLADKGLVEESLGYNAIAAGFQ
GQRHWTDQYPNGDTAEALLNSSFDWNGVREPFVVATENDSLNGVAMLFGHQLTGTAQIFA
DVRTYWSPEAVERVTGQALSGLAEHGIIHLINSGSAALDGACKQRDSEGKPTMKPHWEIS
QQEADACLAATEWCPAIHEYFRGGGYSSRFLTEGGVPFTMTRVNIIKGLGPVLQIAEGWS
VELPKAMHDQLDARTNSTWPTTWFAPRLTGKGPFTDVYSVMANWGANHGVLTIGHVGADF
ITLAAMLRIPVCMHNVEEAKIYRPSAWAAHGMDIEGQDYRACQNYGPLYKR
>EECEBICD_00486 L-fucose-proton symporter
MGNTTIQTQSFRAVDAEQSKSKRYIIPFALLCSLFFLWAVANNLNDILLPQFQQAFTLTN
FQAGLIQSAFYFGYFVIPIPAGILMKKLSYKAGIITGLFLYAVGAALFWPAAEIMNYTLF
LIGLFIIAAGLGCLETAANPFVTVLGPESGGHFRLNLAQTFNSFGAIIAVVFGQSLILSN
VPHQSQEALDKMTPDQLSAYKHSLVLSVQTPYMIIVAIVLVVALLIMLTKFPALQSDDHS
DAKQSSFLSSLSRLIRIRHWRWAVLAQFCYVGAQTACWSYLIRYAIEEIPGMTPGFAANY
LTGTMVCFFIGRFTGTWLISRFAPHKVLAAYALFAMLLCLISAFSGGHIGLLALTLCSAF
MSIQYPTIFSLGIKNLGQDTKYGSSFIVMTIIGGGIVTPVMGFVSDAAGKIPTAELVPAL
CFAVIFIFARFRSQAATN
>EECEBICD_00487 L-fuculose phosphate aldolase
MERNRLARQIIDTCLEMTRLGLNQGTAGNVSVRYQGGLLITPTGIPYEKLTESHIVFIDA
DGQHEQGKLPSSEWRFHMAAYQTRPDANAVVHNHAVHCTAVSILNRPIPAIHYMIAAAGG
NSIPCAPYATFGTRELSDHVAVALKNRKATLLQHHGLIACEENLDKALWLAHEVEVLAQL
YLSTLAIVDPVPVLDDEAIAIVLEKFKTYGLRIEE

>EECEBICD_00488 Lactaldehyde reductase
MANRMILNETAWFGRGAVDALTDEVTRRGYHKALIVTDKTLVQCGVVDKVTSRMDAAGLA
WEIYAGVIPNPTISVVQEGLKVFTQSGADYLIAIGGGSPQDTCKAIGIISNNPEFADVRS
LEGLSPTRKPSVPIMAIPTTAGTAAEVTINYVITDEEKRRKFVCVDPHDIPQAAFIDADM
MDGMPPALKAATGVDALTHAIEGYITRAAWALTDALHIKAIEIIAGALRGAVAGEKEAGE
AMALGQYVAGMGFSNVGLGLVHGMAHPLGAFYNTPHGVANAILLPHVMRFNAGSTNEKFR
DIARAMGVKVEGLSLEEARNAAVEAVFTLNRDVGIPLHLRDVGVRKEDIPALAQAAFDDV
CTGGNPREASLADIVELYHIAW
>EECEBICD_00489 Flap endonuclease Xni
MAAHLLIVDALNLIRRIHAVQGSPCVETCQHALDQLIIHSQPTHAVAVFDDDARSSGWRH
QRLPDYKAGRPPMPDDLHNEMPALRAAFEQRGIRCWASDGNEADDLAATLALKVTEAGHQ
ATIVSTDKGYCQLLSPGLRIRDYFQKRWLDAPFIEKEFGVLPRQLPDYWGLAGISSSKVP
GVAGIGPKSATQLLIQFQNLEGIYAHLDEVPEKWRKKLETHKEMAFLCRDIARLQTDLHI
DGNLQQLRLAR
>EECEBICD_00490 L-serine dehydratase 2
MISVFDIFKIGIGPSSSHTVGPMKAGKQFTDDLIARHILTDVTRVVVDVYGSLSLTGKGH
HTDIAIIMGLAGNLPDTVDIDAIPSFIQDVNTHGRLLLANGQHEVEFPVDKCMNFHADNL
SLHENGMRITALAGDKVLYSQTYYSIGGGFIVDEEHFGLTNSEPVNVPYPYKTAADLQRH
CQETGLSLSGLMMQNELALHSKEALEQHFARVWEVMRSGIERGITTEGVLPGKLRVPRRA
AALRRVLVSQDKTTTDPMAVVDWINMFALAVNEENAAGGRVVTAPTNGACGIVPAVLAYY
DKFIREVNANSLARYMLVASAIGSLYKMNASISGAEVGCQGEVGVACSMAAAGLAELLGG
SPAQVCIAAEIAMEHNLGLTCDPVAGQVQVPCIERNAIASVKAVNAARMALRRTSEPRVC
LDKVIETMYETGKDMNAKYRETSRGGLAMKIVTCD
>EECEBICD_00491 Serine transporter
METTQTSTIASIDSRSAWRKTDTMWMLGLYGTAIGAGVLFLPINAGVGGMIPLIIMAILA
FPMTFFAHRGLTRFVLSGKNPGEDITEVVEEHFGIGAGKLITLLYFFAIYPILLVYSVAI
TNTVESFLTHQLAINPPPRAILSLILIVGMMTIVRFGEQMIVKAMSILVFPFVAALMLLA
LYLIPQWNGAALETLSFDSAASTGNGLWMTLWLAIPVMVFSFNHSPIISSFAVAKREEYG
EGAEKKCSKILAFAHIMMVLTVMFFVFSCVLSLTPADLAAAKEQNISILSYLANHFNAPI
IAWMAPIIAMIAITKSFLGHYLGAREGFNGMVIKSLRGKGKSIEINKLNKITALFMLVTT
WIVATLNPSILGMIETLGGPIIAMILFLMPMYAIQKVPAMRKYSGHISNVFVVIMGLIAI
SAIFYSLFS
>EECEBICD_00492 Pyrimidine/purine nucleotide 5'-monophosphate
nucleosidase
MITHISPLGSMDMLSQLEVDMLKRTASSDLYQLFRNCSLAVLNSGSLTDNSKELLSRFEN
FDINVLRRERGVKLELINPPEDAFVDGRIIRALQANLFAVLRDILFVNGQIHNAGRFQHL
DLESSTHITNLVFSILRNARALHVGEAPNMVVCWGGHSINENEYLYARRVGTQLGLRELN
ICTGCGPGAMEAPMKGAAVGHAQQRYKDSRFIGMTEPSIIAAEPPNPLVNELIIMPDIEK
RLEAFVRIAHGIIIFPGGVGTAEELLYLLGILMNPANKNQVLPLILTGPKESADYFRVLD
EFITHTLGEAARRHYRIIIDDAAEVARLMKKAMPQVKENRRDTGDAYSFNWSMRIAPDLQ
VPFEPSHENMANLKLYPDQPVEILAADLRRAFSGIVAGNVKEVGIRAIEANGPYKIHGDR
EMMRRMDDLLQGFVAQHRMKLPGSAYIPCYEICA
>EECEBICD_00493 NADPH-dependent 7-cyano-7-deazaguanine reductase
MSSYENHQALDGLTLGKSTDYRDNYDASLLQGVPRSLNRDPLGLTADNLPFHGADIWTLY
ELSWLNSQGLPQVAVGHVELDYTSVNLIESKSFKLYLNSFNQTRFDTWETVRQTLERDLR
ACAQGNVSVRLHRLDELEGQPVAHFHGTCIDDQDISIDNYQFTTDYLQHAVSGEKQVEET
LVSHLLKSNCLITHQPDWGSIQIQYRGRKIDREKLLRYLVSFRHHNEFHEQCVERIFNDI
LRFCQPETLSVYARYTRRGGLDINPWRSNTDFVPATGRLARQ
>EECEBICD_00494 Protein Syd
MDELTAQALKAFTTRYCDAWQEKHGSWPLSEELYGVPSPCIISSTRDAVYWQPQPFEGEE
NVNAVERAFDIMVQPALHAFYTTQFAGDMPAQFADEKLTLLQTWSQDDFRRVQENLIGHL
VTQKRLKLPPTLFIATQENELEVISVCNLSGEVIKETLGTRNRTVLAATLAEFLTQLNPL
L
>EECEBICD_00495 putative protein YqcC

MTTHDRVRQQLHALETLLREHRHWRQDAPQAHLFTSTQPFFMDTMEPLEWLQWVLIPRMH
TLLDNAQPLPEAFAVAPYYEMALAADHPQREAILAVLQDLDALFVRDKS
>EECEBICD_00496 tRNA pseudouridine synthase C
MLEILYQDPWLVAVNKPAGWLVHRSWLDRDEKVVVMQTVRDQIGQHVFTAHRLDRPTSGV
LLMGLSSEAGRRLAQQFEQHHIRKRYHAIVRGWLMDDAVLDYPLVEERDKIADKFAREDK
APQPAVTQYRGLATVEMAVPTGRYPTTRYGLVELEPKTGRKHQLRRHLAHLRHPIIGDSK
HGDLRQNRSAAEHFACRRLMLHASRLELTHPFTGQPLMIQAGLDETWMQALTQFGWRGLL
PDNERVEFTAASRQDETHQA
>EECEBICD_00497 putative protein
MAEIGIFVGTMYGNSLLVAEEAEAILARQGHSAIVFEDPELSDWQQYQDKVALVVTSTTG
QGDLPDSIAPLFHGIKDTLGFQPNLRYGVIALGDSSYPNFCNGGKQFDALLQEQSAQRVG
EMLFIDASEHPEPESQSNPWVENWGTLLS
>EECEBICD_00498 putative glucarate transporter
MSSLSHAASSAEKRTNARYWIVVMLFIVTSFNYGDRATLSIAGSEMAKDIGLDPVGMGYV
FSAFSWAYVIGQIPGGWLLDRFGSKRVYFWSIFIWSVFTLLQGFVDIFSGFGIIVALFTL
RFLVGLAESPSFPGNSRIVAAWFPAQERGTAVAIFNSAQYFATVIFAPIMGWLTHEVGWS
HVFFFMGGLGIIISFIWLKVIHEPNNHPGVNKKELEYIAEGGALINMDQKDTKAKVPFSV
KWGQIKQLLGSRMMIGVYIGQYCINALTYFFITWFPVYLVQARGMSILKAGFVASVPAVC
GFIGGVLGGIISDWLMRRTGSLNIARKTPIVLGMLLSMVMVFCNYVNVEWMIIGFMALAF
FGKGIGALGWAVMADTAPKEISGLSGGLFNMFGNISGIVTPIAIGYIVGTTGSFNGALIY
VGVHALVAIISYLVLVGDIKRIELKPVAGQLS
>EECEBICD_00499 Glucarate dehydratase-related protein
MTTQSSPVITDMKVIPVAGHDSMLLNIGGAHNAYFTRNIVVLTDNAGHTGVGEAPGGEVI
YQTLVDAIPMVLGQEVARLNKVVQQVHKGNQAADFDTFGKGAWTFELRVNAVAALEAALL
DLLGQALNVPVCELLGPGKQRDAVTVLGYLFYIGDRTKTDLPYLESTPGSHEWYRLRHQE
ALNSDAVVRLAEASQDRYGFKDFKLKGGVLPGEQEIDTVRALKKRFPDARITVDPNGAWL
LDEAIALCKGLKDVLTYAEYPCGAEQGFSGREVMAEFRRATGLPVATNMIATNWREMGHA
VMLNAVDIPLADPHFWTLTGAVCVAQLCDDWGLTWGCHSNNHFDISLAMFTHVGAAAPGK
PTAIDTHWIWQEGDCRLTKNPLEIKNGKIAVPDAPGLGVELDWEQVRKAHDAYKKLPGGA
RNDAGPMQYLIPGWTFDRKRPVFGRH
>EECEBICD_00500 Glucarate dehydratase
MNTQFTTPVVTEMQVIPVAGHDSMLMNLSGAHAPFFTRNIVIIKDNSGHTGVGEIPGGEK
IRKTLEDAIPLVVGKTLGEYKNVLTAVRNQFADRDAGGRGLQTFDLRTTIHVVTGIEAAM
LDLLGQHLGVNVASLLGDGQQRSEVEMLGYLFFVGNRKATPLPYQSQPDEQCDWYRLRHE
EAMTPETVVRLAEAAYEKYGFNDFKLKGGVLAGEEEAESIVALAKRFPQARVTLDPNGAW
SLNEAISIGKYLKGSLAYAEDPCGAEQGFSGREVMAEFRRATGLPTATNMIATDWRQMGH
TLSLQSVDIPLADPHFWTMQGSVRVAQMCHEFGLTWGSHSNNHFDISLAMFTHVAAAAPG
KITAIDTHWIWQEGNQRLTKEPFEIKGGMVQVPTKPGLGVELDMDQVMKAHELYQKHGLG
ARDDAMGMQYLIPGWTFDNKRPCMVR
>EECEBICD_00501 Glycerate 2-kinase
MKIVIAPDSYKESLSALEVATAIEQGFREIWPDADYLKLPLADGGEGTVEAMVEATAGRI
VHVEVTGPLGHRVNAFYGLSGDARSAFIEMAAASGLEQVPPAQRDPLKTTSWGTGELIRH
ALDAGVEHIIIGIGGSATNDGGAGMVQALGARLRDAQGNDIAQGGIGLETLASIDISGLD
KRLSACHIEVACDVTNPLTGKEGASAVFGPQKGATPEMIERLETALTRYAHLIARDLHVD
VLDLAGGGAAGGMGAALYAFCGAQLRRGIEIVTDALHLEACLADADLVITGEGRIDSQTI
HGKVPIGVANIAKRYNKPVIGIAGSLTEDVGVVHEHGLDAVFSVIYTICTLEDALKNASE
NVRMTARNVAATLKAGQQLR
>EECEBICD_00502 Signal transduction histidine-protein kinase BarA
MTNYSLRARMMILILAPTVLIGLLLSIFFVVHRYNDLQRQLEDAGASIIEPLAVSSEYGM
NLQNRESIGQLISVLHRRHSDIVRAISVYDDHNRLFVTSNFHLAPSQMQLPAGAPFPRRL
SVDRHGDIMILRTPIISESYSPDESAIADAKNTKNMLGYVALELDLKSVRLQQYKEIFIS
SVMMLFCIGIALIFGWRLMRDVTGPIRNMVNTVDRIRRGQLDSRVEGFMLGELDMLKNGI
NSMAMSLAAYHEEMQHNIDQATSDLRETLEQMEIQNVELDLAKKRAQEAARIKSEFLANM
SHELRTPLNGVIGFTRLTLKTELNPTQRDHLNTIERSANNLLAIINDVLDFSKLEAGKLI

LESIPFPLRNTLDEVVTLLAHSSHDKGLELTLNIKNDVPDNVIGDPLRLQQVITNLVGNA
IKFTESGNIDILVEKRALSNTKVQIEVQIRDTGIGIPERDQSRLFQAFRQADASISRRHG
GTGLGLVITQKLVNEMGGDISFHSQPNRGSTFWFHIHLDLNPNVIIDGPSTACLAGKRLA
YIEPNATAAQCTLDLLSDTPVEVVYSPTFSALPLAHYDIMILSVPVTFREPLTMQHERLA
KAASMTDFLLLALPCHAQINAEKLKQGGAAACLLKPLTSTRLLPALTEYCQLNHHPEPLL
MDTSKITMTVMAVDDNPANLKLIGALLEDKVQHVELCDSGHQAVDRAKQMQFDLILMDIQ
MPDMDGIRACELIHQLPHQQQTPVIAVTAHAMAGQKEKLLSAGMNDYLAKPIEEEKLHNL
LLRYKPGANVAARLMAPEPAEFIFNPNATLDWQLALRQAAGKPDLARDMLQMLIDFLPEV
RNKIEEQLVGENPNGLVDLVHKLHGSCGYSGVPRMKNLCQLIEQQLRSGVHEEELEPEFL
ELLDEMDNVAREAKKILG
>EECEBICD_00503 23S rRNA (uracil(1939)-C(5))-methyltransferase RlmD
MAQFYSAKRRVTTRQIITVKVNDLDSFGQGVARHNGKALFIPGLLPEESAEVIITEDKKQ
FARARVSRRLNDSPERETPRCPHFGVCGGCQQQHVSIALQQRSKSAALARLMKHEVNDII
AGAPWGYRRRARLSLNCPPDKPLQMGFRKAGSSDIVNVEQCPVLAPQLAALLPRIRACLA
SLHGTRHLGHVELVQAGSGTLMILRHTAPLSAADKEKLERFSHSEGLSLFLAPFSEILET
VSGEAPWYDSHGLRLAFSPRDFIQVNEAVNQQMVARALEWLDVRAEDRVLDLFCGMGNFT
LPLATRAASVIGVEGVPALVEKGRENAIRNGLHNVTFFHENLEEDVTKQPWAKNGFDKVL
LDPARAGATGVMRHIIKLKPIRIVYVSCNPATLARDSEALVNAGYEVTRLAMLDMFPHTG
HLESMVLFERM
>EECEBICD_00504 GTP pyrophosphokinase
MVAVRSAHINKAGEFDPKKWIASLGISSQQSCERLAETWAYCLQQTQGHPDADLLLWRGV
EMVEILSTLSMDIDTLRAALLFPLADANVVSEDVLRESVGKSIVTLIHGVRDMAAIRQLN
ATHNDSVSSEQVDNVRRMLLAMVDDFRCVAIKLAERIAHLREVKEAPEDERVLAAKECTN
IYAPLANRLGIGQLKWELEDYCFRYLHPAEYKRIAKLLHERRLDREHYIEEFVGHLRAEM
KNEGVQAEVYGRPKHIYSIWRKMQKKHLAFDELFDVRAVRIVAERLQDCYAALGIVHTHY
RHLPDEFDDYVANPKPNGYQSIHTVVLGPGGKTVEIQIRTKQMHEDAELGVAAHWKYKEG
AASGGVRSGHEDRIAWLRKLIAWQEEMADSGEMLDEVRSQVFDDRVYVFTPKGDVVDLPA
GSTPLDFAYHIHSDVGHRCIGAKIGGRIVPFTYQLQMGDQIEIITQKQPNPSRDWLNPNL
GYVTTSRGRSKIHAWFRKQDRDKNIQAGRQILDDELAHLGISLKEAEKHLLPRYNFNELE
ELLAAIGGGDIRLNQMVNFLQSQFNKPSAEEQDAAALKQLQQKTYAPQNRRKDDGRVVVE
GVGNLMHHIARCCQPIPGDEIVGFITQGRGISVHRADCEQLAELRSHAPERIVEAVWGES
YSAGYSLVVRVQANDRSGLLRDITTILANEKVNVLGVASRSDIKQQIATIDMTIEIYNLQ
VLGRVLGKLNQVPDVIDARRLHGG
>EECEBICD_00505 hypothetical protein
MNQKVLFFLSIIAYPGITQAINQNVMIEPLVIRDSTNYLVLPQAQQAEFSQCKSWPHDST
VIPAKYEATLLNGSLISMFIGGITVNLYTTADVKVNTDAQCTLLNGNFPGYYGITCFVTG
YDTDKAVPVRTDNGMSIIESKLYATGTMRYITEIPMDGSGFQGMAIGGAVVINTGAITPT
PNTGVISNITMPPLYDPGFIFTVPGCGQYKYHSGTGSVFTNNNSTYTVASPIGFITASLD
KNSVLNYNELHYNDKAENIELGKIYLMYKEKNVTWGEGFDYTLENSTINVVCADSRIKTN
VDYQCRNGDMGACNNGELGRIIGNWERINVDTNCSVTVILPWQ
>EECEBICD_00506 putative fimbrial-like protein YfcP
MLWAVCNTLPAATPNVHYSGKLVAGACNLVVDNDTMATVDFHTIGSDNFDASGQTTPVPF
TLSLQDCKTALANGVLVTFQGVEDSTLPGLLALEPSSEASGFAIGVETAAQQPVSINATV
GTAFVLKEGITTINLQARLQKYAGEEVMPGEFSGSATVSFEYQ
>EECEBICD_00507 putative fimbrial-like protein YfcQ
MERFIVKRVLILTLLITQFACADNLTFHGKLINPPACTINNGEMLEVSFGSVIIDNIDGV
NYLTEIPWTLTCDSSFRDDALTFTLSYLGTATPYSAKALTTSVPELGIELQQNGTVFPPG
TSLTINESSLPTLKAVPVKQPGKEPAEGDFEAFATLQVDYQ
>EECEBICD_00508 putative fimbrial-like protein YfcR
MLTRWKMLVLLCGGFVTGTEAAGTKTVQLELHLVVTQPPPCTVGGASVEFGDVLTTKVGD
ASQTKPVGYSLNCDGRASDYLKLQIQGTTTTISGEQVLQTSVQGLGIRIQQAGNKQLVPV
GITDWLNFTLSGSNGPELEAVPVKEPTTQLAGGDFNASATLVVDYQ
>EECEBICD_00509 putative fimbrial chaperone YfcS
MNKTNHFKRQALIASVLLAAPLVSHSAIVPDRTRVIFNGNENSITVTLKNGNATLPYLAQ

AWLEDDKFAKDTRYFTALPPLQRIEPKSDGQVKVQPLPAAASLPQDRESLFYFNVREIPP
KSDKPNTLQLALQTRIKFFYRPVAVARQVDKTHPWQTKLTLTYQGDGVIFDNPTPFYLVI
SNAGSKENETASGFKNLLIAPREKVTSPIKGASLGSSPVVGYVDDYGGHRLLVFTCSGNT
CKVNEEKTRDAEKKANK
>EECEBICD_00510 Outer membrane usher protein PapC
MLLSLSPYSASGKDIEFNTDFLDVKNRDNVNIAQFSRKGFILPGVYLLQIKINGQTLPQE
FPVNWVIPEHDPQGSEVCAEPELVTQLGIKPELAEKLVWITHGERQCLAPDSLKGMDFQA
DLGHSTLLVNLPQAYMEYSDVDWDPPARWDNGIPGIILDYNINNQLRHDQESGSEEQSIS
GNGTLGANLGAWRLRADWQASYDHRDDDENTSTLHDQSWSRYYAYRALPTLGAKLTLGES
YLQSDVFDSFNYIGASVVSDDQMLPPKLRGYAPEIVGIARSNAKVKVSWQGRVLYETQVP
AGPFRIQDLNQSVSGTLHVTVEEQNGQTQEFDVNTASVPFLTRPGMVRYKMALGRPQDWD
HHPITGTFASAEASWGVTNGWSLYGGAIGESNYQAVALGSGKDLGVVGAVAVDITHSIAH
MPQDDGFDGETLQGNSYRISYSRDFDEIDSRLTFAGYRFSEKNFMSMSDYLDAKTYHHLN
AGHEKERYTVTYNQNFREQGMSAYFSYSRSTFWDSPDQSNYNLSLSWYFDLGSIKNLSAS
LNGYRSEYNGDKDDGVYISLSVPWGNDSISYNGTFNGSQHRNQLGYSGHSQNGDNWQLHV
GQDEQGAQADGYYSHQGALTDIDLSADYEEGSYRSLGMSLRGGMTLTTQGGALHRGSLAG
STRLLVDTDGIADVPVSGNGSPTSTNIFGKAVIADVGSYSRSLARIDLNKLPEKAEATKS
VVQITLTEGAIGYRHFDVVSGEKMMAVFRLADGDFPPFGAEVKNERQQQLGLVADDGNAW
LAGVKAGETLKVFWDGAAQCEASLPSTFTPELLANALLLPCKMLEGQPPTAPQKSSPLPA
QPLIQEHTQTDGQPAAPVATTTQTPPIPLADNHAVNRKDME
>EECEBICD_00511 Major MR/P fimbria protein
MKSSHFCKLAVTASLVMGIVSGAQAAGSNTAKVTFLGNIVDSPCSVTLDTEDQTVNMGSS
IGNGTLSNGKTTINNARTFHIDLEGCTWATEKNMNVVFTTGSGTTAATGATDNLALMKTD
GTGAISNVSLAIGDAGKNNIKLGDTYTQAIADLDGDTILDEKQSLNFTAWLVGAATGTVG
TGEFSSAANVTISYL
>EECEBICD_00512 Nucleoside triphosphate pyrophosphohydrolase
MTTNHQIDRLLTLMQRLRDPENGCPWDKEQTFASIAPYTLEETYEVLDAIAREDFDDLRG
ELGDLLFQVVFYAQMAQEEGRFDFNDICAAISDKLERRHPHVFGELSADNSEEALVRWEQ
IKTEERAQKAQHSALDDIPRSLPALMRAQKIQKRCSNVGFDWTTLGPVVDKVYEEIDEVM
FEARQAVVDQAKLEEEMGDLLFATVNMARHLGTKAELALQKANDKFERRFREVERIVAAR
GLEMTGVDLETMEEVWQEVKRQEIDL
>EECEBICD_00513 CTP synthase
MTTNYIFVTGGVVSSLGKGIAAASLAAILEARGLNVTIMKLDPYINVDPGTMSPIQHGEV
FVTEDGAETDLDLGHYERFIRTKMSRRNNFTTGRIYSDVLRKERRGDYLGATVQVIPHIT
NAIKERVLEGGEGHDVVLVEIGGTVGDIESLPFLEAIRQLAVDIGREHALFMHLTLVPYL
AAAGEVKTKPTQHSVKELLSIGIQPDILICRSDRAVPANERAKIALFCNVPEKAVISMKD
VDSIYKIPGLLKSQGLDDYICKRFSLNCPEANLSEWEQVIYEEANPAGEVTIGMVGKYIE
LPDAYKSVIEALKHGGLKNRVTVNIKLIDSQDVETRGVEILKDLDAILIPGGFGYRGVEG
KIATARYARENNIPYLGICLGMQVALIEFARNVAGMDNANSTEFVPDCKYPVVALITEWR
DEDGNVEVRSEKSDLGGTMRLGAQQCQLSDDSLVRQLYGASTIVERHRHRYEVNNMLLKQ
IEAAGLRVAGRSGDDQLVEIIEVPNHPWFVACQFHPEFTSTPRDGHPLFAGFVKAANEHQ
KRQAK
>EECEBICD_00514 Enolase
MSKIVKVIGREIIDSRGNPTVEAEVHLEGGFVGMAAAPSGASTGSREALELRDGDKSRFL
GKGVTKAVGAVNGPIAQAILGKDAKDQAGIDKIMIDLDGTENKSNFGANAILAVSLANAK
AAAAAKGMPLYEHIAELNGTPGKYSMPVPMMNIINGGEHADNNVDIQEFMIQPVGAKTVK
EAIRMGSEVFHHLAKVLKGKGMNTAVGDEGGYAPNLGSNAEALAVIAEAVKAAGYELGKD
ITLAMDCAASEFYKDGKYVLAGEGNKAFTSEEFTHFLEELTKQYPIVSIEDGLDESDWDG
FAYQTKVLGDKIQLVGDDLFVTNTKILKEGIEKGIANSILIKFNQIGSLTETLAAIKMAK
DAGYTAVISHRSGETEDATIADLAVGTAAGQIKTGSMSRSDRVAKYNQLIRIEEALGEKA
PYNGRKEIKGQA
>EECEBICD_00515 7-carboxy-7-deazaguanine synthase
MQYPINEMFQTLQGEGYFTGVPAIFIRLQGCPVGCAWCDTKHTWDKLSDREVSLFSILAK
TKESDKWGAASSEDLLAVINRQGYTARHVVITGGEPCIHDLMPLTDLLEKSGFSCQIETS

GTHEVRCTPNTWVTVSPKVNMRGGYDVLSQALERANEIKHPVGRVRDIEALDELLATLSD
DKPRVIALQPISQKEDATRLCIETCIARNWRLSMQTHKYLNIA
>EECEBICD_00516 hypothetical protein
MALRIRVLLENHKGAGADKSLKARPGLSLLVEDESTSILFDTGPDGSFMQNALAMGIDLS
DVSAVVLSHGHYDHCGGVPWLPDNSRIICHPDIARERYAAMTFLGITRKIKKLSCEVDYS
RYRMMYTRDPLPIGKNFIWSGEIPVVAPEAYGIFGGHDAEPDSILDEGVLIYQSTKGLVI
ITGCGHRGIANIVRHCQNITGIKRIYALVGGFHLRCASPFTLWRVRRFLQEQKPEKLCGC
HCTGAWGRLWLPEITAPATGDVLRF
>EECEBICD_00517 6-carboxy-5,6,7,8-tetrahydropterin synthase
MSTTLYKDFTFEAAHRLPHVPEGHKCGRLHGHSFMVRLEITGEVDPHTGWIMDFADLKAA
FKPTYDRLDHYYLNDIPGLSNPTSEVLAKWIWDQVKPVVPLLSAVMVKETCTAGCVYRGE
>EECEBICD_00518 Sulfite reductase [NADPH] flavoprotein alpha-component
MTTPAPLTGLLPLNPEQLARLQAATTDLTPEQLAWVSGYFWGVLNPRSGVVAVTPVPERK
MPGVTLISASQTGNARRVAEALRDDLLAANLNVTLVNAGDYKFKQIASEKLLVIVTSTQG
EGEPPEEAVALHKFLFSKKAPKLENTAFAVFSLGDTSYEFFCQSGKDFDSKLAELGGERL
LDRVDADVEYQAAASEWRARVVDVLKSRAPVAAPSQSVATGAVNDIHTSPYTKDAPLIAT
LSVNQKITGRNSEKDVRHIEIDLGDSGLRYQPGDALGVWYQNDPALVKELVELLWLKGDE
PVTVDGKTLPLAEALEWHFELTVNTANIVENYATLTRSESLLPLVGDKAQLQHYAATTPI
VDMVRFSPAQLDAEALIGLLRPLTPRLYSIASAQAEVESEVHVTVGVVRYDIEGRARAGG
ASSFLADRVEEEGEVRVFIEHNDNFRLPANPQTPVIMIGPGTGIAPFRAFMQQRAADGAE
GKNWLFFGNPHFTEDFLYQVEWQRYVKEGVLSRIDLAWSRDQKEKIYVQDKLREQGAELW
CWINDGAHIYVCGDARRMAADVEKALLEVIAEFGGMDLESADEYLSELRVERRYQRDVY
>EECEBICD_00519 Sulfite reductase [NADPH] hemoprotein beta-component
MSEKHPGPLVVEGKLSDAERMKLESNYLRGTIAEDLNDGLTGGFKGDNFLLIRFHGMYQQ
DDRDIRAERAEQKLEPRHAMLLRCRLPGGVITTTQWQAIDKFAADNTIYGSIRLTNRQTF
QFHGILKKNVKPVHQMLHSVGLDALATANDMNRNVLCTSNPYESQLHAEAYEWAKKISEH
LLPRTRAYAEIWLDQEKVAITDEEPILGQTYLPRKFKTTVVIPPQNDIDLHANDMNFVAI
AENGKLVGFNLLVGGGLSIEHGNKKTYARTASEFGYLPLEHTLAVAEAVVTTQRDWGNRT
DRKNAKTKYTLERVGLETFKAEVERRAGIKFEPIRPYEFTGRGDRIGWVKGIDNNWHLTL
FIENGRILDYPGRPLKTGLLEIAKIHQGEFRITANQNLIIASVPESQKAKIETLARDHGL
MNAVSAQRENSMACVSFPTCPLAMAEAERFLPSFTDKVEAILEKHGIPDEHIVMRVTGCP
NGCGRAMLAEIGLVGKAPGRYNLHLGGNRIGTRIPRMYQENITEPDILASLDELIGRWAK
EREAGEGFGDFTVRAGIIRPVLDPARDFWE
>EECEBICD_00520 Phosphoadenosine phosphosulfate reductase
MSKLDLNALNELPKVDRVLALAETNAQLETLTAEERVAWALENLPGEYVLSSSFGIQAAV
SLHLVNQIRPDIPVILTDTGYLFPETYQFIDELTDKLKLNLKVYRAGESPAWQEARYGKL
WEQGVEGIEKYNEINKVEPMNRALKELKAQTWFAGLRREQSGSRAHLPVLAIQRGVFKVL
PIIDWDNRTVYQYLQKHGLKYHPLWDQGYLSVGDTHTTRKWEPGMAEEETRFFGLKRECG
LHEG
>EECEBICD_00521 Secreted effector protein SopD
MPVTLSFGNHQNYTLNESRLAHLLSADKEKAIHMGGWDKVQDHFRAEKKDHALEVLHSII
HGQGRGEPGEMEVNVEDINKIYAFKRLQHLACPAHQDLFTIKMDASQTQFLLMVGDTVIS
QSNIKDILNISDDAVIESMSREERQLFLQICEVIGAKMTWHPELLQESISTLRKEVTGNA
QIKAAVYEMMRPAEAPDHPLVEWQDSLTEDEKSMLACINAGNFEPTTQFCKIGYQEVQGE
VAFSMMHPCISYLLHTYSPFAEFKPTNSGFLKKLNQDYNDYHAKKMFIDVILEKLYLTHE
RSLHIGKDGCSRNILLT
>EECEBICD_00522 CRISPR-associated endonuclease/helicase Cas3
MSIYHYWGKSRRGETDGGDDYHLLCWHSLDVAAVGYWMVINNIYFIDHYLKKLGIQDKEQ
AAQFFAWILCWHDIGKFAHSFQQLYRHEALNIFNEPTRHYEKIAHTTLGYMLWNSWLSEC
PELFPPSSLSVRKSKRVMALWMPVTTGHHGRPPEAIQELDHFRQQDKDAARDFLLRIKAL
FPLITLPEAWDEDEGIDQFQQLSWFISAAVVLADWTGSASRYFPRTAEKMPVDTYWQQAL
AKAQTAITLFPSAANVSAFTGIETLFPFIQHPTPLQQKALELDINVDGAQLFILEDVTGA
GKTEAALILAHRLMAAGKAQGLYFGLPTMATANAMFERMANTWLALYQPDSRPSLILAHS
ARRLMDRFNQSIWSVTLSGTEEPDEAQPYSQGCAAWFADSNKKALLAEVGVGTLDQAMMA

VMPFKHNNLRLLGLSNKILLADEIHACDAWMSRILEGLIERQASNGNATILLSATLSQQQ
RDKLVAAFSRGVRRSVQAPLLGHDDYPWLTQVTQTELISQRVDTRKEVERCVDIGWLHSE
EACLERIGEAVEKGNCIAWIRNSVDDAIRIYRQLQLSKVVATENLLLFHSRFAFHDRQRI
ESQTLNLFGKQSGAQRAGKVIIATQVIEQSLDIDCDEMISDLAPVDLLIQRAGRLQRHIR
DRNGLVKKSGQDERETPVLRILAPEWDDAPRENWLSSAMRNSAYVYPDHGRMWLTQRILR
EQGTIRMPQSARLLIESVYGEDVNMPVGFAKTEQLQEGKFYCDRAFAGQMLLNFAPGYCA
EISDSLPEKMSTRLAEESVTLWLAKIVDSVVTPYASGEHAWEMSVLRVRQSWWNKHKDEF
EKLDGEPLRKWCAQQHQDKDFATVIVVTDFAACGYSANEGLIGMMGE
>EECEBICD_00523 hypothetical protein
MDNFSLLTTPWLPVRFKDGSTGKLAPVDLADENVVDIAATRADLQGAAWQFLLGLLQCSI
APKRYKNWEDIWFDGLHADVLHKALAPLEHAFQFGAESPSFMQDFEPLSGEKVSIASLLP
EIPGAQTTKFNKDHFVKRGVTERFCPHCAALALFSLQLNAPAGGKGYRTGLRGGGPLTTL
VELQEYQGERQTPIWRKLWLNVMPQDTADLPLPDQCDATVFPWLAATRTSEQANAVTTPE
QVNKLQAYWGMPRRIRLDFATLQSGCCDICGAESDELLGFMTVKNYGVNYDGWRHPLTPY
RAPVKDQNAFFSVKPQPGGLIWRDWLGLSQNNQTEANYESPAQVVKVFNARSLTDVKAGI
RGFGADFDNMKIRCWYEHHFPLLMTEGLIPDLRKAVQTAARLLSLLRSALKEAWFTNAKD
ARGDFSFIDIDFWNLTQGRFLNLIHDLENGHKPDERLNKWQRELWLFTRCYFDDHVFTNP
YESSDLERIMKARKKYFTSSAEKQSAKAAKAKKQEAAE
>EECEBICD_00524 hypothetical protein
MSVVTKDDKATLRQWHDELQEKRGLRASLRRSKTVNDACLAEGLHSLLMQTHSLWKNKAP
WNVTALAITAALAAHIKFIDEQKSFAAQLGQKKGGDTPVMSKLRFSHLLAVKTPDELLRQ
LRRAVKLLDGSVNLFSLADDIFCWCQEQNDLLNHHRRQQRPTEFLRIRWALEYYQAGDTD
NEQD
>EECEBICD_00525 CRISPR system Cascade subunit CasC
MTTFIQLHLLTAYPAANLNRDDTGAPKTVVLGGATRLRISSQSLKRAWRTSELFEQALAG
HIGIRTGRIAREAAQILVDSGIDAKKAVEYVKNIANCFGKVKEDKKPKDELTNAETEQLV
HISPAEFEAVKALARRLAEEKRPATEEEAELLRHDRMAVDIAMFGRMLAKKTDFNVEAAC
QVAHAFGVSETIIEDDFFTAVDDLRQASAEDAGAGHLGETGFGSALFYTYICIDKDLLVK
NLNGNEELANKTLRAFTEAALKVSPTGKQNSFASRAYASWALAEKGTDQPRSLAAAFYEP
INGTDQLNVAVKRITALHENMNEVYAQETAFKNFNVMNQQGSMKDVLDFICA
>EECEBICD_00526 hypothetical protein
MSQYLVFQLHGPMASWGVDAPGEVRHSHELPSRSALLGLLAAALGIRRDEEERLNTFNRH
YQFLLCASGNPRWARDYHTVQMPKEVRKARYFSRREELQDPELLSALISRRDYYTDAWWM
IAVSATPDAPYTLAQLQAALQHPVFPLYLGRKSHPLALPLAPQLLEGNAADVLREAYRWY
QDQFNALKLTLPGLQNECWWEGEHDGLTANKILRRRDMPLSRQQWLFGERSVNQGPWLRK
EDACISQE
>EECEBICD_00527 CRISPR system Cascade subunit CasE
MYLSRITLHTSELSPAQLLHLVERGEYVMHQWLWDLFPGGKERQFLYRREELQGAFRFFV
LSQEQPAASTIFDVQTRPFAPMLSAGQTLRFNLRANPTICKNGKRHDLLMEAKRQRKTQG
DSQDIWSYQQQAALEWLARQGEQNGFTLREASVDAYRQQQIRREKSRQMIQFSSVDYTGV
LVINEPALFLQRLAQGYGKSRAFGCGMMMIKPGDDA
>EECEBICD_00528 CRISPR-associated endonuclease Cas1
MTFVPLNPIPLKDRTSMIFLQYGQIDVLDGAFVLIDKTGIRTHIPVGSVACIMLEPGTRV
SHAAVHLASTVGTLLVWVGEAGVRVYSSGQPGGARADKLLYQAKLALDDDLRLKVVRKMY
ELRFREPPPARRSVEQLRGIEGSRVRATYALLAKQYGVKWHGRNYDPKDWEKGDVVNRCI
SAATSCLYGISEAAILAAGYAPAIGFIHSGKPLSFVYDIADIIKFESVVPKAFEIAARHP
AEPDKEVRLACRDIFRSSKLTGKLIPLIEEVLAAGEIEPPQPAPDMLPPAIPEPESLGDS
GHRGHG
>EECEBICD_00529 CRISPR-associated endoribonuclease Cas2
MSMVVVVTENVPPRLRGRLAVWLLEVRAGVYVGDTSKRIREMIWQQITQLGGVGNVVMAW
ATNTESGFEFQTWGENRRIPVDLDGLRLVSFLPVENQ
>EECEBICD_00530 hypothetical protein
MFSATRRFAVILALGVGFILPAQAASPGPGEIANTQARHIATFFPGRMTGSPAEMLSADY
LRQQFTQMGYQSDIRTFNSRFIYTTKDNRKNWHNVTGSTVIAAHEGRVPQQIIIMAHLDT

YAPQSDADVDANLGGLTLQGMDDNAAGLGVMLELAARLKDIPTHYGIRFIATSGEEEGKL
GAENLLKRMSDAEKKNTLLVINLDNLIVGDKLYFNSGKNTPEAVRTLTRDRALAIARRYG
IAANTNPGRNPSYPKGTGCCNDAEVFDKAGISVLSVEATNWNLGKKDGYQQRVKNASFPN
GNSWHDVRLDNQQHIDKALPGRIERRSRDVVRIMLPLVKELAKAEKTS
>EECEBICD_00531 Sulfate adenylyltransferase subunit 2
MDQKRLTHLRQLEAESIHIIREVAAEFANPVMLYSIGKDSSVMLHLARKAFYPGTLPFPL
LHVDTGWKFREMYAFRDRTANAYGCELLVHKNPEGVAMGINPFVHGSAKHTDIMKTEGLK
QALNKYGFDAAFGGARRDEEKSRAKERIYSFRDRFHRWDPKNQRPELWRNYNGQINKGES
IRVFPLSNWTEQDIWQYIWLENIDIVPLYLAAERPVLERDGMLMMVDDDRIDLQPGEVIK
KRMVRFRTLGCWPLTGAVESHAQTLPEIIEEMLVSTTSERQGRMIDRDQAGSMELKKRQG
YF
>EECEBICD_00532 Sulfate adenylyltransferase subunit 1
MNTILAQQIANEGGVEAWMIAQQHKSLLRFLTCGSVDDGKSTLIGRLLHDTLQIYEDQLS
SLHNDSKRHGTQGEKLDLALLVDGLQAEREQGITIDVAYRYFSTEKRKFIIADTPGHEQY
TRNMATGASTCDLAILLIDARKGVLDQTRRHSFISTLLGIKHLVVAINKMDLVDYCEETF
ARIREDYLTFAEQLPGDLDIRFVPLSALEGDNVAAQSANMRWYSGPTLLEVLETVDIQRA
VDRQPMRFPVQYVNRPNLDFRGYAGTLASGSVKVGERIKVLPSGVESSVARIVTFDGDKE
EACAGEAITLVLNDDIDISRGDLLLAANETLAPARHAAIDVVWMAEQPLAPGQSYDVKLA
GKKTRARIEAIRYQIDINNLTQRDVESLPLNGIGLVEMTFDEPLALDIYQQNPVTGGLIF
IDRLSNVTVGAGMVRELDERGATPPVEYSAFELELNALVRRHFPHWDARDLLGDKHGAA
>EECEBICD_00533 Adenylyl-sulfate kinase
MALHDENVVWHSHPVTVAAREQLHGHRGVVLWFTGLSGSGKSTVAGALEEALHQRGVSTY
LLDGDNVRHGLCRDLGFSDADRQENIRRVGEVASLMADAGLIVLTAFISPHRAERQLVKE
RVGHDRFIEIYVNTPLAICEQRDPKGLYKKSACGRVA
>EECEBICD_00534 Inner membrane protein YgbE
MRNSHNITFTRSDAFMVDDDATSAFPGAVVGFVSWLLLALGIPFLLYGPNTLFFFLYTWPF
FLALMPVSVIIGIALHLLVKGKILFSIMFTLLAVGALFGALFIWLLG
>EECEBICD_00535 Cell division protein FtsB
MGKLTLLLLALLVWLQYSLWFGKNGIHDYSRVNDDVVAQQATNAKLKARNDQLFAEIDDL
NGGQEAIEERARNELSMTKPGETFYRLVPDASKRAATAGQTHR
>EECEBICD_00536 2-C-methyl-D-erythritol 4-phosphate cytidylyltransferase
MAATLLDVCAVVPAAGFGRRMQTECPKQYLSIGNKTILEHSVHALLAHPRVTRVVIAISP
GDHRFAQLPLANHPQITVVDGGNERADSVLAGLQAVAKAQWVLVHDAARPCLHQDDLARL
LTISENSRVGGILASPVRDTMKRGEPGKNAIAHTVERADLWHALTPQFFPRELLHDCLTR
ALNEGATITDEASALEYCGFHPALVEGRADNIKVTRPEDLALAEFYLTRTIHQEKA
>EECEBICD_00537 2-C-methyl-D-erythritol 2,4-cyclodiphosphate synthase
MRIGHGFDVHAFGGEGPIIIGGVRISYEKGLLAHSDGDVALHALTDALLGAAALGDIGKL
FPDTDPAFKGADSRELLREAWRRIQAKGYTLGNVDVTIIAQAPKMLPHIPQMRVFIAEDL
GCHMDDVNVKATTTEKLGFTGRGEGIACEAVALLMKAAK
>EECEBICD_00538 tRNA pseudouridine synthase D
MTEFDNLTWLHGKPQGSGLLKANPEDFVVVEDLGFTPDGEGEHILLRILKNGCNTRFVAD
ALAKFLKIHAREVSFAGQKDKHAVTEQWLCARVPGKEMPDFSAFQLEGCKVLEYARHKRK
LRLGALKGNAFTLVLREISDRRDVETRLQAIRDGGVPNYFGAQRFGIGGSNLQGALRWAQ
SNAPVRDRNKRSFWLSAARSALFNQIVHQRLKKPDFNQVVDGDALQLAGRGSWFVATSEE
LPELQRRVDEKELMITASLPGSGEWGTQRAALAFEQDAIAQETVLQSLLLREKVEASRRA
MLLYPQQLSWNWWDDVTVELRFWLPAGSFATSVVRELINTMGDYAHIAE
>EECEBICD_00539 5'/3'-nucleotidase SurE
MRILLSNDDGVHAPGIQTLAKALREFADVQVVAPDRNRSGASNSLTLESSLRTFTFDNGD
IAVQMGTPTDCVYLGVNALMRPRPDIVVSGINAGPNLGDDVIYSGTVAAAMEGRHLGFPA
LAVSLNGYQHYDTAAAVTCALLRGLSREPLRTGRILNVNVPDLPLAQVKGIRVTRCGSRH
PADKVIPQEDPRGNTLYWIGPPGDKYDAGPDTDFAAVDEGYVSVTPLHVDLTAHSAHDVV
SDWLDSVGVGTQW
>EECEBICD_00540 Protein-L-isoaspartate O-methyltransferase
MVSGRVQALLEQLRAQGIRDEQVLNALAAVPREKFIDEAFEHKAWENIALPIGQGQTISQ

PYMVARMTELLELTPQSRVLEIGTGSGYQTAILAHLVHHVCSVERIKGLQWQARRRLKQL
DLHNVSTRHGDGWQGWQARAPFDAIIVTAAPPEIPTALMAQLDEGGILVLPVGDEQQFLK
RVRRRGGEFIIDTVEAVRFVPLVKGELA
>EECEBICD_00541 Murein hydrolase activator NlpD
MSAGSPKFTVSRIAALSLVSLWLAGCTSSSNPPAPVTSVDSGSSSNTNSGMLITPPPKMG
ATTQQTPQQAPQIQPVQRPVTQPMQTQPVTEQPVQMENGRIVYNRQYGNIPKGSYTGGST
YTVKKGDTLFYIAWITGNDFRDLAQRNSISAPYSLNVGQTLQVGNASGTPITGGNAITQA
DAAQQGVVTRSAQNSTVAVASQPTITYSEGSGEQSANKMLPNNKPAGTVVTAPVTAPTVS
TTEPNASSTSTSAPISAWRWPTDGKVIENFGASEGGNKGIDIAGSKGQAIVATADGRVVY
AGNALRGYGNLIIIKHNDDYLSAYAHNDTMLVREQQEVKAGQKIATMGSTGTSSTRLHFE
IRYKGKSVNPLRYLPQR
>EECEBICD_00542 RNA polymerase sigma factor RpoS
MITTWLKKSCYRKGATQRVLDATQLYLGEIGYSPLLTAEEEVYFARRALRGDVASRRRMI
ESNLRLVVKIARRYGNRGLALLDLIEEGNLGLIRAVEKFDPERGFRFSTYATWWIRQTIE
RAIMNQTRTIRLPIHIVKELNVYLRTARELSHKLDHEPSAEEIAEQLDKPVDDVSRMLRL
NERITSVDTPLGGDSEKALLDILADEKENGPEDTTQDDDMKQSIVKWLFELNAKQREVLA
RRFGLLGYEAATLEDVGREIGLTRERVRQIQVEGLRRLREILQTQGLNIEALFRE
>EECEBICD_00543 Protein VdcD
MICPRCADAHIELMATSPVKGVWTVYQCQHCLYTWRDTEPLRRTSREHYPQAFRMTQKDI
DDAPMVPSIPPLLAEDKR
>EECEBICD_00544 Phenolic acid decarboxylase
MAFDDLRSFLHALDQQGQLLKISEEVNAEPDLAAAANATGRIGDGAPALWFDNIRGFTDA
RVAMNTIGSWQNHAISLGLPPNTPVKKQIDEFIRRWDNFPVAPERRANPGWAENTVDGDA
INLFDILPLFRLNDGDGGFYLDKACVVSRDPLDPDNFGKQNVGIYRMEVKGKRKLGLQPV
PMHDIALHLHKAEERGEDLPIAITLGNDPIITLMGATPLKYDQSEYEMAGALRESPYPIA
TAPLTGFDVPWGSEVILEGVIESRKREIEGPFGEFTGHYSGGRNMTVVRIDKVSYRSKPI
FESLYLGMPWTEIDYLMGPATCVPLYQQLKAEFPEVQAVNAMYTHGLLAIISTKKRYGGF
ARAVGLRAMTTPHGLGYVKMVIMVDEDVDPFNLPQVMWALSSKVNPAGDLVQLPNMSVLE
LDPGSSPAGITDKLIIDATTPVAPDNRGHYSQPVVDLPETKAWAEKLTAMLANRK
>EECEBICD_00545 putative UbiX-like flavin prenyltransferase
MRLIVGMTGATGAPLGVELLQALRAIPDVETHLVMSKWAKTTIELETPYTPAEVAALADY
CHSPADQAATISSGSFRTDGMIIIPCSMKTLAGVRAGYAEGLVGRAADVVLKEGRKLVLV
PREMPLSTIHLENMLALSRMGVAIVPPMPAFYNLPQTVDDIIQHIVARVLDQFGLEHTRT
RRWQGLRQAANFSQENG
>EECEBICD_00546 Transcriptional regulator HosA
MELRNTAFHLLRQLFQQHTARWQHELPELTKPQYAVMRVIAEHPGIEQVDLTEAAVSTKA
TLAEMLSRMENRGLVKRENDPLDKRRRFVYLTVQGQTLLAAAIPLGDRVDDEFLGRLSAE
EREQFTQLVRKMMT
>EECEBICD_00547 HTH-type transcriptional repressor GlcR
MIPTERRQIILDMVAEKGIVSIAELTERMHVSHMTIRRDLQKLEQQGAVIQVSGGVQSST
RVAHEPSHQIKTELATPQKAAIGKLAASLVQPESCIYLDAGTTTLAIARQLVTMNKLTVV
TNDFVIADYLMDNSDCTIIHTGGAVCRENRSCVGEAAATLLRGLMIDQAFISASSWSVRG
ISTPAEDKVTVKRAVASASRQKILVCDATKYGQVATWLALPLAEFNQIVTDDGLPESAIR
ALAKVDISLLMAKQ
>EECEBICD_00548 L-threonate dehydrogenase
MTTGTDFHVGIVGLGSMGMGAARSCLRAGLSTWGADLNPQACANLLAEGACGAAASAREF
AGVVDALVILVVNAAQVRQVLFGEDGVAHLMKPGSAVMVSSTISSADAQEIAAALTALNL
NMLDAPVSGGAVKAAQGEMTVMASGSEAAFTRLKPVLDAVASNVYRISDTPGAGSTVKII
HQLLAGVHIAAAAEAMALAARAGIPLDVMYDVVTHAAGNSWMFENRMQHVVDGDYTPRSA
VDIFVKDLGLVADTAKALRFPLPLASTALNMFTSASNAGYGKEDDSAVIKIFSGITLPGV
TPEEPSC
>EECEBICD_00549 3-oxo-tetronate kinase
MLKIGVIADDFTGATDIASFLVENGMPTVQINDVPTGTQPEGCDAVVISLKTRSCPAQEA
IKQSLAALVWLKKQGCQQVYFKYCSTFDSTAEGNIGPVTDALMVALDTSFTVISPALPVN

GRTVYQGYLFVMNHLLAESGMRHHPINPMTDSYLPRLMEAQAQGRCGVIPAQTLDEGVAA
TRAALSRLQQEGYRYAVLDALNERHLEIQGEVLRDAPLVTGGSGLAMGLARQWAKHGVSQ
ARSAGYPLSGRAVVLSGSCSQMTNQQVAFYRQHAPTRDVDVARCLSSETREAYAEALAQW
VLSQDSELAPMISATASTQALAAIQQQYGATEASHAVEALFSLLAARLAEGGITRFIVAG
GETSGVVTQSLGITGFHIGPCISPGVPWVNALHAPVSLALKSGNFGDESFFIRAQREFQV
>EECEBICD_00550 3-oxo-tetronate 4-phosphate decarboxylase
MTTLAKEEHALREEMVRIAASFFQRGYATGSAGNLSLLLPDGNILATPTGSCLGNLDPQR
LSKVDPQGEWLSGDKPSKEVRFHLALYRNNPCCKAVVHLHSTWSTALSCLEGLDPQNVIR
PFTPYVVMRMGDIPLVPYYRPGDDRIARDLAALAARHQAFLLANHGPVVCGENLQEAANN
TEELEETAKLIFILGERPIRYLTTEEITQLRR
>EECEBICD_00551 2-oxo-tetronate isomerase
MPRFAANLSMMFTEVPFIERFAAAAEAGFQAVEFLFPYGFAASEIKAQLSRHDLTLALFN
TSAGDTAAGEWGRAALPGREHDARADIELALEYALALECEQVHIMAGVVQDGADGARYRA
TFIDNLRYAADRFAAHDKRILIEALSPGVKPGYLFSSQYQALGIAEEVDRPNVFIQLDTF
HAQKVDGNLSHLIREYAGRYAHVQIASLPDRHEPDDGEINYPWLFRLFDDVGYRGWIGCE
YQPRNTTQDGLGWFNAWR
>EECEBICD_00552 D-erythronate dehydrogenase
MQIIITGGGGFLGQKLASALLNSSLAFNELLLVDLKMPARLSDSPRLRCLEADLTQPGVL
ENVITANTSVVYHLAAIVSSHAEDDFDLGWKVNLDLTRQLLETCRRQPQKIRFVFSSSLA
VYGGTLPECVTDTTALTPRSSYGAQKAACELLVNDYTRKGYVDGLALRLPTICVRPGKPN
RAASSFVSAIIREPLQGETTVCPVSESLRLWISSPATVIHNLSLAATLPAPGEASSINLP
GISVTVGEMLETLRQAGGQAARDRVTHQRDEGVEKIVASWPGRIDNQRALALGFVADKRF
DDIIERFRQDDMEGRS
>EECEBICD_00553 High-affinity gluconate transporter
MTSLPTPIIGLIVAVFVLVWLVLRTRVHALIAMLAAACIAGLLGGMGIDKTLSVITSGFG
TTLGSIGLVIGLGVMMGRLLEVSGAAERIAWSFIKWLGKRREEWALAITGYIVSIPIFVD
SAFVILYPVAKALAKSGKRSLLTLGVALAGGLVVTHHTVPPTPGPLGVAGIFNVDIGAML
LTGMALAVPCVIGIVFYAQWLDKRYPDFVPRTLNADEVNAALEQYNKEKEQKDLPSLTLS
LLPIVVPIVLIFLKAICSTLATVEGWSGLATHPVVQAINFVGSPVIALAISVLLAVYTLV
PRMDKHTTAERLEEGLQSAGIILLVTGAGGALGAILRDSGAGQQLAEQVANLPISPILIP
FIVATLVRLIQGSGTVAMITAASISAPILAQIPGINMLLAAQAATMGSLFFGYFNDSLFW
VVNRMMGVSDVKQQMVVWSVPTTIAWAIGGTGVALINLLFGSGGSWLDPLLPIVVLAAIM
LWVRWQAQGIKDKLVVKD
>EECEBICD_00554 HTH-type transcriptional regulator DmlR
MLNLQRMSLFIAVVDSGSFTAAAAASGQTKAVVSFNIRQLEKELGVTLLLRSTRRLTLTD
AGVLFYQKGVNLLNAAKNLQDEVRASHSGLGGELRITTTPEFGEQVIIPLLAQFSQRHPD
LRIRHMSSSHHADLIAERFDVAIRLGSLADSRYRAALISRFTILPVAAPQWLARHPVSSL
ESLAQAEWIIHERLPTPLRWSVTNNHGQHSRLEISKAGKISVDSARSLMAFALAGSGVAL
LPQWLVNTALEDGTLIHVLPDYHFPRQGIYAVYPDARHVTTKVRAFIDFLRSQWDCGEHA
PSL
>EECEBICD_00555 Drug efflux pump JefA
MTYRSKVAVVYLLGFFLDLINLFIASVAFPAMSVELHTSISALAWVSNGYIAGLTLIVPF
SAFLSRYLGARRLIIFSLILFSVAAAAAGFADSLHSLVFWRIVQGAGGGLLIPVGQALTW
QQFKPHERAGVSSVVMMVALLAPACSPAIGGLLVETCGWRWIFFATLPVAVLTLLLAYRW
LNVASTTMVSARLLHLPLLTDKLLRFAMIVYLCVPGMFIGISVVGMFYLQNVAQLSPAAA
GSLMLPWSIASFVAIMLTGRYFNRLGPRPLIIVGCLLQAAGILLLTNVTPATSHRVLMMI
FALMGAGGSLCSSTAQSGAFLTIARRDMPDASALWNLNRQISFFLGATLLTLLLNAFQRV
MSLEVAYRWTFIAAAGITLLPLIYAVCLNNRQALLCLKKERP
>EECEBICD_00556 hypothetical protein
MNPYKEEIIHAHAAIENWLSKGMGSLEALIARFAVDFTMITPGGICLDYPALGAFFQAQR
ACRPGMVIVVEHIDLVAEWPEGAALRYRERQQLPGQAETVRWSTVILKRERGRIVWRHLH
ETTATA
>EECEBICD_00557 DNA mismatch repair protein MutS
MNESFDKDFSNHTPMMQQYLKLKAQHPEILLFYRMGDFYELFYDDAKRASQLLDISLTKR

GASAGEPIPMAGIPHHAVENYLAKLVNQGESVAICEQIGDPATSKGPVERKVVRIVTPGT
ISDEALLQERQDNLLAAIWQDGKGYGYATLDISSGRFRLSEPADRETMAAELQRTNPAEL
LYAEDFAEMALIEGRRGLRRRPLWEFEIDTARQQLNLQFGTRDLVGFGVENASRGLCAAG
CLLQYVKDTQRTSLPHIRSITMERQQDSIIMDAATRRNLEITQNLAGGVENTLAAVLDCT
VTPMGSRMLKRWLHMPVRNTDILRERQQTIGALQDTVSELQPVLRQVGDLERILARLALR
TARPRDLARMRHAFQQLPELHAQLETVDSAPVQALRKKMGDFAELRDLLERAIIDAPPVL
VRDGGVIAPGYHEELDEWRALADGATDYLDRLEIRERERTGLDTLKVGYNAVHGYYIQIS
RGQSHLAPINYVRRQTLKNAERYIIPELKEYEDKVLTSKGKALALEKQLYDELFDLLLPH
LADLQQSANALAELDVLVNLAERAWTLNYTCPTFTDKPGIRITEGRHPVVEQVLNEPFIA
NPLNLSPQRRMLIITGPNMGGKSTYMRQTALIALLAYIGSYVPAQNVEIGPIDRIFTRVG
AADDLASGRSTFMVEMTETANILHNATENSLVLMDEIGRGTSTYDGLSLAWACAENLANK
IKALTLFATHYFELTQLPEKMEGVANVHLDALEHGDTIAFMHSVQDGAASKSYGLAVAAL
AGVPKEVIKRARQKLRELESISPNAAATQVDGTQMSLLAAPEETSPAVEALENLDPDSLT
PRQALEWIYRLKSLV
>EECEBICD_00558 hypothetical protein
MESRQYTRHLSLSELKWFAIGIGFFILSIATATVNYRLSGISLLVGLLFIIWKFSVTVLF
LFTPRRMTLTETALQAGHRVIHYDALESMRLLHQSDKLILRHSGGKKYVIYLDFWNDGNG
IYDRLAAELVRRHGSALGARLAADGRLKFGKVTALADRLEHKNRAVPYAQIASIRTQREE
GAGSSMSYLMISTATGRICKIDRSTIVNEPLLLNFLSQRLPA
>EECEBICD_00559 hypothetical protein
MELYVSKTSQQNMPVEFNIYYSRSKTLVGIVFWICMMGVFIALAVSMATDPEEPKTGGAV
FMGLICCGSLLFIVRLARSFLSAAKPAVTISREGIRFCNGKFAAWTNIAENTWHSQSINF
IPAAAGIKIVLHEGKPIHFATTVIALSSDRYLEMCDLYSSQALG
>EECEBICD_00560 Serine/threonine-protein phosphatase 2
MELIRYADINSDLYRHIWVVGDIHGCYSLLLTRLAQLNFSPDTDLLISTGDNIDRGKENL
ETLRLLNTPWFISVVGNHEAMALDAFETQDGNFWYVNGGYWYDSVTEKDRQEATELLLTF
KQRPHIIEVETSSKKYVIAHADYPDDSYDYGKQVDIDSVLWSRDRLLGSLQGNIHPIRGA
DTFIFGHMIVDYTTTFANQIYIDTGSFCSGNLSFFKIK
>EECEBICD_00561 hypothetical protein
MGYHRRSVVETAMFRIKTLLGGHLSLRNYDAQVGEAMAMVKALNRMTLLAMPSSVRLV
>EECEBICD_00562 hypothetical protein
MMFTDWHEAAIGKTHNRMNFDCGDADLNQFLQRHARQNHEKGTTKTYVALDNSDVTRIHG
FYSVSPASLIYAQVPGAISKGLGRYDVPVFRLGRLAVDKSMQGQGLGAQLLLSAGKRCIQ
AALQVGGVALLIDAKNKQVCDWYKGFGAVPLNDQPLSLLLSFKTLYAALSASGRL
>EECEBICD_00563 hypothetical protein
MPQIAIESNERLSLRVSTDAKKLIVRAAAIQQTNLTDFVVSNVLPVAQKIVDAAERVYLT
ERDTQMIMEILDNPPAPNEKLLAAAFALPDMKK
>EECEBICD_00564 Type 3 secretion system pilotin
MKKFYSCLPVFLLIGCAQVPLPSSVSKPVQQPGAQKEQLANANSIDECQSLPYVPSDLAK
NKSLSNQNADNSASKNSAISSSIFCEKYKQTKEQALTFFQEHPQYMRSKEDEEQLMTEFK
KVLLEPGSKNLSIYQTLLAAHERLQSL
>EECEBICD_00565 Invasion protein InvF
MLNTQEVLKEGEKRKIRSPEAWFIQTCSAQKLHMSFSESRHNENCLIQEGALLFCEQAVV
APVSGDLVFRPLKIEVLSKLLAFIDGAGLVDTTYAESDKWVLLSPEFRAIWQDRKRCEYW
FLQQIITPSPAFNKVLALLRKSESYWLVGYLLAQSTSGNTMRMLGEDYGVSYTHFRRLCS
RALGGKAKSELRNWRMAQSLLNSVEGHENITQLAVNHGYSSPSHFSSEIKELIGVSPRKL
SNIIQLADK
>EECEBICD_00566 Type 3 secretion system secretin
MKTHILLARVLACAALVLVTPGYSSEKIPVTGSGFVAKDDSLRTFFDAMALQLKEPVIVS
KMAARKKITGNFEFHDPNALLEKLSLQLGLIWYFDGQAIYIYDASEMRNAVVSLRNVSLN
EFNNFLKRSGLYNKNYPLRGDNRKGTFYVSGPPVYVDMVVNAATMMDKQNDGIELGRQKI
GVMRLNNTFVGDRTYNLRDQKMVIPGIATAIERLLQGEEQPLGNIVSSEPPAMPAFSANG
EKGKAANYAGGMSLQEALKQNAAAGNIKIVAYPDTNSLLVKGTAEQVHFIEMLVKALDVA
KRHVELSLWIVDLNKSDLERLGTSWSGSITIGDKLGVSLNQSSISTLDGSRFIAAVNALE

EKKQATVVSRPVLLTQENVPAIFDNNRTFYTKLIGERNVALEHVTYGTMIRVLPRFSADG
QIEMSLDIEDGNDKTPQSDTTTSVDALPEVGRTLISTIARVPHGKSLLVGGYTRDANTDT
VQSIPFLGKLPLIGSLFRYSSKNKSNVVRVFMIEPKEIVDPLTPDASESVNNILKQSGAW
SGDDKLQKWVRVYLDRGQEAIK
>EECEBICD_00567 Protein MxiC
MIPGSTSGISFSRILSRQTSHQDATQHTDAQQAEIQQAAEDSSPGAEVQKFVQSTDEMSA
ALAQFRNRRDYEKKSSNLSNSFERVLEDEALPKAKQILKLISVHGGALEDFLRQARSLFP
DPSDLVLVLRELLRRKDLEEIVRKKLESLLKHVEEQTDPKTLKAGINCALKARLFGKTLS
LKPGLLRASYRQFIQSESHEVEIYSDWIASYGYQRRLVVLDFIEGSLLTDIDANDASCSR
LEFGQLLRRLTQLKMLRSADLLFVSTLLSYSFTKAFNAEESSWLLLMLSLLQQPHEVDSL
LADIIGLNALLLSHKEHASFLQIFYQVCKAIPSSLFYEEYWQEELLMALRSMTDIAYKHE
MAEQRRTIEKLS
>EECEBICD_00568 Invasion protein InvA
MLLSLLNSARLRPELLILVLMVMIISMFVIPLPTYLVDFLIALNIVLAILVFMGSFYIDR
ILSFSTFPAVLLITTLFRLALSISTSRLILIEADAGEIIATFGQFVIGDSLAVGFVVFSI
VTVVQFIVITKGSERVAEVAARFSLDGMPGKQMSIDADLKAGIIDADAARERRSVLERES
QLYGSFDGAMKFIKGDAIAGIIIIFVNFIGGISVGMTRHGMDLSSALSTYTMLTIGDGLV
AQIPALLIAISAGFIVTRVNGDSDNMGRNIMTQLLNNPFVLVVTAILTISMGTLPGFPLP
VFVILSVVLSVLFYFKFREAKRSAAKPKTSKGEQPLSIEEKEGSSLGLIGDLDKVSTETV
PLILLVPKSRREDLEKAQLAERLRSQFFIDYGVRLPEVLLRDGEGLDDNSIVLLINEIRV
EQFTVYFDLMRVVNYSDEVVSFGINPTIHQQGSSQYFWVTHEEGEKLRELGYVLRNALDE
LYHCLAVTLARNVNEYFGIQETKHMLDQLEAKFPDLLKEVLRHATVQRISEVLQRLLSER
VSVRNMKLIMEALALWAPREKDVINLVEHIRGAMARYICHKFANGGELRAVMVSAEVEDV
IRKGIRQTSGSTFLSLDPEASANLMDLITLKLDDLLIAHKDLVLLTSVDVRRFIKKMIEG
RFPDLEVLSFGEIADSKSVNVIKTI
>EECEBICD_00569 Surface presentation of antigens protein SpaK
MQHLDIAELVRSALEVSGCDPSLIGGIDSHSTIVLDLFALPSICISVKDDDVWIWAQLGA
DSMVVLQQRAYEILMTIMEGCHFARGGQLLLGEQNGELTLKALVHPDFLSDGEKFSTALN
GFYNYLEVFSRSLMR
>EECEBICD_00570 putative ATP synthase SpaL
MKTPRLLQYLAYPQKITGPIIEAELRDVAIGELCEIRRGWHQKQVVARAQVVGLQRERTV
LSLIGNAQGLSRDVVLYPTGRALSAWVGYSVLGAVLDPTGKIVERFTPEVAPISEERVID
VAPPSYASRVGVREPLITGVRAIDGLLTCGVGQRMGIFASAGCGKTMLMHMLIEQTEADV
FVIGLIGERGREVTEFVDMLRASHKKEKCVLVFATSDFPSVDRCNAAQLATTVAEYFRDQ
GKRVVLFIDSMTRYARALRDVALASGERPARRGYPASVFDNLPRLLERPGATSEGSITAF
YTVLLESEEEADPMADEIRSILDGHLYLSRKLAGQGHYPAIDVLKSVSRVFGQVTTPTHA
EQASAVRKLMTRLEELQLFIDLGEYRPGENIDNDRAMQMRDSLKAWLCQPVAQYSSFDDT
LSGMNAFADQN
>EECEBICD_00571 hypothetical protein
MHSLTRIKVLQRRCTVFHSQCESILLRYQDEDRGLQAEEEAILEQIAGLKLLLDTLRAEN
RQLSREEIYTLLRKQSIVRRQIKDLELQIIQIQEKRSELEKKREEFQKKSKYWLRKEGNY
QRWIIRQKRFYIQREIQQEEAESEEII
>EECEBICD_00572 hypothetical protein
MGDVSAVSSSGNILLPQQDEVGGLSEALKKAVEKHKTEYSGDKKDRDYGDAFVMHKETAL
PVLLAAWRHGAPAKSEHHNGNVSGLHHNGKGELRIAEKLLKVTAEKSVGLISAEAKVDKS
AALLSSKNRPLESVSGKKLSADLKAVESVSEVADNATGISDDNIKALPGDNKAIAGEGVR
KEGAPLARDVAPARMAAANTGKPDDKDHKKVKDVSQLPLQPTTIADLSQLTGGDEKMPLA
AQSKPMMTIFPTADGVKGEDSSLTYRFQRWGNDYSVNIQARQAGEFSLIPSNTQVEHRLH
DQWQNGNPQRWHLTRDDQQNPQQQHRQQSGEEDDA
>EECEBICD_00573 Surface presentation of antigens protein SpaO
MSLRVRQIDRREWLLAQTATECQRHGQEATLEYPTRQGMWVRLSDAEKRWSAWIQPGDWL
EHVSPALAGAAVSAGAEHLVVPWLAATERPFELPVPHLSCRRLCVENPVPGSALPEGKLL
HIMSDRGGLWFEYLPELPAVGGGRPKMLRWPLRFVIGSSDTQRSLLGRIGIGDVLLIRTS
RAEVYCYAKKLGHFNRVEGGIIVETLDIQHIEEENNTTETAETLPGLNQLPVKLEFVLYR

KNVTLAELEAMGQQQLLSLPTNAELNVEIMANGVLLGNGELVQMNDTLGVEIHEWLSESG
NGE
>EECEBICD_00574 Surface presentation of antigens protein SpaP
MGNDISLIALLAFSTLLPFIIASGTCFVKFSIVFVMVRNALGLQQIPSNMTLNGVALLLS
MFVMWPIMHDAYVYFEDEDVTFNDISSLSKHVDEGLDGYRDYLIKYSDRELVQFFENAQL
KRQYGEETETVKRDKDEIEKPSIFALLPAYALSEIKSAFKIGFYLYLPFVVVDLVVSSVL
LALGMMMMSPVTISTPIKLVLFVALDGWTLLSKGLILQYMDIAT
>EECEBICD_00575 Surface presentation of antigens protein SpaQ
MDDLVFAGNKALYLVLILSGWPTIVATIIGLLVGLFQTVTQLQEQTLPFGIKLLGVCLCL
FLLSGWYGEVLLSYGRQVIFLALAKG
>EECEBICD_00576 Surface presentation of antigens protein SpaR
MLYALYFEIHHLVASAALGFARVAPIFFFLPFLNSGVLSGAPRNAIIILVALGVWPHALN
EAPPFLSVAMIPLVLQEAAVGVMLGCLLSWPFWVMHALGCIIDNQRGATLSSSIDPANGI
DTSEMANFLNMFAAVVYLQNGGLVTMVDVLNKSYQLCDPMNECTPSLPPLLTFINQVAQN
ALVLASPVVLVLLLSEVFLGLLSRFAPQMNAFAISLTVKSGIAVLIMLLYFSPVLPDNVL
RLSFQATGLSSWFYERGATHVLE
>EECEBICD_00577 Surface presentation of antigens protein SpaS
MSSNKTEKPTKKRLEDSAKKGQSFKSKDLIIACLTLGGIAYLVSYGSFNEFMGIIKIIIA
DNFDQSMADYSLAVFGIGLKYLIPFMLLCLVCSALPALLQAGFVLATEALKPNLSALNPV
EGAKKLFSMRTVKDTVKTLLYLSSFVVAAIICWKKYKVEIFSQLNGNIVGIAVIWRELLL
ALVLTCLACALIVLLLDAIAEYFLTMKDMKMDKEEVKREMKEQEGNPEVKSKRREVHMEI
LSEQVKSDIENSRLIVANPTHITIGIYFKPELMPIPMISVYETNQRALAVRAYAEKVGVP
VIVDIKLARSLFKTHRRYDLVSLEEIDEVLRLLVWLEEVENAGKDVIQPQENEVRH
>EECEBICD_00578 Chaperone protein SicA
MDYQNNVSEERVAEMIWDAVSEGATLKDVHGIPQDMMDGLYAHAYEFYNQGRLDEAETFF
RFLCIYDFYNPDYTMGLAAVCQLKKQFQKACDLYAVAFTLLKNDYRPVFFTGQCQLLMRK
AAKARQCFELVNERTEDESLRAKALVYLEALKTAETEQHSEQEKE
>EECEBICD_00579 Cell invasion protein SipB
MVNDASSISRSGYTQNPRLAEAAFEGVRKNTDFLKAADKAFKDVVATKAGDLKAGTKSGE
SAINTVGLKPPTDAAREKLSSEGQLTLLLGKLMTLLGDVSLSQLESRLAVWQAMIESQKE
MGIQVSKEFQTALGEAQEATDLYEASIKKTDTAKSVYDAATKKLTQAQNKLQSLDPADPG
YAQAEAAVEQAGKEATEAKEALDKATDATVKAGTDAKAKAEKADNILTKFQGTANAASQN
QVSQGEQDNLSNVARLTMLMAMFIEIVGKNTEESLQNDLALFNALQEGRQAEMEKKSAEF
QEETRKAEETNRIMGCIGKVLGALLTIVSVVAAVFTGGASLALAAVGLAVMVADEIVKAA
TGVSFIQQALNPIMEHVLKPLMELIGKAITKALEGLGVDKKTAEMAGSIVGAIVAAIAMV
AVIVVVAVVGKGAAAKLGNALSKMMGETIKKLVPNVLKQLAQNGSKLFTQGMQRITSGLG
NVGSKMGLQTNALSKELVGNTLNKVALGMEVTNTAAQSAGGVAEGVFIKNASEALADFML
ARFAMDQIQQWLKQSVEIFGENQKVTAELQKAMSSAVQQNADASRFILRQSRA
>EECEBICD_00580 Cell invasion protein SipC
MLISNVGINPAAYLNNHSVENSSQTASQSVSAKDILNSIGISSSKVSDLGLSPTLSAPAP
GVLTQTPGTITSFLKASIQNTDMNQDLNALANNVTTKANEVVQTQLREQQAEVGKFFDIS
GMSSSAVALLAAANTLMLTLNQADSKLSGKLSLVSFDAAKTTASSMMREGMNALSGSISQ
SALQLGITGVGAKLEYKGLQNERGALKHNAAKIDKLTTESHSIKNVLNGQNSVKLGAEGV
DSLKSLNMKKTGTDATKNLNDATLKSNAGTSATESLGIKDSNKQISPEHQAILSKRLESV
ESDIRLEQNTMDMTRIDARKMQMTGDLIMKNSVTVGGIAGASGQYAATQERSEQQISQVN
NRVASTASDEARESSRKSTSLIQEMLKTMESINQSKASALAAIAGNIRA
>EECEBICD_00581 Cell invasion protein SipD
MLNIQNYSASPHPGIVAERPQTPSASEHVETAVVPSTTEHRGTDIISLSQAATKIQQAQQ
TLQSTPPISEENNDERTLARQQLTSSLNALAKSGVSLSAEQNENLRSAFSAPTSALFSAS
PMAQPRTTISDAEIWDMVSQNISAIGDSYLGVYENVVAVYTDFYQAFSDILSKMGGWLLP
GKDGNTVKLDVTSLKNDLNSLVNKYNQINSNTVLFPAQSGSGVKVATEAEARQWLSELNL
PNSCLKSYGSGYVVTVDLTPLQKMVQDIDGLGAPGKDSKLEMDNAKYQAWQSGFKAQEEN
MKTTLQTLTQKYSNANSLYDNLVKVLSSTISSSLETAKSFLQG
>EECEBICD_00582 Cell invasion protein SipA

MVTSVRTQPPVIMPGMQTEIKTQATNLAANLSAVRESATTTLSGEIKGPQLEDFPALIKQ
ASLDALFKCGKDAEALKEVFTNSNNVAGKKAIMEFAGLFRSALNATSDSPEAKTLLMKVG
AEYTAQIIKDGLKEKSAFGPWLPETKKAEAKLENLEKQLLDIIKNNTGGELSKLSTNLVM
QEVMPYIASCIEHNFGCTLDPLTRSNLTHLVDKAAAKAVEALDMCHQKLTQEQGTSVGRE
ARHLEMQTLIPLLLRNVFAQIPADKLPDPKIPEPAAGPVPDGGKKAEPTGINININIDSS
NHSVDNSKHINNSRSHVDNSQRHIDNSNHDNSRKTIDNSRTFIDNSQRNGESHHSTNSSN
VSHSHSRVDSTTHQTETAHSASTGAIDHGIAGKIDVTAHATAEAVTNASSESKDGKVVTS
EKGTTGETTSFDEVDGVTSKSIIGKPVQATVHGVDDNKQQSQTAEIVNVKPLASQLAGVE
NVKTDTLQSDTTVITGNKAGTTDNDNSQTDKTGPFSGLKFKQNSFLSTVPSVTNMHSMHF
DARETFLGVIRKALEPDTSTPFPVRRAFDGLRAEILPNDTIKSAALKAQCSDIDKHPELK
AKMETLKEVITHHPQKEKLAEIALQFAREAGLTRLKGETDYVLSNVLDGLIGDGSWRAGP
AYESYLNKPGVDRVITTVDGLHMQR
>EECEBICD_00583 Acyl carrier protein
MNMDIEARVKKVITSCIAVDVDSINGQTHLVEDLYADSLDLIDIVFGLSEEFDISCNEND
LPDMTTFADICRVVKKSLESRV
>EECEBICD_00584 hypothetical protein
MKIITHVVPGSGMAAIYDDIADSSRFVIKGKLRHVENDPKELLICVPMRSEWLFYWIKGE
KYCARRWARKSIKTLCNQIKFEEAIL
>EECEBICD_00585 Chaperone protein sicP
MQAHQDIIANIGEKLGLPLTFDDNNQCLLLLDSDIFTSIEAKDDIWLLNGMIIPLSPVCG
DSIWRQIMVINGELAANNEGTLAYIDAAETLLLIHAITDLTNTYHIISQLESFVNQQEVL
KNILQEYAKV
>EECEBICD_00586 Secreted effector protein SptP
MNNLTLSSFSKVGVSNDARLYIAKENTDKAYVAPEKFSSKVLTWLGKMPLFKNTEVVQKH
TENIRVQDQKILQTFLHALTEKYGETAVNDALLMSRINMNKPLTQRLAVQITECVKAADE
GFINLIKSKDNVGVRNAALVIKGGDTKVAEKNNDVGAESKQPLLDIALKGLKRTLPQLEQ
MDGNSLRENFQEMASGNGPLRSLMTNLQNLNKIPEAKQLNDYVTTLTNIQVGVARFSQWG
TCGGEVERWVDKASTHELTQAVKKFTLLLKN
>EECEBICD_00587 Secreted effector protein SptP
MPQTMSGPTLGLARFAVSSIPINQQTQVKLSDGMPVPVNTLTFDGKPVALAGSYPKNTPD
ALAAHMKMLLEKECSCLVVLTSEDQMQAKQLPPYFRGSYTFGEVHTNSQKVSSASQGEAI
DQYNMQLSCGEKRYTIPVLHVKNWPDHQPLPSTDQLEYLADRVKNSNQNGAPGRSSSDKH
LPMIHCLGGVGRTGTMAAALVLKDNPHSNLEQVRADFRNSRNNRMLEDASQFVQLKAMQA
QLLMTTAR
>EECEBICD_00588 hypothetical protein
MHYFFIIVIWLLSINTAWADCWLQAEKMFNIESELLYAIAQQESAMKPGAIGHNRDGSTD
LGLMQINSFHMKRLKKMGISEKQLLQDPCISVIVGASILSDMMKIYGYSWEAVGAYNAGT
SPKRSDIRKRYAKKIWENYRKLKGMSAEEKNKRLSIASNK
>EECEBICD_00589 Transcriptional regulator HilA
MQDQVQSESLHYSIVKGLSQYAPFGLSVLPVTITKNCRSVKDILELMDQLRPDYYISGQM
IPDGNDNIVQIEIVRVKGYHLLHQESIKLIEHQPASLLQNKIANLLLRCIPGLRWDTKQI
SELNSIDSTMVYLRGKHELNQYTPYSLQQALKLLTQCVNMSPNSIAPYCALAECYLSMAQ
MGIFDKQNAMIKAKEHAIKATELDHNNPQALGLLGLINTIHSEYIVGSLLFKQANLLSPI
SADIKYYYGWNLFMAGQLEEALQTINECLKLDPTRAAAGITKLWITYYHTGIDDAIRLGD
ELRSQHLQDNPILLSMQVMFLSLKGKHELARKLTKEISTQEITGLIAVNLLYAEYCQNSE
RALPTIREFLESEQRIDNNPGLLPLVLVAHGEAIAEKMWNKFKNEDNIWFKRWKQDPRLI
KLR
>EECEBICD_00590 Transcriptional regulator HilA
MPHFNPVPVSNKKFVFDDFILNMDGSLLRSEKKVNIPPKEYAVLVILLEAAGEIVSKKHL
TGPGMGRRGS
>EECEBICD_00591 HTH-type transcriptional activator RhaR
MENVTFVSNSHQRPAADNLQKLKSLLTNTRQQIKSQTQQVTIKNLYVSSFTLVCFRSGKL
TISNNHDTIYCDEPGMLVLKKEQVVNVTLEEVNGHMDFDILEIPTQRLGALYALIPNEQQ
TKMAVPTEKAQKIFYTPDFPARREVFEHLKTAFSCTKDTSKGCSNCNNKSCIENEELIPY

FLLFLLTAFLRLPESYEIILSSAQITLKERVYNIISSSPSRQWKLTDVADHIFMSTSTLK
RKLAEEGTSFSDIYLSARMNQAAKLLRIGNHNVNAVALKCGYDSTSYFIQCFKKYFKTTP
STFIKMANH
>EECEBICD_00592 Protein PrgH
MSCAVICCYRERQTSLTVYMETSKEKTITSPGPYIVRLLNSSLNGCEFPLLTGRTLFVVG
QSDALTASGQLPDIPADSFFIPLDHGGVNFEIQVDTDTTEIILHELKEGNSESRSVQLNT
PIQVGELLILIRPESEPWVPEQPEKLETSAKKNEPRFKNGIVAALAGFFILGIGTVGTLW
ILNSPQRQAAELDSLLGQEKERFQVLPGRDKMLYVAAQNERDTLWARQVLARGDYDKNAR
VINENEENKRISIWLDTYYPQLAYYRIHFDEPRKPVFWLSRQRNTMSKKELEMLSQKLRA
LMPYADSVNITLMDDVTAAGQAEAGLKQQALPYSRRNHKGGVTFVIQGALDDVEILRARQ
FVDSYYRTWGGRYVQFAIELKDDWLKGRSFQYGAEGYIKMSPGHWYFPSPL
>EECEBICD_00593 Protein PrgI
MATPWSGYLDDVSAKFDTGVDNLQTQVTEALDKLAAKPSDPALLAAYQSKLSEYNLYRNA
QSNTVKVFKDIDAAIIQNFR
>EECEBICD_00594 hypothetical protein
MSIATIVPENAVIGQAVNIRSMETDIVSLDDRLLQAFSGSAIATAVDKQTITNRIEDPNL
VTDPKELAISQEMISDYNLYVSMVSTLTRKGVGAVETLLRS
>EECEBICD_00595 Lipoprotein PrgK
MIRRYLYTFLLVMTLAGCKDKDLLKGLDQEQANEVIAVLQMHNIEANKIDSGKLGYSITV
AEPDFTAAVYWIKTYQLPPRPRVEIAQMFPADSLVSSPRAEKARLYSAIEQRLEQSLQTM
EGVLSARVHISYDIDAGENGRPPKPVHLSALAVYERGSPLAHQISDIKRFLKNSFADVDY
DNISVVLSERSDAQLQAPGTPVKRNSFATSWIVLIILLSVMSAGFGVWYYKNHYARNKKG
ITADDKAKSSNE
>EECEBICD_00596 Oxygen-regulated invasion protein OrgA
MNRQPLPIIWQRIIFDPLSYIHPQRLQIAPEMIVRPAARAAANELILAAWRLKNGEKECI
QNSLTQLWLRQWRRLPQVAYLLGCHKLRADLARQGALLGLPDWAQAFLAMHQGTSLSVCN
KAPNHRFLLSVGYAQLNALNEFLPESLAQRFPLLFPPFIEEALKQDAVEMSILLLALQYA
QKYPNTVPAFAC
>EECEBICD_00597 Oxygen-regulated invasion protein OrgB
MLKNIPIPSPLSPVEGILIKRKTLERYFSIERLEQQAHQRAKRILREAEEEAKTLRMYAY
QEGYEQGMIDALQQVAAYLTDNQTMAWKWMEKIQIYARELFSAAVDHPETLLTVLDEWLR
DFDKPEGQLFLTLPVNAKKDHQKLMVLLMENWPGTFNLKYHQEQRFIMSCGDQIAEFSPE
QFVETAVGVIKHHLDELPQDCRTISDNAINALIDEWKTKTQAEVIR
>EECEBICD_00598 hypothetical protein
MIPGTIPTSYLVPTADTEATGVVSLSARAAMLNNVDSAPLSNGGDVDLYDAFYQRLLALP
ESASSETLKDSIYQEMNAFKDPNSGDSAFVSFEQQTAMLQNMLAKVEPGTHLYEALNGVL
VGTMNAQSQMTSWMQEIILSGGENKEAIDW
>EECEBICD_00599 HTH-type transcriptional activator RhaR
MVLPSMNKSVEAISNNHLQQPNKFPLINGLADVRDYYVANCLLFKLNKGSLRIENEFGEF
IEQSAPCLFLLEKDQTITLSMSEIEGHIDFSSLEVSYDLMQKFYKVFYSTRNYNDRELSL
KTKPKYFFHADLLPGMSDTFDSILHGVACPRVCSNVSIDDHDYSYFSLMYLISAFVRKPG
GFDFLERAIKITTKEKVYNIIISDLTRKWSQAEVAGKLFMSVSSLKRKLAAEEVSFSKIY
LDARMNQAIKLLRMGAGNISQVATMCGYDTPSYFIAIFKRHFKITPLSFMRTMNH
>EECEBICD_00600 hypothetical protein
MRNVIIYGINWTNCYALQSIFKQKYPEKCVKTCNSLTALLHSLSDMPDAGLILALNPHEH
VYLFHALLTRLQNRKVLVVADRLYYIDRCVLQYFGVMDYVLKDELSCAIRSEREKLRLPE
AWLRFCHRPQKKTVAATYAFNAGETPEEVLFNINQYAWWNLPPGVTQAKYALLILLSSGH
PAIELAKKFGLGTKTVSIYRKKVMYRLGMDSSPLSLFRGLKLDAHLQRTAFAHNPAVPDD
NCALPIAVGMN
>EECEBICD_00601 Effector protein YopJ
MLSPTTRNMGASLSPQPDVSGELNTEALTCIVERLESEIIDGSWIHISYEETDLEMMPFL
VAQANKKYPELNLKFVMSVHELVSSIKETRMEGVESARFLVNMGSSGIHISVVDFRVMDG
KTSVILFEPAACSAFGPALLALRTKAALEREQLPDCYFAMVELDIQRSSSECGIFSLALA
KKLQLEFMNLVKIHEDNICERLCGEEPFLPSDKADRYLPVSFYKHTQGVQRLNEYVEANP

AAGSSIVNKKNETLYERFDNNAVMLNDKKLSISAHKKRIAEYKSLLKS
>EECEBICD_00602 Manganese transport system membrane protein MntB
MGIVFSGMFGAGLVLYVSIQSEVHLDHILFGDMLGVSLGDIVQTSVIALGIALIIGLKWK
DLLLHAFDPHQAKASGLNITLLHYGLLCMIALTIVATLKSVGIILSISLLIAPGAIAILL
TRRFARALGLAVSLSVITAFAGVYLSFYLDSAPAPTIVVLFAIVFIAVFIYATWRDRRNE
IVPEAQG
>EECEBICD_00603 hypothetical protein
MFLTTLLEPFQFDFMVNALMVSVIVAIPCALLSVFLVLKGWALMGDAMSHAVFPGVVLAY
IVGIPWRLALSLPDYFALSRPAIWTITAASNVIR
>EECEBICD_00604 Manganese transport system membrane protein MntB
MNWLVEPFGYQYMLNAMWVSAMVGGLCAFLSCYLMLKGWSLIGDALSHSIVPGVAGAWML
GLPFSLGAFLSGGLAAGSMLFLNQRSRLKEDAIIGLIFSSFFGVGLFMVSLNPMSVNIQT
IILGNVLAIAPADIAQLAIIGAVSLTILLLKWKDLMVVFFDETHARSIGLNPGRLKLLFF
TLLSVSTVAALQTVGAFLVICLVVTPGATAWLLTDRFPRLLMIAVVIGSLTSFLGAWLSY
WLDGATGGIIVVMQTLLFITAFIFAPKHGLLANRRRARLQKEPTCS
>EECEBICD_00605 Manganese transport system ATP-binding protein MntB
MSQSAITVDQVTVTYRNGHTALRDATFQVPGGSIAALVGVNGSGKSTLFKALMGFVHLAQ
GDITILQQSVNKALKKNLIAYVPQSEEVDWSFPVLVEDVVMMGRYGHMGWLRRPTAHDHA
CVDAALARVDMQEYRHRQIGELSGGQKKRVFLARAIAQDGQVILLDEPFTGVDVKTEARI
IDLLRELRDEGRTMLVSTHNLGSVTEFCDYTVMIKGTVLASGPTETTFTAANLEQAFSGV
LRHIALSGGEEHIITDDERPFISRRVASGGKSS
>EECEBICD_00606 putative periplasmic iron-binding protein
MTNLHRLKTLLIAGIVAILALSPAYAKEKFKVITTFTVIADMAKNVAGDAAEVSSITKPG
AEIHEYQPTPGDIKRAQGAQLILANGLNLERWFARFYQHLSGVPEVIVSTGVKPMGITEG
PYNGKPNPHAWMSAENALIYVDNIRDALVKYDPDNAQIYKQNAERYKAKIRQMADPLRAE
LEKIPADQRWLVTSEGAFSYLARDNDMKELYLWPINADQQGTPKQVRKVIDTIKKHHIPA
IFSESTVSDKPARQVARESGAHYGGVLYVDSLSAADGPVPTYLDLLRVTTETIVNGINDG
LRSQQ
>EECEBICD_00607 hypothetical protein
MPGKRIAREKLTIKKMIALYESQCPQASAVQGHYDALYAYAQKRLDKCVFGEEKPACKQC
PVHCYQPAKREEMKQIMRWAGPRMLWRHPVLTVRHLIDDKRPVPELPEKYQRKK
>EECEBICD_00608 Formate hydrogenlyase transcriptional activator
MSYTPMSDLGQQGLFDITRTLLQQPDLASLSEALSQLVKRSALADSAGIVLWQALSQRAQ
YYATRENGRPVEYEDETVLAHGPVRRILSRPDALHCNFHEFTETWPQLAASGLYPEFGHY
CLLPLAAEGRIFGGCEFIRQEDRPWSEKEYDRLHTFTQIVGVVAEQIQNRVNNNVDYDLL
CRERDNFRILVAITNAVLSRLDIDELVSEVAKEIHHYFNIDAISIVLRSHRKNKLNIYST
HYLDEHHPAHEQSEVDEAGTLTERVFKSKEMLLINLNERDPLAPYERMLFDTWGNQIQTL
CLLPLMSGKTMLGVLKLAQCEEKVFTTANLKLLRQIAERVAIAVDNALAYQEIHRLKERL
VDENLALTEQLNNVDSEFGEIIGRSEAMYNVLKQVEMVAQSDSTVLILGETGTGKELIAR
AIHNLSGRSGRRMVKMNCAAMPAGLLESDLFGHERGAFTGASAQRIGRFELADKSSLFLD
EVGDMPLELQPKLLRVLQEQEFERLGSNKLIQTDVRLIAATNRDLKKMVADREFRNDLYY
RLNVFPIQLPPLRERPEDIPLLVKAFTFKIARRMGRNIDSIPAETLRTLSSMEWPGNVRE
LENVVERAVLLTRGNVLQLSLPDITAVTPDTSPVATESAKEGEDEYQLIIRVLKETNGVV
AGPKGAAQRLGLKRTTLLSRMKRLGIDKDALA
>EECEBICD_00609 Carbamoyl dehydratase HypE
MNNIQLAHGSGGQAMQQLINSLFMEAFANPWLAEQEDQARLELAQLTAEGDRLAFSTDSY
VIDPLFFPGGNIGKLAICGTANDVAVSGAIPRYLSCGFILEEGLPMETLKSVVNSMAATA
READIAIVTGDTKVVQRGAADKLFINTAGMGAIPADIRWGAQTLSVGDVLLVSGTLGDHG
ATILNLREQLGLDGELASDCAVLTPLIQTLRHIDGVKALRDATRGGVNAVAHEFATSCGY
GIELSESALPLKPAVRGVCELLGLDALNFANEGKLVIAVERQAADRALAALRAHPLGRDA
ALIGEVVERKGVRLAGLYGVKRTLDLPHAEPLPRIC
>EECEBICD_00610 Hydrogenase maturation factor HypD
MRFVDEYRAPEQVMQLIEHLRERAALLPYTAERPLRIMEVCGGHTHAIFKFGLDQLLPEN
VEFIHGPGCPVCVLPMGRIDSCVEIASHPEVIFCTFGDAMRVPGKQGSLLQAKARGADVR

IVYSPMDALKLAQDNPTRKVVFFGLGFETTMPTTAITLQQAKQRDVRNFYFFCQHITLIP
TLRSLLEQPDNGIDAFLAPGHVSMVIGTEAYQFIAADFNRPLVVAGFEPLDLLQGVVMLV
EQKIAALSQVENQYRRVVPDAGNMLAQQAIADVFCVNGDSEWRGLGVIESSGVHLTPEYQ
RFDAEAHFRPAPQQVYDDPRARCGEVLTGRCKPHQCPLFGKTCNPETAFGALMVSSEGAC
AARYQYRQQECEV
>EECEBICD_00611 Hydrogenase maturation factor HypC
MCIGVPGQIRAIDGNQAKVDVCGIQRDVDLTLVGSCDENGQPRLGQWVLVHVGFAMSVIN
EAEARDTLDALQNMFDVEPDVGALLYGEER
>EECEBICD_00612 Hydrogenase maturation factor HypB
MCTTCGCAEGNLYIEGDEHNPHSAFRSAPFAPAARPALNITGIKTPDFAPSQTAEGDLHY
GHGEAGTHAPGMSQRRMLEVEIDVLDKNNRLAERNRARFAARQQLVLNLVSSPGSGKTTL
LTETLMKLKDRVPCAVIEGDQQTVNDAARIRATGTPAIQVNTGKGCHLDAQMIADAAPRL
PLDDHGILFIENVGNLVCPASFDLGEKHKVAVLSVTEGEDKPLKYPHMFAAASLMLLNKV
DLLPYLNFDVEKCIASAREVNPEIEIILISATSGEGMDQWLAWLEAQRCA
>EECEBICD_00613 Hydrogenase maturation factor HypA
MHEITLCQRALELIEQQASAYGAKRVTAVWIKIGAFSCVETSALSFCFDLVCRGTIAEGC
KLHLEEQEAECWCEHCQQYVTLLTHRVRRCPQCHSDTLRIVADDGLQIRRIEIDETED
>EECEBICD_00614 Formate hydrogenlyase regulatory protein HycA
MTIWEISEKADYIAQRHRRLQDQWHIYCNSLVQGITLSKARLHHAMSCAPERDLCFVLFE
HFRIYVALADGFNSHTIEYYVETKDGEDKQLIAQAQLDIDGKVDERVNNRDREQVLEHYL
EKIASVYDSLYTAVETNSPVNLRQLVKGNSPAV
>EECEBICD_00615 Hydrogenase-4 component A
MNRFVIADSTLCIGCHTCEAACSETHRQHGLQSMPRLKVMLNEKESAPQLCHHCEDAPCA
TVCPVNAINRVDGAVQLNESLCVSCKLCGIACPFGAIEFSGSRPLHIPANANTPKAPPAP
PAPARVSTLLDWVPGVRAIAVKCDLCSFDEQGPACVRMCPTKALHLVENTDIARASKRKR
ELTFNTDFGDLTLFQQAQSGDA
>EECEBICD_00616 Hydrogenase-4 component B
MSSLSLITSGVAWFAAAAVLAFLFSFHKALSGWIAGIGGAVGSLCTAGAGFTALTSAVTV
SGVMPFTGHMLQITPLNAIWLITLGLCGLFVSLFNIDWHRHPQVKANGLLINLLMAAAVC
AVVASNLGTMVVMAEIMALCAVFLTGGSKEGKLWFALGRLGTLLLAIACWLVWQRYGTLD
LGLLDQRAQQLPLGSDIWLLGVIGFGLLAGIIPLHGWVPQAHANASAPAAALFSTVVMKI
GLLGILTLSLLGGNAPLWWGVALLVLGMITAFVGGLYALMEHNIQRLLAYHTLENIGIIL
LGLGAGVTGIALNQPVLIALGLTGGLYHLLNHSLFKSVLFLGAGSIWFRTGHRDIEKLGG
IGKRMPVISIAMLVGLMAMAALPPLNGFAGEWVIYQSFFKLGNSGAFVGRLLGPLLAVGL
AITGALAVMCMAKVYGVTFLGAPRTKEAENASCAPILMGVSVVALAICCVIGGVAAPWLL
PMISTAVPLPLETAHTTVSQPMITLLLVACPLLPFIIMAMFKGNRLPSRSRGAAWVCGYD
HEQSMVITAYGFAMPVKEAFAPVLKLRKWLNPVSLVPGWQNAAAAVLFRRLALIELAVLV
VIVVSRGA
>EECEBICD_00617 Formate hydrogenlyase subunit 4
MSVFYPLIQALVLFAVAPLLSGITRVARARLHNRRGPGVLQEYRDIIKLLGRQSIAPSDS
GWVFRLTPFVMVGVMLTIATALPVVTVGSPLPQLGDLITLIYLFAIARFFFSIAGLDTGS
PFTAIGASREAMLGVLVEPILLLGLWVAAQVAGSTHISNIADTIYHWPVARSIPLILALC
ACAFATFIEMGKLPFDLAEAEQELQEGPLTEYSGSGFAVLKWGISLKQLVVLQMFVGVFL
PWGQMETFSAGGLLLALVIAIVKLIVGVLVIALFENSMARLRFCATSRVTWAGFGFAFLA
FVSLLAA
>EECEBICD_00618 Formate hydrogenlyase subunit 5
MSEEKLGQQYLAALHQAFPGVVLDEAWQTKDQLTITVKVNYLPEVVEFLYYQQGGWLSVL
FGNDERQLCGHYAVYYVLSMEQGTKCWITVRVEVDANKLEFPSVTPRVPAAVWGEREVRD
MYGLIPVGLPDERRLVLPDDWPDELYPLRKDSMDYRQRPAPTTDAETYEFINELGDKKNN
VVPIGPLHVTSDEPGHFRLFVDGENIIDADYRLFYVHRGMEKLAETRMGYNEVTFLSDRV
CGICGFAHSTAYTTSVENAMGIQVPERAQMIRAILLEVERLHSHLLNLGLACHFTGFDSG
FMQFFRVRETSMKMAEILTGARKTYGLNLIGGIRRDLLKEDMIQTRQLAQQMRRDVQELV
DMLLSTPNMEQRTVGIGRLDPEIARDFSNVGPMVRASGHARDTRADHPFVGYGLLPMEVH
SEQGCDVISRLKVRINEVYTSLNMIDFGLDNLPGGPLMVEGFTYIPHRFALGFAEAPRGD

DIHWSMTGDNQKLYRWRCRAATYANWPTLRYMLRGNTVSDAPLIIGSLDPCYSCTDRMTV
VDVRKKKSKVVPYKELERYSIERKNSPLK
>EECEBICD_00619 NAD(P)H-quinone oxidoreductase subunit I, chloroplastic
MFTFIKKVIKTGTATSSYPLEPIAVDKNFRGKPEHNPQQCIGCAACVNACPSNALTVETD
LATNELAWQFNLGRCIFCGRCEEVCPTAAIKLSQEYELAVWKKEDFLQQSRFALCNCRVC
NRPFAVQKEIDYAIALLAHNGDSRAENHRESFETCPDCKRQKCLVPSDRIELTRHMKEVS
>EECEBICD_00620 Formate hydrogenlyase subunit 7
MSNLLGPRDANGIPAPMTVDESIASMKASLLKNIKRSAYVYRVDCGGCNGCEIEIFATLS
PLFDAERFGIKVVPSPRHADILLFTGAVTRAMRSPALRAWQSAPDPKICISYGACGNSGG
IFHDLYCVWGGTDKIVPVDVYIPGCPPTPAATLYGFAMALGLLEQKIHARAPGELDDQPA
EILHPDMVQPLRVKVDRAARRLAGYRYGRQIADDYLTQLGQGEQQVARWLEAENDPRLTE
IVTHLNHVVEEARIR
>EECEBICD_00621 hypothetical protein
MSEQVVFSQLSRKFIDENDATPAEAQQVVYYSLAIGHHLGVIDCLEAALTCPWDEYLAWI
ATLEAGSDARRKMEGVPKYGEIVIDFNHVQMLARAFDEARAAQTPQQQEWSKLMLSMLHD
IHQESAIYLMVRRLRD
>EECEBICD_00622 Hydrogenase 3 maturation protease
MTDVLLCVGNSMMGDDGAGPLLAEMCAAQPKGNWVVIDGGSAPENDIVAIRELRPQRLLI
VDATDMGLNPGEIRIIDPDDIAEMFMMTTHNMPLNYLIDQLKEDVGEVIFVGIQPDIVGF
YYPMTQPIKDAVNIVYQRLEGWQGNGGFAALEAPEA
>EECEBICD_00623 hypothetical protein
MLHGKGCKKDVKIIVWSGLLFLCLAARALATGVVGYLPMSDGEYAQKRALKPLLTLPYSV
SPDQTWHFRQVGVSGVTLLPEPKKDNEWRISGKDRAGNSWVVPVGRLINLAGNAQFYRAD
LDRNGIQDLVIWLGNPGLGLAPSAQYIIFTISQEWPPLRL
>EECEBICD_00624 Hydrogenase-4 component A
MNRFIIADASKCIGCRTCEVACVVSHQENQDCASLTPETFLPRIHVIKGVNVSTATLCRQ
CEDAPCANVCPNGAISRDKGFVHVMQERCIGCKTCVVACPYGAMEVVVRPVVRNSGAGLN
VRAEKAEANKCDLCHHREAGPACMAACPTHALICVDRNKLEQLSAEKRRRAALDSTASLL
F
>EECEBICD_00625 Carbamoyltransferase HypF
MAIDTPSGVQLRIRGKVQGVGFRPFVWQLAQQLRLHGDVCNDGDGVVVRLLEEPSQFIAA
LYQDCPPLARIDSVEHASLVWERAPTDFTIRQSAGGSMNTQIVPDAATCPACLAEMNTPG
ERRYRYPFINCTHCGPRFTIIRAMPYDRPFTVMAAFPLCPECDSEYRDPYDRRFHAQPVA
CPSCGPHLEWRSQHERAEKEAALQAAVAQLNAGGIIAVKGLGGFIWPAMRATITQWRCCG
RVNIARRNHWR
>EECEBICD_00626 Carbamoyltransferase HypF
MLPTAQTLPSAARSLLTTPAAPIVLVDKQYVPSLSEGIAPGLTEVGVMLPANPLQHLLLQ
ALNYPLVMTSGNLSGKPPAITNEQALDDLHDIADGFLLHNRDIVQRMDDSVVRDSGEMLR
RSRGYVPDAIALPPGFRDVPPILCLGADLKNTFCLVRGEQAVVSQHLGDLSDDGIQAQWR
EALRLIQSIYDFTPERIVCDAHPGYVSSQWASEMRLPTETVLHHHAHAAACLAEHGWPLD
GGEVIALTVDGIGMGENGALWGGECLRVNYRECEHLGGLPAVALPGGDLAAKHPWRNLLA
QCLRFVPDWQDYPETAGLQQQNWNVLARAIERGVNAPLASSCGRLFDAVAAALRCTPASL
SYEGEAACALEALASQCANVEHPVTMPLNGAQLDVAVFWRQWLNWQATPAQRAWAFHDAL
ACGFATLMRQQATARGITTLVFSGGVIHNRLLRARLAFYLSDFKLLFPQRLPAGDGGLSF
GQGVIAAARALREV
>EECEBICD_00627 Nitric oxide reductase FlRd-NAD(+) reductase
MSRGIIIIGSGFAARQLVKNIRKQDAHVPLTLIAADSMDEYNKPDLSHVISQSQRADDLT
RQLAGEFAEQFNLRLFPHTWVADIDADAHVVKSQDKQWQYDKLVLATGAAAFVPPIAGRE
LMLTLNSQQEYRACETQLRDAQRVLIVGGGLIGSELAMDFCRAGKTVTLMDNAASLLASL
MPPEVSSRLQHHLTDMGVHLLLKSQLQKLEKTEAGIRATLVSQHSIEVDAVIAATGLRPE
TALARRAGVAVNRGVCVDSYLQTSHPDIYAIGDCAEINGQVLPFLQPIQLSAMYLAKNLL
GGNAPLKLPAMLVKVKTPELPLHLAGETQRSDLSWQITAESDGMIAKGMSGEGQLRAFVV
SEDRMKEAFALLKTLSV
>EECEBICD_00628 Anaerobic nitric oxide reductase flavorubredoxin

MSILVKNNIHWVGQRDWEVRDFHGTEYKTLRGSSYNSYLIREEKNVLIDTVDHKFSREFV
QNLRSEIDLADIDYIIINHAEEDHAGALTELMAQIPDTPIYCTANAIDSINGHHHHPEWN
FKVVKTGDTLDIGNGKQLIFVETPMLHWPDSMMTYMTGDAVLFSNDAFGQHYCDERLFND
EVDQTELFEQCQRYYANILTPFSRLVTPKITEILGFNLPVDMIATSHGVVWRDNPTQIVE
LYLKWAADYQEDRITIFYDTMSNNTRMMADAIAQGINEVDPNVAVKIFNVARSDKNEILT
NVFRSKGVLVGTSTMNNVMMPKIAGLVEEMTGLRFRNKRASAFGSHGWSGGAVDRLSTRL
QDAGFEMSLSLKAKWRPDLDALELCRQHGRDIARQWALAPLPETTQKTAPVEETTTCAAA
DFGPKMQCSVCQWIYDPALGEPLQDVAPGTPWNDVPDNFLCPECSLGKDVFDVLATEAK
>EECEBICD_00629 Anaerobic nitric oxide reductase transcription regulator NorR
MSFSVEVLAGIAIELQRGIGHQDRFQRLITTLRQVLACDASALLRYESRQFIPLAIDGLA
QDVLGRRFTLEGHPRLEAIARAGDVVRFPADSDLPDPYDGLIPGQESLKVHACVGLPLFA
GQNLIGALTLDAMTPEQFEVFSDEELRLVAALAAGALSNALLIEQLESQNMLPGSSGVFE
PIKETHMIGLSPAMTQLKKEIEIVAGSDLNVLIGGETGTGKELVAKAIHQGSPRAVNPLV
YLNCAALPESVAESELFGHVKGAFTGAISNRSGKFEMADNGTLFLDEIGELSLALQAKLL
RVLQYGDIQRVGDDRSLRVDVRVLAATNRDLREEVLAGRFRADLFHRLSVFPLFVPPLRE
RGDDVVLLAGYFCEQCRLRLGLSRVVLSPGARRHLLNYGWPGNVRELEHAIHRAVVLARA
TRAGDEVVLEEQHFALSEDVLPAPSAESFLALPACRNLRESTENFQREMIRQALAQNNHN
WAASARALETDVANLHRLAKRLGLKD
>EECEBICD_00630 Arabinose 5-phosphate isomerase GutQ
MSDALLNAGRQTLMLELQEASRLPERLGDDFVRAANIIIHCEGKVIVSGIGKSGHIGKKI
AATLASTGTPAFFVHPAEALHGDLGMIESRDVMLFISYSGGAKELDLIIPRLEDKSVALL
AMTGKPLSPLGRAAKAVLDISVEREACPMHLAPTSSTVNTLMMGDALAMAVMQARGFNEE
DFARSHPAGALGARLLNNVHHLMRQGDAIPQVMLATSVMDAMLELSRTGLGLVAVCDEQH
VVKGVFTDGDLRRWLVGGGALTTPVSEAMTPNGITLQAQSRAIDAKELLMKRKITAAPVV
DENGKLTGAINLQDFYQAGII
>EECEBICD_00631 Glucitol operon repressor
MKPRQRQAAILEHLQKQGKCSVEELAQYFDTTGTTIRKDLVILENAGTVIRTYGGVVLNK
EESDPPIDHKTLINTHKKALIAAAAVKYIHDGDSIILDAGSTVLQMVPLLSRFSNITVMT
NSLHIVNALSELDNEQTILMPGGTFRKKSASFHGQLAENAFEQFSFDKLFMGTDGIDLNV
GVTTFNEVYTVSNAMCNAAREVILMADSSKFGRKSPNVVCSLETVDKLITDAGIDPAFRQ
ALEAKGIEVIITGESNE
>EECEBICD_00632 hypothetical protein
MVSTLITVAVIAWCAQLALGGWQISRFNRAFDKLSQQGRVGVGRSGGRFKPRVVVAVALD
EQQRVTDTLLMKGLTVFARPVKIAAMQGKHLHELQPDVIFPHDSLAQNALSLALKLKHG
>EECEBICD_00633 Sorbitol-6-phosphate 2-dehydrogenase
MNQVAVVIGGGQTLGAFLCRGLAEEGYRVAVVDIQSDKAANVAQEINADFGEGMAYGFGA
DATSEQSVLALSRGVDEIFGRVDLLVYSAGIAKAAFISDFQLGDFDRSLQVNLVGYFLCA
REFSRLMIRDGIQGRIIQINSKSGKVGSKHNSGYSAAKFGGVGLTQSLALDLAEYGITVH
SLMLGNLLKSPMFQSLLPQYATKLGIKPDEVEQYYIDKVPLKRGCDYQDVLNMLLFYASP
KASYCTGQSINVTGGQVMF
>EECEBICD_00634 PTS system glucitol/sorbitol-specific EIIA component
MSVIYQTTITRIGQSAKEALGEQMLITFREGAPADIEEFCFIHCHGELTGALQPGARCEL
GQHCYPVTAVGSVAEQNLRELGHITLRFDGLREAEFPGTVHVAGPVPDDIAPGCILTFVA
>EECEBICD_00635 PTS system glucitol/sorbitol-specific EIIB component
MDGFKEGEPPEAEIGAAIIDCGGTLRCGIYPKRRIPTINIHSTGKSGPLAQYIVEDIYVS
GVKEENITLVGETPASPQPAKTTLGRDYDTSKKITEQSDGLLAKVGMGMGSAVAVLFQSG
RDTIDTVLKTILPFMAFVSALIGIIMASGLGDWIAHGLAPLASHPLGLVTLALICSFPLL
SPFLGPGAVIAQVIGVLIGVQIGLGNIPPHLALPALFAINAQAACDFIPVGLSLAEAKQD
TVRVGVPSVLVGRFLTGAPTVLIAWFVSGFIYQ
>EECEBICD_00636 hypothetical protein
MTRVRIEKGAGGWGGPLELDVTPGKKDRLYHSRYAPGDRRQTGATNRLASGGRL
>EECEBICD_00637 PTS system glucitol/sorbitol-specific EIIC component
MIETITHGAEWFIGLFQKGGEVFTGMVTGILPLLISLLVIMNALINFIGQQRIERFAQRC

AGNPLSRYLLLPFIGTFVFCNPMTLSLGRFMPEKYKPSYYAAASYSCHSMNGLFPHINPG
ELFVYLGIASGLTTLGLPLGPLAVSYLLVGLVTNFFRGWVTDLTTAIFEKKWGFNLSRKF
I
>EECEBICD_00638 Membrane-bound lytic murein transglycosylase B
MFKRRYVALLPLCVLLAACSSTPKSSETQATTGTPSGGFLLEPQHNVMQMGGDFANNPNA
QQFIDKMVNKHGFDRQQLQEILSQSKRLDYVLRLMDRQAPTTQPPAGPNGAWLRYRKQFI
TPDNVQNGVAFWNQYEDALNRAWQVYGVPPEIIVGIIGVETRWGRIMGKTRILDALATLS
FNYPRRAEYFSGELETFLLMARDEQDDPLNLKGSFAGAMGYGQFMPSSYKEYAVDFNGDG
HINLWDPVDAIGSVANYFKAHGWVKGDTIAVPANGQAPGLANGFKTKYSLSQLAAAGLTP
QQSLGNHQEASLLRLDIGTGYQYWYGLPNFYTITRYNHSTHYAMAVWQLGQAVALARVR
>EECEBICD_00639 hypothetical protein
MSLKKTESTQHKLKQTCQLRLWLIVGGVCSLGMGVLNILSSGYFEPYDGGQIILGLGCLG
YSLALSKKISHLQAENQPKKP
>EECEBICD_00640 Nicotinamide-nucleotide amidohydrolase PncC
MTDSELMRLSEQVGLALKARGATVTTAESCTGGWLAKAITDIAGSSAWFERGFVTYSNEA
KAQMIGVREETLAQHGAVSEPVVVEMAIGALKAARADFAVAISGIAGPDGGSEEKPVGTV
WFAFASVSGEGITRRECFSGDRESVRRQATTYALQTLWQQFLQNT
>EECEBICD_00641 Protein RecA
MAIDENKQKALAAALGQIEKQFGKGSIMRLGEDRSMDVETISTGSLSLDIALGAGGLPMG
RIVEIYGPESSGKTTLTLQVIAAAQREGKTCAFIDAEHALDPVYARKLGVDIDNLLCSQP
DTGEQALEICDALARSGAVDVIVVDSVAALTPKAEIEGEIGDSHMGLAARMMSQAMRKLA
GNLKQSNTLLIFINQIRMKIGVMFGNPETTTGGNALKFYASVRLDIRRIGAVKEGDNVVG
SETRVKVVKNKIAAPFKQAEFQILYGEGINFYGELVDLGVKEKLIEKAGAWYSYNGEKIG
QGKANATTWLKENPATAKEIEKRVRELLLSNQNATPDFAVDDSEGVAETNEDF
>EECEBICD_00642 Regulatory protein RecX
MRPFWHFSLLRFFMSEPTSRRPAYARLLDRAVRILAVRDHSEQELRRKLSAPVMGKNGPE
EIDATADDYERVIAWCHEHHYLDDERFVMRFIASRSRKGYGPARIRQELNQKGISRESTE
KAMRECEIDWSEMAREQAVRKYGEPLPSNFSEKVKVQRFLLYRGYLMDDIQQIWRNFAD
>EECEBICD_00643 Alanine--tRNA ligase
MSKSTAEIRQAFLDFFHSKGHQVVASSSLVPNNDPTLLFTNAGMNQFKDVFLGLDKRNYS
RATTSQRCVRAGGKHNDLENVGYTARHHTFFEMLGNFSFGDYFKHDAIQFAWELLTGENW
FALPKERLWVTVYETDDEAYEIWEKEVGIPRERIIRIGDNKGAPYASDNFWQMGDTGPCG
PCTEIFYDHGDHIWGGPPGSPEEDGDRYIEIWNIVFMQFNRQAEGTMEPLPKPSVDTGMG
LERIAAVLQHVNSNYDIDLFRTLIEAVAKVTGATDLGNKSLRVIADHIRSCAFLVADGVL
PSNENRGYVLRRIIRRAVRHGNMLGAKETFFYKLVGPLIEVMGSAGEELKRQQAQVEQVL
KTEEEQFARTLERGLALLDEELAKLQGDTLDGETAFRLYDTYGFPVDLTADVCRERNIKV
DEAGFEAAMEEQRRRAREASGFGADYNAMIRVDSASEFKGYDHLELNGKVTALFVDGKAV
EAINAGQEAVVVLDQTPFYAESGGQVGDKGELKGAGFTFAVDDTQKYGQAIGHLGKLSAG
ALKVGDAVQADVDEARRARIRLNHSATHLMHAALRQVLGTHVAQKGSLVSDKVLRFDFSH
NEAMKPSEIREVEDLVNAQIRRNLPIETNIMDLDAAKAKGAMALFGEKYDERVRVLSMGD
FSTELCGGTHASRTGDIGLFRIISESGTAAGIRRIEAVTGEGAMATVHAQSDRLNDIAHL
LKGDSQNLGDKVRAVLERTRQLEKELQQLKDQAAAQESANLSSKAVDLNGVKLLVSELAG
IEPKMLRTMVDDLKNQLGSTVIVLATVVEGKVSLIAGVSKDVTDRVKAGELIGMVAQQVG
GKGGGRPDMAQAGGTDAAALPAALASVQGWVSAKLQ
>EECEBICD_00644 Carbon storage regulator
MLILTRRVGETLMIGDEVTVTVLGVKGNQVRIGVNAPKEVSVHREEIYQRIQAEKSQQSS
Y
>EECEBICD_00650 Fructose-1-phosphate phosphatase YqaB
MYARYAGLIFDMDGTLLDTEPTHRKAWREVLGRYGLRFDEQAMVALNGSPTWLIAQSIIE
LNHADLDPLSLAREKTDAVKSILLDCVEPLPLVEVVKAWHGRRPMSVGTGSESAIAEALL
AHLGLRRYFDAVVAADHVQHHKPAPDTFLLCAQRMGIMPTQCVVFEDADFGLQAARAAGM
DAVDVRLL
>EECEBICD_00651 Inner membrane protein YqaA
MSDGLSLLLSLFASSFLSATLLPGNSEVVLVAMLVSGVSHPWVLVLTATMGNSLGGVTNVI

LGRFFPLRKTSRWQEKATGWLKRYGAATLLLSWMPVIGDLLCLLAGWMRIPWGRTIFFCV
LVRRCAMLRSQRRRFRA
>EECEBICD_00652 Glutamate--cysteine ligase
MIPDVSQALAWLEKHPQALKGIQRGLERETLRVNADGTLATTGHPEALGSALTHKWITTD
FAEALLEFITPVDGDIQHMLTFMRDLHRYTARKLGDERMWPLSMPCYIAEGQDIELAQYG
TSNTGRFKTLYREGLKNRYGALMQTISGVHYNFSLPMAFWQAKCGVTEGEAAKEKISAGY
FRLIRNYYRFGWVIPYLFGASPAICSSFLQGKPTTLPFEKTDCGMYYLPYATSLRLSDLG
YTNKSQSNLGITFNDLHEYVAGLKRAIKTPSEEYARIGVEKDGKRLQINSNVLQIENELY
APIRPKRVTRSGESPSDALLRGGIEYIEVRSLDINPFSPIGVDEQQVRFLDLFMVWCVLA
DAPEMSSDELLCTRTNWNRVILEGRKPGLTLGIGCETAQFPLPKVGKDLFRDLKRVAQTL
DSIHGGEEYQKVCDELVACFDNPELTFSARILRSMIDEGIGGTGKAFGEAYRNLLREEPL
EILQEEEFIAERDASVRRQQEIEAADTEPFAAWLAKHA
>EECEBICD_00653 S-ribosylhomocysteine lyase
MPLLDSFAVDHTRMQAPAVRVAKTMNTPHGDAITVFDLRFCIPNKEVMPEKGIHTLEHLF
AGFMRDHLNGNGVEIIDISPMGCRTGFYMSLIGTPDEQRVADAWKAAMADVLKVQDQNQI
PELNVYQCGTYQMHSLSEAQDIARHILERDVRVNSNKELALPKEKLQELHI
>EECEBICD_00654 hypothetical protein
MKAKYLALMIGACFSHNLWAANNITIEQRLAELEQRVVNAEKRASDAEAQIRSLKQQQVA
ATPTVNVQSAEPIAADKTPPKLTLSGFSDIKFYGDVEFNMDAASRSGSLTSMRTSANKDW
ASGTNERWDINGRLLLGFDGYQRLDNGNFAGFSVQPLADLTGKMNLDDAVFFFGRENDWK
IKVGRFEAYDMFPLNQDTFIEYSGNTANDLYSDGYGYIYMMKEGRGRSDSGGNFLLSKTI
DNWYFEVNTLLENGSTLYTEKQYHGMDLTNDKNVAYVRPVIAWQNGRFSTAIAMESNVVN
NAYGYYENGKWIDQSDRTGYGFTMTWNGQKTDPEDGAVINLNTAYMDATDETDFTAGVNA
LWHRFELGYIYAHNKIEAFNATNIDAVCEDDCWITDPGNYDIHTIHASYLFPNVMDMKNF
NIYLGAYASWVEANPNNGDNSEDARYGGRLRFKYFF
>EECEBICD_00655 Multidrug export protein EmrB
MQQQKPLEGAQLVIMTIALSLATFMQVLDSTIANVAIPTIAGNLGSSLSQGTWVITSFGV
ANAISIPLTGWLAKRFGEVKLFMWSTVAFAAASWACGVSSSLNMLIFFRVVQGVVAGPLI
PLSQSLLLNNYPPAKRSIALALWSMTVIVAPICGPILGGYISDNYHWGWIFFINVPIGIA
VVLMTLHTLRGRETHTERRRIDAVGLALLVIGIGSLQIMLDRGKELDWFSSQEIIILTVV
AVIAISFLIVWELTDDHPIVDLSLFKSRNFTIGCLCISLAYMLYFGAIVLLPQLLQEVYG
YTATWAGLASAPVGIIPVILSPIIGRFAHKLDMRRLVTFSFIMYAVCFYWRAWTFEPGMD
FGASAWPQFIQGFAVACFFMPLTTITLSGLPPERLAAASSLSNFTRTLAGSIGTSITTTM
WTDRESLHHAQLTESVTAYNPNAQAMYDKLEGLGMTHQQASGWIAQQITNQGLIISANEI
FWMSAGIFLVLLGLVWFAKPPFGAGGGGGGAH
>EECEBICD_00656 Multidrug export protein EmrA
MSANAEIQTPQQPAKKKGKRKTALLLLTLLFVIIAVAYGIYWFLVLRHIEETDDAYVAGN
QVQIMAQVSGSVTKVWADNTDFVKEGDVLVTLDQTDAKQAFERAKTALASSVRQTHQLMI
NSKQLQANIDVQKTALAQAQSDLNRRVPLGNANLIGREELQHARDAVASAQAQLDVAIQQ
YNANQAMILNSNLEDQPAVQQAATEVRNAWLALERTRIVSPMTGYVSRRAVQPGAQISPT
TPLMAVVPATDLWVDANFKETQLANMRIGQPVTVITDIYGDDVKYTGKVVGLDMGTGSAF
SLLPAQNATGNWIKVVQRLPVRVELDARQLEQHPLRIGLSTLVTVDTANRDGQVLASQVR
TTPVAESNAREINLAPVNKLIDDIVQANAG
>EECEBICD_00657 Transcriptional repressor MprA
MDSSFTPIEQMLKFRASRHEDFPYQEILLTRLCMHMQGKLLENRNKMLKAQGINETLFMA
LITLESQENHSIQPSELSCALGSSRTNATRIADELEKRGWIERRESDNDRRCLHLQLTEK
GQAFLQEVLPPQHHCLHQLWSSLSTAEKDQLEHITRKLLTRLDQMEQEGTVLEALR
>EECEBICD_00658 putative transporter
MTKPTHELSPALIVLMSVATGLAVASNYYAQPLLDTIAHHFSLSASSAGFIVTAAQLGYA
AGLLFLVPLGDMFERRTLIVSMTLLAAGGMLITASSQSLSMMILGTALTGLFSVVAQILV
PLAATLATPATRGKVVGTIMSGLLLGILLARTVAGLLANLGGWRTVFWVASALMALMAVA
LWRGLPKLKSDTHLNYPQLLGSVFSLFIHDKLLRTRALLGCLTFANFSILWTSMAFLLAA
PPFSYSEGMIGLFGLAGAAGALGARPAGGFADKGKSHLTTTFGLLLLLLSWLAIWLGHTS
VLALIIGILVLDLTVQGVHITNQTVIYRLHPDARNRLTAGYMTSYFIGGAAGSLISASAW

QHAGWAGVCLAGVTVALLNLLVWWRGFHRQEAVN
>EECEBICD_00659 Glycine betaine/proline betaine-binding periplasmic protein
MRHTVIFASAFATLVTASAFAADLPGKGITVQPIQSTISEETFQTLLVSRALEKLGYTVN
KPSEVDYNVGYTSIASGDATFTAVNWQPLHDDMYAAAGGDNKFYREGVFVSGAAQGYLID
KKTAEQYNITNIAQLKDPKIAKIFDTNGDGKADMMGCSPGWGCEAVINHQNKAFDLQKTV
EVSHGNYAAMMADTITRFKEGKPVLYYTWTPYWVSDVMKPGKDVVWLQVPFSSLPGEQKN
IDTKLPNGANYGFPVNTMHIVANKAWAEKNPAAAKLFAIMKLPLADINAQNAMMHAGKSS
EADVQGHVDGWINAHQQQFDGWVKEALAAQK
>EECEBICD_00660 Vitamin B12 import ATP-binding protein BtuD
MAIKLEVKNLYKIFGEHPQRAFKYIEKGLSKEQILEKTGLSLGVKDASLAIEEGEIFVIM
GLSGSGKSTMVRLLNRLIEPTRGQVLIDGVDIAKISDAELREVRRKKIAMVFQSFALMPH
MTVLDNTAFGMELAGIAAQERREKALDALRQVGLENYAHAYPDELSGGMRQRVGLARALA
INPDILLMDEAFSALDPLIRTEMQDELVKLQAKHQRTIVFISHDLDEAMRIGDRIAIMQN
GEVVQVGTPDEILNNPANDYVRTFFRGVDISQVFSAKDIARRSPVGLIRKTPGFGPRSAL
KLLQDEDREYGYVIERGNKFVGVVSIDSLKAALSQAQGIEAALIDDPLVVDAQTPLSELL
SHVGQAPCAVPVVDEEHQYVGIISKRMLLQALDREGVTMADQTNPWDTAQVADTTTQTAD
AWGTPAGVATDGGSTDWLNSAPAPAPEHFSLLDPFHKTLIPLDSWVTEGIDWVVTHFRPL
FQGIRVPVDYILNGFQQLLLGMPAPVAIILFALIAWQVSGVGMGIATLISLIAIGAIGAW
SQAMITLALVLTALLFCVVIGLPMGIWLARSPRAAKIVRPLLDAMQTTPAFVYLVPIVML
FGIGNVPGVVVTIIFALPPIVRLTILGINQVPADLIEASRSFGASPRQMLFKVQLPLAMP
TIMAGVNQTLMLALSMVVIASMIAVGGLGQMVLRGIGRLDMGLATVGGVGIVILAIILDR
LTQAVGRDSRSRGNRRWYTTGPVGLITRPFVK
>EECEBICD_00661 Ribonucleoside-diphosphate reductase 2 subunit beta
MKLSRISAINWNKIQDDKDLEVWNRLTSNFWLPEKVPLSNDIPAWQTLSAAEQQLTIRVF
TGLTLLDTIQNIAGAPSLMADAITPHEEAVLSNISFMEAVHARSYSSIFSTLCQTKEVDA
AYAWSEENPPLQRKAQIILAHYVSDEPLKKKIASVFLESFLFYSGFWLPMYFSSRGKLTN
TADLIRLIIRDEAVHGYYIGYKYQIALQKLSAIEREELKLFALDLLMELYDNEICYTEAL
YAETGWVNDVKAFLCYNANKALMNLGYEALFPPEMADVNPAILAALSPNADENHDFFSGS
GSSYVMGKTVETEDEDWNF
>EECEBICD_00662 Ribonucleoside-diphosphate reductase 2 subunit alpha
MQETMDYHALNAMLNLYDKAGHIQFDKDQQAIDAFFATHVRPHSVTFASQHERLETLVRE
GYYDDAVLARYNRAFVLRLFEHAHASGFRFQTFLGAWKFYTSYTLKTFDGKRYLEHFEDR
VTMVALTLAQGDETLATQLTDEMLSGRFQPATPTFLNCGKQQRGELVSCFLLRIEDNMES
IGRAVNSALQLSKRGGGVAFLLSNLREAGAPIKRIENQSSGVIPVMKMLEDAFSYANQLG
ARQGAGAVYLHAHHPDILRFLDTKRENADEKIRIKTLSLGVVIPDITFRLAKENAQMALF
SPYDVQRRYGKPFGDIAISERYDELIADPHVRKTYINARDFFQTLAEIQFESGYPYIMFE
DTVNRANPIAGRINMSNLCSEILQVNSASRYDDNLDYTHIGHDISCNLGSLNIAHVMDSP
DIGRTVETAIRGLTAVSDMSHIRSVPSIAAGNAASHAIGLGQMNLHGYLAREGIAYGSPE
ALDFTNLYFYTITWHAVHTSMRLARERGKTFAGFAQSRYASGDYFTQYLQDDWQPKTAKV
RTLFARSGITLPTREMWLKLRDDVMRYGIYNQNLQAVPPTGSISYINHATSSIHPIVAKI
EIRKEGKTGRVYYPAPFMTNENLDMYQDAYDIGPEKIIDTYAEATRHVDQGLSLTLFFPD
TATTRDINKAQIYAWRKGIKSLYYIRLRQLALEGTEIEGCVSCAL
>EECEBICD_00663 Protein NrdI
MSALVYFSSSSENTHRFMQRLGLPATRIPLNERERIQVDEPYILVVPSYGGGGMAGAVPR
QVIRFLNDEHNRARIRGVIASGNRNFGDAWGCAGDVIAQKCGVPWLYRFELMGTQRDIDN
VRKGVNEFWQQLSRSA
>EECEBICD_00664 Glutaredoxin-like protein NrdH
MSITIYTRNNCVQCHATKRAMESRGFEFEMVNVDLVPDAADTLRAQGFRQLPVVIAGDLS
WSGFRPDMINRLHPTPHAANA
>EECEBICD_00665 Alkyl hydroperoxide reductase AhpD
MTTLRQPYYELSPEVYSALVQAKNALEKSTIDPTLMELIYLRVSQINGCAFCLEMHSKAL
RKAGVNQAKLDALAGWRVSHHFNEQECAALAWAESVTHIAETHAEDNVYLPLLDHFSARE
ISDLTFAIGLMNCFNRLAIGMRM

>EECEBICD_00666 HTH-type transcriptional regulatory protein GabR
MPRYQHIARQLKTAIEQGELAPGTRLPSSRTWAQELGVSRATVENAYGELVAQGWLERRG
QAGTFVSNALRFETAPPIPAVFAGESPEPKPFQMGLPALDLFPREKWARVMGRRLRTQTR
FDLALGDVCGEAILRQAIVDYLRVSRSIECLPEQVFITSGYADSMRLILRTLSVPGDSMW
VEDPGFPLIRPVITQEGITLAPIPVDADGLNVAAGMRDCPQGRFALVTPAHQSPLGVALS
LTRRRQLLAWAANVQAWIIEDDYDSEFRYHGKPLPPLKSLDAPQRVIYAGTFSKSLFPAL
RTAWLVVPIKQIEHFRQQASLMPCSVPLLWQHTLADFIRDGHFWRHLKKMRQHYAQRRLW
IEEALAEQGFVVTLQKGGIQLVIEVEGDDKAQVAKANQAGLAVQALSRWRVVSSGKGGIL
LSFTNITSAAMAKQVAWQLRQAIQ
>EECEBICD_00667 putative protein YgaM
MLNKPNRNDVDDGVQDIQNDVNRLADSLEDVLKSWGSDAKDEAEAARRKAQALLKETRAR
MHGRTRVKQAACDAMGCADTFVREKPWCSVGTAAAVGIFIGALLSLRR
>EECEBICD_00668 hypothetical protein
MYLRPDEVARVLEKAGFTVDVVTNKTYGYRRGENYVYVNREARMGRTALIIHPRLKDRSS
SLADPASDIKTCDHYQNFPLYLGGETHEHYGIPHGFSSRIALERYLNGLFGDEKTD
>EECEBICD_00669 L-alanine exporter AlaE
MFSPQSRLRHAVADTFAMVVYCSVVNMLIEIFLSGMSFEQSLSSRLVAIPVNILIAWPYG
VYRDLIMRVARKASPAGWAKNLADVLAYVTFQSPVYIIILLTVGAGWHQIVAAVSSNIVV
SMLMGAVYGYFLDYCRRLFKVSSYHQAKA
>EECEBICD_00670 DNA-binding protein StpA
MNLMLQNLNNIRTLRAMAREFSIDVLEEMLEKFRVVTKERREEEELQQRQLAEKQEKINA
FLELMKADGINPEELFAMDSAMPRSAKKRQPRPAKYRFTDFNGEEKTWTGQGRTPKPIAQ
ALAAGKSLDDFLI
>EECEBICD_00671 hypothetical protein
MFTPGDIVQPRMGGPKLKVIEVNEDHIVAVQVGNEQGEKLILKAADVTPYCEEGDFGVC
>EECEBICD_00672 Inner membrane protein YgaP
MSIGIVSPREAQALIAQGAKLIDVRDADEYLREHIPHAQLAPLSRLEQGDLPANLRAEQI
IFHCQSGKRTSSNAAKLQAIAAPAQVSLLEGGIDGWKAAGLPVTEDKSQPLPLMRQVQIA
AGGLTLLGVILGYTVHGGFFLISGFVGAGLMLAGMTGFCGMARLLEKMPWNTRTH
>EECEBICD_00673 putative HTH-type transcriptional regulator YgaV
MTELEQLQASAEQAAALLKAMSHPKRLLILCMLCGSPKTSAGELARITGLSPSATSQHLA
RMREEGLIDSQRDAQRIHYFIKNEAVNTIIATLKNLYCP
>EECEBICD_00674 hypothetical protein
MGFWRIVFTIILPPLGVLLGKGFGWAFILNILLTLLGYIPGLIHAFWVQMRH
>EECEBICD_00675 Potassium binding protein Kbp
MGLFNFVKDAGEKLWDAVTANHDKDDQAKKVQEHLNKTGIPDADKVNVQIADGKATVTGD
GLSQEAKEKILVAVGNIAGISSVDDQVKTTTPAAESQFYTVKSGDTLSAISKQVYGNANL
YNKIFEANKPMLKSPEKIYPGQVLRIPEE
>EECEBICD_00676 HTH-type transcriptional repressor GlaR
MTALSQPTAIDGYRWLKNDIIRGTYQPDEKLRMSLLTARYALGVGPLREALSQLVAERLV
TVVNQKGYRVASMSEAELLDIFDARANMEAMLVRLAIERGDDAWEAEILARAHMLSKLEA
SDASEHMLDEWDQRHQAFHSAIVAGCGSHYLLQMRERLFDLAARYRFIWLRETVLSVEML
EDKHIQHHTLTEAILAREAARASELMRQHLLTPIPIIRQAMAGKM
>EECEBICD_00677 GABA permease
MGQLSESHALGGGLKSRHVTMLSIAGVIGASLFVGSSVAIAEAGPAVLLAYLFAGLLVVM
IMRMLAEMAVATPDTGSFSTYADKAIGPWAGYTIGWLYWWFWVLVIPLEANIAAIILNSW
IPGIPVWLFSLVITLALTGSNLLSVKNYGEFEFWLALCKVIAILAFIALGATAISGFYPY
AEVSGISRLWDHGGFMPNGFGAVLSAMLITMFSFMGAEIVTIAAAESDTPDKHIVRATNS
VIWRISIFYLCSIFVVVALIPWNMPGLKSVGSYRSVLELLHIPHAKFIMDCVILLSVTSC
LNSALYTASRMLYSLSRRGDAPAIMGKTNRSKTPWVAVLLSTGAAFLTVIVNYYAPAKVF
KFLIDSSGAIALLVYLVIAISQLRMRKILLAQGGEIKLKMWLYPWLTWLVIGFICFVLVV
MLFRPAQQLEVISTGLLGLGIIGTVPIMSRWKKLIRWQKAPLQNLR
>EECEBICD_00678 4-aminobutyrate aminotransferase GabT
MNTNNALMQRRHNAVPRGVGQIHPIFAERAENCRVWDVEGREYLDFAGGIAVLNTGHLHP

GIVSAVEAQLKKLSHTCFQVLAYEPYLALCERMNQKVPGDFAKKTLLVTTGSEAVENAVK
IARAATKRSGAIAFSGAYHGRTHYTLSLTGKVHPYSAGMGLMPGHVYRALYPCPLHNVSD
DDAIASIERIFKNDAAPEDIAAIIIEPVQGEGGFYAASPAFMQRLRALCDQHGIMLIADE
VQSGAGRTGTLFAMEQMGVAADITTFAKSIAGGFPLAGVTGRADVMDAIAPGGLGGTYAG
NPIACAAALAVLDIFEQENLLQKANTLGKTLRDGLMEIAETHREIGDVRGLGAMIAIELF
ENGDPGKPNAALTADIVARAREKGLILLSCGPYYNILRILVPLTIEASQIRQGLEIIAQC
FDEAKQA
>EECEBICD_00679 Succinate-semialdehyde dehydrogenase [NADP(+)] GabD
MQLNDSTLFRQQAFIDGDWRDARGGDVIPVSNPANGKPLGNVPKMGAEETRDAINAANRA
LPAWRALTAKERANILRRWFNLMMEHQDDLAHLMTLEQGKPLAEAKGEISYAASFIEWFA
EEGKRIYGDTIPGHQADKRLLVIKQPIGVTAAITPWNFPSAMITRKAGPALAAGCTMVLK
PASQTPFSALALAELARRAGIPAGVFNVVTGSAGDIGGELTSNPLVRKLSFTGSTEIGRQ
LMEQCAKDIKKVSLELGGNAPFIVFDDADLDKAVEGALASKFRNAGQTCVCANRLYVQDG
VYDRFAEKLNQAVNKLAVGDGLQADVAIGPLIDEKAVAKVQEHIADALEKGARVITGGEA
HKLGGNFFQPTILADVPDNAKVAKEETFGPLAPLFRFSDEADVIRQANDTEFGLAAYFYA
RDLSRVFRVGEALEYGIVGINTGIISNEVAPFGGIKASGLGREGSKYGIEDYLEIKYMCI
GL
>EECEBICD_00680 L-2-hydroxyglutarate dehydrogenase
MYDFVIIGGGIIGMSTAMQLIDVYPDARIALLEKESAPACHQTGHNSGVSHAGVYYTPGS
LKARFCLAGNQATKTFCDQNNIRYDTCGKMLVATSELEMARMRALWERTAANGLEREWLS
AAELREREPNIIGLGGIFVPSSGIVSYRDVATAMANRFQAKGGEIIYHAEVSALTEHAAG
IVIRTSQGREIETATLIGCAGLMADRLVKMLGVEPGFIICPFRGEYFRLAPRHNRIVNHL
IYPIPDPAMPFLGVHLTRMIDGSVTVGPNAVLALKREGYRKRDVSFTDTLEIFRSAGIRR
VLQNHLLSGLGEMKNSLCKSGYLRRVQKYCPSLTVNDLQPWPAGVRAQAVSPDGKLIDDF
LFVTTPRSIHTCNAPSPAATSAIPIGAHIVSKVQALRASQSNPGRTLRAARSVDALHAAF
TR
>EECEBICD_00681 Glutarate 2-hydroxylase
MNALTAVKANTDDLAQRHTGFTLAPSAQSPRLLALTFTADTTRQFLHQVAQWPVQALEYK
SFLRFKIGKILDDLCGNQLQPLLIKTLLNRAQGALLISAEGLDDVAQAEEMVKLATAVAH
LIGRSNYDAMSGQYYARFVVKNVDNSDSYLRQPHRVMELHNDGTYVEEVTDYVLMMKIDE
QNMEGGNSLLLHLDDWEHLESFFTHPLARRVMRWAAPPSKNVSHDVWHPVFDVDQQGRPV
MRYIDQFVQPKDFEEGVWLSELSDALETSQNILSVPVPVGKFLLINNLFWLHGRDRFTPH
PDLRRELMRQRGYFAYAASHYQTHQ
>EECEBICD_00682 hypothetical protein
MGKVMLAGLPGLTHERVGRTSTTYFSSQLTGVNLRFTAVFTEQT
>EECEBICD_00683 hypothetical protein
MDTWIYLSQGFAVAMTPENLVIALIGCFVGTIVGLLPGLGPINGVAILLPLAFALHLPAE
SALILLATVYIGCEYGGRISSILLNVPGDAAAIMTALDGYPMAQQGKGGVALSISAVSSF
FGSLIAIGGIILFAPLLAQWSLAFGPAEYFALMVFAIACLGSMMAQNPLKSFLAALIGLG
LATVGVDANTGVYRFTFDSVHLSDGVQFIVVVIGLFSVSEILLMLEHTSSGQTMVRKTGR
MLFNLKEGAQCIGTTLRSSVIGFFVGVLPGAGATIASAITYMTEKKLSGNSDSFGKGDIR
GVAAPEAANNASACGSFIPMLTLGVPGSGTTAVMMGALTLYNITPGPAMFTEQPDIVWGL
IAALLIANVMLLIMNIPLIGLFTRMLTIPLWFLVPAIAAVSAVGVYAVHSTTFDLVLMVA
LGVLGYILRKMHFPMSPLILGFVLGEMLEQNLRRALSISNGNMAILWQSGVAKALLIMAI
MVIVVPPVLRLLRKHSRKPQVDAG
>EECEBICD_00684 hypothetical protein
MSDRIFAGIWLLLCIAGLFIAWQIQSEYSYEPVGPRPFPLGIIGLMALCALALLLRHPDT
VSWPRRHVLQKLITMIIILLMYAWGFEWLGFPIATALLTMVIGMLFGATIPAAGISGAVL
GILLWYAFDRLLDVTLPLGAWLS
>EECEBICD_00685 hypothetical protein
MKKQLLRTLTASILLMSTSVLAQEAPSRTECIAPAKPGGGFDLTCKLIQVSLLETGAIEK
PMRVTYMPGGVGAVAYNAIVAQRPGEPGTVVAFSGGSLLNLSQGKFGRYGVDDVRWLASV
GTDYGMIAVRADSPWKTLKDLMTAMEKDPNSVVIGAGASIGSQDWMKSALLAQKANVDPH
KMRYVAFEGGGEPVTALMGNHVQVVSGDLSEMVPYLGGDKIRVLAVFSENRLPGQLANVP

TAKEQGYDLVWPIIRGFYVGPKVSDADYQWWVDTFKKLQQTDEFKKQRDLRGLFEFDMTG
QQLDDYVKKQVTDYREQAKAFGLAK
>EECEBICD_00686 Transcriptional regulatory protein tctD
MRLLLAEDNRELAHWLEKALVQNGFAVDCVFDGLAADHLLHSEMYALAVLDINMPGMDGL
EVVQRLRKRGQTLPVLLLTARSAVADRVKGLNVGADDYLPKPFELEELDARLRALLRRSA
GQVHEVQQLGELIFHDEGYFLLQGQPLALTPREQALLTVLMYRRTRPVSRQQLFEQVFSL
NDEVSPESIELYIHRLRKKLQGSDVRITTLRGLGYVLERGDEVG
>EECEBICD_00687 Sensor protein QseC
MKWVKPQSLYLQLLLFLGLPLILLWGLSAFNSYVNALQAATQAYDRTLLSSARTVSERLV
VRNNHLEVNVPWVVLDSFELNMNDRLYYKVVDPSGKVISGYDDLPVMPPATPRTRLYPAL
AWFYHTEYRGEAIRVARLLQPVNEGGIIGMAEIYVAETLQSRRYLAGQLLFSSWISQGLL
VLLTLVLVGWLLRRILRPMRQLSSLMVRREPGLLTPLPELLPWSETRLLIVAFNRYIDRL
RGILSRQERFSADASHQLKTPLAVLKTQAAVALASQHPHHWYESLQAMSVTLDSTIQLTE
RLLQLSAVKHKEQGERRFSPVNLYDIVQSGCFTRLAQARSKHIDLGYEGEQEAMWIEGDE
VLLSELCGNLLDNALKYTPEQGIVTARLERDGDAVTLVVEDSGPGIDDEHIHLALQPFHR
LDNVGNVAGAGIGLALVNDIARLHRTHPHFSRSEALGGLYVRIRFLSLVPQ
>EECEBICD_00688 High-affinity nickel transport protein
MNNHTKRRGIALTVFLVGVNILAWIWAFCVFHHHAVMLSAAILAYSFGLRHAVDADHIAA
IDTVTRKLMQQGKTPLGVGAFFSLGHSTIVVLACLAIVVTSMAFRDRIDVLHQYGSLIGT
AVSAFFLLAMALLNLFILFNVWRQFRSVTRGESVRAHDEAIPGGLMTRIFQRTFRLVTSS
WHMYFVGFLFGLGFDTATEVGLLGISASAANQGLSLWSMMIFPVLFTAGMALVDSLDNFV
MVGAYGWAFSHPLRKLYYNMTITAASVIVALAIGGLEALGLIDDALQLSGTFWQTVSTLN
DHMGNVGFWVVGAFVLFWLLSLLNYRWRGYDKITLNT
>EECEBICD_00689 hypothetical protein
MTHADWLILGYTTVIIIFGIVCYIGLLKLITKGKHEK
>EECEBICD_00690 hypothetical protein
MKIQEVKRILTRWQPSSFTLYREVFTQYGGSINMHPDIVDYFMKRHNWHFKFFHYKEDDK
IKGAYFICNDQNIGILTRRTFPLSSDEILIPMAPDLRCFLPDRTNRLSALHQPQIRNAIW
KLARKKQNCLVKETFSSKFEKTRRNEYQRFLKKGGSVKSVADCSSDELTHIFIELFRSRF
GNTSSCYPADNLANFFSQLHHLLFGHILYIEGIPCAFDIVLKSESQMNVYFDVSNGAIKN
EFRPLSPGSILMWLNINRARHYCQERQKKLLFSIGILKPEWEYKRMWSTPYFTGKSIC
>EECEBICD_00691 hypothetical protein
MTMQQSDMERYNPLLMLKEVMAQTPYRHKRWGERKFRYKFVLRCLINPVTTIKYFNELCH
LSQPRTLIIHRPLLPAKIQRPYLYTGLSIRCRAKAILEHYQFVQSFPESKIKKILLSEEQ
ILLAHLEGKNDALVDIYCGPCGYDREGELTLTLCFNDTPLARLSFSFIRHEGKQIALVAG
LQGPSKHVGPQVIRNATKDCYGLFPKRMLYEAFATLMQACNVDEIYAVSENNHVYRQLRY
LFQKKKTFVASYSEFWESLNGVKKGALYHLPSQVMRKAPESIPSKKRAEYRKRYHILDTI
IQEVNSLSR
>EECEBICD_00692 Secreted effector protein PipB2
MERSLDSLAGMAKSAFGAGTSAAMRQATSPKTILEYIINFFTCGGIRRRNETQYQELIET
MAETLKSTMPDRGAPLPENIILDDMDGCRVEFNLPGENNEAGQVIVRVSKGDHSETREIP
LASFEKICRALLFRCEFSLPQDSVILTAQGGMNLKGAVLTGANLTSENLCDADLSGANLE
GAVLFMADCEGANFKGANLSGTSLGDSNFKNACLEDSIMCGATLDHANLTGANLQHASLL
GCSMIECNCSGANMDHTNLSGATLIRADMSGATLQGATIMAAIMEDAVLTRANLRKASFI
STNLDGADLAEANLNNTCFKDCTLTHLRTEDATMSTSTQTLFNEFYSENI
>EECEBICD_00693 Ferric enterobactin receptor
MKVNKFLWLITVVSTGVNSPLSAAESTDDNGETMVVESTAEQVLKQQPGVSIITRDDIQK
NPPVNDLADIIRKMPGVNLTGNSASGTRGNNRQIDIRGMGPENTLVLIDGVPVTSRNSVR
YSWRGERDTRGDTNWVPPEMVERIEVIRGPAAARYGSGAAGGVVNIITKRPTNDWHGSLS
LYTNQPESSKEGDTRRGNFSLSGPLAGDTLTMRLYGNLNRTDADSWDINSSAGTKNAAGR
EGVTNKDINSVFSWKMTPQQILDFEAGYSRQGNIYAGDTQNSTSNAVTKSLAQSGRETNR
LYRQNYGLTHNGIWDWGQSRLGFYYEKTDNTRMNEGLSGGGEGRITNDQTFTTNRLISYR
TSGEVNVPVIWLFEQTLTVGAEWNRDELNDPSSTSLTVKDSNIAGIPGSAANRSSKNKSE
ISALYVEDNIEPMAGTNIIPGLRFDYLSESGSNFSPSLNLSQELGEYVKVKAGIARAFKA

PNLYQTSEGYLLYSKGNGCPKDITSGGCYLVGNKNLDPEISINKEIGLEFTVDDYHASVT
YFRNDYQNKIVAGDQIIGRSASGAYVLQWQNGGKALIEGIEASMAVPLMPDRLNWNTNAT
YMITSEQKDTGNPLSIIPKYTVNTFLDWTITSALSANVNWTLYGKQKPRTHAESRSEETK
GLSGKALGAYSLVGANVNYDINKNLRLNVGISNIFDKQIYRSAEGANTYNEPGRAYYAGV
TASF
>EECEBICD_00694 hypothetical protein
MYGRQYHNKCYRMALFLVSFCFSALSYAQPDMQPLGPNIADKGSGYYHFRVNDFQSADGA
RHYRVWTAIPNKAAPPSGYPVLYMLDGNAVMDRLPETLLKQLADHSPPVIVAIGYQTNLP
FDLNGRAYDYTPAPGIEGSENNPRFHRKTGGGPAFRQLLERHIAPQVEQGITINPERRGV
WGHSYGGLFVLDSWLSSSFFHIYYSASPSLSRDNFALLNRLTAVKPSPFCHKKLIIMEGS
ASNGDSRQRQMAELLQKVQETVRTLENNGVNAALQHYPGLGHGPMFNASFRSALLDISRE
PASQKPRCH
>EECEBICD_00695 Enterochelin esterase
MNGKSPSCGVPPLLCGGVYVRLNRVTDKDNVAKGMMTQLPTTDIWHLTLRLPASYCGSYT
MVEIPPETPDETVLQLGSRFASLVGKADPLNSTPGINVRGNAQESVLALDHAPAQEEWSG
CRAYAGQLFTSEHRLAGQCRRVRLYLPDVPVVQPLGLLVLTDGEIWFDHLGVSAAIDAAI
RSGRIAPVAVLGIDNINARERVAILGGRRELVLDIAERLLPTLRANYPERRWADRTQTVL
AGQSLGGVTALMAARHTPESFGLVLSHSPSMWWTPDNRNRPPHFSAEERSWVSEHVLSAP
SPAVRTHLCVGALEGSTVPQVKQLHEKLRAAGVESHCSVYTGGHDYAWWRGALIDGLRLL
PR
>EECEBICD_00696 Multidrug efflux ATP-binding/permease protein
MPATHSPMPARAWIVRLARVCWERKTLSIIVIVASVSTILLAALTPLITRQAVNDAIAGD
TTHLPLLACGLLLIALFDFIGNYVRRGYAGELSLWVQHTLRSRAFDSIQKLDGAGQDALR
TGQVISRTNSDLQQVHTLLQMCPVPLAVLTYYVAGIAVMLWMSPSMTLIVICVLAALAIT
ALRARRRVFAQTGLASDRLAHMTEHMREVLEQISVVKSCVAELRETRWLDGQSRQMVRVR
IGAAISQAMPGATMLALPVIGQIVLLCYGGWSVMNGRIDLGTFVAFASFLAMLTGPTRVL
ASFLVIAQRTQASVERVFALIDTRSRMEDGTESVEGQIIGLDVEKMSFHYDNGNRILNEI
SFSIHAGETVAVVGASGSGKSTLLMLLARFYDPTSGGVWLNTTTGQQNIRDLKLTALRRR
VGVVFEDAFLFAGTVAENIAYGHPQATQDDIRRAADAAGASGFINALPQGFNTRLAERGS
NLSGGQRQRIALARALITAPELLILDDTTSAVDAGTEAEINTALGRYADNEHMLLVIARR
RSTLQLADRIVVLDKGRVVDIGTQAELDARCPTFRSLMSGEGDFLALAPAEQRTLWPTTQ
AAKSDDAHERQTPAGKGFVDRMTRVPERAVQMALAGHGRQVSSLLTPVAWMFVIAALLIA
LDSAAGVGVLVLLQRGIDSGVAAGDMSTIGICALLALCLVAIGWCCYALQTIFAARAAES
VQHTVRLRSFSHLLRLSLPWHEKHIDSRLTRMTVDVDSLARFLQNGLASAATSIVTMVAI
AAAMFWLDPILALTALSAVPVVILATWIYRRLSSPAYAQARLEIGKVNSTLQEKVSGMRV
VQSHGQQKQEAARLRALSDNFRATRVRAQKYLAVYFPFLTLCTEAAYAAVLLIGATRVAG
GEMTPGILAAFFLLLGQFYGPVQQLSGIVDSWQQATASGKHINALLATEETENIEPSSIT
PGTGGALRLEALTFRYPEKTQPVLDNLSLTIPPGTVVAVVGRSGAGKSTLIKLLAGLYSP
GSGQIRVGERLIDAASLSDYRRQTGLVTQDVALFSGDIAENIRYSLPDSSDTEVEIAARQ
AGLFETVQHLPLGFRTPVNNGGTDLSAGQRQLIALARAHLAQAHILLLDEATARIDRSAE
ERLMTSLTRVTHTEKRIALIVAHRLTTARRCDVIVVIDKGCIAEYGSHEQLIATHGLYAR
LWRDSIGQTRDTQGEVIG
>EECEBICD_00697 Elloramycin glycosyltransferase ElmGT
MRILFVGPPLYGLLYPVLSLAQAFRVNGHEVLIASGGKFAQKAAEAGLVVFDAAPGFDSE
AGYRRQEALRKENNIGTKMGNFSFFSEEMTDPLVAFVGQWRPDLIVYPPLGVVGPLIAAK
YDIPVVMQTVGFGHTPWHIKGVTKSLSNAYRRHGVSAPPRDLAWIDVTPPSMSILQNDGE
PVISMQYVPYNGGAVWEEWWERTPDRKRLLVSLGTVKPMVDGLDLISWVMDSAGEVDAEI
ILHLPANARSDLRSLPSNVRLVDWLPMGVFLNGADGFIHHGGAGNTLTALHAGIPQIVFG
QGADRPVNARAVVERGCGIIPGKSGLTSSMINTFLGNRALREASQEVAAEMAAQPCPTEV
AKKLIAMLQHG
>EECEBICD_00698 IS200/IS605 family transposase IS200F
MRDTYKAFYGEKRRAVGSILRKLCEWKNVRILEAECCADHIHMLLEIPPKMSVSSFMGYL
KGKSSLMLYEQFGDLKFKYRNREFWCRGYYVDTVGKNTAKIQDYIKHQLEEDKMGEQLSI
PYPGSPFTGRK

>EECEBICD_00699 IS3 family transposase IS1351
MRKARFTEHQIITVIKSVEAGRTVKDVCREAGISEATYYNWKSRYGGMEPSDIKKIKDLE
DENRRLKQMFADLSLENRALKDVIEKKL
>EECEBICD_00700 IS3 family transposase IS1351
MWNHKRIHRIYCLLKLNFRRKGKQRLPVRNPSPLATPEALNQSWSVDFMHDALVCGRRFR
TFNVVDDFNREALSIEIDRNLPAPRVVRVLDRIAANRGYPAMLRMDNGPEFISLALTEWA
EKHAVKLVFIQPGKPTQNAFIERFNRTYRTEILDFYLFRTLNEVREITEKWLSEYNCERP
YESLNNMTPEEYRQHHYLAGNSKNVWN
>EECEBICD_00701 hypothetical protein
MESNDPGKLIWHVACDESGIDGQRFYGFGSLWMKYQRRGDFARIVRELREKHNCSDEIKW
QKAHSKRNAAFYQDLIETFFKHPWLAFHCIVVEKSKVEKSFHGGDYDLAMRKHFGKLIET
KIGNVIKAHPDRECEFRVEVDPLPSRYKKADEEFHVITNHTLARRFGRKDIIKSVVSKDS
KASEHIQIADFLLGAVMCAYQGKATSEAKLAVANNVASYLGWDSLMHDTWPTERKFNIWF
FFDRSKGPRDIVTQEVKLTYALPNTRK
>EECEBICD_00702 hypothetical protein
MPTPESLWKILASPISTKTAVRYGFLSVAIVLSIRFILPILKDIAPLTEQDLPGYSYAAH
FALTILFSLIAETLTFELVAWMYLKLEKFIERRKLRKELARKQEIESKEIKRIQEKFIEN
FILAWPLIAPRYKYFVYELRRGHKGYSDTNSAINYLRQQGWIHPITELPYSHCLYRLDEL
IFEALNRIESAATATDS
>EECEBICD_00704 Type I secretion system membrane fusion protein PrsE
MKINQHDAAMDDPDIQRERAFSGAGRIVLICSLLFLILGIWAWFGRLDEVSTGNGKVIPS
SREQVLQSLDGGILAQLTVREGDRVQANQIVARLDPTRLASNVGESAAKYRASLASSARL
TAEVNDLPLAFPAELNGWPDLIAAETRLYKSRRAQLADTEAELRDALASVNKELAITQRL
EKSGAASHVEVLRLQRQKSDLGLKITDLRSQYYVQAREALSKANAEVDMLSAILKGREDS
VTRLTIRSPVRGIVKNIQVTTIGGVIPPNGEMMEIVPVDDRLLIETRLSPRDIAFIHPGQ
RALVKITAYDYAIYGGLDGVVETISPDTIQDKVKPEIFYYRVFIRTHQDYLQNKSGRRFS
IVPGMIATVDIKTGEKTIVDYLIKPFNRAKEALRER
>EECEBICD_00705 Toxin RTX-I translocation ATP-binding protein
MQAENRFLQQWNSYIRITGESGLRTRKLTQGLISWGMSVQSLVYAAVIMFGAPMVIEGSM
TTGAVVAASMLGSRMIAPMANLCGVLARWQQVKAAKMGLDNIMQLPTETQHDDSLIHRDI
LHGHYLFENAQFRYHNDDQRIPLRLVRLEIMPGERIAILGRNGAGKSTLLQAMAGGLEMI
QGDARLDNLSLSHIDMADLRRNIGFLSQNARLFFGTLRENLTLGAPHANDEQIFDALEVS
GGAVFVRRLAKGLDHPIMEGGNGLSGGQRQSLLLARMLLRSPNIVLLDEPSASLDEHTER
EFIQRLHQWLGNRTLVVATHRVPILELVERVVVLKEGQLVMDAPKAQALNADRMQSHRRE
WKNENQSA
>EECEBICD_00706 Toxin RTX-I translocation ATP-binding protein
MTRAAPDVEEVLSERALSQWAQAISYVAGHYRVACSPGSIQANAPWFRGKSRTTALTQLA
RQAGLSFHAPDIDKTAFSQWRLPLVVELRDGQLLVIEHVNGEDAVDVFVIEEEGQRNRLT
LSELLPEILYVAALRPLSALKDSRVDRYISRFKPDWMRELVLQDIRPYLPVMVAAFLINV
LSLAGIVFSMQVYDRVIPAQSYPTLYVLSFGVLVAVLFGFLLREARTHIMDVLGKRADMR
ISDRVFGHALRLRNSAIPRSTGSFISQLRELEQIREMITSSTLATIVDLPFFFLFMIVLA
IIAPPLAWIAPVAALLMILPGVALQKNWLSSPTRPPTKRRYAMQSWSKAFRGWRILN
>EECEBICD_00707 Outer membrane efflux protein BepC
MGRVAPVAIVLAFALFHHQPRGAEAPPMITSEGLATDQMLPSLDGSAAELPLSAAAPGNL
TLNDAVNRAVNWHPSIREAVGKLLAQNEQIEVAKSKYYPQVSAGVNNGYSNTYTDHGYSP
SLVLSVSQMLYDFGKVASQVRAETAGAAQQQANVLLSIDTVAHETANAIVQTQSWQQMVD
AAEEQLVALDSIGKLIRQRSDEGATSLSDVVQTEARIESARSQLAQYQANLDSAKASLMS
WLGWNSLNGINNDFPAKLARSCETATPDDRLVPAVLAAWAQANVARANLDYASAQMTPTI
SLEPSVQHYLNDKYPSHEVLDKTQYSTWVKVEMPLYQGGGLTARRNAASHAVDAAQSTIQ
RTRLDVRQKLMEARSQAMSLASALQILRRQQQLSERTRELYQQQYLNLGSRPLLDVLNAE
QEVYQARFAELQTESQLHQLQLNCLYNTGALRQAFALNHRSIQSVEIQP
>EECEBICD_00708 hypothetical protein
MRLLAVVSKLTGVSTTVESSAVTLNAPSIVKLSVAREEISQLTRINQDLVVTLHSGETIT
IKNFYVTNDLGASQLVLAENDGTLWWVENPQAGLHFEQIADINELLVTSGASHEAGGAVW

PWVLAGAVAAGGIAAIASSGGGDSHHHSDGDNPPPDNTNPDGNPPDNSNPGGSTPNGNTP
GSSNPVDTTPPLAPGELLISADGKTVSGQAEAGSTITIKDPSGNVVGEGKADSDGKFSID
LTAPQISGEQLTVTATDDAGNTGPSATIDAPNIPLPDTPAITAAIDDAAPLTSTLSNNQF
TNDNTPTLEGTGSAGTVIHIYANGQEIGSTTVDTSGNWHFAITSALADGENHFTAIATNV
KGESSESARFTLTIDTLIPDAPRVELIADNTGLLTGPLQNNDRTDEAKPLFSGQGEAGNT
ITIKEGSTVIGSATVDENGRWTFTPTTPLSDGEHTFTVEQSDKAGNTSRVTTTPTIIVDT
TPPDAAIIDNVAKDGTTVSGTAEAGSTVSIYDPAGNYLGSTITGENNHFSITLNPAQTHG
ERLEARIQDAVGNIGPATEFTASDSQYPAQPTILTVTDDAGAVTGLLKNGDATDDNRPTL
SGTAEPGSTISINDNGFPVATFPPIVADADGKWSFTPSLALADGDHVFTATATNDRGTSG
QSVSFTIDIDTQPPVLEGLAVSDVGDRLTGTTEAGSTVVIKDSLGNTLGSGTAGDDGTFS
IGISPAKINGETLSISVTDKAANSGPVETLNAPDKTAPAAPDGLTVATDGLSVSGQAEAG
ATVTIRDSSNTVLGSAVANGNGQFIVPLNTAQTNGQALIATATDVAKNESAAATVIAPDS
TAPEMPKNVVISEDGTSISGTAEPGSAITIATPDGKPLGSGKADGEGHFTLPLVPAQTNG
EQVTVTATDSANNVSPPTTAQAPDITAPDKPIITQVLDDVESFTGPLVNGQTTNDNRPTL
SGTAEAGARVEVFDNGVSLGLATLQPNGAWTFTPSQNLGEGAHRLTVIATDAKGNASQAA
SFDLVVDTQSPQQPVITFITDDAPGILGSVAHLGLTNDSTPTINGTGEPGSTVHLYQNGA
RIADIIVGNSGVWSYAYTTASPLADDTYTFTVTASDSNGNTTPFSTDFTITIDTQAPAAP
GVIGVADGDGNTIDTNQITQESQPRLSGSGTAGDTIILYDNGNAIGQALVGTDGRWQFTP
PAALGDGDHHLTARANDPAGNESPESISFTLRIDTQAPDAPQIVSAAITGGEGEVLLANG
SITNQRMPTLSGTGEPGTIITLYNNGVELATVQVNPQGSWTYPLTRNLSEGLNILTATAT
DAAGNSSPTSGVFSVTLDTQPPAQPDAPLISDNVAPVIGNIGNNGATNDTTPTFSGTGEI
GSTIILYNNGSEIGRTTVGDNGSWNFTPAALTPETYTITVTETDRAGNISPPSASVTFTL
DTTAPANPVITFAEDNVGEVQDTIVSGATTDDNTPVIHGTGDIGSIITLYNGSSVLGVVT
VDETGTWTLAVTSALPDGVYTLTAIAADAAGNSSGVSNSFTFTVDTVPLQPPVVNEILDD
VAPVTGPLTDGAFTNDRTLTINGSGENGSTVTIYDNGVAIGTALVTDGVWTFNTPELSEA
SHALTFSATDDAGNTTAQTQPITITVDITAPPAPTIQTVDDDGTRVAGLADPYATVEIHH
ADGTLVGSAVANGTGEFVVTLSPAQTDGGTLTAIAIDRAGNNGPATNFPASDSGLPAVPA
ITAIEDDVGSVQGNIAAGGATDDTMPTLRGTTDIGSTVEVFIDGDSAGFATVDASGNWIF
EIATPLSESTHYFTVQATNANGPGGLSAPVGITVDLSAPAQPVITSATDDVPGMTGTLDN
GALTNDSRPTLNGTGEAGATIRILDNGVEIGSATVDQSGNWRFTPNAPLESNAHIFTAVA
TDPAGNSGQPSDGFTLNIDAQAPDVPVITSVIDDNNQPTVPVLPGQSTDDRQPILNGTGE
PGATITIFDNGTPLGTAQVGENGSWTFPVPRNLSEGSHNLTVSATDPAGNTSAVSAPWTI
VVDITPPAIPVLTSVVDDQPGITGNLVSGQLTNDATPTLNGRGEAGATINVYLDGNPASI
GTTTVNSDGTWSFTPQTPLANGSHTFTLSATDPAGNSSSVSSGFVLTIDATPPAAPVIAS
VADNTAPVTGIVPNGGSTNETRPTLSGTGEAGTTISIYNGSALVGTAQVQANGSWSFTPS
TSLGAGVWNLTATATDAAGNTSAASEIRSFTIDTTAPAAPVIDTVYDGTGPITGNLSSGQ
ITDEARPVISGTREANTTIRLYDNGTLLAEIPADNSSSWRYTPDASLATGNHVITVIAVD
AAGNASPVSDSVNFVVDTTPPLTPVITSVSDDQAPGLGTIANGQNTNDPTPTFSGTAEAG
ATITLYENGTVIGTTTAQPDGAWSVSTSTLASGTHVITAVATDAAGNSSPNSTAFTLTVD
TTAPQTPILTSVVDDVAGGVTGNLANGQITNDNRPTLNGTAEAGSVVSIYDGDTLLGVTS
ANASGAWSFTPTTGLNDGTRTLTVTATDPAGNVSPATSGFTIVVDTLAPTVPLITSIVDD
VPNNTGAIGNGQSTNDTQPTLNGTAEANSAVSIFDNSALVATVNANASGNWSWTPTAALG
QGSHAYSVSAADAAGNVSAASPSITIIVDTIAPGAPGNLVINATGNRVTGTAEAGSTVTI
TSDTGVVLGTATADGTGSFTATLTPAQTNGQPLLAFAQDKAGNTGIAAGFTAPDTRVPEA
PIITNVVDDVGIYTGAIANGQVTNDAQPTLNGTAQAGATVSIYNNGALLGTTTANASGNW
SFTPTGNLTEGSHAFTATATNANGTGSVSTAATVIVDTLAPGTPSGTLSADGGSLSGLAE
ANSTVTVTLTGGVTLTTTAGSNGAWSLTLPTKQIEGQLINVTATDAAGNASGTLGITAPV
LPLAARDNITSLDLTSTAVTSTQSYSDYGLLLVGALGNVASVLGNDTAQVEFTIAEGGTG
DVTIDAAATGIVLSLLSTQEIVVQRYDTSLGAWTTIVNTAVGDFANLLTLTGSGVTLNLS
GLGEGQYRVLTYNTSLLATGSYTSLDVDVHQTSAGIISGPTISTGNVMADDTAPTGTTVT
AITNANGVSTPVGAGGVDIQGQYGTLHINQDGSYTYTLAKPTAGYGHKESFTYTITQNGV
GSSAAQLVINLGPAPVPGSVIATDNNASLVFDTHVSYVNNGPSTQSGVTVLSVGLGNVLN
ANLLDDMTNPIIFNVEEGATRTMTLQGTVGGVSLVSTFDLYVYRFNDAIQQYEQFRVEKG
WINTLLLAGQSQPLTLTLPGGEYLFVLNTASGISVLTGYTLAISQDHTYAVDSITANTTG

NVLTNDVVPTDALLTEVNGVAIAATGTTEVNGLYGSLIIDARGNYTYTLKNGVGADSIKT
PDSFIYTVKAPNGDTDTASLNITPTARALDAINDVSDTLSVATLQDTAAWLDSSVGSASW
GLLGKSGSGSGTFDVATGTVLKGASLVFDVSTLITLGNLNISWAIQENGTVIRNGTVPVA
NITLGSATVTVNLSGLELDAGTYTLNFTGTNTLAGAATITPRVIGTTVDLDNFETSGTHT
VLGNIFDGSDAAGAMDQLNTVNTRLSISGYNGSAATLDAAANTTSATIQGHYGTLQINLD
GAYTYTLNNGVAMSSITSKEVFTYQLDDKMGHTDSATLTIDMAPQIVSTNQNDVLIGSAY
GDTLIYHLLNGADATGGNGVDRWQNFSTAQGDKIDIHELLTGWDHQAATLGNFVQVHTSG
ANTVISVDRDGAGSAFKSTDLVTLENVQLTLNDLLQNNHLITGG
>EECEBICD_00709 SsrA-binding protein
MGCFRYQITYEFTTLMTKKKAHKPGSATIALNKRARHEYFIEEEFEAGLALQGWEVKSLR
AGKANIGDSYVILKDGEAWLFGANFTPMAVASTHVVCDPTRTRKLLLNQRELDSLYGRIN
REGYTVVALSLYWKNAWCKVKIGVAKGKKQHDKRSDLKEREWQLDKARIMKNAGR
>EECEBICD_00710 Ribosome association toxin RatA
MPQISRTALVPYSAEQMYQLVNDVQSYPQFLPGCVGSRVLESSPAQMTAAVDVSKAGISK
TFTTRNQLTRNQSILMHLVDGPFKKLIGGWKFTPLSPEACRIEFQLDFEFTNKLIELAFG
RIFKELASNMVQAFTVRAKEVYRAG
>EECEBICD_00711 hypothetical protein
MPDKLVVEVAYALPEKQYLQRVTLEEGATVEEAIRASGLLELRTDIDLAKNKVGIYSRPV
KLTDTVQDGDRVEIYRPLIADPKALRRQRAEKSAGR
>EECEBICD_00712 Outer membrane protein assembly factor BamE
MRCKTLTAAAAVLLMLTAGCSTLERVVYRPDINQGNYLTPTDVAKVRVGMTQQQVAYALG
TPMMTDPFGTNTWFYVFRQQPGHENVTQQTLTLTFNSSGVLTNIDNKPALTK
>EECEBICD_00713 DNA repair protein RecN
MLAQLTISNFAIVRELEIDFQSGMTVITGETGAGKSIAIDALGLCLGGRAEADMVRTGAT
RADLCARFALKDTPAALRWLEENQLEEGRECLLRRVISSDGRSRGFINGTAVPLSQLREL
GQLLIQIHGQHAHQQLTKPEQQKSLLDSYANEAALAQQMAARYQLWHQSCRDLAHHQQQS
QERAARAELLQYQLKELNDFNPQAGEFEQIDEEYKRLANSGQLLTTSQNALALLADGEDV
NLQSQLYSAKQLVSELVGMDSKLSGILDMLEEATIQLTEASDELRHYCERLDLDPNRLFE
LEQRIAKQISLARKHHVSPEALPQLYQSLLEEQQQLDDQADSLETLTLAVNKHHQQALET
AQALHQQRQFYAQELGQLITESMHLLSMPHGLFTIDVKFDEHHLSNDGADRVEFKVTTNP
GQPLQPIAKVASGGELSRIALAIQVITARKMETPALIFDEVDVGISGPTAAVVGKLLRQL
GESTQVMCVTHLPQVAGCGHQHFFVSKETDGAMTETHMQPLDKRARLQELARLLGGSEVT
RNTLANAKELLAA
>EECEBICD_00714 NAD kinase
MEQQIAHELQLKNVPTGTLAEIGQQADLAVVVGGDGNMLGAARTLARYNINVIGINRGNL
GFLTDLDPDNALQQLSDVLEGRYISEKRFLLEAQVCQQDRQKRISTAINEVVLHPGKVAH
MIEFEVYIDETFAFSQRSDGLIISTPTGSTAYSLSAGGPILTPSLDAITLVPMFPHTLSA
RPLVINSSSTIRLRFSHRRSDLEISCDSQIALPIQEGEDVLIRRCDYHLNLIHPKDYSYF
NTLSTKLGWSKKLF
>EECEBICD_00715 Protein GrpE
MCRECGGMTRMPHNADTLEMIIHFSEVLVAKIDDNVSTSLETLNLIPIISEVSEMNAKKN
AEKFMSSKEQKTPEGQAPEEIIMDQHEEVEAVEPNDSAEQVDPRDEKIANLEVQLAEAQT
RERDTVLRIKAEMENLRRRTEQDIEKAHKFALEKFVNELLPVIDSLDRALEVADKANPDM
AAMVEGIELTLKSMLDVVRKFGVEVIAETNVPLDPNVHQAIAMVESEEVPAGNVLGIMQK
GYTLNGRTIRAAMVTVAKAK
>EECEBICD_00716 hypothetical protein
MSEDYVIEWDKNFADDLNVVANVFLSHNPTLWPTIFSQLSTQPEIFEDEDEDEYGLQDVL
DCSGGDLGNNELAQAFLQVLRGERFIHLVDWKGEDEEGELANFAADRFYELTKNLTNSEE
LRNLLVEITQEDEISDVCEAGDRYLDEIFERIQTELNKRGFQIFDLNEGSDTYNVVVLPM
SEYKKIEDFNTPWLEVQDFLS
>EECEBICD_00717 hypothetical protein
MVVISAYFSGSETGMMTLNRYRLRHMAKQGNRSAKRVEKLLRKPDRLISLVLIGNNLVNI
LASALGTIVGMRLYGDAGVAIATGVLTFVVLVFAEVLPKTIAALYPEKVAYPSSFLLAPL
QILMMPLVWLLNTITRLLMRLMGIKTDIVVSGSLSKEELRTIVHESRSQISRRNQDMLLS

VLDLEKVSVDDIMVPRNEIIGIDINDDWKSIERQLTHSPHGRIVLYRDSLDDAISMLRVR
EAWRLMAEKKEFTKEMMLRAADEIYYVPEGTPLSTQLIKFQRNKKKVGLVVNEYGDIQGL
VTVEDILEEIVGDFTTSMSPTLAEEVTPQNDGSVIIDGTANVREINKAFNWHLPEDDART
VNGVILEALEEIPVAGTRVRIEQYDIDILDVQENMIKQVKVVPVKPLRESVAE
>EECEBICD_00718 Inner membrane protein YpjD
MPVFALLALVAYSFSLALIVPGLLQKNSGWRRMAILSAVIALVCHAVALESRILPGGDSG
QNLSLLNVGSLVSLMICTVMTIVASRNRGWLLLPIVYAFALINLAFATFMPNEYITHLEA
TPGMMVHIGLSLFSYATLIIAALYALQLAWIDYQLKNKKLAFSNEMPPLMSIERKMFHIT
QIGVVLLTLTLCTGLFYMHNLFSTENIDKAVLSIVAWFVYIVLLWGHYHEGWRGRRVVWF
NVAGAGILTLAYFGSRILQQFVS
>EECEBICD_00719 Signal recognition particle protein
MFDNLTDRLSRTLRNISGRGRLTEDNVKETLREVRMALLEADVALPVVREFINRVKEKAV
GHEVNKSLTPGQEFVKIVRSELVAAMGEENQTLNLAAQPPAVVLMAGLQGAGKTTSVGKL
GKFLREKHKKKVLVVSADVYRPAAIKQLETLAEQVGVDFFPSDVGQKPVDIVNAALKEAK
LKFYDVLLVDTAGRLHVDEAMMDEIKQVHASIKPVETLFVVDAMTGQDAANTAKAFNEAL
PLTGVVLTKVDGDARGGAALSIRHITGKPIKFLGVGEKTDALEPFHPDRIASRILGMGDV
LSLIEDIESKVDRAQAEKLATKLKKGDGFDLNDFLEQLKQMKNMGGMASLMGKLPGMGQI
PDNVKSQMDDKVLVRMEAIINSMTLKERAKPEIIKGSRKRRIAQGCGMQVQDVNRLLKQF
DDMQRMMKKMKKGGMAKMMRSMKGMMPPGFPGR
>EECEBICD_00720 30S ribosomal protein S16
MVTIRLARHGAKKRPFYQVVVTDSRNARNGRFIERVGFFNPIASEKEEGTRLDLDRIAHW
VGQGATISDRVAALIKEVKKAA
>EECEBICD_00721 Ribosome maturation factor RimM
MSKQLAAQVPAEPVVLGKMGSSYGIRGWLRVFSSTEDAESIFDYQPWFIQKAGQWQQVQL
ESWKHHNQDLIIKLKGVDDRDAANLLTNCEIVVDSSQLPALEEGDYYWKDLMGCQVVTAE
GYDLGKVIDMMETGSNDVLVIKANLKDAFGIKERLVPFLDGQVIKKVDLATRTIEVDWDP
GF
>EECEBICD_00722 tRNA (guanine-N(1)-)-methyltransferase
MFIGIVSLFPEMFRAITDYGVTGRAVKKGLLNIQSWSPRDFAHDRHRTVDDRPYGGGPGM
LMMVQPLRDAIHAAKAAAGEGAKVIYLSPQGRKLDQAGVSELATNQKLILVCGRYEGVDE
RVIQTEIDEEWSIGDYVLSGGELPAMTLIDSVARFIPGVLGHEASAIEDSFADGLLDCPH
YTRPEVLEGMEVPPVLLSGNHAEIRRWRLKQSLGRTWLRRPELLENLALTEEQARLLAEF
KTEHAQQQHKHDGMA
>EECEBICD_00723 50S ribosomal protein L19
MSNIIKQLEQEQMKQNVPSFRPGDTVEVKVWVVEGTKKRLQAFEGVVIAIRNRGLHSAFT
VRKISNGEGVERVFQTHSPVVDSIAVKRRGAVRKAKLYYLRERTGKAARIKERLN
>EECEBICD_00724 Diguanylate cyclase DgcN
MNKEFSLSRPTFKRTLRRISIISVLLTMTLIWLLICVASVLTLKQYAQKNLDLTAATMAH
SLEAALVFSDNAAAAETLATLGRQGQFSAAEVRDKNGRTIASWRYDARAADDKLIGLISH
WLFPLPVSQPVWHNGRAIGEVRLVARDSLIGHFIWLSLAVLTGCILLASGIALLLTRYLH
NGVVDALQNITEVVHDVRTNRNFSRRVPDERIAEFHLFAQDFNSLLDEMEEWQLRLQAKN
AQLLRTALHDPLTGLANRAAFRSCINALMKDNSARSSSALLFLDGDNFKYINDTWGHAAG
DRVLIEVAKRLAEFGGSRYQTYRLGGDEFAMVLYDVHSEYEVQRICAALSQAFNRPFELH
NGQRITMTLSIGFALTWEHATAEKLQELADRNMYQAKHRRAERSLN
>EECEBICD_00725 hypothetical protein
MRFSHRFILLLSLLLASLPLYAQRVTEEEKSVRAIVSGIVSYTHWPALSGPPRLCIFSSA
RFVRVLSEEADWAFPYQPLVIRTTQEALSARCDGFYFGNESPAYQVELTRHYPVNALLLI
AEQNTECIIGSAFCLIINNDEVKFSVNLDSLSHSGVRVNPEVLMLARNQKHE
>EECEBICD_00726 Phospho-2-dehydro-3-deoxyheptonate aldolase, Tyr-sensitive
MQKDALNNVRITDEQVLMTPEQLKAAFPLSLAQEAQIAQSRGIISDIIAGRDPRLLVVCG
PCSIHDPETALEYARRFKALAAEVSDSLYLVMRVYFEKPRTTVGWKGLINDPHMDGSFDV
EAGLKIARQLLVELVNMGLPLATEALDPNSPQYLGDLFSWSAIGARTTESQTHREMASGL
SMPVGFKNGTDGSLATAINAMRAAAQPHRFVGINQAGQVALLQTQGNPHGHVILRGGKAP

NYSPADVAQCEKEMEQAGLRPSLMVDCSHGNSNKDYRRQPAVAESVVAQIKDGNRSIIGL
MIESNIHEGNQSSEQPRSEMKYGVSVTDACISWEMTDALLREIHKDLSGQLAVRVA
>EECEBICD_00727 T-protein
MVAELTALRDQIDDVDKALLNLLAKRLELVAKVGEVKSRFGLPIYVPEREASMLASRRAE
AEAIGVPPDLIEDVLRRVMRESYSSENDKGFKTLCPSLRPVVIVGGGGQMGRLFEKMLTL
SGYQVRILEQQDWPRARDIVADAGMVIVSVPIHVTEQVIAQLPPLPSDCILVDLASVKSD
PLQAMLAAHDGPVLGLHPMFGPDSGSLAKQVVVWCDGRQPEAYQWFLEQIQVWGARLHRI
SAVEHDQNMAFIQALRHFATFAYGLHLAEENVQLEQLLALSSPIYRLELAMVGRLFAQDP
QLYADIIMSSERNLALIKRYYKRFGDAIGLLEQGDKQAFIDSFRKVEHWFGDYARRFQNE
SRVLLRQANDSRP
>EECEBICD_00728 6-deoxy-6-sulfogluconolactonase
MTTPHVLFDYAGHLPECPTWSEDESALYWTDILEQEIHRYHPASGTHSVLAFPEEVGCFA
LREQGGFIVAMRHAIWLADKNGLLQRKVCDNPSNTKLARFNDGGTDGDGRFYAGTFWAPG
DYNGALLMRIDHDLTAKVIQCDIQGHNGLAFSPDNQWMYTSDTPNGVIYRTLLDKHGDPG
KRELFRHFGEGEGLPDGAAMDSEGCYWSAMFDGWCVARFSPQGEQLEEYRLPVRCPTMVC
FGGADMKTLFITTTRENMSAQEVADYPLSGAIFTLQVAVAGMKKSRFIERQAGSTGTTFS
LG
>EECEBICD_00729 Bifunctional chorismate mutase/prephenate dehydratase
MTSENPLLALRDKISALDEELLALLAKRRALAIEVGQAKLLSHRPVRDIDRERALLDRLI
HLGKAHHLDAHYITRLFQLIIEDSVLTQQALLQQHLNNTHPHSARIAFLGPKGSYSHLAA
RQYAARHFEQFIESGCAKFADIFHQVETGQADYAVVPIENTSSGAINDVYDLLQHTSLSI
VGEMTVTIDHCVLVSGATDLNTIETVYSHPQPFQQCSKFLSRYPHWKIDYTESTSAAMEK
VAQANSPRVAALGSEAGGMLHGLQVLERIAANQTQNITRFLVLARKAINVSDQVPAKTTL
LIATGQQAGALVEALLVLRNHNLIMTKLESRPIHGNPWEEMFYLDIQANLESQVMQSALK
ELGEITRSMKVLGCYPSENVVPVEPA
>EECEBICD_00730 Ribosome-associated inhibitor A
MTMNITSKQMEITPAIRQHVADRLAKLEKWQTHLINPHIILSKEPQGFIADATINTPNGH
LVASAKHEDMYTAINELINKLERQLNKVQHKGEARRATASVKDASFVEAEEE
>EECEBICD_00731 Outer membrane protein assembly factor BamD
MTRMKYLVAAATLSLFLAGCSGSKEEVPDNPPNEIYATAQQKLQDGNWKQAITQLEALDN
RYPFGPYSQQVQLDLIYAYYKNADLPLAQAAIDRFMRLNPTHPNIDYVMYMRGLTNMALD
DSALQGFFGVDRSDRDPQHARAAFNDFSKLVRSYPNSQYTTDATKRLVFLKDRLAKYEYS
VAEYYTARGAWVAVVNRVEGMLRNYPDTQATRDALPLMENAYRQMQLNAQADKVAKIIAA
NSKNT
>EECEBICD_00732 Ribosomal large subunit pseudouridine synthase D
MAQRVQLTATVSENQLGQRLDQALAEMFPDYSRSRIKEWILNQRVLVNGQLCDKPKEKVL
GGERVAIDAEIDEEIRFEAQDIPLDIVYEDDDILVINKPRDLVVHPGAGNPDGTVLNALL
HYYPPIADVPRAGIVHRLDKDTTGLMVVAKTVPAQTRLVESLQLREITREYEAVAIGHMT
AGGTVNEPISRHPTKRTHMSVHPMGKPAVTHYRIMEHFRVHTRLRLRLETGRTHQIRVHM
AHITHPLVGDQVYGGRPRPPKGASEEFISTLRKFDRQALHATMLRLYHPVSGIEMEWHAP
IPQDMVDLIDAMRADFEDHKDDVDWL
>EECEBICD_00733 Polyphenol oxidase
MNALIVPQWPLPKGVAACSSTRIGGVSLPPYDSLNLGAHCGDNPEHVEENRKRLFAAGNL
PSKPVWLEQVHGKNVLRLTGEPYASKRADASYSNTPGTVCAVMTADCLPVLFCNREGTEV
AAAHAGWRGLCEGVLEETVTCFADKPENIIAWLGPAIGPTAFEVGPEVRDAFLAKDAQAD
SAFLPHGEKFLADIYQLACQRLANTGVEHVYGGDRCTFSESETFFSYRRDKTTGRMASFI
WLI
>EECEBICD_00734 Chaperone protein ClpB
MRLDRLTNKFQLALADAQSLALGHDNQFIEPLHLMSALLNQEGGSIRPLLTSAGINAGQL
RTAIDQALSRLPQVEGTGGDVQPSSELVRVLNLCDKLAQKRGDNFISSELFVLAALESRG
TLTDLLKSAGATTANITQAIEQMRGGESVNDQGAEDQRQALKKYTVDLTERAEQGKLDPV
IGRDEEIRRTIQVLQRRTKNNPVLIGEPGVGKTAIVEGLAQRIINGEVPEGLKGRRVLAL
DMGALVAGAKYRGEFEERLKGVLNDLAKQEGNVILFIDELHTMVGAGKADGAMDAGNMLK
PALARGELHCVGATTLDEYRQYIEKDAALERRFQKVFVAEPSVEDTIAILRGLKERYELH

HHVQITDPAIVAAATLSHRYIADRQLPDKAIDLIDEAASSIRMQIDSKPEELDRLDRRII
QLKLEQQALMKESDEASKKRLDMLNEELDDKERQYSELEEEWKAEKASLSGTQTIKAELE
QAKIAIEQARRVGDLARMSELQYGKIPELEKQLEAATQSEGKTMRLLRNKVTDAEIAEVL
ARWTGIPVARMLEGEREKLLRMEQELHSRVIGQNEAVEAVSNAIRRSRAGLSDPNRPIGS
FLFLGPTGVGKTELCKALANFMFDSDDAMVRIDMSEFMEKHSVSRLVGAPPGYVGYEEGG
YLTEAVRRRPYSVILLDEVEKAHPDVFNILLQVLDDGRLTDGQGRTVDFRNTVVIMTSNL
GSDLIQERFGELDYGRMKEMVLGVVSQNFRPEFINRIDEVVVFHPLGEQHIASIAQIQLQ
RLYKRLEERGYEIHISDEALKLLSANGYDPVYGARPLKRAIQQQIENPLAQQILSGELVP
GKVIRLEASDDRIVAVQ
>EECEBICD_00739 hypothetical protein
MAIKPFNYQQDFSSIDFRQQPELYQVGRGEQGVLLVEPYKSEILPFWRYKDEASAMKSAE
QVYQLFEAYRQQDDFVGMDMARKFIQMGYTRARRYANYKGGKKYAEDGSLNTRGNDPIKA
AAATVFKGWWDKIRQDEDYLKRKRQHQARWG
>EECEBICD_00740 Alpha-ketoglutarate permease
MAEIIQRIDKNNAEAPEMRRRIWAIVGASSGNLVEWFDFYVYSFCSLYFAHIFFPSGNTT
TQLLQTAGVFAAGFLMRPIGGWLFGRIADRRGRKASMLISVCMMCMGSLVIACLPGYDTI
GTWAPALLLIARLFQGLSVGGEYGTSATYMSEVALEGRKGFFASFQYVTLIGGQLLALLV
VVILQQVLEDAELRAWGWRIPFALGAVLAVVALWLRRQLDETSKHETRALKEAGSLKGLW
RNRKAFLMVLGFTAAGSLCFYTFTTYIQKYLVNTAGMHANVASGIMTVALCVFMFVQPLF
GALSDKIGRRTSMLCFGALATLFTVPILSALQNVTSPYAAFALVMCALLIVSFYTSISGI
LKAEMFPAQVRALGVGLSYAVANALFGGSAEYVALSLKSVGMESSFFWYVTAMAVLAFLV
SLMLHRKGKGLRL
>EECEBICD_00741 hypothetical protein
MRVLIPFTVLFLSGCSHLANDHWSGQDKAQHFMASAMLSAAGNEYARHQGVSPDRSAAIG
LMFSLSLGASKELWDSRPEGSGWSWKDFVWDVAGATTGYAIWQMARY
>EECEBICD_00742 CDP-diacylglycerol--serine O-phosphatidyltransferase
MLSKFKRNKHQQHLAQLPKISQSVDDVDFFYTPATFRETLLEKIASATQRICIVALYLEQ
DDGGKGILDALYAAKRQRPELDVRVLVDWHRAQRGRIGAAASNTNADWYCRLAQENPGID
VPVYGVPINTREALGVLHFKGFIIDDSVLYSGASLNDVYLHQHDKYRYDRYQLIRNRQMA
DIMFDWVTQNLMNGRGVNRLDNTQRPKSPEIKNDIRLYRQELRDASYHFQGDANDEQLSV
TPLVGLGKSSLLNKTIFHLMPCAEHKLTICTPYFNLPAVLVRNIIQLLRDGKKVEIIVGD
KTANDFYIPEDEPFKIIGALPYLYEINLRRFLSRLQYYVNTDQLVVRLWKDDDNTYHLKG
MWVDDKWMLLTGNNLNPRAWRLDLENAILIHDPKQELAPQREKELELIRTHTTIVKHYRD
LQSIADYPIKVRKLIRRLRRIRIDRLISRIL
>EECEBICD_00743 Peptidyl-lysine N-acetyltransferase Pat
MSQQGLEALLRPKSIAVIGASMKPHRAGYLMMRNLLAGGFNGPVLPVTPAWKAVLGVMAW
PDIASLPFTPDLAILCTNASRNLALLDALGAKGCKTCIILSAPTSQHEELLACARHYKMR
LLGPNSLGLLAPWQGLNASFSPVPIKQGKLAFISQSAAVSNTILDWAQQREMGFSYFIAL
GDSLDIDVDELLDYLARDSKTSAILLYLEQLSDARRFVSAARSASRNKPILVIKSGRSPA
AQRLLNTSAGMDPAWDAAIQRAGLLRVQDTHELFSAVETLSHMRPLRGDRLMIISNGAAP
AALALDELWSRNGKLATLSEETCLQLRQALPAHIDIANPLDLCDDASSEHYVKTLDILLA
SQDFDALMVIHSPSAAAPGTESAHALIETIKRHPRGKLVTLLTNWCGEFSSQEARRLFSE
AGLPTYRTPEGTITAFMHMVEYRRNQKQLRETPALPSNLTSNTAEAHNLLQRAIAEGAAS
LDTHEVQPILHAYGLHTLPTWIASDSAEAVHIAEQIGYPVALKLRSPDIPHKSEVQGVML
YLRTASEVQQAANAIFDRVKMAWPQARIHGLLVQSMANRAGAQELRVVVEHDPVFGPLIM
LGEGGVEWRPEEQAVVALPPLNMNLARYLVIQGIKQRKIRARSALRPLDIVGLSQLLVQV
SNLIVDCPEIQRLDIHPLLASASEFTALDVTLDIAPFDGDNESRLAVRSYPHQLEEWVEM
KNGDRCLFRPILPEDEPQLRQFIAQVTKEDLYYRYFSEINEFTHEDLANMTQIDYDREMA
FVAVRRMDNAEEILGVTRAISDPDNVDAEFAVLVRSDLKGLGLGRRLMEKLIAYTRDHGL
KRLNGITMPNNRGMVALARKLGFQVDIQLEEGIVGLTLNLAKCDES
>EECEBICD_00744 hypothetical protein
MTNNAVLQLRAERLARATRPFLARGNRVRRCQRCLLPLKSCLCDTLTPSQAKSRFCLVMF
DTEPMKPSNTGRLIADILPDTAAFQWSRTEPPQALLELVQHPDYQPIVVFPASYAGEARE
VISTPPAGKPPLFIMLDGTWPEARKMFRKSPYLDHLPVISVDLSRLSAYRLREIHAEGQY

CTAEVAIALLDLAGDTEAATSLGEHFTRFKTRYLAGKTQHPGNVTA
>EECEBICD_00745 Thioredoxin 2
MNTVCTHCQAINRIPDDRLQDAAKCGRCGHELFDGEVINATGETLDKLLKDDLPVVIDFW
APWCSPCRNFAPIFEDVAEERSGKVRFVKVNTEAERELSARFGIRSIPTIMIFKHGQVVD
MLNGAVPKAPFDSWLNEAL
>EECEBICD_00746 putative tRNA/rRNA methyltransferase YfiF
MNDELKNKSGKVKVMYVRSDDDSDKRTHNPRTGKGGGRPAKSRTDGGRRPARDERNNQSR
DRKHETSPWRTVSRAPGDETPEKVDHGGISGKSFIDPEVLRRQRAEETRVYGENACQALF
QSRPDAIVRAWFIQSVTPRFKEALRWMAANRKAYHVVDEAELAKASGTEHHGGVCFLIKK
RNGTTVKQWVKQAADQDCVLALEDVANPHNLGGMMRSCAHFGVKGVVVQDAALLESGAAI
RTAEGGAEHVQPITGESIVDVLDDFRQAGYTVVTTSSDRGQALFSTTLPEKMVLVLGREY
DYLPEAAREPDDLCVKINGTGNVESLNVSVATGVLLAEWWRQNKA
>EECEBICD_00747 Uracil-DNA glycosylase
MATELTWHDVLADEKQQPYFINTLHTVAGERQSGITVYPPQKDVFNAFRFTELGDVKVVI
LGQDPYHGPGQAHGLAFSVRPGIAPPPSLVNMYKELEASIPGFVRPAHGYLESWARQGVL
LLNTVLTVRAGQAHSHASLGWETFTDKVISLINQHREGVVFLLWGSHAQKKGAIIDPQRH
HILKAPHPSPLSAHRGFFGCNHFALTNQWLEQHGEKTIDWTPVLPAESE
>EECEBICD_00748 Autonomous glycyl radical cofactor
MITGIQITKAANDDLLNSFWLLDSEKGEARCIVAKSGFAEDEVVAVSKLGEIEYREIPME
VKPEVRVEGGQHLNVNVLRRETLEDAVKHPEKYPQLTIRVSGYAVRFNSLTPEQQRDVIA
RTFTESL
>EECEBICD_00749 Cysteine/O-acetylserine efflux protein
MTPMLLSAFWTYTLITALTPGPNNILALSAATAHGFRQSIRVLAGMSLGFLVVMLLCAGI
AFSLAVIDPAIIHLLSWVGAAYILWLAWKIATSPAADEKVRPKPVGFWVSFGLQFVNVKI
ILYGITALSTFVLPQTQALNWVIGVSILLALIGTFGNVCWALAGHLFQRAFRHYGRQLNI
ILALLLVYCAVRIFY
>EECEBICD_00750 HTH-type transcriptional regulator GltR
MDLRRFITLKTVVEEGSFLRASQKLCCTQSTVTFHIQQLEREFSLQLFEKIGRRMCLTTE
GKKLMPHIHELTRVMELIREAARQDAEPGGELRVATGETLLAYKMPQVLQRFKLRAPNVK
LSLQSLNCYVIRDALLNDEVDLGVFYRVGNDDALTMQQLGEQSLALVASPLLQDADFTQP
DQHIPCSFIINEPQCVFRQLFESTLRQRRITLENTIELWSIESIKQCVAANLGISFLPRF
TVERELSTGQLKELPFGAPSLSIMALCAHHAGKAVSPAMQIFIQCMEACFTVEDKKMPG
>EECEBICD_00751 ATP-dependent RNA helicase SrmB
MTVTTFSELELDESLLDALQDKGFTRPTAIQAAAIPPALDGRDVLGSAPTGTGKTAAYLL
PALQHLLDFPRKKSGPPRILILTPTRELAMQVADHARELAKHTHLDIATITGGVAYMNHA
EVFSENQDIVVATTGRLLQYIKEENFDCRAVETLILDEADRMLDMGFAQDIEHIAGETRW
RKQTMLFSATLEGDAIKDFAERLLEDPVEVSANPSTRERKKIHQWYYRADNFEHKVALLK
HLLKQDDATRSIVFVRKRERVHELAETLRLAGINNCYLEGEMAQIKRNEGIKRLTDGRVN
VLVATDVAARGIDIPDVSHVINFDMPRSGDTYLHRIGRTGRAGRKGTAISLVEAHDHLLL
LKIGRYIEEPLKARVIDELRPTTRAPSEKLTGKPSKKVLAKRAEKKKEKEKEKPRVKKRH
RDTKNIGKRRKPSGTKMQEQSSEE
>EECEBICD_00752 tRNA1(Val) (adenine(37)-N6)-methyltransferase
MSQSGSVLRRNGFTFKQFFVAHDRCAMKVGTDGILLGAWAPVADVKRILDIGTGSGLLAL
MLAQRTDDNVPIDAVELDAGAAMQAQENVAHSPWPHRITVHTDDIQSWAPRQTVRFDLII
SNPPYYEPGVECATPQREQARYTATLDHQTLLAIAADCITEDGFFCVVLPEQIGNAFTQQ
ALNMGWHLRLRTDVAENEARLPHRVLLAFSPQAGECFSDRLVIRGSDQHYSESYTALTQA
FYLFM
>EECEBICD_00753 L-aspartate oxidase
MMTTPELSCDVLIIGSGAAGLSLALRLAEKHKVIVLSKGPVSEGSTFYAQGGIAAVFDET
DSIASHVEDTLIAGAGICDRHAVEFVASNARTCVQWLIDQGVLFDTHVQPNGKESYHLTR
EGGHSHRRILHAADATGKEVETTLVSRAQNHPNIQVLERSNAVDLIISDKMGLPGPRRVV
GAWIWNRNKEWVETCHAKSVVLATGGASKVYQYTTNPDISSGDGIAMAWRAGCRVANLEF
NQFHPTALYHPQARNFLLTEALRGEGAYLKRPDGSRFMPDVDERGELAPRDIVARAIDHE
MKRLGADCMFLDISHKPDDFVRQHFPMIYAKLLDLGMDLTKEPIPIVPAAHYTCGGVVVD

DYGRTDVDGLYAIGEVSYTGLHGANRMASNSLLECLVYGWSAAMDIDRRMPSVHSVDVLP
AWDESRVENADERVVIQHNWHELRLLMWDYVGIVRTTKRLERALRRITMLQQEIDEYYAN
FRVSNNLLELRNLVQVAELIVRCAMMRKESRGLHFTLDYPQQLAESGPSILSPLTPHINR
>EECEBICD_00754 ECF RNA polymerase sigma-E factor
MSEQLTDQVLVERVQKGDQKAFNLLVVRYQHKVASLVSRYVPSGDVPDVVQESFIKAYRA
LDSFRGDSAFYTWLYRIAVNTAKNYLVAQGRRPPSSDVDAIEAENFESGGALKEISNPEN
LMLSEELRQIVFRTIESLPEDLRMAITLRELDGLSYEEIAAIMDCPVGTVRSRIFRAREA
IDNKVQPLIRR
>EECEBICD_00755 Anti-sigma-E factor RseA
MQKEKLSALMDGETLDSELLKALTHDPEMQKTWESYHLIRDSMRGDTPDVLHFDISARVM
AAIENEPVRQVSPLIPEAQPAPQQWQKMPFWKKVRPWAAQLTQMGVAACVSLAVIVGVQH
YNGQSETSQQPETPVFNTLPMMGKASPVSLGVPSEAAPVGSQQQQVQEQRRRINAMLQDY
ELQRRLHSEQLQFEQAQTQQAAVQVPGIQTLGTQSQ
>EECEBICD_00756 Sigma-E factor regulatory protein RseB
MKQLWFAMSLVAASLFFSANASADPASGALLQQMNIASQSLNYELSFVSITKQGVESLRY
RHARLDGRPLAQLLQLDGPRREVVQRGNEISYFEPGLEPFTLNGDYIVDSLPSLIYTDFK
RLAPYYDFISVGRTRIADRLCEVIRVVARDGTRYSYIVWMDMDTKLPMRVDLLDRDGETL
EQFRVIAFTVSQDIGSNMQALAKANLPPLLSVPGGEKTKFNWSPSWVPQGFSEVSSSRRP
LPTMDNLPIESRLYSDGLFSFSVNVNRATQNSSDQMLRTGRRTVYSSVRDNAEITIVGEL
PPQTAKRIADSIKFRAVQ
>EECEBICD_00757 Protein RseC
MIKEWATVISWQNGQAVVSCDVKASCSSCASRAGCGSRVLNKLGPQTTHTIVVPSAEPLA
PGQKVELGIAEKSLLGSALLVYMSPLAGLFLCAALFQMLFGSDLAALSGAVLGGVGGFLV
ARGYSRKLAERDAWQPVILNVALPPDLVRVETTSIETRQ
>EECEBICD_00758 Elongation factor 4
MKNIRNFSIIAHIDHGKSTLSDRIIQICGGLSDREMEAQVLDSMDLERERGITIKAQSVT
LDFKASDGETYQLNFIDTPGHVDFSYEVSRSLAACEGALLVVDAGQGVEAQTLANCYTAM
EMDLEVVPVLNKIDLPAADPERVAEEIEDIVGIDATDAVRCSAKTGVGVTDVLERLVRDI
PPPQGDPDGPLQALIIDSWFDNYLGVVSLVRIKNGTMRKGDKIKVMSTGQTYNADRLGIF
TPKQVDRTELKCGEVGWLVCAIKDILGAPVGDTLTSARNPAEKALPGFKKVKPQVYAGLF
PVSSDDYESFRDALGKLSLNDASLFYEPESSSALGFGFRCGFLGLLHMEIIQERLEREYD
LDLITTAPTVVYEVETTAKETIYVDSPSKLPPLNNIYELREPIAECHMLLPQAYLGNVIT
LCIEKRGVQTNMVYHGNQVALTYEIPMAEVVLDFFDRLKSTSRGYASLDYNFKRFQASDM
VRVDVLINNERVDALALITHRDNSQSRGRELVEKMKDLIPRQQFDIAIQAAIGTHIIARS
TVKQLRKNVLAKCYGGDISRKKKLLQKQKEGKKRMKQIGNVELPQEAFLAILHVGKDNK
>EECEBICD_00759 Signal peptidase I
MASMFALILVIATLVTGILWCVDKFVFAPKRRARQAAAQTASGDALDNATLNKVAPKPGW
LETGASVFPVLAIVLIVRSFLYEPFQIPSGSMMPTLLIGDFILVEKFAYGIKDPIYQKTL
IETGHPKRGDIVVFKYPEDPKLDYIKRAVGLPGDKITYDPVAKEVTIQPGCSSGQACENA
LPVTYSNVEPSDFVQTFARRNGGEATSGFFEVPLNETKENGIRLTERKETLGDVTHRILM
VPIAQDQLGMYYQQPGQPLATWVVPPGQYFMMGDNRDNSADSRYWGFVPEANLVGKAVAI
WMSFDKQEGEWPTGVRLSRIGGIH
>EECEBICD_00760 Ribonuclease 3
MYQVCFVCWIVDAFIYWYRMNPIVINRLQRKLGYTFNHQELLQQALTHRSASSKHNERLE
FLGDSILSFVIANALYHRFPRVDEGDMSRMRATLVRGNTLAELAREFDLGECLRLGPGEL
KSGGFRRESILADTVEALIGGVFLDSNIQTVEQLILNWYKTRLDEISPGDKQKDPKTRLQ
EYLQGRHLPLPSYLVVQVRGEAHDQEFTIHCQVSGLSEPVVGTGSSRRKAEQAAAEQALK
KLELE
>EECEBICD_00761 GTPase Era
MSTDKTYCGFIAIVGRPNVGKSTLLNKLLGQKISITSRKAQTTRHRIVGIHTEGPYQAIY
VDTPGLHMEEKRAINRLMNKAASSSIGDVELVIFVVEGTRWTPDDEMVLNKLRDGKAPVI
LAVNKVDNVQEKADLLPHLQFLASQMNFLDIVPISAETGMNVDTIAGIVRKHLPEAIHHF
PEDYITDRSQRFMASEIIREKLMRFLGAELPYSVTVEIERFVTNERGGYDINGLILVERE
GQKKMVIGNKGAKIKTIGIEARKDMQEMFEAPVHLELWVKVKSGWADDERALRSLGYVDD

L
>EECEBICD_00762 DNA repair protein RecO
MEGWQRAFVLHSRPWSETSLMLDVFTEESGRVRLVAKGARSKRSNLKGALQPFTPLLLRY
SGRGEVKTLRSAEAVSLALPLSGITLYSGLYINELLSRVLEYETRFSELFFDYLNCIQAL
AGTTGSPEPALRRFELALLGHLGYGVNFTHCAGSGERVDDTMTYRYREEKGFFASVVIDN
NTFTGRHLKALEEREFPDVDTLRAAKRFTRMALKPYLGGKPLKSRELFRQFMPKRTVKMK
KD
>EECEBICD_00763 Pyridoxine 5'-phosphate synthase
MAELLLGVNIDHIATLRNARGTDYPDPVQAAFIAEQAGADGITVHLREDRRHITDRDVRI
LRQTLHTRMNLEMAVTEEMLAIAVETRPHFCCLVPEKRQEVTTEGGLDVAGQRDKMRDAC
ARLAAAGIQVSLFIDADETQINAAAEVGAPFIEIHTGCYANAETDAEQAKELARIASAAT
LAARLGLKVNAGHGLTYHNVKAIAALPEMHELNIGHAIIGRAVMTGLKEAVAEMKRLMLE
ARG
>EECEBICD_00764 Holo-[acyl-carrier-protein] synthase
MAILGLGTDIVEIARIEAVISRSGERLARRVLSDNEWAIWETHQQPVRFLAKRFAVKEAA
AKAFGTGIRNGLAFNQFEVFNDELGKPRLRLWGEALTLAEKLGVAHMHVTLADERHYACA
TVILES
>EECEBICD_00765 Ferredoxin YfhL
MALLITKKCINCDMCEPECPNEAISMGDSIYEINSDKCTECVGHYETPTCQKVCPIPNTI
LQDPAHVETEEQLWDKFVLMHHADKL
>EECEBICD_00766 HTH-type transcriptional regulator HdfR
MNIKQLHSFLILCDELHYGRAASRLFITQPSLSQQIKQLESSLKTILFKRKGRGIKLTKA
GIILQRHANKIMLDLKNAENELLPYQDQQRDTISIGVSGSYLVLPAFRNFMAHHPEISLN
VKEFSTEQTIKKLTDSAVDIGIVYRTALPAQLSSTMLFEDEIIAAIPLSHPLATRERLHL
KDLNDQPIIVLNDSLLLRGIITTEFNNRKVVPNVICELDNHYSCLEYAEAQIGIAFITRS
LTHLSTPKNVRLVSLGIPAFFIPVMLVHSNDLSLDNATICLLKQIKNFYTVDNKSAPEKS
WPEQKIGV
>EECEBICD_00767 L-lactate transporter
MSASINRWGMLAAHVCINFVLGGVYAFSYFKTPLMAQYHWDPATLALAFSINMGIIPLPM
IWGGRMIDNGKGKQAIVIGGILFSLGFILSGFVDNLPMLFLTYGVIAGLGSGLAFTGNLN
NILKFFPDRRGLASGIVLAGVGVGTLLCTRLAEYFMAQTHDVSRALLYLGIVYLVVIFIV
QFFIRSAPAKDSGGIKASPLDKDYRHMLKDLRFWLLFMILALGVFSGMVISSSSAQIGMT
QYGLLSGALVVSLVSIFNSIGRLFWGGLTDKLGGYNTLVIVYLFTCLCMLLLFFFNGNTS
VFYFSALGVGFAYAGILVIFPGLTSQNFGMRNQGLNYGFMYFGFAVGAVIAPYVTSAIAK
YTGSYNTVFILTTVLLLIGVVLTLITKKYVATVLAKIH
>EECEBICD_00768 2-dehydropantoate 2-reductase
MKIAIAGAGAMGCRFGYMLLEAGHDVTLIDSWQEHVDAIRSKGLFVETETTQKYYPIPAM
LADESQGEFELVILFTKAMQLDSMLLRIKPLLPAAKVVMILSNGLGNIETLEKYVDRQKI
YAGVTLWSSELEGAGHIMATGTGTIELQPIASQDSAQEAKVIATLNSAGLNAEISPDVLL
SIWKKAAFNSVMNTYCALLDCNVGGFGQRPGALDLAQAVVDEFVLVAASQNIPLTEQMVM
NTVKKVFDPRESGHHYPSMHQDLHKGRLTEIDYLNGAIARIGAQNNIAVPVNTLLTQLIH
AKEAQ
>EECEBICD_00769 HTH-type transcriptional regulator MurR
MNCLIRIRQRYPDLAQSDRKLADYLLAQPDTARHLSSQQLAAEAGVSQSSVVKFAQKLGF
KGFPALKLAISEALASNPNPHSVPVHNQIRGDDPMRLVGEKLIKENVAAMHATLDVNSEE
KLLESVTMLRHARRIVITGIGASGLVAQNFAWKLLKIGINAVVERDMHALLATVQALAPE
DLLLAISYSGERRELNLAADETLRAGAKILAITGFSPNALQQRATRCLYTIAEEQATRSA
AISSTHAQMMLTDLLFMALVQQDLERAPERIRHSEALVKKLV
>EECEBICD_00770 N-acetylmuramic acid 6-phosphate etherase
MNLGTLVSETRNPQTMDLDALPTPELVKRFNEQDTRVAEAVKATLPDVARAVDAAVAALK
SGGRIIYMGAGTSGRLGVLDASECPPTFGVPHGLVVGLIAGGPGALLKAVEGAEDSQQAG
EDDLVALNLQEQDLVVGLAASGRTPYVIGGLRYARQSGCTTVAVSCNPDSPIAREANIAI
SPVVGPEALTGSTRLKSGTAQKMVLNMISTGAMVKFGKVYQNLMVDMKATNVKLVDRACR
MVVEATGIGREEAETLLKQTDFEVKPAILMALTGLDAAAAREKLAAHQGFLRAALEH

>EECEBICD_00771 PTS system EIIBC component
MDKTAALASDILRGIGGEQNILRLENCMTRVRVEVQDDSQLDIPRLKALPGISGYVKQGE
QHQLIVGPGKAAQVVDAMRVQIAVGGVKPDDAMARTKSEAKAKYKAPMSDALRKLANVFI
PLIPAFIASGLITGIINILKRPDIVGDVAVHYPNLLGLMGIFGSAVFAIMNILVGVNTAK
VFGGSQALGGVMAGILSSPQLAQITLFGEALQPGRGGVIAVLLVVALMCWIERQFRKLLP
GSLELILNPLLTTVITGAVAIVALQPLGGWISDAIAHGASWAIDRGGFLVGAVLAGTFLP
LVLTGLHQGLVPIHVELVQAHGYNALFPILAMAGVGQIGAAIAVLMKTRNARLKKVIKGA
LPVGLLGIGEPLIFGVTLPLGKPFIGACLGGAVGGALISYWKVATVITFGISGLPLALTI
VAGKVLFYLLGYLIAVIAGFIFTWLLGFNDPEE
>EECEBICD_00772 Phosphatidylglycerophosphatase C
MVSHERRVVFFDLDGTLHQQDMFGSFLRYLLRRQPLNALLVLPLLPIIGIGLLVKGRAAR
WPMSLLLWGCTFGHSETRLQAHQADFVRWFRANVTAFPVVQERLTTYLLSSDADIWLITG
SPQSLVEQVYFDTPWLPRVNLIASQMARRYGGWVLTVRCLGHEKVAQLERKIGTPLRLYS
GYSDSKQDNPLLYFCQHRWRVTPHGELQQLE
>EECEBICD_00773 tRNA-specific adenosine deaminase
MSDVELDHEYWMRHALTLAKRAWDEREVPVGAVLVHNHRVIGEGWNRPIGRHDPTAHAEI
MALRQGGLVLQNYRLLDTTLYVTLEPCVMCAGAMVHSRIGRVVFGARDAKTGAAGSLIDV
LHHPGMNHRVDIIEGVLRDECATLLSDFFRMRRQEIKALKKADRAEGAGPAV
>EECEBICD_00774 Membrane-bound lytic murein transglycosylase F
MKKLKINYLFIGILTLLLAAALWPSIPWFGKTENHIAAIQARGILRVSTIDSPLTYSVIN
GKKYGLDYELAQQFANYLGVKLKVTVRQNISQLFDDLDNGNADLLAAGLVYDSARVKNYQ
PGPMYYSVSQQLVYRVGQYRPRSLATVNENQLTIAPGHVVVNDLQRLKETKFPDLSWKVD
DKKGSTTLLEEVISGKLDYTIADSVAISLFQRVHPELAVALDVTDEQPVTWFSRLDDDNT
LSAALLDFFNSINEDGSLARIEEKYLGHGDDFDYVDTRSFLRAVDNVLPELEPLFKKYAK
EIDWRLLAAISYQESHWDPLATSPTGVRGLMMLTKNTAQSLGLTDRTDAEQSISGGARYL
EDMMAKVPETVPEDERIWFALAAYNMGYAHMLDARSLTVKTKGNPDSWTDVKQRLPLLSQ
KPYYSKLTYGYARGHEAYAYVENIRKYQISLVGYLQEKEKQEAEAMKLAQDYPAVSPEEL
NKAPFPFLSFLSQSSGYLTHSPSLLFTPQKKEEK
>EECEBICD_00775 Phosphoribosylformylglycinamidine synthase
MMEILRGSPALSAFRINKLLARFQAANLQVHNIYAEYVHFADLNAPLNDSEQAQLTRLLQ
YGPALNSHTPAGKLLLVTPRPGTISPGLQKQRISPTTAACNRLIGWSAA
>EECEBICD_00776 Phosphoribosylformylglycinamidine synthase
MTAEQWRQVAAELHDRMMETVFSSLTDAEKLFIHHQPAPVSSVDLLGEGRQALIDANLRL
GLALAEDEIDYLQEAFTKLGRNPNDIELYMFAQANSEHCRHKIFNADWIIDGKPQPKSLF
KMIKNTFETTPDYVLSAYKDNAAVMEGSAVGRYFADHNTGRYDFHQEPAHILMKVETHNH
PTAISPWPGAATGSGGEIRDEGATGRGAKPKAGLVGFSVSNLRIPGFEQPWEEDFGKPER
IVTALDIMTEGPLGGAAFNNEFGRPALTGYFRTYEEKVNSHNGEELRGYHKPIMLAGGIG
NIRADHVQKGEIVVGAKLIVLGGPAMNIGLGGGAASSMASGQSDADLDFASVQRDNPEME
RRCQEVIDRCWQLGDANPILFIHDVGAGGLSNAMPELVSDGGRGGKFELRDILSDEPGMS
PLEIWCNESQERYVLAVAADQLPLFDELCKRERAPYAVIGDATEEQHLSLHDNHFDNQPI
DLPLDVLLGKTPKMTRDVQTLKAKGDALNRADITIADAVNRVLHLPTVAEKTFLVTIGDR
TVTGMVARDQMVGPWQVPVADCAVTTASLDSYYGEAMSIGERAPVALLDFAASARLAVGE
ALTNIAATQIGDIKRIKLSANWMAAAGHPGEDAGLYDAVKAVGEELCPQLGLTIPVGKDS
MSMKTRWQEGNEQREMTSPLSLVISAFARVEDVRHTLTPQLSTEDNALLLIDLGKGHNAL
GATALAQVYRQLGDKPADVRDVAQLKGFYDAMQALVAARKLLAWHDRSDGGLLVTLAEMA
FAGHCGVQVDIAALGDDHLAALFNEELGGVIQVRAEDRDAVEALLAQYGLADCVHYLGQA
LAGDRFVITANDQTVFSESRTTLRVWWAETTWQMQRLRDNPQCADQEHEAKANDADPGLN
VKLSFDINEDIAAPYIATGARPKVAVLREQGVNSHVEMAAAFHRAGFDAIDVHMSDLLGG
RIGLGNFHALVACGGFSYGDVLGAGEGWAKSILFNHRVRDEFETFFHRPQTLALGVCNGC
QMMSNLRELIPGSELWPRFVRNHSDRFEARFSLVEVTQSPSLLLQGMVGSQMPIAVSHGE
GRVEVRDDAHLAALESKGLVALRYVDNFGKVTETYPANPNGSPNGITAVTTENGRVTIMM
PHPERVFRTVANSWHPENWGEDSPWMRIFRNARKQLG
>EECEBICD_00777 Sensor histidine kinase QseE
MLAFLLILLPLLVLAWQAWQSLNALSDQAAVTNRSTLIDARRSEAMTNVALEMERSYRQY

CVLDDPTLAKVYQSQRKRYSDMLDAHAGVLPDDKLYQALRQDLNALAQLQCKDSGPEAAA
AARLEAFANANTEMVQATRTVVYSRGQQLQQEIAERGQFFGWQALVLFLVSLAMVLLFTR
MIIGPVKGIERMINRLGEGRSLGNTVTFTGPRELRSVGQRIIWLSERLAWLESQRHQFLR
HLSHELKTPLASMREGTELLADQVVGPLTPEQKEVVDILDDSSRNLQKLIEQLLDYNRKL
VDSATELEAVDIAPLVDMVVSAHSLPARAKMMHTDVDLEAERCIAEPMLLMSVLDNLYSN
AVHYGAESGNICIRSRSQGSTVYIDVVNSGEPIPQTEREMIFEPFFQGSHQRKGAVKGSG
LGLSIARDCIRRMQGEIQLVDDKAQEVCFRISLPLPASDKH
>EECEBICD_00778 Quorum-sensing regulator protein G
MNLSLVRMSHVFVQTIKRCLLRWGIPVGISCLALTACVPHASQQLPGSAAQDTLPHYQLA
DYLPTACADIWSLRGQAVETNPLYWLRTIDCADRLMPVQSRAEARALTDDNWQNAFRRGI
LLADAKITPPERRAIVTRLEALSAQIPAQVRPVYQIWHDGQALQLALSAERQRYSKLQQT
SDSELDALRQQQQALQTQLDLTTRKLESLTDIERQLSTRKPAGNYNADTPHTNDKPATSE
DGAAPSPSQDEVTP
>EECEBICD_00779 Transcriptional regulatory protein QseF
MISRKPAHLLLVDDDPGLLKLLGMRLTSEGYSVVTAESGQEGLRVLHREKVDLVISDLRM
DEMDGMQLFTEIQKVQPGMPVIILTAHGSIPDAVAATQKGVFSFLTKPIDRDALYKAIDE
ALEQSAPATDDSWRNAIVTRSPLMLRLLEQARMVAQSDVSVLINGQSGTGKEIFAQAIHN
ASPRSNKPFVAINCGALPEQLLESELFGHARGAFTGAVSNREGLFQAAEGGTLFLDEIGD
MPAPLQVKLLRVLQERKVRPLGSNRDIDIDVRIISATHRDLPKAMARGEFREDLYYRLNV
VSLKIPALAERTEDIPLLANHLLRQSAQRHKPFVRAFSTDAMKRLMTASWPGNVRQLVNV
IEQCVALTSSPVISDALVEQALEGENTALPTFAEARNQFELNYLRKLLQITKGNVTHAAR
MAGRNRTEFYKLLSRHELDANDFKE
>EECEBICD_00780 Nitrogen regulatory protein P-II 1
MKKIDAIIKPFKLDDVREALAEVGITGMTVTEVKGFGRQKGHTELYRGAEYMVDFLPKVK
IEIVVPDDIVDTCVDTIIRTAQTGKIGDGKIFVFDVARVIRIRTGEEDDAAI
>EECEBICD_00781 Dipeptide and tripeptide permease C
MKTPSQPRAIFYIVAIQIWEYFSFYGMRALLILYLTHQLGFNDSHAINLFSAYASLVYVT
PILGGWLADRLLGNRVAVITGALLMTLGHVVLGLESDSTLSLYAALAIIICGYGLFKSNI
SCLLGELYAPDDNRRDGGFSLLYAAGNIGSIAAPIACGLAAQWYGWHVGFALAGVGMFIG
LLIFLSGHRHFQQTRGVNRPALRAVKFALPTWGWLVLMLCIAPVFFTLLLENNWSGYVLA
IVCVFAAQLIARIMVKFPEHRRALWQIVLLMITGTLFWVLAQQGGSSISLFIDRFVNRHW
LNMTVPTALFQSVNAIAVMAAGVVLAWLSSPKESARSVLRVWLKFAVGLVLMGGGFMLLA
LNAHQARLDGQASMGMMIAGLALMGFAELFIDPVAMAQITRLNLPGVTGVLTGIYMLATG
AVANWLAGVVAQQTTESQISDTAVAAYGHFFSQMGEWTLSCVALIVAIVGLRWVCTRTTS
ALPQGD
>EECEBICD_00782 Inducible lysine decarboxylase
MNVIAIMNHMGVYFKEEPIRELHRALEGLNFRIVYPNDREDLLKLIENNSRLCGVIFDWD
KYNLELCEEISKLNEYMPLYAFANSYSTLDVSLNDLRMQVRFFEYALGAAADIAAKIRQN
TDEYIDNILPPLTKALFKYVREGKYTFCTPGHMGGTAFQKSPVGSIFYDFFGPNTMKSDI
SISVSELGSLLDHSGPHKEAEEYIARVFNAERSYMVTNGTSTANKIVGMYSAPAGSTVLI
DRNCHKSLTHLMMMSDITPIYFRPTRNAYGILGGIPQSEFQHATIAKRVKETPNATWPVH
AVITNSTYDGLLYNTDYIKKTLDVKSIHFDSAWVPYTNFSPIYQGKCGMSGDRVEGKIIY
ETQSTHKLLAAFSQASMIHVKGDINEETFNEAYMMHTTTSPHYGIVASTETAAAMMKGNA
GKRLINGSIERAIKFRKEIKRLKSESDGWFFDVWQPEHIDGAECWPLRSDSAWHGFKNID
NEHMYLDPIKVTILTPGMKKDGTMDEFGIPASLVAKYLDERGIIVEKTGPYNLLFLFSIG
IDKTKALSLLRALTEFKRAFDLNLRVKNILPALYREAPEFYENMRIQELAQNIHKLVEHH
NLPDLMYRAFEVLPKMVMTPYTAFQKELHGETEEVYLEEMVGRVNANMILPYPPGVPLVM
PGEMITEESRPVLEFLQMLCEIGAHYPGFETDIHGAYRQADGRYTVKVLKENTK
>EECEBICD_00783 putative cadaverine/lysine antiporter
MSSVKKIGLFACTGVVAGNMMGSGIALLPANLASIGGIAIWGWVISIIGAMSLAYVYARL
ATKNPQQGGPIAYAGEISPAFGFQTGVLYYHANWIGNLAIGITAVSYLSTFFPALNNPIP
AGIACIAIVWLFTFINMLGGAWVSRLTTIGLFLVLIPVVLTAVAGWHWFDIATYHANWNT
STTTDSHAIVKSILLCLWAFVGVESAAVSTGMVKNPKRTVPLATMLGTALAGIIYIAATQ
VIAGMFPASVMASSGAPFAISTSTILGGWAAPLVSAFTAFACLTSLGSWMMLVGQAGVRA

ANDGNFPKIYGEMDKNGIPKKGLLLAAVKMTALMILITIMNSSGGKASDLFGELTGIAVL
LTMLPYFYSCVDLIRFEGFNIRNSVSLICSVLGCAFCFIALMGASSFELSGTFIVSLIIL
MFYGRKMHQRQNNDSDNNTAEAL
>EECEBICD_00784 Transcriptional activator CadC
MQQPVVRIGEWLVTPSVNQISRQGRQITLEPRLIDLLMYFAHHPDEVLSRDNIIDHVWMR
TIVTNHVVTQSISELRKSLRDGGDSNAEYIVTVPKRGYKLTAPVIWCEENSDEIDNSSTS
PPPPIAATNAEPTEGVTAATPVPPASLQTPTKKAKKPRIAAFWTWVMFLLSLATLVVFIV
MSVVDHNAAVTKTRLLLNPRDIDVRFEGGNSCNNWVSQESYAIGLGGLITDLLNTYSTFM
VHDKTNYRVNEPSSSGKTLTIQFVNQRHYRAQQCFMSVVLIDNADGSTMLDKRYFVTNTN
QLSIQDDLFNSLSFVLTQPWPNRMREQLALFRTPQNTALMRFYEARQLILSGDAQALGKA
SDILNGIIKETPDFNYAYEYKVLVDVLRQSQQPFDKEQVAALNEEFKKIDQIPGVEKTSV
YYKIKTVDLLGKGDIDAAYEEINKSIELEMSWFNYVLLGKVYEMKGENRLAADAYLTAFN
LRPGENTLYWIENGVFQTSVQKIVPYLNSFLAED
>EECEBICD_00785 Flavohemoprotein
MLDAQTIATVKATIPLLVETGPKLTAHFYDRMFTHNPELKEIFNMSNQRNGDQREALFNA
IAAYASNIENLPALLPAVEKIAQKHTSFQIKPEQYNIVGTHLLATLDEMFNPGQEVLDAW
GKAYGVLANVFIHREAEIYHENASKDGGWEGTRPFRIVAKTPRSALITSFEFEPVDGGTV
AEYRPGQYLGVWLKPEGFAHQEIRQYSLTRKPDGKGYRIAVKREDGGQVSNWLHHHASVG
DVVHLAAPAGDFFMNVAADTPVSLISAGVGQTPMLAMLDTLAKEQHTAQVNWFHAAENGD
VHAFADEVSELGRTLPRFTAHTWYREPTEADRAQRLFDSEGLMDLSKLEAAISDPAMQFY
LCGPVGFMQFAAKQLVSLGVNHENIHYECFGPHKVL
>EECEBICD_00786 Serine hydroxymethyltransferase
MLKREMNIADYDAELWQAMEQEKVRQEEHIELIASENYTSPRVMQAQGSQLTNKYAEGYP
GKRYYGGCEYVDVVEQLAIDRAKELFGADYANVQPHSGSQANFAVYTALLQPGDTVLGMN
LAQGGHLTHGSPVNFSGKLYNIVPYGIDESGKIDYDEMAKLAKEHKPKMIIGGFSAYSGV
VDWAKMREIADSIGAYLFVDMAHVAGLIAAGVYPNPVPHAHVVTTTTHKTLAGPRGGLIL
AKGGDEELYKKLNSAVFPSAQGGPLMHVIAGKAVALKEAMEPEFKVYQQQVAKNAKAMVE
VFLNRGYKVVSGGTENHLFLLDLVDKNLTGKEADAALGRANITVNKNSVPNDPKSPFVTS
GIRIGSPAVTRRGFKEAEVKELAGWMCDVLDNINDEATIERVKAKVLDICARFPVYA
>EECEBICD_00787 putative 3-phenylpropionic acid transporter
MALHSTRWLALSYFTYFFSYGIFLPFWSVWLKGLGLTPETIGLLLGVGLVARFLGSLLIA
PRVSDPSRLISALRVLALLTLVFALAFWAGTHVAWLMVVMVGFNLFFSPLVPLTDALANT
WQKQITLDYGRVRLWGSIAFVIGSALTGKLVSLYDYQAILALLTLGVASMLLGMLLRPSV
PPQGESRQQESAGWPAWRTLVAQSWRFLACVCLLQGAHAAYYGFSAIYWQGAGYSASAVG
YLWSLGVVAEVIIFALSKKLFRRFSARDLLLLSAVCGVVRWGLMGWSTALPWLIVIQILH
CGTFTVCHLAAMRYIAARQGSEVIRLQAVYSAVAMGGSIAIMTVFAGFLYQHLGGGVFWI
MALVALPAIFLRPKVVAAS
>EECEBICD_00788 hypothetical protein
MMPTIAPPSVLSAPQRRCQVLLTLFQPEPIATVEIFSALNGVDDDTAREDITETSLEIQR
YHRLAITTCQNGCYRIEGTALDQRLCLLHWLRRGLRLCPTFVTQQFTPALKNALKQRGIA
RPLYDDINLHALINLCARRLQKPFEHRDVQFLRLFLQYCLLQHHAGITPEFNPVQQIWAQ
SCAEYPLAQEIGRHWQRHVMQAAPLNEALFMALLFSMIRLPDPIRDTHQRAQQLRLEVAR
LVLRFREKGNVRFSDEQGLNDQLYVHLAQALNRSLFTIGIDNTLPEEFNRLYPRLVRTTR
EALAGFEAEYGIHFSEEETGLVAVIFGAWLMQDNDLHERQIVLLADKNDALETHIEQQLR
ELTLLPLNIRRLSLQAFQKEGCPRGVALIVTPYATPLPLFSPPLIHADRTLTEHQQQQIR
KILES
>EECEBICD_00789 hypothetical protein
MKPVKRSALTLFLAVLSFVAAAHPHSFIRLQTQVVSENEQFVALKMRWTMDALTSADLLY
DAGNAAPGSEIWKKLAAEVMANVLGQHYFTEVWRNGAKVKFKNRPTEYGMTRDAHQAVLT
FTLPLAEPQPLSGQTYTFSTFDPSYYVDMHYDQDSDITMPEPLREKCRIQVYTPAPGEET
LRFAQSLDKEDAPPEDMDLGKQFAQTVTLQCQ
>EECEBICD_00790 hypothetical protein
MSVIFSQRTPGRRWLSLWPLALFLLLMLVGGLWIWQAWPQVMLKSALWQRDVNQQLSALL
NAVATHPERAGGSLLLLSFMYGVLHALGPGHGKVVIATWLATHPSKLKSSIVLTLAAALL

QGLVAIGLVVGVLTVLQLPARQLHLSGFWLEKGSYALVGGLGILLCWRAIKRLRALLRKP
VFIAFTPRHVHHEKCGCGHQHLPTQEQLHSGDDWRARLMIVLSMGMRPCSGAIMVLLFSK
VIGVFSWGMASVLAMAAGTSLTITSLALLVHTFRDLAVKLSGNKAPALWRQVGWSTLALA
GGGILLVAALVMWFSVPQPVGGLRPWRG
>EECEBICD_00791 Anaerobic sulfite reductase subunit C
MSIDIDIIKARAKNEYRLSKVRGEAMISVRIPGGILPAHLLTVARDIAETWGNGQIHLTT
RQKLAMPGIRYEDIDNVNAALEPFLREIEIELCDVQVEDTKAGYLAIGGRNIVACQGNRI
CQKANTDTTGLSRRLEKLVYPSPYHLKTVIVGCPNDCAKASMADLGIIGVAKMRFTADRC
IGCGACVKACSHHAVGCLALKNGKAVKEESACIGCGECVLACPTLAWQRKPDQLWQVRLG
GRTSKKTPRVGKLFLNWVTEDVIKQVIVNLYEFEKEMLGGKPIYLHMGHLIDKGGYLRFK
ERVLRGVQLNPEAMVAERIYWAEDESVARMHLKPAGH
>EECEBICD_00792 Anaerobic sulfite reductase subunit B
MSHCSCHDKPQHSLLPAAYRILSITRHTPLEWNFRVAVDFPAHWGQFVEVSLPRVGEAPI
SVSDYGDGWIDLLIRNVGKVTSALFTLKEGDNVWLRGCYGNGYPVDTLRHKPLLVVAGGT
GVAPVKGLMRYFVENPQEIGQLDMILGYKNRDCVLYKEEMATWRGKHNLVLTLDEGEADD
RYQIGRVTDRLADMTLSDIDTMQAIVVGPPIMITFTVKMLLQKGLKPEQIWVDYERRMAC
SVGKCGHCRMGEVYVCTDGPIFNYAVAQRFAD
>EECEBICD_00793 Anaerobic sulfite reductase subunit A
MAIKITPDEFSLLIQRLNKKWRVFAPSAEFRGGRFSDTDNIIYQRISGWRDLIWHEKSHM
SPNTIIAPITETLFYFDKDTIQIAETDISPIIIFARACDINAMSRLDYMYLSNGNNSDYS
YQLLREHIRFVLIECEESFENCFCVSMGTNKTDCYSAAMRFSDEGALVSIRDPFIEAAIQ
GLGQEADYTPSFVSENRETVVTPDSVCRDPQKIRDILTRHPLWDAYDSRCISCGRCTTGC
PTCTCYSVFDVAYDENPQRGERRRQWASCMVPGFSDMAGGHGFREKPGERLRYRALHKVN
DYKARNGIEHMCVGCGRCDDRCPQYIKFSLIINKMTAAVRQALAEEA
>EECEBICD_00794 putative protein
MTLQHTRRIVKSLFILFIIVVCIYLLPRVAINAFYYPDNKVYGPTPAEAESITFTAKDGT
HLHGWFIPTAFGRPENAVATVIHVHGNAGNMSAHWPLVSWLPERNVNLFMFDYRGFGESE
GTPSQEGLLDDTKSAIDYVRHRADVNPERLVLLGQSLGGNNVLAAVGHCVGCANMRYADQ
AGIRAIILDSTFLSYSSIANQMIPGSGYLLDDRYSADRNIASVSPIPVLILHGTADHVIP
WQDSEKLYALAREPKQKIFIPDGDHIDAFSGRYANLYRDAMIKFIQTALSAK
>EECEBICD_00795 Inositol-1-monophosphatase
MHPMLTIAVRAARKAGNVIAKNYETPDAVEASQKGSNDFVTNVDKAAEAVIIDTIRKSYP
QHTIITEESGEHVGTDQDVQWVIDPLDGTTNFIKRLPHFSVSIAVRIKGRTEVAVVYDPM
RNELFTATRGQGAQLNGYRLRGSTARDLDGTILATGFPFKAKQYATTYINIIGKLFSECA
DFRRTGSAALDLAYVAAGRVDGFFEIGLRPWDFAAGELLVREAGGIVSDFTGGHNYMMTG
NIVAGNPRVVKAMLANMRDELSDALKR
>EECEBICD_00796 tRNA (cytidine/uridine-2'-O-)-methyltransferase TrmJ
MLQNIRIVLVETSHTGNMGSVARAMKTMGLTNLWLVNPLVKPDSQAIALAAGASDVIGNA
QIVDTLDEALAGCSLVVGTSARSRTLPWPMLDPRECGLKSVAEAANTPVALVFGRERVGL
TNDELQKCHYHVAIAANPEYSSLNLAMAVQVIAYEVRMAWLATQENGDAADHEETPYPLV
DDLERFYGHLEQTLLSTGFIRENHPGQVMNKLRRLFTRARPESQELNILRGILASIEQQN
KGK
>EECEBICD_00797 HTH-type transcriptional regulator IscR
MRLTSKGRYAVTAMLDVALNSEAGPVPLADISERQGISLSYLEQLFSRLRKNGLVSSVRG
PGGGYLLGKDAGSIAVGEVISAVDESVDATRCQGKGGCQGGDKCLTHALWRDLSDRLTGF
LNNITLGELVNNQEVLDVSGRQHTHDAPRASGRAQDAIDVKLRA
>EECEBICD_00798 Cysteine desulfurase IscS
MKLPIYLDYSATTPVDPRVAEKMMQFLTLDGTFGNPASRSHRFGWQAEEAVDIARNQIAE
LVGADPREIVFTSGATESDNLAIKGAANFYQKKGKHIITSKTEHKAVLDTCRQLEREGFE
VTYLAPQRNGIIDLNELEAAMRDDTILVSIMHVNNEIGVVQDIATIGEMCRARGIIYHVD
ATQSVGKLPIDLSQLKVDLMSFSGHKIYGPKGIGALYVRRKPRIRIEAQMHGGGHERGMR
SGTLPVHQIVGMGEAYRIAKEEMETEMARLRGLRNRLWNGIKDIEEVYLNGDLEQGAPNI
LNVSFNYVEGESLIMALKDLAVSSGSACTSASLEPSYVLRALGMNDELAHSSIRFSLGRF
TTEEEIDYTIDLVRKSIGRLRDLSPLWEMYKQGVDLNSIEWAHH

>EECEBICD_00799 Iron-sulfur cluster assembly scaffold protein IscU
MAYSEKVIDHYENPRNVGSFDNNDDNVGSGMVGAPACGDVMKLQIKVNDEGIIEDARFKT
YGCGSAIASSSLVTEWVKGKSLDEAQAIKNTDIADELELPPVKIHCSILAEDAIKAAIAD
YKSKREAK
>EECEBICD_00800 Iron-binding protein IscA
MSITLSDSAAARVNTFLANRGKGFGLRLGVRTSGCSGMAYVLEFVDEPTAEDTVFEDKGV
KVVVDGKSLQFLDGTQLDFVKEGLNEGFKFSNPNVKDECGCGESFHV
>EECEBICD_00801 Co-chaperone protein HscB
MDYFTLFGLPARYQIDTQALSLRFQDLQRQYHPDKFANGTQAQQLAAVQQSATINQAWQT
LRHPLTRAEYLLSLHGFDLASEQHTVRDTAFLMEQLTLREELDDIEQSKDDVRLESFIKR
VQKMFDARLQQMVEQLDNAAWDAAADTVRKLRFLDKLRSSAEQLEEKLLDF
>EECEBICD_00802 Chaperone protein HscA
MALLQISEPGLSAAPHQRRLAAGIDLGTTNSLVATVRSGQAETLPDHEGRHLLPSVVHYQ
QQGHTVGYAARDNAAQDTANTISSVKRMMGRSLADIQARYPHLPYRFKASVNGLPMIDTA
AGLLNPVRVSADILKALAARASESLSGELDGVVITVPAYFDDAQRQGTKDAARLAGLHVL
RLLNEPTAAAIAYGLDSGKEGVIAVYDLGGGTFDISILRLSRGVFEVLATGGDSALGGDD
FDHLLADYIREQAGIADRSDNRVQRELLDAAIAAKIALSDADTVRVNVAGWQGEITREQF
NDLISALVKRTLLACRRALKDAGVDPQDVLEVVMVGGSTRVPLVRERVGEFFGRTPLTAI
DPDKVVAIGAAIQADILVGNKPDSEMLLLDVIPLSLGLETMGGLVEKVIPRNTTIPVARA
QDFTTFKDGQTAMSIHVMQGERELVQDCRSLARFALRGIPPLPAGGAHIRVTFQVDADGL
LSVTAMEKSTGVEASIQVKPSYGLTDGEIASMIKDSMSFAEQDVKARMLAEQKVEAARVL
ESLTGALTADAALLSAAERQCIDDAAAHLSAVAQGDDVDAIEQAIKNVDKQTQEFAARRM
DQSVRRALKGHSVDEV
>EECEBICD_00803 2Fe-2S ferredoxin
MPKIVILPHQDLCPDGAVLEAETGETILDVALRNGIEIEHACEKSCACTTCHCIVREGFD
SLPESSEEEDDMLDKAWGLEPESRLSCQARVTDDDLVIEIPRYTINHAREH
>EECEBICD_00804 Protein IscX
MGLKWTDSREIGEALYDAYPDVDPKTVRFTDLHQWICELDDFDDDPNASNEKILEAILLV
WLDEAE
>EECEBICD_00805 Peptidase B
MTEAMKITLSTQPADARWGDKATYSINNDGITLHLNGKDDLGLIQRAARKIDGLGIKQVA
LTGEGWDIERCWAFWAGYKGPKGVRTVMWPDLDDAQRQELDNRLTIIDWVRDTINAPAEE
LGPEQLAQRAVDLLCSVACDSVTYRITKGEDLREQNYMGLHTVGRGSERPPVLLALDYNP
TGDKDAPVYACLVGKGITFDSGGYSIKQSAFMDSMKSDMGGAATVTGALAFAITRGLNKR
VKLFLCCADNLISGNAFKLGDIIRYRNGKNVEVMNTDAEGRLVLADGLIDASAQHPELII
DMATLTGAAKTALGNDYHALFSFDDTLAGRLLTSAAQENEPFWRLPLAEFHRNQLPSNFA
ELNNTGSAAYPAGASTAAGFLSHFVENYREGWLHIDCSATYRKAPVEQWAAGATGLGVRT
IANLLTA
>EECEBICD_00806 hypothetical protein
MYMGCRPDKTHGVAIRQNAGWRFAYPAYKNTAQAVSKFAMVRTPRPVAPAAHCSTGALR
>EECEBICD_00807 hypothetical protein
MSETKNELEILLEKAATEPAHRPAFFRTLLESTVWVPGSAAEGEAIVEDSALDLQHWEKE
DGTTVIPFFTSLEALQQAVEDEQAFVVMPARTLFEMTLGETLFLNAKLPTGKEFMPREIS
LLLAEEGSPLSTQEVLEGGESLILSEVAEPPSQMIDSLTTLFKTIKPVKRAFLCAIKEHA
DAQPNLLIGIEADGEIEEIIHAAGNVATDTLPGDEPIDICQVRKGEQGISHFITEHIAPF
YERRWGGFLRDFKQNRII
>EECEBICD_00808 hypothetical protein
MDISRANLIELVKKVNRNKVPNPMPAEEISRLRVRKYRDPQNTETTELPESLKALLAYDR
DLLSNYNMPVIETLQRSIDKEGVIHSYSPDEEAYYGAGMDSSGIDIEDLMPVWSNDPRLP
ALIRIDHVGDQAIFIYITERDANGEYPIARMERNEFWLAESSLVEYLYNIISGAKDIGFT
EEDLHLSQWKAQQKMNEKRDAALLDLEDYHEAFWAKLDALVD
>EECEBICD_00809 hypothetical protein
MKTKTAVMILLIPGVKYLPIKNRILLRENNARLQDSGKELNKNNVCRSFYISLNGRPDKR
ERHPAWQILSGFNR

>EECEBICD_00810 3-mercaptopyruvate sulfurtransferase
MTTAFFVAADWLAEHIDDPEIQILDARMAPPGQEHRDMAGEYRAGHIPGALFFDIEALSD
RASPLPHMMPRPEAFAVAMRELGVRQDKHLVIYDEGNLFSAPRAWWMLRTFGAEKVSILA
GGLAGWQRDEWLLREGEEAHEGGEFEAKFAPQAVVRLTDVLLASHEKTAQIVDARPAARF
NAQADEPRPGLRRGHIPGALNVPWTELVYEGELKTTDELNEVFFSHGVSFDRPIIASCGS
GVTAAVVVLALATLDVPDVTLYDGAWSEWGARTDLPVEPA
>EECEBICD_00811 Alpha-2-macroglobulin
MKHLRVVACMIMLALAGCDNNDKTAPTTKSEAPAVAQPSPAQDPAQLQKLAQQSQGKALT
LLDASEAQLDGAATLVLTFSIPLDPEQDFSRVVHVVDKKSGSVDGAWELAPNLKELRLRH
LEPERVLVVTVDPAVKALNNATFGKSYEKTITTRDVQPSVGFASRGSLLPGKIAEGLPVM
ALNVNHVDVNFFRVKPGSLASFVSQWEYRSSLSNWESDHLLKMADLVYTGRFDLNPARNT
REKLLLPLSDIKPLQQAGVYYAVMNQAGHYNYSNAATLFTLSDIGVSAHRYHNRLDIFTQ
SLENGAAQSGIEIVLLNDKGQTLAQATSDAQGHVQLEADKAAALLLARKEEQTTLLDLTL
PALDLSEFNVAGAPGYSKQFFMFGPRDLYRPGETVILNGLLRDSDGKTLPDQPVKLEVVK
PDGQVMRTVVSQAENGLYRLNYPLDINAPTGLWHVRANTGDNLLRSWDFHVEDFMPERMA
LNLTAQKTPLAPADEVKFSVVGYYLYGAPANGNTLQGQLFLRPLRDAVAALPGFQFGNIA
EENLSRSLDEVQLTLDKGGRGEVSAASQWQEAHSPLQVILQASLLESGGRPVTRRVEQAI
WPADTLPGIRPQFAAKAVYDYRTDTTVNQPIVDEDSNAAFDIVYANAQGEKKAVSGLQVR
LIRERRDYYWNWSESEGWQSQFDQKDLVEGEQTLDLNADETGKVSFPVEWGAYRLEVKAP
NETVSSVRFWAGYSWQDNSDGSGAARPDRVTLKLDKASYRPGDTMKLHIAAPVAGKGYAM
VESSDGPLWWQAIDVPAQGLELTIPVDKTWNRHDLYLSTLVVRPGDKSRSATPKRAVGLL
HLPLGDENRRLDLALESPAKMRPNQPLTVRVKASVKHGEMPKQINVLVSAVDSGVLNITD
YATPDPWQAFFGQKRYGADIYDIYGQVIEGQGRLAALRFGGDGDDLKRGGKPPVNHVNII
AQQAQPITLNEQGEGVVTLPIGDFNGELRVMAQAWTADDFGRGESKVVVAAPVIAELNMP
RFLAGGDVSRLVLDVTNLTDRPQTLNIALAASGLLELLSQQPQPVNLAPGVRTTLFVPVR
ALEGFGEGEIQATISGLNLPGETLGAQHKQWQIGVRPAWPAQTVNSGIALAPGESWHVPE
QHLANVSPATLQGQLLLSGKPPLNLARYIRELKAYPYGCLEQTTSGLFPALYTNAAQLQS
LGITGDSDEKRRAAVDIGISRILQMQRDNGGFALWDENGAEEPWLTAYAMDFLIRAGEQG
YSVPPEAINRGNERLLRYLQDPGTMLIRYSDNTQASTFAAQAYAALVLARQQKAPLGALR
EIWERRSQAASGLPLMQLGIALNTMGDARRGEEAITLALNTPRQDERQWIADYGSSLRDN
ALMLSLLEENNLRPDAQNALLSSLSEQAFGQRWLSTQENNALFLAAHSRQASAGAWQAQT
SLEAQPLSGDKALTRNLDADQLAALEVTNTGSQPLWLRLDSSGYPSSAPEPASNVLQIER
QILGTDGQRKSLSSLRSGELVLVWLTVVADRNVPDALVVDLLPAGLELENQNLADSSASL
PESGSEVQNLLNQMQQADIQYMEFRDDRFVAAVVVNEGQPVTLVYLARAVTPGTYQLAQP
QVESMYAPQWRATGASEGLLIVTP
>EECEBICD_00812 Penicillin-binding protein 1C
MNGWRGKRGRWLWLAGAPLFIFLALWAADKLWPLPLNEVHPARVVVAHDGTPLWRFADAG
GIWRYPVTIEEVSPRYLEALINYEDRWFWRHPGVNPFSVARAAWQDLTAGRVISGGSTLT
MQVARLLDPHSRTFGGKIRQLWRALQLEWHLSKRDILTLYLNRAPFGGTLQGIGAASWAY
FGKPPARLSYADAALLAVLPQAPSRLRPDRWPARAEAARNKVLDRMAAQGVWPAETVRES
REEPVWLAPRQMPQLAPLFARMMLSKSRSDKIVTTLDAGLQRQLEDLARAWKGRLPARSS
LAMIVVDHTDMSVRGWVGSVDLNDDSRFGHVDMVTAIRSPGSVLKPFVYGLALDDGLIHP
ASLLQDVPRRTGDYRPGNFDSGFHGPVSMSDALVRSLNLPAVQVLEAYGPKRFAAKLRNV
GLPLYLPAGAAPNLSLILGGAGARLDEMAAAYSAFARHGKAAKLRLQPDDPLSERPLMSP
GAAWIIRRIMADEAQPLPDNALPRIVPLAWKTGTSYGYRDAWAIGVNARYIIGIWTGRPD
GTPVVGQFGFASAVPLLNQVNNLLLAHTGRLPEDPRPQTVSRGVICWPGGQTLPAGDSNC
RRRLATWLLDDSQPPTLLLPEQEDINGIRFPVWLDDTGRRVAADCPQACAHTFIVWPRPL
EPWLPPAERRSARLPAASDHCPPLQGSDAAPLMLSGVRDGAVIRQLPGQENVTLPVSTTG
GKGRRWWFLNGEPVNGENNRLSLLLNIAGRYQLVVMDESGQVAAVNFELIR
>EECEBICD_00813 Dimethyl sulfoxide reductase DmsA
MKNIKSQGNGEQPAISRRHFIQASSALIALPFVSSPATAQARAVTATENRPAEKVVQTCS
TFDCGGKCDIRAHVSDGIVTRISTRPDNALDAQMPVMRACVRGRAYRKFVYHPDRLKYPM
KRVGKRGEGKFERISWDEATTLIADNLKSITEKYGPASRYVHVGTAVSGGTFSGDKMARR
LLNLTGGYLESYHSVSMGNTAAATPYTYGIAASGSSLDTLLDTKLVILWGHNPTETIFGH

TNYFLQKMKQNGTRFIVVDPRYSDTVSSLADQWIPLLPTTDNALMDAMMYVIISENLHDR
AFIARYAIGFDEDSMPEGVPANESLVAYLTGAKDGVVKSPEWAEKITHVPAQTIRQLACD
YANTKPAALIQGWGPQRHNCGERTARGSTLLATITGNVGIKGGWAAGYGGCANRKFAAGP
EMPDNPVKAKISVMNWVQAADDASKVTPDMGLKDADKLDSNIRILFSLAGNYLANQNPDL
HQAVRVLEDESKIQFIVASDLFMTPSAKYADLLLPETSFMERWNIGETWGTASYLILSEK
LIEPEFERRSDYDWLREVAAKLGIENEFSQGRDEKAWIEHIWEQTRLAMPDENLPDFATL
QKTRQHLFKSAPFIAFEDNIRDPDNHPFPTPSGKIEIFSKRLYDMQHPEIPALSHYVPAH
EGPEDALVKDFPLQLITWKGKNRANSTQYANPWLIEVQQQTLWINPQDAQKRGITHGDMV
RIHNSRGICEIPAEVTPRIIPGVVAMQAGAWWQPDENGVDKGGCANVLSSARITALAKGN
SHQTMLVEVAKA
>EECEBICD_00814 Anaerobic dimethyl sulfoxide reductase chain B
MSQFTHYPPVSDKQLGFFIDSSRCSGCKACQVACKDKNNLEVGRRFRRVYEVKGGSFIPT
GQGGVSNNVFAYTLSISCNHCADPVCTKNCPTTAMHKRPGDGIVRVDTDKCVGCGYCAWS
CPYGAPQLNEQTGQMSKCDMCVDLLAKGEPPVCVATCPLEAIKFAPIDELRAKYGSVCDV
NGLPDSSITKPNLVVKAHQGAEKEGKRHA
>EECEBICD_00815 Anaerobic dimethyl sulfoxide reductase chain C
MHELPLLIFTLCLQGSVGVTVWLALGRQYAVEGRVPARGALPAMAGAFVLACVGLLASAL
HMGYPLNALNALRHVASSWLSREIVFASLYLAALGLGVVLLFFRKPGWQPLLALAAAFGL
VDVFCMAQVYIHASVATWQHSNTLALFFGTSGIIGSVVIALAYLRNAGAAMRCAVVVVAL
MVLIRLIMQPLWLADINAVDTTVVTFPHHPLQALAQLRDVYLLGWCVSAAGMLCFAAGGL
RNARGTLVAGSVLLLIGEIMLRYVFFSIG
>EECEBICD_00816 Ferredoxin-type protein NapF
MVNLTIDPAVDVTQACVRRRFRFSSCRACADVCPAQAFSLAQGQVSIDTTRCIACGDCLF
VCPVDAITGIKPVKRFVQGDTLVGPFSLQAPTVDELLLWHSQYGIRFIDIAVERSAQWLM
ALAGLNLALRRYGEPGWSFKHVVGAEINASRRTLFHVPRDAITPCAVEPGKRRLRQAFSA
FSECVPEISPQECRMCGACWRSCPENVIQFDDNTLTIAAARCTGCGGCAAVCPHQALRLR
FDVEPASTRHSAAHTLTCDICKRTFHALTPEHTHCVLCQSPEFAVRL
>EECEBICD_00817 Nucleoside diphosphate kinase
MAIERTFSIIKPNAVAKNVIGSIFARFEAAGFKIVGTKMLHLTVEQARGFYAEHDGKPFF
DGLVEFMTSGPIVVSVLESENAVQRHRDLLGATNPANALAGTLRADYADSLTENGTHGSD
SLESAQREIAFFFGEGEVCPRTR
>EECEBICD_00818 Dual-specificity RNA methyltransferase RlmN
MSEQIVTPESSTPVVLNNETKINLLDLNRQQMREFFKNLGEKPFRADQVMKWMYHYCCDN
FDEMTDINKVLRGKLKEVAEIRAPEVVEEQRSSDGTIKWAIAVGDQRVETVYIPEDDRAT
LCVSSQVGCALECKFCSTAQQGFNRNLRVSEIIGQVWRAAKIVGAAKVTGQRPITNVVMM
GMGEPLLNLTNVVPAMEIMLDDFGFGLSKRRVTLSTSGVVPALDKLGDMIDVALAISLHA
PNDTIRDEIVPINKKYNIETFLGAVRRYLEKSNANQGRVTIEYVMLDHVNDGTEHAHQLA
ELLKETPCKINLIPWNPFPGAPYGRSSNSRIDRFSKVLMSYGFTTIVRKTRGDDIDAACG
QLAGDVIDRTKRTLRKRMQGEVIDIKAI
>EECEBICD_00819 Cytoskeleton protein RodZ
MNTEATHDQNEAQTTGVRLRNAREQLGLSQQAVAERLCLKVSTVRDIEEDKAPSDLASTF
LRGYIRSYARLVHVPEEELLPGLEKQAPLRAAKVAPMQSFSLGKRRKKRDGWLMSFTWLV
LFVVVGLTGAWWWQNHKAQQEEITTMADQSTAELNADKDSGQSVPLDTGAVTSQDTTPAQ
TAPAPATPVDSTAATQTPAPTAAATQNTVVAPSQANVDTAATSAAPAATETPSALPTSQA
GVAAPAADPNALVMNFTADCWLEVTDATGKKLFSGMQRKDGNLNLTGQAPYKLKIGAPAA
VQIQYQGKPVDLSRFIRTNQVARLTLNAEPTPAQ
>EECEBICD_00820 4-hydroxy-3-methylbut-2-en-1-yl diphosphate synthase (flavodoxin)
MHNQAPIQRRKSTRIYVGNVPIGDGAPIAVQSMTNTRTTDVEATVNQIKALERVGADIVR
VSVPTMDAAEAFKLIKQQVNVPLVADIHFDYRIALKVAEYGVDCLRINPGNIGNEERIRM
VVDCARDKNIPIRIGVNAGSLEKDLQEKYGEPTPQALLESAMRHVDHLDRLNFDQFKVSV
KASDVFLAVESYRLLAKQIDQPLHLGITEAGGARSGAVKSAIGLGLLLSEGIGDTLRVSL
AADPVEEIKVGFDILKSLRIRARGINFIACPTCSRQEFDVIGTVNALEQRLEDIITPMDV
SIIGCVVNGPGEALVSTLGVTGGNKKSGLYEDGVRKDRLDNDDMIAQLESRIRAKASQLD

EARRIDVLQVEK
>EECEBICD_00821 Histidine--tRNA ligase
MAKNIQAIRGMNDYLPGETAIWQRIEGTLKNVLGSYGYSEIRLPIVEQTPLFKRAIGEVT
DVVEKEMYTFEDRNGDSLTLRPEGTAGCVRAGIEHGLLYNQEQRLWYIGPMFRHERPQKG
RYRQFHQLGAEVFGLQGPDIDAELIMLTARWWRALGISEHVSLELNSIGSLEARANYRDA
LVAFLEQHQETLDEDCKRRMYTNPLRVLDSKNPDVQALLNDAPALGDYLDDDSREHFAGL
CKLLDAAGIAYTVNQRLVRGLDYYNRTVFEWVTNSLGSQGTVCAGGRYDGLVEQLGGRAT
PAVGFAMGLERLVLLVQAVNPEFIASPVVDIYLVAAGAQTQSAAMTLAERLRDEMPGVKL
MTNHGGGNFKKQFARADKWGARIALVLGESEVADGTVVVKDLRSGEQTAVAQDSVAAHLR
TLLG
>EECEBICD_00822 hypothetical protein
MEIYENEHDQVDAIKRFFAENGKALAVGVILGVGALVGWRYWSSHQTESARAASLAYQNA
VTAVSADKPDSIPAAEKFAAENKNTYGALASMELAQQFVDKNELKKAEAQLQQGLAATSD
ENLKAVINLRLARVQLQLKQADAALKTLDAVKGEGWTAIVADLRGEALLSKGDKKGARSA
WEAGVNSDASPALSEMMQMKINNLSI
>EECEBICD_00823 Outer membrane protein assembly factor BamB
MQLRKLLLPGLLSVTLLSGCSLFSGEEDVVKMSPLPQVENQFTPTTVWSTSVGNGIGEFY
SNLHPVMADNVVYAADRAGVVKALNADDGKEIWSVNLGEKDGWFSRSSALLSGGVTVAGG
HVYIGSEKAEVYALNTSDGTTAWQTKVAGEALSRPVVSDGIVLIHTSNGQLQALNQADGA
IKWTVNLDMPSLSLRGESAPATAFGAAIVGGDNGRVSAVLMQQGQMIWQQRISQATGPTE
IDRLSDVDTTPVVVNGVVYALAYNGNLTALDLRSGQIMWKRELGSVNDFIVDGDRIYLVD
QNDRVLALTTDGGVTLWTQSDLLHRLLTSPVLYNGDLVVGDSEGYLHWINVDDGRFVAQQ
KVDSSGFLTEPTVADGKLLIQAKDGTVYAITR
>EECEBICD_00824 GTPase Der
MVPVVALVGRPNVGKSTLFNRLTRTRDALVADFPGLTRDRKYGRAEVEGREFICIDTGGI
DGTEDGVETRMAEQSLLAIEEADVVLFMVDARAGLMPADEAIAKHLRSREKPTFLVANKT
DGLDPDQAVVDFYSLGLGEIYPIAASHGRGVLSLLEHVLLPWMDDVAPQEEVDEDAEYWA
QFEVEQNGEEAPEDDFDPQSLPIKLAIVGRPNVGKSTLTNRILGEERVVVYDMPGTTRDS
IYIPMERDEREYVLIDTAGVRKRGKITDAVEKFSVIKTLQAIEDANVVLLVIDAREGISD
QDLSLLGFILNSGRSLVIVVNKWDGLSQEVKEQVKETLDFRLGFIDFARVHFISALHGSG
VGNLFESVREAYDSSTRRVSTAMLTRIMTMAVEDHQPPLVRGRRVKLKYAHAGGYNPPIV
VIHGNQVKDLPDSYKRYLMNYFRKSLEVMGTPIRIQFKEGENPYANKRNTLTPTQMRKRK
RLMKHIKKSK
>EECEBICD_00825 putative protein YfgJ
MEITCPVCHHALERNGDTAHCETCAKDFSLQALCPDCRQPLQVLKACGAVDYFCQNGHGL
ISKKRVNFVISDQ
>EECEBICD_00826 hypothetical protein
MSPYGKIRTSLSVGEGGDLDGSSLDYFIPWYDNQSTLFFSQISAQRKEDRTIGNFGLGVR
QNVGNWLLGGNAFYDYDFTRGHRRLGLGTEAWTDYLKFSGNYYHPLSDWKDSEDFDFYEE
RPARGWDIRMESWLPFYPQLGAKLVYEQYYGDEVALFGTDNLQKDPHAVTLGLEYTPVPL
VTVGTDYKAGTGDSNDFSVNATVNYQIGTPLAAQLDPENVKIQHSLMGSRTDFVDRNNFI
ILEYREKDPLDVTLWLKADATNEHPECVIEDTPEAAVGLEKCKWTVNALINHHYKIISAS
WQAKNNAARTLVMPVVKANALTEGNNNSWNLVLPAWVNADTEEQRTALNTWKVRMTLEDE
KGNKQNSGVVEITVQQDRKIELIVDNIADTDRSDHSHEASALADGEDGVVMDLLITDSFG
DSTDRNGNELVDDAMTPVLYDSNDKKVTLAQTPCTTETPCVFIASRDKEAGTVTLSSTLP
GTFRWKAKADAYGDSNYVDVTFIGDNLSALNAVIYQVKAANPVNLIGKEDKHPTVNNAYR
FLLWRDKNKDGVFQMSEQLTEEEMALYDYQWEFTGQSTNGHTGALANTMNEDLVLPVTNK
EAAQKFAANVEDGVQGYGIRVTYSQK
>EECEBICD_00827 hypothetical protein
MQATVKRRLTKVALALVVAGYCAAPAVAANGNLKSGGQWQIVSEQTGTIQGTVPWITRAAD
KTADTDKDHVTVTIDRGDRKIVTEGDKQFHVGDKVTVNWAIGDTEGDLDTDNTATKATVK
WVSFSDQNGSDPKDLGTGDSYEIQAADADRYIGIKITPTTTTGDPAVATELLLKDLSTDA
GGGSDDDEIPEGPVVDENVHVVIHEKDSNTNLLKNSGTTLKTNTTYQVLLWSDKNGNGTY
DAGENVTDQYDYRWKFVGTSKIAGTGTGGIVNENWNDKDLVIPLTNAEAKEAFEGAEGGV

TVGSDGVQGFGLSIDYKRK
>EECEBICD_00828 hypothetical protein
MNKVVHFLLTLLIMFAVSIAPAQALLKGGTWQELNSVTGAVNGTAPLADGAIIPLYQGST
LLDPSKNHDIEFSAMPRDFSADATSTSMRAVNSTDTEGDLFSDPPTIAWENRQPPAMGLV
WADAATPDTPLSPQPVPNLTFCAQNLAGRQLVAWAQVEDETNVPALWLFTRTGVPNYATI
PLLSPKVALNIKPAVSDPVSVSGDHVDASFEASKVKVGESITLTVTTKACDGEPAINAPF
VIRREDAINRQGVVNNANPVRVGNTELTTAQTEYHGVTDGQGNATVVVTQENGPGVKTRL
IVSSQNYPTLTDNVDVIFTTITSPDSDKASMYGHMIESATATLNGITYTFTRPKLAAEAS
GADKSVVDTNETWALFDWNGADNHCNILPDAEQLVQMRHEHSTLATYTGWPATDDAEYWS
STKDQMSGYHAAVHMNSASVVRAADSDTLLVSCVDKAQPAAHPQITLSPQGPYKAQVGES
IDLTMTVVDRDTQKPLPYRYMELFIDPAKNRKGEHQDAWDNLRVTVDSEGMSASSPEHYT
GVTDVNGQAHLTLKHNSGLGVETPIRIVMPDDEGGNVELSFSVIFTVVTSPDVDGANMWG
HMRGVVDAGNLYKRPLLAVEASHKDGQFSENNEEWATFNSVASATAQCGVGQVPDQSSLA
HLYSEHAGGQMESEHGWPTEDYFIAADSDASGTVHVNLENGDSGKFTDTPNYLTCSANEM
VAVLDVYFNDDPVIKNADAKVGEQVKMNIHSRNALNGIAIGNTDFTITMANGRRRDGLTT
GFTDTSNGEMQFDDVGYVAGQVYHGITDANGDATIILTQKKGVGLLTPLSIVPVNSYINT
PVSRSVKFTVATSPDTAKAKMWGHMADTITVGDWTFERPKLAGEVSNPLRTQDESNETWA
RVAHSDAVGNPDAGGCAANRLPRIDQLEALYNANSGGKIHSIQGWPTYLNYWSSTYQSAT
TWKLIALTNGSEFANSNVSIYASCLASDNPVAASITIEPVNPSQWYDGSDVHAVKVKKGE
TMQLKVTVKDASGNPIPEAPFVLTRGDGYDRRGEKYTAQDGDDLQGIVTPVVIDGESLAW
TTTKMGSQTGTDGTRIISVTRPDTHGTRTAINATLYENAAVSASIDTIFTVVTSPDVSVA
RMWGHMAPSLTAADGAVYQRPLLYAELSSTDNTASKQETNETWAVFHGPASEGANPARCA
AGYYPAVEALDSLYSKYPSRTINTAQGWPVYYSYWSGSNSTSFSSGAKLDFYYAVDLADG
SRRSENSATSTAWQYQICATTPLPQATQITLTSPQAMDDAIQAVKAKNSESIPLLITTTD
AMGNPVPYATFSLKRDAGKARNTDYNKFVATNGTNMTVTPLTGAQQQFYYATSVLTGATG
ADGTLALTLAEPGGIGLKNQLTANLNDTPTATSSLPVVFTVLTSPDSDKANMYGHMPETF
TASNGAEFKRPLVAGEPSSEAHTDTYFETNENWIMVNSFNTGNYGGCPMNQMAAIDDFTA
LYNDHPSGKVATDIGLPVGKRWWAGDSLLKGSTLYWQYKDLKTGKNYSMSENPGNYYLQL
CLTTSRSGLNIALSSDAWNADKSAAVAKKGETIPMTVTVTNDAGQPQAGVAVLLTRDYAY
SRGAVDKQYIEPGVIGEPVPFTTSPANMMLTPVAPAGTAVAFNNQNGLSTKWSGFTGDDG
KLRFTLTQDKSLGLKTSVTAALANQFDEAASVDAIFTVQTSPDTPYASYWGHMPDTVQVN
GVTLRRPYLKAELSAAPRDTWPFNNEIWGTNYYYQSEHVETSLTHLCGSQENIASLDDLK
ALQSVIGTLQWPTTSSWDYVSQDEGQSNKYYCSFNETTGQTTCTREKATTSGLGSCRVP
>EECEBICD_00829 hypothetical protein
MKSKKCVGFLPWLLLCALSLTPFFAQAVRSSGAWQELTEQTAAINGTPPQADSVTIPVYQ
GSVQLRSDAANPVDYSAKPSQFSTSDAGSALTLTNPHDTEGDIFAVPPLVWQAERAPTVT
LVWADAATPDTPLDPQPVSNISFCAQNLAGRHLVVWSQLDTSTAIPPLWLLTRTGVPYNT
AVEVLDQKFAVDIAPAVGDPVTLAADHLDESLNAAKVKAGESITLTVTTKDCGGEPEGNV
DFVITRGDAQNRQGVVNNAAPVRVGNTELTTTATEYHGTTNAEGVATVVVTQANGPGVKT
PLIAHPSSAPALKASVDVIFTTLTSPDSSSANMYGHMAETSTATVDGASYTFYRPKLAAE
TDGEDRTASINNETWAQFNWGHADAHCDILPDARQLEGLKIARGDLATTLGWPVGLTSGD
EEYWSSSQGSTATDHISIDMRSRSLTQMPDATQSLVSCVDKAPPAVTPKLVISADNFDST
VNAAKVKVGEEINMKITVTDSATNKPLPYRYLNVYLGDEQNRQNQKNADIDSGHQWTDDP
VIITNLEGSDGHYHGVTDANGQFSLVLTQDKGAGVLTPVRVVLFDGTEATQNVIFTVVTS
PDVAQARMWGHMQGVVEAGNIYKRPLLADEAAQDTGSEFENNEYWATFNSVTAATNQCGT
GQVPGQLLLDTLYQAHSGNAMETTYGWPTQKHSYIAADTDGSTTAHVNLATGADSTFSGT
EPNYLSCSGNELVTSLDVYFNGDESLRNAVAKVGEQITMNVHSVNALNGLSVPNASFIVT
MAHGKNRDNATTGFTDSSDGTLVMGGTPFGSSQASMTYQGMTDNEGNATLVIEQPQGVGL
LTPLSVLPVNSLITTPVNRSVKFTVPTSPDTPEAQMWGHMADTLTVGDMEFQRPKLAAEA
TAATRTQEQDNETWARVSHADALNNPNAGGCEAGHLPRADQLAALYASSDGNKIHTVSGW
PTTYDYWSSTFASAATWQAVSLAAGGYTASGDASDYVSCLVSKNPTAASITIEPVDAALW
YNANSEHAVKVKKGDTLQLKVTVKDASGNPLPQAPFVLSRGDGYTRQGEKHIAGSGDGIV
SAVVIDGDSLNDSATQIGGMTGENGSKIINVTRPDAHGTKVAITAALYNNASATASIDTI
FTVVTSPDSDKAKMWGHMPETTTAANGEVFKRPLLSAEIASGVTHGDNTENNEAWGIVDF

EMANDACGAGYVPTLADMQSLYDARPGGAMNTQQGWPLDGKNYQDSTADLSRSTQNRYVK
SINLRDGGVGSLLWNEQLYFVCLQNAHPAATQITLTSPVYNDSDGFAKAKVGETIPVIIT
TRDAQGNPAADTPVIFTRGDSVGRANQEVNSSAAADIQINHSDGRSSGANYYTATGADGT
LTLNISQDSGAGFKTPLTAAIEHNGVTSAPLPVIFTVVTSPDTPKANYWGHMAETLTDSS
GVAYRRPLLASEFSATPGKTLTIANGYYDKGETWGMITVDKAWNGTGGGCGRDTLPTVAN
LQTLYGTYPDNAMRSRNGWPMTSSGNNNVSRYWWAGDYVISSDGTKSLYAAVNLFNDGND
VKTTTSTSMYYMQTCLASPRSAAASLTLTLAGQDETTGSAKAKKGEQMAATIDVKDAAGQ
PMKNVMVKISRGSSYNRANSATSSSSVTDDITLRNVMPSGLATYLLDTSAKYLYAQTDAQ
GQVTFTLAQDSTAGLKTTISAATMDGSNLTDSKDAIFTVVTSPDSDKAKYWGHMPETFTN
SKGV
>EECEBICD_00830 hypothetical protein
MYQDTASPCDRLGLPTVNDLQTLYTDYPNGALTTTLGLPVASGKYWGAGNSVPDATHSDS
QFQYVRLSDNNTLTTKANTATAQLCLAKRRDLSIELTSSAMDADKGAPVAKKGESLPLTV
TVRDGSGTPQPNTTIRLGRTLSIDRAGVVDGSSGGGMVLTSVAPSTGSMTFNCTVSSCTS
YWYGITDEDGKAQLEVTQDDSRGLRTPLQAMLVDDPLTVSDMDVIFTVITSPDSDKAKYW
GHMPETVTNSAGVKFRRPLLAAEMTSNSGTYLVNNETWPLVTAANTEKAGATGCDAEYQP
LSGDLQTLYSDNPNGAIGTNYGWPVAGNKSWWAADRAPNTGYYQFINLNSGGKGTASSST
ATGAQVCLVEPRTSTPASITLTSTAMDSAKNAAVVPKGSAMPLTVTVKDSSGNPVANVGF
TLSRGDSKNRAGMVITDGDVAADAGADDLMLKELTPASASQSMTTTGIVFTGTTGSDGTA
TFTLNQDKSLGLKTPLTVKVTDNTTLHASLDVIFMVLTSPDTDKALFWGNMSDTTSVNGK
TLHRPWLQAEMLSGVTPVFTNGVHANNEYWAMAHTVDNTKWDIAKQCGSLSKAPDNNDLL
TLYHSISSLGWPTLGYPYLSKSTSSSGMYCGVDENTKSQNCAIKPAGTAGYATCVE
>EECEBICD_00831 hypothetical protein
MNRTYSIVWSAVRNMYVVASELARGHSKVKAQVCASEIHSPNQKSEYGQIIKATRAALAC
AVAAALGFFSPLAMADNQVSYADAQTHVLDASTPPMSYSGTDEGAALYVSGVATVGWQPT
TVTGTGLVIETSGGGADDPDGGKYVSNAISLDHYAILELTDAKITTTGIYTQGISAADGS
TLTLTDSTLTIDGNFGVMTLYTGSEATLDGTIVEAANSSSAQVQQGSTLNVLDGSTITLA
QGQINVVAGNTATDEGSTLNLSDSSVSSAGTMSTIQGTNKAALNLTNATITHTNASGAAV
QANNATTLDITGGNITSAGTGVYILASDARIDGATINADGDGIFITSKRKLDGYEDLNAL
TVNKAQVNSDTIALHVDTGTTINAPIVLTDSTFEAPEVIKLGSKAVIQANNTTLIGDVAQ
SDMSSSSLSLSQGPR
>EECEBICD_00832 hypothetical protein
MFTTLSLDDTSQWNMTDPSTVGNLTNDGDITLGNASGSTGTLLTVDNTLTLQDNSQINAT
LDTANSAPIIKAANVTLDGTLNLSSTATFVAPETDEHFGSITLIDSQSAITTDFDSVTLD
ADTSAMPDYLTINAGVDANDNTNYELSTGLSWYAGANSARAAHGTFTVDAGSTFTVTSEL
DETTATSNWNGSKLTKQGDGTLILSNTGNDYGDTEIDGGILAAKDAAALGTGDVTIAESA
TLALSQGTLDNNVTGEGQIVKSGSDELIVTGDNTYSGGTTISGGTLTADHADSLGTGAVA
NSGVLQVGEGELENTLSGSGSLVKTGTGELTLSGDNTYSGGTTITGGTLTADHADSLGSG
DIDNSGVLKVGEGDLENTLSGSGSLVKTGTGELTLSGGNDYSGGTTIIGGTLTADHADSL
GTGAVANSGVLQVGEGELENTLSGSGSLVKTGTGELTLSGDNSYSGGTTIIGGTLTADHA
DLLGTGAVANSGVLQVGEGELENTLSGSGSLVKTGTGELTLSGDNSYSGGTTIIGGTLTA
DHADSLGTGAVANSGVLQVGEGELENTLSGSGSLVKTGTGELTLSGDNSYSGDTTIADGT
LIAANVNALGSGNIDNSGTLMLDANGAFELANITTHTGATTALAAGSTLDAGQLTQEDGS
TLSIDLGAATDDAVITADSVTLGGTLNVTGIGSVTDSWTPEAYTYTLIDSDSAITSDFDD
LTIAGMNREDVDFLTIDGKVDEADNTHYDLTASLSWYADRDNATTDAHGTFTLSDPDGSF
NVAATLTDVDDTLDPGSRWDGKSLTKEGAGTLILSGDNDYSGGTTINEGTLVAASTTALG
TGLVDNNATLVLDVDGEVSAVGGITTHSGATTQLALGTSLDLGDSALIQQDGSTLNVELN
SDSVQPLITGGSATLGGDLVVSDASLQARASDAEFQSFKLMDMDSDISGDFTSLTMNLTD
QPDYLTVTGTINPADASEYLLTEGLSWNATATSATPAHGTFTLGAGDSFEVTSVLGDKTG
NGDWDGKSLTKLGAGKLTLSGANTYTGDTNVQEGTLWLSGDGSIGEMGSQQAVNVASGAT
FGGSNGTTVNGKVTNEGTLVFGDSEETGAIFTLNGDLINMGTIASGSTSSTPGNTLYVDG
NYTGNGGSLYLNTVLGDDDSATDKLVITGDASGTTDLYINGIGDGAQTTNGIEVVDVGGV
STSDAFELKNEVNASLYTYRLYWNESDNDWYLASKAQSDDDDSGGDDSDVTPSDGGDDGG
NVTPPDDGGDGGDVAPQYRADIGAYMGNQWMARNLQMQTLYDREGSQYRNADGSVWARFK

AGKAESEAVSGNIDMDSNYSQFQLGGDILAWGNGQQSVTVGVMASYINADTDSTGNRGAD
GSQFTSSGNVDGYNLGVYATWFADAQTHSGAYVDSWYQYGFYNNSVESGDAGSESYDSTA
NAVSLETGYRYDIALSNGNTVSLMPQAQVVWQNYSADSVKDNYGTRIDGQDGDSWTTRLG
LRVDGKLYKGSRTVIQPFAEANWLHTSDDVSVSFDDATVKQDLPANRAELKVGLQADIDK
QWSVRAQVAGQTGSNDFGDLNGSLNLRYNW
>EECEBICD_00833 Exodeoxyribonuclease 7 large subunit
MLSSQTSSIFTVSRLNQTVRLLLEQEMGQVWISGEISNFTQPASGHWYFTLKDDTAQVRC
AMFRNSNRRVTFRPQHGQQVLVRANITLYEPRGDYQIIAESMQPAGEGLLQQKYEQLKAK
LQAEGLFDQQHKQPLPSPAHCVGVITSKTGAALHDILHVLKRRDPSLPVIIYPTAVQGDD
APGQIVRAIELANARGECDVLIVGRGGGSLEDLWSFNDERVARAIFASRIPVVSAVGHET
DVTIADFVADLRAPTPSAAAEIVSRNQQELLRQIQSAQQRLGMAMDYYLANRSRRFTQIF
HRLQQQHPQLRLARQQTALERLRQRMGFALEARIKQANQRQQRVSQRLSQQNPQPRIHRA
QSRIQQLEYRLTENIRSRLSEQRERFGNAVTHLEAVSPLATLARGYTVSTTTDGKVLKKI
KQVKAGDIMTTRLEDGWLESEVKSVTPGT
>EECEBICD_00834 Inosine-5'-monophosphate dehydrogenase
MLRIAKEALTFDDVLLVPAHSTVLPNTADLSTQLTKTIRLNIPMLSAAMDTVTEARLAIA
LAQEGGIGFIHKNMSIERQAEEVRRVKKHESGVVTDPQTVLPTTTLHEVKALTERNGFAG
YPVVTEDNELVGIITGRDVRFVTDLNQPVSVYMTPKERLVTVREGEAREVVLAKMHEKRV
EKALVVDDNFHLLGMITVKDFQKAERKPNSCKDEQGRLRVGAAVGAGAGNEERVDALVAA
GVDVLLIDSSHGHSEGVLQRIRETRAKYPDLQIIGGNVATGAGARALAEAGCSAVKVGIG
PGSICTTRIVTGVGVPQITAVSDAVEALEGTGIPVIADGGIRFSGDIAKAIAAGASAVMV
GSMLAGTEESPGEIELYQGRSYKSYRGMGSLGAMSKGSSDRYFQSDNAADKLVPEGIEGR
VAYKGRLKEIIHQQMGGLRSCMGLTGCATIDELRTKAEFVRISGAGIQESHVHDVTITKE
SPNYRLGS
>EECEBICD_00835 GMP synthase [glutamine-hydrolyzing]
MTENIHKHRILILDFGSQYTQLVARRVRELGVYCELWAWDVTEAQIRDFNPSGIILSGGP
ESTTEENSPRAPQYVFEAGVPVFGVCYGMQTMAMQLGGHVEGSNEREFGYAQVEVLTDSA
LVRGIEDSLTADGKPLLDVWMSHGDKVTAIPSDFVTVASTESCPFAIMANEEKRFYGVQF
HPEVTHTRQGMRMLERFVRDICQCEALWTPAKIIDDAVARIREQVGDDKVILGLSGGVDS
SVTAMLLHRAIGKNLTCVFVDNGLLRLNEAEQVMDMFGDHFGLNIVHVPAEDRFLSALAG
ENDPEAKRKIIGRVFVEVFDEEALKLEDVKWLAQGTIYPDVIESAASATGKAHVIKSHHN
VGGLPKEMKMGLVEPLKELFKDEVRKIGLELGLPYDMLYRHPFPGPGLGVRVLGEVKKEY
CDLLRRADAIFIEELRKADLYDKVSQAFTVFLPVRSVGVMGDGRKYDWVVSLRAVETIDF
MTAHWAHLPYDFLGRVSNRIINEVNGISRVVYDISGKPPATIEWE
>EECEBICD_00836 hypothetical protein
MTKDIVLNALLMAVWRRHPQKQVLVQSDQGSQYTRYEWLKSHGLEGSMSRRGNCHNAVAE
SFFQLLKRERIKKKIYQRLRSVQVIRGDSAQIRGI
>EECEBICD_00837 hypothetical protein
MRDIEKEIIDFIDQEYNTKKYFLCGPKRTITLDISIRDDLKLVFEDSEELLQEYFKRWNV
DSEGFDILNYLNPEYFGSKEPDPRKPLTVGMLVESAKTGRWLYS
>EECEBICD_00838 hypothetical protein
MTFEYTFTVKHCIDTIAGFLKCVEVEPHPVIAEILTQFESTVRQCPLGFQICPELVPATG
SLTTLKAIGFCIRSRES
>EECEBICD_00839 Protein YfgG
MSQANSMRKRHRFNTRMTRIVLLISFLFFFGRFVYSSIGAWQHHQDKKEAQQSSLSVTTP
GQR
>EECEBICD_00840 Cyclic di-GMP phosphodiesterase PdeF
MKLNKTYINIRDKWWGLPLILPSILLPVLSSANTYALTSTGNVVLFYLPLAFMLSLMLFF
GWAALPGIVLAIFWRRYPQTGLYETLSVTMHFIITIVLSWGGYRVFSPRRNNVSHGDAHL
LFQRIFWQVFCSATLFLVIYQFAAFVGMYESKASLMGVMPFNINTLINYQALLVGNLVGV
PLCYFIIRTLRNPLHLRGYYQQLKLQIDSKATKKEIVIWLAVLTTLMFILCMPLTDNSSI
FSTNYTLSLLLPVMLWGAMRYGYKFISIIWAVVLITSIHYYQRYMPWYSGYDTQLAITSS
SYLVFSFIVNYMSVLATRQRVVSGRARRLAYLDPVVHLPNLRALNRALQNAPWSTICFLH
VPGLELLGKNYGVMLRIQYKQKLSHWITPMLASNECVYQMSGHDLVLRLNTEAHQQRIEA

LDKHIKQFRFIWDGLPLQPPVGVSYCCVRSPVSHLYLLLGELSTSSDLSLTTNAPEDLQR
RGAMHLQRDLKGRIAMMNRLQQALEHDHFFLMAQPIFGVRGDVYHEILLRLRDDNGDVIN
PDNFLPVAHEFGLSSAIDLWVIENTLRFMAASREYMPAHRFAINLSPTSVCRARFPAEVK
QLLTQYQVEPWQLVFEVTESNSLTNAEQAQLTLGQLQQFGCQIAIDDFGTGYASYARLKN
VDADILKIDGSFIRNIVSNSLDYQIVASICHLARMKNMQVVAEYVESEEIRSVVLSLGID
YLQGYLIGEPQLLSEIQ
>EECEBICD_00841 Exopolyphosphatase
MPIYDKSPRPQEFAAVDLGSNSFHMVIARVVDGAMQIIGRLKQRVHLADGLGADNKLSEE
AMERGLSCLSLFAERLQGFSPSSVCIVGTHTLRQAQNAADFLKRAEKVIPYPIEIISGNE
EARLIFMGVEHTQPEKGRKLVIDIGGGSTELVIGENFEPRLVESRRMGCVSFAQLYFPGG
VINKENFQRARMAAAQKLETLTWQYRIQGWNVAMGASGTIKAAHEVLLALGEKDGFITPD
RLDKLKSEVLKHRSFNALSLPGLSEERKAVFVPGLAILCGVFDALAIRELRLSDGALREG
VLYEMEGRFRHQDVRSRTAKSLANQYNIDREQARRVLETTMQMYEQWQAQQPKLAHPQLE
ALLRWAAMLHEVGLNINHSGLHRHSAYILQHSDLPGFNQEQQMMMATLVRYHRKAIKLDD
MPRFTLFKKKQYLPLIQLLRLGVLLNNQRQATTTPPTLRLTTDDSHWTLCFPHDWFSQNA
LVLLDLEKEQQYWEAVTGWRLNIEEESSPEIAA
>EECEBICD_00842 Polyphosphate kinase
MGQEKLYIEKELSWLAFNERVLQEAADKSNPLIERMRFLGIYSNNLDEFYKVRFAELKRR
IIISEEQGSNSHSRHLLGKIQSRVLKADQEFDGLYNELLLEMARNQIFLINERQLSVNQQ
SWLRHYFKHYLRQHITPILINRETDLVQFLKDDYTYLAVEIIRGDTINYALLEIPSDKVP
RFVNLPPETPRRRKPMILLDNILRYCLDDIFKGFFDYDALNAYSMKMTRDAEYDLVHEME
SSLMELMSSSLKQRLTAEPVRFVYQRDMPAALVDVLREKLTISRYDSIVPGGRYHNFKDF
INFPNVGKANLVNKPLPRLRHLWFDKEKFRNGFDAIRERDVLLYYPYHTFEHVLELLRQA
SFDPSVLAIKINIYRVAKDSRIIDSMIHAAHNGKKVTVVVELQARFDEEANIHWAKRLTE
AGVHVIFSAPGLKIHAKLFLISRKEGDDVVRYAHIGTGNFNEKTARLYTDYSLLTADARI
TNEVRRVFNFIENPYRPVTFDYLMVSPQNSRRLLYEMIDREIANAQQGLPSGITLKLNNL
VDKGLVDRLYAASGSGVQVNLLVRGMCSLIPQLEGISDNIRAISIVDRYLEHDRVYIFEN
GGDKQVWLSSADWMTRNIDYRIEVATPILDPRLKQRVLDIIDILFSDTVKARFIDKELSN
RYVPRGNRRKVQAQLAIYDYIKSLEQPD
>EECEBICD_00843 Phosphoribosylglycinamide formyltransferase
MNIVVLISGNGSNLQAIIDACEAKKIKGTLRAVFSNKADAFGLERAREAGIPAQALTADR
FDSRDAFDRELIRKIDAYAPDVVVLAGFMRILSPMFVAHYYGRLLNIHPSLLPKYPGLHT
HRQALENGDEEHGTSVHFVTDELDGGPVILQAKVPVFAGDSEDDITARVQTQEHAIYPLV
ISWFAQGRLKMRDNAAWLDGRRLPPQGYASDE
>EECEBICD_00844 Phosphoribosylformylglycinamidine cyclo-ligase
MTDKTSLSYKDAGVDIDAGNALVDRIKGVVKKTRRPEVMGGLGGFGALCALPQKYREPVL
VSGTDGVGTKLRLAMDLKRHDAIGIDLVAMCVNDLVVQGAEPLFFLDYYATGKLDVDTAA
SVINGIAEGCLQSGCALVGGETAEMPGMYHGEDYDVAGFCVGVVEKSEIIDGSRVAEGDV
LIALGSSGPHSNGYSLVRKIIDVSGCDPQTTLLEGKPLADHLLEPTRIYVKSVLELIENV
DVHAIAHLTGGGFWENIPRVLPENTQAVINESSWQWPAIFTWLQTAGNVSRHEMYRTFNC
GVGMVIALSAPEADKALALLNEKGENAWKIGIIKAFDSEQRVVIE
>EECEBICD_00845 Uracil phosphoribosyltransferase
MKIVEVKHPLVKHKLGLMRENDISTKRFRELASEVGSLLTYEATADLETEKVTIEGWNGP
VEIDQIKGKKITVVPILRAGLGMMEGVLENVPSARISVVGMYRNEETLEPVPYFQKLVSN
IDERMALIVDPMLATGGSVIATIDLLKKAGCSSIKVLVLVAAPEGIAALEKAHPDVELYT
ASIDQGLNEHGYIIPGLGDAGDKIFGTK
>EECEBICD_00846 Uracil permease
MTRRAIGVSERPPLLQTIPLSLQHLFAMFGATVLVPVLFHINPATVLLFNGIGTLLYLFI
CKGKIPAYLGSSFAFISPVLLLLPLGYEVALGGFIMCGVLFCLVSFIVKKAGTGWLDVMF
PPAAMGAIVAVIGLELAGVAAGMAGLLPAQGQSPDTKTIIISMVTLAVTVFGSVLFRGFL
AIIPILIGVLAGYALSFALGVVDTTPIAQAHWFALPTFYTPRFEWFAILTILPAALVVIA
EHVGHLVVTANIVKKDLVRDPGLHRSMFANGLSTIISGFFGSTPNTTYGENIGVMAITRV
YSTWVIGGAAIFAILLSCVGKLAAAIQIIPLPVMGGVSLLLYGVIGASGIRVLIESKVDY
NKAQNLILTSVILIIGVSGAKVHIGAAELKGMALATIVGIGLSLIFKLISLLRPEEVVLE

ANDAEPPHQ
>EECEBICD_00847 DnaA regulatory inactivator Hda
MSSWVEVSLNTPAQLSLPLYLPDDETFASFWPGDNASLLAALQNVLRQEHSGYIYLWARE
GAGRSHLLHAACAELSQRGDAVGYVPLDKRTWFVPEVLDGMEHLSLVCIDNIECVAGDEL
WEMAIFDLYNRILESGKTRLLITGDRPPRQLNLGLPDLASRLDWGQIYKLQPLSDEDKLQ
ALQLRARLRGFELPEDVGRFLLKRLDREMRTLFMTLDQLDHASITAQRKLTIPFVKEILK
L
>EECEBICD_00848 putative protein YfgD
MTDTIKIYHNPRCSKSRDTLNLLKSNGVEPEVVLYLDTPADAATVRELLRMLGMSSAREL
MRQKEDLYKTLHLADSQLSEEALIQALVEHPKLMERPIVVANGQARIGRPPEQVLDILG
>EECEBICD_00849 Beta-barrel assembly-enhancing protease
MFRQLKKNLVATLIAALALGQVAPAFADPADTLPDMGTSAGSTLSIGQEMQMGDFYVRQL
RGSAPLINDPLLVQYINALGMRLVSHADSVKTPFHFFLINNDEINAFAFFGGNVVLHSAL
FRYADNESQLASVMAHEISHVTQRHLARAMEDQKRSAPLTWVGALGSILLAMASPQAGMA
ALTGTLAGTRQGMISFTQQNEQEADRIGIQVLQRAGFDPQAMPSFLEKLLDQARYSTRPP
EILLTHPLPESRLADARNRANQMRPVVVQSSADFYFAKARALGMYNSGRNQLTSDLLDQW
SKGNVRQQHAAQYGRALQAMEASKYDEARKTLQPLLSAEPNNAWYLDLATDIDLGQKRAN
DAINRLKNARDLRVNPVLQLNLANAYLQGGQPKAAETILNRYTFSHKDDGNGWDLLAQAE
AALNNRDQELAARAESYALAGRLDQAISLLSSASAQAKLGSQQQARYDARIDQLRQLQER
FKPYTKM
>EECEBICD_00850 hypothetical protein
MLEMLMQWYRRRFSDPEAIALLVILVAGFSILFFFSGLLAPLLVAIVLAYLLEWPTARLQ
AIGCSRRWAASIVLILFVGILLLMAFVVMPIAWQQGIYLIRDMPGMLNKLSDFAATLPRR
YPALMDAGIIDAMAENMRTRMLNMGDSVVKYSLASLVGLLTLAVYLVLVPLMVFFLVKDK
EQMLNAVRRVLPRNRGLAGQVWNEMNQQITNYIRGKVLEMVVVGVATWLGFLLFGLNYSL
LLAVLVGFSVLIPYIGAFVVTIPVVGVALFQFGLGTEFWSCFAVYLIIQALDGNLLVPVL
FSEAVNLHPLVIILSVVIFGGLWGFWGVFFAIPLATLIKAVVHAWPDGQVTDASS
>EECEBICD_00851 Carbohydrate diacid regulator
MLRKHYLKGYTARQIVQRAMKIIPYSVNVMDEHGVIIASGEPSRLRQRHEGAILALKENR
FVEIDSATANQLKGVRSGINLPISFHEQLIGVVGITGEPEEVRAYAELVKMAAELVIEHM
VLIEQRQWDKRYREELINQLILRENSTESLRSMAAYLGIDLAVPRVVLIIELSQPDREAL
RNVMDYFENHARNHLVTFTEFNELIIIKPITLKEGKWNTRQEMGELQIFKSWAASSGFSR
ILVGGYFAGETGLHRSLLTARATQAMAKRQKLRSQYIFYHDHALPALLSGLSESWQVQEL
SRLWLQLAQHDAKGVLQQTLRTWFEHNCDLTQTAKALHIHVNTLRYRLQRCEDITHIKIN
ELKSTLWLYIGMELQAESVPSDKLPLPGRIEIC
>EECEBICD_00852 High-affinity gluconate transporter
MTSISTLGAIAALVVAIVLILRKVSPAYGMMAGALVGGLIGGADLLQTVSLMVSGAQGIV
NAVLRILAAGVLAGVLIESGAANTIAETIVRKVGETRALLALAIATLCLTAVGVFIDVAV
ITVAPIALSIARNAGLSKSAILLAMVGGGKAGNVMSPNPNAIAASDAFHVPLTSIMLAGV
VPDIVGLIIAYLLAKRLNNKGAGVADHEVTHHDDSVARPGFLVAISAPLVAIFLLSLRPF
AGISIDPLIALPVGGLVGLLLMGRARHCNQYMVAGLMRMAPVAIMLLGTGTLAGIIANSE
LKDVLIHGLTASGLPSWLLAPVSGAMMSMATASTTAGTAVASGVFSPTLLELGVSALAGA
AMIHAGATVLDHLPHGSFFHATGGSVNMQIHERLKLMPYETLVGLAITFISTLMFGFFGF
AG
>EECEBICD_00853 Glycerate 2-kinase
MKIVIAPDSFKESLSAMAVAEAIEKGFREIYPDADYVKVPMADGGEGTVQSMVEASGGRY
IDQWVQGPLGQPVAARWGMLGDSDTAVIEMAAASGLHHVSPELRNPLNTTSYGTGELIVA
ALERGVKRIILGIGGSATNDGGAGMMQALGVILRDKQGRSLSPGGEALAALASIDLSGCH
PLLRKVSITVACDVNNPLCGPQGASAIFGPQKGATAEMVNTLDAALENWGRHIYQATGRE
VINAPGAGAAGGMGAALLGLLNAELRAGVEIVVETLQLEQAVKDADLVITGEGRLDSQSI
CGKTPIGVARVAKRYHKPVIALAGGLQHDHHVVYQQGIDAALSILSHIVTLPEALHEAEY
NLSLSARNVAAIWRLARQA
>EECEBICD_00854 Peroxiredoxin Bcp
MNPLKAGDIAPKFSLPDQDGEQVNLTDFQGQRVLVYFYPKAMTPGCTVQACGLRDNMDEL

KKAGVDVLGISTDKPEKLSRFAEKELLNFTLLSDENHQVCEQFGVWGEKSFMGKTYDGIH
RISFLIDADGKIEHVFNDFKTSNHHDVVLNWLKENA
>EECEBICD_00855 Glycine cleavage system transcriptional repressor
MTPSSQHYLVITALGADRPGIVNTITRHVSSCGCNIEDSRLAMLGDEFTFIMLLSGTWNA
ITLIESTLPLKGAELDLLIVMKRTSDRPRPAMPATVWVQVEVADSPHLIERFTALFNSHE
MNIAELVSRTQPAEGDKAAQLFIQITAHSPASQNSANIEQAFKALCTELNAQGSINVVNY
SQHDEQDGVK
>EECEBICD_00856 4-hydroxy-tetrahydrodipicolinate synthase
MFTGSIVALVTPMDEKGNVSRSCLKKLIDYHVANGTSAIVSVGTTGESATLSHDEHGDVV
MMTLELADGRIPVIAGTGANATAEAISLTQRFNDSGIVGCLTVTPYYNRPTQEGLFQHFK
AIAEHTDLPQILYNVPSRTGCDMLPETVGRLAEIKNIIAIKEATGNLTRVHQIKELVSDD
FILLSGDDASALDFMQLGGHGVISVTANVAARDMADMCKLAAEGQFAEARAINQRLMPLH
NKLFVEPNPIPVKWACKALGLVATDTLRLPMTPITDHGRDIVKAALQHAGLL
>EECEBICD_00857 Outer membrane protein assembly factor BamC
MAYSVQKSRLAKVAGVSLVLLLAACSSDSRYKRQVSGDESYLDAAPLAELHAPAGMILPI
MTGDYVIPVTKGSGAVGKALDIRPPAQPLALVSGARTQFSGDTATLLVENGRSSTLWPQV
VSVIQAKNYPIEKRDDASQTLTTDWVNWNRLDEDEQYRGRYQISVKPQGYQQAVTVKLVN
LEQAGKPVADAASLQRYSTEMMNVISAGLDKTATDAANAAQNRSAATMDVQSAADDTGLP
MLVVRGPFNLVWQRLPAALEKVGMKVTDSTRSQGSMAVTYKPLSDSDWRDLGASDPGLAS
GDYKLQVGDLDNRSSLQFIDPKGHTLTQSQNDALVAVFQAAFNK
>EECEBICD_00858 Phosphoribosylaminoimidazole-succinocarboxamide synthase
MQKQAELYRGKAKTVYSTENPDLLVLEFRNDTSAGDGARIEQFDRKGMVNNKFNHFIMTK
LAEAGIPTQMERLLSDTECLVKKLEMVPVECVVRNRAAGSLVKRLGVEEGMELNPPIFDL
FLKNDALHDPMVNSSYCETFGWVSQENLARMKELTYKANDVLKKLFDDAGLILVDFKLEF
GLYKGEVVLGDEFSPDGSRLWDKETLDKMDKDRFRQSLGGLIEAYEAVAHRLGVKLD
>EECEBICD_00859 putative protein
MRWQGRRESNNVEDRRNRPGGPSLGGPGFRLPRGKGGIILLVVVLVAGYYGVDLTGLLTG
QPVSQQQSTRSISPNDDEAAKFTSVILATTEDTWGQLFQKMGRGYQQPKLVMYRGMTRTG
CGAGQSVMGPFYCPADGTVYIDLSFYDDMKNKLGADGDFAQGYVIAHEVGHHVQKLLGIE
PKVRQLQQNASQTEVNRLSVRMELQADCFAGVWGHSMQQQGVLEAGDLEEALNAAQAIGD
DRLQQQGQGRVVPDSFTHGTSEQRYSWFKRGFDSGDPAQCNTFGKNF
>EECEBICD_00860 tRNA(Met) cytidine acetyltransferase TmcA
MSDIDALQALTSQMTQEGIRRLLVISGDAAWCRERAEAIRAALPGDWLWVAPDAPAQPCC
TPQALQTLLGREFRHAIFDAWQGFDAAAFAALSGTLQAGSWLLLLMPPYETWESRPDTDS
LRWSDCAQPIPTPQFAQHLKRTLSRDPQTLLWRQHQPFCWPSYPFRGRWRPATGEPQPEQ
AAILSRLREMPPGVATVIAPRGRGKSALAGQFISRMAGTAIVTAPAKTATDILAAFAGER
FCFMAPDALLASGARADWLVVDEAAAIPAPLLLQLVSRFPRILLTTTVQGYEGTGRGFLL
KFCARFPQLHRFTLRQPVRWAPECPLENIVSEALIFDDEAFAQAPHGAIAISAFYQQAWV
NTPALPRAVYQLLSGAHYRTSPLDLRRMMDAPGQHFLQATANNRVAGALWLVEEGGLSAE
LSQAVWCGFRRPRGNLVAQSLAAHGSDPLAATLVGRRVSRIAVHPARQREGIGQQLIACA
CMQAAQCDYLSVSFGYTPELWRFWQRCGFVLVRMGNHREASSGCYTAMALLPLSDAGKRL
AQQEHQRLRRDADILTQWNGEAIPLAALDEQALNDEDWRELVGFAFAHRPLLTSLGCLHR
LLQYSALPLPALRGRLEEKASDAELCARLRISGRKALLALQRAQAAQALIALDAGRTQRL
RDVMPGGGEHAG
>EECEBICD_00861 hypothetical protein
MDWLAKYWWILVLVFLVGVLLNVIKDLKRIDHKKFLANKPELPPHRDFNDKWDDEEDWPK
KDQPKK
>EECEBICD_00862 Succinyl-diaminopimelate desuccinylase
MSCPVIELTQQLIRRPSLSPDDAGCQALMIERLRKIGFTIEHMDFGDTQNFWAWRGRGET
LAFAGHTDVVPAGDVDRWINPPFEPTIRDGMLFGRGAADMKGSLAAMVVAAERFVAQHPH
HRGRLAFLITSDEEASAKNGTVKVVEALMARNERLDYCLVGEPSSTEIVGDVVKNGRRGS
LTCNLTIHGVQGHVAYPHLADNPVHRAAPFLNELVAIEWDRGNDFFPATSMQVANIQAGT
GSNNVIPGELFVQFNFRFSTELTDEMIKERVHALLEKHQLRYTVDWWLSGQPFLTARGKL
VDAVVNAIEHYNEIKPQLLTTGGTSDGRFIARMGAQVVELGPVNATIHKINECVNAADLQ

LLARMYQRIMEQLVA
>EECEBICD_00863 Protein YffB
MITLYGIKNCDTIKKARRWLEEHGIDYRFHDYRVDGIDLPLLNTFIAELGWQPLLNTRGT
TWRKLDEGDRSGITDADSAAALMVEQPAIIKRPLLCAPGKPMLLGFSESRYQQFFDEV
>EECEBICD_00864 Multidrug efflux pump subunit AcrB
MANFFIDRPIFAWVLAILLCLTGALAIFSLPVEQYPDLAPPNVRITANYPGASAQTLENT
VTQVIEQNMTGLDNLMYMSSQSSGTGQATITLSFIAGTDPDEAVQQVQNQLQSAMRKLPQ
AVQDQGVTVRKTGDTNILTIAFVSTDGSMDKQDIADYVASNIQDPLSRVNGVGDIDAYGS
QYSMRIWLDPAKLNSFQMTTKDVTDAIESQNAQIAVGQLGGTPSVDKQALNATINAQSLL
QTPQQFRDITLRVNQDGSEVKLGDVATVELGAEKYDYLSRFNGNPASGLGVKLASGANEM
ATAKLVLDRLNELAQYFPHGLEYKIAYETTSFVKASIIDVVKTLLEAIALVFLVMYLFLQ
NFRATLIPTIAVPVVLMGTFSVLYAFGYSINTLTMFAMVLAIGLLVDDAIVVVENVERIM
SEEGLTPREATRKSMGQIQGALVGIAMVLSAVFVPMAFFGGTTGAIYRQFSITIVSAMVL
SVLVAMILTPALCATLLKPLHKGEQHGQRGFFGWFNRTFNRNAERYEKGVAKILHRSLRW
ILIYVLLLGGMVFLFLRLPTSFLPQEDRGMFTTSIQLPSGSTQQQTLKVVEKVENYYFTH
EKNNIMSVFSTVGSGPGGNGQNVARMFVRLKDWDARDPTTGSSFAIIERATKAFNQIKEA
RVFASSPPAISGLGSSAGFDMELQDHAGAGHDALMAARDQLIELAGKNSSLTRVRHNGLD
DSPQLQIDIDQRKAQALGVSIDDINDTLQTAWGSSYVNDFMDRGRVKKVYVQAAAKYRML
PDDINLWYVRNKDGGMVPFSAFATSRWETGSPRLERYNGYSAVEIVGEAAPGVSTGTAMD
VMESLVHQLPGGFGLEWTAMSYQERLSGAQAPALYAISLLVVFLCLAALYESWSVPFSVM
LVVPLGVIGALLATWMRGLENDVYFQVGLLTVIGLSAKNAILIVEFANEMNQKGHALLDA
TLYASRQRLRPILMTSLAFIFGVLPMATSTGAGSGSQHAVGTGVMGGMISATILAIFFVP
LFFVLIRRRFPLKPRPE
>EECEBICD_00865 Nitrate/nitrite sensor protein NarQ
MTVKRPVSASLAKAFFYIVLLSILSTGSALLTLTSSLRDAEAINIAGSLRMQSYRLGYDL
QSRSPQINAHRQLFQHALNSPVLQNLNAWYVPQAVKTRYARLHANWLEMNSRLQDGDIAW
YQTNINNYVDQIDLFVLALQHYAERKVMLVVAISLAGGIGIFTLVFFTLRRIRQQVVRPL
NQLVTASQRIEHGQFAPLPLDTSLPNELGLLAKTFSQMSSELHKLYRSLEASVEEKTHDL
HEAHRRLEVLYQCSQALNTSQIDVHCFRHILQIVREHDAAWYLELTVGDNWRISEGMQSP
DLPMQMLPVTMQDTVYGELHWQSPNVNASTPLLNSVSTMLGRGLYFNQAQKHFQQLLLME
ERATIARELHDSLAQVLSYLRIQLTLLKRAIPEDNAGAQSIMADFSRALNDAYRQLRELL
TTFRLTLQQADLPSALHEMLEDLQSQTPAKLTLDCRLPTLALDAQMQVHLLQIVREAVLN
AIKHANASEIAVSCVTAPDGDHTVYIRDNGIGIGEPHEPAGHYGLNIMRERAERLGGTLN
FSQPSGGGTLVSISFRSSNKEESQLT
>EECEBICD_00866 Ferredoxin--NADP reductase
MNRFIMANSQQCLGCHACEVACVMAHNDERHVLTPQRYQPRITVIKHQRQRSAVTCHHCE
DAPCARSCPNGAIAHINDSVQVNAQKCIGCKSCVVACPFGTMQMVLTPVAPNQFKASAHK
CDLCQGREQGPACVENCPADALQLVTEDSLTRLAKTRRLRTARQEIRPWHTVDTQHSGTA
SSKVERMQATPPRGEPDKLAIEARKTTFEEIYLPFRAAQAEREAARCLTCGEHSICEWTC
PLHNHIPQWIELVKAGDIDAAVELSHQTNCLPEITGRVCPQDRLCEGACTLRDEYGAVTI
GNIERYISDRALSKGWRPDLSDVQKSDKRVAIIGAGPAGLACADVLARHGVSATVYDRHP
EIGGLLTFGIPAFKLDKSLLARRREIFSAMGIRFELNCEVGKDISLETLLESYDAVFVGV
GTYRSMKADLPNEDAPGVYDALPFLIANTKQVMGLPALPDEPFIDTAGLNVVVLGGGDTA
MDCVRTALRHGAANVTCAYRRDEANMPGSKKEVKNAREEGANFEFNVQPVELVLDTHGRA
SGIRFLRTRLGEPDGQGRRRPVPVPDSEFVMPADAVIMAFGFHPHGMSWLESHGVKVDNW
GRIAASVESEFRYQTSNPKIFAGGDAVRGADLVVTAMAEGRHAAQGILDWLAK
>EECEBICD_00867 Putative D-alanyl-D-alanine carboxypeptidase
MKFTLVATVLLTFSLSAFAVEYPVLTIASPDQVGFDSQKLHRLDGWIQNQIDAGYPSINL
LVIKDNHIVLQKAWGYAKKYDGSTLLAHPIRATTNTMYDLASNTKMYATNFALQKLVYEG
KIDVNDLVSKYIPGFKDMPGDKIKGKDKLRIIDILHHVAGFPADPQYPNKNVAGKLFSQS
KSTTLEMIKKTPLEYQPGSKHIYSDVDYMILGFIIESITAMPLDRYVETTIYKPLGLKHT
VFNPLMKGFTPPQIAATELHGNTRDGVIHFPNIRTNTLWGQVHDEKAWYSMGGVSGHAGL
FSDTHDMAVLMQVMLNGGGYGNLKLFDDKTVAQFTRHSPEDATFGLGWRVNGNASMTPTF
GVLASPQTYGHTGWTGTLTSIDPVNHMAIVILGNRPHSPVANPKVNPNVFVSGLLPAATY

GWIVDQIYGSLK
>EECEBICD_00868 GDP-mannose pyrophosphatase NudK
MSQTITLIKDKILSDNYFTLRNITYDLTRRNGEVIRHKREVYDRGNGATILLYNSTKKTV
VLVRQFRVATWVNGNQDGMLIETCAGLLDNDEPEVCIRKEAIEETGYDVGEVRKIFELYM
SPGGVTELIHFFIAEYHDSERASIGGGVEDEEIEVLELPFSRALEMVRSGEIRDGKTVLL
LNYLQTSHLMD
>EECEBICD_00869 hypothetical protein
MTLSRSAGARTDAVLRIDRGGLAPPDAKEAAIAPRLLLDGKPLSFNSPHWRVSPWHLMTG
DPATITAFLQTIQDAQAITLKNGVQTLSLAGLKAALLFIDAQQKRVGSETAWIEKGNEPP
LSVPPAPALKGIAVINPTPVPLSEEERDDLLDYAAWRVNGIRCSLDPLRRETQVSALTDD
KALLIVNCEAGAYNTIDLAWVVSRKKTLVSRAVRLRLPFNRGVESNDMELMNAFFDEKTR
ELVTLAKGRGLTDCGIQTRWRYDGDRFRLVRYAEEPSCDSWHGPDAWPTLWITR
>EECEBICD_00870 hypothetical protein
MKINNGPVLCPHCGCLSAYYEIDRLAAIREKVNKEGGSAAWDSTLQAHKKKAFCLMCHKS
IDEVVIGQSDAPESTK
>EECEBICD_00871 Transketolase 2
MSRKDLANAIRALSMDAVQKANSGHPGAPMGMADIAEVLWNDFLKHNPTDPTWYDRDRFI
LSNGHASMLLYSLLHLTGYDLPLEELKNFRQLHSKTPGHPEIGYTPGVETTTGPLGQGLA
NAVGLAIAERTLGAQFNRPDHEIVDHYTYVFMGDGCLMEGISHEVCSLAGTLGLGKLIGF
YDHNGISIDGETEGWFTDDTAKRFEAYHWHVVYDIDGHDPEAVKKAILEAQSVKDKPSLI
ICRTVIGFGSPNKAGKEESHGAALGEEEVALTRQKLGWHHPAFEIPKEIYRAWDGREKGE
KAQQQWQEKFAAYEKAYPELAAEFTRRMSGGLPEAWESATQKFINDLQANPAKIATRKAS
QNTLNAYGPLLPELLGGSADLAPSNLTIWKGSTSLKEDPAGNYIHYGVREFGMTAIANGI
AHHGGFVPYTATFLMFVEYARNAARMAALMKARQIMVYTHDSIGLGEDGPTHQAVEQLAS
LRLTPNFSTWRPCDQVEAAVGWKLAIERQHGPTALILSRQNLAQVERTPEQVKAIARGGY
ILKDSGGKPDIILIATGSEMEITLQAAEKLTGEGHNVRVVSLPSTDIFDAQDEAYRESVL
PAHVTARVAVEAGIADYWYKYVGLKGAIIGMTGYGESAPADKLFPYFGFTVENIVEKARR
VLNIKG
>EECEBICD_00872 Transaldolase
MNQLDGIKQFTTVVADSGDIESIRHYQPQDATTNPSLLLKAAGLEQYGHLIEDAIAWGKK
HGGTQEQQVAAASDKLAVNFGAEILKSIPGRVSTEVDARLSFDKEKSIEKARHLVDLYQQ
QGVDKSRILIKLAATWEGIRAAGQLEKEGINCNLTLLFSFAQARACAEAGVYLISPFVGR
IYDWYQARSPLEPYVVEEDPGVKSVRNIYDYFKQHRYETIVMGASFRRTEQILALTGCDR
LTISPNLLKELKEKEEPVIRKLVPSSQMFHRPTPMTEAEFRWEHNQDAMAVEKLSEGIRL
FAIDQRKLEDLLAAKL
>EECEBICD_00873 NADP-dependent malic enzyme
MDEQLKQSALDFHEFPVPGKIQVSPTKPLATQRDLALAYSPGVAAPCLEIEKDPLAAYKY
TARGNLVAVISNGTAVLGLGNIGALAGKPVMEGKGVLFKKFAGIDVFDIEVDELDPDKFI
NVVAALEPTFGGINLEDIKAPECFYIEQKLRERMNIPVFHDDQHGTAIISTAAILNGLRV
VEKNISDVRMVVSGAGAAAIACMNLLVALGMQKHNIVVCDSKGVIYKGREPNMAETKAAY
AVDDSGKRTLDEVIDGADIFLGCSGPKVLTQEMVKKMARAPMILALANPEPEILPPLAKE
VRPDAIICTGRSDYPNQVNNVLCFPFIFRGALDVGATAINEEMKLAAVRAIAELAHAEQS
EVVASAYGDQDLSFGPEYIIPKPFDPRLIVKIAPAVAKAAMDSGVATRPIADFDAYIDKL
TEFVYKTNLFMKPIFSQARKDPKRVVLPEGEEARVLHATQELITLGLAKPILIGRPSVIE
MRIQKLGLQIKAGVDFEIVNNESDPRFKEYWSEYYQIMKRRGVTQEQAQRAMIGNHTAIG
AIMVQRGEADAMICGTIGDYHEHFSVVKAVFGYRDGVHTAGAMNALLLPSGNTFIADTYV
NEDPTPEQLAEIAVMAAETVRRFGIEPKVALLSHSNFGSSNSLSASKMRETLERVRERAP
DLMIDGEMHGDAALVESIRNDRMPDSPLKGAANILVMPNMEAARISYNLLRVSSSEGVTV
GPVLMGVSKPVHVLTPIASVRRIVNMVALAVVEAQTTPL
>EECEBICD_00874 Ethanolamine utilization protein EutS
MNKERIIQEFVPGKQVTLAHLIAHPGEELAKKIGVPDAGAIGIMTLTPGETAMIAGDLAM
KAADVHIGFLDRFSGALVIYGTVGAVEEALLQTVSGLGRLLNFTLCELTKS
>EECEBICD_00875 Propanediol utilization protein PduV
MKRIAFVGAVGAGKTTLFNALRGNYSLARKTQAVEFNDHGDIDTPGEYFSHPRWYHALIT

TLQDVDTLIYVHAANDKESRLPAGLLDVGTRKRHIAVISKTDMPDADVAATRQLLCEIGF
REPIFELNGHDPQSVRQLVDYLAALSEQEEEAGEKTYHS
>EECEBICD_00876 Ethanolamine utilization protein EutQ
MKKLITANDIRAAHARGEQAMSVVLRASIITPEAREVAELLGFTITECDESVPASTSAQA
CKNESQRIREAIIAQLPEGQFTESLVAQLMEKVLKEKQSLELGTMQPSFTSVTGKGGVKV
IDGSSVKFGRFDGAEPHCVGLTDLVTEQDGSSMAAGFMQWDNAFFPWTLNYDEIDMVLEG
ELHVRHEGETMIAKAGDVMFIPKGSSIEFGTPTSVRFLYVAWPANWQSV
>EECEBICD_00877 hypothetical protein
MNDFITETWLRANHTLSEGSEIHLPADARLTPSARELLESRRLRIKFLDPQGRLFVDDDE
QQPQPVHGLTSSDTHPQACCELCRQPVVKKPDTLTHLTADKMVAKSDPRLGFRAALDSAI
ALTVWLQIELAEPWQPWLFDIRSRLGNIMRADAIDEPLAAQAIVGLNEDELHRLSHQPLR
YLDHDHLVPEASHGRDAALLNLLRTKVRETETLAAQVFITRSFEVLRPDILQALNRLSST
VYVMMILSVAKHPLTVAQIQQRLGEKP
>EECEBICD_00878 Ethanolamine utilization protein EutD
MIIERARELAVRAPARVVFPDALDERVLKAAHYLQQYGLARPVLVASPFALRQFALSHRM
PMDGIQVIDPHSNLSMRQRFAQRWLARAGEKMPPDAVEKLSDPLMFAAAMVSAGEADVCI
AGNLSSTANVLRAGLRVIGLQPGCKTLSSIFLMLPQYAGPALGFADCSVVPQPTAAQLAD
IAIASADTWRAITGEEPRVAMLSFSSNGSARHPNVANVQQATELVRERAPQLLVDGELQF
DAAFVPEVAAQKAPDSPLQGRANVMIFPSLEAGNIGYKIAQRLGGYRAVGPLIQGLAAPL
HDLSRGCSVQEIIELALVAAVPRQADVSRERSLHTLVE
>EECEBICD_00879 Ethanolamine utilization protein EutM
MEALGMIETRGLVALIEASDAMVKAARVKLVGVKQIGGGLCTAMVRGDVAACKAATDAGA
AAAQRIGELVSVHVIPRPHGDLEEVFPISFKGDSNI
>EECEBICD_00880 Ethanolamine utilization protein EutN
MKLAVVTGQIVCTVRHQGLAHDKLLMVEMIDAQGNPDGQCAVAIDSIGAGTGEWVLLVSG
SSARQAHRSELSPVDLCVIGIVDEVVAGGKVVFHK
>EECEBICD_00881 Aldehyde-alcohol dehydrogenase
MNQQDIEQVVKAVLLKMKDSSQPASTVHEMGVFASLDDAVAAAKRAQQGLKSVAMRQLAI
HAIREAGEKHARELAELAVSETGMGRVDDKFAKNVAQARGTPGVECLSPQVLTGDNGLTL
IENAPWGVVASVTPSTNPAATVINNAISLIAAGNSVVFAPHPAAKKVSQRAITLLNQAVV
AAGGPENLLVTVANPDIETAQRLFKYPGIGLLVVTGGEAVVDAARKHTNKRLIAAGAGNP
PVVVDETADLPRAAQSIVKGASFDNNIICADEKVLIVVDSVADELMRLMEGQHAVKLTAA
QAEQLQPVLLKNIDERGKGTVSRDWVGRDAGKIAAAIGLNVPDQTRLLFVETPANHPFAV
TEMMMPVLPVVRVANVEEAIALAVQLEGGCHHTAAMHSRNIDNMNQMANAIDTSIFVKNG
PCIAGLGLGGEGWTTMTITTPTGEGVTSARTFVRLRRCVLVDAFRIV
>EECEBICD_00882 Cell division protein FtsA
MAHDEQLWLTPRLQKAAALCNQTPAASDAPLWLGVDLGTCDVVSMVVDGNAQPVAVCLDW
ADVVRDGIVWDFFGAVTLVRRHLDTLEQQLGCRFTHAATSFPPGTDPRISINVLESAGLE
VSHVLDEPTAVADLLALDNAGVVDIGGGTTGIAIVKQGKVTYSADEATGGHHISLTLAGN
RRIPLEEAEQYKRSNAQEIWPVVKPVYEKMAEIVACHIAGQGVTDLWLAGGSCMQPGVEA
LFRQRFPELQVHLPQHSLFMTPLAIANSGRAKAEGLYAS
>EECEBICD_00883 NAD-dependent methanol dehydrogenase
MQAELQTALFQAFDTLNLQRVKTFSVPPVTLCGLGALGACGQEAQARGVSHLFVMVDSFL
HQAGMTAPLARSLAMKGVAMTVWPCPPGEPCITDVCAAVAQLREAACDGVVAFGGGSVLD
AAKAVALLVTNPDQTLSAMTERSTLRPRLPLIAVPTTAGTGSETTNVTVIIDAVSGRKQV
LAHASLMPDVAILDAAVTEGVPPNVTAMTGIDALTHAIEAYSALSATPFTDSLAIGAIAM
IGKSLPKAVGYGHDLAARENMLLASCMAGMAFSSAGLGLCHAMAHQPGAALHIPHGQANA
MLLPTVMGFNRMVCRERFSQIGRALTNKKSDDRDAIAAVSELIAEVGQSKRLADAGAKPE
HYSAWAQAALEDICLRSNPRTATQAQIIDLYAAAG
>EECEBICD_00884 hypothetical protein
MGINEIIMYIMMFFMLIAAVDRILSQFGGSARFLGKFGKSIEGAGGQFEEGFMAMGALGL
AMVGMTALAPVLAHVLGPVIIPVYEMLGANPSMFAGTLLACDMGGFFLAKELAGGDVAAW
LYSGLILGSMMGPTIVFSIPVALGIIEPSDRRYLALGVLAGIVTIPIGCIAGGLIAMYSG
VQINGQPVEFTFALILMNMIPVLIVAVLVALGLKFIPEKMINGFQIFAKFLVALITIGLA

AAVIKFLLGWELIPGLDPIFMAPGDKPGEVMRAIEVIGSISCVLLGAYPMVLLLTRWFEK
PLMNVGKLLNVNNIAAAGMVATLANNIPMFGMMKQMDTRGKVINCAFAVSAAFALGDHLG
FAAANMNAMIFPMIVGKLIGGVTAIGVAMMLVPKDDAAQVKTEAEAQS
>EECEBICD_00885 hypothetical protein
MNTRQLLSVGIDIGTTTTQVIFSRLELVNRAAVSQVPRYEFIKRDISWQSPVFFTPVDKQ
GGLKEAELKALILAQYQAAGIAPESVDSGAIIITGESAKTRNARPAVMTLSQSLGDFVVA
SAGPHLESVIAGYGAGAQSLSEQRMCRVLNIDIGGGTSNYALFDAGKVSGTACLNVGGRL
LETDAQGRVVYAHQPGQMIIDEVFGSGTDARALAAAQLGQVARRMADLIVEVITGALSPL
AQSLMQTGLLPADITPEVITLSGGVGECYRNQPADPFCFSDIGPLLATALHEHPRLREMN
VQFPAQTVRATVIGAGAHTLSLSGSTIWLEDVQLPLRNLPVAIPQDDADLVNAWRQALLQ
LDLDPQTDAYVLALPATLPVRYAALLTVINALTAFVARYPNPHPLLVVAEQDFGKALGML
LRPQLPQLPLAVIDEVVVRAGDYIDIGTPLFGGSVVPVTVKSLAFPS
>EECEBICD_00886 Ethanolamine ammonia-lyase heavy chain
MKLKTTLFGNVYQFKDVKEVLAKANELRSGDVLAGVAAASSQERVAAKQVLSEMTVADIR
NNPVIAYEEDCVTRLIQDDVNETAYNRIKNWSISELREYVLSDETSVDDIAFTRKGLTSE
VVAAVAKICSNADLIYGGKKMPVIKKANTTIGIPGTFSCRLQPNDTRDDVQSIAAQIYEG
LSFGAGDAVIGVNPVTDDVENLTRVLDTVYGVIDKFNIPTQGCVLAHVTTQIEAIRRGAP
GGLIFQSICGSEKGLKEFGVELAMLDEARAVGAEFNRIAGENCLYFETGQGSALSAGANF
GADQVTMEARNYGLARHYDPFLVNTVVGFIGPEYLYNDRQIIRAGLEDHFMGKLSGISMG
CDCCYTNHADADQNLNENLMILLATAGCNYIMGMPLGDDIMLNYQTTAFHDTATVRQLLN
LRPSPEFERWLETMGIMANGRLTKRAGDPSLFF
>EECEBICD_00887 Ethanolamine ammonia-lyase light chain
MDQKQIEEIVRSVMASMGQDVPQPVAPSTQEGAKPQCAAPTVTESCALDLGSAEAKAWIG
VENPHRADVLTELRRSTAARVCTGRAGPRPRTQALLRFLADHSRSKDTVLKEVPEEWVKA
QGLLEVRSEISDKNLYLTRPDMGRRLSPEAIDALKSQCVMNPDVQVVVSDGLSTDAITAN
YEEILPPLLAGLKQAGLNVGTPFFVRYGRVKIEDQIGEILGAKVVILLVGERPGLGQSES
LSCYAVYSPRVATTVEADRTCISNIHQGGRRQ
>EECEBICD_00888 Ethanolamine utilization protein EutL
MPALDLIRPSVTAMRVIASVNDGFARELKLPPHIRSLGLITADSDDVTYIAADEATKQAM
VEVVYGRSLYAGAAHGPSPTAGEVLIMLGGPNPAEVRAGLDAMVASIENGAAFQWANDAE
NTAFLAHVVSRTGSYLSSTAGIALGDPMAYLVAPPLEATFGIDAAMKSADVQLVTYVPPP
SETNYSAAFLTGSQAACKAACNAFTDAVLDIARNPVQRA
>EECEBICD_00889 Ethanolamine utilization protein EutK
MINALGLLEVDGMVAAVDAADAMLKAANVRLLSHQVLDPGRLTLVVEGDLAACRAALDAG
SAAAQRTGRVISRKEIGRPEEDTQWLIGGFARATTPTEKAPQAPATPEFAEALLALLASV
RQGMTAGEVAAHFGWPLEQARNVLEQLFSDGALRKRSSRYRIKN
>EECEBICD_00890 hypothetical protein
MKKTRTANLHHLYHEALPEDVKLTPRVEVDNVHQRRTTDVYEHALTITAWQQIYDQLHPG
KFHGEFTEILLDEIQVFREYTGLALRQSCLVWPNSFWFGIPATRGEQGFIGAQGLGSAEI
ATRPGGTEFELSTPDDYTILGVVISEDVISRQATFLHNPERVLHMLRNQLALEVKEQHKA
ALWGFVQQALATFSESPETLHQPAVRKVLSDNLLLAMGTMLEEAKPIHSAESISHQGYRR
LLSRAREYVLENMSEPLTVLDLCNQLHVSRRTLQNAFHAILGIGPNAWLKRIRLNAVRRE
LISPWSQSATVKDAAMQWGFWHLGQFATDYQQLFAEKPSLTLHQRMRQWA
>EECEBICD_00891 hypothetical protein
MQTNTRKVVTSDAKWHPHLGISFIGCLLAITLEIYFEERIFIPHSGGVSFGLIVLLVINM
VTIPVVMALIALLCFIIHIPRKSVNCILLCLLACILTIAGLFIAYPVGR
>EECEBICD_00892 Oxygen-dependent coproporphyrinogen-III oxidase
MKPDAHHVKQFLLRLQDDICQKLSAVDGANFVEDSWRREAGGGGRSRVLRNGGIFEQAGV
NFSHVHGDAMPASATAHRPELAGRSFEAMGVSLVVHPHNPYIPTSHANVRFFIAEKPGAD
PVWWFGGGLDLTPYYGFEEDAVHWHRTARDLCQPFGDNVYPRYKKWCDDYFFLKHRNEQR
GIGGLFFDDLNTPDFDHCFAFMQAVGNGYTEAYLPIVERRKAMVWGERERNFQLYRRGRY
VEFNLVWDRGTLFGLQTGGRTESILMSMPPLVRWEYDWQPEAGSPEAALSEFIQVRDWI
>EECEBICD_00893 N-acetylmuramoyl-L-alanine amidase AmiA
MSTFKLLKTLTSRRQVLKTGLAALTLSGMSHAIAKEETLKTSNGHSKPKTKKTGSKRLVM

LDPGHGGIDTGAIGRNGSQEKHVVLAIAKNVRAILRNHGIDARLTRTGDTFIPLYDRVEI
AHKHGADLFMSIHADGFTNPKAAGASVFALSNRGASSAMAKYLSERENRADEVAGKKATD
RDHLLQQVLFDLVQTDTIKNSLTLGSHILKKIKPIHKLHSRTTEQAAFVVLKSPSIPSVL
VETSFITNPEEERLLGTTAFRQKIATAIANGIISYFHWFDNQKAHTKKR
>EECEBICD_00894 Acetyltransferase YpeA
MEIRVFRQEDFEEVITLWERCDLLRPWNDPEMDIERKVNHDVSLFLVAEVSGEVVGTVMG
GYDGHRGSAYYLGVHPEFRGRGIANALLNRLEKKLIARGCPKIQIMVRDDNDVVLGMYER
LGYEHSDALSLGKRLIEDEEY
>EECEBICD_00895 Inner membrane protein YfeZ
MKSTEFHPADYDVHGRLRLPFLFWCVLLLQARAWVLFVIAGSSRGQGNTLLNFFYPDHDN
FWLGLLPGVPAVVAFLLSGRREAVPGVWRWLRGLLILAQLVSLCWLPVMWLGGDPVNGVG
LALLLADIVALIWLLTNQRLRACFSLEKE
>EECEBICD_00896 putative protein YfeY
MKSLRLTLLALPLALTGCSTLSSVNWSAANPWNWFGSSTEVTEQGVGALTAATPLEEPAI
AEALDGDYRLRSGMKTDNGNVVRFFEAMKGDSVAMVINGEQGTVSRIDVLDSDIPTAAGG
KIGTPFSDLYSKAFGHCEPVSSDSHTSVECKAEGSQHISYVFSGEWSGPEGLMPPDDVLK
NWDVRKIIWRR
>EECEBICD_00897 Dye-decolorizing peroxidase YfeX
MSQVQSGILPEHCRAAIWIEANVKGDLDALRAASRTFADKLATFEVKFPDAHLGAVVAFG
NNTWRALSGGVGAEELKDFIPYGKGLAPATQYDVLIHILSLRHDVNFSVAQAAMEAFGDC
IDVKEEVHGFRWVEERDLSGFVDGTENPAGEETRREVAVIKDGVDAGGSYVFVQRWEHNL
KQLNRMSIHDQEMMIGRTKEANEEIDGDARPATSHLSRVDLKEDGKGLKIVRQSLPYGTA
SGTHGLYFCAYCARLHNIEQQLLSMFGDTDGKRDAMLRFTKPVTGGYYFAPSLDRLLAL
>EECEBICD_00898 Oxidoreductase UcpA
MGKLTGKTALITGASQGIGEGIARVFARHGANLILLDISDEIEKLADELGGRGHRCTAVK
ADVRDFASVQAAVARAKETEGRIDILVNNAGVCRLGNFLDMSEEDRDFHIDINIKGVWNV
TKAVLPEMIKRKDGRIVMMSSVTGDMVADPGETAYALSKAAIVGLTKSLAVEYAQSGIRV
NAICPGYVRTPMAESIARQSNPDDPESVLTEMAKAIPLRRLADPLEVGELAAFLASDESS
YLTGTQNVIDGGSTLPESVSVGV
>EECEBICD_00899 Thiosulfate-binding protein
MAVNLLKKRPLTLAAMLLLAGQAQATELLNSSYDVSRELFAALNPPFEQQWAKDNGGDKL
TIKQSHAGSSKQALAILQGLKADVVTYNQVTDVQILHDKGKLIPADWQSRLPNNSSPFYS
TMGFLVRKGNPKNIHDWSDLVRSDVKLIFPNPKTSGNARYTYLAAWGAADNADGGDKAKT
EQFMTQFLKNVEVFDTGGRGATTTFAERGLGDVLISFESEVNNIRKQYEAQGFEVVIPKT
NILAEFPVAWVDKNVQANGTEKAAKAYLNWLYSPQAQTIITDFYYRVNNPEIMGKQADKF
PQTELFRVEDKFGSWPEVMKTHFASGGELDKLLAAGRK
>EECEBICD_00900 Sulfate transport system permease protein CysT
MLAVSSRRVLPGFTLSLGTSLLFVCLILLLPLSALVMQLSQMSWAQYWDVVTNPQVVAAY
KVTLLAAFVASIFNGVFGLLMAWILTRYRFPGRTLLDALMDLPFALPTAVAGLTLASLFS
VNGFYGQFLAQFDIKMTYTWLGIAVAMAFTSIPFVVRTVQPVLEELGPEYEEAAQTLGAT
RLQSFRKVVLPELSPALIAGVALSFTRSLGEFGAVIFIAGNIAWKTEVTSLMIFVRLQEF
DYPAASAIASVILAASLLLLFSINTLQSRFGRRVVGH
>EECEBICD_00901 Sulfate transport system permease protein CysW
MAEVTQLKRYDVPRINWGKWFLIGVGMLVSAFILLVPMIYIFVQAFSKGLMPVLQNLADP
DMLHAIWLTVLIALIAVPVNLVFGILLAWLVTRFNFPGRQLLLTLLDIPFAVSPVVAGLV
YLLFYGSNGPLGGWLDEHNLQMMFSWPGMVLVTIFVTCPFVVRELVPVMLSQGSQEDEAA
ILLGASGWQMFRRVTLPNIRWALLYGVVLTNARAIGEFGAVSVVSGSIRGETLSLPLQIE
LLEQDYNTVGSFTAAALLTLMAIITLFLKSMLQWRLENQEKRAQQEENHEH
>EECEBICD_00902 Sulfate/thiosulfate import ATP-binding protein CysA
MSIEIARIKKSFGRTQVLNDISLDIPSGQMVALLGPSGSGKTTLLRIIAGLEHQSSGHIR
FHGTDVSRLHARERKVGFVFQHYALFRHMTVFDNIAFGLTVLPRRDRPTAAAIKTKVMQL
LEMVQLAHLADRFPAQLSGGQKQRVALARALAVEPQILLLDEPFGALDAQVRKELRRWLR
QLHEELKFTSVFVTHDQEEATEVADRVVVMSQGNIEQADAPDRVWREPATRFVLEFMGEV
NRLTGTVRGGQFHVGAHRWPLGYTPAYQGPVDLFLRPWEVDISRRTSLDSPLPVQVIEAS

PKGHYTQLVVQPLGWYHDPLTVVMAGEDVPVRGERLFVGLQKARLYNGDQRIETREEELA
LAQSA
>EECEBICD_00903 Cysteine synthase B
MNTLEQTIGNTPLVKLQRLGPDNGSEIWVKLEGNNPAGSVKDRAALSMIVEAEKRGEIKP
GDVLIEATSGNTGIALAMIAALKGYRMKLLMPDNMSQERRAAMRAYGAELILVTKEQGME
GARDLALAMSERGEGKLLDQFNNPDNPYAHYTTTGPEIWRQTSGRITHFVSSMGTTGTIT
GVSRFLREQEKPVTIVGLQPEEGSSIPGIRRWPAEYMPGIFNASLVDEVLDIHQNDAENT
MRELAVREGIFCGVSSGGAVAGALRVARATPGAIVVAIICDRGDRYLSTGVFGEEHFSQG
AGI
>EECEBICD_00904 hypothetical protein
MKFAFRTLIFVGLFYAIAPLAYAETSLDELQQQADRGDMNAQFQLGRKYHIGDGVERDVE
KAVFWYQKAAAQGSAKATNNLGVLYEHGHVAPEDEHRSVEWIMEKSFAYFTDAAKKGQCT
AQQNIGATYAYRHEYVQAWAWLTVAATRGSKWGLIDRGRFENSMSSDERKQAEKLAKEYL
QKYDTKEGRCNEQTIDLSTYKR
>EECEBICD_00905 hypothetical protein
MKKIVCAVVALLLTLPAWAKLNAHEEARINAMLNALAQKKDLTFVRNGDAHNCEEAVSHL
RLKLGNTRNRIDTAEQFIDKVASSSSITGKPYIVKIPGKSDENAQPYLHALIAETDKTVA
P
>EECEBICD_00906 hypothetical protein
MRVHFVVHESFESAGAYLKWAEDRGYTISWSRVYAGEALPPNADEFDMLVVFGGPQSPRT
TREECPYFDSRAEQHLINQAITARRMVIGICLGSQLIGEALGAAVCQSPEKEIGHYPITL
TEAGLRHPLIAHFGSPLTVGHWHNDMPGLTDQATVLAESEGCPRQIVQYGNFVYGFQCHM
EFTVEAVEGLIQHSQQELADAQGKRFIRSVAEMRAWNYQQMNEKLWRFLDELTLAHSQK
>EECEBICD_00907 Vitamin B6 salvage pathway transcriptional repressor PtsJ
MIDGKTANEIFDSIRQHIIAGTLRAEDSLPPVRELASELKVNRNTVAAAYKRLITAGLAQ
SLGRNGTVIKGSPSPVALEGGDPHTPLHDLSGGNPDPQRLPDLSRYFARLSRTPHLYGDA
PVSPELHAWAARWLRDATPVAGEIDITSGAIDAIERLLCAHLLPGDSVAVEDPCFLSSIN
MLRYAGFSASPVSVDSEGMQPEKLEQALNQGARAVILTPRAHNPTGCSLSARRAAALQNI
LARYPQVVVIIDDHFALLSSSPWQPVIAQTTQHWAVIRSVSKTLGPDLRLAIVASDSATS
AKLRLRLNAGSQWVSHLLQDLVYACLTDPEYQHRLAQTRLFYATRQQKLARALQQYGIAI
SPGDGVNAWLPLDTHSQATAFTLAKSGWLVREGEAFGVSAPSHGLRITLSTLNDSEINTL
AADIHQALNR
>EECEBICD_00908 Pyridoxine/pyridoxal/pyridoxamine kinase
MGQESDIQSVLFDDNHRALQTDIVAVQSQVVYGSVGNSIAVPAIKAQGLRVTAVPTVLFS
NTPHYKTFYGGIIPAEWFAGYLTALNERDALRELKAITTGYMGSADQIVLLSKWLMAIRA
SHPEVCILVDPVIGDTDSGMYVQAEIPQAYRTHLLPQAQGLTPNVFELEMLSGKPCRTLE
EAVAAAQSLLSDTLKWVVITSAPGESLETITVAVVTAQVVEVFAHPRVATELKGTGDLFC
AELVSGIVQGKKLTTAAKDAAQRVLEVMTWTQQCGCDELILPPAGEAR
>EECEBICD_00909 hypothetical protein
MKSYRLVVRQQGRIVGHFETSGLDALEDICVARAMFGITGGYQCELQVSDSERRMLESGP
EGMKILMREKCFRPVTSQL
>EECEBICD_00910 PTS system glucose-specific EIIA component
MGLFDKLKSLVSDDKKDTGTIEIVAPLSGEIVNIEDVPDVVFAEKIVGDGIAIKPTGNKM
VAPVDGTIGKIFETNHAFSIESDSGIELFVHFGIDTVELKGEGFKRIAEEGQRVKVGDPV
IEFDLPLLEEKAKSTLTPVVISNMDEIKELIKLSGSVTVGETPVIRIKK
>EECEBICD_00911 Phosphoenolpyruvate-protein phosphotransferase
MISGILASPGIAFGKALLLKEDEIVIDRKKISADKVDQEVERFLSGRAKASAQLEAIKTK
AGETFGEEKEAIFEGHIMLLEDEELEQEIIALIKDKHMTADAAAHEVIEGQATALEELDD
EYLKERAADVRDIGKRLLRNILGLAIIDLSAIQEEVILVASDLTPSETAQLNLQKVLGFI
TDAGGRTSHTSIMARSLELPAIVGTGSVTAQVKNGDYLILDAVNNQVYVNPTNDVIEQLR
AVQEQVATEKAELAKLKDLPAITLDGHQVEVCANIGTVRDVEGAERNGAEGVGLYRTEFL
FMDRDALPTEEEQFAAYKAVAEACGSQAVIVRTMDIGGDKELPYMNFPKEENPFLGWRAV
RIAMDRKEILRDQVRAILRASAFGKLRIMFPMIISVEEVRALRKEIEIYKQELRDEGKAF
DESIEIGVMVETPAAATIARHLAKEVDFFSIGTNDLTQYTLAVDRGNDMISHLYQPMSPS

VLNLIKQVIDASHAEGKWTGMCGELAGDERATLLLLGMGLDEFSMSAISIPRIKKIIRNT
NFEDAKVLAEQALAQPTTDELMTLVNKFIEEKTIC
>EECEBICD_00912 Phosphocarrier protein HPr
MFQQEVTITAPNGLHTRPAAQFVKEAKGFTSEITVTSNGKSASAKSLFKLQTLGLTQGTV
VTISAEGEDEQKAVEHLVKLMAELE
>EECEBICD_00913 Cysteine synthase A
MSKIYEDNSLTIGHTPLVRLNRIGNGRILAKVESRNPSFSVKCRIGANMIWDAEKRGVLK
PGVELVEPTSGNTGIALAYVAAARGYKLTLAMPETMSIERRKLLKALGANLVLTEGAKGM
KGAIQKAEEIVASDPQKYLLLQQFSNPANPEIHEKTTGPEIWEDTDGQVDVFISGVGTGG
TLTGVTRYIKGTKGKTDLITVAVEPTDSPVIAQALAGEEIKPGPHKIQGIGAGFIPGNLD
LKLIDKVVGITNEEAISTARRLMEEEGILAGISSGAAVAAALKLQEDESFTNKNIVVILP
SSGERYLSTALFADLFTEKELQQ
>EECEBICD_00914 Sulfate transporter CysZ
MVSSSTTVPRSGVYYFSQGWKLVTLPGIRRFVILPLLVNIVLMGGAFWWLFTQLDAWIPS
LMSHVPDWLQWLSYLLWPIAVISVLLVFGYFFSTLANWIAAPFNGLLAEQLEARLTGATP
PDTGILGIMKDVPRIMKREWQKLAWYLPRAIVLLVLYFIPGIGQTIAPVLWFLFSAWMLA
IQYCDYPFDNHKVPFKTMRAALRTQKVANMQFGALTSLFTMIPVLNLFIMPVAVCGATAM
WVDCWRAKHALWK
>EECEBICD_00915 Cell division protein ZipA
MMQDLRLILIIVGAIAIIALLVHGFWTSRKERSSMFRDRPLKRMKSKRDDDSYDDDVEED
EGVGEVRVHRVNHAPGQSQEHDAPRQSPQHQYQPPYASAQPRPAAPPQPQAPMQQPVQQP
VQPAPQPQQVQPSAPPVQPPQQQPAPPSQAPQPVAQPAPPPSAQTFQPAEPVVEAEPVVE
EAPVVEKPQRKEAVIIMNVAAHHGSELNGEVLLNSIQQSGFKFGDMNIFHRHLSPDGSGP
ALFSLANMVNPGTFDPEMTDFTTPGVTIFMQVPSYGDALQNFKLMLQSAQHIADEVGGVV
LDDQRRMMTPQKLREYQDRIREVMDANA
>EECEBICD_00916 DNA ligase
MEPIEQQLTELRTTLRHHEYLYHVMDAPEIPDAEYDRLMRELRELEAQRPDLITPDSPTQ
RVGAAPLTAFNQIRHEVPMLSLDNVFDEESFLAFNKRVQDRLKSTENVIWCCELKLDGLA
VSILYENGVLVSAATRGDGTTGEDITSNVRTIRAIPLKLHGDNIPARLEVRGEVFLPQAG
FEKINEDARRTGGKVFANPRNAAAGSLRQLDPRITAKRPLTFFCYGVGILEGGELPDTHL
GRLLQFKAWGLPVSDRVTLCDSPQAVLDFYRNVEKDRPTLGFDIDGVVIKVNSLALQEQL
GFVARAPRWAVAFKFPAQEQMTFVRDVEFQVGRTGAITPVARLEPVQVAGVLVSNATLHN
ADEIERLGLRIGDKVVIRRAGDVIPQVVNVVLSERPEETRPIVFPTHCPVCGSDVERVEG
EAVTRCTGGLICGAQRKESLKHFVSRRAMDVDGMGDKIIDQLVEREYVHTPADLFRLTAG
KLTGLDRMGPKSAQNVVNALEKAKATTFARFLYALGIREVGEATAAGLAAYFGTLEALQT
ATIDELQKVPDVGIVVATHVFNFFAEESNRDVIGQLLAEGVHWPAPVVINVQEVDSPFAG
KTVVLTGSLSQMSRDDAKARLAALGAKVAGSVSKKTDLVIAGEAAGSKLAKAQELGITVI
DEAEMIRLLGA
>EECEBICD_00917 hypothetical protein
MEKEQLVEIANTVMPFGKYQGRRLIDLPEEYLLWFARKDQFPAGKLGELMQITLLIKTEG
LTQLVQPLKRPL
>EECEBICD_00918 hypothetical protein
MANILFPTSVLGMMVLPLMIFHQIQLMVCAGLARRYKRQTEKLQAQQESRAAKA
>EECEBICD_00919 hypothetical protein
MKLFRILDPFTLTLITVVLLASFFPAEGGFVPVVEGITTAAIALLFFMHGAKLSREAIIA
GGSHWRLHLWVMCSTFVLFPVLGVLFAWWAPVNVDPMLYSGFLYLCILPATVQSAIAFTS
LAGGNVAAAVCSASASSLLGIFLSPLLVGLVMNIHGAQGSLEEVGKIMLQLLLPFVLGHL
SRPWIGNWVARNKKWIAKTDQTSILLVVYSAFSEAVVNGIWHKVGWGSLLFIVVVSLILL
AIVITINVFVARKCGFNKADEITIVFCGSKKAWPTGSRWPIFCFRRQCWV
>EECEBICD_00920 Hydrogen peroxide-inducible genes activator
MNYSLRQLRIFITVAQAKSFSRAGDRIGLSQSAVSHSVKELERQTGVRLLDRTTREVVLT
EAGQQLATRLERVLDELNSILRDAGRVGTQLTGTVRVAASQTISAHLIPQCIAQSNSLYP
AIDFVLHDRPQQWVLESIRQGEVDFGIVIDPGAAVDLQCEAILSEPFLLLCRQDHPLAHQ
EWVSWQDLKQASLVLQDYASGSRPLIDAALAHFAIEADIVQEIGHPATLFPMVEAGIGIS

VLPALALPLPQGSHLQVKRLTPVVERQLMLARRKNRSLSTAAQALWDVVRTQASELTAAR
AKDPLYQL
>EECEBICD_00921 hypothetical protein
MKKILTLTLSSLLIIPALTHAEFKGGFADIGLHYLDWTSDTTEKTSKKSHKDDFGYLELE
GGANFSWGEMYGFFDWENFYNGRHAKPGSEQRYTFKNTNRIYLGDTGLNLYLHAYGTYGS
PHRANFHDDMFLYGLGYNFTGNGYWFKPFFAKRYTDQTYYTGDNGYVLGWVAGYSFSLGS
EKFSVTNWNEYEFDRDASYAAGNGGKDGINGAVALWWNATPHLTAGVQYRYADNKLGESF
LQDGIIYSIKYLF
>EECEBICD_00922 Purine nucleoside phosphorylase 2
MPHALFSHNPEYCIDIIHAHKPAFTPRVAFILGSGLGALADQIEDAVAISYEKLPGFPVS
TVHGHAGELVLGHLAGVPVACMKGRGHFYEGRGMAVMTDAIRTLKLLGCEVLFCTNAAGS
LRPEVGPGSLVALSDHINTMPGTPMVGPNDDRYGDRFFSLANAYDADYRAMLQSVAAEEG
FPLTEGVFVSYPGPNFETAAEIRMMQIIGGDVVGMSVVPEVISARHCGLKVVAVSAITNL
AEGLGDVKLSHAQTLAAAELSRQNFINLICGFLRKLA
>EECEBICD_00923 Xanthosine permease
MGITSRLKVMSFLQYFIWGSWLVTLGSYMINTLDFTGANVGMVYSSKGLAAIIMPGIMGI
IADKWLRAERAYMLCHLVCAGALLYATTVTDPQTMFWVMLVNAMAYMPTIALSNSVSYSC
LAKAGQDPVTSFPPVRVFGTIGFIVAMWTVSLMGLELSSAQLYIASGASLLLALYALTLP
KIPVAEKKANTTLASKLGLDAFVLFKNPRMAIFFLFAMMLGAVLQITNVFGNPFLHDFAR
NPEFADSFVVKYPSILLSVSQMAEVGFILTIPFFLKRFGIKTVMLMSMLAWTLRFGFFAF
GDPSPFGFVLLLLSMIVYGCAFDFFNISGSVFVEQEVDSSIRASAQGLFMTMVNGVGAWI
GSLLSGMAVDYFSIDGVKDWQTIWLVFAAYALALAVIFALFFKYQHHPEKLSTKSLAH
>EECEBICD_00924 hypothetical protein
MKEIILSSGIGFGIGAFFTLCRIPIPAPNVLPGVLSIVFMYIGYLVVKSIFY
>EECEBICD_00925 Hca operon transcriptional activator HcaR
MERAHRIDLKLLRYFLAVAEELHFGRAAARLNMSQPPLSIHIKELEQQLGTLLFIRHSRS
VALTHAGKVLMEESRRLLASANQALARVEQIGRGEAGRIELGVVGTALWGRMRPAMRHFL
KANPNVEVLFREKSPGMQMALLERRELDAGIWRMAIEPAVGFTSIRLHESAFMVAVPEDH
DLASRDSVPLSALRNEYFVTLPSVHSDWGFLQRVCQQAGFSPMIIREVVEPQTVLAMISM
GIGITLMADGYAQMSWPGVVFRPLEERIPADLYIVYDQQQATPALEKLVAALTV
>EECEBICD_00930 Glutamate--tRNA ligase
MKIKTRFAPSPTGYLHVGGARTALYSWLFARHHGGEFVLRIEDTDLERSTPEAIEAIMDG
MNWLNLEWDEGPYFQTKRFDRYNAVIDEMLEAGTAYKCYCSKERLEQLREEQMAKGEKPR
YDGRCRHSYEHHADDEPCVVRFANPQDGSVIFDDQIRGPIEFSNQELDDLIIRRTDGSPT
YNFCVVVDDWDMEITHVIRGEDHINNTPRQINILKALNAPVPMYAHVSMINGDDGKKLSK
RHGAVSVMQYRDDGYLPEALLNYLVRLGWSSGDQEIFTREEMIKLFSLGAVSKSASAFNT
DKLLWLNHHYINTLAPEYVATHLQWHIEQENIDTRNGPQLAELVKLLGERCKTLKEMAQS
CRYFYEDFSEFDADAAKKHLRPVARQPLEVVRDKLSAITDWSAENVHHAIQATADELEVG
MGKVGMPLRVAVTGAGQSPALDVTVHAIGKTRSIERINKALGFIAERESQQ
>EECEBICD_00931 hypothetical protein
MTTEELAECLGVARQTVNRWIREQHWKTEKFPGVKGGRARLIHIDASVREFILNIPAFRK
LPAFYQAEEAFAEYANAAHSHAYRQIIDAVENMSAQEQEKLALFLSREGIRGFLTRLGIN
EAD
>EECEBICD_00932 hypothetical protein
MFKERMTPEELANLTGYSRQTINKWVRKEGWATSPKPGVQGGKARLVHVNEQVREYIRSA
ERSVDHHADTFTPASNASLEALLMTLAKEMTSSEQKQFTSLLVREGITGLLQRLGIRDSK
>EECEBICD_00935 putative cyclic di-GMP phosphodiesterase PdeA
MPDKCNVLKNIKIFLLAFCLTVPAILLSRLISPRATIDSSYIFLAWLPLCVMFSVLFLFG
RRGVAPMVGGMMLTNEWNFHLPLPQAMVLLFCQTFPVLLVCAIVRWQLGARWRYGIPNQG
IWLRVFWLGLMTPFGIKISMHLAGHYLAFPVTISTFFGTGTAIFSIVDLLSLISAALIFT
LFFYYPLRMIVSPHYIRIFWRRDVAPYLAKEKRLFTLLWFATLATLLAVLCTPFETKYIA
GYLVPVLFIGFTIGVGKISYPLLNFSWALTALFLLSYNRNFLQGVGSEYSLAFILSVLIS
FSICLLYMARINQRSEWLNRQWHSQALTDPLTQLPNLRALEQFLLQDAGQSVCYLRMENL
EFLSRHYGMQMRVHCEREVFRVLQPLLLEKEKIFHLPGSELLLVLTGPETEARLQYMLNV

LNNRKIYWNNTGLDMEYGASWGTFDGRQETLQPLLGQLSWLAEQSCSHRRVLALTQSIEA
ASGQTTERVLRLQKIRQALERGALVLYAQPIRDSQGKGYDEILTRLRCDDGIMMPNQFIP
LIAQFNLSVRFDMQVMEALLQWLSAHPSAEQGARFSVNLMPLTLLQKETASRIMQLFKRY
GVPPASVIIEITEEQAFSHSEISMHNINQLRKFGLKIAIDDFGTGYANYERLKRLKADII
KIDGCFVKDILTDSLDAMIVKSITDLAKAKSLSVVAEFVETPAQRDLLLQLGVHSLQGYL
IGRPRPLGK
>EECEBICD_00936 Nucleoside permease NupC
MDRVLHFVLALAVVAVLALLVSSDRKKIRIRYVIQLLVIEVLLAWFFLNSDVGLGFVKGF
SEMFEKLLGFANEGTNFVFGSMNDQGLAFFFLKVLCPIVFISALIGILQHIRVLPVVIRA
IGFLLSKVNGMGKLESFNAVSSLILGQSENFIAYKDILGKMSRNRMYTMAATAMSTVSMS
IVGAYMTMLDPKYVVAALVLNMFSTFIVLSLINPYVVDASEENIQMSNLHEGQSFFEMLG
EYILAGFKVAIIVAAMLIGFIALIAALNALFATVTGWFGYSISFQGILGYIFYPVAWVMG
VPSSEALQVGSIMATKLVSNEFVAMMDLQKIAATLSPRAEGIISVFLVSFANFSSIGIIA
GAIKGLNEEQGNVVSRFGLKLVYGSTLVSVLSASIAALVL
>EECEBICD_00937 Divalent metal cation transporter MntH
MTDNRVENSSGRAARKLRLALMGPAFIAAIGYIDPGNFATNIQAGASFGYQLLWVVVWAN
LMAMLIQILSAKLGIATGKNLAEQIRDHYPRPVVWFYWVQAEIIAMATDLAEFIGAAIGF
KLILGVSLLQGAVLTGIATFLILMLQRRGQKPLEKVIGGLLLFVAAAYIVELFFSQPDMA
QLGKGMVIPALPNPEAVFLAAGVLGATIMPHVIYLHSSLTQHLHGGTRQQRYSATKWDVA
IAMTIAGFVNLAMMATAAAAFHFSGHTGIADLDQAYLTLEPLLSHAAATVFGLSLVAAGL
SSTVVGTLAGQVVMQGFVRFHIPLWVRRSITMLPSFIVILMGLDPTRILVMSQVLLSFGI
ALALVPLLIFTSNATLMGELVNTRRVKQIGWIIVVLVVALNIWLLVGTVMGLS
>EECEBICD_00938 hypothetical protein
MFRSLILAAALLAFTPLAANAGEITLLPSIKLQIGDRDDYGNYWDGGRWRDRDYWHNHYE
WRKNRWWRHDNGYHRGWDKRKAYERGYREGWRDRDDHRGRGRGHKHHH
>EECEBICD_00939 L-glyceraldehyde 3-phosphate reductase
MIYQPDENRYHTMEYRRCGRSGVKLPAISLGLWHNFGDTTRVENSRALLQRAFDLGITHF
DLANNYGPPPGSAECNFGRILQEDFLPWRDELIISTKAGYTMWDGPYGDWGSRKYLIASL
DQSLKRMGLEYVDIFYHHRPDPETPLKETMKALDHLVRHGKALYVGISNYPADLARQAID
ILEDLGTPCLIHQPKYSLFERWVEDGLLALLQEKGVGSIAFSPLAGGQLTDRYLNGIPED
SRAASGSRFLKPEQITADKLEKVRRLNELAARRGQKLSQMALAWVLRNDNVTSVLIGASK
PSQIEDAVGMLANRRFSAAECAEIDAILEGRF
>EECEBICD_00940 Indole-3-pyruvate decarboxylase
MQNPYTVADYLLDRLAGCGIGHLFGVPGDYNLQFLDHVIDHPTLRWVGCANELNAAYAAD
GYARMSGAGALLTTFGVGELSAINGIAGSYAEYVPVLHIVGAPCSAAQQRGELMHHTLGD
GDFRHFYRMSQAISAASAILDEQNACFEIDRVLGEMLAARRPGYIMLPADVAKKTAIPPT
QALALPVHEAQSGVETAFRYHARQCLMNSRRIALLADFLAGRFGLRPLLQRWMAETPIAH
ATLLMGKGLFDEQHPNFVGTYSAGASSKEVRQAIEDADRVICVGTRFVDTLTAGFTQQLP
TERTLEIQPYASRIGETWFNLPMAQAVSTLRELCLECAFAPPPTRSAGQPVRIDKGELTQ
ESFWQTLQQYLKPGDIILVDQGTAAFGAAALSLPDGAEVVVQPLWGSIGYSLPAAFGAQT
ACPDRRVILIIGDGAAQLTIQEMGSMLRDGQAPVILLLNNDGYTVERAIHGAAQRYNDIA
SWNWTQIPPALNAAQQAECWRVTQAIQLAEVLERLARPQRLSFIEVMLPKADLPELLRTV
TRALEARNGG
>EECEBICD_00941 Putative ion-transport protein YfeO
MFHPRARTMLLLSLPALIIGVASSLVLIAAMKVASVFQQFLWQRLPTSIGIAYDSPFWIV
GMLTLTGIVVGLIIRYSPGHAGPDPAIEPLISMPVSPSALPGLLLALIIGLAGGVSLGPE
HPIMTINIALAAAFGSRLFPRITALDWTILASAGTIGALFGTPVAAALIFSQTLSGSNDI
PMWDRLFAPLMAAAAGSLTTSLFFHPHFSLPIAHYTQMRLVDIASGAIVAAIAIAAGMVA
VWCLPRLHELLHRLKNPVLILGIGGFILGILGVIGGPLTLFKGLDEMQQMAFSQTLGAGD
YFTLAVVKLAALVIAAASGFRGGRIFPAVFIGAALGLMLHAHVEAVPAAITVSCAILGLV
LVVTRDGWLSLFMAAVVVPDTNLLPLLCIVMLPAWLLLAGKPLLAANRHEP
>EECEBICD_00942 Glucokinase
MTKYALVGDVGGTNARLALCDIASGEISQAKTYSGLDYPSLEAVVRVYLDEHSVSVEDGC
IAIACPITGDWVAMTNHTWAFSIAEMKKISALAIWKSSTISPPCRWRSRC

>EECEBICD_00943 Glucokinase
MAIPMLKKEHLIQFGGGEPVDGKPIAVYGAGTGLGVAHLVHVDKRWISLPGEGGHVDFAP
NSEEEAMILEILRAEIGHVSAERVLSGPGLVNLYRAIVKSDNRLPENLRPKDITERALAD
SCIDCRRALSLFCVIMGRFGGDLALTMGTFGGVYIAGGIVPRFLEFFKASGFRGGFEDKG
RFKDYVHGIPVYLIVHDNPGLLGSGAHLRQTLGHIL
>EECEBICD_00944 Glutamate-pyruvate aminotransferase AlaC
MADFRPERRFTRIDRLPPYVFNITAELKMAARRRGEDIIDFSMGNPDGATPPHIVEKLCT
VAQRPDTHGYSTSRGIPRLRRAISHWYQERYDVDIDPETEAIVTIGSKEGLAHLMLATLD
HGDTVLVPNPSYPIHIYGAVIAGAQVRSVPLVEGVDFFNELERAIRESYPKPKMMILGFP
SNPTAQCVELEFFEKVVALAKRYDVLVVHDLAYADIVYDGWKAPSIMQVPGARDVAVEFF
TLSKSYNMAGWRIGFMVGNKTLVSALARIKSYHDYGTFTPLQVAAIAALEGDQQCVRDIA
EQYKRRRDVLVKGLHEAGWMVEMPKASMYVWAKIPEQYAAMGSLEFAKKLLNEAKVCVSP
GIGFGDYGDTHVRFALIENRDRIRQAVRGIKAMFRADGVLPSHPKPVEASTE
>EECEBICD_00945 Lipid A biosynthesis palmitoleoyltransferase
MFPQSKFSRAFLHPRYWLTWFGVGVLWLLVQLPYPVLRFLGTRTGKLARPFLKRRESIAQ
KNIELCFPTLSREEREKLIAENFHSLGMALLETGMAWFWPDNRVRKWFDVDGLDNLTRAQ
EQNRGVMVVGVHFMSLELGGRVMGLCQPMMATYRPHNNPLMEWVQTRGRMRSNKAMIGRN
NLRGIVGALKKGEAVWFAPDQDYGPKGSSFAPFFAVENVATTNGTYVLSRLSGAAMLTVT
MVRKSDNSGYRLYITPEMEGYPADENQAAAYMNKIIEKEIMRAPEQYLWIHRRFKTRPLG
EASLYI
>EECEBICD_00946 hypothetical protein
MYHQLFTRAQKFFIKKRKYFSRSKNKLIIIFNALFLQ
>EECEBICD_00947 hypothetical protein
MIYLWTFLAISILAVSGYIGQVMGAFSAVSSFTGMVILAALIYLLNVWLQDGDDIVSGLL
LFLAPACGLIIRFMVGYGKR
>EECEBICD_00948 Phosphoglycerate transporter protein
MLTILKTGQSAHKVPPEKVQATYGRYRIQALLSVFLGYLAYYIVRNNFTLSTPYLKEQLD
LSATQIGLLSSCMLIAYGISKGVMSSLADKASPKVFMACGLVLCAIVNVGLGFSSAFWIF
AALVVFNGLFQGMGVGPSFITIANWFPRRERGRVGAFWNISHNVGGGIVAPIVGAAFAIL
GNEHWQSASYIVPACVAVIFALIVLVLGKGSPREEGLPSLEQMMPEEKVVLKTKNTAKAP
ENMSAWQIFCTYVLRNKNAWYISLVDVFVYMVRFGMISWLPIYLLTVKHFSKEQMSVAFL
FFEWAAIPSTLLAGWLSDKLFKGRRMPLAMICMALIFVCLIGYWKSESLLMVTIFAAIVG
CLIYVPQFLASVQTMEIVPSFAVGSAVGLRGFMSYIFGASLGTSLFGVMVDKLGWYGGFY
LLMGGIVCCILFCYLSHRGALELERQRQNALHNQDSLQLADAQ
>EECEBICD_00949 Phosphoglycerate transport regulatory protein PgtC
MNGITNRLFSCCSRFARVGAFTLWLAAMFGSCQAYSRELVMATTFSPSATAWIIQRWQTE
PGSVMIRTLNRTSGSLEQLLDTANAENVDLILTSSPMLLQHLQEHQKLALLDSAPAASQK
LVPRSIRSTSVAVAVSGFGLLINRSALAARHLPPPADWQDMGLPSYQGALLMSSPSRSDT
NHLMVESLLQQKGWTAGWATLLAISGNLVTISSRSFGVADKIKSGLGVAGPVIDNYANLL
LNDPNLAFTYFPYSAVSPTYVAVLKNSRHADEARAFIHYLLSPKGQRILADANTGKYPVA
PLSADNPRAAQQQRLMAQPPLNYRLILKRQQLVQRMFDTAISFRLAQLKDAWRALHSAET
RLKRPLPEIRALLTSVPVDAASSEDETWLAQFDNKSFAEQKMMEWQIWFLNNQRLAIHKL
EELK
>EECEBICD_00950 Phosphoglycerate transport system sensor protein PgtB
MKGRLLQRLRQLSISNSLRGAFLTGALLTLIVSMVSLYSWHEQSSQVRYSLDEYFPRIHS
AFLIEGNLNLAVDQLNEFLLAPNTTVRLQLRTQIIQHLDKIERLSQGLQLAERRQLAVIL
QDSRTLLAELDNALYNMFLVREKVSELSARIDWLHDDFTTELNSLVQDFTWQQGTLLDQI
EANQGDAAQYLQRSREVQNEQQQVYTLARIENQIVDDLRDRLNELKSGNNDGMLVETHIR
YLENLKKTADENIRALDDWPSTITLRQTIDELLEIGMVKNKMPDTMRDYVAAQKALLDAS
RAREATLGRFRTLLEAQLGSSHQQMQMFNQRLEQIVRVSGGLILVATLLALLLAWGLNHY
FIRSRLVKRFTALNQAVVQIGLGRTDSTIPVYGRDELGRIARLLRHTLGQLNMQRRQLEQ
EVAERKEIEADLRAMQDELIQTAKLAVVGQTMTTLAHEINQPLNALSMYLFTAGRAIEQG
QSGQARNTLTKAEGLINRIDAIIRSLRQFTRRAELETPLYPVDLRQTFVAAWELLAMRHQ
SRQGALSLPTDTVWVSGDEVRIQQVLVNVLANALDACSHDAAIAVTWQTQGEALEVYIAD

NGPGWPVALLPSLLKPFTTSKAVGLGIGLSISVSLMAQMKGDLRLASTLTRNACVVLQFS
VTDVDDVE
>EECEBICD_00951 C4-dicarboxylate transport transcriptional regulatory protein DctD
MMLNDECSILLIDDDVDVLDAYTQMLEQAGYRVRGFTHPFEAKEWVEADWEGIVLSDVCM
PGCSGIDLMTLFHQDDDQLPILLITGHGDVPMAVDAVKKGRLGFSAKTRRSGQAVDIN
>EECEBICD_00952 C4-dicarboxylate transport transcriptional regulatory protein DctD
MNQFRQRLQQLAETDIAVWFYGEHGTGRMTGARYLHQLGRNAKGPFVRYELTPENAGQLE
TFIDQAQGGTLVLSHPEYLTREQQHHLARLQSLEHRPFRLVGVGSASLVEQAAANQIAAE
LYYCFAMTQIACQSLSQRPDDIEPLFRHYLRKACLRLNHPVPEIAGELLKGIMRRAWPSN
VRELANAAELFAVGVLPLAETVNPQLLLQEPTPLDRRVEEYERQIITEALNIHQGRINEV
AEYLQIPRKKLYLRMKKYGLSKEHYKF
>EECEBICD_00953 Coagulase/fibrinolysin
MKKHAIAVMMIAVFSESVYAESTLFIPDVSPDSVTTSLSVGVLNGKSRELVYDTDTGRKL
SQLDWKIKNVATLQGDLSWEPYSFMTLDARGWTSLASGSGHMVDHDWMSSEQPGWTDRSI
HPDTSANYANEYDLNVKGWLLQGDNYKAGVTAGYQETRFSWTARGGSYIYDNGRYIGNFP
HGVRGIGYSQRFEMPYIGLAGDYRINDFECNVLFKYSDWVNAHDNDEHYMRKLTFREKTE
NSRYYGASIDAGYYITSNAKIFAEFAYSKYEEGKGGTQIIDKTSGDTAYFGGDAAGIANN
NYTVTAGLQYRF
>EECEBICD_00954 IS256 family transposase ISEc39
MDEKKLTALAAELAKGLKTEADLSQFSHMLTTLTVETALNAELADHLGHEKNAPKTSSNS
HNGYSLKTVLYDGGEIVLNMLRDRENTFELQLIKEHQTRITRWTVRFIPLCQRHDMTTRE
IIATFKEMYNADVSPMLILKITAAVKELVKGASKNGVCRSRTGRWQ
>EECEBICD_00955 hypothetical protein
MEHLKYRPDIDGLRAIAVLSVVIFHYFPSLLPGGFVGVDIFFVISGYLITSIILKSASNK
SFSYLDFYKRRVLRIFPALSIVLVSCLIVGWVYLFQDDYKLLGKHVFSGSFFISNFTLWS
ESGYFDSKSYLKPLLHLWSLGIEEQFYIIWPVVILLCFRSKNHNRNIVLSCATIFIISYA
ISIFTMASDGGANYYSPASRFWELMAGAIISTLRFIGINTSLSKLMSLLGIILIALSITM
IDEKMSFPGYIAIIPVLGASLIIASNGNDLVVSKLLSVRPVVFFGLISYPLYLWHWPIYS
FYRSIFAGSPDYHELILLLLSSFFLAILTYYLIEKPLRNARNKYITAILLALSVFGTGLI
GAFIFHINGVKDREINKSAGEYASVTDVYNYYKYGELLRGGICHSVQLTAAISNGCIKNG
KHNIFIIGDSYAAALFNGLSHYIDNKGSDYIISQMTDGNAPPLFVDGKDDLQRSVITLNN
NRINEIKRVQPEVVLLTWSVRGTNGVHDKKLAIDTLSLTIKKIKEASPDSRIIFIGPVPE
WNANLVKIISNYLSEFKKTPPLYMTYGLNSEISEWDSYFSNNVPKMGIEYISAYKALCNE
SGCLTRVGNGPDFITAVDWGHLTKPGSDFLFNKIGNKIIK
>EECEBICD_00956 Prophage bactoprenol glucosyl transferase
MKISLVVLVFNEEDTIPIFYRTVHEFNELEKYKVEIIFINDGSKDVTESIIKIIAVSDPL
VIPFSFTRNFGNDARCTTILLIFF
>EECEBICD_00957 Prophage bactoprenol-linked glucose translocase
MYGMHTHQALTNFSDFVIAVSFSFYANARFTFNASTTAIRYMMYMGFMGALSAVVGWMAD
QCSLPPLVTLITFSAISLVCGFIYSRFFIFRDKNENLSCRPSF
>EECEBICD_00959 Inner membrane protein YfdC
MDSLNDDKINRQSSDLEVESEEKQSGKEIEVDEDRLPSRAMAIHEHIRQDGEKEMERDAM
ALLWSAIAAGLSMGASLLAKGIFHVQLEGVPGGFLLENLGYTFGFIIVIMARQQLFTENT
VTAVLPVMQNPTLSNVGLLMRLWGVVLLGNLIGTGVAAWAFEYMPIFDEETRDAFVKIGM
EVMKNSPTEMFANAIISGWIIATMVWMFPAAGGAKIVVIILMTWLIALGDTTHIVVGSVE
ILYLVFNGTLPWSDFLWPFALPTLAGNICGGTFIFALMSHAQIRNDMSNKRKEEARLRGE
RLERERKKAEKQR
>EECEBICD_00960 Intermembrane phospholipid transport system lipoprotein MlaA
MKLRLSALALGTTLLVGCASSGTEQQGRSDPFEGFNRTMYNFNFNVLDPYVVRPVAVAWR
DYVPQPARNGLSNFTGNLEEPAIMVNYFLQGDPYQGMVHFTRFFLNTLLGMGGFIDVAGM
ANPKLQRVEPHRFGSTLGHYGVGYGPYMQLPFYGSFTLREDGGDMADTLYPVLSWLTWPM

SIGKWTIEGIETRAQLLDSDGLLRQSSDPYIMVREAYFQRHDFIANGGKLKPQENPNAQA
IQDELKEIDSE
>EECEBICD_00961 Long-chain fatty acid transport protein
MSQKTLFTKSALAVAVAIISTQAWSAGFQLNEFSSSGLGRAYSGEGAIADDAGNVSRNPA
LITMFDRPTFSAGAVYIDPDVNISGTSPSRRTLDADNIAPTAWVPNVHFVAPINDQFGWG
ASITSNYGLATEFNDTYAGGSVGGTTDLETMNLNLSGAYRLNEAWSFGLGFDAVYARAKI
ERFAGDLGQLVAAQNPALAPVAGQIPSDTKIAHLNGNQWGFGWNAGILYELDKNNRYALT
YRSEVKIDFKGNYSSDLPIAINRFNLPIPTATGGATQSGYLTLNLPEMWEVSGYNRVAPQ
WAIHYSLAYTSWSQFQELKAKSTAGDTLFEKHEGFKDAYRIALGTTYYYDDNWTFRTGIA
FDDSPVPAQNRSISIPDQDRFWLSAGTTYAFNKDASVDVGVSYMHGQSVKINEGPYQFES
EGKAWLFGTNFNYAF
>EECEBICD_00962 hypothetical protein
MSKCSADETPVCCCMDVGTIMDNSDCTASYSRVFATRAEAEGTLAALTEKARSVESEPCQ
ITPTFTEESEGVRLDIDFVFACEAETLIFQLGLR
>EECEBICD_00963 3-ketoacyl-CoA thiolase FadI
MRQALPLVTRQGDRIAIVSGLRTPFARQATAFHGIPAVDLGKMVVGELLARSEIPADAIE
QLVFGQVVQMPEAPNIAREIVLGTGMNVHTDAYSVSRACATSFQAVANVAESLMAGTIRA
GIAGGADSSSVLPIGVSKALARVLVDVNKARTTRQRLTLFSRLRLRDLLPVPPAVAEYST
GLRMGDTAEQMAKTYGITREQQDALAHRSHQRAAQAWAEGKLAEEVMTTYVPPYKNPFAE
DNNIRGASTLADYAKLRPAFDRKHGSVTAANSTPLTDGAAAVIMMTESRAKELGLRPLGY
LRSYAFTAIDVWQDMLLGPAWSTPLALERAGLTMADLTLFDMHEAFAAQTLANLQLLGSE
RFAREVLGRAQATGEVDDAKFNVLGGSIAYGHPFAATGARMITQTLHELRRRGGGFGLVT
ACAAGGLGAAMVLEAE
>EECEBICD_00964 Fatty acid oxidation complex subunit alpha
MTTTSAFMLNVRLDNVAVVAIDVPGEKVNTLKAEFAAQVRAILKQIRENKALQGVVFISA
KADNFIAGADINMIGHCQNAQEAETLARQGQQLMAEIQALPVPVIAAIHGACLGGGLEMA
LACHRRICTDDVKTVLGLPEVQLGLLPGSGGTQRLPRLVGVSTALDMILTGKQLRARQAL
KAGLVDDVVPQTILLEAAVELAKKERLAQRTLPVRERILAGPLGRALLFRLVRKKTAQKT
QGNYPATERIIDVIETGLAQGSSSGYDAEARAFGELAMTPQSQALRAVFFASTEVKKDPG
SDAPPGPLNSVGILGGGLMGGGIAWVTACKGGLPVRIKDINTQGINHALKYSWDLLETKV
RRRHIKANERDKQLALISGSTDYRGFSHRDLVIEAVFEDLPLKQQMVAEVEQNCAAHTIF
ASNTSSLPIGDIAANAARPEQVIGLHFFSPVEKMPLVEVIPHASTSAQTIATTVKLAKKQ
GKTPIVVSDKAGFYVNRILAPYINEAIRMLTEGERVEHIDAALVKFGFPVGPIQLLDEVG
IDTGTKIIPVLEAAYGERFSAPANVVASILNDDRKGRKNGRGFYLYGEKGRKSKKQVDPA
IYKLIGVQGQSRLSAQQVAERCVMLMLNEAARCFDEKVIRSARDGDIGAVFGIGFPPFLG
GPFRYMDALGPGEMVATLQRLAALYGPRYAPCEQLVRMAERREHFWTNGETDQGN
>EECEBICD_00965 Phosphohistidine phosphatase SixA
MQVFIMRHGDAALDAASDSVRPLTPCGCDESRLMANWLKGQKVDIERVLVSPFLRAEQTL
DVVGDCMNLPAQVDVLPELTPCGDVGLVSAYLQALANEEIGSVLVISHLPLVGYLVSELC
PGETPPMFTTSAIANVTLDESGKGTFNWQMSPCNLKMAKAI
>EECEBICD_00966 Endonuclease MutS2
MRRGDYSPELFLDLHGLTQLQAKQELGALIAACRREHIFCACVMHGHGKHILKQQTPLWL
AQHPHVMAFHQAPKEYGGDAALLVLIEVEEWQPPELP
>EECEBICD_00967 hypothetical protein
MKKKTSLSEEDQALFRQLMVGTRKIKQDTIVHRPLRKKITEVPTRRLIQEQADASHYFSD
EFQPLLNTEGPVKYVREDVSHFELKKCVAATIRQNCFWIYTA
>EECEBICD_00968 50S ribosomal protein L3 glutamine methyltransferase
MDKIFVDEAVSELHTIQDMLRWAVSRFSAANIWYGHGTDNPWDEAVQLVLPSLYLPLDIP
EDMRTARLTSSEKHRIVERVIRRINERIPVAYLTNKAWFCGHEFYVDERVLVPRSPIGEL
INNHFAGLISQQPKYILDMCTGSGCIAIACAYAFPDAEVDAVDISPDALAVAEHNIEEHG
LIHHVTPIRSDLFRDLPKVQYDLIVTNPPYVDAEDMSDLPNEYRHEPELGLASGTDGLKL
TRRILGNAPDYLSDDGVLICEVGNSMVHLMEQYPDVPFTWLEFDNGGDGVFMLTKAQLLA
AREHFNIYKD
>EECEBICD_00969 Chorismate synthase

MAGNTIGQLFRVTTFGESHGLALGCIVDGVPPGIPLTEADLQHDLDRRRPGTSRYTTQRR
EPDQVKILSGVFDGVTTGTSIGLLIENTDQRSQDYSAIKDVFRPGHADYTYEQKYGLRDY
RGGGRSSARETAMRVAAGAIAKKYLAEKFGIEIRGCLTQMGDIPLEIKDWRQVELNPFFC
PDADKLDALDELMRALKKEGDSIGAKVTVMASGVPAGLGEPVFDRLDADIAHALMSINAV
KGVEIGEGFNVVALRGSQNRDEITAQGFQSNHAGGILGGISSGQHIVAHMALKPTSSITV
PGRTINRMGEEVEMITKGRHDPCVGIRAVPIAEAMLAIVLMDHLLRHRAQNADVKTEIPR
W
>EECEBICD_00970 Penicillin-insensitive murein endopeptidase
MKKTAIALLAWFVSSASLAATPWQKITHPVPGAAQSIGSFANGCIIGADTLPVQSDNYQV
MRTDQRRYFGHPDLVMFIQRLSHQAQQRGLGTVLIGDMGMPAGGRFNGGHASHQTGLDVD
IFLQLPKTRWSQAQLLRPQALDLVSRDGKHVVPSRWSSDIASLIKLAAQDNDVTRIFVNP
AIKQQLCLDAGNDRDWLRKVRPWFQHRAHMHVRLRCPADSLECEDQPLPPPGDGCGAELQ
SWFEPPKPGTTKPEKKTPPPLPPSCQALLDEHVL
>EECEBICD_00971 putative membrane transporter protein YfcA
MDNFYDLFMVSPLLLVVLFFVAVLAGFIDSIAGGGGLLTIPALMAAGMSPANALATNKLQ
ACGGSLSSSLYFIRRKVVNLAEQKLNILMTFIGSMSGALLVQHVQADILRQILPILVIFI
GLYFY
>EECEBICD_00972 putative membrane transporter protein YfcA
MPKLGEEDRQRRLYGLPFALIAGGCVGFYDGFFGPAAGSFYALAFVTLCGYNLAKSTAHA
KVLNATSNVGGLLLFIIGGKVIWATGFVMLVGQFLGARMGSRLVLSKGQKLIRPMIVIVS
AVMSARLLYDSHGQEILHWLGMN
>EECEBICD_00973 Elongation factor P hydroxylase
MNNTHHYEQLIEIFNGCFAEEFNTRLIKGDDEPIYLPADAQVPYHRIVFAHGFYASALHE
ISHWCIAGKARRELVDFGYWYCPDGRDAQTQSQFEDVEVKPQAFDWLFCVAAGYPFNVSC
DNLEGDIEPDRVAFQRRVHAQVMAYLEQGIPERPARFIKALQNYYHTPELKAEQFPWPEA
LN
>EECEBICD_00974 hypothetical protein
MIAEFESRILALIDDMVEHASDDELFASGYLRGHLTLAIAELESGDDHSVEAVYANVSQS
LEKAIGAGELSPRDQALVKAMWDNLFDKAKQ
>EECEBICD_00975 tRNA 5-methylaminomethyl-2-thiouridine biosynthesis bifunctional protein MnmC
MKQYAIQPATLEFNAEGTPVSRDFDDVYFSNDNGLEETRYVFLGGNRLAERFPVHSHPLF
IVAESGFGTGLNFLTLWQAFDSFRSAHPQATLQRLHFISFEKFPLTRDDLALAHQHWPEL
APWAEQLQAQWPLPLPGCHRLLLDRGRVTLDLWFGDINELTDQLDATLNQTVDAWFLDGF
APAKNPDMWTPNLFNAMARLARPGATLATFTSAGFVRRGLQEAGFTMQKRKGFGRKREML
CGVMEQHLMPTLSAPWFYRSGSEKRETAIIGGGIASALLSLALLRRGWQVTLYCADDQPA
QGASGNRQGALYPLLSKHDAAINRFFPTAFTFARRLYDALPVSFDHDWCGVTQLGWDEKS
QQKIAQMLSLALPAELASALNAEEAEQAVGVTTRCGGITYPAGGWLCPEQLTRAVIALAT
EQGLQTRFRHTLTSLVAQESRWQLRFMSGETASHETVVLANGHQINRFDQTRPLPVYAVG
GQVSHIPTTPALSALRQVLCYDGYLTPQNPHNQQHCIGASYHRGDESTVWREEDQRQNRQ
RLLDCFPDAKWATEVDVSGNSARCGVRCATRDHLPMVGNVPDYHATLTHYADLADNKTSA
APAPVYPGLFVLGALGSRGLCSAPLCAEILAAQMSNEPIPLDAGTLAALNPNRLWVRKLL
KGKAVK
>EECEBICD_00976 3-oxoacyl-[acyl-carrier-protein] synthase 1
MKRAVITGLGIVSSIGNNQQEVLASLREGRSGITFSQELKDAGMRSQVWGNVKLDTTGLI
DRKVVRFMSDASIYAYLSMEQAVADAGLAPEVYQNNPRVGLIAGSGGGSPKFQVFGADAM
RSPRGLKAVGPYVVTKAMASGVSACLATPFKIYGVNYSISSACATSAHCIGNAVEQIQLG
KQDIVFAGGGEELCWEMACEFDAMGALSTKYNDTPEKASRTYDAHRDGFVIAGGGGMVVV
EELEHALARGAHIYAEIVGYGATSDGADMVAPSGEGAVRCMQMAMHGVDTPIDYLNSHGT
STPVGDVKELGAIREVFGDNSPAISATKAMTGHSLGAAGVQEAIYSLLMLEHGFIAPSIN
IEELDEQAAGLNIVTETTERELTTVMSNSFGFGGTNATLVMRKL
>EECEBICD_00977 hypothetical protein
MPTNTLDKIRHSLSCVAVLFGLFGIFVFASFSPSYAWLYLGGLAAPFIYSIVFVYAIAAW
SIYSKYYPFLSLGRLSFVECFVPALALVCLTVLYNVFSGPEPWMAELSRQFFLHKFLNTL

AMCFLAPVAEEIIFRGFLLNSSIGWGRYSRASGIIITSLAFAFMHTQYLFAVTFVYLFVF
SSILCVVRMRSRGLMIPIILHILNNAWVIFGLLFSATE
>EECEBICD_00978 hypothetical protein
MKTFSLVALILLLCSCSTPHHDSTQAVKQFYTSWMTTFTNDVNPPDDTTALMQRYVAKEV
IHRLALIQSLYEQEIVGADYFMYAQDYAPEWIPQLRVGKAHPFLGGEKVDVLLATESTPI
HLEVYTRWEEGRWKIYRVRDADRGYEQPIYDAGAITQAEAWSAKVAPEYKKH
>EECEBICD_00979 hypothetical protein
MSWDKRVAVNYAKTHAGSHSQGRCAEFTRKAIQAGGITLGHTYHAKDYGPMLRSAGFTAI
GTYEMPREGDVIIIQPYAGGNPSGHMAIYDGTEWYSDFKQRDMWAGPGYRAARPSYTIYR
KN
>EECEBICD_00980 hypothetical protein
MEPKSQDWHRADIKSALEKRGITLRDLSRQAGLSPDSLRNVFTRSWPRAERIIADALGIT
PQEIWPSRYDDMQIKNDADIAE
>EECEBICD_00981 hypothetical protein
MAKEWFTVKECLGLPGFPGSEPAVRERLYKYSEGKAGVRRKRVKSKAEEFHISVFPLYVH
RYLDDSGEEPTPEPISLQEAEPEDIWEMMFRLLTPEQRKQVTGRFKVRGMKAVFPFLFDD
TPPR
>EECEBICD_00982 putative MFS-type transporter YfcJ
MTAVSQKTTTPSANFSLFRIAFAVFLTYMTVGLPLPVIPLFVHHELGYSNTMVGIAVGIQ
FFATVLTRGYAGRLADQYGAKRSALQGMFACGLAGAAWLLAALLPVSAPIKFALLIVGRL
ILGFGESQLLTGTLTWGLGLVGPTRSGKVMSWNGMAIYGALAAGAPLGLLIHSHGFAALA
GTTMVLPLLAWAFNGTVRKVPAYTGERPSLWSVVGLIWKPGLGLALQGVGFAVIGTFISL
YFVSNGWTMAGFTLTAFGGAFVLMRILFGWMPDRFGGVKVAVVSLLVETAGLLLLWLAPT
AWIALVGAALTGAGCSLIFPALGVEVVKRVPAQVRGTALGGYAAFQDISYGVTGPLAGML
ATSCGYPSVFLAGAISAVVGILVTILSFRRG
>EECEBICD_00983 Flagellar regulator flk
MHPISGAPAQPPGEGRNPLSAASEQPLSMQQRTVLERLITRLISLTQQQSAEVWAGMKHD
LGIKNDAPLLSRHFPAAEQNLTQRLGVAQQNHANRQVLSQLTELLGVGNNRQAVSDFIRQ
QYGQTALSQLTPDQLKNVLTLLQQGQLSIPQPQQRPATDRPLLPAEHNTLNQLVTKLAAA
TGESNKLIWQSMLELSGVKSGELIPAKQFTHLATWLQARQTLSLQHAPTLHTLQAALKQP
LEPDELTAIKEYAQHTYQIQPQTVLTTAQVQDLLNHIFLRRVERETDELEPLSIQPIYRP
FAPMIETVKNLSARPGLLFIALIIVLALFWLVS
>EECEBICD_00984 Erythronate-4-phosphate dehydrogenase
MKILVDENMPYARELFSRLGEVKAVPGRPIPVEELNHADALMVRSVTKVNESLLSGTPIN
FVGTATAGTDHVDEAWLKQAGIGFSAAPGCNAIAVVEYVFSALLMLAERDGFSLRDRTIG
IVGVGNVGSRLQTRLEALGIRTLLCDPPRAARGDEGDFRTLDELVQEADVLTFHTPLYKD
GPYKTLHLADETLIRRLKPGAILINACRGPVVDNAALLARLNAGQPLSVVLDVWEGEPDL
NVALLEAVDIGTSHIAGYTLEGKARGTTQVFEAYSAFIGREQHVALETLLPAPEFGRITL
HGPLDQPTLKKTGAFGV
>EECEBICD_00985 USG-1 protein
MSEGWNIAILGATGAVGEALLETLAERQFPVGEIYALARHESAGEHLRFGGKSVIVQDAA
DFDWTQAQLAFFVAGAEASAAWIDDATNAGCLVIDSSGLFALEPDVPLVVPEVNPYVLAD
YRNRNVIAVADSLTSQLLAALKPLIDQGGLSRIAVTSMLSASAQGKKAVDALAGQSAKLL
NGIPIDEDDFFGRQLAFNMLPLLPDREGSVRQERRIVDEVRKILQDDGVMISASVVQSPV
FYGHAQMVSFEALRPLAAEEAREAFSRGEDIVLSEETDYPTQVGDASGNPQLSIGCVHND
YGMPEQIQFWSVADNVRFGGALMAVKIAEKLVQEYLY
>EECEBICD_00986 tRNA pseudouridine synthase A
MSGQQSSPVYKIALGIEYDGSKYYGWQRQNEVRSVQEKLEKALSQVANEPINVFCAGRTD
AGVHGTGQVVHFETTALRKDAAWTLGVNANLPGDIAVRWVKTVPDDFHARFSATARRYRY
IIYNHRLRPAVLAKGVTHYYEPLDAERMHRAAQCLLGENDFTSFRAVQCQSRTPWRNVMH
INVTRHGPYVVVDIKANAFVHHMVRNIVGSLLEVGAHNQPESWIAELLAARDRTLAAATA
KAEGLYLVAVDYPDRFDLPKPPMGPLFLAD
>EECEBICD_00987 Protein DedA
MDLIYFIIDFILHIDVHLAELVAEYGVWVYAILFLILFCETGLVVTPFLPGDSLLFVAGA

LASLETNDLNVHVMVALMLIAAIVGDAVNYTIGRLFGEKLFSNPNSKIFRRSYLDKTHQF
YEKHGGKTIILARFVPIVRTFAPFVAGMGHMSYRHFAAYNVIGALLWVLLFTYAGYFFGT
IPLIQDNLKLLIVGIIVVSILPGVIEIVRHKRAASRAAK
>EECEBICD_00988 Acetyl-coenzyme A carboxylase carboxyl transferase subunit beta
MSWIERIKSNITPTRKASIPEGVWTKCDSCGQVLYRAELERNLEVCPKCDHHMRMSARNR
LHSLLDEGSLVELGSELEPKDVLKFRDSKKYKDRLASAQKETGEKDALVVMKGTLHGMPV
VAAAFEFAFMGGSMGSVVGARFVRAVEQALEDNCPLVCFSASGGARMQEALMSLMQMAKT
SAALAKMQERGLPYISVLTDPTMGGVSASFAMLGDLNIAEPKALIGFAGPRVIEQTVREK
LPPGFQRSEFLIEKGAIDMIVRRPEMRLKLASILAKLMNLPAPNPDAPREGVVVPPAPDQ
ESEA
>EECEBICD_00989 Dihydrofolate synthase/folylpolyglutamate synthase
MKNKSIPQAASPLASWLSYLENLHSKSIDLGLERVSQVAARLDILKPAPFVFTVAGTNGK
GTTCRTLESVLIAAGYRVGVYSSPHLVRYTERVRVQGKELAESAHTASFAEIEAARGDIS
LTYFEYGTLSALWLFKQANLDVVILEVGLGGRLDATNIVDADVAVITSIALDHTDWLGPD
RESIGREKAGIFRAEKPAIVGEPEMPTTIADVAQETGALLRRRGVDWRYEVTATHWAFTD
GDGTLAGLPLPQVPQPNAATALAALRASRLNIDEQAIRDGIAQATLPGRFQIVSESPRVI
FDVAHNPHAAEYLTGRLKMLPKRGRVLAVIGMLHDKDIAGTLAWLKSVVDDWYCAPLEGP
RGATAEQLLEHLGKGNVYDSVAQAWQAAIDAAQPEDTVLVCGSFHTVAHVMEVIDAGRIG
GE
>EECEBICD_00990 Cell division protein DedD
MASKFQNRLVGTIVLVALGVIVLPGLLDGQKKHYQDEFAAIPLVPKPGDRDEPDMMPAAT
QALPTQPPEGAAEEVRAGDAAAPSLDPSRMASNNVELDPIPAETPKPKPVEKPKPQPKPQ
QPVVAASTPTPAPQPVADDKPAPTGKAYVVQLGALKNADKVNEIVGKLRSAGFRVYTSPS
TPVQGKITRILVGPDASKDKMKGSLGELKQISGLSGVVMGYSPN
>EECEBICD_00991 Colicin V production protein
MVWIDYAIIAVIAFSCLVSLIRGFVREALSLVTWGCAFFVASHYYTYLSVWFTGFEDELV
RNGIAIAVLFIATLIVGAIVNFVIGQLVEKTGLSGTDRVLGICFGALRGALIVAAILFFL
DTFTGLSKSEDWSKSQLIPQFSFIIRWFFDYLQSSSSFLPRT
>EECEBICD_00992 Amidophosphoribosyltransferase
MCGIVGIAGVMPVNQSIYDALTVLQHRGQDAAGIIITIDANNCFRLRKANGLVNDIFEARH
MQRLQGNMGIGHVRYPTAGSSSASEAQPFYVNSPYGITLAHNGNLTNAHELRKKLFEEKR
RHINTTSDSEILLNIFASELDNFRHYPLEADNIFAAIAATNRQIRGAYACVAMIIGHGMV
AFRDPHGIRPLVLGKRDVGDGRTEYMVASESVALDTLGFEFLRDVAPGEAIYITEKGQLF
TRQCADNPVSNPCLFEYVYFARPDSFIDKISVYSARVNMGTKLGEKIAREWEDLDIDVVI
PIPETSCDIALEIARILGKPYRQGFVKNRYVGRTFIMPGQQLRRKSVRRKLNANRAEFRD
KNVLLVDDSIVRGTTSEQIIEMAREAGAKKVYLASAAPEIRFPNVYGIDMPTANELIAHG
REVDEIRQIIGADGLIFQDLNDLIEAVRAENPDIQQFECSVFNGVYVTKDVDQQYLDFLD
SLRNDDAKAVLFQNEMENLEMHNEG
>EECEBICD_00993 Arginine utilization regulatory protein RocR
MASTNQELASALRMFSRFFDLIHQPLAVINERGEYVYYNQESADLDGYSIERAMGKHMLD
VYPGMKETQSTMLSSLKKGVEYIGHYQIYYNARGQAVDYQHTTAPLYASDGGMVGVIEIG
RNMSGVRRLQEQVVELNQLLYADRHEKHHAIITENPEMLSNIAKAKRLAASNIPVTIVGE
TGTGKELFSRLIHQCSKRANKPFIALNCGALPPTLIESTLFGTVRGAYTGAENSQGYLEL
ANGGTLFLDELNAMPIEMQSKLLRFLQDKTFWRLGGQQQLHSDVRIVAAMNEAPVKLIQQ
ERLRADLFYRLSVGMLTLPPLRARPEDIPLLANYFIDKYRNDVPQDIHGLSETARADLLN
HAWPGNVRMLENAIVRSMIMQEKDGLLKHIIFEQDELNLGVPETAPENPLPSSPDPQYEG
SLEVRVANYERHLIETALDTHQGNIAAAARSLNVSRTTLQYKVQKYAIRFGVVRN
>EECEBICD_00994 Diaminopimelate decarboxylase
MTDSIMQNYNQLREQVINGDRRFQHKDGHLCFEGVDLDALARQYPTPFYVFSEPEIIRNI
HEIQQAFAAHKNTKTFFASKTCSVMGVLKAIRDAGICAEANSQYEVRKCLEIGFRGDQIV
FNGVVKKPADLEYAIANDLYLINVDSLYELEHIDAISRKLKKVANVCVRVEPNVPSATHA
ELVTAFHAKSGLDLEQAEETCRRILAMPYVHLRGLHMHVGDQVPESEPFAKATKVLVDES
RRLEEVLGIKFDLINVGGGIPVPYKYDDENGDPLKDNMYAGITAQDFADAVIREVHKWRT

DVEICIEPGRKVTGSAAVLLTEVSCEKRKTNYDLNGNVECHVEWKFVDAGYSVLSDSQHF
DWFFYVYNASRMTAAHDAWVKLAGPLCDGGDYFHMGVKGEEFLLPKETHVGDIVAFLDAG
AYTIESQTVYNNRPRTGVVMIDKNGETRLIRREDSYEDMVKYDIY
>EECEBICD_00995 Glutamate/gamma-aminobutyrate antiporter
MSNTNSGLLGIKDIVFMNVIAILSLRQIPNVAPYGASAMLLWVIAAFCLFFPLAMVCGEL
STGWPKDGGIFVWIKEAFGKRIAWIVVVCFLFSCVLFFPLMLQFGFTALGYMIGGGLAEN
KAFIGIGSAVIFWLLTLMNIRGMEWTKIINSISAWCGVFIPSAILILLAVVWLCTGHQMQ
TDYTTAKNWIPDLGHWDTIVFLSSMMFAFAGLEVAPMIAGRTRNPQRDFPRAMAVSAAVI
VGIYMVGTWALNTLLPAGKTDIVAGVMQAMHAAADTLHMPWLIPVMAICMFFGALGQINS
WLVGPIYMLQEASREDNLLGDRIGKLHPVWKTPAFALTVQAIIVTVLCFSTFISPSVAAA
YWMLTALTTITYFIPYLVMFPAFWRLRKTQPDTPRSFKIPGKVLPAILPALGFLSIAFAV
ALLFIPPSQIDMGGYFQYAGKIIGGAVLAVVVAEYIYHRAQKRNARLSMAGGK
>EECEBICD_00996 Ornithine racemase
MYMPVLEINLRKLEENARTEKALLASSGIDVMAVNKVFDGCVETAQAVFNGGITVIAESR
TYNLKKIRETGCTTCLLRSPCLSEIEDVVRYADISLNSEPVVLRALSQEAQRQGKTHQVL
LMVDMGDLREGIWFGEYQRILETVTLITGLPGLELYGLGTNFNCYGTVLPTVKNGEDFLA
LAARLEADSGIPVRRLSAGNCTSYHLLDKGIWPQGLNHLRIGGLHEFGIEYVDMKYLNEF
HHSAKPVDKACSDMYILEAEIIELNSKPTVPVGELGVDAFLQSKTFVDRGIRRRALLAFG
RQDVPSDNCVPCDDAITILGQTSDHTLVDIEDCRQSLKVGDVVRFELDYTGLLMACQTKG
VAWRFTR
>EECEBICD_00997 Amino-acid permease RocC
MDTLQQSPPAEPEGGLRRNLKKRHLLMMSLGGTIGTGLFIGIAEPLSSVGPAGTLLAYLF
AGTIMLATMMCLGELSCAFPHSGSFQHYALMFMPSPCWSYTIGWLYWFSWVFSLAADLTA
AGFIAHQFFPAVPVYMFCLAILLILTAINLTSAKSFGECEYWLSAIKVFAIVLFICAGGV
MIYSLMGHSDWHPTLKIDGMWFPHGWEQIVVCMTIVIYSFQGWNWSEMRQGRPNRRTLFY
RK
>EECEBICD_00998 Amino-acid permease RocE
MILGIGLRIILFYGLAIAVLALVYPHELTPNGQSPFVWVFSHAGIPGADTLMTLVIFSAA
VSAANSAIYASSRMLWSMAGDRFAPACFGKTNGGGVPVYAILITALLALVSLLTRYIPAQ
QFYLYLIASTGQVGCLAWITIGWCQYRFRQSVRNGTYASDLLRYRSPLFPWTARFVIITN
FAIMVGTWFSEQGVVIMLVELAFMIGILLSWYLFRPTLSRLRNTVG
>EECEBICD_00999 Flavin prenyltransferase UbiX
MKRLIVGISGASGAIYGVRLLQILRDVDSVETHLVMSQAARQTLALETHFSLREVQALAD
VTHDARDIAASISSGSYPTAGMVILPCSIKTLSGIVHSYTDGLLTRAADVILKERRPLVL
CVRETPLHIGHLRLMTQAAEIGAVIMPPVPAFYHLPQTLDDVINQTVNRVLDQFDIPLPH
DLFVRWQGA
>EECEBICD_01000 Lysine/arginine/ornithine-binding periplasmic protein
MKKTVLALSLLIGLGATAASYAALPQTVRIGTDTTYAPFSSKDAKGEFIGFDIDLGNEMC
KRMQVKCTWVASDFDALIPSLKAKKIDAIISSLSITDKRQQEIAFSDKLYAADSRLIAAK
GSPIQPTLESLKGKHVGVLQGSTQEAYANDNWRTKGVDVVAYANQDLIYSDLTAGRLDAA
LQDEVAASEGFLKQPAGKEYAFAGPSVKDKKYFGDGTGVGLRKDDTELKAAFDKALIELR
QDGTYDKMAKKYFDFNVYGD
>EECEBICD_01001 Histidine-binding periplasmic protein
MKKLALSLSLVLAFSSATAAFAAIPQKIRIGTDPTYAPFESKNAQGELVGFDIDLAKELC
KRINTQCTFVENPLDALIPSLKAKKIDAIMSSLSITEKRQQEIAFTDKLYAADSRLVVAK
NSDIQPTVASLKGKRVGVLQGTTQETFGNEHWAPKGIEIVSYQGQDNIYSDLTAGRIDAA
FQDEVAASEGFLKQPVGKDYKFGGPAVKDEKLFGVGTGMGLRKEDNELREALNKAFAEMR
ADGTYEKLAKKYFDFDVYGG
>EECEBICD_01002 Histidine transport system permease protein HisQ
MLYGFSGVILQGAIVTLELALSSVVLAVLIGLVGAGAKLSQNRVTGLIFEGYTTLIRGVP
DLVLMLLIFYGLQIALNVVTDSLGIDQIDIDPMVAGIITLGFIYGAYFTETFRGAFMAVP
KGHIEAATAFGFTHGQTFRRIMFPAMMRYALPGIGNNWQVILKATALVSLLGLEDVVKAT
QLAGKSTWEPFYFAVVCGLIYLVFTTVSNGVLLLLERRYSVGVKRADL
>EECEBICD_01003 Histidine transport system permease protein HisM

MIEIIQEYWKSLLWTDGYRFTGVAITLWLLISSVVMGGLLAVILAVGRVSSNKFIRFPIW
LFTYIFRGTPLYVQLLVFYSGMYTLEIVKGTDLLNAFFRSGLNCTVLALTLNTCAYTTEI
FAGAIRSVPHGEIEAARAYGFSSFKMYRCIILPSALRIALPAYSNEVILMLHSTALAFTA
TVPDLLKIARDINSATYQPFTAFGIAAVLYLLISYVLISLFRRAERRWLQHVSSK
>EECEBICD_01004 Histidine transport ATP-binding protein HisP
MSENKLHVIDLHKRYGGHEVLKGVSLQARAGDVISIIGSSGSGKSTFLRCINFLEKPSEG
AIIVNGQNINLVRDKDGQLKVADKNQLRLLRTRLTMVFQHFNLWSHMTVLENVMEAPIQV
LGLSKHDARERALKYLAKVGIDERAQGKYPVHLSGGQQQRVSIARALAMEPDVLLFDEPT
SALDPELVGEVLRIMQQLAEEGKTMVVVTHEMGFARHVSSHVIFLHQGKIEEEGDPEQVF
GNPQSPRLQQFLKGSLK
>EECEBICD_01005 Epimerase family protein
MQILITGGTGLIGRHLIPRLLTLGHQVTVVTRNPDNARQILDSRVTLWKGLAEREHLNEI
DAIINLAGEPIADKRWTSQQKERLCQSRWAITQKLVDLIHASATPPSVLISGSATGYYGD
LGDVVVTEDEPPHNEFTHKLCARWEQIACRAQSDQTRVCLLRTGVVLAPQGGILGKMVPP
FRLGLGGPVGNGRQYLAWIHIDDMVNGILWLLDNDLRGPFNMVSPYPVHNEQFAHALGRA
LRRPAIIRIPATAIRLLMGESSVLVLGGQRALPKRLEAAGFAFRWYDLEEALADVIR
>EECEBICD_01006 Disulfide-bond oxidoreductase YfcG
MIDLYYAPTPNGHKITLFLEEAELAYRLLKVDISKGNQFRPDFLAISPNNKIPAIVDHAP
ADGGQPLSLFESGEILLYLAEKSGKLLSGELRERHTTLQWLFWQVGGLGPMLGQNHHFNH
FAPQAIPYAIERYQVETQRLYNVLNKRLETSPWLGGDHYSIADIASWPWVNAHQRQRIDL
DTYPAVYNWFERIRTRPATARALLQAQLHCNSTKA
>EECEBICD_01007 Glutathione S-transferase YfcF
MSKPVIVLWSDANFFSPYVLSAWVALQEKGLSFTLKTRDLDQGEHLQPGWRGYALTQRVP
VLEADNFELSESSAIAEYLEERFAPPQWERIYPHDLQKRARARQIQAWLRSDLLPLREER
PTDVVFAGAKKAPLSEAGKASAAKLFATAEALLGQGTQNLFGEWCIADTDLALMINRLAL
HGDDVPTSLAAYATFQWQRASVQRFIALSSKRSG
>EECEBICD_01008 Phosphodiesterase YfcE
MKLMFASDIHGSLPATERVLERFAQSGARWLVILGDVLNHGPRNALPEGYAPAQVAERLN
AVATQIIAVRGNCDSEVDQMLLHFPITAPWQQILTQERRLFLTHGHLFGPTNLPALHTGD
VLVYGHTHLPVAQQQEGLYHFNPGSVSIPKGGYAASYGILDDNVLSVIALNDQSIIAQVA
INS
>EECEBICD_01009 putative Nudix hydrolase YfcD
MVEQRRLASTEWVDIVNEDNEVIAQSSREQMRAQRLRHRATYIVVHDGMGKILVQRRTET
KDFLPGMLDATAGGVVQADEQLLESARREAEEELGIAGVPFAEHGLFYFEDQHCRVWGAL
FSCVSHGPFALQEDEVSEVCWLTPEEITARCDEFTPDSLKALALWMTRNAKNEAALQEKP
EETE
>EECEBICD_01010 HTH-type transcriptional regulator GntR
MSIPRKRRSTGKVTIADVAQLAGVGTMTVSRALRTPEQVSDKLREKIEAAVHELGYMPNL
AASALASASSHTIAMVVPNLAEAGCSEMFAGLQQILQPAGYQIMLAESQHRVEQEEKLLE
TLLASNIAAAILLSVEHSTTVRQWLKNASIPVMEMGAIRSDPIDMNIGIDNVAAMYELTE
MLIQRGYQNIGLLCANQEQWIFQQHLHGWYKAMLRHHMSPNRVINAALPPNFSTGASQLP
EFLLAWPELDALVCVSDELACGALYECQRRRIKVPDDLAVVGFGDSDVSRVCQPPLTTMA
VPHRKIGSEAGRALLERLNQGNWSDRKSIASSLCMRESC
>EECEBICD_01011 Ascorbate-specific PTS system EIIA component
MLGTWLSDATITLRESVETWSQALEICGKPLLDAGVIAPEYITAIVQQHQKLGPYYVLAP
GLAMPHARPEEGAKGLGLSLLKLQHGVSFGADEFDPVDIIIMLAAPDKHSHIEMISALAE
LFSSDVDMEKLHQAKNLEDIKTIIDRF
>EECEBICD_01012 hypothetical protein
MKIMAICGSGLGSSFMVEMNIKKVLKKLNIDAEVEHSDLSSATPGAADLFVMAKDIAASA
SVPESQLVVINNIIDINELETQLRAWFAKQ
>EECEBICD_01013 Ascorbate-specific PTS system EIIC component
MFILETLNFVVDILKVPSVLVGLIALIGLVAQKKAFSDVVKGTIKTILGFIVLGGGATVL
VGSLNPLGGMFEHAFNIQGIIPNNEAIVSIALEKYGASTALIMAFGMVANIVVARFTRLK
YIFLTGHHTFYMACMIGVILTVAGFEGVGLVFTGSLILGLVMAFFPALAQRYMRRITGTD

DIAFGHFGTLGYVLSGWIGSLCGKGSRSTEEMNLPKNLSFLRDSSISISLTMMIIYLIMA
VSAGREYVEATFSGGQNYLVYAIIMAITFAAGVFIILQGVRLILAEIVPAFTGFSEKLVP
NARPALDCPVVYPYAPNAVLIGFLFSFLGGLVGLFLLGQMKLVLILPGVVPHFFTGATAG
VFGNATGGRRGAMIGAFANGLLITFLPVLLLPVLGAIGFANTTFSDADFGVIGILLGNLA
RYLSPMAITGLVVALFALLVAYNVLAKNKKATAEVQENSGAKE
>EECEBICD_01014 Apulose-4-phosphate transketolase subunit A
MNVTEITQLARDIRVATLKSLNHLGFGHYGGSMSVVETLAVLYGAVMKIDPADPDWPERD
YFVLSKGHAGPALYSTLAIKGYFPREELNTLNQNGTRLPSHPDRLKTRGVDATTGSLGQG
ISIAGGMALSHKLARRPNRVFCIVGDGELNEGQCWEAFQFIAHHRLNNLTVFIDWNKQQL
DGELEEIINPFDLEGKFRAFGFDVVTVKGDDIAGLLAVVQPVLPADARPRVVILDSIKGQ
GVPYLEQLTNSHHLRLTDGMKQTLNEAIHQLEVMHD
>EECEBICD_01015 Apulose-4-phosphate transketolase subunit B
MIKLAPAGLKDDIEMRKVYAGFVAGQIEAGSPIIALEADLMSSMAMDSVARDYPQHVINC
GIMEANVIGTAAGLALTGRKPFVHTFTAFASRRCFDQLFMALDYQRNNVKVIASDAGVTA
CHNGGTHMSFEDMGIVRGLAHSVVLEVTDAVMFADILRQLMDLDGFYWLRTIRKQATSIY
APGSTFTIGKGNVLREGDDITLIANGIMVAEALEAARQLEQEGVSAAVIDMFTLKPIDRM
LVKNYAEKTRRIVTCENHSIHNGLGSAVAEVLVENCPVPMRRVGVKERYGQVGTQDFLQQ
EYGLTAEAIVEAAKSLL
>EECEBICD_01016 hypothetical protein
MSVVTESKTARKWAMPDTLVIIFFVAILTSIATWVVPVGMFDSQEVQYQVDGQTKTRKVV
DPHSFRIVTNEAGEAQYHRVQFFTTGDERPGLMNFPFEGLTSGSKFGTAVGIIMFMLVIG
GAFGIVMRTGTVDNGILALIRHTRGNEVLFIPVLFVLFSLGGAVFGMGEEAVAFAIIIAP
LMVRLGYDSITTVLVTYIATQIGFASSWMNPFCVVVAQGIAGVPVLSGSGLRIVVWIVAT
LIGLVFTLVYASRVKKNPLLSRVHESDRYFREQQDEVVQRPFTFGDWLVLLVLTGVMIWV
VWGVIVHAWFIPEIASQFFTMGVVIGLIGVIFRLNGMTVNVMASSFTEGARMMIAPALLV
GFAKGILLLVGNGEAGEPSVLNTLLNSIAHGISGLNNAIAAWFMLLFQAVFNFFVTSGSG
QAALTMPLLAPLGDLVGVNRQVTVLAFQFGDGFSHIIYPTSASLMATLGVCRVDFRNWLK
VGASLLGLLFIMSSVVVIGAQMMGYH
>EECEBICD_01017 Phosphate acetyltransferase
MSRIIMLIPTGTSVGLTSVSLGVIRAMERKGVRLSVFKPIAQPRAGGDAPDQTTTIVRAN
STLPAAEPLKMSHVESLLSSNQKDVLMEEIIANYHANTKDAEVVLVEGLVPTRKHQFAQS
LNYEIAKTLNAEIVFVMSQGTDTPEQLNERIELTRSSFGGAKNTNITGVIINKLNAPVDE
QGRTRPDLSEIFDDSSKAQVIKIDPAKLQESSPLPVLGAVPWSFDLIATRAIDMARHLNA
TIINEGDIKTRRVKSVTFCARSIPHMLEHFRAGSLLVTSADRPDVLVAACLAAMNGVEIG
ALLLTGGYEMDARISKLCERAFATGLPVFMVNTNTWQTSLSLQSFNLEVPVDDHERIEKV
QEYVANYVNAEWIESLTATSERSRRLSPPAFRYQLTELARKAGKRVVLPEGDEPRTVKAA
AICAERGIATCVLLGNPDEINRVAASQGVELGAGIEIVDPEVVRESYVARLVELRKSKGM
TEPVAREQLEDNVVLGTLMLEQDEVDGLVSGAVHTTANTIRPPLQLIKTAPGSSLVSSVF
FMLLPEQVYVYGDCAINPDPTAEQLAEIAIQSADSAIAFGIEPRVAMLSYSTGTSGAGSD
VEKVREATRLAQEKRPDLMIDGPLQYDAAVMADVAKSKAPNSPVAGRATVFIFPDLNTGN
TTYKAVQRSADLISIGPMLQGMRKPVNDLSRGALVDDIVYTIALTAIQASQQQQ
>EECEBICD_01018 Acetate kinase
MSSKLVLVLNCGSSSLKFAIIDAVNGDEYLSGLAECFHLPEARIKWKMDGSKQEAALGAG
AAHSEALNFIVNTILAQKPELSAQLTAIGHRIVHGGEKYTSSVVIDESVIQGIKDSASFA
PLHNPAHLIGIAEALKSFPQLKDKNVAVFDTAFHQTMPEESYLYALPYSLYKEHGVRRYG
AHGTSHFYVTQEAAKMLNKPVEELNIITCHLGNGGSVSAIRNGKCVDTSMGLTPLEGLVM
GTRSGDIDPAIIFHLHDTLGMSVDQINKMLTKESGLLGLTEVTSDCRYVEDNYATKEDAK
RAMDVYCHRLAKYIGSYTALMDGRLDAVVFTGGIGENAAMVRELSLGKLGVLGFEVDHER
NLAARFGKSGFINKEGTRPAVVIPTNEELVIAQDASRLTA
>EECEBICD_01019 hypothetical protein
MSTPDNRSVNFFSLFRRGQHYAKTWPMEKRLAPVFVENRVIRMTRYAIRFMPPVAVFTLC
WQIALGGQLGPAVATALFALSLPMQGLWWLGKRSVTPLPPSILNWFYEVRGKLQEAGQAL
APVEGKPDYQALADTLKRAFKQLDKTFLDDL
>EECEBICD_01020 hypothetical protein

MEMTNAQRLILSNQYKMMTMLDPTNAERYRRLQTIIERGYGLQMRELDREFGELTEETCR
TIIDIMEMYHALHVSWTNLKDTQAIDERRVTFLGFDAATEARYLGYVRFMVNIEGRYTHF
DAGTHGFNAQTPMWEKYQRMLNVWHACPRQYHLSANEINQIINA
>EECEBICD_01021 Hexitol phosphatase A
MQCKGFLFDLDGTLVDSLPAVERAWCSWADRFNLAHDEVLGFIHGKQAITSLRHFMAGKS
EAEIAAEFTRLEQIEATETAGITALPGAVDLLNHLNKAGIPWAIVTSGSMPVARARHQVA
GLPAPEVFVTAERVKRGKPEPDAYLLGAQLLGLAPQECVVVEDAPAGVLSGLAAGCHVIA
VNAPADTPRLADVDFALDSLTQLSVAKQPNGDVVVLRKT
>EECEBICD_01022 hypothetical protein
MNGELIWVLSLLAIAVVLFATGKVRMDAVALFVIVAFVLSGTLTLPEAFSGFSDPNVILI
AALFIIGDGLVRTGVATVVGTWLVKMAGSSEIKMLVLLMITVAGLGAFMSSTGVVAIFIP
VVLSVSMHMQTSPSRLMMPLSFAGLISGMMTLVATPPNLVVNSELLREGLHGFNFFSVTP
LGIVVLALGIVYMLVMRFMLKGDAPGQQAGKRRTFRDLIREYRLTGRARRLAIRPGSPMV
GQRLDDLKLRERYGANVIGVERWRRFRRVIVNVNGVSEFRARDVLLIDMSAAEVDLREFC
AEQLLEPMVLRGEYFSDQALDVGMAEISLIPESELIGKSVREIAFRTRYGLNMVGLKRDG
VALEGSLADEPLLMGDIILVVGNWKLISLLGQKGRDFVVLNMPVEVSEASPAHSQAPHAI
FCLVLMVALMLTDEIPNPIAAIIACLLMGKFRCIDAESAYKAIHWPSIILIVGMMPFALA
LQKTGGVSLVVQGLMDIGGGYGPYMMLGCLFVLCAVIGLFISNTATAVLMAPIALAAAKS
MGVSPYPFAMAVAMAASAAFMTPVSSPVNTLVLGPGNYSFSDFVKLGVPFTLIVMAVCIV
MIPMLFPF
>EECEBICD_01023 5'-deoxynucleotidase YfbR
MKQSHFFAHLSRMKLINRWPLMRNVRTENVSEHSLQVAMVAHALAAIKNRKFGGQLNAER
IALLAMYHDASEVLTGDLPTPVKYFNSQIAQEYKAIEKIAQQKLVDMAPDELRDIFAPLI
DENAWSEEEQAIVKQADALCAYLKCLEELSAGNNEFGLAKTRLEKTLELRRSQEMDYFMA
VFVPSFHLSLDEISQDSPL
>EECEBICD_01024 Glutamate-pyruvate aminotransferase AlaA
MSPIEKSSKLENVCYDIRGPVLKEAKRLEEEGNKVLKLNIGNPAPFGFEAPDEILVDVIR
NLPTAQGYCDSKGLYSARKAIMQHYQARGMRDVTVEDIYIGNGVSELIVQAMQALLNSGD
EMLVPAPDYPLWTAAVSLSSGKAVHYLCDESSDWFPDLDDIRAKITPRTRGIVIINPNNP
TGAVYSKELLMEIVNIAREHNLIIFADEIYDKILYDDAEHHSIAALAPDLLTITFNGLSK
TYRVAGFRQGWMVLNGPKKHAKGYIEGLEMLASMRLCANVPAQHAIQTALGGYQSISEFI
LPGGRLYEQRNRAWELINDIPGVSCVKPRGALYMFPRIDAKRFNIHDDQKMVLDFLLQEK
VLLVQGTAFNWPWPDHFRIVTLPREDDLEMAINRFGRFLSGYHQ
>EECEBICD_01025 HTH-type transcriptional regulator HdfR
MINANRPIINLDLDLLRTFVAVADLNTFAAAAAAVCRTQSAVSQQMQRLEQLVGKELFAR
HGRNKLLTEHGIQLLGYARKILRFNDEACSSLMFSNLQGVLTIGASDESADTILPFLLSR
ISSVYPKLALDVRVKRNAYMVDMVKSQEVDLVVTTNQPHSLDCLNLRTSPTHWYCAAEYV
LQRGEPVPLVLLDDPSPFRDMVLETLNAAGIPWRLAYVASTLPAVRAAVKAGLGVTARPV
EMMSPDLRVLGAADGLPPLPDTEYLLCRDPNSQNELAMVIFHAMESYQNPWHYNQFSAEG
GDDPLMVEGGFE
>EECEBICD_01026 NAD(P)H-quinone oxidoreductase subunit 3
MSMSTSTEVIAHHWAFAIFLIVAIGLCCLMLVGGWFLGGRARARHKNVPFESGIDSVGTA
RLRLSAKFYLVAMFFVIFDVEALYLFAWSTSIRESGWVGFVEAAIFIFVLLAGLVYLARI
GALDWTPARSRRERMNPETNSIANRQR
>EECEBICD_01027 NADH-quinone oxidoreductase subunit B
MDYTLTRIDPNGENDRYPLQKQEIVTDPLEQEVNKNVFMGKLHDMVNWGRKNSIWPYNFG
LSCCYVEMVTSFTAVHDVARFGAEVLRASPRQADLMVVAGTCFTKMAPVIQRLYDQMLEP
KWVISMGACANSGGMYDIYSVVQGVDKFIPVDVYIPGCPPRPEAYMQALMLLQESIGKER
RPLSWVVGDQGVYRANMQPERERKRGERIAVTNLRTPDEI
>EECEBICD_01028 NADH-quinone oxidoreductase subunit C/D
MVNNMTDLTAQDAAWSTRDHLDDPVIGELRNRFGPDAFTVQATRTGIPVVWVKREQLLEV
GDFLKKLPKPYVMLFDLHGMDERLRTHRDGLPAADFSVFYHLISIERNRDIMLKVALSEN
DLRVPTFTKLFPNANWYERETWEMFGIDIEGHPHLTRIMMPQTWEGHPLRKDYPARATEF
DPFELTKAKQDLEMEALTFKPEDWGMKRGTDNEDFMFLNLGPNHPSAHGAFRIILQLDGE

EIVDCVPDIGYHHRGAEKMGERQSWHSYIPYTDRIEYLGGCVNEMPYVLAVEKLAGITVP
DRVNVIRVMLSELFRINSHLLYISTFIQDVGAMTPVFFAFTDRQKIYDLVEAITGFRMHP
AWFRIGGVAHDLPRGWDRLLREFLEWMPKRLDSYEKAALRNTILKGRSQGVAAYGAKEAL
EWGTTGAGLRATGIDFDVRKWRPYSGYENFDFEVPVGGGVSDCYTRVMLKVEELRQSLRI
LQQCLDNMPEGPFKADHPLTTPPPKERTLQHIETLITHFLQVSWGPVMPAQESFQMVEAT
KGINSYYLTSDGSTMSYRTRVRTPSFAHLQQIPSAIRGSLVSDLIVYLGSIDFVMSDVDR
>EECEBICD_01029 NADH-quinone oxidoreductase subunit E
MHENQQPQTEAFELSAAEREAIEHEKHHYEDPRAASIEALKIVQKQRGWVPDGAIYAIAD
VLGIPASDVEGVATFYSQIFRQPVGRHVIRYCDSVVCHITGYQGIQAALEKNLNIKPGQT
TFDGRFTLLPTCCLGNCDKGPNMMIDEDTHSHLTPEAIPELLERYK
>EECEBICD_01030 NADH-quinone oxidoreductase subunit F
MKNVIRTPETHPLTWRLRDDKQPVWLDEYRSKNGYEGARKALTGLSPDEIVSQVKDAGLK
GRGGAGFSTGLKWSLMPKDESMNIRYLLCNADEMEPGTYKDRLLMEQLPHLLVEGMLISA
FALKAYRGYIFLRGEYIEAAVHLRRAIAEATEAGLLGKNIMGTGFDFELFVHTGAGRYIC
GEETALINSLEGRRANPRSKPPFPATSGVWGKPTCVNNVETLCNVPAILANGVEWYQNIS
KSKDAGTKLMGFSGRVKNPGLWELPFGTTAREILEDYAGGMRDGLKFKAWQPGGAGTDFL
TEAHLDLPMEFESIGKAGSRLGTALAMAVDHEIGMVSLVRNLEEFFARESCGWCTPCRDG
LPWSVKILRALERGEGQPGDIETLEQLCRFLGPGKTFCAHAPGAVEPLQSAIKYFREEFE
AGIKQPFSNTHLINGIQPNLLKERW
>EECEBICD_01031 NADH-quinone oxidoreductase subunit G
MATIHVDGKEYEVNGADNLLQACLSLGLDIPYFCWHPALGSVGACRQCAVKQYQNAEDTR
GRLVMSCMTPATDGTFISIDDEEAKQFRESVVEWLMTNHPHDCPVCEEGGNCHLQDMTVM
TGHSFRRYRFTKRTHRNQDLGPFISHEMNRCIACYRCVRYYKDYADGTDLGVYGAHDNVY
FGRPEDGTLESEFSGNLVEICPTGVFTDKTHSERYNRKWDMQFAPSICQQCSIGCNISPG
ERYGELRRIENRYNGTVNHYFLCDRGRFGYGYVNLKDRPRQPVQRRGDDFITLNAEQAMQ
GAADILRQSKKVIGIGSPRASIESNFALRELVGAENFYTGIARGEQERLQLALKVLREGG
IYTPALREIESYDAVLVLGEDVTQTGARVALAVRQAVKGKAREMAAAQKVADWQIAAILN
IGQRAKHPLFVTNVDDTRLDDIAAWTYRAPVEDQARLGFAIAHALDNTAPAVDGIDSDLQ
NKIDVIVQALAGAKKPLIISGTNAGSSEVIQAAANVAKALKGRGADVGITMIARSVNSMG
LGMMGGGSLDDALGELETGSADAVVVLENDLHRHASATRVNAALAKAPLVMVVDHQRTAI
MENAHLVLSAASFAESDGTVINNEGRAQRFFQVYDPAYYDNKTIMLESWRWLHSLHSTVE
NREVDWTQLDHVIDAVIAAMPQFAGIKDAAPDATFRIRGQKLAREPHRYSGRTAMRANIS
VHEPRQPQDKDTMFAFSMEGNNQPTAPRSEIPFAWAPGWNSPQAWNKFQDEVGGKLRHGD
PGVRLIEATEGGLDYFTTVPASFQAQDGQWRIAPYYHLFGSDELSQRSPVFQSRMPQPYI
KLNPADAAKLGVNAGTRVSFSYDGNTVTLPVEISEGLAAGQVGLPMGMPGIAPVLAGARL
EDLREAQQ
>EECEBICD_01032 NADH-quinone oxidoreductase subunit H
MSWITPDLIEILLSILKAVVILLVVVTCGAFMSFGERRLLGLFQNRYGPNRVGWGGSLQL
VADMIKMFFKEDWIPKFSDRVIFTLAPMIAFTSLLLSFAIVPVSPNWVVADLNIGILFFL
MMAGLAVYAVLFAGWSSNNKYSLLGAMRASAQTVSYEVFLGLSLMGVVAQAGSFNMTDIV
NNQAHLWNVIPQFFGFVTFAIAGVAVCHRHPFDQPEAEQELADGYHIEYSGMKFGLFFVG
EYIGIVTVSALMVTLFFGGWHGPFLPPFVWFALKTAFFMMMFILIRASLPRPRYDQVMSF
GWKVCLPLTLINLLVTAAVILWQAQ
>EECEBICD_01033 NAD(P)H-quinone oxidoreductase subunit I, chloroplastic
MTLKELLVGFGTQVRSIWMIGLHAFAKRETRMYPEEPVYLPPRYRGRIVLTRDPDGEERC
VACNLCAVACPVGCISLQKAETKDGRWYPEFSALTSHAASSAACAKKRARPRRFS
>EECEBICD_01034 NADH-quinone oxidoreductase subunit I
MTPDFELGEYKRQDLVYEKEDLLISGPGKYPEYNFYRMAGMAIDGKDKGEAENEAKPIDV
KSLLP
>EECEBICD_01035 NADH-quinone oxidoreductase subunit J
MEFAFYICGLIAILATLRVITHTNPVHALLYLVISLLAISGVFFSLGAYFAGALEIIVYA
GAIMVLFVFVVMMLNLGGSEIEQERQWLKPQVWIGPAVLSAIMLAVIVYAILGVNDQGID
GTPISAKAVGITLFGPYVLAVELASMLLLAGLVVAFHVGREERAGEVLSNRADDRAKRKT
EERA

>EECEBICD_01036 NADH-quinone oxidoreductase subunit K
MIPLTHGLILAAILFVLGLTGLVIRRNLLFMLIGLEIMINASALAFVVAGSYWGQTDGQV
MYILAISLAAAEASIGLALLLQLHRRRQNLNIDSVSEMRG
>EECEBICD_01037 NADH-quinone oxidoreductase subunit L
MNMLALTIILPLIGFVLLAFSRGRWSENLSATIGVGSVGLAALVTAFVGMDFFANGKQAF
SQPLWTWMSVGNFNIGFNLVLDGLSLTMLSVVTGVGFLIHMFASWYMRGEEGYSRFFAYT
NLFIASMVVLVLSDNLLLMYLGWEGVGLCSYLLIGFYYSDPKNGAAAMKAFVVTRVGDVF
LAFALFILYNELGTLNFREMVELAPAHFADGNNMLMWATLMLLGGAVGKSAQLPLQTWLA
DAMAGPTPVSALIHAATMVTAGVYLIARTHGLFLMTPEILHLVGIIGAITLAMAGFAALV
QTDIKRVLAYSTMSQIGYMFLALGVQAWDAAIFHLMTHAFFKALLFLASGSVILACHHEQ
NIFKMGGLRKSIPLVYACFLVGGAALSALPLVTAGFFSKDEILAGAMANGHINLMVAGLV
GAFMTSLYTFRMIFIVFHGKEQIHAHAGKGITHHLPLIVLMILSTFVGALIVPPLQGAAA
DN
>EECEBICD_01038 NADH-quinone oxidoreductase subunit L
MCRLCRGLPQTTELAHGRVLTLEITSGVVAIAGILIAAWLWLGKRTLVTSIANSAPGRLL
GTWWYNAWGFDWLYDKAFVKPFLGIAWLLKRDPLNALMNIPAILSRFAGKGLVLSENGYL
RWYVASMSIGAVVVLALLMVLR
>EECEBICD_01039 NADH-quinone oxidoreductase subunit M
MLLPWLILIPFIGGFLCWQTERFGVKVPRWIALITMGLTLALGLQLWLQGGYSLTQSAGI
PQWQSEFVLPWIPRFGISIHLALDGLSLLMVVLTGLLGVLAVLCSWREIEKYQGFFHLNL
MWILGGVIGVFLAIDMFLFFFFWEMMLVPMYFLIALWGHKASDGKTRITAATKFFIYTQA
SGLVMLIAILALVFVHYNATGVWTFNYEELLNTPMSHGVEYLLMLGFFIAFAVKMPVVPL
HGWLPDAHSQAPTAGSVDLAGILLKTAAYGLLRFSLPLFPNASAEFAPIAMWLGVIGIFY
GAWMAFTQYDIKRLIAYTSVSHMGFVLIAIYTGSQLAYQGAVIQMIAHGLSAAGLFILCG
QLYERLHTRDMRMMGGLWGKMKWLPALSMFFAVATLGMPGTGNFVGEFMILFGSYQVVPM
ITVISTFGLVFASVYSLAMLHRAYFGKAKSQIASQELPGMSLRELFIILLLVVLLVLLGF
YPQPILDTSHSAMSNIQQWFVNSVTTTRP
>EECEBICD_01040 NADH-quinone oxidoreductase subunit N
MTITPQHLIALLPLLIVGLTVVVVMLSIAWRRNHFLNATLSVIGLNAALVSLWFVGLAGA
MDVTPLMRVDGFAMLYTGLVLLASLATCTFAYPWLEGYNDNQEEFYLLVLIASLGGILLA
NANHLAALFLGIELISLPLFGLIGYAFRQKRSLEASIKYTILSAAASSFLLFGMALVYAQ
SGNLSFEALGKSLGDGMLHEPLLLAGFGLMIVGLGFKLSLVPFHLWTPDVYQGAPAPVST
FLATASKIAIFGVVMRLFLYAPVGDSEAVRVVLGIIAFASIIFGNLMALSQTNIKRLLGY
SSISHLGYLLVALIALQSGEMSMEAVGVYLAGYLFSSLGAFGVVSLMSSPFRGPDADSLY
SYRGLFWHRPVLAAVMTVMMLSLAGIPMTLGFIGKFYVLAVGVQASLWWLVAAVVVGSAI
GLYYYLRVAVSLYLHAPQQPGRDAPTNWQYSAGGIVVLISALLVLVLGVWPQPLISLVQL
AMPLM
>EECEBICD_01041 hypothetical protein
MLNGKTLMLLLGGVVLSGCGPEPSDPQGNNPAELKQEQAIQKENSAQAGDDTVQKRQAEA
AQQAAKKAAEYKANTEAKALADAKAASLATAEAPQYEMRTRAAASKAFAAQGGNVMGTAR
YEHYDENPIKQVSQAPLATFSLDVDTGSYANVRRFLNQGQLPPPEAVRVEEMLNYFPAPQ
PVADKQDNTKPIAACIPMPFAVKYELAPSPWNAQRTLLKVDVQARDMQVRDLPPANLVFL
IDTSGSMQPAERLPLIQSALKLLVNDLRAQDNITIVTYAGGTHVALASTAGNNTTAIKAA
IDNLDAYGSTGGEAGLRLAYEQAEKGFIKGGVNRILLTTDGDFNLGITDPKDIEALVKKE
REKGITLSTLGVGDDNFNEAMMVRIADVGNGNYSYIDSLSEAQKVLKDEMHQTLVTVAKD
VKSQIEFNPQWVTEYRQIGYEKRQLRDEDFNNDKVDAGDIGAGKHVTLFFELTLNGQKAS
VDKLRYAQDKAASKPTKSSELAWIKLRWKAPQGSESTLAEFPVVMGKMPIFADASEDFRF
RAAVAAFGQKLRGSETLADTTWPQIIKWGEQARGEDRQGYRAEFIKLVKLAEGLSH
>EECEBICD_01042 Chemotaxis protein CheV
MDNFQKDIDDRANLTLSNRFELLLFRLGTSLHEQKSELFGINVFKLREIVPMPAFTRPAG
MKAPLLGMVNIRDQVIPVIDLPAVAGCKPETGLNILLITEYARSVQAFAVESVENIMRLD
WQQVHTAEKAVNGRYITSIACLDDNKETNNLALVLDVEQILYDIVPSSHDLRATNLKTNK
FYITPGAVAIVAEDSKVARAMLEKGLNAMGIPHQMHVTGKDAWERIQQLAQEAEAEGKPI
SEKIALVLTDLEMPEMDGFTLTRKIKTDERLKKIPVVIHSSLSGSANEDHIRKVKADGYV

AKFEINELSSVIQEVLERAATNSQGPLISRKSA
>EECEBICD_01043 Ribonuclease BN
MELIFLGTSAGVPTRSRNVTAILLHLQHPTQPGVWLFDCGEGTQHQMLNTAFHPGKLERI
FISHLHGDHLFGLPGLLCSRSMAGNPHPLTVYGPQGVREFIATTLRLSGSWTDFPLQIEE
VSAGDILDDGLRKVTAFRLEHPLECYGYRVVEHDKPGALNARALKAAGVTPGPLFQALKA
GKTVTLADGRQINGADYLAPAVAGKSVAIFGDTAPCEAALALAQGVDVMVHETTLDASME
EKANARGHSSTRQTATLAREAAVGRLIMTHISSRYDDKGCQRLLAECRAIFPATELAYDF
SVFPV
>EECEBICD_01044 Protein ElaA
MIDWQDLHHSELTVPQLYALLKLRCAVFVVEQRCPYLDVDGDDLVGDNRHILGWHQDELV
AYARILKSDNESDPVVIGRVIVSDAWRGAKLGQQLMAKTLESCGRHWPDKPLYLGAQAHL
QPFYARFGFIPVTDVYDEDGIPHRGMAREVHQA
>EECEBICD_01045 Protein ElaB
MSYQFGESRVDDDLTLLSETLEEVLRSSGDPADQKYIELKARAEQALEEVKNRVSHASDS
YYYRAKQAVYKADDYVHEKPWQGIGVGAAVGLVLGLLLARR
>EECEBICD_01046 Isochorismate synthase MenF
MHSITTALENLTRQLSQEIPATPGLCVFDAPFPLNDAFDALSWLASQSSFPQFYWQQRNG
DEEAAVLGAITVFSSLDLAQRFLRQHAQPDLRIWGLNAFEPKEGYLLLPRLEWRRSGGTA
TLRLHLHSDVSLRDDARQAKAFLASLVGIKPVPALRLSLTSEQHWPDKDGWVKLIQQATH
TIAQEAFDKVVLARATDLQFSRPVNAAAMMASSRRLNLNCYHFFMAFSADTAFLGSSPER
LWRRRETALRTEALAGTVANHPDNHKAWQLGEWLMKDDKNQRENMLVVEDICQRLQNCTH
SLDVLPPQVLRLRKVQHLRRCIWTALNQADDTLCLHQLQPTAAVAGIPREPARCFIEQHE
PFEREWYAGSAGYLSTRQSEFCVTLRSAKVTANVVRLYAGAGIVRGSDPEQEWQEIDNKA
AGLRTLLQMDA
>EECEBICD_01047 2-succinyl-5-enolpyruvyl-6-hydroxy-3-cyclohexene-1-carboxylate synthase
MSVSAFNRRWAAVILEALTRHGVRHVCIAPGSRSTPLTLAAAENPAFIHHTHFDERGLGH
LALGLAKVSQQPVAVIVTSGTAVANLYPALIEAGLTGEKLILLTADRPPELIDCGANQAI
RQAGMFASHPSQTLSLPRPTQDIPARWLVSTIDNALAMLHAGALHINCPFAEPLYGDMND
TGLVWQQRLGDWWQDEKPWLREARRLESDKQRDWFFWRQKRGVVVAGRMSAEEGKKVAQW
AQTLGWPLIGDVLSQTGQPLPCADLWLGNAKAVTELQQAQIVVQLGSSLTGKRLLQWQAT
CEPEEYWVIDNIEGRLDPAHHRGRRLVAKIADWLELHPAEKRKPWCVEIPRLAELAWQRV
VAQRTPLAKRSWRTVSATICRSRGNCSWAIVWWCV
>EECEBICD_01048 2-succinyl-5-enolpyruvyl-6-hydroxy-3-cyclohexene-1-carboxylate synthase
MFVGNSLVVRLIDALSQLPAGYPVYSNRGASGIDGLLSTAAGVQRASAKSTLAIVGDLSA
LYDLNALALLRQVSAPFVLIVVNNNGGQIFSLLPTPQSKRERFYLMPQNVHFDHAAAMFN
LRYHRPENWEELESALAGAWRTPATTVIELVVNDTDGAQTLQQLLAQVSHL
>EECEBICD_01049 2-succinyl-6-hydroxy-2,4-cyclohexadiene-1-carboxylate synthase
MMLHAQHMPGQPGAPSLVFLHGFSGDCREWQPVGEQFHGCSRLYIDLPGHGGSAAIPVGG
FADVIRLLRATLISYNILKFWLVGYSLGGRVAMMAACQGIPGLCGLVVEGGHPGLQNEQA
RAERRLSDGRWAERFRHEPLTEVFHDWYQQPVFASLTAQQRQALTALRSQNNGETLAAML
EATSLAVQPDLREALNALAFPFYYLCGERDSKFRALAQEVAATCHVIRNAGHNAHRENPA
GVVDSLAQILRL
>EECEBICD_01050 1,4-dihydroxy-2-naphthoyl-CoA synthase
MIYPDETMLYAPVEWHDCSEGYTDIRYEKSTDGIAKITINRPQVRNAFRPLTVKEMIQAL
ADARYDDNVGVIILTGEGDKAFCAGGDQKVRGDYGGYQDDSGVHHLNVLDFQRQIRTCPK
PVVAMVAGYSIGGGHVLHMMCDLTIAAENAIFGQTGPKVGSFDGGWGASYMARIVGQKKA
REIWFLCRQYDAQQALDMGLVNTVVPLADLEKRDRALVPRNVAKQSDGAALPESGAECRL
>EECEBICD_01051 1,4-dihydroxy-2-naphthoyl-CoA synthase
MLQNSPMALRCLKAALNADCDGQAGLQELAGNATMLFYMTEEGQEGRNAFNQKRQPDFSK
FKRNP
>EECEBICD_01052 o-succinylbenzoate synthase

MRSAQVYRWQIPMDAGVVLRDRRLKTRDGLYVCLRDGEREGWGEISPLPGFSQETWEEAQ
TALLTWVNDWLQGSEGLPEMPSVAFGASCALAELTGVLPEAADYRAAPLCTGDPDDLVLR
LADMPGEKIAKVKVGLYEAVRDGMVVNLLLEAIPDLHLRLDANRAWTPLKAQQFAKYVNP
DYRARIAFLEEPCKTRDDSRAFARETGIAIAWDESLREADFTFEAEEGVRAVVIKPTLTG
SLDKVREQVAAAHALGLTAVISSSIESSLGLTQLARIAAWLTPGTLPGLDTLHLMQAQQV
RPWPGSALPCLKRDELERLL
>EECEBICD_01053 2-succinylbenzoate--CoA ligase
MTFTDWPWRHWRQVRSQAPALRLNDEVLSWRALCERIDALAGGFAAQGVREGDGVLLRAG
NQPRTLLAWLALMQCGARVLPVNPQLPQTLLEALVPKLTLRFALTLEGENAFSGLTALQI
QKSTAAYAVDWQPQRLVSMTLTSGSTGLPKAAVHTCQAHLASAQGVLSLMPFGPQDDWLL
SLPLFHVSGQGIMWRWLFAGARMTVRDKQPLEQMLAGCTHASLVPTQLWRLLANRAAVTL
KAVLLGGAVIPVELTDQASKQGIRCWCGYGLTEFASTVCAKEADGSDDVGAPLPGREIRI
VDNEVWLRAASMAEGYWRDGKLIPLVNDEGWFATRDRGDLNHGRLTIAGRLDNLFFSGGE
GIQPEEVERVINAHPLVQQAFVVPVEDKEFGHRPVAVVEYASQAGDVNLAEWVRDKLARF
QQPVRWLTLPSELKNGGIKISRRALQQWVCENCKN
>EECEBICD_01054 Signal transduction protein PmrD
MEWLVKKSHYVKKRACHVLVLCDSGGSLKMIAEANSMILLSPGDILSPLQDAQYCINREK
HQTLKIVDARCYSCDEWQRLTRKPS
>EECEBICD_01055 putative 4-amino-4-deoxy-L-arabinose-phosphoundecaprenol flippase subunit ArnF
MGVMWGLISVAIASLAQLSLGFAMMRLPSIAHPLAFISGLGALNAATLALFAGLAGYLVS
VFCWHKTLHTLALSKAYALLSLSYVLVWVASMLLPGLQGAFSLKAMLGVLCIMAGVMLIF
LPARS
>EECEBICD_01056 putative 4-amino-4-deoxy-L-arabinose-phosphoundecaprenol flippase subunit ArnE
MIGVILVLASLLSVGGQLCQKQATRPLTVGGRRRHLMLWLGLALICMGAAMVLWLLVLQT
LPVGIAYPMLSLNFVWVTLAAWKIWHEQVPPRHWFGVALIISGIIILGSAA
>EECEBICD_01057 Undecaprenyl phosphate-alpha-4-amino-4-deoxy-L-arabinose arabinosyl transferase
MMKSIRYYLAFAAFIALYYVIPVNSRLLWQPDETRYAEISREMLASGDWIVPHFLGLRYF
EKPIAGYWINSLGQWLFGATNFGVRAGAILTTLLAAALVAWLTFRLWRDKRTALLASVIF
LSLFAVYSIGTYAVLDPMIALWLTAGMCCFWQGMQATTRTGKIGMFLLLGATCGLGVLTK
GFLALAVPVVSVLPWVIVQKRWKDFLLYGWLAVLSCFVVVLPWAIAIARREADFWHYFFW
VEHIQRFAMSDAQHKAPFWYYLPVLLAGSLPWLGLLPGALKLGWRERNGAFYLLGWTIMP
LLFFSIAKGKLPTYVLSCFAPIAILMARFVLHNVKEGVAALRVNGGINLVFGIIGIVAAF
VVSSWGPLKSPVWTHIETYKVFCVWGVFTVWAFVGWYSLCHSQQYLLPAFCPLGLALLFG
FSIPDRVMESKQPQFFVEMTQAPLASSRYILADNVGVAAGLAWSLKRDDIMLYGHAGELR
YGLSYPDVQDKFVKADDFNAWLNQHRQEGIITLVLSIAKDEDISALSLPPADNVDYQGRL
VLIQYRPK
>EECEBICD_01058 putative 4-deoxy-4-formamido-L-arabinose-phosphoundecaprenol deformylase ArnD
MTKVGLRIDVDTLRGTREGVPRLLATLHRHGVQASFFFSVGPDNMGRHLWRLIRPRFLWK
MLRSNAASLYGWDILLAGTAWPGKNIGNANAGIIRETATYHETGLHAWDHHAWQTHSGHW
SIRQLEEDIARGITALEAIIGKPVTCSAAAGWRADGRVVRAKEPFNLRYNSDCRGTTLFR
PLLMPGQTGTPQIPVTLPTWDEVIGPAVQAQSFNTWIISRMLQDKGTPVYTIHAEVEGIV
HQPLFEDLLVRARDAGITFCPLGELLPTSPESLPLGQIVRGHIPGREGWLGCQQAASAS
>EECEBICD_01059 Bifunctional polymyxin resistance protein ArnA
MKAVIFAYHDMGCQGVQAVLDAGYEIAAIFTHADNPAENTFFGSVSRLAAELGIPVYAPD
NVNHPIWVDRIAELAPDIIFSFYYRNLLSEEILHLAPAGAFNLHGSLLPAYRGRAPLNWV
LVNGESETGVTLHRMVKRADAGEIVASQRVAIAQDDVALTLHHKLCQAARQLLNSILPTM
KCGDIPSVPQRESDATYYGRRRPEDGLIDWHKPVSTVHNLVRAVAAPWPGAFSYNGSQKF
TIWSSRICPDAQGALPGSVISVSPLRVACADGALEIITGQAGDGITVQGSQLAQTLGLVA
GARLNRPPATSGKRRIRVLILGVNGFIGNHLTERLLNEENYEVYGMDIGSNAISRFLLHP
RFHFVEGDISIHSEWIEYHVKKCDVVLPLVAIATPIEYTRNPLRVFELDFEENLRIIRYC

VKYRKRVVFPSTSEVYGMCTDASFDEDKSNLIVGPVNKPRWIYSVSKQLLDRVIWAYGEK
EGLRFTLFRPFNWMGPRLDSLNAARIGSSRAITQLILNLVEGTPIKLIDGGQQKRCFTDI
RDGIEALFRIIVNDGDRCDGKIINIGNPDNEASIQELATLLLDSFDKHPLRCHFPPFAGF
QVVESRSYYGKGYQDVAHRKPSIDNARRCLGWEPSIAMRDTVEETLDFFLRSVDVAERAS
>EECEBICD_01060 Undecaprenyl-phosphate 4-deoxy-4-formamido-L-arabinose transferase
MFDAAPIKKVSVVIPVYNEQESLPELIRRTTAACESLGKAWEILLIDDGSSDSSAELMVK
ASQEADSHIISILLNRNYGQHAAIMAGFSHVSGDLIITLDADLQNPPEEIPRLVAKADEG
FDVVGTVRQNRQDSLFRKSASKIINLLIQRTTGKAMGDYGCMLRAYRRPIIDTMLRCHER
STFIPILANIFARRATEIPVHHAEREFGDSKYSFMRLINLMYDLVTCLTTTPLRLLSLLG
SVIAIGGFSLSVLLIVLRLALGPQWAAEGVFMLFAVLFTFIGAQFIGMGLLGEYIGRIYN
DVRARPRYFVQQVIYPESTPFTEESHQ
>EECEBICD_01061 UDP-4-amino-4-deoxy-L-arabinose--oxoglutarate aminotransferase
MSDFLPFSRPAMGAEELAAVKTVLDSGWITTGPKNQELEAAFCRLTGNQYAVAVSSATAG
MHIALMALGIGEGDEVITPSMTWVSTLNMIVLLGATPVMVDVDRDTLMVTPEHIEAAITP
QTKAIIPVHYAGAPADLDAIYALGERYGIPVIEDAAHATGTSYKGRHIGARGTAIFSFHA
IKNITCAEGGIVVTDNPQFADKLRSLKFHGLGVDAWDRQSGGRAPQAEVLAPGYKYNLPD
LNAAIALAQLQKLDALNARRAAIAAQYHQAMADLPFQPLSLPSWEHIHAWHLFIIRVDEA
RCGITRDALMASLKTKGIGTGLHFRAAHTQKYYRERFPTLTLPDTEWNSERICSLPLFPD
MTESDFDRVITALHQIAGQ
>EECEBICD_01062 Lipopolysaccharide core heptose(II)-phosphate phosphatase
MLAFTLRFIKNKRYFAILAGALVIIAGLTSQHAWSGNGLPQINGKALAALAKQHPVVVLF
RHAERCDRSDNTCLSDSTGITVKGAQDARALGKAFSADIQNYNLYSSNTVRTIQSATWFS
AGRSLTVDKKMMDCGSGIYASINTLLKKSQNKNIVIFTHNHCLTYIAKNKRGVKFDPDYL
NALVMHAENGKLFLDGEFVPG
>EECEBICD_01063 Nucleoside triphosphatase NudI
MRQRTIVCPLIQNDGCYLLCKMADNRGVFPGQWALSGGGVEPGERIEEALRREIREELGE
QLILSDITPWTFRDDIRVKTYADGRQEEIYMIYLIFDCVSANRDICINDEFQDYAWVKPE
ELALYDLNVATRHTLALKGLL
>EECEBICD_01064 hypothetical protein
MCLLERVKMKKSILLGFAGMLFVSASAQAISISGQAGEDYTNIGVGFGTESTGLALSGNW
MHNDDDGDAAGVGLGLNIPVGPLLATVGGKGIYTNPKDSDEGYAAAVGGGLQWKIGNSFR
LFGEYYYSPDSLSSGIDSYEEANAGARFTIMRPLSIEAGYRYLNLAGKDGNRDNAIADGP
YVGVNASF
>EECEBICD_01065 Nicotinamide-nucleotide amidohydrolase PncC
MLNVEMLSTGDEVLHGQIVDTNAAWLADFFFHQGVPLSRRNTVGDNLDSLVAILRERSQH
ADVLIVNGGLGPTSDDLSALAAATAKGEGLVLHQAWLTEMERYFQQRGRVMAPSNRKQAE
LPASAEFINNPVGTACGFALQLNRCLMFFTPGVPSEFKVMVEKEILPRLRARFSLPEPPL
CLRLTTFGRSESDLAQRLDSLPLPPGVTMGYRSSMPIIELKLTGPATQREAMLALWPEVK
RVAGQNLIFEGTENLPAQIARRLQERQLSLTLSEQYTSGLLALQLSRAGAPVLASEVVPS
QEETLAQTAHWTTERRSNHYAGLALAVSGLENEHLNFALATPDGTYALRVRFSANRYSLA
IRQEVCAMMALNMLRRWLNGEDITSEHGWIDVVESLTS
>EECEBICD_01066 putative HTH-type transcriptional regulator RhmR
MLESSKVPALTRAIDILNLIARIGPCSAAIIIETLRIPKSTAYLLLNELKRQRFISLDHQ
DNYCLWTKLVELSGHALSKMDLRELARPRLTQLMDETGLLCHLGIIDHENAYYILKVESS
STISVRSHEGKSLSLYRSGIGKCLLAWQPAAVRKAIIEQLVWERATLTTITEPQQLNDEL
ERIRQRGWSFDNGEDYPDVRCVAAPVFNARNELTAAISVVGTRLQINEENLDYLAGKAIA
CAKDISRLLGWKSPFDSLAS
>EECEBICD_01067 L-rhamnonate dehydratase
MTLPKIKHVRAWFIGGATAEKGAGGGDYHDQGGNHWIDDHIATPMSKYRDYEQSRQSFGI
NVLGTLIVEVEAENGQTGFAVSTAGEMGCFIVEKHLNRFIEGKCVSDIKLIHDQMLGATM
YYSGSGGLVMNTISCVDLALWDLFGKVVGLPVYKLLGGAVRDEIQFYATGARPDLAKEMG
FIGGKMPTHWGPHDGDAGIRKDAAMVADMREKCGPDFWLMLDCWMSQDVNYATKLAHACA

PFNLKWIEECLPPQQYEGYRELKRNAPAGMMVTSGEHHGTLQSFRTLAETGIDIMQPDVG
WCGGLTTLVEIAALAKSRGQLVVPHGSSVYSHHAVITFTNTPFSEFLMTSPDCSTLRPQF
DPILLDEPVPVNGRIHKSVLDKPGFGVELNRDCHLKRPYSH
>EECEBICD_01068 Inner membrane transport protein RhmT
MSITLLDGVVKKNRARLIPFMLALYVLAFLDRSNIGFAKETYQIDTGLSNEAYALGAGIF
FVVYAFLGVPANLLMRKFGARTWIGTTTLLWGFLSAAMAWADSEAKFLIIRTLLGAAEAG
FFPGMIYLTSQWFPQRNRASIMGLFYMGAPLALTLGSPLSGALLEMHGFMGHPGWFWMFV
IEGLLAIGAGIFTFFWLDDTPQQARFLSLEEKNALIRQLASEEEKKVTSRLADALRNGRV
WQLAIIYLTIQVAVYGLIFFLPT
>EECEBICD_01069 Inner membrane transport protein RhmT
MGTKVGFTASVVTAVPWVAALLGTWLIPRYSDRTGDRRNVAAVTLLAAGIGIGLSGLVSP
VLAILALCVAAVGFIAVQPVFWTMPTQLLSGTALAAGIGFVNLFGAVGGFIAPILRVKAE
TLFASDAAGLLTLAGVAIIGSLIIFTLSVNRPVAQSGAAHH
>EECEBICD_01070 2-keto-3-deoxy-L-rhamnonate aldolase
MNALLSNPFKEGLRKGDTQIGLWLSSTTSYMAEIAATSGYDWLLIDGEHAPNTVQDLYHQ
LQAIAPYASQPVIRPIEGSKALIKQVLDIGAQTLLIPMVDTAEQARQVVSATRYPPLGQR
GVGASVARAARWGRIDNYMAQANESLCLLVQVESKVALENLDAILEVEGIDGVFIGPADL
SASLGYPDNAGHPEVQRIIESCIYRIRAAGKAAGFLAVDPAMAQKCLAWGANFVAVGVDT
MLYTEALDSRLAMFKSVQSVSTAKRSY
>EECEBICD_01071 hypothetical protein
MPDGTYALRMRFSAYRYSLAIRQEVCAVMALNMLRRWLNGEDITSEHGWIDVVESLTA
>EECEBICD_01072 Deubiquitinase SseL
MNICVNSLYRLSTPQFHSLYSEDVSDEALALLIGEVENGNQNCIDLLCNLALRNDDLGHK
VEKLLFDLFSGKRSGSPDIDKKINQACLVLHQIANNDITKNNTEWKKLHAPSRLLYMAGS
ATTDLSKKIGIAHKIMGDQFAQTDQEQVGVENLWCGARMLSSDELAAATQGLVQESPLLS
VNYPIGLIHPTTKENILSTQLLEKIAQSGLSHNEVFLVNTGDHWLLCLFYKLAEKIKCLI
FNTYYDLNENTKQEIIEAAKIAGISENENIDFIETNLQNNVPNGCGLFCYHAIQLLSNAG
QNDPATTLREFAENFLTLSVEEQTLFNTQTRRQIYEYSLQ
>EECEBICD_01073 Anaerobic glycerol-3-phosphate dehydrogenase subunit C
MSDTRFESCIKCTVCTTACPVSRVNPGYPGPKQAGPDGERLRLKDGALYDEALKYCINCK
RCEVACPSDVKIGDIIQRARAKYDTTRPSLRNFILSHTDLMGSVSTPFAPVVNTATALKP
VRQLLDYALKIDHRRTLPKYSFGTFRRWYRSVAAQQAKYKDQVAFFHGCFVNYNHPQLGK
DLIKVLNAMGTGVQLLSKEKCCGVPLIANGFTDKARKQAISNVESLREAIAVKGIPVIAT
SSTCTFALRDEYPEVLDVDNAGLREHIELATRWLWRKLDAGKTLPLNPLPLKVVYHTPCH
MEKMGWTLYTLELLRQIPGLELTVLDSQCCGIAGTYGFKKENYPTSQSIGAPLFRQIEES
GADIVVTDCETCKWQIEMSTSKRCEHPITLLAQALG
>EECEBICD_01074 Anaerobic glycerol-3-phosphate dehydrogenase subunit B
MKFDTVIMGGGLAGLLCGLQLQQHGLRCAIVTRGQSALHFSSGSLDLLSALPDGQPVTDI
TAGLDALCRQAPEHPYSRLGAQKVLTLAQQAQTLLNASGAQLYGDVQQAHQRVTPLGTLR
STWLSSPEVPVWPLSAQRICVVGVSGLLDFQAHLAAASLRQRDLNVETAEIDLPELDVLR
DNPTEFRAVNIARLLDNEEKWPLLYDALSPIATNCDMIIMPACFGLANDTLWRWLNERLP
CALTLLPTLPPSVLGIRLHNQLQRQFVRQGGIWMPGDEVKKVTCRRGTVSEIWTRNHADI
PLRPRFAVLASGSFFSSGLVAEREGIREPILGLDVQQTATRAEWYQQHFFDPQPWQQFGV
VTDDAFRPSLAGNTVENLYAIGSVLAGFDPIAEGCGGGVCAVSALQAAHHIAERAGEQQ
>EECEBICD_01075 Anaerobic glycerol-3-phosphate dehydrogenase subunit A
MKTRDSQASDVIIIGGGATGAGIARDCALRGLRVILVERHDIATGATGRNHGLLHSGARY
AVTDAESARECISENQILKRIARHCVEPTDGLFITLPEDDLAFQATFIRACEAAGIRAEA
IDPQQARIIEPSVNPALIGAVKVPDGTVDPFRLTAANMLDAREHGAIVLTAHEVTGLIRE
GATVCGVHVRNHLTGETQTLHAPVVVNAAGIWGQRIAEYADLSIRMFPAKGSLLIMDHRI
NQHVINRCRKPSDADILVPGDTISLIGTTSTHIDYNEIDSNRVTADEVDILLREGEKLAP
VMAKTRILRAYSGVRPLVASDDDPSGRNVSRGIVLFDHAERDGLDGFITITGGKLMTYRL
MAEWATDAVCRKLGNTRPCITADTPLPGSKESTEHTLKRIISLPAPLRGSAVYRHGDRTP
GWLSEGRQHRSLVCECEAVTAGEVQYAVENLTVNSLLDLRRRTRVGMGTCQGELCACRAA
GLLQRFNVTTAAQSITQLSEFLNERWKGVQPVAWGDALRESEFTRWVYQGLCGLEKEHQD

EI
>EECEBICD_01076 Glycerol-3-phosphate transporter
MLSIFKPAPHKARLPAAEIDPTYRRLRWQIFLGIFFGYAAYYLVRKNFALAMPYLVEQGF
SRGDLGFALSGISIAYGFSKFIMGSVSDRSNPRVFLPAGLILAAAVMLFMGFVPWATSSI
AVMFVLLFLCGWFQGMGWPPCGRTMVHWWSQKERGGIVSVWNCAHNVGGGIPPLLFLLGM
AWFNDWKAALYMPAFGAIVVALFAFAMMRDTPQSCGLPPIEEYKNDYPDDYNEKAEEELT
AKQIFMQYVLPNKLLWYIAIANVFVYLLRYGILDWSPTYLKEVKHFALDKSSWAYFLYEY
AGIPGTLLCGWMSDKVFRGNRGATGVFFMTLVTIATIVYWMNPAGNPNVDMACMIIIGFL
IYGPVMLIGLHALELAPKKAAGTAAGFTGLFGYLGGSVAASAIVGYTVDFFGWDGGFMVM
IGGSILAVILLVVVMIGEKRHHDELQLKRNGG
>EECEBICD_01077 Glycerophosphodiester phosphodiesterase, periplasmic
MKTTLKNLSVALMLAGMTIGSGAVAAEKVVIAHRGASGYLPEHTLPAKAMAYAQGADYLE
QDLVMTKDDHLVVLHDHYLDRVTDVADRFPDRARKDGRYYAIDFTLDEIKSLKFTEGFDI
ENGKKVQTYPGRFPMGKSDFRIHTFEEEIEFVQGLNHSTGKNIGIYPEIKAPWFHHQEGK
DIAAKTLEVLKKYGYTGKQDNVYLQCFDVAELKRIKNELEPKMGMDLNLVQLIAYTDWNE
TQQKQPDGRWVNYNYDWMFKPGAMKQVAEYADGIGPDYHMLVAEGSTKGNIKLTGMVQDA
HQNKMVVHPYTVRADQLPDYATDVNQLYDILYNKAGVDGLFTDFPDKAVMFLQKND
>EECEBICD_01078 hypothetical protein
MYCVPIYRDKFTVVVPENHPLAANSTVTVEELMDEPLIVSKGRYELSIMALFKEKGIEPI
FKYEFNHPDTALNFIRQGLGIALLPELTLKATTGKLCSVALEPTFYRQISLLAKEPPVEG
SPLFLLQKCMETLTDEGLL
>EECEBICD_01079 Hydrogen peroxide-inducible genes activator
MTLTQIHALLAVLEYGGFTEASKRLYMTQSAVSQAISALEEELGVDILIRERRKEIELTA
AGSRIVRHLRAIQRDVNAVKEIAEQEKRTRPVRYGLAVFPAPAPAFYPASFAILKAIIQM
>EECEBICD_01080 Staphylopine export protein
MRHLSGSLRMKEKLWTKDFWAITIVSFIIFFVFYVLLTLLPIYIADRLHASADKAGLLVT
CFLAAAIVIRPFAGQWVGKYSNKKILVLSSLAFLVITALYPVCHSIESLLFIRVLHGITF
GVITTVKGTISARLIPASRRGEGISFFSLAMGLAMVVGPWIGLNMARWNAFTAAFWLCSA
VAALGIVLSLIVTVPPVIRHADGSKPNLGFAAMFDRAALPFALVTFFMTFSYAGVSAFLA
LYARELDLMAAASNFLLCYAIFLMICRTFTGNICDKKGPKYVVFPCLLAFTIGLVALGYT
NGSIMMIISGGLIGIGYGSVTPVFQTQIISSVEPHKIGVANSLFFNAMDAGMAIGAFIMG
MMVESVGYRMIYVAGAVLVVLAGALYAVQMKKRGVMPLVSTSELH
>EECEBICD_01081 hypothetical protein
MGRVTLRITGTQLLCQDEHPSLLAALESHNVEVEYQCREGYCGSCRTRLVAGQVDWITEP
LAFIQPGEILPCCCRAKGDIEIEM
>EECEBICD_01082 Ribonucleoside-diphosphate reductase 1 subunit beta
MAYTTFSQTKNDQLKEPMFFGQPVNVARYDQQKYDIFEKLIEKQLSFFWRPEEVDVSRDR
IDYQALPEHEKHIFISNLKYQTLLDSIQGRSPNVALLPLISIPELETWVETWAFSETIHS
RSYTHIIRNIVNDPAVVFDDIVTNEQIQKRAEGISAYYDELIEMTSYWHLLGEGTHTVNG
KTVVVNLRELKKKLYLCLMSVNALEAIRFYVSFACSFAFAERELMEGNAKIIRLIARDEA
LHLTGTQHMLNLLRSGVDDPEMAEIAEECKQECYDLFVQAAQQEKEWADYLFRDGSMIGL
NKDILCQYVEYITNIRMQAVGLDLPFQTRSNPIPWINTWLVSDNVQVAPQEVEVSSYLVG
QIDSEVDTDDLSNFQL
>EECEBICD_01083 Ribonucleoside-diphosphate reductase 1 subunit alpha
MNQSLLVTKRDGRTERINLDKIHRVLDWAAEGLNNVSVSQVELRSHIQFYDGIKTSDIHE
TIIKAAADLISRDAPDYQYLAARLAIFHLRKKAFGQFEPPALYHHVVKMVELGKYDNHLL
EDYTEEEFKQMDSFIVHDRDMTFSYAAVKQLEGKYLVQNRVTGEIYESAQFLYILVAACL
FSNYPRETRLDYVKRFYDAVSTFKISLPTPIMSGVRTPTRQFSSCVLIECGDSLDSINAT
SSAIVKYVSQRAGIGINAGRIRALGSPIRGGEAFHTGCIPFYKHFQTAVKSCSQGGVRGG
AATLFYPMWHLEVESLLVLKNNRGVEGNRVRHMDYGVQINKLMYTRLLKGGDITLFSPSD
VPGLYDAFFADQDEFERLYVKYEHDDSIRKQRVKAVELFSLMMQERASTGRIYIQNVDHC
NTHSPFDPVVAPVRQSNLCLEIALPTKPLNDVNDENGEIALCTLSAFNLGAIKTLDELEE
LAILAVRALDALLDYQDYPIPAAKRGAMGRRTLGIGVINFAYWLAKNGKRYSDGSANNLT
HKTFEAIQYYLLKASNELAKEQGACPWFNETTYAKGILPIDTYKKDLDAIVNEPLHYDWQ

QLRESIKTHGLRNSTLSALMPSETSSQISNATNGIEPPRGYVSIKASKDGILRQVVPDYE
HLKDAYELLWEMPNNDGYLQLVGIMQKFIDQSISANTNYDPSRFPSGKVPMQQLLKDLLT
AYKFGVKTLYYQNTRDGAEDAQDDLAPSIQDDGCESGACKI
>EECEBICD_01084 Ubiquinone biosynthesis O-methyltransferase
MNTEKPSVAHNVDHNEIAKFEAVASRWWDLEGEFKPLHRINPLRLGYITERSGGLFGKKV
LDVGCGGGILAESMAREGATVTGLDMGFEPLQVAKLHALESGIEVEYMQETVEEHAAKHA
QQYDVVTCMEMLEHVPDPQSVVHACAQLVKPGGEVFFSTLNRNGKSWLMAVVGAEYILRM
VPKGTHDVKKFIKPAELLSWVDETVLKEQHITGLHYNPITNTFKLGPGVDVNYMLHTRAK
KA
>EECEBICD_01085 hypothetical protein
MLRKKQYNSKANVSYLIYQQANLMKYTITTIKYVNFLISDILDPLILNKLITMEPGLTME
EKKMKNRLKRDFSHISIEIPKSITQVIKEKIREMIIHGDFDLGQAISENELSNILMVSKT
PIREAFIWLSYNENLVNIIPRSGTFVFSVTDEDINDLIKMRVILEQGAIREAMEKNANNV
IVELSNILSKSAKINAERDTQAYLKLDHDFHYVFVKYADNKYISQAHLLISARLLAIRYR
LDFTTEYITSSNRGHATILDMLKNNNVEGVCNFITHHIGSGFTERARKLLALKA
>EECEBICD_01086 hypothetical protein
MSSPQENLYDAIRIVKRKIIPLAFILYFFNYMDRVNIGFAALRMNESLGITPEDFANISS
VFFISYLIFQIPSSIGLQKLGARKWISSIIIGWGAVTGLIFLLKILNIFCWLAFFWGYLK
LAFSRAWFIILHVGSRLVNAEKSIVSSCYLLLLHRYWLHRCLVGSSSI
>EECEBICD_01087 Putative tartrate transporter
MSGWIIEHLNTPDYEGWRWLFAIEGIPTVFLGILTFYLLPDSPEKAKWLTPQQISALVNK
LRTDNETAAALNKNTNSSFLSVIKNPVLLQLSFAYMLIQAAALAANYWLPGLVKGFSADF
TDTDVGLIMSIPFIFAMFSMPWWGWHSDKKNERKWHAALPMFLAGCGFLMIALVPSMSLR
MLGLTFYGVGILSYYGPYWALPSALLSPSGLAISIAFINSCSSLGGFLINKSLGFVSTHY
GATGIFIVEAILCFAAVAVLALMKIDVKKEKANKQMLYHEPDKWRNYENYIY
>EECEBICD_01088 D-galactonate dehydratase family member RspA
MSSYNPVLIRVNTDSGLSGIGEVGLAYGAGAKAGVGIIRDLAPLVVGEDPLNIEKIWEFF
FRKTFWGMGGGNVFYAGMSAIDIALWDIKGKYLGVPVYQLLGGKTNEKLRTYASQLQFGW
GDKRQILVTPEEYAEAARAALDDGYDAIKVDPLEIDRNGDDCVFQNKNRNYSGLLLADQL
KMGEARIAAMREAMGDDADIIVEIHSLLGTNSAIQFAKAIEKYRIFLYEEPIHPLNSDNM
QKVSRSTTIPIATGERSYTRWGYRELLEKQSIAVAQPDLCLCGGITEGKKICDYANIYDT
TVQVHVCGGPVSTVAALHMETAIPNFIIHEHHTNAMKASIRELCTHDYQPENGYYVAPEQ
PGLGQELNDEVVKEYLAYVIK
>EECEBICD_01089 DNA gyrase subunit A
MSDLAREITPVNIEEELKSSYLDYAMSVIVGRALPDVRDGLKPVHRRVLYAMNVLGNDWN
KAYKKSARVVGDVIGKYHPHGDSAVYDTIVRMAQPFSLRYMLVDGQGNFGSIDGDSAAAM
RYTEIRLAKIAHELMADLEKETVDFVDNYDGTEKIPDVMPTKIPNLLVNGSSGIAVGMAT
NIPPHNLTEVINGCLAYIDNEDISIEGLMEHIPGPDFPTAAIINGRRGIEEAYRTGRGKV
YIRARAEVEADAKTGRETIIVHEIPYQVNKARLIEKIAELVKDKRVEGISALRDESDKDG
MRIVIEVKRDAVGEVVLNNLYSQTQLQVSFGINMVALHHGQPKIMNLKDIISAFVRHRRE
VVTRRTIFELRKARDRAHILEALAIALANIDPIIELIRRAPTPAEAKAALISRPWDLGNV
AAMLERAGDDAARPEWLEPEFGVRDGQYYLTEQQAQAILDLRLQKLTGLEHEKLLDEYKE
LLEQIAELLHILGSADRLMEVIREEMELIRDQFGDERRTEITANSADINIEDLISQEDVV
VTLSHQGYVKYQPLTDYEAQRRGGKGKSAARIKEEDFIDRLLVANTHDTILCFSSRGRLY
WMKVYQLPEASRGARGRPIVNLLPLEANERITAILPVREYEEGVNVFMATASGTVKKTAL
TEFSRPRSAGIIAVNLNDGDELIGVDLTSGSDEVMLFSAAGKVVRFKEDAVRAMGRTATG
VRGIKLAGDDKVVSLIIPRGEGAILTVTQNGYGKRTAADEYPTKSRATQGVISIKVTERN
GSVVGAVQVDDCDQIMMITDAGTLVRTRVSEISVVGRNTQGVILIRTAEDENVVGLQRVA
EPVDDEELDAIDGSVAEGDEDIAPEAESDDDVADDADE
>EECEBICD_01090 Sensor histidine kinase RcsC
MKYLASFRTTLKVSRYLFRALALLIWLLIAFVSVFYIVNALHQRESEIRQEFNLSSDQAQ
RFIQRTSDVMKELKYIAENRLTAENGVMSSRARDDKMVVPDFEPLFADSDCAAMGSAWRG
SLESLAWFMRYWRDNFSAAYDLNRVFLIGSDNLCMANFGLREMPVERDDALKALHERIMK
YRNAPQEESGNNLFWISQGARQGVGYFYALTPVYLANRLQALLGVEQSIRMENFFTPGSL

PMGVTIIDENGHSLISLTGPDGNIKAEPRWMQERSWFGYTPGFRELVLKKSLPPSSLSIV
YSVPVDLVLERIRILILNAILLNVLVGAGLFTLARMYERRIFIPAESDAQRLEEHEQFNR
KIVASAPVGICILRTIDGVNILSNELAHTYLNMLTHEDRQRLTQIICGQQVNFVDVLTSN
NTNLQISFVHSRYRNENVAICVLVDVSTRVKMEESLQEMAQAAEQASQSKSMFLATVSHE
LRTPLYGIIGNLDLLQTKELPKGVDRLVTAMNNSSSLLLKIISDILDFSKIESEQLKIEP
REFSPREVMNHITANYLPLVVRKQLGLYCFIEPDVPVSLNGDPMRLQQVISNLLSNAIKF
TDIGCIVLHVRCDGDYLSIRVRDTGVGIPAKEVVRLFDPFFQVGTGVQRNFQGTGLGLAI
CEKLISMMDGDISVDSEPGMGSQFTLRIPLYGAQYPVKKSVEGLAGTCCWLAVRNTSLCQ
FIETSLARSGVHTQRYEGQEPAADDILIVDDALEHTWQGRAAVVFCRRHIGIPLERAPGE
WVHSVASVHELPALLARIYSIELDSEALSSALPTTDKTADSNDDMMILVVDDHPINRRLL
ADQLGSLGYQCKTANDGVDALNVLSKNAIDIVLSDVNMPNMDGYRLTQRIRQLGLTLPVV
GVTANALAEEKQRCLESGMDSCLSKPVTLDVLKQTLAVYAERVRKTRA
>EECEBICD_01091 Transcriptional regulatory protein RcsB
MNNMNVIIADDHPIVLFGIRKSLEQIEWVNVVGEFEDSTALINNLPKLDAHVLITDLSMP
GDKYGDGITLIKYIKRHFPSLSIIVLTMNNNPAILSAVLDLDIEGIVLKQGAPTDLPKAL
AALQKGKKFTPESVSRLLEKISAGGYGDKRLSPKESEVLRLFAEGFLVTEIAKKLNRSIK
TISSQKKSAMMKLGVENDIALLNYLSSVTLSPTDKE
>EECEBICD_01092 Phosphotransferase RcsD
MSQSDTTVSTRFSLLPGSITRFFLLLIIVLLVTMGVMVQSAVNAWLKDKSYQIVDITHAI
HKRVDTWRYVTWQIYDNIAATTTPSTGEGLQETRLKQDVYYLEKPRRKTEALIFGSHDSA
TLEMTQRMSTYLDTLWGAENVPWSMYYLNGQDNSLILISTLPLKDLSSGFKESTIGNIVD
SRRAEMLQQANALDERESFSSLRKLAWQNGHYFTLRTTFNQPGHLATVVAFDLPINDLIP
PGMPLDSFRIEPDATQATGRSSEKEAPDSVTISFNGSKIEISSALNSTGMRLIWQVPFGT
LLLDTLQNILLPLLLNIGLLALALFGYATFRHQPGRSTESTSGNAANNELRVLRAINEEI
VSLLPLGLLVYDQEGNRTVISNKIADHLLPHLNLQNITSMAEQHQGVIQATINNELYEIR
LFRSQIAPRTQIFIIRDQDREVLVNKKLKQAQRLYEKNQQGRAAFMQNISNTLKEPVRQL
AVNAAAVTTPESLKLADQADVLVRMIDEIQLANMLENDAWKSEATLFSLQDLIDEVVPEV
LPAIKRKGLQLLINNHLSGNDERWGDRDALRRILLLLIQYAVTTTAMGKITLEVEQDESI
AERLTFRILDTGEGVTLNEIDNLHFPYMNETQGDRYGKANPLTFWLCNQLARKLGGHLNI
KARETLGTRYTVHVKMLPHDQHTQVEERLLDDVSVMVDVTSNEVRAIVLRQLENWGATCI
TPDERQISQEYDLFLTDNPSNLTASGLLLSDDESGVRKIGPGQLRVNFNMSNAMQEAVLQ
LIEEQLAQEEIPASPLGGDENAELHASGYYALFVDTVPDDVKRLYTEAATSDFAALAQTA
HRLKGVFAMLNLVPGKQLCETLEHLIREKDAPGIEKYISDIDAYVKSLL
>EECEBICD_01093 Outer membrane porin C
MKVKVLSLLVPALLVAGAANAAEIYNKDGNKLDLFGKVDGLHYFSDDKGSDGDQTYMRIG
FKGETQVNDQLTGYGQWEYQIQGNQTEGSNDSWTRVAFAGLKFADAGSFDYGRNYGVTYD
VTSWTDVLPEFGGDTYGADNFMQQRGNGYATYRNTDFFGLVDGLDFALQYQGKNGSVSGE
NTNGRSLLNQNGDGYGGSLTYAIGEGFSVGGAITTSKRTADQNNTADEHLYGNGDRATVY
TGGLKYDANNIYLAAQYSQTYNATRFGTSNGNNKSTSYGFANKAQNFEVVAQYQFDFGLR
PSVAYLQSKGKDISNGYGASYGDQDIVKYVDVGATYYFNKNMSTYVDYKINLLDKNDFTR
DAGINTDDIVALGLVYQF
>EECEBICD_01094 FAD:protein FMN transferase
MKMTFCRAVCLAAAFLLMGCDEAPETTTASPAAQVLEGKTMGTLWRVSVVGIDAKRAAEL
QTKIQTQLDADDWLLSTYKNDSALMRFNHSRSLAPWPVSEAMADIVTSALRIGAKTDGAM
DITVGPLVNLWGFGPDRQPLHIPTPAQIDAAKAKTGLQHLQVIDRAGHQFLQKDLPDLYV
DLSTVGEGYAADHLARLMEQEGIARYLVSVGGALSSRGMNAQGQPWRVAIQKPTDRENAV
QAIVDINGHGISTSGSYRNYYELDGKRVSHVIDPQTGRPIEHNLVSVTVIAPTALEADGW
DTGLMVLGTQKAQEVVRREGLAVFMIMKEGEGFKTWMSPQFKTFLVSDKN
>EECEBICD_01095 Bifunctional transcriptional activator/DNA repair enzyme Ada
MKKALLTDDECWLRVQARDASADGRFVFAVRTTGVFCRPSCRSKRALRKNVRFFANAEQA
LDAGFRPCKRCQPDNARAQQRRLDKIACACRLLEQETPVTLASLAQAVAMSPFHLHRLFK
VSTGMTPKGWQQAWRARRLREALAKGEPITAAIYRAGFPDSSSYYRHADQTLGMTAKQFR
KGGDNVSVRYALTDWVYGRCLVAESERGICAILPGDSDDALLAELHTLFPTARHEPADAS

FQQRVRQVVAAINTRDVLLSLPLDIQGTAFQQQVWQALCAIPCGETVSYQQLAATIGKPT
AVRAVASACGANKLAMVIPCHRVVRRDGALSGYRWGVRRKAQLLKREAQKEE
>EECEBICD_01096 Alpha-ketoglutarate-dependent dioxygenase AlkB
MLDLFADEAPWQEPLAPGAVVLRRFAFRAAQSLLDDIGFVASQSPFRQMVTPGGYTMSVA
MTNCSALGWTTDRHGYCYAVRDPLTDKPWPALPLSFASVCRQAAIAAGYASFQPDACLIN
RYAPGAKLSLHQDKDEPDLRAPIVSVSLGVPAVFQFGGLRRSDPLQRILLEHGDIVVWGG
ESRLFYHGIQPLKAGFHPMTGEFRYNLTFRQAAEKE
>EECEBICD_01097 ABC transporter ATP-binding/permease protein YojI
MELLILVWRQYRWPFISVMALSLASAALGIGLIAFINQRLIETVDTNVMVLPEFLGLLLL
LMVVTLGSQLALTTLGHHFVYRLRSEFIKRILDTHVERIEQLGSASLLAGLTSDIRNITI
AFVRLPELVQGIILTVGSAAYLAMLSTKMLLVTAIWMAVTIWGGFVLVARVYQHMATLRE
TEDKLYNDYQTVLEGRKELTLNRERAEQIFQQCYTPDAKEYRHHIIRADTFHLSAVNWSN
IMMLGAIGLVFWMANSLGWADTNVAATYSLTLLFLRTPLLSAVGALPTLLTAQVAFNKLN
KFALAPYKPDFPQPKAFPDWKTLELRNVVFHYQDNAFSVGPINLTIHRGELLFLIGGNGS
GKSTLAMLLTGLYQPQSGTILLDGQPIAAGQPEDYRKLFSAVFTDVWLFDRLLGPQGKPA
NPALVEKWLEHLKMTHKLELNDGRILNLKLSKGQKKRIALLLALAEERDIILLDEWAADQ
DPHFRREFYQVLLPLMQEMGKTIFAISHDDHYFIHADRLLEMRNGQLTELTGDERDAASR
DAVARTA
>EECEBICD_01098 Ecotin
MNNQMKMFVPAVVFAALASASASAWANNGDTAQPLEKIAPYPQAEKGMKRQVITLTPQQDES
TLKVELLIGQTLNVDCNQHRLGGTLETKTLEGWGYDYYVFDNVTSPVSTMMACPEGKKEQ
KFVTAWLGEDGMLRYNSKLPIVVYTPANVDVKYRIWKADANVQNAVAR
>EECEBICD_01099 hypothetical protein
MHTPIEVNAVAGSKECQVKTRKRAFSHISMSYIDAYITKITNS
>EECEBICD_01100 Ferredoxin-type protein NapF
MVDLSRRSMLTGSWRNASNGILPPWARETTYFLAHCLRCDACIQACEADILQRGAGGYPS
VDFKRGECSFCYACAQACTESLFLPRHTRAWDLIFTLTENCLARQSVECHRCQDSCEPMA
ITFRPTLSGIYQPQLDSQACNGCGACVAICPVSAIKAENHHAH
>EECEBICD_01101 Chaperone NapD
MRTNWQVCSLVVQAKSQNIAAIGTQLNTLPGCEVALSDVESGQLIVVAEADQSETLMQTI
ESVRNVEGVLAVSLVYHQQDEQGEETP
>EECEBICD_01102 Periplasmic nitrate reductase
MKLSRRSFMKANAVAAAAAAAGLSVPGVARAVVGQQEAIKWDKAPCRFCGTGCGVLVGTQ
QGRVVACQGDPDAPVNRGLNCIKGYFLPKIMYGKDRLTQPMLRMKDGSYHKDGEFTPVSW
EQAFDVMEEKFKTSLKEKGPEAIGMFGSGQWTIWEGYAAAKLFKAGFRSNNIDPNARHCM
ASAVVGFMRTFGMDEPMGCYDDIEQADAFVLWGSNMAEMHPILWSRITNRRLSDPNVKVA
VLSTFQHRSFELADNGIVFTPQSDLVILNYIANYIIQNNAVNQDFFTKHVNLRKGATDIG
YGLRPTHPLEKAAKNPGSDASEPMSFDEYKAFVAEYTLDKTAEMTGVPKDQLEQLAQLYA
DPNKRVISYWTMGFNQHTRGVWANNLVYNLHLLTGKISQPGCGPFSLTGQPSACGTAREV
GTFSHRLPADMVVTNEKHRDICEKHWQIPAGTIPAKVGLHAVAQDRALKDGKLNVYWVMC
NNNMQAGPNINEDRMPGWRDPRNFIIVSDPYPTVSALSADLILPTAMWVEKEGAYGNAER
RTQFWRQQIKAPGEAKSDLWQLVQFSRRFKTEEVWPEALLAQKPELRGKTLYDVLFATPA
VSKFPLSELKEDQLNDESRELGFYLQKGLFEEYAWFGRGHGHDLAPFDDYHNARGLRWPV
VEGKETQWRYSEGNDPYVKAGEGYKFYGKPDGKAVIFALPFEPAAESPDNEYDLWLSTGR
VLEHWHTGSMTRRVPELHRAFPEAVVFIHPLDAKARDLRRGDKVKVSSRRGEVISIVETR
GRNRPPQGLVYMPFFDAAQLVNNLTLDATDPLSKETDFKKCAVKLAKV
>EECEBICD_01103 Ferredoxin-type protein NapG
MSRTAKPQNGRRRFLRDVVRTAGGLAAVGVALGLQQQTARASGVRLRPPGALNENVFASA
CVRCGQCVQACPYDTLKLATLASGLSAGTPYFVARDIPCEMCEDIPCAKVCPSGALNKDI
ASIDDSRMGLAVLLDQENCLNFQGLRCDVCYRECPKIDEAITLELDRNMRTGKHARFLPT
VHSDACTGCGKCEKVCVLEQPAIKVLPLSLAKGELGHHYRFGWLEGKDGKS
>EECEBICD_01104 Ferredoxin-type protein NapH
MANRKRDAGREARAKKGWWRSHRWLVLRRISQFMVLGMFLSGPWLGFWVLHGNYSSSLLF
DTLPLTDPLITLESLASGHLPATVALTGAVIIAVLYALAGKRLFCSWVCPLNPVTDLANW

MRRKFDLNQSATIPRHIRYVLLVVVLIGSALTGTLLWEWINPVSLLGRSLVMGFSSGALL
IIALFLFDLLVVEHGWCGHICPMGALYGVLGSKGVVTVTAKKREKCNRCMDCFHVCPEPH
VLRAPVLDEQSPAQVTHRDCMTCGRCVDVCSEDVFTITIHNIIRDASRR
>EECEBICD_01105 Periplasmic nitrate reductase, electron transfer subunit
MKSHDLMKALCQWMATLALVVSGAVWAANGVDLSQSPEVSGTQEGAIRMPKEQERMPLNY
VNQPPMIPHSVEGYQVTTNTNRCLQCHGVESYRTTGAPRISPTHFMDSDGKVSGNVAPRR
YFCLQCHVPQSDTAPIIDNTFTPSQGYGK
>EECEBICD_01106 Cytochrome c-type protein NapC
MENSNRKPGWIKRVWRWWRSPSRLALGTLLLIGFIGGIIFWGGFNTGMEKANTEEFCISC
HEMRNTVYEEYMETVHYNNRSGVRATCPDCHVPHEWGPKMIRKIKASKELYAKVFGLIDT
PQKFEAHRLTMAQNEWRRMKDNNSQECRNCHNFDFMDLTAQKGVAAKMHDQAVKDGQTCI
DCHKGIAHKLPDMRDVKPGF
>EECEBICD_01107 Cytochrome c biogenesis ATP-binding export protein CcmA
MLEARDLHCERDERTLFRGLSFTVDAGEWVQVTGGNGAGKTTLLRLLTGLARPDGGEVYW
QGEPLRRVRDSFHRSLLWIGHQPGIKSRLTARENLHFFHPGDGARLPEALAQAGLAGFED
VPVAQLSAGQQRRVALARLWLTRAALWVLDEPFTAIDVNGVARLTRRMAAHTAQGGMVIL
TTHQPLPGAADTVRRLALTGGGAGL
>EECEBICD_01108 Heme exporter protein B
MWRVFCLELRVAFRHGADIAGPLWFFLMVITLFPLSVGPQPQLLARIAPGIIQVAALLAS
LLALERLFRDDLQDGSLEQLMLLPVPLPAVVLAKVLAHWAVTGLPLMMLSPLVALLLGMD
VYGWKIMALTLLLGTPALGFLAAPGVALTAGLRRGGVLLGILVLPLSVPVLIFATAAMDA
ASMHLPVDGYLAVLGALLAGSATLSPFATAAALRLSVQ
>EECEBICD_01109 Heme exporter protein C
MYLHVPAAIWSMGIYAAMAVAAFTGLVWQMKMASLAVAAMAPVGAVYTFIALVTGAAWGK
PMWGTWWVWDARLTSELVLLFLYAGVIALWHAFDDRKMAGRAAGILVLVGVVNLPVIHYS
VEWWNTLHQGSTRMQQSIDPAMRSPLRWAIAGFLLLFMTLALMRMRNLILLMEKRRPWVS
ELILKRGHR
>EECEBICD_01110 Heme exporter protein D
MTVAPLVLLALHTVLQRRAILRGVAQQRAREARMRAAQAQQEAA
>EECEBICD_01111 Cytochrome c-type biogenesis protein CcmE
MNLRRKNRLWVVCAVLAGLALTTALVLYALRANIDLFYTPGEILYGKRETQQLPAVGQRL
RVGGMVMPGSVRRDPDSLKVNFSLYDAEGSVTVSYEGILPDLFREGQGVVVQGTLEKGSH
VLAQEVLAKHDENYTPPEVEKAMQENHRRPQRADKDTSS
>EECEBICD_01112 Cytochrome c-type biogenesis protein CcmF
MMPEYGHALLCLALGVALLLSVYPLWGVARGDARMMASAGVFAWLLFICVAGAFFVLVHA
FVVNDFTVAYVAGNSNTQLPVWYRVAATWGAHEGSLLLWVLLMSGWTLAVAVFSRPVPAD
IVARVLAVMGMVCAGFLAFILFTSGPFARTLPAFPVEGRDLNPLLQDPGLIFHPPLLYMG
YVGFSVAFAFAIAALLSGRLDSAFTRFARPWTLAAWVFLTLGIVLGSAWAYYELGWGGWW
FWDPVENASFMPWLAGTALLHSLAVTEQRAGFKAWTLLLSICAFSLCLLGTFLVRSGVLV
SVHAFASDPARGMFILAFMVLVTGGSLLLFAVRGHRVRSRVNNALWSRESLLLGNNVLLM
AAMLVVLLGTLLPLVHKQLGLGSISVGEPFFNTMFTWLMVPFALLLGVGPLVRWGRDRPR
NIRKLLWAAVVTTLVLSVLLPWLLEDKIIAMTAVGMAMACWIAVLAVAEAVQRVSRGTKT
SLSYWGMVAAHLGLAVTITGIAFSQNYSVERDVRMRAGDSVTIHDYRFTFREVRDITGPN
YRGGVALIGVTRHGEPEAVLHAEKRLYNTSRMVMTEAAIDGGLTRDLYAALGEELDNGAW
AVRLYYKPFVRWIWAGGLLMALGGLLCLVDPRYRRRKPLPEAG
>EECEBICD_01113 Thiol:disulfide interchange protein DsbE
MKRNVLLLPLLIFLLIAAALLWQLTRNAQGDDPTNLESALTGKPVPAFRLESLETPGQYY
EAEVLTQGKPVLLNVWATWCPTCRAEHQYLNQLSAQGIRVVGLNYKDDRAKAVAWLKELG
NPYALSLSDSDGMLGLDLGVYGAPETFLIDGRGIIRYRHAGDLNARVWESELKPLWDRYS
REAAQ
>EECEBICD_01114 Cytochrome c-type biogenesis protein CcmH
MRLLPGMVMLMLVLVISGSARATTDVMPFKDEAQEQQFRQLTEQLRCPKCQNNSIADSNA
MIATDMRRRVYDLMQEGKSRQEIIDYMVARYGNFVTYDPPLTPLTVLLWVLPLAAIVAGG
WIIVARTRRRVRLRREPLPADTPVCGARAGWGVYVPGAVIALAVGAGSYALTGSYQQVRA

WQQATAQTPGLLARALDPAAQPLNEEEMARLALGLRTRLQNDAGNVEGWLMLGRTGMVLG
NAGTATGAYANAYRLAPKNSDAALGYAEALTRSSDPEDNRRGGELLRQLVSRDHTDIRVL
SLYAFNAFEQRRFGEAVAAWEMMLKLLPAGDARRAVIERSIRLAQEK
>EECEBICD_01115 Nitrate/nitrite response regulator protein NarL
MPEVTPFQVLIVDDHPLMRRGIRQLLELDPAFHVVAEAGDGASAIDLANRIEPDLILLDL
NMKGLSGLDTLNALRRDGVTAQIIILTVSDSASDIYALIDAGADGYLLKDSDPEVLLEAI
RKGANGGKVFSDRVNEYLRERERFGAQEDPFSILTERELDVLHELAQGLSNKQIASVLNI
SEQTVKVHIRNLLRKLNVRSRVAATILFLQTRGIQ
>EECEBICD_01116 hypothetical protein
MKRIFIYLLLPCAFACSANDNVFFGKGNKHQISFAAGESIRRGGVEHLYTAFLTYSEPSD
FFFLQARNNLELGGFKAKGSDDCSKHSGSVPCNKYNQGVLGISKDVALVHFAGIYTGIGL
GAYIKSKSRDDMRVNSAFTFGEKAFLGWNFGAFSTEAYIRHFSNGSLTDKNSGHNFVGAS
ISYNF
>EECEBICD_01117 hypothetical protein
MHELILHELIGHLSCAVVNVKSMQANNVNHVCTKTEKAHLHPMLEEMFVVAGK
>EECEBICD_01118 hypothetical protein
MLMTRRLPPFWLCQPTPRWLMPPRLLWSPYLRCSPQMLRMKLPTKIPLENQPVRAGFFYA
YN
>EECEBICD_01119 hypothetical protein
MISLEDASLTKKGIVKLSSATDSDSEALAATPKAVKTVMGEVRTKAPLDSPAFTGTPTTP
TPPGDAKGLQTTNAEFVRKLIAALVGSVLEPLDTLQELADALGNDPNFATTVLNKLAGKQ
PLDETLTALSGKSVDGLIEYVGLRETISRAADAL
>EECEBICD_01120 hypothetical protein
MRRIGAARAFDGAVTIGCDDNPWTTAEFIVWLESQGAFNHPYWMCRGSWSYAYNKIITDT
GCGTICLAGAVIEVMGVRGAMTIRVTTSHSVSGW
>EECEBICD_01121 E3 ubiquitin-protein ligase SspH2
MPFHIGSGCLPATISNRRIYRIAWSDTPPEMSSWEKMKEFFCSTHQTEALECIWTICHPP
AGTTREDVVSRFELLRTLAYAGWEESIHSGQHGENYFCILDEDSQEILSVTLDDAGNYTV
NCQGYSETHRLTLDTAQGEEGTGHAEGASGTFRTSFLPATTAPQTPAEYDAVWSAWRRAA
PAEESRGRAAVVQKMRACLNNGNAVLNVGESGLTTLPDCLPAHITTLVIPDNNLTSLPAL
PPELRTLEVSGNQLTSLPVLPPGLLELSIFSNPLTHLPALPSGLCKLWIFGNQLTSLPVL
PPGLQELSVSDNQLASLPALPSELCKLWAYNNQLTSLPTLPSGLQELSVSDNQLASLPTL
PSELYKLWAYNNRLTSLPALPSGLKELIVSGNRLTSLPVLPSELKELMVSGNRLTSLPML
PSGLLSLSVYRNQLTRLPESLIHLSSETTVNLEGNPLSERTLQALREITSAPGYSGPIIQ
FDMAGASAPRETRALHLAAADWLVPAREGEPAPADRWHMFGQEDNADAFSLFLDRLSETE
NFIKDAGFKAQISSWLAQLAEDEALRANTFAMATEATSSCEDRVTFFLHQMKNVQLVHNA
EKGQYDNDLAALVATGREMFRLGKLEQIAREKVRTLALVDEIEVWLAYQNKLKKSLGLTS
VTAEMRFFDVSGVTVTDLQDAELQVKAAEKSEFREWILQWGPLHRVLERKAPERVNALRE
KQISDYEETYRMLSDTELRPSGLVGNTDAERTIGARAMESAKKTFLDGLRPLVEEMLGSY
LNVQWRRN
>EECEBICD_01122 Protein UmuC
MEVLDHLNAKKGWGKLYFAGEGIQQQWAMKRNILSSRYTTRYEDLLQVK
>EECEBICD_01124 Inner membrane protein YejM
MVTHRQRYREKVSQMVSWGHWFALFNILLATLLGSRYLFVADWPTTLAGRIYSYLSIVGH
FSFLVFATYLLILFPLTFIVMSQRLMRFISAILATAGMTLLLIDSEVFTRFHLHLNPIVW
ELVINPDQNEMARDWQLMFISVPVILLIEMLFATWSWQKLRSLTRRRHFARPLAAFFFVS
FIASHLIYIWADANFYRPITMQRANLPLSYPMTARRFLEKHGLLDAQEYQRRLVEQGNPE
AVSVQYPLSNLHYRDMGTGQNVLLITVDGLNYSRFEKQMPELATFAEQNIDFTRHMSSGN
TTDNGIFGLFYGISPGYMDGVLSTRTPAALITALNQQGYQLGLFSSDGFASPLYRQALLS
DFSMPAAQTQSDAQTASQWIDWLGRYAQEDNRWFSWISFNGTNIDDSNQKNFVKRYASAA
SDVDAQINRVLNALREAGKFENTVVIITAGRGIPLTPEENRFDWSQGHLQVPLVIHWPGT
PAQRINVLTDHTDVMTTLMQRLLHVSTPANEYSQGQDIFTVPRRHNWVTAADGSTLAITT
PQMTLVLNNNGHYQTYDLHGEKIKDQKPQLSLLLQVLTEEKRFIAN
>EECEBICD_01125 hypothetical protein

MPQLSRYSDEHVEQLLSELLSVLEKHKAPTDLSLMVLGNMVTNLINTSVAPAQRQAIANS
FARALQSSISEDNAH
>EECEBICD_01126 Nucleoid-associated protein YejK
MSLDINQIALHQLIKRDEQNLELVLRDSLLEPTTTVVEMVAELHRVYSAKNKAYGLFNEE
SELAQALRLQRQGEEDFLAFSRAATGRLRDELAKYPFADGGIVLFCHYRYLAVEYLLVTV
LNNLSSMRVNENLDINPTHYLDINHADIVARIDLTEWETNPQSTRYLTFLKGRVGRKVAD
FFMDFLGASEGLNAKAQNRGLLQAVDDFTAEAQLDKAERQNVRQQVYSYCNEQLQAGEEI
ELESLSKELSGVSEVSFSEFTAEKGYELEESFPADRSTLRQLTKYAGSGGGLTINFDAML
LGERIFWDPATDTLTIKGTPPNLREQLQRRTSGEK
>EECEBICD_01127 hypothetical protein
MSKGIRQKPPVNFSLFRQVLYAHIVAFLMMLVLGMVFTVLSLVLFYTYGANWLLSLFICP
LFLLSGLFITGFAFKSTWSSIRYYYDKGQLKRYGLNLDATLTHKEKVEIRIDNAKRQVRV
DELELHVLFDFQFDSKTWSCGDLLTNEKVFDALNDGQTIPIRILPWKPESASVRQRALFN
RLKGMNTAAETTDSRLGEALIECGEV
>EECEBICD_01128 50S ribosomal protein L25
MFTINAEVRKEQGKGASRRLRAANKFPAIIYGGSEAPIAIELDHDQVMNMQAKAEFYSEV
LTLVVDGKEVKVKAQAVQRHAYKPKLTHIDFVRA
>EECEBICD_01129 Putative DNA repair helicase RadD
MIFTLRPYQQEAVDATLSHFRRHRTPAVIVLPTGAGKSLVIAELARVARGRVLVLAHVKE
LVAQNHAKYCALGLEADIFAAGLKRKESQGKVVFGSVQSVARNLDAFQEEFSLLIVDECH
RIGDDEDSQYQQILTHLSKVNPHLRLLGLTATPFRLGKGWIYQFHYHGMVRGNDNALFRD
CIYELPLRYMIKHGYLTPPERLDMPVVQYDFSRLQAQSNGLFSEADLNRELKKQQRITPH
IISQIMEFAQTRKGVMIFAATVEHAKEIVGLLPADDAALITGDTPGPERDALIDNFKAQR
FRYLVNVSVLTTGFDAPHVDLIAILRPTESVSLYQQIVGRGLRLAPGKTDCLILDYAGNP
HDLYAPEVGSPKGKSDNVPVQVFCPACGFANTFWGKTTADGTLIEHFGRRCQGWFDDDDG
HREQCDFRFRFKNCPQCNAENDIAARRCRECDAILVDPDDMLKAALRLKDALVLRCSGMT
MQHGQDEKGEWLKITYYDEDGADVSERFRLHTPAQRTAFEQLFIRPHTRTPGVPLRWITA
ADIVAQQALLRHPDFVVARMKGQYWQVREKVFDYEGRFRRAHELRG
>EECEBICD_01130 Ribosomal small subunit pseudouridine synthase A
MRLDKFIAQQLGVSRAIAGREIRGNRVTVDGDIIKNAAFKLLPEHAVAYDGNPLAQQHGP
RYFMLNKPQGYVCSTDDPDHPTVLYFLDEPVAYKLHAAGRLDIDTTGLVLMTDDGQWSHR
ITSPRHHCEKTYLVTLESPVADDTVAQFAKGVQLHNEKDLTKPATLEVITPVQVRLTISE
GRYHQVKRMFAAVGNRVVELHRERIGAITLDENLAPGEYRPLTEEEIASVG
>EECEBICD_01131 Bicyclomycin resistance protein
MTTRQHSSFAIVFILGLLAMLMPLSIDMYLPALPVISAQFGVPAGSTQMTLSTYILGFAL
GQLIYGPMADSLGRKPVILGGTLVFAAAAVACALAQTIDQLIVMRFFHGLAAAAASVVIN
ALMRDIYPKEEFSRMMSFVMLVTTIAPLMAPIVGGWVLVWLSWHYIFWILAIAAILASVM
IFALIKETLPVERRQPFHIRTTIGNFAALFRHKRVLSYMLASGFSFAGMFSFLSAGPFVY
IEINHVPPQDFGYYFALNIVFLFVMTIINSRFVRRVGALNMFRAGLWIQFAMAVWMVFSA
LMGIGFWALVVGVAAFVGCVSMVSSNAMAVILDEFPHMAGTASSLAGTFRFGIGAIVGAL
LSLATFTSAWPMIWSIALCAACSILFYLYASRPKKR
>EECEBICD_01132 putative protein YejG
MNTLQLSIVHRLPQNYRWSAGFAGSKVEPIPQNGQSTENSLVALKLLSPAGDSAWSVMHK
LSQALSDIEVPCSVLECEGEPCLFVNRQDEFAATCRLKNFGVAIAEPFSNNNPF
>EECEBICD_01133 putative ABC transporter ATP-binding protein YejF
MTSPLLAIENLSVGFRQQQHVRPVVNAISLQVNAGETLALVGESGSGKSVTALSILRLLP
TPPAVYLSGDIRFHGESLLHASEQTLRGVRGNKIAMIFQEPMVSLNPLHTLEKQLYEVLS
LHRGMRREAARAEMIGCLDRVGIRQASQRLRDYPHQLSGGERQRVMIAMALLTRPELLIA
DEPTTALDVSVQAQILSLLRELQRELNMGLLFITHNLSIVKKLADSVAVMQHGKCVENQR
ADTLLSAPTHPYTQKLLNSEPTGDPVPLPAGQAPLLEVDKLRVAFPIRKGILKRVVDHNV
VVNNISFTLHPGETLGLVGESGSGKSTTGLALLRLIRSEGRIVFDGQSLDTLNRRQLLPV
RHRIQVVFQDPNSSLNPRLNVLQIIEEGLRVHQPTLSGAQREQQVKAVMMEVGLDPETRH
RYPAEFSGGQRQRIAVARALILKPSLIILDEPTSSLDKTVQAQILALLKSLQQKHRLAYI
FISHDLHVVHALCHQVIVLRQGEVVEQGQCERVFTAPQQAYTRQLLALS

>EECEBICD_01134 Inner membrane ABC transporter permease protein YejE
MGPPYALAPIPLILPPRSLSPPRLRRKNWLGTDANGGDVFARILYGTRISILFGLMLTIC
SSVMGVLAGALQGYYGGKVDLWGQRLIEVWSGMPTLFLIILLSSVVQPNFWWLLAITVLF
GWMSLVGVVRAEFLRTRNFDYIRAAQALGVSDRDIILRHMLPNAMVATLTFLPFILCSSI
TTLTSLDFLGFGLPLGSPSLGELLLQGKNNLQAPWLGIAAFLSVAILLSLLIFIGEAVRD
AFDPAKAV
>EECEBICD_01135 Inner membrane ABC transporter permease protein YejB
MGAYLIRRLLLVIPTLWAIITINFFIVQIAPGGPVDQAIAAIEFGQGGALPGASGEGVRA
SHAQTGVGNISDSHYRGGRGLDPEVIAEITHRYGFDKPLHERYFKMLRDYLSFDFGDSLF
RSASVLTLIKDSLPVSITLGLWSTLIIYLVSIPLGIRKAVHNGSRFDVWSSAFIIIGYAI
PAFLFAILLIVFFAGGSYYDIFPLRGLVSANFDTLPWYQKITDYLWHITLPVLATVIGGF
AALTMLTKNSFLDEVRKQYVVTARAKGVSEKNILWKHVFRNAMLLVIAGFPATFISMFFT
GSLLIEVMFSLNGLGLLGYEATVSRDYPVMFGTLYIFTLIGLLLNILSDISYTLVDPRID
FEGR
>EECEBICD_01136 hypothetical protein
MIARVMLLLVALVSAGAQAQEIKESYAFAVLGEPKYAFNFDHFDYVNPAAPKGGQMTLSA
IGTFDNFNRYSLRGNPGVRTEALYDTLFTTSDDEPGSYYPLIADHARYAADYSWVEISIN
PRARFHDGTPITARDVAFTFHKFMTEGVPQFRLVYKGTTVKAIAPLTVRIELAKPGKEDM
LSLFSLPIMPEKFWKNHKLSDPLSTPPLASGPYRITQWKMGQYIVYSRVKNYWAANLPVN
RGRFNLDTIRYDYYLDDNVAFEAFKAGAFDLRLENDAKNWATRYIGKNFDNHYIIKEEQK
NESAQDTRWLAFNIQRPVFKDRRVREAVTLAFDFEWMNKALFYNACSRTNSYFQNTEYAA
RNYPDADELVLLAPMKKDLPPEVFTQIYQPPVSNGDGYDRENLLKADALLTQAGWVINGQ
QRVNSVTGKPLTFELLLPASSNSQWVLPFQHNLQRLGITMTIRQVDNSQLTNRMRSRDYD
MMPRLWRAMPWPSSDLQISWASEYIDSSYNAPGVQSPVVDKLIAQIIAAQGDKAKLVPLG
RALDRVLTWNYYMLPMWYMAQDRLAWWDKFSHPAIRPVYTIGLDTWWYDVNKAAKLPAAR
R
>EECEBICD_01137 putative cyclic di-GMP phosphodiesterase PdeN
MLTRDSSSGRKILLTCIITGIAAALLIGSLQFFLSWHKRDVKYNTLLSDVQHYLASYFAD
LKATTDILQPLTINTCQQVGAELTSRAAFSLNVRAFLLIKDKKVFCSSATGAMNMPLQQL
VPDIDIRKDVAMAILPGTPMMPNKPTMVIWYRNPLLNDSGVFTSLNINLTPYLLYTTRQD
DFNGIALIVGNTALSTFSSRLLTVAELPGTPSRQATINGLPLKIQLYADSWTYNDLWYAL
MLGCISGIVAGFICYFIYALRTRPGKEILTAIKHEQFYVVYQPVVDTRTLSVTGLEVLLR
WRHPTAGEIPPDAFIHYAEAQQLIVPLTQHLFELIARDAPTLQKVMPVGAKFGINIAPEH
LHSDSFKEDMHRLRDALPAHHFHIVLEITERDMLQQYEAAKLFEWLHSAGFEIAIDDFGT
GHSALIYLERFTLDYLKIDRGFIQAIGTETVTSPVLDTVLTLSRRLNMLTVAEGVETPEQ
ARWLRDHGVNYLQGYWISRPLPLQDFVLWMSAPSVPEW
>EECEBICD_01138 Murein DD-endopeptidase MepS/Murein LD-carboxypeptidase
MVKSQPILRYILRGIPAIAVAVLLSACSTTTNTAKNMHSETHAVGNSDSSSLQASQDEFE
NMVRNLDVKSRIMDQYADWKGVRYRLGGSTKKGVDCSSFVQRTFREQFGLELPRSTYEQQ
EMGKAVSRNNLRTGDLVLFRAGSTGRHVGIYIGNNQFVHASTSSGVIISSMNEPYWKKRY
NEARRVLSRS
>EECEBICD_01139 Lipid A 1-diphosphate synthase
MTMKTRYSLIIILLNAAGLALFLSWYLPVNHGFWFTIDSGIFHFFNQKLVESHAFLWWVAI
TNNRAFDGCSLLAMGGLMLSFWLKEDASGRRRIVIIGLVMLLTAVVLNQLGQALIPVKRA
SPTLSFEHIYRVSELLHIPTKDASKDSFPGDHGMMLLIFSAFMLRYFGKTAGIIALIIFV
VFAFPRVMIGAHWFTDIVVGSLTVILIGLPWWLMTPLSDRAIALFENYLPGGNKQILNK
>EECEBICD_01140 Zinc-binding GTPase YeiR
MTKTNLITGFLGSGKTTSILHLLAHKDPAEKWAVLVNEFGEVGIDGALLADSGALLKEIP
GGCMCCVNGLPMQVGLNTLLRQGKPDRLLIEPTGLGHPKQILDLLTAPVYEPWIDLRATL
CILDPRLLLDQQNVANENFRDQLASADIIIANKTDRATAQSDAALQQWWRQYGGDRQLIH
AEHGQIDGKLLDLPRQNLAELPASAAHSHTHASKKGLAALNLPAQQRWRRSLNSGQGHQA
CGWIFDADTVFDTIGLLEWARLAPVGRVKGVMRIQEGLVRINRQGDDLHIETQSVAPPDS
RVELISNTETDWNTLQTALLKLRLATHA
>EECEBICD_01141 Elongation factor P-like protein

MPRANEIKKGMVLNYNGKLLIVKDIDIQSPTARGAATLYKMRFSDVRTGLKVEERFKGDD
IVDTVTLSRRGVDFSYVDGNEYVFMDKEDYTPYTFTKDQIEEELLFMPEGGMPDMQVLTW
DGQLLALELPQTVDLEIVETAPGIKGASASARNKPATLSTGLVIQVPEYLSAGEKIRIHI
EERRYMGRAD
>EECEBICD_01142 hypothetical protein
MDCRSGCGACCIAPSISSPIPGMPNGKPMNTRCVQLSEDNLCLIFGSPLRPKVCSGLQPT
GDMCLTTREEAIIYLLELEQKTLP
>EECEBICD_01143 hypothetical protein
MRGENSEIDLVDVSLFLIRKWLSIVIVTFIFVAIGYVFVSVQHDTKVVSITIKPNLDTPA
TYALCGYTNDIQCKTTVILNQFSRFLPAEYKNKISFDAKNQLVLFKAQGREESVNNIIST
LKNSVDKMAAWYIDDGDIKRYSVHKNMLQTETYAKLSLLSEAVKNNANIEVIDVAVTPKY
KMRLVLVLCGLMGVIFSITGLLCRRALTEYRFEKKTMNDRA
>EECEBICD_01144 Sugar efflux transporter B
MWGTNSLYIINMPLFIINELHLPEKLAGVMMGTAAGLEIPTMLIAGYFAKRLGKRLLMCI
AVVAGLCFYAGMLLAHAPATLLGLQLLNAIYIGILGGIGMLYFQDLMPGQAGSATTLYTN
TIRVGWIIAGSLAGIAAEIWNYHAVFWFALVMIVATMFCLARIKDV
>EECEBICD_01145 Sugar efflux transporter B
MHNSPAAAAPKSFDLTSTAFLIVAFLTGIAGALQTPTLSIFLTDEVHARPGMVGFFFTGS
AVIGIIVSQFLAGRSDKKGDRKKLIVFCCVLGMLACVLFAWNRNYFILLFIGVFLSSFGS
TANPQMFALAREHADRTGREAVMFSSILRAQVSLAWVIGPPLAYALAMGFSFTVMYLSAA
VAFIVCGVMVWFFLPSMRKRCAAGNRHAGSTAS
>EECEBICD_01146 hypothetical protein
MCFTSKNCYKTGNFSCVLKQKDPDFLKRFSAIWFF
>EECEBICD_01147 Multiphosphoryl transfer protein
MFQLSVQDIHPGEQAGNKEEAIRQIAAALAQAGNVAGGYVDGMLAREQQTSTFLGNGIAI
PHGTTDTRDQVLKTGVQVFQFPQGVTWGEGQVAYVAIGIAASSDEHLGLLRQLTHVLSDD
SVAEQLKSAITAEELRALLMGEKQSEQLKLDNETMTLDVIASSLVTLQALNAARLKEAGA
VDAAFVAKTINDSPMNLGQGIWLNDSAEGNLRSAVAVSRATQAFDVEGEKAALLVTVAMN
DEQPIAVLKRLGDLLLNNKADRLLSADAATLLALLTSDDALTDDVLSAEFVVRNEHGLHA
RPGTMLVNTIKQFNSEITVTNLDGTGKPANGRSLMKVVALGVKKGHRLRFTAQGEDAEQA
LKAIGDAIAAGLGEGA
>EECEBICD_01148 Tagatose-6-phosphate kinase
MSRRVATITLNPAYDLVGFCPEIERGEVNLVKTTGLHAAGKGINVAKVLKDLGIDVTVGG
FLGKDNQDGFQQLFSELGIANRFQVVQGRTRINVKLTEKDGEVTDFNFSGFDVTPADWER
FVNDSLSWLGQFDMVCVSGSLPAGVSPEAFTDWMTRLRSQCPCIIFDSSREALVAGLKAA
PWLVKPNRRELEIWAGRKLPEMKDVIDAAHALREQGIAHVVISLGAEGALWVNASGEWIA
KPPAVDVVSTVGAGDSMVGGLIYGLLMRESSEHTLRLATAVAALAVSQSNVGITDRPQLA
AMMARVDLQPFN
>EECEBICD_01149 PTS system fructose-specific EIIB'BC component
MKTLLIIDANLGQARAYMAKTLLGAAAHKANLEIIDNPNDAELAIVLGESLPNDNALNGK
KVWLGDIGRAVAHPELFLSEAKSHATLYSAPAAAAPAASGGPKRVVAVTACPTGVAHTFM
AAEAIETEAKKRGWWVKVETRGSVGAGNAITPEEVAEADLVIVAADIEVDLAKFAGLPMY
RTSTGLALKKTAQELDKAVAEATPYQPAGKASQAATEGKKESAGAYRHLLTGVSYMLPMV
VAGGLCIALSFAFGIEAFKVPGTLAAALMQIGGGSAFALMVPVLAGYIAFSIADRPGLTP
GLIGGMLAVSTGSGFIGGIIAGFLAGYMAKLISTKLKLPQSMEALKPILIIPLISSLVVG
LAMIYLIGKPVAGILEGLTHWLQTMGTANAVLLGAILGGMMCTDMGGPVNKAAYAFGVGL
LSTQTYAPMAAIMAAGMVPPLALGLATMVARRKFDKAQQEGGKAALVLGLCFITEGAIPF
AARDPMRVLPCCIVGGALTGAISMAVGAKLMAPHGGLFVLLIPGAITPVLGYLLAIVAGT
LVAGLAYAVLKRPETEVAAKAA
>EECEBICD_01150 Endonuclease 4
MKYIGAHVSAAGGLANAPARAAEIGATAFALFTKNQRQWRAAPLTPQVIDDFKIACEKYH
FSAAQILPHDSYLINLGHPVSEALEKSRDAFLDEMQRCEQLGLTLLNFHPGSHLMQIAQE
DCLARIAESINIALAQTEGVTAVIENTAGQGSNLGFEFEQLAAIIDGVEDKSRVGVCIDT
CHAFAAGYDLRTPEACEKTFSEFGKIVGFQYLRGMHLNDAKSAFGSRVDRHHSLGEGNIG

HDAFRWIMQDGRFDGIPLILETINPDIWAEEIAWLKAQQIAEAMA
>EECEBICD_01151 hypothetical protein
MTELTLQNHRRTMWHFIPGLALSAVITGVALWGGAIPAVAGAGFSALTLAILLGMVIGNT
VYPQIWKQCDGGVLFAKQHLLRLGIILYGFRLTFSQIADVGISGIVIDVLTLSSTFMLAC
FLGQKVFGLDRHTSWLIGAGSSICGAAAVLATEPVVKAEASKVTVAVATVVIFGTIAIFL
YPAMYPLLAHWFSPETYGIYIGSTMHEVAQVVAAGHAVSPDAENAAVIAKMLRVMMLAPF
LIILAARVKQLSPATGAEKSKITIPWFAIFFIVVAIFNSFHLLPKAVVDMLVTLDTVLLA
MAMAALGLTTHVSALKKAGAKPLLMALALFAWLIIGGGAINVLIHSLIA
>EECEBICD_01152 HTH-type transcriptional activator CmpR
MHITLRQLEVFAEVLKSGSTTQASVMLSLSQSAVSAALTDLEGQLGVQLFDRVGKRLVVN
EHGRLLYPRALALLEQAVEIEQLFREDNGAIRVYASSTIGNYILPAMIARYRQHYPALPL
ELSVGNSQDVINAVLDFRVDIGLIEGPCHSTEIISEPWLEDELVVFAAPSSPLTQGPVTL
EQLAASPWILRERGSGTREIVDYLLLSHLPRFHMAMELGNSEAIKHAVRHGLGISCLSRR
VIAEQLQAGTLGEVAVPLPRLVRTLWRVHHRQKHLSNALQRFLSYCE
>EECEBICD_01153 Lysine-specific permease
MGSKTKTTEAPALRRELKARHLTMIAIGGSIGTGLFVASGATISQAGPGGALFSYILIGL
MVYFLMTSLGELAAYMPVSGSFATYGQNYVEEGFGFALGWNYWYNWAVTIAVDLVAAQLV
MGWWFPDTPGWIWSALFLCVIFLLNYISVRGFGEAEYWFSLIKVATVIIFIIVGVAMIIG
IFKGVEPVGWSNWTTGDAPFAGGFAAMIGVAMIVGFSFQGTELIGIAAGESENPEKNIPR
AVRQVFWRILLFYVFAILIISLIIPYTDPNLLRNDVKDISVSPFTLVFQHAGLLSAAAIM
NAVILTAVLSAGNSGMYASTRMLYTLACDGKAPRIFAKLSRGGVPRNALYATTVIAGLCF
LTSMFGNQTVYLWLLNTSGMTGFIAWLGIAISHYRFRRGYVLQGYDVNDLPYRSGFFPLG
PIFAFVLCLIITLGQNYEAFLKDTIDWGGVAATYIGIPLFLLIWFGYKLIKGTHFVRYSE
MHFPERVKK
>EECEBICD_01154 Colicin I receptor
MFRFNPFVRVGLCMSAVTLAWPVAAATDDGETMVVTASAIEQNLKDAPASISVITQQDLQ
RRPVQNLKDVLKEVPGVQLTNEGDNRKGVSIRGLDSSYTLILIDGKRVNSRNAVFRHNDF
DLNWIPVDAIERIEVVRGPMSSLYGSDALGGVVNIITKKIGQKWHGSVTVDSTIQEHRDR
GDTYNGQFFTSGPLIDGVLGMKAYGSLAKREKDEQQSSATTATGETPRIEGFTSRDGNVE
FAWTPNENHDVTAGYGFDRQDRDSDSLDKNRLERQNYALSHNGRWDLGNSELKFYGEKVE
NKNPGNSSPITSESNSIDGKYVLPLAAVNQFLTFGGEWRHDKLSDAVNLTGGSSTKTSAS
QYALFLEDEWRIFEPLALTTGIRMDDHETYGDHWSPRAYLVYNATDTLTVKGGWATAFKA
PSLLQLSPDWATNSCRGGCRIVGSPDLKPETSESWELGLYYRGEEGILEGVEASVTTFRN
DVDNRISISRTPDVNAAPGYSNFVGFETNSRGQRVPVFRYYNVNKARIQGVETELKVPFN
EAWKLSLNYTYNDGRDVSNGGNKPLSDLPFHTANGTLDWKPAQLEDWSFYVSGKYTGRKR
ADSATAKTPGGYVVWDTGAAWQATKNVKLRAGVLNVGDKDLKRDDYGYTEDGRRYFMAVD
YRF
>EECEBICD_01155 Membrane sensor protein UhpC
MSIKTFIFSQPDKPIVKEKKEIDQTYKKFRFEIIASVFISYAVFYLTRKNFSAAMPAMLT
ETSLTAEDFAIMSSIFYILYGAMKFVGGMLVDKINPKAMTGPVLIGVGIVNILFGFSDSV
AAFYVLYSLNAILQGTSFPPMAKIMASWFSKNERGRWWAIVEAAHNIGGSLAPLLTSFAI
AFSGSWKMGFYVPGAISLLMGIVALFTIKDRPGTLGLPNVGQWRNDPTELAQVKASPVNL
SFWQIFVKYILTNPLVWIIIIGDMSVYIARTILNDWPQIYYSQVHGWSLIKANSIISWFE
AGGLAGGLLAGYLSDFMFKSNRWMTGLIFALALCICIVLVPLVQDTSYTLTAILFTIMGF
ALYGPHMLFAVGCLDVTHKDAAGSITGFRGLFSYVGAAMAGVPVIMVKNSWAWSGVYIYA
VIAILLTTLSLALLSRLHRL
>EECEBICD_01156 Phosphoserine phosphatase SerB2
MSIIYFITTQDIDTFQKKLQETLFNAVTMPFPLLFDKRYAALINTAYLKLTLPAECLTPE
FYRYLRELSLQWQFDFFIKPQPLPANGIIAFDMDSTFIAEEGVDEIARELGMSTQITAIT
QQAMEGKLDFNASFTRRIGMLKGTPKAVLNAVCDRMTLSPGLLTILPVIKAKGFKTAIIS
GGLDIFTQRLKARYQLDYAFSNTVEIRDNVLTDNITLPIMNAANKKQTLVDLAARLNIAT
ENIIACGDGANDLPMLEHAGTGIAWKAKPVVREKIHHQINYHGFELLLFLIEDEL
>EECEBICD_01157 L-serine dehydratase 1
MESCLDIFKIVIGPSSSRTVGPMRAACHFISLLREQETLPLIREIEIELYGALSLSRKCH

NVDTALYLGLLGCQPENVDLRSHMAVIKRAENENKIELPLSDAGGITIKVKIIANHQAHP
GHPYAMTFRARDDYFTVYEETWFSTGAGQVRKHGEPLTPSLPLRTVSPFEFSHAAQLLAL
CRRNGLSVAALMMKNELCRHSPQTLQNYLAQIWDVMQQAVYRGLHTEGVLPGPYQVPRRA
CALHKTLQANRSASDFLTALNWVNAFAIAVSEENASGGQIVTAPTNGACGIIPAALCWYD
KFVTPLEPGALTRFFLTAAAIAILFKQNASILGSEVGCQGEIGVACSMAAAGLAELMGAS
VEQTLSAAEIAMEHHLGLTCDPLGGQVQIPCIERNAISAVKAINAATMAMSRVSEPCISL
DEIIAAMYETGKDMSAKYRETYHGSLGKIQPRKRG
>EECEBICD_01158 hypothetical protein
MKYNTMNNDEIILSLCARLKETRLSLSMTQQQLADRAHVGIATIKRIEKGEGSILIR
>EECEBICD_01159 S-formylglutathione hydrolase YeiG
MEMLEEHRCFGGWQQRWRHHAATLNCAMTFSIFLPPTQDNEPPPVLYWLSGLTCNDENFT
TKAGAQRIAAELGIVLVMPDTSPRGEQVADDSGYDLGHGAGFYLNATQPPWASHYRMYDY
LRDELPALIQTQFNVSDRCAISGHSMGGHGALIMALKNPGKYTSVSAFAPIVNPSRVPWG
IKALTAYLGEDESAWTEWDSCELMLASEPQDAIPVLIDQGDSDQFLADQLQPAVLAEAAR
QTAWPMTLRIQPGYDHSYYFIASFIEDHLRFHARYLRDERETSPT
>EECEBICD_01160 GTP cyclohydrolase 1
MPSLSKEAALVHDALVARGLETPLRPPMDELDNETRKSLIAGHMTEIMQLLNLDLSDDSL
METPHRIAKMYVDEIFAGLDYANFPKITLIENKMKVDEMVTVRDITLTSTCEHHFVTIDG
KATVAYIPKDSVIGLSKINRIVQFFAQRPQVQERLTQQILTALQTLLGTNNVAVSIDAVH
YCVKARGIRDATSATTTTSLGGLFKSSQNTRQEFLRAVRHHP
>EECEBICD_01161 hypothetical protein
MIKAETMERNVTLDFVRGVAILGILLLNISAFGLPKAAYLNPAWYGAIVPEDAWSWAILG
IVAQAKFLTLFALLFGAGLQMLLPRGKQWIQSRLTLLVLLGFIHALFFWDGDILLAYGLV
GLICWRLIRDAPSVKSLFNTGILLYLVGIGVLLLLGVVSSSETSRAWTPDASAILYEKYW
KLNGGMEAISNRAEMLSNSLLALGAQYGWQLAGMMLLGAALMRSGWLKGQYSLRHYRRTG
ALLVALGLLINLPAVILQWRLDWAYRWCAFLLQAPRELSAPLQTLGYAALMFGFWPQLSR
CRLTLAIACVGRMALTNYLLQTIICTTLFYQFGLFMKFNRLELLFFVVPVWAINLLFSVI
WLRFWRQGPVEWLWRQLTLRASGSLR
>EECEBICD_01162 HTH-type transcriptional regulator GalS
MITIRDVARQAGVSVATVSRVLNNSALVSPDTRDAVMQAVTLLGYRPNANAQALATQVSD
TIGVVVMDVSDAFFGALVKAVDLVAQQHQKYVLIGNSYHEAEKERHAIEVLIRQRCNALI
VHSKALTDRELSDFMDQIPGMVLINRIVPGYAHRCVCLDNVSGARMATRMLLNNGHQRIG
YLASSHRIEDDAMRREGWLHALQEQGIAASESWIGTGTPDMQGGESAMVELLGRNLQLTA
VFAYNDNMAAGALTALKDNGIAIPLHLSVIGFDDIPIARYTDPQLTTVRYPIASMAKIAT
ELALQGAAGTLDITATHCFMPTLVRRHSVAWRQNAVLITN
>EECEBICD_01163 D-galactose-binding periplasmic protein
MNKKVLTLSAVMASLLFGAHAQAADTRIGVTIYKYDDNFMSVVRKAIEKDGKSAPDVQLL
MNDSQNDQSKQNDQIDVLLAKGVKALAINLVDPAAAGTVIEKARGQNVPVVFFNKEPSRK
ALDSYDKAYYVGTDSKESGVIQGDLIAKHWQANQGWDLNKDGKIQYVLLKGEPGHPDAEA
RTTYVVKELNAKGIQTEQLALDTAMWDTAQAKDKMDAWLSGPNANKIEVVIANNDAMAMG
AVEALKAHNKSSIPVFGVDALPEALALVKSGAMAGTVLNDANNQAKATFDLAKNLAEGKG
AADGTNWKIENKIVRVPYVGVDKDNLSEFTQK
>EECEBICD_01164 Galactose/methyl galactoside import ATP-binding protein MglA
MGSTISPSSGEYLLEMRGINKSFPGVKALDNVNLNVRPHSIHALMGENGAGKSTLLKCLF
GIYQKDSGSIVFQGKEVDFHSAKEALENGISMVHQELNLVLQRSVMDNMWLGRYPTKGMF
VDQDKMYQDTKAIFDELDIDIDPRARVGTLSVSQMQMIEIAKAFSYNAKIVIMDEPTSSL
TEKEVNHLFTIIRKLKERGCGIVYISHKMEEIFQLCDEITILRDGQWIATQPLEGLDMDK
IIAMMVGRSLNQRFPDKENKPGDVILEVRHLTSLRQPSIRDVSFDLHKGEILGIAGLVGA
KRTDIVETLFGIREKSSGTITLHGKKINNHTANEAINHGFALVTEERRSTGIYAYLDIGF
NSLISNIRNYKNKVGLLDNSRMKSDTQWVIDSMRVKTPGHRTQIGSLSGGNQQKVIIGRW
LLTQPEILMLDEPTRGIDVGAKFEIYQLIAELAKKGKGIIIISSEMPELLGITDRILVMS
NGLVSGIVDTKTTTQNEILRLASLHL
>EECEBICD_01165 Galactoside transport system permease protein MglC

MSALNKKSFLTWLKEGGIYVVLLVLLAIIIFQDPTFLSLLNLSNILTQSSVRIIIALGVA
GLIVTQGTDLSAGRQVGLAAVVAATLLQSMENANKVFPEMATMPIALVILIVCAIGAVIG
LVNGIIIAYLNVTPFITTLGTMIIVYGINSLYYDFVGASPISGFDSGFSTFAQGFVAMGS
FRLSYITFYALIAVAFVWVLWNKTRFGKNIFAIGGNPEAAKVSGVNVALNLLMIYALSGV
FYAFGGLLEAGRIGSATNNLGFMYELDAIAACVVGGVSFSGGVGTVFGVVTGVIIFTVIN
YGLTYIGVNPYWQYIIKGGIIIFAVALDSLKYARKK
>EECEBICD_01166 NAD-dependent dihydropyrimidine dehydrogenase subunit PreA
MLTKDLSVTFCGVKFPNPFCLSSSPVGNCYEMCAKAYDTGWGGIVFKTIGFFIANEVSPR
FDHLTKEDTGFIGFKNMEQIAEHPLEENLAAIRRLKQDYPDKVLIASIMGENEQQWQELA
RLVEEAGADMIECNFSCPQMTSHAMGSDVGQSPELVEKYCRAVKRGSSLPMLAKMTPNIG
DMCEVALAAKRGGADGIATINTVKSITNIDLNRKIGMPVVNGKSSISGYSGKAVKPIALR
FIQQLRMHPELRDFPISGIGGIETWEDAAEFLLLGAATLQVTTGIMQYGYRIVEDMASGL
SHYLADQGFASLQEMIGLANGNIIPAEDLDRSYIVYPRINQEKCVGCGRCYISCYDGGHQ
AMEWDEHSRTPHCNTEKCVGCLLCGHVCPVACIDLGEVKFKKGEKEHALTL
>EECEBICD_01167 NAD-dependent dihydropyrimidine dehydrogenase subunit PreT
MPQQNYLDELTPGFTPLLAIKEASRCLLCHDAPCSQDCPAQTDPGKFIRSIYFRNFKGAA
ETIRENNALGAVCARVCPTEKLCQRGCTRSGIDKPIDIARLQRFITDFEQQTAMQIYQPG
SKTRGKVAIIGAGPAGLQASVTLTHLGYDVTIYEKQPQPGGWLRHGIPAFRLPQSVLDQE
IARIVEMGVNIKCNCDVGGSLSLAQLKAEYRAVLMTVGMSCGSDLPLFEQASHVEIAVDF
LQRARQADGDISVPRSALIIGGGDVAMDVASTLKILGCPSVTCVAREELAQFPASEKEFT
STQALGVSIIDGFTPVAVSGNKVTFHHVRHSGELTLEAENIILAVGQHARLDTFAEIKAQ
HNIIDTHNYQTDDPAIFAAGDIVKGDKTVVYAVKTGKEAAQAIHHYLEEACSC
>EECEBICD_01168 hypothetical protein
MLKRVFYSLLVLVGLLLLTVLGLDRWMSWKTAPYIYDELQDLPYRQVGVVLGTAKYYRKG
VINQYYRYRIQGALNAYNSGKVNYLLLSGDNALQSYNEPMTMRKDLIAAGVDPADIVLDY
AGFRTLDSIVRTRKVFDTNDFIIITQRFHCERALFIALHMGIQAQCYAVPSPKNMLTVRL
REFGARFSALADLYIFKREPRFLGPLVPIPTQHQVPNDAQGYPAVTPEQLLELEKKKGEM
IMDVQTIFVILAFLLLPLFCFREAWKGWRTGAVDKVVKNARKPVYVYRHADPVQYWSYLF
LYTGCGFLFTGMIIYLLFYR
>EECEBICD_01169 Cytidine deaminase
MNELNSGLDLRIHLPGRAPHTLRDYLPDAFGPKDLEIKTLLMDEQDHGFTLTGDTLTQAA
ITAANKSHMPYSHSPSGVALECKDGRIFTGSYAENAAFNPTLPPLQGALNLLSLNGYDYA
DIQRAILAEKGDAALIQWDATAATLKALGCHNIDRVLLG
>EECEBICD_01170 Cytidine deaminase
MHPRFQTAFAQLADNLQSALAPILADHHFPAMLTAEQVSTLKNTAGLDEDALAFALLPLA
AACARTDLSHFNVGAIARGVSGNWYFGANMEFLGATMQQTVHAEQSAISHAWLRGEKRAR
RRHR
>EECEBICD_01171 Inner membrane protein YohK
MMTYIWWSLPLTLAVFFAARRLAAHFKMPLLNPLLVAMVVIIPFLLLTGIPYEHYFKGSE
VLNDLLQPAVVALAYPLYEQLHQIRARWKSIISICFVGSLVAMITGTSVALLMGATPEIA
ASVLPKSVTTPIAMAVGGSIGGIPAISAVCVIFVGILGAVFGHTLLNAMHIRTKAARGLA
MGTASHALGTARCAELDYQEGAFSSLALVICGIITSLVAPFLFPLILAVMR
>EECEBICD_01172 hypothetical protein
MSKSLNIIWQYIRAFVLIYACLYAGIFLASLLPITIPGSIIGMLILFVLLALQILPAKWV
NPGCYVLIRYMALLFVPIGVGVMQYFDLLRAQFGPVVVSCAISTLVVFVVVSWSSHLIHG
ERKVVGQKGTKK
>EECEBICD_01173 HTH-type transcriptional regulator GbpR
MANWAQKLKLHHLQTLVALGEQGNLTHVARMMNISQPALSKWLSQLEDEIGITLFERHSK
GLRPSEGGKLLLQHAQRLINDLERSQYEIARFKQGGLVGSLKIGCSPVATDCVSQAILSL
LNEMPTLHLNIEEKVMTPLLHDLLAGQVDVVVGRVGGRALQLPLNYQVLYTEPVCFVARP
HHPLAARAQIAWSDLAHWRWIVWPTGTPIRISIDNALVDNGVMLPENTIESASMNVSTNL
LQSSDMISILSLRLAQRYASQGQLAILNLPKIEQKGSVGMFWRKNETPSLALSRFLYFLA

QV
>EECEBICD_01174 3-hydroxybenzoate transporter MhbT
MTQRRDLQALIDAAPVGKMQWRVIICCFLVVMLDGFDTAAIGFIAPDIRTHWQLSASELA
PLFGAGLLGLTAGALLCGPLADRFGRKRVIELCVALFGALSLLSAFSPDIETLVLLRFLT
GLGLGGAMPNTITMTSEYLPARRRGALVTLMFCGFTLGSAMGGIVSAQLVPLIGWHGILA
LGGILPLMLFFGLLFALPESPRWQVRRQLPQAVVARTVSAITGERYHDTQFFLHETAAVA
KGSIRQLFAGRQLVITLMLWVVFFMSLLIIYLLSSWMPTLLNHRGIDLQQASWVTAAFQV
GGTLGALLLGVLMDRLNPFRVLAVSYALGAVCIVMIGLSENGLWLMALAIFGTGIGISGS
QVGLNALTATLYPTQSRATGVSWSNAIGRCGAIVGSLSGGMMMALNFSFDTLFFVIAIPA
AISAVMLTLLTVVVRLSISVPDDLPRASVVNE
>EECEBICD_01175 Gentisate 1,2-dioxygenase
MYEINQNVKDSRQQYYQHISGQNLTPLWESLHHLVPQTPNANCAPAYWNYQEIRPLLMES
GNVIGAKEAIRRVLVLENPALRGQSSITATLYAGLQLILPGEVAPSHRHNQSALRFIVEG
KGAFTAVDGERTPMHTGDFILTPQWRWHDHGNPGSEPVVWLDGLDLPLVNLLGCGFAEDY
PEDQQPVTRKEGDYLPRYAANMLPLRHQRGNSSPIFNYRYDRSREALHDLTRMGDPDEWE
GYKLRYVNPVTGGYPMPSMGAFLQLLPKGFASRVARSTDSTIYHVVEGAGQVTIGNETFH
FSAKDIFVAPTWHEVSFRSSEDTVLFSFSDKPVQEALGLFREARY
>EECEBICD_01176 Fumarylpyruvate hydrolase
MTKYVFQPQAPVTVPVAGSDEQFPVRRVYCVGRNYAAHAREMGFDPDREPPFFFCKPADA
VVPVAAGDTLELPYPAQTDNYHYEIELVVAIGKKGSDIPLEKAHEYVWGYATGLDMTRRD
RQMEMRQMGRPWEIGKAFDLSAPIAPLHKAAETHNVDNAPIWLQVNGEDHQRSDIRHLIW
SVNETISYLSGFFELQPGDLIFTGTPEGVGAVVKGDVITGNVEGLTPIAVKIV
>EECEBICD_01177 Maleylpyruvate isomerase
MKLYSFFNSSASYRVRIALALKGIDYQTVGVNIRIGQQNALAYRRMNPVGLVPTLVTDDG
ESLGQSLAIVDWLDRHFPQTPLLPTSDPARSQALEIVYAIACDIHPINNMRVLRYLTDEL
KVSEEDKKRWYAHWIQQGLSAVEQLLRKSQSGRFCVGDAPGLADCCLIPQWANALRMGCD
LSGYPRCKAVYDACVQLPAFIAAAPENQQDKIPA
>EECEBICD_01178 3-hydroxybenzoate 6-hydroxylase
MTHVTSAIIVGGGIGGAATALSLARQGIKVMLLEKAHEIGEIGAGIQLGPNAFSALDSLG
VGDVARQRAVFTDHITMMDAVNAEEVVCIETGQAFRDHFGGPYAVIHRVDIHATVWEAVL
THPGVEYRTSTHIVDIRQTPDDVTVFDEQGNSWTADILVGCDGVKSVVRQSLLGDAPRVT
GHVVYRAVIDCDDMPDDLRINAPVLWAGPHCHLVHYPLRGGQQYNLVVTFHSRQQEEWGV
KDGSKEEVLSYFAGIHPRPRQMLDKPTSWRRWSTADREPVAKWGTERITLVGDAAHPVAQ
YMAQGACMALEDAVTLGKALERCDGDAQQAFALYESVRIPRTARIVWSTREMGRLYHAAG
VERQVRNLLWKGKSQEAFYRGIEWLYGWKEDNCLEPR
>EECEBICD_01179 tRNA-dihydrouridine(16) synthase
MRVLLAPMEGVLDALVRELLTEVNDYDLCITEFVRVVDQLLPVKVFHRICPELHHASRTP
SGTPVRIQLLGQHPQWLAENAARATALGSYGVDLNCGCPSKVVNGSGGGATLLKDPELIY
QGAKAMRAAVPSHLPVTVKVRLGWDSGDRKFEIADAVQQAGASELVVHGRTKAQGYRAEH
IDWQAIGEIRQRLTIPVIANGEIWDWQSAQACMATSGCDAVMIGRGALNIPNLSRVVKYN
EPRMPWPEVVTLLQKYTRLEKQGDTGLYHVARIKQWLGYLRKEYIEAIELFQSIRALNRS
SEIARAIQAIKI
>EECEBICD_01180 Toluene efflux pump outer membrane protein TtgI
MNRNSFLAATASLPLFILLAGCAPMHDTRQSLTQQTPSSHVDSSLPAALKNGWPDSQWWK
AYHDAQLDALIDNAIQHSPDMQVAEQRIQLAEAQAKAVEAQDGPQLDFSADIERQRMSAE
GLMGPFAITDPAAGTTGPWYTNGTFGLTAGWDLDLWGKNRAEVTARIGAVKAREAEQEQT
RQLLASGVARLYWEWQTQAALKNVLMQIEHEQQNVVAVNRELYQHGITSSVEGVETDIDA
SKTQQQLNDVNGKMKVIEARLSALTNTQSAALKLRQVSLPAVESQLPSQLGYSLLARRAD
LQAAHWYIESSLSSIDAAKAAFYPDINLMAFLQQDALHLSDLFRHSAQQYGITGGLTLPI
FDSGRLNANLDIAKAQSNLSIANYNKAVVDAVNDVARAASQVETLAQKNQHQQIEHDAQ
RVVGLAQARFNAGIIAGSRVSEAKIPALREQCNGLLLQGQWLDASIQLTSALGGGYHS
>EECEBICD_01181 putative oxidoreductase YohF
MTKVAIVTASDSGIGKACALLLAQNGFDIGITWHSDERGAQETAKKAAQFGVRAETIHLD
LSQLPEGAQVIEHLIQRLGRVDVLVNNAGAMTKSAFIDMPFTQWRQIFTVDVDGAFLCAQ

IAARHMIKQGEGGRIINITSVHEHTPLPQASAYTAAKHALGGLTKSMALELIEHHILVNA
VAPGAIATPMNDMDDSDIEPGSEPSIPIARPGSTHEIASLVAWLCSEGASYTTGQSLIVD
GGFMLANPQFNAK
>EECEBICD_01182 Inner membrane protein YohD
MDINTLITHYGYAALVIGSMAEGETVTLLGGVAAHQGLLKFPLVAAAVALGGMMGDQLLY
LLGRCYGGKILRRFPRYHTKIRRAQKMIQRHPYLFVIGTRFMYGFRVVGPLLIGASRLPP
KIFLPLNIVGALIWALLFTTLGYLGGEVIAPWLHDLDQHLRHGVWLILAIVLVVGVRWWL
KRRGKAEAR
>EECEBICD_01183 Inner membrane protein YohC
MIKSENETVSHHYTHHVLLMAAIPVVCAFIGTTQIGWNFGDGNVLQLSLFTAFALAVLFY
GVMLAGVAVMGRVIWWMARNYPQRPSLARCMVFAGYVATPLFLSGLVALYPLVWLCALVG
AVALFYTGYLLYLGIPTFLNINREEGLSFSSSTLAIGVLVLEVLLAITVILWGYGYRLF
>EECEBICD_01184 D-alanyl-D-alanine endopeptidase
MLAVPFAPQAVAKTAATTAASQPEIASGSAMIVDLNTNKVIYSNHPDLVRPIASITKLMT
AMVVLDARLPLDEILKVDISQTPEMKGVYSRVRLNSEISRKNMLLLALMSSENRAAASLA
HYYPGGYNAFIKAMNAKAKALGMTHTRFVEPTGLSIHNVSTARDLTKLLIATKQYPLIGQ
LSTTREDMATFSHPAYTLPFRNTNHLVYRDNWNIQLTKTGFTNAAGHCLVMRTVINNKPV
ALVVMDAFGKYTHFADASRLRTWIETGKVMPVPAAALSYKKQKAAQMAAASASAGAQTAQ
ND
>EECEBICD_01185 Quinone-dependent D-lactate dehydrogenase
MSSITMTDNKTFLNELARLVGHSHLLTDPAKTARYRKGFRSGQGDALAVVFPGSLLELWR
VLNACVNADKIILMQAANTGLTEGSTPNGNDYDRDIVIISTQRLDKLHLLDNGQQVLAWP
GTTLYALEKALKPLGREPHSVIGSSCIGASVIGGICNNSGGSLVQRGPAYTEMSLFARID
DQGKLQLVNHLGIDLGHTPEQILSKLDDERIKDEDVRHDGRHAHDHDYVTRVRDINADTP
ARYNADPDRLFESSGCAGKLAVFAVRLDTFAAEKNQQVFYIGTNQPAVLTEIRRHILANF
DNLPVAGEYMHRDIYDIAEQYGKDTFLMIDKLGTDKMPFFFTLKGRTDAMLEKVKFFRPH
FTDRAMQKFGHLFPSHLPPRMKNWRDKYEHHLLLKMAGDGVAEAQRWLNEFFKSAEGGFF
TCTPEEGSKAFLHRFAAAGAAIRYQAVHADEVEDILALDIALRRNDTDWFEHLPPEIDSQ
LVHKLYYGHFMCHVFHQDYIVKKGVDVHALKAQMLELLQARGAQYPAEHNVGHLYKAPET
LTRFYRQNDPTNSMNPGIGKTSKRKFWQENTPDETH
>EECEBICD_01186 Beta-xylosidase
MKWLCSVGVAVSLAMQPALAENLFGNHPLTPEARDAFVTDLLKKMTVDEKIGQLRLISVG
PDNPKEAIREMIKDGQVGAIFNTVTRQDIRQMQDQVMALSRLKIPLFFAYDVVHGQRTVF
PISLGLASSFNLDAVRTVGRVSAYEAADDGLNMTWAPMVDVSRDPRWGRASEGFGEDTYL
TSIMGETMVKAMQGKSPADRYSVMTSVKHFAAYGAVEGGKEYNTVDMSSQRLFNDYMPPY
KAGLDAGSGAVMVALNSLNGTPATSDSWLLKDVLRDEWGFKGITVSDHGAIKELIKHGTA
ADPEDAVRVALKAGVDMSMADEYYSKYLPGLIKSGKVTMAELDDATRHVLNVKYDMGLFN
DPYSHLGPKESDPVDTNAESRLHRKEAREVARESVVLLKNRLETLPLKKSGTIAVVGPLA
DSQRDVMGSWSAAGVANQSVTVLAGIQNAVDDGAKILYAKGANITNDKDIVDFLNLYEEA
VKIDPRSPQAMIDEAVQAAKQADVVVAVVGESQGMAHEASSRTNITIPQSQRDLITALKA
TGKPLVLVLMNGRPLALVKEDQQADAILETWFAGTEGGNAIADVLFGDYNPSGKLPISFP
RSVGQIPVYYSHLNTGRPYNPEKPNKYTSRYFDEANGPLYPFGYGLSYTTFTVSDVTLSS
PTMQRDGKVTASVEVTNTGKREGATVIQMYLQDVTASMSRPVKQLKGFEKITLKPGERKT
VSFPIDIEALKFWNQQMKYDAEPGKFNVFIGVDSARVKQGSFELL
>EECEBICD_01187 Glycine betaine-binding protein YehZ
MTISKLWVSSLALLATVSLPLQAASPVTVGSKIDTEGALLGNMILQVLESHGVKTVNKIQ
LGTTPVVRGAITAGELDIYPEYTGNGAFFFKDENDPAWKNAKQGFEKVKKLDAEQNKLVW
LTPAPANNTWTIAVRQDIAEKNKLSSLADLSRYLKEGGTFKLAASAEFIERADALPAFEK
AYDFTLNQNQLLSLAGGDTAVTIKAAAQQTSGVNAAMAYGTDGPVAALGLQTLSDPKGVQ
PIYAPAPVVRESVLQAYPQIADWLQPVFASLDEKTLQQLNARIAVEGLDAKKVAADYLRQ
KGWVK
>EECEBICD_01188 Glycine betaine uptake system permease protein YehY
MSDAAKKITVNRVLLLLVVLTLLAVALPFINYAPNRLVSGEGRQLWEIWPATIWMLTGAG
CALFTLCFVPGKRGSVLTLMMAQTLFIVMLWGVGRVATQLAQEGSPLARTSLGSGLWLGL

GLMLLACSDAIRRITTGPLWRWLLHAQIVIVPLVLLFSGTFDNLSLLKEYTNRQDVFDAA
LVQHLMLLAGTVLPALAIGLPLGVWCYFSASRQGPVFTVLNVIQTIPSVALFGLLIAPLA
GLVKQFPWLAEFGVAGTGMTPALIALVLYALLPLVRGVVAGLNQVPRDVLESARAMGMSS
GQRFRHVQLPLALPVFLRSLRVVMVQTVGMAVVAALIGAGGFGALVFQGLLSSAIDLVLL
GVIPVIALAVVIDALFDLWIAFLKGATDD
>EECEBICD_01189 Glycine betaine uptake system ATP-binding protein YehX
MIEFNHVSKTFGDQQAVSDLNLHFSEGSFSVLIGTSGSGKSTTLKMINRLVEHDSGTIRF
AGEEIRSLPVLELRRRMGYAIQSIGLFPHWTVAQNIATVPQLQKWSRARINDRIDELMAL
LGLESALRDRYPHQLSGGQQQRVGVARALAADPQVLLMDEPFGALDPVTRGALQQEMTRI
HQLLGRTIVLVTHDIDEALRLADHLVLMDGGNVIQQGSPLSMLTSPENDFVQAFFGRSEL
GVRLLSLRSVGDYVRRHEQLSGDALVEEMTLRDALSMFVARRCDVLPVANQQGEPCGTLH
FRDLLSETSPRETTV
>EECEBICD_01190 Glycine betaine uptake system permease protein YehW
MKRLCDPLLWLIVLFLLLLFGLPYSQPFFAALFPDLPRPVYQQESFAALALAHFWLVGIS
SLFAVVVGVGAGIAVTRESGKEFRPLVETIAAVGQTFPPVAVLAIAVPVMGFGQQPAIIA
LILYGVLPILQATLAGLGAVPASVMSVASGMGMSRRQQLYQVELPLAAPVILAGIRTSVI
INIGTATIASTVGASTLGTPIIIGLSGFNTAYVIQGALLVALAAIIIDRLFERLTRALTR
HAK
>EECEBICD_01191 hypothetical protein
MRVAKIGVIALFLLMAIGGIGGVMLAGYSFILRAG
>EECEBICD_01192 HTH-type transcriptional regulator MlrA
MALYTIGEVALLCDINPVTLRAWQRRYGLLKPQRTDGGHRLFNDADIDRIREIKRWINNG
VQVSKVKVLLSSDSSEQPNGWREQQEILLHYLQSSNLHSLRLWVKERCQDYPAQTLTTNL
FVPLRRRLQCQQPALQALLGILDGILINYIALCLASARKKQGKDALVIGWNIHDTTRLWL
EGWVASQQGWRIDVLAHSLSQFRPELFDGKTLLVWCGENQTLAQQQQLLAWRAQGRDIHP
LGV
>EECEBICD_01193 Sensor histidine kinase BtsS
MYEFNLVLLLLQQMCVFLVIAWLMSKTRLFIPLMQVTVRLPHKLLCYVTFSIFCIMGTYF
GLHIEDSIANTRAIGAVMGGLLGGPVVGGLVGLTGGLHRYSMGGMTALSCMISTIVEGLL
GGLVHSVLIRRGRPDKVFSPLTAGAITCIAELVQMLIILLIARPFDDALHLVSNIAAPMM
VTNTVGAALFMRILLDKRAMFEKYTSAFSATALKVAASTEGILRQGFNEVNSMKVAQVLY
QELDIGAVAITDREKLLAFTGIGDDHHLPGKPISSGYTLKAIETGEVVYADGNEVPYRCS
LHPQCKLGSTLVIPLRGENQRVMGTIKLYEAKNRLFSSINRTLGEGIAQLLSAQILAGQY
ERQKALLTQSEIKLLHAQVNPHFLFNALNTIKAVIRRDSEQASQLVQYLSTFFRKNLKRP
SEIVTLADEIEHVNAYLQIEKARFQSRLQVQLDVPSTLSRQKLPAFTLQPIVENAIKHGT
SQLLDTGNVAIRARREGQHLMLDIEDNAGLYQPSAGSSGLGMSLVDKRLREHFGDDYGIS
VACEPDCFTRITLRLPLEEDA
>EECEBICD_01194 Transcriptional regulatory protein BtsR
MIKVLIVDDEPLARENLRILLQGQDDIEIVGECANAVEAIGAVHKLRPDVLFLDIQMPRI
SGLEMVGMLDPEHRPYIVFLTAFDEYAIKAFEEHAFDYLLKPIEEKRLEKTLHRLRQERS
KQDVSLLPENQQALKFIPCTGHSRIYLLQMDDVAFVSSRMSGVYVTSSEGKEGFTELTLR
TLESRTPLLRCHRQFLVNMAHLQEIRLEDNGQAELILRNGLTVPVSRRYLKSLKEAIGL
>EECEBICD_01195 hypothetical protein
MLSNDILRSVRYILKANNTDLARILALGNVDATPEQIAIWLRKEEEEGFQRCPDIVLSSF
LNGLIYEKRGKDEAAPALTAERRINNNIVLKKLRIAFSLKTDDILAILTGQLFRVSMSEI
TAMMRAPDHKNFRECGDQFMRYFLRGLAAREHAAK
>EECEBICD_01196 hypothetical protein
MQVLRLMALPLFALSLSVSITGCDQKNDTLQGKQNNMTAFIKKIAASKESEETQRYVGNL
NGIEIKLTYYYKGDIVLRQISEHKLLYKTLKANNKEEAQKMLSQVGEAYQGMPGLTERID
YYDSYATEYVDIDFTQAKISDLCKLPGSSIDNCSAYYLSMIRSQKLLEESGYHRIN
>EECEBICD_01197 putative lipoprotein YehR
MKISGKLLSTALASVLVFSLAGCGDKEESKTFNANLAGTEISITYTYKGDKIIKQTSESK
ISYATVGAKTKEDAAKILDPLSAKYKNIAGVEEKLTYEDTYAQENVSVDMEKVDFKALQQ
ISGTMVSGDTSKGISMKQTQTLLEAAGFKEAK

>EECEBICD_01198 Methionine--tRNA ligase
MTQVAKKILVTCALPYANGSIHLGHMLEHIQADVWVRYQRMRGHEVNFICADDAHGTPIM
LKAQQLGITPEQMIGEMSQEHQTDFAGFNISYDNYHSTHSDENRELSELIYTRLKENGFI
KNRTISQLYDPEKGMFLPDRFVKGTCPKCKSADQYGDNCEVCGATYSPTELIEPKSVVSG
ATPVMRDSEHFFFDLPSFSEMLQAWTRSGALQEQVANKMQEWFESGLQQWDISRDAPYFG
FEIPNAPGKYFYVWLDAPIGYMGSFKNLCDKRGDTTSFDEYWKKDSDAELYHFIGKDIVY
FHSLFWPAMLEGSHFRKPTNLFVHGYVTVNGAKMSKSRGTFIKASTWLKHFDADSLRYYY
TAKLSSRIDDIDLNLEDFVQRVNADIVNKVVNLASRNAGFINKRFDGVLAAELADPQLYK
TFTDAAAVIGEAWESREFGKAIREIMALADVANRYVDEQAPWVVAKQEGRDADLQAICSM
GINLFRVLMTYLKPVLPTLSERVEAFLNSELNWDAIEQPLLSHKVNTFKALYNRIDMKQV
EALVEASKEEVKAAAAPVTGPLADFPIQETITFDDFAKIDLRVALIENAEFVEGSDKLLR
LTLDLGGEKRNVFSGIRSAYPDPQALIGRQTVMVANLAPRKMRFGVSEGMVMAAGPGGKD
IFLLSPDDGAKPGQQVK
>EECEBICD_01199 Iron-sulfur cluster carrier protein
MNEQSQAKSPDTLRAMVAGTLANFQHPTLKHNLTTLKALHHVAWMDDTLHVELVMPFVWN
SAFEVLKEQCSADLLRITGAKAIDWKLSYNIATLKRVKNQPGINGVKNIIAVSSGKGGVG
EIVHGGKPGAGVSG
>EECEBICD_01200 Iron-sulfur cluster carrier protein
MLGAEDQRPTSPDGTHMAPIMSHGLATNSIGYLVTDDNAMVWRGPMASKALMQMLQETLW
PDLDYLVLDMPPGTGDIQLTLAQNIPVTGAVVVTTPQDIALIDAKKGIVMFEKVEVPVLG
IVENMSMHICSNCGHHEPIFGTGGAQKLAEKYHTQLLGQMPLHISLREDLDRGTPTVVSR
PESEFTAIYRELADRVAAQLYWQGEVIPGEIAFRAV
>EECEBICD_01201 hypothetical protein
MKKYLLMGIIVSAYGISVPVFASDTATLTISGKVTAPTCSTEVVNAQLQQRCGNTIHVST
LQTPAATPMRGVTTQLYTVPGDSTRQIVVNHYD
>EECEBICD_01202 putative fimbrial-like protein YehD
MKRSLIAASVLSAVFMSAGVFAADEDMGELKINGEVVGTSCTFEGANSATIELSQVGVDR
LTDLNPGDIYTGYTSPEAILKVRCTNTANPRISFNRSQFVDNMQITKNNATNNGAGFAVY
LDGIQVKPDEAGNYTLNSSKFENGVYTLNFSARYAAVENTVTPGSVESVLTMTVLTD
>EECEBICD_01203 putative fimbrial chaperone YehC
MKWMIALCLACAAMPAWSGIYIYGTRIIYPAQKKDITVQLMNDGKRSSLIQAWIDNGDTS
LPPEKLQVPFIMTPL
>EECEBICD_01204 putative fimbrial chaperone YehC
MPGDRESLFYLNVLDIPPNSDENKDKNIIKFALQNRIKLIYRPPGVQKVDKATFSRLQLL
RAPGSISVKNNSANWITIPEIKAKSKVNKETLLLAPWSSQSITTTVVVNSYTVTLIDDSG
NYLNETVKIEN
>EECEBICD_01205 Outer membrane usher protein YehB
MEKLNAITLSLFAALLISPAWAEEETFDTNFMFGGLKGEKVSRYQIDSTKPMAGVYEMDV
YVNKEWRGTYEVNIQDDPDSTCISPDLIASLGIKFTPQSTTVENECIALKTVVHGGSVSY
DTAAFNLYLSVPQAYVLEYEAGYASPETWDRGINAFYTSYYASEYYSHYKSGGSEKNTYA
NFVSGLNLLGWQLHSNANFSKSENSAGKWQSNTLYLERDFPAVLGTMRLGEQYTSGDMFD
TVRFRGVRFWRDMQMLPHSKQNFAPVVRGVAQSNALVTVEQNGFIVYQKEVPPGPFVFED
LQLAGGSRSGRER
>EECEBICD_01206 Outer membrane usher protein YehB
MYLKIFSWQGGADLDVSVKEADGTVSRFIVPYSSVPNMVQPGVAKYDFAAGRSRIEGASQ
QTDFLQGTYQYGVNNLLTLYGGTMLASDYRSFTLGTGWNTLIGAVSVDGTLSHSKQDNGD
VFDGESYQVAWNKYLPQSATHFSLAAYRYSSRDYRTFNDHVWANNRDNYRRDDDDIYDIA
DYYENDFGRKNTFTLNINQTLPDGWGYFTASALWRDYWGRSGTGKDYQLSYSNTWQRLSF
TLSATQTYDSNNREDKRFNIYLSIPLTWGLKENGGNRDIHLSNSTTFDDQGYEANNTSLS
GTFGNRDQFNYTTNLSQQRQEHQTTFGGSVTWNAPLATVGGSYSQSNKYHQVGGNIQGGL
VAWADGVHLASRLNDTIAIINAPYLEGAAVQGRPYLRTNAKGYAVFEALTPYRQNFISLD
VSESESDVALLGNRKVTVPYRGAVVLVHFETETSKPFYFLARRADGEPLTFGYEVEDDEG
NNVGLVGQGSRVFIRTEKVPISVKIATDKQQGLFCKITFDKQIDENNVYICR
>EECEBICD_01207 hypothetical protein

MKRLLFLLMMMQFTQLGYAACNATLTNTKDYTIQSDSLMLGGRSQPLLQMVSQMPIVLIV
ML
>EECEBICD_01208 putative fimbrial-like protein YehA
MTKLETSDHIIGMGPNDSVKLKLKVEWQSSSAINMRNQNGVIEAPYKITISEINSLEAVT
VSARGGYSVTIDNITVMSGAKNQWSSSDWVVFILRYIGCWGNRECVANVITDLNGKGVYK
ASLTINYNRKETTCAPQNLTITLDPVPMTKLRAKGEVSEFSKQGDIILDCKNQVRNAMLT
SRQIAVNLRSDLVWDENEAVLKPDNDNGVGFILRDSNRSLLKINKGVAINSIFKTYAKGS
AVNSVEAIPVFATYYVLDPAKVKAGPLQSKALIVVSYN
>EECEBICD_01209 Nickel/cobalt homeostasis protein RcnB
MKSKLLPCALLLATSFAWAAPATTGIDQYELKSFIADFTHFKPGDTVPQMYRTDEYNIKQ
WKLRNLPAPDAGTHWTYMGGAYVLINDTDGKIIKAYDGEIFYHR
>EECEBICD_01210 Hydroxyethylthiazole kinase
MQPDLHCRTLAAHTLKHFRALSPLTHCMTNDVVQTFTANTLLALGASPAMVIDPVEARPF
AAIANALLINVGTLTASRADAMRAAVESAYDAKTPWTLDPVAVGALEFRRRFCLDLLSLR
PAAIRGNASEILALSGMALGGRGVDTTEAALAALPAAQALARQIDCIVVVTGEIDYVTNG
QRTLSIPGGDPLMTRIVGTGCALSAVVAASCALPGAALDNVASACCWMKLAGQAAAERSE
GPGSFIPAFLDALYHLDVEAANATN
>EECEBICD_01211 Hydroxymethylpyrimidine/phosphomethylpyrimidine kinase
MQRINALTIAGTDPSGGAGIQADLKTFSALGAYGCSVITALVAQNTCGVQSVYRIEPDFV
AAQLDSVFSDVRIDTTKIGMLAETDIVEAVAERLQRHHVRNVVLDTVMLAKSGDPLLSPS
AIETLRVRLLPQVSLITPNLPEAAALLDAPHARTEQEMLAQGRALLAMGCEAVLMKGGHL
EDAQSPDWLFTREGEQRFSAPRVNTKNTHGTGCTLSAALAALRPRHRCWGETVNEAKAWL
SAALAQADTLEVGKGIGPVHHFHAWW
>EECEBICD_01212 putative fructoselysine utilization operon
transcriptional repressor
MEQAHTRLIAQLNERISAADNTPLYMKFAQTVKDAVRSGILEHGNILPGERDLSQLTGVS
RITVRKAMQALEEEGVVTRARGYGTQINNIFEYSLKEARGFSQQVVLRGKKPDTLWVNKR
VVSCPDEVAQHLAIPAGSDVFLLKRIRYVDEDAVSIEESWVPAHLIHDVDAIGISLYDYF
RRQHIYPQRTRSRVSARMPDDEFQSHIQMDGKVPVLVIKQVALDQQQRPIEYSISYCRSD
LYVFVCEE
>EECEBICD_01213 putative sugar kinase YdjH
MNGARLLTLLPTLHTRRPLTVIGAAVIDIIADAWTLPRRGGDIELQQQSVNVGGCALNIA
VALKRLGIDASNALPLGQGVWAERIRRRLAKEGLSSVIDAVEGDNGWCLALVEPDGERTF
MSFSGVENQWNADWLSQLRTPRGSLVYLSGYQLASSCGEPLIQWLETVDDVTPFIDFGPR
IGDITDDAMARIMACRPIVSLNRQEAIIAAERAGLATDTRTFGEAWVKRFSAPLVVRHDK
DGAWYFSAQSSGVVPAFPATVVDTIGAGDSHAGGVLAGLASGWSLADAVLLGNAVASWVV
GHRGGDCAPTREALLLAHKNV
>EECEBICD_01214 ADP-ribosyl-[dinitrogen reductase] glycohydrolase
MKAERILGALYGQALGDAMGMPSELWPRTRVKAHFGWIDRFLPGPKENNAACYFNRAEFT
DDTAMALCLADALLECEGNIDPEIIGRNILAWAERFDAFNKNVLGPTSKIALNAIRDGKP
IAALENNGVTNGAAMRVSPLGCLLPPTNLTAFIAEVALASSPTHKSDLAVAGAVVVAWAV
SRAVNGDTWQSIADSLPAIAHQAQTARITTFSASLSARLELALNIVRHADGVESASEQLY
QIIGAGTSTIESVPCAIAMVELANTDPNRCAVLCANLGGDTDTIGAMATAICGALHGVSA
INPQLKQTLDKVNQLDFARYAVALASYRQRREAL
>EECEBICD_01215 Putative nucleoside transporter YegT
MKTTFKLSFMMFVEWFIWGAWFVPLWLWLNKSGFSAGEIGWSYACTAIAAILSPILVGSV
TDRFFSAQKVLAVLMFAGAVLMYFAAQQTTFAGFFPLLLAYSLTYMPTIALTNSIAFANV
PDVERDFPRIRVMGTIGWIASGLACGFLPQMLGYNDISPTNIPLLITAASSALLGVFAFC
LPDTPPKSTGKMDIKVMLGLDALVLLRDKNFLVFFFCSFLFAMPLAFYYIFANGYLTEVG
MKNATGWMTLGQFSEIFFMLALPFFTKRFGIKKVLLLGLITAAIRYGFFVYGGAETYFTY
ALLFLGILLHGVSYDFYYVTAYIYVDKKAPVHMRTAAQGLITLCCQGFGSLLGYRLGGVM
MEKMFAYPQPVNGLTFNWAGMWTFGAVMIAVIALLFMIFFRESDKEITAIDDRDIALTQG
EVK
>EECEBICD_01216 Fructose-bisphosphate aldolase class 1

MTDIAQLLGKDADSLLQHRCMTIPSDQLYLPGKDYVDRVMIDNNRPPAVLRNMQTLYNTG
RLAGTGYLSILPVDQGVEHSAGASFAANPRYFDPKNIVELAIEAGCNCVASTYGVLASVS
RRYAHRIPFLVKLNHNETLSYPTEYDQTLYASVEQAFNMGAVAVGATIYFGSEQSRRQIE
EISAAFERAHELGMVTVLWAYLRNSSFKKDGVDYHVSADLTGQANHLAATIGADIVKQKM
AENNGGYKAVNFGYTDDRVYSKLTSDNPIDLVRYQLANCYMGRAGLINSGGADGGDTDLG
DAVRTAVINKRAGGMGLILGRKAFKKSMADGVKLINAVQDVYLDSKVTIA
>EECEBICD_01217 putative lipid kinase YegS
MANFPASLLILNGKSADNQPLREAITLLRDEGIQIHVRVTWEKGDAQRYVDEARRLGVET
VIAGGGDGTINEVSTALIQIRDGVAPALGLLPLGTANDFATSAGIPEALDKALKLAIAGN
AMEIDMARVNDKTCFINMATGGFGTRITTETPEKLKAALGGVSYLIHGLMRMDTLTPDRC
EIRGENFHWQGDALVIGIGNGRQGRRRAAIMSYRAG
>EECEBICD_01218 hypothetical protein
MNVTSGVNAQTPLLPPSERGNDEKPVAEIVDFNAYGNKPRCLMCLGTTALFAGVFSGVCS
GAVASVSSGTAYTTALTVLGASFGMGGIGMMGICAGLYLSANGIRTRPAWP
>EECEBICD_01219 Tir chaperone
MYSRADRLLRQFSLKLNADSIVFDENRLCSFIIDNRYRILLTSTNSEYIMIYGFCGRPPD
NNNLAFEFLNANLWFAENNGPHLCYDNNSQSLLLALNFSLNESSVEKLECEIEVVIRSME
KLYHILQDKGITLDTDYT
>EECEBICD_01220 hypothetical protein
MKNKFSVGDSLELMTPQGNINFTLEQMENAKGDAMPVAPGDGYTVWMPVPQDVTLDYALL
MRNFSGESTRNPHAK
>EECEBICD_01221 23S rRNA 5-hydroxycytidine synthase
MFKPELLSPAGTLKNMRYAFAYGADAVYAGQPRYSLRVRNNEFNHENLQLGINEAHALGK
KFYVVVNIAPHNAKLKTFIRDLKPVVEMGPDALIMSDPGLIMLVREHFPAMPIHLSVQAN
AVNWATVKFWQQMGLTRVILSRELSLEEIEEIRQQVPDMEIEIFVHGALCMAYSGRCLLS
GYINKRDPNQGTCTNACRWEYNVQEGKEDVVGNIVHKHEPIPVQNVEPTLGIGAPTDKVF
MIEEAQRPGEYMTAFEDEHGTYIMNSKDLRAIAHVERLTKMGVHSLKIEGRTKSFYYCAR
TAQVYRKAIDDAAAGKPF
>EECEBICD_01222 hypothetical protein
MEQDDRLLNAMFEMCNHKNPLNDGQREWHIADISGLLREERYDELDERYNQTLTESFTSR
EAEKRYFFAWNQMDNPFYDMDTLVEAGPQGLALIKNWQRARPRSTHAWLAEAQYWNHRAW
LYRSYGWARETTRAMWICAAACNERMVIAVLNAIDCEPRQWMAAALTSTNSKVFGQPDWL
VEFLEGADVAGQPLMEDLAEYHRHSPQEVDALMAHSGLSFADAVCPNLPRPSVLPECNDD
AGQKYWLTVCLAIFPTAFYVLDEYIPFRMPRWGGSHEEIREFLESSVCDHLSAAEREHLE
LLIWWDDHRDLRIKEVDSPAEQERIIAKAEEISLRAHIQESRHNTLKWLRVCYSDLDDND
ALWRTLQRSIVEKVKFNNYFFDDTIKFALRDFPDTWWMYNFLCQNAQQTEFAVPKIRRGY
FQYAGLLGFEKDEAQGLAWLDSVADIQYNHNWRAAIKNFDWFGLPEHFVPLAELGAQRNI
PAALNLLGLEHNNKENNGLLPYDPAIALGYFQRAAEILHRQLALRESPPYKLIDNGGYTD
YENDLQNIHFSIGICNQRLSKQEPDTEKRSAYEKELLDNLWLAHQYGHKEAWGLFLLNIF
EVKDITLAHKHLELVQQEANKGTLHAMVTLSRLHGNKHDRTLFNMKLSARWAHFAFTLYP
DNEIVMDCLDHLHFDSFWKRFRFAWYTVRIPNSELPGQVNSMV
>EECEBICD_01223 hypothetical protein
MFWIVLIIAALFFSWRNYKKNKPLIAARIAEEKEKDRLKDLRRDTRRRQWALALADILAR
RNGLPIKGVELVFKLDDDKRRYLAQQVKKELGLSENLDGAALRHKVEDILRRWPAGIGSS
PRTFYHHLAAQGQVRDALAFDCMRTAFLTRCIAGLGWCNENEAWLVLLLNAQRAQDCFDS
WEDYATAYVRARRVWLTLRDTPTALAGRDLQEATHYLQDPVSRWRQLPWNEFKIFEPI
>EECEBICD_01224 hypothetical protein
MTETSSHRYKPRNIINAPNVKSSIFSRSQQRGDSENIQRWLSNHFYRWIIGDFPHVYPVR
SVADYAVYFSADAEIPAWLAPKLGGDERFYYLNVQHPQLVAMERDLVEFLSRQEGTRLET
KLQRINCFTVLAMREAEHQKMQRLREQGWYPSNSEALKPVMTVNNGVLVELDATNPGLRS
EMAYESWHMQHCDGDFDNKGALSGGYGDYYARQMEQQKLRLFSLRDGNNIPHVTISLVVG
NNGLSIDQIKGKQNRHPIKKYANDVLSLLRHLQPLPERHADCEGMGIVYESTPEYSGWKF
ITHIHDLNFLLNVLHDNFHLMEHFPTPPVALQWLLLHSAPEALRYLQVVDPNVATAAEML
FPQHEWHPTLAGKNTSSEPFEIESLTLQTTRYLPVIKEVQ

>EECEBICD_01225 Transcriptional regulatory protein BaeR
MTELPIDENTPRILIVEDEPKLGQLLIDYLRAASYAPTLINHGDKVLPYVRQTPPDLILL
DLMLPGTDGLTLCREIRRFSDIPIVMVTAKIEEIDRLLGLEIGADDYICKPYSPREVVAR
VKTILRRCKPQRELQQQDAESPLMIDESRFQASWCGKALDLTPAEFRLLKTLSLEPGKVF
SREQLLNHLYDDYRVVTDRTIDSHIKNLRRKLESLDAEQSFIRAVYGVGYRWEADACRLV
>EECEBICD_01226 Signal transduction histidine-protein kinase BaeS
MKVWRPGITGKLFLAIFATCIVLLISMHWAVRVSFERGFIDYIKHGNEQRLQMLGDALGD
QYQQHGNWRFLRNNDRFVFQILRSFEHDNDRDKPGPGMPPHGWRTQFWVVDQNGRVLVGP
RGPVPHDGTRRPILVNGAEVGAIIASPVERLTRNTDINFDMQQRRASWMIVALSTLLAAL
ATFLLARGLLAPVKRLVEGTHRLAAGDFTTRVTPTSADELGKLAQDFNQLASTLEKNQQM
RRDFMADISHELRTPLAVLRGELEAIQDGVRQFTPESVTSLQAEVGTLTKLVDDLHQLSL
SDEGALAYQKTTVDLVPLLEVAGGAFRERFTSRGLTLHYALPDSMTVFGDPDRLMQLFNN
LLENSLRYTDSGGGLHISAEQRDKSLFLTFADSAPGVSDEQLQKLFDRFYRTEVSRNRAS
GGSGLGLAICVNIVHAHNGHLHAAHSPFGGVSITVELPLDRDLQREV
>EECEBICD_01227 Putative multidrug resistance protein MdtD
MTPVFLQIGLGFSPFHAGLMMIPMVLGSMGMKRIVVQIVNRFGYRRVLVATTLGLALVSL
LFMSVALLGWYYLLPLVLLLQGMVNSARFSSMNTLTLKDLPDTLASSGNSLLSMIMQLSM
SIGVTIAGMLLGMFGQQHIGIDSSATHHVFMYTWLCMAVIIALPAIIFARVPNDTQQNMV
ISRRKRSL
>EECEBICD_01228 Putative multidrug resistance protein MdtD
MTELPDNTRWQLWIVAFGFFMQSLDTTIVNTALPSMAKSLGESPLHMHMVVVSYVLTVAV
MLPASGWLADKIGVRNIFFAAIVLFTLGSLFCALSGTLNQLVLARVLQGVGGAMMVPVGR
LTVMKIVPRAQYMAAMTFVTLPGQIGPLLGPALGGVLVEYASWHWIFLINIPVGIVGAMA
TFMLMPNYTIETRRFDLPGFLLLAIGMAVLTLALDGSKSMGISPWTLAGLAAGGAAAILL
YLFHAKKTQARYSACDCSARPRFHWGCWAVLPGVSAAVCCRL
>EECEBICD_01229 Multidrug resistance protein MdtC
MRFFALFIYRPVATILIAAAITLCGILGFRLLPVAPLPQVDFPVIMVSASLPGASPETMA
SSVATPLERSLGRIAGVNEMTSSSSLGSTRIIILEFNFDRDINGAARDVQAAINAAQSLLP
GGMPSRPTYRKANPSDAPIMILTLTSESWSQGKLYDFASTQLAQTIAQIDGVGDVDVGGS
SLPAVRVGLNPQALFNQGVSLDEVREAIDSANVRRPQGAIEDSVHRWQIQTNDELKTAAE
YQPLIIHYNNGAAVRLGDVASVTDSVQDVRNAGMTNAKPAILLMIRKLPEANIIQTVDGI
RAKLPELRAMIPAAIDLQIAQDRSPTIRASLQEVEETLAISVALVILVVFLFLRSGRATL
IPAVAVPVSLIGTFAAMYLCGFSLNNLSLMALTIATGFVVDDAIVVLENIARHLEAGMKP
LQAALQGTREVGFTVISMSLSLVAVFLPLLLMGGLPGRLLREFAVTLSVAIGISLVVSLT
LTPMMCGWMLKSSKPRTQPRKRGVGRLLVALQQGYGTSLKWVLNHTRLVGVVFLGTVALN
IWLYIAIPKTFFPEQDTGVLMGGIQADQSISFQAMRGKLQDFMKIIRDDPAVNNVTGFTG
GSRVNSGMMFITLKPRGERKETAQQIIDRLRVKLAKEPGARLFLMAVQDIRVGGRQANAS
YQYTLLSDSLAALREWEPKIRKALSALPQLADVNSDQQDNGAEMNLIYDRDTMSRLGIDV
QAANSLLNNAFGQRQISTIYQPMNQYKVVMEVDPRYSQDISALEKMFVINRDGKAIPLSY
FAQWRPANAPLSVNHQGLSAASTIAFNLPTGTSLSQATEAINRTMTQLGVPSTVRGSFSG
TAQVFQQTMNSQLILIVAAIATVYIVLGILYESYVHPLTILSTLPSAGVGALLALELFNA
PFSLIALIGIMLLIGIVKKNAIMMVDFALEAQRSGGLTPAQAIFQACLLRFRPIMMTTLA
ALFGALPLVLSGGDGSELRQPLGITIVGGLVMSQLLTLYTTPVVYLFFDRLRLRFSRKNS
KPVVEI
>EECEBICD_01230 Multidrug resistance protein MdtB
MQVLPPGSTGGPSRLFILRPVATTLLMAAILLAGIIGYRFLPVAALPEVDYPTIQVVTLY
PGASPDVMTSAVTAPLERQFGQMSGLKQMSSQSSGGASVVTLQFQLTLPLDVAEQEVQAA
INAATNLLPSDLPNPPIYSKVNPADPPIMTLAVTSNAMPMTQVEDMVETRVAQKISQVSG
VGLVTLAGGQRPAVRVKLNAQAIAALGLTSETVRTAITGANVNSAKGSLDGPERAVTLSA
NDQMQSADEYRKLIIAYQNGAPVRLGDVATVEQGAENSWLGAWANQAPAIVMNVQRQPGA
NIIATADSIRQMLPQLTESLPKSVKVTVLSDRTTNIRASVRDTQFELMLAIALVVMIIYL
FLRNIPATIIPGVAVPLSLIGTFAVMVFLDFSINNLTLMALTIATGFVVDDAIVVIENIS
RYIEKGEKPLAAALKGAGEIGFTIISLTFSLIAVLIPLLFMGDIVGRLFREFAVTLAVAI
LISAVVSLTLTPMMCARMLSQQSLRKQNRFSRACERMFDRVIASYGRGLAKVLNHPWLTL

SVAFATLLLSVMLWIVIPKGFFPVQDNGIIQGTLQAPQSSSYASMAQRQRQVAERILQDP
AVQSLTTFVGVDGANPTLNSARLQINLKPLDARDDRVQQVISRLQTAVATIPGVALYLQP
TQDLTIDTQVSRTQYQFTLQATTLDALSHWVPKLQNALQSLPQLSEVSSDWQDRGLAAWV
NVDRDSASRLGISMADVDNALYNAFGQRLISTIYTQANQYRVVLEHNTASTPGLAALETI
RLTSRDGGTVPLSAIARIEQRFAPLSINHLDQFPVTTFSFNVPEGYSLGDAVQAILDTEK
TLALPADITTQFQGSTLAFQAALGSTVWLIVAAVVAMYIVLGVLYESFIHPITILSTLPT
AGVGALLALIIAGSELDIIAIIGIILLIGIVKKNAIMMIDFALAAEREQGMSPRDAIFQA
CLLRFRPILMTTLAALLGALPLMLSTGVGAELRRPLGIAMVGGLLVSQVLTLFTTPVIYL
LFDRLSLYVKSRFPRHKEEA
>EECEBICD_01231 Multidrug resistance protein MdtA
MKGSNTFRWAIAIGVVVAAAAFWFWHSRSESPTAAPGVAAQAPHTAAAGRRGMRDGPLAP
VQAATATTQAVPRYLSGLGTVTAANTVTVRSRVDGQLISLHFQEGQQVNAGDLLAQIDPS
QFKVALAQAQGQLAKDNATLANARRDLARYQQLAKTNLVSRQELDAQQALVNETQGTIKA
DEANVASAQLQLDWSRITAPVSGRVGLKQVDVGNQISSSDTAGIVVITQTHPIDLIFTLP
ESDIATVVQAQKAGKTLVVEAWDRTNSHKLSEGVLLSLDNQIDPTTGTIKIKARFTNQDD
TLFPNQFVNARMLVDTEQNAVVVPAAAVQMGNEGHFVWVLNDENNVSKKRVKIGIQDNRN
VVISAGLSAGDRVVTDGIDRLTEGAKVEVVEPQTTVADEKSPSRHEGQKGARA
>EECEBICD_01232 hypothetical protein
MMTTFIITLSLFWPLPREGLSRFSVLLKSVAYLHCRAATLPHSGALTIFTDESCVAPRKA
SRHSE
>EECEBICD_01233 Chaperone protein DnaK
MFIGFDYGTANCSVAIMRDGHPQLLTMENNSALLPSMLCAPTREAVSEWLYRHHDVPATD
EETQALLRRAIRYNREEDIEVGAQSVQFGLASLAHYIDDPQEVWFVKSPKSFLGASGLKP
QQVALFEDLVCAMMVHIRHTAHSQLPEAITQAVIGRPINFQGLGGDDANRQAQGILERAA
KRAGFQEVVFQYEPVAAGLDYEATLREEKRVLVVDIGGGTTDCSMLLMGPQWRQRADREN
SLLGHSGCRVGGNDLDIALAFKNLMPLLGMGGETEKGIALPVLPWWNAVAINDVPAQSDF
YSSANGRLLNDLVRNAREADKVALLLKVWRQRLSYRLVRCAEESKIALSGQADVTARLPF
ISDDLAVAISQQGLEAALDQPLARILEQVQLALDSAQEKPDVIYLTGGSARSPLIKKALS
EQLPGIPVAGGDDFGSVTAGLARWAEVVFR
>EECEBICD_01234 DNA-3-methyladenine glycosylase 2
MFTLSWQPPYDWSWMLGFLAARAVDGVETVGEGFYARSLVVGEHRGLISVSPHLPTHTVQ
VSVSAGLLPVAPACLAKVSRLFDLDCQPAQVAAVLGPLGEDRPGLRLPGSVDTFEQGVRA
ILGQLVSVAMAARLTAKVARRYGEALPDAPDYVCFPGPETLALADPLALKALGMPLRRAE
ALIHLAQATLAGKLALAAPPDIEQSVKNLQTFPGIGRWTANYFALRGWQAKDIFLPDDYL
IKQRFAGMTPAQIRRYAERWKPWRSYALLHIWYTHGWQPSMDSEIAGIQ
>EECEBICD_01235 hypothetical protein
MSKPSQHVFVTVPHPLLRLVSLGLVAFVFTLFSLVLSRVGTQLAPLWFPTSIMMVAFYRH
AGRLWPGIAVACSLGSIGASLTLFPAASLNFSWTAINIIEAATGAILLRKLLPSYNPLQN
LNDWFRLAIGSAVIPPLLGGLLFWLIAPEAVASKAFLIWVLSEAIGALTLVPLGLLFKPH
YLLRHRDPHLLLETLLTLVITLALSWLAMRYIPWPFTCVIVLLMWSAVRLPRMEAFLIFL
ATVIVVSLMLANDPTLLATPKTDVMVNMPWLPFLMILLPANMMTMVMYAFRTERKHITES
ESRFRNAMEYSAIGMALVGTEGQWLQVNKSLSHFLGYSQDELRTMTFQQLTWPEDLNNDL
EQLNMLVRGDINSYSMEKRYYTRNGDVVWALLAVSLVRHKDNKPLYFIAQIEDINDLKQS
EQENQRLMERITQANDALFQEKERLHITLDSIGEAVVCIDVAMNITFMNPIAEKMSGWRQ
EDALGAPLLTVLRITSGDKGPLLEDIYRADRSRSDMEQEIVLHCHNGGSYDIHYSITPLS
TLDGDKIGSVLVIQDVTESRKMLRQLSYSATHDALTHLANRASFEKQLQQRLQTIQESPQ
HHALVFIDLDRFKAVNDSAGHAAGDALLRELASLMLSMLRSGDLLARLGGDEFGLLLPDC
NSDSARFIVTRLINAVNEYHFMWEGRLHRIGASAGITMINEHNCQLTEVVSQADIACYAA
KNSGRGRLTVYEPQHALTSSKGMMPLEEQWHMIKNNHLLMLARNVAPPRTPEATSFWLVS
LRLWTSEGEVLEERAFRAGLADSALHHALDRRVFHEFFHHAATAVASKGLSVALPLSAAG
LYSATLIDELLEQLEHSPLPPRLLHLIIPADVIVKQAQTAAATLRKLRQRGCQVILSHVG
RDLQLFNLLPPHIVDYLLLDSDLIANVHESLMDEMLTSIIQGHAQHLDIKTLAGPVQNPQ
VLDTLSCIGVDLIYGDTIAEAQPLDLLLNTSYFAIH
>EECEBICD_01236 Uridine kinase

MTDQSHQCVIIGIAGASASGKSLIASTLYRELREQVGDEHIGVIPEDSYYKDQSHLSMEE
RVKTNYDHPNAMDHSLLFQHLQALKRGSAIELPVYSYVEHTRMQETVRVEPKKVIILEGI
LLLTDARLREEMNFSIFVDTPLDICLMRRIKRDVNERGRSMDSVMAQYQKTVRPMFLQFI
EPSKQYADIIVPRGGKNRIAIDILKAKISQFFE
>EECEBICD_01237 dCTP deaminase
MRLCDRDIEAWLDEGRLSITPRPPVERINGATVDVRLGNKFRTFRGHTAAFIDLSGPKDE
VSAALDRVMSDEIVLPDGEAFYLHPGELALAVTFESVTLPPDLVGWLDGRSSLARLGLMV
HVTAHRIDPGWSGCIVLEFYNSGKLPLALRPDMLIGALSFEPLSGPAARPYNRRQDAKYR
DQQGAVASRIDKD
>EECEBICD_01238 hypothetical protein
MRRFLTTLMILLVVLVAGFSALVLLVNPNDFRAYMVQQVAARSEYQLQLDGPLRWHVWPQ
LSILSGRMTLTARGASEPLVRADNMRLDVALWPLLSHQLHVKQVMLKGGVIQLTPQTEAV
RSDDAPVAPKDNTLPDVAEDRGWSFDIRSLRVADSVLVFQHENDDQVTVRDIRLNMEQDA
EHRGTFDFSGRVNRDQRDLALSFSGTVDASDYPHNLTAGIEQLRWQLQGADLPAQGIEGQ
GQLQAQWQEAQKRLSFNHLNLTANDSSLTGHVQVTLAEQPEWQIDLQSSKLNLDNLLPHH
SAVAQTGGAVSQGQNTLPLTRPVIASRVGAPPYQGLQSFTAEIALQADSVRWRGMDFTQV
STKMSNQAGLLDITELQGKLADGEMSLPGTLDARTASPRIEFHPRLNHVEIGTILKAFNY
PISLTGKMSLVGDFSGADIDAEAFRHSWKGKAHVDMSNTRLEGMNFQQLVQQAVERSGGD
AQQSQENMDNATRLDRFTTDLTLNKGTLTLDDMVGQSSMLALTGSGTLDLVKQSCDTQFN
LRVLGGWNGDSNLITFLKETPVPLRVYGKWQELNYTLQVDQLLRKHLQDEAKRRLNDWAE
RNKDTRNGKDVKKLLDKL
>EECEBICD_01239 hypothetical protein
MEWIADPSIWAGLVTLIVIELVLGIDNLVFIAILAEKLPPGQRDRARITGLILAMIMRLL
LLASISWLVTLTKPLFSVQALSFSARDLIMLFGGFFLLFKATMELNERLEGKDSANPTQR
KGAKFWAVVAQIVVLDAIFSLDSVITAVGMVDHLAVMMAAVIIAISLMLLASKSLTRFVN
NHPTIVILCLSFLLMIGFSLVAEGFGFHIPKGYLYAAIGFSVMIEALNQLAIFNRRRFLS
ANHTLRQRTTEAVMRLISGKKEDAELDAETAAMLADHDDSQIFNPQERRMIKRVLNLNQR
TVSSIMTSRHDIEHIDLNAPEAEIRALLEKNQHTRLVVTGENEQEDLLGVVHVIDLLQQS
LRGEPLNLRALIRQPLVFPETLPLLPALEQFRNARTHFAFVVDEFGSVEGIVTLSDVMET
IAGNLPNEVEEIDARHDIQKNPDGSWTANGHMPLEDLVQYVPLPLDEKREYHTIAGLLME
YLQRIPQTGEEVQVGDYLLKTLQVESHRVQKVQLIPLHNGEPDQEM
>EECEBICD_01240 hypothetical protein
MMKSKMKLMPLLASLSLISGCTVLPGSNMSTMGKDVIKQQDADFDLDRMVNVYPLTPRLV
EQLRPRPNVAQPNMSLDQEIASYQYRVGPGDVLNVTVWDHPELTTPAGQYRSSSDTGNWV
QPDGTMFYPYIGKVSVVGKTLSEIRSDITGRLAKYIADPQVDVNIAAFRSQKAYISGQVN
KSGQQAITNVPLTVLDAINAAGGLTDMADWRNVVLTHNGKEQRISLQALMQNGDLSQNRL
LYPGDILYVPRNDDLKVFVMGEVKKQSTLKMDFSGMTLTEALGNAEGIDLTTSNASGIFV
IRPLKGEGERGGKIANIYQLDMSDATSLVMATEFRLQPYDVVYVTTAPVARWNRLINQLL
PTISGVRYMTDTASDIHSW
>EECEBICD_01241 Low molecular weight protein-tyrosine-phosphatase Wzb
MFNKILVVCVGNVCRSPTAERLLKRFHPSLTVASAGLGALVGKGADPAAASVASAHNLSL
ENHCARQISARLCREYDLILTMEKRHIAALCDIAPEMRGKVMLFGHWDSEREIPDPYRKS
RDAFEAVYTLLERSARQWAQALNAEQGKP
>EECEBICD_01242 Tyrosine-protein kinase wzc
MTEKVKQSAAVTGSDEIDIGRLVGTVIEARWWVLGTTAIFALCAVIYTFFATPIYSADAL
VQIEQNAGNSLVQDINSALANKPPASDAEIQLIRSRLVLGKTVDDLDLDIAVTKNTFPLF
GAGWERLMGRHNEMVKVTTFTRPETMSGQIFTLKVLGDKRYQLVSDGGFSAQGVVGQPLN
KDGVTMQVEAIDARPDTEFTVSKFSTLGMINNLQNNLTVTETGKDTGVLSLTFTGEDRDQ
IREILNSITRNYLQQDIARKSEEAGKSLAFLAKQLPEVRSRLDVAENKLNAFRQDKDSVD
LPLEAKAVLDSMVNIDAQLNELTFKEAEISKLFTKAHPAYRTLLEKRKALEDEKAKLNGR
VTAMPKTQQEIVRLTRDVESGQQVYMQLLNKQQELKITEASTVGDVRIVDPAITQPGVLK
PKKALIILGSIILGLMLSIVGVLLRSLFNRGIESPQALEEHGISVYASIPLSEWQKARDS
VKTIKGIKRYKQSQLLAVGNPTDLAIEAIRSLRTSLHFAMMQAQNNVLMLTGVSPSIGKT
FVCANLAAVISQTHKRVLLIDCDMRKGYTHELLGTNNVDGLSDILAGKGEIASCAKPTAI

ANFDLIPRGQVPPNPSELLMSERFGELIAWASSRYDLVLIDTPPILAVTDAAIVGRHVGT
TLMVARYAVNTLKEVETSLSRFDQNGIQVKGVILNSIFRRATGYQDYGYYEYEYQSDSK
>EECEBICD_01243 hypothetical protein
MARPVTVGRTSVAPCGKTIWGISMTTDNPLISIYMPTWNRQQLAIRAIKSVLRQDYRHWE
MIIVDDCSSSYQQLQQFVEELNDPRVRYTHNARNTGACAVRNQAIMQAQGHYITGIDDDD
EWTPNRLSVFLAHKHQLTTHAFLYANDYVCEGEVYSQPASLPLYPKSPYSRRLFYKRNII
GNQVFTWAWRFKACLFDTELKAAQDYDIFLRMVVEYGEPWKVEEATQILHINHGEMQITS
SPKKFSGYFHFYRKHKDKFDRASKKYQLFTLYQIRNKRMTWRTLLTLLSVRNSKRLAAGL
RGR
>EECEBICD_01244 Serine acetyltransferase
MLEDLRANSWSLRPCCMVLAYRIAHFCSVWRKKNVLNNLWAAPVLVLYRVITECLFGYEI
QAAATIGRRFTIHHGYAVVINKHVVAGDDFTIRHGVTIGNRGADSLACPVIGHGVELGAN
VILLGDITIGNHVTIGAGSVVLDSIPDHALVVGEKARVKVST
>EECEBICD_01245 hypothetical protein
MNILQFNVRLAEGGAAGVALDLHLRALQKGLTSRFVYGYGKGGKKASATTVIRR
>EECEBICD_01246 hypothetical protein
MIKQTPRGTAIANIALFRFLNRDLFGNLDNLYRTVIQTSGPLVLHFHVLHSYWLNLADIV
TFCEKVKAQKPDVTLVWTLHDHWSVTGRCAFTDGCEGWKSGCQKCPTLSNYPPVRVDRAH
QLIGGKRQRFRDMLRLGCQFISPSQHVAEAFNSVYGAGLCRVINNGIDLATEAILAQLSP
VPLNPGKPRIAIVAHDLRYDGKTDQRLVHDMMALGEKIELHTFGKFSPFTGQNVVNHGFE
TDKRKLMSALNEMDALVFSSRVDNYPLILCEALSIGVPVLATHSEAAQEVLAKSGGQTFA
ATDVLRLAQRRKPEIAQAVFGATLDAFRMRSRVAYSGQQMLEEYVSFYQNL
>EECEBICD_01247 hypothetical protein
MSRSIRICSYLLLPLIYLLVNVKIAQLGESFPITIVTFLPLLLLLFVERISVKKLMIALG
IGAGLTAFNFLFGQSLNAGKYVTSTMLFVYIVVIIGMVWSIRFKTISAHNHRKILRFFYL
VVGIVVALAAVEMAQIILTGGSSIMEGISKYLIYSNSYVLNFIKFGGKRTTALYFEPAFF
ALALISIWLSIKQFGIKTPKSDAMILAGIILSGSFSGVMTFILFYLLEWAFQYLNKDAIK
KKLPLALVSLTLFLVGVIIAFPYIATRLGDLGTEGSSSYYRIVGPLVMVGYSLTHIDGVV
RFGSLYEYVASFGIFNGADVGKTIDNGLYLLIIYFSWFAVLMTLWYMGKVLKMALNAFGD
NRNFRVQLYLFTPVSLFFTGSIFSPEYAFLIVCPFILRKALKIS
>EECEBICD_01248 hypothetical protein
MFLSVVTVAFRNYEGVVKTWRSLRNLARDPGLSFEWIVVDGGSEDGTREFLQKRHGEFNL
RFISEKDRGIYDAMNKGIALAQGRYTLFLNSGDAFHDDAALFVRQLARQKGEAMYIGDAL
LDFGGGHKIRRKAKPGYYIYHSLPASHQAIFFPTAALKKYPYDLQYHVSSDYALAARLYK
AGYPFRRINGLVSEFSMGGVSTSNNLELCQDAKNVQRKILRVPGFWAQLSYLLRLKTTGK
TKARYNKV
>EECEBICD_01249 hypothetical protein
MQDLSGFTVPKGFRGANVLKVQLWWAVQATLFAGSPQILYRWRAFLLRLFGAKIGKNVVI
RPSVKITYPWKLTVGDYAWVGDDAVLYTLGEINIGAHAVISQKGYLCTGSHDYTSAHFDI
NAAPIVIGEKCWLATDVFVAPGVTIGHGTVVGARSSVFKSLPANAICRGNPAVVTRQRVQ
KVTP
>EECEBICD_01250 GDP-mannose 4,6-dehydratase
MSKVALITGVTGQDGSYLAEFLLEKGYEVHGIKRRASSFNTERVDHIYQDPHSCNPKFHL
HYGDLTDASNLTRILQEVQPDEVYNLGAMSHVAVSFESPEYTADVDAMGTLRLLEAIRFL
GLEKKTRFYQASTSELYGLVQEIPQKETTPFYPRSPYAVAKLYAYWITVNYRESYGIYAC
NGILFNHESPRRGETFVTRKITRAIANIAQGLESCLYLGNMDSLRDWGHAKDYVRMQWMM
LQQEQPEDFVIATGVQYSVRQFVELAAAQLGIKLRFEGEGINEKGIVVSVTGHDAPGVKP
GDVIVAVDPRYFRPAEVETLLGDPSKAHEKLGWKPEITLSEMVSEMVANDLEAAKKHSLL
KSHGYEVAIALES
>EECEBICD_01251 GDP-L-fucose synthase
MNKQRIFVAGHRGMVGSAIVRQLAPRGDVELVLRTRDELDLLDGRAVQAFFAGAGIDQVY
LAAAKVGGIVANNTYPADFIYENMMIESNIIHAAHLHNVNKLLFLGSSCIYPKLARQPMA
ESELLQGTLEPTNEPYAIAKIAGIKLCESYNRQYGRDYRSVMPTNLYGPHDNFHPDNSHV
IPALLRRFHEAAQSHAPEVVVWGSGTPMREFLHVDDMAAASIHVMELAREVWQENTAPML

SHINVGTGVDCTIRELAQTIAKVVGYQGRVVFDATKPDGTPRKLLDVTRLHQLGWYHEIS
LEAGLAGTYQWFLENQHRFRG
>EECEBICD_01252 GDP-mannose mannosyl hydrolase
MPVLTSGSLRISTGSGGDNVFTSGRFRRRGADHAPHLPRFHRGKRPGEILLGQRLNRPAQ
GYWFVPGGRVCKDETLEAAFARLTQAELGVRLPLAAGTFYGVWQHFYDDNFSGEDFSTHY
IVLGFRLRVTESDLRLPDAQHGSYRWLTPEQLLASDNVHENSRAYFLPDAPAVGL
>EECEBICD_01253 hypothetical protein
MKILVYGINYSPELTGIGKYTGEMVAWMAQEGHEVRVITAPPYYPQWKVGERYSAWRYRR
EEGEATVWRCPLYVPKQPSTLKRLLHLGSFALSSFFPLMAQRRWKPDRIIGVVPTLFCTP
GMRLLATLSGARTVLHIQDYEVDAMLGLGMAGKGKRGSVARLATAFERSALRNVDNVSTI
SRSMMNKAREKGVAAEKILFFPNWSEVARFQDVNDADVTALRQQLGLPEGKKIVLYSGNI
GEKQGLEKVIDAAERLRDRPLIFAIVGQGGGKARLENMARERGLANIKFLPLQPYDALPA
LLKMGDCHLVVQKRGAADAVLPSKLTNILAVGGNAVITAEPHTELGQLCARYPGIAVCVE
PESTDALVNGISQALAMPKNNTTAREYAERTLNKENVLRQFIADIRG
>EECEBICD_01254 Mannose-1-phosphate guanylyltransferase 1
MRQTKLYPVVMAGGSGSRLWPLSRVLYPKQFLCLKGDLTMLQTTICRLNGVECESPVVIC
NEQHRFIVAEQLRQLNKLTENIILEPAGRNTAPAIALAALAATRQHTDCDPLMLVLAADH
AIANEEAFRDAVRGAMPYADAGKLVTFGIVPDLPETGYGYIRRGDVVPGATDAVAFEVAQ
FVEKPGLETAQAYVASGDYYWNSGMFLFRAGRYLEELKKFRPDILAACEQAMRGVDPDLD
FIRVDEEAFLACPEESIDYAVMERTVDAVVMPMDAGWSDVGSWSSLWEISAHTPEGNVHH
GDVISHKTENSYVYAESGLVTTVGVKDLVVVQTKDAVLIADRHAVQDVKKVVEKIKADGR
HEHHMHREVYRPWGKYDSIDAGERYQVKRITVKPGEGLSVQMHHHRAEHWVVVAGTARVT
INGEVKLLGENESIYIPLGATHCLENPGKIPLDLIEVRSGSYLEEDDVVRFEDRYGRV
>EECEBICD_01255 Phosphomannomutase/phosphoglucomutase
MTKLTCFKAYDIRGRLGEELNEDIAWRIGRAYGEYLKPKTIVLGGDVRLTSEALKLALAK
GLQDAGVDVLDIGMSGTEEIYFATFHLGVDGGIEVTASHNPMDYNGMKLVREGARPISGD
TGLRDVQRLVEAGDFPPVNEAARGSYRQISLRDAYIGHLLGYISVNNLTPLKLVFNAGNG
AAGPVIDAIEARLKALGAPVEFIKIHNTPDGTFPNGIPNPLLPECRDDTRKAVIEHGADM
GIAFDGDFDRCFLFDEKGQFIEGYYIVGLLAEAFLEKHPGAKIIHDPRLTWNTEAVVTAA
GGTPVMSKTGHAFIKERMRTEDAIYGGEMSAHHYFRDFAYCDSGMIPWLLVAELVCLKRQ
SLGELVRDRMAAFPASGEINSRLAEPAAAIARVEAHFAEEAQAVDRTDGLSMSFADWRFN
LRSSNTEPVVRLNVESRGDIPLMEARTRTLLALLNQ
>EECEBICD_01256 UDP-glucose:undecaprenyl-phosphate glucose-1-phosphate
transferase
MTNLKKRERAKTNASLISMVQRFSDITIMFGGLWFICEVSGLPFLYMHLLVALITLVVFQ
MLGGMTDFYRSWRGVKMSTELTLLLQNWTLSLIFSAGLVAFNDDFDNRLATWLAWYALSS
LGLVLCRSFIRFGAGWLRNRGYNTRRVAVAGDLPAGQALLDSFRNEPWLGFEVVGVYHDP
KPGGVSADWAGNFEQLIDDAKASRIHNVYIAMSMSDGAMVKKLVRELADTTCSVILIPDV
FTFNILHSRVEEMSGVPVVPLYDTPLSGINRLLKRAEDIVLASLILLLISPVLCCIALAV
KLSSPGPVIFRQTRYGMDGKPIKVWKFRSMKVMENDKVVTQATQNDPRVTRVGNFLRRTS
LDELPQFINVLTGGMSIVGPRPHAVAHNEQYRQLIEGYMLRHKMKPGITGWAQINGWRGE
TDTLEKMEKRVEFDLEYIREWSIWFDIKIVFLTIFKGFVNKAAY
>EECEBICD_01257 Lipopolysaccharide biosynthesis protein WzxC
MSLRQKTISGAKWSAIATIVIIGLGLIQMTVLARIIDNHQFGLLTVSLVIIALADTISDF
GIANSIIQRKTIGHLELTTLYWLNVGLGIVVFAVVFWLSDAIAHVLHNPDLAPLIKTLSL
AFIVIPHGQQFRALMQKELEFNKIGMIETTSVLAGFTFTVISAHYWPLALTAILGYLVNS
AVRTLLFGYFGRKIYRPGLHFSLASVSTNLRFGAWLTADSIVNYINTNLSTLVLARILGA
SVAGGYNLAYNVAVVPPAKLNPIITRVLFPAFAKIQDDTEKLRVNFYKLLSVVGIINFPA
LLGLMVVANNFVPLVFGEKWNSIIPILQLLCVVGLLRSVGNPIGSLLMAKARVDISFKFN
VFKTFLFIPAILIGGHLAGAIGVTLGFLVVQIINTILSYFVMIKPVLGSSYRQYILSLWL
PFYLSLPTFITSYGAGKLADGYLPLSGVFALQVMVGILSFILMIIFSRNALVMEIKNQLV
GSAKMKKLLRVG
>EECEBICD_01258 hypothetical protein
MKLLILGNHTCGNRGDSAIMRGLLDAIRQQAPEAEMDVMSRFPVSSAWLQGRPIIADPLY

QLSQKQQAAAGLNGRVKKVLRRRFQHKILLSKVAQEGSLRNFAIAPEFAEFAQFIAQYDA
VIQVGGSNYVDLYGLTHFEYPLCAFMANKPIYMVGHSVGPFQNPDFNQLANYVFGRTSAL
ILRETVSRDMMTAAQIDTRKVEQGVDTAWLVERRHDEFVASYAVQHWLDVTARRKTVAIT
LRDLEPFDKRLGTTQQAYEQAFAQVVNRLIADGYQVLALSTCTSIDRYNRDDRMVALRMA
KYVNDSENFHIAMDELNDVEIGKILASCVLTIGTRLHSAIISMNFGTPAIAINYEHKSAG
IMQQLGLAELAIDIRHLLNGSLQSVVADTLNQLPALKAKVANAVTEERAKGFTMIKSVLD
RIREAQ
>EECEBICD_01259 UDP-N-acetylglucosamine--peptide N-acetylglucosaminyltransferase GtfA subunit
MKVSFFLLKFPLSSETFVLNQITAFIDMGHEVEIVALQKGDTQHTHAAWEKYGLAAKTRW
LQDEPQGRLAKLRYRACKTLPGLHRAATWKALNFTRYGDESRNLILSAICAQVSQPFGAD
VFIAHFGPAGVTAAKLRELGVLRGKIATIFHGIDISSREVLSHYTPEYQQLFRRGDLMLP
ISELWAGRLKSMGCPPEKIAVSRMGVDMTRFTYRPVKAPGMPLEMISVARLTEKKGLHVA
IEACRQLKAQGVAFRYRILGIGPWERRLRTLIEQYQLEDVIEMPGFKPSHEVKAMLDDAD
VFLLPSITGTDGDMEGIPVALMEAMAVGIPVVSTVHSGIPELVEAGKSGWLVPENDAQAL
AARLAEFSRIDHDTLESVITRAREKVAQDFNQQAINRQLASLLQTI
>EECEBICD_01260 hypothetical protein
MPVNKFSRRTLLTAGSALAVLPFLRALPVQAREPRETVDIKDYPADDGIASFKQAFADGQ
TVVLPPGWVCENINAAITIPAGKTLRVQGAVRGNGRGRFILQDGCQVVGEQGGSLHNVTL
DVRGSDCVIKGVTMSGFGPVAQIFIGGKEPQVMRNLIIDDITVTHANYAILRQGFHNQMD
GARITHSRFSDLQGDAIEWNVAIHDRDILISDHVIERIDCTNGKINWGIGIGLAGSTYDN
SYPEDQAVKNFVVANITGSDCRQLVHVENGKHFVIRNVKAKNITPDFSKNAGIDNATIAI
YGCDNFVIDNIDMTNSAGMLIGYGVVKGKYLSIPQNFKLNAIRLDNRQVAYKLRGIQISS
GNIPSFVAITNVRMTRATLELHNQPQHLFLRNINVMQTSAIGPALKMHFDLRKDVRGQFM
ARQDTLLSLANVHAINENGQSSVDIDRINHQTVNVEAVNFSLPKRGG
>EECEBICD_01261 UTP--glucose-1-phosphate uridylyltransferase
MMNLKAVIPVAGLGMHMLPATKAIPKEMLPIVDKPMIQYIVDEIVAAGIKEIVLVTHASK
NAVENHFDTSYELESLLEQRVKRQLLAEVQSICPPGVTIMNVRQAQPLGLGHSILCARPV
VGDNPFIVVLPDIIIDDATADPLRYNLAAMVARFNETGRSQVLAKRMKGDLSEYSVIQTK
EPLDNEGKVSRIVEFIEKPDQPQTLDSDLMAVGRYVLSADIWAELERTEPGAWGRIQLTD
AIAELAKKQSVDAMLMTGDSYDCGKKMGYMQAFVKYGLRNLKEGAKFRKSIEQLLHE
>EECEBICD_01262 GDP-mannose 4,6-dehydratase
MKILITGGAGFIGSAVVRHIIKNTQDTVVNIDKLTYAGNLESLSDISESNRYNFEHADIC
DSAEITRIFEQYQPDAVMHLAAESHVDRSITGPAAFIETNIVGTYVLLEVARKYWSALGE
DKKIIFVFIIFPLMKFTAIYRILMKLKTALRCRYLLKRRHMRQVAPILRQKHPAII
>EECEBICD_01263 dTDP-glucose 4,6-dehydratase
MPIYGKGDQIRDWLYVEDHARALHMVVTEGKAGETYNIGGHNEKKNLDVVFTICDLLDEI
VPKATSYREQITYVADRPGHDRRYAIDAGKISRELGWKPLETFESGIRKTVEWYLANTQW
VNNVKSGAYQSWIEQNYEGRQ
>EECEBICD_01264 dTDP-4-dehydrorhamnose reductase
MNILLFGKTGQVGWELQRSLAPVGNLIALDVHSKEFCGDFSNPKGVAETVRKLRPDVIVN
AAAHTAVDKAESEPELAQLLNATSVEAIAKAANETGAWVVHYSTDYVFPGTGDIPWQETD
ATSPLNVYGKTKLAGEKALQDNCPKHLIFRTSWVYAGKGNNFAKTMLRLAKERQTLSVIN
DQYGAPTGAELLADCTAHAIRVALKKPEVAGLYHLVAGGTTTWHDYAALVFDEARKAGIT
LALTELNAVPTSAYPTPASRPGNSRLNTEKFQRNFDLILPQWELGVKRMLTEMFTTTTI
>EECEBICD_01265 Glucose-1-phosphate thymidylyltransferase
MKTRKGIILAGGSGTRLYPVTMAVSKQLLPIYDKPMIYYPLSTLMLAGIRDILIISTPQD
TPRFQQLLGDGSQWGLNLQYKVQPSPDGLAQAFIIGEEFIGNDDCALVLGDNIFYGHDLP
KLMEAAVNKESGATVFAYHVNDPERYGVVEFDQSGTAVSLEEKPLQPKSNYAVTGLYFYD
NSVVEMAKNLKPSARGELEITDINRIYMDQGRLSVAMMGRGYAWLDTGTHQSLIEASNFI
ATIEERQGLKVSCPEEIAFRKNFINAQQVIELAGPLSKNDYGKYLLKMVKGL
>EECEBICD_01266 dTDP-4-dehydrorhamnose 3,5-epimerase
MIVIKTAIPDVLILEPKVFGDERGFFFESYNQQTFEELIGRKVTFVQDNHSKSKKNVLRG
LHFQRGENAQGKLVRCAVGEVFDVAVDIRKESPTFGQWVGVNLSAENKRQLWIPEGFAHG

FVTLSEYAEFLYKATNYYSPSSEGSILWNDETIGIEWPFSQLPELSAKDAAAPLLHQALL
TE
>EECEBICD_01267 CDP-6-deoxy-L-threo-D-glycero-4-hexulose-3-dehydrase reductase
MSHIIKIFPSNIEFSGREDESILDAALSAGIHLEHSCKAGDCGICESDLLAGEVVDSKGN
IFGQGDKILTCCCKPKTALELNAHFFPELAGQTKKIVPCKVNSAVLVSGDVMTLKLRTPP
TAKIGFLPGQYINLHYKGVTRSYSIANSDESNGIELHVRNVPNGQMSSLIFGELQENTLM
RIEGPCGTFFIRESDRPIIFLAGGTGFAPVKSMVEHLIQGKCRREIYIYWGMQDSKDFYS
ALPQQWSEQHDNVHYIPVVSGDDAEWGEERDLSIML
>EECEBICD_01268 hypothetical protein
MDDFDSLEFFDIYACGSPVMIDASKKDFMMKNLSVEHFYSDAFTASK
>EECEBICD_01269 Glucose-1-phosphate cytidylyltransferase
MKAVILAGGLGTRLSEETIVKPKPMVEIGGKPILWHIMKMYSVHGIKDFIICCGYKGYVI
KEYFANYFLHMSDVTFHMAENRMEVHHKRVEPWNVTLVDTGDSSMTGGRLKRVAEYVKDD
EAFLFTYGDGVADLDIKATIDFHKAHGKKATLTATFPPGRFGALDIQAGQVRSFQEKPKG
DGAMINGGFFVLNPSVIDLIDNDATTWEQEPLMTLAQQGELMAFEHPGFWQPMDTLRDKV
YLEGLWEKGKAPWKTWE
>EECEBICD_01270 CDP-glucose 4,6-dehydratase
MIDKNFWQGKRVFVTGHTGFKGSWLSLWLTEMGAIVKGYALDAPTVPSLFEIVRLSDLME
SHIGDIRDFEKLRNSIAEFKPEIVFHMAAQPLVRLSYEQPIETYSTNVMGTVHLLEAVKQ
VGNIKAVVNITSDKCYDNREWVWGYRENEPMGGYDPYSNSKGCAELVASAFRNSFFNPAN
YEQHGVGLASVRAGNVIGGGDWAKDRLIPDILRSFENNQQVIIRNPYSIRPWQHVLEPLS
GYIVVAQRLYTEGAKFSEGWNFGPRDEDAKTVEFIVDKMVTLWGDDASWLLDGENHPHEA
HYLKLDCSKANMQLGWHPRWGLTETLGRIVKWHKAWIRGEDMLICSKREISDYMSATTR
>EECEBICD_01271 dTDP-4-dehydro-2,6-dideoxy-D-glucose 3-dehydratase
MTANNLREQISQLVAQYANEALSPKPFVAGTSVVPPSGKVIGAKELQLMVEASLDGWLTT
GRFNDAFEKKLGEFIGVPHVLTTTSGSSANLLALTALTSPKLGERALKPGDEVITVAAGF
PTTVNPAIQNGLIPVFVDVDIPTYNIDASLIEAAVTEKSKAIMIAHTLGNAFNLSEVRRI
ADKYNLWLIEDCCDALGTTYEGQMVGTFGDIGTVSFYPAHHITMGEGGAVFTKSGELKKI
IESFRDWGRDCYCAPGCDNTCGKRFGQQLGSLPQGYDHKYTYSHLGYNLKITDMQAACGL
AQLERVEEFVEQRKANFSYLKQGLQSCTEFLELPEATEKSDPSWFGFPITLKETSGVNRV
ELVKFLDEAKIGTRLLFAGNLIRQPYFANVKYRVVGELTNTDRIMNQTFWIGIYPGLTTE
HLDYVVSKFEEFFGLNF
>EECEBICD_01272 CDP-abequose synthase
MGAFGFLGSRLTSYFESRHTVIGLARKRNNEATINNIIYTTENNWIEKIVEFEPNIIINT
IACYGRHNEPATALIESNILMPIRVLESISSLDAVFINCGTSLPPNTSLYAYTKQKANEL
AAAIIDKVCGKYIELKLEHFYGAFDGDDKFTSMVIRRCLSNQPVKLTSGLQQRDFLYIKD
LLTAFDCIINNVNNFPKFHSIEVGSGKAISIREYVETVKNITKSNSIIEFGVVKERANEL
MYSCADIAELEKIGWKREFSLVDALTEIIEEEGK
>EECEBICD_01273 CDP-paratose 2-epimerase
MKLLITGGCGFLGSNLASFALSQGIDLIVFDNLSRKGATDNLHWLSSLGNFEFVHGDIRN
KNDVTRLITKYMPDSCFHLAGQVAMTTSIDNPCMDFEINVGGTLNLLEAVRQYNSNCNII
YSSTNKVYGDLEQYKYNETETRYTCIDKPNGYDESTQLDFHSPYGCSKGAADQYMLDYAR
IFGLNTVVFRHSSMYGGRQFATYDQGWVGWFCQKAVEIKNGINKPFTISGNGKQVRDVLH
AEDMISLYFTALANVSKIRGNAFNIGGTIVNSLSLLELFKLLEDYCNIDMRFTNLPVRER
DQRVFVADIKKITNAIDWSPKVSAKDGVQKMYDWTSSI
>EECEBICD_01274 hypothetical protein
MVRIPRHLIIAASSWLSKIIIAGVQLVSVKFLLEILGEESYAVFTLLTGLLVWFSIADIG
IGSSLQNYISELKADRKSYDAYIKAAIHILFASLIILSSTLFFLSDKLSSLYLTSFSDEL
KNNSGSYFFIASILFIFIGVGSVVYKILFAELLGWKANIINALSYLLGFLDVVAIHYLMP
DSSITFALVALYAPVAILPIIYISFRYIYVLKTKVNFNTYKLLLSRSSGFLIFSSLSIIV
LQTDYIVMSQKLSAADIIKYTVTMKIFGLMFFIYTAVLQALWPVCAELRVKMQWRKLHRI
IFLNIIGGVFFVGLGTLFIYVLKDYIYSIIANGIDYNISGAVFVLLAVYFSIRVWCDTFA
MLLQSMNQLKILWLIVPCQALIGGVTQWYFAEHYGIVGILYGLILSFSLTVFWGLPVYYM

YKSKRLA
>EECEBICD_01275 Abequosyltransferase RfbV
MKVSFCIPTYNRVKFIEDLLESINNQSSHSLIVEVCISDNASTDGTEESINIWRDRFNFP
ILYQRHNENIGPDRNYLSAVNMGTGDYCWIFGSDDILTKNSLALMEDKLAAGSDIYLCDR
RELDISMTKISNPHRRWLNGGSRLFSFSNEADLIEYFSKCNSVGGLFSYLSSIIVKRNKW
SDVIFDESYIGTAYAHVYILLRIINNMNSTLQYISLPLVDCRGDNDTFESNGKARRIKID
FIGYLKLREDFYNNNTKIYISFGRVLTKERPWFYTSLAMACYGDSTDRAELASFYKKLGY
PKIATNLIFRLKGLASYTKKIKLAKMVIKKIFS
>EECEBICD_01276 D-inositol-3-phosphate glycosyltransferase
MIVNLSRLGKSGTGMWQYSIKFLTALREIADVDAIICSKVHADYFEKLGYAVVTVPNIVS
NTSKTSRLRPLVWYVYSYWLALRVLIKFGNKKLVCTTHHTIPLLRNQTITVHDIRPFYYP
DSFIQKVYFRFLLKMSVKRCKHVLTVSYTVKDSIAKTYNVDSEKISVIYNSVNKSDFIQK
KEKENYFLAVGASWPHKNIHSFIKNKKVWSDSYNLIIVCGRTDYAMSLQQMVVDLELKDK
VTFLHEVSFNELKILYSKAYALVYPSIDEGFGIPPIEAMASNTPVIVSDIPVFHEVLTNG
ALYVNPDDEKSWQSAIKNIEQLPDAISRFNNYVARYDFDNMKQMVGNWLAESK
>EECEBICD_01277 hypothetical protein
MKITLIIPTYNAGSLWPNVLDAIKQQTIYPDKLIVIDSGSKDETVPLASDLKNISIFNID
SKDFNHGGTRNLAVAKTLDADVIIFLTQDAILADSDAIKNLVYYFSDPLIAAVCGRQLPH
KDANPLAVHARNFNYSSKSIVKSKADIEKLGIKTVFMSNSFAAYRRSVFEELSGFPEHTI
LAEDMFMAAKMIQAGYKVAYCAEAVVRHSHNYTPREEFQRYFDTGVFHACSPWIQRDFGG
AGGEGFRFVKSEIQFLLKNAPFWIPRALLTTFAKFLGYKLGKHWQSLPLSTCRYFSMYKS
YWNNIQYSSSKEIK
>EECEBICD_01278 Mannose-1-phosphate guanylyltransferase RfbM
MSFLPVIMAGGTGSRLWPLSREYHPKQFLSVEGKLSMLQNTIKRLASLSTEEPVVICNDR
HRFLVAEQLREIDKLANNIILEPVGRNTAPAIALAAFCALQNADNADPLLLVLAADHVIQ
DEIAFTKAVRHAEEYAANGKLVTFGIVPTHAETGYGYIRRGELIGNDAYAVAEFVEKPDI
DTAGDYFKSGKYYWNSGMFLFRASSYLNELKYLSPEIYKACEKAVGHINPDLDFIRIDKE
EFMSCPSDSIDYAVMEHTQHAVVIPMSAGWSDVGSWSSLWDISNKDHQRNVLKGDIFAHA
CNDNYIYSEDMFISAIGVSNLVIVQTTDALLVANKDTVQDVKKIVDYLKRNDRNEYKHHQ
EVFRPWGKYNVIDSGKNYLVRCITVKPGEKFVAQMHHHRAEHWIVLSGTARVTKGEQIYM
VSENESTFIPPNTIHALENPGMTPLKLIEIQSGTYLGEDDIIRLEQRSGFSKEWTNERS
>EECEBICD_01279 Phosphoglucosamine mutase
MNVVNNSRDVIYSSGIVFGTSGARGLVKDFTPQVCAAFTVSFVAVMQEHFSFDTVALAID
NRPSSYGMAQACAAALADKGVNCIFYGVVPTPALAFQSMSDNMPAIMVTGSHIPFERNGL
KFYRPDGEITKHDEAAILSVEDTCSHLELKELIVSEMAAVNYISRYTSLFSTPF
>EECEBICD_01280 hypothetical protein
MDNIDNKYNPQLCKIFLAISDLIFFNLALWFSLGCVYFIFDQVQRFIPQDQLDTRVITHF
ILSVVCVGWFWIRLRHYTYRKPFWYELKEIFRTIVIFAIFDLALIAFTKWQFSRYVWVFC
WTFALILVPFFRALTKHLLNKLGIWKKKTIILGSGQNARGAYSALQSEEMMGFDVIAFFD
TDASDAEINMLPVIKDTEIIWDLNRTGDVHYILAYEYTELEKTHFWLRELSKHHCRSVTV
VPSFRGLPLYNTDMSFIFSHEVMLLRIQNNLAKRSSRFLKRTFDIVCSIMILIIASPLMI
YLWYKVTRDGGPAIYGHQRVGRHGKLFPCYKFRSMVMNSQEVLKELLANDPIARAEWEKD
FKLKNDPRITAVGRFIRKTSLDELPQLFNVLKGDMSLVGPRPIVSDELERYCDDVDYYLM
AKPGMTGLWQVSGRNDVDYDTRVYFDSWYVKNWTLWNDIAILFKTAKVVLRRDGAY
>EECEBICD_01281 hypothetical protein
MSKQQIGVVGMAVMGRNLALNIESRGYTVSVFNRSREKDRRSDCRESRQKAGALLHGERV
C
>EECEBICD_01282 6-phosphogluconate dehydrogenase, decarboxylating
MIAENPGKKLVPYYTVKEFVESLETPRRILLMVKAGAGTDAAIDSLKPYLEKGDIIIDGG
NTFFQDTIRRNRELSAEGFNFIGTGVSGGEEGALKGPSIMPGGQKDAYELVAPILTKIAA
VAEDGEPCVTYIGADGAGHYVKMVHNGIEYGDMQLIAEAYSLLKGGLNLSNEELANTFTE
WNNGELSSYLIDITKDIFTKKDEDGNYLVDVILDEAANKGTGKWTSQSALDLGEPLSLIT
ESVFARYISSLKAQRVAASKVLSGPKAQPAGDKAEFIEKVRRALYLGKIVSYAQGFSQLR
AASDEYHWDLNYGEIAKIFRAGCIIRAQFLQKITDAYAENAGIANLLLAPYFKKIADEYQ

QALRDVVAYAVQNGIPVPTFSAAVAYYDSYRAAVLPANLIQAQRDYFGAHTYKRTDKEGI
FHTEWLE
>EECEBICD_01283 UDP-glucose 6-dehydrogenase
MKITISGTGYVGLSNGLLIAQHHDVVALDIVPSRVELLNDRISPIVDKEIQQFLKEDNIR
FRATLDKFDAYQNADYVIIATPTDYDPKTNYFNTSSVESVIQDVISINPAAVMIIKSTVP
VGFTAAMRQKFATENIIFSPEFLREGKALYDNLYPSRIVIGEQSERAREFAALLQEGAIK
QEIPTLFTDSTEAEAIKLFANTYLAMRVAYFNELDSYAETLGLNTRQIIEGVCLDPRIGN
HYNNPSFGYGGYCLPKDTKQLLANYQSVPNNIISAIVEANRTRKDFIADAILARKPKVVG
IYRLIMKSGSDNFRASSIQGIMKRIKAKGVEVIIYEPVMEEDTFFNSRLERDLHCFKQQA
DVIISNRMAAELLDVAEKVYTRDLFGSD
>EECEBICD_01284 Chain length determinant protein
MTVDSNTSSGRGNDPEQIDLIELLLQLWRGKMTIIVAVIIAILLAVGYLMIAKEKWTSTA
IITQPDAAQVATYTNALNVLYGGNAPKISEVQANFISRFSSAFSALSEALDNQKEREKLT
IEQSVKGQALPLSVSYVSTTAEGAQRRLAEYIQQVDEEVAKELEVDLKDNITLQTKTLQE
SLETQEVVAQEQKDLRIKQIEEALRYADEAKITQPQIQQTQDVTQDTMFLLGSDALKSMI
QNEATRPLVFSPAYYQTKQTLLDIKNLKVTADTVHVYRYVMKPTLPVRRDSPKKAITLVL
AVLLGGMIGAGIVLGRNALRSYKPKAL
>EECEBICD_01285 Histidine biosynthesis bifunctional protein HisIE
MLTEQQRRELDWEKTDGLMPAIVQHAVSGEVLMLGYMNPQALDKTIESGHVTFFSRTKQR
LWTKGETSGHVLNVVSIAPDCDNDTLLVLANPVGPTCHKGTSCCFGYAGHQWLFLYQLEQ
LLAERKTADPASSYTAKLYASGTKRIAQKVGEEGVETALAATVNDRFELTNEASDLMYHL
LVLLQDQDLNLTTVIDNLRKRHQ
>EECEBICD_01286 Imidazole glycerol phosphate synthase subunit HisF
MLAKRIIPCLDVRDGQVVKGVQFRNHEIIGDIVPLAKRYADEGADELVFYDITASSDGRV
VDKSWVARVAEVIDIPFCVAGGIRSIDDAAKILSFGADKISINSPALADPTLITRLADRF
GVQCIVVGIDTWFDDATGKYHVNQYTGDENRTRVTQWETLDWVQEVQQRGAGEIVLNMMN
QDGVRNGYDLTQLKKVRDVCRVPLIASGGAGTMEHFLEAFRDADVDGALAASVFHKQIIN
IGELKAYLAGQGVEIRIC
>EECEBICD_01287 1-(5-phosphoribosyl)-5-[(5-
phosphoribosylamino)methylideneamino] imidazole-4-carboxamide isomerase
MIIPALDLIDGTVVRLHQGDYARQRDYGNDPLPRLQDYAAQGAGVLHLVDLTGAKDPAKR
QIPLIKTLVAGVNVPVQVGGGVRTEEDVAALLKAGVARVVIGSTAVKSPDVVKGWFERFG
AQALVLALDVRIDEHGNKQVAVSGWQENSGVSLEQLVETYLPVGLKHVLCTDISRDGTLA
GSNVSLYEEICARYPQIAFQSSGGIGDIDDIAALRGTGVRGVIVGRALLEGKFTVKEAIQ
CWQNV
>EECEBICD_01288 Imidazole glycerol phosphate synthase subunit HisH
MNVVILDTGCANLSSVKSAVARHGYTPVVSREAEIVLRADKLFLPGVGTAQAAMDQLRER
ELIDLIKACTQPVLGICLGMQLLGRRSEETRGVDLLNIIEQDVPKMTDFGLPLPHMGWNR
VYPQAGNRLFQGIEDGAYFYFVHSYAMPVNPWTIAQCNYGEPFTAAVQKDNFFGVQFHPE
RSGAAGAQLLKNFLEM
>EECEBICD_01289 Histidine biosynthesis bifunctional protein HisB
MSQKYLFIDRDGTLISEPPSDFQVDRFDKLAFEPEVIPVLLKLQKAGFKLVMITNQDGLG
TQSFPQADFDGPHNLMMQIFTSQGVCFDEVLICPHLPADDCDCRKPKVKLVERYLAEQAM
DSANSYVIGDRATDIQLADNMGITGLRYHRETLNWTMIGEQLTKRDRYAHVIRNTKETQI
DVSVWLDREGNSKINTGVGFFDHMLDQIATHGGFRMEITVKGDLYIDDHHTVEDTGLALG
EALKLALGDKRGICRFGFVLPMDECLARCALDISGRPHLEYKAEFTYQRVGDLSTEMIEH
FFRSLSYTMGVTLHLKTKGKNDHHRVESLFKAFGRTLRQAIRVEGDTLPSSKGVL
>EECEBICD_01290 Histidinol-phosphate aminotransferase
MSTENTLSVADLARENVRNLVPYQSARRLGGNGDVWLNANEFPTAVEFQLTQQTLNRYPE
CQPKAVIENYAQYAGVKPEQVLVSRGADEGIELVIRAFCEPGKDAILYCPPTYGMYSVSA
ETIGVERRTVPALENWQLDLQGISDNLDGTKVVFVCSPNNPTGQLINPQDLRTLLELTRG
KAIVVADEAYIEFCPQATLTGWLVEYPHLVILRTLSKAFALAGLRCGFTLANEEVINLLL
KVIAPYPLSTPVADIAAQALSPQGINAMRDRVAQTVQERQYLVNALQQTACVEHVFDSET
NYILARFTASSSVFKSLWDQGIILRDQNKQPSLSGCLRITVGTRQENQRVIDALRAEPV

>EECEBICD_01291 Histidinol dehydrogenase
MSFNTLIDWNSCSPEQQRALLTRPAISASDSITRTVSDILDNVKTRGDDALREYSAKFDK
TEVTALRVTPEEIAAAGARLSDELKQAMAAAVKNIETFHSAQTLPPVDVETQPGVRCQQV
TRPVASVGLYIPGGSAPLFSTVLMLATPARIAGCQNVVLCSPPPIADEILYAAQLCGVQE
IFNVGGAQAIAALAFGSESVPKVDKIFGPGNAFVTEAKRQVSQRLDGAAIDMPAGPSEVL
VIADSGATPDFVASDLLSQAEHGPDSQVILLTPDADIACKVAEAVERQLAELPRADTARQ
ALSASRLIVTKDLAQCVAISNQYGPEHLIIQTRNARDLVDAITSAGSVFLGDWSPESAGD
YASGTNHVLPTYGYTATCSSLGLADFQKRMTVQELSKAGFSALASTIETLAAAERLTAHK
NAVTLRVNALKEQA
>EECEBICD_01292 ATP phosphoribosyltransferase
MLDNTRLRIAIQKSGRLSDDSRELLARCGIKINLHTQRLIAMAENMPIDILRVRDDDIPG
LVMDGVVDLGIIGENVLEEELLNRRAQGEDPRYFTLRRLDFGGCRLSLATPVDEAWDGPA
ALDGKRIATSYPHLLKRYLDQKGVSFKSCLLNGSVEVAPRAGLADAICDLVSTGATLEAN
GLREVEVIYRSKACLIQRDGEMAQSKQQLIDKLLTRIQGVIQARESKYIMMHAPSERLEE
VIALLPGAERPTILPLAGEQQRVAMHMVSSETLFWETMEKLKALGASSILVLPIEKMME
>EECEBICD_01293 Protein YeeZ
MKKVAIVGLGWLGMPLAMSLAARGWQVTGSKTTLDGVEAARMSGIESYPLRLEPELVCEA
DDLDALLDVDALVITLPARRSGPGEDFYLQAMQELVDSALAYRIPRIIFTSSTSVYGDAQ
GVVKENTPRNPVTASGRTLKELEDWLHNLPGTSVDILRLAGLVGPGRHPGRFFAGKTAPD
GQHGVNLVHLEDVVGAITLLLQAPKGGHIYNICAPAHPARNVFYPQMTRLLGMAPPHFRN
APDNGKGKIIDGSRICNELGFEYQYPDPLVMPME
>EECEBICD_01294 HTH-type transcriptional regulator YhaJ
MKPLLDVLIILDALEKEGSFAAASAKLFKTPSALSYTVHKLENDLNIQLLDRSGHRARFT
RTGKMLLEKGREVLHTVRELEKQAIKLHEGWENELVIGVDDTFPFSLLAPLIDAFYQRHS
VTRLKFVNGVLGGSWETLTQGRADIIVGAMRAPPPDTGFGFARLGDLELVFAVAPHHPLV
HEEEPLSRQTIKRFRAIVVGDSVHSSRSIGSELLDAQEAITVFDFKTKLELQISGLGCGY
LPRYLAQRFLESGALIEKKVVAQIVYEPVWVGWNEQTAGLASGWWRDEILANNAIAGVYA
KSTV
>EECEBICD_01295 Low-affinity putrescine importer PlaP
MSHNVTPNTSRVELRKTLTLVPVVMMGLAYMQPMTLFDTFGIVSGLTDGHVPTAYAFALI
AILFTALSYGKLVRRYPSAGSAYTYAQKSISPTVGFMVGWSSLLDYLFAPMINILLAKIY
FEALVPSIPSWVFVVALVAFMTAFNLRSLKSVANFNTVIVVLQVVLIAVILGMVVYGVFE
GEGAGTLASTRPFWSGDAHVIPMITGATILCFSFTGFDGISNLSEETKDAERVIPRAIFL
TALIGGLIFIFATYFLQLYFPDISRFKDPDASQPEIMLYVAGKTFQVGALIFSTITVLAS
GMAAHAGVARLMYVMGRDGVFPKSFFGYVHPKWRTPAMNIILVGAIALLAINFDLVMATA
LINFGALVAFTFVNLSVISQFWIREKRNKTLKDHFQYLFLPMCGALTVGALWVNLEESSM
VLGLIWAGIGLIYLACVTKSFRNPVPQYEDVA
>EECEBICD_01296 Exodeoxyribonuclease I
MTVKNDSIQSTFLFHDYETFGTHPALDRPAQFAALRTDNDFNVIGEPEVFYCKPADDYLP
QPGAVLITGITPQEAREKGENEAAFARRIHALFTVPKTCVVGYNNVRFDDEVTRNIFYRN
FYDPYAWSWQHDNSRWDLLDVMRACYALRPEGINWPENDDGLPSFRLEHLTQANGIEHSN
AHDAMADVYATIAMAQLVKTRQPRLFDYLYSHRSKHKLAALIDVPQMKPLVHVSGMFGAW
RGNTSWVAPLAWHPENRNAVIMVDLAGDISPLLELDSDTLRERLYTAKADLGDHAAVPVK
LVHINKCPVLAQANTLRPEDADRLGINRQHCLDNLKVLRENPQVRDKVVAIFAEAEPFAA
SDNVDAQLYDGFFSDADRAAMKIVLETEPRNLPALDITFVDKRIEKLLFNYRARNFPGTL
DDAEQQRWLEHRRQVLTPEFLQQYANELQMLSQQYAEDKTKLGLLKSLWQYATEIV
>EECEBICD_01297 E3 ubiquitin-protein ligase SopA
MKISSGAINFSTIPNQVKKLITSIREHTKNGLASKITSVKNTHASLNEKLKTGKSSSIEF
ALPQKIKDFFQPKDKNTLNKTLITVKNIKDTNNAGKKNISAEDVSKMNAAFMRKHIANQT
CDYNYRMTGAAPLPGGVSVSANNRPTVSEGRTPPVSPSLSLQATSSPSSPADWAKKLTDA
VLRQKAGETLTAADRDFSNADFRNITFSKILPPSFMERDGDIIKGFNFSNSKFTYSDISH
LHFDECRFTYSTLSDVVCSNTKFSNSDMNEVFLQYSITTQQQPSFIDTTLKNTLIRHKAN
LSGVILNEPDNSSPPSVSGGGNFIRLGDIWLQMPLLWTENAVDGFLNHEHNNGKSILMTI
DSLPDKYSQEKVQAMEDLVKSLRGGRLTEACIRPVESSLVSVLAHPPYTQSALIREWLGP

VQERFFAHQCQTYNDVPLPTPDTYYQQRILPVLLDSFDRNSAAMTTHSGLFNQVILHCMT
GVDCTDGTRQKAAALYEQYLAHPAVSPHIHNGLFGNYDGSPDWTTRAADNFLLLSSQDSD
TAMMLSTDTLLTMLNPTPDTAWDNFYLLRAGENVSTAQISPVELFRHDFPVFLAAFNQQA
TQRRFGELIDIILSTEEHGELNQQFIAATNQKHSTVKLIDDASVSRLATIFAPLLPEGKL
SPAHYQHILSAYHLTDATPQKQAETLFCLSTAFARYSSSAIFGTEHDSPPALRGYAEALM
QKAWELSPAIFPSSEQFTDWSDRFHGLHGAFTCTSVVADSMQRHARKYFPSVLSSILPLA
WA
>EECEBICD_01298 Thiosulfate reductase molybdopterin-containing subunit PhsA
MSISRRSFLQGVGIGCSACALGAFPPGALARNPIAGINGKTTLTPSLCEMCSFRCPIQAQ
VVNNKTVFIQGNPSAPQQGTRICARGGSGVSLVNDPQRIVKPMKRTGPRGDGEWQVISWQ
QAYQEIAAKMNAIKAQHGPESVAFSSKSGSLSSHLFHLATAFGSPNTFTHASTCPAGKAI
AAKVMMGGDLAMDIANTRYLVSFGHNLYEGIEVADTHELMTAQEKGAKMVSFDPRLSIFS
SKADEWHAIRPGGI
>EECEBICD_01299 Thiosulfate reductase molybdopterin-containing subunit PhsA
MARYSSRGDLAVLLAMCHVMIDEQLYDASFVERYTSGFEQLAQAVKETTPEWAAAQADVP
ADVIVRVTRELAACAPHAIVSPGHRATFSQEEIDMRRMIFTLNVLLGNIEREGGLYQKKN
ASVYNKLAGEKVAPTLAKLNIKNMPKPTAQRIDLVAPQFKYIAAGGGVVQSIIDSALTQK
PYPIKAWIMSRHNPFQTVTCRSDLVKTVEQLDLVVSCDVYLSESAAYADYLLPECTYLER
DEEVSDMSGLHPAYALRQQVVEPIGEARPSWQIWKELGEQLGLGQYYPWQDMQTRQLYQL
NGDHALAKELRQKGYLEWGVPLLLREPESVRQFTARYPGAIATDSDNTYGEQLRFKSPSG
KIELYSATLEELLPGYGVPRVRDFALKKENELYFIQGKVAVHTNGATQYVPLLSELMWDN
AVWVHPQTAQEKGIKTGDEIWLENATGKEKGKALVTPGIRPDTLFVYMGFGAKAGAKTAA
TTHGIHCGNLLPHVTSPVSGTVVHTAGVTLSRA
>EECEBICD_01300 Thiosulfate reductase electron transfer subunit PhsB
MNHLTNQYVMLHDEKRCIGCQACTVACKVLNDVPEGFSRVQVQIRAPEQASNALIHFQFV
RVSCQHCENAPCVSVCPTGASYRDENGIVQVDKSRCIGCDYCVAACPFHVRYLNPQTGVA
DKCNFCADTRLAAGQSPACVSVCPTDALKFGRLDESEIQRWVGQKEVYRQQEARSGAVSL
YRRKEIHQEGKA
>EECEBICD_01301 Thiosulfate reductase cytochrome B subunit PhsC
MNTIWGAELHYAPDYWPLWLIYAGVVVLLMLVGLVIHALLRRMLAPKTAGGEEHRDYLYS
LAIRRWHWGNALLFVLLLLSGLFGHFSLGPVALMVQVHTWCGFALLAFWVGFVLINLTTG
NGRHYRVNFSGLVTRCIRQTRFYLFGIMKGEAHPFAATEQNKFNPLQQLAYLAIMYALVP
LLIITGLLCLYPQVAGLGPVMLVLHMALAIIGLLFICAHLYLCTLGDTPGQIFRSMVDGY
HRHRTAPRGDKSAV
>EECEBICD_01302 D-alanyl-D-alanine carboxypeptidase DacD
MKRRLFIAASLFAMHLSPALAADAVSFAPQPPAIDAGAWVLMDYTTGQVLTAGNEHQQRN
PASLTKLMTGYVVDRAIDSHRISPDDIVTVGRDAWAKDNPVFVGSSLMFLKEGDRVSVRD
LSRGLIVDSGNDACVALADYIAGGQPQFVAMMNSYVKKLNLQDTHFETVHGLDAPGQHSS
AYDLAVLSRAIIHGEPEFYHMYSEKSLTWNGITQQNRNGLLWDKTMHIDGLKTGHTSGAG
FNLIASAVDGQRRLIAVVMGAKSSKGREEQARKLLQWGQQNFATVQILHSGKKVGSERIW
YGDKEKIALGTEQDFWMALPKAEIPHIKAKYVLDRKELEAPIAAHQRVGEIELYDRDKLI
AQWPLVTLESVGKGGMFSRLSDYFQHKA
>EECEBICD_01303 DNA gyrase inhibitor
MDYEIRQEQKRKIAGFHMVGPWEHTVKQGFEQLMTWVDRQRIVPVEWIAVYYDNPDVVPA
EKTPLRYRGVRGREFHPAGQ
>EECEBICD_01304 DNA gyrase inhibitor
MVSVAENFILPDNSEGVIVTAIEGGEYATAVARVEDRDFAKPWERFFDVLEQDSAYQIAS
APCFETYLNNGMEDGYWDIEMYIPVQRK
>EECEBICD_01305 Inner membrane protein YeeA
MRADKSLSPFEIRLYRHYRIVHGIRIALAFILTFLLVRLFSIPEGTWPLITLVVIMGPIS
FWGNVVPRAFERIGGTILGAALGLVALRLELFSLPLMLVWCAIAMFLCGWLALGKKPYQA
LLIGITLAVVVGAPAGDMDTALWRGGDVILGSLLAMLFTGIWPQRAFFALAHPTGSLRHG

V
>EECEBICD_01306 Inner membrane protein YeeA
MDKHLQRLLNDVVKMRGLITPASKETRIQKSIFEAIQTINRNLVCMLELQINAHWATRAS
HFVMLNAHTLRETQQMTQQTLLTIAHALFEGNPQPVLANTGKLNDIAAELRQLMNEQQGD
AVAETPIHGYVWLSMETARQLELLSHLICRALRK
>EECEBICD_01307 hypothetical protein
METTKPSFQDVLEFVRLFRRKNKLQREIQDIEKKIRDNQKRVLLLDNLSDYIKPGMSVEA
IQGIIASMKSDYEDRVDDYIIKNAEISKERRDISKKLKAMGEMKHADVKAE
>EECEBICD_01308 L-threonine kinase
MRAHYSYLKGDNVAVAQCPASCGELIQGWILGSEKLVSCPVDWYSTVAVTAAPPLVNERP
LSRAMVERVLAHWQYPAHWSNEIRVDVRSSIPVAKGMASSTADIAATAVATAHHLGHSLD
ETTLAQLCVSIEPTDSTVFHQLTLFDHNNAVTQIACEPPPPIDLLVLESPVTLRTQDYHR
LPRQQKLIASSATLQQAWNLVQEACITQNPLRLGEAATLSAIASQTLLPKPGFTALLSLV
EECDLYGLNVAHSGSVVGLMLDRKRHDIARLKGKLAEKKTYPTLAKTTFTQDGHRRGQTA
VRA
>EECEBICD_01309 putative propionate kinase
MSYKIMAINAGSSSLKFQLLEMPQGDMLCQGLIERIGMADAQVTIKTHSQKWQETVPVAD
HRDAVTLLLEKLLGYQIINSLRDIDGVGHRVAHGGEFFKDSTLVTDETLAQIERLAELAP
LHNPVNALGIHVFRQLLPDAPSVAVFDTAFHQTLDEPAYIYPLPWHYYAELGIRRYGFHG
TSHKYVSGVLAEKLGVPLSALRVICCHLGNGSSICAIKNGRSVNTSMGFTPQSGVMMGTR
SGDIDPSILPWIAQRESKTPQQLNQLLNNESGLLGVSGVSSDYRDVEQAANTGNRQAKLA
LTLFAERIRATIGSYIMQMGGLDALVFTGGIGENSARARSAVCHNLQFLGLAVDEEKNQR
NATFIQTENALVKVAVINTNEELMIAQDVMRIALPATEGLCVPA
>EECEBICD_01310 Propanediol utilization protein PduV
MKRLMFIGPSQCGKTSLTQSLRGEALHYKKTQAIEWSPMAIDTPGEYLENRCLYSALLTS
ACEADVIALVLNADAQWSPFSPGFTAPMNRPTIGLVTKADLAEPQRISLVAEWLTQAGAR
QIFITSALNNSGLDAVLDFLNSKEPLCLTK
>EECEBICD_01311 Propanediol utilization protein PduU
MERQPTTDRMIQEYVPGKQVTLAHLIANPGKDLFKKLGLQDAVSAIGILTITPSEASIIA
CDIATKSGAVEIGFLDRFTGAVVLTGDVSAVEYALKQVTRTLGEMMQFTTCSITRT
>EECEBICD_01312 hypothetical protein
MSQAIGILELTSIAKGMELGDAMLKSANVDLLVSKTICPGKFLLMLGGDIGAIQQAIETG
TSQAGEMLVDSLVLANIHPSVLPAISGLNSVDKRQAVGIVETWSVAACISAADRAVKGSN
VTLVRVHMAFGIGGKCYMVVAGDVSDVNNAVTVASESAGEKGLLVYRSVIPRPHEAMWRQ
MVEG
>EECEBICD_01313 Ion-translocating oxidoreductase complex subunit C
MSTAINSVEMSLSADEIRERVRAAGVVGAGGAGFPAHVKLQAQVEIFLVNAAECEPMLKV
DQQLMWQQAARLVRGVQYAMTATGAREGVIALKEKYRWAIDALTPLLPAGIRLHILPDVY
PAGDEVLTIWMATGRRVAPAALPASVGVVVNNVQTVLNIARAVEQQFPVTRRTLTVNGAV
ARPLTVTVPIGMSLREVLALAGGATVDDPGFINGGPMMGGLITSLDNPVTKTTGGLLVLP
KSHPLIQRRMQDERTVLSVARTVCEQCRLCTDLCPRHLIGHELSPHLLVRAVNFHQAATP
QLLLSALTCSECNVCESVACPVGISPMRINRMLKRELRAQNQRYEGPLNPSDEMAKYRLV
PVKRLIAKLGLSPWYQEAPLVEEEPSVEKVTLQLRQHIGASAVANVAVGERVTRGQCVAD
VPPGALGAPIHASIDGIVSAISEQAITVVRG
>EECEBICD_01314 Aldehyde-alcohol dehydrogenase
MNTFSLQTRLYSGQGSLAVLKRFTNKHIWIICDGFLARSPLLDTLRNALPADNRISVFSE
ITPDPTIHTVVQGIAQMQALQPQVVIGFGGGSAMDAAKAIVWFSQQSGINIETCVAIPTT
SGTGSEVTSACVISDPDKGIKYPLFNNALYPDMAILDPELVVSVPPQITANTGMDVLTHA
LEAWVSPRASDFTDALAEKAAKLVFQYLPTAVEKGDCVATRGKMHNASTLAGMAFSQAGL
GLNHAIAHQLGGQFHLPHGLANALLLTTVIRFNAGVPRAAKRYARLAKACGFCPAEANDI
AAINALIQQIELLKQRCALPSLAVALKEGRSNFSARIPAMVQAALADVTLRTNPRPASAE
EIRELLEELL
>EECEBICD_01315 Aldehyde-alcohol dehydrogenase
MNTSELETLIRTILSEQLTTPAQTPVQPQGKGIFQSVSEAIDAAHQAFLRYQQCPLKTRS

AIISAMRQELTPLLATLAEESANETGMGNKEDKFLKNKAALDNTPGVEDLTTTALTGDGG
MVLFEYSPFGVIGSVAPSTNPTETIINNSISMLAAGNSVYFSPHPGAKKVSLKLISLIEE
IAFRCCGIRNLVVTVAEPTFEATQQMMAHPRIAVLAITGGPGIVAMGMKSGKKVIGAGAG
NPPCIVDETADLVKAAEDIINGASFDYNLPCIAEKSLIVVESVAERLVQQMQTFGALLLS
PTDTDKLRAVCLPEGQANKKLVGKSPSAMLEAAGIAVPAKAPRLLIAVVNADDPWVTSEQ
LMPMLPVVKVSDFDSALALALKVEEGLHHTAIMHSQNVSRLNLAARTLQTSIFVKNGPSY
AGIGVGGEGFTTFTIATPTGEGTTSARTFARSRRCVLTNGFSIR
>EECEBICD_01316 hypothetical protein
MAIYTRTGDAGTTSLFTGQRVSKTHPRVEAYGTLDELNAALSLCACAAADENHRTLLEAI
QQQLFWFSAELASDSEQPSPKQRYISSEEISALEAAIDRAMARVEPLHSFILPGRCEAAS
RLHFARTLARRAERRLVELATEVNVRQVLMRYINRLSDCLYALARAEDSDAHQANIIREV
SKRYLAASQPTRSKETTPVALSFHDLHQLTRAAVERAQQLQVPVVVSIVDAHGTETVTWR
MPDALLVSSELAPKKAWTAVAMKTATHELSDVVQPGAALYGLESHLQGKVVTFGGGYALW
RDGILIGGSWHQRRQR
>EECEBICD_01317 Carbon dioxide concentrating mechanism protein CcmL
MHLARVTGAVVSTQKSPSLIGKKLLLVRRVSADGELPASPTSGDEVAVDSVGAGIGELVL
LSGGSSARHVFSGPNEAIDLAVVGIVDTLSR
>EECEBICD_01318 hypothetical protein
MNGETLQRIVEEIVSRLQRRAQSTVTLSVAQLRDADCPALFCQHASLRILLVDLPLLGQL
ADAETDDTAARKIHDALAFGIRVQLSLHSQLLPVIPVKKLARLPLVFTDERGLPLVLHAG
SVLSYRDVALLSRGRVVVHRKCIVTALAREAANARNIQLIKQE
>EECEBICD_01319 Phosphate propanoyltransferase
MDKELLQSTVRKVLDEMRQRPIPLGVSNRHIHLSAQDYERLFPGHPISEKKALLQPGQYA
AEQTVTLVGPKGQLNNVRLLGPLRSVSQVEISRTDARTLGIAAPLRMSGNLKGTPGIRLV
SPFGELELPSGVIVAQRHIHMSPLDALILKVSHGDRVSVAIEGDERGLIFNNVAIRVSPD
MRLEMHIDTDEANAAGADNPQAFARLVGPR
>EECEBICD_01320 hypothetical protein
MKQSLGLLEVCGLALAISCADIMAKSASITLLALEKTNGSGWMVIKITGDVASVQAAITT
GAQFAEQRNGLVAHKVIARPGEGILLAETPSPSVIEPEPEASEIADVVSEAPAEEAPQES
ELVSCNLCLDPKCPRQKGEPRTLCIHSGKRGEA
>EECEBICD_01321 Propanediol utilization protein PduA
MNNALGLVETKGLVGAIEAADAMVKSANVQLVGYEKIGSGLVTVMVRGDVGAVKAAVDAG
SAAASVVGEVKSCHVIPRPHSDVEAILPKSA
>EECEBICD_01322 hypothetical protein
MDSNHSAPAIVITVINDCASLWHEVLLGIEEEGIPFLLQHHPAGDVVDSAWQAARSSPLL
VGIACDRHTLVVHYKNLPVSAPLFTLMHHQDSQAQRNTGNNAARLVKGIPFRDLHA
>EECEBICD_01323 Diol dehydratase-reactivating factor alpha subunit
MRYIAGIDIGNSSTEVALARQDETGALTITHSALAETTGIKGTLRNVFGIQEALALVAKR
AGINVSDISLIRINEATPVIGDVAMETITETIITESTMIGHNPKTPGGAGLGVGITITPE
ELLTRPADSSYILVVSSAFDFADIANVINASMRAGYQITGVILQRDDGVLVSNRLEKSLP
IVDEVLYIDRIPLGMLAAIEVAVPGKVIETLSNPYGIATVFNLNADETKNIVPMARALIG
NRSAVVVKTPSGDVKARAIPAGNLELQAQGRTVRVDVAAGAEAIMKAVDGCGKLDNVTGE
AGTNIGGMLEHVRQTMAELTNKPSSEIFIQDLLAVDTSVPVSVTGGLAGEFSLEQAVGIA
SMVKSDRLQMAMIAREIEQKLNIDVQIGGAEAEAAILGALTTPGTTRPLAILDLGAGSTD
ASIINPKGEIIATHLAGAGDMVTMIIARELGLEDRYLAEEIKKYPLAKVESLFHLRHEDG
SVQFFPTPLPPAVFARVCVVKPDELVPLPGDLALEKVRAIRRSAKERVFVTNALRALRQV
SPTGNIRDIPFVVLVGGSSLDFEVPQLVTDALAHYRLVAGRGNIRGSEGPRNAVATGLIL
SWHKEFAHGQ
>EECEBICD_01324 Propanediol dehydratase small subunit
MNTDAIESMVRDVLSRMNSLQGDAPAAAPAAGGTSRSAKVSDYPLANKHPEWVKTATNKT
LDDFTLENVLSNKVTAQDMRITPETLRLQASIAKDAGRDRLAMNFERAAELTAVPDDRIL
EIYNALRPYRSTKEELLAIADDLENRYQAKICAAFVREAAGLYVERKKLKGDD
>EECEBICD_01325 Propanediol dehydratase medium subunit
MEINEKLLRQIIEDVLRDMKGSDKPVSFNAPAVSTAPQTAAPAGDGFLTEVGEARQGTQQ

DEVIIAVGPAFGLAQTVNIVGLPHKSILREVIAGIEEEGIRARVIRCFKSSDVAFVAVEG
NRLSGSGISIGI
>EECEBICD_01326 Propanediol dehydratase large subunit
MRSKRFEALAKRPVNQDGFVKEWIEEGFIAMESPNDPKPSIKIVNGAVTELDGKPVSEFD
LIDHFIARYGINLNRAEEVMAMDSVKLANMLCDPNVKRSEIVPLTTAMTPAKIVEVVSHM
NVVEMMMAMQKMRARRTPSQQAHVTNVKDNPVQIAADAAEGAWRGFDEQETTVAVARYAP
FNAIALLVGSQVGRPGVLTQCSLEEATELKLGMLGHTCYAETISVYGTEPVFTDGDDTPW
SKGFLASSYASRGLKMRFTSGSGSEVQMGYAEGKSMLYLEARCIYITKAAGVQGLQNGSV
SCIGVPSAVPSGIRAVLAENLICSSLDLECASSNDQTFTHSDMRRTARLLMQFLPGTDFI
SSGYSAVPNYDNMFAGSNEDAEDFDDYNVIQRDLKVDGGLRPVREEDVIAIRNKAARALQ
AVFAGMGLPPITDEEVEAATYAHGSKDMPERNIVEDIKFAQEIINKNRNGLEVVKALAQG
GFTDVAQDMLNIQKAKLTGDYLHTSAIIVGDGQVLSAVNDVNDYAGPATGYRLQGERWEE
IKNIPGALDPNEID
>EECEBICD_01327 Propanediol utilization protein PduB
MSSNELVEQIMAQVIARVATPEQQAIPGQPQPIRETAMAEKSCSLTEFVGTAIGDTLGLV
IANVDTALLDAMKLEKRYRSIGILGARTGAGPHIMAADEAVKATNTEVVSIELPRDTKGG
AGHGSLIILGGNDVSDVKRGIEVALKELDRTFGDVYGNEAGHIELQYTARASYALEKAFG
APIGRACGIIVGAPASVGVLMADTALKSANVEVVAYSSPAHGTSFSNEAILVISGDSGAV
RQAVTSAREIGKTVLATLGSEPKNDRPSYI
>EECEBICD_01328 Propanediol utilization protein PduA
MQQEALGMVETKGLTAAIEAADAMVKSANVMLVGYEKIGSGLVTVIVRGDVGAVKAATDA
GAAAARNVGEVKAVHVIPRPHTDVEKILPKGISQ
>EECEBICD_01329 Propanediol diffusion facilitator
MNDSLKAQCGAEFLGTGLFLFFGIGCLSALKVAGASLGLWEICIIWGLGISLAVYLTAGI
SGGHLNPAVTIALWLFACFPKQKVLPYIIAQFAGAFGGALLAYVLYSSLFTEFETAHHMV
RGSVESLQLASIFSTYPAAALNVWQAALVEVVITSILMGMIMALTDDGNGIPKGPLAPLL
IGILVAVIGASTGPLTGFAMNPARDFGPKLFTWLAGWGNMAMSGGREIPYFIVPIVAPVI
GACAGAAIYRYFIGKNLPCNRCEL
>EECEBICD_01330 HTH-type transcriptional activator RhaS
MISASALNSELINKIAQDFAQATGLAVVVVNIHGDEISELFNFTPFCQLMRQHPQHSTRC
RMSDRCGGLEASKSDQPCIYRCHAGLTDFSIPLVIAGHLVGFVLCGQVRLSNDVELVNIL
NVDDRWQADPELLNEFRNVPEMDYSRVIASADLLKLIVENCLKKQLNFVVIKDNPQQSEA
NKTTRGPTPHDSKMKKALRYIDAHLSDDLRLEDVASHVYLSPYYFSKLFKKYQGIGFNAW
VNRQRMVSARELLCHSDWSIASIARNLGFSQTSYFCKVFRQTYQVTPQAYRQQINENSHP
PSL
>EECEBICD_01331 Cobyrinate a,c-diamide synthase
MAARHHAFILAGTGSGCGKTTVTLGLLRLLQKRALRVQPFKVGPDYLDTGWHTAICGVAS
RNLDSFMLPPPVLNALFCEQMRQADIAVIEGVMGLYDGYGVDPNYCSTAAMAKQLGCPVI
LLVDGKAVSTSLAATVMGFQHFDPTLNLAGVIVNRVTSDAHYQLLKNAIEHYCSLPVLGY
VPPCDGVALPERHLGLITARESLVNQQSWHDFAASLEQTVDVDALLSLSVLSALPTGMWP
ERPDNTAGAGLTLALADDEAFNFYYPDNIDFLERAGVNIVRFSPLHDRALPDCQMIWLGG
GYPELYAADLAANTAMLKHLRAAHQRGVAIYAECGGLMYLGSTLEDSGGEIHQMANIIPG
HSKMGKRLTRFGYCEAQAMQPTLLAAPGEIVRGHEFHYSDFIPETPAVMACRKVRDGRVL
QEWTGGWQTGNTFASYLHVHFAQRPEMLQHWLAAARRVL
>EECEBICD_01332 Cobalamin biosynthesis protein CobD
MTILAWCIAWVLDFIIGDPQHWPHPVRWIGRLITFVQRIVRRYCPGDKALRIGGGVMWVV
VVGATWGVAWGVLALAQRIHPWFGWSVEVWMIFTTLAGRSLARAAQEVERPLRENDLAES
RIKLSWIVGRDTSQLQPAQINRGVVETVAENTVDGIIAPLFFLFLGGAPLAMAYKAVNTL
DSMVGYKHEKYRAIGMVSARMDDVANYLPARLSWLLLGIAAGLCRLSGWRALRIGWRDRY
NHSSPNCAWSEACVAGALGIQLGGPNNYFGERVDKPWIGDAQRDISVDDISRTIRLMWVA
STLALALFIAARCGLSGVA
>EECEBICD_01333 Cobalt-precorrin-8 methylmutase
MHYIQQPQTIEANSFTIISDIIRETRPDYRFASPLHEAIIKRVIHTTADFDWLDILWFSA
DALEQLCDALRHPCIIYTDTTMALSGINKRLLATFGGECRCYISDPRVVRAAQTQGITRS

MAAVDIAIAEEEKNKLFVFGNAPTALFRLLEHNVTVSGVVGVPVGFVGAAESKEALTHSH
FPAVAALGRKGGSNVAAAIVNALLYHLREA
>EECEBICD_01334 Cobalt-precorrin-5B C(1)-methyltransferase
MSELSFDAPVWHHGKALRKGYTTGSCATAAAKVAALMVLRQHLIHQVSIVTPSGVTLCLN
VESPHIEGQQAIAAIRKDGGDDVDATHGMLIFARVTLNDSGEITLTGGEGIGTVTRKGIG
LPLGSAAINRTPRHTIESAVREAIGPARGADVEIFAPEGEVRAQKTYNSRLGILGGISII
GTTGIVTPMSEESWKRSLSLELEIKRASGLTRVILVPGNHGERFVREQMGVDTQAVVTMS
NFVGYMIEEAVRLGFCQIVLVGHPGKLIKIAAGIFHTHSHIADARMETLVAHLALLGAPL
ELLTLVGDCDTTEAAMEHIEAYGFGHIYNHLARRICLRVMQMLRFTKTPPVCDAILFSFD
NHILGSNRPVDEIAKELQC
>EECEBICD_01335 Cobalt-precorrin-7 C(5)-methyltransferase
MLTVVGMGPAGRHLMTPAALEAIDHADALAGGKRHLAQFPAFGGERFTLGADIGALLSWI
AARWDKGIVVLASGDPLFYGIGTRLVAHFGIEQVRIIPGISAVQYLCAQAGIDMNDMWLT
SSHGRCVSFDQLASHRKVAMVTDARCGPREIARELVARGKGHRLMVIGENLAMENERIHW
LPVSAVNADYEMNTVVILDER
>EECEBICD_01336 Cobalt-precorrin-6B C(15)-methyltransferase
(decarboxylating)
MKDELFLRGENVPMTKEAVRALALSKLELHRASHLIDVGAGTGSVSIEAALQFPSLQVTA
IERNPAALRLLDENRQRFACGNIDILPGEAPMTITGKADAVFMGGSGGHLTALIDWAMGH
LHPGGRLVMTFILQENLHSALAHLAHIGACRMDCVQLQLSSLTPLGAGHYFKPNNPVFVI
ACQKEENHVRDI
>EECEBICD_01337 Cobalt-precorrin-4 C(11)-methyltransferase
MSETFDPRCVWFVGAGPGDRELITLKGYRLLQQAQVVIYAGSLINTELLDYCPAQAERYD
SAELHLEQIIELMAAGVKAGKTVVRLQTGDVSLYGSVREQGEELTRRGIDWQVVPGVSAF
LGAAAELGVEYTVPEVSQSLIITRLEGRTPVPAREQLEAFASHQTSMAIYLSVQRIHRVA
ERLIAGGYPAATPVAVIYKATWPESQTVRGTLADISDKVRDAGIRKTALILVGNFLGKEY
HYSRLYAADFSHEYRKA
>EECEBICD_01338 Cobalt-precorrin-5A hydrolase
MNTVKPESIALFCLTPGGVALAKRLSAMLPLTCFTSEKLREEGFIPFDGGFANTARQAFT
TYTALIFIGATGIAVRVLAPLVNDKFSDPAVVVIDERGQHVISLLSGHAGGANALTRYLA
GMLGADPVITTATDVNEMSALDTLAFQLNARMSDLRTAVKTVNQMLVSHQRVGLWWDAEL
TEEIGQCDIRGFIPVDDLQRLPELDALICVSLRNDLPELPVLHWKLVPQRVVAGIGCRRD
TPFPLLATLLARQLEAQKLDPLALKAIGSVTLKKGEPGLIQLASCCRVPFKTFTAEALRE
FEHHFPGSGFVRKTVGVGSVSGPAAWLLSQGQLLGETLREQGVTITLGVSH
>EECEBICD_01339 putative cobalt-factor III C(17)-methyltransferase
MLSVIGIGPGSQSMMTMEAIEALQAAEIVVGYKTYTHLVKAFTGDKQVIKTGMCKEIERC
QAAIELAQAGHNVALISSGDAGIYGMAGLVLELVGKQKLDVEVRLIPGMTASIAAASLLG
APLMHDFCHISLSDLLTPWPVIEKRIVAAGEADFVICFYNPRSRGREGHLARAFDLLAAS
KSAQTPVGVVKSAGRKKEEKWLTTLGDMDFEPVDMTSLVIVGNKTTYVQDGLMITPRGYT
L
>EECEBICD_01340 Cobalt-precorrin-6A reductase
MNEGEVLVVGGTSDARALCRQLDAANVAYTLSVATPAGKALAGDIKGQVRCGRLEYGQMV
AWLKENRTRWVIDASHPYAEMVSHNLLRACETAGVLLSRYQRPEQLSNLTHPLLYTARSI
ADACEIARRFGPRVLLTTGSKDLAVWRAGLAEKTLLARVLPVAEVIQRCSELGFGVGEIF
ALCGPFSADFNAAFYHQCRADVVITKASGAEGGYQEKVQPCLDAGIPCIVIARPTPLVTG
DELLESQAAFAQRLSRWLAAAKE
>EECEBICD_01341 Sirohydrochlorin cobaltochelatase
MKKALLVVSFGTSYHDTCEKNIVACERDLAASCPDRDLFRAFTSGMIIRKLRQRDGIDID
TPLQALQKLAAQGYQDVAIQSLHIINGDEYEKIVREVQLLRPLFTRLTLGVPLLSSHNDY
VQLMQALRQQMPSLRQTEKVVFMGHGASHHAFAAYACLDHMMTAQRFPARVGAVESYPEV
DILIDSLRDEGVTGVHLMPLMLVAGDHAINDMASDDGDSWKMRFNAAGIPATPWLSGLGE
NPAIRAMFVAHLRQALNMTVEEAA
>EECEBICD_01342 Cobalt-precorrin-2 C(20)-methyltransferase
MNGKLYALSTGPGAPDLITVRAARILGSLDILYAPAGRKGGDSLALSIVRDYLGEQTEVR

CCHFPMSADGAEKEAVWNEVAAALTTEVEAGKQVGFITLGDAMLFSTWIFLLQRIGCPEW
LEIVPGVTSFAAIAARAKMPLAIERQSLAVISCTAPEAEIAQALQQHDSLVLMKVYGRFA
RIKALLAQAGLLECALMMSEATLLGEQCWRHLHEVNDDRALPYFSTILVNKQWEYAE
>EECEBICD_01343 Cobalt transport protein CbiM
MKLEQQLRQLSFSGLAAALLLMVVPQQAFAMHIMEGFLPPVWALAWWLLFLPCLWYGLVR
LRRIVQEDNHQKVLLALCGAFIFVLSALKIPSVTGSCSHPTGVGLAVILFGPGVVAILGA
VVLLFQALLLAHGGLTTLGANGMSMAVIGPVVGYLVWKMACRAGLRRDVAVFLCAMLADL
ATYFVTSVQLGVAFPDPHAGATGSVVKFMGIFCLTQIPVAIAEGLLTVMIYDQLTKRQVI
TVQGH
>EECEBICD_01344 Cobalt transport protein CbiN
MKKTLMLLAMVVALVILPFFINHGGEYGGSDGEAESQIQAIAPQYKPWFQPLYEPASGEI
ESLLFTLQGSLGAAVIFYILGYCKGKQRRDDRA
>EECEBICD_01345 Cobalt transport protein CbiQ
MTGLDRLSYQSRWAHVAPQRKFLLWLAMIILAFVLPPVGQGIELLIIAGLSCWLLRISLW
RWCRWMAIPFGFLLVGVITIIFSISREPQMLLAGISVGPYWIGITRAGVVTANETFWRSL
TALSATLWLVMNLPFPQLISLLKRAHIPRLLTEQILLTWRFLFILLDEAAAIRRSQTLRF
GYCSLPNGYRSLAMLTGLLFTRVLMRYQQMTTTLDIKLYQGDFHL
>EECEBICD_01346 Cobalt import ATP-binding protein CbiO
MLATSDLWFRYQDEPVLKGLNLDFSLSPVTGLVGANGCGKSTLFMNLSGLLRPQKGAVLW
QGKPLDYSKRGLLALRQQVATVFQDPEQQIFYTDIDSDIAFSLRNLGVPEAEITRRVDEA
LTLVDAQHFRHQPIQCLSHGQKKRVAIAGALVLQARYLLLDEPTAGLDPAGRTQMLAIIR
RIVAQGNHVIISSHDIDLIYEISDAVYVLRQGQILTHGAPGEVFACTEAMEQAGLTQPWL
VKLHTQLGLPLCKTETEFFHRMQKCAFREAS
>EECEBICD_01347 Cobyric acid synthase
MTQAVMLQGTASDVGKSVLAAGLCRIFYQDGLRTAPFKSQNMALNSGITSDGKEMGRAQI
FQAEAAGITPDVRMNPVLLKPTSDRQAQVVLMGKVATNMDAVSYHDYKPRLREQILAVYN
SLAQEYDVIVLEGAGSPAEINLRDRDIVNMGMAEMAQCPVILVADIDRGGVFAAIYGTLA
LLHKQERDRVKGVIINKFRGDVALLYSGIEQIESLTGVPVLGVMPWLDVDLEDEDGVALQ
NDKYRGNAPRDITIAIVQLPHISNFTDFNALAAQPDVRIRYIRRPEALTDADLVILPGSK
NTLSDLAWLRESGMADALLQTHRQGVPVMGICGGYQMLGDTIVDEVESGLGTQPGLGLLN
TITRFAQDKITTQVNATMSGELPSWLAAAAGLPVRGYEIHMGETVLQEGCCTAMTLQKNG
CSVADGAVTADGLAFGTYLHGLFDSDAFTRAVVNGLRARKGLAPWETTFCYAEHKARQFD
LLAEAMRQHIDIDKIYTIMQQHQEPV
>EECEBICD_01348 Bifunctional adenosylcobalamin biosynthesis protein CobU
MMILVTGGARSGKSRHAEALIGDAPQVLYIATSQILDDEMAARIQHHKDGRPAHWRTAEC
WRHLDTLITADLAPDDAILLECITTMVTNLLFALGGENDPEQWDYAAMERAIDDEIQILI
AACQRCPAKVVLVTNEVGMGIVPENRLARHFRDIAGRVNQRLSAAADEVWLVVSGIGVKI
K
>EECEBICD_01349 Adenosylcobinamide-GDP ribazoletransferase
MSKLFWAMLAFISRLPVPSRWSQGLDFEQYSRGIVMFPFIGLILGGVSGLIFILLQPWCG
IPLAALFCILALALLTGGFHLDGLADTCDGIFSARRRERMLEIMRDSRLGTHGGLALIFV
LLAKILVVSELALRGTPMLAALAAACAAGRGSAVLLMYRHRYAREEGLGNVFIGKVSGRQ
TCITLGLAVIVATVLLPGMQGLAAMVVTCAAIFILGQLLKRTLGGQTGDTLGAAIELGEL
IFLLALL
>EECEBICD_01350 Nicotinate-nucleotide--dimethylbenzimidazole
phosphoribosyltransferase
MQTLHALLRDIPAPDAEAMARAQQHIDGLLKPPGSLGRLETLAVQLAGMPGLNGTPQVGE
KAVLVMCADHGVWDEGVAVSPKIVTAIQAANMTRGTTGVCVLAAQAGAKVHVIDVGIDAE
PIPGVVNMRVARGCGNIAVGPAMSRLQAEALLLEVSRYTCDLAQRGVTLFGVGELGMANT
TPAAAMVSVFTGSDAKEVVGIGANLPPSRIDNKVDVVRRAIAINQPNPRDGIDVLSKVGG
FDLVGMTGVMLGAARCGLPVLLDGFLSYSAALAACQIAPAVRPYLIPSHFSAEKGARIAL
AHLSMEPYLHMAMRLGEGSGAALAMPIVEAACAMFHNMGELAASNIVLPEGNANAT
>EECEBICD_01351 putative L,D-transpeptidase ErfK/SrfK
MRRITPFFPFFVLLVSHFSLAISYPLPPEGSRLVGQPVTIAVPQNNTQPLESFAARYGQG

LSNMLEANPGVDVFLPQSGSTLVVPQQLILPDTVREGIVVNVAEMRLYYYPAGTNTVEVL
PIGIGQAGRETPRNWVTAVERKQDGPVWVPTANTRREYAKEGKTLPAMVPAGPDNPMGLY
AIYIGRLYAIHGTNANFGIGLRVSQGCIRLRNDDIKYLFDHVPVGTRVQIIDRPVKFSVE
PDGSRWLEVHEPLSRNRAEFESDKKVPLPVTPVLRTFIKGDDVDTSRVNEVLERRSGMPV
NISAGRPGL
>EECEBICD_01353 putative FMN/FAD exporter YeeO
MLHLQTFFLQKALQPYDFSLIIEMWFQLSSLRSILNVSTALRQAVVRTPWYAKRKSYKVL
FWREITPLAIPIFLENTCVLLMGVLSTFLVSWLGKEAMAGVGLADSFNMVIMAFFAAIDL
GTTVVVAFSLGKRDRRRARAAARQSLVIMTLFAVVLAVVIHYFGSEIINIVAGEATSEVK
GLALTYLELTVLSYPAAAIALIGSGALRGAGNTKIPLMINGGMNILNIIISSILIYGAFS
WQGLGFAGAGLGLTISRYIGAVAIIWVLMIGFNPALRIPLKSYLKPLNFGIIWEVMGIGI
PASIESVLFNGGKLLTQMFVAGMGTNVIAGNFIAFSVAALINLPGNALGSASTIITGKRL
GTGQIGQAERQLRHVFWMSTIVLTAIAWGTAPFAGLFASFYTQEQDVKEVVKVLLWLNAV
FMPIWAAAWVLPSGFKGARDVRFAMWVSMLGMWGCRVVAGYTLGIVLGMGVVGVWLGMFL
DWAVRGALFYWRLVSGRWLWRYPRVKRE
>EECEBICD_01355 hypothetical protein
MVDNIEQQVYELVRPYAGTYLFNIKQVELTPEIDLDTDLSIDELEAEDLMNKFFEKLNVE
RGNFRIETYFPNHPFSWHPFKKTEPVPVPDFTISMLIESAKAGKWLYD
>EECEBICD_01356 AMP nucleosidase
MENKGTNLTPEQALDRLEELYEQSVNALREAIADYVDNGTLPDPHARLNGLFVYPSLSVS
WDGATPNPPKTRAFGRFTHPGCYTTTVTRPALFRAYLLEQLNLVYHDYGAHIAVEASHHE
IPYPYVIDGSALTLDRSMSAGLTRHFPTTELAQIGDETADGLFHPGEFYPLSHFDARRVD
FSLARLRHYTGTPVEHFQPFVLFTNYTRYVDEFVRWGCSQILDPDSPYIALSCAGGIWIT
AETEAPEEAISDLAWKKHQMPAWHLVTADGQGITLVNIGVGPSNAKTICDHLAVLRPDVW
LMIGHCGGLRESQAIGDYVLAHAYLRDDHVLDAVLPPDIPIPSIAEVQRALYDATKAVSG
MPGEEVKQRLRTGTVVTTDDRNWELRYSASALRFNLSRAVAIDMESATIAAQGYRFRVPY
GTLLCVSDKPLHGEIKLPGQANRFYEGAISEHLQIGIRAIDLLRAEGDRLHSRKLRTFNE
PPFR
>EECEBICD_01357 hypothetical protein
MLTQHRLPEDPRWNALHEEFSFDDDDNLLGMNMPPEGGEHQAKVKGDRLLLQGRPPL
>EECEBICD_01358 hypothetical protein
MKFKFATLLTAATVFYSYQSVAQSQQSPDVWSVVEQKLQNAVPLNENTTFKSQAAWFELH
RELFMYGPMSRADALLKKLDTQSNDAKFSLNHEASWIAENNSPDAAMVFLSKIGLDKPSS
ITAYESYVDGWVNRKQPEKALALLSKNADARNFYLATVLKSWHSDPDKTAAIYNENYADK
IVVPYTQLKMLLIIAKQYHAKGDTAKALVYADSALKMFDTAIAQQPSAEAYRYQEYLDLM
EIYYATGNKEKAMALSARLRKATGNKGSYFQYSLPGLLSFYKKNELTQNYQETLSTYVTQ
VDKIFNFAPSPRIEMELIDLLSKLDDVALMNKRIDLLMSAPEYTCYDDRYCYEYKIKALK
NLFQHKQNAVAEKHIATLSAEAQTLTFAEWEDATNEIGEVLKEIGHPEAAQKLAVSAEAI
YLSQAKAWPDEDMSRSFQRLAELYGYGNDTVNAKRVLHQHVPSLEEEAMIDHYMNAKQWS
QARELMINADRVDNKNLMLLRQICSENTPECQEHITFTLKKLTTQASITRQDDTGNQQLY
QIGNIFHRLGIIPGAEQQALIQALYNKAAEPKKATP
>EECEBICD_01359 hypothetical protein
MKKIFITIIVSLYCANICAKQSPSTESKPVNFIAQIENIDFNKTAISSDLKLLLADRYYF
KDKTPCNTNTLSARAESMGLTTEEYINKIRSLRPAILDDRYFYLTVDQCDAGGTPMLTGI
ELCTEALCGAEYMKRSSDLWLDDELQPTVKRQATTVVHMPLPYDKEKKLWKVTGWYLESS
EETGEVMQSKQIAFEGYTNEENFANRQRVSVFKSFYESGNLKNIYHYNAQNKRDGKAETY
FDEKDKIAETLTFKDGQPEGEYIVYHENGAVESKRYFAQGKIKDGECPHFYDNGVLKQKH
SYLNQKLEGPAFEYFPDGKIKGKYSYRKGTIVGTSTEYYSTGKIRGVYHRNNQGENDGTF
EQYSEEGKLLSKATYKNGKQLSAQSWYGNGHPKEESSFDSEGRKHGAVKEWFSNGKPASS
KMYKHDVLDGDSEKWYENGHRESVYPYKNGMLNGDAKHWNEQGKLTYTTEYKDDKKQGAD
RRWSERTGKLVEEVMFANDERNGLKREFNDRTGKVLSALPYVDGDKEGTEEAYDEDGIKY
IRCYHNDEELSELYAPTDVTNKAKQGDSTAQYHLGKYEFECTNYDAAMKWLTQSAEQNHP
GALLFLAYAYNDGDGVTQDSKKYLSYLFKAAELGESDAQLEVGYLNLIGEGMPKNLPEAY
KWIKKSADQGNAQAHYNLGLMYRNGDGVEKDLNKAKLHLTAAVKGGVKPALAALKELTPQ

TK
>EECEBICD_01360 hypothetical protein
MGKLGGEMKALAKHCGGSHKTVNDCIHIVQRFDHHLRALNVHMQRVAQIKVRHIESYIHE
RLAQGIGKRTLQNEMASLRAVLQQAGRKQVAEHEWLTNKSLGLAGASRSGTRQAITPEHY
HHVLETARMKDPGLAAALELARLMGLRSQEAVQSVQWIWGMGTGVGGMWRRCMGKFDSWL
RASSGWSRLPPFTVSERLGGGGKYQAALPSPLQSKPESDRTNVEINERTCPE
>EECEBICD_01361 hypothetical protein
MALTDTAGGTQTDPGTGKPSENPPADPPSDGPASEKPHDPPADPNKPTPPKEETPPGAPE
KYAFTAPEGQELDTSALAQFEPVARELNLTQEQAQKLVDVYPKVLAGVQQQQAESWQKQT
EDWAAAVKADKDIGGDKLASNLGAAQRAIDTFGTKELKEYLDGTCARSLVNAAP
>EECEBICD_01362 hypothetical protein
MTKRLPVAEIVEALSKWFDVSRYDALKNLTLEQIYAELERRMFAYKARQQWETLDDKHRN
AVIHHDAMIHSGRVLLEDKWISDSHMLAHSYAVRPMTRDSLFNYGRAMYRLENTPPEENV
SVSSDYISEYLKQGGLNPANKMLIEIDLEEASSDDLAEHLKVLINQWQKHLKVPKPPEKD
FRFGHKTFQKILDYKIIPLMDLIAWEQLNNQKIKYPVLAGILHPDMRYARGSEQIKDTDY
PLAHGFLNNDNYFKSLNDFFIKNNLVKNSPILDVIAMNDKPETKKKTRDIH
>EECEBICD_01363 hypothetical protein
MVKREIKAKTTFIRLPEVIKRTGFGKTWIYELIKAGRFPSQVKTGIRAVAFIESEIDEWI
EATIRNSRQNAA
>EECEBICD_01364 hypothetical protein
MKIELITTKQFIEQAECYFRNYMDGLRRNAPDDFYYFLNNKYNMNDIMESIIKKTRYYFY
DDTEEGKRNRIYGEVSHCKVKQHLRQLWIIYKCVYR
>EECEBICD_01365 hypothetical protein
MNTFKNKTTEIFYVVSLHIYAELFNSKDKTTSNMIMTHVMDHEFVCRLIDLAMRNAEKHL
LKKAWKKNAAEKLSEVDFKGVKQALAKMHYTVLAESIC
>EECEBICD_01366 hypothetical protein
MSTATDFKTLLDNIKIDNAGQISKRYGRITKALNQYFYNLDSKTANSLQVGSYGRFTGIR
GISDLDMLYFLPATAWPRFRDRQSYLLQVVKTEIKKTFKNTDIRGDGQVVVVKFKNQEVE
VVPVFSNEDGTFTYPDTHDGGSWKVCNPRAEMSSFRALNDDRKGHLRRLSKMIRAWKARH
EVEISGFLIDTLCYNFFSNLTEYDDKSFKSYDQLSLDFFTFLENEGDRVFYYAPGSRSKV
SVKKSFNKVAKLTKEYCEEALSATSENSRNLAWKKVFGRPFPNYTTKALSNVNVSEQFIE
DQYEMNLYGHVSIECEIRKNNLLEALLSNLLGEGHDISTNRKLRFYVDEINNISHPYKIK
WKIKNVGDEAERRGNVRGEILDDEGGSERFETADFSGPHFVECYVIYGNQVVARDRIDVP
IHN
>EECEBICD_01367 hypothetical protein
MDNNILEGQLRECYGRVVYTHKTHEKCADILQKKLGCMKNLQLVLLAITTGSFITTVVGD
AKTGAIIGSVLSAILLFLNSYLKDYDLGSIAQKHRQAAGDMWLIRERYLSLLTDLKMQTK
SIEEILKERDALMIELSAIYIGAPSTNYKAYSMAQKALKELEDMTFSDEEIDKFLPTELK
RK
>EECEBICD_01368 DNA-binding protein H-NS
MSEALKALNNIRTLRAHARETDLATLEEMLEKLSAVVEDRREEESAVRKEQEERQAKLEA
FRQKLLEDGIDPAELLAAVGSSQPKTKSTRAPRPAKYKYTDENGNEQTWTGQGRTPKAIA
AAIEAGKKLEDFAL
>EECEBICD_01369 hypothetical protein
MRKVKTDNSDLIEYVNTVKELKNHISIDEYRNEYRRLRSDDIPLVKSQKFKSAHTELRRL
EKKRESLIEYFIDELNPISSSKANTSARSTGNLDLFNERVLYRKALSEKSDEEIIALVIK
QRTEAAVEFKRSIEQSLNQLSHISSEFAPSSQKRRKMSL
>EECEBICD_01370 hypothetical protein
MKKLPGSLEIKLHEKLSKSDILNILAEQMTMLEETFGIQEFKIFSYLECYIGDKKKALYY
RSRNSAVATFKLKGLESPVNTAKLISKENGQRIVSFDKDLDINRISATVRNIQNNNPYRG
WSEGISVVPASIISKIIEVDIIRAQEEQGRLYRIEEQRKKEEQIRKAKEREEYERPLKAF
ISSKIKESDLSEKDFKKQVCSSCDYLKDRSTKSRYFTERPDLLDKYHNERLIRFSIKGTD
GKVGKIEIYTDTGELIFERYKTK
>EECEBICD_01371 hypothetical protein

MNTPDADLLHLFESLTTEQKIDIARRLLQNKEIKSTTVISSEEDKAISFSQPYVFAFTSN
FRVFSDLDISKNEFRVLTYILEYMEFGNLINLSQSSLCKALNIASGNMSNIFKKLRQKGI
LVEHHGHLYINSNIFAKGMSHQLDQKRKEQLSNARNLCAEIEQYPESYSAMSKSERRTLF
LETETKEIYTDAFVFSKKKKNNAVPNPNNSIHVDKPRKVYKRTKENEEARRNAVDADSHM
QKVS
>EECEBICD_01372 hypothetical protein
MLCLNIGSELKFKRKIELTMIEYIRLLLHSSKIQNVSFRGVAVRKHRDKDLFKKKFNNQ
>EECEBICD_01373 hypothetical protein
MNNIKMITLFHPHDKTPFMICIVSKVEDTEHGLKLTLENGNNICVNNYSHYLLSDSVSRC
DKDRLKNIYIRLVSELTQMSEETIKSQML
>EECEBICD_01374 hypothetical protein
MRKIKTKIYIPTTLTGNMGRHYIPVLEDLRKTIYSDRILSRLADSGNIVIHSSVGYPVAK
YKNTGISIGIEPLNPMIRQDLTLGYIVVIRNGKASQEVNGLLNRSLPKAISTFKDHINEY
EAAKSKML
>EECEBICD_01375 hypothetical protein
MEGQLIFCSDSILRFQSDYDETAAVPLLSIQNVIADTDPFFLLRFFHHTVLIEEGTTLAS
IFLAIEPWKALLAAYLDRDVGAYIDEVRKPSGPTTWDIEWIGIDRRSMVYRAYKRQEMQD
GEDFSDYLNRERVLTDEFEIESGCEASGFIKGDKERWSISGDVHEIKNLPVILYSKQTLM
TSPKDGLLKKNISGVKSSKHSCFIYGDTSFSFSEVMEAIFISGLFFYAPKDAASSLDELK
ASLAELEEERAENPNAESTGNETDEEPTIVVAEGAFDSLAAHMESEKAEWQSIKKLCQQE
GGLPIRIGNIKMSESIQIIP
>EECEBICD_01376 hypothetical protein
MYKEENKNIARKSVLKAAIEALTLCRKDSTLAPKDYIRKVKAFYRKDESDPRAFIVDELS
EETIIRWEEFYDSVIQDRTARSIKVAYLSGPNPENDLTEMTDMGLLPENIWAFESDAKIY
NEAVISALSSKFPFIKLIKANVGDFFEVSPQKFDLIYLDFCGPLPSKKAGQKTLKAITSI
LKYHALSPLGVMITNVSLPSKEQNANEHKNIVNLVASYLYPKSTLESNNPEWNCTDGAIS
EGYSLDEWHKKVECEIEDFYGQYITRLLVDLISVISPYDNFTSSHSLYKNMFKISNYNDL
TKSVNDLFHFDSNGNGGDIIVDSGLFPILWTIASIDKKYNNKDKNYYQDIYCDDDFNDYA
QSFLSQMSANGNAHDLIKNISNMHFLLNEGRTENNFYSDSLRNLNKINWYQKVYPFCDLF
LFHQIKEVLFRQLSVPYHVNMEKTLRWKYKAKDTNMYMDMLVLDECRYLYDWMPSLDMFY
SGMMDIERQFSFRFILDAVAKHRMVYNNEFFYGTASVSKFETDYVEKVLSVRKNII
>EECEBICD_01377 hypothetical protein
MKKLLFAIFIILPLGGCTVHGNKSLTDENHQTVKLKIVKGKTTQREILAAFGEPQTRATN
DGQEMWNYSSMTGESQLSNYIPGLALLTNSSTAHIKSLDIWFKGDVVERYNFSQTASKVT
RGMID
>EECEBICD_01378 hypothetical protein
MEATGIVFLVVLFVIIMTAADIQKKKHYNSFTEVLDGDILSYECQQTGIVIDTQKHTVRI
FNKDKDSTYTFDEIREINYTLSEGGKFYGNGTLRGMNNAAIANWREQLSANKRSGLNILP
MILKIPCGKLMFP
>EECEBICD_01379 hypothetical protein
MKKILLVAGAALALAGCGEKGEFEKAINAKIGQDKYCFSLDNNNTSFPIRLAKPRLDSTG
TGTNSVILDGFIEQGLMVFEQGYDSNVLGITEEGKKAKVWSTTDGACVGRRAVDEIKEWT
EPGNGNQKVVRVSYTWKLVDVPNWIDKKAFASVKGMNEPADGAMNLFKTSNGWKAN
>EECEBICD_01380 hypothetical protein
MENNTIKCPFCFKESQRGVHVCTGCLATVLCGTYPGWYAAVVLLLTFGLSTLIGMAGATI
SLPIIAIVGFVAGKVIFADNVVFRRRM
>EECEBICD_01381 hypothetical protein
MNTIYHYCSPESFFSIIQNQRLWLSSMDHMNDYMEKKWFYSTLKKYLYKNLDANCVDQFI
AHLDDNISIGTPFACCLSKSGDILSQWRAYAKDGFGVSIGFDREKLDVYDGIIGNNLDPK
HRLTLSDISYMDINVIECLAERILSRYSFIKKYYMNEIISTSKFNRYDKCILELISNIIH
LNTTTKNPAFKEEKEVRLVYQTLDTGRYEYPESSSIKDLKYRISNNQIISYYELGFPKDA
VSELILGPNNKFKESDIVNFLQYNGFEHSIKILKSKASYGA
>EECEBICD_01382 hypothetical protein
MAIFHLDFKIVKRSEGMTSVAKAAYHTRTRITDDRIGETYDFSHRTDLHGHIILAPVSAP

AHIVESSSALWNEVERVERQNNGQTARYFDVAIPVELSNDDKKKLVAEYCQKNFVDKGMI
ADIAFHDLDSKNPHAHVMLTLKTIGPEGFGKKDRSWNDKKMVVQWRESWATMSNSYLETA
GREERIDHRSLRTQCADALAQAEEAFSAEEKAFWLAKATETNRPAMQRVHRAKWNNTESQ
EQRATEQALRDQQIEEAKKVYNTFSELPLEIVVDVRSFTITHLAEPEEIVIPDFPATTKQ
QPVMTTHATSRRPATKSYRNPNKVSKVNVSGKKSPVLIVPEPTASTKLKTPSIQNTRAKN
RAPMRSRKQTKPRQNGLFKRFTLLIVGYIREKFIWARRKPDTTDADHDKRIAENYVYDEV
LGINVPRSEFEKRAKFNNDQTSPEASIYGNESDHDKTVRFPSRPKDVQTEIDKYIDLIPS
SPSEYSGKSHVSKLRPLAYRNNNYNE
>EECEBICD_01383 hypothetical protein
MDNETKRSRTEKTLKQKVAFAQLELNRLKSMEKSEQKKVETRLKIILGAEVAKAMNCGIE
QVDKELVMGILLSASELNDIERVKYIKAGRWFLAQMDGRQK
>EECEBICD_01384 hypothetical protein
MVLLNSKRKSKKGFSLLELLLVLGIIAALVVAAFIVYPKVQASQRAQAESNNIATIQAGV
KALYTSASSFTGLTNTVAVQAKIFPDNMLSGTGNAAKPINAFKGNVTLAAAATGPSSAAG
SSFTITYDNVPAAECVKITTAAAGNFYTAKVGSKVVKAADGTLDVAATAAACNNATSNTL
VFTSI
>EECEBICD_01385 hypothetical protein
MIKKRGFTLLEITIVLGIGSLIGFMKFQDMRKEQEAVMAQAVGFQMKQVGEAVNRYISIR
YNKLSTLSSSRSQSSDPGPRTCTANGCEITYQTLVNESLLPSTYAGVNAQKSSYKILLKR
SGVSPNYVINGLIATTIPWVEGGKTRYDLLGKSMQSAGIDSGMTKSPAQASGYNGVWTEK
STDYPAINKAGLLVYRVGYDSSMYSVYLRRDGTLPMTGNLNMGTNDINNAKNITASGTGI
FGGDVSSAGKIIAAQEIIAHNGYGDAIHFGGDAVNNDYEITMSKDKTLSIHMASNRTDLT
TLKISGGLSTIGNGTISGTLDTGKSITSGGQFNGHNGGGDSFSIGGGDANDYEFRLDTVK
PLTIWRNGGTSTETRLQVFGKQTNQGDFAITPGTGSTGSIAASGNIQGHTLQPTSTNTTG
GTCPSIGLISKDNMGNILSCVNGKWSVVSNLPVGSPVPWPSNTAPVGWLICRGQSFNTAL
YPQLAKAYPRGRLPDLRGVFIRGLDSGRGLDSGRVINSYQDDQIQNITGHMAADVSQSGN
IGKYVSGAFADSGALGEGDEGHKSNEVRKYTFDASRVVRAGNETRPKNVAMNYIVQAQ
>EECEBICD_01386 hypothetical protein
MSISTTMSNINRIQKDIASLQKQLSDEQRKEAQLSGKINQKNRVPTPSSFSTPYFSKFLY
PFYYNIKFFKSKAQNVI
>EECEBICD_01387 hypothetical protein
MCIIDYCFMYTIWWEMSNTTDWINTISNVMIAGSAIYGAFKAKSYFKSKNDEEAYNDAKK
LIFELYPQYRIDLHKLVIVLLSMTSNRIPNDILETRLEKITNRLSNTQIEIVTISSNIIE
RHRWKIRDDFKESFEIIKNIYRNKLSFPLINVVDEVSQSTETERQNILRELIGKCHEGID
AIHTFTDNKFRVDEYFVRPFLN
>EECEBICD_01388 hypothetical protein
MNSIRVIERQHEIDMIEGEAKHKAELERIKSESQYNTSELKEKLERLDIIVKNQEARNNE
LLEERKN
>EECEBICD_01389 hypothetical protein
MILSPIYEIIKESKFKDIEKYLEKVGVLENSLKRLRDSERKTFSMDELENNDYNAINAD
>EECEBICD_01390 Prophage integrase IntA
MSLTDTKVKNTRPSEKAVKLTDGFGLYLLVHPNGSKYWQLGYRFDGKQKVFSIGVYPAVS
LADARQRRDEAKRLLTQGIDPNAKKQADEKVLQEKRDKTRSFRVVAKSWFATKTKWSEDY
ADTVWKRLETYVFPDIGDRNVSELDTGDLLVPVKKAETLGYLEIAMRIKQYITAILRHAV
QQKLMRHNPAYDMEGAVQKPETEHRPALELEEIPLLLERIDAYKGRGLTTLAIKLNLLIF
IRSSELRFARWSEIDFKSKLWVIPEQREAIENVKHSTRGAKMKRQHFVPLCRQALKILKE
IRQLTYEEGNEAELIFTGCYDSFKPMSENTINKALRKMGYDTTQDICGHGFRTLACSALI
ESGLWSEDAVELQMSHKESNSVRAAYTHKAKHLDQRRLMLQWWADFLDENRYEMVRPFEF
AQKQ
>EECEBICD_01393 Protein MtfA
MIKWPWKAQEITQNEDWPWDDALAIPLLVNLTAQEQARLIALAERFLQQKRLVALQGFEL
DSLKSARIALIFCLPILELGIEWLDGFHEVLIYPAPFVVDDEWEDDIGLVHSQRVVQSGQ
SWQQGPIILNWLDIQDSFDASGFNLIIHEVAHKLDMRNGDRASGIPFIPLRDVAGWEHDL
HAAMNNIQDEIDLVGESAASIDAYAATDPAECFAVLSEYFFSAPELFAPRFPALWQRFCQ

FYRQDPSQRLRVSAAEGDYGEESEH
>EECEBICD_01395 hypothetical protein
MEIAYLCAARVSDVLKMNWNQILEKGIFIQQGKTGVKQIKSWTDRLRDAVEICREWGEEG
PVIRTMYGERYSYKGFNEAWRKARKAAGDDLGRPLDCTFHDLKAKGISDYEGTAKDKQKY
SGHKTESQVLVYDRKVKMSPTLDRKR
>EECEBICD_01396 hypothetical protein
MIRLDTAFLPTGIHITPDMFWLYCFSFGITKYGKYIFLMCQIIFLRSWGQISIIGTGKKF
SP
>EECEBICD_01397 hypothetical protein
MSDRFLTEEELEDATGASQKSLQKEVLTLNGIYFIERRDGSIRTTWYHINHPVSRLLPPA
GYQPVPGMNFDAIES
>EECEBICD_01398 hypothetical protein
MNNLMVDLETMGKKPNAPVVSIGAVFFDPQSGEIGPEFYTAVSLESAMEQGAVPDGDTIL
WWLRQSPEARAAICADAVSVTTALIEFNDFITCHADDLKYLKVWGNGANFDNVILRGAFE
RASLPCLWNYRNDHDVRTMVTLGRAIGFDPKRDMPFEGDMHNALADARHQAKYVSAIWQK
LIPPTSNNI
>EECEBICD_01399 hypothetical protein
MTTITKEWLQQTIAEFENTRDDIPFGLSDDDAKVLIVLKRALASLEAEPAGYHVIKECGK
VGCSVATLEEAEKTRDFWNKKWTIRPYFYTAQPVQETGVYNDVLNIISLLENNEWAEHCT
STVLGSLLESEITRLVGKEQSAPVVTFYRDGVEAAAKWIDQQREAYDSEHGWSDPDTGAF
EFGNDAQRGYSSTLEELAEGIRALHPNAGNSPVIPDGWISCSERMPEDEQEVIVHNKLGY
RYVSYFDEHSGLFFDMRGGNQMNCIEHIFVTHWMPVPAAPKPEINNE
>EECEBICD_01400 hypothetical protein
MWRGLNRGGSQMILTAYEYDPETEKSQSVYLLRHHSKVKKTTLEQKLTVKNDAFGRFKPF
VELEDFPEGLSEREAMLKLADWLHRLSVAIEDNWSTP
>EECEBICD_01401 hypothetical protein
MCNFHERKVRRTEYYQRFVFGWKLRPCTACNGSGYYDHNGSPKCSSCNGTGKERYKPN
>EECEBICD_01402 hypothetical protein
MSNIDKLNDHELVDLKNAIERELKRRADGPKVTTYYVVSCITDAQHFTDLDCALRCLKSV
TENLMEWVTESPENRDYVNQCTGIVGAKLQVKEMNLDHFNMRVAEKYFDDICYPQETAQ
>EECEBICD_01403 hypothetical protein
MSNRIRNAQVFDARTGEYPVDMYIRWIIGGELDFDANYQRGYVWGHEEQQAFLNAVISGF
PIGSVALAKAPDWCSRELPYIEVVDGKQRLTTLKKLITNEIPIILADGPLYWRDMTRAEQ
LAFGRRPLPAVVLDEVTYKDRLAYFMAVNFTGVPQSEEHKRHVMQLMEAAQ
>EECEBICD_01404 hypothetical protein
MKLEMYTLDGSVIVDSNLVTQFYPDYKSGGELTVIETISATGETFTVRVKHSFLQVTSAL
ATAWSVDEKKAEGAAQ
>EECEBICD_01405 hypothetical protein
MSFIQTLSGKQFDYLSATIDDIDIEDIAVALSNICRFSGHLPEFYSVAQHSVLCSQLVSP
EFAFEALMHDAAEAYCQDIPAPLKALLPDYREIEKRTDQLIRFKFGLPLEEASVVKYADL
TMLATERRDLDIDDSIPWVILEGIPPTDLFEIYPLRPGQAFGLFMARFNELMELRQCAA
>EECEBICD_01406 Recombination-associated protein RdgC
MHNPFFKNMLIYRFSRDFNIDIDSLDKKLELFRFSPCGSQDMAKSGWFSPLVQYSDVLYH
AVNNQLLLVIRREEKIIPKQTIADEINKKVSTLEREQGRRLKKTEKDSIRDEVLHSLLPR
AFTKNSLVRIWINTAAGFIVVDTSSIKRAEDSLALLRKTLGSLPVVPLTMENPIELTLTE
WVRSEAAPSGFSIGDEAVLKAILEDGGTGRFKKQDLACDEILTHIEAGKVVTQISMEWQQ
RISFTLSCDGILKRIKFADQLISQNDDIDSEDVVQRFDADITLMTGELSNLISDLTAALG
GEAKR
>EECEBICD_01407 hypothetical protein
MSQKDDIPVFPVTGWQAGPLPGYDALVVKFQFLSSPMQPIESAQETQFLVLTPEMAESLA
SDLQRHIQDLRNSDVHSPQEGKH
>EECEBICD_01408 hypothetical protein
MNNYYTCSFCGVSELDAKKLIAKGSKDEPAICSECVVSCVNILINYAAVIKPVKLNVTKG
E

>EECEBICD_01409 putative HTH-type transcriptional regulator
MNIGNRVRQLRRAKNMKIAELAEAIGVDAANISRLETGKQKQFTEQTLSRLADCLSVDIA
ELFTSDPKGNTVCKHSDMRKDSANVKDLFRIEILDVSASAGNGLIQGGDVIDVIHAIEYN
KDKALAMFGGRPAAELKVINVRGDSMAPTIEPGDLIFVDISINQFDGDGIYVFGFDDKIY
VKRLQMIPDKLLVISDNTNYREWSITKDNECRFGVFGKVLISQTQSLKRHN
>EECEBICD_01410 hypothetical protein
MQSPLRKLRKSHGYTLQHVAKGVQVDPATLSRVERCEQAPSTELAERLAQFYAGEISEMQ
ILYPNRYQLSDSAI
>EECEBICD_01411 hypothetical protein
MGHEPEWKVEKQPRWLVAAIKKTISSLHGGYEEAAEWLDVTKDALFNRLRTGGDQIFPIG
WALVLQRAGGTYHLAHSVARASGGVFVPLADMEEVDNADINHRLLEAIEQITSYSQQIRV
AIEDGVIEPHEKAVIDEELYQAIAKLQQHSTLVYRVFCVPEKGDARECAAPGAVASNFME
KTNA
>EECEBICD_01412 hypothetical protein
MNSLTVNNRLSQQPGMYEYRPLRHECRLSNSLVVRNHREHSLTVGDESCRNLTAGFGMEG
DFMSMSFAGNQKLSALSICARAIRMSVLALCGNSGVILLSVKRQEHIDSAIPGRYTVQAP
HKAGAGRGNPEFNIEHNRAHAVFSCHEHCYAQIMVGRAGPVSAGPGSMLTGISTPVRLTT
YKVVESLGGEFIEFNIEAATMATVPTLAQPEIRIINGQAVTSSLAVADYFIKRHADVIRK
IESLECSTLFRKRNFAFTSISINQPNGGTRKLPCYQITRDGFAFLAMGFTGKRAAQFKEA
YIDAFNQMEKQLSTPSVLSDAAHNASVLYSYISSIHQVWLQQLYPMLEKAESPLAVSLHD
RINDAAALASLINMTLNRSEVRGRK
>EECEBICD_01413 hypothetical protein
MIRNIFKRFTSQRFHCPRPGQWYSTPEGYVLRISLVDRECQKVVCEPLGRNYRVNMPLIA
FRSGKNMKHLGGAA
>EECEBICD_01414 hypothetical protein
MSSLIQLLDRPIAYNPAFAKLKAGKVKAGPVAAVFLSQLVYWHNRMDGGWMYKTQADIAS
ETALTRDEQETARKRLVALGVLEEARRGVPATMHYRINTARLEALLLETAKPVKKGAQEK
TRLRDFQNVETPQSGLVQPRKPDCGDAANKNVETPQTSTGQPNEQACGDPTIFPTGDYTE
TTQEITQESKTPFCPVAEQPDPEVTLTDQAIEVLTHLNQVSGSRYQKSKTSLENIRARLR
EGYSVADLQLVIDLKHEHWHENDEQYQYMRPETLFGPKKFESYLQSATRWDQKGRPKRAD
WGAKKRDVMAFGPVDTTIPEGFRG
>EECEBICD_01415 hypothetical protein
MSLLAKVQAFIELNPGLTSNEIADAFPEYARFDVQRSASKLYRCKRVNRRLDGDVFRYYA
GKDEAVILTLRQKRSGHTGSGDPMVIAKLVSRAEELESRGLFNRASIVWLEAFSESQFIY
EREEFLRRRQKCLNRIKKRIRPVEQVYLAGRFVGNVE
>EECEBICD_01416 DNA adenine methylase
MTSESVCIESSDVTISVDESASRTWRRPFLKWAGGKYSMLPDLYQVIPAGMRLIEPFVGG
GSVFLNSDKHACFLLADVNTDLINLYQMLAVVPGAVIRHARVMFDRLNDAESYMALREEF
NAQVMDAPERAAAFLFLNRHCFNGLIRYNRNNQFNVGWGKYPSPYFPEEEIRAFTEMAHN
CVFMAAGFRRTLALAGEGDVVYCDPPYEPMPGKDGFTHYAAGGFTWDDHIALAECCVAAH
QRGARVVIGNSTSPRVIDLYSQHGFEIRYISARRSISSKGSTREKAKDLVAIL
>EECEBICD_01417 Crossover junction endodeoxyribonuclease RusA
MKLTLPFPPSVNTYWRAPNKGPLKGRHMVSASGRKYQSEACAAVIEQLRRLPKPSTAPAA
VEITLYPPDKRIRDLDNYNKALFDALTHAGVWEDDSQVKRMLVEWGPVFPKGKVEITITK
FETGAGAAA
>EECEBICD_01418 hypothetical protein
MNNLMVIDGIEVRRDVHGRYCLNDLHRAAGGEQKYRPKYWLDNKQTRELIEQLFTEGGIP
PSEQNQSVSFFQGGSDTRSLARAPVNTVRGGAEQGTYVCKELVFAYAMWISPSFHLKVIR
TFDRITSAPQISSGMAADKMQAGVILLGFMRKELNLSNSSVLGACQKLQEAVGLPNLAPQ
YAIDAPAGAPDGSSRPTLALSALLKQHGIRMTANQAYQQLAKLGVVEHRERYSRSAINGI
KKFWSLTAKGCMFGKNITSPANPRETQPHFFESKFPELLKLLDTVH
>EECEBICD_01419 hypothetical protein
MRALLTPEIAPRMGVVLFRPGSELMPLFMQGRVLLEPEPEQYSSFACGAVPAVSQPLADD
PAVRDVFRNESVIYRAGGLDSLESWLLRGNGCQWPHSVWHSEQMTTMRHAPGAIRLCWHC

DNLLREQFTERLESIAVENTTKWVLSVVCRDLGFDDMHAVTLPELCWWMVRNDLAEVLPE
SAARKALRMPKAIVQSATRESEIVPSVPATSIVQDKAKKVLALRVDPESPESFMLRPKRR
RWINERYTRWVKSQPCACCGKQADDPHHLTGHGQGGMGTKAHDLFVLPLCRTHHNELHAD
TVAFEEKYGSQLELIFRFIDRALAIGVLA
>EECEBICD_01420 hypothetical protein
MNLEALPKYYSPKSPKLSDDAPATTSESLTITDVMAAQGMVQSKAPLGFALFLAKVGIQN
PDFAIEGLIHYAVALDNPTLNKLSEETRLQIVPYLVNFAFADYSRSAASKARCEHCAGTG
FHHVLREVVKHSRNGEPVIKEEWEKELCQHCHGKGEVSTVCRGCKGKGIVLDEKRTRFHG
APVYKICGRCNGNRFSRLPTTLARHHVQKLVPDLTDYQWYKGYADVIDKLVTKCWQEEAY
AEAQLRKVTR
>EECEBICD_01421 hypothetical protein
MDKILPTMGFVCLSLISLKNPPPSGFFICDHFIFCLAKLLYGQELKLSGDIVLSNNERWV
SFFDFAFTPTHAAAPSIPIEDILKKLKVLVSSGSAVKLYNHRSRALRISEMKYSIGDSQA
TLLIQLCDKNGSDPVFGELTTGNLRVEPKLAGEGIAVSCHIVISTDVVKNTADHHKTLVE
SVPGISKSVLEPFLNAMLREAFAGCEFKNPATKGMCQHRPKLEIYSHGSQTLMDALKGAK
IHNVKLVSTRRKGGLDQTAYTELSERSVKYKIIRQPPLKDKERLLEILRKKGQQSGYTKV
SISYSKDGKQASLDLDRNEDAATKLFTKSERVILGNLINQCESTVHLQLETKMIGLL
>EECEBICD_01422 hypothetical protein
MKLFSPLSYLRIKHEEKDWYDYKIPAAVSLIVTIVYYFHASKISLIETNGLLLQVNGLLQ
VLIGFYIAALAAVSTFSSSSIDEVMAGVPPTLVEKFRGQKLTVELTRRRFVCYLFGYLAL
VSFMLFCLGMISILIGKPFHLWLLTFCSPDAILWLKTVFVGVYIFILMNIITTTLLGLYF
LAVRFHQSSL
>EECEBICD_01423 hypothetical protein
MSEPVSSATVLAGGLMGASVFGLATGTDYGVVFGAFAGAVFYVATATNIGRIRLVAYFIT
SFIVGVLGAGLIGTKLAAITHYEKPLDALGAVIISAMCIKFLTFLNSQDLNTLFSILSRI
RGGDQMVANDPSAALNAVICGVIVIVLMFYRRGDATHRPLISLLAYVMVLVYASVPFRFV
FGLYESSHWLVVMVNILICAAVLWARGNVARLVDALRH
>EECEBICD_01424 hypothetical protein
MNQQQFQQAAGISAGLSARWFPHIDAAMKEFGITAVNDQAMFIAQTGHESAGFTVLKESF
NYSVEALKKTFGKRLTPYQCEMLGRIDGRQVAHQPQIANLVYGGRMGNKDAGDGWKYRGR
GLLQITGRENYVKCGAALKLDLISTPELLAQEKHAARSAAWFFTLRGCLMYSGDVVRVTQ
IINGGQNGLADRNSRYNKARAALLV
>EECEBICD_01425 hypothetical protein
MHKHKPLNLLPLLLACLPLLASCVPKQQSLPPAWTPQSTPQILPLPSEAKQPEPTPQCSP
TCSDALQKKLDIMLSDLTKATAQE
>EECEBICD_01426 hypothetical protein
MPPRTPKSCRVRGCRSTTTDPSGYCESHRSEGWKQYKPGQSRHQRGYGSKWDVIRERILK
RDKGLCQLCLRAGVVREAKTVDHIIPKAHGGTDADSNLQSLCWPCHKAKTARERLK
>EECEBICD_01427 hypothetical protein
MGAVVRSSGGGRKRNLPSGQKSKLTRIAPPEELMSDIAIRIWKTQSKILIERGVFDLEDA
PLLLAYCNAFHLMIEAEKVIAEEGLTVSSEMGGEKKHPAVNVRNDSVSQLARLGSLLGLD
PLSRIRMTSGKNDPDDEGNEFDEFD
>EECEBICD_01428 hypothetical protein
MATYPNVNAANQYARDVVNGKILACRLTMLACQRHLDDLERAKDPHWPYRFDKNKAERFL
RFSQKMPHTSGEWARRKLRIEFEPWQKFALGVPFGWVRKDTGFRRFTEIYIEVPRKNGKS
AIAAAVGNYMFCADGEYAAEVYCGATTEKQAWKVFAPALAMVKKLPALRQKFCIKPWAKK
MTRPDGSLFAPIIGDPGDGDSPSCAIIDEYHEHDTDALYTTMTTGMGAREQPITLIITTA
GFDIASPCYEKRTQVVEILERIREGGENEAIFGIIYTLDDDDDWTQPEALIKANPNYNIS
VKEGFLKAKQLLAMSTPGQTNKILTKHFNKWVSSKAAYYNLQKWMTAADKTLRLSDFAGE
ECYPGIDLASKLDLNAVVPVFRREIDGLSHYYCVSPMFWVPEDTVYATDPALKTIADRYQ
SFVNQGVLVPSDGAEVDYRLILEAILKLRETVKIAASPIDPYGATGLSHMLQDEGLEPVT
ITQNYTNMSDPMREIEAAIAAGRFHHDGNPLMTWCISNVVGKYLPGSDDVVRPVKEGAGN
KIDGAVGLMMGVGRAMLNEPKDFLSNLDPDEELLFL
>EECEBICD_01429 hypothetical protein

MLIDAIFRSNSLENPAVPVTVEAVENDGIFNGDVIVNPRTAMKLAAVYACIYVISSNVAQ
MPLHVMRRTGKKVETARDHPAFYLVHDEPNSWQTSYKWRELKQRHILGWGNGYTRVLRHR
RTGEVTGLEACMPWETTLLNTGGRYTYGVYNEEGSFAINPDDMIHVRALGNDQKMGLSPV
LQHAETIGMGMSGQKYTESFFSGNARPAGIVSVKGELNDGSWKRLKEMWQKATAMLRSQE
NRTMLLPAELDYKALTVSPVDAQLIDMMKLNRSMIAGIFNVPAHMINDLEKATFSNISEQ
AIQFVRYTMMPWVTNWEQELNRRLFTRAEREAGYYVRFNLAGLLRGTAKERAEFYHFAIT
DGWMSRNEARAFEDMNPKDGLDEMLVSVNASRPAKSTTQENTQDE
>EECEBICD_01430 hypothetical protein
MSEREIRCYSGEVRAETHDSEPSRIIGYGSVFDSRSELIFGSFREIIRPGAFDEVLNDDV
RALFNHDPNFILGRRSAGTLALTVDERGLRYDITAPETQTIRDLVLAPMQRGDINQSSFA
FRVARDGEEWYQDEDGVVIREITRFSRLLDVSPVTYPAYQEADSAVRSMKAWQEARDSSA
LQKAINQRMARERVLTLLNA
>EECEBICD_01431 hypothetical protein
MKLHELKQKRNTIATDMRALNEKIGDNPWTDEQRTEWNKAKSELEALDERIAREEELRRQ
DQTYVDENEEEQRNNQDPDKDPQQDEKRGQIFDKWMRHGASELSSEERKALRELRAQGVA
PDEKGGYTVPDTFLAKVVEQMKSYGGIASVAQILATSDGRTMEWATADGTAEVGVLLGEN
EEAGEEDTEFGMDSLGALKMTSKIIRVSNELLQDSAIDMEAYLARRIAERIGRGEARYLI
QGTGTGTPKQPKGLKASVTGTTQTAAAGAVKWQEILALKHSIDPAYRRGPKFRLAFNDNT
LKLISEMEDGQGRPLWLPDIVGVAPASVLNVPYVIDQEIDDIGAGKKFMFCGDFDRFIIR
RVRYMILKRLVERYAEFDQTGFLAFHRFDCILEDTSAIKALVGKGSASS
>EECEBICD_01432 hypothetical protein
MLLSPEEIKLQLRLDEDYADEDKFLELLGRAVQARTENFLNRRLYTAEAGVPADDPEGLI
LSDDIRMGMLLLVTHFYENRSTVTEVEKVELPMSFNWLVGPYRYIPL
>EECEBICD_01433 hypothetical protein
MKLRQAQASATYLLPDPGELDQRIVIRRVDVPADDFGVTPTYPEQIRAWAKKAQPGAAA
YQGAVQIENRVTHYFTIRFRRGITADHEVLHDDISYRVKRVRDLNSKRRFLLLECEELGT
DNGSDYAAESIFTR
>EECEBICD_01434 hypothetical protein
MPQKAYLHVDFEQPETLVFNRARMRRAFVSIGQVHMRDARRLVMKRGRSGPGDNPSYRTG
KLARSIGYYVPRASSRRPGLMVKIAPNQKNGEGNRPISGAFYPAFLFYGVRRGAKRKKGH
HRGASGGSGWRVAPRNNYMTEVLDKRRSWTRYVLSRELRKSLRPQRRKKK
>EECEBICD_01435 hypothetical protein
MKLTPIIAALRSRCPRFENRVGGAAQFKAIPEAGKLRLPAAYVVPAEDVTGEQKSQTDYW
QDLTEGFSVIVVLSNERDEKGQWASYDAVHDVRQEIWKALLGWEPDPQAHEIQYAGGMLL
DLNRHELYYQFDFTVKYEITETDTRQQDDLDGLPDLKTLSIDVDFIEPGTGPDGDIEHHT
EITFQE
>EECEBICD_01436 hypothetical protein
MFVKPAKGRSVPDPARGDLLPEGGRNVDENNYWLRREAAGDVRRTNKKVKTNGD
>EECEBICD_01437 hypothetical protein
MAISFNSIPSDTRVPLFYAEMDNSAANTARDSGASLLIGHASNDASIAVNSLVLVSSVDY
ARQICGAGSQLARMVGAYRKTDPFGELYVIAVPESTGAAATVALTVTGEATETGTVNVYT
GRTRVQAPVTSGDDAAAVAVSIKDAVNANPDLPFTATSEAGVVTLTARHKGLYGNEIPVT
LNYYGFGGGEVLPAGVNITVASGVKGAGAPALNDAVAAMGDEPFDYIGLPFNDTASVNTM
ATEMNDSSGRWSYIRQLYGHVYTAKTGTLSELVAAGDQFNLQHITLAGYEKDTQTPADEL
AASRTARAAVFIRNDPARPTQTGELVDMLPAPKGKRFTTTEQQTLLSHGVATAYVESGVL
RIQRDITTYRKNAYGVADNSYLDSETLHTSAYVLRRLKSVITSKYGRHKLANDGTRFGPG
QAIVTPAVIRGELGSTYRQLEREGIVENFDLFQQHLIVERNANDSNRLDVLFPPDYVNQL
RVFAVLNQFRLQYSEEAA
>EECEBICD_01438 hypothetical protein
MGKIAGTTYFKIDGQQLSVTGGIEVPMNTKVRDDVIGLDGSVDYKETSRAPYTKVTAKVP
KNFPVDKITSSDVMTITSELANGQVYVLSNAWLHGEANHNPEEGTVDLEFHGEEGFYQ
>EECEBICD_01439 hypothetical protein
MIKELVLKKPIMAHNEKLHVLELREPSYDEIEAIGFPFTVSGDGGVRLDSSVALKYIPVL
AGIPRSSAAQLAKLDIFKACMLILNFFTRSETEEDSESGSTTPHTSGE

>EECEBICD_01440 hypothetical protein
MADSFQLKAIITAVDQLSGPLKGMQRELKGFQKEMAGLAIGAAAAGTAVLGALALPVNAA
IGFESKMADIRKVVDGLDDKKAFAQMSDDILTLSTQLPMAAEGIAEIVAAGGQAGIARGD
LMQFANDAVKMGVAFDTTAEESGQMMAQWRTAFRLTQEDVVVLADKINYLGNTGPANAKK
ISDIVTRIGPLGGVAGVASGEIAAMGATIAGMGVESEIASTGIKNFMLSLTAGNSATKAQ
KQAMAFLKLNPRKLAEDMQKDSRGAMLKVLDSLAKVPKAKQAAVMNALFGKESLSAIAPL
LTNLDLLRTNFDRVADAQEYGGSMQKEYASRASTTENQLVLLKNSVNAISVTLGDTFLPA
INEAAEAVMPYLEQLRTFVRANPELVQSAAKFGAALLAVGVSIGSLSRAVKILNSVINLS
PAKVAIAALVAGAMLIIENWDDVAPVIKAVWQEVDNVAQEMGGWETVIEGVGLVMAGSFT
VRTIGALQQSVLLAGRLSGLLGKIGRMGAMTLTIGVAVSLFKELKDLEQGAKDAGMDAGA
FAVQKLQTKERERGYNGFIPRLKELLGMDTPIPQGRYQPYVPLTRRSGVLGRAVPPSTQR
SELKVTFENAPQGMRVTDIPKSGNPLMNISHDVGYSPFRTSR
>EECEBICD_01441 hypothetical protein
MAFFSSTGWRGRLRDASFRGVPFSVEDDESTFGRRVQVHEYPNRDKPWTEDLGRATRRLT
INAYLVGDDYADRRDRLIGAIETAGPGTLVHPQYGEMQGSIDGQVRITHSSTEGRMCRVS
FQFVESGELSFPVAGMATAKRLETSGGLFDDAIDSMFSTFSLSGISDFIQNDVIADAASM
LGDVADAFRMVDSGVSAAMRLLQGDLSVILMPPGAASDFVNALQKAWRSGDRLRGSTSDL
VTMIKTMSGITLDPGLSPRGTWPTDSGSAAKQKMQRNMIAAAIRTTAISTAVHAVTTLKQ
PRDVPDVRGVNQPAGTGRDSDIITVMHPALDGVQTVSNGSFPPNYEDLKAIRTALNAAID
QEQLRIRDDVLFQQISVMRTDLNRDISARLAQVERTALRTPDDVLPALVLAAAWYDDAGR
ESDILTRNPVPHPGFIPVEPLRVPVR
>EECEBICD_01442 hypothetical protein
MNNTVFLRVNGRDWGGWTSVRISAGIDRIARDFNVSITRQWPGGEDVPPVKNGDAVEVLI
GDDLVITGWVEALPLRYDAQTIMTGIVGRSKTADLIDCSASPAQHNGKNLFLIASALARP
FGVDVVDAGAPAAAVIEAQPEHGETVVDCLNRLLGQAQALAYDDERGRLVLGRPGSMKAA
TALVLGENILSCDTERSVRERFSSYLVTGQRPGTDDDFGEATIAAIRQSTGDAGVTRYRP
HTIQQSGTATTDSCKSRCEFEARQRAAKTLETTYTVQGWRQGNGELWKPNQAVVVYDPLN
GFDNETLVIAEVTYSQDNNGTLTEIRVGPADAYLPEPFRPKAKKKVSEEADF
>EECEBICD_01443 hypothetical protein
MANHPLQNMITRAVITAIDTVRKCQTAGLKLIAGEKKRMWSILNLTVSPLQHRMAQKRWY
CFPAVAVRTEWLWLWLTAASD
>EECEBICD_01444 hypothetical protein
MVVADRRFRLKGLARGEVALYDDQGQSVTLTRAGIVVNGGGKPVIFTNATKARFEMPIES
TGDIRDNCDSSGKTMAEMRTTYNGHTHKENGDGGGITDKPGQPMS
>EECEBICD_01445 hypothetical protein
MILYVNGIRKDATASLDFLTRAVVISLFTWRRAERDDRTPQPYGWWGDTWPAVQNDRIGS
RLYLLKRRKLTNKTPQDAREYMQQALAWMTDDGVAARIDVTSERTGTDTLAAGVTIYQRD
GVIHNITFDDIWSKLNG
>EECEBICD_01446 hypothetical protein
MADSQFARPELPQLIATIRSDLLTRFQQDVVLRRMDAEVYSRVQAAAVHTLYGYIDYLAR
NMLPDMCDEDWLYRHARIKRCPRKNAVSAKGFARWDGIAGTPEIPAGTQIQRDDQVTFTT
LQTVKASGGLLRVPVIADVAGTAGNTDDGTALRLGTPITGIPSTGYADTLTGGADTEEPE
TWRARVMERYYWIPQGGADPDYVIWAKEIAGITRAWTFRHYKGTGTVGVMVATSNPVNPA
PGDDLVKAVRDHILPLAPVAGGGLFVFAATEKSIPVTVALAKDTPEIRTAIIAELNALML
RDGAPSGKIYVSRISEAISLATGEVAHQLRVPAADVVLGKTELPVLGNITWATYTGENG
>EECEBICD_01447 hypothetical protein
MALQDEYTQLLYHLLPEGPAWDGENPLIEGLAPSLNRVHQRADELMAEIDPARTTELIDR
YEQLYGLPDSCAPEGVQTLQQRQQRLDAKANVAGGINERFYREQLDALGYTAATIEQFQN
LDSTPDPEWGEFWRYYWRVNIPADANISWQTCTSTCDSAIRTWGDTVAECVIDKLCPSHT
VVVFAYPEGKENAQN
>EECEBICD_01448 hypothetical protein
MHRIDTPTAQKDKFGQGKNGFTNGDPATGRRATDLNSDMWDAVQEEVCTVIEAAGIPLSK
GEHTQLHAAIGRLIDEQVKTRLEKNQNGADIPNKPLFLQNVGLGETINLAAGALQKSQNG
GDIPDKKQFARTIGAVTSTTITLGESGWFKIATVVMPQATSTAVIKLYGGAGFNAGSPEQ

AAISELVLRAGNGSPVGITATLWRRSPSAANEVAWVNTSGDTYDIYINIGQYAYWLIAQY
DYTGNANVTLHSTPEYSSVQPGNSTSGQTYTLFNSLMKPTAGDVEALSVNGGRLNGPLGI
GTDNALGGNSIVFGDNDTGFKWHSDGVLGIYANNALVGYIDNSGLHMSVDVLTNGAVRAG
NAKKLSLTSNNNSTMTATFNLWGDANRPTVIELDDDQGWHLYSQRNPDGSIVFTVNGDIT
ANTLRASGAIYQNNGDIFGSLWGNGWLSTWINNNLVLDVQLGAGTSVTTWNNAGSWPNTP
GYVVTSVWKDYQGENIDGINYAPLQKRVGSQWYTVQGGTV
>EECEBICD_01449 hypothetical protein
MKKYQNIKNFRLIDAPVNRDKTQAEINIGAYFLESDDGQDWYECQSLFSDDTAKIMYDHE
GVIWGVVNKPVPQRGNTYSVSMLWPVNMSVAEIDAADCPDDCRGDGTWLYQDGKVVQRGY
SPEELRKKAEAEKVRRLAEAESAIAPLARAVKLKIATDEEIKRLEAWELYSVMVNRVDTS
APDWPDIPR
>EECEBICD_01450 hypothetical protein
MSDFHHGTQVIQFNGGTRVTSTISIEIVSMVFTFSFAETLLAFISYVCK
>EECEBICD_01451 hypothetical protein
MYDKKLSDIYLENIAKIEAQPANVRDEYLLGEIKKSLNEVLKNNPEESLVSSHDKRLGHV
RFDFYRNLFLLKGSNAFLEAGKHGCHHLQPGGGCIYLDADMLLTGKLGTLYLPDGIAVHV
SRKGNSMSLENGIIAVNRSEHPALKKGLEIMHSKPYGDPYIDGVCGGLRHYFNCSIRHNY
EEFCNFIEFKHEHIFMDTSSLTISSWR
>EECEBICD_01452 hypothetical protein
MQLPEQDEFSDFFAANDDEQASLRRKFFLEKHKEPCLSESALEDYQALFMSIYGINIDWK
EGTFSLLEALSDNQEGSLSRSNSIMTVRLKQQR
>EECEBICD_01453 hypothetical protein
MGSDGFDTELVRIEHILANSGYSLRVYQNSTFSDTLSFLLIPSDEWKRVEQHYSPEHISE
YFVPYGKQLVIPEVTAPVVNYVPSVKQEASNVPALFNARGIRICFLSIMLIAFAIYILWN
ILTKIEPLSSGQPAGCENLQNLYSKLRPEVAEPLKEKMRKSLGCK
>EECEBICD_01454 hypothetical protein
MTLINFVAANQKYYAFNLLSVFIMVFWSVQYLLQHLLVQTQVSHQLLQPLILILQLPQTP
QFRHARPGKFFLPVNGMDYFLPLRLTVRNVLTRRITMNIVFLGIDLAKNVFQLCGLNQAG
KPVYTKRTGRKELLQTLANIPACLIGIEASTGAFYWQREFEKLGHKVKVISPQYVRPFVR
GQKNDGNDAQAIAVALMQPTMQFVPPKSPEQQDIQALHRARQRIVNHRTATVCQIRGLLL
DRGIPIGSAVSRARRAIPLILEDAENGLSSRMRRTIAELYDLFNDLGRRIHFFDKEIETV
FRQSEACPRIAKVKGIGPKTATAVVAAIGKGTEFKNGRHFAAWLGLVPRQHSSGDRQVLM
NMTKKGDKHLRTLFIHGARAVVRVATNNNDGHMNQWVNQLKERRGFNKTTVAVANKNARI
IWSMLRNDTGYQVVCN
>EECEBICD_01455 hypothetical protein
MKKTRYTEEQIAFALKQAETGTRVGEVCRKMGISEATFYIYGLPPFCKY
>EECEBICD_01457 Glyoxylate/hydroxypyruvate reductase A
MEIIFYHPTFNTAWWVNALEKALPHARVREWKVGDNNPADYALVWQPPVEMLAGRRLKAV
FALGAGVDAILSKLNAHPEMLDASIPLFRLEDTGMGLQMQEYAVSQVLHWFRRFDDYQAL
KNQALWKPLPEYTREEFSVGIMGAGVLGAKVAESLQAWGFPLRCWSRSRKSWPGVESYVG
REELHAFLNQTRVLINLLPNTAQTVGIINSELLDQLPDGAYVLNLARGVHVQEADLLAAL
DSGKLKGAMLDVFSQEPLPQESPLWRHPRVAMTPHIAAVTRPAEAIDYISRTITQLEKGE
PVTGQVDRARGY
>EECEBICD_01458 putative phosphatase YcdX
MYPVDLHMHTVASTHAYSTLSDYIAEAKRKGIKLFAITDHGPDMEDAPHHWHFINMRIWP
RLVDGVGILRGIEANIKNINGEIDCSGKMFDSLDLIIAGFHEPVFAPHDKETNTQAMIAT
IASGKVHIISHPGNPKYPVEVKAIAQAAAKHHVALEINNSSFLHSRKGSEDNCRAVAAAV
RDAGGWLGSVRL
>EECEBICD_01459 Chaperone protein YcdY
MNEFSILCRVLGSLFYRQPQDPLLVPLFTLIREGKLAANWPLEQDDMLARLQKSCDITQI
STDYNALFVGEECAVAPYRSAWVEGAEESEVRAFLTSRGMPLADTPADHIGTLLLAASWL
EDQSAEDESEALETLFADYLLPWCNTFLGKVEAHAVTPFWRTLAPLTRDAIGAMWDELQE
EDEE
>EECEBICD_01460 Inner membrane protein YcdZ

MMRAMNILLSIAITTGILSGIWGWVAVSLGLLSWAGFLGCTAYFACPQGGFKGLLISVCT
LLSGMVWALVIIHGSALAPHLEIVSYVLTGIVAFLMCIQAKQLLLSFVPGTFIGACATFA
GQGDWRLVLPSLALGLIFGYAMKNSGLWLASRREQHSANTAVTK
>EECEBICD_01461 Curli production assembly/transport component CsgG
MPRLLILVAVLLLSGCLTAPPKQAAKPTLMPRAQSYKDLTHLPAPTGKIFVSVYNIQDET
GQFKPYPASNFSTAVPQSATAMLVTALKDSRWFIPLERQGLQNLLNERKIIRAAQENGTV
AMNNRIPLQSLTAANIMVEGSIIGYESNVKSGGVGARYFGIGADTQYQLDQIAVNLRVVN
VSTGEILSSVNTSKTILSYEVQAGVFRFIDYQRLLEGEIGYTSNEPVMLCLMSAIETGVI
FLINDGIDRGLWDLQNKADRQNDILVKYRELSVPPES
>EECEBICD_01462 Curli production assembly/transport component CsgF
MRVKHAVVLLMLFSPLTWAGNMTFQFRNPNFGGNPNNGSFLLNSAQAQNSYKDPAYDNDF
GIETPSALDNFTQAIQSQILGGLLTNINTGKPGRMVTNDFIIDIANRDGQLQLNVTDRKT
GRTSTIEVSGLQTQSTDF
>EECEBICD_01463 Curli production assembly/transport component CsgE
MKRYLTWIVAAELLFATGNLHANEVEVEVPGLLTDHTVSSIGHEFYRAFSDKWESEYTGN
LTINERPSARWGSWITITVNQDVIFQTFLFPMKRDFEKTVVFALAQTEEALNRRQIDQTL
LSTSDLARDEF
>EECEBICD_01464 putative csgAB operon transcriptional regulatory protein
MFNEVHSSHGHTLLLITKPSLQATALLQHLKQSLAITGKLHNIQRSLEDISAGCIVLMDM
MEADKKLIHYWQDNLSRKNNNIKTLLLNTPDDYPYREIENWPHINGVFYATEDQEHVVSG
LQGILRGECYFSQKLASYLITHSGNYRYNSTESALLTHREKEILNKLRIGASNNEIARSL
FISENTVKTHLYNLFKKIAVKNRTQAVSWANDNLRR
>EECEBICD_01465 Minor curlin subunit
MKNKLLFMMLTILGAPGIATATNYDLARSEYNFAVNELSKSSFNQAAIIGQVGTDNSARV
RQEGSKLLSVISQEGGNNRAKVDQAGNYNFAYIEQTGNANDASISQSAYGNSAAIIQKGS
GNKANITQYGTQKTAVVVQKQSHMAIRVTQR
>EECEBICD_01466 Major curlin subunit
MKLLKVAAFAAIVVSGSALAGVVPQWGGGGNHNGGGNSSGPDSTLSIYQYGSANAALALQ
SDARKSETTITQSGYGNGADVGQGADNSTIELTQNGFRNNATIDQWNAKNSDITVGQYGG
NNAALVNQTASDSSVMVRQVGFGNNATANQY
>EECEBICD_01467 Curli assembly protein CsgC
MHTLLLLAALSNQITFTTTQQGDIYTVIPQVTLNEPCVCQVQILSVRDGVGGQSHTQQKQ
TLSLPANQPIELSRLSVNISSEDSVKIIVTVSDGQSLHLSQQWPPSAQ
>EECEBICD_01468 putative protein YmdA
MSVIKKNIPGIGLCICAFFIHSAVGQQTVQGGVIHFRGAIVEPLCDISTHAENIDLTCLR
EGKKQMHRIDLRQASGLPQDIQSIATVRLHYLDAQKSLAVMNIEYR
>EECEBICD_01469 O-acetyl-ADP-ribose deacetylase
MTSRLQVIQGDITQLSVDAIVNAANASLMGGGGVDGAIHRAAGPALLDACKLIRQQQGEC
QTGHAVITPAGKLSAKAVIHTVGPVWRGGEHQEAELLEEAYRNCLLLAEANHFRSIAFPA
ISTGVYGYPRAQAAEVAVRTVSDFITRYALPEQVYFVCYDEETARLYARLLTQQGDDPA
>EECEBICD_01470 Cardiolipin synthase C
MKKLPGFTQDYLLSKATTLPDKTRLERAVEPLCARHPGECGILALDNSLDAFAARYRLTE
MAARTLDVQYYIWEDDMSGRLLFSVLLSAAKRGVHVRLLLDDNNTPGLDDTLRLLDSHPN
IEVRLFNPFSFRTLRALGYLTDFARLNRRMHNKSYTADGVVTLVGGRNIGDAYFGAGEEP
LFSDLDVMAIGPVVNDVANDFERYWRCSSVSTLQQVLSLSEQELTQRIELPESWYNDEIT
RRYLHKLETSQFMADLDRGTLPLIWAKTRLLSDDPSKGEGKAQRHSLLPQRLFDVMGSPT
ERIDIISAYFVPTRAGVAQLLNLVRKGVKIAILTNSLAANDVAVVHAGYARWRKKLLRYG
VELYELKPTREHETAVHDRGLTGNSGSSLHAKTFSIDGSKVFIGSLNFDPRSTLLNTEMG
FVIESETLATLIHKRFTQSQRDAAWQLRLDRWGRINWIDRQQEEEKVLKKEPATRFWQRV
LVRLAAILPVEWLL
>EECEBICD_01471 Glucans biosynthesis protein C
MSSVPAPREYFLDSIRAWLMLLGIPFHISLIYSTHSWHVNSATPSWWLTLFNDFIHAFRM
QVFFVISGYFSYMLFLRYPLKRWWKVRVERVGIPMLTAIPLLTLPQFILLQYVKEKTENW
PTLSAYEKYNTLAWELISHLWFLLVLVILTTVSIGIFTWFQKRQETSKPRPAAISLVRLS

LIFFLLGMAYAAIRRIIFIVYPAILSDGMFNFIVMQTLFYVPFFILGALAFIHPDLKARF
TTPSRGCTLGAAVAFIAYLLNQRYGSGDAWMYETESVITMVMGLWMVNVVFSLGHRLLNF
QSARVTYFVNASLFIYLVHHPLTLFFGAYITPHISSNLIGFLCGLIFVMGIALILYEIHL
RIPLLKFLFSGKPPVKQESRAAIG
>EECEBICD_01472 Glucans biosynthesis protein G
MKHKRQMMKMRWLGAAIMLTLYASSSWAFSIDDVAKQAQSLAGKGYEAPKSNLPSVFRDM
KYADYQQIQFNSDKAYWNNLKTPFKLEFYHQGMYFDTPVKINEVTATTVKRIKYSPDYFN
FGNVQHDKDTVKDLGFAGFKVLYPINSKDKNDEIVSMLGASYFRVIGAGQVYGLSARGLA
IDTALPSGEEFPRFREFWIERPKPTDKRLTVYALLDSPRATGAYRFVIIPGRDTVVDVQS
KVYLRDKVGKLGVAPLTSMFLFGPNQPSPTTNYRPELHDSNGLSIHAGNGEWIWRPLNNP
KHLAVSSYAMENPQGFGLLQRGREFSRFEDLDDRYDLRPSAWITPKGDWGKGKVELVEIP
TNDETNDNIVAYWTPDQLPEPGKEMNFKYTLTFSRDEDKLHAPDNAWVLQTRRSTGDVKQ
SNLIRQPDGTIAFVVDFVGADMKKLPPDTPVAAQTSIGDNGEIVDSNVRYNPVTKGWRLM
LRVKVKDAKKTTEMRAALVNADQTLSETWSYQLPANE
>EECEBICD_01473 Glucans biosynthesis glucosyltransferase H
MNKTTEYIDALLLSEREKAALPKTDIRAVHQALDAEHRTYSREDDSPQGSVKARLEHAWP
DSLAKGQLIKDDEGRDQLQAMPKATRSSMFPDPWRTNPVGRFWDRLRGRDVTPRYVSRLT
KEEQASEQKWRTVGTIRRYILLILTLAQTVVATWYMKTILPYQGWALINPMDMVGQDIWV
SFMQLLPYMLQTGILILFAVLFCWVSAGFWTALMGFLQLLIGRDKYSISASTVGDEPLNP
EHRTALIMPICNEDVSRVFAGLRATWESVKATGNAAHFDVYILSDSYNPDICVAEQKAWM
ELIAEVQGEGQIFYRRRRRRMKRKSGNIDDFCRRWGNQYSYMVVLDADSVMSGECLSGLV
RLMEANPNAGIIQSSPKASGMDTLYARCQQFATRVYGPLFTAGLHFWQLGESHYWGHNAI
IRVKPFIEHCALAPLPGEGSFAGSILSHDFVEAALMRRAGWGVWIAYDLPGSYEELPPNL
LDELKRDRRWCHGNLMNFRLFLVKGMHPVHRAVFLTGVMSYLSAPLWFMFLALSTALQVV
HALTEPQYFLQPRQLFPVWPQWRPELAIALFASTMVLLFLPKLLSIMLIWCKGTKEYGGF
WRVTLSLLLEVLFSVLLAPVRMLFHTVFVVSAFLGWEVVWNSPQRDDDSTPWGEAFMRHG
SQLLLGLVWAVGMAWLDLRFLFWLAPIVFSLILSPFVSVISSRSTVGLRTKRWKLFLIPE
EYSPPQVLVDTDKYLEMNRRRILDDGFMHAVFNPSLNALATAMATARHRASKVLEIARDR
HVEQALNETPEKLNRDRRLVLLSDPVTMARLHYRVWNAPERYSSWVNHYQSLVLNPQALQ
GRTSSAR
>EECEBICD_01474 hypothetical protein
MRIFAVSIMVITLSGCGSIISRTIPGQGHGNQYYPGVQWDMRDSAWRYITILDLPFSLIF
DTLLLPLDIHHGPYE
>EECEBICD_01475 hypothetical protein
MTMYATLEEAIDAAREEFLADHPGLEQDEANVQQFNVQKYVLQDGDIMWQVEFFADEGED
GECLPMLSGEAAQSVFDGDYDEIEIRQEWQEENTLHEWDEGEFQLEPPLDTEEGRTAADE
WDER
>EECEBICD_01476 Staphyloferrin B transporter
MSPSDVPINWKRNLTVTWLGCFLTGAAFSLVMPFLPLYVEQLGVTGHSALNMWSGLVFSI
TFLFSAIASPFWGGLADRKGRKIMLLRSALGMAIVMLLMGMAQNIWQFLILRALLGLLGG
FIPNANALIATQVPRHKSGWALGTLSTGGVSGALLGPLAGGLLADHYGLRPVFFITASVL
FICFLLTFFFIHENFLPVSKKEMLHVREVVASLKNPRLVLSLFVTTLIIQVATGSIAPIL
TLYVRELAGNVSNIAFISGMIASVPGVAALLSAPRLGKLGDRIGPEKILIVALIISVLLL
IPMSFVQTPWQLALLRFLLGAADGALLPAVQTLLVYNSTNQIAGRIFSYNQSFRDIGNVT
GPLMGAAISASYGFRAVFCVTAGVVLFNAIYSWNSLRRRRLAIE
>EECEBICD_01477 Lipid A biosynthesis lauroyltransferase
MTKLPKFSVALLHPRYWLTWLGIGALWLVVQLPYPVIYKLGCALGHLARRVMKRRAKIAY
RNLELCFPEMTAQERHTMVVKNFESVGMGVMETGMAWFWPDRRVNRWMEASGLEHIREVK
AQGLGFILVGIHFLTLEFGARMFGMHNPGIGVYRPNDNPLLDWLQTWGRLRSNKSMLDRK
DLKGMVKALKSGELIWYAPDHDYGPRASVFVPLFAVDQAATTSGTWMLARMSKACIIPFV
PRRKPDGKGYELIILPAEYSPPLESAEATAAWMNKIVEQCIMMAPEQYMWLHRRFKTRPE
GVPSRY
>EECEBICD_01478 hypothetical protein
MPVLHNRISNDELKAKMLAESEPRTTISFYKYFTIASPQQTRDALYQVFTALDVFGRVYL

AHEGINAQISVPQSKVETFRQQLYTFDPALDGLRLNIALEDDGKSFWVLRMKVRDRIVAD
GIDDPTFDASNVGDYLKAADVNAMLDDPDAVFIDMRNHYEYEVGHFENALEIPADTFREQ
LPKAVEMLREHADKKIVMYCTGGIRCEKASAWMKHNGFNKVWHIEGGIIEYARRAREQGL
PVRFIGKNFVFDERMGERISDEVIAHCHQCGAPCDSHTNCKNDGCHLLFIQCPQCASKFN
GCCSEQCCEELALPEEEQRRRRAGRENGNKIFNKSRGRLNSKLSIPDPAE
>EECEBICD_01479 Protein YceI
MKKNLLGFTLASLLFTTGSAVAAEYKIDKEGQHAFVNFRIQHLGYSWLYGTFKDFDGTFT
FDEKNPSADKVNVTINTNSVDTNHAERDKHLRSAEFLNVAKFPQATFTSTSVKKEGDELD
ITGNLTLNGVTKPVTLEAKLMGQGDDPWGGKRAGFEAEGKIKLKDFNITTDLGPASQEVE
LIISVEGVQQK
>EECEBICD_01480 Cytochrome b561
MQFKNTPQRYGVVSAALHWLTALVVYGMFALGLWMVTLSYYDGWYHQAPEIHKSIGILLM
MALIVRIIWRLYSPPPVALTSYSRLTRAGAAAGHLLLYLLLFAIIISGYLISTADGKPIS
VFGWFEIPATLTDAGAQADIAGTLHLWFAWSLVIISLSHGVMALKHHFIDKDDTLKRMTG
MSSSDYGAQK
>EECEBICD_01481 putative protein YceO
MRRLFHFLMNNIREHFMLYVILWSLLAVMDIVYLLFF
>EECEBICD_01482 N-methyl-L-tryptophan oxidase
MKYDLIIIGSGSVGAAAGYYATRAGLKVLMTDAHMPPHQQGSHHGDTRLIRHAYGEGEKY
VPLMLRAQTLWDELSTHNEEPIFVRSGVVNLGPADSAFLANVARSAQQWQLNVERLDATA
LMTRWPEIRVPDNYIGLFEADSGFLRSELAITTWLRLAREAGCAQLFNSQVSHIHHDDNG
VTIETSEGSYHASKALISAGTWVKALVPELPVQPVRKVFAWFKADGRYSTKNRFPAFTGE
MPNGDQYYDFPAENDELKIGKHNGGQLIQAPEERKPFAAVASDGAEAFPFLRNVLPGIGG
CLHGAACTYDNSPDENFIIDTLPGHENTLVITGLSGHGFKFAPVLGEIAADFALGKTSSF
DLTPFRLSRFSQ
>EECEBICD_01483 Biofilm regulator BssS
MEKNNEVIQTHPLVGWDISTVDSYDALMLRLHYQTPNRPEPEGTEVGQTLWLTTDVARQF
ISILEAGIAKIESGDYQENEYRRH
>EECEBICD_01484 DNA damage-inducible protein I
MRIEVTIAKTSPLPAGAIDALAGELSRRISHHFPENLGNVTVRYATANNLSVIGASKEDK
ERISEILQETWESADDWFINE
>EECEBICD_01485 Dihydroorotase
MTAPSQVLKIRRPDDWHVHLRDGDMLKTVVPYTSEIYGRAIVMPNLASPITTVDAAIAYR
QRILDAVPAGHDFTPLMTCYLTDSLDADELERGFHEGVFTAAKLYPANATTNSSHGVTSV
DAIMPVLERMEKLGMPLLVHGEVTHAEVDIFDREARFIDTVMEPLRQRLTALKVVFEHIT
TKDAAQYVRDGNDYLAATITPQHLMFNRNDMLVGGIRPHLYCLPILKRNIHQQALRELVA
SGFTRAFLGTDSAPHSRHRKETSCGCAGCFNAPSALGSYAAVFEEMNALAHFEAFCSLNG
PQFYGLPVNAGWVELVRDEQQVPENIALADDSLVPFLAGETVRWSVKK
>EECEBICD_01486 putative lipoprotein YceB
MGCNQLTQYTISEQEINQALEKRNNFSKDIGLPGIADAHIVLTNLVSQIGREEPNKVTLT
GDARLDMNSLFGSQKATMKLKLKALPVFDKEKGAIYLQEMEVVDATVTPEKMQSVLQTLL
PYLNQSLRSYFNQRPAYVLREDSSKGEALAKKLAKGIEVKPGEIVIPFTN
>EECEBICD_01487 Glutaredoxin 2
MKLYIYDHCPFCVKARMIFGLKNIPVELNVLQNDDEATPTRMIGQKMVPILQKDDSRYLP
ESMDIVHYVDNLDGKPLLTGKRNPAIEEWLRKVNGYVNQLLLPRFAKSAFDEFSTPAARQ
YFIRKKEASSGSFDNHLAHSAGLIKKIGDDLRLLDKLIVQPNAVNGELSEDDIHLFPLLR
NLTLVAGIHWPTKVADYRDNMAKQTQINLLSSMAI
>EECEBICD_01488 Multidrug resistance protein MdtH
MSRVSQARNLGKYFLLIDNMLVVLGFFVVFPLISIRFVDQMGWAAVMVGIALGLRQFIQQ
GLGIFGGAIADRFGAKPMIVTGMLMRAAGFATMGIAHEPWLLWFSCFLSGLGGTLFDPPR
SALVVKLIRPEQRGRFFSLLMMQDSAGAVIGALLGSWLLQYDFRLVCATGAILFILCALF
NAWLLPAWKLSTVRTPVREGMRRVMSDKRFVTYVLTLAGYYMLAVQVMLMLPIMVNDIAG
SPAAVKWMYAIEACLSLTLLYPIARWSEKRFRLEHRLMAGLLVMSLSMLPIGMVGNLQQL
FTLICAFYIGSVIAEPARETLSASLADARARGSYMGFSRLGLAIGGAIGYIGGGWLFDMG

KALAQPELPWMMLGIIGFITFLALGWQFSHKRTPRRMLEPGA
>EECEBICD_01489 [Ribosomal protein S5]-alanine N-acetyltransferase
MFGYRSNVPKVRLTTDRLVVRLVHERDAWRLADYYAENRHFLKPWEPVRDESHCYPSGWQ
ARLGMIGEFHKQGSAFYFALLDPEEKEIIGVANFSNVVRGSFHACYLGYSIAQKWQGQGL
MFEALTAAIRYMQRTQHIHRIMANYMPHNKRSGALLARLGFEKEGYAKDYLLIDGQWRDH
VLTALTTPLWTPGR
>EECEBICD_01490 hypothetical protein
MKYELTATEARVIGCLLEKQVTTPEQYPLSVNGVVTACNQKTNREPVMNLTEQEVQEQLD
NLVKRHFLRTVSGFGNRVTKYEQRFCNSEFGDLKLSAAEVALVTTLLLRGAQTPGELRSR
ASRMHEFSDMAEVESTLERLASREDGPYVVRLAREPGKRESRYMHLFCGDVDELSLQTSA
PESASGDLQSRVEALESEVAELKQRLDSLLAHLGE
>EECEBICD_01491 Putative oxidoreductase YceM
MRTLRIGIVGLGGIAQKAWLPVLTNTAGWTLQGAWSPSRDKALRICESWSIPYVDSLANL
ASGCDAVFVHSSTASHYAVVSELLNAGVHVCVDKPLAENLRDAERLVALAAQKN
>EECEBICD_01492 Putative oxidoreductase YceM
MVGFNRRFAPLYRELKTRLGTAASLRMDKHRTDSVGPHDLRFTLLDDYLHVVDTALWLAG
GEARLASGTLLTSESGEMCYAEHHFSADKLQITTSMHRRAGSQRESVQAVTDGGLYDVTD
MREWREERGQGILIKPIPSWQTTLEQRGFVGCARHFIDCVQNQTVPETAGEQAILAQRVV
EALWRDAISE
>EECEBICD_01493 putative lipid II flippase MurJ
MQEFYARVWNTKEMNLLKSLAAVSSMTMFSRVLGFARDAIVARIFGAGMATDAFFVAFKL
PNLLRRIFAEGAFSQAFVPILAEYKSKQGEEATRIFVAYVSGLLTLALAVVTVAGMLAAP
WVIMVTAPGFADTADKFALTTQLLRITFPYILLISLASLVGAILNTWNRFSIPAFAPTFL
NISMIGFALFAAPYFNPPVLALAWAVTVGGVLQLVYQLPYLKKIGMLVLPRINFRDTGAM
RVVKQMGPAILGVSVSQISLIINTIFASFLASGSVSWMYYADRLMEFPSGVLGVALGTIL
LPSLSKSFASGNHDEYCRLMDWGLRLCFLLALPSAVALGILAKPLTVSLFQYGKFTAFDA
AMTQRALIAYSVGLIGLIVVKVLAPGFYSRQDIKTPVKIAIVTLIMTQLMNLAFIGPLKH
AGLSLSIGLAACLNASLLYWQLRKQNIFTPQPGWMWFLMRLIISVLVMAAVLFGVLHIMP
EWSQGSMLWRLLRLMAVVIAGIAAYFAALAVLGFKVKEFVRRTA
>EECEBICD_01494 Flagella synthesis protein FlgN
MTRLSEILDQMTTVLNDLKTVMDAEQQQLSVGQINGSQLQRITEEKSSLLATLDYLEQQR
RLEQNAQRSANDDIAERWQAITEKTQHLRDLNQHNGWLLEGQIERNQQALEVLKPHQEPT
LYGADGQTSVSHRGGKKISI
>EECEBICD_01495 Negative regulator of flagellin synthesis
MSIDRTSPLKPVSTVQTRETSDTPVQKTRQEKTSAATSASVTLSDAQAKLMQPGVSDINM
ERVEALKTAIRNGELKMDTGKIADSLIREAQSYLQSK
>EECEBICD_01496 Flagella basal body P-ring formation protein FlgA
MQTLKRGFAVAALLFSPLTMAQDINAQLTTWFSQRLAGFSDEVVVTLRSSPNLLPSCEQP
AFSMTGSAKLWGNVNVVARCANEKRYLQVNVQATGNYVAVAAPVARGGKLTPANVTLKRG
RLDQLPPRTVLDIRQIQDAVSLRDLAPGQPVQLTMIRQAWRVKAGQRVQVIANGEGFSVN
AEGQAMNNAAVAQNARVRMTSGQIVSGTVDSDGNILINL
>EECEBICD_01497 Flagellar basal body rod protein FlgB
MLDRLDAALRFQQEALNLRAQRQEILAANIANADTPGYQARDIDFASELKKVMVRGREET
GGVALTLTSSHHIPAQAVSSPAVDLLYRVPDQPSLDGNTVDMDRERTQFADNSLKYQMGL
TVLGSQLKGMMNVLQGGN
>EECEBICD_01498 Flagellar basal-body rod protein FlgC
MALLNIFDIAGSALAAQSKRLNVAASNLANADSVTGPDGQPYRAKQVVFQVDAAPGQATG
GVKVASVIESQAPEKLVYEPGNPLADANGYVKMPNVDVVGEMVNTMSASRSYQANIEVLN
TVKSMMLKTLTLGQ
>EECEBICD_01499 Basal-body rod modification protein FlgD
MSIAVNMNDPTNTGVKTTTGSGSMTGSNAADLQSSFLTLLVAQLKNQDPTNPLQNNELTT
QLAQISTVSGIEKLNTTLGAISGQIDNSQSLQATTLIGHGVMVPGTTILAGKGAEEGAVT
STTPFGVELQQPADKVTATITDKDGRVVRTLEIGELRAGVHTFTWDGKQTDGTTVPNGSY
NIAITASNGGTQLVAQPLQFALVQGVTKGSNGNLLDLGTYGTTTLDEVRQII

>EECEBICD_01500 Flagellar hook protein FlgE
MSFSQAVSGLNAAATNLDVIGNNIANSATYGFKSGTASFADMFAGSKVGLGVKVAGITQD
FTDGTTTNTGRGLDVAISQNGFFRLVDSNGSVFYSRNGQFKLDENRNLVNMQGMQLTGYP
ATGTPPTIQQGANPAPITIPNTLMAAKSTTTASMQINLNSTDPVPSKTPFSVSDADSYNK
KGTVTVYDSQGNAHDMNVYFVKTKDNEWAVYTHDSSDPAATAPTTASTTLKFNENGILES
GGTVNITTGTINGATAATFSLSFLNSMQQNTGANNIVATNQNGYKPGDLVSYQINNDGTV
VGNYSNEQEQVLGQIVLANFANNEGLASQGDNVWAATQASGVALLGTAGSGNFGKLTNGA
LEASNVDLSKELVNMIVAQRNYQSNAQTIKTQDQILNTLVNLR
>EECEBICD_01501 Flagellar basal-body rod protein FlgF
MDHAIYTAMGAASQTLNQQAVTASNLANASTPGFRAQLNALRAVPVDGLSLATRTLVTAS
TPGADMTPGQLDYTSRPLDVALQQDGWLVVQAADGAEGYTRNGNIQVGPTGQLTIQGHPV
IGEGGPITVPEGSEITIAADGTISALNPGDPPNTVAPVGRLKLVKAEGNEVQRSDDGLFR
LTAEAQAERGAVLAADPSIRIMSGVLEGSNVKPVEAMTDMIANARRFEMQMKVITSVDEN
EGRANQLLSMS
>EECEBICD_01502 Flagellar basal-body rod protein FlgG
MISSLWIAKTGLDAQQTNMDVIANNLANVSTNGFKRQRAVFEDLLYQTIRQPGAQSSEQT
TLPSGLQIGTGVRPVATERLHSQGNLSQTNNSKDVAIKGQGFFQVMLPDGTSAYTRDGSF
QVDQNGQLVTAGGFQVQPAITIPANALSITIGRDGVVSVTQQGQAAPVQVGQLNLTTFMN
DTGLESIGENLYIETQSSGAPNESTPGLNGAGLLYQGYVETSNVNVAEELVNMIQVQRAY
EINSKAVSTTDQMLQKLTQL
>EECEBICD_01503 Flagellar L-ring protein
MANGSIFQSAQPINYGYQPLFEDRRPRNIGDTLTIVLQENVSASKSSSANASRDGKTSFG
FDTVPRYLQGLFGNSRADMEASGGNSFNGKGGANASNTFSGTLTVTVDQVLANGNLHVVG
EKQIAINQGTEFIRFSGVVNPRTISGSNSVPSTQVADARIEYVGNGYINEAQNMGWLQRF
FLNLSPM
>EECEBICD_01504 Flagellar P-ring protein
MFKALAGIVLALVATLAHAERIRDLTSVQGVRENSLIGYGLVVGLDGTGDQTTQTPFTTQ
TLNNMLSQLGITVPTGTNMQLKNVAAVMVTASYPPFARQGQTIDVVVSSMGNAKSLRGGT
LLMTPLKGVDSQVYALAQGNILVGGAGASAGGSSVQVNQLNGGRITNGAIIERELPTQFG
AGNTINLQLNDEDFTMAQQITDAINRARGYGSATALDARTVQVRVPSGNSSQVRFLADIQ
NMEVNVTPQDAKVVINSRTGSVVMNREVTLDSCAVAQGNLSVTVNRQLNVNQPNTPFGGG
QTVVTPQTQIDLRQSGGSLQSVRSSANLNSVVRALNALGATPMDLMSILQSMQSAGCLRA
KLEII
>EECEBICD_01505 Peptidoglycan hydrolase FlgJ
MIGDGKLLASAAWDAQSLNELKAKAGQDPAANIRPVARQVEGMFVQMMLKSMREALPKDG
LFSSDQTRLYTSMYDQQIAQQMTAGKGLGLADMMVKQMTGGQTMPADDAPQVPLKFSLET
VNSYQNQALTQLVRKAIPKTPDSSDAPLSGDSKDFLARLSLPARLASEQSGVPHHLILAQ
AALESGWGQRQILRENGEPSYNVFGVKATASWKGPVTEITTTEYENGEAKKVKAKFRVYS
SYLEALSDYVALLARNPRYAAVTTAATAEQGAVALQNAGYATDPNYARKLTSMIQQLKAM
SEKVSKTYSANLDNLF
>EECEBICD_01506 Flagellar hook-associated protein 1
MSSLINHAMSGLNAAQAALNTVSNNINNYNVAGYTRQTTILAQANSTLGAGGWIGNGVYV
SGVQREYDAFITNQLRGAQNQSSGLTTRYEQMSKIDNLLADKSSSLSGSLQSFFTSLQTL
VSNAEDPAARQALIGKAEGLVNQFKTTDQYLRDQDKQVNIAIGSSVAQINNYAKQIANLN
DQISRMTGVGAGASPNDLLDQRDQLVSELNKIVGVEVSVQDGGTYNLTMANGYTLVQGST
ARQLAAVPSSADPTRTTVAYVDEAAGNIEIPEKLLNTGSLGGLLTFRSQDLDQTRNTLGQ
LALAFADAFNAQHTKGYDADGNKGKDFFSIGSPVVYSNSNNADKTVSLTAKVVDSTKVQA
TDYKIVFDGTDWQVTRTADNTTFTATKDADGKLEIDGLKVTVGTGAQKNDSFLLKPVSNA
IVDMNVKVTNEAEIAMASESKLDPDVDTGDSDNRNGQALLDLQNSNVVGGNKTFNDAYAT
LVSDVGNKTSTLKTSSTTQANVVKQLYKQQQSVSGVNLDEEYGNLQRYQQYYLANAQVLQ
TANALFDALLNIR
>EECEBICD_01507 Flagellar hook-associated protein 3
MRISTQMMYEQNMSGITNSQAEWMKLGEQMSTGKRVTNPSDDPIAASQAVVLSQAQAQNS
QYALARTFATQKVSLEESVLSQVTTAIQTAQEKIVYAGNGTLSDDDRASLATDLQGIRDQ

LMNLANSTDGNGRYIFAGYKTEAAPFDQATGGYHGGEKSVTQQVDSARTMVIGHTGAQIF
NSITSNAVPEPDGSDSEKNLFVMLDTAIAALKTPVEGNDVEKEKAAAAIDKTNRGLKNSL
NNVLTVRAELGTQLSELSTLDSLGSDRALGQKLQMSNLVDVDWNSVISSYVMQQAALQAS
YKTFTDMQGMSLFQLNR
>EECEBICD_01508 Ribonuclease E
MKRMLINATQQEELRVALVDGQRLYDLDIESPGHEQKKANIYKGKITRIEPSLEAAFVDY
GAERHGFLPLKEIAREYFPANYSAHGRPNIKDVLREGQEVIVQIDKEERGNKGAALTTFI
SLAGSYLVLMPNNPRAGGISRRIEGDDRTELKEALASLELPEGMGLIVRTAGVGKSAEAL
QWDLSFRLKHWEAIQKAAESRPAPFLIHQESNVIVRAFRDYLRQDIGEILIDNPKVMEMA
RQHIAALGRPDFSSKIKLYTGEIPLFSHYQIESQIESAFQREVRLPSGGSIVIDSTEALT
AIDINSARATRGGDIEETAFNTNLEAADEIARQLRLRDLGGLIVIDFIDMTPVRHQRAVE
NRLREAVRQDRARIQISHISRFGLLEMSRQRLSPSLGESSHHVCPRCSGTGTVRDNESLS
LSILRLIEEEALKENTQEVHAIVPVPIASYLLNEKRTAVNAIETRQDGVRCVIVPNDQME
TPHYSVLRVRKGEETPTLSYMLPKLHEEAMALPSEEEYAERKRPEQPALATFAMPDVPPA
PTPVEPAVSVATAKKDNVAAAQPAQPGLFSRFLNALKQLFSGEETKAVETAAPKAEEKAE
RQQDRRKPRQNNRRDRNERRDTRDNRAGRDGGESRDDNRRNRRQTQQQNAEARDTRQQET
AEKVKTGDEQQQTPRRERSRRRNDDKRQAQQEVKALNREEQPVQETEQEERVQQVQPRRK
QRQLNQKVRFTNSAVVETVDTPVVVDEPRPVENVEQPVPAPRTELAKVDLPVVADIAPEQ
DDSVEPRDNTGMPRRSRRSPRHLRVSGQRRRRYRDERYPTQSPMPLTVACASPEMASGKV
WIRYPIVRPQETQVVEEQREADLALPQPVVAEPQVIAATVALEPQASVQAVENVAVEPQT
VAEPQAPEVVKVETTHPEVIAAPVDEQPQLIAESDTPEAQEVIADAEPVAETADASITVA
ENVADVVVVEPEEETKAEAAVVEHTAEETVIAPAQVVEKPQDVVCVDDDHNHASAPMTRA
PAPEYVPETPHHSDWQRPSFHFEGKGAAGGHSATRHASAPATRPQPVE
>EECEBICD_01509 Ribosomal large subunit pseudouridine synthase C
MFIHWRRRLYKHRKMCGANLTGYLESPPMKTETPSVKIVAIAADEAGQRIDNFLRTQLKG
VPKSMIYRILRKGEVRVNKKRIKPEYKLEAGDEVRIPPVRVAEREEEAVSPHLQKVAALA
DVILYEDDHILVLNKPSGTAVHGGSGLSFGVIEGLRALRPEARFLELVHRLDRDTSGVLL
VAKKRSALRSLHEQLREKGMQKDYLALVRGQWQSHVKTVQAPLLKNILQSGERIVRVSQE
GKPSETRFKVEERYAFATLVRCSPVTGRTHQIRVHTQYAGHPIAFDDRYGDREFDQQLTE
AGTGLKRLFLHAAALKFTHPGTGEVMRIEAPMDNALKRCLQVLRNAK
>EECEBICD_01510 hypothetical protein
MANTSWMRSFTQRWGKYVIVPLVIAMLTGCEGKGDSSRRAFVEYLNTQIITSPVVGVCEL
TPAQRQAFGHYADDYDVLLAAYGQIFAVVYETGQEDKITGVIQSERDNLVFLNELRVARD
VNNRFILRLKNVSSENRKKYSALKLPSDVKPVFDAAWNKTVTPLDETMTRYYFLRGKRLD
QLVAMGEFLQKQGNKVRFETDGKAVFVDLAAKEHFQDQQFNFFITQNDINHLTQQMVEFI
NSQQDAADAGGVN
>EECEBICD_01511 hypothetical protein
MGNSLTRTYTAVDTLWDKTHRTSVRNTNAFFLVGEWIDQETGPHEESEDRQALNCSQQAL
SSWLELEAVETAPQ
>EECEBICD_01512 7-methyl-GTP pyrophosphatase
MPRLILASTSPWRRALLEKLTIPFECATPDVDETPMPGEAPRQLVLRLAQAKAQSLAARF
PNHLIIGSDQICVLDGEITGKPLTEEKARQQLAKASGNIVTFYTGLALYNSASGHLQTEV
EPFDVHFRHLSEAEIDDYVRKEHPLHCAGSFKSEGLGIALFERLEGRDPNTLIGLPLIAL
CQMLRREGFNPLLQ
>EECEBICD_01513 Large ribosomal RNA subunit accumulation protein YceD
MQKVKLPLTLDPVRTAQKRLDYQGIYTPDQVERVAESVVSVDSDVECSMSFAIDNQRLAV
LTGDAVVTVSLECQRCGKPFTHQVHTTYCFSPVRSDEQAEALPEAYEPIEVNEFGEIDLL
ATVEDEIILALPVVPVHDSEHCEVSEADMVFGELPDEAQKPNPFAVLASLKRK
>EECEBICD_01514 50S ribosomal protein L32
MAVQQNKPTRSKRGMRRSHDALTAVTSLSVDKTSGEKHLRHHITADGYYRGRKVIAK
>EECEBICD_01515 Phosphate acyltransferase
MGGDFGPSVTVPAALQALNANSQLTLLLVGNPDIITPLLAKADFEQRSRLQIIPAQSVIA
SDARPSQAIRASRGTSMRVALELVKEGRAEACVSAGNTGALMGLAKLLLKPLEGIERPAL
VTVLPHQQKGKTVVLDLGANVDCDSTMLVQFAVMGAVLAEEVVGIKNPRVALLNIGEEET

KGLDSIREASLMLKTVPTINYIGYLEANELLTGKTDVLVCDGFTGNVTLKTMEGVVRMFL
SLLKSQGEGKKRSWWLLLLKRWLQKSLTRRFSHLNPDQYNGACLLGLRGTVIKSHGAANQ
RAFAVAIEQAVQAVQRQVPQRIAARLESVYPAGFEPLDDGKGVNLRAHR
>EECEBICD_01516 3-oxoacyl-[acyl-carrier-protein] synthase 3
MYTKIIGTGSYLPEQVRTNADLEKMVETSDEWIVTRTGIRERHIAAPNETVATMGFTAAN
RAIEMAGIDKDQIGLIVVATTSATHAFPSAACQIQSMLGIKGCPAFDVAAACAGFTYALS
IADQYVKSGAVKHALVVGSDVLARTCDPGDRGTIIIFGDGAGAAVLSASEEPGIISTHLH
ADGRYGELLTLPNADRVNPDNPIYLTMAGNEVFKVAVTELAHIVDETLAANNLDRSELDW
LVPHQANLRIISATAKKLGMSMDNVVVTLDRHGNTSAASVPCALDEAVRDGRIKAGQLVL
LEAFGGGFTWGSALIRF
>EECEBICD_01517 Malonyl CoA-acyl carrier protein transacylase
MTQFAFVFPGQGSQSVGMLAEMAANYPIVEETFAEASAALGYDLWALTQQGPAEELNKTW
QTQPALLTASVALWRVWQQQGGKMPALMAGHSLGEYSALVCAGVINFADAVRLVEMRGKF
MQEAVPEGTGGMSAIIGLDDASIAKACEESAEGQVVSPVNFNSPGQVVIAGHKEAVERAG
AACKAAGAKRALPLPVSVPSHCALMKPAADKLAVELAKITFNVPTVPVVNNVDVKCETDA
AAIRDALVRQLYNPVQWTKSVEFIAAQGVEHLYEVGPGKVLTGLTKRIVDTLTASALNEP
AALSAALTQ
>EECEBICD_01518 3-oxoacyl-[acyl-carrier-protein] reductase FabG
MSFEGKIALVTGASRGIGRAIAETLVARGAKVIGTATSENGAKNISDYLGANGKGLMLNV
TDPASIESVLENIRAEFGEVDILVNNAGITRDNLLMRMKDDEWNDIIETNLSSVFRLSKA
VMRAMMKKRCGRIITIGSVVGTMGNAGQANYAAAKAGLIGFSKSLAREVASRGITVNVVA
PGFIETDMTRALSDDQRAGILAQVPAGRLGGAQEIASAVAFLASDEASYITGETLHVNGG
MYMV
>EECEBICD_01519 Acyl carrier protein
MSTIEERVKKIIGEQLGVKQEEVTNNASFVEDLGADSLDTVELVMALEEEFDTEIPDEEA
EKITTVQAAIDYINGHQA
>EECEBICD_01520 3-oxoacyl-[acyl-carrier-protein] synthase 2
MSKRRVVVTGLGMLSPVGNTVESTWKALLAGQSGISLIDHFDTSAYATKFAGLVKDFNCD
DIISRKEQRKMDAFIQYGIVAGVQAMQDSGLEVTEENASRIGAAIGSGIGGLGLIEENHS
SLVKGGPRKISPFFVPSTIVNMVAGHLTIMYGLRGPSISIATACTSGVHNIGHAARIIAY
GDADAMVAGGAEKASTPLGVGGFGAARALSTRNDNPQAASRPWDKERDGFVLGDGAGMLV
LEEYEHAKARGAKIYAEIVGFGMSSDAYHMTSPPENGAGAALAMVNALRDAAIEPAQIGY
VNAHGTSTPAGDKAETQAVKSVFGDAASRVMVSSTKSMTGHLLGAAGAVESIFSILALRD
QAIPPTINLDNPDEGCDLDFVPHEARQVSDLEYALCNSFGFGGTNGSLIFKKI
>EECEBICD_01521 Aminodeoxychorismate lyase
MFLINGHAQDQLAVSDRATQFGDGSFTTARIVDGNICHLEAHLQRLQVACEKLRIAFSHW
ATLRQEMTMLATGHDSGVLKVIISRGSGGRGYSAMNCQAATRILSVSAYPAYYSQWRKQG
ITLTLSPIPLGRNPYLAGLKHLNRLEQVLIRSHLEQTDADEALVLDSEGWVTECCAANLF
WRTGDIVFTPRLDQAGVNGIMRQFCLRQLAQSPFQVLEVQAREEAVRQADEIIICNALMP
IIPIRAYHGTSYSSRTLFQFLAPFCEHPN
>EECEBICD_01522 Endolytic murein transglycosylase
MKKLSGVFLLLLVVLGIAAGVGMWKVRHLANSTLLIKNETIFTLKAGTGRLALGDQLYDE
KIINRPRVFQWLLRVEPELSHFKAGTYRFTPGMTVREMLELLESGKEAQFPLRFVEGMRL
SDYLKQLREAPYIRHTLPDDDYATVAQALKLAHPEWVEGGFWPDTWMYTANTSDVAILKR
AHQKMVKAVDTVWKGRAEGLPYKDQNQLVTMASIIEKETAVASERDQVASVFINRLRIGM
RLQTDPTVIYGMGTSYNGNLSRADLEKPTAYNTYTITGLPPGPIASPSEASLQAAAHPAK
TPYLYFVADGKGGHTFNTNLASHNRSVQEYLKVLKEKNGQ
>EECEBICD_01523 Thymidylate kinase
MGSNYIVIEGLEGAGKTTARDVVVETLEQLGIRNMIFTREPGGTQLAEKLRSLVLDIRSV
GDEVITDKAEVLMFYAARVQLVETVIKPALAQGIWVIGDRHDLSTQAYQGEGAALIKPCW
RHYAMPCWAIFVLT
>EECEBICD_01524 Thymidylate kinase
MLATLRDAVLGDFRPDLTLYLDVTPEVGLKRARARGDLDRIEQESFDFFNRTRARYLELA
AQDSRIRTIDATQPLDAVMRDIRATVTKWVQEQAA

>EECEBICD_01525 DNA polymerase III subunit delta'
MKWYPWLRPAYEKLVESYQAGRGHHALLIQALPGMGDEALCYALSRYLLCQQPEGHKSCG
HCRGCQLMQAGTHPDYYTLTPDKGKSSLGVDAVREVSEKLYEHSRLGGAKVVWIADAALL
TDAAANALLKTLEEPPEQTWFFLASPEPARLLATLRSRCRLHHLAAPSESYAMSWLSREV
TASQEALLTALRLNAGSPGAALALLQSERWAQREALCQALMDSLHTGDWYALLTALNHEQ
APARLHWLATLLVDALKRQHGASYLTNVDADAVVAALAGPLSPARIQAILNDVCHCRDQL
LHVTGLNRELVLTDLILRIEHYLQPGTLLPVPHL
>EECEBICD_01526 putative metal-dependent hydrolase YcfH
MFLVDSHCHLDGLDYQSLHKDVDDVLAKAAARDVKFCLAVATTLPGYRHMRELVGKRDNV
VFSCGVHPLNQDEAYDVEELRLLAAEDDVVAMGETGLDYFYTPETKIRQQASFIHHIQIG
RELHKPVIVHTRDARADTLAILREEKVTDCGGVLHCFTEDRETAGKLLDLGFYISFSGIV
TFRNAEQLRDAARYVPLDRLLVETDSPYLAPVPHRGKENQPAMVRDVAEYMAVLKGVAVE
ELAQITTDNFARLFHIDASRLSSIR
>EECEBICD_01527 PTS system glucose-specific EIICB component
MFKNAFANLQKVGKSLMLPVSVLPIAGILLGVGSANFSWLPAVVSHVMAEAGGSVFANMP
LIFAIGVALGFTNNDGVSALAAVVAYGIMVKTMAVVAPLVLHLPAEEIAAKHLADTGVLG
GIISGAIAAYMFNRFYRIKLPEYLGFFAGKRFVPIISGLAAIFTGVVLSFVWPPIGTAIQ
AFSQWAAYQNPVVAFGIYGFIERCLVPFGLHHIWNVPFQMQIGEYTNAAGQVFHGDIPRY
MAGDRTAGMLSGGFLFKMYGLPAAAIAIWHSAKPENRAKVGGIMISAALTSFLTGITEPI
EFSFMFVAPILYIIHAILAGLAFPICILLGMRDGTSFSHGLIDFIVLSGNSSKLWLFPIV
GAGYAIVYYTVFRVLIKALDLKTPGREDTTDDAKAGATSEMAPALVAAFGGKENITNLDA
CITRLRVSVADVAKVDQAGLKKLGAAGVVVAGSGVQAIFGTKSDNLKTEMDEYIRNS
>EECEBICD_01528 FhuE receptor
MSFIQYRRDKHLPSTAAPSLLAMGMAMAFMPAAFAAEDTVIVEGATTADAVNREEQDYSV
KTTAAGTKMPMTQRDIPQSVSIVSQQRMEDQQLQTLGEVMTNTLGISGSQADSDRISYYS
RGFEIDNYMVDGIPTYFESRWNLGDALTDTALYERVEVVRGANGLMTGTGNPSASINMIR
KHATSREFKGNVSTEYGSWNKQRYVMDLQSPLTADGNVRGRIVAGYQNNDSWLDRYNSEK
AFFSGIVDADLGTTTNLSAGYEYQKIDVNSPTWGGLPRWNTDGSKNSYDRARSTAPDWAY
NNKEINKFFVTLKQRFAESWQATLNATHTEVKFDSKMMYIDALVDKETGTLVSPYGASYP
VVGGTGWNSGKRKVDAIDLFADGAYELFGRQHNMMFGGSYSKQNNRYFSAWANVFPDDIG
NFSAFNGNFPQTHWAPQNLAQDDTTHMKSLYAATRISLADPLHLILGARYTNWRVDTLTY
SMEKNHTTPYAGLIYDINDNWSAYASYTSIFQPQNKRDKAGQYLAPITGNNYEAGLKSDW
MNSRLTTTLSVFRIEQNNVAQATTIPIPGSNGEFAWKSTDGTVSKGVEFEVNGAITDNWQ
MTFGATRYVAEDNEGNAVNPNLPRTSVKLFTRYRLPAIPELTVGGGVNWQNRVYKDTTTP
YGTFRAEQGSYALVDLFTRYQVTKNFSVQGNINNLFDKTYDTNIDGSIVYGAPRNVSLTA
NYQF
>EECEBICD_01529 Purine nucleoside phosphoramidase
MKKENVVAEETIFSKIIRREIPSDIVYQDELVTAFRDISPQAPTHILIIPNILIPTVNDV
TAEHEQALGRMITVAAKIAEQEGIAEDGYRLIMNTNRHGGQEVYHIHMHLLGGRPLGPML
AHKGL
>EECEBICD_01530 hypothetical protein
MIKGCIAVSLGLLLLTGCRSHPEIPVSDAQSLVMESTVLAAGVTAEPPELTASDIQPSAS
SRVYNERQEPITVHYRFYWYDARGLEMHPLEAPRSVTIPARSSVTLFGSANYLGAHKVRL
YLYL
>EECEBICD_01531 Penicillin-binding protein activator LpoB
MTKMHRYAAIAALAIFLSGCMAQRQPAPVEEVKPAPEQPAQPPQPPVVPSVPTIPQQPGP
IEHEDQTGQPAPKVRHYDWNGAMQPLVSKMLQADGVTAGSVLLVDSVNNRTNGSLNANEA
TETLRNALANNGKFTLVSVQQLSMAKQQLGLSPQDSLGTRSKAIGIARNVGAQYVLYSSA
SGNVNAPALQMQLMLVQTGEIIWSGKGAVQQQ
>EECEBICD_01532 Thiamine kinase
MRSNNNNPLTRDEILSRYFPQYRPAVAASQGLSGGSCIIAHDTHRIVLRRHHDPDAPPAH
FLRHYRALSQLPASLAPRALFYTPGWMAVEYLHGVVNSALPDADELAALLYHLHQQPRFG
WRIALSPLLAQYWSCCDPARRTPFWLRRLKQLQKNGEPRPLRLAPLHMDVHGDNIVLTSA
GLRLIDWEYAGDGDIALELAAVWVEDERQHRQLADAYAARARIDARQLWRQIRLWHPWVI

MLKAGWFEYRWRQTGEQQFIRLADETWRQLRMKG
>EECEBICD_01533 Beta-hexosaminidase
MGPVMLNVEGCELDAEEREILAHPLVGGLILFTRNYHDPEQLRELVRQIRAASRNHLVVA
VDQEGGRVQRFREGFTRLPAAQSFLRCMGWKRGATGAGGWVADGQRNDRDGY
>EECEBICD_01534 Beta-hexosaminidase
MASEMIAMDIDISFAPVLDVGHISAAIGERSYHADPAKALAMATRFIDGMHDAGMKTTGK
HFPGHGAVTADSHKETPCDPRPETDIRGKDMSVFRTLISENKLDAIMPAHVIYRAIDPRP
ASGSPYWLKTVLRQELGFDGVIFSDDLSMEGAAIMGSYAERAQASLDAGCDMILVCNNRK
GAVSVLDNLSPIKAERVTRLYHKGSFSRRELMDSARWKTASAQLNQLHERWQEEKAGH
>EECEBICD_01535 hypothetical protein
MIIYLHGFDSNSPGNHEKVLQLQFIDPDVRLVSYSTRHPKHDMQHLLKEVDKMLQLNVDE
RPLICGVGLGGYWAERIGFLCDIRQVVFNPNLFPYENMEGKIDRPEEYADIATKCVTNFR
EKNRDRCLVILSRHDEALDSQRSAQALHPYYEIVWDEEQTHKFKNISPHLQRIKAFKTLG
>EECEBICD_01536 NADH dehydrogenase
MTTPLKKIVIVGGGAGGLEMATQLGHKLGRKKKAKITLVDRNHSHLWKPLLHEVATGSLD
EGVDALSYLAHARNHGFQFQLGSVMDIDREAKTITIAELRDEKGELLVPERKIAYDTLVM
ALGSTSNDFNTPGVKEHCIFLDNPHQARRFHQEMLNLFLKYSANLGANGKVNIAIVGGGA
TGVELSAELHNAVKQLHSYGYKGLTNDALNVTLVEAGERILPALPPRISSAAHNELTKLG
VRVLTQTMVTSADEGGLHTKEGEYIQADLMVWAAGIKAPDFMKEIGGLETNRINQLVVEP
TLQTTRDPDIYAIGDCASCARPEGGFVPPRAQAAHQMATCAMNNILAQMNGKPLKAYQYK
DHGSLVSLSNFSTVGSLMGNLTRGSMMIEGRIARFVYISLYRMHQIALHGYFKTGLMMLV
GSINRVIRPRLKLH
>EECEBICD_01537 hypothetical protein
MNKSMLAGIGIGVAAALGVAAVASLNVFDRSPQYAQVISAKPIKETVKTPRQECRNVTVT
HRRPVQNENRIAGSVLGAVAGGVIGHQFGGGRGKDVATVVGALGGGYAGNQIQGSMQESD
TYTTTQQRCKTVYDKSEKMLGYDVTYKIGDQQGKIRMDKDPGTQIPLDGNGQLVLNNKA
>EECEBICD_01538 HTH-type transcriptional repressor ComR
MTTDTTSCAKKSRGRPKVFDREAALDKAMTLFWQHGYEATSLADLVEATGAKAPTLYAEF
TNKEGLFRAVLDRYITRFASKHEAQLFCEEKSVESALEDYFTEIAACFTSTDTPAGCFMI
NTSATLAASSRDIARTVKSRHAMQEQTLIQFLRQRQERGEIPAHCNPQALAEYINCILQG
MSISAREGATFEQLMQITRTTLRIWPELLKS
>EECEBICD_01539 Multiple stress resistance protein BhsA
MKNVKNLIAAAVLSSLSFASFAAVEVQATPEGQQKFGTISANGGTNLGSLEDQLAQKAQE
MGAKSFRITSVTGPNTLHGTAVIYK
>EECEBICD_01540 putative L,D-transpeptidase YcfS
MFNNTRLSRWWALFALTATIMLALPAQANTWPLPPPGSRLVGENKFHVVEDDGGSLEAIA
KKYNVGFLALLQANPGIDPYVPRAGSVLTIPLQTLLPDAPREGIVINLAELRLYYYQPGK
NTVTVYPIGIGQLGGDTLTPTMVTTISDKRANPTWTPTANIRARYKAQGIDLPAVVPAGP
DNPMGHHAIRLAAYGGVYLLHGTNADFGIGMRVSSGCIRLRDGDIETLFRQVTPGTKVNI
INTPIKASVEPGGVRLVEVHQPLSKNIGDDPQVLPIVLNGPMQAFKDAPQTDAAVMEHVM
EVRSGMPVDVTRQAEAKPQSL
>EECEBICD_01541 Transcription-repair-coupling factor
MPEKQRYTLPTKAGDQRQLGELTGAACATLVAEIAERHAGPVVLIAPDMQNALRLHDEIR
QFTDQMVMNLADWETLPYDSFSPHQEIISSRLSTLYQLPSMQRGVLIVPVNTLMQRVCPH
SYLHGHALVMKKGQRLSRDALRAQLDSAGYRHVDQVMEHGEYATRGALLDLFPMGSEQPY
RLDFFDDEIDSLRLFDADTQRTLEEVEAINLLPAHEFPTDKAAIELFRSQWRDTFEVKRD
AEHIYQQVSKGTLPAGIEYWQPLFFSEPLPPLFSYFPANTLVVNTGSLETSAERFQADTL
ARFENRGVDPMRPLLPPEALWLRVDELFSELKRWPRLQLKTDHLPEKAANTNLGFQKLPD
LAIQAQQKAPLDALRKFLESFSGPVIFSVESEGRREALGELLARIKIAPKRILRQDEAQD
AGRYLMIGAAEHGFIDTQRNLALICESDLLGERVARRRLDSRRTINPDTLIRNLAELHVG
QPVVHLEHGVGRYAGMTTLEAGGIKGEYLMLTYANDAKLYVPVSSLHLISRYAGGAEESA
PLHKLGGDAWSRARQKAAEKVRDVAAELLDIYAQRAAKEGFAFKHDREQYQLFCDSFPFE
TTPDQAQAINAVLSDMCQPLAMDRLVCGDVGFGKTEVAMRAAFLAVENHKQVAVLVPTTL
LAQQHYDNFRDRFANWPVRIEMLSRFRSAKEQTQILAEAAEGKIDILIGTHKLLQSDVKL

RDLGLLIVDEEHRFGVRHKERIKAMRADVDILTLTATPIPRTLNMAMSGMRDLSIIATPP
ARRLAVKTFVREYDSLVVREAILREILRGGQVYYLYNDVENIQKAAERLAELVPEARIAI
GHGQMRERELERVMNDFHHQRFNVLVCTTIIETGIDIPTANTIIIERADHFGLAQLHQLR
GRVGRSHHQAYAWLLTPHPKAMTTDAQKRLEAIASLEDLGAGFALATHDLEIRGAGELLG
EEQSGSMETIGFSLYMELLENAVDALKAGREPSLEDLTSQQTEVELRMPSLLPDDFIPDV
NTRLSFYKRIASAKNENELEEIKVELIDRFGLLPDPARNLLDIARLRQQAQKLGIRKLEG
NEKGGTIEFAEKNHVDPAWLIGLLQKQPQHFRLDGPTRLKFIQDLSERKTRIDWVRQFMQ
QLEENAIA
>EECEBICD_01542 Lipoprotein-releasing system transmembrane protein LolC
MYQPVALFIGLRYMRGRAADRFGRFVSWLSTIGITLGVMALVTVLSVMNGFERELQNNIL
GLMPQAILSAEHGSLNPSQMPEKAVNLQGVNRIAPLTTGDVVLQSARSVAVGVMLGIDPA
QKDPLTPYLVNVKQSELQPGKYNVILGEQLAGQLGVNRGDQIRLMVPSASQFTPMGRLPS
QRLFTVIGTFAANSEVDGYEMLVNIQDASRLMRYPAGNITGWRLWLDEPLQVDTLSQQTL
PQGTKWQDWRERKGELFQAVRMEKNMMGLLLSLIVAVAAFNIITSLGLMVMEKQGEVAIL
QTQGLTPRQIMMVFMVQGASAGIIGALLGAALGALLASQLNNLMPIIGAFLDGAALPVAI
EPLQVIVIALVAMAIALLSTLYPSWRAAATQPAEALRYE
>EECEBICD_01543 Lipoprotein-releasing system ATP-binding protein LolD
MNKILLQCDNLCKRYQEGTVQTDVLHDVSFSIGEGEMMAIVGSSGSGKSTLLHLLGGLDT
PTSGDVIFSGQPMSKLSSAAKAELRNQKLGFIYQFHHLLPDFTALENVAMPLLIGKKKPA
EIDARAREMLHAVGLEHRATHRPSELSGGERQRVAIARALVNNPRLVLADEPTGNLDARN
ADSIFELLGELNRLQGTAFLVVTHDLQLAKRMSRQLEMRDGRLTAELSLMGAE
>EECEBICD_01544 Lipoprotein-releasing system transmembrane protein LolE
MASPLSLLIGLRFSRGRRRGGMVSLISVISTIGIALGVAVLIVGLSAMNGFERELNNRIL
AVVPHGEIEAVNQPWTNWREALAKVQKVPGIAAAAPYINFTGLVESGANLRAIQVKGVDP
KQEQQLSALPSFVQNHAWDHFKAGEQQIIIGKGVADALNVKQGDWVSIMIPNANADHKLL
QPKRVRLHVIGILQLSGQLDHSFAMIPLEDAQQYLDMGSSVSGIALKVHDVFNANKLVRD
AGEVTNSYVYIKSWIGTYGYMYRDIQMIRAIMYLAMILVIGVACFNIVSTLVMAVKDKSG
DIAVLRTLGAKDGLIRAIFVWYGLLAGLLGSLIGVAIGVVVSLQLTAIINGIEKEIGHQF
LSGDIYFIDFLPSELHWLDVVYVLVTALLLSLLASWYPARRASNIDPARVLSGQ
>EECEBICD_01545 N-acetyl-D-glucosamine kinase
MYYGFDIGGTKIALGVFDSTRRLQWEKRVPTPHTSYSAFLDAVCELVAEADQRFGVKGSV
GIGIPGMPETEDGTLYAANVPAASGKPLRADLSARLDRDVRLDNDANCFALSEAWDDEFT
QYPLVMGLILGTGVGGGLVLNGKPITGQSYITGEFGHMRLPVDALTLMGFDFPLRRCGCG
QMGCIENYLSGRGFAWLYQHYYDQSLQAPEIIALWEQGDEQAHAHVERYLDLLAVCLGNI
LTIVDPDLLVIGGGLSNFTAITTQLAERLPRHLLPVARAPRIERARHGDAGGMRGAAFLH
LTD
>EECEBICD_01546 NAD-dependent protein deacylase
MQSRRFHRLSRFRKNKRLLRERLRQRIFFRDRVVPEMMENPRVLVLTGAGISAESGIRTF
RAADGLWEEHRVEDVATPEGFARNPGLVQTFYNARRQQLQQPEIQPNAAHLALAKLEEAL
GDRFLLVTQNIDNLHERAGNRNIIHMHGELLKVRCSQSGQILEWNGDVMPEDKCHCCQFP
APLRPHVVWFGEMPLGMDEIYMALSMADIFIAIGTSGHVYPAAGFVHEAKLHGAHTVELN
LEPSQVGSEFEEKHYGPASQVVPEFVDKFLKGL
>EECEBICD_01547 Spermidine/putrescine-binding periplasmic protein
MKKWSRHLLAAGALALGMSAAHASDNDTLYFYNWTEYVPPGLLEQFTKETGIKVIYSTYE
SNETMYAKLKTYKDGAYDLVVPSTYYVDKMRKEGMIQKIDKSKLTNFHNLDPEMLNKPFD
PNNDYSVPYIWGATAIGVNSDAIDPKTITSWADLWKPEYKNSLLLTDDAREVFQMALRKL
GYSGNTTDPKEIEAAYEELKKLMPNVAAFNSDNPANPYMEGEVNLGMVWNGSAFVARQAG
TPLEVVWPKEGGIFWMDSLAIPANAKNKEGALKLINFLLRPDVAKEVAETIGYPTPNLAA
RKLLSPEVANDKSLYPDAQTISKGEWQNDVGDASAIYEEYYQKLKAGR
>EECEBICD_01548 Inner membrane ABC transporter permease protein YdcV
MIGRLLRGGFMTAIYAYLYIPIIILIVNSFNSSRFGINWQGFTTKWYELLVNNDSLLQAA
QHSLTMAIFSATFATLIGSLTAVALYRYRFRGKPFVSGMLFVVMMSPDIVMAISLLVLFM
LLGIQLGFWSLLFSHITFSLPFVVVTVYSRLKGFDVRMLEAAKDLGASEVTILRKIILPL
AMPAVAAGWLLSFTLSMDDVVVSSFVTGPGYEILPLKIYSMVKVGVSPEVNALATVLLVL

SLVMVIASQLIARDKTKGR
>EECEBICD_01549 Secreted effector protein SifA
MAMCGLKQLEIPPPHTHIPIEKMVKEVLLADKTFQAFLVTDPSTSQSMLAEIIETISDQV
FHAIFRIDPQAIQKMAEEQLTTLHVRSEQQSGCLCCFL
>EECEBICD_01550 Secreted effector protein SifA
MLFAERAPTRERLTEIFFELKELACASQRDRFQVHNPHENDATIILRIMDQNEENELLRI
TQNTDTFSCEVMGNLYFLMKDRPDILKSHPQMTAMIKRRYSEIVDYPSLRHYVSILLARR
YYRFH
>EECEBICD_01551 hypothetical protein
MPITIGNGFLKSEILTNSPRNTKEAWWKVLWEKIKDFFFLLAKQKRTVVYMRCCLPNAPP
HESGLQRFFLS
>EECEBICD_01552 Spermidine/putrescine transport system permease protein PotB
MKNTSKFQNVVIVTIVGWLVLFVFLPNLMIIGTSFLTRDDASFVKMVFTLDNYARLLDPL
YFEVLLHSLNMALIATLSCLVLGYPFAWFLAKLPEKIRPLLLFLLIVPFWTNSLIRIYGL
KIFLSTKGYLNEFLLWLGVIDTPIRIMFTPSAVIIGLVYILLPFMVMPLYSSIEKLDKPL
LEAARDLGASKMQTFIRIIIPLTMPGIVAGCLLVMLPAMGLFYVSDLMGGAKNLLIGNVI
KVQFLNIRDWPFGAATSITLTIVMGLMLLIYWRASRLLNKKVSDISD
>EECEBICD_01553 Spermidine/putrescine import ATP-binding protein PotA
MGQSKKLNNQPRSLSPLVLLSGISKSFDGKEVISQLDLTINNGEFLTLLGPSGCGKTTVL
RLIAGLETVDAGHIMLDNQDITHVPAENRYVNTVFQSYALFPHMTVFENVAFGLRMQKTP
AAEIAPRVTDALRMVQLEEFAQRKPHQLSGGQQQRVAIARAVVNKPRLLLLDESLSALDY
KLRKQMQNELKALQRKLGITFVFVTHDQEEALTMSDRIVVMRNGVIEQDGTPREIYEEPK
NLFVAGFIGEINRFDATVIERLDEQRVRASVEGRECNIYVNFAVEPGQKLNVLLRPEDLR
VEEINDDNHIEGLIGYVRERNYKGMTLESVVELENGKMVMVSEFFNEDDPDFDHSLDQKM
AISWVESWEVVLADEEHK
>EECEBICD_01554 Peptidase T
MDKLLERFLHYVSLDTQSKSGVRQVPSTEGQWKLLRLLKQQLEEMGLVNITLSEKGTLMA
TLPANVEGDIPAIGFISHVDTSPDFSGKNVNPQIVENYRGGDIALGIGDEVLSPVMFPVL
HQLLGQTLITTDGKTLLGADDKAGVAEIMTALAVLKGNPIPHGEIKVAFTPDEEVGKGAK
HFDVEAFGAQWAYTVDGGGVGELEFENFNAASVNIKIVGNNVHPGTAKGVMVNALSLAAR
IHAEVPADETPETTEGYEGFYHLASMKGTVDRAEMHYIIRDFDRKQFEARKRKMMEIAKK
VGKGLHPDCYIELVIEDSYYNMREKVVEHPHILDIAQQAMRDCHITPEMKPIRGGTDGAQ
LSFMGLPCPNLFTGGYNYHGKHEFVTLEGMEKAVQVIVRIAELTAKRGQ
>EECEBICD_01555 hypothetical protein
MLRIVVKRFIIYGGIFSAINFSAWSAEYTPSWSQRQQQSAACFMTGDETCMTFIDDAVRL
ASQQYGKRSIQLVRSLLLQSDIYQWLGKPELTPQMLLRARAIMKTFPAGTYPGDRADMFE
HLAAFYVYSDDRHIEYSSTEQWRYEIKVDYRQRIAWQEQALTWRLKDKKASTEALVYTLN
RMRDAYSDALEERDVECDSARKAYYLAKIDTTERQWLSVILRDKTWDNRERVASFLQQKA
DIAYNAGHISEAINALSQALKIEQTLYGAEFGEMTVDSNNLAGFYAQGHHYKEAKDLYLK
LIAYYQSRLTPMATVISRLRFYLPENIDLDSTSPYLPLLEEYKRRQSDVSMVLYGISLLY
QNNQQLEQAKDFAERAFTLDAVAYPAKMQYERLQRLANIAEGLGDNVLARRYRQLSFRHR
MAHSIYPGDPQYNDFAKPGGDRCG
>EECEBICD_01556 50S ribosomal protein L16 3-hydroxylase
MEYQLTLNWPDFLERHWQKRPVVLKRGFSNFIDPLSPDELAGLAMESEIDSRLVSHQDGK
WQVSHGPFESYDHLGESNWSLLVQAVNHWHEPTAALMRPFRALPDWRIDDLMISFSVPGG
GVGPHLDQYDVFIIQGTGRRRWRVGEKLQMRQHCPHPDLLQVDPFEAIIDEELEPGDILY
IPPGFPHEGYALENAMNYSVGFRAPNSRELISGFADYVLQRELGNTYYSDPDMPSRKHPA
DILPQEMDKLRNMMLDLINQPAHFQQWLGEFLSQSRHELDIAPPEPPYQPDEIYDALKQG
EVLVRLGGLRVLRIGDEVYANGEKIDSPHRPALEALASHIALTAENFGDALEDPSFLAML
AALVNSGYWFFEG
>EECEBICD_01557 Virulence sensor histidine kinase PhoQ
MNKFARHFLPLSLRVRFLLATAGVVLVLSLAYGIVALVGYSVSFDKTTFRLLRGESNLFY
TLAKWENNKISVELPENLDMQSPTMTLIYDETGKLLWTQRNIPWLIKSIQPEWLKTNGFH

EIETNVDATSTLLSEDHSAQEKLKEVREDDDDAEMTHSVAVNIYPATARMPQLTIVVVDT
IPIELKRSYMVWSWFVYVLAANLLLVIPLLWIAAWWSLRPIEALAREVRELEDHHREMLN
PETTRELTSLVRNLNQLLKSERERYNKYRTTLTDLTHSLKTPLAVLQSTLRSLRNEKMSV
SKAEPVMLEQISRISQQIGYYLHRASMRGSGVLLSRELHPVAPLLDNLISALNKVYQRKG
VNISMDISPEISFVGEQNDFVEVMGNVLDNACKYCLEFVEISARQTDDHLHIFVEDDGPG
IPHSKRSLVFDRGQRADTLRPGQGVGLAVAREITEQYAGQIIASDSLLGGARMEVVFGRQ
HPTQKEE
>EECEBICD_01558 Virulence transcriptional regulatory protein PhoP
MMRVLVVEDNALLRHHLKVQLQDSGHQVDAAEDAREADYYLNEHLPDIAIVDLGLPDEDG
LSLIRRWRSSDVSLPVLVLTAREGWQDKVEVLSSGADDYVTKPFHIEEVMARMQALMRRN
SGLASQVINIPPFQVDLSRRELSVNEEVIKLTAFEYTIMETLIRNNGKVVSKDSLMLQLY
PDAELRESHTIDVLMGRLRKKIQAQYPHDVITTVRGQGYLFELR
>EECEBICD_01559 Adenylosuccinate lyase
MELSSLTAVSPVDGRYGDKVSALRGIFSEYGLLKFRVQVEVRWLQKLAAHAAIKEVPAFA
ADANGYLDTLVANFNEEDAARIKTIERTTNHDVKAVEYFLKEKVAAIPALHDVSEFIHFA
CTSEDINNLSHALMLKTARDEVILPYWRQMINAVKDLATQYRDIPLLSRTHGQPATPSTL
GKEMANVAYRMERQFRQLNQVEILGKINGAVGNYNAHIAAYPEVDWHQFSEEFVTSLGIQ
WNPYTTQIEPHDYIAELFDCIARFNTILIDFDRDVWGYIALNHFKQKTIAGEIGSSTMPH
KVNPIDFENSEGNLGLSNAVLHHLANKLPVSRWQRDLTDSTVLRNLGVGIGYALIAYQST
LKGVSKLEVNRDHLLDELDHNWEVLAEPIQTVMRRYGIEKPYEKLKELTRGKRVDAEGMK
QFIDSLALPEAEKTRLKAMTPANYIGRAVTLVDELK
>EECEBICD_01560 High frequency lysogenization protein HflD
MAKNYYDITLALSGICQSARLVQQLAHQGHCDADALHVSLNSVIDMNPSSTLGVFGGSEA
NLRLGLETLLGVLNASSRQGLNAELTRYTLSLMVLERKLSSAKGALNTLGDRINGLQRQL
DHFDLQSDTLMSAMAGIYVDVISPLGPRIQVTGSPAVLQSPQVQAKVRASLLAGIRAAVL
WHQVGGGRLQLMFSRHRLTTQAKQILAHLTPEL
>EECEBICD_01561 tRNA-specific 2-thiouridylase MnmA
MSESPKKVIVGMSGGVDSSVSAWLLQQQGYQVEGLFMKNWEEDDGEEYCTAAADLADAQA
VCDKLGIELHTVNFAAEYWDNVFELFLEEYKAGRTPNPDILCNKEIKFKAFLEFAAEDLG
ADYIATGHYVRRADVNGKSRLLRGLDGNKDQSYFLYTLGHEQIAQSLFPVGELEKPQVRK
IAEDLGLVTAKKKDSTGICFIGERKFRDFLGRYLPAQPGKIITVDGDEIGEHQGLMYHTL
GQRKGLGIGGTKDGSEDPWYVVDKDVENNVLIVAQGHEHPRLMSVGLIAQQLHWVDREPF
TGTLRCTVKTRYRQTDIPCTINALDDDRIEVIFDEPVAAVTPGQSAVFYSGEVCLGGGII
EQRLPLTV
>EECEBICD_01562 Phosphatase NudJ
MFKPHVTVACIVHAEDKFLVVEETINGKSLWNQPAGHLEADETLAQAAARELWEETGITA
QPQYFIRMHQWIAPDKTPFLRFLFAIKLDHICATEPHDNDIDCCRWVSADEILNAPNLRS
PLVAESIRSYQTGQCYPLSIVGEFNWPFTEGVK
>EECEBICD_01563 hypothetical protein
MKRLLIAFALLTPLSVDAASFDCQKAQAADEKAICAHLTLNDKNVEMHTKYQFLKGLFAM
GSRGALQDAQQSWLKQRQQCKADVTCLTKAYNERLKQLDAIYDHIDKPL
>EECEBICD_01564 Ribosomal large subunit pseudouridine synthase E
MFCNEHVNTTLLFAAMKDTHYCAIMRQLISSENTMQKTSFRNHYVKRFSSRQASKSRKEN
QPKRVVLFNKPYDVLPQFTDEAGRRTLKDFIPVQGVYAAGRLDRDSEGLLVLTNDGALQA
RLTQPGKRTGKIYYVQVEGIPDNAALQALRTGVTLNDGPTLPAGIEIVAEPDWLWPRTPP
IRERKNIPTSWLKVTLYEGRNRQVRRMTAHVGHPTLRLIRYSMGDYTLNGLDNGQWREIA
QEKDR
>EECEBICD_01565 Isocitrate dehydrogenase [NADP]
MESKVVVPVEGKKITLQNGKLNVPENPIIPFIEGDGIGVDVTPAMLKVVDAAVEKAYKGE
RKISWMEIYTGEKSTQVYGQDVWLPAETLDLIRDYRVAIKGPLTTPVGGGIRSLNVALRQ
ELDLYVCLRPVRYYQGTPSPVKHPELTDMVIFRENSEDIYAGIEWKADSADAEKVIKFLR
EEMGVKKIRFPEHCGIGIKPCSEEGTKRLVRAAIEYAITNDRDSVTLVHKGNIMKFTEGA
FKDWGYQLARDEFGGELIDGGPWLKIKNPNTGKEIVVKDVIADAFLQQILLRPAEYDVIA
CMNLNGDYISDALAAQVGGIGIAPGANIGDECALFEATHGTAPKYAGQDKVNPGSIILSA

EMMLRHMQWFEAADLIVKGMEGAIAAKTVTYDFERLMEGAKLLKCSEFGDAIIANM
>EECEBICD_01566 hypothetical protein
MLKPICHSGSIKVPEYLETDKEKNAGRTPLSSDIQQVRNVVEDVPPFPESRTARGSVSAA
YRLSFDEVFCGLSNEERKKVYGRLFGKQVLAHIHSRCQRDADIIREKALRRISRECGTEI
DCTLLLNKMVDILQNARLTINFNAAKIDFASLLKNKEYLNSYALGCRPGDLPAYNVGRDS
VETKAFELERLADSPYAPYGQTGGFSVAYTPNSRTFSPTSRPIYAALDFLNGENGGASAY
GKSFFELNDNVKTNCTLSPFDIYGHRFGLDTSKLSTFWHMENLIASCQNDFFGYNCFKSL
VKMAKGEKFLAHSNYGKGYEGNYIEAHIHGDVCLFRDIKHVYLSLQENSYSESQLYDYAK
QINLVLNRDCIILY
>EECEBICD_01567 hypothetical protein
MKNFFKIITDFIADISLDLFAIFLCMLFVYKTGPSIGVISFFIALIIYIILHFVFYSFRE
KIIKKIFK
>EECEBICD_01568 hypothetical protein
MNKIHVTYKNLLLPITFIAATLISACDNDKDAMAEAEKNQEKYMQKIQQKEHQQSMFFYD
KAEMQKAIANINAKGGANLAIIEVRFFKGGYSFIRQSVNTPAKVEMFKFNNGYWGDLRLS
T
>EECEBICD_01569 hypothetical protein
MFVELVYDKRNVEGLPGAREIILNELTKRVHQLFPDAQVKVKPMQANALNSDCTKTEKER
LHRMLEEMFEEADMWLVAE
>EECEBICD_01570 hypothetical protein
MTLLSGKTTLVLCLSSILCGCTTNGLPTPYSINLSFPVITQNQINSGGYYINDAEQIRTT
DGLCLDTGPDQQNRLTLRECKHVQSQLFSFHRDRITQGEKCLDAAGQGTKEGTPIILYSC
TGNDNQRWLTDDNKIKGKQSRKCLGTNSIIVRKGDPVVLADCDFSRALEFTIR
>EECEBICD_01571 hypothetical protein
MKHHAFMLWSLLIFSFHVLASSGHCSGLQQASWDIFIYDFGSKTPQPPTNTDKKQARQIS
SPSCPTTKPMMSAPVNDARKGNTFSRT
>EECEBICD_01572 Attachment invasion locus protein
MKNIILSTLFITTSVLVVNVAQADTNAFSVGYAQSKVQDFKNIRGVNVKYRYEDDSPVSF
ISSLSYLYGDRQASGSVEPEGIHYHDKFEVKYGSLMVGPAYRLSDNFSLYALAGVGTVKA
TFKEHSTQDGDSFSNKISSRKTGFAWGAGVQMNPLENIVVDVGYEGSNISSTKINGFNVG
VGYRF
>EECEBICD_01574 hypothetical protein
MMKRKLIPFTLFLAALSASTTSIAASQEISKSIYTCNDNQVMEVIYVNTEAGNAYAIISQ
VNEMIPMRLMKMASGANYEAIDKNYTYKLYTKGKTAELVEGDDKPVLSNCSLAN
>EECEBICD_01575 hypothetical protein
MVAKNKIHGRRIRQSLQQLHHRCLFQQLAVSPPGAKYVSVTAETKNWLIHLVTSEGR
>EECEBICD_01576 Small heat shock protein IbpB
MLILFFSDRFNRIDRLFSQLTGDTPVTATPAYDLQKRDANNYLLTVSVPGWKEEELEIET
VGGNLNITGKHTEETVEDQTHWIYRGIRKADFQLSFSLPEHAKVNNAKLEQGLLLVEIYQ
EIPESEKPKKIAIESKPKAIEHKS
>EECEBICD_01577 hypothetical protein
MKLVLVNRQVILPESGTESFQCHASTLVRLPCGTLVAAWFAGLREGSEDTAIWLSRYEHN
IWTTPQRVAAREGEAHWNPVLFYPSDKLWLFYKVGSDVHVWKTWFITSSDRGFTWSTPAP
LVNDDILPRGPVKNKLLLASNGAWIAPGSIESPERWRAFVDRSSDEGKHWNISFVPLEPD
NAISGTNVALWDGVKKGMLWECCLENLLRWDGVIQPTLWESSPGHIHMLLRSTRGAIFRS
DSIDYGATWSVARATSLSNNNSGIDLVSMQDGTLILALNLVNGNWGKRYPLSLIASQDNG
ESWLPLLDLESDHGEYSYPAIISEGGVVHITYTWNRKNIVYCRLQTV
>EECEBICD_01578 Cytochrome b561
MAHFSRLQITLHWLTLLLTGIAYAAIELRGWAPKGSSVYLFMKDTHYDMGVLVWALMFLR
LYLKHKYPDPVITPPPPHWQHVAAKLMHIALYLTFLALPLLGVAMMASGGKSWSFFGFTV
PVFLTPDSALKSDIKRIHEMLANIGYFLIAMHAAAALFHHYIQKDDTFSRMLPGKS
>EECEBICD_01579 Oligopeptide-binding protein AppA
MLGWSHGSYLLLHAPLLKQNADMSWGNLLTEKVDTSPVGKIWTLTLKPGLKFSDGSPLTA
EDVVFTYNKAAKSGGKIDMGNFSHARALDARRIEMTLSHPQSTFVNVLGSLGIVPASRYD

EKTFAREPIGAGPYRLVSFQPGQQLIVEANPWYAGKKNDFNRLVFVFLDEDNAYAAARSG
QLGLVRIAPSMAVAPQQDNLKLWVRDSVENRGIVFPMVPAGKKDANGYPVGNDVTADVAI
RRAINYAINRKQLAEQVMEGHAIPAYSAVQGLPWQNPSVIFSDGDIAKARAILEEAGWKI
NRAGVREKAGKEARLTLWYASGDSTRRDLAEAVRAMLQPLGIVVSLQSGSWETVERHMHA
NPTLFGWGSLDPMELFHHYSGKAAGVEYYNPGYYSNPAVEAHLKQAIDAPDWQKAIPFWQ
QVEWDGKQGAGVQGDAAWAWLLNIQHTYLANPCIDLGKGAPEIHGSWSVLNNLDDWTWTC
R
>EECEBICD_01580 Dipeptide transport system permease protein DppB
MKLFFSHFLRLIILLVLVAAGTFILLSFSPVDPIRAYIGNDLLHVPPEQYARIAARWGLD
QPLWERFGHWFWRLLQGDMGYSMLFNMPVASVIRERFATSFALLAGAWLLSGVLGVTLGF
LAGRFLHRWPDKMICRISYLLSSLPTFWIAMLLLALFAVRWPVLPVCCAWDPGNNAGTAL
LSERLRHLVLPVCALSLLGMGQIALHTREKIASVMKSEFIRFARAQGDKGWSLLRHQVLR
HAITPALCLQFASLGELMGGALLAEKVFAYPGLGQATIDAGLRGDVPLLMGIVLFCTLLV
FAGNTISAWLVVVLNRSLERPDAL
>EECEBICD_01581 Dipeptide transport system permease protein DppC
MLYNPAPTLFRLILSVTCLLFIAGYGYATLSQPPEVNLLARHLSPDIQHWFGTDNLGRDV
WLRCFQGAFTSLQIGVGAALCSGVIALVMAAVARIHPRLDLLMRLITDAMLAMPHLLLLI
LICFTLGGGKSGVIAAVALTHWPRLALILRADAERVAQSDYLTLTYRLGHGHLYCWRYHY
FPALLPQWLTGTLLMFPHAVLHSAALSFLGFGLAPHEPSLGLLLADALRFISHGNWWLVL
FPGLMLFTLVMLFDQFARAIQRLWLRSDVC
>EECEBICD_01582 Vitamin B12 import ATP-binding protein BtuD
MLSLQQVTLESARYRWYGTRRWSPLLQNVSFDIAPGEMVALVGGSGEGKSLLLQCLLDLL
PENLRFRGEITLDGNRLDRHTIRQLRGNTFSYVPQGVQALNPMLNIRKHLNRACHLTGRA
WDETQMVQLLQQSDLEPTVLERFPRQLSGGMAKRILACHASLSQARYILADEITAWLDTA
LANQLLEHLRGLCERGCGVLWVTHDLLLAARYADRIVALHQGYITDNIRCEQLQPEEMSE
PLKRQWQALPELNPLFMPTGEGIEC
>EECEBICD_01583 Oligopeptide transport ATP-binding protein OppF
MLSCRDLVIRQGGKVLWQNLTFRISAGERVGIHAPSGTGKTTLGRVLAGWQKPTAGDVLL
DGSPLPLHQYCPVQLVPQHPELTFNPWRSAGDAVRDAWQPDPETMRRLHVQPEWLTRRPM
QLSGGELARIAILRALDPRTRFLIADEMTAQLDPSIQKAIWVYVLEVCRSRSLGMLVISH
QSALLDQVCTRHLQVE
>EECEBICD_01584 hypothetical protein
MEYLARLNIVAMLMSTTFWINLAVVFFVTLITYWLINWLLNVVYKALQRPDKKNDAHGRL
RPIVFEMLKKTSKMLIFFAAFLFSLRFVALPDRLFSTLSHAWFLVVAIQMAIWLDQGVQS
WMRHLLYAPGSNKNPVTLVILGMILRVLVWSMMLLSILANVGVDITALVASLGVGGIAIA
LAVQTVLSDVFASLSIGFDKPFEIGDFVVFNDVAGTIEHIGLKTTRIRSLSGEQIVCANA
QLLQQTIHNYKRMQTRRIVFTFGVATATPPEKLRLIGDMVKKIITDVGETQFDRAHLLAF
GQDRLTYEVVHIVNTADYNKYMDIQQEINIRIIEKLIENDIELALPSLVVRAPVQVEETR
DYSNTADAKNADKRVMQ
>EECEBICD_01585 putative cysteine-rich protein YhjQ
MQQEHRECIEQCYECAAACDICASSCLREDNVEMMKHCIQLDMQCAAICRLAAQFMALES
EYSQKLCRLCADICKACAEECARHDHDHCQNCARACSQCADACLKMAA
>EECEBICD_01587 Metal-binding protein ZinT
MVIHLKKLTMLLGMLLVNSPAFAHGHHAHGAPMTEVEQKAAAGVFDDANVRDRALTDWDG
MWQSVYPYLVSGELDPVFRQKAKKDPEKTFEDIKAYYRKGYVTNVETIGIENGVIEFHRD
NNVASCKYNYAGYKILTYASGKKGVRYLFECKDANSKAPKYVQFSDHIIAPRKSAHFHIF
MGNTSQQALLQEMENWPTYYPYQLKANEVVDEMLHH
>EECEBICD_01588 Streptomycin 3''-adenylyltransferase
MTLSIPPSIQCQTEAACRLITRVTGDTLRAIHLYGSAVAGGLKPNSDIDLLVTICQLLTE
AQRATLMQELLALSSPPGASAEKRALEVTVVLYSQLVPWCFPPAREMQFGEWLREDICQG
IYEPAQQDWDIVLLITQILETSIPLKGERAERLFTPAPAAQLLKALRYPLDLWQSTADVQ
GDEYHIVLTLARIWYTLSTGRFTSKDAAADWLLPQLPEDYAATLRAAQREYLGLEQQDWH
ILLPAVVRFVDFAKAHIPTQFT
>EECEBICD_01589 hypothetical protein

MTGSASSVLKTDTRLLSENLFLNYGLESLFKDVAIIAGNVNYIIFDIDDYYTVQQYITST
FKAVLIGIVTHNEYNFIDEHHAIYRIKVDASIAEWRELLILAAAGQFFPRSEKVRLTANE
RNVLAMLVKGMDIRQISCELNVHLKTIYSVRYHVLTKLGCRTVLDYQILSVSKAFTHWLT
INNVDNVISRVNSKVIA
>EECEBICD_01590 HTH-type transcriptional repressor BluR
MATFSISAMAAQCGVTTANIRSWERYGLLQPTKDEAGNRFYCIEDAIRIQHIIDALSKGF
SLKEIKPALYGEETHCRSGWLFYQEEILAQCRKFEPAKLRKLLWRYGREIPPCIVIESIL
RPLRLWLSAGDDDARRFEKALLDTAIIEYATFQLSSVRKAPAGTMLIAAFSLNDPIELWL
ETIKYCSEGMRVEVIPWQAPMPDLLSTSFEHIVLWHDETLTPAQENLIQALKESGKFSLH
VKNGSGLTLLPAVYRQHEMPESMQHVAPQIHANGYEKSGTAR
>EECEBICD_01591 hypothetical protein
MTTITLVNEQNSTGNPFSAHMLCEQRAIQEITYELLQSQQHVSNKDIIAKLIEKLETEKD
VVQLDIYRNALEAVLFQTPDDI
>EECEBICD_01592 Monofunctional chorismate mutase
MIRHIAIFLCSLLMCSTTFADSVTSVSLGALLTALNERMLLMKDVAAYKMKHHLPIEDFT
REQNVFAEAEEEAKNNGLDPHSITPFIRSLMDASKAIQYRYLAQWRTGSEPSFPIQTLSV
TRQRIRQLDNQMLIIISQRLMVGAFSHDDMVWLRAQFNAPNLNESDISNVLAALSLVRRA
R
>EECEBICD_01593 Leucine efflux protein
MFAEYGVLNYWTYLVGAIFIVLVPGPNTLFVLKNSVGRGVKGGYLAACGVFIGDAILMFL
AYAGVATLIKTTPVLFNIVRYLGAFYLLYLGAKILYATLTSKGRAATETVVPFGAIFKRA
LILSLTNPKAILFYVSFFVQFIDVTAPHTGVSFFILATTLEIVSFCYLSFLILSGAFVTH
YIGTKKKLAKVGNSLIGLLFVGFAARLATLQS
>EECEBICD_01594 putative protein YeaR
MRQIPQNHIHTRSTPFWNKETAPAGIFERHLDKGTRPGVYPRLSVMQGAVKYLGYADEHC
SEPEEIMVINAGEFGVFPPEKWHNIEVMTDDTYFNIDFFVAPEVLMEGATSRKVIHAGRE
>EECEBICD_01595 Protein YoaG
MGKATYTVTVTNNSNGVSVDYETEAPMTLLVPEVAAEVVKDLVNTVRSYDTENEHDVCGW
>EECEBICD_01596 hypothetical protein
MKLDTRLTSSALTLALAAVVIPFTADWQLPLLNGVVVRWIENGQALWLLFGALFTAWYIR
PLSRPEGAKQFWLWAVVWWVVLLGRSTSWGRDYFPDEPRMLFRTISVLLIAALVLPVLFS
AGLRKEIVRRLRDAPLPLWLFTVTACSYLISDTVEHHRWLSPIFLHNARYTDLIEELYEV
PFMIGLFMVTVVFMQQDKQDECTALEMTPYHAK
>EECEBICD_01597 hypothetical protein
MGILSWIIFGLIAGILAKWIMPGKDGGGFFMTIILGIVGAVVGGWISTFFGFGKVDGFNF
GSFAVAVIGAIVVLLIYRKIKS
>EECEBICD_01598 hypothetical protein
MKFTWIALPGVLLLSACSSSQPNAPRPPRIGMPNPAAVYCEQQGGTLMPVQTPQGVRSDC
KLPNGEIIDEWALWRREHPDTKK
>EECEBICD_01599 hypothetical protein
MFQLKPGALAIVIGAKTPAGRRNIGKSVELFCLCQPGDEFINPVNGHVTLLPKEAPRALW
LVTGDVASADGQHGFAWVRAEHLMPLSPDRQPGQAAARQSQLS
>EECEBICD_01600 hypothetical protein
MRIQCKRVYFPAEKDDGYRVLVDRLWPRGIKKSALVYDEWNKAITPSTELRKAFHSELID
FNHFAQQYRAELAQQHHEGKRLADIARHQPLTLLYAAKDTRQNHAIVLAEWLREL
>EECEBICD_01601 2-nitroimidazole transporter
MTTALSPRGKQGALLIAGILMIATTLRVTFTGAAPLLETIRSDYGLSTAQTGLLTTLPLL
AFALVSPLAAGIARRFGMERSLFAAMLLICAGIALRSLPSAALLFAGTAIIGCGIALGNV
LLPGLIKRDFSQHVARLTGAYSLTMGAAAALGSALVVPLALHGFGWRGALLMLMLFPLLA
FLIWLPQWRTTRSANLSSSRALHERGIWRSPLARQVTLFLGLNSLIYYVIIGWLPTILIS
HGYSEAQAGSLHGLLQLATAAPGLAIPLILPRFNDQRWIAALVSLLCAVGAAGLWFVPGQ
AIIWTLLFGFGSGATMILGLTFIGLRASSAHQAAALSGMAQSVGYLLAACGPPVMGKLHD
ASGSWYLPLSGVTVLAIIMAILGLYAGRDREIAS
>EECEBICD_01602 HTH-type transcriptional regulator NimR

MIGLRLDGYDPDLHHDAAVAFCIRARDDELFSPRHQHRKGQLILALHGAITCEVENAMWM
VPPQYAVWLPGSLPHSNHVTAGAELCFLFIEPAAVVMPERCCTLKISPLCRELILSLARR
TDPERAQMPTQRLIQVLFDELPQQPQEQLQLPVSGHPKIRQMVETMAQEPARWNTLGQWA
SVFAMSERNLARLVVKETGLSFRRWRHQLQLILALQALIAGRNVQQTAQMLGYDSTTAFI
TMFKKGLGQTPGQYLTGELLLPHN
>EECEBICD_01603 hypothetical protein
MFDVTLLILLGLAALGFISHNTTVAVSILVLIIVRVTPLNTFFPWIEKQGLTVGIIILTI
GVMAPIASGTLPPSTLIHSFVNWKSLVAIAVGVFVSWLGGRGITLMGNQPQLVAGLLVGT
VLGVALFRGVPVGPLIAAGLVSLIVGKQ
>EECEBICD_01604 hypothetical protein
MYDPPFLEALMITASFFAIFIIIVVSVLLLEGEGD
>EECEBICD_01605 hypothetical protein
MAEMTEVAKGVATHQRLVALLTQENARYRVVNHEAVGKCEAVSEIRGTALGQGAKALVCK
VKGNGVNQHVLAILAADQQADLSQLASHLGGLRASLASPAEVDMLTGCVFGAIPPFSFHP
NLKLVADPLLFERFDEIAFNAGLLEKSVIMNTQDYLRIARPELAVFRRFQSSPTA
>EECEBICD_01606 putative diguanylate cyclase DgcJ
MNLHHKALRHFISASVIVLTSSFLIYELIASDRAMNAYMRYIMERADSSFLYDKYQNQSI
AAHLMRTFEAPGDPVTAEKRRAFCDAFEAINGTHGVNLTRHNYPGLHGTLQTAATQCTDN
LDDALLLPAFDQAVSINRSQDDHSHGLGTLELKFRYYVDLNKHYVHFYDLINSRRFAMHR
WTFLQKGTMGINRKDIDKLFTGRTVISSIYMDDITQENVMSFLTPVYLAGTLKGIVMVDV
NQDNLKNIFYTQDRPLVWRYLNVTLKDMDSGKEIIINQSKNNLFQYVNYSHDIPGGLRVS
LSLDLTYFLVSSWKALAFYLLATALLLNMVRMHFRLYRNVTRENISDAMTGLYNRKILTP
ELEQRLQRLVNAGTPVTFVAIDCDRLKLINDTQGHQEGDRIITLLAKAIKTSIRKSDYAI
RLGGDEFCIILVDYAADLAIHLPERIIRNLQIIAPDKTVHFSAGIYNMQPNDTINDAYQA
SDAQLYLNKQQKQHRSS
>EECEBICD_01607 hypothetical protein
MTWFIDRRLNGKNKSTVNRQRFLRRYKAQIKQSISEAINKRSVTDVDSGESVSIPTDDIS
EPMFHQGRGGLRHRVHPGNDHFIQNDRIERPQGGGGGGSGSGQGQASQDGEGQDEFVFQI
SKDEYLDLLFEDLALPNLKKNQHRQLNEYKTHRAGFTSNGVPANISVVRSLQNSLARRTA
MTAGKRRELHALETELETISHSEPAQLLEEERLRREIAELRAKIERVPFIDTFDLRYKNY
EKRPEPSSQAVMFCLMDVSGSMDQATKDMAKRFYILLYLFLSRTYKNVEVVYIRHHTQAK
EVDEHEFFYSQETGGTIVSSALKLMDEVVKERYDPGQWNIYAAQASDGDNWADDSPLCHE
ILAKKLLPVVRYYSYIEITRRAHQTLWREYEHLQATFDNFAMQHIRDQEDIYPVFRELFQ
KQSANQSV
>EECEBICD_01608 hypothetical protein
MNIFDHYRQRYEAAKDEEFTLQDFLTICRQDRSAYANAAERLLMAIGEPNMVDTAQEPRL
SRLFSNRVIARYPAFEEFYGMEDAIEQIVSYLKHAAQGLEEKKQILYLLGPVGGGKSSLA
ERLKSLMQRVPIYVLSANGERSPVNDHPLCLFNPQEDAQILEKEYGIPRRYLGTIMSPWA
AKRLHEFGGDITKFRVVKVWPSILEQIAIAKTEPGDENNQDISALVGKVDIRKLEHHAQN
DPDAYGYSGALCRANQGIMEFVEMFKAPIKVLHPLLTATQEGNYNGTEGISALPFNGIIL
AHSNESEWVTFRNNKNNEAFLDRVYIVKVPYCLRISEEIKIYEKLLNHSELAHAPCAPGT
LETLSRFSILSRLKEPENSSIYSKMRVYDGESLKDTDPKAKSYQEYRDYAGVDEGMNGLS
TRFAFKILSRVFNFDHAEVAANPVHLFYVLEQQIEREQFPQEQAERYLEFLKGYLIPKYA
EFIGKEIQTAYLESYSEYGQNIFDRYVTYADFWIQDQEYRDPDTGQLFDRESLNAELEKI
EKPAGISNPKDFRNEIVNFVLRARANNSGRNPNWTSYEKLRTVIEKKMFSNTEELLPVIS
FNAKTSTDEQKKHDDFVDRMMEKGYTRKQVRLLCEWYLRVRKSS
>EECEBICD_01609 MltA-interacting protein
MTKLKLLALGVFIATSASVAHAESNLTLGAGVGVVEHPYKDYDSDVYPIPVIAYESENFW
FRGLGGGYYLWNDNADKLSIMAYWSPMYFKPGDSDDHQLRRLDRRKSTMMAGVSYAHHTQ
YGFLRTSLAGDTLDNSNGIVWDLAWLYRYTNGGLTLTPGIGVEWNSENQNDYYYGVSRKE
SSRSGLRGYNPNDSWNPYLELSANYNFAGNWSVYGTARYTRLSDEITDSPMVDKSWTGIL
STGVTYRF
>EECEBICD_01610 Anaerobic sulfatase-maturating enzyme
MSIVFNTVAKPSGSLCNLSCKYCFYLDKPRGQRVISDDVLETYIRRVIDDTPSSEVSFCW

QGGEPTLCGLSFYQKVVRLQQRYANGKTIYNSLQTNGVLINEEWAAFFAQHQFLIGISID
GPQVVHDNYRKTPSGRASFSRVVNAIRLLQANDVEFNTLTVVNDASCRHGNAIYHFLTQE
LESKHLQFIPIVEPLAQKAQRSLTLSDNEDSPSLMPFSVTPEGWGAFMCDVFDQWIRHDV
GRIFVQLFDNLLGVWMGEPATLCTMQSTCGQSLLVEQNGDVFSCDHFVFPAYKLGNLQQH
SLEEMAASPFQQQFGAAKANLSSRCQNCTWRFACHGGCPKHRICMDGGERQNYLCKGYLE
FFQHVTPYMNVMRQLLLNQRPAAHITRIVDMIADDVRQ
>EECEBICD_01611 putative oxidoreductase
MKNVTFCGQVTLPAIGQGTWYMGERADQRQREVSALRAGLDLGLRLIDTAEMYADGGAEK
VVGEALAGRRDEAFLVSKVYPWNAGGQKIVAACEESLRRLKTDYLDLYLLHWAGSFSFEE
TVEGMERLIAQGKIRRWGVSNLDYDDMQALWRIPGGQQCTTNQVLYHLASRGIEYDLLPW
CQQQRMPVMAYSPLAQAGRLRSGLLNHPVVNEIAQAHNATAAQILLTWVIAHPGVMAIPK
AGSTEHVKQNAAALEIALSANELALLDNAWPAPKGKTALDMV
>EECEBICD_01612 Putative glucose-6-phosphate 1-epimerase
MINKIFALPVIEQLTPVLSRRQLDDLDLIVVDHPQVKASFALQGAHLLSWKPVGEEEVLW
LSNNTPFKTGVALRGGVPICWPWFGPAAQQGLPSHGFARNLPWALKAHNEDDNGVMLTFE
LQSSEATRKYWPHDFTLLARFKVGKTCEIELEAHGEFATTSALHSYFNVGDIANVKVSGL
GDRFIDKVNDAKEGVLAGGIQTFPDRTDRVYLNPEACSVIHDATLNRTIDVVHHHHLNVV
GWNPGPALSVSMGDMPDDGYKTFVCVETVYATAPQQATEEKPSRLAQTICVAKR
>EECEBICD_01613 Glyceraldehyde-3-phosphate dehydrogenase A
MTIKVGINGFGRIGRIVFRAAQKRSDIEIVAINDLLDAEYMAYMLKYDSTHGRFDGTVEV
KDGHLIVNGKKIRVTAERDPANLKWDEVGVDVVAEATGIFLTDETARKHITAGAKKVVLT
GPSKDNTPMFVKGANFDKYEGQDIVSNASCTTNCLAPLAKVINDNFGIIEGLMTTVHATT
ATQKTVDGPSHKDWRGGRGASQNIIPSSTGAAKAVGKVLPELNGKLTGMAFRVPTPNVSV
VDLTVRLEKAATYEQIKAAVKAAAEGEMKGVLGYTEDDVVSTDFNGEVCTSVFDAKAGIA
LNDNFVKLVSWYDNETGYSNKVLDLIAHISK
>EECEBICD_01614 Peptide methionine sulfoxide reductase MsrB
MANQPSAEELKKKLSEMQFYVTQDRGTEPPFTGRLLHNKRDGVYHCLVCDTPLFHSHTKY
DSGCGWPSFYQPVSEEAIRYIDDFSHGMQRVEIRCGNCDAHLGHVFPDGPQPTGERYCVN
SASLAFSDEKNGDQLKG
>EECEBICD_01615 hypothetical protein
MNLDEIINSMTPEVYQRLSTAVELGKWPDGVALTPEQKENSLQLVMLWQARHNTEAQHMT
IDTHGQMVMKSKQQLKADFGITSKPIATLKMQ
>EECEBICD_01616 D-arabitol-phosphate dehydrogenase
MKALARFGKAFGGYKMIDVPEPVCGPEDVIIEIKAAAICGADMKHYNVDSGSDEFNSVRG
HEFAGRIARVGEKVKDWKVGQRVVSDNSGHVCGVCPACEQGDFLCCTEKVNLGLDNNTWG
GGFSKYCLVPGEILKIHRHALWEIPEGVDYEEAAVLDPICNAYKSIAQQSKFLPGQDVVV
IGTGPLGLFSVQMARIMGAVNIVVVGLDEDVPVRFPVAKELGATAFVNGSTEDVVARCRE
ICGKDNLGLVIECSGANIALKQAIDMLRPNGEVVRVGMGFKPLDFSINDITSWNKSIIGH
MAYDSTSWRNAIRLLASGAIKVKPMITHRIGLSQWREGFDAMVDKTAIKVIMTYDFDE
>EECEBICD_01617 Inner membrane metabolite transport protein YdjE
MEQITKPHCGARLDRLPDCRWHSSMFAVVAFGLLVCWSNAVGGLILAQLKELGWTDNSTT
ATFSAITTAGMFLGALVGGIIGDKTGRRNAFILYEAIHIASMVVGAFSPNMMFLIACRFV
MGVGLGALLVTLFAGFTEYMPGRNRGTWSSRVSFIGNWSYPLCSLIAMGLTPLISAEWNW
RVQLLIPAVLSLIATALAWRYFPESPRWLESRGRYQEAEKVMQAIEAGIERQTGKPLPPV
VIENSGKPPRSVPYSALLTGVLLKRVILGSCVLIAMNVVQYTLINWLPTIFMTQGINLKD
SIVLNTMSMFGAPFGIFIAMLVMDKIPRKTMGVGLLILIAVLGYIYSLQTSMLLITLIGF
FLITFVYMYVCYASAVYVPEIWPTEAKLRGSGLANAVGRISGIAAPYAVAVLLNGYGVTG
VFVLLGAVSIIVAVAIATIGIETKGVSVESLSIDKVTSK
>EECEBICD_01618 putative zinc-type alcohol dehydrogenase-like protein
YdjJ
MKNSKAILKTPGTMTIIAADIPVPKENEVLIKVEYVGICGSDVHGFESGPFIPPKDPNQE
IGLGHECAGTVVAVGNRVSKFKPGDRVNIEPGVPCGHCRYCLEGKYNICPDVDFMATQPN
YRGALTHYLCHPESFTYKLPDNMDTMEGALVEPAAVGMHAAMLADVKPGKKIVILGAGCI
GLMTLQACKCLGATNIAVVDVLEKRLAMAERLGATTVINGAKEDTVALCQQFTDDMGADI

VFETAGSAVTTQQAPYLVMRGGKIMIVGTVAGDSAINFLKINREVSIQTVFRYANRYPVT
IDAISSGRFDVKSMVTHIYDYKDVQRAFEESVNNKRDIIKGVIKVCD
>EECEBICD_01619 putative protein YdjI
MLADIKYWENDAQNKHYAIAHFNVWNAEMLMGVIDAAEEANSPVIISFGTGFVGNTSFED
FSHMMVSMAKKATVPVITHWDHGRSMDIIHNAWTHGMNSLMRDASSFDFEENIRLTKEAV
DFFHPLGIPVEAELGHVGNETVYEEALAGYHYTDPDQAAEFVARTGCDSLAVAIGNQHGV
YTSKPKLNFEVVERVRQAVSVPLVLHGASGISDADIKKAISLGISKINIHTELCQAAMVA
VKENQDQPFLHLEREVRKAVKARALEKIKLFGSDGKAE
>EECEBICD_01620 putative sugar kinase YdjH
MDEIEVLCIGAAIVDIPLQPVSKNIFDVDSYPLERIAMTTGGDAINEATIISRLGHRTAL
MSRVGDDAAGYFILDHCRKENIDIQSLKQDADIDTSINVGLVTDDGERTFVTNRNGSLWK
LNINDVDFDRFSQVRLLSLASIFNSPLLDGKALTAIFTQAKAHQLVICADMIKPRLNETL
DDIREALSFVDYLFPNIDEAKKLTGKETLDDIADCFLQCGVKTVVIKTGKKGCFIKSADI
KMEVPAVSGITAIDTIGAGDNFASGFIAALLEGKTLRECARFANATAAISVLSVGATTGV
KNRMLVEQLLNEYEG
>EECEBICD_01621 NADH-specific methylglyoxal reductase
MKMIPLGNTDIALSRMGLGTWAIGGGPAWNGNLDMQVCIDTIVEAHRCGINLIDTAPGYN
FGNSEVIVGQALKKITA
>EECEBICD_01622 NADH-specific methylglyoxal reductase
MVVETKCGIVWEREGSLFNKVGDRQLYKNLSPDSIRKEVDASLQRLGIDYIDIYMTHWQS
VPPFFTPIAETVGVLNALKAEGKIRSIGAANVDVSHIREYLKHGELDIIQAKYSILDRAL
ETELLPLCREHGIVVQVYSPLEQGLLTGTITRDYVPGGARANKLWFQRENMLRVIDMLEQ
WQPLCDKYHCAVPTLALAWILRQSELISILSGATSPEQVRENIAALSITLTDDDALLMRQ
MAEALDNP
>EECEBICD_01623 putative HTH-type transcriptional regulator YdjF
MAAKDRIQAIKQMVANDKKVTVSNLSSIFQVTEETIRRDLEKLEDEGFLTRTYGGAVLNS
AVLADNIHFYKRAKSFYEEKQIIARNTLPFIKNKTTMAADSSSTVMELLKLLKDRNDLTL
LTNSAEAFHELAQSDINLVSTGGELNKNTLSLQGRITKEIISRYHVDIMVMSCKGLDMAS
GALDSNEAEAEIKKTMIRQATEVALLVDHSKFDRKAFVRLVDFSHIHYLITNKTPGDEWI
AFCDKNNIQLVY
>EECEBICD_01624 Inner membrane metabolite transport protein YdjE
MEDNDHIGARLDRLPLSRFHFRIFGIISFGLLLTGFLSYSGNVVLAKLINNGWSNNYLNA
AFTSALMLGYFIGSLTGGFIGDYLGRRKAFRINLLLVGISATAAAFVPNMYWLIFFRCLM
GTGMGALIMVGYASFTEFIPPIVRGKWSARLSFVGNWSPMLSAGIGVVVIAFLSWRMMFL
LGGIGMLLAWSLSGKYFIESPRWLAGKGRQREAEQQLLMIESQIEKEKSIVLPPHQESNK
STESRFENGTFWLLFKGQMLRRTLVAITVLIAMNISLYTITVWIPTIFVNSGIDVTKSIF
MTAIIMIGAPVGIFIAALIIDHFPRRLFGSLLLIVIAVVGYFYSIQTEEWAILVYGLVMI
SFLYMYVCFASAVYVPELWPTHLRLRGSGFVNAVGRIVAVFTPYGVAVLLTRYGSVTVFV
VLGMLLAACALILFCFGIETRRVSLEALSSLR
>EECEBICD_01625 Nicotinamidase
MTNRALLLVDLQNDFCAGGALAVAEGDSTIDIANALIDWCQPRQIPVLASQDWHPAQHGS
FASQHQAEPYSQGELDGLPQTLWPDHCVQHTDGAALHPLLNQHAIDACIYKGENPLIDSY
SAFFDNEHRQKTTLDTWLREHDVTELIVMGLATDYCVKFTVLDALELGYAVNVITDGCRG
VNIHPQDSAHAFMEMASAGATLYTLADWEETQGQAVAR
>EECEBICD_01626 L-asparaginase 1
MAASAHSRHPIIINTSFATRLNIIMQKKSIYVAYTGGTIGMQRSDKGYIPVSGHLQRQLA
LMPEFHRPEMPDFTIHEYAPLMDSSDMTPEDWQHIAADIKAHYDEYDGFVILHGTDTMAY
TASALSFMLENLGKPVIVTGSQIPLAELRSDGQINLLNALYVAANYPVNEVTLFFNNRLF
RGNRTTKAHADGFDAFASPNLPPLLEAGIHIRRLNTPPAPYGSGELIVHPITPQPIGVVT
IYPGISAEVVRNFLRQPVKALILRSYGVGNAPQNKAFLQELKEASSRGIVVVNLTQCMSG
RVNMGGYATGNALAHAGVIGGADMTVEATLTKLHYLLSQGLDTQAIRSAMAQNLRGELTP
DD
>EECEBICD_01627 Protease 4
MRTLWRFIAGFFKWTWRVLNFVREMVLNLFFIFLVLVGVGIWMQIGNGSNSEQTARGALL

LDISGVIVDKPSTNHRLGALGRQLFGASSDRLQENSLFDIVNAIRQAKDDRNITGIVLDL
KNFTGADQPSMRYIGKALREFRDSGKPVFAVGENYSQGQYYLASFANKIWLSPQGQVDLH
GFATNGLYYKTLLDKLKVSTHVFRVGTYKSAVEPFIRDDMSPAAREADSRWIGELWQNYL
HTVSANRQISPQQLFPGAQAIIDGLTSVGGDTAKYALDHKLVDALASSADVEKALTKQFG
WSKTENNYRAISYYDYSLKTPADTGGTIAVIFANGAIMDGEETPGNVGGDTTASQIRDAR
LDPKVKAIVLRVNSPGGSVNASEVIRAELAAARAAGKPVVVSMGGMAASGGYWISTPANY
IVASPSTLTGSIGIFGVINTVENSLSSIGVHSDGVSTSPLADISMTKALSPEVQQMMQLS
IEYGYKRFITLVADARKRTPEQIDKIAQGHVWTGEDAKANGLVDSLGDFDDAVAKAAELA
KLKQWHLDYYQDEPTVLDMVMDSMTGSVRAMLPEAIQAMLPAPLVSAANTVKAEGDKLAA
FNDPQNRYAFCLTCANVR
>EECEBICD_01628 Putative NAD(P)H nitroreductase YdjA
MDALELLVNRRSASRLAEPAPVGEQLQNILRAGMRVPDHKSLQPWRFFVIEGEGRDRFSA
VLERGAVAAGGDEKAIEKARNSPFRAPLIITVVAKCEENHKVPVWEQEMSAGCAVMAMQM
AAIAQGFNGIWRSGALTESAIVREAFECRPQDKIVGFLYLGTPQLKASTTISTPDPTPFV
RYF
>EECEBICD_01629 Selenide, water dikinase
MSEQAIRLTQYSHGAGCGCKISPKVLETILHSEQAKFVDPNLLVGNETRDDAAVYDLGNG
TSIISTTDFFMPIVDNPFDFGRIAATNAISDIFAMGGKPIMAIAILGWPINTLSPDIARE
VTEGGAFCLPSGRYRAGGRTLY
>EECEBICD_01630 Selenide, water dikinase
MPTERVKKNSTAQAGCKLFLTKPLGIGVLTTAEKKSLLKPEHQGLATEVMCRMNVAGAAF
ANIDGVKAMTDVTGFGLLGHLSEMCQGAGVQALLCYQDIPKLPGVEEYIALGAVPGGTER
NFASYGHLMGDMSREVRSLLCDPQTSGGLLLAVTPDAEDDVKATAAEFGIDLTAIGELVE
ARGGRAMVEIR
>EECEBICD_01631 DNA topoisomerase 3
MRLFIAEKPSLGRAIADVLPKPHRKGDGFIECGNGQVVTWCIGHLLEQAQPDAYDSRYAR
WNLADLPIVPEKWQLQPRPSVTKQLNVIKRFLHQAGEIIHAGDPDREGQLLVDEVLDYLQ
LPAEKRQQVRRCLINDLNPQAVERAIDRLRANSDFVPLCVSALARARADWLYGINMTRAY
TILGRNAGYQGVLSVGRVQTPVLGLVVRRDEEIENFVAKDFFEVKAHIVTPADERFTAIW
QPSEACEPYQDEEGRLLHRPLAEHVVNRINGQPALVTSYNDKRESESAPLPFSLSTLQIE
AAKRFGLSAQNVLDICQKLYETHKLITYPRSDCRYLPEEHFAGRQAVMNAISVHAPDLLP
QSVVNPDTRNRCWDDKKVDAHHAIIPTARSSSVHLTENEAKVYTLIARQYLMQFCPDAVF
RKCVIELEIAKGKFVAKARFLAEAGWRTLLGSKERDEENDGTPLPVVAKGDELLCEKGEV
VERQTQPPRHFTDATLLSAMTGIARFVQDKDLKKILRATDGLGTEATRAGIIELLFKRSF
LTKKGRYIHSTDAGKALIHSLPEMAARPDMTAHWESVLTQISEKQCRYQDFMQPLVGTLY
QLIDQAKRTPVKRFRGIVAPGGGEKKKSAPRKRAGKKSPPAEETGRQTE
>EECEBICD_01632 NADP-specific glutamate dehydrogenase
MDQTCSLESFLNHVQKRDPHQTEFAQAVREVMTTLWPFLEQNPRYRHMSLLERLVEPERV
IQFRVVWLDDKNQVQVNRAWRVQFNSAIGPYKGGMRFHPSVNLSILKFLGFEQTFKNALT
TLPMGGGKGGSDFDPKGKSEGEVMRFCQALMTELYRHLGPDTDVPAGDIGVGGREVGFMA
GMMRKLSNNSACVFTGKGLSFGGSLIRPEATGYGLVYFTEAMLKRHGLGFEGMRVAVSGS
GNVAQYAIEKAMAFGARVVTASDSSGTVVDESGFTPEKLARLCEIKASRDGRVADYAREF
GLTYLEGQQPWSVPVDIALPCATQNELDVDAARVLIANGVKAVAEGANMPTTIEATDLFL
EAGVLFAPGKAANAGGVATSGLEMAQNAARLSWKAEKVDARLHHIMLDIHHACVEYGGDN
KHTNYVQGANIAGFVKVADAMLAQGVI
>EECEBICD_01633 hypothetical protein
MCRSLVVAVMAIMPMVGLANQNSRPDIQVNVPPEVFSTRGQSSQPCIQCCVYQDQNYSEG
AVIKVEGVVLQCQRDEKTISTNPLVWRRVKP
>EECEBICD_01634 CTP pyrophosphohydrolase
MIKKLDVVAAIIECDGKILLAQRPAHADQAGLWEFAGGKVEPGETQPQALIRELREELGI
DATPGVYIASHQRDVSGRRIHLHAWHVPAFNGLIRALEHQALAWCTPEEALEYPLAPADI
PLLQAFMALRDARLTDSC
>EECEBICD_01635 Exodeoxyribonuclease III
MSGSHPVKFLLPSQHDSPCKVAVTMKFVSFNINGLRARPHQLEAIVEKHQPDVIGLQETK

VHDEMFPLEEVAKLGYNVFYHGQKGHYGVALLTKATPISVRRGFPDDGEEAQRRIIMAEI
PSPLGNITVINGYFPQGENRDHPLKFPAKAQFYQNLQNYLETELKCDNPVLIMGDMNISP
TDLDIGIGEENRKRWLRTGKCSFLPEEREWMSRLLKWGLVDTFRQANPQTMDKFSWFDYR
SKGFVDNRGLRIDLLLASAPLAERCAETGIDYDIRSMEKPSDHAPVWATFRV
>EECEBICD_01636 Succinylornithine transaminase
MSLSVTRENFDEWMVPVYVPAPFIPVRGEGSRLWDQQGKEYIDFAGGIAVNALGHAHPAL
REALNEQANRFWHTGNGYTNEPALRLAKKLIDATFAERVFFCNSGAEANEAALKLARKYA
HDRVGNHKSGIVAFKNAFHGRTLFTVSAGGQPTYSQDFAPLPPDIRHAAYNDLNSASALI
DDNTCAVIVEPVQGEGGVIPATKAFLQGLRELCDRHQALLIFDEVQTGVGRTGELYAYMH
YGVTPDILTTAKALGGGFPIGAMLTTQDYASVMTPGTHGTTYGGNPLATAVAGKVLDIIN
TPEMQNGVRQRHDAFIERLNTINARFGMFSEIRGLGLLLGCVLQTEFAGKAKLIAQEAAK
AGVMVLIAGGDVVRFAPALNVSDEEIATGLDRFALACERLQTGGASCG
>EECEBICD_01637 Arginine N-succinyltransferase
MRVIRPVEHADIAALMQLAGKTGGGLTSLPANEATLAARIERALKTWSGELPKGEQGYVF
VLEDSETGEVGGICAIEVAVGLNDPWYNYRVGTLVHASKELNVYNALPTLFLSNDHTGSS
ELCTLFLDPEWRKEGNGYLLSKSRFMFMAAFRDKFNEKVVAEMRGVIDEHGYSPFWQSLG
KRFFSMDFSRADFLCGTGQKAFIAELMPKHPIYTHFLSEEAQAVIGEVHPQTAPARAVLE
KEGFRYRHYIDIFDGGPTLECDIDRVRAIRKSRLVEVAEGQPAPGDYPACLVANENYHHF
RAALVRADPQTSRLVLTAAQLDALKCRAGDHVRLVRLCAEEKTV
>EECEBICD_01638 N-succinylglutamate 5-semialdehyde dehydrogenase
MTLWINGDWITGQGERRRKTNPVSAEILWQGNDANAAQVAEACQVARAAFPRWARQPFAA
RQAIVEKFAALLEAHKAELTEVIARETGKPRWEAATEVTAMINKIAISIKAYHARTGEQK
SELVDGAATLRHRPHGVLAVFGPYNFPGHLPNGHIVPALLAGNTLIFKPSELTPWTGETV
IKLWERAGLPAGVLNLVQGGRETGQALSSLDDLDGLLFTGSASTGYQLHRQLSGQPEKIL
ALEMGGNNPLIIEDVANTDAAVHLTLQSAFITAGQRCTCARRLLVKQGAQGDAFLARLVD
VAGRLQPGRWDDDPQPFIGGLISAQAAQHVMEAWRQREALGGRTLLAPRKVKEGTSLLTP
GIIELTGVADVPDEEVFGPLLNVWRYAHFDEAIRLANNTRFGLSCGLVSTDRAQFEQLLL
EARAGIVNWNKPLTGAASTAPFGGIGASGNHRPSAWYAADYCAWPMASLESPELTLPATL
SPGLDFSRREAV
>EECEBICD_01639 N-succinylarginine dihydrolase
MTAHEVNFDGLVGLTHHYAGLSFGNEASTRHRFQVSNPRLAVKQGLLKMKALADAGFLQA
VIPPHERPFIPALRQLGFTGSDEQILDKVARQAPRWLSSVSSASPMWVANAATVCPSADA
LDGKVHLTVANLNNKFHRALEAPVTEALLRAIFRDENQFSVHSALPQVALLGDEGAANHN
RLGGEYGSAGVQLFVYGREEENEIRPARYPARQSREASEAVARLNQVNPQQVIFAQQNPE
VIDQGVFHNDVIAVSNRQVLFCHEAAFARQKVLINQLRTRVDGFMAIEVPAEEVSVSDAV
ATYLFNSQLLSRDDGSMLLVLPRECQDHAGVWRYLNKLVAEDNPISAIQVFDLRESMANG
GGPACLRLRVVLTEEERRAVNPAVMMNDALFTALNAWADRYYRDRLTAADLADPLLLREG
REALDVLTRLLDLGSVYPFQQTGAADG
>EECEBICD_01640 Succinylglutamate desuccinylase
MDNFLALTLSGTTPRVRQGKGAGFRWRWLSHGLLELTPDAPVDRALILSAGIHGNETAPV
EMLDKLLSALYSGSLTLTWRVLVVLGNPQALAAGIRYCHSDMNRMFGGRWQSFAESDETR
RARELELSLDTFFSSGQARVRWHLDLHTAIRGSHHLRFGVLPQRDRPWETDFLAWLGAAG
LEALVFHQAPGGTFTHFSSEHFGALSCTLELGKALPFRQNDLTQFNVTSQALSALLSGVE
TSTSFSPPLRYRVVSQITRHSDKFALYMDAQTLNFTAFAKGTLLAEEGDKRVTVTHDVEY
VLFPNPSVACGLRAGLMLERLP
>EECEBICD_01641 hypothetical protein
MIWDTVSCYYGLFLENCFINKRCCVIVLHNFCYIMLLYISLSTLRNLFFFSSQLAKKCFL
YFHCFTVVMTN
>EECEBICD_01642 Periplasmic chaperone Spy
MRKLTALFVASTLAMGAANLAHAAETTTAAPADAKPMMQHKGKFGPHHDMMFKNLNLTDA
QKQQIRDIIKAQREQMKRPLLEERRAMHDIIASDTFDKAKAEAQITKMEAQRKANMLAHM
ETQNKIYNVLTPEQKKQYNANFEKRLTERPAQEGKMPAAAE
>EECEBICD_01643 Excinuclease cho
MVRRQSAPRLEFEAAAIYEYPEHLRPFLSEAPALPGVYVFHSESDTLPLYIGKSVNIRSR

VLSHLRTPDEAAMLRQARRISWICTAGEMGALLLEARLIKEQQPLFNKRLRRNRQLCSLQ
LSEQKIEVVSARSVDFSHEPNLFGLFANRRAALQSLQNLADEQKLCYGLLGLEPVSRGRA
CFRFALKRCAGACCGQETPQAHFLRLQASLERLRVVCWPWKGAIALKESRPQMTQFHIIN
NWLWLGAVPSLDEAATLVRTPAGFDQDGYKILCKPLMSGQYEIIELHTDCRQS
>EECEBICD_01644 NH(3)-dependent NAD(+) synthetase
MTLQQEIIQALGAKPHINPEEEIRRSVDFLKAYLETYPFLKSLVLGISGGQDSTLAGKLS
QMAIAELREETGDNALQFIAVRLPYGVQADEQDCQDAIAFIQPDRVLTVNIKGAVLASEQ
ALREAGIELSDFVRGNEKARERMKAQYSIAGMTHGVVVGTDHAAEAITGFFTKYGDGGTD
INPLHRLNKRQGKQLLAALGCPEHLYKKVPTADLEDDRPSLPDEAALGVTYDNIDDYLEG
KTLDPAIAKTIEGWYVKTEHKRRLPITVFDDFWKR
>EECEBICD_01645 Osmotically-inducible putative lipoprotein OsmE
MNKNVAGILSAAAVMTMLAGCTAYDRTKDQFVEPVVKDVKKGMSRAQVAQIAGKPSSEVS
MIHARGTCQTYILGQRDGKAETYFVALDDTGHVINSGYQTCAEYDTDPQAPKQ
>EECEBICD_01646 PTS system N,N'-diacetylchitobiose-specific EIIB
component
MEKKHIYLFCSAGMSTSLLVSKMRAQAEKYEVPVIIEAFPETLAGEKGPAADVVLLGPQI
AYMLPEIQRLLPGKPVEVIDSMLYGKVDGLGVLKAAVAAIKKAAAN
>EECEBICD_01647 PTS system N,N'-diacetylchitobiose-specific EIIC
component
MSNVIASLEKVLLPFAVKIGKQPHVNAIKNGFIRLMPLTLAGAMFVLINNVFLSFGEGSF
FYSMGIRLDASTIETLNGLKGIGGNVYNGTLGIMSLMAPFFIGMALAEERKVDALAAGLL
SVAAFMTVTPYSVGEAYAVGANWLGGANIISGIIIGLVVAEMFTFIVRRNWVIKLPDSVP
ASVSRSFSALIPGFIILSIMGIIAWALSNYGSNFHQIIMDTISTPLASLGSVVGWAYVIF
VPLLWFFGIHGSLALTALDSGIMTPWALENISIYQQYGSVDAALEAGKTFHIWAKPMLDS
YIFLGGSGATLGLIIAIFLASRRADYRQVAKLALPSGIFQINEPILFGLPIIMNPVMFIP
FILVQPILAAITLVAYYLGIIPPITNIAPWTMPTGLGAFFNTNGSVAALLVALFNLAVAT
LIYLPFVVVANKAQNAIEQEESEEDIANALKF
>EECEBICD_01648 PTS system N,N'-diacetylchitobiose-specific EIIA
component
MFDLENVVDNQTAAEELEEVVMGLIINSGQARSLAYGALRKAKEGDFEAAKAMMDQSRIA
LNEAHLVQTKLIEGDQGEGKMKVSLVLVHAQDHLMTSMLARELIAELIELHEKLK
>EECEBICD_01649 HTH-type transcriptional regulator ChbR
MQLQVNATEIKTVYEQQLFNGKNFHVFIYNKTESVTGLHQHDYYEFTLVLTGRYYQEING
KRVLLERGDFVFIPVGSNHQSFYEFGATRILNVGISKRFFEQHYLPLLPFCFVASQVYRA
NSTFLTYIETVIASLNFRGNGLDEFIEVVTFYIINRLRHYREEQVYDDIPQWLKATVEIM
HDKTQFGEHALENMVRLSAKSQEYLTRATRRYYSKTPMQIINEIRINFAKKQLEMTNYSV
TDIAYEAGYSSPSLFIKTFKKMTSFTPNSYRKRLTEINE
>EECEBICD_01650 6-phospho-beta-glucosidase
MSQKLKVVTIGGGSSYTPELLEGFIKRYHELPVTELWLVDVEDGKEKLGIIYDLCQRMID
KAGVPLKLYKTLDRREALKDANFVTTQLRVGQLKARELDERIPLSHGYLGQETNGAGGLF
KGLRTIPVIFDIIKDVEELCPNAWVINFTNPAGMVTEAVYRHTNFKKFIGVCNIPVGMKM
FIHDVLALNETDDLSIDLFGLNHMVFIKDVLVNGTSRFAELLDGVASGQLKASTVKNIFD
LPFSEGLIRSLNMLPCSYLLYYFKQKEMLAIEMGEYYKGGARAQVVQKVEKQLFDLYKNP
ELNVKPKELEQRGGAYYSDAACEVINAIYNDKQTEHYVNIPHHGHVENIPADWAVEMTCI
LGRNGATPHPRITRFDEKVLGLIHTIKGFEVAASNAALSGNFNDVLLALNLSPLVHSDRD
AEVLARELILAHEKWLPNFAACIEALKGKHH
>EECEBICD_01651 Chitooligosaccharide deacetylase ChbG
MERVLIVNADDFGLSKGQNYGIVEAYRNGVVTSTTALVNGEAIDHAAQLSRELPALGVGM
HFVLTLGKPVSEMPGLTRDGLLGKWIWQMAEEDTLPLDEIAHELACQYQRFIDVFGREPT
HLDSHHHVHMFPQIFPIVAHFAAQRGIALRIDRQTVLNADDLPSDLRSTQGFSSEFYGEE
ITEACFLRILDASAHRGEASLEVMCHPAFVDNIIRQSAYCYPRLTELEVLTSASLKAAIA
ERGYRPGCFLDI
>EECEBICD_01652 Catalase HPII
MSHNEKSPHQSPVHDTRESQPGLDSLAPSDGSHRPTPEPTPPGAQPTAPGSLKAPETAND

KLTALDAFRKGSENYALTTNQGVRIADDQNSLRAGSRGPTLLEDFILREKITHFDHERIP
ERIVHARGSAAHGYFQSYKDLSDITKAAFLCDPQKITPVFVRFSTVQGGAGSADTVRDIR
GFATKFYTEEGIFDLVGNNTPIFFIQDAHKFPDFVHAVKPEPHWAIPQGQSAHDTFWDYV
SLQPETLHNVMWAMSDRGIPRSYRTMEGFGIHTFRLINAQGKATFVRFHWKPLAGKASLV
WDESQKLTGRDPDFHRRDLWEAIEAGDFPEYELGLQLIAEEDEFKFDFDLLDPTKLIPEE
LVPVQRVGKMVLNRNPDNFFAENEQAAFHPGHIVPGIDFTNDPLLQGRLFSYTDTQISRL
GGPNFHEIPINRPTCPYHNFQRDGMHRMDIDTNPANYEPNSINDNWPRETPPAPKRGGFE
SYQERVDGNKIRERSPSFGEYYSHPRLFWLSQTPIEQQHIIDAFSFELGKVARAYIRERV
VDQLAHIDVTLAQGVAHNLGFALTHEQTQIAPPPDVNGLKKDPALSLYAVPDGDVKGRVV
AILLNDKVNAAELLTILQALKAKGVHAKLLYSRMGEVTADDGSTLTIAATFAGAPSLTVD
AVIVPCGNIADIESCGDARYYLLEAYKHLKPIALAGDARRFKALLNIDSQGEEGLVEADN
VDHHFMDTLLTLMAAHRVWSRAGKINAIPA
>EECEBICD_01653 Cell division activator CedA
MMKPLRQQNRQIISYIPRVEPAPPEHAIKMDTFRDVWILRGKYVAFVLTGESFQRSPAFS
VPESAQRWANQVRQENEIAD
>EECEBICD_01654 L-cystine uptake protein TcyP
MNFPLIANIAVFVILLFVLAQARHKQWSLAKKVLVGLVMGVVFGLALHTIYGADSQVLKD
SIQWFNIVGNGYVQLLQMIVMPLVFASILSAVARLHNASQLGKISFLTIGTLLFTTLIAA
LVGVLVTNLFGLTAEGLVQGGAETARLNAIETSYVGKVADLSVPQLVLSFVPKNPFADLT
GANPTSIISVVIFAAFLGVAALKLLKDDAPKGERVLVAIDTLQSWVMKLVRLVMQLTPYG
VLALMTKVVAGSNLQDIIKLGSFVVASYLGLAIMFVVHGILLGVNGISPLKYFRKVWPVL
TFAFTSRSSAASIPLNVEAQTRRLGVPESIASFAASFGATIGQNGCAGLYPAMLAVMVAP
TVGINPLDPVWIATLVGIVTVSSAGVAGVGGGATFAALIVLPAMGLPVTLVALLISVEPL
IDMGRTALNVSGSMTAGTLTSQWLKQTDKTILDSEEDAELAHR
>EECEBICD_01655 Inner membrane protein YdjM
MGFIMTAEGHLLFSIASAVFAKNAELTPVLAHGDWWHIIPSAILTCLLPDIDHPKSFLGQ
RLKWVSKPIARAFGHRGFTHSLLAVFALLATFYLKVPDTWIIPADALQGMVLGYLSHIVA
DMLTPAGVPLLWPCRWRFRLPILAPRKGNQLERFLCMALFAWAVWMPQSMPENNVIRWSS
QMITILQMQFNRFINHQID
>EECEBICD_01656 Hexitol phosphatase B
MSTPRQILAAIFDMDGLLIDSEPLWDRAELDVMASLGVDITRRHELPDTLGLRIDMVVDL
WFAQQPWNGPDRQEVTNRVIARAITLIEETRPLLPGVREAVALCKAQGLLVGLASASPLH
MLEKVLTMFELRDSFDALASAEKLPYSKPHPQVYLDCAAKLGVDPLTCVALEDSVNGLIA
AKAARMRAIVVPAEENQPDPRFALANVKLNSLTELTAAHLLG
>EECEBICD_01657 hypothetical protein
MTYQQAGRIAILKRVVGWVIFIPALLSTLISVLKFMYAHSEKQEGINAVLLDFTHVMIDM
MRVNTPFLNVFWYNSPTPNFQGSLNIGFWLIFILIFVGLAMQDSGARMSRQSRFLREGVE
DQLILEKAKGAEGLTREQIESRIVVPHHTIFLQFFPLYILPVIIIVLGYFFFSLLGFM
>EECEBICD_01658 putative ketoamine kinase
MWQAISRLLSEQVGEGEIELRNELPGGEVHAAWHLRYSGHDFFVKCDEREMLRGFTAEAD
QLELLSRSKTVVVPKVWAVGSDRDYSFLVMDYLSPRPLDAHNAFILGQQLARLHQWSDQP
QFGLDFDNALSTTPQPNTWQRRWSTFFAEQRIGWQLELAAEKGITFGNIDAIVEHVQQRL
ASHQPQPSLLHGDLWSANCALGPDGPYIFDPACYWGDRECDLAMLPLHTDQPPQIYDGYQ
SVSPLPLDFLDRQPIYQLYTLLNRARLFGGQHLATAQKAMDRLLAV
>EECEBICD_01659 hypothetical protein
MASGDITRYVITVTFHEDSLTEINELNNHLTRSGFLLTLTDDEGNVHELGTNTFGFVSAQ
SADEIKALVAGLAKSALDKDVDITVATWEAWSKNAQ
>EECEBICD_01660 ATP-dependent 6-phosphofructokinase isozyme 2
MVRIYTLTLAPSLDSATITPQIYPEGKLRCSAPVFEPGGGGINVARAIAHLGGTATAIFP
AGGATGEHLVALLADENVPVSTVDAKDWTRQNLHVHVESSGEQYRFVMPGATLDDDEFRQ
LEEQVLEIESGAILVISGSLPPGVKVEKLTQLISAAQKQGIRCIIDSSGDALTAALALGD
IELVKPNLKELSALVNRDLTQPDDVRKAAQELVQSGKARRVVVSLGPQGALGIDSENCIQ
VVPPPVKSQSTVGAGDSMVGAMTLKLAQDASLEEMVRFGVAAGSAATLNQGTRLCSRDDT
QKIYAYLSAQ

>EECEBICD_01661 hypothetical protein
MKLLKAVPAVVMLAGGVFASLYAAADDSVFTVMDDPSTAKKPFEGNLNAGYLAQSGNTKS
SSLTADTAMTWYGQRTAWSLWGNASNTSSNDERSSEKYAVGARNRFNMTDYDYTFGQASW
LTDRFNGYRQRDVLTAGYGRQFLNGPVHSFRFEFGPGVRYDEHTDDTTETQPLGYASGSY
AWQLTDNAKFTQGVSVFGAEDTTLNSETALNVAINEHFGLKVGYNLTWNSQPPESAPEHT
DRRTTVTLGYKM
>EECEBICD_01662 hypothetical protein
MNKYSYCATMIAAILSTTTMANASSLAISVANDDAGIFQPSLNALYGHPAADRGDYTAGL
FLGYSHDLTDASQLSFHIAQDIYSPSGANKRKPEAVKGDRAFSAFLHTGLEWNSLATNWL
RYRLGTDIGVIGPDAGGQEVQNRAHRIIGAEKYPAWQDQIENRYGYTAKGMVSLTPAIDI
LGVNVGFYPEVSAVGGNLFQYLGYGATVALGNDKTFNSDNGFGLLSRRGLIHTQKEGLIY
KVFAGVERREVDKNYTLQGKTLQTKMETVDINKTVDEYRVGATIGYSPVAFSLSLNKVTS
EFRTGDDYSYINGDITFFF
>EECEBICD_01663 hypothetical protein
MLKNGVKKHAPDIIEYPFFIRYLSARNYWLHDYAYNTMFFGINMNITLYSFELIFYDVFD
VYLLLIFTVIVLSLMMSASNGW
>EECEBICD_01664 Nuclease
MNKTINLLKLLPVVLLSACTTSYPPQDIISAPELPHRNVLVQQPDNCSVGCPQGGSQQTI
YRHVYTLNNNSATKFANWVAYSVTKTSQASGRPRNWAQDPDLPPSDTLAPSAYKNAHTLL
KVDRGHQAPLAGLGGVSDWPSLNYLSNITPQKSALNQGAWAALENRVRELAKQADVSVVH
VVTGPLFERHIATLPEDATVEIPSGYWKVLFTGTAPSKSEGNYAAFIMDQNTPRSANFCD
YQVTVEAIEHKTKPVLTLWSALPEAVASEVKTTKGSLVQKLGCR
>EECEBICD_01665 hypothetical protein
MLIISYIVLCLLFIVYLYTLSVRIEGKIINVMVPYLIITVPTLYVFEGIFVYLSEVRKYT
VEYLFFYTCYITYIASFVISYLYTQRKPIYNKSNTKNKPRYVFTSLLFTFLAFIIYLPVL
MEFREYILSPRRIYELTRTGYGIYFYPSLMFSLVASICAFFTYKKSKLFCISIVLFNCIL
IFLHGNKGPIFSIFIAFILYLSYIENKKIKFMFLVKSFAVIAVIVTAFFAYTFTDGNPIE
NMANYSDYTRNAVLVASSNFDFMYGKLLMESEVYSRIPRAIWPDKPEDFGALYLAKVFFP
DAFYRNQGAPAFGYGELYADFGLFTPVWLVISGVFKGVLAKYFSNKTQETKSAHYFIMFL
FCIGISVIPVSMGWLFPEHLMIAFIVYIASSFVFSAHIRFVLLRSDK
>EECEBICD_01666 Threonine--tRNA ligase
MPVITLPDGSQRHYDHPVSPMDVALDIGPGLAKATIAGRVNGELVDASDLIENDATLAII
TAKDEEGLEIIRHSCAHLLGHAIKQLWPHTKMAIGPVVDNGFYYDVDLDRTLTQEDVEAL
EKRMHELAEKNYDVIKKKVSWHDARETFVKRGETYKVAILDENIAHDDKPGLYHHEEYVD
MCRGPHVPNMRFCHHFKLMKTAGAYWRGDSNNKMLQRIYGTAWADKKALNAYLQRLEEAA
KRDHRKIGKQLDLYHMQEEAPGMVFWHNDGWTIFRELEVFVRSKLKEYQYQEVKGPFMMD
RVLWEKTGHWDNYKDAMFTTSSENREYCIKPMNCPGHVQIFNQGLKSYRDLPLRMAEFGS
CHRNEPSGALHGLMRVRGFTQDDAHIFCTEEQIRDEVNACIRMVYDMYSTFGFEKIVVKL
STRPDKRIGSDEMWDRAEADLAVALEENNIPFEYQLGEGAFYGPKIEFTLYDCLDRAWQC
GTVQLDFSLPSRLSASYVGEDNERKVPVMIHRAILGSMERFIGILTEEFAGFFPTWLAPV
QVVVMNITDSQSEYVNELTQKLQNAGIRVKADLRNEKIGFKIREHTLRRVPYMLVCGDKE
VEAGKVAVRTRRGKDLGSLDVNDVIEKLQQEIRSRSLQQLEE
>EECEBICD_01667 Translation initiation factor IF-3
MSLREALEKAEEAGVDLVEISPNAEPPVCRIMDYGKFLYEKSKSSKEQKKKQKVIQVKEI
KFRPGTDEGDYQVKLRSLIRFLEEGDKAKITLRFRGREMAHQQIGMEVLNRVKDDLQELA
VVESFPTKIEGRQMIMVLAPKKKQ
>EECEBICD_01668 50S ribosomal protein L35
MPKIKTVRGAAKRFKKTGKGGFKHKHANLRHILTKKATKRKRHLRPKAMVSKGDLGLVIA
CLPYA
>EECEBICD_01669 50S ribosomal protein L20
MARVKRGVIARARHKKILKQAKGYYGARSRVYRVAFQAVIKAGQYAYRDRRQRKRQFRQL
WIARINAAARQNGISYSKFINGLKKASVEIDRKILADIAVFDKVAFTALVEKAKAALA
>EECEBICD_01670 Phenylalanine--tRNA ligase alpha subunit
MSHLAELVANAAAAINQASDVAALDNVRVEYLGKKGHLTLQMTTLRDLPPEERPAAGAVI

NAAKEQVQQALNARKAELESAALNARLAAETIDISLPGRRIENGGLHPVTRTIDRIESFF
GELGFTVATGPEIEDDYHNFDALNIPGHHPARADHDTFWFDATRLLRTQTSGVQIRTMKA
QQPPIRIIAPGRVYRNDYDQTHTPMFHQMEGLIVDTNISFTNLKGTLHDFLRNFFEEDLQ
IRFRPSYFPFTEPSAEVDVMGKNGKWLEVLGCGMVHPNVLRNVGIDPEIYSGFAFGMGME
RLTMLRYGVTDLRSFFENDLRFLKQFK
>EECEBICD_01671 Phenylalanine--tRNA ligase beta subunit
MKFSELWLREWVNPAIDSDALANQITMAGLEVDGVEPVAGSFNGVVVGEVVECAQHPNAD
KLRVTKVNVGGERLLDIVCGAPNCRQGLKVAVATIGAILPGDFKIKAAKLRGEPSEGMLC
SFSELGISDDHSGIIELPADAPLGTDIREYLKLDDNTIEISVTPNRADCLGIIGVARDVA
VLNKAPLQEPEMVPVTATISDTLPITVEAADACPRYLGRVVKGINVNAPTPLWMKEKLRR
CGIRSIDAVVDVTNYVLLELGQPMHAFDKDRIDGGIVVRMAKEGETVVLLDGSEATLNAD
TLVIADHHKALGIAGIFGGEHSGVNGETQNVLLECAYFNPLSITGRARRHGLHTDASHRY
ERGVDPALQYKAIERATRLLLDICGGDAGPIIDVSNEATLPKRATITLRRSKLDRLIGHH
IADEQVSDILRRLGCEVTEGQDEWKAVAPTWRFDMEIEEDLVEEVARVYGYNNIPDEPIQ
AGLIMGTHREADLSLKRVKTMLNDKGYQEVITYSFVDPKVQQLIHPGAEALLLPNPISVE
MSAMRLSLWSGLLATVVYNQNRQQNRVRIFETGLRFVPDTQANLGIRQDLMLAGVICGNR
YDEHWNLAKETVDFYDLKGDLEAVLDLTGKLGDIQFKAEMNPALHPGQSAAIYLKDERIG
FIGVVHPELERKLDLNGRTLVFELEWNKLADRIVPQAREISRFPANRRDIAVVVAENVPA
ADILSECKKVGVNQVVGVNLFDVYRGKGVAEGYKSLAISLILQDTNRTLEEEEIAATVAK
CVEALKERFQASLRD
>EECEBICD_01672 Integration host factor subunit alpha
MALTKAEMSEYLFDKLGLSKRDAKELVELFFEEIRRALENGEQVKLSGFGNFDLRDKNQR
PGRNPKTGEDIPITARRVVTFRPGQKLKSRVENASPKEE
>EECEBICD_01673 Vitamin B12 import system permease protein BtuC
MLTFARQQQRRNVRWLLSLSLLVLLATLLSLCAGEQWIAPGDWLSARGELFVWQIRLPRT
LAVLLVGAALALSGAVMQALFENPLAEPGLLGVSNGAGVGLIAAVLLGQGQLPGWALGLC
AIAGALIITLILLRFARRHLSTSRLLLAGVALGIICSALMTWAIYFSTSFDLRQLMYWMM
GGFGGVDWQQSWLMIALIPVLIWICCQSQPLNMLALGETSARQLGLPLWFWRNLLVIATG
WMVGVSVAMAGAIGFIGLVIPHILRLCGLTDHRVLLPGCALAGAIALLLADVVARLALAS
AELPIGVVTATLGAPVFIWLLLKSAR
>EECEBICD_01674 Thioredoxin/glutathione peroxidase BtuE
MQNSLLNTHVTTIDGEVTTLEKYAGKVLLIVNVASRCGLTPQYEQLENIQKAWADQGFVV
LGFPCNQFMGQEPGSEEEIKTYCASTWGVTFPMFSKIDVNGDARHPLYQKLIAAAPTAVA
PEKSGFYERMVSKGRTPLYPDDILWNFEKFLVGRDGQVVQRFSPDMTPEDPIVMESIKIT
LAK
>EECEBICD_01675 Vitamin B12 import ATP-binding protein BtuD
MSQLMQLKDVAESTRLGPLSGEVSAGEILHLVGPNGAGKSTLLARMAGLTSGEGSIRFGG
APLEAWATATLAQHRAYLAQQQNPPFAMPVWHYLTLHQPDKTRTGQLNEVADMLGLGDKL
GRSVNQLSGGEWQRVRLAAVVLQIHPDANPVGQLLLLDEPMNSLDVAQQNALDRVLHHLC
QAGIAIVMSSHDLNHTLRHAHKAWLLKRGKLIACGRREEVLTPSYLAQAYGLRFRRLDVE
GHPTLISAT
>EECEBICD_01676 Murein DD-endopeptidase MepS/Murein LD-carboxypeptidase
MRFWLLVITALFLAGCSTHRAPAPNARLSDSITVIAGLNDQLQSWRGTPYRYGGMSRRGV
DCSGFVVVTMRDKFDLQLPRDTREQSKIGTRIDKDELLPGDLVFFKTGSGESGLHVGIYD
TNNEFIHASTSRGVMRSSLDNVYWRKNFWQARRI
>EECEBICD_01677 Anti-FlhC(2)FlhD(4) factor YdiV
MIASLDELYHSELFFLPVMDENARLVGLEIIATFAAEDGAVRMPTELVAPRLSVEEQYCL
FVEKLALLETCQHFFIQHKLIAWLNLPPAISDLLLLDSELFSRAARFPFLELAINENYPG
LNQGKNNETLANLAMHFPLMLANFGAGEASTKAIFDGLFKRVMLDKNFIQQRAEMISFEP
FMHAIVAQISSSCESLMIAGIDNEAMFSRAAPLGFSAFQGGLWPPVPVSQLIKLVQR
>EECEBICD_01678 Protein adenylyltransferase SelO
MTLSFTARWRDELPATYTALLPTPLKNARLIWYNDKLAQQLAIPASLFDATNGAGVWGGE
TLLPGMSPVAQVYSGHQFGVWAGQLGDGRGILLGEQLLAYGSTLDWHLKGAGLTPYSRMG
DGRAVLRSTIRESLASEAMHYLGIPTTRALSIVASDTPVQRETQETGAMLMRLAQSHMRF

GHFEHFYYRREPEKVQQLADFAIRHYWPQWQDVPEKYALWFEEVAARTGRLIAEWQTVGF
SHGVMNTDNMSILGLTIDYGPFGFLDDYDPGFIGNHSDHQGRYRFDNQPLVALWNLQRLA
QTLTPFIEIDALNRALDRYQDALLTHYGQRMRQKLGFFTEQKDDNALLNELFSLMAREGS
DYTRTFRMLSHTEQQSASSPLRDTFIDRAAFDAWFDRYRARLRTEAVDDALRQQQMQRVN
PAVVLRNWLAQRAIDAAEQGDMAELHRLHEVLRQPFTDRDDDYASRPPEWGKRLEVSCSS
>EECEBICD_01679 putative protein YdiE
MSRMDNTELPHPKEIDNETLLPAAERRVNSQALLGPDGKVIIDHNGQEYLLRKTQAGKLL
LTK
>EECEBICD_01680 Phospho-2-dehydro-3-deoxyheptonate aldolase, Trp-sensitive
MNRTDELRTARIDSLVTPTELAQRYPVSSSVASHVTDSRRRIEKILNGEDPRLLVVIGPC
SIHDLNAAMEYATQLQAQRQKHQARLEIVMRTYFEKPRTVVGWKGLISDPDLNGSYRVNY
GLELARRLLLQVNELGVPTATEFLDMVTGQFIADLISWGAIGARTTESQIHREMASALSC
PVGFKNGTDGNTRIAVDAIRASRASHMFLSPDKDGQMTIYQTSGNPYGHIIMRGGKKPNY
HAEDIAAACDTLHEFDLPEHLVVDFSHGNCQKQHRRQLEVCDDICQQIRNGSTAIAGIMA
ESFLREGTQKIISGQPLIYGQSITDPCLNWEDTEVLLEKLAAAVDSRF
>EECEBICD_01681 Phosphoenolpyruvate synthase regulatory protein
MDNVVDRHVFYISDGTAITAEVLGHAVMSQFPVTISSITLPFVENESRARAVKDQIDAIY
QQTGVRPLVFYSIVLPEIRAIILQSEGFCQDIVQALVAPLQQEMKLDPTPIAHRTHGLNP
GNLNKYDARIAAIDYTLAHDDGISLRNLDQAQVILLGVSRCGKTPTSLYLAMQFGIRAAN
YPFIADDMDNLTLPTSLKPLQHKLFGLTIDPERLAAIREERRENSRYASLRQCRMEVAEV
EALYRKNQIPCLNSTNYSVEEIATKILDIMGLNRRMY
>EECEBICD_01682 Phosphoenolpyruvate synthase
MSNNGSSPLVLWYNQLGMNDVDRVGGKNASLGEMITNLSGMGVSVPNGFATTADAFNQFL
DQSGVNQRIYELLDKTDIDDVSQLAKAGAQIRQWIIDTPFQPALENAIRDAYAQLSSDDE
NASFAVRSSATAEDMPDASFAGQQETFLNVQGFDAVLVAVKHVFASLFNDRAISYRVHQG
YDHRGVALSAGVQRMVRSDLASSGVMFSIDTESGFDQVVFITSAWGLGEMVVQGAVNPDE
FYVHKPTLAAGRPAIVRRTMGSKKIRMVYAATQEHGKQVKIEDVPQQQRDIFSLTNEEVQ
ELAKQAVQIEQHYGRPMDIEWAKDGHTGKLFIVQARPETVRSRGQVMERYTLHAQGKIIA
EGRAIGHRIGAGTVKVIHDIGEMNRIEPGDVLVTDMTDPDWEPIMKKAAAIVTNRGGRTC
HAAIIARELGIPAVVGCGDATERMKDGEKVTVSCAEGDTGYVYADMLDFSVKSSSVDTMP
ELPLKVMMNVGNPDRAFDFACLPNEGVGLARLEFIINRMIGVHPRALLEFDDQDAALQND
IREMMKGFDSPREFYVGRLTEGIATLGAAFYPKRVIVRLSDFKSNEYANLVGGERYEPHE
ENPMLGFRGAGRYVAESFRDCFALECEAVKRVRNDMGLTNVEIMIPFVRTVDQAKAVIEE
LARQGLKRGENGLKIIMMCEIPSNALLAEQFLEYFDGFSIGSNDMTQLALGLDRDSGVVS
ELFDERNDAVKALLSMAIRAAKKQGKYVGICGQGPSDHEDFAAWLMEEGIDSLSLNPDTV
VQTWLSLAELKK
>EECEBICD_01683 Medium-chain fatty-acid--CoA ligase
MSVTLTFDAARRKTYRESGYWGDASLGDYWRQTARAVPDKIAVVDNHGASWTYAALDYAA
SRLANWLLSQGIQPGDRVAFQLPGWCEFTLIYLACLKTGAVSVPLLPAWREAELVWVLNK
CQAKIFFAPTVFKQNRPVDLILPLQNQLRHLTHIVGVDKLAPATTALALSQIIDRSEPLQ
SDINIHGDELAAVLFTSGTEGMPKGVMLTHNNILASERAYCARLNLTWQDVFLMPAPLGH
ATGFLHGVTAPFLIGARSVLLDIFTPEACLTLLAQQRCTCMSGATPFIYDLLCAVEQQPA
DLSSLRFFLCGGTIIPKKVARDCQQRGIKLLSIYGSTESSPHSMVNLGDSTSRMMNTDGY
AATGVEIKIVDEDRNTLPAGHEGEEASRGPNVFMGYLDEPELTARALDNEGWYYSGDLCR
MDEDGYIKITGRKKDIIIRGGENISSREVEDILLQHPRIHDACVVAMPDERLGERSCAYV
VLKPPHLSLTLEEVIAFFSRKRVAKYKYPERIVIVEKLPRTASGKVQKFLLRQDIIERLR
QEHTAV
>EECEBICD_01684 Ferredoxin-like protein FixX
MSSDNKVNVDVKLGVNKFYVDEGHPHIILRSSPDMQEFNKLIKACPAGLYKLDDAGNIHF
DSAGCLECGTCRVLCGNTLLEKWEYPAGTFGVEFRYG
>EECEBICD_01685 hypothetical protein
MMPELANDGVMIVGDAAGLCLNLGYTVRGMDLAIASAQAAAQTAIDAKARQDFSAASLAG
YKRALEKNGVLRDMRHFRKLPGLMENPRLFTEYPRMAANIVGDLFTVDGGPVPPLRKTIL

SHAKKIGLVNLLKDGIKGATAL
>EECEBICD_01686 Electron transfer flavoprotein-ubiquinone oxidoreductase
MSDDKFDAIVVGAGVAGTVAAYVMAKAGLDVLVIERGNSAGSKNMTGGRLYAHSIESIMP
GFATSAPIERVVTREKISFLTEESAVTLDFHRAPPTPPLTSYTVLRNKLDPWLMAQAEQA
GAQFIPGVRVDALVREGNRVTGVQAGDDILDANIVILADGVNSMLGRSLDMVPASSAHHY
AVGVKELIGLSPALIEERFNLASHEGAAWLFAGAPSNGLMGGGFLYTNRDSVSLGLVCGL
GDIAHATKSVPQMLEDFKQHPAIRPLIQGERCSNTPLIWCPKAA
>EECEBICD_01687 Protein FixB
MNHLANVWVFSDNVERYAELMTGARQWGEKVYAIVQGNTEIDYVKALGADEIVVLESHTD
LQRVENYAETLASLLGDQNGLLLMAATKRCKALGARLSIQLDAMMVNDATSIDLLDGTLH
AEHRMYGGLAFGKEKLNSPQAIITLAPGVLEPAAPLPGHTCPQTTLAYIAPRHEVLCQQR
RAKSLSSVDLSKAKRVIGVGRGLAAQSDLDMVHKLAAVLGAEVGCSRPIAEGENWMERER
YIGVSGVLLKSDLYLTLGISGQIQHMVGGNGAKIIVAINKDKNAPIFNYADYGLVGDIYK
VVPALIDQLSR
>EECEBICD_01688 Protein FixA
MVQDAQLEHALPKETALALASAAEKIGFDLLLFGEGSGDIYAQQVGLLTGELLKLPVINA
VSHIQRDGDKIIVERTLEEDVEIIELTLPAVLCVTSDINVPRIPSMKAILGAGKKAVTSW
LASDIHWTPTTPLAELVAISVPPQTERKHIILDNDSPEAITALADHLKKSLN
>EECEBICD_01689 HTH-type transcriptional activator RhaS
MYQRCFDSAAETIFEQDKTPRFSRFVISDDPNWESGHHMHDNETELIYVKKGIARLIIDS
SLYVAHADDIVVVERGRLHAVASDSNSPATTYTCALYGFCFPGWEENQLLQSHSCPVVSV
VQGKEVIKSIFNELSVLLPQGKNVLASSVYDAFAYTLTALYYENFKNAYRSEQGYIKKDV
LIKDVLVYLNNNYREKITLDQLSKKFRASVSYICHEFAKEYHISPINYVIQRRMTEAKFA
LTNTESPLAEISWRVGYENVDHFAKLFMRHVGCSPNDYRKQFKNSLVEQAYLLPNT
>EECEBICD_01690 Crotonobetainyl-CoA reductase
MDFSLTEEQELLLASIRELITSNFPEEYFRTCDQTGTYPKEFMQALAENGISMLGVPEEF
GGIPADYVTQMLALMEISKCGAPAFLITNGQCIHSMRRFGSAEQLRKTAESTLATGDPAY
ALALTEPGAGSDNNSATTTWTRKNGKVYLNGQKTFITGAKEYPYMLVLARDTEPKDPKKA
FTLWWVDSNKPGIKINPLHKIGWHMLSTCEVYLDNVEVDESDRVGEEGMGFLNVMYNFEM
ERLINAARSTGFAECAFEDAARYANQRIAFGKPIGHNQMIQEKLALMAIKIENMRNMVLK
VAWQADREESLRTSAALAKLYCARTAMEVIDDAIQIMGGLGYTDEARVSRFWRDVRCERI
GGGTDEIMIYVAGRQILKDYQNK
>EECEBICD_01691 Acetate CoA-transferase YdiF
MKINKPSRINGRVPVLSAQEAVNYIPDEATLCILGAGGGILEATTLITALADKYQTTQSP
RDLSIISPTGLGDRADRGISPLAQEGLVKWALCGHWGQSPRISELAEQNKIAAYNYPQGV
LTQTLRASAAHQPGILSDIGIGTFVDPRQQGGKLNDVTKEDLIKLVEIDNKEYLYYKAIA
PNVAFIRATTCDSEGYASFEDEVMYLDALVIAQAVHNNGGIVMMQVQKMVKKATLHPKSV
RIPGYLVDIVVVDADQTQLYGGAPVNRFISGDFTLDDSTQLTLPLNQRKLVARRALFEMR
KGAVGNVGVGIADGIGLVAREEGCADDFVLTVETGPVGGITSQGVAFGANVNTRAILDMT
SQFDFYHGGGLDVCYLSFAEVDQHGNVGVHKFNGKIMGTGGFIDISATSQKIIFCGTLTA
GSLKTEITDGKLNILQEGRVKKIRQRTAGNYVQWKNCA
>EECEBICD_01692 3-dehydroquinate dehydratase
MKTVTVRDLVVGEGAPKIIVSLMGKTITDVKSEALAYREADFDILEWRVDHFANVTTAES
VLEAAGAIREIITDKPLLFTFRSAKEGGEQALTTGQYIALNRAAVDSGLVDMIDLELFTG
DDEVKATVGYAHQHNVAVIMSNHDFHKTPAAEEIVQRLRKMQELGADIPKIAVMPQTKAD
VLTLLTATVEMQERYADRPIITMSMSKTGVISRLAGEVFGSAATFGAVKKASAPGQISVA
DLRTVLTILHQA
>EECEBICD_01693 Quinate/shikimate dehydrogenase
MDVTAKYELIGLMAYPIRHSLSPEMQNKALEKAGLPYTYMAFEVDNTTFASAIEGLKALK
MRGTGVSMPNKQLACEYVDELTPAAKLVGAINTIVNDDGYLRGYNTDGTGHIRAIKESGF
DIRGKTMVLLGAGGAATAIGAQAAIEGIKEIKLFNRKDDFFEKAVAFAKRVNENTDCVVT
VTDLADQHAFTEALASADILTNGTKVGMKPLENESLIGDVSLLRPELLVTECVYNPHMTK
LLQQAQQAGCKTIDGYGMLLWQGAEQFELWTGKAFPLDYVKQVMGFTA
>EECEBICD_01694 Inner membrane transport protein YdiN

MSQNKARNMPYLLAVICIYFSYFLHGMSVITLAQNMTSLAQKFSTDSAGIAYLISGIGLG
RLVSILFFGVLSDKFGRRAIILLGAVLYMLFFFGIPASPNLMIAFILAVCVGVANSALDT
GGYPALMECFPKASGSAVILVKAMVSFGQMIYPLIVSALLVNHIWYGYAVVIPGILFVLI
TLMLLKSRFPSQLVDASIAKELPQMNSTPLVWLEGVASVLFGVAAFSTFYVIVVWMPKYA
MAFAGMAESDALKTISYYSMGSLVCVFIFAALLKKMVRPIWANVFNAGLATLTAAAIYLY
PSPLICNAGAFVIGFSAAGGILQLGVSVMSEFFPKSKAKVTSIYMMMGGVANFIIPLITG
YLSTIGLQYIILLDFAFALLTFITAIIVFIRYYRVFKIPQNDVRFGERYFQ
>EECEBICD_01695 Inner membrane transport protein YdiM
MKNRYFPTAVGLYFNYFVHGMGVILMSLNMSSLEQQWHTSAAGVSIVISSLGIGRLSVLL
IAGMLSDRFGRRPFIILGTACYLIFFIGILYAQTIFVAYACGFLAGMANSFLDAGTYPSL
MEAFPRSPSTANILIKAFVSGGQFLLPIIISLLVWANMWFGWSFLLAGAIMLINALFLLR
CPFPPYPGRILKPKISQAPVAGVHHCSLIDLISYTLYGYISMATFYLISQWLAQYGQFVA
GMSYTQSIKLLSIYTCGSLLCVFITAPLVRKTIRSTTLLMFYTFISFIALLTVCLHPQAY
VVMIFAFVIGFSSAGGVVQIGLTLMAARFPQEKGKATGIYYSAGSIATFTIPLITARISE
MSIAHIMWFDTGIAAVGFLLALFIGYRSRAESQHHAEAASTAQ
>EECEBICD_01696 hypothetical protein
MMNALELQALRRIFDMTIEECTIYITQDNNSTTWQRWEAGDTPISPEIIARLKEMKARRQ
RRINAIVDKINNRIGNNTMRYFPDLSSFQSIYTEGDFIEWKIYQSVAAELFAHDLERLC
>EECEBICD_01697 Putative transport protein YdiK
MVNVRQPRDIAQVLLSVLFLAIMIVACLWIVQPFILGFAWAGTIVIATWPVLLKLQKILW
GRRSLAVLVMTLLLVLLFVIPVALLVNSIVDGSGPLIHAVTGGDMTLPDLAWLNNIPLVG
AKLYAGWHSLLDMGGSAIMAKVRPYIGTTTTWFVGQAAHIGRFMMHCALMLLFSALLYWR
GEQVAMGIRHFACRLAAKRGDAAVLLAAQAIRAVALGVVVTALVQAVLGGVGLAISGVPY
ATLLTVVMILSCLVQLGPLPVLIPAIIWLYWTGDTTWGTVLLVWSAVVGTLDNVIRPVLI
RMGADLPLLLILSGVIGGLIAFGMIGLFIGPVLLAVTWRLFSAWVHEVPAPTNEPEEILE
ELDDIEEANKQS
>EECEBICD_01698 putative protein
MIPQISQAPGVVQLVLNFLQALEQQGFTGDTATSYADRLTMSTDNSIYQLLPDAVVFPRS
TADVALLARLAAEPRFTSLIFTPRGGGTGTNGQALNQGIIVDMSRYMNRIIEINPEEGWV
RVEAGVIKDQLNQALKPYGYFFAPELSTSNRATLGGMINTDASGQGSLVYGKTSDHVLGI
RAVLLGGDILDTQSMPVELARTLGENTTTPGRIYQTVYQSCLENRQLILDSFPKLNRFLT
GYDLRHVFNDDMTRFDLTRILTGSEGTLAFITEARLDITPIPKVRRLVNVKYDSFDSALR
NAPFMVDARALSVETVDSKVLNLAREDIVWHSVSELITDVPDKEMLGLNIVEFAGDDEAL
IDGQVEALCHRLDGLMARAEAGVIGWQLCTDLTGIERIYAMRKKAVGLLGNAKGAAKPIP
FAEDTCVPPEHLADYIAEFRALLDGHGLSYGMFGHVDAGVLHVRPALDMCDPQQELLMKQ
ISDEVVALTARYGGLLWGEHGKGFRAEYSPAFFGEVLYGELRKIKAAFDPHNRLNPGKIC
PPQGIEAPMMKVDAVKRGTWDRQIPLAVRQTWRGAMECNGNGLCFNFDAKSPMCPSMKIS
LNRIHSPKGRATLVREWLRLLADRGVDPLKLEKELPEKRASLRTLIARTRNSWHKRKGEY
DFSHEVKEAMSGCLACKACTTQCPIKIDVPEFRSRFLQLYHTRYLRPVRDHLVATVETYA
PLMARAPKTFNFFINQPVVRNLAKKHIGMVDLPLLSAPSLQQQLVGHPSANMTLEQLERM
SAEQKAKTVLVVQDPFTSYYDARVVADFIRLAEKLGRRPVLLPFSPNGKAQHIKGFLNRF
AKTAKKTSEFLNRIAALGMPMVGVDPALVLCYRDEYKLALGEQRGDFHVLLVNEWLAQEI
NARLSVEVSGEPWYFFGHCTEVTALPGAPAQWAAIFAHFGAKLENVSVGCCGMAGTYGHE
LTNHKNSLGIYELSWHQAMQRLPRNRCLATGYSCRSQVKRIEGTGVRHPLQALLEIIG
>EECEBICD_01699 1,4-dihydroxy-2-naphthoyl-CoA hydrolase
MIWKREVTLDALNAMGEGNMVGLLDIRFERIGDDTLEATMPVDHRTKQPFGLLHGGASVV
LAESIGSVAGYLCTQGEQKVVGLEVNANHVRSARQGRVRGVCKALHTGARHQVWQIEIFD
EQGRLCCSSRLTTAIV
>EECEBICD_01700 hypothetical protein
MSTDLDPTQLAIEFLRRDKTELSPAQYLKRLKQLELEFADLLTLSATELKEEIYFAWRLG
VH
>EECEBICD_01701 L-cystine uptake protein TcyP
MVVTLAYIALFLVFSWVILRINQKSDSLSKSVFIAIFLGAVIGLSLHFISANHTKTIIEW
YSIVGNGYVHLLKLVAIPLIFISILSAINKLENSAGIGKMSLTIVGCMLCLVMVAGFIGL

LTAHVLGLDASAFVHMPSMLTTEEVNKTAAVSIPQLVTSLIPTNIFLDLTGARSVSVIGI
VIFTLIAGIALLKVKKEAPEEGQKLSAGINAIQIWVMKMVRIVIALTPYGVMALMTTVFS
SYHFEQFASLLGFIGACYIAIFMMFIVHAILLILSGNNPARYFSMVWPVLTFAFVSRSSA
ASIPLAISAQEKFGVQSTIANISASFGSSMGQNGCAGIYPAIMVAMIAPTIGIDPLSLHF
LAAMLPAIALGSIGVAGVGGGGTFAALIVLSTLNFPVALVGIFIAIEPIVDMARTALNVN
GSMMSGVLANRILNNHTADDMPAVIDRP
>EECEBICD_01702 Protein SufA
MELHSDTFNPEDFPWQGLTLTPAAAAHIRELAEKQPGMLGVRLSVKQTGCAGFGYVLDTV
REPDKDDLVFEAEGAKLFAPLQAMPFIDGTEVDYVQEGLNQLFKFHNPKAQNECGCGESF
GV
>EECEBICD_01703 FeS cluster assembly protein SufB
MSRNTEATEEVGIWTGGRLNYKEGFFTQLPTDELAKGISEEVVRAISAKRNEPEWMLEFR
LNAYRAWLDMDEPHWLKAHYDKLNYQDYSYYSAPSCGNCDDSCVSQPGAVQQTGANTFLS
KEVEDAFEQLGVPVREGKEVAVDAIFDSVSVATTYREKLAEQGIIFCSFGEAIHDHPDLV
RKYLGTVVPGNDNFFAALNAAVASDGTFIYVPKGVRCPMELSTYFRINAEKTGQFERTIL
VADEGSYVSYIEGCSAPVRDSYQLHAAVVEVIIHKDAEVKYSTVQNWFPGDNNTGGILNF
VTKRALCEGENSKMSWTQSETGSAITWKYPSCILRGDNSIGEFYSVALTSGHQQADTGTK
MIHIGKNTRSTIISKGISAGHSQNSYRGLVKIMPTATNARNFTQCDSMLIGADCGAHTFP
YVECRNNSAQLEHEATTSRIGEDQLFYCLQRGISEEDAISMIVNGFCKDVFSELPLEFAV
EAQKLLTISLEHSVG
>EECEBICD_01704 putative ATP-dependent transporter SufC
MLSIKDLQVSVEEKAILRGLNLEIRPGEVHAIMGPNGSGKSTLSATLAGREDYEVTGGSV
TFKGKDLLELSPEERAGEGIFMAFQYPVEIPGVSNQFFLQTALNAVRAYRGQTSLDRFDF
QDLMEEKIALLKMPEDLLTRSVNVGFSGGEKKRNDILQMAVLEPELCILDESDSGLDIDA
LKIVAEGVNALRDDKRAFIIVTHYQRILDYIKPDYVHVLYQGRIVRSGDFTLVKQLEEQG
YGWLTEQQ
>EECEBICD_01705 FeS cluster assembly protein SufD
MAGLPNSSKALQQWQHLFEEKRESRTEQARQHLQQMLRLGLPTRKHEDWKYTPLEGLTHS
QFIQQCATISAAQRDALALQIDAVRLVFVDGRFMPELSDSTQNSGFDVSVRDERQTLAAP
VQPEVFLHLTESLAQCVTYIQVRRNQRPTRPLLLMHITQGVDGDELNTAHYRHHLALAEG
AEATVIEHYVSLTAAKHFTGARLTMNVADNAQLHHIKLAFENASSYHFAHNDLLLATDAS
AFSHSFLLGAAVLRHHSSSQLNGENATLRLNSLAMPVKNEVCDTRTWLEHNKGYCNSRQS
HKTIVSDKGRAVFNGLINVAQHAIKTDGQMTNNNLLLGKLAEVDTKPQLEIYADDVKCSH
GATIGRIDDEQMFYLQSRGIRQQEARHMILYAFAAELTEAIHDSALKQQVLARIGQRLPG
GLV
>EECEBICD_01706 Cysteine desulfurase
MTFPVEKVRADFPILQREVNGLPLAYLDSAASAQKPNQVIDAESAFYRHGYAAVHRGIHT
LSAQATESMENVRKQASRFINARSAEELVFVRGTTEGINLVANSWGTENIRAGDNIIISE
MEHHANIVPWQMLCERKGAELRVIPLHPDGTLRLETLAALFDDRTRLLAITHVSNVLGTE
NPLPDMIALARQHGAKVLVDGAQAVMHHAVDVQALDCDFYVFSGHKLYGPTGIGILYVKE
ALLQEMPPWEGGGSMISTVSLTQGTTWAKAPWRFEAGTPNTGGIIGLGAAIDYVTSLGLD
KIGDYEQMLMRYALEQLAQVPDITLYGPAQRLGVIAFNLGKHHAYDVGSFLDNYGIAVRT
GHHCAMPLMAWYGVPAMCRASLAMYNTHEEVDRLVAGLTRIHRLLG
>EECEBICD_01707 Cysteine desulfuration protein SufE
MAALPDKEKLLRNFTRCANWEEKYLYIIELGQRLAELNPQDRNPQNTIHGCQSQVWIVMR
RNANGIIELQGDSDAAIVKGLMAVVFILYHQMTAQDIVHFDVRPWFEKMALAQHLTPSRS
QGLEAMIRAIRAKAATLS
>EECEBICD_01708 putative L,D-transpeptidase YnhG
MKRASFITLTIIGAYSALQAAWAVDYPLPPEGSRLIGQNQTYTVQEGDKNLQAIARRFDT
AAMLILEANNTIAPVPKPGTLITIPSQMLLPDAPREGVIVNLAELRLYYYPPGENRVQVY
PIGIGLQGLETPVMDTRIGQKIPNPTWTPTAGIRQRSLERGITLPPVIPAGPNNPLGRYA
LRLAHGNGEYLIHGTSAPDSVGLRVSSGCIRMNAPDIKALFAQVRTGTPVKVINQPVKFS
VEPNGIRYVEVHRPLSPEEEQNVQTMPYVLPAEFTAFRNAQGVDSRLVDKALYRRAGYPV
SVSAGQTPAVNTTAVESAQNGFVGEEGQTRATQ

>EECEBICD_01709 Major outer membrane lipoprotein Lpp
MAHIVRHFFYPYNHAKYNVIVMNRTNQLILGAVVLGSTLLAGCSNNAKIDQLSSDVQTLS
AKVEQLSNDVNAMRSDVQAAKDDAARENQRLDNKVVSHVRK
>EECEBICD_01710 Major outer membrane lipoprotein Lpp 1
MNRTKLVLGAVILGSTLLAGCSSNAKIDQLSSDVQTLNAKVDQLSNDVNAMRSDVQAAKD
DAARANQRLDNQATKYRK
>EECEBICD_01711 Pyruvate kinase I
MKKTKIVCTIGPKTESEEMLSKMLDAGMNVMRLNFSHGDYAEHGQRIQNLRNVMSKTGKK
AAILLDTKGPEIRTIKLEGGNDVSLKAGQTFTFTTDKSVVGNNEIVAVTYEGFTSDLSVG
NTVLVDDGLIGMEVTAIEGNKVICKVLNNGDLGENKGVNLPGVSIALPALAEKDKQDLIF
GCEQGVDFVAASFIRKRSDVVEIREHLKAHGGENIQIISKIENQEGLNNFDEILEASDGI
MVARGDLGVEIPVEEVIFAQKMMIEKCIRARKVVITATQMLDSMIKNPRPTRAEAGDVAN
AILDGTDAVMLSGESAKGKYPLEAVSIMATICERTDRVMNSRLDYNNDSRKLRITEAVCR
GAVETAEKLEAPLIVVATQGGKSARAVRKYFPDATILALTTNEVTARQLVLSKGVVSQLV
KEINSTDDFYRLGKDVALQSGLAQKGDVVVMVSGALVPSGTTNTASVHVL
>EECEBICD_01712 Putrescine importer PuuP
MKKVKKLSLTDLVLYGLVFMVPIAPVSLYGVVYNLSHGMVALVYIIGAIAMFFTAYSYST
LSQHISSSGSAYAYAGVCINPAVGFLTGWILLLDYLLFPTLVAVLGGVAVHAILPQIPVW
VWPLIYVAIGTGVNYLGIQQTAKFDKLLVFIQLGILAIFVLLIVRLMLLDSSQITLSFRP
FFDSQWFTPGLIATAISVAALNFLGFDAISTLSEESEGGGRAVSKATLLALVLATVLFII
VVAFAAFATGNVDRFAEGNATNEAFFTIAGNVGGIWLKVAFSIIVAFVCAVGNIITAQTA
VSRVLFSMGRDRMLPAFLAHVHTTRKTPDYAILFTGGVTLLLSYLFSGKIESISTLVNFG
ALFAFFVVNLCVFILFNFRMKAQRRIFAHVISPIMGMIVIGYVCLNMNIHALILGISWAA
IGIAILCYRKAHNQNIAIDLEGKKLLD
>EECEBICD_01713 L-amino acid amidase
MNDTYLYGEMFSENILTSYRIYGDRKSLLTPLIILHGGPSGGFDYLLNYRRLADDGRMVI
FYDQYGCGRSTHFPHADASFWTIARYLSQLTQLIHHLGIGHGYSILGHSWGGMLAAEHAC
LQPAGLRGTILASSPASIALWQQEAIRLFNALTPMSDDDIKNVIMPAVIYQNPPEQLVAY
YARHVYTLAEEAVHVQRSNAQFAADPTGYHILWGTNELAANGKLADWDITPHLCQIRCPV
LVLRGENDQATERVVSPLLSHISDCRAVTIPGSSHNPHEENIAPCLAAVSAFLRDLA
>EECEBICD_01714 hypothetical protein
MKILFIGESWHIHMIHSKGFDSFTSSKYEEGADYLLSCLRQGNIDVDYMPAHIVQTRFPH
TAEALACYDAIVISDIGSNTFLLQNRTFYNMDIIPDALQLIADYVAEGGGLLMIGGYLSF
TGIEAKANYKNTVLAEVLPVDMLDVDDRVELPQGCKAVNTAVEHVITQPFSEWPPLLGYN
KLIAKENSQVLAEINGDPLLVMGTYHKGKVCCFASDCSPHWGSPQFLQWEHYATFWCNVL
HTIKK
>EECEBICD_01715 Ribokinase
MFKEERRHAIINLLIKDNSVSVSKLSDLYKVSQETIRSDLRYFQKSGMLQRCYGGGILNR
DALSKLITENKIDISSTIATPIHQDAKLRRENPKKAGKVCVLGSFNIDVSATVPWFPQSG
ESILASQFGFYPGGKGANQALAANNAGAAAHFIFKVGKDQFSAFAMNHIIQSGIASYSAY
QTDKAPTGSALIYVSAVDGDNIIAIYPGANMMLTTQEINEQHRYIAESDVMLMQLETNIE
ALTEFIRLGKQENKMIMLNPAPYTKQVTHLLSDIDIITPNETEASFLSGVTITDINDAKK
AGNIILQSGVKKVIITLGARGSLLCEHARTLYIPAWSAVVKDAAGAGDAFNGALAAALAR
QADMVAAIQYASAFASLAVEQVGASSMPQHLQVLHRMRTQSNKVIHIN
>EECEBICD_01716 Tetrathionate reductase subunit A
MANLTRRQWLKVGLAVGGMVTFGLSYRDVAKRAIDGLLNGTSGKVTRDRIFGNALIPEAQ
AQTHWQQNPQQTIAMTQCFGCWTQCGIRARVNADGKVIRIAGNPYHPLSQEHPIDSSVPF
SEAMEQLAGESGLDARSTACARGATLLESLYSPLRLLEPMKRVGKRGEGKWQRISFEQLI
EEAVEGGDLFGEGHVDGLRAIHAPDTPIDAKHPSFGPKTNQLLVTNTSDEGRDAFLRRFA
LNSFGSKNFGAHGAYCGLAYRAGSGALMGDLDKNPHVKPDWENVEFALFMGTSPAQSGNP
FKRQARQLASARLRENFQYVVVAPALPLSTVLADPRGRWQPVMPGSDSALAMGMIRWIMD
NERYNADYLAIPGVQAMQQAGEQSWTNATHLVIADELPTLAGQHLTLRHLTPDGEETPVV
LNTDGELVDASTCRQARLFVTQYVTLADGQRVTVKSGLQRLKEAAEKLSLAQYSEQCGVP
EAQIIALAETFTSHGRKAAVISHGGMMAGNGFYNAWSVMMLNALIGNLSLSGGVFVGGGK

FNGVSDGPRYNMNSFAGKVKPSGLSIARSKTAYEASEEYRDKIAGGQSPYPAKAPWYPFV
AGQLTELLTSALEGYPYPLKAWISNMSNPFYGVPGLRAVAEEKLKDPRRLPLFIAIDAFM
NETTALADYIVPDTHNFESWGFTAPWGGVASKATTARWPVVAPATRRTVDGQPVSMEAFC
IAVAKRLHLPGFGDRAITDPQGNTFPLNRAEDFYLRVAANIAFMGKTPVALANQEDISLT
GVSRILPAIQHTLKADEVGRVAFIYSRGGRFAPEDSGYTEQRLGNAWKKPLQIWNADVAA
HRHAITGERFSGCPVWYPARLSDGRAIDDQFPIGQWPLKLISFKSNTMSSSTAVIPRLHH
VKPANLVALNPQDGERYGLQHGDRVRIITPGGQVVAQISLLNGVMPGVIAIEHGYGHREM
GATQHSLDGVPMPYDPQIRAGINLNDLGFADPTRTITNTWLDWVSGAAVRQGLPAKIERI
>EECEBICD_01717 Tetrathionate reductase subunit C
MTHSLIIEEVLAHPQDISWLPWAVQYFFFIGIAACAALFACYLHWRKKDAATEENRALLI
AITCAITAPLALTADLHQTARVWHFYAWPTPWSWMPWGALFLPLFTGFLALWFLAQQIKR
LFNKSYNVTKWLALASALCAVGLLIYTGREVSVVLARPIWFSYAFPVAMFLSALQAFFAL
MIVAARRDSVRLPKILWGQIWTLAALGLVVAMWVSGDTLSGTAIRQWITVALSAKYYAVG
WLALWVFTLLFCSLTLRHPLSQLRRVLLVLSALALCWLMRWTLLIQVQTVPKFNAQFNPY
SLPGGTDGWLAILGTFGLWIALLIIIRETLNGLTRRLQHG
>EECEBICD_01718 Tetrathionate reductase subunit B
MDSSKRQFLQQLGVLTAGASLVPLAEAKFPFSPERHEGSPRHRYAMLIDLRRCIGCQSCT
VSCTIENQTPQGAFRTTVNQYQVQREGSQEVTNVLLPRLCNHCDNPPCVPVCPVQATFQR
EDGIVVVDNKRCVGCAYCVQACPYDARFINHETQTADKCTFCVHRLEAGLLPACVESCVG
GARIIGDIKDPHSRIATMLHQHRDAIKVLKPENGTSPHVFYLGLDDAFVTPLMGRAQPAL
WQEV
>EECEBICD_01719 Tetrathionate sensor histidine kinase TtrS
MRGKTVRRLAVLAAVGLLCHGAWAGTWNIGILAMRGEASTRSHWQPLAKTLSQQLPGETF
HIQPLDLHQMQEAVNQGTVQFVITNPAQFVQLNSHAPLRWLASLRSTRDGKAVSNVIGSV
ILTRRDSGITTAHDLIGKTVGAIDAQAFGGYLLGYKALSDAGLRPERDFHLRFTGFPGDA
LVYMLREKAVQAAIVPVCLLENMDQEGLINKKDFIALLSRPTPLPCLTSTPLYPDWSFAA
LPAVSDALADRVTRALFNAPAAASFHWGAPASTSQVEALLRDVRQHPQQRRLWLDVKSWL
IQHQLMVGGVILAFLLLTLNYIWVMLLVRRRGKQLERNNVVLHQHERALETARQMSVLGE
MTSGFAHELNQPLSAIRHYAQGGLIRLRAADEQHPLLPALEQIDQQAQRGADTLRNLRHW
VSQAQGNPVLTEAWKAIAIREAIDHVWQLLRMAQQFPTVTLHTEVSAALRVTLPSVLLEQ
VLANIILNAAQAGATHLWVVAERTEHGISIALQDNAGGIDEALLRQAFQPFMTTRKEGMG
LGLAICQRLVRYGRGDISIRNQTAPDGLSGTVVTIHFLHENGGRDGDNSSTG
>EECEBICD_01720 Tetrathionate response regulatory protein TtrR
MATIHLLDDDTAVTNACAFLLESLGYDVKCWTQGADFLAQASLYQAGVVLLDMRMPVLDG
QGVHDALRQCGSTLAVVFLTGHGDVPMAVEQMKRGAVDFLQKPVSVKPLQAALERALTVS
SAAVARREIILCYQQLTPKERELASLVAKGFMNREIAEAMNIAVRTVEVHRARVMEKMQA
GSLAELIRRFEKMASPETRIRTTYEP
>EECEBICD_01721 Fumarase D
MTKPTKDDELYREMCRVVGKVVLEMRDLGQEPKYIVIAGVLRTALANQRIQRSALEKQAM
ETVINALARS
>EECEBICD_01722 hypothetical protein
MNNKPVVGISGCLTGATVRFDGGHKRMDFVMNSLAPWVDYKPICPEVAIGLPVPRPALRL
IQTTCGDIRMRYSHAPHDDVTERMNAFADRFLPTIGELAGFIVCAKSPSCGMERVRLYDE
KGNRGRKAGTGLFTAAMMDKYPWLPIGEDGRLHDPVLRENFIARIFALHELNALRAQGLS
RHSLLAFHSRYKLQLLAHHQAGYREIGPFVARLHEWGDLDAFFVRYREKLMAILRHPASR
KNHTNVLMHIQGYFHRALNSRQRAELREVILGYRAGQLPILAPLTLLKHYLAEHPDDYLR
SQNYFEPYPDTLGLRLAIT
>EECEBICD_01723 HTH-type transcriptional regulator MlrA
MSYSIGEFARLCGINAATLRAWQRRYGLLKPQRTDGGHRLYSDDDIRQALSILDWVRKGV
PISQVKPLLSRPVIRLGDNWITIQETMLQHLHEGRIDALRLLIYDCGREYPRAELVTHLL
RPLRSKVSAHLPAVMTLREILDGIIIAYTSFCLEGDRKAPGNNAFISGWHLSDHCEIWLE
ALTRTGQELRLNVLPSPPAALAPELFAQRKWFLVTTGKLTAGQKKQLAQWRNVVASLEVI
TL
>EECEBICD_01724 Oxygen regulatory protein NreC

MKEYKILLVDDHEIIINGIMNALLPWPHFKIVEHVKNGLEVYNACCAYEPDILILDLSLP
GINGLDIIPQLHQRWPAMNILVYTAYQQEYMTIKTLAAGANGYVLKSSSQQVLLAALQTV
AVNKRYIDPTLNREAILAELNADTTNHQLLTLRERQVLKLIDEGYTNHGISEKLHISIKT
VETHRMNMMRKLQVHKVTELLNCARRMRLIEY
>EECEBICD_01725 hypothetical protein
MTLATLARYISIAAEYQLLRNIELQEQDPSRCSALLATDDMVINSKIFQSLDLLLADIEN
AVSAGQKIDQLIHTLKGCLGQIGQTELVCYVIDIENRVKMGKIIALEELTDLRQKIRMIF
KNYTIT
>EECEBICD_01726 Response regulator receiver protein
MLVSLGQHVTVAASSNEALTLSQQQRFDLVLIDIRMPEIDGIECVQLWHDEPNNLDPDCM
FVALSASVATEDIHRCKKMGFIITLPNQ
>EECEBICD_01727 Sensor histidine kinase RcsC
MNLLNLKNTLQTSLVIRLTFLFLLTTIIIWLLSVLTAAYISMVQKRQHIIEDLSVLSEMN
IVLSNQRFEEAERDAKNLMYQCSLATEIHHNDIFPEVSRHLSVGPSNCTPTLNGEKHRLF
LQSSDIDENSFRRDSFILNHKNEISLLSTDNPSDYSTLQPLTRKSFPLYPTHAGFYWSEP
EYINGKGWHASVAVADQQGVFFGVTVKLPDLITKSHLPLDDSIRVWLDQNNHLLPFSYIP
QKIRTQLENVTLHDGWQQIPGFLILRTTLHGPGWSLVTLYPYGNLHNRILKIILQQIPFT
LTALVLMTSAFCWLLHRSLAKPLWRFVDVINKTATAPLSTRLPAQRLDELDSIAGAFNQL
LDTLQVQYDNLENKVAERTQALNEAKKRAERANKRKSIHLTVISHELRTPMNGVLGAIEL
LQTTPLNIEQQGLADTARNCTLSLLAIINNLLDFSRIESGHFTLHMEETALLPLLDQAMQ
TIQGPAQSKKLSLRTFVGQHVPLYFHTDSIRLRQILVNLLGNAVKFTETGGIRLTVKRHE
EQLIFLVSDSGKGIEIQQQSQIFTAFYQADTNSQGTGIGLTIASSLAKMMGGNLTLKSVP
GVGTCVSLVLPLQEYQPPQPIKGTLSAPFCLHRQLACWGIRGEPPHQQNALLNAELLYFP
GKLYDLAQQLILCTPNIPVINNLLPPGSCRFFWLMMPILIGISSAKCLSAWDNTSLLPPV
VTRL
>EECEBICD_01728 Salmonella pathogenicity island 2 protein C
MLAVLKGIALIQDIKAEGNSRSWIMTIDGHPARGEIFSEAFSISLFLNDLESLPKPCLAY
VTLLLAAHPDVHDYAIQLTADGGWLNGYYTTSSSSELIAIEIEKHLALTCILKNVIRNHH
KLYSGGV
>EECEBICD_01729 Type 3 secretion system secretin
MVVNKRLILILLFILNTAKSDELSWKGNDFTLYARQMPLAEVLHLLSENYDTAITISPLI
TATFSGKIPPGPPVDILNNLAAQYDLLTWFDGSMLYVYPASLLKHQVITFNILSTGRFIH
YLRSQNILSSPGCEVKEITGTKAVEVSGVPSCLTRISQLASVLDNALIKRKDSAVSVSIY
TLKYATAMDTQYQYRDQSVVVPGVVSVLREMSKTSVPASSMNNGSPATQALPMFAADPRQ
NAVIVRDYAANMAGYRKLITELDQRQQMIEISVKIIDVNAGDINQLGIDWGTAVSLGGKK
IAFNTGLNDGGASGFSTVISDTSNFMVRLNALEKSSQAYVLSQPSVVTLNNIQAVLDKNI
TFYTKLQGEKVAKLESITTGSLLRVTPRLLNDNGTQKIMLNLNIQDGQQSDTQSETDPLP
EVQNSEIASQATLLAGQSLLLGGFKQGKQIHSQNKIPLLGDIPVVGHLFRNDTTQVHSVI
RLFLIKASVVNNGISHG
>EECEBICD_01730 hypothetical protein
MAIAFGKQDCELADYQIPVSRSGYWWLAGVFLIFIGGMGVLLSISGQPETVNDLPLRVKF
LLDKSNIHYVRAQWKEDGSLQLSGYCSSSEQMQKVRATLESWGVMYRDGVICDDLLVREV
QDVLIKMGYPHAEVSSEGPGSVLIHDDIQMDQQWRKVQPLLADIPGLLHWQISHSHQSQG
DDIISAIIENGLVGLVNVTPMRRSFVISGVLDESHQRILQETLAALKKKDPALSLIYQDI
APSHDESKYLPAPVAGFVQSRHGNYLLLTNKERLRVGALLPNGGEIVHLSADVVTIKHYD
TLINYPLDFK
>EECEBICD_01731 hypothetical protein
MTTLTRLEDLLLHSREEAKGIILQLRAARKQLEENNGKLQDPQQYQQNTLLLEAIEQAEN
IINIIYYRYHNSALVVSEQE
>EECEBICD_01732 Type III secretion system chaperone SseA
MIKKKAAFSEYRDLEQSYMQLNHCLKKFHQIRAKVSQQLAERAESPKKSRETESILHNLF
PQGVAGVNQEAEKDLKKIVSLFKQLEVRLKQLNAQAPVEIPSGKTKR
>EECEBICD_01733 Secreted effector protein SseB
MSSGNILWGSQNPIVFKNSFGVSNADTGSQDDLSQQNPFAEGYGVLLILLMVIQAIANNK

FIEVQKNAERARNTQEKSNEMDEVIAKAAKGDAKTKEEVPEDVIKYMRDNGILIDGMTID
DYMAKYGDHGKLDKGGLQAIKAALDNDANRNTDLMSQGQITIQKMSQELNAVLTQLTGLI
SKWGEISSMIAQKTYS
>EECEBICD_01734 hypothetical protein
MKKDPTLQQAHDTMRFFRRGGSLRMLLDDDVTQPLNTLYRYAMQLMEVKEFAGAARLFQL
LTIYDAWSFDYWFRLGECCQAQKHWGEAIYAYGRAAQIKIDAPQAPWAAAECYLACDNVC
YAIKALKAVVRICGEVSEHQILRQRAEKMLQQLSDRS
>EECEBICD_01735 Secreted effector protein SseC
MNRIHSNSDSAAGVTALTHHHLSNVSCVSSGSLGKRQHRVNSTFGDGNAACLLSGKISLQ
EASNALKQLLDAVPGNHKRPSLPDFLQTNPAVLSMLMTTLILSVFGNNAQSLCQQLERAT
EVQNALRDKQVKEYQEQIQKAIEQEDKARKAGIFGAIFDWITGIFETVIGALKVVEGFLS
GNPAEMASGVAYIAAGCAGMVKAGAETAMMCGADHDTCQAIIDVTSKIQFGCEAVALALD
VFQIGRAFMATRGLSGAAAKVLDSGFGEEVVERMVGAGEAEIEELAEKFGEEVSESFSKQ
FEPLEREMAMANEMAEEAAEFSRNVENNMTRSAGKSFTKEG
>EECEBICD_01736 Secreted effector protein SseC
MAKEAAKEALEKCVQEGGKFLLKKFRNKVLFNMFKKILYALLRDCSFKGLQAIRCATEGA
SQMNTGMVNTEKAKIEKKIEQLITQQRFLDFIMQQTENQKKIEQKRLEELYKGSGAALRD
VLDTIDHYSSVQARIAGYRA
>EECEBICD_01737 Secreted effector protein SseD
MEASNVALVLPAPSLLTPSSTPSPSGEGMGTESMLLLFDDIWMKLMELAKKLRDIMRSYN
VEKQRLAWELQVNVLQTQMKTIDEAFRASMITAGGAMLSGVLTIGLGAVGGETGLIAGQA
VGHTAGGVMGLGAGVAQRQSDQDKAIADLQQNGAQSYNKSLTEIMEKATEIMQQIIGVGS
SLVTVLAEILRALTR
>EECEBICD_01738 hypothetical protein
MVQEIEQWLRRHQVFTEPAYLGETAILLGQQFILSPYLVIYRIEAKEMIICEFRRLTPGQ
PRPQQLFHLLGLLRGIFVHHPQLTCLKMLIITDVLDEKKPCYAGNYCAS
>EECEBICD_01739 hypothetical protein
MKEDQKNKIPEDILKQLLSVDPETVYASGYASWQEGDYSRAVIDFSWLVMAQPWSWRAHI
ALAGTWMMLKEYTTAINFYGHALMLDASHPEPVYQTGVCLKMMGEPGLAREAFQTAIKMS
YADASWSEIRQNAQIMVDTLIA
>EECEBICD_01740 hypothetical protein
MKIHIPSAASNIVDGNSPPSDIQAKEVSFPPPEIPAPGTPAAPVLLTPEQIRQQRDYAIH
FMQYTIRALGATVVFGLSVAAAVISGGAGLPIAILAGAALVIAIGDACCAYHNYQSICQQ
KEPLQTASDSVALVVSALALKCGASLNCANTLANCLSLLIRSGIAISMLVLPLQFPLPAA
ENIAASLDMGSVITSVSLTAIGAVLDYCLARPSGDDQENSVDELYADPSALLAEQMAALC
QSATAPALMDSSGHTSRGEP
>EECEBICD_01741 hypothetical protein
MKPVSPNAQVGGQRPVNAPEESPPCPSLPHPETNMESGRIGPQQGKERLLAGLAKRVIEC
FPKEIFSWQTVILGGQILCCSAGIALTVLSGGGAPLVALAGIGLAIAIADVACLIYHHKH
HLPMAHDSIGNAVFYIANCFANQRKSMAIAKAVSLGGRLALTATVMTHSYWSGSLGLQPH
LLERLNDITYGLMSFTRFGMDGMAMTGMQVSSPLYRLLAQVTPEQRAPE
>EECEBICD_01742 hypothetical protein
MDIAQLVDMLSHMAHQAGQAINDKMNGNDLLNPESMIKAQFALQQYSTFINYESSLIKMI
KDMLSGIIAKI
>EECEBICD_01743 hypothetical protein
MESLLKSEVISDDVRRLLLEIMFAGVNHSLISQVHAMLPALTVIVPDKKLQLVCLALLLA
GLNEPLKAAKILSDIDLPEAMALRLLFPAPNEGFEN
>EECEBICD_01744 hypothetical protein
MNISDMSVVPVSTQSYVKSSAEPSQEQINFFEQLLKDEASTSNASALLPQVMLTRQMDYM
QLTVGVDYLARISGAASQALNKLDNMA
>EECEBICD_01745 Yop proteins translocation lipoprotein J
MKVHRIVFLTVLTFFLTACDVDLYRSLPEDEANQMLALLMQHHIDAEKKQEEDGVTLRVE
QSQFINAVELLRLNGYPHRQFTTADKMFPANQLVVSPRKNSRRLIF
>EECEBICD_01746 hypothetical protein

MEGVINAKVTIALPTYDEGSNASPSSVAVFIKYSPQVNMEAFRVKIKDLIEMSIPGLQYS
KISILMQPAEFRMVADVPARQTFWIMDVINANKGKVVKWLMKYPYQLMLSLTGLLLGVGI
LIGYFCLRRRF
>EECEBICD_01747 hypothetical protein
MLLQWFSEDEIWQLYGWLGQRDGKLLPPQVMQQTALQIGTAILNREAHDDAVLHALLVLL
PPPQRILWPKTSLTEIIFMEHLL
>EECEBICD_01748 hypothetical protein
MSFTSLPLTEINHKLPARNIIESQWITLQLTLFAQEQQAKRVSHAIVSSAYRKAEKIIRD
AYRYQREQKVEQQQELACLRKNTLEKMEVEWLEQHVKHLQEDENQFRSLVDHAAHHIKNS
IEQVLLAWFDQQSVDSVMCHRLARQATAMAEEGALYLRIHPEKEALMRETFGKRFTLIIE
PGFSPDQAELSSTRYAVEFSLSRHFNALLKWLRNGEDKRGSDEY
>EECEBICD_01749 hypothetical protein
MNIKINEIKMTPPTAFTPGQVIEEQEVISPSMLALQELQETTGAALYETMEEIGMALSGK
LRESNKFTDAEKLERRQQALLRLIKQIQEDNGAALRPLTEENSDPDLQNAYQIIALAMAL
TAGGLSKKKKRDLQSQLDTLTAEEGWELAVFSLLELGEVDTATLSSLKRFMQQAIDNDEM
PLSQWFRRVADWPDRCERVRILLRAIAFELSICIEPSEQSRLAAALVRLRRLLLFLGLEK
ECQREEWICQLPPNTLLPLLLDIICERWLFSDWLLDRLTAIVSSSKMFNRLLQQLDAQFM
LIPDNCFNDEDQREQILETLREVKINQVLF
>EECEBICD_01750 hypothetical protein
MDWDLITERNIQLFIQLAGLAERPLATNMFWRQGQYETYLNYHNGRIHLCQILKQTFLDE
ELLFKALANWKPAAFQGIPQRLFLLRDGLAMSCSPPLSSSAELWLRLHHRQIKFLESQCV
HG
>EECEBICD_01751 Secretion system apparatus protein SsaV
MRSWLGEGVRAQQWLSVCAGRQDMVLATVLLIAIVMMLLPLPTWMVDILITINLMFSVIL
LLIAIYLSDPLDLSVFPSLLLITTLYRLSLTISTSRLVLLQHNAGNIVDAFGKFVVGGNL
TVGLVVFTIITIVQFIVITKGIERVAEVSARFSLDGMPGKQMSIDGDLRAGVIDADHART
LRQHVQQESRFLGAMDGAMKFVKGDTIAGIIVVLVNIIGGIIIAIVQYDMSMSEAVHTYS
VLSIGDGLCGQIPSLLISLSAGIIVTRVPGEKRQNLATELSSQIARQPQSLILTAVVLML
LALIPGFPFITLAFFSALLALPIILIRRKKSVVSANGVEAPEKDSMVPGACPLILRLSPT
LHSADLIRDIDAMRWFLFEDTGVPLPEVNIEVLPEPTEKLTVLLYQEPVFSLSIPAQADY
LLIGADASVVGDSQTLPNGMGQICWLTKDMAHKAQGFGLDVFAGSQRISALLKCVLLRHM
GEFIGVQETRYLMNAMEKNYSELVKELQRQLPINKIAETLQRLVSERVSIRDLRLIFGTL
IDWAPREKDVLMLTEYVRIALRRHILRRLNPEGKPLPILRIGEGIENLVRESIRQTAMGT
YTALSSRHKTQILQLIEQALKQSAKLFIVTSVDTRRFLRKITEATLFDVPILSWQELGEE
SLIQVVESIDLSEEELADNEE
>EECEBICD_01752 putative secretion system apparatus ATP synthase SsaN
MKNELMQRLRLKYPPPDGYCRWGRIQDVSATLLNAWLPGVFMGELCCIKPGEELAEVVGI
NGSKALLSPFTSTIGLHCGQQVMALRRRHQVPVGEALLGRVIDGFGRPLDGRELPDVCWK
DYDAMPPPAMVRQPITQPLMTGIRAIDSVATCGEGQRVGIFSAPGVGKSTLLAMLCNAPD
ADSNVLVLIGERGREVREFIDFTLSEETRKRCVIVVATSDRPALERVRALFVATTIAEFF
RDNGKRVVLLADSLTRYARAAREIALAAGETAVSGEYPPGVFSALPRLLERTGMGEKGSI
TAFYTVLVEGDDMNEPLADEVRSLLDGHIVLSRRLAERGHYPAIDVLATLSRVFPVVTSH
EHRQLAAILRRRLALYQEVELLIRIGEYQRGVDTDTDKAIDTYPDICTFLRQSKDEVCGP
ELLIEKLHQILTE
>EECEBICD_01753 hypothetical protein
METLLEIIARREKQLRGKLTVLDQQQQTIITEQQICQTRALAVSTRLKELMGWQGTLSCH
LLLDKKQQMAGLFTQAQSFLTQRQQLENQYQQLVSRRSELQKNFNALMKKKEKITMVLSD
AYYQS
>EECEBICD_01754 hypothetical protein
MRITKVEGSLGLPCQSYQDDNEAEAERMDFEQLMHQALPIGENNPPAALNKNVVFTQRYR
VSGGYLDGVECEVCESGGLIQLRINVPHHEIYRSMKALKQWLESQLLHMGYIISLEIFYV
KNSE
>EECEBICD_01755 hypothetical protein
MLRIANEERPWMEMLPTQGATIGELTLSMQQYPVQQGTLFTINYHNELGRVWIAEECWQR

WCEGLIGTANRSAIDPELLYGIAEWGLAPLLQASDATLCQNEPPTSCSNLPHQLALHIKW
TVEEHEFHSIIFTWPTGFLRNIVGELSAARQQIYPAPPVVVPVYLGWCQLTLIELESIEI
GMGVRIHCFGDIRLGFFAIQLPGGIYARVLLTEDNTMKFDELVQDIETLLASGSPMSKSD
GTSSVELEQIPQQVLFEIGRASLEIGQLRQLKTGDVLPVGGCFAPEVTIRVNDRIIGQGE
LIACGNEFMVRITRWYLCKNTA
>EECEBICD_01756 Surface presentation of antigens protein SpaP
MSLPDSPLQLIGILFLLSILPLIIVMGTSFLKLAVVFSILRNALGIQQVPPNIALYGLAL
VLSLFIMGPTLLAVKERWHPVQVAGAPFWTSEWDSKALAPYRQFLQKNSEEKEANYFRSL
IKRTWPEDIKRKIKPDSLLLILIPAFTVSQLTQAFRIGLLIYLPFLAIDLLISNILLAMGM
MMVSPMTISLPFKLLIFLLAGGWDLTLAQLVQSFS
>EECEBICD_01757 Surface presentation of antigens protein SpaQ
MNDSELTQFVTQLLWIVLFTSMPVVLVASVVGVIVSLVQALTQIQDQTLQFMIKLLAIAI
TLMVSYPWLSGILLNYTRQIMLRIGEHG
>EECEBICD_01758 Surface presentation of antigens protein SpaR
MAQQVNEWLIALAVAFIRPLSLSLLLPLLKSGSLGSAILRNGVLMSLTFPILPIIYQQKI
MMHIGKDYSWLGLVTGEVIIGFLIGFCAAVPFWAVDMAGFLLDTLRGATMGTIFNSTIEA
ETSLFGLLFSQFLCVIFFISGGMEFILNILYESYQYLPPGRTLLFDQQFLKYIQAEWRTL
YQLCISFSLPAIICMVLADLALGLLNRSAQQLNVFFFSMPLKSILVLLTLLISFPYALHH
YLVESDKFYIYLKDWFPSV
>EECEBICD_01759 Yop proteins translocation protein U
MSEKTEQPTEKKLRDGRKEGQVVKSIEITSLFQLIALYLYFHFFTEKMILILIESITFTL
QLVNKPFSYALTQLSHALIESLTSALLFLGAGVIVATVGSVFLQVGVVIASKAIGFKSEH
INPVSNFKQIFSLHSVVELCKSSLKVIMLSLIFAFFFYYYASTFRALPYCGLACGLLVVS
SLIKWLWVGVMAFYIVVGILDYSFQYYKIRKDLKMSKDDVKQEHKDLEGDPQMKTRRREM
QSEIQSGSLAQSVKQSVAVVRNPTHIAVCLGYHPTDMPIPRVLEKGSDAQANYIVNIAER
NCIPVVENVELARSLFFEVERGDKIPETLFEPVAALLRMVMKIDYAHSTETP
>EECEBICD_01762 Multidrug resistance protein MdtK
MQKYTSEARQLLALAIPVILAQVAQTAMGFVDTVMAGGYSATDMAAVAIGTSIWLPAILF
GHGLLLALTPVIAQLNGSGRRERIAHQVRQGFWLAGFVSVLVMIVLWNAGYIIRSMHNID
PALADKAVGYLRALLWGAPGYLFFQVARNQCEGLAKTKPGMVMGFLGLLVNIPVNYIFIY
GHFGMPELGGIGCGVATAAVYWVMFIAMLSYIKHARSMRDIRNEKGFGKPDSIVMKRLIQ
LGLPIALALFFEVTLFAVVALLVSPLGIVDVAGHQIALNFSSLMFVLPMSLAAAVTIRVG
YRLGQGSTLDAQTAARTGLGVGICMAVVTAIFTVTLRKHIALLYNDNPEVVALAAQLMLL
AAVYQISDSIQVIGSGILRGYKDTRSIFFITFTAYWVLGLPSGYILALTDLVVDRMGPAG
FWMGFIIGLTSAAVLMMLRMRYLQRQPSAIILQRAAR
>EECEBICD_01763 Riboflavin synthase
MFTGIVQGTAKLVSIDEKPNFRTHVVTLPDYMLEGLETGASVAHNGCCLTVTEINGNQIS
FDLMKETLRITNLGTLRVGDEVNVERAAKFSDEIGGHLMSGHIITTAEIAKILTSENNHQ
VWFKVQDASLMKYILYKGFIGVDGISLTVGEVTPTRFCVHLIPETLERTTLGRKKLGERV
NIEIDPQTQAVVDTVERVLAARENAVRNQADIG
>EECEBICD_01764 Cyclopropane-fatty-acyl-phospholipid synthase
MSSSCIEEVSVPDDNWYRIANELLSRADITINGSAPSDIRVKNPDFFKRVLQEGSLGLGE
SYMDGWWECERLDIFFSKVLRAGLENQLPHHVKDTLRILGARLINLQSKKRAWIVGKEHY
DLGNDLFSRMLDPYMQYSCAYWKDADTLEAAQQAKLKLICEKLQLQPGMRVLDIGCGWGG
LSQYMATHYGVSVVGVTISAEQQKMAQTRCEGLDVSILLEDYRDLNDQFDRIVSVGMFEH
VGPKNYNTYFEVVDRNLKPDGLFLLHTIGSKKTDHNVDPWINKYIFPNGCLPSVRQIAEA
SESHFVMEDWHNFGADYDTTLMAWHERFINAWPEIAGNYNERFKRMFSYYLNACAGAFRA
RDIQLWQVVFTRGVENGLRVPR
>EECEBICD_01765 Inner membrane transport protein YdhC
MQPGKGFLVWLAGLSVLGFLATDMYLPAFAAIQADLQTPAAAVSASLSLFLAGFAVAQLL
WGPLSDRYGRKPILLLGLSIFALGSLGMLWVESAAALLTLRFVQAVGVCAATVIWQALVT
DYYPSQKINRIFATIMPLVGLSPALAPLLGSWILTHFSWQAIFATLFVITLLLMLPALRL
KPSVKARTEGQDKLTFATLLRSKTYRGNVLIYAACSASFFAWLTGSPFILSAMGYSPAVI
GLSYVPQTIAFLIGGYGCRAALQKWQGYQLLPWLLGLYALSVIATWGAGLLNNASLVEIL

IPFCVMAIANGAIYPIVVAQALRPFPQATGRAAALQNTLQLGLCFLASLVVSWLISTPLL
TTTSVMLSTVVLAALGYKMQSHADCAETGFPHANVAHDKSH
>EECEBICD_01766 putative HTH-type transcriptional regulator YahB
MWSEYSLEVVDAVARNGSFSAAAQELHRVPSAVSYTVRQLEEWLAVPLFVRRHRDVELTP
AGVWFLKEGRSVIKKMQITRQQCQQIANGWRGQLAIAVDNIVRPERTRQMIVDFYRHFDD
VELLVFQEVFNGVWDALSDGRVELAIGATQAIPVGGRYAFRDMGTLSWSCVVASDHPLAS
MPGPLSDDTLRNWPSLVREDTSRTLPKRITWLLDNQKRVVVPDWESSATCLSAGLCVGMV
PTHFARQWIDSGKWVALTLENPFPDAACCVTWQQNEASPALAWLLDYLGDSETLNREWLR
EPEEAPDSGD
>EECEBICD_01767 HTH-type transcriptional repressor PurR
MATIKDVAKRANVSTTTVSHVINKTRFVAEETRNAVWAAIKELHYSPSAVARSLKVNHTK
SIGLLATSSEAAYFAEIIEAVEKNCFQKGYTLILGNAWNNLEKQRAYLSMMAQKRVDGLL
VMCSEYPEPLLSMLEEYRHIPMVVMDWGEAKADFTDTVIDNAFAGGYMAGRYLVERGHRD
IGVIPGPLERNTGAGRLAGFMKAMEEALINVPDNWIVQGDFEPESGYHAMQQILSQSHRP
TAVFCGGDIMAMGALCAADEMGLRVPQDVSVIGYDNVRNARYFTPALTTIHQPKDSLGET
AFNMLLDRIVNKREESQSIEVHPRLVERRSVADGPFRDYRR
>EECEBICD_01768 putative protein YnhF
MSTDLKFSLITTLIVLGVIVAGGLTAALH
>EECEBICD_01769 Superoxide dismutase [Fe]
MSFELPALPYAKDALAPHISAETLEYHYGKHHQTYVTNLNNLIKGTAFEGKSLEEIVRTS
EGGIFNNAAQVWNHTFYWNCLAPNAGGEPTGKLADAIAASFGSFAEFKAQFTDAAIKNFG
SGWTWLVKSADGKLAIVSTSNAGTPLTTDATPLLTVDVWEHAYYIDYRNARPNYLEHFWA
LVNWEFVAKNLAA
>EECEBICD_01770 Murein DD-endopeptidase MepH
MARINKISITLCALLFTTLPFTPMAHASRQAKATPAVSHVTKTADKKKSTTKKISKTSKK
NVKKTSKTSRSSSASTPKRTTAATKSRHTTHRRNRTAPASIAALDVTEKCTTRKGRKPHC
VKGKGTLPVSIADAHRAKVQKATKTAMSKLMNQIGKPYHWGGASPRTGFDCSGLVYYAYK
DLVKIRIPRTANEMYHLRDAAPIERSELKNGDLVFFRTQGRGTADHVGVYVGNGKFIQSP
RSGQEIKITSLNEEYWQRHYVGARRVMTPKTIR
>EECEBICD_01771 Glutaredoxin 4
MSTTIEKIQRQIAENPILLYMKGSPKLPSCGFSAQAVQALSACGERFAYVDILQNPDIRA
ELPKYANWPTFPQLWVDGELVGGCDIVIEMYQRGELQQLIKETAAKYKTQEPDAE
>EECEBICD_01772 Ribonuclease T
MSDNAQLSGLCDRFRGFYPVVIDVETAGFNAKTDALLEIAAITLKMDEQGWLMPDMTLHF
HVEPFAGANLQPEALAFNGIDPSNPLRGAVSEYEALHAIFKMVRKGIKESGCSRAIMVAH
NATFDHSFMMAAAERASLKRNPFHPFVTFDTAALSGLALGQTVLSKACLAAGMEFDGEKA
HSALYDTERTAVLFCEIVNRWKRLGGWPLPLPTDK
>EECEBICD_01773 Lactoylglutathione lyase
MRLLHTMLRVGDLQRSIAFYTNVLGMKLLRTSENPEYKYSLAFVGYGPETEEAVIELTYN
WGVESYDMGNAYGHIALSVDNAAEACERIRQNGGNVTREAGPVKGGSTIIAFVEDPDGYK
IELIEAKDAGRGLGN
>EECEBICD_01774 N-ethylmaleimide reductase
MAPLTRLRSIEPGDIPTPLMAEYYRQRASAGLIISEATQISAQAKGYAGAPGLHSDEQIA
AWKKITQGVHAQGGHMAVQLWHTGRISHASLQPGGQAPVAPSAINAGTRTSLRDENGQAI
RVETSTPRALDTHEIPGIVNDFRQAIANAREAGFDLVELHSAHGYLLHQFLSPSSNHRTD
RYGGSVENRARLVLEVVDAGIKEWGADRIGIRLSPVGTFQNVDNGPNEEADALYLIEALG
KRGIAYLHMSEPDWAGGEPYSDAFREKVRACFHGPIIGAGAYTPEKAEDLIEKGLIDAVA
FGRDYIANPDLVARLQRKAELNPQRPESFYGGGAEGYTDYPTL
>EECEBICD_01775 HTH-type transcriptional repressor NemR
MNKQTEHDTREHLLATGEQLCMQRGFTGMGLSELLKTAQVPKGSFYHYFRSKEAFGVAML
ERHYACYHQRLTAHFTSGPGNHRDRLLAWYQETLKQFCQQGAISGCLTVKLSAEVCDLSE
DMRSTMDKGAQEIMALLARALEDGRNSHCLHFTGQPLPQAQVLYALWLGANLQAKISRSA
APLENALAHVKTIIATPEQ
>EECEBICD_01776 hypothetical protein

MAEQLEFFPVASPCRGICQSDERGFCRGCMRSRDERFNWQKMSDAEKQNVLRLCRQRFLR
KIRANKPLPSEDPQQPSLF
>EECEBICD_01777 Oxidoreductase YdhF
MVQRVTIAPQGPEFSRFVMGYWRLMDWNMSARQLVSFIEEHLDLGVTTVDHADIYGGYQC
EAAFGEALTLAPHLRERLQIVTKCGIATTARAENKLGHYITDRRHIILSAEQSLKNLATD
YLDMLLIHRPDPLMDADDVAEAFQHLHQSGKVRHFGVSNFTPAQFTLLQSRLPFTLATNQ
VEISPVHQPLLLDGTLDQLQQLRIRPMAWSCLGGGRLFNDEAYQPLRQELSVIAQELNAS
SIEQVVYAWILRLPSQPLPIIGSGKIERVRAALEAETLSLSRQQWFRIRKAALGYDVP
>EECEBICD_01778 Superoxide dismutase [Cu-Zn]
MKRLSLAMVTLLACAGAQAASEKVEMNLVTAQGVGQSIGTVVIDETEGGLKFTPHLKALP
PGEHGFHIHANGSCQPAIKDGQAVAAEAAGGHLDPQNTGKHEGPEGQGHLGDLPVLVVNN
DGIATEPVTAPRLKSLDEVKDKALMIHVGGDNMSDQPKPLGGGGTRYACGVIK
>EECEBICD_01779 p-hydroxybenzoic acid efflux pump subunit AaeB
MKLSLPALRNTPWFKATSGQWRYALRNTIAMCLALTFAYYLNLDEPYWAMTSAAVVSFPT
VGGVISKSLGRIAGSLLGATAALIIAGHTLNEPWLFLFSMAAWIGFCTWACAHFTNNAAY
AFQLSGYTAAIIAFPMVNIVEITQLWDIAQARVCEVIVGILCGGMMMMILPSTSDGTALL
TALKNMHARLLEHASLLWQPETTDAIRSAHEGVIGQILTMNLLRIQAFWSHYRFRRQNAL
LNALLHQQLRLTSVISSLRRMLLNWPTPPENSREVIEQLLAELAKPRADSYTVARIIAPL
RPQDEQDYRHLAFWQRLRYFCQLYLRSSRQLYLIESGAPVDQIHIRRTPGLARHTDNAEA
IWSGVRTFCTLTVIGAWSIGAQWESGPGALTLAAISCVLYSIVATPFKSLSLLMRTLVLL
SLFSFVVKFGLMVQITDLWQFLLFLFPLFVTMQLLKLQMPKLAGLWGQLIVFMGSFIAVT
NPPVYDFADFLNDNTAKIVGVAISWLAFAILRPGSDAVKSRRHIRALRRDFVDQLSRHPS
HNESEFESLTYHHVSQLSNSQDALARRWLLRWGVVLLNCSHVVWQLRAWESRSDPLSRVR
DICISLLRDVMSERGVQQRPLAVTLQELQRICDTLAHHHQPAAHELAAIIWRLHCSLSQL
EQAPAQGTLSPGYLMTPQA
>EECEBICD_01780 p-hydroxybenzoic acid efflux pump subunit AaeA
MSLKTIKYFSTLIVAVVAVLAAWWLWNYYMQSPWTRDGKIRAEQVSVTPQVSGSITQLNI
KDNQFVNAGDVLFVIDKTPFHIAELNAQAQLAKAQSDLAKANNEADRRRHLSRNYISAED
LDSANLNVKAMQASVDVALATLKQAQWQLSQTEVKAPVSGWVTNLSTRTGDYASTGKPLF
ALVDSHSFYVMGYFEETKLRHIREGEPALITLYSGNVKLQGHVGSIGRAIYDQSVESDSG
LVPDIKPNVPWVRLAQRVPVRIEFDALPQDITLVSGTTCSVAIGQR
>EECEBICD_01781 hypothetical protein
MKCILNATGLPLQDLMLGASVYFPPFFKAFALGFVIWLFIHRLLRDRIYSDEIWHPLLMD
LSLFTLCVCLGLVLLIAW
>EECEBICD_01782 Transcriptional regulator SlyA
MESPLGSDLARLVRIWRALIDHRLKPLELTQTHWVTLHNIHQLPPDQSQIQLAKAIGIEQ
PSLVRTLDQLEDKGLISRQTCASDRRAKRIKLTEKAEPLIAEMEAVIHKTRGEILAGISS
EEIELLIKLIAKLEHNIMELHSHD
>EECEBICD_01783 Outer membrane lipoprotein SlyB
MIKRVLAVSLMGLSLAGCVNNDSLSGDVYTASEAKQVQNVTYGTIVNVRPVQIQGGDDSN
VIGAIGGAVLGGFLGNTIGGGTGRSLATAAGAVAGGVAGQGVQSAMNKTQGVELEIRKDD
GNTIMVVQKQGNTRFSAGQRVVLASNGSQVTVSPR
>EECEBICD_01784 Anhydro-N-acetylmuramic acid kinase
MKSGRFIGVMSGTSLDGVDVVLAAIDETMVAQQASLTWPIPVHLKKGILDICQGQPLTLS
QLGQLDTQLGRLFAQAVNALLARQRLQPRDIVAIGCHGQTVWHEPTGEAPHTLQIGDNNH
IVAHTGITVVGDFRRRDIALGGQGAPLVPAFHHALLGHPTEKRMVLNIGGIANLSLLFPG
QAVRGYDTGPGNMLMDAWIWRQCAQPYDKDAAWAKEGQVILPLLQKMLRDPYFAASAPKS
TGREYFNYGWLERHLTAFPGADARDVQATLAELTAVSIAQQVLLNGGCERLMVCGGGSRN
PLVMARLAALLPGIEVSTTDKAGISGDDMEALAFAWLAWRTLAGLPGNLPSVTGATEASV
LGAIYPANPITQS
>EECEBICD_01785 Membrane-bound lysozyme inhibitor of C-type lysozyme
MPFQKQESFRDMPMKKIAIMCLPVLLTGCSVYQQFVERMQTDTLEYQCDEKPLTVKVNNP
REEVSFVYDNKLLTLKQGISASGARYTDGIYVFWSQGESATVYKRDRIVLNNCQLQNPKR
>EECEBICD_01786 Pyridoxine/pyridoxamine 5'-phosphate oxidase

MSDNDQLQQIAHLRREYTKGGLRRRDLPAEPLTLFERWLGQACDARLADPTAMVVATVDD
KGQPYQRIVLLKHYDEKGLVFYTNLGSRKAHQIEHNPRISLLFPWHMLERQVMVTGKAER
LSTLEVVRYFHSRPRDSQIGAWVSKQSSRISARGILESKFLELKQKFQQGEVPLPSFWGG
FRVSIEQMEFWQGGEHRLHDRFLYQRDDGAWKIDRLAP
>EECEBICD_01787 Tyrosine--tRNA ligase
MASSNLIKQLQERGLVAQVTDEDALAERLAQGPVALYCGFDPTADSLHLGHLVPLLCLKR
FQQAGHKPVALVGGATGLIGDPSFKAAERKLNTEETVQEWVAKIRKQVAPFLDFDCGENS
AIAANNYDWFGSMNVLTFLRDIGKHFSVNQMINKEAVKQRLNRDDQGISFTEFSYNLLQG
YDFACLNKLHGVALQIGGSDQWGNITSGIDLTRRLHQNQVFGLTVPLITKADGTKFGKTE
GGAVWLDPKKTSPYKFYQFWINTADADVYRFLKFFTFMDIEEINALEEEDKNSGKAPRAQ
YVLAEQVTRLVHGEEGLVAAKRITECLFSGSLSALSEADFEQLAQDGVPMVEMEKRRRPD
AGAGRRRTPAVAWSGA
>EECEBICD_01788 Tyrosine--tRNA ligase
MQALVDAELQPSRGQARKTIASNAVTINGEKQSDPEYIFNDEDRLFGRYTLLRRGKKNYC
LICWK
>EECEBICD_01789 Pyridoxal kinase PdxY
MKNILAIQSHVVFGHAGNSAAEFPMRRLGANVWPLNTVQFSNHTQYGKWTGCVMPPSHLT
EIVQGIADIGQLAHCDAVLSGYLGSAEQGEHILGIVRQVKAANPQAKYFCDPVMGHPEKG
CIVAPGVAEFHVRYALPASDIIAPNLIELEILSKHSVNNVNDAVQAARELIAQGPEIVLV
KHLARAGYSSERFEMLLVTAQEAWHISRPLVDFGSRQPVGVGDVTSGLLLVKLLQGATLQ
QALEHVTAAVYEIMIATKTMQEYELQVVAAQDRIANPEHYFSATRL
>EECEBICD_01790 Glutathione S-transferase GstA
MKLFYKPGACSLASHITLREIGKDFTLDGVDLAKKRLENGDDYLAVNPKGQVPALLLDDG
TLLTEGVAIMQYLADSVPDRQLLAPVSSLARYHTLEWLNYIATELHKGFTPLFRPDTPET
LKPAVRAALEKKFQYVDESLSDDQWICGQRFTIADAYLFTVLRWAYGVKLNMDGLTHIES
YMQRVAKRPTVAAALKAEGLN
>EECEBICD_01791 Dipeptide and tripeptide permease A
MSTANKKPTESVSLNAFKQPKAFYLIFSIELWERFGYYGLQGIMAVYLVKQLGMSEADSI
TLFSSFSALVYGLVAIGGWLGDKILGTKRVIMLGAVVLAIGYALVAWSGHDAGIVYMGMA
AIAVGNGLFKANPSSLLSTCYAKDDPRLDGAFTMYYMSVNIGSFFSMLATPWLAARYGWS
TAFALSVVGMLITVVNFAFCQRWVKSYGSKPDFEPINFRNLLLTIVGIVVLIAVATWLLH
NQDIARMVLGVIALGIVIIFGKEAFSMHGAARRKMIVAFILMLQAIIFFVLYSQMPTSLN
FFAIRNVEHSILGIAFEPEQYQALNPFWIIIGSPILAAIYNRMGDTLPMPMKFAIGMVLC
SGAFLILPLGAKFANDAGIVSVNWLIASYGLQSIGELMISGLGLAMVAQLVPQRLMGFIM
GSWFLTTAGANIIGGYVANLMAVPSDVTDPLMSLEVYGRVFMQIGIATAVIAVLMLLTAP
KLNRMTQDDDTAEKGSKAATV
>EECEBICD_01792 Endonuclease III
MNKAKRLEILTRLRDNNPHPTTELNFTSPFELLIAVLLSAQATDVSVNKATAKLYPVANT
PAAMLELGVEGVKSYIKTIGLFNSKAENVIKTCRILLDKHNGEVPEDRAALEALPGVGRK
TANVVLNTAFGWPTIAVDTHIFRVCNRTQFAPGKNVEQVEEKLLKVVPNEFKVDCHHWLI
LHGRYTCIARKPRCGSCLIEDLCEYKEKVDI
>EECEBICD_01793 Ion-translocating oxidoreductase complex subunit E
MSEIKDIVVQGLWKNNSALVQLLGLCPLLAVTSTATNALGLGLATTLVLTLTNLTVSALR
RWTPAEIRIPIYVMIIASVVSAVQMLINAYAFGLYQSLGIFIPLIVTNCIVVGRAEAFAA
KKGPWLSALDGFSIGMGATGAMFVLGSLREILGNGTLFDGADSLLGGWAKVLRVEIFHTD
SPFLLAMLPPGAFIGLGLMLAVKYLIDEKMKKRRAETAPSAVPAGETGKV
>EECEBICD_01794 Ion-translocating oxidoreductase complex subunit G
MLKTIRKHGITLALFAAGSTGLTAVINQMTKSTIHEQALQQQHALFDQVLPPDRYNNNLQ
ESCYLVDAPALGKGTHRVFIARKDDKPVAAIIEATAPDGYSGAIQLIVGADFNGTVLGTR
VTEHHETPGLGDKIERRLSDWITHFSGKTISGENDTHWAVKKDGGDFDQFTGATITPRAV
VNAVKRAGLYAESLPAQLPHLTACGE
>EECEBICD_01795 Ion-translocating oxidoreductase complex subunit D
MVFRIASSPYTHNQRQTSRIMLLVVIAALPGIAAQTWFFGWGTLFQIVLAAITALVAEAI
VLRLRKQSVASHLQDYSALLTGLLLAVSIPPLAPWWMVVLGTGFAIIIAKQLYGGLGQNP

FNPAMIGYVVLLISFPVQMTSWLPPYEIAATTPDMLDTLRMIFSGHTASGGDMTLLRIGI
DGISQATPLDTFKTSLRAGHSVEQIMQYPIYSGALAGVGWQWVNLAWLVGGVFLLWQKAI
RWHIPVSFLLTLALCAALGWLFSPATLASPQLHLLSGATMLGAFFILTDPVTASTTNHGR
LIFGALAGVLVWLIRSFGGYPDGVAFAVLLANITVPLIDYYTRPRVYGHRKG
>EECEBICD_01796 Ion-translocating oxidoreductase complex subunit C
MLKLFSAFRKDKIWDFDGGIHPPEMKTQSNGTPLRQVPLAPRFVIPLKQHIGAEGELCVS
VGDRVLRGQALTRGRGRMLPVHAPTSGTVIAIAPHSTAHPSALAELSVIIDADGEDRWIE
REGWSDYRAHSREALIERIHQYGVAGLGGAGFPTGVKLQGGGDKITTLIINAAECEPYIT
ADDRLMQDCAAQIVEGIRILAHILQPREVLIGIEDNKPQAISMLRAVLADAHDISLRVIP
TKYPSGGAKQLTQILTGKQVPHGGRSSDIGVLMQNVGTAYAVKRAVVDGEPITERVVTLT
GEAVSRPGNVWARLGTPVRHLLNDAGFCPSADQMVIMGGPLMGFPLPWLDVPVVKITNCL
LAPSVTEMGAPQEEKSCIRCSACADACPADLLPQQLYWFSKGQQHDKATAHHIADCIECG
ACAWVCPSNIPLVQYFRQEKAEINAIRLEEKRAAEAKARFEARQARLEREKAARLARHKS
AAVQPAAKDQDAIAAALARVKEKQAQATQPVVIQAGSLPDNSAVIAAREARKAQARAKQA
AHPVADSAISGGDPRKAAVEAAIARAKARKQEQQAGSEPAEPVDPRKAAVEAAIARAKAR
KQEQQAGSEPAEPVDPRKAAVEAAIARAKARKQEQQAGGEPAEPVDPRKAAVEAAIARAK
ARKQEQQAGSEPAEPADPRKAAVAAAIARVQAKKAAQQQVVNED
>EECEBICD_01797 Ion-translocating oxidoreductase complex subunit B
MNTIWIAVGALTLLGLVFGAILGYASRRFAVEDDPVVEKIDAILPQSQCGQCGYPGCRPY
AEAVGLQGEKINRCAPGGEAVMLKMAELLNVEPQPCDGEEQQAAPVRMLAVIDENNCIGC
TKCIQACPVDAIVGATRAMHTVMSDLCTGCNLCVDPCPTHCIELRPVNETPDSWKWDLNT
IPVRIIPVEQHA
>EECEBICD_01798 Ion-translocating oxidoreductase complex subunit A
MTDYLLLFVGTVLVNNFVLVKFLGLCPFMGVSKKLETAMGMGLATTFVMTLASICAWLID
TWILIPLDLIYLRTLAFILVIAVVVQFTEMVVRKTSPALYRLLGIFLPLITTNCAVLGVA
LLNINLGHHFLQSALYGFSAAVGFSLVMVLFAAIRERLAVADVPAPFRGNAIALITAGLM
SLAFMGFSGLVKL
>EECEBICD_01799 Inner membrane protein YdgK
MTTTPPQRIGGWLLGPLAWLLVALLSASLALLLYVMALATPQTFKTLSGQETGNLLLWGI
SFITAIAMWYYTLWLTIAFFKRRRCVPKHYIIWLLVSVLLAVKAFAFSPVSDAFAVRQLL
FPLLATALIVPYLKRSARVKTTFVNP
>EECEBICD_01800 Transcription modulator YdgT
MTVQDYLLKFRKISSLESLEKLFDHLNYTLTDDMDIVNMYRAADHRRAELVSGGRLFDVG
QVPQSVWRYVQ
>EECEBICD_01801 putative oxidoreductase YdgJ
MNDKIRVGLIGYGYASKTFHAPLIMGTPGLELAAVSSSDETKVKADWPAVSVVSEPRHLF
NDPHIDLIVIPTPNDTHFPLAKAALEAGKHVVVDKPFTVTLSQARELDALARSTGRVLSV
FHNRRWDSDFLTLKALLADGVLGEVSLFESHFDRFRPQVRDRWREQGGPGSGIWYDLAPH
LLDQAIQLFGLPVSMTVDLAQLRPGAQSTDYFHAILSYPQRRVILHGTMLAAAESARYIV
HGSRGSYVKYGLDPQEERLKNGERLPQEDWGYDMRDGVLTLVEGETRKEENWLTLPGNYP
AYYAAIRDALNGNGENPVPASQAIQIMELIELGMESAKHRATLCLA
>EECEBICD_01802 Adenosine deaminase
MGVKVLASLDACRRVAFENIEDAARNGLHYVELRFSPGYMAMAHQLPIAGVVEAVIDGVR
DGCNTFGVEARLIGIMSRTFGEAACLQELDALLAHRENITALDLAGDELGFPGSLFLSHF
NRARDAGWHITVHAGEAAGPESIWQAIRELGAERIGHGVKAVEDRALMDFLVQQRIGIES
CLTSNIQTSTVASLADHPLKTFLEHGVLASLNTDDPAVQGVDIIHEYHVAAPAAGLSREQ
IRQAQINGLEIAFLSDGEKRALREKVAAA
>EECEBICD_01803 Adenosine deaminase
MIDITLPLTDIHRHLDGNIRAQTILDLGRQFNIALPAQTLETLIPHVQVTSTEPDLVSFL
TKLDWG
>EECEBICD_01804 Protein YdgA
MKKTLVAAGVVIALGIVWTGGAWYTGKKLENHLSEMVTQANEQLKRTAPEAGVELSYQNY
QRGVFSSHLQLVVKPVAGADTTWLKPGQSIVLDESVSHGPFPLAQLKTLNLIPSMASVKT
TLVNNDAAKPLFDIAKGDTPFVINTRIGYGGDTRSDISLKPLNYENAGEKVAFSGGEFQL

NADKDGNVVSLSGEAQSGLVDAVNEYNQKVQLTFNNLKTDGTSKLASFGERVGDQKLTLD
KLSIAIEGKEMAVLEGMEIAGKSDLVNDGKTINSQLDYSLNSLKVQNQDLGSGKLTLKVG
QIDGEAWHQFSQQYHAQTQALLNQPDVAQNPELYQQKVTEAFFSALPVLLKGDPVLTLAP
LSWKNAKGETTLNLSLFLKDPATTTAQPQTLAQEVDRSVKSLDAKLAIPMDMAVEFMTQI
AKLEGYQQDDAEKLAKQQVQGLSAMGQMFRLTTLKDNTIASSLQYANGQITLNGQKMPLE
DFVGLFGMPALSVPDVPALPQQ
>EECEBICD_01805 Mannose-6-phosphate isomerase
MQKLINSVQNYAWGSKTALTELYGIANPQQQPMAELWMGAHPKSSSRITTANGETVSLRD
AIEKNKTAMLGEAVANRFGELPFLFKVLCAAQPLSIQVHPNKRNSEIGFAKENAAGIPMD
AAERNYKDPNHKPELVFALTPFLAMNAFREFSDIVSLLQPVAGAHSAIAHFLQAPNAERL
SQLFASLLNMQGEEKSRALAVLKAALNSQQGEPWQTIRVISEYYPDDSGLFSPLLLNVVK
LNPGEAMFLFAETPHAYLQGVALEVMANSDNVLRAGLTPKYIDIPELVANVKFEPKPAGE
LLTAPVKSGAELDFPIPVDDFAFSLHDLALQETSIGQHSAAILFCVEGEAVLRKDEQRLV
LKPGESAFIGADESPVNASGTGRLARVYNKL
>EECEBICD_01806 Fumarate hydratase class I, aerobic
MSNKPFFYQDPFPLKKDDTEYYLLTSEHVSVAEFEGQEILKVAPEALTLLARQAFHDASF
MLRPAHQQQVADILRDPQASENDKYVALQFLRNSDIAAKGVLPTCQDTGTAIIVGKKGQR
VWTGGGDEAALARGVYNTYIEDNLRYSQNAALDMYKEVNTGTNLPAQIDLYSVDGDEYKF
LCIAKGGGSANKTYLYQETKALLTPGKLKNYLVDKMRTLGTAACPPYHIAFVIGGTSAEA
NLKTVKLASAKYYDALPTEGNEHGQAFRDIELEKELLLEAQNLGLGAQFGGKYFAHDIRV
IRLPRHGASCPVGMGVSCSADRNIKAKINREGIWIEKLERNPGKYIPEALRQAGEGEAVR
VDLNRPMSEILQQLSQYPVSTRLSLNGTIIVGRDIAHAKLKERMDSGEGLPQYIKDHPIY
YAGPAKTPEGYASGSLGPTTAGRMDSYVDQLQSQGGSMIMLAKGNRSQQVTDACKKHGGF
YLGSIGGPAAVLAQGSIKRLECVEYPELGMEAIWKIEVEDFPAFILVDDKGNDFFQQIQS
SQCARCVK
>EECEBICD_01807 Fumarate hydratase class II
MVTVRREKDSMGAIEVPADKLWGAQTQRSLEHFRISTEKMPVSLIHALALTKRAAAKVNQ
DLGLLAAEKASAIIQAADEVLAGKHADEFPLAIWQTGSGTQSNMNMNEVLANRASEILGG
VRGMERKVHPNDDVNKSQSSNDVFPTAMHVAALLALREHLIPQLSALTDTLRDKSHAFAD
IVKIGRTHLQDATPLTLGQEISGWVAMLEHNLRHIEHSLPHVAELALGGTAVGTGLNTHP
EYARRVAEELATITAAPFVTAPNKFEALATCDALVQAHGALKGLAASLMKIANDVRWLAS
GPRCGIGEIAIPENEPGSSIMPGKVNPTQCEAVTMLCCQVMGNDVAINMGGASGNFELNV
YRPMVIHNFLQTVRLLADGMASFNQHCASGIEPNRERITQLLNESLMLVTALNTHIGYDK
AAEIAKKAHKEGLTLKASAVALGYLSDEEFDAWVRPELMVGSMTPGR
>EECEBICD_01808 DNA replication terminus site-binding protein
MSRYDLVERLNGTFRQIEQHLAALTDNLQQHSLLIARVFSLPQVTKEAEHAPLDTIEVTQ
HLGKEAETLALRHYRHLFIQQQSENRSSKAAVRLPGVLCYQVDNATQLDLENQVQRINQL
KTTFEQMVTVESGLPSAARFEWVHRHLPGLITLNAYRTLTLINNPATIRFGWANKHIIKN
LSRDEVLSQLKKSLASPRSVPPWTREQWQFKLEREYQDIAALPQQARLKIKRPVKVQPIA
RIWYKGQQKQVQHACPTPIIALINTDNGAGVPDIGGLENYDADNIQHRFKPQAQPLRLII
PRLHLYVAD
>EECEBICD_01809 Sensor protein RstB
MKKLFVQFYLLLFVCFLVMTLLVGLVYKFTAERAGRQSLDDLMKSSLYLMRSELREIPPR
EWGKTLKEMDLNLSFDLRVEPLNHYKLDAATTQRLREGDIVALDDQYTFIQRIPRSHYVL
AVGPVPYLYFLHQMRLLDIALMALIAFSLAFPVFIWMRPHWQEMLRLESAAQRFGEGHLT
ERLHFDNGSSFERLGVAFNQMADNINALIASKKQLIDGIAHELRTPLVRLRYRLEMSENL
TPPESQALNRDIGQLEALIEELLTYARLDRPQNELMLTEPDLPAWLLAHLQDVQSVTPER
AVNLLTCVIGDYGALDMRLMSRVLDNLLNNALRYSRTTVQVSLLLDGSQATLIVEDDGPG
IEADARERIFEPFVRLDPSRDRATGGCGLGLAIVHSIALAMGGSVVCDESELGGAKFSFR
WPVWHAMPDMTAA
>EECEBICD_01810 hypothetical protein
MGYRIPVQPEAKGHEAQWFSNLMAFRPARADDGQLAFLGLGESLVAGMNDGVRMAPGFSS
SEQDKTALRQVATFAAKRGIPLEIHAYTDDSADAILTIFEQVAQQYDLRPLRWSIAHLNT
GSPQTLERMRKLGLAYTVQMGPYFEGLAIRDANPPGATDNSPPVRLALDKGLVVAGGTDS

TRIGIAGVWHAIEYHITGIASGGSVRKPASERLTRLEALALYTRHAAWLAFAEQHRGQLR
VGKQADLAVLNQPFMTMPEDRIDTIRAVLTLVDGRIVHESPDLNAGQ
>EECEBICD_01811 Adenine deaminase
MISLVPPLLSRTALLFLLTATGAATAARPAADIILHNGNIITLNDAQPQASALVISGSRI
VAIGDDTATDEWRGDHTRTIDLQGKTVIPGLTDTHIHAIRGGQTWTFETYWYDSPSLKDA
LDKLRADANRRPHDQWVAVVGSWIPAQFAENRAPTVAELSHALPDHPAYIQYLYDYALVN
QRGIDVLGLNDTPPPDLAGIRVERDAKGSATGKLFGDIAAFNQLFASISSNADREGGLRQ
FFADMNARGVTGIIDPSAGPAAAYEPLFAMRNQGGFTAARGVSHSGTARSERS
>EECEBICD_01812 Outer membrane porin N
MKRKVLALVIPALLAAGAAHAAEIYNKDGNKLDLYGKVDGLHYFSDDSSKDGDQTYMRVG
FKGETQINDQLTGYGQWEYNVQANTTEGEGANSWTRLAFAGLKFGDYGSFDYGRNYGVLY
DVEGWTDMLPEFGGDSYTYADNYMTGRANGVATYRNTDFFGLVDGLNFALQYQGKNESQS
ADDVNIGTNNRNNGDDIRYDNGDGFGISTTYDIGMGFSAGAAYTTSDRTNEQVNAGGTIA
GGDKADAWTAGLKYDANNIYLATMYSETRNMTPYGKTDAGYAGGVANKTQNFEVTAQYQF
DFGLRPAVSFLMSKGKDLTYNNVNGDDKDLVKYADVGATYYFNKNFSTYVDYKINLLDDD
DQFYKDAGISTDDIVALGMVYQF
>EECEBICD_01813 Transcriptional regulatory protein RstA
MNRIVFVEDDAEVGSLIAAYLAKHDIDVIVEPRGDRAEDLILTTQPDLVLLDIMLPGKDG
MTICRDLRHRWQGPIVLLTSLDSDMNHILALEMGACDYILKTTPPAVLLARLRLHLRQSE
QTQQAKSLQESALTPHKALRFGALTIDPLNRAVQLNGDFISLSTADFELLWELATHAGQI
MDRDALLKTLRGVNYDGLDRSVDVAISRLRKKLLDSAAEPYRIKTIRNKGYLFAPHAWDE
TTG
>EECEBICD_01814 Inner membrane protein YdgC
MGLVIKAALGALVVVLIGLLSKTKNYYIAGLIPLFPTFALIAHYIVASERGIDAMRTTIV
FSMWSIIPYFIYLATLWYFSGVMRLPVALGGAVVCWGLSAWLLIFCWIKWH
>EECEBICD_01815 Putative arginine/ornithine antiporter
MEKKLGLSALTALVLSSMLGAGVFSLPQNMAAVASPAALLIGWAITGVGILLLAFAMLIL
TRIRSELDGGIFTYAREGFGELIGFCSAWGYWLCAVIANVSYLVIVFSALSFFTDTPALR
LFGDGNTWQAIVGASVLLWIVHFLILRGVQTAASINLVATLAKLLPLGAFIVLAIMMFKL
DTFTLDFTGVELGIPVWEQVKNTMLITLWVFIGVEGAVVVSARAKNKRDVGRATLLAVLA
ALGIYLLVTLLSLGVLARPELAEMRNPSMAGLMVKMMGPWGEIIIAAGLIVSVCGAYLSW
TIMAAEVPFLAAAYKSFPGIFARQNAQGAPSASLWLTNICVQICLVLIWLTGSDYNTLLT
IASEMILVPYFLVGAFLLKIATRPLHRAVGIGACIYGLWLLYASGPMHLLLSVVLYAPGL
LVFLYARRTHKHDNVLNRQEVVLIGLLLVAAVPATWMLVG
>EECEBICD_01816 Protein YdgH
MKLKNTLLASALLSAAAFSVHAATELTPEQAAALKPYDRIVITGRFNAIGDAVSAVSRRA
DKEGAASFYVVDTSEFGNSGNWRVVADVYKADAPKADAPKNRVINGIVELPKDQAVQLEP
YDTVTVQGFYRSQPEVNDAITKAAKQKGAYAFYIVRQIDANQGGNQRITAFIYKQDAKKR
IVQSPDAIPADSEAGRAALAQGGEAAKKVEIPGVATSASPSAEVGRFFETQSSKGGRYTV
TLPDGTKVEELNKATAAMMVPFDSVKFTGNYGNMTEISYQVAKRAAKKGAKYYHITRQWQ
ERGNNITISADLYK
>EECEBICD_01817 NAD(P) transhydrogenase subunit alpha
MRIGIPKERLPNETRVAATPKTVEQLLKLGFSVAIESGAGQLASFDDKAFAQAGADIVDG
NAIWQSEIILKVNAPEEEEIALLNPGTTLVSFIWPAQNPGLMEKLAERKVTVMAMDSVPR
ISRAQSLDALSSMANIAGYRAIVEAAHEFGRFFTGQITAAGKVPPAKVMVIGAGVAGLAA
IGAANSLGAIVRAFDTRPEVKEQVQSMGAEFLELDFKEEAGSGDGYAKVMSEAFIKAEMA
LFAAQAKEVDIIVTTALIPGKPAPKLITRDMVDSMKAGSVIVDLAAQNGGNCEYTVANQV
VTTDNGVKVIGYTDLPGRLPTQSSQLYGTNLVNLLKLLCKEKDGNIDVDFDDVVIRGVTV
IRDGDITWPAPPIQVSAQPQAAPKAAPAPKEPEKPASPWRKYALMALAIILFGWLADVAP
KEFLGHFTVFALACVVGYYVVWNVSHALHTPLMSVTNAISGIIVVGALLQIGQGGWVSFL
SFIAVLIASINIFGGFTVTQRMLKMFRKN
>EECEBICD_01818 NAD(P) transhydrogenase subunit beta
MSGGLVTAAYIVAAILFIFSLAGLSKHETSRQGNNFGIAGMAIALLATIFGPDTGNVAWI
LVAMIIGGAIGIRMAKKVEMTEMPELVAILHSFVGLAAVLVGFNSYLYHDASLAPVLVNI

HLTEVFLGIFIGAVTFTGSIAAFGKLRGKISSKPLMLPNRHKMNLAALVVSFLLLLVFVR
TESVGLQVLALLLMTIIALAFGWHLVASIGGADMPVVVSMLNSYSGWAAAAAGFMLSNDL
LIVTGALVGSSGAILSYIMCKAMNRSFISVIAGGFGTDGSSTGDDQEAGEHREITAEETA
EMLKNSHSVIITPGYGMAVAQAQYPVAEITEKLRARGINVRFGIHPVAGRLPGHMNVLLA
EAKVPYDIVLEMDEINDDFADTDTVLVIGANDTVNPAAQDDPRSPIAGMPVLEVWKAQNV
VVFKRSMNTGYAGVQNPLFFKENTQMLFGDAKASVDAILKAL
>EECEBICD_01819 AI-2 transport protein TqsA
MAKPIITLNGLKIVIMLGMLVVILSGIRFAADIIVPFILALFIAVVLNPVVQRMTRCRIP
RVIAVSLLIVIIVMLMVLLLAYLGTSLNELARTLPQYRSSLVIPLQRIEPWLQRAGIGVS
VDELAKYIDPNAAMTLVTNLLTQLSNAMSSIFLLLLTVVFMLLEVPQLPEKLQQMMSRPV
EGMAAIQRALDSVSHYLVLKTAISIVTGLVAWGMLAALDVRFAFVWGLLAFALNYIPNIG
SVLAALPPIIQVLVFNGFYDALLVLAGYLLINLVFGNILEPRIMGRGLGLSTLVVFLSLI
FWGWLLGPVGMLLSVPLTIIVKIALEQTTGGQSIAVLLSDLNKR
>EECEBICD_01820 Spermidine export protein MdtJ
MFYWILLALAIATEITGTLSMKWASVGNGNAGFILMLVMITLSYIFLSFAVKKIALGVAY
ALWEGIGILFITIFSVLLFDEALSTMKIAGLLTLVAGIVLIKSGTRKPGKPVKEATRATI
>EECEBICD_01821 Spermidine export protein MdtI
MQQFEWIHGAWLGLAIMLEIAANVLLKFSDGFRRKCYGILSLAAVLAAFSALSQAVKGID
LSVAYALWGGFGIAATLAAGWVLFGQRLNPKGWVGVILLLAGMVMIKFA
>EECEBICD_01822 hypothetical protein
MFFTGYGESQGAETDATDASDVKTLFFNHDDRVPVADPTQSPWDAIGQLETASGNLCTAT
LISPHIALTAGHCLLTPPKGKPDKAVALRFVARKGVWRYEIHGIEGRVEPSLGRRLKADG
DGWIVPPAAASWDFGLVILRYPPSGITPVPLFTGDKAALTAALKAADRKVTQSGYPEDHL
NALYSHQDCVVTGWAQNAVLSHQCDTLPGDSGSPLLLHTDSGWQLIGVQSSAPAAKDRWR
ADNRAISVTGFRDKLKALAQD
>EECEBICD_01823 Acid shock protein
MKKVLALVVAAAMGLSSAAFAAETATPAKTAAPAKTTQNTQHHKKQHKKTVEQKAQAAKK
HQKKGGKAPAKTPSKPTTQPAA
>EECEBICD_01824 Inner membrane transport protein YnfM
MSRTTILDDATASDIDEQRHSQPVQFIKRGTAPFMRVTLALFSAGLATFALLYCVQPILP
VLSQEFGVSPASSSVSLSISTAMLAIGLLFTGPLSDAIGRKPVMVTALLLASCCTLLSTM
MTSWHGILIMRALIGLSLSGVAAVGMTYLSEEIHPSFVAFSMGLYISGNSIGGMSGRLLS
GVMTDFFNWRIALAAIGCFALASALMFWKILPASQHFRPTSLRPKTLFINFRLHWRDRGL
PLLFAEGFLLMGAFVTLFNYIGYRLMLSPWELSQAVVGLLSVAYLTGTWSSPKAGAMTSR
YGRGPVMLFSTAVMLGGLLLTLFTSLWLIFAGMLLFSAGFFAAHSVASSWIGPRARRAKG
QASSLYLFSYYLGSSIAGTLGGVFWHSYGWNGVGGFIALMLVLAILVGTRLHHRLHA
>EECEBICD_01825 HTH-type transcriptional regulator BenM
MNIELRHLRYFVAVAEELHFGRAAARLNISQPPLSQQIQILEQQVGARLLARTNRSVALT
AAGRQFLVDSRHILSLVNDAAARAERLHQGEAGEIRIGFTSSAPFIRAVSHTFSLFRQSY
PGVHMQTREMNTREQIAPLNEGVLDIGLLRNTPLPDTLNREVILHEPLMAMIPREHRLAQ
KPVVSLTELAEEPFVFFDPHVGTGLYDDILGLMRRYNLSPTITQEVGEAMTIIGLVAAGL
GVSILPASFKRVQMSEMRWVPLAEEDAVSEMWLVWPKHHEQSHAVQRFCQLLLLAARRD
>EECEBICD_01826 Protein mlc
MVADSQPGHIDQIKQTNAGAVYRLIDQLGPVSRIDLSRLAQLAPASITKIVREMLEAHLV
QELEIKEAGSRGRPAVGLMVETEAWHYLSIRISRGEIFLALRDLSSKLVVEECLALPLNE
ATPLLERIITHVDRFFTRHQQKLERLTSIAITLPGIIDTENGVVHRMPYYEDVKEMPLGD
ALERHTGVPVYIQHDISAWTMAEALFGASRGARDVIQVVIDHNVGAGVITDGHLLHAGSS
SLVEIGHTQVDPYGKRCYCGNHGCLETIASVDSVLELTQLRLNQSMSSMLHGQPLTVDSL
CQAAMQGDLLAKDIISGVGAHVGRILAIMVNLFNPQKILIGSPLSKAADILFPAIADSIR
QQALPAYSRNTVVESTQFTNQGTMAGAALVKDAMYNGSLLIRLLQG
>EECEBICD_01827 ATP-dependent dethiobiotin synthetase BioD 1
MLKRFFITGTDTSVGKTVVSRALLQALASSGKSVAGYKPVAKGSKETAEGMRNKDALVLQ
SVSSLELPYEAINPIALSEEESSVAHSCPINYTLLSNGLASLSDKVDHVVVEGTGGWRSL
MNDLRPLSEWVVQEQLPVLMVVGIQEGCINHALLTAQAVANDGLPLIGWVANRINPGLAH

YAEIIDVLGKKLPAPLIGELPYLPRAEQRELGQYIRLSMLGSVLAVDRIMA
>EECEBICD_01828 Voltage-gated ClC-type chloride channel ClcB
MFRRLLIATLIGILAALAVAAFRHAMQLLEWIFLSNDTGSLVNAAEGLSPWRRLITPALG
GLAAGLLLWGWQKMNQQRPHAPTDYMEALQTDGQFDVGASLVKSLASLLVVVSGSAIGRE
GAMILLAALAASSFARRFTPREEWKLWIASGAAAGMAGAYHAPLAGSLFIAEILFGTLML
ASLGPVVVSAVVALLTTHLLNGSDSLLYTVHLTVDLHAREYVMIVSTGLVAGLCGPLLMW
LMTASHNSFLRLKLSPPWQLALGGLIVGLLSLLTPTVWGNGYSVVQSFLLSPPLFSLIGG
IFACKILAVLASSGSGAPGGVFTPTLFVGLSIGMFLGRIWGFWLPGSDEIAILLGLAGMA
TLLAATTHAPIMSTLMICEMTGEYQLLPGLLIACVVASVLSRTLRHDSIYRQHAAEH
>EECEBICD_01829 Osmoprotectant import ATP-binding protein OsmV
MIKLENLTKQFVQKKGQPLKAVDNVNLNVPEGEMCVLLGPSGCGKTTTLKMINRLIAPSS
GNILINGENTNDMDTVTLRRNIGYVIQQIGLFPNMTIEENITVVPRMLGWDKARCKQRAE
ELMDMVALDARKFLHRYPKEMSGGQQQRIGVIRALAADPPVLLMDEPFGAVDPINREVIQ
NQFLDMQRKLKKTVMLVSHDIDEALKLGDRIAVFRQGRIVQCASPDELLAKPANEFVGSF
VGQDRTLKRLLLVSAGDVTDQQPTITARPSMPLSEAFGIMDDHDIRAITVIDNDGKPLGF
VKRREARNASGICADITHPFRITGKAEDNLRIVLSRLYESNTSWMPIVDEDGRYNGEISQ
DYIADYLSSGRTRRALNIHESS
>EECEBICD_01830 Osmoprotectant import permease protein OsmW
MDTIHYMLDNAGYLASLTFQHLWLVALAVGLAIIIGVPLGVLIVRHKWLAMPVLGAATLL
LTIPSIALFGLMIPLFSLIGHGIGVLPAVTAVFLYSLLPIVRNTHTALDSLPPGLREAGR
GIGMTFWQRLRWVEIPMALPVIFGGIRTAVVMNIGVMAIAAVIGAGGLGLLLLNGISGSD
IRMLIAGALMICLLAIVLDWLLHRLQVVLTPKGIR
>EECEBICD_01831 Osmoprotectant-binding protein OsmX
MRFKKHLLGWLAATLLFSSQTQAAPLVLATKSFTEQHILSAMTVQYLQKKGFQVQPQTNI
AAVISRNAMVNKQIDITWEYTGTSLIIFNRIDKRMSPQETYDTVKRLDAKLGLVWLKPAD
MNNTYAFAMQRKRAESENITTISQMVAKIEQVRQNDPDHNWMLGLDLEFAGRSDGMKPLQ
QAYQMQLDRPQIRQMDPGLVYNAVRDGLVDAGLVYTTDGRVKGFDLKVLEDDKGFFPSYA
VTPVVRKEVLEANPGLDDALNTLSGLLNNDVISTLNAQVDIEHRTPQQVAHQFLQDKGLL
>EECEBICD_01832 Osmoprotectant import permease protein OsmY
MHTLTLKRVLGFTIVILLLLALFIWGIGLETLKARQVDLLYLGQRHLMLVFTSMFFALLV
GIPSGILLSRPAAKGFAEYVMQIFNVGNTLPPLAVLALAMVIIGIGIGDTPAIVALFLASLL
PIVRNTYAGLCSVPASLIEAANGIGMTKWQRLRQVELPNAWPVMLSGIRIATAINVGTAP
LAFLIGASSYGELIFPGIYLNDFPTLILGATATALFALILDTLLAWFGRRLSPHTV
>EECEBICD_01833 Tat proofreading chaperone DmsD
MTTFLQRDDFSVTARVLGALFYYSPESHETAPLVQALLTDDWQAQWPLDAEALAPVAAMF
KTHSEESLPQAWQRLFIGPYALPSPPWGSVWLDRESVLFGDSTLALRQWMRENGIQFEMQ
QNEPEDHFGSLLLLAAWLAENDRHHECEQLLAWHLFPWSSRFLDVFIDHAGHPFYQALGQ
LARLTLAQWQAQLIIPVAVKPLFR
>EECEBICD_01834 Anaerobic dimethyl sulfoxide reductase chain C
MGSGWHEWPLMIFTVFGQCVVGALIVSGLGWLTAKDDTIARQRIVRSMFFLWLVMGLGFL
ASIMHLGSPMRAFNSLNRVGASALSNEIAAGSVFFAVGGIWWLVAVLGKMPPALGKVWLL
VSMALGVAFIWAMTLVYQIDTVPTWYNGYTTLAFFLTAFLCGPVFAALLLRIARVPFCSV
TFASISGLALVVCVAVIVLQGLSLSTIHSSVQQASHLAPDYGMLQVWRIVLLAAGLGCWL
CPLIRRREPHTVGLLLGVVLVLAGEIIGRGLFYGLHMTVGMAIAG
>EECEBICD_01835 Anaerobic dimethyl sulfoxide reductase chain B
MTTQYGFFIDSSRCTGCKTCELACKDYKDLTPDVSFRRIYEYAGGDWQEDNGVWHQNVFA
YYLSISCNHCEDPACTKVCPSGAMHKRDDGFVVVNEEVCIGCRYCHMACPYGAPQYNAAK
GHMTKCDGCHDRVADGKKPICVESCPLRALDFGPIDELRKKHGELAAVAPLPRAHFTKPN
IVIKPNANSRPTGDTTGYLANPKEV
>EECEBICD_01836 putative dimethyl sulfoxide reductase chain YnfF
MAASISRRSLVKTSAIGSLALASSAFTLPFSRIAHAAADLASGNVAEKAVWSSCTVNCGS
RCLLRLHVKDDTVYWVESDTTGNDEYGNHQVRACLRGRSIRRRMNHPDRLKYPMKRVGKR
GEGKFERISWDEALDTIGDNLKRILKDYGNEAVHVLYGTGVDGGNITNSNVPYRLMNACG
GYLSRYGSYSTAQISAAMSYMFGGNDGNSPDDIANTKLVVMFGNNPAETRMSGGGVTYYV

EQARERSNARMIVIDPRYNDTAAGREDEWLPIRPGTDGALAAAIAWVLITEDLIDKPFLD
KYCIGYDETTLPASAPRNAHYKAYILGQGDDGIAKTPQWAAQITSIPAEKIIQLAREIGS
AKPAYICQGWGPQRHSNGEQTARAIAMLSVLTGNVGINGGNSGVREGTWDLGVEWFSMLE
NPVKTQISVFTWTDAIDHGAEMTATRDGVRGKDKLDVPIKFLWCYASNTLINQHGDIAHT
HEVLQDDSKCEMIVGIEHFMTASAKYCDILLPDLMPTEQEDLISHESAGNMGYVILGQPA
TSPKFERKPIYWTLSEVAKRLGPDVYQTFTEGRTQHEWVKYLHAKTKARNPEMPDYEEMK
QTGIFKKKCPEEHYVAFRSFREDPAANPLKTPSGKIEIYSERLATLANTWELKKDEIIHP
LPAYTPGFDGWDDPLRQRYPLQLTGFHYKARTHSSYGNIDILQQACPQEIWINPIDAQAR
GIQHGDTVRVFNQNGEMLILAKVTPRILPGVTAIGQGAWLNADMFGDKVDRGGSINILTS
HRPSPLAKGNPSHSNLVQVEKA
>EECEBICD_01837 Putative dimethyl sulfoxide reductase chain YnfE
MPGEKQQTGVSRRTLVKSAALGSLALAAGGVSLPFGMRTAAAAVQQAMRNEEDKIVWGAC
SVNCGSRCALRLHVKDNEVWWVETDNTGDDVYGNHQVRACLRGRSIRRRINHPDRLNYPM
KRVGRRGEGKFERISWQEALDTISASLKKTVETYGNEAVYIHYSSGIVGGNITRSSPAAS
PVKRLMNCYGGSLNQYGSYSTAQISCAMPYTYGSNDGNSTSDIENSKLVVMFGNNPAETR
MSGGGITWFLEQARERSNARMIVIDPRYTDTAAGREDEWIPIRPGTDAALVAGIAWVLIN
ENLVDQPFLDNYCIGYDEKTLPADAPPNGHYKAYILGQGEDGIAKTPQWASHITGIPADR
IIKLAREIGSVKPAYICQGWGPQRQANGEQTARAIAMLPILTGNVGIHGGNSGARESTYT
ITIERLPVLENPVKTTISCFSWTDAIARGPEMTALRDGVRGKDKLDVPIKFLWNYAGNTL
INQHSDINKTHEILQDETKCEMIVVIDNFMTSSAKYADILLPDLMTVEQEDIIPNDYAGN
MGYLIFIQPATTPKFERKPIYWVLSEIARRLGDDVYQRFTEGRTQAQWLQYLYAKMQARD
PALPAYDDLKKMGIYKRKDPNGHFVAYKKFREDPQANPLKTPSGKIEIYSSKLAHIASTW
ELAEGDVISPLPIYTPTFEGWDDPKRSTFPLQLFGFHYKSRTHSSYGNIDVLQAACRQEV
WINPLDAQKRGIANGDKVRVFNDRGEVRLPAKVTPRILPGVSAMGQGAWHNADMAGDKID
HGACINTLTTLRPSPWQKGILNIQIWWKSKKSKPPAPGRFYAPGAPRFANTTTFKTLFWM
SIVSCYENHQP
>EECEBICD_01838 putative protein YnfD
MKIATAIAALALVSAPSLVMAAPGSCERVKSDIEQRIINNGVPADNFTLTIVPNDQADQP
DSQVVGHCANDTHKILYTRTSSGNAPANTSPAQDGSSAEPQ
>EECEBICD_01839 hypothetical protein
MIRVKKPLLLTLLCMILAGCDNPKSLESFTPEMASFSNEFDFDPLRGPVKDFSQTLMSEN
GEVAKQVTGTLSQEGCFDTLELHDLENNTGLALVLDANYYRDAQTLEKKVQLQGKCQLAA
LPSAGVTWETDDNGFVVSATGKEMKVEYRYDSEGYPLGKTTINSQNTLSVTAKPSADPRK
KLDYTAVSRVDDRQVGNVTQSCEYDAYANPVDCRLVIVDESVKPAVFHHYTIKNRIDYY
>EECEBICD_01840 Spermidine N(1)-acetyltransferase
MTSDYKVRLRPLEREDLRFVHQLDNNASVMRYWFEEPYEAFVELSDLYDKHIHDQSERRF
VVECDGENAGLVELVEINHVHRRAEFQIIISPEYQGKGLASRAAKLAMDYGFTVLNLYKL
YLIVDKENEKAIHIYRKLGFRVEGELIHEFFINGEYRNTIRMCIFQQQYLDEHKTSGSTL
LKPTAQ
>EECEBICD_01841 hypothetical protein
MNNTLSKRLCLTAMLTLAAVVYTTSAFAETSKLVIESGDSAQSRQEAAMEKEQWNDTRSL
RQKVNTRAEKEWDKADAAFDNRDKCEQSANINAYWEPNTLRCLDRRTGRVITP
>EECEBICD_01842 hypothetical protein
MNLMLKTTLLFFVTALCEIIGCFLPWLWLKRGASVWWLLPAAASLALFVWLLTLHPAASG
RVYAAYGGVYVCTALLWLRVVDGVRLTVYDWCGALIALCGMLIIVVGWGRT
>EECEBICD_01843 D-galactonate dehydratase family member RspA
MKIVGAEVFVTCPGRNFVTLKITTEDGITGLGDATLNGRELPVASYLTDHLCPQLIGRDA
RRIEDIWQFFYKGAYWRRGPVTMSAISAVDMALWDIKAKAANMPLYQLLGGASREGVMVY
CHTTGHTIDDVLEDYARHKEQGFKAIRVQCGVPGMKTTYGMAKGKGLAYEPATKGQWPEE
QLWSTEKYLDFTPKLFDAVRNTFGFNEHLLHDMHHRLTPIEAARFGKGIEDYRLFWMEDP
TPAENQACFRLIRQHTVTPIAVGEVFNSIWDCKQLIEEQLIDYIRTTLTHAGGITGMRRI
ADFASLYQVRTGSHGPSDLSPVCMAAALHFDLWVPNFGVQEFMGYSEQMLEVFPHNWTFE
GGYMHPGDKPGLGIEFDEKLAAKYPYDPAYLPVARLEDGTLWNW
>EECEBICD_01844 Starvation-sensing protein RspB

MKSIVIEKPNTLTIETRALPQPAPGEVRIKVKLAGICGSDSHIYRGHNPFAKYPRVIGHE
FFGVIDAVGDNINRDRIGERVSVDPVISCGHCYPCSVGKPNVCTSLVVLGVHRDGGFSEY
AVAPARNAHRIPDNIADHHAVMVEPFTIAANVTGQVNPTEQDVALIYGAGPMGLTTVQAL
KGVYQVKTVIVVDRIEERLAMAKQSGADWTLNNGQHSLAEFLQQKALKPTLIVDAACHPA
ILQEAITLASSAARIVLMGFSSDPCEIVQQGITGKELAIYSSRLNANKFPVVIDWLNKGL
IDPDKLITHTFDYQHVTDAIELFEKDQRQCCKVLLTFAK
>EECEBICD_01845 Putative transporter YdfJ
MTQIKHERSTQDLVRAAVSGWLGTALEFMDFQLYSLGAALVFHEIFFPEQSAAMALILAM
GTYGAGYIARIIGAFVFGKMGDRIGRKKVLFITITMMGICTTLIGVLPTYAQIGIFAPVL
LVTLRIIQGLGAGAEISGAGTMLAEYAPKGKRGIISSLVAMGTNCGTLSATAIWAVMFFA
LEREQLIAWGWRIPFLASVVVMIFAIWLRMNLKESPVFEKVSEGEKSPALTPASENTLGA
MFTSKSFWLATGLRFGQAGNSGLIQTFLAGYLVQTLLFNKSIPTDALMISSVIGFITIPL
LGWLSDKYGRRLPYIILNISAIILAWPMLSIVVDKTYSPGVIMAALIVIHNFAVLGLFAL
ENITMAEIFGSRNRFTRMAISKEAGGWWRSVLAPSSQGSFVT
>EECEBICD_01846 hypothetical protein
MAVGFGPVLAGIFCNMTDSWLPILIMLVLYSCIGLISALLMPEVRDRDLSLPEDAAEATA
AEKLRHSATQTS
>EECEBICD_01847 Polyol:NADP oxidoreductase
MQNRLLSAKATLPDYDRAALVARMVHLGFGAFHRAHQGVYTDILAAEQHSDWGYYEVNLI
GGEQQIADLKQQDNLYTVAEMSAEAWTARVVGVVKAALHVQVDGLERVLAAMCEPQIAIV
SLTITEKGYCHSPATGQLLLEHPMIAADLQNPHQPLTAPGIIVEALARRKAAGLPAFTVM
SCDNMPENGHVTRQVVTAYAREVDAELAIWIEQNVTFPSTMVDRIVPAVTPETLDKIEQL
TGVRDPAGVACEPFRQWVIEDTFVAGRPLWENAGATLVADVVPFEEMKLRMLNGSHSFLA
YLGYLAGYQHINDCMADDNYRLTAQALMLREQAPTLKVQGVDLQRYADQLIARYRNPALR
HRTWQIAMDGSQKLPQRMLDSVRWHLANHSDFDLLALGVAGWMRYVGGVDEQGKAIDVSD
PLLPVIQRAVANSEEGASRVKALLGMAEIFGNDLPQAERFTQKVQEAYDSLLTYGAKASV
AKYAERLK
>EECEBICD_01848 Putative selenoprotein YdfZ
MMTYDRNRNAITTGSRVMISGTGHTGIIKAIESEGLDAGQIRRGKTVIVEGCEGKFAPVE
LIRLGMN
>EECEBICD_01849 HTH-type transcriptional repressor RspR
MTVETQLNPTQPVNQQIYRILRRDIVHCLIPPGTPLSEKEVSTRFNVSRQPVREAFIKLA
ENGLIQIRPQRGSYVNKISLSQVQNGCFVRQAIECAVVRRAAAMITDNQCYQLAQNLHLQ
HIAIERKQIDDFFQLDDDFHQKLAQIADCQLAWDTIENIKATIDRVRYMSLDHVSPPEML
LRQHHDIFSALENRDGNAVESAMTQHLQEISESVQLIRQENSGWFSED
>EECEBICD_01850 NADP-dependent 3-hydroxy acid dehydrogenase YdfG
MIVLVTGATAGFGECIARRFVENGHKVIATGRRHERLQALKDELGENVLTAQLDVRNRAA
IEEMMASLPAQWRDIDVLVNNAGLALGLEPAHKASVEDWETMIDTNNKGLIYMTRAVLPG
MVERNRGHIINIGSTAGSWPYAGGNVYGATKAFVRQFSLNLRTDLHGTAVRVTDIEPGLV
GGTEFSSVRFKGDDEKAGKTYENTTALTPEDITEAVWWVATLPAHVNINTVEMMPVTQSF
AGLSVHRS
>EECEBICD_01851 Dipeptidyl carboxypeptidase
MSTNPLLDQSMLPYQAPRFDRIKDCHYRPAFDEGVRQKRVEIEAIVNHPAAPDFTNTLLA
LEQSGALLSRVTSIFFAMTAAHTNDELQRLDEAFSAELAALSNDIYLNSALFARVDAVWQ
QRHSLGLDDESLRLVDVIHQRFVLAGAQLAEEDKAQLKVLNTESATLMSQFNQRLLAASK
AGGLAVDDAHCLAGLSPEEMTVAAEAAREKGLEERWFIPLLNTTQQPALATLRDRQTREN
LFAASWTRAEKGDAHDTRAIVQRLVEIRRCQAKLLGFPNYAAWKMADQMAKTPQAALSFM
RGIVPPARQRVLNEQAEIQNVIDGEQGGYTVQAWDWMFYAEQVRREKYALDEAQLKPYFA
LNTVLQEGVFWTANQLFGITFVERFDIPVYHPDVRVWEIFDSDGVGMALFYGDFFARDSK
SGGAWMGNFVEQSTLNETRPVIYNVCNYQKPVDGQPALLLWDDVITLFHEFGHTLHGLFA
VQRYATLSGTNTPRDFVEFPSQINEHWASHPRVFERYARHVDSGEKMPADLQEKMRKASL
FNKGYDMTELLGAALLDMRWHMLEESVAEQSVAEFEQQALAAEHLDLPAVPPRYRSSYFA
HIFGGGYAAGYYAYLWTQMLADDGYQWFVEQGGLTRENGQRFRDAILSRGNSTDLETLYS
AWRGHEPHIDPMLQYRGLDR

>EECEBICD_01852 hypothetical protein
MAEHRGGSGNFAEDKEKASEAGRKGGQHSGGNFKNDPQRASEAGKKGGQNNHSGGRKSGN
>EECEBICD_01853 Nicotinamide-nucleotide amidohydrolase PncC
MRLEQHDIVTLDEAESVDSLTKKLGDLLVNQKLRLTTAESCTGGKLASALCAAEDTPSFY
GVGYVTFTDEAKAKILRVQRHSLAEHTAVSEAVVTEMAQGAKDQAEVNISIAISGYGGPE
GGEDGTPAGTVWFAWNINNTTFTSRQHFNGDCQEVLEKCVHFALAELLFLLTKKA
>EECEBICD_01854 Protein YgiW
MTIEQAKTLHDGATVSLRGNLIDHKGDDRYVFRDKSGEINVIIPSAVFDGREVQPDQMIN
INGSLDKKAKEPLVRVNRLQK
>EECEBICD_01855 Small protein MgtS
MLGSINLFIVVLGIILFSGFLAAWFSHKWDD
>EECEBICD_01856 hypothetical protein
MVVADSGFAEKVVAVVLPVNAVIVVSLQYAVGRRLTAVNIRPMMTTGTVFFIAGLIGFIF
SGDNLFFWGLSAAVFTIGEIIYAPGEYMLIDNIAPAGMKASYFSAQSLGWLGAAVNPLVS
GVILTTLPAWSLFVVLIIAIVFAWALMLKGMRITPTQQAITC
>EECEBICD_01857 hypothetical protein
MNPGLRRSTLALLASSLLLTIGRGATLPFMTIYLNRQYGLGVDLIGYALTSALTIGVIFS
LGFGILADKFDKKRYMLLAIIAFACGFIAIPMVHNVVLVVLLFALINCAYSVFSTVLKAW
FADNLTATTKTRIFSLNYTVLNIGWTVGPPLGTLLVMQSINLPFWLAAICSAFPLVFIQV
WVTRSVAASEGKNAAIWSPSVLLRDKALLWFTLSAFLASFVGGLCVLYFTVRYGRCR
>EECEBICD_01858 putative amino-acid metabolite efflux pump
MMSRKDGFLALLVVVVWGLNFVVIKVGLHHMPPLLLAGLRFLLVAFPAIFFVARPKVPLT
LLLGYGLTISFGQFAFLFCAIKFGMPAGLASLVLQAQAFFTMALGAFVFSERLQRKQLAG
IALAIIGVLVLIEASLNGQHIAMSGFMLTLAAAFSWACGNIFNKKIMQHSPRPAVMSLVV
WSALIPILPFLLSSLLLEGADHITQSLITIDMTTILSLLYLAFVATILGYGIWGALLGRY
ETWRVAPLSLLVPVVGLASAAVLLGETLTGMQLAGAVLIMAGLYINVFGFRVRRTARVRG
>EECEBICD_01859 hypothetical protein
MLFPALPGLLLIASGYGIAEQTLLPVAQNSRDVMLLPCVGDPPNDLHPVSVNSDKSDELG
VPYYNDQHL
>EECEBICD_01860 Multiple antibiotic resistance protein MarA
MSRRNTDAITIHSILDWIEDNLESPLSLEKVSERSGYSKWHLQRMFKKETGHSLGQYIRS
RKMTEIAQKLKESNEPILYLAERYGFESQQTLTRTFKNYFDVPPHKYRITNMHGESRYML
PLNHGNY
>EECEBICD_01861 Multiple antibiotic resistance protein MarR
MLCSIRCAGCITPVELKKVLSVDLGALTRMLDRLLCKGWIERLPNPNDKRGVLVKLTPDG
AAICEQCHQRPGQDLHQELTKNLTADEVATLEYLLKKILP
>EECEBICD_01862 hypothetical protein
MMDLFKAIGLGLVVLLPLANPLTTVALFLGLAGNMNSAERNRQSYMASVYVFAIMMVAYY
AGQLVMNTFGISIPGLRIAGGLIVAFIGFRMLFPQQKAHESPEAKSKSEELADEPTANIA
FVPLAMPSTAGPGTIAMIISSASTVRHGGEFPDWVIMVAPPIIFLAVAVILWGCLRSSGA
IMRLVGKGGIEAISRLMGFLLVCMGVQFIINGVLEIIKTYH
>EECEBICD_01863 Sugar efflux transporter
MTINPVSRKVAWLRVVTLAIAAFIFNTTEFVPVGLLSDIAESFHMQTAQVGIMLTIYAWV
VAVMSLPFMLLTSQMERRKLLICLFVLFIASHVLSFLAWNFTVLVISRIGIAFAHAIFWS
ITASLAIRLAPAGKRAQALSLIATGTALAMVLGLPIGRVVGQYFGWRTTFFAIGMGALIT
LLCLIKLLPKLPSEHSGSLKSLPLLFRRPALMSLYVLTVVVVTAHYTAYSYIEPFVQNVA
GLSANFATVLLLILGGAGIIGSLVFGKLGNRHASSLVSIAIALLVVCLLLLLPAADSEAH
LAILSIFWGIAIMVIGLGMQVKVLALAPDATDVAMALFSGIFNIGIGAGALVGNQVSLHW
SMSAIGYIGAIPACAALVWAVLIFRKWPVTLEEQPH
>EECEBICD_01864 HTH-type transcriptional regulator GltR
MDLTQLEMFNAVALTGSITQAAAKVHRVPSNLTTRIRQLEADLGVSLFIRENQRLRLSPA
GHNFLRYSQQILALVDEARMVVAGEEPQGLFSLGALESTAAVRIPALLAGYNQRYPKIQF
ALTTGPSGAMLDGVLEGKLNAAFIDGPLMHPGLEGIPAYQEEMMIVAPHGHSVVSRASEV
NGYNIYAFRANCSYRRHFESWFHADGATPGTIHEMESYHGMLACVIAGAGIALMPASMLN

SMPGHHQVEAWPLAEKWRWLNTWLMWRRGAMTRQLEAFIELLNAQLASVD
>EECEBICD_01865 Succinate semialdehyde dehydrogenase [NAD(P)+] Sad
MTMMTATQALSVNPATGQTLAAMPWANAQEIEHALSLAASGFKKWKMTSVAQRAQTLRDI
GQALRAHAEEMAQCITREMGKPIKQARAEVTKSAALCDWYAEHGPAMLNPEPTLVENQQA
VIEYRPLGVILAIMPWNFPLWQVLRGAVPILLAGNSYLLKHAPNVTGCAQMIARILTEAG
TPAGVYGWVNANNEGVSQMINDPRIAAVTVTGSVRAGAAIGAQAGAALKKCVLELGGSDP
FIVLNDADLELAVKAAVAGRYQNTGQVCAAAKRFIVEEGIAQAFTDRFVAAAAALKIGDP
LVEENDLGPMARFDLRDELHQQVQASVAEGARLLLGGEKIAGEGNYYAATVLADVTPDMT
AFRQELFGPVAAITVAKDAAHALALANDSEFGLSATIFTADDTLAAEMAARLECGGVFIN
GYSASDARVAFGGVKKSGFGRELSHFGLHEFCNVQTVWKNRV
>EECEBICD_01866 Glutaminase 2
MAWAMDNAILETILQRVRPLIGQGKVADYIPALASVEGSKLGIAICTVDGQHYQAGDAHE
RFSIQSISKVLSLVVAMRHYPEEEIWQRVGKDPSGSPFNSLVQLEMEQGIPRNPFINAGA
LVVCDMLQGRLSAPRQRMLEVVRALCGVSDITYDATVARSEFEHSARNAAIAWLMKSFGN
FHHDVPTVLQNYFHYCALKMSCMELARTFVFLANQGEAFHLDEPVVTPMQARQINALMAT
SGMYQNAGEFAWRVGLPAKSGVGGGIVAIVPHEMAIAVWSPELDPAGNSLAGIAALEQLT
QTLGRSVY
>EECEBICD_01867 hypothetical protein
MNALEPLFARLARSTFRSRFRLGIKERQYCWDKGAEVIDKHAADFIAQRLAPAHPANDGK
QTPMRGHPVFIAQHATATCCRGCLAKWHQIPQGEPLSEAQQQYIVSVIHYWLVIQMNQR
>EECEBICD_01868 hypothetical protein
MTVLFYLLKIIMIVRPQQHWIRLIFVWHGSVLSKIFSRLLLNFLLSIAIIIMLPWYTMLG
IKFTLAPFSILGVAIAIFLGFRNNACYARYVEARHLWGQLMIASRSILREVKTTLPDERG
IEDFVRLQIAFAHCLRMTLRRQPQTQVLGNYLDQEALQKVVASHSPANRILLLMGEWLAI
RRRSGKLSDILFHSLNNRLNDMSSVLAGCERIANTPVPFAYTLILHRTVYLFCIMLPFAL
VVDLHYMTPFISVLISYTFIALDALAEELEDPFGTENNDLPLDAICNAIEIDLLQMNDER
DIPEKRIPDKRYQLT
>EECEBICD_01869 hypothetical protein
MQKTSLLFSAAAITLTLMVSSASAATPTAAQNEATSTVKQEITEGINRYLYSIDKADPTL
GKQLFYVSPETSFIHPRGHERGWSQIAENFYGTTMGKTFSKRTLKLDAPPAIHVYGNAAV
AEFDWHFTAVRRDNGQTQHTTGRESQVWAKIPNTGWRIVHVHYSGPAKTGVGEGY
>EECEBICD_01870 Outer membrane porin C
MKKVVVLSAVAAAVMMAGAANAAEIYNKDGNKLDLYGKVDGLHYFSSNHSTDGDQSYIRM
GIKGETQITDQLTGFGQWEYQVNANRPEDGDSSGSPQSWTRLGFAGLAFADMGSVDYGRN
YGVLYDIGSWTDVLPEFGNDSYEASDNFMTGRANGVLTYRNNDFFGLVDGLNIALQYQGK
NDGLSKEGDPLSNNARKSIAYQNGDGFGASATYDLGMGVSLGAAYTSSKRTLDQMTQDKY
DNGDRAEAWTGGVKYDANNIYLAANYTRTYDMTYMGDTLGGFAHKTDNWEMVGQYQFDNG
LRPSLAFLQSRANDVDGLGSFDLVKYIDVGSYYYFNKNMSAYVDYKINLLKDGNPSNPNT
DNTVALGLVYEF
>EECEBICD_01871 Hydrogenase maturation factor HybF
MHELALAQNIIELLEEQAVNHQFSKVKQVWLEIGVMACVEVPALHFGLDVAARHTLADNA
RFHITIAPAQGWCLSCNQPFTSQTSTLCCPFCHSGKVQIDDSSRMRIREIEVE
>EECEBICD_01872 hypothetical protein
MQRLTVYSHPLRIIWQEAPIGRLLQGATPVYAKTLISRLFTLCAQAHSAAAALLLFPEKK
PDMQAAQQELARETLRRALTDWLPLFSHRQATAEEWALLRRGELSPLASTIFFDDDPQTW
LAAGVKGWEAWFLQERSETARWLAAVQNIITPTLPMASSPDHTLITHGPLDVSPLAIEYP
LLSACCLSGKTTALRLLARCITLARSLSALPTLRWNRFDDGEWKIAVVETARGWLVHQAR
LTTSGNILDYRIISPTTRHAQPDGVIARELATIPLSLWSQQLQVIDPCVAVNIVE
>EECEBICD_01873 Anaerobic nitric oxide reductase flavorubredoxin
MNYSRTIPVVNIAGPGSQPEEEDFNFLPIPAGINLPLTPVLPEQALPAELRVARHILTTL
IRDMDNPVATLPFPLSYKLNATEQQNSGLLDQLLGEGEISARVLLSDGKEQRIQETVFTG
VWRVREYNADQQRVADEIIIGPIPESIWQTHPQPPITPELPPQPAGLMNGAFIAHEIAER
VKQPVKEPHIINLTLLPVNDADREYLDHFLGEGCSAIFSRGYGKCRIVSTHFPGVWRVNY
FNDMNTLLQDMIEIADIPDIAVAGIDDIEDAYAGLKNTLEWLKEYPVTENEPVVRMECKV

CWWVYDPALGDDVWQIPPGVPFSQLPDYWCCPVCETSKSGFMVIDEGNSSCKD
>EECEBICD_01874 hypothetical protein
MTISPQNPVCPLADKGFLVTDAQTLPSLINAHPTVLVLLQSDPNKHPEVADSWVIIPEIL
KQFPATSYTAAFADSEQSELIAREYRILKYPALLFFRQHRFVGSLAGLYSWQEYSQRVAA
LLTTPGYRQDIPVITQ
>EECEBICD_01875 Hydrogenase 1 maturation protease
MAEVTILGLGNLLWADEGFGVRAAEKLFEQYADNEKVDVVDGGTQGLALLPWLQQTEKLL
IMDAIDFGMAPGSLAMFRDEQVPAYLTAKKLSLHQTSFSEVLALLQLTGGQLSEIVLIGV
QPECLDDYGGSLTPQVKAQLMPAVYLAQEVLAQWGITASSAALPTERLNHYSLCMERYED
ERPDAQSACRVGDIRVLQREKS
>EECEBICD_01876 putative Ni/Fe-hydrogenase 1 B-type cytochrome subunit
MHLKEITSQGYYIYEAPVRLWHWITALSIVVLAVTGYFIGRPLPSIQGEATFMFWMGWIR
LIHFTTAYIFTVALLFRIYWACVGNEYAREMFLVPFWRRAWRKGVISEIRWYFFLEKEAH
RYYGHNPVAGLAVMFYFWMSVLMVCSGFALYGEGLGTDSWAYQWFGWMIRLTGNDSLALH
FWHRLGMWFIIAFVIAHVYTAIREDIMSRQSVISVMISGWRWFR
>EECEBICD_01877 Hydrogenase-1 large chain
MAYPYQTQGFTLDNSGRRIVVDPVTRIEGHMRCEVNIDSNNVITNAVSTGTMWRGLEVIL
KGRDPRDAWAFVERICGVCTGTHALTSIRAVENALGIAIPDNANCIRNMMQATLHVHDHL
VHFYHLHALDWVDVVAALKADPHQTSAIAQSLSAWPLSSPGYFRDLQNRLKRFIESGQLG
PFRNGYWGHPAMKLPPEANLLAVAHYLEALDFQKEIVKIHTVFGGKNPHPNWLVGGVPCA
INLDETGAVGAVNMERLNLVSSIIQKARQFCEQVYLPDVLLIASYYKDWAKIGGGLSSMN
LLAYGEFPDNPNDYSASNLLLPRGAIINGRFDEIHPVDLTAPDEIQEFVTHSWYTYGNGN
NDKGLHPWDGLTEPQLVMGEHYKGTKTFIEQVDESAKYSWIKSPRWKGHAMEVGPLARYL
IGYHQNKPEFKEPVDQLLSVLKLPKEALFSTLGRTAARALESVWAGNTLQYFFDRLMRNL
KSGDTATANVTLWEPDTWPTSAKGVGFSEAPRGALGHWIKIENQKIDSYQCVVPTTWNAG
PRDDKGQIGAYEAALMGTKLAVPDQPLEILRTLHSFDPCLACSTHVIDNHGGELVRVQVR
>EECEBICD_01878 Uptake hydrogenase small subunit
MQTQDTFYQVMRRHGVTRRSFLKFCSLTATSLGLSSSMIPQIAYALENKPRTPVIWLHGL
ECTCCTESFIRSAHPLAKDAILSLISLDYDDTIMAAAGQQAEQALADVMREYKGNYIVAV
EGNAPLNEDGMFCILAGEPFLEKLKRVSADAKAIIAWGACASWGCVQAARPNPTKATPVH
KLITDKPIIKVPGCPPIPEVMSAVITYMLAFDRIPPLDRLGRPKMFYGQRIHDKCYRRAH
FDAGQFVEAWDDEGARKGYCLYKMGCKGPTTYNACSTVRWNDGVSFPIQSGHGCLGCSED
GFWDYGSFYSRATGIPQTGIEATADKIGLGVAGVAGAAAIAHATVSAIKHARNKNNTSSE
NAPEEKK
>EECEBICD_01879 hypothetical protein
MKKSKAILFIAPALFSYAMAAQACTTLAIQDKQGDIFHGRTLEYMQDLPSWLTYYPAGTQ
FDKKTPDGSQGISYQAKYPILAITSTITDGDSRDILEGMNSAGLSFSENMIMNAQLPPLP
ASEYKQAIPVTSLGEWALARFATVGEVKQAIKEGKFWSPELHRFGDLKSPFHYAFYDKKG
GSIVVEVENGKFHVYDNPTRVMTNGPAFPWHLTNLNNYTQLTNVDRSSGTLGGIKVMQPD
SGIAIADLPSSDTSVSRFIRGVYYTTYAPQATSAHDAMNTLAHIMSRFDRPKNITVDYMG
SEGEGNATRKPVSEYTVWTTLSDLTHGEMMVRGYNDINYKTWSLSQFKNATAPVFEKINV
KG
>EECEBICD_01880 Pyruvate dehydrogenase complex repressor
MVIPEVRQERLYQKIANLIIKLINDNIFPPGTFLPPERELAKQLGVSRASLREALIVLEI
SGWIVIQSGNGVIVSDKKHLASDYTIEEILSTRELVDSHCARLAAQNDNVDVINQIEAIY
HRMDQAINDNNVHGFYSLDKEFHLAISEASRNRVLFDMSRMLWEQRINIPYAGLDEQSGD
RNVLLNLNKQHKAIVDAMRQSDADSAYEGSLEHLRYVRKIVGG
>EECEBICD_01881 putative zinc-type alcohol dehydrogenase-like protein
YjmD
MKVKAVFINKPGEVETRWVDYPHKKENEVLIKVDAAGICGSDIGAFRGTNPLVTYPRIIG
HEVTGIVLQEGAGMPDNIKEGDRVIVDPYIYCGHCYPCSVGRTNCCENLNVIGVHVDGAM
QEVVTHPAHLIHKIPDNVPSEMAPLAEPLTIALHALHRANVKAGEYVAIIGAGAIGLMAA
LSARHYKATPILIDIVEERLRYAQKLGVPYVLNAADDQVIDAIKNITHGTMAQVVIEASG
ANSAIRNMLDLASFAGRISFTGWPKQETSLPTNLITYKELDLRGSRTSAGEFDEALRMLS

TLEINPQDVVSKVVNLDEIPDAVKELDRYPERYLKINAVFH
>EECEBICD_01882 Hexuronate transporter
MGDNSGFFQLQLYSFFFLTWFPSYLNHSLHLDIKEISIATVIPWVIGAIGMVLGGVCSDV
IYRITGNALLSRRLILGVCLAGAAVCVAVSGTVSTIGSAITLMSVSLFLLYLTGPIYWAV
IQDVVHKDKVGSVGGAMHGLANISGIIGPLVTGFIVQFSGKYDYAFYLAGAIAIVSSLLV
FVFVKSKGFKANESQSCVH
>EECEBICD_01883 Hexuronate transporter
MKRKTMGWLIVFLLFIVYMLNYMDRSALSITAPLIEKELGFNAAEMGMIFSAFFIGYALF
NFIGGWASDKVGPKTVFLIAALLWSVFCGLTGLVTGLWTMLIVRVLFGMAEGPVSAAGNK
IINNWISRKESATAIGIFSAGSPLGGAVSGPIVGLLALSLGWRPAFGIIFLFGLVWVLLW
YFIVSDKPTMSKRLAPEERIDFENHEDVILSDDGRATPSLGYYMKQPMVWATTLAFFSYN
YILFSS
>EECEBICD_01884 hypothetical protein
MKKTYLVVIVLFFISTKVYTLLHNNIFFCRNSPECDLSHVLPDYREQISGTPLKYTLINT
APLAQVVVRHYELLSQHWSPDDMVTPAQWRHNVDIYIPETAKEHHALVVVNNGINYDKGV
QITGKPGDFPQETLASISRDTNTIVISVSDIPNQYLTFQDDKKPLKEDESVSRSWALFME
APEKRELMPLNIPMVTALSQAMRLAKKELTQWNINSFIITGISKRGWTTWLSAIADPDVE
AIVPFAIDLLDIDASLEHIYQSYGGNWPITFYPYYQQGIDEKIKSPTFTQLRQIIDPLRY
LNTIYQPRLAIPKYIINASGDDFFVPDNTRFYYSKLPGVKSLRIVPNMNHYSINQFAEES
LVPFINRFQSKKTLPQLIGLIHHHLLTVYFSEAPVKVVRWTANNPNARDFRYACGIRYKP
LTIDIPANNKISITLNEPKTGWEATYIEATFNDGYVATSQVYITPDEKYPQTAPPSVNAA
CQTLPGRGLGENDSPD
>EECEBICD_01885 Tetracycline resistance protein, class C
MNTNVYENTDSETITPLNKRRILPVFLLVGLYAASTAAVMSVLPFYIREMGGSPLIIGII
IATEAFSQFCAAPLIGHLSDRVGRKRILIVTLAIAAISLLLLANAQCILFILLARTLFGI
SAGNLSAAAAYIADCTHVRNRRQAIGILTGCIGLGGIVGAGVSGWLSRISLSAPIYAAFI
LVLGSALVAIWGLKDPSTTSRTTDKIAAFSARAILKMPVLRVLIIVMLCHFFAYGMYSSQ
LPVFLSDTFIWNGLPFGPKALSYLLMADGVINIFVQLFLLGWVSQYFSERKLIIILIFALL
CTGFLTAGIATTIPVLVFAIVCISIADALAKPTYLAALSVHVSPARQGIVIGTAQALIAI
ADFISPVLGGFVLGYALYGVWIGIAISVAIIGLVTAMIYLSKSSVLIVKPETE
>EECEBICD_01886 Flavin-dependent monooxygenase
MTRYTTTDVLICGAGVTGLTLAIELARHGVSFRLIEKRTTPFIGSRGKGIQPRTQEIFED
LGILNKVVAAGGLYPRLRTYRHDGSYVDSDIAHHTKPTHAEPYHLPLMVPQNVTETIMRE
QLKAWGHRVEFGCELRHFAQTPRTVTAYVAGPAGEEVIIAHYLIGADGGGSFVRKKLGVS
FPGRTLGIHALVADASLSGLNRDVWHHFNDGDMTRMITICPLAGTQLFQIQALLAPDDSQ
NFSADVLTAFLTERIGRTDVRIHSIPWVSKYQMNARIAEHYRVGKVFLAGDAAHVHPPTG
GQGLNTSIQDAYNLGWKMAASLRGAGEELLDSYEQERRPVAESLLHLSTRLLDSQKQGGI
KRERDVQQLDIQYTDSPLAHTLPERQHGLQAGERAPDAPLLGAGGQSLRLFQLLQGPDWN
LLAYETHGKVIDARRGLRIHHIGEQDELIDTLGHFRESYHLAPGQCVVIRPDGYVGAFFH
GKQSNDIENYLSRFAIGIKDEY
>EECEBICD_01887 hypothetical protein
MSENKNVQDTHISEQMKALHGALIRVVSALNRPRNDEKLIAEAGIQLDRALFSILISIER
LGPIGVVELAERAGRDYTTVSRQVAKLEKLGLIIRQHNAIDRRIREAVISRTGKAMTERI
DAAREQMGKVVFKDWPQDELDVFVRLMQKFADAMDSDASMPE
>EECEBICD_01888 S-adenosylmethionine:tRNA ribosyltransferase-isomerase
MLTEKYDFRITDQMTIPLRPHWIANDSYREKCKMLVLNRSKGEIHKVDFSKLTDYIKEGD
VICFNDSTIINHMFICKTRQNRLIKIVLEGFLPNNRVIISGLLKERLNANDVFYLVDNPE
ISIKIEQRFSEESQYRAVVENHEALICYLASHGERLDEYVDSSLFYKYPDAYRSVFSKKY
GSLEIPSAGIHFTWDLIQKIKDKGGLISFITLHVASTEMLSNRKIQTKCVEEVTINEEYY
EVPQATADIINTAKQNGGRIFAVGTTVTRCLESAYSREHNCLKASSGWTALYIHPGYQLK
VVDCLLTNLHQPKTTHMVLTGQFAGVDLLMKAYASEDIQSCQFDMFGDCMLIIQDEG
>EECEBICD_01889 hypothetical protein
MVSDTLEQRIYELVRSHDGIYLFKKKELTPSTDLDSDLRLEDDEALALMDDFFTTFNVDR
GSFSITTYYPPEPPLKHLLNPFRKNDIPQVADFTIGMLIASARAGRWLYD

>EECEBICD_01890 hypothetical protein
MAAYDIEKDIGPRVQIIQEIHELILAGEMEVAKRMLRTLIKVTFAYPAIFDEVRQECQIN
NAHVVAGCGSGYQSVYSRCQSCRTSVRKSSGPG
>EECEBICD_01891 HTH-type transcriptional regulator GalS
MAGVSTATVSRVINQTAWVEPVTRERVEKAMRDLNYRRNAAAIALAKRSGDMLGLLTGNL
ADPFFARLARGVEDVSRKQQYRLMVCSGGHDEEMEKAGLDFLVNQGCEAIVVHASRLPDK
ELLRYAAHFPALVVVNRYIAGMANRCIWLENRSAAREATRYLLANGHRRIACVTSDLPII
DRQERLDGYRQALEEYGISPDPRWVISVPFNEEGGERAAHQLINSGLPLTAAVTFNDVMA
AGIMRILHQRGVQLPQQLSIVGFDDVVLARYLYPALTTMHYPVEQMARCAAQLAIQLYQG
ITPPPSSNHFNAELVIRDSVAPYFSR
>EECEBICD_01892 hypothetical protein
MSASQNKKKTLSLGLALIPVISMLLLLIIGYGIMGLRIEPLLLCSAAVAAGIAWWQGYCW
EDIINSVVDKLAKAMPVIMILICVGGLIGTWMFSGTIPYMVYWG
>EECEBICD_01893 Malate-2H(+)/Na(+)-lactate antiporter
MIAAFFLTSVVSVCTGTSWGSAGTVGVALMGVAAGLDVSLAAAAGAVVSGAYFGDKISPL
SDSTNFAAIVADTTLFEHIQHLLWTTLPSFLLAAVVYLIAGHSNMLGEVATPQRVTDIIH
SLESLYHFNIILILPPVIVLWGAIRKKACHPINVKRLRTCLVPRRHHAGTEHQTGTGRFH
>EECEBICD_01894 hypothetical protein
MQGLSIKQGLDAFIDGFDIAMFPQGAEGVVADVPRLLNRGGMFSMMGTILLVFCAFSFAG
ALTLTGALTIIINRLLTIIHSVGQLIAATIGTTILVTGATSDGKLALLVPAELFKDAYRR
MGLDTKNLSRTIEDAGTVIEPLLPWTSAGVYMATTLGVSTLDLLPWAIQCYAAIFFALIY
GFSGVGIARTASASEKSPQTSVTK
>EECEBICD_01895 Cystathionine beta-lyase PatB
MDFNQIINRNNTGSVKWDFIERHFGDGAGKLLPMWVSDFDFACPPEVQAALHQRIEHGVF
GYSERDEAYFNALLHWFSSRHQLTLKQEWVCSVEGVVPGLALLVQMLTHPGDGVVVQGPY
YGSFAKIITLNGRKLLENPLSESPDDGYQMDFCQLERLFRHERPPLMILCNPHNPTGRCW
SANELEQLLLLCEAYDVTLISDEIWADLLLPGETFTSVLHLGERWHKRVISATAASKTFG
LSSLRISNFLIPDPTLRQRFLSRLDAHGLDVFNALSVQAATTAWNESRQWLDSLLDYLAE
NRRWFVEQATEHLPWARIVPAQGTYLLWMDCKKLGLDDEQLKWVMADVAGIAPSMGSGFG
PAGSGFIRLNLGCPRTYLEMAIDGLKRISVKTIKGIPTGG
>EECEBICD_01896 Glycogen operon protein GlgX
MVRAFHDAGVEVILDVVYNHTAEGNELGPTLSFKGIDNFSYYRTMPDQHRYYINDTGTGN
TVNTSHPRVLQMVMDSLRYWAESMHVDGFRFDLGTILGREPEGFDQRGGFFDAVTQDPVL
AKLKLIGEPWDIGPGGYQVGGFPPGWGEWNDKYRDTVREYWKGDNVTNDFAARLLGSGDL
YDLRGRRPWSSVNFITAHDGFTLNDLVSYNDKHNEANGEDNNDGHNDNRSCNYGAEGPTD
DEGINAIRERQKRNFLTTLLFSHGTPMLLAGDEFGRSQMGNNNGYCQDSEISWVHWDNLP
ETANALREFTRHLIQLRATQPLLRRESWRDGLEIRWFNAGGGAQQSEQWDEGSTIGVCIS
RPDLQPEAGIWHDALLLFNPFEGSVPFRIPMWGEGGWVLELTTADNAQQGIRITEEMDFD
LAGRSIVLFRRP
>EECEBICD_01897 Glycogen debranching enzyme
MTTNRAFEIRSGYSHTLGANYDGEGVNFAIFSAHAERVELCLYDPSGEHEIARLELPEYT
DEIWHGYVPKLQPGALYGYRVYGPYDPENGHRFNPNKLLIDPYARELVGDIQWNDAHFAY
QLLHDDKDLTFDDQDSAPFTPKCRVIDPAEANWEDRQRPSIPWSNAIIYETHVKGFTQLN
SAIPEPLRGTFEGMGHKASVDYIKSLGITSVELLPVHWFPDDQHLLDKGLKNFWGYNSLG
FLRRRRATMVQKVFRDSAIWCALSMMPASK
>EECEBICD_01898 Maltooligosyl trehalose synthase
MAKSVEDTLFFRQNMGLALNEVGAEPVTHHFSIERFHHEMKTRQARQPDALSGTSTHDTK
RGEDARARLYTLTEAPEQWSECLARWRQMNQTHVKFLNDGTAPKSADTWMLYQALTGVWP
PMLQPQDETGLNALKIRFEAFVEKALREAKLRTDWVDSNEAYETAMLDYARYLLAPDNQT
FLQDFYRSLQPFIRAGLVNSLTQTVIKLTAPGVPDIYQGSEALNFSLVDPDNRREPDFAT
LAQQLDQLTPGVFSREESWLNGQVNQYVTAALLRLRQQNHELFRFGDYIPLRAVGQRADK
VIAYARVNHDDALIVVAPRLVFAECDGLLSQSHSGFWSGTDIIIPGQLNQHRYRNVLTQE
RLMPGERLSLASHQGGVLVLMSD
>EECEBICD_01899 Maltooligosyl trehalose synthase

MIPTATYRLQFRNGMTFDRAAALVPYLKNLGISHLYASPIFTATKASTHGYDVTDANEIE
PSIGGREGFERLVAELKAQGLGLIIDIVPNHMASSLENAWWRDVLEYGKESRYARHFDID
WSRRLTLPFLGDTFDAVLENGEIAIKPDPATGKPAFAYYDNYYPLAPATWQGREAEILAL
TDKAAIADLHERQPWKLMSWRDAARSLSYRRFFEVTGLVGMRVEDKTVFDDTHRLILELV
RTGAVDGLRIDHIDGLADPKAYLARLRQEVGPACYITVEKILAKGEQLPDDWPVSGTTGY
EFIASLAEVLVDDEQIDNLRQAYETVKGAPVDMRAELRAAKLLMVDRNFEGEFTRLLALA
LSIASELQIAQEESIVRQALRELLIAFPVYRTYGTAEGLPPTDICLLHRIVERVKTLEKS
SAARSAYISEPSSYWRCSRLQPGGGDTVSRSVPATHRSADGEIGRGYAFLSSKHGTGVK
>EECEBICD_01900 Malto-oligosyltrehalose trehalohydrolase
MSSKIFCKSWGAEYIAADVVRFRLWATGQQKVMLRLAGKDQEMQASGDGWFTLDVSGVTP
GTEYNFVLNDGMVVPDPASRAQKTDVNGPSYVVDPGSYTWRNTGWKGSRWEQAVVYEMHT
GTFTPEGTFRAAIAKLPYLAELGVTVIEVMPVAQFGGERGWGYDGVLLYAPHSAYGTPDD
FKAFIDAAHGYGLSVVLDIVLNHFGPEGNYLPLLAPAFFHKERMTPWGNGIAYDVDAVRR
YIIEAPLYWLTEYHLDGLRFDAIDQIEDSSARHVLVEIAQRIREDITDRPIHLTTEDSRN
IISLHPRDQDGNAPLFTAEWNDDFHNAVHVFATGETQAYYNDFADTPEKHLARALAEGFA
YQGEISPQTGEPRGVKSTGQPPVAFVDFIQNHDQVGNRAQGDRLITLAGAERTKVLLATL
LLSPHIPLLFMGEEYGESRPFLFLPISMGI
>EECEBICD_01901 hypothetical protein
MKILPLALFIIPFLAGCGANNTPPQTPIPGEKTSAKLRTLETGAAAIQSRPPVDAISTYL
DGFHFYSGDKNGQMEAHHYVTVLNEDVMQAVIYDGNTKNARLMGVEYIISERLFKTLPPE
EKKLWHSHQYEVKSGSLVAPGLPQVADKALMSKIVNTYGKTWHTWHTDRDKTLPMGIPAL
MMGFTGDGQLDPALLADRDRRLGIDTQAIKRERQDLPEHPVVKGANAWEQGEVIQLQRVQ
GSGEHGRGDTAHFGTSEQSRQ
>EECEBICD_01902 Acid stress chaperone HdeB
MNKFSLATAGIIVAALVTSVSVNAATDTTKTNVTPKGMSCQEFVDLNPQTMAPVAFWVLN
EDEDFKGGDYVDFQETETTAVPLAVELCKKNPQSELSKIKDEIKKELSK
>EECEBICD_01903 Peroxiredoxin OsmC
MTIHKKGQAHWEGDIKRGKGTVSTESGVLNQQPYGFNTRFEGAQGTNPEELIGAAHAACF
SMALSLMLGEAGFTPTSIDTTADVSLDKVEAGFAITKIALQSKVAVADIDASTFDQIIQK
AKAGCPVSQVLNAEITLDYQLNA
>EECEBICD_01904 Protein bdm
MFTYHSANTSAAQPALVNAIEQGLRAELGVVTEDDILMELTKWVEASDNDILSDIYQQTI
NYVVSGQHPTL
>EECEBICD_01905 Stationary-phase-induced ribosome-associated protein
MKSNRQARHILGLDYRISNQRKVVTEGDTSSVVNNPTGRKRRADSQK
>EECEBICD_01906 NAD-dependent malic enzyme
METITKKARSLYIPYAGPVLLEFPLLNKGSAFSVEERRNFNLSGLLPEVVESIEEQAERA
WLQYQGFKTEIDKHIYLRNIQDTNETLFYRLVQNHLEEMMPVIYTPTVGAACERFSEIYR
RARGVFISYPNRHNMDDILQNVPNHNIKVIVVTDGERILGLGDQGIGGMGIPIGKLSLYT
ACGGISPAYTLPVVLDVGTNNQQLLNDPLYMGWRHPRITDDEYYAFVDEFIQAVKQRWPD
ILLQFEDFAQKNAMPLLTRYRDEICSFNDDIQGTAAVTVGTLIAASRAAGSQLSEQKIVF
LGAGSAGCGIAEQIIAQTQREGLSEDAARQNVFMVDRFGLLTDRMPNLLPFQAKLVQKCD
NLQHWDTENDVLSLLDVVRNVKPDILIGVSGQTGLFTEEIIREMHKHCPRPIVMPLSNPT
SRVEATPQDIIAWTEGNALVATGSPFSPVIWKDKIYPIAQCNNAYIFPGIGLGVIASGAS
RITDEMLMSASETLAKHSPLVNNGEGLVLPALKDIQVVSRAIAFAVGKMAQQQGVAVKTS
AEALQQAIDDNFWKPEYRDYRRTSI
>EECEBICD_01907 Alcohol dehydrogenase, propanol-preferring
MKAAVVTQDHQVDVTEKTLRPLRHGEALLKMECCGVCHTDLHVKNGDFGDKTGVILGHEG
IGVVAEVGPGVTSLKPGDRASVAWFYEGCGHCEYCNTGNETLCRNVKNAGYTVDGGMAEE
CIVVADYAVKVPEGLDSAAASSITCAGVTTYKAVKISHIKPGQWIAIYGLGGLGNLALQY
AKNVFNAKVIAIDVNDGQLKLAEEMGADLTINSRTEDAAKIVQEKTGGAHAAVVTAVAKA
AFNSAVDAVRAGGRVVAVGLPPEAMNLDIPRLVLDGIQVVGSLVGTRQDLTEAFQFAAEG
KVVPKVALRPLEDINVIFKEMEQGQIRGRMVIDFRR

>EECEBICD_01908 Formate dehydrogenase, nitrate-inducible, cytochrome b556(Fdn) subunit
MSKSKMIVRTKFVDRACHWTVVICFFLVALSGISFFFPTLQWLTQTFGTPQMGRILHPFF
GIAIFIALMFMFVRFVHHNIPDKKDIPWLKNIVEVLKGNEHKVADVGKYNAGQKMMFWSI
MSMIFVLLVTGVIIWRPYFAQYFPMQVVRYSLLIHAAAGIILMHAILIHMYMAFWVKGSI
KGMIEGKVSRRWAKKHHPRWYREIEKAEAKKESEEGIQ
>EECEBICD_01909 Formate dehydrogenase, nitrate-inducible, iron-sulfur subunit
MSMETQDIIKRSATNAITPPPQARDYKAEVAKLIDVSSCVGCKACQVACSEWNDIRDNVG
HCVGVYDNPADLSAKSWTVMRFTETEQNGKLEWLIRKDGCMHCEDPGCLKACPSAGAIIQ
YANGIVDFQSEHCIGCGYCIAGCPFNIPRLNKEDNRVYKCTLCVDRVSVGQEPACVKTCP
TGAIHFGTKKEMLELGEQRVEKLKARGFEHAGIYNPQGVGGTHVMYVLHHANQPELYHGL
PKDPQIDTSINLWKGALKPLAAAGFIATFAGLIYHYIGIGPNKETDDDEEDHHE
>EECEBICD_01910 Formate dehydrogenase, nitrate-inducible, major subunit
MTNHWVDIKNANVVMVMGGNAAEAHPVGFRWAMEAKNNNDATLIVVDPRFTRTASVADIY
APIRSGTDITFLSGVLLYLIENNKINAEYVKHYTNASLLVRDDFAFDDGLFSGYDAQKRQ
YDKSSWNYQFDENGYAKRDETLTHPRCVWNLLKQHVSRYTPDVVENICGTPKADFLKVCE
VLASTSAPDRTTTFLYALGWTQHTVGAQNIRTMAMIQLLLGNMGMAGGGVNALRGHSNIQ
GLTDLGLLSTSLPGYLTLPSEKQADLQTYLAANTPKATLADQVNYWGNYPKFFVSLMKSF
YGDAAQKENDWGFAWLPKWDQSYDVIKYFNMMDSGKVTGYFCQGFNPVASFPDKNKVVQS
LSKLKYLVVIDPLVTETSTFWQNHGESNDVDPTTIQTEVFRLPSTCFAEEDGSIANSGRW
LQWHWKGQDAPGEARNDGEILAGIYHRLREMYRAEGGKGAEPLLKMSWNYKQPDEPHSEE
VAKENNGYALEDLYDANGTLLARKGQLLSSFALLRDDGTTSSSCWIYTGSWTEQGNQMSR
RDNADPSGLGNTLGWAWAWPLNRRVLYNRASADPQGKPWDPKRMLIQWNGAKWTGNDIPD
FNNAAPGSGTNPFIMQPEGLGRLFAIDKMAEGPFPEHYEPMETPLGTNPLHPNVVSNPAA
RLYEEDALRMGKKEQFPYVGTTYRLTEHFHTWTKHALLNAIAQPEQFVEISETLAAAKGI
ANGDYVKVSSKRGFIRAVAVVTRRLRTLHVNGQQVETVGIPIHWGFEGVARKGYIANTLT
PNVGDANSQTPEYKAFLVNIEKA
>EECEBICD_01911 Formate dehydrogenase, nitrate-inducible, major subunit
MDVSRRQFFKICAGGMAGTTVAALGFTPKMALAQARNYKLLRAKEIRNSCTYCSVGCGLL
MYSLGDGAKNAKEAIYHIEGDPDHPVSRGALCPKGAGLLDYVHSEDRLRYPEYRAPGSDK
WQRISWDDAFTRIAKLMKADRDANFIEKNEQGVTVNRWLSTGMLCASAASNETGMLTQKF
ARSLGMLAVDNQARV
>EECEBICD_01912 Methyl viologen resistance protein YddG
MTSQKATLIGLVAIVLWSTMVGLIRGVSEGLGPVGGAAMIYSLSGLLLIFTVGLPDIRRF
PGRYLIAGSVLFVSYEICLALSLGYAATRHQAIEVGMVNYLWPSLTILFAILFNGQKTNW
LIVPGLLIALTGVCWVLGGENGLNPGEIISNVATSPLSYLLAFLGAFIWATYCTVTNKYA
RGFNGITVFVLLTAVALWFHYFLTPQPAMIFSLPVIAKLFTAALTLGFAYAAWNVGILHG
NVTIMAVGSYFTPVMSSALAALLLSSPLSFSFWQGAVMVCVGSLLCWLATRRR
>EECEBICD_01913 Outer membrane porin protein OmpD
MKLKLVAVAVTSLLAAGVVNAAEVYNKDGNKLDLYGKVHAQHYFSDDNGSDGDKTYARLG
FKGETQINDQLTGFGQWEYEFKGNRTESQGADKDKTRLAFAGLKFADYGSFDYGRNYGVA
YDIGAWTDVLPEFGGDTWTQTDVFMTGRTTGVATYRNTDFFGLVEGLNFAAQYQGKNDRD
GAYESNGDGFGLSATYEYEGFGVGAAYAKSDRTNNQVKAASNLNAAGKNAEVWAAGLKYD
ANNIYLATTYSETLNMTTFGEDSAGDAFIANKTQNFEAVAQYQFDFGLRPSIAYLKSKGK
NLGTYGDQDLVEYIDVGATYYFNKNMSTFVDYKINLLDDSDFTKAAKVSTDNIVAVGLNY
QF
>EECEBICD_01914 hypothetical protein
MVGWNSHVSGSLRATTASPLSPEPPYFSSSSRGERYFCSGCGCPLWFRLTDTDRYFIPWT
LLELNEVDRRRLILAAEIYTETQPAFWRLTGQYARLSGKEVEEMDSPAS
>EECEBICD_01915 Methyl viologen resistance protein SmvA
MFRQWLTLVIIVLVYIPVAIDATVLHVAAPTLSMTLGASGNELLWIIDIYSLVMAGMVLP
MGALGDRIGFKRLLMLGGTLFGLASLAAAFSHTASWLIATRVLLAIGAAMIVPATLAGIR
ATFCEEKHRNMALGVWAAVGSGGAAFGPLIGGILLEHFYWGSVFLINVPIVLVVMGLTAR

YVPRQAGRRDQPLNLGHAVMLIIAILLLVYSAKTALKGNLSLWAISLTLLTGTLLLGLFL
RTQLATSRPMIDMRLFTHRIILSGVVMAMTAMITLVGFELLMAQELQFVHGLSPYEAGVF
MLPVMVASGFSGPIAGALVSRLGLRLVATGGMALSALSFYGLAMTDFSTQQWQAWGLMAL
LGFSAASALLASTSAIMAAAPAEKAAAAGAIETMAYELGAGLGIAIFGLLLSRSFSASIR
LPAGLEAQEIARASSSMGEAVQLANSLPPTQGQAILDAARHAFIWSHSVALSSAGSMLLL
LAVGMWFSLAKAQRR
>EECEBICD_01916 hypothetical protein
MSYLNRDERREVILQAAMRVALADGFAAMTVRRIASEADVAAGQVHHHFSSAGELKALAF
VHLIRTLLDAGQVPPPATWRARLHAMLGSEDGGFEPYIKLWREAQILADRDPHIRDAYLL
TMQMWHEETVTIIEQGKQAGEFTFTANATDIAWRLIALVCGLDGMYVLGIPEMADPAFKF
HLDRMITLELFA
>EECEBICD_01917 Nitrate/nitrite transporter NarU
MTRQNENYNRYLLSDWRPENPAFWENKGKGIARRNLLISVSCLLLAFCVWMLFSAVAVNL
NKIGFNFTTDQLFLLTALPSLSGAILRVPYSFMVPLFGGRKWTVLSTVILIIPCTWLGFA
VQNPATPFGVFMLIALLCGFAGANFASSMGNISFFFPKARQGSALGINGGLGNLGVSVMQ
LIAPLVIFLPIFTFLGVRGVPQPDGSLLALTNAAWIWVPLLAVATLAAWFGMNDIGSSKA
SVASQLPVLKRLHLWLLSLLYLATFGSFIGFSAGFAMLAKTQFPDVNILQLAFFGPFIGA
LARSAGGVISDKFGGVRVTLINFIFMALFTALLFLTLPGSGAGSFSAFYLVFMGLFLTAG
LGSGSTFQMIAVIFRQITLYNVKLRGGSDEQAQREAVTDTAAALGFISAIGAVGGFFIPK
AFGTSLALTGSPVGAMKIFLLFYIACVLLTWLVYGRRKPKQQ
>EECEBICD_01918 Respiratory nitrate reductase 2 alpha chain
MSKLLDRFRYFKQKGDTFADGHAQVMHTNRDWEDSYRQRWQFDKIVRSTHGVNCTGSCSW
KIYVKNGLVTWETQQTDYPRTRPDLPNHEPRGCPRGASYSWYLYSANRLKYPLVRKRLIE
LWREALSRHSDPVLAWESIMNDPQKCQSYKQVRGHGGFIRSNWKELNQLIAAANVWTIKT
YGPDRVAGFSPIPAMSMVSYAAGTRYLSLLGGTCLSFYDWYCDLPPASPMTWGEQTDVPE
SADWYNSAYIIAWGSNVPQTRTPDAHFFTEVRYKGTKTIAITPDYSEVAKLCDQWLAPKQ
GTDSALAMAMGHVILKEFHLDNPSDYFLNYCRRYTDMPMLVLLDERADGSYVPGRMMRAS
DLVDGLGEANNPEWKTVALNSTGELVAPNGSIGFRWGEKGKWNLEPVAAGVETELSLSLL
GQHDDVAGVAFPYFGGNENPHFRSVRQEPVLVRQLPVKRLALADGSERMVVSVYDLVLAN
YGLDRGLDDGHSANNYNDVKAYTPAWGEQITGVPRRHIETIAREFAETAHKTHGRSMIIL
GAGVNHWYHMDMNYRGMINMLVFCGCVGQTGGGWAHYVGQEKLRPQTGWLPLAFALDWNR
PPRQMNSTSFFYNHASQWRYEKLTAQELLSPLADPAKFSGHLIDFNVRAERMGWLPSAPQ
LNLNPLSVKASADKAGLSAADYTVQALKSGAIRFACEQPDSGHNHPRNLFVWRSNLLGSS
GKGHEYMLKYLLGTDSGIQGEALGSSEGIKPEEVEWQSAAIEGKLDLLVTLDFRMSSTCL
FSDIVLPTATWYEKDDMNTSDMHPFIHPLSAAVDPAWESKSDWEIYKGIASVFSEVCVGH
LGQETDVVLHPLQHDSPAELAQPFDILDWRKGECELIPGKTAPNIVVVERDYPATYERFT
SLGPLLDKLGNGGKGIAWNTQDEVDFLGKLNYTKHDGPAKGRPRIDTALDASEVILALAP
ETNGQVAVKAWQALGEMTGREHTHLAINKEDEKIRFRDIQAQPRKIISSPTWSGLESEHV
SYNAGYTNVHELIPWRTLSGRQQLYQDHAWMRAFGESLVAYRPPIDTRSVSEMREIPPNG
FPEKALNFLTPHQKWGIHSTYSENLLMLTLSRGGPIVWISEADARELGIEDNDWIEAFNA
NGALTARAVVSQRVPPGMTMMYHAQERIMNIPGSEVTGMRGGIHNSVTRVCPKPTHMIGG
YAQLAYGFNYYGTVGSNRDEFIMIRKMKNINWLDDEGRDQVQEAKK
>EECEBICD_01919 Respiratory nitrate reductase 2 beta chain
MKIRSQVGMVLNLDKCIGCHTCSVTCKNVWTGREGMEYAWFNNVETKPGIGYPKNWEDQQ
EWQGGWVRDVNGKIRPRLGGKMGVISKIFANPVIPQIDDYYEPFTFDYQHLHNAPESKHQ
PTARPRSLIDGKRMDKVIWGPNWEELLGGEFEKRARDRNFDNIQKEMYGQFENTFMMYLP
RLCEHCLNPSCVATCPSGAIYKREEDGIVLIDQDKCRGWRLCISGCPYKKIYFNWKSGKS
EKCIFCYPRIESGQPTVCSETCVGRIRYLGVLLYDADRIEEAASTEHETDLYERQCDVFL
NPHDPAVIEEALKQGIPQNVIDAAQRSPVYKMAMDWKLALPLHPEYRTLPMVWYVPPLSP
IQSYADAGGLPHNGNILPAVETLRIPVQYLANMLSAGDTGPVIRALKRMMAMRHYMRSQT
VEGVTDTRAIDEVGLSVQQVEEMYRYLAIANYEDRFVIPTSHREMARDAFPERNGCGFTF
GDGCHGSDTKFNLFNSSRIDAINITEVRDKAEGE
>EECEBICD_01920 putative nitrate reductase molybdenum cofactor assembly chaperone NarW

MQILKAIGLLMEYPDDELWECRDEALALIQHDAPMLADFTRELLYAPLLDKQAEWCEVFD
RGRATSLLLFEHVHAESRDRGQAMVDLLSQYETVGLQLNCRELPDHLPLYLEYLSVLPEA
EAREGLQNIAPILALLGGRLKQRGAPWYQLFDALLTLAGSTLTSDSVTKQIVQESRDDTR
QALDAVWEEEQVKFIEDNATTCDSSPLHHYQRRFSQDAAPQYVDVSAGGPK
>EECEBICD_01921 Respiratory nitrate reductase 2 gamma chain
MIQYLNVFFYDIYPYICATVFFLGSWLRYDYGQYTWRASSSQMLDKRGMVIWSNLFHIGI
LGIFFGHLFGMLTPHWMYAWFLPIAVKQQMAMILGGVCGVLTLIGGAGLLWRRLTNQRVR
ATSTTPDIIIMSILLIQCLLGLSTIPFSAQYPDGSEMMKLVGWAQSIVTFRGGSSEMLSG
VAFVFRVHLVLGMTIFLLFPFTRLVHVWSAPFEYFTRRYQIVRTRR
>EECEBICD_01922 putative isomerase YddE
MKPQIYHVDAFTSEPFRGNSAGVVLHADTLSDAQMQLIARELRHSETAFLLKSEESDVRI
RYFTPTVEVPICGHATVAAHYVRATVLGLGNTTVWQTSLAGRHRVEIHAEHNDYRITLEQ
GQPSFEPPLVGEIRAAIITALNLTEDDIVPGAPIQVASTGHAKVMILLKPDVDIDALSPN
LAALTAISQQIGCNGFFPFQIRPGKNETDGRMFSPAIGIVEDPVTGNANGPMGAWLVHHG
LMAHDGKTLQIQGHQGRALGKEGTVDVTVTIRDNQPENVTISGQAVILFHAEWAITF
>EECEBICD_01923 Arylamine N-acetyltransferase / N-hydroxyarylamine O-acetyltransferase
MTSFLHAYFTRLHCQPLGVPTVEALRTLHLAHNCAIPFENLDVLLPREIQLDETALEEKL
LYARRGGYCFELNGLFERALRDIGFNVRSLLGRVILSHPASLPPRTHRLLLVDVEDEQWI
ADVGFGGQTLTAPLRLQAEIAQQTPHGEYRLMQEGSTWILQFRHHEHWQSMYCFDLGVQQ
QSDHVMGNFWSAHWPQSHFRHHLLMCRHLPDGGKLTLTNFHFTRYHQGHAVEQVNVPDVP
SLYQLLQQQFGLGVNDVKHGFTEAELAAVMAAFDTHPEAGK
>EECEBICD_01924 hypothetical protein
MAAYNALPQALGAIPCLEYYIARASNMQEAKAQWAADIKARYHNYLDNY
>EECEBICD_01925 hypothetical protein
MPYTSVSTYARALSGNKLPHVAAGDYENKLSTKIMKGILYVLTAGLAYGFTRVIEHYCNV
TPKVDEFCANAGNIHNHLADAVCDGLYTIDVDLSDGRRLTFEQLSLMAGREPIVRISDGE
HTVEVKGSFEEICMRLEEGFLKRQRTMIMILMKNIKPSESEWQHIMPYLRH
>EECEBICD_01926 L-asparagine permease 2
MKTQTTHAAEQHAAKRRWLNAHEEGYHKAMGNRQVQMIAIGGAIGTGLFLGAGARLQMAG
PALALVYLICGIFSFFILRALGELVLHRPSSGSFVSYAREFLGEKAAYVAGWMYFINWAM
TGIVDITAVALYMHYWGAFGDVPQWVFALGALTIVGTMNMIGVKWFAEMEFWFALIKVLA
IVIFLVVGTIFLGTGQPLEGNATGFHLITDNGGFFPHGLLPALVLIQGVVFAFASIELVG
TAAGECKDPQTMVPKAINSVIWRIGLFYVGSVVLLVLLLPWNAYQAGQSPFVTFFSKLGV
PYIGSIMNIVVLTAALSSLNSGLYCTGRILRSMSMGGSAPKFMAKMSRQHVPYAGILATL
VVYVVGVFLNYLVPSRVFEIVLNFASLGIIASWAFIMVCQMRLRQAIKEGKAADVSFKLP
GAPFTSWLTLLFLLSVLVLMAFDYPNGTYTIASLPLIAILLVAGWFGVRRRVAEIHRTAP
VTADSTESVVLKEEAAT
>EECEBICD_01927 putative lipoprotein YgdR
MKKLFICSGLGMMFFMLAGCTTNYVMTTKNGQTIVTQGKPQLDKETGMTSYTDQEGNQRE
INSNDVAQLIKAD
>EECEBICD_01928 putative protein YncE
MHLRHLFSPRLRGSLLLGSLLVASSFSTLAAEDMLRKAVGKGAYEMAWSQQENALWLATS
QSRKLDKGGVVYRLDPVTLEITQAIHNDLKPFGATINAATQTLWFGNTINSAVTAIDAKT
GDVKGRLVLDARKRTEEVRPLQPRELVADAATNTIYISGVGKESAIWVVDGETIKLKTTI
ENTGKMSTGLALDSKAQRLYTTNADGEFITIDTASNKILSRKKLLDDGKEHFFINLSLDT
AGHRAFITDSKATEVLVVDTRNGNILAKIAAPASLAVLFNPTRNEAYVTHRQAGQVSVID
AKTYNVVKTFDTPTYPNSLALSADGKTLYVSVKQKSTREQEATQPDDVIRIAL
>EECEBICD_01929 Fe(3+) dicitrate transport protein FecA
MSRFPLIRLHLSGIFMKIISVRQRLYPALLLPLTFSPVLQAASASNEQTMIVTATPQTVS
ELDTPAAVSVIEGEDMRLATPRVNLSETLTSVPGLQVQNRQNYAQDLQISIRGFGSRSAF
GVRGIRLYVDGIPATMPDGQGQISNIDINSIQDVEVLRGPFSALYGNASGGVINVTTETG
IEASSYYGSYGSWRYGLKATGAMGDGTQPGDVDYTVSTTRFTTHGYRDHSGARKNLANAK
LGVRLDDVSKLSLIFNSVDIKADDPGGLTESEWKADPQQAPRAEQYNTRKTIKQTQAGLR

YERQLSAQDDISVMAYAGERETTQYQSIPLVAQLKPAQAGGVITLQRHYQGIDSRWTHRG
ELGVPVTFTGGLNYENMSENRKGYNNFRLNNGPPEFGHKGDLRRDERNLMWNVDPYLQTQ
WQLTQKLSLDAGVRYSSVWFDSNDHYIAPGNGDDSGDASYHHWLPAGSLKYALTDAWNLY
LAAGRGFETPTINELSYRADGQSGFNFDLKPSTNDTVEVGSKTRIGNGLLTAALFQTDTD
DEIVVASSMGGRTTYKNAGKTRRQGAELALDQRFAGDWRVKASWTWLDATYRSNVCQGQN
CDGNRMPGIARNMGFASLGFIPDEGWYAGTDVRYMGDIMANDENTAKAPSYTVVGLNTGY
KFNYSQLTVDIFGRVDNLFDKEYIGSVIVNESNGRYYEPAPGRNYGVGINLAWQFE
>EECEBICD_01930 HTH-type transcriptional regulator McbR
MPGTEKTQHISLTTQVENRLKHQLSIGALKPGARLITKNIAQELGVSITPVREALLRLVS
SSALAVAPAQAFMVPEISLESLLEINTIRTALEEMAVVAAAGKITPDREQTLNALLDEFQ
QALESGVMEKILLANRAFRFEIYHYADMPTLYAMIEQLWVRLGPSLHFLYDNFKLDDYQN
GVNLYRKLLNALVTGDKEASRHCLQNVLQQNVATIKNQYFM
>EECEBICD_01931 NADPH-dependent curcumin reductase
MKQQTNRNRRWVLASRPHGAPQMDNFRLEEDDVATPGEGQVLLRTVFLSLDPYMRGRMSD
EPSYAPPVEPGCVMVGGTVSRVVASNHPDYRPDEWVLSYNGWQDYDISDGEGLVKLGDNP
QHPSWSLGILGMPGFTAYMGLLDIGQPKEGETLVVAAATGPVGATVGQIGKLKGCRVVGI
AGGAEKCRHATDVLGFDLCLDHHADNFPQQLAQACPQGIDIYYENVGGKVFDAVLPLLNT
SARIPLCGLVSGYNATALPDGPDRLPLLMATLLKKRIRLQGFIINQDYGHRIHEFQQEMG
RWIKEGKIHYREQITDGLENAPEAFMGLLAGKNFGKVVIRLADDA
>EECEBICD_01932 L-methionine sulfoximine/L-methionine sulfone acetyltransferase
MTIRFADKADCAAITEIYNHAVLHTAAIWNDRTVDTDNRLAWYEARQLLGYPVLVSEENG
VVTGYASFGDWRSFDGFRYTVEHSVYVHPAHQGKGLGRKLLSRLIDEARRCGKHVMVAGI
ESQNAASIRLHHSLGFTVTAQMPQVGVKFGRWLDLTFMQLQLDEHAAPDAC
>EECEBICD_01933 Inner membrane protein YdcZ
MNQSLTLIFLIAAGVGLVVQNSIMVRITQTSSTILIAMLLNSLVGIVLFVTILWFKQGAA
GFGELVASVRWWTLIPGLLGSFFVFASISGYQNVGAATTIAVLVASQLIGGLALDIARSH
GVTLRAMVGPAFGALLLVIGAWLIAKRQF
>EECEBICD_01934 putative protein YdcY
MSHLEEVSARVDAAIAESVIAHMNELLIALSDDAELRREDRYVQQQRLRTAIAHHGRQYQ
EDRDARREQLTKGGTIL
>EECEBICD_01935 hypothetical protein
MAKSLLRSGNLDDYQAVGGGGQAVFESALQIRETLRLRKQQAMVDCLAIPQLNDNGDRVD
WYSPIEGQAIAWKAADEETRSRALRYLASTFESAAALSRKSLQSGKTALQLFGSLLEKAT
QFPGENHVFLVNGKPVITFWGFVNLNENTRDDVLDCLRVTEAIPDIPLVEPEPEPEEKPL
VEAAFSQADEPLLTSVIEPPKMPEEPVAPPVIVSEPKPATPIPVAEAKRARRLPLWSLPV
AAVVIAAVATPFFWPSSSVDGSSPVRAAPVATAKTDVTPLPELTAHLPLHRAEVTPAPKA
APLPEAPVIIAAIPKDALVMDSTQMKLGTTRFLNGSWRVSVDVKDPITGKPPSLRYQIQN
NKGIARVVHGDNVVCRAEIFSGLHQTGELMIKSRGNARCTDGSRYPMPEITCKAGVNDVA
TCTARYGDHAAIPLTFKKIGA
>EECEBICD_01936 hypothetical protein
MLVNLCDYKQSVTLIANSGVQFLDFGLTPQESAHYGRFVRKTANGPLLRLDFDLTSGRYT
LPGRAGGQPEVVKPESTQTLHYSLDVLDGIWLPLPFLRFNPPRTFIDGPDNWARIQVRKL
SEPDSAGNTHRITLAFDSQLAKNMPAALAPCENDLLNGTRFALAWQDEEVADFLDQTWID
GWLRESFLQYASQVENRSEQAIQQALRSFEYQAHWLNLLTLLGEQLTVPEVKFVTHTLST
PAIPVDLILDVGNTHTCGVLIEDHGDANDGLRQTAELQVRSLSEPQYLNDPLFTSRVEFS
EARFGKQHFSVESGRDDAFVWPSIVRVGDEARALAMQRVGTEGSSGISSPRRYLWDETPA
LQDWRFSQIHGKTQREALATAFPLMNLMNDDGQPLFRLPHEERLPVFSPQYSRSTLMTHM
LCEILAQALGQINSVATRLRLGFPASPRQLRTLILTLPSAMPKQEREIFRQRMFEALALV
WKAMGWHPQDEDFTTPKQREKSVVPVPEIQMEWDEASCGQLVWLYNEAISHYAGRTESFF
NALARPDRQPEPGVVPGRALRVASIDIGGGTTDMAIVHYQLDDGVGANVKITPHLLFREG
FKVAGDDLLLDIIQRCVLPSLQTALQRAGVTDAAALLATLFGDSGRIDTQAILRQQTALQ
LFMPLGHAVLSAWEQSDINDPFAGLHATFGDLLIRRPTSNVMNYIQQAIDHALPSGSPTF
DIFNVPLQIQFSQLQEALLAGQFTLTTPLHAVCEAISHYHCDILLVTGRPTCLPGVQALI

RHLQPVPVNRIVWMDKYQVHEWYPFSQQGRIGNPKSTAAVGAMLCSLALDLRLPRFNFKA
ADIGAYSTVRYLGVLDNTVNTLRDENIWYHEIDLDKPGATLDARLHFPLRGNVTLGFRQL
ANSRWPATPLYCLSINSAELAKTIAGDGVLNVRLKLRGSSKDSAPESFILSDAWLQDGTP
VAADALTLKLNTLADRRHSGSHYWIDSGSVYLK
>EECEBICD_01937 hypothetical protein
MTKPLNATQAVIEWVNNTRRYATRLDDEADALLAQLTLAAADESALNAACASHGCVGLYG
YAQSAKAHLLTTLCGNENGKLEIITPDRDYDYFSHINPGHAPANMAIRFTRDIFSNESGW
PLRLRLISEAELVQIFIAWTSASPICRQVEKSIITSRLEKWQSLRQPQPVPGVTAEEVAT
IASFWRSCLPSARQHIDDATWQHFASLLPALDLTTRAHAWALLWGEQPEITQQWLALAHM
LQQTGHAGELAAPLSLLVDHFGLPAENFLTQMALTANDTQIDVVVHPVKEGRLLNAVSLS
LDSLALLTRELVLTVENNVLDNVDLLDIPVAPDSHPHPLWRAKLGWMLAHYRQQVQPDVL
VICNALASRSQTSTAARHLLEWVNATQPQHESALPGVVWAITPQDARFATQQNLDEAVQQ
LMGKPGVHWGTLQALDKHSMQRLVEWLSQATSAPQRQARLQALREQLRGRVRDLLPMFDD
ARLPVETVIRRLQAQAARHGDLLAGLLPPVQNFEALLRTRQSREEQVCGLFNDAIDLFAD
EPTRASASEGHETGYQAHKMWINHLRQWAHCRDNAQRLGLEPQMLNAVAEILITASYRLG
LPQQLQKTMQREEVSGAQLHAIIGNFIAWLGYANIEEAQRPASRVQKGAAIFAATPRSTM
LRLTKLDEQPVHAASRYVYDWLVALYTLANENAGYRHPQDVTDVDRAQLIALIA
>EECEBICD_01938 Orphan toxin OrtT
MTLFQKILIFYAVMAVISSLITWFLTHDKKRIRFLSAFLVGATWPISFPVALLFSLF
>EECEBICD_01939 Gamma-aminobutyraldehyde dehydrogenase
MQYQLLINGVLVDGEGERQSVYNPATGEVILEIAEASPAQVDAAVLAADSAFAEWGQTTP
KARAECLLKLADSIEQNALEFARLESQNCGKPLHCVINDEIPAIVDVFRFFAGAARCLSG
LAAGEYLEGHTSMIRRDPIGVVASIAPWNYPLMMAAWKLAPALAAGNCVVIKPSEITPLT
ALKLAALAKDIFPPGGLNVLFGRGQTVGDVLTGHEKVRMVSLTGSIATGEHILRHTAPAI
KRTHMELGGKAPVIVFDDADLDAVAQGVRTFGFYNAGQDCTAACRIYAQRGIYDALVEKL
GNAVSSLKMGAPEDESTELGPLSSLAHLKRVTAAVEEAKALSHIRVITGGSQTEGKGYYF
APTLLADAKQEDAIVQREVFGPVVSITVFDDEDQVLRWANDSRYGLASSVWTQDVGRAHR
LSARLQYGCTWINTHFMLVSEMPHGGQKQSGYGKDMSLYGLEDYTLVRHIMVKH
>EECEBICD_01940 2-aminoadipate transaminase
MKKYQRLAEQIREQIASGVWQPGDRLPSLREQVASSGMSFMTVGHAYQLLESQGRIIARP
QSGYYVAPHPVCRSVATAAHVIRDEAVDINTYIFEMLQASRDTSVVPFASAFPDPRLFPL
PQLNRSLAQVSKTATAMSVIENLPPGNAELRYAIARRYAQQGITVSPDEIVITAGALEAL
NLSLQAVTAPGDWVVVENPCFYGALQALERLRLKALSIPTDVKEGIDLMALEQALQEYPV
KACWLMTNSQNPLGFTLSAEKKARLVALLTHHNVTLIEDDVYSELYFGREKPLPAKAWDR
DDTVLHCSSFSKCLVPGFRIGWVAGGSHARQIQRLQLMSTLSTSSPMQLALVDYLSTRRY
DAHLRRLRRQLAERKQQAWQTLLRHLPAEVKIHHNDSGYFLWLELPEQLDAGELSAKALE
HLISIAPGKMFSTSGAWTRFFRFNTAWHWGEREEQAVKQLGSLIREMLSAKSLV
>EECEBICD_01941 D-alanyl-D-alanine dipeptidase
MVKCVSSFLLFSLLSVQAMSAENHIDLHQPKDFVDITTVAPDVQVDMRYFSSHNFIGRPI
KGYNAPVCLLTRPAANAVKQVADRLRPFGLTLKIYDCYRPQSAVNDFIAWAKDPSQNQMK
NEFYPQVEKNRLFEEGYLAARSGHSRGSTLDLTIVPLDSKIPIYDPGRPLVNCTASAAQR
SPDNSLDFGTGFDCFSPLSHPDNVMLTAQQRANRLLLQTLMRDAGFTPLDTEWWHFSLTH
EPYPNTWFDFPVKQRP
>EECEBICD_01942 hypothetical protein
MKKSDGEIHEKTASWGILQSEWLRKCGRLLLLLLYRFVIGWAFFQLLAMIVAGIFLLGVL
LFHPIIFVQTIAITEKLNHASLDLWHILKLCLWHYGIIAGFIFMAECTLSKSIRQVQRLS
KKFGAQDFSSRP
>EECEBICD_01943 hypothetical protein
MLPLRLTRNVYLIYFARYMNYHLRHAEEISIFSTIKMLSVNIQISLLKMAKIFRYAL
>EECEBICD_01944 hypothetical protein
MIRQDHYYYDIMNRTVLCVDTQPAHLKRYSDITIKASTYVCEELCCLFPERLLLSLSGGI
TFPVDLKNIKETLIAMAEKGNLCDWKEQERKAAISSRINLGIAQADVPPIDDAIKNKIAA
KVIENTNLKNAAFEPNYAQSSVTQIVYSCLFKNEILMNMLEESSFHGLLCLNELTEYVAL
QVHNSLFSEDLSSLVETTKNEAHHQS

>EECEBICD_01945 hypothetical protein
MLTKTLSVVLLTCALFSGQLLAKQQDHAFVWFATGGHQLRHEADSDELRAAAEESAEGLR
EHHNWQKSRKPESYFR
>EECEBICD_01946 23S rRNA 5-hydroxycytidine synthase
MRQQPHYLELLSPARDAAIAREAILHGADAVYIGGPGFGARHNASNSLRDIADLVPFAHR
YGARIFVTLNTILHDDELEPAQRLITDLYDTGVDALIVQDMGILELDIPPIELHASTQCD
IRSVEKAKFLADVGFSQIVLARELNLSQIAAIHQATDATIEFFIHGALCVAYSGQCYISH
AQTGRSANRGDCSQACRLPYTLKDDQGRVVSYEKHLLSMKDNDQTANLGALIDAGVRSFK
IEGRYKDMSYVKNITAHYRQMLDAIIEQRGDLARASVGRTEHFFVPSTEKTFHRGSTDYF
VNARKGDIGAFDSPKFIGLPVGEVLNVAKDYLDVEATEPLANGDGLNVLIKREVVGFRAN
TVEKTGHNRYRVWPNDMPADLHKVRPHHPLNRNLDHNWQQALTKTSSERRVAVDIMLGGW
QEQLILTLTSEDGVCITHTLDGVFEEANNSEKALNNLKAGLAKLGQTPYYARDMQVTLPA
ALFVPNSLLNQFRREAIDMLDAARLAHYQRGRRKPVAQPAPVYPQTHLSFLANVYNHKAR
EFYHRYGVQLIDAAYEAHQEKGEVPVMITKHCLRFAFNLCPKQAKGNIKSWKATPMQLVH
GDEVLTLKFDCRPCEMHVIGKIKNHILKMPQPGSVVASVSPEALMKTLPKRRGV
>EECEBICD_01947 HTH-type transcriptional regulator SutR
MALLGGKMDNLTHYLATTLRMLRHQRGWSLSRLAEISGVSKAMLGQIERNESSPTVATLW
KIATGLNVPFSVFISPPQAEFPPTFDPQQQAMVITPLFPWDPELCFDYFSLLLAPGAVSE
STPHKAGVVEHVVVIQGRLDMCTGGVWQTIDAGKGLRFAGDMAHTYRNSSDQTVHFHSLI
HYPRG
>EECEBICD_01948 Inner membrane protein YdcO
MRLLSLPLPTVLSGLVAVLVGYASSAAIIWQAALAAGATPAEIAGWMTALGIAMGISTLT
LTLWYRAPVLTAWSTPGAALLVTGLQGLSLPDAVGIFIVANALIVLCGVTGLFARLMRII
PHSLAAAMLAGILLRFGLQAFGTLNGEFVMCGGMLLAWLLFKVFAPRYAVIAAMVMGITV
ALIQGKVAMSGIHFAPVWPTFVPPHFSFAQSLSVAVPLFLVTMASQNAPGVATMKASGYQ
LPVSPLMIFTGLLALLLSPFGVYSICIAAITAAICQSPDAHPDPTRRWLAAAAAGVFYLL
AGWFGGSITGLMVALPVSWVQMLAGLALLSTISGSLYQALTHESERDAAVIAFLVTASGL
TLMGIGSAFWGLIAGGIGYAVLTRTRRPSLSG
>EECEBICD_01949 hypothetical protein
MRTHTLFKVAVLTGLLALSGCASKVTQPDKYSGFLKNYSDLQETTSATGKPVLRWVDPHF
NDSNYDSIVYNPITYYPVPKPTTQVGQQVLDKLLAYTNTKVKSAIEQRKPLVTTPGPRSL
IFRGAITGVDTSKEGLQFYEVIPVALIVAGTQMATGHRTMDTHLYFEGELIDAATNKPVV
KVVRQGEGKDLSNSSTPMAFETLKQVVDDMATDTSMFDVNKK
>EECEBICD_01950 Tellurite methyltransferase
MTVRDENYFTEKYGLTRTHSDVLAAAKVVAPGRTLDLGCGNGRNSLYLAANGYDVTAWDK
NPASMANLERIKAAEGLDNLQTDLVDLNTLTFDGEYDFILSTVVMMFLEAQTIPSLIANM
QRCTKPGGYNLIVAAMDTPDFPCTVGFPFAFKEGELRRYYEGWDMLKYNEDVGELHRTDE
NGNRIKLRFATMLARKTA
>EECEBICD_01951 Tellurite resistance protein TehA
MRNHKQSDRVLNLPAGYFGIVLGTIGMGFAWRYASQIWGISHWPGDIMVILAMIIWALLT
LAFLSRLVRFPHSVMAEVRHPVMSSFVSLFPATTMLVAIGFVPWYRPLAVALFSVGVVIQ
LAYAAWQTAGLWRGAHPEEATTPGLYLPTVANNFISAMACGALGYNDAGLVFLGAGVFSW
LSLEPVILQRLRSCGELPAVLRTSLGIQLAPALVACSAWLSVNGGEGDTLAKMLFGYGLL
QLLFMLRLMPWYLSQPFNASFWSFSFGVSALATTGLHLGHGSESGLFHILAVPLFIFTNA
IIALLLVRTFLLLVQGTLLIRTERAALLKTEEKNDRS
>EECEBICD_01952 hypothetical protein
MIKYRLSEEPRAFTYQVDGEKKSVLLRQVIAVTDFNDVKAGTSGGWVDADNVLSQQGDCW
IYDENAMAFAGTEITGNARITLPCTLYNNVRIGDNVWVDRADISDGARISDNVTIQSSSV
RGECAIYGDARVLNQSEILAVQGLTHEHAQILQIYDRATVNHSRIVHQVQLYGDATITHA
FIEHRAEVFDFALIEGNKDNNVWICDCAKVYGHARVIAGTEEDAIPTLRYSSQVAEHALI
EGNCVLKHHVLVGGHAEVRGGPILLDDRVLIEGHACIQGEILIEHQVEISGRAAVIAFDG
NTIHLRGPKVINGEDRITRTPLVGSL
>EECEBICD_01953 Ribosomal-protein-serine acetyltransferase
MVEIIPVSTTLELRAADESHVPALHELVLKNKAWLQQSLDWPQYVTSQEETRKHVQGNIL

LHQRGYAKMYLIFCQNEMAGVLSFNAIEPVNKAAYIGYWLDESLQGQGIMSQSLQALMTH
YARRGDIRRFVIKCRVDNQASNAVARRNHFTLEGCMKQAEYLNGDYHDVNMYARIIDAD
>EECEBICD_01954 Putative aminopeptidase YsdC
MTFSVQETLFSLLRLNGISGHESSIANVMQHAFEQQAKDVWRDRLGNVVARYGSDKSDAL
RLMIFAHMDEVGFMVRKIEPSGFLRFERVGGPAQITMPGSVVTLAGRSGDIMGCIGIKAY
HFAKGDERTQPPALDKLWIDIGAKDKADAERMGIQVGTPVTLYNPPHCLGNDLVCSKALD
DRLGCTALLGVAEALASTPLDIAVFLVASVQEEFNIRGIVPVLRRVRPDLAIGIDITPSC
DTPDLQDYSDVRVNHGVGITCLNYHGRGTLAGLITPPRLLRMLETTAHENNIPVQREVAP
GVITETGYIQVELDGIPCASLSIPCRYTHSPAEVASLRDLADCIRLLTALANMSPEQFPI
EPETGATQEARP
>EECEBICD_01955 PTS system galactitol-specific EIIB component
MMKKILVACGTGMSTSTMIAQKLQDFLAEQGIAATTAQCCLNEIPLNCNGMDLIVTSMRT
HSDYGIPTLNGAALLTGINDDALKQEIKALLTQ
>EECEBICD_01956 PTS system galactitol-specific EIIC component
MFDYILSLGGTVFVPIIMIIIGLIFRIPWLQAVKAGVTVGIGFVGMGLVIVMAIDSLSPP
IKVMIERFGLTLHVFDVGAGPASGVGYATAIGAMIIPVIFLLNVGMLVTRLTKTMNVDIY
NYWHYAITGTVVQLMTGSLIYGVLGAICHAALSLKMADWTAKRVQNIVGLEGISIPQGYG
SSSVPLFVLLDAIYEKIPFMKGRNIDAQEIQKRYGMVGDPVIIGVVLGLIFGLAAGEGFK
GCATLMITVAAIMVLFPRMIRLIVEGLMPISDGARKFFQKHFKGREVFIGLDTAVTLGHP
TTIAVGLLLIPIMLILASILPGNKVLPLADLPVAPFFICMATVIHRGDLIRTLLSGIIVM
ITVLLIATQFAPYFTDMALKGGFSFAAENAQITALSVGNMFGWSISELMSLGMIGVVIVV
GIVASIILVLRKRELPE
>EECEBICD_01957 hypothetical protein
MSWLKEVIGTEKAVIAMCHLRALPGDPGFDTRKGMNWVIDRAHDDLMALQNGGVDAVMFS
NEFSLPYLTKVRPETTAAMARIIGQLMSEIRVPFGVNVLWDPVASFDLAMATDAKFIREI
FTGAYASDFGVWDTNVGETIRHQHRIGAGHVKTLFNIVPEAAVYLGNRDVCSIAKSTVFN
NNPDALCVSGLTAGARTDSAILKRVKETVPDTVVLANTGVCLENVEEQLCIADGCVTATT
FKKDGVFANFVDQARVAKFMEKVRHIRQ
>EECEBICD_01958 Ascorbate-specific PTS system EIIA component
MINDVKWVQAQRHASDWRHALDIAVRPLITFGAAAPCYLDGIIENTLKWGPYYLIAPGIA
LPHARPEQGANHNQISVTTLATPVAFGNADCDPIWLLLCASATDAEAHIRTIQRISQWLD
SPESVAMLQDAPNDAALFAQLTALR
>EECEBICD_01959 Ribulose-phosphate 3-epimerase
MILHPSLASADPLRYAEALTALHDAPLGSLHLDIEDTSFINNITFGMKTIQAVAQHTRHP
LSFHLMVSSPQRWLPWLAAIRPGWIFIHAESVQNPPEILADIRAIGAKAGLALNPATPLL
PYRYLALQLDALMIMTSEPDGRGQQFIAAMCEKVSQGREHFPAAECWADGGITLRAARLL
AAAGAQHLVIGRALFTTANYDVTLSQFTAL
>EECEBICD_01960 Glucitol operon repressor
MSQQRPDRIKQMLHYLWQHRHLSTQQAIELFGYAEATVRRDFHYIASRYPGMVRGHGCID
FDDSTEDKEYVFDVKRTLQSEAKREIAALARTFIKDGDCFFLDSGSTCLELAKCLVDAKV
KVICNDIKIANELGGFPHVESYIIGGLIRPGYFSVGESLALEMINAFAVERAFISCDALS
IETGITNATMFEVGVKTRIIQRSREVILMADHSKFDTVEPHAVATLSCITTILSDSALPS
AIARRYQQAGCRLIMPDPSSGVR
>EECEBICD_01961 Aminoglycoside N(6')-acetyltransferase type 1
MDIRQMNKTHLEHWRGLRKQLWPGHPDDAHLADGEEILQADHLASFVAMADGVAIGFADA
SIRHDYVNGCDSSPVAFLEGIFVLPSFRQRGVAKQLIAAVQRWGTNKGCREMASDTTPEN
TISQKVHLALGFEETERVIFYRKRC
>EECEBICD_01962 D-galactonate dehydratase family member
MSNLKITNVKTILTAPGGIDLAVVKIETNEPGLYGLGCATFTQRIFAVKSAIDEYMAPFL
VGKDPTRIEDIWQSGVVSGYWRNGPIMNNALSGVDMALWDIKGKLAGMPVYDLLGGKCRD
GIPLYCHTDGGDEVEVEDNIRARMEEGYQYVRCQMGMYGGAGTDDLKLIATQLARAKNIQ
PKRSPRSKTPGIYFDPDAYAKSVPRLFDHLRNKLGFGIEFIHDVHERVTPVTAINLAKTL
EQYQLFYLEDPVAPENIDWLKMLRQQSSTPISMGELFVNVNEWKPLIDNRLIDYIRCHVS
TIGGITPARKLAVYSELNGVRTAWHGPGDISPVGVCANMHLDLSSPNFGIQEYTPMNDAL

RDVFPGCPEIDHGYAYLNDKPGLGIDIDEAKAAKYPCEGESHPGQWRAHQTVRPQDRKQ
>EECEBICD_01963 5-keto-D-gluconate 5-reductase
MTALFDLTGKTALVTGSARGLGFAYAEGLAAAGARVILNDIRATLLAESVDTLTRKGYDA
HGVAFDVTDELAIEAAFSKLDAEGIHVDILINNAGIQYRKPMVELELENWQKVIDTNLTS
AFLVSRSAAKRMIARNSGGKIINIGSLTSQAARPTVAPYTAAKGGIKMLTCSMAAEWAQF
NIQTNAIGPGYILTDMNTALIEDKQFDSWVKSSTPSQRWGRPEELIGTAIFLSSKASDYI
NGQIIYVDGGWLAVL
>EECEBICD_01964 D-galactonate transporter
MKASRQRLFILTLLFIVTAINYMDRANLAVAGSNIQNDFSLTPTQLGLLFSMFTWAYAAS
QIPVGYVLDRIGSRILYGGAIILWSIFTFMMGFASHHLFATATASFAMLLACRALIGVAE
APSFPSNTKIIATWFPDHERARATAIYSSAQYIGLALLTPALAFIVANYGWEMSFYLSGG
AGILFGIYWLMYYRDPQHSTAVNQAELDYIKAGGGYGSENQSSVSAKISWQNIKFFLSKK
TIWGLFITQFACSSTLYFFLTWFIVYLEKGLHLSISKAGIGAMLPYIMAMLGVLCGGTLS
DMLLKKGKSRTLARKLPVMAGLCVTMIIGLVNFFENQPVIAIVILSVAFFANAFSNLGWV
VWSDVIPRNFLGTMGGFLNICGNLSGIVSPIVIGVILQRTQNFQYAMWYIAGVAGLGLLA
YIFLVGKIEVILPGKKNADTVDKNAINPATANK
>EECEBICD_01965 L-idonate 5-dehydrogenase (NAD(P)(+))
MPTLWIRMPLTRQLPTNNEVIIKMEKITCNACLAHAEKDVRFESREIEHSEHDVVVKVAC
GGICGSDIHYYQHGRAGMSVLKHPMVIGHEFVGVISKVPAGSDLKVGQTVAVNPSSPCNQ
CEMCLSGHQNLCGSMRFMGSAQFNPHVNGGFSEYVVVKPEQCIPYDRRVPANVMAFSEPL
AVAIHAVKKAGQLTGKRVLVIGAGPIGCLILAAARSAGASELVASDLSPRCLELARQMGA
TAVMDPRDEEQVAHYQQHKGYFDVVFEASGAPIAVASTVDFTRPAGTIVQVGMGASPVSW
PVSTMLVKELNWVGSFRFIGEFITAVRWLEDGRVDPRPLISAEFPPQQIEDALITATDKN
VSAKVLIRFD
>EECEBICD_01966 HTH-type transcriptional repressor NanR
MSIKSIQKQNVVNEIYDQISSKLLDGSWAPGSRLPSEVELTASFNVSRVSVRSAVQRFRD
LGIVVTRQGSGSYVSENFTPQMLSNDPRPIMHLSREEFHDMMIFRQTVEFKCVELAVTHA
TDDDIRQLEEALNNMLIHKGDYKKYSEADYEFHLAIVRASHNSVFYNVMSSIKDIYYYYL
EELNRALGITLESVEAHIKVYMSIKNRDASTAVEVLNEAMSGNIIAIEKIKSTETSGTK
>EECEBICD_01967 Protein YceI
MKKSFLGVVLSLGLLSAAHADVYKFDNTHTNAVFNIDHFQTSTNHGGFYAISGELKYQPE
KQDAEMRVTIPVSALNTGVDAFDNHIRSSDILDAEKYPEMVFKSTKWHFEDNKPVSIDGL
LTMKGVTKPVTLTTTKFGCYMSPIFKAQVCGGDFVTQIDRTQWGVDYLVDMGMTKVVDIK
IQAEAVKQ
>EECEBICD_01968 Glucans biosynthesis protein D
MAAVCGSSGIASLFSQAAFAAESDIADGKIVRFDFAGLQSMAQALAKKPWGGAPGPLPDT
LANLTPQAYNSIQYDAAHSLWNGVANRQLDIQFFHVGMGFRRRVRMFSVDTTTHLAREIH
FRPELFKYNDAGVDTTQLEGQSDLGFAGFRVFKAPELARRDVVSFLGASYFRAVDDTYQY
GLSARGLAIDTYTDGQEEFPDFTAFWFDTAKPGDTTFTVYALLDSASVTGAYKFVIHCEK
SQVIMDVENHLYARKDIKQLGIAPMTSMFSCGNNERRVCDTIHPQIHDSDRLAMWRGNGE
WICRPLNNPQKLQFNAYMDDNPKGFGLLQLDRDFSHYQDVMGWYNKRPSLWVEPRSKWGK
GAVSLMEIPTTGETLDNVVCFWQPEKAIKAGDTLAFNYRLYWSAQPPVQSPLARVMATRT
GMGGFPEGWAPGEHYPDKWARRFAIDFVGGDLKAAAPKGIEPVITLSSGEAKQIEILYVE
PFDGYRIQFDWYPTSDSTAPVDMRMFLRCQGEAISETWLYQYFPPAPDKRRYVDDRIMR
>EECEBICD_01969 Para-nitrobenzyl esterase
MLNPSIPLVATRHGKIVGVVQEETHIWRGIPYAAPPTGELRWRAPQPVTPWQDVRQADCF
SCASWQDITWCRELGGGDPGNFSEDCLYLNVWAPAVRHEPLPVMVWLHGGGYTIGAGSLP
PYDGQALAKRGAIVVTVNYRLGHLGFFAHPALEGEGAECIHNFALLDQIAALRWVQDNIA
AFGGDTQNVTLFGESAGARSVLSLMASPLAKGLFHKAIIQSGYTLPDTPREVALKKGVAL
AEHLGLAHATAEQLRALPAETFWPLDAPFKIAPTPISGDVVLPHPMLETFFAAKQHPIPV
MIGSNSDEASVLAVFGVDIAGQIQKMRRERRVGLGLIRLLYPGVKGDEALGRQVCRDMVF
TTLGYVVMQAQQRIGEPCWRYWFDYVAEAEHNTYANGACHGNEIPYVFDTLTRAEPTCHY
VNENDLAFASQVADYWVNFARHASRTRDVLHGPVRWPASIRGRDRLLRIGLNKLAGFKVE
NRFMRARLALFKRVMKHHVSLE

>EECEBICD_01970 putative protein
MANTITADEIREHFSQAMSAMYQQEVPQYGTLLELVADVNLAVLENNPKLHEQLANADEL
ARLNVERHGAIRVGTAEELSTLRRIFAIMGMYPVSYYDLSQAGVPVHSTAFRPIDEASLS
RNPFRMFTSLLRLELIENAALRQRAAEILSQRDIFTSRCRQLLDEYDEQGGFSAAQAEEF
VRETLETFRWHRQATVDEETYRSLHREHRLIADVVCFPGCHINHLTPRTLDIDRVQAMMP
ECGITPKTLIEGPPRREVPILLRQTSFKALEEQVLFVDEKQGTHTARFGEIEQRGVALTP
KGRRLYDELLHKAGTGKDNFTHQLHLREVFNAFPDSEFLLRQQGLAWFRYRLTPSGEAHR
QAIHPGDDPQPLIERGWVIAQPITYEDFLPVSAAGIFQSNLGNETLARSHGNASRDAFEQ
ALGCAVRDEFSLYQEAEERSKRRCGLL
>EECEBICD_01971 HTH-type transcriptional regulator GbpR
MEKNGLFSQRIRLRHLHTFVAVAQQGTLGRAAETLNLSQPALSKTLNELEQLTGTRLFER
GRLGAQLTVPGEQFLTHAVKVLDALNTAGQALNRKEDASADVVRVGALPTAALGILPAAI
GRFHQQQKSTSLQVATMNNTMLLAGLKSGEIDLGIGRMSDPELMGGLNYELLFLESLKLV
VRPGHPLLQETITLSRVMEWPVVVSPKGTVPRQNAEALLQSQGCKMPAGCIETLSASLSR
QLTVDYDYVWFVPSGAVKEDLRQATLVSLPVPTQSAGEPIGILTRVDIPLSTGAQMLIAA
IRKSMPL
>EECEBICD_01972 Methyl-accepting chemotaxis protein III
MGNTFSMQASHKLGFLHHIRLVPLFSSILGGILLLFALSAGLAGYFLLQADRDQRDVTDE
IQVRMGLSNSANHLRTARINMIHAGAASRIAEMDEMKANIAAAETRIKQSQDGFNAYMSR
AVKTPADDALDNELNARYTAYINGLQPMLKFAKNGMFEAIINHENEQAKQLDAAYNHVLL
KAIELRTERARLLSEQAYQRTRLGMMFMIGAFTLALVLTLMTFMVLRRTVIQPLQQSASR
IERIAAGDLTMADEPTGRSEIGRLSHHLQQMQHALQQTVGAVRQGAEEIYRGTSEITAGN
TDLSSRTEQQAAAIEQTAASMEQLTATVKQNADNAHHASKLAEDASGKASRGGQMVSGVV
QTMGNISTSSKKISEITAVINSIAFQTNILALNAAVEAARAGEQGRGFAVVASEVRTLAS
RSAQAAKEIEGLIGASVSLIEQGSEEVIAAGSTMNEIVDAVKRVTDIMLDIAAASDEQSR
GIVQVSQAISEMDKVTQQNASLVEEASAAAASLEEQAARLTQAVDAFHLQDTGATMRSSF
L
>EECEBICD_01973 S-(hydroxymethyl)glutathione dehydrogenase
MKSRAAVAFGPGQPLKIVEIDVAPPKKGEVLVKITHTGVCHTDAFTLSGDDPEGVFPAVL
GHEGGGVVVEVGEGVTSLKPGDHVIPLYTAECGECKFCKSGKTNLCQAVRATQGKGLMPD
GTTRFSYNGEPVYHYMGTSTFSEYTVCAEISLAKVNPQAPLDKVCLLGCGVTTGIGAVHN
TAKVKAGDTVAVFGLGGIGLAVIQGAVQAKAGRILAVDTNPEKFTLAGEMGATDFINPND
YDKPIQDVIVELTDGGVDFSFECIGNVNVMRAALECCHKGWGESIIIGVAGAGQEIKTRP
FQLVTGRVWRGSAFGGVKGRTQLPGMVEDAMNGKIRLDPFITHRLPLEQINDAFELMHQG
KSIRTVIHFGDN
>EECEBICD_01974 Transcriptional repressor FrmR
MPHSPEDKKRILTRVRRIRGQVEALERALESGEPCLAILQQIAAVRGASNGLMSEMVEIH
LKDELVSGETTPDQRAVRMAEIGHLLRAYLK
>EECEBICD_01975 hypothetical protein
MFQAKPKLNWSAQKPITYITNIGRKLMLWAEYTTAIIQTGC
>EECEBICD_01976 hypothetical protein
MNDDSIIKNSSMLLAYLGGLGWGSAYFYGWGVSAYYGFPWWYVGVGPDNIARSLFHAISL
MAIFIIAWGVGILLFFLVKQKARIHDLSFLRLFLAAVLFFIPIVIEFSLLTESFLWELFF
IILLVALCLSVGMRFYSKLMPVICFTQLSWVRRHCFTIVMLGFIIYFFIFSFFVGIYKPQ
LKKEYEMILYDGGWYYVLARYHDSFILSKSFTKNNNRFIIFRPEDGHSYEITLVKVRL
>EECEBICD_01977 Secreted effector protein SseJ
MPLSVGQGYFTSSISSEKFNAIKESARLPELSLWEKIKAYFFTTHHAEALECIFNLYHHQ
ELNLTPVQVRGAYIKLRALASQGCKEQFIIESQEHADKLIIKDDNGDNILSIEVECHPEA
FGLAKEINKSHPKPKNISLGDITRLVFFGDSLSDSLGRMFEKTHHILPSYGQYFGGRFTN
GFTWTEFLSSPHFLGKEMLNFAEGGSTSASYSCFNCIGDFVSNTDRQVASYTPSHQDLAI
FLLGANDYMTLHKDNVIMVVEQQIDDIEKIISGGVNNVLVMGIPDLSLTPYGKHSDEKRK
LKDESIAHNALLKTNVEELKEKYPQHKICYYETADAFKVIMEAASNIGYDTENPYTHHGY
VHVPGAKDPQLDICPQYVFNDLVHPTQEVHHCFAIMLESFIAHHYSTE
>EECEBICD_01978 hypothetical protein

MYLNRFCYLLLIFAAVGSIFTSHPVAMWFLLANVLTLAIYGIDKTAARKTWRRVPESTLL
VFGVVGGWPGAIVGQQLFRHKTQKQPFKTYFIVSVIVSILVTVAIYRLYPFLSY
>EECEBICD_01979 L-cystine-binding protein FliY
MLSKKFGLSMIVLGIMSSSAFADSIVEGRTLNVAVSPASPPMLFKSADGKLQGIDLELFS
SYCQSRHCKLNITEYAWDGMLGAVASGQADVAFSGISITDKRKKVIDFSEPYYINSFYLV
SMANHKITLNNLNELNQYSIGYPRGMAYSDLIKNDLEPKGYYSLSKVKLYPTYNETMADL
KNGNLDLAFIEEPVYFTFKNKKMPIESRYVFKNVDQLGIAFKKGSPVRDDFNLWLKEQG
PQKISGIVDSWMK
>EECEBICD_01980 putative amino-acid permease protein YxeN
MNLSVGLISGSYLAEVFRGALKLVEPYEITVAKVAGMRQLQVIINIELPQMLRFSVPGII
NEFSSVLKATPFAYTVGIAEITKQAMSLTAITLNGLQIYTLDGVLYFIIYKVFTLLAGVF
EKKYRIS
>EECEBICD_01981 hypothetical protein
MALLKLKEISKEYSGKKVLDTVSLKIQSGDMKVVMGPSGCGKTTLLRCLLRLEEPDYGNI
YFHGKNIYDKEFTILEFRKKLVVSSRITLCIAILTSWIILPLPYVKCLACRDN
>EECEBICD_01982 Histidine transport ATP-binding protein HisP
MDNITLALCKVFGMPGQLAREKALHELQKLDMASHSAKYPSQLSGGQQQRVALARTMVTD
PELIIFDEPTSALDPLMTREVGMLIKQLHDNGVTILCVTHDIRLARLLSDNVTFLNHGKI
RAEGAFTDLALRETDPDIHCFFSEAQR
>EECEBICD_01983 L-cystine transport system permease protein YecS
MIAGWSLFFNDLTEQLPLVVDGIKETCKLALIVSITGFLWGIIIFFLSLSHRPVVKAITR
LYMDFFIGTPLILILFVIYYGLPQSGIHLSSFTVAVTGFTLNVGAYNAAYMTTAYNALNK
YETEAAVVQGLNKRQVFLWIILPQVLLSSIPALTNQVINNLKDSTIVFLIQYTEFFARIQ
EVAATSFKFFHAYLFAAIVYLIGVTFLVGLTRFLEHRLLRHYGQGY
>EECEBICD_01984 hypothetical protein
MALPQLSIWQPGDGLRKIKYKYHVCLIVIFSVLIRLLFLTDRYLWCDEASSVLISRHSVD
NLLFHTAFDVHPPLYYLLLHDWIILLGDSIAAVRSLSLLFGILTVVLVIALTRWLANERT
ALLAGWFAALMPMAVHYSQETRMYALMGMLTLAAALALMMWIKTPTHNRYLIAYALLMTF
SFYTHYFTLFTLIAHWIAVTILSCQSHKTACYLKLPGWWFANLAIAVAYLPGCPYCLIY
>EECEBICD_01985 hypothetical protein
MLVFIISWWMPLFIDRYFFFSSLSIPPLLAVLLTRAKKAVCGFWFILFTLLFGYGSYHNN
PEHIDEFKPLVNYINTRHQPNDAVVVSKMFDYLSYVYYNKRDYRTFLYTPPNAHGTSGRP
NAYGFGSLFYAQADQTYIDTLTTLSKSYHRVWLVSGGNFSQDYPLPSEWQNIANFRSGRF
QVQLFVIPTQQARQMQ
>EECEBICD_01986 hypothetical protein
MIEVFEAFGWTLLLGGLAVAVLFITALDKKR
>EECEBICD_01987 hypothetical protein
MKHTQQSGAKVYNPFTLSLYDWWVLNISNKYAWKCPTDTRLLPFFLHHMGETHLDIGVGT
GYYLKHAPATHAISLMDLNPDSLKIAASRVGNTKIRATLQHDVFDTFPADWHGRFDSVSM
YYLLHCLPGEMTTKAKAIKNAGMALKPGGTLFGATILGKEVPHNAFGKKLMAVYNKKGIF
SNTNDSADTLRSVLDEHFAKVTLEQHGAVALFSATMPRSL
>EECEBICD_01988 Cytochrome b561
MGNKYSGLQIGIHWLVFFLVIVAYAAMELRGFAPSSYRPWFNMTHVSCGITILLLMVARL
IIRLKYPTPPIVPRPKPMMTGMAHLGHLVIYLLFIVLPVIGLVMMYNRGNPWVAFGITMP
HAAEANFERVDMLKSWHVTLANLGYFVIGLHAIAALMHHYFWKDNTLLRMMPRKRN
>EECEBICD_01989 Protein YdcF
MNTTPFPALSAETLLAVNTVGQWLAQNDFSGEQPYSSDCVVLAGNAVIPTIDAACRIAKA
QGVPLLISGGIGHSTPFLYAVIARHPRYHTIRTTGRAEAAILADIANQFWHIPAEKIWLE
DRSTNCGENARFSCALIRQAKENINTAIVVQDPTMQRRTIAAFRRVTNDDTDAPRWLSFP
GFVPVLRHLNGGTRFANVEEGIWTVERYLSLIAGELPRLRDDETGYGPRGKDFIIHVDIP
RDIENAWQVLQADTTLRSALEQRALR
>EECEBICD_01990 hypothetical protein
MLPKSFMTEQSTLTLQVLQQRLDALMLRDKQRFARRLHGVKKVKNPDAQQAIFQTMAKEI
EQAVAQVALREAARPSITYPQNLPVSQKKQDILEAVRDHQVVIVAGETGSGKTTQLPKIC

MELGRGIKGLIGHTQPRRLAARTVANRIAEELQTEPGGCIGYKVRFSDHVSSNTMVKLMT
DGILLAEIQQDRLLMQYDTIIIDEAHERSLNIDFLLGYLKELLPRRPDLKVIITSATIDP
ERFSRHFNNAPIIEVSGRTYPVEVRYRPIVEEADDTERDQLQAIFDAVDELGRESPGDIL
IFMSGEREIRDTADALNKLNLRHTEVLPLYARLSNSEQNRVFQSHSGRRIVLATNVAETS
LTVPGIKYVIDPGTARISRYSYRTKVQRLPIEPISQASANQRKGRCGRVSEGICIRLYSE
DDFLSRPEFTDPEILRTNLASVILQMTALGLGDIAAFPFVEAPDKRNIQDGVRLLEELGA
ITADEQQTAYKLTPLGRQLSQLPVDPRLARMVLEAQKHGCVREAMIITSALSIQDPRERP
VDKQQASDEKHRRFHDKESDFLAFVNLWNYLGEQQKALSSNQFRRLCRTDYLNYLRVREW
QDIYTQLRQVVKELGIPVNSEPAEYREIHVALLTGLLSHIGMKDADKQEYTGARNARFSI
FPGSGLFKKPPKWTMVAELVETSRLWGRIAARIEPEWVEPVAQHLIKRSYSEPHWERAQG
AVMATEKVTVYGLPIVAARKVNYSQIDPALCRELFIRHALVEGDWQTRHAFFRENLKLRA
EVEELEHKSRRRDILVDDDTLFEFYDQRISHDVISARHFDSWWKKISRETPDLLNFEKSM
LIKEGAEKISKLDYPNFWHQGNLKLRLSYQFEPGADADGVTVHIPLPLLNQVDESGFEWQ
IPGLRRELVIALIKSLPKPVRRNFVPAPNYAEAFLGRVTPLELPLLDALERELRRMTGVT
VDREDWHWDQVPGHLKITFRVVNDKNKKLQEGRSLAELKNALKGKVQETLSAVADDGIEQ
SGLHIWSFGELPESYEQKRGNYKVKAWPALVDERDSVAIKLFDNPLEQQQAMWCGLRRLL
LLNIPSPIKYLHEKLPNKAKLGLYFNPYGKVLELIDDCIACGVDKLIDANGGPVWSEAGF
TALHEKVRAELNDTVVDIAKQVERILTTVFNINKRLKGRVDMSMALGLSDIKAQMSGLVY
RGFVTGNGFKRLGDTLRYLQAIEKRLEKLAVDPHRDRAQMLKVESVQQAWQQWINKLPPA
RREDDDVKEIRWMIEELRVSYFAQQLGTPYPISDKRILQAMDQITA
>EECEBICD_01991 FMN-dependent NADH-azoreductase
MSKVLVLKSSILAGYSQSGQLTDYFIEQWREKHVADEITVRDLAANPVPVLDGELVGAMR
PGDAPLTPRQQDALALSDELIAELKAHDVIVIAAPMYNFNIPTQLKNYFDLIARAGITFR
YTEKGPEGLVTGKRAVVLSSRGGIHKDTPTDLIAPYLKVFLGFIGITDVNFVFAEGIAYG
PEVAAKAQADAKAAIDSVVAA
>EECEBICD_01992 hypothetical protein
MTSEKLSAACHCGSVVFTVQLSDGFHTARRCNCSFCRMRGAVAVSAPLSGIKVLKGQDKL
TEYRFNTGKAVHFFCSVCGIYTFHQRRSNPDQYGVNVACIENVSPFDFACVEVNDGVTHP
SDGGSSGVVGYLRYEPETLPPVETGGKNI
>EECEBICD_01993 hypothetical protein
MKKYLMLWVLTLSLLTPSVWALTLDEARTQGRVGETLNGYLVALKNDAETQKLVLDINHA
RRASYQQLADSNHLPVDEVAKMAGQKLVERARPGEYVQGINGKWMRK
>EECEBICD_01994 hypothetical protein
MKMSIAMLSALASFIVVGCTPRIEVAAPEQPITINMNVKIEHEIHIKVDKDVEELLKSRS
DLF
>EECEBICD_01995 hypothetical protein
MKGKYKAALALLLLLILIPLTLLMTLGLWVPTLAGIWLPVGTRIALEQSPRLTRHGLVIP
DLRYLVNDCSLAHITQAELTHPSRWLLNIKSLKLDAACLAKLPATEASPAAPRTLAQWQS
MLPNTWINIDNVILAPWPEWQGKLAISMTPVIQQIRYQGEKVKFQGQLRGQALTVSQLEI
AALANQPPVSLAGEFVLPLVSDGLPVSGHAAATLRLPQEPSLVDAELEWRDNAGQLIVMA
RGNPDPILDLPWAVTRQRLTISDGRWNWPYQGFPLSGRLAFNIDNWQAGLDNAQVSGRLN
ILTQGDAGKANAVLTIGPGKLSMDSSEMPLQLTGEAKQKDLIFYAVLPAMFRGSLADPQL
TFAPGALLRSRGRVIDALDIDEIRWPLAGVKVTPRGVDGRLQAILRAHENEMGDFVLHLD
GLANDFLPDAGRWRWRYWGQGSFTPMRAHWDIAGQGEWHDNTIRLTSLSTGFDQLHYGAM
TVTSPRLALNKPIVWVRDATTPSLQGALSLVAGKTVFTSGSVLPPSTLNFSVEGREPTLF
QFKGDLRAGAIGPVRLNGRWDGERLRGQAWWPKQSLIVFQPLLPPDWKMTLREGSLYAQV
AFSAAQGQGFEAGGHGVLKGGSAWMPDNKINGVDFILPFRFHNGAWQLGTRGPISLRIAG
IVNQVTAKNITADLQGGYPWSESNPLLLSDVSVDVLGGQIIMKQLRMPQHDPALLRVQNI
SSSELISAINPKQFAMSGPVSGALPLWLNNEKWIIKDGWLTNPGPMTLRIDKDTADAVVK
DNVTAGSAINWLRYMEITHSWTKINVDNLGVLTMQAAITGKSRVDGKTAIVNLNYTHEEN
VFTLWRSLRFGDNLQAWLEQNTVLPQPPCRKDKDCEDK
>EECEBICD_01996 D-lactate dehydrogenase
MKLAVYSTKQYDKKYLQQVNEAFGFELEFFDFLLTEKTAKTANGCEAVCIFVNDDGSRPV
LEELKKHGVKYIALRCAGFNNVDLDAAKELGLQVVRVPAYSPEAVAEHAIGMMMTLNRRI

HRAYQRTRDANFSLEGLTGFTMHGKTAGVIGTGKIGVAALRILKGFGMRLLAFDPYPSTA
ALDLGVEYVDLQTLFAESDVISLHCPLTPENYHLLNHAAFDQMKNGVMVINTSRGALIDS
QAAIEALKNQKIGSLGMDVYENERDLFFEDKSNDVIQDDVFRRLSACHNVLFTGHQAFLT
AEALISISETTLQNLSQLEKGEACPNALF
>EECEBICD_01997 Heat shock protein HslJ
MKKSVTLVALSIIMTGCVSSGKVSVKREQLEHHRFVLESVNGKTVTGPELSFGEDMTVSG
KMCNQFTGEGKLSDGELKVKNLAMTRMMCADPQLNALDGTLSKLFSKGAQVDLTANQLTL
ATAETTLMYKLADLAH
>EECEBICD_01998 hypothetical protein
MRAAFWVGCAALLLSACSSEPVQQATAAHVSPGMKAAMSSAGEANCAMIGGSLSAARQLD
GSVIGMCALPNGKRCSEQSLAAGSCGSY
>EECEBICD_01999 Pyruvate-flavodoxin oxidoreductase
MQTIDGNGAVASVAFRTSEVIAIYPITPSSTMAEQADAWAGNGLKNVWGDTPRVVEMQSE
GGAIAAVHGALQTGSLSTSFTSSQGLLLMIPTLYKLAGQLTPFVLHVAARTVATHALSIF
GDHSDVMAVRQTGCAMLCAASVQEAQDFALIAHRATLKSRVPFIHFFDGFRTSHEINKII
PLTNETILNLMPQAEIDAHRARALNPEHPVIRGTSANPDTYFQSREATNPWYNAVYDHVE
EAMKAFGDATGRQYQPFEYYGHPQAERVIIMMGSALGTCEEVVDELLIRGEKVGVLKVRL
FRPFSAKHLLQALPETVRAIAVLDRTKEPGAQAEPLYLDVMTALAEAFNNGERETLPRTI
GGRYGLSSKEFGPACVLAVFNELSRAKPKPRFTVGIYDDVTNLSLPLPENTLPGSAKLEA
LFYGLGSDGSVSATKNNIKIIGNSTPWYAQGYFVYDSKKAGGLTVSHLRVSEKPIRSAYL
IAQADFVGCHQLQFIDKYQMAERLKPGGIFLLNTPYSADEVWSRLPQEVQAVLNQKKARF
YVVNAAKIARECGLGARINTVMQMAFFHLTHILPGDSALVELQGAIAKSYSSKGQDLVER
NWQALALAQESLAEVPLQAVNPHSAHRPPVVSDAAPDFVKTVTAAMLAGLGDALPVSALP
PDGTWPMGTTRWEKRNIAEEIPVWKEELCTQCNHCVAACPHSAIRAKVVSPQAMENAPAS
LHSLDVKSRDMRGQKYVLQVAPEDCTGCNLCVEVCPAKDRQNPQIKAINMMSRLEHVEEE
KVNYDFFLDLPEIDRSKLERIDIRTSQLITPLFEYSGACSGCGETPYIKLLTQLYGDRML
IANATGCSSIYGGNLPSTPYTTDANGRGPAWANSLFEDNAEFGLGFRLSVDQHRARVMRL
LAQFADRIPAELNDALHAEATPDVRREQVAALRQHLKSVAGAEELLKDADALVEKSIWLI
GGDGWAYDIGFGGLDHVLSLTENVNILVLDTQCYSNTGGQASKATPLGAVTKFGEHGKRK
ARKDLGVSMMMYGHVYVAQISLGAQLNQTVKAIQEAEAWPGPSLIIAYSPCEEHGYDLAL
SHDQMRQLTATGFWPLYRFDPRRADEGKPPLALDSRPPSDALAETLLNEQRFRRLNAQQP
EVAEQLWRDAALDLQKRYDFLALLAGKAEKPGAD
>EECEBICD_02000 Universal stress protein F
MNRTILVPIDISDSELTQRVISHVEAEAKIDDAKVHFLTVIPSLPYYASLGLAYSAELPA
MDDLKAEAKSQLEAIIKKFNLPADRVQAHVAEGSPKDKILEMAKKLPADMVIIASHRPDI
TTYLLGSNAAAVVRHAECSVLVVR
>EECEBICD_02001 Multidrug transporter EmrE
MTKEAVIFLFIAIVVEVIATISLKLSDSFTRLVPSLVTIIGYCIAFWCLTIPMRTIPAGI
IYAIWSGVGIVLIGLIGWLFLGQKLDMPAIIGMLLIICGVIVINLFSKSVSH
>EECEBICD_02002 hypothetical protein
MPRMAAKWALVAILVPFLLVGCASLDKARQIFEAAGQSRSERLLGLKSKSPVVADNWGFL
>EECEBICD_02003 hypothetical protein
MKPKDEIFDEILGKEGGYVNHPDDKGGPTKWGITEKVARAHGYRGDMRNLTRGQALEILE
TDYWYGPRFDRVAKASPDVAAELCDTGVNMGPSVAAKMLQRWLNVFNQGGRLYPDMDTDG
RIGPRTLNALRVYLEKRGKDGERVLLVALNCTQGERYLELAEKREADESFVYGWMKERVL
I
>EECEBICD_02004 hypothetical protein
MESNLTGTLNAGLCLVTVLALFLYRRNGARYKPGIAWLSYLLMLGYALVPFRFLAGHYPS
SSWPVVLMNALFCGLVLWARGNVSKILSLLRLR
>EECEBICD_02005 hypothetical protein
MLGIFRKKTRKAIVEVKKMENRDAVEATVWGAYSIAYADGTCDAKEIAVLEKTIAALPAF
APFSGEIAQMSANIRARYEASPRSANAQALRELADVAGTAEAVDVLCLYWPRSCPALLTT
TVNRWMRCVQW
>EECEBICD_02006 hypothetical protein

MIYPTNTGKSGEHLRLSTLESVWIQGKLRMWGRWSYIGGGKTGNMFNQLLASKKLTKTAI
NDALRRMKKAGLEKPELEVFLREMINGKQKSWLAHCTDTEALIIDRVVGEVLTDHPGLLG
ILNQRYVGRGMSKRRMAELLNEQYPEWALITCRRRVEQWLSIAEFILYSPMRKAFDYA
>EECEBICD_02007 Putative nuclease YbcO
MADLRKAARGLMCTVRIPGHCNHNPETSVLAHYRLAGTCGTATKPNDMQAAIACSSCHDI
VDGRVKIDDFTKTEIRLMHAEGVFRTQEIWREKGIL
>EECEBICD_02008 hypothetical protein
MAHLQLVKQTSSGLLLPATPESEDFLRSVKIGAWIHADFKRVRNYAFHKRFFKLLQLGFD
YWTPIGGAILPQEQELITGFVDFLCESAAQGHSPALSDAAEQYLHKVAVNRTLDVALLKS
FDAFREWVTIQAGFYTEHYYPDGSRGRRAKSIAFANMDETEFQQVYKAVLNVLWNWILFR
KFSSPEEVENVAAQLLEFA
>EECEBICD_02009 hypothetical protein
MKKLKDLLELDEDGLYAVRVKNGEISFCTLIPDDHLILSVEAFIDYLIRLGFTVSY
>EECEBICD_02010 hypothetical protein
MLCTCRLASYVPKGKEKQAMKQQKAMLIALIVICLTVIVTALVTRKDLCEVRIRTGQTEV
AVFTAYEPEE
>EECEBICD_02011 hypothetical protein
MHSVNFYSFRVLTHKGSRASKKLNDLGLSNKKTAYELFVDYFTLYKNTPIEFGVSKTKIS
LEQHTKLHFDNTKKIIYGYIKVGKYGESSEIKDVKLKKVHYRTTAYDVTLKERYILIYLP
DNLEEGIIAFHSCDNISARGVLSDSITEYLKKQFQLEARINPLHHKKIPQYILNSELKQI
KAQGYKAPEDIADSFGKNKTNIKTDLIIKANDGIFGSFRDLRNKNIGNIIEIIEDKCDAI
KVSLQLGSRTVVFNYDTILKKGISAELDDNDLKINPLTGIPDLTALHDTIKNLSNDILEE
LHCGNKGVII
>EECEBICD_02012 hypothetical protein
MNKINVLGVIIKHYKTMSDQRGTMLMSDITVHFIVPLSLSFVLCWTYGIMKPAIASVFVN
FGAITTALLMSAVIMIYEQKQKTITKISDIIEGNKSRDKLISLNTNKTIYEQLCHNVAYA
ILTSIVLVIFSVIIYFLPDNAVDLMKWYFRAPAYIVSFLAYTSFFITVITFLMVIKRFST
ILDN
>EECEBICD_02013 hypothetical protein
MNSGLITLTELRRMTGLTIYSTRHYLDKAERCGDVYQAGRRGGIFPSEEAYRAWKKQAKV
DADLIWKLPDGEVRRYDRHHNVICRECRKSEYMQRVLAFYRGNFQEVLL
>EECEBICD_02014 hypothetical protein
MRKKREEIAPPEATQRLRAIWDAKKRDLKLTQEIAADLMGFETQSTVSHYLNGKAPLNTD
AALKFSVLLRVKPEELRPDLADLMNYVRSSGTYDDNFEGGGWRMVSRQQADLLNLFDILP
ESEKEKLIDRLKGQNELYKEAFQNMLAAQKRLKNQ
>EECEBICD_02015 hypothetical protein
MGRNEVIQYLMDSCNVSFSAALQALRDNGWDMFLAQCELQEQYYPG
>EECEBICD_02016 putative protein YdfB
MDFDTIMEKAYEEYFESLDEGEEALSFSEFLLALSANG
>EECEBICD_02017 hypothetical protein
MSETALVIVKFLIGKSVGQFMLTVALFFLIIIFIPRDITELIEARSDLPYAVQIFSFAVA
YLIVLILKVTGYFFVSALPLCQRRGRAKRMLKTLNSLSTEQLFLLEPFLKTHSPTFRASW
DNPDADALVKAGIVRPAGSCIDGVSVMFKIEPEYESLMLSTWNPCTKRFDISR
>EECEBICD_02018 Killing protein KilR
MIAHHFGTDEIPRQCITPGDYVIHDGRTYIASANNIKKRRLYIRDLTTQRCITDCMVKVW
LNRNGLPAKAESW
>EECEBICD_02019 hypothetical protein
MKNVPESVIAELRQLSGKIRTLCIENNMPCVVSYARDCDDESVSRTLVAYTDSETGAYDR
SITAAIMLLKMNEAPPENFISLLKLMECKELVTNAFCSMKNESLH
>EECEBICD_02020 hypothetical protein
MSDNKTEYSYYIKVKNESARKRLGFPFAFWWKTESSEAAATARLAVSMLDAGFEPTDFAK
PVRVNSPAVNELPPEGSFDTTFCQKYELGGEDGKTFMLIPGTPATDAHDEKTEECADDAG
TEESGTDTSDNDECQDCEVSVATLPFPQRVLHIFTYAATDKKYLHHATRAQRRHITVLEM
EQENSYIQNLLMVLRKSEQVHAQDE

>EECEBICD_02021 Putative defective protein IntQ
MSKGKQKPYYSVSSLGARWNAAVKRAGIRRRNPYHTRHTFACWLLTAGANPAFIASQMGH
ETAQMVYEIYGMWIDDMNDEQVAMLNARLS
>EECEBICD_02022 tRNA-cytidine(32) 2-sulfurtransferase
MQEIQKNTKKEQYNLNKLQKRLRRNVGEAIADFNMIEEGDRIMVCLSGGKDSYTMLEILR
NLQQSAPINFSLVAVNLDQKQPGFPEHILPAYLEQLGVEYKIVEENTYGIVKEKIPEGKT
TCSLCSRLRRGILYRTATELGATKIALGHHRDDILQTLFLNMFYGGKMKGMPPKLMSDDG
KHIVIRPLAYCREKDIVRFAEAKAFPIIPCNLCGSQPNLQRQVIADMLRDWDKRYPGRIE
TMFSAMQNVVPSHLCDTNLFDFKGITHGSEVVDGGDLAFDREEIPLQPAGWQPEEDDTAL
EALRLDVIEVK
>EECEBICD_02023 ATP-dependent RNA helicase DbpA
MTAFSTLNVLPAAQLNNLTELGYLEMTPVQAAALPVILAGNDVRVQARTGSGKTAAFGLG
LLHRIDVTLFQTQALVLCPTRELADQVAGELRRLARFLPNTKILTLCGGQPFGAQRDSLQ
HAPHIIVATPGRLLDHLQKETVSLDALHILVMDEADRMLDMGFSDAIDEVIRFAPATRQT
LLFSATWPEAIAAISGRVQQQPIRIEIDTVDALPAIEQQFFETSAHEKISLLQTLLSQHQ
PASCVVFCNTKKDCQAVCDALNAVGQSALALHGDLEQRDRDQTLVRFANGSARILVATDV
AARGLDIKSLELVVNYELAWDPEVHVHRIGRTARAGSSGLAISFCAPEEAQRANILSEML
QLKLNWLNAPARQPSLPLAAEMATLCIDGGKKAKMRPGDILGALTGDIGLDGADIGKINV
HPMHVYVAVRQAVAQKAWKQLQNGKIKGKSCRVRLLK
>EECEBICD_02024 Zinc transport protein ZntB
MADEIAQVMQESLARRTYTMSLMAMVFLPSTFLTGLFGVNLGGIPGGGWRFGFSLFCILL
VVLIGGVTLWLHRSKWL
>EECEBICD_02025 Zinc transport protein ZntB
MEAIKGSDVNVPDAVFAWLLDGRGGVKPLEDNDVIDSQHPCWLHLNYTHPDSARWLASTP
LLPNNVRDALAGESSRPRVSRMGEGTLITLRCINGSTDERPDQLVAMRLYMDERFIVSTR
QRKVLALDDVVSDLQEGTGPVDCGSWLVDVCDALTDHASEFIEELHDKIIDLEDNLLDQQ
IPPRGFLALLRKQLIVMRRYMAPQRDVYARLASERLPWMSDDHRRRMQDIADRLGRGWMK
LTLVSRVLASWRMKLPR
>EECEBICD_02026 Methyl-accepting chemotaxis protein IV
MLRNISVRTCIILFMVCTFLLVDTLQITFLHDLPILITCNIIYLISTLLLWWYMTCYLVV
PINTVKKSIEEVAAGNLSIHISEFGNNCAGRLIPGINSLSENISALVREIRSSSQTAMTL
SEQLAARSMSLSVKTEQQSASLIQTAASMDEMAASTKNNADNTRMASIQADCATQCARKG
GELMVRVTENMRSITDCASQMTEIISLIDGIAFQTNILALNAAVEAARAGDHGKGFSVVA
GEVRNLAHRSAEAAKSIKALIDVTHENVRQGAAIVQEAEKNMQEIVGGSGQLNVLMSEIS
TTTREQEKGINQITLALSDLESATHSNVLMVEALSASSDVLKAQVIELQTKTDKFRLSQP
GYSEHALSRSHVSPLSTITRRGQA
>EECEBICD_02027 putative DNA endonuclease SmrA
MNLDDKALFLDAMEDVQPLKRHTDVHWQPTRNLKTPQRIDTLQLDNFLTTGFLDILPLNE
PLEFRREGLQQGVIDKLRSGKYPQQASLNLLRQPVETCRKMLFRFILEAQKEGLRNVLII
HGKGREAKSHANIVRSYVARWLTEFEDVQAYCSALPHHGGGGACYVALRKTVQAKQDNWE
RHAKRSR
>EECEBICD_02028 Methylated-DNA--protein-cysteine methyltransferase
MLRLLEEKIATPLGPLWVVCDEQFRLRAIEWEQYRDRMEQLLNIHYRHEGYERVSATNPG
GLSDKLTDYFAGNLAVIDTLETATGGTPFQREVWQALRAIPCGQVMHYGQLAAQLGRPGA
ARAVGAANGANPISIVVPCHRVIGRNGTLTGYAGGVQRKEWLLRHEGYLLL
>EECEBICD_02029 Fumarate and nitrate reduction regulatory protein
MIPEKRIIRRIQSGGCAIHCQDCSISQLCIPFTLNEHELDQLDNIIERKKPIQKGQTLFK
AGDELKSLYAIRSGTIKSYTITEQGDEQITGFHLAGDLVGFDAIGSGHHPSFAQALETSM
VCEIPFETLDDLSGKMPNLRQQMMRLMSGEIKGDQDMILLLSKKNAEERLAAFIYNLSRR
FAQRGFSPREFRLTMTRGDIGNYLGLTVETISRLLGRFQKSGMLAVKGKYITIENSDALA
ALAGHTRNVA
>EECEBICD_02030 Universal stress protein E
MAMYQNMLVVIDPNQDDQPALRRAVYLHQRIGGKIKAFLPIYDFSYEMTTLLSPDERTAM
RQGVISQRTAWIREQAKYYLEAGVPIEIKVVWHNRPFEAIIQEVIAGSHDLVLKMAHQHD

RLEAVIFTPTDWHLLRKCPSPVWMVKDQPWPEGGKALVAVNLASEEPYHNALNEKLVKET
LQLAEQVNHTEVHLVGAYPVTPINIAIELPEFDPSVYNDAIRGQHLLAMKALRQKFSIDE
KVTHVEKGLPEEVIPDLAEHLQAGIVVLGTVGRTGLSAAFFGEHGRTGH
>EECEBICD_02031 hypothetical protein
MIMMKLKSAKGKKFLLCLLAVFIVAASVVTRATIGGVIEQYHIPLSEWTSSMYAIQSAMI
FVYSLVFTILLAIPLGIYFLGGDE
>EECEBICD_02032 Low conductance mechanosensitive channel YnaI
MFTEFFLKNAFNLAILFSCGMALLVVRFWLSRNVQWKKGFTFHAAQFFIYAIIIGTIGSI
LNNAIEDYNLRFISSGVIDFICTSLIALILTIKLFLIINQFEKAQVNKGRDVTSTRILAR
VIKITIIVAIVLLYGEHFGMSLSGLLTFGGIGGIAVGMAGKDVLSNFFSGIMLYFDRPFS
IGDWIRSPDRNIEGTVAEIGWRITRINTFDHRPLYVPNSVFSSISVENPGRMTNRRIKTV
IGLRYEDADKIGLIVDAIRNMLQAHRDIDQKQTLLVYFNEFADSSLNIMVYCFTKTTVWQ
EWLAVQQDVYLKIIAIVQENGADFAFPSQTLYIDDPEAAPATK
>EECEBICD_02033 HTH-type transcriptional regulator SutR
MTKKVNLSTTTGADVSTINEAVSQRIKQFRSQKKMSLDELARRSGVSKGMLVEIEGCKAN
PSIALLCKIAAAMGVSVADFVNVASEPMVHLIDRDAIPVLWRGEKGGSAKLMAGTSGPDM
LELWQWIMHPGEQFESAGHPADTCELLFVNQGTLTLTVNGRRFIIREGCSAVARTDMPHA
YVNDTNEVLEFTMTVNEKSR
>EECEBICD_02034 hypothetical protein
MSSMLPSISPELARIAPGFRALSINVIAAPIRDAQVGEIALKEACQAVINGQPTWAQAHI
DAWNAVFKAFGAKPKRTPCSAEALRKRVLKDGTMAALDPVVDLYNAVSLRYAVPVGGENS
AAYCGSPRLVFADGSETFDTLKEGQPVTESPEPGEVIWRDDRGVTCRRWNWRQGVRTRLS
ASDKAMWFILESLPEMPVDELYAAGNMLTDGLEKMMPGLRFESTLMGV
>EECEBICD_02035 hypothetical protein
MSLFLTSFIFEKKSTTKSFINWMAGLSAIHRLLMAILGWKAIPMPPAEEWLIIITGRPAN
QWSY
>EECEBICD_02036 Thiol peroxidase
MTPATTQDYISLGQEHAVTFGKTQLTLKPGILAEGEPLPCTKGLVSHNLLPGYCIPGIKK
RIIVVPSLDTPVCEWQVKDYSNRLKSAGSHSNRAVYVLSMDTPFAQARFILEHDIHPGIT
FVSDYACRQFLDNSGLKINELSIFARALIECDENNVVTRVIVPRDITHLPVY
>EECEBICD_02037 hypothetical protein
MSKHNLIIYSLLLAAAPISVLADSSTTSAATVGMFSPPTAPSVGHRPGTPRDGYELKFNG
LDFSIEKYTPFPGMTIERYARNVGIRDPDNNYQSAETFTTVCTYYQINKDNSQHILKREN
PCEHKFNIQKEHRGSKIKLEIYNETDMASASGYTPAPSVSEFQYIETETISNTPSRDLTV
MSIDKSVLSPGESATITTIVKDIDGNPINEVYINKSVACEILECLWDYGPLKKENAPGKY
TQVITYRGHSNERIDISFKYSAAFTKTISIRGRP
>EECEBICD_02038 Invasin
MFIPVQETTSSLLFGQLGFRDHDNSSFDGRTYVNVGMGYRQEVNGWLLGVNTFLDADIRY
SHLRGGIGGEVYKDSLAFSGNYYFPLTGWKTSAAHELHDERPAYGFDLRTKGTLPDFPWF
SGELTYEQYYGDKVDLLGNGTLSRNPRAAGADLVWNPVPLLEVRAGYRDAGNGGSQAEGG
LRVNYSFGTPLHEQLDYRNVGAPSNTTNRRAFVDRNYDIVMAYREQASKIRITAMPVSGL
SGTLVTLMATVDSRYPIEKVEWSGDAELLAGLQLQGSLGSGLILPQLPLTVTDGQEYSLY
LTVTDSRGTRVTSERIPVRVTQDETSFRSWINVINDDVQVEDGNFVISTPLPAGEEGKVI
EWHYVRERSEEEWASLKPRHIKYQSDMPGLAFKALGGTERDGHWVERVLVTHIGDDTRSL
KLHIEASGPDDKHPVKGTVLLQAQPDSIAQKVTSVEVLFTPGTEEANGSVTAPVVGTEMR
ARTLCVNNTDCTDAFNYQWEISDEMKSWQSVPGATKATWLIPYSLNGESLQNKYIRVRII
SDKENAKSSTAASDAN
>EECEBICD_02039 hypothetical protein
MYFKRSLLALAICASVVSVETVANTRANDMASSATPASVGHRPVATTNAKKIEVSGELTT
GSTLQIADVFIRDEDGDPLSLDKMNNADDIKWYLVDNEAADPSGTPAATGTAFTIPADAG
GKKIKIVYRIKTATGTPDSAFLPATVLLTSASSGVSGDSAADGTISNKLRSVTINVVNES
GKPTDELNGTNDANTPVVGGTLEAVLECAATAAAECEVSNYNFTWQMADAGTPDKFTDLN
NTNAEKHKYIIKGTEQNKLFRVHVTPKTTKATPENKRAIRR
>EECEBICD_02040 Right origin-binding protein

MKKAIIISVLEQIEKNLEERIDIEKLVVITGYSRRSLQDFFKEKCGVSIGKYIRQRKLSR
SATLLKLTSQSVTDIAFRMGFDSVQSYSREFKKTFGVNPNNYRKADFWDLRNLRPPYWLD
CDEHYQFEICQLTSKEIFGFQTSHQIATNDLPKKASPIKWKIIHETLRTWGENVYCLSSF
KPDNTKDQVIAVSSFFGMEHNSVDGGKIPMSRVIKCGKYAKFHFVGHKEQYQQFSNTIYM
CILPKLNLIRREGEDIEYFHLASVQKQQSNESIVDLYYYIPVL
>EECEBICD_02041 hypothetical protein
MYTTIRNTTLAMVACFSYIAHASTHPPLIITRGAGGDASGATVIHDNWRHGTPDLVNLTD
IPIDKIRPEKYRCVLIIGQGAIKEMLLANNASAILSGKTVGLYTHLIDQNTLRLLRQLQN
KVRFNLFFTRSQITLLKLRNISEYNFLSSKVNNVWGQDSLAIETVAPDRGNIPEKTLPLK
TTDYVIWLGGNYTTSSGTQRIFTNDQIVVALKPLHNVISSNASIAIMLSPRFFDNSMSKE
AKVKRLKAVLNTFSRNRVTFYMSKEMLANLKEFDLPVQLSPPYAELMRMPWASATQHFAS
VDQYNLFADLIPKVTPFLLEPNDADQALYATDYLNTRRVSLTQNILNHGCD
>EECEBICD_02042 putative lipoprotein YgdR
MQKCSLITVLSLSVLMLAGCTTTYTMTTRTGEIIETQGKPEVDSATGMTKYADVYGYHRV
IKTSEIVQTTEGASKLDW
>EECEBICD_02043 HTH-type transcriptional repressor BdcR
MTTKHSRTPGRPRQFDPEQAIETAQHLFHSRGYDAVSVADLTKAFGINPPSFYAAFGSKL
GLYTRVLKRYRMTDAIPLGALLRHDRPTAKCLIDVLMEAARRYAADPDATGCLVLEGAHC
NDKPAREAACEFYIAAENLIRTYVAMRYPQEADRTTDFMGTLMAGLSAKARAGYSLERLQ
ESVLLAGDVLERLLPD
>EECEBICD_02044 Cyclic-di-GMP-binding biofilm dispersal mediator protein
MMLFRNKSVLVLGGSRGIGAAIVRRFSADGASVVFSYAGSREAAEKLAAETGSTAIQTDS
ADRDAVISLVREYGPLDILVVNAGVALFGDALEQDSDAIDRLFRINIHAPYHASVEAARN
MPEGGRIIIIGSVNGDRMPVPGMAAYAASKSALQGLARGLARDFGPRGITINVVQPGPID
TDINPEDGPMKELMHSFMAIKRHGRPEEVAGMVAWLAGPEASFVTGAMHTIDGAFGA
>EECEBICD_02045 putative oxidoreductase YtbE
MPMVGFGVFKVTDKEECQQSVLSAIRSGYRLIDTAAVYGNEDAVGDAVRKAIATGLCTRE
ELFITSKLWVQDMANYDLAKAGIEASLKKSGLDYFDLYLLHQAMGDYFSAWRALEDAYEA
GKLKAIGVSNFYAHVLANFCETVRITPMVNQVELHPYFAQPAALETMKHYNVQPEAWAPL
GGGRHKPYENVMLQRIADAHQKTIAQVVLRWNVQRGVTVIPKSTRQERIEENFAIWDFSL
TDNEMAQINALDLGYVGEAVKHFNPEFVRGCLGVKIYD
>EECEBICD_02046 HTH-type transcriptional regulator PgrR
MDKLEAMQVYVCVVDTHSFIRAAEVLGVPRSTVSRVVKELESWLKIQLLQRTTRKLSVTA
EGRRYYEECKRVLADIAAMESSFPGRAAQPKGRFKVGMPQSLARHCIIPTLPAFLCQYPE
LELILCSSDSVEDIILQGYDCVIRAGRIDDSTTLVARPLASFNWVIAASPTYIERYGSPE
NLDDLQKHHAVGYLNHRTGRTTDWFFTREGEDYAMRVQETLVVDDTDAYIQAGIQGLGLI
RVASYLVAPYLHSGALVTCMDNHFYDLPLALVYPQNRFLPPAVRAFYDWCKTALNQPEPL
R
>EECEBICD_02047 hypothetical protein
MKQSNNQVGEKVLVLGAGQLGASVLASLVPAVTQRNGSVCVIVSGRSRDKQSKRRSSIHQ
QLADAGARFIPVDIAGSSVAALKDQFHGFDTIINCMGFVAGAGTQIKITRAVLEAGVKRY
FPGSLA
>EECEBICD_02048 hypothetical protein
MNYDVVGKGSGQPVWDEQYDVRTLLREQNVTEWVIVSTGIFTSFLFEPAFGVVNLAQKTI
NALGGWEMQVTVTSPTDIGRLTTEIYLHQPRITNEVVFVAGETTSYAKLAETVERVTQQT
FTRGVLTLPDLQEQLRLHPHDPMLRYRVAFARGDGMWWPMSDTWNAQHHLPTQDIAAWLK
TQQ
>EECEBICD_02049 Periplasmic murein peptide-binding protein
MRHSVSVTCCALLVSSFSLAYAADVPGGTVLAEKQELVRHIKDEPASLDPAKAVGLPEIQ
VIRDLFEGLVNQNEKGEIIPGVASQWKSNDNRIWTFTLRDNAQWADGTPVTAQDFVYSWQ
RLVDPKTLSPFAWFAALAGITNAQAIIDGKVTPDQLGVSAVDAHTLRVQLDKPLPWFASL
TASFAFYPVQKANVESGKDWMKPGKLIGNGAYVLKERVVNEKLVVVPNTHYWDNAKTVLQ
KVTFLPINQESAATKRYLAGDIDITESFPKNMYQKLLKDIPGQVYTPPQLGTYYYAFNTQ
KGPTADSRVRLALSMTIDRRLMAEKVLGTGEKPAWHFTPDVTAGFKPDPSPFEQMSQEEL

NAQAKTLLRAAGYGSQKPLKLTLLYNTSENHQKIAIAVASMWKKNLGVDVKLQNQEWKTY
IDSRNTGNFDVIRASWVGDYNEPSTFLSLLTSTHTGNISRFTNPTYDKILTQATMENTAE
ARNADYNAAEKILTEQAPIAPIYQYTNGRLIKPWVKGYPITNPEDVAYSRTMYIVKH
>EECEBICD_02050 Murein peptide amidase A
MTVTRPRAERGAFPPGTEHYGRSLLGAPLIWFPAPAANRESGLILAGTHGDETSSVVTLS
CALRTLNPTLRRHHVVLAVNPDGCQLGLRANANGVDLNRNFPAANWKAGETVYRWNSRAQ
ERDVVLLTGDHPGSEPETQALCQLIHRLQPAWVVSFHDPLACIEDPRRSELGEWLADAFA
LPLVTSVGYETPGSFGSWCADLNLHCITAEFPPVSADEASEKYLLAMSNLLRWHPKDGVD
PS
>EECEBICD_02051 L-Ala-D/L-Glu epimerase
MRSVKVYEETWPLHTPFVIARGSRSEAHVVVVELEEEDVKGIGECTPYPRYGESDASVMA
QIMSIVPQLENGLTREALQKLLPAGAARNAVDSALWDLQARQRQSLAGLLGVTLSPVFT
TAQTVVIGTPEQMAASAAALWEKGATLLKIKLDARLISERMVAIRSAVPEATLIVDANES
WRPEGLAARCQLLADLNVAMLEQPLPAQDDAALENFIHPLPICADESCHTRSSLKALTGR
YDMINIKLDKTGGLTEALALATEAREQGFGLMLGCMLCTSRAISAALPLMPQVSFADLDG
PTWLAVDVEPALRFTTGQLHL
>EECEBICD_02052 Thiol peroxidase
MSQTVHFQGNPVTVANVIPQAGSKAQAFTLVAKDLSDVSLSQYAGKRKVLNIFPSIDTGV
CAASVRKFNQLATEVENTVVLCVSADLPFAQSRFCGAEGLSNVITLSTLRNNEFLKNYGV
EIVDGPLKGLAARAVIVLDENDNVIFSQLVDEITHEPDYDAALNVLKA
>EECEBICD_02053 Transcriptional regulatory protein TyrR
MRLEVFCEDRLGLTRELLDLLVLRSIDLRGIEIDPIGRIYLNFAELEFTDFSSLMAEIRR
ISGVTDVRTVPWMPSEREHLALSALLEALPEPVLSLDMKSKVEMANPASCQLFAQSQERM
RHHTAAQLINGFNFQRWLDGNPQSSHNEHVVINGQNFLMEITPVHLQNENDEYVLTGAVV
MLRSTIRMGQQLQNLSTQDLSAFSQIIAVSAKMKHVVEQARKLAMLSAPLLITGDTGTGK
DLFAYACHQASPRSAKPYLALNCASIPEDAVESELFGHAPEGKKGFFEQANGGSVLLDEI
GEMSPRMQAKLLRFLNDGTFRRVGEDHEIHVDVRVICATQKNLVELVQKGLFREDLYYRL
NVLTLNLPPLRDCPQDIMPLTELFVARFADEQGVPRPKLSADLSTVLTRYGWPGNVRQLK
NAIYRALTQLEGYELRPQDILLPDYDAATVAVGEDAMEGSLDDITSRFERSVLTQLYRSY
PSTRKLAKRLGVSHTAIANKLREYGLSQKKGEE
>EECEBICD_02054 hypothetical protein
MSEPLKPRIDFAEPLKEEPTSAFKAQQTFSEAESRTFAPAAIDERPEDEGVAEAAVDAAL
RPKRSLWRKMVMGGLALFGASVVGQGVQWTMNAWQTQDWVALGGCAAGALIIGAGVGSVV
TEWRRLWRLRQRAHERDEARELLHSHSVGKGRAFCEKLAQQAGIDQSHPALQRWYAAIHE
TQNDREIVGLYAHLVQPVLDAQARREISRFAAESTLMIAVSSLALVDMAFIAWRNLRLIN
RIATLYGIELGYYSRLRLFRLVLLNIAFAGASELVREVGMDWMSQDLAARLSTRAAQGIG
AGLLTARLGIKAMELCRPLPWIDNDKPRLGDFRRQLIGQLKETLQKSKSSPEK
>EECEBICD_02055 putative protein YcjX
MKRLKTELNALVNRGVDRHLRLAVTGLSRSGKTAFITAMVNQLLNVHAGARLPLLSAVRE
ERLLGVKRVPQRDFGIPRFTYDEGILQLYGNPPAWPTPTRGVSEIRLALRYRSNDSLLRH
FKDTSTLYLEIVDYPGEWLLDLPMLAQDYLSWSRQMNGLLQGQRAEWAAKWRQLCDGLDP
LAPADENRLAEIAAAWTDYLHQCKSQGLHFIQPGRFVLPGDMAGAPALQFFPWPDVDAFG
ESKLAQADKQTNAGMLRERFNYYCEKVVKGFYKNHFLRFDRQIVLVDCLQPLNSGPQAFN
DMRLALTQLMQSFHYGQRTLFRRLFSPVIDKLLFAATKADHVTLDQHANMVALLQQLIQD
AWQNAAFEGISMDCLGLASVQATTSGVIEVNGEKIPALRGNRLSDGAPLTVYPGEVPSRL
PGQAFWDSQGFQFEAFRPQVMDVDKPLPHIRLDAALEFLIGDKLR
>EECEBICD_02056 Thiosulfate sulfurtransferase PspE
MLKKGIFALALFVAMPLYAAEYWIDVRIPEQYQREHIQGAINIPLKEIKSHIETVVPDRN
DTVKLYCNSGRQSGMAKQMLLDMGYTHAMNMGGISRLDMPKVKK
>EECEBICD_02057 Phage shock protein D
MNTRWQRVGQKVKPGFKIAGKLVLLTALRYGPAGVAGWAVKSVARRPLKMLLAFALEPVL
RKAANKISQRYK
>EECEBICD_02058 Phage shock protein C
MGGINLNKKLWRMPQKGMVRGVCAGIADYLDVPVKLVRILVVLSIFFGLAFFTVVAYIVL

SFVLDPMPDNLAAGKPQPTSGELLDTVDRELAAGEKRLREMERYVTSDTFTLRSRFRQL
>EECEBICD_02059 Phage shock protein B
MSALFLAIPLTIFVLFVLPIWLWLHYSNRAGRGELSQSEQQRLLQLTDDAQRMRERIQAL
EDILDAEHPNWRER
>EECEBICD_02060 Phage shock protein A
MGIFSRFADIVNANINALLEKAEDPQKLVRLMIQEMEDTLVEVRSNSARALAEKKQLSRR
IEQATVQQTEWQEKAELALRKDKDDLARAALIEKQKLTDLIATLEQEVTLVDDTLARMKK
EIGELENKLSETRARQQALMLRHQAASSSRDVRRQLDSGKLDEAMARFESFERRIDQMEA
ESHRFGKQQSLDQQFANLKADDEISEQLAQLKAKMKQDNQ
>EECEBICD_02061 Psp operon transcriptional activator
MAEFKDNLLGEANRFLEVLEQVSRLAPLDKPVLIIGERGTGKELIANRLHYLSSRWQGPL
ISLNCAALNENLLDSELFGHEAGAFTGAQKRHPGRFERADGGTLFLDELATAPMLVQEKL
LRVIEYGELERVGGSQPLQVNVRLVCATNADLPAMVKEGTFRADLLDRLAFDVVQLPPLR
ERQSDIMLIAEHFAIQMCRELRLPLFPGFTDRAKETLLHYAWPGNVRELKNVVERSVYRH
GSSEHPLDEIVIDPFQRHSAEPPTPALPAASATPDLPLKLREFQLQQEKALLQRSLQQAK
FNQKRAADLLALTYHQFRALLKKHQL
>EECEBICD_02062 Peptide transport periplasmic protein SapA
MRLVLSSLIVIAGLLSSQATAAAAPEQTASADIRDSGFVYCVSGQVNTFNPQKASSGLIV
DTLAAQLYDRLLDVDPYTYRLVPELAESWEVLDNGATYRFHLRRDVSFQKTAWFTPTRKL
NADDVVFTFQRIFDRRHPWHNINGSSFPYFDSLQFADNVKSVRKLDNNTVEFRLTQPDAS
FLWHLATHYASVMSAEYAAQLSRKDRQELLDRQPVGTGPFQLSEYRAGQFIRLQRHDGFW
RGKPLMPQVVVDLGSGGTGRLSKLLTGECDVLAWPAASQLTILRDDPRLRLTLRPGMNIA
YLAFNTDKPPLNNPAVRHALALSINNQRLMQSIYYGTAETAASILPRASWAYDNDAKITE
YNPEKSREQLKALGIENLTLHLWVPTSSQAWNPSPLKTAELIQADMAQVGVKVVIVPVEG
RFQEARLMDMNHDLTLSGWATDSNDPDSFFRPLLSCAAINSQTNFAHWCNPEFDSVLRKA
LSSQQLASRIEAYEEAQNILEKELPILPLASSLRLQAYRYDIKGLVLSPFGNASFAGVSR
EKHEEVKKP
>EECEBICD_02063 Peptide transport system permease protein SapB
MIIFTLRRLLLLLVTLFFLTFIGFSLSYFTPHAPLQGASLWNAWVFWFNGLLHWDFGVSS
INGQLISEQLKEVFPATMELCILAFGFALMVGIPVGMLAGVTRSKWPDRFISALALLGFS
IPVFWLALLLTLFFSLTLGWLPVSGRFDLLYEVKPVTGFAIIDAWISDSPWRDEMVMSAI
RHMVLPVLTLSVAPTTEVIRLMRISTIEVYDQNYVKAAATRGLSRFTILRRHVLHNALPP
VIPRLGLQFSTMLTLAMITEMVFSWPGLGRWLIHAIRQQDYAAISAGVMVIGSLVIVVNV
ISDILGAMANHLKHKEWYALR
>EECEBICD_02064 Peptide transport system permease protein SapC
MPYDSVYSEKRPPGTLRTAWRKFYSDAPAMVGLYGCAGLALLCIFGGWIAPYGIDQQFLG
YQLLPPSWSRYGEVSFFLGTDDLGRDVLSRLLSGAAPTVGGAFIVTLAATLCGLVLGVVA
GATHGLRSAVLNHILDTLLSIPSLLLAIIVVAFAGPHLSHAMFAVWLALLPRMVRSVYSM
VHDELEKEYVIAARLDGATTLNILWFAILPNITAGLVTEITRALSMAILDIAALGFLDLG
AQLPSPEWGAMLGDALELIYVAPWTVMLPGAAITLSVLLVNLLGDGIRRAIIAGVE
>EECEBICD_02065 Peptide transport system ATP-binding protein SapD
MPLLDIRNLTIEFKTSEGWVKAVDRVSMTLSEGEIRGLVGESGSGKSLIAKAICGVAKDN
WRVTADRMRFDDIDLLRLSSRERRKLVGHNVSMIFQEPQSCLDPSERVGRQLMQNIPAWT
YKGRWWQRLGWRKRRAIELLHRVGIKDHKDAMRSFPYELTDGECQKVMIAIALANQPRLL
IADEPTNSMEPTTQAQIFRLLTRLNQNSNTTILLISHDLQMLSQWADKINVLYCGQTVET
APSKDLVTTPHHPYTQALIRAIPDFGSAMPHKSRLNTLPGAIPLLEQLPIGCRLGPRCPY
AQRECIITPRLTGAKNHLYACHFPLNMERE
>EECEBICD_02066 Peptide transport system ATP-binding protein SapF
MVETLLEVRNLSKTFRYRTGWFRRQTVDAVKPLSFTLRERQTLAIIGENGSGKSTLAKML
AGMIEPTSGELLIDDHPLHYGDYSFRSQRIRMIFQDPSTSLNPRQRISQILDFPLRLNTD
LEPEQRRKQIVETMRMVGLLPDHVSYYPHMLAPGQKQRLGLARALILRPKVIIADEALAS
LDMSMRSQLINLMLELQEKQGISYIYVTQHIGMMKHISDQVLVMHQGEVVERGSTADVLA
SPLHELTRRLIAGHFGEALTADAWRKDR
>EECEBICD_02067 Putative anti-FlhC(2)FlhD(4) factor YdiV

MRYFFMAEPIRAMEGDLLGVEITTHFASSPARPLHPEFVISSWDNSQKRRFLLDLLRTIA
AKHGWFLRHGLFCIVNIDRGMAQLVLQDKDIRALLHAMLFVELQVAEHFSCQDNALIDPL
IHALHKQPNPLWLGDLGVGNATAVPLVCGCFSGVKLDRSFFVSQIEKMTFPLLVKHIRRY
CDKIVVGGQENTRYLPVLKTAGIWATQGTLFPSVALEEVETLLLGSRMNTLRESN
>EECEBICD_02068 Secreted effector kinase SteC
MTETECSERCVDFLKETMFNYNNGEITLAALGNDNLPPSEAGSNGIYEAFEQRLIGFLTT
PATASGDESGAIDQTDASQPAAIEAFINSPEFQKNIRMRDIEKNKIGSGSYGTVYRLHDD
FVVKIPINERGIKVDVNSQEHRNCHPERVSKYLNMANGDKNFSRSAIMNINGKDVTVLVS
KYIQGQEFDVEDEDNYRMAEALLKSRGVYMHDINILGNILVKEGVLFFVDGDQIVLSQES
RQQRSVSLATRQLEEQIKAHHMIKLKRAETEGNTEDVEYYKSLITDLDALIGEEEQTPAP
GRRFKLAAPEEGTLVAKVLKDELKK
>EECEBICD_02069 hypothetical protein
MPFTFHIGNHSCQISERYLRDIIDNKREHVFSTCEKFIDFFRNIFTRRSLISDYREIYNL
LCQKKSIPILKGLSLQDHFRSEMKTVHDGAPFLDILNLLMPPDLKQ
>EECEBICD_02070 Enoyl-[acyl-carrier-protein] reductase [NADH] FabI
MGFLSGKRILVTGVASKLSIAYGIAQAMHREGAELAFTYQNDKLKGRVEEFAAQLGSSIV
LPCDVAEDASIDAMFAELGNVWPKFDGFVHSIGFAPGDQLDGDYVNAVTREGFKIAHDIS
SYSFVAMAKACRTMLNPGSALLTLSYLGAERAIPNYNVMGLAKASLEANVRYMANAMGPE
GVRVNAISAGPIRTLAASGIKDFRKMLAHCEAVTPIRRTVTIEDVGNSAAFLCSDLSAGI
SGEVVHVDGGFSIAAMNELELK
>EECEBICD_02071 hypothetical protein
MEQCRKAGKSHWYHETQSTMSSQPPLSLMPEAAYVNDRFLLDLTVSETALTPFESWLKPA
HQLADVLFPRTVLNDRLHTFSTYERMSTALTAAQVFGVQRLCRYYAARLAPLPGPDASRE
SNQRLAQITQYARQLAGSPSVINTRAREQLAEVGLTARDTVLINQIIGFIGFQARVAAIF
QAFCRLPVRELPGQEMQRFARAARFQNPQTIWRPAASLVEYPPAHTKVRRQYSSSQCQMM
APVLMRDPSSFALLERILTSTLHTASPPSLLPLITLLTSRINGSAACFNEQATQPGAWRR
AVITLRQEDDDIARWELEPALTQAIQWLTRAPDRFSAAHFFPLLTRGVSSEQAINMLGWC
GVCGWLNRLKIALGETY
>EECEBICD_02072 Exoribonuclease 2
MFQDNPLLAQLKQQLHSQTPRAEGVVKATEKGFGFLEVDAQKSYFIPPPQMKKVMHGDRI
VAVIHTEKERESAEPEELIEPFLTRFVGKVQGKNDRLSIVPDHPLLKDAIPCRAARGVQH
EFKEGDWAVAEMRRHPLKGDRSFYADLTQYITFADDHFVPWWVTLARHNLEKEAPNGVAT
EMLDEGLERQDLTALNFVTIDSASTEDMDDALYAEELADGRLQLTVAIADPTAWIAEGSK
LDNAAKIRAFTNYLPGFNIPMLPRELSDDLCSLRANEVRPALACRMIISADGTIDDDIAF
FAATIESKAKLAYDNVSDWLENNGTWQPDNEGIAHQIRLLHRICLSRSEWRHHHALVFKD
RPDYRFVLGEKGEVLDIVAEPRRIANRIVEESMIAANLCAARVLRDKLGFGIYNVHTGFD
PANADALAALLKTHGLHVDAEEVLTLEGFCKLRRELDAQPSGFLDSRIRRFQSFAEISTE
PGPHFGLGLEAYATWTSPIRKYGDMINHRLLKAVIKGEAIARPQEDITQQMAERRRLNRM
AERDVGDWLYARFLNDKAGTNTRFAAEIIDVSRGGMRVRLVDNGAIAFIPAPFLHAVRDE
LVCSQENGTVQIKGETVYKVTDVIDVTIAEVRMETRSIIARPAA
>EECEBICD_02073 Cyclic di-GMP phosphodiesterase PdeR
MMKQIQEQTALNPLHSHWRLADDRNVLYLSPTGETDTEKTVELSPEQAGRIREITAITSS
LLITLLIEKQVTAVHLVGRKINNREWAGSLATWPDTPAVAPTQAQELSFAEQIVSEANSV
IAILDSRGKICRFNRLCEEYTGLKERDVIGQSVFKLFMSRREAVASRHYIENFFRNGNAY
EIERWIKTRKGQRLFLFRNKFVHNGSGKNEIFLICAGTDITEERRAQERLRILANTDTVT
GLPNRNAIHEFINHAIASAGESQVGIVYLDLDNFKKINDAYGHMFGDQLLQAVSLALLSC
LEEDQLLARLGGDEFIVLAAHTSQAALEAVASRILTRLRQPFRIGLIEVYTGCAIGISLA
PRHGQDSDSLIRTADTAMYNAKEGGRGQFCVFSPEMNQRVFDYHWLDTNLRKALENDQLL
IHYQPKITWRGEVRSLEALVRWQSPERGLIPPLEFISYAEESGLIVPLGRWVILDVVRQI
ARWRDKGINLRVAVNFSARQLADQTLFTALKQALYDLNFEYCPIDVELTESCLIENDTLA
LSVIQQFSQLGAQIHLDDFGTGYSSLSQLARFPIDAVKLDQAFVRDIHKQPLSQSLVRAI
VAVAQALNLQVIAEGVENAKEDAFLTKNGVNERQGFLFAKPMPAVSFERWYKRYQTKKMR
>EECEBICD_02074 hypothetical protein
MSDIEAQRIAARIDTVLDILVAGDYHSAINNLEILRAELLDQVKDGISPSQAPGSPWEI

>EECEBICD_02075 HTH-type transcriptional repressor CsqR
MVVDKGQMSVAELAKITGVSEVTIRQDLNTLEKQSYLRRAHGFAVSLESDDVETRMMTNY
TLKRRLAEFAASLVSPGESVFIENGSSNALLARTLAEQKDVTIITVSSYIAHLLKETPCE
VILLGGIYQKKVKAW
>EECEBICD_02076 hypothetical protein
MVGPLTRQFIHQVHFSKAFIGIDGWQADTGFTGRDMMRSDVVNAVLEKGSEAIVLTDSSK
FGCVHPYPLGPLSRFHRVITDSKISASDQMQLEHAGLLVNVIGSSV
>EECEBICD_02077 Osmotically-inducible lipoprotein B
MFMTSKKMAAAVLAITVAMSLSACSNWSKRDRNTAIGAGAGALGGAVLTDGSTLGTLGGA
AVGGVIGHQVGK
>EECEBICD_02078 putative protein YciH
MSDSNSRLVYSTQTGRIEEPKTAPVRPKGDGIVRIQRQTSGRKGKGVCLITGIEMNDAEL
TKLAAELKKKCGCGGAVKEGIIEIQGDKRDLIKSLLEAKGMKVKLAGG
>EECEBICD_02079 Orotidine 5'-phosphate decarboxylase
MTFTASSSSCAITESPVVVALDYHERDKALAFVDKIDPRDCRLKVGKEMFTLFGPQLVRD
LQQRGFDVFLDLKFHDIPNTTARAVAAAADLGVWMVNVHASGGARMMAAARDALAPFGKD
APLLIAVTVLTSMETSDLHDLGVTLSPAEHAERLARLTQQCGLDGVVCSAQEAVRFKQAF
GAAFKLVTPGIRPAGSEAGDQRRIMTPEQALSAGVDYMVIGRPVTQSVDPAQTLKDINAS
LKREA
>EECEBICD_02080 Lipopolysaccharide assembly protein B
MLELLFLLLPVAAAYGWYMGRRSAQQTKQDEANRLSRDYVAGVNFLLSNQQDKAVDLFLD
MLKEDTGTVEAHLTLGNLFRSRGEVDRAIRIHQTLMESASLTYEQRLLAVQQLGRDYMAA
GLYDRAEDMFNQLTDETEFRVGALQQLLQIYQLTSDWQKAIEVAERLVKLGKDKQRIEIA
HFYCELALQQMGNDDMDRAMALLKKGAAADKNSARVSIMMGRVYMARGDYAKAVESLQRV
IVQDKELVSETLEMLQTCYQQLGKNAEWAEFLRRAVEENTGAGAELMLADILEAREGSDA
AQVYITRQLQRHPTMRVFHKLMDYHLNEAEEGRAKESLMVLRDMVGEQVRSKPRYRCQKC
GFTAYTLYWHCPSCRAWSTIKPIRGLDGQ
>EECEBICD_02081 Lipopolysaccharide assembly protein A
MKYLLIFLLVLAIFVISVTLGAQNDQQVTFNYLLAQGEYRISTLLAVLFAVGFAIGWLIC
GLFWLRVRVSLARAERKIKRLENKLSPATDVAVPADSSVVKE
>EECEBICD_02082 Phosphatidylglycerophosphatase B
MLSIARRTAAGAALLLIMPLAVWVSGWQWQPGHQVWWLKTLFWITETVTKPWGVITHVIL
CGWFLWCLRFRLRAAIMLFAILGGAIIVGQGVKSWVKERVQEPRPFVVWLEKTHHIPVDE
FYTLKRTERGHLVKEQLAGQQNIPVFLRQHWQKETGFAFPSGHTMFAASWALLAVGLLWP
RRRTFTIAFLLVWATGVMGSRLLLGMHWPRDLVVATLISWLLVTLATWLAQRICGPLMPP
REEAQEIAEREQES
>EECEBICD_02083 GTP cyclohydrolase-2
MQLKRVAEAKLPTPLGDFLMVGFEELATGHDHAALVFGDISGKTPVLARVHSECLTGDAL
FSLRCDCGFQLEAALTHIAEEGRGILIYHRQEGRNIGLLNKIRAYALQDQGYDTVEANHQ
LGFAADERDFTLCADMFKLLGVDEVRLLTNNPKKVEILTEAGINIVERVPLIVGRNPNNE
HYLDTKAAKMGHLLSK
>EECEBICD_02084 Aconitate hydratase A
MSSTLREASKDTLQAKDKTYHYYSLPLAAKSLGDIARLPKSLKVLLENLLRWQDGESVTD
EDIQALAGWLKNAHADREIAWRPARVLMQDFTGVPAVVDLAAMREAVKRLGGDTSKVNPL
SPVDLVIDHSVTVDHFGDDDAFEENVRLEMERNHERYMFLKWGKQAFSRFSVVPPGTGIC
HQVNLEYLGKAVWSELQDGEWIAYPDSLVGTDSHTTMINGLGVLGWGVGGIEAEAAMLGQ
PVSMLIPDVVGFKLTGKLREGITATDLVLTVTQMLRKHGVVGKFVEFYGDGLDSLPLADR
ATIANMSPEYGATCGFFPIDAITLEYMRLSGRSDDLVELVEAYAKAQGMWRNPGDEPVFT
STLELDMGDVEASLAGPKRPQDRVALGDVPKAFAASAELELNTAQRDRQPVDYTMNGQPY
QLPDGAVVIAAITSCTNTSNPSVLMAAGLLAKKAVTLGLKRQPWVKASLAPGSKVVSDYL
AQAKLTPYLDELGFNLVGYGCTTCIGNSGPLPEPIETAIKKGDLTVGAVLSGNRNFEGRI
HPLVKTNWLASPPLVVAYALAGNMNINLATDPLGYDRKGDPVYLKDIWPSAQEIARAVEL
VSSDMFRKEYAEVFEGTEEWKSIQVESSDTYGWQSDSTYIRLSPFFDEMQAQPAPVKDIH
GARILAMLGDSVTTDHISPAGSIKPDSPAGRYLQNHGVERKDFNSYGSRRGNHEVMMRGT

FANIRIRNEMLPGVEGGMTRHLPGTEAMSIYDAAMLYQQEKTPLAVIAGKEYGSGSSRDW
AAKGPRLLGIRVVIAESFERIHRSNLIGMGILPLEFPQGVTRKTLGLTGEEVIDIADLQN
LRPGATIPVTLTRSDGSKETVPCRCRIDTATELTYYQNDGILHYVIRNMLN
>EECEBICD_02085 Protein YmiA
MPSENQEPRRDPELKRKAWLAVFVSSALFWVVVALVIWHWWG
>EECEBICD_02086 HTH-type transcriptional regulator CysB
MKLQQLRYIVEVVNHNLNVSSTAEGLYTSQPGISKQVRMLEDELGIQIFARSGKHLTQVT
PAGQEIIRIAREVLSKVDAIKSVAGEHTWPDKGSLYIATTHTQARYALPGVIKGFIERYP
RVSLHMHQGSPTQIAEAVSKGNADFAIATEALHLYDDLVMLPCYHWNRSIVVTPDHPLAA
TSSVTIEALAQYPLVTYTFGFTGRSELDTAFNRAGLTPRIVFTATDADVIKTYVRLGLGV
GVIASMAVDPLADPDLVRIDAHDIFSHSTTKIGFRRSTFLRSYMYDFIQRFAPHLTRDVV
DTAVALRSNEEIEAMFQDIKLPEK
>EECEBICD_02087 DNA topoisomerase 1
MGKALVIVESPAKAKTINKYLGNDYVVKSSVGHIRDLPTSGSAAKKSADSTSTKTAKKPK
KDERGALVNRMGVDPWHNWDAHYEVLPGKEKVVSELKQLAEKADHIYLATDLDREGEAIA
WHLREVIGGDDARYSRVVFNEITKNAIRQAFEQPGELNINRVNAQQARRFMDRVVGYMVS
PLLWKKIARGLSAGRVQSVAVRLVVEREREIKAFVPEEFWEIDANTTTPSGEALPLQVTH
QNDKPFRPVNREQTLAAVSLLEKASYSVLEREDKPTSSKPGAPFITSTLQQAASTRLGFG
VKKTMMMAQRLYEAGYITYMRTDSTNLSQDAVNMVRGYIGDNFGKKYLPDNPNQYASKEN
SQEAHEAIRPSDVAVMAESLKDMEADAQKLYQLIWRQFVACQMTPAQYDSTTLTVGAGEF
RLKARGRILRFDGWTKVMPALRKGDEDRTLPAVNKGDALTLLELTPAQHFTKPPARFSEA
SLVKELEKRGIGRPSTYASIISTIQDRGYVRVENRRFYAEKMGEIVTDRLEENFRELMNY
DFTAQMEDSLDQVANHQAEWKAVLDNFFSDFTQQLDKAEKDPEEGGMRPNQMVLTSIDCP
TCGRKMGIRTASTGVFLGCSGYALSPKERCKTTINLVPENEVLNVLEGDDAETNALRAKR
RCQKCGTAMDSYLIDPKRKLHVCGNNPTCDGYEIEEGEFRIKGYDGPIVECEKCGSEMHL
KMGRFGKYMACTNDECKNTRKILRNGEVAPPKEDPVPLPELPCEKSDAYFVLRDGAAGIF
LAANTFPKSRETRAPLVEELYRFRDRLPEKLRYLADAPQQDPEGNKTVVRFSRKTKQQYV
AAEKDGKATGWSAFFVDGKWVEGKK
>EECEBICD_02088 Protein YciN
MHKETQPIDRETLLLEANKIIREHEDTMAGIVATGVTQRNGVLVFSGDYFLDEQGLPTPK
STAVFNMFKHLAHVLSEKYHLVD
>EECEBICD_02089 putative protease SohB
MIDFKGSMDAHEVNALREEVTAVLAVAKPGDRAVVRLESPGGVVHGYGLAASQLQRLRDK
NIPLTVTVDKVAASGGYMMACVAEKIIAAPFAIVGSIGVVAQIPNFNRFLKSKDIDIELH
TAGQYKRTLTLLGENTEEGRQKFREDLNETHHLFKEFVQRMRPALDIEQVATGEHWYGQQ
ALEKGLVDEINTSDEVILGLMEGREVLNVRYMQRKKLIDRVTGSAAESADRLLLRWWQRG
QKPLM
>EECEBICD_02090 hypothetical protein
MLSEYGLFLAKILTVVVAIAVIVLLIVNATQRKRQRGELRVTNLSEQYQEMKDDLAAALM
DGHQQKLWHKAQKKSISRRRKPPKRKRSWGTLRHRTNRAYG
>EECEBICD_02091 putative oxidoreductase YciK
MHYQPKQDLLQNRIILVTGASDGIGREAALTYARYGATVILLGRNEEKLRRVAQHIADEQ
HVQPQWFTLDLLTCTAEECRQVADRIAAHYPRLDGVLHNAGLLGEIGPMSEQDPQIWQDV
MQVNVNATFMLTQALLPLLLKSDAGSLVFTSSSVGRQGRANWGAYATSKFATEGMMQVLA
DEYQNRSLRVNCINPGGTRTSMRASAFPTEDPQKLKTPADIMPLYLWLMGDDSRRKTGMT
FDAQPGRKPGIAQ
>EECEBICD_02092 Corrinoid adenosyltransferase
MSDERYQQRQQKVKDRVDARVAQAQEERGIIIVFTGNGKGKTTAAFGTAARAVGHGKNVG
VVQFIKGTWPNGERNLLEPHGVEFQVMATGFTWETQNREADTAACMAVWQHGKRMLADPL
LDMVVLDELTYMVAYDYLPLEEVISALNARPGHQTVIITGRGCHRDILDLADTVSELRPV
KHAFDAGVKAQMGIDY
>EECEBICD_02093 Ribosomal large subunit pseudouridine synthase B
MSEKLQKVLARAGHGSRREIESIIEAGRVSVDGKIATLGDRVEVTPGLKIRIDGHLISVK
ESAEQICRVLAYYKPEGELCTRNDPEGRPTVFDRLPKLRGARWIAVGRLDVNTCGLLLFT

TDGELANRLMHPSREVEREYAVRVFGQVDESKLRDLSRGVQLEDGPAAFKTIKFSGGEGI
NQWYNVTLTEGRNREVRRLWEAVGVQVSRLIRVRYGDIPLPKGLPRGGWTELDLAQTNYL
RGLVELPPETSSKVAVEKDRRRMKANQIRRAVKRHSQIAGGRRSGGRNNNG
>EECEBICD_02094 putative protein YciO
MSQFFYIHPDNPQQRLINQAVDIVRKGGVIVYPTDSGYALGCKIEDKGAMERICRIRHLP
DGHNFTLMCRDLSELSTYSYVDNVAFRLMKNNTPGNYTFILKGTKEVPRRLLQEKRKTIG
LRVPSNPIALALLDTLGEPMLSTSLMLPGSDFTESDPEEIKDRLEKQVDLIIHGGYLGQQ
PTTVIDLTNDSPVVLREGVGDVTPFL
>EECEBICD_02095 5'-3' exoribonuclease
MSDTNYAVIYDLHSHTTASDGLLTPETLVHRAVEMRVGTLAITDHDTTAAIPAAREEISR
SGLALNLIPGVEISTVWENHEIHIVGLNIDIAHPAMRDFLAQQTQRRQARGRLIAERLEK
AHIPGAWEGALRLANGGAVTRGHFARFLVECGKAATMADVFKKYLARGKTGYVPPQWCTI
EQAIDVIHHSGGKAVLAHPGRYDLSAKWLKRLVAHFADHHGDAMEVAQCQQSPNERTQLA
TLACQHHLWASLGSDFHQPCPWIELGRKLWLPAGVEGVWQAWEQPQISQ
>EECEBICD_02096 Anthranilate synthase component 1
MQTPKPTLELLTCDAAYRENPTALFHQVCGDRPATLLLESADIDSKDDLKSLLLVDSALR
ITALGDTVTIQALSDNGASLLPLLDTALPAGVENEVLPAGRVLRFPPVSPLLDEDARLCS
LSVFDAFRLLQGVVNIPTQEREAMFFGGLFAYDLVAGFEALPHLEAGNNCPDYCFYLAET
LMVIDHQKKAPVFRPVCSPQATGKNSA
>EECEBICD_02097 Anthranilate synthase component 1
MRCECNQSDDAFGAVVRQLQKAIRAGEIFQVVPSRRFSLPCPSPLAAYYVLKKSNPSPYM
FFMQDNDFTLFGASPESSLKYDAASRQIEIYPIAGTRPRGRRADGTLDRDLDSRIELDMR
TDHKELSEHLMLVDLARNDLARICTPGSRYVADLTKVDRYSYVMHLVSRVVGELRHDLDA
LHAYRACMNMGTLSGAPKVRAMQLIADAEGQRRGSYGGAVGYFTAHGDLDTCIVIRSALV
ENGIATVQAGAGIVLDSVPQSEADETRNKARAVLRAIATAHHAQETF
>EECEBICD_02098 Bifunctional protein TrpGD
MADILLLDNIDSFTWNLADQLRTNGHNVVIYRNHIPAQTLIDRLATMKNPVLMLSPGPGV
PSEAGCMPELLTRLRGKLPIIGICLGHQAIVEAYGGYVGQAGEILHGKASSIEHDGQAMF
AGLANPLPVARYHSLVGSNVPAGLTINAHFNGMVMAVRHDADRVCGFQFHPESILTTQGA
RLLEQTLAWAQQKLEPTNTLQPILEKLYQAQTLTQQESHQLFSAVVRGELKPEQLAAALV
SMKIRGEHPNEIAGAATALLENAAPFPRPDYLFADIVGTGGDGSNSINISTASAFVAAAC
GLKVAKHGNRSVSSKSGSSDLLAAFGINLDMNADKSRQALDELGVCFLFAPKYHAGFRHA
MPVRQQLKTRTLFNVLGPLINPAHPPLALIGVYSPELVLPIAETLRVLGYQRAAVVHSGG
MDEVSLHAPTIVAELHDGEIKSYQLTAEDFGLTPYHQDQLAGGTPEENRDILTRLLQGKG
DAAHEAAVAANVAMLMRLHGQEDLKANAQTVLDVLRNGTAYDRVTALAARG
>EECEBICD_02099 Tryptophan biosynthesis protein TrpCF
MQTVLAKIVADKAIWVEARKQQQPLASFQNEIQPSTRHFYDALQGARTAFILECKKASPS
KGVIRDDFDPARIANIYQHYASAISVLTDEKYFQGSFDFLPVVSQSAPQPILCKDFIIDP
YQIYLARYYQADACLLMLSVLDDEQYRQLSAVAHSLKMGVLTEVSNDEERERAIALGAKV
VGINNRDLRDLSIDLNRTRQLAPKLGHGVTVISESGINTYGQVRELSHFANGFLIGSALM
AHDDLNAAVRRVLLGENKVCGLTRAQDAKAACDAGAIYGGLIFVPSSPRAVSVEQAREVI
SGAPLQYVGVFQNADIADVCQKAAVLSLSAVQLHGSEDQAYVNALREALPRNVQIWKALS
VSNALPARDYHHVDKYIFDNGQGGSGQRFDWSLLQGQPLDDVLLAGGLAADNCVQAAQVG
CAGLDFNSGVESQPGIKDARLLASVFQTLRAY
>EECEBICD_02100 Tryptophan synthase beta chain
MTTLLNPYFGEFGGMYVPQILMPALNQLEEAFVSAQKDPEFQAQFADLLKNYAGRPTALT
KCQNITAGTRTTLYLKREDLLHGGAHKTNQVLGQALLAKRMGKSEIIAETGAGQHGVASA
LASALLGLKCRIYMGAKDVERQSPNVFRMRLMGAEVIPVHSGSATLKDACNEALRDWSGS
YETAHYMLGTAAGPHPYPTIVREFQRMIGEETKAQILDKEGRLPDAVIACVGGGSNAIGM
FADFINDTSVGLIGVEPGGHGIETGEHGAPLKHGRVGIYFGMKAPMMQTADGQIEESYSI
SAGLDFPSVGPQHAYLNSIGRADYVSITDDEALEAFKTLCRHEGIIPALESSHALAHALK
MMREQPEKEQLLVVNLSGRGDKDIFTVHDILKARGEI
>EECEBICD_02101 Tryptophan synthase alpha chain
MERYENLFAQLNDRREGAFVPFVTLGDPGIEQSLKIIDTLIDAGADALELGVPFSDPLAD

GPTIQNANLRAFAAGVTPAQCFEMLALIREKHPTIPIGLLMYANLVFNNGIDAFYARCEQ
VGVDSVLVADVPVEESAPFRQAALRHNIAPIFICPPNADDDLLRQVASYGRGYTYLLSRS
GVTGAENRGALPLHHLIEKLKEYHAAPALQGFGISSPEQVSAAVRAGAAGAISGSAIVKI
IEKNLASPEQMLAELRSFVSAMKAASRA
>EECEBICD_02102 hypothetical protein
MAEHRGGSGNFAENREKASEAGRKGGQHSGGNFKNDPQRASEAGKKGGQNSHGGRKSDNS
>EECEBICD_02103 Protein YciF
MNIKTVEDLFIHLLSDTYSAEKQLTKALSKLARATSNEKLSQAFQSHLEETQGQIERIDQ
IVESESGIKLKRMKCVAMEGLIEEANEVIESTEKNEVRDAALIAAAQKVEHYEIASYGTL
ATLAEQLGYSKALKLLKETLDEEKQTDLKLTDLAVSNVNKSAERKSK
>EECEBICD_02104 Protein YciE
MNYTEHYHDWLRDAHAMEKQAESMLESMASRIENYPDIKARIEQHISETKHQITMLEEVL
DRNGISRSVLKDSMSKMAAMGQSIGGMFPSDEIVKGSISGYVFEQFEIACYTSLLAAAKK
AGDTASIPTIEAILKEEMQMADWLIKHIPQTTEQFLLRSEADGVEAKK
>EECEBICD_02105 Manganese catalase
MFRHVKQLQYTVRVSEPNPGLANLLLEQFGGPQGELAAACRYFTQGLSDDDAGRREMLMD
IATEELSHLEIIGSLVGMLNKGAKGELAEGTENEAELYRSLTQNGNDSHITSLLYGGGPA
LTNSGGVPWTAAYIDTIGEVTADLRSNIAAEARAKIIYERLINLTDDPGVKDTLSFLMTR
EVAHQLSFEKALYSIRNNFPPGKLPPVEQYTDVYYNMSQGDDPRGSWNSDENFNYVAEPM
PAVDGGDGLATVKLPREQMALLKAMAERTKSDPTVDPLTGAELGCGEPKEDK
>EECEBICD_02106 Outer membrane protein W
MKKFTVAALALTTLLSGSAFAHEAGEFFMRAGPATVRPTEGAGGTLGHLNGFDVSNNTQL
GLTFTYMATDNIGVELLAATPFRHKVGTGATGDIATVHLLPPTLMAQWYFGDSSSKVRPY
VGVGVNYTTFFDNDFNDNGKNAGLSDLSFKDSWGAAGQVGVDYLINRDWLIGASVWYMDI
DTTANYKMGGVQHHDSVRLDPWVFMFSAGYRF
>EECEBICD_02107 hypothetical protein
MSVLNPCMTCGACCAYFRVSFYWAEGDDASGRVPASLTEPVTPFLRCMAGTNQKQPHCKA
LIGTPGENVSCAIYENRPSTCREFSISGEGGEVNEACNRARARYGLPPLYKDMLFHTTAD
AATVELSRVQLPAN
>EECEBICD_02108 hypothetical protein
MSITAKSVYRDAGNFFRNQFITILLVSLLCAFITVVLGHAFSPSDAQIAQLSEGEHLAGS
AGLFELVQNMTPEQQQILLRASAASTFSGLIGNAILAGGIILMIQLVSAGHRVSALRAIG
ASAPALPKLFILIFLTTLLVQIGIMLIVVPGIIMAIVLALAPVMLVEEKMGVFAAMRSSM
RLAWANMRLVAPAVIGWLLAKTLLLLFAPSFAVLTPNVGAVLANTLSNLISAVLLIYLFR
LYMLIRQ
>EECEBICD_02109 putative intracellular septation protein A
MKQFLDFLPLVVFFAFYKLYDIYAATSALIVATAIVLIYSWVRYRKIEKMALITFVLVAV
FGGLTLFFHNDEFIKWKVTVIYALFAGALLISQWVMKKPLIQRMLGKELALPQQVWSKLN
LAWALFFIACGLANIYIAFWLPQNIWVNFKVFGLTALTLIFTLLSGVYIYRHLPQEDKS
>EECEBICD_02110 putative acyl-CoA thioester hydrolase
MTTMDNTPQGELVLRTLAMPADTNANGDIFGGWLMSQMDIGGAILAKEIAHGRVVTVRVE
GMTFLRPVAVGDVVCCYARCVKRGTTSISINIEVWVKKVASEPIGQRYKATEALFIYVAV
DPDGKPRPLPVQG
>EECEBICD_02111 Protein TonB
MTLDLPRRFPWPTLLSVGIHGAVVAGLLYTSVHQVIELPAPAQPITVTMVSPADLEPPQA
VQPPPEPVVEPEPEPEPIPEPPKEAPVVIEKPKPKPKPKPKPKPEPVKKVEEQPKREVKP
AAPRPASPFENSAPARPTSSTASATSKPAVSVPTGPRALSRNQPQYPARAQALRIEGRVK
VKFDVTSAGRVENVQILSAQPANMFEREVKNAMRKWRYEAGKPGSGLVVNIIFRLNGTAQ
IE
>EECEBICD_02112 Protein YciI
MLYVIYAQDNANSLEKRLSVRPAHLARLQLLHDEGRLLTAGPMPAVDSNDPGVAGFTGST
VIAEFESLEAAQSWADADPYVAAGVYAQVSVKPYKKIF
>EECEBICD_02113 hypothetical protein
MKRSRTEVGRWRMLRQASRRKARWLEGQSRRNMRIHTIRKCIFNRQRNSLLFAIHGV

>EECEBICD_02114 Cardiolipin synthase A
MTTFYTVVSWLVILGYWVLIAGVTLRILMKRRAVPSAMAWLLIIYILPLVGIIAYLSVGE
LHLGKRRAERARAMWPSTAKWLNDLKACKHIFAQENSSVASSLFKLCERRQGIAGVKGNQ
LQLLTDSDDVMQALIRDIQLARHNIEMVFYIWQPGGMADQVAESLMAAARRGIHCRLMLD
SAGSVAFFRSPWAAMMRNAGIEVVEALKVNLMRVFLRRMDLRQHRKMVMIDNYIAYTGSM
NMVDPRFFKQDAGVGQWVDLMARMEGPVATAMGIVYSCDWEIETGKRILPPPPDVNIMPF
EQASGHTIHTIASGPGFPEDLIHQALLTATYAAREYLIMTTPYFVPSDDLLHAICTAAQR
GVDVSIILPRKNDSLLVGWASRAFFSELLAAGVKIYQFEGGLLHTKSVLVDGELSLVGTV
NLDMRSLWLNFEITLVIDDTGFGADLAAVQDDYISRSRLLDARLWVKRPLWQRITERLFY
FFSPLL
>EECEBICD_02115 hypothetical protein
MEMDLNNRLTEDETLEQAYDIFLELAADNLDPADIILFNLQFEERGGAELFDPAEDWQEH
VDFDLNPDFFAEVVIGLADTEDGEINDIFARVLLCREKDHKLCHILWRE
>EECEBICD_02116 hypothetical protein
MSLSLSSVRRRLYHNLFDLTTRSGRRFEGLCALFALLSVLVIFVESGVGTEYHLTFDEWH
IFVWLELCVTLIFTGEYLLRLFSWPEPAKYVFSFWGFIDLVTILPLYVMWLWPEISLNYM
FAWRAMRAIRVLRILKLLRFMPSLRVFWSAIISARHQLILFYSFIAIVMIIFGALMYLIE
GPKYGFTTLNASVYWAIVTVTTVGYGDITPHTPLGRIVASVLILIGYSVIAIPTGLITTH
MSSAFQKRHWQRKCPQCQQSQHEHSAQYCNRCGSKLPD
>EECEBICD_02117 Vitamin B12 import ATP-binding protein BtuD
MNAVIEQRKVLLEIADLKVHFDIKEGKQWFWQPPKTLKAVDGVTLRLYEGETLGVVGESG
CGKSTFARAIIGLVKATDGKVAWLGKDLLGMKADEWREVRSDIQMIFQDPLASLNPRMTI
GEIIAEPLRTYHPKLSRQDVRDRVKAMMLKVGLLPNLINRYPHEFSGGQCQRIGIARALI
LEPKLIICDEPVSALDVSIQAQVVNLLQQLQREMGLSLIFIAHDLAVVKHISDRVLVMYL
GHAVELGTYDEVYHNPLHPYTKALMSAVPIPDPDLERNKKIQLLEGELPSPINPPSGCVF
RTRCPLAGPECAQTRPVLEGSFRHAVSCLKVDPL
>EECEBICD_02118 Oligopeptide transport ATP-binding protein OppD
MSLSETATQAPQPANVLLEVNDLRVTFATPDGDVTAVNDLNFTLRAGETLGIVGESGSGK
SQTAFALMGLLATNGRIGGSATFNGREILNLPERELNTLRAEQISMIFQDPMTSLNPYMR
VGEQLMEVLMLHKGMSKAEAFEESVRMLDAVKMPEARKRMKMYPHEFSGGMRQRVMIAMA
LLCRPKLLIADEPTTALDVTVQAQIMTLLNELKREFNTAIIMITHDLGVVAGICDKVLVM
YAGRTMEYGKARDVFYQPVHPYSIGLLNAVPRLDSEGAEMLTIPGNPPNLLRLPKGCPFQ
PRCPHAMEICNNAPPLEAFSPGRLRACFKPVEELL
>EECEBICD_02119 Oligopeptide transport system permease protein OppC
MMLSKKNSETLENFSEKLEVEGRSLWQDARRRFMHNRAAVASLIVLFLIALFVTVAPMLS
QFTYFDTDWGMMSSAPDMASGHYFGTDSSGRDLLVRVAIGGRISLMVGIAAALVAVIVGT
LYGSLSGYLGGKIDSVMMRLLEILNSFPFMFFVILLVTFFGQNILLIFVAIGMVSWLDMA
RIVRGQTLSLKRKEFIEAAQVGGVSTASIVIRHIVPNVLGVVVVYASLLVPSMILFESFL
SFLGLGTQEPLSSWGALLSDGANSMEVSPWLLLFPAGFLVVTLFCFNFIGDGLRDALDPK
DR
>EECEBICD_02120 Oligopeptide transport system permease protein OppB
MLKFILRRCLEAIPTLFILITISFFMMRLAPGSPFTGERALPPEVLANIEAKYHLNDPIM
TQYFSYLKQLAHGDFGPSFKYKDYTVNDLVAASFPVSAKLGAAAFLLAVIIGVSAGVIAA
LKQNTRWDYTVMGFAMTGVVIPSFVVAPLLVMVFAITLQWLPGGGWNGGALKFMILPMVA
LSLAYIASIARITRGSMIEVLHSNFIRTARAKGLPMRRIIFRHALKPALLPVLSYMGPAF
VGIITGSMVIETIYGLPGIGQLFVNGALNRDYSLVLSLTILVGALTILFNAIVDVLYAVI
DPKIRY
>EECEBICD_02121 Periplasmic oligopeptide-binding protein
MGILTALIAASAATAADVPAGVQLADKQTLVRNNGSEVQSLDPHKIEGVPESNVSRDLFE
GLLISDVEGHPSPGVAEKWENKDFKVWTFHLRENAKWSDGTPVTAHDFVYSWQRLADPNT
ASPYASYLQYGHIANIDDIIAGKKPATDLGVKALDDHTFEVTLSEPVPYFYKLLVHPSVS
PVPKSAVEKFGDKWTQPANIVTNGAYKLKNWVVNERIVLERNPQYWDNDKTVINQVTYLP
ISSEVTDVNRYRSGEIDMTYNNMPIELFQKLKKEIPNEVRVDPYLCTYYYEINNQKAPFN
DVRVRTALKLALDRDIIVNKVKNQGDLPAYSYTPPYTDGAKLVEPEWFKWSQQKRNEEAK

KLLAEAGFTADKPLTFDLLYNTSDLHKKLAIAVASIWKKNLGVNVNLENQEWKTFLDTRH
QGTFDVARAGWCADYNEPTSFLNTMLSDSSNNTAHYNSPAFDKLIADTLKVADDTQRSEL
YAKAEQQLDKDSAIVPVYYYVNARLVKPWVGGYTGKDPLDNIYVKNLYIIKH
>EECEBICD_02122 hypothetical protein
MIQPLFDFPVYFKFFIGLFALVNPVGIIPVFISMTSYQTATARNKTNLTANLSVAIILWS
SLFLGDGILQLFGISIDSFRIAGGILVVTIAMSMISGKLGEDKQNKQEKSETAIRESIGV
VPLALPLMAGPGAISSTIVWGTRYHSIAHLLGFSVAIALFALCCWGVFRMAPWLVRLLGQ
TGINVITRIMGLLLMALGIEFIVTGIKGIFPGLLN
>EECEBICD_02123 hypothetical protein
MAVTNVAELNALVERVKKPSVNMPVSLKNRSTKSSAPPLWQPLMPAFRWPKWPSPNPVWA
SWKTK
>EECEBICD_02124 Aldehyde-alcohol dehydrogenase
MAVAESGMGIVEDKVIKNHFASEYIYNAYKDEKTCGVLSEDDTFGTITIAEPIGIICGIV
PTTNPTSTAIFKSLISLKTRNAIIFSPHPRAKEATNKAADIVLQAAIAAGAPKDLIGWID
QPSVELSNALMHHPDINLILATGGPGMVKAAYSSGKPAIGVGAGNTPVVIDETADIKRAV
ASVLMSKTFDNGVICASEQSVVVVDSVYDAVRERFASHGGYMLQGQELKAVQNVILKNGA
LNAAIVGQPAYKIAELAGFSVPETTKILIGEVTVVDESEPFAHEKLSPTLAMYRAKDFEE
AVEKAEKLVAMGGIGHTSCLYTDQDNQPERVAYFGQMMKTARILINTPASQGGIGDLYNF
KLAPSLTLGCGSWGGNSISENVGPKHLINKKTVAKRAENMLWHKLPKSIYFRRGSLPIAL
DEVITDGHKRALIVTDRFLFNNGYADQITSVLKAAGVETEVFFEVEADPTLSVVRKGAEL
ANSFKPDVIIALGGGSPMDAAKIMWVMYEHPETHFEELALRFMDIRKRIYKFPKMGVKAK
MIAVTTTSGTGSEVTPFAVVTDDATGQKYPLADYALTPDMAIVDANLVMDMPKSLCAFGG
LDAVTHALEAYVSVLASEFSDGQALQALKLLKENLPASYHEGSKNPVARERVHSAATIAG
IAFANAFLGVCHSMAHKLGSQFHIPHGLANALLICNVIRYNANDNPTKQTAFSQYDRPQA
RRRYAEIADHLGLSAPGDRTAAKIEKLLAWLESIKAELGIPKSIREAGVQEADFLAHVDK
LSEDAFDDQCTGANPRYPLISELKQILLDTYYGRDFTEGEVAAKKDVVAAPKAEKKAKKS
A
>EECEBICD_02125 Thymidine kinase
MAQLYFYYSAMNAGKSTALLQSSYNYQERGMRTVVYTAEIDDRFGAGKVSSRIGLSSPAK
LFNQNTSLFEEIRAESARQTIHCVLVDESQFLTRQQVYQLSEVVDKLDIPVLCYGLRTDF
RGELFVGSQYLLAWSDKLVELKTICFCGRKASMVLRLDQDGRPYNEGEQVVIGGNERYVS
VCRKHYKDALEEDSLTAIQERHRHI
>EECEBICD_02126 DNA-binding protein H-NS
MSEALKILNNIRTLRAQARECTLETLEEMLEKLEVVVNERREEESAAAAEVEERTRKLQQ
YREMLIADGIDPNELLNSMAAAKSGTKAKRAARPAKYSYVDENGETKTWTGQGRTPAVIK
KAMEEQGKQLEDFLIKE
>EECEBICD_02127 UTP--glucose-1-phosphate uridylyltransferase
MAALNSKVKKAVIPVAGLGTRMLPATKAIPKEMLPLVDKPLIQYVVNECIAAGITEIVLV
THSSKNSIENHFDTSFELEAMLEKRVKRQLLEEVQSICPPHVTIMQVRQGLAKGLGHAVL
CAHPVVGNEPVAVILPDVILDEYESDLSQDNLAEMIRRFDETGNSQIMVEPVEDVTAYGV
VDCKGVELAPGESVPMVGVVEKPKADVAPSNLAIVGRYVLSADIWALLAKTPPGAGDEIQ
LTDAIDMLIEKETVEAYHMKGKSHDCGNKLGYMQAFVEYGIRHNSLGAEFKAWLEEEMGI
KK
>EECEBICD_02128 Regulator of RpoS
MICDIAMPRMNGLKLVENLRNRGDQTPILVISATENMADIAKALRLGVEDVLLKPVKDLN
RLRETVFACLYPNMFNSRVEEEERLFRDWDAMVSNPTAAAQLLQELQPPVQQVISHCRIN
YRQLVSADQPGLVLDIAPLSDNELAFYCLDVTRAGDNGVLAALLLRALFNGLLQDQLGQQ
KHRLPELGALLKQVNHLLRQANLPGQFPLFVGYYHSELKNLILVSAGLNATLNTGAHQVQ
ISSGVPLGTLGNAYLNQLSQRCDSWQCQIWGAGGRLRLMLSAE
>EECEBICD_02129 Regulator of RpoS
MTQPLVGKQILIVEDEPVFRSLLDSWFSSLGATTALAGDGVDALELMGRFTLIS
>EECEBICD_02130 putative NTE family protein
MKIGLALGSGAARGWSHIGVIKALKQTGIDIDIVAGCSIGSLVGAAYACNKLSALEQWVC
SFRYWDVLRLMDVSWGRGGLLRGERVFNHYRDIMPVTDFDHCSRRFGAVATNLSTGRELW

FTEGDLHLAVRASCSMPGLMSPVEHNGYWLVDGAVVNPVPVSLTRALGADIVIAVDLQHD
AHLMQQDLLSVNVGNINEESDDALPWHKRLKERFSSLTSRRGVSSPGAMEIMTTSIQVLE
NRLKRNRMAGDPPDILIQPFCPQISTLDFHRAHAAIAAGQLAVEKKMDELIPLVRTDV
>EECEBICD_02131 hypothetical protein
MSQPCPCGSADEYSLCCGRIVSGERVAPDPSHLMRSRYCAFVMKDADYLIKSWHPTCNAA
AFRDDIIAGFANTRWLGLTIFEHTWSEAENTGYVSFIARFSEQGKTGAIIERSRFIKENG
QWYYIDGTRPQLGRNDPCPCGSGKKFKKCCGQ
>EECEBICD_02132 Formyltetrahydrofolate deformylase
MQSLQRKVLRTICPDQKGLIARITNICYKHELNIVQNNEFVDHRTGRFFMRTELEGIFND
STLLADLDSALPEGSVRELNPAGRRRVVILVTKEAHCLGDLLMKANYGGLDVEIAAVIGN
HETLRSLVERFEIPFELVSHEGLTREEHDTKMADAIDAHQPDYVVLAKYMRVLTPGFVAR
FPNKIINIHHSFLPAFIGARPYHQAYERGVKIIGATAHYVNDNLDEGPIIMQDVIHVDHT
YTAEDMMRAGRDVEKNVLSRALYQVLAQRVFVYGNRTIIL
>EECEBICD_02135 hypothetical protein
MRFSSIFKIIILNIFISKMAHSAVCEERYPPDSEECQYVQELEQKAEQGDESAQFALGSW
YAEGRYVKPDYKLAIKWLEKAGKQGSDFSYFILGYHYNYGENFPLSRQKALEWYRKAAEL
GDSSTQEILGDAYMYGDGFPQNTQLALEWYRKAASPTNDAGVVRGQGSASSAQFKLGVMY
AHGQGVPQDYQQTAILMRKAAENMYYPAQLYLGVAYFYGEGVPQDYRQAVYWLNEGIPSS
YTPGHIPLNALYDKAHPADQVHSQTWYRKTAQRVMAKVQYNFGVWYYNGYHLLKDHNLAL
EWYRRAAAQGLAEAQDAIGVMFMQGEGVSQDYQQALAWYRKAARQGLPAAQTHLGIMSAF
GRGVAQSDRQAIAWYRKAAKQDFAKAQYQLGVAYSTGRGVPENSRNALKWYLKAAEQGFT
PAQLALGEIYAHGRQGVPKDNKQAYIWYYMASIYTEKSKDDCSALIAERNRLKGTLTPDQ
LSETYAAFDLIRRKINQSKEAKKIARKKY
>EECEBICD_02136 Respiratory nitrate reductase 1 gamma chain
MHFLNMFFFDIYPYIAGSVFLIGSWLRYDYGQYTWRAASSQMLDRKGMNLASNLFHIGIL
GIFAGHFLGMLTPHWMYEAFLPVDVKQKMAMIAGGACGVLTLVGGILLLKRRLFSPRVRA
TTTGADILILSLLVIQCALGLLTIPFSAQHMDGSEMMKLVGWAQSVVTFHGGASAHLDGV
AFIFRMHLVLGMTLFLLFPFSRLVHIWSVPVEYLTRKYQIVRARH
>EECEBICD_02137 Nitrate reductase molybdenum cofactor assembly chaperone NarJ
MIELVIVSRLLEYPDAALWQHQQELFDALASSENLEKEDAQTLGVFLRDLTAQDLLDVQA
AYSELFDRGRATSLLLFEHVHGESRDRGQAMVDLMAQYEQHGLQLDSRELPDHLPLYLEY
LAQLPKSEALGGLQDIAPILALLSARLQQRDSRYAVLFDLLLKLANTVIDSDKVAEKIAN
EARDDTPQALDAVWEEEQVKFFADQGCGESEISAHQRRFAGAVAPQYLNITTGGQH
>EECEBICD_02138 Respiratory nitrate reductase 1 beta chain
MKIRSQVGMVLNLDKCIGCHTCSVTCKNVWTSREGMEYAWFNNVESKPGVGFPNDWENQE
KWKGGWIRKINGKLQPRMGNRALLLGKIFANPHLPGIDDYYEPFDYDYQNLHTAPEGSKH
QPIARPRSLITGQRMDKITSGPNWEEILGGEFEKRAKDQNFENMQKAMYGQFENTFMMYL
PRLCEHCLNPACVATCPSGAIYKREEDGIVLIDQDKCRGWRMCITGCPYKKIYFNWKSGK
SEKCIFCYPRIEAGQPTVCSETCVGRIRYLGVLLYDADAIESAASTENEKDLYQRQLDVF
LDPNDPAVIEQALKDGVPQSVIDAAQQSPVYKMAMDWKLALPLHPEYRTLPMVWYVPPLS
PIQSAADAGELGSNGILPDVDSLRIPVQYLANLLTAGDTQPVLLALKRMLAMRHYKRAET
VDGKVDTRALEEVGLSEAQAQEMYRYLAIANYEDRFVVPSSHRELARDAFPEKSGCGFTF
GDGCHGSDTKFNLFNSRRIDAVDVTSKTEPHA
>EECEBICD_02139 Respiratory nitrate reductase 1 alpha chain
MNELIAASNVYTVKTYGPDRVAGFSPIPAMSMVSYASGARYLSLIGGTCLSFYDWYCDLP
PASPQTWGEQTDVPESADWYNSSYIIAWGSNVPQTRTPDAHFFTEVRYKGTKTVAVTPDY
AEIAKLCDLWLAPKQGTDAAMALAMGHVMLREFHLDNPRQYFTDYVRRYTDMPMLVMLEE
RDGYYAAGRMLRAADLVDSLGQENNPEWKTVAINSNGEMVAPNGSIGFRWGEKGKWNLEQ
RDGTTGAETELQLSLLGSQDEIADVGFPYFGGEGSEYFNHVALDNVLLHKLPAKRLQLAD
GSSALVTTVYDLTMANYGLERGLNDENCAASYDDTKAYTPAWAEQITGVPRAQIIRIARE
FADNADKTHGRSMIIVGAGLNHWYHLDMNYRGLINMLVFCGCVGQSGGGWAHYVGQEKLR
PQTGWQPLAFALDWQRPARHMNSTSYFYNHSSQWRYETVTAEELLSPMADKSRYSGHLID
FNVRAERMGWLPSAPQLGTNPLYIAREAEKAGMTPVDYTVKSLKEGSIRFAAEQPENGKN

HPRNLFIWRSNLLGSSGKGHEYMLKYLLGTEHGIQGLDLGKQGGVKPEEVEWRDNGLDGK
LDLVVTLDFRLSSTCLYSDIVLPTATWYEKDDMNTSDMHPFIHPLSAAVDPAWESKSDWE
IYKGIAKKFSEVCVGHLGKETDVVTLPIQHDSAAELAQPLDVKDWKKGECDLIPGKTAPH
IMTVERDYPATYERFTSIGPLMEKIGNGGKGIAWNTQSEMDLLRKLNYTKADGPAKGQPM
LNTAIDAAEMILTLAPETNGQVAVKAWAALSEFTGRDHTHLATNKEEEKIRFRDIQAQPR
KIISSPTWSGLEDEHVSYNAGYTNVHELIPWRTLSGRQQLYQDHQWMRDFGESLLVYRPP
IDTRSVKAVMGRKSNGNPEKALNFLTPHQKWGIHSTYSDNLLMLTLSRGGPIVWMSETDA
KDLGIEDNDWIEVFNSNGALTARAVVSQRVPTGMTMMYHAQERIVNLPGSEITQQRGGIH
NSVTRITPKPTHMIGGYAQLAYGFNYYGTVGSNRDEFVVVRKMKNINWLDGEGNDQVQES
VK
>EECEBICD_02140 Respiratory nitrate reductase 1 alpha chain
MSKFLDRFRYFKQKGETFADGHGQLLETNRDWEDGYRQRWQHDKIVRSTHGVNCTGSCSW
KIYVKNGLVTWETQQTDYPRTRPDMPNHEPRGCPRGASYSWYLYSANRLKYPLMRKRLMK
MWREAKKTASRSGRGMGLHH
>EECEBICD_02141 Nitrate/nitrite transporter NarK
MAIFSGLLFLTLPTGGVGGSFIAFYGVFLALFLTAGLGSGSTFQMISVIFRKLTMDRVKA
EGGSDERAMREAATDTAAALGFISAIGAIGGFFIPKAFGSSLALTGSPVGAMKVFLIFYI
ACVVITWAVYGRHSKNKK
>EECEBICD_02142 Nitrate/nitrite transporter NarK
MSHSSAPERATGAVITEWRPEDPAFWQQHGHRVASRNLWISVPCLLLAFCVWMLFSAVAV
NLPKVGFNFTTDQLFMLTALPSVSGALLRVPYSFMVPLFGGRRWTAFSTGILIVPCVWLG
FAVQDTSTPFSTFIIISLLCGFAGANFASSMANISFFFPKQKQGGALGLNGGLGNMGVSV
MQLVAPLVVSLSIFAAFGSHGVEQPDGSQLYLANAAWIWVPFLAIFTLAAWFGMNELATS
KASLKEQLPVLKRGHLWIMSLLYLATFGSFIGFSAGFAMLSKTQFPDVQILHYAFFGPFI
GALARSAGGAISDRLGGLASRLSTLS
>EECEBICD_02143 Nitrate/nitrite sensor protein NarX
MFKRCFSPLTLVNQLALIVMLSTAIGVAGMAVSGWLVQGVQGSAHAINKAGSLRMQSYRL
LAAVPLDAKDQKLLDEMEQTAFSPELTRAAERDGQQKQLKALQDYWHNELSPGLQHAQNA
HAVAEDVTRFVAGLDRLVTSFDHTTELRIERVVLVHRVMAIFMALLLVFTIIWLRVRLLQ
PWKQLLSMARAVSQRDFTQRANISGRNEMAALGSALNNMSEELAESYAVLEQRVQEKTAG
LEHKNQILSFLWQANRRLHSQAPLCERLSPVLNGLQNLTQLHDIELRVYDLEDEDNHQEF
TCQSDISCDDKGCHLCPRSALPMINGGTTLKWRLTDSHTQYGILLATLPYGRHLSHDQQQ
LVDTLVEQLTATLALDRHQERQQQLIVMEERATIARELHDSIAQSLSCMKMQVSCLQMQG
DVLPESSRELLSQIRNELNCSWAQLRELLTTFRLQLTEPGLRPALEASCQEFSARFGFTV
KLDYQLPPRLVPSHQAIHLLQIAREALSNALKHSHADDVVVTVTQCGKQVKLKVQDNGCG
VPENAERSNHYGMIIMRDRAQSLRGDCQVRRRETGGTEVTVTFIPETNFTETQGDTHE
>EECEBICD_02144 Nitrate/nitrite response regulator protein NarL
MNNQEPATILLIDDHPMLRTGVKQLVSMAPDISVVGEASNGEQGIDLAESLDPDLILLDL
NMPGMNGLETLDKLREKALSGRIVVFSVSNHEEDVVTALKRGADGYLLKDMEPEDLLKAL
QQAAAGEMVLSEALTPVLAASLRANRATTDRDVTQLTPRERDILKLIAQGLPNKMIARRL
DITESTVKVHVKHMLKKMKLKSRVEAAVWVHQERIF
>EECEBICD_02145 Invasin
MPFLLSFVVEVADTLSRIVFRSFSLSLLLLAASGTIRAQAQDPFTQNRLPDLGMMPESHE
GEKHFAEMAKAFGEASMKNNDLDTGEQARQFAFGQVRDVVSEQVNQQLESWLSAWGSASV
DINVDNEGHFNGSHGSWFIPLQDKQRYLTWSQLGLTQQTDGLVSNVGIGQRWAQDGWLLG
YNTFYDNLLDENLQRAGFGAEAWGEYLRLSANYYQPFADWQTHTATLEQRMARGYDINAQ
MRLPFYQHINTSVSLEQYFGDSVDLFDSGTGYHNPVALKLGLNYTPVPLLTMTAQHKQGE
SGVSQNNLGLTLNYRFGVPLKKQLAASEVAQSQSLRGSRYDTPQRNSLPTMEYRQRKTLT
VFLATPPWDLTPGETVALKLQVRSVHGIRHLSWQGDTQALSLTAGTDTRSTEGWTIIMPA
WDHREGAANRWRLSVVVEDEKDQRVSSNEITLALTEPFITMPDDNPHWQPFQEQ
>EECEBICD_02146 Protein YchN
MQNIVIIANGAAYGSESLFNSLRLAIALREQESHLDLRLFLMSDAVTAGLRGQKPAEGYN
IQQMLEILTAQNVPVKLCKTCADGRGITPLPLIDGVEIGTLVELAQWTLAADKVLTF
>EECEBICD_02147 Putative cation transport regulator ChaB

MPYKAKSDLPDNVRNVLPTHAQDIYKEAFNSAWEQYKDKADRRDDASREETAHKVAWAAV
KNDYEKGENDKWHKKK
>EECEBICD_02148 Sodium-potassium/proton antiporter ChaA
MTHACEAVKTRHKETSLIFPVLALVVLFLWGSSQSLPVVIGINILALIGILSSAFSVVRH
ADVLAHRLGEPYGSLILSLSVVILEVSLISALMATGDAAPTLMRDTLYSIIMIVTGGLVG
FSLLLGGRKFATQYMNLFGIKQYLIALFPLAIIVLVFPMALPQANFSTSQALLVALISAA
MYGVFLLIQTKTHQSLFIYEHEDEGDDDNPHHGKPSAHSSGWHTVWLLIHLIAVIAVTKM
NASPLEALLTSMNAPVAFTGFLVALLILSPEGLGALKAVLNNQVQRAMNLFFGSVLATIS
LTVPVVTLIAWATGNDLVFGLGAPEMVVMVASLVLCHISFSTGRTNVLNGAAHLALFAAY
LMTIFA
>EECEBICD_02149 2-dehydro-3-deoxyphosphooctonate aldolase
MKQKVVNIGDIKVANDLPFVLFGGMNVLESRDLAMRICEHYVTVTQKLGIPYVFKASFDK
ANRSSIHSYRGPGLEEGMKIFQELKQTFGVKVITDVHEASQAQPVADVVDVIQLPAFLAR
QTDLVEAMAKTGAVINVKKPQFVSPGQMGNIVDKFHEGGNDKVILCDRGANFGYDNLVVD
MLGFSVMKKVSGNSPVIFDVTHALQCRDPFGAASGGRRGQVTELARAGMAVGLAGLFLES
HPDPANAKCDGPSALPLAKLEQFLTQIKAIDDLVKSFDELDTEN
>EECEBICD_02150 hypothetical protein
MRSLADFEFNNAPLCDGMILASEMIRLDFPTQFVYDELERLVSLAQEEISQLLSQDEQLE
KLLALFYGEWGFTDSRGVYRLSDALWLDKVLKKRQGSAVSLGAILLWIANRLDLPLVPVI
FPTQLILRIESLEGEMWLINPFNGETLDEHTLEVWLKGNISPVAELFNEDLDEADNAEVI
RKLLDTLKSSLMEERQMELALRVSEALLQFNPEDPYEIRDRGLIYAQLECEHVALTDLSY
FVEQCPEDPISEMIRAQINTIAHKQIVLH
>EECEBICD_02151 Protein YchQ
MTIAMLLTLHLICVALSVSLFVARYWWRYCGHALAAARWTRIVPPVIDTLLLLSGIGLIV
KTHILPFTESGSWLTGKLFGVIIYIVLGFIALDYRQARSQQARFIAFPLALVVLYIIIKL
ATTKIPLLG
>EECEBICD_02152 Release factor glutamine methyltransferase
MDFQHWLHEAVNQLRDSDSPRRDAEILLEYVTGKGRTYIMAFGETPLTDVQQQQLADLLQ
RRKQGEPIAYLTGLREFWSLPLFVSPATLIPRPDTECLVEQALARLPVKTCRILDLGTGT
GAIALALACERPDCEVTAVDRMPDAVALAIRNAEHLAIRNVRILQSCWFSALSGQQFDMI
VSNPPYIDAQDPHLSEGDVRFEPRSALVADENGMADLTHIIDNARQMLTPGGFLLLEHGW
QQGEAVRAVFRRSGYSDVETCRDYGGNERVTCGRFTP
>EECEBICD_02153 Peptide chain release factor RF1
MKPSIVAKLEALHERHEEVQALLGDAGIIADQDRFRALSREYAQLSDVSRCFTDWQQVQD
DIETAQMMLDDPEMREMAQEELREAKEKSEQLEQQLQVLLLPKDPDDERNAFLEVRAGTG
GDEAALFAGDLFRMYSRYAEARRWRVEIMSMSEGEHGGYKEIIAKISGDGVYGRLKFESG
GHRVQRVPATESQGRIHTSACTVAVMPELPEAELPDINPADLRIDTFRSSGAGGQHVNTT
DSAIRITHLPTGIVVECQDERSQHKNKAKALSVLGARIHAAETAKRQQAEASTRRNLLGS
GDRSDRNRTYNFPQGRVTDHRINLTLYRLDETMEGKLDMLIEPIVQEHQADLLAALSEQE
>EECEBICD_02154 Glutamyl-tRNA reductase
MTLLALGINHKTAPVSLRERVTFSPDTLDQALDSLLAQPMVQGGVVLSTCNRTELYLSVE
EQDNLQEALIRWLCDYHNLNEDDLRNSLYWHQDNDAVSHLMRVASGLDSLVLGEPQILGQ
VKKAFADSQKGHLNASALERMFQKSFSVAKRVRTETDIGASAVSVAFAACTLARQIFESL
STVTVLLVGAGETIELVARHLREHKVQKMIIANRTRERAQALADEVGAEVISLSDIDARL
QDADIIISSTASPLPIIGKGMVERALKSRRNQPMLLVDIAVPRDVEPEVGKLANAYLYSV
DDLQSIISHNLAQRQAAAVEAETIVEQEASEFMAWLRAQGASETIREYRSQSEQIRDELT
TKALSALQQGGDAQAILQDLAWKLTNRLIHAPTKSLQQAARDGDDERLNILRDSLGLE
>EECEBICD_02155 Outer-membrane lipoprotein LolB
MTLPDFRLIRLLPLASLVLTACTLPGHKGPGKSPDSPQWRQHQQEVRHLNQYQTRGAFAY
ISDDQKVYARFFWQQTGQDRYRLLLTNPLGSTELELNAQPGNVQLVDNKGQRYTADDAEE
MIGKLTGMPIPLNSLRQWILGLPGDATDYKLDDQYRLSEVNYRQDGKNWKVVYGGYDSKT
QPAMPANMELSDGSQRIKLKMDNWIVK
>EECEBICD_02156 4-diphosphocytidyl-2-C-methyl-D-erythritol kinase
MMTHWPSPAKLNLFLYITGQRADGYHTLQTLFQFLDYGDTLHIEPRRDGEIHLLTPVTGV

ENEDNLIVRAARLLMKVASESGRLPAGSGADISIEKRLPMGGGLGGGSSNAATVLVALNH
LWQCGLSIDELATLGLTLGADVPVFVRGHAAFAEGVGEILTPVNPPEKWYLVAHPGVSIP
TPVIFKDPQLPRNTPKRSIDTLLKCEFSNDCEVIARKRFREVDAALSWLLEYAPSRLTGT
GACVFAEFDTESCARQVLEQAPEWLNAFVAKGVNLSPLHRELL
>EECEBICD_02157 Ribose-phosphate pyrophosphokinase
MPDMKLFAGNATPELAQRIANRLYTSLGDAAVGRFSDGEVSVQINENVRGGDIFIIQSTC
APTNDNLMELVVMVDALRRASAGRITAVIPYFGYARQDRRVRSARVPITAKVVADFLSSV
GVDRVLTVDLHAEQIQGFFDVPVDNVFGSPILLEDMLQLNLDNPIVVSPDIGGVVRARAI
AKLLNDTDMAIIDKRRPRANVSQVMHIIGDVAGRDCVLVDDMIDTGGTLCKAAEALKERG
AKRVFAYATHPIFSGNAANNLRNSVIDEVVVCDTIPLTDEIKALPNVRTLTLSGMLAEAI
RRISNEESISAMFEH
>EECEBICD_02158 C4-dicarboxylic acid transporter DauA
MPFRALIDACWKEKYTASRFTRDVIAGITVGIIAIPLAMALAIGSGVAPQYGLYTSAVAG
IVIALTGGSRFSVSGPTAAFVVILYPVSQQFGLAGLLVATLMSGFFLILFGLARLGRLIE
YIPVSVTLGFTSGIGITIGTMQIKDFLGLQMAHVPEHYLQKVGALFMALPTVNIGDAAIG
VVTLGTLIFWPRLGIRLPGHLPALLAGCAVMGIVNLLGGNVATIGSQFHYVLADGTQGNG
IPQLLPQLMLPWSLPGSDFTLSWDSLRALLPAAFSMAMLGAIESLLCAVVLDGMTGTKHK
ANSELIGQGLGNMVAPFFGGITATAAIARSAANVRAGATSPISAVIHAILVILALLVLAP
LLSWLPLSAMAALLLMVAWNMSEAHKVVDLLRHAPKDDIIVMLLCMSLTVLFDMVIAISV
GIVLASLLFMRRIARMTRLAPVNVDVPEDVLVLRVIGPLFFAAAEGLFTDLESRIKGKRI
VVLKWDAVPVLDAGGLDAFQRFVKRLPEGCELRISNLEFQPLRTMARAGIKPIPGRLTFF
PNRTEALADLLS
>EECEBICD_02159 hypothetical protein
MKRKNASLFGNVLMGLGLVVMVVGVGYSILNQLPQFNLPQFFAHGAILSIFVGAVLWLAG
ARVGGHEQVSDKYWWVRHYDKRCRRNDNRRHS
>EECEBICD_02160 Peptidyl-tRNA hydrolase
MAIKLIVGLANPGAEYAATRHNAGAWYVDLLAERLRAPLREEPKFFGYTSRITLEGEDVR
LLVPTTFMNLSGKAVGAMASFYRIQPDEILVAHDELDLPPGVAKFKLGGGHGGHNGLKDI
ISKLGNNPNFHRLRVGIGHPGDKNKVVGFVLGKPPVSEQKLIDEAIDEAARCTELWFKEG
LAKATSRLHTFKAQ
>EECEBICD_02161 Ribosome-binding ATPase YchF
MGFKCGIVGLPNVGKSTLFNALTKAGIEAANFPFCTIEPNTGVVPMPDPRLDQLAEIVKP
QRILPTTMEFVDIAGLVKGASKGEGLGNQFLTNIRETEAIGHVVRCFENDNIIHVAGKVN
PAEDIDVINTELALADLDTCERAIHRVQKKAKGGDKDAKAELAALEKCLPHLAEAGMLRS
LDLTDEDKAAIRYLSFLTLKPTMYIANVNEDGFENNPYLDQVREIAAKEGSVVVPVCAAV
EADIAELDDDERDEFMAELGLEEPGLNRVIRAGYRLLNLQTYFTAGVKEVRAWTIPVGAT
APQAAGKIHTDFEKGFIRAQTIAFDDFITYKGEQGAKEAGKMRAEGKDYIVKDGDIMNFL
FNV
>EECEBICD_02162 hypothetical protein
MDSFIYNLIIFLFIAFLITFFIFTQRIKAKAIDKIDNSLTLYSIIFNKGVQDHVTSPQNI
ISHLKIDRFYPLVTYLPAIPLFFLVLYCQEGLSLAILTISIIALLSFAALSLTMSLDIYE
NAIVHNTLLRKRVFLFSQLDSIDEKIDYDGVPNTKEEALMYPRYYAIVKNGRVIFCIPKT
LSNYSSFKKISMNRLFL
>EECEBICD_02163 hypothetical protein
MFQNSGEVIMYFGCFLFSLPFILVLIRKVFFVGLQYNFLHSHKAGVAFGLLLIYGLIIAY
IGQSYKDRICNDVMLSYYEQGINYSELTPSQRINILYASIHMPIDFKKGNDVSKYLPALE
KYTYQSKIYKHKSIEKAKEETNQFMKTFTQ
>EECEBICD_02164 Hydrogenase-1 small chain
MNNEETFYQAMRRKGVTRRSFLKFCSLAATSLGLGAGMTPKIAWALENKPRIPVVWIHGL
ECTCCTESFIRSSHPLAKDVILSLISLDYDDTLMAAAGAQAEEVFDDITTRYAGKYILAV
EGNPPLGEQGMFCISGGRPFIEKLKKAAAGASAIIAWGNCASWGCVQAARPNPTQATPID
KVITDKPIVKVPGCPPIPDVMSAIITYMVTFDRLPELDRMGRPLMFYGQRIHDKCYRRAH
FDAGEFVESWDDAAARKGYCLYKMGCKGPTTYNACSSTRWNDGVSFPIQSGHGCLGCSEN
GFWDRGSFYSRVVDIPQMGTHSTADTVGLTALGVVAAGVGGHAVASALNQRKRHKQQLAQ

AEQQPDNEDKQA
>EECEBICD_02165 Hydrogenase-1 large chain
MSNQYQTQGYTVNDAGRRLIVDPITRIEGHMRCEVNIDEQNVITNAVSCGTMFRGLEIIL
QGRDPRDAWAFVERICGVCTGVHALASVYAIEDAIGIQVPDNANIIRNIMLATLWCHDHL
VHFYQLAGMDWIDVLNALKADPRATSQLAQSLSAWPMSSPGYFFDVQNRLKKFVDGGQLG
IFRNGYWGHPQYKLSPEANLMGFAHYLEALDFQREIVKIHTIFGGKNPHPNWIVGGMPCA
INLDQSGAVGAINMERLNLVQSIITRTADFINNVMVPDALAIGQFNKAWSQIGTGLSDKC
VLSYGAFPDIANDFSQQSLLMPGGAVVNGDFKNVMPVDLADPQQIQEFVDHAWYRYPDDR
LGRHPFDGITDPWYNPGDVKGSDTHIQQLNEQERYSWIKAPRWRGHAMEVGPLARTLIAY
HKGDAATIESVDRMMSALKLPLSGIQSTLGRILCRAHEAQWAVGKLQYFFDRLMTNLKNG
DLATANTEKWEPASWPQHCRGIGFTEAPRGALGHWASIRDQKIELYQCVVPTTWNASPRD
PKKQIGAYEAALMGTQMAIPDQPLEILRTLHSFDPCLACSTHVLGDDGSELIAVQVR
>EECEBICD_02166 putative Ni/Fe-hydrogenase 1 B-type cytochrome subunit
MTGKLSPRVGEARDTAVNHYVFEAPVRLWHWLTVACMLVLMVTGYFIGRPLPSVSGEATY
LFYMGYIRLIHFAAAMIFTVLLLGRIYWACVGNRYSRELFIVPVWRRSWWQGAFSVVRWY
LFLEKKPGSDIGHNPVAQAAMFGYFLLSVFMILTGFALFGEHSQYAIFAPFRYVVEFFYW
TGGNSIDIHSWHRLGMWLIAAFIVGHVYLAIREDIMSDDTVISTMINGYRSHKFPKPHDK
EPS
>EECEBICD_02167 Hydrogenase 1 maturation protease
MNAQRVVVMGLGNLLWADEGFGIRVAERLYARYHWPEEVEIVDGGTQGLNLLGYVEQASH
LLLLDAIDYGLAPGSLRTYAGEKIPAYLSAKKMSLHQNSFSEVLALADIRGHLPCHIALV
GLQPAHLDDYGGSLSEIARGQLPAAEQAALAQLAAWGIVPQANEVARCLNYKCLSMENYE
GVRIRQYQTRLEGKRSGE
>EECEBICD_02168 Hydrogenase-1 operon protein HyaE
MANDTPFSALWQRLLTRGWQPVEASTVDDWIKRVGDGVILLSSDPRRTPEVSDNPVMIAE
LLREFPQFDWQVAVADLEQSEAIGDRFNVRRFPATLVFTDGELRGALSGIHPWAELLTLM
RSMVDTPAAQETVQ
>EECEBICD_02169 hypothetical protein
MSNAFFHLLGPGTQPDDASFSMNPLPLTCQVNGDPSMAALERCAHSPAVMALLTDLRGQL
ARRIPEVGDVLGWELSPLNADDLSFLNTLLGEGEVSVRIQHPDGSESEIQETIFCGLWRV
RHLHNRRLLTDRLEAGSAPLTLWQAATADTLPDDSLLPPPVAGLMNGLPLAHELLAHVRD
PALQPHSINLTQLPLSEADRLFLARLCGHGNIQIRISGYGESQINATALRHLWHVRCLDA
LKGPLLDSYEICLLPELVLAAPEDLADSRQRLDEVCRWLETR
>EECEBICD_02170 Cytochrome bd-II ubiquinol oxidase subunit 1
MWDVIDLSRWQFALTALYHFLFVPLTLGLIFLLAVMETIYVVTGKTVYRDMTRFWGKLFG
INFALGVATGLTMEFQFGTNWSLYSNYVGDIFGAPLAMEALLAFFLESTFVGLFFFGWQR
LNKYQHLLVTWLVAFGSNISALWILNANGWMQYPTGAHFNIDTLRMEMSSFSDLVFNPVS
QVKFVHTVMSGYVTGAMFIMSISAWYLLRGREREVALRSFAIGSVFGTLAILGTLQLGDS
SAYEVAQIQPVKLAAMEGEWQTEPAPAPFHLIAWPQQEQERNAFAVKIPALLGILATHSL
DTPVPGLKNLMDDALPRLKRGREAWLLMQEIAQGNRSPQVLNAFHAVEGDLGYGILLAKY
APDMNHVTPEQYRAAQRGAIPQVAPVFWSFRIMVGCGSLLLVVMLIALIQTLRMRIDQHR
WVLRMTLWSLPLPWIAIEAGWFMTEFGRQPWAIQDILPTWYAHSALTPGQLAFSMGLILG
LYTLFLIAEVYLMQKYARLGPSAMQHQQQAQQQG
>EECEBICD_02171 Cytochrome bd-II ubiquinol oxidase subunit 2
MLDYETLRFIWWLLIGVILVAFMVTDGFDMGVGCLLPLIARNDDERRVLINSVGAHWEGN
QVWLILAGGALFAAWPRVYAAAFSGFYVAMILVLCALFFRPLAFDYRGKIANARWRALWD
TGLVIGSLVPPVVFGIAFGNLFLGVPFAFTPQLHVDYFGTFWQLLSPFALLCGLLSLSLV
IMQGGVWLQLKTEGVIRQRALSATRHSALLIVICFLLAGYWLWAGVDGFVLLTQDANGPS
NPLLKGVAILPGAWMNHFIRSPLLLIIPLLGMILPILAFYACLRGQTIRGFLFASLTQAC
VIFTAGITLFPFVMPSSVSPLSSLTVWDSTSSQMTLEIMLVIVLIFLPIVLLYTLWSYYK
MLGRINLETLRRNDHELY
>EECEBICD_02172 Putative cytochrome bd-II ubiquinol oxidase subunit AppX
MWYLLWFVGILLMCSLSTLALVWLESRQQ
>EECEBICD_02173 Glutamate dehydrogenase

MDKLSYASDSSTSAWNTYLQQIERVAPYLGELSPWVDTLRHPKRALIVDIPVQMDDGTIR
HFEGYRVQHNLSRGPGKGGVRYHPDVDLNEVMALSAWMTIKCAALNLPYGGAKGGIRVDP
FSLSEGELERLTRRYTSEIGIIIGPQKDIPAPDVGTNGKVMAWMMDTYSMNHGTTVTGVV
TGKPIHLGGSLGREKATGRGVFVSGLEAARRANIAVEGARVAVQGFGNVGSEAARLFAGA
GARVVAIQDHTATLFNATGIDMKALTAWQTEHKQIAGFPGAETIASDAFWRLEMDILIPA
ALEGQITRQRAEALTCKLVLEGANGPTYPDADDVLASRGILVVPDVVCNAGGVTVSYFEW
VQDMASFFWSEEEINARMDKIMTDAIVHVWEKAAEKSCSLRTAAYIVACERILLARKDRG
IYPG
>EECEBICD_02174 Periplasmic trehalase
MIPPEIRRSVLLQKAIKLALAGTLLTFASFSATAADPSSDTETPQPPDILLGPLFNDVQN
AKLFPDQKTFADAIPNSDPLMILADYRMQRNQSGFDLRHFVDVNFTLPKAGEKYVPPAGQ
SLREHIDGLWPVLTRSTKNVEKWDSLLPLPESYVVPGGRFREIYYWDSYFTMLGLAESGH
WDKVADMVANFGYEIDAWGHIPNGNRTYYLSRSQPPFFAFMVELLAQHEGDDALKEYLPQ
LQKEYAYWMEGVETLQPGQQNQRVVKLEDGSVLNRYWDDRDTPRPESWVEDIATVKSNPN
RPATEIYRDLRSAAASGWDFSSRWMDNPQQLSTIRTTTIVPVDLNALLYQLEKTLARASA
AAGDRAKASHYDALANARQKAIEMHLWNNKEGWYADYDLKNNKIRDQLTAAALFPLYVNA
AAKDRAAKVAAAAQAHLLQPGGLATTSVKSGQQWDAPNGWAPLQWVAAEGLQNYGQDDVA
MEVTWRFLTNVQHTYDREKKLVEKYDVSSTGTGGGGGEYPLQDGFGWTNGVTLKMLDLIC
PQEKPCDSVPSTRPASLSATPTKTPSAATQ
>EECEBICD_02175 hypothetical protein
MGIIAWIVFGLIAGVIAKLLMPGRDGGGFILTCILGIVGAVVGGWLATMFGIGGSISGFN
LHSFLVAVVGAIMVLVIFRLLRRG
>EECEBICD_02176 Flagellar brake protein YcgR
MSGYNEQFLKKNPLAILGVLRDLNKNQVPLRISWAHGQFISKILAVDPEKLIVDYGSQEY
ENSAVLRAGQVAIIAETQGAKVEFTLPQLVTGEYQRLPAFITPLPSSLWFVQRREYFRIG
APLYPPYYGVTTLPDTRTLRFRLFDLSLGGMGALLESAIPDGLIEGARFSQVELNMGPWG
IFHVDAQLIAISERKVIDGKNETITTPRLSFRFLNVSPAVERELQRIIFSLEREARERAN
KVRE
>EECEBICD_02177 Endo-type membrane-bound lytic murein transglycosylase A
MKLRWFAFLVVILAGCSSKQDYRNPPWNAEVPVKRAMQWMPISEKAGAAWGVDPHLITAI
IAIESGGNPNAVSKSNAIGLMQLKASTSGRDVYRRMGWRGEPTTSELKNPERNISMGAAY
LSILENGPLAGIKDPQVMQYALVVSYANGAGALLRTFSSDRKKAIEKINDLDADEFFEHV
VDNHPAPQAPRYIWKLQQALDAM
>EECEBICD_02178 Murein tetrapeptide carboxypeptidase
MSLFHLIAPSGYCINQQAALRGVQRLTDAGHQVENDEVIRRRFQRFAGTDAERLADVNSL
ASLTSPDTIVMPVRGGYGASRLLDRIDWQALASRQQRDPLLICGHSDFTAIQAGLLAQAN
VITFSGPMLAANFGAETLNTFTEQHFWLALRKAQFTVEWQGDGPQCDVQGTLWGGNLAML
ISLIGTPWMPTIDKGILVLEDVNEHPFRVERMLLQLEYAGILNRQSAIVLGSFSGAAPNE
YDAGYSLESVYAFLRSRLSVPLITGLDFGHEQRTVTLPIGANATLKNTRQGTQLTLSGHP
TLQL
>EECEBICD_02179 K(+)/H(+) antiporter NhaP2
MDAATIISLFILGSILVTSSILLSSFSSRLGIPILVIFLAIGMLAGVDGIGGIPFDNYPF
AYMVSNLALAIILLDGGMRTQASSFRVALGPALSLATLGVLITSGLTGMMAAWLFHLDLI
EGLLIGAIVGSTDAAAVFSLLGGKGLNERVGSTLEIESGSNDPMAVFLTITLIEMIQKHE
TGLDWMFAVHIIQQFGLGIVFGLGGGYLLQQMINRISLPSGLYPMLALSGGILIFALTTA
LEGSGILAVYLCGFLLGNRPIRNRYGILQNFDGLAWLAQIAMFLVLGLLVTPSDLWPIAV
PALILSIWMIFFARPLSVFTGLLPFRGFNLRERIFISWVGLRGAVPIILAVFPMMAGLEN
ARLFFNVAFFVVLVSLLLQGTSLSWAAKRAKVVVPPVGWPVSRVGLDIHPDNPWEQFIYQ
LSADKWCVGAALRDLHMPNETRIAALFRNNELFHPTGSTRLQEGDVLCVIGRERDLPALG
KLFSQSPPVSLDQRFFGDFILEANAKFADVALIYGLEEGTEYRDKQQTLGEIIQQLLGAA
PVVGDQVEFGGMIWTVAEKEDNVVRKIGVRVAEDEAE
>EECEBICD_02180 Alanine racemase
MAEQAGVTFRFNTPVEKLLYENDQIYGVKCADEIIKADAYVMAFGSYSTAMLKGIVDIPV
YPLKGYSLTIPIVEPDGAPVSTILDETYKIAITRFDKRIRVGGMAEIVGFNTDLLQPRRE

TLEMVVRDLFPRGGHIEQATFWTGLRPMTPDGTPVVGRTRYKNLWLNTGHGTLGWTMACG
SGQLLSDILSGRTPAIPYDDLSVARYRSDFTPTPRNVCIARITDKGSEMTRPIQASLDLQ
VMKQNLAIVRRAAPEARVWSVVKANAYGHGIERVWSALGATDGFAMLNLEEAITLRERGW
KGPILMLEGFFHAQDLEAYDTYRLTTCIHSNWQLKALQNARLNAPLDIYVKVNSGMNRLG
FQPERAQTVWQQLRAMRNVGEMTLMSHFAQADHPEGIGEAMRRIALATEGLQCSYSLSNS
AATLWHPQAHYDWVRPGIILYGASPSGQWRDIADTGLKPVMTLSSEIIGVQTLNAGERVG
YGGGYSVTQEQRIGIVAAGYADGYPRHAPTGTPVLVDGIRTRTVGTVSMDMLAVDLTPCP
QAGIGTPVELWGKEIKVDDVASAAGTLGYELLCAVAPRVPFVTT
>EECEBICD_02181 D-amino acid dehydrogenase
MRVVILGSGVVGVTSAWYLSQAGHDVTVIDRESGPAQETSAANAGQISPGYAAPWAAPGV
PLKAIKWMFQRHAPLAVRLDGTPFQLKWMWQMLRNCDTRHYMENKGRMVRLAEYSRDCLK
TLRAATGIEYEGRQGERYNSSAPRNSMRTPPVISPCWKMPGCRISSWNPADWRKLNPLWL
RLRIN
>EECEBICD_02182 hypothetical protein
MAMIDSMNKDTTRLSDGPDWTFDLLDVYLAEIDRVAKLYQLDTYPHQIEVITSEQMMDAY
SSVGMPINYPHWSFGKKFIETERLYKHGQQGLAYEIVINSNPCIAYLMEENTITMQALVM
AHACYGHNSFFKNNYLFRSWTDASSIVDYLIFARNYITRCEERYGVDEVEKLLDSCHALM
NYGVDRYKRPQKISLQEEKARQKSREEYLQSQVNMLWRTLPKREEEKTVAEARRFPAEPQ
ENLLYFMEKNAPLLEPWQREILRIVRKVSQYFYPQKQTQVMNEGWATFWHYTILNHLYDE
GKVTERFMLEFLHSHTNVVFQPPYNSPWYSGINPYALGFAMFQDIKRICQSPTDEDKYWF
PDIAGSDWLETLHFAMRDFKDESFISQFLSPKVMRDFRFFTVLDDDRHNYLEISAIHNEE
GYREIRSKLSSQYNLSNLEPNIQVWNVDLRGDRSLTLRYIPHNRAPLDKGRKEVLKHVHR
LWGFDVMLEQQNEDGSVELLERCPPRMNTL
>EECEBICD_02183 Fatty acid metabolism regulator protein
MVIKAQSPAGFAEEYIIESIWNNRFPPGTILPAERELSELIGVTRTTLREVLQRLARDGW
LTIQHGKPTKVNNFWETSGLNILETLARLDHESVPQLIDNLLSVRTNISTIFIRTALRQH
PDKAQEVLATAHEVADHADAFADLDYNIFRGLAFASGNPIYGLILNGMKGLYTRIGRHYF
ANPEARSLALGFYHKLSSLCEQGAHDQVYETVRRYGHDSGEIWHRMQKNLPGDLAIQGR
>EECEBICD_02184 Na(+)/H(+) antiporter NhaB
MEISWGRAMWRNFLGQSPDWYKLALLVFLIVNPFIFLANPFIAGWLLVAEFIFTLAMALK
CYPLLPGGLLAIEAVIIGMTSAAHVREEVAANLEVLLLLMFMVAGIYFMKQLLLFIFTRL
LLSIRSKMVLSLAFCVAAAFLSAFLDALTVVAVVISVAVGFYGIYHRVASSRGEENDMLD
DSHIDPHYKTVLEQFRGFLRSLMMHAGVGTALGGVMTMVGEPQNLIIAKAAGWHFGDFFL
RMSPVTVPVLVCGLLTCMLVEKMRWFGYGETLPEKVRDVLQQFDDQSRKKRTRQDKIKLI
VQAVIGVWLVTALALHLAEVGLIGLSVIILATALTGVTDEHAIGKAFTESLPFTALLTVF
FSIVAVIIDQHLFAPIIQFVLQASEHAQLTLFYLFNGLLSSISDNVFVGTIYINEAKAAM
ENGAISLKQFELLAVAINTGTNLPSVATPNGQAAFLFLLTSALAPLIRLSYGRMVWMALP
YTIVLTLIGLLCVEFTLAPATEWMTQAGWLATLS
>EECEBICD_02185 Disulfide bond formation protein B
MLRFLNQCSRGRGAWLLMAFTALALEMVALWFQHVMLLKPCVLCIYERCALFGVMGAGLV
GAIAPKTPLRYVAMVIWIYSAWRGLQLAYEHTMIQLHPSPFMTCDFMARFPDWLPLGKWL
PQVFVASGDCAERQWSFLTLEMPQWLLGIFAAYLVVAIAVVIAQAFKPKKRDLFGR
>EECEBICD_02186 putative protein YeaR
MSHLRIPANWKVKRSTPFFTKENVPAALLSHHNTAAGVFGQLCVMEGTVTYYGFANETAT
EPEVKVVINAGQFATSPPQYWHRVELSDDARFNIHFWVEEDHQGEEMYQQKKA
>EECEBICD_02187 Protein GnsA
MNSEELTHKAEEEIAALISKKVAELRKKTGQEVSEIEFAPRETMKGLEGYHVKIKLL
>EECEBICD_02188 hypothetical protein
MSERPDLVDPLPEDEPLPGENIPDTPEGDDPLDPDTQNEVEDPRR
>EECEBICD_02189 hypothetical protein
MSDTPFWQRKTLDEMTDAEWESLCDGCGQCCLHKLMDEDTDEIYFTNVACRQLNIKTCQC
RHYERRFEFEPDCIKLTRENLPDFEWLPMTCAYRLLAEGKPLPTWHPLLTGSKAAMHGER
ISVRHIAVKESEVRDWQDHILNKPSWAE
>EECEBICD_02190 putative protein YcgM

MYQHRNWQGALLDYPVSKVVCVGSNYANHIKEMGSATPEEPVLFIKPETALCDIRQPLAI
PADMGAVHHEVELAVLIGATLRQATEDHVRKAIAGYGVALDLTLRDIQGKMKKAGQPWEK
AKGFDNACPISGFIPVAAFCGDPQNTVIGLNVNGEVRQQGSTADMIHPIVPLIAYMSRYF
TLKAGDVVLTGTPAGVGPLLSGDELDIRFNGETLSTRVL
>EECEBICD_02191 Protein YcgL
MFCVIYRSSKRDQTYLYVEKKDDFSRVPEALMKGFGQPQLAMMLPLDGRKKLVNAELEKV
KQALSEQGYYLQLPPPPEDLLKQHLSSVGQNTSPADR
>EECEBICD_02192 Septum site-determining protein MinC
MSNTPIELKGSSFTLSVVHLHEAEPEVIRQALEDKIAQAPAFLKHAPVVINVSGLESPVN
WPELHKIVTSTGLRIIGVSGCKDTSLKVEIDRMGLPLLTEGKEKAVRPAPVEPATPSEPP
QNANPITKTRLIDVPVRSGQRIYAPQCDLIVTSHVSAGAELIADGNIHVYGMMRGRALAG
ASGDREAQIFCTHLTAELVSIAGVYWLSDKIPAEFYGKAARLRLADNALTVQPLN
>EECEBICD_02193 Septum site-determining protein MinD
MARIIVVTSGKGGVGKTTSSAAIATGLAQKGKKTVVIDFDIGLRNLDLIMGCERRVVYDF
VNVIQGDATLNQALIKDKRTENLFILPASQTRDKDALTREGVAKVLDSLKAMDFEFIVCD
SPAGIETGALMALYFADEAIITTNPEVSSVRDSDRILGILASKSRRAENGEEPIKEHLLL
TRYNPGRVNKGDMLSMEDVLEILRIKLVGVIPEDQSVLRASNQGEPVILDATADAGKAYA
DTVDRLLGEERPFRFIEEEKKGFLKRLFGG
>EECEBICD_02194 Cell division topological specificity factor
MALLDFFLSRKKSTANIAKERLQIIVAERRRSDAEPHYLPQLRKDILEVICKYVQIDPEM
VTVQLEQKDGDISILELNVTLPEAEESK
>EECEBICD_02195 Ribonuclease D
MIETDDALASLCEAVRACPAIALDTEFVRTRTYYPQLGLIQLFDGANVALIDPLGISDWS
PLKAVLRDTGITKFLHAGSEDLEVFLNAFGELPEPLIDTQILAAFCGRPLSWGFAAMVEE
YTGVALDKSESRTDWLARPLSERQCEYAAADVWYLLPIAKKLMIETEAAGWLPAALDECR
LMQQRRQEIQAPEEAWRDITNAWQLRTRQLACLQLLADWRIRKARERDMAVNFVVREENL
WAVARYMPGSLGELDSLGLSGSEIRFHGKTLISLVAKAQALPEEALPEPLLNLMDMPGYR
KAFKAIKALVAEVSASHHVSGELLASRRQINQLLNWHWKLKPQNGQPELISGWRAELMAE
KLTLLLQEYPR
>EECEBICD_02196 Long-chain-fatty-acid--CoA ligase
MLTTCLLTGVVMTTNTHFRGDALKKVWLNRYPADVPAEINPDRYQSLVELFEHAATRYAD
QPAFVNMGEVMTFRKLEERSRAFAAYLQQGLGLKKGDRVALMMPNLLQYPVALFGILRAG
MIVVNVNPLYTPREPEHQLNDSGAAAIIIVSNFAHTLEKVVEKTSVQHVILTRMGDQLST
AKGTVVNFVVKYIKRLVPKYHLPDAISFRSALQHGYRMQYVKPEVVAEDLAFLQYTGGTT
GVAKGAMLTHRNMLANLEQVKATYGPLLHPGKELVVTALPLYHIFALTMNCLLFIELGGQ
NLLITNPRDIPGLVKELAKYPFTAMTGVNTLFNALLNNKEFQQLDFSSLHLSAGGGMPVQ
NVVAERWVKLTGQYLLEGYGLTECAPLVSVNPHDIDYHSGSIGLPVPSTEAKLVDDDDNE
VAPGEAGELCVKGPQVMLGYWQRPDATDEIIKDGWLHTGDIAVMDEDGFLRIVDRKKDMI
LVSGFNVYPNEIEDVVMQHSGVQEVAAVGVPSGSSGEAVKLFVVKKDPALTDDALITFCR
RHLTGYKVPKQVEFREELPKSNVGKILRRELRDEARGKVDNKA
>EECEBICD_02197 Outer membrane protein Slp
MAVQKRLIKGALACAFALMLSGCVTIPDAIKGSSPTPQQDLVRVMNAPQLYIGQEARFGG
KVVAVQNQQGKTRLEIATVPLDSGARPVLGEASRGRIFADVNGFLDPVDFRGQLVTVVGP
ITGTVDGKIGNTPYKFMLMQATGYKRWHLVQQVVMPPQPIDPWFYGGRAWPYGYGGWGWY
NPGPAEVQTIVTE
>EECEBICD_02198 tRNA threonylcarbamoyladenosine biosynthesis protein TsaB
MRILAIDTATEACSVALWNNGTINAHFELCPREHTQRILPMVQEILAASGVSLNEIDALA
FGRGPGSFTGVRIGIGIAQGLALGANLPMIGVSTLATMAQGAWRKTGATRVLAAIDARMG
EVYWAEYQRDAQGVWQGEETEAVLKPERVGERLKQLSGEWATVGTGWSAWPDLAKECGLT
LHDGEVSLPAAEDMLPIASQKLAAGETVAVEHAEPVYLRNEVAWKKLPGKE
>EECEBICD_02199 putative ATP-dependent DNA helicase YoaA
MQDQLYSRDLPTVAKALAFTGKTALLKGRSNYLCLERLEQQALAGGDLPVQTLSDVILLR
SWSNQTRDGDISTCVSVAEDSQAWPLVTSTNDNCLGSDCPLYKECFVVKARKKAMDADVV
IVNHHLFLADMVVKESGFGELIPQAEVMIFDEAHQLPDIASQYFGQSLSSRQLLDLAKDI

TIAYRTELKDTQQLQKCADRLAQSAQDFRLQLGEPGYRGNLRELLADSRIQRAFLLLDDT
LELCYDVAKLSLGRSALLDAAFERATLYRSRLKRLKEINQPGYSYWYECTSRHFTLALTP
LTVADKFKEVMEQKPGSWIFTSATLSVNDDLHHFTARLGIEQAESMLLPSPFDYTRQALL
CVPRNLPQTNQPGAARQLAAMLRPIIEANNGRCFMLCTSHAMMRDLAEQFRATMTLPVLL
QGETSKGQLLQQFVSAGNALLVATSSFWEGVDVRGDTLSLVIIDKLPFTSPDDPLLKARM
EDCRLRGGDPFDEVQLPDAVITLKQGVGRLIRDADDRGVLVICDNRLVMRPYGATFLASL
PPAPRTRDIARAVRFLAIPSAE
>EECEBICD_02200 2-iminobutanoate/2-iminopropanoate deaminase
MSIVRIDAEDRWSDVVIYNNTLWYTGVPENLDADAFEQTANTLAQIDAVLEKQGSSKSRI
LDATIFLSDKADFAAMNKAWDAWVVAGHAPVRCTVQAGLMNPKYKVEIKIVAAV
>EECEBICD_02201 hypothetical protein
MFAGLPSLSHEQQQKAVERIQELMSQGMSSGEAIAQVAGELRANHTGERIVARFEDEDE
>EECEBICD_02202 Aminodeoxychorismate synthase component 1
MMKTLSPTVITLPWRPDAAEHYFAPVNHLPWAMLLHSGDAIHPYNRFDILVADPVTTLTT
RAQETTVCTARTTTVTFDDPLHVLQTQLEALPFHPQPDPDLPFQGGALGLFGYDLGRRFE
ILPDTAARDIALPDMAIGLYDWALIVDHQKQVVSLISYHDADARYRWLTSQHAPTRTPFR
LTSAWQSNMTRCEYGEKFRQVQAWLHSGDCYQVNLSQRFQASYEGDEWQAFEHLNRANRA
PFSAFLRLHDGAILSLSPERFIQLENGHIQTRPIKGTLPRLNDPQADRQQAQKLANSMKD
RAENLMIVDLMRNDIGRVAVPGSVKVPELFVVEPFPAVHHLVSTITARLPDSLHATDLLR
AAFPGGSITGAPKVRAMEIIDELEPQRRNAWCGSIGYLSFCGKMDTSITIRTVTATQGQL
YCSAGGGIVADSKEEAEYQETFDKVNRILHPLEN
>EECEBICD_02203 putative Nudix hydrolase NudL
MDTSRLTLDHFLSRFQLLRPQMTHETLNQRQAAVLIPVVRRPQPGLLLTQRAIHLRKHAG
QVAFPGGAVDSTDASLIAAALREAQEEVAIPPQAVEVIGVLPPVDSVTGFQVTPVVGIIP
PNLPWRASEDEVSAVFEMPLAQALQLGRYHPLDVYRRGNSHRVWLSWYEHYFVWGMTANI
LRELALQIGVKP
>EECEBICD_02204 L-serine dehydratase 1
MISLFDMFKVGIGPSSSHTVGPMKAGKQFVDDLVEKGLLDNVTRVAVDVYGSLSLTGKGH
HTDIAIIMGLAGNEPATVDIDSIPGFIRDVETRERLLLAQGRHEVDFPKDDGMRFHNGNL
PLHENGMQIHAWHDDTVIYSKTYYSIGGGFIVDEAHFGQEATNEVTVPYPFKSAKEMLEY
CNSTGLSLSGMVMQNELALHSKKEIEEYFGHVWQTMQACIDRGMNTEGVLPGPLRVPRRA
SALRRMLVSSDKLSNDPMNVIDWVNMFALAVNEENAAGGRVVTAPTNGACGIVPAVLAYY
DHFIESVSPDIYTRYFLAAGAIGALYKMNASISGAEVGCQGEVGVACSMAAAGLAELLGA
SPEQVCVAAEIGMEHNLGLTCDPVAGQVQVPCIERNAIASVKAINAARMAMRRTSAPRVS
LDKVIETMYETGKDMNAKYRETSRGGLAIKVQCD
>EECEBICD_02205 putative cyclic di-GMP phosphodiesterase PdeD
MQTAQRIINHYRRNRFIVCTICALVTLILTLSIRFISERNLNHHRTVAFANHAVDALDNV
LHPLQVGRNVLLPLLELPCATAHLPLRKQAARLQTVRSIGLVKEGILYCSSIFGARNTPI
RQLQPDLPAVGDLLLLSTDQSLLKGSPILIQWYPASADGQDGVMEIVNIDLLTTMLLEPQ
QPQITSASLTVGKRHLLYGRGVVDTLPELKKDEERYQLSSRHFPFTISVSGPSAGVLAFK
HLPTQLPLAVLLSLLIGYIAWLATASRMSFSWEINLALAEREFELFCQPLLNARTQQCTG
VEILLRWNNPRQGWISPDVFIPIAEEHNLIAPLTRYVIAETIRQRHYFPINHQFHIGINV
AASHFRHGVLLKDLNQYWFSAEPVQQLVLELTERDALLDVDYRLMRELHRKGVKLAIDDF
GTGNSSLSWLEKLRPDILKIDKSFTAAIGTDAVNSTVTDIIIALGQRLNIELVAEGVENQ
TQAQHLRQHGVQMLQGYLYAKPMPISEFPQWLAGSAPPPARHNGQIMSAMPHL
>EECEBICD_02206 hypothetical protein
MELLMDPSIWVGLLTLVVLEIVLGIDNLVFIAILADKLPPKQRDKARLIGLSLALIMRLA
LLSIISWLVTLTKPLFTVWDFTFSGRDLIMLLGGIFLLFKATTELHERLENREHDTGHGK
GYASFWVVVTQIVILDAVFSLDAVITAVGMVNHLPVMMAAVVIAMAVMLLASKPLTRFVN
QHPTVVVLCLSFLLMIGLSLVAEGFGFHIPKGYLYAAIGFSIVIEVFNQIARRNFIRHQS
TLPLRARTADAILRLMGGKRQASTQHETDSPAAISVSEGAFAEEERYMINGVLTLASRSL
RGIMTPRGEISWVDANLSVAEIRQQLLSSPHSLFPVCRGELDEIIGIVRAKELLVALEEG
ADVAAIAASSPAIVVPETLDPINLLGVLRRARGSFVIVTNEFGVVQGLVTPLDVLEAIAG
EFPDADETLEIVADAEGWIVKGGTDLHALQQALEVDHLVNEEGDIATVAGLVIAANGHIP

RVGDVIDVPPLRITIVEANDYRVDLVRIVKEQPAHDEEE
>EECEBICD_02207 PTS system mannose-specific EIIAB component
MTIAIVIGTHGWAAEQLLKTAEMLLGEQENVGWIDFVPGENAETLIEKYNAQLAKLDTSK
GVLFLVDTWGGSPFNAASRIVVDKERYEVIAGVNIPMLVETFMARDDDPSFDELVALAVE
TGREGVKALKAKPVEKAAPAPVAAAPKAATPAKPMGPNDYMVIGLARIDDRLIHGQVATR
WTKETNVSRIIVVSDEVAADTVRKTLLTQVAPPGVTAHVVDVAKMIRVYNNPKYAGERVM
LLFTNPTDVERIVEGGVKVTSVNIGGMAYRQGKTQVNNAVSVDEKDIEAFKKLNERGIEL
EVRKVSTDPKLKMMDLIAKVAK
>EECEBICD_02208 PTS system mannose-specific EIIC component
MEITTLQIVLVFIVACIAGMESVLDEFQFHRPLIACTLIGAVLGDMKTGIIIGGTLEMIA
LGWMNIGAAVAPDAALASIISTVLVIAGHQSIGAGIALAIPLAAAGQVLTIIVRTITVAF
QHAADKAAENGNLTALSWLHVSSLFLQAMRIAIPAVIVAISVGTSEVQGMLNAIPEVVTG
GLNIAGGMIVVVGYAMVINMMRAGYLMPFFYLGFVTAAFTNFNLVALGVIGAVMAILYIQ
LSPKYNRVAGAPATAAGNNDLDNELD
>EECEBICD_02209 PTS system mannose-specific EIID component
MVDMTKTTTEKKLTPSDIRGVFLRSNLFQGSWNFERMQALGFCFSMVPAIRRLYPENNDA
RKQAIKRHLEFFNTHPYVAAPVLGVTLAMEEKRANGAEIDDGAINGIKVGLMGPLAGVGD
PIFWGTVRPVFAALGAGIAMSGSLLGPLLFFILFNLVRLATRYYGVAYGYRKGVDIVKDM
GGGFLQKLTEGASILGLFVMGALVNKWTHVNIPLVVSTITGQDGQTRVTTVQTILDQLMP
GLVPLLLTFACMWLLRKKVNPLWIIVGFFVIGIAGYSVGLLGQ
>EECEBICD_02210 hypothetical protein
MTITDLVLILFIAALLAYALYDQFIMPRRNGPTLLSIALLRRGRVDSVIFVGLVAILIYN
NVTSHGAQMTTWLLSALALMGFYIFWIRTPRIIFKQRGFFFANVWIEYNRIKEMNLSEDG
VLVMQLEQRRLLIRVRNIDNLEKIYKLLIENQ
>EECEBICD_02211 putative manganese efflux pump MntP
MHFTATVLLAFGMSMDAFAASIGKGATLHKPKFSEALRTGLIFGAVETLTPLIGWGLGIL
ASKFVLEWNHWIAFVLLIFLGGRMIIEGIRGGSDEDETPLRRHSFWLLVTTAIATSLDAM
AVGVGLAFLQVNIIATALAIGCATLIMSTLGMMIGRFIGPMLGKRAEILGGVVLIGIGVQ
ILWTHFHG
>EECEBICD_02212 23S rRNA (guanine(745)-N(1))-methyltransferase
MSFTCPLCHQPLTQINNSVICPQRHQFDVAKEGYINLLPVQHKRSRDPGDSAEMMQARRA
FLDAGHYQPLRDAVINLLRERLDQSATAILDIGCGEGYYTHAFAEALPGVTTFGLDVAKT
AIKAAAKRYSQVKFCVASSHRLPFADASMDAVIRIYAPCKAQELARVVKPGGWVVTATPG
PHHLMELKGLIYDEVRLHAPYTEQLDGFTLQQSTRLAYHMQLTAEAAVALLQMTPFAWRA
RPDVWEQLAASAGLSCQTDFNLHLWQRNR
>EECEBICD_02213 Peptidoglycan D,D-transpeptidase FtsI
MKKKSDGDTRNFTPIRFALLCTAILLSMALLLGRVAWLQIVTPSKLVKQEDMRSLREVTT
ASPRGMITDREGRPLAVSVPVNAVWADPKTIISKGGVGYNERWQALASALHLSLSTLAER
VNSNPAGRFIYLARQVSPQQAEWIDKLNLPGINLREESRRFYPAGHVAANLIGFTNIDGQ
GIEGIEKSFNAQLTGKPGSRLVRKDKFGHVIENITEVNPVPAHELQLSIDERLQTVTEDA
LDNAVIWNKAESGAAVLINIPTGEILSMASYPDFNPNNREGAQLDDFRNRAISDTFEPGS
TVKPLVIMTALQQGIVQPDSVIDTHPFTLDGHRIRDVGYYPELTLTGILQKSSDTGVSHL
SLAMPVQKLMDTYKSFGFGVPTGLGLTGESSGLLPKRRYWSDLDRATFAFGYGLMVTPLQ
LAHVYATIGSFGIYRPLSITKVDPPVIGTRVMPEELVHEVEHMMESVALPGGGGTKAAVR
DYRIAVKTGTAKKIGDDGKYVDKYVAYTAGVAPASNPRFALVVVINNPQNGAYYGGAVSA
PVFSQIMGDVLRLENVEPDGMPADSDHLLVMHGSHVAVPGS
>EECEBICD_02214 Cold shock-like protein CspC
MAKIKGQVKWFNESKGFGFITPADGSKDVFVHFSAIQGNGFKTLAEGQNVEFEIQDGQKG
PAAVNVTAI
>EECEBICD_02215 hypothetical protein
MNDVLNSGAFSLASLIVSMVVLVVGLALWFFVNRASSRANEQIELLEALLDQQKRQNALL
RRLCEANEPEKEAEPATAASEPKEDEDIIRLVAER
>EECEBICD_02216 hypothetical protein
MRLIIRAIVLFALVWIGLLMSGYGILVGSKVNAAGLGLQCHYLTARGTSTAQYLHTNSGI

IGFSDCPIFRKIATVVDNG
>EECEBICD_02217 Transcriptional regulator KdgR
MANADLDKQPDSVSSVLKVFGILQALGEEREIGITELSQRVMMSKSTVYRFLQTMKTLGY
VAQEGESEKYSLTLKLFELGARALQNVDLIRSADIQMRELSRLTKETIHLGALDEDSIVY
IHKIDSMYNLRMYSRIGRRNPLYSTAIGKVLLAWRDRDEVKQILDGVEYKQSTGRTITST
EALLPLLDEVRAQGYGEDNEEQEEGLRCIGVPVFDRFGVVIAGLSISFPTLRFSEERLQE
YVAMLHAAARKISEQMGYNDYPF
>EECEBICD_02218 Riboflavin transporter RibZ
MEKTRADGLPLPQRYGAILTIIIGISMAVLDGAIANVALPTIATDLHASPASSIWIVNAY
QIAIVVSLLSLSFLGDMFGYRRIYKCGLVVFLLSSLFCALSDSLQMLTLARIAQGFGGAA
LMSVNTALIRLIYPQRHLGRGMGINSFIVAVSSAAGPTIAAAILSISSWKWLFLINVPLG
IIALILAIRFLPANIAHDTKPRFDLPSAVMNALTFGLLITALSGFAQGQSLTLIGAELLV
LVVVGFFFVRRQLSLPVPLLPIDLLRIPLFSLSIGTSICSFCAQMLAMVSLPFYLQTVLG
RSEVETGLLLTPWPLATMVMAPLAGYLIERLHAGLLGALGMVIMAAGLFALVMLPASPLR
SEYYLADDPLWRRVWPVPVAE
>EECEBICD_02219 hypothetical protein
MILCGAGFGLFQSPNNHTIITSAPRERSGGASGMLGTARLLGQSTGAALVALMLNQFGDS
GTHLSLLAAAILATLAAVVSGLRITQPRVQA
>EECEBICD_02220 Protease HtpX
MMRIALFLLTNLAVMVVFGLVLSLTGIQSSSVQGLLIMALLFGFGGSFISLLMSKWMALK
SVGGEVIEQPRNERERWLMNTVATQARQAGIAMPQVAIYHAPDINAFATGARRDASLVAV
STGLLQNMSPDEAEAVIAHEISHIANGDMVTMTLIQGVVNTFVIFISRIIAQIAAGFLGG
NRDEGEGSNGNPLIYFAVATVLELVFGILASIITMWFSRYREFHADAGSAKLVGREKMIA
ALQRLKTSYEPQEATSMMAFCINGKSKSLSELFMTHPPLDKRIEALRSGEYLK
>EECEBICD_02221 Tail-specific protease
MNTFFRLTALAGLLALAGQSFAVEDITRADQIPVLKEETQHATVSERVTSRFTRSHYRQF
DLDEAFSAKIFDRYLNLLDYSHNVLLASDVEQFAKKKTVLGDELRTGKLDVFYDLYNLAQ
KRRFERYQYALKVLERPMDFTGNDTFNLDRSKAPWPKDEAELNALWDGKVKFDELSLKLT
GKSDKEIRETLTRRYKFAIRRLAQTNSEDVFSLAMTAFAREIDPHTNYLSPRNTEQFNTE
MSLSLEGIGAVLQMDDDYTVINSLVAGGPAAKSKSISVGDRIVGVGQAGKPMVDVIGWRL
DDVVALIKGPKGSKVRLEILPAGKGTKTRIITLTRERIRLEDRAVKMSVKTVGKEKVGVL
DIPGFYVGLTDDVKVQLQKLEKQNVNSIVIDLRSNGGGALTEAVSLSGLFIPSGPIVQVR
DNNGKVREDSDTDGVVYYKGPLVVLVDRFSASASEIFAAAMQDYGRALIVGEPTFGKGTV
QQYRSLNRIYDQMLRPEWPALGSVQYTIQKFYRVNGGSTQRKGVTPDIIMPTGNEETETG
EKFEDNALPWDSIDAAKYVKSDDLAPFGPELLKEHNARIAKDPEFQYIMKDIARFNAMKD
KRNIVSLNYAQREKENNEEDALRLARINDRFKREGKPLLKKLDDLPKDYQEPDPYLDETV
KIALDLAHLEKEKPAEQAAANK
>EECEBICD_02222 RNA chaperone ProQ
MENQPKLNSSKEVIAFLAERFPHCFSAEGEARPLKIGIFQDLVERVGGEMNLSKTQLRSA
LRLYTSSWRYLYGVKPGATRVDLDGNPCGELEEQHVEHARKQLEEAKARVQAQRAEQQAK
KREAAAAAGEKEDAPRRERKPRPVARRKEGAERKPRADKPTTKAPRAPREEKHTPVSDIS
VLTVGQSLKVKAGNNAMDATVLEITKDGVRVQLNSGMSLIVRAEHLVF
>EECEBICD_02223 Free methionine-R-sulfoxide reductase
MSKNAFNAYFNSLCLGVRPRSDYIMSKTELYAALNRDFQSLMAGETSFLATLANTSALLF
ERLTEVNWAGFYLLEGDTLVLGPFQGRIACVRIPVGRGVCGAAVAQNKVQRIDDVHAFDG
HIACDAASNAEIVLPVTVGERIIGVLDIDSTAFGRFTEEDEHGLRTLVAQLETVLATTDY
KKFFASVAG
>EECEBICD_02224 Intermembrane transport protein YebS
MALNTSHVTPTKKLTIRSISEALPRSHYQRCPECDMLFSLPEMSAHQSAYCPRCQAKIRD
GRDWSLTRLTAMAVTMLLLMPFAWSEPLLHIYLLGVRIDANVMHGIWQMTQQGDPLTAAM
VLFCVVGAPLILVFSIAYLWFGSLLGMNLRPVLLMLEKLKEWVMLDIYLVGIGVASIKVQ
DYAFLQPGIGLLAFVSLVVLSILTMIHLNVEQLWERFYPQRPAQRADERLRVCLGCHFSG
YPDAKGRCPRCHIPLRLRRKQSIQKCWAALLASIVFLLPANLLPISVIYINGGRQEDTIL
SGIMSLASSNIAVAAVVFIASILVPFTKVIVMFTLLLSIHFKCQQGLRTRILLLRLVTWI

GRWSMLDLFVISLTMSLINRDQILAFTMGPAAFYFGAAVILTILAVEWLDSRLLWDAHES
GNARFED
>EECEBICD_02225 Intermembrane transport protein YebT
MSQETPASKTEAQIKTKRRISPFWLLPLIALMIAGWLVWDSYQDRGNSVTIDFMSADGIV
PGRTPVRYQGVEVGTVEDVSLSKDLRKIEVRVSIKSDMEDALREETQFWLVTPKASLAGV
SGLDALVGGNYIGMMPGKGKPRDHFVALDTQPKYRLSNGDLMIHLNAPDLGSLNSGSLVY
FRKIPVGRVYDYSINPNKQGVTIDVLIERRFTDLVKKGSRFWNVSGIDADLSLSGAKVKL
ESLAALVNGAIAFDSPDNSKPAAQDDTFGLYKDLAHSQRGVIVKLELPSGDGLKAESTPL
MYQGLEVGELSKLTLNPGGKVTGEMTVDPSVVPLMRENTRIELRNPKLSLSDANISSLLT
GKTFELVPGDGEPRSEFVVVPGEKALLHEANALTLTLTAPESYGIEPGQPLILHGVKIGQ
VIERNLSSKGVSFTVAIEPQHRDLVQGDSKFVVNSRVDVKVGLDGVEFLGASASEWIDGG
IRILPGTSGKMKSTYPLYANLEKALENSLSDLPTTTLTLTAETLPDVQAGSVVLYRKFEV
GEVITVRPRANTFDIDLHIKPEYRHLLTSNSVFWAEGGAKVQLNGSGLTVQASPLSRALK
GAISFDNLSGASASRRKGDKRILYASETSARAVGGQITLHAFDAGKLAEGMPIRYLGIDI
GQIQTLELITARNEVQAKAVLYPEYVQTFARAGTRFSVITPQISAAGVEHLDTILQPYIN
VEPGRGAARRDFELQEATITDSRYLDGLSIVVEAPEAGSLNIGTPVLFRGIEVGTVTGMS
LGSLSDRVMITLRISKRYQYLVRNNSVFWLASGYSLDFGLTGGVVKTGTFNQFIRGGIAF
ATPPGTPLAPKAQAGKHFLLQESEPKEWREWGTALPR
>EECEBICD_02226 Ribosomal RNA small subunit methyltransferase F
MGYRSATLNTRLRRTRAGAFYATLRACFFAGDTPVAQHAVYFPDAFLTQMREAMPSTLSF
DEFISACQRPLRRSIRINTLKISVADFLALIAPYGWSLTPIPWCHEGFWIERDDEEALPL
GSTAEHLSGLFYIQEASSMLPVAALFADDNHPQRVMDMAAAPGSKTTQIAARMGNRGAIL
ANEFSASRVKVLHANISRCGIANTALTHFDGRVFGAALPEMFDAILLDAPCSGEGVVRKD
PDALKNWSPESNLDIAATQRELLNSAFHALRPGGTLVYSTCTLNRQENEEVCLWLKETYA
DAVEFLPLGDLFPDADRALTPEGFLHVFPQIYDCEGFFVARLRKMSSLPAMPAPGYKVGA
FPFTPSKAAKHYTLPRRPTPSDCYGMKISIFGSVKKRSGCSRQKLNH
>EECEBICD_02227 putative protein YebV
MMKTSVRIGAFEIDDAELHGESPGERTLTIPCKSDPDLCMQLDAWDAETSVPAILNGEHS
VLFRTHYDPKSDAWVMRLA
>EECEBICD_02228 hypothetical protein
MFALVLFVCYLDGGCEDIVVDIYDTEQQCLYSMDDQRIRHGGCFPVEDFIDGFWRPAQQY
SDF
>EECEBICD_02229 Serine/threonine-protein phosphatase 1
MMRPEEIYQRIEAKNWRHVWVVGDIHGCFSMLMKRLRECRFDPQQDLLVSVGDLIDRGPD
SLGCLALLRESWMMAVRGNHEQMALDARASLQSTLWLMNGGDWFTRLTAEHAAQAEALFI
LCQRLPWILEVRCRHSTHVIAHADYPASTYQWQRKVDLHQVLWSRERLINKRGGISGADH
FWFGHTPLRRRMDFANVHYIDTGAVFGGQLTLARIQ
>EECEBICD_02230 hypothetical protein
MPPLKKIVLRLFVGAMVATVTTPALALVCLEDHSAKECAISCAEAMWFMFPICF
>EECEBICD_02231 Guanine nucleotide exchange factor sopE2
MTNITLSTQHYRIHRSDVEPVKEKTTEKDIFAKSITAVRNSFISLSTSLSDRFSLHQQTD
IPTTHFHRGNASEGRAVLTSKTVKDFMLQKLNSLDIKGNASKDPAYARQTCEAILSAVYS
NNKDQCCKLLISKGVSITPFLKEIGEAAQNAELPGEIKNGVFTPGGAGANPFVVPLIASA
SIKYPHMFINHNQQVSFKAYAEKIVMKEVTPLFNKGTMPTPQQFQLTIENIANKYLQNAS
>EECEBICD_02232 putative protein YdfO
MELQLMLNHFFERVRKDANFNAFLIDLEYNNIAYYIYFVATGNVKIITHAGHFISIKSNR
KLIKVNSTPNTQLIKLISAKHFSGEHSYEKYCTDLATAGVFKWIVELNQKTRQYWSKDNQ
LLYIENVVMPL
>EECEBICD_02233 hypothetical protein
MSKDEISYQILYRYSLEKLYSTLARRVDNVLSFALVFLGVGVTINVGSPFILGPGIVGIA
ILKRVLRFGTRSAQADRQSRAWLKLFNTQHRFPSDKTLFLAFTSLEQDASEVWSMLIGPA
IVMTETALGKTPIEPLTAGEKLCAFLSGATKSQLADRN
>EECEBICD_02234 Acetyltransferase
MNIQITHQVTEFDKEELLAGLRSYNAQFVDFSKNGQLGVYCRNESGEMVGGLIADRKGPW

LCIDYLWVSESARNCGLGSKLMAMAEKEGLRKGCVHGLVDTFSFQALPFYEKQGYILQMS
LPDFPKVGSQRHYLIKLGL
>EECEBICD_02235 hypothetical protein
MRTPAFADLRIFIVPGFRERIHRHQCRLFGRGLIDHLPAAGDGFVVFPGYVFQAVTHHMD
NAQLDMGLREDAVYRIREALQTIHTGNQDVLNAPGFQLRQHA
>EECEBICD_02236 IS256 family transposase IS285
MDEKKLKALAVELAKGLNTEADLNPFSRMLTKLTVETALNAELADHPGYEKNAPKTGSNT
RNGYSSKTVLCDDGEIELNTPRDRENTFEPQLIKKHQTRITQMDSQILSLYAKGMTTREI
VATFKEMYDANVSPSLISKVTDAVKEQVTERQNRQPDALYPIVYMDCIVVKVRQNGSVIN
KAVFLALGINTEGQKELLGIWLAENEGEKVLAKRADGAENPGRSGHPDCLCGWSEGLPGC
DKQRLPANPYPAVHYPYGA
>EECEBICD_02237 hypothetical protein
MRKVSISILFMLVSLTWGTTWLAMRIAVETIPPVFATGMRFMFAAPFLIIIAWLRKKHCC
FLPDNDYSSL
>EECEBICD_02238 hypothetical protein
MIYGETYVNSGLAAIIFANMPVAVLIASVLFLNEKAKLMQIAGLTIAITALTGILLEETN
TSTESHWQGITALISAVLIHAIIYTQCKKRSCTVSVITFNALPCLLAGLILSATGWFFER
PQVSTFSVHSILATLYLGAFAGVFGILCYFALQQKANAFQASLVFLIFPLIAVSLEDYIY
GYAISTHSMLLIIPLVIGIFLTLVARNIPVTSRCRDNSSQK
>EECEBICD_02239 hypothetical protein
MFMFLPFLLALSVAMGAINRKDKVSYILWAVLLIVTILSFIHHMTNSLTLSF
>EECEBICD_02240 hypothetical protein
MKTHHHPTTLVHLINQVGLLGICVALVVAFYYQLVRHELPCPICLLQRAGLIIAGFGFLF
NLCFGLRGIHYGMVIIGSILTGVMASRQICLHIMPGDTGYGSAFFGLHFYTWTLITSILI
IIAVAVVLAISSMNVAFRSLNINPDLFSIVGWVFLLLITANLISTVLECGGGECAANPVT
YKLLSKQDIAFLKTGLLTRAVLRL
>EECEBICD_02241 hypothetical protein
MFVIHLEGYLADSIKRQLWPLLQYPVCHYSATTLVAVGQN
>EECEBICD_02242 Prophage tail fiber assembly protein TfaE
MWSGPPGQRRPANCTDIAPPDIPSSHIAVFDAETQTWSLQEDHRGETVYDTTTGNQVYIS
DLGPLPENVTSVSPGGGYKKWDSKAQVWMNDEAAEAAARLREAEGTKNRLLQITSEKIAP
LQDAVDLDEATNKEKASLLAWRKYRVQVNRVDTLKPVWPEKPASSL
>EECEBICD_02243 hypothetical protein
MDGNKSHSYTARAQDIDLGTKTTSSFDYGTKSTNTTGNHTNQFGGYINSYWGDSNHTSFQ
LGGGAWTQAAGDHAHTVYIGGHEHTMYIGPHGHVVIVVADGNAETFGLMDGGVDAAITAY
FGSQLQERVQQNIIREYLGEQPVGTAFVIETGNSKHPWLVHAPTMRVPLIIDGTDAVYNA
TRAALLAIFQHNKSAGEDRKITSVALPAMGAGCGQVPPDSVARQIVLI
>EECEBICD_02244 hypothetical protein
MGEVQTKAPLDSLALTGTPTAPMPETTAAGIEIATAAFVAAKVAQLVGSAPEALDTLQEL
ADALGNDPNFAITVLNKLAGKQPLDETLTALSGKSADGFIEYVGLRETINHAADALHKSQ
NGGDIPEKPLFVQNIGALPASGTAVAANRLASRGGLPALTGTTRGSDSGLIMGEVYNNGY
PTQYGNILRLTGTGDGEY
>EECEBICD_02245 hypothetical protein
MFRDGSFLQIGWPSITVFSSSDYKRVALTDYDRFPEDIDGEGDGFSLASKRTTTFMSAGM
TPAESLPGREITDVKWRRSSPHEAPPTTGILSLYNRGDRRRWYWPCPHCGDWFQSAMENM
VGYG
>EECEBICD_02246 hypothetical protein
MTGNKYGSAAAVRREVAEYLRPPRRMPVAEGIKQFMFVPRGANTAVPWDDTLASQSFPK
>EECEBICD_02247 hypothetical protein
MDGELKNLKCNICQLAAITGLHRQTVVSRLSGVPLAPGSNEKNKLYLLTDVIRVLMETPV
SQAAEHQDPNKMTPKERKNWFDSEKGRFWLEKEMKQVVPLPEVRQQMAAIVKAITQVLEV
WSDKLERDKGWSADQLNEAQDVVDEARILLVKAIQETADDDGE
>EECEBICD_02248 hypothetical protein
MKNIITIIVAIIIVFYAGMWSQKFLMEDECLDSGGSYNENGICNIAGSHQDVPLSKQNAF

>EECEBICD_02249 hypothetical protein
MHKRKPLNLLPLLLACLPLLASCVPKQKNLPPAWTPQSTPQILPLPSEAKQPTPTPECLP
TCSEILQKKLNIMLESLTSATGQE
>EECEBICD_02250 hypothetical protein
MNGKKAFAPRSAKIRITGLENYTRCGVALKLDLVANPGQLELERHAARSAAWLFVTKGCL
KYSGDLVRVTQIINGG
>EECEBICD_02251 hypothetical protein
MPDNTPTTHEENAARLSQAGKCLRDIEAGRFQCDEEKPQPTGELADEPATPEAVEQDTTE
HHPAPQPLENEPPVSQTEAGYQKIRAELHEARKNIPPKNPVDVGKQLAAARGEYVEGISD
PNDPKWVHNNYSASNQGEKEEVVQEEKQPAAEPEAVTRNADGTFDVSALFPPPSNQTEKT
EARTERDGETPKESNQQETAGDTGQEITTDGGSGTTS
>EECEBICD_02252 hypothetical protein
MSAWIDRYEVLLQRRSLSVNTYKIRSNQLATVREKMGEMILAEVTTRHIAEFLESWIAEG
KNTMAGAMRSVLSDMFREAIVEGRITTNPVEPTRAPEIKVARERLQLETYNATRTAAEYL
PVWFPLAMDLALVTGQRREDIVNMKFSDIVDGRLHVTQIKTGMKIAFPLSLTLQAPGLRP
GRLSIAADW
>EECEBICD_02253 hypothetical protein
MISAGIRKNSPTGNIHPDGLTKKFVKARKISDVKCSDNPPTFHEIRSLLGRLYKDERGEE
FAQKLLGHTSENTTKLYLDERDNKAYVML
>EECEBICD_02254 hypothetical protein
MGYVFPLEIPRNFLEFLYSGLFGYITSKSSLTFLLQQNQSIT
>EECEBICD_02255 hypothetical protein
MVVFLTFTDSKGISEYILDPWLDARIFKKEESYEIYKNNSSEYINENHCFEAYDKYTAIM
NSAEYIDAITKTINLLYRVNLDEIQLTNPFKFI
>EECEBICD_02256 Guanine nucleotide exchange factor SopE
MTKITLFPHNFRIQKQETTPLKEKSTEKNSLAKSILAVKNHFIKLNSKLSERFISHKNTE
SSATHFHRGSASEGRAVLTNKVVKNFMLQTLHDIDIRGSASKDPAYASQTREAILSAVYS
KYKDQYCNLLISKGIDIAPFLKEIGEAAQNAGLPGATKNDVFTPSGAGANPFITPLITSA
YSKYPHMFTSQHQKASFNIYAEKIIMTEVVPLFNECAMPTPQQFQQILENIANKYIQNTP
>EECEBICD_02257 hypothetical protein
MKDEAAEKAAQLRQAEETKSRLLQMASEKIAPLQDAVDLGLATDDEKAQLDEWKKYRVLV
NRVDTLNPDWPEKPS
>EECEBICD_02258 hypothetical protein
MEDTPDGKISQFLVAADRIAYINPANGNETPGFVMQGDQIIMNEAFLKYLSAPTITSGGN
PPAFSLTPDGKLTAKNADISGHINAVSGSFTGEINATSGKFSGVIEAREFVGDICGSKVM
QGVSIRATNDERSTSTRYTDSATYQIGKTITVMANCERNGGTGAITVTININGQVKTAEV
MPYTAGIPAMYQTVVFSVYTTSPVVDISVSLRVRGQYTTSASVWPLVMVSRSGSNFTN
>EECEBICD_02259 hypothetical protein
MTLNAYITTQRKAYDVLADFCSVMRCMPVWNGRKMTFIQDRPSDKAWTYTNGNVVGGRFK
YSFSALKDRHNAIEVRYTDPLNGWQTSTELVEDHASQARYGRNLLKMDAFGCTSRGQAHR
TGLWVMMTELLETQTVDFSVGAEGLRHTPGDIIEVCDNDYAGASVGGRITDLDISTRTLT
LDREITLPESGATTLNIVGPDGKPFSTEIQSQPAPDRVVTKVLPETVQPYSIWGLKLPSL
KRRLFRCVRIKENDDGTYAITALQHVPEKESIVDNGAHFDPLPGTTNSIIPPAVQHLTVS
TDNDSTLYQAKAKWGTPRVVKDVRFVVRLTTGSGNEGDPVRLVTTATTSETEYAFHELPL
GDYTLTVRAINGYGQQGEPASVAFSIQAPEAPSTIEMTPGYFQITVTPHQTVYDASVQYE
FWYSATQLATAADIQSKAQYLGVGSFWIKDGLKPLHDAWFYVRSVNLAGKSVFAEASGRP
GDDAKGYLDFFKGLITETYLGTELLKKN
>EECEBICD_02260 hypothetical protein
MLINNTPVVDADGNSNIHGVTVVYQVGETPQAPLEGFEASGAETVLGVEVKHDNPVTRTV
VSENVDRLRFTFGVQMLQETMDKGDRNPSSVNLLIQFQRSGIWNTEFDITINGKITTQYL
ASVVADNLPPRPFSVRMVRVTPDSTTDRLQNKTLWSSYTEIIDIRQGYPGTAVAGLLVDA
EQFGSQQVTRNYHLRGRIFQVPSNYDPDTRTYTGLWDGAFKPAYTNNPAWCTMDKLTHPR
YGLGRRIGGRMWINGRCTPSRSTAINRCRTDLAARNPA
>EECEBICD_02261 hypothetical protein

MINDDILAHARQCAPAESCGYVVRTAQGERYFPCENLSAEPTMYFRISPEDYLNARNRGD
IVALVHSHPDGKPCLSSADRTLQIQSGTVCRAC
>EECEBICD_02262 hypothetical protein
MQDIPQETLSETTKAEQSAKVDLWEFDLTAIGGERFFFCNEPNEKGEPLTWQGRQYEPYP
IQVQDFEMNGKGASPRPNLVVANLFGLVTGMAEDLQSLVGASVVRHQVYSKFLDAVNFSN
GNPGADPEQEAVARYNVEQLSELDSSTATIILASPAETDGSVVPGRTMLADSCPWDYRDE
NCGYDGPPVADEFDKPTSDPKKDKCSHCMKGCEMRNNLVNAGFFASINKLS
>EECEBICD_02263 Superoxide dismutase [Cu-Zn] 1
MKYTILSLVAGALISCSAMAENTLTVKMNDALSSGTGENIGEITVSETPYGLLFTPHLNG
LTPGIHGFHVHTNPSCMPGMKDGKEVPALMAGGHLDPEKTGKHLGPYNDKGHLGDLPGLV
VNADGTATYPLLAPRLKSLSELKGHSLMIHKGGDNYSDKPAPLGGGGARFACGVIEK
>EECEBICD_02264 hypothetical protein
MLIRRINGNIFNRVKLNTCPISKGGFIVRISPVILNMSVCCTNHHVSIQIVVKTV
>EECEBICD_02265 hypothetical protein
MFDTERHFHRIQEKSTTVDQEIKSLELNITQLSAITGAHRQTIASRLKGVKTSGGNGSNL
KIYRLVDILTAMMTMPAVTGENDPNKMKPSDRRAWFQSEMTRIELEKEMRTLIPASEVLS
V
>EECEBICD_02266 hypothetical protein
MMFNWKTMFVGLLLVSLIVAGRLANHYRNNAITYKEQRDTVTHRLTLANATITDMTKRQR
DVAALDEKYTKELADAKAENDALRDDVAAGRRRLYVNATCPAVPTGKSTSTARMDNAASP
RLADSAQRDYFALKERVKTMQKQLEGAQAYIRTQCHGNAGKTSNQW
>EECEBICD_02267 Lysozyme RrrD
MMRISEKGITLIKEFEGCSLTAYPDPGTGGDPWTIGYGWTHSVDGKPVKPGMMIDEATAE
RLLNTGLVGYENDVSRLVKVKLTQGQFDALVSFAYNLGARTLSSSTLLRKLNAGDYAGAA
DEFLRWNKAGGKVLNGLTRRREAERALFLS
>EECEBICD_02268 hypothetical protein
MKMNDKTPEFWAAVLTGLKNAWPQILGALMAGLIAYGRLIYDGATRKNKWLEGVLCGALS
LCVTSALDVVGLPVSISPFVGGIIGFVGVDKLREIAISALKKRAGVNDENQ
>EECEBICD_02269 hypothetical protein
MDPDIEIDDDTYDECREVLSRILEDAYTQSGTFRRLMNYAYDQELRDVEQRWLLGAGENF
GTTVTDEDLESSEGRKVIALNLDDTDDDSIPEYYESNDGPQQFDTTRSFIHEVVHALTHL
QDKEDSNPRGPVVEYTNIILKEMGHTSPPRIAYEFSN
>EECEBICD_02270 hypothetical protein
MSESISGTGLAGGILTGASVYGLLTCISSDLNWLLCCSIVGFCIF
>EECEBICD_02271 hypothetical protein
MKNTALGKFIFIVGTALLLGGCSGMVMPPYATHGTSVGIIAPAGGYSEWHTDSRNHTTGD
SHSQSQGNCTQSEDSQLSENSLTRTHQSNCNTRSQTHSSSTSKTRSSSVGFSVGGPVGAS
IGLIKQMESMNRAPANDMSSNEMFKNFGF
>EECEBICD_02272 hypothetical protein
MFYRDLFQVFGPDPLYKEEEGIAILREQYGIEAPEQIFKQIYCGLSNNSEFQTLYGHLNL
KSLKWDLVRLKTAEFTKFGRNATYPDYMLEISEDFNACGSKFCIDAREEVANHWLKFGTW
AEPPMFIERSLIIPGESGLHLMEGHTRLGTLLGAIKYKFVQLADTHELYIASQK
>EECEBICD_02273 hypothetical protein
MNTQYLEYVRQQLIVATADLSGATKGQLQAWLENAQLYTKNYPRKKQRIRDEVTGKMITL
NNPPIAGKQSLAKGSAIPLVQPVEYSTSSWRRALLSLEEHNKAWLLWNYSENTCWEYQVT
VTRWAWEKFSQQLEGKRVAKKTLARLRQLIWLAAQDVKAELARRETYEYQTLAELMGVAK
STWTETYMSHWLVMRNSFKRLDSDALISVTRSRSQQKATNLDISLAKPN
>EECEBICD_02274 hypothetical protein
MKMTWFQHPVCTTEEADELVAGYRRRGVKVERYGEAEVLELESNNTPQRWTVEELKEIRI
AALADLRALKKLEAA
>EECEBICD_02275 hypothetical protein
MAHLQLVKQTSSGLLLPATPESGDFLRSVKIGEWIHADFKRVRNYAFHKRFFKLLQLGFD
YWMPTGGTVTSREQKLISGFVNFLCDSAGQEYTPALNEAAEQYLHNVATLRTGDVALLKS
FDAFREWVTVQAGFYTEHFYPDGSRGRRAKSIAFASMDETEFQQVYKAVLNVLWNWILFR

KFSSLEEVENVAAHLLEFA
>EECEBICD_02276 hypothetical protein
MMIINSLLYIHVENEDALKMLKFVIYGDTVKNLNKTFTCKYAVIRRDDMTVIAEMDFFPD
CNRSLMYRDGRYVRFLPLLQNDIMGSDTLINELTIRAGYHE
>EECEBICD_02277 Putative alkyl/aryl-sulfatase YjcS
MRLKLIVKSFALAGLLSSTALTPLFAQEAPKGATASTKQANDALYNQLPFSDNTDFTNAH
KGFIAGLPEEVIKGEQGNVIWNPQQYAFIKEGEKSPDTVNPSLWRQSQLINISGLFEVTD
GVYQIRNLDLSNMTIIEGKEGITVVDPLVSAETAKAGMDLYFKNRGNKPVVAIIYTHSHV
DHYGGVRGVVDEADVKSGKVKVYAPAGFMEAAVAENIMAGNVMSRRASYMYGNLLKPDAS
GQVGAGLGTTTSAGTVTLIAPTNIIDKDGQKEVIDGLTYDFMLAPGSEAPSEMLWFIEEK
KLIEAAEDVTHTLHNTYSLRGAKIREPLPWSKYINEAIVRWGDKAEIIMAQHHWPTWGNE
NVVGLLKSQRDLYRYINDQTLRMANEGLTRDEIAANFKLPDSLAKTWANRGYYGSISHDV
KATYVLYLGWFDGNPATLDELPPEEAAKKFVEYMGGADAILQKAKADFDQGNYRWVAQVV
SKVVFADPNNQNARNLEADALEQLGYQAESGPWRNFYLTGAQELRNGVVKGPTPNTASPD
TVRAMTPEMFFDFLAVHINGEKAGNARAVFNIDLGSDGGKYKLELENGVLNHTANAEAKD
ADATITLNRDTLNKIILKEETLKQAQDKGEVNVTGNAAKLDEMLGYMDKFEFWFNIVTP
>EECEBICD_02278 hypothetical protein
MSRKKYDANLPRNLTYRKASKSFFWRNPLTDKEFPLGQIARRDAITQAIEANNFIAQNHT
PVALIEKLKGTDSFTVSAWIDRYEVLLQRRSLSVNTYKIRGNQLATVREKMGEIILAEVT
TRHIAKFLESWITEGKNTMAGAMRSVLSDMFREAIVEGHIVKNPVEATRIPEIKVARERL
QLETYNATRAAAEHMPAWFPLAMDLALVTGQRREDIVNMKFSDVFDNRLYVTQIKTGMKI
AIPLSLTLRATGLRLGTVIDRCRLVSRTDFMISAGIRKNSPTGNIHPDGLTKTFVKARKA
SGVNFSNNPPTFHEIRSLAGRLYKNEHGEVFAQKLLGHTSANTTKLYLDERDDKAYMML
>EECEBICD_02279 hypothetical protein
MKKILLPALLLATSGVALAAPQVITVSRFEVGKDKWAFNREEVMLTCRPGQALYVINPST
LVQYPLNAIAEQQVAEGKTRAQPIAVIRIDNPAKPGEKMSLAPFIERAQKLCDPSNS
>EECEBICD_02280 Inner membrane protein YebZ
MMLTFVWITLRFIHFASLMLVYGCALYGAWLAPASIRRLMTRRFLHLQRHAAAWSVISAA
FMLAIQGGLMGGGWPDVFSVSVWGAVLQTRFGAVWIWQIILALVTLAVVVIAPVKMQRRL
LILTVAQFILLAGVGHATMRDGVVGTLQQINHALHLLCAAAWFGGLLPVVYCMRMAQGRW
RQHAISAMMRFSRYGHFFVAGVLLTGIGNTLFITGFTAIWQTTYGQLLLLKCALVVLMVA
IALTNRYVLVPRMRQENPRTDLWFVRMTQIEWGVGGIVLAIVSLFATLEPF
>EECEBICD_02281 Protein YobA
MASSAPSRRLALLLLASTFATPAAWAHAHLTHQYPAANAAVTASPQALTLNFSEGIEPGF
SGATITGPQQELIKTRPAKRNEQDKTQLIIPLEQPLKSGAYTVDWHVVSVDGHKTKGKYT
FSVK
>EECEBICD_02282 DNA polymerase III subunit theta
MKTNLAQLEQAEMDKVNVDLAAAGVAFKERYNMPVVAEAVEREQPEHLRAWFRERLIAHR
LASVSLSRLPYEPKVK
>EECEBICD_02283 hypothetical protein
MSSWKIAAAQYAPLNASPAEHVAHHLEYIELAARQQCELLVFPSLSLLGCDERNKSLPAP
PDEALLQPLTHAADTHHMTIIVGMPVEHNCRFVKGIAIFAPWLTSPLMFHKSHGACIARQ
RSAINVVDEQPEGGDIDPSFTLFTTSQCLNEPELHASTSRLQRFSHKYALAVLMANACGS
SALWDESGQLIVRADCGSLLLTGLRTTEGWQGDIIPLR
>EECEBICD_02284 Exodeoxyribonuclease 10
MLRIIDTETCGLQGGIVEIASVDVIDGNIVNPMSHLIRPDRPITPQAMAIHRITEAMVAD
KPWIEDVIPLYYGSEWYVAHNASFDRRVLPELPGEWICTMKLSRRLWPGIKYSNMSLYKS
RKLSVQTPPGLHHHRALYDCYITAALLIDIMRTTGWTAEEMVNITGRPALLTTFPFGKYR
GKAVSEVAKRDPGYLRWLFNNLDNMSPELRLTLKHYLEDVQAGEQRSNGTPQ
>EECEBICD_02285 Protease 2
MLPKANRIPYAMTVHGDTRIDNYYWLRDDTRSQPEVLDYLHQENEYGRKVMSSQQALQDR
ILKEIIDRIPPREVSAPYVKNGYRYRYIYEPGCEYAIYQRQSALSEEWDVWETLLDANQR
AAHSEFYTLGGLAITPDNTIMALAEDYLSRRQYGLRFRNLESGNWYPELLDNVAPEFVWA
NDSLTLYYVRKHKKTLLPYQVWRHTIGTPSSQDELVYEEKDDTFYVSLHKTTSQHYVVIH

LASATTSEVLLLDAELADAEPFSFLPRRKDHEYSLDHYQHKFYLRSNRNGKNFGLYRTRV
RNENAWEELIPPREHIMLEGFTLFTDWLVVEERQRGLTSLRQINRKTREVIGIAFDDPAY
VTWLAYNPEPETSRLRYGYSSMTTPDTLFELDMDTGERRVLKQTEVPGFDSGCYQSEHLW
ITARDGVEVPVSLVYHQKYFRKGQNPLLVYGYGSYGSSIDADFSSSRLSLLDRGFVYAIV
HVRGGGELGQQWYEDGKFLKKRNTFNDYLDACDALLKLGYGSPSLCYGMGGSAGGMLMGV
AINERPELFHGVIAQVPFVDVLTTMLDESIPLTTGEFEEWGNPQDIEYYDYMKSYSPYDN
VKAQDYPHLLVTTGLHDSQVQYWEPAKWVAKLRELKTDQRLLLLCTDMDSGHGGKSGRFK
SYEGVALEFAFLIGLAQGTLHSA
>EECEBICD_02286 Inner membrane protein YebE
MANWLNQLQSLLGQKGASASSSGEQGLNKLLVPGALGGLAGLLVANKSSRKLLTKYGTGA
LLVGGGAVAGSVLWNKYKDKVRAAHQGEPQFGSQSTPLDVRTERLILALVFAAKSDGHID
AKERAAIEHQLRESGVEEQGRVFIEKAIEQPLDPQRLAQGVRNEEEALEIYFLSCAAIDI
DHFMERSYLNALGDALKIPQEVRDGIEQDLQQQKQALPG
>EECEBICD_02287 Protein YebF
MNKRGALLSLLLLSASVSAFAASTESKSVKFPQCEGLDAAGIAASVKRDYQQNRIVRWAD
DQKKVGQADPVAWVNVQDVVGQNDKWTVPLTVRGKSADIHYQVIVDCKAGKAEYKPR
>EECEBICD_02288 hypothetical protein
MAVEVKYVVIREGEEKMSFTSKKEADAWDKMLDTADLLDTWLEQSPVVLEDGQREALSLW
LAEHKEVLSTILKTGKLPSPQAVEKDAASKTKKQAA
>EECEBICD_02289 Formate-dependent phosphoribosylglycinamide formyltransferase
MTLLGTALRPAATRVMLLGAGELGKEVAIECQRLGIEVIAVDRYPDAPAMHVAHRSHVIN
MLDGEALRHVITEEKPHYIVPEIEAIATDTLRELEDEGLNVVPCARATQLTMNREGIRRL
AAEELGLPTSTYRFADSEASFHDAVAAVGFPCIVKPVMSSSGKGQSFIRSAEQLAQAWEY
AQQGGRAGAGRVIVEGVVKFDFEITLLTVSAVDGVHFCAPVGHRQQDGDYRESWQPQQMS
ELALKRAQEIARHVVLALGGHGLFGVELFVCGDEVIFSEVSPRPHDTGMVTLISQDLSEF
ALHVRAFLGMPIGAIRQYGPAASAVILPQLTSQNVTFDNVHTAVGAGVQVRLFGKPEIDG
SRRLGVALATGENVEEAVIRAKKAASRVTVKG
>EECEBICD_02290 KHG/KDPG aldolase
MKNWKTSAEAILTTGPVVPVIVVNKLEHAVPMAKALVAGGVRVLEVTLRTACAMDAIRAI
AKDVPEAIVGAGTVLNPQQLAEVTEAGAQFAISPGLTEPLLKAATAGTIPLIPGISTVSE
LMLGMDYGLKEFKFFPAEANGGTKALQAIAGPFSQVRFCPTGGISPANYRDYLALKSVLC
IGGSWLVPADALEAGDYDRITKLAREAVEGAKQ
>EECEBICD_02291 Phosphogluconate dehydratase
MNPNLLRVTQRIVERSQQTREAYLARIEQAKTATVHRSQLACGNLAHGFAACQPEDKASL
KSMLRNNIAIITSYNDMLSAHQPYEHYPQIIRQALHSVNAVGQVAGGVPAMCDGVTQGQD
GMELSLLSREVIAMSAAVGLSHNMFDGALFLGVCDKIVPGLAMAALSFGHLPAIFVPSGP
MASGLPNKEKVRIRQLYAEGKVDRMALLESEAASYHAPGTCTFYGTANTNQMVVEFMGMQ
LPGSSFVHPDAPLREALTAAAARQVTRLTGNGNTWMPLGKMIDEKVVVNGIVALLATGGS
TNHTMHLVAMARAAGILINWDDFSDLSEVVPLMARLYPNGPADINHFQAAGGVPVLMREL
LNAGLLHEDVNTVAGFGLKRYTLEPWLNNGELDWREGAERSLDNDVIASFDKPFSPHGGT
KVLSGNLGRAVMKTSAVPVENQIIEAPAMVFESQHDVLPAFDAGLLDRDCVVVVRHQGPK
ANGMPELHKLMPPLGVLLDRRFKIALVTDGRLSGASGKVPSAIHVTPEAYDGGLLAKVRD
GDIIRVNGQTGELTLLVDEAELAARQPHIPDLSASRVGTGRELFGALREKLSGAEQGATC
ITF
>EECEBICD_02292 Glucose-6-phosphate 1-dehydrogenase
MAVTQTAQACDLVIFGAKGDLARRKLLPSLYQLEKAGQIHPDTRIIGVGRADWDKEAYTH
VVREALETFMKEKIDEGLWDTLSGRLDFCNLDVNDTPAFSRLGDMLDQKNRTTINYFAMP
PSTFGAICKGLGEAKLNAKPARVVMEKPLGTSLATSREINDRVGEYFEECQVYRIDHYLG
KETVLNLLALRFANSLFVNNWDNRTIDHVEITVAEEVGIEGRWGYFDQAGQMRDMIQNHL
LQILCMIAMSPPSDLSADSIRDEKVKVLKSLRRIDRSNVREKTVRGQYTAGFAQGQKVPG
YLEEEGANKSSNTETFVAIRVDIDNWRWAGVPFYLRTGKRLPTKCSEVVVYFKTPELNLF
KESWQDLPQNKLTIRLQPDEGVDIQVLNKVPGLDHKHNLQITKLDLSYSETFNQTHLADA
YERLLLETMRGIQALFVRRDEVEEAWKWVDSITEAWAMDNDAPKPYQAGTWGPVASVAMI

TRDGRSWNEFE
>EECEBICD_02293 HTH-type transcriptional regulator HexR
MNMLEKVQSQLEHLSKSERKVADVILAAPGRSIHLSIAMLAQEANVSEPTVNRFCRSMNT
RGFPDFKLHLAQSLANGTPYVNRNVDEDDSVEAYTGKIFESAMASLDHVRQSLDKSAVNR
AVDLLTQAKKSPFLVLVPPPRSRMTP
>EECEBICD_02294 HTH-type transcriptional regulator HexR
MNKFFRFNVPVIYSDDIVLQRMSCMNCSDDDVVVLISHTGRTKSLVELAQLARENDAMVI
ALTSAGTPLAREATLAITLDVPEDTDIYMPMVSRLAQLTVIDVLATGFTLRRGAKFRDNL
KRVKEALKESRFDKELLIKSDDR
>EECEBICD_02295 Pyruvate kinase II
MSRRLRRTKIVTTLGPATDRDNNLEKVIAAGANVVRMNFSHGSPEDHKMRADKVREIAAK
LGRHVAILGDLQGPKIRVSTFKEGKVFLNIGDKFLLDANLGKGEGDKEKVGIDYKGLPAD
VVPGDILLLDDGRVQLKVLEVQGMKVFTEVTVGGPLSNNKGINKLGGGLSAEALTEKDKA
DIQTAALIGVDYLAVSFPRCGEDLNYARRLARDAGCDAKIVAKVERAEAVCDQNAMDDII
LASDVVMVARGDLGVEIGDPELVGIQKALIRRARQLNRAVITATQMMESMITNPMPTRAE
VMDVANAVLDGTDAVMLSAETAAGQYPSETVAAMARVCLGAEKIPSINVSKHRLDVQFDN
VEEAIAMSAMYAANHLKGVTAIITMTESGRTALMTSRISSGLPIFAMSRHERTLNLTALY
RGVTPVHFDSAADGVVAAHEAVNLLRDKGYLVSGDLVIVTQGDVMSTVGSTNTTRILTVE
>EECEBICD_02296 Lipid A biosynthesis myristoyltransferase
MCAGFFVLCAAQTSLHYSQFLFASAEPWKSMETKKNNSEYIPEFEKSFRYPQYWGAWLGA
AAMAGIALTPASFRDPLLATLGRFAGRLGKSSRRRALINLSLCFPQRSEAEREAIVDEMF
ATAPQAMAMMAELAMRGPKKIQQRVDWEGLEIIEEMRRNDEKVIFLVPHGWGVDIPAMLM
ASQGQKMAAMFHNQGNPVFDYIWNTVRRRFGGRLHARNDGIKPFIQSVRQGYWGYYLPDQ
DHGPEHSEFVDFFATYKATLPAIGRLMKVCRARVIPLFPVYNGKTHRLTIQIRPPMDDLL
TADDHTIARRMNEEVEIFVGPHPEQYTWILKLLKTRKPGEIQPYKRKDLYPIK
>EECEBICD_02297 Murein DD-endopeptidase MepM
MQQIARSVALAFNNLPRPHRVMLGSLTVLTLAVAVWRPYVYHPESAPIVKTIELEKSEIR
SLLPEASEPIDQAAQEDEAIPQDELDDKTAGEVGVHEYVVSTGDTLSSILNQYGIDMSDI
SRLAASDKELRNLKIGQQLSWTLTADGDLQRLTWEVSRRETRTYDRTANGFKMSSEMQQG
DWVNSLLKGTVGGSFVASAKEAGLTSSEISAVIKAMQWQMDFRKLKKGDEFSVLMSREML
DGKREQSQLLGVRMRSDGKDYYAIRAADGKFYDRNGVGLAKGFLRFPTAKQFRISSNFNP
RRLNPVTGRVAPHRGVDFAMPQGTPVLSVGDGEVVVAKRSGAAGYYIAIRHGRTYTTRYM
HLRKLLVKPGQKVKRGDRIALSGNTGRSTGPHLHYEVWINQQAVNPLTAKLPRTEGLTGS
DRREYLAQVKEVLPQLRFD
>EECEBICD_02298 High-affinity zinc uptake system protein ZnuA
MIECYNITFHTFITMISRIMLQKNTLLFAALSAALWGSATQAADAAVVASLKPLGFIASA
IADGVTDTQVLLPDGASEHDYSLRPSDVKRLQGADLVVWVGPEMEAFMEKSVRNIPDNKQ
VTIAQLADVKPLLMKGADDDEDEHAHTGADEEKGDVHHHHGEYNMHLWLSPEIARATAVA
IHEKLVELMPQSRAKLDANLKDFEAQLAATDKQVGNELAPLKGKGYFVFHDAYGYYEKHY
GLTPLGHFTVNPEIQPGAQRLHEIRTQLVEQKATCVFAEPQFRPAVVEAVARGTSVRMGT
LDPLGTNIKLGKTSYSAFLSQLANQYASCLKGD
>EECEBICD_02299 Zinc import ATP-binding protein ZnuC
MVLGLVAPDEGVIKRNGQLRIGYVPQKLYLDTTLPLTVNRFLRLRPGTQKTDILPALKRV
QAGHLIDAPMQKLSGGETQRVLLARALLNRPQLLVLDEPTQGVDVNGQVALYDLIDQLRR
ELDCAVLMVSHDLHLVMAKTDEVLCLNHHICCSGAPEVVSMHPEFISMFGPRGAEQLGIY
RHHHNHRHDLQGRIVLRRGNGHS
>EECEBICD_02300 High-affinity zinc uptake system membrane protein ZnuB
MIELLLPGWLAGMMLACAAGPLGSFVVWRRMSYFGDTLAHASLLGVAFGLLLDVNPFYAV
IAVTLLLAAGLVWLEKRPHLAIDTLLGIMAHSALSLGLVVVSLMSNVRVDLMAYLFGDLL
AVTPEDLISIAIGVVIVLAILFWQWRNLLSMTISPDLAFVDGVKLQRVKLLLMLVTALTI
GVAMKFVGALIITSLLIIPAATARRFARTPEQMAGVAVGVGMIAVTGGLTFSAFYDTPAG
PSVVLCAALLFIFSMMKKQAS
>EECEBICD_02301 Holliday junction ATP-dependent DNA helicase RuvB
MIEADRLISAGATIAEDVADRAIRPKLLAEYVGQPQVRSQMEIFIQAAKRRGDALDHLLI

FGPPGLGKTTLANIVANEMGVNLRTTSGPVLEKAGDLAAMLTNLEPHDVLFIDEIHRLSP
VVEEVLYPAMEDYQLDIMIGEGPAARSIKIDLPPFTLIGATTRAGSLTSPLRDRFGIVQR
LEFYQVPDLQHIVGRSARHMGLEMSDDGALEVARRARGTPRIANRLLRRVRDFAEVKHDG
AISAEIAAQALDMLNVDAEGFDYMDRKLLLAVIDKFFGGPVGLDNLAAAIGEERETIEDV
LEPYLIQQGFLQRTPRGRMATVRAWNHFGITPPEMP
>EECEBICD_02302 Holliday junction ATP-dependent DNA helicase RuvA
MIGRLRGIILEKQPPIVLLETGGVGYEVHMPMTCFYELPEAGQEAIVFTHFVVREDAQLL
YGFNNKQERTLFKELIKTNGVGPKLALAILSGMSAQQFVNAVEREELGALVKLPGIGKKT
AERLIVEMKDRFKGLHGDLFTPAVDLVLTSPASPTSEDAEQEAVAALVALGYKPQEASRM
VSKIARPDASSETLIRDALRAAL
>EECEBICD_02303 hypothetical protein
MVDDKPMRIGWVFYFSYIFVAIIFHLFISFCYCLSIDMAEANTVVFLLKPGTISLLFLLL
PARRFRTRLLATLSSVFITLVFNQWHLVAGNKELVLCLQAACFMAFLAMTSVKKSGWMIS
ASLFLVCAAGTIRQCWLEQLFNAADIYIVDDGRSSGASGHCFQYIAAKGRGLAAKRQALF
SSEEYVNIYYSYSEGMPAVNFDGMKNEFVQYLLCHGELKSVARDDKTTCD
>EECEBICD_02304 hypothetical protein
MKKHYSVQYETGDTVFTCIGAALFGQISTASQCWSNHVGIIIGHNGDDYLVAESRVPLST
VTTLSLFIQRSAGQRYAVRRLRGGLTVEQKLAIMEQVPARLNKFYHTGFKYESSRQFCSK
FVFDIYKEALCIPVGDIETFEELLHSNPDAKLTFWKFWFLGSIPWDRKTVTPASLWHHPN
LELISACGIETPQREAEGE
>EECEBICD_02305 Crossover junction endodeoxyribonuclease RuvC
MSIILGIDPGSRITGYGVIRQVGRQLTYLGSGCIRTKVDDLPSRLKLIYAGVTEIITQFQ
PDYFAIEQVFMAKNADSALKLGQARGVAIVAAVNQELPVFEYAARQVKQTVVGIGSAEKS
QVQHMVRTLLKLPANPQADAADALAIAITHCHVSQNAMQMSESRLNLARGRLR
>EECEBICD_02306 putative transcriptional regulatory protein YebC
MAGHSKWANTRHRKAAQDAKRGKIFTKIIRELVTAAKLGGGDPDANPRLRAAVDKALANN
MTRDTLNRAIARGVGGDEDSNMETIIYEGYGPGGTAIMIECLSDNRNRTVAEVRHAFSKC
GGNLGTDGSVAYLFSKKGVISFEKGDEDTIMEAALEAGAEDVVTYDDGAIDVYTAWEEMG
KVRDALEAAGLKADSAEVSMIPSTKADMDAETAPKLLRLIDMLEDCDDVQEVYHNGEISD
EVAATL
>EECEBICD_02307 Dihydroneopterin triphosphate diphosphatase
MKDKVYKRPVSVLVVIFAQDTKRVLMLQRRDDPDFWQSVTGSIEEGETALQAAVREVKEE
VTIDVAAEQLTLIDCQRTVEFEIFSHLRHRYAPGVMHNTEFWFCLALPHERQVIFTEHLT
YQWLDAPDAAALTKSWSNRQAIEEFVINVA
>EECEBICD_02308 Aspartate--tRNA ligase
MRTEYCGQLRLSHVGQQVTLCGWVNRRRDLGSLIFIDMRDREGIVQVFFDPDRADALKLA
SELRNEFCIQVTGTVRARDAKNVNADMATGEIEVLASSLTIINRADSLPLDANHVNTEEA
RLKYRYLDLRRPEMAQRLKTRAKITSLVRRFMDDHGFLDIETPMLTKATPEGARDYLVPS
RVHKGKFYALPQSPQLFKQLLMMSGFDRYYQIVKCFRDEDLRADRQPEFTQIDVETSFMT
APQVREVMEALVRHLWLEVKGVDLGDFPVMTFAEAERRYGSDKPDLRNPMELVDVADLLK
SVEFAVFAGPANDPKGRVAALRVPGGAQLSRKQIDDYGNFVKIYGAKGLAYIKVNERAKG
LDGINSPVAKFLTADIVDAILERTGAQDGDMIFFGADNKKVVADALGALRLKLGKDLSLT
DEDKWAPLWVIDFPMFEDDGEGGLTAMHHPFTAPRDMTASELKTAPEEAVANAYDMVING
YEVGGGSVRIHNGEMQQTVFGILGINEQEQREKFGFLLDALKYGTPPHAGLAFGLDRLTM
LLTGTDNIRDVIAFPKTTAAACLMTEAPSFANQAALTELGIQVVKKAENN
>EECEBICD_02309 Isochorismatase family protein YecD
MLKLNATTTALVVIDLQEGILPFAGGPYTANEVVARAARLAEKCRANGSPVVMVRVGWSD
DYAEALKQPVDAATPAHALPENWWTWPTALGKKDSDLEVTKRQWGAFYGTDLELQLRRRG
IDTIILCGISTNIGVESTARNAWELGFNLIIAEDACSAASSEQHQSSMTHIFPRIGRVRS
VEDILNAL
>EECEBICD_02310 hypothetical protein
MIYIGLPQWSHPKWARLGITSLEEYARHFNCVEGNTTLYALPKAEIVDRWYAQTTDDFRF
CFKFPATISHQAALRHCDDLVQAFFTRLAPLETRIGQYWLQLPAAFGPRDLPALWQFLDA
LPATFTYGVEVRHPCFFDKGEDEQRLNRGLHARGVNRVILDSRPVHAAHPHSEAVRDAQR

KKPKVPVHAVVTASHPMVRFIGSDNMAQNREFFAAWLQKLPQWRQTTTPFLFLHTPDIAQ
APELVNTLWHDLRSVLPEIGTAPSIPQQSSLF
>EECEBICD_02311 Inner membrane protein YecN
MVSALYAVLGALLLMKFSFNVVRLRMQYRVAYGDGGFSELQSAIRIHGNAVEYIPVALVL
LLFMEMNGAETWMVHICGIILIAGRLMHYYGFHHRLFRWRRAGMSATWCALLLMVLANLW
YMPWELVFSLY
>EECEBICD_02312 Carboxy-S-adenosyl-L-methionine synthase
MSHRDTLFSAPIARLGDWTFDERVAEVFPDMIQRSVPGYSNIISMIGMLAERFVQPNTQV
YDLGCSLGAATLSVRRNIRHEHCRIIAVDNSPAMIERCRRHIDAYKAPTPVEVVEGDIRD
ITIENASMVVLNFTLQFLEPAERQALLDKIYLGLNPGGALVLSEKFSFEDAKVGELLFNM
HHDFKRANGYSELEISQKRSMLENVMLTDSVETHKARLRKAGFEHSELWFQCFNFGSLVA
LKAGVAA
>EECEBICD_02313 tRNA U34 carboxymethyltransferase
MIEFGNFYQLIAKNHLSHWLETLPAQIAAWQREQQHGLFKQWSNAVEFLPEMTPWRLDLL
HSVTAESETPLSEGQLKRIDTLLRNLMPWRKGPFSLYGVDIDTEWRSDWKWDRVLPHLSD
LTGRTILDVGCGSGYHLWRMIGAGAHLAVGIDPTQLFLCQFEAVRKLLGNDQRAHLLPLG
IEQLPALKAFDTVFSMGVLYHRRSPLEHLWQLKDQLVNEGELVLETLVVDGDENTVLVPG
DRYAQMRNVYFIPSAPALKKWLEKCGFIDVRIADVCVTTTEEQRRTEWMVTESLADFLDP
NDRSKTVEGYPAPQRAVLIARKP
>EECEBICD_02314 Copper homeostasis protein CutC
MALLEICCYSMECALTAQRNGADRIELCAAPKEGGLTPSLGVLRSVREHITIPVHPIIRP
RGGDFYYTDGEFAAMLEDIRLVRELGFPGLVTGVLTVDGDVDMSRMEKIMAAAGPLAVTF
HRAFDMCANPFNALKNLADAGVARVLTSGQKADAAQGLSIIMELIAQGDAPTIMAGAGVR
ANNLQNFLDAGVREVHSSAGVLLPSPMRYRNQGLSMSADIQADEYSRYRVEGAAVAEMKG
IIVRHQAK
>EECEBICD_02315 Protein YecM
MANWQHIDELHDISADLPRFTLAFRELSTRLGLQISALEADHISLRCHQNTTAERWRRGF
EQCGELLSENIINGRPICLFKLHEPVCVEHWRFSVIELPWPGEKRYPHEGWEHIEIVLPG
EPETLNARALALLSDEGLSQPGIVVKTSSPQGEHERLPNPTLAVTDGRITVKFHPWSIEA
IVASEQAAH
>EECEBICD_02316 Arginine--tRNA ligase
MNIQALLSEKVSQAMIAAGAPADCEPQVRQSAKVQFGDYQANGMMAVAKKLGMAPRQLAE
QVLTHLDLSGIASKVEIAGPGFINIFLEPAFLAEQVQQALTSDRLGVSQPTRQTIVVDYS
APNVAKEMHVGHLRSTIIGDAAVRTLEFLGHHVIRANHVGDWGTQFGMLIAWLEKQQQEN
AGDMALADLEGFYRDAKKHYDEDEAFAERARNYVVKLQSGDTYFREMWRKLVDITMTQNQ
ITYDRLNVTLTRDDVMGESLYNPMLPGIVADLKAKGLAVESEGATVVFLDEFKNKEGDPM
GVIIQKKDGGYLYTTTDIACAKYRYETLHADRVLYYIDSRQHQHLMQAWTIVRKAGYVPD
SVPLEHHMFGMMLGKDGKPFKTRAGGTVKLADLLDEALERARRLVAEKNPDMSADELEKL
ANAVGIGAVKYADLSKNRTTDYIFDWDNMLAFEGNTAPYMQYAYTRVLSVFRKADIDEQA
LASAPVIISEDREAQLAARLLQFEETLTVVAREGTPHVMCAYLYDVAGLFSGFYEHCPIL
SAENDAVRNSRLKLAQLTAKTLKLGLDTLGIETVERM
>EECEBICD_02317 Peptidoglycan D,D-transpeptidase MrdA
MTFKDFDAEEKLFLRRVIVAFGVVVVCFGILIFNLYNLQIRQHHYYTTRSNENDIKMLPV
APTRGIIYDRNGIPLVRNVTWYDIAVTPYKIADMDALLKQLTPIVDLSPDDIADFRRALK
SSSRYRPVVLKNALTDVEIARFAVNQFHFNGVTINSYQDRQYPYGAELAHVLGYVSKIND
NDLKALDKKGLAENYAADHNIGKQGIERYYENDLHGKTGYQEVEVDNHGRIVRLLKDVPP
IAGKNIHLTLDLHLQEYIESLLAGQRAAVLVEDPHDGSVLAMVSMPSYDPNPFVKGISYQ
DYGKLLHDKNLPLINRVTQGLYPPASTVKPYMAMSALLCGIITPQTTFFGAPTWTLPGTQ
RHYRDWKKTGHGMLDVTKAIEESADTFFYQVAYMMGIDRIDTMLSQFGYGKPTGIDLNEE
YDGLLPSRAWKQRVHKKAWYQGDTISVGIGQGYWIATPIQMVKAMVALINNGKVIAPHLL
LNEESGKTVVPYRPSGTPAQIADPASPYWGLVRQAMYGMANAPNGTGYKFFHTAPYGIAA
KSGTSQVFSLKENQTYNAKMIPIRLRDHVFYTAFAPYKNPKVAIALILENGGSDGVTAAP
IMRKILDHLFDPQADTTQSGQAP
>EECEBICD_02318 Unsaturated rhamnogalacturonyl hydrolase YesR

MMVYPVKHSPLLRQPEHFIARDELKALVQKVTHNLVNIKDETGEFLLRLDDGRVIDTKGW
AGWEWTHGVGLYGMYHYYQQTGDQTMRKIIDDWFADRFAEGATTKNVNTMAPFLTLAYRY
EETRNPAYLPWLETWAEWAMNEMPRTDHGGMQHITLAEENHQQMWDDTLMMTVLPLAKIG
KLLNRPEYVEEATYQFLLHVQNLMDKETGLWFHGWSYDGHHNFANARWARGNSWLTIVIP
DFLELLDLPENNAVRRYLVQVLNAQIAALAKCQDESGLWHTLLDDPHSYLEASATAGFAY
GILKAVRKRYVGRHYAQVAEKAIRGIVKHISPEGELLQTSFGTGMGHDLDFYRHIPLTSM
PYGQAMAMLCLTEYLRNYF
>EECEBICD_02319 Flagellar protein FlhE
MRKWLALLLFPLTVQAAGEGAWQDSGMGVTLNYRGVSASSSPLSARQPVSGVMTLVAWRY
ELNGPTPAGLRVRLCSQSRCVELDGQSGTTHGFAHVPAVEPLRFVWEVPGGGRLIPALKV
RSNQVIVNYR
>EECEBICD_02320 Flagellar biosynthesis protein FlhA
MANLVAMLRLPSNLKSTQWQILAGPILILLILSMMVLPLPAFILDLLFTFNIALSIMVLL
VAMFTQRTLDFAAFPTILLFTTLLRLALNVASTRIILMEGHTGAAAAGKVVEAFGHFLVG
GNFAIGIVVFIILVIINFMVITKGAGRIAEVGARFVLDGMPGKQMAIDADLNAGLIGEDE
AKKRRSEVTQEADFYGSMDGASKFVRGDAIAGILIMVINVVGGLLVGVLQHGMSIGSAAE
SYTLLTIGDGLVAQIPALVISTAAGVIVTRVSTDQDVGEQMVGQLFSNPRVMLLAAAVLG
LLGMVPGMPNMVFLLFTAALLGLAWWLRGREEKAPEEPQPVKMPENNSVVEATWNDVQLE
DSLGMEVGYRLIPMVDFQQDGELLGRIRSIRKKFAQDMGFLPPVVHIRDNMDLQPARYRI
LMKGVEIGSGDAYPGRWLAINPGTAAGTLPGEKTVDPAFGLDAIWIESALKEQAQIQGFT
VVEASTVVATHLNHLIGQFSAELFGRQEAQQLLDRVSQEMPKLTEDLVPGVVTLTTLHKV
LQNLLAEKVPIRDMRTILETLAEHAPLQSDPHELTAVVRVALGRAITQQWFPGNEEVQVI
GLDTALERLLLQALQGGGGLEPGLADRLLAQTQEALSRQEMLGAPPVLLVNHALRPLLSR
FLRRSLPQLVVLSNLELSDNRHIRMTATIGGK
>EECEBICD_02321 Flagellar biosynthetic protein FlhB
MAEESDDDKTEAPTPHRLEKAREEGQIPRSRELTSLLILLVGVCIIWFGGESLARQLAGM
LSAGLHFDHRMVNDPNLILGQIILLIKAAMMALLPLIAGVVLVALISPVMLGGLIFSGKS
LQPKFSKLNPLPGIKRMFSAQTGAELLKAVLKSTLVGCVTGFYLWYHWPQMMRLMAESPI
VAMGNALDLVGLCALLVVLGVIPMVGFDVFFQIFSHLKKLRMSRQDIRDEFKESEGDPHV
KGKIRQMQRAAAQRRMMEDVPKADVIVTNPTHYSVALQYDENKMSAPKVVAKGAGLIALR
IREIGAEHRVPTLEAPPLARALYRHAEIGQQIPGQLYAAVAEVLAWVWQLKRWRLAGGQR
PPQPENLPVPEALDFMNEKNTDG
>EECEBICD_02322 hypothetical protein
MALIEIAGYDKKVSYPAGLTDSNPISAAPSGQKSLKPKTVQQVVHLILTGDDASFRGIHL
RAVE
>EECEBICD_02323 Protein phosphatase CheZ
MMMQPSIKPADEGSAGDIIARIGSLTRMLRDSLRELGLDQAIAEAAEAIPDARDRLDYVV
QMTAQAAERALNSVEASQPHQDAMEKEAKALTQRWDEWFDNPIELADARELVTDTRQFLR
DVPGHTSFTNAQLLDIMMAQDFQDLTGQVIKRMMDVIQEIERQLLMVLLENIPEQSARPK
RENESLLNGPQVDTSKAGVVASQDQVDDLLDSLGF
>EECEBICD_02324 Chemotaxis protein CheY
MADKELKFLVVDDFSTMRRIVRNLLKELGFNNVEEAEDGVDALNKLQAGGFGFIISDWNM
PNMDGLELLKTIRADSAMSALPVLMVTAEAKKENIIAAAQAGASGYVVKPFTAATLEEKL
NKIFEKLGM
>EECEBICD_02325 Protein-glutamate methylesterase/protein-glutamine glutaminase
MSKIRVLSVDDSALMRQIMTEIINSHSDMEMVATAPDPLVARDLIKKFNPDVLTLDVEMP
RMDGLDFLEKLMRLRPMPVVMVSSLTGKGSEVTLRALELGAIDFVTKPQLGIREGMLAYS
EMIAEKVRTAARARIAAHKPMAAPATLKAGPLLSSEKLIAIGASTGGTEAIRHVLQPLPL
SSPAVIITQHMPPGFTRSFAERLNKLCQISVKEAEDGERVLPGHAYIAPGDKHMELARSG
ANYQIKIHDGPPVNRHRPSVDVLFHSVAKHAGRNAVGVILTGMGNDGAAGMLAMYQAGAW
TIAQNEASCVVFGMPREAINMGGVSEVVDLSQVSQQMLAKISAGQAIRI
>EECEBICD_02326 Chemotaxis protein methyltransferase
MTSSLPSGQTSVLLQMTQRLALSDAHFRRICQLIYQRAGIVLADHKRDMVYNRLVRRLRA

LGLDDFGRYLSMLEANQNSAEWQAFINALTTNLTAFFREAHHFPILAEHARRRHGEYRVW
SAAASTGEEPYSIAITLADALGMAPGRWKVFASDIDTEVLEKARSGIYRLSELKTLSPQQ
LQRYFMRGTGPHEGLVRVRQELANYVEFSSVNLLEKQYNVPGPFDAIFCRNVMIYFDKTT
QEDILRRFVPLLKPDGLLFAGHSENFSNLVREFSLRGQTVYALSKDKA
>EECEBICD_02327 Methyl-accepting chemotaxis protein II
MATGGLAVVLVLILMVVWFGIRHALLNPLARVITHIREIASGDLTKTLTVSGRNEIGELA
GTVEHMQRSLIDTVTQVREGSDAIYSGTSEIAAGNTDLSSRTEQQASALEETAASMEQLT
ATVKQNADNARQASQLAQSASETARHGGKVVDGVVNTMHEIADSSKKIADIISVIDGIAF
QTNILALNAAVEAARAGEQGRGFAVVAGEVRNLASRSAQAAKEIKALIEDSVSRVDTGSV
LVESAGETMTDIVNAVTRVTDIMGEIASASDEQSRGIDQVALAVSEMDRVTQQNASLVQE
SAAAAAALEEQASRLTQAVSAFRLASRPLVANKPEMRLSVDAQSGNTPRSVAAGDDANWE
TF
>EECEBICD_02328 Methyl-accepting chemotaxis protein II
MFNRIRVVTMLMMVLGVFALLQLVSGGLLFSSLQHNQQGFVISNELRQQQSELTSTWDLM
LQTRINLSRSAARMMMDASNQQSSAKTDLLQNAKTTLAQAAAHYANFKNMTPLPAMAEAS
ANVDEKYQRYQAALAELIQFLDNGNMDAYFAQPTQGMQNALGEALGNYARVSENLYRQTF
DQSAHDYRFAQWQLGGLRSCWC
>EECEBICD_02329 Chemotaxis protein CheW
MTGMSNVSKLAGEPSGQEFLVFTLGNEEYGIDILKVQEIRGYDQVTRIANTPAFIKGVTN
LRGVIVPIVDLRVKFCEGDVEYDDNTVVIVLNLGQRVVGIVVDGVSDVLSLTAEQIRPAP
EFAVTLSTEYLTGLGALGERMLILVNIEKLLNSEEMALLDIAASHVA
>EECEBICD_02330 Chemotaxis protein CheA
MSMDISDFYQTFFDEADELLADMEQHLLDLVPESPDAEQLNAIFRAAHSIKGGAGTFGFT
ILQETTHLMENLLDEARRGEMQLNTDIINLFLETKDIMQEQLDAYKNSEEPDAASFEYIC
NALRQLALEAKGETTPAVVETAALSAAIQEESVAETESPRDESKLRIVLSRLKASEVDLL
EEELSNLATLTDVVKGADSLSATLDGSVAEDDIVAVLCFVIEADQIAFEKVVAAPVEKAQ
EKTEVAPVAPPAVVAPAAKSAAHEHHAGREKPARERESTSIRVAVEKVDQLINLVGELVI
TQSMLAQRSNELDPVNHGDLITSMGQLQRNARDLQESVMSIRMMPMEYVFSRFPRLVRDL
AGKLGKQVELTLVGSSTELDKSLIERIIDPLTHLVRNSLDHGIEMPEKRLEAGKNVVGNL
ILSAEHQGGNICIEVTDDGAGLNRERILAKAMSQGMAVNENMTDDEVGMLIFAPGFSTAE
QVTDVSGRGVGMDVVKRNIQEMGGHVEIQSKQGSGTTIRILLPLTLAILDGMSVRVAGEV
FILPLNAVMESLQPREEDLHPLAGGERVLEVRGEYLPLVELWKVFDVDGAKTEATQGIVV
ILQSAGRRYALLVDQLIGQHQVVVKNLESNYRKVPGISAATILGDGSVALIVDVTALQGL
NREQRMAITAA
>EECEBICD_02331 Motility protein B
MEKQPNIDELKKRMEQSRLNKLRGDLDQLIESDPKLRALRPHLKIDLVQEGLRIQIIDSQ
NRPMFKTGSAEVEPYMRDILRAIAPVLNGIPNRISLAGHTDDFPYANGEKGYSNWELSAD
RANASRRELVAGGLDNGKVLRVVGMAATMRLSDRGPDDAINRRISLLVLNKQAEQAILHE
NAESQNEPVSVLQQPAAAPPASVPTSPKAEPR
>EECEBICD_02332 Motility protein B
MKNQAHPIVVVKRRRHKPHGGGAHGSWKIAYADFMTAMMAFFW
>EECEBICD_02333 Motility protein A
MLILLGYLVVIGTVFGGYVMTGGHLGALYQPAELVIIGGAGIGAFIVGNNGKAIKGTMKA
IPLLFRRSKYTKSMYMDLLALLYRLMAKSRQQGMFSLERDIENPKESEIFASYPRILADA
VMLDFIVDYLRLIISGNMNTFEIEALMDEEIETHESEAEVPANSLAMVGDSLPAFGIVAA
VMGVVHALASADRPAAELGALIAHAMVGTFLGILLAYGFISPLATVLRQKSAETTKMMQC
VKITLLSNLNGYAPPIAVEFGRKTLYSSERPSFIELEEHVRAVRNPNQQQTTEEA
>EECEBICD_02334 Flagellar transcriptional regulator FlhC
MSEKSIVQEARDIQLAMELINLGARLQMLESETQLSRGRLIRLYKELRGSPPPKGMLPFS
TDWFMTWEQNIHASMFCNAWQFLLKTGLCSGVDAVIKAYRLYLEQCPQPPEGPLLALTRA
WTLVRFVESGLLELSSCNCCGGNFITHAHQPVGSFACSLCQPPSRAVKRRKLSRDAADII
PQLLDEQIEQAV
>EECEBICD_02335 Flagellar transcriptional regulator FlhD
MHTSELLKHIYDINLSYLLLAQRLIVQDKASAMFRLGINEEMANTLGALTLPQMVKLAET

NQLVCHFRFDDHQTITRLTQDSRVDDLQQIHTGIMLSTRLLNEVDDTARKKRA
>EECEBICD_02336 Universal stress protein C
MSYTHILVAVAVTPESHQLLAKAVSIARPVQAKVSLITLASDPELYNQFAAPMMEDLRAV
MHEETENFLKMLGEKADYPIEQTFIASGELSQHILAVCRKHHVDLVICGNHNHSFFSRAS
CSAKSVVSASQVDVLLVPLAGD
>EECEBICD_02337 Trehalose-6-phosphate synthase
MSRLVVVSNRIAPPDNKGGAGGLAVGVLGALKAAGGLWFGWSGETGNEDEPLKKVTKGNI
TWASFNLSEQDYEDYYCQFSNAVLWPAFHYRLDLVQFQRPAWEGYMRVNALLADKLLPLI
KENDIIWVHDYHLLPFASELRKRGVNNRIGFFLHIPFPTPEIFNALPPHDELLEQLCDFD
LLGFQTENDRLAFLDSLSSQTRVTTRSGKQHIAWGKDFQTEVYPIGIEPDEIALQAAGPL
PPKLAQLKAELKNVKNIFSVERLDYSKGLPERFLAYEALLENYPQHRGKIRYTQIAPTSR
GEVQAYQDIRHQLETEAGRINGKYGQLGWTPLYYLNQHFDRKLLMKIFRYSDVGLVTPLR
DGMNLVAKEFVAAQDPANPGVLVLSQFAGAANELTSALIVNPYDRDDVAAALNRALTMPL
AERISRHAEMLDVIVKNDINRWQERFIHDLKEVTPRSPERQQQNNVATFPKLA
>EECEBICD_02338 Trehalose-6-phosphate phosphatase
MVFLNGEQRHTVYTYMTNETVCESQYDDKEETRVAEPLTVSPELTANYAYFFDLDGTLAE
IKPHPDQVVVPHKILQLLDRLAAHNAGALALISGRSMTELDALAKPFRFPLAGVHGAERR
DINGKTHIVRLPEAVVREVEALLRSTLVALPGTELETKGMAFALHYRQAPEHEAALLALA
QHVTQHWPQLALQPGKCVVEIKPKGTNKGEAIAAFMQEAPFAGRIPVFVGDDLTDEAGFG
VVNHAGGISVKVGVGATQAAWRLESVPDVWRWLEQINYPQQEQQVMNNRRDGYESFSRSI
>EECEBICD_02339 Protein/nucleic acid deglycase 3
MKKVAVLLAPGFEEAEAIVTLDILRRLHIDVETLACAESRAVVSYHDIPMVADSTLSERQ
QALFDAVVLPGGPQGSANLAANPAVIAFVARHDAAGKFICPICSAAARVLGAHGLLKGRR
YVCSGDLWKAVPEGVYVDAPVVEDGNLISGKGLGHVFDFALTLSARLLGDDAPVREQAEH
IYYPW
>EECEBICD_02340 Bacterial non-heme ferritin
MAAVGMVQKLNTPMNLEFYASNLYLHLSEWCSEHSLTGTATFLRTQAQGNVTQMMRMFNF
MKNAGATPIVKAIDVPGEELCSLEELFQKTLEDYQQRASTLSRLADEAKALNDASTLDFL
HTLEKEQQQDGVLLQTILEEVRSAKRAGLCLAQTDQHLLNVVTYQHH
>EECEBICD_02341 hypothetical protein
MKIALMMENSQASKNAIIYNELSAVANEKGFPVFNVGMCDENDHHLTYIHLGIMASILLN
ANAVDFVVTGCGTGQGALMSLNIHPGVICGYCIDPADAFLFAQINNGNALSLPFAKGFGW
GAELNVRFIFEKAFTGRKGEGYPPERKAPQVRNAGILNQVKAAVVKENYLDTLRAIDPEL
VKTAVSGPRFQQCLFENGQNKEIEAFVREMLG
>EECEBICD_02342 hypothetical protein
MKPLIFTLSLLALTGCTITRQAQVSEASPISGIVRLTYNQPLFFTSRTDDYVSHGTATRE
CQQMGYADAVSFGQPVGTCSIYAGSLCLNTRFTLSWQCRGVAVPQIMPLYY
>EECEBICD_02343 Bacterial non-heme ferritin
MLKTEMIDKLNEQMNLELYSSLLYQQMSAWCSYHSFEGAAAFLRRHAQEEMTHMQRLFDY
LTDTGSLPRIHTVSSPFAEYASLDELFRATYEHEQLITQKINELAHAAMTSQDYPTFNFL
QWYVAEQHEEEKLFKSIIDKLTLAGKSGEGLYFIDKELSTLDTQN
>EECEBICD_02344 hypothetical protein
MDSIHGHEVLNMMIESGEQYTHTSLEAAIKARFGERARFHTCSASDMTAAELVAFLAAKG
KFIAVEDGFSTHESKICRH
>EECEBICD_02345 Tyrosine-specific transport protein
MPYPAPRISPFFVTGVVESVKNRTLGSIFIVAGTTIGAGMLAMPLAAAGVGFSVTLGLLI
GLWALMCYTALLLLEVYQHVPADTGLGSLAKRYLGRYGQWLTGFSMMFLMYALTAAYISG
AGELLASSINNWLGATLSPAAGVLLFTFVAGGVVCVGTSLVDLFNRFLFSAKIIFLVIML
ALLTPHIHKVNLLTLPLQQGLALSAIPVIFTSFGFHGSVPSIVSYMNGNIRRLRWVFMTG
SAIPLVAYIFWQLATQGSIDSPTFRGLLASHAGLNGLLQALREVVASPHVELAVHLFADL
ALATSFLGVALGLFDYLADLFQRRSTVSGRLQTGLITFLPPLAFALFYPRGFVMALGYAG
VALAVLALLIPAMLVWQCRKQSPQAGYRVAGGTPALALVFICGIVVIGVQFSIALGFLPD
PG
>EECEBICD_02346 hypothetical protein

MKMEPLNENELEWLDDVLTKYNTDQAILDVAELDGLITAVLSSPRPIEPEQWLVAIWGGP
AYVPRWTSEKEMTRFMDLVFQHMADTAARLEDYPEQFEPLFGLREVDGHELTIVEEWCFG
YMRGVSLSDWSDLPDTLKPALEAIALHGTEENFALLDKMSPEAFDKSVDAIRIAALELHA
WWMAHPHTTPLQMPIKAEVKVGRNDPCPCGSGKKFKQCCLH
>EECEBICD_02347 hypothetical protein
MVKRRNLLHFNNGARSERDLGDLVTKVFEKAAKKEPQPLYTFSLPLLSVQDEIRVYCKKK
NIKIGYDTLFMEITFSADREAVDELIKHFFTENKLYLRGRFYLSAAVV
>EECEBICD_02348 hypothetical protein
MQKKTWAVLLIPLCLTACHQTTYRSTAKDEILTGSYIDPTKTRFPLADYSQSVDKWIPPD
SADYTIPVIDSATQQRYFNALKSHYFGMDSEAHSPWNGFYITALLKKNAAQARDASIKQF
LSDGSAYWGENFRLYTSRWKEEVRGNTDTQIDNIYNASRRGIMVRESLVRALPTDDPLFN
DPRQAGEGYPFDNLQMSSLRPGTPVYTLTKSKDQRWQYVVSPAVTGWVHSEDIASTDQKF
ITQWVLLAHKQLGAFINAPVSVHAAGVYYFTGRPGTILPFRHQRAGQFLIAAPVRDSNGR
AFIHWVWLSGNEFTAMPWKMTPENIAVLMKAMHGAPYGWGNFNFYNDCSAEVRSLLMPFG
IFLPRHSSAQVEAAGRVVDLSHKNPQMRIDYLTRYGKPFTTLVYIPGHIMLYIGNTTMNG
QVMPMTYQNIWGLRPNHANSRSIIGEAVFLPLLRFYPENPELISLAGKVLFKLGYIE
>EECEBICD_02352 CDP-diacylglycerol--glycerol-3-phosphate 3-
phosphatidyltransferase
MQFNIPTLLTLFRVILIPFFVVVFYLPFAWAPMVSALIFCIAAITDWFDGFLARRWNQST
RFGAFLDPVADKVLVAIAMVLVTEHYHSWWVTLPAATMIAREIIISALREWMAELGKRSS
VAVSWIGKVKTTAQMVALAWLLWRPNIWVEYAGIALFFVAAVLTLWSMLQYLSAARGDLL
DQ
>EECEBICD_02353 UvrABC system protein C
MSEIFDAKAFLKTVTSQPGVYRMYDAGGTVIYVGKAKDLKKRLSSYFRSNLASRKTEALV
AQIQHIDVTVTHTETEALLLEHNYIKLYQPRYNVLLRDDKSYPFIFLSGDTHPRLAMHRG
AKHAKGEYFGPFPNGYAVRETLALLQKIFPIRQCENSVYRNRSRPCLQYQIGRCLGPCVA
GLVSEEEYAQQVEYVRLFLSGKDDQVLTQLIARMEKASQDLAFEEAARIRDQIQAVRRVT
EKQFVSNAGDDLDVIGVAFDAGMACVHVLFIRQGKVLGSRSYFPKVPGGTELGEVVETFV
GQFYLQGSQMRTLPGEILLDFNLSDKTLLADSLSELAGRRIHVQTKPRGDRARYLKLART
NAATALITKLSQQSTITQRLTALAAVLKLPAIKRMECFDISHTMGEQTVASCVVFDANGP
LRAEYRRYNIAGITPGDDYAAMNQVLRRRYGKAIEESKIPDVILIDGGKGQLAQAKAVFA
ELDVPWDKHRPLLLGVAKGADRKAGLEILFFEPEGEGFSLPPDSPALHVIQHIRDESHDH
AIGGHRKKRAKVKNTSTLETIEGVGPKRRQMLLKYMGGLQGLRNASVEEIAKVPGISQGL
AEKIFWSLKH
>EECEBICD_02354 Response regulator UvrY
MINVLLVDDHELVRAGIRRILEDIKGIKVVGEACCGEDAVKWCRTNAVDVVLMDMNMPGI
GGLEATRKIARSTADIKVIMLTVHTENPLPAKVMQAGAAGYLSKGAAPQEVVSAIRSVYS
GQRYIASDIAQQMALSQIEPAKTETPFASLSERELQIMLMITKGQKVNEISEQLNLSPKT
VNSYRYRMFSKLNIHGDVELTHLAIRHGLCNAETLTSQ
>EECEBICD_02355 hypothetical protein
MSTPDFSTAENNQELATEVNCLKAMLTLMLQAMGQADAGRVILKMEKQIAQMDDEAQAAV
FSSTVKQIKQAYRQ
>EECEBICD_02356 Regulatory protein SdiA
MQENDFFTWRRAMLLRFQEMAAAEDVYTELQYQTQRLEFDYYALCVRHPVPFTRPKISLR
TTYPPAWVTHYQSENYFAIDPVLKPENFRQGHLHWDDVLFHEAQAMWDAAQRFGLRRGVT
QCVMLPNRALGFLSFSRSSLRCSSFTYDEVELRLQLLARESLSALTRFEDDMVMAPEMRF
SKREKEILKWTAEGKTSSEIAIILSISENTVNFHQKNMQKKFNAPNKTQIACYAAATGLI
>EECEBICD_02357 Arginine transport ATP-binding protein ArtM
MSAIEVTNLVKKFHGQTVLHGIDLEVKPGEVVAIIGPSGSGKTTLLRCINLLEQPEAGTI
KVGDIIIDTARPFSQQKGLIRQLRQHVGFVFQSFNLFPHRTVLENIIEGPVIVKGEPKAE
ATALARELLAKVGLAGKETRYPRRLSGGQQQRVAIARALAMRPEVILFDEPTSALDPELV
GEVLNTIRQLAQEKRTMVIVTHEMGFARDVADRAIFMDQGQIVEQGPAKTLFAEPQHPRT
RQFLEKFLVK
>EECEBICD_02358 L-cystine transport system permease protein YecS

MQESIQLVIESLPYLLKGAVFTLQLSIGGMFFGLLLGFVLALMRMSPLWLVRWLARFYIS
IFRGTPLIAQLFMIYYGLPQFGIELDPIPAAMIGLSLNTAAYTSETLRAAISAIDKGQWE
AAASIGMTPWQTLRRAILPQAARVALPPLSNSFISLVKDTSLAATIQVPELFRQAQLITS
RTLEVFTMYLAASLIYWVMATVLSALQNYFENQLNRQEREPK
>EECEBICD_02359 D-cysteine desulfhydrase
MPLHHLTRFPRLELIGAPTPLEYLPRLSDYLGREIYIKRDDVTPIAMGGNKLRKLEFLVA
DALREGADTLITAGAIQSNHVRQTAAVAAKLGLHCVALLENPIGTTAENYLTNGNRLLLD
LFNTQIEMCDALTDPDAQLQTLATRIEAQGFRPYVIPVGGSSALGAMGYVESALEIAQQC
EEVVGLSSVVVASGSAGTHAGLAVGLEHLMPDVELIGVTVSRSVAEQKPKVIALQQAIAG
QLALTATADIHLWDDYFAPGYGVPNDAGMEAVKLLASLEGVLLDPVYTGKAMAGLIDGIS
QKRFNDDGPILFIHTGGAPALFAYHPHV
>EECEBICD_02360 L-cystine-binding protein FliY
MKLALLGRQALMGVMAVALVAGMSAKSFADEGLLNKVKERGTLLVGLEGTYPPFSFQGDD
GKLTGFEVDFAEALAKHLGVKASLKPTKWDGMLASLDSKRIDVVINQVTISDVRKKKYDF
STPYTVSGVQALVKKGNEGTIKTAADLQGKKVGVGLGTNYEEWLRQHVQGVDIRTYDDDP
TKYQDLRVGRIDAILVDRLAALDLVKKTKGTLAVTGDAFSRQESGVALRKGNEDLLKAVD
NAIAEMQKDGTLKALSEKWFGADVTQ
>EECEBICD_02361 Regulator of sigma S factor FliZ
MTVQQPKRRPLSRYLKDFKHSQTHCAHCHKLLDRITLVRRGKIVNKIAISQLDMLLDDAA
WQREQKEWVALCRFCGDLHCKKQSDFFDIIGFKQYLFEQTEMSHGTVREYVVRLRRLGNY
LSEQNISHDLLQDGFLDESLAPWLPETSTNNYRIALRKYQQYKAHQQIAPRQKSPFTASS
DIY
>EECEBICD_02362 RNA polymerase sigma factor FliA
MNSLYTAEGVMDKHSLWQRYVPLVRHEALRLQVRLPASVELDDLLQAGGIGLLNAVDRYD
ALQGTAFTTYAVQRIRGAMLDELRSRDWVPRSVRRNAREVAQAMGQLEQELGRNATETEV
AERLGIPVAEYRQMLLDTNNSQLFSYDEWREEHGDSIELVTEEHQQENPLHQLLEGDLRQ
RVMDAIESLPEREQLVLTLYYQEELNLKEIGAVLEVGESRVSQLHSQAIKRLRTKLGKL
>EECEBICD_02363 hypothetical protein
MKEITVTEPAFVTRFSCSGSACRDHCCKGWKITLDKTTVKKYLTSKDVTIREIAKNSIIL
LKKDPNNWGEIKLPSGTGSCPYLDDDRLCKVQKTLGAKALSYTCTTFPRVFHTYKNEVRH
SLSLACPEVTAHILNDTDAVTLNEKAIIQQKYNTAPLFSPQQKLLNLFCLSLINHAASNP
DAALYALIKFVMYVQKFPRIDDAALGEIEQVYGTLVSQLQSGSLTQELANITPDKKFKTS
LVLLMQDYFRALPPSRGSYVLDHYIQCLLRVLTAEEGVSMEQKVSDIESSLARCLQADEQ
QKNWAFRNLILYKIWENNFPNQPNVDPLRALYIIVAEYAFIKLLTAASVHERGRLEWDDV
TNIVYSFHSRSQHNSEVAANFHRHIETVRTGDDLSMIHLLT
>EECEBICD_02364 Flagellin
MAQVINTNSLSLLTQNNLNKSQSSLSSAIERLSSGLRINSAKDDAAGQAIANRFTSNIKG
LTQASRNANDGISIAQTTEGALNEINNNLQRVRELSVQATNGTNSDSDLKSIQDEIQQRL
EEIDRVSNQTQFNGVKVLSQDNQMKIQVGANDGETITIDLQKIDVKSLGLDGFNVNGPKE
ATVGDLKSSFKNVTGYDTYAAGADKYRVDINSGAVVTDAAAPDKVYVNAANGQLTTDDAE
NNTAVDLFKTTKSTAGTAEAKAIAGAIKGGKEGDTFDYKGVTFTIDTKTGDDGNGKVSTT
INGEKVTLTVADIATGATDVNAATLQSSKNVYTSVVNGQFTFDDKTKNESAKLSDLEANN
AVKGESKITVNGAEYTANATGDKITLAGKTMFIDKTASGVSTLINEDAAAAKKSTANPLA
SIDSALSKVDAVRSSLGAIQNRFDSAITNLGNTVTNLNSARSRIEDADYATEVSNMSKAQ
ILQQAGTSVLAQANQVPQNVLSLLR
>EECEBICD_02365 Flagellar hook-associated protein 2
MASISSLGVGSNLPLDSLLTKLTNAEKGRLTPITQQQSANTARLTAYGTLKSALEKFQTA
NTALNKADLFKSTNVTSSTEDLKVSTEAGAAPGTYVVSVTQLAQAQSLSTATKITSTKEV
LGDTTSDSRTIKIEQKGRKEPLEIKLTKDQTSLEGIRDAINDADSGISASIVKVKEGDYQ
LVLTADSGTDNQMTISVEGDSKLSDLLSYDSSTGTGKMKQLVAADNALLTVNGIDIERPS
NKITDAPQGVTLELTKEVKDARITVTKDNEKATEAVKGWVDAYNSLLDTFSSLTKYTEVD
PGAEEQDKNNGALLGDTVVRTIQTGIRAQFANGASTGTFKTLNEIGITSDGTTGKLKIDD
TKLKKALDENTASVRELLVGDGKETGITTKIATEVKSYLADDGIIDSAQDSINATLKKLT
KQYLTVSSSIDDTVARYKAQFTQLDTMMSKLNNTSTYLTQQFNAMNKS

>EECEBICD_02366 Flagellar secretion chaperone FliS
MYTASGIKAYAQVSVESAVMSASPHQLIEMLFDGANSALVRARLFLEQGDVVAKGEALSK
AINIIDNGLKAGLDQEKGGEIATNLSELYDYMIRRLLQANLRNDAQAIEEVEGLLSNIAE
AWKQISPKASFQESR
>EECEBICD_02367 Flagellar protein FliT
MTSTVEFINRWQRIALLSQSLLELAQRGEWDLLLQQEVSYLQSIETVMEKQTPPGITRSI
QDMVAGYIKQTLDNEQLLKGLLQQRLDELSSLIGQSTRQKSLNNAYGRLSGMLLVPDAPG
AS
>EECEBICD_02368 Cytoplasmic alpha-amylase
MKNPTLLQYFHWYYPDGGKLWSELAERADGLNDIGINMVWLPPACKGASGGYSVGYDTYD
LFDLGEFDQKGTIATKYGDKRQLLTAIDALKKNNIAVLLDVVVNHKLGADEKERIRVQRV
NQDDRTQIDDNIIECEGWTRYTFPARAGQYSNFIWDYHCFSGIDHIENPDEDGIFKIVND
YTGDGWNDQVDDELGNFDYLMGENIDFRNHAVTEEIKYWARWVMEQTHCDGFRLDAVKHI
PAWFYKEWIEHVQAVAPKPLFIVAEYWSHEVDKLQTYIDQVDGKTMLFDAPLQMKFHEAS
RQGAEYDMRHIFTGTLVEADPFHAVTLVANHDTQPLQALEAPVEPWFKPLAYALILLREN
GVPSVFYPDLYGASYEDNGENGETCRVDMPVINQLDRLILARQRFAHGIQTLFFDHPNCI
AFSRSGTEENPGCVVVLSNGDDGEKTLLLGDNYANKTWRDFLGNRSQHVVTNDQGEATFF
CNAGSVSVWVIEDV
>EECEBICD_02369 hypothetical protein
MKKVAIVGALLVLAGCAEVENYNDVVKTPAPAGLEGYWQSKGPQRKLVSPEAIASLVVTK
EGDTLDCRQWQRVIALPGKLTMLSDDLTNVTVKRELYEIERDGNTLEYDGMTLQRVARPT
PECAAALEKNAAADAAAVISLPGSALPPGTLITRPQ
>EECEBICD_02370 hypothetical protein
MSWQHFKQTWLIKFWAPAPAVIAAGILSTYYFGITGTFWAVTGEFTRWGGQILQLFGVHA
EQWGYYKLIHLEGTPLTRIDGMMILGMFGGCFAAALWANNVKLRMPRSRIRIVQAVVGGM
IAGFGARLAMGCNLAAFFTGIPQFSLHAWFFALATAIGSWFGARFTLLPIFRIPVKMQKV
SAASPLTQKPDQARRRFRLGMLVFIGMIGWALLTAMHQPKLGLAMLFGVGFGLLIERAQI
CFTSAFRDLWISGRAHMAKAIIFGMAVSAIGIFSYVQLGVAPKIMWAGPNAVIGGLLFGF
GIVLAGGCETGWMYRAVEGQVHYWWVGLGNVIGSTILAYYWDDFAPALATSWDKVNLLNT
FGPLGGLLVTYLLLFAALMLIIGWEKRFFRRAGLTPAKESV
>EECEBICD_02371 Putative sulfur carrier protein YedF
MKNIVPDYRLDMVGEPCPYPAVATLEAMPQLKKGEILEVVSDCPQSINNIPLDARNHGYT
VLDIQQDGPTIRYLIQK
>EECEBICD_02372 Flagellar hook-basal body complex protein FliE
MAAIQGIEGVISQLQATAMAARGQDTHSQSTVSFAGQLHAALDRISDRQTAARVQAEKFT
LGEPGIALNDVMADMQKASVSMQMGIQVRNKLVAAYQEVMSMQV
>EECEBICD_02373 Flagellar M-ring protein
MSATASTATQPKPLEWLNRLRANPRIPLIVAGSAAVAIVVAMVLWAKTPDYRTLFSNLSD
QDGGAIVAQLTQMNIPYRFANGSGAIEVPADKVHELRLRLAQQGLPKGGAVGFELLDQEK
FGISQFSEQVNYQRALEGELARTIETLGPVKSARVHLAMPKPSLFVREQKSPSASVTVTL
EPGRALDEGQISAVVHLVSSAVAGLPPGNVTLVDQSGHLLTQSNTSGRDLNDAQLKFAND
VESRIQRRIEAILSPIVGNGNVHAQVTAQLDFANKEQTEEHYSPNGDASKATLRSRQLNI
SEQVGAGYPGGVPGALSNQPAPPNEAPIATPPTNQQNAQNTPQTSTSTNSNSAGPRNTQR
NETSNYEVDRTIRHTKMNVGDIERLSVAVVVNYKTLADGKPLPLTADQMKQIEDLTREAM
GFSDKRGDTLNVVNSPFSAVDNTGGELPFWQQQFFIDQLLAAGRWLLVLVVAWILWRKAV
RPQLTRRVEEAKAAQEQAQVRQETEEAVEVRLSKDEQLQQRRANQRLGAEVMSQRIREMS
DNDPRVVALVIRQWMSNDHE
>EECEBICD_02374 Flagellar motor switch protein FliG
MSNLSGTDKSVILLMTIGEDRAAEVFKHLSTREVQALSTAMANVRQISNKQLTDVLSEFE
QEAEQFAALNINANEYLRSVLVKALGEERASSLLEDILETRDTTSGIETLNFMEPQSAAD
LIRDEHPQIIATILVHLKRSQAADILALFDERLRHDVMLRIATFGGVQPAALAELTEVLN
GLLDGQNLKRSKMGGVRTAAEIINLMKTQQEEAVITAVREFDGELAQKIIDEMFLFENLV
DVDDRSIQRLLQEVDSESLLIALKGAEPPLREKFLRNMSQRAADILRDDLANRGPVRLSQ
VENEQKAILLIVRRLAETGEMVIGSGEDTYV

>EECEBICD_02375 Flagellar assembly protein FliH
MSNELPWQVWTPDDLAPPPETFVPVEADNVTLTDDTPEPELTAEQQLEQELAQLKIQAHE
QGYNAGLAEGRQKGHAQGYQEGLAQGLEQGQAQAQTQQAPIHARMQQLVSEFQNTLDALD
SVIASRLMQMALEAARQVIGQTPAVDNSALIKQIQQLLQQEPLFSGKPQLRVHPDDLQRV
EEMLGATLSLHGWRLRGDPTLHHGGCKVSADEGDLDASVATRWQELCRLAAPGVL
>EECEBICD_02376 Flagellum-specific ATP synthase
MTTRLTRWLTALDNFEAKMALLPAVRRYGRLTRATGLVLEATGLQLPLGATCIIERQDGP
ETKEVESEVVGFNGQRLFLMPLEEVEGILPGARVYARNGHGDGLQSGKQLPLGPALLGRV
LDGGGKPLDGLPAPDTLETGALITPPFNPLQRTPIEHVLDTGVRAINALLTVGRGQRMGL
FAGSGVGKSVLLGMMARYTRADVIVVGLIGERGREVKDFIENILGPDGRARSVVIAAPAD
VSPLLRMQGAAYATRIAEDFRDRGQHVLLIMDSLTRYAMAQREIALAIGEPPATKGYPPS
VFAKLPALVERAGNGIHGGGSITAFYTVLTEGDDQQDPIADSARAILDGHIVLSRRLAEA
GHYPAIDIEASISRAMTALITEQHYARVRLFKQLLSSFQRNRDLVSVGAYAKGSDPMLDK
AITLWPQLEAFLQQGIFERADWEDSLQALDLIFPTV
>EECEBICD_02377 Flagellar FliJ protein
MAQHGALETLKDLAEKEVDDAARLLGEMRRGCQQAEEQLKMLIDYQNEYRSNLNTDMGNG
IASNRWINYQQFIQTLEKAIEQHRLQLTQWTQKVDLALKSWREKKQRLQAWQTLQDRQTA
AALLAENRMDQKKMDEFAQRAAMRKPE
>EECEBICD_02378 Flagellar hook-length control protein
MITLPQLITTDTDMTAGLTSGKTTGSAEDFLALLAGALGADGAQGKDARITLADLQAAGG
KLSKELLTQHGEPGQAVKLADLLAQKANATDETLTDLTQAQHLLSTLTPSLKTSALAALS
KTAQHDEKTPALSDEDLASLSALFAMLPGQPVATPVAGETPAENHIALPSLLRGDMPSAP
QEETHTLSFSEHEKGKTEASLARASDDRATGPSLTPLVVAAAATSAKVEVDSPPAPVTHG
AAMPTLSSATAQPLPVASAPVLSAPLGSHEWQQTFSQQVMLFTRQGQQSAQLRLHPEELG
QVHISLKLDDNQAQLQMVSPHSHVRAALEAALPMLRTQLAESGIQLGQSSISSESFAGQQ
QSSSQQQSGRAQHTDAFGAEDDIALAAPASLQAAARGNGAVDIFA
>EECEBICD_02379 hypothetical protein
MTDSAINKKSKRSIWIPLLVLITLAACATAGYSYWRMQQQPTTNAKAEPAPPPAPVFFAL
DTFTVNLGDADRVLYIGVTLRLKDEATRARLNEYLPEVRSRLLLLFSRQNAAELSTEAGK
QKLIAAIKETLAAPLVAGQPKQVVTDVLYTAFILR
>EECEBICD_02380 Flagellar motor switch protein FliM
MGDSILSQAEIDALLNGDSDTKDEPTPGIASDSDIRPYDPNTQRRVVRERLQALEIINER
FARQFRMGLFNLLRRSPDITVGAIRIQPYHEFARNLPVPTNLNLIHLKPLRGTGLVVFSP
SLVFIAVDNLFGGDGRFPTKVEGREFTHTEQRVINRMLKLALEGYSDAWKAINPLEVEYV
RSEMQVKFTNITTSPNDIVVNTPFHVEIGNLTGEFNICLPFSMIEPLRELLVNPPLENSR
HEDQNWRDNLVRQVQHSELELVANFADIPLRLSQILKLKPGDVLPIEKPDRIIAHVDGVP
VLTSQYGTVNGQYALRVEHLINPILNSLNEEQPK
>EECEBICD_02381 Flagellar motor switch protein FliN
MSDMNNPSDENTGALDDLWADALNEQKATTTKSAADAVFQQLGGGDVSGAMQDIDLIMDI
PVKLTVELGRTRMTIKELLRLTQGSVVALDGLAGEPLDILINGYLIAQGEVVVVADKYGV
RITDIITPSERMRRLSR
>EECEBICD_02382 Flagellar protein FliO
MMKTEATVSQPTAPAGSPLMQVSGALIGIIALILAAAWVIKRMGFAPKGNSVRGLKVSAS
ASLGPRERVVIVEVENARLVLGVTASQINLLHTLPPAENDTEAPVAPPADFQNMMKSLLK
RSGRS
>EECEBICD_02383 Flagellar biosynthetic protein FliP
MRRLLFLSLAGLWLFSPAAAAQLPGLISQPLAGGGQSWSLSVQTLVFITSLTFLPAILLM
MTSFTRIIIVFGLLRNALGTPSAPPNQVLLGLALFLTFFIMSPVIDKIYVDAYQPFSEQK
ISMQEALDKGAQPLRAFMLRQTREADLALFARLANSGPLQGPEAVPMRILLPAYVTSELK
TAFQIGFTIFIPFLIIDLVIASVLMALGMMMVPPATIALPFKLMLFVLVDGWQLLVGSLA
QSFYS
>EECEBICD_02384 Flagellar biosynthetic protein FliQ
MTPESVMMMGTEAMKVALALAAPLLLVALITGLIISILQAATQINEMTLSFIPKIVAVFI
AIIVAGPWMLNLLLDYVRTLFSNLPYIIG

>EECEBICD_02385 Flagellar biosynthetic protein FliR
MIQVTSEQWLYWLHLYFWPLLRVLALISTAPILSERAIPKRVKLGLGIMITLVIAPSLPA
NDTPLFSIAALWLAMQQILIGIALGFTMQFAFAAVRTAGEFIGLQMGLSFATFVDPGSHL
NMPVLARIMDMLAMLLFLTFNGHLWLISLLVDTFHTLPIGSNPVNSNAFMALARAGGLIF
LNGLMLALPVITLLLTLNLALGLLNRMAPQLSIFVIGFPLTLTVGIMLMAALMPLIAPFC
EHLFSEIFNLLADIVSEMPINNNP
>EECEBICD_02386 Transcriptional regulatory protein RcsA
MSTIIMDLCSYTRLGLSGYLVSRGVKKREINDIETVDELAIACGAHQPSVVFINEDCFIH
TPSDSQQIKQIINQHPDTLFIVFMAIANVHFDEYLLVRKNLLISSKSIKPDSLDTILGDI
LKKESGISGTINLPTLSLSRTESSMLRMWMEGQGTIQISDRMNIKAKTVSSHKGNIKRKI
KTHNKQVIYHVVRLTDNVTNGIFVNMR
>EECEBICD_02387 Protein DsrB
MKVNDRVTVKTDGGPRRPGVVLAVEEFSEGTMYLVSLEDYPLGIWFFNESGHQDGIFVEK
AEQD
>EECEBICD_02388 hypothetical protein
MKTAKEYSDTAKREVSVDVDALLAAINEISESEVHRSQEDPERVSVDGREYHTWHELAEA
FELDIHDFSVTEVNR
>EECEBICD_02389 Mannosyl-3-phosphoglycerate phosphatase
MLSIHDPLLIFTDLDGTLLNSHTFEWQPAAPWLTRLHESGVPVILCSSKTAAEMLQLQTT
LNLQGLPLIAENGAVIQLDVHWEDHPNYPRLIAGISHNEIRLVLHKLREKEQFKFTTFDD
VDDQVISEWTGLNRAQSALTRLHEASVSLIWRDSDERMAQFVAHLNDLGLQFVHGARFWH
VLDASAGKDQAANWLIEAYRRQWRARPLTLGLGDGPNDAPLLDVMDYAVVVKGLNREGVH
LRNDDPQRVYRSQNEGPDGWREGMDYFFSRS
>EECEBICD_02390 putative diguanylate cyclase DgcQ
MPHETLLDNQGWFKKLARRFGPGHVVNTCFLIVMLFSTLLTWREVMILKDAYVASQRNHL
GSVANVLDRQLQFNMDRLIFLRNGMHEALVAPLAFSALQSAVTQFEQRRVRHFWQLELDK
RRTLPLYGVSDQFVARTTLLSRESRDLANELTATLELGYLARLARSSAMLTLETMYVSRS
GFYLSTLPTAYGSDIVSRYYQYVTQPWFIEQSQRRNPQRGVRWFTSAQPYVADEQKKVTA
SLPLDHDNYWYGVLAMDIPVASLQRFLRDAAEKDIEGEYQLYDNHLRLLTDSAPEQQTAN
TLNDRERALLAREIEKDTLGGLRLGTHYVSWERLDHFDGILLRVHTLREGIQGNFGSISI
ALTLLWVLFTAMLLISWGVIRHMVKNMFVLQNSLQWQAWHDPLTRLYNRGALFEKASRLA
KRYREARQPFSVIQLDLDYFKSVNDRFGHQAGDRVLSHAAGLIGSTIRAHDIAGRVGGEE
FCIVLPGATKAQALQIAERIRQRINDKEILVTKSTTLRISASMGISSAEEYGDYDFEQLQ
SLADKRLYYAKQSGRNRICASDATQEREKK
>EECEBICD_02391 hypothetical protein
MVFSVSEEVTVKEGGPRMIVTGYSSGMVECRWYDGFGVKREAFHENELVPGKERRGRDEA
R
>EECEBICD_02392 Inner membrane protein YedI
MAGSSLLTLLDDIATLLDDISVMGKLAAKKTAGVLGDDLSLNAQQVTGVRANRELPVVWS
VAKGSLINKVILVPLALLISAFIPWAITPLLMLGGAFLCFEGVEKVLHTFEARKHKEDPA
ERQKRLEALAAQDPLAFEKDKVKGAVRTDFILSAEIVAITLGIVAQAPLLNQVLVLAGIA
LVVTIGVYGLVGIIVKLDDMGYWLAEKRSVLAQGVGKGLLIIAPWLMKALSIVGTLAMFL
VGGGIVVHGIAPLHHAIEHFAQQQGAFMAHTIPAGLNLVLGFIIGAIVVALVKGVAKIRG
VSH
>EECEBICD_02393 putative inner membrane transporter YedA
MRFRQLLPLFGALFALYIIWGSTYFVIRIGVESWPPLMMAGVRFLSAGMLLMAFLLLRGE
KLPPLRQTINAALIGLLLLAVGNGLVTVAEHQNVPSGIAAVVVATVPLFTLCFSYFFGIK
ARKLEWVGIAIGLAGIIILLNSGGNLSGNPWGAILILIGSMSWAFGSVYGSRIALPVGMMA
GAIEMLAAGVVLLCAAFLSGEKLATLPGLSGFMAVGYLALFGSIIAINAYMYLIRNVSPA
LATSYAYVNPVVAVLLGTGLGGERLSPVEWAALGVIVFAVVLVTLGKYLFPARAVVTPCK
TEDSRQ
>EECEBICD_02394 Very short patch repair protein
MADVHDKATRSKNMRAIATRDTAIEKRLAGLLSAQGITFHTQDATLPGKPDFVVNDYDCV
IFTHGCFWHHHHCYLFKVPATRTAFWLEKIGKNVERDERDIQRLQALGWRVLIVWECALR

GRAKLSDAALAERLEEWICGGGASAQIDTQGIHLLA
>EECEBICD_02395 DNA-cytosine methyltransferase
MQENISVTHARNLIADDAGSEIQAMLSQLLEIYDVKTLVAHLNGLGEQHWSPAILKRVMM
NAAWHRLSDNELTCLKTGLPTPPAHHPHYAFRFIDLFAGIGGIRRGFEAIGGQCVFTSEW
NKHAVRTYKANYFCDPLQHRFNEDIRDITLSHREGVSDDEAAEHIRQHIPQHDVLLAGFP
CQPFSLAGVSKKNALGRAHGFACETQGTLFFDVVRIIDARRPALFVLENVKNLKSHDQGN
TFRIIMQTLDELGYDVADAADNGPDDPKIIDGQHFLPQHRERIVLVGFRRDLNLKTDFTL
RNIARCYPPRRPTLAELLEPVVEAKYILTPVLWKYLYRYAKKHQARGNGFGYGMVYPDNP
ESVARTLSARYYKDGAEILIDRGWDMAKGEVNFDDAGNQQHRPRRLTPRECARLMGFEAP
QTYQFRIPVSDTQAYRQFGNSVVVPVFAAVAKLLEPKIHQAVTLRQRETVDGGRSR
>EECEBICD_02396 putative protein YedJ
MDSGYWQSQFEDWLRHHHQEQDAAHDIFHFSRVWATAQTLGENSPVDWLVVLSACYFHDI
VSLAKNHPQRHRSSILAAAETRRIFLRDFPDFPAEKLAGICHAIEAHSFSAKIAPTTPEA
KIVQDADRLEALGAIGLARVFAVSGALGVALFDADDPFADRRPLNDKQFALDHFQTKLLK
LPLTMQTERGKYLAQRNADFLVSYMAKLSAELKGDYETRDEAVIQMFATHQ
>EECEBICD_02397 Inner membrane protein YedR
MSTREDNATRSMGGKLALWVFYTFCGYFIWAMARCVWLMSAIQTEPVLGPISTPGSATEK
WLNALSLGVVWLILGSIAWYTRPRKNRGYPADTQPETRKHARM
>EECEBICD_02398 Outer membrane porin N
MNRKVLALLVPALLVAGAANAAEIYNKNGNKLDLYGKVDGLRYFSDNAGDDGDQSYARIG
FKGETQINDMLTGYGQWEYNIKVNTTEGEGANSWTRLGFAGLKFGEYGSFDYGRNYGVIY
DIEAWTDALPEFGGDTYTQTDVYMLGRTNGVATYRNTDFFGLVEGLNFALQYQGNNEDPG
AGEGTTNGSDAGSGSRKLARENGDGFGMSTSYDFDFGLSLGAAYSSSDRTDNQVTRGYGD
GHHYYANSYAGGETAEAWTVGAKYDAYNVYLAAMYAETRNMTYYGGGDGGNGGIANKTQN
FEVVAQYQFDFGLRPSIAYLQSKGKDLGGQDKDSRGNYRYTDKDLVKYVDVGMTYYFNKN
MSTYVDYKINLLDEDDDFYANNGIATDDIVGVGLVYQF
>EECEBICD_02399 Cold shock-like protein CspJ
MTTKITGLVKWFNPEKGFGFITPKDGSKDVFVHFSAIQSNEFRTLNENQEVEFSVEQGPK
GPSAVNVVAL
>EECEBICD_02400 hypothetical protein
MSNSWRVKTEGDYISPPSHIMDTPLLGYEYVMLQRLLAHSLNLPLPESGQCNIYCGVLVS
DGSNIFQIFEYQSLEQRIFQQLQLIPVDKASLCHDEINKIQFDRTKKDYFPVYHFPGDAC
FVVKM
>EECEBICD_02401 hypothetical protein
MSSVPYHSNNSVMPVRVTIYEAVNIIKKQANEEITASEIWRYALYGHPTLSIYFQSPVIF
RRIKTRKNKIFLMKGKDDPVNRLCYLNSDIIL
>EECEBICD_02402 hypothetical protein
MKICSMIYLCTTIFLLSACVRSPIDAKKKDVPRTESSISKNMASQLTDKDIFGNETTLAV
SEEDIQAALEGEKFSVPLNSAVILVQSGNRAPEAIMQQEMSKYYTVATFSGIPDRQKTVT
CSKDKIKDGNQDIATAENMNWMQALRYVAAKGHQKAIIVYQDTLQSGKYDAMTKNTVWSD
FKNEKLTDSVSLRYLVRFTLVNVATGEWATWSPLNYENTVLPPQPGKKDSAEATTEQQIS
QLRQRTYASMVKDLVNRYQ
>EECEBICD_02403 IS3 family transposase IS1230B
MTAQLACDALQMALWRRKCPENVIVHTDRGGQYCSTDYQSLLKRHNLRGSMSARGCCYDN
ACAESFFHTLKVECIHGEDFVSREIMRTAVFNYIECDYNRWRRHSACGGLSPEQFENQNL
A
>EECEBICD_02404 hypothetical protein
MKYVFIENHQAEFNIKAMCRVLQIARSGWYVWHQRRHQINRRQRFRLVCDNVVREAFSDA
KQRYGASRLTDELRAQDYQFNVKTVAASLRRQGLRAKASRRFRPVSYRKHGLPVSENLLK
QDFYASGPNQKWVGDITYLRTGIWPWLSICGRGQSLAGRCLRG
>EECEBICD_02405 IS3 family transposase ISSty2
MTKPASTTKKPRKQHTPEFRQEAMKLAERIGVAAAARELNLYESQLYNWRSKQQNQLSSS
EREQEMSAEIARLKRQLAERDEELAIIQKAATYFAKRLK
>EECEBICD_02406 Secretory immunoglobulin A-binding protein EsiB

MKKNILMMVFTVIVLFGGVIAWLYHSNDSLENVSANSDIAHLTKLAEQGDMKAQTDLGLA
YGSGNSIPQDYTKAMYWYNQAAKQGYAPAQFNLGLFYENGWGGSRDLQLAKEFYRKAANQ
GFTNAQINLGILFMDGKGGGLIMFKPENYL
>EECEBICD_02407 hypothetical protein
MLQRNHGKVLALLSPHGNKCYLFDDENIRIYIYEDNHEYFIDNKGVVLFKNKKNYKCSKN
KINENEIVMLLNDGNVDTWEKDE
>EECEBICD_02408 hypothetical protein
MIKISPPCLMKFYVFSLHLQKIIKNTLLLLVCILPFFCDAKMLFFRIPFRIQTYTPITRG
ELKDLQ
>EECEBICD_02409 hypothetical protein
MEHTCYDPSEYTFYIEIDSDHAACQYQGKGKACKLTPTPLSVVIAHEMGHAMGENDDGPG
HMNNVKKHENPVRKEMGIPPRMKY
>EECEBICD_02411 hypothetical protein
MSQDALTHIIVTGQDPRGLPEFSALREEINKSSHPSQPELNWKLVESLALAIFKAHGVDL
HTATYYTLARTRTHGLAGFCEGVELLAAMISHDWDKFWPQDESARTGMLDWLNSRTGNIL
RQQLSFSGADLPLLYRTERALQLICDKLQQVELKRQPHVENLLYFVQNTRKRLEPPPKSS
VDIPPQTTVRTLIYAPESTQPLTAENLPPLPELPEMKVEVHHGAVEDPEPVARATSGRTI
KGFMAGVVCAAAVAAALWWWQVYPMQQQLAQVRDTAQGAAMLWLASPELDSYGQRLQQLP
DASPLQLLEAGMQMMHTADSRWPESLQQQQATAQWNEILKTRAQSSPQMRGWQQARQNLR
DFADLMMQRETEKQGFTLSYIKTVTWQAERLLNQETPLESLLTQYQDARAQGRNAEVLEK
QINERLDGVLSRWLLLKNNVVPETATKAPRK
>EECEBICD_02412 Major exported protein
MANLIYLTLNGEKQGLISAGCCSLDSIGNKAQLLHLDHIMVYELTHGLSRDQNVNHHSVT
IKKPVDKSSPLLGKAINDNEILTCTFDFYRTNRFGINEKYYKLELKNARISDINFSIAHV
VIDSEAQPQESISFIYESINWEHCIAGTSAFSLWDERVF
>EECEBICD_02413 hypothetical protein
MVNRTASAHKGIPTTENPRERPQVNTRTTGKGSGHPPKKLSPLDEWKAGREKAIGNPDWY
IYDNTIRKLVSEINRHLSTSKNIEKYKPLDWKLIKAMIWTETGAAVTAWKTRPIQIGNTG
DEGIKEVVIPARPRKYNIIIPKTWNTYLINKTDLIRSNPEYNIRAGIALLMIKMSETEKD
KIVYDNENEDTYEVVEGDRGYSSIAKKIGTTQSVLTKLNGVKVIHPGDKLKYKKAHLEQY
IPGWLLFTPENIQKQYNIDPTKAQPGHRGDHTYADKIRFTYALIVADESK
>EECEBICD_02414 hypothetical protein
MNIKKFYVFFLYVVQPMRITSILRNAIMRYNRMTSIGALWINTSKVLSL
>EECEBICD_02415 hypothetical protein
MRKKFLIIINFIMAFFYCNYLFAENVNHIVIYDMPQDLRDFFETADSCEGWIRDFDVRQE
KLTYQFVEDSIKRDCSNIENKLLSMKNKYKNNKDYSARLTVYDDTIIIYDEYKKTQIKNE
SNE
>EECEBICD_02416 hypothetical protein
MAEEILSMQKNCLMYMHYLPLQQEIKIND
>EECEBICD_02417 hypothetical protein
MISIARIFIILATLIFSFSSIHSTYANNVYYPSKNNCESGEEAELVDCLRNEVLESTITL
RKAEENIRLRINKWSEVKNAALDDAIPIALYRLDKSNQEFIRYRLAQCSFAESWGNGVGS
FTRIYFCMAELNKQRAEQLMHADIPNYTEDTK
>EECEBICD_02418 hypothetical protein
MTRLLLCFLLFVCNSSYSREEPEYSKTYTPDEYYYLSSKIPKEVRQLIMNVNMAPPLAAI
FKKELVQKYGLFAYISPKPRGDENHRPGFDTKTPGAFLNRACSGPEGARSRDGLSSGAAS
WTDDEPARRLPEGRAKRVNSPLPASSKKEPVRKYQTTDKP
>EECEBICD_02419 hypothetical protein
MLALYAETRKILMSGIMETTFLSGPFFYAPGDAASNLDELYVRLAELQYAKKAELDDKPV
VRLHIIRRGNVG
>EECEBICD_02420 hypothetical protein
MSVLCVVVLTYVKPLHEIDAQIPAHVEWLKKGYADGLFLASGRKIPRTGGVILANYDDAD
VLQNYLAQDPFRLSGVAKVDLIPFEPTMMVTELKAVL
>EECEBICD_02421 putative oxidoreductase YjhC

MTKYGVVGTGYFGAELARFMSKVEGAKITAIYDPVNAAPIAKELNCVATATMEALCSHPD
VDCVIIASPNYLHKAPVIAAAKAGKHVFCEKPIALNYQDCKDMVDACKEAGVTFMAGHVM
NFFHGVRHAKALIKAGEIGEVTQVHTKRNGFEDVQDEISWKKIRAKSGGHLYHHIHELDC
TLFIMDETPSLVSMAAGNVAHKGEKFGDEDDVVLITLEFESGRFATLQWGSSFHYPEHYV
LIEGTTGAILIDMQNTAGYLIKAGKKTHFLVHESQAEDDDRRNGNISSEMDGAIAYGKPG
KRTPMWLSSIMKLEMQYLHDVINGLEPSEEFAKLLTGEAATNAIATADAATLSSNEGRKV
KLTEILG
>EECEBICD_02422 Putative metabolite transport protein YjhB
MLIFYIMYLIKADLGLTDMEGAFLATAAFIGRPFGGALFGLLADKFGRKPLMMWSIVAYS
VGTGLSGLASGVIMLTLSRFIVGMGMAGEYACASTYAVESWPKHLKSKASAFLVSGFGIG
NIIAAYFMPSFAEAYGWRAAFFVGLLPVLLVIYIRARAPESKEWEEAKLSGPGKHSQSAW
SVFSLSMKGLFNRAQFPLTLCVFIVLFSIFGANWPIFGLLPTYLAGEGFDTGVVSNLMTA
AAFGTVLGNIVWGLCADRIGLKKTFSIGLLMSFLFIFPLFRIPQDNYLLLGACLFGLMAT
NVGVGGLVPKFLYDYFPLEVRGLGTGLIYNLAATSGTFNSMAATWLGITMGLGAALTFIV
AFWTATILLIIGLSIPDRLKARRESFQSTKEF
>EECEBICD_02423 Oligogalacturonate-specific porin KdgM
MKINKYLLGMVSFIAFSSYLQAATLDYRHEYADRTRINKDRIAIIEKLPNGIGFYVDASV
KSGGVDGEQDKHLSDLVANAIELGVSYNYKVTDNFVLQPGFIFESGPDTSIYKPYLRGQY
NFDSGVYMAGRYRYDYARKTANYSDDEKTNRFDTYIGYLFDELKLEYNFTWMDSDQIKFD
NKKTNYEHNVALAWKLNKSFTPYVEVGNVAVRNNTDERQTRYRVGLQYHF
>EECEBICD_02424 N-acetylneuraminate epimerase
MKNFKKMMTLMALCLSVAITTSGYATTLPDIPEPLKNGTGAIDNNGVIYVGLGTAGTSWY
KIDLKKQHKDWERIKSFPGGAREQSVSVFLNDELYVFGGVGKKNSESPLQVYSDVYKYSP
VKNTWQKVDTISPVGLTGHTGVKLNETMVLITGGVNEHIFDKYFIDIEAADESEKNKVIY
NYFNKPAKDYFFNKIVFIYNAKENTWKNAGELPGAGTAGSSSVMENNFLMLINGELKPGL
RTDVIYRAMWDNDKLTWLKNSQLPPSPGEQQQEGLAGAFSGYSHGVLLVGGGANFPGAKQ
NYTNGKFYSHEGINKKWRDEVYGLVNGHWQYMGKMKQPLGYGVSVSYGDEVFLIGGENAK
GKPVSSVTSFTMRDGNLLIK
>EECEBICD_02425 Putative N-acetylmannosamine-6-phosphate 2-epimerase
MSLLARLEQSVHENGGLIVSCQPVPGSPMDKPEIVAAMAQAAASAGAVAVRIEGIENLRA
VRPHLSVPIIGIIKRDLTGSPVRITPYLQDVDALAQAGADIIAFDASFRSRPVDIDSLLT
RIRLHGLLAMADCSTVNEGISCHQKGIEFIGTTLSGYTGPITPVEPDLAMVTQLSHAGCR
VIAEGRYNTPALAANAIEHGAWAVTVGSAITRIEHICQWFSHAVKR
>EECEBICD_02426 Sodium/glucose cotransporter
MITHSFGIVNYLVLFGYLLAMMLVGVYFSRRQKTADDYFRGGGRVPGWAAGVSVFATTLS
SITFMSIPAKAFTSDWTFIIGQYLAIAILPLVFYFYIPFFRKLKVTSAYEYLEARFDVRC
RLFASMSFMLFHIGRIAIITFLTVLALRPFIAIDPVILVLLISVMCIIYTWMGGIEGVIW
TDVIQGLLLSGSAILIFIVICLKVQGGIGEIFTVTQQADKFFPATQFHWSWTESTVPVLM
IGFLFANIQQFTASQDVVQRYIVTDSIEETKKTLLTNAKLVAVIPVFFFAIGSALFVYYQ
QHPQLLPAGFNTGGILPLFVVTEMPVGIAGLIIAAIFAAAQSSISSSLNSISSCFNSDIY
QRLSHKKRTPENRMKIAKLVILVAGLISSAASVWLVMADESEIWDAFNSLIGLMGGPMTG
LFMLGIFFKRANAGSAVLGIIISVITVLGARYATDLNFFFYGVIGSLSVVISGVIFAPLF
APAPPLTLDEKPEPKVTL
>EECEBICD_02427 putative HTH-type transcriptional regulator YbbH
MEPQPPRLKPGKILDTLGAMQKSLTRASQRIAQYILAFPRQVTQSSIADLSRDTQAGEAT
VIRFCRTLGYKGFQDFKMDLAIELATTESDDSSPLLDAEVSESDDAHAIGLKLQNTISNV
LSETLNLLDMQQVLGVVDALRHCHSVYIFGVGSSGITALDMKHKLMRIGLRGDAVSNNHF
MYMQATLLKAGDVAMGVSHSGTSPETVHSLRLARQAGATTVAITHNLGSPLCEEADFCLI
NGNRQGMLQGDSIGTKAAQLFVFDLLYTLLVQSSPEQARESKLRTMNALDMTK
>EECEBICD_02428 Protein PhoH
MGRQKAVIKARREAKRVLRRDSRSHKQREEESVTSLVQMGGVEAIGMARDSRDTSPVKAR
NEAQAHYLNAIDSKQLIFATGEAGCGKTWISAAKAAEALIHKDVERIIVTRPVLQADEDL
GFLPGDIAEKFAPYFRPVYDVLLKRLGTSFMQYCLRPEIGKVEIAPFAYMRGRTFENAVV
ILDEAQNVTAAQMKMFLTRLGENVTVIVNGDITQCDLPRGVRSGLSDALERFEEDEMVGI

VHFNKDDCVRSALCQRTLHAYS
>EECEBICD_02429 hypothetical protein
MVMSAPGHIVYSSYNTLYGHSLSGGGLVILKALIISLTVHTHDVICGARSRVWRRFKKQA
KAYKEANPQMCVRIIAFKRTRVMYTYNSRCYPWEDKKQ
>EECEBICD_02430 Sodium/proline symporter
MAISTPMLVTFCVYIFGMILIGFIAWRSTKNFDDYILGGRSLGPFVTALSAGASDMSGWL
LMGLPGAIFLSGISESWIAIGLTLGAWINWKLVAGRLRVHTEFNNNALTLPDYFTGRFED
KSRVLRIISALVILLFFTIYCASGIVAGARLFESTFGMSYETALWAGAAATIIYTFIGGF
LAVSWTDTVQASLMIFALILTPVMVIVGVGGFSESLEVIKQKSIENVDMLKGLNFVAIIS
LMGWGLGYFGQPHILARFMAADSHHSIVHARRISMTWMILCLAGAVAVGFFGIAYFNNNP
ALAGAVNQNSERVFIELAQILFNPWIAGVLLSAILAAVMSTLSCQLLVCSSAITEDLYKA
FLRKSASQQELVWVGRVMVLVVALIAIALAANPDNRVLGLVSYAWAGFGAAFGPVVLFSV
MWSRMTRNGALAGMIIGAVTVIVWKQYGWLDLYEIIPGFIFGSLGIVIFSLLGKAPTAAM
QERFAKADAHYHSAPPSKLQAE
>EECEBICD_02431 Bifunctional protein PutA
MGTTTMGVKLDDATRERIKMAASRIDRTPHWLIKQAIFSYLDKLENSDTLPELPALFAGA
ANESEEPVAPQDEPHQPFLEFAEQILPQSVSRAAITAAWRRPETDAVSMLMEQARLSPPV
AEQTHKLAYQLAEKLRNQKSASGRAGMVQGLLQEFSLSSQEGVALMCLAEALLRIPDKAT
RDALIRDKISNGNWQSHIGRSPSLFVNAATWGLLFTGRLVSTHNEANLSRSLNRIIGKSG
EPLIRKGVDMAMRLMGEQFVTGETIAQALANARKLEEKGFRYSYDMLGEAALTAADAQAY
MVSYQQAIHAIGKASNGRGIYEGPGISIKLSALHPRYSRAQYDRVMEELYPRLKSLTLLA
RQYDIGLNIDAEEADRLEISLDLLEKLCFEPELAGWNGIGFVIQAYQKRCPLVIDYLVDL
ASRSRRRLMIRLVKGAYWDSEIKRAQMEGLEGYPVYTRKVYTDVSYLACAKKLLAVPNLI
YPQFATHNAHTLAAIYHLAGQNYYPGQYEFQCLHGMGEPLYEQVTGKVADGKLNRPCRIY
APVGTHETLLAYLVRRLLENGANTSFVNRIADATLPLDELVADPVEAVEKLAQQEGQAGI
PHPKIPLPRDLYGEGRINSAGLDLANEHRLASLSSALLSNAMQKWQAKPVLEQPVADGEM
TPVINPAEPKDIVGWGREATESEVEQALQNAVNQAPVWFATPPQERAAILQRAAVLMEDQ
MQQLIGLLVREAGKTFSNAIAEVREAVDFLHYYAGQVRDDFDNETHRPLGPVVCISPWNF
PLAIFTGQIAAALAAGNSVLAKPAEQTSLIAAQGIAILLEAGVPPGVVQLLPGRGETVGA
QLTADARVRGVMFTGSTEVATLLQRNIATRLDAQGRPIPLIAETGGMNAMIVDSSALTEQ
VVVDVLASSFDSAGQRCSALRVLCLQDDIAEHTLKMLRGAMAECRMGNPGRLTTDIGPVI
DSEAKANIERHIQTMRAKGRPVFQAARENSDDAQEWQTGTFVMPTLIELENFAELEKEVF
GPVLHVVRYNRNQLAELIEQINASGYGLTLGVHTRIDETIAQVTGSAHVGNLYVNRNMVG
AVVGVQPFGGEGLSGTGPKAGGPLYLYRLLAHRPPNALNTTLTRQDARYPVDAQLKTTLL
APLTALTQWAADRPALQTLCRQFADLAQAGTQRLLPGPTGERNTWTLLPRERVLCLADDE
QDALTQLAAVLAVGSQALWSDDAFHRDLAKRLPAAVAARVQFAKAETLMAQPFDAVIFHG
DSDKLRTVCEAVAAREAVAAREGAIVSVQGFARGESNILLERLYIERSLSVNTAAAGGNA
SLMTIG
>EECEBICD_02432 hypothetical protein
MKQIFLTCAALWFSSQALADECASASTQLEMNRCAAAQYQAADKKLNETYQSAIKRAQPP
QRELLQKAQVAWIALRDADCALIRSGTEGGSVQPMIASQCLTDKTNEREAFLASLLQCEE
GDLSCPLPPAG
>EECEBICD_02433 HTH-type transcriptional regulator RutR
MSQRTEKKIGKRSQATGAKRQLILTAALAVFSQYGIHGARLEQVAERAGVSKTNLLYYYP
SKEALYVAVMRQILDVWLAPLKAFRAEFSPLEAIKEYIRLKLEVSRDYPQASRLFCMEML
AGAPLLMEELTGDLKALIDEKSALIAGWVHSGKLAPVSPHHLIFMIWAATQHYADFAPQV
EAVTGATLRDEAFFNQTVESVQRIIIEGIRVR
>EECEBICD_02434 hypothetical protein
MANHRGGSGNFAEDRERASEAGRKGGQHSGGNFKNDPQRASEAGKKGGKSSNRNS
>EECEBICD_02435 NAD(P)H dehydrogenase (quinone)
MAKILVLYYSMYGHIETMAHAVAEGAKKVDGAEVIIKRVPETMPPEIFAKAGGKTQNAPV
ATPQELADYDAIIFGTPTRFGNMSGQMRTFLDQTGGLWASGALYGKLGSVFSSTGTGGGQ
EQTITSTWTTLAHHGMVIVPIGYAAQELFDVSQVRGGTPYGATTIAGGDGSRQPSQEELS
IARYQGEYVAGLAVKLNG

>EECEBICD_02436 putative protein YccJ
MPTQEAKAHRVGEWASLRNTSPEIAEAIFEVAHYDEKLAEKIWEEGSDEVLIKAFEKTDK
DSLFWGEQIIERKNV
>EECEBICD_02437 Glucose-1-phosphatase
MGTMDPTFNPVITDDSAAFRQQAVQAMEKARSQLHLDESYKLLEQITHYQDSPSCKEKHQ
CSLIDAKDTFSANYQQEPGVQGPLKVGNSLVDAFTLQYYEGFPMDQVAWGGIHTDRQWKV
LSKLKNGYQDSLFTSPTVARNVAAPLVKYIDKVLVADRVSAPKVTVLVGHDSNIASLLTA
LDFKPYQLHDQYERTPIGGQLVFQRWHDGNANRDLMKIEYVYQSARQLRNAEALTLKSPA
QRVTLELKGCPVDANGFCPLDKFDNVMNTAAK
>EECEBICD_02438 Glucose-1-phosphatase
MKKSLLAVAVAGAVLLSSAVQAQTTPEGYQLQQVLMMSRHNLRAPLANNGSVLAQSTPNA
WPAWDVPGGQLTTKGGVLEVYMGHYTREWLVAQGLIPSGECPAPDTVYAYANSLQRTVAT
AQFFITGAFPGCDIPVHH
>EECEBICD_02439 Thiol-disulfide oxidoreductase ResA
MAAKLRRWLREAAVFLALLIAIMVVMDVWRAPQAPPAFAATPLHTLTGESTTLATLSEER
PVLLYFWASWCGVCRFTTPAVARLAAEGENVMTVALRSGDDAEVARWLARKGVDFPVVND
AKGALSAGWEISVTPTLVVVSQGRVVFTTSGWTSYWGMKLRLWWAKTF
>EECEBICD_02440 hypothetical protein
MKYMIVLLLALFSTLSIAQETAPFTPDQEKQIENLIHAALFNDPASPRIGAKHPKLTLVN
FTDYNCPYCKQLDPMLEKIVQKYPDVAVIIKPLPFKGESSVLAARIALTTWREHPQQFLA
LHEKLMQKRGYHTDDSIKQAQQKAGATPVTLDEKSMETIRTNLQLARLVGVQGTPATIIG
DELIPGAVPWDTLEAVVKEKLAAANGG
>EECEBICD_02441 Thiol:disulfide interchange protein DsbD
MMILFRRILFCLLWLWLPVSWAAESGWLRSPDNDHASIRLRADTSANGETRLLLDVKLEN
GWKTYWRAPGEGGVAPSIAWKGDMPEVSWFWPTPSRFDVANITTQGYHDEVTFPMIVRGT
PPATLSGVLTLSTCSNVCLLTDYPFSVTATVQNADFAHDYARAMGKVPLRSGLTDSLDVG
YRPGELVVTATRAAGWSSPGLYLDTIDDVDFAKPRLRVEGDRLQATVPVTDSWGEKAPDL
RNKSLTLVLADGAIAQESTQTIGAAPAQTPDNAALPFWQVVMMALIGGLILNLMPCVLPV
LGMKLGSILLVEEKSRSHIRRQFLASVAGIIASFMALAAFMTLLRLSNHALAWGVQFQNV
WFIGFMALVMLLFSASLFGLFEFRLPSSMTTKLATYGGNGMSGHFWQGAFATLLATPCSA
PFLGTAVAVALTASLPTLWGLFLALGLGMSAPWLLVAIRPGLALRLPRPGRWMNVLRRIL
GLMMLGSAIWLATLLLPHFGFTASKSAQDTVQWQPLSEQAIQSALAQHKRVFVDVTADWC
ITCKVNKYNVLQKEDVQAALQQPDVVALRGDWTLPSDAITDFLKTRGQVAVPFNQVYGPG
LPEGEALPTLLTRDAVLQTLKKAKGITQ
>EECEBICD_02442 hypothetical protein
MAKQQRMGWWFLCLACVVVMVCTAQRMAGLHALQMQATASAAVISAPSSTDDGSPVTPCE
LSAKSLLAAPPVLFEGAILALCLLLSLLAPVRVMRLPFSPPRAISPPTLRVHLRFCVFRE
>EECEBICD_02443 Curved DNA-binding protein
MELKDYYAIMGVKPTDDLKTIKTAYRRLARKYHPDVSKEPDAEARFKEVAEAWEVLSDEQ
RRAEYDQLWQHRNDPQFNRQFQQHEGQPYNAEDFDDIFSSIFGQHGRHSHHRHAARGHDI
EIEVAVFLEETLEEHQRTISYSVPVYNAFGLVEREIPKTLNVKIPAGVSNGQRIRLKGQG
TPGENGGPNGDLWLVIHIAPHPLFDIVNQDLEVVLPLAPWEAALGAKVSVPTLKERILLT
IPPGSQAGQRLRIKGKGLASKKHTGDLYAIIKIVMPPKPDEKTAALWQQLADAQSSFDPR
QQWGKA
>EECEBICD_02444 Chaperone modulatory protein CbpM
MANITVTFTITEFCLHTGVTEEELNEIVGLGVIEPYEDDNADWQFDDRAASVVQRALRLR
EELALDWPGIAVALTLLEENSRLREENRLLLQRLSRFISHP
>EECEBICD_02445 Anti-adapter protein IraM
MEWKVVDTVISPSTGVSFSCIHSLKNLRLTLWYQADVYMPPGSIIIPFNKGVLINDKLYP
VTVYNVTRFNPVLWKSLKENSHCPGNCNPKPEACSYPFECLVSVCPFGLTRNIQIDNKKV
>EECEBICD_02446 Ribonuclease Z
MLTVLIPRSKRIVFSGDTSWFPPLATFAQGADILVHEAVHVPSVAKLANSIGNGKTLAEA
IASHHTTIEDVGKIAREAHVKKLVLSHLVPATVADDVWQQEAMKNYPGPVIVGHDNMTIS
VP

>EECEBICD_02447 Ribonuclease Z
MMKKSVAMLAVCMLAQSHLAIAASAPAPQEINIVLLGTKGGPSLLNTARLPQATALTIGD
KIWLIDAGYGASLQLVKNGIPLRNINTILLTHLHSDHILDYPSLLMNAWASGLKDHTIQV
YGPPGTQAMTKASWKVFDRDITLRMEEEGKPDPRNLVKATDIGQGVIYKDELVTISALKV
PHSPFPDGEAFAYRFDTQE
>EECEBICD_02448 HTH-type transcriptional activator RhaR
MCQRAIANIDISKEYDESMGSNDVHYQSFARMADFFGRDMQAHRHDQFFQMHFLDTGQIE
LQLDDHRYSVQAPLFVLTPPSVPHAFITESDSDGHVLTVREELVWPLLEVLYPGTREAFG
LPGICLSLADKPNELAALKHYWQLIERESTEQLAGCEHTLVLLAQAVFTLLLRNAKLDDH
AATGMRGELKLFQRFTLLIDNHFHQHWTVPDYACELHITESRLTDICRRFANRPPKRLIF
DRQLREAKRLLLFSDNAVNEIAWQLGFKDPAYFARFFNRLAGCSPSQFRQREVPSFLN
>EECEBICD_02449 Putative tartrate transporter
MLFIFSFLDRINIGFAGLTMGQDLGLSATMFGLATTLFYATYVIFGIPSNVMLSIVGARR
WIATIMVLWGIASTATMFAVGPKSLYVLRMLVGITEAGFLPGILLYLTYWFPAFFRARAN
ALFMIAMPATTALGSIVSGYILSLDGIFNLHGWQWLFLLEGFPSVLLGIMVWFYLDDTPA
KAKWLTAEDKKCLQEMMDNDRLTLVQPEGAISHNAMQQRSLWREVFTPIVLMYTLAYFCL
TNTLSAISIWTPQILKSFNEGSSNITIGLLAAIPQICTILGMIYWSRHSDKHQERKHHTA
LPFLFAAAGWLLASATDHNLIQLLGIVMASTGSFSAMAIFWTTPDQSISLRARAIGIAVI
NATGNIGSALSPFMIGWLKDITGSFNSGLWFVASLLVVGAAIIWLIPMKASRPRATP
>EECEBICD_02450 4-hydroxy-2-oxo-heptane-1,7-dioate aldolase
MKNAFKDALKAGRPQIGLWLGLANSYSAELLAGAGFDWLLIDGEHAPNNVQTVLTQLQAI
APYPSQPVVRPSWNDPVQIKQLLDVGAQTLLIPMVQNADEARNAVAATRYPPAGIRGVGS
ALARASRWNRIPDYLHQANDAMCVLVQIETREAMSNLASILDVDGIDGVFIGPADLSADM
GFAGNPQHPEVQAAIENAIVQIRAAGKAPGILMANEALAKRYLELGALFVAVGVDTTLLA
RGAEALAARFGAEKNCPVRPASIKPGPVSAALPGNRSTLQNSHQRKKNERHIICTSGKPR
VCPFAQRAQHGSTNRHK
>EECEBICD_02451 2-oxo-hept-4-ene-1,7-dioate hydratase
MLDKQTHTLIAQRLNQAEKQREQIRAVSLDYPNITIEDAYAVQREWVNIKIAEGRTLKGH
KIGLTSKAMQASSQISEPDYGALLDDMFFHDGGDIPTDRFIVPRIEVELAFVLAKPLRGP
HCTLFDVYNATDYVIPALELIDARSHNIDPETQRPRKVFDTISDNAANAGVILGGRPIKP
DELDLRWISALLYRNGVIEETGVAAGVLNHPANGVAWLANKLAPYDVQLEAGQIILGGSF
TRPVPASKGDTFHVDYGNMGAISCRFV
>EECEBICD_02452 5-carboxymethyl-2-hydroxymuconate Delta-isomerase
MPHFIAECTENIREQADLPGLFSKVNEALAASGIFPIGGIRSRAHWLDTWQMADGKHDYA
FVHMTLKIGTGRSLESRQEVGEMLFGLIKAHFADLMENRYLALSFEIAELHPTLNYKQNN
VHALFK
>EECEBICD_02453 3,4-dihydroxyphenylacetate 2,3-dioxygenase
MGKLALAAKITHVPSMYLSELPGKNHGCRQAAIDGHIEIGKRCREMGVDTIIVFDTHWLV
NSAYHINCADHFQGVYTSNELPHFIRDMTYDYDGNPELGHLIADEAVKLGVRAKAHNIPS
LKLEYGTLVPMRYMNSDKHFKVVSISAFCTVHDFADSRRLGEAILKAIEKYDGTVAVFAS
GSLSHRFIDDQRAEEGMNSYTREFDHQMDERVVKLWREGKFKEFCTMLPEYADYCYGEGN
MHDTVMLLGMLGWDKYDGKVEFITDLFASSGTGQVNAVFPLPA
>EECEBICD_02454 NAD/NADP-dependent betaine aldehyde dehydrogenase
MKKINHWINGKNVAGNDYFQTTNPATGDVLAEVASGGEAEVNQAVAAAKEAFPKWANLPM
KERARLMRRLGDLIDQNVPEIAAMETADTGLPIHQTKNVLIPRASHNFEFFAEVCQQMNG
KTYPVDDKMLNYTLVQPVGVCALVSPWNVPFMTATWKVAPCLALGNTAVLKMSELSPLTA
DRLGELALEAGIPAGVLNVVQGYGATAGDALVRHHDVRAVSFTGGTATGRNIMKNAGLKK
YSMELGGKSPVLIFEDADIERALDAALFTIFSINGERCTAGSRIFIQQSIYPEFVKRFAE
RANRLRVGDPTDPNTQVGALISQQHWEKVSGYIRLGIEEGATLLAGGAEKPTDLPAHLKG
GNFLRPTVLADVDNRMRVAQEEIFGPVACLLPFKDEAEGLRLANDVEYGLASYIWTQDVS
KVLRLARGIEAGMVFVNTQNVRDLRQPFGGVKASGTGREGGEYSFEVFAEMKNVCISMGD
HPIPKWGV
>EECEBICD_02455 Homoprotocatechuate catabolism bifunctional
isomerase/decarboxylase

MKGTVFAVALNHRSQLDAWQEAFSQPPYNAPPKTAVWFIKPRNTVIRHGEPIPYPQGEKV
LSGATVALIVGKTASRIRPEAAADYIAGYALANEVSLPEESFYRPAIKAKCRDGFCPLGE
MAPLSDVDNLTIITEINGREADHWNTADLQRSAAQLLSALSEFATLNPGDAILLGTPQNR
VALRPGDRVRILAKGLPALENPVVAEDEFARHQTFTWPLSATGTLFALGLNYADHASELA
FTPPKEPLVFIKAPNTFTEHHQTSVRPNNVEYMHYEAELVVVIGKTARKVSEAEAMEYVA
GYTVCNDYAIRDYLENYYRPNLRVKSRDGLTPIGPWIVDKEAVSDPHNLTLRTFVNGELR
QEGTTADLIFSIPFLISYLSEFMTLQPGDMIATGTPKGLSDVVPGDEVVVEVEGVGRLVN
RIVSEESAK
>EECEBICD_02456 HTH-type transcriptional regulator FarR
MHDSLTIALLQAREAAMTYFRPIVKSHNLTDQQWRIVRILADSPSMDFHELAFRTCILRP
SLTGILTRMERDGLVLRLKPVNDQRKLYVMLTEQGQTLYARARSQVEEAYRKIEADFTPE
KTQQLMLLLDDLIALGRQHPDSEAEA
>EECEBICD_02457 4-hydroxyphenylacetate 3-monooxygenase oxygenase component
MKPEDFRTDNKRPLTGEEYLKSLQDGREIYIYGERVKDVTTHPAFRNAAASVAQLYDALH
KPSMQDTLCWNTDTGSGGYTHKFFRVAKSADDLRQQRDAIAEWSRLSYGWMGRTPDYKAA
FGCALGANPAFYGQFEQNARNWYTRIQETGLYFNHAIVNPPIDRHKPADEVKDVYIKLEK
ETDAGIIVSGAKVVATNSALTHYNMIGFGSAQVMGENPDFALMFVAPMDAEGVKLISRAS
YEMVAGATGSPFDYPLSSRFDENDAILVMDKVLIPWENVLIYRDFDRCRRWTMEGGFARM
YPLQACVRLAVKLDFITALLKKSLECTGTVEFRGVQADLGEVVAWRNMFWALSDSMCSEA
TPWVNGAWLPDHAALQTYRVMAPMAYAKIKNIIERNVTSGLIYLPSSARDLNNPQIDQYL
AKYVRGSNGMDHVERIKILKLMWDAIGSEFGGRHELYEINYSGSQDEIRLQCLRQAQSSG
NMDKMMAMVDRCLSEYDQNGWTVSHLHNNDDINQLDKLLK
>EECEBICD_02458 4-hydroxyphenylacetate 3-monooxygenase reductase component
MQVDEQRLRFRDAMASLAAAVNIVTTAGHAGRCGITATAVCSVTDTPPSVMVCINANSAM
NPVFQGNGKLCINVLNHEQELMARHFAGMTGMAMEERFHQPCWQNGPLGQPVLNGALAGL
EGEISEVQTIGTHLVYLVAIKNIILSQDGHGLIYFKRRFHPVRLEMEAPV
>EECEBICD_02459 5-hydroxyisourate hydrolase
MKRHILATVIASLVAAPAMALAAGNNILSVHILDQQTGKPAPGVEVVLEQKKDNGWTQLN
TRHTDQDGRIKALWPEKAAAPGDYRVIFKTGQYFESKKLDTFFPEIPVEFHISKTNEHYH
VPLLLSQYGYSTYRGS
>EECEBICD_02460 Transcriptional regulatory protein HprR
MSSCWRFTDSLTSLWHTALMKILLIEDNQKTIEWVRQGLTEAGYVVDYACDGRDGLHLAL
QEHYSLIIILDIMLPGLDGWQVLRALRTAHQSPVICLTARDSVEDRVKGLEAGANDYLVKP
FSFAELLARVRAQLRQHVPVFTRLTINGLDMDATKQSVSRNGKPISLTRKEFLLLWLLAS
RAGEIVPRTAIASEVWGINFDSETNTVDVAIRRLRAKVDDPFEKKLIMTVRGMGYRLQAE
TSQNG
>EECEBICD_02461 Sensor histidine kinase HprS
MVKLSMTLRLTISFIAILILACTGISWTLYNALSKELTYRDDMTLINRAAQMQQLLLDGA
RPENLPLYFNRMVDTKQDILLIHSATGHNVAINHSGIPDQRFNEIPLAKNITRETLFRQA
VQGTELTAVRVNARSGDDPLTLTIARLATERRQMLTQYRRNSLLMSLIAILVCSALSPLV
IRNGLRAITSLSRLTAATDSGTLRQPLAEQALPVELRPLGQALNTMRQKLSDDFERLNQF
ADDLAHELRTPVNILLGKNQVMLSQERSAEEYQQALVDNIEELEGLSRLTENILFLARAE
HQNIAVKKQPVSLNALVENMLDYLSPLAEEKRICFINQCQGTVWADEILLQRVLSNLLTN
AIRYSDENAVIRIESAYDDNVAEIRVANPGSHPADADKLFRRFWRGDNARHTAGFGLGLS
LVNAIALLHGGSASYRYADEHNIFSVRLPDSGDS
>EECEBICD_02462 hypothetical protein
MPAKNIAGSITFSVSNPGSIHPITRYSVGTFMKKYLAFAVTLLGMGKVIACTTLLVGNQA
SADGSFIIARNEDGSANNAKHKVIHPVAFHQQGEYKAHRNNFSWPLPETAMRYTAIHDFD
TNDNAMGEAGFNSAGVGMSATETIYNGRAALAADPYVTKTGITEDAIESVILPVAQSARQ
GAKLLGDIIEQKGAGEGFGVAFIDSKEIWYLETGSGHQWLAVRLPADSYFVSANQGRLRH
YDPNDNANYMASPTLVSFAKKQGLYDPARGEFDFHQAYSQDNKNDTTYNYPRVWTLQHQF
NPHLDTAVSEGETFPVFLTPITKISVAAVKNALRNHYQGTSHDPYASHNPQEPWRPISVF

RTQESHILQVRPKLPQAIGNVEYIAYGMPSLSVYLPYYQGMRHYQPGDDKGTDRASNDST
YWTFRTLQTLVMQDYNAFAPDVQHAWKTFEQQTAKQQYKMEQSYLRLYASHPKEAQRLLQ
NFEDKTMQNAQTLARRLTNNIITTMTYRTDMKYHFSSTQP
>EECEBICD_02463 hypothetical protein
MTLQANISRETKAAKTQEVYKHVVLFKMAAPSYIRR
>EECEBICD_02464 Inositol phosphate phosphatase SopB
MQIQSFYHSASLKTQEAFKSLQKTLYNGMQILSGQGKAPAKAPDARPEIIVLREPGATWG
NYLQHQKTSNHSLHNLYNLQRDLLTVAATVLGKQDPVLTSMANQMELAKVKADRPATKQE
EAAAKALKKNLIELIAARTQQQDGLPAKEAHRFAAVAFRDAQVKQLNNQPWQTIKNTLTH
NGHHYTNTQLPAAEMKIGAKDIFPSAYEGKGVCSWDTKNIHHANNLWMSTVSVHEDGKDK
TLFCGIRHGVLSPYHEKDPLLRHVGAENKAKEVLTAALFSKPELLNKALAGEAVSLKLVS
VGLLTASNIFGKEGTMVEDQMRAWQSLTQPGKMIHLKIRNKDGDLQTVKIKPDVAAFNVG
VNELALKLGFGLKASDSYNAEALHQLLGNDLRPEARPGGWVGEWLAQYPDNYEVVNTLAR
QIKDIWKNNQHHKDGGEPYKLAQRLAMLAHEIDAVPAWNCKSGKDRTGMMDSEIKREHIS
LHQTHMLSAPGSLPDSGGQKIFQKVLLNSGNLEIQKQNTGGAGNKVMKNLSPEVLNLSYQ
KRVGDENIWQSVKGISSLITS
>EECEBICD_02465 Chaperone protein SigE
MESLLNRLYDALGLDAPEDEPLLIIDDGIQVYFNESDHTLEMCCPFMPLPDDILTLQHFL
RLNYTSAVTIGADADNTALVALYRLPQTSTEEEALTGFELFISNVKQLKEHYA
>EECEBICD_02466 Secreted effector protein PipB
MPITNASPENILRYLHAAGTGTKEAMKSATSPRGILEWFVNFFTCGGVRRSNERCFQEVI
GKLTTSLLYVNKDAFFDGNKIFLEDVNGCTICLSCGAASENTAPMVIIEVNKNGKTVTDK
VDSERFWNVCRMLKLMSKHNIQQPDSLITEDGFLNLRGVNLAHKDFRGEDLSDIDASDAD
FRETTLSNVNLVGANLCCANLHAVNLMGSNMTKANLTHANLTCANMSGVNLTAAILFGSD
LTDTKLNGAKLDKIALTLAKSLTGADLTGSQHTPTPLPDYNDRTLFPHPIF
>EECEBICD_02467 hypothetical protein
MLPVTYRLIPQSGVSTYGLNTADTPVFPDIPEHAPNPSRLRLAHDSLAINSEFRLEPECV
VEYLISGAGGIDPDTEIDDDTYDECYDELSSVLQNAYTQSETFRRLMSYAYEKELHDVEQ
RWLLGAGEAFETTVAQEHFKLSEGRKVICLNLDDSDDSYTEHYESNEGRQLFDTKRSFIH
EVVHALTHLQDKEENHPRGPVVEYTNIILKEMGHPSPPRMVYIFNK
>EECEBICD_02469 Modulator of FtsH protease YccA
MDRIITSSRDRSSLLSTHKVLRNTYFLLSLTLAFSAITATASTVLMLPSPGLILTLVGMY
GLMFLTYKTANKPVGILSAFAFTGFLGYILGPILNAYLSAGMGDVIGLALGGTALVFFCC
SAYVLTTRKDMSFLGGMLMAGIVVVLIGMVANIFLQLPALHLAISAVFILISSGAILYET
SNIIHGGETNYIRATVSLYVSLYNIFVSLLSILGFASRD
>EECEBICD_02470 Sulfurtransferase TusE
MLIFEGKEISTDSEGYLKETTQWSETLAVAIAANEGIELSAEHWEVVRFVREFYLEFNTS
PAIRMLVKAMANKFGEEKGNSRYLYRLFPKGPAKQATKIAGLPKPVKCI
>EECEBICD_02471 Acylphosphatase
MSNVCIIAWVYGRVQGVGFRYTTQHEAQRLGLTGYAKNMDDGSVEVVACGDAAQVEKLIK
WLKEGGPRSARVDKILTEPHSPRETLTGFSIRY
>EECEBICD_02472 hypothetical protein
MQEMHIRHQDLTTAEVRSSHLHRLHRVTLFSAAICHITQGSKVIIQDDSRLVAGPGELII
IPANTPLEIINQPAQNGFRSDLLLLSPEIIARFKTMYVQDYPPANLTSLCTPMSRSLTFM
WENVLDAVRQGLPVGLQEHQAMGLLLALLHDGAAGPLLIERRYTLTEQVRQLIMLSPAKL
WTAQEIARRLAMGTSTLRRRLQRESQSYRQIIEEVRMSCALSQLQSTTLPIGEIALRCGY
LSGSRFTARFRQHYGCLPSQVR
>EECEBICD_02473 hypothetical protein
MKTVFSLTAAAMMALSGGVSAASAFSLSSADIPADFRLTQQHVFKGFGCSGENISPQLSW
RNPPAGTKSYAITVFDPDAPTGSGWWHWTMVNIPAQIHDLPTGADKKTLPAGVVQGRNDF
GYAGFGGACPPPGDKPHRYQFTVWALNTATLPLDSESSGALVGFMLNAHVIAKAKFTATY
GR
>EECEBICD_02474 Ribosomal RNA large subunit methyltransferase I
MTESTFPQYPRLVLSKGREKSLLRRHPWVFSGAVSRLEGKANLGETIDIVDHQGKWLARG

AWSPASQIRARVWTFDKAESIDIAFFTRRLRQAQQWRDWLAKKDGLDSYRLIAGESDGLP
GVTIDRFGHFLVLQLLSAGAEYQRAALISALQTCDPDCAIYDRSDVAVRKKEGMALTQGP
VTGELPPALLPIEEHGMKLLVDIQGGHKTGYYLDQRDSRLATRRYVENQRVLNCFSYTGG
FAVSALMGGCRQVVSVDTSQDALDIARQNVELNQLDLSKAEFVRDDVFKLLRAYREHGEK
FDVIIMDPPKFVENKSQLMGACRGYKDINMLAIQLLNPGGILLTFSCSGLMTSDLFQKII
ADAAIDAGRDVQFIEQFRQAADHPVIATYPEGLYLKGFACRVM
>EECEBICD_02475 Heat shock protein HspQ
MIASKFGIGQQVRHSLLGYLGVVVDIDPEYSLDEPSPDELAVNDELRAAPWYHVVMEDDD
GQPVHTYLAEAQLRSEMRDEHPEQPSMDELARTIRKQLQAPRLRN
>EECEBICD_02476 putative protein YccU
MKETDIADVLTSTRTIALVGASDKPDRPSYRVMKYLLEQGYHVIPVAPKVAGKTLLGQQG
YDTLADIPEKVDMVDVFRNSEAAWGVAQEAIAIGAKTLWLQLGVINEQAAVLARDAGMTV
VMDRCPAIEIPRLGLAK
>EECEBICD_02477 hypothetical protein
MKTGALATFLALCLPVTVFATTLRLSNEVDLLVLDGKKVSSSLLRGAESIELENGPHQLV
FRVEKTIRLPGNEERLYISPPLVISFDTQLISQVNFQLPRLENEREASHFNAAPRLALLD
GDAMPIPVKLDILAITSTAKVVDYEIETERYNKSAKRASLPQFATMMADDSTLLSDVSEL
DTVPPQSQTLTEQRLKYWFRLADPQTRHHFLQWAEKQPPS
>EECEBICD_02478 Methylglyoxal synthase
MELTTRTLPTRKHIALVAHDHCKQMLMNWVERHQPLLEKHVLYATGTTGNLIQRATGMDV
NAMLSGPMGGDQQVGALISEGKIDVLIFFWDPLNAVPHDPDVKALLRLATVWNIPVATNV
STADFIIQSPHFNDAVDILIPDYARYLAERLK
>EECEBICD_02479 DNA helicase IV
MELKATSLGKRLAQHPYDRAEILNAGVKVSGDRHEYLIPFNQLLAIHCKRGLVWGELEFV
LPEDKVVRLHGTEWSETQQFHRYLDAHWRRWSQEMSDVAAQALQEQWARISERTGGNQWL
TRERVRGLEHEIRQTFAALPLPVSRLEEFAHCREIWRKCLAWLQDSEGSRQQHNQAYADA
MLEAHADFFTQIESSPLNPSQARAVVNGESSLLVLAGAGSGKTSVLVARAGWLLARGQAD
AGQILLLAFGRKAAEEMDERIRERLHTEEITARTFHSLALYIIQQGSKKAPVVSKLESDA
TARHQLFLRTWRQQCSEKKAQAKGWRQWLEEEMQWVVPEGNFWDDETLQRRLAPRLDRWV
SLMRMHGGAQAEMIAGAPEECRELFGKRIKLMAPLLKAWKSALKAENAVDFSGLIHQAMV
ILEKGRFISPWKHILVDEFQDISPQRAALLEALRKQNSQTTLFAVGDDWQAIYRFSGAQL
SLTTAFHQTFGEGEHCHLDTTYRFNSRIGDIANRFVQQNPHQLKKPLNSLTPGDKKAVTL
LDESQLDALLDKLSGYAKEDERILVLARYHHLKPASLQKAATRWPKLQIDFMTIHASKGQ
QADYVILVGLQEGNDGFPAPARESIMESALLPQVEDFPDAEERRLLYVALTRARARVWLL
FNKDNPSRFVEALKQLDVPVARKP
>EECEBICD_02480 Inner membrane protein YccF
MRTVLNILNFVLGGFATTLAWLLATLVSIVLIFTLPLTRSCWEITKLSLFPYGNEAIHVD
ELNPAAKSVLMNTGGTLLNIFWLLFFGWWLCLMHIASGIAQCVTIIGIPVGIANFKIAAI
ALWPVGRRVVSVETARAAREANARRRFE
>EECEBICD_02481 Inner membrane protein YccS
MLSPLIRRYTWNSTWLYYIRIFIALCGTTALPWWLGDVKLTIPLTLGMVAAALTDLDDRL
AGRLRNLIITLICFFIASASVELLFPWPWLFALGLTLSTSGFILLGGLGQRYATIAFGAL
LIAIYTMLGTSLYDHWYQQPLLLLAGAVWYNLLTLTGHLLFPIRPLQDNLARSYEQLAHY
LELKSRLFDPDIEDESQAPLYDLALANGQLMATLNQTKVSLLSRLRGDRGQRGTRRTLHY
YFVAQDIHERASSSHIQYQTLRDYFRHSDVMFRFQRLMSMQAQACTQLARCILLRTPYQH
DPRFERVFTHIDAALERMRASGASLELLNTLGFLLTNLRAIDAQLATIESEQAQAMPRNE
SENQLADDSLHGFSDIWLRLSRNFTPESALFRHAVRMSLVLCIGYALIQITGMRHGYWIL
LTSLFVCQPNYNATRHRLALRIIGTLVGVAIGLPILWFVPSLEGQLVLLVITGVLFFAFR
NVQYAHATMFITLLVLLCFNLLGEGFEVALPRVVDTLIGCAIAWAAVSFIWPDWRFRNLP
RVLQRATDANCRYLDAILEQYHQGRDNRLAYRIARRDAHNRDAELASVVSNMSSEPDVTA
ETREAAFRLLCLNHTFTSYISALGAHREKLSNPDVLGLLDDAVCYVDDALHHQPEDEQRV
HQALEGLKQRVQSLETRPDSKEPLVVQQIGLLIALLPEIGRLQRQISPPTSTLITQP
>EECEBICD_02482 Protein Sxy
MRALSYDRIYKSQEYLASLGTIQYRSLFGSYSLTVEDTVFAMVANGELYLRACEESVPYC

VKHPPAWLMFMKCGRPVMLNYYRVDESLWRDQQQLVRLSKYSLDAAMKEKHSRILQHRLK
DLPNMTFHLETLLNESGIKDENMLRILGAKMCWLRLRQSNPLLTVKVLYALEGAIVGVHE
AALPASRRQELADWAHSLTAG
>EECEBICD_02483 Cell division inhibitor SulA
MYTSGYANRSSSFPTTTHNAARTATENAAAGLVSEVVYHEDQPMMAQLLLLPLLRQLGQQ
SRWQLWLTPQQKLSREWVQSSGLPLTKVMQISQLAPRHTLESMIRALRTGNYSVVIGWMT
EELTEEEHASLVEAAKVGNAVGFIMRPVRAHALSRRQHSGLKIHSNLYH
>EECEBICD_02484 Outer membrane protein A
MKKTAIAIAVALAGFATVAQAAPKDNTWYAGAKLGWSQYHDTGFIHNDGPTHENQLGAGA
FGGYQVNPYVGFEMGYDWLGRMPYKGDNINGAYKAQGVQLTAKLGYPITDDLDVYTRLGG
MVWRADTKSNVPGGPSTKDHDTGVSPVFAGGIEYAITPEIATRLEYQWTNNIGDANTIGT
RPDNGLLSVGVSYRFGQQEAAPVVAPAPAPAPEVQTKHFTLKSDVLFNFNKSTLKPEGQQ
ALDQLYSQLSNLDPKDGSVVVLGFTDRIGSDAYNQGLSEKRAQSVVDYLISKGIPSDKIS
ARGMGESNPVTGNTCDNVKPRAALIDCLAPDRRVEIEVKGVKDVVTQPQA
>EECEBICD_02485 Macrodomain Ter protein
MKYQQLENLESGWKWKYLVKKHREGELITRYVEASAAQEAVNLLLALENEPVRVNVWIDR
HMNPALLNRMKQTIRARRKRHFNAEHQHTRKKSIDLEFMVWQRLAGLAQRRGKTLSETIV
QLIEDAEHKEKYATQMTTLKQDLQALLGKK
>EECEBICD_02486 Lon protease
MTITKLAWRDLVPDSESYQEIFAQPHATDENDTLLSDTQPRLQFALEQLIQPWASSSFML
TKAPEEQEYLTLLSDAVRALQTDAGQLTGGHYDVSGHTVHYRAAQNAQDNFATVTQVVSA
DWVEAEQLFGCLRQYNGDIILQPGLVHQANGGVLIISLRTLLAQPLLWMRLKAIVSRERF
DWVAFDESRPLPVSVPSMPLKLKVILVGERESLADFQEMEPELAEQAIYSEFEDNLQIAD
AEAMTLWCQWVTRIALRDNLPPPAPDAWPVLIREAVRYTGEQDTLPLCPLWIARQFKEAS
PLCEGDTCGAEALSLMLARREWREGFLAERMQDEILQEQILIETEGERVGQINALSVIEF
PGHPRAFGEPSRISCVVHIGDGEFNDIERKAELGGNIHAKGMMIMQAFLMSELQLEQQIP
FSASLTFEQSYSEVDGDSASMAELCALISALANVPVNQNIAITGSVDQFGRAQPVGGLNE
KIEGFFAICEQRELNGKQGVIIPAANVRHLSLKSELLQAVKEEKFTIWAVDDVTDALPLL
LNLVWDGEGQTTLMQTIQERIAQATQQEGRHRFPWPLRWLNAFIPN
>EECEBICD_02487 3-hydroxydecanoyl-[acyl-carrier-protein] dehydratase
MVDKRESYTKEDLLASGRGELFGAKGPQLPAPNMLMMDRVVKMTETGGNFDKGYVEAELD
INPDLWFFGCHFIGDPVMPGCLGLDAMWQLVGFYLGWLGGEGKGRALGVGEVKFTGQVLP
TARKVTYRIHFKRIVNRRLIMGLADGEVLVDGRLIYTAHDLKVGLFQDTSAF
>EECEBICD_02488 Intermembrane transport lipoprotein PqiC
MKKWLVVIMAFWLASCSSGGENKSYYQLPIAQSGVQSTASQGNRLLWVEQVSVPDYLAGN
GVVYQTSDVQYVIANNNLWASPLDQQLRNTLVANLSARLPGWVVASQPLGTTQDTLNVTV
TGFHGRYDGKVIVSGEWLLNHNGQLIKRPFHIEASQQKDGYDEMVKVLASAWSQEAAAIA
DEIKRLP
>EECEBICD_02489 Intermembrane transport protein PqiB
MEPKKGEAKVQKVKNWSPVWIFPIVTALIGAWILFYHYSHQGPEVTLITTNAEGIEGGKT
TIKSRSVDVGVVESATLTDDLTHVQIKARLHSGMEKLLHKDSVFWVVKPQVGREGISGLG
TLLSGAYIELQPGSKGSQPESYQLLDSPPLAPPDAKGIRVILDSKKAGQLSPGDPVLFRG
YRVGSVETSSFDPQKRTMSYQLFIKAPNDRLVTSNVRFWKDSGIAVDLTSAGMRVEMGSL
TTLFGGGVSFDVPEGLEQGQPVAEKTAFNLYDDQKSIQDSLYTDHIDYLMFFKDSVRGLQ
PGAPLEFRGIRLGTVSKVPFFASKMRQVFNDDYRIPVLVRIEPERLKAQLGENADVGAHL
TELLKRGLRASLKTGNLVTGALYVDLDFYPKEPPITGLREFDGYEIIPTVSSGLAQIQQR
LVETLDKINNLPLNPMIEQATNTLSESQRTMRRLQTTLDNMNKITSSQSMQQLPADMQTT
LRELNRSMQGFQPGSAAYNKMVADMQRLDQVLRELQPVLKTLNEKSNALVFEAKDKKDPE
PKRAKQ
>EECEBICD_02490 Intermembrane transport protein PqiA
MCEHHHAAKHILCPQCDMLVALPRLSHGQKAACPRCGATLTTEWDAPRQRPTACALAALF
MLLLSNLFPFVNMNVAGVTSEVTLLEIPGVMFSEDYASLGTFFLLFVQLVPAFCLVTILL
LVNRASLPLSVKKTLARIFFLLKSWGMAEIFLAGVLVSFVKLMAYGDIGIGSSFIPWCLF
CLVQLRAFQCVDRRWLWDDIAPQPALAQPLTPGITGIRQSLRSCACCTAILPAESLVCPR

CHTKGYVRRKNSLQWTLALLFTSIMLYLPANILPIMITDLLGSKMPSTILAGVILLWSEG
SYPVAAVIFLASIMVPTLKMIAIAWLCWDAKGHGKRDSERMHFIYEVVEFVGRWSMIDVF
VIAVLSALVRMGGLMNIYPAMGALMFALVVIMTMFSAMTFDPRLSWDREYEPGHEES
>EECEBICD_02491 ABC transporter ATP-binding protein uup
MSLISMHGAWLSFSDAPLLDNAELHIEDNERVCLVGRNGAGKSTLMKILNREQGLDDGRI
IYEQDLIVARLQQDPPRNIAGSVYDFVAEGIEEQAEYLKRYHEISRLVMTDPSEKNLNEM
ARVQEQLDHHNLWQLENRINEVLAQLGLDPNAALSSLSGGWLRKAALGRALVSNPRVLLL
DEPTNHLDIETIDWLEGFLKTFNGTIIFISHDRSFIRNMATRIVDLDRGKLVTYPGNYDQ
YLLEKEEALRVEELQNAEFDRKLAQEEVWIRQGIKARRTRNEGRVRALKAMRRERSERRE
VMGTAKMQVEEATRSGKIVFEMENVDYQVEGKQLVKDFSAQVQRGDKIALIGPNGCGKTT
LLKLMLGQLQADSGRIHVGTKLEVAYFDQHRAELDPEKTVMDNLAEGKQEVMVNGKPRHV
LGYLQDFLFHPKRAMTPVRALSGGERNRLLLARLFLKPSNLLILDEPTNDLDVETLELLE
ELIDGYQGTVLLVSHDRQFVDNTVTECWIFEGGGKIGRYIGGYHDARAQQEQHLATKQPM
AKKMRKLLPLKQKL
>EECEBICD_02492 Ribosomal RNA large subunit methyltransferase K/L
MNSLFASTARGLEELLKTELEKLGAVGCQVVQGGVHFQGDTRLIYQSLMWSRLASRIILP
MGECKVYSDLDLYLGVQAINWTEIFNPGATFAVHFSGLNDTIRNSQYGAMKVKDAIVDAF
TRKNLPRPNVDRESPDLRINVWLNKETASIALDLSGDGLHLRGYRDRTGLAPIKETLAAA
IVMRSGWQPGTPLLDPMCGSGTLLIEAAMWATDRAPGLHRGHWGFSGWAQHDETIWQEVK
AEAQTRARKGLAEYSSHFYGSDSDARVIERARSNARRAGIGELITFEVKDVAQLSNPLPK
GPYGTVISNPPYGERLDSEPALIALHSLLGRTMKNQFGGWNLSLFSASPDLLGSLQLRAD
KQFKAKNGPLDCVQKNYHIAETTADSKPATVAEDYANRLRKNLKKLEKWARQEGIECYRL
YDADLPEYNVAVDRYGDWAVIQEYAPPKTVDAQKARQRLFDIIAATLSVLGIPPNKLVLK
TRERQKGKNQYQKMSEKGEFLEVSEYNARLWVNLTDYLDTGLFLDHRIARRMLGEMSKGK
DFLNLFSYTGSASVHAGLGGARSTTTVDMSRTYLEWAERNLRLNGLSGRAHRLIQADCLG
WLREANEQFDLIFIDPPTFSNSKRMEESFDVQRDHVALMKDLKRLLRKGGTIMFSNNKRG
FRMDLEGLAELGLTAQEITQKTLSPDFARNRQIHNCWLIRAA
>EECEBICD_02493 putative protein YcbX
MATLSRLFIHPVKSMRGIGLTHALADISGLAFDRIFMVTEPDGTFITARQFPQMVRFTPS
PLHDGLHLTAPDGSSALVRFTDFTPQDAPTEVWGNHFTARVAPTAINQWLSGFFSRDVQL
RWVGPQLTRRVKRHNAVPLGFADGYPYLLTNEASLRDLQQRCPAGVQMEQFRPNLVVSGV
AAWEEDSWKVLRIGDVIFDVVKPCSRCIFTTVSPEKGQKHPSGEPLATLQAFRTAQDNGD
VDFGQNLIARNSGVVRVGDEVEILATAPAKAYGATTVDDSVTPDKHPDASVTIDWQGQTF
CGNNQQVLLEQLENQGIRIPYSCRAGICGCCRIRLLEGEVSPLKKSAMGDDGTILSCSCV
PKTALRLEN
>EECEBICD_02494 Cell division protein ZapC
MRIKPDDNWRWYYDEEHDRMMLDLANGILFRSRFSRKMLTPDAFCPTGFCVDDAALYFSF
EEKCRDFELTKEQRAELVLNALVAIRYLKPQMPKSWHFVAHGEMWTPGTGDAASVWLSDT
AEQVNLLVVEPGENAALCLLAQPGVVIAGRTMQLGDAIKIMNDRLKPQVHCHSFSLEQAV
>EECEBICD_02495 Dihydroorotate dehydrogenase (quinone)
MYYPFVRKALFQLDPERAHEFTFQQLRRITGTPLEALVRQKVPTKPVTCMGLTFKNPLGL
AAGLDKDGECIDALGAMGFGSLEIGTVTPRPQPGNDKPRLFRLVDAEGLINRMGFNNLGV
DNLVENVKKAHFDGILGINIGKNKDTPVENGKDDYLICMEKVYAYAGYIAINISSPNTPG
LRTLQYGDALDDLLTAIKNKQNDLQAIHHKYVPVAVKIAPDLCEEELIQVADSLLRHNID
GVIATNTTLDRSLVQGMKNCQQTGGLSGRPLQLKSTEIIRRLSQELKGQLPIIGVGGIDS
VIAAREKIAAGATLVQIYSGFIFKGPPLIKEIVTHI
>EECEBICD_02496 Aminopeptidase N
MTQQPQAKYRHDYRAPDYQITDIDLTFDLDAEKTVVTAISQAVRHSAPDAPLRLDGEDLT
LVSIHVNDAPWTAYKEEEGALIISDLPERFTLRIVNEISPAANTALEGLYQSGDALCTQC
EAEGFRHITWYLDRPDVLARFTTKIIADKSKYPFLLSNGNRVAQGELENGRHWVQWQDPF
PKPCYLFALVAGDFDVLRDTFTTRSGRDVALELYVDRGNLDRAPWAMTSLKNSMKWDEAR
FGLEYDLDIYMIVAVDFFNMGAMENKGLNIFNSKYVLARTDTATDKDYLDIERVIGHEYF
HNWTGNRVTCRDWFQLSLKEGLTVFRDQEFSSDLGSRAVNRISNVRTMRGLQFAEDASPM
AHPIRPDKVIEMNNFYTLTVYEKGAEVIRMIHTLLGEENFQKGMQLYFERHDGSAATCDD

FVQAMEDASNVDLSHFRRWYSQSGTPIVTVKDDYNPETEQYTLTISQRTPATADQAEKQP
LHIPFAIELYDNEGNVIPLQKGGHPVNAVLNVTQAEQTFTFDNVYFQPVPALLCEFSAPV
KLEYKWSDQQLTFLMRHARNDFSRWDAAQSLLATYIKLNVARHQQGQPLSLPVHVADAFR
AVLLDEKIDPALAAEILTLPSANEIAELFEVIDPIAIAQVREALTRTLAAELADEFLAIY
NANHLDEYRVDHGDIGKRTLRNACLRFLAFGETELANTLVSKQYRDANNMTDALAALSAA
VAAQLPCRDTLMQEYDDKWHQDGLVMDKWFILQSTSPAENVLETVRGLLKHRSFSMSNPN
RVRSLIGAFAGSNPAAFHAQDGSGYQFLVEMLTDLNSRNPQVASRLIEPLIRLKRYDEKR
QEKMRAALEQLKGLENLSGDLYEKITKALA
>EECEBICD_02497 hypothetical protein
MTDKTAKAKILYIQTVISETRMFVELVYDKRNVEGLEGASEIILAELTKQVHQIFPDAEV
RVKPMQANCLNSDTNKSDRENLNR
>EECEBICD_02498 hypothetical protein
MLRHIQNSLGSVYRSNTATPQGQIIHHRNFQSQFDTTGNTLYNNCWVCSLNVIKSRDGNN
YSALEDITSDNQAFNNILEGIDIIECENLLKEMNVQKIPESSLFTNIKEALQAEVFNSTV
EDDFESFISYELQNHGPLMLIRPSLGSECLHAECIVGYDSEVKKVLIYDSMNTSPEWQSN
IDVYDKLTLAFNDKYKNEDCSICGLYYDGVYEPKPLHSSSWKDWCTIL
>EECEBICD_02499 hypothetical protein
MGGRGSEVQILSCRPKFPLKTSLSGLFFLWLNFLRGSYGVKLGNKAVEIRRNLRLIHQFA
HCSVSEFINTQIFISNYCINSL
>EECEBICD_02500 SOS response-associated protein YedK
MCGRFAQAQTREEYLAYLADEADRNIAYDPQPIGRYNVAPGTKVLLLSERDEQLHLDPVI
WGYAPGWWDKAPLINARVATAASSRMFKPLWQHGRAICFADRWFEWKKEGDKKQPYFIHR
KDGKPIFMAAIGSTPFERGDEAEGFLIVTSAADKGLVDIHDRRPLALTPETARVWMRQFL
EPHSKSITYRVIPALTRPMMRKDTNPCQ
>EECEBICD_02501 IS66 family transposase ISSfl3
MEIDNNICENALRCVALGRRNYLFFGSDRSGEAAAIIYSLLGTCKLNGVESEAWLRDVLW
KISDWSSNRVHELLPWNLETVK
>EECEBICD_02502 Secreted effector protein SseI
MPFHIGSGCLPAIISNRRIYRIAWSDTPPEMSSWEKMKEFFCSTHQTEALECIWTICHPP
AGTTREDVVSRFELLRTLAYDGWEENIHSGLHGENYFCILDEDSQEILSVTLDDVGNYTV
NCQGYSETHHLTMATEPGVERTDITYNLTSDIDAAAYLEELKQNPIINNKIMNPVGQCES
LMTPVSNFMNEKGFDNIRYRGIFIWDKPTEEIPTNHFAVVGNKEGKDYVFDVSAHQFENR
GMSNLNGPLILSADEWVCKYRMATRRKLIYYTDFSNSSIAANAYDALPRELESESMAGKV
FVTSPRWFNTFKKQKYSLIGKM
>EECEBICD_02503 Prophage tail fiber assembly protein TfaE
MTFKMSEQAQTIKIFNLRSDTNEFIGAGDAYIPPHTGLPANCTDIAPPDIPASHIAIFDA
ETQTWSLHEDHRGEMVYDTTTGNQVYISAPGPLPENVTSVSPGGEYQKWDGKAKVWVKDE
AAEKAAQLRQAEETKSRLLQMASEKIAPLQDAVDLGIATDDEKARLDEWKKYRVLVNRMD
TAAPDWPERPASQ
>EECEBICD_02504 hypothetical protein
MIIGFGNNVVSSLAADITASQTTIQVMPGVGAMFANLLTSDYANSSNPLKTYAKITLTDA
KETVFEVCHLTAVNNDMLTVIRGQEGTTAKGWSLNDVIANFATRGSENQFVQIEELQSGH
YVAGVAGGTENNLTLELPATYFVNGGVDWTLRTPLVVIPALNNTGASTLQLTMGGRVLGI
FPLYKGNKAELSANDIIKDIPVLCVLDNTKTYFSVLNPLEIYLGSRYLQKDQNLSDVPDK
AKGRSSLEVYSKTESDENYMAKSQCGADIPNKPLFVQNIGALPASGTAVAANRLASRGAL
PALTGTTRGSDSGLIMGEVYNNGYPTQYGNILRLTGTGDGEILIGWSGTNGAPAPAYIRS
HRDTADAEWSEWAMLYTTLNPPPDSHPVGAAIAWPSDATPAGYALMQGQSFDKSAYPLLA
IAYPSGVIPDMRGWTIKGKPISGRAVLSQEMDGNKSHSHTARAQVTDLGTKSTSSFDYGT
KSTNTTGNHTHQFGGYINSYWGDSNHTSFQPGGGAWTQAAGDHAHTVYIGGHEHTMYIGP
HGHVVIVDADGNAETTVKNIAFNYIVRLA
>EECEBICD_02505 hypothetical protein
MAALLESIIPAYPYTQYNDDPDIVAFFDAYNKLAQGYLDYFNNLNLPCWTSPAITGELLD
WIAAGIYGESRPLLQISEDAIARGAYNTIEYNNVAYAKLRNYVPGSASYVPDDYFKRILT
WNFYKGDGSHFCINWFKRRLARFIHGANGIDPPVQSTFDISVMPDKGIFFVSIPDYGDGV

GHFLKDAIDQSLVKLPFIYTYSVTVVEQ
>EECEBICD_02506 hypothetical protein
MYGVQGTPDCYRIELKNVYGVQENLISYRQASLGAWVAIAGGGDPYEVAYAIYKAVPDIS
VLTNDVVNPSGAAVDKKTIPIIVYPDTYHVPFVVPSSQNVTLLITWNTASTSYIDPTGIE
KAVQQSIADYINGIATGEPINIFLIRDIFLNQVKGLVSSNLVSMIDIQVGINGKIVPPAT
DSSLVYGDTYAYFSTSSSQIQVKQYGSSS
>EECEBICD_02507 hypothetical protein
MENTNIVTTEQQAPNTISASNAIFNVQALGQLTAFANLMADSQVTVPAHLAGKPADCMAI
VMQAMQWGMNPYACWSLSAGANPNFIATQMGHTDAQMVYKVYGKWMSEKSAEQVSLLNQA
LSRYAPSLPQSMVAAQ
>EECEBICD_02508 Nicotinate phosphoribosyltransferase
MTQFASPVLHSLLDTDAYKLHMQQAVFHHYYDVQVAAEFRCRGDDLLGIYADAIREQVDA
MQHLRLQEDEFQWLSGLPFFKPDYLNWLREFRYNPAQVCVTNDNGKLNIRLTGPWREVIM
WEVPLLAVISELVHHYRSPNAGVDQALDALESKLVDFTALTANLDMSRFHLMDFGTRRRF
SREVQQAIVKRLQQESWFVGTSNYDLARRLALTPMGTQAHEWFQAHQQISPDLATSQRAA
LAAWLNEYPDQLGIALTDCITMDAFLRDFGIEFASRYQGLRHDSGDPVAWGEKAIAHYEK
LGIDPLTKTLVFSDNLDLPKAVELYRHFASRVQLSFGIGTRLTCDIPQVKPLNIVIKLVE
CNGKPVAKLSDSPGKTICHDKAFVRALRKAFDLPQVRKAS
>EECEBICD_02509 Amino-acid carrier protein AlsT
MWIIAVVGMMTKMVEVTLAVKYRSKGENGEYYGGPMHYIKKGLNKKWHPLAGLYAFALMI
LVITDACFVQTNTMAAVIHYTFEIPTSVIGGFIVIVGALVILKGLSSLGKFCTIALPPIT
IAYFIGAAGVVVLNIEAIPQVIKSIFYYAFAPAPAVGGFVGSTIMMAISKGASRGIFTNE
AGMGTSATVHATANVDYAFRQGMWGAVEVFFVSMITCNFTAFAVLASGMWTDASYQGIQI
IFAALKETWHPIIVQVLCLGVALILFTSYLGSYIKFRTSINYIFGDKLERIIKWLYFLPP
LIAVNMEIPVIWLMADIAVGFLVIPNVIALFLLRKEFISEFNLFRTRTQRDTHSEKTTQI
THVNMSKSEGKEE
>EECEBICD_02510 hypothetical protein
MLEQLSQLFEFLWGGPLFLCVIGIGFYFTVRLKFFQIINLKEIYRNTIGTLAGKNKQNTT
GEAASKKILKINRGGCHCAIRLIRRGNDRRCCCGYCGWRTWGNFLDVDHCGRGYDDQNG
>EECEBICD_02511 Diaminopropionate ammonia-lyase
MHELIKYQFNTRRKKYGTGAALSLLNGNVGREVLAFHKKLPNYAVTPLHNLAHLSRRLGL
GSIHIKDESWRFGLNAFKGLGGSYAVGKYLADKLQCDINSLSFAALNTPEIKEKIKDCVF
VTATDGNHGRGVAWAAEQLGLKAVVYMPKGSSLIRAENIRHHGAECTITDLNYDDAVRLA
HRMAQTKGWVLLQDTAWTGYEEIPTWIMQGYMTLAVEAYDQLAETNSPLPTHLILQAGVG
SFAGSVMGYFVEKMQESIPNIIVVEPHQANCLYQSAVMNDGQPHCVTGDMATIMAGLACG
EPNIISWPIIRDNTSCFISADDCLAAKGMRISAAPRPGTDTPFISGESGAIGVGLLYELM
NNMHYQDLANRLQLDANAHVLLISTEGDTSPDIYEDIVWNGRSA
>EECEBICD_02512 Leucine-responsive regulatory protein
MKLDAWDKNILTLLQRDNRLSQREIAEQVNLSPSAVNRRIAALEEAGIIKGNVSLIDAGK
VGRPVTIVVQVVIENERFNLLEEARQRFVSCPQVQQVYYVTGDFDFLLVLNVRDMAEYEA
LTRELFFSSGNIKSFKTIVVMQNAKQEMRVLIE
>EECEBICD_02513 Asparagine--tRNA ligase
MSVVPVADVLQGRVAVDQEVTVRGWVRTRRDSKAGISFLAVYDGSCFDPVQAVINNSLPN
YNEEVLHLTTGCSVVVTGKVVASPGQGQSFEIQATKVEVAGWVEDPDTYPMAAKRHSIEY
LREVAHLRPRTNLIGAVARVRHTLAQALHRFFDEQGFFWVSTPLITASDTEGAGEMFRVS
TLDLENLPRNDQGRVDFDKDFFGKESFLTVSGQLNGETYACALSKIYTFGPTFRAENSNT
SRHLAEFWMLEPEVAFADLEDNARLAEAMLKYVFKAVLEERADDMKFFAERVDKDAIARL
ERFVSTDFAQVDYTDAVAILERCGKTFENPVFWGVDLSSEHERYLAEEHFKAPVVVKNYP
KEIKAFYMRLNEDGKTVAAMDVLAPGIGEIIGGSQREERLDVLDARMAEMGLNKEDYWWY
RDLRRYGTVPHSGFGLGFERLIAYVTGVQNVRDVIPFPRTPRNASF
>EECEBICD_02514 Outer membrane porin F
MMKRKILAAVIPALLAAATANAAEIYNKDGNKLDLYGKAVGRHVWTTTGDSKNADQTYAQ
IGFKGETQINTDLTGFGQWEYRTKADRAEGEQQNSNLVRLAFAGLKYAEVGSIDYGRNYG
IVYDVESYTDMAPYFSGETWGGAYTDNYMTSRAGGLLTYRNSDFFGLVDGLSFGIQYQGK

NQDNHSINSQNGDGVGYTMAYEFDGFGVTAAYSNSKRTNDQQDRDGNGDRAESWAVGAKY
DANNVYLAAVYAETRNMSIVENTVTDTVEMANKTQNLEVVAQYQFDFGLRPAISYVQSKG
KQLNGADGSADLAKYIQAGATYYFNKNMNVWVDYRFNLLDENDYSSSYVGTDDQAAVGIT
YQF
>EECEBICD_02515 Aspartate aminotransferase
MFENITAAPADPILGLADLFRADDRPGKINLGIGVYKDETGKTPVLTSVKKAEQYLLENE
TTKNYLGIDGIPEFARCTQELLFGKGSALINDKRARTAQTPGGTGALRIAADFLAKNTPV
KRVWVSNPSWPNHKSVFNAAGLEVREYAYYDAENHTLDFEALQASLSEAQAGDVVLFHGC
CHNPTGIDPTLEQWQVLAELSVEKGWLPLFDFAYQGFARGLEEDAEGLRAFAALHKELIV
ASSYSKNFGLYNERVGACTLVAADAETVDRAFSQMKSAIRANYSNPPAHGASIVATILSN
DALRAIWEQELTDMRQRIQRMRQLFVNTLQEKGANRDFSFIIKQNGMFSFSGLTKDQVLR
LREEFGVYAVASGRVNVAGMTPDNMAPLCEAIVAVL
>EECEBICD_02516 Hydroxyacylglutathione hydrolase GloC
MNYRIIPVTAFSQNCSLIWCEQTRLAALVDPGGDAEKIKQEVDASGVTLMQILLTHGHLD
HVGAASELAQHYGVPVIGPEKEDEFWLQGLPAQSRMFGLDECQPLTPDRWLNDGDRVSVG
NVTLQVLHCPGHTPGHVVFFDEQSQLLISGDVIFKGGVGRSDFPRGDHTQLIDAIKRKLL
PLGDDVTFIPGHGPLSTLGYERLHNPFLQDEMPVW
>EECEBICD_02517 hypothetical protein
MDKFDANRRKLLALGGVALGAAILPAPAFATLSTPRPRILTLNNLHTGESIKAEFFDGRA
YIQDELAKLNHFFRDYRANKVRSIDPRLFDQLYRLQGLLGTRKPVQLISGYRSLDTNNEL
RARSSGVAKKSYHTKGQAMDFHIEGVALSNIRKAALSMRAGGVGYYPRSNFVHIDTGPAR
HW
>EECEBICD_02518 putative L,D-transpeptidase YcbB
MLLNKMCGRCLSAISLCLAVTFAPLFNAQADEPEMIPGDSAVAAPDLAGPQKQSAATAIM
AGIQPLPEGVSAEKVRADLQSQLPSGYTPVYMSQLTLLYAARDMKPMWDNRDAVKVFQQQ
LAEVAIAGFQPQFTAWVALLTDPAVNGMARDVVLSDAMMGYLHFIANIPVKGQRWLYSNK
PYALATPPVSVINQWQIALEEGQLPMFVASLAPQHPQYAPMHDALLKLVADSRPWPQLAN
TATLRPGQWSNDVPALREILQRTGMLDGGPKIALPGDNTADSAVVSPSAVVDETSVAQPV
DEQTARRSKPAPAVRAAYDNELVEAVKRFQAWQGLGADGVIGPATRNWLNMTPAQRAGVL
ALNIQRLRLLPAELSTGIMVNIPAYSLVYYQNGNQVLASRVIVGRPDRKTPMMSSALNNV
VVNPPWNVPPTLARKDILPKVWNDPGYLERHGYTVMRGWNSKEAIDPWQVDWTTITPSNL
PFRFQQAPGAHNSLGRYKFNMPSSDAIYLHDTPNHTLFQRDARALSSGCVRVNKASELAN
MLLQDAGWNDARISGALKQGDTRYVNIRQNIPVNLYYLTAFVGADGRMQYRTDIYNYDLT
ARSSAQIVPKVEQLIR
>EECEBICD_02519 Chromosome partition protein MukB
MIERGKFRSLTLINWNGFFARTFDLDELVTTLSGGNGAGKSTTMAAFVTALIPDLTLLHF
RNTTEAGATSGSRDKGLHGKLKAGVCYSMLDTINSRHQRVVVGVRLQQVAGRDRKVDIKP
FAIQGLPMSVQPTQLVTETLNERQARVLSLAELKDKLDEMEGVQFKQFNSITDYHSLMFD
LGIIARRLRSASDRSKFYRLIEASLYGGISSAITRSLRDYLLPENSGVRKAFQDMEAALR
ENRLTLEAIRVTQSDRDLFKHLISEATDYVAADYMRHANERRVHLDQALAFRRELYTSRK
QLAAEQYKHVDMARELGEHNGAEGSLEADYQAASDHLNLVQTALRQQEKIERYEADLEEL
QIRLEEQNEVVAEAAEMQDENEARAEAAELEVDELKSQLADYQQALDVQQTRAIQYNQAI
SALARAKELCHLPDLTPESAAEWLDTFQAKEQEATEKLLSLEQKMSVAQTAHSQFEQAYQ
LVAAINGPLARSEAWDVARELLRDGVNQRHLAEQVQPLRMRLSELEQRLREQQEAERLLA
EFCKRQGKNFDIDELEALHQELEARIASLSESVSSASEQRMALRQEQEQLQSRIQHLMQR
APVWLAAQNSLNQLSEQCGEEFTSSQEVTEYLQQLLEREREAIVERDEVGARKNAVDEEI
ERLSQPGGAEDQRLNALAERFGGVLLSEIYDDVSLEDAPYFSALYGPSRHAIVVPDLSQI
AEQLEGLTDCPEDLYLIEGDPQSFDDSVFSVDELEKAVVVKIADRQWRYSRFPSLPIFGR
AARENRIESLHAEREVLSERFATLSFDVQKTQRLHQAFSRFIGSHLSVAFEDDPEAEIRR
LNGRRVELERALATHESDNQQQRLQFEQAKEGVSALNRLLPRLNLLADETLADRVDEIQE
RLDEAQEAARFVQQYGNQLAKLEPVVSVLQSDPEQFEQLKEDYAWSQQMQRDARQQAFAL
AEVVERRAHFSYSDSAEMLSGNSDLNEKLRQRLEQAEAERTRAREALRSHAAQLSQYSQV
LASLKSSYDTKKELLNDLQRELQDIGVRADSGAEERARQRRDELHAQLSNNRSRRNQLEK
ALTFCEAEMENLTRKLRKLERDYHEMREQVVTAKAGWCAVMRMVKDNGVERRLHRRELAY

LSADELRSMSDKALGALRLAVADNEHLRDVLRLSEDPKRPERKIQFFVAVYQHLRERIRQ
DIIRTDDPVEAIEQMEIELSRLTEELTSREQKLAISSRSVANIIRKTIQREQNRIRMLNQ
GLQSVSFGQVNSVRLNVNVRETHATLLDVLSEQQEQHQDLFNSNRLTFSEALAKLYQRLN
PQIDMGQRTPQTIGEELLDYRNYLEMEVEVNRGSDGWLRAESGALSTGEAIGTGMSILVM
VVQSWEDEARRLRGKDISPCRLLFLDEAARLDARSIATLFELCERLQMQLIIAAPENISP
EKGTTYKLVRKVFQNTEHVHVVGLRGFAPQLPETLPGTQTEDTPSEAS
>EECEBICD_02520 Chromosome partition protein MukE
MSLTNIEQVMPVKLAQALANPLFPALDSALRSGRHIGLDELDNHAFLMDFQEYLEEFYAR
YNVELIRAPEGFFYLRPRSTTLIPRSVLSELDMMVGKILCYLYLSPERLANEGIFTQQEL
YDELLTLADEAKLLKLVNNRSTGSDVDRQKLQEKVRSSLNRLRRLGMVWFMGHDSSKFRI
TESVFRFGADVRAGDDPREAQRRLIRDGEAMPIENHLQLNDETEESQPDSGEEE
>EECEBICD_02521 Chromosome partition protein MukF
MSEFSQTVPELVAWARKNDFSISLPVDRLSFLLAVATLNGERLDGEMSEGELVDAFRHVS
DAFEQTSETIGVRANNAINDMVRQRLLNRFTSEQAEGNAIYRLTPLGIGITDYYIRQREF
STLRLSMQLSIVAGELKRAADAAAEGGDEFHWHRNVYAPLKYSVAEIFDSIDLTQRIMDE
QQQQVKDDIAQLLNKDWRAAISSCELLLSETSGTLRELQDTLEAAGDKLQANLLRIQDAT
MTHDDLHFVDRLVFDLQSKLDRIISWGQQSIDLWIGYDRHVHKFIRTAIDMDKNRVFAQR
LRQSVQTYFDDPWALTYANADRLLDMRDEEMALRDDEVTGELPPDLEYEEFNEIREQLAA
IIEEQLAIYKTRQTPLDLGLVVREYLAQYPRARHFDVARIVIDQAVRLGVAQADFTGLPA
KWQPINDYGAKVQAHVIDKY
>EECEBICD_02522 tRNA 5-carboxymethoxyuridine methyltransferase
MQDRNFDDIAEKFSRNIYGTTKGQLRQAILWQDLDRVLEEIGGRKLRVLDAGGGEGQTAI
KMAERGHQVTLCDLSGEMIARARQAAEAKGVSKDMHFIQCPAQDVASHLESPVDLILFHA
VLEWVADPVGVLETLWSVLRPGGALSLMFYNANGLLMHNMVAGNFDYVQAGMPKRKKRTL
SPDYPRDPAQVYQWLEAIGWQITGKTGVRVFHDYLREKHQQRDCYETLVELETRYCRQEP
YISLGRYIHVTAIKSPALAADARITYE
>EECEBICD_02523 hypothetical protein
MLFTLKKVIGGMLLPLPLMLLIIGVGLALLWFSRFQKTGKVFISVGWLALLLLSLQPVSD
HLLRPIENRYPTWQGPQKVEYIVVLGGGYTWNPQWAPSSNLINNSLPRLAEGIRLWRANP
GARLIFTGGIAKTNTVSTAEVGARVAQSLGVPRSDIITLDKPKDTEEEAAAVKQAIGDAP
FLLVTSASHLPRAMIFFQHAGLNPLPAPANQLAIDSPLNPWERAIPSPVWLMHSDRAGYE
TLGRLWQWLKGISGKPGE
>EECEBICD_02524 hypothetical protein
MEQLRAELSHLLGEKLSRIECVNEKADSALWSLYDSQGIPMPLMARSFTTPGVAQQLAWK
TSMLARSGTVRMPVIYGVLTHEEHPGPDVLLLERLRGVPVEAPARTPERWEQLKDQIVEG
LLAWHRQDSRGCVGAVDHTQENIWPSWYRQRVEVLWTTLNQFSNTGLTMQDRRILFRTRE
CLPSLFEGFNDNCVLVHGNFCLRSMLKDARSDQLLAMVGPGLMLWAPREFELFRLMDNPL
AEGLLWHYLQRAPVAESFIWRRWLYVLWDEVAQLVNTGRFNRTNFDLAAKSLLPWLA
>EECEBICD_02525 3-deoxy-manno-octulosonate cytidylyltransferase
MSFVVIIPARFSSTRLPGKPLVDINGKPMIVHVLERARESGAERIIVATDHEDVARAVEA
AGGEVCMTRADHQSGTERLAEVVEKCGFSDDTVIVNVQGDEPMIPAVIIRQVAENLAQRQ
VGMATLAVPIHSAEEAFNPNAVKVVLDAEGYALYFSRATIPWDRDRFAKSLETVGDTCLR
HLGIYGYRAGFIRRYVSWQPSQLEHIEMLEQLRVLWYGEKIHVAVAKAVPGTGVDTADDL
ERVRAEMR
>EECEBICD_02526 hypothetical protein
MDHRLLEIIACPVCNGKLWYNQEQQELICKLDNLAFPLRDGIPVLLENEARALTSDESKS
>EECEBICD_02527 hypothetical protein
MSLPYLSLSQARCLHLAAQGLLKKPRRNVLPGDVLAAISRMALLQIDTINVVARSPYLVL
FSRLGSYPQAWLDEALRRGELMEYWAHEACFLPRRDFKLIRHRMLSPEKMGWKYRAAWMH
EHAEEIEQLVRHIQEHGPVRSADFEHARKGVSGGWWEWKPHKRHLEGLFTAGKVMVVERRN
FQRVYDLTPRVMPHWDDERDGLSQSQAENLMLDNSARSLGIFREQWLADYYRLKRPDLKG
WRESRAEQQQIIPVEVETLGRMWLHADLLSQLEPALNNALKATHSAVLSPFDPVVWDRKR
AAQLFGFNYRLECYTPAAKRQYGYFVLPLLYQGRLVGRMDAKMHRKTGVLEIISLYLEDD
IRPGVSLQKGIWQAISAFAAWQRASRVTLGQCPPGLFSAMRHGWEIDPAP

>EECEBICD_02528 Tetraacyldisaccharide 4'-kinase
MIARIWSGESPLWRLLLPLSWLYGLVSGAIRLSYKLGFKRAWRAPVPVVVVGNLTAGGNG
KTPVVIWLVEKLQQRGVRVGVVSRGYGGKAAAYPLLLTPETTTAEAGDEPVLIYQRTGAP
VAVAPERAAAVKAILAAHNLQIIITDDGLQHYRLARDIEIVVIDGVRRFGNGWWLPAGPM
RERASRLKTVDAAIVNGGVARAGEIPMQLAPGLAVNLRTGARCDVAQLSNIVAMAGIGHP
PRFFATLEACGAHPQKCVPLADHQTLAPADVQALVGEGQTLVMTEKDAVKCRAFAEDNWW
FLPVDARLSGEQPDKLLEHITSLVR
>EECEBICD_02529 Lipid A export ATP-binding/permease protein MsbA
MHNDKDLSTWQTFRRLWPTIAPFKAGLIVAGIALILNAASDTFMLSLLKPLLDDGFGKTD
RSVLLWMPLVVIGLMILRGITSYISSYCISWVSGKVVMTMRRRLFGHMMGMPVAFFDKQS
TGTLLSRITYDSEQVASSSSGALITVVREGASIIGLFIMMFYYSWQLSIILVVLAPIVSI
AIRVVSKRFRSISKNMQNTMGQVTTSAEQMLKGHKEVLIFGGQEVETKRFDKVSNKMRLQ
GMKMVSASSISDPIIQLIASLALAFVLYAASFPSVMDSLTAGTITVVFSSMIALMRPLKS
LTNVNAQFQRGMAACQTLFAILDSEQEKDEGKRVIDRATGDLEFRNVTFTYPGREVPALR
NINLKIPAGKTVALVGRSGSGKSTIASLITRFYDIDEGHILMDGHDLREYTLASLRNQVA
LVSQNVHLFNDTVANNIAYARTEEYSREQIEEAARMAYAMDFINKMDNGLDTIIGENGVL
LSGGQRQRIAIARALLRDSPILILDEATSALDTESERAIQAALDELQKNRTSLVIAHRLS
TIEQADEIVVVEDGIIVERGTHSELLAQHGVYAQLHKMQFGQ
>EECEBICD_02530 hypothetical protein
MKITTVSVCVVCGILPLMILPVLPDTWILAVLFCLACLLCLIPHHYARYAALTLLFFMWG
IFAARQAIWAGNVLPAATQEATVVITATDHMTTHYGRITHLHGKPLFPAVGIVLHGQYLP
TEVCAGQQWAMTLKVRAVHGQLNEGGFDSQRYALAQHQPLTGRFLQAKAINPECSLRGRY
LASLRATLAPYPWQQVILALGMGERGAVSQEVKAVMRDTGTAHLMAISGLHIAFAALLAA
GLIRGGQFFLPVRWIRWQTPLLGGILCAICYAWLTGLQPPALRTVAALSVWGGLKLSGRQ
WSGWQVWCCCLAAIIFADPVAVISQSLWLSAFAVAGLLFWYQWFPAPNGNFPRGLRWLLN
LLHLQAGITLLLLPLQVALFHGISVTAMLANLFAVPWVTFVTVPLILAGMILHLTGPFFL
EEWVWYLTDRALAALFYLLNALPQGWVNIDNRWQWLTLLPWLTLIAWRLNVWRTCPAVCL
CGLLLMSWPLWRPINASGWQVHMLDVGQGLAIAIVRGDKVILYDTGRAWPEGDSGQQVII
PWLRWHNLTPEGVILSHEHLDHRGGLRSLQQVWPSMWIRSPLGWQGHLPCFRGEQWQWQG
LTFQAHWPLRESADRGNNRSCVVKVDDGVHSILLTGDIEAGAEQKMLSRYWRHLAATFIQ
VPHHGSNTSSSLPFIQRVHGEAALASASRYNAWRLPSRKVKQRYRQQAYQWFDTPHQGQI
SLLFSPQGWRIQGLRDQILPRWYHQWFGVSEDNG
>EECEBICD_02531 Integration host factor subunit beta
MTKSELIERLATQQSHIPAKAVEDAVKEMLEHMASTLAQGERIEIRGFGSFSLHYRAPRT
GRNPKTGDKVELEGKYVPHFKPGKELRDRANIYG
>EECEBICD_02532 30S ribosomal protein S1
MTESFAQLFEESLKEIETRPGSIVRGVVVAIDKDVVLVDAGLKSESAIPAEQFKNAQGEL
EIQVGDEVDVALDAVEDGFGETLLSREKAKRHEAWITLEKAYEDAETVTGVINGKVKGGF
TVELNGIRAFLPGSLVDVRPVRDTLHLEGKELEFKVIKLDQKRNNVVVSRRAVIESENSA
ERDQLLENLQEGMEVKGIVKNLTDYGAFVDLGGVDGLLHITDMAWKRVKHPSEIVNVGDE
INVKVLKFDRERTRVSLGLKQLGEDPWVAIAKRYPEGTKLTGRVTNLTDYGCFVEIEEGV
EGLVHVSEMDWTNKNIHPSKVVNVGDVVEVMVLDIDEERRRISLGLKQCKSNPWQQFAET
HNKGDRVEGKIKSITDFGIFIGLDGGIDGLVHLSDISWNVAGEEAVREYKKGDEIAAVVL
QVDAERERISLGVKQLAEDPFNNWVALNKKGAIVTGKVTAVDAKGATVELADGVEGYLRA
SEASRDRVEDATLVLSVGDDVEAKFTGVDRKNRAISLSVRAKDEADEKDAIATVNKQEDA
NFSNNAMAEAFKAAKGE
>EECEBICD_02533 Cytidylate kinase
MTAIAPVITIDGPSGAGKGTLCKAMAEALQWHLLDSGAIYRVLALAALHHHVDLASEDAL
VPLASHLDVRFVSTDGNLEVILEGEDVSGEIRTQEVANAASQVAAFPRVREALLRRQRAF
REAPGLIADGRDMGTVVFPDAPVKIFLDASSEERAHRRMLQLQENGFSVNFERLLAEIKE
RDDRDRNRAVAPLVPAADALVLDSTRLSIEQVIEKALQYARQKLALA
>EECEBICD_02534 Metalloprotease YcaL
MKNKSLLLAVAISATLLAGCKNGVNGNLIASSGMSAYKAATLSDADVKALSNNACKQMDS
ENQLAGSKSKYTKRLSKIAKALGNNIDGTPVSYKVYMTSDINAWAMANGCVRVYSGLMDL

MTDNEIEGVLGHELGHVSLGHSRKAMQTAYATLAARDAISATSGVAAQLSQSQLGDLAEG
VINSAFSRSQESDADDFSYDLLKKRGINTQGLVTAFDKFATMDAGHAKSLMDSHPASADR
AQHMRDRIAEDKK
>EECEBICD_02535 3-phosphoshikimate 1-carboxyvinyltransferase
MESLTLQPIARVDGAINLPGSKSVSNRALLLAALACGKTVLTNLLDSDDVRHMLNALSAL
GINYTLSADRTRCDITGNGGPLRAPGALELFLGNAGTAMRPLAAALCLGQNEIVLTGEPR
MKERPIGHLVDSLRQGGANIDYLEQENYPPLRLRGGFIGGDIEVDGSVSSQFLTALLMTA
PLAPKDTIIRVKGELVSKPYIDITLNLMKTFGVEIANHHYQQFVVKGGQQYHSPGRYLVE
GDASSASYFLAAGAIKGGTVKVTGIGRKSMQGDIRFADVLEKMGATITWGDDFIACTRGE
LHAIDMDMNHIPDAAMTIATTALFAKGTTTLRNIYNWRVKETDRLFAMATELRKVGAEVE
EGHDYIRITPPAKLQHADIGTYNDHRMAMCFSLVALSDTPVTILDPKCTAKTFPDYFEQL
ARMSTPA
>EECEBICD_02536 Phosphoserine aminotransferase
MAQVFNFSSGPAMLPAEVLKLAQQELRDWHGLGTSVMEISHRGKEFIQVAEEAEQDFRDL
LNIPSNYKVLFCHGGGRGQFAGVPLNLLGDKTTADYVDAGYWAASAIKEAKKYCAPQIID
AKITVDGKRAVKPMREWQLSDNAAYLHYCPNETIDGIAIDETPDFGPEVVVTADFSSTIL
SAPLDVSRYGVIYAGAQKNIGPAGLTLVIVREDLLGKAHESCPSILDYTVLNDNDSMFNT
PPTFAWYLSGLVFKWLKAQGGVAAMHKINQQKAELLYGVIDNSDFYRNDVAQANRSRMNV
PFQLADNALDKVFLEESFAAGLHALKGHRVVGGMRASIYNAMPIEGVKALTDFMIDFERR
HG
>EECEBICD_02537 hypothetical protein
MKAFDLQRMALDNVPVAFLGEVALRSFYTFVLVFLFLKVTGRRGVRQMSLFEVLIIILTLG
SAAGDVAFYDDVPMLPVLVVFITLALLYRLVMWLMAHSEKLEDLLEGKSVVIVEDGELAW
EKLQRSNMTEFEFFMELRLNGVEQLGQVRLAILETNGQISVYFFENKDVKPGLSILPEHC
TPRFIVAPEAGDYACVRCSEVIRMNIGEKQLCPRCANPEWTKASRAKRVV
>EECEBICD_02538 Ribosomal protein S12 methylthiotransferase accessory factor YcaO
MSRRDSARHIKPVSLRAFRTTKVIFMTQTFIPGKDAALEDSIARFQQKLLDLGFHIEEAS
WLNPVPNVWSVHIRDKECALCFTNGKGATKKAALASALGEYFERLSTNYFFADFWLGETV
ANGPFVHYPNEKWFPLTENDDVPEGLLDARLRAFYDPENELTGSQLIDLQSGNEARGVCG
LPFTRQSDNQTVYIPMNIIGNLYVSNGMSAGNTRNEARVQGLSEVFERYVKNRIIAESIS
LPEIPAEVMARYPAVMESIATLEAEGFPIFAYDGSLGGKYPVICVVLFNPANGTCFASFG
AHPDFGVALERTVTELLQGRGLKDLDVFTPPTFDDEEVAEHTNLETHFIDSSGLISWDLF
KQDADYPFTDWSFSGTTEEEFATLMAIFAAEDKEVYIADYEHLGVYACRIIVPGMSDIYP
AEDLWLANNNMGSHLRETLLSLPGSAWNKEDYLNLIEQLDEEGFDDFTRVRELLGLATGA
DNGWYTLRVGELKAMLALAGGDLEQALIWTEWTMEFNSSVFSPTRANYYRCLQTLLLLSQ
EDARQPLQYLNAFIKMYGAEAVEAASAALSGEAAFYGLPAVDHDLQAFPAHQSLLKAYDK
LQRAKAAYWSK
>EECEBICD_02539 putative formate transporter 1
MKADNPFDLLLPAAMAKVAEEAGVYKATKHPLKTFYLAITAGVFISIAFVFYITATTGTG
AMPYGMAKLIGGICFSLGLILCVICGADLFTSTVLIVVAKASGRITWGQLAKNWLNVYFG
NLIGALLFVLLMWLSGEYMTANGQWGLNVLQTADHKMHHTFIEAVCLGILANLMVCLAVW
MSYSGRSLMDKAFIMVLPVAMFVASGFEHSIANMFMIPMGIVIRDFATPEFWTAVGSSPE
SFFSPDRDELHH
>EECEBICD_02540 Formate acetyltransferase 1
MSELNEKLATAWEGFTKGDWQNEVNVRDFIQKNYTPYEGDESFLAGATDATTKLWDSVME
GVKQENRTHAPVDFDTSVASTITSHDAGYINKALEKIVGLQTEAPLKRAIIPFGGIKMVE
GSCKAYNRELDPMIKKIFTEYRKTHNQGVFDVYTPDILRCRKSGVLTGLPDAYGRGRIIG
DYRRVALYGIDYLMKDKFAQFTSLQSDLENGVNLEATIRLREEIAEQHRALGQIKEMAAK
YGCDISGPATNAQEAIQWTYFGYLAAVKSQNGAAMSFGRVSTFLDAYIERDLKAGKITEQ
DAQEMIDHLVMKLRMVRFLRTPEYDELFSGDPIWATESIGGMGVDGRTLVTKNSFRFLNT
LYTMGPSPEPNITVLWSEKLPLNFKKFAAKVSIDTSSLQYENDDLMRPDFNNDDYAIACC
VSPMIVGKQMQFFGARANLAKTMLYAINGGVDEKLKMQVGPKSEPIKGDVLNFDEVMDRM
DHFMDWLAKQYVTALNVIHYMHDKYSYEASLMALHDRDVIRTMACGIAGLSVAADSLSAI

KYAKVKPIRDEDGLAIDFEIEGEYPQFGNNDARVDDMAVDLVERFMKKIQKLTTYRGAIP
TQSVLTITSNVVYGKKTGNTPDGRRAGAPFGPGANPMHGRDQKGAVASLTSVAKLPFAYA
KDGISYTFSIVPNALGKDDEVRKTNLAGLMDGYFHHEASIEGGQHLNVNVMNREMLLDAM
EHPEKYPQLTIRVSGYAVRFNSLTKEQQQDVITRTFTQTM
>EECEBICD_02541 Secreted effector protein sopD2
MPVTLSFGNRHNYEINHSRLARLMSPDKEEALYMGVWDRFKDCFRTHKKQEVLEVLYTLI
HGCERENQAELNVDITGMEKIHAFTQLKQYANPSQQDRFVMRFDMNQTQVLFEIDGKVID
KCNLHRLLNVSENCIFKVMEEDEEELFFKVCIKYGEKIARYPELLEGFANKLKDAVNEDD
DVKDEVYKLMRSGEDRKMECVEWNGTLTEEEKNKLRCLQMGSFNITTQFFKIGYWELEGE
VLFDMVHPTLSYLLQAYKPSLSSDLIETNTMLFSDVLNKDYDDYQNNKREIDAILRRIYR
SHNNTLFISEKSSCRNMLI
>EECEBICD_02542 hypothetical protein
MIHLQYVLRLRRYELMNEPEFLAVRVMQHRFAVYFILNRGKIAPLQHILEQPHIL
>EECEBICD_02543 Pyruvate formate-lyase 1-activating enzyme
MSNLTNCITNESVAVTADKKPVIGRIHSFESCGTVDGPGIRFITFFQGCLMRCLYCHNRD
TWDTHGGKEITVEDLMKEVVTYRHFMNASGGGVTASGGEAILQAEFVRDWFRACKKEGIH
TCLDTNGFVRRYDPVIDELLDVTDLVMLDLKQMNDEIHQNLVGVSNHRTLEFAQYLSKKN
VKVWIRYVVVPGWSDDDDSAHRLGEFTRDMGNVEKIELLPYHELGKHKWVAMGEEYKLDG
VKPPKKETMERVKGILEQYGHKVMY
>EECEBICD_02544 Inner membrane transporter YcaM
MPNVQEKQLRWYNIALMSFITVWGFGNVVNNYANQGLVVVFSWVFIFALYFIPYALIVGQ
LGSTFKEGKGGVSTWIKHTMGPGLAYLAAWTYWVVHIPYLAQKPQAILIALGWALKGDGS
LIKEYTVVALQGLTLALFVFFMWVASRGMKSLKVVGSVAGIAMFIMSILYVVMAVTAPAI
TNVEIATTNITWQSFIPHIDFTYITTISMLVFAVGGAEKISPYVNQTRNPGKEFPKGMLF
LAVMVAVCAILGSLAMGMMFDSRHIPDDLMTNGQYYAFQKLGEYYHMGNSLMVIYAIANT
LGQIAALVFSIDAPLKVLLGDADTKYIPQRLCRTNASGTPVNGYLLTLVLVAILIMLPTL
GIGDMNNLYKWLLNLNSVVMPLRYLWVFVAFIAVIRLAQKYKPEYVFIRNRALALTVGIW
CFAFTAFACLTGIFPKMEAFTPEWTFQLTLNIVTPFVLVGLGLIFPLLARRNT
>EECEBICD_02545 putative MFS-type transporter YcaD
MSTYTRPVMLLLCGLLLLTLAIAVLNTLVPLWLAQANLPTWQVGMVSSSYFTGNLVGTLF
TGYLIKRIGFNRSYYLASLIFAAGCVGLGVMVGFWSWMSWRFIAGIGCAMIWVVVESALM
CSGTSHNRGRLLAAYMMAYYMGTFLGQLLVSKVSGELLHVLPWVTGMILAGILPLLFTRI
VNQQTQARHSSSISAMLKLRQARLGVNGCIISGIVLGSLYGLMPLYLKHQGMANASIGFW
MAVLVSAGILGQWPMGRLADKFGRLLVLRVQVFVVILGSIAMLTQAAMAPALFILGAAGF
TLYPVAMAWACEKVEHHQLVAMNQALLLSYTVGSLLGPSFAAMLMQNYSDNLLFIMIASV
SFIYLLMLLRNAGQTPNPVAHI
>EECEBICD_02546 Anaerobic dimethyl sulfoxide reductase chain C
MGSGWHEWPLMIFTVFGQCVAGGFIVLALALMKGDLRAETQQRVIACMFGLWVLMGIGFI
ASMLHLGSPMRAFNSLNRVGASALSNEIASGSVFFAVGGIGWLLAVLKKLPPALRTLWLI
ITMVLGVVFVWMMVRVYNSIDTVPTWYSVWTPLGFFLTLFIGGPLLGYLLLRIAGVNGWA
MRLLPAVSVLALVVIAIMVAMQGAELATIHSSIQQASALVPDYGSLMAWRMVLLAAALCC
WIVPQLKGYQPAVPLLSVAFILMLAGELIGRGVFYGLHMTVGMAVAS
>EECEBICD_02547 Anaerobic dimethyl sulfoxide reductase chain B
MTTQYGFFIDSSRCTGCKTCELACKDYKDLTPDVSFRRIYEYAGGDWQEDNGVWHQNVFA
YYLSISCNHCEDPACTKVCPSGAMHKRDDGFVVVNEEVCIGCRYCHMACPYGAPQYNAAK
GHMTKCDGCYDRVAEGKKPICVESCPLRALDFGPIDELRKKHGELAAVAPLPRAHFTKPN
IVIKPNANSRPTGDTTGYLANPKEV
>EECEBICD_02548 Dimethyl sulfoxide reductase DmsA
MKTKIPDAVLAAEVSRRGLVKTTAIGGLAMASSAFTLPFTRIANAAEAISPTKTDEKVVW
SACTVNCGSRCPLRMHVVDGEIKYVETDNTGNDDYDGLHQVRACLRGRSMRRRVYNPDRL
KYPMKRVGARGEGKFERISWDEAYDIIATNMQRLIKEYGNESIYLNYGTGTLGGTLTRSW
PPGKTLVARLMNCCGGYLNHYGDYSSAQIAAGLNYTYGGWADGNSPSDIENSKLVVLFGN
NPGETRMSGGGVTYYLEQARQKSNARMIIIDPRYTDTGAGREDEWIPIRPGTDAALVNGL
AYVLITENMVDQPFLDKYCVGYDEKTLPASAPKNGHYKAYILGQGKDGIAKTPEWAAQIT

GIPADRIIKLAREIGSAKPAYICQGWGPQRHANGEIATRAISMLAILTGNVGINGGNSGA
REGSYALPFERMPTLENPVETSISMFMWTDAIERGPEMTALRDGVRGKDKLDVPIKMIWN
YAGNCLINQHSEINRTHEILQDDKKCEMIVVIDCHMTSSAKYADILLPDCTASEQMDFAL
DASCGNMSYVIFTDQAIKPRFECKTIYEMTSELAKRLGVEQQFTEGRTQEEWMRHLYEQS
RKSIPNLPTFEEFRKQGIFKQRDPEGHHVAYKDFREDPQANPLTTPSGKIEIYSQALADI
AATWELPEGDVIDPLPIYTPGFENYNDPLTDKFPLQLTGFHYKARVHSTYGNVDVLKAAC
RQEMWINPMDAQKRGINNGDKVRIFNDRGEVHIEAKVTPRMMPGVVALGEGAWYDPDAKR
VDQGGCINVLTTQRPSPLAKGNPSHTNLVQVEKA
>EECEBICD_02549 Serine--tRNA ligase
MLDPNLLRNEPDAVAEKLARRGFKLDVDKLRALEERRKVLQVNTENLQAERNSRSKSIGQ
AKARGEDIEPLRLEVNKLGEELDAAKAELDTLLAEIRDIALTIPNLPADEVPVGKDENDN
VEVSRWGTPREFDFEIRDHVTLGEMHSGLDFAAAVKLTGSRFVVMKGQIARMHRALSQFM
LDLHTEQHGYSENYVPYLVNHDTLYGTGQLPKFAGDLFHTRPLEEEADSSNYALIPTAEV
PLTNLVRDEIIDEDQLPIKMTAHTPCFRSEAGSYGRDTRGLIRMHQFDKVEMVQIVRPED
SMAALEEMTGHAEKVLQLLGLPYRKIILCTGDMGFGACKTYDLEVWVPAQNTYREISSCS
NVWDFQARRMQARCRSKSDKKTRLVHTLNGSGLAVGRTLVAVMENYQQADGRIEVPEVLR
PYMNGLEYIG
>EECEBICD_02550 Replication-associated recombination protein A
MSNLSLDFSDNTFQPLAARMRPENLAQYIGQQHLLAAGKPLPRAIEAGHLHSMILWGPPG
TGKTTLAEVIARYASADVERISAVTSGVKEIREAIERARQNRNAGRRTILFVDEVHRFNK
SQQDAFLPHIEDGTITFIGATTENPSFELNSALLSRARVYLLKSLTTDDIEQVLDQAMQD
KTRGYGDQDIVLPNETRRAIAELVNGDARRALNTLEMMADMAEADDSGKRVLLPALLTEI
AGERSARFDNKGDRFYDLISALHKSVRGSAPDAALYWYARIITAGGDPLYVARRCLAIAS
EDVGNADPRAMQVAIAAWDCFTRVGPAEGERAIAQAIVYLACAPKSNAVYTAFKAALADA
RERPDYDVPVHLRNAPTKLMKEMGYGQEYRYAHDEPNAYAAGEVYFPPEIAQTRYYHPTN
RGLEGKIGEKLAWLAEQDQNSPTKRYR
>EECEBICD_02551 Outer-membrane lipoprotein carrier protein
MKKMAIACALLSSVVASSVWADAASSLKSRLDKVSSFHATFTQKVTDGSGAAVQEGQGDL
WVKRPNLFNWHMTQPDESILVSDGKTLWFYNPFVEQATATWLKDATGNTPFMLIARNQAS
DWQQYNIKQDGDNFVLTPKASNGNLKQFTINVGRDGTIHQFSAVEQDDQRSAYQLKSQQN
GAVDPSKFTFTPPQGVTIDDQRK
>EECEBICD_02552 hypothetical protein
MSQEYTEDKDVTLTKLSSGRRLLEALLILIALFAVWLMAALLSFNPSDPSWSQTAWHEPI
HNLGGAPGAWLADTLFFIFGVMAYTIPVIIVGGCWFAWRHQSTDDYIDYFAVSLRLIGVL
ALILTSCGLAAINADDIWYFASGGVIGSLLSTTLQPLLHSSGGTIMLLCIWAAGLTLFTG
WSWVSIAEKLGGWLLNILTFASNRTRRDDTWVDDEEYDDEYDEETDGVQRESRRARILRG
ALARRKRLAEKFSNPRGRQTDAALFSGKRMDDDEDIQYSARGVAADPDDVLFSGNRATQP
EYDEYDPLLNGHSVTEPVAAAAAATAVTQTWAASADPIMQTPPMPGAEPVVAQPTVEWQP
VPGPQTGEPVIAPAPEGYQPHPQYAQPQEAQSAPWQQPVPVASAPQYAATPATAAEYDSL
APQETQPQWQAPDAEQHWQPEPTHQPEPVYQPEPIAAEPSNMPPPVIEQPVATEPEPDTE
ETRPARPPLYYFEEVEEKRAREREQLAAWYQPIPEPVKENVPVKPTVSVAPSIPPVEAVA
AAASLDAGIKSGALAAGAAAAAPAFSLATGGAPRPQVKEGIGPQLPRPNRVRVPTRRELA
SYGIKLPSQRIAEEKAREAERNQYETGAQLTDEEIDAMHQDELARQFAQSQQHRYGETYQ
HDTQQAEDDETAAEAELARQFAASQQQRYSGEQPAGAQPFSLDDLDFSPMKVLVDEGPHE
PLFTPGVMPESTPVQQPVAPQPQYQQPQQPVAPQPQPQYQQPQQPVAPQPQYQQPQQPVA
PQPQYQQPQQPVAPQPQYQQPQQPVAPQPQYQQPQQPVAPQPQYQQPQQPVAPQPQYQQP
QQPVAPQPQYQQPQQPTAPQDSLIHPLLMRNGDSRPLQRPTTPLPSLDLLTPPPSEVEPV
DTFALEQMARLVEARLADFRIKADVVNYSPGPVITRFELNLAPGVKAARISNLSRDLARS
LSTVAVRVVEVIPGKPYVGLELPNKKRQTVYLREVLDNAKFRENPSPLTVVLGKDIAGDP
VVADLAKMPHLLVAGTTGSGKSVGVNAMILSMLYKAQPEDVRFIMIDPKMLELSVYEGIP
HLLTEVVTDMKDAANALRWSVNEMERRYKLMSALGVRNLAGYNEKIAEAARMGRPIPDPY
WKPGDSMDVQHPVLEKLPYIVVLVDEFADLMMTVGKKVEELIARLAQKARAAGIHLVLAT
QRPSVDVITGLIKANIPTRIAFTVSSKIDSRTILDQGGAESLLGMGDMLYSGPNSTMPVR
VHGAFVRDQEVHAVVQDWKARGRPQYVDGITSDSESEGGGGGFDGGEELDALFDQAVNFV

TQKRKASISGVQRQFRIGYNRAARIIEQMEAQGIVSAQGHNGNREVLAPPPFE
>EECEBICD_02553 Leucine-responsive regulatory protein
MVDSKKRPGKDLDRIDRNILNELQKDGRISNVELSKRVGLSPTPCLERVRRLERQGFIQG
YTALLNPHYLDASLLVFVEITLNRGAPDVFEQFNAAVQKLEEIQECHLVSGDFDYLLKTR
VPDMSAYRKLLGETLLRLPGVNDTRTYVVMEEVKQSNRLVIKTR
>EECEBICD_02554 Thioredoxin reductase
MGTTKHSKLLILGSGPAGYTAAVYAARANLQPVLITGMEKGGQLTTTTEVENWPGDPNDL
TGPLLMERMHEHAAKFETEIIFDHINNVDLQNRPFRLTGDSAEYTCDALIIATGASARYL
GLPSEEAFKGRGVSACATCDGFFYRNQKVAVIGGGNTAVEEALYLSNIASEVHLIHRRDG
FRAEKILIKRLMDKVENGNIILHTNRTLEEVTGDQMGVTGLRLRDTQQSDNIETLDIAGL
FVAIGHSPNTALFEGQLELENGYIKVQSGTHGNATQTSIPGVFAAGDVMDHIYRQAITSA
GTGCMAALDAERYLDGLADASK
>EECEBICD_02555 ATP-binding/permease protein CydD
MNKTRQKELTRWLKQQSVISQRWLNISRLLGFMSGVLIVAQAWIMARILQHMIMENIPRE
ALLLPFILLILVFVLRAWVVWLRERVGFHAGQHIRFEIRRQVLDRLQQAGPAWIQGKPAG
SWATLVLEQIDDMHDYYARYLPQMALAVCVPLLIVAAIFPSNWAAALILLGTAPLIPLFM
AMVGMGAADANRRNFLALARLSGHFLDRLRGMETLRIFGRGEAETESIRAASQDFRQRTM
EVLRLAFLSSGVLEFFTSLSIALVAVYFGFSYLGELNFGHYGTGVTLAAGFLALILAPEF
FQPLRDLGTFYHAKAQAVGAADSLKTFLETPLAHPARGEVELAENEPVTIDAVNLIITSP
EGKTLAGPLNFSLAAGERAVLVGRSGSGKSSLLNVLSGFLSYQGSLRINGVELRDLSPES
WRQHLSWVGQNPQLPAATLRENVLLARPDASEQELYAALDAAWVSEFLPLLPQGVDTPLG
NHASRLSVGQAQRVAVARALLNPCQLLLLDEPAASLDAHSEQRVMQALKAASKRQTTLMV
THQLEDLADWDAIWVMQDGAIVEQGSYAELSAANGAFATLLAHRQEDI
>EECEBICD_02556 putative multidrug resistance ABC transporter ATP-binding/permease protein YheH
MRALLPYLTLYKRHKWMLTLGIALAILTLLASIGLLTLSGWFLSASAIAGFAGIYSFNYM
LPAAGVRGAAITRTAGRYFERLVSHDATFRVLQHLRIYTFSKLLPLSPAGLARYQQGELL
NRIVADVDTLDHLYLRVISPLVGAFVVIMIVTLGLSVLDVTLAITLGGIMLLTLFIMPPV
FYRAGKNTGQNLTHLRGQYRQQLTSWLQGQAELTIFGASKRYRAQMEATELQWHEAQRRQ
SELTALSQALMLLIGALAVMLMLWMASGGVGGNTQPGALIALFVFCALAAFEALAPVTGA
FQHLGQVIASALRITELTEQKPEVTFPQSETPAPEKVTLTLRDVSFRYPDQPINALNALS
LQANPGEHVAILGRTGCGKSTLLQLLTRAWDPQQGEILLNDLPLSSLSESALRRTISVVP
QRVHLFSATLRDNLLLAAPNASDEALSDMLRHVGLENLLEDSGLNSWLGEGGRLLSGGEL
RRLAIARALLHDAPLMLLDEPTEGLDATTESEMLELLADVMREKTVLMVTHRLRGLARFD
QIIVMDDGRIIERGTHAELLAGQGRYYQFKQRL
>EECEBICD_02557 Leucyl/phenylalanyl-tRNA--protein transferase
MRLVQLSRHSIAFPSPEGALREPNGLLALGGDLSPARLLMAYQHGIFPWFSPGDPILWWS
PDPRAVLWPEKFHLSRSMKRFHNASPYRVTLNYAFDRVIDGCANHRDEGTWITRGIEEAY
RRLHELGHAHSIEVWRDRELVGGMYGVSQGALFCGESMFSRQENASKTALLVFCAEFIRH
GGKLIDCQVLNSHTASLGAIEIPRRDYLDHLAALRQQPLASRFWVPRTLFLPRK
>EECEBICD_02558 Translation initiation factor IF-1
MAKEDNIEMQGTVLETLPNTMFRVELENGHVVTAHISGKMRKNYIRILTGDKVTVELTPY
DLSKGRIVFRSR
>EECEBICD_02559 HTH-type transcriptional regulator DmlR
MIKTDLNDFAWFVHVVEEGGFAAAGRALDEPKSKLSRRIAQLEERLGVRLIQRTTRQFNV
TEVGQTFYEHCKAMLVEAQAAQDAIAALQVEPRGIVKLTCPVTLLHVHIGPMLAKFMARY
PDVSLQLEATNRRVDVVGEGVDVAIRVRPRPFEDSDLVMRVLADRGHRLFASPDLIARMG
IPSAPAELSHWPGLSLASGKHIHRWELYGPQGARAEVHFTPRMITTDMLALREAAMAGVG
LVQLPVLMVKEQLAAGELVAVLEEWEPRREVIHAVFPSRRGLLPSVRALVDFLTEEYARM
VEE
>EECEBICD_02560 hypothetical protein
MKQITGVYTAPRPHWVGDGFPVRSLFSYQSHAQQLSPFLLLDYAGPHTFTPGNEKRGVGE
HPHRGFETVTIVYSGEVEHRDSTGRGGVIGPGDVQWMTAGAGILHEEFHSDAFTRRGGEL
EMVQLWVNLPMKDKMTTPGYQSITHDVIPTVTLPDDAGVVRVIAGRYEETKGPAHTFSPL

NVWDMRLQRNRQLTLAQPEGWSTALVVLKGNITVNGTTPVNEAQLVVLSQQGKTLHLEAS
SDASVLLLSGEPLNEPIVGYGPFVMNTKQEIAEAVRDFNSGRFGQI
>EECEBICD_02561 putative hydrolase YcaC
MSSPANFNGLRPVIDVNDSVMLLIDHQSGLFQTVGDMPMPELRARAAALAKMATLAKMPV
ITTASVPQGPNGPLIPEIHANAPHAQYIARKGEINAWDNEDFVKAVKATGRKTLIIAGTI
TSVCMAFPAISAVAEGYKVFAVIDASGTYSKMAQEITLARIVQAGVVPMDTAAVASELQG
TWNREDAAAWADVYTQVFPAYQLLIESYSKAQDVVKNNEMLDSQR
>EECEBICD_02563 hypothetical protein
MPGLIGYWKQLPTKDEYIKKHNMSKISCYSCGHEKFSDVGLIQVWDNHRRILCAKCKTTL
FREED
>EECEBICD_02564 ATP-dependent Clp protease ATP-binding subunit ClpA
MLNQELELSLNMAFARAREHRHEFMTVEHLLLALLSNPSAREALEACSVDLVALRQELEA
FIEQTTPVLPASEEERDTQPTLSFQRVLQRAVFHVQSSGRSEVTGANVLVAIFSEQESQA
AYLLRKHEVSRLDIVNFISHGTRKDEPSQSSDLGNQPTGDEQAGGEERMENFTTNLNQLA
RVGGIDPLIGREKELERAIQVLCRRRKNNPLLVGESGVGKTAIAEGLAWRIVQGDVPEVM
ADCTIYSLDIGSLLAGTKYRGDFEKRFKALLKQLEQDTNSILFIDEIHTIIGAGAASGGQ
VDAANLIKPLLSSGKIRVIGSTTYQEFSNIFEKDRALARRFQKIDITEPSVEETVQIING
LKPKYEAHHDVRYTAKAVRAAVELAVKYINDRHLPDKAIDVIDEAGARARLMPVSKRKKT
VNVADIESVVARIARIPEKSVSQSDRDTLKNLGDRLKMLVFGQDNAIEALTEAIKMSRAG
LGHEHKPVGSFLFAGPTGVGKTEVTVQLSKALGIELLRFDMSEYMERHTVSRLIGAPPGY
VGFDQGGLLTDAVIKHPHAVLLLDEIEKAHPDVFNLLLQVMDNGTLTDNNGRKADFRNVV
LVMTTNAGVRETERKSIGLIHQDNSTDAMGEIKKVFTPEFRNRLDNIIWFDHLSGEVIHQ
VVDKFIVELQAQLDQKGVSLEVSQEARDWLAEKGYDRAMGARPMARVIQDNLKKPLANEL
LFGSLVDGGQVTVALDKEKNALTYGFQSAQKHKPEAAH
>EECEBICD_02565 ATP-dependent Clp protease adapter protein ClpS
MGKTNDWLDFDQLVEDSVRDALKPPSMYKVILVNDDYTPMEFVIDVLQKFFSYDVERATQ
LMLAVHYQGKAICGVFTAEVAETKVAMVNKYARENEHPLLCTLEKA
>EECEBICD_02566 Cold shock-like protein CspD
METGTVKWFNNAKGFGFICPEGGGEDIFAHYSTIQMDGYRTLKAGQSVRFDVHQGPKGNH
ASVIVPIEAEAVA
>EECEBICD_02567 Macrolide export ATP-binding/permease protein MacB
MTALLELCNVSRSYPSGEEQVAVLKDISLQIHAGEMVAIVGVSGSGKSTLMNILGCLDKP
TSGTYRVAGRDVSTLDPDALAQLRREHFGFIFQRYHLLSHLTAAQNVEIPAVYAGIERKK
RQARARELLLRLGLSDRVDYPPSQLSGGQQQRVSIARALMNGGQVILADEPTGALDSHSG
EEVMAILRQLRDRGHTVIIVTHDPLIAAQAERIIEIHDGKIVHNPPAEEKKREQGVDAAV
VNTAPGWRQFASSFREALSMAWLAMAANKMRTLLTMLGIIIGIASVVSIVVVGDAAKQMV
LADIRAMGTNTIDIHPGKDFGDDNPQYRQALKYDDLVAIQKQPWVNSATPSVSKSLRLRY
GNIDIAVNANGVSGDYFNVYGMSFREGNTFNSVQQQDRAQVVVLDANTRRQLFPNKANVV
GEVVLVGNMPVIVIGVAEEKPSMYGNSNLLQVWLPYSTMSDRIMGQSWLNSITVRVKDGV
DSDQAEQQLTRLLTLRHGKKDFFTWNMDSVLKTAEKTTYTLQLFLTLVAVISLVVGGIGV
MNIMLVSVTERTREIGIRMAVGARASDVLQQFLIEAVLVCLVGGALGISLSMFIAFMLQL
FLPGWEIGFSLTALASAFLCSTFTGILFGWLPARNAARLDPVDALARE
>EECEBICD_02568 Macrolide export protein MacA
MRAKGKKFKKRYLVIIILILLVGGMAGWRMINAPLPTYQTLIVRPGDLEQSVLATGKLDAL
RKVDVGAQVSGQLKTLLVSIGDNVKKDQLLGVIDPDQAENQIKEVEATLMELNAERQQAA
AELKLARVTLARQQQLAKTQAVSQQDLDTAATEMAVKQARIGTIDAQIKRNRASLDTAKT
NLEYTRIVAPMAGEVTQITTLQGQTVIAAQQAPNILTLADMSTMLVKAQVSEADVIHLRA
GQKAWFTIAGDPQTRYEGVLKDILPTPEKINDAIFYYARFEVPNPKRILRLDMTAQVYIQ
LMDVKNVLIIPLAALGEPVGGNRYKVALLRNGEKREREVVIGERNDTDVEVVKGLEAGDE
VIIGESRPGATP
>EECEBICD_02569 hypothetical protein
MTQLTDNTWYTSDYISPLQLFIRLTRGQLQPGKFWRKASFRRKFLIRSLVMPRATSQLLT
NLTQWPELNTLLARQPRLPIRLHRPYMAVNIKRDFALDALCFHYQQMRQLLSREQQVSYL
SQYGLNLAKFETKTGELFQLDLVSLVSLDKEGESTIVVRDAQLRILAEITFTLCRFNQQR

TLFIGGLQGAANDVPHEIIQQATKACHGLFPKRIVMEALCQFAQVFQAEQIIAVSNDAHV
YRSWRYMDKKTQMHADYDAFWESLGGERIKGNYYALPLAIARKSESEIASKKRAEYRRRY
ALLDSVVEQVPVTFKR
>EECEBICD_02570 hypothetical protein
MILERVEIVGFRGINRLSLMLEQNNVLIGENAWGKSSLLDALTLLLSPELDLYHFVREDF
WFPPGDIKGREHHLHIILTFRETQPGRHRVRRYRALEACWSLCEDDFHRIFYRLEGECGA
DGSVMTLRSFLNSDGQPLLVENINDQVRHLVRLMPVLRLRDARFMRRIRNGTVPNVPDVE
VTARQLDFLARELATRPQNLTDGQIRQGLSAMVQLLEHYFSEQGAGQARHRLMRRRSQNE
QRSWRYLDIINRMIDKPGSRSHRVILLGLFSTLLQAKGTLRLHKDARPLLLVEDPETRLH
PIMLSVAWQLLNLLPLQRIATTNSGELLSLTPVEHVVRLVRESSRVAAWRLGPGGLSAED
GRRIAFHIRFNRASSLFARCWLLVEGETETWVINELARQCGHHFDAEGIKVIEFAQSGLK
PLVKFARRMGIEWHVLVDGDEAGKKYAATVRSLLNNDREEEREHLTALPALDMEHFMYRQ
GFADVFHRVAQLPPNVPMNTRKIITKAIHRSSKPDLAIEVAMEAGRRGIDAVPPLFRKMF
SRVVWLARGRAD
>EECEBICD_02571 Lysine exporter LysO
MFSGLLIIILVPLIVGYLIPLRHKAALQLINRLLSWIVYLILFFMGISLAFLDNLASNLVA
IFHYSAVSITIIILLCNIAALLWLERILPWRHHHQQEKLPSRIAMALESLQLCGVVVLGFV
IGLSGLSVLQHATEASEYTLIFLLFLVGIQLRNSGMTLKQIVLNRRGMMVAVVVVASSLL
GGVINAFILDLPLKTALAMASGFGWYSLSGILLTESFGPVIGSAAFFNDLARELLAIMLI
PGLVRRSRSTALGLCGATSMDFTLPVLQRSGGVEIVPAAIVHGFILSLLVPLLMAFFSA
>EECEBICD_02572 Hydroxylamine reductase
MFCVQCEQTIRTPAGNGCSYAQGMCGKTAETSDLQDLLIAALQGLSAWAVKAREYGIINH
DVDNFAPRAFFSTLTNVNFDSPRIVGYAREAIALREALKAQCLSVDANAHCDNPMADLQL
VSDDLGELQRQAAEFTPNKDKAAIGENILGLRLLCLYGLKGAAAYMEHAHVLGQYDNDIY
AQYHKIMAWLGTWPADMNALLECAMEIGQMNFKVMSILDAGETTKYGHPTPTQVNVKATE
GKCILISGHDLKDLYNLLEQTEGTGVNVYTHGEMLPAHGYPELRKFKHLVGNYGSGWQNQ
QVEFARFPGPIVMTSNCIIDPTVGSYDDRIWTRSIVGWPGVSHLEGDDFGPVIAQAQQMA
GFPYSEIPHLITVGFGRQTLLGAADTLIDLVSREKLRHIFLVGGCDGARGERNYFTDFAT
SVPDDCLILTLACGKYRFNKLEFGDIEGLPRLVDAGQCNDAYSAIILAVTLAEKLGCGVN
DLPLSLVLSWFEQKAIVILLTLLSLGVKNIVTGPTAPGFFTPDLLAVLNEKFGLRSVTTV
EEDMKQLLSA
>EECEBICD_02573 NADH oxidoreductase HCR
MTMPTSQCPWRMQVHHIRQETPDVWTIALLCHDYYPYRAGQYALVSVRNSAETLRAYTLS
STPGVSEYITLTVRRIDDGTGSQWLTHDIKRGDYIWLSDAMGDFTCDDKTEDKFLLLAAG
CGVTPIMSMRRWLAKYRPQADVQVIFNVRSPDDVIFADEWRQYPVTLVAENHATEGFVAG
RLTTELLQRVPDLASRTIMTCGPAPYMDFVEQGVKALGVTRFFKEKFFTPVAETATSGLK
FTKLQPAQEFYAPIGTTLLEALESNKVPVAAACRAGVCGCCKTKVVSGDYTVSSTMTLSE
AEIAEGYVLACSCHPQSDLVLA
>EECEBICD_02574 Pyruvate dehydrogenase [ubiquinone]
MKQTVAAFIAKTLEQAGVKQIWGVTGDSLNGLSDSLNRMGTIEWMPTRHEEVAAFAAGAQ
AQLTGELAVCAGSCGPGNLHLINGLFDCHRNHVPVLAIAAHIPSSEIGSGYFQETHPQEL
FRECSHYCELVSSPEQIPQVLAIAMRKAVLNRGVSVVVLPGDVALKPAPENAVTHWYHAP
HPVVTPAEEELKKLAQLLRYSSNIALMCGSGCAGAHEELVALAAKLKAPIVHALRGKEHV
EYDNPYDVGMTGLIGFSSGFHTMMNADTLILLGTQFPYRAFYPSDAKIIQIDINPGSIGA
HSKVDMALVGDIKATLRALLPLVEEKSNRKFLDKALEHYRDARKGLDDLAKLSDKAIHPQ
YLAQQISHFAADDAIFTCDVGTPTVWAARYLKMNGKRRLLGSFNHGSMANAMPQALGAQA
TAPGRQVIAMCGDGGFSMLMGDFLSVVQMKLPIKIVVFNNSVLGFVAMEMKAGGYLTDGT
ELHDTNFARIAEACGITGIRVEKAADVDGALQRAFSIDGPVLVDVVVAKEELAIPPQIKL
EQAKGFSLYMLRAIISGRGDEVIELAKTNWLR
>EECEBICD_02575 Low specificity L-threonine aldolase
MIDLRSDTVTRPGRAMLEAMMTAPVGDDVYGDDPTVNALQRYAADLSGKEAALFLPTGTQ
ANLVALLSHCERGEEYIVGQGAHNYLYEAGGAAVLGSIQPQPIDAAADGTLPLENVAAKI
KADDIHFARTRLLSLENTHNGKVLPRAYLKDAWTFTRERGLALHVDGARIFNAVVAYGCE
LKEITQYCDSFTICLSKGLGTPVGSLLVGNRDYIKRATRWRKMVGGGMRQAGILAAAGLY

ALKHNVARLQEDHDNAAWLAQQLREAGAEVMRHETNMLFVRVGEAQAAALGDYLRERNIL
INAAPSVRLVTHLDVSREQLADVVAHWRAFLAR
>EECEBICD_02576 hypothetical protein
MAQRILVLGASGYIGQHLVFALSQQGHQVRAAARRVERLEKQRLANVSCHKVDLHWPENL
PALLRDIDTVYYLVHGMGEGGDFIAHERQAALNVRDALRQTPVKQLIFLSSLQAPEHEQS
DHLRARQLTADTLRDAGVPVTELRAGIIVGAGSAAFEVMRDMVYNLPILTPPRWVRSRTT
PIALENLLYYLVGLLDHPAHEHRILEAAGPQVLSYQQQFERFMAVSGKRRPLIPVPFPTR
WISVWFLNVITSVPPTTAKALIQGLRHDLLADDAALKKLIPQTLITFDDAVRRTLKEEEK
LVNSSDWGYDALAFARWRPEYGYFPKQAGFTAQTPASLSALWQVVNRLGGKEGYFFGNIL
WQTRAAMDRLVGHKLAKGRPSHTLLKPGDTVDSWKVIIVEPEKQLTLLFGMKAPGLGRLS
FTLHDKGRYREIDVRAWWHPHGMPGLIYWLLMIPAHLFIFRGMARRIARFAEQITEK
>EECEBICD_02577 2-alkyl-3-oxoalkanoate reductase
MKVLVTGATSGLGRNAVEFLRNKGISVRATGRNEAMGKLLEKMGAEFVHADLTELVSSQA
KVMLAGIDTLWHCSSFTSPWGTQQAFDLANVRATRRLGEWAVAWGVRNFIHISSPSLYFD
YHHHRDIKEDFRPHRFANEFARSKAAGEEVINLLAQANPQTRFTVLRPQSLFGPHDKVFI
PRLAHMMHHYGSVLLPHGGSALVDMTYYENAIHAMWLASQPGCDHLPSGRAYNITNGENR
TLRSIVQKLIDELTIDCRIRSVPYPMLDMIARSMERFGKKSAKEPPLTHYGVSKLNFDFT
LDTTRAQEELGYQPIITLDEGIERTAAWLRDHGNLPR
>EECEBICD_02578 N-acetylmuramoyl-L-alanine amidase AmiD
MKALLWLVGLALLLTGCASEKGIIDKEGYQLDTRHRAQAAYPRIKVLVIHYTAENFDVSL
ATLTGRNVSSHYLIPATPPLYGGKPRIWQLVPEQDQAWHAGVSFWRGATRLNDTSIGIEL
ENRGWRMSGGVKSFAPFESAQIQALIPLAKDIIARYDIKPQNVVAHADIAPQRKDDPGPR
FPWRELAAQGIGAWPDAQRVAFYLAGRAPYTPVDTATVLALLSRYGYEVKADMTAREQQR
VIMAFQMHFRPAQWNGIADAETQAIAEALLEKYGQD
>EECEBICD_02579 hypothetical protein
MQFSTTPTLEGQSIVEYCGVVTGEAILGANIFRDFFAGIRDIVGGRSGAYEKELRKAREI
AFQELGEQAKALGADAVVGIDIDYETVGKDGSMLMVSVSGTAVKTRR
>EECEBICD_02580 putative lipoprotein YbjP
MRYSKLSLLIPCALLLSACTTVTPAYKDNGPRIGACVEGGPDSVAQQFYDYRIQHHSNDI
AALRPYLSDNLAKLLTDASRDTEHRQLMSGDLFSSRATLPDSANVASASTIPNRDARNIP
LRVALKQGDQSWQDEVLMIHEGSCWVIDDVRYLGGDVHAPAGTLRQSIENR
>EECEBICD_02581 Arginine transport ATP-binding protein ArtP
MSIQLNGINCFYGAHQALFDITLDCPEGETLVLLGPSGAGKSSLLRVLNLLEMPRSGTLT
IAGNHFDFTKTPSDKAIRELRQNVGMVFQQYNLWPHLTVVQNLIEAPCRVLGLTKDQALA
RAEKLLERLRLKPYSDRYPLHLSGGQQQRVAIARALMMEPQVLLFDEPTAALDPEITAQI
VSIIHELAETNITQVIVTHEVEVARKTASRVIYMENGHIVEHGDAGCFAHPQTEAFKNYL
SH
>EECEBICD_02582 Putative ABC transporter arginine-binding protein 2
MKKVLIAALIAGFSLSATAAQTIRFATEASYPPFESMDANNKIVGFDVDLANALCKEIDA
SCTFTNQAFDGLIPSLKFRRFNAVMAGMDITPEREKQVLFTTPYYDNSALFVGQQGKYTS
VDQLKGKKVGVQNGTTHQKFIMDKHPEITTVPYDSYQNAKLDLQNGRIDAVFGDTAVVTE
WLKANPKLAPVGDKVTDKDYFGTGLGIAVRQGNTELQQKFNTALEKVKKDGTYETIYNKW
FQK
>EECEBICD_02583 Arginine ABC transporter permease protein ArtQ
MNEFFPLASAAGMTVGLAVCALIIGLALAMFFAVWESVKWRPIAWIGSALVTILRGLPEI
LVVLFIYFGSSQLLLTLSDGFTLHLGFTQIPVQMEIENFDVSPFLCGVIALSLLYAAYAS
QTLRGALKAVPSGQWESGQALGLSKTAIFFRLVMPQMWRHALPGLGNQWLVLLKDTALVS
LISVNDLMLQTKSIATRTQEPFNWYIIAAAIYLVITLISQYILKRIDLRATRFERRPD
>EECEBICD_02584 Arginine ABC transporter permease protein ArtM
MLEYLPELLKGLHTSLTLTVASIAVALVLALVFTIILTLKTPFVVWLVRGYITLFTGTPL
LVQIFLIYYGPGQFPTLQEYPLLWHLLSEPWLCALIALSLNSAAYTTQLFYGAIRAIPEG
QWQSCGALGMSKKDTLAILLPYAFKRALSSYSNEVVLVFKSTSLAYTITLMEVMGYSQLL
YGRTYDVVVFGAAGVIYLVVNGLLTLMMRLIERKALAFERRN
>EECEBICD_02585 ABC transporter arginine-binding protein 1

MKKLVLAALLASFATGSIAAEKINFGVSATYPPFESLDASNKIVGFDIDLATALCKQMQA
ECTFTNHAFDSLIPALKFRKYDAVISGMDITPERSKQVAFSNPYYANSALVIAKKDTYKT
FTDLKGKRIGMENGTTHQKYLQDKHPEVKTVAYDSYQNAIIDLKNGRIDGVFGDTAVVNE
WLKTNPQLGPATEKVTDPQYFGTGLGIAVRPDNKALLEKLNNALAAIKADGTYQKISDQW
FPQ
>EECEBICD_02586 hypothetical protein
MKAIILLFDSLNKNYLPPYGDLLTKAPNFQRLAAHAATFDNSYVGSMPCMPARRELHTGR
YNFLHREWGPLEPFDDSMPELLKKAGIYTHLISDHLHYWEDGGGNYHNRYSSWDVVRGQE
GDHWKASVGEPPIPEVLRVPQKQTGGGVSGLWRHDWANREYIQQEADFPQTKVFDAGCDF
IHKNHAEDNWLLQVETFDPHEPFYTTEEYLSLYDDEWQGPHYDWPRGKVSESEEAIAHIR
CRYRALVSMCDRNLGRILDLMDEHDLWRDTMLIVGTDHGFLLGEHGWWAKNQMPYYNEVA
NNPLFIWDPRSAVCGARRQSLVQMIDWAPTLLDYFQQPIPADMQGQPLAKVIASDEPVRE
GALFGVFSGHVNVTDGRYVYMRAAQPGREHDIANYTLMPIKMNARYDVDELGKLSLAPPF
NFTKGLQVLRIPAREKYKGVNSFGHLLFDLRDDPQQQHPIHDEAIEARMINLLIRLMKEN
DAPAEQYRRLGLDVV
>EECEBICD_02587 hypothetical protein
MKDSVNILFVCGYGVGSSVMLQTVVKKALAKYDFSFDMEHTAAGEVGGFTDWADIYAISK
KLLDVVSLDPKHGQYLIPIENIMDGESIGKQIYDVVEKNFPHLLNK
>EECEBICD_02588 Ascorbate-specific PTS system EIIC component
MEGVQTMFAKFIDVIQTFLTEPAILIGILVGVGYALDKKTPIKIITGMISAMVGLMMVLF
GGFQFSATFKPVAEAVSKAYGVHGYLMDSYAMKAATQIALGDNFGYVGYVFVLAFFTNLL
LVLFGRYTGAKGIFLTGNTGVSHSQAVLWLIVFWLGFGWVQSIVIAGVLTGVFWAFSTTL
IVKPIAKVTNNAGFTIAHNQMLGLWFFSKFAHKFGDPEKHDAENLKLPGWLAIFNHNVTA
IAIVMTLFVGGFLLATGIDNVQLMAKGKPWYIYIINLGLQFSMYMVILLQGVRMMVGEIN
GSFKGWQDRFIPNAIPAVDVAALLPFSPNAATLGFVFCTFGTIFSMGILLLIHSPIMVLP
GFVPLFFSGGPIGVLANRMGGYRSVIICTFLLGIIQTFGTVWAIPLTGLAKEGVGWTGIF
DWATLWPAICELLKFIASTFHLGPYSI
>EECEBICD_02589 23S rRNA (uracil(747)-C(5))-methyltransferase RlmC
MCIIVAPDNMKVFGMQCALYDAGRCRSCQWITQSVNEQLSAKTADLHRLLAGLPVEQWCA
PIGGPEQHFRNKAKMVVSGSVEKPLFGMLHRDGTPVDLCGCPLYPASFAPVFSALKPFIA
RAGLTPYNVARKRGELKYLLLTESQFDGGMMLRFVLRSETKLTQLRAALPWLRAQLPQLR
VITANIQPVHMAIMEGETEIYLTDQQALAERFNDVPLWIRPQSFFQTNPTVASRLYATAR
DWVGQLPVRHMWDLFCGVGGFGLHCATPQMQLTGIEIAPEAIACAKQSAAELGLTRLHFQ
ALDSTQFATAQGETPDLVLVNPPRRGIGKPLCDYLAQMAPRFIIYSSCNAQTMAQDIRHL
PNYRIQRVQLFDMFPHTAHYEVLALLRRL
>EECEBICD_02590 Inner membrane protein YbjO
MAIILIRGLDVLMIMNTLGIRGMGEFIHRSVQTWSLTLVFLASLVLVFVEIYCAFSLVKG
RSWARWVYLATQIIVSGYLWAASLGYGYPELFSIAGESKRDILHSLVMQKLPDLLILFLL
FIPAPSRRFFRLQ
>EECEBICD_02591 Inner membrane ABC transporter permease protein YdcV
MNNLPVVRSPWRILILVLGFTFLYAPMLMLVIYSFNSSKLVTVWAGWSTRWYGELFRDTA
MMSAVGLSLTIAACAATMAVVLGTIAAVVMVRFGRFRGANGFAFMITAPLVMPDVITGLS
LLLLFVALGHAIGWPSDRGMLTIWLAHVTFCTAYVAVVIASRLRELDRSIEEAAMDLGAA
PLKVFFVITLPMIMPAVISGWLLAFTLSLDDLVIASFVSGPGATTLPMLVFSSVRMGVNP
EINALATLILGVVGIVGFIAWYLMARAEKQRIRDIQRARRG
>EECEBICD_02592 Putrescine transport system permease protein PotH
MSTLEPPARISKPGGAALWLARMQMKHGRKLVIALPYIWLILLFLLPFLIVFKISLAEMA
RAIPPYTDLLEWADGQLSITLNLGNFLQLTDDPLYFEAYLQSLQIAGISTICCLLLGYPL
AWAVAHSKPSTRNILLLLVILPSWTSFLIRVYAWMGILKNNGVLNNVLLWLGVIDQPLTI
LHTNLAVYIGIVYAYLPFMVLPIYTALTRIDYSLVEASLDLGARPLKTFFSIIVPLTKGG
IIAGSMLVFIPAVGEFVIPELLGGPDSIMIGRVLWQEFFNNRDWPVASAVAIIMLLLLIV
PIMWFHKHQQKSVGEHG
>EECEBICD_02593 Spermidine/putrescine import ATP-binding protein PotA
MNDVPHRPQVKTRKALTPLLEIRNLTKSFDGQHAVDDVSLTIYKGEIFALLGASGCGKST

LLRMLAGFEPPSSGQIMLDGIDLAHVPPYQRPINMMFQSYALFPHMTVEQNIAFGLKQDK
LPKAEITSRVNEMLGLVHMQEFAKRKPHQLSGGQRQRVALARSLAKRPKLLLLDEPMGAL
DKKLRDRMQLEVVDILERVGVTCVMVTHDQEEAMTMAGRIAIMNRGKFVQIGEPEEIYEH
PTTRYSAEFIGSVNVFEGLLKAREEDGLVIDAPGLVHPLKVDADASVVDNVPVYVALRPE
KIMLCESPPADGYNFAVGEVAHIAYLGDLSIYHVRLKSGQMISAQLQNAHRYRKGLPTWG
DEVRLCWEADSCVVLTV
>EECEBICD_02594 Putrescine-binding periplasmic protein
MMTALKKKWLSGLVAGALMVVSVGTLAAEQKTLHIYNWSDYIAPDTVANFEKETGINVIY
DVFDSNEVLEGKLMAGSTGFDLVVPSASFLERQLTAGVFQPLDKSKLPGWKNLDPELLKL
VAKHDPDNKYAMPYMWATTGIGYNVDKVKAVLGDDAPVNSWDLVLKPENLEKLKSCGVSF
LDAPEEIFATVLNYLGKDPNSSKADDYTGPATDLLLKLRPNIRYFHSSQYINDLANGDTC
VAIGWAGDVWQAANRAKEAKNGVNISFSIPKEGAMAFFDVFAMPADAKNKDEAYQFLNYL
LRPDVIAHISDHVFYANANKEATALVSQQVLDNPGIYPPADVRAKLFTLKVQEPKIDRVR
TRAWTKVKSGK
>EECEBICD_02595 putative protein YbjN
MISLVVPTLDTLRQWLDDLGMNFFECDTCQALHLPHMQNFDGVYDAKIDLVDNTILFSAM
AEVRPSALLPLAADLSAINASSLTVKAFLDMQDDNLPKLVVCQSLSVMQGVTYEQFEWFV
RQSEEQISMVILEAGAHQLLFNAEEDAQKTSAVDHFLH
>EECEBICD_02596 Ribosomal protein S6--L-glutamate ligase
MKIAILSRDGTLYSCKRLREAAMRRGHLVEILDPLSCYMNINPAASSIHYKGRRLPHFDA
VIPRIGSAITFYGTAALRQFELLGSYPLNESVAITRARDKLRSLQLLARQGIDLPITGIA
HSPDDTSDLIKMVGGAPLVVKLVEGTQGIGVVLAETRQAAESVIDAFRGLNAHILVQEYI
AEAKGCDIRCLVVGNEVVAAIERCAKAGDFRSNLHRGGVASIATITPRERDIAIKAAQTL
GLDVAGVDILRAARGPLVMEVNASPGLEGIEKTTGVDIAGRMIQWIERHATPEFCLKIGG
>EECEBICD_02597 Oxygen-insensitive NADPH nitroreductase
MSPTIELLCGHRSIRHFTDEPVTDAQREAIIAAARSTSSSSFLQCSSIIRITDRALREAL
VPLTGGQKHVAQAAEFWVFCADFNRHLQICPDAQLGLAEQLLLGVVDTAMMGQNALTAAE
SLGLGGVYIGGIRNNIESVTELLKLPKHVLPLFGLCLGWPADNPDLKPRLPAELVVHEN
>EECEBICD_02598 hypothetical protein
MRTIGVLPKSVLILECLGMILLALALLSLNHYLTLPAPFNTPLAGVLMVFLGVVLILPAA
VAMMWRIAQLLAPQLMKRPPDISSRSDREKHNESDH
>EECEBICD_02599 Glutaredoxin 1
MFTVIFGRPGCPYCVRAKELAEKLSKERDDFNYRYIDIHAEGITKADLEKTVGKPVETVP
QIFVDQKHIGGCTDFEAWAKENLNLFA
>EECEBICD_02600 Inner membrane protein YbjM
MKHKHGWASVVCCFVLFIVVCLSLTMHVQGAFRAAGHPEIGLLFFTLPGAVASFCSHRRE
VIRPLIGAMLAAPFCLVLMRVVFMPTRSFWQELAWLFSAVFWCALGALCYLFISSLFSHR
RKKRQ
>EECEBICD_02601 Aspartate/alanine antiporter
MNINVADLLNGNYILLLFVVLALGLCLGKLRLGSVQLGNSIGVLVVSLLLGQQHFSINTD
ALNLGFMLFIFCVGVEAGPNFFRFFSRR
>EECEBICD_02602 Aspartate/alanine antiporter
MLALVMVGSALLIALGLGKLFGWDIGLTAGMLAGSMTSTPVLVGAGDTLRHSGIASTQLS
SALDNLSLGYALTYLIGLVSLIVGARYLPKLQHQDLQTSAQQIARERGLDTDANRKVYLP
VIRAYRVGPELVAWTDGKNLRELGIYRQTGCYIERIRRNGILANPDGDAVLQMGDEIALV
GYPDAHARLDPSFRNGKEVFDRDLLDMRIVTEEIVVKNHNAVGRRLAQLKLTDHGCFLNR
VIRSQIEMPIDDNVVLNKGDVLQVSGDARRVKTIADRIGFISIHSQVTDLLAFCAFFIIG
LMIGMITFQFSNFSFGIGNAAGLLFAGIMLGFLRANHPTFGYIPQGALNMVKEFGLMVFM
AGVGLSAGSGISNGLGAVGGQMLIAGLVVSLVPVVICFLFGAYVLRMNRALLFGAMMGAR
TCAPAMEIISDTARSNIPALGYAGTYAIANVLLTLAGTLIVIIWPGLG
>EECEBICD_02603 HTH-type transcriptional regulator RcdA
MKENAVRRANDPKRREKIIQATLEAVKTYGVHAVTHRKIAAIAQVPLGSMTYYFAGMDAL
LSEAFTLFTENMSRQYQDFFAQVTDAEGACQAITEMIYGSQVTTPDNMALMYQLYAYASD
KPALKIVMQNWMQRSQNTLAQWFDAQTARALDAFIEGMTLHFVTDRTPLAREAILQMVKR

IAGESMSCHSASGNKKPTNLEPKWRG
>EECEBICD_02604 Inner membrane protein YbjJ
MTVLSSRNALKRRTWALFMFFFLPGLLMASWATRTPAIRDILSVSTAEMGAVLFGLSIGS
MSGILCSAWLVKRFGTRKVIRTTMTCAVGGMVILSVALWCASPLIFALGLAVFGASFGAA
EVAINVEGAAVERELNKTVLPMMHGFYSFGTLAGAGVGMALTALSVPPNIHIILAAAVAI
APIFIAIRAIPDGTGKNASEDAHLQEKGLPFYRDIQLLLIGVVVLAMAFAEGSANDWLPL
LMVDGHGFSPTSGSLIYAGFTLGMTVGRFTGGWFIDRYSRVTVVRASALMGALGIGLIIF
VDSDWVAGVSVILWGLGASLGFPLTISAASDTGPDAPTRVSVVATTGYLAFLVGPPLLGY
LGEHYGLRSAMMVVLALVILAALVAKAVAKPVSTPQPVMEHNA
>EECEBICD_02605 5-amino-6-(5-phospho-D-ribitylamino)uracil phosphatase YbjI
MSIKLIAVDMDGTFLSDQKTYNRDRFMAQYQQMKRQGIRFVVASGNQYYQLISFFPEIAH
EIAFVAENGGWVVSEGEDIFNGELTKADFQTVVEHLLTRADVEIIACGKNSAYTLKRYND
ALKAVAAMYYHRLEFVDNFNNINDVFFKFGLNITDERIPEVQAALHDAIGDIMVPVHTGY
GSIDLIIPGVHKANGLRLLQQRWGIHDSEVVVFGDGGNDIEMLRQAGFSFAMSHASEAVA
AAAKYRAGSNNQEGVLDIIDSVLNNEPPFNV
>EECEBICD_02606 Multidrug transporter MdfA
MQNRLQSGGRLGRQALLFPLCLVLYEFSTYIGNDMIQPGMLAVVEQYQACLDWVPTSMTA
YLAGGMFLQWLLGPLSDRIGRRPVMLAGVVWFIVTCLATLLAKNIEQFTFLRFLQGISLC
FIGAVGYAAIQESFEEAVCIKITALMANVALIAPLLGPLVGAAWVHVLPWEGMFILFAAL
AAIAFFGLQRAMPETATRRGETLSFKALGRDYRLVIKNRRFVAGALALGFVSLPLLAWIA
QSPIIIISGEQLSSYEYGLLQVPVFGALIAGNLVLARLTSRRTVRSLIVMGGWPIVAGLI
IAAAATVVSSHAYLWMTAGLSVYAFGIGLANAGLVRLTLFSSDMSKGTVSAAMGMLQMLI
FTVGIEVSKHAWLSGGNGLFSLFNLANGILWLLLMLVFLKDKRTGNSQTV
>EECEBICD_02607 Putative undecaprenyl-diphosphatase YbjG
MLSLHGDELAIPFDFIAPSLDVMLENLNNSLFYFINATPDSAQWIISLAIFIAKDVINIV
PLLAVVLWLWGPRDRVCAQRQLIVKIGIALIISLAVSWTMGHLFPHDRPFVDHIGYNFLH
HAADDSFPSDHGTVIFTFALAFLFWHRLWSGIVMMAIAMTIAWSRVYLGVHWPLDMIGAL
LVAMLGCLSAQLIWQWYGAPLYQALQRCYRFCFALPIRKGWVRD
>EECEBICD_02608 Deoxyribose operon repressor
METRRDERIGQLLQALKRSDKLHLKEAATLLGVSEMTIRRDLNHKSAPVVLLGGYIVLEP
RSASHYLLSDQKSRLVEEKRRAAQLAAGLVQAHQTVFIDCGTTTPWIIEAIDNDLPFTAV
CYSLNTFLALQDKPHCRAILSGGEFHASNAIFKPLDFHETLNNICPDIAFYSAAGVHTSK
GATCFNLEELPVKHWAMTMAQCHVLVVDHSKFGKVRPARMGELSRFDTIISDRRPDEAFV
AYAKAQQITLMY
>EECEBICD_02609 D-alanyl-D-alanine carboxypeptidase DacC
MDYASGKVLAEGNADEKLDPASLTKIMTSYVVGQALKAGKIKLTDMVTVGKDAWATGNPA
LRGSSVMFLKPGDQVSVADLNKGIIIQSGNDACIALADYVAGSQESFIGLMNAYAKRLGL
TNTTFQTVHGLDAPGQFSTARDMALLGKALIHDVPDEYAIHKEKEFTFNNIRQPNRNRLL
WSTNLHVDGMKTGTTAGAGYNLVASATQGDMRLISVVLGAKTDRIRFNESEKLLTWGFRF
FETVTPIKPDATFVTQRVWFGDKREVNLGAGDAGSVTIPRGQLKNLKASFTLNEPQLTAP
LKKGQVVGTIDFQLNGKSIEQRPLMVMENVEEGDFSAGCGILC
>EECEBICD_02610 Glutathione S-transferase GstB
MITLWGRNNSTNVKKVLWTLEELELPYDQILAGGKFGVNQDADYLAMNPNGLVPLLKDDE
TDLLLWESNAIVRYLAAQYGQNRLWVDNPARRAEGEKWMDWANQTLSPAHRVILMGLVRT
PPEKRDQAAIEAGIEKCDGLFALLDDALARQPWFSGDNFGTGDIAIAPFVYNLLNVGLKW
TPRPNLERWYQQLTERPAFRKVVMIPVT
>EECEBICD_02611 Aldose sugar dehydrogenase YliI
MRVDGNTVTEEGRILGDRKQRIRDVRVGPDGYLYVLTDESDGQLLKVSPAATR
>EECEBICD_02612 hypothetical protein
MAKVDVKCPFCAQTASVKKYGPGSAGHQHYRCQACCRSFQVDYEYRACQPGMKGQVVDLA
MYNAGIRNPQGLAINPWSGALWLHEHGPRGGDEINIPEKGKNYGWPLATWGVNYSGLKVS
EAKGKIVAGAEQPVYYWKDSPAISGMAFISATFLCRDGTNCLSAR
>EECEBICD_02613 hypothetical protein

MYSLLMLVAYRGYSVILFAPLVALGAVLFTRPVTVAPVFSDIFMDKLIGFVKLYFPVFLL
GAVFGKLIEFAGFYYSIVSAVTCMVGQNKAMPMIILVCALLTYGGVSLFVVAFANLFFTW
LIPHIYGDQFMINLPGLKKPL
>EECEBICD_02614 HTH-type transcriptional regulator CysL
MHFDIKDLKLFLAISEEGSLTRGAEKTYMSAASGSVRIRNLEQSINLRLLYRSSKGITLS
SAGETFKHHASLIVNQMEMLRSDMQEHVKGIKGHVRIFSSITAIAEYLPPLLSHFLTLYN
DVNIDLREYPSPEIVRSVIEGKTDLGIVAGNITTDNLEILPFRTDRLVLITATDHPFADR
KSVTFHELIQYEQISLPEGTGMFEFIRKKAELVPSKLKIRIQVGNFETFCRMVEAKVGIG
IMPHSAASRLRQLHEFSIIDLDEFWSLRKLQICARSFDKLPGFTQKLVTMLTAQTE
>EECEBICD_02615 Electron transfer flavoprotein-ubiquinone oxidoreductase
MNDTLSNTQQSRETIECDVLIVGAGPAGLSAALKLKLQANDAGKELSIIVLDKGAEPGSH
ILSGAVMDPRALNELIPDWQERGAPIKQPVTQDKLLLLTNEKCINLPDALIPDNFRNHGN
FIVSLGNLIKWLAGQAETSGVDIYAGFSATEILYDQDTNIIGVVTGDMGRHRDGSKKEGF
QSGIEILAKYTIFAEGARGSLAKELIKKFHLDTGKAPQSFSIGIKELWEVPSDQSHPGLV
IHTTGWPLDKESFGGGFLYHLNDNKIALGLVVGLDYSNPWLSPFQEMQRLKTHPSIRKYI
DRGKRIGYGARAINNGGIASMPDPCLPGGLLIGCNAGTLNASRIKGIHTAIKSGMIAADA
IFNALLDNRKNDILTEYQTLLRQSWLWQELENGSNFKPWFKKGRVIGFIMTGIEHWLLPR
MGIKKSPGV
>EECEBICD_02616 hypothetical protein
MKQLPLSGVIIVSIEHVIATPFCTRQFADYFSRVTIIECQENRDFSCNYDERLKNDLCIF
FFI
>EECEBICD_02617 Acyl-CoA dehydrogenase
MKSIERDKYQDIRDAVRSLCSEFDDKYHRKIDEAKAYPEEFVNKLTKAGWLAALIPEEFG
GSGLGLTEASIIMEEINRSGGNSGACHGQMYNMNTLLRHGSDKQKEFWLPRIATGEWRLQ
SMGVTEPTAGTDTTRIKTTAVKREDRYVINGQKVWISRVQHSDWMILLVRTTPLANVKRK
SEGMSIFMVDIKEAMKNGMTVRPIPNMVNHETNELFFDNLEIPEENLIGEEGMGFHYILD
GLNAERTLIAAECIGDGYWFMDRVTRYTCEREVFGRPIGQNQGVQFPIAEAFIAVEAANL
MRFKACELFDQHLDCGAQANMAKYLAAKASWEAANACLQFHGGYGFACEYDIERKFRETR
LYQVAPISTNLILSYIAEHVLEMPRSF
>EECEBICD_02618 hypothetical protein
MQYTDIQIWQPGILRNTDYLNPGPAKLLAATLDKDIKIFKEGGVLPELWHWLYFLPVDRQ
SDLSADGHPIKGHFIPPVGAGLLRKKL
>EECEBICD_02619 Electron transfer flavoprotein subunit alpha
MASLVIAEHNGNTLLPSTLSTITAAKAINSDIDILMLGYGIESIAVKASHIQGISTVFVA
DSPLFEHLLAENVEKQISYFLNSGKNHYQSILFPASSFGKNCAPRLAAKLDVSQISDITR
VIDQHTFERPIYAGNAIATVHSDDEYKVITVRPTSFAAAAAVAEDEGKAPIEFTEVVLGS
DQVKFISQHVNKSNRPDLQSARVVVSGGRGVGSAQAFKELVDNLADKLGAAVGASRAAVD
AGYAPNDYQIGQTGKIVAPQLYIALGISGAIQHLAGMKESGVIVAINKDPDAPIFSIADY
GLVADIFKAVPELIDKL
>EECEBICD_02620 Electron transfer flavoprotein subunit beta
MKALVCVKQVVDHNVRIRIKRDHSDVDISDSKLSINPFDEIAVEEAVRLKERGLISEVVV
VSIGNTGVGDVLRTALAAGADRAIYILTKNSLTPLIVAKTITAITRNEKPDIILLGKQAI
DDDCNQVGQMLAALLDLPQATNVSEITISDNSLTAVREVDGGLETLNLSLPAVLTTDLRL
NTPRNISLPNVIKAKKKPVKEIDFDSLGINPSSRLTIIKVDEPARRKAGIIVPDINTLLD
KLKNEEKVI
>EECEBICD_02621 hypothetical protein
MQTLERFFLFVTGDQPERFEKANSSCADSVILDLENAVSSEKKIIARENALNFMSNDEKV
LIAVRAKIVITSRLAGSYPSVDGITTEFMKNELTIQNAIHSCKMGFSGKVCIHPPQISHV
NRAFSYLKQEIEWVPQIMRLAQYPHGAFSHEGQMVDKPLLEKAKRILAHSI
>EECEBICD_02622 hypothetical protein
MVVDRLRTDLLNKLINARIDLAAYLQLRKAKGYMSVSESDTLRDNFFELNRELHDHALRQ
GLHLDQEEWNALRRAEGALAAAAVCLMSGHHDCPTFIAVNADKLENCLTTLTLSIQSLKA
HSPLTQV
>EECEBICD_02623 Ribosomal protein S12 methylthiotransferase RimO

MSNVTHQPKIGFVSLGCPKNLVDSERILTELRTEGYDVVPRYDDADMVIVNTCGFIDSAV
QESLEAIGEALNENGKVIVTGCLGAKEDQIREVHPKVLEITGPHSYEQVLQHVHHYVPKP
KHNPFLSLVPEQGVKLTPRHYAYLKISEGCNHRCTFCIIPSMRGDLVSRPIGDVLSEAKR
LVDAGVKEILVISQDTSAYGVDVKHRTGFHNGEPVKTSMVSLCEQLSKLGVWTRLHYVYP
YPHVDDVIPLMAEGKILPYLDIPLQHASPRILKLMKRPGSVDRQLARIKQWREICPELTL
RSTFIVGFPGETEEDFQMLLDFLKEARLDRVGCFKYSPVEGAGANELPDQVPEEVKEERW
NRFMQLQQQISAERLQEKVGREILVIVDEVDEEGAIGRSMADAPEIDGAVYLNGETNVKP
GDIVRVKVENADEYDLWGSRV
>EECEBICD_02624 Glutathione transport system permease protein GsiD
MRLFNWRRQAILHAMPVVKPDQIRTPWREFWRRFRRQHVALVAGGFVLALILVAIFARWL
TPYDAENYFDYDSLNNGPSLQHWFGVDSLGRDIFSRVLVGAQISLAAGVFAVFIGAIIGT
VLGLLAGYYEGWWDRFIMRICDVLFAFPGILLAIAVVAVLGSGIANVIVAVAIFSIPAFA
RLVRGNTLVLKQQTFIESARSIGASDTTILFSHILPGTVSSIVVFFTMRIGTSIISAASL
SFLGLGAQPPTPEWGAMLNEARADMVIAPHVALFPAVAIFLTVLAFNLLGDGLRDALDPK
IKG
>EECEBICD_02625 Glutathione transport system permease protein GsiC
MGLDGARVVRHPVKVRVMLNYVLKRLLGLIPTLLIVAVLVFLFVHLLPGDPARLIAGPEA
DAQVIALVRQQLGLDQPLHVQFWRYITHVLQGDFGTSMVSRRPVSEEIASRFLPTLWLTI
TSMIWAVLFGMAIGIAAAVWRNRWPDRLGMTLAVTGISFPAFALGMLLMQIFSVDLGWLP
TVGADSWQHYILPSLTLGAAVASVMARFTRSSFVDVLSEDYMRTARAKGVSETWVVLKHG
LRNAMIPVVTMMGLQFGFLLGGSIVVEKVFNWPGLGRLLVDSVDMRDYPVIQAEVLLFSL
EFILINLVVDVLYAAINPAIRYK
>EECEBICD_02626 Glutathione-binding protein GsiB
MTQFITHKWLAALGLASSIAAFPALAAKDVVVAVGSNFTTLDPYDANDTLSQAVAKSFYQ
GLFGLDKDMKVKNVLAEGYTVSDDGLTYTITLRQGVKFQDGADFDAAAVKANLDRASNPD
NHLKRYNLYKNIAKTEVVDPATVKITLKQPFSAFINILAHPATAMISPQALEKYGKDIGF
HPVGTGPYQLETWNQTDFVKVKKFAGYWQQGLPKLDSITWRPVTDNNTRAAMLQTGEAQF
AFPIPYEQAALLAKNKNLELVASPSIMQRYISMNVTQKPFDNPKVREALNYAINRQALVK
VAFAGYATPATGVVPPSIAYAQSYQPWPYNPAKARELLKEAGYPDGFSTTLWSSHNHSTA
QKVLQFTQQQLAQIGIKARITAMDAGQRAAEVEGKGQKESGVRMFYTGWSASTGEADWAL
SPLFASQNWPPTQFNTAFYSNKQVDSDLAAALKTNDPQEKTRLYKEAQDIIWKESPWIPL
VVEKLVSAHSKNLTGFWIMPDTGFSFDDADLK
>EECEBICD_02627 Glutathione import ATP-binding protein GsiA
MPHSDELDSRDVLSVSGLNIAFHHEGQQVDAVRNVSLRLKRGETLAIVGESGSGKSVTAL
ALMRLIEQSGANVRCGEMLLRRRNRQVIELSEQSDAQMRRVRGADIAMIFQEPMTSLNPV
FTVGEQIAESIRLHQGASHEEALAEAKRMLDQVRIPESQAILSRYPHQLSGGMRQRVMIA
MALSCRPAVLIADEPTTALDVTIQAQILQLIKVLQQEMSMGVIFITHDMGVVADIADRVL
VMYQGEAVETGSVEQIFHAPTHPYTQTLLAAVPQLGAMRGHSLPRRFPLISADEPALYES
QIEQDTVVEGEPILQVRGLVTRFPLRSGLFNRVTREVHAVENISFDLWPGETLSLVGESG
SGKSTTGRALLRLVESRQGEIIFNGQRIDTLSAGKLQPLRRDIQCIFQDPYASLDPRQTV
GYSIMEPLRIHGLGQGDAAAKRVAWLLERVGLRPEHAWRYPHEFSGGQRQRICIARALAL
NPKVIIADEAVSALDVSVRGQIINLLLDLQREMGIAYLFISHDMAAVERISHRVAVMYLG
QIVEMGPRRAVFENPQHPYTRKLMAAVPVADPSRHRPRRVLLSDDIPSNIHKRGEETPAV
SLQLVGPGHYVARPQQDNALSRL
>EECEBICD_02628 Isoaspartyl peptidase
MNKAVIAIHGGAGAIARAQMSHEQELRYIQALSEIVESGQKMLEAGDSALDVVTEAVRLL
EACPLFNAGIGAVYTRDGTHELDACVMDGNTLKAGAVAGVSHVRHPVLAARLVMERSPHV
LMVGEGAENFAFSQGMARVSPDIFSTPARYEQLLAARAAGEMALDHSGAPLDETKKMGTV
GAVARDKFGNLAAATSTGGMTNKLPGRVGDSPLVGAGCYANNASVAVSCTGTGEVFIRTL
AAYDIAALMEYGGLSLADACERVVMEKLPALGGSGGLIAVDHEGNVALPFNSEGMYRAWG
YAGDTPTTGIYRE
>EECEBICD_02629 Molybdopterin molybdenumtransferase
MEFSAGLMPLETALTQMLSRITPLTAVETLPLVNCFGRILATDIVSPLDVPGFDNSAMDG
YAVRMADLSADKPLPVAGKAFAGQPYQGEWPAGTCIRIMTGAPVPTGCEAVVMQEQTEQT

DDGVRFTADVRCGQNIRRRGEDIRQDAVVFPAGTRLTTAELPVLASLGIADAQVVRKVRV
ALFSTGDELQLPGQPLEAGQIYDTNRLTIHLMLQQLGCEVINLGIIPDDPGKLRAAFVDA
DSQADVVISSGGVSVGEADYTKTILEELGEIAFWKLAIKPGKPFAFGKLSNSWFCGLPGN
PVSAALTFYQLVQPLLAKLGGNTASAVPPRQRVRTASRLKKTPGRLDFQRGILQRSANGE
LEVTTTGHQGSHIFSSFSLGNCFIVLERERGNVEPGEWVEVEPFNALFGGL
>EECEBICD_02630 Molybdopterin-synthase adenylyltransferase
MAELSDQEMLRYNRQIILRGFDFEGQETLKDARVLVVGLGGLGCAATQYLAGAGVGQLTL
LDFDTVSVSNLQRQTLHSDATVGQPKVESARDALARINPHITITPVNARLDDDAMTSLIA
GHSLVLDCTDNVSVRNQLNAGCYTAKVPLISGAAIRMEGQVTVFTYRENEPCYRCLSRLF
GENALTCVEAGVMAPLIGVIGSLQAMEAIKLLAHYGQPASGKIVMYDAMTCQFREMKLMR
NPGCEVCGQ
>EECEBICD_02631 Choline trimethylamine-lyase activating enzyme
MIFNIQRYSTHDGPGIRTVVFLKGCSLGCRWCQNPESRARAQDLLYDARLCLEGCDLCAQ
AAPDVIERALNGLLIHREKLTDAHFSVLAHCCPTQALTVCGEIKSVDEIMATVLRDKPFY
DRSGGGLTLSGGEPFMQPELAAELFKASHDAGIHTAVETCLHVPWKYIAPSLPYIDLFLA
DLKHVADGPFKQWTDGSASRVLENLRKLAAAGKKMVIRVPLIQGFNADEEAIKAITDFAA
DELHVGEIHFLPYHTLGINKYHLLSQPYHAPDKPLDAPALLDFAQKYACQKGLTATLRG
>EECEBICD_02632 Trans-4-hydroxy-L-proline dehydratase
MTQLKLDTLSDRIKAHKTALVHIVKPPVCTERAQHYTEMYQQHLDKPIPVRRALALAHHL
AERTIWIKHDELIVGNQASEVRAAPIFPEYTVSWIEKEIDDLADRPGAGFSVSEENKRIL
HDVCPWWRGQTVQDRCYGMFTDEQKGLLATGIIKAEGNMTSGDAHLAVNFPLLLEKGLDG
LRDKVAERRSRINLTVLEDLHGEQFLKAIDIVLDAVSQHITRFAALARQMAGEESRESRR
KELLTIAENCEVIAHQPPQTFWQALQLCYFIQLILQIESNGHSVSFGRMDQYLYPYYRRD
VELNQTLDREHAIELLHSCWLKLLEVNKIRSGSHSKASAGSPLYQNVTIGGQNLINGQPM
DAVNPLSYAILESCGRLRSTQPNLSVRYHAGMSNDFLDACVQVIRCGFGMPAFNNDEIVI
PEFIKLGIEPQDAYDYAAIGCIETAVGGKWGYRCTGMSFINFARVMLAALEGGRDATSGK
VFLPQEKALSAGNFNNFDEVMAAWDTQIRYYTRKSIEIEYVVDTMLEENVHDILCSALVD
DCIERAKSIKQGGAKYDWVSGLQVGIANLGNSLAAVKKLVFEQGVIGQQQLAAALADDFD
GLTHEQLRQRLINGAPKYGNDDDSVDTLLARAYQTYIDELKQYHNPRYGRGPVGGNYYAG
TSSISANVPFGAATMATPDGRKAHTPLAEGASPASGTDHLGPTAVIGSVGKLPTGSILGG
VLLNQKLNPTTLENESDKQKLMVLLRTFFEVHKGWHIQYNIVSRETLLDAKKHPDQYRDL
VVRVAGYSAFFTALSPDAQDDIIARTEHML
>EECEBICD_02633 Sugar phosphatase YbiV
MTVNVVVTDMDGTFLDDAKQYDRVRFMAQYQELKKRNIEFVVASGNQYYQLISFFPELKD
EISFVAENGALVYEHGKQLFHGELTQHESRIVIGELLKDKQLNFVACGLKSAYVSKNAPE
TFVALMAKHYHRLQPVNDYHDIDDILFKFSLNLPDEQIPLVIDKLHVSLDGIMKPVTSGF
GFIDLIIPGLHKANGISRLLKRWNRSPQNVVAIGDSGNDAEMLKMAHYSFAMGNAADNIK
ALSRYHTDDNNHQGALNVIQAVLDGTDPF
>EECEBICD_02634 putative protein YbiU
MTTPFTHETLPADPKAAIRQMKQALRAQIGDVQAVFDRLSATIAARVAEINDLKAQGQPV
WPIIPFSELAMGNISDATRAEVKRRGCAVIKGHFPREQALAWDQSMLDYLDKNHFDEVYK
GPGDNFFGTLSASRPEIYPVYWSQAQMQARQSEEMALAQSFLNRLWQVEHDGKRWFNPDI
SIIYPDRIRRRPPGTTSKGLGAHTDSGALERWLLPAYQRVFASVFNGNVEQYDPWNAAHR
TEVEEYTVDNTTKCSVFRTFQGWTALSDMLPGQGLLHVVPIPEAMAYILLRPLLDDVPED
ELCGVAPGRVLPISEQWHPLLMAALTSIPPLEAGDSVWWHCDVIHSVASVENQQGWGNVM
YIPAAPMCEKNLAYARKVKAALETGASPGDFPREDYETTWEGRFTLRDLNIHGKRALGMD
V
>EECEBICD_02635 Sugar phosphatase YbiV
MIGELLKDKQLNFVACGLQSAYVSENAPEAFVTLMAKHYHRLKPVKDYQEIDDVQFKFSL
NLPDEQIPLVIDKLHVTLDGIMKPATSGFDFIDLIIPGLHKANGISRLLKRWDLSPQNVV
AIGDSGNDAEMLKMARYSFAMDNAAENIK
>EECEBICD_02636 hypothetical protein
MLIEKSGQLPRLSLKCKRHFIFQHSLNHIQCAFMIIIIGSVTGDLFNVFCGIVHRKGITR
HFKHLCVIAAVANGDDILWRQIPAFQ

>EECEBICD_02637 hypothetical protein
MSILERFFGRKEKNSSNIQGVLVANAEIDNPLSLVVVFDTLPENNSKELTRLLRNYHSSM
KQATCELVPELDFFGLAGWGKHVISMVGFKTPYPQESIEQCVAPAHYPQEVKEQVRATSA
NIILYYKGYDTSPLEQYVALAVVAGALSSMGAVAVLNESAHTSLPAGVFKSQELGKHSLE
ILREGFPLTSLFCGFVKYEVEDIEGVWMRTYGADCFGLPDFAAHAQGHHEGQKYSDIFNN
VLRYLLESGAEMAAGHTMQVGKTTFMKLRDPLDDEYYLQGPGTTLVVELIEEDECNAH
>EECEBICD_02638 putative ABC transporter ATP-binding protein YbiT
MLVSSNVTMQFGSKPLFENISVKFGGGNRYGLIGANGSGKSTFMKILGGDLEPTLGNVSL
DPNERIGKLRQDQFAFETFTVLDTVIMGHGELWEVKQERDRIYALPEMSEEDGYKVADLE
VKYGEMDGYSAEARAGELLLGVGIPLEQHYGLMSEVAPGWKLRVLLAQALFSNPDILLLD
EPTNNLDIDTIRWLEQVLNERDSTMIIISHDRHFLNMVCTHMADLDYGDLRVYPGNYDEY
MTAATQARERLLADNAKKKAQIAELQSFVSRFSANASKSRQATSRARQIDKIKLEEVKAS
SRQNPFIRFEQDKKLFRNALEVEALTKGFDNGPLFKNVNLLLEVGEKLAVLGTNGVGKST
LLKTLVGDLDADHGTVKWSENARIGYYAQDHEYEFENDLTVFEWMSQWKQEGDDEQAVRS
ILGRLLFSQDDIKKPAKVLSGGEKGRMLFGKLMMQKPNILIMDEPTNHLDMESIESLNMA
LELYQGTLIFVSHDREFVSSLATRVIEITPERVVDFSGNYDDYLRSKGIE
>EECEBICD_02639 putative L,D-transpeptidase YbiS
MNMKLTTLFAAAFAVVGFCKTASAVTYPLPTDGSRLIGQNQVITVPEGNTQPLEYFAAEY
QMGLSNMLEANPGVDTFLPKGGTVLNIPQQLILPDTVHEGIIINSAEMRLYYYPKGTNTV
IVLPIGIGQLGKDTPINWTTKVERKKAGPTWTPTAKMHAEYRAAGEPLPAVVPAGPDNPM
GLYALYIGRLYAIHGTNANFGIGLRVSHGCVRLRNDDIKFLFENVPVGTRVQFIDEPVKA
TTEPDGSRYIEVHNPLSTTEAQFEGKEAVPITLNKSILAVTNEPDVDQTVVQQAVQDRSG
MPVRLN
>EECEBICD_02640 Inner membrane protein YbiR
MNLAFLRALRRDRFFQLLIIVGMALSLFVPFAPRAWPGAIDWHTIITLSGLMLLTKGVEL
SGYFDVLGRKMTRRFHTERQLAMFLVLAAAALSTFLTNDVALFIVVPLTITLKKLCAIPV
NRLIIFEALAVNAGSLLTPVGNPQNILLWGRSGLTFAEFSGQMAPLAVMMMLTLLLLCWF
CFPGRALQYHTGTRSPQWQPRLVWSCLALYVVFLTALELRQELWGLVLVAAGFIVLARRV
IVSVDWTLLLVFMAMFIDVHLLTQLPALQGVFNQVGALSHLGLWLTAIGLSQVISNVPST
ILLLNYVPASTLLAWAVNIGGFGLLPGSLANLIALRMANDRRIWWRFHFYSLPMLAWAAL
VGYGLLQLMP
>EECEBICD_02641 Transcriptional regulator MntR
MGRRAGTPTTKKVTQLVNVEEHVEGFRQVREAHRRELIDDYVELISDLIIEVGEARQVDM
AARLGVSQPTVAKMLKRLASLGFIQMIPWRGVFLTPEGEKLAQESRERHQIVENFLLVLG
VSPEIARRDAEGMEHHVSQETLDAFLAFTQQHGTSAE
>EECEBICD_02642 Small protein MntS
MNEFKRCIRVFSHSPFKVRLMLLSMLCDMLNGKPEQDNPSTK
>EECEBICD_02643 Phosphoethanolamine transferase OpgE
MNSTLIDSLLARRRAVSPWAGLYFLQSLLINLALGYPFSLLYTAAFTCLLLLLWRYLPRG
QKALLGICSLVAACYFPFGQAYGAPNFNTLLALHSTNMEESSEILTIFPWYSYLTGLFIF
ALGIIALRRRKEEVRPRWNSLDSLCLLISVAAFFVAPIQNLAWGGVFKVIDTGYPVFRFA
KDVIVNNNEVIEEQHRMAQLSGIKDSWTVTAVKPRYHIYVVVIGESARRDAMGAFGGHWD
NTPFASHVNGDFFMDYIAASGSTQKSLGLTLNRVVVNKPQYQDNFVTLANRAGFQTWWFS
NQGQIGEYDTAIASIAKRADEAHFLKNGDFEADKNTRDDALLTMTAQVLATERTQPQLIV
LHLMGSHPQACDRTQGKYATFVQSKETSCYLYTMTQTDNLLRQLYTQLRHSGDSFSLVYF
SDHGLAFKERGKAVQYLAHDDKFQQNFQVPFMVLSSDSKAHRIIKARRSANDFLSFFSQW
TGISAKEIKNRYRFISEQKAGPVYITNFKLQKVDYNHLGSDIFSLK
>EECEBICD_02644 Outer membrane protein X
MKKIACLSALAAVLAFSAGTAVAATSTVTGGYAQSDAQGVANKMSGFNLKYRYEQDDNPL
GVIGSFTYTEKDRTNGAGDYNKGQYYGITAGPAYRLNDWASIYGVVGVGYGKFQTTDYPT
YKHDTSDYGFSYGAGLQFNPMENVALDFSYEQSRIRSVDVGTWIAGVGYRF
>EECEBICD_02645 Threonine/homoserine exporter RhtA
MPGSTRKLPVWLPILVLLIAMSSIQSGASLAKSLFPLVGAPGVTALRLALGTLILIAFFK
PWRLRFAKEQRLPLLFYGLSLGGMNYLFYLSIQTVPLGIAVALEFTGPLAVALFSSRRPV

DFIWVVLAVLGLWFLLPLGQDMSHVDLTGAALALGAGACWAVYILTGQRAGAEHGPATVA
VGSLIAAIIFVPIGAVQAGDALWHWSILPLGLAVAVLSTALPYSLEMIALTRLPTRTFGT
LMSMEPALAAVSGMIFLGETLTGIQILALCAIIAASMGSTLTIRREPQIKQVDVK
>EECEBICD_02646 DNA protection during starvation protein
MSTAKLVKTKASNLLYTRNDVSESDKKATVELLNRQVIQFIDLSLITKQAHWNMRGANFI
AVHEMLDGFRTALTDHLDTMAERAVQLGGVALGTTQVINSKTPLKSYPLDIHNVQDHLKE
LADRYAVVANDVRKAIGEAKDEDTADIFTAASRDLDKFLWFIESNIE
>EECEBICD_02647 Glutamine-binding periplasmic protein
MKSLLKVSLAALTLAFAVSSHAADKKLVVATDTAFVPFEFKQGDKYVGFDVDLWDAIAKE
LKLDYTLKPMDFSGIIPALQTKNIDLALAGITITDERKKAIDFSDGYYKSGLLVMVKANN
NDIKSVKDLDGKVVAVKSGTGSVDYAKANIKTKDLRQFPNIDNAYMELGTNRADAVLHDT
PNILYFIKTAGNGQFKAVGESLEAQQYGVAFPKGSDELREKVNGALKTLRENGTYNEIYK
KWFGTEPK
>EECEBICD_02648 Glutamine transport system permease protein GlnP
MQFDWSAIWPAIPLLLEGAKMTLWISVLGLAGGLVIGLAAGFARTFGGWFANHIALVFIE
IIRGTPIVVQVMFIYFALPMAFNDLRIDPFSAAVVTIMINSGAYIAEITRGAVLSIHKGF
REAGLALGLSRRETIRHVILPLALRRMLPPLGNQWIISIKDTSLFIVIGVAELTRQGQEI
IAGNFRALEIWSAVAVFYLIITLVLSFILRRLERRMKIL
>EECEBICD_02649 Glutamine transport ATP-binding protein GlnQ
MIEFKNVSKHFGPTQVLHNIDLNIRQGEVVVIIGPSGSGKSTLLRCINKLEEITSGDLIV
DGLKVNDPKVDERLIRQEAGMVFQQFYLFPHLTALENVMFGPLRVRGVKKEEAEKQAKAL
LAKVGLAERAHHYPSELSGGQQQRVAIARALAVKPKMMLFDEPTSALDPELRHEVLKVMQ
DLAEEGMTMVIVTHEIGFAEKVASRLIFIDKGRIAEDGSPQALIENPPSPRLQEFLQHVS
>EECEBICD_02650 Moderate conductance mechanosensitive channel YbiO
MRWTLFILFCLLGAPAHAVAIPGVTTGTSTSQQAAPEPSPEQKKAAYAALADVLENEASR
NELIAQLREASTTPPTEPAPALAPPAAKDEKTVLENVTTVTRQYGDAFATRFAQLYRNIT
DAPHKPFNSQTFTNALTHFLFLAVAVFGFYSVIRLCALPLYRKMGLWARKKNRERSNWLQ
LPAMIVGAFIIDLLLLALTLFIGQMLSDNFHAGSRTIAFQQSLFLNAFALIEFFKAILRL
IFCPNVPELRPFAIQDATARYWNRRMSSLSSLIGYGLIVAVPIISNQVNVQVGAMANVAI
MLCITIWALYLIFKNKAEITQHLLSLAERSLAFFSLFIRAFALVWHWLASAYFIVLFFFS
LFDPGNSLKFMMGATVRSLAIIGIAAFISGMFTRWIAKTITLSPHTQRNYPELQKRLNGW
LSSALKVARFLTVCVAVMLLLNAWGLFDFWHWLHYGAGEKMVDILIRIALILFFSAVGWT
ILASLIENRLASDIHGRPLPSARTRTLLTLFRNALAVIISTITIMIVLSEIGVNIAPLLA
GAGALGLAISFGSQTLVKDIITGVFIQFENGMNTGDLVTIGPLTGTVERMSIRSVGVRQD
TGAYHIIPWSSITTFANFVRGIGSVVANYDVDRHEDADKANQALKDAVTALMETEDIRGL
IIGEPTFAGIVGLTNTAFTLRVSFTTLPLKQWTVRFALDSQVKKHFDLANVRAPVQTYQV
LPAPAGGPPPDSLPPREPTI
>EECEBICD_02651 Ribosomal RNA large subunit methyltransferase F
MSAQKPGLHPRNRHQHRYDLAALCQTTPELTSFLIRTPAGEQSVDFANPQAVKALNKALL
AHFYAVTHWDIPPGFLCPPVPGRADYIHHLADLLGETTGSIPAQATILDVGVGANCIYPL
IGVHEYGWRFTGSEVSDAAMSSAQAIIQANTGLSRAIRLRRQKDPAAIFTGIIHKNEFYD
ATLCNPPFHDSAAAARAGSERKRRNLGQNKDDALNFGGQQQELWCEGGEVAFIKKMIAES
QSFRRQVLWFTTLVSRGENLPPLYRALTEAGAVKVVKKEMAQGQKQSRFIAWTFMDDDQR
RRFITRKR
>EECEBICD_02652 putative protein YbiI
MASGWANDDAVNEQINNTIEDAVARARGELPHGESRDECESCGDPIPEARRKAVPGVRLC
ISCQQEKDLHNATFSGYNRRGSKDSQLR
>EECEBICD_02653 putative protein McbA
MKTIKYAVAAIALSTLSFGAFAAEPVSASQTQNLHKIGVVSADGATTLDGLEAKLAEKAA
AAGASAYNITSAVGNDKMSGTAVIYK
>EECEBICD_02654 putative protein YbiB
MDYRKIIKEIGRGKNHARDLDLDTARGLYTRMLNGDIPDLEMGSILMALRIKGEGEAEML
GFYEAMQNHTIKLTPPADRPLPVVIPSYNGARKQANLTPLLAILLHKLGFPVVVHGVSED
PTRVLTETIFELMGIIPTLHGGQAQAKLDGRQPVFIPVSALCPPLEKQLAMRWRMGVRNS

AHSLAKLATPFAEDAALRLSSVSHPEYVPRVATFFSRIGGRALLMHGTEGEVYANPQRCP
QISLIDSRGVQVLHERQSDTHDEPLSLPATKDPEITARWIERCLAGHEPVPQSLKTQMAC
CLVATREAATLEDGLARVEQAFSE
>EECEBICD_02655 ATP-dependent DNA helicase DinG
MALTAALKAQIAAWYKALQDQIPDFIPRAPQRQMIADVARTLAGEEGRHLAIEAPTGVGK
TLSYLIPGIAIAREEQKTLVVSTANVALQDQIFSKDLPLLRKIIPDLRFTAAFGRGRYVC
PRNLAALASSEPTQQDLLAFLDDELTPNNQEEQKRCARLKGDLDGYKWDGLRDHTDIAID
DDLWRRLSTDKASCLNRNCHYYRECPFFVARREIQEAEVVVANHALVMAAMESEAVLPEP
KHLLLVLDEGHHLPDVARDALEMSAEITASWYRLQLDLFSKLVATCMEQFRPKTTPPLAN
PERLNAHCEEVYELIASLNAILNLYMPAAQEAEHRFAMGELPDEVMEICQRLAKLTETLR
GLAESFLNDLSEKTGSHDIVRLHRVILQMNRALGMFEAQSKLWRLASMAQSSGAPVSKWA
TREIREGQLHVWFHCVGIRVSEQLERLLWRSVPHIIVTSATLRSLNSFSRLQEMSGLKEK
AGDRFVALDSPFNHVEQGKLVIPQMRYEPTIDNEEQHIAEMAAYFREQLESKKHHGMLVL
FASGRAMQRFLEHVADVRLLLLVQGDQPRYRLVELHRKRVESGERSVLVGLQSFAEGLDL
KGELLTQVHIHKIAFPPIDSPVVITEGEWLKSLNRYPFEVQSLPSASFNLIQQVGRLIRS
HACRGEVVIYDKRLLTKNYGQRLLNALPVFPIEQPAVPDVIVKPKAKPARRRRR
>EECEBICD_02656 ATP-dependent RNA helicase RhlE
MSFDSLGLNPDILRAIAEQGYREPTPIQQQAIPAVLEGRDLMASAQTGTGKTAGFTLPLL
QHLITHQPHAKGRRPVRALILTPTRELAAQIGENVRDYSKYLNIRSLVVFGGVSINPQMM
KLRGGVDVLVATPGRLLDLEHQNAVKLDQIEILVLDEADRMLDMGFIHDIRRVLAKLPAK
RQNLLFSATFSDDIKALAEKLLRNPLEIEVARRNTASEQVTQHVHFVDKKRKRELLSQMI
GQGNWQQVLVFTRTKHGANHLAEQLNKDGIRSAAIHGNKSQGARTRALADFKSGDIRVLV
ATDIAARGLDIEELPHVVNYELPNVPEDYVHRIGRTGRAAATGEALSLVCVDEHKLLRDI
EKLLKKEIPRITTPGYEPDPSIKAEPIQNGRQQRGGGGRGRGQNQGGAGRSQQPRRQDSG
AAKAKPKPRTGEGKPAGDKARPPRRPRRITPAQ
>EECEBICD_02657 HTH-type transcriptional dual regulator CecR
MNIPTTTTKGEQAKSQLIAAALAQFGEYGLHATTRDIAALAGQNIAAITYYFGSKEDLYL
ACAQWIADFLGEKFRPHAEKAERLFSQPAPDRDAIRELILLACKNMIMLLTQEDTVNLSK
FISREQLSPTSAYQLVHEQVIDPLHTHLTRLVAAYTGCDANDTRMILHTHALLGEVLAFR
LGKETILLRTGWPQFDEEKAELIYQTVTCHIDLILHGLTQRSLD
>EECEBICD_02658 hypothetical protein
MKKPVVIGLAIAAIVAVIAGGTWWYQSRQDDGLTLYGNVDIRTVNISFRVGGRLASLNVD
EGDTIKAGQVLGELDHAPYENALMQAKAGVSVAQAQYDLMLAGYRDEEIAQAAAAVRQAQ
AAYDYAQNFYNRQQGLWKSRTISANDLENARSSRDQAQATLKSAQDKLSQYRTGNREQDI
AQAKASLEQAKAQLAQAQLDLQDTTLIAPANGTLLTRAVEPGSMLNAGSTVLTLSLTRPV
WVRAYVDERNLSQTQPGRDILLYTDGRPDKPYHGKIGFVSPTAEFTPKTVETPDLRTDLV
YRLRIIVTDADDALRQGMPVTVKFNDEARHE
>EECEBICD_02659 putative multidrug ABC transporter ATP-binding protein
YbhF
MSEAVITLHGLTKRFAGMDKPAVAPLDCTIHSGYVTGLVGPDGAGKTTLMRMLAGLLKAD
GGNASVLGFDPIQNDSELHAVLGYMPQKFGLYEDLTVMENLNLYADLRSVTGEIREKTFA
RLLEFTALGPFTGRLAGKLSGGMKQKLGLACTLVGEPKVLLLDEPGVGVDPISRRELWQM
VHELAGDGMLILWSTSYLDEAEQCRDVLLMNEGELLYQGEPKALTQTMAGRSFLMSSPQE
NNRKLLQRALRLPQVSDGMIQGRSVRLILKKEATAADIRQAEGMPEITLNETAPRFEDAF
IDLLGGAGTSESPLGSILHTVEGTAGETVIEAQELTKKFGDFAATDHVNFVVQRGEIFGL
LGPNGAGKSTTFKMMCGLLVPTSGKALVLDMDLKVSSGKARQHLGYMAQKFSLYGNLTVE
QNLRFFSGVYGLRGRAQNEKIQRMSEAFGLKSIASHPTDALPLGFKQRLALACSLMHEPD
ILFLDEPTSGVDPLTRREFWLHINSMVEKGVTVMVTTHFMDEAEYCDRIGLVYRGKLIAS
GTPDDLKAQAADEQQADPTMEQAFITLINDWDKEHTHE
>EECEBICD_02660 putative multidrug ABC transporter permease YbhS
MSKSLSWRRVRALCVKETRQIVRDPSSWLIAVVIPLLLLFIFGYGINLDSSKLRVGILLE
QQSQEALDFTHAMTGSPYIDATISDNRHELIEKMQAGRIRGLVVIPVDFAQQMARDGDSA
PIQVITDGSEPNTANFVQGYVEGIWQIWQQQRAEDRGDTFEPLIDVQTRYWFNPAAISQH
FIIPGAVTIIMTVIGAILTSLVVAREWERGTMEALLSTEVTRVELLLCKLIPYYFLGMLA

MLLCMLVSVFILGVPYRGSLVILFIITSLFLLSTLGMGLLISTITRNQFNAAQVALNAAF
LPSIMLSGFIFQIDSMPAVIRAVTYIIPARYFVSTLQSLFLAGNIPVVLGVNVLFLIASA
VMFIGLTWLKTKRRLD
>EECEBICD_02661 putative multidrug ABC transporter permease YbhR
MFHRLWTLIRKELQSLLREPQTRAILILPVLIQVILFPFAATLEVTNATIAIYNEDNGKH
SVELTQRFARAKAFTHVLLLNSPQEIQPTIDTQKALLLVRFPADFSRNLDTFQPAPMQLI
LDGRNSNSAQIAANYLQQIVKEYQQTLMDGKPKPNNSELVVRNWYNPNLDYKWFVVPSLI
AMITTIGVMIVTSLSVAREREQGTLDQLLVSPLTTWQIFVGKAVPALIVATFQATIVLAI
GIWAYQIPFAGSLALFYFTMVIYGLSLVGFGLLISSLCATQQQAFIGVFVFMMPAILLSG
YVSPVENMPVWLQNLTWINPIRHFTDITKQIYLKDASLGIVWGSLWPLLVIAATTGSAAY
AMFRRKVM
>EECEBICD_02662 Inner membrane protein YbhQ
MKWQQRVRVATGLGCWQIMLHLLVVAMLVIGWMSGTLVRVGLGLCVLYGVTVLLMLALQR
HHEQRWRDVADVLEELTTTWYFGTALIVLWLLSRVLQNNVLLALAGLAILAGPAVVSLLA
KDKKLHHFASKHRIRR
>EECEBICD_02663 hypothetical protein
MTQHTRNFSFKVLTINTHKGFTAFNKRFILPELRDAVRTVGADIVCLQEVMGAHEVHPLH
IENWPDTTHYEFLADTMWSDFAYGRNAVYPEGHHGNAVLSRYPIEHYENRDVSVGGSEKR
GVLYCRITPPMLNRPIHVMCVHLGLRESHRQAQLTMLAGWVNALPESEPVLVAGDFNDWR
QKAGPPLNAAGLEEIFTRAHGRPARTFPVSMPLLRLDRIYVKNANASSPTALPLRNWRHL
SDHAPLSAEIHL
>EECEBICD_02664 Cardiolipin synthase B
MKCGWREGNQIQLLENGDQFYPAVFEAIAQAQQKIILETFILFEDEVGKKLHAALLKAAQ
RGVKAEVLLDGYGSPDLSDAFVGELTSAGVIFRYYDPRPRLLGLRTNIFRRMHRKIVVID
DRIAFVGGINYSAEHMSDYGPQAKQDYAVRVEGPVVADILQFEVENLPGQSPARRWWKRH
HQAEENRHPGEAQALFVWRDNEEHRDDIERHYLKMLTQAKREVIIANAYFFPGYRLLHAM
RKAARRGVSVKLIVQGNQTCR
>EECEBICD_02665 Cardiolipin synthase B
MPIVKVGARLLYNYLVKGGVQVYEYRRRPLHGKVALMDDHWATVGSSNLDPLSLSLNLEA
NLIIHDRTFNQTLRDNLQGIIVNDCKQVDESMLPKRTWWNLTKSVLAFHFLRHFPALVGW
LPAHTPHLAQVPPPAQPEMETQDRVDPENTGVKP
>EECEBICD_02666 Inner membrane protein YbhN
MAKSHPRWRLAKKVLTWLFFIAVVVLLVVYAKKVDWGDVWTVIRDYNRTALLSAVGLVIV
SYLLYGCYDLLGRLYCGHKLAKRQVMLVSFVCYAFNLTLSTWVGGIGMRYRLYSRLGLPG
STITRIFSLSITTNWLGYILLGGIIFTFGIVQLPDHWYIDEGTLRILGIVLLLIIAVYLW
FCAFAKRRHMTIKGQKLVLPSWKFALAQMAISSANWMAMGAIIWLLMGQDVNYFFVLGVL
LVSSIAGVIVHIPAGIGVLEAVFLALLAGEHTSQGTIIAALLAYRVLYYFIPLLLALVCY
LILESRAKKLRAKNERAMAK
>EECEBICD_02667 hypothetical protein
MNLMTWLTGKTLAELVKKGQLIPPEIKLPVYPASHYKKNWYEQGIPHGGVIGLTAIMMTM
TWIVSINMVIAQFELHNKKHLELVFFLAVGYIAFLTPSNQLLLKGRMSGVIIYRYALLLA
IISVTLATFCTLSSVFMGEFNEVLLLLFSWSLLLFSRLQMNTSFFSAYVYFYRIKRVVLE
SENASA
>EECEBICD_02668 hypothetical protein
MMGIMSKLCGKQFWLAFCEAKDVTRAIKNGGEDFNKKCPPALISHFITGFSWEHSLPNPA
PVCAFSFMLLVGGVCSAHFVFDKFILYWPLNIIIYFGSAIGMMLCVMAEVWLIAKGFILG
LRLCVLLFLLNIMLCGTGLFDVLCHQNSELKSDLVIAIIMVVLCRWVMNGETFTMMIRFC
LGRRLVRVALQKYLTSQTRK
>EECEBICD_02669 Inner membrane protein YbhL
MLGLGYGAVTCLSRFGTVVATLIYIAIITLTGVSLAYLFSSGATIFVIVGIMFSLNALFI
FYLNISSGLFRLLIFMAVSGIIAAIVVNSLVASSTMVWIVSMLTVLVWTLITALEKSTLH
GYARMLYHSEFSSLSRCALFGALTLYLGIINAVVTLCRYIILMILEILLSFRP
>EECEBICD_02670 Inner membrane protein YbhL
MDRFPRSDSIVQARSGLQTYMAQVYGWMTVGLLLTAFIAWYAANTPAVMMFVFSSKITFF

GLIIAQLALVFVLSGLVHKLSAGMATTLFMLYSALTGLTLSSIFIVYTYSSIASTFVVTG
GMFGAMSLYGYTTKRDLSGFGNMLFMALIGIVLASLVNFWLKSEALMWAVTYIGVVVFVG
LTAYDTQKLKNIGEQIDTRDSANLRKYSILGALTLYLDFINLFLMLLRIFGNRR
>EECEBICD_02671 Molybdopterin synthase catalytic subunit
MRETRIVVGPAPFSVGEEYSWLAARDEDGAVVTFTGKVRNHNLGDSVKALTLEHYPGMTE
KALAEIVVEARSRWPLGRVTVIHRVGELWPGDEIVFVGVTSAHRSSAFDAGQFIMDYLKT
RAPFWKREATPEGDRWVEARDSDQQLAKRW
>EECEBICD_02672 Molybdopterin synthase sulfur carrier subunit
MIKVLFFAQVRELTGTDALDLPADFSTVESLRQHLATKSDRWALALEDGKLLAAVNQTLV
SFDHPLTAGDEVAFFPPVTGG
>EECEBICD_02673 Cyclic pyranopterin monophosphate synthase
MVDVSAKAETVREARAEAFVTMRSETLAMIVDGKHHKGDVFATARIAGIQAAKRTWELIP
LCHPLLLSKVEIQLQAEPEYNRVRIESLCRLTGKTGVEMEALTAASVAALTIYDMCKAVQ
KDMVIGPVRLLAKSGGKSGDFKVDAHD
>EECEBICD_02674 Molybdenum cofactor biosynthesis protein B
MSQVSAEFIPTRIVILTVSSRRGEEDDTSGHYLRDSAQEAGHEVVAKAIVKENRYAIRAQ
VSAWIASDEVQVVLITGGTGLTEGDQAPEALLPLFDREVEGFGEVFRMLSFEEIGTATLQ
SRAVAGVANNTLIFAMPGSTKACRTAWENIIAPQLDARTRPCNFHPHLKK
>EECEBICD_02675 GTP 3',8-cyclase
MTPPPVFGKVYMASQLTDAFARKFYYLRLSITDVCNFRCTYCLPDGYKPGGVTNNGFLTV
DEIRRVTRAFASLGTEKVRLTGGEPSLRRDFTDIIAAVGENDAIRQIAVTTNGYRLARDA
ANWREAGLTGVNVSVDSLDARQFHAITGQDKFRQVMAGIDAAFDAGFEKVKVNTVLMRDV
NHHQLDTFLAWIQPRPIQLRFIELMETGEGSDLFRKHHISGQVLRDELIKRGWIHQLRQR
SDGPAQVFCHPDYAGEIGLIMPYEKDFCATCNRLRVSSVGKLHLCLFGDGGVSLRDLLQD
DAQQYALEKRISDALREKKQTHFLHQSNTGITQNLSYIGG
>EECEBICD_02676 Putative gluconeogenesis factor
MRNRTLADLDRVVALGGGHGLGRVLSSLSSLGSRLTGIVTTTDNGGSTGRIRRSEGGIAW
GDMRNCLNQLITEPSVASAMFEYRFGGNGELSGHNLGNLMLKALDHLSVRPLEAINLIRN
LLKVDAQLIPMSELPVDLMAIDEQGHEIYGEVNIDQLATPPQEIMLTPNVPATREAVQAI
NDADLILIGPGSFYTSLMPCLLLDELAQALRRTPAPMVYIGNLGRELSLPAASLTLVDKL
AMMEQYIGKKVIDAVVVGPRVDVSAVNDRLVIQEVLEASDIPYRHDRQLLHNALEKALQA
LG
>EECEBICD_02677 E3 ubiquitin-protein ligase SlrP
MVHDAERGAFDSHLAELIMAGREIFRLEQIESLAREKVKRLFFIDEVEVFLGFQNQLRES
LSLTTMTQDMRFYNVSGITESDLDEAEIRIKIAENRDFHKWFALWGPWHKVLERIAPEEW
REMMAKRAECIETDEYQSRVNAELEALGIAGDPDAERMAGMRIMEEINQTLFTEIMENIL
LKKEVSSLMSAYWR
>EECEBICD_02678 E3 ubiquitin-protein ligase SlrP
MNVQSNSLTALPETLPPGLKTLEAGENALTSLPASLPPELQVLDVSKNQITVLPETLPPT
ITTLDVSRNALTNLPENLPAALQIMQASRNNLVRLPESLPHFRGEGPQPTRIIVEYNPFS
ERTIQNMQRLMSSVDYQGPRVLFAMGDFSIVRVTRPLHQAVQGWLTNLEEEDVNQWRAFE
TEVNAAAFSMFLDRLSDTQNTRHPDFKEQVSAWLKIAH
>EECEBICD_02679 E3 ubiquitin-protein ligase SlrP
MFNITNIQSTARHQSISNEASTEVPLKEEIWNKISAFFSSEHQVEAQSCIAYLCHPPETA
SPEEIKSKFECLRMLAFPAYADNIQYSRGGADQYCILSENSQEILSIVFNTEGYTVEGGG
KSVTYTRVTESEQASSASGSKDAVNYELIWSEWVKEAPAKEAANREEAVQRMRDCLKNNK
TELRLKILGLTTIPAYIPEQITTLILDNNELKSLPENLQGNIKTLYANSNQLTSIPATLP
DTIQEMELSINRITELPERLPSALQSLDLFHNKISCLP
>EECEBICD_02680 UvrABC system protein B
MSKPFKLNSAFKPSGDQPDAIRRLEEGLEDGLAHQTLLGVTGSGKTFTIANVIADLQRPT
MVLAPNKTLAAQLYGEMKEFFPENAVEYFVSYYDYYQPEAYVPSSDTFIEKDASVNEHIE
QMRLSATKALLERRDVVVVASVSAIYGLGDPDLYLKMMLHLTVGMLIDQRAILRRLAELQ
YTRNDQAFQRGTFRVRGEVIDIFPAESDDIALRVELFDEEVERLSLFDPLTGQVESTVPR
YTIYPKTHYVTPRERILQAMEEIKDELADRRKVLLANNKLLEEQRLSQRTQFDLEMMNEL

GYCSGIENYSRFLSGRGPGEPPPTLFDYLPADGLLVVDESHVTIPQIGGMYRGDRARKET
LVEYGFRLPSALDNRPLKFEEFEALAPQTIYVSATPGNYELEKSGDEVVDQVVRPTGLLD
PIIEVRPVATQVDDLLSEIRQRAAINERVLVTTLTKRMAEDLTEYLEEHGERVRYLHSDI
DTVERMEIIRDLRLGEFDVLVGINLLREGLDMPEVSLVAILDADKEGFLRSERSLIQTIG
RAARNVNGKAILYGDKITPSMAKAIGETERRREKQQKYNEEHGITPQGLNKKVVDILALG
QNIAKTKAKGKGKGRSTAKAGIVELDMTPKALQQKIHELEGQMMQHAQNLEFEEAAQIRD
QLHQLRELFIAAS
>EECEBICD_02681 ATP-dependent dethiobiotin synthetase BioD 1
MTKRYFVTGTDTEVGKTVASCALLQAATQLGYQTVGYKPVASGSEMTTDGLRNSDALALQ
RNSSLPQPYSAINPYTFAEPTSPHIASADEGRAIDAAVLSRGLRTLEAQADWVLTEGAGG
WFTPLSATLTFADWVQTEQLPVILVVGVKLGCINHAMLTALAVEQAGLPLVGWIANDMQP
PGARHGEYLATLRRVIPAPLLGEIPWLGVSPSQAATGQYLDLSPLERA
>EECEBICD_02682 Malonyl-[acyl-carrier protein] O-methyltransferase
MAQVNKQAIAAAFGRAASQYEQHASLQQHSADALLTLLTGRQFASVLDAGCGPGRMSRYW
RERGSEVTALDLSLPMLQQARDRQAAHHYLLADIEAIPHDAGVFDLAWSNLAVQWCGDLR
DALSELYRVVRPGGVVAFTTLCQGSLPELRQAWQAVDNRAHANSFLPEEAIDHALRGWRA
SRHTQAMTLWFEDALSAMRSLKGIGATHLHEGRETDVLTRARLRQIQLAWPQRQGKYPLT
YHLFMGVIERD
>EECEBICD_02683 8-amino-7-oxononanoate synthase
MADRLSHASLLEAANLSPAQLRRFIHNDTQHLSRLLQSPCVGQQLVVTEGVYSMDGDSAP
LAEIQHIARRHHAWLLVDDAHGIGVTGDEGRGTCWQRGVKPELLVVTFGKGFGVSGAAVL
CSESVADYLLQFARHLVYSTSMPPAQAQALSASLAVIRSDEGRERREKLAALVQRFRAGV
NASRFTLLNAHSAIQPLIVGDNSRALRLAEALRQQGCWATAIRPPTVPVGTARLRLTLTQ
AHEACDIDRLLEVLHGAGE
>EECEBICD_02684 8-amino-7-oxononanoate synthase
MSWQQRVDDALTARRATDTLRRRYVVSQGAGRWLVANGRQYLNFSSNDYLGLSQHPQIIR
AWQQAATRFGVGSGGSGHISGYSVAHQALEEELAQWLGYPRALLFISGFAANQAVITALM
KKMTGLWPTG
>EECEBICD_02685 Biotin synthase
MARHPRWTLSQVTELFEKPLLELLFEAQQIHRQHFDPQQVQVSTLLSIKTGACPEDCKYC
PQSSRYKTGLEAERLMEVEQVLDSARKAKNAGSTRFCMGAAWKNPHERDMPYLEQIVQGV
KAMGLETCMTLGMLNESQAQRLANAGLDYYNHNLDTSPEFYGNIITTRTYQERLDTLEKV
REAGIKVCSGGIVGLGETVTDRAGLLLQLANLPTPPESVPINMLVKVKGTPLADNDDVDA
FDFIRTIAVARIMMPTSYVRLSAGREQMNEQTQAMCFMAGANSIFYGCKLLTTPNPAEDK
DLQLFRKLGLNPQQTRVLAGDNEQQQRLEQTLMTPDTDDYYNAAAL
>EECEBICD_02686 Adenosylmethionine-8-amino-7-oxononanoate
aminotransferase
MTTDDLAFDKRHIWHPYTSMTSPLPVYPVERAEGCELVLASGERLIEGMSSWWAAIHGYN
HPQLNAAMKAQIDAMSHVMFGGITHTPAVNLCRKLVAITPEPLECVFLADSGSVSVEVAM
KMALQYWQARGESRQRFLTFRHGYHGDTFGAMSVCDPDNSMHSLWKGYLPENLFAPAPQS
RMDGEWDESDIAPFARLMAAHRHEIAAVILEPIVQGAGGMRIYHPQWLRRIRNMCDREGI
LLIADEIATGFGRTGKLFACEHAGIAPDILCLGKALTGGTMTLSATLTTRQVAETISNGE
AGCFMHGPTFMGNPLACAVACASLTLLESGEWRQQVASIESQLRAELAPAQSSPWVADVR
VLGAIGVVETTHTVNMAALQRFFVGQGVWIRPFGKLIYLMPPYIIRPDQLRRLTQAVNDA
VHNETFFSH
>EECEBICD_02687 hypothetical protein
MKLISNDLRDGDKLPHRHVFNGMGYDGDNISPHLAWDDVPMGTKSFVVTCYDPDAPTGSG
WWHWVVVNLPADTRVLTQGFGSGLVAVPDGVIQTRTDFGKAGYGGAAPPKGETHRYIFTV
HALDVERLDVDEDASGAMVGFNVHFHSLASASITAMFS
>EECEBICD_02688 Histidine ammonia-lyase
MNTMTLTPGQLSLSQLYDVWRHPVQLRLDASAIDGINASVACVNDIVAEGRTAYGINTGF
GLLAQTRIADEDLQNLQRSLVLSHAAGVGDPLDDAMVRLIMVLKINSLARGFSGIRLSVI
EALIALVNAGVYPLIPAKGSVGASGDLAPLAHLSLTLLGEGKARWQGEWLPAQTALKKAG
LEPVALAAKEGLALLNGTQASTAFALRGLFEAQELFASAVVCGALTTEAVLGSRRPFDAR

IHAARGQQGQIDVARLFRHLLTDTSAIAESHHHCHKVQDPYSLRCQPQVMGACLTQLRQT
KEVLLAEANAVSDNPLVFADAGEVISGGNFHAEPVAMAADNLALAIAEIGALSERRIALM
MDKHMSQLPPFLVKNGGVNSGFMIAQVTAAALASENKALAHPHSVDSLPTSANQEDHVSM
APAAGRRLWEMAANTRGIIAVEWLAACQGIDLREGLTSSPLLEQARQTLREQVAHYTQDR
FFAPDIECATALLAQGALQRLVPDFM
>EECEBICD_02689 Urocanate hydratase
MPESKYRQQTIRAPRGTVLTAKSWLTEAPLRMLMNNLDPDVAENPHELVVYGGIGRAARN
WECYDAIVDALTRLEADETLLIQSGKPVGVFKTHDNAPRVLIANSNLVPHWATWEHFNEL
DAKGLAMYGQMTAGSWIYIGSQGIVQGTYETFVEAGCQHYNGTLAGRWVLTAGLGGMGGA
QPLAATLAGACSLTIECQQSRIDFRLRTRYVDEQAATLDDALARITRYTREGKAVSVALC
ANAADILPELVNRGVRPDLVTDQTSAHDPLHGYLPSGWRWEEYQKNAQSDPHGTMQAAKR
SMAAHVRAMLAFSKMGVPTFDYGNNIRQMAKEMGVENAFDFPGFVPAYIRPLFCRGIGPF
RWVALSGDPQDIYKTDAKVKEIVAEDKHLHHWLDMARERIHFQGLPARICWVGLEWRQKL
GLAFNEMVRCGEVSAPIVIGRDHLDSGSVASPNRETEAMRDGSDAVSDWPLLNALLNTAS
GATWVSLHHGGGVGMGFSQHAGMVIVCDGTDEAAARIRRVLHNDPATGVMRHADAGYDLA
VECAVEQGLNLPMVAATQGKG
>EECEBICD_02690 HTH-type transcriptional repressor NagR
MYSSRSRSAPAPFYETVKQDICKKIAGGVWQPHDRIPSEAELVAQYGFSRMTINRALREL
TDEGWLVRLQGVGTFVAEPKGQSALFEVRSIAEEIAARRHQHRCEVLTLERVRANAIQAS
ALNVNESDVIFHSIMVHYENDLPVQIEDRCVNADIAVDYLTQDYRQTTPHAYLSRIAPLT
EGEHIVEAVRATAQECAWLTIKEHEPCLLIRRTTWSASRIVSHARLLFPGSRYRLQGRFI
S
>EECEBICD_02691 Formimidoylglutamase
MTQWYPASPALWQGRDDSIEAPDARRLFQTVTRSETFSPENWQQKIALMGFACDEGVKRN
AGRPGAAGAPDALRKALANMASHQGHERLVDLGNWVAPTPDLEGAQQALRDAVSRCLRAG
MRTLVLGGGHETAFGHGAGVLDAFAQESVGIINLDAHLDLRQTDRATSGTPFRQLAQLCD
AQSRAFHYACFGVSRAANTQALWREAQWRNVTVVEDLDCHDALAQMTQFIDKVDKIYLTI
DLDVLPVWEMPAVSAPAALGVPLIQVLRLIEPVCRSGKLQAADLVEFNPRFDEDGAAARV
AARLGWQIAHWWR
>EECEBICD_02692 Imidazolonepropionase
MDPQRQAPYGLVDNQALIVREGHICDIVPETQLPVSGDNIHDMQGRLVTPGLIDCHTHLV
FAGNRAAEWEQRLNGASYQHISAQGGGINATVSATRACAEETLYLLARERMMRLASEGVT
LLEIKSGYGLELATEEKLLRVAAKLAAENAIDISPTLLAAHATPAEYRDDPDGYITLVCE
TMIPQLWQKGLFDAVDLFCESVGFNVAQSERVLQTAKALGIPVKGHVEQLSLLGGAQLVS
RYQGLSADHIEYLDEAGVAAMRDGGTVGVLLPGAFYFLRETQRPPVELLRRYQVPVAVAS
DFNPGTSPFCSLHLAMNMACVQFGLTSEEAWAGVTRHAARALGRQATHGQLRADYRADFV
VWDAEQPVEVVYEPGRNPLYQRVYRGQIS
>EECEBICD_02693 Putative acyl-CoA thioester hydrolase YbhC
MNTLSVSRLALALAFGVTLSACSSTPPDQIPSDQTAPGTASRPILSANEAKNFVAARYFA
SLTPNTAPWSPSPITLPAQPDFVVGPAGTPGVTHTSIQAAVDAAMVKRTNKRQYIAIMPG
DYQGTVYVPAAPGSLTLYGTGEKPIDVKIGMAIDGEMSVADWRRAVNPGGKYMPGKPAWY
MFDNCQSKHAATIGVMCSAAFWSQNNGLQLQNLTIENTLGDSVDAGNHPAVALRTDGDKV
QINKVNILGRQNTFFVTNSGVQNRLQTDRQPRTLVTNSYIEGDVDMVSGRGAVVFDNTNF
QVVNSRTQQEAYVFAPATLSNIYYGFLAINSRFNASGDGVAQLGRSLDVDANTNGQVVIR
DSVINEGFNVAKPWADAVISKRPFAGNTGTVDDKDEVQRNLNDTNYNRMWEYNNRGVGSK
VVAEPKQ
>EECEBICD_02694 6-phosphogluconolactonase
MKQTVYTASPESQQIHVWSLNHEGTLTLVQVVDVPGQVQPMVVSPDKRYLYVGVRPEFRV
LAYRIAPDDGALTFAAESALPGSPTHISTDHHGRFVFVGSYNAGNVSVTRLQDGLPVELV
DVVEGLDGCHSANITPDNRTLWVPALKQDRICLFTLSDDGHLVAQEPAEVNTVEGAGPRH
MVFHPNRQYAYCVNELNSSVDVWQLKNPHGEIECVQTLDMMPADFSDTRWAADIHITPDG
RHLYACDRTASLITVFSVSEDGSVLSVEGFQPTEAQPRGFNIDNSGKYLIAAGQKSHHIA
VYEITGTQGLLTEKGRYAVGQGPMWVVVNAY
>EECEBICD_02695 Pyridoxal phosphate phosphatase YbhA

MTARVIALDLDGTLLTPHKTLLPSSLEALSRAKEAGFQLIIVTGRHHVAIHPFYQALALE
TPAICCNGTYLYDYQAKTVLDADPMPVDKALQLIDLLDEHQIHGLMYVDDAMLYEHPTGH
VVRTSRWAQTLPPEQRPTFTQVSSLAQAARDVNAVWKFALTDEDIPRLQRFGQHVEQALG
LECEWSWHDQVDIARKGNSKGKRLTQWIEAQGGSMKNVIAFGDNYNDISMLEAAGTGVAM
GNADEAVKARADVVIGDNTTDSIAKFIYTHLL
>EECEBICD_02696 Vitamin B12 import ATP-binding protein BtuD
MLELNFSQTLGTHCLTLNETLPASGITAIFGVSGAGKTSLINAISGLTRPQKGRIALNGR
VLHDAENGICLTPEKRRIGYVFQDARLFPHYKVRGNLRYGMAKSMTGQFDKLVSLLGIEA
LLDRLPGSLSGGEKQRVAIGRALLTAPELLLLDEPLASLDIPRKRELLPYLQRLAREINI
PMLYVSHSLDEILHLADKVMVLEDGQVKAFGPLEEVWGSSVMHPWLPKEQQSSILKVSVL
EHHPHYAMTALALGDQHLWVNKLNQPLQSTLRIRIQASDVSLVLQPPQQTSIRNVLRAKV
ANCYDDNGQVEVQLEIGGRTLWARISPWARDELNIKPGLWLYAQVKSVSITA
>EECEBICD_02697 Molybdenum transport system permease protein ModB
MILTDPEWQAVLLSLKVSSLAVLFSLPFGIFFAWLLVRCRFPGKALLDSVLHLPLVLPPV
VVGYLLLVSMGRRGFIGQWLYDWFGLTFAFSWRGAVLAAAVMSFPLMVRAIRLALEGVDI
KLEQAARTLGAGRWRVFLTITLPLTLPGIIVGTVLAFARSLGEFGATITFVSNIPGETRT
IPSAMYTLIQTPGGESAAARLCVISIVLALISLLISEWLARISRERTGR
>EECEBICD_02698 Molybdate-binding protein ModA
MAHSWLRLVAGATLSFVIAGHALADEGKITVFAAASLTNAMQDIAAEYKKEKNVDVVSSF
ASSSTLARQIEAGAPADLFISADQKWMDYAADKKAVDTTTRETLLGNSLVVVAPKASEQK
PFTIDNKTDWIRLLNGGRLAVGDPQHVPAGIYAKEALQKLGAWQTLEPKLAPGEDVRGAL
ALVERNEAPLGIVYGSDAVASKGVNVVATFPEDSHKKVEYPIAIVEGHKNATVSAFYDYL
KGPQASAIFKRYGFTTK
>EECEBICD_02699 Multidrug efflux pump accessory protein AcrZ
MLELLKSLVFAVIMVPVVMAIILGLIYGLGEVFNIFSGIGQKDQSRQNR
>EECEBICD_02700 DNA-binding transcriptional dual regulator ModE
MQAEILLTLKLQQKLFADPRRISLLKHIALSGSISQGAKDAGISYKSAWDAINDMNQLSE
HMLVERATGGKGGGGAVLTRYGQRLIQLYDLLGQIQQKAFDVLSDDDALPLDSLLAAISR
FSLQTSARNQWFGTITARDRDQVQQHVDVLLADGKTRLKVALTAQSGERLGLEEGKEVLI
LLKAPWVGITRDAAVARAADNQLSGTISHIERGAEQCEVLMALPDGQTLCATIPTSDAAT
LKEGDDVIAWFNADRVIIATLC
>EECEBICD_02701 ABC transporter ATP-binding protein ModF
MSSLQISQGTFRLSDTKTLHLDSLTLNAGDSWAFVGANGSGKSALARALAGELPLLTGER
QCRFTRITRLSFEQLQKLVSDEWQRNNTDMLSPGEDDIGRTTAEIIQDEVHHPARCAMLA
QQFGISTLLNRRFKYLSTGETRKALLCQALMSEPELLILDEPFDGLDVTARQQLAQRLTA
LNQAGMTLALVLNRFDEIPDFVQFAGVLADCTLAETGTTAELLQRALVAQLAHSERLTDV
QLPEPDEPSARHALPDGEPRIVLNDGVVSYNDRPILHHLSWRVNPGEHWQIVGPNGAGKS
TLLSLITGDHPQGYSNDLTLFGRRRGSGETIWDIKKHIGYVSSSLHLDYRVSTTVRNVIL
SGYFDSIGIYQAVSDRQRKLAQQWLDILGIDKRTADAPFHSLSWGQQRLALIVRALVKHP
TVLILDEPLQGLDPLNRQLVRRFVDVLIRQGETQLLFVSHHAEDAPACITHRLEFVPDGE
RYKYVSGRCNN
>EECEBICD_02702 hypothetical protein
MAFRFPQIILFLLAAMLFCPSSYAEQKPTAAQEARKTAVEVAVEGMSRAAVAGPTKISLG
DKATLNLPEGFTWIPAKEAAVFMREIGNYVDDEYFYGLVFKKEMNGFISIEYDDSGYVKD
DDAKNWDADELMDNLRKGTKEANKDRIAKGIEPIEIIGWIEKPTYDATNHRLIWSAAIHD
IGTNEPLNEQGVNYNTYLLGREGYFSLNLVTDRGSVDHEIPLAKRILSSVKFNAGQRYAD
FNESTDKIAEYGLAALIGGIAAKKVGLLAMLGIALLKFWKVTAIGVVAVGALARKLLSRK
KD
>EECEBICD_02703 UDP-glucose 4-epimerase
MRVLVTGGSGYIGSHTCVQLLQNGHDVVILDNLCNSKRSVLPVIERLGGKHPTFVEGDIR
NEALITEILHDHAIDTVIHFAGLKAVGESVARPLEYYDNNVNGTLRLVSAMRAANVKNLI
FSSSATVYGDQPKIPYVESFPTGTPQSPYGKSKLMVEQILTDLQKAQPEWSIALLRYFNP
VGAHPSGDMGEDPQGIPNNLMPYIAQVAVGRRESLAVFGNDYPTEDGTGVRDYIHVMDLA
DGHVVAMEKLADKSGVHIYNLGAGVGSSVLDVVNAFSKACGKPINYHFAPRRDGDLPAYW

ADASKADRELNWRVTRTLDEMAQDTWHWQSRHPQGYPD
>EECEBICD_02704 Galactose-1-phosphate uridylyltransferase
MTPFNPIDHPHRRYNPLTGQWVLVSPHRAKRPWQGAQETPSQQMLPAHDPDCFLCAGNTR
VTGDKNPDYKGTYVFTNDFAALMADTPDAPDSHDPLMRCQSARGTSRVICFSPDHSKTLP
ELSLPALTEIVRTWQTQTAELGKTYPWVQVFENKGAAMGCSNPHPHGQVWANSFLPNEAE
REDRLQKAYFAEQRSPMLVDYVQRELADGSRTVVETEHWLAVVPYWAAWPFETLLLPKTH
VLRITDLSDEQRDSLALALKKLTSRYDNLFQCSFPYSMGWHGAPFNGEENAHWQLHAHFY
PPLLRSATVRKFMVGYEMLAETQRDLTAEQAAERLRAVSDIHFRESGV
>EECEBICD_02705 Galactokinase
MNLKEKTRALFAEIFGYPATHTIQAPGRVNLIGEHTDYNDGFVLPCAIDYQTVISCAPRD
DRTVRVIAADYDNQVDEFSLDAPIVTHDSQQWSNYVRGVVKHLQQRNNAFGGVDMVISGN
VPQGAGLSSSASLEVAVGTVFQQLYHLPLDGAQIALNGQEAENQFVGCNCGIMDQLISAL
GKKDHALLIDCRTLGAKAVSMPKGVAVVIINSNFKRTLVGSEYNTRREQCETGARFFQQP
ALRDVSLEAFNAVASELDPVVAKRVRHVLSENARTVEAASALEKGDLQRMGQLMAESHAS
MRDDFEITVPQIDTLVDIVKATIGDQGGVRMTGGGFGGCVVALIPEDLVPAVQQAVAQQY
EAKTGIKETFYVCKPSQGAGQC
>EECEBICD_02706 Aldose 1-epimerase
MLNETPALAPDGQPYRLLTLRNSAGMVVTLMDWGATLLSARIPLSDGSVREALLGCASPE
HYPEQTSFLGASIGRYANRIANSRYTFAGETVQLSPSQGENQLHGGPEGFDKRRWQIVNQ
NDRQVLFALTSDDGDQGFPGHLCATAQYRLTDDNRISITYRATVNKPCPVNLTNHVYFNL
DGDRTDVRQHKLQILADEYLPVDESGIPRQGLKSVANTSFDFRMPKVIASEFLADDDQRK
VKGYDHAFLLQTQGDGKKPAARLWSQDGKLQMMVYTTAPALQFYSGNYLAGTPSRGPEPY
ADWQGLALESELLPDSPNHPEWPQPDCILRPGEEYASLTEYQFIPF
>EECEBICD_02707 2,3-bisphosphoglycerate-dependent phosphoglycerate mutase
MAVTKLVLVRHGESQWNKENRFTGWYDVDLSEKGVSEAKAAGKLLKEEGFSFDFAYTSVL
KRAIHTLWNVLDELDQAWLPVEKSWKLNERHYGALQGLNKAETAEKYGDEQVKQWRRGFA
VTPPELTKDDERYPGHDPRYAKLSEKELPLTESLALTIDRVIPYWNDTILPRMKSGERVI
IAAHGNSLRALVKYLDNMSEDEILELNIPTGVPLVYEFDENFKPLKHYYLGNADEIAAKA
AAVANQGKAK
>EECEBICD_02708 putative siderophore transport system ATP-binding protein YusV
MRGLMLRSFSAGYSTQPVIADLNVPLLPCGKITILLGPNGCGKSTLLRSLAGLNNADGEA
LLDGEDLMSLSFAERAQKVVFLPQSLPQGVHLHVLESIIVVLRASGGRDNAQGRAQILAI
LEQLGITHLALQYLDQLSGGQRQLVGLAQSLIRRPELLLLDEPLSALDLNYQFHVMDLVR
RDTQAQNRVTIVVAHDINIALRHGDHVLMLKDGRLVASGAPETVITAERLAEVYRVRGRV
ERCSQGKLQVVLDGVIAV
>EECEBICD_02709 Hemin transport system permease protein HmuU
MTCQDKSLLAPALASTPVIPRYRQIIRKRAVLMLAIALAMMTSLMVDVTCGSSGLPLSAL
WQALFQPEKVNAGIHVIVWDIRLPYALMALLVGMALGLAGAEMQTILNNPLATPFTLGVS
SAAAFGAALAIVLGIGIPGIPAAWFIPANAFIFALLSALLLDGITRWTGVAASGVVLFGI
ALVFTFNALVAIMQFVADEDTLQGLVFWTMGSLVRASWEKLGVLAVVFIAVLFCALRSAW
QLTALRLGEERAMSFGIHVRRLRLLSLLRISLLSALAVAFVGPIGFIGLVAPHIARILLG
EDHRFYLPGSVLIGGLVLSLASIAAKNSIPGVMVPVGIVTSLVGVPFFLSIVLRHRGSL
>EECEBICD_02710 2-(5''-triphosphoribosyl)-3'-dephosphocoenzyme-A synthase
MNETIAFRPEGRASIKDVVVNYTLASLDELEQLHPALNLLAHPSIVRGSHDFFVCCYNAG
VQHAQAGAAETVAVLPDSLPQERQRSGLLCAAAGRLDALRQPLTHNRLCDLASQFCAGMA
DVDSETRSGFYTVRSISLPVYRRLLRDQHSHGVCLQQALLHLLAWKSDSPWARQQAQRLL
WQGGVLGDKGEFALMTLDDELRERQIEWPGLWSLLAVTGFLAKFPAGPIFAD
>EECEBICD_02711 Oxaloacetate decarboxylase beta chain
MESLNALLQGMGLMHLGAGQAIMLLVSLLLLWLAIAKKFEPLLLLPIGFGGLLSNIPEAG
MALTALESLLAHHDAGQLAVIAAKLNCAPDVHAIKEALALALPSVQSQMENLAVDMGYTP
GVLALFYKVAIGSGVAPLVIFMGVGAMTDFGPLLANPRTLLLGAAAQFGIFATVLGALTL
NYFGLISFTLPQAAAIGIIGGADGPTAIYLSGKLAPELLGAIAVAAYSYMALVPLIQPPI
MKALTTETERKIRMVQLRTVSKREKILFPVVLLLLVALLLPDAAPLLGMFCFGNLMRESG

VVERLSDTVQNGLINIVTIFLGLSVGAKLVADKFLQPQTLGILLLGVIAFGIGTAAGVLM
AKLLNLCSKNKINPLIGSAGVSAVPMAARVSNKVGLESDPQNFLLMHAMGPNVAGVIGSA
IAAGVMLKYVLAM
>EECEBICD_02712 Oxaloacetate decarboxylase alpha chain
MTIAITDVVLRDAHQSLFATRLRLDDMLPIAAALDDVGYGSLECWGGATFDACIRFLGED
PWLRLRELKKAMPKTPLQMLLRGQNLLGYRHYADDVVERFVERAVKNGMDVFRVFDAMND
PRNMKAALQAVRSHGAHAQGTLSYTTSPAHTLQTWLDLTEQLLETGVDSIAIKDMSGILT
PMAAFELVSEIKKRYDVRLHLHCHATTGMAEMALLKAIEAGVDGVDTAISSMSATYGHPA
TEALVATLAGTEHDTGLDILKLENIAAYFREVRKKYHAFEGQLKGYDSRILVAQVPGGML
TNLESQLKQQNAADKLDQVLAEIPRVREDLGFIPLVTPTSQIVGTQAVLNVLTGERYKTI
AKETAGILKGEYGHTPVPVNAALQARVLEGGAPVTCRPADLLKPELAELEADVRRQAQEK
GIQLAGNAIDDVLTVALFPQIGLKFLENRHNPAAFEPLPQAEAAQPVTKAEKPAASGIYT
VEVEGKAFVVKVSDGGDISQLTAAAPAASSAPATAPAGAGTPVTAPLAGNIWKVIATEGQ
TVAEGDVLLILEAMKMETEIRAAQAGTVRGIAVKSGDAVSVGDTLMTLA
>EECEBICD_02713 Oxaloacetate decarboxylase gamma chain
MNSSVLLGEGFTLMFLGMGFVLAFLFLLIFAIRGMSAAVNRFFPEPAPAPKLAPAAAAPA
DDFARLKPVIAAAIHHHRRLNP
>EECEBICD_02714 hypothetical protein
MSPAIITLLVLVVAIIIFVSDRLPMGLVAFMVPMALYFTGVIDAKDIFASIVNANVILIV
AMCLLGAAFFKTGLAWQSSKILLKYAKTERSLSVLIFLIGGVMSAFVSNSGTVAVLIPIV
LGIAASSQIKPIKLLMPLVFGATIGADISIIGSPGNLIAKNTIETFSKGSLSVPFFEYAK
IGIPLLIACSIFLYFFGSKLIADRDGNTQSDSQMDYSQIPAWHRNLTLAVFILSIAGMVA
TDYIKFLPPMHIIACCAAIVLVFAGVLTQKETFNSFETLTVFMLAFMMPLGAALNSTGAG
EMIANAVISVTGDSGVMIIMASLWILTWALTQVMSNTAACTLLCPVG
>EECEBICD_02715 HTH-type transcriptional regulator YjiE
MQYRHHLELTWLEDCLALKETLNFSKASASRYVTQPAFSRRIQSLEEWVGTPLFERSKRG
VTLTKAGEVFTDQLPELIHSLYTLKSDTLEAAGNKQPSLVFSATHALSFSFVPHLLKQSD
KIAKFGSFRLLSDSLNACEKMMRQGDSQFLLCHHHPHMHLNLNKNNFMSIRLGFDTLIPF
SKPDSETLKPLWNINNKIQFPYLSFSSQSGLGRIIANTASINRITHNINVAFVADLAATL
LAMVRSGDGVAWIPQSLARQDIEAKTIVTAAEKESNLWVPIEIRLYRPAKRMPPDAEELW
EIFVEEQI
>EECEBICD_02716 HTH-type transcriptional regulator CynR
MEIRQLEYFVSASLLGNLTRVAERHFVSQPNITIAIKKLETELGVTLFDRKKNKLVLTEE
GMFFLQKIEPILIALKNSVAEMKDYRNENGGIVTLGIPPMISLFLFSPLFKHFREIYPEM
ELSLVEEGTFGLHDKLSAGELDLAIVIINDCPKELVTVPLMTQQHVVCMSDKHPLAKKDS
IDWEDLQYEPLILMKKDSWHRKTIIEECTKRGIHTHIFLSSNRVQTNIDLVAKNEGISFI
LDAVDLKAEKVITKAMTEPTYVTIGLAWKKDKYLSYATRALIKFIEDYIKEHFTIK
>EECEBICD_02717 L(+)-tartrate dehydratase subunit alpha
MSRILNTEIIISVIEKLVKKACYELDDNLMCSFRKAYDKEESKIGKETIKILIDNGEYAK
KEQLACCHDTGTCIVIMEIGQHVCWEGKPLKDQVNQGVRQGYENGYLRKSMVADPLERIN
TNDNTPAILHTEIVDGDRVTITVMPKGGGSENMGTFKTLLPGDGIDGIKDFVLETVRRVG
GNPCPPYIIGIGVGGTMDHCSWMAKKALLRPLGEFNAKPLYAQLEAELLEAVNNTGIGPL
GMGGRITALGVHVDYYPCHITALPVAINFQCNASRHASEII
>EECEBICD_02718 Fumarate hydratase class I, anaerobic
MMELTLPLTKEKAKTLKVGDILFLTGFAYTCRDAAHKKIEVALSNGEKSPVDFQNQTIYY
AGPCPARPGLPIGSNGPTTAARMDPFVEMMFQQGATAFLGKGDRADYVAELCKKYGGVSL
LGIGGASAINTKHVKSVEIVAYEELGTESIKKLYFDRYRVIVGIDSEGNTLQKQEVPKYA
K
>EECEBICD_02719 Phospho-2-dehydro-3-deoxyheptonate aldolase, Phe-
sensitive
MNYQNDDLRIKEINELLPPVALLEKFPATENAANTVAHARKAIHKILKGNDDRLLVVIGP
CSIHDPAAAKEYAARLLALRDELQGELEIVMRVYFEKPRTTVGWKGLINDPHMDNSFQIN
DGLRIARKLLLDINDSGLPAAGEFLDMITPQYLADLMSWGAIGARTTESQVHRELASGLS
CPVGFKNGTDGTIKVAIDAINAAGAPHCFLSVTKWGHSAIVNTSGNGDCHIILRGGKAPN

YSAQHVAEVKEGLIKAGLTPQVMIDFSHANSCKQFQKQMEVCADVCQQIAGGEKAIIGVM
VESHLVEGNQSLESGQPLTYGKSITDACIGWEDTDALLRQLSAAVKARRG
>EECEBICD_02720 hypothetical protein
MKMTKLTTLLLTATLGLASGTALAAESNAQSSNGQANSAANAGQVAPDARQNVAPNDVNN
NDINTNGNTNSTMQHPDGSTMNHDGMTKDEEHKNTMCKDGRCPDINKKVETGNGVNNDVN
TKTDGTTQ
>EECEBICD_02721 Zinc transporter ZitB
MAHSHSHADSHLPKDNNARRLLFAFIVTAGFMLLEVVGGILSGSLALLADAGHMLTDAAA
LLFALLAVQFSRRPPTVRHTFGWLRLTTLAAFVNAIALVVITLLIVWEAIERFYTPRPVA
GNLMMVIAVAGLLANLFAFWILHRGSDEKNLNVRAAALHVMGDLLGSVGAIVAALIIIWT
GWTPADPILSILVSVLVLRSAWRLLKDSVNELLEGAPVSLDINALQRHLSREIPEVRNVH
HVHVWMVGEKPVMTLHAQVIPPHDHDALLERIQDFLMHEYHIAHATIQMEYQVCHGPDCH
LNQTPSGHVHHH
>EECEBICD_02722 Nicotinamide riboside transporter PnuC
MDFFSTHNILIHIPIGAGGYDLSWIEAVGTIAGLLCIWLASLEKISNYFFGLVNVTLFAI
IFFQIQLYASLLLQLFFFAANIYGWYAWSRQTKDNEAELKIRWLPLPKAMAWLAICVIAI
GLMTRYIDPVFAVLTRVAVAIMQMLGLQVTMPVLQPDAFPFWDSCMMVLSIVAMILMTRK
YVENWLLWVIINVISVVIFALQGVYAMSLEYLILTFIAVNGSRLWINSARERGSRALSR
>EECEBICD_02723 Quinolinate synthase A
MSVMFDPQAAIYPFPPKPTPLNDDEKQFYREKIKRLLKERNAVMVAHYYTDPEIQQLAEE
TGGCISDSLEMARFGAKHAASTLLVAGVRFMGETAKILSPEKNILMPTLAAECSLDLGCP
IDEFSAFCDAHPDRTVVVYANTSAAVKARADWVVTSSIAVELIEHLDSLGEKIIWAPDRH
LGNYVQKQTGADVLCWQGACIVHDEFKTQALTRLKKSIPMPLFWFTLNRRSPLSRWPMRW
APPVSLLRRQKRCRTDSLSWRPIAASSTKCSRRCLKKNCLKRPRLARGRPAVAARTVRGW
R
>EECEBICD_02729 Cell division coordinator CpoB
MERQKQIMLQLGSLNNGGAAQPAAGDQSGTATTATPAPDAGTATSGAPVQSGDANTDYNA
AIALVQDKSRQDDAIVAFQNFIKKYPDSTYQPNANYWLGQLNYNKGKKDDAAYYFASVVK
NYPKSPKAADAMYKVGVIMQDKGDTAKAKAVYQQVINKYPGTDGAKQAQKRLNAM
>EECEBICD_02730 Cell division coordinator CpoB
MSSNFRHHLLSLSLLVGIAAPWAAFAQAPISSVGSGSVEDRVTQLERISNAHSQL
>EECEBICD_02731 Peptidoglycan-associated lipoprotein
MQLNKVLKGLMIALPVMAIAACSSNKNASNDGSEGGMLNGAGTGMDANGNGNMSSEEQAR
LQMQQLQQNNIVYFDLDKYDIRSDFAAMLDAHANFLRSNPSYKVTVEGHADERGTPEYNI
SLGERRANAVKMYLQGKGVSADQISIVSYGKEKPAVLGHDEAAYAKNRRAVLVY
>EECEBICD_02732 Tol-Pal system protein TolB
MKQALRVAFGFLMLWAAVLHAEVRIEITQGVDSARPIGVVPFKWAGPGAAPEDIGGIVAA
DLRNSGKFNPLDRSRLPQQPATAQEVQPTAWSALGIDAVVVGQVTPNPDGSYNVAYQLVD
TGGAPGTVLAQNSYKVNKQWLRYAGHTASDEVFEKLTGIKGAFRTRIAYVVQTNGGQFPY
ELRVSDYDGYNQFVVHRSPQPLMSPAWSPDGSKLAYVTFESGRSALVIQTLANGAVRQVA
SFPRHNGAPAFSPDGTKLAFALSKTGSLNLYVMDLASGQIRQITDGRSNNTEPTWFPDSQ
TLAFTSDQAGRPQVYKMNINGGAAQRITWEGSQNQDADVSSDGKFMVMVSSNNGQQHIAK
QDLVTGGVQVLSSTFLDETPSLAPNGTMVIYSSSQGMGSVLNLVSTDGRFKARLPATDGQ
VKSPAWSPYL
>EECEBICD_02733 hypothetical protein
MSKATEQNDKLKRAIIISAVLHIILFAVLIWSSFDEHIEASAGGGGGSAIDAVMVDPGAV
VQQYNRQQDQQASARRAEEERKKLQQQQAEELQQKQAAEQERLKQLEKERLAAQEQQKQA
EEAAKLAQQQQQQAEEAAKAAADAKKKAEAEAAKAAADAKKKAEAEAAKAAADAKKKAEA
EAVKAAADAKKKAEAEAAKAAAEAKKKAEAEAAKAAAEAKKKADAEAAKAAAEAKKKADA
AAAKAAADAKKKAAAEKAAAAEGVDDLLGDLSSGKNAPKTGGGAKGNGQPSKDSGTSGAN
GGATGADISAYAKQIQVAIQSRLYDASLYQGKQCVLHISLAPDGSLKSITSEGGDPALCQ
AALMAAKTAKIPKPPSQAVYEKIKDAKLDFKL
>EECEBICD_02734 Tol-Pal system protein TolR
MARTRGRGRRELKSEINIVPLLDVLLVLLLIFMATAPIITQSVEVDLPEANQSQAVSSND

DPPVIIEVSGVGQYSVVVDKDRMDQLPSEQVIAEVKRHLQANPKTVFLIGGAKEVPYDEI
IKALNLLHSAGVKSVGLMTQPI
>EECEBICD_02735 Tol-Pal system protein TolQ
MTDMNILDLFLKASLLVKLIMLILIGFSIASWAIIIQRTRILNAAAREAEAFEDKFWSGI
ELSRLYQESQGRRDSLSGSEQIFYSGFKEFVRLHRANSHAPEAVVEGASRAMRISMNREL
ETLETHIPFLGTVGSISPYIGLFGTVWGIMHAFIALGAVKQATLQMVAPGIAEALIATAI
GLFAAIPAVMAYNRLNQRVNKLELNYDNFMEEFTAILHRQAFTVSESNKG
>EECEBICD_02736 Acyl-CoA thioester hydrolase YbgC
MNKYMFRWPIRVYYEDTDAGGVVYHASYVAFYERARTEMLRHHHFSQQVLLAERVAFVVR
KMTLEYYAPARLDDMLEVQTEITSMRGTALVFTQRIVNAENTLLNEAEVLIVCVDPLKMK
PRALPKSIVAEFKQ
>EECEBICD_02737 hypothetical protein
MTHIIAKLYAVMDKRPLRALSFVMAIVLAGCMFWDPSRFAARTSTLEIWHGLLLMWAVCA
GIIHGVGFRPESVHWQGIFCPLLADIVLIVGLIFFFL
>EECEBICD_02738 Cytochrome bd-I ubiquinol oxidase subunit X
MWYFAWILGTLLACAFGIITALALEHVEAGKTGQEES
>EECEBICD_02739 Cytochrome bd-I ubiquinol oxidase subunit 2
MIDYEVLRFIWWLLVGILLIGFAVTDGFDMGVGMLTRFLGRNDTERRIMINSIAPHWDGN
QVWLITAGGALFAAWPMVYAAAFSGFYVAMILVLASLFFRPVGFDYRSKIEDPRWRNMWD
WGVFIGSFVPPLVIGVAFGNLLQGVPFHVDEYLRLYYTGNFFQLLNPFGLLAGIVSVGMI
ITQGATYLQMRTVGELHLRARATSQIAALVTLVCFALAGVWVMYGIDGYVVTSAIDHHAA
SNPLTKEVAREAGAWLVNFNNAPILWLVPALGVVLPLLTILTSRMEKGAWAFLFSSLTLA
CIILTAGIAMFPFVMPSSTMMNASLTMWDATSSQMTLNLMTWVAAVFVPIILIYTSWCYW
KMFGRITKEHIESNTHSLY
>EECEBICD_02740 Cytochrome bd-I ubiquinol oxidase subunit 1
MLDIVELSRLQFALTAMYHFLFVPLTLGMAFLLAIMETVYVLSGKQIYKDMTKFWGKLFG
INFALGVATGLTMEFQFGTNWSYYSHYVGDIFGAPLAIEGLMAFFLESTFVGLFFFGWDR
LSKVQHMCVTWLVALGSNLSALWILVANGWMQNPIAADFNFETMRMEMVSFSELVLNPVA
QVKFVHTVASGYVTGAMFILGISAYYMLKGRDFAFAKRSFAIAASFGMAAVLSVIVLGDE
SGYEMGDVQKTKLAAIEAEWETQPAPASFTLFGIPDQDKQENHLAIQIPYALGIIATRSV
DTPVIGLKDLMVQHEERIRNGMKAYELLEQLRAGSTDQAVRDQFNSMKKDLGYGLLLKRY
TPNVTDATEAQIQQATKDSIPRVAPLYFAFRIMVACGFLLLAIIALSFWSVIRNRIGEKK
WLLRAALYGIPLPWIAVEAGWFVAEYGRQPWAIGEVLPTAVANSSLTVGDLLFSMLLICG
LYTLFLVAELFLMFKFARLGPSSLKTGRYHFEQSTVTSQPAR
>EECEBICD_02741 hypothetical protein
MSVFDMVGHLYGGLFYGFIVCGKVRVKHPGYKIIGFVNIEL
>EECEBICD_02742 Succinate--CoA ligase [ADP-forming] subunit alpha
MSVLINKDTKVICQGFTGSQGTFHSEQAIAYGTQMVGGVTPGKGGTTHLGLPVFNTVREA
VEATGATASVIYVPAPFCKDSILEAIDAGIKLIITITEGIPTLDMLTVKVKLDEAGVRMI
GPNCPGVITPGECKIGIMPGHIHKPGKVGIVSRSGTLTYEAVKQTTDYGFGQSTCVGIGG
DPIPGSNFIDILKLFQEDPQTEAIVMIGEIGGSAEEEAAAYIKEHVTKPVVGYIAGVTAP
KGKRMGHAGAIIAGGKGTADEKFAALEAAGVKTVRSLADIGEALKAIIK
>EECEBICD_02743 Succinate--CoA ligase [ADP-forming] subunit beta
MNLHEYQAKQLFARYGLPAPVGYACTTPREAEEAASKIGAGPWVVKCQVHAGGRGKAGGV
KVVKSKEEIRAFAENWLGKRLVTYQTDANGQPVNQILVEAATDIGKELYLGAVVDRSSRR
VVFMASTEGGVEIEKVAEETPHLIHKVALDPLTGPMPYQGRELAFKLGLEGKLVQQFTKI
FMGLATIFLERDLALIEINPLVITKQGDLICLDGKLGADGNALFRQPDLREMRDQSQEDP
REAQAAQWELNYVALDGNIGCMVNGAGLAMGTMDIVKLHGGEPANFLDVGGGATKERVTE
AFKIILSDDNVKAVLVNIFGGIVRCDLIADGIIGAVEEVGVNVPVVVRLEGNNAELGAKK
LADSGLNIIAAKSLTDAAQQVVAAVEGK
>EECEBICD_02744 Dihydrolipoyllysine-residue succinyltransferase component of 2-oxoglutarate dehydrogenase complex
MSSVDILVPDLPESVADATVATWHKKPGDAVVRDEVLVEIETDKVVLEVPASADGILDAV
LEEEGTTVTSRQILGRLREGNSAGKETSAKSEEKASTPAQRQQASLEEQNNDALSPAIRR

LLAEHNLEASAIKGTGVGGRLTREDVEKHLAKGESKAPAVEPAAQPALGARGEKRVPMTR
LRKRVAERLLDAKNSTAMLTTFNEVNMKPIMDLRKQYGEVFEKRHGIRLGFMSFYVKAVV
EALKRYPEVNASIDGDDVVYHNYFDVSMAVSTPRGLVTPVLRDVDTLGMADIEKKIKELA
VKGRDGKLTVEDLTGGNFTITNGGVFGSLMSTPIINPPQSAILGMHAIKDRPMAVDGKVE
ILPMMYLALSYDHRLIDGRESVGFLVTIKELLEDPTRLLLDV
>EECEBICD_02745 2-oxoglutarate dehydrogenase E1 component
MQNSALKAWLDSSYLSGSNQSWIEQLYEDFLTDPDSVDANWRLTFQQLPGTGVKPDQLHS
KTREYFRRQALAGSRHSSTISDPDTNVKQVKVLQLINAYRFRGHQHANLDPLGLWKQERV
ADLDPSFHDLTEADFQETFNVGSFASGKETMKLGELLDALKQTYCGPIGAEYMHITSTEE
KRWIQQRIESGRAAFSADEKKRFLNELTAAEGLERYLGAKFPGAKRFSLEGGDALIPMLK
EMVRHAGNSGTREVVLGMAHRGRLNVLINVLGKKPQDLFDEFAGKHKEHLGTGDVKYHMG
FSSDIETEGGLVHLALAFNPSHLEIVSPVVMGSVRARLDRLDEPSSNKVLPITIHGDAAV
TGQGVVQETLNMSKARGYEVGGTVRIVINNQVGFTTSNPLDARSTPYCTDIGKMVQAPIF
HVNADDPEAVAFVTRLALDFRNTFKRDVFIDLVCYRRHGHNEADEPSATQPLMYQKIKKH
PTPRKIYADKLEADKVATLEDATEMVNLYRDALDAGECVVKEWRPMNMHSFTWSPYLNHE
WDEAYPNKVEMKRLQELAKRISTVPEAIEMQSRVAKIYGDRQAMAAGEKLFDWGGAENLA
YATLVDEGIPVRLSGEDSGRGTFFHRHAVIHNQTNGSTYTPLQHIHSGQGQFKVWDSVLS
EEAVLAFEYGYATAEPRTLTIWEAQFGDFANGAQVVIDQFISSGEQKWGRMCGLVMLLPH
GYEGQGPEHSSARLERYLQLCAEQNMQVCVPSTPAQVYHMLRRQALRGMRRPLVVMSPKS
LLRHPLAVSTLDELANGSFQPAIGEIDELDPKAVKRVVMCSGKVYYDLLEQRRKNDQKDV
AIVRIEQLYPFPHKAVQEALQPYAHVHDFVWCQEEPLNQGAWYCSQHHFREVIPFGAALR
YAGRPASASPAVGYMSVHQKQQQDLVNDALNVD
>EECEBICD_02746 Succinate dehydrogenase iron-sulfur subunit
MKLEFSIYRYNPDVDNAPRMQDYTLEGEEGRDMMLLDALIQLKEKDPSLSFRRSCREGVC
GSDGLNMNGKNGLACITPISALTQPGKKIVIRPLPGLPVIRDLVVDMGQFYAQYEKIKPY
LLNNGQNPPAREHLQMPEQREKLDGLYECILCACCSTSCPSFWWNPDKFIGPAGLLAAYR
FLIDSRDTETDSRLEGMSDAFSVFRCHSIMNCVSVCPKGLNPTRAIGHIKSMLLQRSA
>EECEBICD_02747 Succinate dehydrogenase flavoprotein subunit
MKLPVREFDAVVIGAGGAGMRAALQISQSGQTCALLSKVFPTRSHTVSAQGGITVALGNT
HEDNWEWHMYDTVKGSDYIGDQDAIEYMCKTGPEAILELEHMGLPFSRLDDGRIYQRPFG
GQSKNFGGEQAARTAAAADRTGHALLHTLYQQNLKNHTTIFSEWYALDLVKNQDGAVVGC
TALCIETGEVVYFKARATVLATGGAGRIYQSTTNAHINTGDGVGMALRAGVPVQDMEMWQ
FHPTGIAGAGVLVTEGCRGEGGYLLNKHGERFMERYAPNAKDLAGRDVVARSIMIEIREG
RGCDGPWGPHAKLKLDHLGKEVLESRLPGILELSRTFAHVDPVKEPIPVIPTCHYMMGGI
PTKVTGQALTVNEQGEDVVIPGLFAVGEIACVSVHGANRLGGNSLLDLVVFGRAAGLHLQ
ESIAEQGVLRDASESDVEGSLERLNRWNNNRNGEDPVAIRKALQECMQHNFSVFREGDAM
AKGLEQLKVIRERLKNARLDDTSSEFNTQRVECLELDNLMETAYATAVSANFRTESRGAH
SRFDFPERDDANWLCHTLYQPQTESMTRRSVNMEPKLRPAFPPKIRTY
>EECEBICD_02748 Succinate dehydrogenase hydrophobic membrane anchor subunit
MVSNASALGRNGVHDFILVRATAIVLTLYIIYMVGFFATSGELTFEAWTGFFSSAFTKVF
TLLALFSILIHAWIGMWQVLTDYVKPLAVRLILQLVIVVALVVYVIYGFVVVWGV
>EECEBICD_02749 Succinate dehydrogenase cytochrome b556 subunit
MWALFMIRNVKKQRPVNLDLQTIRFPITAIASILHRVSGVITFIAVGILLWLLGTSLSSP
EGFQQAADIMDGFIVKFIMWGILTALAYHVIVGIRHMLMDFGYLEETFEAGQRSAKISFV
ITVVLSLLAGVLVW
>EECEBICD_02750 Citrate synthase
MADTKAKITLTGDTAIELDVLKGTLGQDVIDIRSLGSKGVFTFDPGFTSTASCESKITFI
DGDEGILLHRGFPIDQLATDSNYLEVCYILLYGEKPTQEEYDEFRTTVTRHTMIHEQITR
LFHAFRRDSHPMAVMCGITGALAAFYHDSLDVNNPRHREIAAFRLLSKMPTMAAMCYKYS
IGQPFVYPRNDLSYAGNFLNMMFSTPCETYEVNPVLERAMDRILILHADHEQNASTSTVR
TAGSSGANPFACIAAGIASLWGPAHGGANEAALKMLEEISSVKHIPEFVRRAKDKNDSFR
LMGFGHRVYKNYDPRATVMRETCHEVLKELGTKDDLLEVAMELEHIALNDPYFIEKKLYP
NVDFYSGIILKAMGIPSSMFTVIFAMARTVGWIAHWNEMHTDGMKIARPRQLYTGYDKRD

FKSALKR
>EECEBICD_02751 hypothetical protein
MPILQWFLLFLLSLLISLIFLWLHLPAAMLLGPMIAGIVLSLRGSTLQLPRGIFLAAQAI
LGCMIAQNLTGSILTTLALYWPVVLLVLLVTLLSSAIVGWLLVRYSSLPGNTGAWGSSPG
GAAAMVAMAQDYGADIRLVAFMQYLRVLFVAGAAVLVTRLILGDSAAAVSQQVIWFPPLS
GNLLTTLLLAIVAGIAGRLLRIPSGTMLLPMLAGAILHSQGIIEIELPEWLLAVAYMAIG
WRIGLGFDRQVFFMALRPLPQILLSIFALMAICAAMAWGMAYYMQIDFMTAYLATSPGGL
DTVAAIAAGSSADMALIMAMQTLRLFSILLTGPAVARFISTYAPRQPL
>EECEBICD_02752 Endonuclease 8
MPEGPEIRRAADNLEAAIKGKPLTDVWFAFAQLKPYESQLTGQLVTRIETRGKALLTHFS
NGLTLYSHNQLYGVWRVIDTGEIPQTTRILRVRLQTADKTILLYSASDIEMLTAEQLTTH
PFLQRVGPDVLDARLTPEEVKARLLSPRFRNRQFSGLLLDQAFLAGLGNYLRVEILWQVG
LTGQHKAKDLNEAQLNALSHALLDIPRLSYTTRGQSDENKHHGALFRFKVFHRDGEACER
CGGIIEKTTLSSRPFYWCPHCQK
>EECEBICD_02753 5-oxoprolinase subunit A
MNIDLNADVGEGCASDSELLTLVSSANIACGFHAGDAQTMLTSVREALKNGVAIGAHPSF
PDRDNFGRTAMALPPETVYAQTLYQIGALGAIVQAQGSVMRHVKPHGMLYNQAAKDPHLA
QAIAKAVHDYDPSLILVGLAGSELIRAGERHRLVTRQEVFADRGYQADGSLVPRMQPGAL
IHDEEQALAQTLDMVQAGRVKSVTGVWTTVTAQTVCIHGDGEYALAFARRLRAAFNARNI
HVIA
>EECEBICD_02754 5-oxoprolinase subunit C
MLNIIRAGIYTSVQDSGRHGFRQSGLSHCGALDKPAFQTANLLVGNDANAPALEITLGQL
VVEFENETWFALTGAGCEAQLDDQPVWTGWRLPVKAGQRLTLHRPLHGMRSYLAVAGGIA
VPEVMGSCSTDLKSGIGGLEGRLLKDGDRLAMGKPSRQFSGPQGVKQLLWGNRIRALPGP
EYREFDRASQEAFWRSPWQLSPQSNRMGYRLQGQSLTRTTDRELLSHGLLPGVVQVPYNG
QPIVLMNDAQTTGGYPRIACIIEADMYHLAQIPLGQPIHFVQCSLEEALNARRERQRYLE
QLTWRLQHEH
>EECEBICD_02755 5-oxoprolinase subunit B
MQRARCYLLGETAVVLELEPPITLASQKRIWRLTQRLVDMPNVVEAIPGMNNITVILREP
QTLALDAIERLQRWWEESEALEPDSRSVEIPVIYGGAGGPDLAAVARHSGLSEKQVVELH
ASVEYVVWFLGFQPGFPYLGNLPEPLHMPRRAEPRLQVPAGSVGIGGAQTGIYPLSTPGG
WQLIGLTPLKLFDPMREPPVLLRPGDSVRFVPQKEGIC
>EECEBICD_02756 GTP cyclohydrolase 1 type 2
MKNTELEQLINDKLNSAAISDYAPNGLQVEGKETVQKIVTGVTASQALLDEAVRLQADAV
IVHHGYFWKGESPVIRGMKRRRLKTLLANDINLYGWHLPLDAHPELGNNAQLAALLGITV
KGEIEPLVPWGELSMPVPGLELASWIEARLGRKPLWCGDTGPENVQRVAWCTGGGQSFID
SAARFGVDAFITGEVSEQTIHSAREQGLHFYAAGHHATERGGIRALSEWLNENTALDVTF
IDIPNPA
>EECEBICD_02757 Dipeptide permease D
MPAFLPQRLYPGNTMNKQASQPRAIYYVVALQIWEYFSFYGMRALLILYLTNQLKYDDNH
AYELFSAYCSLVYVTPILGGYLADKVLGNRMAVMLGAFLMAIGHLVLGASEIAPTFLYLS
LAIIVCGYGLFKSNISCLLGELYQPEDPRRDGGFSLLYAAGNIGSIVAPIACGYVQEEYS
WAMGFALAAIGMLAGLVIFLCGNRHFTHTTGVNKAVLCARNYLLPNWGWLLILLVAAPLL
ITVLFWKEWSVYALIVATAIGLVVLAKIYHQAQTAKQRKELGLIVTLTLFSMLFWAFAQQ
GGSSISLYIDRFVNRDILGYSVPTAMFQSVNAFAVMLCGVVLAWLVKESVSGNRTVRIWG
KFALGLGLMSAGFCILTLSARWSAAYGHSSMPLMVLGLAVMGFAELFIDPVAMSQITRID
IPGVTGVLTGIYMLLSGAIANYLAGVIADQTSQSAFDASGAVNYAINAYVDVFEQITWGA
LACVGVVLLIWLYQSFKFKSRALAVES
>EECEBICD_02758 Deoxyribodipyrimidine photo-lyase
MPTHLVWFRRDLRLQDNLALAAACRDASARVLALYISTPAQWQAHDMAPRQAAFISAQLN
ALQTALAEKGIPLLFHEVADFNASIETVKNVCRQHDVSHLFYNYQYEFNERQRDAAVEKT
LPSVICEGFDDSVILAPGAVMTGNHEMYKVFTPFKNAWLKRLKEDIPPCVPAPKIRVSGA
LSTPLTPVSLNYPQQAFDAALFPVEENAVIAQLRQFCAQGADEYALRRDFPAVDGTSRLS
ASLATGGLSPRQCLHRLLAEQPQALDGGPGSVWLNELIWREFYRHLMTWYPALCKHQPFI

RWTKRVAWQENPHYFQAWQKGETGYPIVDAAMRQLNATGWMHNRLRMITASFLVKDLLID
WRLGERYFMSQLIDGDLAANNGGWQWAASTGTDAAPYFRIFNPTTQGERFDRDGEFIRQW
LPALRDIPGKAIHEPWRWAEKAGVVLDYPRPIVEHKQARIATLSAYEAARKGA
>EECEBICD_02759 hypothetical protein
MEHYKDYPAHVIFARRAFAVTAGVLALPVMLFWKDRARFYSYLHRVWSKTSDKPVWMAQA
EKATCDFY
>EECEBICD_02760 Potassium-transporting ATPase potassium-binding subunit
MAAQGFLLIASFLLILLVLAKPLGSGLARLIAAVPLPGVAGVERILWRTLGITDHEMNWR
QYLLALLTLNLLGLGILFCLLFWQEWLPLNPQRLPGLSWDLALNTAVSFVTNTNWQAYSG
ESTLSYFSQMAGLTVQNFLSAATGIAVVFALIRAFTRQNVHTLGNAWQDLVRITLWILFP
VALIIALFFIQQGVPQNLSAYQPITTLEGAKQLLPMGPVASQEAIKMLGTNGGGFFNANS
SHPFENPTALTNLAQMLAIFLIPAALCFAFGEAAGDRRQGRALLWAMSFIFVVCVAVVMW
AEVQGNPHLLAAGADSSVNMEGKETRFGVLASSLFAVVTTAASCGAVNAMHDSFTALGGM
VPMWLMQIGEVVFGGVGSGLYGMLLFVLLAVFIAGLMIGRTPEYLGKKIDVREMKMTALA
ILVTPMLVLLGSALAMMTDAGRSAMLNPGPHGFSEVLYAVSSAANNNGSAFAGLSANSPF
WNCLLAFCMFVGRFGVIIPVMAIAGSLVSKKVQPASQGTLATHGALFIGLLIGTVLLVGA
LTFIPALALGPVAEHFSLP
>EECEBICD_02761 Potassium-transporting ATPase ATP-binding subunit
MSRKQLALFEPVLLVQALTDAVKKLSPRAQWRNPVMFVVWAGSVLTTLLTLAMVTGQIAG
SALFTGIISLWLWFTVLFANFAEALAEGRSKAQANSLKGVKKTAFARRLRAPRHDAQADN
VPAAELRKGDIVLVKAGDIIPCDGEVIEGGASVDESAITGESAPVIRESGGDFASVTGGT
RILSDWLVIACSVNPGETFLDRMIAMVEGAQRRKTPNEIALTILLIALTIVFLLATATLW
PFSAWGGNAVSVTVLVALLVCLIPTTIGGLLSAIGVAGMSRMLGANVIATSGRAVEAAGD
VDVLLLDKTGTITLGNRQASDFIPARGVDERTLADAAQLASLADETPEGRSIVILAKQRF
NLRERDVQSLHATFVPFTAQSRMSGINIDNRMIRKGSVDAIRRHVESNGGHFPADVEQNV
ENVARLGATPLVVVEGARVLGVIALKDIVKGGIKERFAQLRKMGIKTVMITGDNRLTAAA
IAAEAGVDDFLAEATPEAKLALIRQYQAEGRLVAMTGDGTNDAPALAQADVAVAMNSGTQ
AAKEAGNMVDLDSNPTKLIEVVHIGKQMLMTRGSLTTFSIANDVAKYFAIIPAAFAATYP
QLNALNVMGLHSPNSAILSAVIFNALIIIFLIPLALKGVSYKPLSASAMLRRNLWIYGLG
GLVVPFIGIKVIDVLLTLLGLA
>EECEBICD_02762 Potassium-transporting ATPase KdpC subunit
MIGLRPAFSTMLFLLLLTGGVYPLLTTALGQWWFPWQANGSLIHKDNVIRGSALIGQSFT
AAGYFHGRPSATADTPYNPLASGGSNLAASNPELDAQIQSRVAALRAANPQASSAVPVEL
ATASASGLDNNLTPGAAAWQIPRVAAARQLPVEQVAQLVAEYTHRPLARFLGQPVVNIVE
LNLALDALQGHRAK
>EECEBICD_02763 Sensor protein KdpD
MNDEPLRPDPDRLLEQTPPPHRGKLKIFFGACAGVGKTWAMLAQAQRLRAQGLDVVIGVV
ETHGREETAALLDGLTILPPKRHSHRGRQIREFDLDAALARRPALILMDELAHSNAPGSR
HPKRWQDIEELLEAGIDVFTTVNVQHLESLNDVVSGVTGIQVRETVPDPFFDAADEVVLV
DLPPDDLRQRLNEGKVYIAGQAERAIEHFFRKGNLIALRELALRRTADRVDDQMRAWRAH
PGEEKVWHTRDAILLCIGHNTGSEKLVRTAARLASRLGSVWHAVYVETPTLHRLPEKQRR
AILSALRLAQELGAETATLSDPAEEKAVVRYAREHNLGKIVMGRPASRRWWRRDAFADRL
ARRAPDLDQVIVALEEPPARALAQTPDNRPFKEKWRGQIQGCLVAVALCAITTLIAMQWL
VTFDAANLVMLYLLGVVVIALLYGRWPSVVATVINVASFDLFFIAPRGTLAVSDVQYLLT
FAVMLTVGLVIGNLTAGVRYQARVARYREQRTRHLYEMSKALAVGRSEHDIAATSERFIA
STFQARSQILLPDANGKLLPLTHQQGMTPWDDAIARWSFDKGQPAGAGTDTLPGVPYQIL
PLKSADKTYGLAIVEPGNLRQLMVPEQQRLLETFTLLVANALERLTLTASEEQARLASER
ESIRNALLAALSHDLRTPLTVLFGQAEILTLDLASAGSPHARQASEIRQHILNTTRLVNN
LLDMARIQSGGFNLKKEWLTLEEVVGSALQMLEPGLLHPITLSLPQQLTLIHVDGPLFER
VLINLLENAVKYAGPQASIGIDAAVKDDRLQLDVWDNGPGIPAGQEQKIFDKFARGNKES
AVPGVGLGLAICHAIVEVHGGTLTAYNRPQGGACFRVTLPQGKPPELDDFHEDM
>EECEBICD_02764 KDP operon transcriptional regulatory protein KdpE
MTNVLIVEDEQAIRRFLRAALEGDGLRVYEAETLQRGLLEAATRKPDLIILDLGLPDGDG
IDFIRDLRQWSAIPVIVLSARSEESDKIAALDAGADDYLSKPFGIGELQARLRVALRRHA

ASPSADPIVRFSGVTVDLAARLIHRGDEEIHLTPIEFRLLAVLLNNTGKVLTQRQLLNQV
WGPNAVEHSHYLRIYMGHLRQKLEQDPTRPRHFITETGIGYRFMP
>EECEBICD_02765 hypothetical protein
MENNNRFMPHIRRTTHIMMFAHRNSFDFHFFNAR
>EECEBICD_02766 Inducible ornithine decarboxylase
MSELKIAVSRHCPDCFSTHRNIVNVDESRFIDVAAIVLSIDDIEHGKLDEIDATGYGIPV
FVATHDEGRVPPEYLPRISGVFEYNESRTAFYGRQLETAASHYETQLRPPFFRALVDYVN
QGNSAFDCPGHQGGEFFRRHPAGNQFVEYFGETLFRSDLCNADVAMGDLLIHEGAPCIAQ
QHAAKVFNADKTYFVLNGTSSSNKVVLNALLTPGDLVLFDRNNHKSNHHGALLQAGATPV
YLETARNPYGFIGGIDAHCFEEDYLRELISEVAPQRAREARPFRLAVIQLGTYDGTIYNA
RQVVDKIGHLCDYILFDSAWVGYEQFIPMMADCSPLLLELNENDPGILVTQSVHKQQAGF
SQTSQIHKKDSHIKGQSRYVPHKRMNNAFMMHASTSPFYPLFAALDVNAKMHEGVSGRNM
WMDCVVNGIDARKLILENCHHIRPFVPELIDGKPWQSYPTSEIASDLRFFHFVPGEHWHA
FEGYAEHQYFVDPCKLLLTTPGINAASGEYEDFGVPATILANFLRENGVVPEKCDLNSIL
FLLTPAEDMAKLQQLVALLARFEKLLEADAPLAEVLPSIYKQHEARYAGYTLRQLCQEMH
DLYARHNVKQLQKEMFRKSHFPKVSMNPQEANYAYLRGEVELVRLPEAEGRIAAEGALPY
PPGVLCVVPGEIWGGSVLRYFSALEEGINLLPGFAPELQGVYIEEHDGRKQVWCYVIKPR
DAQRSLLKEEKL
>EECEBICD_02767 Putrescine transporter PotE
MSKPKSNKMGVVQLTILTMVNMMGSGIIMLPTKLSEVGTISIISWLVTAVGSMALAWAFA
KCGMFSRKSGGMGGYAEYAFGKSGNFMANYTYGVSLLIANVAIAISAVGYGTELFGAALT
PLQIGLATIAVLWVCTIANFGGARITGQISSVTVWGVIIPVVGLCIIGWFWFSPSMYVAS
WNPHHVPFFSAVGSSIAMTLWAFLGLESACANADVVENPEKNVPIAVLGGTLGAAVIYIV
STNVIAGIVPNMDLANSTAPFGLAFAQMFTPAVGKVIMALMVMSCCGSLLGWQFTIAQVF
KSSADEGYFPKVFSRVTKVDAPVQGMLIIVIIQTGLSLMTISPSLNSQFNVLVNLAVVTN
IIPYILSMAALVIIQKMANVPSSKAKVANFVAFVGAMYSFYALYSSGEEAMLYGSIVTFL
GWTLYGLVSPRFELKNKHG
>EECEBICD_02768 hypothetical protein
MSNPEREVTDIAEILAILSKHEVCRIGFNDNLCPYVVPVNFGYEYQNGRWIFYFHGARTG
KKMRLLRKNPAVCIEMDGDHALLRADDPCDYSYAYTSVFATGLASILQTREEMRYGLDVI
MRQTDPGKTFSYREDMMKAVCVVRIECDALTCRRHLATQSE
>EECEBICD_02769 Phosphoglucomutase
MAIHNRAGQPAQQSDLINVAQLTAQYYVLKPEAGNAEHAVKFGTSGHRGSAGRHSFNEPH
ILAIAQAIAEERAKNGITGPCYVGKDTHALSEPAFISVLEVLAANGVDVIVQENNGFTPT
PAVSNAILVHNKKGGPLADGIVITPSHNPPEDGGIKYNPPNGGPADTNVTKVVEDRANAL
LAGGLQGVKRISLDAAMASGHVKAVDLVQPFVEGLADIVDIAAIQKAGLTLGVDPLGGSG
IEYWKRIAEHYKLNLTLVNDQVDQTFRFMHLDKDGAIRMDCSSECAMAGLLALRDKFDLA
FANDPDYDRHGIVTPAGLMNPNHYLAVAINYLFQHRPLWGKDVAVGKTLVSSAMIDRVVN
DLGRKLVEVPVGFKWFVDGLFDGSFGFGGEESAGASFLRFDGTPWSTDKDGIIMCLLAAE
ITAVTGKNPQEHYNELAARFGAPSYNRLQAAATSAQKAALSKLSPEMVSASTLAGDPITA
RLTAAPGNGASIGGLKVMTDNGWFAARPSGTEDAYKIYCESFLGEEHRKQIEKEAVEIVS
EVLKNA
>EECEBICD_02770 Negative modulator of initiation of replication
MKTIEVDDELYSYIASHTKHIGESASDILRRMLKFSATTQPTASAVKGTPAAQPVAEAKP
VNPVKDKVRAMRELLLSDEYAEQKKAVNRFMLILTTLYSLDHHAFAEATESLHGRTRVYF
AADEQTLLKNGNQTKPKHVPGTPYWVITNTNTGRKCSMIEHIMQSMQFPAELIEKVCGTI
>EECEBICD_02771 Esterase YbfF
MKLNIRAQSAQNLHNNSPIVLVHGLFGSLDNLGVLARDLVTDHDIIQVDMRNHGLSPRDP
VMDYPAMAQDLLDTLDAQQIEKATFIGHSMGGKAVMALTALAPDRIDRLVAIDIAPVDYH
VRRHDRIFAAINAVSESDATSRQQAAGIMRQHLNEEGVIQFLLKSWAEGEWRFNVPVLWE
QYPHIVGWETIPPWEHPALFIPGGNSPYVTEAYRDALLAQFPLARAHVIAGAGHWVHAEK
PEAVLRAIRRYLHDKR
>EECEBICD_02772 putative protein YbfE
MAKEQTDRTTLDLFAHERRPGRPKTNPLSRDEQLRINKRNQLKRDKVRGLKRVELKLNAQ

AVDALNELAEARNISRSELIEEILMKQLMALRGQGLV
>EECEBICD_02773 Flavodoxin 1
MAITGIFFGSDTGNTENIAKMIQKQLGKDVADVHDIAKSSKEDLEGYDILLLGIPTWYYG
EAQCDWDDFFPTLEEIDFNGKLVALFGCGDQEDYAEYFCDALGTIRDIIEPRGATIVGHW
PTAGYHFEASKGLADDDHFVGLAIDEDRQPELTAERVEKWVKQVSAELHLDDILNA
>EECEBICD_02774 Ferric uptake regulation protein
MTDNNTALKKAGLKVTLPRLKILEVLQEPDNHHVSAEDLYKRLIDMGEEIGLATVYRVLN
QFDDAGIVTRHNFEGGKSVFELTQQHHHDHLICLDCGKVIEFSDDSIEARQREIAAKHGI
RLTNHSLYLYGHCAEGDCREDEHAHDDVTK
>EECEBICD_02775 HTH-type transcriptional regulator CatM
MELRQLRYFVRIIETGSMGSAAQDLDIGVSALSQQMSRLENELAIRLLQRTSRGVTPTNA
GLAFYSQAQLALRHADDAILAAREARLSGHVSVGMAPSTASILGIPFIHAMQENYADVRL
HVVESLSGNLERMINTRQIDLAVVFQKDKILRWSARPILEEQLFLIGSHALLAALPDNPI
TPEQLAGIPLIMPSQGHGLRGRLDAVCQEHALNVEIVAEIDGLALLMRAVRDGLGATLQP
GAAISHLDNDALRVIGVHNPVLSRPNFLVSLSDDELTPAGLAARVVLTKVMRQLVDAGEW
PGATLYAY
>EECEBICD_02776 Fumarate reductase flavoprotein subunit
MVDVLVIGGGNAALCAALTAREAGASVLLLEAAPREWRGGNSQHTRNLRCMHDAPQDVLV
ESYPEEEFWQDLWRVTDGNTNEALARLVIRTSSQCRDWMRKHGVNFQPPLSGALHVARTN
AFFMGGGKALVNAYYRSAERLGVQVRYNTPVHALELHDGEFVAALAGSERITAKACVLAA
GGFESNREWLREAWGENARGEWPADNFLIRGTRFNQGVLLKFMMDAGADIIGDPSQSHCV
AIDARAPLYDGGICTRVDCVSLGIVVNRDAERFYDEGEDFWPKRYAIWGRLVAQQPGQIG
YSIIDSKAIGHFMPPVFPGAQANTLAELACQLGLDAEKFTHTVTQYNQACQPGHFDHTLL
DDCATKNLTPAKTHWARPLDAPPYYGYALRPGITFTYLGLKVNERAAVHFAGHPSRNLFV
AGEMMAGNVLGKGYTAGVGMSIGTTFGRIAGIEAARAAHKEAQHETA
>EECEBICD_02777 hypothetical protein
MKQLEKLIIEATVLTEPEAEVERVMQVCNACRYCEGFCAVFPAMTQRLEFGKADIHYLAN
LCHNCGACLHACQYAPPHEFAINVPKAMAQARLETYQQYAQPAAFGALYRRAGITVALAL
IVGLTLFLLLAMALKGSLIHPPLAGDFYQIFPHSLLAWMFGSVFVLAIGLLMAGVIRFWR
EISPGVPRSAEIAEASHNALTLKYLDGGHGKGCNEADDAFTLLRRRFHHFTFYGFMLCFA
ATVVATGYHYVAGWEAPYPFFSLPVMLGTLGGIGLLIGPAGLLWLNLRRSPLHGDARQKP
MDRGFILLLFLTSLTGLALLAGRDTSGMGILLALHLGVVMALFLTLPYGKFAHGFFRCAA
LLKWAVEKRRGKHAGDTGN
>EECEBICD_02778 Proline/betaine transporter
MAQHTPATSRAGTFGAILRVTSGNFLEQFDFFLFGFYATYIARTFFPAESEFASLMLTFA
VFGSGFLMRPVGAIVLGAYIDRIGRRKGLMVTLAIMGCGTLLIALVPGYQTIGLAAPALV
LLGRLLQGFSAGVELGGVSVYLSEIATPGNKGFYTSWQSASQQVAIVVAALIGYSLNITL
GHDAISEWGWRIPFFIGCMIIPLIFVLRRSLQETEAFLQRKHRPDTREIFATIAKNWRII
TAGTLLVAMTTTTFYFITVYTPTYGRTVLNLSARDSLIVTMLVGVSNFIWLPIGGAISDR
IGRRAVLMGITLLALITTWPVMQWLTAAPDFTRMTLVLLWFSFFFGMYNGAMVAALTEVM
PVYVRTVGFSLAFSLATAIFGGLTPAISTALVKLTGDKSSPGWWLMCAALCGLAATAMLF
VRLSRGYIAAENKA
>EECEBICD_02779 putative lipoprotein ChiQ
MKKILLIASMTAGLTACASSPAPEEDSRLKEAYSACINTAQGSPEKIEACQSVLNVLKKD
RKHQQFANEESVRVLDYQQCIQATRTGNDQAVKADCDKVWQEIRSHNNVQ
>EECEBICD_02780 Chitoporin
MYDLKNWNLPGWAVGASYVYAWDAKPATWQSNPDAYYDKNRTIEESSYSLDAVYTLQEGR
AKGTMFKLHFTEYDNHSNIPSWGGGYGNIFQDERDVKFIVIAPFTIF
>EECEBICD_02781 Chitoporin
MRTFSGKRSTLALAIAGITAMSGWIVVPQAQASGFFDDSTLTGGIYYWQRERDRKDVTDG
DKYKTNLSHATWNANLDFQSGYAADMFGLDIAAFTAIEMAENGDSGHPNEIAFSKKNKGY
DEDYSGDKSGISLYKAAAKFKYGPVWARAGYIQPTGQTLLAPHWSFMPGTYQGAEAGASF
DYGDAGALSFSYMWTNEYKAPWHTEMDKFYQADKKTNVDYLHSIGAKYDFKNDLVLEAAF
GQSEGYVDQYFAKASYKFDLGGNPFTTSYQFYGARDKVDDRSVNDIYDGTAWLQALTFGY

KVAEVVDLRLEGTWVKADGQQGYFLQRMTPTYASSNGRLDIWWDNRSDFNANGEKGGFLR
RDV
>EECEBICD_02782 Glutamine--tRNA ligase
MSEAEARPTNFIRQIIDEDLASGKHTTVHTRFPPEPNGYLHIGHAKSICLNFGIAQDYQG
QCNLRFDDTNPVKEDIEYVDSIKNDVEWLGFHWSGDIRYSSDYFDQLHAYAVELINKGLA
YVDELTPEQIREYRGTLTAPGKNSPFRDRSVEENLALFEKMRTGGFEEGKACLRAKIDMA
SPFIVMRDPVLYRIKFAEHHQTGNKWCIYPMYDFTHCISDALEGITHSLCTLEFQDNRRL
YDWVLDNITIPVHPRQYEFSRLNLEYTVMSKRKLNLLVTDKHVEGWDDPRMPTISGLRRR
GYTAASIREFCKRIGVTKQDNTIEMASLESCIREDLNENAPRAMAVIDPVKLVIENYPQG
ESEMVTMPNHPNKPEMGSREVPFSGEIWIDRADFREEANKQYKRLVMGKEVRLRNAYAIK
AERVEKDAEGNITTIFCTYDADTLSKDPADGRKVKGVIHWVSAAHALPIEIRLYDRLFSV
PNPGAAEDFLSVINPESLVIKQGYGEPSLKAAVAGKAFQFEREGYFCLDSRYATADKLVF
NRTVGLRDTWAKAGE
>EECEBICD_02783 PTS system N-acetylglucosamine-specific EIICBA component
MNILGFFQRLGRALQLPIAVLPVAALLLRFGQPDLLNMPFIAQAGGSIFDNLALVFAIGV
ASSWSKDSAGAAALAGAVGYFVMTKAMVTINPEINMGVLAGIITGLVGGAVYNRWSGIKL
PDFLSFFGGKRFVPIATGFFCLVLAAIFGYVWPPVQHGIHAGGEWIVSAGALGSGIFGFI
NRLLIPTGLHQVLNTIAWFQIGEFTNAAGTVFHGDINRFYAGDGTAGMFMSGFFPIMMFG
LPGAALAMYFAAPKERRPMVGGMLLSVAITAFLTGVTEPLEFLFMFLAPLLYLLHAILTG
ISLFVATLLGIHAGFSFSAGAIDYVLMYNLPAASNNVWMLLVMGVVFFIIYFLLFSAVIR
MFNLKTPGREDKVDEMVTEEANSNTEEGLTQLATSYIAAVGGTDNLKAIDACITRLRLTV
NDSARVNDAACKRLGASGVVKLNKQTIQVIVGAKAESIGDEMKKVVARGPVAAASADAAH
VATPAPAAKPQAVPNAVTIAELVSPITGEVVALDQVPDEAFASKAVGDGVAVKPTDKTVV
SPAAGTIVKIFNTNHAFCLETEKGAEIVVHMGIDTVALNGQGFKRLVEEGAEVTAGQPVL
ELDLDFLNANARSMISPVVCSNSDDFSALVIKADGHVVAGQTPLYEIKSK
>EECEBICD_02784 Glucosamine-6-phosphate deaminase
MRLIPLSTAEQVGKWAARHIVNRINAFKPTADRPFVLGLPTGGTPLTAYKALVEMHKAGE
VSFKHVVTFNMDEYVGLPKEHPESYHSFMHRNFFDHVDIPAENINLLNGNAPDIDAECRQ
YEEKIRSYGKIHLFMGGVGNDGHIAFNEPASSLASRTRIKTLTHDTRVANSRFFDGDVNQ
VPKYALTVGVGTLLDAEEVMILVLGHQKAQALQAAVEGNVNHMWTISCLQLHPKAVVVCD
EPSTMELKVKTLKYFNELEAENIKGL
>EECEBICD_02785 N-acetylglucosamine-6-phosphate deacetylase
MLSPPRQSMRGGKFLISGVGMYALTQGRIFTGHEILDDHALVVANGLIDRVCPMAELPPG
IEQRSLNGAILSPGFIDVQLNGCGGVQFNDTAEAVSVETLEIMQKANEKSGCTNFLPTLI
TTSDELMKQGIRVMREYLEKHPHQALGLHLEGPWLNLVKKGTHNPDFVRKPDAALVDFLC
DNADVITKVTLAPEMVPADVIAKLANAGIVVSAGHSNATLKEAKAGFRAGITFATHLFNA
MPYITGREPGLAGAVLDEADIYCGVIADGLHVDYANIRNAKRLKGDKLCLVTDATAPAGA
NIEQFIFAGKTIYYRNGLCVDENGTLSGSSLTMIEGVRNLVAHCGIALDEVLRMATLYPA
RAIGVDKHLGSIAPGKVANLTAFTHDFKIIKTIVNGDEVVDLSK
>EECEBICD_02786 N-acetylglucosamine repressor
MTPGGQAQIGNVDLVKQLNSAAVYRLIDQHGPISRIQIAEQSQLAPASVTKITRQLIERG
LIKEVDQQASTGGRRAISIVTETRNFHAIGVRLGRHDTTLTLYDLSSKVVAEEHYPLPER
TQETLEHALLNTIAVFIDSCQRKIRELIAISVILPGLVDPESGVIRYMPHIQVENWGLVE
ALEKRFHVTCFVGHDIRSLALAEHYFGASQDCEDSILVRVHRGTGAGIISNGRIFIGRNG
NVGEIGHIQVEPLGERCHCGNFGCLETIAANAAIEQRVLNLLKQGYQSRVPLDDCTIKTI
CKAANRGDSLASEVIEHVGRHLGKTIAIAINLFNPQKIVIAGEIIEADKVLLPAIESCIN
TQALKAFRKNLPVVRSTLDHRSAIGAFALVKRAMLNGTLLQRLLES
>EECEBICD_02787 Ribonucleotide monophosphatase NagD
MTIKNVICDIDGVLMHDNVAVPGAAEFLTGILEKGLPLVLLTNYPSQTGQDLANRFATAG
VNVPDSVFYTSAMATADFLRRQEGKKAYVVGEGALIHELYKAGFTITDVNPDFVIVGETR
SYNWDMMHKAAFFVANGARFIATNPDTHGRGFYPACGALCAGIEKISGRKPFYVGKPSPW
IIRAALNKMQAHSEETVIVGDNLRTDILAGFQAGLETILVLSGVSTINDIDSMPFRPSWI
YPSVAEIDVI
>EECEBICD_02788 Asparagine synthetase B [glutamine-hydrolyzing]

MCSIFGVFDIKTDAAELRKKALELSRLMRHRGPDWSGIYACDNAILAHERLSIVDVNAGA
QPLYNARKTHVLAVNGEIYNHQTLRAEYGDRYAFQTGSDCEVILALYQEKGPDFLDDLQG
MFAFALYDSEKDAYLIGRDHIGIIPLYMGYDEFGNFYVASEMKALTPVCRTIKEFPAGSY
LWSKDGEIRQYYQRDWFDYDAVKDNVTGKNALRQALEESVKSHLMSDVPYGVLLSGGLDS
SVISAITKKFAARRVEDQERSEAWWPQLHSFAVGLEGSPDLKAAQEVANHLGTVHHEIHF
TVQEGLDAIRDVIYHIETYDVTTIRASTPMYLMSRKIKAMGIKMVLSGEGSDEVFGGYLY
FHKAPNAKELHEETVRKLQALHMYDCARANKAMSAWGVEARVPFLDKKFLDVAMRINPQD
KMCGNGKMEKHVLRECFESYLPASVAWRQKEQFSDGVGYSWIDTLKEVTAKQISDQQLET
ARFRFPYNTPSSKEAYLYREIFEELFPVPSAAECVPGGPSVACSSAKAIEWDEAFKTMDD
PSGRAVGVHQSAYQ
>EECEBICD_02796 hypothetical protein
MVVLLCAFTFIDITASAAWLIILGLVYPVAVSMRLHILHQKKAVMLRQNSAEWIVYLPGI
PVQEKQSALINVAFSSKRALRGFYIRALSSKVILHIITFYILWFDVQHATLTWYRILAAL
ITLAILVKSMSSTLLMLHKVVRCQYNVCTIEMASPWYEIDFKGKLGLCALFAVK
>EECEBICD_02797 3-demethoxyubiquinol 3-hydroxylase
MTNQPTEIAIVGGGMVGGALALGLAQQGFTVMVIEHAAPAPFVADSQPDVRISAISAASV
ALLKGLGVWEAVQGMRSHPYRRLETWEWENAHVVFDAAELKLPLLGYMVENNVLQQALWQ
ALEAHPGVTLRVPASLAALHRRHDGYALELADGEWVTPKLVIGADGANSQVRQMAGIGIH
AWQYAQSCMLITVKCENAPGDSTWQQFTPTGPRAFLPLFDDWASLVWYDAPARIRQLQSL
SMTQLQVEINQHFPARLGAVMPVAAGAFPLTRRHALQYAQPGLALVGDAAHTIHPLAGQG
VNLGYRDVDALIDVLASARSYGESWASHSVLKRYQTRRMADNFMMQSGMDLFYAGFSNEL
PPLRILRNMGLMAAQRAGVLKRQALKYALGL
>EECEBICD_02798 tRNA-2-methylthio-N(6)-dimethylallyladenosine synthase
MTKKLHIKTWGCQMNEYDSSKMADLLDATHGYQLTDVAEEADVLLLNTCSIREKAQEKVF
HQLGRWRLLKEKNPDLIIGVGGCVASQEGEHIRQRAHYVDIIFGPQTLHRLPEMINSVRG
DRSPVVDISFPEIEKFDRLPEPRAEGPTAFVSIMEGCNKYCTYCVVPYTRGEEVSRPSDD
ILFEIAQLAAQGVREVNLLGQNVNAWRGENYDGTTGTFADLLRLVAAIDGIDRIRFTTSH
PIEFTDDIIEVYRDTPELVSFLHLPVQSGSDRVLNLMGRTHTALEYKAIIRKLRAARPDI
QISSDFIVGFPGETNDDFEKTMKLIADVNFDMSYSFIFSARPGTPAADMVDDVPEEEKKQ
RLYILQERINQQAMAWSRRMLGTTQRILVEGTSRKNIMELSGRTENNRVVNFEGTPEMIG
KFVDVEITDVYPNSLRGKVVRTEDEMGLRVAETPESVIARTRKENELGVGFYQP
>EECEBICD_02799 PhoH-like protein
MNIDTREITLEPADNARLLSLCGPFDDNIKQLERRLGIEINRRDNHFKLTGRPICVTAAT
DILRSLYVDTAPMRGQTQDIEPEQIHLAIKEARVLEQSAESVPDYGKAINIKTKRGVIKP
RTPNQAQYIANILDHDITFGVGPAGTGKTYLAVAAAVDALERQEIRRILLTRPAVEAGEK
LGFLPGDLSQKVDPYLRPLYDALFEMLGFEKVEKLIERNVIEVAPLAYMRGRTLNDAFII
LDESQNTTIEQMKMFLTRIGFNSKAVITGDVTQIDLPRNTKSGLRHAIEVLAEVDEISFN
FFHSEDVVRHPVVARIVNAYEAWEEAEQKRKAALAAERKREAQEQEQK
>EECEBICD_02800 Endoribonuclease YbeY
MSQVILDLQLACENHAGLPDEAQFQRWLDGVIPQFQEEAEVTIRLVDETESHDLNLTYRG
KDKPTNVLSFPFEAPPGIEMPLLGDLIICRQVVEQEAQEQSKPLEAHWAHMVVHGSLHLL
GYDHIDDDEAEEMESLETEIMLAMGYEDPYIAEKIAE
>EECEBICD_02801 Magnesium and cobalt efflux protein CorC
MSDDNSHSSDTVNSKKGFFSLLLSQLFHGEPKNRDELLALIRDSGQNELIDEDTRDMLEG
VMDIADQRVRDIMIPRSQMITLKRNQTLDECLDVIIESAHSRFPVISEDKDHIEGILMAK
DLLPFMRSDAEAFSMDKVLRTAVVVPESKRVDRMLKEFRSQRYHMAIVIDEFGGVSGLVT
IEDILELIVGEIEDEYDEEDDIDFRQLSRHTWTIRALASIEDFNDAFGTHFSDEEVDTIG
GLVMQAFGHLPARGETIDIDGYQFKVAMADSRRIIQVHVRIPDDSPQPKLDE
>EECEBICD_02802 Apolipoprotein N-acyltransferase
MTRPSQNWTNNSNSGLKMAFASLIERQRIRLLLALLFGACGTLAFSPYDVWPAAIVSLIG
LQALTFNRRPLQSAAIGYCWGLGLFGSGINWVYVSIAQFGGMPGPVNVFLVVLLAAYLSL
YTGLFAGILSRLWPKTNWLRVAIAAPAIWQITEFLRGWVLTGFPWLQFGYSQVDGPLKGL
APVMGVEAINFLLMMVSGLLALALATRNWRPLAVAVILFALPFPLRYIQWFTLEPAKATQ
VSLVQGDIPQSLKWDENQLLNTLKIYLNETRPELGKSQIIIWPESAIPDLEINQQPFLRS

LDEMLREKNSTLITGIVDARLNKQNRYDTYNTIITLGKDNPYSYDSPNRYNKNHLVPFGE
FVPLESILRPLAPFFDLPMSSFSRGPYIQPQLHAHDYKLTAAICYEIILGEQVRDNFRPD
TDYLLTISNDAWFGKSIGPWQHFQMARMRSLELARPLLRSTNNGITAVIGPQGEIQAMIP
QFTRQVLTTNVTPTTGLTPYARTGNWPLWVLTALFAFGAVVMSLRQRRK
>EECEBICD_02803 Glutamate/aspartate import solute-binding protein
MQLRKLTTAMLVMGLSAGLAHAEDGAPAAGSTLDRIAKNGVIVVGHRESSVPFSYYDNQQ
KVVGYSQDYSNAIVEAVKKKLNKPDLQVKLIPITSQNRIPLLQNGTFDFECGSTTNNLER
QKQAAFSDTIFVVGTRLLTKKGGDIKDFPDLKGKAVVVTSGTTSEILLHKLNEEQKMGMR
IISAKDHGDSFRTLESGRAVAFMMDDALLAGERAKAKKPDNWEIVGKPQSQEAYGCMLRK
NDPEFKKLMDDTIAQAQTSGEAEKWFDKWFKNPIPPKNLNMNFELSDEMKALFKAPNDKA
LN
>EECEBICD_02804 Glutamate/aspartate import permease protein GltJ
MSIDWNWGIFLQEAPFGNTTYLGWLWSGFQVTVALSITAWIIAFLVGSIFGILRTVPNRF
LSGLGTLYVELFRNVPLIVQFFTWYLVVPELLPEDLGMWFKAELDPNIQFFLSSMICLGL
FTAARVCEQVRAAIQSRLAGRRTRRWRWG
>EECEBICD_02805 Glutamate/aspartate import permease protein GltJ
MGLTLPQAYRYVLLPNAYRVIVPPMTSEMMNLVKNSAIASTIGLVDMAAQAGKLLDYSAH
AWESFTAITLAYVLINAVIMLVMSLVERKVRLPGNVGSK
>EECEBICD_02806 Glutamate/aspartate import permease protein GltK
MYEFDWSSIIPSLPYLLAGLVITLKITVTAVVVGIVWGTILAVMRLSSFAPIAWFAKAYV
NVFRSIPLVMVLLWFYLIVPGFLQNVLGLSPKTDIRLISAMVAFSMFEAAYYSEIIRAGI
QSISRGQSSAALALGMTHWQSMKLIILPQAFRAMVPLLLTQGIVLFQDTSLVYVLSLADF
FRTASTIGERDGTQVEMILFAGAVYFVISLSASLLVSYLKKRTV
>EECEBICD_02807 Glutamine transport ATP-binding protein GlnQ
MITLKNVSKWYGHFQVLTDCSTEVKKGEVVVVCGPSGSGKSTLIKTVNGLEPVQKGEITV
NGIIVNDKKTDLAKLRSRVGMVFQHFELFPHLSIIENLTLAQVKVLKRDKAAARDKGLKL
LERVGLSAHANKFPAQLSGGQQQRVAIARALCMDPIAMLFDEPTSALDPEMINEVLDVMV
ELANEGMTMMVVTHEMGFARKVANRVIFMDEGKIVEDSPKEEFFANPSSDRAKDFLAKIL
H
>EECEBICD_02808 Pyrimidine-specific ribonucleoside hydrolase RihA
MALPIIIDCDPGHDDAIALVLALASPELEVKAITSSAGNQTPEKTLRNVLRMLTLLKRPD
IPVAGGAVKPLMRELIIADNVHGESGLNGPALPEPSFAPQSGTAVELMAKTLRESAQPVT
IVSTGPQTNVALLLNSHPELHTKIARIVIMGGAMALGNWTPAAEFNIYVDPEAAEIVFQS
GIPVVMAGLDVTHKAQIHAADIERFRAIGNPISTIVAELLDFFMEYHKDEKWGFVGAPLH
DPCTIAWLLKPEIFTTVERWVGVETKGKYTQGMTVVDYYFLTGNKPNATVMVDVDRQGFV
DLLAERLQYYA
>EECEBICD_02809 hypothetical protein
MFWRNNRPEISLLQHDVAHITFSVRNGKALLRPCVIHAPDSDAGIHTLSWHGSPLIRFYT
EAWCPTCAEFVYAGFSNDDEGAAQFLSSLAEWNQTGVGLNEAFTALTPLFSLFADGYYRL
EERELYPTDGNGHFFWAVGNEKQPNPATTGQWIADVDYHYQSGEPCFLLPG
>EECEBICD_02810 hypothetical protein
MNDTWLCVLLDGHHKATAAALEGRPVKTWVISQPVAMTCYETRQQYLRFYDGERLEEAQF
QRRIPLKIQYEKLPPSLWEDYFTRHDERYTRVNWPNALANCAANYPNLAACTDIIAAGDL
SEAGLNKIMAQGITEEGFPAVLLRALFYTHSPLLIDFVRFLTRTPDYACHYPLAFRLLAQ
KRTPQADAFFLDFAINDDGERPELTNIMDEYFRQA
>EECEBICD_02811 Chaperone protein HscC
MDNATLAIGIDLGTTNSLIAVWQDGAAQLIPNKFGEYLTPSIISMDENKQILVGKPAAAR
KTSHPDKTAALFKRAMGSNTHWHLGEESFNAPELSSLVLRSLKEDAEDYLQQPIKDVVIS
VPAYFSDEQRKHTRLAAELAGLNAVRLINEPTAAAMAYGLHTQQNSRSLVFDLGGGTFDV
TVLEYATPIIEVHASAGDNYLGGEDFTHLLLDEVLKRWNLDKSALTDSDLAALYACVEAA
KCASSSPLRMSWLYQESVLESTFYDDELEALWLPLLNRLRTPIEQALRDSRLKPEQIDSL
VLVGGASQMPLVQRIAVRLFGKLPYQSYDPSTIVALGAATQAACRLRHEDVEEVILTDIC
PYSLGVEVNRQGVPGIFSPIIERNTTVPVSKVETYSTMHPEQDSICVRVYQGESHKVKNN
ILIDSFDVMLKPNGHIQAIDIRFSYDINGLLEVDVLLEDGKSESRIISHNATSLTTQQID

ASRERLLALKIYPRDMLINRTFKAQLEEQWSRALGDEREMLGEIITDFDAALLSNDMQRV
DDVRRRACEYLGIDEPKAP
>EECEBICD_02812 hypothetical protein
MLEAFPKEKPFFRLLIRLEEGQSCPLEALITQALSSKIAMKPGDIRDKKELAEELRLAAE
KEETKLSSEAKTEAPRQAESGGVMKIMKIVGLIALIVCALNRFFHFF
>EECEBICD_02813 hypothetical protein
MNICWEILGIEPTTDLECIRQAYLALLPSFHPESDPQGFKRLRQAYEEAQHWAASPAEDA
KTEEVGDEHEILVAFRELLASERDRFQPSAWQKFIQQLNQCSMDEIDKLRWQLCDIAMKT
ETISLSCLALLAQRLNWQPQEAEDDADGEELEAFLETVKRGDAFDLLSLAKLPLVVQDQT
DAYFFKLERIWRFYPEHFADALQVQGAWIIPDDKQLHRKLLHWFSAEQWGMAELVDIARR
WQAAEPDNEDAHYYLCKQRLLCGEGESLLADLCDFWQRYPSTQADDLLLGWCRQHRPDFF
PLAVMAVEARCMADAEYVPGESARTRLLWADILHSGRLSPLGRSFVDSLFYKRKAMAWAN
SRLSTQGNPKRRCWICTGRRSRWCWKRFPKKSPFFAC
>EECEBICD_02814 hypothetical protein
MDKEEQYLLFALSTPMEVLYIGNEPSHTSPAMYTGIPAVDLSDSWGIDNREDLIQTIYRM
TDDGHAADLAPFYIRWFTLSPRQWREFTAQFGEQGQIYARFVAETALCCGRGGIKAWDYV
RMGFLCRMGVLNQWLTEEESLWLQSRIYARAYYFYDGWTQYFAAYSLGRLYWQAKGDTIQ
AYFAHLKYDASGARMFNELASTTESYYAQLPWRPLNEQPTCPETLKGVSDL
>EECEBICD_02815 hypothetical protein
MKTCWQVLGIEATTDTDAIRQAYLALLPSFHPETDPQGFKQLREAYENALQGTTSPAITV
VDEDPDTPWVNYLREIFGDLLADGERRFQPQAWQEFIQQINKLSILQVEKSRWWLCAIAM
KTFPISYSCLKLLSDRLAWEQGSDNRDVDAEEVEDLLFEIRRGDLFDYSLLLHLPVAVQN
QTIAFYDALDNTFFEHPLYFAQLMAQHGPWIIPDNLRFQRRLLRWFSTLHWGIAELLPVA
LAWQEAEPDNATPGYYYLAQRVFCGEGDSLLPELYAQWWAHPSTQLDDLLLRWCRQHRPD
FFPLLVMAVEGREQVDINAEQLLYIPGSSARSCLLWAEALHSGALSPLSESFIARRLNYG
APAVSEAHSQHPYWLLYQVADSLACAEEPSAALLQSLVTKLDAPDICPLEALVIRGLLGR
AAAVATPCEAVVADEEPVTTVTSPSTEESGGGFGLWQVIKIILFIGVVGHVLRQFMH
>EECEBICD_02816 hypothetical protein
MDKESQYLLFALSSPMEVLNEDCLPSHSSPKMYLGVKRFDLESSWGIENRDELLQTISRM
TDDGHATQLEWLYRRWFRYAPQEWQEYTDALDEGDRIYARFVADTAVCCGEGGIRSWDYV
RMGFLCRMGVLNEWLTEEESLWLQSRIQLRALSYYSGWLPYFSAYYTGRLYWQLRNGDNL
PLLRETFARKEFDDAGRRMMNKLIAGKDSFYATLPWRYLPHYPECPDTLQEVSDL
>EECEBICD_02817 Secretory immunoglobulin A-binding protein EsiB
MNHSITSHPCDNVSLAQLTELAQSGNSEAQYILGRLYNDECIDGSEEDKLSFYWLQQAAE
QGHCEAQYWLGLRYKDTPTDMKDNTLALFWSEKAAQQGHRHAFNTLGWVQEGETGMAPDY
AQAVAWYRKGAEQSHNLAQYNLGRMYHSGTGVEQNDTQALYWFKQAALQGHCASQERLAY
MYGNGKGCRKNLSLAALWYKKSALQESSYSQYQMGYCYYIGKGIKQDYQQAIYWFRKAAD
QGDNDAYNSIGWMYKCGHGVEQNYSLALEWFHKSAECNNSSGWYNLGCMYRDGHGTAQDL
QQALYWFKKAQPTGKWNVDEEIRKLEAQLHA
>EECEBICD_02818 hypothetical protein
MNKVAQYYRELVASLSERLRNGERDIDALVEQARQRVMQTGELTRTEVDELTRAVRRDLE
EFAMSYEESQDDSVFLRVIKESLWQELADITDKTQLEWREVFQDLNHHGVYHSGEVVGLG
NLVCEKCHYHLAVYTPDVLPLCPKCGHDQFQRRPFEP
>EECEBICD_02819 Anaerobic nitric oxide reductase transcription regulator
NorR
MNKTKDIAASPLCFVSPYPQLAKAAEALVAQLDYAVTIHQTTLNRILDELPLLESRGHQV
LISRGGCAEILKKHSKLPVVEIKMSGYDILDALIPFKGQKGTVGIVGFSSVIKGCARVAE
QLNINYKIFTLQGNDKETISCLKRQLASTPLDCIVGDTVCQDYFSPLGSQFRLLDSSPAS
ITEALEEARSLYLAFRSQLLERHHLQLILDQFDKAVITLDDTGALLHYNKYASQLFKINA
SGEIYDASFLKQVLHQERHTLREGKTVSAKVVDTPQGAMVVNLYPVFAARQLSRVVLTMQ
TVSSLQGAEHHVRRQELSRRGLSARYHFDDLLTENPEMLRRLAIIKNYAGTDATILINGE
SGTGKEVLAQSIHNASQRVNGPFVAINCGAMAPQILESELFGYVAGAFTGASPKGKIGLF
ELAHHGTIFLDEISELDKPLQTRLLRVLQERQIMRLGSDQMIPVDIRVIAATNQTLTKLI
ADGTFREDLYYRLNVLKVTTIPLRKRPEDIKAIGLSLLTSFSQHYKRPALTLTPALWQEL

QRFAWPGNVRQLSNIIERLVLSIDHSPATLDEGRLLLDDLEEGSRREPTTCHDCQMLAGD
YKTIRLRILRKLLEAERDNKSLVAKRLNVDRTSLTRWIRESA
>EECEBICD_02820 2-keto-3-deoxygluconate permease
MKIKKTLERFPGGMMVVPLIIGALFKTFAPEALEIGGFVTSISHGAMAILGMFLVCMGAD
IQFKAAPKALKKGAAITFAKFASGVIIGILVGKFCGPDGLLGLSALAIISAMTNSNSGLY
AALVGEYGDETDGGAIAVISLNDGPFFTMLALGSAGMVSIPFMNLVAVIIPIIIGMILGN
LDEDMRKFLKQGSVVTIPFFAFGLGYGIDFARLITAGSSGILLGLMTVAIGGFFNIFADR
VTGGSGVAGAAVSTTSGNAVATPAAIALLDPHFTDLASTAAAQVAASTIITALCAPFLTV
WIKKRYDRKLNPAAAGG
>EECEBICD_02821 (2R)-sulfolactate sulfo-lyase subunit beta
MATMCTSTMWKANADVVTGNNIKQGKMIMSNETFLGFRRPDGRFGIRNYVLILPTSVCAN
KVAQDIARQVKGATWVNNDFGCCQVAGDARLTEKTLINVANNPNVGAIVVVGLGCEGAEP
LRIAEEITAFGKPTSCITIQEEGGTLKCQARGISLARDYAQQLSMQKPQQAPVSELLLAM
ECGGSDTTSGLASNPSCGVASDKLIRCGGSSILSETTEFIGAEHVMAKRAVTPEVGQQLI
DLVVGCEARAKALGEDIRGGQPTPGNIKGGLTTIEEKSLGCMHKAGHAPLQGVLEYADSP
THPGLWIMDTPGQDIESISGMVAGGAQIVIFTTGRGTPAGNPIAPVIKITGNKATWEMMQ
DNIDIDVSAIMSGEASITQMGEEIYQEILRVANGKTTKSEDLGHNEFSIYKIAPTF
>EECEBICD_02822 Altronate dehydratase
MKKNAVVIHPQDCVATAITALKAGETATMFIGEREISVVLHQDVPFGHKFAICDVPFHGE
VYKYGESIGRATQEIKSGDYVHVHNVESERGRGDWK
>EECEBICD_02823 Leucine--tRNA ligase
MQEQYRPEEIESKVQLHWDEKRTFEVTEDESKEKYYCLSMLPYPSGRLHMGHVRNYTIGD
VVARYQRMLGKNVLQPIGWDAFGLPAEGAAVKNNTAPAPWTYDNIAYMKNQLKTLGFGYD
WSREIATCTPEYYRWEQKFFTELYKKGLVYKKTSAVNWCPNDQTVLANEQVIDGCCWRCD
TKVERKEIPQWFIKITAYADELLRDLDKLDHWPDTVKTMQRNWIGRSEGVEITFDVKGYD
NTLTVYTTRPDTFMGATYLAVAAGHPLAQKAAANNAELAAFIDECRNTKVAEAEMATMEK
KGVDTGFKATHPLTGEEIPVWAANFVLMEYGTGAVMAVPGHDQRDYEFASKYGLTIKPVI
LAADGSEPDLSEQALTEKGVLFNSGEFDGLAFEAAFNAIADKLAEKGVGERKVNYRLRDW
GVSRQRYWGAPIPMVTLEDGTVLPTPEDQLPVILPEDVVMDGITSPIKADPEWAKTTVNG
MPALRETDTFDTFMESSWYYARYTCPQYQEGMLDSKAANYWLPVDIYIGGIEHAIMHLLY
FRFFHKLMRDAGMVTSDEPAKQLLCQGMVLADAFYYVGENGERNWVSPVDAIVERDEKGR
IVKAKDAAGHELVYTGMSKMSKSKNNGIDPQVMVERYGADTVRLFMMFASPADMTLEWQE
SGVEGANRFIKRVWKLVYEHTAKGPVAALNVDALSEDQKALRRDVHKTIAKVTDDIGRRQ
TFNTAIAAIMELMNKLAKAPQEGEQDRALLQEALQAVVRMLNPFTPHVCFTLWQELGGEG
DIDNAPWPVADEQAMVENTTLVVVQVNGKVRGKITVAVDATEEQVRERAGQEHLVAKYLD
GVTVRKVIYVPGKLLNLVVG
>EECEBICD_02824 LPS-assembly lipoprotein LptE
MRYLVTLLLSLAVLVTAGCGWHLRSTTQVPASMKTMILDSGDPNGPLSRAVRNQLRLNNV
NLLDKDTTRKDVPSLRLGTVTISQDTASVFQDGQTAEYQMVMTVNASVLIPGHDIYPIST
KVYRSFFDNPQMALAKDNEQAMIVQEMYDKAAEQLIRKLTSVRAADIQATKEEATADNET
AAPASTPARVSTTLSN
>EECEBICD_02825 DNA polymerase III subunit delta
MIRLYPEQLRAQLNEGLRAAYLLLGNDPLLLQESQDAIRLAAASQGFEEHHAFTLDPSTD
WGSLFSLCQAMSLFASRQTLVLQLPENGPNAAMNEQLATLSELLHDDLLLIVRGNKLTKA
QENAAWYTALADRSVQVSCQTPEQAQLPRWVAARAKAQNLQLDDAANQLLCYCYEGNLLA
LAQALERLSLLWPDGKLTLPRVEQAVNDATHFTPFHWVDALLMGKSKRALHILQQLRLEG
SEPVILLRTLQRELLLLVNLKRQSAHTPLRALFDKHRVWQNRRPMIGDALLRLHPAQLRQ
AVQLLTRTEITLKQDYGQSVWADLEGLSLLLCHKALADVFIDG
>EECEBICD_02826 Nicotinate-nucleotide adenylyltransferase
MGDMKSLQALFGGTFDPVHYGHLKPVETLANLIGLSRVIIMPNNVPPHRPQPEASSAQRK
YMLELAIDDKPLFTLDERELQRNAPSYTAQTLKAWREEQGPEAPLAFIIGQDSLLNFPTW
HDYDTILDNTHLIVCRRPGYPLEMTQAQHQQWLEQHLTHTPDDLHQLPAGKIYLAETPWL
NISATLIRERLEKGESCDDLLPENVLNYINQQGLYR
>EECEBICD_02827 Threonine-phosphate decarboxylase

MALFNSAHGGNIREAATVLGISPDQLLDFSANINPLGMPVSVKRALIDNLDCIERYPDAD
YFHLHQALARHHQVPASWILAGNGETESIFTVASGLKPRRAMIVTPGFAEYGRALAQSGC
EIRRWSLREADGWQLTDAILEALTPDLDCLFLCTPNNPTGLLPERQLLQAIADRCKSLNI
NLILDEAFIDFIPHEAGFIPALKDNPHIWVLRSLTKFYAIPGLRLGYLVNSDDAAVARMR
RQQMPWSINALAALAGEVALQDSAWQQATWHWLREEGARFYQALCQLPLLTVYPGRANYL
LLRCEREDIDLQRRLLTQRILIRSCANYPGLDSRYYRVAIRSAAQNERLLAALRNVLTGI
APAD
>EECEBICD_02828 Adenosylcobalamin/alpha-ribazole phosphatase
MRLWLVRHGETEANVAGLYSGHAPTPLTEKGIGQAKTLHTLLRHAPFDRVLCSELERARH
TARLVLEGRDVPQHILPELNEMYFGDWEMRHHRDLTHEDAESYAAWCTDWQNAVPTNGEG
FQAFTRRVERFISRLDAFSDCQNLLIVSHQGVLSLLIARLLAMPAASLWHFRVEQGCWSA
IDICEGFATLKVLNSRAVWRPE
>EECEBICD_02829 Ribosomal silencing factor RsfS
MQGKALQDFVIDKIDDLKGQDIIALDVQGKSSITDCMIICTGTSSRHVMSIADHVVQSSR
AAGMLPLGVEGENAADWIVVDLGDVIVHVMQEESRRLYELEKLWS
>EECEBICD_02830 Ribosomal RNA large subunit methyltransferase H
MKLQLVAVGTKMPDWVQTGFTEYLRRFPKDMPFELIEIPAGKRGKNADIKRILDKEGEQM
LAAAGKNRIVTLDIPGKPWDTPQLANELERWKQDGRDVSLLIGGPEGLSPACKAAAEQSW
SLSALTLPHPLVRVLVAESLYRAWSITTNHPYHRE
>EECEBICD_02831 Peptidoglycan D,D-transpeptidase MrdA
MKRQNSFRDYTAESALFVRRALVAFLGILLLTGVLIANLYNLQILRFTDYQTRSNENRIK
LVPIAPSRGIIYDRNGIPLALNRTIYQIEMMPEKVDNVQQTLDALRSVVDLNDDDIAAFK
KERARSHRFTSIPVKTNLTEVQVARFAVNQYRFPGVEVKGYKRRYYPYGSALTHVIGYVS
KINDKDVERLDRENKLANYAATHDIGKLGIERYYEDILHGQTGYEEVEVNNRGRVIRQLK
EVPPQAGHDIYLTLDLKLQQYIETLLAGSRAAVIVTDPRTGGVLSLVSMPSYDPNLFVDG
ISSKDYSGLLNDPNTPLVNRATQGVYPPASTVKPYVAVSALSAGVITRNTSLFDPGWWQL
PGSEKRYRDWKKWGHGHLNITKSLEESADTFFYQVAYDMGIDRLSEWMGKFGYGHYTGID
LAEERSGNMPTREWKQKRFKKPWYQGDTIPVGIGQGYWTATPIQMSKALMILINDGVVKV
PHLLMSTAENGKQVPWVQPHEPPVGDIHSGYWEIAKDGMYGVANRPNGTAHKYFASAPYK
IAAKSGTAQVFGLKANETYNAHKIAERLRDHKLMTAFAPYNNPQVAVAIILENGGAGPAV
GTIMRQILDHIMLGDNNTHLPAENPVVAAAEDQ
>EECEBICD_02832 Peptidoglycan glycosyltransferase MrdB
MTDNPNKKTFWDKIHIDPTMLLILLALLVYSALVIWSASGQDIGMMERKIGQIAMGLVVM
VVMAQIPPRVYEGWAPYLYIICIILLVAVDAFGAISKGAQRWLDLGIVRFQPSEIAKIAV
PLMVARFINRDVCPPSLKNTAIALVLIFMPTLLVAAQPDLGTSILVALSGLFVLFLSGLS
WRLIGVAIVLIAAFIPILWFFLMHDYQRQRVMMLLDPETDPLGAGYHIIQSKIAIGSGGL
RGKGWLHGPQSQLEFLPERHTDFIFAVLAEELGLVGILILLALYILLIMRGLWIAARAQT
TFGRVMAGGLMLILFVYVFVNIGMVSGILPVVGVPLPLVSYGGSALIVLMAGFGIVMSIH
THRKMLSKSV
>EECEBICD_02833 Endolytic peptidoglycan transglycosylase RlpA
MRKQLPVICVAAGIVLLAACTNDGGQQQTTVAPQPAVCNGPTVEISGAEPRYEPLNPTAN
QDYQRDGKSYKIVQDPSRFSQAGLAAIYDAEPGSNLTASGEMFDPMQLTAAHPTLPIPSY
ARITNLANGRMIVVRINDRGPYGTDRVISLSRAAADRLNTSNNTKVRIDPIIVAPDGSLS
GPGMACTTVAKQTYALPPRPDLSGGMGSASSAPAQPQGDVLPVSNSTLKSDDTTGAPVSS
SGFLGAPTTLAPGVLESNEPTPTPQTAPVSAPVTAPATATPVSAPAAAAPVSAPVSAPAA
AASGRFVVQVGAVSDQTRAQQYQQRLSQQFSVPGRVIQNGAVWRIQLGPFASKAEASALQ
QRLQTEAQLQSFIASAQ
>EECEBICD_02834 D-alanyl-D-alanine carboxypeptidase DacA
MKTTFSARFMQRMALTTALCAAFISTAHADDLNIKTMIPGVPQIDAESYILIDYNSGKVL
AEQNADERRDPASLTKMMTSYVIGQAMKAGKFKETDLVTVGNDAWATGNPVFKGSSLMFL
KPGMQVPVSQLIRGINLQSGNDACVAMADFAAGSQDAFVGLMNSYVNALGLKNTHFQTVH
GLDADGQYSSARDMALIGQALIRDVPNEYAVYKEKEFTFNGIRQLNRNGLLWDNSLNVDG
IKTGHTSKAGYNLVASATEGQMRLISAVMGGRTYKGRETESKKLLTWGFRFFETVNPLKA
GKEFASEPAWFGNTDRASLGVDKDVYLTIPRGRMKDLKASYVLNTAELHAPLQKNQVVGT

INFQLDGKTIEQRPLVVLQEIPEGNFFGKIIDYIKLMFHHWFG
>EECEBICD_02835 hypothetical protein
MKTKLNELLEFPTPFTYKVMGQALPELVDQVVEVVQRHAPGDYSPTVKPSSKGNYHSVSI
TINATHIEQVETLYEELGNIDIVRMVL
>EECEBICD_02836 Octanoyltransferase
MYQDKILVRQLGLQPYEAISQAMHNFTDMRDENSHDEIWLVEHYPVFTQGQAGKAEHILM
PGDIPVVQSDRGGQVTYHGPGQQVMYVLLNLKRRKLGVRDLVTLLEQTVVNTLAEIGIEA
HPRADAPGVYVGEKKICSLGLRIRRGCSFHGLALNVNMDLSPFLRINPCGYAGMEMAKIT
QWKEDATTDNIAPRLLANILALLNNPPYEYIAA
>EECEBICD_02837 putative HTH-type transcriptional regulator YbdO
MSDNNQAERQPADQHLQDKPQIFRTLRNIDLNLLTIFEAVYVHKGIVNAAKILNLTPSAI
SQSIQKLRTIFPDPLFIRKGQGVTPTTYATHLHEYISQGLESILGALDLTGSYDKQRTIT
IGTTPSVGALVMPVIYQAIRPRYPQLLLRNIPVDDAESQLSQFQTDLIINTHSYNNRSIQ
HHVLFSDTLEIVCRQHHPCLANAITEERLNSADHTFLMLEGQHLSLLRQRLQETFPDQQI
SFSSYNIFTIASLIASSDLLGLMPTRFYDLFSRCWPLERLPYAPVNSEQVEFSLHYNKLS
LRDPVLENVIDIIREAF
>EECEBICD_02838 Lipoyl synthase
MSKPIVMERGVKYRDADKMALIPVKNVVTERDALLRKPEWMKIKLPADSTRIQGIKAAMR
KNGLHSVCEEASCPNLAECFNHGTATFMILGAICTRRCPFCDVAHGRPVAPDAEEPQKLA
QTIADMALRYVVITSVDRDDLRDGGAQHFADCITAIRAKSPEIKIETLVPDFRGRMDRAL
DILNATPPDVFNHNLENVPRIYRQVRPGADYNWSLKLLERFKEAHPEIPTKSGLMVGLGE
TNAEIIEVMRDLRRHGVTMLTLGQYLQPSRHHLPVQRYVSPEEFDEMKAEALAMGFTHAA
CGPFVRSSYHADLQAKGMEVK
>EECEBICD_02839 Sec-independent protein translocase protein TatE
MGEISITKLLVVAALVVLLFGTKKLRTLGGDLGTAIKGFKKAMNDEDAGVKKDVDGSVQA
EKLSHKE
>EECEBICD_02840 Deaminated glutathione amidase
MFVAAGQFVVSSVWEENAQVCVSLMAQAAGRGVSLLVLPEGILARNDIDLDLPIRAAQPL
DGAFMTRLLEESAHNNMTTIFTILVPSTPGRAVNMLVALRAGHIVARYAKLHLYDAFSMQ
ESQSIDAGTVIAPVLDVEGVKVGLMTCYDLRFPDMALALALQGADVLALPTGWVRGPLKE
QQWSTLLAARALDTTCYMIAAGECGNRNIGQSRIIDPLGVTIAAAADRPALIVAEIFRER
IDQVREQLPLLQQRRFAPPQLL
>EECEBICD_02841 Putative fluoride ion transporter CrcB
MLQLLLAVFIGGGTGSVARWMLSMRFNPLHQAIPIGTLTANLLGAFIIGMGFAWFNRMTH
IDPMWKVLITTGFCGGLTTFSTFSAEVVFLLQEGRFGWALLNVLINLLGSFAMTALAFWL
FSAAAAR
>EECEBICD_02842 Cold shock-like protein CspE
MSKIKGNVKWFNESKGFGFITPEDGSKDVFVHFSAIQTNGFKTLAEGQRVEFEITNGAKG
PSAANVTAL
>EECEBICD_02843 Lipid A palmitoyltransferase PagP
MYVAMIIRKYFLIIALLVMPWLAIPSVSAADKGWFNTFTDNVAETWRQPEHYDLYVPAIT
WHARFAYDKEKTDRYNERPWGVGFGQSRWDDKGNWHGLYMMAFKDSFNKWEPIGGYGWEK
TWRPLEDDNFRLGLGFTAGVTARDNWNYIPIPVLLPLASIGYGPATFQMTYIPGSYNNGN
VYFAWMRFQF
>EECEBICD_02844 Putative cryptic C4-dicarboxylate transporter DcuD
MLTVIELLIGVVVIVGVARYIIKGYSATGVLFVGGLVLLIISALMGHKVLPASETSTGYT
ATDIVEYIKILLMSRGGDLGMMIMMLCGFAAYMTHIGANDMVVKLASKPLQYINSPYLLM
IAAYFVACLMSLAVSSATGLGVLLMATLFPVMVNVGISRGAAAAICASPAAIILSPTSGD
VVLAAKAAEMPLIDFAFKTTLPISIAAIIGMAIAHFFWQRYLDKKENISHEMLDVAEITT
TAPAFYALLPFTPIIGVLIFDGKWGPQLHIITILVICMLLAAVLEFVRGFNTQNVFSGLE
VAYRGMADAFAGVVMLLVAAGVFAQGLSTIGFIQSLISIATSFGSASIILMLVLVILTML
AAMTTGSGNAPFYAFVEMIPKLAHSSGINPAYLSIPMLQASNLGRTISPVSGVVVAVAGM
AKISPFEVVKRTSVPVIVGLLIVIIATEIMVPGASSAVTGG
>EECEBICD_02845 Transcriptional regulatory protein DpiA

MTEPLTLLIVEDETLLAEMHAEYIRHIPGFNQIWLAGNLAQARMMIDRFKPGLILLDNYL
PDGKGITLLHELMQSRYPGGVVFTTAASDMETVAEAVRSGAFDYLVKPIAYERLGQTLTR
YQQRRRMLASADSASQKQIDEMFNAYARGEPKGDLPTGIDALTLNAVMKLFADPTVRHTA
ETVAQALTISRTTSRRYLEYCASRHLIVAEIIHGKVGRPQRIYHGG
>EECEBICD_02846 Sensor histidine kinase DpiB
MSYQKNKKLFPFFRQLAFPLRIFLLILVVSVFIVAALAQYLSASFEDYLASHVRDMAMNQ
AKIIASNDSIIAAVKNRDYKRLAIIANKLQRGTDFDYVVIGDRHSIRLYHPNPEKIGYPM
QFTKPGALERGESYFITGKGSIGMAMRAKTPIFDNKGNVIGVVSIGYLVSKIDSWRLDFL
LPMAGVFVLLLVVLMLLSWFFAAHIRRQMLGMEPKQIARVVRQQEALFSSVYEGLIAVDP
EGHITAINRSARKMLGLPSPGRQWLGKLIAEVVNPADFFTCQIAERRQDVMANFNGLSVI
ANREAIRSGEELLGAIISFRSKDEIATLNAQLTQIKQYVESLRTLRHEHLNWMSTLNGLL
QMKEYDRVREMVQGESQAQQQLIDSLRGAFADRQVAGLLFGKVQRARELGLKMVIVPGSQ
LHQLPEGLDSTEFAAIVGNLLDNAFEASLRTQEGDNTIELFLSDEGDEVVIEVADQGCGV
PEALREKIFEQGVSTRTDEPGEHGIGLYLIASYVGRCGGVITLEDNDPCGTLFSLFLPKV
KKNDDRTINPIDR
>EECEBICD_02847 [Citrate [pro-3S]-lyase] ligase
MFGNNVFTRVKRSENKKMAEIAHFLKENDLSVDTTVEVFITVSRDDRLIACGGIAGNIIK
CVAISESVRGEGLALTLATELINLAWERHCTHLFIYTKTEYEALFKQCSFSTIASVPGVM
VLMENSATRLKRYAESLATLRHEGKKIGCIVMNANPFTHGHRYLIQQAAAQCDWLHLFLV
KEDTSRFPYEDRLDLVLKGTTDIPRLTVHRGSEYIISRATFPCYFIKEQSVINHCYTEID
LKIFRQYLAPALGITHRFVGTEPFCTVTAQYNLDMRFWLETPTLPAPPITLVEIERLCFQ
ETPISASWVRKLLVKHDLTAIAPLVPDATLRYLQGMVERHPGSAAARQKSPVLATGEK
>EECEBICD_02848 Citrate lyase acyl carrier protein
MKINQLAVAGTLESGDVMIRIAPLDTQDIDLQINSSVEKQFGEAIRATILEVLSRYDVRG
VQLNVDDKGALDCILRARLETLLARASGIAALPWEDRQ
>EECEBICD_02849 Citrate lyase subunit beta
MISASLQQRKTRTRRSMLFVPGANAAMVSNSFIYPADALMFDLEDSVALREKDAARRLVY
HALQHPLYRDVETIVRVNALDSEWGVNDLEAVVRGGADVVRLPKTDTAQDVIDIENEILR
IENACGREPGSTGLLAAVESPLGITRAVEIAHASERLIGIALGAEDYVRNLRTERSPEGT
ELLFARCAILQAARSAGIQAFDTVYSDANNEAGFLQEAAHIKQLGFDGKSLINPRQIELL
HNLYAPTRKEVAHARLVVEAAEAAAREGLGVVSLNGKMVDSPVIERARLVLSRAELSGIR
EE
>EECEBICD_02850 Citrate lyase alpha chain
MTQKNESQRQDRVAAWSRHAESELSAYQSAAKLDLQAQKPRDHKLCASLEEAIRRSGLRD
GMTLSFHHAFRGGDLTINLVMETIAKMGFKNLTLASSSLSDCHAPLVEHIRNGVVSRIYT
SGLRGPLAEEISRGLLAEPVQIHSHGGRVHLVQSGELNIDVAFLGVPSCDEFGNANGYTG
KACCGSLGYAMVDADSAKQVVMLTEQLLPYPHNPASIAQDQVDLIVQIEEVGDANKIGAG
ATRMTTNPRELLIARSAAEVIAHSGYFNEGFSLQTGTGGASLAVTRFLEDKMRSRNIVAN
FALGGITATMVDLHEKGLIRKLLDVQSFDRNAAQSLARNPNHIEISANQYANWGSKGASV
DRLDVVVLSALEVDTHFNVNVLTGSDGVLRGASGGHCDTAVAAALSIIVAPLVRGRIPTL
VDNVTTCVTPGSSVDILVTDHGIAVNPARPELAERLQAAGMKVVSIEWLRERAQLLTGQP
RPIEFTDRVIAVVRYRDGSVIDVVHQVKE
>EECEBICD_02851 Apo-citrate lyase phosphoribosyl-dephospho-CoA transferase
MRLFPELATCHDVSIPELLASRDERQARQRAWLTRHATPLVSFTVVAPGPIKDSALTRRI
FNHGVTALHTLAEEYGWTIREQAALASASGPEGLLAIDAPAQALKQATIALEQRYPLGRL
WDIDVLTAEGEILSRRHFALPARRCLLCGQSAAECARGKTHALTDLLIHMEALLHDADSR
QPD
>EECEBICD_02852 2-(5''-triphosphoribosyl)-3'-dephosphocoenzyme-A synthase
MMPIPANPTNASIQPQSLYDAWADLAWRAMLTEVNLSPKPGLVDRLNCGAHKDMALADFH
RSAEAIRHWLPRFMEYGASCTRLPPESVLAGLRPLGMACEAAMFRATAGVNTHKGSIFSL
GLLCAAIGRLYQLRQPIAAETLCATAADFCRGLTTRELRQNNLQLTAGQRLYQQLGLTGA
RGEAEAGYPLVIRHALPHYRALLAQGRDPELALLDTLLLLMSLNGDTNVASRGGADGLRW
LQQQAAVLLHQGGIRTPDDLVYLHRFDQQCIERNLSPGGSADLLIVTWFLAQISQVNH

>EECEBICD_02853 L-tartrate/succinate antiporter
MSLSKDNIWKLLAPLVVMGVMLLIPVPDGMPPQAWHYFAVFVAMIVGMILEPIPATAISF
IAVTICVIGSNYLLFDASELADPAFNASKQALKWGLAGFSSTTVWLVFGAFIFALGYEVT
GLGRRIALFLVKFMGKRTLTLGYAIVIIDILLAPFTPSNTARTGGTVFPVIKNLPPLFKS
FPNDPSARRIGGYLMWMMVISTSLSSSMFVTGAAPNVLGLEFVSKIAGVQISWLQWFLSF
LPVGIILLIVAPWLSYVLYKPEVTHSAEVAAWAGGELKNMGRLSRKEWTLIGLVLLSLGL
WVFGGKIINATAVGLLAVSLMLALHVVPWKDITRYNSAWNTLVNLATLVVMANGLTRSGF
IDWFANTMSTHLEGFSPDATVIVLVLVFYFAHYLFASLSAHTATMLPVILAVGKGIPGVP
MEQLCILLVLSIGIMGCLTPYATGPGVIIYGCGYVKSRDYWRLGAIFGVIYIAMLLLVGW
PILAMWN
>EECEBICD_02854 Ribonuclease I
MNTIWHYSPLLAALLTPIFAANADELQAQQYGDFTDYVLALSWQTGFCQSQHERRHREPD
ECRLQKEPANKADFLTVHGLWPGLPKSIAARGVDQRRWQRFGCATRPIPNLPEVKASRKC
SAPTPGLSPDIATALKEVMPGAGGIPVWSATNMPNMAPVSGLIPMPISAP
>EECEBICD_02855 Ribonuclease I
MIRLDKAIKDSALGQFLTDNYGKTVSRAEFDSVVAQMWGQDNVKAVKVNCHGNPAYLTEI
QFSLKASMINAPLSSASFQPQPHPGNCGKQFIIDKAGY
>EECEBICD_02856 Regulator of nucleoside diphosphate kinase
MSRPTIIINDLDAERIDRLLEQPAYADLPIADALNAELDRAQMCSPQEMPNDVVTMNSRV
KFRNLSDGETRVRTLVYPANMTDSSTQLSVMAPVGAALLGLRVGDTIHWELPGGASTHLE
VLELEYQPEAAGDFLR
>EECEBICD_02857 putative zinc-binding alcohol dehydrogenase
MKALTYHGPHHVKVENVPDPGIEQPDDIILRVTATAICGSDLHLYRGKIPQVKHGDIFGH
EFMGEVVETGHDVKNIRKGDRVVIPFVIACGDCFFCRLQQYSACENTNDGHGATMNKKQI
PPPAALFGYSHLYGGVPGGQAEYVRVPKGNVGPFKVPPLLSDDKALFLSDILPTAWQAAK
NAQIQQGSSVAVFGAGPVGLLTIACARLLGAEQIFVVDHHAWRLRFAEERYGAIPINFDD
ENDPAEKIIEQTAGHRGVDAVIDAVGFEAKGSLTETVLTNLKLEGSSGKALRQCIAAVRR
GGVVSVPGVYAGFIHGFLFGDAFDKGLTFKMGQTHVHAWLGELLPLIEKGLLKPEEIVTH
YMPFEEAARGYEIFEKREEECRKVILVPGATSAQAAQKQATGVINPMPGGVV
>EECEBICD_02858 Universal stress protein UP12
MYKTIIMPVDVFEMELSDKAIRHAEFLAQQDGVIHLLHVLPGSASMSLHRFAADVRRFEE
HLQHEAETRLQTMVGHFSIDPSRIKTHVRFGSVRDVVNEMGEELDADVVVIGSRNPSITT
HLLGSNASSVVRHATLPVLVVR
>EECEBICD_02859 hypothetical protein
MEKYELPLVFFTVLSQMSVGMALVLTWRTLRGEVEGQRFYWLATGLVLALASIAAILHLA
HPDRAYDALINLRHAWLSREILGATLFGAAVGVTFLAKGHKAMALIASVFGVLLVAVQGM
TYAAPAMVAIANGFTMLLFFITVWVMGCAAIPLLRLRPAVPALRQGIVVCTAVLIAAPLV
WLSGGTVMQMTARSWLASPFYLASLVCLALAFVASRHGDSRPKLLFVLLFVGVFLSRLVF
FGDTVSTIVNIGHLY
>EECEBICD_02860 Ion-translocating oxidoreductase complex subunit B
MRRAFLVNSDKCIGCRGCAMACKSFNQLEPDRFWRYVYPLDKDIYPHEERAFYSLACNHC
EHPACVAACPVEAYTKREDGVVVHNPERCIGCKNCIRNCPYGAPRFNEETRKAEKCSMCY
ERLDIGMNPACVNACPVGALSIIDLDSDPVPDNAVQYPPGFPHMPQLNPGTRFILARQPK
QPEDK
>EECEBICD_02861 Putative dimethyl sulfoxide reductase chain YnfE
MSNETGHLNRRSFLKGIVALGAVAALPGGLLTSRCALAQPPVPFNPKTYKIYRNACPRNC
YDTCSLKTWVKDGVITFVEGAPESTFTHGTPCVKGLSYPRRVYSPDRIKYPMAQDVRGSG
NWRRISWEEAMQRIAAKMLEIKKKDGSMLGLALTKYSGKFGVLNYAVEGMMSSLGYTTRF
AGTPCWPAGIDAQNYDMGDMWCNDPEDMVKAKYIIIWGANPAWCSMHSMKYIYQAREKGA
KVVVIDPLLSQTAAKADLYLRVRPGSDGALALGMARHLVDKGLVDQDFVNNDAHGYPEFE
AYLRNNVTVEWAAEICGLSADVIRQLAEEFTAVKPATVWIGYGMQRHVNGGANVRAIDAF
VAMSGNIGVEGGGARYGHLHTWGFNYNAMLQKPPVGAIGIPGAAGTTSEFGAAAGSDAVQ
YSDRSLNINQTAQGILEANDPPVRMLWVACKNPFAQDFDRSKMKKAFEKLEMVVCADQFF
NETVQHADIVLPVTTAFEEWNVDASYWHYWLSINQPAIKPMYEAKCDLEIATMLSRTINK

MAPGSCTFPQEFDHKRWLDQEFNDGMAKMFGISSWDDLLDGPKKAILPSSAAWYDRKFKT
PSGKYEFKSELAEKNGHTALPEYKPEAESKLPFHLFTPHVQFGLHSQFINLDWMQVFYPE
PFVYIHPSSAQKKGISENDLVKVFNGTGEVELRAKVTTNVPEDFLVMYEAWFPKRQYNVQ
NVVADTPADMGLMKTGAPGAAIHSQFADVVLIGKGESVA
>EECEBICD_02862 Chaperone protein TorD
MDSIYESLTELEKTGLTLRDFFNSHDSHSLKSAYVRLNPSAAVNLTDRAWLEAEYDFNAL
FVGPGKLLAAPFASVYLEDDALVMGKATLEIREFMAALGLSVNQESNIPDDHISCVLELT
TLLLANTRQTSPYRSTLTQYINNYLTKWVPLYIEKIKTHAQTTTLYTVADILFYWLDELK
REYQYE
>EECEBICD_02863 Alkyl hydroperoxide reductase subunit F
MLDTNMKTQLRAYLEKLTKPVELIATLDDSAKSAEIKELLAEIAELSDKVTFKEDNTLPV
RKPSFLITNPGSQQGPRFAGSPLGHEFTSLVLALLWTGGHPSKEAQSLLEQIRDIDGDFE
FETYYSLSCHNCPDVVQALNLMAVLNPRIKHTAIDGGTFQNEITERNVMGVPAVFVNGKE
FGQGRMTLTEIVAKVDTGAEKRAAEALNKRDAYDVLIVGSGPAGAAAAVYSARKGIRTGL
MGERFGGQVLDTVDIENYISVPKTEGQKLAGALKAHVSDYDVDVIDSQSASKLVPAAAEG
GLHQIETASGAVLKARSIIIATGAKWRNMNVPGEDQYRTKGVTYCPHCDGPLFKGKRVAV
IGGGNSGVEAAIDLAGIVEHVTLLEFAPEMKADQVLQDKVRSLKNVDIILNAQTTEVKGD
GSKVVGLEYRDRVSGDIHSVALAGIFVQIGLLPNTHWLEGALERNRMGEIIIDAKCETSV
KGVFAAGDCTTVPYKQIIIATGEGAKASLSAFDYLIRTKIA
>EECEBICD_02864 Alkyl hydroperoxide reductase C
MSLINTKIKPFKNQAFKNGEFIEVTEKDTEGRWSVFFFYPADFTFVCPTELGDVADHYEE
LQKLGVDVYSVSTDTHFTHKAWHSSSETIAKIKYAMIGDPTGALTRNFDNMREDEGLADR
ATFVVDPQGIIQAIEVTAEGIGRDASDLLRKIKAAQYVAAHPGEVCPAKWKEGEATLAPS
LDLVGKI
>EECEBICD_02865 Thiol:disulfide interchange protein DsbG
MIKRTLLLAMLPILAHAEELPAPVKAIEKQGITILKSFEAPGGMKGYLGKYQDMGVTIYL
TPDGKHAISGYMYNEKGENLSNALIEKEIYAPAGREMWQKMEKASWILDGKKDAPVVLYV
FADPFCPYCKQFWQQARPWVESGKVQLRTLLVGVIKPESPATAAAILAAKDPAKTWHDYE
ASAGKMKLEVPASIPPAQMKVINQNQQLMDDLGANATPAIYYMNKDKILQQVVGLPEKAQ
LDAMMGQP
>EECEBICD_02866 putative HTH-type transcriptional regulator YbdO
MANLYDLKKFDLNLLVIFECIYQHLSISKAAETLYITPSAVSQSLQRLRTQFNDPLFIRS
GKGITPTVTGINLHYHLENNLNSLEQTINIMNQSSLKKKFIIYSPQLLITQYVMKLVKYI
RKDPQVEIEHHDILTTDELPEDLLAYRKADIVVSMAPLNNRSIVCTHFNRLECILVCSEN
HPRLGDTATIDEILQESFTQLVSRATGMKEYHSLMDDVLGERIIGFRSKSLMTIANSISS
TELIGFLPQTFFDYYSSSIKLKKVTIPFTIAPIQFYLMYNRASLNNSGFAELIEHITKKH
>EECEBICD_02867 Phosphoadenosine phosphosulfate reductase
MSVYKVPLEQNVLEAAQERIMWTLETLPRVCVSFSGGKDSGLMLHLTATLARKMNKKIHV
LFIDWEAQFSCTIAYIESLREYYADVIERFYWVALPLTTQNSLSQYQPEWQCWQPGTDWV
RQPPEDAITDPAFFSFYQHGMTFEQFVRDFADWFSEKRPAAMLVGIRSDESYNRFAAIAN
SHKQRFADDKPWTTLAPKGHTWYIYPIYDWKTADIWTWFAKTGKPCNPLYNLMYQAGVPP
RYMRICEPFGPEQRQGLWLYHVIEPERWAAMCARVSGALSGGIYAGQDSHFYGHRKILKP
DHLNWEEYALLLLHSMPEVTAEHYRNKIAVYLQWYKKKGMHTISQTQHGDIGSRDIPSWR
RICKVLLNNDYWCRALSFSPTKPKNYQRYNERMKAKRQEWGILCNTDSQPK
>EECEBICD_02868 putative protein YbdM
MQHRLTTEITHFLSELPEEERIAAINEFRMAIHSVSPFRDEPVDCVLWVKNDHISPNDYN
PNNVAPPEKKLLLKSIEQDGFTQPIVVVKANTEEYEIVDGFHRHELGKGKAALKRRLKGY
LPITCLDRERHERMAATIRHNRARGRHQIHAMSEIVRELSLLGWDESKIGQELGMDADEV
LRLKQINGLQELFADRRFSRAWTVK
>EECEBICD_02869 Methionine aminotransferase
MRNNPLIPKSKLPNLGTTIFTQMSALAQKHQAINLSQGFPDFDGPRYLHERLAYHVAQGA
NQYAPMTGAQALREAIADKTAEIYGYRPDDVSDITVTAGATEALYAAITALVRAGDEVIC
FDPSYDSYAPAVALSGGVLKRIALTPPHFRVDWQAFSALLSERTRLVILNTPHNPTATVW
RQADIEALWQAIGEREIYVLSDEVYEHICFAAEGHASVLAHPQLRERAVAVSSFGKTFHM

TGWKIGYCVAPAAISAEIRKVHQYLTFCVNTPAQLALADMLRAAPEHYRALPDFYRKKRD
VLVNALAQSRLKVLPCEGTYFLLIDYSAVSTLNDVEFCQWLTEEVGVAAIPLSVFCAAPF
PHQLIRLCFAKQESTLPAAAERLCKL
>EECEBICD_02870 Hydroxycarboxylate dehydrogenase A
MNHTEIRVVTGPANYFSHAGSLERLTDFFTPEQLSHAVWVYGERAIAAARPYLPEAFERA
GAKHLPFTGHCSERHVAQLAHACNDDRQVVIGVGGGALLDTAKALARRLALPFVAIPTIA
ATCAAWTPLSVWYNDAGQALQFEIFDDANFLVLVEPRIILQAPDDYLLAGIGDTLAKWYE
AVVLAPQPETLPLTVRLGINSACAIRDLLLTSSEQALADKQQRRLTQAFCDVVDAIIAGG
GMVGGLGERYTRVAAAHAVHNGLTVLPQTEKFLHGTKVAYGILVQSALLGQDDVLAQLIT
AYRRFHLPARLSELDVDIHNTAEIDRVIAHTLRPVESIHYLPVTLTPDTLRAAFEKVEFF
RI
>EECEBICD_02871 hypothetical protein
MFDTLSKAGKYLGQAAKMMIGVPDYDNYVEHMRITHPDQTPMTYEEFFRERQDARYGGKG
GARCC
>EECEBICD_02872 Peptide transporter CstA
MNKSGKYLVWTALSVLGAFALGYIALNRGEQINALWIVVASVCVYLIAYRFYGLYIAKKV
LAVDPTRMTPAVRHNDGLDYVPTDKKVLFGHHFAAIAGAGPLVGPVLAAQMGYLPGMIWL
LAGVVLAGAVQDFMVLFVSTRRDGRSLGELVKEEMGATAGVIALVACFMIMVIILAVLAM
IVVKALTHSPWGTYTVAFTIPLAIFMGIYLRYLRPGRIGEVSVIGLVFLIFAIISGGWVA
ASPTWAPYFDFTGVQLTWMLVGYGFVAAVLPVWLLLAPRDYLSTFLKIGTIVGLAVGILI
MRPTLTMPALTKFVDGTGPVWTGDLFPFLFITIACGAVSGFHALISSGTTPKMLANEGQA
CFIGYGGMLMESFVAIMALVSACIIDPGVYFAMNSPMAVLAPAGTADVVASAAQVVSSWG
FAITPDTLHQIANEVGEQSIISRAGGAPTLAVGMAYILHGALGGMMDVAFWYHFAILFEA
LFILTAVDAGTRAARFMLQDLLGVVSPGLKRTDSLPANLLATALCVLAWGYFLHQGVVDP
LGGINTLWPLFGIANQMLAGMALMLCAVVLFKMKRQRYAWVALVPTAWLLICTLTAGWQK
AFSPDAKIGFLAIANKFQAMIDSGNIPPQYTESQLAQLVFNNRLDAGLTIFFMVVVVVLA
VFSIKTALAALKIDKPTANETPYEPMPENVDEIVTQAKGAH
>EECEBICD_02873 Proofreading thioesterase EntH
MIWKRHLTLDELNATSQNTLVAHLGIVYTRLGDEVLEAEMPVDARTHQPFGLLHGGASAA
LAETLGSMAGYLMTRDGQCVVGTELNATHHRAVSQGKVRGVCLPLHLGRQNQSWEITLFD
EQGRRCCTCRLGTAVMG
>EECEBICD_02874 2,3-dihydro-2,3-dihydroxybenzoate dehydrogenase
MACIDFSDKTVWVTGAGKGIGYATALAFVDAGARVIGFDREFTQENYPFATEVMDVADAA
QVAQVCQRVLQKTPRLDVLVNAAGILRMGATDALSVDDWQQTFAVNVGGAFNLFSQTMAQ
FRRQQGGAIVTVASDAAHTPRIGMSAYGASKAALKSLALTVGLELAGCGVRCNVVSPGST
DTDMQRTLWVSEDAEQQRIRGFGEQFKLGIPLGKIARPQEIANTILFLASDLASHITLQD
IVVDGGSTLGA
>EECEBICD_02875 Enterobactin synthase component B
MAIPKLQSYALPTALDIPTNKVNWAFEPERAALLIHDMQDYFVSFWGRNCPMMDQVIANI
AALRQYCKEHHIPVYYTAQPKEQSDEDRALLNDMWGPGLTRSPEQQKVVEALTPDEADTV
LVKWRYSAFHRSPLEQMLKDTGRNQLIITGVYAHIGCMTTATDAFMRDIKPFMVADALAD
FSREEHLMALNYVAGRSGRVVMTESLLPTPVPASKAALRALILPLLDETDEPLDDENLID
YGLDSVRMMGLAARWRKVHGDIDFVMLAKNPTIDAWWALLSRGVE
>EECEBICD_02876 Enterobactin synthase component E
MRIPFTRWPDEFARRYREKGYWQDVPLTDILTRHAGSDKTAVIEGERAFSYRQLNQAADN
LACSLRRQGIKPGETALVQLGNVPELYITFFALLKLGVAPVLALFSHQRTELNAYAMQIA
PTLVIADRQHTLFAGEDFLNTFVAEHRSVRVVLLRNDDGDHSLDAAMRQAAEDFTATPSP
ADEVAYFQLSGGTTGTPKLIPRTHNDYYYSVRRSNEICGFNEETRFLCAIPAAHNYAMSS
PGALGVFLAKGTVVLATDPSATLCFPLIEKHQINATALVPPAVSLWLQAIQEWGGNAPLA
SLRLLQVGGARLSATLAARIPAEIGCQLQQVFGMAEGLVNYTRLDDSPERIINTQGRPMC
PDDEVWVADADGNPLPPGEIGRLMTRGPYTFRGYFNSPLHNASAFDANGFYCSGDLISID
QDGYITVHGREKDQINRGGEKIAAEEIENLLLRHPAVIHVALVSMEDELLGEKSCAYLVV
KEPLRAVQVRRFLREQGVAEFKLPDRVECVASLPLTPVGKVDKKQLRQRLASRSPL
>EECEBICD_02877 Isochorismate synthase EntC

MDMSLAEDAQETMATLAPDRFFFMSPYRSFTTSGCFARYTEPAVAGDSPDSPFQQKLRQQ
FAEAKSQGIANPILVGAIPFDTRQPSSLFIPMAWQSFSRQQKQRTARYFTDHQSLTVTAR
KAIPEQDAFEAMVARAAMLTATPDVDKVVLSRLVDITTDVAVDSGALLERLVAQNPVSYN
FHVPLADGGVLLGASPELLLRKEGERFSSLPLAGSARRQPDDVLDREAGNRLLASQKDRH
EHELVTQAMKQILRDRSTELQLPSSPQLITTPTLWHLGTPFEGKANAGENALTLACLLHP
TPALSGFPHQVAKKLIAELEPFDRELFGGIVGWCDAEGNGEWVVTIRCAKLHGNQVRLFA
GAGIVPASSPVGEWRETGVKLSTMLNVFGLH
>EECEBICD_02878 Ferrienterobactin-binding periplasmic protein
MRLPAFYRWLLLVVGISISGISLAQDAGWPRQIQDSRGVHTLDHKPARIVSTSVTLTGSL
LAIDAPVVASGATTPNNRFADDQGFMRQWSDVAKARHVARLYIGGPNAETVAAQMPDLIL
ISATGGDSALALYDQLSAIAPTLVINYDDKSWQSLLTQLGEITGQEKQAAARIAEFEAQL
TTVKQRIALPPQPVSALVYTPAAHSANLWTPESAQGKLLTQLGFTLATLPRGLQTSKSQG
KRHDIIQLGGENLAAGLNGESLFLFAGDNNDVAALYANPLLAHLPAVQNKRVYALGTETF
RLDYYSATLLLNRLAALF
>EECEBICD_02879 Enterobactin exporter EntS
MNRQSWLLNLSLLKTHPAFRAVFLARFISIVSLGLLGVAVPVQIQMMTHSTWQVGLSVTL
TGGAMFIGLMVGGVLADRYERKKVILLARGTCGIGFIGLCVNALLPEPSLLAIYLLGLWD
GFFASLGVTALLAATPALVGRENLMQAGAITMLTVRLGSVISPMLGGILLASGGVAWNYG
LAAAGTFITLLPLLTLPRLPVPPQPRENPFIALLAAFRFLLASPLIGGIALLGGLVTMAS
AVRVLYPALAMSWQMSAAQIGLLYAAIPLGAAIGALTSGQLAHSVRPGLIMLVSTVGSFL
AVGLFAIMPVWIAGVICLALFGWLSAISSLLQYTLLQTQTPENMLGRMNGLWTAQNVTGD
AIGAALLGGLGAMMTPVASASVSGFGLVIIGLLLLLVLGELRRFRQTPPVSDAG
>EECEBICD_02880 Ferric enterobactin transport system permease protein FepD
MSCSFSVTRAFAVPGLLLLLSLAAVLSLVIGAKPLPAAVVLEAFTGVCQSADCTIVLDAR
LPRTLAGLLAGGALGLAGALMQTLTRNPLADPGILGVNNGASFAIVLGAALFGFSTPLEQ
LFMAFSGALIASLIVAFTGSQGGGQLSPVRLTLAGVALGAVLEGLTSGIALLNPEVYDQL
RFWQAGSLDIRSLQTLKVALAPVVIAGIVALFLSRALNSLSLGSDTATALGSKVARTQLI
GLLAITVLCGSATALVGPIAFIGLMMPHMARWLVGADHRWSLPVTLLATPALLLFADIIG
RLLVPGELRVSVVSAFIGAPVLIFLVRRKSNGGGL
>EECEBICD_02881 Ferric enterobactin transport system permease protein FepG
MFLSRRLIVSCLLLAAGCFAIGLWSLQSGAVPLETGQIIAALLGDAPRNITLVVTEWRLP
RVLMALLIGAALGVSGAIFQSLMRNPLGSPDVMGFNTGAWSGVLVAMVLFGQHLTAIALA
AMAGGIVTSLIVWLLAWRNGIETFRLIIIGIGIRAMLVAFNTWLLLQASLETALTAGLWN
AGSLNGLTWAKTWPSAPLIIVMLTGAALLVRRMRLLEMGDDSACALGVSVERSRLMLMLV
AVSLTAAATALAGPISFIALVAPHIARRLSGTARWGLTQSALCGALLLLAADACAQQLFM
PYQLPVGVVTVSLGGIYLIVLLIQESRKK
>EECEBICD_02882 Ferric enterobactin transport ATP-binding protein FepC
MTESVARLRGDQLTLGYGSYTVAKNLNVSIPDGHFTAIIGPNGCGKSTLLRTLSRLMTPV
DGHVWLDGEQIQRYASKEVARRIGLLAQNATTPGDITVQELVARGRYPHQPLFTRWRKED
ADAVASAMRATGITSLAAQSVDTLSGGQRQRAWIAMVLAQETSIMLLDEPTTWLDISHQI
DLLELLSDLNREKGYTLAAVLHDLNQACRYATHLIALREGNIVAQGAPKEIVTAELIEKI
YGLRCMIIDDPVAGTPLVVPLGRR
>EECEBICD_02883 Ferric enterobactin transport protein FepE
MPSLNVKQEKNQSLAGYSLPPTNSHEIDLFSLIEVLWQAKRRILATVFAFACVGLLLSFL
LPQKWTSQAIVTPAESVQWQGLERTLTALRVLDMEVSVDRGSVFNLFIKKFSSPSLLEEY
LRSSPYVMDQLKGAQIDEQDLHRAIVLLSEKMKAVDSNVGKKNETSLFTSWTLSFTAPTR
EEAQKVLAGYIQYISDIVVKETLENIRNQLEIKTRYEQEKLAMDRVRLKNQLDANIQRLH
YSLEIANAAGIKRPVYSNGQAVKDDPDFSISLGADGISRKLEIEKGVTDVAEIDGDLRNR
QYHVEQLAAMNVSDVKFTPFKYQLSPSLPVKKDGPGKAVIIILAALIGGMMACGGVLLRH
AMVSRKMENALAIDERLV
>EECEBICD_02884 Enterobactin synthase component F
MKLNAPAGAFRQLAAHMPEIPRADLALALVTLWLGRLCGRMDYAAGFIFMRRMGSAALTA

TGPVLNVLPLAINLHATEDLPTLAKRLAAQLKKMRRHQRYDAEQIVRDSGRAAGETPLFG
PVLNIKVFDYHLDLPGIQAQTHTLATGPVNDLELALFPDENGGLDIELLANAQRYDDATL
SRHALRLMALITQFADNPALRCGDAQMLLAEEQTQLAHLNNTAVTIPAATLSDLVAQQAQ
KTPEASALADAHYHFTYREMREQVVALAYALRERGVQPGDSVAVALPRSVFLTLALHGIV
EAGAAWLPLDTGYPDDRLRMMLEDAQPKLLITTQAQLARFHDIPGMEYLCYSQPLPVSDA
TPLGLSLPHHTAYIIFTSGSTGRPKGVMVGQTAIVNRLLWMQAHYPLTADDVVAQKTPCS
FDVSVWEFFWPFIAGAKLVMAEPEAHRDPLAMQRFFAQYGVTTTHFVPSMLAAFIASLTP
ASAGKSCASLTRVFCSGEALPTALCREWETLTNAPLHNLYGPTEAAVDVSWYPACGDELA
AVDGNSIPIGYPVWNTGLRILDAHMQPVPPGVAGDLYLTGIQLAQGYLGRPDLTASRFIA
DPFAPGERMYRTGDVARWLDSGAVEYLGRSDDQLKIRGQRIELGEIDRVMQTLPDVEQAV
AHACVFNQAAATGGDARQLVGYLVSHSGLPLDLPALQEKLRQKLPAHMVPVVLLQLAGLP
LSANGKLDRKALPLPDLTPRVKGRAPQSATEIAVAAAFSRLLGCEINDVESDFFALGGHS
LLAMKLAAQLSQTFNRQVTPGQVMVASDVAQLSKLLDTDDDERSRNLGFGPLLPLRESDG
PTLFCFHPASGFAWQFSVLSRYLSPSWSIMGIQSPRPAGPMQTATTLDEVCEHHLATLLA
RQPHGPYYLLGYSLGGTLAQGIAARLCARGETVAFLGLLDTWPPETQNWREKEANGLNPD
VLAEIERERAAFVAAQQGNASDALFTAIEGNYADAVRLLTTAHSVPFDGHATLFVADKTV
PEGVSPEQSWSPWIASLAIYRQPCAHVDIISPSAFETIGPIISELINK
>EECEBICD_02885 hypothetical protein
MTQRLPLVAAQPGIWMAEKLSDLPSAWSVAHYVELNGELDAALLAKAVAVGMQQADTLRM
RFTEENGEVWQWIDPEHTFGEPPIADLRDHPDPHHAALALMQADLRQNLRADSGKPLAFH
QLIRIDDTRWYWYQRYHHLLVDGFSFPAITRQIAAIYRAWQSDAPTPESPFTPLRMWSRN
ISAIARARPGSVTAPSGRSSAASCRRRRQCLPRRCRGGRRARIFYV
>EECEBICD_02886 Enterobactin biosynthesis protein YbdZ
MEFSNPFDNPQGQFYILQNAQRQFSLWPAACTLPAGWDVVCEPQSQDACQQWLNTRWTTL
NPAHYADKQEAK
>EECEBICD_02887 Enterochelin esterase
MASSHHPAGGEVITRLKTGALCARGLRIVLEAGVREPIVFQANQALYAQLNTSQQSIFWR
QVDGGHDALCWRGGLTQGLMLLWQPLIDTL
>EECEBICD_02888 Enterochelin esterase
MARIAGTDVWRWSTALSANWRGSYCFIPTERDDVFAAFAPGETPDRNALREGWRQLLPQA
IADPLNSQSWRGGRGHAVSALEMPDAPLQPGWDRPETPYSPPLMMQWHSARLGNSRRVWI
LTTGDEAPEERPLAILLDGQFWAENMPVWPALASLTHQRLLPGAVYLLIDAIDTHHRSQE
LTCNADFWLAVQQELLPQVRAVTPFSDDAGRTVVAGQSFGGLSALYAGLNWPTRFGCVLS
QSGSFWWPHRITPPEGKSLPG
>EECEBICD_02889 hypothetical protein
MKEALATGSKAWWRTKTGPEWIREKRRKLSGHFLVARPAGKRNTLPDTACLGLHHWRNRS
SSKRAASDDGAYCRNGCLALEYGA
>EECEBICD_02890 Ferrienterobactin receptor
MNKKIHSLTLLVNLGIYGAALPVMAEDKTDSAALTNEDTIVVTAAQQNLQAPGVSTITAD
EIRKNPPARDVSEIIRTMPGVNLTGNSTSGQRGNNRQIDIRGMGPENTLILIDGKPVTSR
NSVRLGWRGERDTRGDTAWVPPEMIERIEVLRGPAAARYGNGAAGGVVNIITKKGGSEWH
GSWNTYFNAPEHKDEGATKRTNFSLNGPLGGDFSFRLYGNLDKTQADARNINQGHQSERT
GSYADTLPAGREGVINKDINGVVRWDFAPLQSLELEAGYSRQGNLYAGDTQNTNTNQLVK
DNYGKETNRLYRQNYSLTWNGGWNNGVTTSNWVQYEHTRNSRMPEGLAGGTEGIFDPKAS
QKYADADLNDVTLHSEVSLPFDLLVNQNLTLGTEWAQQRMKDELSNSQTFMGGNIPGYSS
TNRSPYSKAEIFSLFAENNMELTDSTMLTPGLRFDHHSIVGDNWSPSLNLSQGLGDDFTL
KMGIARAYKAPSLYQTNPNYILYSKGQGCYATGAGTGIGCYMMGNDDLKAETSINKEIGL
EFKRDGWLAGITWFRNDYRNKIEAGTVPLQRINNGKTDVYQWENVPKAVVEGLEGTLNVP
VSDTVNWTNNVTYMLQSKNKETGERLSIIPQYTLNSTLSWQVRQDVSLQSTFTWYGKQEP
KKYDYQGNPVTGTDKQAVSPYSIVGLSATWDVTKNVSLTGGVDNLFDKRLWREGNAQTVR
DTQTGAYMAGAGAYTYNEPGRTWYMSINTHF
>EECEBICD_02891 hypothetical protein
MLTSHFPLPFAGHRLHIVDFDASSFREHDLLWLPHHDRLRSAGRKRKAEHLAGRIAAVHA
LREVGVRTVPGMGDKRQPLWPDGLFGSISHCATTALAVISQQRIGIDIEKIMSQHTATEL

APSIIDSDERQILQASSLPFPLALTLAFSAKESVYKAFSDRVTLPGFDSAKVTSLTATHI
SLHLLPAFAAMMAERTVRTEWFQRSNSVITLVSAITRFPHDRSAPASILSAIPR
>EECEBICD_02892 Putative glutamate--cysteine ligase 2
MALNDFHVSEPYTLGIELEMQVINPPGYDLSQDSSTLIDAVKPQLTAGEIKHDITESMLE
MATGVCRDIDQAAAQLSAMQHVILQAASEHHLGICGGGTHPFQKWQRQEVCDNERYQRTL
ENFGYLIQQATVFGQHVHVGCANGDDAIYLLHGLSHFVPHFIALSAASPYMQGSDTRFAC
ARLNIFSAFPDNGPMPWVSNWQEFAGLFRRLSYTTMIDSIKDLHWDIRPNPAFGTVEVRV
MDTPLTLDHAINMAGLIQATAHWLLTERPFKPQEQDYLLYKFNRFQACRYGLEGVITDAY
TGDRRRLADDTLRLLDNVTPSARKLGADSAIDALRLQVKKGGNEAQYMREFIADGGSLIG
LVQKHCEIWAGQ
>EECEBICD_02893 hypothetical protein
MKHPLESLMTAAGILLMALLSSLLLPAPSLGLALAQKLVGIFHLMDLNQLYTLLFCLWFL
LLGALEYYVIRFIWRRWFSLAD
>EECEBICD_02894 Multiple antibiotic resistance protein MarA
MTISAQVIDTIVEWIDDNLNQPLRIDDIARHAGYSKWHLQRLFMQYKGESLGRYVRERKL
KLAARDLRDTDQKVYDICLKYGFDSQQTFTRIFTRTFNLPPGAYRKEKHGRTH
>EECEBICD_02895 HTH-type transcriptional regulator BetI
MARPKSEDKKQALLEAATQAIAQSGIAASTAVIARNAGVAEGTLFRYFATKDELINTLYL
HLKQDLCQSMIMELDRSITDAKTMTRFIWNSYISWGLNHPARHRAIRQLAVSEKLTKETE
QRADDMFPELRDLCHRSVLMVFMSDEYRAFGDGLFLALAETTMDFAARDPARAGEYIALG
FEAMWRALTREEQ
>EECEBICD_02896 hypothetical protein
MDGQTLHACAKRFALELPFTEHCWPFGPQYDVFKVGGKIFMLFTEHHCRPVVNLKSDPQK
SLVNQQIYPSIAPGYHMNKKHWISVYAGEDITVSLLNDLINDSWNLVVDGLPKREQLRLR
PR
>EECEBICD_02897 Oxygen-insensitive NAD(P)H nitroreductase
MDIVSVALQRYSTKAFDPSKKLTAEEADKIKTLLQYSPSSTNSQPWHFIVASTEEGKARV
AKSAAGNYTFNERKMLDASHVVVFCAKTAMDDAWLERVVDQEDADGRFATPEAKAENDKG
RRFFADMHRVSLKDDHQWMAKQVYLNVGNFLLGVAAMGLDAVPIEGFDAEVLDAEFGLKE
KGYTSLVVVPVGHHSVEDFNAGLPKSRLPLETTLTEV
>EECEBICD_02898 Lipase 1
MRAGQAWLDGDLHYLSAKFSNIQRSITLGALRRVEEGETNGRLWGARLTSGYDFVMMPWL
TTGPMLQYAWDYSHVNGYSEKLNTSTSMRFGDQNAHSQVGSAGWRLDLRHSIIHSWAQIN
YRRQFGDDTYVANGGLKSTALTFSRDGKAQDKNWVDIAIGADFPLSATVSAFAGLAQTAG
LSDGNQTRYNVGFSARF
>EECEBICD_02899 Lipase 1
MTQKRTLLKYGILSLALAAPLSACAFDSLTVIGDSLSDTGNNGRWTWDSGQNKLYDEQLA
ELYGLALSPSSNGGSNYAAGGATATPELNPQDNTADQVRQWLAKTGGKADHNGLYIHWVG
GNDLAAAIARPAMAQQIAGNSATSAAAQVGLLLDAGAGLVVVPNVPDISATPMLLEAVIT
AGLGAAAPPALKAALEALAEGATPDFASRQQAIRKALLAAAATVSSNPFIQQLLVEQLLA
GYEAAAGQASALTDDYNRMEEKGLEQHGGNIARADINGLFKEILAHPQAFGLTNTVGMAC
PPGVSASACSSAMPGFNASQDYLFADHLHPGPQVHTIIAQYIQSIIAAPVQATYLNQSVQ
SMAQGSRTTLDSRYQQLRQGKPLSVRWACSADTAGDINVMIIMRPTGTVIIII
>EECEBICD_02900 Miniconductance mechanosensitive channel YbdG
MQELISQVEDLAGIEMNHTTSLVVIFGIIFLTAVVVHIILHWIVLRAFEKRASASSRLWL
QIITQNKLFHRLAFTLQGIIVNIQAALWLQKGSEAADILTTCAQLWIMTYALLSLFSLLD
VIFNLSQKWPAASQLPLKGIFQGIKLIGAIIVGILMISLLIGQSPAILISGLGAMAAVLM
LVFKDPILGLVAGIQLSANDMLKLGDWLEMPKYGADGAVIDIGLTTVKVRNWDNTITTIP
TWSLVSDSFKNWSGMSASGGRRIKRSINIDATSIHFLDDDEKQRLLTAQLLKPYLTSRHQ
EIDEWNKQLDAPESALNHRRMTNIGTFRAYLNEYLRHHPRIRKDMTLMVRQLAPDDHGLP
IEIYAFTNTVVWLEYESIQADIFDHIFAVVEEFGLRIHQTPTGSDIRALSGTLRH
>EECEBICD_02901 Phenylalanine-specific permease
MKNASTASGASVSDAASKNEPTLQRGLQNRHIQLIALGGAIGTGLFLGIGPAIQMAGPAV
LLGYGVAGIIAFLIMRQLGEMVVEEPVSGSFAHFAWKYWGPFAGFLSGWNYWVMFVLVGM

AELTAAGIYMQYWLPDVPTWVWAAAFFIIINAVNLVNVRLYGETEFWFALIKVLAIIGMI
GFGLWLLFSGHGGEHASIDNLWRYNGFFATGWHGLLLSLAVIMFSFGGLELIGITAAEAR
DPQKSIPKAVNQVVYRILLFYIGSLVVLLALYPWVEVKSNSSPFVMIFHNLDSNVVASAL
NFVILVASLSVYNSGVYSNSRMLFGLSVQGNAPKFLTRVSHRGVPVNSLILSGAITSLVV
LINYLLPQKAFSLLMALVVATLLLNWIMICLAHLRFRAAMRRKGRETQFKALFYPAGNYL
CIAFLALILVLMCTMDDMRLSAILLPVWIVFLFIAFTVLRRKAH
>EECEBICD_02902 hypothetical protein
MNKIIKRLEIIKSAIELEDEEIIRQQLIYLKNEPQDAVISAIAQAIEARRFSDAMQEIAA
WLQAQRALSTWQDPSIAASKLELKALEAQLRDLIDKRNARVQILDDFNDLYHLRLGPLMS
RILELRKQLAVSMQRKQEAEIKRREKDYQSCLQFISQAVDQLATLKQQWTGLNAASREAV
GIRQRIQQQTELITALLAEIRELEADFSHQDDSAFRQAQENAEQDYHQYREQQQEAQFRY
ARDQRLSADERNELKRLWRQASRLCHPDVVADELKEKAHQMMVQLNQARQNADLAAIRAL
LTQLQSGLEPMMASDRLNNLEHLRHKIRQLRTQIDALLKEITQLETENAWRLASSVADKE
AYFSEQERALTEIRNTLEAQVQQVEQELLSG
>EECEBICD_02903 Inner membrane protein RclC
MDKYLRLLSQGDRLGLTLIRLSIAIVFIWIGLLKFVPYEADSITPFVANSPFMSFFYEHP
EEYRQHLTHEGELKPEERAWQTANNTYAFSDGLGVVELIIAVLVLANPVSRWLGLAGGVL
AFLTPFVTLSFLITTPEAWVMPLGDAHYGFPYLSGAGRLVLKDTLMLAGAVMIMADSARS
LLLQRQ
>EECEBICD_02904 putative protein RclB
MLKVTTLIASLLAAPLAFSVSAQPLTDVEYISVSAVSATPSMLEDAIARLAQSKQASSWK
ITSMRIDNTGYATAILYK
>EECEBICD_02905 putative pyridine nucleotide-disulfide oxidoreductase RclA
MTQYQALIIGFGKAGKTLAATLAKTGWRVAIIEQSASMFGGTCINIGCIPTKTLVHDAER
EGDFSVAMQRKAAVVNFLRDKNFHNLADLDNVDVIEGRAEFIDNHTLRVFQADGERVLRG
EKIFINTGAESVIPAITGLTTTAGVFDSTGLLSLNQRPARLGILGGGYIGLEFASMFANF
GTKVTIFEAAPQFLPREDQDIAQAITHILQEKGVELILNANVQAVSSKEGAVQVETPEGA
HLVDALLVASGRKPATAGLQLQNAGVAVNERGGIIVDDYLRTTADNIWAMGDVTGGLQFT
YISLDDFRIVRDGLLGDGKRSTRDRQNVPYSVFMTPPLSRVGLTEEQARASGATVQVVTL
PVAAIPRARVMNDTRGVLKAVVDVNTQRIVGVSLLCVDSHEMINIVKTVMDADLPYTVLR
DQIFTHPTMSESLNDLFSLIK
>EECEBICD_02906 RCS-specific HTH-type transcriptional activator RclR
MDALSRLLTLNAPQGSIDKNCPLGGDWQLPHAAGELSVIRWHTVTQGEAQLEMPTGDAMT
LTPGKVVILPQNSAHRLRQSGEAPTHIVCGSLRLHTTSRYFLTALPEVLCLAPPLHSPAS
IWLNAAILLLQQESERHLPGADVLYSQQCATLFTLAVRDWLSQAGTAKSVLNLLLHPRLG
RVILHMLETPAHPWTVETLAQRVHMSRASFAQLFRDVSATTPLAVLTTLRLQIAAQTLSR
EALPVMVIAESVGYASESSFHKAFVREFGCTPGEYRKRVSALER
>EECEBICD_02907 Sensor histidine kinase CusS
MLRRAISNLLSNTMRYTPPGKTITLRIKEADDQIHIIVENPGTPIAPEHLPRLFDRFYHV
TPSRQRKGEGRGIGLAIVKSIVTAHQGKVSVTSDTRATRFILTLPKYLR
>EECEBICD_02908 putative copper-binding protein PcoE
MESRQLSATAHEMMNNGDAFAHQQMAQTQKTSAPATVENATSFSEMDGYEKAMVIHQSMN
NAHSFSHEIQAEEHHKQIKRN
>EECEBICD_02909 Prophage bactoprenol-linked glucose translocase
MLKLFAKYTSIGVLNTLIHWGVFAFCVYGMHTHQALANFSGFVIAVSFSFYANARFTFNA
STTTLRYMMYVGFMGTLSAVVGWMADKCSLPPLLTLVTFSAISLICGFIYSKFIVFRDAK
>EECEBICD_02910 Prophage bactoprenol glucosyl transferase
MKISLVVPVFNEEATIPIFYKTVREFEELKPYEVEIVFINDGSKDATESIINKIAASDPL
VIPLSFTRNFGKEPALFAGLDHATGDTVIPIDVDLQDPIEVIPHLIEKWQAGADMVLAKR
SDRSTDGRMKRKTAEWFYKLHNKISNPKIEENVGDFRLMSREVVENIKQMPERNLFMKGV
LSWVGGKTDIVEYARAERVAGDSKFNGWKLWNLALEGITSFSTFPLRIWTYIGLFIAGMS
FLYGAWMIIDKLIFGNNVPGYPSLLVSVLFLGGVQLIGIGILGEYIGRIYIETKKRPKYI
IKNEKKNG

>EECEBICD_02911 hypothetical protein
MVNNRLKMVIAILIVFSLVYSIGFITPMNSDDYTYALRELSLSSVKMHYLGWSGRVVSDT
ISTSLLKFFSPHIYNAINSAALTLMVLCWTMIPATLTKSSPSPYVMIFLFFLYFIANPAL
GQTNFWLVGSANYLWTNMFIAIYILISIYLSNGKKSNLILFVYAISSIFAGCSNENTSLV
VVLISVAYFFIMNRNKYLLIGVFGSAIGAGVLLLAPGNLSRASTIQDWYNQPLAWRVLEH
FSERLPSAMGAYWQVYIAFIILLISVVLSRNSSSKLMFGSFLFILGAIAANVAFLASPAM
PSRALNGALCFMILSISFVAHSAFTKFNKASIYLSVTTYAMAFLYFIPSYILYYSSIKSI
SKQTEIREEIIDRAKHNKQDQAIIPDYYFPPVLHAGPSLDTFNSEAMSRYYGIDLKITAP
GFFDYSRAFNFKPLNINAKICNNVYIKSLWIYKQQMDIKTFVIFEFNKNPADSLDEKTAM
FISFKTKDGKIINADVDKKTFQIDGRWLSGRAINDIDSNELESITSGTWDVRTGARTNEN
ITEIIK
>EECEBICD_02912 hypothetical protein
MGFGHMRILACIGQLPESGLMHYGSVGFFFGTDGALRLLAKKPDGAFVTYDM
>EECEBICD_02913 hypothetical protein
MSGKRYPEEFKIEAVRQVVERGHSVSSVATHLDITTHSLYARIKKYGSNSSTNKEIKSSQ
MIRPKSADSRKS
>EECEBICD_02914 hypothetical protein
MSRRGNCHDNAVAESFFQLLKRERIKEKIYGTRDEARSDIFDYIEMFYNSRRRHGSSEQM
PPAEYENLYHQRLRSVQVIRGDSQICSPCA
>EECEBICD_02916 hypothetical protein
MLRIAIKEQNSHFEHGLKIIMTRLANQWQQKIDFLPPEEIDNADIAFLALDDDWFSAGCY
QIPMHTQHQLRVIICNKCDKEKLMFRPCLYMLPHIYRKDDVEEITRKMILILHKRALRHS
VPSGICHYCTTRHFSVTERHLLKLIASGYHLSETGALLSLSEEQTKSLRRSIMRKLHVKT
EQQFLKYIRVNLHFLLSK
>EECEBICD_02917 hypothetical protein
MRSVPRRERHRRLRNAKDCACRYHSPTPQIFDRLELLDQQLNYALPVGIISQAIITTDNY
LGYSLSHYLFSGKPTAAFRSLDDISLWIEKGSLRQLIVDMEALPVSCIEALNQLRALSWQ
QSDIQIYLLVSDKTPAITQFIRMAGRFFVLSRRQNLASVREALLSAAKPRLSESFSRTDW
LMIETLAQGASLKEIARQQSVPYHRVVYRLKQLITLLNLPHRQSFLRLIQQLNVTFHDIF
>EECEBICD_02918 Virulence factors putative positive transcription regulator BvgA
MKPASVIIMDEHPIVRMSIEVLLGKNSNIQVVLKTDDSRTAIEYLRTYPVDLVILDIELP
GTDGFTLLKRIKSIQEHTRILFLSSKSEAFYAGRAIRAGANGFVSKRKDLNDIYNAVKMI
LSGYSFFPSETLNFISNTRTPKGGHHDMPLSNREVTVLRYLANGMSNKEIAEQLLLSNKT
ISAHKANIFSKLGLHSIVELIDYAKSHELL
>EECEBICD_02919 putative fimbrial-like protein SfmF
MIFRRVFIAIGCVLFSPLSQANSSLGEVNIELRGNVVDFTCAVVAGDSNKSVNLGTWPTK
QLHAAGDATQPVAFSLKLEGCPPGSASITFSGTPAPGTALLALADTAMAQKLAIEIRDGD
QRRLPLEQASKAVDIDNNGNATLKFYANYIALADGVQPGLANADATFLINYN
>EECEBICD_02920 putative fimbrial-like protein SfmH
MKIYSALLLAGTALFFTHPALATVCRNSNGTATDIFYDLSDVFTSGNNQPGQVVTLPEKS
GWVGVNATCPAGTTVNYTYRSYVSELPVQSTEGNFKYLKLNDYLLGAMSITDSVAGVFYP
PRNYIRMGVDSNVSQQMPFGVQDSKLVFKLKVIRPFINMVTIPRQTMFTVYVTTSTGDAL
STPVYTISYSGKVEVPQNCEVNAGQVVEFDFGDIGASLFSQAGAGNRPQGVTPQTKTIAI
KCTNVAAQAYLSMRLEAEKASGQAMVSDNPDLGFVVANSNGTPLTPNNLSSKIPFHLDDN
AAARVGIRAWPISVTGNKPAEGPFTARGYLRVDYD
>EECEBICD_02921 Outer membrane usher protein SfmD
MKKTTWFAGRFPGYVSPLSGVALSVLAALCPLTSRGESYFNPAFLSADTASVADLSRFEK
GNHQPPGIYRVDIWRNDEFVATQDIRFEAGAVGAGDKSGGLMPCFTPEWIKRLGVNTAVF
PVSDKGVDTTCIHLPEKIPGAEVAFDFASMRLNISLPQASLLNSARGYIPPEEWDEGIPA
ALINYSFTGSRGTDSDSYFLSLLSGLNYGPWRLRNNGAWSYSKGDGYHSQRWNNIGTWVQ
RAIIPLKSELVMGDSNTGNDVFDSVGFRGARLYSSDNMYPDSLQGYAPTVRGIARTAAKL
TIRQNGYVIYQSYVSPGAFAITDLNPTSSSGDLEVTVDEKDGSQQRYTVPYSTVPLLQRE
GRVKYDLVAGDFRSGNSQQSSPFFFQGTVIAGLPAGLTAYGGTQLADRYRAVVVGAGRNL

GDWGAVSVDVTHARSQLADDSTHQGQSLRFLYAKSLNNYGTNFQLLGYRYSTRGFYTLDD
VAYRSMEGYDYEYDSDGRRHKVPVAQSYHNLRYSKKGRFQVNISQNLGDYGSLYLSGSQQ
NYWNTADTNTWYQLGYASGWQGISYSLSWSWNESVGISGADRILAFNMSVPFSVLTGRRY
ARDTILDRTYATFNANRNRDGDNSWQTGVGGTLLEGRNLSYSVTQGRSSSNGYSGSASAS
WQATYGTLGVGYNYDRDQHDYNWQLSGGVVGHADGITFSQPLGDTNVLIKAPGAKGVRIE
NQTGVKTDWRGYAVMPYATVYRYNRVALDTNTMDNHTDVENNVSSVVPTEGALVRAAFDT
RIGVRAIITARLGGRPLPFGAIVRETASGITSMVGDDGQIYLSGLPLKGELFIQWGEGKN
ARCIAPYALAEDSLKQAITIASATCIRPSS
>EECEBICD_02922 putative fimbrial chaperone SfmC
MNVKAIPSVDKSHIEGKNVLQLAILSRIKLFVRPANLPQTPEDAPTLLKFSRVGNHLKIT
NPSAYYLTLVNISVGAKKIDNVMIAPKSDMQIPLPTGAQGSVTFQTVNDYGALTSATTAS
LG
>EECEBICD_02923 Type-1 fimbrial protein, A chain
MIRKGAALVGLVLMSPVIAQPVMVESGRVHLRGQLVNGGCAVATESQDLRVLMGQYRTNA
FTGPGSFAPVSVPFSLRLISCSAEVWRHVGIAFAGVTPAEDPHVFLASGEGIGNAGIGLA
LFDDQQRQIIPNTLPLHYTPILTSEMTLHFTARYRAISENMTPGRIHSEVWFTLVYP
>EECEBICD_02924 Type-1 fimbrial protein, A chain
MKHKLMTSTIASLMFVAGAAVAADPTPVSVSGGTIHFEGKLVNAACAVSTKSADQTVTLG
QYRTASFTAIGNTTAQVPFSIVLNDCDPKVAATAAVAFSGQADNTNPNLLAVSSADNSTT
ATGVGIEILDNTSSPLKPDGATFSAKQALVEGTNTLRFTARYKATATATTPGQANADATF
IMKYE
>EECEBICD_02925 Bifunctional protein FolD protein
MAAKIIDGKTIAQQVRSEVAQKVQARVAAGLRAPGLAVVLVGSNPASQIYVASKRKACDE
VGFVSRSYDLPETTSEAELLALIDTLNADNTIDGILVQLPLPAGIDNVKVLERIAPDKDV
DGFHPYNVGRLCQRAPRLRPCTPRGIVTLLERYNIDTYGLNAVVIGASNIVGRPMSMELL
LAGCTTTVTHRFTKDLRHHVEHADLLIVAVGKPGFIPGEWIKEGAIVIDVGINRLENGKI
VGDVVFDEAAARASYITPVPGGVGPMTVATLIENTLQACIEYHDPQGK
>EECEBICD_02926 putative protein YbcJ
MATFSLGKHPHVELCDLLKLEGWSESGAQAKIAIADGLVKVDGVVETRKRCKIVAGQTVS
FEGQSVNVVA
>EECEBICD_02927 Inner membrane protein YbcI
MPTVITHAAVPLCLGAGLGLKVIPPRLLFAGVVLAMLPDADVLAFKFGVAYGNVFGHRGF
THSLLFAFVLPLLCVLAGRRWFRAGQVRCWLFLTVSLLSHSLLDSITTGGKGVGWLWPWS
DERFFAPWQVIKVAPFALSSYITPYGHQVILSELLWVWLPGSILVLFLWVLKTHIKRNQY
E
>EECEBICD_02928 hypothetical protein
MNKMTFAAAVICFSFSHASYSEEMSATGWYAASSAVVSVGSSALVSGIILSPVLLPLNLS
IGSVVENKKQKTALLTTKSPDNKDIKMQVPLNVVKEGNLKAGDKVTLEKTQEGTGAYLKK
EGKVLAHMVNQDDAGLSSNQTVPAK
>EECEBICD_02929 hypothetical protein
MKRFAFALWLSAISLNAYADSANCHQKANTPESIAATMDQALQLKQQLNSQSDPVVILVR
QGQDMSSRHLTWSHAGYAMRQPNGDWRVYHNLNTCGTAESALYIQGLYEFLADDLVNQSI
AVLRPRSDIATALQTLLHSAIKLNLFHSPRYNLIAWPFSGPYQNSNGWLLEVFARANDAQ
IWSRNDARRWLQLQGYQPSIVSAGTFERLGAKLFTPNVFTDDQPAELLRKGNVGLNSGDS
VIRFIAHYSRAMPGCEHQNLGESVCVYLSPGAKK
>EECEBICD_02930 Cysteine--tRNA ligase
MLKIFNTLTRQKEEFKPIHAGEVGMYVCGITVYDLCHIGHGRTFVAFDVVARYLRFLGYK
LKYVRNITDIDDKIIKRANENGESFVALVDRMIAEMHQDFDALNILRPDSEPRATHHIQE
IIELTRTLIEKGHAYVADNGDVMFDVPTDPTYGQLSRQDLEQLQAGARVDVVDVKRNPMD
FVLWKMSKEGEPSWPSPWGEGRPGWHIECSAMNCKQLGNHFDIHGGGSDLMFPHHENEIA
QSTCAHDGEYVNYWMHSGMVMVDREKMSKSLGNFFTVRDVLKYYDAETVRYFLMSGHYRS
QLNYSEENLKQARASLERLYTALRGTDKSAAPAGGEAFEARFVEAMNDDFNTPEAYSVLF
DMAREVNRLKGEDMTAANAMASHLRKISGVLGLLEQEPDVFLQSGAQADDGEVAEIEALI
QQRLDARKAKDWAAADAARDRLAEMGIILEDGPQGTTWRRK

>EECEBICD_02931 Peptidyl-prolyl cis-trans isomerase B
MVTFHTNHGDIVIKTFDDKAPETVKNFLDYCREGFYNNTIFHRVINGFMIQGGGFEPGMK
QKATKEAIKNEANNGLKNTRGTLAMARTQAPHSATAQFFINVADNDFLNFSGESLQGWGY
CVFAEVVEGMDVVDKIKGVATGRSGMHQDVPKEDVIIENVTVSE
>EECEBICD_02932 UDP-2,3-diacylglucosamine hydrolase
MATLFIADLHLQTEEPAIVAGFLRFLAVEARQADALYILGDLFEAWIGDDDPNPLHREMA
VAIKSLVDSGVPCFFIHGNRDFLIGKRFARESGMTLLPQEKVLDLYGRNVLIMHGDTLCT
DDAGYQAFRAKVHNPWVQRLFLTLPLFIRRRIAARMRAGSKAANSSKSLDIMDVNAQTVV
AEMEKHRVQWLIHGHTHRPAVHELSANDQPAFRVVLGAWHHEGSMVKVTPDNVELIAFPL
>EECEBICD_02933 N5-carboxyaminoimidazole ribonucleotide mutase
MSSRNNPARVAIVMGSKSDWATMQFAAEIFEILDVPHHVEVVSAHRTPDKLFSFAETAEE
NGYQVIIAGAGGAAHLPGMIAAKTLVPVLGVPVQSAALSGVDSLYSIVQMPRGIPVGTLA
IGKAGAANAALLAAQILAQHDAELHQRIADWRKAQTDEVLENPDPRGTL
>EECEBICD_02934 N5-carboxyaminoimidazole ribonucleotide synthase
MKQVCVLGNGQLGRMLRQAGEPLGIAVWPVGLDAEPTAVPVQQSVITAEIERWPETALTR
ELARHPAFVNRDVFPIIADRLTQKQLFDKLGLATAPWQLLTSTDEWSGIFDRLGELAIIK
RRVGGYDGRGQWRLRADETGQLPDDCYGECIVERGIHFSGEVSLVGARAHDGSTVFYPLT
HNLHQDGILRTSVAFPQANAEQQEQAESMLSAIMQALNYVGVMAMECFITPEGLLINELA
PRVHNSGHWTQNGASISQFELHLRAITGLPLPAPVINAPSVMINLIGSELNYDWLKLPLV
HLHWYDKAVRPGRKVGHLNLTDSDTSRLSATLEALSPLLPGEYASGIIWAQSKLK
>EECEBICD_02935 Carbamate kinase 2
MKTLVVALGGNALLQRGEALTAENQYRNIADAVPALARLARSYRLAIVHGNGPQVGLLAL
QNLAWKAVEPYPLDVLVAESQGMIGYMLAQRLALEPDMPPVTTVLTRIKVSADDPAFLEP
EKFIGPVYSPEEQMALEATYGWHMKRDGKYLRRVVASPAPRQIIESAAIELLLKEGHVVI
CSGGGGVPVAGEGEGVEAVIDKDLAAALLAEQIAADGLIILTDADAVYEHWGTPQQRAIR
QASPDELAPFAKADGAMGPKVTAVSGYVKRCGKPAWIGALSRIDDTLAGRAGTCIRL
>EECEBICD_02936 hypothetical protein
MKRIHPLMASRQSADYRQPWQMSGVWRRSINFVAKSGELLTLHRRGSGFSPGGWILRGGD
FDALRDVLTDGEKPQSTAAGIQIGEFLLCEPERRCSLRVPRYLASPRLNATYMQRSEETG
LFGPLTVAAAQPLCPELRQFCHCFQSALAGAATDWRLWLGKGPGLTPSHDDTLTGMLLAA
WYFGAIDERVGRNFFAQSGSLDRATTLVSVSYLRYAAMGYFASPLLHFIHALRRDARTET
AIDGLLALGHTSGADTLLGFWLGQQIVKG
>EECEBICD_02937 hypothetical protein
MFTSVAQANAAVIEQIRRARPHWLDVKPASSLISVLNQGKTLLHAGPPMRWQEMTGPMKG
ACIGACLFEGWAKDEMSALALLEQGKVNFIPCHHVNAVGPMGGITSASMPMLVVENITDG
NRAYCNLNEGIGKVMRFGAYGEDVQQRLRWMRDVLMPVLSAALGRLERGLDLTAMMAQGI
TMGDEFHQRNIASSALLMRTLAPHIARLEHDKQQIAEVMDFLSVTDQFFLNLAMAYCKAA
MDAGAQIRAGSIVTAMTRNGDMFGIRVSGLGDRWFTAPVNTPQGLFFTGFSQDQANPDMG
DSAITETFGIGGAAMIAAPGVTRFVGAGGMEAAKSVSEEMAEIYLERNMQLQIPGWDFQG
ACLGLDIRRVVETGITPLINTGIAHKEAGIGQIGAGTVRAPLACFEQALEALAESMGVS
>EECEBICD_02938 Protein FdrA
MIHAFIKKGCFQDSVSLMIISRKLSESENVDDVSVMMGTPANKSLLETTGFWHDDFHGAT
PNDICVAIRTEAVDDGITQVIVQQLEEALQQLAQGSSGSQSLIQVRRWESACQKLPEANL
ALVSVAGEYAAELAEQALDRSLNVMIFSDNVTLDDEIRLKRRARDKGLLVMGPDCGTAMI
ANTPLAFANVMPEGNIGVIGASGTGIQELCSQIALAGEGITHAIGLGGRDLSAEVGGISA
LTALEILSADEKSQVLAFVSKPPADAVRQHIIAAMKATGKPVVALFLGFSSPVAREGNVW
FASTLDEAARLACLLSRVTSQRKEMVSTGGVIRGLYTGGTLAAEAAGLLAGYLGVAADTE
HHHGMMLDADGHQIIDLGDDFYTVGRPHPMIDPALRNQLIAGLGAQPQVRVLLVDVVIGF
GATADPAASLIQAWQKACAARASDQPLFAIATVTGTERDPQCRSQQIAALEDAGITVVDS
LPEATLLAAELIRPTLSSTHPSAPRLLEAVAVINAGLRSFALDLQAAGMPVVHYQWAPVA
GGNKKLARLLERLQ
>EECEBICD_02939 Ureidoglycolate dehydrogenase (NAD(+))
MKISRETLHQLIENKLYKAGLKREHAATVADVLVYADARGIHSHGAVRVEYYAERISKGG
TNREPTFRIENTGPCTAILHADNAAGQVAAKMGMEHAIEIAKKNGVAVVGISRMGHSGAI

SYFVRQAAREGLIGLSICQSDPMVVPFGGADIYYGTNPLAFAAPGEGDDIITFDMATTVQ
AWGKVLDARSRNESIPESWAVDKNGAPTHDPFAVNALLPAAGPKGYGLMMMIDILSGILL
GLPFGRQVSSMYEDLHAGRNLGQLHLVINPAFFSSCELFRKHISQTMQELNSVKPAPGFK
QVYYPGQDQDIKQKNADMNGIDIVDDIYQYLISDALYLKSYETKNPFAQ
>EECEBICD_02940 Allantoate amidohydrolase
MIKHFRHAIEETLPWLSSIGADPTGGMTRLLYSPEWLETQQQFKKRMAESGLETRFDDVG
NLYGRLCGTQFPEQVILSGSHIDTVVNGGNLDGQFGALAAWLAIDWLKATYGAPLRTVEV
VSMAEEEGSRFPYVFWGSKNIFGLANPEEVRHIQDAKGTGFVDAMQACGFTLPAAPLAAR
TDIRAFVELHIEQGCVLESNGQSIGVVNAIVGQRRYTVTLNGESNHAGTTPMGYRRDTVH
AFSRICSQSIEKAKQHGDPLVLTFGKVEPQPNTVNVVPRKTTFTIDCRHTDAAVLREFTE
QLENDMRAICDEMDISIDIDLWMDEAPVPMNAELVAALTRLCETEQLNYRIMHSGAGHDA
QIFAPRVPTCMIFVPSINGISHNPAERTNINDLAEGVKTLALMLHQLAWQK
>EECEBICD_02941 (S)-ureidoglycine aminohydrolase
MGYLNNVTGYRDDLLANRAIVKHGNYALLTPDGLVKNIIPGFENCDVTILSTPKLGASFV
DYLVTLHQNGGNQQGFGGEGIETFLYVITGNIEAKAEGKTFSLTQGGYLYCPPGEMMTFS
NAQTEDSQLFLYKRRYTPVEGHAPWLVSGNASQLERIHYEGMEDVILLDFLPKELGFDMN
MHILSFEPGASHGYIETHVQEHGAYILSGQGVYNLDNNWVPVKKGDYIFMGAYSLQAGYG
VGRGEAFSYIYSKDCNRDVMI
>EECEBICD_02942 Glycerate 3-kinase
MKIVIAPDSFKESLSADKCCQAIKAGFSTVFPDARYVCLPIADGGEGTVDAMVAATGGKR
VSVDVSGPMGEKVNGFYGLTGDGKTAIIEMAAASGLMLVAPEARNPLLASSFGTGELIRH
ALDAGIRHIILGIGGSATVDGGMGVAQALGVRFLDAQGMLLGAGGGNLSRLASIDLQGCD
PRISECRIEVACDVDNPLVGPRGAAAVFGPQKGATPEMVETLENGLRNYARVLHALTGRD
MSQIPGGGAAGGMGIAAIVFLEAEMKPGIEIVMQAVKLEEAVKEASLVITGEGRIDSQTA
GGKAPIGVASVAKRHHVPVIGIAGVLGDGVEVVHRHGIDAVFSILPRLAPLPEVLANGEQ
NLYHSACNIARVIKLGQDIGTR
>EECEBICD_02943 Putative purine permease YbbY
MFPVINRESVLSGFQWFFFIFCNTVVVPPTLLSAFHLPADNLLMLTQYAFLTTALACLVQ
AFCGHRRAIMEGPTGLWWGAILTITLGEASRGTPLNDIASSLSVGIAISAMVTIFIGISG
LGHRLARLFTPAVMVFFMLLLGAQLTSIFFKGMLGLPFGIADTSARIKLAPFGLAVAVMC
FILAMIIFLQQRISRYALLVGTVVGWGLWRLCFPSSHLPPGELSWRWFPLGSHGTLMPGI
IVTAVMTGLVNISNTYGAIRGTDIFYLHQGSGSSRYRRSFMISGLMTLVTVPFAVVPFSP
FVSSIGLLTQTGDSSRKSFICGSALCLLVAIITPLTRLFCAIPLAISSAVMLVSYLPLLY
SGLVFSQQITFTARNIYRLALPLFVGIFLMGLPPVYLQDLPLMIRPLLSNGLLVGILLAV
LMENLIPWERIK
>EECEBICD_02944 Allantoinase
MSFDLIIKNGTVILENEARVIDIAVQGGKIAAIGENLGEAKNVLDATGLIVSPGMVDAHT
HISEPGRTHWEGYETGTRAAAKGGITTMIEMPLNQLPATVDRETIELKFDAAKGKLTIDA
AQLGGLVSYNLDRLHELDEVGVVGFKCFVATCGDRGIDNDFRDVNDWQFYKGAQKLGEMD
QTVLVHCENALICDELGEEAKREGRVTAHDYVASRPVFTEVEAIRRVLYLAKAAGCRLHV
CHISSPEGVEEVTRARQEGQDVTCESCPHYFVLDTDQFEEIGTLAKCSPPIRDQENQKGM
WEKLFNGEIDCLVSDHSPCPPEMKAGNIMQAWGGIAGLQNCMDVMFDEAVQKRGMSLPMF
GKLMATNAADIFGLKHKGRIAPGKDADLVFIQPDSSYVLKNEDLEYRHKVSPYVGRTIGA
RITKTILRGDVIYDIEHGFPVPPKGQFILKHQQ
>EECEBICD_02945 Putative allantoin permease
MEHQRELYQQRGYSEDLLPKTETQRNWKAFNYFTLWMGSVHNVPNYVMVGGFFILGLSTF
NIMLAIIISALFIAAAMVMNGAAGSKYGVPFAMILRGSYGVRGALFPGLLRGGIAAIMWF
GLQCYAGSLAFLILIGKIWPGFLTLGGDFKLLGLSLPGLITFLIFWIINVGIGFGGGKVL
NKFTAILNPCIYIVFGGMAIWAISLVGIGPILDYLPSGVQKAEHSGFLFLVVINAVVAVW
AAPAVSASDFTQNAHSFRAQALGQTLGLIVAYILFAVASVCIIAGASIHYGMDTWNVLDI
VQRWDSLFASFFAVLVILMTTISTNATGNIIPAGYQIAALAPTKLNYKNGVMIASIISLL
ICPWKLMENQDSIYLFLDIIGGMLGPVIGVMLAHYFVVMRGKINLDELYTASGDYKYYDN
GFNLTAFSVTLVAVILSLGGKFIPFLEPLSRVSWFVGVIVAFVTYALLKKRTGFENTGEK
KLAG

>EECEBICD_02946 putative sulfoacetate transporter SauU
MSETKKSGIDYWKQIVVVMSLGWVAIWIYRTVLTPIYPEIQASLDNVSNAEIGAIASFYF
FAYCSMQIPCGILVDKFGQKIMLMAGFTLFIIGTLCIAKANGLTMIYIGSLMAGGGCASF
FSSAYSLSSANVPQARRALANAIINSGSAIGMGIGLIGSSVLVKNMSMAWQNVLYIVAAI
LVIMLCVFTLIIRGKAKSDSAQAEKQTQTVTEDEKRAPLFSGLLCSVYFLYFCTCYGYYL
IVTWLPSYLQTERGFDGGAIGLASALVAVVGVPGALFFSHLSDKFRNSKVKVILGLEIVA
AAMLAFTVLSPNTTMLMVSLTLYGLLGKMAVDPILISFVSEQASAKSLGRAFSLFNFFGM
SSAVVAPTLTGFISDVTGSKEISFVISACLVVTGTLIFAVVTLYKKKATQRIASA
>EECEBICD_02947 2-hydroxy-3-oxopropionate reductase
MKLGFIGLGIMGSPMAINLARAGHQLHVTTIGPVADELLSLGAVNVETARQVTEFADIIF
IMVPDTPQVEEVLFGEHGCAKTSLQGKTIVDMSSISPIETKRFAQRVNEMGADYLDAPVS
GGEIGAREGTLSIMVGGEQKVFDRVKPLFDILGKNITLVGGNGDGQTCKVANQIIVALNI
EAVSEALVFASKAGADPVRVRQALMGGFASSRILEVHGERMINRTFEPGFKIALHQKDLN
LALQSAKALALNLPNTATCQELFNTCAANGGSQLDHSAMVQALELMANHKLS
>EECEBICD_02948 Hydroxypyruvate isomerase
MLRFSANLSMLFTEYDFLERFDKAALSGFRGVEFMFPYDNDIEVLKRKLRDNNLEHTLHN
LPAGDWAAGERGIACIPGREEEFRDGVAAAIRYARALGNKKINCLVGKTPSGFSATEIHD
TLVENLRYAANMLAKEDILLLIEPINHFDMPGFHLTGTQQALALIKDIGSDNIKIQYDIY
HMQRMEGELTQTMTAWADKIGHLQIADNPRRGEPGTGEINYDFIFNVIKQSGYDGWVGCE
YKPLTTTEAGLSWINQYR
>EECEBICD_02949 Glyoxylate carboligase
MAKMRAVDAAMYVLEKEGITTAFGVPGAAINPFYSAMRKHGGIRHILARHVEGASHMAEG
YTRAAAGNIGVCLGTSGPAGTDMITALYSASADSIPILCITGQAPRARLHKEDFQAVDIE
AIAKPVSKMAVTVREAALVPRVLQQAFHLMRSGRPGPVLVDLPFDVQVAEIEFDPDMYEP
LPVYKPAASRVQIEKALEMLIQSERPVIVAGGGVINADAAPLLQQFAELTNVPVIPTLMG
WGCIPDDHPLMAGMVGLQTAHRYGNATLLASDMVFGIGNRFANRHTGSVEKYTQGRKIIH
IDIEPTQIGRVLCPDLGIVSDAKAALTLLIDVAQEMQKAGRLPCRKTWVDECQQRKRTLL
RKTHFDNVPVKPQRVYEEMNKAFGRDVCYVTTIGLSQIAAAQMLHVFKDRHWINCGQAGP
LGWTIPAALGVCAADPQRNVVAISGDFDFQFLIEELAVGAQFNIPYIHVLVNNAYLGLIR
QSQRAFDMDYCVQLAFENINSSEVNGYGVDHVKVAEGLGCKAIRVFKPEDIAPAFEQAKA
LMAQYRVPVVVEVILERVTNISMGSELDNVTEFEEVADSAKDAPTETCFMKYE
>EECEBICD_02950 HTH-type transcriptional repressor AllR
MTEVRRRGRPGQAEPTAQKGAQALERGIAILQYLERSGGSSSVSDISGSLDLPLSTTFRL
LKVLQAADFVYQDSQLGWWHIGLGVFNVGSAYIHNRDVLSVAGPFMHRLMLLSGETVNVA
IRNGNEAVLIGQKECKSMVRMCAPLGSRLPLHASGAGKALLYPLTEEELVGIVVNTGLRR
FTPTTLVDLPILLKNLEQAREQGYTVDQEEHVVGLNCIASAIYDDAGSVVAAISISGPAS
RLTEDRFISQGELVRDTAKDISTALGLKPPVA
>EECEBICD_02951 Ureidoglycolate lyase
MKLEVLPLDQKTFSAYGDVIETQERDFFHINNGLVERYHDLAKVEVLEQDRTLISINRAQ
PAAMPIVVHELERHPLGTQAFVPMNGEAFVVIVALGDDKPELSTLRAFISNGRQGVNYHR
NVWHHPLFAWQTVTDFLTVDRGGSDNCDVESIPTHELCFA
>EECEBICD_02952 HTH-type transcriptional activator AllS
MFDPETLRTFISVAETGSFSKAAERLCKTTATISYRIKLLEENTGVGLFFRTTRSVSLTA
AGSHLLSQAKDWLAWLDSMPDELRQVNDGVERQVNIVVNNLLYSPQAVASLLSWLNARYP
FTQFHFSRQIYMGVWDSLLYEGFSLAIGVTGTEPLANTFMLDPLGSVQWRFVMSADHPLA
HVSGPLTEAQLRRFPAINIEDSARTLTKRVAWRLPGQKEIIVPDMETKIAAHLAGVGIGF
VPQPLCQTLIDKNELVSCTIPTMRPPSPLSLAWHKFGGGKAVEDIVKLFTQRQPEIAGFL
SIFNTVRC
>EECEBICD_02953 tRNA 2-selenouridine synthase
MQDRQKAQDYRALLLADTPLIDVRAPIEFEQGAMPGAINLPLMMDDERAAVGTCYKRQGA
DAALALGHRLVCGDIRQQRLEAWKAAYQRFPNGYLCCARGGQRSHIVQRWLQETGIDCPL
IEGGYKALRQTAIQATWQLAQKPILLIGGCTGSGKTQLVRQQPNGVDLEGLARHRGSSFG
RTLNPQLSQASFENKLAVELLKINARQTLKRWVLEDEGRTIGANHLPECLRERMAQAPIA
VVEDPFALRLERLREEYFIRMHHDFTHAYGDEAGWQAYSEYLHHGLFAIRRRLGLQRFAE

LTDTLDRALAEQLSSGSTDGHMAWLVPLLNEYYDPMYRYQLEKKAANIVFRGTWQEVANW
LKAQ
>EECEBICD_02954 Methionine import system permease protein MetP
MDDLLPDLTLAFNETFQMLSISTVLAILGGLPLGFLIFVTDRHLFWQNRFIYLAASVLVN
IIRSVPFVILLVLLLPLTQLLLGNTIGPIAASVPLSVAAIAFYARLVDSALREVDKGIIE
AALAFGASPMRIICTVLLPEASAGLLRGLTITLVSLIGYSAMAGIVGGGGVGDLAIRYGY
YRYETEVMVVTVVALIVLVQVVQMLGDWLAKRADKRDRH
>EECEBICD_02955 Methionine import ATP-binding protein MetN
MIEIEKVCVDFTAGRGTLTRAVDDVSLHIAAGEIFGIVGTSGAGKSTLLRTLNALTRPSQ
GRVNVNGVEISALDGKALRQARQRIGMIFQHFNLMHTRTVAQNVAFSLKAAGWERSKIAP
RVAEILTLVGLADKANRFPVQLSGGQKQRVGIARAIANHPDVLLCDEPTSALDLETSATI
LALLRQINAQLGITIVLITHEMNVIKSICDRVAVMSGGKVVESGEVFDVFAHPQHAFTQQ
LVSHTLNLTLPERLREHLPGQLLKILFIGDSAEQPVLSEVAIKFGVAVNILHGKIEYIGE
RALGILVVQLTAPHNPTAVAAAVEHIRQRTAQVEVIRG
>EECEBICD_02956 D-methionine-binding lipoprotein MetQ
MGLRQSLRIAASTLLLACGLQFAHADSSPQTIVFGVAPGPYGDMVKQAIAPTLKEKGYKV
VVREFSDYVQPNMALANGSIDANLFQHTLYFDKFTADKGLKLSKLIVVPTAGMGFYSRKI
NSLDALKKGDIITLSNDPTNLARGLRFLQSLGLITIKDNIDPTKASERDIASNPKGLVFK
PLEAAQLPRTLDGVTGALVNGNFAVAAGLDLSSAIKQEHLDENLKNIIAVRSEDADKPFA
KDIVEAVKSPAYRAVIDDPKNIYSAFQKPEWMTATP
>EECEBICD_02957 hypothetical protein
MTIKITALAASIGAAVAFMPFATQAEITVLKQDPQAGNPLSRLNFTVGGSIRPQFQNMTG
NDGANGYKRNGFDGGTRFRFAADYYLFDDISWISYYELGVNIPAVFDWDNHYAKGATDTT
RRMLYTGFKSATWGTLTFGQQNSIYYDVVGVKTDIWDYDMIGQAPGNGINGDYDGSYRTR
KSLKYKKSIGNVDLYASYLFSDDYNANNGLNYKRKGGGSLGADYHLTDDLTWGTAWNYTR
AEMRGNGNKTYDQNIVGTALSWTPENWTIAAGGGWYQNFMTTKKVSVNDYFAGDAWGLEY
FVGYAFPIGQYAVKAIQPYFMGDRIEYVNGRDYLRTDNGVGITFQLDYGFRVDYEHVFTS
STDNLGDMNLVRLRYDF
>EECEBICD_02958 hypothetical protein
MIHDVNNFAPVFKTARTFLVAQIANSLIFSSTNH
>EECEBICD_02959 hypothetical protein
MIARWFWREWRSPSLIIVWLALSLAVACVLALGSISDRMEKGLSQQSREFMAGDRTLQSS
REVPKAWLEEARKRGLNVGEQLTFATMTFAGDTPQLASVKAVDSVYPMYGELQTRPGGVK
PQPGSVLLAPRLMALLNLKTGDTIDVGDVTLRIAGEVVQEPDSGFNPFQMAPRLMMNMAD
VAKTGAIQPGSRVMWRYKFGGTPQQLAGYESWLLPQLKPEHRWYGLEQDDGALGKSLERS
QQFLLLSALLTLLLAVAAVAVAMSHYCRSRYDLVAILKTLGAGRAQLRKLIIGQWLMVLG
LSAVTGGAIGLLFENILLVLLKPVLPADLPPASLWPWLWALGAMTVISLLVGLRPYRLLL
ATQPLRVLRRDVVARVWPLKIYLPVACAVVVALLAGLMGGSMLLWAVLAGAVILALLCGL
VGWMLLNVLRGMTLTSLPLRLAVSRLLRQPWSTLSQLSAFSLSFMLLALLLVLRGDLLDR
WQQQLPPESPNYFLINIASEQVAPLKAFLAEHQVIPQTFYPIVRARLTRINGNPTEGQQD
ESLNRELNLTWQDTRPAHNPLVAGHWPPKPGEVSMEEGLAKRLNVKLGDSVTFMGDTQAF
SAKVTSLRQVDWESLRPNFFFIFPSGALDGQPQSWLTSFRWENGNGMLTQLNREFPTVSL
LDIGAILKQVGQVLEQVSRALEVMVVLVTLCGMLLLLAQVQVGMRQRHQELVVWRTLGAG
KKLLRTTLWCEFAMLGLVAGLVAAIGAETALAILQINVFDFPWEPDWRLWGALPFCGALL
LSVCGGWLGVRLLKGKALFRQFSG
>EECEBICD_02960 Lipoprotein-releasing system ATP-binding protein LolD
MPAENSVEVHRLRKSVGQGEHELSILTGIELVVKRGETIALIGESGSGKSTLLAILAGLD
DGSSGEVSLVGKPLHQMDEEARAQLRAQHVGFVFQSFMLIPTLNALENVELPALLRGENS
GQSKAGAKALLEQLGLGKRLDHLPAQLSGGEQQRVALARAFNGRPDVLFADEPTGNLDRQ
TGDKIADLLFSLNREHGTTLILVTHDPALAARCDRRLRLVNGQLQEEA
>EECEBICD_02961 Thioesterase 1/protease 1/lysophospholipase L1
MPFLFLILLTFRAAAADTLLILGDSLSAGYRMAASAAWPSLLNDKWQNKTSVVNASISGD
TSQQGLARLPALLQQHHPRWVVVELGGNDGLRGFAPAQTEQTLRKIIQTVKAADAQPLLM
QIHLPANYGRRYNESFSAIYPKLAKEFDIPLLPFFMEEIYLKPQWMQDDGIHPNRDAQPF

IADWMAKQLTPFLS
>EECEBICD_02962 hypothetical protein
MQKSVLITGCSSGIGLDSALELKRQGFQILAGCRKPDDVARMNSMGFTGVLIDLDSPESV
DRAADEVIALTDNRLYGIFNNAGYGVYGPLSTISREQMEQQFSSNFFGAHQLTMRLLPAM
LPHGEGRIVMTSSVMGLISTPGRGAYAASKYALEAWSDALRMELRHTGIKVSLIEPGPIR
TRFTDNVNQTQRDKPVENPGIAARFTRDPDAVVAKVRHAFASDKPRLRYPVTLVTWAVMA
LKRLLPGRMLDKILQS
>EECEBICD_02963 Chaperedoxin
MSVQNIVNINESNLQQTLEQSMTTPVLFYFWSERSQHCLQLTPVLESLAAQYHGQFILAK
LDCDAEQMIAAQFGLRAIPTVYLFQNGQPVDGFQGPQPEEAIRALLDKVLPREEELKAQQ
AMQLMQEGNYIDALPLLKDAWQLSSQNGEIGLLLAEAQIALNRSEEAEAVLKTIPLQDQD
TRYQGLVAQIELLKQAADTPEIQQLQQQVAENPQDAALATQLALQLHQVGRNEEALELLF
SHLRKDLAAAEGQTRKTFQEILAALGTGDALASKYRRQLYALLY
>EECEBICD_02964 putative iron export permease protein FetB
MNEHNITNTSLALSMLLVVVAMLISHKEKLALEKDILWSVCRAVIQLIIVGYVLKYIFGV
NHAALTLLMVLFICFNAAWNAQKRSKYIDKAFLSSFIAITVGAGLTLTVLVLTGSIEFAP
MQVIPIAGMVAGNAMVAVGLCYNQLGLRFHSEQQQIQEKLSLGATPKMASAGLIRDSIRA
SLIPTIDSAKTVGLVSLPGMMSGLIFAGIDPVKAIKYQIMVTFMLLSTASLSTIIACYLT
YRKFYNSRHQLVVMPLKKS
>EECEBICD_02965 putative iron export ATP-binding protein FetA
MTERSMLLELKQVGYRTATTKIVNNISFTLQRGEFKLITGPSGCGKSTLLKMVASLISPD
IGMILFEGQDITTLSPEAYRQQVSYCAQTPALFGDTVYDNLIFPWQIRHQRPQPTAFADD
LARFELPDTILQKPIAALSGGEKQRIALIRNLQFLPKILLLDEITSALDEHNKRNVNDII
HRYVRDKQVAVLWVTHDKDEIQHADKVITLTPSAGEMQEARYERA
>EECEBICD_02966 Protein QmcA
MLILIPILIFVALVIVGAGVKIVPQGYQWTVERFGRYTKTLQPGLSLVVPFMDRIGRKIN
MMEQVLDIPSQEVISKDNANVTIDAVCFIQVIDAPRAAYEVSNLELAIINLTMTNIRTVL
GSMELDEMLSQRDSINTRLLHIVDEATNPWGIKVTRIEIRDVRPPAELISSMNAQMKAER
TKRAYILEAEGVRQAEILKAEGEKQSQILKAEGERQSAFLQAEARERSAEAEARATQMVS
EAIAAGDIQAINYFVAQKYTEALQQIGSANNSKVVMMPLDASSLMGAIAGISELVKDSAS
ERKKA
>EECEBICD_02967 Inner membrane protein YbbJ
MIAMILVHPHIFWLSLGGLLLAAEMLGGNGYLLWSGVAAVITGLTVWLLPLGWEWQGVLF
AALTLLAAWLWWRWLARRVREQKPTDSRLNQRGQQLVGRRFMLDAALVNGRGHMRVGDSS
WPVCADEDLRAGTHVEVIAVEGITLRIRAV
>EECEBICD_02968 HTH-type transcriptional regulator CueR
MNISDVAKKTGLTSKAIRFYEEKGLVTPPLRSENGYRTYTQKHLNELTLLRQARQVGFNL
EECGELVNLFNDPRRHSADVKKRTLEKVAEIERHISELQSMRDQLLALAESCPGDDSADC
PIIDNLSGCCHHKAQKPR
>EECEBICD_02969 Copper-exporting P-type ATPase
MSQTIDLTLDGLSCGHCVKRVKESLEQRPDVELADVTVTEAHVTGTASADALIETIKQAG
YGATLSHPKAKPLTESSIPSEALAAVPHELPVATADEESQQLLLSGMSCASCVTRVQHAL
QSVPGVTQARVNLAERTALVMGSASAADLVQAVEKAGYGAEAIEDDIKRRERQQETAIAT
MKRFRWQAIVALAVGIPVMVWGMIGDNMMVTGDNRSLWLAIGLITLAVMVFAGGHFYRNA
WKSLLNGTATMDTLVALGTGVAWLYSMSVNLWPQWFPMEARHLYYEASAMIIGLINLGHM
LEARARQRSSKALEKLLDLTPPTARVVTEDGEKSVPLADVQPGMLLRLTTGDRVPVDGEI
TQGEAWLDEAMLTGEPIPQQKGEGDSVHAGTVVQDGSVLFRASAVGSHTTLSRIIRMVRQ
AQSSKPEIGQLADKISAVFVPVVVAIALFSAAIWYFFGPAPQIVYTLVIATTVLIIACPC
ALGLATPMSIISGVGRAAEFGVLVRDADALQRASTLDTLVFDKTGTLTEGKPQVVAIKTF
NGVEEAQALRLAAALEQGSSHPLAHAILEKAGDDKLPQVNGFRTLRGLGVSGEAEGHQLL
LGNQALLNEQHVATDDMTAEITAQASQGSTPVLLAIDGKAAALLAVRDPLRSDSIAALER
LHNAGYRLVMLTGDNPTTANAIAKEAGIDEVIAGVLPDGKADAIKRLQSQGRQVAMVGDG
INDAPALAQADVGIAMGGGSDVAIEIAAITLMRHSLMGVADALAISRATLRNMKQNLLGA
FIYNSIGIPVAAGILWPFTGTLLNPVVAGAAMALSSITVVSNANRLLRFKPKA

>EECEBICD_02970 hypothetical protein
MFRKAVTLLFLLSPAVSYAGLFDSTPELKCGNDNAVTAAKEWIYNEALGRLQQDYIKEPS
TLFFDIPQTQYEQQLRAIPVQFSDVITQNPQAENANLRTCSATVAMGIPQPLFKLMKDLP
NTLFYISQGDGQVINNTVTWKQVNYNIQLADNNKDIVVTPVPKTDKLARSIYVMARMTVS
GDSIIKKKITH
>EECEBICD_02971 hypothetical protein
MKLLNQLKRLWRALRGTPNSWPAIDLSLPGGRHLHLVGSIHMGTRDMAPLPAKLVKKLRQ
ADALVVEADISGNETPFSNLPKCPPLVERLSAGQLSALEKRVSELGMPLIHFDNQPLWQI
AMVLQATQAQRLGLRPDYGIDYQLLQAAREMSLPVQELEGAKHQLELLCDLPDGGMALLD
DTLTHWHTNARLLQVMIGWWLEQPPTSVGASLPRTFSQPLYDVLMVKRNEAWRDALLALP
PGRYVVAVGALHLYGEGNLPQILK
>EECEBICD_02972 Cys-tRNA(Pro)/Cys-tRNA(Cys) deacylase YbaK
MTPAVKLLEKNKIPFNIHTYDHDPNETNFGDEVVRKLGLNADQVYKTLLVAVNGDMKQLA
VAVTPVAGQLDLKKVAKALGAKKVDMADPMVAQRTTGYLVGGISPLGQKKRLPTLIDAPA
RTFATIYVSGGKRGLDIELAADDLAAILGAKFADIARRD
>EECEBICD_02973 Protein UshA
MKFLKRGVALALLAAFALTTQPAQAYEKDKTYKITILHTNDHHGHFWRSEYGEYGEYGLA
AQKTLVDSIRKEVAQEGGSVLLLSGGDINTGVPESDLQDAEPDFRGMNLIGYDAMAVGNH
EFDNPLTVLRQQEKWAKFPFLSANIYQKSTGERLFKPWAIFTRQDIKIAVIGLTTDDTAK
IGNPEYFTDIEFRKPAEEAKVVIQELNMNEKPDVIIATTHMGHYDNGDHGSNAPGDVEMA
RSLPAGSLAMIVGGHSQDPVCMASENKKQVNYVPGTPCAPDKQNGIWIVQAHEWGKYVGR
ADFEFRNGEMKMVNYQLIPVNLKKKVTWDNGKSERVLYTPEIAENPQMLLLLTPFQNKGK
AQLEVKIGSVNGLLEGDRSKVRFVQTNMGRVILAAQIARTGADFGVMSGGGIRDSIEAGD
ITYKSVLKVQPFGNIVVYADMSGKEVVDYLTAVAQMKPDSGAYPQFANVSFVAKEGKLTE
LKIKGEPVDPAKTYRMATLSFNATGGDGYPRIDNKPGYVNTGFIDAEVLKEFIQQNSPLD
AAAFTPKGEVSWL
>EECEBICD_02974 Fosmidomycin resistance protein
MAMSEPTQPLAGAPAAAAKARTSFGILGAISLSHLLNDMIQSLILAIYPLLQAEFSLTFV
QIGLITLTFQLASSLLQPVVGYWTDKYPMPWSLPVGMCFTLSGLVLLAMAGSFGGVLLAA
ALVGTGSSVFHPESSRVARMASGGRHGLAQSIFQVGGNFGSSLGPLLAAVIIAPYGKGNV
AWFVLAALLAIVVLAQISRWYAAQHRIAKGKPKAIIANPLPRNKVVLAVSVLLLLIFSKY
FYMASISSYYTFYLMQKFGLSIQNAQLHLFAFLFAVAAGTVIGGPVGDKIGRKYVIWGSI
LGVAPFTLVLPYATLYWTGILTVIIGFILASAFSAILVYAQELLPGRIGMVSGLFFGFAF
GMGGLGAAVLGLIADHTSIYLVYKICAFLPLLGILTIFLPDNRHKS
>EECEBICD_02975 hypothetical protein
MVVFYKKIRSEGKKSTERKSDKYGMDKARWHQDVQAVKAYL
>EECEBICD_02976 Putative cation/proton antiporter YbaL
MHHATPLITTIVGGLVLAFILGMIANKLRISPLVGYLLAGVLAGPFTPGFVADTKLAPEL
AELGVILLMFGVGLHFSLKDLMAVKSIAIPGAVAQIAVATLLGMALSAVLGWSLMTGIVF
GLCLSTASTVVLLRALEERQLLDSQRGQIAIGWLIVEDLVMVLTLVLLPAVAGMVEKGDV
GIASLAVDMGITIGKVVAFIAIMMLVGRRLVPWIMARSAATGSRELFTLSVLALALGIAF
GAVELFDVSFALGAFFAGMVLNESELSHRAAHDTLPLRDAFAVLFFVSVGMLFDPLVLIQ
QPLAVLATLAIIVFGKSIAAFFLVRMFGHSPRTALTIAASLAQIGEFAFILAGLGMALNL
LPQAGQNLVLAGAILSIMLNPVLFTLLEKYLAKTETLEEQTLEEAIEEEKQIPVDICNHA
LLVGFGRVGSLLGEKLLAAGIPLVVIETSRTRVDELRERGIRAVLGNAANEEIMNLAHLD
CARWLLLTIPNGYEAGEIVASAREKSPDIEIIARAHYDDEVKYITERGANQVVMGEREIA
RAMLELLETPPAGEVVAS
>EECEBICD_02977 Inosine-guanosine kinase
MCYHAALRYSSVSTIMKFPGKRKSKHYFPVNARDPLLQQIQPEQETNASWVVGIDQTLVD
IEAKVDDDFITRYGLSAGHSLVIEDEVAEKLYQELTRENLITHQFAGGTIGNTMHNYSVL
ADDRSVLLGVMCSNIEIGSYAYRYLCNTSSRTDLNYLQAVDGPIGRCFTLIGESGERTFA
ISPGHMNQLRAESIPEAVIAGASALVLTSYLVRCKPGEPMPDATMKAIEYAKKHNVPVVM
TLGTKFVIADNPQWWQAFLKENVSILAMNEEEAEALTGENDPLLAADKALDWVDLVLCTA
GPIGLYMAGFTEEEAKRKTQHPLLPGAIAEFNQYEFSRAMRHKDCINPLRVYSHIAPYMG

GPEKIMNTNGAGDGALAALLHDITANSYHRSNVPNSSKHKFTWLTYSSLAQVCKYANRVS
YQVLNQHSPRLTRGLPEREDSLEESYWDR
>EECEBICD_02978 Acetyl esterase
MKPENKIPVLTRLSDEMKAVVNFQQPGLPPWPADGDIETQRQYYLLERRFWNADAPSMTT
RTCAVPTPYGDVTTRLYSPQPTSQATLYYLHGGGFILGNLDTHDRIMRLLARYTGCTVIG
IDYSLSPQARYPQAIEETVAVCSYFSQHADEYSLNVEKIGFAGDSAGAMLALASALWLRD
KHIRCGNVIAILLWYGLYGLQDSVSRRLFGGAWDGLTREDLDMYEKAYLRNDEDRESPWY
CLFNNDLTRDVPPCFIASAEFDPLIDDSRLLHKTLQAHQQPCEYKMYPGTLHAFLHYSRM
MTIADDALQDGARFFMARMKTPR
>EECEBICD_02979 Ferrochelatase
MRQTKTGILLANLGTPDAPTPEAVKRYLKQFLSDRRVVDTPRLLWWPLLRGVILPLRSPR
VAKLYQSIWMDGGSPLMVYSREQQQALAARLPDTPVALGMSYGSPSLESAVDELLASDVD
HIVVLPLYPQYSCSTVGAVWDELGRILARKRRIPGISFIRDYADDSAYIDALAKSARESF
ARHGEPDVLLLSYHGIPQRYADEGDDYPQRCRDTTRELVSALGLPPEKVMMTFQSRFGRE
PWLTPYTDETLKMLGEKGTGHIQVMCPGFAADCLETLEEIAEQNREIFLEAGGKKYAYIP
ALNATPEHIDMMLKLTAPYR
>EECEBICD_02980 Adenylate kinase
MRIILLGAPGAGKGTQAQFIMEKYGIPQISTGDMLRAAVKSGSELGKQAKDIMDAGKLVT
DELVIALVKERIAQEDCRNGFLLDGFPRTIPQADAMKEAGIVVDYVLEFDVPDELIVDRI
VGRRVHAASGRVYHVKFNPPKVEGKDDVTGEDLTTRKDDQEETVRKRLVEYHQMTAPLIG
YYQKEAEAGNTKYAKVDGTQAVADVRAALEKILG
>EECEBICD_02981 Chaperone protein HtpG
MKGQETRGFQSEVKQLLHLMIHSLYSNKEIFLRELISNASDAADKLRFRALSNPDLYEGD
GELRVRVSFDKDKRTLTIADNGVGMNRDEVIDHLGTIAKSGTKSFLESMGSDQAKDSQLI
GQFGVGFYSAFIVADKVTVRTRAAGDKPENGVFWESAGEGEYTVADITKNDRGTEITLHL
REGEDEFLDDWRVRSIISKYSDHIALPVEIEKREEKDGETVISWEKINKAQALWTRNKSE
IKDDEYNEFYKHIAHDFTDPLTWSHNRVEGKQEYTSLLYIPSQAPWDLWNRDHKHGLKLY
VQRVFIMDDAEQFMPNYLRFVRGLIDSNDLPLNVSREILQDSTVTRNLRSALTKRVLQML
EKLAKDDAEKYQTFWKQFGLVLKEGPAEDHANQEAIAKLLRFASTHTDSSAQTVSLEDYV
SRMKEGQEKIYYITADSYAAAKNSPHLELLRKKGIEVLLLSDRIDEWMMNYLTEFDGKAF
QSVAKADESIEKLADEVDENAKEAEKALEPFVERVKTLLGDRVKEVRLTHRLTDTPAIVT
TDADEMSTQMAKLFAAAGQSVPEVKYIFELNPDHVLVKRTADTEDEAQFKEWVELLLDQA
LFAERGTLEDPNQFIRRMNQLLVS
>EECEBICD_02982 Recombination protein RecR
MQTSPLLTQLMEALRCLPGVGPKSAQRMAFTLLQRDRSGGMRLAQALTRAMSEIGHCADC
RTFTEQDVCNICSNPRRQENGQICVVESPADIYAIEQTGQFSGRYFVLMGHLSPLDGIGP
DDIGLDRLEQRLASEKISELILATNPTVEGEATANYIAELCAEAGVEASRIAHGVPVGGE
LEMVDGTTLSHSLAGRHKIIF
>EECEBICD_02983 Nucleoid-associated protein YbaB
MFGKGGLGNLMKQAQQMQEKMQKMQEEIAQLEVTGESGAGLVKVTINGAHNCRRVEIDPS
LLEDDKEMLEDLVAAAFNDAARRIEETQKEKMASVSSGMQLPPGFKMPF
>EECEBICD_02984 DNA polymerase III subunit tau
MSYQVLARKWRPQTFADVVGQEHVLTALANGLSLGRIHHAYLFSGTRGVGKTSIARLLAK
GLNCETGITATPCGVCDNCREIEQGRFVDLIEIDAASRTKVEDTRDLLDNVQYAPARGRF
KVYLIDEVHMLSRHSFNALLKTLEEPPAHVKFLLATTDPQKLPVTILSRCLQFHLKALDV
EQIRHQLEHILNEEHIAHEPRALQLLSRAADGSLRDALSLTDQAIASGDGQVSTQAVSAM
LGTLDDDQALSLVEAVVDANGERVMSLINEAAARGIEWEALLVEMLSLLHRIAMVQLSPA
ALGSDMAAIEQRMRELARTVPPGDLQLYYQTLLIGRKELPWAPDRRMGVEMTLLRALAFH
PRMPLPEPETPRQSFAPVAPTAVMTPPQVQQSSAPAPQTSPAPLPASTSQVLAARNQLQR
AQGVTKTKKSEPAAASRARPVNNSALERLASVSERVQARPAPSALETAPVKKEAYRWKAT
TPVVQTKEVVATPKALKKALEHEKTPELAAKLAAEAIERDPWAAQVSQLSLPKLVEQVAL
NAWKEQNGNAVCLHLRSTQRHLNSSGAQQKLAQALSDLTGTTVELTIVEDDNPAVRTPLE
WRQAIYEEKLAQARESIIADNNIQTLRRFFDAELDEESIRPI
>EECEBICD_02985 Adenine phosphoribosyltransferase

MTATAQQLEFLKNSIKSIQDYPKPGILFRDVTSLLEDPKAYALSIELLVERYKNAGITKV
VGTEARGFLFGAPVALGLGVGFVPVRKPRKLPRETIAETYELEYGTDQLEIHVDAIKPGD
NVLVVDDLLATGGTIEATVKLIRRLGGKVTDAAFIINLFDLGGEQRLEKQGITCYSLVPF
PGH
>EECEBICD_02986 Inner membrane protein YbaN
MQRTILIIIGWLAVVLGTLGVVLPLLPTTPFILLAAWCFARSSPRFHAWLLYRSWFGGYL
RHWQRYRAMPPGAKPRAIALILLTFGISLWLVNMMWVRVLLLVILACLLIFMWRIPVIDE
KQQKR
>EECEBICD_02987 Primosomal replication protein N''
MLLQTLEERLATLRQRCAPLAQHATLSARFDRHLFRTRSTLLQGYLEEADANLAALRQAV
KHEQLPQVAWLAEHLASQLEAISHETAAWSLRQWDAAAPGLGRWQRRRIQHQEFERRLLA
MTQERKIRLAQATGLVEQQTLQKEVEIYEGRLARCRHALEKIENVLARLTR
>EECEBICD_02988 hypothetical protein
MSLENAPDEVKLAVDLIVLLEENRLPARTVLRALEIVMRDYENKLKSTEDDSQTE
>EECEBICD_02989 ISNCY family transposase ISRor2
MERLPTTPHDAVFRQMLMQKEVARDFLAIHLPEAFLAICDLDSLKLESGSFVEDNLRSRY
SDILYSLHTQHGPGYVYALIEHQSKSDRLMAFRLMRYAIAAMQRHLDAGHDTLPLVVPIL
FYHGPESPWPYSLNWHNMFVKPDMAKALYSCEFALVDLTTMPDNQLLQHRRIAMLELLQK
HIRQRDLSELLDPLITLLTQDHLTDTQLSVLINYMLKAGNAAEPGALIRQLAQGAPQYKE
QLMTIAEWLEEKGRTEGLQKGLQKGLEQGLAQGREAEARAIARKMLANGLEPGLIASVTG
ITPEELSTLSH
>EECEBICD_02990 Mechanosensitive channel MscK
MTMLQLYKRSQHFVFITISVLIILLSCQSLAFARGQTNGDLPSKADVQNQLDTLNKQKDL
SAQDKLVQQDLIDTLATLEKIERVKEETVQLRQKVAQAPEKMRQATAALNALSDVDNDDE
MRKTLSALSLRQLELRVAQVLDDLQNSQNDLAAYNSQLVSLQTQPERVQNAMYTASQQIQ
QIRNRLDGNNVGEAALRPSQQVLLQAQQALLNAQIDQQRKSLEGNTVLQDTLQKQRDYVT
ANSNRLEHQLQLLQEAVNSKRLTLTEKTAQEAISPDETARIQANPLVKQELDINHQLSQR
LIVATENGNMLMQQNIKVKNWLDRALQSERNIKEQIAVLKGSLLLSRILYQQQQTLPSAD
ELEDMTNRIADLRLEQFEINQQRDALFQSDAFVDKLEEGHTSEVNDEVHDALLQVVEMRR
ELLDQLNKQLGNQLMMAINLQVNQQQLMSVSKNLKAILTQQIFWVNSNRPMDWDWLKAFP
QTLKEQFSAMKITVNWQKAWPAVFIAFLAGLPLLLIAGLIRWRLKWLKAYQQKLAAAVGA
LRNDSQLNTPKAILIDLIRALPVCLIIILALGLILLTMQLNISDLLWAFSKKLAMFWLVFG
LCWKVLEKEGVAIRHFGMPAQLTSHWRRQIVRISLALLPLHFWSVVAELSPLNLMDDVLG
QAVIFLNLLVITLLVWPLCRESWRDKESHGIRLVTVTILSIIPVALMVLTATGYFYTTLR
LAGRWIETVYLVIIWNLLYQTVLRGLSVAARRIAWRRALARRQNLVKEGAEGAEPQEEPA
IALEQINQQTLRITMLLMLALFGVMFWAIWSDLITVFSYLDSITLWHYNGSEAGAAVVKS
VTMGSLLFAIIAAMVAWALIRNLPGLLEVLVLSRLNMRQGASYAITTILNYVIIAVGAMT
VFGSLGVSWDKLQWLAAALSVGLGFGLQEIFGNFVSGLIIILFERPVRIGDTVTIGTYSGT
VSKIRIRATTITDFDRKEVIIPNKAFVTERLINWSLSDTTTRLVIRLGVAYGSDLEKVKR
VLLQAAMEHPKVMHDPEPAVFFTTFGASTLDHELRLYVRELRDRSHTVDELNRAIDRLCR
ENDINIAFNQLEVHLHNAKGDEVTEVKRDLNGGDLAPTAS
>EECEBICD_02991 HTH-type transcriptional regulator AcrR
MARKTKQQALETRQHILDVALRLFSQQGVSATSLAEIANAAGVTRGAIYWHFKNKSDLFS
EIWELSESNIGELEIEYQAKFPDDPLSVLREILVHILEATVTEERRRLLMEIIFHKCEFV
GEMVVVQQAQRSLCLESYDRIEQTLKHCINAKMLPENLLTRRAAILMRSFISGLMENWLF
APQSFDLKKEARAYVTILLEMYQLCPTLRASTFNGSP
>EECEBICD_02992 Multidrug efflux pump subunit AcrA
MNKNRGLTPLAVVLMLSGSLALTGCDDKQDQQGGQQMPEVGVVTLKTEPLQITTELPGRT
VAYRIAEVRPQVSGIILKRNFVEGSDIEAGVSLYQIDPATYQATYDSAKGDLAKAQAAAN
IAELTVKRYQKLLGTQYISKQEYDQALADAQQATAAVVAAKAAVETARINLAYTKVTSPI
SGRIGKSSVTEGALVQNGQASALATVQQLDPIYVDVTQSSNDFLRLKQELANGSLKQENG
KAKVDLVTSDGIKFPQSGTLEFSDVTVDQTTGSITLRAIFPNPDHTLLPGMFVRARLQEG
TKPTALLVPQQGVTRTPRGDATVLVVGADNKVETRQIVASQAIGDKWLVTDGLKAGDRVV
VSGLQKVRPGAQVKVQEITADNKQQAASGDQPAQPRS

>EECEBICD_02993 Multidrug efflux pump subunit AcrB
MPNFFIDRPIFAWVIAIIIMLAGGLAILKLPVAQYPTIAPPAVTISATYPGADAKTVQDT
VTQVIEQNMNGIDNLMYMSSNSDSTGTVQITLTFESGTDADIAQVQVQNKLQLAMPLLPQ
EVQQQGVSVEKSSSSFLMVVGVINTDGTMTQEDISDYVAANMKDPISRTSGVGDVQLFGS
QYAMRIWMNPTELTKYQLTPVDVINAIKAQNAQVAAGQLGGTPPVKGQQLNASIIAQTRL
TSTDEFGKILLKVNQDGSQVRLRDVAKIELGGENYDVIAKFNGQPASGLGIKLATGANAL
DTATAIRAELKKMEPFFPPGMKIVYPYDTTPFVKISIHEVVKTLVEAIILVFLVMYLFLQ
NFRATLIPTIAVPVVLLGTFAVLAAFGFSINTLTMFGMVLAIGLLVDDAIVVVENVERVM
TEEGLPPKEATRKSMGQIQGALVGIAMVLSAVFIPMAFFGGSTGAIYRQFSITIVSAMAL
SVLVALILTPALCATMLKPVAKGDHGEGKKGFFGWFNRLFDKSTHHYTDSVGNILRSTGR
YLLLYLIIVVGMAYLFVRLPSSFLPDEDQGVFLTMVQLPAGATQERTQKVLDEVTDYYLN
KEKANVESVFAVNGFGFAGRGQNTGIAFVSLKDWADRPGEKNKVEAITQRATAAFSQIKD
AMVFAFNLPAIVELGTATGFDFELIDQAGLGHEKLTQARNQLFGEVAKYPDLLVGVRPNG
LEDTPQFKIDIDQEKAQALGVSISDINTTLGAAWGGSYVNDFIDRGRVKKVYVMSEAKYR
MLPDDINDWYVRGSDGQMVPFSAFSSSRWEYGSPRLERYNGLPSMEILGQAAPGKSTGEA
MAMMEELASKLPSGIGYDWTGMSYQERLSGNQAPALYAISLIVVFLCLAALYESWSIPFS
VMLVVPLGVIGALLAATFRGLTNDVYFQVGLLTTIGLSAKNAILIVEFAKDLMDKEGKGL
VEATLEAVRMRLRPILMTSLAFMLGVMPLVISSGAGSGAQNAVGTGVLGGMVTATVLAIF
FVPVFFVVVRRRFSRKSEDIEHSHSTEHR
>EECEBICD_02994 Hha toxicity modulator TomB
MDEYSPKRHDIAQLKFLCETLYHDCLANLEESNHGWVNDPTSAVNLQLNELIEHIATFAL
NYKIKYNEDNKLIAQIDEYLDDTFMLFSSYGINTQDLQKWRKSGNRLFRCFVNATRANPV
SLSC
>EECEBICD_02995 Hemolysin expression-modulating protein Hha
MSDKPLTKTDYLMRLRRCQTIDTLERVIEKNKYELSDNELAVFYSAADHRLAELTMNKLY
DKIPSSVWKFIR
>EECEBICD_02996 Maltose O-acetyltransferase
MSDEKQKMIAGALYCPTDETLCQDRLRARQLIHQYNYTTPDEINKRQAILRDLLGRCEDA
YIEPSFRCDYGYNIFLGHSFYANFDCVMLDVCPIHIGDNCMLAPGVHIYTATHPLDAVER
NSGRELGKPVTIGNNVWIGGRAVVNPGVTIGDNVVVASGAVVTKNVPPDVVVGGNPARII
KKL
>EECEBICD_02997 Inner membrane protein YlaC
MTEIQRLLSETIDDLNVREKRDNRPRFSISFIRKHPGLFIAMYAAWFATLAVMLQSETLV
GSVWLLVVLFIAFNGFFFFDIAPRYHYNDIDVLDLRVCYNGEWYNTRFVPPTLIETILQS
PQVDNEHKVQLQKMVARKGELSFYDIFTLARAEASR
>EECEBICD_02998 50S ribosomal protein L31 type B
MKPDIHPVYRTVVFHDTSANEYVKVGSTIKTEREIELDGVTYPYVTIDVSSKSHPFYTGR
QKTFDSESSAARFQKRFGHFIGAKRG
>EECEBICD_02999 putative cyclic di-GMP phosphodiesterase PdeB
MTTRHLVSLVTGVLILSVLFPIGLSLWLAHRQVEKRFNEELDIFSSRVALRTERVSDQAK
AALTHIASFKGEPCSSAHLLAMRRVSYSFRYVQEVLYLKNSIPVCSSLEKESHIPAFPPP
MKITRDGYRAWFTAQNDLGIKRYMAALGSDSYIVMIDPASFIDVIPFGAWPIDVAIIGRE
RNTVVASSGNLDPAILPLIHHETPLRLENRGIQYAIHPFPEMGITIVSWAPTAPLERSWY
RQAFIWLPAGIVTGLLAAAFILRILRRLQSPRHRLQDAIDNREINVHYQPIVSLSSGKIV
GAEALARWQQADGTFLSPEIFIALAEQTGLTEPLTRLIIDTVFEDMGSWLKSHPEQHISI
NLEASDLASETLPTLLSTLLNRSQISPSQIALELTEREFADPKTSAPIVARYRNAGHSIY
IDDFGTGYSSLSYLQNLDVDVLKIDKSFVDALEYKNVTPHIIEMAKTLKLKMVAEGIETT
RQEQWLRQHGVHYGQGWLYSKPLPATAFILWAEQRL
>EECEBICD_03000 DNA base-flipping protein
MDIQDTFPQRVWQIVASIPEGFVTTYGDVARLAGSPRAARQVGGVLKRLPEGSTLPWHRV
VNRQGAISLTGPDLQRQRQALLAEGVVVSGSGQIDLQRYRWVY
>EECEBICD_03001 putative lipoprotein YbaY
MKLVHIVSGLAVAISLAACADKSADIQTPAPNPNMSITANQSTIQQPNVSGTVWIRQKVA
LPPDAVLTVTLSDASLADAPSKVLSQKAVRTEGKQAPFSFVLPFNPSDIQPNARILLSAA

ITVDNKLVFITDSVKPVINNGGTKADLTLVPVQQTAVPVQASGGATTTVPSTSPTQVNPS
SAVPAPTQY
>EECEBICD_03002 Acyl-CoA thioesterase 2
MSQTLNNLLTLLNLEKIEEGLFRGQSEDLGLRQVFGGQVVGQALYAAKETVPEARLVHSF
HSYFLRPGDSQKPIIYDVEVLRDGNSFSARRVAAIQNGKPIFYMTASFQAPEPGFEHQKT
MPTAVGPEGLPSETEIAQSLAHLLPPILKEKFLCDRPLEIRPVEFHNPLKGHVAAPVRQV
WIRANGTVPDDIRVHQYLLGYASDLNFLPVALQPHGIGFLEKGIQIATIDHSMWFHRPFN
FNEWLLYSVESTSASSARGFVRGEFYTQDGALVASTVQEGVMRNHN
>EECEBICD_03003 Ammonia channel
MKIAMIKTTLASLALLPGLTIAAPAVADKADSAFMMICTALVLFMTIPGIALFYGGLIRA
KNVLSMLTQVTVTFALVCILWVVYGYSLAFGEGNHFFGNTDGAMLKNIALTAVTGTIYQY
IHVAFQGSFACITVGLIVGALAERIRFSAVLIFVVVWFTLSYIPIAHMVWGGGLLAAHGA
LDFAGGTVVHINAAIAGLVGAYLIGKRVGFGKEAFKPHNLPMVFTGTAILYIGWFGFNAG
SAGSANEIAALAFVNTVVATAAAILGWIIGEWTLRGKPSLLGACSGAIAGLVGVTPACGY
VGVGGALVIGVIAGLAGLWGVTMLKRLLRVDDPCDVFGVHGVCGIVGCILTGVFAASSLG
GVGFVDGVTMGHQVMVQLESIAITVVWSGVVAFVGYKLADILVGLRVPEEQEREGLDVNS
HGENAYNA
>EECEBICD_03004 Nitrogen regulatory protein P-II 2
MKLVTVIIKPFKLEDVREALSSIGIQGLTVTEVKGFGRQKGHAELYRGAEYSVNFLPKVK
IDVAIADDQLEEVIDIISKAAYTGKIGDGKIFVAELMRVIRIRTGEADEAAL
>EECEBICD_03005 Multidrug resistance-like ATP-binding protein MdlB
MRSFGQLWPTLKRLLAYGSPWRKPLSVAVMMLWIAAAAEVSGPLLISYFIDNMVARHHLP
LGKVAGLAAAYVGLQFLAAGLHYAQSLLFNRAAVGVVQSLRTDVMDAALRQPLSAFDTQP
VGQLISRVTNDTEVIRDLYVTVVATVLRSAALIGAMLVAMFSLDWRMALVAVLIFPAVLT
VMVIYQRYSTPIVRRVRAYLADINDGFNEIINGMSVIQQFRQQARFGERMGEASRSHYMA
RMQTLRLDGFLLRPLLSLFSALILCGLLMLFSFTSAGTIEVGVLYAFISYLSRLNEPLIE
LTTQQSMLQQAVVAGERVFELMDRPRQRYGSDDRPLQSGAIDIDHLSFAYRDDNLVLQDI
TLSVPSRSFVALVGHTGSGKSTLASLLMGYYPLTQGEIRLDGREIASLNHRVLRQGVAMV
QQDPVVMADTFLANVTLGRDVSEAQVWQALETVQLADLARGLSDGLHTHLGEQGNTLSVG
QKQLLALARVLVDAPQILILDEATASIDSGTEQAIQQALAAIRERTTLVVIAHRLSTIVE
ADTILVLHRGQAVERGTHQQLLAAQGRYWQMYQLQLVGEELAASVHEEPGPAA
>EECEBICD_03006 putative multidrug resistance ABC transporter ATP-binding/permease protein YheI
MRLFAQLSWYFRREWRRYLGAVALLMLIAMLQLIPPKVVGIVVDGVTAQHFTPGRIAMWI
GTIALIAVVVYLLRYVWRVLLFGASYQLAVELREDYYRQLSRQHPEFYQRHRTGDLIARA
TNDVDRVVFAAGEGVLTLVDSLVMGCAVLIVMSTQISWQLTLLALLPMPIMALMIKRYGD
RLHDYFKLAQAAFSSLNDRTQESLTSIRMIKAFGLEDRQSSLFAADAEDTGKKNMRVARI
DARFDPTIYIAIGMANLLAISGGSWMVVNGSLTLGELTSFMMYLGLMIWPMLALAWMFNI
VERGSAAYSRIRAMLAEAPVVKDGEEPVPAGRGELTAAIREFCYPQTTHPALENVNFRLK
PGQMLGICGPTGAGKSTILSLIQRHFDVTQGEIRFHDVPLTHLQLDSWRSRLAVVSQTPF
LFSDSIANNIALGRPEATQEEIEQVARLASVHEDILRLPQGYDTQVGERGVMLSGGQKQR
ISIARALLLNAEILLLDDALSAVDGRTEHQILHNLRQWGEGRTVIISAHRLSALTDANEI
IVMQHGHVVQRGDHDQLAQQIGWYRDMYRYQQLEAALDDAPERGEEAVNA
>EECEBICD_03007 DNA-binding transcriptional activator DecR
MLDKIDRKLLCLLQQDCTLSLQALADAVNLTTTPCWKRLKRLEDDGILLGRVALLDPEKL
GLGLTAFVLIKTQHHSSEWYCRFVTVVSEMPEVLGFWRMAGEYDYLMRVQVADMKRYDDF
YKRLVNSVPGLSDVTSSFAMEQIKYTTSLPIE
>EECEBICD_03008 N-(2-amino-2-carboxyethyl)-L-glutamate synthase
MMSSNWVKNAINEINADHQRSADTHLIRLPLSAFPGIQLYLKDESTHPTGSLKHRLARSL
FLYGLCNGWIKEGTPIIEASSGSTAISEAWFARLLGLPFIAVMPACTAKRKIEQIQFYGG
HCHFVESACEIYAASERLAHELNGHYMDQFTYAERATDWRGNNNIADSIFRQMRNEPHPV
PRFIVMSAGTGGTSATIGRYIRCQGYDTQLMVVDPENSVFLPYWQDRDASLRSPVGSKIE
GIGRPRVEPSFIPDVVDEMLRVPDAASVATAHWLETQLGRKVGASTGTNMWGALQLAARM
REAGETGAIVTLLCDSGDRYLDTYYHPAWVSDHIGDLTPWSAAIAKLLTGD

>EECEBICD_03009 HMP-PP phosphatase
MARLAAFDMDGTLLMPDHHLGRETIATLARLRERDITLTFATGRHVLEMRHILGTLSLDA
YLITGNGTRIHSLEGDVLHRQDLDPQVADTVMHHAWDTRASMHVFNDNGWFTGQEIPALL
QAHVYSGFRYQVIDIKSIPAHQVTKICFCGDHDDLIRLRIQLNEALEERAHLCFSAVDCL
EVLPLGCNKGSALAVLSNHLGLSLADCMAFGDAMNDREMLGSVGRGLIMGNAMPQLIAAL
PHLSVIGHCGNQAVSHFLTHWLDNPHLPYSPE
>EECEBICD_03010 HTH-type transcriptional regulator SgrR
MRLLNRLNQYQRLWQPSAGETQHVTVSELAERCFCSERHLRTLLRQAQQAGWLRWEAQSG
RGKRGRLQFLVTPESLRTAMMEQALEKGQQLNVLELAQLAPGELRAMLQPFMGGQWQNDT
PTLRIPYYRPLDPLQPGFLPGRAEQHLAGQVFSGLTRFDRDSQYPCGDLAHHWEVSADGL
RWDFYIRSTLHWHNGDAVDTAQLHERLERLLTLPALSKLFISVARIEVTHPQCLTFLLHR
PDYWLAHRLASYCSGLAHPDLPLIGTGPFRLALFTPELVRLESHDHYHLSHPLLKAIEFW
ITPQLFAQDLGTSCRHPVQIAIGKPEELATLSQVSSGISLGFCYLTLKKGSRLNVQQARR
LIHIIHHTSLLKTLPVDENLIMPSQGLLPGWTIPQWQDVDETPLPKKLTLAYHLPVELHT
MAEQLRHYLATLGCELTLIFHNAKNWDNCPALAQADLMMGDRLIGEAPEYTLEQWLRCDQ
IWSHVLDAPAFSHLQATLDALQIQPNEKDRRAALQQVFANLMDDATLTPLFNYHYRISAP
PGVNGVRLTPRGWFEFSEAWLPPPSP
>EECEBICD_03011 7-cyano-7-deazaguanine synthase
MKRAVVVFSGGQDSTTCLAQARHQYDEVHCVTFDYGQRHRAEIDVARALALKLGARAHKV
LDVTLLNELAVSSLTRDSIPVPDYEPNADGIPNTFVPGRNILFLTLAAIYAYQVKAEAVI
TGVCETDFSGYPDCRDEFVKALNHAVNLGMAKDIRFETPLMWIDKAETWALADYWGQLDL
VREETLTCYNGIKGDGCGHCAACNLRANGLNHYLSNKAAVMAAMKQKTGLR
>EECEBICD_03012 Long-chain acyl-CoA thioesterase FadM
MQTQIKVRGYHLDVYQHVNNARYLEFLEEARWDGLENSDSFQWMTARNIAFVVVNININY
RRPAVLSDLLTVTSQVQQLNGKSGVLSQTITLEPEGQVVADALITFVCIDLKTQKALPLE
GELREKLEQMVQ
>EECEBICD_03013 putative protein
MKHGIKALLITLSLTCAGMAHSALAATPVAKNAAVETKADTPSSSSAKAALPAKASDDEG
TRVSINTASAEDLARVMNGVGLKKAQAIVSYREEYGPFKTVDDLKQVPGMGSALVERNLA
VLTL
>EECEBICD_03014 Peptidyl-prolyl cis-trans isomerase D
MMDSLRTAANSLVLKIIFVIIIVSFILTGVSGYLIGGSNNYAAKVNGQEISRAQFENAFN
NERNRMQQQLGDRYSELAANEGYMKSLRQQVLNRLIDEALLDQYSRELKLGISDEQVKQA
IFATPAFQVDGKFDNNRYNAIVNQMGMTADQYAQALRNQLTTQQLINGVAGTDFMLKGET
DELAALVSQQRVVREAVIDVNALAAKQQVTDQEVSSYYDQNKVRFMTPEQFRVSYIKLDA
ATMQAPVSDADIQAYYDQHVDQFTQPERIRYSIIQTKTEDDAKAVLDALNKGEDFATLAK
EKSTDIISARNGGDMGWLEESATVPELKNAGLKEKGQISGVIKSSVGFLVARLDDIQPAK
VKPLSEVRDDIAAKVKQEKALDAYYALQQKVSDAASNDNESLAGAEQVAGVKAVETGWFS
RDTLPEELNFKPVADAIFNGGLVGENGTPGPNSDIITVDGDRAFVVRVSEHKPEAVKPLA
EVKEQVTALVKHNKAEQQAKLDAEKLLVELKAGKGAEAMKAAGLSFGEPKTLSRTSQDPV
SQAAFALSLPTKDKPVYGVANDMQGNVVLLAFDEVKAGAMPEAQKKAMVQGITQNNAQIV
FEALMSNLRKEAKIKIGDALEQQ
>EECEBICD_03015 DNA-binding protein HU-beta
MNKSQLIEKIAAGADISKAAAGRALDAIIASVTESLKEGDDVALVGFGTFAVKERAARTG
RNPQTGKEITIAAAKVPSFRAGKALKDAVN
>EECEBICD_03016 Lon protease
MNPERSERIEIPVLPLRDVVVYPHMVIPLFVGREKSIRCLEAAMDHDKKIMLVAQKEAST
DEPGVNDLFTVGTVASILQMLKLPDGTVKVLVEGLQRARISALSDNGEHFSAKAEYLDSP
AIDEREQEVLVRTAISQFEGYIKLNKKIPPEVLTSLNSIDDPARLADTIAAHMPLKLADK
QSVLEMSDVNERLEYLMAMMESEIDLLQVEKRIRNRVKKQMEKSQREYYLNEQMKAIQKE
LGEMDDAPDENEALKRKIDAAKMPKEAKEKAEAELQKLKMMSPMSAEATVVRGYIDWMVQ
VPWNARSKVKKDLRQAQEILDTDHYGLERVKDRILEYLAVQSRVNKIKGPILCLVGPPGV
GKTSLGQSIAKATGRKYIRMALGGVRDEAEIRGHRRTYIGSMPGKLIQKMAKVGVKNPLF
LLDEIDKMSSDMRGDPASALLEVLDPEQNVAFSDHYLEVDYDLSDVMFVATSNSMNIPAP

LLDRMEVIRLSGYTEDEKLNIAKRHLLPKQIERNALKKGELTVDDSAIIGIIRYYTREAG
VRSLEREISKLCRKAVKQLLLDKSLKHIEINGDNLHDYLGVQRFDYGRADSENRVGQVTG
LAWTEVGGDLLTIETACVPGKGKLTYTGSLGEVMQESIQAALTVVRARAEKLGINPDFYE
KRDIHVHVPEGATPKDGPSAGIAMCTALVSCLTGNPVRADVAMTGEITLRGQVLPIGGLK
EKLLAAHRGGIKTVLIPFENKRDLEEIPDNVIADLDIHPVKRIEEVLTLALQNEPSGMQV
VTAK
>EECEBICD_03017 ATP-dependent Clp protease ATP-binding subunit ClpX
MTDKRKDGSGKLLYCSFCGKSQHEVRKLIAGPSVYICDECVDLCNDIIREEIKEVAPHRE
RSALPTPHEIRTHLDDYVIGQEQAKKVLALAVYNHYKRLRNGDTSNGVELGKSNILLIGP
TGSGKTLLAETLARLLDVPFTMADATTLTEAGYVGEDVENIIQKLLQKCDYDVQKAQRGI
VYIDEIDKISRKSDNPSITRDVSGEGVQQALLKLIEGTVAAVPPQGGRKHPQQEFLQVDT
SKILFICGGAFAGLDKVIANRVETGSGIGFGATVKAKSDKASEGELLSQVEPEDLIKFGL
IPEFIGRLPVVATLNELSEEALIQILKEPKNALTKQYQALFNLEGVDLEFRDEALDAIAR
KAMARKTGARGLRSIVEAALLDTMYDLPSMEDVEKVVIDESVIAGQSKPLLIYGKPEAQA
SGE
>EECEBICD_03018 ATP-dependent Clp protease proteolytic subunit
MSYSGERDNLAPHMALVPMVIEQTSRGERSFDIYSRLLKERVIFLTGQVEDHMANLIVAQ
MLFLEAENPEKDIYLYINSPGGVITAGMSIYDTMQFIKPDVSTICMGQAASMGAFLLTAG
AKGKRFCLPNSRVMIHQPLGGYQGQATDIEIHAREILKVKGRMNELMAHHTGQSLEQIER
DTERDRFLSAPEAVEYGLVDSILTHRN
>EECEBICD_03019 Trigger factor
MQVSVETTQGLGRRVTITIAADSIETAVKSELVNVAKKVRIDGFRKGKVPMNIVAQRYGA
SVRQDVLGDLMSRNFVDAIIKEKINPAGAPNYVPGEYKVGEDFTYSVEFEVYPEVELTGL
ESIEVEKPVVEVTDADVDVMLDTLRKQQATWKEKDGAADAEDRVTIDFTGSVDGEEFEGG
KATDFVLAMGQGRMIPGFEDGVKGHKAGEEFTIDVTFPEEYHAENLKGKAAKFVINLKKV
EERELPELTEEFIKRFGVEDGSVAGLRAEVRKNMERELKGAVRNRVKSQAIEGLVKANDI
DVPAALIDSEIDVLRRQAAQRFGGNEKQALELPRELFEEQAKRRVVVGLLLGEVIRTNEL
KADEERVKGLIEEMASAYEDPKEVIEFYSKNKELMDNMRNVALEEQAVEAVLAKAKVSEK
ATSFNELMNQQA
>EECEBICD_03020 DNA-binding transcriptional regulator BolA
MIREQIEEKLRTAFDPVFLEVVDESYRHNVPAGSESHFKVVLVSDRFTGERFLNRHRMIY
GTLTAELSTTVHALALHTYTLKEWEGLQDTIFASPPCRGAGSIA
>EECEBICD_03021 hypothetical protein
MMAGIFTYHVLSVIESLNMLKKLLFPLVALFMLAGCATPPTTIDVAPKITLPQQDPSLMG
VTVSITGADQRQDQALAKVTRNNQLVTLTASRDLRFLLQEVLEKQMTARGYMIGPNGAVN
LQIIVNQLYADVSQGSVRYNIATKADIAIIATAANGSKMTKNYRANYSIEGAFQASNQNI
ADAVNSVLTDTIADMSQDTSIHDFIKQNAR
>EECEBICD_03022 Anhydromuropeptide permease
MPMSSQYLRIFQQPRSAILLILGFASGLPLALTSGTLQAWMTVENIDLKTIGFFSLVGQA
YVFKFLWSPLMDRYTPPFLGRRRGWLLATQLLLLAAIAAMGFLEPGTQLRWMAALAVVIA
FCSASQDIVFDAWKTDVLPAEERGAGAAISVLGYRLGMLVSGGLALWLADRWLGWQGMYW
LMAALLIPCIIATLLAPEPTDTIPSPKSLEQAVVAPLRDFFGRNNAWLILLLIVLYKLGD
AFAMSLTTTFLIRGVGFDAGEVGVVNKTLGLLATIVGALYGGILMQRLSLFRALLIFGIL
QGASNAGYWLLSITDKNMFSMGAAVFFENLCGGMGTAAFVALLMTLCNKSFSATQFALLS
ALSAVGRVYVGPVAGWFVEAHGWPTFYLFSVVAAVPGLLLLLVCRQTLEYSWQNERFIPR
TQYRGAYNFALSILLAGVALLAVWVLLLTMNALDYTNFSFLSGLLETAVAVAVCGIVFGG
LLDYLALRKTRLL
>EECEBICD_03023 Cytochrome bo(3) ubiquinol oxidase subunit 2
MRLRKYNKSLGWLSLIAGTALLSGCNSALLDPKGQIGLEQRSLILTAFGLMLIVVIPAIL
MAVGFAWKYRASNKDAKYSPNWSHSNKVEAVVWTVPILIIIFLAVLTWKTTHALEPSKPL
AHDEKPITIEVVSMDWKWFFIYPEQGIATVNEIAFPANTPVYFKVTSNSVMNSFFIPRLG
SQIYAMAGMQTRLHLIANEPGTYDGISASYSGPGFSGMKFKAIATKDRAEFDQWVAKAKQ
SPNTMNDMAAFEKVAMPSEYNKVEYFSSVKPDLFKDVINKFMDHGKSMDMTQPEGEHSSH
EGMEGMDMSHAESANSKG

>EECEBICD_03024 Cytochrome bo(3) ubiquinol oxidase subunit 1
MFGKLSLDAVPFHEPIVMVTIAAIIVGGLAILAAITYFGKWTYLWKEWLTSVDHKRLGIM
YIIVAIVMLLRGFADAIMMRSQQALASAGEAGFLPPHHYDQIFTAHGVIMIFFVAMPFVI
GLMNLVVPLQIGARDVAFPFLNNLSFWFTVVGVILVNLSLGVGEFAQTGWLAYPPLSGIE
YSPSVGVDYWIWALQLSGIGTTLTGINFFVTILKMRAPGMTMFKMPVFTWASLCANVLII
ASFPILTVTVALLTLDRYLGTHFFTNDMGGNMMMYINLIWAWGHPEVYILILPVFGVFSE
IAATFSRKRLFGYTSLVWATVCITVLSFIVWLHHFFTMGAGANVNAFFGITTMIIAIPTG
VKIFNWLFTMYQGRIVFHSAMMWTIGFIVTFSVGGMTGVLLAVPGADFVLHNSLFLIAHF
HNVIIGGVVFGCFAGMTYWWPKAFGFKLNETWGKRAFWFWIIGFFVAFMPLYVLGFMGMT
RRLSQQIDPQFHTMLMVAAAGAALIALGILCQLIQIFVSIRDRDQNRDLTGDPWGGRTLE
WSTSSPPPFYNFAVVPHVHERDAFWEMKEKGEAYQQPGQYEEIHMPKNSGAGIVIAAFAT
VFGFAMIWHIWWLAIVGFAGMIISWIVKSFDEDVDYYVPVPEVEKLENQHFDEITKAGLK
NGN
>EECEBICD_03025 Cytochrome bo(3) ubiquinol oxidase subunit 3
MATDTLTHATAHAHEHGHHDAGQNKIFGFWVYLMSDCILFSILFATYAVLVNGTAGGPTG
KDIFELPFVLVETFLLLFSSITYGMAAIAMHKNNKSQVISWLALTFLFGAGFIGMELYEF
HHLIVNGMGPDRSGFLSAFFALVGTHGLHVTSGLIWMAVLMVQVARRGLTSTNRTRIMCL
SLFWHFLDVIWICVFTVVYLMGAM
>EECEBICD_03026 Cytochrome bo(3) ubiquinol oxidase subunit 4
MSHSTESGGASHGSVKTYMTGFILSVILTVIPFWMVMTGAASPAVILGSILAMAVVQILV
HLVCFLHMNTKSDEGWNMTAFIFTVLIIAILVVGSIWIMWNLNYNMMMH
>EECEBICD_03027 Protoheme IX farnesyltransferase
MMFKQYLQVTKPGIIFGNLISVIGGFLLASKGSIDYPLFIYTLVGVSLVVASGCVFNNYI
DRDIDRKMERTKNRVLVKGLISPGVSLVYATLLGIAGFMLLWFGANPLACWLGVMGFVVY
VGVYSLYMKRHSVYGTLIGSLSGAAPPVIGYCAVTGDFDSGAAILLAIFSLWQMPHSYAI
AIFRFKDYQAANIPVLPVVKGISVAKNHITLYIIAFAVATLMLTLGGYAGYKYLVVAAAV
SVWWLGMALRGYKVEDDKVWARKLFGFSIIAITALSIMMSVDFMVPNSQNLLTYVW
>EECEBICD_03028 hypothetical protein
MAAQQGNGEALYTLGRMYYSGVMVNVDYDKALYFFKKAYEKELQAAADYLAQMYFNGQSV
DVDCQQSWHYYDNSYIKKMTQRDYLDYCEKDRKRRNDFSQQLPELTLEKYAGLFGRIDNI
PLCQIGFVVNTNKLIHVANLRVELILKNDAGVSDERIVAFPPLGLNTLGAEQGMGDSFKS
MGYLLMKNGDLCDYHKLTFTVKSATATINGKKVDLLKTDNLHIIQNR
>EECEBICD_03029 hypothetical protein
MIKRKWSILFLLGLSFASVCQAGGDIDALYSRIAQKDIQALNELKALAKDNNAQALAELG
FIYEYGVSVSVDIPQAIKYYEQACDLDGDYGCFNAAYFYEYGIGTQKDITQAKTLAKQLR
EKINLSNINLDKKVSERIIGSVYSNKLEAYRDLSFRPHFIQGLSFYFYAPQSDERKLLSR
IGFDSSHLARIAILWAREGDPEVAY
>EECEBICD_03030 hypothetical protein
MKWFRAAAEKGVVEAQSLLGGIYSGGEGDEWGIKPDIQEAQKWYGQAAKQGDSDAQIALG
KIYYSGATGRTDYAKALALFTQVENDGTNSRSTMPLSWMYYNGLGTAPDCDKAWSYYKKA
SRYVGKKVEEKIFLSKCAADIQSRKNNADALPKVTLKKERIFSRGITAKPKECALIFQIG
TDKIRNMANLHITLELKNADGMATEETLMIPPFGLNTLGIDMQNHDVDPLVTPYDLPLYT
QDFCHGIDDIHFTLKSATATINGKNVDLLKADSVRFLDKE
>EECEBICD_03031 hypothetical protein
MEIKFLSLCILAGVLFVSQVNASANNGKDDVKYAALTQKDLDALPVEKRASFLDELGFIH
EYGLNVPMNREQALQYYKQACELGGNYGCYNVKYAYQYGDGVAKDSAQANKYAKKMNLDN
LLIEQEYIDKFSQEIFTAKVLADTDKSQRPEFINALFNFLNNRPESDALFLVVSDLIRKK
RSALLRYGCRTAIHKWIINWGYSPLMISLVIMWMSLIKPGRPR
>EECEBICD_03032 Inner membrane transport protein YajR
MNDYKMTPGELRATWGLGTVFSLRMLGMFMVLPVLTTYGMALQGASEALIGIAIGIYGLA
QAIFQIPFGLLSDRIGRKPLIVGGLAVFVAGSVIAALSHSIWGIILGRALQGSGAIAAAV
MALLSDLTREQNRTKAMAFIGVSFGITFAIAMVLGPIVTHSLGLNALFWMIAALATLGII
LTIWVVPNSTNHVLNRESGMVKGSFSKVLAEPRLLKLNLGIMCLHILLMSTFVALPGQLA
DAGFPAAEHWKVYLATMVIAFAAVVPFIIYAEVKRRMKQVFLFCVGLIVVAEIVLWGAGQ

HFWELVIGVQLFFLAFNLMEALLPSLISKESPAGYKGTAMGVYSTSQFLGVALGGSLGGW
IDGTFDGQTVFLAGAVLAMVWLAVASTMKEPPYVSSLRVEIPADIVADDRLKQRLLEMKG
VSEALIVAEEHSAYVKIDSKVTNRFEVEQLISKG
>EECEBICD_03033 hypothetical protein
MPSFDIVSEVDLQEARNGVDNAVREVESRFDFRGVEATIELNDANKTIKVLSESDFQVNQ
LLDILRAKLLKRGIEGASLDVPDEFVHSGKTWYVEAKLKQGIESAVQKKIVKLIKDSKLK
VQAQIQGEEIRVTGKSRDDLQSVMALVRGGDLGQPFQFKNFRD
>EECEBICD_03034 2-dehydropantoate 2-reductase
MKITVLGCGALGQLWLSALCKHGHDVQGWLRVPQPYCSVNLIDTDGSFFNESLTANDPDF
LAKSELLLVTLKAWQVSDAVRTLASTLPVTSPILLIHNGMGTIEELQNIQQPMLMGTITH
AARRDGNIIIHVANGTTHIGPAREQDGDYSYLADILQGVLPDVAWHNNIRAEMWRKLAVN
CVINPLTALWNCPNGELRHHTDEINAICEEVAAVIEREGYHTSADDLRYYVEQVIDSTAE
NISSMLQDVRAMRHTEIDYITGYLLKRARVHGLAVPENSRLFEMVKRKESEYERSGTGMP
RPW
>EECEBICD_03035 Protein/nucleic acid deglycase 3
MSAQALVCLAPGSEETEAVTTIDLLVRGGIHVTTASVASDGNLTIVCSRGVKLLADAPLV
EVADGDYDIIVLPGGIKGAECFRDSPLLVETVKQFHRSGRIVAAICAAAATVLVPHDIFP
IGNMTGFPALKDKIPAEQWQDKRVVWDARVKLLTSQGPGTAIDFGLKIIDLLVGREKAHE
VASQLVMAAGIYNYYE
>EECEBICD_03036 Phosphonoacetaldehyde hydrolase
MNRIHAVILDWAGTTVDFGSFAPTQIFVEAFRQAFDVEITLAEARVPMGLGKWQHIEALG
KLPAVDARWQAKFGRSMSAADIDAIYAAFMPLQIAKVVDFSSPIAGVIDTIAALRAEGIK
IGSCSGYPRAVMERLVPAAAEHGYRPDHWVATDDLAAGGRPGPWMALQNVIALGIDAVAH
CVKVDDAAPGISEGLNAGMWTVGLAVSGNEFGATWDAYQTMSKEDVAVRREHAASKLYAA
GAHYVVDSLADLSGVIAHINARLAQGERP
>EECEBICD_03037 2-aminoethylphosphonate--pyruvate transaminase
MTSRNYLLLTPGPLTTSRTVKEAMLFDSCTWDDDYNIGVVEQIRQQLTELATASEGYTSV
LLQGSGSYAVEAVLGSALGPQDKVLIVSNGAYGARMVEMAGLMGIAHHAYDCGEVARPDV
QAIDAILNADPTISHIAMVHSETTTGMLNPIDEVGTLAHRYGKTYIVDAMSSFGGIPMDI
AALHIDYLISSANKCIQGVPGFAFVIAREQKLAACKGRSRSLSLDLYAQWRCMEDNHGKW
RFTSPTHTVLAFAQALKELAKEGGVAARHQRYQQNQRSLVAGMRALGFNTLLDDELHSPI
ITAFYSPEDPQYRFSEFYRRLKEQGFVIYPGKVSQSDCFRIGNIGEVYAADITALLTAIR
TAMYWTK
>EECEBICD_03038 Putative transcriptional regulator of 2-
aminoethylphosphonate degradation operons
MKSIPGDIPQYLLIKAQLQARIQSGALKSGDKLPSERELCAIFNTTRITIRESLAQLESS
GLIWRADRRGWFVTPERLWLDPTQNTNFHKLCREQGREPKTALLSGVLTTVPVEVMEPLQ
LQPFDQIYLLTRLRYADGRAVCYCENHCLPARVPELLQYDLNGSLTEVYESHYNLVYTSM
HLSFYPTAMPAQAAQALGVMEGRPALLLRRLNYDQHGRVLDLDIEYWRHDSLRIEVDTH
>EECEBICD_03039 Putative 2-aminoethylphosphonate-binding periplasmic
protein
MKLSRLALLSVFALASAPSWAESVVTVYSIDGLHDGDNSWYQVQFDAFTKATGITVRYVE
GGGGVVVERLAKERTNPQADVLVTAPPFIQRAAAEKLLANFNTDAASAIPDANNLYSPLV
KNYLSFIYNSKLLKTAPASWQDLLDGKFKNKLQYSTPGQAADGTAVMLQAFHSFGSKDAG
FAYLGKLQANNVGPSASTGKLTALVNKGEIYVANGDLQMNLAQMERNPNVKIFWPANDKG
ERSALAIPYVIGLVQGAPQSENGKKLINFLLSKEAQTRVSELSWGMPVRSDVTPSDEHYK
AATAALEGVQSWQPNWDDVAVSLSADISRWHKVTESE
>EECEBICD_03040 Putative 2-aminoethylphosphonate import ATP-binding
protein PhnT
MLMKTTAVHVPASQGTSGIVLDSLRVAYHGNVVLKPLSLTIELGEVLALIGPSGSGKTTV
LRAVAGFVQPAGGRILIGDTDVTHLPPYKRGLAMVVQNYALFPHLKVEDNVAFGLRAQKQ
PKALINERVTQALKTVGMSDYAARYPHQLSGGQQQRVAIARAIAVRPRVLLLDEPLSALD
AQIRHNMVEEIACLHRELPELTILYVTHDQTEALTLADKIGIMKDGSLIAHGETRALYQH
PPNRFAAEFLGRANILSAIALGITEAPGLVDVSCGGAVIRAFSRGSHHGYNKLLCIRPQH

LSLTPRSAYSNRFNATLQSVHWQGDLTHLLCDVAGETVRMVLTHVNPLPRVGDKLALWFE
PDDAVLIEV
>EECEBICD_03041 Putative 2-aminoethylphosphonate transport system permease protein PhnU
MSLILPLEKPALNLRPLLWLLLPLLVLATLFFWPLSLIVEQALRGANGEIGLETFRQVVD
SKRFVGALLNTLQIAFFATAGCLLLGSVMSLILVFIPFPGSELIGRVVDTFIALPTFLIT
VAFTFIYGSAGLLNGTLMSLFAFELPPVDFLYSMQGVILAEITVFTPLVMRPLMAALRQI
DKSQLEAASILGAHPLRVIGQVIFPAALPALMAGGSLCLLLTTNEFGIVLFIGAKGVNTL
PMMVYSKAILESDYTVACMIALINIVLSLGLFSLYRLAASRTGVRSQPC
>EECEBICD_03042 Putative 2-aminoethylphosphonate transport system permease protein PhnV
MLIWSPKGRAAAGVVASVLFIVFFFLPLAVILMSSLSQQWNGILPSGFTLNHFVNALHGA
AWDALLASLTIGFCASLFALLCGVWAALALRQYGVKTQKWLSMVFYLPSAIPSVSVGLGI
LVAFSQVPLQMNGTLWIVLTAHFVLISAFTFSNVSTGLARISADIENVASSLGASPWYRL
RHVTLPLLMPWMVSALALSLSLSMGELGATMMIYPPGWTTLPVAIFSLTDRGNIADGAAL
TIVLVAITLLLMMKLERIAKRLGQK
>EECEBICD_03043 tRNA sulfurtransferase
MKFIIKLFPEITIKSQSVRLRFIKILTGNIRNVLKHYDETLAVVRHWDNIEVRAKDENQR
LAIRDALTRIPGIHHILEVEDVPFTDMHDIFEKALAQYREQLEGKTFCVRVKRRGKHEFS
SIEVERYVGGGLNQHIESARVKLTNPDVTVHLEVEDDRLLLIKGRYEGIGGFPIGTQEDV
LSLISGGFDSGVSSYMLMRRGCRVHYCFFNLGGAAHEIGVRQVAHYLWNRFGSSHRVRFV
AINFEPVVGEILEKVDDGQMGVVLKRMMVRAASKVAERYGVQALVTGEALGQVSSQTLTN
LRLIDNVSDTLILRPLISYDKEHIINLARQIGTEDFARTMPEYCGVISKSPTVKAIKAKI
EAEEENFDFSILDKVVEEANNVDIREIAQQTQQEVVEVETVSGFGPNDVILDIRSVDEQD
DKPLKVEGVDVVSLPFYKLSTKFGDLDQSKTWLLWCERGVMSRLQALYLREQGFENVKVY
RP
>EECEBICD_03044 Exodeoxyribonuclease 7 small subunit
MPKKNEAPASFETALSELEHIVTRLESGDLPLEDALNEFERGVQLARQGQAKLQQAEQRV
QILLSDNEEASPEPFIADNE
>EECEBICD_03045 Farnesyl diphosphate synthase
MDFTQQLQACVTQANQALSRFIAPLPFQNTPVVEAMQYGALLGGKRLRPFLVYATGQMFG
VSTATLDAPAAAVECIHAYSLIHDDLPAMDDDDLRRGLPTCHIKFGEANAILAGDALQTL
AFAILSDAPMPEVTDRDRIAMIAELANASGIAGMCGGQALDLAAEGQRITLDALERIHRH
KTGALIRAAVRLGALSAGDKGRNTLPILDRYAESIGLAFQVQDDILDVVGDTATLGKRQG
ADQQLGKSTYPALLGLEQARNKARDLIEDARQSLHQLAAQSLDTSALEALANYIIQRDK
>EECEBICD_03046 1-deoxy-D-xylulose-5-phosphate synthase
MSFDIAKYPTLALVDSTQELRLLPKESLPKLCDELRRYLLDSVSRSSGHFASGLGTVELT
VALHYVYNTPFDQLIWDVGHQAYPHKILTGRRDKIGTIRQKGGLHPFPWRGESEYDVLSV
GHSSTSISAGIGIAVAAEKEGKDRRTVCVIGDGAITAGMAFEAMNHAGDIRPDMLVILND
NEMSISENVGALNNHLAQLLSGKLYSSLREGGKKVFSGVPPIKELLKRTEEHIKGMVVPG
TLFEELGFNYIGPVDGHDVMGLISTLKNMRDLKGPQFLHIMTKKGRGYEPAEKDPITFHA
VPKFDPSSGCLPKSSGGLPGYSKIFGDWLCETAAKDSKLMAITPAMREGSGMVEFSRKFP
DRYFDVAIAEQHAVTFAAGLAIGGYKPVVAIYSTFLQRAYDQVIHDVAIQKLPVMFAIDR
AGIVGADGQTHQGAFDLSYLRCIPDMVIMTPSDENECRQMLFTGYHYNDGPTAVRYPRGN
AQGVALTPLEKLPIGKGLVKRHGEKLAILNFGTLMPEAAKVAEALNATLVDMRFVKPLDD
TLILEMAAQHDALVTLEENAIMGGAGSGVNEVLMAHRKPVPVLNIGLPDFFIPQGTQEEA
RAELGLDAAGIEAKIKAWLA
>EECEBICD_03047 1-deoxyxylulose-5-phosphate synthase YajO
MQYNTLGNTDLRVSRLCLGCMTFGEPSRGNHAWTLPEESSRPIIKRALEGGINFFDTANS
YSDGSSEEIVGRALRDFARRDEVVVATKVFHRVGDLPEGLSRAQILRSIDDSLTRLGMEY
VDILQIHRWDYTTPIEETLEALNDVVKAGKARYIGASSMHASQFAQALALQKQHGWAPFV
TMQDHYNLIYREEEREMLPLCWQEGVAVIPWSPLARGRLTRPWGETTARLVSDDVGKNLY
AESDKNDAQIAERLAGVSKALGATRAQVALAWLLSKRGVAAPIIGASREEQLDELLNAVD
LTLKPEQIAELETPYKQHPVVGFK

>EECEBICD_03048 Phosphatidylglycerophosphatase A
MTILPRHKDVAKSRLNLRNPWHLLATGFGSGLSPVVPGTMGSLAAIPFWYLMTFLPWQLY
SLVVMFGICIGVYLCHQTAKDMGVHDHGSIVWDEFIGMWITLMALPTTDWQWVAAGFVIF
RILDMWKPWPIRWFDRNVHGGMGIMIDDIVAGVISAGILYFIGHHWPLGII
>EECEBICD_03049 Thiamine-monophosphate kinase
MACGEFSLIARYFDRVKSSRLDVETGIGDDCALLNIPEKQTLAISTDTLVAGIHFLPDID
PADLAYKALAVNLSDLAAMGADPAWLTLALTLPDVDEAWLAAFSDSLFEQLDYYDMQLIG
GDTTRGPLSMTLGIHGLVPVGRALKRSGAKPGDWIYVTGTLGDSAAGLAILRGDFRVGSW
GDADYLVKRHLRPTPRILQGQALRDLASSAIDLSDGLISDLGHILQASNCGARIDLEALP
DSEELWGHANDPEQKLRWMLSGGEDYELCFTVPELNRGALDVALGHLGVPFTCIGQMTAD
IEGIAFVRDGEPVTFDWKGYDHFATP
>EECEBICD_03050 Transcription antitermination protein NusB
MKPAARRRARECAVQALYSWQLSQNDIADVEYQFLAEQDVKDVDVLYFRELLSGVATNSA
YLDGLMKPYLSRLLEELGQVEKAVLRIALFELSKRSDVPYKVAINEAIELAKTFGAEDSH
KFVNGVLDKAAPVIRPNKK
>EECEBICD_03051 6,7-dimethyl-8-ribityllumazine synthase
MNIIKANVAAPDARVAITIARFNQFINDSLLDGAVDALTRIGQVKDDNITVVWVPGAYEL
PLATEALAKSGKYDAVVALGTVIRGGTAHFEYVAGGASNGLASVAQDSGVPVAFGVLTTE
SIEQAIERAGTKAGNKGAEAALTALEMINVLKAIKA
>EECEBICD_03052 Riboflavin biosynthesis protein RibD
MQDEFYMARALKLAQRGRFTTHPNPNVGCVIVNNGDIVGEGYHQRAGEPHAEVHALRMAG
AKAKGATAYVTLEPCSHHGRTPPCCDALIAAGVARVVAAMQDPNPQVAGRGLYRLQQAGI
AVSHGLMMSEAEALNKGFLKRMRTGFPYLQLKLGASVDGRTAMASGESQWITSPQARRDV
QRLRAQSHAILTSSATVLADDPALTVRWAELDASTQASYPQENLRQPVRIVIDSQNRVTP
AHRIVQQPGETWIARTQEDSRAWPDSVRTISVPAHNGHLDLVVLMMQLGRQQINSIWVEA
GPGLAGALLQAGLVDELIVYVAPKLLGTQARGLCELPGLEKLADAPQFTFSEIRHVGPDV
CLHMVSA
>EECEBICD_03053 Transcriptional repressor NrdR
MHCPFCFAVDTKVIDSRLVGEGSSVRRRRQCLVCNERFTTFEVAELVMPRVIKSNDVREP
FNEDKLRSGMLRALEKRPVSADDVEMALNHIKSQLRATGEREVPSKMIGNLVMEQLKKLD
KVAYIRFASVYRSFEDIKDFGEEIARLQD
>EECEBICD_03054 putative lipoprotein YajI
MTRRYLRILLVGSLLSLTACAPQSEVRQMHQSISTLNKEMTQLNQETVKITQQNKLNAES
TRGVYLLPGANTPARLESQIGTLRMTLLEITPVADGAHATLHIQGESRDPLPAFSATVEY
GQLQGTTENYQEVNAQSLLVNAPASLLAPSDVNISLPLKGITPAQLGFIRIHDIQPVNQ
>EECEBICD_03055 Nucleoside-specific channel-forming protein Tsx
MKKTLLAVSAALALTSSFTANAAENDQPQYLSDWWHQSVNVVGSYHTRFSPKLNNDVYLE
YEAFAKKDWFDFYGYIDIPKTFDWGNGNDKGIWSDGSPLFMEIEPRFSIDKLTGADLSFG
PFKEWYFANNYIYDMGDNKASRQSTWYMGLGTDIDTGLPMGLSLNVYAKYQWQNYGASNE
NEWDGYRFKVKYFVPITDLWGGKLSYIGFTNFDWGSDLGDDPNRTSNSIASSHILALNYD
HWHYSVVARYFHNGGQWQNGAKLNWGDGDFSAKSTGWGGYLVVGYNF
>EECEBICD_03056 hypothetical protein
MLRGLDPRWPCRWKEKTPAHCSVFTGNITGANLTLDKLRIAANRCHCNKGGNMTLTELSI
TIWHDLAAPTLVGIATGLFLGWWHRRK
>EECEBICD_03057 hypothetical protein
MAIIPKNYARLESGYREKALKLFPWVCGRCSREFVYSNLRELTVHHIDHDHTNNPEDGSN
WELLCLYCHDHEHSKYTEADQYGSTVIAGEDAQKDVGEATYNPFADLKAMMNKKK
>EECEBICD_03058 hypothetical protein
MTRRADRLFQIVQILRGRRLTTAALLAQRLAVSERTIYRDIRDLSLSGVPVEGEAGSGYR
LMAGFDLPPLMLTHRESEALMVAIRLLKTWGGDSLSRELESAQEKVLAILPEESRRKAEQ
TRIYAPDLGTSPHSRSAFDLIHQAVSAQQVLALHYRDEAGHLSSRDIQPLGLFFWGEHWL
LVAWCERREDYRCFRLDRCLQITPLNRRFRETIDRSLRDFLRKVEHEKMP
>EECEBICD_03059 hypothetical protein
MKNLINWFEIPVTDMARAIAFYEQVMQISLRRETMDGADLAVFPYAEPGPGGALIKQDDV

APVAQGVVIYLHTADLLASIERVVLAGGKCVLEPMVPGDDIGTIALFIDSEGNRIGLHQP
A
>EECEBICD_03060 Protein translocase subunit SecF
MPPTEGETGGQVLGSKVVTIINEATNQNAAVKRIEFVGPSVGADLAQTGAMALLVALISI
LVYVGFRFEWRLAAGVVIALAHDVIITLGILSLFHIEIDLTIVASLMSVIGYSLNDSIVV
SDRIRENFRKIRRGTPYEIFNVSLTQTLHRTLITSGTTLVVILMLYLFGGPVLEGFSLTM
LIGVSIGTASSIYVASALALKLGMKREHMLQQKVEKEGADQPSILP
>EECEBICD_03061 Protein-export membrane protein SecF
MAQEYTVEQLNHGRKVYDFMRWDFWAFGISGLLLIAAIVIMGVRGFNWGLDFTGGTVIEI
TLEKPAEMDVMREALQKRAMKSRSCRTLAAVTTLWCVCRQPKVKPAVRYWVAKW
>EECEBICD_03062 Protein translocase subunit SecD
MLVVVIIVGLLYALPNLYGEDPAVQITGVRGVAASEQTLIQVQKTLQEEKIPAKSVALEE
GAILARFDTTDTQLRAREALMSVLGDKYVVALNLAPATPRWLAAIHADPMKLGLDLRGGV
HFLMEVDMDTALGKLQEQNIDSLRSDLREKGIPYTTVRKENNYGLSITFRDSKARDEAIA
YLTPRHRDLVISSQSGNQLRAVMTDARLSEAREYAVQQNINILRNRVNQLGVAEPVVQRQ
GADRIVVELPGIQDTARAKEILGATATLEFRLVNTNVDQAAAAAGRVPGDSEVKQTREGQ
PVVLYKRVILTGDHITDSTSSQDEYNQPQVNISLDSAGGNIMSNFTKDNIGKPMATLFVE
YKDSGKKDANGRAVLVKQEEVINIANIQSRLGNSFRITGISNPNEARQLSLLLRAGALIA
PIQIVEERTIGPTLGMQNIKQGLEACLAGLVVSILFMIFFYKKFGLIATSALVANLVLIV
GIMSLLPGATLSMPGIAGIVLTLAVAVDANVLINERIKEELSNGRTVQQAINEGYAGAFS
SIFDANITTLIKVIILYAVGTGAIKGFAITTGIGVATSMFTAIIGTRAIVNLLYGGKRVT
KLSI
>EECEBICD_03063 Sec translocon accessory complex subunit YajC
MSFFISDAVAATGAPAQGSPMSLILMLVVFGLIFYFMILRPQQKRTKEHKKLMDSIAKGD
EVLTNGGLVGRVTKVAEAGYIAIALNDTTEVVIKRDFVAAVLPKGTMKAL
>EECEBICD_03064 Queuine tRNA-ribosyltransferase
MKFELDTTDGRARRGRLVFDRGIVETPAFMPVGTYGTVKGMTPEEVEATGAQIILGNTFH
LWLRPGQEIMKLHGDLHDFMQWKGPILTDSGGFQVFSLGDIRKITEQGVHFRNPINGDPI
FLDPEKSMEIQYDLGSDIVMIFDECTPYPADWDYAKRSMEMSLRWAKRSRDRFDSLGNKN
ALFGIIQGSVYEDLRDISVKGLVEIGFDGYAVGGLAVGEPKADMHRILEHVCPQIPADKP
RYLMGVGKPEDLVEGVRRGIDMFDCVMPTRNARNGHLFVTDGVVKIRNAKHKSDTSPLDA
ECDCYTCRNYSRAYLHHLDRCNEILGARLNTIHNLRYYQRLMAGLRKAIEEGKLESFVTE
FYQRQGRPVPPLNVD
>EECEBICD_03065 S-adenosylmethionine:tRNA ribosyltransferase-isomerase
MRVTDFSFELPESLIAHYPQPERSRCRLLSLEGPTGALTHGTFTDLLDKLNPGDLLVFNN
TRVIPARLFGRKASGGKIEVLVERMLDDKRILAHIRASKAPKPGTELLLGDDESIHATMT
ARHGALFEVEFNDPRPVLDILNAIGHMPLPPYIDRPDEDADRELYQTVYSEKPGAVAAPT
AGLHFDEPLLAALREKGIEMAFVTLHVGAGTFQPVRVDTIEDHIMHSEYAEVPQEVVDAV
LAAKARGNRVIAVGTTSVRSLESAAQAAKNDLIEPFFGDTQIFIYPGYQYKVIDALITNF
HLPESTLIMLVSAFAGYQHTMNAYKTAVEQKYRFFSYGDAMFITYNPQAISERP
>EECEBICD_03066 Acyl carrier protein phosphodiesterase
MNFLAHLHLAHLADSSLSGNLLADFVRGNPATHYPPDVVEGIYMHRRIDVMTDNLPEVRE
AREWFRHETRRVAPITLDVMWDHFLSRHWTQISPDFPLQAFVGYAHAQVATILPDSPPRF
VNLNDYLWSEKWLERYRDMDFIQNVLNGMANRRPRLDALRDSWYDLDAHYDALEERFWHF
YPRMMAQAARKAL
>EECEBICD_03067 Alkyl hydroperoxide reductase C
MVLVTRQAPDFTAAAVLGSGEIVDKFNFKQHTNGKTTVLFFWPMDFTFVCPSELIAFDKR
YEEFQKRGVEVVGVSFDSEFVHNAWRNTPVDKGGIGPVKYAMVADVKREIQKAYGIEHPD
EGVALRGSFLIDANGIVRHQVVNDLPLGRNIDEMLRMVDALQFHEEHGDVCPAQWEKGKE
GMNASPDGVAKYLAENISSL
>EECEBICD_03068 Maltodextrin glucosidase
MLNAWHLPVAPFVKQHNDKLTITLWLSGENPPSRVTLRSEFDNEEISLAMRKQRRQPQPG
VTAWRATLNIALGQPRRRYSFKLLWHDRQLWFTPQGFSCFPPARLEQFAVDVPDSGPQWV
ADQIFYQIFPDRFARSQSREAGQDKVYYHHAAGHDIVRKEWDEPLTPQAGGSTFYGGCLN

GICEKLPYLKKLGVTALYLNPVFTAPSVHKYDTEDYRHVDPQFGGDRALLRLRRETQAQG
MRLVLDGVFNHSGDSHAWFDRHNRSTGGACHHPDSPWRDWYSFSPQGVALDWLGYPSLPK
LDYQSETLVDEIYRGEDSIVRHWLKAPWNMDGWRLDVVHMLGEAGGARHNLQHVAGITRA
AKEAQPEAYIVGEHFGDARQWLQADAEDAAMNYRGFTFPLWGFLANTDIAYEPQHIDART
CIAWMDNYRAGLSHQQQLRMFNQLDSHDTARFKTLLGKDAARLPLAVVWLFTWPGVPCIY
YGDEVGVDGANDPMCRKPFPWDERKQDGNLLALYQRMAKLRQSSLALRRGGCQALYAEGD
VVVFVRVYQQQRALVAINRGEACEVALEALPLLNVAGWQCKTGSGDIREGRLSLPAISAT
VWMNR
>EECEBICD_03069 Proline-specific permease ProY
MESNNKLKRGLSTRHIRFMALGSAIGTGLFYGSADAIKMAGPSVLLAYIIGGVAAYIIMR
ALGEMSVHNPAASSFSRYAQENLGPLAGYITGWTYCFEILIVAIADVTAFGIYMGVWFPA
VPHWIWVLSVVLIICAINLMSVKVFGELEFWFSFFKVATIIIMIVAGIGIIVWGIGNGGQ
PTGIHNLWSNGGFFSNGWLGMIMSLQMVMFAYGGIEIIGITAGEAKDPEKSIPRAINSVP
MRILVFYVGTLFVIMSIYPWNQVGTNGSPFVLTFQHMGITFAASILNFVVLTASLSAINS
DVFGVGRMLHGMAEQGSAPKVFAKTSRRGIPWVTVLVMTIALLFAVYLNYIMPENVFLVI
ASLATFATVWVWIMILLSQIAFRRRLPPEEVKALKFKVPGGVVTTIAGLIFLVFIIALIG
YHPDTRISLYVGFAWIVLLLIGWMFKRRRDRQLAQA
>EECEBICD_03070 Branched-chain amino acid transport system 2 carrier protein
MTHQLKSRDIIALGFMTFALFVGAGNIIFPPMVGLQAGEHVWTAAIGFLITAVGLPVLTV
VALAKVGGGVDSLSTPIGKVAGLLLATVCYLAVGPLFATPRTATVSFEVGIAPLTGDSAM
PLLIYSVVYFAIVILVSLYPGKLLDTVGNFLAPLKIIALVILSVAAIVWPAGPISNALDA
YQNAAFSNGFVNGYLTMDTLGAMVFGIVIVNAARSRGVTEARLLTRYTVWAGLMAGVGLT
LLYLALFRLGSDSATLVDQSANGAAILHAYVQHTFGGAGSFLLAALIFIACLVTAVGLTC
ACAEFFAQYIPLSYRTLVFILGGFSMVVSNLGLSHLIQISIPVLTAIYPPCIALVVLSFT
RSWWHNSTRIIAPAMFISLLFGILDGIKASAFGDMLPAWSQRLPLAEQGLAWLMPTVVMV
ILAIIWDRAAGRQVTSSAH
>EECEBICD_03071 Phosphate regulon sensor protein PhoR
MLERLSWKRLVLELVLCCIPALILSAFFGYLPWFLLAAVTGLLVWHFWNLLRLSWWLWVD
RSMTPPPGRGSWEPLLYGLHQMQLRNKKRRRELGNLIKRFRSGAESLPDAVVLTTEEGGI
FWCNGLAQQILGLRWPDDNGQNILNLLRYPEFTQYLKTRDFTRPLHLVLNTGRHLEIRVM
PYTDKQLLMVARDVTQMHQLEGARRNFFANVSHELRTPLTVLQGYLEMMQEQALEGATRE
KALHTMREQTYRMEGLVKQLLTLSKIEAAPALLLNEKVDVPMMLRVVEREAQTLSQQKHT
FTFEVDDSLSVLGNEEQLRSAISNLVYNAVNHTPAGTHITVSWRRVVHGAEFCIQDNGPG
IAAEHIPRLTERFYRVDKARSRQTGGSGLGLAIVKHALNHHESRLEIDSSPGKGTRFSFV
LPERLIAKNSD
>EECEBICD_03072 Phosphate regulon transcriptional regulatory protein PhoB
MARRILVVEDEAPIREMVCFVLEQNGFQPVEAEDYDSAVNKLNEPWPDLILLDWMLPGGS
GLQFIKHLKREAMTRDIPVVMLTARGEEEDRVRGLETGADDYITKPFSPKELVARIKAVM
RRISPMAVEEVIEMQGLSLDPGSHRVMTGDSPLDMGPTEFKLLHFFMTHPERVYSREQLL
NHVWGTNVYVEDRTVDVHIRRLRKALEHSGHDRMVQTVRGTGYRFSTRF
>EECEBICD_03073 Nuclease SbcCD subunit D
MRILHTSDWHLGQNFYSKSRAAEHQAFLDWLLETAQAHQVDAIIVAGDIFDTGSPPSYAR
ELYNRFVVNLQQTGCHLVVLAGNHDSVATLNESRDILAFLNTTVIASAGYAPRLLHRRDG
SPGAVLCPIPFLRPRDIITSQAGLSGSEKQQQLLHAIADYYQQQYQEACQLRGERKLPVI
ATGHLTTVGASKSDAVRDIYIGTLDAFPAQHFPPQIISH
>EECEBICD_03074 Nuclease SbcCD subunit D
MHLVTFDQGKWQSTESLAVPVTQPLAVLKGDLASITEQLEQWRGVEQSPPVWLDIEITTD
DYLHDIQRRIQTLTESLPVEVLLVRRSREQRERSLANERRETLSELSVEEVFARRLALEA
LDTPQRERLNQLFSSTLYALNEEHEA
>EECEBICD_03075 Nuclease SbcCD subunit C
MKILSLRLKNLNSLKGEWKVDFTAEPFASNGLFAITGPTGAGKTTLLDAICLALYHETPR
LNTVSQSQNDLMTRDTAECLAEVEFEVKGEAWRAFWSQNRARNQPDGNLQAPRVELARCS
DGKIFADKVKDKLEMIATLTGLDYGRFTRSMLLSQGQFAAFLNAKAKERAELLEELTGTE

IYGQISAQVFEKHKSARLELEKLQAQASGVALLADEQLQQLEASLQALTDEEKRLLADQQ
VQQQHLHWLTRKNELHTELHARQQALYAAQEAREKAQPQLAALTLAQPARQLRPHWERIQ
EQTRAVERVRQHSDEVNARLQSAYRLRRRIRACAHRQFTQLNTTGQRLKTWLAEHDGIRI
WRSELAGWRALLTQQSHDRAQLSQWQQQLLSDTRQRDALPPLTLDLTPQALAEARALHTR
QRPLRHRLAALQGQILPKQKRQAQLQAAIARHHQEQAQYTQRLADKRLSYKTKAQELADV
RTICEQEARIKDLESQRAHLQSGQPCPLCGSTTHPAIAAYQALELSANQTRRDALEKEVK
TLAEEGAALRGQLDALTQQLQRDESEAQSLLQEEQALTEEWQTLCATLGVQLQPQEDLAG
WLTAAEEHEQQLDQLSQRHALQTQIAAHTEQVARFTAQIAQRQASLTADLAQYTLSLPAP
EDEASWLNERADEAKIWQQRQTEFADLQMQIDRLALLLETLPQTDTADSDDDVPLDNWRQ
AHDECVSLQSQLQTLQEQTTQEQQRAAEAIAHFDAALKNSPFDSQATFLAALLDEETVTR
LEKQQQTLESQLQQAKALSAQSAQALADHQQQPPAGLDPTCTAEQLAQRLTQLAQQLREN
TTRQGEIRQQIKQDADNRQRQRALMAEMKQASQQVEDWGYLNALIGSKEGDKFRKFAQGL
TLDNLVWLANHQLTRLHGRYLLQRKASDALELEVVDTWQADAVRDTRTLSGGESFLVSLA
LALALSDLVSHKTRIDSLFLDEGFGTLDSETLDAALDALDALNASGKTIGVISHVEAMKE
RIPVQIKVKKINGLGYSKLDKAFAVE
>EECEBICD_03076 Putative transporter AraJ
MKKVIFSLALGTFGLGMAEFGIMGVLTELARDVGITIPAAGHMISFYAFGVVLGAPVMAL
FSSRFSLKHILLFLVTLCVMGNAIFTFSSSYLMLAVGRLVSGFPHGAFFGVGAIVLSKII
RPGKVTAAVAGMVSGMTVANLVGIPFGTYLSQEFSWRYTFLLIAVFNIAVLTAIFFWVPD
IRDKAQGSLREQFHFLRSPAPWLIFAATMFGNAGVFAWFSYIKPFMMYISGFSETSMTFI
MMLVGLGMVLGNLLSGKLSGRYTPLRIAVVTDLVIVLSLMALFFFSGYKTASLTFAFICC
AGLFALSAPLQILLLQNAKGGELLGAAGGQIAFNLGSAIGAWCGGLMLTLGFAYHYVALP
AALLSFSAMSSLLVYGRLKHKQPSVTPVAG
>EECEBICD_03077 Fructokinase
MRIGIDLGGTKTEVIALDDAGEQRFRHRLPTPREDYQQTIETIATLVDMAEQATGQTGSV
GIGIPGSLSPYTGVVKNANSTWLNGQPFDSDVSRRLKREVRLANDANCLAVSEAVDGAAA
GAQTVFAVIIGTGCGAGVALNGRAHIGGNGTAGEWGHNPLPWMDDDELRYREEIPCYCGK
QGCIETFISGTGFATDYQRLSGKTLKGDEIIRLVDAQDAVAELAISRYELRLAKALSHVV
NILDPDVIVLGGGMSNVERLYKTVPSLMKSFVFGGECETPVRKARHGDSSGVRGAAWLWP
LA
>EECEBICD_03078 Recombination-associated protein RdgC
MLWFKNLMVYRLSRDITLRAEEMEKQLASMTFTPCGSQDMAKMGWVPPMGSHSDALTHTA
NGQIIICARKEEKILPSPVIKQALEAKIQKLEADQGRKLKKTEKDSLKDEVLHSLLPRAF
SRFSQTMMWIDTVNGLIMVDCASAKKAEDTLALLRKSLGSLPVVPLALENPIELTLTEWV
RSGTVAQGFQLLDEAELKAMLEDGGVIRAKKQDLVSDEIAVHIEAGKVVTKLALDWQQRI
QFVMCDDGSIKRLKFCDELRDQNEDIDREDFAQRFDADFILMTGELAALIQSLVEGLGGE
AQR
>EECEBICD_03079 Pyrimidine/purine nucleoside phosphorylase
MGVNLIMRRGLIMLQSNEYFSGKVKSIGFTSSSTGRASVGVMAEGEYTFGTAEPEEMTVV
SGALKVLLPGTVEWKVYTAGEVFNVPGHSEFHLQVAEPTSYLCRYL
>EECEBICD_03080 hypothetical protein
MSASLAILTIGVVPMSEVLPLLTEYIDEQHITHHSLLGKMSREDVMADYAVEPGDDPLLT
LLNDNQIAHVSRQKVERDLQSVVEVLDNQGYDVIILMSTAAIKSMAARNSILLEPLRIIP
PLVASIVDGHQVGVIVPVAELLAAQEKKWQVLQMPPVYSLANPVHGSEQQLIDAGQALLD
QGADVIMLDCLGFHQRHRDILQQALDVPVLLSNVLIARLASELLV
>EECEBICD_03081 putative protein YaiA
MPTRPPYPREAYIVTIEKGTPGQTVTWYQLRADHPKPDSLISEHPTAEEAMDAKKRYEDP
DKS
>EECEBICD_03082 Shikimate kinase 2
MMQPLYLVGPRGCGKTTIGMALAQATGFRFADTDRWLQSHVQMSVADIVEKEGWGGFRAR
ETAALEAVSAPSTVVATGGGIILTEYNRRYMHRVGVVIYLCAPVSTLVNRLEAEPEADLR
PTLTGKPLSEEVREVLEQRDALYRETAHYIIDATKAPAQVVSEIIAALPPSTQRLQGDVY
T
>EECEBICD_03083 hypothetical protein

MTIWVDADACPNVIKEILYRAAERMQLPLILVANQALRVPPSRFIRTLRVAAGFDVADNE
IVRQCKAGDLVITADIPLAAEVLEKGAAVLNPRGERYSDATIRERLTMRDFMDTLRASGV
QTGGPNTLSPRDRQHFAAELDKWWLESQRKK
>EECEBICD_03084 Pyrroline-5-carboxylate reductase
MEKKIGFIGCGNMGKAILGGLIASGQVLPGQIWVYTPSPDKVAALHDQYGINAAQSAQEV
AQVADIVFGAVKPGIMVKVLSEISSSLNKDSLVVSIAAGVTLDQLARALGHDRKIIRAMP
NTPSLVNAGMTSVTPNALVTPEDTADVLNIFRCFGEAEVIAEPMIHPVVGVSGSSPAYVF
MFIEAMADAAVLGGMPRAQAYKFAAQAVMGSAKMVLETGKHPGELKDMVCSPGGTTIEAV
RVLEERGFRAAVIEAMTKCMEKSEAISKS
>EECEBICD_03085 putative diguanylate cyclase DgcC
MFLPVAAVLVSQPVFGGWWLLLVGWSFVWPHLAWQWAAKALDPLRQEIYNLKVDAILSGM
WIALMGVNMLPAAALFMMMSMNLMGAGGRRLFTVGMGLLLASCLVTLQLTGLPVAMRSSS
LEVTLSLPVIMLYPLLFAWVSYQTAIKLAEHKRRLQAMSSRDGMTGVYNRRHWEILLRNE
FDHSRRHHREATLLIIDIDHFKSINDTWGHDVGDEAIIALTRQLQITLRGSDIIGRFGGD
EFAVIMCGTPADSAITAMSRVHERLNTLRLPGAPQVMLRISVGVAPLTPQIGHYREWLKS
ADMALYKAKNAGRNRTEVAA
>EECEBICD_03086 Phosphate starvation-inducible protein PsiF
MKITLLVTLLFGLVFLTAVGATEKPLTPQQQRMTTCNQQATAQALKGDARKTYLSDCLKN
SQSGPGEKSLTPQQQKMRECNVQATEQSLKGDDRSKFMSACLKKAA
>EECEBICD_03087 Anti-adapter protein IraP
MKNLIAELLLKLAQKEEESKELVAQVEALEIIVTAMLRNMAQNEQEMLIRQVEGALEGVK
PDASVPDHDTELLRQYVKKLLRHPRH
>EECEBICD_03088 Staphyloferrin B transporter
MESWKVNLISVWFGCFFTGLAISQILPFLPLYISQLGVSSHEALSMWSGLTFSITFLISA
IVSPMWGSLADRKGRKLMLLRASLGMAIAILLQAFATHVWQLFLLRGIMGLTSGYIPNAM
ALVASQVPRERSGWALSTLSTAQISGVIGGPLMGGFVADHIGLRAVFLITAMLLVVSFLV
TLFLIKEGVRPVIRKSERLSGKAVFASLPYPALVISLFFTTMVIQLCNGSISPILALFIK
SMMPDSNNIAFLSGVIASVPGISALISAPRLGKLGDRIGTERILMATLICAVLLFFAMSW
VTTPFQLGLLRFLLGFADGAMLPAVQTLLVKYSSDQITGRIFGYNQSFMYLGNVVGPLMG
ATVSAMAGFRWVFIATAAIVLINIGQLTLALRRRRNAQKAKGQ
>EECEBICD_03089 hypothetical protein
MRGKGFLIIVLLGGIGGLGYRYLPSYYNPFAPLQLADPPGWITTFKLQRLMPSQCRELLT
AANQQGLISSQPVADSAGECPLSHVVRVRDFGQVKLSSSFLASCPLALRSALFVEQQAKP
LTETWMKRRLTRIEHLGSYACRNIYHRPDARRSEHASAEALDISGFQLSDGRKITVLRGW
GRQETGPWLRAMLNASCHYYGNGLGPDYNAAHANHFHLGMRGYGVCR
>EECEBICD_03090 D-alanine--D-alanine ligase A
MEWVFKMAKLRVGIVFGGKSAEHEVSLQSAKNIVDAIDKTRFDVVLLGIDKAGQWHVNDA
ENYLQNADDPAHIALRPSAISLAQVPGKHQHQLINAQNGQPLPTVDVIFPIVHGTLGEDG
SLQGMLRVANLPFVGSDVLSSAACMDKDIAKRLLRDAGLNIAPFITLTRTNRHAFSFAEV
ESRLGLPLFVKPANQGSSVGVSKVANEAQYQQAVALAFEFDHKVVVEQGIKGREIECAVL
GNDNPQASTCGEIVLNSEFYAYDTKYIDDNGAQVVVPAQLPSGVNDKIRAIAIQAYQTLG
CAGMARVDVFLTADNEVVINEINTLPGFTNISMYPKLWQASGLGYTDLISRLIELALERH
TANNALKTTM
>EECEBICD_03091 hypothetical protein
MRLPVKIRRDWHYYAFSIGLIFILNGVVGLLGFEAKGWQTYGVGLVTWIISFWLAGFIIR
RREDDEVKDAR
>EECEBICD_03092 Inner membrane protein YaiY
MADFTLSKSLFSGKHRETSSTPGNIAYAIFVLFCFWAGAQLLNLLVHAPGIYEHLMQVQD
TGRPRVEIGLGVGTIFGLVPFLTGCLIFAVIAAFLRWRHRHQ
>EECEBICD_03093 hypothetical protein
MSAASRLYPLPFLAVAILAGCSSQSGQPVSKGEKPVDVASVVRQKMPASVKDREAWAKDI
ATTFKSQGLAPTVENICSVLAVAQQESGYQADPVVPGLSKIAWQEIDRRAERLHIPLFLV
HTALKINSPNGKSYSERLDTVKTEKQLSAIFDDFINMVPMGQTLFGSYNPVHTGGPMQVS
IAFAEQHAKGYPWKMAGTVRQEVFTRRGGLWFGTYHLLNYPANYSAPVFRFADFNAGWYA

SRNAAFQNAVSKASGVKLALDGDLIRYNSKEPGKTELAVRKLAGQLGMSEREIRSQLEKG
DSLAFEKTALYKKVYKLAEAKTGKTLAREMLPGIQLESPKITRKLTTAWFAKRVDERRAR
CMGR
>EECEBICD_03094 Peptide antibiotic transporter SbmA
MFKSFFPKPGPFFMSAFVWALIAVIFWQAGGGDWVARLVGASDEVPISAARFWSLDYLIF
YAYYLICVGLFATFWFIYSPHRWQYWSILGTSLIIFVTWFLVEVGVAVNAWYAPFYDLIQ
TALSSPHKVTLGQFYHEVGVFLGIALIAVVIGVLNNFFVSHYVFRWRTAMNEHYMAHWQY
LRHIEGAAQRVQEDTMRFASTLENMGVSFINAIMTLIAFLPVLVTLSAHVPNLPIVGHIP
YGLVIAAIVWSLMGTGLLAVVGIKLPGLEFKNQRVEAAYRKELVYGEDDASRATPPTVRE
LFSAVRHNYFRLYFHYMYFNIARILYLQVDNVFGLFLLFPSIVAGTITLGLMTQITNVFG
QVRGSFQYLINSWTTLVELMSIYKRLRSFERQLDGQPVQEVTHSFS
>EECEBICD_03095 D-alanyl-D-alanine-carboxypeptidase/endopeptidase AmpH
MKRSLLFTAMLFAASLTSVQAAQPIADPVFASDIVDRYAEHIFYGSGATGMALVVIDGNQ
RVFRSFGDTRPGNNVRPQLDSVIRIASLTKLMTSEMLVKLLDQGTVKLNDPLSKYAPPGA
HVPTYQGTPITLVNLATHTSALPREQPGGAAHRPVFVWPTREQRWNWLSTATLKVAPGSQ
AAYSNLAFDLLADALATASGKPYTQLFEEKITRPLGMKDTTFTPSPDQCKRLIVAEKGAS
PCNNTLAAIGSGGVYSTPGDMMRWMQQYLSSDFYHRSQQADRMQTLIYQRTQLKKIIGMD
VPGKADALGLGWVYMAPKDGRPGIIQKTGGGGGFITYMAMIPQYNIGAFVVVTRSPLTRF
TNMSDGINDLVTELSGNKPIAIPAS
>EECEBICD_03096 Inhibitor of hydrogen peroxide resistance
MLSLSKPLQEFYRLDKCLSKHGTRFEFVNDKEIICSPDESNTHTFVILEGVVSLVRGDKV
LIGIVQAPFIFGLADGVAKKEAQYKLIAESGCIGYRLSSSQTLAIIEQNQLWREAFCWIV
WKSQVLELRDKQLIGNNSYDQIRATLMTMIEWDEELRSRIGVMNYIHQRTRVSRSVVAEV
LAALRKGNYIEMNKGKLISINRLPSEY
>EECEBICD_03097 hypothetical protein
MHSWKKKLVVSQLALACTLAITSQANATTNDISGTTYNTFHHYNDATYADDVYYDGYVGW
NNYATDSYYNGDIYPVINNARVNGVISTYYLDDGVTTNTNANSLTIKNSTIHGMITSACL
EGTDCVNRDTGYVYDRLALTVDNSTIDDNYEHYTYNGTYTDGTADTHVVDVYGMGTAITL
DQEVDLSIVNNSHVAGITLTQGYEWEDIDDNTVSTGVNSSEVFNNTINVVDSTVTSGSLS
DEGTSGWFGNTNNASDYSGSYTADDIAIAAIAHPYADNAMQTTVNLSNSTLMSDVVFSSN
FDENFFPNGADSYRDTDADRDTNGWDGTDRMDVTLNNGSKWVGAAMSVHMTDTGTDADGD
GVFEYTVDTNGDGILDATSPDGVYDSYAVGTEATATLIDIAANSLWPLSTVGVDNIYTDY
SEYNHIVGNEVYQSGLFNVTLNTGSQWDTTKTSLIDTLSINSGSVVNVADSTLVSDTIGL
TGGSALNINEDGHVATDTLSIDNSTVTIADDVSAGWSVNDAALYANTINVTNNGVLDVGN
SASNALQVDTLNLTSYTDANGYVNAGVFNIESNRFVLDADLTNDRTNDTTKSNYGYGVIA
MNSDGHLTINGNGDTYDTDQTEAGQDEVDNVGDNVAAATGNYKVRIDNATGAGSVADYKG
NELIYVNDKNSTATFSAANKADLGAYTYQAKQEGNTVVLEQMELTDYANMALSIPSANTN
IWNLEQDTVGTRLTNARHGLADNGGAWVSYFGGNFNGDNGTINYDQDVNGIMVGVDTKVD
GNNAKWIVGAAAGFAKGDLSDRTGQVDQDSQSAYIYSSARFANNIFVDGNLSYSHFNNDL
SANMSDGTYVDGNTSSDAWGFGLKLGYDLKLGDAGYVTPYGSVSGLFQSGDDYQLSNDMK
VDGQSYDSMRYELGVDAGYTFTYSEDQALTPYFKLAYVYDDSNNDADVNGDSIDNGVEGS
AVRVGLGTQFSFTKNFSAYTDANYLGGGDVDQDWSANVGVKYTW
>EECEBICD_03098 Delta-aminolevulinic acid dehydratase
MTDLIHRPRRLRKSAALRAMFEETTLSLNDLVLPIFVEEELDDYKAIDAMPGVMRIPEKQ
LAREIERIANAGIRSVMTFGISHHTDDTGSDTWKEDGLVARMSRICKQTVPEMIVMSDTC
FCEYTSHGHCGVLCEHGVDNDATLANLGKQAVIAAAAGADFIAPSAAMDGQVQAIRQALD
AAGFTDTAIMSYSTKFASSFYGPFREAAGTALKGDRKTYQMNPMNRREAIRESLLDEAQG
ADCLMVKPAGAYLDVLREIRERTELPLGAYQVSGEYAMIKFAAMAGAIDEEKVVLESLGS
IKRAGADLIFSYFALDLAEKNILR
>EECEBICD_03099 Acetyl-coenzyme A synthetase
MSFSEFYQRSINEPEAFWAEQARRIDWRQPFTQTLDHSRPPFARWFCGGTTNLCHNAVDR
WLDKQPEALALIAVSSETDEERTFTFSQLHDEVNAVAAMLLSLGVQRGDRVLVYMPMIAE
AQITLLACARIGAIHSVVFGGFASHSVAARIDDARPALIVSADAGARGGKILPYKKLLDD
AIAQAQHQPKHVLLVDRGLAKMAWVDGRDLDFATLRQQHLGASVPVAWLESNETSCILYT

SGTTGKPKGVQRDVGGYAVALATSMDTIFGGKAGGVFFCASDIGWVVGHSYIVYAPLLAG
MATIVYEGLPTYPDCGVWWKIVEKYQVNRMFSAPTAIRVLKKFPTAQIRNHDLSSLEALY
LAGEPLDEPTASWVTETLGVPVIDNYWQTESGWPIMALARALDDRPSRLGSPGVPMYGYN
VQLLNEVTGEPCGINEKGMLVIEGPLPPGCIQTIWGDDARFVKTYWSLFTRQVYATFDWG
IRDAEGYYFILGRTDDVINIAGHRLGTREIEESISSYPNVAEVAVVGIKDALKGQVAVAF
VIPKQSDTLADREAARDEENAIMALVDNQIGHFGRPAHVWFVSQLPKTRSGKMLRRTIQA
ICEGRDPGDLTTIDDPASLQQIRQAIEE
>EECEBICD_03100 2-methylcitrate dehydratase
MSAHISNIRPDFDREIVDIVDYVMNYEITSKVAYDTAHYCLLDTLGCGLEALEYPACKKL
LGPIVPGTVVPNGARVPGTQFQLDPVQAAFNIGAMIRWLDFNDTWLAAEWGHPSDNLGGI
LAIADWLSRNAVAAGKAPLTMKQVLSGMIKAHEIQGCIALENAFNRVGLDHVLLVKVAST
AVVAEMLGLTRDEILNAVSLAWVDGQSLRTYRHAPNTGTRKSWAAGDATSRAVRLALMAK
TGEMGYPSALTAKTWGFYDVSFKGETFRFQRPYGSYVMENVLFKISFPAEFHSQTAVEAV
MTLYEQMQAAGKTAADIEKVTIRTHEACLRIIDKKGPLNNPADRDHCIQYMVAVPLLFGR
LTAADYEDEVAQDKRIDALREKIVCYEDPAFTADYHDPEKRAIGNAITVEFTDGSRFGEV
VVEYPIGHARRRADGIPKLIEKFKINLARQFPTRQQQRILDVSLDRARLEQMPVNEYLDL
YVI
>EECEBICD_03101 2-methylcitrate synthase
MTDTTILQNNTHVIKPKKSVALSGVPAGNTALCTVGKSGNDLHYRGYDILDLAEHCEFEE
VAHLLIHGKLPTRDELNAYKSKLKALRGLPANVRTVLEALPAASHPMDVMRTGVSALGCT
LPEKEGHTVSGARDIADKLLASLSSILLYWYHYSHNGERIQPETDDDSIGGHFLHLLHGE
KPTQSWEKAMHISLVLYAEHEFNASTFTSRVIAGTGSDVYSAIIGAIGALRGPKHGGANE
VSLEIQQRYETPDEAEADIRKRVENKEVVIGFGHPVYTIADPRHQVIKRVAKQLSEEGGS
LKMYHIADRLETVMWETKKMFPNLDWFSAVSYNMMGVPTEMFTPLFVIARVTGWAAHIIE
QRQDNKIIRPSANYTGPDDRQFVPIEKRC
>EECEBICD_03102 2-methylisocitrate lyase
MSLHSPGQAFRAALSKENPLQIVGAINANHALLAQRAGYQAIYLSGGGVAAGSLGLPDLG
ISTLDDVLTDIRRITDVCPLPLLVDADIGFGSSAFNVARTVKSIAKAGAAALHIEDQVGA
KRCGHRPNKAIVSKEEMVDRIRAAVDARTDPNFVIMARTDALAVEGLEAALDRAQAYVDA
GADMLFPEAITELSMYRRFADVAQVPILANITEFGATPLFTTDELRSANVAMALYPLSAF
RAMNRAAEKVYTVLRQEGTQKNVIDIMQTRNELYESINYYQFEEKLDALYRNKKS
>EECEBICD_03103 Propionate catabolism operon regulatory protein
MTTAHSAPRDNSDKPVIWTVSVTRLFELFRDISLEFDHLATITPIQLGFEKAVTYIRKKL
ATERCDAIIAAGSNGAYLKSRLSIPVILIKPSGFDVLQALAKAGKLTSSIGIVTYQETIP
ALLAFQKTFHLRLEQRSYVTEEDARGQINELKANGIEAVVGAGLITDLAEEAGMTAIFIY
SAATVRQAFHDALDMTRLTRRQRVDYSSGKGLQTRYELGDIRGQSPQMEQLRQTITLYAR
SRAAVLIQGETGTGKELAAQAIHQTFFHRQPHRQNKPSPPFVAVNCGAITESLLEAELFG
YEEGAFTGSRRGGRAGLFEIAHGGTLLLDEIGEMPLPLQTRLLRVLEEKAVTRVGGHQPI
PVDVRVISATHCDLDREIMQGRFRPDLFYRLSILRLTLPPLRERQADILPLAESFLKQSL
AAMEIPFTESIRHGLTQCQPLLLAWRWPGNIRELRNMMERLALFLSVDPAPTLDRQFMRQ
LLPELMVNTAELTPSTMDAHTLQDVLARFKGDKTAAARYLGISRTTLWRRLKAGAKDQSD
N
>EECEBICD_03104 hypothetical protein
MKTGYKVMLGALAFVVTNVYAAEIMKKTDFDKVASEYTKIGTISTTGEMSPLDAREDLIK
KADEKGADVVVLTSGQTENKIHGTADIYKKK
>EECEBICD_03105 Threonine efflux protein
MEPFHAVVLTVSLFVLTFFNPGANLFVVVQTSLASGRRAGVITGLGVATGDAFYSGLGLF
GLATLITQCEAVFSLIKIVGGAYLLWFAWNSIRHQATPQMSTLQTPIAAPWTIFFRRGLM
TDLSNPQTVLFFISIFSVTLSAETPTWARLMAWAGIVLSSVIWRIFLSQAFSLPAVRRAY
GRIQRIASRVIGAIIGMFALRLLYEGVTHR
>EECEBICD_03106 Ferrichrome outer membrane transporter/phage receptor
MFATTRMALLIGGAIGGATFPLFAQETTKNDTVIVTSPVQSGATKLATPDIETPQSVSII
TRQQFEEQGATSVRQAVSYTPGVYSNQIGASNRFDYIVLRGFSDGSLDNVYLDGLKMMGD
TNSHSSLVVDPWFLEDIEVVRGPASVLYGRSSPGGIVALTSRKPAFDVGGEVKLFAGNHN

QRGAAFDVTGPLDDNERVAARLSGMTRYADSQFTPLKEERYALMPSLTWRITDRTRLDLM
AYLHRDPEGGSHSGLPYQGTVVPYNGRKISNTFFEGEDDYDKYDRRENMVGYNIEHLFDN
GWSVRQKLRYLHTKVTLNQVYAAGWLNETALNRGYSGSGEKMSAIALDNQLDGSVDTGAI
NHRLLVGIDYQDRSNHTTGYYGAFPPIDAFNPVYGAQPDYITLYSREKHKLRQTGYYLQD
QMSWDRWRFTLGGRYDRVSVSNIDKLHDSRSDLDKNNVSTRAALLYLFDNGVAPYLSYST
AFTPTSFADENGNVLEPMKGKQWEAGVKYEPLGGIASLAPPCIVLTRQISRPKKNLPIRT
ARLVKLSLKGWNWRPLAICRIASVCRRRIPILISVIRKVARKSRVSARFMLHGTRPAPG
>EECEBICD_03107 HTH-type transcriptional activator RhaR
MMSPISCHSSAAPAMKKIFSVSDFIAFGERYGIDYRFPALPQYTQSSPVLHGDIEEIALP
GGICITRSDVHVLQPYETTSRHSSPLYMLVVLEGNVALAVNEQTFLLSAGMAFCSQLSEQ
QTIRAHHGADSKLRTLSLGMYPDGGWRERLPVSLADEWENSATSARVWQVPEFLLSGLRY
AQQPGPHAASRQLMLEGIMLQLLGYALNLCQPATQKRGLPVTGEYQRLELIRRLLEQTPE
KAYTLNELARRAAMSPSSLRCKFRHAYGCTVFDYLRDCRLARARRYLMEGYSVQQAAWMS
GYQHATNFATAFRRRYGCSPGELRDASLTASRHCA
>EECEBICD_03108 hypothetical protein
MVKVKKWSRRVVWMVAIWCASVLLLAGVGMLFRLLMTSAGFRS
>EECEBICD_03109 Cytochrome bd-II ubiquinol oxidase subunit 2
MTIDLPVIWFAIIVFATLMYIVMDGFDLGVGILFPFIRDKHDRDVMVNSVAPVWDGNETW
LVLGGAGLFGAFPLAYAVITDALTIPLVVMLLGLIFRGVAFEFRFKATESHRAFWDKSFI
VGSILATFAQGVVVGAVVSGFQVEGRTFSGSQLDWLTPFNLFCGVGLVVTYALLGATWLI
MKSEDPLHSRMCALSRPLLIVLLLVMGGVSVWTPLTHDDIAERWFTLPNLYYFLPVPVLV
LAFSIWLWRSVKKPESHARPFILTLGLIFLGFSGLGISIWPNIIPPDISLYAAAAPPQSQ
SFMLVGALIIIPIILAYTFWSYYVFRGKVRHGEGYH
>EECEBICD_03110 hypothetical protein
MEFDAFFLARLQFAFTVSFHIIFPAITIGLASYLVVLEGLWLKTRNPVWRSLYQFWLKIF
AVNFGMGVVSGLVMAYQFGTNWSGFSQFAGSITGPLLTYEVLTAFFLEAGFLGVMLFGWN
KVGPGLHFLSTCMVALGTLMSTFWILASNSWMHTPQGFEIHNGQVVPVDWFAVIFNPSFP
YRLLHMSVAAFLSSAMFVGASAAWHLLKGNDTPAIRRMFSMALWMAVVVAPVQALIGDMH
GLNTLKHQPVKIAAIEGHWENTPGEPTPLTLVGWPDMEAERTRYALEIPALGSLILTHSL
DKQVPALKDYPKEDRPNSTVVFWSFRLMVGMGVLMIFLGLASLWLRYRRRLYHSRPFMHF
ALWMGPSGLIAILAGWVTTEVGRQPWVVYGLLRTRDAVSAHSTLQMSISLLAFFVVYSLV
FGVGYIYMIRLIQKGPQPAETPTAETDGRPARPISAVGESLEQEKRE
>EECEBICD_03111 hypothetical protein
MLNRYYRFAVEKDDPSEAPESGDKPDSQPQKKS
>EECEBICD_03112 hypothetical protein
MMNILLEELPHQEQALAAILASFTGIDHAQADHNHYANPLIKERYDDKANIDVKMETGTG
KTYVYTRLMYELHQKYGLFKFVLVVPTPAIKEGARNFITSDYARQHFSQFYENTRMELCT
INAGDFKVKSGRKNFPAQLLSFTDASRRDSHTIQVLLINAQMLNSASMTRDDYDQTLLGG
LTSPVKGLQMTRPVVIIDEPHRFARDNKFYRAIQAIQPQMIVRFGATFPDIVEGKGKNKC
VRKDYYRRQPQFDLNAVDSFNDGLVKGIDIYYPNLPEEQANNRYIVDSVTAKKLILRRGS
KIAEVGVGENLADVDAGFEGSIEYAGSKMLSNDLELEAGMALVPGTFGASYQELIIQDAI
DKHFDTEQANFLRSNEPENNAPRIKTLSLFFIDSIKSYRDDEGWLKVTFERLLKKKLTQL
IDDYQRKTLPREVEYLSFLQATLASLHSDNQNVHAGYFGEDRGSGDEAIQAEVDDILKNK
EKLLSFSDHHGNWETRRFLFSKWTLREGWDNPNVFVIAKLRSSGSESSKIQEVGRGLRLP
VDENGHRVHQEEWPSRLSFLIGYDEKAFASMLVDEINRDSKVQLNEQKLDEAMITLIVTE
RQKVDPAFTELRLLEDLDDKKLINRSNEFKPSVTLNGETKSGFAWLLEFYPELTQARVRA
DRIRDNKPASRLRVRLRKENWEQLSSIWEQFSRRYMLQFERSGASLEQIAAEVLRDPALY
IRQKPSQVQQRLVSNEDNGRFEVAQREGELAASEFMVGMKYGHFLKQLALRTSLPVNVLH
PVLMAMLRDVLHGDSRYLSEISLDNMTRALQTRINAHFAQRHDYLPLDFQASTSVFDSTA
RQFREEISAEIVGKNVDENAIDDPRSLYQIPPLRYDSVDPELPLLKYDYPQQVSVFGKLP
KRAIQIPKYTGGSTTPDFVYRIERQDADSVYLLVETKAENMRVGDQVILDAQRKFFDMLR
RQNINVEFAEATSAPAVFSTINGLIEGKAN
>EECEBICD_03113 hypothetical protein
MLKDNQKHNESVAPNSAFLSELQRALPEFFTADRYNEQGELIAKGGFDLARFERALKARN

IDELTSGYQIDFIGKDYAKKQAGEKSVTVIVPDVEHNTLAENKNSHNLFLTGDNLDVLRH
LQNNYADTVDMIYIDPPYNTGSDGFVYPDHFEYSDRALQDMFGLNDTELARLKSIQGKST
HSAWLSFMYPRLFLARKLLKDTGFIFISIDDNEYANLKLMMDEIFGEGGFVTNVMWKRKK
EISNDSDNVSIQGEYILVYAKTGQGALRLEPLSKEYIQKSYKEPTEQFPEGKWRPVPLTV
SKGLSGGGYTYKITTPNGTVHERLWAYPEASYQKLVADNLVYFGKDNGGIPQRVMYAHHS
KGQPTTNYWDNVASNKEGKKEILDLFGDNVFDTPKPTALLKKIIKLAIDKDGVVLDFFAG
SGTTAHAVMALNEEDGGQRTFILCTIDQALSNNTIAKKAGYNTIDEISRERITRVAAKIR
ANNPATNSDLGFKHYRFATPPQQTLDDLDSFDIATGHFINTSGQLAAFTESGFTDMINPF
SARGLGVPGGASGEETLLTTWLVADGYKMDIDVQTIDFSGYCARYVDNTRLYLIDERWGT
EQTRDLLNYIGTHQLPVQTIVIYGYSFDLESIRELEIGLKQLDQKVNLVKRY
>EECEBICD_03114 Putative metabolite transport protein YjhB
MYTPLNWTTTQRHVAFASFTSWMLDAFDFFILVFVLSDLAEWFHASVSDVSIAIMLTLAV
RPIGALLFGRMAEKYGRRPILMLNILFFTVFELLSAWSPTFMAFLIFRVMYGVAMGGIWG
VASSLAMETIPDRSRGLMSGIFQAGYPCGYLFASVIFGLFYSMVGWRGMFLIGALPVVLL
PYIWFKVPESPVWLAARARKGNTALLPVLRKQWKLCLYLVLVMAFFNFFSHGTQDLYPTF
LKMQHGFDPHLISIIAIFYNIAAMLGGIFYGTLSERIGRKKAIMIAAFLALPVLPLWAFS
SGSFTIGLGAFLMQFMVQGAWGVVPTWLNELVPANARAVLPGFVYQLGNLLASVNATLQA
RIAETHGGNYGLAMAIVAGAVAILICVFVAFGRETRGVVISGTDSDALPESAIGHRT
>EECEBICD_03115 hypothetical protein
MQFHIDDMTCGGCASTVKKTILTLDANATVRTDPATRLVDVETSLSAEQIAAALQKAGFP
PRER
>EECEBICD_03116 HTH-type transcriptional regulator HmrR
MNIGKAAKASKVSAKMIRYYEQIGLIPAASRTDSGYRAYTQADVNQLHFIRRARDLGFSV
AEISDLLNLWNNQSRQSADVKRLAQTHIDELDRRIQNMQHMAQTLKALIHCCAGDALPDC
PILHTLGQPDDSEPEARTGAVLRRPRRHGLAKRL
>EECEBICD_03117 Copper-transporting P-type ATPase
MEQAQGSKLPIQAVVDKVTLWFVPMVMLIAALTFVVWLAFGPSPALTFALINGVAVLIIA
CPCAMGLATPTSIMVGTGRGAEMGVLFRKGEALQLLKDAKVVAVDKTGTLTEGRPVLTDL
DVASGFERREVLAKVAAVESRSEHPIARAIVVSAEEEGIALPGMNGFESVTGMGVYATVD
GTRVDVGADRYMREIGVDISGFATTAERLGQEGKSPLYAAIDGQLAAIIAVADPIKPSTP
AAINALHQLGIKVAMITGDNARTAQAIARQLGIDDVVAEVLPEGKVEAIRRLKAAYGQVA
FVGDGINDAPALAESDVGLAIGTGTDVAVESADVVLMSGNLQGVPNAIALSKATIRNIHQ
NLFWAFAYNTALIPVAAGALFPVWGILLSPVFAAGAMAMSSVFVLGNALRLRRFRAPMAT
PSDTSTTRGGAS
>EECEBICD_03118 Copper-transporting P-type ATPase
MSQSENRHDTISLLIEGMTCASCVARVEKGIKAVPGVTDATVNLATERATVRGTASAEAV
IAAIEKTGYEARPIETAGQGEDDSEEKKEAERVRLKRDLILASVLALPVFVLEMGSHLIP
GMHEWVIKTIGLQQSWYWQFALTLLVLTIPGRRFYLKGFPALARLAPDMNSLVAVGTAAA
FGYSLVATFTPDLLPEGTVNVYYEAAAVIVALILLGRFLEARAKGRTSEAIKRLVGLQAR
VAHVLREGRIEDIPVDEVVLGDCVEVRPGERIPVDGEVTEGRSFVDESMITGEPIPVEKS
AGSAVVGGTVNQKARSRCGRPPSADRPCWRKSFVWWNRRRVQNCRFRPSWIK
>EECEBICD_03119 Multidrug resistance protein MexA
MGGTLDAVSVPEGRLVSRGQLLFQIDPRPFEVALDTAVAQLRQAEVLARQAQADFDRIQR
LVASGAVSRKNADDVTATRNARQAQMQSAKAAVAAARLELSWTRITAPIAGRVDRILVTR
GNLVSGGVAGNATLLTTIVSHNPMYVYFDIDEATWLKALRHTRSDKNSPVVNMGLTTDNG
LPYQGVLDFMGNQMNRSTGTIRARAVIPDPNGMLSPGLFARISLPIGEPRETVLIDDMAV
SADQGKNYVLIVGKENQVEYRPVELGQMVDGLRVVTQGVQPGEKIILKGLVRPGMTVAPR
LVPMRQNVTDKQTATLTKADGDSAPKAVRQ
>EECEBICD_03120 multidrug efflux RND transporter permease subunit OqxB15
MKFTHFFIARPIFAIVLSLLMLLAGAIAFLKLPLSEYPAVTPPTVQVSASYPGANPQVIA
DTVAAPLEQVINGVDGMLYMNTQMAIDGRMVISIAFEQGTDPDMAQIQVQNRVSRALPRL
PEEVQRIGVVTEKTSPDMLMVVHLVSPQKRYDSLYLSNFAIRQVRDELARLPGVGDVLVW
GAGEYAMRVWLDPAKIANRGLTASDIVTALREQNVQVAAGSVGQQPEASAAFQMTVNTLG
RLTSEEQFGEIVVKIGADGEVTRLRDVARVTLGADAYTLRSLLNGEAAPALQIIQSPGAN

AIDVSNAIRGKMDELQQNFPQDIEYRIAYDPTVFVRASLQSVAITLLEALVLVVLVVVLF
LQTWRASIIPLVAVPVSLVGTFALMHLFGFSLNTLSLFGLVLSIGIVVDDAIVVVENVER
HISQGKSPGEAAKKAMDEVTGPILSITSVLTAVFIPSAFLAGLQGEFYRQFALTIAISTI
LSAINSLTLSPALAAILLRPHHDTAKADWLTRLMGTVTGGFFHRFNRFFDSASNRYVSAV
RRAVRGSVIVMVLYAGFVGLTWLGFHQVPNGFVPAQDKYYLVGIAQLPSGASLDRTEAVV
KQMSAIALAEPGVESVVVFPGLSVNGPVNVPNSALMFAMLKPFDEREDPSLSANAIAGKL
MHKFSHIPDGFIGIFPPPPVPGLGATGGFKLQIEDRAELGFEAMTKVQSEIMSKAMQTPE
LANMLASFQTNAPQLQVDIDRVKAKSMGVSLTDIFETLQINLGSLYVNDFNRFGRTWRVM
AQADAPFRMQQEDIGLLKVRNAKGEMIPLSAFVTIMRQSGPDRIIHYNGFPSVDISGGPA
PGFSSGQATDAIEKIVRETLPEGMVFEWTDLVYQEKQAGNSALAIFALAVLLAFLILAAQ
YNSWSLPFAVLLIAPMSLLSAIVGVWVSGGDNNIFTQIGFVVLVGLAAKNAILIVEFARA
KEHDGADPLTAVLEASRLRLRPILMTSFAFIAGVVPLVLATGAGAEMRHAMGIAVFAGML
GVTLFGLLLTPVFYVVVRRMALKRENRVDSHDQQA
>EECEBICD_03121 Outer membrane protein OprM
MKITFTGYRQTATLATLAFVTTLAGCTMAPKHERPASPTAMVYPYATSTVSGAPDAADIG
WRDFFHDPLLQELIAIALRNNRDLRKAGLNVEAARALYRIQRAEMLPTLGIATAMDAGRT
PADLSVTDEPEINRRYEMAGATTAWELDLWGRVRSLSDQALAAYMALDETYIAARMSLVS
EVASAWLTLRADRELLRLTEDTLAAQKSSYTLTTQLARTGNATQLDLRMAEIALRSAEIN
RAAYTRQLARDRNALELLLGQPLTPELSRRLNEAVTLTEVAIPTTLPGGLPSDLLVRRPD
IRAAEYRLRGANARIGAARAAFFPTISLTGTAGTASASLSGLFEPGSGSWRFLPQITLPL
FHGGALRADLDRAHVQKQIEIARYENVIQQAFRDVADGLAGQRTLNDQVQSEQRAVEASQ
IAYELAGLRFQEGVDDYLTLLDTHRMLYGAQQRLVRTRLMQQLNIINLYKALGGGWREYS
EKKQG
>EECEBICD_03122 hypothetical protein
MKKILVCFAGLALTACSANSLNYGAEQVRVMTSEPGKECSYLGDITGSQGNFFTGGWTSN
SNLETGARNDLKNKAYKMGGNTVVLLTQRAGQTGSSWHGSGSSKQTNVTLSGNVYRCPR
>EECEBICD_03123 hypothetical protein
MFPLNGEYNSCQISASEDIIIIIDGRVLIQGVWKGFKALMQHYPHARRIWLTRRDIGKLY
PHGCDRDPDIDPDLAKPVFIEHFIKYACANEVAVGELAPLTKKEEELLWHFLYKKSMSQI
ASSYGMSRKTLYIHRLRICRKYGFKRFFHLLFIYQRSRHIFASKICRVDKNADQA
>EECEBICD_03124 Attachment invasion locus protein
MKRRSSFLVFLGLLLASPLALANDQHTVSFGYAQTHLSSLKNSDSKDLRGFNFKYRYEFN
ETWGMLGSFTATRNEMENYTWKEGKLHKNGSDSVDYGSLMFGPTYRFNDYVSLYGNAGIA
TMKFNKHSKEESFAYGAGVIFNPVKSISIDASWEASRFFAVDTNTFGVSVGYRF
>EECEBICD_03125 hypothetical protein
MHFHKRTLLSVATLGMLAVSVVLMVVWVRNSNHSSINTNEFLCTTRTVTTIQPKDIHADG
SLVLDFKMKRITLQYEIKTKDNGVKILYRDVYMKNLHRTAPGVYTFEVSQVKVFATDTAG
DLLSYLRVLHPEAANEIRISKVGEKTFFYSLNRQLYNVCSAQ
>EECEBICD_03126 hypothetical protein
MSQNNYLIDKRVILDCERMTLSCAGESITISESERSLLIAFHEGLFKKDDLINYVWGRKG
VVVSDASYYKLINQLRGSFDKIGLKGASVVTRPRVGVLLSVSIEPLSDEAQNTPLPAVAE
TEVSTVEVRHDDTIITPIPGAVNKKDWLYFCLAALLMFFMMYKVKGENIDYFTLQGSYDG
YTFYSVGHDKTHLTDIVEAYSEMSNEIHKQNGKIIYYIRVPNTNIFVQCLNQLDIAEPKC
ISIKERY
>EECEBICD_03127 putative cyclic di-GMP phosphodiesterase PdeN
MKVRKLFLMLFIAVPLIVMILVGLLAGIFQLKRDIAVSANTLLRFSADISAASWQVAGKA
ARLAESSCTDTLKELSRTRAFTPYVRDIGFLENGDITCSFVTGTERYHFSRLAGLSLPAS
YPERWLRSIGSMAEGPDRLVVVYVKKVAANKAAFVIVDSQYVQELIEILAAERASAFSLT
FGAGEAITSAATLRGKAFLTQRFTSTDHTLQLMVRTPFSTLSAYWLQNLFIFVPLSLCLS
VGMMLFYRRWYLKRLSLAREIARGITHNEFTVHYQPVFNVKHGSCGGVEALMRWPQPDGR
FITPDIFITAAENEGMIIPLSRHLFELIAHDVINWTVPDDFYISVNISPAHLMDDGFIQD
IEALRTRLGTITLMLELTERSLIVEPSQVAEKLSTLREKGVLIAIDDFGTGYCSLSYLQQ
LPVDSLKIDRTFIDTIDTSSDDVPVLDTIITLSQRLGLNVVAEGVSTQHQLRYILSHGVG
FVQGFLYARPMGANDFMSWLGKSAQRQNGLLKGENESASGLVAE

>EECEBICD_03128 hypothetical protein
MLLPIRKRNGFFRDICISKLRRTGMKTFTFKVNSVKTFESDTAGDLFSWLRLLQPGTINE
LKMVKIGKNTYMFSLNRHLYNVCTTSSNVEL
>EECEBICD_03129 hypothetical protein
MYYSKRTLLIIFVIFIAIVAIGSVIALEWVNRDSYRTNNLICTTKSEAYIEPRKLYMDGG
LVLDLKSGRITLHYDVTTDTKEKRLFS
>EECEBICD_03130 hypothetical protein
MNESIFLLDKRVVFDSTKMTLSHGNEIIRISEAETHLLLAFWHGLYKKEDIIHLVWENRG
GCVSESSYYKLINQMRNDFSSIGLQPSDIVTRPRVGVSLSVAIEPIKKITSLKVSDENVK
GPSTREKIFYKIKRHSVFVVLTGAILLALLYGVFTIYKAPVRNSPDSFFTYLGEYNDYAI
YKTKEDKVTLSEVVFAFNSLKIKIYRQNGRHLYYIREPNMNIFLQCLNPVEMAVPKCITV
KERY
>EECEBICD_03131 S-fimbrial protein subunit SfaA
MSMKKYLAMITGSLLVSSSAMAVSDNTITFQGEVSDETCSVVINGNQAKPVVLLPTVSTK
DLSEQGKTAGPITFDIGLSGCTGSQDKTTKISTVFVGNQVTSNGNLGNTGSAKNVEVQLL
DTSGEPINLTGGFTGDGDLQLEPNASEASATYTARYYSTGKAEAGTVAATLQYAVSYK
>EECEBICD_03132 putative fimbrial chaperone YadV
MKMDTRHSALYYLIVFLLLALPATASWASVTILGSRIIYPSTASSVDVQLKNNDAIPYIV
QTWFDDGDMNIQPGK
>EECEBICD_03133 putative fimbrial chaperone YadV
MPFIATPPVFRIQPKAGQVVRVIYNNTKKLPQDRESVFWFNVLQVPPTNIGSDSGQNKML
VMLRSRIKLFYRPDGLGKPDNLAKKLQIKTVNKGSGKSGIVIVNPQPWFASLSNLNVKVN
GASYNLDADMIAPFSSQTWWLPGKRSLKSFSGTVTVTLVNDLGARISESYDVPHH
>EECEBICD_03134 Outer membrane usher protein FimD
MSPTINWNRKSLALLIAIVCSGSAQGEEYYFAPALLQGATYGQNIARFNEQQTPSGDYLA
DVYVNGTLVTSSTNIRFNAVKEGQQTEPCLPLSVMKAAQIKSLPATDAATECRPLREWVP
HAGWQFDSATLRLLLTIPMTELTHKPRGYISPSEWDSGALALFLRHNTNWTHTENTDSHY
RYQYLWSGLNMGVNLGLWQVRHQSNLRYANSNQSGSAWRYNSVRTWVQRPVASINSILSL
GDSYTDSSLFGSLSFNGAKLVTDERMRPQGKRGYAPEVRGVAASSAHVVVKQLGKVIYET
NVPPGPFYIDDLYNTRYQGDLEVEVIEASGKTSRFTVPYSSVPDSVRPGNWHYSLAFGRV
RQYYDIENRFFEGTFQHGVNNTITLNLGSRIAQRYQAWLAGGVWATGMGAFGLNATWSNA
RAEHNDRQQGWRAELSYSKTFTTGTNLVLAAYRYSTNGFRDLQDVLGVRREAKTGIDYYS
DTLHQRNRLSATVSQPLGRLGTLNLSASTADYYNNQSRITQLQMGYSNQWRNISYGVNIA
RQRTTWDYDRFYHGVNEPLDVSSRQKYTETTMSFNVSIPLDWGENRTSVAMNYNQSSQSR
SSTVSMTGSSGENSDLSWSVYGGYERYRNSNSDSSAPTTFGGNLQQNTRFGALRANYDQG
DNYRQEGLGASGTLVLHPGGLTAGPYTSDTFALIHADGAQGAIVQNGQGAVVDRFGYAIL
PSLSPYRVNNVTLDTRKMRSDAELTGGSQQIVPYAGAIARVNFATISGKAVLISVKMPDG
GIPPMGADVFNGEGTNIGMVGQSGQIYARIAHPSGSLLVRWGTGANQRCRVAYQLDLHTK
EPFLYLNKICEKE
>EECEBICD_03135 hypothetical protein
MLFSFRTLLFITSLFVSAGTWSSCIKVTDKSALSDAAIKAGYTAQNWIGATDTNTGNIGL
PTVISVSNSETFQPSGTLLASGIGNFLTAATGTPYSSKQVLYRCDSADAGKLYEMYSTNG
DSAFAGAFFTPEVEGAYYDVERNVAVRMTNLSTGEYYSRFWKERQLTADSWFQDDKYIYI
PASAFSNVLYEMFKIDSRKYFAYQNPMDRDTWTQPRGYIAFKGPGLITERIKAGLDHASD
YYGWPSYWPGAWSTYNSVTYVRGALCKITDYPAIVKIPPVAVGILAAGGNSQAPFHVSLE
CESGAVSSALPSTSAANVAMGFVVNQPTAVAAARRLGLTTSAGGLSWLLDTHYGEPGVAS
GVGIRIYNDAGTPINLLPDRIKTGTGNARGWYGYKDLTTRVSSGSVETYSGDFTASLEAI
GGQTVTAGSVNAQLQAVVSFQ
>EECEBICD_03136 Chaperone protein FimC
MRNYRQWLVFSKVILTLLGLTGWYGPAQAAVNIDRTRIIFASDDVAQSLTLSNDNTTPML
LQVWTDAGNIDASPDNSKTPLVALPPVFKMQPGELRTLRLLLSSRQQLATDRESLFWLNI
YQIPPVTQDIKNHPRKLVLPLRLRLKILIRPTELKAPTEAEEKKLRFIAKENTIRIVNPT
SWYMSLTLTMDNKKSIGDIMVAPKTALDVPLTRSPTPGASINYAVINDSGNWRQYTAILE
KYISKSDFIPEF

>EECEBICD_03137 hypothetical protein
MKLIIILLLALFPLCSSASNHYALVFENNTILLVLNLKCSPCDLNCANIQYQIFNKETES
VISGVAKPVTTGIENNFRGYMMRNNNTFYTLIESDNENVWDMSVEEKGTQQNKDAVQKVK
YQLNAIFDNGTC
>EECEBICD_03138 hypothetical protein
MTLKPGEKSGNNGGIYVEVGPRGGIKDNFATIAETKPRHQRNALKATGCWQKEHLTVNVS
QSIWRHDSWRRLITVRPVLYRLASIVQDAP
>EECEBICD_03139 HTH-type transcriptional regulator DmlR
MRLTPEGENALGGAMQVVSEMDGFVSRLDNQRGVLAGNITINCSFGFGHKYVAEALSSFM
IAYPDITVKLTLTDREVDLVEEGIDIEIRVGDDINELYIARQLATNRRILCASPEYLERH
GKPESVSALVQHDCLMIQEKNSAFGNWILTDGKQQTHCRLNGFHSSNSGSVVLIWALKGH
GITLRSEWDVAQYIERGELVRVLPQWYQEANIWAVYTRRSSSSDRIKICIDFLTEHLAQC
LPGGKAPGVL
>EECEBICD_03140 Haloacetate dehalogenase H-1
MSTLIECGASPFIPGVALKDVRLENGLTVRVAIGGSGSPLVLLHGHPQNHTTWRKVAPTL
AQNHTVILPDLRGYGDSDKPTSDPAHRTYSKRTMAQDIVMLMDALGFSRFAFVGHDRGGR
VGHRLALDYPDRVTCCTFIDIAPTATMYALTDKSFATRYFWWFFLIQPFPLPETMIAHDP
AFFLRKHISGQLKIEGATSQEAFNEYLRCYQNPEMIHAICEDYRAAATIDLDDDAADTSA
RIRCPLQLLWGGLGTVGQLYNVVGTWKEKALNVQGEALPCGHSPQEECPEYFIQKLQSFL
HSVL
>EECEBICD_03141 hypothetical protein
MKLVQYLVNGGKRYGIMQETGIIDLSQRLGDKYPTLKSLLCANALTDAALWCDEPADYYY
QEVTFLPVIDDPQKIICVGMNYADKRIEFNETNPAPTLFVRFADSQTGHNGLLLKPENTN
EFDYEGELAVVIGRPCSRVSAEDALDYVAGYSCYMDGSVRDWQHSWFTAGKNWPSTGSFG
PCLVTTDDIPDPQMLRLLTRLNGREVQNESTANMIHPIASLIAYISTFTLLSPGDTILTG
SPGGVGKKRVPPLFLHDGDVIEVEIEHIGTLRNVVRDSRYLTSSVSWHDGRK
>EECEBICD_03142 3-isopropylmalate dehydratase small subunit
MDTFKQISGRIAPMLEPNIDTDVIMPKQFLKGIDRQGLDKGVFFDRRFMAGGQPNPDFIL
NMPGWQSATFLLVGPNFGCGSSREHAVWGLKQLGIRGLIGSTFAGIFDDNCQRNGILTVS
LDEPALARLAQLAASADTNSITVSLDRCEITTAEETISFVISELKRAMLAAGEDAIAWTL
QYLPEIENFEVAHYSRRPWLKRPASPRG
>EECEBICD_03143 3-isopropylmalate dehydratase large subunit
MSAKTLYEKLVESHTIRELDNEGHVLLYIDRSILNEYTSPQAFSGLRERGRTVRHTDTFL
LNIDHVNPTRSQRDDLMTDPGGQLQVDYFRENAADFGITLFDVLDPRQGIEHVVAHEQGL
VMPGMVIAAGDSHTTTYGAFGALGFGIGTSEIEHLLATQTLVYRKLKTMRVSVQGELPFA
CSAKDIVLELLERIGADGATGYAIEFVGEAISALSVEGRMTLCNMAVEAGARGAIIAPDK
KVFDYIYGKPQMPVGELWQQALLEWSQLSSDADAVFDKTVAINCHDLEPKVTWGISPDQT
GSITGRVPFPEQETNPLKRLALEKALHYMGLTAGMLLKDIRISHAFIGSCTNGRIEDLRA
VAKVLEGRKIASHVRGIIVPGSTMVRRQAEEEGLAKIFIAAGFEWRQSGCSMCLAMNEDV
LSPGDRCASGTNRNFPGRQGAGARTHLMSPAMVAAAAVAGHLVDVRSLLQAGE
>EECEBICD_03144 Inner membrane transport protein YnfM
MKNNIEETIGKYLPILMILPLAGLAELASLYSIQALLPKLSEVYNIPLNQVGMILSAEVG
FLALAMLFSGTLSDRFGRKPIIFYSLLAGGILTLLCATASSWPMLVVYRALLGIAVSGIT
AAVTVYISEEVSPALAGIVTGYFIFGNSLGSMSGRVFATLMMEHVSIDTIFFIFGGVLIA
MALAVKLFLPTSRQFVPTPSLQLGAVLKGGLEHFKNIRVSLCFVIGFILFGSFTSIFNFL
AFYLHRPPYELSYTWIGLIPVSFSLTFFLAPYAARVALNIGSMNALSMLIICMMVGAFLT
LIAPSLWVFISGIVLLSVAFFSAHSTVLAWVSSRSPNAKGQATSFYLLCYYSGGAVMGYL
NGYLFSWQGWNAIAASCLMMLGIGLFICRFIFAKYEKQPQIKKQSVQESF
>EECEBICD_03145 hypothetical protein
MPTAIEKALDFIGGMNTSAPVPHSMDESTAKGILKYLHDLGVPASPEVVMARGEQEGWNP
EFTKKVAGWAEKVASGNRILIKNPEYFSTYMQEQLKELV
>EECEBICD_03146 hypothetical protein
MPPLSITMAQYGVVAGQGNIRGTEGPRNAVATGLVLAGEAKK
>EECEBICD_03147 Diol dehydratase-reactivating factor alpha subunit

MASLTSHDMNAVHIQDLLAVDTFIPRAVQGGIAGECSMENAVGIAAMVKSDRLQMQAIAS
ELSARLNTPVEVGGVEANMAVAGALTTHGCAVPLAILDLGAGSTDAAIINNDGAVRRCRR
AG
>EECEBICD_03148 IS3 family transposase ISSty2
MTKPASTTKKPRKQHTPEFRQEALKLAERIGVAAAARELNLYESQLYNWRSKQQNQLSSS
EREQEMSAEIARLKRQLAERDEELAILQKAATYFAKRLK
>EECEBICD_03149 hypothetical protein
MFAVTDRTEPARGFRPPPLPTQAGRHGFHVELVTLSTKLIRQAWRTIALFCVTECFPDDV
ITDKAEPLTLVYLMTTLMPDVPAATGNLKYTAHGFDIELSLMFFDEDILHFRRFAKYVAA
FWRMASSSSRSAS
>EECEBICD_03150 Prophage integrase IntA
MPLNDMQIRRAKPETKAYTLGDGQGLSLLIEPNGSKSWRFRYRFAGKPKMISLGVYPTIT
LADASSRRDVTHPLLIRAAGVKVLLQPIL
>EECEBICD_03152 Gamma-glutamyl phosphate reductase
MLEQMGIAAKAASYKLALLSSGEKNRVLEKIADELEAQMESILSANVQDVEQARANGLSE
AMLDRLTLTPARLKAIADDVRQVCNLADPVGQVIDGGLLDSGLRMERRRVPLGVVGVIYE
ARPNVTVDVASLCLKTGNAVILRGGKETYRTNAATVRVIQKALKACGLPEAAVQAIDNPD
RSLVNEMLRMDKYIDMLIPRGGAGLHKLCREQSTIPVITGGIGVCHIFVDSSADIAPALK
IIVNAKTQRPSTCNTVETLLVHQDIAERFLPALSKQMAESGVTLHGDETVMQLHGPAKLV
PLKPEKLDNEFLSLDLNVVVVENMDGAIAHIREHGTQHSDAILTSDMHNAARFVNEVDSA
AVYVNASTRFTDGGQFGLGAEVAVSTQKLHARGPMGLEALTTYKWIGFGDGTIRA
>EECEBICD_03153 Glutamate 5-kinase
MSDSQTLVVKLGTSVLTGGSRRLNRAHIVELVRQCAQLHAAGHRIVIVTSGAIAAGREHL
GYPELPATIASKQLLAAVGQSRLIQLWEQLFSIYGIHIGQMLLTRADMEDRERFLNARDT
LRALLDNHIVPVINENDAVATAEIKVGDNDNLSALAAILAGADKLLLLTDQQGLFTADPR
SNPQAELIKDVYGVDDALRSIAGDSVSGLGTGGMSTKLQAADVACRAGIDTIIASGSKPG
VIGDVMEGISVGTRFHAQASPLENRKRWIFGAPPAGEITVDEGATAAMLERGSSLLPKGI
KSVTGNFSRGEVIRICNLQGRDIAHGVSRYNSDALRRIAGHHSQQIDAILGYEYGPVAVH
RDDMITR
>EECEBICD_03154 Outer membrane porin PhoE
MNKSTLAIVVSIIASASVHAAEVYNKNGNKLDVYGKVKAMHYMSDYDSKDGDQSYVRFGF
KGETQINDQLTGYGRWEAEFAGNKAESDSSQQKTRLAFAGLKLKDIGSFDYGRNLGALYD
VEAWTDMFPEFGGDSSAQTDNFMTKRASGLATYRNTDFFGIVDGLDLTLQYQGKNEDRDV
KKQNGDGFGTSVSYDFGGSDFAVSGAYTLSDRTREQNLQRRGTGDKAEAWATGVKYDAND
IYIATFYSETRNMTPVSGGFANKTQNFEAVIQYQFDFGLRPSLGYVLSKGKDIEGVGSED
LVNYIDVGATYYFNKNMSAFVDYKINQLDSDNTLGINDDDIVAIGLTYQF
>EECEBICD_03155 Sigma factor-binding protein Crl
MTLPSGHPKSRLIKKFTALGPYIREGQCEDNRFFFDCLAVCVNVKPAPEKREFWGWWMEL
EAQEKRFTYRYQFGLFDKEGNWTAVPINETEVVERLEYTLREFHEKLRDLLISMELALEP
SDDFNDEPVKLSA
>EECEBICD_03156 hypothetical protein
MTQANLSETLFKPRFKHTETSTLVRRFNRGSQPPMQSALDGKNVPHWYRMINRLMWIWRG
IDPREILDVQARIVMSDAERTDDDLYDTVIGYRGGNWIYEWAKQAMDWQQKACQEQDAMR
SGRYWLHASTLYNIAAYPHLKGDELAEQAQALANRAYEEAAQRLPGSLREMEFAVPGGSP
VTAFLHMPKGDGPFPTVLMCGGLDAMQTDYYTLYERYFAPRGIAMLTLDMPSVGFSSKWK
LTQDSSLLHQHVLKALPNVPWVDHTRVAAFGFRFGANVAVRLAYLEAPRLKAVACLGPVV
HALLSDPQRQSTVPEMYLDVLASRLGMHDASDEALRVELNRYSLKVQGLLGRRCPTPMLS
GFWKNDPFSPEEESRLITTSSSDGKLIEIPFNPVYRNFDRALQEITDWINHRLC
>EECEBICD_03157 Xanthine phosphoribosyltransferase
MSEKYVVTWDMLQIHARKLASRLMPSEQWKGIIAVSRGGLVPGALLARELGIRHVDTVCI
SSYDHDNQRELKVLKRAEGDGEGFIVIDDLVDTGGTAVAIREMYPKAHFVTIFAKPAGRP
LVDDYVIDIPQNTWIEQPWDMGVVFVPPISGR
>EECEBICD_03158 Cytosol non-specific dipeptidase
MSELSQLSPQPLWDIFAKICSIPHPSYHEEQLAEHIVSWAKEKGLYVDRDQVGNILIRKP

ATAGMENRKPVVLQAHLDMVPQKNSDTVHDFTTDPIQPYIDGEWVKARGTTLGADNGIGM
ASALAVLADDNVVHGPLEVLLTMTEEAGMDGAFGLQSGWLQADILINTDSEEEGEIYMGC
AGGIDFTSNLPLTREAVPAGFACFKLTLKGLKGGHSGGEIHLGLGNANKLLARFLAGHAE
ELDLRLIDFNGGTLRNAIPREAFATLAVAADNVGALKTLVNAYQDILKNELAEKEKNLTL
QLNEVASDKAALTAPSRDTFVRLLNATPNGVIRNSDVAKGVVETSLNVGVVTMSDANVEI
HCLIRSLIDSGKDYVVSMLDSLGKLAGAKTEAKGSYPGWQPDANSPVMHLVRETYQRLFN
KTPNIQIIHAGLECGLFKKPYPDMDMVSIGPTITGPHSPDEQVHIESVGHYWTLLTELLK
AIPAK
>EECEBICD_03159 Peptide chain release factor RF2
MILLQLSSAQGPDECCLAVKKALDCLTKEAAREKVSLTRLETEPGRLPDTLRSALVSLDG
EKAMAFSERWCGTLLWICTSPYRPHHGRKNWYVGIGRFSADEHIQSDEIRFETLRSSGPG
GQHVNKTDSAVRATHLASGISVKVQSERSQHANKRLARLLIAWRLEQQRQNECAALKSER
RLFHHQIERGNPLRIFKGMAFTPQ
>EECEBICD_03160 RNA-splicing ligase RtcB
MGNYIRPLSDVVFSIASDNLWIEDSAIQQLYTTAKLTGMKRVIGMPDLHPGRGYPIGAAF
FSRGRFYPALVGNDIGCGMALWQTDILGRKYNADKLEKRLASLPDVADAQWLEENVPAAM
QHHSWRSALGSIGGGNHFAELQQVDRIVDADSFALSGLQKAQLLLLVHSGSRGLGQAILR
RHVEAFSHNGLPEDSDDARHYPAEHDDALAFARSNRALIARRILQQLRAEGEPRLDVAHN
FVEPCTVAGEAGWLHRKGATPDGQGLVIIPGSRGDYSWLVKPVVSEESLFSLAHGAGRKW
MRTECKERLSAKFTPRQLCRTGMGSRVICRDRQLIYEEAPQAYKSIDSVVDCLADAGLIT
PVACLRPVLTLKTSGEKSA
>EECEBICD_03161 DNA polymerase IV
MRKIIHVDMDCFFAAVEMRDNPALRDIPIAIGGSRERRGVISTANYPARQFGVRSAMPTA
MALKLCPHLTLLPGRFDAYKEASRHVRDIFSRYTSLIEPLSLDEAWLDVTDSPHCYGSAT
LIAREIRQTIFNELQLTASAGVAPVKFLAKIASDLNKPNGQYVITPADVPGFLKTLPLAK
IPGVGKVSAAKLENMGLRTCGDIQQCDLAMLLKRFGKFGRVLWERSQGIDERDVNSERLR
KSVGVERTLAEDIHEWSDCEAIIEHLYPELERRLAIVKPDLLIARQGVKLKFNDFQQTTQ
EHVWPQLNKEDLITTARKTWDERRGERGVRLVGLHVTLLDPQLERQLVLGL
>EECEBICD_03162 hypothetical protein
MRKIAFFLAMLLMPCVSFAGLLSSSSPVTPVSKEYKQQLMGSPVYIQIFKEERTLDLYVK
MGEQYQLLDSYKICNYSGGLGPKRRQGDFKSPEGFYSVQRNQLKPDSRFYKAINIGFPNA
YDRAHGYDGKYLMIHGACVSVGCYAMTDSGIDEIFQFVTAALVFGQPSVQVSIYPFRMTD
ANMQRHKYSYYKDFWAQLKPGYDYFEQTHKPPTVSIVDGRYVVSKPLSHEVVQPQLASNY
TLSEAK
>EECEBICD_03163 Putative glutamine amidotransferase YafJ
MCELLGMSANVPTDICFSFTGLVQRGGGTGPHKDGWGITFYEGKGCRTFKDPQPSYHSPI
AKLVQNYPIKSCSVIAHIRQANRGEVALENTHPFTRELWGRNWTYAHNGQLNGYKSLETG
NFRPVGETDSEKAFCWLLHKLTQRYPRTPGNMTAVFKYIATLATVLREKGVFNMLLSDGR
YVMAFCSTHLHWITRRAPFGVATLVDQDMEIDFSSQTTPNDVVTVIATQPLTGNETWQKI
MPGEWALFCLGERII
>EECEBICD_03164 Phosphoheptose isomerase
MYQDLIRNELNEAAETLANFLKDDANIHAIQRAAVLLADSFKAGGKVLSCGNGGSHCDAM
HFAEELTGRYRENRPGYPAIAISDVSHISCVSNDFGYDYIFSRYVEAVGREGDVLLGIST
SGNSGNVIKAIAAAREKGMKVITLTGKDGGKMAGTADIEIRVPHFGYADRIQEIHIKVIH
ILIQLIEKEMVK
>EECEBICD_03165 Acyl-coenzyme A dehydrogenase
MMILSIIATVVLLGALFYHRVSLFLSSLILLAWTAALGVAGLWSIWLLVPLAIILVPFNL
TPMRKSMISAPVFRGFRKVMPPMSRTEKEAIDAGTTWWEGDLFQGKPDWKKLHNYPQPQL
TAEEQAFLDGPVEEACRMANDFQITHELADLPPELWAYLKEHRFFAMIIKKEYGGLEFSA
YAQSRVLQKLSGVSGILAITVGVPNSLGPGELLQHYGTEEQKNHYLPRLARGQEIPCFAL
TSPEAGSDAGAIPDTGVVCMGEWQGQQVLGMRLTWNKRYITLAPIATVLGLAFKLSDPDR
LLGGEEELGITCALIPTSTPGVEIGRRHFPLNVPFQNGPTRGNDIFVPIDYIIGGPKMAG
QGWRMLVECLSVGRGITLPSNSTGGVKSVALATGAYAHIRRQFKISIGKMEGIEEPLARI
AGNAYVMDAAASLITYGIMLGEKPAVLSAIVKYHCTHRGQQSIIDAMDITGGKGIMLGES

NFLARAYQGAPIAITVEGANILTRSMMIFGQGAIRCHPYVLEEMAAAQNNDVNAFDKLLF
KHIGHVGSNTVRSFWLGLTRGLTSHTPTGDATKRYYQHLNRLSANLALLSDVSMAVLGGS
LKRRERISARLGDVLSQLYLASAVLKRYDDEGRHEADLPLVHWGVQDALYRAEQAMDDLL
QNFPNRVVAGLLTAMIFPTGRHYLAPSDKLDHAVAKILQVPNATRSRIGRGQYLTPAEHN
PVGLLEEALRDVIAADPIHQRICKELGKNLPFTRLDELARNALAKGLIDKDEAAILAKAE
ESRLRSINVDDFEPEALATKPVKLPEKVRKVEAA
>EECEBICD_03166 Omega-amidase YafV
MSGLKITLLQQPLVWMDGPANLRHFDRQLELVSGRDVIVLPEMFTTGFAMEAANNSLSQD
SVITWMQAKARQTDALIAGSAALQTERGAVNRFLLVEPEGKVHLYDKRHLFRMADEHQHY
AAGDKRIIVQWRGWRILPLLCYDLRFPIWSRNRNDYDLALYVANWPAPRSLHWQTLLTAR
AIENQAYVAGCNRVGTDGNGLHYRGDSRIINPQGDIIATAEPHQATRIDADLSLVALQDY
REKFPAWRDADPFTL
>EECEBICD_03167 hypothetical protein
MKNFVRTTLLAATLAGVSFGAFATAVPNPPLPAQDPIVQHLKLTNDQITRIKKLHQQLET
DVSQISMKGIKDGALIEVIKSGKWDDAAVKQQLAAFSNIEQQARYYRVKYYFDLSKVLTP
EQRQQVQQDLAQALE
>EECEBICD_03168 Outer membrane protein PagN
MKNFFAVCIIPLVVAWSATASAKEGIYITGKAGTSVVNVYGINSTFSQDEIVNGHATLPD
RTKGVFGGGVAIGYDFYDPFQLPVRLELDTTFRGETDAKGGQDIIAFGDPVHINVKNQVR
MTTYMVNGYYDFHNSTAFTPYISAGVGLAHVKLSNNTIPVGFGINETLSASKNNFAWGAG
IGAKYAVTDNIMIDASYKYINAGKVSISKNHYAGDEHTAYDADTKAASNDFMLGITYAF
>EECEBICD_03169 HTH-type transcriptional regulator DmlR
MMELINNRGMRDWMIFIKVAEVGNLSRAARELDISISAVSKSLSRLENSIEVTLLRRDSH
HLELTGAGQTAYASMKRITSSFQSLLDELRNPDKIIRGSIKFSAPALVCEFLANKWIWEF
TASYPDTKIYLDSRERSDFFSKSLEFDELVFKSGIIESEDLVYRKISPLKLVLCASPKYI
RKYGRISHPGDLENHIIVGLHNHGLSGPLTLFRQDESYTISGAVNVHLSSNNLLSVLNLV
LEGKGINLITPAWLATKYLKNNELEIILPEWRVPDLPIYLVWRHRQYYSPLFQRFLSFIE
DKWNNRPQIDFLNDD
>EECEBICD_03170 hypothetical protein
MHRSPNKVSRGNKQNIARLFLLESPTRLDIICESGGNRTDGLNKTLTQNINLCAGSSHFV
TSLISSFTGARGRYLRKAIIPAQQQPLPLGLCTFKISHDQKPAGRSPAPVTCCLQILRNR
VYHGLRCYLISAQNQHHHLGYSLQPLPADDGGGFGMQHFHPGLRLSQLSDVPPYAGPAAK
KIRAWCDKTDDVSASPHSFAPLC
>EECEBICD_03171 hypothetical protein
MSYARHLPVLMYHHVSDKPGQITLSPRTFRAQMKWLAESGWKTVTAAEVEAFYHGARLPR
KSVMLTFDGGWLDNWLQVFPVLQEFNLHAHLFLVTSLISDGPVRIPAGELVYSHDECQKL
VKQGRADEVMLRWSEVREMHLSGLVEFHSHTHTHRRWDQKPVSRNPSDLLRVDILLSRKR
MREMLGYCSQHLCWPEGWYCSDYIHVAEELGFTYLYTTERRMNNPVIGSQRIGRINTKER
KNVGWLKRRLFYHTTPGFSSLLARHKGARRIAD
>EECEBICD_03172 hypothetical protein
MWMKIQRMKTVIYSVTLLVAASSLMPIANAAEKLQTTLRVGAYFRAGHVPDGMVLAQGWV
TYHGSHSGFRVWSDEQKAGNTPTVLLLSGQQDPRHHIQVRLEGEGWQPDTVSGRGAILRT
AADNASFSVVVDGNQEVPADTWTLDFKACALTHEEA
>EECEBICD_03173 F1 capsule-anchoring protein
MQPSSRTTLMLSQSWGRHLGNLSLTGSRTDWRNRPGHDDSYGLSWGTSIGGGSLSLNWNQ
NRTLWRNGAHRKENITSLWFSMPLSRWTGNNVSASWQMTSPSHGGQTQQVGVNGEAFSQQ
LDWEVRQSYRADAPPGGGNNSALHLAWNGDYGLLGGDYSYSRAMRQMGANIAGGIVIHHH
GVTLGQPLQGSVALVEAPGASGVPVGGWPGVKTDFRGDTTVGNLSVYQENTVSLDPSRLP
DDAEVTQTDVRVVPTEGAVVEAKFHTRIGARALMTLKREDGSAIPFGAQVTVNGQDGSAA
LVDTDSQVYLTGLADKGELTVKWGAQQCRVNYQLPAHKGIAGLYQMSGLCR
>EECEBICD_03174 F1 capsule-anchoring protein
MKFKQPALLLFIAGVVHCANAHAYTFDASMLGDAAKGVDMSLFNLGVQQPGTYRVDVMVN
GKRVDTRDVVFKLEKDGQGTPFLAPCLTVSQLSRYGVKTEDYPQLWKAAKTPDECADLTA
IPQAQAVLDINNQQLQLSISQLALRPEFKGIAPEDLWDDGIPAFLMNYSARTTQTDYKMD

MERRDNSSWVQLQPGINIGAWRVRNATSWQRSSQLSGKWQAAYTYAERGLYSLKSRLTLG
QKTSQGEIFDSVPFTGVMLASDDNMVPYSERQFAPVVRGIARTQARVEVKQNGYTIYNTT
VAPGPFALRDLSVADSSGDLHVTVWEADGSTQMFVVPYQTPAIALHQGYLKYSLLAGRYR
SSDSATDKAQIAQATLMYGLPWNLTAYGGIQSATHYQAASLGLGGSLGRWGSLSVDGSDT
HSQRQGEAVQQGASWRLRYSNQLTATGTNFF
>EECEBICD_03175 Chaperone protein caf1M
MKTINFVLMAVALFASNSMVSVYAARQQLNSATKSFSVKLGATRVIYHAGTPGATLSVSN
PQNYPILVQSSVKAEDKNSPAPFMVMPPLFRLEANQQSQLRIIRTGGDMPTDRETLQWVC
IKAVPPENEPSDTQAKGATLDLNLSISTCDKLIFRPDAVKGTPEDVAGNLRWVETGNKLK
VENPTPFYMNLASVTVGGKPITGLEYIPPLLTKH
>EECEBICD_03176 hypothetical protein
MKSIKKLIIASALSMMAASCYASSFLPNTEQEKSVNVSFAAPENLTISFDQVPGLMAGQK
PAGMNIAKLTVDSASIKEYGARGVANTTLDAAGSAWKITGKNSGTILTVGFSNNNMSRGH
GAQMWNGRSWFTFDTNAPLDIVTIGAQNIPPDTYPITVDVVGYQP
>EECEBICD_03177 IS3 family transposase IS1351
MLRMDKGPEFISLALAEWAKKHAVKLAFIQPGKPKKNVFITRFNRTYRTEILNSYLFRTL
NEVREITDKGLSEYNCERPYESRNNMIPKEYR
>EECEBICD_03178 hypothetical protein
MPQAGFINGMPVKVRVMRDCIVITPQHTHGLFVCIEGMGVTRINKRSAEQWLKKFLGALN
NTGDIPVIKRKRTEQELMSN
>EECEBICD_03179 hypothetical protein
MVNDKDTAILISDLMLRFGKELDESVAVVQSRCDEDEFKVYREAVGLIMGEMLIKIMNPL
YEKHPEIKPKGLK
>EECEBICD_03180 hypothetical protein
MVLIVLFVILFFTWLGRENIKNDSAIREVAKEEVDKLFSLYNKGEYAEIYDLSCDSFKNA
TARKDFLTVMGTKMKILGEFKGRKLQYSNVINSKSVELYYRVDYINYSLIEEFNYIKNDG
QKICLQAMFTDDAGKHGEVIKLH
>EECEBICD_03181 hypothetical protein
MAGQLEPEYTPQRKERFVCIIHCVIIAVQVLLLVCYSHYQPLMLIYHQEYQSLEGILMGH
FLREWLIQMLVIKMIVINIQKV
>EECEBICD_03182 Endoribonuclease SymE
MNASGVSVRHINSKTHMTTYYSQIPSLHLKGDWLEEAGFKTGRGVTVKISQGCIVLMADS
NEEQKLREQLYQAKQVVKGIKDVLV
>EECEBICD_03183 hypothetical protein
MTLDDEIKEKILQLSDSLLIIDSWNSIADELSDSFEWIGSKINWSKTSKHESLNLKGNYF
DWIDQINNFIHANNIDSEILHSDNIYYINDSSLDFSVSIKPKQFYQFLKMAINNIPQHHY
FFNREKNGVL
>EECEBICD_03184 putative deoxyribonuclease RhsA
MLYEVCLERTPDNPDENCMTYVAPMGRQIDLQAVEPGSGFYSPGEGLAVRRSEQGHWLIS
SDDGVYRLFEADPFSPQRRRLKMLGDRNSNCQHLTYDNHGRLVEISGDRQRPCIRLHYEL
AAHPQRVTRIFRHYPEGEPELLRRYRYDEAGRLNGVVDNAGQYQREFAYDDNDCMTMHRE
PGGERYYYTWAWFEGPDDAAWRVTGHHTDSGEQYRLDWNLAERSLCVTDSLGRTRCHWWD
AQGLVTAYRDEAGQMTTFRWSDEERLLLGMTDAQGGKWRYVYDRLGHLTETHDPLGRVEQ
TQWHPVWHQPETEVDAAGAAWRYEYDERGNLQAVSDPLHQRTVYGYDRHGQVVRITDARG
GDKYLQWNEDGQLMRHTDCSGSQTAWFYDERTRLERVTDAESNSTRYSYDGNGHLTEVMF
ADGRTERYQPDAAGRLVKYTSPAGQITRWQRDGQGRVRRQTDATGRRTAYEYDAYGRLTT
LTNENGESYRFRYDVLDRVTEQTDPGGSRRAYGYNALNAVTAVIYGGERGGEIRHGLERD
AAGRLTAKITPETRTEYRYDAADRLLEIRRRRHDAAEGGEPEVIRFSYDSAGNLLSEETA
QGVLQNRYDVQGNRTETQMPDGRTLRYLYYGSGHLQQINLGRDVISEFTRDHLHREVQRS
QGRLDTRRMYDRTGRLTRKLTCKGMRGVVPETFIDREYAYSGQDELLKKRHSRQGVTDYF
YDTTGRITACRNEAYLDSWQYDAAANLLDRRQGETAQAGAGSVVPFNRITSYRGLHYRYD
EYGRVVEKRGRNGTQHYRWDAEHRLTEVAVIRGSTVRRYGYVYDAPGRRVEKHELDAEGK
PYNRTTFLWDGMRLAQECRPGRSSSLYIYSDQGSHEPLARVDRAAPGEADEVLYYHTDVN
GAPEEMTDGRGNIVWEAGYQVWGNLTHEKETRPVQQNLRFQGQYLDRETGLHYNLYRFYD

PDIGKFISGDPISLKGGINLYAYAPNPLSWIDPLGLKCGSSYEQARNKALKWLEERGFKA
ERVNIGKFGSTRGKPVGMTTADGKTGFRIEYDERSGAHINVFSGKDKGEHFLSDASESIV
TKLQKLFDLPSKPQRPIS
>EECEBICD_03185 hypothetical protein
MSFVSTNNKSGIGGLTTTTPPITGESGGVTADSVAGSVADAAEAAVEQAAGSLFGALPEP
SGLVKAAVAAAQAAAAGMAQDAVSAIVSAVAGGPGAHNVTVSGSAVPPGALLFASLDGGE
TLSELFSYVVQLKTPDTLNLGYVSPAANLPLKPMVGKDLCVNIELDGGGTSRWSGSGPTA
AVMNGRRGCSGGAGACCMKCAWSVRRTTRMKTA
>EECEBICD_03186 hypothetical protein
MAVRLTFDGQKLTWPGIGIFKATTGLPDLQWPDKQCVPDAAIPEGNYKLFIQFQGEAPIR
NAADCDLGPSWGWSTIPRGQAAGTCEIYWANWGYNRIRLESADEKTRKACGGKRGGFYIH
DSTKGYSHGCIEVEPVFFRILKQETEKENGEKTFTVNVKYVSGQQTNGGTKQ
>EECEBICD_03187 hypothetical protein
MDNLTILKTTVTLKKSTGECGCFSTLINYTSLLAQDVEGYERGSAYSLQEGNISLAKMQG
RYPFSFVLSVDNQSVRDQKLALMIRCTPPL
>EECEBICD_03188 hypothetical protein
MPLHYTELALNRSESRHDPTLVHFSNIFHHRWLTQFWQAWRSAQSAGGGLDKPEHDRFAF
YIASLSGQDLRESAESPLSDHVRLAASAHLVRESRNPDGLAATLAHYFSVPFAVKEFALH
WITVANDEITRLGTPAQSSVLGNGALIGQAVPDMQYKFRLIIGPLTLEDYLRFLPGGNNL
PVLTELVRTFIGFEYCWEIELQLKPHAAPPAVIGGAQRLGWTAWAGKAMHDRPVTGMIFE
PEHGLTN
>EECEBICD_03189 hypothetical protein
MDMSLTKPVSAEQPCGPDPEYDPEYLLLFSRAAPQAEAQYGDFVSTPEAVNWPEIERDTR
RLLTRSKDIRVLILLLRSRIQQAGAQGLAEMLTQLAELSVIWSDALHPQLLTGEGAPAES
IEDAALARSNALAALLDHEGVMADIRGITLSNSAVLRLQVRDVERALSAPRPADALAPES
VRQQLADLEARGALPLAAFREAQESAERLQVWARETLGDLAPDFSRLRQLLALLPDVVQT
TTPEIPPSPAPVPDPAGTVMDNAVPARAEDVPPVNAPPGISLPHNAEPGPIRDRNDALER
LRCIRRWFESSEPSSPTIPLLRQAERLVGKRFSEVINEIPVELLEKWDALE
>EECEBICD_03191 DNA polymerase III subunit epsilon
MTDMSTAITRQIVLDTETTGMNQIGAHYEGHKIIEIGAVEVINRRLTGNNFHVYLKPDRL
VDPEAFGVHGIADEFLLDKPVFADVVDEFLDYIRGAELVIHNASFDIGFMDYEFGLLKRD
IPKTNTFCKVTDSLALARKMFPGKRNSLDALCSRYEIDNSKRTLHGALLDAQILAEVYLA
MTGGQTSMTFAMEGETQRQQGEATIQRIVRQASRLRVVFASEEELAAHESRLDLVQKKGG
SCLWRA
>EECEBICD_03192 Ribonuclease HI
MLKQVEIFTDGSCLGNPGPGGYGAILRYRGHEKTFSEGYTLTTNNRMELMAAIVALEALK
EHCEVTLSTDSQYVRQGITQWIHNWKKRGWKTAEKKPVKNVDLWKRLDAALGQHQIKWVW
VKGHAGHPENERCDELARAAAMNPTQEDSGYQAEA
>EECEBICD_03193 hypothetical protein
MKPARLSQTVVAPGCWGELPWGNYYREALEQQLNPWFAKMYGFHLLKIGNLSAEINSEAC
AVSHQVNVSSQGSPMQVLADPLHLPFADKSVDVCLLAHTLPWCTDPHRLLREADRVLIDD
GWLVISGFNPLSLMGLRKLVPVLRKTPPYNSRMFTLMRQLDWLSLLNFEVLHYSRFHVLP
WKKQGGGF
>EECEBICD_03194 Hydroxyacylglutathione hydrolase
MNLNSIPAFQDNYIWVLTNDEGRCVIVDPGEAAPVLKAIAEHKWMPEAIFLTHHHHDHVG
GVKELLQHFPQMTVYGPAETQDKGATHLVGDGDTIRVLGEKFTLFATPGHTLGHVCYFSH
PYLFCGDTLFSGGCGRLFEGTPSQMYQSLMKINSLPDDTLICSAHEYTLANIKFALSILP
HDSFINEYYRKVKELRVKKQMTLPVILKNERKINLFLRTEDIDLINEINKETILQQPEAR
FAWLRSKKDTF
>EECEBICD_03195 Membrane-bound lytic murein transglycosylase D
MDDGTSIAPDQDLWAFIGDELKMGIPENSRIREQKQKYLRNKSYLHDVTLRAEPYMYWIA
GQVKKRNMPMELVLLPIVESAFDPHATSGANAAGIWQIIPSTGRNYGLKQTRNYDARRDV
VASTTAALDMMQRLNKMFDGDWLLTVAAYNSGEGRVMRAIKANKARGKPTDFWSLSLPRE
TKLYVPKMLALSEILKNSKRYGVRLPTTDESRALARVRLNSPVEMAQVADMAGMSISKLK

TFNAGVKGSTLGVSGPQYVMVPKKHAEQLRESLASGEIDAVQSQLIADNTPLNSRSYKVR
SGDTLSGIASRLGVSTKDLQQWNNLRGSRLKIGQSLTVGAGSSAQRLANNSDSIIYRVRK
GDSLSSIARRHGVNIKDVMRWNHDTDNLQPGDQLTLFVKDNSTPDS
>EECEBICD_03196 putative methyltransferase YcgJ
MTTHSHHDNVEKQFGSQANAYLHSAVHASGRDLARLAQRLSDFSHANVLDMGCGAGHASF
VAAQHANSVVAYDLSASMLEVVAGAAEERHLSNITLRQGYAEKLPFEDASFEVVISRYSA
HHWHDVGQALREVYRVLKPGGVLIIMDVMSPGHPVSDIWLQTVEALRDTSHVRNYSSGEW
LTMVNNAMLVTNTVITDRLSLEFRSWVTRMRTPAPLVEAIRLYQASAPVEVKRYFELQDD
GSFSSDTIMLEAHKAV
>EECEBICD_03197 hypothetical protein
MRKNTYAMRYVAGQPAERILPPGSFASIGQALPAGEPLSSEERIRILVWNIFKQQRAEWL
SVLKNYGKDAHLVLLQEAQTTPELVQFATANYLAADQVPAFVLPQHPSGVMTLSAAHPVY
CCPLREREPILRLAKSALVTVYPLPDTRLLMVVNVHAVNFSLGVDVYSKQLLPIGDQIAH
HSGPVIMAGDFNAWSRPRMNALYRFAREMSLRQVRFTDDQRRRAFGRPLDFVFYRGLNVN
EASVLVTRASDHNPLLVEFSPGKPEQ
>EECEBICD_03198 Inner membrane transport protein YdhP
MPLALFALTISAFAIGTTEFVIVGLVPTIAQQLAISLPSAGMLVSIYALGVAIGAPVLTA
LTGRLPRKQLLVALMVLFTAGNLLAWQAPGYMTLIAARLLTGLAHGVFFSIGSTIATSLV
PKEKAASAIAIMFGGLTVALVTGVPLGTFIGQHFGWRETFLAVSLLGVIALMSSQLLIPA
NIPGRAAASIRDQVKVLTHPRLLLIYAVTALGYGGVFTAFTFLAPMMQDLAGFSPAAVSW
ILLGYGVSVAIGNIWGGKLADKHGAVPALKFIFAALFVLLMVFQVTASTQYAALATILVM
GVFAFGNVPGLQVYVVQKAEQFTPNAVDVASGLNIAAFNIGIALGSVIGGQTVAHYGLAQ
TPWIGALIVLVAFLLMGVSGRLDKTVRIALE
>EECEBICD_03199 HTH-type transcriptional regulator DmlR
MKATSEELAIFVAVVESGSFSRAAEQLGQANSAVSRAVKKLEMKLGVSLLNRTTRQLSLT
EEGERYFRRVQQILQEMAAAETEIMESRNTPRGLLRIDAATPVMLHFLMPLIKPFRERYP
EITLSLVSSETFINLIERKVDVAIRAGTLTDSSLRARPLFTSYRKIIASPDYIARFGKPE
SVEELKQHLCLGFSEPISLNTWPIACSDGQLHEIECGISSNSGETLKQLCLSGNGIACLS
DYMIDKEIARGELVELLADKRLPVEMPFSAVYYSDRAVSTRIRAFIDFLSEYIRTAPAGA
VKEG
>EECEBICD_03200 2,5-diketo-D-gluconic acid reductase B
MTIPAFGLGTFRLKDDVVIASVKTALELGYRAVDTAQIYDNEAAVGQAIAESGVPRNELY
ITTKIWIENLSKDKLIPSLKESLKKLRTDYVDLTLIHWPSPGDAVSVEEFMQALLEAKKQ
GLTREIGISNFTIPLMEKAIAAVGADHIATNQIELSPYLQNRKVVDWAKAHGIHITSYMT
LAYGKALKDEVIARIAAKHNATPAQVILAWAMGEGYSVIPSSTRRENLASNLLAQDLHLD
AEDKNAIAALDCNDRLVSPEGLAPAWD
>EECEBICD_03207 D-glycero-beta-D-manno-heptose-1,7-bisphosphate 7-phosphatase
MAKFVPAIFLDRDGTINMDHGYVHEIDAFEFIDGVIDAMRELKKMGYALVVVTNQSGIAR
GKFTEAQFETLTEWMDWSLADRDVDLDGIYYCPHHPQGSIEEFRQVCDCRKPHPGMLISA
RDFLHIDMAASYMVGDKLEDMQAAAAANVGTKVLVRTGKPVTAEAENAADWVLNSLADLP
SAIKKQQK
>EECEBICD_03208 Methionine import ATP-binding protein MetN
MIKLSNITKVFQQGTRTIQALNNVSLHVPAGQIYGVIGASGAGKSTLIRCVNLLERPTEG
SVQVGGQELTTLSESELTKARRQIGMIFQHFNLLSSRTVFGNVALPLELDNTPKEEIKRR
VTELLDLVGLGDKHDSYPANLSGGQKQRVAIARALASNPKVLLCDEATSALDPATTRSIL
ELLKDINRRLGLTILLITHEMDVVKRICDCVAVISNGELIEQDTVSEVFSHPKTPLAQKF
IQSTLHLDIPEDYQARLKASPETDSVPMLRMEFTGQSVDAPLLSETARRFNVNNNIISAQ
MDYAGGVKFGIMLTEMHGTQEETQAAIAWLQDHHVKVEVLGYV
>EECEBICD_03209 D-methionine transport system permease protein MetI
MSEAMMWLLVRGVWETLAMTFVSGFFGFVIGLPVGVLLYVTRPGQIMENARLYRSLSAVV
NIFRSIPFIILLVWMIPFTRIIVGTSIGLQAAIVPLTVGAAPFIARMVENALLEIPAGLI
EASRAMGATPLQIVRKILLPEALPGLVNAATITLITLVGYSAMGGAVGAGGLGQIGYQYG
YIGYNATVMNTVLVLLVVLVYLIQLSGDRIVRAVTHK

>EECEBICD_03210 D-methionine-binding lipoprotein MetQ
MAFKFKTFAAVGALIGSLALAGCGQDEKDPNHIKVGVIVGAEQQVAEVAQKVAKEKYGLD
VELVTFNDYVLPNEALSKGDIDANAFQHKPYLDQQIKDRGYKLVSVGKTFVYPIAGYSKK
IKSLDELKDGSQVAVPNDPTNLGRSLLLLQKVGLIKLKDGVGLLPTSLDIVENPKNLKIV
ELEAPQLPRSLDDAQIALAVINTTYASQIGLTPAKDGIFVEDKDSPYVNLIVTREDNKDA
ENVKKFVQAYQSDEVYEAANKVFNGGAVKGW
>EECEBICD_03211 Outer membrane lipoprotein RcsF
MRALPICLLALMLGGCSMLSRSPVEPVQSTATPPKAEPEKPKAPRAAPVRIYTNAEDLVG
KPFRDLGEVSGESCQATNQDSPPNIPTARKRMQINASKMKANAVLLHSCEITSGTPGCYR
QAVCIGSALNISAK
>EECEBICD_03212 tRNA (adenine(37)-N6)-methyltransferase
MSSFQFEQIGVIRSPYKEKFAVPRQPGLVKSACGELHLIAPYNQADAVRGLEAFSHLWVL
FVFHQTMDGGWRPTVRPPRLGGNARMGVFATRSTFRPNPIGMSLVALKGIECRKESVILK
LGSLDLVDGTPVVDIKPYLPFAEALPDAAASYAQQAPIAEMPVSFTAEVAQQLTTLERRY
PQLRTFICDVLAQDPRPAYRKGEETGKTYAVWLHDFNVRWRVVNTGFEVFALEPR
>EECEBICD_03213 Proline--tRNA ligase
MRTSQYLLSTLKETPADAEVISHQLMLRAGMIRKLASGLYTWLPTGLRVLKKVENIVREE
MNNAGAIEVSMPVVQPADLWQESGRWEQYGPELLRFVDRGERPFVLGPTHEEVITDLVRN
ELSSYKQLPLNFFQIQTKFRDEVRPRFGVMRSREFLMKDAYSFHTSQESLQETYDAMYAA
YSRIFSRMGLDFRAVQADTGSIGGNASHEFQVLAQSGEDDIVFSDVSDYAANIELAEAIA
PQTPRAAATQEMTLVDTPNAKTIAELVEQFNLPIEKTVKTLLVKAVKDSKSPLVALLVRG
DHELNEVKAEKLPHVASPLTFATEEEIRAVINAGPGSLGPVNMPIPVIIDRTVAAMSDFA
AGANIDGKHYFGINWDRDVATPVVADIRNVVAGDPSPDGQGTLLIKRGIEVGHIFQLGTK
YSEALKASVQGEDGRNQILTMGCYGIGVTRVVAAAIEQNFDERGIVWPDAIAPFQVAILP
MNMHKSFRVQELAEKLYSELRAQGIEVLMDDRKERPGVMFADMELIGIPHTIVIGDRNLD
NDDIEYKYRRSGEKSLIKTGDIVDYLVKAIKG
>EECEBICD_03214 Lipoprotein NlpE
MVRTAILSVAAACTLFALIGCNNRAEVDALQPAQAAELKPMQQSWRGVLPCADCEGIETS
LFLEKDGTWVMNERYQGVREEPSSFASYGTWARTADKLVLTDSNGEKSYYRAKGNELEML
DREGNPVASQLNYTLVPVTASLPVTPMPLRGMYVYRADAATFTDCATGKRLPVASNAQLE
RGYLAAKGEAEKPVLLTVEGHFVLAANPDTGEPVKTLIADKNVKFAPGKDCTH
>EECEBICD_03215 Peptidyl-tRNA hydrolase ArfB
MIAISRTVSIADNELEITAIRAQGAGGQHVNKTSSAIHLRFDIRASGLPEYYKQRLLTAS
HHLISDDGVIIIKAQEFRSQELNREAAIARLVAVIKELTAEQKSRRATRPTRASKERRLS
SKAQKSSVKALRGKVRRPLD
>EECEBICD_03216 putative protein YaeQ
MALKATIYKAVVNVADLDRNRFLDAALTLARHPSETQERMMLRLLAWIKYADERLQFTRG
LSAEDEPEAWLRNDHLGIDLWIELGLPDERRIKKACTQASDVALFAYNSRAAQIWWQQHQ
SKCAQFANLSVWYLDDGQLAQLSEFADRTMTLQATIQDGAIWLSDARNNLEIQLTAWQQP
S
>EECEBICD_03217 hypothetical protein
MEKYCELVRKRYAEIASGDLGYVPDALGCVLKVLNEVAADSALSESVREKAAYAAANLLV
SDYVNE
>EECEBICD_03218 Protein rof
MSMNETYQPINCDDYDSLELACMHHLILTLTLKDGEVLQAKANDLILRKNVEYLLAEVSG
ESCELRLDKIASFSHPEIGTVVVSES
>EECEBICD_03219 tRNA(Ile)-lysidine synthase
MTTLTLNTSLLSSRRILAAFSGGLDSTVLLHQLVLWLERHPDVTLRAIHIHHGLSPHAES
WVRHCETVCARWQVPLVVERVTLADNGLGIEAHAREARYRAFAQTLLPGEVLATAQHLDD
QCETFLLALKRGSGPAGLSAMGERSPFAGTLLLRPLLRETRKTLEQWAVRHGLCWIEDES
NQDDAYDRNFLRLRALPLLQQRWPHFPAAVARSATLCAEQERLLDELLASDLTDCITTEG
TLRLSPLMSMSDVRRAAILRRWLAMRNAPMPSRDALERIWQEVALARDDASPCLRFGDHE
IRRYQSQLWWIKSVAGQHETTVAWPVWQTPLALPAGLGTVQLVPGGELRRPREEESVSIR
FKAPGLLHIVGRHGGRKLKKIWQEQGIPPWRRDTTPLLFYGETLIAAAGVFVTREGAAED

KEGVSLVWHA
>EECEBICD_03220 hypothetical protein
MLGLKQVHHIAIIATDYAVSKAFYCDILGFDLLSEVWREERDSWKGDLALNGQYVIELFS
FPFPPARPSRPEACGLRHLAFSVENVENAVAHLEKHQVKCEPIRIDPYTGKRFTFFNDPD
GLPLELYEQ
>EECEBICD_03221 Constitutive lysine decarboxylase
MNIIAIMGPHGVYYKDEPIKELERALQSLGFQIIWPQNSVDLLKFIEHNPRICGVIFDWD
EYSLDLCSEINQLNEYLPLYAFINTNSTLDVSVHDMRMALWFFEYALGLAEDIATRIHQY
TNEYLDNITPPFTKALFTYAKEGKYTFCTPGHMAGTAYQKSPPGCLFYDFFGGNTLKADV
SISVTELGSLLDHTGPHLEAEEYIARTFGAEQSYMVTNGTSTSNKIVGMYAAPAGSTLLI
DRNCHKSLAHLLMMSDVVPLWLKPTRNALGILGGIPKREFTRDSITRKVAETAQAQWPVH
AVITNSTYDGLLYNTNWIKQMLDVPSIHFDSAWVPYTHFHPIYQGKSGMSGDRVPGKVIF
ETQSTHKMLAAFSQASLIHIKGEYDEETFNEAFMMHTSTSPSYPIVASIETAAAMLRGNP
GKRLINRSVERALHFRKEVQRLREESDSWFFDIWQPEEVDEAECWPVTPGETWHGFADAD
DDHMFLDPVKVTILTPGMDEQGNMSEEGIPAALVAKFLDERGVVVEKTGPYNLLFLFSIG
IDKTRAMGLLRGLTEFKRAYDLNLRVKNMLPDLYAEDPDFYRNMRIQDLAQGIHKLIRQH
DLPGLMLRAFEVLPEMIMTPHQAWQRQIKGEVETVALDQLPGRVSANMILPYPPGVPLLM
PGERITQQSRAVLDFLLMLCSIGQHYPGFETDIHGAKRNEDGVYQVRVLKHAC
>EECEBICD_03222 hypothetical protein
MGLQKTLALSAVAAGIMLSLSGAQAAPLLSSSEPMTINASDLAAKEKALTDFPLMEAVKS
SIQTLDNSAVEQIEPGRAANPANVKRVESILKEADWDYLFPMRAPEYTYSNFLKAIGKFP
AVCGTYTDGRDSDAICRKTLATMFAHFAQETGGHESWRDIPEWRQALVYLREVGWTEGQK
GGYNGECNPDVWQGQTWPCGKDKDGDFLSYFGRGAKQLSYNYNYGPFSDAMYGDVRPLLD
KPELVADTWMNLASAVFFFVYPQPPKPSMLHVIDGTWQPNDRDKANGLVSGFGVTIQIIN
GGVECGGADENAQSLNRIAYYKEFANYLKVPVPADEVLGCKKMKQFDEGGAGALPIYWEQ
DWGWSADTADGKTYSCQLVGYQTPYTAFKEGDYTKCVQHYFNVNVVDDNGTTEPDVTPTP
APVTDENVAPVARIAGPVGAVEAGSPVSLSAEGSTDANGDKLTYTWMSQDGKTLSGQDKA
VVIFNAPDVTQNTQYVVNLTVSDGTLSSTAVYTLNVKAKAAAADDEDKTTSYPAWSSSQK
WNPGDIVNSNGALYQCKPFPEGSWCNVAPAYYEPGVGIAWADAWNAL
>EECEBICD_03223 Acetyl-coenzyme A carboxylase carboxyl transferase subunit alpha
MSLNFLDFEQPIAELEAKIDSLTAVSRQDEKLDINIDEEVHRLREKSVELTRKIFADLGA
WQVAQLARHPQRPYTLDYVRLAFDEFDELAGDRAYADDKAIVGGIARLEGRPVMIIGHQK
GRETKEKIRRNFGMPAPEGYRKALRLMEMAERFNMPIITFIDTPGAYPGVGAEERGQSEA
IARNLREMSRLNVPVICTVIGEGGSGGALAIGVGDKVNMLQYSTYSVISPEGCASILWKS
ADKAPLAAEAMGIIAPRLKELKLIDSIIPEPLGGAHRNPEAMAASLKAQLLEDLADLDVL
STDDLKNRRYQRLMSYGYA
>EECEBICD_03224 DNA polymerase III subunit alpha
MSEPRFVHLRVHSDYSMIDGLAKTGPLVKKAASLGMPALAITDFTNLCGLVKFYGAGHGA
GIKPIVGADFNVHNELLGDELTHLTVLAANNTGYQNLTLLISKAYQRGYGAAGPIIERDW
LVELKEGLILLSGGRMGDVGRCLLRGNQALVEECVAFYEAHFPDRYFLELIRTGRQDEET
YLHAAVELAEARGLPVVATNDVRFLESDDFDAHEIRVAIHDGFTLDDPKRPRNYSPQQYM
RSEEEMCELFSDIPESLENTVEIAKRCNVTVRLGEYFLPQFPTGDMTTEDYLVKKAKEGL
EERLAFLFPDEEERKKRRPEYDERLDIELQVINQMGFPGYFLIVMEFIQWSKDNGVPVGP
GRGSGAGSLVAYALKITDLDPLEFDLLFERFLNPERVSMPDFDVDFCMEKRDQVIEHVAD
MYGRDAVSQIITFGTMAAKAVIRDVGRVLGHPYGFVDRISKLVPPDPGMTLAKAFEAEPQ
LPEIYEADEEVRALIDMARKLEGVTRNAGKHAGGVVIAPTKITDFAPLYCDEEGKHPVTQ
FDKSDVEYAGLVKFDFLGLRTLTIINWALEMINKRREKNGEPPLDIAAIPLDDKKSFDML
QRSETTAVFQLESRGMKDLIKRLQPDCFEDMIALVALFRPGPLQSGMVDNFIDRKHGREE
LSYPDVQWQHESLKPVLEPTYGIILYQEQVMQIAQVLSGYTLGGADMLRRAMGKKKPEEM
AKQRSVFEEGAKKNGIDGELAMKIFDLVEKFAGYGFNKSHSAAYALVSYQTLWLKAHYPA
EFMAAVMTADMDNTEKVVGLVDECWRMGLKILPPDINSGLYHFHVNDEGEIVYGIGAIKG
VGEGPIEAIIDARNQGGYFRELFDLCARTDTKKLNRRVLEKLIMSGAFDRLGPHRAALMN
SLGDALKAADQHAKAEAIGQADMFGVLAEEPEQIEQSYASCQPWPEQVVLDGERETLGLY

LTGHPINQYLKEIERYVGGVRLKDMHPTERGKVTTAAGLVIAARVMVTKRGNRIGICTLD
DRSGRLEVMLFTDALDKYQQLLEKDRILIVSGQVSFDDFSGGLKMTAREVMDIDETREKY
ARGLAISLTDRQIDDQLLNRLRQSLEPHRSGTIPVHLYYQRADARARLRFGATWRVSPSD
RLLNDLRGLIGSEQVELEFD
>EECEBICD_03225 Ribonuclease HII
MIEFVYPHTHLVAGVDEVGRGPLVGAVVTAAVILDPARPIVGLNDSKKLSEKRRLSLYDE
IKEKALSWSLGRAEAHEIDELNILHATMLAMQRAVAGLHIAPEYVLIDGNRCPELPVPSM
AVVKGDSRVAEISAASILAKVTRDAEMAALDIVFPQYGFAQHKGYPTAFHLEKLAQYGAT
AHHRRSFAPVKRALGLVS
>EECEBICD_03226 Lipid-A-disaccharide synthase
MAAQRPLTIALVAGETSGDILGAGLIRALKARVPNARFVGVAGPRMQAEGCEAWYEMEEL
AVMGIVEVLGRLRRLLHIRADLTRRFTELKPDVFVGIDAPDFNITLEGNLKKQGIKTIHY
VSPSVWAWRQKRVFKIGRSTHMVLAFLPFEKAFYDKFNVPCRFIGHTMADAMPLDPDKNA
ARDVLGIPHDAHCLALLPGSRGAEVEMLSADFLKTAQLLRQRYPDLEVVVPLVNAKRREQ
FEKIKAEVAPDLAVHLLDGMAREAMIASDAALLASGTAALECMLAKCPMVVGYRMKPFTF
WLAKRLVKTEYVSLPNLLAGRELVKELLQEECEPQKLAEALLPLLANGKTSHAMHDTFRE
LHQQIRCNADEQAADAVLELAQ
>EECEBICD_03227 Acyl-[acyl-carrier-protein]--UDP-N-acetylglucosamine O-acyltransferase
MIDKSAFIHPTAIVEDGAVIGANAHIGPFCIVGPQVEIGEGTVLKSHVVVNGQTKIGRDN
EIYQFASIGEVNQDLKYAGEPTRVEIGDRNRIRESVTIHRGTVQGGGLTKVGSDNLLMIN
AHVAHDCTVGNRCILANNATLAGHVSVDDFAIIGGMTAVHQFCIIGAHVMVGGCSGVAQD
VPPYVIAQGNHATPFGVNIEGLKRRGFSREGLVAIRNAYKLLYRSGKTLDEAKLEIAELA
EKHPEVKAFTEFFERSTRGPIR
>EECEBICD_03228 3-hydroxyacyl-[acyl-carrier-protein] dehydratase FabZ
MTTNTHTLQIEEILELLPHRFPFLLVDRVLDFEEGRFLRAVKNVSVNEPFFQGHFPGKPI
LPGVLILEAMAQATGILAFKSVGKLEPGELYYFAGIDEARFKRPVVPGDQMIMEVTFEKT
RRGLTRFKGVALVDGKVVCEATMMCARSREA
>EECEBICD_03229 UDP-3-O-(3-hydroxymyristoyl)glucosamine N-acyltransferase
MPSIRLADLAEQLDAELHGDGDIVITGVASMQSATTGHITFMVNPKYREHLGLCQASAVV
MTQDDLPFAKSAALVVKNPYLTYARMAQILDTTPQPAQNIAPSAVIDATATLGSNVSVGA
NAVIESGVQLGDNVVIGAGCFVGKNSKIGAGSRLWANVTIYHDIQIGENCLIQSSTVIGA
DGFGYANDRGNWVKIPQLGRVIIGDRVEIGACTTIDRGALDDTVIGNGVIIDNQCQIAHN
VVIGDNTAVAGGVIMAGSLKIGRYCMIGGASVINGHMEICDKVTVTGMGMVMRPITEPGV
YSSGIPLQPNKVWRKTAALVMNIDDMSKRLKAIERKVNQQD
>EECEBICD_03230 Chaperone protein Skp
MKKWLLAAGLGLAMVTSAQAADKIAIVNMGNLFQQVAQKTGVSNTLENEFKGRAAELQKM
ETDLQSKMQRLQSMKAGSDRTKLEKDVMSQRQTFAQKAQAFEKDRARRSNEERNKLVTRI
QTAVKKVANDQSIDLVVDANTVAYNSSDVKDITADVLKQVK
>EECEBICD_03231 Outer membrane protein assembly factor BamA
MAMKKLLIASLLFSSATVYGAEGFVVKDIHFEGLQRVAVGAALLSMPVRTGDTVNDEDIS
NTIRALFATGNFEDVRVLRDGNTLLVQVKERPTIASITFSGNKSVKDDMLKQNLEASGVR
VGESLDRTTLSDIEKGLEDFYYSVGKYSASVKAVVTPLPRNRVDLKLVFQEGVSAKIQQI
NIVGNHAFSTEELISHFQLRDEVPWWNVVGDRKYQKQKLAGDLETLRSYYLDRGYARFNI
DSTQVSLTPDKKGIYITVNITEGDQYKLSGVQVSGNLAGHSAEIEKLTKIEPGELYNGTK
VTKMEDDIKKLLGRYGYAYPRVQSQPEINDADKTVKLRVNVDAGNRFYVRKIRFEGNDTS
KDSVLRREMRQMEGAWLGSDLVDQGKERLNRLGFFETVDTDTQRVPGSPDQVDVVYKVKE
RNTGSFNFGIGYGTESGVSFQAGVQQDNWLGTGYSVGINGTKNDYQTYSELSVTNPYFTV
DGVSLGGRIFYNDFQADDADLSDYTNKSYGTDVTLGFPINEYNTLRAGLGYVHNKLSNMQ
PQIAMDRYLESMGQSADTSSFAADDFTFNYGWTYNKLDRGYFPTDGSRVNLTGKVTIPGS
DNEYYKVSLDTATYVPIDNDHKWVVLGRTRWGYGDGLGGKEMPFYENFYAGGSSTVRGFQ
SNTIGPKAVYKNGAHTSWDDNDDYEDCTQESGCKSDDAVGGNAMAVASLEFITPTPFISE
KYANSVRTSFFWDMGTVWDTNWDPSSAPSDVPDYSDPGNIRMSAGIALQWMSPLGPLVFS
YAQPFKKYDGDKAEQFQFNIGKTW

>EECEBICD_03232 Regulator of sigma-E protease RseP
MLSILWNLAAFIIALGVLITVHEFGHFWVARRCGVRVERFSIGFGKALWRRTDRYGTEYV
IALIPLGGYVKMLDERAELVAPELRRHAFNNKTVGQRAAIIAAGPVANFIFAIFAYWLVF
IIGVPGVRPVIGEITPNSIAAQAQIAPGTELKAVDGIETPDWDAVRLQLVSKIGDQQTTV
SVAPFGSDQRQDKTLDLRHWAFEPDKQDPVSSLGIRPRGPQIEPVLSEVQANSAASKAGL
QAGDRIVKVDGQPLTQWMKFVTFVRDNPGKPLALEIERQGSALSLTLTPDTKSVNGKAEG
FAGVVPKIIPLPEEYKTIRQYGPFSAILEATDKTWQLMKLTVSMLGKLITGDVKLNNLSG
PISIAQGAGMSAEFGVIYYLMFLALISVNLGIINLFPLPVLDGGHLLFLAIEKLKGGPVS
ERVQDFSYRIGSILLVLLMGLALFNDFSRL
>EECEBICD_03233 Phosphatidate cytidylyltransferase
MFGKLFGKHKLAPKVSPGKTWQGFIGGLATAAVISWGYGMWANLNVAPVILLICSVVAAL
ASVLGDLTESMFKREAGIKDSGHLIPGHGGILDRIDSLTAAVPVFACLLLLVFRTL
>EECEBICD_03234 Phosphatidate cytidylyltransferase
MLKYRLISAFVLIPAVIAALFLLPPVGFAIITLVVCMLAAWEWGQLSGFAARSQRVWLAV
LCGLLLALMLFLLPEYHHNIRQPLVEMSLWASLGWWVVALLLVLFYPGSAAIWRNSKTLR
LIFGLLTIVPFFWGMLALRAWHYDENHYSGAIWLLYVMILVWGSGLWRVYVW
>EECEBICD_03235 Ditrans,polycis-undecaprenyl-diphosphate synthase
((2E,6E)-farnesyl-diphosphate specific)
MDGNGRWAKKQGKIRAFGHKAGAKSVRRAVSFAANNGIDALTLYAFSSENWNRPAQEVSA
LMELFVWALDSEVKSLHRHNVRLRIIGDISRFNSRLQERIRKSEALTAHNTGLTLNIAAN
YGGRWDIVQGVRQLAEQVQAGVLRPDQIDEERLGQQICMHELAPVDLVIRTGGEHRISNF
LLWQIAYAELYFTDVLWPDFDEQDFEGALHAFANRERRFGGTEPGDDKA
>EECEBICD_03236 1-deoxy-D-xylulose 5-phosphate reductoisomerase
MKQLTILGSTGSIGCSTLDVVRHNPDSFRVIALVAGKNVARMAEQCLEFSPRYAVMDDTS
SAEQLKIMLQQHGSRTEVLSGQQAACEMAALDEVGHVMAAIVGAAGLLPTLAAIRAGKTI
LLANKESLVTCGRLFMDEVKRSNARLLPVDSEHNAIFQSLPQSIQHNLGYADLEQNGVTS
ILLTGSGGPFRETPMCDLAAMTPDQACRHPNWSMGRKISVDSATMMNKGLEYIEARWLFN
ASARQMEVLIHPQSVIHSMVRYQDGSVLAQLGEPDMRTPIAHTMAWPNRVTSGAQPLDFC
KLSALTFSAPDYQRYPCLKLAMEAFEQGQAATTALNAANEITVAAFLAQQIRFTDIAGLN
LAVLERMDLHEPASVEDVLQVDAIAREVARKQVIRLSR
>EECEBICD_03237 Ribosome-recycling factor
MISDIRKDAEVRMEKCVEAFKTQISKVRTGRASPSLLDGIVVEYYGTPTPLRQLASVTVE
DSRTLKINVFDRSMGPAVEKAIMASDLGLNPSSAGTDIRVPLPPLTEERRKDLTKIVRGE
AEQARVAVRNVRRDANDKVKALLKDKAISEDDDRRSQEEVQKMTDAAIKKVDAALADKEA
ELMQF
>EECEBICD_03238 Uridylate kinase
MATNAKPVYKRILLKLSGEALQGTEGFGIDASILDRMAQEIKELVELGIQVGVVIGGGNL
FRGAGLAKAGMNRVVGDHMGMLATVMNGLAMRDALHRAYVNARLMSAIPLNGVCDNYSWA
EAISLLRNNRVVILSAGTGNPFFTTDSAACLRGIEIEADVVLKATKVDGVFTADPAKDPS
ATMYDQLTYSEVLDKELKVMDLAAFTLARDHKLPIRVFNMNKPGALRRVVMGEKEGTLIT
E
>EECEBICD_03239 Elongation factor Ts
MAPFFTFISVRFDAGQIKSFEDFRMAEITASLVKELRERTGAGMMDCKKALTEANGDIEL
AIENMRKSGAIKAAKKAGNVAADGVIKTKIDGNVAFILEVNCQTDFVAKDAGFQAFADKV
LDAAVAGKITDVEVLKAQFEEERVALVAKIGENINIRRVASLEGDVLGSYQHGARIGVLV
AAKGADEELVKQLAMHVAASKPEFVKPEDVSADVVEKEYQVQLDIAMQSGKPKEIAEKMV
EGRMKKFTGEVSLTGQPFVMEPSKSVGQLLKEHNADVTGFIRFEVGEGIEKVETDFAAEV
AAMSKQS
>EECEBICD_03240 30S ribosomal protein S2
MATVSMRDMLKAGVHFGHQTRYWNPKMKPFIFGARNKVHIINLEKTVPMFNEALAELNKI
SARKGKILFVGTKRAASEAVKEAANSCDQFFVNHRWLGGMLTNWKTVRQSIKRLKDLETQ
SQDGTFEKLTKKEALMRTRELEKLENSLGGIKDMGGLPDALFVIDADHEHIAIKEANNLG
IPVFAIVDTNSDPDGVDFVIPGNDDAIRAVSLYLGAVAATVREGRSQDLASQAEESFVEA
E

>EECEBICD_03241 Methionine aminopeptidase
MAISIKTSEDIEKMRVAGRLAAEVLEMIEPYIKPGVTTGELDRICNDYIVNEQHAISACL
GYHGYPKSVCISINEVVCHGIPDDAKHLKDGDIVNIDVTVIKDEFHGDTSKMFIVGKPTI
LGERLCRVTQESLYLGIKMVKPGIRLRTIGAAIQKYAEGEGFSVVREYCGHGIGRGFHEE
PQVLHYDADDGGVVLQPGMTFTIEPMLNAGDYRIRTMKDGWTVKTKDRSLSAQYEHTIVV
TENGCEILTLRKDDTIPAIITHDE
>EECEBICD_03242 Bifunctional uridylyltransferase/uridylyl-removing enzyme
MNTLPEQHANTALPTLPDQPQNPGVWPRAELTVAGIKARIDIFQHWLGEAFDSGICAEQL
IEARTEFIDQLLQRLWIEAGFGQIADLALVAVGGYGRGELHPLSDIDLLILSRKKLPDEQ
AQKVGELLTLLWDVKLDVGHSVRTLEECLLEGLSDLTVATNLIETRLLIGDVALFLALQK
HIFSEGFWPSDKFYAAKVEEQNQRHQRYHGTSYNLEPDIKSSPGGLRDIHTLQWVARRHF
GATSLDEMVGFGFLTPAERAELNECLHILWRIRFALHLVVSRYDNRLLFDRQLSVAQRLN
YSGEGNDPVERMMKDYFRVTRRVSELNQMLLQLFDEAILALPADEKPRPVDDEFQLRGTL
IDLRDDTLFIREPQAILRMFYMMVRNSAITGIYSTTLRHLRHARRHLSQPLCYIPEARTL
FLSMLRHPGAVSRGLLPMHRHSVLWAYMPQWSHIVGQMQFDLFHAYTVDEHTIRVMLKLE
SFAKEETRQRHPLCVDLWPRLPHPELILIAALFHDIAKGRGGDHSVLGAQDVLTFAELHG
LNSRETQLVAWLVRQHLLMSVTAQRRDIQDPEVIKQFAEEVQTETRLRFLVCLTVADICA
TNETLWNSWKQSLLRELYFATEKQLRRGMQNTPDMRERVRHHQLQALALLRMDNIDEAAL
HKIWTRCRANYFVRHSPNQLAWHARHLLQHDLRQPLVLLSPQATRGGTEIFIWSPDRPYL
FAAVCAELDRRNLSVHDAQIFTTRDGMAMDTFIVLEPDGSPLAADRHDVIRTGLEQTITQ
RSWQPPQPRRQPAKLRHFTVETEVNFLPTHTDRKSFMELIALDQPGLLARVGQIFADLGI
SLHGARITTIGERVEDLFIIATADRRALNNVLQLEVQQRLTAALNPNDKG
>EECEBICD_03243 2,3,4,5-tetrahydropyridine-2,6-dicarboxylate N-succinyltransferase
MQQLQNVIETAFERRADITPANVDTVTREAVKQVISLLDSGALRVAEKIDGQWVTHQWLK
KAVLLSFRINDNQVIDGAESRYFDKVPMKFADYDEARFQKEGFRVVPPAAVRQGAFIARN
TVLMPSYVNIGAYVDEGTMVDTWATVGSCAQIGKNVHLSGGVGIGGVLEPLQANPTIIED
NCFIGARSEVVEGVIVEEGSVISMGVYLGQSTKIYDRETGEVHYGRVPAGSVVVSGNLPS
KDGKYSLYCAVIVKKVDAKTRGKVGINELLRTID
>EECEBICD_03244 Fosfomycin resistance protein AbaF
MTATLSSFASFGVGFISRPLGALFFGNRGDTLGRKNTLLITLGGMGAVTFLIGCLPSYAS
IGALAPALLVILRFLQGFMVGGEWGGAMLMVVEYAAGKHRGRLSALSQTGGLTGQLLATG
VFIVVTQLPKEALLSWGWRIPFLLSALLVLPGLYMRHRLDETPVFRAFKKQQAINHRQQR
EERPVVKVVREQWRSILLIIILRFAESVPFFLATVFAVSWATTQLGIASLTILYIVMFTC
LLAYPMHVLFGIMSDRRGCRQVYIFGALFVAAMAFPFFWLLESRSLILMTMGYVLLINIG
HNSLNAVQPSFFAGLFHPPVRYSGSSIGAQLGAVVAGGFTPFIAKALSAVYDNSWTLVAG
YVVLTALASAFAAKIAPETVLPHSP
>EECEBICD_03245 hypothetical protein
MSGTYNATIRRVVISAWIGNSIEYYDFLLYGLASALVFGPLFSLAPAH
>EECEBICD_03246 hypothetical protein
MYDNLKSLGITNPEEIDRYSLRQEANNDILKIYFQKDRGEFFAKSVKFKYPRQRKTVVAD
GIGQGYKEVQEISPNLRYVIDELDQICQRDRSELDLKRKILDDLRHLESVVANKISEIEA
DLDKLTRK
>EECEBICD_03247 hypothetical protein
MTQEITLTCYSLPAAPGLDNIKFEKGREHDRQALGFILPANTQLQIRQPNNNAGNARLRL
LCNDSACEKSLTLNGNWQTISTTVDSVPFIDTLFFAQGGEFSVIYRQPTSNKNLPHWRKG
QSEDAFFQTWEEQASPFALLELDRVRFLLPWADRANVINAGITALDAYYTRVIDAYNGWT
GLSDSPASPLNQNVANRYFIKADKHGVGAAYYLPWWCAQTAATLSQGWIDNVATQWTILH
EIGHGYQGVFMNDVDLPVGEVWNNIYAAFFQQLNLNQGNHLYTDGWLYNYGRQSEQELQF
ITYLRNRTPVNAWGVRPRLQFLMLMLFKGGTEAFRVFNQNYRELGADENFLPCEHRLTDL
LADAIATASGYDVAPFIQLCGLPVDAFTREQIAAQAVKPVWPLYDLLPEREWESARQQLG
LDSFVWLVENAELAELNKTGTLTLTLNIDQPEQLYGRALTLHDNAGSTYTLPVNDSTLTL
TPLPIGIYHLTLPKGRSQKYRPDTDYVVIREGENALTVNFTALQDSAAYNEQLIFLGYGD
MPFARLAVDHEARQLVLDITNATQHSYFANTLYASITVLTASGEKVFERKMNGINCATGK

IVVPFSDHYHLYLYHAEPGRLKASPGYLTLVSSTKYQLLRLDSEGLYHFSLNNDPAADLQ
AMFTHRADAIRACPLLMAQPYAACKNDLWLMLSHIEEPTRSALMRDSVDVLPADNSEPGE
GIGKGVTLQLRGQGDRTFCQLAYDNRQQRMTIETLAGQPHPYYTATYSTLTIKEESGEVI
YSRHYDGITHYSADSDTVVLQAGMYIELFHDEPYRCSAINETTGQNVRLKKHNRWRVVSD
GLEVDSPEQTEEKNTSDAAALYGDKFSWQLIGKEENGFASMEIDIRAQQFIFTAYPIAPH
SDFATEYAAVTIYNTRGTVVYRQSIKGSVQLGGYTDVCGLDEDYTIEVFHAEGADQSVIR
NPLNGESWPQPQHVIWQVTARGLQRLTTNYPPITASSGAVRLRCRMAALPNPAYHFAQFP
CSGQRSAAGLNAVHPIVAPHTATADRQNFPNPARSVPKYDLADIPACYDE
>EECEBICD_03248 Carbohydrate diacid regulator
MAGWHLDTKMAQDIVARTMRIIDTNINVMDARGRIIGSGDRERIGELHEGALLVLSQGRV
VDIDNAVARHLHGVRQGINLPLRLEGEIVGVIGLTGEPEHLRKYGELVCMTAEMMLEQSR
LMHLLAQDSRLREELVMNLIQAEENTPALVEWAQRLGIDLNQPRVAAVVEVDSGQLGVDS
AMAELQQLQNALTTPERNNLIAIVSLTEMVVLKPALNSFGRWDAEDHRKRVEQLISRMKE
NGQLRFRVALGNYFTGPGSIARSYRTARTTMMVGKQRMPESRSYFYQDLMLPVLLDSLRG
GWQANELARPLVKLKAMDNNGLLRRTLTAWFRHNVQPLATSKALFIHRNTLEYRLNRISE
LTGLDLGNFDDRLLLYVELQLDEQR
>EECEBICD_03249 Periplasmic serine endoprotease DegP
MKKTTLAMSALALSLGLALSPLSATAAETSSSAMTAQQMPSLAPMLEKVMPSVVSINVEG
STTVNTPRMPRNFQQFFGDDSPFCQDGSPFQNSPFCQGGGNGGNGGQQQKFMALGSGVII
DAAKGYVVTNNHVVDNASVIKVQLSDGRKFDAKVVGKDPRSDIALIQIQNPKNLTAIKLA
DSDALRVGDYTVAIGNPFGLGETVTSGIVSALGRSGLNVENYENFIQTDAAINRGNSGGA
LVNLNGELIGINTAILAPDGGNIGIGFAIPSNMVKNLTSQMVEYGQVKRGELGIMGTELN
SELAKAMKVDAQRGAFVSQVMPNSSAAKAGIKAGDVITSLNGKPISSFAALRAQVGTMPV
GSKISLGLLREGKAITVNLELQQSSQSQVDSSTIFSGIEGAEMSNKGQDKGVVVSSVKAN
SPAAQIGLKKGDVIIGANQQPVKNIAELRKILDSKPSVLALNIQRGDSSIYLLMQ
>EECEBICD_03250 Deoxyguanosinetriphosphate triphosphohydrolase
MASIDFRNKINWHRRYRSPQGVKTEHEILRIFESDRGRIINSPAIRRLQQKTQVFPLERN
AAVRTRLTHSMEVQQVGRYIAKEILSRLKEQNRLEEYGLDALTGPFESIVEMACLMHDIG
NPPFGHFGEAAINDWFRQRLHPEDAESQPLTHDRCVVSSLRLQEGEENLNDIRRKVRQDI
CHFEGNAQGIRLVHTLMRMNLTWAQVGGILKYTRPAWWRGPVPDSHRYLMKKPGYYLSEE
KYIARLRKELQLAPYSRFPLTWIMEAADDISYCVADLEDAVEKRIFSVEQLYHHLYHAWG
HHEKDSLFELVVGNAWEKSRANTLSRSTEDQFFMYLRVNTLNKLVPYAAQRFIDNLPQIF
AGTFNQALLEDASGFSRLLGLYKNVAVEHVFSHPDVEQLELQGYRVISGLLDIYQPLLSL
SLNDFRELVEKERLKRFPIESRLFQKLSTRHRLAYVEVVSKLPTDSAEYPVLEYYYRCRL
IQDYISGMTDLYAWDEYRRLMAVEQ
>EECEBICD_03251 5'-methylthioadenosine/S-adenosylhomocysteine
nucleosidase
MKIGIIGAMEEEVTLLRDKIDNRQTITLGGCEIYTGQLNGTEVALLKSGIGKVAAALGAT
LLLEHCKPDVIINTGSAGGLASTLKVGDIVVSDEARYHDADVTAFGYEYGQLPGCPAGFK
ADDKLIAAAESCIRELNLNAVRGLIVSGDAFINGSVGLAKIRHNFPDAVAVEMEATAIAH
VCYNFSVPFVVVRAISDVADQQSHLSFDEFLAVAAKQSTLMVETLVQKLAHG
>EECEBICD_03252 Vitamin B12-binding protein
MAKQMFRALVALLLTLPVWLYAAPRVITLSPANTELAFAAGITPVGVSSYSDYPPEAQKI
EQVSTWQGMNLERIVALKPDLVVAWRGGNAERQVNQLTSLGIKVMWVDAVSIEQIADTLR
QLAAWSPQPEKAQQAAQTLLNEYAALNAEYAGKAKKRVFLQFGMNPLFTSGKGSIQHQVL
TTCGGENVFADSRVPWPQVSREQVLARHPQAIIVAGKAGEILKIEQYWGNLLKIPVIPLN
SDWFERASPRIILAAKQLCNALSQVN
>EECEBICD_03253 hypothetical protein
MLVYWLDIVGTAVFAISGVLLAGKLRMDPFGVLVLGVVTAVGGGTIRDMALDNGPVFWVK
DPTDLVVAMVTSMLTILLVRQPRRLPKWMLPVLDAVGLAVFVGIGVNKAFLAETGPLVAV
CMGVITGVGGGIIRDVLAREIPMILRTEIYATACIIGGIVHATAFYTFSVPLESASMMGM
VVTLFIRLAAIRWHLKLPTFALDENGR
>EECEBICD_03254 Iron-sulfur cluster insertion protein ErpA
MSDDVALPLQFTDAAANKVKSLIADEDNPNLKLRVYITGGGCSGFQYGFTFDDQVNEGDM

TIEKQGVGLVVDPMSLQYLVGGSVDYTEGLEGSRFIVTNPNAKSTCGCGSSFSI
>EECEBICD_03255 H(+)/Cl(-) exchange transporter ClcA
MKTDTSTFLAQQIVRLRRRDQIRRLMQRDKTPLAILLMAAVVGTLTGLVGVAFEKAVSWV
QNMRIGALVQVADHAFLLWPLAFILSALLAMVGYFLVRKFAPEAGGSGIPEIEGALEELR
PVRWWRVLPVKFIGGMGTLGAGMVLGREGPTVQIGGNLGRMVLDVFRMRSAEARHTLLAT
GAAAGLSAAFNAPLAGILFIIEEMRPQFRYNLISIKAVFTGVIMSSIVFRIFNGEAPIIE
VGKLSDAPVNTLWLYLILGIIFGCVGPVFNSLVLRTQDMFQRFHGGEIKKWVLMGGAIGG
LCGILGLIEPEAAGGGFNLIPIAAAGNFSVGLLLFIFITRVVTTLLCFSSGAPGGIFAPM
LALGTLLGTAFGMAAAVLFPQYHLEAGTFAIAGMGALMAASVRAPLTGIVLVLEMTDNYQ
LILPMIITCLGATLLAQFLGGKPLYSTILARTLAKQDAEQAAKNQNAPAGENT
>EECEBICD_03256 Glutamate-1-semialdehyde 2,1-aminomutase
MSKSENLYSAARELIPGGVNSPVRAFTGVGGTPLFIEKADGAYLYDVDGKAYIDYVGSWG
PMVLGHNHPAIRNAVIEAAERGLSFGAPTEMEVKMAELVTNLVPTMDMVRMVNSGTEATM
SAIRLARGFTGRDKIIKFEGCYHGHADCLLVKAGSGALTLGQPNSPGVPADFAKHTLTCT
YNDLTSVRAAFEQYPQEIACIIVEPVAGNMNCIPPLPEFLPGLRALCDEFGALLIIDEVM
TGFRVALAGAQDYYGVVPDLTCLGKIIGGGMPVGAFGGRRDVMDALAPTGPVYQAGTLSG
NPIAMAAGFACLNEVAQPGIHETLDELTTRLAEGLLEAAEEANIPLVVNHVGGMFGIFFT
DAESVTCYQDVMACDVERFKRFFHLMLEEGVYLAPSAFEAGFMSVAHSEEDINNTIDAAR
RVFAKL
>EECEBICD_03257 putative protein YfcO
MMRSIQILFLLLAVVCCRSIAAGPMLKATFSSTTMYYGIGPNSDKSIVAEVTIATPEGVY
YGSWNLSGHRKGETLTADSWSGPEPAPKVVLKDFDNTVSRSACKNLPSNWRGCGSFTLEI
TVQSDDYGCPWLASSHIVATAFITNETYSPPDTRSSVCPKVPVDTFDISWDANVSKQKTT
LMLDATGGTVNRTLHTYLMEGGNCAMAANLTTVALIAVLSHRVLRSMCWAATSRASRPAP
LIIR
>EECEBICD_03258 putative fimbrial-like protein YfcP
MAKRQALLHGAACILCGALILPVSAADNLHFSGSLVASPCTLTMQGTGIAEVDFSSLDSS
DFTPDGQSARKPLVFELTDCDSALSNGVQVTFTGTEATGMRGILAIDSHSGAFGIGIGIE
TLSGVPVGMNDKEGAIFTLVTGNNALNLNAWVQRLPGEDLVPGTFFASALVTFEYL
>EECEBICD_03259 putative fimbrial-like protein YfcQ
MKKMALTMLMGGSLAAQAADNLKFHGTLISPPNCTINNDQTIDVKFGNLLINKIDGTRYA
QNVPYEITCDSTVRDETMAMTLTLSGSVSDFNPAAVNTSVAGLGIELRQNDQPFTLGSTI
TVNEQSIPVLKAIPVKKSGASLKEGGFDATATLQVDYQ
>EECEBICD_03260 putative fimbrial-like protein YfcR
MRQWRLRLGTVTCAAMLSAVSMPLAAGSKTVNMTLTIVVNAAPPCTVTGGEVEFGNVLTT
KVDGVNYRQAVGYRLSCNGRVSDYLKLQIQGNAVTINGESVLQTDVDGLGIRLQTATDGE
LISPGNTQWLSFQYSGGSGPAIEAIPVKNNGVTLTGGAFNAGATLVVDYQ
>EECEBICD_03261 putative fimbrial chaperone YfcS
MMSLKRYNLAAVLLLLLGGYGSAQAAIAPDRTRLVFRGEDKSISVDLKNANSKLPYLAQS
WVEDEKGVKITSPLIVVPPVQRIEPSAIGQVKIQGMPALASLPQDRETLFYYNVREIPPQ
SDKPNTLQIALQTRIKVFYRPQALSKIDMQHPWQYKITLQRQGNGYQVSNPTGYYVVLSN
ASNRMDGTPARGFSPLVIAPKSNVTLGGDASELGHSPVLTYVNDYGARLPLIFNCTDNSC
AVDEAKSRKS
>EECEBICD_03262 Outer membrane usher protein PapC
MAHYKKFRLSTLAAVVGIVLAVGPENSYAEAPIQFNTRFLDVKDDASLDLSRFSRKGYIM
PGSYHLQVLVNQSQIAQDNVITYSVDNNDPDNTYPCLSPELVSLLGLKPEIADKMIWINA
GQCLQPDQLEGMETQTDLSQSTLTVIIPQAYLEYSDEEWDPPSRWDEGIPGVLFDYNVNS
QWRHAEHDDGDEYDISGNGTVGANLGAWRLRADWQANYRHENDSEDKDNFGSSSEQNWDW
NRYYAWRAIPQLRAQLTLGEGSLESDIFDGFNYVGGSLITDDQMLPPNLRGYAPDISGVA
RTNAKVTVTQRGRVIYESQVPAGPFRIQDINETVSGDLHVKIEEQSGQVQEYDVSTASIP
FLTRPGQVRYKLAAGRPQDWDHNMEGGFFTSAEASWGIANGWSLYGGAIGEQDYQALALG
LGRDLALLGAFSVDVTHSRATLPEGSAYGDGTIQGNSFRASYAKDFDDIDSRLTFAGYRF
SEENYMTMDEFIDTHNDDNDRQRTGHDKEMYTLTYSQNFSAINVNAYINYTHRTYWNQPN
QDSYNLTLSHYFDVGEVRGISLSVNGFRNEYDNERDDGVYVSLSIPWGNNRTLSYNGSFS

DDNNSNQVGYYERIDDRNNYQINAGRADNGATLDGYYRYQASYADIDVSANYQEGDYTSG
GLNIQGGATLTAKGGALHRTSVNGGSRLLVDVGDEANVPISGYSTPVYTNAFGKAVIVDV
NDYYRNQVKIDITQLPEDAEAILSIAQATLTEGAIGYRRMEVLSGKKAMGSIRLRDGGTP
PFGAEVYNSRQQQLGIVGEDGSVYLIGINPGERLQVTWEGKTQCEAALPDPLPGDLFSGL
LLPCIGDASSPEATQPEEKPLLQLHTQRRTSSTQPEALSSRYPTH
>EECEBICD_03263 Fimbria A protein
MNTAVKAAVAAALVMGVSSFANAAGSNTGTVTFTGTIEDSPCSIVVGDEHQTVNLGHIGT
GSLMGGKESSKVDFHIGLENCAFTTEKEVSTVFSAIGNESSANPGSVALMRIGGGEMAGS
SIVIGNHLGSAIKLGDAYSENLTMNGSVAAAKQTLNFKAWVKGDSAASTIDTGEFSSTVN
FTISYL
>EECEBICD_03264 hypothetical protein
MLNPAYKLHNINMLWQYFVGLKIAERYQVRELSCHVLSRELCFYFQQEAGVIFVSISHEL
FQWRAAVS
>EECEBICD_03265 Iron(3+)-hydroxamate import system permease protein FhuB
MSRKREMPDGGVKNALSDLRFGRFVGRIRRSRHPALLLLALFVAACWLTWVNFSVALPRS
QWQQAIWSPDIDIIEQMIFHYSLLPRLAISLLVGAGLGLVGVLFQQVLRNPLAEPTTLGV
ATGAQLGITVTTLWAIPGALATQFAALTGACIVGALVFGVAWGKRLSPVTLILAGLVVSL
YCGAINQLLVIFHHDQLQSMFLWSTGTLTQTDWSGVQRLWPQLLGGVMLTLLLLRPMTLM
GLDDGVARNLGLALSLARLAALSLAIVLSALLVNAVGIIGFIGLFAPLLAKMLGARRLLA
RLMLAPLIGALILWLSDQIILWLTRVWMEVSTGSVTALIGAPLLLWLLPRLKSMSAPDMN
ASDRVAAERRHVLAFAVAGGALLLLATWVALSFGRDAHGWTWASGTLLEELMPWRWPRIL
AALMAGVMLAVAGCIIQRLTGNPMASPEVLGISSGAAFGVVLMLFLVPGNAFGWLLPAGS
LGAAATLLIIMIAAGRGGFSPQRMLLAGMALSTAFTMLLMMLQASGDPRMAEVLTWLSGS
TYNATGGQVTRTAIVMVILLALVPLCRRWLTILPLGGDAARAVGMALTPSRIALLALAAC
LTATATMTIGPLSFVGLMAPHIARMLGFRRTMPHMVISALAGGVLLVFADWCGRMALFPY
QIPAGLLSSFIGAPYFIYLLRKQSR
>EECEBICD_03266 Iron(3+)-hydroxamate-binding protein FhuD
MRDLYPLTRRRLLTAMALSPLLWQMNTAQAAAIDPRRIVALEWLPVELLLALGITPYGVA
DVPNYKLWVSEPPLPDSVIDVGLRTEPNLELLTEMKPSFMVWSAGYGPSPEKLARIAPGR
GFDFSDGKKPLAVARRSLVELAQTLNLEAAAEKHLAQYDRFIASQKPHFIRRGGRPLLMT
TLIDPRHMLVLGPNCLFQEVLDEYSIVNAWQGETNFWGSTAVSIDRLAMYKEADVICFDH
GNSTDMNALMATPLWQAMPFVRAGRFHRVPAVWFYGATLSTMHFVRILNNVLGGKA
>EECEBICD_03267 Iron(3+)-hydroxamate import ATP-binding protein FhuC
MQENHIHSDTTFALRSVAFRVPGRTLLHPLSLTFPAGRVTGLIGHNGSGKSTLLKMLGRH
QLPSEGDILLDNQPLASWSSKAFARKVAYLPQQLPQAEGMTVRELVAIGRYPWHGALGRF
GVADREKVDEAITLVGLKPLAHRLVDSLSGGERQRAWIAMLVAQDSRCLLLDEPTSALDI
AHQVDVLALVHRLSQQRGLTVVAVLHDINMAARYCDYLVALRGGEMIAQGTPAELMRSDT
LEQIYGIPMGILPHPAGAAPVSFVY
>EECEBICD_03268 Ferrichrome outer membrane transporter/phage receptor
MARLKTAQPNSSLRKIAVVVATAVSGMSVYAQAAVQPKEETITVTAAPAAQESAWGPAAT
IAARQSATATKTDTPIQKVPQSISVVTAEEMALHQPKSVKEALSYTPGVAVGTRGASNTY
DYLIIRGFAADGQSQNNYLNGLKMQGNFYNDAVIDPYMLERAEVMRGPVSVLYGKSSPGG
LLNMVSKRPTTEPLKEIQFKMGTDSLFQTGFDFSDALDEEGVYSYRLTGLARSANAQQDR
AEEQRYAIAPAFTWRPDDKTNFTFLSYFQNEPETGYYGWLPKEGTVEPLPNGKRLPTDFN
EGAKNNTYSRNEKMVGYSFDHEFNDTFTVRQNLRYAENKVSQNSVYGYGVCSDPANRDSK
QCAALAPADKGHYLARKYVVDDEKLQNFSVDTQLQSKFATGEVDHTLLTGVDFMRMRNDI
NAWFGYDDSVPLLDLYNPVYTDFDFASRDPATSGPYQILNKQKQTGLYVQDQAQWDKVLV
TLGGRYDWADQESLNRTTGITSKRDDKQFTWRGGVNYLFDNGITPYFSYSESFEPASQTG
ENGKIFAPSKGKQYEAGVKYVPNDRPIVITGAVYQLTKTNNLMADPAGSFFSVEGGEIRA
RGVELEAKAALSASINVVGSYTYTDAEYTTDTTYKGNTPAQVPKHMASLWGDYTFFDGPL
SGLTLGTGGRFTGSSYGDPANSFKVGSYTVVDALVRYDLARVGMAGSNVALHVNNLFDRE
YVASCFQTYGCFWGAERQVVATATFRF
>EECEBICD_03269 Penicillin-binding protein 1B
MAGNDREPIGRKGKPSRPVKQKVSRRRQHDDDYDDDYEDEEPMPRKGKGKGRKPRGKRGW

LWLLLKLFIVFVVLFAIYGVYLDQKIRSRIDGKVWQLPAAVYGRMVNLEPDMPVSKNEMV
KLLEATQYRLVTKMTRPGEFTVQANSIEMIRRPFDFPDSKEGQVRARLTFSDGRLETIVN
LDNNRQFGFFRLDPRLITMLSSPNGEQRLFVPRSGFPDLLVDTLLATEDRHFYEHDGISL
YSIGRAVLANLTAGRTVQGASTLTQQLVKNLFLSSERSYWRKANEAYMALIMDARYSKDR
ILELYMNEVYLGQSGDNEIRGFPLASLYYFGRPVEELSLDQQALLVGMVKGASIYNPWRN
PKLALERRNLVLRLLQQQKIIDQELYDMLSARPLGVQPRGGVISPQPAFMQMVRQELQAK
LGDKIKDLSGVKIFTTFDSVAQDAAEKAVVEGIPALKKQRKLSDLETAMVVVDRFSGEVR
AMVGGAEPQYAGYNRAMQARRSIGSLAKPATYLTALSQPNLYRLNTWIADAPISLRQPNG
QVWSPQNDDRRYSESGKVMLVDALTRSMNVPTVNLGMALGLPAVTDTWTKLGVPKDQLNP
VPAMLLGALNLTPIEVAQAFQTIASGGNRAPLSALRSVIAEDGKVLYQSYPQAERAVPAQ
AAYLTLWTMQQVVQRGTGRQLGAKYPGLHLAGKTGTTNNNVDTWFAGIDGSQVTITWVGR
DNNQPTKLYGASGAMAIYQRYLANQTPTPLVLTPPEDVVDMGVDYDGNFVCSGGMRTLPV
WTDDPNTLCQQGEMMQQQQQPSGNPFDQSSQPQQPAQQQPPKEEKSDGVAGWIKEMFGGN
>EECEBICD_03270 ATP-dependent RNA helicase HrpB
MLQCGAKKVNPVEPFVTSLPVAAVLPELLTALKTAPQVLLSAPTGAGKSTWLPLQLLQQG
PVAGKILLLEPRRLAARNVAQRLAEALNEKPGETVGYRMRAQSCVGPRTRLEVVTEGVLT
RMIQRDPELRGVGLVILDEFHERSLQADLALALLLDIQQGLRDDLRLLIMSATLDNDRLC
QRLPDAPTIVSEGRAFPVERRYQPLAAHLRFDEAVAMATAELLRNENGSLLLFLPGVGEI
QRVHEHLASRVGSDVLLCPLYGALSLEAQRKAIVPAPAGMRKVVLATNIAETSLTIEGIR
LVVDSAQERVARFDARTGLTRLVTQRISQASMTQRAGRAGRLAPGICLHLLAKEQAERAA
AQSDPEILHSDLSGLLMEVLQWGCHDPASLFWLDRPPEVNLQAARRLLLMLGALEGERLS
ARGRKMAATGNDPRLAAMLVNAGEGDSAATAAMLAAILEDPPRGGGTDLSVVFSRRQPGW
QQRSQQLLKRLQVRNGEPDSALIMPLLARAFSDRIARRRGQEGRYQLANGMGAMLDADDA
LGRHEWLIAPLLLQGSASPDARILLAQPLDIASLIQACPDLLRQSDTVEWDEAQGTLKAW
RRMRIGQLTVSVQPLAKPSEEELHQAMLNGIRDKGLSVLNWTPEAEQFRLRLHCAAKWLP
EYDWPAVDEASLLATLENWLLPHMTGVQSLRSLKSLNVTQALRGLLDYAMLQRLDSELPG
HYTVPTGSRITIRYHEDNPPALAVRMQEMFGEAKTPTIAQGRVPLVLELLSPAQRPLQIT
RDLSAFWQGAYREVQKEMKGRYPKHVWPDDPANTAPTRRTKKYS
>EECEBICD_03271 RNA 2',3'-cyclic phosphodiesterase
MSEPKRLFFAIDLPDDARAQIIAWRAAHFASEDGRPVAAANLHLTLAFLGDVSSDKQRAL
AQLAGRIRQPGFTLHLDDAGQWLRSRVVWLGMRQPPRGLLQLANMLRAQAARSGCYQSPQ
PFHPHITLLRDASHTVAIPPPGFCWSFPVTSFALYASSYGQGRTRYAELQRWTLGE
>EECEBICD_03272 Sugar fermentation stimulation protein A
MLFSPPLQRATLIQRYKRFLADVITPDGTTLTLHCPNTGAMTGCATPGDTVWYSTSENTK
RKYPHTWELTETQSGAFICVNTLRANQLTKEAIQENRLPALAGYNILKSEVKYGAERSRI
DFMLQADFRPDCYIEVKSVTLAEKENGYFPDAITERGQKHLRELMGVAAAGHRAVVVFAV
LHSAITRFSPARHIDIKYAQLLSEAQNKGVEVLAYKAELSAQKMELNEPVPITL
>EECEBICD_03273 RNA polymerase-binding transcription factor DksA
MQEGQNRKTSSLSILAIAGVEPYQEKPGEEYMNEAQLSHFKRILEAWRNQLRDEVDRTVT
HMQDEAANFPDPVDRAAQEEEFSLELRNRDRERKLIKKIEKTLKKVEDEDFGYCESCGVE
IGIRRLEARPTADLCIDCKTLAEIREKQMAG
>EECEBICD_03274 Glutamyl-Q tRNA(Asp) synthetase
MTGGSFPALFFIARSMTDSHYIGRFAPSPSGELHFGSLIAALGSYLQARAQRGIWRVRIE
DIDPPREVPGAAATILRQLEHYGLHWDGEVLWQSQRHEAYREALAWLHEQGLSYYCTCPR
SRIQRLGGIYDGHCRTLCHGPENAAVRIKQQHPVMHFHDALRGDIQADPQLASEDFIIHR
RDGLFAYNLAVVVDDHFQGVTEIVRGADLIEPTVRQLSLYKQFGWRVPGYVHLPLALNEQ
GAKLSKQNHAPALATGDPRPVLVQALRFLGQRAVVAWQEMSVEELLRFAVAHWRLTAVPT
SANVNPAFSNASR
>EECEBICD_03275 Poly(A) polymerase I
MTLPRCAIFTRVANFCRKVLSREESEAEQAVARPHMTIIPREQHAISRKDISENALKVLY
RLNKAGYEAYLVGGGVRDLLLGKKPKDFDVTTNATPDQVRKLFRNCRLVGRRFRLAHVMF
GPEIIEVATFRGHHEGSESDRTTSQRGQNGMLLRDNIFGSIEEDAQRRDFTINSLYYSVA
DFTVRDYVGGMQDLQEGVIRLIGNPETRYREDPVRMLRAVRFAAKLNMRISPETAEPIPR
LATLLNDIPPARLFEESLKLLQAGNGYETYQQLREYHLFQPLFPTITRYFTENGDSAMER

IIAQVLKNTDNRIRNEMRVNPAFLFAAMFWYPLLEMAQKIAQESGLAYYDAFALAMNDVL
DEACRSLAIPKRLTTLTRDIWQLQLRMSRRQGKRAWKLMEHPKFRAAFDLLELRAQVENN
TELQRLAQWWAEFQASAPPEQKGMLNELDDDPAPRRRRSRPRKRAPRREGTV
>EECEBICD_03276 2-amino-4-hydroxy-6-hydroxymethyldihydropteridine pyrophosphokinase
MTIAYIALGSNLASPLEQVNAALKAIADIPDSRIVAVSSFYRTPPLGPQDQPDYLNAAVA
LDTALAPEELLNHTQRIELQQGRVRKAERWGPRTLDLDIMLFGDEVINTDHLTVPHYDMK
NRGFMLWPLFEIAPDLIFPDGISLHQHLTHLGAAKPAHW
>EECEBICD_03277 3-methyl-2-oxobutanoate hydroxymethyltransferase
MKPTTISLLQKCKQEKKRFATITAYDYSFAKLFADEGINVMLVGDSLGMTIQGHDSTLPV
TVEDIAYHTRAVRRGAPNCLLLSDLPFMAYATPEQACENAAIVMRAGANMVKIEGGAWLV
DTVKMLTERAVPVCGHLGLTPQSVNIFGGYKIQGRGDAGQVLLDDALALEAAGAQLLVLE
CVPVELAKRVTEALSIPVIGIGAGNVTDGQILVMHDAFGITGGHIPKFAKNFLAEAGDMR
AAVQQYMAEVESGVYPGEEHSFH
>EECEBICD_03278 Pantothenate synthetase
MLIIETLPLLRQHIRRLRQEGKRVALVPTMGNLHDGHMKLVDEAKARADVVIVSIFVNPM
QFDRPDDLVRYPRTLQEDCEKLNKRKVDYVFAPAVEEIYPQGLEGQTYVDVPGLSTMLEG
ASRPGHFRGVSTIVSKLFNLIQPDIACFGEKDFQQLALIRKMVADMSYDIEIVGVPIIRA
KDGLALSSRNAYLTAEQRKIAPGLYNVMNSIAEKLIAGNRELQEIIAIAEQELNEKGFRA
DDIQIRDADTLQELTETSKRAVILAAAWLGQARLIDNQSVTLAQ
>EECEBICD_03279 Aspartate 1-decarboxylase
MIRTMLQGKLHRVKVTQADLHYEGSCAIDQDFLDASGILENEAIDIWNVTNGKRFSTYAI
AAERGSRIISVNGAAAHCAEVGDIVIIASFVTMSDEEARTWRPKVAYFEGDNEMKRTAKA
IPVQVA
>EECEBICD_03280 hypothetical protein
MVMRVVLILLFFFAGNVLAALPARYMQTTKDAAIWSQIGDKMVTVGNIRAGQILSVTPVA
ADYYAFKFGFGVGFIDKGHLESVQGKQKVEDGLGDLNKPLSNQNLVTWKDTPVYNAPDIS
SAPFGVLVDNLRYPIISKLKGRLHQTWYQIRIGDRLAYVSAMDAQEDNGIPILTYHHILR
DEENTRFRHTSTTTSVRAFSNQMTWLRDRGYATLTMYQLEDYIYNRANFPARAVAITFDD
GLKSVSRYAYPVLKQYDMKATAFIISSRIKRHPQKWNPRSLQFMSVSELRKISDVFDFQS
HTHFLHRVDGHRRPILYSRSYHNILFDFERSRRALTQFTPHVFYLSYPFGGYNATAIKAA
KDAGFHLAVTTVRGKVKPGDNPMLLKRLYILRTDSLETMSRLISNQPQG
>EECEBICD_03281 hypothetical protein
MLGWVISCHDDRAQEMLEGLEKKYGPLAQCRAVNFWRGLSVNMLSRMMCDALHATDSGEG
VIFLTDISGAAPYRVASLMSHKHSQCEVISGVSYSLMEEMITWRESMSSSAFRDQIVALG
APDVTSLWHQQQKNPPFVLLHDSYEF
>EECEBICD_03282 Type-1 fimbrial protein, A chain
MKLSLKTLTVALAAITLSPAALADTAKDGTVHITGLIKQNACTVKTDSVEVTLQEEFASL
FTAAGQTAGDTDFTIELENCDANVYSSVQARFEGTLDGTDATILKNEDDAENIGVQILDK
TSTPMTFNDLQAWSAAVALPTAEGVTELSMPFTARYIATAVPVKSGTVDATATFYLQYN
>EECEBICD_03283 Chaperone protein FimC
MNSLAKAGLLCCLLCGSLAHAAGINIGTTRVIFHGDAKDASISISNSDNVPYLIQSWAQS
ISETGASGDAPFMVTPPLFRLNGGQKNVLRIIRTGGNLPEDRESMYWLDIKSIPSSNPDN
KHNTLMLAVKAEFKLIYRPKALTQKPEEVADRLTWSRQGRTLTVKNPTPYYMNFATLSVG
SQKVKAPRYVAPFGNAQYTLPAAASGPIAWSIINDFGGTGPEHKQTP
>EECEBICD_03284 Outer membrane usher protein FimD
MRVPMRKRQHRVFYLSRCAVCIALGSTFTHVAWARDYFNPAFIENHGQASRTSVDLSTFD
NDKSQLPGTYYVTININKTEIGARNVDFQLATLSDGQQALQGCLRLQELKDNGVKTDLFP
TLESEKGCVDLSVIPGASQRFDFQQQALSVSIPQLYIANNARGYVPPEKWQEGITALMLN
YSFSGYKEYGSSEDSDDAESKYLALQPGFNLGPWRFRNYSNWSSNNGESGSWNSVYNYLQ
RDIIALKSQFTAGDSNTPSDVFDSVPFRGLQLTSDDQMQPNSQRGYAPTIRGIARSNAQV
IVRQNGYIAYQTAVSPGEFEINDMFPTGSNGDYDVTVKETDGSEQHFIVPYSSLPILQRT
GRAKYSVTVGKYRDYNNHTLDDFGQATLLYGLPWGITLYGGSQIAGDKYQSVAFGVGQNM
QMLGAISLDGIWSHAKFDYGRKEIGQSWRVRYSKGVVSTGTTFSLAGYRYASENYNSLSE

VINPDDDFYDNYGKRHNRFEASVNQQISNTLGSLTLSWVKEDYWHSAQQMESLSASYNNS
WGPVSYTLSYSYNKNTYQYRSDNDDDDNDDDRYNQNDRLFTLSLHVPFTVFDSRLYASYM
LNTRKHDATVNSTTLSGTALRDRNLNWSLQQSHSTQDGDSGGVNASYKGTYANLNAGYNQ
SPDSQQVSYGISGGILAHENGITLSQPITGAAILIKAPGASGVSVENQTGVATDFRGYTV
IPNVTPYYRYDISLDSSTFADNVDIPLNNQTVYPTRNAVVRAAYDTHKGYRVLLTLTRSN
GEPVPFGATASVDGQDANLASIVGDKGQVFLSGLPEEGLLLVNWGSASCRADYHLDISKN
MNGIVMANAVCQ
>EECEBICD_03285 putative minor fimbrial subunit LpfD
MKNLWMLLALSLFSGHALADGTMGNGSGWCQPTSGTHNFFFPLDQTITDTDENQAGKIVK
ESWSVGGEYSARCDCDNKDYQGVNYFTATTGDLTQKGTYSEAGSNGQQMDFYVLVAGKLE
IGTETYIVGNLKQYIPVPFSAISNQDPTAGGCTGADINKMSAGNKGNVRIYITHPLVGEI
TIPETTIMNLYLSKTPGSSGDNIPPSVPPMAHVTMSGTITVPQSCSINAGQVIEVRLPDI
EGKDIRHLGDSPQNSHVTTQVNFTCSNVADGTNLSMSLNGATDPHNPDYLKTDNENLGIR
ISDKYDNTIVPGGSAELPIEDYADGKGSTEFTAAPVNTTGHVPHTGEYQATATLEIQIR
>EECEBICD_03286 Inner membrane transport permease YadH
MMQLYWVALKSIWAKEIHRFMRIWVQTLVPPVITMTLYFIIFGNLIGSRIGEMHGFSYMQ
FIVPGLIMMAVITNSYANVASSFFSAKFQRNIEELLVAPVPTHVIIAGFVGGGVARGLCV
GILVTAISLFFVPFQVHSWVFVALTLILTAILFSLAGLLNAVFAKTFDDISLIPTFVLTP
LTYLGGVFYSLTLLPPFWQGLSHLNPIVYMISGFRYGFLGIHDVPLVTTFGVLVIFIAAF
YLLCWSLIQRGRGLRS
>EECEBICD_03287 putative ABC transporter ATP-binding protein YadG
MTIALELQQLKKTYPGGVQALRGIDLQVEAGDFYALLGPNGAGKSTTIGIISSLVNKTSG
RVNVFGYDLEKDVVNAKRQLGLVPQEFNFNPFETVQQIVVNQAGYYGVEHKEAVLRSEKY
LKQLDLWEKRHERARMLSGGMKRRLMIARALMHEPKLLILDEPTAGVDIELRRSMWGFLK
DLNAKGTTIILTTHYLEEAEMLCRNIGIIQHGELVENTSMKNLLSKLKSETFILDLAPKS
PLPKLTGYQYRLVDTSTLEVEVLREQGVNSVFSQLSEQGVQVLSMRNKANRLEELFVSLV
HEKQGDRA
>EECEBICD_03288 Carbonic anhydrase 2
MKDIDTLISNNALWSKMLVEEDPGFFEKLAQAQKPRFLWIGCSDSRVPAERLTGLEPGEL
FVHRNVANLVIHTDLNCLSVVQYAVDVLEVEHIIICGHSGCGGIKAAVENPELGLINNWL
LHIRDIWLKHSSLLGKMPEEQRLDALYELNVMEQVYNLGHSTIMQSAWKRGQNVTIHGWA
YSINDGLLRDLDVTATNRETLENGYHKGISALSLKYIPHQ
>EECEBICD_03289 Hypoxanthine phosphoribosyltransferase
MKHTVEVMIPEAEIKARIAELGRQITERYKDSGSEMVLVGLLRGSFMFMADLCREVQVPH
EVDFMTASSYGSGMSTTRDVKILKDLDEDIRGKDVLIVEDIIDSGNTLSKVREILGLREP
KSLAICTLLDKPSRREVDVPVEFVGFSIPDEFVVGYGIDYAQRYRHLPYVGKVVVLDE
>EECEBICD_03290 Quinoprotein glucose dehydrogenase
MAENNARSPRLLVTLTALFAALCGLYLLIGGVWLVAIGGSWYYPIAGLVMLVVAGLLWRS
KRAALWLYAALLLATMIWGVWEVGFDFWALTPRSDILVFFGIWLILPFVWHRLVVPSSGA
VAALVVALLISGGILTWAGFNDPQEINGTLRADATPAATSSSIADEDWPAYGRNQEGQRY
SPLKQITADNVHQLKEAWVFRTGDLKQPNDPGEITNEVTPIKVGDTLYLCTAHQRLFALD
AASGKEKWHFDPQLKTDSSFQHVTCRGVSYHEAKADTASPEVIADCPRRIILPVNDGRLF
AVNAETGKLCETFANKGVLNLQTNMPDTTPGLYEPTSPPIITDKTIVIAGSVTDNFSTRE
TSGVIRGFDVNSGKLLWAFDPGAKDPNAIPADEHAFTFNSPNSWAPAAYDAKLDLVYLPM
GVTTPDIWGGNRTPEQERYASSILALNATTGKLAWSYQTVHHDLWDMDLPAQPTLADITV
DGTTVPVIYAPAKTGNIFVLDRRNGELVVPAPEKPVPQGAAKGDYVAKTQPFSDLTFRPK
KDLSGADMWGATMFDQLVCRVMFHQLRYEGIFTPPSEQGTLVFPGNLGMFEWGGISVDPD
RQVAIANPMALPFVSKLIPRGPGNPMEPPKDAKGTGTEAGIQPQYGVPFGVTLNPFLSPF
GLPCKQPAWGYISALDLKTNEIVWKKRIGTPRDSMPFPMPVPVPFNMGMPMLGGPISTAG
NVLFIAATADNYLRAYNMSNGEKLWQGRLPAGGQATPMTYEVNGKQYVVVSAGGHGSFGT
KMGDYIVAYALPDDAK
>EECEBICD_03291 Blue copper oxidase CueO
MLRRDFLKYSVALGVASALPLWSRAAFAAERPALPIPDLLTADASNRMQLIVKAGQSTFA
GKNATTWGYNGNLLGPAVQLHKGKSVTVDIHNQLAEDTTLHWHGLEIPGIVDGGPQGIIP

AGGTRTVTFTPQQRAATCWIHPHKHGKTGRQVAMGLAGLVLIEDDEIRKLRLPKQWGIDD
VPVIIQDKRFSADGQIDYQLDIMTAAVGWFGDTLLTNGAIYPQHSAPKGWLRLRLLNGCN
ARSLNIAASDNRPLYVIASDGGLLAEPVKVTELPLLMGERFEVLVDISDGKAFDLVTLPV
SQMGMAIAPFDKPHPVMRIQPLRITASGTLPDTLTTMLALPSLEGLTVRNLKLSMDPRLD
MMGMQMLMKKYGAQAMSGMDHDSMNAHMQGGNMGHGEMDHGNMDHSGMNHGAMGNMNHGG
KFDFHNANFINGQVFDMNKPMFAAQKGRHERWVISGVGDMMLHPFHIHGTQFRILSENGK
APAAHRTGWKDTVRVEGGISEVLVKFDHDAPKEHAYMAHCHLLEHEDTGMMLGFTV
>EECEBICD_03292 hypothetical protein
MLTLTFPDAVVEAMKTFFRPVLFGSLMALCANSYALTESEAEDMADLTAVFVFLKNDCGY
QNLPNSQIRRALVFFAQQNQWDLSNYDTFDMKSLGEDSYRDLSGIGIPVAKKCKALARDS
LSLLAYVK
>EECEBICD_03293 Polyamine aminopropyltransferase
MAENTMWHETLHDQFGQYFAVDNVLYHEKTDHQDLIIFENAAFGRVMALDGVVQTTERDE
FIYHEMMTHVPLLAHGHAKHVLIIGGGDGAMLREVTRHKNVETITMVEIDAGVVSFCRQY
LPNHNAGSYDDPRFTLVIDDGVNFVNQTHQTFDVIISDCTDPIGPGESLFTSAFYEGCKR
CLNPGGIFVAQNGVCFLQQDEALDSHRKLSHYFSDVGFYQAAIPTYYGGIMTFAWATDND
ALRHLSSEIIQARFHAAGLKCRYYNPAIHAAAFALPQYLHDALSAQ
>EECEBICD_03294 S-adenosylmethionine decarboxylase proenzyme
MKKLKLHGFNNLTKSLSFCIYDICYAKTAEERDGYIAYIDELYNANRLTEILSETCSIIG
ANILNIARQDYEPQGASVTILVSEEPVDPKLIDQTEHPGPLPETVVAHLDKSHICVHTYP
ESHPEGGLCTFRADIEVSTCGVISPLKALNYLIHQLESDIVTIDYRVRGFTRDVNGMKHF
IDHEINSIQNFMSEDMKSLYDMVDVNVYQENIFHTKMLLKEFDLKHYMFHTKPEDLTETE
RQQITAALWKEMREIYYGRNISAV
>EECEBICD_03295 HTH-type transcriptional repressor GlcR
MKGQHRLDLIVAYLKNHTLVTVEQLVEAVDASPATIRRDLIKLDEQGVISRSHGGVALRR
FEPAQPTTNEKQLRSPAEKRAIARVAASLVNAGDAVVLDAGTTMMELAKCLTHLPLRVIT
VDLHIALFLAEFRQIEVTIVGGRIDDSSQSCIGEHGRKLLRSVYPNIAFMSCNSWGVKTG
VTTPTEDKAGLKQEIIANAQRKVLLADSSKYGAHSLFNVVPLERFNDVITDVNLPPSAQV
ELKGRAFALTLVQPE
>EECEBICD_03296 D-threonate 4-phosphate dehydrogenase
METKTVAITMGDPAGIGPEIIVKALSEDGLNGAPLVVIGCLATLKRLQAKGITPNVELRA
IERVAEARFAPGIIHVIDEPLAQPEALEAGKVQAQAGDLAYRCVKRATELALRGDVQAIA
TAPLNKEALHLAGHNYPGHTELLATLTHSRDYAMVLYTDKLKVIHVSTHIALRKFLDTLS
TARVETVIGIADTFLKRVGYVKPRIAVAGVNPHAGENGLFGDEETRILTPAITDARAKGM
DVYGPCPPDTVFLQAYEGQYDMVVAMYHDQGHIPLKLLGFYDGVNITAGLPFIRTSADHG
TAFDIAWTGKAKSESMAVSIKLAMQLA
>EECEBICD_03297 D-threonate kinase
MKMMVIADDFTGSNDTGVQLAKKGARTEVMLSASQKPSRRADVLVINTESRAMPADQAAS
AVYAALSPWCETSPAPLVYKKIDSTFRGNIGAEVTAAMRASQRKLAVIAAAIPAAGRTTL
EGKCLVNGVPLLETEFASDPKTPIVSSRIAEIVALQSEIPVYEVFLQDVRRGGLSALLTA
YAAEGEGIIVVDAVEERDLTLIAQAACEQPSMPLLVGAAGLANALPVELFMQDRQRLPVL
VVAGSMSEATRRQVDNALCRGRAEVVDIDAARMVSDSAEQEIASVVEQACALLSQHRHTI
LRTSRRAEDRQLIDALCEKSAMSRQQLGERLSQRLGVVTLNIIEQARIGGLFLTGGDIAT
AVAGALGAEGYRIQSEVAPCIPCGTFVNSEIDDLPVITKAGGFGSDSTLCDALYYIEEMY
CGD
>EECEBICD_03298 2-keto-3-deoxygluconate permease
MNIKKAIERVPGGMMVVPLVIGAVINTFAPQALEIGGFTTALFKNGAAPLIGAFLLCMGA
GISVKAAPQALLQGGTITLTKLLVTIGIGLGVEHLFGAEGIFGLSGVAIIAAMSNSNGGL
YAALVGEFGNERDVGAISILSLNDGPFFTMIALGAAGMANIPIMALVAVLVPLVIGMILG
NLDPHMRDFLTKGGPLLIPFFAFALGAGINLEMLLQGGLAGILLGVLTTFVGGFFNIRAD
RLVGGTGIAGAAASSTAGNAVATPLAIAQADPSLAEVAAAAAPLIAASVITTAILTPVLT
SWVAKKQARQASLEKNA
>EECEBICD_03299 hypothetical protein
MDYEFLRDVTGGVKVRMSMGHEVVGHWFNEEVKDNLSLLDEVEQAARTVKGSERSWQRAG

HEYTIWMDGEEVMIRANQLDFSGDEMEEGMSYYDEESLSLCGMEDFLRVVAAYREFVSKA
>EECEBICD_03300 hypothetical protein
MNVLFICSRNQWRSPTAEQVFRRYPGLSVRSAGTSRNAKKSVSCGLLQWADVICVMEQKH
KDRLMAEYRRIIENKPLHVLDIPDDYRYMDPELVRQLEELVPEVLGI
>EECEBICD_03301 hypothetical protein
MSVFRRIIYHSGWFILFGPLIGAAIAIGIINFLPAIIGGPDSFLLYFCRTIPELVIITGW
IYSLLPAWLTGVACALIPLKLYQKIINRMILCAIAGGLITTLFNLVRYGFDISILPLIYG
EVLIMLIPAIVAGAIMGGVITYLPGLREVKRNNK
>EECEBICD_03302 Aconitate hydratase B
MLEEYRKHVAERAAQGIVPKPLDATQMAALVELLKTPPVGEEEFLLDLLINRVPPGVDEA
AYVKAGFLAAVAKGDTTSPLVSPEKAIELLGTMQGGYNIHPLIDALDDAKLAPIAAKALS
HTLLMFDNFYDVEEKAKAGNEYAKQVMQSWADAEWFLSRPPLAEKITVTVFKVTGETNTD
DLSPAPDAWSRPDIPLHAQAMLKNAREGIEPDQPGVVGPIKQIEALQKKGYPLAYVGDVV
GTGSSRKSATNSVLWFMGDDIPNVPNKRGGGLCLGGKIAPIFFNTMEDAGALPIEVDVSN
LNMGDVIDVYPYKGEVRNHETDELLATFELKTDVLIDEVRAGGRIPLIIGRGLTTKAREA
LGLPHSDVFRQAKDVAESSRGFSLAQKMVGRACGVKGIRPGAYCEPKMTSVGSQDTTGPM
TRDELKDLACLGFSADLVMQSFCHTAAYPKPVDVTTHHTLPDFIMNRGGVSLRPGDGVIH
SWLNRMLLPDTVGTGGDSHTRFPIGISFPAGSGLVAFAAATGVMPLDMPESVLVRFKGKM
QPGITLRDLVHAIPLYAIKQGLLTVEKKGKKNIFSGRILEIEGLPDLKVEQAFELTDASA
ERSAAGCTIKLNKEPIVEYLTSNIVLLKWMIAEGYGDRRTLERRIQGMEKWLADPQLLEA
DADAEYAAVIDIDLADIKEPILCAPNDPDDARLLSDVQGEKIDEVFIGSCMTNIGHFRAA
GKLLDNHKGQLPTRLWVAPPTRMDAAQLTEEGYYSVFGKSGARIEIPGCSLCMGNQARVA
DGATVVSTSTRNFPNRLGTGANVFLASAELAAVAALIGKLPTPEEYQTYVAQVDKTAVDT
YRYLNFDQLSQYTEKADGVIFQTAV
>EECEBICD_03303 hypothetical protein
MTLPFKPHLIALVCSAGLFAASGVLYVKSRAPEAPAQAATPAPEQTQTAVPAPVAKTTFT
TAQIDQWVAPVALYPDALLSQVLMASTYPANVVQAVQWSRDNPTLQGDAAIQAVASQPWD
PSVKSLVAFPQLMALMGENPQWVQNLGDAFLAQPQDVMDAVQRLRLLAQQTGSLKSTPQQ
TVTSVPKSSASTAVTTTTTTSASTPATPSTVIKIEPANPQVVYVPNYNPATVYGAWPNTA
YPPVYLPPPPGQQFADSFVKGFGYSLGVATTYALFSSIDWDDDDHHHHDDDHHDDDYHHG
GNGYQHNGDNININVNNFNRISGQNLPGQTMGWQHNPAWRNGVPYPNNTVAQRFHPTNVS
GGLSATQQAPVSRDSQRQAAMTQFQQRSHTANASLPAPGTRDAQRKAASQQLNQIAQRNN
YRGYDGTQNSSRREAAQQTLNKSTTQQHRSELKAKAQQHPVSQQQRDTARQRIESSTPQQ
RQAFRQNMHANVFSGNDSRSPSWQSQQLRGLESRRGSHLNTEQRAAAREHFSEHHEFHRR
>EECEBICD_03304 hypothetical protein
MKKVMLSALLLSLPLLGYAQERFPSPEAAASAFAAAVAGKNETQLTALLGDDWRQFLPPE
GADPEAVARFNRDWREGHRIVQKDNTAHLNVGREDWQLPVPMMKETGGWRFDMAAAGNEI
LTRTIGRNELSTLQAMHAYVDAQQDYYLQNHRWAHRIISSEGQKDGLYWPTKAGDVPSPL
GPNFSPAAPDEGYHGYHFRIISDNDGHGAALLAWPMHYGETGVMSFMVNQDDRIYQADLG
KETESKVQAITRFAPDAQWQVAE
>EECEBICD_03305 hypothetical protein
MKLFLTTAALTATLISGMTFASDPVIPWATNSGGTESTHIAAMGEDLNAQHQQITHTHEG
VWAANSGSIQADEAALTSNKPPVQGHPELMPHQG
>EECEBICD_03306 Dihydrolipoyl dehydrogenase
MSTEIKTQVVVLGAGPAGYSAAFRCADLGLETVIVERYNTLGGVCLNVGCIPSKALLHVA
KVIEEAKALAEHGIVFGEPKTDIDKIRTWKEKVITQLTGGLAGMAKGRKVKVVNGLGKFT
GANTLEVEGENGKTVINFDNAIIAAGSRPIQLPFIPHEDPRVWDSTDALELKEVPKRMLV
MGGGIIGLEMGTVYHALGSEIDVVEMFDQVIPAADKDIVKVFTKRISKKFNLMLETKVTA
VEAKEDGIYVSMEGKKAPAEAQRYDAVLVAIGRVPNGKNLDAGKAGVEVDDRGFIRVDKQ
LRTNVPHIFAIGDIVGQPMLAHKGVHEGHVAAEVIAGKKHYFDPKVIPSIAYTEPEVAWV
GLTEKEAKEKGISYETATFPWAASGRAIASDCADGMTKLIFDKESHRVIGGAIVGTNGGE
LLGEIGLAIEMGCDAEDIALTIHAHPTLHESVGLAAEVFEGSITDLPNPKAKKK
>EECEBICD_03307 Dihydrolipoyllysine-residue acetyltransferase component of pyruvate dehydrogenase complex

MVKVGDTVEAEQSLITVEGDKASMEVPAPFAGTVKEIKVNTGDKVSTGSLIMVFEVAGAA
PAAAPAKAEAAPAAPAAATGVKDVNVPDIGGDEVEVTEVMVKVGDKVAAEQSLITVEGDK
ASMEVPAPFAGTVKEIKISTGDKVKTGSLIMVFEVEGAAPAAAPAKQEAAAPAPAAKAEK
PAAPAAKAEGKSEFAENDAYVHATPLIRRLAREFGVNLAKVKGTGRKGRILREDVQAYVK
DAIKRAEAAPAAAGGGIPGMLPWPKVDFSKFGEVEEVELGRIQKISGANLSRNWVMIPHV
THFDKTDITDLEAFRKQQNAEAEKRKLDVKYTPVVFIMKAVAAALEQMPRFNSSLSEDGQ
RLTLKKYINIGVAVDTPNGLVVPVFKDVNKKSVTELSRELTTISKKARDGKLTAGEMQGG
CFTISSIGGLGTTHFAPIVNAPEVAILGVSKSAMEPVWNGKEFVPRLMMPISLSFDHRVI
DGADGARFITIINNMLSDIRRLVM
>EECEBICD_03308 hypothetical protein
MAIEIKVPDIGTDEVEITEILVKVGDKVEAEQSLITVEGDKASMEVPSPQAGVVKEIKVS
VGDKTETGALIMIFDSADGAAGRCACSGRREERSDSGSSTCCGSGERRSRSGYRQ
>EECEBICD_03309 Pyruvate dehydrogenase E1 component
MSERFQNDVDPIETRDWLQAIESVIREEGVERAQYLIDQLLSEARKGGVKVAAGAGASNY
INTIAVEDEPEYPGNLELERRIRSAIRWNAIMTVLRASKKDLELGGHMASFQSSATIYDV
CFNHFFRARNEQDGGDLVYFQGHISPGIYARAFLEGRLTQEQMDNFRQEVHGNGLSSYPH
PKLMPEFWQFPTVSMGLGPIGAIYQAKFLKYLEHRGLKDTSKQTVYAFLGDGEMDEPESK
GAITIATREKLDNLVFVINCNLQRLDGPVTGNGKIVNELEGIFAGAGWNVIKVMWGGRWD
ELLRKDTSGKLIQLMNETVDGDYQTFKSKDGAYVREHFFGKYPETAALVADWTDEQIWAL
NRGGHDPKKVYAALKKAQETKGKATVILAHTIKGYGMGDTAEGKNIAHQVKKMNMDGVRY
VRDRFNVPVADADLEKLPYITFPEGSEEHKYLHERRQALHGYLPSRQPNFTEKLELPQLS
DFGALLEEQSKEISTTIAFVRALNVMLKNKSIKDRLVPIIADEARTFGMEGLFRQIGIYS
PNGQQYTPQDREQVAYYKEDEKGQILQEGINELGAGASWLAAATSYSTNDLPMIPFYIYY
SMFGFQRIGDLCWQAGDQQARGFLIGGTSGRTTLNGEGLQHEDGHSHIQSLTIPNCISYD
PAYAYEVAVIMHDGLERMYGEKQENVYYYITTLNENYHMPAMPAGAEEGIRKGIYKLETL
EGSKGKVQLLGSGSILRHVREAAEILAKDYGVGSDVYSVTSFTELARDGQDCERWNMLHP
LETPRVPYIAQVMNDAPAVASTDYMKLFAEQVRTYVPADDYRVLGTDGFGRSDSRENLRH
HFEVDASYVVVAALGELAKRGEIDKKVVADAITKFNIDADKVNPRLA
>EECEBICD_03310 Pyruvate dehydrogenase complex repressor
MAYSKIRQPKLSDVIEQQLEFLILEGTLRPGEKLPPERELAKQFDVSRPSLREAIQRLEA
KGLLLRRQGGGTFVQSSLWQSFSDPLVELLSDHPESQFDLLETRHALEGIAAYYAALRST
DEDKDRIRELHHAIELAQESGDLDAESEAVLQYQIAVTEAAHNVVLLHLLRCMEPMLAQN
VRQNFELLYARREMLPLVSTHRTRIFEAIMAGKPEEAREASHRHLAFIEEIMLDRSREES
RRERALRRLEQRKN
>EECEBICD_03311 Aromatic amino acid transport protein AroP
MDSQQHGEQLKRGLKNRHIQLIALGGAIGTGLFLGSASVIQSAGPGIILGYAIAGFIAFL
IMRQLGEMVVEEPVAGSFSHFAYKYWGGFAGFASGWNYWVLYVLVAMAELTAVGKYIQFW
YPEIPTWASAAAFFVIINAINLTNVKVFGEMEFWFAIIKVIAVIAMILFGAWLLFSDTAG
PQATVRNLWEQGGFLPHGWTGLVMMMAIIMFSFGGLELVGITAAEADNPEQSIPKATNQV
IYRILIFYIGSLAVLLSLLPWTRVTADTSPFVLIFHELGDTFVANALNIVVLTAALSVYN
SCVYCNSRMLFGLAQQGNAPKALLNVDKRGVPVSSILVSAVVTALCVLLNYLAPESAFGL
LMALVVSALVINWAMISLAHMMFRRAKQQQGVKTRFPALFYPFGNVLCLLFMAAVLIIML
MTPGMAISVWLIPVWLLILGVGYLCKEKTAKTVKAH
>EECEBICD_03312 Inner membrane symporter YicJ
MDKGRLSVREKIGYGMGDAGCNIIFGAIMLFVNYFYTDIFGLAPALVGVLLLSVRVIDAV
TDPVMGALADRTQSKYGRFRPWLLWIAFPYALFSVLMFTTPDWGYNSKVVYAFVTYFLLS
VTYTAINIPYCSLGGVITNDPKERVACQAYRFVLVGIATLLLSLTLLPMVDWFGGGDKAK
GYQLAMTVLAIIGMGMFLFCFASVRERVRPAVPTHDDMKNDFKDVWKNDQWVRILLLTLC
NVCPGFIRMAATMYYVTWVMGQSTHFATLFISLGVVGMMIGSMLAKVLTDRWCKLKVFFW
TNIALAIFSCAFYFFDPKATVMIVALYFLLNILHQIPSPLHWSLMADVDDYGEWKTGKRI
TGISFSGNLFFLKLGLAIAGAMVGFLLSWYGYDASAKAQSASAMNGIMLLFTVIPGVGYL
ITAGVVRLLKVDRELMKKIQDDLEKRRTNYRELSELQDLKAAESVRKA
>EECEBICD_03313 Extracellular exo-alpha-(1->5)-L-arabinofuranosidase
MANWPNPFIEQRADPFILRDGSDYYFIASVPEYDRLEIRRADSLEGLRAADPVVVWRKPE

SGPMSQLIWAPEMHRINGKWYIYFAATHTQALDKLGIFQHRMFALECADADPLTGKWTEK
GQIKTPFDTFALDATTFYHQGKQWYLWAQKAPDIAGNSNIYLAELENPWTIKGEPVRLSK
PEYDWECRGFWVNEGPAVVVHGDKLFISYSASATDENYCMGLLWINVNDDPRDPANWHKS
PRPVFTTSYENRQYGPGHNSFTQTPEGEDVLVYHARNYTEIEGDPLYDPNRHTRLKRVRW
DENGMPDFGVPPADTI
>EECEBICD_03314 Protein AmpE
MTLFTTLLVLIVERLFKLGEHWQLDHRLEALFRRITHFSMLRTMGMTAIAMVVTFLLLQA
LKGLLFNVPTLVVWILLGVLCIGAGKVRVHYHAWLKAASRNDPHPCEAMASELTLIHGVP
PDCNEREFLRELQNALLWINFRFYLAPLFWFIVGGPWGPVTLVGYAFLRAWQTWLARYQT
PHQRLQSGIDAILHVLDWIPVRLAGVVYALLGHGEKALPAWFASLADLHTSQYQVLTRLA
QFSLAREPHTDKVETPKAAVSMAKKASFVVVVIIALLTIYGALV
>EECEBICD_03315 1,6-anhydro-N-acetylmuramyl-L-alanine amidase AmpD
MLPDKGWLVEARRVPSPHYDCRPDDEKPSLLVVHNISLPPGEFGGPWIDALFTGTIDPDA
HPFFAEIAHLRVSAHCLIRRDGEIVQYVPFDKRAWHAGVSNYQGRERCNDFSIGIELEGT
DTLAYTDAQYQQLAAVTRTLIACYPAIADNMTGHCNIAPDRKTDPGPAFDWPRFRALVAL
SSHKEMT
>EECEBICD_03316 Nicotinate-nucleotide pyrophosphorylase [carboxylating]
MPPRRYNPDDRRDALLERINLDIPAAVAQALREDLGGEVDAGNDITAQLLPADTQAHATV
ITREDGVFCGKRWVEEVFIQLAGDDVRLTWHVDDGDAIHANQTVFELNGPARVLLTGERT
ALNFVQTLSGVASEVRRYVGLLAGTQTQLLDTRKTLPGLRTALKYAVLCGGGANHRLGLT
DAFLIKENHIIASGSVRQAVEKAFWLHPDVPVEVEVENLDELDDALKAGADIIMLDNFNT
DQMREAVKRVNGQARLEVSGNVTAETLREFAETGVDFISVGALTKHVRALDLSMRFC
>EECEBICD_03317 hypothetical protein
MEKQRGFTLIELMVVIGIIAILSAIGIPAYQNYLRKAALTDMLQTFVPYRTAVELCALEH
GGTSTCDAGVNGIPSPVITRYVSGMSVEKGVITLTGQESLSGLSVIMTPAWDNANGITGW
TRNCNIQSDSALQQACEDVFRFDAN
>EECEBICD_03318 Type II secretion system protein E
MKDAQLNTLCQRHQAVLINSASNSITVAVVDAPSHALLDALHFATQKQIDIVCWTRQQME
NHRHKPDQAPSANAAKGGETAAQLLNQTLRSAMAKRASDIHLEPGASRYRIRLRIDGVLH
ILQDIAKETGLALTARLKVLGNLDIAEHRLPQDGQFTVDLAGDSISFRIATLPCKEGEKV
VLRLLHQVEQTLDLDTLGMYGAQLTAFRQALQQPQGLVLVTGPTGSGKTVTLYSALQTRN
TPGINLCSVEDPIEIPLDGINQTQIHPRAGLTFQNVLRALLRQDPDIIMVGEIRDGDTAE
IAIKAAQTGHLVLSTLHTNSTSETLIRLQQMGVARWMISSALTLVVAQRLVRKLCPHCKQ
RLSDPVVLSPNLWPSALPRWQANGCQHCYHGFYGRTALFEVLTVTPALRQLIASGASAQA
LEAHLQQTGTGTLFENGCRAVEQGMTSFEEILRVLGMPHER
>EECEBICD_03319 Type II secretion system protein F
MSVKQLWRWQGVNDKGQLEQDVVWVDNRLALIITLQHQRIMPLRIKRIGVNAALWKEEQS
AEIIHQLATLIHAGLTLSEGLELLAKQHPHRQWQALLRTLAHELEQGVPFSSALVSWPQV
FPPLYQTMIRTGELTGKLAECCFELARQQKAQRQITVSVKKALRYPAIILTMAALVVFAM
LHFVLPEFAAIYRSFNTPLPLLTRGIIAIAQWGSAWGWLILFLTMLVAIAHRRVKQKPSW
QAQRQRLLLRLPVMGRLIRGQKLAQIFTVLALTQSAGIPFLQGLESAIESLGCPYWSQRL
TQVHQEIAAGNPVWLALKNTQEFSPLCLQLVRTGEASGSLDIMLHNLARHHSESTLALAD
NLASLLEPALLIITGLIIGTLVVAMYLPIFHLGDAMSGMG
>EECEBICD_03320 GMP reductase
MRIEEDLKLGFKDVLIRPKRSTLKSRSDVELERQFTFKHSGQTWSGVPIIAANMDTVGTF
EMAQALAGFDILTAVHKHYTVEEWAAFINTASADVLKHVMVSTGTSDADFEKTVQILALN
PALNFVCIDVANGYSEHFVQFVAKAREAWPTKTICAGNVVTGEMCEELILSGADIVKVGI
GPGSVCTTRVKTGVGYPQLSAVIECADAAHGLGGMIVSDGGCTMPGDVAKAFGGGADFVM
LGGMLAGHEESGGSVVEENGEKFMLFYGMSSESAMNRHVGGVAKYRAAEGKTVKLPLRGP
VGNTARDILGGLRSACTYVGASRLKELTKRTTFIRVQEQENRIFNSL
>EECEBICD_03321 Dephospho-CoA kinase
MRYTVALTGGIGSGKSTVADAFADLGITVIDADIIARQMVEPGQPALNAIAEHFGSELIA
ADGTLRRRALRERIFSHPEEKAWLNALLHPLIQQETQRQFQQATSPYVLWVVPLLVENRL
YQKANRVLVVDVTPETQLIRTMQRDDVTREHVEHILAAQATREARLAVADDVIDNNGAPD

AIASDVARLHASYLKLASQFVSQEKP
>EECEBICD_03322 Cell division protein ZapD
MHTQVLFEHPLNEKMRTWLRIEFLIQQLSINLPIADHAGALHFFRNISDLLDVFERGEVR
TELLKELERQQRKLQAWVEVPGVDQDRIEALRQQLKSAGSVLISAPRIGQQLREDRLIAL
VRQRLSIPGGCCSFDLPTLHIWLHLQQAQRDAQIESWLASLNPLTQALTLVLDLIRNSAP
FRKQTSLNGFYQDNGDDADLLRLMLTLDSQLYPQISGHKSRFAIRFMPLDSENGLVPERL
DFELACC
>EECEBICD_03323 DNA gyrase inhibitor YacG
MSDVTVVNCPTCGKPVVWGEISPFRPFCSKRCQLIDLGEWAAEEKRIASSGDQSDSDDWS
EER
>EECEBICD_03324 HTH-type transcriptional regulator PgrR
MRHDVMSILHAFCMIAENHSFTRAAAQLHISTSALSQNMRQLEDELGVKLLNRTTRRVGL
TEAGHAFLASIRPALNHIDEAMEHARQSRDIPGGTLRLTVPRTAATLLLQPNLAGFMRDY
PDIRLDIDVNDGFVDLINERFDAGIRFGDAVQLDMNVVPLGDVLRPAIVASPAYLARFGT
PLHPQELINHRCLCHRFTRESGLYRWEFVHGAQRLEITPEAALMVNDKALRLSAARDGAG
LTYVFEQDVHEDLQDGRLCSVLEEWLPAADRFYLYYPGRAHMAPKLRVFIDYFCHKAILP
SQQSHHR
>EECEBICD_03325 Aldo-keto reductase YhdN
MQYRTLGANGPRVSAIGLGCMGMSAFYGAHDDSTSIKTLHYALDQGVTLLDTADMYGPYT
NERLVGRAIADRRDRVFLATKFGIVLDPANPMARGVNGRPEYVRRSCEQSLQRLGVDHID
LYYQHRVDPSVPIEETVGAMADLVREGKVRYLGLSEASTQTLEPPITFTLLPRCKVSIRF
GPAKRKFQHFPPVNGWV
>EECEBICD_03326 hypothetical protein
MGENFSRNLRLAEAIKQMAREKECTPAQLALAWLLARNRHIVPIPGTRHCARVDENLGAL
SLTLSPQELTAIEAVFPHDAAAGPRYWPEIMSTLNR
>EECEBICD_03327 8-oxo-dGTP diphosphatase
MKKLQIAVGIIRNPNDEIFITQRAADAHMANKLEFPGGKIEAGETPEQALIRELQEEVGI
TPTQVTLFDTLEYQFPDRHITLWFWLVERWDGEPWGKEGQPGRWIAQNALSTDDFPPANE
PIIRKLRQFAL
>EECEBICD_03328 Protein translocase subunit SecA
MLIKLLTKVFGSRNDRTLRRMRKAVSLINAMEPEMEKLSDDELKAKTNEFRARIEKGESV
ESLIPEAFAVVREASKRVFGMRHFDVQLLGGMVLNDRCIAEMRTGEGKTLTATLPAYLNA
LSGKGVHVVTVNDYLAQRDAENNRPLFEFLGMSVGINLPGMPAPAKREAYAADITYGTNN
EYGFDYLRDNMAFSPEERVQRKLHYALVDEVDSILIDEARTPLIISGPAEDSSEMYKKVN
KIIPHLIRQEKEDSDTFQGEGHFSVDEKARQVNLTERGLVLIEELLVQEGIMDEGESLYS
PGNIMLMHHVTAALRAHALFTRDVDYIVKDGEVIIVDEHTGRTMQGRRWSDGLHQAVEAK
EGVEIQNENQTLASITFQNYFRLYEKLAGMTGTADTEAFEFSSIYKLDTVVVPTNRPMIR
KDLPDLVYMTEAEKIQAIIEDIKERTANGQPVLVGTISIEKSEVVSRELTKAGIKHNVLN
AKFHANEAGIVAQAGYPAAVTIATNMAGRGTDIMLGGSWQAEVAALEAPTEEQIAQIKAD
WQVRHDAVLAAGGLHIIGTERHESRRIDNQLRGRSGRQGDPGSSRFYLSMEDALMRIFAS
DRVSGMMRKLGMKPGEAIEHPWVTKAIANAQRKVESRNFDIRKQLLEYDDVANDQRRAIY
TQRNELLDVSDVSDTINSIREDVFKETIDAYIPPQSLEEMWDIPGLQERLKNDFDLEMPI
AEWLDKEPELHEETLRERILAQSIEVYQRKEEVVGAEMMRHFEKGVMLQTLDSLWKEHLA
AMDYLRQGIHLRGYAQKDPKQEYKRESFAMFAAMLESLKYEVISTLSKVQVRMPEEVEAM
EMQRREEAERLAQMQQLSHQDDDAAVAADLAAQTGERKIGRNDPCPCGSGKKYKQCHGRL
S
>EECEBICD_03329 Secretion monitor
MSGILTRWRQLGRRYFWPHLLLGMVAASFGLPALSNAAETNTPARTTASTASKVNFSHFA
LLEASNRRPNFTVDYWHQHAIRTVIRHLSFAMAPQTLPVADAPSPLQAHHIALLNTLSAM
LTQEGTPPAIVRRLSLAYFAPQTAFSIPAWISQAQGIRAGPQRLS
>EECEBICD_03330 UDP-3-O-acyl-N-acetylglucosamine deacetylase
MIKQRTLKRIVQATGVGLHTGKKVTLTLRPAPANTGVIYRRTDLNPPVDFPADAKSVRDT
MLCTCLVNEHDVRISTVEHLNAALAGLGIDNIVIEVNAPEIPIMDGSAAPFVYLLLDAGI
DELNCAKKFVRIKETVRVEDGDKWAEFRPYNGFTLDFTIDFNHPAIDSSSQRYAMNFSAD

AFMRQISRARTFGFMRDIEYLQSRGLCLGGSFDCAIVVDDYRVLNEDGLRFEDEFVRHKM
LDAIGDLFMCGHNIIGAFTAYKSGHALNNKLLQAVLAKQEAWEFVTFQDDAELPLAFKAP
STVLA
>EECEBICD_03331 Cell division protein FtsZ
MFEPMELTNDAVIKVIGVGGGGGNAVEHMVRERIEGVEFFAVNTDAQALRKTAVGQTIQI
GSGITKGLGAGANPEVGRNAADEDREALRAALEGADMVFIAAGMGGGTGTGAAPVVAEVA
KDLGILTVAVVTKPFNFEGKKRMAFAEQGITELSKHVDSLITIPNDKLLKVLGRGISLLD
AFGAANDVLKGAVQGIAELITRPGLMNVDFADVRTVMSEMGYAMMGSGVASGEDRAEEAA
EMAISSPLLEDIDLSGARGVLVNITAGFDLRLDEFETVGNTIRAFASDNATVVIGTSLDP
DMNDELRVTVVATGIGMDKRPEITLVTNKQVQQPVLDRYQQHGMAPLTQEQKTVAKVVND
NTPQAAKEPDYLDIPAFLRKQAD
>EECEBICD_03332 Cell division protein FtsA
MVNIIGVGSCPSRGMDKGGVNDLESVVKCVQRAIDQAELMADCQISSVYLALSGKHISCQ
NEIGMVPISEEEVTQEDVENVVHTAKSVRVRDEHRVLHVIPQEYAIDYQEGIKNPVGLSG
VRMQAKVHLITCHNDMAKNIVKAVERCGLKVDQLIFAGLAASYSVLTEDERELGVCVVDI
GGGTMDIAVYTGGALRHTKVIPYAGNVVTSDIAYAFGTPPSDAEAIKVRHGCALGSIVGK
DESVEVPSVGGRPPRSLQRQTLAEVIEPRYTELLNLVNEEILQLQEQLRQQGVKHHLAAG
IVLTGGAAQIEGLAACAQRVFHTQVRIGAPLNITGLTDYAQEPYYSTAVGLLHYGKESHL
NGEAEVEKRVTASVGSWIKRLNSWLRKEF
>EECEBICD_03333 Cell division protein FtsQ
MSQAALNTRNSDEETPSSRRNNGTRLAGILFLLTVLCTVFVSGWVVLGWMEDAQRLPLSK
LVLTGERHYTRNDDIRQAILALGAPGTFMTQDVNIIQSQIERLPWIKQASVRKQWPDELK
IHLVEYVPIARWNDQHMVDAEGNTFSVPSDRIGKQVLPMLYGPEGSASEVLQGYREMGQV
LAKDKFTLKEAAMTARRSWQLTLNNGIKLNLGRGDTMKRLARFVELYPVLQQQAQTDGKR
ISYVDLRYDSGAAVGWAPLPLRNLISNRIRHRQNNNDQGDGQKTGSRTGDWHREGCRFSR
GSSARRYGQYHWRGQLPIAGYG
>EECEBICD_03334 D-alanine--D-alanine ligase B
MADKIAVLLGGTSAERDVSLNSGAAVLAGLREGGIDAHPVDPQEVDVAQLKAMGFQKVFI
ALHGRGGEDGTLQGMLELLGLPYTGSGVMASALSMDKLRSKLLWQGAGLPVAPWVALTRA
EFEKGLSEEQKARISALGLPLIVKPSREGSSVGMTKVVEENALQGALSLAFQHDDEILIE
KWLCGPEFTVAIVGEEILPSIRIQPAGTFYDYEAKYLSDETQYFCPAGLDASQEAALQSL
VLQAWKALGCTGWGRIDVMLDSDGQFYLLEANTSPGMTSHSLVPMAARQAGMSFSQLVVR
ILELAD
>EECEBICD_03335 UDP-N-acetylmuramate--L-alanine ligase
MNTQQLAKLRSIVPEMRRVRHIHFVGIGGAGMGGIAEVLANEGYQISGSDLAPNPVTQQL
TSLGATIFFNHRPENVRDASVVVVSSAISADNPEIVAAHEARIPVIRRAEMLAELMRFRH
GIAIAGTHGKTTTTAMVSSIYAEAGLDPTFVNGGLVKAAGVHARLGHSRYLIAEADESDA
SFLHLQPMVAIVTNIEADHMDTYHGDFENLKQTFINFLHNLPFYGRAVMCVDDPVIRELL
PRVGRQTTTYGFSEDADVRVEDYQQIGPQGHFTLLRQGMPDLHVTLNAPGRHNALNAAAA
VAVATEEGIDDDAILRALESFQGTGRRFDFLGEFPLEPVNGKAGTAMLVDDYGHHPTEVD
ATIKAARAGWPDKNLVMLFQPHRYTRTRDLYDDFANVLTQVDALLMLDVYPAGEAPIPGA
DSRSLCRTIRNRGKIDPILVSDPAQVATMLAPVLTGNDLILVQGAGNVGKIARYLSEIKL
KPQIQEEEQHG
>EECEBICD_03336 UDP-N-acetylglucosamine--N-acetylmuramyl-(pentapeptide) pyrophosphoryl-undecaprenol N-acetylglucosamine transferase
MSGQPKRLMVMAGGTGGHVFPGLAVAHHLMSQGWQVRWLGTADRMEADLVPKHGIDIDFI
RISGLRGKGVKALLAAPLRIFNAWRQARAIMKRFKPDVVLGMGGYVSGPGGLAAWSLGIP
VVLHEQNGIAGLTNQWLAKIATTVMQAFPGAFPNAEVVGNPVRTDVLALPLPQVRLAGRD
GPIRVLVVGGSQGARVLNQTMPQVAARLGDTVTIWHQSGKGVQHTVEQAYAGVGQPQHKV
TEFIDDMAAAYAWADVVVCRSGALTVSEIAAAGLPAIFVPFQHKDRQQYWNALPLENAGA
AKIFEQPQFTVEAVADTLAGWSREALLTMAERARAVSIPDATERVASEVSRVART
>EECEBICD_03337 putative peptidoglycan glycosyltransferase FtsW
MRLSLPRLRMPRVPGFGLLAWLFAALKGWVMASRDKDADSLIMYDRTLLWLTFGLAAIGF
VMVTSASMPVGQRLANDPFLFAKRDALYIFLAFCLAMVTLRLPMTFWQKYSTTMLIASII

MLLIVLVVGSSVNGASRWIALGPLRIQPAEFTKLSLFCYLANYLVRKVDEVRNNLRGFLK
PMGVILVLAVLLLAQPDLGTVVVLFVTTLAMLFLAGAKLWQFIAIIGMGISAVILLILAE
PYRIRRVTSFWNPWEDPFGSGYQLTQSLMAFGRGEIWGQGLGNSVQKLEYLPEAHTDFIF
AIIGEELGYIGVVLALLMVFFVAFRAMSIGRKALEIDHRFSGFLACSIGIWFSFQALVNV
GAAAGMLPTKGLTLPLISYGGSSLLIMSTAIMFLLRIDYETRLEKAQAFTRGSR
>EECEBICD_03338 UDP-N-acetylmuramoylalanine--D-glutamate ligase
MADYQDKNVVIIGLGLTGLSCVDFFLARGVTPRVMDTRVTPPGLDKLPQEVERHVGGLND
EWLLAADLIVASPGIALAHPSLSAAASAGVEIVGDIELFCREAQAPIVAITGSNGKSTVT
TLVGEMAKAAGVNVGVGGNIGLPALMLLDADRELYVLELSSFQLETTSSLQAAAATVLNV
TEDHMDRYPFGLQQYRAAKLRVYEKAKVCVVNADDALTMPVRGADERCVSFGVNMGDYHL
NRQQGETWLRVKGEKVLNVKEMKLSGQHNYTNALAALALADAVGLPRASSLKALTTFTGL
AHRFQLALEHNGVRWINDSKATNVGSTEAALNGLHVDGTLHLLLGGDGKSADFSPLTRYL
TGDRIRLYCFGRDGAQLAALRPEIAQQTETMEEAMRLLAPRVQPGDMVLLSPACASLDQF
KNFEQRGDVFTRLAKELG
>EECEBICD_03339 Phospho-N-acetylmuramoyl-pentapeptide-transferase
MLVWLAEHLVKYYSGFNVFSYLTFRAIVSLLTALFISLWMGPRMIARLQKLSFGQVVRND
GPESHFSKRGTPTMGGIMILTSIVISVLLWAYPSNPYVWCVLVVLIGYGIIGFVDDYRKV
VRKDTKGLIARWKYFWMSVIALGVAFALYLVGKDTPATQLVVPFFKDVMPQLGLFYILLS
YFVIVGTGNAVNLTDGLDGLAIMPTVFVAAGFALVAWATGNMNFANYLHIPYLRHAGELV
IVCTAIVGAGLGFLWFNTYPAQVFMGDVGSLALGGALGIIAVLLRQEFLLVIMGGVFVVE
TLSVILQVGSFKLRGQRIFRMAPIHHHYELKGWPEPRVIVRFWIISLMLVLIGLATLKVR
>EECEBICD_03340 UDP-N-acetylmuramoyl-tripeptide--D-alanyl-D-alanine ligase
MISVTLSKIADVLGAEHRGADLTLDTVITDTRKVTPGCLFVALKGERFDAHDFADKAKAN
GAGALLVSRPLDIDLPQVIVKDTRQAFGQLAAWVRMQVPARVVALTGSSGKTSVKEMTAA
ILSQCGNTLYTAGNFNNDIGVPITLLRLNHDYDYAVIELGANHQGEIAWTVSLTRPEAAL
VNNLAAAHLEGFGSLAGVAKAKGEIYTGLPENGIAIMNADNNDWLNWQSIIGDRLVWRFS
PNAANSDFTAANIHVTSHGTEFTLQTPMGSIDVLLPLPGRHNIANALAAAALSMAVGATL
TAIKAGLAALKAVPGRLFPIQLSENQLVLDDAYNANVGSMTAAVQVLSEMPGYRVLVVGD
MAELGAESEACHLQVGEAAKAAGIDRVLSTGKLSQAISHASGVGEHFADKAALIARLHAL
LQEQPMMTILVKGSRSAAMEDVVHALQEKGSC
>EECEBICD_03341 UDP-N-acetylmuramoyl-L-alanyl-D-glutamate--2,6-diaminopimelate ligase
MADRNLRDLLAPWVAGLPARELREMTLDSRVAAAGDLFVAVVGHQADGRRYIPQAIAQGV
AAIIAEAKDEASDGEIREMHGVPVVYLSQLNERLSALAGRFYHEPSENMRLVAVTGTNGK
TTTTQLLAQWSQLLGETSAVMGTVGNGLLGKVIPTENTTGSAVDVQHVLASLVAQGATFG
AMEVSSHGLVQHRVAALKFAASVFTNLSRDHLDYHGDMAHYEAAKWMLYSTHHHGQAIVN
ADDEVGRRWLASLPDAVAVSMEGHINPNCHGRWLKAEAVEYHDRGATIRFASSWGDGEIE
SRLMGAFNVSNLLLALATLLALGYPLTDLLKTAARLQPVCGRMEVFTAPGKPTVVVDYAH
TPDALEKALQAARLHCAGKLWCVFGCGGDRDKGKRPLMGAIAEEFADIVVVTDDNPRTEE
PRAIINDILAGMLDAGQVRVMEGRAEAVTNAIMQAKDNDVVLIAGKGHEDYQIVGTQRLD
YSDRVTAARLLGVIA
>EECEBICD_03342 Peptidoglycan D,D-transpeptidase FtsI
MKAAAKTQKSKRQEEQTNFISWRFALLCGCILLALVFLLGRAAWLQIIAPDMLVRQGDMR
SLRVQEVSTSRGMITDRSGRPLAVSVPVKAIWADPKEVHDAGGISVGDRWKALSTALNIP
LDQLSARINANPKGRFIYLARQVNPDMADYIKKLKLPGIHLREESRRYYPSGEVTAHLIG
FTNVDSQGIEGVEKSFDKWLTGQPGERIVRKDRYGRVIEDISSTDSQAAHNLALSIDERL
QALVYRELNNAVAFNKAESGSAVLVDVNTGEVLAMANSPSYNPNNLAGTPKDAMRNRTIT
DVFEPGSTVKPMVVMTALQRGIVNENTVLNTVPYRINGHEIKDVARYSELTLTGVLQKSS
NVGVSKLALAMPSSALVDTYSRFGLGKATNLGLVGERSGLYPQKQRWSDIERATFSFGYG
LMVTPLQLARVYATIGSYGIYRPLSITKVDPPVPGERIFPESTVRTVVHMMESVALPGGG
GVKAAIKGYRIAIKTGTAKKVGPDGRYINKYIAYTAGVAPASQPRFALVVVINDPQAGKY
YGGAVSAPVFGAIMGGVLRTMNIEPDALATGEKNEFVINQGEGTGGRS
>EECEBICD_03343 Cell division protein FtsL

MISRVTEALSKVKGSIGSNERHALPGVIGDDLLRFGKLPLCLFICIILTAVTVVTTAHHT
RLLTAQREQLVLERDALDIEWRNLILEENALGDHSRVERIATEKLQMQHVDPSQENIVVQ
K
>EECEBICD_03344 Ribosomal RNA small subunit methyltransferase H
MMENFKHTTVLLDEAVNGLNIRPDGIYIDGTFGRGGHSRLILSQLGEEGRLLAIDRDPQA
IAVAQTINDPRFSIIHGPFSALADYVAERELTGKIDGILLDLGVSSPQLDDAERGFSFMR
DGPLDMRMDPTRGQSAAEWLQTAEEADIAWVLKTFGEERFAKRIARAIVERNREQPMTRT
KELAEVVAAATPVKDKFKHPATRTFQAVRIWVNSELEEIEQALKSSLSVLAPGGRLSIIS
FHSLEDRIVKRFMREQSRGPQVPAGLPMTEAQLKKLGGRELRALGKLMPGEKEVAENPRA
RSSVLRIAERTNA
>EECEBICD_03345 Transcriptional regulator MraZ
MFRGATLVNLDSKGRLTVPTRYREQLIESATGQMVCTIDIHHPCLLLYPLPEWEIIEQKL
SRLSSMNPVERRVQRLLLGHASECQMDGAGRLLIAPVLRQHAGLTKEVMLVGQFNKFELW
DETTWYQQVKEDIDAEQSATETLSERLQDLSL
>EECEBICD_03346 Catabolite repressor/activator
MKLDEIARLAGVSRTTASYVINGKAKQYRVSDKTVEKVMAVVREHNYHPNAVAAGLRAGR
TRSIGLVIPDLENTSYTRIANYLERQARQRGYQLLIACSEDQPDNEMRCIEHLLQRQVDA
IIVSTSLPPEHPFYQRWANDPFPIVALDRALDREHFTSVVGADQDDAEMLAEELRKFPAE
TVLYLGALPELSVSFLREQGFRTAWKDDPREVNFLYANSYEREAAAQLFEKWLETHPMPQ
ALFTTSFALLQGVMDVTLRRDGKLPSDLAIATFGDHELLDFLQCPVLAVAQRHRDVAERV
LEIVLASLDEPRKPKPGLTRIRRNLYRRGILSRS
>EECEBICD_03347 Acetolactate synthase isozyme 3 small subunit
MMRRILSVLLENESGALSRVIGLFSQRGYNIESLTVAPTDDPTLSRMTIQTVGDEKVLEQ
IEKQLHKLVDVLRVSELGQGAHVEREIMLVKIQASGYGREEVKRNTEIFRGQIIDVTPTL
YTVQLAGTSDKLDAFLASLRDVAKIVEVARSGVVGLSRGDKIMR
>EECEBICD_03348 Acetolactate synthase isozyme 3 large subunit
MVVDLPKDILNPAKKMPYAWPETVSMRSYNPTTSGHKGQIKRALQTLASAKKPVVYVGGG
AISAACYAPLRHIIETFNLPVVSSLMGLGVFPATHRQSLGMLGMHGTYEANMTMHNADVI
FAVGVRFDDRTTNNLAKYCPNATVLHIDIDPTSISKTVNADIPVVGDARLVLEQMLELLA
QDAPSQPQDDIRDWWQQIESWRARQCLKYDAESESIKPQAVIETLWRLTKGDAYVTSDVG
QHQMFAALYYPFDKPRRWINSGGLGTMGFGLPAALGVKMALPKEMVVCVTGDGSIQMNIQ
ELSTALQYELPVLVLNLNNRYLGMVKQWQDMIYSGRHSQSYMQSLPDFVRLAEAYGHVGL
QINHPDELESKLSEALEHVRNNRLVFVDVTVDGSEHVYPMQIRGGGMDEMWLSKTERT
>EECEBICD_03349 Acetolactate synthase isozyme 3 large subunit
MEMLSGAEMVVRSLIDQGVKQVFGYPGGAVLDIYDALHTVGGIDHVLVRHEQAAVHMADG
LARATGDVGVVLVTSGPGATNAITGIATAYMDSIPLVILSGQVATSLIGYDAFQECDMVG
ISRPVVKHSFLVKQTEDIPWCLKRPSGWRQADVLGRWSWICRKIF
>EECEBICD_03350 HTH-type transcriptional regulator LeuO
MPEVKTEKPHLLDMGKPQLRMVDLNLLTVFDAVMQEQNITRAAHTLGMSQPAVSNAVARL
KVMFNDELFVRYGRGIQPTARAFQLFGSVRQALQLVQNELPGSGFEPTSSERVFNLCVCS
PLDNILTSQIYNRVEKIAPNIHVVFKASLNQNTEHQLRYQETEFVISYEEFRRPEFTSVP
LFKDEMVLVASRKHPRISGPLLEGDVYNEQHAVVSLDRYASFSQPWYDTPDKQSSVAYQG
MVLISVLNVVSQTHLVAIAPRWLAEEFAESLDLQILPLPLKLNSRTCYLSWHEAAGRDKG
HQWMEDLLVSVCKR
>EECEBICD_03351 2-isopropylmalate synthase
MSQQVIIFDTTLRDGEQALQASLSAKEKLQIALALERMGVDVMEVGFPVSSPGDFESVQT
IARTIKNSRVCALARCVEKDIDVAAQALKVADAFRIHTFIATSPMHIATKLRSTLDEVIE
RAVYMVKRARNYTDDVEFSCEDAGRTPVDDLARVVEAAINAGARTINIPDTVGYTMPFEF
AGIISGLYERVPNIDKAIISVHTHDDLGIAVGNSLAAVHAGARQVEGAMNGIGERAGNCA
LEEVIMAIKVRKDIMNVHTNINHHEIWRTSQTVSQICNMPIPANKAIVGSGAFAHSSGIH
QDGVLKNRENYEIMTPESIGLNQIQLNLTSRSGRAAVKHRMEEMGYKDTDYNMDHLYDAF
LKLADKKGQVFDYDLEALAFINKQQEEPEHFRLDYFSVQSGSSDIATASVKLACGEEIKA
EAANGNGPVDAIYQAINRITGYDVELVKYDLNAKGQGKDALGQVDIVVNHHGRRFHGVGL
ATDIVESSAKAMVHVLNNIWRAAEVEKELQRKAQNKENNKETV

>EECEBICD_03352 3-isopropylmalate dehydrogenase
MSKNYHIAVLPGDGIGPEVMAQALKVMDAVRSRFDMRITTSHYDVGGIAIDNHGHPLPKT
TVEGCEQADAILFGSVGGPKWENLPPESQPERGALLPLRKHFKLFSNLRPAKLYQGLEAF
CPLRADIAANGFDILCVRELTGGIYFGQPKGREGSGQYEKAFDTEVYHRFEIERIARIAF
ESARKRRRKVTSIDKANVLQSSILWREIVNDVAKTYPDVELAHMYIDNATMQLIKDPSQF
DVLLCSNLFGDILSDECAMITGSMGMLPSASLNEQGFGLYEPAGGSAPDIAGKNIANPIA
QILSLALLLRYSLDANDAATAIEQAINRALEEGVRTGDLARGAAAVSTDEMGDIIARYVA
EGV
>EECEBICD_03353 3-isopropylmalate dehydratase large subunit 1
MAKTLYEKLFDAHVVFEAPNETPLLYIDRHLVHEVTSPQAFDGLRAHHRPVRQPGKTFAT
MDHNVSTQTKDINASGEMARIQMQELIKNCNEFGVELYDLNHPYQGIVHVMGPEQGVTLP
GMTIVCGDSHTATHGAFGALAFGIGTSEVEHVLATQTLKQGRAKTMKIEVTGNAAPGITA
KDIVLAIIGKTGSAGGTGHVVEFCGDAIRALSMEGRMTLCNMAIEMGAKAGLVAPDETTF
NYVKGRLHAPKGRDFDEAVEYWKTLKTDDGATFDTVVTLRAEEIAPQVTWGTNPGQVISV
TDIIPDPASFSDPVERASAEKALAYMGLQPGVPLTDVAIDKVFIGSCTNSRIEDLRAAAE
VAKGRKVAPGVQALVVPGSGPVKAQAEAEGLDKIFIEAGFEWRLPGCSMCLAMNNDRLNP
GERCASTSNRNFEGRQGRGGRTHLVSPAMAAAAAVTGHFADIRSIK
>EECEBICD_03354 3-isopropylmalate dehydratase small subunit 1
MAEKFTQHTGLVVPLDAANVDTDAILPKQFLQKVTRTGFGAHLFNDWRFLDEKGQQPNPE
FVLNFPEYQGASILLARENFGCGSSREHAPWALTDYGFKVVIAPSFADIFYGNSFNNQLL
PVTLSDAQVDELFALVKANPGIKFEVDLEAQVVKAGDKTYSFKIDDFRRHCMLNGLDSIG
LTLQHEDAIAEYEDKQPEFMR
>EECEBICD_03355 hypothetical protein
MKKVFLLAALFAMSNSSFAAMAESCQQYFNDIDALVEQAAATSEQAKQQFDAMKPQLEAS
KKQLETVAASDQDAACKQALASMVQIKQALGLK
>EECEBICD_03356 Putative inhibitor of glucose uptake transporter SgrT
MRQFWLRYFAATEKTSWLACLSAPQRLKMLAELMQWEATD
>EECEBICD_03357 HTH-type transcriptional regulator SgrR
MPSGRLQQQFIRLWQCCDGKTQDTTLNELADLLNCSRRHMRTLLNTMQARGWLTWEAEVG
RGKRSRLTFLYTGLALQQQRAEDLLEQDRIDQLVQLVGDKSAVRQMLISHLGRSFRQGRH
ILRVLYYRPMHNLLPGTALRRSETHIARQIFSSLTRVNEENGELEADIAHHWQQISPLLW
RFYLRPGIHFHHGRELEMEDVITSLTRINTLPLYSHITKIDSPTAWTLDIHLSQPDRWLP
WLLGQVPAMILPREWETLANFASHPIGTGPYAVRRNTPNQLKILAFDDYFGYRALIDEVN
VWVLPDISEEPACGLMLEGPIQGGEKAIESRLEEGCYYLLFDARTPRGAHLQVREWVSHV
LSPTNLLYHADEPLQQLWFPAYGLLPRWHHARPGPGEKPAGLETLTLTFYREHIEHRVIA
RIMSALLAEHQVHLHIQEIDYDQWHAGDIESDIWLNSANFTLPLDFSLFAHLCEVPLLQN
CIPRDWQDDAAQWRAGEMNLANWCQQLLANKAIVPLIHHWLIIQGQRSMRGLRMNTLGWF
DFKSAWFAPPDP
>EECEBICD_03358 Thiamine-binding periplasmic protein
MLKKYLPLLLLCAAPAFAKPVLTVYTYDSFAADWGPGPAVKKAFEADCNCELKLVALEDG
VSLLNRLRMEGKNSKADVVLGLDNNLLEAATQTKLFAKSGVANEAVKVPGGWKNDTFVPF
DYGYFAFVYDKSKLKNPPKSLKELVESDQKWRVIYQDPRTSTPGLGLLLWMRKVYGDNAP
QAWQKLAAKTVTVTKGWSEAYGLFLKGESDLVLSYTTSPAYHIIEEKKDNYAAANFSEGH
YLQVEVAARTVASKQPELAEKFLKFMVSPAFQNAIPTGNWMYPVADVALPAGFESLAKPA
TTLEFTPQQVAAQRQAWISEWQRAVSR
>EECEBICD_03359 hypothetical protein
MATRRQPLIPGWLIPGLCAAALMITVSLAAFLALWLNAPSGAWSTIWRDSYLWHVVRFSF
WQAFLSAVLSVVPAVFLARALYRRRFPGRLALLRLCAMTLILPVLVAVFGILSVYGRQGW
LASLWQMLGLQWTFSPYGLQGILLAHVFFNLPMASRLLLQSLESIPGEQRQLAAQLGMRG
WHFFRFVEWPWLRRQIPPVAALIFMLCFASFATVLSLGGGPQATTIELAIFQALSYDYDP
ARAAMLALIQMVCCLALVLLSQRLSKAIAPGMTLTQGWRDPDDRLHSRLTDALLIVLALL
LLLPPLVAVVVDGVNRSLPEVLAQPILWQAVWTSLRIALAAGVLCVVLTMMLLWSSRELR
QRQQVFAGQTLELSGMLILAMPGIVLATGFFLLLNSSVGLPESADGIVIFTNALMAIPYA
LKVLENPMRDITARYGMLCQSLGIEGWSRLKVVELRALKRPLAQALAFACVLSIGDFGVV

ALFGNDNFRTLPFYLYQQIGSYRSQDGAVTALILLLLCFTLFTLIEKLPGRHAKTD
>EECEBICD_03360 Thiamine import ATP-binding protein ThiQ
MLKLIDITWLYHHLPMRFTLAVERGEQVAILGPSGAGKSTLLNLIAGFLAPASGTLLIAG
DDHTLTPPSRRPVSMLFQENNLFSHLNVQQNIGLGLNPGLTLNASQREKRDAIAHQMGIE
SLMTRLPGELSGGQRQRVALARCLVREQPVLLLDEPFSALDPALRQEMLTLVSDICRERQ
LTLLMVSHSVEDAARIAPRSIVVADGRIAWQGKTDELLSGQASASALLGIKSHLL
>EECEBICD_03361 Inner membrane protein YabI
MQALLEHFITQSTLYSLIAVLLVAFLESLALVGLILPGTVLMAGLGALIGSGELNFWHAW
LVGIIGCLMGDWISFWLGWRFKKPLHRWSFMKKNKSLLDKTEHALHQHSMFTILVGRFVG
PTRPLVPMVAGMLDLPVAKFIGPNLIGCLLWPPFYFLPGILAGAAIDIPSDMQSGDFKWL
LLATALLLWVGGWLCWRLWRSGKAAVDRLTAYLPRSRLLYLAPLTLGSGVVALVVLIRHP
LMPVYIDILRKVVGY
>EECEBICD_03362 Arabinose operon regulatory protein
MAETQNDPLLPGYSFNAHLVAGLTPIEANGYLDFFIDRPLGMKGYILNLTIRGEGVINNN
GEQFVCRPGDILLFPPGEIHHYGRHPDASEWYHQWVYFRPRAYWQEWLTWPTIFAQTGFF
RPDEARQPHFSELFGQIISAGQGEGRYSELLAINLLEQLLLRRMAVINESLHPPMDSRVR
DACQYISDHLADSHFDIASVAQHVCLSPSRLSHLFRQQLGISVLSWREDQRISQAKLLLS
TTRMPIATVGRNVGFDDQLYFSRVFKKCTGASPSEFRAGCE
>EECEBICD_03363 Ribulokinase
MAIAIGLDFGSDSVRALAVDCATGDEIATSVEWYPRWQEGRYCDGPNNQFRHHPRDYMES
MEAALKAVLAQLSAAQRANVVGIGVDSTGSTPAPIDADGNVLALRPEFAENPNAMFVLWK
DHTAVEEADEITRLCHKPGKVDYSRYIGGIYSSEWFWAKILHVTRQDSAVAQAAVSWIEL
CDWVPALLSGTTRPQDIRRGRCSAGHKTLWHASWGGLPPASFFDELDPCINRHLHYPLFS
ETFTADLPVGTLCAEWAQRLDLPESVVISGGAFDCHMGAVGAGAQSNTLVKVIGTSTCDI
LIADKQSVGDRAVKGICGQVDGSVVPNFIGLEAGQSAFGDIYAWFSRVLSWPLEQLAAQH
PELKTQINASQKQLLPALTDAWAKNPSLDHLPVVLDWFNGRRTPNANQRLKGVITDLNLA
TDAPALFGGLVASTAFGARAIQECFTEQGIAVNNVMALGGIARKNQVIMQVCCDVLNRPL
QIVASDQCCALGAAIFAAVAAKVHADIPAAQQSMASAVERTLRPRPEQAQRFERLYRRYQ
QWALSAEQHYLPTAAPAPTTPANQAILTH
>EECEBICD_03364 L-arabinose isomerase
MTIFDNYEVWFVIGSQHLYGAETLRQVTQHAEHVVNALNTEAKLPCKLVLKPLGTSPDEI
TAICRDANYDDRCAGLVVWLHTFSPAKMWINGLSILNKPLLQFHTQFNAALPWDSIDMDF
MNLNQTAHGGREFGFIGARMRQQHSVVTGHWQDKEAHTRIGAWMRQAVSKQDTRQLKVCR
FGDNMREVAVTDGDKVAAQIKFGFSVNTWAVGDLVQVVNAISDGDINALIDEYESSYTLT
PATRIHGDKRQNVREAARIELGMKRFLEQGGFHAFTTTFEDLHGLKQLPGLAVQRLMQQG
YGFAGEGDWKTAALLRIMKVMSTGLQGGTSFMEDYTYHFEKGNDLVLGSHMLEVCPSIAV
EEKPILDVQHLGIGGKEDPARLIFNTQTGPAIVASLIDLGDRYRLLVNCIDTVKTPHSLP
KLPVANALWKAQPDLPTASEAWILAGGAHHTVFSHALDLNDMRQFAEIHDIEIAVIDNDT
RLPAFKDALRWNEVYYGFKR
>EECEBICD_03365 L-ribulose-5-phosphate 4-epimerase AraD
MLEDLKRQVLEANLALPKHNLVTLTWGNVSAVNRERGVLVIKPSGVDYSVMTADDMVVVS
LETGEVVEGHKKPSSDTPTHRLLYQAFPTIGGIVHTHSRHATIWAQAGRPIPATGTTHAD
YFYGTIPCTRKMTEAEINGEYEWETGNVIVETFEKQGIDAAQMPGVLVHSHGPFAWGKNA
EDAVHNAIVLEEVAYMGIFCRQLAPQLPDMQQSLLDKHYLRKHGAKAYYGQ
>EECEBICD_03366 hypothetical protein
MNVSMQARLVGSDRYLFVNTQRANPSIKTVSRFFEYKTWTEQIWRTEIIENGNAFFHWQG
HDRKNGHRDTIINYLLNGQRWQSTIEDYIFFHALEGKAWQGHYDNIIEYVSSDHYVYQSA
FAEYITDQIHQRAPHGTRF
>EECEBICD_03367 hypothetical protein
MKYRYIIRIAAGIILIMATVFIHNAIAIYQFSQQNQTRVADCAIVAGAGVSGKTPSPVFQ
ARLDHAIWLYQQHIVRALVLTGGYGAGATVSDAAIARQYVLEKGIPAKAIFIEERSTVTR
ENIRYAKQILRQQQWRTALLVSDPLHMLRLKTIAQDNGIDSRSSPTRSTRYKSWWAQTRF
LLRETFYYSGYRLLRYMPVQNNSITP
>EECEBICD_03368 DNA polymerase II

MAQAGFILTRHWRDTPQGTEVSFWLATDNGPVQATLAPQESVAFIPTSQTSRAASLLQAE
KDYRLTPLQLRDFHRQPVSGLYCRTHRQLMRMEKLLRENGVTVYEADVRPPERYLMERFI
TSPVWVDGEMRNGVIRNARLKPHPDYRPPLKWVSLDIETTRHGELYCIGLEGCGQRTVYM
LGPANGDDRQLDFELVYVASRPQLLEKLNAWFAEHDPDVIIGWNVVQFDLRMLQKHAERY
RIPLRLGRDNSELEWREHGFKNGVFFAQARGRVIIDGIDALKSAFWNFSSFSLEAVSQEL
LGEGKSIDNPWDRMDEIDRRFAQDKPALATYNLKDCELVTRIFHKTEIMPFLLERATING
LPVDRHGGSVAAFGHLYFPRMHRAGYVAPNLGEVPPLASPGGYVMDSQPGLYDSVLVLDY
KSLYPSIIRTFLIDPVGLVEGMAQPDPEHSTEGFLDAWFSREKHCLPEIVSQIWHWRDEA
KRQGNKPLSQALKIIMNAFYGVLGTTACRFFDPRLASSITMRGHAIMRQTKALIEAQGYD
VIYGDTDSTFVWLRRAHSEADAAEIGHRLVRHVNEWWAQTLQQQNLTSALELEFETHFCR
FLMPTIRGADTGSKKRYAGLIQEGDSQRMVFKGLETVRTDWTPLAQRFQQELYLRVFRNE
PYQDYVRETIDKLMAGELDAQLVYRKRLRRPLHEYQRNVPPHVRAARLADEQNLKQGRPT
QYQNRGAIKYVWTVNGPEPVDYQQSPLDYEHYLTRQLQPVAEGILPFVEDNFATLLTGQL
GLF
>EECEBICD_03369 RNA polymerase-associated protein RapA
MPFTLGQRWISDTESELGLGTVVAMDARTVTLLFPSTGENRLYARSDSPVTRVMFNPGDT
ITSHEGWQLHIDEVKEENGLLVYVGTRLDTEETNVTLREVLLDSKLVFSKPQDRLFAGQI
DRMDRFALRYRARKFQSEQYRMPYSGLRGQRTNLIPHQLNIAHDVGRRHAPRVLLADEVG
LGKTIEAGMILHQQLLSGAAERVLIIVPETLQHQWLVEMLRRFNLRFALFDDERYTEAQH
DAYNPFETEQLVICSLDFARRNKQRLEHLCDAEWDLLVVDEAHHLVWSTDAPSREYMAIE
QLAERVPGVLLLTATPEQLGMESHFARLRLLDPNRFHDFEQFVEEQKNYRPVADAVAMLL
AGNKLSNDELNRLGDLIGEQDIEPLLQAANSDRDDAQAARDELVSMLMDRHGTSRVLFRN
TRNGVKGFPKRELHTVKLPLPTQYQTAIKVSGIMGARKSAEDRARDMLYPEQIYQEFEGD
TGTWWNFDPRVEWLMGYLTSHRSQKVLVICAKATTALQLEQVLREREGIRAAVFHEGMSI
IERDRAAAWFAEEDTGAQVLLCSEIGSEGRNFQFASNLVMFDLPFNPDLLEQRIGRLDRI
GQAHDIQIHVPYLEKTAQSVLVRWYHEGLDAFEHTCPTGRAIYDSAYASLINYLAAPEET
DGFDDLIKSCREQHEALKAQLEQGRDRLLEIHSNGGEKAQQLAQSIEEQDDDTNLIAFAM
NLFDIVGINQDDRGDNLIVLTPSDHMLVPDFPGLPEDGCTITFERDVALSREDAQFITWE
HPLIRNGLDLILSGDTGSSTISLLKNKALPVGTLLVELVYVVEAQAPKQLQLNRFLPPTP
VRMLLDKNGNNLAAQVEFETFNRQLSAVNRHTGSKLVNAVQQDVHAILQLGETQIEKSAR
ALIDNARREADEKLSGELSRLEALRAVNPNIRDDELAAIDSNRQQVLESLNQAGWRLDAL
RLIVVTHQ
>EECEBICD_03370 Dual-specificity RNA pseudouridine synthase RluA
MGMENYNPPQEPWLVILYQDEHIMVVNKPSGLLSVPGRLEAHKDSIMTRIQRDYPQAESV
HRLDMATSGVIVVALTKAAERELKRQFREREPKKQYVARVWGHPSPAEGLVDLPLICDWP
NRPKQKVCYETGKPAQTEYNVVEFAADNTARVVLKPITGRSHQLRVHMLALGHPILGDRF
YASPEALSLAPRLQLHAEMLTITHPAYGTNMTFKVPADF
>EECEBICD_03371 Co-chaperone protein DjlA
MQYWGKIIGVAVALMMGGGFWGVVLGLLVGHMFDKARSRKMAWFANQRERQALFFATTFE
VMGHLTKSKGRVTEADIHIASQLMDRMNLHGDSRTAAQNAFRVGKADNYPLREKMRQFRS
VCFGRFDLIRMFLEIQIQAAFADGSLHPNEREVLYVIAEELGISRVQFDQFLRMMQGGAQ
FGGGYHQQSGGGWQQAQRGPTLEDACNVLGVKTTDDATTIKRAYRKLMSEHHPDKLVAKG
LPPEMMEMAKQKAQEIQKAYELIKEQKGFK
>EECEBICD_03372 LPS-assembly protein LptD
MKKRIPTLLATMIASALYSHQGLAADLASQCMLGVPSYDRPLVKGDTNDLPVTINADNAK
GNYPDDAVFTGNVDIMQGNSRLQADEVQLHQKQAEGQPEPVRTVDALGNVHYDDNQVILK
GPKGWANLNTKDTNVWEGDYQMVGRQGRGKADLMKQRGENRYTILENGSFTSCLPGSDTW
SVVGSEVIHDREEQVAEIWNARFKVGPVPIFYSPYLQLPVGDKRRSGFLIPNAKYTTKNY
FEFYLPYYWNIAPNMDATITPHYMHRRGNIMWENEFRYLTQAGAGLMELDYLPSDKVYED
DHPKEGDKHRWLFYWQHSGVMDQVWRFNVDYTKVSDSSYFNDFDSKYGSSTDGYATQKFS
VGYAVQNFDATVSTKQFQVFNDQNTSSYSAEPQLDVNYYHNDLGPFDTRIYGQAVHFVNT
KDNMPEATRVHLEPTINLPLSNRWGSLNTEAKLMATHYQQTNLDSYNSDPNNKNKLEDSV
NRVMPQFKVDGKLIFERDMAMLAPGYTQTLEPRVQYLYVPYRDQSGIYNYDSSLLQSDYN
GLFRDRTYGGLDRIASANQVTTGVTTRIYDDAAVERFNVSVGQIYYFTESRTGDDNIKWE

NDDKTGSLVWAGDTYWRISERWGLRSGVQYDTRLDSVATSSSSLEYRRDQDRLVQLNYRY
ASPEYIQATLPSYYSTAEQYKNGINQVGAVASWPIADRWSIVGAYYFDTNSSKPADQMLG
LQYNSCCYAIRVGYERKLNGWDNDKQHAIYDNAIGFNIELRGLSSNYGLGTQEMLRSNIL
PYQSSM
>EECEBICD_03373 Chaperone SurA
MKNWKTLLLGIAMIANTSFAAPQVVDKVAAVVNNGVVLESDVDGLMQSVKLNAGQAGQQL
PDDATLRHQILERLIMDQIILQMGQKMGVKITDEQLDQAIANIAKQNNMTMDQMRSRLAY
DGLNYSTYRNQIRKEMIISEVRNNEVRRRITVLPQEVDALAKQIGTQNDASTELNLSHIL
IALPENPTSEQVNDAQRQAESIVEEARNGADFGKLAITYSADQQALKGGQMGWGRIQELP
GIFAQALSTAKKGDIVGPIRSGVGFHILKVNDLRGQSQSISVTEVHARHILLKPSPIMND
QQARLKLEEIAADIKSGKTTFAAAAKEYSQDPGSANQGGDLGWATPDIFDPAFRDALTKL
HKGQISAPVHSSFGWHLIELLDTRKVDKTDAAQKDRAYRMLMNRKFSEEAATWMQEQRAS
AYVKILSN
>EECEBICD_03374 4-hydroxythreonine-4-phosphate dehydrogenase
MSSAQRVVITPGEPAGIGPDLVVQLAQRAWPIELVVCADGALLTERAAMLGLPLSLLPYS
PDVPAAPQPAGTLTLLPVSLRAPAIPGQLTVENGPYVVETLARACDGCLNGEFAALITGP
VHKGVINDAGIPFTGHTEFFEERSQAKKVVMMLATEELRVALATTHLPLRAIADAITPAL
LHEVIAILHHDLRTKFGIAEPRILVCGLNPHAGEGGHMGTEEIDTIIPVLDELRAQGMKL
NGPLPADTLFQPKYLDNADAVLAMYHDQGLPVLKYQGFGRGVNITLGLPFIRTSVDHGTA
LELAGRGKADVGSFITALNLAIKMIVNTQ
>EECEBICD_03375 Ribosomal RNA small subunit methyltransferase A
MNNRVHQGHLARKRFGQNFLNDRFVIDSIVSAINPQKGQAMVEIGPGLAALTEPVGERLD
KLTVIELDRDLAARLQTHPFLGPKLTIYQQDAMTMNFGELSAQLGQPLRVFGNLPYNIST
PLMFHLFSYTDAIADMHFMLQKEVVNRLVAGPNSKAYGRLSVMAQYYCQVIPVLEVPPSA
FTPPPKVDSAVVRLVPHATMPYPVKDIRVLSRITTEAFNQRRKTIRNSLGNLFSVETLTE
MGIDPAMRAENISVAQYCQMANYLSENAPLKES
>EECEBICD_03376 Protein ApaG
MINSPRVCIQVQSVYIEAQSSPDDERYVFAYTVTIRNLGRAPVQLLGRYWLITNGHGRET
EVQGEGVVGVQPRIAPGEEYQYTSGAVIETPLGTMQGHYEMIDENGDAFTIDIPVFRLAV
PTLIH
>EECEBICD_03377 Bis(5'-nucleosyl)-tetraphosphatase, symmetrical
MATYLIGDVHGCYDELIALLQQVEFTPDTDTLWLTGDLVARGPGSLDVLRYVKSLGNSVR
LVLGNHDLHLLAVFAGISRNKPKDRLTPLLEAPDADELLNWLRRQPLLQVDEEKKLVMAH
AGITPQWDLQTAKECARDVEAVLSSDSYPFFLDAMYGDMPNNWSPELSGLARLRFITNAF
TRMRYCFPNGQLDMYSKASPENAPAPLKPWFAIPGPVSEAYSIAFGHWASLEGKGTPEGI
YALDTGCCWGGELTCLRWEDKQYFVQPSNRQMDMGEGEAVNA
>EECEBICD_03378 IS1595 family transposase ISSsu9
MISLIAALAVDRVIGMENAMPWNLPADLAWFKRNTLNKPVVMGRHTWESIGRPLPGRKNI
VISSQPGTDDRVQWVKSVDEAIAACGDAPEIMVIGGGRVYEQFLPKAQKLYLTHIDAEVE
GDTHFPDYEPDDWESVFSEFHDADAQNSHSYCFEILERR
>EECEBICD_03379 Glutathione-regulated potassium-efflux system protein
KefC
MDSHTLLQALIYLGSAALIVPIAVRLGLGSVLGYLIAGCIIGPWGLRLVTDAESILHFAE
IGVVLMLFVIGLELDPQRLWKLRASVFGGGALQMVVCGGLIGLFCMFLGLRWQVAELIGM
TLALSSTAIAMQAMNERNLTVSQVGRSAFAVLLFQDIAAIPLVAMIPLLAASGASTTLGA
FALSALKVAGALALVVLLGRYVTRPALRFVARSGLREVFSAVALFLVFGFGLLLEEVGLS
MAMGAFLAGVLLASSEYRHALESDIEPFKGLLLGLFFIGVGMSIDFGTLVENPLRILLLL
AGFLAIKIVMLWLVARPLGVPAKQRRWFAVLLGQGSEFAFVVFGAAQMADVLEPEWAKAL
TLAVALSMAATPIFLVLLTRMEKTATGEAREADEIDEEQPRVIVAGFGRFGQIAGRLLLS
SGVKMVVLDHDPDHIETLRKFGMKVFYGDATRMDLLESAGAAKAEVLINAIDDPQTNLQL
SELVKSHFPHLQIIARARDVDHYIRLRQAGVAMPERETFEGALKSGRQALEALGLGRYEA
RERADLFRHFNTRMVEEMAKGENDPLSRAAAYKRTSAMLSEIITEDREHLSLIQRHGWQG
TAEGKHSGEAADEPEVKPSI

>EECEBICD_03380 Glutathione-regulated potassium-efflux system ancillary protein KefF
MILIIYAHPYPHHSHANKQMLEQAGTLENVEIRSLYHLYPDFNIDVAAEQEALSRASLIV
WQHPMQWYSVPPLLKLWMDKVLTHGWAYGHGGTALHGKHLLWAVTTGGGENHFTIGSHPG
FDVLSQPLQATALYCGLKWLPPFSMHCTFICDDDTLQAQARQYKQRLLAWQEVNHG
>EECEBICD_03381 hypothetical protein
MLSGAVKADEKCAVKAKPAWDKPFTGEIPEKLPEGYNILLVVTDQERFFPTFPFPVPGRE
RLMKTGVTFCNHQNTSNVCTPSRSVLYTGLHMPQTKMFDNLGLPWMPYDLDPALGTTGHM
MRELGYYTAYKGKWHLTEKLEKPLPDEKDEDIDVGDIPEPELHKIMEKYGFADYHGIGDI
IGHSKGGYFYDSTTTSQTINWLRCKGQPLNDQHKPWFLAVNLVNPHDVMFIDTDKEGEKV
QWRGELDQDDNTLAPTQPPENELYQASWPNYPLPANRHQSFNEQGRPPAHLEYQTARAAL
EGQFPDEDRRWRKLLDYYFNCIRDCDTHLDRILNELDALKLTDKTIVVFTADHGELGGSH
QMHGKGASVYKEQIHVPMIISHPAYPGNKKCQALTCHLDIAPTLVGLTGLPEEKQHQALG
NRKGVNFSGLLKNPESVAVNAVRNASLYCYGMILYTDAHYLHRVIALQRDKQKTVAQIKQ
EISHLHPDFSHRSGTRMINDGRYKFARYFSLREHNTPETWEDLIKYNDLELYDLKNDPDE
NHNLAADKQKYQDLILTMNEKLNKIIKDEIGVDDGSFMPDAAHEPWDLTIEQFNRMAKD
>EECEBICD_03382 hypothetical protein
MKKRIIAAALLATVASFSTLAAEQVSKQEISHFKLVKVGTINVSQSGGQISSPSDLREKL
SELADAKGGKYYHIIAAREHGPNFEAVAEVYNDATK
>EECEBICD_03383 hypothetical protein
MKLKPIPFYLLALFSAASGATEINACKDLIGTWKTTADNPPYTMTILPPVESCGEKCVKL
NVQYELDVTHRNALYCHEGHEGVKGQGPMVIAFEGAYGGHAIGTYNRQLQLLWAGVIPKN
KKGKWITKMENYWFRQVKAH
>EECEBICD_03384 putative lipoprotein YgdR
MQKRLMTALFTAATLFTVAGCSSNQAVETTDGKTIVTDGKPEVDNDTGMVSYKNAATGKT
EQINRDQLKNMSELDN
>EECEBICD_03385 Sialic acid transporter NanT
MQQPRNFDDLKFSSIHRRIMLWGSGGPFLDGYVLVIIGVALEQLTPLLHLDAEWIGALGA
ATLAGLFIGTSLFGYICDKVGRRKMFLLDIIAIGVISVATMFVSTPLELLVMRVLIGIVI
GADYPIATSMITEFSNTRQRAFSIGFIAAMWYVGATCANLVGYWLYDMEGGWRWMLGSAF
IPCLIILIGRFDLPESPRWLLRKGRVKECEQMMIKLFGEPVCFDDEPPQETRFLQLFNRR
HFPFVLFVAAIWTCQVIPMFAIYTFGPQIVGLLGWEQGRNAALGNVVISLFFYAGLYTGD
VLAKQYWPSPSAYWQLRHDDYRAGAAGAGVKSGHHTGRGGVCGIRLLFRRTGHTAMALS
>EECEBICD_03386 Ferredoxin-like protein FixX
MTSPVNVDVKLGVNKFNVDEDSPHIILKTDPDKQALEVLIKACPAGLYKKQDDGSVRFDY
AGCLECGTCRILGLDTALEKWEYPRGTFGVEFRYG
>EECEBICD_03387 Thiamine thiazole synthase
MSEDIFDAIIVGAGLAGSVAALVLAREGAQVLVIERGNSAGAKNVTGGRLYAHSLERIIP
GFADQAPIERMITHEKLAFMTDKGAMTMDYCNGEDAASSQVSYSVLRSKFDAWLMEQAEE
AGAQLITGIRVDNVVQRDGRVVGVEADGDILEAKVVILADGVNSLLAEKLGMAKRVEASH
VAVGVKELIELPKSVIEDRFQLQGNEGAACLFAGAPTDGLMGGGFLYTNETTLSLGLVCG
LHHLKDAKKSVPQMLEDFKQHPAVAPLIAGGKLVEYAAHVVPEAGMNMQPELVGDGVLIA
GDAAGMCMNLGFTIRGMDLAISAGEAAAKTVLSAMKSDDFSKQSLDEYRQHLDEGPMRDM
RMYQKLPAFLDNPRMFTAYPEMAVSIAHDLFTVDGSAPVPMRKKILRHAKKVGFINLMKD
GLKGVTVL
>EECEBICD_03388 Protein FixB
MNKFSSVWVFSDTPSRLPELMSGAQAVGEKVNAFVLNEADSATACHLGADHVWLLSGKPE
DRMIEDYAAAMAETIRQHSEGGAVLLPNTRRGKLLAAKLGYRLSAAVSNDASEVSLQDGK
AAVKHMVYGGLAIGAETIASPFAVITLSSGTFDAQDPDTSRSGEMHTVQWQAPATAVTRT
ATQARQSNSVDLDKARLVVSVGRGIGSKENISLAEALCQTIGAELACSRPVAENEKWMEH
ERYVGISNLMLKPELYLAVGISGQIQHMVGANGAQTIFAINKDKNAPIFQYADFGIVGDA
LKILPALTAALAR
>EECEBICD_03389 Protein FixA
MKIITCYKCVPDEQDIAINNADGTLDFSKADSKISQYDLNAIEAACQLKQQLGDAQVVAM

SVGGKALTNAKGRKDVLSRGPDELIVVIDDQFEQALPQHTATALAAAAQKSGFDLLICGD
GSSDLYAQQVGLLVGEALNIPAINGVSKILSLTDSTLTVERELEDEVETLSIPLPAVIAV
STDINTPQIPSMKAILGAAKKPVQVWSPADIGLNSVSAYSTQQVAAPKQRKRQRVVIEGD
GEEQIAAFVENLRKII
>EECEBICD_03390 L-carnitine/gamma-butyrobetaine antiporter
MKNEKKKSGIEPKVFFPPLIIVGILCWLTVRDLDAANVVINAVFSYVTNVWGWAFEWYMV
VMLFGWFWLVFGPYAKKEIR
>EECEBICD_03391 L-carnitine/gamma-butyrobetaine antiporter
MMFASCTSAAVLFWGSIEIYYYISTPPFGLEPNSTGAKEIGLAYSLFHWGPLPWATYSFL
SVAFAYFFFVRKMDVIRPSSTLVPLVGEKHAKGLFGTIVDNFYLVALIFAMGTSLGLATP
LVTECMQWLFGIPHTLQLDAIIITCWIILNAICVACGLQKGVRIASDVRSYLSFLMLGWV
FIVSGASFIMNYFTDSVGMLLMHLPRMLFYTDAIGKGGFPQGWTVFYWAWWVIYAIQMSI
FLARISRGRTVRELCFGMVMGLTASTWILWTVLGSNTLLLMDKNILNIPQLIEQHGVARA
IIETWAALPLSTATMWGFFILCFIATVTLINACSYTLAMSTCREVRDGEEPPLLVRIGWS
VLVGIIGIVLLALGGLKPIQTAIIAGGCPLFFVNIMVTLSFIKDAKVHWKDK
>EECEBICD_03392 Crotonobetainyl-CoA reductase
MDFNLNDEQELFVAGIRELMASENWEAYFAECDRDSVYPERFVKALADMGIDSLLIPEEH
GGLEAGFVTVAAVWMELGRLGAPTYVLYQLPGGFNTFLREGTQEQIDKIMAFRGTGKQMW
NSAITEPGAGSDVGSLKTTYTRKNGKVYLNGSKCFITSSAYTPYIVVMARDGASPDKPVY
TEWFVDMSKAGIKVNKLEKLGLRMDSCCEITFDDVELDEKDMFGREGNGFNRVKEEFDHE
RFLVALTNYGTAMCAFEDAARYANQRVQFGEAIGRFQLIQEKFAHMAIKLNSMKNMLLEA
AWKADNGTITSGDAAMCKYFCANAAFEVVDTAMQVLGGVGIAGNHRITRFWRDLRVDRVS
GGSDEMQILTLGRAVLKQYR
>EECEBICD_03393 L-carnitine CoA-transferase
MNHLPMPTFGPLAGVRVVFSGIEIAGPFAGQMFAEWGAEVIWIENVAWADTIRVQPNYPQ
LSRRNLHALSLNIFKDEGREAFLKLMETTDIFIEASKGPAFARRGITDEVLWEHNPKLVI
AHLSGFGQYGTEEYTNLPAYNTIAQAFSGYLIQNGDVDQPMPAFPYTADYFSGMTATTAA
LAALHKVRETGKGESIDIAMYEVMLRMGQYFMMDYFNGGEICPRMTKGKDPYYAGCGLYK
CADGYIVMELVGITQINECFKDIGLAHILGTPEVPEGTQLIHRVECPYGPLVEEKLDAWL
ATHTIAEVQARFAELNIACAKVLTIPELEGNPQYVARESITQWQTMDGRTCKGPNIMPKF
KNNPGKIWRGMPSHGMDTAAILKNIGYSEADIKELVGKGLAKVED
>EECEBICD_03394 Crotonobetaine/carnitine--CoA ligase
MDIVGGQNLRQMWDDLAGVYGDKTALIFESCEGIVRQFSYASLNEEINRTANLFYSLGIR
KGDRVALHLDNCPEFIFCWFGLAKIGAIMVPINARLLGEESAWILQNSQVSLLVTSVQFY
PMYREIRQDNSTPLNHICLIGEQLPADDGVSHFSQLQARQSATLCYTPALSTDDTAEILF
TSGTTSRPKGVVITHYNLRFAGYYSAWQIALRDDDVYMTVMPAFHIDCQCTAAMPAFSAG
STFVLLEKYSARAFWDQVRKYQATVTECIPMMIRTLMVQPAAPTDRQHHLREVMFYLNLS
EQEKDDFTERFGVRLLTSYGMTETIVGIIGDRPGDKRRWPSIGRVGFSYEAEIRDDQNRP
LPAGEIGEICIKGIPGKTIFKEYYMQPEATARALEPEGWLHTGDSGYQDEDGYFYFVDRR
CNMIKRGGENVSCVELENIISAHPKIQDIVVVGIKDAIRDEAIKAFIVLNEGETLSEAEF
FSFCENNMAKFKVPSFMEIRTDLPRNCSGKIIKKNLK
>EECEBICD_03395 Carnitinyl-CoA dehydratase
MSESLHLTRNGPILEITLDRPKANAIDAKTSFAMGEAFLNFRDDPELRVAIITGGGEKFF
SAGWDLKAAAEGEAPDADFGSGGFAGLTEIFDLDKPVIAAVNGYAFGGGFELALAADFIV
CAENASFALPEAKLGIVPDSGGVLRLPKLLPPAIVNEMVMTGRRMSAEEALRWGVVNRVV
SQSELMDSARELAQQLVNSAPLAIAALKEIYRATSEMPVEEGYRYIRSGVLKHYPSVLHS
EDALEGPQAFAEKRDPVWKGR
>EECEBICD_03396 Carnitine operon protein CaiE
MSYYAFEGLIPVVHPDAFVHPSAVLIGDVIVGAGVYIGPLASLRGDYGRLILEAGSNLQD
GCIMHGYCDTDTIVHENGHIGHGAILHGCVVGRDALVGMNSVIMDGAVIGEESIVAAMSF
VKAGFQGEARQLLVGSPARVLRQVTDQELHWKRLNTKEYQDLAIRCRTGLSETKPLTQVE
ENRPRLKGTTDVKPKSAQ
>EECEBICD_03397 hypothetical protein
MCEKYVERPLYLLIADWMMAENRWITAREISRQFDIEHCKAINTLSYILSEVGEIVCEVK

MIPNQIAGRGCQCQRLVKVVSIDSQLYRRLNHNLQERKVSVAKAPRLSAVPPTELNREQK
WQMMLSKSMRR
>EECEBICD_03398 Carbamoyl-phosphate synthase large chain
MPKRTDIKSILILGAGPIVIGQACEFDYSGAQACKALREEGYRVILVNSNPATIMTDPEM
ADATYIEPIHWEVVRKIIEKERPDAVLPTMGGQTALNCALELERQGVLEEFGVTMIGATA
DAIDKAEDRRRFDIAMKKIGLDTARSGIAHTMEEALAVAADVGFPCIIRPSFTMGGTGGG
IAYNREEFEEICERGLDLSPTNELLIDESLIGWKEYEMEVVRDKNDNCIIVCSIENFDAM
GIHTGDSITVAPAQTLTDKEYQIMRNASMAVLREIGVETGGSNVQFAVNPKNGRLIVIEM
NPRVSRSSALASKATGFPIAKVAAKLAVGYTLDELMNDITGGRTPASFEPSIDYVVTKIP
RFNFEKFAGANDRLTTQMKSVGEVMAIGRTQQESLQEALRGLEVGATGFDPKVSLDDPEA
LTKIRRELKDAGADRIWYIADAFRAGLSVDGVFNLTNIDRWFLVQIEELVRLEEKVAEVG
ITGLNADFLRQLKRKGFADARLAKLAGVREAEIRKLRDQYDLHPVYKRVDTCAAEFATDT
AYMYSTYEDECEANPSVDRDKIMVLGGGPNRIGQGIEFDYCCVHASLALREDGYETIMVN
CNPETVSTDYDTSDRLYFEPVTLEDVLEIVRIEKPKGVIVQYGGQTPLKLARALEAAGVP
VIGTSPDAIDRAEDRERFQHAVDRLKLKQPANATVTAIEQAVEKAKEIGYPLVVRPSYVL
GGRAMEIVYDEADLRRYFQTAVSVSNDAPVLLDRFLDDAVEVDVDAICDGEMVLIGGIME
HIEQAGVHSGDSACSLPAYTLSQEIQDVMRQQVQKLAFELQVRGLMNVQFAVKDNEVYLI
EVNPRAARTVPFVSKATGVPLAKVAARVMAGKSLTEQGVTQEIIPPYYSVKEVVLPFNKF
PGVDPLLGPEMRSTGEVMGVGRTFAEAFAKAQLGSNSTMKKQGRALLSVREGDKERVVDL
AAKLLKQGFELDATHGTAIVLGEAGINPRLVNKVHEGRPHIQDRIKNGEYTYIINTTAGR
RAIEDSRVIRRSALQYKVHYDTTLNGGFATTMALNADATEKVTSVQEMHAQIKKS
>EECEBICD_03399 Carbamoyl-phosphate synthase small chain
MIKSALLVLEDGTQFHGRAIGATGSAVGEVVFNTSMTGYQEILTDPSYSRQIVTLTYPHI
GNVGTNEADEESSQVHAQGLVIRDLPLIASNFRNTEDLSSYLKRHNIVAIADIDTRKLTR
LLREKGAQNGCIIAGDSPDAKLALEKAKAFPGLNGMDLAKEVTTAETYRWTQGSWTLKDG
LPEAKSEDDLPFHVVAYDFGAKRNILRMLVDRGCRLTVVPAQTSAEEVLKMNPDGIFLSN
GPGDPAPCDYAITAIQKFLETDIPLFGICLGHQLLALASGAKTVKMKFGHHGGNHPVKDM
DRNVVMITAQNHGFAVDEDSLPANLRVTHKSLFDGTLQGIHRTDKPALSFQGHPEASPGP
HDAAPLFDHFIELIEQYRQSAK
>EECEBICD_03400 4-hydroxy-tetrahydrodipicolinate reductase
MIVQSSLEAVKDDFDVFIDFTRPEGTLTHLAFCRQHGKGMVIGTTGFDDAGKQAIREASQ
EIAIVFAANFSVGVNVMLKLLEKAAKVMGDYSDIEIIEAHHRHKVDAPSGTALAMGEAIA
GALDKNLKDCAVYSREGYTGERVPGTIGFATVRAGDIVGEHTAMFADIGERVEITHKASS
RMTFANGALRSALWLKTKKNGLFDMRDVLGLDVL
>EECEBICD_03401 4-hydroxy-tetrahydrodipicolinate reductase
MHEAQIRVAIAGAGGRMGRQLIQAAMAMEGVQLGAALEREGSSLLGSDAGELAGGESPA
>EECEBICD_03402 2-(5''-triphosphoribosyl)-3'-dephosphocoenzyme-A synthase
MNVSVVTERRTPAYSSLAAGELNGLVARALLTEARLTPKPGLVDIRNSGAHRDMDLAAFE
RSTTAIAPWMEKFFIMGNNTAALAAENVLVMLRPLGMACENDMLQATNGVNTHRGAIFAF
GLLSAAIGRLLARGEPLEQNRICDQVARLSRNIVAHELSAKKAGKLTKSETHFQCYGLSG
ARGEAESGFRTVRTQALPVFNRVVQEHDDTHLALLQTLLHLMAWNDDTNLVSRGGLEGLY
YVQQQAQKLLWQGGVLVEGGIEAMQSLDDELILRNLSPGGSADLLAVTWFLSHFPAGSLY
PE
>EECEBICD_03403 Apo-citrate lyase phosphoribosyl-dephospho-CoA
transferase
MKGTDLLYQGQAVTLEEMLQARDKRAARQRQALNCYRLPLISLTLVAPGAVKNSAVWRRV
ADYAIAEILALCEQKEWVNVWEMQVNERSGPEWMAAVCAPAMALKQHMSTLEMSHPLGRL
WDIDIIDSDGKSLSRRELGHPARPCLICQQDAHLCARGKHHTLDLLLDEIARRIECYERE
RCD
>EECEBICD_03404 Citrate lyase alpha chain
MKETVTMLNQQYVVPEGLQPYQGVTANSPWLASETEKRRRKICDSLEEAIRRSGLKNGMT
ISFHHAFRGGDKVVNMVMAKLAEMGFRDLTLASSSLIDAHWPLIEHIKNGVVRQIYTSGL
RGKLGEEISAGLMENPVQIHSHGGRVKLIQSGELNIDVAFLGVPCCDEFGNANGFSGKSR
CGSLGYAQVDAQYAKCVVLLTEEWVEFPNYPASIAQDQVDLIVQVDEVGDPEKITAGAIR

LSSNPRELLIARQAANVIEHSGYFCDGFSLQTGTGGASLAVTRFLEDKMRRHNITASFGL
GGITGTMVDLHEKGLIKALLDTQSFDGDAARSLAQNPHHIEISTNQYANPASKGAACERL
NVVMLSALEIDVNFNVNVMTGSNGVLRGASGGHSDTAAGADLTIITAPLVRGRIPCVVEK
VLTTVTPGASVDVLVTDHGIAVNPARQDLLDNLRAAGVALMTIEQLQQRAEQLTGKPQPI
EFTDRVVAVVRYRDGSVIDVIRQVKG
>EECEBICD_03405 Citrate lyase subunit beta
MRPRRSMLFIPGANAAMLSTSFVYGADAVMFDLEDAVALLEKDTARLLVHHALQHPFYQD
VEKVVRINPLNTPFGLADLEAVVRGGADIVRLPKTDSKNDVLELEAQVERIERECGREVG
STRLMAAIESAKGVVNAVDIATSSPRMVAIALAAFDYVMDMGTSRGDGTELFYARCAVLH
AARVAGIAAYDVVWSDINNEEGFLKEVQLAKGLGFNGKSLVNPRQIELLHQAYSPTRKEV
EHAYEVIAAAEEAESRGLGVVSLNGKMIDGPIIDHARKVVALSASGIRD
>EECEBICD_03406 Citrate lyase acyl carrier protein
MKIIKDALAGTLESSDVMIRIGPSSEPGIRLELESLVKQQFGAAIEQVVRETLAKLGVER
ALVSVDDKGALECILRARVQAAALRAAEQTEIQWSAL
>EECEBICD_03407 [Citrate [pro-3S]-lyase] ligase
MFLEGVGLALEDDCEIAVCAYWQGEIVGCGSLAGNVLKCIAVSPVLQGEGLSLKLLTELL
TLAYELNRSELFLFTKPQNRLLFSGAGFWPIAQAGELAVLMENSSERLARFCRQLALYRQ
PGKTIGAIVMNANPFTLGHRYLVEQAAAACDWLHLFVVKEDASFFSYTDRWALIEQGIGG
IDNVTLHSGSAYMISRATFPGYFLKEKGVVDDCHCQIDLQLFREHLAPALGITHRFVGSE
PFCPLTCAYNQRMHDILHDPKRSGPVIEVVELARVEKNGAAISASRVRKLYSERNWPAIS
ALVPAGTLAYLQRHAARHTETI
>EECEBICD_03408 Citrate-sodium symporter
MTNMTQASATEKKGASDLLRFKIFGMPLPLYAFALITLLLSHFYNAIPTDLVGGFALMFV
MGAIFGEIGKRLPIFNKYIGGAPVMIFLVAAYFVYAGIFTQKEIDAISNVMDKSNFLNLF
IAVLITGAILSVNRKLLLKSLLGYIPTILAGIVGASLFGIVIGLCFGIPVDRIMMLYVLP
IMGGGNGAGAVPLSEIYHSVTGRSREEYYSTAIAILTIANIFAIIFAALLDMIGKKYTWL
SGEGELVRKASFKTEDDEKAGQITHRETAVGMVLSTTCFLLAYVVAKKILPSIGGVSIHY
FAWMVLIVAALNASGLCSPEIKAGAKRLSDFFSKQLLWVLMVGVGVCYTDLQEIIDALTF
ANVVIAAIIVVGAVVGAAIGGWLIGFYPIESSITAGLCMANRGGSGDLEVLSACNRMNLI
SYAQISSRLGGGIVLVIASIVFSMMV
>EECEBICD_03409 Oxaloacetate decarboxylase gamma chain
MNEAVLLGEGFTLMFLGMGFVLSFLFLLIFAIRGMSAVITRFFPEPVAAPAPRAVPVVDD
FTRLKPVIAAAIHHHRHHV
>EECEBICD_03410 Oxaloacetate decarboxylase alpha chain
MTIAITDVVLRDAHQSLFATRLRLDDMLPIAAALDDVGYGSLECWGGATFDACIRFLGED
PWLRLRELKKAMPKTPLQMLLRGQNLLGYRHYADDVVERFVERAVKNGMDVFRVFDAMND
PRNMKAALQAVRSHGAHAQGTLSYTTSPAHTLQTWLDLTEQLLETGVDSIAIKDMSGILT
PMAAFELVSEIKKRYDVRLHLHCHATTGMAEMALLKAIEAGVDGVDTAISSMSATYGHPA
TEALVATLAGTEHDTGLDILKLENIAAYFREVRKKYHAFEGQLKGYDSRILVAQVPGGML
TNLESQLKQQNAADKLDQVLAEIPRVREDLGFIPLVTPTSQIVGTQAVLNVLTGERYKTI
AKETAGILKGEYGHTPVPVNAALQARVLEGGAPVTCRPADLLKPELAELEADVRRQAQEK
GIQLAGNAIDDVLTVALFPQIGLKFLENRHNPAAFEPLPQAEAAQPVTKAEKPAASGIYT
VEVEGKAFVVKVSDGGDISQLTAAAPAASSAPATAPAGAGTPVTAPLAGNIWKVIATEGQ
TVAEGDVLLILEAMKMETEIRAAQAGTVRGIAVKSGDAVSVGDTLMTLA
>EECEBICD_03411 Oxaloacetate decarboxylase beta chain
MESLNALLQGMGLMHLGAGQAIMLLVSLLLLWLAIAKKFEPLLLLPIGFGGLLSNIPEAG
MALTALESLLAHHDAGQLAVIAAKLNCAPDVHAIKEALALALPSVQSQMENLAVDMGYTP
GVLALFYKVAIGSGVAPLVIFMGVGAMTDFGPLLANPRTLLLGAAAQFGIFATVLGALTL
NYFGLISFTLPQAAAIGIIGGADGPTAIYLSGKLAPELLGAIAVAAYSYMALVPLIQPPI
MKALTTETERKIRMVQLRTVSKREKILFPVVLLLLVALLLPDAAPLLGMFCFGNLMRESG
VVERLSDTVQNGLINIVTIFLGLSVGAKLVADKFLQPQTLGILLLGVIAFGIGTAAGVLM
AKLLNLCSKNKINPLIGSAGVSAVPMAARVSNKVGLESDPQNFLLMHAMGPNVAGVIGSA
IAAGVMLKYVLAM
>EECEBICD_03412 Sensor histidine kinase CitA

MQENTVIKYLNTNMFIREITRWFARHSFQNRIFLLLLFTSTIVMLAITWYLTDITKERLH
YQVGQRALIQAMQISAMPELVEAVEAHDLSRIKALIDPMRSFSDATYITVGNEKGQRLYH
VNPDEIGKYMEGGDSDDALYNAKSYVSVRKGSLGSSLRGKSPIQDSTGKVIGIVSVGYTL
EQLESWLNLQISSLLFPMALLLLVLLYCARRFSLHIKKQMLNMEPQQLSQLLIQQSVLFE
SVFEGLIAIDSHHRITAINQTARRLLNLSQPESELIGYAIHHVVLPEAFFYDAPQKNKKD
EIVTFNQMKVIASRMAVIIDNQPQGWVISFRNKDDINTLSLQLSQVRQYADNLRAVQHEH
RNLISTIAGLLFLKRYDNALALIQQQSESHQKVLDFISHNFRDHHLAGLLIGKYYRAKEL
GLELIFDPSSFVEHLPSSLSHNEWISIVGNLLDNAYNATLLNREGSRQIECLINSEGNEV
IIEVADQGCGIDENIRQHIFERGVTTHNNGDHGIGLWLVHSYVKQANGDIIVDNNNPFGT
IFTLYIPFTREE
>EECEBICD_03413 Transcriptional regulatory protein CitB
MDSITTLIVEDEPMLAEILVDTIKIFPQFSIVGIADKLESAKKQIRLYQPQLILLDNFLP
DGKGIDLIRHTISTNYTGRIIFITADNHMDTISDALRMGVFDYLIKPVHYQRLQHTLERF
TRYRSSLRSSEQANQTHVDALFNIQAKEQTAEPNSGLRGIDENTFNRVRQIFADPQAVHT
ADTLAQILGSSKTTARRYLEQGVKNNFLEAEISYGKVGRPERLYRGKV
>EECEBICD_03414 Non-specific ribonucleoside hydrolase RihC
MTASLHIILDTDPGIDDAAAIAAALFAPQLDLQLITTVAGNVSVEKTTRNALQLLHFWDA
DVPLAQGAATPLLRPLRDAAYVHGESGMEGYDFVDHQRQPLAKPAFIAIRDVLMNAPEPM
TLVAIGPLTNIALLLMHYPECACNIRRLVLMGGSAGRGNFTPNAEFNIAVDPEAAALVFR
SGLEIVMCGLDVTNQAMLSPDFLNKLPALNRTGKMLHSLFNHYRSGSMRTGVRMHDLCAI
AWLVRPELFTLQSCFVAVETQGEYTAGTTVVDIEGRLGQPANAQVALALDVDGFRQWVAE
VFAYAP
>EECEBICD_03415 hypothetical protein
MGRVNLFWLTGNVRTVIQAEKPGAEPESLEIQWQENKASPGRFCPLVPFVEGDKLSPRLV
TDDDVPDTCISRAEYEDIKQ
>EECEBICD_03416 hypothetical protein
MGKIKYGLPTLLAIGFWMDATSATVLELPAWERNYTGTIAGKPVNVNITRFGNSLNGYYC
YEPCNQHKAVIVLRGSLQDKEVHLEERVKALSGYWNAEISSSEIKGEWTSADKKRHFPVA
LIYYKPKNSPDIVLVTNTNDAGGYDPSKEIDCGNTPAISAIKLYRDGKLIQTLDTASVGT
CSPFMPQWGDVNFDGYPDLSIVTELLAGPDAPVQTWLYDPAKQRYVDAPASYQEITSPEI
DAEHKQIVSYWRGGCCSHGVNVYRWKGKTIELIDRGESYFQPVISKGKMYNCYMTPSYAD
GRIIYPLVRKNGHLTPPLSLDGTCQSFLANR
>EECEBICD_03417 4-hydroxy-3-methylbut-2-enyl diphosphate reductase
MYLVESPEDVWTLNVKNEGKLSFMTQTTLSVDDTSDVIDALRKRFPKIVGPRKDDICYAT
TNRQEAVRALAEQADVVLVVGSKNSSNSNRLAELAQRMGRTAFLIDDAADIQEAWVKEAA
CVGVTAGASAPDILVQNVIARLREFGGGEAVTLEGREENIVFEVPKELRVDVREVE
>EECEBICD_03418 4-hydroxy-3-methylbut-2-enyl diphosphate reductase
MQILLANPRGFCAGVDRAISIVENALAIYGAPIYVRHEVVHNRYVVDSLRQRGAIFIEQI
SEVPDGAILIFSAHGVSQAVRNEAKSRDLTVFDATCPLVTKVHMEVARASRRGEESILIG
HAGHPEVEGTMGQYSNPEGGCTWWSRQRTSGR
>EECEBICD_03419 FKBP-type 16 kDa peptidyl-prolyl cis-trans isomerase
MSKSVQSNSAILVHFTLKLDDGSTAESTRNNGKPALFRLGDTSLSEGLEQQLLGLKEGEK
KAFSLEPDAAFGVPSPDLIQYFSRREFMAAGEPEVGAIMLFTAMDGSEMPGVIREINGDS
ITVDFNHPLAGHTVHFDIEVLEIDPALEA
>EECEBICD_03420 Lipoprotein signal peptidase
MSKPLCSTGLRWLWLVVVVLIIDLGSKYLILQNFALGDTVGLFPSLNLHYARNYGAAFSF
LADSGGWQRWFFAGIAIGICVILLVMMYRSKATQKLNNIAYALIIGGALGNLFDRLWHGF
VVDMIDFYVGNWHFATFNLADSAICIGAALIVLEGFLPKPTAKEQA
>EECEBICD_03421 Isoleucine--tRNA ligase
MSDYKSTLNLPETGFPMRGDLAKREPGMLARWTDDDLYGIIRAAKKGKKTFILHDGPPYA
NGSIHIGHSVNKILKDIIVKSKGLSGFDSPYVPGWDCHGLPIELKVEQEFGKPGEKFTAA
EFRAKCREYAATQVDGQRKDFIRLGVLGDWSHPYLTMDFKTEANIIRALGRIIKNGHLHK
GAKPVHWCVDCRSALAEAEVEYYDKTSPSIDVAFRAVDQDAVKAKFGLPGVSGPVSLVIW
TTTPWTLPANRAISLAPDFDYALVQIDGQAVILAKDLVESVMQRIGAAEYTILGTVKGAE

LELLRFTHPFMGFDVPAILGDHVTLDAGTGAVHTAPGHGPDDYVIGQKYGLETANPVGPD
GAYLPGTYPTLDGVNVFKANDIVIELLKEKGALLHVEKMQHSYPCCWRHKTPIIFRATPQ
WFVSMDKEGLRQQSLKEIKGVQWIPDWGQARIESMVANRPDWCISRQRTWGVPMSLFVHK
ETQELLPIERTLAAMEEVAKRVEVDGIQAWWDLDPKEILGEDADQYEKVPDTLDVWFDSG
STSYSVVDARPEFAGHAADMYLEGSDQHRGWFMSSLMISVAMKGKAPYRQVLTHGFTVDG
QGRKMSKSIGNTVSPQDVMNKLGADILRLWVASTDYTGEMAVSDEILKRAADSYRRIRNT
ARFLLANLNGFNPATDMVKPEEMVVLDRWAVGCAKTAQQEILKAYEAYDFHEVVQRLMRF
CSVEMGSFYLDIIKDRQYTAKADSVARRSCQTALYHIAEALVRWMAPIMSFTADEIWGYL
PGEREKYVFTGEWYDGLFGLEENEEFNDAFWDDVRYIKDQVNKELENQKANGIKSNLEAK
VTLKYADDANGTIKKLKLLGEEVRFIFITSQFVISEQAGGIDDENIQYNAGNTTVQAVVT
RAEGDKCPRCWHYTTDVGKVAEHADICGRCVSNIAGNGEQRKFA
>EECEBICD_03422 Bifunctional riboflavin kinase/FMN adenylyltransferase
MKLIRGIHNLSQAPHGCVLTIGNFDGVHRGHRALLQGLQEEGRRRGLPVVVMIFEPQPLE
LFATDKAPARLTRLREKLHYLAQCGVDYVLCVKFDRRFAALTAQTFISELLVERLGVQFL
AVGDDFRFGASRAGDFLLLQKAGAEYGFAVSSTQTFCEGGVRISSTAVRQALAEDNLALA
ESLLGHPFTISGRVVHGDELGRTIGFPTANLPLRRQVSPVKGVYAVEVTGLGDKPLPGVA
NIGTRPTVAGVRQQLEVHLLDVVMDLYGRHIDVILRKKIRNEQRFASLDELKAQIARDEL
TARKFFGLAGQV
>EECEBICD_03423 hypothetical protein
MTCSFVFVDQTFSSLTVHDRLHFVKCFQCSSFVASFDSCVYFLDESTHHRATACVVLTSL
FRLNGALLS
>EECEBICD_03424 Isoprimeverose transporter
MSSVIEDTQPSGSASLSLLQRISYGSLDVAGNLLYCFGSTYILYFYTDVAGISLAVAGVI
LLLARIIDGIDAPIWGIIIDKTRSRYGKCRPWFLWLPLPFAVFSALSFWSPDISMTGKAI
YAAISYMIASILFTGLNTPLSAILPLMTLSPKERLVLNSWRMTGGQIGVLLMNATALPLV
AFLGNGDDHAGFIYTAITFAIISCALTLFAFKNIREMDTDKIQHEPKLPMKKSFAAMKGN
WPWILMVLANLIFWIALQQRNTTIVYYLTYNLDRKDLVPLINSLATIQILFIIAIPFFSK
YLAKTWIWVGGLLVATFGGVMMWLAADNITFLIAAWILGNIGSGIACSMPFAMLGFAVDF
GAWKTGIKATGILIAFGSTFCIKMGSGLGTAFAAWIMNSFGYVPNHAQSAAGLEGIIWAF
IWAPALLFALAAIPLLFFRKYEAMEEKIRHDLETVNS
>EECEBICD_03425 hypothetical protein
MPFMQQDPRRLVWQQNDRYLWIEPWGENSLRVRSGRHLPVMRNEDWALTEPVAESQCHID
YEHHQATLTNGKIIAIVNQKGQVTFYRHPHKPLLQEFWRLRGEIGEDESSHGQYVSALNL
EGREFRPIQGGKYSLKARFEATEGEKIYGMGQYQQANLDLKGCVLELAQRNSQASVPFML
SSLGYGFLWNNPAVGRVTFAQNVTEWEAQVSEQLDYWITAGDTPAEISRAYALATGTPPM
MPDYAMGFWQCKLRYRTQEELLEVAREYKRRNLPISVIVIDFFHWPNQGDWMFDARDWPD
PDAMIAELKSLGIELMVSVWPTVDNRTESYREMRENGWLVQTERGLPINMDFLGNTTYFD
ATHPGARDYVWGKAKRNYYDKGVKLFWLDEAEPEFSVYDYDNYRYHAGPVLEVGNIYPRM
YAKTFFDGMKADGEDQVINLLRCAWAGSQKYGALVWSGDIHSSFRSLRNQFAAGLNMGIA
GIPWWTTDIGGFHGGNIHDPKFHELLIRWFQWGVFSPVMRLHGNRDPQILPAQPYRDGIA
QCPTGAPNEVWSYGEEVCDVLTGCLALREKLKPYIKALMEETHKHNTPVMRPLFFEFPEQ
ETSWTITDQYCFGPDLLIAPVMHEGMRERDVWLPEGETWTDLATGESYSGGQTLHYATPL
NRIPVFIREGGQYRSLLNL
>EECEBICD_03426 Transcriptional activator protein NhaR
MSHINYNHLYYFWHVYKEGSVVGAAEALFLTPQTITGQIKALEERLQGKLFKRKGRGLEP
SELGELVFRYADKMFTLSQEMLDIVNYRKESNLLFDVGVADALSKRLVSSVLDAAVVVDE
PIHLRCFESTHEMLLEQLSQHKLDMIISDCPIDSTQQEGLFSVKIGECSVSFWCTNPLPE
KAFPACLEERRLLVPGRRSMLGRKLLNWFNSQGLQVEILGEFDDAALMKAFGATHDAIFV
APSLYSLDFYADESVIEIGRVENVMEEYHAIFAERMIQHPAVQRICNADYSALFKLQ
>EECEBICD_03427 Na(+)/H(+) antiporter NhaA
MKHLHRFFSSDASGGIILIIAAALAMLMANMGATSGWYHDFLETPVQLRVGALEINKNML
LWINDALMAVFFLLIGLEVKRELMQGSLASLRQAAFPVIAAIGGMIVPALLYLAFNYSDP
VTREGWAIPAATDIAFALGVLALLGSRVPLALKIFLMALAIIDDLGAIVIIALFYTSDLS
IVSLGVAAFAIAVLALLNLCGVRRTGVYILVGAVLWTAVLKSGVHATLAGVIVGFFIPLK

EKHGRSPAKRLEHVLHPWVAYLILPLFAFANAGVSLQGVTIDGLTSMLPLGIIAGLLIGK
PLGISLFCWLALRFKLAHLPQGTTYQQIMAVGILCGIGFTMSIFIASLAFGNVDPELINW
AKLGILIGSLLSAVVGYSWLRARLNAPA
>EECEBICD_03428 Arylsulfate sulfotransferase AssT
MNTLTTTSVVLPAPRPAINQGIDINNEMVLNHTAIYENCLAQVTQENTVENALMLLDPYG
TAPLSAYAGVWSLEPAEIIVTVQDAAKTAMPVEHLYTLTAGANLLPVLGLVADTENRIVF
SQADTPLAVYTLITQPLPPVDSAEVVLGFPIINVTQPATDADKMAPGFYFITHFDRYNYA
LDQNGLVRWYVTQDYPSYNFVRIDNGHFLTTSEAKNTYLDMYEFDMMGRLHTFYNLDNQF
HHSIWPWDSNTIVAPSEYTSGRPDDLKTNEDGVSVVDLTTGLETAYYDMAKVLDTTRVSR
PSGTAPGEDPTVKDWLHINQSYVNETNQLLIASGRHQSAVFGVDLQTQALRFILSTHEDW
DDAYQPYLLTPVDSEGVALYDFSKQEDIDAADRDFWTWGQHNVVEIANNTPGIVEFMVFD
NGNYRSRDDSKSLLPPDNYSRIVHFVVNMNEMTVMRPFEYGKELGARGYSSCVSAKAIQQ
NGNIVVHFADCTFDENGRAISCQPGESDIIDPQAGSEAMGLLILQEIAPTEKTVLFEATM
TSGYYKNAETNGEGYRYDITSFRVYKMDLYA
>EECEBICD_03429 hypothetical protein
MFSHINVDACKTPGCKNLGILGSPDYLPQGKNVLCRACGFLFPIISARSLNLFRQAANQP
WKGLVKSCPHCGGTSLKKYGFSTKGERRMYCRQCNKTFISYTAVRSDARQENLATLIGEG
ASLVEIRAALAIDSTGFSRELQKLSRRANQAERDFVFPAFDIAMSTRAFRVKFNGGDSSL
YVLVTAEEESGKVVAISTNYSAQPVEADYQYHSDYEERLPSGTLAHLVQRKEALTMRRNV
LFDVDYGPAVLYKNDPGMLVKPVLPAYRHFELVQALTDERSLNVQHYLDHECFILGGCMM
ANFSYLRQGRCHISFVRERGVTPPKRDLPPRLFLSGGIRNNVWRTFSTRDYAMAVCNLTG
NKKVSLLRHATLNSATAFIRYVHNHPFLPHLNRMSPGNVVAVLDYLKFEYNASRN
>EECEBICD_03430 Anaerobic sulfatase-maturating enzyme
MMFGKSCQVMVKPTGSVCNLDCKYCFYLEKEKLYPDRKNHYKMSEETLELFIRQQIAAQD
IDEVIFAWQGGEPTLMGIPFYRKAVEFQQRYCGGKTIVNTFQTNGILINDDWATFFREHD
FLVGVSIDGDAALHDEWRVTRSGKPTHEKVENAVRCLAQHDVEFNTLTVVNRTNMHHPVQ
VYRYLKSIGSRYMQFIPLVERCGENGLAQPQDKHIAMTPWSVDSLQFGQFLNAVFDIWIR
EDIGDIGIQLFEQTLAAWCGLPPQVCVFAPTCGSAFAMEMNGDVYNCDHFVYPQFKLGNI
HQKTLRQMNQGEQNRQFGSDKQRSMAQECHRCQWKFACYGGCPKHRFLPSASGATNHNYL
CAGYQAFFSHTATAMSAMRTLYEKGISPAEIKSIFV
>EECEBICD_03431 N-acetylgalactosamine-6-O-sulfatase
MKRTVVASMIGLALCAGCVLSTAQAATAKRPNLVIILADDLGYGDLATYGHRIVKTPNID
KLAQEGVKFTDYYAPAPLCSPSRAGLLTGRMPFRTGIRSWIPEGKDVALGRNELTIANLL
KQQGYDTAMMGKLHLNAGGDRTDQPQAKDMGFDYTLVNPAGFVTDATLDNAKERPRYGVV
HPTGWIRNGQHIGRADKMSGEFVSSEVVNWLDNKKDDNPFFLYVAFTEVHSPLASPKKYL
DMYSQYMTDYQKQHPDLFYGDWADKPWRGTGEYYANISYMDEQVGKVLDKIKAMGEEDNT
IVIFTSDNGPVTREARKVYELNLAGETDGLRGRKDNLWEGGIRVPAIIKYGKHIPQGMVT
DTPVYGLDWLPTLANMMDFKLPTDRTYDGQSLVPLLKDKTLKRQKPLIFGIDMPFQDDPT
DEWAIRDGDWKMIIDRQNKPKYLYNLKTDRFETLNQIGKQPQIEKQLYGKFLKYKKDIDN
DSLMKARGDKPTPVTWG
>EECEBICD_03432 hypothetical protein
MKNKKYINSLLAACVLFSCFNGQAAELKRVYGKLSFGYGDWNKGFVNVDRGEVWKAVADF
GAVFDRGEFASFYEMNVLNHPVEGRNHVTQFLGHYRVVEGSNFTAMMKLYMSMENKFGDE
LNMMYGVGYLGLTSPSGFIKPYFAVHNLSNDYTSKKYGQATGFNGYVLGWVAAYNFDMFN
EKFVISNWNEVEMDRNDAYAEQQGGTTGLNGAVTFTWKFMPRWTASVSYRYFNNKLGYDG
YGDRMNYLIGFEF
>EECEBICD_03433 Mannosylglucosyl-3-phosphoglycerate phosphatase
MNKKFSISLLSLCIGLSSAISFSADARDITIYYTNDLHAHVTPEIIPYVSKTRPVGGFAP
ISKIVKDAKAKEKDVFFFDAGDYFTGPFISTLTKGEAIIDILNTMPYDAVSVGNHEFDHG
HENLVKQLSKLQFPVLLDNVFYSGTDTPLIKEPYTIVEKDGFKIGVIGMHGVSAFYEAIA
AGVREGVDCRDPIPYVKKQLEELKGKVDLTVLLAHEGVPGMQSSAGEADVARALKTDVDM
AKSLEGYGLNVLITGHAHKGTPEPIKVGDTLVVSTDAYTIELGKLVLDWNPETKKVDSYN
GKLITMYADTYKPDPVTQAKIDEWDNKVKKITDEVVAHSPEVLTRSYGESAPTGNLITDA
LMATVPGADASFYNAGGIRTELPKGNITYGDVLSMYPFTNDVMSMEISGKDLKSIMSHAA

DLKNGMLHVSKTVQFKYDSTKPLGQRIVEFDIKGKPVEDNKLYTVALDSFIGKGGGGFTF
TKGKNIKYIGVQTAPALVNYMKQVNNIQPDHTMRVDDISK
>EECEBICD_03434 Arylsulfate sulfotransferase AssT
MNKKSSSMVNMPAPREPINQKIDTNNALVLNHNAIYEQRLAEITQSNTCDKAIVTVNPYG
TAPLSLYLGVWMDEAAALEINVVDSEATTEAVRYQYDVHPGANLIPVCGMVSAVNNQITL
RLASQIVGQYTVMTDALPPTDSANVSLGFPIISVSCPAQQASLMEEGLYFSTYFDRYNLA
FDHNGIVRWYVSQEIPSYNFVRMDNGHFLATSQGINHCLNMYEFDIMGRVYTVYLLDNEF
HHSILPIENNLAIAPSEYSNGRPDGYSTGKDGVSIINLSTGLEVAYYDMLYVMDYSRSPR
PSGSAPGQDVSMDDWLHINQSYINEPNNLLICSGRHQSAIFGVNVDSGELRFIMANHEDW
SDEFKQYLLTPVDDDGVPLYDLTSPGGIDAADKNFWTWGQHNIVEIPNDEPGILEFMVFD
NGNYRSREDAKSLLPLDNFSRVVQFKINLNTMTVTRPYEYGKTEVGNRGYSSFVSAKHLL
TNGHLVIHFGATTVDEFEHTITAQPGSSDLVDPDEGQQALGRLVLQEINKETKEVLFEAM
VTSGYFKNEETNGTNYRYDISAFRVYKMPLFA
>EECEBICD_03435 hypothetical protein
MTIYLINSTHTYNDKTNELKNIKTGKMIKIAAMRIKCLEYMLNHAQQEIIYKKQLTNELW
GERSQFISDANLTQILYLLRRDLKGFGLSQFFSTVPRTGIKVDANIIISNENKSCLPSSL
KKEAYKYMALFFALLTMVITVIHLIR
>EECEBICD_03436 HTH-type transcriptional regulator LeuO
MRPIKNAKKIDYNLIKVFDTVITEGNATRAARKLDVTPAAISQALLRLQNLYGEELFIRT
RKGLVPSSKGKSLHQVFRQAIESIESTLCDKTDAQESNELIVLGGDITENYYFPALLDTV
LMNRYIIKHYAIKKTGEYSPASMLTHGYADVIMGILEIKNEMIESYLIDNLSDFVCVCGE
KSPLVGLEKISLYNFYAARHAVYHSDMFSSFTADSIDLFKSSTPYAGRREIGYYSDSLFG
VIGVVEKSDMVAILPGKIATYFRDVRRYNIKILRMPDEIIFRMLPVYAYLATNSTHYKNA
KKLISTFQSTFLFSQEKQPDALVEGSTSLCDLSV
>EECEBICD_03437 hypothetical protein
MRQYTINNEFIYNESLREIISLHDKKVLKVTLMRARCLSYLFENAYKKLITREMISHAVW
GERSQFVSDANLTQLLYLLRRDLQQIGLFELFVTLPRQGIKIDERFIIDAADIPPQAIQY
HTHRCNKIISIGIPTLFLLIVLFFLAPFI
>EECEBICD_03438 hypothetical protein
MKSMRCVIPVILLSFIVHEGTAKPTAQIHFMGSVVEAGCWNDVGTLEIQCHNKEGVERYI
IVENIITPISSPHATVKRDYLDEDKQLTVLRIVYD
>EECEBICD_03439 hypothetical protein
MYYHALKLSRLAMLTLAGVAVSASAIAADSAPTSQIGPTAEAYIVSHPDKVGEVVATYLA
EHPEFLVAASETLHQRQQIAQQQAYVQLALQYRAELLSSSSPSVGPNEAKAAVVMFFDYQ
CSWCSKMAPVVENLIKANPDTRFIFKEFPIFSSRWPVSGLAARVGEQVWLTQGGAKYLDW
HNALYATGKVEGALTEHDVYTLAQHYLTPTQLAAVKEAQSSGAVHDALLTNQALAQHMDF
SGTPAFVVMPQTQDGDVKRVTVIPGSTTQDMLQMAIQKAKG
>EECEBICD_03440 putative fimbrial chaperone YcbF
MPPQPSFLPMNKFFLRCAIYWCLLPISWAQAGVVIGGTRFIYHAGAPALSVPVSNHSEAS
WLIDTHILPGGRWPGTKNEGNIMPFVVTPPLFMLSARQENSMRVVYTGAPLPADRESLFT
LSIAAIPSGKPEANRVQMAFRSALKLLYRPEGLAGNPQQAYRHLIWSLTPDGATVRNPTP
YYVTLFLLRANERAQDNAGVVAPFATRQTDWCRHTVRCTVRWQSINDYGRVMTAQTVDLT
RIH
>EECEBICD_03441 Protein FimF
MRNDILYGIGMLLAASGVQAHDGRVYVSGTITDNTCSLSPGSENINVAMGAVSQRQFYRA
GDGSAWQPFAIDLQNCGSTASGVTVSFSGAADSRNTDLLALTAGESDASGIGIALYDQNK
TLIPLGQESDVVTLSPGQASAHLQFYARYLADGGAVTPGDANASATFILAYE
>EECEBICD_03442 putative fimbrial-like protein YcbU
MNRSHCALFAMGLMLCPQINASADDLAYNLEFTGTIVAQTCDIDISSLSQSIDLGQFAVG
DFPSTGTTTKFKPFNINLKNCSRGIAGAKIWFTGEPDPDNPALLAITDTGMGSGNMLASG
VGVEILNDDQDTISINNADSVVYPLKAGRNTLSFYIRYKSTRPTVTSGNATAVMYFDMQY
E
>EECEBICD_03443 putative fimbrial-like protein SfmH
MKIPLLFALLAGSVVSQYAFADVCKNVNGVPSSINYDLTTTLTAEQNQVGKTVQLEKSQE

VNVQAVCPAGASTYSQTYRSYVSPYPVVETSGNWKYLKLDPDYLEGGMRIEDSSAGDIYP
PMNNVLMGYDENVKAGQPFYVRDSNLEFQLKIVKPFVGTVNISPKTMFNVYVMTAAGDPL
TDVVYSILYSGTVTVPQSCEINAGQTILVNFGALYSGNFNHAGQKPEGVRAKKFSVPVKC
SGLDSQVNLTMRLIATPDSHVPQAIASDNADVGVVVETDEGNALIPNDAQSVAPFITDSA
GRANITLQAYPVSTTGETPAEGAFTALASLRVDFD
>EECEBICD_03444 putative outer membrane usher protein ElfC
MRNQLFMTRYYSSVTKPVLTPLALAIALAPAPGWAENYFNPAFLSDDPSAVADLSTFSRN
AQAAGMYRVDVYLNNTFLATRDIAFQAVKTTGKSAPTDDSGLRACLTPEMLKNMGVNTGA
FPLLAKAAAGSCPDLASAIPAARTRFDFAQQRLDISIPQAAMVASARGYIPPKYWDEGIN
ALLLNYTFTGANSQDRSPGGSAENSYFLGLNSGLNLGAWRLRDYSTWNANSGDQNSDSDW
QHISTYLERDVVFLQGELTAGDSYTPSALFDSLPFRGLQLASDDNMLPDSMKGFAPTIHG
IARSNAQVTIRQNGYIINQRYVPPGAFTINDLYPTAASGDLTVEVKESDGSINRYNVPYS
AVPILQREGRLKYAATVAEYRSESSQKEKVKFSQATLIWGLPHGFTLYGGTQLSSHYHAL
AIGSGANLGDWGAVSLDVTQATSTLADNNTYQGQSLRFLYAKSLAQSGTNLQLMGYRYST
SGFYTLDDTTWKRMSGYDDDSRTDSDKSRPEWADYYNLYYTRRGKVQLDINQQLGGLGSL
FITGSQQSYWHTDEKDSLLQVGYSDTLAGIAWSVSYNNNKSAGDAERDQIFALNISVPLS
QWLQHDDEVTRHHNVYATFSTSTDKQHNVTQNAGLSGTLLDENNLSYNIQQGYQNHGIGE
SGAARLEYDGAKGNANIGYNVSDNGDYQQVNYGLSGGLVAHAHGVTLSQPLGNTNILIAA
PGAANVGVVDQPGIHTDARGYAVVPYATTYRQNRMALDVNAMADDVDIDDAVTRVVPTEG
ALVLARFKARVGARALVTLNHNGKPVPFGATVTVNDRHAEAIVDEAGEVYLSGLSAQGVL
HVRWGNLPDQQCVASYHLSSSRQILSRQHAECH
>EECEBICD_03445 putative fimbrial chaperone SfmC
MKKNVPIFLRLLLLLSAAGLSFAVQAGGIALGATRVIYPQGSKQTSLPIINSSASNVFLI
QSWVANADGSRSTDFIITPPLFVIQPKKENILRIMYVGPSLPTDRESVFYLNSKAIPSVD
KNKLTGNSLQIATQSVIKLFIRPKNLAEAPAHAPSTLRCRNERGQLTITNPSPYYVSMVE
LYSAGKKLPNTMVPPKGAITLPATPGQVSLRTVNDFGATTPARVCPAS
>EECEBICD_03446 Laminin-binding fimbrial subunit ElfA
MKKPVLALMVSAIAFGGMLSTAQADTTTVTGGTVNFVGQVVDAACSVSADSVDQTVTLGQ
VRASKLTEAGMVANQKEDFTIKLEDCDTQTSQNAAVIFNGQQDANQPGSLANTAGAGSAT
NVALQLYGPDGQALNIGESSSTVTLNDGENVIPLSVDYIATGTATAGNVTATATFSMVYS
>EECEBICD_03447 Inhibitor of hydrogen peroxide resistance
MLPFGQPFTLGIGDEFYLQANDEHYIVLLESGVVSFCHDDKRLHISSSFAPSVVGMVDSY
GATYNVPARPEHFLLAETVCTGRFVRLPDFIKIADECDLWHDVARCLAYRLMVMSARDRE
LVGVDSYLKVRALLTEIWAYPQAYRESIIVLNFIQRRTGISRSRTMKILSELKKGGYIHI
DNGRLTALGKLPVAY
>EECEBICD_03448 hypothetical protein
MTISIHASAFDVNSWYQKITLTFINESGNAVDMNHAAISFTASGHIDPWGNSGGTLKGNL
PLTLNESSYGTLETNNIIINNSDALLLQPGERGTLSFSLAATQVPVKMSAITLTLASSSS
EDAESETPSDQETPAIPAADEQLAEPDVPEKDNDLQEHGLTLNVSELNTASWYQRVTFTL
TNLYAQAVDLNQLQLNFTASAHPDPYSPFQGTMLGNQAVTLASDGGWPIEKNTITINHDG
ALMLAAGDTAELQCYLAATQTPVAISDLNATLAHDPARQGKVCVHFPAMTQTVALKPVIE
LLFPAGETRRFVGEWGEVLTIGDLSAGTYRLTVPVLANDEMQIAPVESSFTVTLQSGDAA
AQVQVSCLPIVRYASARLMIDAPALGNAKLTVEIADATQADERTVTLIANQPQLITRLLA
GHHYTVNLQPAMINNRFISAPIQLTGFIPAAAQIAEVAVAYQQSALDTASFVTVDATILG
LPDGVAPQRYLFSSGKYQYSLMLESGSDRQTLALRFAPGLYDVQTDDIFIDSVPWRCEQA
GPLRLLQKVNHVALEFLPGVTLQVKGWPDYLAHGGVTVNAPETVSLYRDIPFSALFKYDG
FDGGGDPVPAAEVDVNGDGFLDYATLPIHKTVALVRQIEKEAGRSVMPVMVIYTANASGG
SALADLQDAQKLRNHFGNFITQCLAAQSYKDETHPVPATFVLNPDFLGALQQGPYGYTVV
RQKNSVPVNAQLAAAIQALPAMAGFIVPSLPTFSDDLYGYIQAVNYLVRQFAPDVAFGWQ
TNVWATGTADWVLRDTADPVAEGQAIAGFIHELGVYSGEYAPDFIAFDKFERDCFSPDAL
AHYGWNATCWLNYLAMVKQVTKALLTPAMLWQIPGGHMPTVEEGVSKISAAHFASGGTFF
MGDARIGSDPDTLSLQLLNTALNSATYGVPTVGDFLRKDKGYDWGQMQALNLPDFNVFSI
LWGGGSTISITTIHSNGEDGGWLADKMVEYYAAPRYFR
>EECEBICD_03449 hypothetical protein

MATSKLIQGDTITETTHAANGFDPATSDDKISYTSARVAKPVYNKYKNSTTKPKVFGYYT
DWSQYDSRLQGNMSQPGRGYDLTKVSPTAYDKLIFGFVGITGFRKIDTEDRDVVAEAAAL
CGKVKYEPTFLDPWGDFQSYINLGFDVSGWDVDPKTVTQSNAKGLLGALRDMQAKAKAAG
HTLALSMSIGGWSMSNGFHETAASDSSRKIFAKGVVKLFKQFPMFSEVDIDWEYPNNEGA
GNPFGPEDGANYALLIAELRKQLDSAGLSNVKISIAASAVTTIFDYAKVKDLMAAGLYGI
NLMTYDFFGTPWAETLGHHTNRKALEEGGWAVETIVDHLLAEGFSADRINIGYAGYTRNA
RQVEIESLSPLKGSYNPGSGPTTGSFESGTSEWYDVIYSYLDLENQKGRNGFNVYTDQVA
DADYLYSPESKLFLSLDTPRTVKAKGEYAAKLGLGGVFTWTIDQDNGVLVNAVREGLGYE
IESEVIDMEPFYFEGINVEKDEEQSDSDDAQKVNHAPKAAIELLVVGGSTVQLSGAGSSD
EDNDELSFSWGVPSQITVADKTAEIIEVVVPEVSEKTAFQFTLFVRDCYNEPSSQQRFVL
TAVPALSQVQPEPEEEEDIIVPVPDEEEDTTPAEDDTPADDKTSPYAQWDASTVYGANWG
SFEIVSWKGHNYQVKWWSMGNQPDLNCGVGGAWTDLGAY
>EECEBICD_03450 hypothetical protein
MKTYIINSNCIYNEGKYELRTVSNSQVIKMTAMRAKCLSFIIENAHLEIIERQKITTALW
GSRSHYVNDANLTQILYLIRRDLKALGINDLFITIPRQGLKVNSDIAIIATDSETKGRKK
QIVRQTIAALTTVFSVTLGSLMYLHIH
>EECEBICD_03451 hypothetical protein
MNPIIDGIIALEGGYVFNPKDKGGATHWGITEATARAHGYAGDMRDLTHAEAYAILEEDY
WIKPGFDVISTLSWPVSFELCDAAVNIGAYHPSAWLQRWLNVFNHEGKRYPDIHVDGNIG
PRTLAALEHYLAWRGQEGEAVLVKALNCSQGTYYLNVAEKNHNNEQFIYGWIKNRVT
>EECEBICD_03452 hypothetical protein
MSIPNHVSTTEVVLLELEILLTIISIGAWGGFVSYLLRKDKTEYNSSHESIKYCLTQIVI
SCFTSFLLSAIAIEKECSFNIVLLAAGLGGVFASPILKILGRRIKKIIEGNNSD
>EECEBICD_03453 putative HTH-type transcriptional regulator YbdO
MGSKGANKSFDYNLIKILDAVILSGNAAMAAKKLGITPAAVSLALKRLQSYYPEELFSRG
KGGLIPTAKAVDIHQNFSQVMKLVDDTFLCNSKKDEAFQITLLGSDIVESYYLSQLYNSD
IFDRILINHFTVRNMSREHISELLFTAQGDLLISAEPLLESGIENQIIDSFKSFVCICSS
KHMLSTLSQLSLHHFYSSRHALYQPGMGASVIYHDSELFKDELYYTGRRIVGYRSDSLNG
LMSMIERTSLIALMPLKLALFYKNHRKYDIKFIQPPPELALKSVQVYYASWNKNSRNISTI
NEMVSMLQTLSSFRR
>EECEBICD_03454 hypothetical protein
MRLTEAWARTGRKKPGYTPGLKKNTTGKRLASQIVKEFFHAVKEAF
>EECEBICD_03455 Chaperone protein DnaJ
MAKRDYYEILGVSKTAEEREIKKAYKRLAMKYHPDRNQGDKEAEAKFKEIKEAYEVLTDA
QKRAAYDQYGHAAFEQGGMGGGFGGGFNGGADFSDIFGDVFGDIFGGGRGRQRAARGADL
RYNMDLTLEEAVRGVTKEIRIPTLEECDVCHGSGAKAGTQPQTCPTCHGSGQVQMRQGFF
AVQQTCPHCQGRGTLIKDPCHKCHGHGRVEKSKTLSVKIPAGVDTGDRIRLAGEGEAGEH
GAPAGDLYVQVQVKQHPIFEREGNNLYCEVPINFAMAALGGEIEVPTLDGRVMLKVPSET
QTGKLFRMRGKGVKSVRGGAQGDLLCRVVVETPVGLSEKQKQLLKDLQESFGGPTGEKNS
PRSKSFFDGVKKFFDDLTR
>EECEBICD_03456 Chaperone protein DnaK
MGKIIGIDLGTTNSCVAIMDGTQARVLENAEGDRTTPSIIAYTQDGETLVGQPAKRQAVT
NPQNTLFAIKRLIGRRFQDEEVQRDVSIMPYKIIGADNGDAWLDVKGQKMAPPQISAEVL
KKMKKTAEDYLGEPVTEAVITVPAYFNDAQRQATKDAGRIAGLEVKRIINEPTAAALAYG
LDKEVGNRTIAVYDLGGGTFDISIIEIDEVDGEKTFEVLATNGDTHLGGEDFDTRLINYL
VDEFKKDQGIDLRNDPLAMQRLKEAAEKAKIELSSAQQTDVNLPYITADATGPKHMNIKV
TRAKLESLVEDLVNRSIEPLKVALQDAGLSVSDINDVILVGGQTRMPMVQKKVAEFFGKE
PRKDVNPDEAVAIGAAVQGGVLTGDVKDVLLLDVTPLSLGIETMGGVMTPLITKNTTIPT
KHSQVFSTAEDNQSAVTIHVLQGERKRASDNKSLGQFNLDGINPAPRGMPQIEVTFDIDA
DGILHVSAKDKNSGKEQKITIKASSGLNEEEIQKMVRDAEANAESDRKFEELVQTRNQGD
HLLHSTRKQVEEAGDKLPVDDKTAIESALNALETALKGEDKAAIEAKMQELAQVSQKLME
IAQQQHAQQQAGSADASANNAKDDDVVDAEFEEVKDKK
>EECEBICD_03457 hypothetical protein
MRSVLTISAGLLFGLALSSVAHANDHKILGVIAMPRNETNDLALKIPVCRIVKRIQLTAD

HGDIELSGASVYFKTARSASQSLNVPSSIKEGQTTGWININSDNDNKRCVSKITFSGHTV
NSSDMARLKVIGDD
>EECEBICD_03458 hypothetical protein
MNVTYLHDEDLDFLQHCSEEQLADFARLLTHNEKGKARLSSVLSHNELFKAMEGHPEQHR
RNWQLIAGEFQHYGGDSIANKLRGHGKQYRAILLDVAKRLKLKADKSMSTFEIEQQLLEH
FLRHTWQKMDAAHKQEFLQAVDAKVSELEELLPLLMKDRSLAKGVSHLLSTQLTRILRTH
AAMSILGHGLLRGAGLGGPVGAALNGVKAMSGSAYRVTIPAVLQIACLRRMMAAVQA
>EECEBICD_03459 Succinate-acetate/proton symporter SatP
MGNTKLANPAPLGLMGFGMTTILLNLHNAGFFALDGIILAMGIFYGGIAQIFAGLLEYKK
GNTFGLTAFTSYGSFWLTLVAILLMPKMGLTDAPDAQLLGAYLGLWGVFTLFMFFGTLKA
ARALQFVFLSLTVLFALLAVGNITGNEAIIHIAGWVGLVCGASAIYLAMGEVLNEQFGRT
ILPIGEAH
>EECEBICD_03460 Molybdopterin adenylyltransferase
MDTLRIGLVSISDRASSGVYQDKGIPALEEWLASALTTPFEVQRRLIPDEQEIIEQTLCE
LVDEMSCHLVLTTGGTGPARRDVTPDATLAIADREMPGFGEQMRQISLRFVPTAILSRQV
GVIRKQALILNLPGQPKSIKETLEGVKADDGSVSVPGIFASVPYCIQLLDGPYVETAPEV
VAAFRPKSARRENMSS
>EECEBICD_03461 Transaldolase B
MTDKLTSLRQFTTVVADTGDIAAMKLYQPQDATTNPSLILNAAQIPEYRKLIDDAVAWAK
QQSSDRAQQVVDATDKLAVNIGLEILKLVPGRISTEVDARLSYDTEASIAKAKRIIKLYN
DAGISNDRILIKLASTWQGIRAAEQLEKEGINCNLTLLFSFAQARACAEAGVYLISPFVG
RILDWYKANTDKKDYAPAEDPGVVSVTEIYEYYKQHGYETVVMGASFRNVGEILELAGCD
RLTIAPALLKELAESEGAIERKLSFSGEVKARPERITEAEFLWQHHQDPMAVDKLADGIR
KFAVDQEKLEKMIGDLL
>EECEBICD_03462 Amino-acid carrier protein AlsT
MPEFFSFINEILWGSVMIYLLLGAGCWFTWRTGFIQFRYIRQFSRSLKGSLSPQPGGLTS
FQALCTSLAARIGSGNLAGVALAIAAGGPGAVFWMWVSAIIGMATSFAECSLAQLYKERD
PTGQFRGGPAWYMARGLGMRWMGVVFALFLLVAYGLIFNSVQANAVSRALHFAFNIPPLI
SGIALAFCALLIIIRGIKGVARLMQWLIPIIALLWVAGSVFICLWHIEQMPGVIASIVKS
AFGWQEAAAGAAGYTLTQAITSGFQRGMFSNEAGMGSTPNAAAAATSYPPHPVAQGIVQM
IGVFSDTIIICTASAMIILLAGNHASHSSTEGIQLLQHAMVSLTGEWGASFVALIVILFA
FSSIVANYIYAENNLFFLRLHNAKAIWLLRLATLGMVIAGTLISFPLIWQLADMIMACMA
ITNLTAILLLSPVVYTLAGDYLRQRKLGVRPQFDPRRFPDIEPQLAPDTWDAASRD
>EECEBICD_03463 Peroxide stress resistance protein YaaA
MLILISPAKTLDYQSPLATTRYTQPELLDHSQQLIQQARQLSAPQISRLMGISDKLADLN
ATRFHDWQPHFTPDNARQAILAFKGDVYTGLQAETFNDADFDFAQQHLRMLSGLYGVLRP
LDLMQPYRLEMGIRLENPRGKDLYQFWGDIITDKLNEALEAQGDRVVVNLASEEYFKSVK
PKKLNAELIKPVFLDEKNGKFKVVSFYAKKARGLMSRFIIENRLTKPEQLTAFDREGYFF
DEETSTQDELVFKRYEQ
>EECEBICD_03464 Threonine synthase
MLNQDFVSRSAKILSAFIGDEIPQQILEERVRAAFAFPAPVAQVESDVGCLELFHGPTLA
FKDFGGRFMAQMLTHISGDKPVTILTATSGDTGAAVAHAFYGLENVRVVILYPRGKISPL
QEKLFCTLGGNIETVAIDGDFDACQALVKQAFDDEELKTALGLNSANSINISRLLAQICY
YFEAVAQLPQGARNQLVISVPSGNFGDLTAGLLAKSLGLPVKRFIAATNINDTVPRFLQD
GKWAPKATQATLSNAMDVSQPNNWPRVEELFRRKIWRLTELGYAAVDDTTTQQTMRELKA
KGYISEPHAAVAYRALRDQLNPGEYGLFLGTAHPAKFKESVESILGETLALPEALAERAD
LPLLSHHLPADFAALRKLMMTRQ
>EECEBICD_03465 hypothetical protein
MKLYNLKDHNEQVSFAQAVTQGLGKQQGLFFRTNCRSLA
>EECEBICD_03466 Homoserine kinase
MVKVYAPASSANMSVGFDVLGAAVTPVDGTLLGDVVSVEAADHFRLHNLGRFADKLPPEP
RENIVYQCWERFCQALGKTIPVAMTLEKNMPIGSGLGSSACSVVAALVAMNEHCGKPLND
TRLLALMGELEGRISGSIHYDNVAPCFLGGMQLMIEENGIISQQVPGFDEWLWVLAYPGI
KVSTAEARAILPAQYRRQDCIAHGRHLAGFIHACYSRQPQLAAALMKDVIAEPYRARLLP

GFSQARQAVSEIGALASGISGSGPTLFALCDKPETAQRVADWLSKHYLQNQEGFVHICRL
DTAGARVVG
>EECEBICD_03467 Bifunctional aspartokinase/homoserine dehydrogenase 1
MRVLKFGGTSVANAERFLRVADILESNARQGQVATVLSAPAKITNHLVAMIEKTIGGQDA
LPNISDAERIFSDLLAGLASAQPGFPLARLKMVVEQEFAQIKHVLHGISLLGQCPDSINA
ALICRGEKMSIAIMAGLLEARGHRVTVIDPVEKLLAVGHYLESTVDIAESTRRIAASQIP
ADHMILMAGFTAGNEKGELVVLGRNGSDYSAAVLAACLRADCCEIWTDVDGVYTCDPRQV
PDARLLKSMSYQEAMELSYFGAKVLHPRTITPIAQFQIPCLIKNTGNPQAPGTLIGASSD
DDNLPVKGISNLNNMAMFSVSGPGMKGMIGMAARVFAAMSRAGISVVLITQSSSEYSISF
CVPQSDCARARRAMQDEFYLELKEGLLEPLAVTERLAIISVVGDGMRTLRGISAKFFAAL
ARANINIVAIAQGSSERSISVVVNNDDATTGVRVTHQMLFNTDQVIEVFVIGVGGVGGAL
LEQLKRQQTWLKNKHIDLRVCGVANSKALLTNVHGLNLDNWQAELAQANAPFNLGRLIRL
VKEYHLLNPVIVDCTSSQAVADQYADFLREGFHVVTPNKKANTSSMDYYHQLRFAAAQSR
RKFLYDTNVGAGLPVIENLQNLLNAGDELQKFSGILSGSLSFIFGKLEEGMSLSQATALA
REMGYTEPDPRDDLSGMDVARKLLILARETGRELELSDIVIEPVLPDEFDASGDVTAFMA
HLPQLDDAFAARVAKARDEGKVLRYVGNIEEDGVCRVKIAEVDGNDPLFKVKNGENALAF
YSHYYQPLPLVLRGYGAGNDVTAAGVFADLLRTLSWKLGV
>EECEBICD_03468 tRNA (cytidine(34)-2'-O)-methyltransferase
MRVTIVLVAPARAENIGAAARAMKTMGFTDLRIVDSQAHLEPATRWVAHGSGDIIDNIEV
FHTLADALHDVDFTVATTARSRAKFHYYASPAELVPLLQEKSRWMRHAALVFGREDSGLT
NDELALADVLTGVPMAADYPSLNLGQAVMVYCYQLAGLMQQTTESVDIADESQLQALRAR
LLRLLTTLEATDDHKLTDWLQQRIGLLGQRDTVMLHRLVHDIEKKLTK
>EECEBICD_03469 Aerobic respiration control protein ArcA
MQTPHILIVEDELVTRNTLKSIFEAEGYDVFEATDGAEMHQILSEYDINLVIMDINLPGK
NGLLLARELREQANVALMFLTGRDNEVDKILGLEIGADDYITKPFNPRELTIRARNLLSR
TMNLGTASEERRSVESYKFNGWELDINSRSLIGPDGEQYKLPRSEFRAMLHFCENPGKIQ
SRAELLKKMTGRELKPHDRTVDVTIRRIRKHFESTPDTPEIIATIHGEGYRFCGDLQD
>EECEBICD_03470 hypothetical protein
MKKSPLCCYLFCAAMSASSAALAATAPVSAGVIHFKGQIVEYGCNLAPHDRNIEVSCLRN
NIPHLQTVATPSGNAISLMNGIATVRHVSLKDHPEIQSLIVQYR
>EECEBICD_03471 Inhibitor of hydrogen peroxide resistance
MKQLNGGNVYRPTGKEKNLQAILNIHRHVIQNISPVTITAGKTLDIKIDNQSHILLITKG
AIKVCRRIDSIVMGCGVSPMILGLVDAYADLYQLEENSDVFFIAETTCEYYPVPVKDFVE
IADEKHLWRDISNILMYRIAFMTNRDRNLIGQDAYSQIKALLLELWSYPESSRFEINAQN
FIQQRTGLSRSRIMQVLSELKAGDYIKILHGRLLELRNLPVSF
>EECEBICD_03472 Protein FimF
MFNKKIIILAMLTAVASTPVFAQNTITFNGKIYDQACTVQVNGSTDTTIDLGNYSKERIA
EKGATTDYVPFTVSLVSCPEAGTGVPTQAMFRFHGATDDSNPTYFKNADEGSETGAAGVG
ISIQNASHSDVTDNTDDAAVNLPTDGSKADFIYYAAMANDGSATVTGGDVSTQVTYTVSY
Q
>EECEBICD_03473 putative fimbrial chaperone LpfB
MKHIKKSVLVVLLTSHVAHASIVVGGTRLVFDGNNDESSINVENKDSKANLVQSWLSVAD
PQVTNKQAFIITPPLFRLDAGQKNSIRVIRSGAPLPADRESMYWLNIKGIPSIDDNASAN
RVEISINTQIKLIYRPPALTKSTPDSQSQQLKWQTAGDVITVNNPTPYYMNFASVTLNSH
EVKSATFVPPKSSASFKLGSTAAPHGTVTWRLISDYGMSLEPHSGSF
>EECEBICD_03474 Outer membrane usher protein FimD
MIADPREFVPTSEWDEGINAFLLNYSFTGSQDHDIDENRTENSEYANLRPGINIGAWRFR
NYSTWNHDSDGQNSWDSAYTYVSRDIEFLKGQLIAGENNTPADVFDSISFKGVQISSDDD
MLPDSMKGFAPVIRGVAKSSAQVTVEQNGYTIYKTNVPAGPFAINDLYPTGGSGDLYVTI
KESDGSEQHFIVPYASVPVLQREGHLKYDLTVGRTRSSDTHSAQQNFAELTALYGLAGGI
TAYGGIESTLSNDVYHAALIGTGLNLGDLGALSLDVTNSWSKIKAGDVVSDTLTGQSWRI
RYSKDIQSTGTNFTVAGYRYSTKDYYALEDVLDTYSDNSHYDHVRNRTDLSLSQDIIYGS
ISLTLYNEDYWNDTHTTSLGIGYNNTWHNVSYGINYSYTLNADNSQDEDDDTEDSNDQQI
SINISIPLDAFMPSTYATYNMNSAKDGDTTHTVGLNGTALAQKNLSWSVQEGYSSQEKAT

SGNVSATYNGTYADINGGYSYDNHMRRLNYGVQGGVLLHRNGLTLSQPMDDTIILVKAPG
AAGVPVNNETGVDTDFRGYAVVPYASPYHRNEVSLDTTGIRKNIELIDTSKTLVPTRGAV
VRAEYKTNIGYKALMVLTRINNLPVPFGATVSSLTKPDNHSSFVGDAGQAWLTGLEKQGR
LLVKWGPTAADRCQVSYRIPSSPSASGVEILHEQCQ
>EECEBICD_03475 hypothetical protein
MNMNRFAALAALLTFTGLALWSPSISAAYNTADAVDINVTGLIYADPCTVTVPDTLSLGQ
YARADIAVAGGNTATIPFTIKMTNCPATTTKAAISFNGVPYPDPNYASAIYANSAANPAQ
DLGLQLFNLDGKSLVNLANNVTYTVDINSNTHEANLPVAARMYTPYGKVTAGDFTSVVTI
TIAWQ
>EECEBICD_03476 hypothetical protein
MRRLYLALILLFAYSGHSYASCKRSGNEGAITITPPSQLVVDSHAYTAGEVLWQSGWVST
SEVTMDGCSRDYKVGFLYEPGSAQSNTSATINANDGNNTPVFSTGISGVGVAIKTQTNAG
PYDNVMPIDNTYHNGDGNKTHHAMAPAYNVELVALGGPITSGTATFQSPLARVSFRDSAT
EDSGGDILTHLYLGNTQLIMKAMGCRVETPAITVDLGSVNLGSFANSQTAGTGEQDILLT
CEQGTAISASLSAQPASGNNPDNSVIQLSNASAPTSATGVGVQLGIQAPDAGFFTDSLPI
NQKIDLFTHTITTNADGSQTVNGGTMNMSTTLKISARYYKTAATVTAGQANATATLNLTY
N
>EECEBICD_03477 Inner membrane protein CreD
MILLLIPLMMVRHTIVERADYRSHVENAIRQSTSGPQKVVGPLVAIPVTELYTVLEEEKE
VQYKRSYLYFWLPESLLVEGNQNVEARKIGIYQGQVWHTDMAIKAEFDVARLHELNRPNI
TLGKPFIVVGVGDARGISAVKAPQVNGETLTVEPGTGLPESREGIHIPLPDSQWATRNLT
LAMSLNLSGTGSFSLVPVGRSSEMTLTSNWPHPNFVGDFLPGKREISGSGFQAQWQTSRF
ATNLGEQFADAQKVDWDNLPAFSVAVSTPADQYQLTDRATKYAILLITLTFMAFFVFETL
TGQRLHPMQYLLVGLSLVMFYLLLLALSEHIGFTPAWIAASLVGALMNSVYLQAVLKGWR
NSVLFTLALLALDGVMWGLLRSEDSSLLLGTGVLLLALGGVMFLTRHLDWYSLSCQQRKS
LPPVKDDELRLWK
>EECEBICD_03478 Sensor protein CreC
MRIGMRLLLGYFLIVAVAAVFVLSIFVQEIKPGVRRATEGTLIDTATLLAALARDDLLSG
NPTDGQLAKAFAQLQHRPFRANISGIVKVRNEYHVYMTDAQGKVLFDSENKAVGQDYSRW
NDVWLTLRGQYGARSTAKDSADPNSTVMYVAAPIISDGRIIGVLSVGKPNAAMAPVIKRS
ERRILWASAVLLGIALAIGAGMVWWINRSIARLTRYADSVTENRPLALPALGSSELRKLA
QALESMRIKLEGKNDIEQYVYALTHELKSPLAAIRGAAEILREGPPADVVTRFTENILAQ
NTRMQALVETLLRQARLENRQDIPLALVAVDELFTQLSEARSIQLAAKNITLTLRPSSLV
VVADAELLAQALGNVLDNAIDFTPENGVITLSAQPMGEKAILQVTDSGCGIPDFALPRIF
DRFYSLPRENGRKSSGLGLAFVSEAARLLNGEVTLCNRPEGGVLASLTLHRHFT
>EECEBICD_03479 Transcriptional regulatory protein CreB
MQQPQVWLVEDEQGIADTLIYTLQLEGFTVELFARGLPALEKARQQRPDAVILDVGLPDI
SGFELCRQLLERHPALPILFLTARSDEVDRLLGLEIGADDYVAKPFSPREVSARVRTLLR
RVKKFAAPSPVVRTGHFELNEPAAQIAWFGTPLSLTRYEFLLLKTLLLSPERVYSRQQLM
DIVWSDAQETFDRTVDTHIKTLRAKLRAINPDLSPINTHRGMGYSLRSV
>EECEBICD_03480 hypothetical protein
MNGNNMKYKSLVLFSILLLLGQSARAEQIGSVDTVFKMFGPDHKIVVEAFDDPDVKNVTC
YVSRAKTGGIKGGLGLAEDTSDAAISCQQVGPIELSDKIKNGKAQGEVVFKKRTSLIFKS
LQVVRFYDEKRNTLAYLAYSDKVVDGSPKNAISAVPVMPWRQ
>EECEBICD_03481 hypothetical protein
MLFLGNVTGNYSNPVIYNHFILECLIITQRNFYEW
>EECEBICD_03482 Right origin-binding protein
MDQAGIIRDLLIWLEGHLDQPLSLDNVAAKAGYSKWHLQRMFKDVTGHAIGAYIRARRLS
KSAVALRLTARPILDIALQYRFDSQQTFTRAFKKQFSQTPALYRRSSEWSAFGIRPPLRL
GEFTVPEHQFVTLEDTPLLGVTQSYSCSLEQISDFRHEMRVQFWHDFLGHSPTIPPVLYG
LNETRPSMEKDDEQEVFYTTALPQEQADGYVQSAHPVLLQGGEYVMFTYEGLGTGVQDFI
LTVYGTCMPMLNLTRRKGQDIERYYPSEDTKTGDRPINLRCEFLIPIRR
>EECEBICD_03484 Inosine/xanthosine triphosphatase
MHQVISATTNPAKIQAILQAFEEIFGEGSCHITPVAVESGVPEQPFGSEETRAGARNRVD

NARRLHPQADFWVAIEAGIDDDATFSWVVIDNGVQRGEARSATLPLPAVILDRVRQGEAL
GPVMSHYTGIDEIGRKEGAIGVFTAGKLTRSSVYYQAVILALSPFHNAVYR
>EECEBICD_03485 Trp operon repressor
MTQRSPYSSAIAEQRNQEWLRFVELLRQAYAEDLHLPLLQLMLTPDEREALGTRVRIIEE
LLRGEMSQRELKTELGAGIATITRGSNSLKSAPVELRHWLENVLLKNA
>EECEBICD_03486 Soluble lytic murein transglycosylase
MDRAKPFVWRLVAASVCLLTFCHLARADSLEEQRNRYAQIKQAWDNRQMDVVEQMMPGLK
DYPLYPYLEYRKITDDLMNQPAIAVTQFVRANPTLPPARTLQSRFVNELARREDWRGLLA
FSPEKPGTTEAQCNYYYAKWSTGQTEAAWQGAKDLWLTGKSQPNACDKLFSVWRASGKQD
PLAYLERIRLAMKAGNTGLVTVLAGQMPAEYQTIASAIITLANDPNNVLTFARTTGATDF
TRQMAEVAFASVARQDAENARLMIPSLVQAQKLNEEQTQALRDIVAWRLMGNDVTDAQAK
WRDDAIMRSQSTSLIERRVRMALGMGDRRGLNTWLARLPMEAKEKDEWRYWQADLLLERG
RDAEAKEILHALMQKRGFYPMVAAQRLGEEYTLKIDKAPANVNSALTQGPEMARVRELMY
WNLDNTARSEWANLVKSRSKSEQAQLARYAFNQHWWDLSVQATIAGKLWDHLEERFPLAY
NDLFTRYTRGKDISQSYAMAIARQESAWNPKVKSPVGASGLMQIMPGTATHTVKMFSIPD
YRGPGQLLEPETNINIGTSYLQYVYQQFGNNRIFASAAYNAGPGRVRTWLGNSAGRIDAV
AFVESIPFSETRGYVKNVLAYDAYYRHFMGQKEALMSDSEWQRRY
>EECEBICD_03487 Energy-dependent translational throttle protein EttA
MAQFVYTMHRVGKVVPPKRHILKNISLSFFPGAKIGVLGLNGAGKSTLLRIMAGLDKDIE
GEARPQPGIKIGYLPQEPQLNPEHTVRESIEEAVSEVVNALKRLDEVYALYADPDADFDK
LAAEQGRLEEIIQAHDGHNLNVQLERAADALRLPDWDAKVEKLSGGERRRVALCRLLLEK
PDMLLLDEPTNHLDAESVAWLERFLHDFEGTVVAITHDRYFLDNVAGWILELDRGEGIPW
EGNYSSWLEQKDQRLAQEASAEAARRKSIEKELEWVRQGAKGRQSKGKARLARFEELNSV
EYQKRNETNELFIPPGPRLGDKVIEVSNLRKSYGDRVLIDDLSFSVPKGAIVGIIGPNGA
GKSTLFRMMSGQEQPDSGTITLGETVKLASVDQFRDAMDNSKTVWEEVSGGLDIMKIGNT
EMPSRAYVGRFNFKGVDQGKRVGELSGGERGRLHLAKLLQVGGNVLLLDEPTNDLDIETL
RALENALLEFPGCAMVISHDRWFLDRIATHILDYQDEGKVEFFEGNFTEYEEYKKRTLGA
EALEPKRIKYKRIAK
>EECEBICD_03488 Trifunctional NAD biosynthesis/regulator protein NadR
MSSFDYLKTAIKQQGCTLQQVADASGMTKGYLSQLLNAKIKSPSAQKLEALHRFLGLEFP
RRQKNIGVVFGKFYPLHTGHIYLIQRACSQVDELHIIMGYDDTRDRGLFEDSAMSQQPTV
SDRLRWLLQTFKYQKNIRIHAFNEEGMEPYPHGWDVWSNGIKAFMAEKGIQPSWIYTSEE
ADAPQYLEHLGIETVLVDPERTFMNISGAQIRENPFRYWEYIPTEVKPFFVRTVAILGGE
SSGKSTLVNKLANIFNTTSAWEYGRDYVFSHLGGDEMALQYSDYDKIALGHAQYIDFAVK
YANKVAFIDTDFVTTQAFCKKYEGREHPFVQALIDEYRFDLVILLENNTPWVADGLRSLG
SSVDRKAFQSLLVEMLKENNIEFVHVKEADYDGRFLRCVELVKEMMGEQG
>EECEBICD_03489 DNA repair protein RadA
MAKAPKRAFVCNECGADYPRWQGQCSACHAWNTITEVRLAASPTVARNERLSGYAGSAGV
SKVQKLSDISLEELPRFSTGFKEFDRVLGGGVVPGSAILIGGNPGAGKSTLLLQTLCKLA
EQMKALYVTGEESLQQVAMRAHRLGLPTANLNMLSETSIEQICLIAEEEQPKLMVIDSIQ
VMHMADIQSSPGSVAQVRETAAYLTRFAKTRGVAIVMVGHVTKDGSLAGPKVLEHCIDCS
VLLDGDADSRFRTLRSHKNRFGAVNELGVFAMTEQGLREVSNPSAIFLSRGDEVTSGSSV
MVVWEGTRPLLVEIQALVDHSMMANPRRVAVGLEQNRLAILLAVLHRHGGLQMSDQDVFV
NVVGGVKVTETSADLALLLAMVSSLRDRPLPQDLVVFGEVGLAGEIRPVPSGQERISEAA
KHGFRRAIVPAANVPKKPPEGMQVFGVKKLADALSVFDDL
>EECEBICD_03490 Phosphoserine phosphatase
MPNITWCDLPEDVSLWPGLPLSLSGDEVMPLDYHAGRSGWLLYGRGLDKQRLTQYQTKLG
AAMVIVAAWCVEDYQVIRLAGSLTPRATRLAHEAQLDVAPLGKIPHLRTPGLLVMDMDST
AIQIECIDEIAKLAGTGEKVAEVTERAMRGELDFTASLRSRVATLKGADADILRQVRGNL
PLMPGLTQLVLKLEALGWKIAIASGGFTFFADYLRDQLRLTAAVANELEIMDGKFTGHVI
GDIVDAEYKANTLLRLAQEHDIPLAQTVAIGDGANDLPMIKAAGLGIAFHAKPKVNEKTE
ITIRHADLMGVFCILSGSMNQK
>EECEBICD_03491 putative inner membrane protein Smp
MARAKLKFRLHRAVIVLFCLTLLVALMQGASWFSQNHQRQRNPQLEELARTLARQVTLNI

APLMRSETPDENRINAVLRQLTQESRILDAGVYDEQGDLITRAGESVNVRDRLALDGKKA
GSYFNQQIVEPIQGKNGPLGYLRLTLDTHTLATEAKQVDNTTNILRLMLLLSLAIGVVLT
RTLLQGKRTRWQQSPFLLTASKPVPEEEEREEKE
>EECEBICD_03492 Lipoate-protein ligase A
MTTLRLLISDSYDPWFNLAVEECIFRQMPATQRVLFLWRNADTVVIGRAQNPWKECNTRR
MEEDNVRLARRSSGGGAVFHDLGNTCFTFMAGKPEYDKTISTHIVLAALNSLGVMADASG
RNDLVVKTPDGDRKVSGSAYRETKDRGFHHGTLLLNADLSRLANYLNPDKKKLAAKGITS
VRSRVANLTELLPGITHEQVCQAVTEAFFAHYGERVDAEVISPDKTPDLPNFAETFARQS
SWEWNFGQAPAFSHLLDERFTWGGVELHFDVEKGVITRAQVFTDSLNPAPLEALGERLQG
CQYRVDVLEQACESLIAEFPAQKGELRELAAWMAQAVR
>EECEBICD_03493 Toxin YjjJ
MSALTDLLLQGPSSAAELRSSLAVSQATFSRLVSAHQDVIQFGKARATRYALVRPVRGVA
AFPLWQVNAQGQAAKFGVLYPCWPQGSCLVALDTGEWQWFDSLPWYLTDLRPQGFLGRAW
GRTVAGYAHLPDDIRLWQEDEVLYALRAYQGENAGGWIVGEENYQRWVTAPDATAITQSE
KLNRYQQLAEDALAGEVIGSSAGGEQPKFACFAETRWGNQNVLVKFSARARNTVSERWSD
LLIAESIALAVLSAASINASLATAICSEGGQVFLESIRFDCTGIRGRRQIVSLEALQGEY
ASSPGIWPVVTHRLAGQGIIDAVAHQQIEKIWAFGRLIANSDMHAGNLSFYYSDAPMALA
PVYDMLPMAYAPAASGMLRYAPVDIRVEATIDKKAWEFALPLAERFWVLVADDKRISAGF
QEIARAMVGKLKLAAQTIGRLA
>EECEBICD_03494 Purine nucleoside phosphorylase DeoD-type
MATPHINAEMGDFADVVLMPGDPLRAKHIAETFLEDVREVNNVRGMLGFTGTYKGRKISV
MGHGMGIPSCSIYTKELITDFGVKKIIRVGSCGAVRMDVKLRDVVIGMGACTDSKVNRIR
FKDHDFAAIADFDMVRNAVDAAKALGVDARVGNLFSADLFYSPDGEMFDVMEKYGVLGVE
MEAAGIYGVAAEFGAKALTICTVSDHIRTHEQTTAAERQTTFNDMIKIALESVLLGDQE
>EECEBICD_03495 Phosphopentomutase
MKRAFIMVLDSFGIGATEDADRFGDVGSDTLGHIAEACAKGEADNGRKGPLNLPNLTRLG
LVKAHEGSTGKIAAGMDGNADVIGAYAWAHELSSGKDTPSGHWEIAGVPVLFDWGYFSDH
ENSFPQELLDKLVKRANLPGYLGNCHSSGTVILDQLGEEHMKTGKPIFYTSADSVFQIAC
HEETFGLDKLYELCEIAREELTEGGYNIGRVIARPFIGDKAGNFQRTGNRHDLAVEPPAP
TVLQKLVDEKQGHVVSVGKIADIYANCGITKKVKATGLDALFDATLKEMKEAGDKTIVFT
NFVDFDSSWGHRRDIAGYAAGLELFDRRLPELMELVGEDDILILTADHGCDPSWTGTDHT
REHIPVLIYGPKVKPGSLGHRETFADIGQTLATYFGTSPMDYGKNML
>EECEBICD_03496 Thymidine phosphorylase
MFLAQEIIRKKRDGHALSDEEIRFFINGIRDNTISEGQIAALAMTIFFHDMTMPERVSLT
MAMRDSGTVLDWKSLNLNGPIVDKHSTGGVGDVTSLMLGPMVAACGGYVPMISGRGLGHT
GGTLDKLEAIPGFDIFPDDNRFREIIQDVGVAIIGQTSSLAPADKRFYATRDITATVDSI
PLITGSILAKKLAEGLDALVMDVKVGSGAFMPTYELSEALAEAIVGVANGAGVRTTALLT
DMNQVLASSAGNAVEVREAVQFLTGEYRNPRLFDVTMALCVEMLISGQLAKDDAEARAKL
QAVLDNGKAAEVFGRMVAAQKGPSDFVENYDKYLPTAMLSKAVYADTEGFISAMDTRALG
MAVVSMGGGRRQASDTIDYSVGFTDMARLGDSIDGQRPLAVIHAKDEASWQEAAKAVKAA
IILDDKAPASTPSVYRRITE
>EECEBICD_03497 Deoxyribose-phosphate aldolase
MTDLKASSLRALKLMDLTTLNDDDTNEKVIALCHQAKTPVGNTAAICIYPRFIPIARKTL
KEQGTPDIRIATVTNFPHGNDDIDIALAETRAAIAYGADEVDVVFPYRALIAGNEQVGFD
LVKACKDACAAANVLLKVIIETGELKEEALIRKASEISIKAGADFIKTSTGKVPVNATPE
SARIMMEVIRDMGVSKTVGFKPAGGVRTAEDAQKFLAIADELFGADWADSRHYRFGASSL
LASLLKALGHGDGKSASSY
>EECEBICD_03498 putative protein YjjI
MFEGHAPFKPRYVLPDYARFLANGSQWLELEGAKDLDDALSLLTILYHHVPSVTSMPVYL
GQLDALLQPYVRILTQDAIDIRIKRFWRYLDRTLPDAFMHANIGPADTPVTRAILRADAE
LKQVTPNLTFIYDAEITPDDLLLEVAKNICECSKPHISNGPVNDKIFTKGHYGIVSCYNS
LPLGGGGSTLVRLNLKAVAERSTSVDDFFSRTLPHYCRQQIAIINSRCEFLYEKSHFFEN
SFLVQEGLIDPERFAPMFGMYGLAEAVNLLCENAGLNARYGKNETANELGYRISAQLADF
VENTPVKYGWKQRALLHAQSGISSDIGTTPGARLPYGDEPDPITHLQTVAPHHAFYHAGI

SDILTLDETIKRNPQALVQLCLGAFKAGMREFTANVSGNDLVRVTGYMVRLSDLAKFRAE
GSRTNTTWLGEEAARNTRILERQPRVVSHEQQMRFSQ
>EECEBICD_03499 Putative glycyl-radical enzyme activating enzyme YjjW
MNNRCALVSKIIAFSCVDGPGSRLALFLQGCNLRCKNCHNPWTMGRCNDCGECVSQCPHH
ALSVDGGKVVWRSDVCEQCDTCLKMCPQQATPMAQTMSVDDVLRHIRKASLFIEGITVSG
GEATTQLPFIVALFTAIKADPQLQRLTCLVDSNGQLSETGWQKLLPVCDGVMLDLKAWKS
ECHHRLTGRDNTHIKHSIRFLAARGKLAELRLLVIPNQVDYAAHIDSLAAFITSLGEVPV
RLNAFHAHGVYGEAKAWPGATPQEVERLADGLRARGVAKLILPALYL
>EECEBICD_03500 putative metal-dependent hydrolase YjjV
MSWRFIDTHCHFDFPPFTGDERASIQRACEAGVEKIIVPATEAAHFPRVLALAARFPSLY
AALGLYPIVIERHADDDPDKLQQALAQQQNVVAVGEIGLDLYRDDPQFARQERFLDVQLQ
LAKRYDLPVILHSRRTHDKLAMHLKRQDLPRTGVVHGFAGSLQQAERFVRLGYKIGVGGT
ITYPRASKTRDVMARLPLDALLLETDAPDMPLKGFQGQPNRPEQAARVFDALCELRPEPA
EVIADTLYRNTITLFRL
>EECEBICD_03501 hypothetical protein
MKNRKAEVGQRIPVTLGNIAPLSLTPFRPGRMALVCEGGGQRGIFTAGVLDEFMRAQFNP
FHLYFGTSAGAQNLSAYLCNQPGYGRKVIMRYTTRREFFDPLRFVRGGNLIDLDWLVEST
AARMPLQMDTAARLFDSGKSFYMCACRGDDYTPGYFSPTKQNWLDIIRASSAIPGFYRTG
VTLEGINYLDGGISDAIPVQEAAKRGAKTIVVIRTVPSQMYYTPQWFKRMERWLGESSLQ
PLVNLVQHHETSYSAIQQFIEKPPGKLRIIEIYPPKPLHSMALGSRIPALREDYKTGQLC
GRYFLATVGKLLAATPPLLRHSSRIAVPETVVVPPAPVANDTHVAEVISAPQANDTTFTD
EDLA
>EECEBICD_03502 hypothetical protein
MFRWGIIFLVIALIAAALGFGGLAGTAAGAAKIVFVVGIVLFLVSLFMGRKRP
>EECEBICD_03503 Osmotically-inducible protein Y
MTMTRLKISKTLLAVMLTSAVATGSAFAENATTDKAQSGTETAGQKVDSSMNKVGNFMDD
SAITAKVKAALVDHDNIKSTDISVETNQKVVTLSGFVESQAQAEAAVKVAKGVEGVTSVS
DKLHVRDNKEGSVKGYAGDTATTSEVKAKLLADDLVPSRKVKVETTDGVVQLSGTVETQE
QSDRAESIAKAVDGVKSVKNDLKVQ
>EECEBICD_03504 Peptide chain release factor RF3
MTLSPYLQEVAKRRTFAIISHPDAGKTTITEKVLLFGQAIQTAGTVKGRGSSQHAKSDWM
EMEKQRGISITTSVMQFPYHDCLVNLLDTPGHEDFSEDTYRTLTAVDCCLMVIDAAKGVE
DRTRKLMEVTRLRDTPILTFMNKLDRDIRDPMELLDEVENELKIGCAPITWPIGCGKLFK
GVYHLYKDETYLYQTGKGHTIQEVRIVKGLNNPDLDAAVGEDLAQQLRDELELVQGASNE
FDEELFLAGEITPVFFGTALGNFGVDHMLDGLVAWAPAPMPRQTDTRTVEASEEKFTGFV
FKIQANMDPKHRDRVAFMRVVSGKYEKGMKLRQVRTGKDVVISDALTFMAGDRSHVEEAY
PGDILGLHNHGTIQIGDTFTQGEMMKFTGIPNFAPELFRRIRLKDPLKQKQLLKGLVQLS
EEGAVQVFRPISNNDLIVGAVGVLQFDVVVARLKSEYNVEAIYESVNVATARWVESADAK
KFEEFKRKNETQLALDGGDNLTYIAPTMVNLNLTQERYPDVQFRKTREH
>EECEBICD_03505 Pyrimidine 5'-nucleotidase YjjG
MMKWDWIFFDADETLFTFDSFTGLQRMFLDYSVTFTAEDFQDYQAVNKPLWVDYQNGAIT
SLQLQHARFQSWAERLNVAPGLLNDAFISAMAEICSPLPGAVSLLNAIRGQAKIGIITNG
FTALQQTRLERTGLREYFDLLVISEQVGVAKPDPKIFNYALEQAGNPDRSRVLMVGDTAE
SDILGGINAGLSTCWLNAHHREQPAGIHPTWTVASLSELEQLLCKH
>EECEBICD_03506 [Ribosomal protein S18]-alanine N-acetyltransferase
MNTISILSTTDLPAAWQIEQRAHAFPWSEKTFFGNLGERYLNLKLTADDRMAAFAITQVV
LDEATLFNIAVDPDFQRRGLGRMLLEHLIDELEKRGVVTLWLEVRASNAAAIALYESLGF
NEATIRRNYYPTAQGHEDAIIMALPISM
>EECEBICD_03507 DNA polymerase III subunit psi
MTSRRDWQLQQLGITQWALRRPGALQGEIAISLPAHVRLIVVAEELPALNEPLMRDILRA
LTVSPDQVLPLAPERVAMLPQGSRCNSWRLGTDAPLQLEGAQVTTPAFNELRANPAARAA
LWQQICEHEHDFYPQHDRSPRSLAD
>EECEBICD_03508 Ribosomal RNA small subunit methyltransferase C
MSAFTPASEVLLRHSDDFEQSRILFAGDLQDDLPARFECAASRAHTQQFHHWQVLSRQMG

DNVRFSLVAQASDVADCDTLIYYWPKNKPEAQFQLMNILSLMPSGVDVFVVGENRSGVRS
AEPMLADYAPLNKVDSARRCGLYHGRLEKQPQFSLESWWAEYNIDGLTIKTLPGVFSRDG
LDVGSQLLLSTLTPHTKGKVLDVGCGAGVLSAALASHSPKVRLTLCDVSAPAVEASRATL
AANGLEGEVFASNVFSEVKGRFDMIISNPPFHDGMQTSLDAAQTLIRGAVRHLNSGGELR
IVANAFLPYPKILDETFGFHEVIAQTGRFKVYRTVMTRQAKK
>EECEBICD_03512 hypothetical protein
MLQRTLGSGWGVLLPGVIIVGLAFIGLSADALKLLIVSGLLLSALMLYHKQLRHFVLLPS
CIALIGGMMLAMTNWNQG
>EECEBICD_03513 hypothetical protein
MTTPSWRSLRIKKYQFSLRLFLFLNAVSALFTLVFPLYQINVFCTPMIGIVVLSVLLLIW
HGKYGQKRINLPFISLLFGGIWAAHIALKYPALGHYDFSFLLISLLSVLFIGSIAFAANI
VAFTLYSLPPVAVCLWLNDNEQGLRILYLLALPMVGIAIQHVIQKRYDNFAQQLMFKLLA
ERETLNGLSMLDPLTGLYNRRGLQNRLDTLQAPGSHEHYVLLLDIDHFKAYNDHYGHMMG
DQALIRVSAAIRDAVRSRDVVCRFGGEEFLVLLTTAEPQQARATAERIRQEVYDLKIPHM
FNESVATNVTVSIGIAPLTDRNIGDAIEKADKALYEAKHLGRNHILVSDDMRAL
>EECEBICD_03514 Ferric iron reductase protein FhuF
MAYRSAPIADDIIWRAAPQPEDASLADAVRETIASTRKHLLDFIRLDETPPPTAMTLAQW
TRPATFRSLLAAYSDHIYRHTPGLPRENKPLLSLWAQWYIGLMAPPLMLALLTQARAINV
SAEHIHVEFHETGRAACFWLDVHQDNLATERSPEERMETLIVSALQPVIQALEATGYINA
KLIWSNTGYLINWYLTEMKPLLGEALLATLRQRCFFEKQLSDGQDNPLWRTVVMRDGLLV
RRTCCQRYRLPDVQQCGDCTLK
>EECEBICD_03515 hypothetical protein
MSLQSVRQFLADHAPDIEIIELNQSTATVELAAKAHNVEPGQIAKTLSLKVKDTIILVVA
KGDARLDNKKLKTTFGAKARMLSSDEVVNATGHPVGGVCPFGLEHPLPVYCDISLKGYNE
VLPAAGATHSAVRITPERMAELTSATWVDVCQ
>EECEBICD_03516 Transcriptional activator protein BglJ
MRRLVIADDDAEARLISALSPLPLDGVISKASPLDVLQDALFTSLNSARRTTERTDNLWE
LHQNCMLSPTEREILRFMSNGYSMPQIAVQLERNIKTIRAHKFNVMSKLGVSSDAGLLDA
ADILLYLRAGDATALQHRV
>EECEBICD_03517 hypothetical protein
MENRISKRNVALVEKCVMSTIGIKGLFDAMPDCRHTLHIFQNHPRFIRLR
>EECEBICD_03518 Putative transcription factor YjjQ
MLPGCCKNGLIISKTPLIQEGLKGVITGNFPDYKLAYCRTIEELTLLQLRRSNLVIADLA
VNNASPRAICEYFYSLLSQYRDIHWVFLVPKSCYPHAVDLLMGPVSTLLSDEEPIENLIS
VIHAGNARSERISKTLLSPQVPSEIQQSHDRPIVLTLSERKVLRLLGKGWGINQIAALLK
KSNKTISAQKNSAMRRLSIHSNAEMYAWINSSQGARELNLPSVYGETMEWKTESAREMLR
S
>EECEBICD_03519 Inner membrane protein YjjP
MTKRTSQEAYALRLAADVFINKKLILTNAIMPALRVKKEASVIVAAHRMSFWTGRVMQEE
QSIQRAVTRLCIQCGLFLLQHGAESALVDELSTRLGLALGMDSVESAISSNAIVLTTIKD
GQCLTSTRKNQDRGINMHVVTEVQHIVILAEHRLLDYKGVEKRFSQLRPLRYPRWLVAFM
VGLSCSCFCKLNNGGWDGAVITFFASMIAMYIRQMLAQRHLHPQINFCITAFVATTISGL
MLTLPAFSQTPTIAMAASVLLLVPGFPLINSVADMFKGHINTGLARWAIASLLTLATCVG
VVMSMTVWGLRGWV
>EECEBICD_03520 hypothetical protein
MGIIDFLLALMQDMILSAIPAVGFAMVFNVPHRALPWCALLGALGHGSRMLMMSAGFNIE
WSTFMASLLVGSIGIQWSRWYLAHPKVFTVAAVIPMFPGISAYTAMISAVKISHLGYSEP
MMITLLTNFLKASSIVGALSIGLSVPGLWLYRKRPRV
>EECEBICD_03521 Primosomal protein 1
MSSRILTSDVIGIDALLHDHHAVLAKSTGGAVAVFANNAPAFYAVTPARMAELLALEEKL
SRPGSDVALDAQFYEEPEAAPVAIPCGKFAMYPAWQPDADFQRQAALWGVALREPVTAEE
LAAFIAYWQAEGKVFHHIQWQQKLARSVQISRSSNGGMPQRDINSVSEPDNHIPPGFRG
>EECEBICD_03522 DNA replication protein DnaC
MKNVGDLMQRLQKMMPAHITPAFKTGEELLAWQKEQGEIGAAALARENRAMKMQRTFNRS

GIRPLHQNCSFDNYRVECDGQMNALSKARQYVDEFDGNIASFVFSGKPGTGKNHLAAAIC
NELLLRGKSVLIITVADIMSAMKDTFSNRETSEEQLLNDLSNVDLLVIDEIGVQTESRYE
KVIINQIVDRRSSSKRPTGMLTNSNMEEMTKMLGERVMDRMRLGNSLWVNFTWDSYRSRV
TGKEY
>EECEBICD_03523 hypothetical protein
MNTAKHVLCCAAIASVLISTSGIAASQLSAQGANAQQGGMSLSALTGLLSRGAQSLSADN
MNNAAGILQYCAKQKLASATNVENVKNQILNKLGLDTTQQEQDTNYLNGLQGLLKTKDGQ
QLNLNNIGSTPLAEKVKTKACDLVLQQGLNFLS
>EECEBICD_03524 Phosphoglycerol transferase I
MSELLSVALFLASVLIYAWKAGRNTWWFAATLTVLGLFVILNITLYASDYFTGDGINDAV
LYTLTNSLTGAGVGKYILPGIGIALALVAVFGALGWVLRRRRHHPHHVGYSLLALLLALG
SVDASPAFRQITELVKSQMRDGDPDFAVYYKEPAKTIPNPKLNLVYIYGESLERTYFDND
AFPNLTPELGALKNEGLDFSHTMQLPGTDYTIAGMVASQCGIPLFAPFEGNASASVSSFF
PQNICLGDILKNSGYQNYFVQGANLRFAGKDVFLKSHGFDHLYGAEELKTVVADPSYRND
WGFYDDTVLDEAWKKFEALSRSGQRFSLFTLTVDTHHPDGFISRTCNRKRYDYDGKPNQS
FSAVSCSQENIAEFINKIKASPWFKDTVIVVSSDHLAMNNTAWKYLNKQDRNNLFFILRG
DKPQQETLAVKRNTMDNGATVLDILGGDNFIGLGRSSLSGQSLSEVFLNVKEKVLAMKPD
IIRLWNFPKEIKDFTVDRDKNMIAFSGSHFRLPLLLRVSDKRVEPLPESEYSAPLRFQLA
DFAPRDNFVWIDRCYKMAQLWAPALALSTDWCVSQGQLGGQQTVQHVDKAQWKGKTAFKE
TVIDVTRYQGNVDTLKIVDNDIRYKADSFIFNVAGAPEEVKQFSGISRPESWGRWSNAQL
GDEVKIEYKAPLPKKFDLVITAKAFGDNANRPIPVRVGNEEQTLVLGHDVSTITLHFNNP
TDANTLVIAPPAPVSTNEGNILGHSPRKLGIGMVEIKVVNVES
>EECEBICD_03525 Fructosamine deglycase FrlB
MLGFNQDEYLTSAREIIAARQKAEQVADEIYQAGFSSLFFASVGGSLAPMMAINEFAKEL
TTLPVYVEQAAELIHKGNKRLNKDSVVITLSKSGDTKESVAIAEWCKAQGIRVVAITKNA
DSPLAQAATWHIPMRHKNGVEYEYMLLYWLFFRVLSRNNEFASYDRFASQLEILPANLLK
AKQKFDPQADAIASRYHNSDYMMWVGGAEMWGEVYLFSMCILEEMQWKRTRPVSSAEFFH
GALELLEKDVPLILVKGEGKCRALDERVERFASKITDNLVVIDPKAYALDGIDDEFRWIM
APCVVSTLLVDRLAAHFEKYTGHSLDIRRYYRQFDY
>EECEBICD_03526 Glutamine--fructose-6-phosphate aminotransferase
[isomerizing]
MSPTMLTYINEESDVLANIIRCHRQSLEEVSRFASQKTLRRILILATGSSLNAAFCARYF
FERCGISIDIKEPYTFSQYENSDPQADMVIAISQSGKSASTLEAMRKVQAQGRPVFALTA
DPQSPLGKASDYPLDILTGIESVGFVTRGFSATVLNLLLIALLVARQQQLTESQVEEYVA
QLQRIAATLPLVIVRTEAFIHQHQAVLRNGTRFVATGYGALVGVAKELETKFTETVRVPS
SGFELEAYMHGPYLEANAEHVMFFFEDRPDARSRALREYMTPAVAKTFTLTLAKAAQDDQ
TLALDVAVDHHFSPLLLIVPVQLMAFHIASLKGIDLSVRIFDDFDRVLKSKI
>EECEBICD_03527 PTS system mannose-specific EIID component
MTMKMISDKDRQQAETTGLVTARDLRRVFWRSFQMEFSWNYERQMNLAFVYTLIPVLKKL
YSRKEDLAEALKRHLAFFNTTPHIVTLILGITVAMEEKNSQQKEMDASSIDNVKASLMGP
LAGIGDSFFWGTLRLIATGIGTSLALKGNILGPILFLLVFNVPHILARWFFTRWGYVLGT
GVLQRIQQSGMMESLTYGASIIGLMVVGAMTASMIDITIPITFGTGEAKTHVQDIINDIM
PCLLPLISFAIVYWLLGKKVKPLTIIGGIALVGIFGSWIGLF
>EECEBICD_03528 N-acetylgalactosamine permease IIC component 1
MVEALLLGLVAFIAQSEYALGTSLISRPIVTGLLTGLVLGDMETGIVMGATLELAFIGSF
SVGASLPPDVVTGGILGVAFAINSGAGAETALLLGLPIATLALIVKNIYNGMFIPLLCHK
ADAYAEVGDTRGIERMHLISGIGLSLMLGIIVTVSYLAGVNMVKGFLDAIPEFIKHGLSV
ATGIIPALGFAMLARLLINKKVAPYFFLGFVLMAYLKIPVTGIAILGAIVAVVMVNIPKF
AASQPAPAQGASHDDEDDF
>EECEBICD_03529 PTS system sorbose-specific EIIB component
MIVFLRVDHRLLHGQVAFSWTQYVGADCILIANDSVPNDDLRKTTIKMAKPPAVKLVIKN
IADSIEAIKSGVTDKYKLFIVVESVADAYRLARELPDIKSINLGGTKVREGSQNIAKAIN
LLADEMTQLRELAAGGVEIEIRLVPNDRKQLFA
>EECEBICD_03530 hypothetical protein

MKRHYIFASHGTLASGVLNSVELILGKRSNVWTLCAYVEESVDLSQQVDTLIARIPPEDE
IIALTDIFAGSVNNEFVRYLSRENFHLLAGINLPLVIDLFMSENDGNTTHTIMTALADSK
ENIQYCNQTITSAMQSDKDF
>EECEBICD_03531 Transcriptional regulatory protein DagR
MRKDALLAFLMNQTDFFDPENVSEVFTARYLAQRFNMQRNTASHYLNQLVAQGVLCKINT
RPVYFLHKQAFSQQFFTLSRNEYASVAELLAHGEQECAPDDHFSLLIGHNESLKRPIEQL
KTALFYPDGGLPLLMTGESGTGKSYLAQLMHEYAIAQALLSPDAPFISFNCAQYASNPEL
LAANLFGYVKGAFTGAQSDRPGAFEAADGGMLFLDEVHRLSAEGQEKLFTWLDRGEIYRV
GDTAQGHPVSVRLVFATTEEIHSTFLTTFLRRIPIQVNLPDLQHRSRQEKEALILLFFWT
EAKKLSATLILKPRLLQILNQYVYRGNVGELKNVVKYAVATAWAKKPGQETVTVSLHDLP
DAMLSALPSLNEPLADDTPVSISPDTNLTWLLRARDEMQGMIHDTQCHVLALYELVRSGK
EGWETVQKRMGDEIETLFDRLIFTGDDNVHSQRLLLITSQVREEFYRLEKRFNMQLNGNC
IYALSHYLIHRTALAPSRLNSEQIRQLDAFLAQKYPLLYSFCLQILETLGQKLDLEPRRI
DMLLLALWLHKQGANNQKQVTHAVILAHGYATASSIANVANRLLKNTIFESFDMPLDVTP
EAIAQQVMRYLEEHPLASGLMILVDMGSLKAIHRHFDRALSTPVTIINNVSTSMALYVGE
RILQGHFIEEIARDIARDVPVEYQLYWPKSNKPRAILTTCATGIGVATNLCALLSASIPQ
ALEIDVVACDYAMLANNKTQEPVFMRYDVLAIVGTLDPHIASVPWISLDSLISGEGNHYL
MRLFGSLTTPEQVAEINNLLLKNFSLRRVIESVTILDTSKVINHVEQFLLRYEHLAGVTV
SNERKVALYVHISCLIERLIRHAGITAWSGQQCPEQELNRLREAFSVIESNYSVKIPTAE
LGYIHNILTFETELIEQDQQF
>EECEBICD_03532 Methyl-accepting chemotaxis protein I
MLKRIKIVTSLLLVLALFGLLQLTSGGLFFNSLKNDKENFTVLQTIRQQQSALNATWVEL
LQTRNTLNRAGIRWMMDQSNIGSGATVAELMQGATNTLKLTEKNWAQYEALPRDPRQSEA
SFLEIKRTYDIYHGALAELIQLLGAGKINEFFDQPTQSYQDAFEKQYMAYMQQNDRLYDI
AVEDNNSSYNQAMWVLVSVLIAVLVVIIAVWFGIKLSLIAPMNRLIESIRHIASGDLVKR
IDVEGSNEMGQLAENLRHMQSELMRTVGDVRNGANAIYSGASEIAMGNNDLSSRTEQQAA
SLEETAASMEQLTATVKQNAENARQASHLALSASETAQKGGKVVDNVVQTMRDIASSSQK
IADIISVIDGIAFQTNILALNAAVEAARAGEQGRGFAVVAGEVRNLAQRSAQAAREIKSL
IEDSVSRVDVGSTLVESAGETMDEIVNAVTRVTDIMGEIASASDEQSRGIDQVGLAVAEM
DRVTQQNASLVEESAAAAAALEEQASRLTQAVAVFRIQQQQQRARDVAAVKTPAAVSSPK
AAVADGSDNWETF
>EECEBICD_03533 Pyruvate/proton symporter BtsT
MDTKKIFKHIPWVILGIIGAFCLSVVALRRGEHVSALWIVVASVSVYLVAYRYYSLYIAQ
KVMKLDPTRATPAVINNDGLNYVPTNRYVLFGHHFAAIAGAGPLVGPVLAAQMGYLPGTL
WLLAGVVLAGAVQDFMVLFISSRRNGASLGEMIKEEMGTVPGTIALFGCFLIMIIILAVL
ALIVVKALAESPWGVFTVCSTVPIALFMGIYMRFLRPGRVGEVSVIGIVLLVASIYFGGV
IAHDPYWGPALTFKDTTITFALIGYAFVSALLPVWLILAPRDYLATFLKIGVIVGLALGI
VILNPELKMPALTQYVDGTGPLWKGALFPFLFITIACGAVSGFHALISSGTTPKLLACET
DARFIGYGAMLMESFVAVMALVAASIIEPGLYFAMNTPPAGLGITMPNLHEMGGENAPLI
MAQLKDVTAHAAATVSSWGFVISPEQILQTAKDIGEPSVLNRAGGAPTLAVGIAHVFHKV
LPMADMGFWYHFGILFEALFILTALDAGTRSGRFMLQDLLGNFVPFLKKTDSLVAGIIGT
AGCVGLWGYLLYQGVVDPLGGVKSLWPLFGISNQMLAAVALVLSTVVLIKMQRTKYIWVT
VIPAVWLLICTTWALGLKLFSANPQMEGFFYMANLYKEKIANGTNLTAQQIANMNHIVVN
NYTNAGLSILFLVVVYSIIFYGFTTWMKVRNSDKRTDKETPYVPVPEGGVKISSHH
>EECEBICD_03534 hypothetical protein
MFDTLGQAKKYLGQAAKMLIGIPDYDNYVEHMKTNHPDKPYMTYEEFFRERQQARYGGDG
KGGMRCC
>EECEBICD_03535 P-loop guanosine triphosphatase YjiA
MTPIAVTLLTGFLGAGKTTLLRHILNEQHGFKIAVIENEFGEVSVDDQLIGDRATQIKTL
TNGCICCTRSNELEDALLDLLDSRDRGDIAFDRLVIECTGMADPGPIIQTFFSHDVLCER
YLLDGVIALVDAVHANEQMNQFTIAQSQIGYADRILLTKTDVAGDSEKLRERLARINARA
PVYTVVHGDIDLSQLFNTSGFMLEESVLASQPRFHFIADKQNDVSSIVVELDYPVDISEV
SRVMENLLLESADKLLRYKGMLWIDGEPNRLLFQGVQRLYSADWDRPWGDETPHSTLVFI
GIQLPEDEIRAAFAGLRK

>EECEBICD_03536 hypothetical protein
MSMVKHKRGNASALSAQHEAELKALAKKSDDEIDYSDIPASEDGQWSEAVRGKFFRPLKT
QASVRIDADVMEWLKRPGKGYQTRLNAILREAMLREQNKK
>EECEBICD_03537 hypothetical protein
MPMEFEWDANKAKSNLRKHGVRFEDAVLVFDDPRHLSRQERYENGEYRWQTLGLVHGIVV
ILVAHSVRFESGFEVIRIISARKADRKERNRYEHG
>EECEBICD_03538 hypothetical protein
MDGGDRRLMPFCTKRCCESKIRNSQKMPDAGCRMPDAGCAFCGLLFWSIFFYLEYANCEA
ISNNPGIIVR
>EECEBICD_03539 ATP-dependent DNA helicase Rep
MAIIIPTVSSCSEKITAGEKRLARLLERGLSDSCTCWYDTPMGKQHSHPDFVILTPDKGI
LFIEVKDWFITKIKGANKTYVQYETKNGIESLKNPIEQVRQYAFQTINSLKTDPQLRQQE
GQYQGSFVMPYGYGVYLSNISRSQLEKAFSPTELADILPTDKVICKDELNEFMSQEKVAA
RLALLAKYNFAHQTTPQQLDRIRWHLYPDIRIHKPIKQADVKNFTLHTPSIISIMDRQQE
QLARSMGSGHRVIHGVAGSGKTLILLHRCLELANNIENEKPVLVICYNITLARKLKALIE
KHTLRLPVEVTHFHLWCHQQLQSYGRLPPKSKNFVELMENALSIAFEDGIIQPEQYSAVL
IDEGHDFNPEWLRILTRMADTKNNTLLFLYDDAQSIYQKKKALDFTLSSVGIKAQGRTTI
LNINYRNTQQILHFASSIAFNYLNNHIEDALKYQQPDSGGIAGSYPELTCFDNQDEEITH
ILNWVIEQHQRGIPWFDIAILCPSTYSIKDVLGPQLTARNIPYQMIITSADKKTGRHSKN
GCVLCLCRAVKDSNFSQSLSWTQPDPKMKKT
>EECEBICD_03540 hypothetical protein
MLAECIFSIHSYQQIIASPCEANVKPFDIIAQVFFIFGSGCVHDSD
>EECEBICD_03541 hypothetical protein
MICLETTVLCHLLNNASRQLIHTDFTSIFSIYEKKIINDNSYIKLNQKEFKLIFSNITLY
DFSQSRDIKNYISRITEICNEYINTLSIHSILDLFTSLIEENRPPTQKHYTPHEIVTFMG
NIIQAQKGESFFDPACGSGEFISEIIKNQVAISGSEYDVDRLKISKMKMLVNDLSPSNIS
PSYFTEGHNLKKNFDIILSNPPFSLKIPFDMEMHFCMYGKPPTSNADFAFLQYCIFMLKD
NGRAAIILPDGILFREGKEYEIRKKIIKNNHISAIIYLPKGMFKTTAIATNIIVFKKKQK
TNDILMINVRKKNNLNVNLLLELITKRSTTEISRLTSLNEISAHDYNLSASLYFRPQVKK
TDLKQLIMKQKELEEKLHSLQYAFQHKLTSLNL
>EECEBICD_03542 hypothetical protein
MNMENILGKPSVSFLAILIEPGIGLACKTMFIFFLIQYRQWAID
>EECEBICD_03543 hypothetical protein
MKNYKVITPLFQHTNMTLAMMKAVARCSVKAVRNMITAIQEQTGPPQNPVDWSEPDKWIK
ERLSGEDASIAWQIWNMDNYILNPRHSYGCYMFINNPLFDLMQTAEDGGWYPSEQGRLLM
DHDEATLHKLGDIEGMLHATGVTGWP
>EECEBICD_03544 hypothetical protein
MLLALLAGRKQARRADLLPKWQTSLHQHSKFASPSSVKTTLYARLYNLVERRLVARKGMS
YKITDAVRKWLNKALPERRTDPRKELLDAVKRYNEQPKEVLREQLSTMNPYKFEHLVGQL
LEAMGYEQVEVTKPLVTKACMSSVKCRSASPPLPK
>EECEBICD_03545 Mrr restriction system protein
MYVIGKVQVSITTITEVVQVKRMQNTITHLYIDQLRGALPYHKAIRGTLITTDKFAAKCA
EAALFPGAAPITLIDGDRLLELLIENNVGIRRSNAVELLDVDLQLFDELEID
>EECEBICD_03546 hypothetical protein
MAKAPTKKAKAKKGFEDTLWDTANQLRGSVESSEYKHVVLSLVFLKFISDKFEARRKQME
DEGQGDFLEMEVFYQQDNIFYLPEEARWSFIKQNAKQDDIAVRIDTALSTIEKRNPSLKG
ALPDNYFSRQNLETKKLASLIDTIDNIETLAHETDVEALSKEDLVGRVYEYFLGKFAATE
GKGGGEFYTPKCVVTLLTEMLEPFQGKIYDPCCGSAGMFVQSVKFVESHQGKSRDIALYG
QELTATTYKLAKMNLAIRGLSANLGERPADTFFSDQHPDLKADYILANPPFNLKDWRNDA
ELTKDPRFAGYRTPPTGNANYGWILHMLSKLSANGTAGFVLANGSMSSNTSGEAEIRAQM
IENDLIDCMIALPGQLFFTTQIPVCLWFMTKSKAADPAKGYRNRQGETLFIDARNLGTMI
NRTTKELTADDIATIADTYHAWRSTPEELVERVKRGDSQLAQYEDQAGFCKVATIAEIKA
NDSVLTPGRYVGAAEQEDDGVAFETKMRELSQTLFAQMKQAEELDNAIRQNLEVLGYGI
>EECEBICD_03547 hypothetical protein

MAFEKTIPLNEFITLQRGFDLPQDKRVMGDIPVVASTGVVGYHNEEKVLAPGVVIGRSGS
IGGGQYITTNFWPLNTTLWVKDFKGHHPRFVYYLLRSIDFSQFNVGSGVPTLNRNHLSGI
LVADTSYSYEKEASDIIGILDDKIKLNKELNHTLEQISQTLFKSWFVDFDPVIDNALDAG
NPIPEALQSRAELRQKIRNSADFKPLPADIRALFPAEFEETELGWMPKGWITTSFNDLIE
LIGGGTPKTSVEEFWNGDIPWFSVVDAPSESDVYVLTTEKKITIEGLNNSSAKLLRKGTT
IISARGTVGKCAMVAVPMAMNQSCYGVIGKNNISDEYIYFQLKNAVQTLQQMGHGSVFNT
ITRDTFKNIKVPFCNEELTNSYSLLVKNYFSKILNNNYQNIALTNLRDTLLPKLISGELS
LEDLPNLAKQTEPA
>EECEBICD_03548 hypothetical protein
MHLSFSEAKLEQAIIELLQDQGYQHLIGDNVPRSSLDQVIIEDDLRHYLAARYQADGITE
EEIQRLIKQFTTLPASDLYESNKTFCAWLANGFLFKRDDRQQKDLYIELLDTRHLPAALR
ELFDTEDVPLQQAAETPASYHLIQPLNRVKIVNQLTITGKENQPRIPDAILYINGLPLVV
FEFKSAVREQEANIGDAWKQPCTRYRRDIPQLFVYNALCIISDGVNNRMGNLFAPYEYFY
SWRKVTGNENREQNGIPSLHSMIQGLFHPVRLLDVIKNFICFPDKAKHEVKICCRYPQYY
AARKLYYSIERARKPFGDGKGGTYFGATGCGKSYTMQFLTRLLMKSVEFASPTIVLITDR
TDLDDQLSAQMCNAKNYIGDDTVVPVTSRDDLREKLAGRNSGGVFLTTIHKFTEDTELLS
ERSNIICISDEAHRSQVNLDQKVIVDKESGRVRKTYGFAKYLHDSLPRATYVGFTGTPID
ATMDVFGVIVDSYTMTESVQDEITVRIVYEGRAAKVILDAGKLEEVEKYYEECANAGTNE
WQIDESKKASATMNAILGDEDRLKALAEDFAKHYEKRVAEGSTVKGKAMFVCASREIAWD
FYRQLKDFRPAWFEVKQAPEGVELTGQEEKELPPSEMVKMVMTRGKDDDAKLYDLLGSKE
YRKELDKQFKNAKSNFKIAIVVDMWLTGFDVPELDTIYIDKPLQKHNLIQTISRVNRKME
GKSKGLVVDYIGIKRQMNQALAMYSRIDATNFEDIQQSVIEVKNHLDLLAQVFHEFDSRP
YFSGEPQAQLACLNFAAEFVMRTQKLERRFMGLVKRLKAAYDVCCGSEALSQAERDHIHF
YIAVRSIVFKLTKGDAPDLTQMNARVREMIAEALKADGVEELFFLGDKKAESIDIFDDDY
LARINKIKLPATKIQLLQKLLEKAISDFKQINQLQGINFTRRFQSIMDKYNERREDDVLN
GEEFDNFSQEMTDIIYDIKTEMGTYAEMGIDIEEKAFYDILAHMRDKYQFTYEDDKMLAL
AKEMKAVVDNTSKYPDWSKRDDIKAKLKVELILLLHKHKFPPVANDDVYMGVLSQAENFK
QHHSHTLH
>EECEBICD_03549 hypothetical protein
MSDIQLFRLGGGKVQELPGKAAAIEKDLQMLIESHMETFLGVRFLETEYHTGKTHRGRID
SLGLDENNCPVIIEYKRHSNENVINQGLFYLDWLLDHKAEFQLLVMEKISKTAAKAIDWS
GTRLICIAADFNKYDEHAVQQINRNINLIRYKLFADDLLMLELVNAVVENSPQYIIANGS
VSSGKRHTRTQREQLSSASPALLSLYEQLKSYVLSLSDEVQFKELKLYDAFRLIRNFLCV
AVYPVTDPHLRLWLKINPQHIQLEEGFSRDVTNIGHWGTGDVELIVRNEHDLDKAKLLIE
KAWQEN
>EECEBICD_03550 hypothetical protein
MEFYENRPRQPFIGFVQLRRALKRWWLRKRACQALRRMSDEQLRDIGLEKKDVE
>EECEBICD_03551 Succinate semialdehyde dehydrogenase [NAD(P)+] Sad
MIDSRVEELAKIASQEMGKLIEQSRGEVKLCAQIARYYADNAKQFLAPVPYKTEFGDAWV
EHHPIGVIMAVEPWNFPYYQLMRVLAPNLAAGNPVLAKHASIVPHCAETFAHLVREAGAP
EGAWTNLFISSDQVANIIADPRVQGAALTGSEKAGSAVAAQAAKHIKKSTLELGGNDVFV
VLDDADLEKAVKIGVQARLTNAGQVCTAAKRFILHEKIADQFLSQFTEAFRKVKVGDQMD
ASTELGPLSSKDALDTLTRQVEEAVKNGATLHVGGTPLESKGNFFEPTILTNITRDNPAY
FEEFFGPVAQMYVVKDDDEAVKLANDSHYGLGGAVFSQNIERAKRMASRIETGMVYINWL
TDTAAELPFGGVKRSGFGRELSDLGIKEFVNQKLVVVRR
>EECEBICD_03552 hypothetical protein
MAYQTVNPANNQLIKEYPRTRARILKPRCKKLTRSITPTGPRERLTNVCRYCISWLT
>EECEBICD_03553 ISNCY family transposase ISRor2
MKKSTTSTPHDAVFKTFLRHPETARDFMEIHLPVSLRQLCDLRTLKLEPGSFIEKNLRAY
YSDVLWSLKTREGDGYIYVVIEHQSTPDAHMAFRLMRYAMAAMQQHLDAGHKHLPLVVPM
LFYHGVDSPYPFSLCWLDEFANPEVARRLYAAAFPLVDITVVSDDDIMQHRRIALLELIQ
KHIRQRDLLGLVERIASLLVTGCANDRQLKALFNYLMIQHGHTPRFTTFIRDVVGHVPHT
KERLMTLIERIRAADRRKGERQGRQVGLEEGLEKGLEKGQHVAALRIARQMLADGLDRET
VQRFTGLTAEELQDVSH

>EECEBICD_03554 Multidrug resistance protein MdtM
MQRIIQFFSQRATTLFFPMALILYDFAAYLTTDLIQPGIINVVRDFNADVSLAPASVSLY
LAGGMALQWLLGPLSDRIGRRPVLIAGALIFTLACAATLLTTSMTQFLVARFVQGTSICF
IATVGYVTVQEAFGQTKAIKLMAIITSIVLVAPVIGPLSGAALMHFVHWKVLFGIIAVMG
LLALCGLLLAMPETVQRGAVPFSAVSVLRDFRNVFRNPIFLTGAATLSLSYIPMMSWVAV
SPVILIDAGGMSTSQFAWAQVPVFGAVIVANMIVVRLVKDPTRPRFIWRAVPIQLSGLAT
LLLGNLLLPHVWLWSVLGTSLYAFGIGMIFPTLFRFTLFSNNLPKGTVSASLNMVILTVM
AVSVEVGRWLWFHGGRLPFHLLAAVAGVIVVFTLATLLQRVRQHEAAELAAEK
>EECEBICD_03555 hypothetical protein
MNKIAELKRAKRLALSLLLIAAATFVTTLFLPPSFWVLGVKAIAEAAMVGALADWFAVVA
LFRRIPIPFISRHTAIIPRNKDRIGENLGQFVQEKFLDTQSLIALIRRHEPALLIGNWFS
QPDNASRVGQHLLQIMSGFLELTDDARIQRLLKRAVHKAIDKVDLSGTSALMLESMTKND
RHQVLLDTLIAQLIALLQRDSSRTFIARQIIRWLETEHPLKAKILPTEWLGEHSAELVSD
AVNSLLDDISHDRAHQIRHAFDRATYKLIDKLKHDPEMAARAENIKSYLKEDEAFNRYLG
EIWADLRQWLKTDINAEDSKGKQRIAHAGQWFGETLIADDALRASLNGHLEQAAHRVAPE
FAAFLTRHISDTVKGWDARDMSQQIELNIGKDLQFIRVNGTLVGGAIGLVLYLLSQIPAL
LSI
>EECEBICD_03556 hypothetical protein
MVHAQSASPRHPIFTALFGMMVLTLGMGVGRFLYTPMLPVMLAEKQLTFNQLSWIASANY
AGYLAGSLLFSFGLFHLPSRLRPMLLASAVATGILILSMAIFTQPAVVMLVRFLAGVASA
GMMIFGSMIVLHHTRHPFVIAALFSGVGAGIALGNEYVIGGLHYALSAHSLWLGAGALAG
ILLLIVAMLIPPALMRCRLRL
>EECEBICD_03557 hypothetical protein
MPWWQLALLYGFAGFGYIIVATYLPLMAKSAGSPLLTAHLWSLVGLAIIPGCFGWLWAAK
HWGVLPCLTANLLIQSACVLLSLASDSLLLLILSSIGFGATFMGTTSLVMPLARQLSAPG
NINLLGLVTLTYGIGQILGPLAASLSGNGASAIINATLCGAAALFFCRADQRRAADKTKT
VCDT
>EECEBICD_03558 hypothetical protein
MAQQVDGAAMPLDTEKVGIKGYLAFFLTIIFFSGVFSGSEGWWRVFDFTVLNGSFGHVTG
TQTFRGAGGTGAKDGFLFALELAPSVILSLGIIAITDGLGGLRAAQQLMTPILKPLLGIP
GICSLALIANLQNTDAAAGMTKELAQEGEITERDKVIFAAWQTSGSAIITNYFSSGVAVF
AFLGTSVIIPLVVILLFKFIGANLLRVWINIEERRHPAQGAQS
>EECEBICD_03559 Inner membrane protein YjiG
MTTQVRKNVMDMFIDGARRGFTIATTNLLPNVVMAFVIIQALKITGLLDWVGHICQPVMA
LWGLPGEAATVLLASLMSMGGAVGVAASLATAGALSGHDVTVLLPAIYLMGNPVQNVGRC
LGTAEVNAKYYPHIIAVCAINALLSIWVMQLIV
>EECEBICD_03560 Isoaspartyl dipeptidase
MPDLSAAEFTLLQGAHLFAPEDRGICDVLLANGKIIAVGADIPGDIVPDCAVINLSGRML
CPGFIDQHVHLIGGGGEAGPTTRTPEVSLSRLTEAGITTVVGLLGTDSVSRHPASLLAKT
RALNEEGITAWMLTGAYHVPSPTITGSVEKDVALIDRVIGVKCAVSDHRSAAPSGNQLAS
MAAESRVGGLLGGKPGVSVFHMGSSKKGLQPLYDILENSDVPIGKLLPTHVNRSESLFEQ
ALAFALKGGVIDMTTSIPDPVAPAEGIARAVKAGVPLSRVTLSSDGNGSQPLFDAAGNLT
GIGVAGFESLLETLQTLVNHYGFSLTDALRPLTTSVAAFLSLDGKGEIRPGNDADLLVFS
ADLRIEQVYARGKRMVNEGKACVKGTFEPA
>EECEBICD_03561 HTH-type transcriptional regulator YjiE
MDVTGAGLHNIETKWLYDFLTLEKCRNFSQAAIIRNVSQPAFSRRIRALEHAVGVELFNR
QVSPLQLSEQGKIFHSQVRHLLQQLESNLTELRGGSDYTLRKIKIAAAHSLSLGLLPTIV
KQMPTQFTYAVEAIDVDQAVDMLREGQSDFIFSYHDENLQQAPFDNIRLFESRLFPVCAN
NGRGEPRYTLEQPHFPLLNYSQNSYMGRLINRTLTRHAELSFSTFFVSSMSELLKQVAMD
GCGIAWLPEYAIRQEITDGRLIVLDADELVIPIQAYAYRMNTRMSQVAETFWRDLRGLQA
AL
>EECEBICD_03562 Broad specificity amino-acid racemase RacX
MKHTIGILGGMGPAATADMLEKFVELRHASCDQQHIPLIVSSIPDIPDRTACLLSGGPSP
YRYLERYLHMLEDAGAECIVIPCNTAHYWFDDLQNVAKARMISILDATLGDIPPSARHVG

LLATNATLATGLYQKKALARGLTLIQPEEAGQALVMQAIYTLKRGDKTVAQALLLPQIDS
LIARGAQAIIMGCTEIPLIVAGHERAIACPMIDSTASLVRAAIRWYESWPDTRASLTGEQ
RLTA
>EECEBICD_03563 hypothetical protein
MDAIYLKLDGIEGESRIKGFENQIKLIAYNHNPTKRESGEARGTYIGGLTLTKPVDLATP
GLYEHYCNGKTVKEGVLTLCRRDKGAMLPFIIYTLTNVRISRMSNHGDAEGSATETVDLV
YSHIRWDIPALASKSKTRRPLHRQALWR
>EECEBICD_03564 Tryptophan--tRNA ligase 2
MNTLPIILTGDRPTGSLHLGHYVGSLRQRVALQQDHQQYVLIADLQGLTDNGSNPQKIRD
NIPQVLADYLAVGIDPTLTTICLQSALPALAELTVLYMNIVTVARVERNPTVKNEIAQKG
FTRSLPVGFMAYPISQAADITAFKAEMVPVGDDQLPMIEQTNEIVHKMNSLFSSPVLRPC
QALLSDMGRLPGIDGSAKMSKSLGNTLLLSASEETIHRAVSAMYTDPNHLKISDPGKIEG
NVVFTWLDAFHPDKAKVAAMKAHYQQGGLGDRVCKNELETCLQELIAPIRERRATFIADK
GMLMELLKKGSERAHEVTQKTLQEVKRGLGLPTLFQV
>EECEBICD_03565 Putative L-lactate dehydrogenase operon regulatory
protein
MIRDLIVQTPYRPGERLPPEREIAERLNVTRTVVREALIMLEIKGLVEVRRGAGIYVLDS
ADNNEMEGADVNHCNDAGPFELLQARQLLESNIAEFAALQATREDIIKMRQALQLEEREL
ASSAPGGSESGDMQFHLAIAEATHNSMLVELFRQSWQWRENNPMWLQLHSHLGDTLYRKE
WLVDHKQILAALIKKDARAAKLAMWQHLENVKQRLLEFSNVDDIYFDGYLFESWPLDNVD
A
>EECEBICD_03566 hypothetical protein
MTRGFDIYIPALFPHHTPFRYEQQEEAYRHFSANVGFDPAVVTTLLHDLRTQYETLIVVG
YSVGATLAWLSAASGLCDGVICYYGSRIRQYLHLAPLCPTLVIIARYEPAFDPLAMRQTL
EKRPWVRCKMYDARHGFCDADSATFDAALSHQVMDDVSAFIRDITRANTSPD
>EECEBICD_03567 hypothetical protein
MSTILKPFLLAVTLMLILIRLGFATEDGAITMVKTYSAYDYLQTRARLMHAVADHGLVLF
GEFDHARAAQNVNLKMPPTTVLVFGNPKGGTPLMLAHPELALDLPFRVLISQQADGRTLV
SYHPAETLQRYGLDAAAIQALKKLEQLVEKSLH
>EECEBICD_03568 hypothetical protein
MHTFRPYSLRHSDLLYEDIPLEIREQIILLIINTLGNCSSFYDMTLYCYHNSHSDEVYRR
ICKTLRKEYGLFTLYAHSTSYLDEMSNLLLKTDDKRKHIDTIELAFNYIDTYLRTYEVTL
GLEPDKAISELNNIFHEHSLKYRYENGRIVRLRRIKRLKNICYYLYSPGEYGFVEYDLME
AYNRLILNDFACVVRECHAAFRSVLLRIHERKGIVYHEQDSLNTLMANLMARGVISAEYV
HKFHFLSDVLESEIFLPMAPEKSHHHYAMMLRISEELACSIYYLTERSIFFLTQRAEEDS
VAP
>EECEBICD_03569 hypothetical protein
MKVIWTVTPVGYQRIAKRCPSCSVKRDFTPSGAFRVNSQKKVLDVWSIYKCTHCDYTWNI
SLFSRLPVSKINRDLYGRLMANDAATVQYFAYDNAILKRNNAELSGQPDFHIQERWLVSI
ASHKQVSVSVRISRSFQVSLLSILKKQLLLSAAEIKRRIETGQISGVTMKMLKSRKLKNA
KYDLQLSVETLYDRRRIVLTRR
>EECEBICD_03570 hypothetical protein
MFMYEKYLSDIFQWVKKRFPTQQPCQWLKRYAEIIKAAIIGYMNLINIHYGSGGIYSTDL
LNA
>EECEBICD_03571 hypothetical protein
MNLSRQEQRTLHVLAKGGRITHIRDASGRVTAVECYSREGLLLADCTLAVFKKLKTKKLI
KSVNGQPYRINTTGLNNVRAQPDNR
>EECEBICD_03572 Ubiquinone biosynthesis O-methyltransferase,
mitochondrial
MDISAVIFATHRIRLLPDEGKIPWDEPAFSQRMLENHLSQDHDWASRRLTVIEQQVTWIT
RQLPAGARILDLGCGPGFYTRLLAERGFHCTGVDFSPASITWARQQAQAANLNIDYIQQD
IRTYHPTEPFDFIMMTFGELNVFSAADAQSLISHCAQWLKPDGKLLVEVHTFDEVKRQGM
AQPCWQRCPHGLFLAEPHLLLTENAWDEEAQTSSTQFWAIAENGCTTRFGSQMKAWRDDE
YISLLGDAGFTVLQRPDSHTWPVGETFAGKLFALLAEKAPPAEGITLSKRNNPMDIPRIF

TISESEHRIHNPFTAEKYATLGHALRMKPGTRILDLGSGSGEMLCTWARDYGVTGTGIDI
SPLFTTQAKQRAEELGVSEYVHFIHNDAAGYIDEEKYDVAACVGATWIAGGVAGTIDLLA
KSLKPGGIILIGEPYWRQIPATEEIAHACGVSSVADFRPLPELVAFFDQLGYDVVEMVLA
DPQGWDRYEAAKWMTMRRWLEENPDDEFAQEVRTELTVAPKRHVTWTREYFGWGVFALIA
R
>EECEBICD_03573 putative transcriptional regulatory protein YeeN
MGRKWANIVAKKTAKDGATSKVYAKFGVEIYAAAKQGEPDPESNSALKFVIERAKQAQVP
KHVIDKAIDKAKGGGDETFVQGRYEGFGPNGSMVIAETLTSNVNRTIANIRTIFNKKGGN
IGAAGAVSYMFDNTGVIVFKGTDPDHIFEILLDAEVDVRDVTEEEGNIVIYTEATDLHKG
IAALKAAGITEFSTTELEMIAQSEVELSPEDLEIFEGLVDALEDDDDVQKVYHNVANL
>EECEBICD_03574 hypothetical protein
MPLKMMMMCKKYIITLQTSNYKLNKSVSMTDLFNL
>EECEBICD_03575 hypothetical protein
MLDLLERYGPQRREQRPDMPFWRLKGDGFWELQNAEFCSTSGSRQPPKRELIEYNVAGGF
DAVNFALVTKKRKLIDTLAQQILEAHFPTSIQEDIADEMGFDIRTSLRQRDPKFRQAVLR
AYNYQCAVCGFNMRHDNAPIALEAAHIRWKQHHGPCEVPNGLALCAIHHKAFDRGSIGLD
ENMRVVVSDAVNGGGVVQRLFWDFAGKEIALPQMKENYPGERFVEWHKREVFRGGH
>EECEBICD_03576 hypothetical protein
MTSDKTLKQAISNITIWRKGEQRAPHKPLLLLYVLSHYRQGHDRLFDYGSEIHE
>EECEBICD_03577 IS3 family transposase IS1351
MPVRNPSPLATPEALNQSWSVDFMHDDLVCGRRFRTFNVVDDFNREALSIEIDLNLPALR
VVRVLDRTAANRGYPVMLRMDNGPEFISLALAEWAEQHAVKLEFIQPGKPT
>EECEBICD_03578 hypothetical protein
MDPFVIPRVSLPPQNLEKFWKTVPWVSLCHCLQRDNNGFVTRVIRTVMVNRSAQVQTPAG
LTDTESKCRYQMSDQLTLKGWF
>EECEBICD_03579 IS3 family transposase IS1400
MRKARFTEHQIIAVIKSVEAGRTVKDVCREAGISEATWYNWKSRYGGMEASDIKKIKDLE
DENRRLKQMFADLSLENRALKDVIEKSFKTSL
>EECEBICD_03580 putative protein YjiK
MFRYLCNQKAALLTAILLMAAGVLTLCFPESWYPQETEWQLTAEKEITGIHGGLSGLTWN
PDSRTLFAVTDHPSSVVELDTEGNVLRVIPSDGDHDFEAIEYLGGNRYALSRERERTLTT
HCIDSSTTVLPPATYSLTLDVNRHSDNAGFEGLAQGRGEHALMVAQEKKPLRLYVTDQSP
DALSVSDSLTHRASLPWFLKDISGLHYDRNNGLLYVLSHESDVVVVSDLDGGRKVMSLRR
GHYGLRRDIPQAEGIASDDRDTLWIVSEPNLFYRFTRTASS
>EECEBICD_03581 hypothetical protein
MLRWSGNCWPVTLMPTDDKGTIQAGDILPLRALILLLLTSVFISGFLTGVLAMLWLICRG
A
>EECEBICD_03582 Thiol:disulfide interchange protein DsbA
MIAQEWEFITPLVADAPAEVELFSFYCPPCYAFSQTMGVAQAIRHVLPHGDRMIKYHVNL
LGPLGHELTRARALAMMMKETDVVEKAFFMADMVEKRLHSPDDVHRVFMSATGISRGEYD
RSIKSPAVNDMVALQERLFKEYGVRGTPSVYVRGRYHINNAAFSAFSVEDFRSRYAAVVR
KLLAGNPDAD
>EECEBICD_03583 hypothetical protein
MWVGSRISITESYFIMFMKFVYYACLAVVILMAMVFCVSVLFILENS
>EECEBICD_03584 Transcriptional regulatory protein RcsB
MASALTVFPEDGDINRIWYDRFCIEFNSDGGLSPAELRVIRLLLQGYNCGQIAALCFRSV
KTISAQKVLAFRRLGVRNDATLLPTLMLLGMVTLYTDLNRCHSLEEGGPECGSEAG
>EECEBICD_03585 hypothetical protein
MGESIITNIISIIRERQSADNAPVKIRDIADAAGLSIYQVRSYLEQLRAVGVLEKVNAGK
GAPGLWRLL
>EECEBICD_03586 Virulence regulon transcriptional activator VirF
MLKKNAIKIKLYRYAILHSKNCIVTIKNKSKPEEIKITRGNIALIEKNIEAVVEIEYMDD
IESFDIITLPDELLSRVLCLFEASNCSESLSPIRYRTFSDKVFIITDNGINGILFEYLKK
RKNNNNDIYEIACLFSKVNNIEQLYTSLCISVSRSFSDIVRKTIDNDISTKWRLKTLSEK

LNLSEVTIRKKLENENTNFYRILLDARMQKAARLVLDSDTHINKVSYAVGMSSVSYFIKL
FSDYYGLTPKQFHLKYKHRNTGEKAAFMLYN
>EECEBICD_03587 hypothetical protein
MNQYNSSIPKFIVSVFLIVTGFFSSTIKAQELKLMIKINEAVFYDRITSNKIIGTGHLFN
REGKKILISSSLEKIKNTPGAYIIRGQNNSAHKLRIRIGGEDWQPDNSGIGMVSHSDFTN
EFNIYYFGNGDIPVDTYLISIYATEIEL
>EECEBICD_03588 F1 capsule-anchoring protein
MPYSTPVLSLPDGYSKYSVTIGRYREVNNDYIDPVFFEGTYIYGLPYGFTLFGGVQWVNI
YNSYAIGASKDIGEYGALSFDWKTSVSKTDTSNENGHAYGIRYNKNIAQTNTEVSLASHY
YYSKNYRTFSEAIHSSEHDEFYDKNKKSTTSMLLSQALGSLGSVNLSYNYDKYWKHEGKK
SIIASYGKNLNGVSLSLSYTKSTSKISEENEDLFSFLLSVPLQKLTNHEMYATYQNSSSS
KHDMNHDLGITGVAFNSQLTWQARGQIEDKSKNQKATFLNASWRGTYGEIGANYSHNEIN
RDIGMNVSGGVIAHSSGITFGQSISDTAALVEAKGVSGAKVLGLPGVRTDFRGYTISSYL
TPYMNNFISIDPTTLPINTDIRQTDIQVVPTEGAIVKAVYKTSVGTNALIRITRTNGKPL
ALGTVLSLKNNDGVIQSTSIVGEDGQAYVSGLSGVQKLIASWGNKPSDTCTVFYSLPDKN
KGQISFLNGVCK
>EECEBICD_03589 F1 capsule-anchoring protein
MKKTTITLFVLTSVFHSGNVFSRQYNFDYGSLSLPPGENASFLSVETLPGNYVVDVYLNN
QLKETTELYFKSMTQTLEPCLTKEKLIKYGIAIQELHGLQFDNEQCVLLEHSPLKYTYNA
ANQSLLLNAPSKILSPIDSEIADENIWDDGINAFLLNYRANYLHSKVGGEDSYFGQIQPG
FNFGPWRLRNLSSWQNLSSEKKFESAYIYAERGLKK
>EECEBICD_03590 Chaperone protein caf1M
MNEKEYPILVQTQVYNDDKSSKAPFIVTPPILKVESNARTRLKVIPTSNLFNKNEESLYW
LCVKGVPPLNDNESNNKNNITTNLNVNVVTNSCIKLIYRPKTIDLTTMEIADKLKLERKG
NSIVIKNPTSSYVNIANIKSGNLSFNIPNGYIEPFGYAQLPGGVHSKITLTILDDNGAEI
IRDY
>EECEBICD_03591 Fimbrial protein
MRKSASAVAVLALIACGSAHAAGFVGNKAEVQAAVTIAAQNTTSANWSQDPGFTGPAVAA
GQKVGTLSITATGPHNSVSIAGKGASVSGGVATVPFVDGQGQPVFRGRIQGANINDQANT
GIDGLAGWRVASSQETLNVPVTTFGKSTLPAGTFTATFYVQQYQN
>EECEBICD_03592 IS3 family transposase IS1230B
MAAATSELNLYESQLYNWRSKQQNQLSSSEREQEMSAEIVRLKRQLAERDEELTILQNGR
DILRETPEMKYVFIEKHQAEFSIKAICRVLQVARNSWYVRRQQFRLVCDNVVREAFSDAK
QRYGTPRLTDELRAQGRVYNIKTVAASLRRQVLRAKASRKISPVSYREHGLPVQRIC
>EECEBICD_03593 hypothetical protein
MALTDIKVRTTKPSDKPFKLTDGQGMHLLINPNGSKYWRLQYCFVGKLILPA
>EECEBICD_03594 Toxin CcdB
MQYMVYRNKGNSKAYPYLLDVQSDIIDELHTRMVIPLFPVSRLVNNPVKRLTPTLNVEGN
DYLVMTHEMASIRLSQIGDEVMDVRSHRQTIKNALDFIFDGF
>EECEBICD_03595 hypothetical protein
MPGMSVRHKKTVSVTLEPALLQQARDAGINLSAVLTAALKEEISATGVERWKAENRAGLQ
ELNRITDEHGLLSDDYRTF
>EECEBICD_03596 hypothetical protein
MASSAKQTISAQIPVELAAAVENLAIELDRSKSWIIKEALTSMLAERERRHQSIQAGLAD
VDAGRVVSHSDMVDFDNRLKET
>EECEBICD_03597 hypothetical protein
MLMLHRGDCVSDVARTLCCARSSVGRWINWFTLSGIEGLKSLSAGRTRRWPFEHICTLLR
ELVKHSPGDFGYQRSRWSTELLAIKINEITGCQLHAGTVRRWLPSAGLVWRRAAPTLRIR
DPHKDEKISIRYFQKGSGHITFKRLDLVEKMNDIVAKHYPGMLPVK
>EECEBICD_03599 Aldehyde reductase Ahr
MTIIKSYAAKEAGGELELYEYDAGELQPEDVEVRVDYCGICHSDLSMIDNEWGFSQYPLV
AGHEVIGRVAALGSAAQDKGLKVGQRVGIGWTARSCGHCDACISGNQINCLEGAVPTILN
RGGFAEKLRAGWQWVIPLPENMDMASAGPLLCGGITVFKPLLMHHITATSRVGVIGIGGL
GHIAIKLLHAMGCEVTAFSSNPSKEQEVLAMGANNVVNSRDPEALKALAGQFDLIINTVN

VDLDWQPYFEALTYGGNFHTVGAVLKPLPVPAFTLIAGDRSISGSATGTPYELRKLMKFA
GRSKVAPTTELFAMSQINEAIQHVRDGKARYRVVLKADF
>EECEBICD_03600 Thermoresistant gluconokinase
MAGESYILMGVSGSGKSLIGSKIATLFSAKFIDGDDLHPAKNIDKMSQGIPLTDEDRLPW
LERLNDASYSLYKKNETGFIVCSSLKKQYRDILRKSSPNVHFLWLDGDYATILQRMQRRA
GHFMPPDLLQSQFDALERPCADEHDIARIDVNHDIEHVTEQCRQAVQAFRQALSAS
>EECEBICD_03601 L-idonate 5-dehydrogenase (NAD(P)(+))
MEVKTQSCVVAGKRAVAVTEQNIEWNNKGTLVQITRGGICGSDLHYYQEGKVGNFTVKAP
MILGHEVIGKIVHSDSDLLREGQPVAINPSKPCGHCKYCLQHEENHCTEMRFFGSAMYFP
HVDGGFTRFKAVDTAQCIPWPEQADEKAMAFAEPLAVAIHAAHEAGDLQGKRVFISGVGP
IGCLIVSAVKTLGAAEVVCADISARSLSLARQMGADTLVNPQHDSLDGWKAEKGYFDISF
EVSGHPSSISTCLEVTRAKGVMVQVGMGGAVPNFPMMMVISKEISLKGSFRFTTEFNTAV
SWLANRVINPLPLLSAEYPFTDLEAALIFAGDKTQAAKVQLIF
>EECEBICD_03602 5-keto-D-gluconate 5-reductase
MNDLFSLAGKNILITGAAQGIGYLLATGLGRYGARIIVNDITPERAETAVTKLQQEGIKA
IAAPFNVTHKQDIEAAIEHIEKDIGVIDVLINNAGIQRRHPFTEFPEQEWNDVIAVNQTA
VFLVSQAVTRRMVARKAGKVINICSMQSELGRDTITPYAASKGAVKMLTRGMCVELARHN
IQVNGIAPGYFKTEMTKALVEDEAFTSWLCKRTPAARWGDPQELIGAAVFLSSKASDFVN
GHLLFVDGGMLVAV
>EECEBICD_03603 Gnt-II system L-idonate transporter
MPLIIIAAGVALLLVLMIGFKVNGFIALVLVAAVVGFAEGMGAQDVLHSIQNGIGGTLGG
LAMILGFGAMLGRLISDTGAAQRIATTLINTFGKKRVQWALVITGLIVGLAMFFEVGFVL
LLPLVFTIVASSGLPLLYVGVPMVAALSVTHCFLPPHPGPTAIATIFEANLGTTLLYGLI
ITIPTVIVAGPLFSKLLARFEKAPPEGLFNPHLFSEEEMPSFWNSIFAAVIPVILMAIAA
VCEITLPKTNAVRVFFEFIGNPAVALFIAIIIAIFTLGRRNGRTVEQVMDIVGESIGAIA
MIVFIIAGGGAFKQVLVDSGVGQYISQLMTGTSLSPLLMCWTVAAVLRIALGSATVAAIT
TAGVVLPIINVTHADPALMVLATGAGSVIASHVNDPGFWLFKGYFNLSVGETLRTWTVME
TLISVMGLLGVLALNAVLH
>EECEBICD_03604 HTH-type transcriptional regulator GntR
MRNHRISLQDIATLAGVTKMTVSRYIRSPKKVARETGERIAQIMEEINYIPNRAPAMLLN
AQSYTLGVLIPSFQNQLFADILAGIESVTSGHNYQTLIANYNYNRDSEEESVINLLSYNI
DGIILSEKYHTLRTVKFLRSATLPIVELMDIQGDRLDMEVGFDNRQAAFDMVSTMLDKRQ
RRKILYLGSKDDIRDEQRYHGYCDAMTRRGLAPLRINPRAISSIHLGIQLMRDALTAHPD
VDGVFCTNDDIAMGALLWCRERQLAVPEQISIAGFHGLEMGRQMIPSLASVITPRFEIGR
RAAQMLLNKIKNNDLNHNTIDLGYQIYHGNTL
>EECEBICD_03605 Lipopolysaccharide export system permease protein LptG
MQPFGVLDRYIGKTIFTTIMMTLFMLVSLSGIIKFVDQLKKAGQGNYDALGAGMYTLLSV
PKDIQIFFPMAALLGALLGLGMLAQRSELVVMQASGFTRMQVALSVMKTAIPLVLLTMAI
GEWVAPQGEQMARNYRAQAMYGGSLLSTQQGLWAKDGNNFVYIERVKGDEELAGISIYAF
NDQRRLQSVRYAASAKFDPEHKVWRLSQVDESDLQNPKQITGSQTVSGTWKTNLTPDKLG
VVALDPDALSISGLHNYVKYLKSSGQDAGRYQLNMWSKIFQPLSVAVMMLMALSFIFGPL
RSVPMGVRVVTGISFGFVFYVLDQIFGPLTLVYGIPPIVGALLPSASFFLISLWLMLRKS
>EECEBICD_03606 Lipopolysaccharide export system permease protein LptF
MIIIRYLVRETLKSQLAILFILLLIFFCQKLVRILGAAVDGDIPANLVLSLLGLGVPEMA
QLILPLSLFLGLLMTLGKLYTESEITVMHACGLSKAVLVKAAMVLALFTGILAAVNVMWA
GPWSSKHQDEVLAEAKANPGMAALAQGQFQQATNGSSVLFIESVDGSDFHDVFLAQIRPK
GNARPSVVVADSGHLTQLRDGSQVVTLNKGTRFEGTALLRDFRITDFQNYQAIIGHQAVA
LDPNDTDQMDMRTLWNTDNDRARAELHWRITLVFTVFMMALMVVPLSVVNPRQGRVLSML
PAMLLYLLFFLIQTSIKSNGGKGKLDPVIWMWAVNLIYLALAIGLNLWDTVPVRRLRARF
LRKGAV
>EECEBICD_03607 Cytosol aminopeptidase
MEFSVKSGSPEKQRSACIVVGVFEPRRLSPIAEQLDKISDGYISALLRRGELEGKPGQTL
LLHHVPNVLSERILLIGCGKERELDERQYKQVIQKTINTLNDTGSMEAVCFLTELHVKGR
NNYWKVRQAVETAKETLYSFDQLKTNKSEPRRPLRKMVFNVPTRRELTSGERAIQHGLAI

AAGIKAAKDLGNMPPNICNAAYLASQARQLADSYSKNVITRVIGEQQMRELGMNAYLAVG
HGSQNESLMSVIEYKGNPSEDARPIVLVGKGLTFDSGGISIKPSEGMDEMKYDMCGAAAV
YGVMRMVAELQLPINVIGVLAGCENMPGGRAYRPGDVLTTMSGQTVEVLNTDAEGRLVLC
DVLTYVERFEPEAVIDVATLTGACVIALGHHITGLMSNHNPLAHELIGASEQAGDRAWRL
PLGDEFQEQLESNFADMANIGGRPGGAITAGCFLSRFTRKYNWAHLDIAGTAWRSGKAKG
ATGRPVALLSQFLLNRAGFNGEE
>EECEBICD_03608 DNA polymerase III subunit chi
MTTHLNPPQEAPYMKNATFYLLDNDTTVNGLSAVEQLVCEIAAERWRAGKRVLIACEDEK
QAIRLDEALWARPAESFVPHNLAGEGPRGGAPVEIAWPQKRNSSPRDILISLRTSFADFA
TAFTEVVDFVPYEETLKQLARERYKAYRVAGFNLNTATWK
>EECEBICD_03609 Valine--tRNA ligase
MEKTYNPQDIEQPLYEHWEKQGYFKPNGDESKESFCIMIPPPNVTGSLHMGHAFQQTIMD
TMIRYQRMQGKNTLWQVGTDHAGIATQMVVERKIAAEEGKTRHDYGRDAFIDKIWQWKAE
SGGTITRQMRRLGNSVDWERERFTMDEGLSNAVKEVFVRLYKEDLIYRGKRLVNWDPKLR
TAISDLEVENRESKGSMWHIRYPLADGAKTADGKDYLVVATTRPETVLGDTGVAVNPEDP
RYKDLIGKFVILPLVNRRIPIVGDEHADMEKGTGCVKITPAHDFNDYEVGKRHALPMINI
LTFDGDIRESAEVFDTKGEESDVYSSEIPAEFQKLERFAARKAVVAAVDALGLLEEIKPH
DLTVPYGDRGGVVIEPMLTDQWYVRADVLAKPAVEAVENGDIQFVPKQYENMYFSWMRDI
QDWCISRQLWWGHRIPAWYDNDGNVYVGRTEDEVRQENNLGADIQLRQDEDVLDTWFSSA
LWTFSTLGWPENTDALRQFHPTSVMVSGFDIIFFWIARMIMMTMHFIKDENGKPQVPFHT
VYMTGLIRDDEGQKMSKSKGNVIDPLDMVDGISLPELLEKRTGNMMQPQMAEKIRKRTEK
QFPNGIEPHGTDALRFTLAALASTGRDINWDMKRLEGYRNFCNKLWNASRFVLMNTEEQD
CGFNGGEMTLSLADRWILAEFNQTVKAYREALDNFRFDIAAGILYEFTWNQFCDWYLELT
KPVMTGGSESELRGTRHTLVTVLEGLLRLAHPIIPFITETIWQRVKVICGITADTIMLQP
FPEYNAAQVDEAALADTEWLKQAIVAVRNIRAEMNIAPGKPLELLLRGCSEEAVRRVNDN
RSFLQTLARLESITVLPADDKGPVSVTKIIDGAELLIPMAGLINKDDELARLAKEVAKIE
GEIARIEGKLSNEGFVARAPEAVIAKEREKLEGYAEAKAKLIEQQAVISAL
>EECEBICD_03610 Inner membrane protein YjgN
MNNVISSKDNHNHTLVFTGKGGKYFVICLVNFLLTCITLGIYAPWAMVKCRRYIYTNMTL
NNQPFAYKATGGALFISVLLVFIIYIVSLSLIEHGHPGLGFTLFGLLIAIIPFMAVKGLQ
YQAMMTSLNGVHFGFQCSMRRAWWYMFALPVLLMVALYIVLYIISLVTIAVGGLVFSIVF
LGLLAIIGIGVINGITYSKWMTLFGNGANFGIHRFSIQVNVKTCIRGCVLAMLTLFPFAV
VIGYLIAPVFTDMILLSMMGNAQAGGALILQYYGQIMACYFLYFLAIIVVTSYLYVALRN
LFLNNLSLANDSIRFHSSVTAHGMLWRLLVVFVISGVTLGLAYPWLKIWLVSWLAQNTQV
QGDLDSLELTNDEKPLENSPLMWISRGIMPYFPFI
>EECEBICD_03611 hypothetical protein
MNNVASPTLTVRRITTADNAAIARVIRQVSAEYGLTADKGYTVADPNLDELYQVYSQPGA
AYWVVEQNGCVVGGGGVAPLSCSEPDICELQKMYFLPVIRGQGLAKKLALMALDHAREQG
FKRCYLETTAFLREAIALYERLGFEHISEPLGCTGHVDCEVRMLKDL
>EECEBICD_03612 hypothetical protein
MKRLLSAIVFPAMFISISNVYALDIQPGEWKMENIEMRTINPDTKEVLMDEKKFGHSDAY
VLYP
>EECEBICD_03613 hypothetical protein
MKKNSGIATLMCYTPKMSEDSKKMVKGFSTSAGGCTTTFVESTDTKLINETVCNNPDVKS
HSIVETTKISDTEFAMTMKSDVDAGGNKTTSINKIKQTFVGKTCSEASKGVKQ
>EECEBICD_03614 hypothetical protein
MNYPQILSPVLNFLHCPTPQAWIVQARDPQNLPLLLTDHLICELKAAQTALLLVRKYVAD
KSGADALLSWLQPYEAFAFRQGPEPDFVALHKQISKSAMPQTDDPWGRQLIDRMVLLIKE
ELHHFWQVREVMQARNIPYVKITASRYAKGMLKAVRTHEPLTLIDKLICGAYIEARSCER
FAALAPWLDEDLQTFYLSLLRSEARHYQDYLALAQQISAEDISARVRYFGEVEADLILSP
DREFRFHSGVPAAG
>EECEBICD_03615 Regulator of ribonuclease activity B
MANPELLEEQREETRLIIEELLEDGSDPDALYTIEHHLSADDFETLEKAAVEAFKLGYEV
TEPEELEVEEGDTVICCDILSECALNAELIDAQVEQLMNLAEKYDVEYDGWGTYFEDPNG

EEGDDDDYVDEDDDGVRH
>EECEBICD_03616 Ornithine carbamoyltransferase subunit I
MSTFYQKPFLKLLDFTASELTALLQLAAKLKADKKNGKEEQKLVGKNIALIFEKDSTRTR
CSFEVAAYDQGARVTYLGSSGSQIGHKESIKDTARVLGRMFDGIQYRGYGQEIVETLAEY
SGVPVWNGLTDEYHPTQLLADLLTMQEHLPGKAFNEMTLVYAGDARNNMGNSMLEAAALT
GLDLRLVAPKACWPQAALVAECSAMAKKNGGAITLTEDIASGVKGADFIYTDVWVSMGEP
KEKWAERIALLRDYQVNSQMMALTGNPQVKFLHCLPAFHDDETTLGKKMAEEYGLHGGME
VTDEVFESAASIVFDEAENRMHTIKAVMVATLSK
>EECEBICD_03617 Toxin-antitoxin biofilm protein TabA
MIIGNIEYLEAWLPTALRQAIEHVNAHVTATTAPGKYDIDGDRLFYMISENMTEPGESRS
AEYHARYLDIQIVLQGQEGMAFSTRPAGTPHTDWLADKDIAFLPTSVDEKTVVLNEGDFV
VFYPGEVHKPLCAVGEPARVRKAVVKMLMA
>EECEBICD_03618 Arginine deiminase
MEKHFVGSEIGQLRSVMLHRPNLSLKRLTPSNCQELLFDDVLSVERAGEEHDIFANTLRQ
QGIEVLLLTDLLTQTLDVADAKAWLLDTQISDYRLGPTFAADIRAWLADMPHRELARHLS
GGLTYGEIPASIKNMVVDTHDINDFIMKPLPNHLFTRDTSCWIYNGVSINPMAKPARQRE
TNNLRAIYRWHPQFAGGDFIKYFGDEDINYDHATLEGGDVLVIGRGAVLIGMSERTTPQG
VEFLAQALFKHRQAERVIAVELPKHRSCMHLDTVMTHIDIDTFSVYPEVVRPDVQCWTLT
PDGRGGLKRTQESTLVHALEKALGIDQVRLITTGGDAFEAEREQWNDANNVLTLRPGVVV
GYERNIWTNEKYDKAGITVLPIPGDELGRGRGGARCMSCPLERDGI
>EECEBICD_03619 Carbamate kinase 1
MENKRTLVVALGGNALLKRGEPLEADIQRKNIELAARTIAQLTRQWRVVLVHGNGPQVGL
LALQNSAYANVTPYPLDILGAESQGMIGYMLQQALKNHLPEREISVLLTQVEVDANDPAF
LNPTKYIGPIYDEAQARALQAEKGWVFKADGNAFRRVVPSPQPKRIVENDAIRALISRDH
LVICNGGGGVPVVEKADGYHGIEAVIDKDLSAALLASQIHADALLILTDADAVYLDWGKP
TQRPLAQVTPELLREMQFDAGSMGPKVTACAEFVSHCRGIAGIGSLADGQAILAGEKGTL
IRCETADVDA
>EECEBICD_03620 Ornithine carbamoyltransferase
MVISLKNRNFLKLLDYTPAEIQHLIDLAIELKAAKKAGCEKQTLIGKNIALIFEKTSTRT
RCAFEVAAFDQGAQVTYLGPSGSQIGHKESMKDTARVLGRMYDGIEYRGFGQHIVEELGE
YAGVPVWNGLTDEFHPTQILADLMTMLEHAPGKTLPELSFAYLGDARNNMGNSLMVGAAK
MGMDIRLIAPKSFWPDAALVAQCREIASVTGARITLTESVEDGVHGVDFLYTDVWVSMGE
PKEAWAERVSLMTPYQVNQQVVNATGNPDVKFMHCLPAFHNEHTKVGREIEMAYGLKGLE
VTEEVFESAGSIVFDEAENRMHTIKAVMVATLGD
>EECEBICD_03621 hypothetical protein
MGKFKFPTAYTILFILIALVAVMTWIVPAGKYQMAMNATLGKEVPVAGTYAPVDAHPQGI
TAVLLAPIDGLYNHETYTAGAIDVALFVLIIGGFLGVVNKTGAIDAGIERVTIKLNGKDE
WMIPILMALFAAGGTIYGMAEESLPFYTLLVPVMMAARFDPLVAAATVLLGAGIGTLGST
INPFATVIAANAAGIPFTQGMLMRVLLLIVGYVLCVFWVMRYARKVRAHPELSIVADKME
ENRAHFLGNRANTLLEFTATRKWVLLIFAASFAVMIYGVAVLGWWMAEISGVFLAAAIIV
GVITRMGEEAFTSTFIDGARDLLGVALIIGIARGIVVVMDNGMITHTILHSAESLVSGLS
TTIFINVTYWLEVLLSFLVPSSSGLAVLTMPIMAPLADFAHVQRDLVVTAYQSASGIVNL
VTPTSAVVMGGLAIARVPYVRYLKWVAPLLLILTLLNMAVLSIGAMM
>EECEBICD_03622 Arginine repressor
MKEYDDYSAKEQQQLAVCQRLISEKSYLSQEEIRRDLQNEGFEGISQSTVSRLLKLLGAI
KIRNTKGQKIYSVNPQRRPSPDAGRSIAEMVVSVEHNSEFILIHTAAGYGRAVARILDYH
ALPEILGVVAGSSIVWVAPRVVQRTALVHKQINYLLKMNLNS
>EECEBICD_03623 hypothetical protein
MVQCVRHSVLPRLKKDAGLPFFFPLKTNTKPLN
>EECEBICD_03624 Aspartate carbamoyltransferase catalytic subunit
MANPLYQKHIISINDLSRDDLNLVLATAAKLKANPQPELLKHKVIASCFFEASTRTRLSF
ETSMHRLGASVVGFSDSANTSLGKKGETLADTISVISTYVDAIVMRHPQEGAARLATEFS
GQVPVLNAGDGSNQHPTQTLLDLFTIQETQGRLDNLHIAMVGDLKYGRTVHSLTQALAKF
SGNRFYFIAPDALAMPQYILDMLDEKGMAWSLHGSIEEVMADVDILYMTRVQKERLDPSE

YANVKAQFVLRASDLNGARENMKVLHPLPRIDEITTDVDKTPHAWYFQQAGNGIFARQAL
LALVLNSELSL
>EECEBICD_03625 Aspartate carbamoyltransferase regulatory chain
MTHDNKLQVEAIKCGTVIDHIPAQVGFKLLSLFKLTETDQRITIGLNLPSGEMGRKDLIK
IENTFLTEEQVNQLALYAPQATVNRIDNYDVVGKSRPSLPERINNVLVCPNSNCISHAEP
VSSSFAVKKRANDIALKCKYCEKEFSHYVVLAN
>EECEBICD_03626 2-iminobutanoate/2-iminopropanoate deaminase
MSKTIATENAPAAIGPYVQGVDLGSMVITSGQIPVDPKTGAVAEDVSAQARQSLENVKAI
VEAAGLKVGDIVKTTVFVKDLNDFATVNATYEAFFTEHNATFPARSCVEVARLPKDVKIE
IEAIAVRR
>EECEBICD_03627 hypothetical protein
MVRARFTEEQIADFLQQSKNGVPNKALCEEYGFSNSTLRRWQEKHAESIRQELKQIESTA
KIVFLCFIVAAILLTLMFPKPTAALAIPPYLVYCISYIRRFRRISAKHIRRWDISSSRSG
LGAENVFYKLSWTFLFFMPAYSILQLLE
>EECEBICD_03628 Magnesium-transporting ATPase, P-type 1
MLKTITRQLFARLNRHLPYRLVHRDPLPGAQTAVNATIPPSLSERCLKVAAMEQETLWRV
FDTHPEGLNAAEVTRAREKHGENRLPAQKPSPWWVHLWVCYRNPFNILLTILGGISYATE
DLFAAGVIALMVGISTLLNFVQEARSTKAADALKAMVSNTATVLRVINENGENAWLELPI
DQLVPGDIIKLAAGDMIPADLRIIQARDLFVAQASLTGESLPVEKVAATREPRQNNPLEC
DTLCFMGTNVVSGTAQAVVMATGADTWFGQLAGRVSEQDNEQNAFQKGISRVSMLLIRFM
LVMAPVVLIINGYTKGDWWEAALFALSVAVGLTPEMLPMIVTSTLARGAVKLSKQKVIVK
HLDAIQNFGAMDILCTDKTGTLTQDKIVLENHTDISGKPSEHVLHCAWLNSHYQTGLKNL
LDTAVLEGVDETAARQLSGRWQKIDEIPFDFERRRMSVVVAEDSSVHQLVCKGALQEILN
VCTQVRHNGDIVPLDDNMLRRVKRVTDTLNRQGLRVVAVATKYLPAREGDYQRIDESDLI
LEGYIAFLDPPKETTAPALKALKASGITVKILTGDSELVAAKVCHEVGLDAGDVIIGSDI
EGLSDDALAALAARTTLFARLTPMHKERIVTLLKREGHVVGFMGDGINDAPALRAADIGI
SVDGAVDIAREAADIILLEKSLMVLEEGVIEGRRTFSNMLKYIKMTASSNFGNVFSVLVA
SAFLPFLPMLPLHLLIQNLLYDVSQVAIPFDNVDEEQIQKPQRWNPADLGRFMVFFGPIS
SIFDILTFCLMWWVFHANTPETQTLFQSGWFVVGLLSQTLIVHMIRTRRLPFIQSRAAWP
LMAMTLLVMVVGVSLPFSPLASYLQLQALPLSYFPWLIAILAGYMTLTQLVKGFYSRRYG
WQ
>EECEBICD_03629 HTH-type transcriptional regulator TreR
MQNRLTIKDIARLSGVGKSTVSRVLNNESGVSERTRERVEAVMNQHGFSPSRSARAMRGQ
SDKVVAIIVTRLDSLSENLAVQTMLPAFYEQGYDPIMMESQFSPTLVMEHLGMLRRRNID
GVVLFGFTGITEELIAPWKASLVLLARDAQGFASVCYDDEGAIHILMQRLYEQGHRNISF
LGVPHSDITTGKRRHDAYLAFCKKHKLHPVAALPGLAMKQGYEHTASVIMPDTTALVCAT
DTLALGASKYLQEQRIETLQLASVGNTPLIKFLHPEIVTVDPGYAEAGRQAASQLIEQIN
GRCDPRRIVIPSTLA
>EECEBICD_03630 PTS system trehalose-specific EIIBC component
MSKVKQADIDRLIDLVGGRDNIATVSRCITRLRFVLHQPANARPKKIEQLPMVKGCFTNA
GQFQVVIGTDVGDYYNALLETTGKAYADKEQAKKAARQNMKWHEQLISHFAEIFFPLLPA
LISGGLILGFRNVIGDVPMSNGQTLAQMHPALKTLYDFLWLIGEAIFFYLPVGICWSAVK
KMGGTPILGIVLGVTLVSPQLMNAYLLGQQTPDVWNFGLFSIEKVGYQAQVIPALLAGLA
LGFIETRLKRIVPDYLYLVVVPVCSLILAVFLAHTFIGPFGRMIGDGVAFAVRYLMTGSF
APIGAALFGFLYAPLVITGVHQTTLAIDMQMIQSMGGTPVWPLIALSNIAQASAVVGIII
SSRKHNEREISVPAAISAYLGVTEPAMYGINLKYRFPMLCAMIGSGLAGLLCGLNGVIAN
GIGVGGLPGILSIPPRYWQVYSMAMVIAIVIPVILTTFIYQRKHRQGTLQIV
>EECEBICD_03631 Trehalose-6-phosphate hydrolase
MTIPLWWQNGVIYQIYPKSFQDTTGSGTGDLRGVTQRLDYLQRLGVDAIWLTPFYISPQV
DNGYDVANYTAIDPTYGTLDDFDELVAQAKARGIRIILDMVFNHTSTQHAWFREALNKES
PYRQFYIWRDGTPDVCPNNWQSKFGGSAWRWHSQSEQYYLHLFAPEQADLNWENPAVRAE
LKKVCEFWADRGVDGLRLDVVNLIAKDQDFPDDPTGDGRRFYTDGPRAHTFLREMNRDVF
TPRNLMTVGEMSSTTLENCQQYAALSGDELSMTFNFHHLKVDYPNGEKWTLAKPDYVALK
ALFRHWQQGMHNVAWNALFWCNHDQPRIVSRFGDEGEYRVPAAKMLAMALHGMQGTPYIY

QGEEIGMTNPHFTRITDYRDVESHNMFAALRAAGRDPDELLAILASKSRDNSRTPMQWDN
GKNAGFTQGEPWISLCDNYTEVNVAAALRDENSVFYTYQKLIALRKTQPVLIWGDYQDLL
PDSPSVWCYRRQWQGQILLVVANLSNQCQEWHPPHIKGQWQALLHNYGEVTSQPAAMTLR
PFEAIWWLQR
>EECEBICD_03632 Anaerobic ribonucleoside-triphosphate reductase
MTPHVMKRDGCKVPFKSERIKEAILRAAKAAGVDDADYCATVAEVVSSQMNARSQVDINE
IQTAVENQLMSGPYKQLARAYIEYRHDRDIQREKRGRLNQEIRGLVEQTNSALLNENANK
DSKVIPTQRDLLAGIVAKHYARQHLLPRDVVQAHERGDIHYHDLDYSPFFPMFNCMLIDL
KGMLTQGFKMGNAEIEPPKSISTATAVTAQIIAQVASHIYGGTTINRIDEVLAPFVTESY
NKHRKTADEWQIPDAEGYARSRTEKECYDAFQSLEYEVNTLHTANGQTPFVTFGFGLGTS
WESRLIQASILRNRIAGLGKNRKTAVFPKLVFAIRDGLNHKFGDPNYDIKQLALECASKR
MYPDILNYDQVVKVTGSFKTPMGCRSFLGVWENENGEQIHDGRNNLGVISLNLPRIALEA
KGDETAFWKLLDERLALARKALMTRIARLEGVKARVAPILYMEGACGVRLKADDDVSEIF
KNGRASISLGYIGIHETINALFGGEHLYDSEQLRAKGIAIVERLRQAVDQWKDETGYGFS
LYSTPSENLCDRFCRLDTAEFGVVPGVTDKGYYTNSFHLDVEKKVNPYDKIDFEAPYPPL
ANGGFICYGEYPNIQHNLKALEDVWDYSYQHVPYYGTNTPIDECYECGFTGEFECTSKGF
TCPKCGNHDAARVSVTRRVCGYLGSPDARPFNAGKQEEVKRRVKHLGNGQIG
>EECEBICD_03633 Anaerobic ribonucleoside-triphosphate reductase-activating protein
MRYHQYYPVDIVNGPGTRCTLFVSGCVHECPGCYNKSTWRLNSGQPFTKEMEDKIIADLN
DTRIHRQGISLSGGDPLHPQNVPDILALVQRIHAECPGKDIWVWTGYKLDELNAAQMQVV
DLINVLVDGKFVQDLKDPALIWRGSSNQVVHHLR
>EECEBICD_03634 mRNA interferase toxin RelE
MTYELEFDPRALKEWHKLGDTVQAQLKKKLADVLLNPRIDSARLNDLPDCYKIKLKSSGY
RLVYQVRDDVVIVFVVAVGKREHSAVYHDANKRL
>EECEBICD_03635 Antitoxin RelB
MATLNVRLDDKLKNEAYAVLEKLNITPTEAVRLLFQYVAETGRMPVKTVTLSDSEDALIQ
TVRERLSSPQKGIKVSLDDL
>EECEBICD_03636 putative licABCH operon regulator
MRFPNQRLAQLFAMLQNETLPQDELAQRLSVSTRTVRADIAALNMLLTPHGAQFTLSRGN
GYQLKIDDPARYQSLQTQQSPALARGPRTSQERIHYLLVRFLTSAFSLKLEDLADEWFVS
RATLQNDMADVREHLLRYHLTLETRPRHGMKLFGGEMAIRACLTDLLWTLAQQEPSHPLI
VSTTLNTEVSQRLRSLLPDIFSHCQIRLTDEGELFLRLYCAVAVRRIREGYPLSECVAEE
VDEKVRHAAHEIAELLQQLADKPLSEPEVSWLKVHIAARQVQEIAPSAINADDEEALVRY
ILNFINTQYNYNLLNDKQLHADLLTHIKTMITRVRYQIMLPNPLLENIKQHYPMAWDMTL
AAISSWGKYTPYTISENEIGFLVLHIGVGLERSYNIGYQRQPQVLLVCDAGNAMVRMIEA
VLARKYPQIEIARTLTLRDYEARDSIVEDFVISTARIGEKDKPVIMIAPFPTDYQLEQIG
KLVLVDRTRPWMLDKYFDASHFRIVEGEIDQQTLFKTLCDQLHEEGFVDAAFLDSVIERE
AIVSTLLGDGIALPHALGLLAKKTVVYTVLAPQGIAWGDETAHVIFLLAISKSEYEEAMA
IYDIFVTFLRERAMTRLCACQNFTQFKTVAMECVSRF
>EECEBICD_03637 2-dehydro-3-deoxy-phosphogluconate aldolase
MKLTPNFYRDRVCLNVLAGSKDNAREIYAAAEGHVLVGVLSKNYPDVASAVADMREYAAL
IDNALSVGLGAGDPNQSAMVSEISRQVQPQHVNQVFTGVGASRALLGQNETVVNGLVSPT
GTPGRVKISTGPLSSRAPDGIVPIETAIALLKDMGGSSVKYFPMGGLTCRDEYKAVADAC
ARHDFWLEPTGGIDLENFAEILHIALDAGVSKIIPHIYSSIIDKVSGNTRADDVRQLLAI
VRSRVG
>EECEBICD_03638 D-glucosaminate-6-phosphate ammonia lyase
MPSIFEKYQLKQVINASGRMTALGVSTPRPEVIDAAMAGMNQYFEMKDLVNKTGEYIAKL
LEVEGATVVSCASAGLAQSVAAVLVQDSDWLLENLHVTPIENNEIVLPKGHNVNFGAPVG
TMVALGGGKLVEAGYANECSAAQLAAAITPRTAAILYIKSHHCVQKSMLNVEQAAVVART
HNLPLIVDAAAEEDLQCYYRVGADLVIYSGAKAIEGPTSGLVIGKTQYVEWVKRQSAGIG
RAMKVGKEGIVGLTCAIELYLRAQKESGAEMVEKMAPFIDTLNTFNGVSARVVWDSAGRD
IARTEIKFDEAVTGIATGELVDALKQGEYAIYFRGYKANEGIIEADVRSVDRAQLAIVAR
RIGEVINQEKQA

>EECEBICD_03639 Deacetylase
MFDLLLRHARLVDDTLTNIALQDGKIAVLGDVDGPAVKTIDLRGECYVSAGWIDSHVHCY
PTSPIYHDEPDSVGIATGVTTVVDAGSTGADDIDDFYTLTRQATTDVYALLNVSRVGLIA
QNELANMANIDADAVRQAVKRHPDFIVGLKARMSSSVVGVNGITPLERAKAMQQENGNLP
LMVHIGNNPPDLDEIAERLTAGDIITHCYNGKPNRILRPDGELRASVTRALARGVRLDVG
HGTASLSFAVAKRAISLGILPHTISSDIYCRNRINGPVHSLANVMSKFLAIGMSLPQVIA
CVTANAADSLNLKTKGRLQLGLDADLTLFTLKRQPTVLVDAENDSLQAEELLTPLAAIRA
GKGYMTEQGSAEHAFDF
>EECEBICD_03640 hypothetical protein
MEENKGFWYADWSFPIFVGLLSSGVFAGTHMYYLYGIGIAFNEVAFVAMLKAGMDTGVYGA
VAAFGASFLFARIIEGSLVGILDIGGAIQTGVGLGVPALLLGAGFVFPVANFIASLITGL
VIGLAIGYIIILARKFTINQSDSTYGADVMMGAGNTSGRFLGPLIILSAMTASIPIGLGS
LVGALLFYIWQKPITGGAILGAMILGSIFPIAIS
>EECEBICD_03641 hypothetical protein
MFLIIILIKSLIIGALVGVGVGAGAARMFHAPTTQGMGAFRTLGELNSCEGDPASHFSFGL
GFFFNAWASSVAAGSFTQDVDHRIIPNWGAAALMIKNRNVGETLHDPKKMAIACAVIGMI
VVTFLNLTASSVPEALQVTAVKVLVPAANLLVNIVMPVIFWLAAIDAGKKSGFWATVFGG
AAQLIMGNAVPGLVLGILIGKGVEESGWNHVTKVMMVAIVLLFVLSGFFRGFDMKMIESF
NMTVPNWLELIHNSLSGK
>EECEBICD_03642 hypothetical protein
MMKEQFTTTVRVKGKGDAKARAFADALNHVQSAVMRESPYILLRIEPQDVRIVQAHESVR
KEAFLFFFLRRERRTYSVELDVTVNVTAINLDRVDFVAKR
>EECEBICD_03643 hypothetical protein
MEQITVVIGDRLGKGQKVAAGVEKAGGRAVVVPGVAADMKLGDVMKAENATFGISFCGSG
GAGAITAQNKHGYKAKYGMRSVDEGVTAINEGCNVLGFGFMDKEELGERLVQAWQKKYGA
>EECEBICD_03644 hypothetical protein
MDNGAVKHDAGERINELAEQVLTQVDSLLGRHHIVPNAVQTQMLTSHVRAMAHRSITGEP
LPEVDASLFDEISAESMALAREIVAAFGNLPDEEAWLLSVHFEVAKDNL
>EECEBICD_03645 Soluble cytochrome b562
MRKSLLAILAVSSLVFGSAVFAADLEDNMDILNDNLKVVEKTDSAPELKAALTKMRAAAL
DAQKATPPKLEDKAPDSPEMKDFRHGFDILVGQIDGALKLANEGNVKEAKAAAEALKTTR
NTYHKKYR
>EECEBICD_03646 Metalloprotease PmbA
MAFAMKVISQVEAQRKILEEAVSTALELASGKSDGAEVAVSKTTGISVSTRYGEVENVEF
NSDGALGITVYHQNRKGSASSTDLSPQAIARTVQAALDIARYTSPDPCAGVADKELLAFD
APDLDLFHPAEVTPDEAIELAARAEQASLKADKRITNTEGGSFNSHYGIKVFGNSHGMLQ
GYCSTRHSLSSCVIAEENGDMERDYAYTIGRAMADLQAPEWVGAECARRTLSRLSPRKLS
TMKAPVIFANEVATGLFGHLVGAIAGGAVYRKSTFLLDSLGKQILPEWLTIEEHPHLLKG
LASSPFDSEGVRTERRDIVKDGVLMQWLLTNYSARKLGLKSTGHAGGIHNWRIAGQGLNF
EQLLKEMGTGLVVTELMGQGVSGITGDYSRGAAGFWVENGEIQYPVSEITIAGNLKEMWR
NIVTVGDDIETRSNIQCGSVLLPEMKIAGQ
>EECEBICD_03647 hypothetical protein
MTKQPEDWLDDVPGDDIEDEDDEIIWVSKSEIKRDAEELKRLGAELVDLGKNALDKIPLD
ADLRDAIELAQRIKMEGRRRQLQLIGKMLRQRDVEPIRQALDKLKNRHNQQVVLFHKLEH
LRDRLIVEGDDAVAEVLTLWPHADRQQLRSLIRNAKKEKEGNKPPKSARQIFQYLRELAE
NEG
>EECEBICD_03648 UDP-N-acetylmuramate--L-alanyl-gamma-D-glutamyl-meso-2,6-diaminoheptandioate ligase
MRIHILGICGTFMGGLAMLARSLGHEVTGSDANVYPPMSTLLEKQGIDLIQGYDASQLDP
QPDLVIIGNAMTRGNPCVEAVLEKNIPFMSGPQWLHDFVLRDRWVLAVAGTHGKTTTAGM
ATWILEACGYKPGFVIGGVPGNFEVSARLGESPFFVIEADEYDCAFFDKRSKFVHYCPRT
LILNNLEFDHADIFDDLKAIQKQFHHLVRIVPGQGRIIWPENDINLKQTMALGCWSEQEL
VGEQGHWQAKKLTTDASEWEVWLDGEKVGDVKWGLVGEHNMHNGLMAIAAARHVGVAPAE
AASALGSFINARRRLELRGEANGVTVYDDFAHHPTAILATLAALRGKVGGTARIIAVLEP

RSNTMKMGLCKDDLAPSLGRADEVFLLQPPHIPWQVSEVADACVQPAHWSGDVDTLAEMV
VKTAQPGDHILVMSNGGFGGIHQKLLDGLAKKALVVE
>EECEBICD_03649 Fructose-1,6-bisphosphatase class 1
MKTLGEFIVEKQHEFSQATGELTALLSAIKLGAKIIHRDINKAGLVDILGASGAENVQGE
VQQKLDLFANEKLKAALKARDIVAGIASEEEDEIVVFEGCEHAKYVVLMDPLDGSSNIDV
NVSVGTIFSIYRRVTPVGTPVTEEDFLQPGNKQVAAGYVVYGSSTMLVYTTGCGVHAFTY
DPSLGVFCLCQERMRFPEKGKTYSINEGNYIKFPNGVKKYIKFCQEEDSSTSRPYTSRYI
GSLVADFHRNLLKGGIYLYPSTASHPQGKLRLLYECNPMAFLAEQAGGKASDGKERILDI
IPESLHQRRSFFVGNRHMVDDVERFIREYPDA
>EECEBICD_03650 Inorganic pyrophosphatase
MSLLNVPAGKELPEDIYVVIEIPANADPIKYEVDKESGALFVDRFMSTAMFYPCNYGYIN
HTLSLDGDPVDVLVPTPYPLQPGAVIRCRPVGVLKMTDESGEDAKLVAVPHTKLSKEYDH
IKDVNDLPELLKAQITHFFEHYKDLEKGKWVKVDGWDNAEAAKAEIVASFERAAKK
>EECEBICD_03651 Deacetylase
MKNDILITGGHIIDPARNINEINNLRIINDIIVDADKYPVTSETRIIHADGMIVTPGLID
YHAHVFYDATEGGVRPDMYMPPNGVTTVVDAGSAGTANFDAFYRTVICASKVRIKAFLTV
SPPGQTWSQENYDPDNIDENKIHALFRQYRNVLQGLKLKVQTEDIAEYGLKPLTESLRIA
NDLRCPVAIHSTHPVVPMKELVSLLRRGDIIAHAFHGKGSTILTDEGAVLAEVRQARERG
VIFDAANGRSHFSMNTARRAIANGFLPDIISSDLSTITKLAWPVYALPWILSKYLALGVA
LTDVINACTHTPAVLMGMAAEIGTLAPGAFADIAIFKLKNRHVEFADIHGETLTGTHVLV
PQMTIKSGEILFRQIDFGARPNGVEK
>EECEBICD_03652 putative sulfoacetate transporter SauU
MTSIQKWLRIGATLMFGLFVAYLDRSNLSVTLPTITHDLNIDGATASIVLTIFLIGYAFS
NIFGGVFTQRYDPKKIVILMVLIWSIATVFVGFTSSVYVILICRLVLGITEGIYWPQQSR
FASDWFSDKERTQANSIIQYYGQFLALGLGFMILSPLDAAFGWRNVFIITGVIGIVVVVP
LYITMLKKQEEAPYYRAPAPTEKTKLTLESLGGTPFLLLIFTYITQGMLFWGITLWIPMV
VNSLGYTGFSKALVSSLPYLTAVILAIPISWISDKTQKRVLIASLGLLIPGVMLFLLPFV
DAPGFKITLITLAMGYYAASFTPNIWSIIQSNVKPHAIGPASGIINGIGAGGGGTLAGLM
VGYFYRTTGSYMQGFMVLGCIVILGGASLLIYGRIRAHHARR
>EECEBICD_03653 Gamma-glutamylcyclotransferase family protein YtfP
MRIFVYGSLRQKQGNSHWMTNAQLLGDYRIDNYQLYSLGHYPGAVPGNGAVYGEVYRIDN
ATLAELDALRTRGGEYARQLIQTPYGSAWMYVYQRPVDGLTLIESGDWLDRNQP
>EECEBICD_03654 Translocation and assembly module subunit TamB
MSLWKKISLGVLIFIVVLLASVAFLVGTTTGLHLVFSAANRWVPGLEIGQVTGGWRDLSL
KNIRYEQPGVAVNAGEIHLAVGLDCLWRSSLCVNDLALKDINVAIDSKKMPPSEPAQEEE
ESGPLNLSTPWPITLSRVALNNINIKIDDTTVSVLDFTSGLAWQEKNLTLKPTRLQGLLI
ALPKVADVAQEEVVEPKIEKPQPDEKPLGETLKDLFAKPVMPEMTDVHLPLNLNIESFRG
EQLRITGDTDLTVRTMLLKVSSIDGNMKLDTLDIDANQGTVKASGTAQLANNWPVDITLN
STLNIDPLKGEKIKLKVGGALREQLEVGVNLSGPMDVALRAQTRLAEAGLPLNLEVVSQR
IAWPLTGDTQFQADDLKLKLSGKMTDYTLSMRTAVKGQDIPPATITLDAKGNERQINLDK
LTVAALEGKTELKALVDWQQAISWRGELTLNGINTAKEIPDWPAKLNGVMKTKGSLYGGT
WQMDVPELKLTGNVKQNSVNVNGTLKGNSYMQWTIPGLHLALGPNSADIKGELGVKELNL
DAAIDAPGLDNALPGLGGMAKGIVKVRGTVEAPQLLADITARGLRWQELSVAQARIEGDI
KSTDQIAGHLNVRVERISQPDVNINLVTLDAKGSEKQHQLQLRVQGEPVSGQLSLTGSFD
REAARWKGTVSDTRFQTPVGPWSLTRAIALDYRNKEQKISIGPHCWLNPNAELCIPQTID
AGAAGRAVVNLNRFDLAMLKPFMPDTTQASGIFSGKADVSWDTTQEGLPQGKVTLSGRNV
KVTQTVNDAPLPVAFETLNLSADLHNNRAELGWLIRLTNNGQFDGLVQVTDPQGRRNLGG
NVNMRNVNLAMVNPVFSRGEKAAGMLNARLRLGGDVQSPQLFGQLQLSALDIDGNFMPFE
MQPSQLTMNFSGTRSTLAGIVRTQQGQINLNGNADWSQIDNWRARVTAKGSRVRITVPPM
VRLDVSPDVVFDATPSLFTLDGRVDVPWARIVVHDLPESAVGVSSDVVMLNNDLQPETPQ
TASIPINSNLTVHVGNNVRIDAFGLKARLTGDLKVAQDKQGLGLNGQINIPDGRFRAYGQ
DLLVRKGELLFSGPPDQPLLNIEAIRNPDATEDDVIAGVRVTGTADEPKAEIFSDPAMPQ
AEALSYLLRGQGLDSNQSDSAAMTSMLIGLGVAQSGQVVGKIGETFGVSNLALDTQGVGD
SSQVVVSGYVLPGLQVKYGVGIFDSLATLTLRYRLMPKLYLEAVSGVDQALDLLYQFEF

>EECEBICD_03655 Translocation and assembly module subunit TamA
MPHIRQLCWVSLLCLSSSAVAANVRLKVEGLSGELEKNVRAQLSTIQSDEVTPDRRFRAR
VDDAIREGLKALGYYEPTIKFDLLPPPAKGRQVLIARVTPGQPVLIGGTEVILRGGARTD
KDYLALLKTRPAIGTVLNQGDYDNFKKSLTSVSLRKGYFDSEFVKSQLGIALGCHQAFWD
IDYDSGERYRFGPVTFEGSQIRDEYLQNLLPFKEGDEYESKDLAELNRRLSATGWFNSVV
VAPEFEKSRKTKILPLKGVVSPRTENTIETGVGYSTDVGPRVKASWKKPWMNSYGHSLTT
STSISAPEQVLDFSYKMPLLKNPLEQYYLVQGGFKRTDLNDTEQDSTTLAVSRYWDLSSG
WQRAINLRWSFDHFTQGNVTNTTMLFYPGVMISRTRSRGGLMPTWGDSQRYSVDYSNTAW
GSDVDFSVLQAQNVWIRTLYDRHRFVMRANLGWIETGDFDKVPPDLRFFAGGDRSIRGYK
YKSISPKDSDGNLKGASKLATGSLEYQYNVTGKWWGAVFVDSGEAVSDIRRSDFKTGTGV
GVRWASPVGPIKLDFAVPVGDKDEHGLQFYIGLGPEL
>EECEBICD_03656 Peptide methionine sulfoxide reductase MsrA
MSLFDKKHLVTQADALPGRNTPMPIATLHAVNEHSMTNVPAGMEIAYFAMGCFWGVERLF
WQLPGVYSTAAGYAGGYTPNPTYREVCSGQTGHAEAVRIVYDPAVIRYEQLLQTFWENHD
PTQGMQQGNDHGTQYRSAIYPLTPEQNAAAHASRERFQSAMAAAGDHRPITTEIAHATPF
YYAEDEHQQYLHKNPYGYCGIGGIGVCLPPDA
>EECEBICD_03657 hypothetical protein
MLNSIFIIFCLIAVSAFFSISEISLAASRKIKLKLLADEGSINAQRVLKMQENPGMFFTV
VQIGLNAVAILGGIVGDAAFSPAFSALFSHYMSPELSEQLSFILSFSLVTGLFILFADLT
PKRIGMIAPEAVALRIINPMRFCLFVFRPLVWLFNGMANNIFRLFKIPMVRKDDITSDDI
YAVVEAGALAGVLRKQEHELIENVFELESRTVPSSMTSRESIIWFDLHEDEQSLKKKVAE
HPHSKFLVCNEDIDHIIGYVDSKDLLNRVLANQSMALNSGVQIRNTLIVPDTLTLSEALE
SFKTAGEDFAVIMNEYALVVGIITLNDVMTTLMGDLVGQGLEEQIVARDENSWLVDGGTP
IDDVMRVLDIDEFPQSGNYETIGGFMMFMLRKIPKRTDSVKFSGYKFEVVDIDNYRIDQL
LVTRLDNKSNVPAPKLPDAKDKEDSAA
>EECEBICD_03658 hypothetical protein
MKIFQRYNPLQVAKYVKILFRGRLYIKDVGAFEFDKGKILIPKVRDKQHLSVMSEVNRQV
MRLQTEMA
>EECEBICD_03659 putative protein YtfJ
MTLRKILAMSCLLLPIMACAHNLEDNQRVPPVGIADRGELTLNKDEFSYKNWNSAQLAGK
VRVLLHIAGRTSAKEKNATLIEAIKSAGLPHDRYQTTTIVNTDDAIPGSGMFVRSSIESN
KKLYPWSQFIVDSNGVARNAWQLKEEGSAVIVLDKDGRVQWVKDGALTQQEVQQVVDLLH
KLLSK
>EECEBICD_03660 3'(2'),5'-bisphosphate nucleotidase CysQ
MLEQVCQLARNAGDAIMQVYDGAKPMEYARKQDDSPVTAADIAAHTVILEGLRTLTPDIP
VLSEEDPPAWEVRQHWQRYWLVDPLDGTKEFIKRNGEFTVNIALIEQGKPVLGVVYAPVL
KVMYYAAEGKAWKEECGVRKQIQVRDARPPLVVISRSHTDDELTEYLQQLGEHQTTSIGS
SLKFCLVAEGQAQLYPRFGPTSVWDTAAGHAIAVAAGAHVHDWQGKTLDYTPRESFLNPG
FRVTIY
>EECEBICD_03661 2',3'-cyclic-nucleotide 2'-phosphodiesterase/3'-nucleotidase
MIKFSATLLATLIAASVNAATVDLRIMETTDLHSNMMDFDYYKDTATEKFGLVRTASLIH
AARNEVKNSVLVDNGDLIQGSPLGDYMAAKGLKDGDVHPVYKALNTLDYAVGNLGNHEFN
YGLDYLHNALAGAKFPYVNANIIDVKTQKPLFTPYLIKETSVIDKDGHPQTLKIGYIGFV
PPQIMIWDKANLSGKVTVNDITETARKYVPEMREKGADIVVVIAHSGLSADPYHSMAENS
VYYLSEVPGVDAIMFGHAHAVFPGKDFADIKGADIAKGTLNGIPAVMPGMWGDHLGVVDL
VLNNDSGKWQVTQAKAEARPIYDAAAKKSLAAEDSRLVGILKADHDATSEFVSKPIGKSA
DNMYSYLALVQDDPTVQVVNNAQKAYVEHFIQGDPDLAKLPVLSAAAPFKVGGRKNDPAS
FVEVEKGQLTFRNAADLYLYPNTLVVVKASGKEVKEWLECSAGQFNQIDIHSNKPQSLIN
WDGFRTYNFDVIDGVNYQIDVSQPARYDGECQMVNPQAERIKNLTFNGKPVDPNATFLVA
TNNYRAYGDKFAGTGDSHIAFASPDENRAVLAAWIGAESKRAGEIHPAADNNWRLAPIHS
DTTLDIRFETSPGDKAAAFIKEKGQYPMKKVAVDDIGFAIYQVDLSK
>EECEBICD_03662 hypothetical protein
MRAHTLSRQLREGNLFAEQCPSREVLKHVTSRWGVLILVALRDGTHRFSDLRRKMGGVSE

KMLAQSLQALEQDGFLNRVSYPVVPPHVEYSLTPLGEQVSDKVAALADWIELNLPQVLAQ
RERLSDGG
>EECEBICD_03663 Quinone oxidoreductase 2
MIAITGATGQLGQHVIENLLKTTPASHLVAIARNPKKAAPLSQRGIAVRQADYANEAALT
TALQGVDKLLLISSSEVGQRTAQHRNVIQAAIAAKVKFIAYTSLLHADKSPLALADEHIE
TEKMLAESGIPHTLLRNGWYTENYLASVPAALKHGVFIGAAGQGKIASAMRADYAAAAAR
VISEEGHAGNVYELAGDDAWTLRQLADELTHQSGKKIVYQNLSEVDFAAALKGAGLPDGL
ADMLANSDAGAAKGGLFDDSHTLRKLIGRPTTTLTESLRSVL
>EECEBICD_03664 Inner membrane protein YtfF
MISGVLYALLAGLMWGLIFVGPLIVPDYPALLQSMGRYLALGLIALPIAWLGRTRLRQLT
RKDWFTALSLTLMGNVIYYACLASAIQRTGAPVSTMIIGTLPVVIPLFANVLYSQRDGRL
SWRRLAPALALIGFGLLCVNIAELSQGLSDVSGWRYGSGIVLALISVACWAWYALRNARW
LRENPDKHPMMWATAQALVTLPVSLIGYAAVCLWLSIQKPLFTLPFGPHPALFIGLMIAI
AVLCSWVGTLCWNIASQKLPTVILGPLIVFETLAGLLYTFILRQEIPPFLTLSGIALLVA
GVVIAVRAKPEKATVVPVTER
>EECEBICD_03665 Iron-sulfur cluster repair protein YtfE
MAYRDQPLGELALSIPRASALFRQYDMDYCCGGKQTLARAAARHDVDIDIIEAQLAQLAE
QPIEKDWRAVPLADIIDHIVVRYHDRHREQLPELILQATKVERVHADKPNVPRGLTKYLT
ALHEELSSHMMKEEQILFPMIKQGMGRQATGPISVMESEHDEAGELVDVIKHVTQNVTPP
PEACTTWKAMYNGINEMIDDLMEHISLENNVLFPRALAGE
>EECEBICD_03666 hypothetical protein
MLRIKQFCLSILLMLAPVLALACSGIPYALFHYQPWGQGTKYVVEEDYDYGINFKDIEIR
EVKFSQLRHVPDRIVNEYNDRGKMSNDFVHVAAFSSLIKNDFWQFYWLTDGQHILWVGKI
VQNPPGKPPVDAATFRAYGRFAADKDSLYFDGERTDDNHGEKRVDIDSLQQVGGNIKLND
SGDVLKDRRNLYFQGRWLASAQGYSILGVKSWEQRNILFSPTSCDTTSNPGPWDTILRTA
TQVFVNGVAIDADPNSFHVVRWVAGVQLLYRDKAGVKRYYFGKDCLNTFNVQRDKVTWMT
REASRRGDDCRIATIPNVDPEYFHPIHYNWTVAQYKDLLYQVKYVSPVDRVLTITRLPDP
QVQLKAGANLVGKQIYFIGDGGVQIIDPIGPLVWFKNPDGSFSDVFAHDDRYLYFFGDTL
VDSGQRRNVTRTLDTAHIDKSGYYLVTEEGKYEVINNAFTSR
>EECEBICD_03667 hypothetical protein
MTIRRWILSATLLLLPVPAFADFQYQQDKDGVFYTADDEPQILSRLPDVSYSHLRRIADL
SHPQDPRPLIEINPDSHNCDDNHICQHAYLSDGRFILWAGKIVQNTGDEPAVDVASFQSF
GAFAADKHGLYFDGKRRDSNAGEKRVDMATLAETKIWNLLRDKNNLYYEGRWLGRADGFR
VLRLDSTSAREFIVTTAQRVIVNGIPITADANTFQIIRWMPGEVLIYRDKTGKHDYEIDN
SSRYCGYFNIGLREVTWLKHEATNAGSSCKVETLPGVDPEYFFRLNGNTGWYKDRIYQVS
TNALGEGVLRIFTSQEKLPALKIDRVTYNYYHLALSADGQLYRQISRDQWQRYNPILTEW
TTVSPAPTDVISLLPSDYH
>EECEBICD_03668 D-serine/D-alanine/glycine transporter
MVDQVKVAADEQAPAEQSLRRNLTNRHIQLIAIGGAIGTGLFMGSGKTISLAGPSIIFVY
MIIGFMLFFVMRAMGELLLSNLEYKSFSDFASDLLGPWAGYFTGWTYWFCWVVTGMADVV
AITAYAQFWFPGLSDWVASLAVVILLLSLNLATVKMFGEMEFWFAMVKIVAIVALIVVGL
VMIAMHFKSPTGVEASFAHLWNNGGWFPKGISGFFAGFQIAVFAFVGIELVGTTAAETKD
PEKSLPRAINSIPIRIIMFYVFALIVIMSVTPWSSVVPDKSPFVELFVLVGLPAAASVIN
FVVLTSAASSANSGVFSTSRMLFGLAQEGVAPKAFAKLSKRAVPAKGLTFSCICLLGGVV
MLMVNPSVIGAFTMITTVSAILFMFVWTIILCSYLVYRKKRPHLHEKSIYKMPLGKLMCW
VCMAFFAFVLVLLTLEDDTRQALIVTPLWFIALGLGWLFLRKNKLAK
>EECEBICD_03669 FKBP-type 22 kDa peptidyl-prolyl cis-trans isomerase
MATPTFDTIEAQASYGIGLQVGQQLSESGLEGLLPEALVAGIADALEGKHPAVPVDVVHR
ALREIHERADAVRRERFKAMAAEGVKYLEENREKDGVNSTESGLQFRVLTQGEGAIPART
DRVRVHYTGKLIDGTVFDSSVARGEPAEFPVNGVIAGWIEALTLMPVGSKWELTIPQELA
YGERGAGASIPPFSTLVFEVELLEIL
>EECEBICD_03670 hypothetical protein
MPGRFELKPTLAKIWHAPDNFRIMEPLPPMHRRGIIIAAIVLVIGFLLPASETSDSPVVT
REAQLDLQSQSQPPTEAQLQAQLVAPQNDPDQVAPVAPEPIQEGQPEEQNQPQTQPFQQD

SGIGQQWRSYRVESGKTLAQLFRDHGLPPTDVYAMAQVEGAGKPLSNLKNGQMIKIRQNA
SGVVTGLTIDGDNGQQVLFTRQPDGSFIRAQ
>EECEBICD_03671 hypothetical protein
MDTQRQASPFARKNVVYVCAAFCCLLWGSAYPAIKSGYDLFQIATDDIPSKIVFAGYRFL
FAGGLLLLFALLQRKPIGRFRPRQFAQLTLLGLTQTSLQYLFFYIGLAFTSGVKGSIMNA
TGTFFSVLLAHFIYQNDRLSYNKTLGCILGFAGVMVVNVSNGLDFSFNLPGEGSVVLAAF
ILSAATLYGKRLSQTVDPMVMTGYQLGIGGLVLVIGGYVFGGTLTIHGFSSVAILVYLTL
LSSVAFALWSILLKYNRVGMIAPFNFLIPFSGAALSAIFLGENILEWKYMIALVLVCSGI
WWVNKVKR
>EECEBICD_03672 50S ribosomal protein L9
MQVILLDKVANLGSLGDQVNVKAGYARNFLVPKGKAVPATKKNVEYFEARRAELEAKLAD
VLAAANARAEKINALETVTIASKAGDEGKLFGSIGTRDIADAVTAAGVDVAKSEVRLPNG
VLRTTGEHEVNFQVHSEVFAKVIINVVAE
>EECEBICD_03673 30S ribosomal protein S18
MARYFRRRKFCRFTAEGVQEIDYKDIATLKNYITESGKIVPSRITGTRAKYQRQLARAIK
RARYLSLLPYTDRHQ
>EECEBICD_03674 Primosomal replication protein N
MTNRLALSGTVCRAPLRKVSPSGIPHCQFVLEHRSVQEEAGFHRQAWCQMPVIVSGHENQ
AITHSITVGSRITVQGFISCHKAKNGLSKMVLHAEQIELIDSGD
>EECEBICD_03675 30S ribosomal protein S6
MRHYEIVFMVHPDQSEQVPGMIERYSAAITGAEGKIHRLEDWGRRQLAYPINKLHKAHYV
LMNVEAPQEVIDELETTFRFNDAVIRSMVMRTKHAVTEASPMVKAKDERRERRDDFANET
ADDAEAGDSEE
>EECEBICD_03676 hypothetical protein
MRARIMLFLAALLPGVTATAAIELNNHQARNMDDVRSLGVIYINHKFATESEANLALNDE
ADARNAMYYHVILIREPGSNGNIHASANIYR
>EECEBICD_03677 L-ribulose-5-phosphate 4-epimerase UlaF
MQKLKQQVFDANMDLPRYGLVTFTWGNVSAIDRERGLVVIKPSGVAYETMKVDDMVVVDM
DGKVVEGGYRPSSDTATHLALYQRYPSLGGVVHTHSTHATAWAQAGMAIPALGTTHADYF
FGDIPCTRALSEEEVQGEYELNTGKVIIETLGEVEPLHTPGIVVYQHGPFAWGKDAHDAV
HNAVVMEEVARMAWIARGINPGLNPIDDYLMNKHFMRKHGPNAYYGQK
>EECEBICD_03678 L-ribulose-5-phosphate 3-epimerase UlaE
MLSKQIPLGIYEKALPAGECWLERLRLAKTLGFDFVEMSVDETDARLARLDWSREQRLAL
VSAVAETGVRVPSMCLSAHRRFPLGSEDDAVRAQGLEIMRKAIQFAQDVGIRVIQLAGYD
VYYQQANDETRCRFRDGLKESVDMASRAQVTLAMEIMDYPLMNSISKALGYAHYLNNPWF
QLYPDIGNLSAWDNDVQMELQAGIGHIVAVHVKDTKPGVFKNVPFGEGVVDFERCFETLK
QSGYCGPYLIEMWSETAENPAAEVAKARDWVKARMASAGLVEAA
>EECEBICD_03679 3-keto-L-gulonate-6-phosphate decarboxylase UlaD
MSLPMLQVALDNQTMDSAYETTRLIAEEVDIIEVGTILCVGEGVRAVRDLKALYPHKIVL
ADAKIADAGKILSRMCFEANADWVTVICCADINTAKGALDVAKEFNGDVQIELTGYWTWE
QAQQWRDAGIQQVVYHRSRDAQAAGVAWGEADITAIKRLSDMGFKVTVTGGLALEDLPLF
KGIPIHVFIAGRSIRDAESPVEAARQFKRSIAQLWG
>EECEBICD_03680 Ascorbate-specific PTS system EIIA component
MKLRDSLAENNSIRLQAEANTWQEAVKIGVDLLVAADVVEPRYYQAILDGVEQFGPYFVI
APGLAMPHGRPEEGVKKTGFSLVTLKKPLEFNHEDNDPVDILITMAAVDANTHQEVGIMQ
IVNLFEDEANFDRLRACRTAQEVLDLIDRTNAAA
>EECEBICD_03681 Ascorbate-specific PTS system EIIB component
MTVRILAVCGNGQGSSMIMKMKVDQFLTQSNIDHTVNSCAVGEYKSELSGADIIIASTHI
AGEITVTGNKYVVGVRNMLSPADFGPKLLEVIKEHFPQDVK
>EECEBICD_03682 Ascorbate-specific PTS system EIIC component
MEILYNIFTIFFNQVMTNAPLLLGIVTCLGYILLRKSVSVIIKGTIKTIIGFMLLQAGSG
ILTSTFKPVVAKMSEVYGINGAISDTYASMMATIERMGDAYSWVGYAVLLALALNICYVL
LRRITGIRTIMLTGHIMFQQAGLIAVSFYIFGYSMWTTIICTAILVSLYWGITSNMMYKP
TQEVTDGCGFSIGHQQQFASWIAYKVAPFLGKKEESVEDLKLPGWLNIFHDNIVSTAIVM

TIFFGAILLSFGIDTVQAMAGKTHWTIYILQTGFSFAVAIFIITQGVRMFVAELSEAFNG
ISQRLIPGAVLAIDCAAIYSFAPNAVVWGFMWGTIGQLIAVGILVACGSSILIIPGFIPM
FFSNATIGVFANHFGGWRAALKICLVMGMIEIFGCVWAVKLTGMSAWMGMADWSILAPPM
MQGFFSLGIAFMAVIIVIALAYMFFAGRALRAEEDAEKQLAEQSA
>EECEBICD_03683 putative L-ascorbate-6-phosphate lactonase UlaG
MSKVQSITRESWILSTFPEWGSWLNEEIEQEQVAPGTFAMWWLGCTGIWLKSEGGTNVCV
DFWCGTGKQSHGNPLMKTGHQMQRMAGVKKLQPNLRTTPFVLDPFAIRQIDAVLATHDHN
DHIDVNVAAAVMQNCADDVPFIGPQTCVDLWVGWGVPKERCIVVKPGDVVKVKDIEIHAL
DAFDRTALITLPADQKAAGVLPDGMDVRAVNYLFKTPGGNLYHSGDSHYSNYYAKHGNEH
QIDVALGSYGENPRGITDKMTSADILRMAESLNTKVVIPFHHDIWSNFQADPQEIRVLWE
MKKDRLKYGFKPFIWQVGGKFTWPLDKDNFEYHYPRGFDDCFTIEPDLPFKSFL
>EECEBICD_03684 HTH-type transcriptional regulator UlaR
MTEAQRHQILLDMLAQLGFVTVENVIERLGISPATARRDINKLDESGKLKKVRNGAEAIT
QQRPRWTPMNLHQAQNHDEKVRIAKAASQLVNPGESVVINCGSTAFLLGREMCGKPVQII
TNYLPLANYLIDQEHDSVIIMGGQYNKSQSITLSPQGSENSLYAGHWMFTSGKGLTADGL
YKTDMLTAMAEQKMLSVVGKLVALVDSSKIGERAGMLFSRADQIAMLITGKNANPQVLQQ
LEAQGVSILRV
>EECEBICD_03685 hypothetical protein
MIAIETRQLAGGVVLHAFPEGKRAVPLPCVVFYHGFTSSSLVYSYFAVALAQAGFRVVMP
DAPEHGARFGGDSQGRIHRFWQILHQNMQEFTTLRAAIQAENWLLDGRLAVGGASMGGMT
ALGIMTRHREVKCGASLMGSGYFTGLARTLFPPLSPQNPAQQAEFDNIIAPLREWEVTHQ
LERLADRPLLLWHGQEDDVVPAIETFRLQQALAAAKLDKHVTCLWAAGVRHRITPEALSA
TVAFFRQHL
>EECEBICD_03686 Lipoprotein BsmA
MRRFASLIAALLLSACSVLQGTPQPAPPIADHPQEIRRDQTQGLQRMGTVSALVRGSPDD
AIDEIRAKAVAAKADYYVILMVDETVVTGQWYSQAILYRQ
>EECEBICD_03687 hypothetical protein
MKQELALSSLLLSAGLVSTTAQSAEFASADCVTGLNEIGLISVSNVAGNPQDVERIIALK
ADEQGASWYRIIQMYEEQQSDNWRVQAILYA
>EECEBICD_03688 Putative acyl-CoA dehydrogenase AidB
MSWQTHTVFNQPAPLNNSNLFLSDGALCEAVSREGAGWDSDLLASIGQQLGTAESLELGR
LANAHPPELLRYDPQGQRLDDVRFHPAWHLLMQGLCANRVHNLAWEEEARAGSFVARAAR
FVLHAQVEAGTLCPVTMTFAATPLLLQMLPTTFHDWLAPLRSDRYDSHLLPGGQKRGLLI
GMGMTEKQGGSDVLSNTTHAERLADDSYRLVGHKWFFSVPQSDAHLVLAQAKGGLSCFFV
PRFLPDGQRNSVRLERLKDKLGNRSNASAEVEFQDAVGWRLGEEGEGIRHILKMGGMTRL
DCALGSHGLMRRAFSVAIYHAHQRQAFGKPLIEQPLMRQTLSRMALCLEGQTALLFRLAR
AWEQRREAKEALWARLFTPAAKFAICKQGIPFVAEAMEVLGGMGYCEESELPRLYREMPV
NSIWEGSGNIMCLDVLRVLTKQHGVYDVLSEAFAEVKGQDRHYDRAVRQLQQRLRKPDEA
MGREITQQLFLLGCGAEMLRHASPPLAQAWCQMMLDTRGEMPLSAQVQNDLLLRATGGLR
>EECEBICD_03689 Putative acid--amine ligase YjfC
MLRHNVPIRRDLDKIACDHGFDFHVIDNEIYWDESRAYRFTLRQIEEQIEKPTAELHQMC
LEIVERAVRDEQIMQQLAIPPLYWDVIAESWRSRDPSLYGRMDFVWCGKDPVKLLEYNAD
TPTSLYESSYFQWLWLEDARRSGAIPRDADQYNAIQERLIARFSELYSREPLYFCCCEDT
DEDRTTVLYLQDCAQQAGQETRFIYIEELGLGVGGVLTDLDDNVIRRAFKLYPLEWMMRD
DNGPLLRKRREQWIEPLWKSILSNKGLLPLLWRFFPGHPNLLPAWFEGEKSLAAPGESSV
RKPIYSREGGNVTIFDERQNVIDHADGDYADEPMIYQAFQPLPRFGDSYTLIGSWIIDDE
ASGMGIREDNTLITKDTSRFVPHYIAG
>EECEBICD_03690 hypothetical protein
MAKKRKSRSTSGVGHSAIRRIAEPVNPFERQRNRYTPKCLTLAIMGGAAFFLLKGCGDGG
NTDNDGDGTFYATVNDCIDDGNSASVCADGWNNAKTEFYANVPKQLTQERCQTQFGDCYF
DITERSWVPVLSGFLLSRAIRQNRDEQYIYSSGGSSYVSRPVWRTTSGDYAWRSGTGKSD
TVTSPGYITRKASTVSRGGYGRSSSARGHWGG
>EECEBICD_03691 hypothetical protein
MHILDALLAFCAYFFIGAAMVIVFLFIYSKITPHNEWQLIKNNNTAASLAFSGTLLGYVI

PLSSAAINSVSIPDYFAWGGIALVIQLLIYGCVRLYMPTLSEKIIHHNVAAGLFMGTAAL
AGGIFNAACMTW
>EECEBICD_03692 hypothetical protein
MMQFFQRLLGKTSAPAPIRGPLGLHLNAGFTLDTLAFRLLESSLLVALPGEKYTVAAASR
IDLGGGSQIFRYYTSGDEFLQINTTGGTDVDDIDDIKLFVYEESFGINEERHWRSAIAPA
AIGPMTLNWQERRWQRFFNHEEPGNIEPVYMLEKVENQQAEKWDVHNFTMGFQRQVTDDA
WEYLLLNGEESFNERGEPEWVFSRALGVDIPLTSLTVIG
>EECEBICD_03693 hypothetical protein
MLFTWIVKTCQRHLSRLTWPALLGLFIGQYLLCYLVLRLLRESALVSQLSDFIYYCSVVG
STLGFGDLSPQTAPGRLFTALWQIPVSVGLFGALMGKVIAQVQGMLAKGITGMGDYRHLH
HHMLVIGWRGHQTEKMISLLLYDKRRAFERVLLCELEDIQHPLPGNNWVDFIRISNFNDP
QEHQRMGLEQCQSVIIFARTDEQTFTAALSLADYVPAQCHIVVYLEDERYAGLLETHCPR
MEIVRNLSAEQLSRSIQDPGSSQSVASIMNPMLGDTGFALPVPASVAPMRYGALMRYMKL
HHDATVLGISHCKNGRGMELNPVISTEVRGGMWLHLVGNNRILAEEVAWQEIEVPL
>EECEBICD_03694 hypothetical protein
MGILKSLFTLGKSFVAQAEDAIDEAQGVRMLEQHIRDAKAELDKAGKSRVDLLARVKLSH
DKLNDLRERKASLETRALAAMSKNVDAALLNEVAEEIARLENAILAEEQVLTNLEASRDA
VEKAVTATGQRIAQFEQQLEVVKATEAMQRAQQAVTTSTVGAASNVSTAAESLKRLQTRQ
AERQARLDAAAQLEKVADGRDLDEKLAQAGIGATNKSNAQDVLARLQRQQGE
>EECEBICD_03695 hypothetical protein
MAWTPRTLADALNNIAELDIDIENNESSLIIKMNDYGDLPLAVLFTSQQIVIETYICPVN
TIRDTAEFNLFLLRNQKVLPLSSVGITQVKQEEYYVAFGALSLNSSLADVTLEITTLVEN
ALDIAEITQVYSQE
>EECEBICD_03696 23S rRNA (guanosine-2'-O-)-methyltransferase RlmB
MSEMIYGIHAVQALLERAPERFQEVFILKGREDKRLLPLIHALESQGVVIQLANRQYLDE
KSDGAVHQGIIARVKPGRQYQENDLPDLIALHERPFLLILDGVTDPHNLGACLRSADAAG
VHAVIVPKDRSAQLNATAKKVACGAAESVPLIRVTNLARTMRMLQEENIWIVGTAGEADH
TLYQSKMPGRMALVMGAEGEGMRRLTREHCDELISIPMAGSVSSLNVSVATGICLFEAVR
QRT
>EECEBICD_03697 Ribonuclease R
MSQDPFQEREAEKYANPIPSREFILEHLTKREKPASREELAVELNIEGEEQLEALRRRLR
AMERDGQLVFTRRQCYALPERLDLLKGTVIGHRDGYGFLRVEGRKDDLYLSSEQMKTCIH
GDQVLAQPLGADRKGRREARIVRVLVPKTSQIVGRYFTDAGVGFVVPDDSRLSFDILIPP
EDVMGARMGFVVVVELTQRPTRRTKAVGKIVEVLGDNMGTGMAVDMALRTHEIPYIWPQA
VEQQVAGLKEEVPEEAKVGRVDLRDLPLVTIDGEDARDFDDAVYCEKKRGGGWRLWVAIA
DVSYYVRPPTPLDREARNRGTSVYFPSQVVPMLPEVLSNGLCSLNPQVDRLCMVCEMTIS
AKGRLTGYKFYEAVMSSHARLTYTKVWHMLQGDQDLREQYAPLVKHIEELHNLYKVLDKA
REERGGISFESEEAKFIFNAERRIERIEQTQRNDAHKLIEECMIMANISAARFVEKAKEP
ALFRIHDKPTTEAITSFRSVLAELGLELPGGNKPEPRDYAELLESIADRPDAEMLQTMLL
RSMKQAIYDPENRGHFGLALQSYAHFTSPIRRYPDLSLHRAIKYLLAKEQGNKGNTTETG
GYHYSMEEMLQLGQHCSMAERRADEATRDVSDWLKCDFMLDQVGNVFKGVIASVTGFGFF
VRLDELFIDGLVHVSSLDNDYYRFDQVGQRLIGESGGQTYRLGDRVEVRVEAVNMDERKI
DFSLISSERAPRNVGKTAREKAKKGESKNAGKRRQVGKKVNFEPDSAFRGEKKAKPKAAK
KDARKAKKPSAKTQKIAAATKAKRAAKKKAAE
>EECEBICD_03698 HTH-type transcriptional repressor NsrR
MQLTSFTDYGLRALIYMASLPDGRMTSISEVTEVYGVSRNHMVKIINQLSRAGFVTAVRG
KNGGIRLGKPANTICIGDVVRELEPLSLVNCSSEFCHITPACRLKQALSKAVQSFLKELD
NYTLADLVEENQPLYKLLLVE
>EECEBICD_03699 Adenylosuccinate synthetase
MGNNVVVLGTQWGDEGKGKIVDLLTERAKYVVRYQGGHNAGHTLVINGEKTVLHLIPSGI
LRENVTSIIGNGVVLSPSALMKEMKELEDRGIPVRERLLLSEACPLILDYHVALDNAREK
ARGAKAIGTTGRGIGPAYEDKVARRGLRVGDLFDKETFAEKLKEVMEYHNFQLVNYYKVE
AVDYQKVLDDTMAVADILTSMVVDVSDLLDQARQRGDFVMFEGAQGTLLDIDHGTYPYVT
SSNTTAGGVATGSGLGPRYVDYVLGILKAYSTRVGAGPFPTELFDETGEFLCKQGNEYGA

TTGRRRRTGWLDTVAVRRAVQLNSLSGFCLTKLDVLDGLKEVKLCVAYRMPDGREVTTTP
LAADDWKGVEPIYETMPGWSESTFGVKDRSGLPQAALNYIKRIEELTGVPIDIISTGPDR
TETMILRDPFDA
>EECEBICD_03700 hypothetical protein
MNSTIWLALALVLVLEGLGPMLYPGAWKKMVSALAQLPENVLRRFGGGLVVAGVVVYYML
RKTIG
>EECEBICD_03701 Modulator of FtsH protease HflC
MRKSVIAIIIIVLVVLYMSVFVVKEGERGITLRFGKVLRDDENKPLVYAPGLHFKIPFIE
SVKMLDARIQTMDNQADRFVTKEKKDLIVDSYIKWRISDFSRYYLATGGGDISQAEVLLK
RKFSDRLRSEIGRLDVKDIVTDSRGRLTLEVRDALNSGSAGTDDEVATPAADDAIAEAAE
RVTAETKGKVPVINPNSMAALGIEVVDVRIKQINLPTEVSEAIYNRMRAEREAVARRHRS
QGQEEAEKLRAAADYEVTKTLAEAERQGRIMRGEGDAEAAKLFADAFSQDPDFYAFIRSL
RAYEKSFEGNQDVMVLSPDSDFFRYMKTPSSTTR
>EECEBICD_03702 Modulator of FtsH protease HflK
MAWNQPGNNGQDRDPWGSSKPGSNSGGNGNKGGRDQGPPDLDDIFRKLSKKLGGFGGGKG
TGSGGGSSSQGPRPQLGGRIVAIAAAAVVIIWAASGFYTIKEAERGVVTRFGKFSHLVEP
GLNWKPTFIDDVTPVNVEAVRELAASGVMLTSDENVVRVEMNVQYRVTDPQKYLFSVTSP
DDSLRQATDSALRGVIGKYTMDRILTEGRTVIRSDTQRELEETIKPYNMGITLLDVNFQA
ARPPEEVKAAFDDAIAARENEQQYIREAEAYTNEVQPRANGQAQRILEEARAYKTQTILE
AQGEVARFAKILPEYKAAPQITRERLYIETMEKVLSHTRKVLVNDKSGNLMVLPLDQMLK
GGNAPAAKSDNSASNLLRLPPSTKSNASGASNTSSSNQGDIMDQRRANAQRNDYQRQGE
>EECEBICD_03703 GTPase HflX
MFDRYDAGEQAVLVHIYFSQDKDMEDLQEFESLVSSAGVEAMQVITGSRKAPHPKYFVGE
GKAVEIAEAVKATGAAVVLFDHALSPAQERNLERLCECRVIDRTGLILDIFAQRARTHEG
KLQVELAQLRHLATRLVRGWTHLERQKGGIGLRGPGETQLETDRRLLRNRIVQIQSRLEK
VEKQREQGRQSRIKADVPTVSLVGYTNAGKSTLFNQITEARVYAADQLFATLDPTLRRID
VADVGETVLADTVGFIRHLPHDLVAAFKATLQETRQATLLLHVVDAADVRVQENIEAVNT
VLEEIDAHEIPTLMVMNKIDMLDDFEPRIDRDEENKPIRVWLSAQSGVGIPQLFQALTER
LSGEVAQHTLRLPPQEGRLRSRFYQLQAIEKEWMEEDGSVSLQVRMPIVDWRRLCKQEPA
LIEYVI
>EECEBICD_03704 RNA-binding protein Hfq
MAKGQSLQDPFLNALRRERVPVSIYLVNGIKLQGQIESFDQFVILLKNTVSQMVYKHAIS
TVVPSRPVSHHSNNAGGGASNNYHHGSNAQGSTAQQDSEETE
>EECEBICD_03705 tRNA dimethylallyltransferase
MNDVSKASLPKAIFLMGPTASGKTALAIELRKVLPVELISVDSALIYRGMDIGTAKPNAD
ELKAAPHRLLDIRDPSQAYSAADFRRDALAQMAEITSAGRIPLLVGGTMLYFKALLEGLS
PLPSADPEVRSRIEQQAAELGWEALHQQLQEIDPVAAARIHPNDPQRLSRALEVFFISGK
TLTELTQTSGDALPYQVHQFAIAPASRELLHQRIELRFHQMLASGFEAEVRALFARGDLH
TDLPSIRCVGYRQMWSYIEGEISYDEMVYRGVCATRQLAKRQMTWLRGWEGVRWLDSENP
DRARKEVLQVVGAIAD
>EECEBICD_03706 DNA mismatch repair protein MutL
MPIQVLPPQLANQIAAGEVVERPASVVKELVENSLDAGATRVDIDIERGGAKLIRIRDNG
CGIKKEELALALARHATSKIASLDDLEAIISLGFRGEALASISSVSRLTLTSRTAEQAEA
WQAYAEGRDMDVTVKPAAHPVGTTLEVLDLFYNTPARRKFMRTEKTEFNHIDEIIRRIAL
ARFDVTLNLSHNGKLVRQYRAVAKDGQKERRLGAICGTPFLEQALAIEWQHGDLTLRGWV
ADPNHTTTALTEIQYCYVNGRMMRDRLINHAIRQACEDKLGADQQPAFVLYLEIDPHQVD
VNVHPAKHEVRFHQSRLVHDFIYQGVLSVLQQQTETTLPLEEIAPAPRHVPENRIAAGRN
HFAVPAEPTAAREPATPRYSGGASGGNGGRQSAGGWPHAQPGYQKQQGEVYRALLQTPAT
SPAPEPVAPALDGHSQSFGRVLTIVGGDCALLEHAGTIQLLSLPVAERWLRQAQLTPGQS
PVCAQPLLIPLRLKVSADEKAALQKAQSLLGELGIEFQSDAQHVTIRAVPLPLRQQNLQI
LIPELIGYLAQQTTFATVNIAQWIARNVQSEHPQWSMAQAISLLADVERLCPQLVKAPPG
GLLQPVDLHSAMNALKHE
>EECEBICD_03707 N-acetylmuramoyl-L-alanine amidase AmiB
MIYRIKNAVIAVLILLCAQAGAASLSDIQVSNGEQQARITLSFIGEPEYAYSQDGKRTVA

LDIRQTGVIQGLPLQFSGNNLVKTIRAGTPKDAQSLRLLVDLTENGKTEAVKRQNGGNYT
VIFTINADVPPPPPPVVAKRVESAPRPTEPARNPFKSSDDRLTGVTSSNTVTRPAARASA
GAGDKVVIAIDAGHGGQDPGAIGPGGTREKNVTIAIARKLRTLLNNDPMFKGVLTRDGDY
FISVMGRSDVARKQNANFLVSIHADAAPNRDATGASVWVLSNRRANSEMANWLEQHEKQS
ELLGGAGDVLANSQSDPYLSQAVLDLQFGHSQRVGYDVATNVLSQLDGVGSLHKRRPEHA
SLGVLRSPDIPSILVETGFISNHGEERLLASDRYQQQIADAIYRGLRKYFAAHPIQSAPQ
GGPGQTASTNQPGTITAAN
>EECEBICD_03708 tRNA threonylcarbamoyladenosine biosynthesis protein TsaE
MMNRVIPLPDEQATLDLGQRVANACDGATVIYLYGDLGAGKTTFSRGFLQALGHNGNVKS
PTYTLVEPYALDNMMVYHFDLYRLADPEELEFMGIRDYFANDAICLVEWPQQGKGVLPDP
DVEIHIDYQAQGREARVSAVSSSGHSLLARLAD
>EECEBICD_03709 Bifunctional NAD(P)H-hydrate repair enzyme Nnr
MDHNMKKNPVSIPHSIWLADDIKRLERDAADAFGLTLYELMLRAGDAAFRVARDSYPDTR
HWLVLCGHGNNGGDGYVVARLAQAAGISVTLLAQESDKPLPEEAAQARDAWLNAGGIIHA
ADIIWPEATDLIIDALLGTGIAQAPRDPVAGLIEQANAHPAPVVAVDIPSGLLAQTGATP
GAVISAAHTVTFIALKPGLLTGKARDVTGILHYDALGLEGWLASQTPPLRRFDATQLGQW
LTPRRPTSHKGDHGRLAIIGGDQGTAGAIRMAGEAALRTGAGLVRVLTRGENIAPLLTAR
PELMVHELTPQSLEESLTWADVVVIGPGLGQQEWGKKALQKVENVRKPMLWDADALNLLA
INPDKRHNRVITPHPGEAARLLGCSVAEIESDRLLSAQRLVKRYGGVVVLKGAGTIIAAE
HHPLAIIDAGNAGMASGGMGDVLSGIIGALLGQKFTPYDAACVGCVAHGAAADLLAARYG
ARGMLATDLFTTLRRIVNPDVIDVNHDESSNSAT
>EECEBICD_03710 Epoxyqueuosine reductase
MSKPLDLNQLAQNIKQWGLELGFQQVGITDTDLRASEPALQAWLDKQYHGEMAWMARHGM
MRARPHELLPGTLRVISVRMNYLPANAAFASTLKDPTLGYVSRYALGRDYHKLLRSRLKK
LGEQIQQYCGSLNFRPFVDSAPILERPLAEKAGLGWTGKHSLILNREAGSFFFLGELLID
LPLPVDQPVEEGCGKCVACMTICPTGAIVEPYTVDARRCISYLTIELEGAIPEAFRPLIG
NRIYGCDDCQLICPWNRYSQLTDEADFSPRKALHNPDLVELFSWSEAQFLKVTEGTAIRR
IGHLRWLRNVAVALGNAPWSNAVITALESRKGEHPLLDEHIEWAIAQQIEKRNACIIEVQ
LPKKQRLVRVIEKGLVRDA
>EECEBICD_03714 ABC transporter arginine-binding protein 1
MLLASLSVHAASVSARTLHFGTSATYAPYEFVDADNKIVGFDIDVANAVCKEMQAECSFT
NQSFDSLIPSLRFKKFDAVIAGMDMTPKREQQVSFSQPYYEGLSAVVVTRKGAYHTFADL
KGKKVGLENGTTHQRYLQDKQQAITPVAYDSYLNAFTDLKNNRLEGVFGDVAAIGKWLKN
NPDYAIMDERASDPDYYGKGLGIAVRKGNDALLQEINAALDKVKASPEYAQMQEKWFTQ
>EECEBICD_03715 Oligoribonuclease
MSADENNLIWIDLEMTGLDPERDRIIEIATLVTDASLNILAEGPTIAVHQSDAQLALMDD
WNVRTHTGSGLVDRVKASTMGERDAELATIEFLKTWVPAGKSPICGNSIGQDRRFLFKYM
PELEAYFHYRYLDVSTLKELARRWKPEILAGFTKQGTHQAMDDIRESVAELAYYREHFIK
L
>EECEBICD_03716 Small ribosomal subunit biogenesis GTPase RsgA
MSKNKLSKGQQRRVNANHQRRLKTSAEKADYDDNLFGEPAEGIVISRLGMHADVESADGE
VHRCNIRRTIRSLVTGDRVVWRPGKAAAEGVNVKGIVEAVHERTSVLTRPDFYDGVKPIA
ANIDQIVIVSAILPELSLNIIDRYLVGCETLQVEPLIVLNKIDLLDDEGMDFVNEQMDIY
RNIGYRVLMVSSHTQDGLKPLEEALTGRISIFAGQSGVGKSSLLNALLGLQNEILTNDVS
NVSGLGQHTTTAARLYHFPHGGDVIDSPGVREFGLWHLEPEQITQGFVEFHDYLGHCKYR
DCKHDADPGCAIREAVENGAIAETRFENYHRILESMAQVKTRKNFSDTDD
>EECEBICD_03717 Phosphatidylserine decarboxylase proenzyme
MLNSFKLSLQYILPKLWLTRLAGWGASKRAGWLTKLVIDLFVKYYKVDMTEAQKPDTASY
RTFNDFFVRPLRDDVRPLNTDPNILVMPADGVISQLGRIEEDKILQAKGHNYSLEALLAG
NYLMADKFRNGTFVTTYLSPRDYHRVHMPCNGILREMIYVPGDLFSVNHLTAQNVPNLFA
RNERVICLFDTEFGPMAQILVGATIVGSIETVWAGTITPPREGIIKRWTWPEGEHEGSVA
LLKGQEMGRFKLGSTVINLFAPGKVNLIASLASLSVTKIGQPLATSTETFVAPEVEPAPL
PAEEIKAEHDASPLVDNKKDDT
>EECEBICD_03718 Miniconductance mechanosensitive channel MscM

MRLIIAFLMAWCLSTGAFAATAPDAKQITQELEQAKAAKPAQPEAVEALQTALNALEERK
GSLERAKQYQHVIDNFPKLSATLRAQLNNLRDEPRSVPPEMSTEALNQEILQVSSQLLDK
TREAQQEQERVREIADSLSQLPQQQNDARRQLNEIERRLGAAGGSAALSQAQSLSMQAES
AKLKALVDELELAQLSANNRQELARLRSELAEKQSQQLDAYLQALRNQLNSLRQREAERA
LESTELLAENSAGLPEGIVEQFKVNRELSQALNQQAQRMDLVASQQRQATSQTLQVRQAL
NTLREQSQWLGVSNMLGEALRAQVARLPEMPKPQQLDTEMAQLRVHRMRYEELLNKQPQL
RQIRQANGQPLTAEQNQILDAQLRTQRELLNSLLQGGDTLILELTKLKVSNSQLEDALKE
VNEATHRYLFWTADVSPLSLSWPVDLVQDLRRLISLDTFNQLGKASIMMLTSKETLLPLF
GALALVGFSLYSRQHFNRFLERSASRVGKVTQDHFSLTLRTVFWSILVASPLPVLWATLG
YGLQEAWPYPLAVAIGDGVTATVPLLWVVMICAAFARPNGLFVAHFGWPRNRVAKAMRYY
LMSIGLIVPLIMAVIMFDNLNDREFSGSLGRLCFILICGALALVTLSLKKAGIPLYLDKE
GNGDNMVNSLLWNMLMGAPLIAILAAAVGYLATAQALLARLETSVAIWFLLLVIYHVIRR
WMLIQRRRLAFDRAKHRRAEMLAQRARGEEEPAHSSSLEGAVDIDESEIDLDAISAQSLR
LVRSILMLIALLSVIVLWSEIHSAFGFLENISLWDVTSTVQGVESLEPITLGAVLIAILV
FIITTQLVRNLPALLELALLQHLDLTPGTGYAITTITKYLLMLIGGLVGFSMIGIEWSKL
QWLVAALGVGLGFGLQEIFANFISGLIILFEKPIRIGDTVTIRDLTGSVTKINTRATTIS
DWDRKEIIVPNKAFITEQFINWSLSDSVTRVVLTIPAPADANSEEVTQILLTAAQRCSLV
LDNPPPEIFLVDLQQGIQIFELRIYAAEMGHRMPLRHEIHQLILAGFREHGIDMPFPPFQ
MRLESLGGKQTGRTLTSAGKTSRPAGSL
>EECEBICD_03719 Inner membrane protein YjeO
MSPKMFALCAIWLLLAIPLIAVFSVLDKEWMIGESGINNICDVMRTVENDDSRGFGAMMT
LPLFFPFFYVTVYKKIRSWFLYCVALVIFAYWSWQFFLRYQFCV
>EECEBICD_03720 Inner membrane transporter YjeM
MTHTIKKMSLIGLILMIFTSVFGFANSPSAFYLMGYSAIPWYIFSALLFFIPFALMMAEM
GSAYRKEEGGIYSWMNNSVGPRYAFIGTFMWFSSYVIWMVSTAAKIWVPFSTFVFGADMT
QHWRIAGLEPTQVVGLLAVGWMILVTCVAARGINKIARITAVGGIAVMCLNLVLLLVSVA
ILLLNGGHFAQEINFTSSPNPGYHSGLAMLSFVVFAIFAYGGIEAVGGLVDKTEKPEKNF
AKGIVFAAIVISIGYSLAIFLWGVSTNWQQILSNSAVNLGNITYILMSSLGTTLGNALNL
SPDAAMTVGVWFARITGLSMFLAYTGAFFTLSYSPLKAIIQGTPKALWPAPMTTLNANGM
PATAMWLQCVLVSLFILLVSFGGDTASAFYNKLTLMANVSMTLPYLFLALAFPFFKARQD
LERPFVLFKTKASTLVATGVVVLVVTFANVFTIIQPVIEAGDWDSALWMIGGPIFFSLLA
MAIYQNYSSRMSADPEWAAE
>EECEBICD_03721 Elongation factor P--(R)-beta-lysine ligase
MSETATWQPSASIPNLLKRAAIMAEIRRFFADRGVLEVETPCMSQATVTDIHLFPFETRF
VGPGHSQGINLYLMTSPEYHMKRLLAAGCGPVFQLCRSFRNEEMGRHHNPEFTMLEWYRP
HYDMYRLMNEVDDLLQQVLDCQPAESLSYQQAFQRHLEIDPLSADKTQLREAAAKLDLSN
IADTEEDRDTLLQLLFTMGVEPHIGKEKPTFIYHFPASQASLAQISTEDHRVAERFEVYY
KGIELANGFHELTDAREQQQRFEQDNRKRAARGLPQQPIDQNLLDALAAGLPDCSGVALG
VDRLVMLALGAESLADVIAFTVDRA
>EECEBICD_03722 Fumarate reductase flavoprotein subunit
MQTFQADLAIIGAGGAGLRAAIAAAQANPNAKIALISKVYPMRSHTVAAEGGSAAVAQDH
DSFDYHFHDTVAGGDWLCEQDVVDYFVHHCPTEMTQLEQWGCPWSRRPDGSVNVRRFGGM
KIERTWFAADKTGFHMLHTLFQTSLQFPQIQRFDEHFVLDILVDDNHARGLVAMNMMEGT
LVQIRANAVVMATGGAGRVYRYNTNGGIVTGDGMGMALSHGVPLRDMEFVQYHPTGLPGS
GILMTEGCRGEGGILVNKNGYRYLQDYGMGPETPLGEPKNKYMELGPRDKVSQAFWHEWR
KGNTISTPRGDVVHLDLRHLGEKKLHERLPFICELAKAYVGVDPVKEPIPVRPTAHYTMG
GIETDQNCESRVKGLFAVGECSSVGLHGANRLGSNSLAELVVFGRLAGEQAMERAATAGA
ANSAALDAQVADIEQRLKNLVNQEGNENWSKIRDEMGLSMEEGCGIYRTPELMQKTVDKL
AELQERFKRVRISDTSSVFNTDLLYTIELGHGLNVAECMAHSALARKESRGAHQRLDEGC
TERDDVNFLKHTLAFRDADGTTRLEYSDVKITTLPPAKRVYGGEAEAADKKEKANG
>EECEBICD_03723 Fumarate reductase iron-sulfur subunit
MAEMKNLKVEVVRYNPETDTAPHSAFYEVPYDETTSLLDALGYIKDNLSPDLSYRWSCRM
AICGSCGMMVNNVPKLACKTFLRDYTNGMKVEALANFPIERDLVVDMTHFIESLEAIKPY
IIGNSRTPDQGPNVQTPAQMAKYHQFSGCINCGLCYAACPQFGLNPEFIGPAAITLAHRY

NEDSRDHGKKERMAQLNSPNGVWTCTFVGYCSEVCPKHVDPAAAIQQGKVESSKDFLIAT
LKPR
>EECEBICD_03724 Fumarate reductase subunit C
MTTKRKPYVRPMTSTWWKKLPFYRFYMLREGTAVPAVWFSIELIFGLFALKHGAESWMGF
VGFLQNPVVVILNLITLAAALLHTKTWFELAPKAANIIVKDEKMGPEPIIKGLWVVTAVV
TVVILYVALFW
>EECEBICD_03725 Fumarate reductase subunit D
MINPNPKRSDEPVFWGLFGAGGMWGAIIAPVIVLLVGIMLPLGLFPGDALSFERVLTFAQ
SFIGRVFLFLMIVLPLWCGLHRMHHAMHDLKIHVPAGKWVFYGLAAILTVVTAIGVITL
>EECEBICD_03726 Outer membrane lipoprotein Blc
MRILPVVAAVTAAFLVVACSSPTPPKGVTVVNNFDAKRYLGTWYEIARLDHRFERGLEQV
TATYSLRDDGGINVINKGYNPDRGMWQKTEGKAYFTGSPNRAALKVSFFGPFYGGYNVIA
LDREYRHALVCGPDRDYLWILSRTPTLSEEIKQQMLAVATREGFDVNKLIWVKQSGS
>EECEBICD_03727 Guanidinium exporter
MSWSILLIAGLLEVVWAVGLKYTHGFSRLTPSIITITAMVISMALLSWAMKTLPVGTAYA
IWTGIGAVGAAITGILLLGESASPARLLSLGLIVAGIIGLKLSAH
>EECEBICD_03728 Transcriptional regulatory protein RcsA
MLKILVIDRCHFTRTGMEAWLNHTDVLSSSLLVSGMNNLILAKEHILQWKPHLVIADLYG
FLNDALPEQPINAFFAACRMTPLVLLQSGNMPYRPQYASHAVLSKHTPLHELAQRIKGAL
HTHPLQEAPESATPLLSPQEEKVLAMWTEGTSNKEIAYALGIHGKTVYTYKRNIRMKLHM
DNRYSPFLMLPEKID
>EECEBICD_03729 hypothetical protein
MLRHPFFRASMMKRFISLVALVLLTSTLLTACNTARGFGEDIQHLGHAISRAAS
>EECEBICD_03730 Elongation factor P
MATYYSNDFRSGLKIMLDGEPYAVESSEFVKPGKGQAFARVKLRRLLTGTRVEKTFKSTD
SAEGADVVDMNLTYLYNDGEFWHFMNNETFEQLSADAKAIGDNAKWLLDQAECIVTLWNG
QPISVTPPNFVELEIVDTDPGLKGDTAGTGGKPATLSTGAVVKVPLFVQIGEVIKVDTRS
GEYVSRVK
>EECEBICD_03731 L-lysine 2,3-aminomutase
MAHIVTLNTPLREDWLAQLADVVTNPDELLHLLQIEADENLRAGQDARRLFALRVPRAFI
ARMEKGNPDDPLLRQVLTSRDEFIVAPGFSTDPLEEQHSVVPGLLHKYQNRALLLVKGGC
AVNCRYCFRRHFPYAENQGNKRNWTVALEYIAAHPELDEIIFSGGDPLMAKDHELDWLLT
QLEAIKHVKRLRIHSRLPIVIPARITDELVARFDQSRLQILLVNHINHANEVDEAFCLAM
KKLRHVGVTLLNQSVLLRGVNDNARTLANLSNALFDAGVMPYYLHVLDKVQGAAHFMVTD
DEARQIMRELLTLVSGYMVPRLAREIGGEPSKTPLDLQLRQC
>EECEBICD_03732 hypothetical protein
MALTIKGLNTGVIRHNDKFIALALKVKSLRNKETLLFFPVLALRDLLIGLEHRLYLQHSL
PEQEQEKRQKAKSSHVLKMHENIPAILREELENADVNQRVESLALSDNTEKVLTFTLKLH
NGSHLDLQVGEWQVEVLVMAIIHAINNAEMRELALRISSMLDFLPLYDADCLENGNIEFD
TYNQPDWKHNLYNHYLALVYRYTDEAGQSHDCGTIIKTRSQSGSKEAEAISRRLLNFSPR
LKKLEGKPCKVFVRTLGTGKAARLTQDQCMRALHNLRMASSQGKR
>EECEBICD_03733 hypothetical protein
MHVKYLAGIVGAALLMAGCSSSNELTAAGQNVRFVEDKPGAECQLIGTATGKQSNWFSGQ
HGEEGGSMRGAANDLRNQAAAMGGNVLYGVSSPSQGMLSSFVPTASEMNGQVYKCPN
>EECEBICD_03734 60 kDa chaperonin
MAAKDVKFGNDARVKMLRGVNVLADAVKVTLGPKGRNVVLDKSFGAPTITKDGVSVAREI
ELEDKFENMGAQMVKEVASKANDAAGDGTTTATVLAQSIITEGLKAVAAGMNPMDLKRGI
DKAVAAAVEELKALSVPCSDSKAIAQVGTISANSDETVGKLIAEAMDKVGKEGVITVEDG
TGLQDELDVVEGMQFDRGYLSPYFINKPETGAVELESPFILLADKKISNIREMLPVLEAV
AKAGKPLLIIAEDVEGEALATLVVNTMRGIVKVAAVKAPGFGDRRKAMLQDIATLTGGTV
ISEEIGMELEKATLEDLGQAKRVVINKDTTTIIDGVGEEAAIQGRVAQIRQQIEEATSDY
DREKLQERVAKLAGGVAVIKVGAATEVEMKEKKARVEDALHATRAAVEEGVVAGGGVALI
RVASKIADLKGQNEDQNVGIKVALRAMEAPLRQIVLNCGEEPSVVANTVKGGDGNYGYNA
ATEEYGNMIDMGILDPTKVTRSALQYAASVAGLMITTECMVTDLPKSDAPDLGAAGGMGG

MGGMGGMM
>EECEBICD_03735 10 kDa chaperonin
MSIRPLHDRVIVKRKEVESKSAGGIVLTGSAAGKSTRGEIIAVGKGRILDNGTVQPLDVK
VGDIVIFNDGYGVKSEKIDNEEVLIMSESDILAIVEA
>EECEBICD_03736 L-methionine/branched-chain amino acid exporter YjeH
MNELKKELGLVQGVILLTTSLLGTGVFALPELAALAAGDISLWAWPLLIILIFPIAIVFA
VLGRHFPHAGGVAHFVGMAFGPRLQRVISWLFLSVIPVSFPAALHIAVGFGQALFGWQSE
QLLFGELGTLGLLWFMGSRGASSSANLQAIIAGLIIALIAAILWKGAIKPADITFPAANE
ITFSRLCTALAIMFWGFVGIEAFTHLSSEFKNPERDFPRALIIGLMLAGSIYWTCTAVVL
HFGVYSDKIAATASLPLIIVHLFGIQALWIACIIGYLTCFASLNVYAQSFARLIWTQMQY
QPDHYLAQLSPGRLPLHALNVILACCCVSSLVVYALKINLNALIVYANGIFIMLYLLCML
AGCRLLKGRCYALAVTGCLLCLLLLVMLGWKSLYAIIMLAALWLFLPKRKRMA
>EECEBICD_03737 hypothetical protein
MRWIPFLAVFLYVYIEISIFIQVAHVLGVLMTLILVIFTSVIGMSLVRNQGFKNFLLMQQ
KMAAGESPAAEMIKSVSLIIAGLLLLLPGFFTDFLGLLLLLPPVQKHLTLKLLPHLRFSR
MPGGGFSAGTGGGETFDGEYQRKDDDRDRIEHKDDRRD
>EECEBICD_03738 Aspartate ammonia-lyase
MSNNIRIEEDLLGTREVPAEAYYGVHTLRAIENFYISNNKISDIPEFVRGMVMVKKAAAL
ANKELQTIPKSVANAIIAACDEVLNNGKCMDQFPVDVYQGGAGTSVNMNTNEVLANIGLE
LMGHQKGEYQYLNPNDHVNKCQSTNDAYPTGFRVAVYASIVKLIDAINQLREGFERKAVE
FQDILKMGRTQLQDAVPMTLGQEFRAFSILLKEEVKSIERTAELLLEVNLGATAIGTGLN
TPKEYSPLAVKKLAEVTGFACVPAEDLIEATSDCGAYVMVHSALKRLAVKMSKICNDLRL
LSSGPRAGLNEINLPELQAGSSIMPAKVNPVVPEVVNQVCFKVIGNDTTVTMAAEAGQLQ
LNVMEPVIGQAMFESIHILSNACYNLLEKCVNGITANKEVCEGYVYNSIGIVTYLNPFIG
HHNGDIVGKICAETGKSVREVVLERGLLTEAELDDIFSVQNLMHPAYKAKRYTDESEQ
>EECEBICD_03739 Anaerobic C4-dicarboxylate transporter DcuA
MIVVELIIVLLAIFLGARLGGIGIGSAGGLGVLVLAAIGVKPGTIPFDVISIIMAVIAAI
SAMQVAGGLDYLVNQTEKLLRKNPKYITILAPIVTYFLTIFAGTGNISLATLPVIAEVAK
EQGIKPCRPLSTAVVSAQIAITASPISAAVVYMSSVMEGHGISYIHLLSVVIPSTLLAVL
VMSFLVTMLFNSKLSDDPIYRKRLEEGLIELRGEKQIEIKPMAKNSVWLFLLGVICVVVY
AIVNSPSLGLVEKPLMNTTNAILIIMLSVATLTTILCKVETDAILNSSTFKAGMSACICI
LGVAWLGDTFVSANIDWIKDTAGSVIQGHPWLLAVIFFFASALLYSQAATAKALMPMALA
LNVSPLTAVASFAAVSGLFILPTYPTLVAAVQMDDTGTTRIGKFVFNHPFFIPGTLGVVL
AVCFGFLLGSFML
>EECEBICD_03740 Divalent-cation tolerance protein CutA
MLDVKSQDISIPEAVVVLCTAPDEATAQDLAAKVLAEKLAACATLLPGATSLYYWEGKLE
QEYEVQMILKTTVSHQQALIDCLKSHHPYQTPELLVLPVTHGDTDYLSWLNASLR
>EECEBICD_03741 Thiol:disulfide interchange protein DsbD
MAQRIFTLILLLCSTSAFAGLFDAPGRSQFVPADRAFVFDFQQNQHDLTLSWQVKEGYYL
YRKQISITPTKADIAAVQLPAGVWHEDEFYGKSEIYRKRLNVPVTVNQAAAGATLTITYQ
GCADAGFCYPPETKTVPLSEVAAAIDATPTPAVTQTSETSKPAAQLPFSALWALLIGIGI
AFTPCVLPMYPLISGIVLGGRQRLSTGRALLLAFIYVQGMALTYTALGLVVAAAGLQFQA
ALQHPYVLIGLAIVFTLLALSMFGLFTLQLPSSLQTRLTLMSNRQQGGSPGGVFVMGAIA
GLICSPCTTAPLSAILLYIAQSGNMWLGGGTLYLYALGMGLPLMLVTVFGNRLLPKSGPW
MAHVKTAFGFVILALPVFLLERIIGEAWGLRLWSLLGVAFFGWAFITSLQARRAWMRIVQ
IILLAAALISVRPLQDWAFGSPSAQAPAHLNFTAISTVDELNQALAQAKGKPVMLDFYAD
WCVACKEFEKYTFSDPRVQQALGDTVLLQANVTANNAQDVALLKHLQVLGLPTILFFNAQ
GQEQPQSRVTGFMDAATFSAHLHDRQP
>EECEBICD_03742 hypothetical protein
MQREDILGEALKLLETQGIADTTLEMVAERVNRPLDTLQRFWPDKEAILYDALRYLSQQV
DIWRRQLLLDETLSAEQKLLARYSALSECVSNNRYPGCLFIAACTFYPDPTHPIHQLANQ
QKRAAHDFTHELLTTLEIDDPAMVARQMELVLEGCLSRMLVNRSQADVDTAQRLAEDILR
FAQCRQGGALT
>EECEBICD_03744 Major phosphate-irrepressible acid phosphatase

MKSRYLVFFLPLIVAKYTSAETVQPFHSPEESVNSQFYLPPPPGNDDPAFRYDKEAYFKG
YAIKSSPRWKQAAEDADVSVENIARIFSPVVGAKINPKDTPETWNMLQNLLTMGGYYATA
SAKKYYMRTRPFVLFNHSTCRPEDENTLRKDGSYPSGHTAYGTLLALVLSEARPERAQEL
ARRGWEFGQSRVICGAHWQSDVDAGRYVGAVEFARLQTIPAFQKSLAKVREELNDKNNLL
SKEDHPELNY
>EECEBICD_03745 hypothetical protein
MISTPEPLHAGHILTPFCCGVDSIDNWLKQRAMKNQTTGASRTFVCCGSDSNVLAYYSLA
SSAVTTNTSPGRFRRNMPDPIPVVVLGRLAVDKSLHGQGVARALVRDAGLRVIQVAETIG
IRGMLVHALSDEAREFYQRVGFVPSPMDPMMLMVTLGDLVESV
>EECEBICD_03746 hypothetical protein
MPAANSMAMKRETLNLRIKPAERDLIDRAAKARGKNRTDFVLEAARAAAEEALIEQRIIM
ADPEAYQEFLVRLDQTPSPNAALRKTMQTPAPWEQEK
>EECEBICD_03747 HTH-type transcriptional regulator GadX
MLKVFNPSPVQVGSIECLQSAQNWQRKSLSLQGLNLLQSVLIKLTTGKISITTSSGEYIT
ASGPMLIFLAKDQTIHITMEETHEQLNYNLIELDSASIKNAYNFFLYEHADFSAPLTKPT
TKHLLAPIETGVARVFNLLHSSNKSQKLSQDKKEYLIRFLLSEFIYEPEAFALFRELSQN
TLAENIYNIIISDISRKWALKDISDSLYMSCSTLKRKLKQENTSFSEVYLNARMNKATKL
LRNSEYNITRVAYMCGYDSASYFTCVFKKHFKTTPSEFLAFLSSSRHQYVN
>EECEBICD_03748 hypothetical protein
MQYKNKAKHINIKDGILLLAKKFDLTLSEKKLFITWQQDYLLKVAQIY
>EECEBICD_03749 hypothetical protein
MDNSSLSIGWLTIDSTPPVRSMKDNSDIGARGDNITNINTPTFIGSSEAHATIKLTIDTQ
PSAEPTLGSTVNSLDNTVTLKGVVEAHQDIQVKVTVNDTDYTASIDNDISN
>EECEBICD_03750 putative protein YjiK
MRLLKRVIVVVLLGVILFMVRDDIRYVYQLILKYGDKPSALALSSYKAVIQQKPVAGVKS
NLSGLTYSAEDRMLFAVINNPPELVWLTTEGQLVGRMPLQGIHDPESIAWSGGNQFQIGS
EKDGAVYKTQVDIQRGTMQIISMVKLEGYDKAKNKGLEGTAWDAKNERLYAAKERKPIMI
KEVEMSKNGITRALPSAITASVSDVSGLEYHAPTDSLLVLSDESKMILEVSSEWRVRDRL
FLTAEWSGLRDDIPQPEGIAMDNENNLYIVSEPNLFYKFSCDIQND
>EECEBICD_03751 hypothetical protein
MKLNLITVSLATLVAAGAFPAHAGPQAHVVCGYHHTLGDDAIMMFGKANQAMWHDFFGNT
HTDAVSTYQTLRAQPDTTCDNKADSSAYWAPSMKLPDGEIVNPTYQKTYYQSTNVAQYPL
HPFPAGLELLAGDHHGTGPSTAITFLCANGKGYTNKVGEICGLRKAGDAVQFNIGIAFPN
CWDGVNLKPTHSHNNAIYADHGKCSADYPVKIPTVNMNIAWVLPQISSLDTSKVELSMDP
VMHGETREERWGSLYTAHADFMNGWTEDGAQFMTDLCMNQGLDCGTTVPYAYSKAEENTW
VSSEDDKPHASVDTLYVQDDWTNGGRTQHPETLTLVKFKIPPLPANMDTSLFKYRIRLFG
GKTETNGADQIFFYPTSSDWHVSTVSWNNKPAINYRSDAVLYLDHSHEYRMVDVDKAVRK
ALAEGKTEISWYIGGDRQGNHYDFTPADSKQSLVLMLTGFKKTPEL
>EECEBICD_03752 Tat proofreading chaperone DmsD
MPSRAVLPRILGALFYYSPERPEVKALFDCLPTLPELYPWRDRGPIESLCASWSLPDDDA
LTWQFSVLFEGQGKMPVPPWGSVYLEKDNLLMGETTADYRAFLQSQGMVFTDREREPEDQ
FGLMLLACSDLLARGDNVAANRLLEAHLLPWGFRYLELLQRNTVSAFYARLAVIATCYLQ
DVQQQQGLQPENKRLFF
>EECEBICD_03753 Anaerobic dimethyl sulfoxide reductase chain C
MHELPLVFFTVFTQSAVGAFILLLIGGAMGLVAPRRKAIGLFSVMCLFGIGVVVGTFHVG
QPLRALNMLLRVGHSPMSNEIALSAAFAALGGLGALGLFLNRATPLCNALVWLAAIVGVV
FLYAVPQIYQLPTVATWRSSYTTAMMILTPLIGGGALAALFGVRRLGLLVSVLAILVSFC
LRPGYMATLMSADSALTAAQHSWFTAQAILLAAGVVGVVVCARLKSSAAVLAMTAVVVIA
AELAGRIAFYNLWTLPM
>EECEBICD_03754 Anaerobic dimethyl sulfoxide reductase chain B
MKQYGFYVDSSRCSGCKTCQVSCKDNKDLDVGPKLRRVYEYGGGSWVKEGESWHHDTFTY
YLSIACNHCDEPVCVSGCPTGAMHKRKEDGLVVVDDSVCVGCRYCEMRCPYGAPQFDTQA
NVMRKCDGCLDRLENNLRPICVDSCPQRALDFGPVDELRAKYGTENQIAPLPSASFTHPN
LIIKPHPKARPTGDTEGAIMNIREVRHA

>EECEBICD_03755 Putative dimethyl sulfoxide reductase chain YnfE
MKNDRIVRIEPEDAKDDAVFGEHQIRPCLRGRSSRWRVYSPDRIKYPMKRVGKRGEGKFK
RISWDEATAFIAAELTRVSEKYGREAIYYNYQSGAYYHTQGRPAWIRLLNLTGGYLNYHN
TYSTAQIATATPYTHGDYVGSHFTQIAHSDLVVLFGLNLSETRMSGGGQVEELRRALETS
KARVIIIDPRYTDSVITEHAEWLPIRPTTDAALVAGIAHTLITEQLLDEALVNRYCVGYD
RSTLPDTAAPNASYKDYVLGTGDDGIAKTPEWAADITGIPATRIRQLAREIASARACYIC
QGWGPQRHANGEQTVRAIQTLPALTGHFGRPGTNNGNWPYGTPYGVPLLPVGKNPITTSI
PCYLWTDAIQHPEKMTATTMGVKGADRLKTGIKLFFNQAGNTLLNQHGETNRTRQILADE
SLCETIIVIENHMTPSAMYADLLLPETSYLEAEDLVDSSYAAGSHNYMIAIQKTVKPMWE
VRSTYDICADIAGHLGLREQYTEGRTQAQWAEMHYQQIREKRPYLPEWSVAKEMGVIDQR
IATEQQSLAFADFRADAEANPLSTPSGKIEIYSQALADLAQTWTLPEGERIPALPEFCPA
KESHLNKALTAKYPLQLSGFHTKGHTHSTYSNVLMLHEAVPDEVWINPIDASARQLKSGD
RVHVFNDRGVVELPCKVTQRILPGVAAMPQGAWTRLDGNGVDVGGCINTLTSHHPSPLAK
GNPQHTNLVEIKRA
>EECEBICD_03756 Sensor histidine kinase DcuS
MKHSLPCRALRKRPMKLGTTVILMVSAVLFSVLVVVHLIYFSQISSMTRDALADKALAVA
RSLADSPAIREGLKKPPAESGIQPLAEAVSQHNGFLFIVVTNMQGIRYSHPETQRIGQPF
KGDDILLALQGKENVAINRGFLAKALRVFTPVYDERHRQIGVVAIGLELSHVTQQINNSR
GSIIWSILFGALVGLLGTYALVKVLKRILFGLEPYEISTLFEQRQAMLQSIKEGVIAVDD
SGEVTLINHAAQALLDYRKTQDDARLSTLSHAWSQVVDISEVLRDGTPRRDEEIIVKGRL
LLVNTVPVRSNGEIIGAISTFRDKTEVRQLMQRLDGMVNYADALRERSHEFMNKLHVILG
LLHLKSYKQLEAYIIKTANNYQEEIGLLLGKIKSPIIAGFLLSKITRASDFGHSLVISSD
SQLPDNNNEDQVTVLITALGNLIENALEALSQESGGEISVSLHYRHGWLHCEVSDDGPGI
EPERIEAIFEKGVSSKGAERGVGLALVKQQVEALGGVISVESEPGIFTQFFVQLPWDGKR
TSL
>EECEBICD_03757 Transcriptional regulatory protein DcuR
MINVLIVDDDAMVAELNRRYVAQISGFHCCGTASTLEKAKEFIFNSENHIDLILLDIYMQ
QENGLDLLPVLHSAGCKSDVIVISSAADAATIKDSLHYGVVDYLIKPFQATRFEEALTGW
LRKKTAMEKRQYYEQSELDLLIHGNSSSEQEPRRLPKGLTQQTLRTLCQWIDAHQEQEFS
TDELANEVNISRVSCRKYLIWLVNCHILFTSIHYGVTGRPVYRYRVQPEHYSLLKQYCQ
>EECEBICD_03758 hypothetical protein
MDNTYIGEQIRVSTRHTNNLKKWRDNDHISHILFDLSRDLPDGETQKLNNKRLFLKQTNH
NLLLSLTPPAHRQRVRPSEHHYRKKIIT
>EECEBICD_03759 Anaerobic C4-dicarboxylate transporter DcuB
MLFSIQLLIILICLFYGARKGGIALGLLGGIGLVILVFVFHLQPGKPPVDVMLVIIAVVA
ASATLQASGGLDVMLQIAEKLLRRNPKYVSIVAPFVTCTLTILCGTGHVVYTILPIIYDV
AIKNNIRPERPMAASSIGAQMGIIASPVSVAVVSLVAMLGNVTFDGKHLEFLDLLSITIP
STLLGILAIGIFSWFRGKDLDKDEAFQKFISVPENRQYVYGDTATLLDKKLPKSNWLAMW
IFLAAIAVVALLGADSDLRPTFGGKPLSMVLVIQMFMLLTGALIIILTKTNPASISKNEV
FRSGMIAIVAVYGIAWMAETMFGAHMSEIQGVLGEMVKEYPWAYAIVLLLVSKFVNSQAA
ALAAIVPVALAIGVDPAYIVASAPACYGYYILPTYPSDLAAIQFDRSGTTRIGRFVINHS
FILPGLIGVSVSCVFGWIFAAMYGFL
>EECEBICD_03760 Fumarate hydratase class I, anaerobic
MSKKEFIYQAPFPMGEDKTEYYLLTSDYVSVSEFNGESILNVEPQALTLLAQQAFHDASF
MLRPEHQQQVAAILHDPEASENDKYVALQFLRNSEIAAKGVLPTCQDTGTAIIMGKKGQR
VWTGGGDEAALSKGVFNTYIEDNLRYSQNAPLDMYKEVNTGSNLPAQIDLYAVDGDEYKF
LCVAKGGGSANKTYLYQETKALLTPGKLKNFLVEKMRTLGTAACPPYHIAFVIGGTSAES
TLKTVKLASTHYYDALPTEGNEHGQAFRDLHLEQELLEEAQKLGLGAQFGGKYFAHDIRV
IRLPRHGASCPVGMGVSCSADRNIKAKINREGIWIEKLEHNPGQYIPPALRQAGEGDAVK
VDLNRPMKEILAQLSQYPVSTRLSLTGTIIVGRDIAHAKLKERIESGEDLPQYIKDHPIY
YAGPAKTPAGYPSGSLGPTTAGRMDSYVDLLQSHGGSMIMLAKGNRSQQVTDACKKHGGF
YLGSIGGPAAVLAQQSIKHLECVEYPELGMEAIWKIEVEDFPAFILVDDKGNDFFQQIVS
KQCANCAK
>EECEBICD_03761 Melibiose carrier protein

MSISLTTKLSYGFGAFGKDFAIGIVYMYLMYYYTDVVGLSVGLVGTLFLVARIWDAINDP
IMGWIVNATRSRWGKFKPWILIGTLTNSLVLFLLFSAHLFEGTAQVVFVCVTYILWGMTY
TIMDIPFWSLVPTITLDKREREQLVPFPRFFASLAGFVTAGITLPFVNYVGGADRGFGFQ
MFTLVLIAFFIASTIVTLCNVHEVYSSDNGVTAGRPHLTLKTIVGLIYKNDQLSCLLGMA
LAYNIASNIINGFAIYYFTYVIGDADLFPYYLSYAGAANLLTLIVFPRLVKMLSRRILWA
GASVMPVLSCAGLFAMALADVHNAALIVAAGIFLNIGTALFWVLQVIMVADTVDYGEFKL
NIRCESIAYSVQTMVVKGGSAFAAFFIALVLGLIGYTPNVAQSAQTLQGMQFIMIVLPVL
FFMMTLVLYFRYYRLNGDMLRKIQIHLLDKYRKTPPFVEQPDSPAISVVATSDVKA
>EECEBICD_03762 Alpha-galactosidase
MTAPKITFIGAGSTIFVKNILGDVFHREALKSAHVALMDIDETRLEESHIVVRKLMDSAG
ASGRITCHTNQKAALQDADFVVVAFQIGGYEPCTVTDFEVCKRHGLEQTIADTLGPGGIM
RALRTIPHLWRICEDMTEVCPKATMLNYVNPMAMNTWAMYARYPHIKQVGLCHSVQGTAE
ELARDLNIDPTSLRYRCAGINHMAFYLELERKTADGTYVNLYPELLAAYDAGQAPKPNIH
GNERCQNIVRYEMFKKLGYFVTESSEHFAEYTPWFIKPGREDLIARYKVPLDEYPKRCVE
QLANWHKELEEYKTAERIDIKPSREYASTIMNALWTGEPSVIYGNVRNEGLIDNLPQGSC
VEVACLVDANGIQPTKVGTIPSHLAAMMQTNINVQTLLTEAILTENRDRVYHAAMMDPHT
AAVLGIEEIYALVDDLIAAHGDWLPAWLRR
>EECEBICD_03763 Melibiose operon regulatory protein
MSTQAISLLPPDPHMCNGDEKQTRSPLSLYSEYQRLDVELRPPHRMASSHWHGQVEVNVP
FDGDVEYLINNEVVQIKQGHITLFWACTPHQLTRPGNCRQMAIFSLPMHLFLSWPLDRDL
INHVTHGMVVKSLATQQLSTFEVLRWQQETSSPNEQIRQLAIDEIGLMLKRFSLSGWQPI
LLNKTSRTHKNSVSRHAQFYVSQMLGFIADNYDQALTINDVAEHVKLNANYAMGIFQRVM
QLTMKQYITAMRINHVRALLSDTDKTILDVALTAGFRSSSRFYSTFSKFVGMSPQQYRKL
SQQRRQTMPG
>EECEBICD_03764 Biodegradative arginine decarboxylase
MMKVLIVESEFLHQDTWVGNAVERLADALSQQNVTVIKSTSFDDGYAILSANEAIDCLMF
SYQMEQPDEHLSVRQLIGKLHERQQNVPVFLLGDREKATASLDRDLLELVDEFAWILEDT
ADFIAGRAVAAMTRYRQQLLPPLFNALMKYSDIHEYSWAAPGHQGGVGFTKTPAGRFYHD
YYGENLFRSDMGIERTTLGSLLDHTGAFGESEKNAARVFGADRSWSVVVGTSGSNRTIMQ
ACMTDNDVVVLDRNCHKSIEQGLILTGAKPVYMVPSRNRYGIIGPIYPQEMQPETLQKKI
SASPLTKTKAGQKPSYSVVTNCTYDGVCYNAKEAQDLLAKTSDRIHFDEAWYGYARFNPI
YCDHYAMRGEPGDHNGPTVFATHSTHKLLNALSQASYIHVREGRGAVNFSRFNQAYMMHA
TTSPLYAICASNDVAVSMMDGNSGLSLTQEVIDEAVDFRQAMARLYKEFTDEGDWFFKPW
NKDVVTDPQTGKTYDFADAPAKLLATDQNCWVMRPGETWHGFKDLPDNWSMLDPIKVSIL
APGMGDDGELEASGVPAALVTAWLGRHGIVPTRTTDFQIMFLFSMGVTRGKWGTLINTLC
SFKHHYDANTPLAQVMPELVQDYPDTYANMGIHDLGDKMFAWLRKNNPGARLNAAYSTLP
VAEITPRDAYNAIVNNNIEMVAIENLPGRIAANSVIPYPPGIPMLLSGENFGDENSPQVG
YLRSLQSWDHHFPGFEHETEGTEIIDGVYHVMCVKA
>EECEBICD_03765 HTH-type transcriptional regulator GadX
MRICSNEPCIVLLTEKDTWLRVNGKEPISLKANHMAILACENNVIDISSLNSVLVIQVSR
NNIKDYLQFLNKDLSHLPVWQRNADPLLTANCLTPDIFRVAARYSAMEAPDEIVSERTRA
LLFTVLSRFLDHKKFISLLMHMLRSRISDSVYHIIQSDIHKDWNLSAVASCLCLSPSLLK
KKLKNENTSYSQIITTCRMRYAVNQLLMDGKNISQVSQLCGYNSTSYFISVFKEFYGMTP
LHYVSQHRERSAA
>EECEBICD_03766 Arginine/agmatine antiporter
MSSDADAHKVGLIPVTLMVSGNIMGSGVFLLPANLAATGGIAIYGWLVTIIGALALSMVY
AKMSSLDPSPGGSYAYARRCFGPFLGYQTNVLYWLACWIGNIAMVVIGVGYLSYFFPILK
DPLVLTLTCVAVLWIFVLLNIVGPKMITRVQAVATVLALVPIVGIAVFGWFWFKGETYMA
AWNVSGMNTFGAIQSTLNVTLWSFIGVESASVAAGVVKNPKRNVPIATIGGVLIAAVCYV
LSTTAIMGMIPNAALRVSASPFGDAARMALGDTAGAIVSFCAAAGCLGSLGGWTLLAGQT
AKAAADDGLFPPIFARVNKAGTPVAGLLIVGVLMTIFQFSSMSPNAAKEFGLVSSVSVIF
TLVPYLYTCAALLLLGHGHFGKARPLYLLITFVAFVYCIWAVIGSGAKEVMWSFVTLMVI
TALYALNYNRIHKNPYPLDAPVKQD
>EECEBICD_03767 Phosphoethanolamine transferase EptA

MLKRFLKRPVLGQIAWLLLFSFYIAVCLNIAFYKQVLQDLPLNSLRNVLVFISMPVVAFS
VVNSVLTLASFIWLNRPLACVFILVGAAAQYFILTYGIIIDRSMIANMMDTTPAETFALM
TPQMVLTLGLSGVLAAVIAFWVKIRPATPRLRSGLYRLVSVLISILLVILVAAFFYKDYA
SLFRNNKQLIKALSPSNSIVASWSWYSHQRLANLPLVRIGEDAHRNPLMLKSDRKNLTIL
IVGETSRGDDFSLGGYPRDTNPRLAKDDVIYFPHTTSCGTATAISVPCMFSDMPRKHYDE
ELAHHQEGLLDIIQRAGINVLWNDNDGGCKGACDRVPHQNVTELNLPGQCIDGECYDEVL
FHGLEDYIDHLKGDGVIVLHTIGSHGPTYYNRYPPQFKKFTPTCDTNEIQNCSQQQLINT
YDNTVLYVDYIVDKAINLLKSHQDKFTTSLVYLSDHGESLGENGVYLHGLPYSIAPDTQK
HVPMLIWLSKDYQQRYQVDQACLQKRASTLDYSQDNLFSTMLGLTGVQTTYYQAADDILQ
PCRRLSE
>EECEBICD_03768 Transcriptional regulatory protein BasR
MKILIVEDDTLLLQGLILAAQTEGYACDGVSTARAAEHSLESGHYSLMVLDLGLPDEDGL
HFLTRIRQKKYTLPVLILTARDTLNDRITGLDVGADDYLVKPFALEELHARIRALLRRHN
NQGESELTVGNLTLNIGRHQAWRDGQELTLTPKEYALLSRLMLKAGSPVHREILYNDIYN
WDNEPSTNTLEVHIHNLRDKVGKSRIRTVRGFGYMLVATEES
>EECEBICD_03769 Sensor protein BasS
MSLMRFQRRAMTLRQRLMLTIGLILLVFQLISTFWLWHESTEQIQLFEQALRDNRNNDRH
IMHEIREAVASLIVPGVFMVSLTLLICYQAVRRITRPLAELQKELEARTADNLAPIAIHS
STLEIESVVSAINQLVTRLTTTLDNERLFTADVAHELRTPLSGVRLHLELLSKTHNVDVA
PLIARLDQMMDSVSQLLQLARVGQSFSSGNYQEVKLLEDVILPSYDELNTMLETRQQTLL
LPESAADVVVRGDATLLRMLLRNLVENAHRYSPEGTHITIHISADPDAIMAVEDEGPGID
ESKCGKLSEAFVRMDSRYGGIGLGLSIVSRITQLHQGQFFLQNRTERTGTRAWVLLKKA
>EECEBICD_03770 hypothetical protein
MKSYIYKSLTTLCSVLIVSSFIYVWVTTY
>EECEBICD_03771 Proline/betaine transporter
MLKRKKIKPITLGDVTIIDDGKLRKAITAASLGNAMEWFDFGVYGFVAYALGKVFFPGAD
PSVQMIAALATFSVPFLIRPLGGLFFGMLGDKYGRQKILAITIVIMSISTFCIGLIPSYA
TIGIWAPILLLLCKMAQGFSVGGEYTGASIFVAEYSPDRKRGFMGSWLDFGSIAGFVLGA
GVVVLISTIVGEENFLEWGWRIPFFIALPLGIIGLYLRHALEETPAFQQHVDKLEQGDRE
GLQDGPKVSFKEIATKHWRSLLSCIGLVIATNVTYYMLLTYMPSYLSHNLHYSEDHGVLI
IIAIMIGMLFVQPVMGLLSDRFGRRPFVIMGSIALFALAIPAFILINSNVIGLIFAGLLM
LAVILNCFTGVMASTLPAMFPTHIRYSALAAAFNISVLIAGLTPTLAAWLVESSQDLMMP
AYYLMVIAVIGLITGISMKETANRPLKGATPAASDIQEAKEILGEHYDNIEQKIDDIDQE
IAELQVKRSRLVQQHPRIDE
>EECEBICD_03772 hypothetical protein
MSLPHCPQCNSEYTYEDNGMFICPECAHEWNDAEPAQESDELIVKDANGNLLAEGDSVTI
VKDLKVKGSSSMLKIGTKVKNIRLVEGDHNIDCKIDGFGPMKLKSEFVKKN
>EECEBICD_03773 hypothetical protein
MPLSPYLSFAGNCADAIAYYQQTLGAELLYKLSFGEMPPSAADNEEGCPSGMRFPDTAIA
HANMRIAGSDIMMSDASPSGTAHYSGFTLVLDTQDVAEGKRWFDNLAAQGQIEMDWQETF
WAQGFGKVSDRFGVPWMINVVKHQPAT
>EECEBICD_03774 Aminoalkylphosphonate N-acetyltransferase
MPVCELRHATTEDTDSVYALICELLKNELDYQAFRDGFAANLLDPNFHYRLALRNGEVVG
MISLHMQFHLHHANWIGEIQELVVLPPMRGQKIGSQLLAWAEEEARQAGAELTELSTNIK
RRDAHRFYLREGYKQSHFRFTKAL
>EECEBICD_03775 hypothetical protein
MFAAIIIGIFIISVIYAHSRGVEKQKLSRQLFDHSTFMAPINMFMTRFSTLPAKQPYFDT
TAFPELQKLTENWQVIREEALRLQHHIKAAQANNDAGFNTFFKRGWKRFYLKWYSDAHPS
AETLCPITTKLVNSIPSIKAAMFAELPPGAYLGKHRDPYAGSVRYHLGLSTPNDDRCFIE
VDRQRHSWRDGKAVIFDETYVHWAENKTEQTRIILFCDIERPMKWRWAQSVNHWVGASLM
SAASSPNDENDRTGAINRIFKYVHAARDAGQRLKKKNRTLYYALKYLVIAAIFAAIILFS
LL
>EECEBICD_03776 Formate dehydrogenase H
MKKVVTVCPYCASGCKINLVVDNGKIVRAEAAQGKTNQGTLCLKGYYGWDFINDTQILTP

RLKTPMIRRQRGGKLESVSWDEALNYVAERLSAIKAKYGPDAIQTTGSSRGTGNETNYVM
QKFARAVIGTNNVDCCARV
>EECEBICD_03777 Formate dehydrogenase H
MSNAINEIDNTDLVFVFGYNPADSHPIVANHVINAKRNGAKIIVCDPRKIETARIADMHI
ALKNGSNIALLNAMGHVIIEENLYDKAFVASRTEGFEEYSKIVEGYTPESVEEITGVSAQ
EIRQAARMYASAKSAAILWGMGVTQFYQGVETVRSLTSLAMLTGNLGKPSAGVNPVRGQN
NVQGACDMGALPDTYPGYQYVKFPENREKFAKAWGVESLPAHTGYRISELPHRAAHGEVR
AAYIMGEDPLQTDAELSAVRKAFEDLELVIVQDIFMTKTASAADVILPSTSWGEHEGVFS
AADRGFQRFFKAVEPKWDLKTDWQIISEIATRMGYPMHYNNTQEIWDELRHLCPDFYGAT
YEKMGELGYIQWPCRDTSDADQGTSYLFKEKFDTPNGLAQFFTCDWVAPIDKLTEEYPMV
LSTVREVGHYSCRSMTGNCAALAALADEPGYAQINTADAARLGIEDEALVWVHSRKGKII
TRAQVSDRPNKGAIYMTYQWWIGACNELVTENLSPITKTPEYKYCAVRVEPIADQRAAEQ
YVIDEYNKLKTRLRESAMG
>EECEBICD_03778 hypothetical protein
MKLLLALMLFMTFFAHAADPEPGSQYLQAAEAGDRRAQYFLADSWLSYGDLNKAEYWAQK
AADSGDADACALLAQIKITNPVSLDYPDAKKLAEKAANAGSKAGEITLARILINTQAGRP
DYPKAISLLQKASEDLDNDSAVDAQMLLGLIYANGVGIAADDEKAAWYFKRSSAISRTGY
SEYWAGMMFLNGEPGFIEKNKQKALHWLNLSCLEGFDTGCEEFETLTNG
>EECEBICD_03779 Proton/glutamate-aspartate symporter
MKNLKVSLAWQILLAMVLGILLGSYLHYHSDSREWLIANLLSPAGDIFIHLIKMIVVPIV
ISTLIVGIAGVGDAKQLGRIGAKTILYFELITTVAIILGITLANVFQPGSGIDMSQLATV
DISKYQNTTAEVQSHAHGLMGTILSLVPTNIIASMAKGDMLPIIFFSVLFGLGLSSLPAT
HREPLVTVFRSISETMFKVTHMVMRYAPVGVFALIAVTVANFGFASLWPLAKLVLLVHFA
IIFFALVVLGIVARLCGLSIWILIRILKDELILAYSTASSESVLPRIIEKMEAYGAPASI
TSFVVPTGYSFNLDGSTLYQSIAAIFIAQLYGIDLSLWQEIVLVLTLMVTSKGIAGVPGV
SFVVLLATLGSVGIPLEGLAFIAGVDRILDMARTALNVVGNALAVLVIAKWEHKFDRKKA
LAYERDVLGKFDKTAQ
>EECEBICD_03780 Formate-dependent nitrite reductase complex subunit NrfG
MSQSEHSTVPLRPMPVKRLAAAAVLMVAACVGGYLLTPKWQAVRSEQQRLADPLRDFTNP
QTPEAQLSRLQEKIRANPQDSEQWARLGEYYLYRNAYDNALLAYRQALRLRGDNAQLFAA
LATVLYYQAGQHMTPATREMINKALALDATEVTAQMLLAADAFMQADYAQAVSLWQTLLD
ANSPRVNRAQLVEAINLTKLLQNRQK
>EECEBICD_03781 hypothetical protein
MRKLLTALPDGGVNALSGLQDAQTVGRISVAPSGRKFLLLTIVLFLITFATHAQVVDTWR
FDNPQQQEKALSIASQLRCPQCQNQNLLESNAPVAVSMRHQVYSMVAEGKSEAEITAWMT
DRYGDFVRYNPPLNEQTLLLWALPYLLLLLVGVVAWRVRKRQHAGEGEQ
>EECEBICD_03782 Cytochrome c-type biogenesis protein CcmF
MHNLDIFLPEAGFLALLLSLGVNVLTPLATWCGMGLHWRGVMRLTTCGAWTAFTLLLLAF
AILVSCFLTSDFSVVYVAQHSHSQLSWGLKLAAVWGGHEGSLLLWALLLSGWTALFAWRS
RHESDALFPLTLSILSLIMASLLLFIVLWSDPFLRIFPPAMEGRDLNPMLQHLGLILHPP
LLYLGYGGLMTAASVALASLLCGGFNAATAWVCWRWALPGWCALTLGIILGSWWAYCELG
WGGWWFWDPVENASLLPWLAASALLHSLYVSRQRGSFRHWSLLLAILTLILSLLGTLIVR
SGILVSVHAFALDNVRAVPLFTLFAALSLASLVLYGWRAREPYPATRLGGGKCETLILAT
LLLFSAVLLIVLVGTLYPMIYGLMGWGRLSVGAPYFNRVTLPFGLLMLVVIVLATIRSRK
VSVRCQLPALLAHTGVFIFAAGIMVSSGSRHEISLNLSPGQQVVLAGYTFRFERLDLEAK
GNYTSEKARITLWRNEKRIGSLQPERRFYAARRQQMMEPGIHWNLLHDWYAVMGEKNGPD
RYAMRLYVQTGVRWIWGGGLLMVCGALLSGWRGRKRDA
>EECEBICD_03783 hypothetical protein
MTSASPFHFASLVWDWPIAIYLFLIGISAGLVTLAILLRRFHPEAGGSDSTLLRTTLVLG
PGAIIFGLLILVFHLTRPWTFWKLMFHYSFTSVMSMGVMLFQLYMVVLVLWLAKIFEKEV
IALQQRWLPRLGIVQKVLALITPFHRALETLMLVLAVLLGAYTGFLLSALKSYPFLNNPI
LPALFLFSGISSGAAVALIAMALRHRSNPHSTEARFVHRIETPVVWLEIFLLAAFFIGLA
LGDDGKMRALAAALGGGFWSWWFWLGVVGLGLIIPLLLKPWANRSVTFHGVLAVCGASLT
GVLLLRFFILYAGQLTVA

>EECEBICD_03784 putative ferredoxin-like protein YdhX
MSCTRRQFITRVGSLAAVSGMAGRVVANTLNINGVRYGMVHDESLCIGCTACMDACREVN
NVPEGVSRLTIIRSEPQGTFPDVKYRFFRHSCQHCDHAPCVDVCPTGASFRDAASGIVDV
NPDLCVGCQYCIAACPYRVRFIHPVSKTADKCDFCRKINLKAGKQPACVESCPTKALTFG
NLDDPNSDISRLLRQKTTYRYKLALGTKPKVYRVPFNYGEVSQ
>EECEBICD_03785 Cytochrome c-type protein NrfB
MSVLRSLLTAGVLASGLFWSLSGITATPTPQESDQRWTVTQQRNPDAACLDCHKPDTEGM
HGKHTGAINPNNKLPITCTNCHGQPSLHHREGVKDVMRFNDPMYTVEQQNSVCMSCHLPE
QLQKAFWPHDVHVTKVTCASCHSLHPQQDTMQTLSEKGRIKICVDCHSDQRTNPHFNPAS
VPLLKEQP
>EECEBICD_03786 Cytochrome c-552
MARKTLRARRFFSLIFPFFFITSVYAEQTPESAKTVTVEAKNEMFAPQHPDQYQSWKATS
EQSAREDALAEDPRLVILWAGYPFSRDYNKPRGHAYAVTDVRETLRTGAPKTAEDGPLPM
ACWSCKSPDVARLIQQEGEDGYFHGKWARGGPEIVNDLGCADCHNTASDDFAQGKPALTL
SRPYAERAMEAIGKPFDKAGRFDQQSMVCGQCHVEYYFDGKNKAVKFPWDEGMKVENMEQ
YYDAIAFSDWTNSLSKTPMLKAQHPEYETWSAGIHGKNNVTCIDCHMPKVQNAEGKLYTD
HKIGNPFDNFAQTCANCHTQDKASLQKVVAERKQAIHDLKIKVEDQLVHAHFEAKAAWDA
GATDAEMKPILNDIRHAQWRWDLAIASHGIHMHAPEEGLRMLGSAMDKAADARTKLARLL
ATKGITHEIPLPDISTKEKAQKAIGLNMQQINAEKQDFLKTVVPQWEDQARKNGLLSQ
>EECEBICD_03787 Acetyl-coenzyme A synthetase
MSQTHKHAIPANIADRCLINPEQYETKYKQSINDPDTFWGEQGKILDWITPYQKVKNTSF
APGNVSIKWYEDGTLNLAANCLDRHLQENGDRTAIIWEGDDASQSKHISYRELHRDVCRF
ANTLLDLGIKKGDVVAIYMPMVPEAAVAMLACARIGAVHSVIFGGFSPEAVAGRIIDSSS
RLVITADEGVRAGRSIPLKKNVDDALKNPNVTSVEHVIVLKSTDSDIDWQEGRDLWWRDL
IEKASPEHQPEAMNAEDPLFILYTSGSTGKPKGVLHTTGGYLVYAATTFKYVFDYHPGDI
YWCTADVGWVTGHSYLLYGPLACGATTLMFEGVPNWPTPARMCQVVDKHQVNILYTAPTA
IRALMAEGDKAIEGTDRSSLRILGSVGEPINPEAWEWYWKKIGKEKCPVVDTWWQTETGG
FMITPLPGAIELKAGSATRPFFGVQPALVDNEGHPQEGATEGNLVITDSWPGQARTLFGD
HERFEQTYFSTFKNMYFSGDGARRDEDGYYWITGRVDDVLNVSGHRLGTAEIESALVAHP
KIAEAAVVGIPHAIKGQAIYAYVTLNHGEEPSPELYAEVRNWVRKEIGPLATPDVLHWTD
SLPKTRSGKIMRRILRKIAAGDTSNLGDTSTLADPGVVEKLLEEKQAIAMPS
>EECEBICD_03788 Inner membrane protein YjcH
MNDQIYQRIEDSARFRELVEKRQRFATILSVIMLTVYISFILLIAFAPGWLGTPLHAGTS
VTRGIPIGVGVIVISFVLTGIYIWRANGEFDRLNNAVLHEVKAL
>EECEBICD_03789 Cation/acetate symporter ActP
MKRVLTALAAALPFAAHAADAISGAVERQPTNWQAIIMFLIFVVFTLGITYWASKRVRSR
SDYYTAGGNITGFQNGLAIAGDYMSAASFLGISALVFTSGYDGLIYSLGFLVGWPIILFL
IAERLRNLGRYTFADVASYRLKQGPIRILSACGSLVVVALYLIAQMVGAGKLIELLFGLN
YHIAVVLVGVLMMMYVLFGGMLATTWVQIIKAVLLLFGASFMAFMVMKHVGFSFNNLFTE
AMAVHPKGTAIMSPGGLVQDPISALSLGLGLMFGTAGLPHILMRFFTVSDAREARKSVFY
ATGFMGYFYILTFIIGFGAIMLVGANPAYKDAAGALIGGNNMAAVHLANAVGGNLFLGFI
SAVAFATILAVVAGLTLAGASAVSHDLYANVFRKGATEREELKVSKITVLVLGVIAIILG
VLFENQNIAFMVGLAFAIAASCNFPIILLSMYWSKLTTRGAMLGGWLGLLTAVVLMILGP
TIWVQILGHEKAIFPYEYPALFSISVAFLGIWFFSATDNSAEGNREREQFRAQFIRSQTG
FGVEQGRAH
>EECEBICD_03790 Inner membrane protein YohK
MNNVQLSVLCLVITLGIYFANKRLYRRFRKLPLMPLVLTPALLVLMLVFGHISWQNYIGE
SHWLLWLLGPATIAFAVPVYDNLAIIKRHWMSLTAGVVTATVVAVTSSVWLARLFTLSDE
IQRSLAVRSVTTPFALAAAEPLGGQPDLVALFVVVTGVFGMAVGDALFLRLSIREGMAKG
AGFGAASHGAGTARSYELGQQEGVVASLVMMLSGVVMVLAAPLVARVMF
>EECEBICD_03791 Holin-like protein CidA
MAVAISRVTPAVVQRLQVPVQVLLYAGLFIFSQYLVSWLHLPLPANLVGMVLMLALIVCR
IIPLSWVRAGARWLLAEMLLFFVPAVVAVVNYAHLLLVDGWRIFSVIAISTLMVLGATAW
VVDKVYRYEMSRLNRE

>EECEBICD_03792 Hydrogen peroxide-inducible genes activator
MDIRTLRYFVEVVRQQSFTRAAEKLFVTQPTISKMLKNLEDELNCTLLIRDGRKLLLTDT
GRVVFERGLAILAEFRQLEAELSDINHLNKGVLRLGIPPMVGMLMAGPISLFRQRYPGVE
LKISEFGGLTVQQAVMNGELDVAMTALPVEEASGLTTLSLFSHPLCVLVPRSGQWTTCDS
IAPEALAEHPLLIYNEDFALSRQLMTLFSQHDVKPRIAVRSGQWDFLAAMVQAGVGIAIL
PEPICQRLDKATLRWLPLESDLRWQLGMIWREGVYLSHSARAWLTCCEGFWLKQP
>EECEBICD_03793 Sodium, potassium, lithium and rubidium/H(+) antiporter
MEIFFTILIMTLVVSLSGVFTRVLPFQLPLPLMQIAIGALLAWPTFGLHVEFDPELFLVL
FIPPLLFADGWKTPTREFLEHGREIFGLALALVLVTVVGIGFLIYWLVPGIPLIPAFALA
AVLSPTDAVALSGIVGEGRIPKKIMGILQGEALMNDASGLVSLKFAVAVAMGTMVFTVGG
ATLEFFKVAIGGILAGFVVSWLYGRSLRFLSRWGGDEPATQIVLLFLLPFASYLIAEHIG
VSGILAAVAAGMTITRSGVMRRAPLAMRLRANSTWAMLEFVFNGMVFLLLGLQLPGILES
SLAAAEADPNVETWMLFTDIVLIYAALMLVRFGWLWTMKKFSLRFLKKKPMEFASWTSRE
ILIASFAGVRGAITLAGVLSIPLLLPDGSGFPARYELVFLAAGVILFSLFVGVIMLPLLL
QHLEVADHAQQLKEERIARAATAEVAIVAIQKMEERLAADTEENIDNQLLTEVSSRVIGN
LRRRADGRNDVESSIQEENLERRFRLAALRSERAELYHLRATREISNETLQKLLHDLDLM
EALLIENQ
>EECEBICD_03794 Guanine/hypoxanthine permease GhxP
MSTPSARTGGSLDAWFKISQRGSTVRQEVVAGLTTFLAMVYSVIVVPGMLGKAGFPPAAV
FVATCLVAGVGSIVMGLWANLPLAIGCAISLTAFTAFSLVLGQHISVPVALGAVFLMGVL
FTVISATGIRSWILRNLPQGVAHGTGIGIGLFLLLIAANGVGLVIKNPLDGLPVALGDFD
TFPVIMSLVGLAVILGLEKLKVPGGILLTIIGISIVGLLFDPNVHFSGIFAMPSLSDENG
NSLIGSLDIMGALNPVVLPSVLALVMTAVFDATGTIRAVAGQANLLDKDGQIIDGGKALT
TDSLSSVFSGLVGAAPAAVYIESAAGTAAGGKTGLTAITVGVLFLLILFLSPLSYLVPVY
ATAPALMYVGLLMLSNVAKIDFADFVDAMAGLVTAVFIVLTCNIVTGIMIGFATLVVGRL
VSGEWRKLNIGTVVIAVALVAFYAGGWAI
>EECEBICD_03795 hypothetical protein
MLTVHHLNQSRSQRILWALEELALPYQIVRYQREKTMLAPPALKKVHPLGKSPVIEDHGM
VIAESGAILEYLQDTYDNIGRFKPADAQGKQHYRFWLHYAEGSLMPLLLMRLLFTSLGKP
PVPLGLRTLGGVIGKGAQKAYLNPQLETHARFIDGHLANHPWFAGEQLSMADIQMSFPLF
ALLARGGIAHLDHINAWKARVEMRPAWQRAIQQGGPFTISGG
>EECEBICD_03796 Redox-sensitive transcriptional activator SoxR
MSALHFYESKGLITSIRNSGNQRRYKRDVLRYVAIIKIAQRIGIPLATIGDAFGILPEGH
TLSAKEWKQLSSQWREELDRRIHTLVALRDELDGCIGCGCLSRSDCPLRNPGDRLGEHGT
GARLLEDD
>EECEBICD_03797 Regulatory protein SoxS
MSHQQIIQTLIEWIDEHIDQPLNIDVVAKKSGYSKWYLQRMFRTVTHQTLGEYIRQRRLL
LAAVELRTTERPIFDIAMDLGYVSQQTFSRVFRREFDRTPSDYRHRL
>EECEBICD_03798 putative cyclic di-GMP phosphodiesterase PdeC
MSQSTRRKVLRFLGTIIVVLLPILLAIWFAHIRAVSETRNQLHSFAQLVLDKTERVILQA
DLARDAAEQYQGQACTPAHQQRMLNIIRGRLYINELIYAKGQRFLCSTVMTPTSPYFIPR
ADYKRKPDIAIYYYRDTPFFNGYKMTYMQRGNYVAVINPLSYSEVMSDDPALAWGMYDTV
TNTFFSLSEQAQADQLLPLVRRSEPVFQQGERFYTLVKSAKRPVATIVSTSKQRFYQNLF
HQLTLTLPLGIICSIIVLFMWSRSRQAYYSPRRLLQRAITRHQLCLHYQPIIDIRNGTCV
GAEALLRWPGYHGPVMSPCEFIPLAEKEGMIEQITDYVVEEVFNDLGGFLAAHPHLYVSI
NLSAADFLSSRLIVMIHEKTRQHSVLAQQIKVEVTERGFIDVPKMTPIIQAFRQAGYEVA
IDDFGTGYSNLHNLYSLNVDLLKIDKSFVDTLTTNSASHLIVEHIIEMAQSLRLKIIAEG
VETAEQVSWLLKRGVQYCQGWHFAKALPPQEFIAWLQQTPAPLTIRGQRPYRR
>EECEBICD_03799 hypothetical protein
MAAITTGVVLLRWQLLSAVLMFLASTLNIRFRKSDYIGLAVISSGLGVVSACWFATGLLG
ITMMDLAAIWHNIEAVMVETMSQTPPEWPMVLT
>EECEBICD_03800 Lipid A export ATP-binding/permease protein MsbA
MVTTHDVLFTGTIESNFALKPQNDRGRVLKALQLANCGFILQHPMGLKFPVNFMAKNLSS
GQQQQLLLARSLSSDASVFLWDEPTSNLDENTEKQIFDNLDEFIHGKTLIMVTHRRYLIK

YFDRVLVMKGGKIIRDCSPDKLLM
>EECEBICD_03801 Leukotoxin export ATP-binding protein LtxB
MAIFALTIPVFSNLFYDKLVPSASVSSLFGVAIIVAVFIVFEFILRTSKDIYQSITARQD
DVDIDIAFLEAVLYSKKKNGRSMSSAFVLWNEFQKIKPVLLNSIFQRIADIPIFIIFLIV
IYVNLGLVVIVPITMFIVSIIISLVNHHYTNELMNKQKEGQKNRNIFISEVFLSIKMIHT
LNNQGLLFDWVNTSNEQSYLNLKIRKLNLIYQSILGSMSSITQITIMVIAFFMVIKGDVT
TGAIVSSVIVSGRISGIISNFSSTLISILSAEKTGKDLLSFFDEDQAEKTPALQSISKCN
GDISIRGVSYQYDAQSPMIINRLSIDIPAGQRVAVVGECGAGKSSLLGMLSGYLSPTDGA
ILYDGYNLGHLSQNFFLSI
>EECEBICD_03802 hypothetical protein
MDKKLEPYYLSAETALSIVSKKFNIKIDIKEDDINLRFKKYDRNNTDDSIQMKNFFLSLG
LSLQDILFNNGEDLLNEPMPILLLTPEMKWMVCVSGGQKIKLVNARGELCYVEIEDEYLK
ELSAFSILPLNKVVDSIRVKNIIKNSLSMNKIFYTKYFFHLFLWQFLR
>EECEBICD_03803 hypothetical protein
MTSVLVSFDGVNWTPISKNAAGQWEFTAGSALPDGHYTLHVQATDRAGNTANSTLGFTVD
TQIDGLSVVMLDDAGKDSTDGITNITSPRFEISAREPLQSVTVILNGKSSTLTQGAGNKW
LFTPDTPLVDGTYKIEIVAEDIAGNKISKEVSFTIDTIVSDPSIDLLDADDTGESAVDNI
TSVTKPRFVIGNVPADIDTVVIRINGVSYPVTANGNNLWEFQVPVALNDGVYEAVVVFRD
IAGNTSETKLPFTIDTTTSVSVRMEPASDTGNSNSDNLTNKQNPKFEGTAEPNAKLVITI
VDDKSGREVLKQTITVGADGNWSVTPNILPDGMYTINVVATDVAGNTAQTQERFTIDTVT
IDPTIRLSDPSIDDQHEATSLRPEFKGFAEAFSTIMIQWDGKVVGSANANANGEWSWTPP
SVLAPGSYVVSIVAKDKAGNESSQVDFPVVIPVIDVTPPTIKLSEESDSGALGDFTTNNK
TPTLVGNTLPNAIVSIYVDGVKVGEATADTAGRYTFQLSEMKDGHYVVQVGIVNPRDNSE
LRSTAVDVTIDTEVAELVWNISGMHEGGYINTVTPEIGGTSEPNSKITIFVNGVEKAIAY
TTGAGHWGVVLPALGNDGNYVLTFKVEDVAGNIREFGPQNVILDTVISPLTVVLREADDS
GKVGDWITNKSHVTIDGTAEAGSTLTIRSPQGVVIATLVVGNDGRWSAELDLREGSNAFV
VVSEDKAGNSQQKDILIEHDTQIEISDISLSRDTNSGDKYDLITNNKSPVLVAMTDPGAT
VQVYINGVLQGTVEASSSGNISYTMPANSADGEYQVQFVATDTAGNRVESAITTVTIDSQ
IAVFDIDEDSLPALSNNRALSVSGVGEAGSQVSIFVDGKLVNVVMVEADGTWRAPILLQD
DGTFNIHFSITDVAGNTEVSKDYSVDVDSSTDFPTLNLEDASNSGSLDDLITNHNKPVLV
GTAEAGATIHIYVDEKIVANVLVLEDGTWSYQFDNVLKDGEYSIRVVAEDPAGNTAESPR
LLVTIDTSTFIDNPAMVAGSDNGIFSNDSITSQTRPTFSISGEMNQSVQIFIDGVLVDTI
TVTDRNQVYRPESPLGDGSHSIYYVITDKAGNTATSKTLNFTIDTFNTTPVAIDSIGGQT
LAEMTGSDGKIYITDTTRNLLFSGSAEPNSKIEIIINGLNVGEVWVNEKGHWQMPVNPLY
FTEGQLDITVKSTDRAGNVNQEKYSIWVDTHIQVFTSELDDNKSSSKTDWWSNSSTITMR
GMGEIGATVSLIVAGVTLATAVVAANGQWELSTDQLPEGKYDITLSIEDNAGNRKEEVHE
IFIDRTPPNAPVVTYSDIVNDLIIMQGTAEAKSQLIITDSNGNTYTLTVPDNGKWSMAIP
YPSEGKFTITSVDAIGNRSDDVSLDIMKEVPVISLSPDSDSGTVGDNITRDKQPTFIIGN
LESDVVVVQVDINGTVYNAEKNADGVWFFTPGTPLADGSYTISVIASDAAGNQKNSLPIT
VTIDSTLTVPEIALAAGEDNGVSDSDNVTNHTQPKFTLQHIDADVTGVTVNVTHNGVTDT
YQATQGADGWTFTPPAAWNDGTYTLSVTVVDRAGNSQQSASLAVTVDSTVTVTADSQHDD
ASDDATATAVTPPESETVNAESATHLRTVPSAAEESVVKETAYSITLLNADSGDEIDRSI
SQTPSFEISVPENIVNVSIMFEGEEFTLPITNQKAIFEVPLSLEDGEYTMDVKFIDKDND
FLIKEKTFSVDHSSADIVNAMNARGKTEDDINDSPSTSSVGHNNNGAIDVFAVNEVTLPV
DNQEEHA
>EECEBICD_03804 hypothetical protein
MGNKSIQKFFADQNSVIDLSSLGNAKGAKVSLSGPDMNITTPRGSVIIVNGALYSSIKGN
NLAVKFKDKTITGAKILGSVDLKDIQLERIDSSLVDSAQVEKKGNGKRRNKKEEEELKKQ
LDDAENAKKEADKAKEEAEKAKEAAEKALNEAFEVQNSSKQIEEMLQNFLADNVAKDNLA
QQSDASQQNTQAKATQASKQNDAEKVLPQPINKNTSTGKSNSSKNEENKLDAESVKEPLK
VTLALAAESNSGSKDDSITNFTKPQFVGSTAPNATVIIKINGIAVGQAVADSLGNFTFTA
PETLTDGAYNLEAEAKTADGSGSAKLVITIDSVTDKPTFELSPESSVSGHKGLTPTLTPS
IVGTAEENAKVDIYVDNKLVASVDVDKDGNWSYEFKDNELSEGENSIKVVAVDKAGNKNE
TTDSIITDTIPPEKPTIELDDSSDSGIKNDNITNSTLPTFIGVAEPGSTVSIYLGLKHLG

EVIVAKDGTWSYTLTTPLKDGEYNITATATDIAGHTSATANLPFTIDTRISYFSAEIETT
NDSGIVGDNVTNNTRPTFTGKTEPNAIISVINSETGEEVIFKANDKGEWTFNFTSDSVEG
INNLTFTVEDVAGNKKDFSFSYVIDTIAPVPPTVSLEDFVVLPNGIILSGNDLPALVGTA
EPKSTILLMRDGKLYDSIEVDSNGTWNYQFSNKFLQGAYDIEIISQDAAGNKSSTVKYSF
TIQTEVVPPKAELDASDDSGAKGDWITNKHNALTLLGTADRFATVNILIDGKTIGVTTAD
ADGNWNFDISRNLSDNVYKITVESIDPLGRTSSVDYQLTIDSFTPIPTVMLHDSADSGVK
GDMITKINTPLFTGMAEANAKVSIYVDGVLSGEAIAGDDGVWNFQFTTALSDGSHDVTVK
VEDIAGNTASSSAYNFQIVTQTQKPTIELVNDTGVDNTDHIINEKNPALTGTAAPYSTVK
LYIDGALIAEVRTNKDGRWEYTLKADQGLVDGDHRITASVEDIAGNIAHSDPFLISVDTA
ISIPIVSLSPDSDSGIADDNLTNIVKPTLHLKDIDPDIISVQVWDAMSDTQIGVATQQPD
GSWAYTFTSDLTEGLHQVYVKVEDIAGNKANSAVFDFTIDTTVSTPVISLLSKDDTGVTG
DNLTNINKPGFAISGVDADAHRVVVQVMHNGVSEEIELSHLNGSWLFTPGNTWADGSYTL
TVKVEDKAGNTSYSAPLTVVIDTQIAIDGVELVNDSGVKGDNMTNDDRPHFRVTVPTDVN
EVRLSIDGGNSWVQATPGVAGSWEYIWPTDLADGQYTLTVEATDKAGNTVTKTIDFAVDT
TLSVPVIVLNSADDTGVQGDNMTNRTQPTFALQHIDDDAVRVTVSVEHGGVTTTFDATKG
TGGWTFTPPTSWADGDYTLSVSVEDKAGNTSHSASLTVTVDTQIAINNIELVNDSGIPDD
NLTNNVRPHFQVTVPTDVNVVRLSIDGGKTWFNATQSATPGAWDYIWPDDVADGGYTLTV
EATDKAGNKTTQELDFTIDTTLSVPTLSLDSADDSGIAGDNITNVKTPGFTLNNIDTDVS
RVIVEVMHNGIKQEVPLVQTGGQWRFAPTSDWADGDYILTVKVEDRAGNVKQSAPLTVTV
DTHIAIDRIELVNDSGIPGDNLTNEARPHFQVTVPADVNGVRLSIDGGKTWFDATQSATS
GVWDYTWLTNVANGPHTLMVEASDKAGNKTTQKLDFIIDTLLSEPTITLDSADDSAAGDN
ITNVKMPGFTLGNIDADVTKVVVTVAHDGKNQQIELIKNGGVWRFTPGAAWTDGDYTLTV
KVEDKAGNTNYSAPLTVTIDTQTSIDRIELLNDTGIVGDNLTNEARPQFHITVPTDVNSV
QLSLDGGINWVNATLTSDGVWEYIWPTDLVENTYTLTVKATDVAGNTATETLNFIIDTTL
STPTITLDSADDSGTANDNKTNVKTPGFIIGGIDSDVTQVVVQVMRDGHSEEVELTQTNG
QWRFVPGSAWTDGDYTLTVTVKDEAGNIRHSAPLTVTIDTQITIDHIELVNDSGIPDDNL
TNNVRPHFQVTVPTDVNVVRLSIDGGKTWFNATQSATPGVWDYTWLADVGEGKHTLTVEA
TDKAGNKTTQQLDFIIDTLLSEPTIVLDSTDDSGTKGDNLTNVNKPTFLLGNIDADARYV
TVEVQHGGTKEVLTATKDATGNWSVTPTGTWADGDYTLTVRVEDEAGNEKHSASLTVTVD
TQITIDVIELVNDNGIPGDNMTNDAHPQFRVTVPGDVNEVSLSIDGGVTWVKATQSATPG
VWNYTWPGTVPDGDYTLNVKATDNAGNTVTETLHFTIDTTLSVPVIVLNSADDTGVQGDN
MTNSTQPTFALQHIDDDAVRVTVSVEHGGVTTTFDATKGVGGWSFTPTGAWADGDYTLSV
SVEDKAGNTSHSASLTVTVDTQIAINNIELVNDSGIPDDNLTNNVRPHFQVKVPTDVNEV
RLSIDGGKTWFNATQSATPGVWDYTWLADVGEGKHTLTVEATDKAGNQTTQKLDFIIDTM
LSEPTIVLDSTDDSGTKGDNLTNANKPTFILGNIDADARYVTVEVQYGGTKEVLTATKGA
TGIWSVTPTGTWADGDYTLTVRVEDDAGNVKYSAPLTVTVDTQITIDVIELVNDNGIPGD
NLTNDVRPHFRVTVPGDVNEVRLSIDGGNTWVRATQGTAGIWDYTWPKDVTDGLHTLTVE
ATDKAGNKTTQTLDFTIDTRLSTPTITMDSRDDTGAIGDHITSVKRPGFTIGNIDSDAQS
VILRITQGGNSQEVTLTQVGGQWRFTPDADWADGSYTLTVEVTDNAGNVRQSTPLIVTVD
TQTSITDITLVNDHGVPDDNLTNSTRPQFEITVPADVNSVQLSIDGGANWVSAAQGIEGV
WGYTWPTDMGDGKHTLTVMVTDRAGNTATQTLEFFIDTRLSTPTIALDSTDDTGTPGDDM
TNRTRPTFILQNIDSDVINVTVSVTHNGTTTSFTATQGAGGWSFTPPAPWGDGDYTLTVT
VEDRAGNTRPSTPLTVTVDTQIAIDHIELVNDSGVPGDNVTKHVRPQFQISVPDDVEKVL
LSIDGGTTWVTAIKSSTAGIWDYTWPTDMPEGQHTLTVEVTDGAGNKMTETLNFTIDITL
MTPTIELAPDQDTGQNKNDNLTSVTQPVFVLGSIDKDVRHVELSIEHNGTFKTVVLTESA
DGWRYRPDSALADGSYTFTVTVTDVAGNQQTSAPLKVTIDGTLTTPVIELAAGEDSGTVG
DRLTNHDRPVFDIRQVDSDVTRVMVKVTYNGKTHEEAAVFTNGQWRFTPSASWADGSYQL
AVVVEDLAGNVKESAPFEVRIDTTTTINNIVLLNDTGVQNDQLTNVAKPSFRIDVPGDVV
QVRVTLDGGANWNVIRKNADGQWIFDSPNTLVDGTYTLRVEATDEAGNIANKDLVFNIDT
NIQVPTIALDAGQDTGANTADNITNISRPTFTIGNVDPDVIKVVVTIDGHDYNATKVGAG
WQFTPGNAIPDGSYNITVTVEDKAGNTATSKPLPVVIDTTAEIESVTLVTDSGDSDVDNI
TKVDKPQFSIVTADDITHVRVKIDNAANWIELTKGGDGRWIFNVGSALPDGQHTLLVDVT
DIAGNVAQETLQFTIDTTLREPTIVLDPTHDTGDDTNDNLTRINKPVFIIGNVDNDVSHI
VVHIDGRDYTIENTGGI

>EECEBICD_03805 hypothetical protein
MLAEEINILSPLVKKGISPYTNFLNKKQAYIKVKSEINDIESSITLKKDDIELVVNDIEA
LNNELRLSLSKIISKNLQELEVVNSTLKVIEKQINEEDIYSPVDGVIYKINKSATTHGGV
IQAADLLFEIKPKVRTMLADVKILPKYRDQIYVDEAVKLDVQSIIQPKIKSYNATIDNIS
PDSYEENTGGTIQRYYKVIIAFDVNEDDLRWLKPGMTVDASVITGKHSIMEYLLSPLMKG
VDKAFSEPVNTKRLDTP
>EECEBICD_03806 hypothetical protein
MNRRQSDHLMMIIISLTILIIILTYFIEINSVVHGQGVITTKDNAQLISLSKGGTIQDIY
VAEGDTVKKGELLAKVVNLDLQKEYQRYRTQKGYLDKDVNEISFILDKENESGLITLDGT
RSLSNKEVKANIELVHSQIRAKELKKPLLILRLADYKRS
>EECEBICD_03807 Outer membrane protein TolC
MKIKMFFLTTAFITQSTYASELPVIPLRDLVNAALTHQPSVAVSYYETEKKNSDLDLSRA
ALYPTLDLTSGLNNNRKESSGTERNVENKVSLSYRITDFGVRGANIRKSEYERDNSKTDY
EKTKNIVSQEVVTTYYNISKYREMIDGVNLEKEFYKKMLETFSLLVSSGVAMQSDMRKVQ
VSIDALNTRSIMYQSMLDDEMYKMQNMTGLNLSPVQIQSDEKFNLFKKYIFVESPEKLMD
MVMKYNDDYKMLVNTRKAATEDINAAKSSYFPTVDLVSSYVQNNPSGSAKKSDYEDEFKT
GINVSFNIFNGFRNSAQERKMVASYSQAKLQIDDFLIKTRYNIDSQLSRYAAAKETYSVA
ERSHTNALQLTELYEQEFQLGQKSLLDLISSRNEAFQAYVSMIDSKYSLYILKLQQLSLI
FHLMDYLKGNTESELNVMK
>EECEBICD_03808 hypothetical protein
MKYINHYRYLFVCFFLAILPFFALSFPGIREYVFDNFMVSAIYNGVIIAIYITGSLCALF
TILKNISAKDILIAQDASRKNSILSNLNQVLFAGEPKQCDFNLLMELDDNVSTARNQRLS
FIMSCSNVSTLVGLLGTFAGLSITIGSIGNLLSSPSDVGGDNASNTLNMIVTMVASLSEP
LKGMNTAFVSSIYGVVCAILLTSQSVFVRSSYSLVSTEIKKLKIISNRSNNKQRSLRVES
ETLVEFKELFKAFFDNYLTVENLRTQDEEKKRVMLSDSFVTLQNRLLDNSAKLEQLSTLI
DGYLVSNNENTKKLVDGVITITSRLSEGNTLLADNNARLEAMSTIQNIIDKKNDSIMTSV
DKCYQESLSHGKTINDIAAGSADISHTLDGLRKEMDEDMNNVHLALSDLSATDKKIIANT
KEISAEMVSYRDTYMPLMEKITSMHQEIVKQRLLNKEEKNED
>EECEBICD_03809 hypothetical protein
MEDESNPWPSFVDTFSTVLCIFIFLMLVFALNNMIIMYDNSIKVYKANIENKTKSTAQNS
GANDNSNDNEIVNKEVNTQDVSDGMTTMSGKEVGVYDIADGQKIDITSTKNELVITYHGR
LRSFSEEDTHKIEAWLEDKINSNLLIEMVIPQADISFSDSLRLGYERGIILMKEIKKIYP
DVVIDMSVNSAASSTTSKAIITTINKKVSE
>EECEBICD_03810 Single-stranded DNA-binding protein
MASRGVNKVILVGNLGQDPEVRYMPSGGAVANLTLATSESWRDKQTGEMKEQTEWHRVVM
FGKLAEVAGEYLRKGSQVYIEGQLRTRKWTDQSGQERYTTEINVPQIGGVMQMLGGRQGG
GAPAGGQQQGGWGQPQQPQQPQGGNQFSGGAQSRPQQSAPAPSNEPPMDFDDDIPF
>EECEBICD_03811 UvrABC system protein A
MDKIEVRGARTHNLKNINLVIPRDKLIVVTGLSGSGKSSLAFDTLYAEGQRRYVESLSAY
ARQFLSLMEKPDVDHIEGLSPAISIEQKSTSHNPRSTVGTITEIHDYLRLLFARVGEPRC
PDHDVPLAAQTVSQMVDNVLSQPEGKRLMLLAPIIKERKGEHTKTLENLASQGYIRARID
GEVCDLSDPPKLELQKKHTIEVVIDRFKVRNDLSQRLAESFETALELSGGTAVVADMDDE
KAEELLFSANFACPICGYSMRELEPRLFSFNNPAGACPTCDGLGVQQYFDPDRVIQNPDL
SLAGGAIRGWDRRNFYYFQMLKSLAEHYKFDVDAPWASLSANVHKVVLYGSGGKENIEFKY
MNDRGDTSVRRHPFEGVLHNMERRYKETESSAVREELAKFISNRPCASCEGTRLNREARH
VFVENTPLPAISDMSIGHAMDFFTNLKLSGQRAKIAEKVLKEIGDRLKFLVNVGLNYLTL
SRSAETLSGGEAQRIRLASQIGAGLVGVMYVLDEPSIGLHQRDNERLLGTLIHLRNLGNT
VIVVEHDEDAIRAADHVIDIGPGAGVHGGEVVAEGPLEAIMAVPESLTGQYMSGKRKIEV
PKQRVPANPEKVLKLTGARGNNLKDVTLTLPVGLFTCITGVSGSGKSTLINDTLFPIAQR
QLNGATIAEPAPYRDIQGLEHFDKVIDIDQSPIGRTPRSNPATYTGVFTPVRELFAGVPE
SRSRGYTPGRFSFNVRGGRCEACQGDGVIKVEMHFLPDIYVPCDQCKGKRYNRETLEIKY
KGKTIHEVLDMTIEEAREFFDAVPALARKLQTLMDVGLTYIRLGQSATTLSGGEAQRVKL
ARELSKRGTGQTLYILDEPTTGLHFADIQQLLDVLHQLRDQGNTIVVIEHNLDVIKTADW
IVDLGPEGGSGGGEILVSGTPETVAECEASHTARFLKPMLK

>EECEBICD_03812 hypothetical protein
MKLPYAITLLLCLFLAACTLPDRFSAVAFQQLTLLQTRSTRFLQDAARIPWQKETLLKDD
RDIRQTFFQAERVARQGGDKHRLDNLALLKNHYLRLYARVMQRKQPLTHIQAERYQQQNN
QVWKLAIQGECLHWGAHCTQGDENGVY
>EECEBICD_03813 hypothetical protein
MASINEIHYLMTTARAEHPVASSAIAEFIQTYKQAREDSDDAIRESAAFIARALQEHARG
WLDDDDMIILLEGQRDLARLRANNAQIALGSRIRSTVIRLIDIALALLVGAL
>EECEBICD_03814 putative protein YjbR
MTISELLQYCMAKPGAEQSVHSDWKATQIKVEDVLFAMVKEVEGRPAASLKTSPELAELL
RQQHSDVRPSRHLNKAHWSTVYLDGSLPDSQLYYLVDASYQQAVNALSEDKRKQLPQS
>EECEBICD_03815 hypothetical protein
MWYQRTITLSEKPRGFHLITDEIIDKLSGLPPVETGLLHLLLLHTSASLTLNENCDPTVR
ADMERHFLKTVPDNAAYEHDYEGADDMPSHIKSSVLGVSLLLPVRQGRLQLGTWQGIWLG
EHRIHGGPRKIIATLQGE
>EECEBICD_03816 Class B acid phosphatase
MKKITLALSAVCLLFTLNHSANALVSSPSTLNPGTNVAKLAEQAPVHWVSVAQIENSLTG
RPPMAVGFDIDDTVLFSSPGFWRGKKTYSPDSDDYLKNPAFWEKMNNGWDEFSIPKEVAR
QLIDMHVRRGDSIYFVTGRSQTKTETVSKTLADNFHIPAANMNPVIFAGDKPEQNTKVQW
LQEKNMRIFYGDSDNDITAARDCGIRGIRILRAANSTYKPLPQAGAFGEEVIVNSEY
>EECEBICD_03817 Aromatic-amino-acid aminotransferase
MFQKVDAYAGDPILSLMERFKDDSRHDKVNLSIGLYYNEDGIIPQLKTVAEAEARLNAQP
HGASLYLPMEGLNTYRHTIAPLLFGADHPVLQQQRVATIQTLGGSGALKVGADFLKRYFP
DAGVWVSDPTWENHIAIFAGAGFEVSTYPWYDDATNGIRFNDLLATLNTLPARSIVLLHP
CCHNPTGADLTPSQWDAVIEIVKARDLIPFLDIAYQGFGAGMDEDAYAIRAIASAGLPAL
VSNSFSKIFSLYGERVGGLSVVCEDAEIAARVLGQLKATVRRIYSSPPCFGAQVVATVLG
DEALKAGWLAEVDAMRNRIISMRQTLVKELKAEMPDRNFDYLLQQRGMFSYTGLSAEQVE
RLRDEFGVYLIASGRMCVAGLNASNVHRVAKAFAAVM
>EECEBICD_03818 Alanine racemase, biosynthetic
MQAATVVINRRALRHNLQRLRELAPASKLVAVVKANAYGHGLLETARTLPDADAFGVARL
EEALRLRAGGITQPILLLEGFFDAADLPTISAQCLHTAVHNQEQLAALEAVELAEPVTVW
MKLDTGMHRLGVRPEEAEAFYQRLTHCKNVRQPVNIVSHFARADEPECGATEHQLDIFNA
FCQGKPGQRSIAASGGILLWPQSHFDWARPGIILYGVSPLEHKPWGPDFGFQPVMSLTSS
LIAVRDHKAGEPVGYGGTWVSERDTRLGVVAMGYGDGYPRAAPSGTPVLVNGREVPIVGR
VAMDMICVDLGPNAQDNAGDPVVLWGEGLPVERIAEMTKVSAYELITRLTSRVAMKYID
>EECEBICD_03819 Replicative DNA helicase
MAGNKPFNKPQTDARDRDPQVAGIKVPPHSIEAEQSVLGGLMLDNERWDDVAERVVAEDF
YTRPHRHIFTEMGRLQESGSPIDLITLAESLERQGQLDSVGGFAYLAELSKNTPSAANIS
AYADIVRERAVVRDMIAVAHEIADAGYDPQGRNSDELLDLAESRVFQIAENRANKDEGPK
SIDQILDATVARIEQLFQQPHDGVTGVDTGYQDLNKKTAGLQRSDLIIVAARPSMGKTTF
AMNLCENAAMLQDKPVLIFSLEMPGEQIMMRMLASLSRVDQTRIRTGQLDDEDWARISGT
MGILLEKRNMYIDDSSGLTPTEVRSRARRIFREHGGLSLIMIDYLQLMRVPSLSDNRTLE
IAEISRSLKALAKELQVPVVALSQLNRSLEQRADKRPVNSDLRESGSIEQDADLIMFIYR
DEVYHENSDLKGIAEIIIGKQRNGPIGTVRLTFNGQWSRFDNYAGPQYDDE
>EECEBICD_03820 Quinone oxidoreductase 1
MATRIEFHKHGGPEVLQTVEFTPTEPAEHEIQVENKAIGINFIDTYIRSGLYPPPSLPAG
LGTEAAGVVSKVGNGVEHIRVGDRVVYAQSTLGAYSSVHNVTADKAAILPDAISFEQAAA
SFLKGLTVFYLLRKTYEVKPDEPFLFHAAAGGVGLIACQWAKALGAKLIGTVGSAQKAQR
ALDAGAWQVINYREESIVERVKEITGGKKVRVVYDSVGKDTWEASLDCLQRRGLMVSFGN
ASGPVTGVNLGILNQKGSLYATRPSLQGYITTREELTEASNELFSLIASGVIKVDVAENQ
RYALKDARRAHEVLESRATQGSSLLIP
>EECEBICD_03821 Phage shock protein G
MLELLFVLGFFLMLMVTGVSLLGILAALVVATAVMFLGGMFALMIKLLPWLLLAVAVVWV
IKAVKTPKIPQYQRNNRRFY
>EECEBICD_03822 tRNA-dihydrouridine(20/20a) synthase

MQPETQSSAFPAYRFSIAPMLDWTDRHCRYFLRLLSRQTLLYTEMVTTGAIIHGKGDYLA
YSEEEHPVALQLGGSDPAQLAHCAKLAEARGYDEINLNVGCPSDRVQNGMFGACLMGNAQ
LVADCVKAMRDVVSIPVTVKTRIGIDDQDSYAFLCDFIDTVSGRGECEMFIIHARKAWLS
GLSPKENREIPPLDYPRVYQLKRDFPHLTMSINGGIKSLEEAKEHLRHMDGVMVGREAYQ
NPGILAAVDREIFGADTTDADPVAVVRAMYPYIERELSQGAYLGHITRHMLGLFQGIPGA
RQWRRYLSENAHKAGADVAVLEQALKLVADKR
>EECEBICD_03823 hypothetical protein
MNKNVKLSLIAIAVSLFMAKQASAANTWTEARNDAMGGTGVASANYGSGVLLNPALLAKA
KPEDNITVVLPAVGVQITDKDNLQDEIDDISDKVDYYDEVVDNLTLGQILLNPRGVLNQF
QGAARDLADELEYLNGKTARANAGAGLAVSIPGQTLSVAFIAKGYAHGRVSSSIDQNDIQ
YLRDIQHDERVALREAGRAALLGSDEITKHLNSTASGRVAIVSDYGIALAKQFVVGEVPV
SIGVTPKLQKTWLYNYTTSIYNYDSSDWNSSRYRNDDTGFNIDAGLAADIGENWTLGLSG
QNLVSRDIDTKDIYITNGMTGETTNYKDTYQIRPLVTAGVAWHNDLLTVSADGDLTETKG
FKSEDNSQYVGVGAEVRPLSWLAVRAGYRADVKNNDSNVVTGGLGFAPFNRVHLDLMGLY
GEDETWGAGAQLTMTF
>EECEBICD_03824 Zinc uptake regulation protein
MEKTTTQELLAQAEKLCAQRNVRLTPQRLEVLRLMSLQQGAISAYDLLDLLRETEPQAKP
PTIYRALDFLLEQGFVHKVESTNSYVVCHLFDQPTHSSAMFICDRCGVVKEECAEGVEDI
MHTLAAKMGFALRHNVIEAHGLCPACVEVEACRHPGDCGHDHSVLVKKKPR
>EECEBICD_03825 hypothetical protein
MMNKDEAGGNWKQFKGKMKEQWGKLTDDDMTVIEGKRDQLVGKIQERYGYQKDQAEKEVV
DWETRNNYRW
>EECEBICD_03826 hypothetical protein
MVCQIVTVKNSEYKTEGRIPAYNYYHVQQKLQCIWANNTCYS
>EECEBICD_03827 DNA damage-inducible protein F
MPLFTSSDKALWHLALPMIFSNITVPLLGLVDTAVIGHLDSPVFLGGVAVGATATSFLFM
LLLFLRMSTTGLTAQAFGAKNPQALARALIQPLLLALGAGVMIVLFRTPLIELALYIVGG
NDAVLVQARRFLEIRWLSAPASLANLVLLGWLLGVQYARAPVILLVVGNILNIALDLWLV
MGLHMNVQGAALATVIAEYVTLLIGLMMVRKVLHLRGVSLDMLKQAWRGNVRRLLALNRD
IMLRSLLLQLCFGAITVSGARLGSDIIAVNAVLMTLLTFTAYALDGFAYAVEAHSGQAYG
ARDGSKLLDVWRAACRQSGIVALLFSTVYALAGEHIVALLTSLPQIQLLADRYLIWQVVL
PLVGVWCYLLDGMFIGATRAAEMRNSMAVAAGGFALTLFALPVLGNHGLWLALTVFLALR
GLSLSLIWRRHWREGTWFARS
>EECEBICD_03828 LexA repressor
MKALTARQQEVFDLIRDHISQTGMPPTRAEIAQRLGFRSPNAAEEHLKALARKGVLEIVS
GASRGIRLLQEEEDGLPLVGRVAAGEPLLAQQHIEGHYQVDPSLFKPSADFLLRVSGMSM
KDIGIMDGDLLAVHKTQDVRNGQVVVARIDDEVTVKRLKKQGNKVELLPENSEFTPIVVD
LREQSFTIEGLAVGVIRNGEWL
>EECEBICD_03829 Diacylglycerol kinase
MANNTTGFTRIIKAAGYSWKGFRAAWTHEAAFRQESIAVLLGVIIACWLDVDAITRVLLI
GSVLLIMIVEILNSAIEAVVDRIGSEYHELSGRAKDMGSAAVLLSIFVALMTWGILLWSH
FR
>EECEBICD_03830 Glycerol-3-phosphate acyltransferase
MSGWPRIYYKLLNLPLSILVKSKSIPAEPAQELGLDTSRPIMYVLPYNSKADLLTLRAQC
LAHDLPDPLEPLEIDGALLPRYVFIHGGPRVFTYYTPKEESVKLFHDYLDLHRSNPALDV
QMVPVSVMFGRAPGREKGEDNPPLRMLNGVQKFFAISWLGRDSFVRFSPSVSLRRMADEH
GTDKIIAQKLARVARMHFARQRLAAVGPRLPARQDLFNKLLASKAIARAVEDEARSKKIS
HEKAQQNAIALMEEIAANFSYEMIRLTDRILGFTWNRLYQGINVHNAERVRQLAHDGHEI
VYVPCHRSHMDYLLLSYVLYHQGLVPPHIAAGINLNFWPAGPIFRRLGAFFIRRTFKGNK
LYSTVFREYLGELFSRGYSVEYFVEGGRSRTGRLLDPKTGTLSMTIQAMLRGGTRPITLV
PIYIGYEHVMEVGTYAKELRGATKEKESLPQMLKGLSKLRNLGQGYVNFGEPMPLMTYLN
QHVPEWRESIDPIEAIRPAWLTPTVNSIAADLMVRINNAGAANAMNLCCTALLASRQRSL
TREQLTEQLDCYLDLMRNVPYSTDSTVPAASAGELIAHALQMNKFEVEKDTIGDIIILPR
EQAVLMTYYRNNIAHMLIMPSLMAAIITQHRRISRDALQQHVEALYPMLKAELFLRWERE

ELASVIDALASEMQRQGLITLQDDELHINPTHSRTLQLLAAGARETLQRYAITFWLLSAN
PSINRSTLEKESRTVAQRLSVLHGINAPEFFDKAVFSSLVLTLRDEGYISDTGDAEPAET
MKIYQMLADLITSDVRLTIESATQGE
>EECEBICD_03831 4-hydroxybenzoate octaprenyltransferase
MEWSLTQSKLLAFHRLMRTDKPIGALLLLWPTLWALWVATPGMPQLWILAVFVAGVWLMR
AAGCVVNDYADRKFDGHVKRTVNRPLPSGAVTEKEARNLFVVLVLLAFLLVLTLNAMTIL
LSVAALALAWVYPFMKRYTHLPQVVLGAAFGWSIPMAFAAVSESLPLSCWLMFLANILWA
VAYDTQYAMVDRDDDIKIGIKSTAILFGRYDTLIIGILQLGVMALMALIGWLNGLGWGYY
WAVLVAGALFVYQQKLIANREREACFKAFMNNNYVGLVLFLGLAMSYWHF
>EECEBICD_03832 Chorismate pyruvate-lyase
MSHPALTQLRALRYFDAIPALEPHLLDWLLLEDSMTKRFEQQGKRVSVTLIREAFVGQSE
VEEASGLLPSESRYWLREILLCADGEPWLAGRTVVPESTLCGPEQVLQHLGKTPLGRYLF
TSSTLTRDFIEIGRDATLWGRRSRLRLSGKPLLLTELFLPASPLY
>EECEBICD_03833 hypothetical protein
MKMKKSLVALCLTAGLFASVPGISLAEVNYVPQNTSAAPAIPAAALQQLTWTPVDQSKTQ
STQLATGGQRLDVAGITGPVAAYSVPANIGELTLTLTSEVNKQASVFAPNVLILDQNMTP
SAFFPSSYFTYQQPGVMSADRLEGVMRLTPALGQQKLYVLVFTTEKDLQQTTTLLDPAKA
YAKGVGNSIPDIPDPVARHTTDGVVKLKVKTNSSSSVLVGPLFGSSGTGPVTVGNTAAPV
AAPAPVAPKKSEPMLNDTESYFNKAIKDAVAKGDVDKALKLLDEAERLGSTSARSTFISS
VKGKG
>EECEBICD_03834 Maltoporin
MMITLRKLPLAVAVAAGVMSAQAMAVDFHGYARSGIGWTGSGGEQQCFQATGAQSKYRLG
NECETYAELKLGQEVWKEGDKSFYFDTNVAYSVNQQNDWESTDPAFREANVQGKNLIEWL
PGSTIWAGKRFYQRHDVHMIDFYYWDISGPGAGIENIDLGFGKLSLAATRSTEAGGSYTF
SSQNIYDEVKDTANDVFDVRLAGLQTNPDGVLELGVDYGRANTTDGYKLADGASKDGWMF
TAEHTQSMLKGYNKFVVQYATDAMTTQGKGQARGSDGSSSFTEELSDGTKINYANKVINN
NGNMWRILDHGAISLGDKWDLMYVGMYQNIDWDNNLGTEWWTVGVRPMYKWTPIMSTLLE
VGYDNVKSQQTGDRNNQYKITLAQQWQAGDSIWSRPAIRIFATYAKWDEKWGYIKDGDNI
SRYAAATNSGISTNSRGDSDEWTFGAQMEIWW
>EECEBICD_03835 Maltose/maltodextrin import ATP-binding protein MalK
MASVQLRNVTKAWGDVVVSKDINLDIHDGEFVVFVGPSGCGKSTLLRMIAGLETITSGDL
FIGETRMNDIPPAERGVGMVFQSYALYPHLSVAENMSFGLKLAGAKKEVMNQRVNQVAEV
LQLAHLLERKPKALSGGQRQRVAIGRTLVAEPRVFLLDEPLSNLDAALRVQMRIEISRLH
KRLGRTMIYVTHDQVEAMTLADKIVVLDAGRVAQVGKPLELYHYPADRFVAGFIGSPKMN
FLPVKVTATAIEQVQVELPNRQQIWLPVESRGVQVGANMSLGIRPEHLLPSDIADVTLEG
EVQVVEQLGHETQIHIQIPAIRQNLVYRQNDVVLVEEGATFAIGLPPERCHLFREDGSAC
RRLHQEPGV
>EECEBICD_03836 Maltose/maltodextrin-binding periplasmic protein
MKIKTGVGILALSALTTMMISAPALAKIEEGKLVIWINGDKGYNGLAEVGKKFEQDTGIK
VAVEHPDKLEEKFPQVAATGDGPDIIFWAHDRFGGYAQSGLLAEVTPDKAFQDKLYPFTW
DAVRYNGKLIAYPIAVEALSLIYNKDLVPNPPKTWEEIPALDKELKAKGKSAIMFNLQEP
YFTWPLIAADGGYAFKFENGKYDVKDVGVDNAGAKAGLTFLIDMIKNKNMSADTDYSIAE
AAFNKGETAMTINGPWAWSNIDKSKVNYGVTLLPTFKGKPSKPFVGVLSAGINAASPNKE
LAKEFLENYLLTDQGLEAVNKDKPLGAVALKSFQEQLAKDPRIAATMDNAQKGEIMPNIP
QMSAFWYAVRTAVINAASGRQTVDAALKDAQSRITK
>EECEBICD_03837 Maltose/maltodextrin transport system permease protein
MalF
MDVIKKKHWWQSDQLKWSVIGLLGLLVGYLVVLMYVQGEYLFAIMTLILSSAGLYIFANR
KTYAWRYVYPGLAGMGLFVLFPLVCTIAIAFTNYSSTNQLTFERAQQVLMDRSYQAGKTY
NFGLYPSGDEWQLALTDGETGKHYLSDAFSFGGEQKLQLKETDALPGGERANLRIITQNR
LALNQITAVLPDESKVIMSSLRQFSGTRPLYTLADDGLLTNNQSGVKYRPNNDSGYYQSI
NADGSWGDEKLSPGYTVTIGAKNFTRVFTDEGIQKPFFAIFVWTVVFSVLTVVLTVAVGM
VLACLVQWEALKGKAIYRVLLILPYAVPSFISILIFKGLFNQSFGEINMMLSALFGIKPA
WFSDPNTARAMVIIVNTWLGYPYMMILCMGLLKAIPDDLYEASAMDGAGPFQNFFKITLP

LLIKPLTPLMIASFAFNFNNFVLIQLLTNGGPDRLGTTTPAGYTDLLVSYTYRIAFEGGG
GQDFGLAAAIATLIFLLVGALAIVNLKATRMKFD
>EECEBICD_03838 Maltose/maltodextrin transport system permease protein MalG
MAMVQPKSQKLRLLITHLGLLIFIAAIMFPLLMVIAISLREGNFATGSLIPDKISWEHWR
LALGFSVEHADGRVTPPPFPVLLWLWNSVKIAGITAIGIVALSTTCAYAFARMRFPGKAT
LLKGMLIFQMFPAVLSLVALYALFDRLGQYIPFIGLNTHGGVIFAYLGGIALHVWTIKGY
FETIDSSLEEAAALDGATPWQAFRLVLLPLSVPILAVVFILSFIAAITEVPVASLLLRDV
DSYTLAVGMQQYLNPQNYLWGDFAAAAVLSAIPITLVFLLAQRWLVNGLTAGGVKG
>EECEBICD_03839 Protein PsiE
MMPLSRSRLEFIATILQNVLNLGLLTLGLILVVFLGKETVHLADALFVPEQASKYELVEG
LVIYFLYFEFIALIVKYFKSGLHFPLRYFVYIGITAIVRLIIVDHKTPMDVLLYSAAILL
LVITLWLCNSNRLRRE
>EECEBICD_03840 hypothetical protein
MKKTHLLSVLALGISAACHAETYPAPVGPSQSDFGGVGLLQTPTARMAREGEMSLNYRDN
DQYRYYSASVQLFPWLETTLRYTDVRTKKYSSVESFSGDQTYKDKAFDVKLRLWEESYWM
PQVAVGARDIGGTGLFDAEYIVASKAWGPFDFSLGLGWGYLGTSGNVSNPFCSYSDKFCS
RDNSYKEAGSVDGSDMFHGPASLFGGVEYQTPWQPLRLKLEYEGNNYQQDFAGKLEQKSK
FNVGAIYRVTDWADVNLSYERGNTFMFGVTLRTNFNDLRPAYHDNSRPQYRPQPQDAILQ
HSVVANQLTLLKYNAGLADPKIQVKGDTLYVTGEQVKYRDSREGIVRANRIVMNDLPEGI
RTIRVTENRLNLPQVTTETDVASLKRHLEGEPLGHETPLAQKRVEPIVPESTEQGWYIDK
SRVDFHLDPVLNQSVGGPENFYMYQLGVMGTADLWVTDHLLTTGSVFANIANNYDKFNYT
NPPKDSHLPRVRTHVREYVQNDVYVNNLQANYFQYFGNGFYGQVYGGYLETMFGGAGAEV
LYRPVDSNWAFGLDANYVKQRDWRSAQDMMKFTDYSVKTGHLTAYWTPSFAQDVLVKASV
GQYLAGDKGGTLEIAKRFDSGVVVGGYATITDASPDEYGEGDFTKGVYVSVPLDLFSSGP
TRSRAAIGWTPLTRDGGQQLGRKFGLYDMTSDRSVNFR
>EECEBICD_03841 hypothetical protein
MMKRMISALALAFIASSVFASGTVTVFTQGNSEPKTLTDAERLLDLVGQPRLATSWWPAA
VIGEEQATVTARQQQQELLGRLAALGAEEDGDAAAAINTLRRQIQAVKVTGRQLVNLDPD
VVRVSERGNPPLQGHYTLWVGPQPTQVTLFGLISQPGSQPFVPGRDVASYLEGQRLLSGA
DRSYAWVVYPDGRSQKAPVAYWNKRHIEPMPGSIIFVGFADSLWRGTPKAINADILHTLT
QRIPE
>EECEBICD_03842 putative lipoprotein GfcB
MKRLALILICLLLQACSATTKGLGDSLWDSLFGTPGVQLTDDDIQNMPYASQYMQLNGGP
QLFVVLAFSENGQQKWVTQDGATIVTQHGRLVKTLLSGDNLIDVNNLAADPLAKPGQIID
GATWTRTLGWTEHRQVRYATARSVFTWRGTDSVNVGSEETAVRVLDEEVTTDQTRWRNRY
WVDSEGQIRQTEQYLGANYFPVKTTLIKAAKS
>EECEBICD_03843 hypothetical protein
MMKKVLYGIFAITALAATSVSAAPVQVGEAAGSAATSVSAGSSSATSVSTVSSAVGVALA
ATGGGDGSNTGTTTTTTTSTQ
>EECEBICD_03844 Glucose-6-phosphate isomerase
MKNINPTQTSAWQALQKHYDEMKDVTIAELFANDSDRFAKFSATFDDLMLVDFSKNRITE
ETLAKLQDLAKETDLAGAIKSMFSGEKINRTEDRAVLHVALRNRSNTPIIVDGKDVMPEV
NAVLEKMKTFSQAIISGQWKGYTGKAITDVVNIGIGGSDLGPFMVTEALRPYKNHLTMHF
VSNVDGTHIAEVLKKVNPETTLFLVASKTFTTQETMTNAHSARDWFLKTAGDEKHVAKHF
AALSTNAKAVGEFGIDTANMFEFWDWVGGRYSLWSAIGLSIILSVGFDNFVELLSGAHAM
DKHFSTTPAEKNLPILLALIGIWYNNFFGAETEAILPYDQYMHRFAAYFQQGNMESNGKY
VDRNGNAVDYQTGPIIWGEPGTNGQHAFYQLIHQGTKMVPCDFIAPAITHNPLSDHHQKL
LSNFFAQTEALAFGKSREVVEQEYRDQGKDPAQLEHVVPFKVFEGNRPTNSILLREITPF
SLGALIALYEHKIFTQGAILNIFTFDQWGVELGKQLANRILPELGDDKAISSHDSSTNGL
INRYKAWRA
>EECEBICD_03845 Lysine-sensitive aspartokinase 3
MTEIVVSKFGGTSVADFDAMNRSADIVLSDANVRLVVLSASAGITNLLVALAEGMEPGER
FATLDAIRKIQFDILDRLRHPNVIREEIERLLENITILAEAASLATSAALTDELVSHGEL

MSTLLFVEILRERDVQAHWFDVRKVMRTSDRFGRAEPDVAALAELAAQQLLPRLSETLVI
TQGFIGSESKGRTTTLGRGGSDYTAALLAEALHAARVDIWTDVPGIYTTDPRVVPVAQRI
DEIDFEEAAEMATFGAKVLHPATLLPAVRSDIPVFVGSSKDPQAGGTLVCNKTQNPPLFR
ALALRRNQTLLTLHSLNMLHSRGFLAEVFGILARHNISVDLITTSEVSVALTLDTTGSTS
TGDTLLTQSLLMELSALCRVEVEEGLALVALIGNNLSKACGVGKEVFGVLEPFNIRMICY
GASSHNLCFLVPGDDAEKVVQKLHQNLFE
>EECEBICD_03846 hypothetical protein
MIQKSKEMVRLPEIINDLAFHASQVLIESMNIDSASAENAGQAIADRMMRNWGGQSIYFP
KGISGRASERDYQIYSECDGRNYAELAKKYNLTLQWIYKIVKRVHTEKQHQRRML
>EECEBICD_03847 UDP-3-O-(3-hydroxymyristoyl)glucosamine N-acyltransferase
MYIYSSKKQKKTGLWINRKLNSKFGIDIELGAVIGYGLDIPHHMGIVITKKARIGCNLSL
KQNTTVGNKQGLKEDDFIIIGNNVDIGANTCIIGSITIGDNVTIGAMSFVNKSIPANSIY
ITKKTSEVIPVINHKKWD
>EECEBICD_03848 hypothetical protein
MIIVSVLRQSKDFTTKHAQWLHKQLKGYDSVCLTDALKIKGVNTAPLLYDWPGWWAKLEL
FNPLHPVLGNEDILYIDIDSVVVGDITPLTTMKKITLLNDFSQHGASVAPATGIMFIPAP
AKKNVWDEFMKNPEKEINAIRTPPYHGDQGFIGRICQDAERWQNILPGRIISYKANIATP
KMIGFNPELYDGTGNGKLPDGVSIVCFHGSPRPWNTALPWVPYFSLKNTIQSKVKQYKLS
LR
>EECEBICD_03849 hypothetical protein
MKKIAIIGSSGGNLYNLGGAEPEKLLQEIYQQCEAAGVNVAAVQFIAAEASMDVAKPDTP
ASVYALTTENQQKPQRIFQGKLSEVNTSVVESDRQIAEMIRRGEIDGIVVMSADPVKANQ
AVFAAAVEMKTPIVGTGGTSMALVAAKGANVVATSGTTGTTSRTRAVSFVASLCKHWGIK
YKPQLGSASPSQSGSGKSLLKRFNIRSIMIPALPGFIAMAIVLALSHIPGLEKLNDIFEI
LLKGLPVLVAVLAAKQISELDEVSIVAGVVAGVLSVEGGLIGGIIGGVMAGIFVRWLFEL
CLNWRFPMTTVNIVAGGISGLAAGLVMHYLLSPLALSAGNYIKLAIESTLAFSPILAGLL
AGLVIWPAILGGVYHAVILPLVLLEMEKSGVSFLGAVDMVGLVMVAAGINLANVIAPREK
SEAAVATPGLLINLGFGTFVESAYPFMFANKIVFGSAIFWAGMGGMMLGFFNVKGVAYVP
AFASPFLSSNALQMAIVMIATMAMTCLTTIIANRFKPVVQSESTTTAVN
>EECEBICD_03850 hypothetical protein
MFKGDMNKKRAKALQKVKEAITLHGGQTILSTGITGDDARLAKAVCEAGVKLLEPNHPAL
ALARGHKGVSNMHAAEQIRHEITNSQMAEAVHGVRNVVPDDIFITVGIAGGFTETLPVPL
SETEILEIARAGADGLHTHKSDWDDLQDIVNLAHQYGLTVDAYIGHPDDLHTFGIPARTP
EEVASVAKRMQDIGVDMIGLMTGMSYEGVAAGDIPQMIKDRLRALVGAVDVPTLAEGGIN
LANAKAFKDTGVNILVVGTAIDNMVCNAAKEAVTPFIVH
>EECEBICD_03851 Sugar phosphatase YidA
MLLITDLDGTLLTSQKTISPRTRQALIAFRQDGGLLAACSARPVSSMVRLLRQQQVDRLF
SWCAGFNCGHLLEMAGQRIIHAAPLTATDLWNIDQHISLSRYHHHFFSAEAIHHRDDRLI
AHWTTYEARLFGLPLITETAENIFNRRNIYKITLVAASPEIDNLCTEVNNHLPCGYYAVV
TGENYIDIQRSDINKGCIIEQLIHYLNISSDKVVAIGDQQNDVSMFAAAGISIAMGNAPD
AVKRQAGYVTATNDEEGIVHALEWLRCLTHPVTMRQRLTAAKDNESN
>EECEBICD_03852 HTH-type transcriptional repressor GlcR
MNPIKRRKHILDELNKFGEVTVIKLSEMLNVTSETIRRDLSLLESEGKITKIHGGAVKKQ
VVQEDAFSARIEAHREEKIAIGKLAAEMVSERDTLFIDSCTTNLILAEQLPPLAFSVITN
SALIADKIKEHNLQARVYVLGGEYDYHFRANLGVSVCQQINAIHADICFIGAGGISPQHG
VLVKSFEEAYVAKAMIAMSKKSVILADHTKFGQDGVMCIASVKEIDNIITDKGFTSTGYT
PQDFAGKLRIAD
>EECEBICD_03853 hypothetical protein
MALPRITQKEMTEREQRELKTLLDRARIAHGRPLTNSETNSVKKEYIDKLMALREAEAKK
ARQLKKKQAYKPDTEASFSWSANTPTRGRR
>EECEBICD_03854 Dual-specificity RNA pseudouridine synthase RluF
MLTDTSIRLNKYISESGICSRREADRFIEQGNVFINGKRAAIGDQVVAGDVVKVNGRLIE
PREADDLVLIALNKPVGIVSTTEDGERDNIVDFVNHSKRIFPIGRLDKDSQGLIFLTNHG
DLVNKILRAGNDHEKEYLVTVDKPITDEFIRGMGAGVPILGTVTKKCKVKKEAPFVFRIT

LVQGLNRQIRRMCEYFGYEVTKLERTRIMNVSLSGIPLGEWRDLTDDELIDLFKLIERSS
SEAKPKAKAKPKTTGIKRPVIAIEKSNEKARPASSGKRFTSPGRKKKGR
>EECEBICD_03855 hypothetical protein
MAKLTLQEQLLQAGLVTSKKMAKVQRTAKKSRVQAREAREAVEKNKKAQLERDKQLSEQQ
KQAVLAKEFKAQVKQLIEMNRINVSKGDIGFNFTDNNVIKKIDVDKLTQTQLINGRLAIA
RLVINNSGECEYAIIPASVADKIAQRDADSIVVNSALSQEEQDEDDPYADFKVPDDLMW
>EECEBICD_03856 Peptidase E
MFIPFAGVTQTWDEYTDKTAEVLAPLGINVTGIHRVADPLAAIEKAEIIIVGGGNTFQLL
KESRERGLLAPMADRVKRGALYIGWSAGANLACPTIRTTNDMPIVDPNGFDALDLFPLQI
NPHFTNALPEGHKGETREQRIRELLVVAPELTVIGLPEGNWIQVSNGQAVLGGPNTTWVF
KAGEEAVALEAVIAFNPWPH
>EECEBICD_03857 hypothetical protein
MLTLLHLLSAVALLVWGTHIVRTGVMRVFGARLRTVLSRSVEKKPLAFCAGIGVTALVQS
SNATTLLVTSFVAQDLVALTPALVIVLGADVGTALMARILTFDLSWLSPLLIFIGVIFFL
GRKQSRAGQLGRVGIGLGLILLALELIVQAVTPITQANGVQVIFASLTGDIMLDALIGAM
FAIISYSSLAAVLLTATLTAAGIISFPVALCLVIGANLGSGLLAMLNNSAANAAARRVAL
GSLLFKLIGSLVILPFVHPLANLMDELSLPKSELVIYFHVFYNLVRCLAMVPFAELMARF
CKRIIRDEPELDTHLKPKHLDVSALDTPTLALANAAREVLRIGDAMEQMMEGLKKVMHGE
PREEKALRKLADDVNVLYTAIKLYLARMPKDELAAEESRRWAEIIEMALNLEQASDIIER
MGSEIADKSLAARRAFSEEGLKELDALYDQLLSNLQLAMSVFFSGDVTSARRLRRSKHRF
RILNRRYSHAHVDRLHQQNVQSIETSSLHLGLLGDMKRLNSLFCSVAYSVLEQPDQDEER
GEY
>EECEBICD_03858 Methionine synthase
MSSKVEQLRAQLNERILVLDGGMGTMIQSYRLHEEDFRGERFADWPCDLKGNNDLLVLSK
PEVIAAIHNAYFEAGADIIETNTFNSTTIAMADYRMESLSAEINYAAAKLARACADEWTA
RTPEKPRFVAGVLGPTNRTASISPDVNDPAFRNITFDQLVAAYRESTKALVEGGADLILI
ETVFDTLNAKAAVFAVKEEFEALGVDLPIMISGTITDASGRTLSGQTTEAFYNSLRHAEA
LTFGLNCALGPDELRQYVQELSRIAECYVTAHPNAGLPNAFGEYDLDADTMAKQIREWAE
AGFLNIVGGCCGTTPEHIAAMSRAVAGLLPRQLPDIPVACRLSGLEPLNIGDDSLFVNVG
ERTNVTGSAKFKRLIKEEKYSEALDVARQQVESGAQIIDINMDEGMLDAEAAMVRFLSLI
AGEPDIARVPIMIDSSKWEVIEKGLKCIQGKGIVNSISMKEGVEAFIHHAKLLRRYGAAV
VVMAFDEQGQADTRARKIEICRRAYKILTEEVGFPPEDIIFDPNIFAVATGIEEHNNYAQ
DFIGACEDIKRELPHALISGGVSNVSFSFRGNDPVREAIHAVFLYYAIRNGMDMGIVNAG
QLAIYDDLPAELRDAVEDVILNRRDDGTERLLDLAEKYRGSKTDEAANAQQAEWRSWDVK
KRLEYSLVKGITEFIEQDTEEARQQAARPIEVIEGPLMDGMNVVGDLFGEGKMFLPQVVK
SARVMKQAVAYLEPFIEASKEKGSSNGKMVIATVKGDVHDIGKNIVGVVLQCNNYEIVDL
GVMVPAEKILRTAREVNADLIGLSGLITPSLDEMVNVAKEMERQGFTIPLLIGGATTSKA
HTAVKIEQNYSGPTVYVQNASRTVGVVAALLSDNQRDDFVARTRKEYETVRIQHARKKPR
TPPVTLEAARDNDLAFDWERYTPPVAHRLGVQGVEASIETLRNYIDWTPFFMTWSLAGKY
PRILEDEVVGVEAQRLFKDANDMLDKLSAEKLLNPRGVVGLFPANRVGDDIEIYRDATRT
HVLTVSHHLRQQTEKVGFANYCLADFVAPKLSGKADYIGAFAVTGGLEEDALADAFEAQH
DDYNKIMVKAIADRLAEAFAEYLHERVRKVYWGYAPNESLSNDELIRENYQGIRPAPGYP
ACPEHTEKGTIWQLLDVEKHTGMKLTESFAMWPGASVSGWYFSHPESKYFAVAQIQRDQV
TDYAFRKGMSVEDVERWLAPNLGYDAD
>EECEBICD_03859 Transcriptional repressor IclR
MVAPVPAKRGRKPAATTAPVTGQVQSLTRGLKLLEWIAESNGSVALTELAQQAGLPNSTT
HRLLTTMQQQGFVRQVGELGHWAVGAHAFIVGSSFLQSRNLLAIVHPILRKLMEDSGETV
NLAVLDQSDHQAIIIDQVQCTQLMRMSAPIGGKLPMHASGAGKAFLSQLSEEQVTSLLHR
KGLHAYTHATLVSPLHLKDDLAQTRKRGYSFDDEEHALGLRCVASCIYDEHREPFAALSI
SGPCSRITDDRVTELGAMVIKAAKEVTLAYGGTR
>EECEBICD_03860 hypothetical protein
MVELAEMSSLKLYWQEKYPQAFCWSFGDSPALADELAALVVAGKKRGTCSSLVSYQKEQP
PVTPGSYHIVLNGTGDAVCVIRTLALRLIRFNEMSADLAALEGEGDLSLAYWQAAHRAFF
EREGNWSPEMELVYEEFAVLEIAP

>EECEBICD_03861 Isocitrate dehydrogenase kinase/phosphatase
MPRGLELLIAQTILQGFDAQYGRFLEVTSGAQQRFEQADWHAVQQAMKSRIHLYDHHVGL
VVEQLRCITDGKSTDADFLLRVKEHYTRLLPDYPRFEIAESFFNSVYCRLFDHRSLTPER
LFIFSSQPERRFRTIPRPLAKDFFPDHGWEPLLMRILSDLPLRLPWQNKSRDIRYIIAHL
TETLGEDALPRCHVQVANELFYRNKAAWLVGKLTTPDGTLPFLLPIHRTDEGELFVDTCL
TTTAEASIVFGFARSYFMVYAPLPAALVEWLREILPGKTTAELYMAIGCQKHAKTESYRE
YLCYLAESDEKFIEAPGIRGMVMLVFTLPGFDRVFKIIKDKFAPQKEMSAAHVRACYQLV
KEHDRVGRMADTQEFENFVLDKRQIDPALMALLRQEVPEKITDLGEHIVIRHLYIERRMV
PLNIWLEQVEGQQLRDAIEEYGNAIRQLAAANIFPGDMLFKNFGVTRHGRVVFYDYDEIC
YMTEVNFRDIPPARYPEDELASEPWYSVSPGDVFPEEFRHWLCADPRIGPLFEEMHADLF
RADYWRALQTRIKEGHVEDVYAYRRRQRFSVRYGAISSTANSS
>EECEBICD_03862 Isocitrate lyase
MKTRTQQIEELQKEWTQPRWEGITRPYSAEEVVKLRGSVNPECTLAQLGAAKIWRLLHGE
AKKGYINSLGALTGGQALQQAKAGIEAIYLSGWQVAADANLASSMYPDQSLYPANSVPAV
VDRINNTFRRADQIQWASGIEPNDPRYVDYFLPIVADAEAGFGGVLNAFELMKSMIEAGA
AAVHFEDQLASVKKCGHMGGKVLVPTQEAIQKLVAARLAADVMGVPTLVIARTDADAADL
ITSDCDPYDSGFITGERTSEGFYRTHAGIEQAISRGLAYAPYADLVWCETSTPDLELARR
FADAIHAKYPGKLLAYNCSPSFNWQKNLDDKTIASFQQQLSDMGYKYQFITLAGIHSMWF
NMFDLAHAYAQGEGMKHYVEKVQQPEFAAAKDGYTFVSHQQEVGTGYFDKVTTIIQGGAS
SVTALTGSTEESQF
>EECEBICD_03863 Malate synthase A
MNPQATTTDELTFTRPQGELEKQVLTAEAVEFLTELVTRFTPKRNKLLAARIQQQQDIDN
GKLPDFISETTSIRESNWQIRGIPADLQDRRVEITGPVERKMVINALNANVKVFMADFED
SLAPDWNKVIDGQINLRDAVNGTISYTNEAGKIYQLKPDPAVLICRVRGLHLPEKHVTWR
GEAIPGSLFDFALYFFHNYKALLAKGSGPYFYLPKTQAWQEAAWWSEVFSYAEDRFNLPR
GTIKATLLIETLPAVFQMDEILHALRDHIVGLNCGRWDYIFSYIKTLKNHPDRVLPDRQV
VTMDKPFLSAYSRLLIKTCHKRGAFAMGGMAAFIPSKDVERNNQVLAKVKADKALEANNG
HDGTWIAHPGLADTAMAVFNEVLGEHKNQLFITRDEDAPITAEQLLEPCEGERTEAGMRA
NIRVAVQYIEAWISGNGCVPIYGLMEDAATAEISRTSIWQWIHHEKTLSNGKPVTKALFR
EMLAEEMRVIQDELGEHRYSSGRFDDAARLMEQITTSDDLIDFLTLPGYRLLA
>EECEBICD_03864 Homoserine O-succinyltransferase
MPIRVLDELPAVNFLREENVFVMTTSRASGQEIRPLKVLILNLMPKKIETENQFLRLLSN
SPLQVDIQLLRIDARESRNTPAEHLNNFYCNFDDICDQNFDGLIVTGAPLGLVEFNDVAY
WPQIRQVLEWAKDHVTSTLFVCWAVQAALNILYGIPKQTRTDKLSGVYEHHILHPHALLT
RGFDDSFLAPHSRYADFPAALIRDYTDLEILAETEEGDAYLFASKDKRIAFVTGHPEYDA
HTLAGEYFRDVEAGLNPEVPYNYFPKNDPQNIPRATWRSHGNLLFTNWLNYYVYQITPYD
LRHMNPTLD
>EECEBICD_03865 Peptidyl-lysine N-acetyltransferase YjaB
MMINIRRSRHEEGEKLIAIWRRSVDTTHDFLSNAYRAELEELVSDFLPEAPLWVAVTDQD
EPVGFMLLTGEHMDALFVDPDVRGQGIGKRLVEHALTLAPGLTTNVNEQNTQAVGFYKKM
GFKVTGRSEVDDLGKPYPLLNLIYP
>EECEBICD_03870 Bifunctional purine biosynthesis protein PurH
MQQRRPVRRALLSVSDKAGIIEFAQALSARGVELLSTGGTARLLAEKGLAVTEVSDYTGF
PEMMDGRVKTLHPKVHGGILGRRGQDDAIMEQHHIAPIDMVVVNLYPFAETVARVGCSLE
DAVENIDIGGPTMVRSAAKNHKDVAIVVKSSDYDAIIKEMDANEGSLTLDTRFDLSIKAF
EHTAAYDSMIANYFGSMVPAYHGESKEAAGRFPRTLNLNFIKKQDMRYGENSHQQAAFYI
EENVKEASVATAQQVQGKALSYNNIADTDAALECVKAFNEPACVIVKHANPCGVAVSTSI
LDAYDRAYKTDPTSAFGGIIAFNRELDAETAQAIISRQFVEVIIAPSATEEALKITAAKQ
NVRVLTCGQWAQRVPGLDFKRVNGGLLVQDRDLGMVSEAELRVVSKRQPTEQELRDALFC
WKVAKFVKSNAIVYAKENMTIGIGAGQMSRVYSAKIAGIKAADEGLEVKGSAMASDAFFP
FRDGIDAAAAVGVSCVIQPGGSIRDDEVIAAADEHGIAMIFTDMRHFRH
>EECEBICD_03871 Phosphoribosylamine--glycine ligase
MKVLVIGNGGREHALAWKAAQSPLVDTVFVAPGNAGTALEPALQNVAIGVTDIPALLSFA
QHEKIDLTIVGPEAPLVIGVVDAFRAAGLKIFGPTEGAAQLEGSKAFTKDFLARHQIPTA

EYQNFTEIEPALAYLREKGAPIVIKADGLAAGKGVIVAMTLEEAEAAVHDMLAGNAFGDA
GHRIVIEEFLDGEEASFIVMVDGEHVLPMATSQDHKRVGNGDTGPNTGGMGAYSPAPVVT
DEVHQRTMERIIWPTVKGMAAEGNTYTGFLYAGLMIDKQGNPKVIEFNCRFGDPETQPIM
LRMKSDLVDLCLAACDGKLDEKTSEWDERASLGVVIAAGGYPGNYNTGDEIHGLPLEEVA
DGKVFHAGTKLADDDRVLTSGGRVLCATALGHTVAEAQKRAYALMTDIRWDGSFSRNDIG
WRAIEREQN
>EECEBICD_03872 Transcriptional regulatory protein ZraR
MIRGKIDILVVDDDVSHCTILQALLRGWGYNVALAYSGHDALAQVREKVFDLVLCDVRMA
EMDGIATLKEIKALNPAIPILIMTAFSSVETAVEALKAGALDYLIKPLDFDRLQETLEKA
LAHTRETGAELPSASAAQFGMIGSSPAMQHLLNEIAMVAPSDATVLIHGDSGTGKELVAR
ALHACSARSDKPLVTLNCAALNESLLESELFGHEKGAFTGADKRREGRFVEADGGTLFLD
EIGDISPLMQVRLLRAIQEREVQRVGSNQTISVDVRLIAATHRDLAEEVSAGRFRQDLYY
RLNVVAIEMPSLRQRREDIPLLADHFLRRFAERNRKAVKGFTPQAMDLLIHYDWPGNIRE
LENAIERAVVLLTGEYISERELPLAIAATPIKTEYSGEIQPLVDVEKEVILAALEKTGGN
KTEAARQLGITRKTLLAKLSR
>EECEBICD_03873 Sensor protein ZraS
MSGSELTRFRRQKASKNDSHDPPGESFLPIRIRKGVRQRAVMRSEEWHDLCLSKENKNEP
GKPVSFIRLHKDAAATWLSRLLPAAIFILVGLFSIMVIRDYGRESAAARQTLLEKGNVLI
RALESGTRVGMGMRMHHAQQQTLLEEMAGQPGVLWFAVTDAQGVIITHSNPGMVGKSLYS
ASEMHQLNPGPQERWRRVDVAANGETVPALEIYRQFQPLFGMRGHGMRGHGMARSANDDE
PAKQTIFIAFDASELAATQAREWRNTLIVLSALATVLLATLLAFFWHQRYQRSHRELLDA
MKRKEKLVAMGHLAAGVAHEIRNPLSSIKGLAKYFAERTPAGGESHELAQVMAKEADRLN
RVVSELLELVKPAHLTLQAVNLNDIITHSLNLVSQDAQSREIQLRFTANETLKRIQADPN
RLTQVLLNLYLNAIHAIGRQGTITVEAKESGTDRVIITVTDSGKGIAPDQLEAIFTPYFT
TKADGTGLGLAVVQNIIEQHGGAIKVKSIEGKGAVFTIWLPVIARQQD
>EECEBICD_03874 Zinc resistance-associated protein
MKRNNKSAIALIALSLLALSSGAAFAGHHWGNNDGMWQQGGSPLTTEQQATAQKIYDDYY
TQTSALRQQLISKRYEYNALLTASSPDTAKINAVAKEMESLGQKLDEQRVKRDVAMAQAG
IPRGAGMGYGGCGGYGGGYHRGGGHMGMGNW
>EECEBICD_03875 hypothetical protein
MNSFIEGARQPLLSVWRRAFLFSGALLLTACSHNASPPPFTASGFAGDHGAVRIWRKDTN
DEVHLLSVFSPWHSGSTTTSEYRWQGDTLSLIELNIYSKPPEHIRARFDAHGELSFMQRE
VGGQKQQLSNDQIALYRYRAEQIRQTSDALRLGRVILRQGRWHADHTVTTCEGETLKPDL
DSWAISHIERRQNHSSVEVSVAWLEAPEGSQLLLVANSDFCHWQPQAKTF
>EECEBICD_03876 DNA-binding protein HU-alpha
MNKTQLIDVIADKAELSKTQAKAALESTLAAITESLKEGDAVQLVGFGTFKVNHRAERTG
RNPQTGKEIKIAAANVPAFVSGKALKDAVK
>EECEBICD_03877 putative protein
MLQNPIHLRLERLESWQHVTFMACLCERMYPNYAMFCKQTEFGDGQIYRRILDLIWETLT
VKDAKVNFDSQLEKFEEAIPAADDYDLYGVYPAIDACVALSELMHSRLSGETLEHAIEVS
KTSITTVAMLEMTQAGREMTDEELKTNPAVEQEWDIQWEIFRLLADCEERDIELIKGLRA
DLREAGESNIGINFQQ
>EECEBICD_03878 Endonuclease V
MDLASLRAQQIELASSVCREDRLDKDPPAFIGGADVGFEQGGEVTRAAMVLLKYPSLELV
EYKVARIATTMPYIPGFLSFREYPALLAAWEQLSQKPDLLFVDGHGISHPRRLGVASHFG
LLVDVPTIGVAKKRLCGKFEPLSTEPGALSPLMDKGEQLAWVWRSKARCNPLFIATGHRV
STDSALAWVQRCMKGYRLPEPTRWADAVASGRPAFVRWQEIQR
>EECEBICD_03879 Uroporphyrinogen decarboxylase
MTELKNDRYLRALLRQPVDVTPVWMMRQAGRYLPEYKATRAQAGDFMSLCKNAELACEVT
LQPLRRYPLDAAILFSDILTIPDAMGLGLYFEAGEGPRFTAPVTCKADVDKLPIPDPEDE
LGYVMNAVRTIRRELKGEVPLIGFSGSPWTLATYMVEGGSSKAFTVIKKMMYADPQALHL
LLDKLAKSVTLYLNAQIKAGAQSVMIFDTWGGVLTGRDYQQFSLYYMHKIVDGLLRENDG
RRVPVTLFTKGGGQWLEAMAETGCDALGLDWTTDIADARRRVGHKVALQGNMDPSMLYAP
PARIEDEVATILAGFGQGEGHVFNLGHGIHQDVPPEHAGAFVEAVHRLSAQYHN

>EECEBICD_03880 NADH pyrophosphatase
MDRIIEKLESGWWIVSHEQKLWLPYGELPHGLAANFDLVGQRALRIGEWQGEPVWLVLQH
RRHDMGSVRQVIDQDAGLFQLAGRGVQLAEFYRSHKFCGYCGHPMHPSKTEWAMLCSHCR
ERYYPQIAPCIIVAIRREDSILLAQHVRHRNGVHTVLAGFVEVGETLEQAVAREVMEESG
IKVKNLRYVTSQPWPFPQSLMTAFMAEYDSGEIVIDPKELLEANWYRYDDLPLLPPPGTV
ARRLIEDTVAMCRAEYD
>EECEBICD_03881 Regulator of sigma D
MLNQLENLTERVGGSNKLVDRWLDVRKHLLVAYYNLVGIKLGKESYMRLNEKALDDFCQS
LVDYLSAGHFSIYERILHKLEGNGQLLHAAKIWPLLEDNTQRIMDYYDTSLETAIDHDNC
LEFQQALSDIGEALEARFVLEDKLIMLVFDAMHDGARVKRPA
>EECEBICD_03882 Phosphomethylpyrimidine synthase
MSTTTLTRREQRAKAQHFIDTLEGTAFPNSKRIYVTGSQHDIRVPMREIQLSPTLIGGSK
DNQQFEENEAVPVYDTSGPYGDPEVAINVQQGLAKLRQPWIDARNDSEELDDRSSAYTRE
RLADDGLDDLRFTGLLTPKRAKAGKRITQLHYARQGIVTPEMEFIAIRENMGRERICSEV
LRHQHPGMSFGARLPENITPEFVRDEVAAGRAIIPANINHPESEPMIIGRNFLVKVNANI
GNSAVTSSIEEEVEKLVWSTRWGADTVMDLSTGRYIHETREWILRNSPVPIGTVPIYQAL
EKVNGIAEDLTWEAFRDTLLEQAEQGVDYFTIHAGVLLRYVPMTAKRLTGIVSRGGSIMA
KWCLSHHKENFLFEHFREICEICAAYDVSLSLGDGLRPGSIQDANDEAQFSELHTLGELT
KIAWEYDVQVMIEGPGHVPMHMIQRNMTEELESCHEAPFYTLGPLTTDIAPGYDHFTSGI
GAAMIGWFGCAMLCYVTPKEHLGLPNKEDVKQGLITYKIAAHAADLAKGHPGAQIRDNAM
SKARFEFRWEDQFNLALDPFTARAYHDETLPQESGKVAHFCSMCGPKFCSMKISQEVRDY
AAAQAIEVGMADMSENFRAKGGEIYLKREEA
>EECEBICD_03883 Thiamine-phosphate synthase
MYQPDFPTVPFRLGLYPVVDSVAWIERLLEAGVRTIQLRIKDKRDEEVEADVIAAIALGR
RYNARLFINDYWRLAIKHRAYGVHLGQEDLETTDLKAIQAAGLRLGVSTHDDMEIDVALA
AKPSYIALGHVFPTQTKQMPSAPQGLAQLASHIERLADYPTVAIGGISLERATAVLATGV
GSIAVVSAITQAADWRAATAQLLDIAGVGDE
>EECEBICD_03884 Sulfur carrier protein ThiS adenylyltransferase
MNDRDFMRYSRQILLGDIAIEGQQKLLASHVLIVGLGGLGSPAALYLAGAGIGKLTLADD
DDIHLSNLQRQILFTTDDIARSKSQVARQRLTQLNPDIELVSLQRRLKGDALRHAVSRAD
VVLDCTDNMSTRQEINAACVALNTPLITASAVGFGGQLMVLMPPWEQGCYRCLWPDDVEP
ERNCRTAGVLGPVVGVMGTLQALEAIKLLSGIETPNGQLRLFDGKTSQWRSLALRRASGC
PVCGGQHANSIQ
>EECEBICD_03885 Sulfur carrier protein ThiS
MQCAAGQTVSGLLIQLNQLKPGAALALNQQILPREQWEQQIVQEGDQILLFQVIAGG
>EECEBICD_03886 Thiazole synthase
MLRIADKTFDSHLFTGTGKFASSQLMVEAIRASGSQLVTLAMKRVDLRQHNDAILAPLIE
AGVTLLPNTSGAKTAEEAIFAAQLAREALGTNWLKLEIHPDARWLLPDPIETLKAAEALV
KQGFVVLPYCGADPVLCKRLEEVGCAAVMPLGAPIGSNQGLETKTMLEIIIQQATVPVVV
DAGIGVPSHAAQALEMGADAVLVNTAIAVADDPVMMATAFRLAVEAGLLARQAVPGNRST
YASATSPLTGFLEALA
>EECEBICD_03887 2-iminoacetate synthase
MKTFTDRWRQLEWDDIRLRINGKTAADVERALNAAHLSRDDLMALLSPAAADYLEPIAQR
AQRLTRQRFGNTVSFYVPLYLSNLCANDCTYCGFSMSNRIKRKTLDEAEIARECSAIREL
GFEHLLLVTGEHQAKVGMDYFRRHLPAIRRQFSSLQMEVQPLSQENYAELKTLGIDGVMV
YQETYHEAIYAQHHLKGKKQDFFWRLETPDRLGRAGIDKIGLGALIGLSDSWRVDCYMVA
EHLLWMQKHYWQSRYSVSFPRLRPCTGGVEPASVMDEKQLVQTICAFRLLAPEIELSLST
RESPWFRDHVIPLAINNVSAFSKTQPGGYADDHPELEQFSPHDARRPETVASALSAQGLQ
PVWKDWDSWLGRASQIR
>EECEBICD_03888 hypothetical protein
MIPPLNRYVPALSKNELVKTVTNRDIQFTSFNGKDYPLCFLDEKTPLLFQWFERNPARFG
KNDIPIINTEKNPYLNNIIKAATIEKERLIGIFVDGDFFPGQKDAFSKLEYDYENIKVIY
RNDIDFSMYDKKLSEIYMENISKQESMPEEKRDCHLLQLLKKELSDIQEGNDSLIKSYLL
DKGHGWFDFYRNMAMLKAGQLFLEADKVGCYDLSTNSGCIYLDADMIITEKLGGIYIPDG

IAVHVERIDGRASMENGIIAVDRNNHPALLAGLEIMHTKFDADPYSDGVCNGIRKHFNYS
LNEDYNSFCDFIEFKHDNIIMNTSQFTQSSWARHVQ
>EECEBICD_03889 hypothetical protein
MYEQQVFYNGEPIDFNNLDLWDKFKVTFGINVTEKNKPNDKKFQF
>EECEBICD_03890 hypothetical protein
MPAIISLIYLMFSLLVFTKKITISNVLSAFLLFLSLYILHHKSLLIFIYCLIIIVATAIL
HRVKTSADKKAVFYFSMPCWLLVLSVIININE
>EECEBICD_03891 DNA-directed RNA polymerase subunit beta'
MKDLLKFLKAQTKTEEFDAIKIALASPDMIRSWSFGEVKKPETINYRTFKPERDGLFCAR
IFGPVKDYECLCGKYKRLKHRGVICEKCGVEVTQTKVRRERMGHIELASPTAHIWFLKSL
PSRIGLLLDMPLRDIERVLYFESYVVIEGGMTNLERQQILTEEQYLDALEEFGDEFDAKM
GAEAIQALLKSMDLEQECETLREELNETNSETKRKKLTKRIKLLEAFVQSGNKPEWMILT
VLPVLPPDLRPLVPLDGGRFATSDLNDLYRRVINRNNRLKRLLDLAAPDIIVRNEKRMLQ
EAVDALLDNGRRGRAITGSNKRPLKSLADMIKGKQGRFRQNLLGKRVDYSGRSVITVGPY
LRLHQCGLPKKMALELFKPFIYGKLELRGLATTIKAAKKMVEREEAVVWDILDEVIREHP
VLLNRAPTLHRLGIQAFEPVLIEGKAIQLHPLVCAAYNADFDGDQMAVHVPLTLEAQLEA
RALMMSTNNILSPANGEPIIVPSQDVVLGLYYMTRDCVNAKGEGMVLTGPKEAERIYRAG
LASLHARVKVRITEYEKDENGEFVAHTSLKDTTVGRAILWMIVPKGLPFSIVNQALGKKA
ISKMLNTCYRILGLKPTVIFADQTMYTGFAYAARSGASVGIDDMVIPEKKHEIISEAEAE
VAEIQEQFQSGLVTAGERYNKVIDIWAAANDRVSKAMMDNLQTETVINRDGQEEQQVSFN
SIYMMADSGARGSAAQIRQLAGMRGLMAKPDGSIIETPITANFREGLNVLQYFISTHGAR
KGLADTALKTANSGYLTRRLVDVAQDLVVTEDDCGTHEGILMTPVIEGGDVKEPLRDRVL
GRVTAEDVLKPGTADILVPRNTLLHEQWCDLLEANSVDAVKVRSVVSCDTDFGVCAHCYG
RDLARGHIINKGEAIGVIAAQSIGEPGTQLTMRTFHIGGAASRAAAESSIQVKNKGSIKL
SNVKSVVNSSGKLVITSRNTELKLIDEFGRTKESYKVPYGAVMAKGDGEQVAGGETVANW
DPHTMPVITEVSGFIRFTDMIDGQTITRQTDELTGLSSLVVLDSAERTTGGKDLRPALKI
VDAQGNDVLIPGTDMPAQYFLPGKAIVQLEDGVQISSGDTLARIPQESGGTKDITGGLPR
VADLFEARRPKEPAILAEIAGIVSFGKETKGKRRLVITPVDGSDPYEEMIPKWRQLNVFE
GERVERGDVISDGPEAPHDILRLRGVHAVTRYIVNEVQDVYRLQGVKINDKHIEVIVRQM
LRKATIESAGSSDFLEGEQVEYSRVKIANRELEANGKVGATFSRDLLGITKASLATESFI
SAASFQETTRVLTEAAVAGKRDELRGLKENVIVGRLIPAGTGYAYHQDRMRRRAAGEQLS
TEWKRLGVSNTAGPCGRCICKPGRTAERRSGRF
>EECEBICD_03892 DNA-directed RNA polymerase subunit beta
MVYSYTEKKRIRKDFGKRPQVLDVPYLLSIQLDSFQKFIEQDPEGQYGLEAAFRSVFPIQ
SYSGNSELQYVSYRLGEPVFDVQECQIRGVTYSAPLRVKLRLVIYEREAPEGTVKDIKEQ
EVYMGEIPLMTDNGTFVINGTERVIVSQLHRSPGVFFDSDKGKTHSSGKVLYNARIIPYR
GSWLDFEFDPKDNLFVRIDRRRKLPATIILRALNYTTEQILDLFFEKVVFEIRDNKLQME
LIPERLRGETASFDIEANGKVYVEKGRRITARHIRQLEKDDIKHIEVPVEYIAGKVVSKD
YVDESTGELICAANMELSLDLLAKLSQSGHKRIETLFTNDLDHGPYISETVRVDPTNDRL
SALVEIYRMMRPGEPPTREAAESLFENLFFSEDRYDLSAVGRMKFNRSLLRDEIEGSGIL
SKDDIIDVMKKLIDIRNGKGEVDDIDHLGNRRIRSVGEMAENQFRVGLVRVERAVKERLS
LGDLDTLMPQDMINAKPISAAVKEFFGSSQLSQFMDQNNPLSEITHKRRISALGPGGLTR
ERAGFEVRDVHPTHYGRVCPIETPEGPNIGLINSLSVYAQTNEYGFLETPYRRVVDGVVT
DEIHYLSAIEEGNYVIAQANSNLDDEGHFVEDLVTCRSKGESSLFSRDQVDYMDVSTQQV
VSVGASLIPFLEHDDANRALMGANMQRQAVPTLRADKPLVGTGMERAVAVDSGVTAVAKR
GGTVQYVDASRIVIKVNEDEMYPGEAGIDIYNLTKYTRSNQNTCINQMPCVSLGEPVERG
DVLADGPSTDLGELALGQNMRVAFMPWNGYNFEDSILVSERVVQEDRFTTIHIQELACVS
RDTKLGPEEITADIPNVGEAALSKLDESGIVYIGAEVTGGDILVGKVTPKGETQLTPEEK
LLRAIFGEKASDVKDSSLRVPNGVSGTVIDVQVFTRDGVEKDKRALEIEEMQLKQAKKDL
SEELQILEAGLFSRIRAVLVSGGVEAEKLDKLPRDRWLELGLTDEEKQNQLEQLAEQYDE
LKHEFEKKLEAKRRKITQGDDLAPGVLKIVKVYLAVKRRIQPGDKMAGRHGNKGVISKIN
PIEDMPYDENGTPVDIVLNPLGVPSRMNIGQILETHLGMAAKGIGDKINAMLKQQQEVAK
LREFIQRAYDLGADVRQKVDLSTFSDDEVLRLAENLRKGMPIATPVFDGAKEAEIKELLK
LGDLPTSGQITLFDGRTGEQFERPVTVGYMYMLKLNHLVDDKMHARSTGSYSLVTQQPLG

GKAQFGGQRFGEMEVWALEAYGAAYTLQEMLTVKSDDVNGRTKMYKNIVDGNHQMEPGMP
ESFNVLLKEIRSLGINIELEDE
>EECEBICD_03893 50S ribosomal protein L7/L12
MSITKDQIIEAVSAMSVMDVVELISAMEEKFGVSAAAAVAVAAGPAEAAEEKTEFDVILK
AAGANKVAVIKAVRGATGLGLKEAKDLVESAPAALKEGVSKDDAEALKKSLEEAGAEVEV
K
>EECEBICD_03894 50S ribosomal protein L10
MALNLQDKQAIVAEVSEVAKGALSAVVADSRGVTVDKMTELRKAGREAGVYMRVVRNTLL
RRVVEGTQFECLKDTFVGPTLIAYSMEHPGAAARLFKEFAKANAKFEVKAAAFEGELIPA
SQIDRLATLPTYEEAIARLMATMKEASAGKLVRTLAAVRDAKEAA
>EECEBICD_03895 50S ribosomal protein L1
MAKLTKRMRVIREKVDATKQYDINEAIALLKELATAKFNESVDVAVNLGIDARKSDQNVR
GATVLPHGTGRSVRVAVFTQGPNAEAAKAAGAELVGMEDLADQIKKGEMNFDVVIASPDA
MRVVGQLGQVLGPRGLMPNPKVGTVTPNVAEAVKNAKAGQVRYRNDKNGIIHTTIGKVDF
DADKLKENLEALLVALKKAKPSQAKGVYIKKVSISTTMGAGVAVDQAGLSASAN
>EECEBICD_03896 50S ribosomal protein L11
MAKKVQAYVKLQVAAGMANPSPPVGPALGQQGVNIMEFCKAFNAKTDSIEKGLPIPVVIT
VYADRSFTFVTKTPPAAVLLKKAAGIKSGSGKPNKDKVGKISRAQLQEIAQTKAADMTGA
DIEAMTRSIEGTARSMGLVVED
>EECEBICD_03897 Transcription termination/antitermination protein NusG
MSEAPKKRWYVVQAFSGFEGRVATSLREHIKLHNMEELFGEVMVPTEEVVEIRGGQRRKS
ERKFFPGYVLVQMVMNDASWHLVRSVPRVMGFIGGTSDRPAPISDKEVDAIMNRLQQVGD
KPRPKTLFEPGEMVRVNDGPFADFNGVVEEVDYEKSRLKVSVSIFGRATPVELDFSQVEK
A
>EECEBICD_03898 Protein translocase subunit SecE
MSANTEAQGSGRGLEAMKWIVVAILLIVAIVGNYLYRDMMLPLRALAVVILIAAAGGVAL
LTTKGKATVAFAREARTEVRKVIWPTRQETLHTTLIVAAVTAVMSLILWGLDGILVRLVS
FITGLRF
>EECEBICD_03899 Elongation factor Tu 1
MSKEKFERTKPHVNVGTIGHVDHGKTTLTAAITTVLAKTYGGAARAFDQIDNAPEEKARG
ITINTSHVEYDTPTRHYAHVDCPGHADYVKNMITGAAQMDGAILVVAATDGPMPQTREHI
LLGRQVGVPYIIVFLNKCDMVDDEELLELVEMEVRELLSQYDFPGDDTPIVRGSALKALE
GDAEWEAKIIELAGFLDSYIPEPERAIDKPFLLPIEDVFSISGRGTVVTGRVERGIIKVG
EEVEIVGIKETQKSTCTGVEMFRKLLDEGRAGENVGVLLRGIKREEIERGQVLAKPGTIK
PHTKFESEVYILSKDEGGRHTPFFKGYRPQFYFRTTDVTGTIELPEGVEMVMPGDNIKMV
VTLIHPIAMDDGLRFAIREGGRTVGAGVVAKVLG
>EECEBICD_03904 Pantothenate kinase
MTPYLQFDRSQWAALRDSVPMTLTEDEIAQLKGINEDLSLEEVAEIYLPLSRLLNFYISS
NLRRQAVLEQFLGTNGQRIPYIISIAGSVAVGKSTTARVLQALLSRWPEHRRVELITTDG
FLHPNQVLKERGLMKKKGFPESYDMHRLVKFVSDLKSGVPNVTAPVYSHLIYDVIPEGDK
TVAQPDILILEGLNVLQSGMDYPHDPHHVFVSDFVDFSIYVDAPEELLQTWYINRFLKFR
EGAFTDPDSYFHNYAKLSKEEAVNTATSLWKEINWLNLKQNILPTRERASLIMTKSANHA
VEQVRLRK
>EECEBICD_03905 Bifunctional ligase/repressor BirA
MKDTTVPLTLISLLADGEFHSGEQLGERLGMSRAAINKHIQTLRDWGVDVFTVPGKGYSL
PEPIQLLDADRIHSQLDSGNVAVLPVIDSTNQYLLDRIGELRSGDACVAEYQQAGRGRRG
RKWFSPFGANLYLSMYWRLEQGPAAAIGLSLVIGIVMAEVLRELGADKVRVKWPNDLYLL
DRKLAGILVELTGKTGDAAQIVIGAGINMAMRRVEEDVINQGWITLQEAGITLDRNMLAA
KLIYKLRAALELFEQEGLSPYLSRWKKLDNFIDRPVKLIIGDKEIFGISRGIDTQGALLL
EQDGVIKPWMGGEISLRSAE
>EECEBICD_03906 UDP-N-acetylenolpyruvoylglucosamine reductase
MTHSLKPWNTFGIDHCAKHIVCAENEQQLLSAWQQATREGLPVMILGEGSNVLFLENYAG
TVILNRLKGIEVNETADAWHLHVGAGENWHQLVRYALDNNMPGLENLALIPGCVGSSPIQ
NIGAYGVELQRVCDYVDCVELETGKRLRLSAAECRFGYRDSIFKNEYQDRVAIVAVGLRL

SKQWQPVLTYGDLTRLDPKTVTAQQVFDAVCHMRTTKLPDPKVNGNAGSFFKNPVVAADI
AMELLERFPNAPHYPQADGSVKLAAGWLIDQCQLKGVTIGGAAVHRQQALVLINANDATS
KDVVALAHHVRQKVGEKFNVWLEPEVRFIGRSGEVNAVESIA
>EECEBICD_03912 Glutamate racemase
MATKLQDENTPCLAATPSEPRPTVLVFDSGVGGLSVYDEIRRLLPDLHYIYAFDNVAFPY
GEKSETFIVERVVEIVTAVQQRYPLSLAVIACNTASTVSLPALREKFAFPVVGVVPAIKP
AARLTANGVVGLLATRATVKRPYTHELIARFANECQIAMLGSAELVELAEAKLHGDSVSL
EELRRILRPWLRMPEPPDTVVLGCTHFPLLRDELLQVLPEGTRLVDSGAAIARRTAWLLE
HEAPDAKSTDANIAYCMAMTPGAEQLLPVLQRYGFETLEKLPV
>EECEBICD_03913 Vitamin B12 transporter BtuB
MIKKATLLTAFSVTAFSAWAQDTSPDTLVVTANRFQQPRSAVLAPVTIVTRQDIERWQST
SVNDVLRRLPGVDIAQSGGAGQNSSIFIRGTNSSHVLVLIDGVRLNLAGVSGSADLSQFP
VSLVQRIEYIRGPRSAIYGSDAIGGVVNIITTRDNPGTELTAGWGSNSYQNYDISTQQQL
GENTRATLIGDYEYTKGFDVVAKGGTGMQAQPDRDGFLSKTLYGALEHTFSDRWSGFVRG
YGYDNRTDYDAYYSPGSPLIDTRKLYSQSWDAGLRFNGERIQSQLVSSYSHSKDYNYDPH
YGRYDTSATLDEMKQYNVQWTNSVVVGHGNVGAGVDWQKQTTTPGTGYVPEGYDQRNTGV
YLTGLQQLGDFTLEAAARSDDNSQFGRHGTWQTSAGWEFIEGYRFIASYGTSYKAPNLGQ
LYGYYGNPNLNPEKSKQWEGAFEGLTAGVSWRISGYRNDINDMIDYDDHLQKYYNEGKAR
IKGIEATANFDTGPLTHTVSYDYVDARNAVTDTPLPRRSKQMAKYQLDWDVYDFDWGVTY
QYLGSRYDSDYSAYPYRTVKMGGVSLWDLTVAYPVTSHLTVRGKIANLFDKDYETVYGYQ
TAGREYTLSGSYTF
>EECEBICD_03914 tRNA/tmRNA (uracil-C(5))-methyltransferase
MTPEHLPTEQYEAQLAEKVARLQSMMAPFSGLVPEVFRSPVSHYRMRAEFRLWHDGDDLY
HIMFDQQTKSRIRVDTFPAASQLINTLMKAMIAGVRDNHALRHKLFQIDYLTTLSNQAVV
SLLYHKKLDEEWREAATALRDALRAQGLNVHLIGRATKTKIELDQDYIDERLPVAGKEMI
YRQVENSFTQPNAAMNIQMLEWALEVTKDSKGDLLELYCGNGNFSLALARNFNRVLATEI
AKPSVAAAQYNIAANHIDNVQIIRMAAEEFTQAMNGVREFNRLQGIDLKRYQCETIFVDP
PRSGLDSETEKMVQAYPRILYISCNPETLCKNLETLSQTHTVSRLALFDQFPYTHHMECG
VLLTAR
>EECEBICD_03915 Inner membrane protein YijD
MKQSGQDKGTLLLALIAGLSINGTFAALFSAIVPFSVFPVISLVLTVYCLHQRYQNRTMP
VGLPGLAAACFILGVLLYSTVVRAEYPDIGSNFFPAVLSVILVFWIGFKLRNRKQDTAE
>EECEBICD_03916 HTH-type transcriptional repressor FabR
MMGVRAQQKEKTRRSLVEAAFSQLSAERSFASLSLREVAREAGIAPTSFYRHFRDVDELG
LTMVDESGLMLRQLMRQARQRIAKGGSVIRTSVSTFMEFIGNNPNAFRLLLRERSGTSAA
FRAAVAREIQHFIAELADYLELENHMPRAFTEAQAEAMVTIVFSAGAEALDIGAEQRRQL
EERLVLQLRMIAKGAYYWYRREQEKIAHHSE
>EECEBICD_03917 Soluble pyridine nucleotide transhydrogenase
MPHSWDYDAVVIGSGPGGEGAAMGLVKQGARVAVIERYHNVGGGCTHWGTIPSKALRHAV
SRIIEFNQNPLYSDHSRLLRSSFADILNHADNVINQQTRMRQGFYERNHCEILQGNAHFI
DEHTLALECHDGTVETLTAEKFVIACGSRPYHPNDVDFSHPRIYDSDSILSLHHEPRHVI
IYGAGVIGCEYASIFRGMDVKVDLINTRDRLLAFLDQEMSDSLSYHFWNSGVVIRHNEEY
EKIEGCDDGVIMHLKSGKKLKADCLLYANGRTGNTDSLALENIGLETDSRGQLKVNSMYQ
TALPHVYAVGDVIGYPSLASAAYDQGRIAAQALVKGEATAHLIEDIPTGIYTIPEISSVG
KTEQQLTAMKVPYEVGRAQFKHLARAQIVGMNVGTLKILFHRETKEILGIHCFGERAAEI
IHIGQAIMEQKGGGNTIEYFVNTTFNYPTMAEAYRVAALNGLNRLF
>EECEBICD_03918 Hydrogen peroxide-inducible genes activator
MNIRDLEYLVALAEHRHFRRAADSCHVSQPTLSGQIRKLEDELGVMLLERTSRKVLFTQA
GLLLVDQARTVLREVKVLKEMASQQGETMSGPLHIGLIPTIGPYLLPLIIPMLHQTFPKL
EMYLHEAQTHQLLAQLDSGKLDCAILALVKESEAFIEVPLFDEPMMLAIYEDHPWANRDR
VPMSDLAGEKLLMLEDGHCLRDQAMGFCFEAGADEDTHFRATSLETLRNMVAAGSGITLL
PALAVPQERKRDGVVYLPCIKPEPRRTVGLVYRPGSPLRSRYEQLAEAIRGAMDGHFDKA
LKQAV
>EECEBICD_03919 Argininosuccinate lyase

MALWGGRFTQAADQRFKQFNDSLRFDYRLAEQDIVGSVAWSKALVTVGVLTADEQRQLEE
ALNVLLEEVRVNPQQILQSDAEDIHSWVEGKLIDKVGQLGKKLHTGRSRNDQVATDLKLW
CKETVRELLTANRQLQSALVETAQANQDAVMPGYTHLQRAQPVTFAHWCLAYVEMLARDE
SRLQDTLKRLDVSPLGCGALAGTAYEIDREQLAGWLGFASATRNSLDSVSDRDHVLELLS
DAAIGMVHLSRFAEDLIFFNSGEAGFVELSDRVTSGSSLMPQKKNPDALELIRGKCGRVQ
GALTGMMMTLKGLPLAYNKDMQEDKEGLFDALDTWLDCLHMAALVLDGIQVKRPRCQDAA
QQGYANATELADYLVAKGVPFREAHHIVGEAVVEAIRQGKPLEALPLADLQKFSHVIGDD
VYPMLSLQSCLDKRAAKGGVSPQQVAQAIDDARARLAL
>EECEBICD_03920 Acetylglutamate kinase
MMNPLIIKLGGVLLDSEEALERLFTALVNYRESHQRPLVIVHGGGCVVDELMKGLNLPVK
KKDGLRVTPADQIGIITGALAGTANKTLLAWAKKHHIASVGLFLGDGDSVNVTQLDEALG
HVGLAQPGSPKLINMLLENGFLPVVSSIGVTDDGQLMNVNADQAATALAATLGADLILLS
DVSGILDGKGQRIAEMTASKAEQLIDQGIITDGMIVKVNAALDAARALGRPVDIASWRHA
EQLPALFNGTPIGTRILA
>EECEBICD_03921 N-acetyl-gamma-glutamyl-phosphate reductase
MLNTLIVGASGYAGAELVSYVNRHPHMTITALTVSAQSNDAGKLISDLHPQLKGIVDLPL
QPMSDVRDFSADVDVVFLATAHEVSHDLAPQFLQAGCVVFDLSGAFRVNDRAFYEKYYGF
THQYPELLEQAVYGLAEWNADKLNTANLIAVPGCYPTAAQLSLKPLIDGGLLDLTQWPVI
NATSGVSGAGRKAAISNSFCEVSLQPYGVFTHRHQPEIAAHLGAEVIFTPHLGNFPRGIM
ETITCRLKAGVTHAQVADVLQKAYGDKPLVRLYDKGVPALKNVVGLPFCDIGFAVQGEHL
IVVATEDNLLKGAAAQAVQCANIRFGFAETQSLI
>EECEBICD_03922 Acetylornithine deacetylase
MKNVLPPFIEIYRALIATPSISATEESLDQSNASLITLLAGWFSDLGFNVEVQPVPGTRN
KFNMLASTGHGAGGLLLTGHTDTVPFDDGRWTRDPFTLTEHDNKLYGLGTADMKGFFAFI
LDALRDVDVTKLKKPLYILATADEETSMAGARYFSETTALRPDCAIIGEPTSLQPIRAHK
GHISNVVRVLGQSGHSSDPARGVNAIELMHDAIGHIMQLRDSLKARYHYEAFTVPYPTLN
LGHIHGGDASNRICACCELHMDIRPLPGMTLNDLNGLLNDALAPVSERWPGRLTVAELHP
PIPGYECPPDHQLVEVVEKLLGTKTDVVNYCTEAPFMQTLCPTLVLGPGSINQAHQPDEY
LETRFIKPTRELITQVVHHFCWH
>EECEBICD_03923 Phosphoenolpyruvate carboxylase
MNEQYSALRSNVSMLGKVLGETIKDALGEHILDRVETIRKLSKSSRAGNEANRQELLTTL
QNLSNDELLPVARAFSQFLNLANTAEQYHSISPKGEAASNPEVIARTLRKLKNQPDLNDA
TIKKAVESLSLELVLTAHPTEITRRTLIHKMGEINNCLKQLDNTDIADYERHQVMRRLRQ
LIAQSWHTDEIRKQRPSPVDEAKWGFAVVENSLWQGVPNYLRELNEQLEENLGYKLPVDF
VPVRFTSWMGGDRDGNPNVTADITRHVLLLSRWKATDLFLKDIHVLVSELSMVDATPELL
ALVGEEGASEPYRYLMKKLRARLMATQSWLEARLKGEKLPKPAGLLTQNEQLWEPLYACY
QSLQACGMGIIANGELLDTLRRVKCFGVPLVRIDIRQESTRHTEALGEITRYLGIGDYES
WSEADKQAFLIRELNSKRPLLPRNWEPSNDTREVLETCKVIAEAPKGSIAAYVISMAKTP
SDVLAVHLLLKEAGIGFAMPVAPLFETLDDLNNADDVMTQLLNIDWYRGLIQGKQMVMIG
YSDSAKDAGVMAASWAQYQAQDALIKTCEKAGIELTLFHGRGGSIGRGGAPAHAALLSQP
PGSLKGGLRVTEQGEMIRFKYGLPEVTVSSLSLYTSAILEANLLPPPEPKDSWRHIMDEL
SVISCETYRGYVRENKDFVPYFRSATPEQELGKLPLGSRPAKRRPTGGVESLRAIPWIFA
WTQNRLMLPAWLGAGTALQKVVEDGKQSELEAMCRDWPFFSTRLGMLEMVFSKADLWLAD
YYDQRLVAKTLWPLGKELRDLLEEDIKVVLAIANDSHLMADLPWIAESIQLRNVYTDPLN
VLQAELLYRSRLTEEQGKSPDPRVEQALMVTIAGVAAGMRNTG
>EECEBICD_03924 Phosphoethanolamine transferase CptA
MQSTLLQTKPAFSWKALGWALLYFWFFSTLLQAIIYLTGYSGTNGLRDSLLYSSLWLIPV
FLFPGRIRVIAAVIGVVLWAASLAALSYYVIYGQEFSQSVLFVMFETNANEASEYLSQYF
SLKIVLVALAYTVAAILLWTRLRPVYIPSPWRYLVSFALLYGLILHPIAMNTFIKHKSME
KTLDSLASRMEPAAPWQFITGYYQYRLQLASLNKLLNENDALPPLANFQDHSGDAPRTLV
LVIGESTQRGRMSLYGYPRETTPELDALHKTDPGLTVFNNVVTSRPYTIEILQQALTFAD
EKNPDWYLTKPSLMNMMKQAGYKTFWITNQQTMTARNTMLTVFSKQTDKQFYMNQQRTQS
AREYDSNVLEPFKAVLADPAPKKFIIVHLLGTHIKYKFRYPENQGKFDGKTDHVPPGLSS
DELESYNDYDNANLYNDYVVASLIKDYKATDPNGFLLYFSDHGEEVYDTPPHKTQGRNED

SPTRHMYTVPFLLWTSEKWQAAHPRDFSQDVDRKYSSSELIHTWSDLAGLTYDGYDPTRS
ITNPQFKETTRWIGNPYKKNALIDYDTLPYGDQVGNQ
>EECEBICD_03925 HTH-type transcriptional activator RhaR
MYHDVSHLLSRLINGPLPLRQIYFASASGPAPELAYQVDFPRLEIVLEGELTDMSITAPL
IPCDVLYVPAGGWNIPQWQTPVTTLSILFGKQQLGFSVVHWDGQQHQNLTKQHVARRGPR
IGSFLLQTLNEMQMQPQEQQTARLIVASLLSHCRDLLGSQIQTASRSRALFEAIREYIDE
RYAAPLTRESVAQAFYISPNYLSHLFQKTGAIGFNEYLNHTRLEHAKTLLKGYDLKVKEV
AHRCGFVDSNYFCRLFRKNTERSPSEYRRQYHSQLTEKQITPG
>EECEBICD_03926 PTS system fructose-like EIIB component 3
MAVPVRHLVAVTACVSGVAHTYMAAERLEKLCQQEKWSIKIETQGALGTENRLTEEDIRR
ADVVLLITDIELAGVERFTRSRYVQSGISAFLREPQRVMSAVRKLLSAPQHTHLILE
>EECEBICD_03927 Benzylsuccinate synthase activating enzyme
MTLFAAPRISCEVVGARDEETARIFNIQRYSLNDGQGIRTVVFFKGCPHTCPWCANPESI
SPRIETVRRENKCLRCTPCLRDADECPSGAFERIGRDITLDELEREVLKDDIFFRTSGGG
VTLSGGEVLMQAPFATRFLQRLRRWGVPCAIETAGDTSASRLLPLARACDEVLFDLKIMD
AERAREVINMNLPRVLENLRLLVSEGITVIPRLPLIPGFTLNAENLQCALTLLRSLGIKQ
VHLLPFHQYGEPKYRLLGKSWMMKDIPAPSVQEIALFREMAEQAGFQVTTGG
>EECEBICD_03928 Choline trimethylamine-lyase
MTHRIQRLKAALFQNHREISLERALLYTASHQQTEGEPVILRRAKATAYILEHVEISIRD
EELIAGNRTVKPRAGIMSPEMDPYWLLNELDRFPTRPQDRFDISEEDKRLYREVLFPYWE
KRSMKDFINGQMTDEVKAAVSTQIFSINQTDKGQGHIIIDYPRLLNNGLGELAAQMRARC
EQQPENDFYQAALLLLEASQRHILRYAVLAEHQAERCPDARRRQELLTIAANSRHNAQHK
PQTFWQACQLFWYMNIILQYESNASSLSLGRFDQYMLPFYQTSLTQGDDPAFLKELLESL
WVKCNDIVLLRSTSSARYFAGFPTGYTALLGGLTESGRSAVNVLSFLCLDAYQSVQLPQP
NLGVRTNALIDTPFLLKTAETIRLGTGIPQIFNDEVVVPAFLNRGVSLEDARDYAVVGCV
ELSIPGRTYGLHDIAMFNLLKVMEISLYENEGNDTLTYEALLAHIRAKISHYITLMVEGS
NICDIGHRDWAPVPLLSSFISDCLEKGRDITDGGARYNFSGVQGIGIANLSDSLHALNGL
VFDQQRLSFDALLSILKNNFATPEGEKIRARLINRFEKYGNDIDNVDNISAELLRYYCKE
VEKYQNPRGGQFTPGSYTVSAHVPLGAVVGATPDGRFAGEQLADGGLSPMLGQDMQGPTA
VLKSVSKLDNTLLSNGTLLNVKFTPATLEGEAGLRKLADFLRAFTQLKLQHIQFNVVNAD
TLREAQLRPQDFAGLVVRVAGYSAFFVELSKEIQDDIIRRTAHQL
>EECEBICD_03929 PTS system fructose-like EIIB component 1
MTKIIAVTACPSGVAHTYMAAEALESAAKAKGWDVKVETQGSIGLENELTAEDVASADMV
ILTKDIGIKFEERFAGKTIVRVNISDAVKRADAIMNKIDAHLSQTA
>EECEBICD_03930 PTS system mannose-specific EIIBCA component
MKELVQILKNTRQHLMTGVSHMIPFVVAGGILLAVSVMLYGKGAVPDAATDPNLKKLFDI
GVAGLTLMVPFLAAYIGYSIAERSALAPCAIGAWVGNSFGAGFFGALIAGLIGGIVVHYL
KKIPVHKVLRSVMPIFVIPIVGTFITAGIMMWGLGEPIGALTSSLTQWLQGMQQGSIVLL
AVIMGLMLAFDMGGPVNKVAYAFMLICVAQGVYTVVAIAAVSICVPPLGLGLATLIGRKN
FSVEEREAGKAALVMGCVGVTEGAIPFAAADPLRVIPSIMVGSACGAVMAALFGAQCYAG
WGGLIVLPVVEGKLGYVAAVAVGAVVTAVCVNVLKSLTRKNVSQVDEKEDDLDLDFEMN
>EECEBICD_03931 Multiphosphoryl transfer protein 1
MPLIVEFTCELPNGVHARPASHVETLCNTFTSQIEWHNLRTDRKGSAKSALALIGTDTLA
GDHCQLVISGADEQVACQRLSQWLRDEFPLCDAPLAEIKNSELEPLPASLTQLNPQIYRA
RSVCSGSAGGVLTPLSSLDLNALGELPTANDTETEQAALDNGLAMLIKHIEFRQLDSDGA
ASAILEAHRSLAGDASLRQHLLDGVLRGLSCAQAIVESANHFCNEFARASSSYLQERALD
VRDVCFQLLQHIYGEQRFPAPGQLTRPSICMAEELTPSQFLELDKTFLKGLLLKSGGNTS
HTVILARSFNIPTLVGVEIEALTPWRQQTVYIDGNAGAIVVAPDEPVTRYYQQEARVQDA
LREQQRIWLTQEARTADGIRMEVAANIAHSVEAQAAFSNGAEAVGLFRTEMLYMDRACAP
DENELYNIFCQALESAKGRSIIVRTMDIGGDKPVDYLNIPAEANPFLGYRAVRIYEEYAS
LFTTQLRSILRASAHGNLKIMIPMISSMEEILWVKEKLAEAKQQLRNEHIPFDEKIPLGI
MLEVPSVMFIIDQCCEEIDFFSIGSNDLTQYLLAVDRDNAKVTRHYNSLNPAFLRALDFA
VQAVHRQGKWIGLCGELGAKGSVLPLLVGLGLDEISMGAPSIPAAKARMAQLDSRACRQL
LNQAMACRTSLEVEHLLAQFRMSQQDAPLVTAQCITLDSDWRSKEEVIKGMTDNLLLAGR

CRYPRKLEADLWAREAVFSTGLGFSFAIPHSKSEHIEQSTISVARLNAPVRWGDDEAQFI
IMLTLNKHAAGDQHMRIFSRLARRIMHEEFRNTLVNAASADAIASLLQHELEL
>EECEBICD_03932 Fructose-6-phosphate aldolase 2
MELYLDTANVAEVERLARIFPIAGVTTNPSIVAASKESIWDVLPRLQNAIGEEGTLFAQT
MSRDAKGMVEEAKRLNNAIPGIVVKIPVTAEGLAAIKLLKKEGIVTLGTAVYSASQGLLA
ALAGAKYVAPYVNRVDAQGGDGIRMVQELQTLLEHHAPDSMVLAASFKTPRQALDCLLAG
CQAITLPLDVAQQMLNTPAVESAIEKFEQDWKNAFGNLNL
>EECEBICD_03933 Glycerol dehydrogenase
MDRIIQSPGKYIQGANVIARLGDYLKPMANNWLVVGDKFVLGFAEETLRKSLTDAGLSVE
IAPFGGECSQNEIDRLRAVAEKSQCGAVLGIGGGKTLDTAKALAHFMNVPVAIAPTIAST
DAPCSALSVIYTDAGEFDRYLLLPHNPNMVIVDTQIVAGAPARLLAAGIGDALATWFEAR
ACSRSGATTMAGGKCTQAALALAELCYNTLIEEGEKAMLAAEQHVVTPALERVIEANTYL
SGVGFESGGLAAAHAIHNGLTAIPDAHHYYHGEKVAFGTLTQLVLENAPVEEIETVAALC
HSVGLPITLAQLDIKQDIPAKMRTVAEASCAEGETIHNMPGGATPDEVYAALLVADQYGQ
RFLQEWE
>EECEBICD_03934 hypothetical protein
MKFAQALIGILAAFFISHISHHSPRSDTRPITVQAQTNAAIAQSAGTQIGKTLFYDAAYV
RLDYPNGDLPLERGVCADVVIRALRSQQVDLQKRVHEDMQAHFSAYPNNWKLKRPDSNID
HRRVPNLETWFQRQNKALPVTDKYSDYQPGDIVSWRLDNGLAHIGVVSLNVTPEGVPLVV
HNIGAGAQEEDVLFNWKVTGHFRYFSH
>EECEBICD_03935 Catalase-peroxidase
MNQHSNRSNPLGEDFDYRKEFSKLDYSALKGDLKALLTDSQPWWPADWGSYVGLFIRMAW
HGAGTYRSIDGRGGAGRGQQRFAPLNSWPDNVSLDKARRLLWPIKQKYGQKISWADLFIL
AGNVALENSGFRTFGFGAGREDVWEPDLDVNWGDEKAWLTHRHPEALAKAPLGATEMGLI
YVNPEGPDHSGEPLSAAAAIRATFGNMGMNDEETVALIAGGHTLGKTHGAAAASHVGADP
EAAPIEAQGLGWASSYGSGVGADAITSGLEVVWTQTPTQWSNYFFENLFKYEWVQTRSPA
GAIQFEAVDAPDIIPDPFDPSKKRKPTMLVTDLTLRFDPEFEKISRRFLNDPQAFNEAFA
RAWFKLTHRDMGPKARYIGPEVPKEDLIWQDPLPQPLYQPTQEDIINLKAAIAASGLSIS
EMVSVAWASASTFRGGDKRGGANGARLALAPQRDWDVNAVAARVLPVLEEIQKTTNKASL
ADIIVLAGVVGIEQAAAAAGVSISVPFAPGRVDARQDQTDIEMFSLLEPIADGFRNYRAR
LDVSTTESLLIDKAQQLTLTAPEMTVLVGGMRVLGTNFDGSQNGVFTDRPGVLSTDFFAN
LLDMRYEWKPTDESNELFEGRDRLTGEVKYTATRADLVFGSNSVLRALAEVYACSDAHEK
FVKDFVAAWVKVMNLDRFDLQ
>EECEBICD_03936 hypothetical protein
MSTTDDTHNTLSTGKCPFHQGGMTEAQAQGLPAATGGRTSFAWIF
>EECEBICD_03937 5,10-methylenetetrahydrofolate reductase
MSFFHANQREALNQSLAEVQGQINVSFEFFPPRTSEMEQTLWNSIDRLSSLKPKFVSVTY
GANSGERDRTHSVIKGIKERTGLEAAPHLTCIDATRDELRTIARDYWNNGIRHIVALRGD
LPPDSGKPEMYAADLVGLLKEVADFDISVAAYPEVHPEAKSAQADLLNLKRKVDAGANRA
ITQFFFDVESYLRFRDRCVSAGIDVEIIPGILPVSNFKQAKKFADMTNVRIPSWMSLMFE
GLDNDAETRKLVGANIAMDMVKILSREGVKDFHFYTLNRAEMSYAICHTLGVRPGL
>EECEBICD_03938 HTH-type transcriptional regulatory protein GabR
MNTANFESIHIMIDAQVWRNLPLHLRLYHAFRQLVISGALVDGAKLPSARALAQQLRLSR
DTVENAYSQLQRDGFMTRRQGAGSFASRQGIPHIQGQHSLPPSPRQTPTLSARWQTIADR
AVSFDARRVQTFTLGLAETRNFPQTIWQNFARQIQRERAHTLMLHGDPQGEPELRQQIAH
YLNFERGAKVDAQQIVIVSGARQAFWLCGQLLAEAGQSVVMENPGYPGARQAFSAAQLNL
LPIEVDRDGIRVDKLPPAPLVYVTPSHQFPSGHLLSLARRHALIDWARTHNAWIIEDDYD
SEFHYNGAPVACMQGLDREQRTLYVGTFSKTLYPGLRIGYLVVPASLAAHFAAARNLLDG
ATPALTQLTLARFIASGHFAAHIRTMRSMYAVRQACLAQAINQHLSDWVTPVVTPGGLQM
PCWLHEGIDEQQTLNAARRAGLNIAGMRQFWLTHPAPTGWLLGFAAFTPYEIENGVKLLR
AALCAENARQAGS
>EECEBICD_03939 Alanine racemase, biosynthetic
MMKLAEIQAACGVLCVDLAAVVDNYQTLARHVAPAQCGAVLKANGYGLGAEAIAPALYAA
NCRIFFVAQLSEGVALRKILSADAMVVLLNGVMPQAMPLCCAQQITPLLNSVDQVMAWLA

LQEARSQRRPVLIQLDSGMSRLGVTPEQLARLAAIFRQRGWAAPDYIISHLANADRPDHA
LNVYQHTLLQQAKEAFPTSRYSLANSCGMFLHPAWREDLCRPGVALFGVAQPWFSTPLKP
AFTLTLSILRVQDVPVGTPIGYGSTVTTTRPLRIATVSAGYADGIPRNLRPPAGVCWRGV
RLPVLGRVCMDSFMVDASAIMPTPGDVVEFIGVSQTLEEVAAACDTIPYEIMTRLGGRFR
RIMQPAEA
>EECEBICD_03940 Serine hydroxymethyltransferase
MINNDPLFDLLNKEQQRQQHSLELIASENFASPAVLAAQGSVLTNKYAEGYYQHRYYGGC
KFIDEVEMLAITRAQQLFGARYVNVQPHSGSQANQAVYLALLKPGDKILGMSLQCGGHLT
HGSPVNQSGKWFNAFHYGVDAHSGLIDMDEVETIAKRERPRLIIAGGSAYPRHYDFARFR
RIADAVGAILLVDMAHFAGLVAGGCFPSPLAYADVITTTTHKTLRGPRGGMILANDARLA
KKIDSAIFPGLQGGPLMHVIAAKAVALGEALQPEFKRYAGQVIENAQAMCQQLAQRGLTL
LTGGTDCHLGIIDLRPQGLTGAQVEYFLELAGITVNKNTLPGDPQPPSITSGIRIGSAAC
ATRGMNADDFTLIADWISEIIFAIKTPNITDICADIRQKVTKLTTNYPLPYQ
>EECEBICD_03941 Trifunctional nucleotide phosphoesterase protein YfkN
MKVKLLAAGILFTLPFWACAKDVTIIYTNDLHAHVEPYKVPWIADGKRDIGGWANITTLV
KQEKAKNKATWFFDAGDYFTGPYISSLTKGKAIIDILNTMQYDAATIGNHEFDHGWDNTL
LQLSRAKFPIVQGNIFYEDSSKSFWDKPYTIVEKDGVKIGVIGLHGVFAFNDTVSAATRV
GIEARDEIKWLQRYIDELKGKVDLTVALIHEGTPARQSSMGNTDVRRALDKDIQTASQVK
GLDILITGHAHVGTPEPIKVGNTLILSTDSGGIDVGKLVLDYQEKPHQFTVKNFELKTIF
ADEWKPDPQTKQVIDGWNKKLDKVVQQTVAQSPVELTRAYGESSSLGNLAADALLFTAGK
DTQLALTNSGGIRNEIPAGAVTMGAVISTFPFPNELVTMDLTGKQLRSLMEHGAGLSNGV
LQVSKGLEMKYDSSKPVGQRVTVLTLNGKPIDDAAVYHIATNSFLADGGDGFAAFTEGQA
RNTSGGYYVSNAIVDYFKAGNTITDEQLKGMRVADVKK
>EECEBICD_03942 hypothetical protein
MPPLFTINACKSAGCRNLGLPDSPDYVWPDYRLGYPALHCRACGSYPPLFNEGEFRRWAS
AYIAQYAKEHGHFCPDCYQKTWIRYGRNPGGTQRLQCQYCKKVWTPKQHALNVAETPEQI
CSIPLLVPFQGANAFQQLYFLFSFDAVRGNILHLSSNFTLLSAGKSLHYHWKGIAPPEGE
KGDIIHRIAIKERQFLQRSQFDEIQYGPAALKRNAQGTILRPVITAHGHFRVLKNRFPDV
TTHIIAHECFLRGAVITAWAERFRQRLSSLWFVEEEINDDDCRAEWQLLGKTWQGWWQNQ
WQLWGQGHNRKMVCSLTGSHLEQGVAVNLAASRRFVTWLWQQPEFQQSAHYSAKRVTQIL
YLLTEKYNSQWNHI
>EECEBICD_03943 hypothetical protein
MEITLPSMSDKKWTCADLLSSYRFWGITFFFLVLSFISSLGSSYGVYFWSKSLSIPADRI
GVILVCGQLGYLLGMVASWFVCRIKNCYPFYFVALLLIIGVVCSFCINGPSDVVRLIIAQ
FLINLGMGIVTLLVPMLLFSAIGSAQTFVILFSLCLLFKFIIAGYLSIFIYSIPYIGITV
ITLAVIALLLLLPLKKELFYIAPPKRFSSTQRPECAEPVVVALLAFFIPFYIIYWFFRIH
REIQFVAPSPRLMTACGAGWLSAIMPFGTAILCLTLSDEIRALLANKNEDGGIRTGWTLF
WALLLPPVGAAIIQAKMNRFITANTADTADRG
>EECEBICD_03944 Small-conductance mechanosensitive channel
MEGFELFPKIKGAIKWMAEHSDSVIHFGWNVVAAIILLFIGKLIARLLSRGLEKLLLRRQ
VDATIVHFFSALVRYITIAFTAVAALGRVGIETSSIIAVIGAAGLAIGLALQGALSNFAA
GVLLVSLRPFRAGEIVQIGLVIGTVEKVHIFSTTLLTADSKEVVIPNGKIIADNIINYSR
HPYRRIDLIIGVDYQSRIADVKSVIHRIIEQDHRIDKTRDITVRLGELAPSSLNFYVRVW
VPNAQYWSTYYDLLENIKEAMDENGINIPYPRMDVRVENVKSITP
>EECEBICD_03945 Bifunctional aspartokinase/homoserine dehydrogenase 2
MSVIAQAGAKGRQLHKFGGSSLADVKCYLRVAGIMAEYSQPDDMMVVSAAGSTTNQLISW
LKLSQTDRLSAHQVLQTLRRYQCDLISGLLPADAADDLTSAFISDLERLAALLDGGVTDA
VYAEIVGHGEIWSARLMSAVLNQQGLDAAWLDARAFLRAERAAQPQVDEGLSYPLLQQLL
AQHPGKRLVVTGFISRNHDGETVLLGRNGSDYSATQIGALAGVSRVTIWSDVAGVYSADP
RKVKDACLLPLLRLDEASELARLAAPVLHARTLQPVSGSDIDLQLRCSYTPDQGSTRIER
VLASGTGARIVTSHDDICLIEFQVPASQDFRLAHKELDQILKRAQARPLAVGVHRDRQLL
QFCYTAEVADSVLKLLDDVGLPGELRLRQGLALVAMVGAGVTRNPLHCHRFWQQLKGQPV
EFTWQSEEGISLVAVLRTGPTESLIQGLHQSVFRAEKRIGLMLFGKGNIGSRWLELFARE
QSTLSARTGFEFVLAGVVDSRRSLLNYEGLDASRALAFFDDEAVEQDEESLFLWMRAHPY

DDLVVLDVTASEQLADQYLDFASHGFHVISANKLAGASASDKYRQIHDAFEKTGRYWLYN
ATVGAGLPINHTVRDLIDSGDTILSISGIFSGTLSWLFLQFDGTVPFTDLVDQAWQQGLT
EPDPRVDLSGKDVMRKLVILAREAGYYIEPDQVRVESLVPAHCEEGSIDHFFENGDALNE
QMVQRLEAARELGLVLRYVARFDANGKARVGVEAVRPEHPLAALLPCDNVFAIESRWYRD
NPLVIRGPGAGRDVTAGAIQSDINRLAQLL
>EECEBICD_03946 Cystathionine gamma-synthase
MTRKQATIAVRSGLNDDEQYGCVVPPIHLSSTYNFTGFNEPRAHDYSRRGNPTRDVVQRA
LAELEGGAGAVLTNTGMSAIHLVTTVFLKPGDLLVAPHDCYGGSYRLFDSLATRGCYRVR
FVDQGDERALQAALEEKPKLVLVESPSNPLLRVVDIAKICRLAREAGAVSVVDNTFLSPA
LQNPLALGADLVLHSCTKYLNGHSDVVAGVVIAKDPEVVTELAWWANNIGVTGGAFDSYL
LLRGLRTLVPRMELAQRNAQAIVKYLQTQPLVKKLYHPSLPENQGHEIAARQQKGFGAML
SFELDGDEETLRRFLGGLSLFTLAESLGGVESLISHAATMTHAGMSPQARAAAGISETLL
RISTGIEDGEDLIADLENGFRAANKG
>EECEBICD_03947 Met repressor
MAEWSGEYISPYAEHGKKSEQVKKITVSIPLKVLKILTDERTRRQVNNLRHATNSELLCE
AFLHAFTGQPLPDDADLRKERSDEIPEAAKEIMREMGIDPETWEY
>EECEBICD_03948 hypothetical protein
MTSADATIGLFGSKTPEAKTLPLKHRQHNTGKTFLVNNIFISPFDIIDKMNIY
>EECEBICD_03949 Arylsulfate sulfotransferase AssT
MVDVAPYGGVFPLTAIINKANHNVQDVKVTVLGKGEKGIPISYDVGPQAINTHDGIPVFG
LYPDYVNKVKVDWTEEGKKQTYTWSIYAAPVSLPSTTGQTAVLPTVEPVKVDSSLKNRLY
LFNHITGMPRAGHIMHVAGGAANWDYTGINWISDTNGDVRGYMNIDKFRNQDDITRFGSM
MSFHQVNDGNLIFGQGQRYFKYDFLGRVISDKRLPKGFIDFSHAITETPKGTYLLRVAKE
NYPLNGKYTINTVRDHILEVDQNGDTVDYWDLPKILDPYRDDVILAMDQGAVCLSVDAEH
SGQVMTKEQLAKQPFGDIAGSGPGRNWAHVNSVSYDPRDDSIIISSRHQSAIIKIGRDKK
VKWILSDPSGWKGELAKKVLKPVDSNGKPLTCEAHHCDGGFDWTWTQHTGWLVPSKSTGG
KTVVTAFDNGDARGMEQPAMPSMKYSRGVEYQIDEKNMTVSQMWEYGKERGFDWYSAITS
VTEYRPETKTMFMYSATAGMSGTKPIVSVLDEVKDGTQDVMLELKVHSNRAGMLGYRALI
IDPEQMFKK
>EECEBICD_03950 hypothetical protein
MKNLFIAWLSLLLYGCTTFPQAVKPTTGGQPYPTEIYIVSHGWHTGIIAPAHDVNTVLPQ
LKKRFAQETQWYEIGWGDKGFYQSQEITTALTLQAMFWSSGAVMHIVAFSGKPERYFAGS
EIKSLLLHTNQRNSLMRYLGRSFARDEEGNLIPLKQGIYGDSQFYAANGRYGILNTCNKW
TAKGLESAGLTINPSLKLTAGSVMKAITDHRMCLNKYCYP
>EECEBICD_03951 50S ribosomal protein L31
MKKGIHPNYVEITATCSCGNVIKTHSTVGHDLNLDVCGKCHPFFTGKQRVVDTGGRVERF
NKRFSIPGSK
>EECEBICD_03952 Primosomal protein N'
MSVAHVALPVPLPRTFDYLLPEGGVAKAGCRVRVPFGKQQERVGIVVSVSDHSELPLDEL
KSVIDILDNEPIFSPSIWRLLLWAADYYHHPLGDVLFHALPVLLRQGKPASNAPLWYWFA
TEEGQAVDINSLKRSAKQQQALAALRQGKIWRYQVAELDFTDATLQTLRRKGLCELASEA
PAFTDWREHYAIAGERLRLNTEQATAVGAIHSASDGFSAWLLAGVTGSGKTEVYLSVLEN
VLAQGKQALVMVPEIGLTPQTIARFRERFNAPVEVLHSGLNDSERLSAWLKAKNGEAAIV
IGTRSSLFTPFKNLGVIVIDEEHDSSYKQQEGWRYHARDLAVYRAHSEQIPIILGSATPA
LETLCNVRQKKYRMLRLTRRAGNARPALQHVLDLKGQRLQAGLAPALIARMRQHLQADNQ
VILFLNRRGFAPALLCHDCGWIAECPRCDHYYTLHQAQHHLRCHHCDSQRPVPRQCPSCG
STHMLPVGLGTEQLEQVLAPFFPGVPISRIDRDTTSRKGALEQHLAEVHRGGARILIGTQ
MLAKGHHFPDVTLVALLDVDGALFSADFRSAERFAQLYTQVSGRAGRAGKQGEVVLQTHH
PEHPLLQTLLYKGYDAFAEQALAERQTLQLPPWTSHVIIRAEDHNNQQAPVFLQQLRNLI
QASPLSDDKLWILGPVPALAPKRGGRYRWQILLQHPSRIRLQHIISGTLALINTLPEARK
VKWVLDVDPIEG
>EECEBICD_03953 HTH-type transcriptional repressor CytR
MKSNKQVTAATMKDVALKAKVSTATVSRALMNPDKVSQSTRSRVEQAALEVGYFPQSMGR
NVKRNESRTILVIVPDICDPFFSEIIRGIEVTAAEQGYLVLIGDCAHQNQKEKTFLNLII

TKQIDGMVLLSSRLPFDASVEEQRNLPPMVMSNEFAPELELPTVHIDNLTAAFNAMNYLL
DLGHKRIGCIAGPEDMPLCHYRLQGYVQALRRSGIVVDPHYIARGDFTFEAGANALKQLL
EQPLPPTAVFCHSDVMALGALSWAKRQGLKVPDDLSIIGFDNIALAEFCDPPLTTVAQPR
FDIGREAMLLLLLDQMQGQNVSSGSRLMDCELIIRGSTRALP
>EECEBICD_03954 Cell division protein FtsN
MPAVSPAMVAIAAAVLVTFIGGLYFITHHKKEESETLQNQKVTGNGLPPKPEERWRYIKE
LESRQPGVRTPTEPSAGGEVMNPNQLTSEQRQLLEQMQADMRQQPTQLNEVPWNEQTPEQ
RQQTLQRQRQAQQQQWAQTQPVQQPRTQPRVNEQSQTRTVQSAPAQPARQSQPPKQTASQ
QPYQDLLQTPAHTSAAAPKAAPITRAPEAPKATAEKKDERRWMVQCGSFKGAEQAESVRA
QLAFEGFDSKITTNNGWNRVVIGPVKGKENADSTINRLKMAGHTNCIRLATGG
>EECEBICD_03955 ATP-dependent protease subunit HslV
MTTIVSVRRNGHVVIAGDGQATLGNTVMKGNVKKVRRLYNDKVIAGFAGGTADAFTLFEL
FERKLEMHQGHLVKAAVELAKDWRTDRMLRKLEALLAVADETASLIITGNGDVVQPENDL
IAIGSGGPYAQAAARALLENTELGAREIAEKALDIAGDICIYTNHFHTIEELTAKA
>EECEBICD_03956 ATP-dependent protease ATPase subunit HslU
MSEMTPREIVSELNKHIIGQDNAKRSVAIALRNRWRRMQLDEELRHEVTPKNILMIGPTG
VGKTEIARRLAKLANAPFIKVEATKFTEVGYVGKEVDSIIRDLTDAAVKMVRVQAIEKNR
YRAEELAEERILDVLIPPAKNNWGQAEQQQEPSAARQTFRKKLREGQLDDKEIEINLAAA
PMGVEIMAPPGMEEMTSQLQSMFQNLGGQKQKPRKLKIKDAMKLLVEEEAAKLVNPEELK
QDAIDAVEQHGIVFIDEIDKICKRGETSGPDVSREGVQRDLLPLVEGCTVSTKHGMVKTD
HILFIASGAFQVAKPSDLIPELQGRLPIRVELQALTTSDFERILTEPNASVTVQYKALMA
TEGVNIEFTDSGIKRIAEAAWQVNETTENIGARRLHTVLERLMEEISYNASDLHGQNITI
DAEYVSKHLDALVADEDLSRFIL
>EECEBICD_03957 1,4-dihydroxy-2-naphthoate octaprenyltransferase
MTEQQQISRTQAWLESLRPKTLPLAFAAIIVGTALAWWQGYFDPLVALLALITAGLLQIL
SNLANDYGDAVKGSDKPDRIGPLRGMQKGVITRQEMKRALIITVVLICISGLALVAVACH
TLTDFIGFLILGGLSIIAAITYTVGNRPYGYIGLGDISVLVFFGWLSVMGSWYLQAHTLI
PALILPATACGLLATAVLNINNLRDINSDRENGKNTLVVRLGDVNARRYHACLLLGALLC
LALFNLLSLHSPWGWLFILAAPLLIKQARYVMREREPAAMRPMLERTVKGALLTNLLFVI
GILLSQWAV
>EECEBICD_03958 Regulator of ribonuclease activity A
MKYDTSELCDIYQEDVNVVEPLFSNFGGRSSFGGQIITVKCFEDNGLLYDLLEQNGRGRV
LLVDGGGSVRRALVDAELARLATQNEWEGLVIYGAVRQVDDLEELDIGIQAIAAIPVGAA
GEGIGESDVRVNFGGVTFFSGDHLYADNTGIILSEDPLDIE
>EECEBICD_03959 Cell division protein ZapB
MSLEVFEKLEAKVQQAIDTITLLQMEIEELKEKNNSLTQEVQSAQHQREELERENNSLKE
QQSGWQERLQALLGRMEEV
>EECEBICD_03960 Glycerol uptake facilitator protein
MSQTSTLKGQCIAEFLGTGLLIFFGVGCVAALKVAGATFGQWEISVIWGLGVAMAIYLTA
GVSGAHLNPAVTIALWLFACFDKRKVVPFIISQVAGAFCAAALVYGLYYNLFVDFEQTHH
IVRGSVESLDLAGIFSTYPNPHINFVQAFAVEMVITAILMGLILALTDDGNGVPRGPLAP
LLIGLLIAVIGASMGPLTGFAMNPARDFGPKMFAGLAGWGEIAFTGGRDIPYFLVPLFGP
IVGAILGAFAYRKFIGRHLPCDTCVVEEKDSTATTQQNASL
>EECEBICD_03961 Glycerol kinase
MTEKKYIVALDQGTTSSRAVVMDHDANIVSVSQREFEQIYPKPGWVEHDPMEIWASQSST
LVEVLAKADISSDQIAAIGITNQRETAIVWERETGKPIYNAIVWQCRRTADICEQLKRDG
LEDYIRDNTGLVVDPYFSGTKVKWILDHVEGSRERAKRGELLFGTVDTWLIWKMTQGRVH
VTDYTNASRTMLFNIHDLDWDDKMLDVLDIPRAMLPQVRKSSEVYGQTNIGGKGGTRIPI
AGIAGDQQAALFGQLCVKEGMAKNTYGTGCFMLMNTGEKAVKSENGLLTTIACGPSGEVN
YALEGAVFMAGASIQWLRDEMKLISDAFDSEYFATKVKDTNGVYVVPAFTGLGAPYWDPY
ARGAIFGLTRGVNSNHIIRATLESIAYQTRDVLEAMQADSGIRLHALRVDGGAVANNFLM
QFQSDILGTRVERPEVREVTALGAAYLAGLAVGYWQNLDELQEKAVIEREFRPGIETTER
NYRYSGWKKAVKRAMAWEEHDK
>EECEBICD_03962 Fructose-1,6-bisphosphatase 1 class 2

MRRELAIEFSRVTEAAALAGYKWLGRGDKNTADGAAVNAMRIMLNQVNIDGTIVIGEGEI
DEAPMLYIGEKVGTGHGDAVDIAVDPIEGTRMTAMGQANALAVLAVGDKGCFLNAPDMYM
EKLIVGPGAKGAIDLNLPLADNLRNIADALGKPLGDLTVTILAKPRHDEVIAEMAKLGVR
VFAIPDGDVAASILTCMPDSEVDVLYGIGGAPEGVVSAAVIRALDGDMQGRLLARHDVKG
DSEENRRIGEQELARCKAMGIEAGKALCLGDMARSDNVIFSATGITKGDLLEGISRKGHI
ATTETLLIRGKSRTIRRIQSIHYLDRKDPDVQAHIL
>EECEBICD_03963 Flavodoxin/ferredoxin--NADP reductase
MADWVTGKVTKVQNWTDALFSLTVHAPINPFTAGQFTKLGLEIDGERVQRAYSYVNAPDN
PNLEFYLVTVPQGKLSPRLAALKPGDEVQVVSDASGFFVLDEVPDCETLWMLATGTAIGP
YLSILQYGQDVARFKNLVLVHAARFAADLSYLPLMLELQQRYEGKLRIQTVVSRESVPGS
LTGRVPALIENGELEKAVGLPMDKETSHVMLCGNPQMVRDTQQLLKETRQMTKHLRRRPG
HMTAEHYW
>EECEBICD_03964 hypothetical protein
MTIQQWLFSFKGRIGRRDFWIWIGLWFAGMLALFSLAGKNLLDIQTAAFCLVCLLWPTAA
VMVKRLHDRGRSGAWALLMILAWMLLAGNWAMLPGMWQWVVGRFIPTLILVMMLIDLGAF
VGVQGENKFGKATQDVKFKAEL
>EECEBICD_03965 hypothetical protein
MKPGCTLFLLLFSALTASITAHAQLSSSTTTAPYLLAGSPTFDLSISQFRENFNRQNPDL
PLNEFRAIENSRDKANLTRAASKINENLYASTALERGTLKVKSMQITWLPIQGPEQKAAK
AKALEYMAAIIRTVAPLLTKEQSQKKLQKMLIAGKGKHYYAETEGAVRYVVADNGEKGLT
FAVEPIKLALSENLEGAN
>EECEBICD_03966 Triosephosphate isomerase
MRHPLVMGNWKLNGSRHMVNELVANLRKELAGVAGCDVAIAPPEMYIDLAKRAAAGSHIM
LGAQNVDLNLSGAFTGETSAEMLKDIGAQYIIIGHSERRTYHKESDELIAKKFAVLKEQG
LTPVLCIGETEAENEAGKTEEVCARQIDAVLKTQGAAAFEGAVIAYEPVWAIGTGKSATP
AQAQAVHKFIRDHIAKADAKIAEQVIIQYGGSVNASNAAELFAQPDIDGALVGGASLKAD
AFAVIVKAAEAAKQA
>EECEBICD_03967 Putative epimerase LsrE
MNSQFAGLTREACVALLASYPLSVGILAGQWIALHRYLQQLEALNQPLLHLDLMDGQFCP
QFTVGPWAVGQLPQTFIKDVHLMVADQWTAAQACVKAGAHCITLQAEGDIHLHHTLSWLG
QQTVPVIGGEMPVIRGISLCPATPLDVIIPILSDVEVIQLLAVNPGYGSKMRSSDLHERV
AQLLCLLGDKREGKIIVIDGSLTQDQLPSLIAQGINRVVSGSALFRDDRLVENTRSWRAM
FKVAGDTTFLPSTA
>EECEBICD_03968 (4S)-4-hydroxy-5-phosphonooxypentane-2,3-dione isomerase
MMHVTLVEINVHDDKVEQFIDVFRQNHLGSIKEPGNLRFDVLQDPQVPTRFYIYEAYVDE
QAVAFHKTTPHYKTCVEQLEPLMTGPRTKKVFMGLMP
>EECEBICD_03969 3-hydroxy-5-phosphonooxypentane-2,4-dione thiolase
MADLDDIKDGKDFHTDKPQTNTLFALKGCGALDWGMQSRLARIFNPKTRKTVMLAFDHGY
FQGPTTGLERIDINIAPLFEYADVLMCTRGILRSVVPPAINKPVVLRASGANSILTELSN
EAVAVAMDDAVRLNSCAAAAQVYIGSEHEHQSIKNIIQLIDAGLRVGMPIMAVTGVGKDM
ARDQRYFSLATRIAAEMGAQIIKTYYVDKGFERIAAGCPVPIVIAGGKKLPEREALEMCY
QAIDQGASGVDMGRNIFQSEDPVAMIKAVHAVVHHNETAERAYELFLSEKG
>EECEBICD_03970 Autoinducer 2-binding protein LsrB
MARHSIKMIALLTAFGLTSAVMTVQAAERIAFIPKLVGVGFFTSGGNGAQEAGKALGIDV
TYDGPTEPSVSGQVQLVNNFVNQGYDAIIVSAVSPDGLCPALKRAMQRGVKILTWDSDTK
PECRSYYINQGTPKQLGSMLVEMVAHQVDKEKAKVAFFYSSPTVTDQNQWVKEAKAKISQ
EHPGWEIVTTQFGYNDATKSLQTAEGIIKAYPDLDAIIAPDANALPAAAQAAENLKRNNL
AIVGFSTPNVMRPYVQRGTVKEFGLWDVVQQGKISVYVANALLKNMPMNVGDSLDIPGIG
KVTVSPNSEQGYHYEAKGNGIVLLPERVIFNKDNIDKYDF
>EECEBICD_03971 Autoinducer 2 import system permease protein LsrD
MHRTHMGRNVFLIGQSARVAQYSAIPVNRTLYTVYAMTGCASAIAAVLLVSYFGSARSDL
GASFLMPAITAVVLGGANIYGGSGSIMGSALAALLVGFLQQGLQMAGVPNQISSALSGAL
LIVVVVGRSVSLHRHQILEWYSRRRNAHQA
>EECEBICD_03972 Autoinducer 2 import system permease protein LsrD

MNPWRRYSWEIALAALLIFEILAFGVINPRLLDINVLLFSTSDFICIGIVALPLTMVIVS
GGMDISFGSTIGLCAITLGVLFQLGMPLPLAIIITLLLGAICGLINAGLIIYTGVNPLVI
TLGTMYLFGGSALLLSGMAGATGYEGIGGFPTAFTDFANISFLGIPMPLIFFLCAVCFSG
CSCIARIWDATFS
>EECEBICD_03973 Autoinducer 2 import system permease protein LsrC
MLKFIQNNREATALLAIVCLFVFPGALDSQYLSVQTLTMVFSSAQILMLLAIGATMVMLT
RNIDVSVGSTTGMCAVLLGVMLNAGYSLPVACLATLILGIVAGFFNGVLVAWLKIPAIVA
TLGTLGLYRGIMLLWTGGKWIEGLPAGLKQLSAPVFLGISAIGWFTLVLALLMAWLLAKT
AFGRNFYATGDNLQGARQLGVRTEMVRIMAFSLNGGMAALAGIVFASQIGFIPNQTGTGL
EMKAIAACVLGGISLLGGSGTVIGAILGAYFLTQIDSVLVLLRIPAWWNDFIAGLVLLGV
LVFDGRLRCALQRNLRRQKYARFISPPTPLQTEAKTHAQQNKNKEVA
>EECEBICD_03974 Autoinducer 2 import ATP-binding protein LsrA
MQISHNTASPLICVQNIYKSYSGVEVLKGIDFTLHAGEVHALLGGNGAGKSTLMKIIAGI
VPPDGGTIDIAGVRCSHLTPLKAHQYGIYLVPQEPLLFPSLSVRENILFGLQGRQASTEK
MQQLLKAMGCQLDPASAAGTLDVADRQIVEIMRGLMRDSRILILDEPTASLTPAETDRLF
TRLQELLKKGVGIVFISHKLPEIRQLAHCVSVMRDGKIALFGKTHDLSTDEIIQAITPAT
QGVSLSANQKLWLELPGSRPQNERGATVLALESLTGEGFMNINLEVRAGEILGLAGLVGA
GRTELAETLYGIRPVNAGRMLFNGEEINALTTQQRLQLGLVYLPEDRQSSGLYLDASLAW
NVCSLTHNQKGFWIKPQRDNATLERYHRALNIKLNNAEQAARTLSGGNQQKVLIAKCLEA
SPQLLIVDEPTRGVDVSARSDIYQLLRSIAQQNVAVLFISSDLEEIEQMADRVYVMHQGE
LGGPALCGEEINVDTIMHVAFGEHGASEATC
>EECEBICD_03975 Transcriptional regulator LsrR
MSDNTLVSDYGMCEEEQVARIAWFYYHDGLTQSEISERLGLTRLKVSRLLEKGHQSGIIR
VQINSRFEGCLEYENALRNHFALQNIRVLPALPDADIGLRLGIGAAHMLMESLRPQQLLA
VGFGEATMTTLKRLSGFISAQQIRLVTLSGGVGPYMTGIGQLDAACSVSIMPAPLRASSQ
EIACTLRNENSVRDVMLTAQAADAAIVGIGAINQKDQASILKSGYITQGEQLMIGRKGAV
GDILGYFFDAHGEIIPDIKIHNELIGLKLNSLSTIPTVIGVAGGEQKAEAIIAAMRGNYI
NALVTDQKTAGKIIQLIEK
>EECEBICD_03976 Autoinducer-2 kinase
MARLCTHTESGHYLMALDAGTGSVRAVIFDLQGKQIAVGQAEWQHLAVPDVPGSMEFDLA
KNWQLACQCIRQALQKAAIPATAIAAVSACSMREGIVIYDSNGEPIWACANVDARAAHEV
SELKELHDNTFEEEVYRCSGQTLALSAIPRLLWLAHHRPDIYHRASTVTMISDWMAFMLS
GELAVDPSNAGTTGLLDLVTRNWKRSLLQMAGLRSDILSPVKETGTLLGHISQKAAEQCD
LQAGTPVIVGGGDVQLGCLGLGVVRPAQTAVLGGTFWQQVVNLPAPVTDPNMNVRINPHV
IPGMVQTESISFFTGLTMRWFRDAFCAEEKLIAERLGIDAYSLLEDMASRVPPGAYGVMP
IFSDVMRFKRWYHAAPSFINLSIDPEKCNKATLFRALEENAAIVSACNLQQIAAFSGVQA
DSLVFAGGGSKGKLWSQILADVTGLTVHVPVVKEATALGCAIAAGVGVGVWPSLAETGEK
LVRWDREHKPNPENFAVYQQAREKWQAVYQDQRALVDGGLTTSLWKAPGL
>EECEBICD_03977 hypothetical protein
MQKTNEKNHEYRFGDNGPKYLIRGPGCDMGVVILQPGQSFPNHRHNTACEVFYTLSGEVC
LYLEGTPHILQTGDVLQCEPGEAHYLINNGDKPWKGVFIKSPHLENDSHPADPPAW
>EECEBICD_03978 hypothetical protein
MSLWLSHPLFLPSIIVGVTILLWATSLLPEFMTALLFFAAVVAMGPGKVPARAGML
>EECEBICD_03979 Mannosyl-D-glycerate transport/metabolism system
repressor MngR
MQVDKTSFTPLYKQLFFIICQQIQNGSLPLGSQLPTQKEIARAYNVSLIVVKQAWSELIN
AGIISSQRGSGSVVCSVPEGVSYGHTFRGITRDLQDASVAIENRILEIAPRRARDAQADG
LSLPAQHHYLYISRIRCLNNRPFNHEKIYLDLSFFPDLQLTPQALEHTSLYSLLNVTSDS
AIEKVEAILPSADLCEKLQIAANKPLLSVARQTFQAGKDSPFEYCRYYVLSEYFGEIHYH
>EECEBICD_03980 hypothetical protein
MHVDQNKILGCLVGAAAADAMGAATEVRTQQQIKDYFGGWVTTFQKPPADTFGRCNEAGM
CTDDFIQAKYIMDALLRHQRQVSDEAMREAFQQWLDYPYYANFTGPTTRAAMKAIFNDNR
ASLQGELEGEKQSVQIINKGNAEATNGAAMKIWPAAVLHPGDIDAAIDCALQICRFTHNN
VLAMSGAAAMAAATSEALRAQTNADSIIAAGIYGAQRGYLLAQEQGAMMVAGPSVARRIE

LAVDIGKRHRHWETAVVELADIIGSGLHVSEAVPAAFGLFACCPNSAVDAIISGVNIGND
TDTVATMVGAISGAFHGVEAFPADYLTTLDRMNHFDLAELARQIAG
>EECEBICD_03981 Fructokinase
MKAMNKVWVIGDASVDLVPEKQNSYLKCPGGASANVGVCVARLGGECGFIGCLGDDDAGR
FLRQVFQDNGVDVTFLRMDPALTSAVLIVNLTADGERSFTYLVHPGADIYVSPQDLPPFR
QYEWFYFSSIGLTDSPAREACLEGARRMREAGGYVLFDVNLRSKMWRNTDEIPDLIARSA
ALASICKVSADELCQLSGASHWQDARYYLRDLGCDTTIISLGADGALLITAEGEFHFPAP
RVDVVDTTGAGDAFVGGLLFTLSRANCWNHALLAEAISNANACGAMAVTAKGAMTALPFP
DQLNTFLSSHSLAQAMTVK
>EECEBICD_03982 Putative glycoside/cation symporter YagG
MKLSIVEKIGFGAGDMAINVVIIAMQLLLAYFYTDIYGLSAADVGVLFVVVRMIDAIIDP
AMGVLTDKLNTRWGRYRPWLLWFAIPFGFAVYLMFITPDMAYMAKLAWAYGTYILMTLVY
TAITIPYISMIGVITSDPVERLSANGYRFVMTKIAAFLVTIVVPMLAVWLGQGNKALGYQ
FSMGLMGAMGALLFIFCFLTTRERSEPEITSLSVGKQFKYLLRNDQWIILGVVILLLMCG
YVIRGSVAAYYAKYYLNGGDSLISPFLTTGVVASILAMIATTWITKFWDKIKMFRYTQII
TFILSALMYFSVGRENLILAFAFYFLINFFCDMQMPVFWSSIAEAVDYGEKKTGLRVSGL
AFGGILFFQKFGMGIAGGILGFLLSHFGYQADVEQTARSLTGIALMMTLIPALFHLAVGL
LMKKYLINNEYYRDIQLALAQKQA
>EECEBICD_03983 CDP-diacylglycerol pyrophosphatase
MAVRSTKLANAKPFINPVTYYVTGFFLCHNFALLFPVYGSQGTHIMKKTGYFLLAVIVIV
AAAGVGYWKFSGNPDALREIVLEQCLPDQLQHQNPAPCAEVKPRAGYVVFKDRHGPLQYL
LMPTYRINGTESPLLLEPATPNFFWLAWQARGYMSKKYGHDIPDSAVSLAINSRLGRSQD
HLHIHISCIRPDVREQLDNDLTRISTRWLPLPGGLMGHEYLARRVTESELAQRSPFMMLA
EEVPEARDHMGRYALAVVRQSDDSFVLLATERNLLTLNRASAEEIQDHSCAILSSR
>EECEBICD_03984 Sulfate-binding protein
MKKWGVGFTLLLASTSILAKDIQLLNVSYDPTRELYEQYNKAFSAHWKQETGDNVVIRQS
HGGSGKQATSVINGIEADVVTLALAYDVDAIAERGRIDKNWIKRLPDNSAPYTSTIVFLV
RKGNPKQIHDWNDLIKPGVSVITPNPKSSGGARWNYLAAWGYALHHNNNDQAKAQDFVKA
LFKNVEVLDSGARGSTNTFVERGIGDVLIAWENEALLATNELGKDKFEIVTPSESILAEP
TVSVVDKVVEKKGTNAVAEAYLKYLYSPEGQEIAAKNFYRPRDADVAKKYDDAFPKLKLF
TIDEVFGGWAKAQKDHFANGGTFDQISKR
>EECEBICD_03985 ATP-dependent 6-phosphofructokinase isozyme 1
MIKKIGVLTSGGDAPGMNAAIRGVVRAALTEGLEVMGIYDGYLGLYEDRMVQLDRYSVSD
MINRGGTFLGSARFPEFRDENIRAVAIENLKKRGIDALVVIGGDGSYMGAKRLTEMGFPC
IGLPGTIDNDIKGTDYTIGYFTALGTVVEAIDRLRDTSSSHQRISIVEVMGRYCGDLTLA
AAIAGGCEFIVVPEVEFNREDLVAEIKAGIAKGKKHAIVAITEHMCDVDELAHFIEKETG
RETRATVLGHIQRGGSPVPYDRILASRMGAYAIDLLLEGHGGRCVGIQNEQLVHHDIIDA
IENMKRPFKSDWMECAKKLY
>EECEBICD_03986 Ferrous-iron efflux pump FieF
MNQTYGRLVSRAAIAATAMASALLLIKIFAWWYTGSVSILAALVDSLVDIAASLTNLLVV
RYSLQPADDEHTFGHGKAESLAALAQSMFISGSALFLFLTSIQNLIKPTPMNDPGVGIGV
TVIALICTIILVTFQRWVVRKTQSQAVRADMLHYQSDVMMNGAILIALGLSWYGWHRADA
LFALGIGIYILYSALRMGYEAVQSLLDRALPDAERQEIIDIVTSWPGVSGAHDLRTRQSG
PTRFIQIHLEMEDNLPLVQAHFVADQVEQAILQRFPGSDVIIHQDPCSVVPREGRKFELV
>EECEBICD_03987 Periplasmic protein CpxP
MRKVTAAVMASTLAFSFLSHAAEVVTSDNWHPGDGATQRSAQNHMFDGISLTEHQRQQMR
DLMQQARHEQPPVNVSEMETMHRLVTAEKFDESAVRAQAEKMAQEQVARQVEMARVRNQM
YRLLTPEQQAVLNEKHQQRMEQLRDVAQWQKSSSLKLLSSSNSRSQ
>EECEBICD_03988 Transcriptional regulatory protein CpxR
MNKILLVDDDRELTSLLKELLEMEGFNVLVAHDGEQALELLDDSIDLLLLDVMMPKKNGI
DTLKALRQTHQTPVIMLTARGSELDRVLGLELGADDYLPKPFNDRELVARIRAILRRSHW
SEQQQSSDNGSPTLEVDALSLNPGRQEASFDGQTLELTGTEFTLLYLLAQHLGQVVSREH
LSQEVLGKRLTPFDRAIDMHISNLRRKLPERKDGHPWFKTLRGRGYLMVSAS
>EECEBICD_03989 Sensor histidine kinase CpxA

MIGSLTARIFAIFWLTLALVLMLVLMLPKLDSRQMTELLDSEQRQGLMIEQHVEAELAND
PPNDLMWWRRLFRAIDKWAPPGQRLLLVTSEGRVIGAERSEMQIIRNFIGQADNADHPQK
KKYGRVEMVGPFSVRDGEDNYQLYLIRPASSSQSDFINLLFDRPLLLLIVTMLVSSPLLL
WLAWSLAKPARKLKNAADEVAQGNLRQHPELEAGPQEFLAAGASFNQMVTALERMMTSQQ
RLLSDISHELRTPLTRLQLGTALLRRRGGESKELERIETEAQRLDSMINDLLVMSRNQQK
NALVSETMKANQLWGEVLDNAAFEAEQMGKSLTVNYPPGPWPLYGNPNALESALENIVRN
ALRYSHTKIEVGFSVDKDGITITVDDDGPGVSPEDREQIFRPFYRTDEARDRESGGTGLG
LAIVESAMQQHRGWVKADDSPLGGLRLTLWLPLYKRT
>EECEBICD_03990 hypothetical protein
MDAHILEACTEEVKHILFEEYHLKSIEELWERSLDEDFLAELYQQFKFITEKYGVNGDEL
DSILDTL
>EECEBICD_03991 hypothetical protein
MTEAENAVAIVKEFLVASMIPDAERAATYMHPEVKITFTGGRAMAGAADIAQFNGARYKW
VKKALGEFDAVQHDDYVVIYSNGTLYGEWPDGRPFADNRFIDRFEVRDGKITRMDVWNDS
AEWILAPEISR
>EECEBICD_03992 Protein YiiM
MHYPVDVFIGKIRDYDGSRPSAIAKVQADGELMLTELGLAGDEQAEKKIHGGPDRALCHY
PREHYADWIRQFPEQATLFCAPAFGENLSTDGMTEHNVFIGDIYRWGEVLIQVTQPRSPC
FKLNFHFAISDMALLMQNSGKTGWLYRVIAPGKVSSDAPLELASRLSDVSVHEAGVIAWS
MPFDDEQYHRLLSAAGLSASWSRTMQKRRLSGKIEDSARRLWGDKTPPK
>EECEBICD_03993 Superoxide dismutase [Mn]
MSYTLPSLPYAYDALEPHFDKQTMEIHHTKHHQTYVNNANAALENLPEFADLPVEELITK
LDQVPADKKTVLRNNAGGHANHSFFWKGLKKGTTLQGDLKAAIERDFGSVDNFKAEFEKA
AATRFGSGWAWLVLKGDKLAVVSTANQDSPLMGEAISGASGFPILGLDVWEHAYYLKFQN
RRPDYIKEFWNVVNWDEAAARFAAKK
>EECEBICD_03994 hypothetical protein
MKQPGFIRLATLALLSTLSFFSHGETILRLAYAENSQPVKDALHFLGQQVEEKTGGDIKV
QYFPDGQLGGERELVELTQVGVVDITKVSSGLMESFSPEYGVFSLPYLFATVEEYYRVMD
NPQVMEPVYQSTAAQGFVGVGWYDSGARNFYMSKAPVKRIEDLRGKKIRVMQSETAIQTL
KLLGASPIAMSQAEVYTSLQQGILDGAENNEFALTIARHGEVARYRLFQHHDAGTAFRYF
DVRHRAKNVLQPGQFCLAGRAVLRFVRGDHE
>EECEBICD_03995 C4-dicarboxylate TRAP transporter large permease protein
DctM
MLAVPFFVLSGVIMNSGGIAARLVNFAKLFTGKLPGSLSYTNIVGNMMFGAISGSAIAAS
TSIGGVMVPMSAREGYDRGFAAAVNIASAPTGMLIPPTTAFILYALASGGTSIAALFAGG
LVAGVLWGVGCMLVTLVVAKRRNYRVFFTVQKGMALKVAVEAIPSLLLIVIIVGGIVQGI
FTAIEASAIAVVYTLLLTMVFYRTLKIKDLPSILLQTVVMTGVIMFLLATSSAMSFSMSI
TNIPAALSDMILGISANKLVILLVITVFLLIIGAFMDIGPAILIFTPILLPIMAKLGVDP
VHFGIIMIYNLAIGTITPPVGSGLYVGASVGKVKVEEVIKPLLPFYGAIIGVLLLITYIP
EITLFLPRLLGIM
>EECEBICD_03996 Oligogalacturonate-specific porin KdgM
MRKSLFLLLPLVVTNAHAVYVDVRHEYLDDSKANYDRAYISHRFANGVGFAIEAISKSGG
DDTNKAFNDLETQGNEYTISYQFKTGDVAWQPGFVLETGNGYSTYKPYFRATWTLNESWW
VGARYRFEYVRRSSDIRDDDTINRMDVWAGYKWNNFDWTIEGIYKKADKYDLYDGGKDNY
EYNFRTAYIIDQWSPFVEVGNVSVNSNSDERQTRFRVGIGYTF
>EECEBICD_03997 L-rhamnose-proton symporter
MSNAITMGIFWHLIGAASAACFYAPFKQVKQWSWETMWSVGGIVSWLILPWAISALLLPD
FWAYYGQFNLSTLLPVFLFGAMWGIGNINYGLTMRYLGMSMGIGIAIGITLIVGTLMTPI
INGNFDVLIHTEGGRMTLLGVFVALIGVGIVTRAGQLKERKMGIKAEEFNLKKGLLLAVM
CGIFSAGMSFAMNAAKPMHEAAAALGVDPLYVALPSYVVIMGGGALVNLGFCFIRLAKVQ
NLSIKADFSLARPLIISNILLSALGGLMWYLQFFFYAWGHARIPAQYDYMSWMLHMSFYV
LCGGLVGLVLKEWKNAGRRPVAVLSLGCVVIIIAANIVGLGMAS
>EECEBICD_03998 HTH-type transcriptional activator RhaR
MANQLILLKKDFFTDEQQAVTVADRYPQDVFAEHTHEFCELVMVWRGNGLHVLNERPYRI

TRGDLFYIRAEDKHSYTSVNDLVLQNIIYCPERLKLNVNWQAMIPGFQGAQWHPHWRLGS
MGMNQARQVINQLEHESNGRDPLANEMAELLFGQLVMTLKRHRYATDDLPATSRETLLDK
LITALANSLECPFALDAFCQQEQCSERVLRQQFRAQTGMTINQYLRQVRICHAQYLLQHS
PLMISEISMQCGFEDSNYFSVVFTRETGMTPSQWRHLSNQSD
>EECEBICD_03999 HTH-type transcriptional activator RhaS
MTVLHSVDFFPSGKAPVAIEPRLPQAAFPEHHHDFHEIVIVEHGTGIHVFNGQPYTISGG
TVCFVRDHDRHLYEHTDNLCLTNVLWRSPDAFQFLAGLDQLLPQEQDGYYPSHWRGNQSV
LQQVRQLVGLMERAGDGMDAPAVANREILFMQLLVLLRRSSLMEGATNNDAKLNQLMAWL
EDHFAEEVCWEAVAEQFSLSLRTLHRQLKQHTGLTPQRYLNRLRLIKARHLLRHSDHSVT
EIAYRCGFGDSNHFSTLFRREFNWSPRDIRQGRDAIIQ
>EECEBICD_04000 Rhamnulokinase
MTFRHCVAVDLGASSGRVMLARYDSKHRTLTLREIHRFVNCLQKTDGFDTWDIDSLEKDI
RLGLKKVCNEGILIDSIGIDTWGVDYVLLDKQGQRVGLPVSYRDNRTTGIMPQALVQIGK
SEIYRRSGIQFLPFNTIYQLRALTKQQPELTAQVAHALLMPDYFSYRLTGEMNWEYTNAT
TTQLVNINTDDWDDTLLAWTGAKKSWFGRPSHPGNVIGDWICPQGNRIPVVAVASHDTAS
AVIASPLANKHSAYLSSGTWSLMGFESKMPYTTDEALAANITNEGGAEGRYRVLKNIMGL
WLLQRVLKERRITDLPALIAQTEALPACRFLINPNDDRFINPDDMRAEIQAACRETDQPV
PVSDAELARCIFDSLALLYADILHELANLRGEKFTQLHIVGGGCQNSLLNQLCADACGIR
VMAGPVEASTLGNIGIQLMTLDELNNVDDFRQVVSANYDLTTYIPNPDSEIARHVAQFQP
KRQTKELCA
>EECEBICD_04001 L-rhamnose isomerase
MTTQLEQAWELAKQRFAAVGIDVEEALRQLDRLPVSMHCWQGDDVAGFENPEGSLTGGIQ
STGNYPGKARNATELRADLEQALRLIPGPKRLNLHAIYLESDTPVARDQIKPEHFKNWVE
WAKANRLGLDFNPTCFSHPLSADGFTLSHPDAKIRQFWIDHCKASRRVSAYFGEQLGTPS
VMNIWIPDGMKDITVDRLAPRQRLLEALDEVISEKFDPAHHIDAVESKLFGIGAESYTVG
SNEFYMGYATSRQTALCLDAGHFHPTEVISDKISAAMLYVPRLLLHVSRPVRWDSDHVVL
LDDETQAIASEIVRHNLFDRVHIGLDFFDASINRVAAWVIGTRNMKKALLRALLEPTDQL
RQLEASGDYTVRLALLEEQKSLPWQAVWEMYCQRHDTPTGSQWLDSVRTYEKEILSKRS
>EECEBICD_04002 Rhamnulose-1-phosphate aldolase
MQNITDSWFVQGMIKATSDAWLKGWDERNGGNLTLRLDEADIAPFATNFHEKPRYIALSQ
PMPLLANTPFIVTGSGKFFRNVQLDPAANLGVVKIDSDGAGYHILWGLTHDAVPTSELPA
HFLSHCERIKATHGKDRVIMHCHATNLIALTYVLENNTALITRKLWEGSTECLVVFPDGV
GILPWMVPGTDEIGQATAQEMQKHSLVLWPFHGVFGSGPTLDETFGLIDTAEKSAEVLVK
IYSMGGMKQTITREELVALGKRFGVTPLASAVALY
>EECEBICD_04003 NAD-dependent methanol dehydrogenase
MSFMLALPKISLHGAGAIADMVNLVANKQWGKALIVTDGQLVKQGLLDSLFSALDEHQMS
YHLFDEVFPNPTEELVQKGFAAYQSAECDYIIAFGGGSPIDTAKAVKILTANPGPSTAYS
GVGKVKNAGVPLVAINTTAGTAAEMTSNAVIIDSSRKVKEVIIDPNIIPDIAVDDASVML
EIPASVTAATGMDALTHAVEAYVSVGAHPLTDANALEAIRLINLWLPKAVDDGHNLEARE
QMAFGQYLAGMAFNSAGLGLVHALAHQPGATHNLPHGVCNAILLPIVENFNRPNAVARFA
RIAQAMGVETRGMSDEAASQEAINAIRTLSKRVGIPEGFSKLGVTKEDIEGWLDKALADP
CAPCNPRTASRDEVRGLYLEAL
>EECEBICD_04004 L-rhamnose mutarotase
MIRKAFVMQVNADAHEEYQRRHNPIWPELEAVLKSHGAHHYAIYLDQERNLLFATVEIES
EERWNAVASTDVCQRWWKHMRDVMPANPDNSPVSAELKEVFYLQ
>EECEBICD_04005 HTH-type transcriptional regulator SutR
MTQPISVIARSLVRERQRTGLSLAEIARRAGIAKSTLSQLEAGNGNPSLETLWSLCVALD
IPFARLLEPQVQKTQVIRRGEGTKVVAEQAHYQAILLAACPPGARRDIYLLLTQPGADRI
SHPHPPGSVEHIIVTQGKALVGLTEAPEELAEGDYICYPGDQAHIFKALEPDTQAILVAE
QN
>EECEBICD_04006 hypothetical protein
MKHYFSCLKGDTIKAIFLVCLAVGVVGMSYGSLAMAYGFPLWVPFVLSIFVLAGASEFMF
IGIVASGGNPLAAAAAGLLVNARHVPFGVTVRDLVGTRAASFFGCHIMNDESVVFGLSQK
TPEQRKAAYWLCGLGVALFWPIGTLIGAGVGKLLPAPETIGLDAVFPAILLALVIPAFKN

RTTLIRGCSGAALSLAAVPFVAAGLPVLLSLLGLLARKK
>EECEBICD_04007 hypothetical protein
MGNMTLFIIGIALLSAGTYLMRLGGAKLGSRLALSERSQALLSDAATVLLFSVALATTFY
EGEHFAGMARVLGVGFAVFLAWRKMPLIVVIIAAAVVTALLRMAGIN
>EECEBICD_04008 hypothetical protein
MKRNLLSSAIIIALMTLGATGCDNNNVKTEATPAASSQPATPAPSQTPETQSDESPAQPS
AAKPETATQPPAAKPETPAQPEVDAEEVYSEKMDVYIDCFNKLQLPVQHSLARYADWVKD
FKKGPTGKESLVYGIYGITESYITNCQKEMKQVAALTPLLEPIDGVAVSYIDSAAALGNT
INDMEKYYTQENYKDDAFAKGKALHQTLLKNIEDFKPVSEKYHEAIQEINDRRQLTQLKR
IEEAEGKTFNYYSLAVMISAKQINKVISADTFDAEAMMKKVAELETMIAQLKEVNTDGRN
SSFISSAADYQLQAKKYIRRIRDNVEYSDFEKKRVQDPATGWMVADSYPASLRSYNEMVD
DYNRLR
>EECEBICD_04009 hypothetical protein
MKKDLLSSIIIAMLMTAGLSACDEKKRTNNL
>EECEBICD_04010 hypothetical protein
MSGQHAANEIKATEKKEGKSIKYYTLLTMQEAETLNDAVADDSFDVAAVSKQLADFEEHT
QKLNEKINVDIDKHRSFPGFISELEKFQGKVKKRIRRVRDNVAYTSHEQDYLNSGSGDMV
DGSYEAVVKAYNELIDTYNGYHLEREF
>EECEBICD_04011 Sulfur carrier protein FdhD
MNNILSEEVLNVTDFTTSRQLTLWKREDLQSPQLDDVAEEVPVALVYNGISHVVMMASPK
DLTHFAMGFSLSEGIIDSPREIYGMDVVPSCNGLEVQIDLSSRRFMGLKARRRALAGRTG
CGVCGVEQLNDIGKPVQPLPFSQTFNLGNLDRALKHLNDFQPTGKLTGCTHAAAWVMPSG
ELAGGHEDVGRHVALDKLLGRRATEGEEWRQGAALVSSRASYEMVQKSAMCGVEILFAVS
AATTLAVDVAERCNLTLVGFCKPGRATIYTHPQRLIAD
>EECEBICD_04012 Formate dehydrogenase-O major subunit
MQVSRRQFFKICAGGMAGTTAAALGFAPGVALAETRQYKLLRTRETRNTCTYCSVGCGLL
MYSLGDGAKNAKASIFHIEGDPDHPVSRGALCPKGAGLVDFIHSESRLKFPQYRAPGSDK
WQQISWEEAFDRIAKLMKEDRDANYQAQNAEGVTVNRWLTTGMLCASASSNETGYLTQKF
SRALGMLAVDNQARV
>EECEBICD_04013 Formate dehydrogenase-O major subunit
MTNHWVDIKNANLVVVMGGNAAEAHPVGFRWAMEAKIHNGAKLIVIDPRFTRTASVADFY
APIRSGTDIAFLSGVMLYLLTNEKYNREYTEAYTNASLIVREDFGFDDGLFTGYDADKRQ
YDKTSWHYELDENGFAKRDTTLQHPRCVWNLLKQHVSRYTPDVVENICGTPKADFLKVCE
YIAETSAKDKTASFLYALGWTQHSIGAQNIRTMAMIQLLLGNMGMAGGGVNALRGHSNIQ
GLTDLGLLSQSLPGYLTLPSEKQTDLQTYLAANTPKPLLKDQVNYWGNYPKFFVSMMKAF
FGDKATAENSWGFDWLPKWDKGYDVLQYFEMMKQGKVNGYICQGFNPVASFPNKNKVVAS
LSKLKYLVTIDPLNTETSTFWQNHGESNDVDPAKIQTEVFRLPSTCFAEENGSIVNSGRW
LQWHWKGADAPGIAMTDGEILAGIFLRLRKMYSEQGGANPEQVLNMTWNYTKPYEPASEE
VAMESNGKALADLIDPATGAVVVKKGQQLSSFAQLRDDGTTSSGCWIFAGSWTPEGNMMA
RRDNADPSGLGNTLGWAWAWPLNRRILYNRASADPQGNPWDPKRQLLKWEGGKWAGWDIP
DYSAAAPGSDVGPFIMQPEGMGRLFAIDKMAEGPFPEHYEPFETPLGTNPLHPNVISNPA
ARIFKDDADALGKADKFPYVGTTYRLTEHFHYWTKHALLNAIAQPEQFVEIGEKLANKLG
IAHGDTVKVSSNRGYIKAKAVVTKRIRTLKADGKDIDTIGIPIHWGYEGVAKKGFIANTL
TPFVGDANTQTPEFKSFLVNVEKV
>EECEBICD_04014 Formate dehydrogenase-O iron-sulfur subunit
MAYQSQDIIRRSATNGFTPAPQARDHQEEVAKLIDVTTCIGCKACQVACSEWNDIRDEVG
NNVGVYDNPADLTAKSWTVMRFSEVEQNDKLEWLIRKDGCMHCADPGCLKACPAEGAIIQ
YANGIVDFQSEQCIGCGYCIAGCPFNVPRLNPEDNRVYKCTLCVDRVVVGQEPACVKTCP
TGAIHFGSKEDMKTLAGERVAELKTRGYDNAGLYDPAGVGGTHVMYVLHHADKPNLYHGL
PENPEISETVKFWKGVWKPLAAFGFAATFAASVFHYVGVGPNRAEEEDDNLHEEKDEVRK
>EECEBICD_04015 Formate dehydrogenase, cytochrome b556(fdo) subunit
MKRRDTIVRYTAPERINHWIVAFCFVLAAVSGLGFLFPSFNWLMHILGTPQLARILHPFV
GVVMFASFIIMFFRYWHHNLINRDDIFWAKNIRKIVVNEEVGDTGRYNFGQKCVFWAAII
FLVLLLVSGVIIWRPYFAPAFSIPVIRFALMLHSFAAVALIVVIMVHIYAALWVKGTITA

MVEGWVTRSWAKKHHPRWYREVRKTTEKETE
>EECEBICD_04016 Protein FdhE
MSIRIIPQDELGSSEKRTADMIPPLLFPRLKNVYNRRAERLRELAENNPLGDYLRFAALI
AHAQEVVLYDHPLRMDLTARIKDANDQGKPPLDIHVLPRDKHWHTLLHSMIAELKPEMSG
PALAVIENLEKASEQELEQMASALFASDFASVSSDKAPFIWAALSLYWAQMASLIPGKAR
AEYGEARQYCPVCGSMPVSSMVQIGTTQGLRYLHCNLCETEWHVVRVKCSNCEQSRDLHY
WSLENEQAAVKAESCGDCGTYLKILYQEKDPKVEAVADDLASLVLDARMEQEGFARSSIN
PFLFPGEGE
>EECEBICD_04017 hypothetical protein
MDKVLFERLTQSMSQMNEIIEGTREPSRTFHIDAMKIKEIRQASGLSQSKFAELISVNVD
TLRNWEQGRRSPTGPAKALLRAIANDPRNVIQALRY
>EECEBICD_04018 Toxin HigB-2
MQFIETELFTEDVKKLLDEDEYHKLQVFMAQHPDCGDVIQETGGLRKMRWGARGKGKRSG
VRIIYFHRSQRYEIRLLLIYQKGIKDDLTPQEKAVLRMLNERW
>EECEBICD_04019 Carboxylesterase NlhH
MPLEKGIAELVAGFIAAGRPSSREQNIDDRRAGYIASTTLAGETETRVQVEDIELDAMTF
RVVSPLNATGKLPCIIYYHGGCFVSGGFATHDNQLRQLAFYSRCRVIAAQYRLAPEHTFP
AAHNDAETGANTIWKYAQKLGIDRENITLAGDSAGGHLALVTALRLKAARQWQPAQLILI
YPMLDATARFASYTCNGLDYIITRDTLLSGYEMYMPRTDPLHPEASPLWREDFAGLPSTH
IITAEFDPLRDEGEALYQRFQEQGVECTCQRYLGVIHGFFQLGGVSQAARSAMRDVAWRV
VSPSTGKMT
>EECEBICD_04020 mRNA interferase HigB
MHVISRKPFNEAMLMYPNHELALTELLNVLEKKTLTQPEEMKRYIPSLDNFKHRDKWWVI
DVSGNSLRLISYIDFRLHKIFVKHIVSHAEYDKLTAYDRGNKE
>EECEBICD_04021 Antitoxin HigA
MRTHRQMDATSAKKIVDTFSDAVKSVPLMGEDRNDNEYRRALALVEFLVDHDDLENPLFE
LLCARISEYEKHAPEFKALNQHLEKTPPGVSVLRTLMDQYGLKAADLANELGSKSNVSNI
LNGRRALTVNHIKALTQRFKLPADAFIE
>EECEBICD_04022 hypothetical protein
MYHLRVPQTEEELERYYQFRWEMLRKPLHQPKGSERDGWDALAHHQMVVDEEGNLVAVGR
LYINADNEASIRFMAVHPSVQDKGLGTLMAMTLESVARQEGVKRVTCSAREDAVEFFAKL
GFVNQGEITTPTTTPIRHFLMIKPVASLDDILHRGDWCGQLQQAWYEHIPLSEKMGVRIQ
QYTGQKFITTMPETGNQNPHHTVFAGSLFSLATLTGWGLIWLMLRERHLGGTIILADAHI
RYSKPISGKPNAVADLGSLSGDLDRLARGKKARVQLQVELLGGETAGAIFEGIYIVLPAK
PFGPYEEGGNEEE
>EECEBICD_04023 D-aminoacyl-tRNA deacylase
MIALIQRVTRASVTVEDEVTGEIGPGLLVLLGVEKEDDEQKANRLCERVLGYRIFSDADG
KMNLNVQQAGGSVLVVSQFTLAADTERGMRPSFSGGAAPDRAQALYEYFVERCRQQAIDT
QTGRFAADMQVELVNDGPVTFWLQV
>EECEBICD_04024 hypothetical protein
MTTLAGNLAYVSLLSLVPLIAVVFALFAAFPMFSDVSIQLRHFIFANFMPATGDVIQRYI
EQFVANSNKMTAVGACGLIVTALLLMYAIDSALNTIWRSKRTRPKVYSFAVYWMILTLGP
LLAGASLAISSYLLSLRWASDLNTVIDNVLRILPLLLSWISFWLLYSIVPTTRVPNRDAL
VGAFVAALLFEAGKKGFALYITMFPSYQLIYGVLAVIPILFVWVYWTWCIVLLGAEITVT
LGEYRKLKQAAEQEEADQP
>EECEBICD_04025 Alpha-D-glucose 1-phosphate phosphatase YihX
MLYIFDLGNVIVDIDFNRVLGVWSDLSRVPLASLKQKFTMGETFHQHERGEITDEAFAEA
FCHEMALSLSYEQFAHGWQAVFVGLRPEVIAIMHKLREQGHRVVVLSNTNRLHTHFWPEE
YPEVRAAADHIYLSQDLGMRKPEARIYQHVLQKEGFSAADAVFFDDNADNIEGANQLGIT
SILVKDKATIPDYFAKLLC
>EECEBICD_04026 HTH-type transcriptional repressor CsqR
MSLTELTGNPRHDRLLMLIDERGYMNIDELASLLEVSTQTVRRDIRKLSEQGLITRHHGG
AGRASSVVNTAFEQREVSWTQEKKAIAEAVADYIPDGSTIFITIGTTVEQVARALLNHNH
LRIITNSLRVAHILYNNPRFEVMVPGGTLRPHNSGIIGPSAAAFVAGFRADYLVTSVGAI

ESDGALLEFDVNEASVVKTMMAHSRHILLAADHTKYHASAAVEIGNVSQITALFTDEYPG
PALQNLLQSQQIEVVQVSPSLDDAVSA
>EECEBICD_04027 Sulfofructose kinase
MVRIACVGITVMDRIYYVEGLPTEGGKYVAKRYTEVGGGPAATAAVAAAKLGAQVDFIGR
VGDDDTGNSLLAELESLGVNTRYTRRYTQAMSSQSAIMVDAKGERIIVNYPSPDLLPDAD
WLNDIDFSQWDVVLADVRWHDGAKQAFTLARQAGVMTVLDGDITPQDISELVALSDHAAF
SEPGLARLTGMSEAIDALKKAQMLTNGHVYVTRGSEGCNWLEKAAVRHQPGFTVEVVDTT
GAGDVFHGALAFGLASGYAIEEAVRFASGVAALKCTRPGGRAGIPDCEQTRSFLSLFV
>EECEBICD_04028 3-sulfolactaldehyde reductase
MAVIAFIGLGQMGSPMASNLLKQGHQLSVFDVNPDAVQRLVDKGAQPASSPAQATIGAEF
VITMLPNGDLVRSVLFGEQGVCETLSHEALVIDMSTIHPLQTDKLIADMQSKGFSMMDVP
VGRTSDNAITGTLLLLAGGTAEQVERATPVLMAMGNELVNTGGPGMGIRLKLINNYMSIA
LNALSAEAAVLCEALGLSFDVALKVMSGTAAGKGHFTTTWPNKVMKGDLSPAFMIDLAHK
DLGIALDVANQLHVPMPLGAASREVYNLARAAGRGREDWSAILEQVRISAGLTANVKK
>EECEBICD_04029 Sulfofructosephosphate aldolase
MNNYTIKDITRASGGFAMLAVDQREAMRLMFAAAGAKTPVADSVLTDFKVNAAKILSPYA
SAVLLDQQFCYRQAVEQNAVAKSCAMIVAADDFIPGNGIPVDNVVIDKKINAQAVKRDGA
KALKLLVLWRSDEDAQQRLDMVKEFNELCHSNGLLSIIEPVVRPPRCGDKFDREQAIIDA
AKELGDSGADLYKVEMPLYGKGARSDLLTASQRLNGHINMPWVILSSGVDEKLFPRAVRV
AMEAGASGFLAGRAVWSSVIGLPDTELMLRDVSAPKLQRLGEIVDEMMAKRR
>EECEBICD_04030 Sulfoquinovose isomerase
MKWFNTLSHNHWLEQETDRIFNFGKNAVVPTGFGWLGNKGQIKEEMGTHLWITARMLHVY
SVAASMGRPGAYDLVDHGIKAMNGALRDKKYGGWYACVNDQGVVDASKQGYQHFFALLGA
ASAVTTGHPEARKLLDYTIEVIEKYFWSEEEQMCLESWDEAFSQTEDYRGGNANMHAVEA
FLIVYDVTHDKKWLDRALRIASVIIHDVARNGDYRVNEHFDSQWNPIRDYNKDNPAHRFR
AYGGTPGHWIEWGRLMLHLHAALEARFETPPAWLLEDAKGLFHATIRDAWAPDGADGFVY
SVDWDGKPIVRERVRWPIVEAMGTAYALHTLTGDSQHEEWYQKWWDYCIKYLMDYENGSW
WQELDADNKVTTKVWDGKQDIYHLLHCLVIPRLPLAPGLAPAVAAGLLDINAK
>EECEBICD_04031 putative protein YihR
MHSGGKTIQLNAGHYQAKIVTVGAGLAELTHHGRHVVIPHKPEEIPMAHLGKVLIPWPNR
VTNGCYSYNGKVFQLAVNDPVSQTAIHGLLAWRDWQINYQSTTEASLTIFLPPSYGYPFA
LISEVIYRLDAASGLHVLIRTQNIGDESAPYGAGAHPYLTCNLQSIDSCVLTLPASEELP
AGRDFSASCLLGETRLDHAVKTATTPAEWEVRLTSPTQNMSTFLRSTQPWLQIYTGEKLS
RKGLAVEPMSCPPDAFNSGIALIHLAPKAIHQLHFSIGCD
>EECEBICD_04032 Sulfoquinovosidase
MNSLPQRSTDFELTTSQDGFALSWQQRLILRHSTENPCLWIGAGVADIDMFRGNFSIKDK
LNEKIALTEATVSELPDGWLVQFSRGATISATLRISADEAGRLKLDLQNDDLHHNRIWLR
LAANPDDHIYGCGEQFSYFDLRGKPFPLWTSEQGVGRNKTSYVTWQADCKENAGGDYYWT
FFPQPTFVSTQKYYCHVDNSCYMNFDFSAPEYHELALWEDKTTLRFECADTYIALLEKLT
ALLGRQPELPDWVYDGVTLGIQGGTEVCQQKLDTMRNAGVKVNGIWAQDWSGIRMTSFGK
RVMWNWKWNSDNYPQLDSRIKQWKEEGVQFLSYINPYVASDKDLCAEAAKHGYLAKDATG
GDYLVEFGEFYGGVVDLTNPEAYDWFKDVIKKNMIALGCSGWMADFGEYLPTDTYLHNGV
SAEIMHNAWPALWAKCNYEALQETGKLGEILFFMRAGYTGSQKYSTMMWAGDQNVDWSLD
DGLASVVPAALSLAMTGHGLHHSDIGGYTTLFDMKRSKELLLRWCDFSAFTPMMRTHEGN
RPGDNWQFDGDEETIAHFARMTTVFTTLKPYLKQAVAQNADTGLPVMRPLFLHYENDAAT
YTLKYQYLLGQDLLVAPVHEQGRCDWTLYLPEDHWVNIWTGEVHHGGEITVDAPIGKPPV
FYRAKSEWASLFASLRNI
>EECEBICD_04033 Putative 2,3-dihydroxypropane-1-sulfonate exporter
MSQTSSNPATLRLPFKEKLAYGLGDLGSNILLDIGTLYLLKFYTDVLGLPGTYGGIIFLI
AKFFTAFTDMGTGIMLDSRRKIGPKGKFRPFVLYAAFPVTLLAIANFVGTPFEVTGKTVV
ATMLFMLYGLVFSMMNCSYGAMVPAITKNPDERASLAAWRQGGATLGLLLCTVGFVPVMN
LIEGNAQLSYIFAATLFSLFGLLFMWLCYAGVKERYVEVKPVDSAQKPGLLQSFRAIAGN
RPLFILCIANLCTLGAFNVKLAIQVYYTQYVLNDPILLSWMGFFSMGCIFIGVFLMPGAV
RRFGKKKVYIGGLLIWVAGDLLNYFFGGGSVSFVAFSCLAFFGSAFVNSLNWALVSDTVE

YGEWRTGVRSEGTVYTGFTFFRKVSQALAGFFPGWMLTQIGYIPNVVQSAGTVEGLRQLI
FIYPCVLAVITIIAMGCFYNLNEKMYVRIVEEIEARKHTV
>EECEBICD_04034 Putative 2,3-dihydroxypropane-1-sulfonate exporter
MSNHDPLTLKLSLREKCAYGVGDFGSNLMLCIGTLYLLKFYTDELGMPAYYGGIIFLVAK
FFTAFTDMLTGVLLDSRRNIGTKGKFRPFILYASFPVALVATAQFFATHFTLPVKTAFAT
VLFMLFGLFYSLMNCSYGAMVPAITKNPHERAQLAAWRQGGATIGLLLCTVGFMPIQALF
TRSPSLGYLIAAVIFSVCGLFSMWWCFSGVKERYIETVPDTHKPSILKSFCAIFRNPPLL
VLCVANLCTLAAFNIKLAIQVYYTQYVLNDIHLLSWMGFSAWAVS
>EECEBICD_04035 Putative 2,3-dihydroxypropane-1-sulfonate exporter
MDGFFSMGCILIGVLLVPAAVKRFGKKQVYLGGLILWAVGDILNFIWGGTSFLFVIFSCI
AFFGTAFVNSLNWALVPDTVDYGEWKTGIRAEGSVYTGYTFSRKISAALAGFLPGIMLTQ
IGYIPNIAQSDTTLLGLRQLIFLWPCGLAIIAALTMGFFYKLNEQRFAFIIEEIAQRKKT
GNQIVATNNKQSISTVNN
>EECEBICD_04036 Porin OmpL
MKSLNTLVILTSVISTSVFAGAYVENREAYNLASDQMEFMLRVGYNSDMGAGIMLTNTYT
LQRDDELKHGYNEIEGWYPLFKPTDKLTIQPGGLINDKSIGSGGAVYLDVNYKFTPWFNL
TVRNRYNHNNYSSTDLNGELDNNDSYEIGNYWNFIITDKFSYTFEPHYFYNVNDFNSSNG
TKHHWEITNTFRYRINEHWLPYFELRWLDRNVGPYHREQNQIRIGAKYFF
>EECEBICD_04037 hypothetical protein
MNKRFNIDWDNELTQEQLINLILTDEDLPKLRSLTIGNWGDCWEDETCQPIIDMIVENAS
RFTHLESLFIGDMESEDCEISWIKQGDYSRLYAALPNLKELIIKGASDLRLGAIHHEKLE
HLEIISGGIPSNVLAELQNAQLPALKTLKLFLGVEEYGFDGSLDDVMALASKDLFPQLTH
LGLMNSEEQDDIARRVLESNILPQLNVLELSCGTLTDNGAEALLEHKDRIAHLETLDLHH
HYLTPEMQEKLKATLPINLNLSEALEPDDYDGDIYMNAMYTE
>EECEBICD_04038 hypothetical protein
MITLIGPRQSKRTHYFMQAAAVLGEPVTALTHPQALDASLTSGIVKIDPPVHSETDIRKI
NAIGLDYIHFLQNMAARPGVTWLNHPAGLIHLLDKKLCKRRLLEHGIPTPPLVAEDVKSF
VQLTAIMEEKRVASVFIKPSLGSGAAGVIALRRHPDGIKQVLYSAIAISGQQLFNSKKIQ
QYRQKEDIQLIIDGVLQQENLVEQWLPKANVKHKTYDLRIVCLDGEIIWRVVRTSSQPIT
NLHLQNQAYCFESLELSAAKVTEIDTLCQNAMRLFPGIRLAGIDVLLTTSLTPYVIEING
QGDLIYQDAQQNNLIYQAQIRAMRKQNV
>EECEBICD_04039 hypothetical protein
MYNSMDEVPVSLHASIDTGDGEFDMNTLISNNAHILFIVLDSLRYDIALQEQTAGNTPNL
NHYGQWTKCEAAGNFTWPSHHAMFSGFMPKPIDDTVNQTMLFFPKDIGLGRKGPKNAFAF
DDATWIKSLENKGYQTICIGGVSFFNNRSGMGKVFPSMFKESYWHPRFACTVKESMDNQI
HYIQKIMAERAGSQPVMMYINIDTIHYPNHFYVEGAGPGDTVETHAAALRYIDARIDGLL
NIFRQTGGETFVIVCSDHGTCYGEDGKYFHSFNHPIVNTVPYMHFLLSCNH
>EECEBICD_04040 Heme chaperone HemW
MNNNDIYRQYMYSYPHKTAYREVENVSFSEVKPRIYEHDTHLYVHMPFCQSRCGFCNLFT
CTGADNTFIDSYIDAIITQGRQMALAPAHWASFTVGGGTPLLLNIAQLEKLFFSVFDVLN
VDWNIYKTIETSPTDTTAEKVALLNAFSVNRVSIGVQSFHDSELHTLHRRHNAASAHQAL
EWLKAGHFPSLNIDIIYGIPGQTHASLTESLHQALVYQPEELFLYPLYNMPRQENMHSYY
VLARDLLRNAGYTQTSMRRFVLNPAPSAAAESCGFENSLALGAGGRSYLGNLHYCSPWSS
NLQTSRKIIQAFIDSPDKTVINHGYLLPPDEMKRRFIIKNLFFWQGLSLSDYQQYFASDA
LRDFPDLERFIRQGYCYQNGSRLRLTETGMALSDCLAPVFVSPEVMLRENRQR
>EECEBICD_04041 hypothetical protein
MSAPALRDIHTLFYRGYLKSCNYHCGYCPFHKHARNDKKRDEAALWRMVDHLPLLGTPLN
VMITPYGEALRLRYYMAALAEISRYPHVQAVGIQTNASFSLMSLLESFRAGGGDISKLRL
WCSFHPSQITAERFLQQCLALSAAGITWCAGAVASMKDIAQFRWLRQQLPDQNYFWFNAN
ECANTRHTVEETIAFSELDPLYVVETQQWPAQLDICTAGRKSIFMNAEGDLFACHISKIK
MGNLYQQRLNSPACQAKQCHCFLAYQHRLDIPLLNHLDTSRCFRIGRSCDIV
>EECEBICD_04042 Pyridoxal phosphate phosphatase YbhA
MRYRLIAFDLDGTLLNRNKQILPESLTAIRQMQDMGCRIMLVTGRSHREAYAYYQTLALT
EPMICCNGSYIYQPAQQQILHPLPLTHLQTEKILARVYPLKPTIRADDKIIFQADELSSR

ENIWQISVVHRHIKQLQNIAEFIQHELQLSCTWSWHHQLDILQKGCSKGQSLARYAQQQH
IAMREVMAFGDNDNDAEMLRLAGLGVAMGNASARAKVYADSVIGRHNTPAIADFLATLSL
SQRE
>EECEBICD_04043 GTP-binding protein TypA/BipA
MIENLRNIAIIAHVDHGKTTLVDKLLQQSGTFDARAETQERVMDSNDLEKERGITILAKN
TAIKWNDYRINIVDTPGHADFGGEVERVMSMVDSVLLVVDAFDGPMPQTRFVTKKAFAHG
LKPIVVINKVDRPGARPDWVVDQVFDLFVNLDATDEQLDFPIIYASALNGIAGLDHEDMA
EDMTPLYQAIVDHVPAPDVDLDGPLQMQISQLDYNNYVGVIGIGRIKRGKVKPNQQVTII
DSEGKTRNAKVGKVLTHLGLERIDSDIAEAGDIIAITGLGELNISDTICDPQNVEALPAL
SVDEPTVSMFFCVNTSPFCGKEGKFVTSRQILDRLNKELVHNVALRVEETEDADAFRVSG
RGELHLSVLIENMRREGFELAVSRPKVIFREIDGRKQEPYENVTLDVEEQHQGSVMQALG
ERKGDLKNMNPDGKGRVRLDYVIPSRGLIGFRSEFMTMTSGTGLLYSTFSHYDDIRPGEV
GQRQNGVLISNGQGKAVAFALFGLQDRGKLFLGHGAEVYEGQIIGIHSRSNDLTVNCLTG
KKLTNMRASGTDEAVILVPPIKMSLEQALEFIDDDELVEVTPTSIRIRKRHLTENDRRRA
NRGQKEE
>EECEBICD_04044 Glutamine synthetase
MSAEHVLTMLNEHEVKFVDLRFTDTKGKEQHVTIPAHQVNAEFFEEGKMFDGSSIGGWKG
INESDMVLMPDASTAVIDPFFADSTLIIRCDILEPGTLQGYDRDPRSIAKRAEDYLRATG
IADTVLFGPEPEFFLFDDIRFGASISGSHVAIDDIEGAWNSSTKYEGGNKGHRPGVKGGY
FPVPPVDSAQDIRSEMCLVMEQMGLVVEAHHHEVATAGQNEVATRFNTMTKKADEIQIYK
YVVHNVAHRFGKTATFMPKPMFGDNGSGMHCHMSLAKNGTNLFSGDKYAGLSEQALYYIG
GVIKHAKAINALANPTTNSYKRLVPGYEAPVMLAYSARNRSASIRIPVVASPKARRIEVR
FPDPAANPYLCFAALLMAGLDGIKNKIHPGEAMDKNLYDLPPEEAKEIPQVAGSLEEALN
ALDLDREFLKAGGVFTDEAIDAYIALRREEDDRVRMTPHPVEFELYYSV
>EECEBICD_04045 Sensory histidine kinase/phosphatase NtrB
MASGIQPDAGQILNSLINSVLVVDDALAIHYANPAAQQLLAQSSRKLFGTPLPELLSYFS
LNIDLMRESLAAGQGFTDNEVTLVIDSRSHILSLTAQRLPDDFILLEMAPMDNQRRLSQE
QLQHAQQIAARDLVRGLAHEIKNPLGGLRGAAQLLSKALPDPALTEYTKVIIEQADRLRN
LVDRLLGPQHPGMHITESIHKVAERVVALVSMELPDNVRLIRDYDPSLPELPHDPEQIEQ
VLLNIVRNALQALGPDGGEITLRTRTAFQLTLHGERYRLAARIDVEDNGPGIPPHLQDTL
FYPMVSGREGGTGLGLSIARNLIDQHAGKIEFTSWPGHTEFSVYLPIRK
>EECEBICD_04046 DNA-binding transcriptional regulator NtrC
MQRGIVWVVDDDSSIRWVLERALAGAGLTCTTFENGNEVLAALASKTPDVLLSDIRMPGM
DGLALLKQIKQRHPMLPVIIMTAHSDLDAAVSAYQQGAFDYLPKPFDIDEAVALVERAIS
HYQEQQQPRNIEVNGPTTDMIGEAPAMQDVFRIIGRLSRSSISVLINGESGTGKELVAHA
LHRHSPRAKAPFIALNMAAIPKDLIESELFGHEKGAFTGANTIRQGRFEQADGGTLFLDE
IGDMPLDVQTRLLRVLADGQFYRVGGYAPVKVDVRIIAATHQNLERRVQEGKFREDLFHR
LNVIRIHLPPLRERREDIPRLARHFLQVAARELGVEAKLLHPETETALTRLAWPGNVRQL
ENTCRWLTVMAAGQEVLIQDLPGELFEASTPDSPSHLPPDSWATLLAQWADRALRSGHQN
LLSEAQPELERTLLTTALRHTQGHKQEAARLLGWGRNTLTRKLKELGME
>EECEBICD_04047 putative protein YshB
MLESIINLVSSGAVDSHTPQTALAAVLCAALVGLFS
>EECEBICD_04048 Oxygen-independent coproporphyrinogen III oxidase
MSEQQIDWDLALIQKYNYSGPRYTSYPTALEFSEDFEDAAFLQAVARYPERPLSLYVHIP
FCHKLCYFCGCNKIVTRQQHKADQYLDALEQEIRHRAPLFADRHVSQLHWGGGTPTYLNK
TQISRLMTLLRENFHFNTDAEISIEVDPREIELDVLDHLRAEGFNRLSMGVQDFNKEVQR
LVNREQDEEFIFALLNHARDIGFTSTNIDLIYGLPKQTPESFAFTLKRVTELNPDRLSVF
NYAHLPTLFAAQRKIKDADLPSAQQKLDILQETIVSLTQAGYQFIGMDHFARPDDELAVA
QREGVLHRNFQGYTTQGDTDLLGMGVSAISMIGDGYMQNQKELKRYYQQVDERGNALWRG
ITLTRDDCIRRDVIKALICNFRLDFNAVEQQWGLHFAEYFAEDLQLLSPLAKDGLVDISE
KGIQVTAKGRLLIRNICMCFDAYLRQKARMQQFSRVI
>EECEBICD_04049 Der GTPase-activating protein YihI
MKKPTSAPRSKAFGKQRRKTREELNQEARDRKRLKKHRGHAPGSRAAGGNSASGGGNQNQ
QKDPRIGSKTPVPLGVTEKVTQQHKPKSEKPMLSPQAELDLLETDERLDALLERLEAGET

LSAEDQAWVDAKLDRIDELMQKLGLSYDDDEEDDEEDEKQEDMMRLLRGGN
>EECEBICD_04050 hypothetical protein
MHIARTLTGSTTQDEGQERQEAKTQDCQEDKRPETLVKGNGNNMECSRLREKQGVLIANR
DGGTR
>EECEBICD_04051 putative GTP-binding protein EngB
MTNLNYQQTHFVMSAPDIRHLPSDCGIEVAFAGRSNAGKSSALNTLTNQKSLARTSKTPG
RTQLINLFEVVEGKRLVDLPGYGYAEVPEEMKRKWQRALGEYLEKRQSLQGLVVLMDIRH
PLKDLDQQMIQWAVESNIQVLVLLTKADKLASGARKAQLNMVREAVLAFNGDVQVEAFSS
LKKQGVDKLRQKLDSWFSELAPVEEIQDGE
>EECEBICD_04052 DNA polymerase I
MVQIPENPLILVDGSSYLYRAYHAFPPLTNSAGEPTGAMYGVLNMLRSLIMQYQPTHAAV
VFDAKGKTFRDELFEHYKSHRPPMPDDLRAQIEPLHAMVKAMGLPLLAVSGVEADDVIGT
LAREAEKVGRPVLISTGDKDMAQLVTPNITLINTMTNTILGPDEVVNKYGVPPELIIDFL
ALMGDSSDNIPGVPGVGEKTAQALLQGLGGLDTLYAEPEKIAGLTFRGAKTMAGKLAQNK
DVAYLSYKLATIKTDVELELTCEQLEVQQPIADELLGLFKKYEFKRWTADVEAGKWLQAK
GAKPAAKPQETVVIDESPSEPAAALSYENYVTILDDVTLESWIEKLKKAPVFAFDTETDS
LDNIAANLVGLSFAIEPGVAAYVPVAHDYLDAPDQISRQRALELLKPLLEDEKVRKVGQN
LKYDRGVLQNYGIELRGIAFDTMLESYILNSVAGRHDMDSLSDRWLKHKTITFEDIAGKG
KNQLTFNQIALEEAGRYAAEDADVTLQLHLKMWPELQQHKGPLNVFENIEMPLVPVLSRV
ERNGVKIDPAVLHKHSEEITLRLAELEKKAHDIAGEAFNLSSTKQLQTILFEKQGIKPLK
KTPGGAPSTSEEVLEELALDYPLPKVILEYRGLAKLKSTYTDKLPLMINPKTGRVHTSYH
QAVTATGRLSSTDPNLQNIPVRNEEGRRIRQAFIAPEDYLIVSADYSQIELRIMAHLSRD
KGLLTAFAEGKDIHRATAAEVFGLPLDSVTGEQRRSAKAINFGLIYGMSAFGLSRQLNIP
RKEAQKYMDLYFERYPGVLEYMERTRAQAKEQGYVETLEGRRLYLPDIKSSNAARRAGAE
RAAINAPMQGTAADIIKRAMIAVDAWLQAEQPRVRMIMQVHDELVFEVHKDDLDAVAKRI
HQLMENCTRIDVPLLVEVGSGENWDQAH
>EECEBICD_04053 putative acyltransferase YihG
MTRILAAITLPLSIVLTILVTIVCSVPIIVAGMVKLLLPVPCIWRKVSIFCNFMMYCWCA
GLAALLRLNPHLQWDVEGLEGLSKKNWYLLICNHRSWADIVVLCVLFRKHIPMNKYFLKQ
QLAWVPFIGLACWALDMPFMKRYSRSYLLRHPDRRGKDVETTRRSCEKFRRYPTTIVNFV
EGSRFTHEKRQQTHSPYQHLLPPKAAGIAMAINVLGSQFDKLLNITLCYPNNDRHPFYDM
LSGRLTRIVVRVQLEPINEELHGDYVNDKTFKRRFQRWLNTLWDKKDIQIEEIKTSYKNA
GQ
>EECEBICD_04054 Thiol:disulfide interchange protein DsbA
MKKIWLALAGMVLAFSASAAQISDGKQYITLDKPVAGEPQVLEFFSFYCPHCYQFEEVLH
VSDNVKKKLPEGTKMTKYHVEFLGPLGKELTQAWAVAMALGVEDKVTVPLFEAVQKTQTV
QSAADIRKVFVDAGVKGEDYDAAWNSFVVKSLVAQQEKAAADLQLQGVPAMFVNGKYQIN
PQGMDTSSMDVFVQQYADTVKYLVDKK
>EECEBICD_04055 Serine/threonine protein kinase RdoA
MNDNAFTFQTLHPETIMDALFEQGIRVDSGLTPLNSYENRVYQFQDEDRRRFVVKFYRPE
RWSVDQIREEHQFALELVKDEVPVAAPLAFNGQTLLAHQGYHYAIFPSVGGRQFEADNID
QMEAVGRYLGRLHQTGRKRPFTFRPDIGLAEYLFEPRQVFEDAALIPSGQKAAFLKATDT
LLSAVTECWRTDFATLRLHGDCHAGNILWRDGPLFVDLDDARNGPAIQDLWMLLNGDKAE
QRMQLETIIEAYEEVSEFDTAEIGLIEPLRAMRLVYYLAWLIRRWGDPAFPKNFPWLTGE
DYWQRQTTTFIEQTKILHEPPLQLTPMY
>EECEBICD_04056 Protein YihD
MKCKRLNEVIELLQPAWQKEPDLNLTQFLQKLAKESGFDGKLEDLTDDILIYHLKMRDSA
KDAAIPGIQKDYEEDFKTALLRARGVIKE
>EECEBICD_04057 Molybdenum cofactor guanylyltransferase
MNLDEVITGVVLAGGKARRMGGADKGLLELNGKPLWRHVADALAPQLATVVISANRHLDI
YQASGLKVIPDSIADFPGPLAGMLSVFQQVASDWFLFCPCDNPCIPHNLVDRLAAQRHGA
PVVWVHDGERDHPTIALINRAVEPQLTAYLQAGERRVMIFMRQVGGHAVDFSDCKEAFVN
VNTPEELAQWQKRP

>EECEBICD_04058 Molybdopterin-guanine dinucleotide biosynthesis adapter protein
MAKETMIPLLAIAAWSGTGKTTLLKKLIPALCEHGIRPGLIKHTHHDMDVDKPGKDSYEL
RKAGAAQTLVASQQRWALMTETPGQEELDLAWLVSRMDASKLDMVLVEGFKHEAVAKILL
FRQGCGHSEEELILDEHVIAVASDIPLAVAVPVLDLNDVSQISAFILRWLEKARSL
>EECEBICD_04064 Protoporphyrinogen IX dehydrogenase [menaquinone]
MKTLILFSTRDGQTREIASYLASELKEMGIWADVVNLHRAEEPDWDSYDRVVIGASIRYG
HYHSAFQEFVKKYATRLNGMPSAFYSVNLVARKAEKRTPQTNSYARKFLMSSPWRPDYCA
VIAGALRYPRYRWYDRLMIKLIMKMSGGETDTSKEVVYTDWEQVAHFAREIAHLTNKSSA
K
>EECEBICD_04065 Trk system potassium uptake protein TrkH
MHFRAITRIVGLLVILFSGTMILPGLVALIYRDGAGRAFTQTFFVALAIGSILWWPNRRE
KGELKSREGFLIVVLFWTVLGSVGALPFIFSESPNLTITDAFFESFSGLTTTGATTLVGL
DSLPHAILFYRQMLQWFGGMGIIVLAVAILPILGVGGMQLYRAEMPGPLKDNKMRPRIAE
TAKTLWLIYVLLTVACALALWFAGMPAFDAIGHSFSTIAIGGFSTHDASVGYFDSPTINT
IIAIFLLISGCNYGLHFSLLSGRSLKVYWRDPEFRMFIGVQLTLVVICTLVLWFHNIYDS
ALTTLNQAFFQVVSMATTAGFTTDSIARWPLFLPVLLLCSAFIGGCAGSTGGGLKVIRIL
LLFKQGNRELKRLVHPNAVYSIKLGNRALPERILEAVWGFFSAYALVFIVSMLAIIATGV
DDFSAFASVVATLNNLGPGLGVVADNFASMNPVAKWILIANMLFGRLEVFTLLVLFTPTF
WRE
>EECEBICD_04066 IMPACT family member YigZ
MDSWLIPAAPVTVVEEIKKSRFITLLAHTDGVDAAKAFVESVRAEHPDARHHCVAWVAGA
PDDSQQLGFSDDGEPAGTAGKPMLAQLMGSGVGEITAVVVRYYGGILLGTGGLVKAYGGG
VNQALRQLATQRKTPLTEYTLQCEYGQLAGIEALLGQFAGKIVSSDYQASVRLRVALPFA
HVNAFSTKLADFSRGSLQLLAIEE
>EECEBICD_04067 Xaa-Pro dipeptidase
MESLAALYKNHIVTLQERTRDVLARFKLDALLIHSGELFNVFLDDHPYPFKVNPQFKAWV
PVTQVPNCWLLVDGVNKPKLWFYLPVDYWHNVEPLPTSFWTEEVEVVALPKADGIGSQLP
AARGNIGYIGPVPERALQLDIAASNINPKGVIDYLHYYRAYKTDYELACMREAQKMAVSG
HRAAEEAFRSGMSEFDINLAYLTATGHRDTDVPYSNIVALNEHAAVLHYTKLDHQAPSEM
RSFLLDAGAEYNGYAADLTRTWSAKSDNDYAHLVKDVNDEQLALIATMKAGVSYVDYHIQ
FHQRIAKLLRKHQIITDMSEEAMVENDLTGPFMPHGIGHPLGLQVHDVAGFMQDDSGTHL
AAPSKYPYLRCTRVLQPRMVLTIEPGIYFIESLLAPWREGPFSKHFNWQKIEALKPFGGI
RIEDNVVIHENGVENMTRDLKLA
>EECEBICD_04068 Fatty acid oxidation complex subunit alpha
MLYKGDTLYLDWLEDGIAELVFDAPGSVNKLDTATVASLGQALEVLEKQHDLKGLLLRSN
KAAFIVGADITEFLSLFLVPEEQLSQWLHFANSVFNRLEDLPVPTLAAVNGYALGGGCEC
VLATDYRLATPDLRIGLPETKLGIMPGFGGSVRLPRMLGADSALEIIAAGKDVGAEHALK
IGLVDGVVKQEKLIEGAIAVLRQAITGDLDWRAKRQPKLEPLKLSKIEAAMSFTIAKGMV
AQTAGKHYPAPMTAVKTIEAAARFGREEALNLENKSFVPLAHTNEARALVGIFLNDQYVK
GKAKKLTKDIETPKQAAVLGAGIMGGGIAYQSAWKGVPVIMKDINDKSLNLGMTEAAKLL
NKQLERGKIDGLKLAGVISTIHPTLDYAGFDRVDVVVEAVVENPKVKKAVLAETEQKVRP
ETVLASNTSTIPIGELASALERPENFCGMHFFNPVHRMPLVEIIRGEKSSDETIAKVVAW
ASKMGKTPIVVNDCPGFFVNRVLFPYFAGFSQLLRDGADFRKVDKVMEKQFGWPMGPAYL
LDVVGIDTAHHAQAVMAAGFPQRMQKEYRDAIDALFDASRFGQKNGLGFWRYKEDSKGKP
KKEEDAAVDDLLASVSQTKRDFSDDEIIARMMIPMINEVVRCLEEGIIASPAEADAGLRP
GLPSVPRRRIPLAGYTGQREIS
>EECEBICD_04069 3-ketoacyl-CoA thiolase
MEQVVIVDAIRTPMGRSKGGAFRNVRAEDLSAHLMRSLLARNPSLTAATLDDIYWGCVQQ
TLEQGFNIARNAALLAEIPHSVPAVTVNRLCGSSMQALHDAARMIMTGDAQVCLVGGVEH
MGHVPMSHGVDFHPGLSRNVAKAAGMMGLTAEMLSRLHGISREMQDQFAARSHARAWAAT
QSGAFKTEIIPTGGHDADGVLKQFSYDEVIRPETTVEALSTLRPAFDPVSGTVTAGTSSA
LSDGAAAMLVMSESRARELGLKPRARIRSMAVVGCDPSIMGYGPVPASKLALKKAGLSAS
DIDVFEMNEAFAAQILPCIKDLGLMEQIDEKINLNGGAIALGHPLGCSGARISTTLINLM

ERKDAQFGLATMCIGLGQGIATVFERV
>EECEBICD_04070 Arylsulfate sulfotransferase AssT
MKFKYALTSLALSVAILSSVPSTAFAIGGASGAKVDYQVQGKIGEVVMNPYDIAPLTAVI
RNGGYQLRDVHVRIVPKENGQEIAYKVNNKYLLTYGGIPVFGLYPDYVNTVEVEYTRIQG
SKTENIKESYKMYAPPAYIESAGTKEEQSALFTIDVKKVSPEFKDRLYLLNNTKDKSGNG
TRTVWNNPTGGALEWNFTTANAIIDTSGDIRWFMNPSSIYDLKSIYRAGVMMGFKQNQDG
ALSWGYGQRYVKYDIMGREIFNRRLPDNYNDFSHSMDNAANGHYFLRVASSNYKRPDGKN
VRTVRDVIAEVDQNGVVVDEWRLFDILDPYRDVIMKTLDQGAVCLNIDASQSGHTLSEED
LAALDSSDKFGDIVGSGAGRNWAHVNSVDYDSEDDSIIISSRHQSAIIKIGRDKKVKWIL
GTPAGWKAPFNAAILTPVDSKGQKIACQDSGCEGDFDWTWTQHTAFKIDSKSKGDILYLS
AFDNGDGRGLEQPAMQSMKYSRSVIYKIDQKNKTVQQIWQYGKERGNEWFSPVTSITEYQ
TDKNSVFVYSATAGGAFDLSVGAFTSLPNPYLEEFKWGEKEPAVEMQIHGARGYQAMPFS
LTKALTE
>EECEBICD_04071 NAD(P)H-flavin reductase
MTTLSCKVTSVEAITDTVYRVRLVPDAAFSFRAGQYLMVVMDERDKRPFSMASTPDEKGF
IELHIGASELNLYAMAVMDRILKDREIVVDIPHGDAWLRDDEERPLILIAGGTGFSYVRS
ILLTALARNPNRDVTIYWGGREEKHLYDLSELEALSVNHPNLRIEPVVEQPEEGWRGRTG
TVLTAVLQDYGTLAGHDIYIAGRFEMAKIARDLFCHERNAREDRLFGDAFAFI
>EECEBICD_04072 3-octaprenyl-4-hydroxybenzoate carboxy-lyase
MDAMKYHDLRDFLTLLELQGELKRITLPVDPHLEITEIADRTLRAGGPALLFENPKGYAM
PVLCNLFGTPKRVAMGMGQDDVSALREVGKLLAFLKEPEPPKGFRDLFDKLPQFKQVLNM
PTKRLRGAPCQQKIASGDDVDLTRLPVMTCWPDDAAPLITWGLTVTRGPHKERQNLGIYR
QQLIGKNKLIMRWLSHRGGALDFQEWLAARPGERFPVSVALGADPATILGAVTPVPDTLS
EYAFAGLLRGTKTEVVKCLSNDLEVPASAEIILEGYIEPGEMAPEGPYGDHTGYYNEVDN
FPVFTVTHITQREDAIYHSTYTGRPPDEPAVLGVALNEVFVPILQKQFPEIVDFYLPPEG
CSYRLAVVTMKKQYAGHAKRVMMGVWSFLRQFMYTKFVIVCDDDVNARDWNDVIWAITTR
MDPARDTVLVENTPIDYLDFASPVSGLGSKMGLDATNKWPGETQREWGRPIVKDPEVTAR
IDAIWDELAIFK
>EECEBICD_04073 Transcription antitermination protein RfaH
MQSWYLLYCKRGQLQRAQEHLERQAVSCLTPMITLEKMVRGKRTSVSEPLFPNYLFVEFD
PEVIHTTTINATRGVSHFVRFGAHPAIVPSSVIHQLSIYKPEGVVDPETPYPGDSVIITE
GAFEGLKAIFTEPDGETRSMLLLNLLNKEVKQSVKNTGFRKI
>EECEBICD_04074 3'-5' ssDNA/RNA exonuclease TatD
MGASMFDIGVNLTSSQFAKDRDDVVARAFAAGVKGMLLTGTNIHESQQALKLARRYPHCW
STAGVHPHDSSQWSPASEDAIIALANQPEVVAIGECGLDFNRNFSTPQEQERAFQAQLQI
AAELQMPIFMHCRDAHERFLALLDPWLDSLPGAILHCFTGSRQQMQACVDRGLYIGITGW
VCDERRGLELRELLPFIPAEKLLIETDAPYLLPRDLTPKPTSRRNEPAYLPHILERIALW
RGEDPQWLAAMTDANARTLFEVVF
>EECEBICD_04075 Sec-independent protein translocase protein TatC
MAVEDTQPLITHLIELRKRLLNCIVAVLLIFLALIYFANDIYHLVAAPLIKQMPQGATMI
ATDVASPFFTPIKLTFMVSLILSAPVILYQVWAFIAPALYKHERRLVVPLLVSSSLLFYI
GMAFAYFVVFPLAFGFLTHTAPEGVQVSTDIASYLSFVMALFMAFGVAFEVPVAIVLLCW
MGITTPEDLRKKRPYILVGAFIVGMLLTPPDVFSQTLLAIPMYCLFEIGVFCSRFYVGKR
RTRDEDNEAETEKAEHTED
>EECEBICD_04076 Sec-independent protein translocase protein TatB
MFDIGFSELLLVFVIGLIVLGPQRLPVAVKTVAGWIRALRSLATTVQNELTQELKLQEFQ
DSLKKVEKASLENLTPELKASMDELRQAAESMKRTYSANDPEQASDEAHTIHNPVVKGNE
TQHEGVTPAAAETQASAPEQKPEPVKANVPESTETASVATIDAEKKSAAPVVESSPSSSD
KP
>EECEBICD_04077 Sec-independent protein translocase protein TatA
MGGISIWQLLIVAVIVVLLFGTKKLGSIGSDLGASIKGFKKAMSDDDAKQDKTSQDADFT
AKSIADKQGEAKKEDAKSQDKEQV
>EECEBICD_04078 putative protein kinase UbiB
MTPGEVRRLYFIIRTFLSYGLDELIPRMRLTLPLRLWRYSLFWMPNRHKDKLLGERLRLA

LQELGPVWIKFGQMLSTRRDLFPPQIADQLALLQDKVAPFDGRLAKAQIEEAMGGLPVDA
WFDDFDIQPLASASIAQVHTARLKSNGKEVVIKVIRPDILPVIQADLKLIYRLARWVPRL
LPDGRRLRPTEVVREYEKTLIDELNLLRESANAIQLRRNFENSPMLYIPEVYSDYCSQNM
MVMERIYGIPVSDVAALEKNGTNMKLLAERGVKVFFTQVFRDSFFHADMHPGNIFVSHEH
PENPQYIGIDCGIVGSLNKEDKRYLAENFIAFFNRDYRKVAELHVDSGWVPPDTNVEDFE
FAIRTVCEPIFEKPLAEISFGHVLLNLFNTARRFNMEVQPQLVLLQKTLLYVEGVGRQLY
PQLDLWKTAKPFLESWIKDQVGIPALTRALKEKAPFWVEKMPEIPELVYDSLRQGKYLQH
SVDKIARELQVNHVRQSQSRYLLGIGATLLLSGSFLLVNRPEWGLMPGWLMVGGVVVWLV
GWRKTR
>EECEBICD_04079 Ubiquinone biosynthesis accessory factor UbiJ
MPFKPLVTAGIEGLLNTFLYRSPALKSARTRLQGKVLCVKLKGFSTPLVLVFSERQVDVL
GAWEGEADCTVITQASVLPKLRDRQQLAALIRSGELEVQGDIQVVQNFVALADLAEFDPA
ELLAPYTGDIAAESIGKAVRGGAKFLRHGFQRQQRYAAEAITEEWRMAPGPLEVAWFAEE
TAAVERAVDSLTTRLEKLEAK
>EECEBICD_04080 Ubiquinone/menaquinone biosynthesis C-methyltransferase UbiE
MVEDSQETTHFGFQTVAKEQKADMVAHVFHSVASKYDVMNDLMSFGIHRLWKRFTIDCSG
VRRGQTVLDLAGGTGDLTAKFSRMVGETGKVILADINDSMLKMGREKLRNIGVIGNVEYV
QANAEALPFPDNTFDCITISFGLRNVTEKEKALRSMFRVLKPGGRLLVLEFSKPIIEPLS
KAYDAYSFHILPRIGSMVANDADSYRYLAESIRMHPDQDTLKAMMQDAGFESVDYYNLTA
GVVALHRGYKF
>EECEBICD_04081 hypothetical protein
MNITLMISAVVALAAGAVIGWLATKAHADQIRADLIEERRELDIELSAARQQLAQEAHWR
SECELLNNELRSLHSINTSLEADLREVTTRLEATQQHAEDKIRQMINSEQRLSEQFENLA
NRIFEHSNRRVDEQNRQSLNSLLTPLREQLDGFRRQVQESFGKEAQERHTLAHEIRNLQQ
LNAQMAQEAINLTRALKGDNKAQGNWGEVVLARVLEASGLREGYEYETQVSIENDARSRM
QPDVIVRLPQGKDVVIDAKMTLVAYERYFNAEDDYTREAALQEHIASVRNHIRLLGRKDY
QQLPGLRSLDYVLMFIPVEPAFLLALDKQPELITEALKNNIMLVSPTTLLVALRTIANLW
RYEHQSRNAQHIADRASKLYDKMRLFVDDMSAIGQSLDKAQDNYRQAMKKLASGRGNVLA
QAEAFRGLGVEIKREINPDLAEQAVTQDEEYRLRSIPEGRQDEHYPNDERVKQQLS
>EECEBICD_04082 Uridine phosphorylase
MSKSDVFHLGLTKNDLQGAQLAIVPGDPERVEKIAALMDKPVKLASHREFTSWRAELDGK
AVIVCSTGIGGPSTSIAVEELAQLGIRTFLRIGTTGAIQPHINVGDVLVTTASVRLDGAS
LHFAPMEFPAVADFACTTALVEAAKSIGATTHVGVTASSDTFYPGQERYDTYSGRVVRRF
KGSMEEWQAMGVMNYEMESATLLTMCASQGLRAGMVAGVIVNRTQQEIPNAETMKQTESH
AVKIVVEAARRLL
>EECEBICD_04083 hypothetical protein
MTTTHPSGFAPAASPLAPTMIHTPDGAISAGITSIPSQGDDMPAYYARPKASDGALPVVI
VVQEIFGVHEHIRDICRRLALEGYLAIAPELYFREGDPNDFADIPTLLSGLVAKVPDSQV
LADLDHVASWASRNGGDAHRLMITGFCWGGRITWLYAAHNPQLKAAVAWYGKLVGDTSLN
SPKHPVDIATDLNAPVLGLYGGQDTSIPQESVETMRQALRAANAKAEIVVYPDAGHAFNA
DYRPGYHEASAKDGWQRMLEWFAQYGGKKG
>EECEBICD_04084 Anaerobic sulfatase-maturating enzyme
MAVAGCHVMAKPGGAICNIDCTYCFYLEKEALYPERNKNWRMSDETLEQFIRQHIAAQSG
DRIDFAWQGGEPTMMGLPFFRRVVALCEKYGDGRKITHALQTNGILVNDEWARFFAEQHF
LIGLSIDGPASLHNHYRLNRAGKGTHEQVVAAMARLKAHHVDFNTLTVVGKHNVGHAADV
YEFLLAAGSRFIQFIPLVERMSTDNSSVLNLVMPGESAAKLAPWTVPSWQYGEFLNQIFD
IWVRRDVDRVYVQMFDVALAAWTAQQPVLCVHSETCGHAFALESNGDLYNCDHFVYPEHL
LGNIHQHSIKTLNNSERAIAFGEAKRETLTADCRRCDYRFACHGGCPKHRFAVSPSGHPA
HNYLCAGYKHFFQHVTPYMNVWRELLAQGYPMASIMRWLAQDARKDTGAVSRNHLCPCGS
GKKYKKCCGKA
>EECEBICD_04085 5-methyltetrahydropteroyltriglutamate--homocysteine methyltransferase
MTILTHTLGFPRVGLRRELKKAQESYWAGNSTREALLAVGRELRARHWEQQKQAGIDLLP

VGDFAWYDHVLTTSLLLGNVPARHQNNDGSVDIDTLFRIGRGRAPTGEPAAAAEMTKWFN
TNYHYIVPEFSKGQQFRLTWTQLLEEVDEALALGHKIKPVLLGPVTYLWLGKVKGEPFDR
LTLLKDILPVYQHVLAELAKRGVEWVQIDEPALVLELPQAWLDAFKPAYDALAGQVKLLL
TTYFEGVTPNLDTIIALPVQGLHVDLIHGKDDVAELHQRLPVDWLLSAGLINGRNVWRAD
LTEKYAQINAIVGKRALWVASSCSLLHSPIDLSVETRLDTEVKSWFAFALQKCGELALLR
DALNSGETAALEEWSAPIQARRHSRRVHNAAVEKRLAAITAQASQRENPYEVRAEAQRAR
FKLPAWPTTTIGSFPQTTEIRGLRLDFKKGNLDANNYRTGIAEHIKQAIIEQERLGLDVL
VHGEAERNDMVEYFGEHLDGFVFTQNGWVQSYGSRCVKPPVVIGDISRPAPITVEWAKYA
QSLTDKPVKGMLTGPVTILCWSFPREDVTRETIAKQIALALRDEVADLEAAGIGIIQIDE
PALREGLPLRRSDWDAYLEWGVEAFRINAAVAKDETQIHTHMCYCEFNDIMDSIAALDAD
VITIETSRSDMELLESFEAFDYPNEIGPGVYDIHSPNVPSVEWIEALLKKAAQRIPAQRL
WVNPDCGLKTRGWPETRAALANMVKAAHNLRQAK
>EECEBICD_04086 HTH-type transcriptional regulator MetR
MIEIKHLKTLQALRNSGSLAAAAAVLHQTQSALSHQFSDLEQRLGFRLFVRKSQPLRFTP
QGEVLLQLANQVLPQISRALQACNEPQQTRLRIAIECHSCIQWLTPALENFRASWPQVEM
DFTSGVTFDPQPALQQGELDLVMTSDILPRSGLHYSPMFDFEVRLVLAPDHPLASKTQIT
PEDLASETLLIYPVQRSRLDVWRHFLQPAGTSPLLKSVDNTLLLIQMVAARMGIAALPHW
VVESVERQGLVVTKTLGDGLWSRLYAAVRDGDQRQAVTEAFIRSTRDHACDHLPFVRSAE
RPIFDAPTAKPGSQPRL
>EECEBICD_04087 Biotin transporter
MALLIITTILWAFSFSLFGEYLAGHVDSYFAVLIRVGLAALVFLPFLRSRGHNLKTISLY
MLVGAMQLGIMYMLSFHAYLYLTVSELLLFTVLTPLYITLIYDVMSQRRLRWGYAFSALL
AVIGAGIIRYDRVTDHFWVGLLLVQLSNISFAIGMVGYKRLMETRPMPQHNAFAWFYLGA
FLVAAVAWSLLGNAQKLPETTLQWSILVFLGVVASGIGYFMWNYGATQVDAGTLGIMNNM
HVPAGLLVNLAIWHQQPHWPSFITGAAVILASLWVHRKWVAPRSAQTADDRRRDPASSE
>EECEBICD_04088 Pyridoxal phosphate phosphatase YigL
MYQVVASDLDGTLLSPDHTLSPYAKETLKLLTARGIHFVFATGRHHVDVGQIRDNLEIKS
YMITSNGARVHDTDGNLVFTHNLDSDIASDLFGVVNANPDIVTNVYRDDEWFMNRHRPDE
MRFFKEAVFNYSLFEPALLEPEGVSKVFFTSDTHEKLLPLEQAINARWGDRVNVSFSTLT
CLEVMAGGVSKGHALEAVAQAMGYSLKECIAFGDGMNDAEMLTMAGKGCIMGNAHQRLKD
LYPELEVIGINADNAVPHYLRDLFLR
>EECEBICD_04089 Lysophospholipase L2
MFQQQNDWETRENAFAAFAMGPLTDFWRQREEAEFIGVGNIPVRFVRFRNDSNDRTIVIC
PGRIESYVKYAELAYDLFHLGFDIFIIDHRGQGRSGRMLSDPHRGHVDHFNDYVEDLAAF
WQQEIELGPWRKRYILAHSMGGAIATLFLQRHRVRCDAIALTAPMFGIVIRLPSFMVRHI
LDWAEGHQRIREDYAIGTGQWRALPFGMNALTHSRQRYQRNLRFYADEPQLRVGGPTWHW
VREGILAGEQVLAGASDDTTPTLLIQAEEERVVDNRTHDRFCEIRAAAGYPCEGGKPLVI
KGAYHEILFEKDAMRSVALNAIVEFFNKPNLSSGNRFA
>EECEBICD_04090 Homoserine/homoserine lactone efflux protein
MTFEWWFAYLLTSTLLSLSPGSGAINTMTTSINHGYRGAVASIAGLQTGLGIHIVLVGVG
LGTLFSRSLLAFEILKWAGAAYLIWLGIQQWRAAGAIDLHTLAQTQSRGRLFKRAIFVNL
TNPKSIVFLAALFPQFIMPQQPQLAQYLILGVTTIVVDMVVMTGYATLAQRIAAWIKGPK
QMKALNKAFGSLFMLVGALLASARHA
>EECEBICD_04091 Threonine efflux protein
MMMLFFTVAMVHIVALMSPGPDFFFVSQTAVSRSRKEAMMGVLGITCGVMVWAGVALLGL
HLIIEKMAWLHTIIMVGGGLYLCWMGYQMLRGALKKQDAAASSPHIELAQSGRSFLKGLL
TNLSNPKAIIYFGSVFSLFVGDNVGAAARWGIFALITLETLAWFTVVASLFALPKMRRGY
QRLAKWIDGFAGALFAGFGIHLIISR
>EECEBICD_04092 ATP-dependent DNA helicase RecQ
MVNGVNVAQAEVLNLESGAKQVLQETFGYQQFRPGQEAIIDTALSGRDCLVVMPTGGGKS
LCYQIPALLLDGLTVVVSPLISLMKDQVDQLLANGVAAACLNSTQSREQQLEVMAGCRTG
QIRLLYIAPERLMLDNFLDHLAHWNPVLLAVDEAHCISQWGHDFRPEYAALGQLRQRFPA
LPFMALTATADDTTRLDIIRLLGLNDPLIQISSFDRPNIRYMLMEKFKPLDQLMRYVQEQ
RGKSGIIYCNSRAKVEDTAARLQSRGISAAAYHAGLENAIRADVQEKFQRDDLQIVVATV

AFGMGINKPNVRFVVHFDIPRNIESYYQETGRAGRDGLPAEAMLFYDPADMAWLRRCLEE
KPAGQLQDIERHKLNAMGAFAEAQTCRRLVLLNYFGEGRQEPCGNCDICLDPPKQYDGLN
DAQIALSTIGRVNQRFGMGYVVEVIRGANNQRIRDFGHDKLKVYGMGREKSHEHWVSVIR
QLIHLGLVMQNIAQHSALQLTDAARPVLRGDVPLKLAVPRIVALKPRVMQKSFGGNYDRK
LFAKLRKLRKAIADEENIPPYVVFNDATLIEMAEQMPVSASEMLSVNGVGMRKLERFGKE
FMALIRAHVDGDDEE
>EECEBICD_04093 Phospholipase A1
MRAILRGLLPATLLPLAAYAQEATIKEVHDAPAVRGSIIANMLQEHDNPFTLYPYDTNYL
IYTNTSDLNKEAISTYNWSENARKDEVKFQLSLAFPLWRGILGPNSVLGASYTQKSWWQL
SNSKESSPFRETNYEPQLFLGFATDYRFAGWTLRDVEMGYNHDSNGRSDPTSRSWNRLYT
RLMAENGNWLVEVKPWYVIGSTDDNPDITKYMGYYQLKIGYHLGEAVLSAKGQYNWNTGY
GGAEVGLSYPVTKHVRLYTQVYSGYGESLIDYNFNQTRVGVGVMLNDIF
>EECEBICD_04094 hypothetical protein
MSAVLTAEQALKLVGEMFVYHMPFNRALGLELERYEKAFAQLAFNNQPMMVGNWAQSILH
GGVIASALDVAAGLVCVGSTLTRHETISEDELRQRLSRMGTIDLRVDYLRPGRGNRFTAT
SSLLRAGNKVAVARVELHNEDQLYIASATATYMVG
>EECEBICD_04095 Protein RarD
MDAKQTRQGVLLALAAYFIWGIAPAYFKLIYYVRADEILTHRVIWSFFFMVALLSVSRQW
RQVKRLLKTPKKIFLLALSAVLVGGNWLLFIWAVNNHHMLEASLGYFINPLVNILLGMIF
LGERFRRMQWLAVILAVCGVLVQLWTFGSLPIIALGLAFSFAFYGLVRKKIAVEAQTGML
VETLWLLPVAAIYLFGIADSPTSHMGQNALSLNLLLMAAGVVTTIPLLCFTGAATRLRLS
TLGFFQYIGPTLMFLLAVTFYGEVPGADKMVTFAFIWVALAIFVMDAIYTQRRKH
>EECEBICD_04096 hypothetical protein
MQKKNFWQIYFGKDPFSKAEFIKAWIGWWVIAIVLLGVLSICTSLNLRGIGIALGFGFCQ
IHARYLTFVGWNKFIGVLGVFPVTNIILFIVLLFTKKK
>EECEBICD_04097 hypothetical protein
MTLITIAVIRKFSVIYLHVRSEVGYQYGKNNMIRIIKDIFHPSPRDTLKRAKFVIYTLSS
WIILFTIMPKNQYLNLIFIIANTYIHINIGAKRIRDSGCRALYFIALSIGVNVFCTVSVA
LNGADCDNIGIQLNIAWVILTTLLLSFIASSKQELSS
>EECEBICD_04098 Magnesium transport protein CorA
MLSAFQLEKNRLTRLEVEESQSLIDAVWVDLVEPDDDERLRVQSELGQSLATRPELEDIE
ASARFFEDEDGLHIHSFFFFEDAEDHAGNSTVAFTIRDGRLFTLRERELPAFRLYRMRAR
SQAMVDGNAYELLLDLFETKIEQLADEIENIYSDLEKLSRVIMEGHQGDEYDEALSTLAE
LEDIGWKVRLCLMDTQRALNFLVRKARLPGGQLEQAREILRDIESLLPHNESLFQKVNFL
MQAAMGFINIEQNRIIKIFSVVSVVFLPPTLVASSYGMNFEFMPELKWSFGYPGAIIFMI
LAGLAPYLYFKRKNWL
>EECEBICD_04099 DNA helicase II
MDVSYLLDSLNDKQREAVAAPRSNMLVLAGAGSGKTRVLVHRIAWLLSVENNSPYSIMAV
TFTNKAAAEMRHRIGQLMGTSQGGMWVGTFHGLAHRLLRAHHMDANLPQDFQILDSEDQM
RLLKRLIKAMNLDEKQWPPRQAMWYINSQKDEGLRPHHIQSYGNPVEQTWQKVYQAYQEA
CDRAGLVDFAELLLRAHELWLNKPHILQHYRERFTNILVDEFQDTNNIQYAWVRLLAGDT
GKVMIVGDDDQSIYGWRGAQVENIQRFLNDFPGAQTIRLEQNYRSTSNILSAANALIENN
NGRLGKKLWTDGVDGEPISLYCAFNELDEARFVVNRIKTWQDNGGALAQCAILYRSNAQS
RVLEEALLQASMPYRIYGGMRFFERQEIKDALSYLRLIANRNDDAAFERVVNTPTRGIGD
RTLDVVRQTSRDRQLTLWQACRELLQEKALAGRAASALQRFMELIDALAQETADMPLHVQ
TDRVIKDSGLRTMYEQEKGEKGQTRIENLEELVTATRQFSYNDEDEDLMPLQAFLSHAAL
EAGEGQADTWQDAVQLMTLHSAKGLEFPQVFIVGMEEGMFPSQMSLDEGGRLEEERRLAY
VGVTRAMQKLTLTYAETRRLYGKEVYHRPSRFIGELPEECVEEVRLRATVSRPVSHQRMG
TPLAENDTGYKLGQRVRHAKFGEGTIVNLEGSGEHSRLQVAFQGQGIKWLVAAYAKLETV
>EECEBICD_04100 5-amino-6-(5-phospho-D-ribitylamino)uracil phosphatase
YigB
MRFYRPLGRIAALTFDLDDTLYDNRPVILRTEQEALAFMQNYHPSLRSFQNVDLQRIRQA
VREAEPEIYHDVTRWRHRAIEQAMRDAGLSAQEAIAGANAAMMHFAKWRSQIEVPQATHE
TLQQLAKKWPLVAITNGNAQPELFGLGDYFKFVLRAGPDGRSKPFSDMYFLAAEKLHVPI

GEILHVGDDLTTDVAGAIRCGMQACWIKPENADLMRTQDSRLLPHIEISRLASLTSLI
>EECEBICD_04101 Tyrosine recombinase XerC
MTDVALSQDVSRFLRYLGVERQLSPITVQNYQRQLDAIIALAGETGLKSWQQCDAAIVRS
FAVRSRRKGLGPASLALRLSALRSFFDWLVSQGELKANPAKGVSAPKAPRHLPKNIDVDD
VNRLLDIDLNDPLAVRDRAMLEVMYGAGLRLSELVGLDIKHLDLDTGEVWVMGKGSKERR
LPIGRNAVTWIEHWLDLRGLFASDEEALFLSKLGKRISARNVQKRFAEWGIKQGLNSHVH
PHKLRHSFATHMLESSGDLRGVQELLGHANLSTTQIYTHLDFQHLASVYDAAHPRAKRGK
>EECEBICD_04102 hypothetical protein
MKQPEEELQETLTELDDRAVVDYLRHHPEFFIRNAHAVEAMRVPHPVRGTVSLVEWHMAR
ARNHINVLEENMTLLMEQAHANESLFYRLLHLQSRLVAADSLDEMLVRFHRWARDLGLAG
ATLRLFPDRWRLGAPSRYTHLALNRQAFEPLRIQRLGQSQHYLGPLNGPELLVVLPEAKA
VGSVAMSMMGSDGDLGVILFSSRDPHHYQPGQGTQLLQEIALMLPELLERWIKRV
>EECEBICD_04103 Diaminopimelate epimerase
MQFSKMHGLGNDFMVVDAVTQNVFFSPELIRRLSDRHLGVGFDQLLVVEPPYDPELDFHY
RIFNADGSEVSQCGNGARCFARFVRLKGLTNKRDIRVSTANGRMVLSVTEDELVRVNMGE
PNFEPAQVPFRANKAEKTYIMRAAEQTILCGVVSMGNPHCVIQVDNVDTAAVETLGPVLE
SHERFPERANIGFMQVVRREHIRLRVYERGAGETRACGSGACAAVAVGIQQGLLAEEVRV
ELPGGRLDIAWKGPGHPLYMTGPAAHIYDGFIHL
>EECEBICD_04104 hypothetical protein
MKNVFKTLAVLLTLFSLTGCGLKGPLYFPPADKNAPPPTKKVDSQTQSTMPDKNDRATGD
GPSQVNY
>EECEBICD_04105 hypothetical protein
MQYRNLFCLFSLSLLLPPAWGCRLSESPHNVYQRQGSGVVYLQPEEENNLSLPEVNFKRL
RRLPNSLINPANQGWNWIAQPPS
>EECEBICD_04106 hypothetical protein
MRDSQQGQGIHVFSINGPLKVTERFAYDNRYVYTWIGSQLYRTASPCPDKSHTLNRNVMY
TNNKDIIIITQAQECQK
>EECEBICD_04107 hypothetical protein
MTPLNRFARLNKHLKNALRGVGIGSCIYVIVESVMHGTINGVEAAGVLTISAVLGLLSAL
LDIKAKPRAPLILLHFLLCGGCVALMVAMLSALNHFPVNASVILITSALFIVIYLPLSFI
LYRSQR
>EECEBICD_04108 Iron-sulfur cluster assembly protein CyaY
MNDSEFHRLADALWLTIEERLDNWDGDSDIDCEINGGVLTLSFENGSKIIINRQEPLHQV
WLATKQGGYHFDLKGDEWVCDRSGETFWDLLEQAATQQAGEKVSFR
>EECEBICD_04109 Adenylate cyclase
MYLYIETLKQRLDAINQLRVDRALAAMGPAFQQVYSLLPTLLHYHHPLMPGYLDGNVPSG
ICFYTPDETQRHYLNELELYRGMTPQDPPKGELPITGVYTMGSTSSVGQSCSSDLDIWVC
HQSWLDGEERQLLQRKCSLLESWAASLGVEVSFFLIDENRFRHNESGSLGGEDCGSTQHI
LLLDEFYRTAVRLAGKRILWSMVPCDEEEHYDDYVMTLYAQGVLTPNEWLDLGGLSSLSA
EEYFGASLWQLYKSIDSPYKAVLKTLLLEAYSWEYPNPRLLAKDIKQRLHDGEIVSFGLD
PYCMMLERVTEYLTAIEDPTRLDLVRRCFYLKVCEKLSRERACVGWRREVLSQLVSEWGW
DDARLTMLDNRANWKIDQVREAHNELLDAMMQSYRNLIRFARRNNLSVSASPQDIGVLTR
KLYAAFEALPGKVTLVNPQISPDLSEPNLTFIHVPPGRANRSGWYLYNRAPNMDSIISHQ
PLEYNRYLNKLVAWAWFNGLLTSRTHLFIKGNGIVDLPKLQEMVADVSHHFPLRLPAPTP
KALYSPCEIRHLAIIVNLEYDPTAAFRNKVVHFDFRKLDVFSFGEEQNCLIGSIDLLYRN
SWNEVRTLHFNGEQAMIEALKTILGKMHQDAAPPDSVEVFCYSQHLRGLIRTRVQQLVSE
CIELRLSSTRQETGRFKALRVSGQTWGLFFERLNVSVQKLENAIEFYGAISHNKLHGLSV
QVETNQVKLPSVVDGFASEGIIQFFFEETGDEKGFNIYILDESNRAEVYHHCEGSKEELV
RDVSRFYSSSHDRFTYGSSFINFNLPQFYQIVKTDGRAQVIPFRTQPINTVPPANQDHDA
PLLQQYFS
>EECEBICD_04110 Porphobilinogen deaminase
MIMTVTSMLDNVLRIATRQSPLALWQAHYVKDALMATHPGLTVELVPMVTRGDVILDTPL
AKVGGKGLFVKELEIALLEKRADIAVHSMKDVPVAFPDGLGLVTICEREDPRDAFVSNKY
HSLDDLPAGSIVGTSSLRRQCQLAERRPDLIIRSLRGNVGTRLGKLDNGDYDAIILAVAG

LKRLGLESRIRTALPPDVSLPAVGQGAVGIECRLDDARTQALLAPLNHSQTALRVTAERA
MNTRLEGGCQVPIGSYAEIINGEIWLRALVGAPDGSVMVRGERRGSPEQAEQMGISLAEE
LLENGARAILTEVYNGETPA
>EECEBICD_04111 Uroporphyrinogen-III synthase
MSILITRPSPAGEALVSRLRALGQVAWSFPLIEFVAGRELPTLADRLATLTENDLVFALS
QHAVAFAHAQLQRDGRNWPASPRYFAIGRTTALALHTVSGFDIRYPLDREISEALLQLPE
LQNIAGKRALILRGNGGRELLGETLTARGAEVSFCECYQRCAKHYDGAEEAMRWHTRGVT
TLVVTSGEMLQRLWSLTPEWYREHWLLRCRLLVVSERLAHLARELGWQDIKVADNADNDA
LLRALQ
>EECEBICD_04112 hypothetical protein
MTEQEKSSAVVDETRESVETTPQPVNTEKKVRTAPRWY
>EECEBICD_04113 Protein HemX
MAIAIALAAGIGLYGWGKQQATAQTETSDALATQLTALQKAQESQKAELEGIIKKQAAQL
DDANRQQAALAKQLDEVQQKVATISGSDAKTWLLAQADFLVKLAGRKLWSDQDVTTAAAL
LKSADASLADMNDPSLITARRAITDDIASLSSVAQIDYDGIILKLNQLSNQIDNLRLADN
DTDGSPMDSDSSELSSSLSEWRVNLQKSWQNFMDSFITIRRRDDTAVPLLAPNQDVYLRE
NIRSRLLVAAQAVPRHQEETYRQALDNVSTWVRAYYDTDDAATKAFLEEVDKLSQQNITM
DLPETLGSQAILEKLMQTRVRNLLAQPTVSTAAPATQTDAPAAAPQGE
>EECEBICD_04114 Protein HemY
MMLKVLLLFVLLLAGIVVGPMIAGHQGYVLIQTDNYNIETSVTGLAIIILIVAMVVLFAIE
WLLRRLFRTGAHTRGWFAGRKRRRARKQTEQALLKLAEGDYQQVEKLMSKNADHAEQPVV
NYLLAAEAAQQRGDEARANQHLERAAELAGNDTIPVEITRVRLQLARNENHAARHGVDKL
LEVTPRHPEVLRLAEQAYIRTSAWSSLLDIIPSMAKAHVGDEAHRAMLEQQAWIGLMDQA
RAEQGSEGLRTWWKNQSRKTRHQVALQVAMAEHLIECDDHDMAQQIIIDGLKRQYDDRLV
LPIPRLRTNNPEQLEKVLRQQIKTVGDRPLLWSTLGQSLMKHGEWQEATLAFRAALKQRP
DAYDYAWLADALDRLHQPEEAAAMRRDGLMLTLQNNPPQ
>EECEBICD_04119 putative transport protein YifK
MAEKKPELQRGLEARHIELIALGGTIGVGLFMGAASTLKWAGPSVLLAYIIAGLFVFFIM
RSMGEMLFLEPVTGSFAVYAHRYMSPFFGYLTAWSYWFMWMAVGISEITAIGVYVQFWFP
EMAQWIPALIAVGLVALANLAAVRLYGEIEFWFAMIKVTTIIVMIIIGLGVIFFGFGNGG
QAIGFGNLTEHGGFFAGGWKGFLTALCIVVASYQGVELIGITAGEAKNPQVTLRSAVGKV
LWRILIFYVGAIFVIVTIFPWNEIGSNGSPFVLTFAKIGITAAAGIINFVVLTAALSGCN
SGMYSCGRMLYALAKNRQLPAAVAKVSRHGVPVAGVALSILILLVGSCLNYIIPNPQRVF
VYVYSASVLPGMVPWFVILISQLRFRRAHKEAIADHPFRSIMFPWANYLTMAFLVCVLIG
MYFNEDTRMSLFVGVIFLLAVTLVYKVFGLNRHGTAHKVGE
>EECEBICD_04120 UDP-N-acetyl-D-mannosaminuronic acid transferase
MTNNAAAPLYSLRGLPLIGWRDMSHALNYLFADGQLKQGTLVAINAEKLLTAEDNPEVRA
LIAAAEFKYADGISVVRSIRKKFPQAQVSRVAGADLWEALMARAGKEGTPVFLVGGKPEV
LAQTEAKLRTQWNVNIVGSQDGYFTPEQRQALFARIHASGAKIVTVAMGSPKQELLMRDC
REVHPHALYMGVGGTYDVFTGHVKRAPKIWQNLGLEWLYRLLSQPRRITRQMRLLRYLRW
HYTGDL
>EECEBICD_04121 putative ECA polymerase
MSLMQFSGLLVVWLLSTLFIATLTWFEFRRVRFNFNVFFSLLFLLTFFFGFPLTSVLVFR
FDVGVAPPEILLQALLSAACFYGVYYVTYKTRLRKRVVDVPRKPLFTMNRVETHLTWVIL
MGIALVSVAIFFMHNGFLLFRLHSYSQIFSSEVSGVALKRFFYFFIPAMLVVYFLRQDSK
AWLFFLVSTVAFGLLTYMIVGGTRANIIIAFAIFLFIGIIRGWISLWMLAAAGVLGIVGM
FWLALKRYGLNVSGDEAFYTFLYLTRDTFSPWENLALLLQNYHNIDFQGLAPIVRDFYVF
IPTWLWPGRPSIVLNSANYFTWEVLNNHSGLAISPTLIGSLVVMGGALFIPLGAIVVGLI
IKWFDWLYELGNREPNRYKAAILHSFCFGAIFNMIVLAREGLDSFVSRVVFFLVVFGASL
LVAKLLFWLFDSAGLIHKRTTSLPQAQVEGKL
>EECEBICD_04122 TDP-N-acetylfucosamine:lipid II N-
acetylfucosaminyltransferase
MTVLIHVLGSDIPHHNHTVLRFFNDTLAATSEHAREFMVAGEDNGFTESCPALSLRFYGS
KKALAQAVIAKAKANRRQRFFFHGQFNTSLWLALLSGGIKPAQFYWHIWGADLYEVSNGL

KFRLFYPLRRIAQGRVGCVFATRGDLSYFARQHPNVRGELLYFPTRMDPSLNAMAKECQR
AGKLTILVGNSGDRSNQHIAALRAVYQQFGDTVNVVVPMGYPANNQAYIDEVRQAGLALF
SAENLQILSEKMEFDAYLALLRQCDLGYFIFARQQGIGTLCLLIQADIPCVLNRDNPFWQ
DMAEQHLPVLFTTDDLNEQVVREAQRQLASVDKSGITFFSPNYLQPWHNALRIAAGEAE
>EECEBICD_04123 Lipid III flippase
MSLAKASLWTAVSTLVKIGAGLLVVKLLAVSFGPSGVGQAGNFRQMITVLGVLAGAGIFN
GVTKLVAQHHDNPAQLRTVVGTSSAMVLGFSTLLALVFLLAAAPISQGLFGHTDYQGLVR
LVALVQMGIAWANLLLALMKGFRDASGNALSLIVGSFLGVAAWYLCYRVGGYEGALLGLA
LVPALVVVPAGMILIKRGTVPLRYLKPSWDNGLAGQLSKFTLMALITSVTMPVAYVMMRN
QLAAHYSWSDVGIWQGVSSISDAYLQFITASFSVYLLPTLSRLTEKQDITREVVKALKFV
LPAVAAASFTVWLLRDFAIWLLFSAKFTAMRDLFAWQLVGDVLKVGAYVFGYLVIAKASL
RFYILAEISQFILLTAFAHWLIPAHGALGAAQAYMVTYIVYFSLCCGVFLLWRRRA
>EECEBICD_04124 dTDP-4-amino-4,6-dideoxygalactose transaminase
MIPFNAPPVVGTELEYMQSAMSSGKLCGDGGFTRRCQQWLEQHFGSAKVLLTPSCTASLE
MAALLLDIQPGDEVIMPSFTFVSTANAFVLRGAKIVFVDIRPDTMNIDETLIEAAITDKT
RAIVPVHYAGVACEMDVIMALADKYNLFVVEDAAQGVMSTYKGRALGTIGHIGCFSFHET
KNYTAGGEGGATLINDRTLIERAEIIREKGTNRSQFFRGQVDKYTWRDIGSSYLMSDLQA
AYLWAQLEAADRINQQRLSLWQTYYDALTPLARAGRIELPSIPENCGHNAHMFYIKLRDI
ADRSALINFLKEAEIMAVFHYIPLHDCPAGDKFGEFIGDDVYTTKESERLLRLPLFYNLA
PVDQRTVITTLLNYFS
>EECEBICD_04125 dTDP-fucosamine acetyltransferase
MPVRASIEPLLWENTFFGVNSGIVRIDASAPELTPEALQAWQRVQVKVPAENIAWLSALQ
SLGFSLVEGEVDFALPVKGHRDQHGAEIAHLTDIPALRQLAGEAFTQSRFRAPWYAPDAS
ARFYAQWIENAVRGTFDHQCLVLRTETGAIRGYVSLRELNDTDARIGLLAGRGAGAELMQ
AAICWAQSRGKATLRVATQLGNTAALKRYIQSGANIESTAYWLYR
>EECEBICD_04126 hypothetical protein
MKGIILAGGSGTRLHPITRGVSKRQGFKIACLEEIAWRNGWLDDDGLKRAASQLEKTGYG
QYLLELLRARPRQY
>EECEBICD_04127 dTDP-glucose 4,6-dehydratase 2
MKRILVTGGAGFIGSAVVRHIIHETADAVVVVDKLTYAGNLMSLASVAQSDRFAFEKVDI
CDRASLERVFQQYQPDSVMHLAAESHVDRSIDGPAAFIETNIVGTYTLLEAARAYWSALD
ADAKAAFRFHHISTDEVYGDLHTADDFFTETTPYAPSSPYSASKASSDHLVRAWLRTYGL
PTLVTNCSNNYGPYHFPEKLIPLMILNALAGKPLPVYGNGQQIRDWLYVEDHARALYHVV
TNGAVGETYNIGGHNERKNLDVVRTICALLEELAPQKPQGVANYHDLITFVDDRPGHDLR
YAIDASKIARELGWTPQETFESGMRKTVQWYLANEAWWKPVQDGSYQGERLGLKR
>EECEBICD_04128 UDP-N-acetyl-D-mannosamine dehydrogenase
MSFTTISVIGLGYIGLPTAAAFASRQKQVIGVDINQHAVDTINRGEIHIVEPALGNVVKM
AVEGGFLRATTTPVEADAYLIAVPTPFKGDHDPDMAYVEAAAKSIAPVLKKGALVILEST
SPVGATEQMAGWLAGMRPDLTFPQQAGEQADVNIAYCPERVLPGQVMVELIKNDRVIGGM
TPVCSARASALYKIFLEGECVVTNSRTAEMCKLTENSFRDVNIAFANELSLICAEQGINV
WELIRLANRHPRVNILQPGPGVGGHCIAVDPWFIVAQNPQQARLIRTAREVNDGKPHWVV
DQVKAAVADCLAATDKRASEVKIACFGLAFKPNIDDLRESPAMGIAQSIARWHSGETLVV
EPNIRQLPKKLDGLCTLAKLDAALTAADVLVMLVDHDEFKAIPGDAVHQRYVVDTKGVWR
>EECEBICD_04129 UDP-N-acetylglucosamine 2-epimerase
MYHFAPTENSRQNLLRENIPDERIFVTGNTVIDALIWVRDRVLTSDTLQAELAEQYPFLN
ANKKMILVTGHRRESFGQGFEHICQALAEIAAANQNVQIVYPVHLNPNVSEPVNRILGHV
ENVVLIEPQDYLPFVWLMNHAWLILTDSGGIQEEAPSLGKPVLVMRETTERPEAITAGTV
RLIGTDSRRIVAEVMRLLHDENEYQTMSRAHNPYGDGQSCARILQALKSYRVSL
>EECEBICD_04130 UDP-N-acetylglucosamine 2-epimerase
MAPLVHALEKDPHFEAKVCVTAQHREMLDQVLTLFSIVPDYDLNIMQPGQGLTEITCRIL
EGLKPILADFKPDVVLVHGDTTTTIATSLAAFYQRIPVGHVEAGLRTGDLYSPGRKRQTA
R
>EECEBICD_04131 ECA polysaccharide chain length modulation protein
MTQPLPGARAVSAENELDIRGLFRTLWAGKFWIIGIGLLFALIALAYTFFARQEWSATAI

TDRPTVNMLGGYYSQQQFLRNLDIKTDPASSDKPSVMDEAYKEFIMQLASWDTRRDFWLQ
TDYYKQRMVGNSKADAAMLDELINNIQFTPGDFTRAINDNVKLIAETAPDANNLLRQYVA
FASQRAASHLNDELKGAWAARTVQMKAQVKRQEEVAKAIYSRRVNSIEQALKIAEQHNIS
RSATDVPADELPDSELFLLGRPMLQARLENLQAVGPAFDLDYFQNRAMLNTLNVGPTLDP
RFQTYRYLRTPEEPVKRDSPRRAFLMIMWGIVGALIGAGVALTRRRTI
>EECEBICD_04132 Undecaprenyl-phosphate alpha-N-acetylglucosaminyl 1-phosphate transferase
MRAGFTSRQAFVLITLAAAILAGVGVTAEYSHFVPEWVMLVLFLLAFFLYGYCIKRAWKV
ARFIKRVKRRLRRQRENRPNLTK
>EECEBICD_04133 Undecaprenyl-phosphate alpha-N-acetylglucosaminyl 1-phosphate transferase
MKLLTALSELISIFLFTTIFIFLARKVAIKIGLVDKPNFRKRHQGVIPLVGGISVFAGIC
FMFGLSDYYIPHLSLYLICAGVLVFVGAMDDRFDISVKIRAVVQAVIAVVMMVIAKLHLG
SLGYIFGPWELVLGPFGYFLTLFAVWAAINAFNMVDGIDGLLGGLSSVSFAAMGLILWFD
GQTSLAMWCFAMIAAILPYIMLNLGILGRRYKVFMGDAGSTLIGFTVIWLLLETTQGKTH
SISPVTALWIIAIPLMDMVAIMYRRLRKGMSPFFP
>EECEBICD_04134 Transcription termination factor Rho
MNLTELKNTPVSELITLGESMGLENLARMRKQDIIFAILKQHAKSGEDIFGDGVLEILQD
GFGFLRSADSSYLAGPDDIYVSPSQIRRFNLRTGDTISGKIRPPKEGERYFALLKVNEVN
YDKPENARNKILFENLTPLHANSRLRMERGNGSTEDLTARVLDLASPIGRGQRGLIVAPP
KAGKTMLLQNIAQSIAYNHPDCVLMVLLIDERPEEVTEMQRLVKGEVVASTFDEPASRHV
QVAEMVIEKAKRLVEHKKDVIILLDSITRLARAYNTVVPASGKVLTGGVDANALHRPKRF
FGAARNVEEGGSLTIIATALIDTGSKMDEVIYEEFKGTGNMELHLSRKIAEKRVFPAIDY
NRSGTRKEELLTTQEELQKMWILRKIIHPMGEIDAMEFLINKLAMTKTNDDFFEMMKRS
>EECEBICD_04135 Thioredoxin 1
MSDKIIHLTDDSFDTDVLKADGAILVDFWAEWCGPCKMIAPILDEIADEYQGKLTVAKLN
IDQNPGTAPKYGIRGIPTLLLFKNGEVAATKVGALSKGQLKEFLDANLA
>EECEBICD_04136 ATP-dependent RNA helicase RhlB
MSKTHLTEQKFSDFALHPQVVEALEKKGFYNCTPIQALALPLTLAGRDVAGQAQTGTGKT
MAFLTSTFHYLLSHPAIDDRKVNQPRALIMAPTRELAVQIHADAEPLAQATGLKLGLAYG
GDGYDKQLKVLESGVDILIGTTGRLIDYAKQNHINLGAIQVVVLDEADRMYDLGFIKDIR
WLFRRMPPAAQRLNMLFSATLSYRVRELAFEQMNNAEYVEVEPEQKTGHRIKEELFYPSN
EEKMRLLQTLIEEEWPDRAIIFANTKHRCEDIWGHLAADGHRVGLLTGDVAQKKRLRILD
EFTRGDLDILVATDVAARGLHIPAVTHVFNYDLPDDCEDYVHRIGRTGRAGASGHSISLA
CEEYALNLPAIESYIGHSIPVSKYNPEALMNDLPKPLRLTRSRPGNGPRRAGAPRNRRRS
G
>EECEBICD_04137 Guanosine-5'-triphosphate,3'-diphosphate pyrophosphatase
MNSTSLYAAIDLGSNSFHMLVVREAAGSIQTLTRIKRKVRLAAGLNNDNHLSAEAMERGW
QCLRLFAERLQDIPQPQIRVVATATLRLAVNAGEFIAKAQTILGCPVQVISGEEEARLIY
QGVAHTTGGADQRLVVDIGGASTELVTGTGAQTTSLFSLSMGCVTWLERYFSDRNLAQEN
FDDAEKAARDVLRPVADELRFHGWKVCVGASGTVQALQEIMMAQGMDERITLAKLQQLKQ
RAIQCGRLEELEIEGLTLERALVFPSGLAILIAIFTELNIQSMTLAGGALREGLVYGMLH
LAVDQDIRSRTLRNIQRRFIVDTDQANRVAKLADNFLKQVENAWHIEPISRELLLSACQL
HEIGLSVDFKQAPYHAAYLVRHLDLPGYTPAQKKLLATLLLNQTNPVDLSSLHQQNAVPP
RVAEQLCRLLRLAILFAGRRRDDLVPEITLQALNENLTLTLPGDWLAHHPLGKELIDQES
QWQSYVHWPLDVR
>EECEBICD_04138 ATP-dependent DNA helicase Rep
MRLNPGQQHAVEFVTGPCLVLAGAGSGKTRVITNKIAHLIRGCGYQARHIAAVTFTNKAA
REMKERVGQTLGRKEARGLMISTFHTLGLDIIKREYAALGMKSNFSLFDDTDQVALLKEL
TEGLIEDDKVLLQQLISTISNWKNDLKTPAQAAAGAKGERDRIFAHCYGLYDAHMKACNV
LDFDDLILLPTLLLQRNDEVRERWQNKIRYLLVDEYQDTNTSQYELVKLLVGQRARFTVV
GDDDQSIYSWRGARPQNLVLLSQDFPALQVIKLEQNYRSSGRILKAANILIANNPHVFEK
RLFSELGYGAELKVLSANNEEHEAERVTGELIAHHFVNKTQYKDYAILYRGNHQSRVFEK
FLMQNRIPYKISGGTSFFSRPEIKDLLAYLRVLTNPDDDSAFLRIVNTPKREIGPATLQK

LGEWAMTRNKSLFTASFDMGLSQKLTGRGYDSLTRFTHWLGEIQRLAEREPVAAVRDLIH
GIDYESWLYETSPSPKAAEMRMKNVNQLFSWMTEMLEGNELDEPMTLTQVVTRFTLRDMM
ERGESEEELDQVQLMTLHASKGLEFPYVYMVGMEEGFLPHQSSIDEDNIEEERRLAYVGI
TRAQKELTFTLCKERRQYGELVRPEPSRFLLELPQDDLIWEQERKVVSAEERMQKGQSHL
ANLKAMMAAKRAKS
>EECEBICD_04139 Peptidyl-prolyl cis-trans isomerase C
MAKMAAALHILVKEEKLALDLLEQIKNGGDFEKLAKKHSICPSGKKGGHLGEFRQGQMVP
AFDKVVFSCPVLEPTGPLHTQFGYHIIKVLYRK
>EECEBICD_04140 Ketol-acid reductoisomerase (NADP(+))
MANYFNTLNLRQQLAQLGKCRFMGRDEFADGASYLQGKKVVIVGCGAQGLNQGLNMRDSG
LDISYALRKEAIAEKRASWRKATENGFKVGTYEELIPQADLVVNLTPDKQHSDVVRSVQP
LMKDGAALGYSHGFNIVEVGEQIRKDITVVMVAPKCPGTEVREEYKRGFGVPTLIAVHPE
NDPKGEGMAIAKAWAAATGGHRAGVLESSFVAEVKSDLMGEQTILCGMLQAGSLLCFDKL
VAEGTDPAYAEKLIQFGWETITEALKQGGITLMMDRLSNPAKLRAYALSEQLKEIMAPLF
QKHMDDIISGEFSSGMMADWANDDKKLLTWREETGKTAFETAPQFEGKIGEQEYFDKGVL
MIAMVKAGVELAFETMVDSGIIEESAYYESLHELPLIANTIARKRLYEMNVVISDTAEYG
NYLFSYACVPLLKPFMAELQPGDLGSAIPEGAVDNAQLRDVNDAIRSHAIEQVGKKLRGY
MTDMKRIAVAG
>EECEBICD_04141 Hydrogen peroxide-inducible genes activator
MDLRDLKTFLHLAESRHFGRSARAMHVSPSTLSRQIQRLEEDLGQPLFVRDNRTVTLTEA
GEELRVFAQQTLLQYQQLRHTLDQQGPSLSGELHIFCSVTAAYSHLPPILDRFRAEHPSV
EIKLTTGDAADAMDKVVTGEADLAIAGKPETLPGAVAFSMLENLAVVLIAPALPCPVRNQ
VSVDKPDWSTVPFIMADQGPVRRRIELWFRRHKISNPQIYATVGGHEAMVSMVALGCGVA
LLPEVVLENSPEPVRNRVMILERSDEKTPFELGVCAQKKRLHEPLIDAFWKILPN
>EECEBICD_04142 hypothetical protein
MIAKTDSDIRHVTPSGGNVFADLGFHKQDAEKFYADSLNEIENTLAIKQQLMEEITLWIT
QNQMKQAEVATVLHISRPRVSDVVNKKCSKFTIDALVNMLSRIGKPVRVMVGP
>EECEBICD_04143 hypothetical protein
MLLTRALSNEKEIRWVGSSLEDLLAFPITVRKAVGYQLHKIQYGIEPDDWKPFSEIGAGV
NEIRIRDNNGIYRVMYVAKFEEALYVLHSFQKQTQQTSQHDKNIARTRYNRVVQQRRNSL
>EECEBICD_04144 L-threonine dehydratase biosynthetic IlvA
MAESQPLSVAPEGAEYLRAVLRAPVYEAAQVTPLQKMEKLSSRLDNVILVKREDRQPVHS
FKLRGAYAMMAGLTEEQKAHGVITASAGNHAQGVAFSSARLGVKSLIVMPKATADIKVDA
VRGFGGEVLLHGANFDEAKAKAIELAQQQGFTWVPPFDHPMVIAGQGTLALELLQQDSHL
DRVFVPVGGGGLAAGVAVLIKQLMPQIKVIAVEAEDSACLKAALEAGHPVDLPRVGLFAE
GVAVKRIGDETFRLCQEYLDDIITVDSDAICAAMKDLFEDVRAVAEPSGALALAGMKKYI
ALHNIRGERLAHVLSGANVNFHGLRYVSERCELGEQREALLAVTIPEEKGSFLKFCQLLG
GRMVTEFNYRFADAKNACIFVGVRVSQGLEERKEIITQLCDGGYSVVDLSDDEMAKLHVR
YMVGGRPSHPLQERLYSFEFPESPGALLKFLHTLGTHWNISLFHYRSHGTDYGRVLAAFE
LGDHEPDFETRLHELGYECHDESNNPAFRFFLAG
>EECEBICD_04145 Dihydroxy-acid dehydratase
MPKYRSATTTHGRNMAGARALWRATGMTDSDFGKPIIAVVNSFTQFVPGHVHLRDLGKLV
AEQIEASGGVAKEFNTIAVDDGIAMGHGGMLYSLPSRELIADSVEYMVNAHCADAMVCIS
NCDKITPGMLMASLRLNIPVIFVSGGPMEAGKTKLSDKIIKLDLVDAMIQGADPKVSDDQ
SNQVERSACPTCGSCSGMFTANSMNCLTEALGLSQPGNGSLLATHADRKQLFLNAGKRIV
ELTKRYYEQNDESALPRNIASKAAFENAMTLDIAMGGSTNTVLHLLAAAQEAEIDFTMSD
IDKLSRKVPQLCKVAPSTQKYHMEDVHRAGGVLGILGELDRAGLLNCNVKNVLGLTLPQT
LEQYDITVTQDEAVKKMFRAGPAGIRTTQAFSQDCRWDSLDDDRAAGCIRSLEYAYSKDG
GLAVLYGNFAENGCIVKTAGVDDSILKFTGPAKVYESQDDAVEAILGGKVVEGDVVVIRY
EGPKGGPGMQEMLYPTSFLKSMGLGKACALITDGRFSGGTSGLSIGHVSPEAASGGTIAL
IEDGDTIAIDIPNRSIQLQLNEAEIAARREAQEARGDKAWTPKNRQRQVSFALRAYASLA
TSADKGAVRDKSKLGG
>EECEBICD_04146 Branched-chain-amino-acid aminotransferase
MTTKKADYIWFNGEMVRWEDAKVHVMSHALHYGTSVFEGIRCYDSHKGPVVFRHREHMQR

LRDSAKIYRFPVSQSIDELMEACREVIRKNNLTSAYIRPLVFVGDVGMGVNPPPGYTTDV
IIAAFPWGAYLGAEALDQGIDAMVSSWNRAAPNTIPTAAKAGGNYLSSLLVGSEARRHGY
QEGIALDVNGYISEGAGENLFEVKDGVLFTPPFTSSALPGITRDAIIKLAKELGIEVREQ
VLSRESLYLADEVFMSGTAAEITPVRSVDGIQVGEGRCGPVTKRIQQAFFGLFTGETEDK
WGWLDPVNS
>EECEBICD_04147 Acetolactate synthase isozyme 2 small subunit
MMQHQVNVSARFNPETLERVLRVVRHRGFQVCSMNMEAATDAQNINIELTVASPRSVDLL
FSQLSKLVDVAHVAICQSAATSQQIRA
>EECEBICD_04148 Acetolactate synthase isozyme 2 large subunit
MNGAQWVVHALRAQGVKTVFGYPGGAIMPVYDALYDGGVEHLLCRHEQGAAMAAIGYARS
TGKTGVCIATSGPGATNLITGLADALLDSVPVVAITGQVSAPFIGTDAFQEVDVLGLSLA
CTKHSFLVQSLAELPRIMAEAFEVANAGRPGPVLVDIPKDIQLASGELEPWFTTVDNEAT
FPQADVEQVRQMLEQAKKPMLYVGGGVGMAQAVPALRKFIAVTQMPVTCTLKGLGAVEAD
YPYYLGMLGMHGTKAANFAVQECDLLIAVGARFDDRVTGKLNTFAPNASVIHMDIDPAEM
NKLRQAHVALQGDLNSLLPALQQPLKIDAWRQSCAELRAEHAWRYDHPGETIYAPLLLKQ
LSERKPADSVVTTDVGQHQMWSAQHMTYTRPENFITSSGLGTMGFGLPAAVGAQVARPND
TVICISGDGSFMMNVQELGTVKRKQLPLKIVLLDNQRLGMVRQWQQLFFQERYSETTLTD
NPDFLMLASAFGIPGQHITRKDQVEAALDTMLASEGPYLLHVSIDELENVWPLVPPGASN
SEMLEKLS
>EECEBICD_04149 Competence protein ComM
MSLAIVHTRAALGVNAPPITIEVHISNGLPGLTMVGLPETTVKEARDRVRSAIINSGYEF
PAKKITINLAPADLPKEGGRYDLPIAVALLAASEQLTASNLEAYELVGELALTGALRGVP
GAISSATEAIRAGRNIIVATENAAEVGLISKEGCFIADHLQTVCAFLEGKHALERPLAQD
MASSTATADLRDVIGQEQGKRGLEITAAGGHNLLLIGPPGTGKTMLASRLSGILPPLSNE
EALESAAILSLVNADTVQKRWQQRPFRSPHHSASLTAMVGGGAIPAPGEISLAHNGILFL
DELPEFERRTLDALREPIESGQIHLSRTRAKITYPARFQLIAAMNPSPTGHYQGNHNRCT
PEQTLRYLNRLSGPFLDRFDLSLEIPLPPPGILSQHASKGESSATVKKRVIAAHERQYRR
QKKLNARLEGREIQKYCVLHHDDARWLEDTLVHLGLSIRAWQRLLKVARTIADIELADQI
SRQHLQEAVSYRAIDRLLIHLQKLLA
>EECEBICD_04150 hypothetical protein
MAESFTTTNRYFDNKHYPRGFSRHGDFTIKEAQLLERHGHAFNDLDLGKREPVTEEEKLF
VAVCRGEREPVTDAERVWSKYMTRIKRPKRFHTLSGGKPQVEGAEDYTEADD
>EECEBICD_04151 HTH-type transcriptional regulator HdfR
MDTELLKTFLEVSRTRHFGRAAEALYLTQSAVSFRIRQLENQLGVNLFTRHRNNIRLTTA
GEKLLPYAETLMNTWQAARKEVAHTSRHNEFSIGASASLWECMLNAWLGRLYQLQEPQSG
LQFEARIAQRQSLVKQLHERQLDLLITTEAPKMDEFSSQLLGHFTLALYCSSPARKKSEL
NYLRLEWGPDFQQHETGLIAADEVPVLTTSSAELARQQLSALNGCSWLPVNWANEKGGLH
TVADSATLSRPLYAIWLQNSDKYSLICDLLKTDVLDEQ
>EECEBICD_04158 Putative L-lactate dehydrogenase operon regulatory protein
MPLSAQQLAAQKNLSYVLAEKLAQRILKGDYAPGTILPGEIELGEQYGVSRTAVREAVKT
LTAKGMVLPRPRIGTRVMPQGNWNFLDQELLTWWMTEENFHQVVEHFLVMRISLEPQACL
LAATLGTPEQKARLNALMEEMVALKKHFKRERWIEVDMAWHEHIYEMSANPFLISFATLF
HSVYHTYFTSITYNEVVKLDLHQAIVDAIADGDGERAFQACQALLIAPNERPDN
>EECEBICD_04159 putative transport protein HsrA
MTSKKARSMAGLPWIAAMAFFMQALDATILNTALPAIAHSLNRSPLAMQSAIISYTLTVA
MLIPVSGWLTALVRVAFLWLP
>EECEBICD_04160 putative transport protein HsrA
MVAVSLFTLGSLACALSSSLSELVIFRVVQGVGGAMMMPVARLALLRAYPRSELLPVLNF
VTMPGLVGPILGPVLGGVLVTWASWHWIFLINIPIGVAGILYARKYMPNFTTPRRKFDMT
GFFLFGLSLVLFSSGMELFGEKIVATWIASAIIFCSIVLLLAYIRHARRHPTPLISLSLF
KTRTFSVGIAGNLATRLGTGCVPFLMPLMLQVGFGYPALIAGCMMAPTALGSIIAKSTVT
QVLRRLGYRKTLVGITVFIGLMIAQFSFQSPAMPIWMLVLPLFILGMAMSTQFTAMNTIT
LADLTDDNASSGNSVLAVTQQLSISLGVAISAAVLRIYEGFAGTSTVEQFHYTFITMGAI

TIVSALMFMLLRAKDGNNLIKERHKSKPTHAPSKPE
>EECEBICD_04161 Ribose operon repressor
MKDVARLAGVSTSTVSHVINKDRFVSETITEKVEAAIKSLNYAPSALARSLKLNQTRTIG
MLITASTNPFYSELVRGVERSCFERGYSLVLCNTEGDEQRMNRNLETLMQKRVDGLLLLC
TETHQPSREIMERYPSIPTVMMDWSPFDGEGDSDLIQDNSLLGGDLATQHLIDKGFTRIA
CITGPLDKTPARLRLEGYRAAMKRAGLAIPQGYEITGDFEFGGGFDAMQALLAHQQRPQA
VFTGNDAMAVGAYQALYQAGLRIPQDMAVIGYDDIELARYMTPPLTTIHQPKDELGELAI
DVLIHRMAQPALQQQRLQLTPVLTVRGSV
>EECEBICD_04162 Ribokinase
MKTAGNLIVLGSINADHILNLESFPTPGETVTGSHYQVAFGGKGANQAVAAGRSGANIAF
IACTGDDDIGDSIRKQLVSDRIDIAPVSVIKGESTGVALIFVNGEGENVIGIHAGANAAL
SPALVDAQRERIAQADALLMQLESPLESVLAAAKIAHQHHTTVALNPAPARELPDELLAL
VDIITPNETEAEKLTGVRVENDDDAAKAAQVLHDKGIRTVLITLGSRGVWASVDGEGRRV
PGFSVEAVDTIAAGDTFNGAFITAILEETPLPEAIRFAHAAAAIAVTRKGAQPSVPWREE
IEAFLHQQG
>EECEBICD_04163 Ribose import binding protein RbsB
MNMKKLATLVSAVALSATVSANAMAKDTIALVISTLNNPFFVSLKDGAQKEADKLGYNLV
VLDSQNNPAKELANVQDLTVRGTKILLINPTDSDAVGNAVKMANQAKIPVITLDRQATKG
DVVSHIASDNVLGGKIAGDYIAKKAGEGAKVIELQGIAGTSAARERGEGFQQAVAAHKFN
VLASQPADFDRTKGLNVMQNLLTAHPDVQAVFAQNDEMALGALRALQTAGKADVMVVGFD
GTPDGEKAVKDGKLAATIAQLPDQIGAKGVEVADKVLKGEKVQAKYPVDLKLVIKQ
>EECEBICD_04164 Ribose import permease protein RbsC
MTTQAVSGRRYFTKAWLLEQKSLIALLVLIAIVSTLSPNFFTVNNLFNILQQTSVNAIMA
VGMTLVILTSGIDLSVGSLLALTGAVAASIVGVEVNALVAVAAALALGAAIGAVTGVIVA
KGRVQAFIATLVMMLLLRGVTMVYTDGSPVNTGFTDNADLFGWFGIGRPLGVPTPVWIMA
IVFIAAWYMLHHTRLGRYIYALGGNEAATRLSGISVSKVKIIVYSLCGLTASLAGIIEVA
RLSSAQPTAGTGYELDAIAAVVLGGTSLAGGKGRIVGTLIGALILGFLNNGLNLLGVSSY
YQMIVKAVVILLAVLVDNKKQ
>EECEBICD_04165 Ribose import ATP-binding protein RbsA
MRTSFFALALRSEAAMDALLQLKGIDKAFPGVKALSGAALNVYPGRVMALVGENGAGKST
MMKVLTGIYTRDAGSLLWLGKETTFNGPKSSQEAGIGIIHQELNLIPQLTIAENIFLGRE
FVNRFGKIDWKKMYAEADHLLAKLNLRFKSDKLVGELSIGDQQMVEIAKVLSFESKVIIM
DEPTDALTDTETDSLFRVIRELKSQGRGIVYISHRMKEIFEICDDVTVFRDGQFIAEREV
ATLTEDSLIEMMVGRKLEDQYPHLDNAPGEIRLKVDNLCGPGVNDVSFVLRKGEILGISG
LMGAGRTELMKVLYGAMPRTSGYVTLDGHEVVTRSPQDGLANGIVYISEDRKRDGLVLGM
SVKENMSLTALDYFSRAGGSLKHKDEQQAVGDFIRLFNVKTPSMEQAIGLLSGGNQQKVA
IARGLMTRPKVLILDEPTRGVDVGAKKEIYQLINQFKADGLSIILVSSEMPEVLGMSDRI
IVMHEGHLSGEFTREQATQEVLMAAAVGKLNRVNQE
>EECEBICD_04166 D-ribose pyranase
MKKGTVLNSEISSVISRLGHTDTLVVCDAGLPIPNSTARIDMALTQGVPSFMQVVDVVTR
EMQVEAAILATEIKQQNPQLHETLLTHLEQLQQHQGNTIKISYTTHEQFKKLTADSQAVI
RSGECSPYANVILCAGVTF
>EECEBICD_04167 Low affinity potassium transport system protein kup
MSTDNKQSLPAITLAAIGVVYGDIGTSPLYTLRECLSGQFGFGVERDAVFGFLSLIFWLL
IFVVSIKYLTFVMRADNAGEGGILTLMSLAGRNTSARTTSMLVIMGLIGGSFFYGEVVIT
PAISVMSAIEGLEIVAPQLDTWIVPLSIIVLTLLFMIQKHGTGMVGKLFAPIMLTWFLIL
AVLGLRSIIANPEVLHALNPVWAVRFFLEYKTVSFIALGAVVLSITGVEALYADMGHFGK
FPIRLAWFTVVLPSLVLNYFGQGALLLKHPEAIKNPFFLLAPDWALIPLLILAALATVIA
SQAVISGVFSLTRQAVRLGYLSPMRIIHTSEMESGQIYIPFVNWLLYFAVVVVIVSFEHS
SNLAAAYGIAVTGTMVLTSILSTTVARKNWHWNKYFVALILIAFLCVDIPLFSANLDKLL
SGGWLPLSLGLIMFTIMTTWKSERFRLLRRMHEHGNSLEAMIASLEKSPPVRVPGTAVYM
SRALSVIPFALLHNLKHNKVLHERVILLTLRTEDAPYVHNVRRVQIEQLSPTFWRVVASY
GWRETPNVEEVFHRCGLEGLSCRMMETSFFMSHESLIVGKRPWYLRLRGKLYLLLQRNAL
RAPDQFEIPPNRVIELGTQVEI

>EECEBICD_04168 ATPase RavA
MAHPHLLAERISRLSSALEKGLYERSHAIRLCLLAALSGESVFLLGPPGIAKSLIARRLK
FAFQRARAFEYLMTRFSTPEEVFGPLSIQALKDEGRYERLTTGYLPEAEIVFLDEIWKAG
PAILNTLLTAINERHFRNGAFEEKIPMRLLVAASNELPEADSSLEALYDRMLIRLWLDKV
QDKANFRSMLVSQQDESDNPVPASLQVSDEEYQQWQKDIGAISLPDPVFELIFTLRQQLD
NLPNAPYVSDRRWKKAIRLLQASAFFSGRDAVAPIDLILLKDCLWYDAQSLNLMQQQLEI
LMTGHAWQQQAMLTRLGGIVQRRLQLQQQQSDKTAFTVIKEGGMFSRRPHYTLPPEASAS
TLTLLLQKPLKLHDMEVIHITFDRSALELWLTKGGEIRGKLNGIGFAQTLNMEVDNAQHL
VVRDISLQGTRLALPGAAEDSMPAEIKQQLETLENDWRQQHTRFSEQQHCLFIHSDWLGR
IEASLQDVGEQIRQAQQC
>EECEBICD_04169 Protein ViaA
MLTLDTLNTMLAVSEEGMVEEMILALLASPQLVIFFEKFPRLKNAVTADLPRWREALRSR
LKDARVPPELTEEVMCYQQSQLLSTPQFIVQLPQILALLHRLHSPYAAQAKQLTESNSTF
TPALHTLFLQRWRLSLVVQATTLNQQLLEEEREQLLSDVQERMTLSGQLEPTLAENDNAA
GRLWDMSAGQLKRGDYQLIVKYGEFLAAQPELMQLAEQLGRSREAKSVPKKDAPMETFRT
LVREPATVPEQVDGIQQGDDILRLLPPELATLGITELEYEFYRRLVEKQLLTYRLHGEAW
REKVTERPVVHQDVDEQPRGPFIVCVDTSGSMGGFNEQCAKAFCLALMRVALADNRRCFI
MLFSTDVVRYELSGPEGIEQAIRFLSQRFRGGTDIASCFRAIIERMQGREWFDADAVVIS
DFIAQRLPDDVVSKVGELQRLHQHRFHAVAMSAHGKPGIMRIFDHIWRFDTGMRSRLLRR
WRR
>EECEBICD_04170 Aspartate--ammonia ligase
MKTAYIAKQRQISFVKSHFSRQLEERLGLIEVQAPILSRVGDGTQDNLSGCEKAVQVKVK
ALPDAQFEVVHSLAKWKRQTLGQHDFSAGEGLYTHMKALRPDEDRLSPLHSVYVDQWDWE
RVMGDGERQFSTLKSTVEAIWAGIKATEAEVHKQFGLAPFLPEQIQFVHSQELLSRYPDL
DAKGRERAIAKELGAVFLVGIGGKLSDGHRHDVRAPDYDDWSSASELGYAGLNGDILVWN
PVLEDAFELSSMGIRVDADTLMRQLALTGDEDRLQLEWHQALLRGEMPQTIGGGIGQSRL
TMLLLQLPHIGQVQCGVWPAQVRESIPAIL
>EECEBICD_04171 Regulatory protein AsnC
MENYQIDNLDRGILDALMENARTAYAELAKQFGVSPGTIHVRVEKMKQAGIITGARIDVS
PKQLGYDVGCFIGIILKSAKDYPSALARLESLDEVTEAYYTTGHYSIFIKVMCKSIDALQ
HVLINKIQTIDEIQSTETLIVLQNPIMRTIKP
>EECEBICD_04172 Protein MioC
MADITLISGSTLGGAEYVAEHLAEKLEAAGFSTETVHGPLLEDLSASGIWLIISSTHGAG
DIPDNLTPFYEDLQTQKPDLSAVRFGAIGIGSREYDTFCGAIEKIEAELKDAGAKQVGET
LKINILEHEIPEDPAEIWLGSWINLLK
>EECEBICD_04173 tRNA uridine 5-carboxymethylaminomethyl modification
enzyme MnmG
MFYQDPFDVIIIGGGHAGTEAAMAAARMGQQTLLLTHNIDTLGQMSCNPAIGGIGKGHLV
KEVDALGGLMAKAIDQAGIQFRILNASKGPAVRATRAQADRVLYRQAVRTALENQPNLMI
FQQAVEDLIVENDRVVGAVTQMGLKFRAKAVVLTVGTFLDGKIHIGLDNYSGGRAGDPPS
IPLSRRLRELPLRVSRLKTGTPPRIDARTIDFSVLAQQHGDNPMPVFSFMGNASQHPQQV
PCYITHTNEKTHDVIRNNLDRSPMYAGVIEGIGPRYCPSIEDKVMRFADRNQHQIFLEPE
GLTSNEIYPNGISTSLPFDVQMQIVRSMQGMENAKIVRPGYAIEYDFFDPRDLKPTLESK
FIHGLFFAGQINGTTGYEEAAAQGLLAGLNAARLSADKEGWAPARSQAYLGVLVDDLCTL
GTKEPYRMFTSRAEYRLMLREDNADLRLTEMGRELGLVDDERWARFNEKLESIERERQRL
KSTWVTPSAESADEVNAHLTTPLSREASGEDLLRRPEMTYAQLTSLAAFAPALEDEQAAE
QVEIQVKYEGYIARQQDEIEKQLRNENTLLPATLDYRQVSGLSNEVIAKLNDHKPASIGQ
ASRISGVTPAAISILLVWLKKQGMLRRSA
>EECEBICD_04174 Ribosomal RNA small subunit methyltransferase G
MLNKLSRLLADAGISLTDHQKTLLVAYVDMLHKWNKAYNLTSVRDPNEMLVRHILDSIVV
APYLQGQRFIDVGTGPGLPGIPLAIVLPDAHFTLLDSLGKRVRFLRQVQHELKLENITPV
QSRVEAYPSEPPFDGVISRAFASLNDMVSWCHHLPGEKGRFYALKGQLPGDEIASLPDNF
SVESVEKLRVPQLEGERHLVIIKSNKV
>EECEBICD_04175 ATP synthase protein I

MSVSLVSRNVARKLLFIQFLAVIASGLLFCLKDPFWGISAVCGGLAVALPNMLFMIFAWR
HQAHTPAKGRVSWTFAFGEAFKVLAMLVLLVVALAVLKAVFLPLIVTWVLVLVVQILAPA
VINNKG
>EECEBICD_04176 ATP synthase subunit a
MASENMTPQEYIGHHLNNLQLDLRTFSLVDPQNPPATFWTLNIDSMFFSVVLGLLFLVMF
RSVAKKATSGVPGKFQTAIELIVGFVHGSVKDMYHGKSKLIAPLALTIFVWVFLMNLMDL
LPIDLLPYIAEHWLGLPATRVVPSADVNITLSMALGVFILILFYSIKMKGIGGFAKELTL
QPFNHWAFIPVNLILEGVSLLSKPVSLGLRLFGNMYAGELIFILIAGLLPWWSQWILNVP
WAIFHILIITLQAFIFMVLTIVYLSMASEEH
>EECEBICD_04177 ATP synthase subunit c
MENLNMDLLYMAAAVMMGLAAIGAAIGIGILGGKFLEGAARQPDLIPLLRTQFFIVMGLV
DAIPMIAVGLGLYVMFAVA
>EECEBICD_04178 ATP synthase subunit b
MNLNATILGQAIAFILFVWFCMKYVWPPLMAAIEKRQKEIADGLASAERAHKDLDLAKAS
ATDQLKKAKAEAQVIIEQANKRRAQILDEAKTEAEQERTKIVAQAQAEIEAERKRAREEL
RKQVAILAVAGAEKIIERSVDEAANSDIVDKLVAEL
>EECEBICD_04179 ATP synthase subunit delta
MSEFVTVARPYAKAAFDFAVEHQSVERWQDMLAFAAEVTKNEQMAELLSGALAPETLAES
FIAVCGEQLDENGQNLIRVMAENNRLNALPDVLEQFIHLRAASEATSEVEVTSATALSEE
QLSKISAAMEKRLSRKVKLNCKIDKSVMAGVIIRAGDMVIDGSVRGRLERLADVLQS
>EECEBICD_04180 ATP synthase subunit alpha
MQLNSTEISELIKQRIAQFNVVSEAHNEGTIVSVSDGVIRIHGLADCMQGEMISLPGNRY
AIALNLERDSVGAVVMGPYADLAEGMKVKCTGRILEVPVGRGLLGRVVNTLGAPIDGKGP
VDNDGFSAVEAIAPGVIDRQSVDQPVQTGYKAVDSMIPIGRGQRELIIGDRQTGKTALAI
DAIINQRDSGIKCIYVAIGQKASTISNVVRKLEEHGALANTIVVVATASESAALQYLAPY
AGCAMGEYFRDRGEDALIIYDDLSKQAVAYRQISLLLRRPPGREAFPGDVFYLHSRLLER
AARVNADYVEAFTKGEVKGKTGSLTALPIIETQAGDVSAFVPTNVISITDGQIFLESNLF
NAGIRPAVNPGISVSRVGGAAQTKIMKKLSGGIRTALAQYRELAAFSQFASDLDDATRKQ
LDHGQKVTELLKQKQYAPMSVAQQSLVLFAAERGYLADVELAKIGSFEAALLAYVDRDHA
PLMQEINQSGGYNDEIEGKLKGILDSFKATQSW
>EECEBICD_04181 ATP synthase gamma chain
MAGAKEIRSKIASVQNTQKITKAMEMVAASKMRKSQDRMAASRPYAETMRKVIGHLANGN
LEYKHPYLEERDVKRVGYLVVSTDRGLCGGLNINLFKKLLADMKAWSDKGVQCELAMIGS
KGVSFFNSVGGNVVAQVTGMGDNPSLSELIGPVKVMLQAYDEGRLDKLYIVSNKFINTMS
QVPTITQLLPLPASEDDDLKRKAWDYLYEPDPKALLDTLLRRYVESQVYQGVVENLASEQ
AARMVAMKAATDNGGSLIKELQLVYNKARQASITQELTEIVSGAAAV
>EECEBICD_04182 ATP synthase subunit beta
MATGKIVQVIGAVVDVEFPQDAVPRVYDALEVQNGNEKLVLEVQQQLGGGIVRTIAMGSS
DGLRRGLDVKDLEHPIEVPVGKATLGRIMNVLGEPVDMKGEIGEEERWAIHRAAPSYEEL
SNSQELLETGIKVIDLMCPFAKGGKVGLFGGAGVGKTVNMMELIRNIAIEHSGYSVFAGV
GERTREGNDFYHEMTDSNVIDKVSLVYGQMNEPPGNRLRVALTGLTMAEKFRDEGRDVLL
FVDNIYRYTLAGTEVSALLGRMPSAVGYQPTLAEEMGVLQERITSTKTGSITSVQAVYVP
ADDLTDPSPATTFAHLDATVVLSRQIASLGIYPAVDPLDSTSRQLDPLVVGQEHYDTARG
VQSILQRYQELKDIIAILGMDELSEEDKLVVARARKIQRFLSQPFFVAEVFTGSPGKYVS
LKDTIRGFKGIMEGEYDHLPEQAFYMVGSIDEAVEKAKKL
>EECEBICD_04183 ATP synthase epsilon chain
MAMTYHLDVVSAEQQMFSGLVEKIQVTGSEGELGIYPGHAPLLTAIKPGMIRIVKQHGHE
EFIYLSGGILEVQPGSVTVLADTAIRGQDLDEARALEAKRKAEEHIKSSHGDVDYAQASA
ELAKAIAKLRVIELTKKAM
>EECEBICD_04184 hypothetical protein
MSFQPSFAGPQPDSRIDRTTFIRRAYLHLAVAIVGFIVLSAAWSFIGVGEYALDVLLAGG
RYSWLVVLGAFMLVGMLATRLADNAGTNQTQLIGLGIYVLAESLIFAPLLTVAAYINPSS
IGAAAITTLLLVGGLTFTAFSIKKDFSFLRSFLTMAGFIAFGAIIASVICGFSLGVWFSA
LMVLLCAGFILYDTSNIIHHYPTDRPAGAALHLFASIATMFWYILRIFMSRN

>EECEBICD_04185 Bifunctional protein GlmU
MLNSAMSVVILAAGKGTRMYSDIPKVLHTLAGKPMVQHVIDAATKLGAAQVHLVYGHGGE
LLKQTLKDDKLNWVLQAEQLGTGHAMQQAAPFFSDDEDILMLYGDVPLISVETLQRLRDA
KPQGGIGLLTVKLDDPSGYGRITRENGKVTGIVEHKDATDEQRQIQEINTGILIANGADL
KRWLSKLTNNNAQGEYYITDIIALAYQEGREIAAVHPARISETDGVNNRLQLSRLERIYQ
AEQAEKLLLSGVMLRDPARFDLRGTLHCGMDVEIDANVIIEGYVTLGHRVKIGAGCIIKN
SVIGDDCEISPYSVVEDAHLEAACTIGPFARLRPGAELLAGAHVGNFVEMKKARLGKGSK
AGHLTYLGDAEIGDNVNIGAGTITCNYDGANKFKTVIGDDVFVGSDTQLVAPVTVGKGAT
IAAGTTVTRNVADNELVLSRVPQVHKQGWQRPVKKK
>EECEBICD_04186 Glutamine--fructose-6-phosphate aminotransferase [isomerizing]
MCGIVGAIAQRDVAEILLEGLRRLEYRGYDSAGLAVVDAEGHMTRLRRLGKVQMLAQAAE
EHPLHGGTGIAHTRWATHGEPSEANAHPHVSEHIVVVHNGIIENHEPLREALKARGYTFV
SETDTEVIAHLVNWELKQGGTLREAVLRAIPQLRGAYGTVIMDTRHPDTLLAARSGSPLV
IGLGMGENFIASDQLALLPVTRRFIFLEEGDIAEITRRSVNIFDNTGAEVKRQDIESNLQ
YDAGDKGIYRHYMQKEIYEQPNAIKNTLTGRISHGEVDLSELGPNADDLLSKVEHIQILA
CGTSYNSGMVSRYWFESLAGIPCDVEIASEFRYRKSAVRRNSLMITLSQSGETADTLAGL
RLSKELGYLGSLAICNVPGSSLVRESDLALMTNAGTEIGVASTKAFTTQLTVLLMLVAKL
ARLKGLDTSIEHDIVHGLQVLPSRIEQMLSQDKRIEALAEDFSDKHHALFLGRGDQYPIA
LEGALKLKEISYIHAEAYAAGELKHGPLALIDADMPVIVVAPNNELLEKLKSNIEEVRAR
GGQLYVFADQDAGFVSNDNMHIIEMPHVEEVIAPIFYTVPLQLLAYHVALIKGTDVDQPR
NLAKSVTVE
>EECEBICD_04187 HTH-type transcriptional regulator SgrR
MLIPVREISPFMTTRHTEQKYLKLLQHYGDKPVSVTLQELADVLFCTRRHMRNLLLQMQE
AKWLIWQSQAGRGHRARLHLRYKPEQLLSEKAEQLLESGHIDQAIQLLGKNKHQVAQLLR
SKLGYSVRADYQRLCIPYYRTMPSLCPGIPLRRSEQHLVRQIFSGLTRINEEKGEVEADL
AHHWRQIDPLRWRFYLRPAVLWHDGQELTIDAVIASLTRSAKLPLFSHLQTIQATGPLSL
EITLAHPDNRLPLLLSHIDAMILPPDHTQRADFPAHPVGTGPYEVVENNGFHLQMKAFDH
YFGLRGLLDEVEVFIWPNLTETDNLAESLSDNDTAAWLSSSLSDEDYVSGRLSQVSGKPS
DNLREMFLERGGYFLLCDSRSPHWHTAEHRRWLRETLSPYALLQHLSEAIRPFWVPGGSL
LSSWFHTIEAGPACSPFISSSPYAKLRLAYHDQHPEFPMLLDIMQEIMRQQGILLEGVEL
NYDDWANGKANVDLWLGTVNFPIPEEWNVGTWLLGSPLLRHAISGGDDALLAQWETQWHA
ETISAEQLVRETTRSGWLQPLFHHWMRLKGPDRARGIHLNNLGWFDFRSTWIEPGP
>EECEBICD_04188 Shikimate dehydrogenase-like protein
MTKRINKDTQVCMSLAARPSNFGTRFHNYLYEALDLNYLYKAFSPTDLTQAISGVRGLGI
RGCAISMPFKEACIPLVDELDASAQAIHSVNTIVNTNGHLKAYNTDYIAIARLLASYKVS
PDLVFALRGSGGMAKAVACALKDAGFTRGYIVAIDEASGKQLAELYGYEWRPDTDGIEAD
LLINATPIGMAGGNDADKLSYSEQEVDKASVIFDVVALPPVTPLIRYARERGKTVITGSE
VFAIQAVEQFVLYTGIRPDDALFQKAAAWSRL
>EECEBICD_04189 PTS system fructose-specific EIIB'BC component
MTKKIVAVTACPTGVAHTFMAAEALEIEARKRGDWIKVETRGSVGAKNTLTAEEIAQADV
VIIAADIELDLSGFVGKRLYRTSTGAALKKSAQEMDNAFNSAEVYQGSAGRSSSAGKTEL
PGVYKHLMTGVSHMLPLVVAGGLCIALSFVFGIQAFNEPGTLAAALFQIGGKAAFALMVP
VLAGFIAFSIADRPGLAPGLIGGMLASLCGAGFLGGIVAGFLAGYSVRFLAQNIKLPASM
EALKPVLVLPLLSTLITGLIMIYVVGGPVSAVMEGLTTFLGNMTSTNAILLGMLLGAMQG
FDLGGPVNKAAYTFGVGLLASHSYMPMAAIMAAGMVPALGMGVATWAARAKFTASEHEAG
NASFILGLCFISEGAIPFAARDPMRVIPSTMVGGAIAGGLSMYFGCTLMAPHGGLFVLAI
PHAVEHVMQYLLSIALGTIVCGLMYALLKPSAVAQTV
>EECEBICD_04190 Phosphate-binding protein PstS
MKVMRTTVATVVAATLSMSAFSAFAAASLTGAGATFPAPVYAKWADTYQKETGNKVNYQG
IGSSGGVKQITANTVDFGASDAPLSDEKLNQEGLFQFPTVIGGVVLAVNIPGLKSGELVL
DGKTLGDIYLGKIKKWDDEAITKLNPGVKLPSQNIAVVRRADGSGTSFVFTSYLAKVNDE
WKSKVGAGSTVNWPTGLGGKGNDGIAAFVQRLPGAIGYVEYAYAKQNNLAYTKLVSADGK
PVSPTEESFSNAAKGADWSKTFAQDLTNQKGDDVWPITSTTFILVHKAQKKPEQGAEVLK

FFDWAYKNGAKQANDLDYASLPDNVVEQVRAAWKTSIKDSNGKALY
>EECEBICD_04191 Phosphate transport system permease protein PstC
MAATKPAFNPPGKKGDMIFSALVKLAALIVLLMLGGIIVSLIISSWPSIQKFGFSFLWTK
EWDAPNDIYGALVPIYGTLVTSFIALLIAVPVSFGIALFLTELAPGWLKRPLGIAIELLA
AIPSIVYGMWGLFIFAPLFATYFQEPVGNILSNIPFVGALFSGPAFGIGILAAGVILAIM
IIPYIAAVMRDVFEQTPVMMKESAYGIGCTTWEVIWRIVLPFTKNGVIGGIMLGLGRALG
ETMAVTFIIGNTYQLDSASLYMPGNSITSALANEFAEAESGLHVAALMELGLILFVITFI
VLAASKFMIMRLAKNEGAR
>EECEBICD_04192 Phosphate transport system permease protein PstA
MATLDMQNTAQLAESRRKMQARRRMKNRIALTLSMATMAFGLFWLIWILMSTITRGIDGM
SLALFTEMTPPPNTAGGGLANALAGSGLLILWATVLGTPLGIMAGIYLAEYGRKSWLAEI
IRFINDILLSAPSIVVGLFVYTIVVAQMQHFSGWAGVIALALLQVPIVIRTTENMLKLVP
DSLREAAYALGTPKWKMISAITLKASISGIMTGILLAIARIAGETAPLLFTALSNQFWST
DMMQPIANLPVTIFKFAMSPFAEWQQLAWAGVLIITLCVLLLNILARVVFAKKKHG
>EECEBICD_04193 Phosphate import ATP-binding protein PstB
MSMVETAPSKIQVRDLNFYYGKFHALKNINLDIAKNQVTAFIGPSGCGKSTLLRTFNKMY
SLYPEQRAEGEILLDGDNILTNTQDIALLRAKVGMVFQKPTPFPMSIYDNIAFGVRLFEK
LSRADMDERVQWALTKAALWNETKDKLHQSGYSLSGGQQQRLCIARGIAIRPEVLLLDEP
CSALDPISTGRIEELITELKQDYTVVIVTHNMQQAARCSDHTAFMYLGELIEFSNTDDLF
TKPAKKQTEDYITGRYG
>EECEBICD_04194 Phosphate-specific transport system accessory protein PhoU
MDSLNLNKHISGQFNAELESIRTQVMTMGGMVEQQLSDAITAMHNQDSELAKRVVEGDHQ
VNMMEVAIDEACVRIIAKRQPTASDLRLVMAIIKTIAELERIGDVADKICRTALEKFSQQ
HQPLLVSLESLGRHTVQMLHDVLDAFARMDLDEAVRIYREDKKVDQEYEGIVRQLMTYMM
EDSRTIPSVLTALFCARSIERIGDRCQNICEYIFYFVKGQDFRHVGGDELDKLLAGKDPK
E
>EECEBICD_04195 6-phosphogluconate phosphatase
MSQIEAVFFDCDGTLVDSEVICSRAYVTMFRQFGITLKLTEVFRRFKGVKLYEIIDTISK
EHGVALAKAELEPVYRAEVARLFDTELEAIAGANTLLESIKTPMCVVSNGPVSKMQHSLG
KLGMLHYFPDLLFSGYDIQRWKPDPALMLHAANAMKVNVEKCILVDDSSAGAQSGIAAGM
EVFYFCADPHNKPIDHPNVTTFTDLAQLPGLWKARGWEITR
>EECEBICD_04196 Adenine permease AdeP
MSQQHTTQASGQGMLERVFKLREHGTTARTEVIAGFTTFLTMVYIVFVNPQILGVAGMDT
SAVFVTTCLIAAFGSILMGLFANLPVALAPAMGLNAFFAFVVVQAMGLPWQVGMGAIFWG
AVGLLLLTIFRVRYWMIANIPVSLRVGITSGIGLFIGMMGLKNAGVIVANPETLVSIGNL
TSHSVLLGVLGFFIIAILASRNIHAAVLVSIIVTTLLGWMMGDVHYNGIVSAPPSVTSVV
GHVDLAGSFNLGLAGVIFSFMLVNLFDSSGTLIGVTDKAGLADAKGKFPRMKQALFVDSI
SSVTGAFVGTSSVTAYIESSSGVSVGGRTGLTAVVVGILFLLVIFLSPLAGMVPPYAAAG
ALIYVGVLMTSSLARVNWQDLTESVPAFITAVMMPFSFSITEGIALGFISYCVMKIGTGR
LRDLSPCVVIVALLFVLKIVFIDAH
>EECEBICD_04197 Quinone reductase
MKKGARMSETLNVVTLLGSLRKGSFNGMVARTLPKVAPAGMTVSPLPSIGDIPLYDADIQ
QDEGFPASVEALAEQIRNADGVVIVTPEYNYSVPGGLKNAIDWLSRLPEQPLAGKPVLIQ
TSSMGAIGGARCQYHLRQILVFLDAMVMNKPEFMGGVIQNKVDPQTGEVVDQGTLDHLTG
QLTAFGEYIQRVKA
>EECEBICD_04198 hypothetical protein
MATHFARGILTEGQLVSIRLSSSCHIEARNLPAHRRTRFLASRGLLAELMFMLYGISELP
EIIIQAKGKPAFRDKNLPGFSISYAGNMVGVALTTEGECGLDMELQRTSRGFHHPHSLER
HPFSRNENLWVANQNDPNEARAQLITLRQSVLKLTGDVMNDDPRELQLLPVAGRLKCAHV
TQLEAVCDAEDVLVWSVTVTPAIEKLKVWEFDGKLGWKSLPDIQTRANEPTGRLMRFAQL
PAAKSYTLNRS
>EECEBICD_04199 HTH-type transcriptional regulator YidZ
MKKSLTNLDLNLLLCLQLLMQERSVTKAAKRMNVTPSAVSKSLAKLRAWFDDPLFVNTPL

GLAPTPLMVSMEQSLADWMQMGNQLLDKPHHQTPRGLKFELAAESPLMMIMFNSLSQQIY
QRYPQATIKVRNWDYDSLEAITRGEVDIGFTGRESHPRSRELLSLLPLAIDFEVLFSDLP
WVWLREDHPALREAWDLDTFLRYPHISICWEQSDTWALDDVLQEMGRKRHIALSLPGFEQ
SLFMAAQPDHTLIATAPRYCQHYNQLHQLPLVARPLPFDAQQREKLMVPFTLLWHKRNSH
NPKIVWLRQAINTLCRRLI
>EECEBICD_04200 Multidrug resistance protein MdtL
MKRFLLCSFALVLLYPAGIDMYLVGLPRIAADLNASEAQLHIAFSVYLAGMATAMLFAGK
IADQSGRKPVAIVGAIVFMMASLLCSRASEGSLFLSGRFLQGIGAGGCYVVAFAILRDTL
DEHRRAKVLSLLNGITCIVPVLAPVVGHLIMLRFPWQSLFYTMSAMGIIVGLLSLFILRE
TRPVRLAPRDLSRSSPAAESLINRFFVSRLAITTLSVSVILTFVNASPVLLMEVMGFSRG
DYAITMALTAGVSMVVSFSTPFALGLFKPRTLMLVSQGLFLTAGVTLSLAHTNTVTLFGL
TLICAGFSVGFGVAMSQALGPFSLRAGVASSTLGIAQVCGSSLWIWLAAILGISAMNMLI
GILIGCSIVSILLIFSVTPNRSVAEHEEIPYQSRP
>EECEBICD_04201 tRNA modification GTPase MnmE
MSHNDTIVAQATPPGRGGVGILRISGLKARDVAQEVLGKLPKPRYADYLPFKDVDGSALD
QGIALWFPGPNSFTGEDVLELQGHGGPVILDLLLKRILTLPGVRIARPGEFSERAFLNDK
LDLAQAEAIADLIDASSEQAARSALNSLQGAFSARVNHLVEALTHLRIYVEAAIDFPDEE
IDFLSDGKIEAQLNGVIADLDAVRTEARQGSLLREGMKVVIAGRPNAGKSSLLNALAGRE
AAIVTDIAGTTRDVLREHIHIDGMPLHIIDTAGLRDASDEVERIGIERAWQEIEQADRVL
FMVDGTTTDAVDPADIWPDFIARLPKNLPITVVRNKADITGETLGISEVNGHSLVRLSAR
TGEGVDVLRNHLKQSMGFDTNMEGGFLARRRHLQALAEAAEHLEQGKAQLLGAWAGELLA
EELRLAQQSLSEITGEFTSDDLLGRIFSSFCIGK
>EECEBICD_04202 Membrane protein insertase YidC
MDSQRNLLLVIALLFVSFMIWQAWEQDKNPQPQTQQTTQTTTTAAGSAADQGVPASGQGKM
ITVKTDVLDLTINTRGGDVEQALLPAYPKELGSNEPFQLLETTPQFIYQAQSGLTGRDGP
DNPANGPRPLYNVEKDAFVLADGQNELQVPMTYTDAAGNTFTKTFVFKRGDYAVNVNYSV
QNAGEKPLEVSTFGQLKQSVNLPPHRDTGSSNFALHTFRGAAYSTPDEKYEKYKFDTIAD
NENLNVSSKGGWVAMLQQYFATAWIPRNDGTNNFYTANLGNGIVAIGYKAQPVLVQPGQT
GAMTSTLWVGPEIQDKMAAVAPHLDLTVDYGWLWFISQPLFKLLKWIHSFVGNWGFSIII
ITFIVRGIMYPLTKAQYTSMAKMRMLQPKIQAMRERLGDDKQRQSQEMMALYKAEKVNPL
GGCFPLIIQMPIFLALYYMLMGSIELRHAPFALWIHDLSAQDPYYILPILMGVTMFFIQK
MSPTTVTDPMQQKIMTFMPVIFTVFFLWFPSGLVLYYIVSNLVTIIQQQLIYRGLEKRGL
HSREKKKS
>EECEBICD_04203 hypothetical protein
MLAQTEAFTRQTFNPVAFMRTSNIFLGDGKTDTGMPQRIQAAEDGDLRRASPLRLLEDEC
EMSGS
>EECEBICD_04204 Chromosomal replication initiator protein DnaA
MSLSLWQQCLARLQDELPATEFSMWIRPLQAELSDNTLALYAPNRFVLDWVRDKYLNNIN
GLLNTFCGADAPQLRFEVGTKPVTQTLKTPVHNVVAPTQTTTAQPQRVAPAARSGWDNVP
APAEPTYRSNVNVKHTFDNFVEGKSNQLARAAARQVADNPGGAYNPLFLYGGTGLGKTHL
LHAVGNGIMARKPNAKVVYMHSERFVQDMVKALQNNAIEEFKRYYRSVDALLIDDIQFFA
NKERSQEEFFHTFNALLEGNQQIILTSDRYPKEINGVEDRLKSRFGWGLTVAIEPPELET
RVAILMKKADENDIRLPGEVAFFIAKRLRSNVRELEGALNRVIANANFTGRAITIDFVRE
ALRDLLALQEKLVTIDNIQKTVAEYYKIKIADLLSKRRSRSVARPRQMAMALAKELTNHS
LPEIGDAFGGRDHTTVLHACRKIEQLREESHDIKEDFSNLIRTLSS
>EECEBICD_04205 Beta sliding clamp
MKFTVEREHLLKPLQQVSGPLGGRPTLPILGNLLLQVADGTLSLTGTDLEMEMVARVTLS
QPHEPGATTVPARKFFDICRGLPEGAEIAVQLEGDRMLVRSGRSRFSLSTLPAADFPNLD
DWQSEVEFTLPQATMKRLIEATQFSMAHQDVRYYLNGMLFETEGSELRTVATDGHRLAVC
SMPLEASLPSHSVIVPRKGVIELMRMLDGGENPLRVQIGSNNIRAHVGDFIFTSKLVDGR
FPDYRRVLPKNPDKHLEAGCDILKQAFARAAILSNEKFRGVRLYVSENQLKITANNPEQE
EAEEILDVSYGGTEMEIGFNVSYVLDVLNALKCETVRIMLTDSVSSVQIEDAASQSAAYV
VMPMRL
>EECEBICD_04206 DNA replication and repair protein RecF

MSLTRLLIKDFRNIENADLALSPGFNFLVGANGSGKTSVLEAIYTLGHGRAFRSLQPGRV
IRHEQEAFVLHGRLQGEERETSIGLTKDKQGDSKVRIDGTDGHKIAELAHLMPMQLITPE
GFTLLNGGPKYRRAFLDWGCFHNEAGFFTAWSNLKRLLKQRNAALRQVSRYEQLRPWDKE
LIPLAEQISTWRAEYSSAIAQDMADTCQQFLPEFSLTFSFQRGWEKETDYADVLERSFER
DRMLTYTAHGPHKADFRIRADGAPVEDTLSRGQLKLLMCALRLAQGEFLTRESGRRCLYL
IDDFASELDDARRGLLASRLKATQSQVFVSAISAEHVIDMSDENSKMFTVEKGKITD
>EECEBICD_04207 DNA gyrase subunit B
MSNSYDSSSIKVLKGLDAVRKRPGMYIGDTDDGTGLHHMVFEVVDNAIDEALAGHCKDIV
VTIHADNSVSVTDDGRGIPTGIHPEEGVSAAEVIMTVLHAGGKFDDNSYKVSGGLHGVGV
SVVNALSQKLELVIQRDGKIHRQIYEHGVPQAPLAVTGDTDKTGTMVRFWPSHETFTNVT
EFEYEILAKRLRELSFLNSGVSIRLRDKRDGKEDHFHYEGGIKAFVEYLNKNKTPIHPNI
FYFSTEKDGIGVEVALQWNDGFQENIYCFTNNIPQRDGGTHLAGFRAAMTRTLNAYMDKE
GYSKKAKVSATGDDAREGLIAVVSVKVPDPKFSSQTKDKLVSSEVKSAVEQQMNELLSEY
LLENPSDAKIVVGKIIDAARAREAARRAREMTRRKGALDLAGLPGKLADCQERDPALSEL
YLVEGDSAGGSAKQGRNRKNQAILPLKGKILNVEKARFDKMLSSQEVATLITALGCGIGR
DEYNPDKLRYHSIIIMTDADVDGSHIRTLLLTFFYRQMPEIVERGHVYIAQPPLYKVKKG
KQEQYIKDDEAMDQYQISIALDGATLHANAHAPALSGEALEKLVSEYNATQKMIGRMERR
FPKALLKELVYQPTLTEADLSDEQTVTRWVNALITELNEKEQHGSQWKFDVHTNTEQNLF
EPIVRVRTHGVDTDYPLDHEFVTGAEYRRICTLGEKLRGLIEEDAFIERGERRQPVTSFE
QALEWLVKESRRGLAIQRYKGLGEMNPDQLWETTMDPESRRMLRVTVKDAIAADQLFTTL
MGDAVEPRRAFIEENALKAANIDI
>EECEBICD_04208 HTH-type transcriptional regulator BenM
MNLTIRQFRAFLAVADLRSFTAASKYLCITQGAVSGLINEMESQMEVSLFDRTSRVVKLS
PDGEKFLPAAMRVVEEFQRAENYARDLKEIKKSLVRVVGAPLIACAFLPQIIHAFKRQHP
HTHVQLIDKPMSQLQKSIMLGEADFGIGPERPLEPEIDKQTLFTTEIALYCHPDYRLHKK
TVRWEEIQSEELIAVGNESIPMISATAGVRIKPKMTVEHMATAMSLAAQCEGVVIAGTFS
RHYAKSYQLATCSLTPPLRRNMNLYFHAWRAQTPATQRFRDFLFSYVDEHHDAPANQR
>EECEBICD_04209 D-galactonate dehydratase
MKITSVDIIDVANDFASATSKWRPVVVKINTDEGISGFGEVGLAYGVGASAGIGMAKDLA
AIIIGMDPMNNEAIWEKMLKKTFWGQGGGGIFSAAMSGIDIALWDIKGKAWGVPLYKMLG
GKSREKIRTYASQLQFGWGDGSDKDMLTEPEQYAQAALTAVSEGYDAIKVDTVAMDRHGN
WNQQNLNGPLTDKILRLGYDRMAAIRDAVGPDVDIIAEMHAFTDTTSAIQFGRMIEELGV
FYYEEPVMPLNPAQMKQVADKVNIPLAAGERIYWRWGYRPFLENGSLSVIQPDICTCGGI
TEVKKICDMAHVYDKTVQIHVCGGPISTAVALHMETVIPNFVIHELHRYALLEPNTQTCK
YNYLPKNGMYEVPELPGIGQELTEETMKKSPTITVK
>EECEBICD_04210 putative sulfoacetate transporter SauU
MSMALTAPPTRKRFLIVACLFIGIFIAYLDRVNVSVLAANEPFLAYMGIEGMPLQIGMMM
TVFLAAYGIANVVLSPLGDYLGPRKAMMLCILIWTIALMIGGVATSFALIIICRILLGIG
EGFYYPLQSVFIKNWFPKQERGRANAAWIVGQSVAPAIAMPFFTWWIGTHGWRSNFFLCA
ALGLIPLWLLWRYVADKPEQHKSISEQELAYIKAGQETESAGSSESFMLRVKPVITNYSY
WLLVLWYLCLQCLYWGMITWLPTYLKSARGFSWAEMGWLASLPFVLSIFAKAAAGVFVDK
IGRSAPILMVLMFFAGVSIYFGTITEHKYMSAVLLSFAVAFCTMGTPVAWTLLQGMIPGK
SISAASGVMNGVANGLSSLSPVFIGLFISITGTYTGGLLCLVFISAIAVVSALILTIKKY
>EECEBICD_04211 Sugar phosphatase YidA
MAIKLIAIDMDGTLLLPDHTISPAVKNAIAAAREKGVNVVLTTGRPYAGVHSYLKELHME
QPGDYCITYNGALVQKAGDGSTVAQTALSYDDYRYLEKLSREVGSHFHALDRNTLYTANR
DISYYTVHESYVATIPLVFCEAEKMDPNTQFLKVMMIDEPAVLDRAIARIPAEVKEKYTV
LKSAPYFLEILDKRVNKGTGVKSLAEALGIKPEEVMAIGDQENDIAMIEYAGMGVAMDNA
IPSVKEVANFVTKSNLEDGVAWAIEKFVLNPDHSSGHFPAR
>EECEBICD_04212 HTH-type transcriptional regulator LutR
MTLNKTDRIVITLGKQIVSGKYVPGSALPAEADLCEEFETSRNIIREVFRSLMAKRLIEM
KRYRGAFIAPRNQWNYLDTDVLQWVLENDYDPRLISAMSEIRNLVEPAIARWAAERATSS
DLAEIESALNDMIANNQDREAFNEADIRYHEAVLQSVHNPVLQQLNVAISSLQRAVFERT
WMGDAANMPKTLQEHKALFDAIRHQDGDAAEQAALTMIASSTRRLKEIT

>EECEBICD_04213 putative 2-dehydro-3-deoxygalactonokinase DgoK1
MTARYIAIDWGSTNLRAWLYQGDKCLESRQSEAGVTRLNGKSPDAVLAEVTTHWRDSATP
VVMAGMIGSNVGWQNAPYLPVPALFSAIGEQLTAVGDNIWIIPGLCVSREDNHNVMRGEE
TQLLGARELSPSSVYVMPGTHCKWVQTDTQQIHDFRTVMTGELHHLLLRHSLVGAGLPEQ
EVSGDAYAAGLERGLNSPAVLPSLFEVRASHVLGHLAREQVSDFLSGLLIGAEVASMSES
FAAQQAITLVAGPALISRYQQAFSAIGRDVSTVDGDMAFQAGIRSIAHAVAN
>EECEBICD_04214 2-dehydro-3-deoxy-6-phosphogalactonate aldolase
MQWQTNLPLIAILRGITPDDALAHVGAVVDAGFDAIEIPLNSPQWEKSISFVVKAYGGRA
LIGAGTVLKPEQVDQLAGMGCKLIVTPNIQPEVIRRAVSYGMTVCPGCATATEAFSALDA
GAQALKIFPSSAFGPGYISALKAVLPPDVPLFAVGGVTPENLAQWIKAGCVGAGLGSDLY
RAGQSVERTAQQAAAFVNAYREAVK
>EECEBICD_04215 D-galactonate dehydratase
MKITHITTYRLPPRWMFLKIETDEGVVGWGEPVIEGRARTVEAAVHEFADYLIGKDPARI
NDLWQVMYRAGFYRGGPIMMSAIAGIDQALWDIKGKVLNAPVWQLMGGLVRDKIKAYSWV
GGDRPADVIDGIEKLRGIGFDTFKLNGCEEMGVIDNSRAVDAAVNTVAQIREAFGSEIEF
GLDFHGRVSAPMAKVLIKELEPYRPLFIEEPVLAEQAEYYPRLAAQTHIPIAAGERMFSR
FEFKRVLDAGGLAILQPDLSHAGGITECYKIAGMAEAYDVALAPHCPLGPIALAACLHID
FVSRNAVFQEQSMGIHYNKGAELLDFVKNKEDFSMDGGFFKPLTKPGLGVDIDEARVIEL
SKSAPDWRNPLWRHADGSVAEW
>EECEBICD_04216 D-galactonate transporter
MDISVTAAQPGRRRYLTLVMIFITVVICYVDRANLAVASMHIQKEFGITKAEMGYVFSAF
AWLYTLCQIPGGWFLDRIGSRLTYFIAIFGWSVATLLQGFATGLLSLIGLRAITGIFEAP
AFPANNRMVTSWFPEHERASAVGFYTSGQFVGLAFLTPLLIWIQEMLSWHWVFIVTGGIG
IIWSLVWFKVYQPPRLTKSLSQAELEYIRDGGGLVDGDAPAKKEARQPLTKADWKLVFHR
KLVGVYLGQFAVNSTLWFFLTWFPNYLTQEKGITALKAGFMTTVPFLAAFFGVLLSGWLA
DKLVKKGFSLGVARKTPIICGLLISTCIMGANYTNDPLWIMALMAIAFFGNGFASITWSL
ISSLAPMRLIGLTGGMFNFIGGLGGISVPLVIGYLAQSYGFAPALVYISVVALLGALSYI
LLVGDVKRVG
>EECEBICD_04217 Sensor protein TorS
MSHEIRTPLYGILGTVQLLADKPLMANYRDDLQAINDSGESLLAILNDILDYSAIEVGGT
NVSISEEPFEPRQLLNSALHLMHSRVQVALIADFSEQLPSTLQGDPRRIRQIVINLLSNA
AKFTDRGSIVLRTFCDDQSWFIEVEDTGCGIPEAKLTAIFKPFVQATGRRGGTGLGLAIS
ASLAEAMGGTLTVTSTLHVGSCFRLQLPVRHPKPASKSAFRKPINLNGLRLLLIEDNMLT
QRITAEMLTGKGVKVSVAESANDALRCLAEGESFDVALVDFDLPDYDGLTLAQQLMSQYP
AMKRIGFSAHVIDDNLRQRTAGLFCGIIQKPVPREELYRMIAHYLQGKSHNARAMLNEHQ
LAGDMASVGPEKLRQWIALFKDSALPLVEEIEAARAMNDDVNIKRLAHKLKSGCASLGMT
QATEACRELELQPLSDIDIKTIVTQGVTALDAWIADHPSP
>EECEBICD_04218 Sensor protein TorS
MSTPSLTRRLWLAFALMAALTLLSTVIGWISLRVISQVEQTNTQALLPTMNMARQLSEAS
AYELFSAQNLTNADSEGVWLAQGKMLKAQSLKINHLLQALSEQGFNTSAIARQEKEIAQT
LGQQGTLVGEILTLRAQQQQLSRQIAEAAESIAAQAHGQANNAATSAGATQAGIYDLIES
GKGDQAERALDRLIDIDLEYVNQMNELRVNALRFKLLIVTLKDAQGLSDAEDTDEKLNQL
VKILSRRQQRIEDPTVRAQIADALEKINQYTTLVTLFRKENAIRDQLQTLMANNLFQFTR
FSTEVSQLVNAIEKRNEAGLARLTHASQRGQIGLVILGILALCSLSFILWRVVYRSVSRP
LAQQTQALQRLLEGDIDSPFPEAAGVSELDTISRLMEAFRANVRKLNRHREDLAEQVRSQ
TAELHALVLEHRQARAEAEKGQRGEIDVPCRHEP
>EECEBICD_04219 Periplasmic protein TorT
MIIIVSNVETASAETGLLHWTRADRAIPWRQTSVNASKPWKLCALYPSLKDSYWLSVNYG
MQKAAKLYGVDLKVLEANGYRQLATQQQQMTQCREWGADAILLGSSTIRFPELERYAGNV
PVIEVANVIRDASVATHVGLPWFQMGYLPGRFLVQWSKGKTLNVLLFPGPEEAGGSQEMV
AGFRQAIKGSAINIVDIAWGDNDIEVQRNLLQEMLERHPDANVVAGSAIAAEEAAMGEGRN
LTTPLTIVSFYLTHQVYRGLKRGHILMALSDQMAWQGELAITQSIKVLQGQPVPENISPP
VLILTHNNADSARVRRSLSPPGFRPVYLYQYTSEAKK
>EECEBICD_04220 TorCAD operon transcriptional regulatory protein TorR

MNRYEQKETARRMSHHIVIVEDEPVTQARLQAYFEQEGYRVSVTDSGAGLRDIMEHEHVS
LILLDINLPDENGLMLTRALRERSTVGIILVTGRCDQIDRIVGLEMGADDYVTKPLELRE
LVVRVKNLLWRIDLARPTPQNASENCYMFSGYCLNVMNHTLEHNGEAIKLTRAEYELLLA
FVTNPGKVLHRERLLRMLSARRVETPDLRTIDVLVRRLRHKITPELLVTQHGEGYFLASE
VY
>EECEBICD_04221 Cytochrome c-type protein TorC
MRKLWRALLRPSARWSILALVIVGIVIGVALIVLPHVGIKLTSTTEFCVSCHSMQPVYQE
YKQSVHFQNASGVRAECHDCHIPPDIPGMVKRKLEASNDLYQTFIAHSIDTPEKFEAKRA
ELAEREWARMKENNSATCRSCHNYDAMDHAKQNPEAARQMKIAAKENQSCIDCHKGIAHQ
LPDMSSGFRKQFDELRASASTHNDGDTLYSLDIKPIYAAKGDKEPAGSLLPASEVKVLKR
DGDWLQVQIEGWTETDGRQRVLTQLPGKRIFVASIRGDVQQHVKTLEETTVAATNTQWSK
LQATAWMQKGDMVNDIKPIWAYADSLYNGTCNQCHGAPDKAHFDANGWIGTLNGMIGFTS
LDKREERTLLKYLQMNASDTTNTPHSDKGEHNEK
>EECEBICD_04222 Trimethylamine-N-oxide reductase 1
MKNKDPLHVSRRRFLAQLGGLTVAGMLGPSLLTPRSAHAADAVAPGAATKEGILTGSHWG
AIRATVVDGRFVAAKPFEQDKYPSKMIAGLPDHVHNAARIRYPMVRVDWMRKGHQSDTSQ
RGDNRFVRVSWDEALDLFYQELERVQKTYGPSALLTASGWQSTGMFHNASGMLARAIALH
GNSVSTGGDYSTGAAQVILPRVVGSMEVYEQQTSWPLVLQNSKTIVLWGSDMVKNQQANW
WCPDHDVYQYYEQLKEKVASGAISVISIDPVVTSTHDYLGRDKVKHIAINPQTDVPLQLA
LAHTLYSEKLYDKNFLDNYCVGFDQFLPYLLGEKDGQPKDAAWAEKLCGIDADTIRALAR
QMAGDRTQIIAGWCVQRMQHGEQWSWMVVVLAAMLGQIGLPGGGFGFGWHYNGAGTPGRK
GIILSGFSGSTTVPPVHDSTDYKGYSSTIPIARFMDAILEPGKDINWNGKSVKLPPLKMC
VFAGTNPFHRHQQINRIIEGWRKLETVIAIDNQWTSTCRFADIVLPATTQFERNDLDQFG
NHSNRGIIAMKQVVSPQFEARNDFDIFRDLCRRFNREAAFTEGLDEMGWLKRIWQEGSQQ
GKGRGIHLPTFEVFWNQQEYIEFDHPQMFVRHQAFREDPDLEPLGTPSGLIEIYSKTIAD
MQYDDCQGHPMWFEKIERSHGGPGSQRWPLHLQSVHPDFRLHSQLCESETLRQQYAVGGK
EPVFINPQDASARGIRNGDIVRVFNARGQVLAGAVVSDRYAPGVARIHEGAWYDPDKGGD
LNALCKYGNPNVLTLDIGTSQLAQATSAHTTLVEIEKYTGPMDNVTAFNGPVEMVAQCEY
VPASQGNPHD
>EECEBICD_04223 Chaperone protein TorD
MIKQPALAQEQYACVYAWLALLFFREVDDEGLIQLQSAEIADWLALLKRQPALAASVALL
EQKIAALSLRQDAQLELAADFCGLFLMTDKKSALPYASQYPQQEPGMIKHLLLEAGMEVN
DDFKEPADHLAIYLELLSHLHFSLGESFQQRRMNKLRQKTLSSLLEWLPEFTNNCLKHDP
YGFYAALSQLLLAIVRFDDGKEDLSIVAVE
>EECEBICD_04224 hypothetical protein
MKKITLYATTVITVGLLCYLGLSGYVWYYDKQRSKKSDVQASVVGENNKILGYFREKGCD
YCHTPSAELPFYSSFPVAKQLMDYDIQLGYKSFNLEAVRAALIADTPVPQSELNKIEWVM
QHQTMPPTRYVALHWAGGVSDKERTDILNWIADQRERNYASADTDPAHRNEPVQPIPRNI
PVDAKKVDLGFRLYHDERLSGDSTISCAHCHALNAGGVDGRKTSIGVGGAVGPINAPTVF
NSVFNIEQFWDGRAATLQEQAGGPPLNPIEMASKSWDEIISKLDKDPALKKDFQAVYPQG
FTGENITDAIAEFEKTLITPDSAFDKWLRGDENALTAQQKHGYQLFKENKCATCHGGIIL
GGRSFEPLGLKRDFNFGEITAADIGRMNVTKEVRDKLRQKVPGLRNVALTAPYFHRGDVP
TLDGAVKLMLRYQVGTDLPQNDIDDIVAFLESLTGVYTPYQPEYAQ
>EECEBICD_04225 Cytochrome c biogenesis ATP-binding export protein CcmA
MTMMLEARDLYCERDERTLFRGLSFTVEAGEWVQVTGGNGAGKTTLLRLLTGLARPDGGE
VYWQGEPLRRVRDSFHRSLLWIGHQPGIKSRLTARENLHFFHPGDGARLPEALAQAGLAG
FEDVPVAQLSAGQQRRVALARLWLTRAALWVLDEPFTAIDVNGVARLTRRMAAHTAQGGM
VILTTHQPLPGAADTVRRLALTGGGAGL
>EECEBICD_04226 Heme exporter protein B
MWRVFCLELRVAFRHGADIAGPLWFFLMVITLFPLSVGPQPQLLARIAPGIIQVAALLAS
LLALERLFRDDLQDGSLEQLMLLPVPLPAVVLAKVLAHWAVTGLPLMMLSPLVALLLGMD
VYGWKIMALTLLLGTPALGFLAAPGVALTAGLRRGGVLLGILVLPLSVPVLIFATAAMDA
ASMHLPVDGYLAVLGALLAGSATLSPFATAAALRLSVQ
>EECEBICD_04227 Heme exporter protein C

MTTMWKTLHQLAAPPRLYQICGRLVPWLAAAGIIALATGWVRGFGFAPADYQQGESYRIM
YLHVPAAIWSMGIYAAMAVAAFTGLVWQMKMASLAVAAMAPVGAVYTFIALVTGAAWGKP
MWGTWWVWDARLTSELVLLFLYAGVIALWHAFDDRKMAGRAAGILVLVGVVNLPVIHYSV
EWWNTLHQGSTRMQQSIDPAMRSPLRWAIAGFLLLFMTLALMRMRNLILLMEKRRPWVSE
LILKRGHR
>EECEBICD_04228 Heme exporter protein D
MGGYAFFVWLAVAMTVAPLVLLALHTVLQRRAILRGVAQQRAREARMRAAQAQQEAA
>EECEBICD_04229 Cytochrome c-type biogenesis protein CcmE
MNLRRKNRLWVVCAVLAGLALTTALVLYALRANIDLFYTPGEILYGKRETQQLPAVGQRL
RVGGMVMPGSVRRDPDSLKVNFSLYDAEGSVTVSYEGILPDLFREGQGWWCRAPWRRAAT
SWRRRCWPSMTRTTPRRKWKRRCRKTTAARNALIRTPHHDA
>EECEBICD_04230 Cytochrome c-type biogenesis protein CcmF
MMPEYGHALLCLALGVALLLSVYPLWGVARGDARMMASAGVFAWLLFICVAGAFFVLVHA
FVVNDFTVAYVAGNSNTQLPVWYRVAATWGAHEGSLLLWVLLMSGWTLAVAVFSRPVPAD
IVARVLAVMGMVCAGFLAFILFTSGPFARTLPAFPVEGRDLNPLLQDPGLIFHPPLLYMG
YVGFSVAFAFAIAALLSGRLDSAFTRFARPWTLAAWVFLTLGIVLGSAWAYYELGWGGWW
FWDPVENASFMPWLAGTALLHSLAVTEQRAGFKAWTLLLSICAFSLCLLGTFLVRSGVLV
SVHAFASDPARGMFILAFMVLVTGGSLLLFAVRGHRVRSRVNNALWSRESLLLGNNVLLM
AAMLVVLLGTLLPLVHKQLGLGSISVGEPFFNTMFTWLMVPFALLLGVGPLVRWGRDRPR
NIRKLLWAAVVTTLVLSVLLPWLLEDKIIAMTAVGMAMACWIAVLAVAEAVQRVSRGTKT
SLSYWGMVAAHLGLAVTITGIAFSQNYSVERDVRMRAGDSVTIHDYRFTFREVRDITGPN
YRGGVALIGVTRHGEPEAVLHAEKRLYNTSRMVMTEAAIDGGLTRDLYAALGEELDNGAW
AVRLYYKPFVRWIWAGGAADGAGRAAVPGGPALPPP
>EECEBICD_04231 Thiol:disulfide interchange protein DsbE
MKRNVLLLPLLIFLLIAAALLWQLTRNAQGDDPTNLESALTGKPVPAFRLESLETPGQYY
EAEVLTQGKPVLLNVWATWCPTCRAEHQYLNQLSAQGIRVVGLNYKDDRAKAVAWLKELG
NPYALSLSDSDGMLGLDLGVYGAPETFLIDGRGIIRYRHAGDLNARVWESELKPLWDRYS
REAAQ
>EECEBICD_04232 Cytochrome c-type biogenesis protein CcmH
MRLLPGMVMLMLVLVISGSARATTDVMPFKDEAQEQQFRQLTEQLRCPKCQNNSIADSNA
MIATDMRRRVYDLMQEGKSRQEIIDYMVARYGNFVTYDPPLTPLTVLLWVLPLAAIVAGG
WIIVARTRRRVRLRREPLPADTPVCGARAGWGVYVPGAVIALAVGAGSYALTGSYQQVRA
WQQATAQTPGLLARALDPAAQPLNEEEMARLALGLRTRLQNDAGNVEGWLMLGRTGMVLG
NAGTATGAYANAYRLAPKNSDAALGYAEALTRSSDPEDNRRGGELLRQLVSRDHTDIRVL
SLYAFNAFEQRRFGEAVAAWEMMLKLLPADDTRRAVIERSIRLAQEK
>EECEBICD_04233 hypothetical protein
MKQITFTPRHHQLTNTNTWTPDSQWLVFDVRPSGASFTGKTIERVNVHTGDVEVIYRAVQ
GAHVGVVTVHPADNHYVFIHGPENPDETWHYDFHHRRGVIATSEGVTNLDAMDITAPYTP
GALRGGSHVHVFSPNGELVSFTYNDHVLHERDPALDLRNVGVAAPYGPVTVPVQHPREYS
GSYWCVLVSRTTPAPRPGSDDINRAYEEGWVGNRQIAFIGDTLSLTGKKVPELFIVDLPC
HENGWKQAGDTPLTGTESTMPSPPLGVVQRRLTFTHQRVYPGLTNEPRHWVRSNPQATAI
AFLMRDDNGVAQLWLISPQGGEPRQLTHHATGVQSAFNWHPSGKWLGLVLENRIACCDAQ
SGKIDFLTARHDNPPSADAVVFSPDGRHVAWMEEVKGFRQLWVTETGR
>EECEBICD_04234 hypothetical protein
MLLLGGCSSVMSHTGGKEGTYPGTRASATMIGNDETNWGTKSLAILDMPFTAVMDTILLP
WDIFRKDSSVRSRVEKSEEETKMTNAVIPPAKMPPP
>EECEBICD_04235 Small heat shock protein IbpA
MRNFDLSPLYRSAIGFDRLFNLLENNQSQSNGGYPPYNVELVDENHYRIAIAVAGFAESE
LEITAQDNLLVVKGAHADEQKERTYLYQGIAERNFERKFQLAENIHVRGANLVNGLLYIE
LERVIPEANKPRRIEIN
>EECEBICD_04236 Small heat shock protein IbpB
MRNYDLSPLLRQWIGFDKLANALQNSGESQSFPPYNIEKSDDNHYRITLALAGFRQEDLD
IQLEGTRLTVKGTPEQPENEPKWLHQGLVMQPFSLSFTLAENMEVSGATFTNGLLHIDLT
RNEPETIAPQRIAINERSALNS

>EECEBICD_04237 Aspartate/alanine antiporter
MSDIALTVSVLALVAVVGLWIGNIKVRGVGFGIGGVLFGGIIVGHFVDQAGVTLSGDMLH
FIQEFGLILFVYTIGIQVGPGFFASLRVSGLRLNLFAVLIVIMGGLVTAILHKIFAIPLP
VVLGIFSGAVTNTPALGAGQQILRDLGTPVDLVDQMGMSYAMAYPFGICGILLTMWLMRL
IFRVNVEAEAQKHESSLANGHSLIQTMNIRVENPNLNNMAIQDVPILNSDKIICSRLKRD
DTLMVPSPGTIIQAGDLLHLVGQPTDLHNAQLVIGKEVDTSLSTRGTDLRVERVVVTNEK
VLGKRIRDLHFKERYDVVISRLNRAGVELVASSDASLQFGDILNLVGRPASIDAVANVVG
NAQQKLQQVQMLPVFIGIGLGVLLGSIPLFVPGFPVALKLGLAGGPLIMALILGRIGSIG
KLYWFMPPSANLALRELGIVLFLAVVGLKSGGDFVDTLTQGEGLSWIGYGIFITAIPLIT
VGLLARIFAKMNYLTLCGMLAGSMTDPPALAFANNLHATSGAAALSYATVYPLVMFLRII
TPQLLAVIFWGMG
>EECEBICD_04238 Inner membrane protein YidH
MKISRLGEAPDYRFSLANERTFLAWIRTSLGFLAAGVGLDQLAPDFATPVIRELLALLLC
LFAGGLAIYGYLRWLRNEKAMRLKEDLPYTHSLLIISVILMIVAVIVMGLVLYAG
>EECEBICD_04239 Inner membrane protein YidG
MPDSRKARRQNDPGLQPERTSLAWFRTLLGYGALMALAVRHHWHQAGFLFWVSISVLAIV
AVILWRYTLSRNLMDVAHSDFSETHVMRDKFLISLAVLSLAILFAVTHIRQLIAFIGNFI
>EECEBICD_04240 hypothetical protein
MTGRQDIVVSDDQIQVVVNRQNSQRPQQLYRNLQRLGIRNVHFIPLLEHDRNGMLTEDSL
CSADWGRFLNSVFDIWVREDIQRISVRLFDETLQQWCGGRNGAEAPDKVPLSAECQKCSL
LRFCGGGCPEHRDSQGKNQLCEGYQTFFNYSSPHMRVMRDLLKQHRSPEELMAMLR
>EECEBICD_04241 D-serine dehydratase
MENIQKLIARYPLVEDLVALKETTWFNPGATSLAQGLPYVGLTEQDVNAAHDRLARFAPY
LAKAFPQTAAAGGMIESDVVAIPAMQKRLEKEYGQTIDGEMLLKKDSHLAISGSIKARGG
IYEVLTHAEKLALEAGLLTTDDDYSVLLSPEFKQFFSQYSIAVGSTGNLGLSIGIMSACI
GFKVTVHMSADARAWKKAKLRSHGVTVVEYEDDYGVAVEQGRKAAQSDPNCFFIDDENSR
TLFLGYAVAGQRLKAQFAQQGRVVDASHPLFVYLPCGVGGGPGGVAFGLKLAFGDNVHCF
FAEPTHSPCMLLGVYTGLHDAISVQDIGIDNLTAADGLAVGRASGFVGRAMERLLDGLYT
LDDQTMYDMLGWLAQEEGIRLEPSALAGMAGPQRICAAAAYQQRHGFSQTQLGNATHLVW
ATGGGMVPEDEMEQYLAKGR
>EECEBICD_04242 D-serine transporter DsdX
MHAQIWVVSTLLISIVLIVLTIVKFKFHPFLALLLASFFVGAMMGMGPLEMVTAIENGIG
GTLGFLAAVIGLGTILGKMMEVSGAAERIGLTLQRCRWLSADVIMVLVGLICGITLFVEV
GVVLLIPLAFSIAKKTHTSLLKLAIPLCTALMAVHCVVPPHPAALFVANKLGADIGSVIV
YGLLVGLMASLVGGPLFLKFLGNRLPFKPVPTEFADLEVRAENTLPSLGATLFTVLLPIG
LMLVKTVAELNMAKDGTLYTLLEFIGNPITAMFIAVFVAYYILGLRQHMGMNALLTHTEN
GFGAIANILLIIGAGGAFNAILKSSGLADTLAVILSNMHMHPILLAWLVALILHAAVGSA
TVAMMGATAIVAPMLPLYPDVSPEIIAIAIGSGAIGCTIVTDSLFWLVKQYCGATLNETF
KYYTTATFIASVVALAGTFLLSFII
>EECEBICD_04243 HTH-type transcriptional regulator DsdC
MEPLRDTRNRLLNGWQLSKLYTFEVAARHQSFALAADELSLSPSAVSHRINQLEAELGIQ
LFVRSHRKVELTHEGKRVFWALKSSLDTLNQEILDIKNQELSGTLTVYSRPSIAQCWLVP
ALGDFTRRYPSISLTMLTGNDNVNLQRAGIDLAIYFDDAPSAQLTHHFLMDEAILPVCSP
DYARRFALQDNLNNLRHCTLLHDRQAWSNDSGTDEWHSWAQHFAVNLPDSSGIGFDRSDL
AVIAAMNHIGVAMGRKRLVQKRLDSGELIAPFGDMRLKCHQHYYVTTLPGRQWPKIEAFI
RWLQEQV
>EECEBICD_04244 hypothetical protein
MFKPLIASLMLTSAVVYAKPMPLTAARYAQQLGVGMDVDWARTERGIREFDPLVVRDFKA
KGLTHVRIRVAGAPTEARLIHLRKLVEACEYYGVIPIIAYQADAYKTDPSASHEKELINW
WSVVARYFGQTSPLLGFDLIYEPADKLNHNMASLNRVYDKTIRLIHAIDPQRMIFVAPRM
RAAPEDLSALKLPAQSQNYVLAEWHIFPWGPLKSGGKYPWTSGTAAEKAAIRARINAAVR
WQHKTGHASWVGGWAPGESIKMTPVASQFAFARFMACELKKAHIPYAINADTQFYDGEEG
AWRPAQEPLLNAMIAPECETPGKKPGEGNVKPSVPDAISVTPAAASTPGSAIP
>EECEBICD_04245 Multidrug resistance protein D

MKRQRNVNLLLMLVLLVAVGQMAQTIYIPAIADMAQALNVREGAVQSVMAAYLLTYGVSQ
LFYGPLSDRVGRRPVILVGMSIFMVATLFAMTTHSLTVLIAASAMQGMGTGVGGVMARTL
PRDLYEGTQLRHANSLLNMGILVSPLLAPLIGGLLDTLWNWRACYAFLLVLCAGVTFSMA
RWMPETRPAAAPRTRLIASYKTLFGNGAFNCYLLMLIGGLAGIAVFEACSGVLMGAVLGL
SSMVVSILFILPIPAAFFGAWFAGRPNKRFSALMWQSVICCLLAGLMMWIPGWFGVMNVW
TLLIPAALFFFGAGMLFPLATSGAMEPFPFLAGTAGALVGGLQNIGSGVLAWLSAMLPQN
GQGSLGLLMTLMGLLILACWLPLASRISHQGQTV
>EECEBICD_04246 hypothetical protein
MTLSVFCILLFAALLHASWNAIVKAGNDKLYAAIGVSGSAAVMALILLPFSPQPAHASIP
FLAASTALQVVYTVLVAKTYQVSDMSQTYPLMRGTAPLLVALISVLFLGDSLSSMAWVGI
AVICMAILGMACNGRASSQRGVVLALTNACFIAGYTLVDGTGVRLSETALGYTLWSFFLN
GACLLTWAMIARRREASRYLAQQWKKGIFGGIGTMGSYGLALWAMTQAPLAVVAALRETS
ILFGALIAWLLLKEKVAGLRLVAAGGIALGAILLRLS
>EECEBICD_04247 Small toxic protein TisB
MSVVDITILILKLIVAALQLLDAVLKYLK
>EECEBICD_04248 hypothetical protein
MNPSMLNATLLTTAPSRAVVVVRVVVVVGNAP
>EECEBICD_04249 Acetolactate synthase isozyme 1 large subunit
MASSGTTSTPQRFTGAEFIVHFLERQGIRIVTGIPGGSILPVYDALSQSTQIRHILARHE
QGAGFIAQGMARTEGKPAVCMACSGPGATNLVTAIADARLDSIPLICITGQVPASMIGTD
AFQEVDTYGISIPITKHNYLVRNINELPQVMSDAFRLAQSGRPGPVWIDVPKDVQTATIE
LDALPSPAEKSPPPEFSAESIREAAAMINAAKRPVLYLGGGVINAPTRVRELAEKAQLPT
TMTLMALGMLPKAHPLSLGMLGMHGARSTNFILQEADLLIVLGARFDDRAIGKTEQFCPN
AKIIHVDIDRAELGKIKQPHVAIQADVDDVLAQLIPHIEAQPREAWHQLVADLQQEFPCS
IPQENNPLSHYGLINAVAACVDDNAIITTDVGQHQMWTAQAYPLNRPRQWLTSGGLGTMG
FGLPAAIGAALANPGNKVLCFSGDGSLMMNIQEMATASENQLDVKIILMNNDALGLVHQQ
QSLFYKQGVFAATYPGSINFMQIAAGFGLDTCDLNNEADPQAALQAIIRRPGPALIHVRI
DAEEKVYPMVPPGAANTEMVGE
>EECEBICD_04250 Acetolactate synthase isozyme 1 small subunit
MQKQSHDNVILELTVRNHPGVMTHVCGLFARRAFNVEGILCLPIQGSDQSRIWLLVNDDQ
RLEQMISQIDKLEDVAKVVRNQSDPTMFNKIAVFFE
>EECEBICD_04251 Deoxyribose operon repressor
METKQKERIRRLMELLKKTDRIHLKDAARMLEVSVMTIRRDLSQDDDPLPLTLLGGYIVM
VNKPASSTNMPLPAKKTHHRDDLPVAILAAGLVRENDLVFFDNGPEMPLVISMIPDDITF
TGICYSHRVFIALNEKPNATAILCGGTYRAKSDAFYDANNPSALDSLNPRKVFISASGVH
EHFGVSWFNPDDLAAKRKAMERGLRKILLARHALFDEVAPASIGPLSAFDVLISDRPLPT
DYAVHCRNGSVKVITPDSESE
>EECEBICD_04252 Ribokinase
MDIAVIGSNMVDLITYTNQMPKEGETLEAPAFKIGCGGKGANQAVAAAKLNSKVLMLTKV
GDDIFADNTIRNLESWGINTTYVEKVPCTSSGVAPIFVNANSSNSILIIKGANKFLSPED
IDRAAEDLKKCQLIVLQLEVQLETVYHAIEFGKKHGIEVLLNPAPALRELDMSYACKCDF
FVPNETELEILTGMPVDTYDHIRAAARSLVDKGLNNIIVTMGEKGALWMTRDQEVHVPAF
RVNAVDTSGAGDAFIGCFAHYYVQSGDVEAAMKKAVLFAAFSVTGKGTQSSYPSIEQFNE
YLSLNE
>EECEBICD_04253 L-fucose-proton symporter
MNDKNIVQMPDGYLNKTPLFQFILLSCLFPLWGCAAALNDILITQFKSVFSLSNFASALV
QSAFYGGYFLIAIPASLVIKKTSYKVAILIGLTLYIVGCTLFFPASHMATYTMFLAAIFA
IAIGLSFLETAANTYSSMIGPKAYATLRLNISQTFYPIGAAAGILLGKYLVFSEGESLEK
QMAGMNAEQVHNFKVLMLENTLEPYKYMIMVLVVVMVLFLLTRFPTCKVAQTASHKRPSA
LDTLRYLASNARFRRGIVAQFLYVGMQVAVWSFTIRLALELGDINERDASTFMVYSFACF
FIGKFIANILMTRFNPEKVLILYSVIGALFLAYVALAPSFSAVYVAVLVSVLFGPCWATI
YAGTLDTVDNEHTEMAGAVIVMAIVGAAVVPAIQGYVADMFHSLQLSFLVSMLCFVYVGV
YFWRESKVRGNLAEVAAS
>EECEBICD_04254 hypothetical protein

MTIRITLWRELFSEQPRILLENDDFTVTAFRYASGVEGLKIQNSRGHLVILPWMGQMIWD
AQFDGHDLTMRNMFRQPKPAAEVVATYGCFAFHSGLLANGCPSPEDTHPLHGEMPCAAMD
DAWLELEGDSLRVTGRYEYVMGFGHHYQAQPAVVMRKSSALFDIQMTVTNLASVAMPLQY
MCHMNYAYVPNATFRQNIPDTALKLRESVPAHVKPTAQWLAFNQRLLQGEASLATLNEPG
FYDPEIVFFADELDRYTDTPEFSMIAPDGTTFVTRFASAELNYVTRWILYNGDQQVAAFA
LPATCRPEGFLAAQRNGTLLQLEPQQTRTFTVTTGIV
>EECEBICD_04255 Transcriptional regulatory protein UhpA
MITVALIDDHLIVRSGFAQLLGLEPDLQVVAEFGSGREALAGLPGRGVQVCICDISMPDI
SGLELLSQLPKGMATIMLSVHDSPALVEQALNAGARGFLSKRCSPDELIAAVHTVATGGC
YLTPDIAVKLAAGRQDPLTKRERQVAEKLAQGMAVKEIAAELGLSPKTVHVHRANLLEKL
GVSNDVELAHRMFDGW
>EECEBICD_04256 Signal transduction histidine-protein kinase/phosphatase UhpB
MTLNWRIACLTVGNEHLFSRLITVVACFFIFSAAWFCLWSISLHLVERPELAALLFPFGL
RLGLMLQCPRGYWPVLLGAEWLLVYWLAQEVALAHLPLLMIGSVLTLLPVALTSRYRHQR
DWRTLLLQGAALTAAALLQSLPWLGQGEAAWNALLLTLTGGLTLAPICLVFWHYLTSTTW
LPLGPSLVSQPVNWRGRHLIWYLLLFIVSLWLQLGLPAELSRFTPFCLALPIIALAWHYG
WQGALIATLMNAIALIASQTWHDHPVDLLLSLLAQSLTGLLLGAGIQRLRELNQSLQKEL
ARNHRLAERLLETEESVRRDVARELHDDIGQTITAIRTQAGIVQRLAADNGGVKQSGQLI
EQLSLGVYDAVRRLLGRLRPRQLDDLTLAQAIRSLLREMELESRGIVSHLDWRIDETALS
ESQRVTLFRVCQEGLNNIVKHANASAVTLQGWQQDERLMLVIEDDGSGLPPGSHQQGFGL
TGMRERVSALGGTLTISCTHGTRVSVSLPQRYV
>EECEBICD_04257 Membrane sensor protein UhpC
MLSFLKAPANAPLITDKHEVDARYRYWRRHILITIWLGYALFYFTRKSFNAAAPEILASG
ILTRSDIGLLATLFYITYGVSKFVSGIVSDRSNARYFMGIGLIATGVVNILFGFSTSLWA
FALLWALNAFFQGFGSPVCARLLTAWYSRTERGGWWALWNTAHNVGGALIPLVMAAVALH
YGWRVGMMVAGLLAIGVGMVLCWRLRDRPQAIGLPPVGDWRHDALEVAQQQEGAGLSRKE
ILAKYVLLNPYIWLLSLCYVLVYVVRAAINDWGNLYMSETLGVDLVTANTAVSMFELGGF
IGALVAGWGSDKLFNGNRGPMNLIFAAGILLSVGSLWLMPFASYVMQAACFFTTGFFVFG
PQMLIGMAAAECSHKEAAGAATGFVGLFAYLGASLSGWPLAKVLEIWHWTGFFAVIAIAA
GISALLLLPFLNAQAPRETSEA
>EECEBICD_04258 Hexose-6-phosphate:phosphate antiporter
MLAFLNQVRKPTLDLPLDVRRKMWFKPFMQSYLVVFIGYLTMYLIRKNFNIAQNDMISTY
GLSMTELGMIGLGFSITYGVGKTLVSYYADGKNTKQFLPFMLILSAICMLGFSASMGAGS
TSLFLMIAFYALSGFFQSTGGSCSYSTITKWTPRRKRGTFLGFWNISHNLGGAGAAGVAL
FGANYLFDGHVIGMFIFPSIIALIVGFIGLRFGSDSPESYGLGKAEELFGEEISEEDKET
EENEMTKWQIFVEYVLKNKVIWLLCFSNIFLYVVRIGIDQWSTVYAFQELKLSKEVAIQG
FTLFEVGALVGTLLWGWLSDLANGRRALVACVALALIIATLGVYQHASNQYVYLASLFAL
GFLVFGPQLLIGVAAVGFVPKKAIGAADGIKGTFAYLIGDSFAKLGLGMIADGTPVFGLT
GWAGTFAALDAAAIGCICLMAMVAVMEERKIRREKKIQQVNIA
>EECEBICD_04259 hypothetical protein
MIWLILATFVVVFIVGFRVLTSDTRRAIRRLSERLNIDVVPIESMIDQMGKTAGGEFLQY
LHRPDESHLQNAAQVLLIWQMVIVDGGDQNLQRWHRLLQKARLAAPITDTQVRLALGFLR
EMEPDMQEINAFQLRYNAFFQPEEGVHWLH
>EECEBICD_04260 hypothetical protein
MSAKTKFKSPAFEAIHSAASGLFSVDAIPQETMRSFDTACLSSIKDLQPLEIKALREELN
VSQPVFARYLNTSVSTVQKWESGAKRPSGMSLKLLNVVQKHGLKVLV
>EECEBICD_04261 hypothetical protein
MRTFKTRWFNREAKPHTIKDDELSEAINAVLQGKADNLGGGVYKKRLNQNRDRAIVLAKG
GEHWFYTFLYAKQDMANIRYRELAGFRELAKHYACLTEDQITALINNKELVEVRHVSKN
>EECEBICD_04262 Purine ribonucleoside efflux pump NepI
MNENIAEKFRADGVARPNWSAVFAVAFCVACLITVEFLPVSLLTPMAQDLGISEGIAGQS
VTVTAFVAMFSSLFITQIIQATDRRYIVILFAVLLTASCLMVSFANSFTLLLLGRACLGL
ALGGFWAMSASLTMRLVPARTVPKALSVIFGAVSIALVIAAPLGSFLGGIIGWRNVFNAA

AVMGVLCVIWVVKSLPSLPGEPSHQKQNMFSLLQRPGVMAGMIAIFMSFAGQFAFFTYIR
PVYMNLAGFDVDGLTLVLLSFGIASFVGTSFSSYVLKRSVKLALAGAPLLLALSALTLIV
WGSDKTVAAVIAIIWGLAFALVPVGWSTWITRSLADQAEKAGSIQVAVIQLANTCGAAVG
GYALDNFGLLSPLALSGCLMLLTALVVAAKVRITPMS
>EECEBICD_04263 6-phospho-beta-glucosidase GmuD
MRYRFPDNFWWGSACSALQTEGDSLNGGKSQTTWDVWFERQPDRFHQGVGPAETSTFYRH
WKQDIALLKQLKHNSFRTSLSWARLIPDGVGEVNPQAVSFYNHVIDELLAQGITPFITLF
HFDMPMVMQEKGGWENRDVVEAFGRYAQTCFTLFGDRVKHWFTFNEPIVPVEGGYLYDFH
YPNVVDFKRAATVAYHTVLAHSTAVRAWRAGRYDGEIGVVLNLTPSYPRSQHPADVQAAH
HADLLFNRSFLDPVLKGEYPADLVALLKTYDQLPACQPGDRQLIADGKIDLLGINYYQPR
RVKCRDTAVNPQAPFMPEWLFDYYDMPGRKMNPYRGWEIYAPGIYDIITNLRDNYGNPRC
FISENGMGVENEQRFVQAGQIHDDYRIDFISEHLKWLHKGISEGCHCLGYHMWTFIDNWS
WLNGYKNRYGFVQLDLATQTRTVKKSGEWFAATAEHNGFD
>EECEBICD_04264 hypothetical protein
MKRKTLLLIAALVALPGVTYADSPFSSLQSAHEKNTILKDLRKMCTPKGALTDEAWEKKI
MASEGNQQHIREAMIAIERNNQHNYWQALGKVECPEM
>EECEBICD_04265 Transcriptional regulatory protein DagR
MRRIEIVLGELERLTRGLCLADLAQETAFTAEAIGFNLGLARNSVSKDLNQLWNDGLAIK
SRGRPVYFLHRQALETLLGRQLEESEREVRSVADVLPHEEHYAPDDPFTSLIGYDRSLRD
AVEKGRAAVLYPHGLHVLLTGPSGVGKTFFAELMHRFACEQASGAIPPLVYFNCAEYAHN
PELLSSHLFGHRQGAFTGANEHKTGLVEQADGGYLLLDEVHRLSYEGQEKLFSILDKGEY
RPLGVSSQPRSISVRLICATTEPVGSALLRTFQRRIQVCIDLPGIRQRSVEEQIELIVGF
LQRESRKIERTVSIDKPLLLWLLNKPLEGNIGQLKSDIQFLCAQAWASGMTEHNDTLQLD
KRLAEMSVNPTPEQRLLVDTLFDGKARLNIDARTLPALKTSLATGAEIEESDLFYSFLTR
EYVNLRNSNVPPAETLAILKNKLSSIFEYGLYSRDSVAHPPRYGDQIEERVTLLIGCVEQ
VLGFSLPENLVNPLRKHFLALIGYVQRGLIPQLYSSSLILDRCKDEYDNATLLCRKINEL
LHIQCPATEVVWLCLFLKECRHYRQRIDASPDCGVILIAHGATTATSQAQYVNRVLEREL
FSAIDMPFEQSVHDTLETLTQMIQTRQYRRLILLVDIGSLIHFGSTISKLFQIDVLLMPN
ITLTSLLEVGLDLSYETSDLPQLTALLQSKNIPCQLCTPQQENGGKVLVISCITGMGTAE
KIKKVLEESFGELMSQDTRMVILDYNEVRSLERVQQALNASERLAGIVGTFQPGLPDIPF
ISLEELFSEQGPELVLSLLTPDLSNAERRLEMERSAMRFISALTMESIINHISVLNPQRI
LKEMEGVFNHLTSSLSLKPSRQVTLRFLIHCCCMVERIVINRKPLQMALESQPNLDARAF
SVIKSAFLPIEDAYAIRLSDAEYFYIYELLYS
>EECEBICD_04266 hypothetical protein
MTTTQPLPHILLLTHGGWGQPLCNSLRMVTGEIKGVTEIALMPVDTLGEFYQRVEAVVKT
LPEGSLILTDFVGGTTSNVAARLSADYPVAVIAGLNASLLLEALDRREQGMLTACVDELV
EAGRSSCLDVVAHVRQLQQSL
>EECEBICD_04267 PTS system fructose-specific EIIB component
MANIVLCRIDSRLIHGQVVTKWVGQSQANRIAVVSDELDADPFMKNIYLMAAPPNIKVDC
FGNQSFAAAWKENQLGDGNVLVLFPSLAAVQDAIQLGFDVTRIQVGGLGGGPNRKAVFQN
ITLDEKDVGILNDLKNRGVQVFFQTIPEDKPQPLDDILKKF
>EECEBICD_04268 N-acetylgalactosamine permease IIC component 1
MDTLLFASLMGLYYWFARLRLGYTFSAMLLQPVVIAVFVGLLLGNMQTAMIIGAGMQLVY
LGVTSTPGGNVPSDPALAACISIPIAVKAGMDPNLAIALAIPFGVIGVFLDQLRRTLNAA
WVHMADKHAETANMAGIMRCAFLYPALLGLVLRFPVVFAANYFGQDVVESFLKLMPHWLT
HSFEIMGGILPALGFAITIMVIGKKSLLPWFIGGFFAVLYLKVDIMAMAIFGTCVAFLIK
GLAKNEGAA
>EECEBICD_04269 PTS system mannose-specific EIID component
MNSDVMQHELVERARESGALTKADITKAWFIYWLGAEVSSSYERLQSLIFCASMTPIIKK
LYPQKEEQVEALKRHLNFFNSEQTFGAVIQGISIAMEEQKTRGEPINDSSITGIKTGLMG
PLAGMGDSIIWAAVMPLLIAIFIPFAANGSAMGGIIPLILYPAITLAISYGMVHKGYTLG
RDSIIGLLQGGRIKELIYGANVLGLIMMGALSASYVKITTPLKISALKGSEVVVQQILDS
IAPGLLPLAAVFAIYFYLVKKGPRYTTILLSIVALSIVCSLLGVL
>EECEBICD_04270 D-glucosaminate-6-phosphate ammonia lyase

MTPNIYQQLGLKKVINACGKMTILGVSSVAPEVMQATARAASAFVEIDALVEKTGELVSR
YTGAEDSYITSCASAGIAIAVAAAITHGERARVALMPDSSGMANEVVMLRGHNVDYGAPV
TSAIRLGGGRIVEVGSSNLAARWQLESAINEKTAALLYVKSHHCVQKGMLSIDDFVQVAQ
ANHLPLIVDAAAEEDLRGWVASGADMVIYSGAKAFNAPTSGFITGKKTWIAACKAQHQGI
ARAMKIGKENMVGLVYALENYHQGQTTVTAAQLQPVAEAISAIHGLYADIEQDEAGRAIW
RIRVRVNASELGLNAQDVEAQLRGGEIAIYARKYQLHQGVFSLDPRTVAEGEMALIVARL
REIAEHAAD
>EECEBICD_04271 2-dehydro-3-deoxy-phosphogluconate aldolase
MQQINFYRQRVAINVLAKDIANAKAIYEAAEGHAVIGVLSAQFATVEEGVPEVKRWMAEV
PSISVGLGAGDPAQYYKAAMIAAHTHPAHVNQTFTGSGFAAGALAATGGEQTHINALVSP
TGTPGEVLISTGVSSSQGTPARVSCEAAVRMMQDMGAHAAKFFPMGGEKSLPELYALATT
AARHGMTLIEPTGGISLDNFGIILQTCLEAGVPRVMPHVYSSIIDPQTGNTRPEDVIRLM
EIVKALV
>EECEBICD_04272 ISNCY family transposase ISRor2
MKKSPTSTPHDAVFKTFLRHPDTARDFLNIHLPHSLRIRCDLTTLKLAPDSFIEKNLRAF
YSDVLWSLKTCEGDGYIYVVIEHQSTPDAHMAFRLMRYATAAMQRHLDAGHKTLPLVIPM
LFYHGAKSPYPFSLCWLDEFDDPALARQLYATAFPLVDITVVPDNEIMQHRRIAMLELVQ
KHIRQRDLMGLVERLAVLLITGNANDSQLKALFNYLLIQHGSTPRFGKFIREVARRVPQH
KERLMTIVDRIRESGRRKGKREGVQQGIQQGIHQGKQEEALRIAHTMLEQGIEREMVLMI
TGLSDEEIKAKRH
>EECEBICD_04273 putative inner membrane transporter YicL
MGSTRKGMLNVLIAAVLWGSSGVCAQYIMEQSRMSSQFLTMIRLLFAGLILVTFSFMHGD
KIFSILKNRKDALSLLIFSVVGALTVQLTFLLTIEKSNAATATVLQFLSPTIIVAWFALA
RRTRPGILVLTAILTSLIGTFLLVTHGNPTSLSISSGALFWGIASAFAAAFYTTWPSRLI
AQYGTLPVVGWSMSFGGLILLPFYAKEGTHFAVSGSLILAFFYLVVIGTSLTFSLYLKGA
QLIGGPKASILSCAEPLSSALLSLLLLGISFTLPDWLGTLLILSSVILISLDSRRRARAA
>EECEBICD_04274 Protein MgtC
MLMFPYILNLLAAMLLGALIGAERQWRQRMAGLRTNALVATGAAVFILSSMTTSPDSPGR
IAAQIVSGIGFLGAGVIMREGMNVRGLNTAATLWCSAGIGVLCGLGQFKNALAATIIILC
ANILLREAAQRINQLPVSAEGEKRYILKVTCNKEDESEVRQWLLNIVKEAAICLQGLGSV
PAQEQGYKEIRAELVGHADYRKTRELIISRIGDNDNITAIHWSIDSQ
>EECEBICD_04275 Magnesium-transporting ATPase, P-type 1
MTDMNIENRKLNRPASENDKQHKKVFPIEAEAFHSPEETLARLNSHRQGLTIEEASERLK
VYGRNEVAHEQVPPALIQLLQAFNNPFIYVLMALAGVSFITDYWLPLRRGEETDLTGVLI
ILTMVSLSGLLRFWQEFRTNRAAQALKKMVRTTATVLRRGPGNIGAVQEEIPIEELVPGD
VVFLAAGDLVPADVRLLASRDLFISQSILSGESLPVEKYDVMADVAGKDSEQLPDKDKSL
LDLGNICLMGTNVTSGRAQAVVVATGSRTWFGSLAKSIVGTRTQTAFDRGVNSVSWLLIR
FMLIMVPVVLLINGFSKGDWVEASLFALAVAVGLTPEMLPMIVSSNLAKGAIAMSRRKVI
VKRLNAIQNFGAMDVLCTDKTGTLTQDNIFLEHHLDVSGVKSSRVLMLAWLNSSSQSGAR
NVMDRAILRFGEGRIAPSTKARFIKRDELPFDFVRRRVSVLVEDAQHGDRCLICKGAVEE
MMMVATHLREGDRVVALTETRRELLLAKTEDYNAQGFRVLLIATRKLDGSGNNPTLSVED
ENELTIEGMLTFLDPPKESAGKAIAALRDNGVAVKVLTGDNPVVTARICLEVGIDTHDIL
TGTQVEAMSDAELAAEVEKRAVFARLTPLQKTRILQALQKNGHTVGFLGDGINDAPALRD
ADVGISVDSAADIAKESSDIILLEKDLMVLEEGVIKGRETFGNIIKYLNMTASSNFGNVF
SVLVASAFIPFLPMLAIHLLIQNLMYDISQLSLPWDKMDKEFLRKPRKWDAKNIGRFMLW
IGPTSSIFDITTFALMWYVFAANNVEAQALFQSGWFIEGLLSQTLVVHMLRTQKIPFIQS
RATLPVLLTTGLIMAIGIYIPFSPLGAMVGLEPLPLSYFPWLVATLLSYCLVAQGMKRFY
IKRFGQWF
>EECEBICD_04276 hypothetical protein
MNRSPDKIIALIFLLISLLVLCLALWQIVF
>EECEBICD_04277 hypothetical protein
MNNRRGLTAVLATLITFSLSATPVLANPGNGNGGGHGNNAANQGNNGNGHKGNAGQKTEH
RKNGGKPDHVESDISYAVARQLAVNLGLTGYQSLPPGIAKNLARGKPLPPGIAKKTVPAS
MLGQLPYYPGYEWKIVGDNLVLIALSTAVVTAIINGVFD

>EECEBICD_04278 putative hydrolase YcaC
MSTPANFNGQRPAIDANDAVMLLIDHQSGLFQTVGDMPMPELRARAAALAKIATLCNMPV
ITTASVPQGPNGPLIPEIHANAPHAQYVARKGEINAWDNADFVQAVKATGRKTLIVAGTI
TSVCMAFPAISAVAEGYKVFAVIDASGTYSKMAQEITMARVVQAGVVPMDTAAVASELQR
TWNREDAAEWADVYTKIFPAYQLLIESYTKAQEVVKNNELLDSQRA
>EECEBICD_04279 hypothetical protein
MSCYAATASDYTQWCLLTLSNYVMFWHGNNAMLWIINDNIEFNPEMNRLASLSRPDLNII
LTTPASRCLRLLLENAPSVVSQQTFFQKVWEEDGMVVSANTLYQNISIIRRGLRTVGENE
DTLIITVPRRGFQIEPGVSLMTIRKDFAQTIEKKGETPPRMSGRWFKHYVPVLWMTGTFA
VGILLGTISWQTVPDKDFYDRYTLVETTQGCHFFSRNEDIESGSRFASYKSMILKTGMDC
QKYPWVYFPSSSRTPAVTALICQQPYKTRGDTGCVTLFFRGVTHG
>EECEBICD_04280 hypothetical protein
MLACLTLLFLGVGLGHLFHLYTEKNRDPEKCTAPVIVFYNNTQANLTLDFMYSLKKRTGV
VSISGTYYVDNKMSGVIRRDVSYVWSENKDSTHFISTDINKVTRDETLSDAVIETVLPDF
YVYPGKSISYTILTQGHRGFMFTIGKRPIFFCTH
>EECEBICD_04281 hypothetical protein
MPTPQNYSFIAIAVSAALASMVFPSQAAWVDVDSLPSSGLVSQLPPELQAIIPAQASTGF
TKVGTMPNYVYQWNTGTIPVYGNGLTLNGPGAEAFEHTVTVIQNSGTGNPGVIFGNDLTI
RTQSANAANNGRDVDGIRTHGANTPDNPVFIITGDRTNIYVDGQDGDGINAGYNSLGQGW
TGSANIYVGDDLYIKTTGSQGRGITANAMRDASRAKNTIVVGNRAHIVTTGDSSEGLRTG
QSGSLIRLGDDATIETSGASSTGIYAASSSRTELGNNATITVNGASAHAVYATNATVNLG
ENATISVNSASKAASYSKAPAGLYALSRGAINLAGGAAITMAGDNSSESYAISTETGGIV
DGSSGGRFVIDGDIRAAGATAASGTLPQQNSTIKLNMTDNSRWDGASYITSATAGTGVIS
VQMSDATWNMTSSSTLTDLTLNSGATINFSHEDGEPWQTLTINEDYVGNGGKLVFNTVLN
DDDSETDRLQVLGNTSGNTFVAVNNIGGAGAQTIEGIEIVNVAGNSNGTFEKASRIVAGA
YDYNVVQKGKNWYLTSYIEPDEPIIPDPVDPVIPDPVVPDPVDPDPVDPVIPDPVIPDPV
DPDPVDPEPVDPVIPDPTIPDIGQSDTPPITEHQFRPEVGSYLANNYAANTLFMTRLHDR
LGETQYTDMLTGEKKVTSLWMRNVGAHTRFNDGSGQLKTRINSYVLQLGGDLAQWSTDGL
DRWHIGAMAGYANSQNRTLSSVSDYHSRGQVTGYSVGLYGTWYANNIDRSGAYVDTWMLY
NWFDNKVMGQDQAAEKYKSKGITASVEAGYSFRLGESAHQSYWLQPKAQVVWMGVQADDN
REANGTLVKDDTAGNLLTRMGVKAYINGHNAIDDNKSREFQPFVEANWIHNTQPASVKMD
DVSSDMRGTKNIGELKVGIEGQVTPRLNVWGNVAQQVGDTGYSDTRGMLGMKYSF
>EECEBICD_04282 HTH-type transcriptional regulator EcpR
MMRKPSQIVHCISCDLSCQLFPDSAVRVQYCHNAAFSIWPDGNAFLKKGFIEKLLLDRHN
HLSSGFIFVDFSFPNLRRFTDLQWADSLADSGMHIVLISDRSLTPLANYWILKSNKIQGI
IYSDDDDIVQQQKMHRLFTGRLANSKRGRTLNYTEFILLKRFVSGISIQQIVNIDNIDIK
KLYVHKLRLENKLGHSIHKIISNIL
>EECEBICD_04283 hypothetical protein
MSGKRYPEEFIIKAVKQVIERGHSVSSVATRLDITTHSLYAWIKPPYSRRYHAITGV
>EECEBICD_04284 hypothetical protein
MADKYLTQSPAGEFVMFASDDGEVRVECRFEQETLWLPQATIANLYQITPQAVTQHIKAI
YEEGELEQNATCKSYLQVQQEGSRQVSRNRLHYSLPVILAVGYRVRSPRGTQFRQWATQT
LQKYLIKGFVMDDERLKNPPVGSSAVPDYFDEMLERIRDIRASERRVYLRVREIFALAAD
YQPSLKETTQFFQTIQNKLHFACTGHTAAELIHQRADASQPHMGLTSYKGEEVRKGDVTV
AKNYLTQDEVSELNRVVNMWLDFAEDQARRRQQIFLRDWQDKLDQFLQFNDREVLQGAGK
VTKKMADEKAQAEYSQFAEQQRRLKEAEGEKDIAGLLQWKTEPKK
>EECEBICD_04285 hypothetical protein
MGVDPQPPVKEQDVFERGIINVFKGLSQEYKTNNPCYFGKKIIVNNLVKHDRWGYSLNWG
WRRDQLADLERMLYLLDSKTIPDNRHDVSIRFMDFVRDNPREQVFEDDMFTIRYF
>EECEBICD_04286 hypothetical protein
MKEAIQAIQQTINCLTKDVTGWRDLEYSSSHNQQLVMNHPQHGKIPLAQLSDGLRNAVAM
VADIAFRCVKLNPHLRDEAALKTQGIVLIDEVDMFLHPAWQQQIIQSLRSAFPLIQFIVT
THSPQVISTVKRESVRLLEQDEKGNGLASIPPGRPTASQVMMYCTA
>EECEBICD_04287 hypothetical protein

MIRTEDVRLVFRPEALNMEMSSPVMITATGEWESGKTATWMLDKRGEQPPHEDKTAAQLT
RWGEQLQTLVREEHNLLQVELLLMLYLGTARLWYQERYEAQPTEQRLDNSAFSRLSGYDD
CLSATSNYKQFEQWYSWLWHSYREHQITQLESPSTKLKEDVRIRG
>EECEBICD_04288 DNA replication and repair protein RecF
MRIDKLSLLHFRCFRQLDITFDEHITILVAPNGAGKTTVLDAVRLALFPFIRGLPASM
>EECEBICD_04289 hypothetical protein
MSYKYVGKHGCDVALRMGYKECPDENAYGDAYYIKDGLKWIFNITGLKKRLGVYSDDDLR
KQNYDVDTYYRVENQPEESADDEMQSLYHNLAVEEGEPVYLEGGMYLYPDGSIR
>EECEBICD_04291 Inner membrane symporter YicJ
MKSEILSVKEKIGYGMGDAASHIIFDNVMLYMMFFYTDIFGIPAGFVGTMFLLARALDAI
SDPCMGLLADRTRSRWGKFRPWVLFGALPFGIVCVLAYSTPDLSLNGKMIYAAITYTLLT
LLYTVVNIPYCALGGVITNDPTQRISLQSWRFVLATAGGMLSTVLMMPLVKLIGGENKAL
GFQGVSRRSRWWRS
>EECEBICD_04292 Inner membrane symporter YicJ
MLAFCFFTTKERVEAPATHTSMREDLRDIWHNDQWRIVGLLTILNILAVCVRGGAMMYYV
TWILGKPGVFVAFLTTYCVGNLIGSALAKPLTDWKCKVSVFWWTNALLAVISVAMFFVPM
HATIAMFVFIFVIGVLHQLVTPIQWVMMSDTVDYGEWCNGKRLTGISFAGTLFVLKLGLA
LGGALIGWMLAGGGYDAAAKTQNSATISIIIALFTIVPAICYLLSAAIAKRYYTLKSPFL
KTILEQLAQGAHRNEQEFTHKKLQN
>EECEBICD_04293 Alpha-xylosidase
MKISDGNWLIQPGLNLIHPVQVFDVEQHGNEMVIYAAPRDVRERTWQLDTPLFTLRFFSP
QEGVIGVRMEHFQGALDNGPHYPLNVLQDINVEMQNNAEFAELKSGSLSVRVTKGEIWSL
DFLRNGVRITGSQLKNNGYVQDTNSGRNYMFERLDLGVGETVYGLGERFTALVRNGQTVE
TWNRDGGTSTEQSYKNIPFYITNRGYGVLVNHPQCVSFEIGSEKVSKVQFSVESEYLEYF
VIDGPTPKDVLNRYTQFTGRPALPPAWSFGLWLTTSFTTNYDEATVNSFIDGMAERNLPL
HVFHFDCFWMKAFQWCDFEWDPVTFPDPKGMIRRLKAKGLKVCVWINPYIGQKSPVFQEL
KEKGYLLKRPDGSLWQWDKWQPGLAIYDFTNPQACEWYADKLKGLVEMGVDCFKTDFGER
IPTDVQWFDGSDPQKMHNHYAYIYNELVWNVLKETVGVEEAVLFARSASVGAQQFPVHWG
GDCYANYESMAESLRGGLSIGLSGFGFWSHDIGGFENTAPAHVYKRWCAFGLLSSHSRLH
GSKSYRVPWAYDDESCDVVRFFTEQKCRMMPYLYREAARANEAGTPMMRAMMLEFPDDPA
CDYLDRQYMLGDAVMVAPVFSEAGDVEFYLPEGRWTHLWRNDEVQGSRWHKQQHDFLSLP
VYVRDNTLLALGNNSQKPDYAWHEGTAFQLFHLDDGCEAVCEVPATDGSTIFTLQAKRTG
NTITVSGEGEARNWTLCLRNITQISGTKCGSYAGSELGVVVTPLGNEVVITL
>EECEBICD_04294 hypothetical protein
MKLIGRLLLYVLIACLVVIFGFYFLLQTRWGADHVSNWVSENSGYHLTFDVMDHRFSAPS
HLLLENVTFGRDGQPATLVAKTVDIGLSIRQLTAPLHVDTILLQDGTLNISVQTAPFPFE
ADRLQLRNMALNSPGSEWRLSAQRVNGGVMPWHPEAGRVLGNKAQIQLSAGSLTLNDVPA
TNVLIEGSIDHDQVMLNTVGADMARGALTGVARRNADGSWVVENLRLNDIRLQSDKSLSE
FFAPLTTVPSLQIGRLEVTDSSLQGPDWAVTDLDLSLRNLTLRKEDWQSQEGKLSMNASE
FIYGSLHLLDPILNAEFSPQGVALRQFTTRWEGGMVRTSGAWLREGKALILDDTAIAGLE
YTLPENWKQLWMKPLPDWLNSLTLKKFSASRNLVIDIDPAFPWQITALDGYGANLELVQH
HQWGVWSGNATLNAAAATFNRVDVRRPSLSLTANASTVNISDLSAFTGKGMLEATASVSQ
LPQRQTQISLNGRGVPMDVLQQWGWPALPIAGDGNIQLTASGNIQADAPLKPTVNGQLHA
VNAQKQQITQTMQAGVVSGGEVTSTEPAL
>EECEBICD_04295 Xanthine permease XanP
MSVNAIESADAQPVAQTQNSELIYRLEDRPPLPQTLFAACQHLLAMFVAVITPALLICQA
LGLPAQDTQHIISMSLFASGVASIIQIKAWGPVGSGLLSIQGTSFNFVAPLIMGGTALKT
GGADVPTMMAALFGTLMLASCTEMVLSRILHLARRIITPLVSGVVVMIIGLSLIQVGLTS
IGGGYAAMSDNTFGAPKNLLLAGVVLAIIILLNRQRNPYLRVASLVIAMAAGYLLAWFMG
MLPENNAPVSQDILMVPTPLYYGLGIDWNLLLPLMLVFMITSLETIGDITATSDVSEQPV
SGPLYMKRLKGGVLANGLNSFVSAVFNTFPNSCFGQNNGVIQLTGVASRYVGFVVALMLI
VLGLFPAVSGFVQHIPEPVLGGATLVMFGTIAASGVRIVSREPLNRRAILIIALSLAVGL
GVSQQPQILQFAPEWVKNLLSSGIAAGGITAIVLNLILPPEKP
>EECEBICD_04296 Sodium/glutamate symporter

MFELDTLSTLVAATLVLLLGRKLVQSVSLLKKYTIPEPVAGGLLVAVALLVLKKSVGWEV
NFDMTLRDPLMLAFFATIGLNANLASLRKGDALLASSWSSWSDYC
>EECEBICD_04297 Sodium/glutamate symporter
MQNAIGIGMASLLGLDPLMGLIAGSITLSGGHGTGAAWSKLFVERYGFANATEVAMACAT
FGLVLGGLIGGPVARYLVKHSTTPDGMPDDQAVPTAFEKPDVGRMITSLVLIETIALIAI
CLTVGKIVAQLLAGTVLELPVFVCVLFVGVILSNGLALVGFYRVFERAVSVLGNVSLSLF
LAMALMSLKLWELASLALPMLAILVVQTIFMALYAIFVTWRMMGKNYDAAVLAAGHCGFG
LGATPTAIANMQAITDRFGPSHMAFLVVPMVGAFFIDIVNALVIKLYLMLPIFAQ
>EECEBICD_04298 ATP-dependent DNA helicase RecG
MSGRLLDAVPLNSLTGVGAAQSSKLAKIGLHTVQDLLLHLPLRYEDRTHLYPIGELLPGI
YATVEGEVLNCNITFGGRRMMTCQISDGSGILTMRFFNFNAAMKNSLATGRRVLAYGEAK
RGKYGAEMIHPEYRLQGDLSTPELQETLTPVYPTTEGVKQATLRKLTDQALELLDTCAIA
ELLPPELAQGMMSLPEALRTLHRPPPTLQLADLETGKHPAQRRLILEELLAHNLSMLALR
AGAQRYHAQPLSTNNILKDKLLASLPFKPTSAQARVVAEIERDMALDVPMMRLVQGDVGS
GKTLVAALAALRAIAHGKQVALMAPTELLAEQHANNFRNWFEPLGVEVGWLAGKQKGKAR
QAQQEAIASGQVQMIVGTHAIFQEQVQFNGLALVIIDEQHRFGVHQRLALWEKGQQQGFH
PHQLIMTATPIPRTLAMTAYADLDTSVIDELPPGRTPVTTVAIPDTRRHEIIDRVRNACT
TEGRQAYWVCTLIEESDLLEAQAAEATWEELKLALPELNIGLVHGRMKPAEKQAVMQAFK
QGELHLLVATTVIEVGVDVPNASLMIIENPERLGLAQLHQLRGRVGRGAVASHCVLLYKS
PLSKTAQKRLQVLRDSNDGFVIAQKDLEIRGPGELLGTRQTGNAEFKVADLLRDQAIIPE
VQRIARHIHERYPQQAQALIERWMPETERYSNA
>EECEBICD_04299 tRNA (guanosine(18)-2'-O)-methyltransferase
MNPKRYARICEMLARRQPDLTVCMEQVHKPHNVSAIIRTADAVGVHEVHAIWPGSRMRTM
ASAAAGSNSWVQVKTHRTIGDAVAHLKGRGMQILATHLSDKAVDFREIDYTRPTCILMGQ
EKTGITQEALDLADRDIIIPMIGMVQSLNVSVASALILYEAQRQRQNAGMYLRENSMLPE
DEQQRLLFEGGYPVLAKVAKRKGLPYPRVNQQGEIDADADWWATMQAAG
>EECEBICD_04300 Bifunctional (p)ppGpp synthase/hydrolase SpoT
MYLFESLNQLIQTYLPEDQIKRLRQAYLVARDAHEGQTRSSGEPYITHPVAVACILAEMK
LDYETLMAALLHDVIEDTPATYQDMEQLFGKSVAELVEGVSKLDKLKFRDKKEAQAENFR
KMIMAMVQDIRVILIKLADRTHNMRTLGSLRPDKRRRIARETLEIYSPLAHRLGIHHIKT
ELEELGFEALYPNRYRVIKEVVKAARGNRKEMIQKILSEIEGRLQEAGIPCRVSGREKHL
YSIYCKMVLKEQRFHSIMDIYAFRVIVHDSDTCYRVLGQMHSLYKPRPGRVKDYIAIPKA
NGYQSLHTSMIGPHGVPVEVQIRTEDMDQMAEMGVAAHWAYKEHGETSTTAQIRAQRWMQ
SLLELQQSAGSSFEFIESVKSDLFPDEIYVFTPEGRIVELPAGATPVDFAYAVHTDIGHA
CVGARVDRQPYPLSQPLSSGQTVEIITAPGARPNAAWLNFVVSSKARAKIRQLLKNLKRD
DSVSLGRRLLNHALGGSRKLAEIPQENIQRELDRMKLATLDDLLAEIGLGNAMSVVVAKN
LQQGEAVVPTVAQSNHGHLPIKGADGVLITFAKCCRPIPGDPIIAHVSPGKGLVIHHESC
RNIRGYQKEPEKFMAVEWDKETEQEFITEIKVEMFNHQGALANLTAAINTTTSNIQSLNT
EEKDGRVYSTFIRLTARDRVHLANIMRKIRVMPDVIKVTRNRN
>EECEBICD_04301 DNA-directed RNA polymerase subunit omega
MARVTVQDAVEKIGNRFDLVLVAARRARQMQVGGKDPLVPEENDKTTVIALREIEEGLIN
NQILDVRERQEQQEQEAAELQAVTAIAEGRR
>EECEBICD_04302 Guanylate kinase
MAQGTLYIVSAPSGAGKSSLIQALLKTQPLYDTQVSVSHTTRAPRPGEVHGEHYFFVNHD
EFKTMIGREAFLEHAEVFGNYYGTSRETIEQVLATGVDVFLDIDWQGAQQIREKMPQARS
IFILPPSKIELDRRLRGRGQDSEEVIAKRMAQAVAEMSHYAEYDYLIVNDDFDTALSDLK
TIIRAERLRMSRQKQRHDALISKLLAD
>EECEBICD_04303 DNA ligase B
MRLWKSMAWGILLWHSQSGALCPAWPPARAAEEITRLQQQLADWNDIYWKQGVSAVDDSV
YDQLSARLVQWQRCVGQDVSSTPVSPPLNGTTMHPVAHTGVRKLADRQAVEQWMRGRSEL
WVQPKVDGVAVTLVYQNGKLTRAISRGNGLQGEDWTPKIRLIPSIPQTTQGALANAVLQG
EIFLQREGHIQQRMGGMNARSKVAGMLMRQDNASALNSLGIFIWAWPDGPANMPERLSQL
AKAGFSLTNKYTLAVKDASEVERARQSWLTSALPFVTDGVVIRMAKEPASQHWRPGQGDW
LAAWKYPPVAQVAQVSAIQFSVGKSGKITVVASLVPVILDDKRVQRVNIDSVKRWEAWDI

APGDQILVSLAGQGIPRLDEVVWRSRERSKPVPPDSHFNSLTCFYASETCQEQFISRLVW
LGSRSALGLDGMGEASWRALHQTHRFKHIFSWLALTSAQIANTPGFAKGKSEQIWRQFNL
ARRQSFTRWIMAMDIPLTQAALQASGDRSWEQLLMRTEQHWRQLPATGERRAGRVIDWRN
NPQIKTLSRWLAAQHIPGFGS
>EECEBICD_04304 hypothetical protein
MLLHVLYLIGITAEAMTGALAAGRRRMDTFGVIIIATATAIGGGSVRDILLGHYPLGWVK
HPEYVIIVATAAVLTTIVAPVMPYLRKLFLVLDALGLVVFSIIGAQIALDMGEGPIIAVV
AAVTTGVFGGVLRDMFCKRIPLVFQKELYAGISFASAVLYIALQHYVMNPDVVVISTLLF
GFTARLLALRLKLGLPVFYYRHEGH
>EECEBICD_04305 hypothetical protein
MNRYALFFVCIFSTSALPAMAALDPSQPLSPTPPLSLFKAWAKPIKPFQITEGVWYVGTK
NLSSILLTTPAGHILIDAGLDESAPQIKANIEAAGFRLTDVRYLLNSHARLDQAGGMARL
KAWSGAQLVASQPNADQMARGGRQDFALGDALPFPPVTTDKIIHHQESIILGGITVTALF
TPGHLPGSTSWRVTLRNGKTLIYADSLATPDYLLINNKNYPDLVTDIQGSFKTLAAQHVD
IFIANKGDRFGLLEKRQQLRNGDTQAFFDSNGLQQYVERSRQRFITQLTAQQP
>EECEBICD_04306 HTH-type transcriptional regulator YidZ
MNLLQMDMNLLKVLYVLLETSSTGKTAQKLALSPSAVSHALMRLRDALNDPLFRREGNRQ
IATPYAQALRDKLAPVFVSLNEELFGDKENGSRCFRVVLPPALNALLTPVLAEKGHLHQA
VIECLPFARRPWRDELLDSSVDLVLAIGDHQKQVSALHYERVGTTRLIAVYGAPLRSRLE
NATALNFADLQHYQHIYCLPWTKENNELDRQQARAGFERPLAFVCHDYSQLAPAVQSAPL
MAIVPRPWYENLSDKRGLFVLPLAGEQAEGAIFMQYRASTVEWKRRLIDAIRRKLKTYYR
>EECEBICD_04307 hypothetical protein
MIRSMTAYARREIKGEWGSATWEMRSVNQRYLETYFRLPEQFRSLEPVVRERIRTRLTRG
KVECMLRFEPDASAQGELILNEKLAKQLVSAANWVKMQSDEGEINPVDILRWPGVMAAQE
QDLDAIAAEILAALDGTLDDFIVARETEGQALKALIEQRLEGVSAEVAKVRAHMPEILQW
QRERLVAKLEDAQVQLENNRLEQELVMMAQRIDVAEELDRLEAHVKETYNILKKKEAVGR
RLDFMMQEFNRESNTLASKSINADVTNSAIELKVLIEQMREQIQNIE
>EECEBICD_04308 Ribonuclease PH
MRPAGRSANQVRPVTLTRNYTKHAEGSVLVEFGDTKVLCTASIEEGVPRFLKGQGQGWIT
AEYGMLPRATHTRNAREAAKGKQGGRTMEIQRLIARALRAAVDLKTLGEFTITLDCDVIQ
ADGGTRTASITGACVALADALNKLVANGKLKTNPMKGMVAAVSVGIVNGEAICDLEYVED
SAAETDMNVVMTEDGRIIEVQGTAEGEPFSHEELLTLLALARGGIESIVATQKAALEN
>EECEBICD_04309 Orotate phosphoribosyltransferase
MKPYQRQFIEFALNKQVLKFGEFTLKSGRKSPYFFNAGLFNTGRDLALLGRFYAEALVDS
GIEFDLLFGPAYKGIPIATTTAVALAEHHDKDLPYCFNRKEAKDHGEGGSLVGSALQGRV
MLVDDVITAGTAIRESMEIIQAHGATLAGVLISLDRQERGRGEISAIQEVERDYGCKVIS
IITLKDLIAYLEEKPDMAEHLAAVRAYREEFGV
>EECEBICD_04310 Nucleoid occlusion factor SlmA
MAEKQTAKRNRREEILQSLALMLESSDGSQRITTAKLAASVGVSEAALYRHFPSKTRMFD
SLIEFIEDSLITRINLILKDEKNTSTRLRLIVLLILGFGERNPGLTRILTGHALMFEQDR
LQGRINQLFERIEAQLRQVLREKRMREGEGYTTDENLLASQLLAFCEGMLSRFVRSEFKY
RPTDDFDARWPLIAAQLQ
>EECEBICD_04311 Deoxyuridine 5'-triphosphate nucleotidohydrolase
MMKKIDVKILDPRVGQQFPLPTYATSGSAGLDLRACLDDAVELAPGATTLVPTGLAIHIA
DPSLAAVMLPRSGLGHKHGIVLGNLVGLIDSDYQGQLMVSIWNRGQDSFTIEPGERIAQM
VFVPVVQAEFNLVEAFDATERGEGGFGHSGRK
>EECEBICD_04312 Coenzyme A biosynthesis bifunctional protein CoaBC
MMSLAGKKIVLGVSGGIAAYKTPELVRRLRERGADVRVAMTEAAKAFITPLSLQAVSGYP
VSDSLLDPAAEAAMGHIELGKWADLVILAPATADLIARVASGMANDLVSTICLATPAPVA
VLPAMNQQMYRAAATQHNLEVLASRGLFIWGPDSGSQACGDVGPGRMLDPLVIVDKAAAQ
IAAVNDLRHINIMITAGPTREPLDPVRYISNHSSGKMGFAIAAAAARRGANVTLVSGPVS
LPTPPFVKRVDVITALDMEAAVQAAVQQQHIFISCAAVADYRAETIASEKIKKQAAQSDE
LIIKMVKNPDIVAGVAALSDNRPYVVGFAAETNNVEEYARQKRIRKNLDLICANDVSLSN
QGFNSDKNALHLFWQDGDKVLPLERKELLGQLLLDEIVTRYDEKNRR

>EECEBICD_04313 hypothetical protein
MDTLDELLPREKMLRSGIASLSDVELLALFYVPARREKML
>EECEBICD_04314 hypothetical protein
MTLAKEILQHFGSLYGLLSADFAQFRGVNGIGLAKFAQLKGIAELARRYYSVRMNEESAL
LSPEMTREFLQSQLTGEEREIFLVIFLDAQHRVLQHSRLFSGTLNHVEVHPREIVREAIK
LNASAVILAHNHPSGCAEPSKADKLITERVIKCCQFMDIRVLDHLIIGRGEYVSFAERGW
I
>EECEBICD_04315 50S ribosomal protein L28
MSRVCQVTGKRPVTGNNRSHALNATKRRFLPNLHSHRFWVESEKRFVTLRVSAKGMRIID
KKGIETVLSELRARGEKY
>EECEBICD_04316 50S ribosomal protein L33
MAKGIREKIKLVSSAGTGHFYTTTKNKRTKPEKLELKKFDPVVRQHVIYKEAKIK
>EECEBICD_04317 Formamidopyrimidine-DNA glycosylase
MPELPEVETSRRGIEPHLVGATILHAHIRNGRLRWPVSDEIYRLSDTPVLSVQRRAKYLL
LELPDGWIIIHLGMSGSLRILSEALPAEKHDHVDLVMSNGKILRYTDPRRFGAWLWTKEL
EGHNVLAHLGPEPLSDEFNGEYLQQKCAKKKTAIKPWLMDNKLVVGVGNIYASESLFAAG
IHPDRLASSLSTEECDLLARVIKAVLLRSIEQGGTTLKDFLQSDGKPGYFAQELQVYGRK
GEPCRVCGTPIVATKHAQRATFYCRHCQK
>EECEBICD_04318 Phosphopantetheine adenylyltransferase
MQKRAIYPGTFDPITNGHLDIVTRATQMFDHVILAIAASPGKKPMFTLDERVALAQKATA
HLGNVEVVGFSDLMANFARDRQANILIRGLRAVADFEYEMQLAHMNRHLMPQLESVFLMP
SKEWSFISSSLVKEVARHQGDVTHFLPDNVHQALMDKLK
>EECEBICD_04319 3-deoxy-D-manno-octulosonic acid transferase
MLELLYTALLYLIQPLIWIRLWVRGRKAPAYRKRWGERYGFYRRPLKPGGIMLHSVSVGE
TLAAIPLVRALRHRYPDLPITVTTMTPTGSERVQSAFGNDVQHVYLPYDLPDALNRFLNK
IDPKLVLIMETELWPNLIAALHKRHIPLVIANARLSARSAAGYAKLGKFVRTLLRRITLI
AAQNEEDGERFVALGAKNNQVTVTGSLKFDISVTPQLAAKAVTLRRQWAPHRPVWIATST
HDGEESIVIAAHQALLHQFPNLLLILVPRHPERFPDAINLVRQAGLSYITRSSGEVPSAS
TQVVVGDTMGELMLLYGIADLAFVGGSLVERGGHNPLEAAAHAIPVLMGPHTFNFKDICA
RLDQASGLITITDAATLAKEVSSLLTDADYRNFYGRHAVEVLYQNQGALQRLLQLLEPYL
PPKTH
>EECEBICD_04320 Lipopolysaccharide core heptosyltransferase RfaQ
MEKPFRRILIIKMRFHGDMLLTTPVISTLKQNYPDAKIDVLLYQNTIPILSENPEINALY
GISNKGAGTKEKIKNALSLIKKLRANSYDLVVNLTDQWSVALIVRFLNAKIKISQDFGNR
QSALWKKSFTHLVPYAGEHAVERTLSALKPLALKQYVTETTMSYRPEHWENMRQQLKQLG
VTRQYVVIQPTARQLFKCWDNDKFSQVIDAVQRRGYQVVLTSGPAADEMACVDAIARGCE
TKPVTGLAGKTRFPELGALIDHADLFIGVDSAPGHIAAAVKTPVICLFGATDHVFWRPWT
DDIIQFWAGNYQPMPERHELDRNKKYLSVIPAEDVIAATEKMLPHEARPIDLDSLL
>EECEBICD_04321 Lipopolysaccharide core biosynthesis protein RfaG
MRVAFCLYKYFPFGGLQRDFMRIAQTVAARGHQVRVYTQSWEGECPDNFELIRVPVKSRT
NHGRNAEYYAWVQHHLRDHPVDRVVGFNKMPGLDVYYAADVCYAEKVAQEKGFFYRLTSR
YRHYAAFERATFEHGKPTQLLMLTNKQIADFQKHYQTEAERFHILPPGIYPDRKYSQQIP
NSRQIYRQKNGISEQQKLLLQVGSDFTRKGVDRSIEALASLPESLRQNTVLYVVGQDKPK
KFAALAERSGVGTNVHFFSGRNDIAELMAAADLLLHPAYQEAAGIVLLEAITAGLPVLTT
AVCGYAHYIVDANCGEAMTEPFRQDALNEVLLKALTQPSLRNAWAENARYYADTQDLYSL
PEKAADIITGDLDG
>EECEBICD_04322 Lipopolysaccharide core heptose(I) kinase RfaP
MVELKAPLTTLWRGKDAFEEVKTLQGEVFRELETRRTLRFELDGKSYFLKWHKGTSLKEI
VKNLISLRMPVLGADREWHAIHRLHELGVDTMHGVGFGEKGVNPLTRTSFIITEDLTPTI
SLEDYCADWAVNPPDAQVKWMIIKRVATMVRKMHAGGINHRDCYICHFLLHLPFTGREED
LKISVIDLHRAQIRQRVPLRWRDKDLIGLYFSSMNIGLTQRDIFRFMREYFSLPLREILQ
KESGLIHQADVKAARIKERTIRKNL
>EECEBICD_04323 hypothetical protein
MSDIKSYISNQKNIIQHDDFFGRGLDIALCFDHGFIMPAGVAIYSIIENNKDIEYALKQK

KRCQVKITP
>EECEBICD_04324 Lipopolysaccharide 1,6-galactosyltransferase
MKIAFIGEAVSGFGGMETVISNVIHTFENSSPKINCEMFFFCRNDKMDKAWLKEIKYAQS
FSNIKLSFLRRAKHVYNFSQWLKETSPDIVICIDIISCLYANKARKKSGKQFTIFSWPHF
SLDHKKHAECITYADYHLAISSGIKEQMMARGISAQNISVVYNPVSIKTIIVPPPERDKP
AVFLYVGRLKFEGQKRVKDLFDGLTRTTGEWQLHIIGDGSDFEKCQAYSRELGIEQRVIW
YGWQSAPWQVVQQKIKNVTALLLTSAFEGFPMTLLEAMSYGIPCISSDCMSGPRDMIKPG
LNGELYTPGAIDDFVGHLNRVISGEVKYQHDIIPGTIERFYDVLYFKNFNNAIFSKLQK
>EECEBICD_04325 hypothetical protein
MSRKYFEEEVIQQTLDYNYAQHSDAAKFNIAYGIDKNFLFGCGVSIASVLLANPEKALAF
HVFTDFFGSEDQQRFEALAKQYATQIVVYLIDCERLKSLPSTKNWTYATYFRFIIADYFS
DKTDRVLYLDADIACKGSIQELIDLNFAENEIAAVVAEGELEWWTKRSVSLATPGLVSGY
FNAGFILINIPLWTAENISKKAIEMLKDPEVVQRITHLDQDVLNILLVNKARFVDKKFNT
QFSLNYELKDSVINPVDAETVFVHYIGPTKPWHSWGAYPVSQYFLQAKSNSPWSHCALLN
PVTSHQLRYAAKHMFNQKHYTSGINYYIAYFKRKLLE
>EECEBICD_04326 hypothetical protein
MDSFPEIEIAEYKVFDESNNNNDDNVLNISYGVDENYLDGVGVSIASVVLNNNIPLAFHI
ICDSYSPCFVKYIERLAVQHHIKISLYLIKVESLEVLPQTKVWSRAMYFRLFAFDYLSKK
VNTLLYLDADVVCKGSLQDLLRLDLTEKIAAVVKDVDSIQNKVNERLSAFNLQGGYFNSG
VVFVNLKLWKENALTKKAFLLLAGKEADSFKYPDQDVLNILLQDKVIFLPRPYNTIYTIK
SELKDKSHKKYSNIINDNTVLIHYTGATKPWHAWANYPSVIYYKNARLNSPWKDSPAKDA
RTIVEFKKRYKHLLVQGHYFKGLMAGSAYLYRKLFHK
>EECEBICD_04327 Lipopolysaccharide core heptose(II) kinase RfaY
MIIEKKIKNYTVFVKKDGEKYIEIFKDFLSYNHQVIKVFRNIEDTKVVLINTDYGKYILK
VFSPKVKNTERFFKSLVKGDYYEKLFHQTDRVRREGFTALNDFYLLAEIKTLRYVKTYVM
IIEYIEGIELVDMPEISDEVRGKIKQSIYSLHQHGMVSGDPHKGNFILQGNEIRIIDLSG
KRPSRQRKAKDRIDLERHYGIKNNVRDIGFYLLIYKKKLRNFLRRIKGKEKR
>EECEBICD_04328 hypothetical protein
MFCNLLRKERLKIVLFFLSGPTSRKTPLSLLRMKDVIAVNGSVQYLLNNNVKPFLYLLTD
VRFLHRRREDFYNFSRNSQFTIVNLDVYEQASVDDQKYIEENCLIIRSFYRREKGGFLKK
IKFNILKRVHKALLISVPLSKRGRLAGFCKDISIGYCSCHTIAYTAIQVAYSLKYGRIIC
SGLDLTGSCPRFYDESTSPMPSELSKDLFKILPFFTFMRKNVSDLNIFNLSDDTAIHYDI
IPYITASELEDEIYYDKIV
>EECEBICD_04329 D-inositol-3-phosphate glycosyltransferase
MIKKIIFTVTPIFSIPPRGAAAVETWIYQVAKRLSIPNAIACIKNAGYPEYNKINDNCDI
HYIGFSKVYKRLFQKWTRLDPLPYSQRVLNIRDKVTTQEDSVIVIHNSMKLYRQIRERNP
NAKLVMHMHNAFEPELPDKDAKIIVPSQFLKAFYEERLPAAAVSIVPNGFCAETYKRNPQ
NNLRQQLNIAEDATVLLYAGRISPDKGILLLLQAFKKLRTLRSNIKLVVVGDPYASRKGE
KAEYQKKVLDAAKEIGTDCIMAGGQSPDQMHNFYHIADLVIVPSQVEEAFCMVAVEAMAA
GKAVLASKKGGISEFVLDGITGYHLAEPMSSDSIINDINRALADKERHQIAEKAKSLVFS
KYSWENVAQRFEEQMKSWFDK
>EECEBICD_04330 hypothetical protein
MLTTSLTLNKEKWKPIWNKALVFLFVATYFLDGITRYKHLIIILMVITAIYQVSRSPKSF
PPLFKNSVFYSVAVLSLILVYSILISPDMKESFKEFENTVLEGFLLYTLLIPVLLKDETK
ETVAKIVLFSFLTSLGLRCLAESILYIEDYNKGIMPFISYAHRHMSDSMVFLFPALLNIW
LFRKNAIKLVFLVLSAIYLFFILGTLSRGAWLAVLIVGVLWAILNRQWKLIGVGAILLAI
IGALVITQHNNKPDPEHLLYKLQQTDSSYRYTNGTQGTAWILIQENPIKGYGYGNDVYDG
VYNKRVVDYPTWTFKESIGPHNTILYIWFSAGILGLASLAYLYGAIIRETASSTFRKVEI
SPYNAHLLLFLSFVGFYIVRGNFEQVDIAQIGIITGFLLALRNR
>EECEBICD_04331 Lipopolysaccharide heptosyltransferase 1
MRVLIVKTSSMGDVLHTLPALTDAQQAIPGIQFDWVVEEGFAQIPSWHSAVDRVIPVAIR
RWRKAWFSAPIKAERTAFHRAVCAYQYDAVIDAQGLVKSAALVTRLAHGIKHGMDWSTAR
EPLASLFYNRKHHIAKQQHAVERTRELFAKSLGYDKPQSQGDYAIAKHFLHCQQAVSDPY
AVFLHATTRDDKHWPEANWRELIGMVGNTGLRIKLPWGAPHEEARAKRLAEGFDYVDVLP

RMSLEEVARVLAGAKFVVSVDTGLSHLTAALDRPNITLYGPTDPGLIGGYGKNQMACCSP
EKNLANLDATSVFGKIH
>EECEBICD_04332 ADP-heptose--LPS heptosyltransferase 2
MKILVIGPSWVGDMMMSQSLYRTLKARYPQAIIDVMAPAWCRPLLSRMPEVNEAIPMPLG
HGALEIGERRRLGHSLREKRYDRAWVLPNTFKSALVPFFANIPHRTGWRGEMRYGLLNDA
RVLDKDAWPLMVERYVALAYDKGVMRTAKDLPQPLLWPQLLVSEGEKSLIRSDFSLSSER
PLIGFCPGAEFGPAKRWPHYHYAELAKQLINEGYQVVLFGSAKDHEAGNEILAALNSEQQ
AWCRNLAGETQLEQAVILIAACKAIVTNDSGLMHVAAALDRPLVALYGPSSPDFTPPLSH
KARVIRLITGYHKVRKGDTAQGYHQSLIDITPQRVLEELHSLLSEEGV
>EECEBICD_04333 ADP-L-glycero-D-manno-heptose-6-epimerase
MIIVTGGAGFIGSNIVKALNDKGITDILVVDNLKDGTKFVNLVDLNIADYMDKEDFLIQI
MSGEELGDIEAIFHEGACSSTTEWDGKYMMDNNYQYSKELLHYCLEREIPFLYASSAATY
GGRTSDFIESREYEKPLNVYGYSKFLFDEYVRQILPEANSQIVGFRYFNVYGPREGHKGS
MASVAFHLNTQLNNGESPKLFEGSENFKRDFVYVGDVAAVNLWFLESGKSGIFNLGTGRA
ESFQAVADATLAYHKKSSIEYIPFPDKLKGRYQAFTQADLTNLRNAGYDKPFKTVAEGVT
EYMAWLNRDA
>EECEBICD_04334 2-amino-3-ketobutyrate coenzyme A ligase
MRGDFYKQLTNDLETARAEGLFKEERIITSAQQADITVADGSHVINFCANNYLGLANHPE
LINAAKAGMDSHGFGMASVRFICGTQDSHKALEQKLASFLGMEDAILYSSCFDANGGLFE
TLLGAEDAIISDALNHASIIDGVRLCKAKRYRYANNDMAELEARLKEAREAGARHVLIAT
DGVFSMDGVIANLKGVCDLADKYDALVMVDDSHAVGFVGENGRGSHEYCDVMGRVDIITG
TLGKALGGASGGYTAARKEVVEWLRQRSRPYLFSNSLAPAIVAASIKVLEMVEAGAELRD
RLWANARQFREQMSAAGFTLAGADHAIIPVMLGDAVVAQKFARELQKEGIYVTGFFYPVV
PKGQARIRTQMSAAHTPEQITRAVDAFTRIGKQLGVIA
>EECEBICD_04335 L-threonine 3-dehydrogenase
MKALSKLKAEEGIWMTDVPEPEVGHNDLLIKIRKTAICGTDVHIYNWDDWSQKTIPVPMV
VGHEYVGEVVGIGQEVKGFKIGDRVSGEGHITCGHCRNCRGGRTHLCRNTTGVGVNRPGC
FAEYLVIPAFNAFKIPDNISDDLASIFDPFGNAVHTALSFDLVGEDVLVSGAGPIGVMAA
AVAKHVGARHVVITDVNEYRLELARKMGVTRAVNVAKESLNDVMAELGMTEGFDVGLEMS
GAPPAFRTMLDTMNHGGRIAMLGIPPSDMSIDWTKVIFKGLFIKGIYGREMFETWYKMAA
LIQSGLDLSPIITHRFSIDDFQKGFDAMRSGQSGKVILSWD
>EECEBICD_04336 Undecaprenyl-phosphate 4-deoxy-4-formamido-L-arabinose
transferase
MKNSKTKVSIIVPLYNAGADFNACMASLIAQTWSALEIIIVNDGSTDHSVEIAKHYAEHY
PHVRLLHQANAGASVARNLGLQAATGDYVAFVDADDLVYPKMYETLMTMALNDDLDVAQC
NADWCVRKTGHAWQSIPTDRLRSTGVLSGPDWLRMALASRRWTHVVWMGVYRRALITDNN
ITFVPGLHHQDILWSTEVMFNATRVRYTEQSLYKYFLHDNSVSRLQRQGSKNLNYQRHYI
KITRLLEKLNRDYARRIPIYPEFRQQITWEALRVCHAVRKEPDILTRQRMIAEIFTSGMY
RRMMANVRSAKAAYQTLLWSFRLWQWRDKTLSHRRMARKALNLS
>EECEBICD_04337 hypothetical protein
MPQFRRSILTLATLLAFAHPVFAGKLAIVIDDFGYRPHTENQVLALPPNISVAVLPNAPH
AREMATKAHNSGHEVLIHLPMAPLSKQPLEKDTLRPDMSSDEIERIIREAVNNVPYAVGL
NNHMGSAMTSSLFGMQKVMQALEHYNLYFLDSMTIGNSQAMRAASGTGVKVIKRKVFLDD
TQNEADIRRQFNRAIELARRNGSAIAIGHPHPATVRVLQQMVYRLPADITLVRPGSLLNE
PQVDTSRPGVTPQKIDAPRNPFRGVKMCKPKKPLQPVYATRFFSVIGESITQSSVVVWFQ
HQWQGWGKIAAPKNVSAKTD
>EECEBICD_04338 Murein hydrolase activator EnvC
MRGKAINTIQQAVKLKTFSVRPLFYASVLSAGVLLCAFSAHADDRDQLKSIQADIAAKER
DVRQQQQQRASLLAQLKAQEEAISAAARKLRETQSTLDQLNAQIDEMNASIAKLEQQKAS
QERNLAAQLDAAFRQGEHTGIQLILSGEESQRGQRLQAYFGYLNQARQETIAELKQTREQ
VATQKAELEEKQSQQQTLLYEQRAQQAKLEQARNERKKTLAGLESSIQQGQQQLSELRAN
ESRLRNSIARAEAAAKARAEREAREAQAVRDKQQEASRKGSTYKPTESERSLMSRTGGLG
APRGQAYWPVRGPILHRYGERLQGELRWKGMVIGASEGTEVKAIADGRVILADWLQGYGL
VVVVEHGKGDMSLYGYNQSALVSVGAQVRAGQPIALVGSSGGQGRPSLYFEIRRQGQAVN

PQPWLGR
>EECEBICD_04339 2,3-bisphosphoglycerate-independent phosphoglycerate mutase
MSVSKKPMVLVILDGYGYREEQQDNAILNAKTPVMDALWAKRPHTLIDASGLEVGLPDRQ
MGNSEVGHVNLGAGRIVYQDLTRLDVEIKERTFFANPVLTNAVDQAKNAGKAVHIMGLLS
AGGVHSHEDHIMAMVELAAERGAEKIYLHAFLDGRDTPPRSAEASLKKFEDKFAALGKGR
VASIVGRYYAMDRDNRWDRVEKAYDLMTLAQGEFQADTAVAGLQAAYARDENDEFVKATV
IRAEGQADAAMEDGDTLIFMNFRADRAREITRAFVNADFDGFARKKVVNLNFVMLTEYAA
DIKTAVAYPPASLANTFGEWMAKNDKTQLRISETEKYAHVTFFFNGGVEEPFAGEERILI
NSPKVATYDLQPEMSSAELTEKLVAAIESGKYDTIICNYPNGDMVGHTGVMEAAIKAVEA
LDNCIEQVTKAVESVGGQLLITADHGNAEQMRDPATGQAHTAHTNLPVPLIYVGEKNVKA
VEGGKLSDIAPTMLSLMGMEIPQEMTGKPLFIVE
>EECEBICD_04340 putative protein YibN
MQEIMQFVGRHPILSIAWIALLVAVLFTTFKGLTSKVKVITRGEATRLINKEDAVVVDLR
QRDDFRKGHIAGATNLLPSEIKANNVGELEKHKDKPIIVVDGSGMQCQESANALTKAGFE
KVFVLKEGVAGWSGENLPLVRGK
>EECEBICD_04341 Glutaredoxin 3
MANIEIYTKATCPFCHRAKALLNSKGVSFQEIAIDGDAVKREEMIKRSGRTTVPQIFIDA
QHIGGCDDLYALDARGGLDPLLR
>EECEBICD_04342 Protein-export protein SecB
MSEQNNTEMAFQIQRIYTKDVSFEAPNAPHVFQKDWQPEVKLDLDTASSQLADDVYEVVL
RVTVTASLGEETAFLCEVQQAGIFSISGIEGTQMAHCLGAYCPNILFPYARECITSLVSR
GTFPQLNLAPVNFDALFMNYLQQQAGEGTEEHQDA
>EECEBICD_04343 Glycerol-3-phosphate dehydrogenase [NAD(P)+]
MNQSNASMTVIGAGSYGTALAITLARNGHQVVLWGHDPKHIATLEHDRCNVAFLPDVPFP
DTLHLESDLATALAASRNILVVVPSHVFSDVLRQIKPLMRPDARLVWATKGLEAETGRLL
QDVAREALGDQIPLAVISGPTFAKELAAGLPTAISLASTDETFADDLQQLLHCGKSFRVY
INADFIGVQLGGAVKNVIAIGAGMSDGIGFGANARTALITRGLTEMSRLGAALGADPATF
MGMAGLGDLVLTCTDNQSRNRRFGMMLGQGMDVKGAQDKIGQVVEGYRNTKEVRELAHRF
GVEMPITEEIYQVLYCGKNAREAALTLLGRARKEELSRH
>EECEBICD_04344 Serine acetyltransferase
MPCEELEIVWKNIKAEARALADCEPMLASFYHATLLKHENLGSALSYMLANKLASPIMPA
IAIREVVEEAYAADPEMIASAACDIQAVRTRDPAVDKYSTPLLYLKGFHALQAYRIGHWL
WNKGRRALAIFLQNQVSVSFQVDIHPAAKIGRGIMLDHATGIVVGETAVIEDDVSILQSV
TLGGTGKTSGDRHPKIREGVMIGAGAKILGNIEVGRGAKIGAGSVVLQPVPPHTTAAGVP
ARIVGKPGSDKPSMDMDQHFNGLHHTFEYGDGI
>EECEBICD_04345 putative glucarate transporter
MNTIIKRTKVRHTILIFLFLATVFNYADRATLSVVAPIMNKELGFDPEAMGLAFSSFGIA
YVIMQLPGGWLLDRYGSRLVYGCALIGWSLVTMFQGTIYLYGSPLIVLVILRLLMGAIEA
PAFPANSRLSVQWFPNNERGFVTSVYQAAQYISLGIITPLMTIILHNLSWHFVFYYIGAI
GVMLGIFWLMKVKDPMHHPKVNQAEIDYIRSGGGEPSLGCKKEPQKITFAQIKTVCVNRM
MIGVYIGQFCVTSITWFFLTWFPTYLYQAKGMSILKVGFVASIPAIAGFIGGLLGGVFSD
WLLKRGYSLTVARKLPVICGMLLSCVIVIANYTSSEFVVIAAMSLVFFAKGFGNLGWCVL
SDTSPKEVLGIAGGVFNMCGNMASIVTPLVIGVILANTQSFDFAILYVGSMGLIGLISYL
FIVGPLDRITLTSSAA
>EECEBICD_04346 L-talarate/galactarate dehydratase
MALSANSDAVTYAKAANTRTAAETGDRIEWVKLSLAFLPLATPVSDAKVLTGRQKPLTEV
AIIIAEIRSRDGFEGVGFSYSKRAGGQGIYAHAKEIADNLLGEDPNDIDKIYTKLLWAGA
SVGRSGMAVQAISPIDIALWDMKAKRAGLPLAKLLGAHRDSVQCYNTSGGFLHTPLDQVL
KNVVISRENGIGGIKLKVGQPNCAEDIRRLTAVREALGDEFPLMVDANQQWDRETAIRMG
RKMEQFNLIWIEEPLDAYDIEGHAQLAAALDTPIATGEMLTSFREHEQLILGNASDFVQP
DAPRVGGISPFLKIMDLAAKHGRKLAPHFAMEVHLHLSAAYPLEPWLEHFEWLNPLFNEQ
LELRDGRMWISDRHGLGFTLSEQARRWTQLTCEFGKRP
>EECEBICD_04347 HTH-type transcriptional regulator GntR

MKRTKSPRAPTLEDVARSAGLSPMTVSRALNSPQLVRPKTVEKVMQAVRVTGYIPNALAG
GLASRRSKLIAVVVPQINNNMFVDTIQSLSDELARRGYHILLCVAGYTEQTEAELVATLL
SRRPDGVVLTGIHHTIELKKVILNAAIPVVEIWDLTPTPLDMLVGFSHEKVGQATGEYLL
SKGYRRPGLLWTADRRAAQRKQGLCSVLQRHAIHAVPQVDVPLPASLSLGRSGLSQLFDE
GTFDVIVCSSDTLAQGAMMEAESRGLRIPHDLAVIGFGDLDFAASNRPSITTVSVDRRAI
GQRAATLLADRIEQKPCAEAIVDIGFHLIERESA
>EECEBICD_04348 tRNA (cytidine(34)-2'-O)-methyltransferase
MLNIVLFEPEIPPNTGNIIRLCANTGFRLHIIEPMGFTWDDKRLRRAGLDYHEFAAVQRH
HDYAAFVAAENPQRLFALTTKGTPAHSAVSYQDGDYLMFGPETRGLPASILDALPKEQKI
RIPMMPDSRSMNLSNAVSVVVYEAWRQLGYSGAVLRS
>EECEBICD_04349 L-lactate dehydrogenase
MIISAASDYRAAAQRTLPPFLFHYIDGGAYAEYTLRRNVEDLSQVALRQRVLKNMSDLSL
ETTLFNETLSMPVALAPVGLCGMYARRGEVQAAAAADAKGIPFTLSTVSVCPIEEVAPTI
KRPMWFQLYVLRDRGFMRNALERAKAAGCSTLVFTVDMPTPGARYRDAHSGMSGPNAAMR
RYWQAVMHPKWAWDVGLNGRPHDLGNISAYLGKPTGLEDYIGWLANNFDPSISWKDLEWI
REFWDGPMVIKGILDPEDARDAVRFGADGIVVSNHGGRQLDGVLSSARALPAIADAVKGD
IAILADSGIRNGLDVVRMIALGADTVLLGRAYLYALATAGKAGVANLLDLIEKEMKVAMT
LTGAKTISEISGDSLVQELGKSLPAALAPMSKGDTA
>EECEBICD_04350 Putative L-lactate dehydrogenase operon regulatory protein
MIVMPKRLSDEIASRVRALIEEQNLEAGMKLPAERQLALQLGVSRNSLREALAKLVSEGV
LVSRRGGGTFVRWQHETWSEQNIVQPLKMLMANDPDYSFDILEARHAIEASTAWHAAMRA
TAADKEKIRLCFDATLSEDPDLASQADVRFHLAIAEASHNVVLLQTMRGFFDVLQSSVKQ
SRQRMYLVPPVFSKLTEQHQAVMDAILDGNAEGARKAMMAHLSFVHTTIKRFDEDQARQA
RITRLPGDHNEMTRENKS
>EECEBICD_04351 L-lactate permease
MNLWQQNYDPAGNIWLSSLIASLPILFFFFALIKLKLKGYVAASWTVVIALAVALLFYKM
PVDHALASVVYGFFYGLWPIAWIIIAAVFVYKISVKTGQFDIIRSSILSITPDQRLQMLI
VGFCFGAFLEGAAGFGAPVAITAALLVGLGFNPLYAAGLCLIVNTAPVAFGAMGIPILVA
GQVTGLDSFEIGQMVGRQLPFLTIIVLFWIMAIMDGWRGVKETWPAVMVAGGSFAIAQYL
SSNFIGPELPDIISSLVSLVCLTLFLKRWQPVRIFRFGDMGASQVDQTLARTRYTTGQIV
RAWSPFLFLTATVTLWSVPPFKALFAPGGALYDWVINVPVPYLDKLVARMPPVVHEATAY
AAVYKFDWFSATGTAILFAALLSIVWLKMKPSAAIQTFGSTLKELALPIYSIGMVLAFAF
ISNYSGLSSTLALALAHTGSAFTFFSPFLGWLGVFLTGSDTSSNALFASLQATAAQQIGV
SDVLMVAANTTGGVTGKMISPQSIAIACAAVGLVGKESDLFRFTVKHSLIFTCMVGVITT
LQAYVLTWMIP
>EECEBICD_04352 Autotransporter adhesin SadA
MNRIFKVLWNAATGTFVVTSETAKSRGKKNGRRKLAVSALIGLSSIMVSADALANAGNDT
GDGVTPTGTQTGGKGWIAIGTDATANTYTNVDGASAAMGYKASAMGKWSTAIGSYSQSTG
DSSLALGVKSVSAGDRAIAMGASSSASGSYSMAMGVYANSSGAKSVALGYKSVASGATSS
ALGYQATASGDDSAAFGNGAKAVGTNSVALGSGSIAQEDNSVAVGNSTTQRQITYVAKGD
INSTSTDAVTGAQIYSLSQSVADRLGGGASVNSDGTVNAPLYEVGTGIYNNVGSALSALN
TSITNTEASVAGLAEDALLWDDSTSAFSASHTGNASKITNLAAGTLAADSTDAVNGSQLF
DTNEKVDQNTADITANTDSINQNTADITANTDSINQNTTDIAANTTSINQNTTDIATNTT
NINSLSDSVTTLTDDALLWDAASGAFSAKHNGNDSKITNLAAGTLAADSTDAVNGSQLFD
TNEKVDQNTADITTNTNSINQNTTDIATNTTNINNLSDSITTLTDDALLWDAASGAFSAN
HNGSASKITNLAAGTLAADSTDAVNGSQLFATNENVSQNTADITTNTNSINQNTTDIATN
TTNINNLSDSITTLTDDALLWDAASGTFSASRSGSASKITNLAAGTLAADSTDAVNGSQL
YETNQRVDQNTSAIADINTSITNLSSDNLSWNETTSSFSASHGSSTTNKITNVAAGELSE
ESTDAVNGSQLFETNEKVDQNTTDIAANTTNITQNSTAIENLNTSVSDINTSITGLTDNA
LLWDEDIGAFSANHGGSTSKITNVAAGALSEDSTDAVNGSQLYETNQKVDQNTSAIADIN
TSITNLGTDALSWDDEEGAFSASHGTSGTNKITNVAAGEIASDSTDAVNGSQLYETNMLI
SQYSESISQLAGDTSETYITENGTGVKYIRTNDNGLEGQDAYATGNGATAVGYDAVASGA
GSLALGQNSSSSIEGSIALGSGSTSNRAITTGIRETSATSDGVVIGYNTTDRELLGALSL

GTDGESYRQITNVADGSEAQDAVTVRQLQNAIGAVTTTPTKYYHANSTEEDSLAVGTDSL
AMGAKTIVNADAGIGIGLNTLVMADAINGIAIGSNARANHANSIAMGNGSQTTRGAQTDY
TAYNMDTPQNSVGEFSVGSEDGQRQITNVAAGSADTDAVNVSQLKVTDAQVSRNTQSITN
LNTQVSNLDTRVTNIENGIGDIVTTGSTKYFKTNTDGADANAQGADSVAIGSGSIAAAEN
SVALGTNSVADEANTVSVGSSTQQRRITNVAAGVNNTDAVNVAQLKASEAGSVRYETNAD
GSVNYSVLNLGDGSGGTTRIGNVSAAVNDTDAVNYAQLKRSVEEANTYTDQKMGEMNSKI
KGVENKMSGGIASAMAMAGLPQAYAPGANMTSIAGGTFNGESAVAIGVSMVSESGGWVYK
LQGTSNSQGDYSAAIGAGFQW
>EECEBICD_04353 Inner membrane lipoprotein SadB
MHKNGKFIPLLALGFTFFLSGCDYFADKHLVEEMKEQQKEQETKINLLEKQQKEQEAKIN
LLEKQQATIINTTKKVTEVVGRVERKQRLFDYTELDPSQTHYFIINNGNIGLAGRILSIE
PIDNGSVIHLDLVNLLSIPVSNLAFNMTWGTKKPSEAKDLPRWKQLLLNTKMDSTIELLP
GAWTNVTLTLKGVSPNNLKYLKIGIDMENVIFDSIQPINDTKKKPKK
>EECEBICD_04354 putative protein YibL
MKEVEKNEIKRLSDRLDAIRHQQAGLSLVESADKYAELEKEKETLEAEIIRLREVHSQKL
SKEAQKLMNLPFRRAITKKEQADMGKLKKRGRARFVVPPPPAAARGGGPKKRRVSPRGGF
>EECEBICD_04355 hypothetical protein
MAKIGENVPLLIDKAVDFMASSQAFREYLNKTPPRDYVPSEVPSESAPIYLQRLEYYRRL
YRPKEEERG
>EECEBICD_04356 Mannitol operon repressor
MTQNPAQVSLRPNNRLSDMQAIMEQTQAFENRVLERLNAGKTVRSFLITAVELLTEAVNI
LVLQVFRKDDYAVKYAVEPLLDGDGPLGDLSVRLKLIYGLGVLSRTEYEDAELLMALREE
LNHDGNEYAFTDDEILGPFGELHCVMALPPPPHFDTSDAALYAMQIQRYQQAVRSTMVLS
LTELISKISLKKAFQK
>EECEBICD_04357 Mannitol-1-phosphate 5-dehydrogenase
MKALHFGAGNIGRGFIGKLLADAGIQLTFADVNQVVLDALNARHSYQVHVVGENEQVDTV
SGVNAVSSIGDDVVDLIAHVDLITTAVGPVVLERIAPAIAKGLAKRKAQGVDAPLNIIAC
ENMVRGTTQLKGHVMNALADGDKAWVEQHVGFVDSAVDRIVPPSASATHDPLEVTVETFS
EWIVDKTQFKGALPNIPGMELTDNLMAFVERKLFTLNTGHAITAYLGKLAGHQTIRDAIL
DESIRAVVKGAMEESGAVLIKRYGFDADKHAAYIQKILGRFENPYLKDDVERVGRQPLRK
LSAGDRLIKPLLGTLEYGLPHVNLVKGIAAAMHFRSDEDPQAQELAALITEKGPQAALAQ
ISGLDANSDVVAEAVNAYNATK
>EECEBICD_04358 PTS system mannitol-specific EIICBA component
MSSDIKIKVQSFGRFLSNMVMPNIGAFIAWGIITALFIPTGWLPNETLAKLVGPMITYLL
PLLIGYTGGRLVGGERGGVVGAITTMGVIVGADMPMFLGSMIAGPLGGYCIKKFDSWVDG
KIKSGFEMLVNNFSAGIIGMILAILAFLGIGPAVEVLSKILAAGVNFMVAHDMLPLASIF
VEPAKILFLNNAINHGIFSPLGIQQSHEMGKSIFFLIEANPGPGMGVLLAYMFFGRGSAK
QSAGGAAIIHFLGGIHEIYFPYVLMNPRLILAVILGGMTGVFTLTILNGGLVSPASPGSI
LAVLAMTPKGAYFANIAAIVAAMAVSFVVSAILLKTSKVKEEDDIEAATRRMQDMKAESK
GASPLAAGDVTNDLSHVRKIIVACDAGMGSSAMGAGVLRKKVQDAGLSQISVTNSAINNL
PPDVDLVITHRDLTERAMRQVPQAQHISLTNFLDSGLYTSLTERLVAAQRHNTNEEKVRD
HLKDSFEEGDNNLFKLGAENIFLGRKAANKEEAIRFAGEQLVKGGYVEPEYVEAMLDREK
LTPTYLGESIAVPHGTVEAKDRVLKTGVVFCQYPEGVRFGEEEDDIARLVIGIAARNNEH
IQVITSLTNALDDESVIERLAHTTSVDEVLELLAGKKA
>EECEBICD_04359 putative GST-like protein YibF
MKLIGSYTSPFVRKISVLLLEKGITFEFINELPYEADNGVAQYNPLGKVPALVTEKGEYW
FDSPIIAEYIELLDIAPAMLPRDPMAALKVRQLEALADGIMDAALVSVREQARPAAQQSE
TELLRQREKINRSLDMLESYLSDGTLKTDTVNLATIAIACAVGYLNFRRVAPGWCVSRPH
LVKLVETLFQRESFARTEAPKA
>EECEBICD_04360 L-seryl-tRNA(Sec) selenium transferase
MTSETRTLYSQLPAIDRLLHDSAFLSLRDRYGHTQVVDLLRRMLDDARDVIRNTQTLPDW
YADWAQEAKLRLENAAQSALRPVINLTGTVLHTNLGRALQAQEAVEAVTQAMRAPVTLEY
DLDGAGRGHRDRALATLLCRITGAEDACIVNNNAAAVLLMLAATASGKEVVVSRGELVEI
GGAFRIPDVMRQAGCTLHEVGTTNRTHAKDYRQAVNENTGLLMKVHTSNYSIEGFTKTVE

EAELAEIGRELDIPVVADLGSGSLVDLSQYGLPKEPMPQQLIAAGVSLVSFSGDKLLGGP
QAGIIVGKKAMIAQLQSHPLKRALRADKMTLAALEATLRLYLHPEALAEKLPTLRLLTRS
EASIREQAQRLQARLAARYGDEFALEVKPCLSQIGSGSLPVDRLPSAAMTFTPHDGRGSR
LEALAARWRMLPVPVIGRIYDGRLWLDMRCLEDESRFMEMMLK
>EECEBICD_04361 Selenocysteine-specific elongation factor
MIIATAGHVDHGKTTLLQAITGVNADRLPEEKKRGMTIDLGYAYWPQPDGRVLGFIDVPG
HEKFLSNMLAGVGGIDHALLVVACDDGVMAQTREHLQILQLTGNLQLTVALTKADRVDEA
RIGEVREEVLAALDNYGFADTVLFVTAANEGRGIAELRAHLQQLPARLHAAQHRFRLAID
RAFTVKGAGLVVTGTALSGEVNVGDTLWLTGVNTPMRVRSLHAQNQPTDHAYAGQRIALN
IAGDAEKEQLNRGDWLLSDAPVGEAFSRVIVSLALHAPLSQWQPLHIHHAASHVTGRVSL
LEGGLAELIFDTPLWLADNDRLVLRDISARATLAGARVVTLKAPRRGKRKPDYLHWLSTL
AAAQDDSAALAIHLERGAVNLPDFGWARQLNPVGMRQLIEQHGFIQAGDNLLSAPVAARW
QRKILDTLATYHEQHRDEPGPGRERLRRMALPMEDEALVLMLIERMRDDGLIHSHHGWLH
LPDHKAGFSDEQQAVWQKVEPLFGDEPWWVRDLAKETGTEEQLMRLVLRQAAQQGIITAI
VKDRYYRNDRIVAFANMIRELDQERGSTCAADFRDRLNVGRKLAIQILEYFDRIGFTRRR
GNDHLLRDALLFPQKE
>EECEBICD_04362 Beta-glucoside kinase
MQQYIGIDVGGTHVKYGVINSDGEELTHHQFDTPEDASTFTRKWQDVVARCQQDYDIAAI
GVSFPGHINPHNGHAAKAGALAYLDDVNLMELFSGLTDLPLVVENDANCAALGEMWRGAG
QHYDNLVCITIGTGIGGGIIVGRELYRGAHFHAGEFGVMPVGNNGESMHKIASTSGLMAS
CRQALALPAEEMPPADVIFERMATDVHLREAVNDWARYLSRGVYSVISMFDPGVVLIGGG
ISEQEKLYPLLTRHLETFEMWEALQVPIQPCQLGNQAGRLGAVWLAQQKLARS
>EECEBICD_04363 Aldehyde dehydrogenase B
MTNNPPSTRIQPGEYGYPLKLKARYDNFIGGDWVAPADGEYYQNLTPVTGQPLCEVASSG
KKDIDLALDAAHKAKDKWAHTSVQDRAAILFKIADRMEQNLELLATAETWDNGKPIRETS
AADVPLAIDHFRYFASCIRAQEGGISEVDSETVAYHFHEPLGVVGQIIPWNFPLLMASWK
MAPALAAGNCVVLKPARLTPLSVLLLMEVIGDLLPPGVVNVVNGAGGEIGEYLATSKRIA
KVAFTGSTEVGQQIMQYATQNIIPVTLELGGKSPNIFFADVMDEEDAFFDKALEGFALFA
FNQGEVCTCPSRALVQESIYERFMERAIRRVESIRSGNPLDSGTQMGAQVSHGQLETILN
YIDIGKKEGADILTGGRRKELDGELKEGYYLEPTILFGKNNMRVFQEEIFGPVLAVTTFK
TMEEALEIANDTQYGLGAGVWSRNGNLAYKMGRGIQAGRVWTNCYHAYPAHAAFGGYKQS
GIGRETHKMMLEHYQQTKCLLVSYSDKPLGLF
>EECEBICD_04364 Non-reducing end beta-L-arabinofuranosidase
MNVLEVDLHKLTVSDPFLGQYQQLVRDVVIPYQWNALNDRIPEAEPSHAIENFRIAAGQQ
TGDFYGMVFQDSDVAKWLEAVAWSLCQKPDPALEKTADEVIELVAAAQCDDGYLNTYFTA
KAPQERWSNLAECHELYCAGHLIEAGVAFFQATGKRRLLDVVCRLADHIDSTFGPGENQL
HGYPGHPEIELALMRLYEVTEQPRYMALASYFIGQRGVQPHFYDEEYEKRGQTSYWHTYG
PAWMVKDKAYSQAHLPISQQQTAIGHAVRFVYLMTGVAHLARLSNDEGKRQDCLRLWKNM
AQRQLYITGGIGSQSSGEAFSSDYDLPNDSVYAESCASIGLMMFARRMLEMEADSQYADV
MERALYNTVLGGMALDGKHFFYVNPLEVHPKSLKFNHIYDHVKPIRQRWFGCACCPPNIA
RVLTSLGHYIYTPRADALYINMYVGNSMEIPVGNGALKLRIGGNYPWQEQVKIAIDSVQP
VRHTLALRLPDWCPEAKVTLNGLEVEQDIRKGYLHIRRTWQEGDTITLTLPMPVRRVYGN
PLARHVAGKVAIQRGPLVYCLEQADNGEELHNLWLPKESEFRVFEGKGLFAHKMLIQAEG
EKQSAPDAQHQALWHYDNAPSSRQPQTLTFIPWFSWANRGEGEMRIWVNER
>EECEBICD_04365 HTH-type transcriptional activator RhaR
MLELSMTLPIKVQNGGLFISRGIGRHPARRLQSWEIIFVEKGCLKIQEEECVFCVEAGES
LLLWPHRRHIGVEEFPADLKFYWLHFEVKAPDSNPRWLTHLSVPQHTQVADPQALIALFR
QFMNEQEKHQRSPALEFIVLLILQQLTVDARQNENAEAAGVSLAWKVQQLIRTHYHLPLS
SSVLAKELHCNVDYLGRVYRRVFHLTLTEAIHRQRVREAEKLLISDARSLKEVAERCGFN
DVGYFRQIFRKHTGLTPTAWKRRYSKEHVNS
>EECEBICD_04366 L-ribulose-5-phosphate 4-epimerase SgbE
MLEQLKAEVLAANLALPAHGLVTFTWGNVSAVDETRKLMVIKPSGVEYEVMTADDMVVVE
IASGKVVEGNKKPSSDTATHLALYRRYPQIGGIVHTHSRHATIWSQAGLDLPAWGTTHAD
YFYGAIPCTRLMTVEEINGEYEYQTGEVIIKTFEERGLDPAQIPAVLVHSHGPFAWGKNA

ADAVHNAVVLEECAYMGLFSRQLAPQLPDMQPELLDKHYLRKHGANAYYGQ
>EECEBICD_04367 L-ribulose-5-phosphate 3-epimerase UlaE
MTKAIRLARDLGIRTIQLAGYDVYYEEHDEGTQQRFAEGLAWAVEQAAAAQVMLAVEIMD
TAFMNSISKWKKWDEMLSSPWFTVYPDVGNLSAWGNNVTAELKLGIDRIAAIHLKDTLPV
TGDSPGQFRDVPFGEGCVDFVGIFKTLHELNYRGSFLIEMWTEKASEPVLEIIQARRWIE
SRMQEGGFTC
>EECEBICD_04368 L-ribulose-5-phosphate 3-epimerase UlaE
MRNHPLGIYEKALAKDLSWPERLVLAKSCGFDFVEMSVDETDERLSRLEWTSAQRASLVN
AMLESGVAIPSMCLSAHRRFPFWQPRRSGTPAGA
>EECEBICD_04369 3-keto-L-gulonate-6-phosphate decarboxylase SgbH
MSRPLLQLALDHTSLEAAQRDVALLQDHVDIVEAGTILCLTEGLSAVKALRAQCPEKIIV
ADWKVADAGETLAQQAFGAGANWMTIICAAPLATVEKGHAVAQSCGGEIQMELFGNWTLD
DARDWYRTGVRQAIYHRGRDAQASGQQWGEADLARMKALSDIGLELSITGGITPADLPLF
RDINVKAFIAGRALAGAAHPAQVAAEFHAQIDAIWGEKHA
>EECEBICD_04370 L-xylulose/3-keto-L-gulonate kinase
MMTHDYLRWCLTGVKGCEESNISESNLYNMATGQYDPRLTEWLGISEIDSALPPVVGSAE
ICGEITAQAAAITGLAAGTPVVGGLFDVVSTALCAGIEDESTLNAVMGTWAVTSGIAHGL
SDHEAHPYVYGRYVNDGQYIVHEASPTSSGNLEWFTAQWGDLSFDEINQAVASLPKAGSD
LFFLPFLYGSNAGLEMTCGFYGMQALHTRAHLLQAIYEGVVFSHMTHLNRMRERFTDVCA
LRVTGGPAHSDVWMQMLADVSGLRIELPQVEETGCFGAALAARVGTGVYRDFREAQRDLQ
HPVRTLLPDMTAHALYQRKYRQYQDLIEALQGYHARIKEHAL
>EECEBICD_04371 L-xylulose/3-keto-L-gulonate kinase
MSNYWLGLDCGGSWLKAGLYDGAGREVAVQRLPLHALSPQPGWVERDMTELWQQCGSVIS
KLLTHTGVSGSQIRGLGISAQGKGLFLLDKSDRPLGKAILSSDRRAMEIVQRWQKEAVPQ
KLYPLTRQTLWTGHPVSLLRWVKRMSRSATRR
>EECEBICD_04372 2,3-diketo-L-gulonate-binding periplasmic protein YiaO
MKLHVIARSLLIAGLTVFSVSSLAAQSLRFGYETPQTDSQHIAAKKFHELLKEKTNGELT
LKLFPDSTLGNAQAMISGVRGGTIDMEMSGSNNFTGLAPVFNLLDVPFLFRDTAHAHKTL
DGKVGDELKKSLDSKGLKVLAYWENGWRDVTNSRAPVKTPGDLKGLKIRTNNSPMNIAAF
KIFGANPIPMPFSEVYTGLETRTIDAQEHPINVVWSAKFYEVQKYLSLTHHAYSPLLLVI
NKAKFDALSPQFQEALLSSAKEAGDYQRKLVAEDQQKIIDGMKEAGVEVLTDIDRKAFSD
ALGSQVRDMFLKDNPQGADLLKAVDEVQ
>EECEBICD_04373 Sialic acid TRAP transporter large permease protein SiaM
MAVVIFLCCLLGGIAIGLPIAWSLLLCGAALMAYLDMFDVQIMAQTLVNGADSFSLLAIP
FFVLAGEIMNAGGLSKRIVDLPMKLVGHKPGGLGYVGVIAAMIMASLSGSAVADTAAVAA
LLVPMMRSANYPINRSVGLIASGGIIAPIIPPSIPFIIFGVSSGLSISKLFMAGIAPGIM
MGAALMLTWWWQAGRLNLPSQPKATPREIWQSLVSGIWALFLPVIIIGGFRSGLFTPTEA
GAVAAFYALFVAVVIYRELTFSSLYHVLVNAAKTTSVVMFLVAAAQVSAWLITIAELPMM
VSDLLQPLVDSPRLLFIVIMISIMVVGMVMDLTPTVLILTPVLLPLVKEANIDPIYFGVM
FIINCSIGLITPPVGNVLNVISGVAKLKFDDAVRGVFPYVVVLMSLLVLFIFIPELIITP
LKWIN
>EECEBICD_04374 2,3-diketo-L-gulonate TRAP transporter small permease
protein YiaM
MKRVLEGILAVLIAVLSCIVFINVILRYGFESSILSVDELSRYLFVWLTFIGAIVAFMDN
AHVQVTFVVEKLSPANQRRLSLLTHSLILLLCLALGWGSLQKALQDWSDHSPILGLPIGL
MYIACLPTSVAIALIELRRLYHLITRNDSFQQPQQGA
>EECEBICD_04375 putative protein YiaX1
MKNNTGYIIGAYPCAPSFHQKSEEEETEFWRQLSDTPDIRGLEQPCLEHLHPLGDEWLLR
HTPGNWQIVVTAIMETMRRRSENGGFGLASSDEEQRKACVEYYRHLHQKINKINGNNTGK
VIALELHAAPLGGNPNVAQATDAFARSLKEIANWDWSCDLVLEHCDAMTGPAPRKGFLPL
VNVLETIADYDISVCINWARSAIEGRDTSLPLIHTQQAKQAGKLGALMFSGTTLDGEYGE
WQDLHAPFAPFCPQSLMTEKHVQELITAAAPELLQFTGIKLLEINASADINHRINILRDG
INIMKKATRR
>EECEBICD_04376 putative protein YhcH

MIFGHIAQPNPCRLPSAIEQALDFLRNTDFRTLEPGVVEIDGKNIFAQIIDMTTRDAAEN
RPEVHRRYLDIQFLAWGEETIGVAIDTGNNQISESLLEQRDIIFYHDSEHESFIEMIPGS
YALFFPQDVHRPGCNKSIATPIRKIVVKVAIDVL
>EECEBICD_04377 2,3-diketo-L-gulonate reductase
MKVTFEELKGAFYRVLRSRNIAEDTADACAEMFARTTESGVYSHGVNRFPRFIQQLDNGD
IIPDAKPQRVTSLGAIEQWDAQRAIGNLTAKKMMDRAIELASDHGIGLVALRNANHWMRG
GSYGWQAAEKGYIGICWTNSIAVMPPWGAKECRIGTNPLIVAIPSTPITMVDMSMSMFSY
GMLEVNRLAGRELPVDGGFDDNGQLTKEPGVIEKNRRILPMGYWKGSGLSIVLDMIATLL
SNGSSVAEVTQENSDEYGVSQIFIAIEVDKLIDGATRDAKLQRIMDFITTAERADDNVAI
RLPGHEFTKLLDDNRRHGITIDDSVWAKIQAL
>EECEBICD_04378 DNA-binding transcriptional repressor YiaJ
MSQNNDKEKPAGSQSLFRGLMLIEILSNYPNGCPLAHLSELAGLNKSTVHRLLQGLQSCG
YVTPAPAAGSYRLTTKFIAVGQKALSSLNIIHVAAPHLEALNLATGETVNFSSREDDHAI
LIYKLEPTTGMLRTRAYIGQHMPLYCSAMGKIYMAFGQPDYVASYWESHKDQIQPLTRNT
ITDLPAMYDELAQIRETSMAMDREENELGVSCIAVPVFDIHHRVPYAISISLSTSRLKQI
GEKNLLKPLRETAQAISNELGFTVRE
>EECEBICD_04379 Hydrogenase-4 component A
MNRFIMADASACIGCRTCEVACVVSHQEQQNSAAVTTADFVPRIRVIKEDSFTTATVCHQ
CEDAPCANVCPVQAIRRDRGHIFVTSSRCIGCKSCMLACPFGAMTVVASASGAQAIKCDL
CWHREAGPACVEACPTSALQCVDATNVQRQRLYSQPF
>EECEBICD_04380 Valine--pyruvate aminotransferase
MTFSLFGDKFTRHSGITRLMEDLNDGLRTPGAIMLGGGNPAHIPAMQDYFQTLLTDMVES
GKAADALCNYDGPQGKTALLNALAVLLRETLGWDIEPQNIALTNGSQSAFFYLFNLFAGR
RADGSTKKVLFPLAPEYIGYADSGLEDDLFVSARPNIELLPEGQFKYHVDFEHLHIGEET
GMICVSRPTNPTGNVITDEELMKLDRLANQHNIPLVIDNAYGVPFPGIIFSEARPLWNPN
IILCMSLSKLGLPGSRCGIIIANDKTITAIANMNGIISLAPGGMGPAMMCEMIKRNDLLR
LSETVIKPFYYQRVQQTIAIIRRYLSEERCLIHKPEGAIFLWLWFKDLPITTELLYQRLK
ARGVLMVPGHYFFPGLDKPWPHTHQCMRMNYVPEPDKIEAGVKILAEEIERAWREG
>EECEBICD_04381 Periplasmic alpha-amylase
MKLAAFALTLIPGIAIASSWTSPGFPTFSTQETGRFTSHAALTKGTRALTLHIDQQCWQP
SGAIKLNQMLSLKPCEGAPPQWRLFKDGDYTITVDTRSGTPTLLLSIKTEPERTAQLAYQ
CPVWDGSPLTLDVRQTFPEGTVVRDYYSGQTDTVQNGQITLQPADSHGLLLLERAETHAS
APFNWRNATVYFVLTDRFRNGDPTNDHSYGRHKDGMQEIGTFHGGDLRGLTSKLDYLQQL
GVNALWISSPFEQIHGWVGGGAKGDFPHYAYHGYYTQDWTTLDANMGNEADLRALVDGAH
QRGIRILFDVVMNHAGYATLEDMQEYQFGALYLSGAERQKILGDRWTNWRPAAGQSWHSF
NDYINFSDSAAWEKWWGKKWIRTDIGDYDSPGFDDLTLSLAFLPDIKTESTTPSGLPAFY
ANKPDTKAKFIEGYTPRDYLTHWLSQWVHDYGIDGFRVDTAKNVELPAWQQLKTQASAAL
HEWKQANPDKALDDSPFWMTGEAWGHGVMKSDYYRYGFDAMINFDYQEQAAKAVDCLAEM
GPVWQQMADKMQDFNVLSYLSSHDTRLFREGGDKAAELLLLSPGAVQIFYGDESARPFGP
TGSDPLQGTRSDMNWQDVSGKSAAAVAHWQRISQFRARHPAIGAGQQTTLTLKHGYGFVR
QYGDDTVMVVWAGRR
>EECEBICD_04382 hypothetical protein
MILTPMRRYGAMILMLLTIAFSGEVLAKTHATQTSQKSHITETSYKQVSSKQEYSRNSAK
SSSLPDLRKYPSGTPRKKAFLRTVMPYITSQNAAITADRNWLISKQYQNRWSPSERARMK
DIAKRYKVSWSGNTRRIPWNTLLERVDIIPTSMVATMAAAESGWGTSKLARSNNNLFGMK
CTKGRCTNTPGKVKGYSQFASVEESVSAYVANLNTHPAYSSFRKSRAQLRKADQEVTATA
MIHKLKGYSTQGSRYNNYLFAMYQDNQRLIAAHM
>EECEBICD_04383 Xylose operon regulatory protein
MFDKRHRITLLFNANKAYDRQVVEGVGEYLQASQSEWDIFIEEDFRARIDNIKEWLGDGV
IADYDDDDIAQLLADVDVPIVGVGGSYHLAENYPAVHYIATDNHALVESAFLHLKEKGVN
RFAFYGLPDSSRKHWAAEREYAFRQLVAEEKYRGVVYQGLETAPENWQHAQNRLADWLQT
LPPQTGIIAVTDARARHVLQACEHLHIPVPEKLCVIGIDNEELTRYLSRVALSSVAQGAR
QMGYQAAKLLHRLLAREEMPLQRILVPPVRVIARRSTDYRSLTDPAVIQAMHFIRNHACK
GIKVEQVLDAVGISRSNLEKRFKEEVGETIHALIHAEKLEKARSLLISTTLAINEISQMC

GYPSLQYFYSVFKKEYVTTPKEYRDQHSEALL
>EECEBICD_04384 Xylose isomerase
MQAYFDQLDRVRYEGPQSTNPLAFRHYNPDELVLGKRMEDHLRFAACYWHTFCWNGADMF
GVGAFNRPWQQPGEALELAKRKADVAFEFFHKLNVPFYCFHDVDVSPEGASLKEYKNNFA
QMVDVLAAKQEQSGVKLLWGTANCFTNPRYGAGAATNPDPEVFSWAATQVVTAMNATHKL
GGENYVLWGGREGYETLLNTDLRQEREQIGRFMQMVVEHKHKMGFQGTLLIEPKPQEPTK
HQYDYDVATVYGFLKQFGLEKEIKVNIEANHATLAGHSFHHEIATAIALGIFGSVDANRG
DAQLGWDTDQFPISVEENALVMYEILKAGGFTTGGLNFDAKVRRQSTDKYDLFYGHIGAM
DTMALSLKIAARMVEDGELDKRVAKRYAGWNSELGQQILKGQLSLGELAQYAEQHNLAPV
HQSGHQELLENLVNRYLFDK
>EECEBICD_04385 Xylulose kinase
MYIGIDLGTSGVKAILLNEQGDVLATHTEKLTVSRPHPLWSEQEPEQWWQATDRAVKGLG
RQQSLSGVRALGIAGQMHGATLLDSRQQVLRPAILWNDGRCSEECAWLEKQVPQSRAITG
NLMMPGFTAPKLVWVQRHEPDIFYQIDKVLLPKDFLRLRMTGVFASDMSDAAGTMWLDVK
KRDWSDVMLNACHLTRQQMPALFEGSDITGTLLPEVASAWGMPTVPVVAGGGDNAAGAVG
VGMIDAGQAMLSLGTSGVYFAVSDGFLSKPESAVHSFCHALPERWHLMSVMLSAASCLDW
AAKLTGQENVPALIAAAQQADEHADSIWFLPYLSGERTPHNNPQAKGVFFGLTHQHGPAE
LARAVLEGVGYALADGMDVVHACGVKPASVTLIGGGARSEYWRQMLSDISGLQLDYRTGG
DVGPALGAARLAQIAVNKQTPLADVLPQLPLEQAHYPDAQHHAVYQQRRETFRRLYQQLL
PLMS
>EECEBICD_04386 hypothetical protein
MDDHVARRKRIFGLGMLVVGAVVYLVGLWPGCHTLSEKGYFFAAIVMCGFPVLIRQEHTG
NGRLLSRCKSLLLLGIGMVAVGVFNLALAGALKILCLVALGVSIYGTDLYASYSDDE
>EECEBICD_04387 O-acetyltransferase WecH
MQPKINWIDNLRGIACLMVVMIHTTTWYITNAHSVSPLNWDIANVLNSASRVSVPLFFMI
SGYLFFGERCAQPRHFLRIALCLIFYSVVALAYISLFTSINVELSLKNVLQKPVFYHLWF
FFAIAVIYLVSPLIQVKNVSGKMLLMLMVIIGIIANPNTVPQKIGGVEWLPINLYISGDT
FYYILYGILGRAIGMMETQKPSLTLICAALFIIAVFVISRGTLHELRWRGNFADTWYLYC
GPMVFICAVSLFTVVKNKLNARTLPGLGLISRHSLGIYGFHALIIHALRTNGLELKRWPP
LDIVWVFAATVTGSLLLSMLLQRIDKRKWVS
>EECEBICD_04388 hypothetical protein
MIMKYFCTVMIAIALVGCTATPPPTQKAQQSKVSPTRTLDMEALCKAQAAQRYNTGAQKI
AVTGFEQFQGSYEMRGNTFRKESFVCSFDADGQFLHLSMR
>EECEBICD_04389 Glycine--tRNA ligase alpha subunit
MQKFDTRTFQGLILTLQDYWARQGCTIVQPLDMEVGAGTSHPMTCLRALGPEPMATAYVQ
PSRRPTDGRYGENPNRLQHYYQFQVVIKPSPDNIQELYLGSLKELGMDPTIHDIRFVEDN
WENPTLGAWGLGWEVWLNGMEVTQFTYFQQVGGLECKPVTGEITYGLERLAMYIQGVDSV
YDLVWSDGPLGKTTYGDVFHQNEVEQSTYNFEYADVDFLFTCFEQYEKEAQQLLALENPL
PLPAYERILKAAHSFNLLDARKAISVTERQRYILRIRTLTKAVAEAYYASREALGFPMCN
KDK
>EECEBICD_04390 Glycine--tRNA ligase beta subunit
MSEKTFLVEIGTEELPPKALRSLAESFAANFTAELDNAGLAHGNVEWFAAPRRLALKVAN
LAESQPDREVEKRGPAIAQAFDAEGKPSKAAEGWARGCGITVDQAERLKTDKGEWLLYRA
HVKGESTEALVPNMVATSLAKLPIPKLMRWGASDVHFVRPVHTVTLLLGDKVIPATILGI
QSDRVIRGHRFMGEPEFTIDNADQYPQILLERGKVIADYEARKAKIKADAEEAARKIGGN
ADLSESLLEEVASLVEWPVVLTAKFEEKFLSVPAEALVYTMKGDQKYFPVYDNAGKLLPN
FIFVANIESKDPTQIISGNEKVVRPRLADAEFFFNTDRKKRLEDHLPRLQTVLFQQQLGT
LRDKTDRIQALAGWIAGQIGADVNHATRAGLLSKCDLMTNMVFEFTDTQGVMGMHYARHD
GEAEDVAVALNEQYQPRFAGDDLPSNPVACALAIADKMDTLAGIFGIGQHPKGDKDPFAL
RRAALGVLRIIVEKNLALDLQTLTEEAVRLYGDKLTNANVVDDVIDFMLGRFRAWYQDEG
YTVDTIQAVLARRPTRPADFDARMKAVSHFRTLEEASALAAANKRVSNILAKATEPLNDI
VHASVLKEAAEIELARHLVVLRDKLQPYFADGRYQEALIELAALRAPVDEFFENVMVNAE
EKDIRINRLTLLSKLRELFLQVADISLLQ
>EECEBICD_04391 putative N-acetyltransferase YafP

MKIRRFSNGDEISLFRVFFSSVHTIASHYYTREQIDAWAPADIDLERWANHIKELQPFVV
ELDGEIAGYADVQPNGYIDHFFVSGTYSRQGVGTLLMNCIHEEARQRGISELTSNVSKAA
EVFFLRHGFHIVERGFPICRGVTLQNALMRKCLAK
>EECEBICD_04392 hypothetical protein
MKSDVQLNLRAKESQRALIDAAAEILHKSRTDFILETACQAAEKVILDRRVFNFNDEQYE
EFINLLDAPVADDPVIEKLLARKPQWDV
>EECEBICD_04393 hypothetical protein
MGRVTAPEPLSAFHQVAEFVSGEAVLDDWLKQKGLKNQALGAARTFVVCKKDTKQVAGFY
SLATGSVNHTEVTGNLRRNMPDPIPVIILARLAVDLSFHGKGLGADLLHDAVLRCYRVAE
NIGVRAIMVHALTEEAKNFYIHHGFKSSQTQQRTLFLRLPQ
>EECEBICD_04394 hypothetical protein
MSKSSWLLLLGLCASGSALAASSESAFLAQHGLAGKTVEQIVDTIDQTPQSRPLPYSASI
TSTELKLSDGEQIYTLPLGDKFYLSFAPYEWRTHPCFNHSLSGCQGEMPNKPFTVKVTDS
KGAVIVQKEMQSYRNGFIGVWLPRNMEGTLEVSYNGKTASHAIATKDDSQTCLTELPLR
>EECEBICD_04395 Cold shock protein CspA
MSGKMTGIVKWFNADKGFGFITPDDGSKDVFVHFSAIQNDGYKSLDEGQKVSFTIESGAK
GPAAGNVTSL
>EECEBICD_04396 hypothetical protein
MHFTIPLCDLQEFSMEYKDPMFELLSSLEQIVFKDETRKITLTQKPNPFTEFEQLRRGTG
LKTDEFARALGVTVAMVQEWESKREKPTPAELKLMRLIQANPRLSKQLME
>EECEBICD_04397 hypothetical protein
MATGKSCSRWFAPVVALLMVFSLSGCFDKEGDQRKAFVDFLQNTAMRSGERLPTLTADQK
KQFGPFVSDYAILYGYSQQVNQAMDSGLRPVVDSVNAIRVPQDYMTQREPLRQANGSLGV
LAQQLQNAKLQADAAHGALKQADDLKPVFDQVYKKVVTVPADALQPLIPAAQIFTQQLVQ
VGDYIAQQGEQVSFVANGIQFPTSQQASQYNALIGPLASQHQAFNQAWTAAVNATQ
>EECEBICD_04398 Glyoxylate/hydroxypyruvate reductase B
MKPSIILYKTLPDDLLHRLEAHFTVTQVPNLHPETVARHAQAFASAQGLLGASETVNRAL
LEKMPALRAASTISVGYDNVEVDALTARKIVLMHTPAVLTETVADTVMALMLATARRVVD
VAERVKAGEWTESIGPAWFGVDVHHKTLGIVGMGRIGMALAQRAHFGFTMPVLYHARRRH
QEAEDRFNARYCDLDTLLQEADFVCVILPLTAETRHLFGATQFARMKSSAIFINAGRGPV
VDENALIAALQNGEIYAAGLDVFEQEPLSVDSPLLNMSNVVAVPHIGSATHETRYNMMAC
AVDNLIDALQGKIEKNCVNPQAAG
>EECEBICD_04399 putative lipoprotein YiaD
MDVQEAKLRDKMRGTGVSVTRSGDNIILNMPNNVTFDSSSATLKPAGANTLTGVAMVLKE
YPKTAVNVVGYTDSTGSHDLNMRLSQQRADSVASSLITQGVDASRIRTSGMGPANPIASN
STAEGKAQNRRVEITLSPLQ
>EECEBICD_04400 putative lipoprotein YiaD
MKKRVFVIAAIVSGALAVSGCTTNPYTGEREAGKSGIGAGIGSLVGAGIGALSSSKKIAV
KAR
>EECEBICD_04401 Biotin sulfoxide reductase
MTHAPSRHSVLTAAHWGPVRVETDGERIFASYGELPTAHQNSLQTVVHDQVHSKTRVRFP
MVRKGFLASPDKPQGIRGQDEFVRVSWDDALDLIHAQHKRIRESYGPSSIFAGSYGWRSN
GVLHKAATLLQRYMALAGGYTGHLGDYSTGAAQAIMPYVVGGNEVYQQQTSWPVVLEHSE
VVVLWSANPLNTLKIAWNASDEQGLDYFAALRQSGKRLICIDPMRSESVDFFGDKMEWIA
PHMGTDVALMLGIAHTLVENGWQDEAFLARCTTGYDRFADYLLGTTDGTAKTAEWAAEIC
GVSAVKIRELAEIFHHNTTMLMAGWGMQRQQFGEQKHWMIVTLAAMLGQIGTPGGGFGFS
YHFANGGNPTRRAAVLASMQGSIPGGVDAVDKIPVARIVEALENPGGFYQHNGMDRRFPD
IRFIWWAGGANFTHHQDTNRLIRAWQKPELVVISECFWTAAAKHADIVLPATTSYERNDL
TMTGDYSNQHLAPMKQVVSPRWEARNDFDVFAELSERWEAGGYARFTEGKSELAWLETFY
NIAAQRGASQGVTLPPFAAFWQANRLLEMPENPANAQFVRFADFRRDPDNHPLKTASGKI
EIYSARIASYGYADCPGHPMWLAPDEWHGNADAGQVQLLSAHPAHRLHSQLNYSSLRERY
AVAGREPVTIHPQDATTRGIVDGDTVRVWNHRGQVLAGAVVTDGIRPGVICIHEGAWPDP
EPTAGGICKNGAVNVLTKDLPSSRLGNGCAGNTALVWFEKYTGPALPLTAFDPPANS
>EECEBICD_04402 Peptidyl-lysine N-acetyltransferase YiaC

MIRKSQSEDMASILALWMKSTIYAHPFIEERYWHESEAIVRDVYLPAAQTWVWEENGQLK
GFVSVLEARFVGALFVAPDALRHGIGKALLEYVQQRFPLLSLEVYQKNQSAVNFYHALGF
RIEDSAWQEDTAHPTWIMSWQADQTP
>EECEBICD_04403 DNA-3-methyladenine glycosylase 1
MQEEDVERLLQNTGIIRHRGKIQAIISNARAWLAMEQNGESFADFVWSFVDGQPQITQAA
SLDKIPTSTPASDALAKALKKRGFKFVGTTICYSFMQACGLVNDHITGCFCHPGEKHDSQ
IPE
>EECEBICD_04404 DNA-3-methyladenine glycosylase 1
MQRCDWVSQDPLYIAYHDNEWGVPETDSRKLFEMICLEGQQAGLSWITVLKKERELSRLF
SSV
>EECEBICD_04405 hypothetical protein
MIVRKRRGRRTLCCLAGLMACSFFINTTYAWQQEYIAEAAPGHTTERYTWDSDHQPNYND
ILAERIQSTQNTVGPVLSLADETPLDATSGISMGWNFPLSRRVTTGPVAALHYDGSTSSM
YNEYGDSATTLAFTDPLWHASVSTLGWRVNSQFGDVRPWAQISYNQQFGENIWKAQSGLS
RMTAGNQAGNWLDVTVGADVLLNPHLAAYAAFSQAENSATDSDYLYTLGVSARF
>EECEBICD_04406 hypothetical protein
MGALYAWCCMCYADASLTPSHCGWRIVVESASEIINSAKITQKVSTLLPPQELLSLRFDY
NF
>EECEBICD_04407 putative major fimbrial subunit LpfA
MKKVVFALSALAVVSTSAFAAESGDGTIKFTGEIVDAPCVVSTDSQNQEVVLGQVKKNIF
KAIGDKSSSKPFQIKLEDCDITSNTKVNVSFNGVGDTDDATLVSVNTEAGAATGVGIGIY
DNANKLVEMNTGKSTTTLAAGQTVLYYTANYVATKDTVTTGYGNAEVDFNLSYE
>EECEBICD_04408 putative fimbrial chaperone LpfB
MNRSRLISCTALVLALIAQNSFAGGVALSSTRVIYDGSRKEASLTVNNKSTTDEFLIQSW
IDDANGNKKTPFIITPPLFKLSPTKNNVLRIVNTTNTLPQDRESVYWINVKAIPAKSEDA
EAKNVLQIAVRTRLKLFYRPAGLKGNSMDGWNKLQFTSAGANQIKVENPSAFNLTFNKFY
ANGRDIEKTGMVPAKGSLNIELPAGTGKVSEVKYNIINDFGTAGDMLTQRVN
>EECEBICD_04409 putative outer membrane usher protein LpfC
MTWTHLPLGNKTSRFTQSALALMIAGTLPAYAGTFNPRFLEDVPGIDQHVDLSMYESNKA
EHLPGKYRVSVVVNEKKWSLAPWSLRQRQRRSAQKWVNPWCRA
>EECEBICD_04410 hypothetical protein
MGESLVPCLSRVQLEDMGVRIDSFPALKMAPPEACVAFDDIIPQAASHFDFADQTLIMSF
PQAAMTQTARGTVPESQWDEGVNALLVDYNFSGSNASYDAHDSETSYNSDSYYLNLRSGM
NLGAWRLRNYSTWTRNDGNNTWDNIGTSLSRAIVPLKSQLTLGDTSTAGDIFDSVQMRGV
QLTSDEEMLPDSQRGFAPVIRGIAKSNAEVTVEQNNYVIYRTFVQPGAFEINDLYPTSNS
GDLTVTIKESDGSEQKFVQPFSSVALLQREGHLKYSLSAGEYRAGNYNSAEPKFGQLDAM
YGLPYGFTVYGGAIFSDNYYSLAGGLGKKLRLYRRDLHRCNPGKKQAGK
>EECEBICD_04411 putative outer membrane usher protein LpfC'
MRSDADSSYSQYHKRSQIQGNVTQQLGAWGSVYFNVTQQDYWNDEGKQRSLNAGYNGRIG
RVNYSVAYTWTKSPEWDESDRLLSFSMSIPLGRVWSNYHLTTDQHGRTNQQLGVSGTALE
DRNLNYSVQEGYGSNGVGNSGSVNLDYQGGVGSASLGYNYNRDGQQVNYGLRGGVIAHSE
GITLSQPLGESMAIISAPGARGAHVINNGGVEVDWMGNAVVPYLTPYRETEVSLRSDSLN
NQVDLDTASVNVVPTRGAIVRARFDTRVGYRVLMNLTQANGKAVPFGATATLLDTTKESS
SIVGEDGQLYISGMPEKGALQVNWGKDQAQQCRVAFTLPEQQDNTGVVMANAVCR
>EECEBICD_04412 putative minor fimbrial subunit LpfD
MLKKLIMFTGLLGGSVLFSGQALAAADFGPCTPEGGTHIFSATINKTVSDTSKNTTGATF
VDFDSWNLGGTYAMSCECPDDTSLINDTLFKAVVPLAFVTNIESRSYYQINNNIAIASDV
LISGGRGEYVNTPFENVGNLTNNRSQCSQNASSKDAIWTSGGKGHLSLYILHPFVGESII
PSTKIMDLFVTKKPSVYGSIPASSVYISGSITVPQGCELSSGSTLEIPFGEFKATDFKDR
KGQVAKNATKFTKELQFKCTNISDGIKIFLRIEGMPNANDSNAIDMGNPDIGAVIEGANG
KILVPNDASVNQELSVSGLVDDTHRTASTTISAYPISTTGKLPAAGDFEGIATMRIDVE
>EECEBICD_04413 putative fimbrial subunit LpfE
MKNLHALMPACLLLTASAMAAPSNIGSAGDIHFTITIKAATCELENDSIDVNMETVVLQR
PVKVGKELNQKNFSIGLKDCAYATKASVTMDGSPDPTDPSLFALDSGGATGVALKIKTSG

GEQQYPSSTDSTPVEHTVWFDGTNKLNYIASYVPVKPDATVGTANATVNFSVTYE
>EECEBICD_04414 Kdo(2)-lipid A phosphoethanolamine 7''-transferase
MRYIKSMTQQKLSFLLALYIGLFMNCAVFYRRFGSYAQEFTIWKGLSAVVELGATVLVTF
FLLRLLSLFGRRVWRVLATLVVLFSAGASYYMTFLNVVIGYGIIASVMTTDIDLSKEVVG
LHFVLWLIAVSVLPLIFIWSNHCRYTLLRQLRTPGQRFRSAAVVVLAGVMVWAPIRLLDI
QQKKVERATGIDLPSYGGVVANSYLPSNWLSALGLYAWAQVDESSDNNSLINPARKFTYV
APKDGDDTYVVFIIGETTRWDHMGIFGYERNTTPKLAQEKNLAAFRGYSCDTATKLSLRC
MFVREGGADNNPQRTLKEQNVFAVLKQLGFSSDLYAMQSEMWFYSNTMADNISYREQIGA
EPRNRGKTVDDMLLIDEMQNSLAQNPEGKHLIILHTKGSHFNYTQRYPRSYAQWKPECIG
VDSGCTKAQMINSYDNSVTYVDHFITSVFDQLRDKKAIVFYAADHGESINEREHLHGTPR
NMAPPEQFRVPMLVWMSDKYLASPQHAQMFAHLKQQAEIKVPRRHVELYDTIMGCLGYTS
PNGGINQNNNWCHIPDAQKVAAK
>EECEBICD_04416 HTH-type transcriptional regulator MalR
MAVQNKKRAKLIDVARHAGVSPGTVSNALHNTRFVEPQTRRRIEEAIVALNYTPNIRARQ
LRTGKTNTIALLSSVPLAIASGASRLGFMMEVALTSAMMALEKQHALILVPPGANPLDAV
SFDAAILIEPAENDPQLQALAQAGIPCVTIGRTPGTDTPVPWVELHSAATAQLLLTHLEA
SGASKCALFVGNTRRTSVLESEAAYQRWCAGRQAPVVYSLNESEGENAGYQAAQQLLQAH
PDVDGVLVLIDTFASGAVRAFQEQDIAIPEQMRVVTRYDGIRARESLPPLTAVNMHLDEV
ARQAITLLFAVLSGEKVSYSDGIMPELVVRASTCR
>EECEBICD_04417 hypothetical protein
MAHLTQDSTFTLGRRPAGLIYADKAKSFGGYTLFAPQTAEGRVYLVDEQGEVAHQWQLPV
RAGRDAVLLPNGNLGYNGSHRTSANLYPAWDLWHGGDFYEVTPDNEIVWHYEDIFHHHDA
QWLENGNLLYTAASPLPADIAARVTGGDPRRDASDGIIQRDVVKEVNRDGEVVWEWRAWE
HLNPEDFPIHDIFDRRHWPMINGLSVTRDGLVLMSLRTTSGVIAVDKESGKVIWHAGPEV
VAQQHTPVEMENGSILVFDNGNLRPGVTSPHSTVLEFDPQTKAITWQYRDIFPPAFFSPY
MGSAQRLANGNTFICESAFGRLFEVTPEGETVWEYIIPFFNEYPEHLSKGIIPGKQNSAF
RAHRYAADAISWLK
>EECEBICD_04418 Uric acid permease PucK
MPQPGLTTPDNDALPWQTTLLLALQHVLVVAATPITSVFLIAKALHFTDTVTASVLSATF
LMCGLGAILQSLGVKGVGARLPFIMVPGGAPIAIFVAIALQTNIQTAIGAVILTSLFYFI
ALPIFRRCLHHFPPFIIGIMLLMVSINLIRLYGGLIIGQPGSADFAHPTSIILSLGTILI
TLIFALAFSGILRQLAVMFGLLAGTLLGMALGSTDFSGVSHGPLFSFPQLLPFGWPIFDL
SASLPLLIYAVISMAEATGQTIATAEIVNSTQNVQQAIPRTIRGDAVMSLLGGFLAPP
>EECEBICD_04419 Nucleobase transporter PlUacP
MVRTTNVKSRFVTAAAGGLLILIAIFAPLVRLATCLPGSVVCGTAVIVFSIIGVIGIDMI
AREPLHTPGKTYALAMGLAMGMLPILVPGLYQNFPAGVQMVFGNGMAAGTLTAILVNSLF
NWSEKRTQARVKS
>EECEBICD_04420 Periplasmic dipeptide transport protein
MSISLKKSGMLKLGLSLVAMTVAASVQAKTLVYCSEGSPEGFNPQLFTSGTTYDASSVPI
YNRLVEFKTGTTEVIPGLAEKWDISEDGKTYTFHLRKGVKWQSSKDFKPTRELNADDVVF
SFDRQKNEQNPYHKVSGGSYEYFEGMGLPDLISEVKKVDDHTVQFVLTRPEAPFLADLAM
DFASILSKEYADNMLKAGTPEKVDLNPVGTGPFQLVQYQKDSRILYKAFDGYWGTKPQID
RLVFSITPDASVRYAKLQKNECQVMPYPNPADIARMKEDKNINLMEQAGLNVGYLSYNVQ
KKPLDDVKVRQALTYAVNKEAIIKAVYQGAGVAAKNLIPPTMWGYNDDIKDYGYDPEKAK
VLLKEAGLEKGFTIDLWAMPVQRPYNPNARRMAEMIQADWAKIGVQAKIVTYEWGEYLKR
AKDGEHQTVMMGWTGDNGDPDNFFATLFSCDAAQQGSNYSKWCYKPFEDLIQPARATDDH
NKRIELYKQAQVVMHDQAPALIIAHSTVYEPVRKEVKGYVVDPLGKHHFENVSVE
>EECEBICD_04421 Dipeptide transport system permease protein DppB
MLQFILRRLGLVIPTFIGITLLTFAFVHMIPGDPVMIMAGERGISPERHAQLLAELGLDK
PMWQQYLHYIWGVMHGDLGISLKSRIPVWDEFVPRFKATLELGVCAMIFAVAVGIPVGVL
AAVKRGSIFDHTAVGLALTGYSMPIFWWGMMLIMLVSVHWNLTPVSGRVSDMVFLDDTNP
LTGFMLIDTAIWGEEGNFIDALAHMILPAMVLGTIPLAVIVRMTRSSMLEVLGEDYIRTA
RAKGLTRMRVIIVHALRNAMLPVVTVIGLQVGTLLAGAILTETIFSWPGLGRWLIDALQR
RDYPVVQGGVLLVATMIILVNLLVDLLYGVVNPRIRHKK

>EECEBICD_04422 Dipeptide transport system permease protein DppC
MSQVTENNVNAAPAPMTPLREFWHYFKRNKGAVVGLAYVLIVILIAVFANFIAPYNPAEQ
FRDALLAPPVWQEGGSWAHILGTDDVGRDVLSRLMYGARLSLLVGCLVVVLSLVMGIILG
LVAGYFGGLVDNIIMRVVDIMLALPSLLLALVLVAIFGPSIGNAALALTFVALPHYVRLT
RAAVLVEVNRDYVTASRVAGAGAMRQMFVNIFPNCLAPLIVQASLGFSNAILDMAALGFL
GMGAQPPTPEWGTMLSDVLQFAQSAWWVVTFPGLAILLTVLAFNLMGDGLRDALDPKLKQ
>EECEBICD_04423 Dipeptide transport ATP-binding protein DppD
MALLNVDQLSVHFGDEGTPFKAVDRISYSVKQGEVVGIVGESGSGKSVSSLAIMGLIDYP
GRVMAENLLFNGQDLKRISEKERRNLVGAEVAMIFQDPMTSLNPCYTVGFQIMEAIKVHQ
GGNKKTRRQRAIDLLNQVGIPDPASRLDVYPHQLSGGMSQRVMIAMAIACRPKLLIADEP
TTALDVTIQAQIIELLLELQQKENMALVLITHDLALVAEAAHKIIVMYAGQVVETGAAQD
IFRAPRHPYTQALLRALPEFAQDKARLASLPGVVPGKYDRPTGCLLNPRCPYATDRCRAE
EPALNQLDDGRQSKCHYPLDDAGRPTL
>EECEBICD_04424 Oligopeptide transport ATP-binding protein OppF
MSTHEATLQQPLLRAIDLKKHYPVKKGIFSPERLVKALDGVSFNLERGKTLAVVGESGCG
KSTLGRLLTMIETPTGGELYYQGQDLLKHDPHAQKLRRQKIQIVFQNPYGSLNPRKKVGQ
ILEEPLLINTSLSKAQRREKALAMMAKVGLKTEHYDRYPHMFSGGQRQRIAIARGLMLDP
DVVIADEPVSALDVSVRAQVLNLMMDLQQDMGLSYVFISHDLSVVEHIADEVMVMYLGRC
VEKGTKEQIFNNPRHPYTQALLSATPRLNPDDRRERIKLTGELPSPLNPPPGCAFNARCS
RRFGPCTQLQPQLKEYDGQLVACFAVDQDENPQKPLS
>EECEBICD_04425 Inner membrane transport protein YhjV
MQDDTLPLNNSNATTTPLSTRLPFTKYDFGWVLLCIGMAIGAGTVLMPVQIGLKGIWVFI
TAFIIAYPATYIVQDIYLKTLSESETCDDYTDIISHYLGKNWGIFLGVIYFLMIIHGVFI
YSLSVVFDSASYIKTFGLTEVDLSQSIIYKVAIFAVLVAIASGGEKLLFKISGPMVVVKV
GIILIFGFAMIPHWNLDNISAFPAASVFFRDVLLTIPFCFFSAVFIQVLNPMNIAYRKRE
PDRVLATRMAIRTHRISYITLIAIILFFSFSFTFSISHEEAVSAFEQNISALALAAQVIP
GHIIHITSTILNIFAVLTAFFGIYLGFHEALKGIVLNVLSRIMDVKNVNPLLLTSGICVF
IVVTLVIWVSFRVSVLVFFQLGSPLYGIVACIIPFFLIYKVAQLEKLRGLKTWLILLYGI
LLCLSPLLKLIE
>EECEBICD_04426 Small toxic polypeptide LdrD
MTLTQLGVVFWHDLAAPIIAGIIASVIVNWLRDRK
>EECEBICD_04427 Cellulose biosynthesis protein BcsG
MKAPHEGAPIDINQPSSYLAISELVVRAVDGKLFTEDSVNWNKLTSNLPQTAPVSENANA
VVIQYQGKPYVRLNGGDWVPYPQ
>EECEBICD_04428 Cellulose biosynthesis protein BcsG
MTQHTQTPSMPSPLWQYWRGLSGWNFYFLVKFGLLWAGYLNFHPLLNLVFMAFLLMPIPK
YRLHRLRHWIAIPVGFALFWHDTWLPGPQSIMSQGTQVAEFSSGYLLDLIARFINWQMIG
AIFVLLVAWLFLSQWIRVTVFVVAIMVWLNVLTLTGPVFTLWPAGQPTDTVTTTGGNAAA
TVATAGDKPVIGDMPAQTAPPTTANLNAWLNTFYAAEEKRKTTFPAQLPPDAQPFDLLVI
NICSLSWSDVEAAGLMSHPLWSHFDILFKHFNSGTSYSGPAAIRLLRASCGQPSHTRLYQ
PANNECYLFDNLAKLGFTQHLMMDHNGEFGGFLKEVRENGGMQSELMNQSGLPTALLSFD
GSPVYDDLAVLNRWLTGEEREANSRSATFFNLLPLHDGNHFPGVSKTADYKIRAQKLFDE
LDAFFTELEKSGRKVMVVVVPEHGGALKGDRMQISGLRDIPSPSITNVPAGVKFFWHESP
A
>EECEBICD_04429 hypothetical protein
MMTISDIVQIILFCALIFFPLGYLARHSLRRISDTTRLLFAKPRYVKPAGTLRRATKVKA
DKK
>EECEBICD_04430 Cyclic di-GMP binding protein BcsE
MDPVFSLGISSLWDELRHMPTGGVWWVNADRQQDAISLVNQTIASQTENANVAVIGMEGD
PGKVIKLDESHGPEKIRLFTMPDSEKGLYSLPHDLLCSVNPTHYFFILICANNTWRNITS
ESLHKWLEKMNKWTRFHHCSLLVINPCNNSDKQSSLLMGEYRSLFGLASLRFQGDQHLFD
IAFWCNEKGVSARQQLLLCQQDERWTLSHQEETAIQPRSDEKRILSHVAVLEGAPPLSEH
WTLFDNNEALFNDARTAQAATIIFSLTQNNQIEPLARRIHTLRRQRGSALKIVVRENIAS
LRATDERLLLGCGANMIIPWNAPLSRCLTLIESVQGQQFSRYVPEDITTLLSMTQPLKLR

GFQPWDIFCDAIHTMMSNTLLPADGKGVLVALRPVPGIRVEQALTLCRPNRTGDIMTIGG
NRLVLFLSFCRVNDLDTALNHIFPLPTGDIFSNRMVWFEDKQISAELVQMRLLSPELWGT
PLPLAKRADPVINAEHDGRIWRRIPEPLRLLDDTAERAS
>EECEBICD_04431 Protein YhjR
MYNNEPGAQSDPTLGYTFQNDFLALSQAFSLPEIDYTDISQREQLAAAIKRWPLLAEFAQ
PHSLRKP
>EECEBICD_04432 Cellulose biosynthesis protein BcsQ
MAILGLQGVRGGVGTTSLTAALAWALQILGENVLVIDASPDNLLRMSFNVDFVHQGGWAR
SLLDGQDWRDAGLRYTSQLDLLPFGQLTAQERENPQSWQETLGEIGSAIQALKASGRYSW
ILLDLPYGASPLTRQLVSLCDHTLAVARVDANCHIRLHQQALPAGAHILINDLRIGSQLQ
DDLYQVWLQSQRRLLPIVIHRDEAMAECMASKQPLGEYRSDSLAAEEVLTLANWCLLHDA
GDKTSAGSLR
>EECEBICD_04433 Cellulose synthase catalytic subunit [UDP-forming]
MSALSRWLLIPPVSARLSERYQGYRRHGASPFSAALGCLWMILAWIVFPLEHPRWQRIRD
EHKALYPHINAARPRPLDPARYLIQTLWLVMISSTKERHEPRWRSFARLKDVRGRYHQWM
DTLPERVRQKTTHLEKEKELGHLSNGARRFILGVIVTFSLILALICITQPFNPLSQFIFL
LLLWGVALLVRRMPGRFSALMLIVLSLTVSCRYIWWRYTSTLNWDDPVSLVCGLILLFAE
TYAWIVLVLGYFQVVWPLNRQPVPLPKEMSQWPTVDIFVPTYNEDLNVVKNTIYASLGID
WPKDKLNIWILDDGGRESFRHFARHVGVHYIARATHEHAKAGNINNALKHAKGEFVAIFD
CDHVPTRSFLQMTMGWFLKEKQLAMMQTPHHFFSPDPFERNLGRFRKTPNEGTLFYGLVQ
DGNDMWDATFFCGSCAVIRRKPLDEIGGIAVETVTEDAHTSLRLHRRGYTSAYMRIPQAA
GLATESLSAHIGQRIRWARGMVQIFRLDNPLFGKGLKLAQRLCYLNAMFHFLSGIPRLIF
LTAPLAFLLLHAYIIYAPALMIALFVIPHMVHASLTNSKIQGKYRHSFWSEIYETVLAWY
IAPPTLVALINPHKGKFNVTAKGGLVEEKYVDWVISRPYIFLVLLNLLGVAAGVWRYYYG
PENETLTVIVSLVWVFYNLVILGGAVAVSVESKQVRRAHRVEIAMPGAIAREDGHLFSCT
VHDFSDGGLGIKINGQAQVLEGQKVNLLLKRGQQEYVFPTQVVRVTGNEVGLQLMPLTTK
QHIDFVQCTFARADTWALWQDSFPEDKPLESLLDILKLGFRGYRHLAEFAPPSVKVIFRS
LTALIAWIVSFIPRRPERQAAIQPSDRVMAQAQQ
>EECEBICD_04434 Cyclic di-GMP-binding protein
MAQTAPSREVKLTFAQIAPPPGSMALRGVNPNGGIEFGMRSDEVASKAVLNLEYTPSPSL
LPVQSQLKVYLNDELMGVLPVTKEQLGKKTLAQVPINPLFITDFNRVRLEFVGHYRDVCE
NPASSTLWLDIGRNSALDLTYNMLAVNNDLSHFPVPFFDPRDNRPVTLPIVFADMPDLAQ
QQAASIVASWFGSRAGWRGQRFPVLYNHLPDRNAIVFATNDRRPDFLRDHPAVNAPVIEM
MSHPDNPYVKLLVVFGRDDKDLLQAAKGIAQGNILFRGSSVVVNDVKPLLARKPYDAPNW
VRTDRPVTFGELKTYEEQLQSSGLEPAPINVSLNLPPDLYLLRSNGIDMDLNYRYTSPPT
KDSSRLDISLNNQFLQAFSLNSTQETNRLLLRLPVLQGLLDGKTDVSIPALKLGAMNQLR
FDFRYMNPMPGGSVDNCITFQPVPNHVVIGDDSTIDFSKYYHFIAMPDLRAFANAGFPFS
RMADLSDTLAVMPKTPTEAQMETLLNTVGAIGGQTGFPAINLTITDDSAQIADKDADLLI
IGAIPGKLKDDKRIDLLVQATQSWVKTPMRQTAFPSIMPDEADRAADAQSTVTASGPMAA
VVGFQSPFNDQRSVIALLADSPRGYQLLNDAVNDSGKRAAMFGSVAVIRESGVHSLRVGD
IYYVGHLPWFERLWYALANHPVLLAVLAALSVVLLAWVLWRLLRILSRRRLDPDHE
>EECEBICD_04435 Endoglucanase
MMTMLRGWITMIVMLTAINAQAACSWPAWEQFKKDYISQQGRVIDPGDARKITTSEGQSY
AMFFALAANDRPAFAQLFNWTQNNLAQGSLREHLPAWLWGQKDPDTWSVLDSNSASDGDI
WMAWSLLEAGRLWKETRYTEVGTALLKRIAREEVVNVPGLGSMLLPGKIGFAEANSWRFN
PSYLPPQLAQYFSRFGAPWSTLRETNLRLLLETAPKGFSPDWVRYESKQGWQLKAEKTLI
SSYDAIRVYLWTGMMHDGDPQKARLLARFKPMATLTMKNGVPPEKVDVVSGNAQGTGPVG
FSAALLPFLQNRDAQAVQRQRVADHFPGSDAYYNYVLTLFGQGWDQHRFRFTVKGELLPD
WGQECVSSR
>EECEBICD_04436 Cellulose synthase operon protein C
MRKFTLSLMHAFLPAGGRNALPGKRGVSRALLGLSLGMALTPLAGAATSAQQQLLEQVRL
GEATHREDLVRQSLYRLELIDPNDPQVIAARFRYLLRQGDSDGAQKLLDRLAQLAPESTA
YQSSRTAMLLSTPQGRQSLQEARLLATTGHTEQAIASYDKLFKGYPPEGELAVEYWTTVA
KLPARRHEAINQLQKINAVSPGNNALQNALAQLLFASGRRDEGFAVLKQMAKSSTGRSAA

SAIWYQQIKDLPVSDASVKALQDYLTQFSEGDSVSAARAQLSEQQKQLADPAFRARSQGI
AAVNAGEGGKAIAQLQQAVSARQDDSEAVGALGQAYSQRGDRARAVAQFEKALAMAPHSS
SRDKWESLLKVNRYWLLIQQGDAALKANNLAQAERFYQQARAVDNTDSYAVLGLGDVAMA
RKDNAAAERYYQQTLRMDSGNTNAVRGLANLYRQQSPQKAAAFIASLSASQRRSIDDIER
SLENDRLAQQAETLESEGKWAQAAELHRRRLALDPGSVWVTYRLSRDLWQAGQHAQADAQ
MRSLAQQKPNDPEQVYAYGLYLSGSDRDRAALAHLNTLPTSQWNSNIQELAGRLQSNQVL
ESANRLRDSGKEREAEALLRQQPPSTRIALTLADWAQQRGDNAAARAAYDAVLAREPGNV
DAMLGRVEIDIAQGDNAAARAQLAALPASQITSINMQRRVALAQLQLGDITAAARTFNRI
TPQAKAQPPSMESAMVLRDAAAFQAQTGEPQRALETYKEAMVAAAITPVRPLDNDTFTRL
TRNDEKDDWLKRGVRSDAAELYRQQDLNVTLAHDYWGSSGTGGYSDLKAHTTMLQVDAPW
SDGRAFFRTDMVNMDVGRFSTDTDGKYDNNWGTCTLEKCSGHRSQADTGASVAVGWQNET
WRWDIGTTPMGFNVVDVVGGVSYSDDIGPLGYTLNAHRRPISSSLLAFGGQKDASSNTGT
KWGGVRANGGGVSLSYDKGEANGVWASLSGDQLSGKNVEDNWRVRWMTGYYYKVINENNR
RVTVGLNNMIWHYDKDLSGYSLGQGGYYSPQEYLSFAVPVMWRQRTENWSWELGGSVSWS
HSRNRTMPRYPLMNLIPADYQEDARDQTNGGGSSQGFGYTARALIERRVTANWFVGTAVD
IQQAKDYTPSHLLLYVRYSAAGWQGDMDLPPQPLVPYADW
>EECEBICD_04437 putative cyclic di-GMP phosphodiesterase PdeK
MRVSRSLTIKQMAMVAAVVMVFVFVFCTVLLFHLVQQNRYNTATQLESIARSVREPLSSA
ILKADLPGAETILESIKPAGVVSRADVVLPNQFQALRKRFIPERPVPVMVTRLFELPVQI
SLPVYSLERPANPQPLAYLVLQADSYRMYKFVMSALSTLVTIYLLLSLILTVAIAWCVNR
LIVHPLRKIARELNDIPQQELIGHQLALPRLHQDDEIGMLVRSYNLNQQLMQRQREEQTD
NAMRFPVSELPNKAFLMALLEQVIARQQTTALIIVTCETLRDTAGVLQETQREILLLTLV
EKLKSVLAPRMVLTQVSGYDFAIIAHGVKEPWHAITLGQQILTIINERLPIQGIQLRPSC
SIGIAMYYGDLTAEALYGRAVSAAFTARRKGKNQIQFFDPAQMEAAQQRLTEESDILTAL
DNHQFAIWLQPQVEMRSGNVLSAEALLRMQQPDGSWELPEGLIERIESCGLMVTVGHWVL
EESCRQLAAWQERGVTLPLSVNLSALQLMHPGMVSDLLELLNRYRIQPGTLILEVTESRR
IDDPHAAVAILRPLRNAGVRIALDDFGMGYAGLRQLQHMKSLPVDILKIDKMFVDGLPDD
HSMVTAIILMARSLNLQLIAEGVENEAQRAWLEQAGVNVAQGFLFARPVPADIFEERYLS
HENSDYKS
>EECEBICD_04438 Aerobic C4-dicarboxylate transport protein
MKTSLFKSLYFQVLTAIAIGILLGHYYPELGAQMKPLGDAFVKLIKMIIAPVIFCTVVTG
IAGMESMKAVGRTGAVALLYFEIVSTIALIIGLIIVNVVQPGAGMNVDPATLDAQAVAVY
AAQAKEQGIIAFLMDVIPGSVIGAFASGNILQVLLFAVLFGFALHRLGSKGQLIFNVIES
FSQVIFGIINMIMRLAPIGAFGAMAFTIGKYGVGSLVQLGQLIICFYITCILFVVVVLGT
IARVTGFSIFKFIRYIREELLIVLGTSSSESALPRMLDKMEKLGCRKSVVGLVIPTGYSF
NLDGTSIYLTMAAVFIAQATNSHMDIFHQITLLVVLLLSSKGAAGVTGSGFIVLAATISA
VGHLPVAGLALILGIDRFMSEARALTNLVGNGVATVVVAKWVKELDHQKLDDVLNNRAPD
GKTHEISS
>EECEBICD_04439 Protein YhjJ
MQGTKIRLLAGSLLMLASAGYVQADALQPDPAWQQGTLANGLQWQVLATPQRPSDRIEVR
LQVNTGSLTESTQQSGFSHAIPRIALTQSGGLDAAQARSLWQQGFDPKRPMPPVIVSYDS
TLYNLSLPNNRNDLLKEALTYLANVSGKLTITPETVNHALSSEDMVATWPADTKEGWWRY
RLKGSALLGHDPAEPLKQPVDAAKIQAFYEKWYTPDAMTLIVVGNIDARSVAEQINKTFG
TLKGKRETPAPVPTLSPLRAESVSIMTDAVRQDRLSIMWDTPWQPIRESAALLRYWQADL
AREALFWHIQQELTKNNAKDIGLGFDCRVLFLRAQCAINIESPNDKLNTNLSLVANELAK
VRDKGLSEEEFTALVAQKNLELQKLFATYARTDTDILTGQRMRSLQNQVVDIAPEQYQKL
RQNFLNSLTVDMLNQNLRQQLSQEMALILLQPQGEPEFNMKALKATWDEIMVPTTAAAVE
ADEAHPEVTETPAAQ
>EECEBICD_04440 2-dehydro-3-deoxygluconokinase
MSKKIAVIGECMIELSQKGADVQRGFGGDTLNTSVYIARQVDSAALAVHYVTALGTDSFS
QQMLEAWQHENVDTSLTQRMENRLPGLYYIETDDTGERTFYYWRNEAAAKFWLESEQSAA
ICKALATFDYLYLSGISLAILSPTSRDKLLSLLRECRANGGKVIFDNNYRPRLWTSREET
QQVYQKMLECTDIAFLTLDDEDALWGQQPVEEVIARTHAAGVQEVVVKRGADSCLVSIQG
EALIDVPAVKLPKEKVIDTTAAGDSFSAGYLAVRLTGGSATDAAKRGHLTASTVIQYRGA

IIPHDAMPQ
>EECEBICD_04441 Cyclic di-GMP phosphodiesterase PdeH
MIKQVIQQLRVPDAGIENLQERRYWLQCERAYTYQPIYQTDGRLMAVELLTVVTHPDNPS
RRIAPDRYFAELAVRHRIDVVKEQLHQLEQKADFFTRHHLLASVNVDGPTLIAMRRQPDI
LAAMERLPWLRFELVEHIRLPKDSSFASMCEFGPLWLDDFGTGMANFSALSEVRYDYIKV
ARELFVMLRQSAEGRNLFTLLLQLMNRYCRGVIVEGVETLEEWRDVQRSPAFAAQGYFLS
RPVPLISLEEVILTL
>EECEBICD_04442 hypothetical protein
MTKAGKITAAITGTFLLLIAIVILLIATFDWNRLKPTINQKVSTELNRPFAIRGDLGVVW
ERQKQETGWRSWVPWPHVHAEDVILGNPPDIPEVTMVHLPRVEATLAPLALLTKTVWLPW
IKLVKPDARLIRLSEKTNNWTFNLTQDKNTDPNAKPSAWSFRLDNILFDQGRIAIDDKVS
KADITILVDPLGKPLPFSEVTGTKGKADKSTVGDYVFGLKAQGRYNGEPLTGTGKIGGML
ALRSESTPFPVQADFRSGNTRVAFSGVVNEPMKMGGVDLRLKFSGDSLGDLYDLTGVLLP
DTPPFETDGRLVAKIDAEKSSVFDYRGFNGRIGDSDIHGSLTYTTGKPRPKLEGDVESRQ
LRLADLGPLIGVDSGKDAEQSKRSEQRKGEKNVQPADKVLPYDRFETDKWDVMDADVRFK
GRRIEHGGSLPISDLSTHIILKNADLRLQPLKFGLAGGSIVSNIHLEGDKKPMQGRADIQ
ARRLKLKELMPDVELMQKTLGELNGDADIRGTGNSVAALLGNSNGNLKLLMNDGLISRNL
MEIVGLNVGNYIVGQIFGDDEVRVNCAAANLNIANGVARPQIFAFDTENALINVTGTASF
ASEQLDLTIDPESKGIRIITLRSPLYVRGTFKNPQAGVKPGPLIARGAVAAALATLVTPA
AALLALISPSEGEANQCRTILTQMKQ
>EECEBICD_04443 Inner membrane metabolite transport protein YhjE
MQATATTLDHEQEHVPVNSRNKVVIASLIGTAIEFFDFYIYATAAVIVFPLIFFPQGDPT
AATLQSLATFAIAFVARPIGSALFGHFGDRVGRKVTLVASLLTMGISTVIIGLLPGYATI
GIFAPLLLALARFGQGLGLGGEWGGAALLATENAPPRKRALYGSFPQLGAPIGFFFANGT
FLLLSWLLTDEQFMSWGWRVPFIFSAVLVIIGLYVRVSLHETPVFAKVAAAKKQVKIPLG
TLLTKHVRVTILGTFIMLATYTLFYIMTVYSMTYSTAAAPVGLGLPRNEILWMLMMAVIG
FGVMVPVAGLLADAFGRRKSMVIITTLIILFALFAFTPLLGSGNPALVFVFLLLGLSLMG
LTFGPMGALLPELFPTEVRYTGASFSYNVSSILGASVAPYIAAWLQSHYGLAAVGVYLAS
MAALTLIALLLTHETRHQAL
>EECEBICD_04444 Inner membrane protein YhjD
MIRGTFIAAIGFEVIKIIMTYTLPSLVKSPSGAAFGSVLGLMAFFYFFARLTLFCAAWIA
TAEYKDDPRMPGKTQR
>EECEBICD_04445 Inner membrane protein YhjD
MTQENDVKRPIQELEHDPIQKIETQPHDAPEKNEKANQALHSVTTLMQKIQRQPMVAHLI
RATERFNDRLGNQFGAAITYFSFLSMIPIMMVSFAAAGFILASHPNLLEDIFSKILMNVS
DPTLASTLKNTINTAVQQRTTVGLVGLGIALYSGVNWMGNLREAIRAQSRDVWERKPQDQ
EKIWLKYLRDFISLIGLLIALIITLSITSIAGSAQQMIISALYLDSIEWLKPAWHLIGLA
ISIFANYLLFSGFSGDCLAIVPVKKR
>EECEBICD_04446 HTH-type transcriptional regulator DmlR
MDKIYAMKLFVRVAERESFSRAAEDIGLPKGSVSRQIQALEHQLGIRLLHRTTRRVQLTQ
DGMVYYERAKDLLSNLDELEGLFQPDPASISGKIRVDIPPCLANSLIMPRLPTFLHQYPG
IALELCSSDRQVDLLREDFDCVVRTEPLHAPGILTRPLGKLRRVNCASPQYLARFGYPEN
LDDLASHAVVHYSLTPGVSSPGFAFETPHGMQWVKTGGMLTVNSTETWHTACLAGLGIIQ
TPRIAVREALRAGTLIEILPQYRAAPLPMTLHYPHRRNLSRRVHLFMVWLTESIKEAAE
>EECEBICD_04447 Putative HTH-type transcriptional regulator YhjB
MQVIMFDRQSIFIHGMKISLQQHIPGISIQSVGQAEELWQKIESAPDALVMLDSGLDAEF
CREVLQRTVQQFPEVKIIITAMDGSQKWLHEVMQFNVQAVVPRDSDAETFVLALNAVARG
MMFLPGDWLNSTELESRDIKALSARQREILQMLAAGESNKQIGRALNISTGTVKAHLESL
YRRLDVKNRTQAAMMLNESN
>EECEBICD_04448 hypothetical protein
MPHISSRFSSACIAFIKQWQGLSLEKYRDRQGNWVIGYGHMLTPDETLTFITPDQAEAFL
LDDLNSCDILLQNCLPELNDRFQRETLIALMFSIGHQRFLSLINTGDISQPEISGLRI
>EECEBICD_04449 hypothetical protein
MTNSASQATHAPFEHSLGIIRQASIEILLLLGIHTTEGKEPRWFMEQLEQARLNLGGWGA

VAKKLRINDAQLSQFMLQLRHLQQHVPQYDSGQEVSENQLLAALRFVTSLEHLRQQQPLL
TYQTELEEPDQEAHLEAQRQLRAIELTLKALIARAWPDRASLNHYLKQHFGPDRLRQWLK
QGEDQHALEGMLFSELALMVVDKKLFARHYVRIFNDASALTLFAESRTTLRMFLDDCRLA
RNEVIARQPLTSAQLMLLNVQYQQIVRPIQRAYAEKRTRVNPASFLLADERELRQFWETA
RLKDRQAGGDKHEISESIEPPRKRPPRTPEEREQLISGALWGAVGVMTLAILAGAFWLFS
SSSPGSDNGQAPAMAQDEPPREAPSARETLNHMGITWDAFTMRAAIERNDTRVTALFLQG
GMNWQLAWTEQAFAAGHTEVLQLLLRYPALMDEVKPCRRFITTLSHDMSSGAPLTAMHKT
YLQTFCTVPAVVTRQQHDTEQARLRAQARPSADNKKWLKIQSAIYDAIH
>EECEBICD_04450 Cytoplasmic trehalase
MLNQKLNPTPSEDLTIDVDLLYETDPCELKLDEMIEAEPEPEMIEGLPASDALTPADRYL
ELFEHVQSTKLFPDSKTFPDCAPKMDPLDILIRYRKVRRHRDFDLRRFVENHFWLPETLS
SEYVSNPENSLKEHIDQLWPILTREPQDHIPWSSLLALPQSYIVPGGRFSETYYWDSYFT
MLGLAESGREDLLKCMADNFAWMIENYGHIPNGNRTYYLSRSQPPVFALMVELFEEDGVR
GARRYLDHLKMEYAFWMDGAESLALNQAYRHVVRMPDGSLLNRYWDDRDTPRDESWLEDV
ETAKHSGRPPNEVYRDLRAGAASGWDYSSRWLRDAGRLASIRTTQFIPIDLNAFLYKLES
AIANISALKGERDTEALFRQKASDRRAAVNHYLWDDENGCYRDYDWRREEMALFSAASIV
PLYVGMANHEQADRLANVVRSRLLTPGGIMATEYETGEQWDKPNGWAPLQWMAIQGFKRY
GDDMLGDEIAHNWLKTVNHFYQEHHKLIEKYHISGGTPREGGGGEYPLQDGFGWTNGVVR
RLIGLYGEP
>EECEBICD_04451 HTH-type transcriptional repressor NagR
MIEQPDSKSAKPLYKQLEAALKEAIARGEYKPGQQIPTENELSARWQVSRVTVRKALDAL
TRENLLTRVSGKGTFVSGEKFQRSMTGIMSFSELCQSQGRRPGSRTIKSVFESVDDETKA
LLNMNDGEKAVVIERIRYADDVAVSLETVHLPPRFAFLLDEDLNNHSLYECLREKYHLWF
THSRKMIELVYASFEVAHYLGVNEGYPLILIKSEMIDNKGELSCVSQQLIVGDKIRFTV
>EECEBICD_04452 Fructosamine deglycase FrlB
MMGMKETVSNIVTSQAEKGGIKHVYYVACGGSYAAFYPAKAFLEKEAKALTVGLYNSGEF
INNPPVALGENAVVVVASHKGNTPETIKAAEIARQHGAPVIGLTWVMDSPLVAHCDYVET
YTFGDGKDIAGEKTMKGLLSAVELLQQTEGYAHYDDFQDGVSKINRIVWRACEQVAERAQ
AFAQEYKDDKVIYTVASGAGYGAAYLQSICIFMEMQWIHSACIHSGEFFHGPFEITDANT
PFLFQFSEGNTRAVDERALNFLKKYGRRIEVVDAKELGLSTIKTTVIDYFNHSLFNNVYP
VYNRALAEARQHPLTTRRYMWKVEY
>EECEBICD_04453 Fructosamine kinase FrlD
MSISVLGIGDNVVDKYLHSGIMYPGGNALNFAVYAKLADIPSAFMGAFGNDDAAQHVQDV
LHQLQIDISHSRHYTGENGYACIRLSHGDRQFVASNKNGVLREHPFSLSDDDLRYISQFT
LVHSSINGHLESELEKIKQQTVLLSFDFSGRGTDDYFKKVCPWVDYGFISCSGLSPDEIK
IKLNKLYRYGCRHIIATCGHEKVYYFSGADYLEWQPAYIEPVDTLGAGDAFLTGFLLSIL
QSGMAEPDKESVLRAMRQGGKSAAQVLSHYGAFGFGKPFAQ
>EECEBICD_04454 Anaerobic C4-dicarboxylate transporter DcuA
MFWTELCFILVALMIGARIGGVFLGMVGGLGVGVMVFIFGLTPSTPPIDVILIIILSVVLA
AASLQASGGLDLLVKLAEKILRRHPRYITLLAPFICYIFTFMSGTGHVVYSLLPVISEVA
RDSGIRPERPLSISVIASQQAITASPISAAMAAMIGLMAPLGVSISTIMMICVPATLIGV
AMGAIATFNKGKELKDDPEYQRRLAEGLIKPAQKESKNTVVTSRAKLSVALFLTSAIVIV
LLGLIPALRPMVETAKGLQPLSMSAAIQITMLSFACLIVLLCRPQVDQIISGTVFRAGAL
AIVCAFGLAWMSETFVNGHIALIKAEVQTLLQQHTWLIAIMMFFVSAMVSSQAATTLILL
PLGLALGVPAYALIGSWPAVNGYFFIPVAGQCLAALAFDDTGTTRIGKYVLNHSFMRPGL
VNVIVSVIVGLLIGKMVLA
>EECEBICD_04455 L-asparaginase
MKIRVFMATVLLLISHCVFSTTSLPHIVILATGGTIAGTAANNTQTAGYKSGELGVQTLI
NAVPEMNNIARVDGEQVANIGSENMTSDIILKLSQKVNALLARDDVDGVVITHGTDTLDE
TAYFLNLTVKSDKPVVFTAAMRPASAISADGAMNLLEAVTVAADPNAKGRGVMVVLNDRI
GSARFVTKTNATTLDTFKAPEEGYLGVIVNGKPQFETRVEKIHTLRSVFDVRNIKKLPNV
VIIYGYQDDPEYMYDAAIAHHADGIIYAGTGAGSVSVRSDAGIKKAEKAGIIVVRASRTG
NGVVPLDKGQPGLVSDSLNPAKARVLLMTALTQTRNPELIQSYFSTY
>EECEBICD_04456 Glutathione reductase

MTKHYDYIAIGGGSGGIASINRAAMYGQKCALIEAKELGGTCVNVGCVPKKVMWHAAQIR
EAIHLYGPDYGFDTTINQFDWSKLIASRTAYIDRIHTSYDNVLGKNNVDVIKGFARFVDA
KTIEVNGETITADHILIATGGRPSHPSIPGVEYGIDSDGFFALSALPERVAVVGAGYIAV
ELAGVINALGAKTHLFVRKHAPLRSFDPMISETLVEVMNAEGPQLHTHAVPKAVVKNADG
SLTLELEDGRSETVDCLIWAIGREPSTDNINLAAAGVKTNEKGYIIVDKFQNTNVEGIYA
VGDNTGAVELTPVAVAAGRRLSERLFNNKPDEHLDYSNIPTVVFSHPPIGTVGLSEPQAR
EQYGDEQVKVYKSSFTAMYTAVTTHRQPCRMKLVCVGPEEKIVGIHGIGFGMDEMLQGFA
VALKMGATKKDFDNTVAIHPTASEEFVTMR
>EECEBICD_04457 Ribosomal RNA large subunit methyltransferase J
MLSYRHSFHAGNHADVLKHTVQSLIIESLKEKEKPFLYLDTHAGAGRYQLGSEHAERTGE
YLEGIARIWQQDDLPAELEPYISVVKHFNRSGQLRYYPGSPLIARQLLREQDSLQLTELH
PSDFPLLRAEFQKDNRARVERADGYQQLKAKLPPVSRRGLILIDPPYEMKTDYQAVVSGI
SEGYKRFATGTYALWYPVVLRQQIKRMVHDLEATGIRKILQIELAIRPDSDQRGMTASGM
IVVNPPWKLEQQMNNVLPWLHSRLAPNGHGHTSVSWIVPE
>EECEBICD_04458 hypothetical protein
MKLHITVLASSLALAMPALAKDIPLSQAESIAKSVTPDSASVAFNDLEAQWLTQLRKALQ
GDTAALTRDAMAQMRQNSIQADNAWLQASGYDFHTTENQQMGITLLSAFNTLPEAVLKDN
LATVTAINHDADVNTRHQALADAESVEYLYFLSDAMGPRLGRAFLAAYDKGELGKAAALI
KASEVSTGAAKKYFHYPRPFQVPGNTIHLTPDDVVVKDGHPYTAGGGAFPSGHTNTGYTD
ALLMAEMIPERFDALVIRGARYGYSRLVLGVHYPLDVMGARMVAQRNVAHYLNDPYYRTL
FNEARAQLREALVKECGTTIVECAASTGKDDPYRDPAMHTFYRFTMTYNLPQQKGEHQPL
KIPKGADVLLQTALPNLSSAQCQALMEETALPAGYPLSGETEDQQFWQRLDLSAAYEMAR
KTR
>EECEBICD_04459 Oligopeptidase A
MTNPLLTSFSLPPFSAIKPEHVVPAVTKALADCRAAVEGVVAHGAPYSWENLCQPLAEAD
NVLGRIFSPISHLNSVKNSPELREAYEQTLPLLSEYSTWVGQHEGLYNAYRDLRDGDHYA
TLNTAQKKAVDNALRDFELSGIGLPKEKQQRYGEIATRLSELGNQYSNNVLDATMGWTKL
ITDEAELAGMPESALAAAKAQAEAKEQEGYLLTLDIPSYLPVMTYCDNQALREEMYRAYS
TRASDQGPNAGKWDNSPVMEEILALRHELAQLLGFENYAHESLATKMAENPQQVLDFLTD
LAKRARPQGEKELAQLRAFAKAEFGVEELQPWDIAYYSEKQKQHLYSISDEQLRPYFPEN
KAVNGLFEVVKRIYGITAKERTDVDVWHPEVRFFELYDENNELRGSFYLDLYAREHKRGG
AWMDDCVGQMRKADGTLQKPVAYLTCNFNRPVNGKPALFTHDEVITLFHEFGHGLHHMLT
RIETAGVSGISGVPWDAVELPSQFMENWCWEPEALAFISGHYETGEPLPKELLDKMLAAK
NYQAALFILRQLEFGLFDFRLHAEFNPQQGAKILETLFEIKKQVAVVPSPTWGRFPHAFS
HIFAGGYAAGYYSYLWADVLAADAYSRFEEEGIFNRETGQSFLDNILTRGGSEEPMELFK
RFRGREPQLDAMLEHYGIKG
>EECEBICD_04460 Ribosomal RNA small subunit methyltransferase J
MQICLMDETGATDGALSVLAARWGLEHDEDNPMALVLTPQHLELRKRDEPKLGGIFVDFV
GGAMAHRRKFGGGRGEAVAKAVGIKGDYLPDVVDATAGLGRDAFVLASVGCRVRMLERNP
VVAALLDDGLTRGYADADIGGWLQERLQLIHASSLTALTDITPRPQVVYLDPMFPHRQKS
ALVKKEMRVFQSLVGPDLDADGLLEPARQLATKRVVVKRPDYAPPLADVATPNAIVTKGH
RFDIYAGTPLTE
>EECEBICD_04461 Dipeptide and tripeptide permease B
MNTTAPTGLLQQPRPFFMIFFVELWERFGYYGVQGILAVFFVKQLGFSQEQAFITFGAFA
ALVYGLISIGGYVGDHLLGTKRTLVLGAIVLAIGYFMTGMSLLNPDLIFIALGTIAVGNG
LFKANPASLLSKCYQPKDPRLDGAFTLFYMSINIGSLLSLSLAPVIADKFGYAVTYNLCG
AGLIVALLVYFACRGMVKNIGSEPDHKPLRFRNLLLVLLGTVVMIFLCAWLMHNVKIANL
VLIVLSIVVTIFFFREAFRLDKTGRNKMFVAFILMIEAVLFYILYAQMPTSLNFFAINNV
HHEILGFAINPVSFQALNPFWVVVASPVLAAIYTRLGSKGKDLTMPMKFTLGMFLCALGF
LTAAAAGMWFADAQGLTSPWFIVLVYLFQSLGELLISALGLAMVAALVPQHLMGFILGMW
FLTQAAAFLLGGYVATFTAVPENITDPLQTLPIYTGVFSKIGLVTLAVTVVMAIMVPWLN
RMINTPGTEQ
>EECEBICD_04462 Universal stress protein A
MAYKHILIAVDLSPESKVLVEKAVSMARPYNAKISLIHVDVNYSDLYTGLIDVNLGDMQK

RISEETHHALTELSTNAGYPITETLSGSGDLGQVLVDAIKKYDMDLVVCGHHQDFWSKLM
SSARQLINTVHVDMLIVPLRDEEE
>EECEBICD_04463 Universal stress protein B
MISTVSLFWALCVVCIVNMARYFSSLRALLVVLRGCDPLLYQYVDGGGFFTTHGQPNKQV
RLVWYIYAQRYRDHHDEEFIRRCERVRRQFLLTSALCGLVVVSLIALMIWH
>EECEBICD_04464 Low-affinity inorganic phosphate transporter 1
MLHLFAGLDLHTGLLLLLALAFVLFYEAINGFHDTANAVATVIYTRAMRSQLAVVMAAVF
NFFGVLLGGLSVAYAIVHMLPTDLLLNMGSAHGLAMVFSMLLAAIIWNLGTWYFGLPASS
SHTLIGAIIGIGLTNAMMTGTSVVDALNIPKVINIFGSLIISPIVGLVFAGGLIFLLRRY
WSGTKKRARIHLTPAEREKKDGKKKPPFWTRIALILSAIGVAFSHGANDGQKGIGLVMLV
LIGVAPAGFVVNMNASSYEITRTRDAINNVETYFEQRPDLLKAVTGVDQLIPSPEPGATE
PTEFHCHPANTINALNRAKGMLANVESYDKLSVEQRSQLRRIMLCISDTTDKVVKLPGVS
SDDQRLLKKLKTDMLSTIEYAPVWIIMAVALALGIGTMIGWRRVATTIGEKIGKKGMTYA
QGMSAQMTAAVSIGLASYTGMPVSTTHVLSSSVAGTMVVDGGGLQRKTVTSILMAWVFTL
PAAIILSGVLYWLSLKII
>EECEBICD_04465 putative protein
MERFDAVIIGAGAAGMFCAAQAGQAGSRVLLIDNGKKPGRKILMSGGGRCNFTNLYVEPA
AYLSQNPHFCKSALARYTQWDFIDLVGRYGIAWYEKTLGQLFCDDSAQRIVDMLVAECDK
GGVTMRLRSEVLSVERDESGFILALNGETVTTQKLVIASGGLSMPGLGASPFGYKIAEQF
GLKVLPTRAGLVPFTLHKPLLEQLQTLSGVSVPCVITARNGTVFRENLLFTHRGLSGPAV
LQISSYWQPGELVSINLLPDLSLEDVLNEQRNAHPNQSLKNTLAMHLPKRLVECLQQLGQ
IPDVSLRQLNVRDQQALVDTLTAWQVQPNGTEGYRTAEVTLGGVDTNELSSRTMEARRVP
GLYFIGEVMDVTGWLGGYNFQWAWSSAWACAQDLAAKR
>EECEBICD_04466 hypothetical protein
MDKMKRHLVWWGAGILVAVAAIAWWMLRPAGIPEGFAASNGRIEATEVDIATKIAGRIDT
ILVSEGQFVRQGEVLAKMDTRVLQEQRLEAIAQIKEAESAVAAARALLEQRQSEMRAAQS
VVKQREAELDSVSKRHVRSRSLSQRGAVSVQQLDDDRAAAESARAALETAKAQVSAAKAA
IEAARTSIIQAQTRVEAAQATERRIVADIDDSELKAPRDGRVQYRVAEPGEVLSAGGRVL
NMVDLSDVYMTFFLPTEQAGLLKIGGEARLVLDAAPDLRIPATISFVASVAQFTPKTVET
HDERLKLMFRVKARIPPELLRQHLEYVKTGLPGMAWVRLDERVPWPDALSVRLSQ
>EECEBICD_04467 Ribosome-associated ATPase
MTSLKLVPVPPVAQLEGVSQHYGKTAALNNITLDIPARSMVGLIGPDGVGKSSLLSLISG
ARVIEQGNVIVLGGDMRDAKHRRDVCPRIAWMPQGLGKNLYHTLSVYENVDFFARLFGHD
KAEREARITELLNSTGLAPFRDRPAGKLSGGMKQKLGLCCALIHDPELLILDEPTTGVDP
LSRAQFWDLIDSIRQRQTNMSVLVATAYMEEAERFDWLVAMNAGEILATGSAQQLRAKTH
SATLEQAFIALLPEAQRQAHKPVVIPPYHAEQEEIAIEAKDLTMRFGKFVAVDHVNFRIP
RGEIFGFLGSNGCGKSTTMKMLTGLLPASEGQAWLFGQPVDPNDIDTRRRVGYMSQAFSL
YNELTVRQNLELHARLFHIPPAEIPARVAQMIERFMLTEVEDTLPASLPLGIRQRLSLAV
AVIHRPEMLILDEPTSGVDPVARDMFWQLMVDLSRQDKVTIFISTHFMNEAERCDRMSLM
HAGKVLASGTPQELVQQRGAANLEAAFISWLQEAAGAAPETPIPPSQTPAASGKPSRQGL
SFRRLFSYSRREALELRRDPVRSTLALLGTVILMLIMGYGISMDVENLRFAVLDRDQTVS
SQAWSLNLAGSRYFIEQPPLASYDELDRRMRSGELAVAIEIPPNFGRDIARGTPAQIGVW
VDGAMPSRAETVKGYVQAMHQSWLQEAASRQPNPVKQTGLLNIETRYRYNPDVKSLPAIV
PAVIPLLLMMIPSMLSALSVVREKELGSIINLYVTPTTRSEFLLGKQLPYIALGMLNFLL
LCALSVFVFGVPLKGSFLTLTLAALLYVIIATGLGLLISTFMKSQIAAIFGTSIITLIPA
TQFSGMIDPVASLEGPGRWIGEIYPTSHFLTIARGTFSKALDLSDLWPLFMPLLIAVPVV
MGLSILLLKKQEG
>EECEBICD_04468 Inner membrane transport permease YhhJ
MRGLRNIYNLGVKELRSLLGDKAMLSLIVFAFTISVYSSATVLPGSLHLAPIAIADMDQS
QLSNRIVNSFYRPWFLPPEMITATEMDAGLDAGRYTFAVNIPPNFQRDVLAGRQPDIQVN
VDATRMSQAFTGNSYIQNIISGEVNSFVARARGDNVQPVSLEIRMRFNPNLDPAWFGGVM
AIINNITMLAIVLTGSALIREREHGTVEHLLVMPVTPFEIMMAKVWSMGLVVLVVSGLSL
MLMVKGVLGVPIEGSIPLFMLGVALSLFATTSIGIFMGTIARSMPQLGLLMILVLLPLQM
LSGGSTPRESMPQAVQDIMLTMPTTHFVSLAQAILYRGAGLSIVWPQFLTLLAIGGVFFL

IALLRFRKTIGTMA
>EECEBICD_04469 Nickel-responsive regulator
MRRRAIPKGLSQMQRVTITLDDDLLETLDSLSQRRGYNNRSEAIRDILRGALAQEATQEH
GTQGFAVLSYVYEHEKRDLASRIVSTQHHHHDLSVATLHVHINHDDCLEIAVLKGDMGDV
QHFADDVIAQRGVRHGHLQCLPKED
>EECEBICD_04470 hypothetical protein
MYQIVLGKVSTLSAGQLPDALIAQAPQGVRRASWLAGRVLLSRALSPLPEMVYGEQGKPA
FSAGTPLWFNLSHSGDTIALLLSDEGEVGCDIEVIRPRDNWRSLANAVFSLGEHAEMEAE
RPEQQLAAFWRIWTRKEAIVKQRGGSAWQIVSVDSTLPSALSVSQCQLDTLSLAVCTPTP
FTLTPQTITKAL
>EECEBICD_04471 Putative transport protein YhhT
MATAQPDKTGMHILLKLASLVIILAGIHAAAGIIVQLLLALFFAIVLNPLVTWFIRRGVK
RPLAITIVVVVMLIVLTALVGVLAASLNEFIAMLPKYSKELTRKVLHLQELMPFLNLHMS
PERMLRGMDSDKIMLFTTTLMTGVSGAMASIVLLVMTVVFMLFEVRHVPYKLRFALNNPQ
IHIAGLHRALKGVSHYLALKTLLSLWTGAIIWLGLALMDIQFALMWGVLAFLLNYVPNIG
SVISAVPPMIQALLFNGFYECVLVGALFLLVHMVIGNIMEPRMMGHRLGMSTLVVFLSLL
VWGWLLGPVGMLLSVPLTSVCKIWMETTKGGSKLAILLGPGRPKSRLPG
>EECEBICD_04472 putative MFS-type transporter YhhS
MIEKKTCCKNAVILMPEPVAEPALNGLRLNLRIVSIVMFNFASYLTIGLPLAVLPGYVHD
AMGFSAFWAGLIISLQYFATLLSRPHAGRYADVLGPKKIVVFGLCGCFLSGLGYLLADIA
SAWPMISLLLLGLGRVILGIGQSFAGTGSTLWGVGVVGSLHIGRVISWNGIVTYGAMAMG
APLGVLCYAWGGLQGLALTVMGVALLAVLLALPRPSVKANKGKPLPFRAVLGRVWLYGMA
LALASAGFGVIATFITLFYDAKGWDGAAFALTLFSVAFVGTRLLFPNGINRLGGLNVAMI
CFGVEIIGLLLVGTAAMPWMAKIGVLLTGMGFSLVFPALGVVAVKAVPPQNQGAALATYT
VFMDMSLGVTGPLAGLVMTWAGVPVIYLAAAGLVAMALLLTWRLKKRPPSALPEAASSS
>EECEBICD_04473 hypothetical protein
MRNLVKYAGIGLLVMGLAACDNNDTKASAESAAAESQASGQPISLMDGKLSFSLPADMTD
QSGKLGTQANNMHVYSDPTGQKAVIVIVGDNTDEALPVLANRLLEQQRSRDPQLQVVTNK
SIELKGHTLQQLDSIISAKGQTAYSSIVLGKVDNQLLTIQVTLPADNQQKAQTTAENIIN
TLVIK
>EECEBICD_04474 Queuosine precursor transporter
MTPFTQSQRVKALFWLSLFHLLVIISSNYLVQLPITIFGFHTTWGAFSFPFIFLATDLTV
RIFGAPLARRIIFAVMIPALLVSYVVSSLFYMGAWQGFAALANFNLFVARIAAASFMAYA
LGQILDVHVFNRLRQNRRWWLAPTASTLFGNISDTLAFFFIAFWRSPDAFMAEHWMEIAL
VDYCFKVLISIIFFLPMYGVLLNMLLKKLADKSEISPLPAS
>EECEBICD_04475 Sulfur carrier protein TusA
MANFHAIVNLPGLLTKMGVKYRRFSTIETKMSDLFSSPDHTLDALGLRCPEPVMMVRKTV
RNMQTGETLLIIADDPATTRDIPGFCTFMEHDLLAQETEGLPYRYLLRKAH
>EECEBICD_04476 Methyl-accepting chemotaxis protein I
MKNIKVITGVIATLGIFSALLLVTGILFYSAVSSDRLNFQNASALSYQQQELGGSFQTLI
ETRVTINRVAIRMLKNQRDPASLDAMNTLLTNAGASLNEAEKHFNNYVNSEAIAGKDPAL
DAQAEASFKQMYDVLQQSIHYLKADNYAAYGNLDAQKAQDDMEQVYDQWLSQNAQLIKLA
SDQNQSSFTQMQWTLGIILLIVLIVLAFIWLGLQRVLLRPLQRIMAHIQTIADGDLTHEI
EAEGRSEMGQLAAGLKTMQQSLIRTVSAVRDNADSIYTGAGEISAGSSDLSSRTEQQASA
LEETAASMEQLTATVRQNTDNARQATGLAKTASETARKGGRVVDNVVNTMNDIAESSEKI
VDITSVIDGIAFQTNILALNAAVEAARAGEQGRGFAVVAGEVRTLASRSAQAAKEIKVLI
ENSVSRIDTGSTQVREAGETMKEIVNAVTRVTDIMGEIASASDEQSKGIEQVAQAVSEMD
SVTQQNASLVEESAAAAAALEDQANELRQAVAAFRIQKQPRREASPTPLSKGLTPQPAAE
QANWESF
>EECEBICD_04477 Zinc/cadmium/lead-transporting P-type ATPase
MSTPDTDGKKVPPFTSFRLAPAIQKADSCCCEEHCASSAPVSTAVAGTRYTWKISGMDCA
ACARKVENAVRQIRGVNQAQVLFATEKLVVDADADLRAQIERAVQKAGYTLRSEDSRDAA
PESRLKENLPLITLIIMMAISWGLEQFNHPFGQLAFIVTTLVGLYPIARQALRLMKSGSW
FAIETLMSVAAIGALFIGATAEAAMVLLLFLIGERLEGWAASRARKGVSALMALKPETAT

RLRDGVREEVAINTLRPGDIIEVAAGGRLPADGKLVSGFASFDESALTGESIPVERATGD
KVPAGATSVDRLVTLEVLSEPGASAIDRILTLIEEAEERRAPIERFIDRFSRIYTPVIMV
IALLVTLIPPLMFDGGWQEWIYKGLTLLLIGCPCALVISTPAAITSGLAAAARRGALIKG
GAALEQLGRITQVAFDKTGTLTIGKPRVTAIHPASGISEAELLALAAAVEQGATHPLAQA
IIREAQTRDLAIPTAESQRTLAGTGIEAQVNGERILICAAGKQPAAAFAGQISELENAGQ
TVVLVLRNETVLGVLALQDTLRDDARDAIRDLHQLGVNGVILTGDNPRAAAAIAGELDLA
FKAGLLPEDKVRAVTALNQQAPLAMVGDGINDAPAMKAASIGIAMGSGTDVALETADAAL
THNRLRGLAQMITLARATHANIRQNITIALGLKAIFLVTTLLGFTGLWLAILADTGATVL
VTANALRLLRKN
>EECEBICD_04478 hypothetical protein
MLWSFIAVCLSAWLYVDASYRGPAWQRWVFKPVTLLLLLLLAWQAPMFDAISYLVLAGLC
ASLVGDALTLLPRQRLLYAVGAFFLSHLLYTIYFASQMTLSFFWPLPLALLIVGALLIAV
IWTRLEELRWPVCTFIAMTLVMVWLAGELWFFRPTAPALSAFTGATLLFIGNIVWLGSHY
RRRFRADNAIAAACYFAGHFLIVRSLYL
>EECEBICD_04479 hypothetical protein
MSKPPLFFIIIIALIVVAASFRFVQQRREKAANEAAPLQQKLVVVSSKREKPVNDRRSRQ
QEVSPAGTSMRYEASFKPLNGGLEKTFRLQAQQYHALTVGDQGTLSYKGTRFVGFVSRTP
DNE
>EECEBICD_04480 hypothetical protein
MLINLGRLLMLFVWAFLILNLVHPFPRPLNIFVNVALVFMALMHGMQLALLKSTIPKEGP
QMTTGEKIRIFLFGVFELLVWQKKFKNKK
>EECEBICD_04481 Ribosomal RNA small subunit methyltransferase D
MKKPTHSGSGQIRIIGGQWRGRKLPVPDSPGLRPTTDRVRETLFNWLAPVMVDAHCLDCF
AGSGALGLEALSRYAAQATLLEMDRAVSQQLQKNLATLKANNARVVNTNTLTFLSQPGTP
HHVVFVDPPFRKGLLEETLQLLETQGWLADDALIYVESEVENGLPPVPANWALYREKVAG
QVAYRLYQRDAQGENDVD
>EECEBICD_04482 Signal recognition particle receptor FtsY
MAKQKKRGFFSWLGFGDKEQKQEQTEEQQIVEEQRPVEPPVETAADVDAQTPAHSKAETE
AFAEEVVDVTEKVQESEKPQPVEPEPAAAIEMAAPQIAVEREELPLPEEVKDEAISPEEW
QAEAETVEVIAAVEEEGENAAKFTDEELEAQALAAQAAEEAVMVVPPAEEDEPVEAIVQE
QEKPTKEGFFARLKRSLLKTKENLGSGFISLFRGKKIDDDLFEELEEQLLIADVGVETTR
KIIANLTEGASRKQLKDAEALYGLLKDEMGEILAKVDEPLNIEGKTPFVILMVGVNGVGK
TTTIGKLARQFEQQGKSVMLAAGDTFRAAAVEQLQVWGQRNNIPVIAQHTGADSASVIFD
AIQAAKARNVDVLIADTAGRLQNKSHLMEELKKIVRVMKKLDEEAPHEVMLTIDASTGQN
AVSQAKLFHEAVGLTGITLTKLDGTAKGGVIFSVADQFGIPIRYIGVGERIEDLRPFKAD
DFIEALFARED
>EECEBICD_04483 Cell division ATP-binding protein FtsE
MIRFEHVSKAYLGGRQALQGVTFHMRPGEMAFLTGHSGAGKSTLLKLICGIERPSAGKIF
FSGHDITRLKNREVPFLRRQIGMIFQDHHLLMDRTVYDNVAIPLIIAGASGDDIRRRVSA
ALDKVGLLDKARNFPIQLSGGEQQRVGIARAVVNKPAVLLADEPTGNLDDALSEGILRLF
EEFNRVGVTVLMATHDIGLISRRSYRQLVLSDGHLHGGLANE
>EECEBICD_04484 Cell division protein FtsX
MNRRDAINQIRQFGGRLDRLRKSRGGTGGGRNAPGRQKPTPKPNSRKTNVFNEQVRYAWH
GALQDLKSKPLATFLTVMVIAISLTLPSVCYMVYKNVNQAATQYYPSPQITVYLQKTLDD
DAAARVVGQLQAEQGVEKVNYLSREDALGEFRNWSGFGGALDMLEENPLPAVAVVIPKLD
FQSTASLNTLRDRISQINGIDEVRMDDSWFARLAALTGLVGRVSAMIGVLMVAAVFLVIG
NSVRLSIFARRDTINVQKLIGATDGFILRPFLYGGALLGFSGAFLSLILSEILVMRLSSA
VTEVAQVFGTKFDLNGLSFDECLLLLLVCSMIGWIAAWLATVQHLRHFTPD
>EECEBICD_04485 RNA polymerase sigma factor RpoH
MTKEMQNLALAPVGNLESYIRAANAWPMLSADEERALAERLHYQGDLEAAKTLILSHLRF
VVHIARNYAGYGLPQADLIQEGNIGLMKAVRRFNPEVGVRLVSFAVHWIKAEIHEYVLRN
WRIVKVATTKAQRKLFFNLRKTKQRLGWFNQDEVEMVARELGVSSKDVREMESRMAAQDM
TFDMSSDDESDSQPMAPVLYLQDKSSNFADGIEDDNWEEQAANRLTDAMQGLDERSQDII
RARWLDEDNKSTLQELADRYGVSAERVRQLEKNAMKKLRAAIEA

>EECEBICD_04486 Leu/Ile/Val/Thr-binding protein
MKGKTLLAGCIALSLSHMAFAEDIKVAVVGAMSGPVAQYGDQEFTGAEQAIADINAKGGI
KGDKLVAVKYDDACDPKQAVAVANKVVNDGIRYVIGHLCSSSTQPASDIYEDEGILMITP
AATAPELTARGYKLVLRTTGLDSDQGPTAAKYILEKVKPQRIAIIHDKQQYGEGLARAVQ
DGLKKGGVNVVFFDGITAGEKDFSTLVARLKKENIDFVYYGGYHPEMGQILRQSRAAGLK
TQFMGPEGVANVSLSNIAGESAEGLLVTKPKNYDQVPANKPIVDAIKAKKQDPSGAFVWT
TYAALQSLQAGLNQSDDPAEIAKYLKGATVDTVMGPLSWDEKGDLKGFEFGVFDWHANGT
ATDAK
>EECEBICD_04487 PanD regulatory factor
MKLTILRLEHFSAQDQIDLGKIWPEYSASSLSVDETHRIYAARFNERLLGAVRVTLSGTQ
GALDSLRVREITRRRGVGQYLVEEVIRDNPNVSSWWMADVGVEDRSVMAAFMQALGFTAQ
HDGWEKR
>EECEBICD_04488 Leucine-specific-binding protein
MKRKAKTIIAGIVALAVSQGAMADDIKVAIVGAMSGPVAQWGDMEFNGARQAIKDINAKG
GIKGDKLVGVEYDDACDPKQAVAVANKIVNDGIQYVIGHLCSSSTQPASDIYEDEGILMI
SPGATNPELTQRGYQYIMRTAGLDSSQGPTAAKYILETVKPQRIAIIHDKQQYGEGLARS
VQDGLKQGNANIVFFDGITAGEKDFSALIARLQKENIDFVYYGGYYPEMGQMLRQARANG
LKTQFMGPEGVGNASLSNIAGGAAEGMLVTMPKRYDQDPANKAIVEALKADKKDPSGPYV
WITYATVQSLATAMTRSASHAPLDLVKDLKANGADTVIGPLKWDEKGDLKGFEFGVFQWH
ADGSSTVAK
>EECEBICD_04489 High-affinity branched-chain amino acid transport system permease protein LivH
MSEQFLYFLQQMFNGVTLGSTYALIAIGYTMVYGIIGMINFAHGEVYMIGSYVSFMIIAA
LMMMGIDTSWLLVAAGFIGAIIIASAYGWSIERVAYRPVRNSKRLIALISAIGMSIFLQN
YVSLTEGSRDVALPSLFNGQWIVGSSENFSASITTMQAVIWIVTFLAMLALTIFIRYSRM
GRACRACAEDLKMASLLGINTDRVIALTFVIGAAMAAVAGVLLGQFYGVINPYIGFMAGM
KAFTAAVLGGIGSIPGAMIGGLILGVAEALSSAYLSTEYKDVVSFALLILVLLVMPTGIL
GRPEVEKV
>EECEBICD_04490 hypothetical protein
MKPMHIAMALFSAAMFFVLAGVFMGVQLELDGTKLVVDTAADIRWQWIFIGTAVVFFFQL
LRPMFQKAVKHVSGPKFILPAIDGSTVKQKLFLMALLVIAVAWPFMVSRGSVDIATMTMI
YIILGLGLNVVVGLSGLLVLGYGGFYAIGAYTFALLNHYYGLGFWTCLPLAGLVSAAAGF
LLGFPVLRLRGDYLAIVTLGFGEIVRILLLNNTEITGGPNGISQIPKPTLFGLEFSRNTR
EGGWDTFSNFFGVKYDPSDRVIFLYLVALLLVVLSLFVINRLLRMPLGRAWEALREDEIA
CRSLGLSPTRIKLTAFTISAAFAGFAGTLFAARQGFVSPESFTFAESAFVLAIVVLGGMG
SQFAVILAAVLLVVSRELMRDFNEYSMLMLGGLMVLMMIWRPQGLLPMTRPQLKLKSGQA
KGEQA
>EECEBICD_04491 Lipopolysaccharide export system ATP-binding protein LptB
MSQPLLAVNGLMMRFGGLLAVNNVSLELREREIVSLIGPNGAGKTTVFNCLTGFYKPTGG
TIMLRERHLEGLPGQQIARMGVVRTFQHVRLFREMTVIENLLVAQHQQLKTGLFSGLLKT
PAFRRAQSEALDRAATWLERIGLLEHANRQASNLAYGDQRRLEIARCMVTQPEILMLDEP
AAGLNPKETKELDELIAELRNHHNTTILLIEHDMKLVMGISDRIYVVNQGTPLANGTPEE
IRNNPDVIRAYLGEA
>EECEBICD_04492 High-affinity branched-chain amino acid transport ATP-binding protein LivF
MEKTMLTFEKVSAHYGKIQALHDVSLHINQGEIVTLIGANGAGKTTLLGTLCGDPRASSG
RVVFDGKDITDWQTAKIMREAVAIVPEGRRVFSRMTVEENLAMGGFFAERDRFQERIKWV
YELFPRLHERRIQRAGTMSGGEQQMLAIGRALMSQPRLLLLDEPSLGLAPIIIQQIFDTI
EQLREQGMTIFLVEQNANQALKLADRGYVLENGHVVLSDTGDALLANEAVRSAYLGG
>EECEBICD_04493 hypothetical protein
MRTVNYSEARQNLAEVLESAVTAGPVTITRRGHKSAVIISAEEFERYQTARMDDEFAAIM
AVHGNELRELADK
>EECEBICD_04494 hypothetical protein
MTLQLISAEEIIQFHDRLLRVTPGVTGMPDPGRAEALMYRVLNQIEYEGVTDVWLLAAMH

LLAISRGHIFNDGNKRTALFITLLFLKRNGISLAANPDFVDMTVDAAAGRLTLEQIVVRL
RA
>EECEBICD_04495 sn-glycerol-3-phosphate-binding periplasmic protein UgpB
MISLRHTALGLALSLAFTGQTLAVTTIPFWHSMEGELGKEVDSLAQRFNQANPDYKIVPV
YKGNYEQNLSAGIAAFRTGNAPAILQVYEVGTATMMASKAIKPVYEVFKDAGINFDESQF
VPTVAGYYTDAKSGHLLSQPFNSSTPVLYYNKDAFKKAGLDPEQPPKTWQELADYTAKLR
AAGMKCGYASGWQGWIQLENFSAWNGLPFASKNNGFDGTDAVLEFNKPEQVKHIALLEEM
NKKGDFSYVGRKDESTEKFYNGDCAMTTASSGSLANIRQYAKFNYGVGMMPYDADIKGAP
QNAIIGGASLWVMQGKDKETYTGVAKFLDFLAKPENAAEWHQKTGYLPITTAAYELTREQ
GYYDKNPGADIATRQMLNKPPLPFTKGLRLGNMPQIRTIVDEELESVWTGKKTPQQALDT
AVDRGNQLLRRFEKASKS
>EECEBICD_04496 sn-glycerol-3-phosphate transport system permease protein UgpA
MSSSRPVFRSRWLPYLLVAPQLVITVIFFIWPAGEALWYSLQSVDPFGFSSQFVGLENFV
ALFHDSYYLDAFWTTIKFSALVTFSGLLVSLFFAALVDYVVRGSRFYQTLMLLPYAVAPA
VAAVLWIFLFNPGRGLITHFLGEFGYDWNHAQNSGQAMFLVVFASVWKQISYNFLFFFAA
LQSIPRSLVEAAAIDGAGPIRRFFRLSLPLIAPVSFFLLVVNLVYAFFDTFPVIDAATAG
GPVQATTTLIYKIYREGFTGLDLSASAAQSVVLMFLVIILTVVQFRYVESKVRYQ
>EECEBICD_04497 Lactose transport system permease protein LacG
MIENRRGLTIFSHTMLILGIAVILFPLYVAFVAATLDDRAVFETPMTLLPGTQLLENIKT
IWVNGVGVNSAPFWLMMLNSFIMAFSITVGKITVSMLSAFAIVWFRFPLRNLFFWMIFIT
LMLPVEVRIFPTVEVIANLKMLDSYAGLTLPLMASATATFLFRQFFMTLPDELVEAARID
GASPMRFFRDIVLPLSKTNLAALFVITFIYGWNQYLWPLLIITDVNLGTAVAGIKGMIAT
GEGTTQWNQVMAAMLLTLIPPVVIVLAMQRAFVRGLVDSEK
>EECEBICD_04498 sn-glycerol-3-phosphate import ATP-binding protein UgpC
MAGLKLQAVTKSWDGKTQVIQPLTLDVADGEFIVMVGPSGCGKSTLLRMVAGLERVTSGD
IWIDRKRVTEMEPKDRGIAMVFQNYALYPHMSVEENMAWGLKIRGMSKAHIEERVREAAR
ILELDGLLKRRPRELSGGQRQRVAMGRAIVREPAVFLFDEPLSNLDAKLRVQMRLELQHL
HRRLRTTSLYVTHDQVEAMTLAQRVMVMNKGVAEQIGTPVEVYEKPASRFVASFIGSPAM
NLLDGVISASGDRFELPGGLALPIGAGYRGHAGRKMTLGIRPEHIALSSQAEGGVPLTVD
TLEILGADNLAHGRWGDQKLVVRLAHQQRPAAGSMLWLHLPEHQRHLFDGETGQRV
>EECEBICD_04499 Glycerophosphodiester phosphodiesterase, cytoplasmic
MSNWPYPHIVAHRGGGKLAPENTLAAIDVGARYGHTMIEFDAKLSKDGEIFLLHDDNLER
TSNGWGVAGELNWQDLLRVDAGGWFSGEFKGEPLPLLSQVAERCRKHGMMANIEIKPTTG
SGRLTGRVVASAARELWAGMTPPLLSSFEIEALEAAQAAAPELPRGLLLDKWREDWRELT
TRLGCVSLHLNHKLLDETRVQEIKAAGLRILVYTVNQSQRAAELLRWGVDCICTDRIDDI
GPHFQF
>EECEBICD_04500 putative protein YhhA
MKRLLLITALLPFAVLAQPINTMNNPNQPGYQIPSQQRMQTQMQTQQIQQQGMLKQQMQT
QARSQQQNLQSQLNANTQRVQQGQPGNGMLGQQTLPNTQGGMLSGSGNPDQMLNHSQPML
QQDSGTPQPDIPLKTISP
>EECEBICD_04501 Glutathione hydrolase proenzyme
MKPTFMRWVAIAALLAGGTFSAVANPPVAPPVSYGVEEDVFHPVRATQGMVASVDAMATQ
VGVDILKQGGNAVDAAVAVGYALAVTHPQAGNLGGGGFMLLRTKDGNTTAIDFREMAPAG
ATRDMFLDDQGNADAKKSLTSHLASGTPGTVAGFSLALEKYGTMPLNKVVRPAMKLAEEG
FVVNDALADDLKTYGSEVIPNHENSKAIFWKDGEPLKKGDKLIQKNLAKSLEMIAENGPD
AFYKGAIADQIAGEMQKNGGLMTKEDLANYKAVERTPISGDYRGYQVFSMPPPSSGGIHI
VQILNILENFDMKKYGFGSADAMQIMAEAEKYAYADRSEYLGDPDFVKVPWQALTHKAYA
KTLADQIDINKAKPSSQIKPGKLAPYESNQTTHFSVVDKDGNAVAVTYTLNTTFGTGIVA
GNTGILLNNQMDDFSAKPGVPNVYGLVGGDANAVGPKKRPLSSMSPTIVVKDGKTWLVTG
SPGGSRIITTVLQMVVNSIDFGMNVAEATNAPRFHHQWLPDELRVEKGFSPDTLKLLEQK
GQKVALKEAMGSTQSIMVGPDGELYGASDPRSVDDLTAGY
>EECEBICD_04502 Phosphotriesterase homology protein
MKGFLQTVTGPVAHTDMGLTLPHEHLFNDLSSVVDEPHYAFSQQLVGKKVSADLQWGLKH

DPYCCADNMDRKEIDDVIFEINNFMSLGGRTIVDATGSESIGRDASALREVALKTGLNIV
ASSGPYLEKFESTRIHKPVELLASLIDKELNQGIGETDIRAGMIGEIGVSPAFTQAERNS
LRAASLAQCNNPHTAMNIHMPGWLRLGDEVLDIVLEEMNVSPAKVSLAHSDPSGKDVVYQ
RKMLDRGVWLEFDMIGLDITFPKEGVAPGVQETADAVANLIVLGYADQIVLSHDVFLKQM
WAKNGGNGWGFVPNVFLAYLAARGVDNDTLRKLCIDNPARLLTA
>EECEBICD_04503 hypothetical protein
MNKNKYSTPLLMLATILAGMLSPMQSAVNGQLGHWLQDGNACAVISFASGLVVMFFIIIA
RKETRQQFASIPTLIKKRKIPLWNWFAGLCGAMVVFSEGASASALGVATFQTALISALLL
SGLLCDRFGIGVEEKKYFTPWRITGALFAVIATIFVVSPQWHSTSFILLAILPFLAGLLA
GWQPAGNAKVAEATGSMLVSITWNFIVGFCVLGAALAIRIALGHVTIQLPDTWWMYLGGP
LGLLSIGLMAILVRGLGLLMLGVASTAGQLLGSVLIDELIPSLGNTVYLVTIIGTLFALV
GAIVTTIPEYRASKMAQRIEVSE
>EECEBICD_04504 hypothetical protein
MNNTQKKLKVLFIGESWHIHMIHSKGYDSFTSSKYEEGATWLLECLRKGGVDIDYMPAHT
VQIAFPESIDELNRYDVIVISDIGSNTFLLQNETFYQLKIKPNALESIKEYVKNGGGLLM
IGGYLSFMGIEAKANYKNTVLAEVLPVIMLDGDDRVEKPDGICAEAVSPEHPVVNGFSDY
PVFLGYNQAVARDDADVVLTINNDPLLVFGEYQQGKTACFMSDCSPHWGTQQFMSWPFYT
DLWVNTLQFIARK
>EECEBICD_04505 Ribokinase
MKFERHHRILKELSISGVVKVSNLAKSLKVTKETIRSDLNELAGQGYLTRCHGGAFITLD
SLDNVAKNEIAYVLEKYESAQKIKKGLSAMKNNVCVIGSFNVDIISYLPRLPSTGESLLA
DKFIFSPGGKGCNQALAASYADSDVHFITKVGSDHFSDYAINFINSSKIHKSVIYQTKET
QTGTATIMVNGDTGDNVIAIYPGANMTISPDEITIQKEAIVHSDIVLVQLETNYEALQQT
IRLAQKNDIPVIINPAPYNDMVNTIIDNIDYITPNETEAGLLANMAVNDIESAKCAAKII
HQKGVKNTIITLGSKGSLAYDGTQFIYSPAFPAVVKNTAGAGDAFNGALASGLAKGKSLA
SALCYASAFASLAVETPNASDMPENDSVLHRIQGSHYKQTISTH
>EECEBICD_04506 L-amino acid N-acetyltransferase AaaT
MSEIVIRHAEPKDYDAIRQIHAQPEVYHNTLQVPHPSLEMWQARLTEQAGVKQLVACIDD
IVVGHLSIQVTQRPRRSHVADFGICVDARWHNRGIASALIHTMIDMCDNWLRVERIELTV
FVDNEPAVAVYKKYGFEIEGTGKKYGLRNGEYVDAYFMARVK
>EECEBICD_04507 putative oxidoreductase YhhX
MTLHCAFIGFGKSTTRYHLPYVLNRKDTWHVAHIFRRHAKPEEQAPQYSHIHFTNDLDEV
LGDPQVKLVIVCTHADSHFEYAKRALEAGKNVLVEKPFTPKLAEAKVLFDLARSKGLTVT
PYQNRRFDSCFLTAKKAIESGKLGKIVEIESHFDYYRPVAETKPGLPQDGAFYGLGVHTM
DQIISLFGRPDHVAYDIRSLRNKANPDDTFEAQLFYGDLKAIVKTSHLVKIDYPKFIVHG
TKGSFVKYGIDQQETSLKANIMPGEPGFAADESVGVLEYVNDDGVTVKEEVKPETGDYGR
VYDALYQTLTVGTPNYVKESEVLTNLEILERAFEQASPATITLAK
>EECEBICD_04508 Quercetin 2,3-dioxygenase
MIYLRKANDRGHANHGWLDSWHTFSFADYYDPNFMGFSALRVINDDVIDAGQGFGTHPHK
DMEILTYVLEGAVEHQDSMGNKEQVPAGEFQIMSAGTGVRHSEYNPSKTDRLRLYQIWII
PQETGITPRYEQRRFDAAQGKQLVLSPDARDGSLKVHQDMELYRWALLKDEQSVHQIAAE
RRVWIQVVKGNVTINGTKATTSDGLAIWDEQAISIHADSDSEVLLFDLPPV
>EECEBICD_04509 HTH-type transcriptional regulator GntR
MKKKRPVLQDVADRVGVTKMTVSRFLRNPEQVSVALRGKIAAALDELGYIPNRAPDILSN
ATSRAIGVLLPSLTNQVFAEVLRGIEAVTDAHGYQTMLAHYGYKPEMEQERLESMLSWNI
DGLILTERTHTPRTLKMIEVAGIPVVELMDSQSPCLDIAVGFDNFEAARQMTAAIIARGH
RHIAYLGARLDERTIIKQKGYEQAMRDSGLVPYSVMMEQSSSYSSGIELMRQARREYPQL
DGIFCTNDDLAVGAAFECQRLGLKIPDDMAIAGFHGHDIGQVMEPRLASVLTPRERMGSI
GAERLLARIRGETVTPKMLDLGFTLSPGGSI
>EECEBICD_04510 Thermoresistant gluconokinase
MSTTNHDHHVYVLMGVSGSGKSAVASAVAHQLHAAFLDGDFLHPRCNIEKMASGEPLNDD
DRKPWLQALNDAAFAMQRTNKISLIVCSALKKHYRDLLREGNPNLSFIYLKGDFDVIESR
LKARKGHFFKTQMLVTQFETLQEPGADERDVLVVDIDQPLEDVVASTIEVINKGSTL
>EECEBICD_04511 Low-affinity gluconate transporter

MSTLTLVLTAVGSVLLLLFLVMKARMHAFVALMVVSMGAGLFSGMPLDKIAATMEKGMGG
TLGFLAIVVALGAMFGKILHETGAVDQIAVKMLKSFGHNRAHYAIGLAGLICALPLFFEV
AIVLLISVAFSMARHTGTNLVKLVIPLFAGVAAAAAFLLPGPAPMLLASQMHADFGWMIL
IGLCAAIPGMIIAGPLWGNFISRYVELRIPDDITEPHLGEGKMPSFGFSLSLILLPLVLV
GLKTIAARFVPEGSTAYEWFEFIGHPFTAILVACLVAIYGLAMRQGMPKDKVMEICGHAL
QPAGIILLVIGAGGVFKQVLVDSGVGPALGEALTGMGLPIAITCFVLAAAVRIIQGSATV
ACLTAVGLVMPVIEQLNFSGAQMAALSICIAGGSIVVSHVNDAGFWLFGKFTGATEAQTL
KTWTMMETILGTVGAIVGMIAFQLLS
>EECEBICD_04512 hypothetical protein
MIAKPSVMRLILPYRASRKSSGDYQGICVRKGIISVAVLLILIMDQRRNLPAFMRVLKHG
EAKCRWAIAMHTSIKKPGSDYALPGLRYRYVPVSRPGKHSATGQQDSAQQLESNHPDNRA
DRAEDSFHHRPGLQRLRFGRAGKFTEQPEAGVVNMRNHN
>EECEBICD_04513 Aspartate-semialdehyde dehydrogenase
MKNVGFIGWRGMVGSVLMQRMVEERDFDAIRPVFFSTSQFGQAAPTFGDTSTGTLQDAFD
LDALKALDIIVTCQGGDYTNEIYPKLRESGWQGYWIDAASTLRMKDDAIIILDPVNQDVI
TDGLNNGVKTFVGGNCTVSLMLMSLGGLFAHNLVDWVSVATYQAASGGGARHMRELLTQM
GQLYGHVADELATPSSAILDIERKVTALTRSGELPVDNFGVPLAGSLIPWIDKQLDNGQS
REEWKGQAETNKILNTASVIPVDGLCVRVGALRCHSQAFTIKLKKEVSIPTVEELLAAHN
PWAKVVPNDRDITMRELTPAAVTGTLTTPVGRLRKLNMGPEFLSAFTVGDQLLWGAAEPL
RRMLRQLA
>EECEBICD_04514 1,4-alpha-glucan branching enzyme GlgB
MSSRIDRDVINALIAGHFADPFSVLGMHQTQAGLEVRALLPDATDVWVIEPKTGRKVGKL
ECLDARGFFCGVLPRRKNFFRYQLAVTWHGQQNLIDDPYRFGPLIQEMDAWLLSEGTHLR
PYETLGAHADTMDGVTGTRFSVWAPNARRVSVVGQFNYWDGRRHPMRLRKESGIWELFIP
GAHNGQLYKFELLDANGNLRIKADPYAFEAQMRPETASMICGLPEKVTPSEERQKANQFD
APISIYEVHLGSWRRHTDNNFWLSYRELADQLVPYAKWMGFTHLELLPVNEHPFDGSWGY
QPTGLYAPTRRFGTRDDFRYFINAAHAAGLNVILDWVPGHFPSDEFSLAEFDGTHLYEHS
DPREGYHQDWNTLIYNYGRREVSNYLVGNALYWMERFGIDALRVDAVASMIYRDYSRKEG
EWIPNEFGGRENLEAIEFLRNTNRIIGEQVPGAVSMAEESTDFSGVTRPPETGGLGFWYK
WNLGWMHDTLDYMKLDPVYRQYHHDKLTFGMLYNHTENFVLPLSHDEVVHGKKSILDRMP
GDAWQKFANLRAYYGWMWAFPGKKLLFMGNEFAQGREWNHDASLDWHLLEGGDNWHHGVQ
SLVRDLNHTYRHHKALHELDFDAYGFEWLVVDDNERSVLIFVRRDKAGNEIIVASNFTPV
PRHDYRFGINQPGRWREILNTDSMHYHGSNTGNGGVVHSDEIESHGRQHSLNLTLPPLAT
IWLMREGE
>EECEBICD_04515 Glycogen debranching enzyme
MTQLAIGEATPHGATYDGHGVNFTLFSAHAERVELCVFDSRGNERRYDLPGRRGDVWHGY
LAGARPGLRYGYRVHGPWQPAQGHRFNPVKLLLDPYARRVEGELKDHPLLHGGHDEPDYR
DNAAVAPKSVVISDHYDWEDDAAPRTPWGKTVIYEAHVKGLTYLHPELPQEIRGTYKALG
HPVMVAYFKQLGITALELLPVAQFASEPRLQRMGLTNYWGYNPMAMFALHPAWASSPEMA
LDEFRDAVKALHRAGIEVILDIVLNHSAELDLDGPTFSLRGIDNRSYYWIRDDGDYHNWT
GCGNTLNLSHPGVVEYACECLRYWVETCHVDGFRFDLASVMGRTPTFRQDAPLFAAIKAC
PVLSTVKLIAEPWDIGEGGYQVGNFPPPFAEWNDHFRDAARRFWLPRNLTTGEFACRFAA
SSDVFKRNGRAPGASVNLLTAHDGFTLRDCVCFNQKHNEANGEENRDGTNSNYSDNHGKE
GLGGPLDLMERRRDSIHALLATLLLSQGTPMLLAGDEHGHSQHGNNNAYCQDNALTWLDW
QQANRGLTTFTAALIRLRQQIPALTGNSWWEEGDGNVRWLNKNAQPLSADEWQNGPKLMQ
ILLSDRFLIAINATLEVTDIVLPEGEWRAVPPFAGEDNPVITAVWQGPAHGLCVFQRG
>EECEBICD_04516 Glucose-1-phosphate adenylyltransferase
MVSLEKNDRVMLARQLPLKSVALILAGGRGTRLKDLTNKRAKPAVHFGGKFRIIDFALSN
CLNSGIRRIGVITQYQSHTLVQHIQRGWSLFSEEMNEFVDLLPAQQRMKGENWYRGTADA
VTQNLDIIRRYKAEYVVILAGDHIYKQDYSRMLIDHVEKGARCTVACMPVPIKEATAFGV
MAVDESDKIIDFVEKPANPPAMPGDASKSLASMGIYVFDADYLYELLAADDKDDASSHDF
GKDIIPKITREGMAYAHPFPLSCVQSDPQAEPYWRDVGTLEAYWKANLDLASVTPELDMY
DQNWPIRTHMESLPPAKFVQDRSGSHGMTLNSLVSGGCIISGSVVVQSVLFPRVRINSFC
NIDSAVLLPEVWVGRSCRLRRCVIDRACIIPEGMVIGENAEEDARRFYRSEEGIVLVTRE

```
MLRKLQVKQER
>EECEBICD_04517 Glycogen synthase
MQVLHVCSEMFPLLKTGGLADVIGALPAAQIADGVDVRVLLPGFPDIRRGIPDAHVVSRR
DTFAGKISLLFGHYNGVGIYLIDAPHLYERPGSPYHDTNLYAYTDNVLRFALLGWVGCEM
ACGLDPFWRPDVVHAHDWHAGLAPAYLAARGRPAKSVFTVHNLAYQGMFYAKHMDDIELP
WSFFNMHGLEFNGQLSFLKAGLYYADHITAVSPTYAREITEPQFAYGMEGLLRQRHLEGR
LSGILNGVDEKIWNPESDLLLASRYTRDTLEEKAENKRQLQIAMGLKVNDKVPLFAVVSR
LTNQKGLDLVLEALPGLLEQGGQLALLGAGDPVLQEGFLAAAAEHPGQVGVQIGYHEAFS
HRIMGGADVILVPSRFEPCGLTQLYGLKYGTLPLVRRTGGLADTVSDSSLENLADGIASG
FVFEDSNAWSLLRAIRRAFVLWSRPSLWRFVQRQAMAMDFSWQVAAKSYRELYYRLK
>EECEBICD_04518 Maltodextrin phosphorylase
MNAPFTYASPTLSVEALKHSIAYKLMFTIGKDPVIANKHEWLNATLFAVRDRLVERWLRS
NRAQLSQETRQVYYLSMEFLIGRTLSNALLSLGIYDDVKGALEAMGLDLEELIDEENDPG
LGNGGLGRLAACFLDSLATLGLPGRGYGIRYDYGMFKQNIVDGRQKESPDYWLEYGNPWE
FKRHNTRYKVLFGGRIQQEGKKARWIETEEILAVAYDQIIPGYDTDATNTLRLWNAQASS
EINLGKFNQGDYFAAVEDKNHSENVSRVLYPDDSTYSGRELRLRQEYFLVSATVQDILHR
HYQLHKTYENLADKIAIHLNDTHPVLSIPELMRLLIDEHKFSWDDAFEVCCQVFSYTNHT
LMSEALETWPVDMLGKILPRHLQIIFEINDYFLKTLQEQYPNDTSLLGRASIIDESNGRR
VRMAWLAVVVSHKVNGVSELHSNLMVQSLFADFAKIFPTRFCNVTNGVTPRRWLALANPP
LSDVLDENIGRTWRTDLSQLSELKQHCDYPLVNHAVRQAKLENKKRLAVVIAQQLNVVVN
PKALFDVQIKRIHEYKRQLMNVLHVITRYNRIKENPEADWVPRVNIFAGKAASAYYMAKH
IIHLINDVAKVINNDPQIGDKLKVVFIPNYSVSLAQVIIPAADLSEQISLAGTEASGTSN
MKFALNGALTIGTLDGANVEMQEHVGEENIFIFGNTAEEVEALRRQGYKPRDYYEKDEEL
HQVLTQIGSGVFNPEEPGRYRDLVDSLINFGDHYQVLADYRSYVDCQDKVDELYRRPEEW
TTKAMLNIANMGYFSSDRTIKEYAENIWHIDPVRL
>EECEBICD_04519 putative HTH-type transcriptional regulator RhmR
MKKITTSIPALDKIMRVFAYLLECDGATFTQIHQNSGIAKSSTSSLLNGMVEHGLLRQEK
DKYYLGLRLYELGNKAAEQYDIKKIALPILEEIRDNTGLTCHLGVLEGDAPIYLLKVESP
QAIVIRSWEGKRLSLHSSGLGKVLIAWLSGEELEELLPPDQILTRFTDTTITDVNILKQE
LAGIRRRGWGYDNEEDSLGVRCIAVPVFNTQGKVIAALSVSGVTFQIPDDKRESLATLMM
DASRSLTRLMC
>EECEBICD_04520 putative 2-dehydro-3-deoxy-D-pentonate aldolase YjhH
MTQITKFHGVFPPVPTIVNAQGELDKVGMATLLDHLIASKVNGVLLLGSGGEFCHMTNAL
RLETAEFCVRHINRRIPVLLGISSTSTQEVIEYGQHADRLEVDAVLVLNPYYARLTDEYI
YHHFKTVANNIKSPVILYNFPALTGQDLSVDLITRLAQDHPNIIGIKDTVDNISHIREII
NNVRPVRPDFVIFSGYDEYMMDTLILGGNGGIPATANFAPQLTCGIYRAWCEKEYETMFG
LQRRLSALSTIYSLDSPFFGIIKKAIQLSGVDISTHVMPPVLPANEEHISNLKQILQRAG
L
>EECEBICD_04521 Dihydroxy-acid dehydratase
MSQKCQHARDLWSQLDALRLGMNYSKEDVNKLQVLVDDCYGESHPGSFHLNQLGDEAVLG
VHESGGRAVRHHVTDICDGWGQGHDGMNYILASREAIANMVEIHASVVPYDAGILISSCD
KSIPAHLIAAARLNLPLLHIPGGSMRPAPNMSTSDLGGITAKLKKGEIGIQQVEAMQQCG
CPTAGACQFMGTASTMQCMSEALGLTLPGSALLPSTLAEIRRVARTAGHQALYLAEKNIT
THKILTPAAFENAIKVHAAIGGSTNAMIHLPAIAHELGWELKPELFDRINNEIPYLTNIQ
PSGEYVTEMMWFAGGVPMVQWYLRDYLDLDVLTVTGRTLGDNLEMLHQSGFFTRNHGYLN
NYKVSPEEVIRKPENATKKGSIAVLKGNIAPEGAVIKYAACAPDMHHHTGPARVFNSEED
AQQAIIHNHIEPGDVIFIRYEGAKGSGAPEMLMTTDAIVYDKRLDGKVALITDGRFSGAT
SGPCVGHVSPEAADGGPIALVEDGDLIEMDVKGRKLNIVGIDGVFKTEEEIRRCLEERRA
SWKKPDYSNRHGVFKQFTANATSLMAGAWLK
>EECEBICD_04522 Inner membrane transport protein RhmT
MYPAILALIGHWFPNEERARAIAYFQMNLAVASIITGPLSGWLIETYGWREMFIIEGLLS
LGLLFVWLPLVSDHPHQAKWLDPKERAWIEQKLLADRALSIGGEQSSIRGVLKSINLWKL
VGIYFFVQVGFYGFALWMPNLIKHLTGSGMTIVGVLTAAPYVLCIIGQYYIAKWCDKTMN
RRLYTAIPLLGFAVCLALSLLLKDNVWLAYGMMVICGFFLQAYAGPFWTLPPLLFAPNVL
```

GGVRGTINALGNIGGFIGPYLVGLLTVTFSQTAGMTVLVAALLIAVGLLFSLPSVTARPA
GSSNTPNTSTPGASLKQEGIAK
>EECEBICD_04523 Putative tartrate transporter
MMSIEIEKPTTRGRWLHIIPATILVYIVAYMDRTNIAIGIAGGMDEDLGMTASFAGLVAG
IFFIGYIFLQIPGGQIAERLSAKKLIAWTIVAWGALPC
>EECEBICD_04524 Glycerol dehydrogenase
MVTTAIFPSRYVQGKGALTTHLPQELAALGHKALILQDPVVHNTYRDTVATALHGVIEFD
IEVFSSECSDEEIARISARAQEIGADVIVGMGGGKTLNTAKATGASLRLPIAVVPTLAST
DAPCSSLVVIYTPEGKFKRYLMIPRNPTLVLVDSSIIAAAPVRFLVSGIGDALATWFEAE
DCRIKGAGNMTTRPGPMTAFELARFCYTTLMRYGRLAKLACEQHQVTPALEHVIEANTLL
SGLGFESGGLAAAHAIHNGLTVLPATHKYWHGEKVAFGTLAMLMLTDRAPELIEEVYQFC
EDIGLPTTLADIGLAGVSDDELLAVARASCQTGETMHNEPFTITPEAVQAALRAADAVGQ
ARKKIDLAG
>EECEBICD_04525 hypothetical protein
MKNAKWLFLSIMMAFIGVPLLVNIITIIVGGMWAYISVLENSIPIAQSIEKATIIPVALL
GAGLIFLCGRIWGKGNASEVNPEWRDCCLLMPALVVLMGWVILMFLTDLNIRAIGRKPIA
QVVQTLWLVPSVISFFNGWVWAVLLIPVGSQLCFALGYGGARWGVLRGGAGYSCRNLLVI
CLLFFGSCAVYQSHLYAVKYPAPESSEYLYFRDYQAHTWGNKLTGLRDEPTLLLTQNWPR
LDGATAGLPLYASAFYALTRFPDDICPEDYLENQGTIEAYQNILNGNADLIFVAQPSTAQ
KQLAEASGVKLVYTPFAREAFVFIVNQNNPVSSLSDVQIRGIFSGRITHWNEVGGEAIDI
KPWQRPENSGSQTAMLAKVMKETKLMPAQTTSVATAMGDMVDIVAEYRNTHNAIGYTFRY
YATQMHSNKEIKLLAINDVAPTVENIRNGSYPYTVDVYMVTREHPTAETQKLVDWFLSPQ
GQQLVQDVGYVPLYPAAK
>EECEBICD_04526 hypothetical protein
MEEVKLYVERVGIDPASGADIRIARRESTSWHKDIVAELINQVLRCGAALIIIAVLTACD
DRQVSYFPDYAAIPEQSGIWTWLPVFFPSSASEIEMTFNLDSNLFYADFSVADGDKRAHF
ESVLGEESVVHYANFSHKSWCKTGKTLWNSEARAKPFFITQISVDRYRITNHMPGCTKPG
E
>EECEBICD_04527 Aerobic glycerol-3-phosphate dehydrogenase
METKDLIVIGGGINGAGIAADAAGRGLSVLMLEAQDLACATSSASSKLIHGGLRYLEHYE
FRLVSEALAEREVLLKMAPHIAFPMRFRLPHRPHLRPAWMIRIGLFMYDHLGKRTSLPGS
TGVRFGADSVLKPEIVRGFEYSDCWVDDARLVLANAQMVVRKGGEVLTRTRATAARRENG
LWIVEAEDIDTGKKYVWQARGLVNATGPWVKQFFDEGMHLPSPYGIRLIKGSHIVVPRVH
NQKQAYILQNEDKRIVFVIPWMEEFSIIGTTDVEYKGDPKAVKIEESEINYLLKVYNAHF
QKQLGRDDIVWTYSGVRPLCDDESDSPQAITRDYTLDIHDENGKAPLLSVFGGKLTTYRK
LAEHAMEKLASYYPGIGPAWTKTCVLPGGDIDGSREDYAAKLRRRYPFLTESLARHYSRT
YGSNTEWILGEATSLLDLGEDFGHEFYEAELKYLVDHEWVRRTEDAIWRRTKEGMWLTAE
QQSRITQWLAAYVEKHQLSMAS
>EECEBICD_04528 Thiosulfate sulfurtransferase GlpE
MEQFECITVEEAYQKLHQGAAVLVDIRDPQSYAMGHAPQAFHLTNDTLGAFMREHGFDTA
VMVMCYHGNSSKGAAQYLLQQGYDAVYSIDGGFEAWHRRFPADVANGA
>EECEBICD_04529 Rhomboid protease GlpG
MLMITSFANPRVAQAFVDYMATQGVILTIQQHNQSDIWLADESQAERVRGELARFIENPG
DPRYLAASWQSGQTNSGLRYRRFPFLATLRERAGPVTWIVMLACVLVYIAMSLIGDQTVM
VWLAWPFDPVLKFEVWRYFTHIFMHFSLMHILFNLLWWWYLGGAVEKRLGSGKLIVITVI
SALLSGYVQQKFSGPWFGGLSGVVYALMGYVWLRGERDPQSGIYLQRGLIIFALLWIVAG
WFDWFGMSMANGAHIAGLIVGLAMAFVDTLNARKRT
>EECEBICD_04530 Glycerol-3-phosphate regulon repressor
MKQTQRHDAIIELVKKQGYVSTEELVEHFSVSPQTIRRDLNDLAEQNMILRHHGGAALPS
SSVNTPWHDRKATQTEEKERIARKVAAQIPNGSTLFIDIGTTPEAVAHALLGHSNLRIVT
NNLNVANTLMAKEDFRIILAGGELRSRDGGIIGEATQDFIAQFRLDFGILGISGIDSDGS
LLEFDYHEVRTKRAIIENSRHVMLVVDHSKFGRNAMVNMGSISMVDAVYTDTLPPPGVMQ
VLTENHIQLELC

>EECEBICD_04531 Anaerobic nitric oxide reductase transcription regulator NorR
MRKTVAFGFVGTVLDYAGRGSQRWEKWRPTLCLCQQETLVVHRLELLYDARSRSLFEGLK
KDIASVSPETEVVGVEIAIRNPWDFEEVYACLHDFARSHTFHPEDEDYLIHITTGTHVAQ
ICWFLLAEARYLPARLAQTSPPRKKDKPHSTGDVTIIDLDLSRYNDIATRFAQEREETLN
FLKSGIATRNPCFNRMIEQIERVAIRSRSPILLNGPTGAGKSFLARRIYELKLARHQFSG
PFVEVNCATLRGDTAMSALFGHVKGAFTGAREERAGLLRSADGGMLFLDEVGELGADEQA
MLLKAIEEKRFYPFGSDQQVSSDFQLIAGTVRDLRQRVAEGTFREDLYARINLWTFELPG
LRQRQEDIEPNLDYELERHAALTGDSVRFNTEARRAWLSFATSPQAAWRGNFRELSASVT
RMATLADNGRITVETVDDEIARLRYSWNDHRPSALDGLPGIDATALDLFDRMQLENVVAI
CRQAKTLSDAGRQLFNVSRQGKATVNDADRLRKYLARFGLTWDVLQN
>EECEBICD_04532 hypothetical protein
MANPLLFRSLLRDAPLANASNQQGAAAFAFTPRHTLAQMVMTGCMNETFYVSGQAQLNDV
LATAKDLDDLFLAQLAIYGRERGMMKDMPALLTAILAARGSALLPVVFARVINNGRMLRN
FVQMLRSGVTGRRSLGTRPKKLVQRWLQNASEERLLQASVGNMPSLADIVKMVHPRPQAA
WQEAFFAWLIGKPCDKAQLPEKTRALLAFREGNMGAALPDVPFLLLTNAPLSREQWAQLA
QRMSWQTLRMNLNTLARHDVFENATLAASVAQRLADRAQVRQSWVYPYQLLSAWSNLQCS
VPQVIREALAQAMEYALENIPPFRGNVVVCPDVSGSMKSSITGYRKGATSKIRCVDVAGL
IAAAVLRNHPQARVLPFECDVVDVQLDARQSVMHNAQKLAAVGGGSTNCSAPLRRLLNER
ARVDLVIMVSDNESWVDKSRHGSTATMECWIELKKRNPQARLVCIDLLPYGTTQAAERSD
ILNVGGFSDEVFTVIDNFVNGRYGSAHWLEEIEAVTL
>EECEBICD_04533 RNA-splicing ligase RtcB
MMNYELMTTQNAPVKMWTKGVPVEDDARQQLINTAKMPFIFKHIAVMPDVHLGKGSTIGS
VIPTKGAIIPAAVGVDIGCGMNALRTSLTAADLPENLAELRSAIEAAVPHGRTTGRGRRD
VGAWGNPPANVDEKWAQLEAGYQWLTQKYPRFLNTNSYKHLGTLGTGNHFIEICLDETDR
VWIMLHSGSRGIGNAIGTYFIGLAQQEMQEQLETLPSRDLAYFNEGSEYFDDYLKAVHWA
QQFASLNREAMMENALAALQRCVEKSIVLDMDEINCHHNYVQKEQHFGEEIYVTRKGAVS
ARRGEFGIIPGSMGAKSFIVRGLGNEESFCSCSHGAGRVMSRTKAKKLFSVDDQIRATAH
VECRKDADVIDEIPMAYKDIDAVMAAQSDLVEIMYTLRQVVCVKG
>EECEBICD_04534 RNA 3'-terminal phosphate cyclase
MARIIALDGAQGEGGGQILRSALSLSMITGQPFEMSDIRAGRAKPGLLRQHLTAVRAATE
ICGAQVNGDELGSQQLRFTPGPIRGGEYRFAIGSAGSCMLVLQTVLPALWFADGSSRVEV
HGGTHNQAAPSADFICRVWEPLLARMGISQRTTLIKHGFYPAGGGAAATVVEPATSLRGL
TLISRGETLRTTAEALLAAVPYHVGEREVATLEAHFPLAEKNVVALEGGCGPGNALLLMI
QSEQLTELFAAFGVKGTSAEAVANQVAHEARRYLASPAAVGEHLADQLILPLALAGEGAF
TVARASAHLLTNIAVVERFLPVRFSCEATESGYLVRVSD
>EECEBICD_04535 Antitoxin DinJ
MAANALVRARIDETLKDQAADVLAEMGLTISDLIRITLTKVAREKALPFDLRIPNELTSR
TIENSEAGVDIHKAKDADDLFDQLGI
>EECEBICD_04536 mRNA interferase toxin YafQ
MGQREIEYSGQFQKDVKRAQKRHKDVGKLKTLMTLLIHHPFPLPAIYKDHPLQGSYSGYR
DAHIEPDWILIYKITDECLRFERTGTHADLF
>EECEBICD_04537 HTH-type transcriptional regulator MalT
MLIPSKLSRPVRLDHTVVRERLLAKLSGANNFRLALVTSPAGYGKTTLVSQWAAGKNELG
WYSLDEGDNQQERFASYLIAAIQQATGGHCSTSEAMAQKRQYASLTSLFAQLFIELAQWH
RPLYLVIDDYHLITNPVIHDAMRFFLRHQPENFTLVVLSRNLPQLGIANLRVRDQLLEIG
SQQLAFNHQEAKQFFDRRLSSPIEAAESSRMCDDVAGWATALQLIALSARQNHTSAHHSA
RRLAGINASHLSDYLVDEVLDNVDVSTRHFLLKSAILRSMNDALIVRVTGEENGQMRLEE
IERQGLFLQRMDDTGEWFSYHPLFGSFLRQRCQWELAAELPEIHRAAAESWMEQGFPSEA
IHHALAAGDAQMLRDILLNHAWGLFNHSELALLEESLKALPWESLLENPRLVLLQAWLMQ
SQHRYSEVNTLLARAEQEIKGVMDGTLHAEFNALRAQVAINDGNPEEAERLAKLALDELP
LAWFYSRIVATSVHGEVLHCKGDLSQSLSLMQQTEQMARHHDVWHYALWSLIQQSEIQFA
QGFLQAAWETQERAFQLIKEQHLEQLPMHEFLVRIRAQLLWAWARLDEAEASARSGIAVL
STFQPQQQLQCLTLLVQCSLARGDLDNARSQLNRLENLLGNGRYHCDWISNADKVRVIYW

QLTGDKKSAANWLRHTPKPAFANNHFLQGQWRNIARAQILLGEFEPAEIVLEELNENARS
LRLMSDLNRNLLLLNQLYWQSGRKNDAQRVLLDALQLANRTGFISHFVIEGEAMAQQLRQ
LIQLNTLPEMEQHRAQRILREINQHHRHKFAHFDEGFVERLLNHPDVPELIRTSPLTQRE
WQVLGLIYSGYSNEQIAGELAVAATTIKTHIRNLYQKLGVAHRQDAVQHAQQLLKMMGYG
V
>EECEBICD_04538 Maltodextrin phosphorylase
MSQPTFNKDQFQAALTRQWQRFGLLSASDMTPRQWWQAVSGALAELLSAQPVAQPTKGQR
HVNYISMEFLIGRLTGNNLLNLGWYQDVSDVLKAHDINLTNLLEEEVDPALGNGGLGRLA
ACFLDSMATVGQSATGYGLNYQYGLFRQSFVEGKQVEAPDDWHRGSYPWFRHNEALDVQV
GIGGKVTKEGRWEPGFVITGQAWDLPVLGYRNGVAQPLRLWQATHAHPFDLTKFNDGAFL
RAEQQGIDAEKLTKVLYPNDNHTAGKKLRLMQQYFQCACSVADILRRHHLAGRKLHELAD
YEVIQLNDTHPTIAIPELLRVLLDEHQMSWDDAWAITSKTFAYTNHTLMPEALECWDERL
IKALLPRHMQIIKQINDRFKTLVDKTWPGDKQVWAKLAVVHDRQVRMANMCVVSGFAVNG
VAALHSDLVVKDLFPEYHQLWPNKFHNVTNGITPRRWIKQCNPQLAALLDKTLKKEWAND
LDQLINLEKYADDAKFRQQYRDIKRANKERLVKFIKARTGIEISSNAIFDIQIKRLHEYK
RQHLNLLHILALYKEIRENPQADRVPRVFLFGAKAAPGYYLAKNIIFAINKVAEAINNDP
AVGDKLKVVFLPDYCVSAAEMLIPAADISEQISTAGKEASGTGNMKLALNGALTVGTLDG
ANVEIAEKVGEENIFIFGHTVEEVKALKAKGYDPVKWRKKDKVLDAVLKELESGQYSDGD
KHAFDQMLHSLGKQGGDPYLVMADFAAYVEAQKQVDALYRDQEAWTRAAILNTARCGMFS
SDRSIRDYQARIWQAKR
>EECEBICD_04539 4-alpha-glucanotransferase
MENKRLDSAALAAGISPSYINAHGKPQSIGAETKRRLLAAMHGTTTGPQAVVPNVKVYTA
GKKMALPVEGRGEFAWLLTTEEGVHYKGRVTGGKKLNLPATLPEGYHTLTLTQDEQRTHC
RIIVAPPRCYEPQALLEGKNCGGPASSFILCVQKKTGVLAISAI
>EECEBICD_04540 4-alpha-glucanotransferase
MLVDVATRGGAFIGLNPIHALYPANPESASPYSPSSRRWLNVIYIDVNAVEDFRLSEEAQ
AWWQMPATQQQLRQARDAQWVDYAIVTALKITALRMAWTRFAARDDAQMAEFRHFIAREG
ESLYWQAAFDALHAYQVKEDEQRWGWPAWPEAYQSVESPAVKQFCEAHREEVEFYLWLQW
LAWRQFAACWDTCQSFKLPIGLYRDLAVGVAEGGAETWCDRELYCLKASVGAPPDILGPL
GQNWGLPPMDPHIIVARAYEPFIDLLRANMQNCGALRIDHVMSLLRLWWIPYGETADQGA
YVHYPVDDLLSILALESQRHRCMVIGEDLGTVPVEIVGKLRDSGVYSYKVLWFENDLEKN
FRAPGAYPQQSMAVASTHDLPTLRGYWECGDLTLGKALGLYPDEVILRGLYEDRERAKQG
LLDALHKYGCLPKRAGHKASLMSMTPTLNRGLQRYIADSNSGLLGLQPEDWLDMADPVNV
PGTSDQYKNWRRKLTATLEQMFADEGVNKLIKDLDKRRKAAAKKK
>EECEBICD_04541 High-affinity gluconate transporter
MPLVIVAIGVILLLLLLMIRFKMNGFIALVLVALAVGLMQGMPLDKVIGSIKAGVGGTLGS
LALIMGFGAMLGKMLADCGGAQRIATTLIAKFGKKHIQWAVVLTGFTVGFALFYEVGFVL
MLPLVFTIAAAANIPLLYVGVPMAAALSVTHGFLPPHPGPTAIATIFHADMGKTLLFGTI
LAIPTVILAGPVYARFLKGIDKPIPEGLYSAKTFTEEEMPGFGVSVWTSLVPVILMAMRA
IAEMILPKGHAFLPVAEFLGDPVMATLIAVLIAMFTFGLNRGRSMDQINDTLVSSIKIIA
MMLLIIGGGGAFKQVLVDSGVDKYIASMMHETNVSPLLMAWSIAAVLRIALGSATVAAIT
AGGIAAPLIATTGVSPELMVIAVGSGSVIFSHVNDPGFWLFKEYFNLTIGETIKSWSMLE
TIISVCGLIGCLLLGMVV
>EECEBICD_04542 Fe/S biogenesis protein NfuA
MIRISDAAQAHFAKLLANQEEGTQIRVFVINPGTPNAECGVSYCPPDAVEATDTALKFDL
LTAYVDELSAPYLEDAEIDFVTDQLGSQLTLKAPNAKMRKVADDAPLMERVEYALQSQIN
PQLAGHGGRVSLMEITDEGYAILQFGGGCNGCSMVDVTLKEGIEKQLLNEFPELKGVRDL
TEHQRGEHSYY
>EECEBICD_04543 Putative ribose-phosphate pyrophosphokinase
MLTVPGLCWLCRMPLALSHWGICSVCARAVRQRVSVCPQCGLPARHPSLPCGRCLQKPPP
WQRLVSVSVSNYTPPLSLLVHQLKFTRRNEIAAALARLLLQEVLMARRSTGLQLPDRIVSVP
LWSRRHWRRGFNQSDLLCQPLAHWLGCAWDSQTITRVRATATQHHLSARLRKRNLKNAFR
LELPVQGLHMVIVDDVVTTGSTVAEIAQLLLRNGAATVQVWCLCRTL
>EECEBICD_04544 Pimeloyl-[acyl-carrier protein] methyl ester esterase

MNDIWWQTYGEGNCHLVLLHGWGLNAEVWHCIREELGSHFTLHLVDLPGYGRSSGFGAMT
LEEMTAQVAKNAPDQAIWLGWSLGGLVASQMALTHPERVQALVTVASSPCFSAREGWPGI
KPEILGGFQQQLSDDFQRTVERFLALQTLGTETARQDARTLKSVVLAQPMPDVEVLNGGL
EILKTVDLREALKNVNMPFLRLYGYLDGLVPRKIAPLLDTLWPHSTSQIMAKAAHAPFIS
HPAAFCQALMTLKSSL
>EECEBICD_04545 ISNCY family transposase ISSen7
MATSTTSTPHDAVFKQFLCHPDTARDFLEIHLPTTLRQICNLNTLRLESGSFIEEDLRPH
YSDILWSLETSEGEGYIYVVIEHQSTPDAHMAFRLMRYAMAAMQRHLEAGHKTLPLVVPM
LFYHGNRSPYPFSLCWLDEFADPVMARKLYATAFPLVDITVVPDDEIMRHRRVALLELIQ
KHIRQRDLMGLVEQLVALLVKGYANDTQLQSLFNYMMHTGDAARFNTFIRQVAMRIPQHK
EKIMTIAERLRQEGHRNGLQKGLQQGKQEGQRLAALRIARSMLNDGFDRDTVLRVTGLAP
ADLASENH
>EECEBICD_04546 putative [Fe-S]-dependent transcriptional repressor FeoC
MASLIQVRDLLALRGRMEATQISHTLHAPQPMIDAMLNQLEIMGKAVRIPEEADGCLSGS
CKSCPEGKACLREWWALR
>EECEBICD_04547 Fe(2+) transporter FeoB
MKKLTIGLIGNPNSGKTTLFNQLTGARQRVGNWAGVTVERKEGQFATTDHQVTLVDLPGT
YSLTTISSQTSLDEQIACHYILSGDADLLINVVDASNLERNLYLTLQLLELGIPCIVALN
MLDIAEKQQVRIDVDALSTRLGCPVVPLVSTRGRGIEALKLAIDRHNANDNVELVHYAQP
LLREAGFLADAMAQEMPLQQRRWLGLQMLEGDIYSRAYADEAAQNLDTSLARLKDEMDDP
ALHIADARYQCIAAICDVVSNTLTAEPSRFTRAVDKIILNRFLGLPIFLFVMYLMFLLAI
NIGGALQPLFDAGSVAIFIHGIQWIGYTLHFPDWLTIFLAQGLGGGINTVLPLVPQIGMM
YLFLSFLEDSGYMARAAFVMDRLMQALGLPGKSFVPLIVGFGCNVPSVMGARTLDAPRER
LMTIMMAPFMSCGARLAIFAVFAAAFFGQNGALAVFSLYVLGIVMAVLTGLMLKHTIMRG
EASPFVMELPVYHVPHIKSLIIQTWQRLKGFVLRAGKVIIIVSIFLSAFNSFSLSGKIVD
NINDSALASVSRVITPVFKPIGVHEDNWQATVGLFTGAMAKEVVVGTLNTLYTAENIQDE
AFNPADFHLGDELLGAVDDTWQSLKDTFSLSVLANPIEASKGDGEMATGAMGVMDQKFGS
AAAAYSYLIFVLLYVPCISVMGAIARESSRGWMGFSILWGLNIAYSLATLFYQVTSFSQH
PTYSLICILAVIVFNVVVLSLLRRARSRVDIELLATRKNVSSCCSGTAGNCH
>EECEBICD_04548 Fe(2+) transport protein A
MQFTPDTAWKITGFAREISPAYRQKLLSLGMLPGSSFNVVRVAPLGDPIHIETRRVSLVL
RKKDLALIEVEAVSC
>EECEBICD_04549 Protein YhgF
MMNDSFCRIIAGEIQANAGQVEAAVRLLDEGNTVPFIARYRKEITGGLDDTQLRNLETRL
GYLRELEDRRQAILKSISEQGKLTDELAGAINATLSKTELEDLYLPYKPKRRTRGQIAIE
AGLEPLADLLWNEPSHDPDVEAAKYIDGDKGVADTKAALDGARYILMERFAEDAALLAKV
RDYLWKNAHLVATVVSGKEEEGAKFRDYFDHHEPIANVPSHRALAMFRGRNEGILQLSLN
ADPQFDEPPKESYCEQIIMDHLGLRLNNAPADSWRKGVVSWTWRIKVLMHLETELMGTVR
ERAEDEAINVFARNLHDLLMAAPAGLRATMGLDPGLRTGVKVAVVDGTGKLVATDTIYPH
TGQAAKAATVIAALCEKYHVELVAIGNGTASRETERFYLDVQKQFPNVTAQKVIVSEAGA
SVYSASELAAQEFPDLDVSLRGAVSIARRLQDPLAELVKIDPKSIGVGQYQHDVSQTQLA
RKLDAVVEDCVNAVGVDLNTASVPLLTRVAGLTRMMAQNIVAWRDENGQFQNRQQLLKVS
RLGPKAFEQCAGFLRINHGDNPLDASTVHPEAYPVVERILAATQQALKDLMGNSNELRHL
KAADFTDDKFGVPTVSDIIKELEKPGRDPRPEFKTAQFADGVETMNDLLPGMILEGAVTN
VTNFGAFVDIGVHQDGLVHISSLSNKFVDDPHTVVKAGDIVKVKVLEVDLQRKRIALTMR
LDEQPGETAARRGGGADRAQGNRPASKAAKPRGRDAQPAGNSAMMDALAAAMGKKR
>EECEBICD_04550 Transcription elongation factor GreB
MKTPLITREGYETLKQELNYLWREERPEVTKKVTWAASLGDRSENADYQYNKKRLREIDR
RVRYLTKCMENLKIVDYSPQQEGKVFFGAWVEIENDDGDTLKFRIVGYDEIFGRKDYISI
DSPMARALLKKEVGDLAVVNTPVGEANWYVNAIEYVK
>EECEBICD_04551 Transcriptional regulatory protein OmpR
MQENYKILVVDDDMRLRALLERYLTEQGFQVRSVANAEQMDRLLTRESFHLMVLDLMLPG
EDGLSICRRLRSQSNPMPIIMVTAKGEEVDRIVGLEIGADDYIPKPFNPRELLARIRAVL
RRQANELPGAPSQEEAVIAFGKFKLNLGTREMFREDEPMPLTSGEFAVLKALVSHPREPL

SRDKLMNLARGREYSAMERSIDVQISRLRRMVEEDPAHPRYIQTVWGLGYVFVPDGSKA
>EECEBICD_04552 Osmolarity sensor protein EnvZ
MRRMRFSPRSSFARTLLLIVTLLFVSLVTTYLVVLNFAILPSLQQFNKVLAYEVRMLMTD
KLQLEDGTQLVVPPAFRREIYRELGISLYTNEAAEEAGLRWAQHYEFLSHQMAQQLGGPT
EVRVEVNKSSPVVWLKTWLSPNIWVRVPLTEIHQGDFSPLFRYTLAIMLLAIGGAWLFIR
IQNRPLVDLEHAALQVGKGIIPPPLREYGASEVRSVTRAFNHMAAGVKQLADDRTLLMAG
VSHDLRTPLTRIRLATEMMGEEDGYLAESINKDIEECNAIIEQFIDYLRTGQEMPMEMAD
LNSVLGEVIAAESGYEREINTALQAGSIQVKMHPLSIKRAVANMVVNAARYGNGWIKVSS
GTESHRAWFQVEDDGPGIKPEQRKHLFQPFVRGDSARSTSGTGLGLAIVQRIIDNHNGML
EIGTSERGGLSIRAWLPVPVARVQGTTKEA
>EECEBICD_04553 Phosphoenolpyruvate carboxykinase (ATP)
MRVNNLTPQDLKAYGINDVQDIVYNPSYDTLYQEELNPGLEGYERGVLTNLGAVAVDTGI
FTGRSPKDKYIVRDDTTRDTLWWSDKGKGKNDNKPLSQETWQHLKGLVTHQLSGKRLFIV
DAFCGANADTRLSVRFITEVAWQAHFVKNMFIRPTDEELVGFKPDFIVMNGAKCTNPQWK
EQGLNSENFVAFNLTERIQLIGGTWYGGEMKKGMFSVMNYLLPLKGIASMHCSANVGEKG
DVAVFFGLSGTGKTTLSTDPKRRLIGDDEHGWDDDGVFNFEGGCYAKTIKLSKEAEPEIY
HAIRRDALLENVTVREDGTVDFDDGSKTENTRVSYPIYHIDNIVKPVSKAGHATKVIFLT
ADAFGVLPPVSRLTANQTQYHFLSGFTAKLAGTERGVTEPTPTFSACFGAAFLTLHPTQY
AEVLVKRMQAAGAQAYLVNTGWNGTGKRISIKDTRAIIDAILNGSLDNAETFRLPLFDLA
IPTELPGVDTHILDPRNTYASPEQWQEKATALAKLFIENFEKYTDTPAGEALVSAGPKL
>EECEBICD_04554 hypothetical protein
MESVALSRTTRWGMLLTGLLQGVLCYLLMAWLVPQNSDWLFYGMPATIALSSMLLLTVVS
FKQRALWGWLGLTFVVVLAMSGWLKWQVETVEKWRLAELLWLYGLRLVLMAMLVLPWMQY
QLHSQTGSARYPQFYMRLWHNVLTLFIVLVANGLFWLVLLLWSALFRLVGIRFFSTLFFE
TEAFIYVTIGLITALAVILARTQSRLVAAVQKLLTLIATGLLPVVSLLALLFIVTLPFTG
LEAISARVSAAGLLSTLTLMLLLLVAIVNEPQKRILPYPRVLRGMISASLCVAPIYMLLA
GWALWVRIQQYGWTPDRLYGALTASVLLVWSFGYLIGLLRRGRDPGEWQGKVILSVSLLT
LVILLLLASPVLDVWRISVNSHMARYHSGKITADQISLYMLDHSGKPGQEALKSLRDDEA
FTQNRKRNRELMTFLQRNKVSPTADDLARVVMIAPGSQKPDAAFWAFVKEQSYSDDSCLE
PDACVLVSQDLNGDGQPEQVLYNFIVAESQVYGLKEGKWTQKAFARLPDGFSKTQLLHAI
AGHRLDSAPKAWRDIIVDGQRLDVDYYNE
>EECEBICD_04555 33 kDa chaperonin
MPQHDQLHRYLFENFAVRGELVTVSETLQQILDNHTYPQPVKTVLAELLVATSLLTATLK
FAGDITVQLQGDGPLSLAVINGNNQQQMRGVARVQGDIPDNADLKTLVGNGYLVITITPE
EGERYQGVVGLEGDTLAACLEDYFLRSEQLPTRLFIRTGDVDGKPAAGGMLLQVMPAQNA
QAEDFDHLAMLTETIKSEELLTLPANDVLWRLYHEEEVTLYDPQDVEFKCTCSRERCAGA
LKTLPDEEVDSILAEEGEIDMHCDYCGNHYLFNAMDIAEIRNNASPADPQVH
>EECEBICD_04556 Heat shock protein 15
MKEKSSVEVRLDKWLWAARFYKTRALAREMIEGGKVHYNGQRSKPSKIVELNATLTLRQG
NDERTVIVKAITEQRRPASEAVALYEETAESVEKREKMAQARKLNALTMPHPDRRPDKKE
RRDLLRFKHGDSE
>EECEBICD_04557 GMP/IMP nucleotidase YrfG
MHIDIDWQNVDTVLLDMDGTLLDLAFDNYFWQKLVPETYGAQQGISPQDAQEYIRQQYHA
VQHTLNWYCLDYWSERLGLDICAMTTAQGPRAVLRDDTVPFLNALKASGKRRILLTNAHP
HNLAVKLEHTGLASHLDLLLSTHTFGYPKEDQRLWRAVTEETGISAEKTLFIDDSEPILD
AAARFGIRYCLGVTNPDSGLAEKHYTRHPSLNDYRRLIPSLM
>EECEBICD_04558 Intracellular growth attenuator protein igaA
MSTILIFIAALLACSLLAIWRFRVKSRRGSLPWISAFQDAQTRKLLPEERSAVENYLDNL
SQIQQVPGPTGASAAPISLTLNAESNSVVILTHSITRYGITTDDPNKWRYYLDSVEVHLP
PFWEQYINDENNVELILTDTLPLVISLNGHTLQEYMQESRGYALQNTASTQASIRGEESE
QIELLNIRQETHEEYALSRPAGLREALLIVASFLLFFFCLITPDVFVPWMIGGAILLLAA
GLWGLFAPPSKSALREIHCLRGTPRRWGLFGENNQEQINNISLGIIDLIYPAHWQPYITQ
DLGQQTDIDIYLDRHVARQGRFLSLHDEVKNFPLQHWLRSTVIAIGSLLVLFMLLFWIPL
DMPIKFTLSWMKGAQTIEATTVKQLEKAGVRVGDTLHLSGKGMCNIHSGATWSGQSNSPF

MPFDCSQIIWNDAPALPLPESDLVNKAMALSQAVNRQLHPKPEDDSRVSASLRSAIQKSG
MVLLDDFGDIVLKTADLCAAEDECVRLKNALVNLGNSKDWNALVKRANAGKLDGVNVLLR
PVSAESLENLVTTSTAPFISRETARAAQSLNSPAPGGFLIASDEGSELVDQAWPSTPLYD
YPAQEQWSAFQRLAQTLMQTPFSAEGIVTSVYTDANGTQHISLHRIPDKSGWWRYLGTTL
LMLAMIVSAVYNGIQAFRRYQRHRTRMADIQEYYESCLNPRLTVSPENLI
>EECEBICD_04559 ADP compounds hydrolase NudE
MSKSLQKPTILKVETVAQSRLFNVESVDLEFSNGVRRVYERMRPSTREAVMIVPIVDEHL
ILIREYAVGTESYELGFSKGLIDPGETVFEAANRELKEEVGFGAHNLTFLKKLSMAPSYF
SSKMNIVVAEDLYPESLEGDEPEPLPQVRWPLAHLMDLLEDPDFNEARNVSALFLVREWL
KAQGRIA
>EECEBICD_04560 Penicillin-binding protein 1A
MKFVKYLLVLAVCCILLGAGSIYGLYRYIEPQLPDVATLKDVRLQIPMQVYSADGELIAQ
YGEKRRIPVTLDQIPPEMINAFIATEDSRFYEHHGIDPVGIFRAASVALFSGHASQGAST
ITQQLARNFFLSPERTLMRKIKEAFLAIRIEQLLNKNEILELYLNKIYLGYRAYGVGAAA
QVYFGKTVDQLSLSEMAVIAGLPKAPSTFNPLYSMDRAIARRNVVLSRMLSEGYITQAQY
DQARSEPIDANYHAPEIAFSAPYLSEMVRQEMYNRYGESAYEDGYRIYTTITRKVQQAAQ
QAVRNNVLDYDMRHGYRGPANVLWKVGETAWDSKKITDTLKALPTYGPLLPTVVTSANPQ
EATAALADGTSVSLHMEGMRWARPYRSDTQQGPTPRKVTDVVQTGQQIWVRQVDNDWWLA
QVPEVNSALVSLNPQTGAVLALVGGFDFNQSKFNRATQALRQVGSNIKPFLYTAAMDKGL
TLASMLNDVPISRWDAGAGSDWRPKNSPPQYAGPIRLRQGLGQSKNVVMVRAMRAMGVDY
AAEYLQRFGFPAQNIVHTESLALGSASFTPMQVARGYAVMANGGFLIDPYFISKIENDQG
GVIFEAKPKIACPECDIPVIYGNTQKSDVLENTNVEEVAVSQEQQNSAVPMPELEQANQA
LVAQNGTQEYAPHVINTPLAFLIKSALNTNIFGEPGWMGTGWRAARDLKRRDIGGKTGTT
NSSKDAWFSGYGPGVVTSVWIGFDDHRRDLGRTTASGAIKDQISGYEGGAKSAQPAWDAY
MKAVLEGVPEQPLTPPPGIVTVNIDRSTGQLASGGNSREEYFIEGTQPTQQAVHEVGTTI
IDNGETHELF
>EECEBICD_04561 hypothetical protein
MAFKTWQIGLHIQQHEALAIAVIRGASGWSLQRWWRLPLMNASTAEGTIPDPQSLAHVLR
PWSRELPLRHRIYLSFPANRTLQRAFPHPPMRLREREQVAWLSQTMARELDMDPDLLRFD
FQDDALSPAFNVTAVQSKEISALLTLAQTLNVRIAAVTPDACALQRLLPFIPSGRQCLVW
RDESQWLWATRYAWGRKSAREATTLHDLAATLSVVPEHISLCAEGEFDPWRAVTVRQPPV
PPDGYRFAIALGLAIGEIR
>EECEBICD_04562 hypothetical protein
MAHSVNLLPWRRQHYVARLRLWCVVWGASLLLIASLATIARLVFWQEGRINELLLTAENG
HTTALAANIPQLQQRQRQQQARLQRQAQRELTQAWQSILTDLANLLPEQAWLTSLNYQQE
TLELEGLARTFDALLTLETSLRHYVSFPLNRTGATQQDAQGRWQFHYQLTRSAARERAL
>EECEBICD_04563 hypothetical protein
MNALFDIWYGMSRRGRVFGWCAGVLCLTLTVTLSVGYPGWKTLHTQQTRLSQQREAARQQ
WRHLRRLSVAAEPLFGRTVENPRPFSPLDFQAPPLRLLHWQPSAQGEKWR
>EECEBICD_04564 hypothetical protein
MALKTSWDAVPSLFVRLAESEMSVSRFSLRKEGAELLMTLQLERLANEG
>EECEBICD_04565 hypothetical protein
MKASRSVLLCFCLLMLTGMRDPFRPPEDRCRIAELSQWRYQGAVRKGERWTGILKDSQQK
WRRVEEGQTLENGWTIVRLTAEALTLTTGKNCAPPQWRWLRQGADNEAMDSHNTDSLDAR
RAGGKSGESDAGG
>EECEBICD_04566 Type IV pilus biogenesis and competence protein PilQ
MPAAQAGKAAKVTLVVDDVPVVQVLQTLAEQERQNLVVSPDVSGTLSLHLTDVPWKQALQ
TVVNSAGLVLRQEGNILHVHSQAWQKEHSARQDAERLRLQANLPLENRSISLQYADAAEL
AKAGEKLLSAKGTIMVDKRTNRMLLRDNRAALAELEKWVSQMDLPVAQVELAAHIVTINE
KSLRELGVKWTLADATQAGSVGDVTTLSSDLSVAAATSRVGFNIGRINGRLLDLELSALE
QKQQLDIIASPRLLASHLQPASIKQGSEIPYQVSSGESGATSVEFKEAVLGMEVTPTVLQ
KGRIRLKLHISQNVPGQVLQQADGEVLAIDKQEIETQVEVKSGETLALGGIFSRKNKSGS
DSVPLLGDIPWLGQLFRHDGKEDERRELVVFITPRLVATE
>EECEBICD_04567 Shikimate kinase 1

MAEKRNIFLVGPMGAGKSTIGRQLAQQLNMEFYDSDQEIEKRTGADVGWVFDVEGEDGFR
NREEKVINELTEKQGIVLATGGGSVKSRETRNRLSARGVVVYLETTIEKQLARTQRDKKR
PLLQVEAPPREVLEALANERNPLYEEIADVTIRTDDQSAKVVANQIIHMLESN
>EECEBICD_04568 3-dehydroquinate synthase
MERITVTLGERSYPITIAAGLFNEPASFLPLKSGDQVMLVTNETLAPLYLDKVRGVLERA
GVNVDSVILPDGEQYKSLTVLDTVFTALLKKPHGRDTTLVALGGGVIGDLTGFAAASYQR
GVRFIQVPTTLLSQVDSSVGGKTAVNHPLGKNMIGAFYQPASVVVDLDCLKTLPARELAS
GLAEVIKYGIILDADFFTWLEGNLDALLRLDGPAMAYCIRRCCELKAEVVAADEREAGLR
ALLNLGHTFGHAIEAEMGYGNWLHGEAVAAGIVMAARASERLGQFSSTDTQRIIALLERA
GLPVNGPCEMSAQDYLPHMLRDKKVLAGELRLVLPLAIGKSEVRGGVSHEVVLSAIADCQ
QA
>EECEBICD_04569 Cell division protein DamX
MDEFKPEDELKPDPSDRRTGRSRQSSERDNEPQINFDDVDLDADDRRPTRTRKARSEEPE
VEEEYESDEDDTVDEERVERRPRKRKKAAHKPASRQYMMMGVGVLVLLLLIIGIGSALKA
PSTSSSEPSASGEKSIDLSGNAADQANATQPAPGATSAEQTAGNTSQDISLPPISSTPTQ
GQSPVVADGQQRVEVQGDLNNALTQNPEQMNNVAVNSTLPTEPATVAPVRNGSTTRQAAV
SEPTERHTTRPERKQAVIEPKKPQTTAKTTTAEPKKPVAPVKRTEPAAPAATPKATTTTA
APTATASAAPVQTAKPAQASTTPVAGGGKSAGNVGALKSAPSSHYTLQLSSSSNYDNLNG
WAKKENLKNYVVYETTRNGQPWYVLVTGMYASKEDAKRAVSTLPADVQAKNPWAKPLHQV
QADLK
>EECEBICD_04570 DNA adenine methylase
MKKNRAFLKWAGGKYPLLDDIKRHLPKGECLVEPFVGAGSVFLNTDFSRYILADINSDLI
SLYNIVKLRTDEYVQASRELFMPETNQAEVYYQLREEFNTCQDPFRRAVLFLYLNRYGYN
GLCRYNLRGEFNVPFGRYKRPYFPEAELYHFAEKAQNAFFYCESYADSMARADKSSVVYC
DPPYAPLSATANFTAYHTNSFSLTQQAHLAEIAENLVSNRIPVLISNHDTALTREWYQLA
KLHVVKVRRSISSNGGTRKKVDELLALYQPGVATPARK
>EECEBICD_04571 Ribulose-phosphate 3-epimerase
MKQYLIAPSILSADFARLGEDTAKALAAGADVVHFDVMDNHYVPNLTIGPMVLKSLRQYG
ITAPIDVHLMVKPVDRIVPDFAAAGASIITFHPEASEHVDRTLQLIKEHGCKAGLVFNPA
TPLSYLDYVMDKLDVILLMSVNPGFGGQSFIPQTLDKLREVRRRIDASGYDIRLEVDGGV
KVNNIGEIAAAGADMFVAGSAIFDKPDYKKVIDEMRSELAKVTHG
>EECEBICD_04572 Phosphoglycolate phosphatase
MDKLQNIRGVAFDLDGTLVDSAPGLAAAVDMALYALELPVAGEERVITWIGNGADVLMER
ALAWAREERATLRKTMGKPPVDEDIPAEEQVRILRKLFDRYYGEVAEEGTFLFPHVADTL
GALHASGLSLGLVTNKPTPFVAPLLESLDIAKYFSVVIGGDDVQNKKPHPEPLLLVASRL
GMMPEQMLFVGDSRNDIQAAKAAGCPSVGLTYGYNYGEAIALSEPDVIYDSFNDLLPALG
LPHSDNQEIKND
>EECEBICD_04573 Tryptophan--tRNA ligase
MTKPIVFSGAQPSGELTIGNYMGALRQWVNMQDDYHCIYCIVDQHAITVRQDAQQLRKAT
LDTLALYLACGIDPEKSTIFVQSHVPEHAQLGWALNCYTYFGELSRMTQFKDKSARYAEN
INAGLFDYPVLMAADILLYQTNLVPVGEDQKQHLELSRDIAQRFNGLYGDIFKVPEPFIP
KSGARVMSLLEPTKKMSKSDDNRNNVIGLLEDPKSVVKKIKRAVTDSDEPPVVRYDVKEK
AGVSNLLDILSAVTGQSIPELEKQFEGKMYGHLKGEVAEAVSGMLSELQERYHRFRNDEA
FLQKVMKDGAEKASARAAETLKAVYEAIGFVAKP
>EECEBICD_04574 hypothetical protein
MFKFVKIAVVAGVLATLTACTGHIENKKNNCSYDYLLHPAISISKIIGGCGPAADQ
>EECEBICD_04575 hypothetical protein
MIPPDDSGDDDVTPPDDSGDDDVTPPDDSGDDDVTPPDDSGDDDVTPPDDSGDDDVTPPD
DSGDDDVTPPDDSGDDDVTPPDDSGDDDVTPPDDSGDDDVTPPDDSGDDDDTPPDDSVIT
FSNGVTIDKGKDTLTFDSFKLDNGSVLEGAVWNYSEQDNQWQLTTADGKTLNVTGWDVTD
ANAAVIEGTQENGLYWKYDSRGYLIIADDNTTVISGDDQAHNSDRGMDISGQDRTGVIIS
GDRTVNTLTGDSSVTDGATGMVISGDGTTNTISGHSTVDNATGALISGNGTTTNFAGDIA
VSGGGTAIIIDGDNATIKNTGTSNISGAGSTGTVIDGNNARVNNDGDMTITDGGTGGHIT
GDNVVIDNAGSTTVSGADATALYIEGDNALVINEGNQTISGGAVGTRIDGDDAHTTNTGD

IAVDGAGSAAVIINGDNGSLTQAGDLLVTDGAMGIITYGTGNEAKNTGNATVRDADSVGF
VVAGEKNTFKNKGDIDVSLNGTGALVSGDMSQVTLDGDINVVSVQDSEGVFSSATGVSVS
GDSNAVDITGNVNISADYGQDDLAAGAPPLTGVVVGGNGNTVTLNGALNIDDNDLSAASG
QYLDVVGLSVTGDDNDVEIDGGINITHSEDPLDGTSADITGISVSGNSTVTLNGHSTIDT
NTVVGGHVVLARVNNGGSLILGDDSVVDVNVSYIPTGYYTYNALLMADGEGTSIENKGDI
TSHGVYSVIRADNGSEVSNSGDILVYATSSNSSEDRAAITRASGEGSAVHNKAGGDITLI
SDQTPQGSGGIEVYPLKWYTHTFYAMMASDYGDVVNDEGATIHLQGAGVYGVTASRGKAL
NEGDIYLDGLVPTLDDENNITSTSYWQPSSLYLTSSGMVAGSTDADGDATAINTGNITVN
NAGFGMMALNGGTAINQGVITLTADDGVTGQADELVGMAALNGGVVINDTSGVINIDADY
GQAFLSDSSSYIINNGSINLNGSPMDDTDSHMGGTPTDKIWIQSLPGSGDSDTRTSDTGF
FTAGTLANYGTETLNGDVDVNGGWLYNEAGASLTVNGTVTINGGANALANYGTLDADAIS
TWHSLFNEADGSITTDLLTLNGDVTFYNNGDFTGSIAGTSYQQEIVNTGDMTVAEDGKSL
VSGSFYFYNEEDATLTNSGSAVEGGENTIINLTRANDSLTQVNSGTITATNGYSAITTAN
GSNDPKWIWNTATGVINGINPDAPLINLGRGYNFGNQGTINVQGDNAVAISGGTSSYVIN
LVNSGTINVGTEQGKEDGTNGTGLIGIKGNGNATTINNTADGVINVYADDSYAFGGKTKA
IINNGEINLLCDSGCDIYAPGTTGTQNDHNGTADIVIPDATTAPTEGSIPTPPADPNAPQ
QLSNYIVGTNADGSSGTLKANNLVIGDNVKVDTGFTSGTADTTVVVDNAFTGSNIQGADN
ITSTSVVWNAQGSQDADGNVDVTMTKNAYADVATDSSVSDVASALDAGYTNNELYTSLNV
GTTAELNSALKQVSGAQATTVFREARVLSNRFTMLADAAPQIKDGLAFNVVAKGDPRAEL
GNDTQYDMLALRQTLDLTASQNLTLEYGIARLDGDGSKTAGDNGLTGGYSQFFGLKHSMA
FDEGLAWNNSLRYDVHNLDSSRSVAYGDVNKIADSDMRQQYLEFRSEGAKTFTMMSDTLK
VTPYAGVKFRHTMEDGYKERSAGDFNLSMNSGNETAVDSIVGLKLDYAGKDGWSATATLE
GGPNLSYSKSQRTASLQGAAGQSFGVDDGQKGGGINGLATIGVKYSSNDTALHLDAYQWK
EDGISDKGFMLNVKKTFR
>EECEBICD_04576 hypothetical protein
MQKKKLISIAIALTLQSYYIPAIAAENNDDEKECPSNISSLPKEKRAKLSPTCLATPEND
NHWGWVAGGVAALVAGVAIGVENNGGGDSNHSYTPLRPIMAAMTM
>EECEBICD_04577 Siroheme synthase
MDHLPIFCQLRDRDCLIVGGGDVAERKARLLLEAGARLTVNALTFIPQFTVWANEGMLTL
VEGPFDETLLDSCWLAIAATDDDTVNQRVSDAAESRRIFCNVVDAPKAASFIMPSIIDRS
PLMVAVSSGGTSPVLARLLREKLESLLPQHLGQVARYAGQLRARVKKQFATMGERRRFWE
KFFVNDRLAQSLANADEKAVNATTERLFSEPLDHRGEVVLVGAGPGDAGLLTLKGLQQIQ
QADIVVYDRLVSDDIMNLVRRDADRVFVGKRAGYHCVPQEEINQILLREAQKGKRVVRLK
GGDPFIFGRGGEELETLCHAGIPFSVVPGITAASGCSAYSGIPLTHRDYAQSVRLVTGHL
KTGGELDWENLAAEKQTLVFYMGLNQAATIQEKLIAFGMQADMPVALVENGTSVKQRVVH
GVLTQLGELAQQVESPALIIVGRVVGLRDKLNWFSNY
>EECEBICD_04578 Nitrite transporter NirC
MFTDTINKCAANAARIARLSANNPLGFWVSSAMAGAYVGLGIILIFTLGNLLDPSVRPLV
MGATFGIALTLVIIAGSELFTGHTMFLTLGVKAGTISHGQMWAILPQTWLGNLVGSVFVA
LLYSWGGGSLLPVDTSIVHSVALAKTTAPATVLFFKGALCNWLVCLAIWMAIRTEGTAKF
LAIWWCLLAFIASGYEHSVANMTLFALSWFGHHSDAYTLSGIGHNLLWVTLGNTLSGVVF
MGLGYWYATPKSERPVPQKTNQIKVTANH
>EECEBICD_04579 Nitrite reductase (NADH) small subunit
MSQWQNICKIDDILPGTGVCALLGTEQVAIFRPYHSDQVFAISNIDPFFEASVLSRGLIA
EHQGELWVASPLKKQRFRLSDGLCMEDEQFSVKQYDARVKDGVVQLRG
>EECEBICD_04580 Nitrite reductase [NAD(P)H]
MSKVRLAIIGNGMVGHRFIEDLLDKSDASLFDITVFCEEPRKAYDRVHLSSYFSHHTAEE
LSLVREGFYEKHGVKVLVGERAITINRQEKVIHSSAGRTVYYDKLIMATGSYPWIPPIKG
SETQDCFVYRTIEDLNAIEACARRSKRGAVVGGGLLGLEAAGALKNLGVETHVIEFAPML
MAEQLDQMGGEQLRRKIESMGVRVHTSKNTQEIVQQGNEARKTMRFADGSELEVDFIVFS
TGIRPRDKLATQCGLAVAQRGGIVINDTCQTSDPDIYAIGECASWNNRVYGLVAPGYKMA
QVAVDHILGSENAFEGADLSAKLKLLGVDVGGIGDAHGRTPGARSYVYLDESKEVYKRLI
VSEDNKTLLGAVLVGDTSDYGNLLQLVLNAIELPENPDSLILPSHAGSGKPAIGVDKLPD
SAQICSCFDVTKGDLIAAINKGCHTVAALKAETKAGTGCGGCIPLVTQVLNAELAKQGIE

VNNNLCEHFAYSRQELFHLIRVEGIKTFEELLAKHGKGYGCEVCKPTVGSLLASCWNEYI
LKPQHTPLQDTNDNFLANIQKDGTYSVIPRSAGGEITPEGLVAVGRIAREFNLYTKITGS
QRIGLFGAQKDDLPEIWRQLIEAGFETGHAYAKALRMAKTCVGSTWCRYGVGDSVGFGVE
LENRYKGIRTPHKMKFGVSGCTRECAEAQGKDVGIIATEKGWNLYVCGNGGMKPRHADLL
AADLDRDTLIKYLDRFMMFYIRTADKLTRTAPWLDNLEGGIEYLKSVIIDDKLGLNEHLE
EEMARLRAAVVCEWTETVNTPSAQVRFKHFINSDKRDPNVQVVPEREQHRPATPYERIPV
TLVEENA
>EECEBICD_04581 Protein TsgA
MTNSNRIKLTWISFLSYALTGALVIVTGMVMGNIADYFHLPVSSMSNTFTFLNAGILISI
FLNAWLMEIVPLKTQLRFGFILMVLAVAGLMFSHSLALFSAAMFVLGLVSGITMSIGTFL
ITQLYEGRQRGSRLLFTDSFFSMAGMIFPMVAAFLLARSIEWYWVYACIGLVYLAIFILT
FGCEFPALGKHAQHSQAPVAKEKWGIGVLFLAVAALCYILGQLGFISWVPEYAKGLGMSL
NDAGALVSDFWMSYMFGMWAFSFILRFFDLQRILTVLAGMAAVLMYLFITGTQAHMPWFI
LTLGFFSSAIYTSIITLGSQQTKVASPKLVNFILTCGTIGTMLTFVVTGPIVAHSGPQAA
LLTANGLYAVVFVMCFALGFVSRHRQHSAPATH
>EECEBICD_04582 Peptidyl-prolyl cis-trans isomerase A
MLKSTLAAVAAVFALSALSPVALAAKGDPHVLLTTSAGNIELELNSQKALISVKNFVDYV
NSGFYNNTTFHRVIPGFMIQGGGFNEQMQQKKPNPPIKNEADNGLRNTRGTIAMARTADK
DSATSQFFINVADNAFLDHGQRDFGYAVFGKVVKGMDVADKISQVPTHDVGPYQNVPTKP
VVILSAKVLP
>EECEBICD_04583 hypothetical protein
MPARARAARVFSLSQRNLKLLLILVVKHRHIQGGPVKKLTDKQKSRFWEQRRNVNFQQSR
RLEGIEIPLVTLTADEALARLDELRRHYER
>EECEBICD_04584 putative protein adenylyltransferase Fic
MSDKFGEGRDPYLYPGLNVMRNRLGIHQAQRLAQAAYEMTALRAATIELGPLIRGLPHLC
AIHRQLYQDIFDWAGQLREVDIYQGDTRFCHFAYIEKEGNALMQDLEEEGYLVGLAHEKF
VERLAHYYCEINVLHPFRLGSGLAQRIFFEQLALHAGYALSWQGIAVETWKQANQSGAMG
DLSALQAIFQKAISEARETE
>EECEBICD_04585 Aminodeoxychorismate synthase component 2
MILLIDNYDSFTWNLYQYFCELGAEVQVRRNDALTLAHIDALNPQKIVISPGPCTPNDAG
ISLAVIRHYAGRIPMLGVCLGHQAMAQAFGASVVRAAKVMHGKTSPVTHNGQGVFRGLPS
PLTVTRYHSLIVDPATLPECFEITAWSETQEIMGIRHREWDLEGVQFHPESILSEQGHAL
LKNFLRR
>EECEBICD_04586 Acetylornithine/succinyldiaminopimelate aminotransferase
MATEQTAITRATFDEVILPVYAPADFIPVKGKGSRVWDQQGKEYIDFAGGIAVTVLGHCH
PALVEALKSQGETLWHTSNVFTNEPALRLGRKLIDATFAERVLFMNSGTEANETAFKLAR
HYACVRHSPFKTKIIAFHNAFHGRSLFTVSVGGQPKYSDGFGPKPADIIHVPFNDLHAVK
AVMDDHTCAVVVEPIQGEGGVQAATPEFLKGLRDLCDEHQALLVFDEVQCGMGRTGDLFA
YMHYGVTPDILTSAKALGGGFPVSAMLTTQEIASAFHVGSHGSTYGGNPLACAVAGAAFD
IINTPEVLQGIHTKRQQFVQHLQAIDEQFDIFSDIRGMGLLIGAELKPKYKDRARDFLYA
GAEAGVMVLNAGADVMRFAPSLVVEDADINEGMQRFAQAVGKVVA
>EECEBICD_04587 hypothetical protein
MWRRLIYHPEINYALRQTLVLCLPVAVGLLIGQLHLGLLFSLVPACCNIAGLDTPHKRFF
KRLIIGASLFAGCSLVTQLLLAESIPLPLILTGLTLVLGVTAEISPLHARLLPASLIAAI
FTLSLAGYMPVWEPLLIYALGTLWYGVFNWFWFWLWREQPLRESLSLLYRELADYCEAKY
SLLTQHIDPEKALPPLLIRQQKAVDLITQCYQQMHMLSAHNNNDYKRLLRAFQEAMDLQE
HISVSLHQPEEVQKLVERSHAEEVIRWNAQTVAARLRVLADDILYHRLPTRFSMEKQIGA
LEKIANQHPENPVGQFCYWHFSRIARVLRTQRPLYARDLMADKQRRLPLLPALKNYMSLK
SPALRNAGRISVMMSIASLMGSALHLPKPYWILMTVLFVTQNGYGATRVRILHRSVGTLV
GLVIAGVTLHLHIPESITLAVMLVLTLASYLIIRKNYGWATVGFTVTAVYTIQLLTLNGE
QFIVPRLIDTLIGCLIAFGGMVWLWPQWQSGLLRKNAHDALEADQEAIRLILSNDPQATP
LAYQRMRVNQAHNTLFNSLNQAMQEPGFNTHYLSDMKLWVTHSQFIVEHINAMTTLAREH
TMLTPDLAQRYLESCEIAIQRCQQRLEYDRPGGSGDVNILESPDMPSHGLLSTLEQHLQR
IIGHLNTMHTISSMAWRQRPHHGIWLSKRLRDTKS

>EECEBICD_04588 cAMP-activated global transcriptional regulator CRP
MVLGKPQTDPTLEWFLSHCHIHKYPSKSTLIHQGEKAETLYYIVKGSVAVLIKDEEGKEM
ILSYLNQGDFIGELGLFEEGQERSAWVRAKTACEVAEISYKKFRQLIQVNPDILMRLSSQ
MARRLQVTSEKVGNLAFLDVTGRIAQTLLNLAKQPDAMTHPDGMQIKITRQEIGQIVGCS
RETVGRILKMLEDQNLISAHGKTIVVYGTR
>EECEBICD_04589 Protein YhfA
MQARVKWVEGLTFLGESSSGHQILMDGNSGDKAPSPMEMVLMAAGGCSAIDVVSILQKGR
QNVTNCEVKLTSERREDAPRLFTHINLHFIVTGSDLKEAAVARAVDLSAEKYCSVALMLE
KAVNITHSYEVIAA
>EECEBICD_04590 Phosphoribulokinase, plasmid
MSAKHPVIAVTGSSGAGTTTTSLAFRKIFAQLNLHAAEVEGDSFHRYTRPEMDMAIRKAR
DAGRHISYFGPEANDFSLLEHTFIEYGQTGKGQSRKYLHTYDEAVPWNQVPGTFTPWQPL
PEPTDVLFYEGLHGGVVTPQHDVARHVDLLVGVVPIVNLEWIQKLIRDTSERGHSREAVM
DSVVRSMDDYINYITPQFSRTHINFQRVPTVDTSNPFAAKGIPSLDESFVVIHFRNLEGI
DFPWLLAMLQGSFISHINTLVVPGGKMGLAMELIMLPLVQRLMEGKKIE
>EECEBICD_04591 hypothetical protein
MIIPWQGLAPDTLDNLIESFVLREGTDYGEHERSLEQKVADVKLQLQSGEAVLVWSELHE
TVNIMPKKQFRE
>EECEBICD_04592 hypothetical protein
MVEITSTEMTPPVNDSHEFIPMRGIRNRHLQTMLPRLIRRKVKFNAHWQRLELPDGDFVD
LAWSEEPQQAKHKPRLVVFHGLEGSLNSPYAHGLIEAAQKRGWLGVVMHFRGCSGEPNRL
NRIYHSGETEDGAWFLRWLQREFGAVPTAAVGYSLGGNMLACLLAKEGRDIPIEAAVIVS
APFVLEACSYHMDKGFSRVYQRYLLNLLKANASRKLAAYPGSLPVNLAQLKSMRRIREFD
DLITAKIHGFADAIDYYRQCSAMPLLNQIAKPTLIIHAKDDPFMDHHVIPKAEDLPPQVE
YQLTEHGGHVGFIGGTPLRPEMWLERRIPDWLTTYLEASS
>EECEBICD_04593 Putative monooxygenase YdhR
MSKTLLQIHFNFSGPFGEEMTQQLVGLAESINEEPGFIWKIWTESEKNQQAGGIYLFESE
ETARAYIKKHTARLKNLGVDEVTFKLFGVNDALTKINHGNLCR
>EECEBICD_04594 hypothetical protein
MNLSITDDITFRKLTILMMLVEKGNIARTAEALHTLKENVRCPLFVHKGLFGTRPDIKVT
VAVGSTGTEGCVM
>EECEBICD_04595 putative ABC transporter ATP-binding protein YheS
MIVFSSLQIRRGVRVLLDNASAIINPGQKVGLVGKNGCGKSTLLALLKNEISADAGSFTL
PGTWQLAWVNQETPALPQPAIEYVIDGDREYRQLEAQLNDANEHNDGHAIASIHGKLDAI
DAWTVRSRAASLLHGLGFSNDQLERPVSDFSGGWRMRLNLAQALICRSDLLLLDEPTNHL
DLDAVIWLEKWLKSYPGTLILISHDRDFLDPIVDKIIHIEQQTLFEYTGNYSAFEVQRAT
RLAQQQAMYESQQERVAHLQSYIDRFRAKATKAKQAQSRIKMLERMELIAPAHVDNPFHF
SFRAPESLPNPLLKMEKVSAGYGDRIILESIKLNLVPGSRIGLLGRNGAGKSTLIKLLAG
ELEPLHGEIGLAKGIKLGYFAQHQLEFLRADESPLQHMARLAPQELEQKLRDYLGGFGFQ
GDKVTEETQRFSGGEKARLVLALIVWQRPNLLLLDEPTNHLDLDMRQALTEALIDFEGAL
VVVSHDRHLIRSTTDDLYLVHDKKVEPFDGDLEDYQQWLSDVQKQENQADNAPKENNANS
AQSRKDQKRREAELRTLTQPLRKEITRLEKEMEKLNAQLAQAEEKLGDSSLYDPSRKAEM
TECLQLQASAKSGLEACEMAWLEAQEQLEQMMQND
>EECEBICD_04596 General stress protein 14
MSQPAKVLLLYAHPESQDSVANRVLLKPAIQHNNVTVHDLYARYPDFFIDTPYEQALLRE
HDVIVFQHPLYTYSCPALLKEWLDRVLSRGFASGPGGNQLVGKYWRSVITTGEPESAYRY
DALNRYPMSDVLRPFELTAAMCRMHWMPPIIVYWARRQSPQTLASHAKAYGEWLANPVSA
GGY
>EECEBICD_04597 Glutathione-regulated potassium-efflux system protein KefC
MEGADLLTAGVLFLFAAVAAVPLAARLGIGAVLGYLLAGIAIGPWGLGFISDVDEILHFS
ELGVVFLMFIIGLELNPSRLWQLRRSIFGVGAAQVLLSAAVLAGLLMLADFLWQAAVVGG
IGLAMSSTAMALQLMREKGMNRSESGQLGFSVLLFQDLAVIPALALVPLLAGSADEHFDW
FKVAMKVLAFAVMLIGGRYLLRPVFRFIAASGVREVFTAATLLLVLSAALFMDALGLSMA

LGTFIAGVLLAESEYRHELENAIDPFKGLLLGLFFISVGMSLNLGVLYTHLLWVAASVVI
LVVIKMLTLYLLARLYGIRSSERMQFASVLSQGGEFAFVLFSTASSQRLFQGDQMALLLV
TVTLSMMTTPLLMKGIDKWLSRRLNGPEENDEKPWVEDDKPQVIVVGFGRFGQVIARLLM
ANKMRITVLERDIGAVNLMRKYGYKVYYGDATQVELLRSAGAEAAESIVITCNEPEDTMK
LVELCQQHFPHLHILARARGRVEAHELLQAGVTQFSRETFSSALELGRKTLVSLGMHPHQ
AQRAQLHFRRLDMRMLRELIPEHSDMVQISRAREARRELEEIFQREMQQERRQLDGWDEF
E
>EECEBICD_04598 hypothetical protein
MAIRKRFIAGAKCPACQAQDTMAMWRENNVDIVECVKCGHQMREADKDVREHVRKEEQVI
GIFHPD
>EECEBICD_04599 FKBP-type peptidyl-prolyl cis-trans isomerase SlyD
MKVAKDLVVSLAYQVRTEDGVLVDESPVSAPLDYLHGHGSLISGLETALEGHEVGDKFDV
AVGANDAYGQYDENLVQRVPKDVFMGVDELQVGMRFLAETDQGPVPVEITEVEDDHVVVD
GNHMLAGQNLKFNVEVVAIREATEEELAHGHVHGAHDHHHDHGEDGCCGGHGHDHGHEHG
GEGCCGGGGKGGCGCH
>EECEBICD_04600 Protein SlyX
MQDITMEARLAELESRLAFQEITIEELNLTVTAHEMEMAKLRDHLRLLTEKLKASQPSNI
ASQAEETPPPHY
>EECEBICD_04601 FKBP-type peptidyl-prolyl cis-trans isomerase FkpA
MKSLFKATLLATTMAVAMHAPITFAADAAKPAATADSKAAFKNDDQKAAYALGASLGRYM
ENSLKEQEKLGIKLDKDQLIAGVQDAFADKSKLSDQEIEQTLQTFEARVKSAAQAKMEKD
AADNEAKGKTFRDAFAKEKGVKTSSTGLLYKVEKEGTGEAPKDSDTVVVNYKGTLIDGKE
FDNSYTRGEPLSFRLDGVIPGWTEGLKNIKKGGKIKLVIPPALAYGKTGVPGIPANSTLV
FDVELLDIKPAPKADAKPADAADAKAADAAKK
>EECEBICD_04602 Transcriptional regulator DauR
MSRSLLTNETSELDLLDQRPFEQTDFDILKSYEAVVDGLAMLIGSHCEIVLHSLQDLKCS
AIRIANGEHTGRKIGSPITDLALRMLHDMTGADSSVSKCYFTRAKSGVLMKSLTIAIRNR
EQRVIGLLCINMNLDVPFSQIMNTFIPPETPEVGSAVNFASSVEDLVTQTLEFTIEEVNA
DRNVSNNAKNRQIVLNLYEKGIFDIKDAINQVADRLNISKHTVYLYIRQFKSGDFQGQDK
>EECEBICD_04603 Sulfurtransferase TusD
MRYAIMVTGPAYGTQQASSALQFAHALLNEGHELASVFFYREGVYNANLLTSPASDEYDL
VRAWQKLNTQHGVALNICVAAALRRGIIDETEAGRLELPSANLQPGFTLSGLGALAEASL
TCDRVVQF
>EECEBICD_04604 Protein TusC
MKRIAFVFSTAPHGSASGREGLDALLATSALTEALGVFFISDGVFQLLPGQKPDAVLARD
YIATFKLFDLYDIDQCWICAASLRERGLENVNFVVDATPLEPVALRRELGNYDVILRF
>EECEBICD_04605 Protein TusB
MLHTLPHCASSVDFPALLRLLKEGDALLLLQDGVTVAIEGNRFLESLRDAPITVYALKED
IDARGLGGQISDSVVRVDYTDFVRLTVKYANQMAW
>EECEBICD_04606 30S ribosomal protein S12
MATVNQLVRKPRARKVAKSNVPALEACPQKRGVCTRVYTTTPKKPNSALRKVCRVRLTNG
FEVTSYIGGEGHNLQEHSVILIRGGRVKDLPGVRYHTVRGALDCSGVKDRKQARSKYGVK
RPKA
>EECEBICD_04607 30S ribosomal protein S7
MPRRRVIGQRKILPDPKFGSELLAKFVNILMVDGKKSTAESIVYSALETLAQRSGKSELE
AFEVALENVRPTVEVKSRRVGGSTYQVPVEVRPVRRNALAMRWIVEAARKRGDKSMALRL
ANELSDAAENKGTAVKKREDVHRMAEANKAFAHYRW
>EECEBICD_04608 Elongation factor G
MARTTPIARYRNIGISAHIDAGKTTTTERILFYTGVNHKIGEVHDGAATMDWMEQEQERG
ITITSAATTAFWSGMAKQYEPHRINIIDTPGHVDFTIEVERSMRVLDGAVMVYCAVGGVQ
PQSETVWRQANKYKVPRIAFVNKMDRMGANFLKVVGQIKTRLGANPVPLQLAIGAEEGFT
GVVDLVKMKAINWNDADQGVTFEYEDIPADMQDLANEWHQNLIESAAEASEELMEKYLGG
EELTEEEIKQALRQRVLNNEIILVTCGSAFKNKGVQAMLDAVIDYLPSPVDVPAINGILD
DGKDTPAERHASDDEPFSALAFKIATDPFVGNLTFFRVYSGVVNSGDTVLNSVKTARERF

GRIVQMHANKREEIKEVRAGDIAAAIGLKDVTTGDTLCDPENPIILERMEFPEPVISIAV
EPKTKADQEKMGLALGRLAKEDPSFRVWTDEESNQTIIAGMGELHLDIIVDRMKREFNVE
ANVGKPQVAYREAIRAKVTDIEGKHAKQSGGRGQYGHVVIDMYPLEPGSNPKGYEFINDI
KGGVIPGEYIPAVDKGIQEQLKSGPLAGYPVVDLGVRLHFGSYHDVDSSELAFKLAASIA
FKEGFKKAKPVLLEPIMKVEVETPEENTGDVIGDLSRRRGMLKGQESEVTGVKIHAEVPL
SEMFGYATQLRSLTKGRASYTMEFLKYDDAPNNVAQAVIEARGK
