# Supplementary material for: Genome sequencing and analysis of Salmonella enterica subsp. enterica serotype Enteritidis PT4 578: insights into pathogenicity and virulence
Source: Access Microbiol. 2024 Nov 4;6(11):000828.v3. doi: 10.1099/acmi.0.000828.v3 (PMC11649194; doi:10.1099/acmi.0.000828.v3)
Supplement: Uncited Supplementary Material 4. [file acmi-6-00828-s004.pdf]

Supplementary Tables

Table S1. Virulence factors present in the *Salmonella* Enteritidis PT4 genome.

Virulence factor class	Virulence factors	Related genes	*Salmonella* Enteritidis PT4 578
Capsule	Vi antigen	*tviA*	-
		tviB	-
		tviC	-
		tviD	-
		tviE	-
		vexA	-
		vexB	-
		vexC	-
		vexD	-
		vexE	-
Fimbrial adherence determinants	Agf/Csg	*csgA*	PT4578_01467
		csgB	PT4578_01466
		csgC	PT4578_01468
		csgD	PT4578_01465
		csgE	PT4578_01464
		csgF	PT4578_01463
		csgG	PT4578_01462
	Bcf	*bcfA*	PT4578_03450
		bcfB	PT4578_03449
		bcfC	PT4578_03448
		bcfD	PT4578_03447
		bcfE	PT4578_03446
		bcfF	PT4578_03445
		bcfG	PT4578_03444
	Fim	*fimA*	PT4578_02927
		fimC	PT4578_02925
		fimD	PT4578_02924
		fimF	PT4578_02922
		fimH	PT4578_02923
		fimI	PT4578_02926
		fimW	PT4578_02919
		fimY	PT4578_02920
		fimZ	PT4578_02921
	Lpf	*lpfA*	PT4578_04416
		lpfB	PT4578_04417
		lpfC	PT4578_04419; PT4578_04420
		lpfD	PT4578_04421
		lpfE	PT4578_04422
	Pef	*pefA*	-
		pefB	-
		pefC	-
		pefD	-
	Peg	*pegA*	PT4578_01203
		pegB	-
		pegC	-
		pegD	PT4578_01209
	Saf	*safA*	PT4578_03179
		safB	PT4578_03178
		safC	PT4578_03177

	safD	PT4578_03175
Sef	*sefA*	PT4578_03596
	sefB	PT4578_03595
	sefC	PT4578_03593
	sefD	PT4578_03592
Sta	*staA*	-
	staB	-
	staC	-
	staD	-
	staE	-
	staF	-
	staG	-
Stb	*stbA*	PT4578_03134
	stbB	PT4578_03136
	stbC	PT4578_03137
	stbD	PT4578_03138
	stbE	PT4578_03139
Stc	*stcA*	-
	stcB	-
	stcC	-
	stcD	-
Std	*stdA*	PT4578_00430
	stdB	PT4578_00431
	stdC	PT4578_00432
Ste	*steA*	PT4578_00512
	steB	PT4578_00511
	steC	PT4578_00510
	steD	PT4578_00509
	steE	PT4578_00508
	steF	PT4578_00507
Stf	*stfA*	PT4578_03267
	stfC	PT4578_03266
	stfD	PT4578_03265
	stfE	PT4578_03264
	stfF	PT4578_03263
	stfG	PT4578_03262
Stg	*stgA*	-
	stgB	-
	stgC	-
	stgD	-
Sth	*sthA*	PT4578_03476
	sthB	PT4578_03477
	sthC	PT4578_03478
	sthD	PT4578_03479
	sthE	PT4578_03480
Sti	*stiA*	PT4578_03286
	stiB	PT4578_03287
	stiC	PT4578_03288
	stiH	PT4578_03289
Stj	*Undetermined*	-
	Undetermined	-

		Undetermined	-
		stjB	-
		stjC	-
	Stk	*stkA*	-
		stkB	-
		stkC	-
		stkD	-
		stkE	-
		stkF	-
		stkG	-
	Tcf	*tcfA*	-
		tcfB	-
		tcfC	-
		tcfD	-
Macrophage inducible genes	Mig-14	*mig-14*	PT4578_00691
	Mig-5	*mig-5*	-
Magnesium uptake	Mg2+ transport	*mgtB*	PT4578_04284
		mgtC	PT4578_04283
Nonfimbrial adherence determinants	MisL	*misL*	PT4578_04290
	RatB	*ratB*	PT4578_00830
	ShdA	*shdA*	PT4578_00832; PT4578_00833
	SinH	*sinH*	PT4578_00827
Regulation	PhoPQ	*phoP*	PT4578_01559
		phoQ	PT4578_01558
Secretion system	TTSS (SPI-1 encode)	*hilA*	PT4578_00590
		hilC	PT4578_00600
		hilD	PT4578_00592
		iacP	PT4578_00584
		iagB	PT4578_00589
		invA	PT4578_00569
		invB	PT4578_00570
		invC	PT4578_00571
		invE	PT4578_00568
		invF	PT4578_00566
		invG	PT4578_00567
		invH	PT4578_00565
		invI	PT4578_00572
		invJ	PT4578_00573
		orgA	PT4578_00597
		orgB	PT4578_00598
		orgC	PT4578_00599
		prgH	PT4578_00593
		prgI	PT4578_00594
		prgJ	PT4578_00595
		prgK	PT4578_00596
		sicA	PT4578_00579
		sicP	PT4578_00586
		sipD	PT4578_00582
		spaO	PT4578_00574
		spaP	PT4578_00575
		spaQ	PT4578_00576
		spaR	PT4578_00577
		spaS	PT4578_00578

	sprB	PT4578_00601
TTSS (SPI-2 encode)	*ssaC*	PT4578_01730
	ssaD	PT4578_01731
	ssaE	PT4578_01732
	ssaG	PT4578_01743
	ssaH	PT4578_01744
	ssaI	PT4578_01745
	ssaJ	PT4578_01747
	ssaK	PT4578_01749
	ssaL	PT4578_01750
	ssaM	PT4578_01751
	ssaN	PT4578_01753
	ssaO	PT4578_01754
	ssaP	PT4578_01755
	ssaQ	PT4578_01756
	ssaR	PT4578_01757
	ssaS	PT4578_01758
	ssaT	PT4578_01759
	ssaU	PT4578_01760
	ssaV	PT4578_01752
	sscA	PT4578_01735
	sscB	PT4578_01740
	sseA	PT4578_01733
	sseB	PT4578_01734
	sseC	PT4578_01736
	sseD	PT4578_01738
	sseE	PT4578_01739
	ssrA	PT4578_01728
	ssrB	PT4578_01725
TTSS effectors translocated via both systems	*slrP*	PT4578_02679; PT4578_02680; PT4578_02681
	sspH1	-
TTSS-1 translocated effectors	*avrA*	PT4578_00602
	sipA	PT4578_00583
	sipB	PT4578_00580
	sipC	PT4578_00581
	sopA	PT4578_01298
	sopB/sigD	PT4578_02466
	sopD	PT4578_00522
	sopE2	PT4578_02232
	sopE	PT4578_02257
	sptP	PT4578_00587; PT4578_00588
TTSS-2 translocated effectors	*gogB*	-
	pipB2	PT4578_00693
	pipB	PT4578_02468
	sifA	-
	sifB	PT4578_01945
	sopD2	PT4578_02543
	spiC/ssaB	PT4578_01729
	spvC	-
	spvD	-
	sseF	PT4578_01741
	sseG	PT4578_01742

		sseI/srfH	PT4578_02504
		sseJ	PT4578_01978
		sseK1	PT4578_03895
		sseK2	PT4578_01452
		sseL	PT4578_01073
		sspH2	PT4578_01122
Serum resistance	Rck	*rck*	-
Stress adaptation	SodCI	*sodCI*	PT4578_02264
Toxin	SpvB	*spvB*	-
	Typhoid toxin	*cdtB*	-
		pltA	-
		pltB	-
Anaerobic respiration	Fused nitrate reductase(Mycobacterium)	*narX*	PT4578_02141

Table S2. Identified protospacers in the CRISPR cluster of *Salmonella* Enteritidis PT4 578.

CRISPR	Spacer nº	Prophage/plasmid	Accession number
1	6	Plasmid pYRKMM821_2 of *Yersinia ruckeri* KMM821	NZ_CP071804.1
		Plasmid p17Y0153.1 of *Yersinia ruckeri* 17Y0153	NZ_CP084651.1
		Plasmid p17Y0189.1 of *Yersinia ruckeri* 17Y0189	NZ_CP084640.1
		Plasmid p16Y0180.1 of *Yersinia ruckeri* 16Y0180	NZ_CP084653.1
		Plasmid p17Y0155.1of *Yersinia ruckeri* 17Y0155	NZ_CP084649.1
		Plasmid pYR4 of *Yersinia ruckeri* NHV_3758	NZ_CP032236.1
2	1	*Escherichia* phage vB_EcoM-783R5	ON470627
		Escherichia phage vB_EcoM-705R4	ON470624
		Escherichia phage vB_EcoM-720R5	ON470604
		Escherichia phage vB_EcoM-569R10	ON470596
	6	Plasmid of *Salmonella* Senftenberg NCTC10384	NZ_LN868944.1
