# Supplementary material for: Genome sequencing and analysis of Salmonella enterica subsp. enterica serotype Enteritidis PT4 578: insights into pathogenicity and virulence
Source: Access Microbiol. 2024 Nov 4;6(11):000828.v3. doi: 10.1099/acmi.0.000828.v3 (PMC11649194; doi:10.1099/acmi.0.000828.v3)
Supplement: Uncited Supplementary Material 2. [file acmi-6-00828-s002.pdf]

```
>EECEBICD_00001 hypothetical protein
ATGTGTATGTGTGTGTGTGTGTGTGTGTATGTGTATCATGTGTATATGTTGTATGTGTAT
GTGTACATCCATGTGTGTCTAATGTGTGTGTGCCTGTCTTTATGTGTGTGTGTGTTCTTT
TATTTTGTGTATGTTGTTGTGTGTGTATGTGTCATCAATGTGTGTGTGTGTGTGTGTATG
TGTGTGTGTGTGTGTGTATGTGTGTGTGTGTATGTGTGTATGTGTGTGTGTGTGTGTGTG
TGTGTGTGTGTGTATGTGTATGTGTATGTGTGTGTGTGTGTGTGTGTGTGTATGTATGTG
TATGTGTATGTATGTGTGTGTATGTGTGTGTGTGTGTGTGTGTGTATGTGTGTGTGTGTG
TGTGTGTGTGTGTGTGTGTGTGTATATATATATATATGTATATATATGTATGTGTATATG
TATATATGTGTATGTGTATGTGTATGTATATATGTGTGTGTGTAA
>EECEBICD_00002 hypothetical protein
ATGTGTGTGTGTGTGTGTGTGTGTGTGTGTGTGTGTGTGTGTGTGTGTGTGTGTGTGTGT
GTGTGTGTGTGTGTGTGTGTGTGTGTGTGTGTGTGTGTGTGTGTGTGTGTGTGTGTGTGT
GTGTGTGTGTGTGTGTATGTGTGTGTATGTGTGTGTGTGTGTGTGTGTGTGTGTGTGTGT
GTGTGTGTGTGTGTGTGTGTGTGTGTGTGTGTGTGTGTGTGTGTGTGCGTGTGTGTATGT
GTGTGTGTGTGTGTGAGTGTGTGTGTGTATGTGTGTGTATGTATGTATGTATGTGTGTGT
GTGTATGTGTGTTGTGTGTGTGTGTGTATGTGTGTGTGTGTGTGTGTGTGTGTGTGTGTG
TGTGTGTGTGTGTGTGTGTGTGTGTGTGTGTGTGTGTGTGTGTGTGTGTGTGTGTGTGTG
TGTGTGTGTGTGTGTGTGTGTGTGTGTGTGTGTGTGTGTGTGTGTTGTGTATGTGTGTGT
GTGTGTGTGTGTGTGTGTGTGTGTGTGTGTGTGTGTGTGTGTGTGTGTATTTGTATGTTT
TTATTGTTGTGTGTGTGTGTGTGTGTATATGTGTGTGTGTATGTGTGTATGTGTGTATAT
GTATATATATATGTTGCTATGTCTGTATCGTCTATATATATGTTTGTGTATATATTGTAT
ATATATGTATATATATATATATGTTCTTTTGTGTGTGTGTGTGTGTGTGTGTGTGTGTGT
GTGTGTGTGTGTGTGTGTGTGTGTGTGTGTGTTGGTGTGTGTGTGTGTGTGTGTGTGTGT
GTGTGTGTATGTGTGTGTATGTGTATGTGTGTGTGTGTGTGTGTGTGTGTGTGTGTGTGT
GTGTGTGTGTGTGTGTGTGTGTGTGTGTGTGTGTGTGTGTGTGTGTGTGTATGTGTGTGT
GTGTGTGTGTATGTGTGTGTGTGTGTGTGTGTGTGTGTGTGTGTGTATGTATGTGTGTGT
GTGTGTGTGTGTGGGGGGGGGGGGGGGGGGGGGGGGGGGGGTGTATGTGTGTGTGTGTGT
GTGTGTGTGTGTGTGTGTGTGTGTGTGTGTATGTGTGTGTGTGTGTGTGTGTGTGTGTGT
GTGTATGTGTATGTGTGTGTGTGTGTGTATGTATGTGTATGTGTATGTGTGTGTGTGTGT
GTGTGTGTGTGTGTGTGTGTGTGTGTGTGTGTGTGTATGTGTGTGTGTATATGTGTGTGT
GTGTGTGTGTGTATGTATGTGTGTGTGTCAGTGTGTGTGTGTGTGTGTTGTGTGTGTG
TGTGTGTGTGTGTGTGTGTGTGTGTGTGTGTGTGTGTGTGTGTGTGTGTGTGTGTGTGTA
TGTGTGTGTGTAAAAAAAAATACAAAATTAA
>EECEBICD_00003 Elongation factor Tu 1
ATGGACGGCGCGATCCTGGTTGTTGCTGCGACTGACGGCCCGATGCCGCAGACCCGTGAG
CACATCCTGCTGGGTCGTCAGGTAGGCGTTCCGTACATCATCGTGTTCCTGAACAAATGC
GACATGGTTGATGACGAAGAGCTGCTGGAACTGGTTGAGATGGAAGTTCGCGAACTGCTG
TCTCAGTACGACTTCCCGGGCGACGACACTCCGATCGTTCGTGGTTCTGCTCTGAAAGCG
CTGGAAGGCGACGCAGAGTGGGAAGCGAAAATCATCGAACTGGCTGGCTTCCTGGATTCT
TACATCCCGGAACCAGAGCGTGCGATTGACAAGCCGTTCCTGCTGCCGATCGAAGACGTA
TTCTCCATCTCCGGTCGTGGTACCGTTGTTACCGGTCGTGTAGAACGCGGTATCATCAAA
GTGGGCGAAGAAGTTGAAATCGTTGGTATCAAAGAGACTCAGAAGTCTACCTGTACTGGC
GTTGAAATGTTCCGCAAACTGCTGGACGAAGGCCGTGCCGGTGAGAACGTAGGTGTTCTG
CTGCGTGGTATCAAACGTGAAGAAATCGAACGTGGTCAGGTACTGGCTAAGCCGGGCACC
ATCAAGCCGCACACCAAGTTCGAATCTGAAGTGTACATTCTGTCCAAAGATGAAGGCGGC
CGTCATACTCCGTTCTTCAAAGGCTACCGTCCGCAGTTCTACTTCCGTACTACTGACGTG
ACTGGCACCATCGAACTGCCGGAAGGCGTAGAGATGGTAATGCCGGGCGACAACATCAAA
ATGGTTGTTACCCTGATCCACCCGATCGCGATGGACGACGGTCTGCGTTTCGCAATCCGT
GAAGGCGGCCGTACCGTTGGCGCGGGCGTTGTTGCTAAAGTTCTGGGCTAA
>EECEBICD_00004 Bacterioferritin-associated ferredoxin
ATGTACGTTTGTTTGTGTAATGGTATAAGCGATAAAAAAATCCGCCAGGCTGTACGACAA
TTTCATCCACAGTCATTTCAACAGTTGCGTAAATTTATTCCTGTGGGAAATCAATGTGGT
AAGTGTATTCGCGCCGCGCGAGAAGTGATGCAGGATGAGTTAACGCAAATGCCGGAATTT
AAAGAGATCGCCTGA
```

>EECEBICD_00005 Bacterioferritin
ATGAAAGGTGATGTTAAAATCATAAATTATCTCAATAAACTATTGGGAAATGAGCTTGTC
GCAATTAATCAGTATTTTCTTCATGCCCGTATGTTCAAAAACTGGGGGCTGACCCGGCTT
AATGATGTTGAGTACCATGAATCCATTGATGAGATGAAACACGCCGATAAGTATATTGAG
CGTATTTTATTTCTGGAAGGCATACCGAACCTACAGGATCTGGGCAAGCTGGGCATCGGT
GAAGATGTCGAAGAGATGCTACGATCGGATCTCCGGCTCGAACTGGAAGGCGCAAAAGAT
CTACGTGAGGCAATAGCCTATGCCGATAGCGTTCATGACTATGTTAGCCGGGATATGATG
ATTGAAATTCTTGCCGACGAGGAAGGCCACATCGACTGGCTGGAAACTGAGCTGGATTTG
ATTGCCAAACTTGGTATGCAAAATTATCTGCAATCACAAATTAAGGTTACCGATTAA
>EECEBICD_00006 Type 4 prepilin-like proteins leader peptide-processing enzyme
ATGTTTGTTGCCCTCCCCTTTCTCATTTTTTATGCGTCGTTCTCTTTATTACTTGGCATA
TACGACGCGCGTACCGGTCTGCTGCCTGACCGGTTTACCTGTCCATTACTGTGGGGTGGT
CTGCTTTATCACCAGATTTGCCTGCCGGAGCGTTTACCGGATGCGCTTTGGGGTGCTATC
GCCGGCTATGGCGGATTCGCGTTAATTTACTGGGGCTATCGCCTGCGTTATCAGAAAGAG
GGGCTTGGATATGGCGACGTCAAGTACCTCGCTGCCTTAGGGGCATGGCATTGTTGGGAA
ACACTGCCGTTACTGGTTTTCCTTGCCGCTATGTTGGCCTGTGGCGGGTTTGGCGTCGCG
CTACTGGTGAGAGGTAAATCGGCATTAATAAACCCGCTACCTTTTGGGCCATGGCTGGCG
GTGGCGGGTTTCATAACAGGTTGGAAAGTTTTCTTTCCAGACGGTTAA
>EECEBICD_00007 30S ribosomal protein S10
ATGCAGAACCAAAGAATCCGTATCCGCCTGAAAGCGTTTGATCATCGTCTGATCGATCAA
TCAACCGCGGAAATCGTCGAGACTGCCAAGCGCACTGGTGCGCAGGTTCGCGGTCCGATC
CCGCTGCCGACCCGCAAAGAGCGCTTTACCGTTCTGATCTCTCCGCACGTTAACAAAGAT
GCGCGCGATCAGTACGAAATTCGCACTCACAAGCGTCTGGTTGACATCGTTGAGCCAACC
GAGAAAACCGTTGATGCTCTGATGCGTCTGGACCTGGCTGCCGGTGTAGACGTGCAGATC
AGCCTGGGTTAA
>EECEBICD_00008 50S ribosomal protein L3
ATGATTGGTTTAGTCGGTAAAAAAGTGGGTATGACCCGCATCTTCACTGAAGATGGCGTC
TCTATCCCAGTAACCGTAATCGAAGTTGAAGCAAACCGTGTTACTCAGGTTAAAGACCTG
GCTAACGATGGCTACCGCGCTGTTCAGGTTACTACGGGTGCTAAAAAAGCTAACCGTGTC
ACCAAGCCGGAAGCGGGTCACTTTGCTAAAGCTGGCGTAGAAGCTGGCCGTGGTCTGTGG
GAATTCCGTCTGGCTGAAGGCGAAGAATACACCGTAGGTCAGAGCATCAGCGTTGAACTG
TTTGCTGACGTTAAAAAAGTTGACGTAACCGGTACCTCTAAAGGTAAAGGTTTCGCTGGT
ACCGTTAAGCGCTGGAACTTCCGTACCCAGGACGCTACTCACGGTAACTCCTTGTCTCAC
CGCGTTCCGGGTTCTATCGGTCAGAACCAGACTCCGGGCAAAGTGTTCAAAGGCAAGAAA
ATGGCAGGTCAGATGGGTAATGAGCGCGTAACCGTTCAGAGCCTTGACGTAGTACGCGTT
GACGCTGAGCGCAACCTGCTGCTGGTTAAGGGTGGTGTTCCGGGTGCAACCGGTTGCGAC
CTGATCGTTAAACCAGCTGTGAAGGCGTAA
>EECEBICD_00009 50S ribosomal protein L4
ATGGAATTAGTATTGAAAGACGCGCAGAGCGCGCTGACTGTTTCCGAAACTACCTTCGGT
CGTGATTTCAACGAAGCGCTGGTTCACCAGGTTGTTGTTGCTTATGCAGCTGGTGCTCGT
CAGGGTACTCGTGCTCAGAAGACTCGTGCTGAAGTAACTGGTTCCGGTAAAAAACCGTGG
CGCCAGAAAGGCACTGGCCGTGCGCGTTCAGGTTCTATCAAGAGCCCGATCTGGCGTTCC
GGTGGCGTGACCTTCGCTGCTCGTCCGCAGGACCACAGTCAAAAAGTTAACAAGAAGATG
TACCGCGGCGCGCTGAAAAGCATCCTGTCCGAACTGGTACGTCAGGATCGTCTGATCGTT
GTCGAGAAGTTTTCTGTAGAAGCGCCGAAAACTAAGCTGCTGGCACAGAAACTGAAAGAC
ATGGCTCTGGAAGATGTGCTGATCATCACCGGTGAGCTGGACGAGAACCTGTTCCTGGCC
GCACGTAACCTGCACAAGGTTGACGTACGCGATGCGACTGGTATCGACCCGGTTAGCCTG
ATCGCCTTCGACAAAGTCGTAATGACTGCTGATGCTGTTAAGCAAGTTGAGGAGATGCTG
GCATGA
>EECEBICD_00010 50S ribosomal protein L23
ATGATTCGTGAAGAACGTCTGCTGAAGGTGCTGCGCGCACCGCACGTTTCTGAAAAAGCG
TCTACTGCGATGGAAAAAACTAACACCATCGTTCTCAAAGTTGCTAAAGACGCGACCAAA
GCAGAAATCAAAGCTGCTGTGCAGAAACTGTTTGAAGTCGAAGTCGAAGTCGTTAACACC

CTGGTCGTTAAAGGGAAAGTTAAACGTCACGGACAGCGTATCGGTCGTCGTAGCGACTGG
AAAAAAGCTTACGTCACCCTGAAAGAAGGCCAGAATCTGGACTTCGTCGGTGGCGCTGAG
TAA
>EECEBICD_00011 50S ribosomal protein L2
ATGGCAGTTGTTAAATGTAAACCGACATCTCCGGGTCGTCGCCACGTCGTTAAAGTGGTC
AACCCAGAGCTGCACAAGGGCAAACCTTTTGCTCCGCTGGTTGAAAAAAACAGCAAATCC
GGTGGTCGTAACAACAATGGCCGTATCACCACTCGTCATATCGGTGGTGGTCACAAACAG
GCTTATCGTATTGTTGACTTCAAACGCAACAAAGACGGTATTCCGGCAGTTGTTGAACGT
CTTGAGTACGATCCGAACCGTTCCGCGAACATCGCGCTGGTTCTGTACAAAGACGGCGAA
CGCCGTTACATCCTGGCCCCGAAAGGCCTGAAAGCTGGCGACCAGATTCAGTCTGGCGTT
GATGCTGCAATCAAAGCAGGCAACACCCTGCCGATGCGCAATATCCCGGTTGGTTCTACC
GTTCATAACGTAGAAATGAAACCAGGTAAAGGCGGTCAGCTGGCGCGTTCCGCTGGTACT
TACGTTCAGATCGTTGCTCGCGATGGCGCTTATGTCACCCTGCGTCTGCGTTCTGGTGAA
ATGCGTAAAGTCGAAGCAGACTGCCGTGCAACCCTGGGCGAAGTTGGCAATGCTGAGCAT
ATGCTGCGCGTTCTGGGTAAAGCAGGTGCTGCACGCTGGCGTGGCGTTCGTCCTACCGTT
CGCGGTACTGCGATGAACCCAGTAGACCACCCACATGGTGGTGGTGAAGGTCGTAACTTT
GGTAAGCACCCGGTAACTCCGTGGGGCGTTCAGACCAAAGGTAAGAAGACCCGCAGCAAC
AAGCGTACTGATAAATTCATCGTACGTCGCCGTAGCAAATAA
>EECEBICD_00012 30S ribosomal protein S19
ATGCCACGTTCTCTCAAGAAAGGTCCTTTTATTGACCTGCACTTGCTGAAGAAGGTAGAG
AAAGCGGTGGAAAGCGGAGACAAGAAGCCCCTGCGCACCTGGTCCCGTCGTTCAACGATC
TTTCCTAACATGATCGGTTTGACCATCGCTGTCCATAATGGTCGTCAGCACGTTCCGGTA
TTTGTTTCCGACGAAATGGTCGGTCACAAACTGGGTGAATTCGCACCGACTCGTACTTAT
CGCGGCCACGCTGCTGATAAAAAAGCGAAGAAGAAATAA
>EECEBICD_00013 50S ribosomal protein L22
ATGGAAACTATCGCTAAACATCGCCATGCTCGTTCTTCTGCTCAGAAGGTTCGCCTTGTT
GCTGACCTGATTCGCGGTAAGAAAGTGTCGCAGGCTCTGGATATTCTGACCTACACCAAC
AAGAAAGCGGCTGTACTGGTCAAGAAAGTTCTGGAATCTGCCATTGCTAACGCTGAACAC
AACGATGGCGCTGACATTGACGATCTGAAAGTTACGAAAATTTTCGTAGACGAAGGCCCG
AGCATGAAGCGCATTATGCCGCGTGCAAAAGGTCGTGCAGATCGCATCCTGAAGCGCACC
AGCCACATCACTGTGGTTGTGTCCGATCGCTGA
>EECEBICD_00014 30S ribosomal protein S3
ATGGGTCAGAAAGTACATCCTAATGGTATTCGCCTGGGTATTGTAAAACCATGGAACTCT
ACCTGGTTTGCGAACACCAAAGAATTCGCTGACAACCTGGACAGCGATTTTAAAGTACGT
CAGTACCTGACGAAGGAACTGGCTAAAGCGTCTGTATCTCGTATCGTTATCGAGCGTCCG
GCTAAGAGCATCCGTGTGACTATTCACACCGCTCGCCCGGGTATCGTTATCGGTAAAAAA
GGTGAAGACGTAGAAAAACTGCGTAAGGTCGTAGCGGATATCGCTGGCGTTCCTGCACAG
ATCAATATCGCCGAAGTTCGTAAACCTGAACTGGACGCTAAACTGGTTGCTGACAGCATC
ACTTCTCAGCTGGAACGTCGCGTGATGTTCCGTCGTGCGATGAAGCGTGCTGTACAGAAC
GCCATGCGTCTGGGCGCTAAAGGTATCAAAGTTGAAGTTAGCGGCCGTCTGGGCGGCGCG
GAAATCGCACGTACCGAATGGTACCGCGAAGGTCGCGTACCTCTGCACACTCTGCGTGCT
GACATCGACTACAACACCTCTGAAGCGCACACCACTTATGGTGTAATCGGCGTTAAAGTG
TGGATCTTCAAAGGCGAGATCCTGGGTGGTATGGCTGCTGTTGAACAACCGGAAAAACCG
GCTGCTCAACCGAAAAAGCAGCAGCGTAAAGGCCGTAAATAA
>EECEBICD_00015 50S ribosomal protein L16
ATGTTACAACCAAAGCGTACAAAATTCCGTAAAATGCACAAAGGCCGCAACCGTGGTCTG
GCTGCAGGCGCGGATGTTAGCTTCGGCAGCTTCGGCCTGAAAGCTGTTGGCCGTGGTCGT
CTGACTGCCCGTCAGATCGAAGCAGCACGTCGTGCTATGACCCGTGCAGTTAAGCGTCAA
GGTAAGATCTGGATCCGTGTATTCCCGGACAAACCGATCACTGAAAAGCCGCTGGCAGTG
CGTATGGGTAAAGGTAAAGGTAACGTGGAGTATTGGGTTGCCTTGATTCAGCCGGGTAAA
GTCCTGTATGAAATGGACGGCGTACCGGAAGAGCTGGCCCGTGAAGCATTCAAGCTGGCA
GCAGCGAAACTGCCGATTAAAACCACCTTTGTAACTAAGACGGTGATGTAA
>EECEBICD_00016 50S ribosomal protein L29
ATGAAAGCAAAAGAGCTGCGTGAGAAGAGCGTTGAAGAGCTGAACACCGAGCTACTGAAC

CTGCTGCGTGAGCAGTTCAACCTGCGTATGCAGGCTGCAAGTGGCCAGCTGCAACAGTCT
CACCTGTTGAAGCAAGTGCGTCGTGATGTCGCACGCGTTAAGACTTTACTGACTGAGAAG
GCGGGTGCGTAA
>EECEBICD_00017 30S ribosomal protein S17
ATGACCGATAAAATCCGTACTCTGCAAGGTCGCGTGGTTAGCGACAAAATGGAGAAATCC
ATTGTTGTTGCTATCGAACGTTTTGTGAAACACCCGATCTACGGTAAATTCATCAAGCGT
ACGACCAAAATGCACGTACATGACGAGAACAACGAATGCGGTATCGGCGACGTGGTTGAA
ATCCGCGAATGCCGTCCGCTGTCCAAGACTAAATCCTGGACTCTGGTTCGCGTTGTAGAG
AAAGCGGTTCTGTAA
>EECEBICD_00018 50S ribosomal protein L14
ATGATCCAAGAACAGACTATGCTGAACGTCGCCGACAACTCCGGTGCACGTCGCGTAATG
TGTATCAAGGTTCTGGGTGGCTCGCACCGTCGCTACGCAGGCGTAGGCGACATCATTAAG
ATCACCATCAAAGAAGCGATTCCGCGTGGTAAGGTCAAAAAAGGTGATGTGCTGAAGGCG
GTAGTGGTGCGCACCAAGAAGGGTGTTCGTCGCCCGGACGGTTCTGTCATTCGCTTCGAT
GGTAATGCATGCGTTATTTTAAACAATAACAGCGAGCAGCCTATCGGTACGCGTATTTTT
GGGCCGGTAACTCGTGAACTTCGTAACGAGAAGTTCATGAAAATTATCTCTCTGGCACCA
GAAGTACTCTAA
>EECEBICD_00019 50S ribosomal protein L24
ATGGCAGCGAAAATCCGTCGTGATGACGAAGTTATCGTGTTAACCGGTAAAGATAAAGGT
AAACGCGGTAAAGTTAAGAATGTCCTGTCTTCCGGCAAGGTCATTGTTGAAGGTATCAAC
CTGGTTAAGAAACACCAGAAGCCGGTTCCGGCTCTGAACCAACCGGGCGGCATCGTTGAA
AAAGAAGCTGCAATTCAGGTTTCTAACGTTGCAATCTTCAATGCGGCAACCGGCAAGGCT
GACCGTGTAGGCTTTAGATTCGAAGACGGCAAAAAAGTCCGTTTCTTCAAGTCTAACAGC
GAAACTATCAAGTAA
>EECEBICD_00020 50S ribosomal protein L5
ATGGCGAAACTGCATGATTACTACAAAGACGAAGTAGTTAACAAACTCATGACTGAGTTT
AACTACAATTCTGTCATGCAAGTCCCTCGGGTCGAGAAGATCACCCTGAACATGGGTGTT
GGTGAAGCGATCGCTGACAAGAAACTGCTGGATAACGCAGCAGCAGACCTGACAGCAATC
TCCGGTCAAAAACCGTTGATCACCAAAGCACGCAAATCTGTTGCAGGCTTCAAAATCCGT
CAGGGCTATCCGATCGGCTGTAAAGTAACTCTGCGTGGCGAACGCATGTGGGAGTTCTTT
GAGCGCCTGATCACTATTGCTGTTCCTCGTATCCGTGACTTCCGTGGCTTGTCCGCTAAG
TCATTCGACGGTCGTGGTAACTACAGCATGGGTGTCCGTGAGCAGATCATCTTCCCAGAA
ATCGACTACGATAAAGTCGACCGCGTTCGTGGTTTGGATATTACCATTACCACTACTGCG
AAATCTGACGAAGAAGGCCGTGCTCTGCTGGCTGCCTTTGACTTCCCGTTCCGCAAGTAA
>EECEBICD_00021 30S ribosomal protein S14
ATGGCTAAGCAATCAATGAAAGCACGCGAAGTAAAACGCGTAGCTTTAGCTGATAAATAC
TTCGCGAAACGCGCTGAACTGAAAGCGATCATCTCTGATGTGAACGCAACCGACGAAGAT
CGTTGGAATGCGGTTCTTAAGCTGCAGACTCTGCCGCGTGATTCCAGCCCGTCACGTCAG
CGTAACCGCTGCCGTCAAACTGGTCGTCCGCACGCTTTCCTGCGGAAGTTCGGGTTGAGC
CGTATTAAGGTCCGTGAAGCCGCTATGCGCGGTGAAATTCCGGGTCTGAAAAAGGCTAGC
TGGTAA
>EECEBICD_00022 30S ribosomal protein S8
ATGAGCATGCAAGATCCGATCGCGGATATGCTGACCCGTATCCGTAACGGTCAGGCCGCG
AACAAAGCTGCGGTCACCATGCCTTCCTCCAAGCTGAAAGTGGCAATTGCCAACGTGCTG
AAGGAAGAAGGTTTTATTGAAGATTTTAAAGTTGAAGGCGACACCAAGCCGGAACTGGAA
CTGACTCTTAAGTATTTCCAGGGTAAAGCTGTTGTAGAAAGCATTCAGCGTGTCAGCCGC
CCAGGTCTGCGCATCTACAAACGTAAAGATGAGCTGCCGAAAGTTATGGCCGGTCTGGGT
ATCGCAGTTGTTTCTACCTCTAAAGGTGTTATGACTGATCGTGCAGCGCGCCAGGCTGGT
CTTGGTGGCGAAATTATCTGCTACGTAGCCTAA
>EECEBICD_00023 50S ribosomal protein L6
ATGTCTCGTGTTGCTAAAGCACCGGTCGTTGTTCCTGCCGGCGTTGATGTCAAAATCAAC
GGTCAGGTTATTACGATCAAAGGTAAAAACGGCGAGCTGACTCGTACTCTCAACGATGCT
GTTGAAGTTAAACATGCAGATAATGCACTGACCTTCGGTCCGCGTGATGGTTACGCAGAC
GGTTGGGCACAGGCTGGTACCGCGCGTGCCCTGCTGAACTCAATGGTTATCGGTGTTACC

GAAGGCTTCACTAAGAAGCTGCAGCTGGTTGGTGTAGGTTATCGTGCAGCGGTTAAGGGG
AATGTAGTAAACCTGTCTTTAGGTTTCTCTCACCCGGTTGACCATCAGCTGCCGGCAGGT
ATTACTGCTGAATGTCCGACTCAAACTGAAATCGTGCTGAAAGGCGCTGATAAGCAGGTG
ATCGGCCAGGTTGCAGCAGATCTGCGCGCCTACCGTCGTCCTGAGCCTTACAAAGGCAAG
GGTGTTCGTTACGCCGACGAAGTCGTGCGTACCAAAGAGGCTAAGAAGAAGTAA
>EECEBICD_00024 50S ribosomal protein L18
ATGGATAAGAAATCTGCTCGTATCCGTCGTGCGACCCGCGCACGCCGCAAGCTCAAAGAG
CTGGGCGCAACTCGCCTGGTGGTACATCGTACCCCGCGTCATATTTACGCACAGGTAATT
GCACCGAACGGTTCTGAAGTTCTGGTAGCTGCTTCTACTGTAGAAAAAGCTATCGCTGAA
CAACTGAAGTACACCGGTAACAAAGACGCGGCTGCAGCTGTGGGTAAAGCTGTCGCTGAA
CGCGCTCTGGAAAAAGGCATCAAAGATGTGTCCTTTGACCGTTCCGGGTTCCAATATCAT
GGTCGTGTCCAGGCACTGGCAGATGCTGCCCGTGAAGCTGGCCTTCAGTTCTAA
>EECEBICD_00025 30S ribosomal protein S5
ATGGCTCACATCGAAAAACAAGCTGGCGAACTGCAGGAAAAGCTGATCGCGGTAAACCGC
GTATCTAAAACCGTTAAAGGTGGTCGTATTTTCTCCTTCACAGCTCTGACTGTAGTAGGC
GATGGTAACGGTCGCGTAGGTTTTGGTTACGGTAAAGCGCGTGAAGTTCCAGCAGCGATC
CAGAAAGCGATGGAAAAAGCCCGTCGCAATATGATTAACGTCGCGCTGAACAACGGCACC
CTGCAGCACCCGGTTAAAGGTGTTCACACGGGGTCTCGTGTATTCATGCAGCCAGCTTCC
GAAGGTACCGGTATCATCGCCGGTGGTGCAATGCGCGCCGTTCTGGAAGTTGCTGGGGTT
CATAACGTTCTGGCTAAAGCATACGGTTCCACCAACCCGATCAACGTGGTTCGTGCAACT
ATTGATGGCCTGGAAAATATGAATTCTCCAGAAATGGTCGCTGCCAAGCGTGGTAAATCC
GTTGAAGAAATTCTGGGGAAATAA
>EECEBICD_00026 50S ribosomal protein L30
ATGGCAAAGACTATTAAAATTACTCAAACCCGCAGTGCAATCGGTCGTCTGCCGAAACAC
AAGGCAACGCTGCTTGGCCTGGGTCTGCGTCGTATTGGTCACACCGTAGAGCGCGAGGAT
ACTCCTGCTGTTCGTGGTATGGTCAACGCGGTTTCCTTCATGGTTAAAGTTGAGGAGTAA
>EECEBICD_00027 50S ribosomal protein L15
ATGCGTTTAAATACTCTGTCTCCGGCCGAAGGCTCCAAAAAGGCGGGTAAACGCCTGGGT
CGTGGTATCGGTTCTGGCCTCGGTAAAACCGGTGGTCGTGGTCACAAAGGTCAGAAGTCT
CGTTCTGGCGGTGGCGTACGTCGCGGTTTCGAGGGTGGTCAGATGCCTCTGTACCGTCGT
CTGCCGAAATTCGGCTTCACTTCTCGCAAAGCAGCGATTACAGCCGAAGTTCGTCTGTCT
GACTTGGCGAAAGTAGAAGGCGGCGTTGTAGACCTGAACACGCTGAAAGCGGCAAACATT
ATCGGTATCCAGATCGAGTTCGCGAAAGTGATCTTGGCTGGCGAAGTCACTACTCCGGTA
ACTGTTCGTGGCCTGCGTGTTACTAAAGGCGCTCGTGCTGCTATCGAAGCTGCTGGCGGT
AAAATCGAGGAATAA
>EECEBICD_00028 Protein translocase subunit SecY
ATGGCTAAACAACCGGGATTAGATTTTCAAAGTGCCAAAGGTGGCTTAGGTGAGCTGAAA
CGCAGACTGCTGTTTGTTATCGGCGCGCTGATTGTGTTCCGTATTGGCTCTTTTATTCCG
ATCCCTGGTATTGATGCCGCTGTACTTGCCAAACTGCTTGAGCAACAGCGAGGCACCATC
ATTGAAATGTTTAACATGTTCTCTGGTGGTGCTCTCAGCCGTGCTTCTATCTTTGCCCTG
GGGATCATGCCGTATATTTCGGCGTCGATCATTATCCAGCTGTTGACGGTGGTTCATCCA
ACGCTGGCAGAAATTAAGAAAGAAGGGGAGTCTGGTCGTCGTAAGATCAGCCAGTACACC
CGTTACGGTACTCTGGTGCTGGCGATATTCCAGTCGATCGGTATTGCTACCGGTTTGCCG
AATATGCCTGGTATGCAGGGCCTGGTGATGAATCCAGGCTTTGCATTCTATTTCACCGCT
GTTGTAAGTCTGGTTACAGGGACGATGTTCCTGATGTGGCTCGGCGAACAGATTACTGAG
CGCGGTATCGGTAACGGTATCTCAATCATCATCTTCGCTGGTATCGTTGCGGGACTCCCG
CCAGCCATTGCCCATACTATCGAGCAAGCGCGTCAAGGCGACCTGCACTTCCTCGTGTTG
CTGTTGGTTGCAGTATTAGTATTTGCAGTGACGTTCTTTGTAGTATTCGTTGAACGTGGT
CAACGCCGTATTGTGGTAAACTACGCCAAACGTCAGCAAGGTCGTCGTGTCTATGCTGCA
CAGAGCACACATTTACCGCTGAAAGTGAATATGGCGGGGGTAATCCCGGCAATCTTCGCT
TCCAGTATTATTCTGTTCCCGGCGACCATCGCGTCATGGTTCGGGGGCGGTACTGGTTGG
AACTGGCTGACAACAATTTCGCTGTATTTGCAGCCTGGGCAACCGCTTTATGTGTTACTC
TATGCGTCTGCAATCATCTTCTTCTGTTTCTTCTACACGGCGTTGGTTTTCAACCCGCGT
GAAACAGCAGATAACCTGAAGAAGTCCGGTGCATTTGTACCAGGAATTCGTCCGGGAGAG

CAAACGGCGAAGTATATCGATAAAGTAATGACCCGCCTGACCTTGGTTGGTGCGCTGTAT
ATTACCTTTATCTGCCTGATCCCGGAGTTCATGCGTGATGCAATGAAAGTGCCGTTCTAC
TTCGGTGGGACCTCACTGCTTATCGTTGTTGTCGTGATTATGGACTTTATGGCTCAAGTG
CAAACTCTGATGATGTCCAGTCAGTATGAGTCTGCATTGAAGAAGGCGAACCTGAAAGGC
TACGGCCGTTAA
>EECEBICD_00029 50S ribosomal protein L36
ATGAAAGTTCGTGCTTCCGTCAAGAAATTATGCCGTAACTGCAAAATCGTTAAGCGTGAT
GGTGTCATCCGTGTGATTTGCAGTGCCGAGCCGAAGCATAAACAGCGCCAAGGCTGA
>EECEBICD_00030 30S ribosomal protein S13
GTGGCCCGTATAGCAGGCATTAACATTCCTGATCAGAAACACGCCGTGATCGCGTTAACT
TCGATCTACGGTGTCGGCAAGACCCGTTCTAAAGCCATCCTGGCTGCAGCGGGTATCGCT
GAAAATGTTAAGATCAGTGAGCTGTCTGAAGAACAAATCGACACGCTGCGTGACGAAGTT
GCCAAATTTGTCGTTGAAGGTGATCTGCGCCGTGAAATCAGCATGAGCATCAAGCGCCTG
ATGGATCTTGGTTGCTATCGCGGTTTGCGTCATCGTCGTGGTCTCCCGGTTCGCGGTCAG
CGTACCAAGACCAACGCTCGTACCCGTAAGGGTCCGCGCAAACCGATCAAGAAATAA
>EECEBICD_00031 hypothetical protein
ATGAGGGATCGGAGTCACATCAGTAATATTAGTGATGCGGAAACCAGCGGCGTTCAGAGC
ACGAATAGTAGATTCGCGGCCTGGACCCGGACCTTTAACCATAACTTCCAGATTCTTGAT
ACCGTATTCTTTCACGGCGTCAGCGCAACGCTCTGCTGCAACCTGAGCTGCAAACGGAGT
GGATTTGCGAGAACCACGGAAACCGGAACCACCGGCTGTTGCCCAACCCAGCGCATTACC
CTGACGATCGGTAATAGTCACGATGGTGTTGTTGAAAGAAGCATGGATATGAGCCACGCC
GTCAGAGACTTGTTTTCTTACACGTTTACGTGCGCGAACTGGTGCCTTTGCCATTATTCA
ATCACCCCGATTATTTCTTGA
>EECEBICD_00032 30S ribosomal protein S4
ATGGCAAGATATTTGGGTCCTAAGCTCAAGCTGAGCCGTCGTGAGGGCACCGACTTATTC
CTTAAGTCTGGCGTTCGCGCGATCGATACCAAGTGTAAAATTGAACAAGCTCCTGGCCAG
CACGGTGCGCGTAAGCCGCGTCTGTCTGACTATGGTGTGCAGTTGCGTGAAAAGCAAAAA
GTTCGCCGCATTTACGGTGTGCTGGAGCGTCAGTTCCGTAACTACTACAAAGAAGCAGCA
CGTCTGAAAGGCAACACCGGTGAAAACCTGTTGGCTCTGCTGGAAGGTCGTCTGGACAAC
GTTGTATACCGTATGGGCTTCGGCGCCACTCGTGCAGAAGCACGTCAGCTGGTTAGCCAT
AAAGCAATTATGGTAAACGGTCGTGTTGTTAACATCGCTTCTTATCAGGTTAGTCCGAAT
GACGTTGTTAGCATTCGTGAGAAAGCGAAGAAGCAGTCTCGCGTGAAAGCCGCTCTGGAG
CTGGCTGAGCAGCGTGAAAAGCCAACCTGGCTGGAAGTTGATGCTGGCAAGATGGAAGGT
ACGTACAAGCGTAAGCCTGAGCGTTCTGATCTGTCTGCGGACATTAACGAACACCTGATC
GTCGAGCTTTACTCCAAGTAA
>EECEBICD_00033 DNA-directed RNA polymerase subunit alpha
ATGCAGGGTTCTGTGACAGAGTTTCTAAAACCGCGCCTGGTAGATATCGAGCAAGTGAGT
TCGACGCACGCCAAGGTGACCCTTGAGCCTTTAGAGCGTGGCTTCGGCCATACTCTGGGT
AACGCACTGCGCCGTATTCTGCTCTCATCGATGCCGGGTTGCGCGGTGACCGAGGTTGAG
ATTGATGGTGTACTACATGAGTACAGCACCAAAGAAGGCGTTCAGGAAGACATCCTGGAA
ATCCTGCTCAACCTGAAAGGGCTGGCGGTGAGAGTTCAGGGTAAAGATGAAGTTATTCTT
ACCTTGAATAAATCTGGCATTGGCCCTGTGACTGCAGCCGATATCACCCATGATGGGGAT
GTCGAAATCGTCAAGCCGCAGCACGTGATCTGCCACCTGACCGATGAAAACGCGTCTATT
AGTATGCGTATCAAAGTTCAGCGCGGTCGTGGTTATGTGCCGGCTTCTACCCGAATTCAT
TCGGAAGAAGATGAGCGCCCAATCGGCCGTCTGCTGGTCGACGCCTGCTACAGCCCTGTA
GAGCGTATTGCCTACAATGTTGAAGCAGCGCGTGTAGAACAGCGTACCGACCTGGACAAG
CTGGTCATCGAAATGGAAACCAACGGCACAATCGATCCTGAAGAGGCGATTCGTCGTGCG
GCAACCATCCTGGCTGAACAACTGGAAGCTTTCGTTGATTTACGTGATGTACGTCAACCG
GAAGTGAAAGAAGAGAAACCAGAATTCGATCCGATCCTGCTGCGCCCTGTTGACGATCTG
GAATTGACTGTCCGCTCTGCTAACTGCCTCAAGGCAGAAGCTATCCACTATATCGGTGAT
CTGGTACAGCGTACCGAGGTTGAGCTTCTTAAGACGCCTAACCTGGGTAAAAAATCTCTT
ACCGAGATTAAAGACGTGCTGGCTTCCCGTGGACTGTCTCTGGGTATGCGCCTAGAAAAC
TGGCCACCGGCAAGCATCGCTGACGAGTAA
>EECEBICD_00034 50S ribosomal protein L17

ATGCGCCATCGTAAGAGTGGTCGTCAACTGAACCGCAACAGCAGCCATCGCCAGGCTATG
TTCCGCAACATGGCAGGTTCACTGGTTCGTCATGAAATCATCAAGACGACCCTGCCTAAA
GCGAAAGAGCTGCGTCGCGTAGTTGAGCCGCTGATTACTCTTGCCAAGACTGATAGCGTT
GCTAATCGTCGTCTGGCATTCGCCCGTACTCGTGATAACGAGATCGTGGCAAAACTGTTT
AACGAGCTGGGCCCGCGTTTCGCGAGCCGCGCCGGTGGTTACACTCGCATTCTGAAGTGT
GGCTTCCGTGCAGGCGACAACGCGCCGATGGCATACATCGAGCTGGTTGATCGTTCAGAG
AAAACAGAAGCTGCTGCAGAGTAA
>EECEBICD_00035 hypothetical protein
ATGTGGTTACTTGACCAGTGGGCAGAGCGTCATATTATCGAGGCACAGCGTAAAGGCGAG
TTTGATAATTTGCCTGGCCGTGGCGAGCCGCTTATTCTGGATGATGATTCTCATGTGCCG
GCGGAACTTCGTGCGGGTTATCGCTTACTCAAGAATGCGGGCTGTCTTCCCCCTGAACTG
GAGCAGCGCAGAGACGCTATTCAGTTACTTGATATCCTCAACAGTATCCGGGAAGATGAC
CCTCGATACCATCAGGTTAGTCGCCAGCTCTCCCTGCTTGAACTAAAACTTCGGCAGGCT
GGGTTGAGTACCGACTTTTTACACGGTGAGTATGCAGAAAAACTGCTGCATAAAATCAAC
GATAATTAA
>EECEBICD_00036 HTH-type transcriptional regulator ZntR
ATGTATCGCATTGGTGAGCTGGCAAAGCTGGCAGACGTAACGCCTGATACCATTCGCTAC
TATGAAAAACAGCAGATGATGGATCATGAGGTACGTACTGAAGGCGGGTTTCGTCTGTAT
ACCGAAACCGATCTGCAGCGTCTCAAGTTTATTCGTTATGCGCGACAGTTGGGTTTCACG
CTCGACTCGATCCGCGAGCTTTTATCGATCCGAATTGATCCTGAGCACCATACCTGTCAG
GAGTCAAAAAGTATTGTGCAAGAGCGATTGCAGGAGGTTGAAGCCAGGATTGCGGAACTG
CAAACGATGCAGCGTTCGTTACAAAGGCTTAATGATGCTTGTTGCGGAACGGCCCACAGT
AGCGTCTATTGTTCCATTCTGGAAGCCCTGGAACAGGGGGCCAGTGGAGCGAAATCAGGA
TGTTGA
>EECEBICD_00037 hypothetical protein
ATGAGTCGTTATCAACATAAGAAAGGGCAGATAAAGGACAATGCCATTGAGGCATTACTG
CACGATCCCCTTTTCAGGCAACGCGTTGAAAAAAATAAGAAAGGCAAAGGAAGCTATCTG
CGCAAAGGTAAACACGGTAATCGAGGGAACTGGGAGGCCAGTGGCAAGAAAGTAAGTAAC
TTTTTTACCACTGGCCTTCTGCTTATTAAGAACGGTTATTTTGTTCTTTCAGCAGATCGC
GGATTTCGCCCAATAGCACTTCCTCTTTAG
>EECEBICD_00038 Large-conductance mechanosensitive channel
ATGAGTTTTATTAAAGAATTTCGCGAATTCGCGATGCGCGGGAATGTTGTGGATTTGGCA
GTGGGTGTCATTATTGGCGCGGCATTCGGTAAGATTGTTTCTTCACTGGTTGCCGATATC
ATTATGCCGCCGTTGGGATTGTTAATCGGCGGGATCGACTTTAAACAATTTGCTTTTACG
TTACGCGAAGCGCAGGGAGATATTCCGGCGGTCGTCATGCATTACGGCGTGTTTATCCAG
AACGTATTTGATTTTGTGATTGTGGCCTTTGCTATCTTTGTTGCCATTAAGCTTATCAAC
AGACTCAACCGTAAAAAAGCAGAAGAGCCGGCAGCTCCGCCAGCGCCTTCTAAAGAGGAA
GTGCTATTGGGCGAAATCCGCGATCTGCTGAAAGAACAAAATAACCGTTCTTAA
>EECEBICD_00039 Trk system potassium uptake protein TrkA
ATGAAAATTATCATACTGGGCGCAGGGCAAGTTGGCGGAACACTGGCGGAAAACCTGGTC
GGCGAAAACAACGACATCACGGTGGTTGATACCAACGGCGAACGGTTACGCAGTTTGCAA
GATAAGTTCGATCTACGCGTAGTTCAGGGGCATGGTTCGCATCCCCGCGTGTTGCGCGAA
GCGGGCGCTGACGATGCGGATATGCTGGTCGCTGTCACCAGTTCAGATGAAACTAATATG
GTTGCCTGTCAGGTAGCCTATTCACTCTTCAACACTCCAAACCGTATCGCCCGCATTCGC
TCCCCGGACTATGTTCGCGATGCGGATAAACTCTTTCATTCTGAAGCGGTTCCGATTGAT
CATCTGATCGCGCCGGAGCAGTTGGTTATCGATAATATTTATCGTCTGATCGAGTATCCG
GGAGCATTACAGGTTGTAAACTTTGCTGAAGGGAAAGTCAGTCTGGCAGTAGTAAAAGCC
TACTATGGCGGTCCACTAATAGGTAACGCGCTCTCGACAATGCGAGAACATATGCCGCAT
ATCGATACTCGCGTTGCCGCAATTTTCCGCCATGACAGACCAATCCGTCCTCAGGGCTCG
ACTATCGTGGAAGCAGGGGATGAAGTCTTCTTTATTGCGGCTTCACAGCATATCCGTGCA
GTAATGAGCGAATTACAGCGTCTGGAAAAACCCTACAAGCGCATCATGCTGGTGGGCGGC
GGCAATATCGGCGCCGGGCTGGCAAGGCGTCTGGAAAAAGACTATAGCGTTAAATTGATC
GAACGCGATCAACAACGCGCCGCAGAACTGGCTGAGAAACTGCAAAACACGATCGTCTTT
TTTGGCGACGCCTCCGATCAGGAATTGCTGGCGGAGGAGCATATTGATCAGGTCGATCTC

TTTATCGCGGTCACTAACGATGATGAAGCCAATATCATGTCCGCGATGTTGGCCAAGCGG
ATGGGCGCCAAAAAAGTGATGGTGTTGATTCAGCGTCGAGCTTATGTCGATCTTGTGCAG
GGTAGCGTTATAGATATTGCGATTTCTCCGCAGCAGGCCACCATCTCTGCATTACTGAGC
CATGTGCGTAAAGCGGATATCGTCGGCGTTTCCTCACTACGTCGCGGAGTTGCCGAAGCC
ATCGAAGCCGTCGCGCACGGCGATGAAAGCACTTCCCGCGTGGTTGGCCGCGTCATTGAC
GAAATAAAACTGCCGCCAGGCACCATTATTGGCGCGGTCGTTCGTGGTAATGATGTCATG
ATTGCTAATGACAATTTGCGCATTGAACAAGGCGATCACGTCATTATGTTTTTGACCGAT
AAAAAGTTTATTACCGACGTCGAACGTCTGTTCCAGCCAAGTCCTTTCTTCCTCTAA
>EECEBICD_00040 Ribosomal RNA small subunit methyltransferase B
ATGAAAAAACAAAACAATTTACGTAGTCTGGCAGCACAGGCCGTTGAACTGGTCGTCGAA
CAGGGACAATCATTAAGTAATGTGCTCCCGCCGCTGCAACAAAAAGTCGCCGATAAAGAC
AAAGCGCTACTTCAGGAGCTGTGCTTTGGCGTCCTGCGCACCCTTTCACAGCTGGAATGG
CTGATCAATAAGCTGATGTCTCGTCCAATGACCGGCAAGCAGCGTACAGTGCATTACTTA
ATTATGGTCGGCTTTTATCAGTTGCTGTACACCCGCGTTCCTCCCCATGCGGCGCTGGCT
GAAACCGTTGAAGGCGCGGTCGCCATCAAACGTCCGCAGTTGAAAGGGTTAATTAACGGC
GTCCTGCGCCAGTTTCAGCGCCAGCAGGAAACGTTGCTTAATGAGTTTGCGACCAGCGAT
GCGCGCTTTTTACATCCAGGTTGGCTGGTAAAGCGTTTGCAAAATGCTTACCCCACACAG
TGGCAACACATTATTGAAGCGAATAACCAACGTCCACCGATGTGGCTACGTGTAAATCGT
ACACACCACACCCGCGACGGTTGGCTTGGCTTACTTGAAGACGCAGGCATGAAGGGATAT
CCCCATCCCGATTATCCCGACGCTGTACGTCTGGAAACGCCAGCTCCGGTACATGCTTTA
CCCGGTTTTGCTGAGGGTTGGGTAACTGTCCAGGATGCCTCTGCGCAGGGATGTGCGGTT
TTTCTCGCTCCTCAGAACGGTGAGCACATTTTAGACTTGTGCGCCGCGCCCGGCGGTAAA
ACCACGCATATTCTCGAAGTGGCCCCCGAAGCAGACGTGTTGGCGGTTGATATTGATGAA
CAACGACTCTCCCGCGTTTACGACAACCTAAAGCGTCTTGGGATGAAAGCGACAGTTAAA
CAAGGGGATGGACGTTATCCCGCCCAATGGTGCGGCGAGCAGCAGTTTGACCGTATTCTG
CTGGATGCGCCCTGCTCCGCAACCGGCGTCATTCGCCGTCATCCAGATATCAAATGGCTG
CGTCGCGATCGCGATATCGCGGAACTGGCGCAATTACAGGCGGAAATTCTTGATGCCGTC
TGGCCGCGCCTGAAGCCTGGCGGGACGCTGGTTTACGCCACCTGTTCCGTACTGCCGGAA
GAAAACCGCGATCAGATCAAAACCTTTTTGCAACGCACTCCCGATGCCGCGCTCAGCGAA
ACGGGAACACCTGATCAGCCAGGACAGCAAAATCTGCCGGGCGGCGAAGAAGGTGATGGG
TTCTTTTACGCTAAGCTAATCAAGAAGTGA
>EECEBICD_00041 Methionyl-tRNA formyltransferase
GTGTCAGATTCACTACGTATTATTTTTGCGGGTACACCTGACTTTGCAGCGCGTCATCTC
GACGCGCTGTTAACTTCTGGACATAACGTTGTCGGCGTATTTACCCAACCCGATCGTCCA
GCAGGTCGCGGCAAAAAGTTGATGCCCAGCCCGGTAAAAGTGCTGGCGGAAGAAAAAGGC
CTGCCCGTTTTCCAGCCCGTGTCGCTTCGTCCTCAGGAAAACCAGCAACTGGTTGCTGAC
TTACATGCCGACGTCATGGTTGTCGTAGCATATGGTCTGATTCTCCCAAAGGCAGTGCTG
GATATGCCCCGTCTCGGATGTATCAACGTTCACGGTTCGCTGCTTCCACGCTGGCGCGGC
GCGGCGCCTATTCAGCGCTCACTCTGGGCAGGCGATGCGGAAACCGGCGTCACAATTATG
CAGATGGATGTAGGGCTGGATACCGGCGACATGCTGTATAAACTTGCGTGCCCGATTACC
GCAGAAGATACCAGTGGCTCACTGTATAATAAGTTGGCTGAGCTTGGCCCGCAGGGGTTA
ATTACCACACTAAAACAACTGGCGGACGGCACAGCGGCGCCTGAAGCGCAGAATGAAGCG
CTGGTCACTCATGCGGAAAAGCTCAGTAAAGAAGAAGCGCGTATCGACTGGTCTCTCTCC
GCAGTACAGCTGGAGCGTTGCATTCGCGCCTTTAATCCGTGGCCAATGAGCTGGCTGGAA
ATCGACGGCCAGCCGGTAAAAGTCTGGCAGGCGTCCGTCATTGAGGACGCCACGCAGTCG
CTGCCGGGAACCATTCTGGCCGCGACGAAACAGGGCATTCAGGTTGCAACAGGCAAGGGG
ATATTAAATTTGCTTTCGCTTCAGCCTGCCGGCAAAAAAGCAATGAGCGCCCAGGATCTG
TTAAATTCACGCCGGGAATGGTTTATTCCTGGTAACCGTCTTGCCTGA
>EECEBICD_00042 Peptide deformylase
ATGTCAGTTTTGCAAGTGTTACATATTCCGGACGAGCGCCTTCGCAAAGTCGCAAAGCCG
GTTGAAGAAGTGAATGCAGAAATTCAGCGTATCGTCGATGATATGTTCGAAACGATGTAC
GCGGAAGAAGGTATCGGTCTGGCCGCAACGCAGGTCGATATCCACCAGCGGATCATCGTG
ATTGACGTTTCGGAAAACCGCGACGAGCGCCTGGTACTCATTAACCCGGAATTGCTGGAG
AAAAGTGGCGAAACGGGTATAGAAGAAGGCTGTCTGTCGATTCCGGAACAGCGCGCTTTA

GTGCCGCGCGCCGAGAAAGTGAAAATCCGCGCGCTGGATCGCGACGGCAACCCGTTTGAG
CTTGAAGCGGATGGTTTGCTGGCAATTTGCATTCAGCATGAGATGGATCATCTGGTCGGT
AAACTGTTTATCGATTATTTGTCGCCGCTGAAGCAACAACGTATTCGTCAGAAAGTAGAA
AAACTCGACCGCCTGAACGCCCGGGCTTAA
>EECEBICD_00043 hypothetical protein
ATGGCCCGTACCGAAATTTGGTTACGTTTAATGTACGTCGGCGACCTTTATGGCGAGGCG
ATGTTGAACATGGCGAACTCGCTTATTCGTCAGCCTCAGATAAATCGCACGCACCTTCAG
GAGGCTGGTCTTACCGCGCGGCAAGCTGAACGTTTTTTACAGCTTCCAGCAGGTGTGCTT
GATGAGACGTTACGCTGGCTTGAACTACCGCAGCATCATTTTCTGTGTGCGGATAGTGAG
ATTTATCCTCCCCAACTGCGTGCTATTGATGATTATCCCGGCGCTATTTTTATTGACGGC
GATCCTGCCTGTCTGCATACCTGCCAACTTGCCGTCGTAGGGAGCCGGAGCCACTCCTGG
TATGGAGAACGCTGGGGACGTCTGCTTTGCGAAAGCCTTGCGAAAAGCGGTTTGACGATC
ACCAGCGGCCTTGCCCGGGGAATTGATGGCGTAGCACACAACGCCGCGGTGAGTATGGGG
GGAAAAAGTGTAGCGGTGTTAGGGAATGGCTTGGCAAAGATTTATCCTCGCCGACATGCC
ATGCTGGCTGAAAACTTGATTGCTACCGGCGGCGCAGTGGTCTCAGAATTTCCGCTTTCA
ACGCCTCCGCTACCGCAAAATTTTCCTCGCAGAAACCGTATCATCAGCGGACTGAGCAAA
GGCGTGCTGGTGATTGAGGCGGCGTTGCGCAGCGGTTCGTTGGTGACGGCGCGCTGCGCG
CTTGAACAGGGGCGGGACGTTTTTGCATTACCAGGTCCTATCGGTAGTCCGGGAAGCGAA
GGCACGCACTGGTTAATTAAACAAGGGGCCACGCTTGTGACGACGCCGGAGGATATTCTG
GAAAATTTGCAATACGGCTTACACTGGTTGCCAACTACGGCGGAAAATTCACTTTATTCA
CTAAATCAGGATGAGGCGGCATTGCCATTTCCTGAGCTCCTGGCTAACGTAGGAGATGAG
GTAACACCTGTTGACGTCGTCGCTGAACGTGCCGGCCAACCTGTGCCAGCGGTAGTGGCT
CAGCTACTCGAACTGGAGTTAGCAGGATGGATCGCAGCTGTACCCGGCGGCTATGTCCGA
TTAAGGAGGGCAAGCCATGTTCGACGTACTAATGTATTTGTTTGA
>EECEBICD_00044 Protein Smg
ATGTTCGACGTACTAATGTATTTGTTTGAAACATACATCCATAACGAAGCCGAGCTGCGC
GTGGATCAGGACAGACTGGAGCGTGATCTCACCGACGCTGGGTTTGATCGCGAAGACATC
TACAATGCGCTGCTGTGGCTGGAAAAACTCGCTGACTATCAGGATGGCCTCGCGGAACCC
ATGCAACTTGCTTCCGATCCTCTTTCAATGCGCATTTATACGGTTGAAGAGTGTGAAAGG
CTTGATGCCAGTTGCCGGGGGTTCCTGTTATTCCTTGAGCAGATTCAGGTGCTAAACCTC
GAAACACGAGAAATGGTGATTGAGCGTGTGCTGGCGCTGGATACCGCAGAGTTTGACCTG
GAAGACTTGAAATGGGTTATTTTGATGGTGTTATTCAACATTCCGGGCTGTGAAAACGCC
TATCAGCAAATGGAAGAATTACTCTTTGAGGTAAATGAAGGTATGCTGCATTGA
>EECEBICD_00045 DNA topoisomerase 1
ATGGCTAAATCAGCACTGTTTTCGGTGCGTAAAAATGAGCCCTGCCCGCAGTGCGGGGCC
GAACTGGTAATTCGCTCCGGGAAACATGGTCCGTTTCTCGGCTGTTCTCGCTATCCGGAA
TGCGATTACGTTCGTCCGCTGAAAAGTCAGGCGGACGGACATATCGTTAAAATTCTGGAG
GGGCAACCGTGCCCTGAGTGTGGCGCCGTGCTGGTGCTGCGTCAGGGGCGCTTTGGCATG
TTCATCGGATGCAGTCAGTATCCGCAATGTGAACATACAGTGGTTATTGATAAACCGGAT
GAAACGGCGATTGCCTGTCCAGCCTGCCAGCAAGGACATTTGGTCCAGCGCCGTTCTCGT
TACGGTAAAATCTTTCATTCCTGCGATCGCTACCCGGAATGTCAGTTCGTCATTAACTTC
ACACCGGTTGCGGGGGAATGCCCCGAGTGCCATTATCCGCTACTCATCGAAAAGAAAACC
GCGCAGGGCGTGAAGCGCTTTTGCGCCAGTAAACAATGTGGAAAGCCGGTTCCGGTGGAA
TAA
>EECEBICD_00046 Threonylcarbamoyl-AMP synthase
GTGAATAATAGCCTGCCAACAGGATCTATCGCCGCAGCGGTAGATCTCCTGAATAAAGAA
AATGTCATCGCTTATCCAACAGAAGCCGTTTTCGGCGTCGGCTGCGATCCCGATAGTGAA
ACAGCCGTTACGCGCCTGCTGGCGTTAAAACAACGGCCCGTCGATAAGGGACTCATTTTG
ATTGCCGCCAGCTTTGAGCAGTTAAAACCTTATATTGATGACAGCATACTTACCGCCGCA
CAGCGTAAAGCGGTATTTGATTGCTGGCCCGGGCCGGTAACGTTTGTTTTTCCTGCGCCC
GCGACAACGCCGCGGTGGCTCACGGGACGTTTTGATTCCCTGGCCGTTCGTGTTACCAAC
CACCCGCTGGTGGTCGCGCTATGCAATGCGTATGGCAAACCGCTGGTTTCCACCAGCGCC
AATCTGAGCGGATTACCGCCATGTCGTACCATTGAAGAGGTGCGTGCGCAATTTGGCGAT
GATTTTCCGGTGGTTGAAGGCGCAACGGGTGGGCGTTTAAATCCTTCAGAAATTCGCGAT

GCTCTGACGGGCGAACTGTTTCGACAGGGGTAA
>EECEBICD_00047 Shikimate dehydrogenase (NADP(+))
ATGATGGAAACCTATGCTGTTTTTGGAAATCCGATTGCGCACAGCAAATCGCCATTTATT
CATCAGCAATTTGCTCAGCAGCTAGATATTGTTCACCCCTATGGTCGCGTGCTGGCCCCT
ATTAATAATTTCATTAATACGCTTGATGCCTTTTTCGCGGCAGGGGGAAAAGGCGCAAAC
ATCACAGTACCTTTTAAAGAGGAGGCGTTTGCGCGTTCGGATGAGTTAACGGAACGAGCA
TCGCTGGCGGGAGCCGTCAATACATTAAAACGGCTGGAAGATGGTCGTTTGCTTGGCGAC
AATACTGACGGTATCGGTTTATTAAGCGATCTCGAACGTTTAAATTTTATCCGCCCAGGA
TTGCGTATTTTGCTGATTGGCGCGGGCGGCGCATCCCGGGGCGTGCTGTTACCCCTGCTT
TCTTTGGATTGCGCGGTCACTATCACTAACCGTACAGCTTCACGTGCCGAAGCGTTGGCG
AAAATCTTTGCTCATACCGGCAGCGTTCATGCCACGGATATGGACAAGCTGGATGGTTGT
GAGTTTGACCTGATTATTAATGCGACCTCCAGCGGCATACGGGGCGAAATCCCGGCGATT
CCGGCGTCACTTATTCACCCTTCCCTCTGTTGCTATGACATGTTCTATCAAAAAGGGAAT
ACGCCATTTCTCTCCTGGTGTGTACAACAGGGCGCAAAACGCTACGCAGATGGGCTGGGA
ATGCTGGTGGGGCAGGCTGCACATGCCGTTTTGCTTTGGCACGGTGTATTACCGCAGGTC
GAGCCAGTGATTGAGCTGCTACAGCAGGAATTATTAGCGTGA
>EECEBICD_00048 hypothetical protein
GTGAACCAGGCCATACAATTTCCTGACCGCGAAGAATGGGATACTGCCGCCAGTGCTATC
ATCTTTCCCGCCCTGGTTAATGGGATGCAACTCACGTGTGCAATAAAAAAAGATGTACTG
GCGTATCGCTTTGGTGGAGAAACAGCGGAGCAATGGCTGGCCATTTTTCGTGAGTATCGC
TGGGACCTGGAAGAAGAGGCGGAAGCATTAATCCTGGCCCAGCAGGAAGATGATCACGGT
TGGATATGGTTATCCTGA
>EECEBICD_00049 Protein YrdA
ATGTCTGACACATTACGTCCTTATAAGGATCTTTTTCCTGAAATCGGTCAGCGCGTGATG
ATCGATACCAGCAGCGTGGTTATTGGTGATGTCCGTCTGGCGGATGATATCGGGATCTGG
CCGCTTGTGGTGATTCGCGGCGATGTGAATTACGTTGCGATAGGGGCTCGTACGAACATT
CAGGATGGCAGCGTTCTTCACGTAACCCACAAGTCTTCATCCAATCCACACGGCAACCCA
TTAATCATTGGCGAAGATGTTACCGTTGGCCATAAAGTTATGCTGCACGGCTGTACCATC
GGTAACCGCGTACTGGTCGGTATGGGCTCCATTGTGTTGGATGGCGCGATAATAGAAGAT
GACGTAATGATTGGCGCGGGAAGCCTGGTGCCGCAGCACAAACGGCTGGAAAGCGGCTAT
TTATATCTTGGCAGTCCGGTAAAACAGATCCGCCCCTTGAGCGATGCTGAGAGATCGGGC
CTGCAGTACTCCGCCAATAACTATGTAAAGTGGAAAGACGACTATCTTTCTCAGGATAAC
CATATCCAACCGTGA
>EECEBICD_00050 16S ribosomal RNA
TTGAAGAGTTTGATCATGGCTCAGATTGAACGCTGGCGGCAGGCCTAACACATGCAAGTC
GAACGGTAACAGGAAGCAGCTTGCTGCTTCGCTGACGAGTGGCGGACGGGTGAGTAATGT
CTGGGAAACTGCCTGATGGAGGGGGATAACTACTGGAAACGGTGGCTAATACCGCATAAC
GTCGCAAGACCAAAGAGGGGGACCTTCGGGCCTCTTGCCATCAGATGTGCCCAGATGGGA
TTAGCTTGTTGGTGAGGTAACGGCTCACCAAGGCGACGATCCCTAGCTGGTCTGAGAGGA
TGACCAGCCACACTGGAACTGAGACACGGTCCAGACTCCTACGGGAGGCAGCAGTGGGGA
ATATTGCACAATGGGCGCAAGCCTGATGCAGCCATGCCGCGTGTATGAAGAAGGCCTTCG
GGTTGTAAAGTACTTTCAGCGGGGAGGAAGGTGTTGTGGTTAATAACCGCAGCAATTGAC
GTTACCCGCAGAAGAAGCACCGGCTAACTCCGTGCCAGCAGCCGCGGTAATACGGAGGGT
GCAAGCGTTAATCGGAATTACTGGGCGTAAAGCGCACGCAGGCGGTCTGTCAAGTCGGAT
GTGAAATCCCCGGGCTCAACCTGGGAACTGCATTCGAAACTGGCAGGCTTGAGTCTTGTA
GAGGGGGGTAGAATTCCAGGTGTAGCGGTGAAATGCGTAGAGATCTGGAGGAATACCGGT
GGCGAAGGCGGCCCCCTGGACAAAGACTGACGCTCAGGTGCGAAAGCGTGGGGAGCAAAC
AGGATTAGATACCCTGGTAGTCCACGCCGTAAACGATGTCTACTTGGAGGTTGTGCCCTT
GAGGCGTGGCTTCCGGAGCTAACGCGTTAAGTAGACCGCCTGGGGAGTACGGCCGCAAGG
TTAAAACTCAAATGAATTGACGGGGGCCCGCACAAGCGGTGGAGCATGTGGTTTAATTCG
ATGCAACGCGAAGAACCTTACCTGGTCTTGACATCCACAGAAGAATCCAGAGATGGATTT
GTGCCTTCGGGAACTGTGAGACAGGTGCTGCATGGCTGTCGTCAGCTCGTGTTGTGAAAT
GTTGGGTTAAGTCCCGCAACGAGCGCAACCCTTATCCTTTGTTGCCAGCGGTTAGGCCGG
GAACTCAAAGGAGACTGCCAGTGATAAACTGGAGGAAGGTGGGGATGACGTCAAGTCATC

ATGGCCCTTACGACCAGGGCTACACACGTGCTACAATGGCGCATACAAAGAGAAGCGACC
TCGCGAGAGCAAGCGGACCTCATAAAGTGCGTCGTAGTCCGGATTGGAGTCTGCAACTCG
ACTCCATGAAGTCGGAATCGCTAGTAATCGTGGATCAGAATGCCACGGTGAATACGTTCC
CGGGCCTTGTACACACCGCCCGTCACACCATGGGAGTGGGTTGCAAAAGAAGTAGGTAGC
TTAACCTTCGGGAGGGCGCTTACCACTTTGTGATTCATGACTGGGGTGAAGTCGTAACAA
GGTAACCGTAGGGGAACCTGCGGTTGGATCACCTCCTT
>EECEBICD_00051 tRNA-Glu(ttc)
GTCCCCTTCGTCTAGAGGCCCAGGACACCGCCCTTTCACGGCGGTAACAGGGGTTCGAAT
CCCCTAGGGGACGCCA
>EECEBICD_00052 23S ribosomal RNA
TTAAGCGACTAAGCGTACACGGTGGATGCCCTGGCAGTCAGAGGCGATGAAGGGCGTGCT
AATCTGCGATAAGCGCCGGTAAGGTGATATGAACCGTTATAACCGGCGATACCCGAATGG
GGAAACCCAGTGTGATTCGTCACACTATCATTAACTGAATCCATAGGTTAATGAGGCGAA
CCGGGGGAACTGAAACATCTAAGTACCCCGAGGAAAAGAAATCAACCGAGATTCCCCCAG
TAGCGGCGAGCGAACGGGGAGGAGCCCAGAGCCTGAATCAGCATGTGTGTTAGTGGAAGC
GTCTGGAAAGGCGCGCGATACAGGGTGACAGCCCCGTACACAAAAGCGCATGTGCTGTGA
GCTCGATGAGTAGGGCGGGACACGTGGTATCCTGTCTGAATATGGGGGGACCATCCTCCA
AGGCTAAATACTCCTGACTGACCGATAGTGAACCAGTACCGTGAGGGAAAGGCGAAAAGA
ACCCCGGCGAGGGGAGTGAAAAAGAACCTGAAACCGTGTACGTACAAGCAGTGGGAGCAC
AGGTTTACCTGTGTGACTGCGTACCTTTTGTATAATGGGTCAGCGACTTATATTCTGTAG
CAAGGTTAACCGAATAGGGGAGCCGGAGGGAAACCGAGTCTTAATTGGGCGTTAAGTTGC
AGGGTATAGACCCGAAACCCGGTGATCTAGCCATGGGCAGGTTGAAGGTTGGGTAACACT
AACTGGAGGACCGAACCGACTAATGTTGAAAAATTAGCGGATGACCTGTGGCTGGGGGTG
AAAGGCCAATCAAACCGGGAGATAGCTGGTTCTCCCCGAAAGCTATTTAGGTAGCGCCTC
GTGAATTCATCTCCGGGGGTAGAGCACTGTTTCGGCTAGGGGGCCATCCCGGCTTACCAA
CCCGATGCAAACTGCGAATACCGGAGAATGTTATCACGGGAGACACACGGCGGGTGCTAA
CGTCCGTCGTGAAGAGGGAAACAACCCAGACCGCCAGCTAAGGTCCCAAAGTCATGGTTA
AGTGGGAAACGATGTGGGAAGGCCCAGACAGCCAGGATGTTGGCTTAGAAGCAGCCATCA
TTTAAAGAAAGCGTAATAGCTCACTGGTCGAGTCGGCCTGCGCGGAAGATGTAACGGGGC
TAAACCATGCACCGAAGCTGCGGCAGCGACACTGTGTGTTGTTGGGTAGGGGAGCGTTCT
GTAAGCCTGTGAAGGTGTGTCGTGAGGCATGCTGGAGGTATCAGAAGTGCGAATGCTGAC
ATAAGTAACGATAAAGCGGGTGAAAAGCCCGCTCGCCGGAAGACCAAGGGTTCCTGTCCA
ACGTTAATCGGGGCAGGGTGAGTCGACCCCTAAGGCGAGGCCGAAAGGCGTAGTCGATGG
GAAACGGGTTAATATTCCCGTACTTGGTGTTACTGCGAAGGGGGGACGGAGAAGGCTATG
TTGGCCGGGCGACGGTTGTCCCGGTTTAAGCGTGTAGGTGTGTGTTCCAGGTAAATCCGG
TTCACTTTAACACTGAGGCGTGACGACGAGGCACTACGGTGCTGAAGCAACAAATGCCCT
GCTTCCAGGAAAAGCCTCTAAGCATCAGGTAACATCAAATCGTACCCCAAACCGACACAG
GTGGTCAGGTAGAGAATACCAAGGCGCTTGAGAGAACTCGGGTGAAGGAACTAGGCAAAA
TGGTGCCGTAACTTCGGGAGAAGGCACGCTGACACGTAGGTGAAGTGATTTACTCATGGA
GCTGAAGTCAGTCGAAGATACCAGCTGGCTGCAACTGTTTATTAAAAACACAGCACTGTG
CAAACACGAAAGTGGACGTATACGGTGTGACGCCTGCCCGGTGCCGGAAGGTTAATTGAT
GGGGTCAGCGCAAGCGAAGCTCCTGATCGAAGCCCCGGTAAACGGCGGCCGTAACTATAA
CGGTCCTAAGGTAGCGAAATTCCTTGTCGGGTAAGTTCCGACCTGCACGAATGGCGTAAT
GATGGCCAGGCTGTCTCCACCCGAGACTCAGTGAAATTGAACTCGCTGTGAAGATGCAGT
GTACCCGCGGCAAGACGGAAAGACCCCGTGAACCTTTACTATAGCTTGACACTGAACATT
GAGCCTTGATGTGTAGGATAGGTGGGAGGCTTTGAAGTGTGGACGCCAGTCTGCATGGAG
CCGACCTTGAAATACCACCCTTTAATGTTTGATGTTCTAACGTGGACCCGTTACCCGGGT
TGCGGACAGTGTCTGGTGGGTAGTTTGACTGGGGCGGTCTCCTCCTAAAGAGTAACGGAG
GAGCACGAAGGTTGGCTAATCCTGGTCGGACATCAGGAGGTTAGTGCAATGGCATAAGCC
AGCTTGACTGCGAGCGTGACGGCGCGAGCAGGTGCGAAAGCAGGTCATAGTGATCCGGTG
GTTCTGAATGGAAGGGCCATCGCTCAACGGATAAAAGGTACTCCGGGGATAACAGGCTGA
TACCGCCCAAGAGTTCATATCGACGGCGGTGTTTGGCACCTCGATGTCGGCTCATCACAT
CCTGGGGCTGAAGTAGGTCCCAAGGGTATGGCTGTTCGCCATTTAAAGTGGTACGCGAGC
TGGGTTTAGAACGTCGTGAGACAGTTCGGTCCCTATCTGCCGTGGGCGCTGGAGAACTGA

GGGGGGCTGCTCCTAGTACGAGAGGACCGGAGTGGACGCATCACTGGTGTTCGGGTTGTC
ATGCCAATGGCACTGCCCGGTAGCTAAATGCGGAAGAGATAAGTGCTGAAAGCATCTAAG
CACGAAACTTGCCCCGAGATGAGTTCTCCCTGAGACTTAGAGTCTCCTGAAGGAACGTTG
AAGACGACGACGTTGATAGGCCGGGTGTGTAAGCGCAGCGATGCGTTGAGCTAACCGGTA
CTAATGAACCGTGAGGCTTAACCT
>EECEBICD_00053 5S ribosomal RNA
TGGCGGCAGTAGCGCGGTGGTCCCACCTGACCCCATGCCGAACTCAGAAGTGAAACGCCG
TAGCGCCGATGGTAGTGTGGGGTCTCCCCATGCGAGAGTAGGGAACTGCCA
>EECEBICD_00054 tRNA-Thr(ggt)
GCTGATATGGCTCAGTTGGTAGAGCGCACCCTTGGTAAGGGTGAGGTCCCCAGTTCGACT
CTGGGTATCAGCACCA
>EECEBICD_00055 5S ribosomal RNA
TGGCGGCAGTAGCGCGGTGGTCCCACCTGACCCCATGCCGAACTCAGAAGTGAAACGCCG
TAGCGCCGATGGTAGTGTGGGGTCTCCCCATGCGAGAGTAGGGAACTGCCA
>EECEBICD_00056 hypothetical protein
ATGAAAAGACTAATTCCAGTTGCATTGCTTACCACCTTATTGGCAGGTTGTGCACATGAT
TCTCCCTGCGTACCGGTTTATGACGATCAAGGGCGGCTGGTTCATACCAATACATGTATG
AAAGGTACAACGCAGGATAACTGGGAGACAGCAGGCGCTATCGCTGGCGGCGCTGCGGCG
GTAGCAGGGTTGACGATGGGTATCATTGCGCTTTCTAAATGA
>EECEBICD_00057 Multidrug export protein AcrF
ATGGCAAACTTTTTTATTAGACGTCCTATTTTCGCCTGGGTTCTGGCCATTATCCTGATG
ATGGCTGGCGCACTGGCAATAATGCAACTTCCCGTTGCGCAGTATCCAACCATTGCGCCG
CCAGCGGTTTCTATTTCTGCAACCTATCCTGGCGCGGATGCGCAGACGGTACAGGATACG
GTTACTCAGGTTATCGAACAAAATATGAACGGTATCGATAACCTGATGTATATGTCCTCT
ACCAGCGACTCTGCTGGTAGCGTGACCATCACCCTGACCTTCCAGTCCGGAACCGATCCG
GATATCGCGCAGGTTCAGGTGCAAAATAAATTGCAGCTCGCCACGCCTTTACTGCCGCAA
GAAGTCCAGCAGCAGGGGATTAGCGTTGAAAAATCCAGCAGCAGCTTTTTGATGGTCGCC
GGGTTCGTCTCAGATAATCCGAACACTACCCAGGACGACATCTCTGACTATGTCGCCTCT
AACATTAAGGATTCTATCAGCCGTCTGAATGGTGTGGGCGACGTTCAGCTATTTGGCGCA
CAGTACGCCATGCGTATCTGGCTGGATGCGAATCTGCTAAATAAATACCAGCTCACGCCA
GTTGACGTCATCAACCAGTTAAAAGTACAGAACGACCAGATTGCGGCAGGCCAACTGGGC
GGCACGCCAGCATTACCGGGCCAGCAGCTTAACGCCTCAATCATCGCCCAAACGCGTCTG
AAAGATCCGGAAGAGTTCGGCAAAGTTACGTTGCGCGTCAATACCGACGGCTCTGTCGTC
CATCTCAAAGATGTCGCGCGTATTGAGCTTGGCGGTGAAAACTATAACGTTGTAGCGCGC
ATTAACGGTAAACCGGCCTCCGGTCTCGGTATTAAACTGGCGACCGGCGCTAACGCGCTG
GATACCGCAACCGCAATCAAAGCGAAACTGGCGGAGCTGCAGCCTTTCTTCCCCCAGGGA
ATGAAGGTGGTTTATCCTTATGACACAACGCCCTTCGTAAAAATATCTATCCACGAAGTG
GTAAAAACGCTGTTTGAAGCGATTATTCTGGTGTTCCTGGTAATGTATCTGTTCTTACAG
AATATCCGGGCAACCCTGATTCCTACCATCGCTGTTCCTGTCGTGTTGCTAGGCACTTTT
GCGGTACTCGCCGCCTTTGGCTATTCCATCAATACCCTGACGATGTTCGGCATGGTACTG
GCGATAGGGCTGTTGGTTGACGATGCGATAGTGGTCGTAGAAAACGTTGAACGTGTAATG
ATGGAGGATAACCTTCCTCCCCGAGAGGCGACGGAAAAATCCATGTCGCAGATTCAGGGA
GCGCTGGTTGGTATCGCGATGGTACTGTCTGCGGTATTTATCCCGATGGCCTTTTTTGGC
GGCTCGACCGGGGCAATTTATCGCCAGTTCTCTATTACTATTGTTTCAGCAATGGCGCTA
TCCGTTCTGGTTGCGTTGATTCTGACGCCAGCGCTGTGCGCTACGCTGCTTAAACCCGTA
TCTGCTGAACATCACGAGAAAAAAAGCGGCTTCTTTGGCTGGTTCAATACCAGGTTTGAC
CACAGCGTTAACCACTATACTAACAGCGTAAGCGGCATCGTGCGTAATACGGGTCGCTAT
CTCATTATCTATCTACTTATTGTAGTCGGAATGGCGGTTCTGTTTTTACGCCTCCCGACC
TCCTTCCTGCCGGAAGAAGATCAGGGGGTATTCCTGACCATGATTCAGCTCCCCTCTGGC
GCTACGCAAGAACGTACGCAGAAAGTGCTGGATCAAGTCACTCATTACTACCTGAATAAT
GAAAAAGCGAACGTCGAAAGCGTGTTTACCGTAAACGGCTTTAGCTTTAGCGGTCAGGGA
CAAAACTCAGGGATGGCATTTGTCAGCCTTAAACCCTGGGAAGAGCGTAACGGTGAAGAA
AATAGCGTCGAAGCCGTTATCGCCAGAGCGACACGCGCCTTCAGCCAGATTCGCGACGGG
TTGGTGTTCCCCTTCAACATGCCAGCAATTGTCGAGTTAGGTACCGCGACAGGTTTCGAC

TTTGAACTGATTGATCAGGGAGGACTCGGTCACGATGCGTTAACAAAAGCGCGTAATCAA
CTCCTGGGTATGGTCGCGAAGCATCCTGACCTATTAGTGCGCGTACGCCCGAACGGGCTG
GAAGACACGCCACAGTTCAAGCTGGATGTCGATCAAGAAAAAGCGCAGGCGCTCGGTGTT
TCGCTGTCTGATATCAACGAAACCATCTCCGCGGCGTTGGGCGGCTATTACGTTAACGAC
TTTATCGATCGCGGACGAGTGAAAAAAGTATACGTTCAGGCTGACGCTCAGTTCCGTATG
CTGCCGGGAGATATCAACAATCTTTATGTACGCAGCGCCAATGGCGAGATGGTGCCCTTC
TCTACCTTTAGCTCAGCACGGTGGATTTATGGTTCGCCACGCCTGGAACGCTATAACGGG
ATGCCGTCAATGGAACTGCTCGGTGAAGCAGCGCCCGGACGAAGTACCGGTGAAGCCATG
TCGTTAATGGAAAACCTGGCTTCACAGCTGCCAAACGGTATTGGCTATGACTGGACAGGT
ATGTCGTATCAGGAACGGTTGTCAGGTAACCAGGCGCCGGCGCTGTACGCAATCTCACTC
ATTGTCGTTTTCCTCTGCCTTGCCGCTCTGTATGAAAGCTGGTCAATTCCGTTCTCGGTA
ATGCTGGTCGTACCGCTCGGCGTGGTTGGCGCTCTGCTTGCAGCGTCATTGCGCGGTCTG
AACAATGACGTTTACTTCCAGGTTGGCTTGTTAACCACTATTGGCCTTTCTGCTAAAAAC
GCCATCCTGATTGTCGAGTTCGCCAAAGATCTCATGGAAAAAGAAGGACGTGGATTGATT
GAAGCGACGCTGGAAGCATCCCGTATGCGTTTACGTCCTATTCTAATGACCTCGCTGGCC
TTTATTCTCGGGGTAATGCCGTTAGTTATCAGTCGTGGCGCAGGTAGTGGGGCACAGAAC
GCAGTAGGCACAGGGGTTATGGGGGGAATGTTAACCGCAACCTTATTAGCTATCTTCTTC
GTGCCAGTATTCTTCGTTGTTGTAAAACGCCGATTTAATCGCCATCATGATTAA
>EECEBICD_00058 Multidrug export protein AcrE
ATGACGAAACATGCCAGGTTTTCACTCCTGCCCTCATTCATCATATTCTCTGCTGCGCTG
CTGGCCGGTTGTAATGACCAGGGAGATACCCAGGCTCATGCCGGCGAGCCGCAAGTCACC
GTCCATGTGGTCGAAACAGCGCCGCTAGCCGTAACGACCGAACTTCCCGGACGTACGTCC
GCATTTCGCATTGCGGAGGTTCGCCCCCAGGTGAGCGGGATCGTGCTTAAAAGAAACTTC
ACCGAAGGTAGCGATGTAGAGGCCGGGCAGTCGCTCTATCAGATCGATCCTGCCACTTAT
CAGGCTGATTATGACAGCGCTAAAGGCGAACTTGCTAAAAGCGAAGCGGCTGCGGCTATC
GCGCACCTGACGGTCAAACGCTATGTTCCACTGGTCGGCACAAAATATATCAGCCAACAG
GAATATGATCAGGCGATTGCCGACGCCCGCCAGGCCGATGCCGCCGTTGTGGCGGCAAAA
GCCGCTGTTGAAAGCGCGCGTATTAACCTTGCGTATACCAAAGTCACCTCACCCATCAGC
GGGCGTATAGGAAAATCTAATGTGACTGAAGGCGCGCTGGTGACTAATGGTCAGTCAACT
GAACTGGCTACCGTGCAACAACTCGATCCGATTTATGTCGACGTGACGCAATCAAGCAAC
GACTTTATGCGACTCAAGCAATCCGTCGAACAAGGTAACCTGCATAAAGACAGCGCCAGT
AGCACGGTTCAACTGGTAATGGAAAATGGTCAGGTCTACCCGATTAAAGGCACGCTGCAA
TTTTCCGACGTTACCGTAGATGAAAGCACCGGCTCTATCACGCTCAGGGCGGTGTTCCCT
AACCCGCAACACAGTCTGCTTCCCGGTATGTTTGTTCGCGCCCGCATTGATGAAGGCGTC
CAGCCCAATGCCATCCTTGTCCCCCAGCAGGGCGTAACCCGCACGCCGCGCGGCGACGCA
ATGGTGATGGTGGTTAACGATAAAAGCCAGGTCGAAGCCCGCAATGTCGTGGCGGCGCAG
GCTATTGGCGATAAATGGCTCATCAGCGAAGGGTTAAAACCGGGCGATAAGGTCATCGTC
AGCGGCTTACAAAAAGCGCGACCGGGCGTCCAGGTGAAAGCCACTACCGATGCTCCTGCA
GCGAAAACGGCGCAATAA
>EECEBICD_00059 HTH-type transcriptional regulator AcrR
ATGGCGAAGAAAACGAAGGCGGATGCGCTTAAAACGCGGCAACATTTGATTGAAACCGCT
ATTGCGCAGTTCGCTTTGCGCGGCGTTGCCAACACGACGTTGAATGATATCGCTGATGCC
GCTGATGTCACGCGGGGCGCCATCTACTGGCACTTTGAGAATAAAACGCAGTTGTTTAAT
GAGGTTTGGTTACAGCAACCACCTTTACGCGAACTTATTCAGGATAGGCTTACGGGATGC
TGGAATGATAATCCTTTACAGGATCTACGAGAGAAATTTATTGCAGCCTTACAATATATT
GCGGCAGTGCCTCGTCAGCAGGCATTAATGCAGATTTTATATCATAAATGTGAATTCCAT
AATGGTATGATATCGGAACAGGCCATTCGGGAGAAGATGGGTTTCCATCATCAGTCGCTG
CTTGAAGTATTACAGCGATGTATGGATAAAAAACTGATCTCAGGCAGTCTTGATCTGGAT
GTCATATTAATTATTCTGCATGGTAGTTTTAGCGGAATAGTAAAAAACTGGTTAATGAAT
CCCACAAGTTATGATCTTTATAAGCAGGCGCCTGCATTAGTCGATAATGTATTAAAAATG
CTAAGCCCGGATGGCAGTGTGAGGCAATTAATGCCAAATGAACAACAGGCGGAAGAAGCC
TGA
>EECEBICD_00060 putative signaling protein
ATGCCGGTTAGTGAGTACAACCACATCCTTGTGGCGGTTTCATTTGCTGTCGCTATTTTT

GCGTCCTATACCGCATTAAATATGGCCGGACGCGTAGCAGGAAGCGCCCGATCAAACGCC
AGAATATGGCTAATGGGCGGCGGCTTCGCGCTGGGCGTCGGTATTTGGGCGATGCATTTT
GTCGGGATGCTGGCGATGGATCACGCGATGAACATGCGCTTCGATCCGTTCCTCACCGGC
CTCTCCATGCTGATCGCGATCGGCTCCTCCCTGTTTGCGCTATGGCTGGTCAGCGCTGAA
AAACTGCGCCTGCGCCGTTTACTGCCGGGCGCGCTGGTGATGGGACTGGGCATCAGCGCA
ATGCATTATACCGGGATGGCGGCGCTGCAATTTGCTTCCATTATCGTCTGGAATAGCGCG
TGGGTTGCCCTCTCTATCATTATCGCGCTACTGGCGTCCTGCGGCGCGCTGTGGCTCACG
TTCCGCCTGCGTCATGAGGGAACAGATGTCGCCCTGAGGCGAGCAGGCGCCGCGGTATTA
ATGGGGATAGCTATCGCCGGCATGCATTACGCCGGCATGAAGGCCGCACACTTTCCCCAA
AACTGGCCGATGGAGCATCGTGGCGTCGATAGCAACTGGCTGGCTGTTCTTGTCAGCGTG
GTCGCGCTGACTATCCTGGGTATTACTCTGCTGGTTTCTTTATTTGACGCCCGGCTTCAG
GCCCGTACCGCACTACTTGCCTCTTCGCTGGCGCAGGCGAACCAGGAACTGGCGCAGTTG
GCGCTGCATGATACGCTCACCCGTTTACCAAACCGGGTGCTTCTTGAGGATCGTCTGGAA
CAGGCCATCAGCAAAGCGAATCGGGAAAACACGTCTTTCGCGCTACTGTTTATGGATCTG
GATGGCTTTAAAACCGTTAATGACGCTTATGGTCATGATATCGGCGATAAGCTACTGGTA
GCGGTCACGCATCGTCTGAACCAACCGCTGAGCGGGCAGTTTACCCTGGCGCGGATTGGC
GGCGACGAATTTGTGCTGCTTGCAGAAGTCAGCGCGCCTGACGAAGCGGCTTCCCTTGCC
AGCGCGCTGGTACATTCGATAGATGCGCCCTTTACAATAGATCCGTACGAACTGGTCGTC
ACCCTTAGCGTGGGAATAGCGCTCTATCCGCTGGACGGAAAAAACGAACGGGAACTGATG
TTCAACGCCGATGCCGCAATGTACCACACGAAACATACGGGGCGTAACGGATACCATTTC
TTTCAGCCATCAATGAATATGCTGGCGCAAACCCAACTTCAGCTAATGAATGACCTGTGG
CTGGCGCTGGAAAGACAAGAGCTCAGGTTAGTGTATCAGCCTAAATTTCAGGCTTCTGCC
GGCCCTATTGTAGGATTTGAGGCCCTGTTGCGCTGGTATCATCCAAAACAAGGCGTACTG
AATCCTGACCAGTTTTTACCGCTGGCGGAAAAAACGGGTCTTATCGTCACTATCGGTAGC
TGGGTGATAGACGAGGCGTGTCGACAGCTCCGCGAGTGGCACTTACAGGGATACGACTTA
TGGTCAGTGGCGGTAAATCTGTCCGCCTTACAATTTGAGCAGCCCGGGCTTGTGGACACC
ATTACGCGGAGCCTGGCCCGACACAGTATCCGTCCCGATTTATTGATTCTGGAAATCACC
GAAACCACGGCGATGAACAAGCCAGAGCAAAGCGTCGCTATACTCACCAGATTAACGGAA
ATGGGGGTTAAGGCCTCTATTGACGACTTTGGCACCGGTTATTCCAGCCTTCTCTATCTG
AAACGTCTTCCGGCGTGCGAACTAAAAATTGATCGCGCTTTCGTACATGAACTCAGCGAG
GCAGGCGACGGCGCGACCATTGTTGCGGCTATTGTCGCGCTGGCGAAAGCGTTAAATCTG
CAAATTGTGGCAGAAGGGGTAGAGAATGAAACGCAGCAACAATTTTTGACCCAGTTAGGA
TGCCATACGCTTCAGGGGTTCCTGCTTGGCAAGCCGCGTACGGCAGAGGAAATTGCTCGC
GATATTCGGGATCCTGCCAATATATTCACAAGGTCAATATATAATATCAACTCAAAATAA
>EECEBICD_00061 hypothetical protein
ATGATACGTAAGTATTGGTGGCTGGTTGTTTTTGCCGTCTCCGTTTTTCTTTTTGATGCG
CTTTTGATGCAATGGATCGAATTGATGGCAACTGAACAGGATAAGTGCCGAAACATGGAT
TCGGTTAACCCTCTCAAATTGATCAATTGTACCGATCTGGAGTAG
>EECEBICD_00062 DNA adenine methyltransferase YhdJ
ATGAAAGCGGAATGTGAACCTCAGTACTTTGGCGATGAATCGAAGAAGATTATTCACGGT
GACGCGCTAACAGAACTTAAAAAACTGCCTTCTGAAAGCATTGACTTAATTTTTGCCGAT
CCGCCTTACAATATCGGAAAAGATTTCGACGGTATGGTGGAGTCCTGGGACGAAGCGTCT
TTTCTGGCCTGGCTGTATGAATGCATTGATGAGTGCCACCGCGTACTGAAGAAACACGGC
ACCATGTACATCATGAATAGCACAGAAAATATGCCGTATATCGATCTCAAATGCCGAACG
CTTTTTACCATCAAAAGCCGTATCGTCTGGTCCTATGATAGTTCCGGGGTGCAGGCGAAA
AAATACTTTGGTTCTATGTATGAACCGATCCTGATGATGGTAAAAAACCCGAAAAGCTAT
ACCTTTAACCGTGATGCGATTCTGGTCGAAACCACTACGGGCGCTAAGCGCGCGCTAATC
GATTATCGAAAAAACCCGCCCCAACCATACAATCAGAAAAAAGTGCCGGGCAACGTCTGG
TCATTTCCTCGCGTACGTTATCTGATGGATGAATATGAAAACCATCCTACGCAAAAGCCC
AGCGCGCTGTTAAAACGGATCATACTGGCCTCTTCTAACCCGAGCGATACCGTATTGGAC
CCTTTCGCGGGCAGTTTCACCACAGGCGCTGTCGCAGCGGCGTCAGGTCGTAAGTTTATC
GGTATTGAACTCAATAATGAGTACGTAAAAATGGGGCTCAGAAGATTGAGCGTCACTTCG
CATTATTCAGAAAATGAACTTGCAAAAGTCAAAAAGAGAAAAACACAAAACCTGTCCAAA
AAGCAGCGAAATGTCGGGATAAACGCGCTATCTTCAGAGAAGTAA

```
>EECEBICD_00063 DNA-binding protein Fis
ATGTTCGAACAACGCGTAAATTCTGACGTACTGACCGTTTCTACCGTTAACTCTCAGGAT
CAGGTAACCCAAAAACCCCTGCGTGACTCGGTTAAACAGGCACTGAAGAACTATTTTGCT
CAACTGAATGGTCAGGACGTTAATGACCTGTATGAGCTGGTACTGGCTGAAGTAGAACAG
CCCCTGTTGGACATGGTGATGCAATACACCCGTGGTAACCAGACCCGTGCTGCTCTGATG
ATGGGCATCAACCGTGGTACGCTGCGTAAAAAATTAAAAAAATACGGCATGAACTAA
>EECEBICD_00064 tRNA-dihydrouridine synthase B
ATGCGCATCGGACAATATCAGCTCAGAAATCGCCTGATCGCAGCGCCCATGGCTGGCATT
ACTGACAGACCTTTCCGGACGCTGTGCTATGAGATGGGAGCAGGATTGACAGTATCCGAG
ATGATGTCTTCTAACCCGCAAGTGTGGGAAAGCGATAAATCTCGTTTACGGATGGTGCAC
GTTGATGAGCCAGGAATTCGCACGGTGCAAATTGCCGGTAGCGACCCTGTTGAGATGGCC
GATGCCGCACGTATTAACGTGGAAAGCGGCGCCCAGATTATTGATATCAATATGGGGTGC
CCGGCGAAGAAAGTGAATCGTAAGCTGGCAGGTTCAGCCCTCTTGCAGTACCCGGATTTA
GTGAAGTCTATACTCATCGGGGTCGTAAACGCAGTGGACGTTCCTGTGACTCTCAAGATT
CGCACCGGCTGGGCGCCGGAACACCGTAACTGCGTAGAGATTGCCCAACTGGCTGAAGAT
TGTGGTATTCAGGCTCTGACCATCCATGGCCGCACCCGCGCCTGTTTGTTCAACGGAGAA
GCTGAATACGACAGTATTCGGGCAGTTAAGCAGAAAGTTTCCATTCCGATTATCGCGAAT
GGCGACATTACTAACCCGCATAAAGCCAGAGCTGTTCTCGACTATACAGGGGCGGATGCC
CTGATGATAGGCCGCGCAGCTCAGGGAAGACCCTGGATCTTTCGGGAAATCCAGCATTAT
CTGGACACTGGGGAGCTGCTTCCGCCGCTGCCGCTGGCAGAGGTAAAGCGCTTGCTTTGC
ACGCATGTTCGGGAACTGCATGACTTTTACGGTCAGGCAAAAGGGTACCGAATCGCGCGT
AAACACGTCTCCTGGTATCTCCAGGAGCACGCTCCAGACGACCAGTTTCGGCGCACATTC
AACGCTATTGAGGACGCCAGCGAACAGCTGGAGGCGTTGGAGGCATACTTCGAAAATTTT
GCGTAA
>EECEBICD_00065 hypothetical protein
ATGCTGCAGAACAACCCGGACAGCATTACTAAGTACGAGGGCGATGGCTGCGACCCGTTA
AAACGCCAGTATCTTTTGTCCGCTGGTAGCAGCTGTTTATCCAGACGGATAGTGACAAAG
AAGTTATTTCAGGCATTCATTTGA
>EECEBICD_00066 Ribosomal protein L11 methyltransferase
ATGCCATGGATCCAATTGAAACTGAATACCACCGGCGCGAATGCTGAAGAGCTGAGTGAT
GCGCTGATGGAAGCGGGCGCCGTTTCCATTACCTTTCAGGATACGCACGATACGCCGGTT
TTTGAGCCGTTGCCGGGCGAAACCCGTCTGTGGGGCGATACCGATGTGATCGGTCTGTTC
GACGCCGAAACCGACATGAAAGACGTTGTCGCGATTCTGGAGCAACATCCGCTGCTGGGT
GCCGGATTTGCCCATAAAATCGAACAACTGGAAGATAAAGACTGGGAACGCGAATGGATG
GATAATTTCCATCCGATGCGTTTTGGCGAGCGTCTGTGGATTTGTCCCAGCTGGCGTGAC
ATTCCGGACGAGAATGCCGTCAACGTGATGCTCGACCCGGGACTGGCGTTCGGTACGGGT
ACGCACCCCACCACCTCTTTATGTCTGCAATGGCTGGACGGGCTCGATCTCAACGGCAAA
ACCGTGATCGACTTTGGCTGCGGTTCAGGCATTCTGGCGATTGCCGCGCTGAAACTGGGA
GCGGCAAAGGCGATTGGTATTGATATCGATCCGCAAGCTATTCAGGCCAGTCGCGATAAT
GCTGAACGCAACGGCGTGTCTGACCGGCTGGAGCTGTACCTGCCGAAAGACCAGCCAGAA
GCCATGAAAGCCGACGTGGTCGTCGCTAACATCCTGGCAGGCCCATTACGCGAACTGGCC
CCGTTAATCAGCGTACTGCCCGTTGAAGGCGGTCTGCTGGGGCTTTCAGGTATTCTCGCC
AGCCAGGCGGAAAGCGTCTGCGATGCTTACGCCGAGCTTTTCACTCTCGACCCGGTCGTC
GAAAAAGAAGAATGGTGCCGCATTACTGGCCGTAAGAAATAA
>EECEBICD_00067 Sodium/pantothenate symporter
ATGCTATTCGCGCGTTACCAAAGCCGCCTGCTGGTGTGGTTGGCAAGCCTGAGTTTATTG
GTCGCGTTTATTGGCGCAATGACCGTACAATTTATCGGCGGCGCGCGACTGCTGGAAACC
GCGGCGGGAATTCCCTACGAAACCGGCCTGCTAATATTTGGCGTCAGTATCGCACTCTAT
ACGGCGTTTGGCGGCTTTCGCGCCAGCGTACTTAACGATACGCTACAGGGCCTGGTGATG
CTGGTCGGCACGATAGTGCTGCTGGTGGGCGTGATCCATGCGGCGGGCGGTCTAAGTCAG
GCCGTTGACACGCTGCACGCTCTCGACCCCAAACTGGTAACGCCGCAGGGCGCCGATGAC
ATACTTTCTCCCGCCTTTATGACCTCTTTTTGGGTACTGGTCTGTTTTGGTGTGATTGGC
CTGCCGCATACGGCGGTGCGCTGCATCTCCTATAAAGACAGTAAAGCGGTACACCGGGGC
ATTATTATCGGTACGATTGTCGTGGCGATTTTAATGTTCGGGATGCACCTTGCCGGCGCG
```

CTGGGTCGCGCGGTGCTTCCGGATCTGACGGTGCCGGATCTGGTGATCCCAACGCTAATG
GTGAAAGTTTTGCCGCCGTTTGCCGCCGGGATCTTCCTCGCTGCGCCAATGGCGGCGATC
ATGTCAACAATTAACGCCCAACTGCTGCAAAGTTCCGCGACGATCATTAAAGATCTCTAT
CTCAACCTGCGCCCCGATCAGATGCAAAATGAGATCCGGCTGAAGCGTATGTCGGCAGCG
ATTACATTACTGCTAGGCGCGCTGTTGCTGCTGGCCGCATGGAAACCGCCAGAGATGATC
ATTTGGCTCAACCTGCTGGCGTTTGGCGGCCTGGAAGCGGTGTTCTTATGGCCGCTGGTA
TTGGGCCTTTACTGGGAGCGGGCAAACGCGGCAGGCGCATTAAGCGCCATGATCGTGGGC
GGCGTGCTATATGCCCTGCTTGCCACATTTAATATTCAGTACCTGGGCTTTCATCCGATT
GTGCCCGCGTTACTGTTAAGTTTATTGGCTTTTCTGATCGGAAACCGATTCGGCTCTTCC
GCTTCGCAAGCCACCGTATTGTCTACTGATAAATAA
>EECEBICD_00068 hypothetical protein
ATGCAGCTTGAGGTCATTCTACCGCTGGTAGCCTATCTGGTCGTGGTCTTCGGGGTCTCG
ATTTACGCTATGCGCAAGCGTACCGCCGGCACTTTTTTAAACGAATATTTTCTTGGCAGC
CGTTCGATGGGCGGGATCGTGCTCGCGATGACGCTTACCGCCACCTATATTAGCGCCAGT
TCATTTATCGGCGGTCCCGGCGCGGCTTATAAATACGGTCTGGGCTGGGTCTTGCTGGCG
ATGATCCAGCTTCCCGCCGTCTGGCTTTCACTGGGTATTCTGGGTAAAAATTCGCTATCC
TGGCGCGTCGCTATAATGCCGTCACGCTTAACGATATGCTATTCGCGCGTTACCAAAGCC
GCCTGCTGGTGTGGTTGGCAAGCCTGA
>EECEBICD_00069 putative membrane protein YhdT
ATGGACGCTCGTTTTGTTCAGGCCCATAAAGAGGCGCGCTGGGCGCTGTGGCTGACCCTT
TGCTATCTGGCGGCATGGTTAGTGGCTGCTTACTTACCTGGAGACTCGCCGGGAATTACC
GGTCTGCCGCACTGGTTCGAGATGGCCTGCTTATTGACGCCGCTGGTCTTTATTTTGCTG
TGCTGGGCGATGGTGAAATTCATTTATCGCGATATTTCGCTGGAGGATGACGATGCAGCT
TGA
>EECEBICD_00070 Biotin carboxylase
ATGTTGGATAAAATTGTCATCGCCAACCGCGGCGAGATCGCACTACGTATTCTTCGAGCC
TGTAAAGAACTCGGCATCAAGACCGTCGCTGTGCACTCAAGCGCGGATCGCGATTTAAAA
CACGTATTGCTGGCGGATGAGACGGTCTGTATTGGCCCGGCACCGTCCGTAAAAAGTTAT
CTGAACATCCCGGCTATCATTAGCGCCGCTGAAATCACCGGCGCGGTGGCAATCCACCCG
GGTTACGGCTTCCTTTCTGAGAACGCCAATTTTGCCGAGCAGGTTGAACGCTCCGGCTTT
ATCTTTATCGGCCCGAAAGCGGACACCATCCGCCTGATGGGCGATAAAGTGTCCGCGATT
ACCGCGATGAAAAAAGCGGGCGTGCCGACCGTACCAGGATCTGACGGCCCGCTGGGCGAC
GATATGAATGCGAACCGCGCTCATGCCAAACGTATTGGCTATCCGGTGATCATCAAAGCG
TCCGGCGGCGGCGGCGGCCGCGGTATGCGCGTGGTTCGTAGCGATGCTGAACTGGCGCAG
TCCATCTCCATGACCAAAGCGGAAGCGAAAGCGGCTTTCAGCAACGACATGGTATACATG
GAAAAATACCTGGAAAATCCACGCCACATCGAAATTCAGGTGCTGGCGGACGGCCAGGGC
AACGCTATCTATCTGGCGGAACGCGACTGTTCCATGCAGCGTCGCCACCAGAAAGTGGTT
GAAGAAGCCCCGGCGCCAGGCATTACGCCGGAACTGCGTCGCTATATCGGCGAACGCTGC
GCGAAAGCGTGCGTAGACATCGGCTATCGCGGCGCAGGGACGTTCGAATTCCTGTTCGAA
AACGGCGAGTTCTATTTCATCGAAATGAACACCCGTATTCAGGTTGAACACCCGGTGACT
GAAATGATTACCGGCGTCGATTTGATCAAAGAGCAGTTGCGCATCGCGGCGGGTCAGCCG
CTGTCGATCACACAGGACGAAGTTGTCGTTCGAGGCCATGCGGTAGAATGCCGTATCAAT
GCCGAAGATCCGAACACCTTCCTGCCAAGCCCAGGCAAAATCACGCGCTTCCATGCGCCT
GGCGGCTTTGGCGTTCGCTGGGAATCTCATATCTACGCGGGCTACACGGTGCCGCCGTAC
TATGATTCCATGATCGGCAAACTCATCTGCTACGGTGAAAACCGCGACGTGGCGATTGCC
CGTATGAAAAATGCCCTGCAGGAACTGATTATCGATGGTATCAAAACCAATATCGATCTG
CAGACCCGCATCATGAATGACGAGCACTTCCAGCACGGTGGAACCAACATCCACTATCTG
GAGAAAAAACTCGGTCTTCAGGAAAAGTAA
>EECEBICD_00071 Biotin carboxyl carrier protein of acetyl-CoA carboxylase
ATGGATATTCGTAAGATTAAAAAACTGATCGAGCTGGTTGAAGAATCAGGTATCTCCGAA
CTGGAAATTTCTGAAGGCGAAGAATCTGTTCGCATCAGCCGCACAACGGCAAACGCCGGT
TTTCCGGTGATGCAGCAGGCTTATGCGGCGCCGATGATGCAGCAACCCGCTCTGTCTAAC
GCTGTCGCGCCAGCGGCTACTCCAGCAATGGAAGCGCCTGCCGCAGCGGAAATCAGTGGT
CACATCGTACGCTCCCCAATGGTTGGTACTTTCTACCGCACCCCAAGCCCGGACGCGAAA

GCGTTCATCGAAGTGGGCCAGAAAGTGAACGTGGGCGATACCCTGTGCATCGTTGAAGCC
ATGAAAATGATGAACCAGATCGAAGCTGACAAAGCAGGTACTGTGAAAGCGATTCTGGTC
GAAAGTGGTCAACCGGTAGAATTTGACGAGCCACTGGTCGTCATCGAGTAA
>EECEBICD_00072 Protein-methionine-sulfoxide reductase heme-binding subunit MsrQ
GTGCGTCTGACAGCAAAACAGATAACCTGGCTAAAAGTTTGTCTGCATCTTGCCGGTTTT
TTGCCGCTTCTGTGGCTCTTTTGGGCGATAAATCACGGTGGACTCAGCGCAGACCCGGTA
AAGGATATCCAACACTTTACCGGCAGAACCGCGCTCAAATTTTTGCTCGCCACCTTGCTG
GTTTCACCGCTGGCGCGCTACGCTAAACAGCCATTATTGATACGCACACGCCGCCTGTTG
GGGTTATGGTGTTTCGTTTGGGCTACGCTGCATTTAACCAGCTATGCCTTACTGGAACTG
GGGATACATAACCTGGCGTTGTTAGGAAGCGAGTTGATTTCACGCCCTTATTTGACGCTT
GGGATCATTAGCTGGCTGGTGTTATTGGCGCTGACGTTGACCTCAACGCAATTTGCGCAG
CGTAAATTGGGCAAACGCTGGCAAACACTGCATAACGTCGTCTATCTTGTCGCGATCCTG
GCTCCCATACATTACCTGTGGTCGGTAAAGATTTTATCACCGCAACCGGTCATCTACGCG
GCATTAGCGTTAGCCCTTTTAGCGTTGCGTTACCGGAGGTTTCGCCAGTGGTGGCGCTAG
>EECEBICD_00073 Protein-methionine-sulfoxide reductase catalytic subunit MsrP
ATGAAAAAGATACGTCCATTAACAGAAGCCGATGTGACTGCGGAATCGGCTTTTTTTATG
CAGCGCCGACAGGTGCTAAAAGCATTAGGCATCAGCGCGGCCGCCTTATCCTTACCCTCA
ACGGCGCAGGCCGATCTCTTCAGTTGGTTTAAAGGCAACGATCGTCCGAAAGCGCCTGCC
GGTAAACCGCTTGAGTTTAGTCAGCCTGCCGCCTGGCGAAGCGATTTAGCGTTAACGCCG
GAAGATAAGGTGACGGGCTACAACAATTTCTATGAGTTTGGCCTTGATAAAGCCGACCCG
GCGGCCAATGCCGGAAGTCTGAAAACCGAACCGTGGACGTTGAAAATCAGCGGGGAAGTC
GCGAAGCCATTTACGCTGGATTATGACGATTTAACACATCGTTTCCCATTAGAAGAGCGT
ATCTATCGAATGCGCTGCGTCGAAGCGTGGTCCATGGTCGTGCCGTGGATTGGTTTCCCT
TTATATAAGCTACTCGCGCAGGCACAGCCCACCAGCCACGCTAAATATGTGGCATTCGAA
ACGCTATACGCGCCGGATGATATGCCAGGACAGAAAGATCGTTTTATTGGCGGCGGACTG
AAATACCCTTATGTCGAAGGGCTACGTCTGGACGAAGCCATGCATCCGCTGACTCTGATG
ACCGTTGGCGTCTATGGTAAGGCGTTACCCCCGCAAAACGGCGCGCCCATTCGACTCATC
GTTCCATGGAAGTATGGTTTTAAAGGTATTAAATCTATTGTCAGCATTAAACTCACCCGC
GAACGTCCGCTAACCACCTGGAATTTGTCGGCTCCCAACGAATATGGTTTTTACGCCAAT
GTGAACCCGCATGTGGATCATCCACGCTGGTCTCAGGCTACCGAACGCTTTATTGGTTCA
GGCGGTATCCTCGATGTGCAAAGGCAGCCGACGCTGCTGTTTAACGGCTATGCCAATGAA
GTCGCTTCGCTGTATCGCGGTCTCAATTTGCGGGAGAATTTTTAA
>EECEBICD_00074 putative acrylyl-CoA reductase AcuI
ATGCAGGCGTTGATCTTAGAACAGCAGGACGGTAAAACCCTCGCATCCGTGCAACATCTC
GAAGAGAGTCAACTGCCGGCAGGTGATGTGACGGTGGATGTCCACTGGTCCAGCCTGAAT
TATAAAGATGCTCTGGCTATCACCGGAAAAGGGAAAATTATCCGTCATTTTCCGATGATT
CCTGGTATTGATTTCGCCGGCACCGTTCACGCAAGCGAAGATCCTCGCTTTCATGCAGGC
CAGGAAGTACTACTGACTGGCTGGGGCGTGGGCGAAAACCACTGGGGCGGCCTGGCCGAA
CGCGCACGCGTTAAAGGCGACTGGCTGGTCGCGCTGCCTGCGGGACTGAGCAGCCGTAAC
GCCATGATCATCGGCACCGCCGGTTTTACCGCCATGCTGTGCGTCATGGCGCTTGAAGAT
GCAGGTATTCGCCCGCAAGACGGTGAGGTTGTCGTCACCGGTGCCAGCGGCGGCGTGGGC
AGCACTGCCGTTGCATTACTGCATAAGCTGGGCTATCAGGTTGCAGCAGTATCAGGACGT
GAAAGCACCTACGGCTATCTTAAGAGCCTGGGAGCTAACCGTATTCTCAGCCGCGATGAG
TTTGCTGAATCTCGCCCGTTGGAAAAACAGCTATGGGCCGGTGCAATTGATACCGTTGGC
GATAAAGTGCTGGCGAAAGTGCTGGCACAGATGAACTATGGCGGTTGCGTGGCCGCCTGC
GGCCTGGCGGGCGGCTTCGCGCTGCCGACCACCGTTATGCCATTCATTCTGCGTAATGTG
CGTTTGCAAGGGGTGGACTCGGTGATGACCCCACCGGCACGTCGCGCAGAAGCCTGGGCG
CGTTTGGTTAAGGATCTGCCGGAATCGTTCTATGCTCAGGCCGCCACAGAGATTACGCTG
GCGGATGCGCCGAAGTTCGCCGATGCCATCATCAATAATCAGGTTCAGGGCCGTACGCTG
GTGAAGATAAAATAA
>EECEBICD_00075 RNase E specificity factor CsrD
ATGCGATTAACGACGAAATTCTCAGCTTTTATCACGCTGCTCACGGGGTTAACGATCTTC

GTGACTCTGATAGGTTGCTCGCTAAGCTTTTATAACGCCGTACAGTATAAGTATGTCAGC
CGTGTGCAGGCGACGGCGACAGCCATTGATACGCACCTGGTGACCCATGATATTGTCTCG
CTGACGCCGCAAATCGACGAACTCATGATCGCTTCCGATATTGTGCGTGTCGATCTGTTG
CAGGGGGAGCGCAGCGTCTACAGCCATTCTCGTGCCAGAGGTTATCGGCCAGCCGGCACC
AGCGACATGTATCGCGAGCTGGTCGTACCGCTGATAAAACATCCAGGAATGTCGCTACGT
CTGGCTTATCAGGACCCGATGGGTAACTATTTTCACTCACTGATCACCACGGCGCCGCTA
ACGCTGGCGATCGGTTTTATCGTTCTGATTTTGTTTTTATCGGTGCGCTGGCTGCAGCGG
CAGCTTTCCGGGCAGGAGCTTCTGGAAATTCGCTCAACGCGTATTCTGAACGGCGAACGC
GGGGCGAATGTCCGCGGTTCTGTTTACGAATGGCCAGCGCGCACCAGTAGCGCGCTTGAT
GTATTGCTTTCCGAAATTCAGTTTGCTCACGAACAGCGTAGCCGCCTGGACACCCTCATT
CGCTCCTACGCCGCGCAAGACACCAAAACCGGACTCGGTAACCGTCTGTTCTTTGATAAC
CAACTGGCGACGCTGCTGGAAGATCAGGAAAAAGTGGGAGTGCATGGCGTGGTGATGATG
ATCCGCTTGCCAGACTTCAATCTTCTGCGCGACAGCCTGGGAGGCAATCAGGCGGAAGAG
CAGATGTTTATGCTTATCAATCTGCTATCGACATTTATTATGCGTTACCCAGGCTCGCTG
CTGGCGCGTTATCACCGCAGCGACTTTGCCGTGCTGCTACCGCATCGAACGCTGAAAGAA
GCGGAAAGTATTGCCGGACAACTTTTAAAAGCTGTCGATGCGCTACCGGCTAACAAAATG
CTTGATCGTGACGATATGGTACATATCGGCATTTGCGCCTGGCGCAGCGGACAGTCCACC
GAGCAGGTGATAGAACATGCTGAAGCCGCGGCGCGGAACGCCGCCCTGCAGGGGGGAAAT
AGCTGGGCTATCTACGACGACACGCTGCCGGAAAAAGGGCGCGGCAATGTGCGCTGGCGC
ACATTGATTGAGCAGATGCTGAACCGGGGCGGACCGCGACTTTATCAAAAACCGGCGGTC
ACGCGAGAAGGTCGCGTACATCATCGGGAATTGATGTGTCGCATCTATGACGGTAAAGAA
GAGGTGAGCTCGGCGGAGTATATGCCGATGGTGTTGCAATTTGGTTTATCGGAAGAGTAT
GACAGATTACAAATTAGCCGCCTGATTACGTTGCTGGGCTACTGGCCGGATGAAAATCTG
GCAATGCAGTTGACGGTCGAGTCGCTGATTCGTCCGCGTTTTCAGCGCTGGTTGCGCGAT
ACGTTAATGCAGTGTGAAAAATCACAGCGAAATCGCATAATTATTGAACTTGCGGAGGCA
GATGTTTGTCAACACATCTGTCGGTTACAACCCATCCTTCGCTTAGTCAATGCGCTTGGC
GTTCGGGTGGCGGTGACACAGGCGGGTTTGACGCTGGTTAGCACGAGCTGGATTAAAGCG
CTCAATGTGGAATTACTCAAGCTGCATCCGAGTCTGGTCAGAAACATTGAGAAGCGAACG
GAAAACCAGCTTCTGGTTCAAAGTCTGGTAGAGGCCTGCGCGGGTACGCCTACGCAGGTT
TACGCGACGGGCGTCCGTTCGCGCGGCGAGTGGCAGACGCTGACAAAACGCGGCGTGGCT
GGCGGGCAGGGTGATTTTTTTGCCTCCTCCCAACTACTTGACACAAACGTGAAAAAATAT
TCGCAAAGATACTCGGTTTAA
>EECEBICD_00076 Cell shape-determining protein MreB
ATGTTGAAAAAATTTCGTGGCATGTTTTCCAATGACCTGTCCATTGACCTGGGTACCGCG
AATACCCTCATTTATGTAAAAGGACAAGGCATCGTATTGAATGAGCCTTCCGTTGTGGCC
ATTCGTCAGGATCGTGCCGGTTCGCCGAAAAGCGTGGCTGCAGTCGGTCATGATGCGAAG
CAGATGCTGGGCCGTACGCCGGGCAATATCGCTGCGATCCGTCCGATGAAAGACGGCGTT
ATCGCTGACTTCTTCGTGACTGAAAAAATGCTCCAGCACTTTATTAAACAAGTGCACAGC
AACAGCTTTATGCGCCCAAGCCCGCGCGTACTGGTATGCGTGCCGGTCGGCGCGACGCAG
GTAGAACGCCGCGCAATTCGTGAATCCGCTCAGGGCGCAGGCGCCCGTGAAGTCTTTTTG
ATCGAAGAGCCGATGGCGGCCGCGATCGGCGCAGGTCTGCCAGTTTCTGAAGCAACCGGT
TCAATGGTTGTGGATATCGGTGGTGGTACCACTGAAGTTGCCGTTATCTCCCTGAACGGC
GTGGTTTACTCTTCTTCTGTCCGTATTGGTGGTGATCGCTTCGACGAAGCCATCATTAAT
TACGTTCGTCGTAACTACGGTTCTCTGATCGGTGAAGCCACCGCAGAACGTATTAAACAC
GAAATCGGTTCCGCTTATCCGGGCGACGAAGTCCGCGAGATCGAAGTGCGTGGCCGTAAC
CTGGCGGAAGGCGTTCCACGCGGCTTTACCCTGAACTCAAACGAGATTCTGGAAGCGTTG
CAGGAACCGTTGACCGGTATCGTCAGCGCGGTAATGGTGGCGCTGGAACAGTGTCCGCCG
GAGCTGGCGTCAGACATCTCCGAGCGCGGTATGGTGCTCACCGGCGGCGGCGCGCTGTTG
CGTAACCTTGACCGTCTGTTAATGGAAGAGACGGGCATTCCTGTCGTGGTTGCTGAAGAT
CCGCTGACCTGTGTGGCGCGCGGCGGCGGCAAGGCGCTGGAAATGATCGATATGCACGGC
GGCGACCTGTTCAGCGAAGAGTAG
>EECEBICD_00077 Cell shape-determining protein MreC
ATGAAGCCAATTTTTAGCCGTGGCCCGTCGCTACAGATTCGCCTTATTCTGGCGGTGCTG
GTGGCGCTCGGCGTTATCATTGCCGACAGCCGCCTGGGGACGTTCAGTCAAATCCGAACG

TACATGGACACTGCCGTCAGTCCTTTCTATTTATTTCAAACGGCCCTCGTGAACTGCTC
GACAGCGTGTCGCAAACGCTGGCTTCTCGCGACCAGCTTGAGCTGGAAAATCGGGCGCTG
CGCCAGGAATTACTGCTAAAAAACAGCGACCTGCTGATGCTGGGGCAGTACAAACAGGAG
AACGCGCGGTTGCGCGAGCTGCTGGGGTCGCCGCTGCGTCAGGACGAGCAGAAAATGGTG
ACTCAGGTTATTTCAACGGTTAACGATCCTTACAGCGATCAGGTCGTTATCGATAAAGGC
AGCGTTAATGGCGTATATGAAGGTCAACCGGTCATTAGCGACAAAGGCGTCGTAGGGCAG
GTTGTCGCCGTGGCGAAACTGACCAGTCGCGTACTGCTGATTTGCGATGCGACTCATGCG
CTGCCGATTCAGGTACTGCGCAATGACATCCGCGTGATTGCCGCCGGTAACGGCTGCACT
GACGATCTTCAGCTTGAACATCTGCCCGCCAATACCGATATTCGCGTTGGCGATGTGCTG
GTAACGTCCGGACTTGGTGGGCGTTTCCCGGAAGGGTATCCGGTGGCGGTGGTCTCCTCC
GTCAAGCTGGACACTCAGCGCGCCTATACCGTCATTCAGGCACGTCCAACCGCGGGTTTG
CAGCGTTTACGCTATCTGCTGCTGTTGTGGGGAGCCGATCGTAACGGCGCCAATCCGATG
ACGCCGGAAGAGGTTCACCGCGTCGCCAATGAGCGCCTGATGCAGATGATGCCGCAGGTG
TTGCCTTCTCCGGATGCGATGGGGCCGCCGGCGCCAGTGCCGGACCCGGCGACGGGGATT
ACTCAGCCATCTGCGGGCCAGACGGCGCCGGTATCAACGCAACCATCGCCTTCGGGCGCG
ACCACGCCGCCTGCGCGTGCGCCGGGAGGGTAA
>EECEBICD_00078 Rod shape-determining protein MreD
ATGGTGGCGAGCTATCGTAGCCAGGGGCGCTGGGTTATCTGGCTCTCGTTTCTTATTGCG
CTGTTGCTGCAAATCATGCCCTGGCCGGACGACATCATTGTTTTCCGGCCAAACTGGGTT
TTGCTCATCTTACTGTACTGGATTCTGGCCCTGCCGCACCGCGTAAATGTGGGGACGGGA
TTTGTGATGGGTGCCATACTTGATCTCATCAGCGGTTCCACACTTGGCGTGCGCGCTCTG
TCGATGAGTATTGTCGCCTATCTGGTCGCTCTGAAATTCCAGCTCTTCCGTAACCTGGCG
CTCTGGCAGCAGGCGCTGGTGGTAATGTTGCTTTCACTGGCCGTGGACATTATTGTTTTC
TGGGCCGAGTTTTTAGTGATCAACGTCTCTTTCAGACCGGAAGTGTTCTGGAGTAGTGTA
GTCAATGGCGTGCTGTGGCCCTGGCTCTTTTTGCTGATGCGTAAAGTACGCCAGCAGTTT
GCTGTGCAATAA
>EECEBICD_00079 dTTP/UTP pyrophosphatase
ATGACAACTCTGTATCTTGCTTCCGGTTCCCCGCGTCGCCAGGAATTATTGACTCAACTT
GGATTTTCATTCGAGCAGGTCGTGCCTGGTATTGAAGAGCAACGGCGTGCTCAGGAAAGC
GCGCAGCAGTATGTTGTTCGGCTGGCACGCGAAAAGGCGCAGGCTGGCGTGGCGCTGGTT
CCCCGTGATCTGCCGGTGTTGGGCGCGGATACGATAGTCGTGTTAAACGGCGAGGTGCTG
GAAAAGCCGTGCGACGCCGCTCATGCCGCAGAAATGCTGCGTTTGCTTTCCGGAAATACG
CATCAGGTGATGACTGCTGTTGCGCTTGCGGACTCGCAGCAGACGCTGGATTGTGTGGTG
GTAACGGAGGTGACTTTCCGCACGCTTTCTGCGCAGGATATTACTGGCTATGTCGCCAGC
GGCGAGCCTTTAGATAAAGCAGGTGCATACGGTATTCAGGGGCGGGGTGGCTGTTTTGTC
AGGAAGATAAATGGCAGCTATCACGCCGTGGTCGGCTTACCGCTGGTTGAAACGTATGAG
TTGTTGAGTCATTTTAACGCACTGCGTGATAAAAGGGATAAACATGACGGCTGA
>EECEBICD_00080 Ribonuclease G
ATGACGGCTGAATTGTTGGTAAACGTAACGCCATCGGAAACGCGCGTGGCGTACATTGAT
GGCGGTATTCTTCAGGAAATCCATATTGAGCGCGAAGCGCGACGCGGAATCGTAGGCAAT
ATCTACAAAGGTCGTGTAAGTCGTGTACTTCCAGGTATGCAGGCGGCTTTTGTAGATATT
GGGCTGGATAAAGCCGCATTTCTTCACGCTTCCGACATTATGCCGCATACCGAATGCGTG
GCGGGCGACGAGCAAAAACAGTTTACCGTGCGCGATATCTCCGAACTGGTGCGTCAGGGA
CAGGATCTGATGGTGCAGGTAGTGAAAGATCCGCTCGGCACGAAAGGCGCGCGTTTGACC
ACGGACATCACGTTGCCCTCCCGTTACCTTGTTTTTATGCCCGGCGCGTCACACGTTGGC
GTTTCCCAGCGTATCGAAAGCGAAAGTGAACGCGAGCGCCTGAAAAAGGTGGTGGCGGAA
TACTGCGACGAACAGGGCGGGTTTATTATCCGCACCGCGGCGGAAGGCGTGTGTGAAGAG
GATCTCGCGTCTGACGCCGCCTATCTCAAGCGCGTCTGGACGAAAGTCATGGAGCGCAAA
AAGCGTCCGCAAACGCGCTACCAGATGTATGGCGAACTGGCGCTGGCGCAGCGCGTGCTG
CGCGATTTCGCCGATGCGCAGTTGGACCGAATTCGCGTCGACTCACGTCTGACCTATGAG
TCGCTGCTGGAGTTTACCGCAGAATACATCCCGGAAATGACCAGTAAGCTGGAGCATTAC
AGTGGCCATCAGCCGATCTTCGATCTTTATGACGTGGAAAATGAGATCCAGCGCGCGCTG
GAGCGTAAGGTTGAACTCAAGTCTGGCGGTTATCTGATTATCGACCAAACCGAAGCGATG
ACGACGGTAGATATCAATACCGGCGCGTTTGTCGGACACCGTAATCTCGACGACACCATT

TTTAATACCAATATTGAAGCGACTCAGGCCATTGCCCGCCAGCTACGCCTGCGTAATCTG
GGCGGCATTATCATTATCGATTTTATCGACATGAATAATGAAGATCATCGCCGCCGGGTG
CTGCATTCGCTGGAACAGGCGTTAAGTAAGGATCGCGTGAAAACCAGTATTAACGGTTTT
TCGCCGCTGGGGCTGGTGGAAATGACCCGTAAACGGACCCGTGAAAGCGTGGAACATGTG
CTTTGTAACGAGTGTCCAACCTGTCATGGCCGCGGGACGGTAAAAACGGTGGAAACCGTC
TGTTATGAGATCATGCGTGAAATTGTACGTGTTCATCACGCTTATGACTCCGATCGCTTC
CTGGTTTATGCTTCTCCTGCGGTGGCGGAAGCGCTGAAAGGCGAAGAATCACACGCGCTG
GCGGAAGTCGAAATCTTTGTCGGCAAACAGGTTAAAGTTCAGGTCGAACCGCTTTATAAC
CAGGAGCAGTTTGACGTCGTAATGATGTAA
>EECEBICD_00081 hypothetical protein
GTGAGGCGATTGCCGGGGATTTTATTGCTCACTGGAGCCGCGCTCATCGTCATTGCAGCG
CTGCTGGTTAGCGGGCTGCGCCTGGCGTTGCCTCATCTTGACGCCTGGCGTCCAGCTATC
CTGAATAAAATTGAGTCCGTGACCGGCGTGCCGGTGGCCGCCAGCCAACTCTCCGCAAGC
TGGCAAAATTTTGGCCCTACGCTGGAAGCGCATAATATTCATGCCGCCCTGAAAGATGGC
GGCGAGCTGTCGATAAAACGCGTTACGCTGGCGCTGGACGTCTGGCAAAGCCTGTTGCAT
ATGCGCTGGCAGTTTCGCGACCTGACCTTCTGGCAACTCAATTTCCGCACCAACACGCCT
CTTCAAAGCAGCGACGGCAAAGGCATCGAAACCAGCCGTTTAAGCGATCTTTTCCTGCGT
CAGTTCGATCATTTCGATCTGCGCGATAGCCAAATCAGTTTTCTGACGCTTTCCGGACAA
CGCGCGGAGCTGGCGATCCCACAGCTTACCTGGCTAAACGGCAAAGAGCGGCACCGCGCC
GAAGGCGAGGTCAGCCTCTCCAGCCTGACCGGGCAGCACGGCGTGATGCAGGTGCGGATG
GATTTACGCGATGACGACGGTTTGCTGAATAACGGCAGAGTCTGGTTACAGGCCGACGAT
ATTGACGTCAAGCCGTGGCTGGGCAAATGGATGCAGGATAATGTGGCGCTCCAGACGGCG
CGTTTCAGTCTGGAAGGCTGGATGACGCTCAGTAAAGGCGAAATCGCCGGAGGAGACGTC
TGGCTGAAACAGGGCGGCGCAAGCTGGCTGGGCGATAACACGACGCACACGCTGTCTGTC
GATAACCTGACGGCGCAGATTAGCCGCGAGCAGCCGGGCTGGCAGTTTTATATTCCGGAC
ACACGGATTACGCTTGATGGTAAACCCTGGCCGAGCGGAGCCTTAACCGTGGCCTGGCTC
CCGCAGCAGGACGTCGGCGGCGAGAATCACACACGTAGCGATGAGCTACGCATTCGCGCC
AGTAATCTTGAACTGGCGGGGCTGGAAGCATTACGTCCGCTGGCGGCTAAACTGTCGCCT
GTGCTGGGCGAAATATGGCAGGCTACGCAGCCGAGCGGAAAGATCGCCACACTGGCGCTG
GATATTCCGCTACAGGCGACCGAAAAAACGCGTTTTCAGGCATCGTGGGAAAATCTTGCC
TGGAAACAGTGGAAGCTGTTGCCCGGCGCGGAACATTTTTCCGGTACGCTGGCGGGAAGC
GTAGAAGACGGGCAGATGAAGGTTGCGATGCAGCAAGCTAAAATGCCCTATGAAACCGTT
TTTCGCGCGCCGCTGGAAATTGAAAACGGCGTCGCGACGCTTAGCTGGTTGAAGAATGAG
AACGGCTTTCAGCTTGATGGCCGCGACATTGACGTCAAAGCAAAGGCGGTACATGCGCGC
GGCGGGTTCCGCTATCTGCAACCGACGGGCGACGAACCGTGGCTGGGTATTCTGGCCGGA
ATCAGTACCGATGATGGATCTCAGGCGTGGCGCTATTTTCCGGAAAACCTGATGGGCAAA
GCGCTGGTCGATTACCTTAGCGGCGCGATTCAGGGCGGCGAGGCGGATAACGCCACGTTG
GTTTACGGTGGTAACCCGCACCTCTTTCCCTATAAGCATAACGAAGGTCAGTTTGAAGTG
CTGGTGCCGCTACGCAATGCGACGTTTGCCTTTCAACCCGACTGGCCGGCGCTAAAAAAT
CTCAACATTGAACTGGATTTCCTGAACGACGGTCTGTGGATGCGTTCGGATAGCGTCGAT
TTGGGCGGGGTGAAGGCCAGCAAACTCGCGGCGACGATTCCGGATTATTCCAAAGAGAAA
CTGCTCATTGATGCCGATATTAACGGGCCGGGAAAAGCCGTTGGGCCTTATTTTGACGAG
ACGCCGCTAAAAGACTCGCTTGGCTCGACGCTGGCGGAGCTCCAGTTAGATGGCGATGTG
AATGCTCGCTTACATCTTGATATTCCGCTGGACGGAGAACAGGTTACCGCCGAGGGCGAT
GTCTCGTTGCGTAATAACAGTCTGTTTATTAAGCCGCTAAACAGCACGCTCAAAAATCTG
AACGGTAAATTCAGCTTTGTGAATGGCGCGCTAAAAAGCGGGCCGCTGACGGCAAACTGG
TTTAATCAGCCGCTTAACCTGGATTTCAGTACGACGGAAGGGGCAAAAGCCTATCAGGTC
GCCGTCAACCTGAACGGTAACTGGCAACCAACGCGTATGGGCGTCTTACCGCCGCAACTG
AATGACGCCCTGAGCGGCAGCGTGACGTGGAATGGTAAAGTCGGCATCGATCTTCCGTAT
CACGCTGACACCACTTATCACATCGAGCTGAACGGCGATCTGAGAAATGTGAGCAGTCAC
TTACCTTCTCCGCTAAATAAACCCGCAGGCGAAGCCATTCCGGTGAACATTCAGGCTGAC
GGCAACCTGAAAAGTTTTGCGCTAACGGGGAGCGCAGGGAGTAAAAATCACTTTAACAGC
CGCTGGTTATTAAATCAGAAGCTGACGTTGGATCGGGCTATCTGGACGACGGACAGCCGG
ACGATTCCGCCATTACCTGCTCAACAAGGCGTTGAACTCAATCTGCCGGCGCTGGATGGC

GCACAATGGCTGGCGTTATTCCAGAAAGGCGCGGCGGATAACGTGAGCAGTTCAGCCGAG
TTTCCTCAACGCGTCACGTTGCGTACTCCCGCGCTATCGCTGGGTGGCCAGCAGTGGAAC
AATTTGAGCGTCGTTTCAGCCCCCTCGCTGAACGGAACAAAAATTGAAGCGCAGGGCCGT
GAGGTGAACGCCACGCTGCTCATGCGCAACCATGCGCCGTGGCTGGCGAATATTAAGTAC
CTGTACTACAACCCTGGCGTCGCAAAAACGCACGCCTCGTCACCAACGCCGACATCGCCG
TTGGCTTCGGCGAACACGATTAGCTTCCGCGGCTGGCCGGACTTACAGCTTCGCTGCGAA
GAGTGCTGGCTGTGGGGGCAAAAATATGGGCGTATTGATGGCGATTTCGCCATCAAAGGC
AATACGCTGACTCTGGCGAATGGCCTGATCGATACCGGATTCGCCCGTTTGAAAGCGAAC
GGCGAGTGGGTGAATGCGCCGGGTAATGAACGAACCTCGCTGAAAGGTAGTCTGCATGGT
AGTAACCTCGACACGGCTGCCGGGTTCTTCGGTATCTCGACGCCAATCCAGAACGCGTCT
TTTAACGTAGATTACGATCTGCACTGGCGAAACCCGCCCTGGCAACCTGAGGAAGCCACG
CTCAACGGGATTTTACGTACGCGTCTGGGTAAAGGCGAGTTTACTGATCTCAGTAGCGGT
CATGCCGGACAGTTACTGCGGCTGCTCAGTTTTGATGCGTTGCTGCGTAAGCTGCGGTTT
GACTTCAGAGATACCTTTAGCGAGGGCTTCTATTTCGACTCTATTCATAGTACCGCGTGG
ATTAAAGATGGCGTCCTGCATACTGACGATACGCTGGTGGATGGGCTGGAAGCGGATATT
GCAATGAAAGGTTCTGTTGATCTGGTGCGTCGTCGTCTTGATATGGAGGCCGTTGTCGCG
CCGGAAATTTCCGCCACAGTGGGCGTCGCCGCCGCGTTTGCCGTTAACCCGATTGTCGGC
GCGGCGGTATTTGCCGCCAGTAAAGTGTTGGGGCCGCTATGGAGCAAGGTCTCCATTTTG
CGATATCGCATTACCGGTCCGGTCGATGCGCCGCAGATCAACGAAGTTCTGCGCCAACCG
ATAAAAGAAAGCCAGCAATGA
>EECEBICD_00082 Metalloprotease TldD
ATGAGTCTGAACCTGGTAAGTGAACAATTGCTAGCGGCGAATGGCCTGAACCATCAGGAT
CTGTTCGCTATTTTGGGCCAACTGGCCGAACGCCGTCTTGATTATGGCGACCTCTATTTT
CAGTCGAGCTATCACGAATCCTGGGTTTTAGAAGACCGCATCATTAAAGATGGTTCATAT
AATATCGACCAGGGCGTTGGCGTTCGCGCCATTAGCGGCGAAAAAACCGGTTTTGCTTAT
GCTGACCAGATAAGCCTCCTGGCGCTGGAGCAGAGTGCGCAGGCAGCGCGAACCATTGTA
CGCGAGAACGGCGAAGGCAAGGTAAAAACGCTCGCCGCCGTAGCGCATCAGCCGCTCTAC
ACCACCCTTGATCCGCTGCAAAGTATGAGCCGCGAAGAGAAGCTGGATATCCTCAGACGC
GTTGACAAAGTGGCGCGAGAAGCCGATAAACGCGTGCAGGAAGTTAACGCCAGCCTGACC
GGCGTATATGAATTAATCCTCGTGGCGGCGACCGACGGGACGCTGGCGGCGGATGTCCGT
CCACTGGTGCGGTTGTCCGTTAGCGTGCAGGTGGAAGAAGACGGTAAACGCGAGCGCGGC
GCCAGCGGCGGCGGCGGTCGCTTTGGTTATGAGTATTTTCTTGCCGATCTCGACGGCGAG
GTTCGCGCCGACGCGTGGGCGAAAGAAGCGGTACGCATGGCGCTGGTTAATCTCTCCGCG
GTCGCTGCGCCAGCGGGGACGTTACCGGTGGTTCTGGGCGCCGGGTGGCCGGGCGTATTG
CTGCACGAAGCGGTCGGTCACGGGCTGGAAGGTGATTTTAACCGTCGTGGGACATCTGTG
TTTAGCGGTCAGATCGGCGAGCAGGTTGCCTCCGCGCTTTGCACCGTAGTGGACGACGGC
ACAATGATGAACCGTCGCGGCTCCGTTGCTATCGATGATGAAGGTACGCCAGGCCAGTAC
AACGTATTGATTGAAAATGGCGTGCTGAAAGGATACATGCAGGACAAGCTGAACGCTCGC
CTGATGGGCGCTGCGCCGACCGGTAACGGGCGTCGCGAATCTTACGCGCATCTGCCGATG
CCGCGTATGACGAATACCTATATGTTGGCGGGGCAGTCAACGCCGCAGGAAATTATCGAA
TCCGTTGAGTACGGCATCTATGCGCCTAACTTTGGCGGTGGTCAGGTGGATATCACCTCC
GGCAAGTTTGTGTTCTCTACCTCGGAAGCGTATCTGATTGAAAATGGCAAAGTCACGACG
CCGGTGAAGGGCGCGACGTTGATTGGATCAGGCATTGAAACGATGCAACAGATCTCCATG
GTCGGCAACGACCTTAAGCTGGATAACGGGGTGGGGGTCTGCGGTAAAGAGGGCCAAAGC
CTGCCGGTAGGCGTAGGCCAGCCGACGCTGAAAGTCGATAACCTGACGGTTGGCGGCACC
GCATAA
>EECEBICD_00083 HTH-type transcriptional regulator DmlR
ATGGAACGATTAAAACGAATGTCGGTATTCGCCAAAGTTGTCGAGTTTGGCTCTTTTACC
GCCGCAGCCAGACAGCTGCAAATGAGCGTATCGTCCATCAGCCAGACTGTGGCGAAACTG
GAAGATGAGCTTCAGGTTAAATTGCTCAACCGCAGCACACGCAGCATTGGGCTCACCGAG
GCTGGAAAAAATCTATTATCAGGGCTGTCGTCGTATGCTGCATGAAGTGCAGGATGTTCAT
GAGCAGCTTTACGCTTTTAATAACACGCCCATCGGTACATTGCGTATCGGCTGTTCTTCA
ACCATGGCGCAAAATGTGCTGGCCGGGCTTACCGCAAAATTATTAAAAGAGTATCCCGGT
CTGGCGGTCAATCTTGTGACAGGAATTCCGGCTCCGGATTTAATTGCCGACGGACTGGAC

GTGGTGATCCGCGTGGGCGCGCTGCAAGACTCCAGCCTGTTTTCCCGTCGACTCGGCGCG
ATGCCGATGGTTGTGTGCGCCGCCAAGCCCTACCTCGCTCAGTACGGCGTCCCGGAAAAA
CCCGCCGATCTCAGTAGCCATTCCTGGCTGGAATATAGCGTGCGCCCGGATAATGAGTTT
GAACTTATCGCGCCGGAAGGAATTTCTACCCGACTGATTCCACAGGGGCGGTTCGTCACT
AACGATCCGATGACGCTGGTACGCTGGCTGACCGCCGGAACCGGAATCGCCTATGTGCCG
CTGATGTGGGTGATCGATGAGATCAATCGTGGCGATCTGGAGATCCTGCTGCCTCGCTAT
CAGTCCGATCCCCGTCCGGTGTACGCGCTGTACACCGAAAAAGATAAGCTGCCGCTTAAA
GTGCAGGTAGTGATTAACGCGTTAACTGACTATTTTGTCGATGTCGCACACCTGTTTCAG
GGGATGCACGGGCGGGGAAAAGAAAAATAA
>EECEBICD_00084 Protein AaeX
ATGAGTCTGTTTCCCGTTATCGTGGTGTTTGGTCTTTCCTTCCCACCGATATTTTTCGAA
TTGCTTTTGTCACTGGCTATTTTTTGGCTGGTGCGTCGGATGCTTGTGCCGACGGGTATC
TATGATTTTGTCTGGCATCCGGCTTTGTTCAATACCGCGCTGTATTGTTGTCTGTTTTAT
TTAATATCGCGCCTGTTCGTTTGA
>EECEBICD_00085 p-hydroxybenzoic acid efflux pump subunit AaeA
GTGAAAACACTAACAAGAAAACTCTCCCGTACGGCCATTACCCTCGTACTGGTTATTCTG
GCCTTTATCGCCATTTTCCGCGCCTGGGTGTATTACACCGAATCTCCCTGGACGCGCGAC
GCGCGTTTTAGCGCCGATGTCGTCGCTATTGCCCCGGATGTCGCGGGCCTGATTACCCAT
GTAAACGTGCATGATAACCAACTGGTGAAAAAGGATCAGGTCCTGTTTACCATCGATCAG
CCACGCTATCAAAAAGCGCTGGCGGAAGCGGAAGCTGATGTAGCCTATTATCAGGTACTG
GCGCAGGAAAAACGCCAGGAAGCCGGTCGCCGTAATCGCCTCGGCGTACAGGCGATGTCC
CGGGAAGAGATCGATCAGGCGAACAACGTCTTGCAGACGGTACTGCATCAACTGGCTAAA
GCCCAGGCGACGCGCGATCTGGCTAAACTCGATCTGGAACGCACCGTCATTCGCGCGCCT
GCCGACGGCTGGGTGACTAACCTTAATGTTTATGCCGGTGAATTTATTACCCGCGGTTCC
ACAGCCGTCGCCCTGGTGAAAAAGAACTCTTTTTACGTGCAGGCCTATATGGAAGAGACC
AAGCTGGAAGGCGTGCGTCCGGGATATCGTGCCGAAATCACGCCGCTTGGCAGCAACCGG
GTATTAAAAGGTACCGTCGATAGCGTGGCGGCAGGGGTAACGAATGCCAGCAGTACCAGC
GATGCGAAAGGGATGGCGACGATAGACTCTAACCTCGAATGGGTGCGTCTGGCCCAGCGT
GTCCCGGTGCGTATCCGACTCGACGAGCAGCAGGGGAATTTGTGGCCTGCCGGTACGACG
GCAACCGTCGTGATCACCGGTAAGCAGGATCGTGACGCAAGCCAGGACTCCTTCTTCCGT
AAACTGGCGCACCGGCTGCGCGAATTCGGTTAA
>EECEBICD_00086 p-hydroxybenzoic acid efflux pump subunit AaeB
ATGGGCATTTTCTCCATTGCCAACCAGCACATTCGTTTTGCGGTAAAGCTGGCGTGCGCC
ATTGTGCTGGCATTATTTATCGGCTTCCATTTTCAACTGGAGACGCCGCGTTGGGCGGTG
CTGACCGCCGCGATCGTGGCGGCAGGCCCGGCATTTGCCGCCGGCGGGGAACCCTATTCC
GGCGCTATCCGTTATCGCGGAATGTTGCGTATTATCGGGACGTTTATTGGCTGTATCGCC
GCGCTTATCATTATTATCTCTATGATCCGCGCGCCGTTATTGATGATTCTGGTGTGCTGT
GTCTGGGCCGGTTTTTGTACCTGGATCTCCTCTTTAGTACGGATCGAAAACTCTTATGCG
TGGGGACTGTCAGGTTATACTGCGCTGATCATTGTGATTACCATTCAGACGGAGCCGCTG
CTTACGCCGCAATTTGCGCTTGAGCGCTGTAGCGAAATCGTCATCGGCATTGGCTGCGCT
ATTTTAGCGGATCTGCTTTTCTCGCCGCGATCGATAAAGCAGGAGGTTGATCGCGAACTG
GACAGTTTACTGGTAGCGCAATATCAGCTAATGCAACTTTGTATTAAGCATGGCGACAGC
GAAGAAGTCGACAACGCCTGGGGCGATCTGGTGCGACGCACGGCGGCGCTGGAGGGAATG
CGAAGTAATCTGAATATGGAATCTTCGCGCTGGGTACGCGCTAACCGCCGCCTGAAAGCG
TTGAATACGCTTTCGCTCACGCTGATTACGCAATCCTGTGAAACTTATCTTATTCAAAAC
ACGCGGCCGGAACTGATTACCGATACATTCCGGGAACTGTTCGAGACGCCGGTTGAAACG
GTACAGGACGTTCACAGGCAGTTAAAACGGATGCGGCGTGTCATTGTCTGGACCGGCGAG
CGCGAAACGCCCGTCACGCTTTATAGCTGGGTAGGCGCAGCGACGCGCTATCTGCTGCTT
AAACGCGGCGTGATCAGCAACACCAAAATCAGCGCGACGGAAGAAGAGATCCTGCAAGGC
GAGCCCGTAGTTAAAGTGGAATCCGCTGAACGTCACCATGCGATGGTGAACTTCTGGCGC
ACCACGCTTTCCTGCATACTGGGGACATTATTCTGGCTATGGACCGGCTGGACATCGGGT
AATGGCGCGATGGTGATGATTGCCGTGGTGACCTCGCTGGCGATGCGTTTACCCAATCCG
CGCATGGTCTGCATTGATTTTATTTATGGCACTCTGGCGGCGTTGCCGCTGGGATTGCTC
TATTTCCTGGTGATTATTCCCAATACGCAGCAAAGTATGCTGTTGTTATGTTTGAGCCTG

GCGGTATTGGGGTTCTTTATCGGCATTGAGGTCCAGAAACGGCGTCTGGGGTCGATGGGG
GCGCTGGCCAGTACGATTAACATTATTGTGTTGGATAATCCGATGACGTTCCATTTCAGC
CAGTTCCTCGACAGCGCGTTGGGGCAAATTGTCGGCTGTATGCTGGCGTTTATCGTCATC
CTGCTGGTGCGTGATAAATCCAAAGATCGAACCGGTCGCGTGCTGCTTAATCAGTTTGTT
TCCGCTGCGGTCTCGGCAATGACGACCAACGTGGTACGTCGTAAAGAAAACCGTCTGCCT
GCGTTATATCAGCAACTCTTTTTGCTGATGAATAAATTCCCTGGCGATTTGCCGAAGTTT
CGCCTGGCGCTAACGATGATCATTGCTCATCAACGTCTGCGCGACGCGCCGATACCGGTT
AATGAGGATCTGTCAGTGTTCCACCGTCAGCTTCGTCGTACCGCAGACCATGTTATTTCC
GCCGGAAGCGATGATAAACGGCGACGCTACTTTGGTCAGTTGCTGGATGAGCTGGATATC
TATCAGGAGAAATTACGTATCTGGGAGGCGCCGACGCAGGTGACGGAACCGGTTAAACGC
CTGACGGGCATGTTACATAAATACCAGAACGCGCTTACCGATAGCTAA
>EECEBICD_00087 putative protein YhcO
ATGAATGTCTATACTTTTGATTTTAATGATATTAAAAACCAAAGCGATTTTTATCGCGAG
TTTACGCAGACGTTTGGTCTTGCCAGTGAGAAAGTAAGCGATCTTGATACATTATGGGAT
GCGGTAATGAGCGATATCTTACCTCTACCGCTAGAGATTGAGTTCGTTCACCTGCCTGAT
AAACTGCGCAGGCGCTACGGGGCGCTGATTTTACTCTTTGATGAAGCGGAAGAAGAGCTG
GAGGGGCGGCTACGGTTTAATGTCCGGCATTGA
>EECEBICD_00088 hypothetical protein
ATGAAAACGAAATACATTATCGCATCACTCGGCCTGGCCACTCTGCTCTCTTTCGGCGCA
AATGCCGCAGTACATCAGGTTAACGCGGAACAGGCGCAGAATCTGCAACCGATGGGCACC
ATTTCCGTCTCGCAGATTGGCAGTACGCCAATGGATATGCGCCAGGAGATTGTCGCTAAA
GCCGAAAAAGCAGGCGCTAATAGCTATCGCATCATCGAATTAAAAGAAGGTGATAACTGG
CACGCCACCGCCGAGTTGTATAAGTAA
>EECEBICD_00089 hypothetical protein
ATGAAAATCAAAACCACTGTTGCAACATTAAGTGTTCTGTCCGTTCTCTCTTTTGGCGCT
TTCGCCGCAGAACCTATCAGCGCAGAGCAAGCGCAAAATCGTGAAGCAATCGGATCGGTT
TCAGTCAGCGCTATCGGCTCATCGCCTATGGATATGAACGCCATGCTGAGCAAAAAAGCG
GATGAACAAGGCGCTACGGCCTACCACATTACCGAAGCGCGTAGCGGTAGCAACTGGCAC
GCCACTGCCGAACTGTACAAATAA
>EECEBICD_00090 Arginine repressor
ATGCGAAGCTCGGCTAAACAAGAAGAACTAGTAAGAGCGTTTAAAGCGCTCCTCAAAGAA
GAAAAATTCAGCTCACAGGGCGAAATCGTCCTCGCGTTGCAGGATCAGGGCTTTGAAAAT
ATCAATCAGTCCAAGGTCTCGCGCATGTTGACTAAGTTCGGCGCAGTGCGTACACGTAAC
GCAAAAATGGAAATGGTGTACTGTCTTCCGGCTGAACTGGGCGTCCCAACAACATCCAGT
CCGTTGAAAAATCTGGTGCTGGATATCGACTATAACGACGCTGTCGTGGTGATTCATACC
AGCCCCGGCGCGGCACAACTCATCGCGCGCCTGCTGGATTCACTGGGGAAAGCAGAAGGT
ATTCTCGGCACGATTGCCGGCGACGATACTATCTTCACTACGCCTGCCAGCGGCTTTTCC
GTAAGAGATCTGTACGAAGCCATTCTGGAACTTTTTGAACAAGAGCTTTAG
>EECEBICD_00091 Malate dehydrogenase
ATGAAAGTCGCAGTCCTCGGCGCTGCTGGTGGTATCGGTCAGGCGCTGGCATTACTTTTA
AAAAACCAACTGCCTTCAGGTTCAGAACTCTCCCTGTACGACATCGCTCCAGTGACTCCC
GGTGTGGCCGTTGATTTGAGCCACATCCCCACCGCTGTAAAAATCAAAGGTTTCTCCGGT
GAAGACGCAACCCCGGCGCTTGAAGGCGCTGACGTAGTACTGATTTCTGCGGGTGTGGCG
CGTAAGCCGGGTATGGACCGTTCCGACCTGTTTAACGTTAACGCTGGCATCGTGAAAAAC
CTGGTGCAGCAGATCGCTAAAACCTGCCCGAAAGCGTGCGTGGGCATTATCACCAACCCG
GTGAACACCACCGTTGCGATTGCGGCGGAAGTGCTGAAAAAAGCAGGCGTATACGACAAA
AACAAACTGTTTGGCGTTACCACGCTGGATATCATCCGCTCTAATACCTTTGTCGCAGAG
CTGAAAGGTAAGCTGCCAACGGAAGTTGAAGTTCCGGTGATTGGCGGACACTCCGGCGTG
ACGATTCTGCCGCTGCTGTCGCAGATTCCAGGCGTAAGTTTTACCGAACAAGAAGCGGCC
GAGCTGACTAAACGTATTCAGAACGCCGGTACTGAAGTCGTCGAGGCGAAAGCCGGCGGC
GGATCGGCAACCCTCTCTATGGGCCAGGCTGCCGCGCGTTTCGGTCTTTCTCTGGTTCGC
GCTCTGCAGGGCGAAAAAGGCGTGGTGGAATGCGCCTATGTGGAAGGCGACGGTCAGTAT
GCCCGTTTCTTCTCTCAGCCGCTGCTGCTGGGTAAAAACGGTGTAGAAGAGCGTAAATCC
ATCGGCACACTGAGCGCTTTCGAGCAACATTCGCTGGACGCTATGCTGGATACGCTGAAA

AAAGATATTCAGTTGGGTGAAGATTTTATTAATAAATAA
>EECEBICD_00092 hypothetical protein
GTGAAAAAGATCCAAAGAACGCAAACCCGCGATCACATTACGCAAATGCTTCGCTATGAA
ATTCTTTCCGGCAATATAAAAGCGGGAGAAGAACTCGCGCAGGAAAGTATTGCGGAGCAG
CTTGGTCTTTCACGAATGCCGGTTCGAGAAGCGCTACAGTCACTCGAACAGGAAGGTTTT
TTAATCCGACTGCCCAACAGGCACATGCAGGTCGCGCATCTTGAGGCCGATCGCGTGAGT
CATATTTTTCGCGTAATTGCCGCGATGGCCGCCGAAATGTTTTCGCTGATACCCAGCGAA
GTAGGCGATGCCCTCCTGATACGTGCGCAAGCGCTGGCTGTCGCAGAAGACAAATCCTGC
GAACTGGAGTGTCATGCGATGCTGATTTCTTATGTGAATAACCGCTATCTGGAAAAGGTG
TATCAGCAGTTCCTGGACGGTTATGTCTCTTATGTTATTTTGCATCTGAAAAAAGATAAT
CAGGAGTCGGCGCAGCTTTTCGCTGAACTGGCTGACGTCATACGCCAGGGGCGGCGTGAT
GAGATTGGGCAAGTTATGCAGCGTTATTTCCTCTCTTTAGCAGAAATAATGCGTCAACAT
ATGAAGGATTGGGAAAGTGCAGAAGCCTAA
>EECEBICD_00093 hypothetical protein
GTGCAGAAGCCTAAGTTAGGGAAGATTAAGCTTCTTTCAGCGAAAGAACAGGTCGCGGCA
GTGCTGCGTAAAGCGATTCTTTCGAGGGAGTTAGTAGAAGGGCAGGAGATTACGTTAGAA
GGCATTGCCAGAATGGTCGGCGTGTCCAGTATGCCAGTGCGCGAGGCGTTTCAGATTCTG
GCGGCGGATGGGCTGATAAAAGTGCGCCCTAATAAAGGGGCGGTGGTGTTGGGCATCAAT
GAACAAACGATCCGTGAACACTATGAAATTCGCGCGCTGTTAGAAAGTGAGGCGGTGGCT
AAAGCTTCTCGTCCTGGCACCGATATCTCGCGGATAGCCCAGGTGCATTATGCCGCTGAA
AAGGCTCTCGCGGAAAATAACTCGGCGGAATACTCCGACCTGAATCAGGCATTTCATATG
GAAATATGGAATGTTGCTGGCAACGAAAAAATGAAAATGTTGCTCTGTAATATGTGGAAC
GGCCTGTCGATGGGCCATAAGGTCACCGAAGAAGAGTATGCCGTGATTTCCATTCAGGAA
CATAAAAGTATTCTGCAAGCTCTTGAACTTCATGATGAAACATTGGCACGCCAGCGCATG
CGCGAACACATTATTCGCTCAATGGAGAATATGCTGACCCGCTATGTTGGTGACCCTTCT
GCCTGA
>EECEBICD_00094 Sodium-dependent dicarboxylate transporter SdcS
ATGGAACCCATTACCCTTACCTTATGTTTATTGGTGTTCGCTATCGTCATGTTTGTATGG
GAAAAAGTGCCACTTGCCGTCACTTCCATGATTGTCTGCGTGGCCTTAGTGATCACCGGC
GTTCTGAATATCAAACAGGCCTTTGCTGGCTTTATCGACACTAACGTTATCCTCTTTGTC
GCGATGTTCATCGTGGGCGGTGCGTTGTTCGAGACCGGTATGGCAAACAAAGTGGGCGGC
GTCATCACGCGCTTCGCGAAAACAGAAAAACAGCTGATCTTCACCATTATGGTGGTGGTG
GGTTTGATGTCTGGCGTCCTCTCTAACACCGGTACCGCTGCGGTTTTGATTCCGGTGGTG
ATCGGCGTGGCGGCAAAATCGGGCTTCTCGCGTTCTCGTCTACTGATGCCGCTGGTTTTC
GCCGCTGCGTTGGGCGGTAACCTGTCGTTAATTGGCGCGCCGGGAAATCTTATCGCGCAG
TCTGCGCTACAGAATATTGGCGGCGGCTTTGGCTTTTTTGAATACGCAAAAATCGGTCTC
CCGATGCTCATCTGCGGCATTCTCTACTTCCTGACTATTGGCTATCGCTTTCTGCCGAAT
AATGCGACCGGCGGTGAGGTGGGGAGCGTCGGGGAGCAACGTGACTACAGCCATGTTCCA
CAGTGGAAACAACGGCTCTCACTGGTGGTGTTGATCGCAACGATCCTCGGCATGATCTTT
GAGAAGAAGATCGGTGTCAGCCTGGCCGTGACCGGTTGTATTGGCGCTCTTGTGCTGGTA
GTTACCGGCGTACTCACGGAAAAACAGGCCTATAAAGCCATTGATTCACAGACTATTTTT
ATCTTTGGCGGCACCCTGGCGCTGGCAAAAGCGCTGGAAATGACCGGGGCAGGCAAGCTG
GTTGCGGACTATGTAATCGGCATGCTGGGGCAAAACTCTTCACCGTTCATGTTGCTGATC
GCCGTTTTCGCCTTATCCGTTGTGATGACCAACTTTATGTCCAATACCGCAACGACAGCG
CTATTGGTGCCGGTGAGTTTGTCCATTGCCGCAGGTATGGGGGCCGACCCGCGTGCGGTT
CTGATGGCGACGGTCATTGGCGGTTCCTGCGCCTACGCAACCCCTATCGGTATGCCTGCC
AACATGATGGTGCTCTCCGCGGGTGGCTATAAATTTGTCGACTACGCTAAGGCCGGTATC
CCCCTGATTATCGTTTCAACCATTGTCAGCTTGATCCTGCTGCCAATCCTTTTCCCTTTT
CATCCGTAA
>EECEBICD_00095 L(+)-tartrate dehydratase subunit alpha
ATGAGCAAAAGTGAACAGATATCCCATATGACCGACGTTATGGCCAAGTTTGTGGGATAT
ACCGGCAAAGTTTTGCCTGATGATGTCACTGCAAAACTGGAAGATTTGCATAAGAAAGAG
ACCAGTAAACTGGCCGACGTCATCTTTACTACCATGATTGAGAACCAGCGTCTGGCGAAA
GAGCTGGATCGACCTTCCTGCCAGGACACCGGTGTCATTCAGTTTCTGGTGGAGTGCGGG

ACGAACTTTCCGCTGATTGGCGAGCTGGAAGCGTTGTTGCGTGAGGCGGTGATCAAAGCA
ACTGTCGATTCTCCGCTGCGCCACAACAGCGTAGAAACTTTTGATGAATACAACACCGGT
AAAAACGTGGGTAAAGGTACGCCGACGGTCTTCTGGGAGATCGTTCCCAATTCCGATCAG
TGCAGCATTTATACCTATATGGCGGGCGGCGGTTGTTCCCTGCCGGGGAAAGCGATGGTG
CTGATGCCGGGTGCAGGCTATGAAGGTGTGACCCGCTTTGTACTGGATGTGATGACCAGC
TACGGCCTCAACGCGTGTCCGCCGCTGCTGGTTGGCGTGGGCGTAGCGACCTCTGTTGAA
ACGGCGGCACTGCTATCGAAAAAAGCGCTGATGCGTCCGATCGGTTCGCATAACGAGAAT
GAGCGTGCAGCCTCGCTGGAAAAAATGCTGGAAGAAGGCATTAACAAAATTGGCCTGGGG
CCGCAGGGGATGTCCGGTAATACCTCCGTTATGGGCGTGAACATTGAAAACACCGCGCGT
CATCCTTCTACCATCGGCGTGGCGGTAAACGTCGGCTGCTGGTCGCACCGTAAAGGGCAT
ATCGTTTTCGATAAAGATTTGAATTACACCATTACGTCTCACAGCGGAGTGAATTTCTGA
>EECEBICD_00096 L(+)-tartrate dehydratase subunit beta
ATGACTAAAAAAATCCTGACTACGCCAATTAAAGATGAAGATTTAGCGGATATTAAGGCT
GGCGATATTATTTATCTCAATGGTCATATTGTTACCTGCCGTGACGTAGCCCATCGCCGT
TTAATTGAAGGTGGTCGCGAGCTTCCGGTAGATGTTCGCGGCGGCGCTATTTTACACGCT
GGCCCCATTGTCCGCCCAATTAAAGGCGAAGACGATAAGTTTGAAATGGTCTCCGTTGGA
CCAACCACCAGCATGCGTATGGAGAAATTCGAAAAAGAATTTATTGCGCAGACCGGCGTG
AAATTAATTGTGGGTAAAGGCGGTATGGGTAAAGGTACCGAAGAGGGCTGTGCTGAACAC
AAAGCGCTGCACTGCGTATTCCCTGCCGGTTGTGCGGTTGTAGCCGCCGTGTGCGTGGAA
GAAATTGAAGATGCCCAATGGCGCGATCTGGGGATGCCGGAAACTCTGTGGCTCTGTCGG
GTTAAAGAGTTTGGCCCGCTGATTGTTTCTATTGATACCCATGGGAATAATCTGTTTGAA
CAGAACAAAATTATTTTTAATCAGCGTAAAGAGATCGTCGCTGATGAAATATGCCAGAAC
GTAAGTTTCATCAAATAA
>EECEBICD_00097 Oxaloacetate decarboxylase gamma chain
ATGACTAATGCAGCTCTGCTATTAGGTGAAGGCTTCACCCTGATGTTCCTCGGCATGGGG
TTCGTGCTGGCGTTTCTGTTCCTGCTGATTTTCGCCATCCGCGGCATGTCTGCCGTGATT
ACTCGCTTCTTCCCGGAACCAGTCGCCGCGCCCGCACCGCGCGCCGTTCCCGTCGTGGAC
GACTTCACCCGCTTAAAGCCGGTGATTGCCGCCGCCATTCACCATCACCGCCTTAACGCT
TAA
>EECEBICD_00098 Oxaloacetate decarboxylase alpha chain
ATGACCATTGCCATTACCGACGTCGTCCTGCGTGACGCCCACCAGTCCCTGTTCGCCACC
CGCCTGCGCCTTGATGACATGCTGCCGATTGCCGCCGCGCTCGATGACGTGGGCTACGGC
TCGCTGGAGTGCTGGGGCGGCGCCACCTTTGACGCCTGTATCCGCTTTCTCGGCGAAGAC
CCGTGGCTGCGCCTGCGCGAGCTTAAAAAGGCCATGCCGAAGACTCCCCTGCAGATGCTG
CTGCGCGGCCAGAACCTGCTCGGCTACCGCCATTACGCCGATGATGTGGTGGAACGCTTC
GTTGAGCGGGCGGTGAAAAACGGCATGGACGTGTTCCGCGTCTTCGATGCCATGAACGAC
CCGCGCAATATGAAGGCCGCCCTGCAGGCGGTGCGCAGCCACGGCGCGCACGCCCAGGGC
ACGCTGAGCTATACCACCAGCCCGGCGCACACCCTGCAGACCTGGCTGGATTTAACCGAA
CAGTTGTTAGAGACCGGCGTCGATTCCATCGCCATCAAGGATATGTCCGGCATTCTCACG
CCGATGGCGGCGTATGAGCTGGTGAGCGAAATCAAAAAACGCTATGACGTGCGCCTGCAC
CTGCACTGCCATGCCACCACCGGGATGGCGGAGATGGCGCTGCTGAAGGCCATCGAGGCG
GGCGTCGACGGCGTGGACACGGCGATTTCCTCCATGAGCGCCACCTACGGCCACCCGGCC
ACCGAAGCGCTGGTGGCGACGTTGGCAGGTACTGAGCACGACACCGGGCTGGATATCCTG
AAGCTGGAAAATATCGCCGCGTACTTCCGCGAGGTGCGCAAAAAGTATCACGCCTTTGAA
GGCCAGCTGAAGGGCTACGACAGCCGTATTCTGGTGGCGCAGGTGCCGGGCGGAATGCTC
GCCAACCTCGAAAGCCAGCTGAAGCAGCAGAACGCGGCGGACAAACTCGACCAGGTGCTG
GCGGAAATCCCCCGCGTGCGCGAGGACCTCGGCTTTATCCCGCTGGTGACCCCGACCTCG
CAGATTGTCGGCACCCAGGCGGTGCTCAATGTCCTGACCGGCGAGCGCTACAAAACCATT
GCCAAAGAAACGGCGGGCATTCTGAAAGGCGAATACGGCCACACCCCGGTGCCGGTGAAC
GCCGCGTTACAGGCCCGCGTGCTGGAAGGGGGCGCTCCGGTGACCTGCCGCCCGGCGGAC
CTGCTGAAGCCGGAACTGGCTGAACTGGAAGCGGACGTCAGGCGCCAGGCGCAGGAGAAG
GGCATTCAGCTTGCGGGAAACGCCATCGACGACGTGCTCACCGTGGCGCTGTTCCCGCAG
ATTGGCCTCAAATTCCTCGAAAACCGCCACAACCCGGCGGCGTTTGAGCCGGTACCGCAG
GCGGAAGCCGCGCAGCCGGTGGCAAAAGCAGAGAAGCCTGCCGCCTCCGGTATCTACACC

GTGGAAGTGGAAGGCAAAGCCTTTGTGGTGAAGGTCAGCGACGGCGGCGATATCAGCCAG
CTGACGGCGGCTTCGTCTGCGCCGGTTCAGGCGGCATCCCCGGTTGCCCCGGCAGGCGCC
GGCACCCCGGTCACCGCGCCGCTGGCGGGCAACATCTGGAAGGTGATTGCCACCGAGGGC
CAGAGCGTGGCCGAAGGCGACGTGCTGCTGATTCTGGAAGCCATGAAGATGGAAACCGAA
ATCCGCGCCGCGCAGGCCGGGACGGTACGCGGTATCGCGGTGAAGTCCGGGGACGCGGTC
TCCGTGGGCGACACCCTGATGACGCTGGCGTAA
>EECEBICD_00099 Oxaloacetate decarboxylase beta chain
ATGGAAAGTCTGAACGCCCTGCTTCAGGGCATGGGGCTGATGCACCTTGGCGCAGGCCAG
GCCATCATGCTGCTGGTCAGCCTGCTGCTGCTGTGGCTGGCGATTGCGAAGAAGTTCGAA
CCGTTACTGCTGCTGCCGATTGGCTTCGGCGGCCTGCTCTCCAATATCCCGGAAGCGGGT
ATGGCGCTGACCGCGCTGGAGAGCCTGCTGGCGCACCACGACGCCGGGCAACTGGCGGTG
ATTGCCGCGAAGCTCAACTGCGCGCCGGACGTGCACGCCATTAAAGAGGCATTAGCGCTG
GCGCTGCCGTCGGTGCAGAGCCAGATGGAGAACCTGGCGGTGGACATGGGCTACACGCCG
GGGGTGCTGGCGCTGTTCTATAAAGTGGCGATTGGCTCCGGTGTCGCGCCGCTGGTCATC
TTTATGGGCGTGGGCGCGATGACCGACTTCGGCCCGCTGCTGGCCAACCCGCGCACCCTG
CTGCTGGGGGCGGCGGCGCAGTTCGGCATCTTCGCCACCGTGCTGGGGGCGCTGACGCTG
AACTACTTCGGCCTGATTTCCTTCACCCTGCCGCAGGCGGCGGCAATCGGCATTATCGGC
GGCGCGGACGGCCCGACGGCGATTTATCTGTCGGGCAAGCTGGCCCCGGAGCTGCTGGGG
GCCATCGCGGTGGCGGCGTACTCGTATATGGCGCTGGTGCCGTTAATCCAGCCGCCGATT
ATGCGCGCGCTGACCAGCGAGAAAGAGCGCAAAATCCGCATGGTGCAGCTGCGCACGGTG
AGTAAGCGGGAAAAAATCCTCTTCCCGGTGGTGCTGTTACTACTGGTGGCCCTGCTGCTG
CCGGACGCCGCGCCGCTGCTGGGGATGTTCTGCTTTGGCAACCTGATGCGTGAAAGCGGC
GTGGTGGAGCGTCTGAGCGACACGGTACAGAACGGGCTGATTAATATCGTGACCATCTTC
CTCGGGCTGTCGGTGGGCGCGAAGCTGGTGGCGGATAAGTTTTTGCAGCCGCAGACGCTG
GGCATTCTGCTGCTGGGTGTGATTGCGTTCGGGATTGGGACGGCGGCCGGGGTGCTGATG
GCGAAGCTGATGAACCTGTGCAGTAAGAACAAAATCAACCCGCTTATCGGTTCGGCGGGG
GTGTCGGCGGTGCCGATGGCGGCACGCGTATCGAACAAGGTGGGCCTGGAGTCGGACCCG
CAGAACTTCCTGCTGATGCACGCGATGGGCCCGAACGTGGCGGGGGTTATCGGTTCGGCC
ATTGCCGCGGGCGTGATGCTGAAGTACGTGCTGGCAATGTAA
>EECEBICD_00100 2-(5''-triphosphoribosyl)-3'-dephosphocoenzyme-A synthase
ATGAGTGAAATGGTTGCGTTTCGGCAAGGTACATCCATGCCATCCAGAGAAACCATCTTG
CGTTATGTTGTTGAGACGGTGAATCAGATAACTGAGCTTGAGCCAGCACTGCATTTGCTG
CCGTGGTCTGGCGTCAACTCAGCAATATATGAGCAACGTTTTGCACAGTGTTACGACGAG
GGGCTTTGTGCCGCACAGACGTCGGCACCGAATGTTCCGCAGGGGATTCTGCCATCGACC
GATTGGGCTCAGGGTATCGGGTTACTCTGTTTTGCCGCAGGGTATATGAGCGCAGGCGAG
CGGCCATTAACACACAATCAGTTATGCGATTTCGTCAAACAGGCGGCTGTTGGTTTATCG
CCGATTGAGGGGGAGGCGGCGAGCGGTTTCTCGACGGTACGTAGTATTGCATTACCCGTA
TTCAGGCGATTACAACGCGATGGTCACGCGTCTCGGATCCTGCTGTTACAAACGTTGCTG
CATCTGGTTGCCTGGAAAAGCGCATCGCAGTATGCGCGTCAACAGGCGCAACGACTGCTC
TGGATGGGCGGCATTTTGGGGGAAGGGGGGAGCATAGCCTGCTTGTACTGGATAAGGCGC
TGCGTGAAGAAGCCGTCGGGGAGAAAAGTCTCCCCGCTCTGCTGA
>EECEBICD_00101 Serine endoprotease DegS
ATGTTTGTGAAGCTCTTACGTTCGGTCGCAATAGGTTTAATTGTCGGCGCTATTCTGTTG
GCCGTCATGCCTTCTTTGCGCAAAATTAATCCTATCGCCGTCCCGCAATTCGACAGTACC
GATGAGACGCCAGCCAGTTATAATTTTGCGGTTCGCCGCGCCGCGCCTGCCGTCGTCAAC
GTCTATAACCGCAGTATGAACAGTACCGCGCATAATCAACTGGAGATCCGCACGCTAGGT
TCCGGCGTGATCATGGATCAACGCGGTTATATTATTACCAACAAGCACGTGATTAACGAT
GCCGATCAGATTATCGTCGCGCTACAGGATGGCCGCGTCTTTGAAGCGCTACTGGTTGGC
TCCGATTCGCTTACCGATCTGGCGGTGCTGAAGATCAACGCCACTGGCGGGCTGCCTACC
ATCCCGATTAATACAAAGCGTACACCGCATATTGGCGACGTCGTACTGGCTATCGGCAAC
CCATATAATCTGGGACAGACCATCACCCAGGGGATCATCAGCGCAACGGGTCGTATCGGC
CTGAACCCGACGGGGCGACAGAATTTTCTCCAGACCGACGCCTCGATTAACCACGGTAAT
TCCGGCGGCGCGCTGGTCAACTCGTTAGGCGAACTGATGGGGATCAACACCCTCTCTTTT
GATAAGAGTAACGATGGCGAAACGCCGGAAGGCCTTGGTTTTGCGATTCCCTTCCAGCTA

GCCACGAAAATTATGGATAAGCTTATCCGCGACGGTCGCGTGATTCGCGGCTATATCGGT
ATTGGCGGACGAGAAATCGCGCCGCTGCACGCGCAGCAGGGTAGCGGCATGGACCCGATT
CAGGGCATTGTCGTTAATGAAGTGACGCCAAACGGCCCCGCCGCGCTTGCCGGTATTCAG
GTTAATGATTTGATTATTTCGGTCAATAATAAACCCGCTGTGTCCGCGCTGGAGACGATG
GATCAGGTGGCGGAAATCCGCCCGGGCTCCGTCATTCCGGTCGTGGTAATGCGGGATGAT
AAGCAGCTCACGTTCCAGGTGACGGTGCAGGAATACCCGGCGTCGAACTAA
>EECEBICD_00102 Periplasmic pH-dependent serine endoprotease DegQ
ATGAAAAAACACACCCAGCTGTTAAGTGCATTAGCGTTAAGTGTCGGGTTAACTCTTTCG
GCGCCGTTTCCAGCCCTTGCATCGATACCAGGCCAGGTGCCAGGCCAGGCGACGCTGCCA
AGCCTTGCCCCTATGCTGGAGAAAGTGCTGCCTGCTGTCGTCAGCGTAAAAGTCGAGGGA
ACCGCCGCCCAGAGCCAAAAAGTGCCGGAGGAGTTTAAAAAATTCTTTGGCGAGGATCTG
CCAGACCAGCCGTCCCAGCCGTTTGAAGGACTCGGTTCGGGGGTGATTATCGATGCCGCG
AAAGGCTATGTATTAACCAATAATCATGTGATTAATCAGGCACAGAAGATCAGTATTCAA
CTGAATGACGGACGCGAATTCGACGCGAAGCTGATCGGCGGCGACGACCAGAGCGATATC
GCTCTGTTACAAATTCAGAATCCCAGCAAGTTAACGCAAATTGCCATCGCCGATTCCGAC
AAACTCCGCGTCGGCGATTTCGCCGTGGCGGTCGGTAATCCGTTTGGTCTAGGACAAACC
GCCACCTCCGGGATTATTTCAGCGCTGGGACGCAGCGGGCTTAATCTGGAAGGGCTTGAG
AACTTTATTCAAACCGATGCCTCTATTAACCGCGGCAACTCCGGCGGCGCGCTGCTTAAC
CTGAACGGCGAGCTGATCGGGATTAATACCGCGATCCTCGCGCCAGGGGGCGGGAGCATC
GGCATTGGCTTTGCTATTCCTTCCAATATGGCGCAGACGCTGGCGCAGCAGTTGATTCAG
TTCGGCGAAATCAAACGCGGATTGCTGGGAATTAAAGGCACTGAAATGACCGCTGATATC
GCTAAGGCATTCAAACTGAACGTTCAGCGTGGCGCTTTTGTCAGCGAGGTTTTACCCAAT
TCAGGTTCGGCGAAGGCCGGGGTGAAATCCGGAGACGTGATTATCAGTCTTAACGGTAAG
CCGCTGAATAGCTTTGCCGAACTGCGTTCGCGTATCGCCACCACCGAACCGGGCACGAAA
GTGAAGCTGGGCCTGCTGCGCGATGGTAAGCCGCTGGAGGTGGAAGTCACGCTGGATTCC
AATACCTCTTCTTCCGCCAGCGCCGAAATGATCGCCCCGGCGTTGCAAGGCGCGACGTTG
AGCGACGGCCAGCTGAAAGACGGGACGAAAGGCGTTAAGGTTGATAGCGTCGAAAAAAGC
AGTCCTGCCGCGCAGGCCGGTTTGCAAAAAGATGATGTTATCATCGGCGTTAACCGCGAT
CGCATCAGTTCTATCGCCGAAATGCGCAAAGTGATGGCGGCAAAACCGTCCATCATTGCT
CTTCAGGTAGTACGCGGCAACGAGAACATTTATCTATTGCTGCGCTAA
>EECEBICD_00103 Inner membrane protein YhcB
ATGACCTGGGAATATGCGCTAATTGGATTAGTCGTCGGTATCATCATTGGTGCCGTGGCC
ATGCGTTTTGGTAATCGTAAGTTACGTCAACAGCAGGCATTGCAGTACGAACTGGAAAAA
AACAAAGCTGAGCTGGAAGAGTACCGTGAAGAGCTAGTTAGCCACTTTGCCCGCAGCGCG
GAACTGCTGGATACCATGGCGCATGATTACCGCCAGCTGTATCAGCATATGGCGAAAAGC
TCCAGCAGCCTGCTGCCGGAAATGTCTGCGGAGTCGAATCCGTTCCGTAACCGTCTGGCG
GAGTCTGAGGCGAGCAACGATCAGGCGCCAGTACAAATGCCACGTGATTATTCTGAAGGC
GCGTCTGGCCTGTTACGTAGCGGCGCAAAGCGCGATTAA
>EECEBICD_00104 Cell division protein ZapE
ATGCAAAGCCTTTCCCCTACATCGCGTTATCTCCAGGCACTCAACGAAGGCACCCATCAG
CCTGACGACGTACAGAAAGAGGCGGTCGACCGTCTGGAAACCCTTTATCAGGCGCTCACC
GCGAAAAAAATCGTCGGCTACGCCACCCGGCGGACTGATAGCGCGACTGGGGAAATTATTA
GGCAAAAACGAACCGGACGCGCAAATACCGGTTCGCGGGCTGTATATGTGGGGGGGGCGT
GGGTCGCGGCAAAACCTGGCTCATGGATCTCTTCTACCACAGCCTGCCGGGTGA
>EECEBICD_00105 Cell division protein ZapE
GTGGGTCGCGGCAAAACCTGGCTCATGGATCTCTTCTACCACAGCCTGCCGGGTGAGCGA
AAATTACGCCTGCATTTTCATCGGTTTATGCTGCGCGTGCATGAAGAACTTACCGCGCTA
CAGGGACAGATCGATCCGTTAGACATTATTGCCGATCGGTTTAAAACCGAGACGGACGTA
CTGTGTTTCGATGAGTTTTTCGTCACCGATATTACCGACGCCATGCTGCTGGGCGGTTTA
ATGAAAGCGCTGTTTGCACGCGGCATTACGCTGGTCGCGACCTCCAATATTCCGCCGGAT
GAACTCTACCGCAACGGTTTGCAACGCGCCCGCTTCCTGCCGGCTATCGATGCCATTAAA
CAGCACTGCGACATTATGAATGTGGACGCCGGCGTCGATTACCGGCTGCGGACGCTGACG
CAGGCCCATTTATGGCTAACGCCGCTTAATGATGAAACCCGGCGGCAAATGGATAAACTG
TGGCTGGCGTTAGCGGGGGCCGCGCGCGAGCACGCTCCGACGCTGGAAATCAATCATCGC

TCTTTATCCACCCTGGGCGTTGAAAACCAAACGCTAGCGGTCTCTTTCGCTACGCTGTGC
GTCGAGGCCCGCAGCCAGCATGATTACATTGCGCTCTCAAGGTTATTTCACACGGTTCTG
CTGTTTGACGTGCCGGTCATGACGCCGCTGATGGAGAACGAAGCGCGCCGCTTTATTGCG
CTGGTGGATGAGTTTTACGAGCGCCACGTTAAGCTGGTGGTCAGCGCCGCCGCACCGTTA
TATGAAATTTATCAGGGGGAACAGCTCAAGTTTGAATTTCAGCGCTGTCTGTCACGCCTG
CAAGAGATGCAGAGTGCGGAGTACCTCAAGCGGGAGCATATGCCGTAA
>EECEBICD_00106 50S ribosomal protein L13
ATGAAAACTTTTACAGCTAAACCAGAAACCGTAAAACGCGACTGGTATGTTGTTGACGCG
ACCGGTAAAACTCTGGGCCGTCTGGCTACTGAACTGGCTCGTCGCCTGCGCGGTAAGCAC
AAAGCGGAATACACTCCGCACGTAGATACTGGTGATTACATCATCGTTCTGAACGCTGAC
AAAGTTGCTGTAACCGGCAACAAGCGTACTGACAAAGTGTACTATCACCACACCGGCCAC
ATCGGTGGTATCAAACAAGCGACCTTTGAAGAGATGATTGCCCGCCGTCCTGAGCGTGTG
ATTGAAATCGCGGTTAAAGGCATGCTGCCAAAAGGCCCGCTGGGTCGTGCTATGTTCCGT
AAACTGAAAGTTTACGCGGGCAACGAGCACAACCACGCGGCACAGCAACCGCAAGTTCTT
GACATCTAA
>EECEBICD_00107 30S ribosomal protein S9
ATGGCTGAAAATCAATACTACGGCACTGGTCGCCGCAAAAGTTCCGCAGCTCGCGTGTTC
ATCAAACCGGGCAACGGTAAAATCGTTATCAACCAACGTTCTCTGGAACAGTACTTCGGT
CGTGAAACTGCCCGCATGGTAGTTCGTCAGCCGCTGGAACTGGTCGACATGGTTGAGAAA
CTGGATCTGTACATCACTGTTAAAGGTGGTGGTATCTCTGGTCAGGCTGGTGCGATCCGT
CACGGTATCACCCGCGCTCTGATGGAGTACGACGAGTCCCTGCGTGGCGAACTGCGTAAA
GCTGGCTTCGTTACTCGTGACGCTCGTCAGGTTGAACGTAAGAAAGTCGGCCTGCGTAAA
GCACGTCGTCGTCCGCAGTTCTCCAAACGTTAA
>EECEBICD_00108 hypothetical protein
GTGGGATTTTTTAACTCATTGTTTACTAAGAAAGAGTCGTTAATGCTGTCGAATAAATGG
GAATTCTTTATAACAACGGTGGATGACCATGTTACGGGTATACGTGTGGATATCGGCGCC
ATTCAGGATGAAAAAATTTGATCGCCTTATCCATACCGGGTTCTTACGTGTTCATTACACC
AACTGTTATGAAAATGGTTTACCGCAGCCCGATGAAACACAGCGTTTAAATCGTATAGAG
GACTGGCTTGATGAAAAGGGGAAGACGTTTCCGATCTGGCTGGTGGGTGTGGTTACCCAA
CAAGGCTGGCGCGATTTTGTGTTCATGTCGGAAGAGGATCTTAACTGGGAAAAGACGCTG
GATAAATTGCTCGCTGGTGGCCCGGAAATCTCTTTTAGTTATAGAGAGAGTCACAATGAT
AAAGGAAACTTTTACCGCCAGTTCTTGTATCCAACCCGATACGACTGGAACTGGATACAC
GATTCCCGTGTGTGCCGGGGATTACAGGAACAAGGTGATGATCTGACGCTTCCGCGCGCT
ATTGATTATTATGCGACATTGCCTACAGAGGTCGCAGCGCGCGACTTGGCGCAAGATATT
GCTGCATTACCGTATGGCATAACGCTGGTCAGTATTCGAATGAATGACCCGCAACAGGGG
TTTATGGCGTCTTTTATCAGCACCGATGCTCCGCAACAATGGCATATGACAGAAATCACT
TGCCAGCTTACCGATCTGGCTGAAAAACATGGCGGCAGTTTTGATGGCTGGGGCGCACCG
GTAGTACAGGCATAA
>EECEBICD_00109 Stringent starvation protein A
ATGGCTGTCGCTGCCAACAAACGTTCGGTAATGACGCTGTTTTCTGGTCCTACTGACATC
TATAGCCATCAGGTCCGCATTGTGTTGGCTGAAAAGGGTGTTAGTTTTGAGATCGAACAT
GTGGAAAAGGACAATCCACCTCAGGATCTGATTGACCTCAACCCGAATCAAAGCGTTCCG
ACACTGGTGGATCGTGAGCTGACCCTGTGGGAATCTCGCATCATTATGGAATATCTGGAT
GAGCGTTTCCCTCATCCGCCGCTGATGCCGGTTTATCCGGTCGCGCGCGGTGAAAGCCGT
CTGTACATGCACCGTATTGAAAAAGACTGGTATACGCTGATGAACGTCATCGTAAACGGG
TCTGCTTCCGAGGCCGACTCTGCGCGTAAACAACTGCGTGAAGAGCTGCTGGCGATTGCG
CCGGTATTTGGTCAGAAACCGTATTTCCTGAGCGACGAATTCAGCCTGGTTGATTGCTAC
CTGGCGCCTCTCTTGTGGCGTTTACCGCAGTTGGGTATTGAATTCAGCGGCGCAGGGGCG
AAAGAGCTGAAAGGCTATATGACCCGCGTCTTTGAACGTGATTCTTTCCTGGCTTCTTTA
ACCGAAGCTGAACGTGAAATGCGCCTCGGTCGGGGTTAA
>EECEBICD_00110 Stringent starvation protein B
ATGGATTTGTCACAGTTGACGCCGCGTCGTCCCTATCTGTTGCGTGCTTTCTACGAGTGG
TTGCTGGATAACCAGTTGACTCCGCATCTGGTGGTGGATGTGATGCTGCCTGGCGTGCAT
GTCCCTATGGAATATGCGCGCGATGGGCAAATCGTCCTCAATATTGCGCCGCGGGCGGTA

GGTAATCTGGAGCTGTCTAATGATGAAGTGCGTTTTAATGCGCGCTTCGGCGGCGTTCCC
CGTCAGGTTTCTGTGCCTTTGGCTGCGGTGCTGGCGATTTACGCCCGTGAAAACGGCGCA
GGCACCATGTTTGAGCCTGAAGCCGCCTATGATGAAGACGTTGTCAGCTTAAATGATGAT
GACAATACCGCAGGCGCGGAAAGCGAGACCGTGATGTCCGTCATTGATGGCGATAAGCCC
GACCACGATGACGACAGTAGTCCTGATGACGAGCCGCCTCCGCCGCGTGGCGGACGTCCG
GCACTGCGCGTAGTAAAATAA
>EECEBICD_00111 HTH-type transcriptional repressor NanR
ATGAACGCATTTGATTCGCAAGCAGAAGATTCACCGACATCCCTCGGGCGTAGTTTGCGC
CGCCGCCCGCTGGCGCGTAAAAAACTGTCTGAAATGGTTGAGGAAGAGCTGGAGCAGATG
ATCCGCCGTCATGAGTTTGGCGAGGGAGAACAGCTACCGTCTGAACGTGAATTAATGGCC
TTCTTCAATGTGGGACGTCCCTCCGTGCGTGAAGCGTTAGCTGCGCTGAAGCGTAAAGGT
CTGGTGCAAATCAATAACGGCGAACGCGCCCGCGTCTCGCGCCCCTCTGCTGATACCATC
ATCAGCGAACTGTCGGGTATGGCGAAAGATTTTCTTACTCATCCTGGCGGCATCGCTCAC
TTTGAACAGCTACGGCTTTTCTTTGAATCCAGCCTGGTACGCTATGCGGCGGAACATGCC
ACCGACGAGCAGATCGCGTTGTTAACGAAAGCGCTGGAAATTAATAGCCAGTCGCTGGAT
GACAACGCGCTGTTTATTCGTTCGGATGTCGAGTTTCACCGGGTGTTGGCTGAAATTCCC
GGCAATCCGATCTTTATGGCTATTCATGTTGCTCTGCTGGACTGGCTAATCGCCGCTCGC
CCAAGCGTTCCCGATCGTGAGCTGCATGAACATAATAACGTGAGTTATCAACAGCATATT
GTGATTGTTGATGCCATTCGCCAGCGAGATCCCGATAAAGCGGATCGCGCTCTGCAAACC
CACCTCAATAGCGTTTCCGCTACCTGGCACGCACTCGGTAAAAAAAGTCAAAAAATGCGG
TAG
>EECEBICD_00112 N-acetylneuraminate lyase
ATGGCAAAAGCATTGCAAGGCGTAATGGCGGCGTTGTTGACCCCATTTGATCATCAGCAG
CAACTGGATAGCGAAAGCCTGCGCCGTCTGGTGCGTTTTAACATTGGGCAGGGTATCGAC
GGGCTGTACGTTGGCGGTTCCACCGGGGAAGCATTCGTACAGAGCCTTGCTGAAAGAGAG
CAGGTACTGGAGATTGTCGCCGAAGAGGCGAAGGGAAAAATCACGTTGATCGCCCATGTC
GGGACAGTAAGCACCGCAGAAAGCCAGCAGCTTGCCAGCGCCGCAAAGCGTTACGGTTTT
GATGCGGTCTCTGCGGTGACGCCTTTTTATTACCCTTTCAGTTTTGAAGAGCACTGTGAC
CATTATCGGGCAATCATTGATTCCGCAGATGGATTGCCGATGGTGGTATACAACATTCCG
GCGTTAAGCGGCGTAAAACTTACCCTCGATCAGATCAATACGCTGGTGACATTACCGGGC
GTGAGCGCGCTGAAGCAAACCTCCGGCGATCTCTTCCAGATGGAGCAGATCCGCCGCGCG
CATCCGGATCTGGTGCTGTACAACGGTTATGACGAAATCTTCGCTTCCGGTCTGCTGGCG
GGCGCGGACGGCGGTATCGGCAGCACATACAACATTATGGGCTGGCGTTATCAGGGGATT
GTTCAGGCATTACGTGAGGGCGATGTGGCGAAAGCGCAGCGCCTGCAAACCGAGTGCAAT
AAGGTTATTGATTTACTGATTAAAACCGGCGTATTCCGCGGCCTGAAAACGGTCCTGCAT
TATATGGACGTGCTATCGGTGCCGCTGTGTCGTAAACCGTTTGCGCCTGTTGATGAAAAA
TACCTGCCGGCGCTCAAGGCGCTGGCTCAACAACTGATGGAAGAAAAGGCGTAA
>EECEBICD_00113 Sialic acid transporter NanT
ATGAGTACTTCTACCCAGAACATCCCGTGGTATCGCCATCTCAACCGGGCGCAGTGGCGG
GCATTTTCCGCTGCCTGGCTGGGATATCTGCTTGATGGTTTTGATTTTGTGTTGATTGCT
CTTGTACTGACTGAGGTACAAAGCGAATTTGGGCTGACGACGGTACAGGCGGCAAGCCTG
ATTTCTGCGGCTTTTATCTCTCGCTGGTTCGGCGGGTTATTACTGGGCGCGATGGGCGAT
CGCTATGGGCGTCGTCTGGCGATGGTCAGCAGCATCATTCTGTTTTCGGTGGGAACCCTG
GCATGCGGGTTTGCGCCCGGTTACACCACCATGTTCATCGCCCGACTGGTGATTGGTATG
GGCATGGCGGGCGAATATGGTTCCAGCGCGACCTATGTGATTGAAAGCTGGCCAAAACAT
TTACGCAATAAAGCCAGCGGTTTTCTGATTTCCGGCTTCTCCGTCGGCGCGGTCGTTGCC
GCGCAGGTGTACAGCCTGGTGGTGCCTGTCTGGGGCTGGCGCGCGCTGTTTTTCATTGGC
ATTTTGCCAATTATCTTCGCTCTCTGGCTGCGGAAAAACATTCCGGAAGCGGAAGACTGG
AAAGAGAAACACGCGGGTAAAGCGCCGGTACGCACGATGGTCGACATTCTTTATCGGGGC
GAGCATCGCATCATCAACATTTTAATGACTTTCGCCGCCGCCGCTGCGCTGTGGTTCTGT
TTTGCCGGTAACCTACAAAATGCTGCGATTGTGGCGGGGCTGGGACTACTGTGCGCGGTT
ATCTTTATCAGCTTTATGGTGCAGAGCAGCGGTAAACGCTGGCCCACTGGCGTCATGCTG
ATGCTGGTGGTACTGTTTGCTTTCCTCTATTCCTGGCCGATTCAGGCGCTATTACCCACT
TATCTGAAAACCGAGCTGGCCTACGATCCGCATACGGTGGCGAATGTCCTGTTCTTTAGC

GGATTTGGCGCGGCGGTTGGTTGCTGCGTAGGCGGTTTTCTTGGCGACTGGCTGGGAACG
CGTAAAGCATATGTCTGTAGCCTGCTGGCCTCGCAAATCCTCATTATTCCGGTCTTTGCG
ATTGGCGGCACAAACGTCTGGGTTCTGGGTCTGCTACTGTTTTTCCAACAGATGTTGGGG
CAGGGGATTGCCGGGATTCTACCGAAACTGATCGGCGGTTACTTCGATACCGATCAGCGC
GCGGCGGGGCTGGGCTTTACTTATAACGTCGGCGCGCTCGGCGGCGCGCTGGCGCCGATC
CTGGGAGCGCTGATCGCTCAACGTCTGGATTTGGGCACTGCGCTGGCATCGCTCTCTTTC
AGCCTGACGTTTGTCGTGATCCTGCTTATTGGGCTTGATATGCCGTCTCGCGTACAGCGT
TGGCTACGTCCGGAAGCGTTACGCACCCACGATGCTATTGACGACAAACCGTTCAGCGGA
GCCGTACCGCTTGGCAGTGGTAAAGGTGCCTTTGTAAAAACGAAAAGTTAA
>EECEBICD_00114 Putative N-acetylmannosamine-6-phosphate 2-epimerase 2
ATGTCGTTACTTGAACAACTGGATAAAAATATTGCCGCTTCTGGCGGCCTGATTGTCTCC
TGCCAGCCCGTTCCGGGCAGTCCGCTGGATAAGCCGGAGATTGTAGCGGCAATGGCGTTG
GCAGCGGAACAGGCGGGCGCGGTTGCGGTGCGTATTGAAGGCATAGACAACCTGCGTATG
ACCCGCTCGCTCGTTTCGGTACCCATTATCGGCATTATCAAACGCGATCTGGACGACTCT
CCAGTGCGCATTACCCCTTTTCTCGACGATGTAGATGCGCTGGCACAGGCCGGAGCAGCT
ATTATCGCCGTTGATGGAACCGCTCGGCAACGGCCGGTAGCCGTTGAGGCGTTGTTGGCG
CGCATTCATCACCACCATCTACTGGCGATGGCCGACTGCTCATCCGTGGACGATGGTCTC
GCCTGTCAACGGTTGGGCGCGGACATTATCGGCACGACAATGTCCGGCTACACCACCCCC
GATACGCCGGAGGAACCCGATCTGCCCTTAGTCAAAGCGTTGCATGACGCCGGATGCCGG
GTAATAGCCGAAGGGCGCTATAACTCGCCAGCACTGGCAGCGGAGGCGATCCGCTATGGC
GCATGGGCGGTGACGGTGGGGTCCGCCATTACCCGTCTGGAACATATCTGTGGATGGTAT
AACGACGCATTAAAAAAGGCGGCGTCATGA
>EECEBICD_00115 N-acetylmannosamine kinase
ATGACTACGTTAGCAATCGATATTGGCGGTACTAAACTTGCCGCCGCGCTCATCGACAAA
AACTTGCGCATTAGCCAGCGTCGCGAGCTACCGACCCCCGCGAGCAAAACGCCGGATGCG
TTACGTGAAGCATTAAAGGCGCTGGTTGAACCACTACGGGCAGAGGCGCGTCAGGTAGCG
ATAGCTTCTACCGGGATCATTCAGGAAGGGATGCTGCTGGCGCTAAACCCCCATAATCTG
GGCGGATTGCTACACTTTCCGTTAGTACAAACACTGGAAACGATCGCTGGCCTGCCGACG
CTGGCGGTGAACGACGCGCAGGCGGCGGCCTGGGCGGAGTATCACGCGCTCCCTGATGAT
ATCCGCGATATGGTATTTATCACCGTTTCAACCGGCGTTGGCGGCGGCGTTGTCTGCGAC
GGCAAGCTCCTTACCGGAAAAGGCGGTCTGGCCGGGCACCTGGGACACACCCTGGCGGAC
CCACACGGGCCCGTATGCGGCTGTGGACGCGTTGGCTGCGTAGAGGCCATCGCCTCTGGA
CGAGGGATGGCCGCGGCGGCGCGGGACGATCTGGCGGGATGCGACGCCAAAACCCTATTT
ATTCGCGCTGGCGAAGGTCATCAGCAGGCGCGACATCTGGTAAGCCAATCCGCGCAGGTA
ATAGCGCGAATGATCGCCGATGTGAAAGCGACTACCGACTGCCAGTGTGTGGTCATTGGC
GGCAGCGTAGGGTTAGCTGAGGGATACCTGGAGCAGGTTCGCGCGTTTTTGATGCAAGAG
CCTGCGCCTTATCACGTGGCGCTGAGCGCCGCTCGCTATCGGCATGACGCAGGATTATTA
GGCGCTGCGTTGTTAGCTCAAGGAGACACATTATGA
>EECEBICD_00116 putative protein YhcH
ATGATGATGGGGGAAGTACAGTCGTTGCCTTCTTGCGGGTTGCATCCGCGATTGTTGGAT
GCCTTGACTCTCGCGCTGGCGGCAAGGCCGCAGGAGAAAGCGCCGGGGCGCTATGAATTG
CAGGGCGACAATGTCTTTATGAATGTAATGCAGTTAACGACGCAAATGCCGGCCGAGAAG
AAAGCGGAATTGCATGAACAATACATTGATATTCAGCTACTACTAACCGGCGTCGAGCGT
ATTGCGTTTGGGATGTCCGGTGCCGCTCGCCAGTGCGAGGAGATGCACGTTGAAGAGGAT
TACCAGCTTTGTAGTCAAATTGTCGACGAGCAGACTATTACGCTGCAAGCGGGGATGTTT
GCTGTGTTTATGCCAGGAGAACCGCATAAACCGGGATGTGCAGTGGGTGAGCCGGATGAC
ATCAAGAAAGTGGTAGTGAAAGTACGCGCCAGTCTGCTGGCGGCCTGA
>EECEBICD_00117 Cytosine deaminase
ATGCAGAACAATAACATCACTATTCGTCAGACGCGTCTGCAGGGACACGAAGGATTATGG
CAGATTACGATTGAAAACGGGCGCTTTAGCCGGATTGAGCCTCAGGAAGCCGCATCGTTA
CCGCAGGGCGAAGTGCTTGATGCCGAAGGCGGCCTGGCTATTCCACCGTTTGTTGAGCCA
CATATCCACCTGGATACCACGCAAACGGCGGGTGAACCGAGCTGGAACCAGTCCGGCACA
CTGTTTGAAGGGATTGAGCGCTGGGCAGAACGTAAAGCAATGCTCACGCATGAAGATGTG
AAAGCGCGCGCGATGCAGACCCTGAAATGGCAGATGGCTAACGGCATCCAGTACGTTCGT

ACTCACGTTGACGTTTCAGATCCTACGCTCACCGCGCTGAAAGCCATGCTGGAAGTGAAG
CAAGAGGTTGCGCCGTGGGTAGACCTGCAAATTGTCGCCTTTCCGCAAGAAGGTATTCTG
TCTTATCCCAACGGTGAAGCGCTGTTAGAGGAAGCCGTGCGTTTGGGCGTTGACGTTATT
GGCGCCATTCCGCACTTTGAATTTACGCGTGAATATGGCGTTGAATCGCTGCACAAAACC
TTCGCCCTGGCGCAAAAATACGATCGCTTGATCGATGTGCACTGCGACGAAATTGATGAC
GAGCAGTCTCGCTTTGTCGAAACGGTCGCGGCGCTGGCGCATCGCGATGGCATGGGGGCG
CGCGTGACCGCCAGCCACACCACGGCTATGCACTCGTACAACGGCGCGTATGCGTCACGC
CTGTTCCGTCTGTTGAAAATGTCGGGGATTAACTTCGTCGCCAACCCGCTGGTGAATATT
CATCTGCAAGGACGGTTTGACACTTACCCGAAACGTCGTGGCGTCACGCGAGTGAAAGAG
ATGCTGGAAGCGGGGATCAACGTCTGCTTTGGCCATGATGACGTCTTCGATCCGTGGTAT
CCATTAGGCACGGCAAATATGCTGCAGGTGCTGCATATGGGGTTACACGTTTGCCAGTTG
ATGGGGTATGGGCAAATCAACGACGGGTTAAATCTGATCACTACCCACAGCGCAAAAACC
CTGCATTTACAGGACTACGGTCTGAGCGTCGGCAATGCCGCGAATCTGGTTATCTTACCC
GCTGAGAATGGATTCGATGCGGTACGTCGCCAGACGCCTGCGCGTTACTCGATTCGCCAC
GGGCGAGTGATTGCCGAGACGGTGCCGAGCCAGACGACGCTGCACCTGACCCAGCCGGAA
GCCGTGACGTTTAAGCGTTAA
>EECEBICD_00118 Cytosine permease
GTGTCGCAGGACAACAATTATAGCCAGGGCCCCGTCCCTCTGGCGGCGCGGAAGGGCGTG
ATTCCACTGACGTTTGTCATGTTGGGTTTAACGTTTTTTTCCGCCAGTATGTGGACCGGA
GGGACACTCGGCACCGGTCTTACCTATCACGATTTCTTCCTCGCAGTTCTCTTCGGTAAT
CTCCTCCTCGGTATCTACACTGCATTTCTTGGTTACATCGGCGCAAAAACCGGACTCTCC
ACCCACCTCCTTGCACGTTACTCCTTTGGCGTTAAAGGATCATGGCTTCCCTCGCTGCTG
CTAGGCGGCACACAGGTAGGCTGGTTTGGCGTTGGCGTAGCGATGTTCGCTATTCCGGTC
AGTAAAGCGACGGGCATTGATGCCAATATTCTGATTGCCATTTCGGGTCTACTGATGACC
CTGACCATTTTTTTCGGCATCTCGGCGTTGACCATTTTGTCTATCATTGCCGTACCCGCG
ATCGTTATACTGGGCAGCTACTCCGTCTGGCTGGCGGTCAGCGGCGTGGGTGGGCTGGAG
CATTTAAAAACGATAGTGCCGCAGACGCCGCTGGATTTTTCCAGCGCGCTGGCGCTGGTG
GTGGGCTCGTTTGTCAGCGCCGGTACATTGACCGCCGACTTCGTCCGCTTCGGGCGTCAT
GCCAAAAGCGCCGTACTGATTGCGATGGTCGCTTTTTTCCTCGGCAACTCGCTGATGTTT
ATCTTTGGCGCGGCAGGCGCTGCCGCCGTCGGTCAGGCGGATATCTCTGACGTGATGATA
GCGCAGGGGCTGCTGCTGCCCGCGATTGTGGTGCTTGGCCTGAATATCTGGACCACCAAC
GATAACGCGCTGTACGCATCGGGTCTGGGCTTCGCCAATATTACCGGTCTTTCCAGCCGT
ACGCTGTCGGTGGTGAACGGGATTATCGGTACCGTGTGCGCGCTGTGGCTTTACAATAAT
TTTGTCGGCTGGCTGACGTTCCTGTCATCTGCCATCCCACCGATTGGCGGAGTGATTATT
GCCGACTATCTGTTGAACCGCCGCCGCTATGCCGACTTCAACACCGTGCGCTTTATTCCC
GTTAACTGGATTGCTATTCTTTCCGTCGCGCTGGGCATCGCCGCCGGACATTATGTTCCG
GGTATTGTGCCCGTCAACGCCGTACTCGGCGGCGTCTTCAGCTATATCCTGCTGAATCCA
CTTTTCAACCGCAGCCTTGCTAAATCACCAGAGGTCAGCCATGCAGAACAATAA
>EECEBICD_00119 Putative nuclease YhcG
ATGACGAATCCAACTTTAGTGCCACAGTCTGATGAATATCAGCAAATTCATGACGGTATT
ATTCGTCTGGTCGATACGGCACGTACCGAGACCGTGCGCAGCATCAATGCCATTATGACT
GCCACCTACTGGGAAATTGGCCGACGCATTGTCGAATTTGAACAGGGAGGAGAAGCGCGA
GCGGCCTATGGTACTCAGCTTATCGAACGATTATCGGTAGATTTAAGTCAGCGCTATAAG
CGCGGTTTTTCCACCAGAAACCTATGGCAAATTCGTACATTTTATCTTTGTTTTCAACAC
ATTGAAATTCCGCAGACACTGTCTGCGGAATTTTCAAATCTCATACCGCTCGCCAAAACA
TTCCCTCTTCCCTGGTCAGCGTATGTCCGCCTCTTGTCGGTAAAAAATCCCGATGCCCGC
ACTTTTTACGAGAAAGAGACGCTACGTAACGGCTGGTCAGTTAGGCAGCTCGATCGGCAA
ATCGCCACTCAGTTTTACGAACGAACGCTACTGTCCCACGACAAATCCGCCATGTTGCAG
CAGCCTGCACCCGCCGAGCCAAACGTTTTGCCGGAGCAAGCCATACGCGACCCGTTCATT
CTGGAATTTCTTAACCTGAAGGACGAGTATTCCGAATCAGATCTCGAAGACGCGCTGCTC
AGCCACCTGATGGACTTTATGCTGGAGCTCGGTAACGATTTTGCTTTTGTTGGCCGCCAA
CGCCGACTACGCATAGACGATAGCTGGTTCCGCGTCGATCTGCTGTTCTTCCATCGTCGC
TTGCGCTGCCTGTTGCTCGTTGACCTGAAAGTAGGCAAATTCGGTTATGCGGATGCCGGG
CAGATGAATATGTACCTGAACTATGCCAAAGAGCACTGGACAATGCCCGGAGAAAACCCG

CCAGTCGGCCTGGTTTTGTGCGCAGGGAAAGGCGCGGGGGAAGCGCATTACGCGCTGACT
GGCCTGCCAAACACCATCATGGCGAGCGAGTACAAAGTGCAGTTGCCCGACGAAAAATTA
CTCACAGACGAACTTATTCGTTCGCAAACCATGCTGGAAACTCAACTCACACGCGGTGGT
TCACTGACAACGGAGAAAAACTAA
>EECEBICD_00120 Glutamate synthase [NADPH] small chain
ATGAGCCAGAATGTTTATCAATTTATCGACCTGCAGCGCGTTGATCCGCCGAAGAAACCG
CTGAAGCTCCGTAAGATCGAGTTTGTTGAAATCTACGAACCGTTTTCTGAAGGTCAGGCG
AAAGCCCAGGCGGATCGCTGTTTGTCCTGCGGGAACCCCTACTGCGAGTGGAAATGTCCG
GTACATAACTACATCCCGAACTGGCTGAAACTGGCTAACGAAGGGCGTATTTTTGAGGCG
GCTGAGCTATCGCACCAGACCAACACCCTGCCGGAAGTGTGTGGCCGCGTCTGCCCGCAG
GACCGCCTGTGCGAAGGCTCCTGTACGCTGCACGATGAGTTCGGCGCAGTGACCATCGGT
AACATTGAGCGCTATATCAACGATAAAGCGTTTGAGATGGGCTGGCGCCCGGATATGACC
GGCGTACGCCAGACGGACAAACGGGTAGCGATAATCGGCGCAGGTCCGGCAGGGCTGGCG
TGCGCCGACGTGCTGACCCGCAACGGCGTGAAGGCGGTGGTCTTTGATCGCCATCCGGAA
ATCGGTGGTCTGCTGACCTTCGGTATCCCGGCCTTCAAGCTGGAGAAAGAAGTCATGACC
CGCCGCCGTGAGATCTTCACCGGCATGGGTATTGAGTTCAAACTTAATACCGAAGTGGGT
CGTGATGTACAGCTTGAAGATCTGCTGAAAGATTACGATGCGGTATTCCTTGGCGTCGGC
ACCTATCAGTCGATGCGCGGCGGGCTGGAAAATGAAGATGCGGACGGGGTTTTCGACGCT
CTGCCGTTCCTGATCGCCAATACGAAGCAGATCATGGGGTTTGGCGAAACCAGCGACGAA
CCATATGTCAGCATGGAAGGCAAACGCGTGGTGGTGCTGGGCGGCGGCGATACCGCAATG
GACTGCGTACGTACCTCCATTCGTCAGGGCGCAACGCACGTAACCTGTGCTTATCGTCGT
GATGAAGAGAACATGCCGGGCTCCAGACGCGAAGTGAAGAACGCGCGCGAGGAAGGTGTC
GAATTTCAGTTTAACGTTCAACCGCTGGGCATTGAAGTCAATGCCAACGGCAAAGTGAGC
GGCGTAAAAATGGTGCGCACTGAAATGGGCGAACCGGATGCCAAAGGCCGTCGGCGGGCA
GAGATCGTCGCCGGTTCCGAGCATGTGGTTCCGGCTGATGCGGTGGTGATGGCGTTTGGT
TTTCGTCCACACAGCATGGAATGGCTGGCGAAACACAGCGTGGAACTGGACTCGCAGGGA
CGAATTATCGCGCCGGAACGCAGCGATAATGCTTTCCAGACCAGCAACCCGAAAATCTTC
GCCGGTGGCGACATCGTGCGCGGTTCCGATCTGGTGGTGACGGCGATTGCCGAAGGCCGT
AAAGCGGCTGACGGGATTATGAACTACCTCGAAGTATAA
>EECEBICD_00121 Glutamate synthase [NADPH] large chain
ATGTTGTACGATAAATCCCTTGAGAAGGATAACTGTGGTTTCGGCCTGATCGCCCACATA
GAAGGCGAACCTAGCCACAAGGTAGTGCGTACCGCCATACACGCGCTGGCCCGGATGCAG
CACCGTGGCGCGATCCTTGCCGACGGTAAAACCGGCGACGGTTGCGGCTTGCTGTTACAA
AAACCTGACCGTTTCTTTCGCATCGTGGCCGAAGAGCGCGGCTGGCGCTTAGCCAAAAAC
TACGCCGTCGGGATGATCTTTTTGAATAAAGATCCTGAACTGGCCGCCGCGTCACGCCAT
ATTGTCGAAGAAGAATTACAGCAGGAAACCCTGTCGATTGTCGGCTGGCGCGATGTGCCC
ACCAATGAAGGCGTGCTCGGTGAAATCGCCCTCTCCTCCCTGCCGCGTATTGAGCAGATT
TTTGTCAACGCTCCGGCGGGCTGGCGTCCGCGCGATATGGAGCGCCGTCTGTTTATCGCC
CGCCGACGCATTGAAAAACGCCTGCAGAACGACAAGGATTTCTACGTATGCAGCCTGTCG
AACCTGGTCAACATTTATAAAGGTCTGTGTATGCCGGCGGATCTGCCGCGTTTCTACCTG
GATCTTGCGGATCTGCGTCTGGAATCGGCCATCTGTCTGTTCCACCAGCGCTTTTCCACT
AACACCGTGCCGCGTTGGCCGCTGGCGCAGCCGTTCCGCTACCTGGCGCATAACGGCGAG
ATTAACACTATCACCGGCAACCGCCAGTGGGCGCGCGCCCGTACCTATAAATTCCAGACG
CCGCTGATCCCGGATTTACAGTCCGCTGCGCCGTTTGTTAACGAAACCGGTTCGGATTCC
AGCTCGCTGGACAACATGCTGGAACTGCTGCTGGCAGGCGGGATGGACATCATCCGCGCC
ATGCGCCTGTTGGTGCCGCCCGCCTGGCAGAACAACCCCGATATGGACCAGGATCTGCGC
GCCTTCTTTGACTTTAACTCCATGCATATGGAGCCGTGGGACGGTCCGGCAGGCATCGTG
ATGTCCGACGGGCGCTTCGCCGCCTGTAACCTTGACCGTAACGGCCTGCGCCCGGCACGC
TACGTCATTACCAAAGATAAGCTGATCACCTGCGCCTCCGAAGTCGGTATCTGGGACTAT
CAGCCGGATGAAGTGGTCGAGAAAGGCCGCGTCGGCCCAGGCGAGCTGATGGTGATCGAC
ACCCGCGGCGGACGTATTCTGCATTCCGCGGAAACCGATGACGATCTGAAAAGCCGCCAT
CCATATAAAGAGTGGATGGAGAAAAACGTCCGCCGCCTGGTGCCGTTTGAAGAGCTGCCT
GATGAAGAAGTTGGTAGCCGCGAACTGGACGATGACCTGCTCGCCAGCTATCAGAAACAG
TTTAACTACAGCGCCGAAGAGCTGGACTCGGTTATCCGCGTATTGGGCGAAAACGGCCAG

GAAGCCGTCGGTTCAATGGGCGACGATACCCCCTTTGCCGTGCTCTCCAGCCAGCCGCGT
ATTATTTACGACTACTTCCGCCAGCAGTTCGCGCAGGTCACTAACCCGCCTATCGACCCG
CTGCGTGAAGCGCACGTCATGTCGCTCGCCACCAGCATCGGTCGCGAAATGAACGTCTTC
TGCGAAGCGGAAGGTCAGGCCCATCGCCTGAGTTTTAAATCGCCAATTCTGCTGTACTCC
GATTTCAAACAGCTCACCACCATGAAAGAGGAGCATTATCGCGCTGACCGTCTGGATATC
ACCTTCGACGTGACCGAAACCACACTGAACGCGACGGTTAAGGCGCTGTGCGATAAAGCG
GAACAGATGGTGCGCAACGGCACCGTGCTGCTGGTCCTCTCGGATCGCAATATCGGTAAG
AATCGCCTGCCGGTCCCCGCGCCGATGGCGGTGGGCGCGGTGCAAACGCGTCTGGTAGAA
CAAAGCCTGCGCTGCGACGCCAACATCATCGTAGAAACCGGAAGCGCCCGCGATCCGCAT
CACTTTGCGGTACTGCTCGGCTTTGGCGCAACGGCTATCTATCCGTACCTGGCCTACGAA
ACGCTGGGGCGTCTGATTGATACCCAGGCCATCGCCAAAAACTACCGTACCGTGATGCAG
AACTACCGTAACGGCATCAACAAGGGGCTGTACAAGATTATGTCCAAAATGGGCATTTCG
ACCATTGCCTCTTACCGCTGCTCCAAACTGTTTGAAGCGGTCGGACTGCACGATGATGTG
GTAAACCTGTGCTTCCAGGGCGTGGTCAGCCGTATCGGCGGCGCGAGCTTTGATGACTTC
CAGCAGGATCTGCTGAATCTCTCCAAACGCGCCTGGCTGGCGCGCAAACCCATTAGCCCG
GGCGGCCTGCTGAAGTACGTCCACGGCGGCGAATATCACGCCTATAACCCGGATGTCGTG
CGTACCCTGCAACAGGCGGTACAGAGCGGCGAATACAGCGATTATCAGGAATACGCCAAA
CTGGTCAATGAGCGTCCGGCGGCAACGCTGCGCGATCTGCTGGCGATACATCCAGACGGC
GAGGCGGTGACTATCGACGAGGTTGAGCCCGCCAGTGAGCTGTTTAAACGCTTTGATACC
GCAGCCATGTCTATCGGCGCATTAAGTCCGGAAGCGCACGAAGCGCTGGCGGAAGCGATG
AACAGCCTCGGCGGCAACTCCAACTCCGGCGAAGGCGGCGAAGACCCGGCGCGCTACGGC
ACCAATAAAGTGTCGCGTATCAAGCAGGTGGCTTCCGGCCGTTTTGGCGTGACGCCAGCG
TACCTGGTCAATGCCGATGTGATTCAGATTAAAGTCGCGCAGGGCGCGAAACCGGGCGAA
GGCGGTCAGTTGCCGGGGGATAAAGTCACTCCGTATATCGCTAAACTACGCTATTCAGTG
CCGGGCGTGACGCTGATCTCCCCGCCGCCGCACCACGATATTTACTCTATCGAGGACTTA
GCGCAGCTCATTTTCGACCTCAAACAGGTCAACCCGAAGGCGATGATCTCCGTGAAGCTG
GTGTCCGAACCGGGCGTCGGCACCATCGCGACCGGCGTGGCGAAAGCCTATGCGGATCTG
ATCACTATCGCCGGCTACGACGGCGGTACCGGCGCCAGCCCGCTCTCTTCGGTGAAATAT
GCGGGCTGTCCGTGGGAACTCGGACTTGTTGAAACCCAGCAGGCGCTGGTCGCCAACGGT
CTGCGTCACAAGATTCGTCTGCAGGTGGACGGCGGTCTGAAAACCGGCGTCGATATCATT
AAAGCGGCAATTCTCGGTGCGGAAAGTTTCGGCTTCGGCACCGGCCCGATGGTGGCGCTG
GGCTGTAAATACCTGCGCATTTGCCATCTGAACAACTGCGCAACCGGCGTGGCGACGCAG
GATGAAAAACTGCGTAAGAACCACTATCACGGCCTGCCGTTCAAGGTAACCAACTACTTT
GAATTTATCGCCCGCGAAGTGCGCGAGCTGATGGCTTCGCTTGGCGTGACCCGCCTGGTG
GATTTGATTGGCCGCACCGACCTGCTGAAAGAGCTGGAGGGCTTCACCGCTAAACAGCAG
AAGCTGGCGCTGTCTCGTCTACTGGAAACCGCTGAACCTCATCCGGGTAAAGCGCTGTAC
TGCACCGAAAATAACCCGCCGTTTGATAACGGCGTGCTGAATGCGCAACTGTTGCAGCAG
GCGAAACCGTTTGTCGATGCGCGCCAGAGCAAAACCTTCTGGTTCGACATCCGCAACACC
GATCGTTCCGTCGGCGCGTCGCTTTCCGGCTACATCGCACAGACCCACGGCGATCAGGGG
CTGGCTTCCGATCCGATTAAAGCGCACTTCAGCGGTACCGCAGGCCAGAGCTTTGGCGTG
TGGAACGCGGGCGGCGTAGAGTTATATCTGACTGGCGATGCCAACGACTATGTCGGTAAA
GGTATGGCAGGCGGTTTGATCGCCATCAGACCACCGGTAGGTTCCGCCTTCCTCAGCCAT
AAAGCCAGCATTATCGGCAACACCTGCCTGTATGGAGCCACCGGTGGCCGTCTGTACGCG
GCGGGCCGCGCGGGCGAACGTTTCGGCGTGCGTAACTCCGGGGCGATTACCGTAGTAGAG
GGCATCGGCGACAACGGCTGTGAATACATGACCGGCGGCATCGTCTGCGTGCTGGGTAAA
ACCGGCGTTAACTTCGGCGCGGGGATGACCGGCGGCTTCGCCTATGTGCTGGACGAAGAT
GGCGAATTCCGTAAACGCGTGAACCCGGAACTGGTAGAAGTACTGGACGTTGATTCGCTG
GCTATCCACGAAGAGCATCTGCGCGGCCTGATCACCGAGCACGTGCAGCATACTGGTTCG
CAACGCGGCGAAGAGATCCTGTCCAGGTGGTCAAGCTTCTCAACCCAATTCGCGTTAGTT
AAGCCCAAGTCCAGCGATGTCAAAGCCCTGTTGGGCCACCGTAGTCGTAGTGCGGCAGAA
TTGCGCGTGCAGGCGCAGTAA
>EECEBICD_00122 hypothetical protein
ATGCAGTTACAGAAATTAGTCAATATGTTTGGTGGGGATCTTTTACGTCGGTATGGGCAA
AAGGTTCATAAGCTGACGCTTCATGGCGGTTTTAGCTGCCCTAACCGCGACGGCACCATC

GGGCGTGGCGGTTGCACCTTCTGTAATGTCGCCTCTTTTGCCGATGAGGCGCAGCAGCAT
CATTCGATTGCCGAGCAGCTTGCGCATCAGGCGCATCTGGTGAACCGCGCTAAACGTTAT
CTGGCCTATTTCCAGGCCTACACCAGCACCTTTGCGGAAGTGCAGGTGCTGCGTTCCATG
TATCAACAGGCGGTCAGCCAGGCCAGTATCGTCGGGTTATGCGTCGGTACGCGTCCGGAT
TGTGTCCCGCAGGCAGTAGTAGATTTACTGTGTGAATATAAAGATCAAGGCTACGAGGTA
TGGCTGGAACTGGGATTGCAAACCGCGCACGATAAAACCCTGCACCGAATTAACCGCGGG
CATGACTTCGCCTGCTATCAGCGTACCACCCGGATTGCCCGGGAGCGCGGGCTGAAAGTG
TGTGCGCATCTGATTGTGGGGCTGCCGGGAGAAGGGCAGGCAGAATGCCTGCAAACCATG
GAGCGTGTGGTGGAGACGGGCGTTGACGGTATTAAACTGCATCCGCTGCATATTGTGAAA
GGCAGTACGATGGCCAAAGCCTGGGAAGCGGGGCGGCTGAACGGCATCGAACTGGACGAC
TACACGCTAACGGCAGGTGAGATGATTCGTCATACGCCGCCGGAGGTGATTTATCACCGA
ATTTCAGCCAGCGCTCGCCGTCCGACGCTGCTTGCCCCGCTGTGGTGCGAGAATCGCTGG
ACGGGGATGGTTGAGCTGGACAAATACCTGAATGAGCATGGCGTACAGGGCTCTGCGCTG
GCCCGTCCCTGGATACCACCTGTAGCGTAG
>EECEBICD_00123 Aerobic respiration control sensor protein ArcB
ATGAAGCAAATTCGTATGCTGGCGCAATACTATGTCGACCTGATGATGAAATTGGGTCTG
GTGCGCTTTTCCATGCTCCTGGCGTTGGCATTGGTGGTGCTGGCGATTGTCGTGCAGATG
GCGGTGACCATGGTGCTGCATGGTCAGGTTGAAAGTATCGACGTTATTCGTTCCATCTTT
TTTGGCCTGTTAATTACGCCCTGGGCGGTCTATTTCTTATCCGTTGTTGTTGAACAACTG
GAAGAGTCAAGGCAGCGACTGTCGCGTCTGGTGCAAAAGCTGGAGGAGATGCGCGAGCGC
GATCTCAAGCTTAACGTACAGTTGAAGGACAATATTGCCCAGCTTAATCAGGAAATTGCC
GATCGCGAAAAAGCGGAAGCGGAGCTGCAAGAGACATTTGAACAGCTAAAAGTAGAAATT
AAAGAGCGTGAAGAAGCGCAAATCCAGCTAGAACAGCAATCCTCTTTTCTGCGCTCCTTT
CTGGACGCCTCGCCGGATTTGGTTTTTATCGTAATGAGGATAAAGAATTTTCCGGCTGT
AACCGCGCAATGGAATTGCTAACCGGCAAGAGCGAAAAACAGCTGGTGCATTTGAAGCCG
GAAGATGTGTACTCTCCCGAAGCGGCGGAGAAAGTCATTGAAACCGATGAAAAAGTATTC
CGCCATAACGTCTCCCTGACCTACGAACAGTGGCTGGATTACCCGGACGGACGAAAGGCC
TGCTTTGAAATTCGTAAAGTGCCTTATTACGACCGTGTCGGTAAACGTCATGGCCTGATG
GGATTTGGTCGTGATATTACCGAACGTAAGCGTTATCAGGACGCGCTGGAACGCGCCAGC
CGCGACAAGACCACATTTATCTCTACCATCAGTCATGAGCTGCGCACCCCGCTTAACGGC
ATCGTCGGTTTAAGCCGCATCCTGCTGGATACCGATCTTACCGCCGAACAGGAAAAATAC
CTTAAGACAATCCATGTCTCCGCCGTGACGTTAGGCAATATTTTCAATGATATCATTGAT
ATGGATAAGATGGAGCGGCGTAAGGTTCAGCTTGATAACCAACCGGTTGATTTCACCAGC
TTTATGGCCGATCTGGAAAACCTTTCCGGTTTGCAGGCGCAGCAAAAAGGGCTACGTTTT
GTGCTTGAGCCGACGCTGCCGCTGCCGCACAAAGTCATTACCGACGGAACGCGTCTGCGC
CAGATTTTATGGAATTTGATCAGCAATGCGGTGAAATTTACTCAGCAGGGCCAGGTGACG
GTGCGGGCGCGCTATGATGAAGGCGACATGCTGCACTTTGAAGTGGAAGATTCCGGGATC
GGCATTCCGCAGGATGAGCAGGATAAAATTTTCGCCATGTATTATCAGGTGAAAGACAGT
CATGGCGGTAAACCGGCGACGGGTACCGGTATCGGGCTGGCGGTCTCCCGTCGGCTGGCG
AAAAATATGGGCGGCGATATTACCGTTTCCAGTCTGCCGGGCAAAGGATCGACCTTTACG
CTAACGGTTCATGCGCCAGCGGTGGCGGAAGAGGTGGAGGACGCCTTTGATGAAGACGAC
ATGCCGCTGCCGGCGCTGCATGTTCTGCTGGTCGAAGATATTGAACTGAACGTGATCGTA
GCGCGTTCGGTACTGGAAAAACTGGGTAACAGCGTGGATGTCGCGATGACCGGCAAGGCG
GCGCTGGAGATGTTCGCGCCAGGCGAATATGACCTGGTCTTGCTGGATATTCAGCTACCG
GATATGACGGGTCTGGATATTGCCAGAGAACTGACCCGTCGCCACACGCGGGAAGATTTA
CCGCCGCTGGTAGCGCTTACGGCTAACGTTCTGAAAGATAAAAAAGAGTATCTGGACGCT
GGTATGGACGATGTGCTGAGTAAGCCGCTGTCGGTGCCAGCCTTAACCGCGATGATTAAA
AAATTCTGGGATGCCACCGATAAAGAGGAGAGTACCGTGACGCCTGAAGAGAGCGATAAA
GCGCAAGCGCTGCTGGATATCCCGATGCTGGAACAGTACATCGAGTTGGTTGGGCCTAAA
TTGATCACCGACGGTCTCGCGGTGTTTGAAAAAATGATGCCCGGCTATTTAAGCGTGCTG
GAGTCCAATCTGACGGCGCGTGACAAAAAAGGCGTCGTTGAGGAAGGGCATAAGATTAAA
GGCGCAGCCGGTTCGGTAGGGCTGCGTCATCTGCAACAGCTTGGCCAGCAAATCCAGTCG
CCGGATCTTCCTGCCTGGGAAGATAATGTCGCGGAATGGATTGAAGAGATGAAGCAGGAG
TGGCAGCACGATGTCGCGGTGCTGAAAGCCTGGGTGGCAAACGCGGAAAAAAAATGA

>EECEBICD_00124 Glyoxalase ElbB
ATGAAAAAAATTGGCGTAGTGCTCAGCGGATGCGGCGTCTATGACGGCACCGAAATTCAT
GAGGCCGTTCTGACGCTGTTGGCCATTGCCCGCAGCGGCGCGCAGGCTGTCTGCTTCGCG
CCGGATAAACCACAAGCGGATGTGATTAATCACCTGACCGGCGAAGCGATGGCGGAAACG
CGTAACGTCCTGATTGAGGCGGCGCGTATTACGCGGGGCGATATTCGTCCCCTGTCCCAG
GCGCAGCCAGAGGAACTGGATGCGCTCATTGTGCCCGGTGGTTTCGGCGCGGCGAAGAAC
CTGAGTAATTTCGCCAGCCAGGGGAGCGAATGCCGGGTGGATAGCGATGTCGTCGCGCTG
GCGAAGGCCATGCACCAGTCCGGCAAGCCGCTGGGATTCATCTGTATCGCGCCCGCAATG
TTGCCGAAAATCTTTGACTTCCCGCTACGTCTGACAATAGGGACGGATATTGATACCGCT
GAAGTACTTGAAGAGATGGGCGCCGAACACGTACCTTGCCCGGTTGATGATATCGTTGTG
GACGAGGATAACAAGGTCGTGACCACCCCTGCCTATATGCTGGCGCAGGATATCGCCCAG
GCGGCAAGCGGCATTGATAAATTAGTGTCGCGCGTGCTGGTTCTGGCGGAATGA
>EECEBICD_00125 Biosynthetic peptidoglycan transglycosylase
ATGAGCAAACGACGCATCGCGCCGTTAACGTTTTTGCGCCGTCTACTTTTACGTATCCTT
GCTGCGCTCGCCGTCTTTTGGGGCGGCGGCATTGCGCTGTTTAGCGTGGTTCCGGTGCCT
TTTTCGGCGGTGATGGCGGAACGGCAGATTAGCGCCTGGCTCGGCGGAGAGTTCGGTTAT
GTGGCGCATTCTGACTGGGTCAGTATGGCGGATATCTCGCCGTGGATGGGGCTGGCGGTA
ATTGCGGCGGAAGACCAGAAGTTCCCTGAGCACTGGGGCTTTGATGTTCCGGCGATTGAA
AAAGCGCTGGCGCATAATGAGCGGAATGAGAGCCGTATTCGCGGCGCCTCTACGCTTTCG
CAGCAAACGGCGAAAAATCTCTTTTTATGGGATGGACGCAGTTGGCTGCGCAAAGGTCTG
GAGGCCGGGCTAACGCTGGGCATTGAAACTGTCTGGAGCAAAAAGCGCATTCTCACGGTT
TATCTGAATATCGCGGAGTTTGGCGATGGTATTTTTGGCGTGGAGGCTGCGGCACAACGG
TATTTTCATAAACCTGCCAGTCGCTTGAGTGTGTCTGAAGCCGCGTTGCTGGCGGCGGTA
TTGCCAAATCCGTTACGCTATAAGGCGAATGCGCCGTCTGGCTATGTGCGTAGCCGGCAG
GCGTGGATTATGCGCCAGATGCGACAACTGGGCGGGGAATCTTTTATGACGCGCAACCAG
CTTAATTAA
>EECEBICD_00126 putative protein YrbL
ATGATCCTTTTATCCGAACAGACTCCGCTGGGCGCTGGTCGACATCGTAAATGCTATACG
CATCCGGATAATGCCCGGCGTTGCATAAAAGTTATTTACAACCGTGACCACGGCGGTGAT
AAAGAGATTCGGCGTGAACTGAGCTACTATGCGCATCTGTCCCGCTACCTGACGGACTGG
AGCGCTATCCCCCGTTATTACGGCACGGTAGAAACCGATTGCGGCACCGGCTATGTCTAC
GACATGATTACCGATTTCAACGGCGCGCCCTCTATTACCCTGACCGAGTTCGCCCGCGCAA
TGTCGTTATGAAGAGGATGTCGCTGTCCTGCGGCGGCTACTGAAAAAACTGAAACGTTAC
CTGCTGGATAACCATATTGTGACGATGTCGCTGAAGCCGCAAAATATCCTGTGCCAGCGT
ATCAGCGAGTCAGAAGTGGTTCCGGTGGTTTGCGACAATCTGGGGGAAAGTACCTTTATC
CCGCTGGCGACCTGGTCAACATGGTGCTGTGAACGCAAACTGGAGAGAGTGTGGCAGCGG
TTTATCGCCCAGCCTGCGCTGGCCGTTGCCCTGGAGAGGGACGAGCAGCCAAAAGACAAG
AAACGGCTGGCGCTTACTTCTCACGAAGCTTAA
>EECEBICD_00127 hypothetical protein
ATGTTTTTATGCATAGGTAAAGCGCACGAAATGTACAAATCTTACGAAAGGGTGGGGCTG
CGGGACAAGTGGCAGGCAGGCGTAACACGCCCGGTTTTGGGGTGA
>EECEBICD_00128 Phosphocarrier protein NPr
ATGACCGTAAAGCAAACTGTTGAAGTCACCAATAAGCTGGGCATGCATGCCCGGCCTGCC
ATGAAGCTTTTCGAACTGATGCAGGGGTTTGACGCCGAGGTGCTATTACGCAATGACGAA
GGCACCGAAGCGGAAGCAAACAGCGTCATCGCTCTGTTAATGCTCGACTCCGCCAAAGGG
CGTCAGATCGAAATTGAAGCCACCGGCCCGCAAGAAGTTGAAGCGCTGGCGGCGGTTATC
GCGCTGTTCAATTCCGGCTTTGACGAAGACTAA
>EECEBICD_00129 RNase adapter protein RapZ
ATGGTACTGATGATCGTCAGCGGGCGTTCCGGATCAGGTAAATCTGTCGCTCTGCGCGCG
CTGGAAGATATGGGATTTTACTGCGTGGATAACCTCCCCGTCGTGCTGTTGCCCGATCTG
GCGCGAACGCTGGCCGATCGCCAGATTTCCGCCGCCGTCAGCATTGATGTGCGCAATATG
CCTGAATCACCTGAGATTTTTGAACAGGCGATGAATAATCTGCCTGGCGCTTTCTCGCCG
CAATTGCTGTTTCTTGATGCCGACCGCAATACCTTGATTCGCCGCTACAGCGACACGCGC
CGTTTGCATCCGCTTTCCAGCAAAAATCTTTCTTTAGAGAGTGCGATCGATAAAGAGAGT

GACTTGCTGGAGCCGTTACGCTCACGTGCGGATCTGATCGTCGACACTTCAGAAATGTCC
GTCCATGAGCTGGCGGAGATGCTGCGTACCCGGCTGTTGGGCAAACGCGAGCGCGAACTG
ACGATGGTATTTGAGTCCTTCGGCTTTAAACATGGTATCCCGATTGATGCGGACTATGTG
TTTGATGTCCGCTTTCTGCCCAACCCGCACTGGGATCCAAAACTGCGCCCCATGACCGGG
CTTGATAAGCCGGTAGCCGCGTTTCTTGACCGACACACAGAAGTTCACAATTTTATCTAT
CAGACTCGCAGCTATCTTGAGCTATGGTTACCGATGCTGGAGACCAACAACCGCAGCTAC
CTTACCGTCGCTATCGGTTGTACCGGCGGGAAACACCGTTCGGTGTATATTGCAGAACAG
CTGGCAGACTACTTCCGCTCGCGCGGTAAAAATGTCCAGTCTCGCCATCGCACGCTGGAA
AAACGCAAAACATGA
>EECEBICD_00130 Nitrogen regulatory protein
ATGATAAATAACGATACGACTCTACAACTGAGCAGTGTACTTAACCAGGAGTGTACGCGT
AGCGGCGTTCATTGTCAGAGTAAGAAACGCGCGCTGGAAATCATCAGCGAACTGGCGGCA
AAACAGCTCAGTTTACCTCCTCAGGTCGTGTTTGAAGCCATTCTGACACGCGAAAAAATG
GGCAGTACCGGTATTGGTAATGGTATCGCCATCCCGCACGGAAACTGGAAGAAGATACC
TTACGCGCCGTCGGCGTGTTCGTTCAGCTCGAAACGCCGATCGCGTTCGACGCCATCGAT
AACCAGCCGGTCGATCTCCTCTTCGCCCTGCTGGTGCCCGCCGATCAAACTAAAACGCAT
CTGCACACGCTGTCGCTGGTGGCGAAACGTCTGGCGGATAAAACGATCTGCCGCCGCCTG
CGCGCGGCGCTGAATGATGAAGAGCTGTATCAAATCATTACTGACACCGAAGGTGAGCAG
AATGAGGCATAA
>EECEBICD_00131 Ribosome hibernation promoting factor
ATGCAGCTCAATATCACCGGACATAATGTCGAAATTACTGAAGCCCTGCGTGAATTTGTG
ACGACAAAATTCGCCAAACTTGAACAGTATTTTGAGCGGATTAATCAGGTATACGTGGTG
TTGAAAGTGGAGAAGGTCACGCATATCTCGGATGCGACGCTACACGTTAACGGCGGCGAA
ATTCACGCCAGCGCGGAAGGCCAGGATATGTATGCCGCCATTGACGGCTTGATTGATAAA
CTGGCAAGGCAGTTAACCAGGCATAAAGATAAACTGAAACAACACTAA
>EECEBICD_00132 RNA polymerase sigma-54 factor
ATGAAGCAAGGTTTGCAACTCAGGCTTAGCCAACAACTGGCAATGACGCCTCAGCTACAA
CAGGCCATCCGTCTGTTGCAGTTGTCTACGCTGGAACTTCAGCAGGAACTCCAGCAAGCG
CTGGAAAATAACCCGCTGCTTGAGCAAACCGATCTTCATGACGAAATCGACACTCAGCAA
CCTCAGGATAACGATCCTCTCGATACCGCCGACGCGCTCGAACAAAAAGAGATGCCGGAA
GAGCTGCCGCTTGACGCCAGTTGGGATGAAATTTACACCGCCGGGACGCCGTCCGGCCCC
AGCGGCGATTATATCGACGATGAGCTGCCCGTCTATCAGGGTGAAACGACGCAGTCGTTG
CAGGATTATCTGATGTGGCAGGTTGAGCTAACGCCCTTCTCGGACACCGATCGCGCTATT
GCGACATCCATTGTCGATGCGGTAGATGATACCGGCTATCTCACCGTATCCCTGGACGAA
ATTCGCGAAAGCATGGGCGATGTAGAGGTGGATCTCGATGAGGTCGAAGCCGTCCTGAAG
CGTATTCAGCGTTTTGATCCGGTAGGCGTCGCGGCAAAAGATCTTCGCGACTGTCTGCTG
ATCCAGCTTTCACAATTCGACAAATCCACGCCGTGGCTGGAAGAGGCGCGGCTCATTATC
TGCGATCATCTTGATCTGCTGGCCAACCACGATTTCCGCACGTTGATGCGCGTTACCCGA
CTAAAAGAAGAGGTGCTGAAAGAGGCGGTAAACCTGATTCAGTCGCTCGATCCCAGACCC
GGTCAATCGATCCAGACCGGCGAGCCGGAGTACGTTATCCCGGATGTGCTGGTACGCAAG
CATAACGGTCGCTGGACGGTGGAGCTTAATAGCGACAGTATTCCCCGTTTACAGATTAAC
CAGCACTATGCCGCCATGTGCAATAGCGCGCGCAACGATGCCGACAGCCAGTTCATCCGC
AGTAATTTACAGGATGCGAAATGGCTGATAAAAAGCCTTGAAAGCCGCAACGACACGCTG
CTGCGCGTCAGTCGCTGCATCGTCGAGCAACAGCAAGCCTTTTTTGAACAGGGCGAAGAA
TATATGAAACCGATGGTACTGGCGGATATCGCCCAGGCCGTCGAGATGCACGAATCCACT
ATATCCCGCGTGACCACGCAGAAGTATTTGCACAGCCCGCGCGGTATTTTTGAACTCAAA
TATTTCTTTTCCAGCCACGTCAATACCGAAGGCGGAGGCGAAGCCTCTTCCACCGCGATT
CGCGCGCTGGTGAAGAAGTTAATTGCGGCGGAAAACCCCGCGAAACCGCTGAGCGACAGC
AAGTTAACCTCTCTGCTGTCAGAACAAGGTATCATGGTGGCGCGCCGCACTGTTGCGAAG
TACCGAGAGTCTTTATCCATTCCGCCGTCAAACCAACGCAAACAGCTGGTTTGA
>EECEBICD_00133 Lipopolysaccharide export system ATP-binding protein LptB
ATGGCAACATTAACTGCAAAGAATCTGGCGAAGGCCTATAAAGGCCGTCGCGTAGTGGAA
GATGTCAGTCTGACCGTTAACTCCGGGGAGATTGTCGGGCTGCTCGGTCCAAACGGCGCG
GGTAAAACCACCACCTTTTATATGGTCGTCGGTATCGTGCCGCGTGACGCAGGCAATATC

ATCATCGATGATGAAGATATCAGTCTGCTGCCGCTGCACGCGCGCGCGCGCCGCGGTATC
GGCTATTTGCCGCAGGAAGCTTCTATTTTCCGCCGTCTCAGCGTATTTGATAACCTGATG
GCGGTACTGCAAATTCGTGACGATTTGACCGCAGAACAGCGAGAAGACCGCGCCAACGAG
CTGATGGAAGAGTTTCATATCGAGCATCTGCGCGACAGCATGGGACAGGCGCTGTCCGGT
GGTGAACGCCGTCGTGTAGAAATAGCCCGCGCGTTGGCCGCTAATCCCAAATTTATCCTG
CTGGATGAACCCTTTGCCGGCGTTGACCCGATTTCCGTTATCGACATCAAACGCATTATC
GAGCATCTGCGCGATAGCGGGCTCGGCGTGCTGATCACCGACCATAACGTCCGTGAGACG
CTGGCAGTCTGTGAACGCGCCTATATCGTGAGCCAGGGCCACCTTATTGCCCACGGTACG
CCGACAGAAATCCTGCAAGACGAACACGTTAAGCGTGTATATCTTGGGGAAGACTTCAGA
CTCTGA
>EECEBICD_00134 Lipopolysaccharide export system protein LptA
ATGAAATTCAAAACAAACAAACTCAGCCTTAATCTTATGCTTGCCGGCTCGCTGCTGGCC
GCCAGCATTCCGGCTTTCGCCGTCACGGGCGATACCGAACAGCCTATTCATATTGACTCG
GATCAACAGTCGCTGGATATGCAAGGCAACGTCGTGACCTTTACGGGCAACGTGGTGATG
ACCCAGGGCACGATTAAGATCAACGCCGATAAAGTCGTGGTTACCCGCCCTGGCGGCGAA
CAGGGGAAAGAGGTGATCGACGGTTACGGCAATCCGGCCACCTTCTACCAGATGCAGGAC
AACGGTAAGCCGGTGAAAGGCCACGCTTCCCATATGCATTACGAGCTGGCGAAAGATTTT
GTGGTGCTGACCGGCAACGCTTATCTGGAGCAGCTTGACAGCAACATTACCGGCGACAAA
ATCACCTACCTGGTGAAAGAGCAGAAAATGCAGGCCTTCAGCGAGAAAGGCAAACGCGTC
ACCACCGTTCTGGTGCCGTCGCAGTTGCAAGACAAAAACAAAGGCCAGACTCCGGCGCAA
AAGAAGAGTAACTAA
>EECEBICD_00135 Lipopolysaccharide export system protein LptC
ATGAGTAAAACCAGACGTTGGGTTATCATTCTACTGTCGCTGGCCATTCTGGTACTGATT
GGTATCAATCTGGCGGATAAAGACGATCCTGCTGCCGTGATGGTCAACAGCAACGATCCC
ACGTATAAAAGCGAGCATACGGATACCGTCGTGTACAGTCCGGAAGGCGCGTTGAGCTAT
CGTCTGATCGCGCAGCACGTTGAATATTTTTCCGATCAGGCGGTCTCATGGTTTACGCAA
CCGGTATTAACCACCTTTGATAAGGATAAGGTGCCGACATGGTCGATCAAGGCCGATAAA
GCCAAATTGACTAACGATCGGATGCTCTATCTGTATGGTCATGTTGAAGTCAACGCCCTG
GTGCCTGACGCTCAATTACGCAGAATTACCACCGATAACGCGCAGATTAATCTGGTGACG
CAGGATGTTACCTCGAACGATCTGGTGACGTTATACGGCACAACATTTAACTCCAGCGGA
CTGAAAATGCGCGGTAACTTACGCAGCAAGAACGCCGAGCTGATTGAAAAGGTTAGAACC
TCTTATGAAATTCAAAACAAACAAACTCAGCCTTAA
>EECEBICD_00136 3-deoxy-D-manno-octulosonate 8-phosphate phosphatase KdsC
ATGAGTAAAGCAGGTGCGTCGCTTGCGACCTGTTATGGCCCCGTCAGCACCCATGTCATG
ACCAAAGCGGAGAATATCCGCCTGCTCATCCTGGACGTGGATGGCGTATTGTCTGACGGC
CTGATTTATATGGGCAATAACGGCGAAGAGCTGAAGGCTTTCAACGTTCGTGACGGCTAT
GGTATTCGCTGTGCTTTGACCTCCAATATCGAGGTTGCCATTATTACCGGACGAAAGGCT
AAACTTGTAGAAGATCGCTGCGCCACGCTGGGGATTGTCCATCTTTATCAGGGACAGTCG
AATAAGCTGATCGCCTTTAGCGATCTGCTGGAAAAACTGGTCATTGCGCCGGAAAATGTT
GCCTATGTCGGCGATGATCTGATTGACTGGCCAGTGATGGAAAAAGTGGGGTTAAGCGTT
GCCGTTGCGGATGCGCATCCGCTGCTGATCCCCCGCGCTGACTATGTGACGCACATTGCC
GGCGGGCGCGGCGCGGTACGTGAGGTGTGCGACTTGTTGCTGCTGGCGCAGGGCAAGCTG
GATGAGGCCAAAGGGCAATCGATATGA
>EECEBICD_00137 Arabinose 5-phosphate isomerase KdsD
ATGTCGCATTTAGCGTTGCAACCGGGGTTTGACTTTCAGCAGGCAGGCAAAGAGGTCCTG
GAAATTGAACGTGAAGGCCTGGCGGAGCTTGATCAATATATCAACCAGCATTTCACGCTG
GCCTGTGAAAAAATGTTCAACTGCACCGGAAAAGTGGTGGTCATGGGGATGGGAAAATCC
GGTCATATCGGACGGAAAATGGCCGCCACGTTCGCCAGTACCGGCACCTCTTCCTTTTTT
GTACATCCAGGCGAAGCCGCGCACGGCGATTTGGGGATGGTTACGCCGCAGGATGTGGTT
ATCGCTATCTCCAACTCCGGCGAATCCAGCGAGATCGCGGCGCTAATACCGGTACTTAAA
CGTCTGCACGTGCCGTTAATTTGCATTACCGGGCGACCGGAAAGCAGCATGGCGCGTGCG
GCAGATGTGCATCTGTGTGTTAAAGTGCCCAAAGAAGCGTGTCCGTTAGGACTGGCGCCT
ACCAGTAGTACCACCGCAACGCTGGTGATGGGCGACGCGCTCGCTGTGGCGTTGTTAAAA
GCGCGCGGCTTTACCGCCGAGGATTTCGCTCTGTCGCATCCTGGCGGCGCGCTGGGACGT

AAGCTACTGCTGCGAGTAAGCGATATTATGCATACTGGCGATGAGATCCCGCATGTTAAT
AAACATGCCACCCTGCGTGATGCGCTGTTAGAAATTACGCGTAAAAATCTCGGTATGACC
GTCATTTGCGATGAATCCATGAAGATCGACGGTATCTTCACCGACGGCGATTTACGTCGC
GTATTCGATATGGGCGGCGATATGCGCCAGTTGGGGATCGCCGAGGTGATGACGCCAGGG
GGCATTCGCGTGCGTCCCGGTATTCTCGCTGTCGATGCGCTGAATTTAATGCAGTCCCGC
CATATCACCTCCGTACTGGTTGCTGATGGCGACCAGTTACTCGGTGTGTTACATATGCAT
GATCTCCTGCGTGCAGGCGTCGTGTAG
>EECEBICD_00138 Inner membrane protein YrbG
ATGCTTTTAGCGATGGCGCTACTCATAATTGGTTTGCTTCTGGTGGCCTACGGCGCCGAT
CGTCTGGTGTTTGCGGCCTCTATTTTATGCCGTACATTCGGTATTCCCCCGCTGATCATC
GGGATGACGGTCGTGAGTATCGGCACCTCGCTGCCGGAAATTATTGTGTCGGTCGCCGCA
TCGCTACACGGTCAGCTCGATTTGGCCGTCGGCGCGGCGCTCGGTTCTAACATCACCAAT
ATTTTGCTGATCTTAGGGCTGGCGGCGCTGGTTCGTCCATTTACCGTGCATTCCGATGTT
TTACGTCGCGAATTGCCATTAATGTTATTTGTTAGCGTGGTAGCCGGTTCCGTACTGCAT
GACGGACAGTTAAGCCGCAGTGATGGTATCTTTCTCCTGCTTCTGGCTGTGCTATGGCTG
TTATTCATTGTTAAAATCGCGCGTCTGGCCGAACGCCAGGGGAATGACAGCCTCACCCGC
GAACAACTGGCGGAGCTGCCGCGCGAAGGCGGGCTCCCCGTCGCGTTCTTATGGCTGGGC
ATTGCGCTGGTCATCATGCCGATGGCGACGCGCATGGTAATCGATAACGCTACGGTGCTG
GCGAACTATTTCGCCATGAGTGAATTAACGCTCGGTCTGACAGTCATCGCCGTCGGCACC
AGTCTGCCAGAACTGGCGACGGCGATTGCCGGCGTGCGAAAAGGCGAAAATGATATTGCC
GTTGGTAATCTCATTGGCGCCAATATTTTTAATCTCGCCATCGTGCTGGGACTGCCTGCG
CTGATCGCGCCGGGGGAGATCAACCCGCTGGCATTTGGGCGTGATTATAGCGTTATGTTG
CTGGTTAGCGTGGTTTTCGCTTTACTTTGCTGGCGGCATCCGCGTCAGATTGGTCGGGGC
GCAGGTATACTGCTGACCGGCGGGTTTATCGTATGGCTGGCGATGCTCTATTGGCTATCG
CCACTTCTTGTTGGATAA
>EECEBICD_00139 Intermembrane phospholipid transport system ATP-binding protein MlaF
ATGGGTCAGTCTGCGGCCAATTTAGTCGATATGCGCGACGTCAGCTTTTCCCGTGGCGAA
CGCTGCATTTTCGATAATATTTCCCTGACGGTGCCGCGCGGGAAGATCACGGCGATCATG
GGGCCGTCGGGCATCGGTAAAACCACGCTGTTGCGCCTGATTGGCGGACAGATCCCGCCG
GATAAGGGCGAGATCTTATTTGATGGCGAGAATGTGCCGGCCATGTCCCGTTCGCGGCTC
TACACCGTCCGCAAACGTATGAGTATGCTGTTTCAGTCGGGAGCGCTGTTTACCGACATG
AACGTGTTTGACAATGTGGCCTATCCGCTACGGGAGCACACCAATTTACCCGCGCCGCTG
CTAAAAAGCGTCGTGATGATGAAACTGGAAGCTGTCGGGCTGCGCGGCGCGGCAAAACTG
ATGCCTTCCGAGCTCTCCGGTGGGATGGCGCGCCGCGCCGCGTTGGCGCGCGCCATCGCT
CTGGAACCGGATCTCATCATGTTCGATGAGCCGTTTGTCGGCCAGGACCCGATTACCATG
GGCGTTCTGGTGAAGCTGATTTCAGAATTGAACAGCGCGCTGGGCGTGACCTGCGTGGTG
GTCTCGCATGATGTGCCAGAGGTGCTCAGTATTGCTGATCACGCCTGGATCATGGCGGAC
AAAAAAATCGTCGCTCACGGTAGCGCTCAGGCGTTGCAGGAGAATACGGACCCGCGCGTG
CGTCAGTTCCTTGACGGCATTGCCGACGGGCCGGTTCCGTTCCGCTATCCGGCGGGCGAC
TATCACCTTGATTTACTCGAAACAGGGAGTTAA
>EECEBICD_00140 Intermembrane phospholipid transport system permease protein MlaE
ATGCTGTTAAATGCGCTGGCAGCACTCGGACACAGCGGGATCAAAACCGTCAGGACGTTC
GGGCGCGCCGGGTTAATGTTATTCAATGCGATTATCGGTAAGCCGGAGTTTCGCAAACAT
GCGCCGCTGCTGGTGCGCCAGCTTTATAATGTGGGCGTCTTGTCGATGCTTATCATTATT
GTCTCCGGCGTATTCATCGGTATGGTGCTGGGGCTGCAAGGCTATCTGGTTCTGACCACT
TACAGCGCGGAGACCAGTCTTGGCATGTTAGTGGCGCTTTCGCTGCTGCGCGAGCTGGGG
CCGGTGGTCGCGGCGTTGCTGTTTGCCGGGCGGGCCGGGTCGGCGTTGACGGCGGAAATC
GGCCTGATGCGGGCGACGGAGCAGCTCTCCAGTATGGAGATGATGGCGGTCGATCCATTG
CGCCGGGTGATTTCGCCGCGCTTTTGGGCGGGCGTGATTTCGTTGCCGCTACTGACCATT
ATCTTTGTGGCGGTCGGTATCTGGGGCGGCTCGCTGGTCGGCGTAAGCTGGAAAGGAATC
GACGCCGGGTTCTTCTGGTCCGCGATGCAGAATGCCGTCGACTGGCGTATGGACCTGGTG
AACTGTCTGATAAAGAGCGTGGTGTTCGCCATTACGGTAACATGGATTGCGTTATTCAAC

GGTTATGACGCAATACCAACTTCTGCCGGGATTAGCCGGGCAACGACGCGTACCGTGGTT
CACGCATCGTTGGCCGTTCTGGGGCTGGATTTTGTGCTGACCGCACTGATGTTTGGGAAT
TGA
>EECEBICD_00141 Intermembrane phospholipid transport system binding protein MlaD
ATGCAAACGAAAAAAAATGAAATTTGGGTAGGCGTTTTTTTGCTGGTAGCGTTGCTGGCG
GCGCTGTTTGTTTGCCTGAAGGCGGCAAATGTGACCTCTATGCGCACGGAGCCGACCTAT
AAGGTTTATGCGACCTTCGATAACATCGGCGGTCTGAAAGTGCGTTCCCCGGTACGCATC
GGCGGGGTGGTTGTCGGGCGGGTTGAAGATATCTCGCTTGATCCGAAAACATACCTGCCG
CGCGTAACGCTCGACATCGAAGAGCGTTATAACCATATTCCCGATACCAGCTCGTTGAGT
ATTCGTACTTCCGGGCTGCTGGGGGAGCAATATCTGGCATTAAACGTCGGTTTTGAAGAT
CCTGAGCTGGGAACGTCTATCCTCAAAGATGGGAGTACGATTCAGGACACCAAGTCTGCA
ATGGTGCTGGAAGATATGATTGGCCAGTTCCTTTATAACAGCAAAGGCGACGACAATAAG
AATTCTGGCGATGCGCCAGCCGCAACGGAAGGCCATACTGAAGCCACAACGCCCGCGGGC
GAAACGAAATAA
>EECEBICD_00142 Intermembrane phospholipid transport system binding protein MlaC
ATGTTTAAACGACTGATGATGGTGGCCCTGCTGGTCATTGCGCCATTAAGCGCAGCAACG
GCAGCGGATCAGAGCAATCCTTATAAACTGATGAACGAGGCGGCGCAGAAAACGTTTGAT
CGCCTGAAAAACGAACAGCCGAAAATTCGTGCCAATCCGGATTACCTGCGAGACGTTGTT
GATCAGGAACTTCTGCCGTACGTGCAGGTAAAATATGCCGGCGCGCTGGTGTTGGGCCGC
TACTACAAAGAGGCGACCCCGGCGCAGCGTGAAGCCTATTTTGCCGCGTTTCGCGAATAC
CTGAAACAGGCATACGGTCAGGCGCTGGCGATGTACCACGGTCAGACTTACCAGATAGCG
CCTGAGCAACCGCTGGGCGATGCGACCATTGTGCCTATCCGTGTAACGATTATCGATCCC
AACGGTCGTCCGCCGGTACGCCTGGATTTCCAGTGGCGCAAAAACACTCAGACCGGCAAC
TGGCAGGCGTATGACATGATTGCGGAAGGCGTCAGCATGATCACCACCAAGCAAAATGAG
TGGAGCGATCTGCTGCGTACTAAAGGCATCGACGGCCTGACCGCACAACTGAAATCTATC
TCTCAACAGAAAATTACTTTGGACGAGAAACAGTAA
>EECEBICD_00143 Intermembrane phospholipid transport system binding protein MlaB
ATGACGCCGCAACTCACCTGGACGCGTGAGGCGGACACTCTGGTGCTGGCGGGAGAGCTG
GATCAGGACGTGTTGGCGCCGCTATGGGACGCGCGCGTAGAAGCGATGACTGGCGTTACC
CGTATCGACCTGAGCCAGATCTCCAGGGTGGATACGGGGGGACTGGCGCTGCTGGCGCAC
CTTGTGAACCAGGCAAAAAAGCAGGGCAATGCCGTGTCGCTGTCTGGCGTTAACGACAAG
GTTTACGCGCTGGCGCAATTATATAACTTGCCCGAAGACGTACTTCCCCGTATGTAA
>EECEBICD_00144 Acid stress protein IbaG
ATGTTGGGCTGTTTTCACTATTTGACGAATAAAGAACCCATGGAAAATCATGAAATTCAG
AGTGTGCTGATGAATGCCCTCTCCCTCCAGGAAGTCCACGTCTCCGGCGATGGCAGTCAC
TTTCAGGTTATTGCCGTGGGCGAGATGTTTGACGGCATGAGCCGGGTCAAGAAACAGCAA
ACGGTCTATGGGCCGCTAATGGAGTACATTGCGGACAACCGTATCCATGCCGTATCGATC
AAAGCATATACCCCGGCGGAATGGGCGCGCGATCGTAAACTTAACGGATTTTAA
>EECEBICD_00145 UDP-N-acetylglucosamine 1-carboxyvinyltransferase
ATGGATAAATTTCGTGTACAGGGGCCAACGACGCTCCAGGGCGAAGTCACAATTTCCGGC
GCCAAAAACGCCGCGCTGCCGATCCTGTTCGCCGCTCTGCTGGCAGAAGAACCGGTAGAG
ATCCAAAACGTTCCAAAACTGAAAGACGTGGATACGTCGATGAAGCTCCTGAGCCAATTA
GGCGCGAAAGTGGAACGCAATGGATCGGTACACATTGACGCCAGCCAGGTGAACGTGTTC
TGCGCGCCTTATGATCTGGTTAAGACCATGCGCGCCTCGATATGGGCGCTGGGGCCGCTA
GTTGCCCGTTTTGGTCAGGGACAAGTCTCTCTGCCGGGGGGCTGTACTATCGGCGCGCGT
CCGGTTGATTTGCATATTACCGGTCTGGAACAACTGGGTGCGACCATCAAGCTGGAAGAG
GGCTACGTAAAGGCCTCCGTCGAAGGACGTCTTAAAGGGGCGCATATCGTGATGGATAAG
GTCAGCGTCGGCGCGACCGTCACCATTATGTGCGCCGCCACGCTGGCGGAAGGGACGACC
ATCATTGAAAACGCCGCGCGCGAGCCGGAAATTGTCGACACCGCTAACTTCCTGGTTACG
CTGGGGGCGAAGATCGCCGGGCAGGGCACCGATCGTATTACGATCGAGGGCGTAGAGCGT
CTGGGCGGCGGCGTTTATCGTGTGCTGCCGGATCGTATCGAAACGGGGACTTTCCTGGTG

GCGGCAGCGATCTCTCGCGGCAAAATCCTCTGCCGTAACGCGCAACCGGACACTCTGGAC
GCAGTACTGGCGAAACTGCGCGATGCTGGCGCGGATATCGAAGTGGGCGAGGACTGGATT
AGTCTGGACATGCACGGCAAACGACCGAAGGCGGTCAATGTCCGTACCGCGCCGCACCCG
GCATTCCCGACCGATATGCAGGCGCAGTTCACGTTGCTGAACCTGGTGGCGGAAGGGACG
GGCTTTATCACTGAAACTGTCTTTGAAAACCGTTTTATGCACGTGCCTGAACTCAGCCGC
ATGGGCGCGCGTGCGGAAATTGAAAGCAATACCGTCATATGCCATGGCATAGAAACGCTC
TCTGGCGCCCAGGTTATGGCGACAGATCTGCGGGCATCTGCGAGCCTGGTGCTGGCGGGC
TGTATTGCGGAAGGCACGACGATTGTGGATCGCATTTATCACATCGATCGCGGTTATGAG
CGCATCGAAGACAAACTGCGCGCGCTAGGCGCGAATATTGAACGCGTGAAAGGCGAATAA
>EECEBICD_00146 hypothetical protein
ATGGAAAGCAAGCTTATTGACTGGCATCCGGCCGATATCATTGCCGGACTACGTAAAAAA
GGGACTTCAATGGCCGCTGAGTCACGTCGGAATGGATTGAGTTCATCCACGCTGGCCAAT
GCATTAACGCGCCCGTGGCCGAAAGGAGAGTTGATTATCGCGAAAGCATTGGGAACGGAA
CCCTGGGTTATTTGGCCATCGCGTTATCACGATCCGCGCACGCATGAGTTTATCGACAGA
ACGCGGCTTATGAGAGCGCGTAATAAAGACAAACAGAATGTAGAGTAA
>EECEBICD_00147 Octaprenyl diphosphate synthase
ATGAATTTAGAAAAAATCAACGAGTTAACCGCGCAAGATATGGCGGGTGTCAATGCGACA
ATCCTTGAACAGCTTAATTCCGACGTTCAACTGATTAATCAGCTAGGATATTACATTATT
AGCGGCGGCGGAAAACGCATTCGTCCGATGATTGCCGTTCTTGCCGCACGCGCGGTGGGT
TATCAGGGTAACGCGCACGTGACTATCGCCGCTCTGATCGAGTTTATCCACACGGCGACG
CTGCTACATGATGATGTGGTGGATGAATCTGATATGCGTCGCGGCAAAGCGACGGCAAAT
GCCGCTTTCGGTAATGCCGCCAGCGTGTTGGTCGGCGACTTTATCTATACCCGCGCCTTC
CAGATGATGACCAGCCTCGGCTCGTTAAAAGTGCTGGAAGTGATGTCGGAAGCGGTCAAC
GTGATTGCAGAAGGTGAAGTGCTGCAATTGATGAACGTGAATGACCCGGACATTACCGAA
GAAAATTACATGCGGGTGATTTACAGTAAAACGGCCCGTCTGTTCGAAGCCGCCGCCCAG
TGCTCCGGTATTCTTGCCGGTTGTACGCCTGAGCAAGAGAAAGGGTTGCAGGACTATGGC
CGCTACCTTGGTACGGCTTTCCAGCTCATTGACGATCTGCTGGATTACAGTGCCGACGGC
GAGCATCTCGGTAAAAATGTGGGTGATGACCTCAATGAGGGCAAACCTACCTTACCGTTG
CTTCACGCCATGCGCCACGGTACGCCAGAACAGTCAGCGATGATCCGTACCGCTATTGAA
CAAGGCAATGGCCGTCATCTTCTGGAACCGGTTCTGGAAGCGATGACCACCTGCGGTTCG
CTGGAATGGACGCGTCAGCGAGCGGAAGAAGAAGCCGACAAAGCGATATCCGCGTTGCAG
ATATTGCCGGATACCCCATGGCGTGAGGCGCTAATCGGTCTCGCACACATCGCGGTGCAG
CGCGATCGTTAA
>EECEBICD_00148 50S ribosomal protein L21
ATGTACGCGGTTTTCCAAAGTGGTGGTAAACAACACCGAGTAAGCGAAGGTCAGACCGTT
CGCCTGGAAAAGCTGGACATCGCAACTGGCGAAACTATCGAGTTCGCTGAAGTTCTGATG
ATCGCAAACGGTGAAGAAGTCAAAATCGGCGTTCCTTTCGTTGATGGCGGCGTAATCAAA
GCTGAAGTTGTTGCCCACGGTCGTGGCGAGAAAGTTAAAATCGTTAAGTTTCGTCGCCGT
AAACACTACCGTAAGCAGCAGGGCCATCGTCAGTGGTTCACTGATGTGAAAATTACCGGC
ATCAGCGCTTAA
>EECEBICD_00149 50S ribosomal protein L27
ATGGCACATAAAAAGGCTGGCGGCTCGACTCGTAACGGTCGCGATTCTGAAGCTAAACGT
CTGGGCGTAAAACGCTTTGGCGGTGAAGCAGTTCTGGCAGGCAGCATCATCGTTCGTCAA
CGTGGTACTAAATTCCACGCTGGCACCAACGTTGGTTGCGGTCGTGACCACACTCTGTTT
GCTAAAGCAGACGGTAAAGTGAAATTCGAAGTTAAAGGCCCGAAAAACCGTAAATACATC
AGCATCGTTGCTGAGTAA
>EECEBICD_00150 putative inner membrane transporter YhbE
ATGAAGCAGCAGGCAGGCATTGGCATTCTTTTAGCGCTCACTACCGCGATGTGCTGGGGA
GCGTTGCCAATAGCAATGAAGCAAGTGCTGGAGGTGATGGAGCCTTCAACCATTGTCTTC
TACCGCTTCTTAATGGCCAGCATTGGCCTCGGGGCGATACTGGCAGTAAAAAGAAAGCTG
CCGCCGCTGCGTATTTTTCGTAAGCCGCGTTGGTTGGTGTTATTGGCGATAGCGACCTGC
GGACTGTTCGGTAATTTCATTTTGTTCAGTTCTTCCCTGCAATATTTAAGCCCAACCGCG
TCGCAGGTTATCGGCCAGCTCTCGCCGGTTGGCATGATGGTCGCCAGCGTCTTTATTCTG
AAAGAAAAGATGCGCGGTACGCAGGTGATTGGCGCGCTGATGTTATTGAGCGGGCTGGTG

ATGTTTTTTAACACCAGTCTGATCGAGATTTTTACCCGACTGACCGACTACACCTGGGGC
GTTATTTTTGGCGTAGGCGCGGCGATGGTGTGGGTGAGCTATGGCGTGGCGCAAAAAGTA
TTATTGCGACGTCTGGCCTCGCAGCAGATCCTGTTTTTACTGTACACTTTATGTACGATA
GCGCTCTTGCCGCTGGCAAAACCGATGGTGATAGCGCAACTGAGCGACTGGCAGCTCGCC
TGTTTGATTTTCTGCGGGCTAAATACGCTGGTAGGATATGGCGCCCTGGCGGAAGCGATG
GCGCGTTGGCAGGCGGCGCAGGTGAGCGCGATAATCACGCTTACACCGCTATTTACCCTG
CTGTTTTCAGATCTTTTATCTATGGCCTGGCCCGATTTCTTCGCCAGACCGATGTTAAAC
CTTTTAGGTTATCTCGGTGCGTTTGTCGTGGTTGCGGGCGCGATGTATTCCGCCATTGGT
CATCGTATTTGGGGCGGTTTACGCAAGCATGAAACGGTGGTTTCGCAACCCCGCTCAGGC
GAATGA
>EECEBICD_00151 GTPase Obg
ATGAAGTTTGTTGATGAAGCATCGATTCTGGTCGTTGCAGGCGATGGCGGTAATGGCTGT
GTGAGCTTTCGCCGCGAAAAATATATCCCGAAAGGCGGCCCGGACGGCGGTGATGGCGGC
GACGGCGGCGACGTCTGGATGGAAGCCGATGAAAACCTGAATACGCTGATCGATTACCGC
TTTGAAAAATCGTTCCGCGCTGAACGTGGTCAGAATGGCGCGAGCCGCGACTGTACCGGT
AAACGCGGTAAAGACGTGACCATCAAAGTGCCGGTCGGTACGCGCGTTATCGACCAGGGA
ACCGGTGAAACGATGGGCGACATGACCAAACACGGTCAGCGTCTGTTGGTCGCGAAGGGC
GGCTGGCATGGGCTAGGCAATACCCGTTTTAAATCCTCGGTTAACCGTACGCCGCGCCAG
AAAACCAATGGTACGCCGGGCGATAAGCGCGACCTGCTGCTGGAACTGATGCTGCTGGCG
GATGTCGGGATGCTGGGGATGCCAAACGCCGGTAAATCAACGTTCATTCGCGCGGTATCG
GCAGCAAAACCGAAAGTCGCGGACTATCCGTTTACCACGCTGGTGCCGAGCCTGGGCGTG
GTACGTATGGACAGCGAAAAGAGTTTTGTGGTGGCCGACATTCCGGGGTTGATCGAAGGC
GCGGCGGAAGGCGCGGGCCTGGGGATTCGCTTCCTGAAGCACCTCGAACGTTGCCGCGTA
CTGCTGCACCTCATTGATATCGATCCGATCGACGGTTCCGATCCGGTGGAAAACGCCCGT
ATCATTATTGGCGAACTGGAAAAATACAGCCAGGATCTGGCGGCTAAACCGCGCTGGTTA
GTGTTCAACAAGATCGATCTGATGGATAAAACCGAAGCGGAAGAGAAAGCGAAAGCGATC
GCCGAAGCGTTGGGCTGGGAAGGGAAATACTATCTGATTTCCGCCGCCAGTCAGCTAGGG
GTGAAAGATCTCTGCTGGGATGTGATGACCTTTATCATCGAGAATCCGATCGCGCAGGCT
GAAGAAGCAAAACAGCCGGAAAAAGTCGAGTTCATGTGGGACGATTATCATCGCCAGCAG
TTGGCTGAAGTGGAAGAAGACGCAGACGATGACTGGGATGACGACTGGGATGAAGACGAC
GAAGAAGGCGTCGAATTCATCTACAAACGTTAA
>EECEBICD_00152 D-alanyl-D-alanine carboxypeptidase DacB
ATGCGATTTTCCAGATTTATCATCGGATTGACCACCAGTATAGCGTTCAGCGTTCAGGCC
GCGAATATTGACGAATACATCAAACAGCTTCCGGCCGGCGCCAACCTCGCGCTGATGGTG
CAAAAGATTGGCGCGCCCGCGCCGGCTATTGATTATCATAGTCAGCAGATGGCGCTTCCC
GCCAGTACCCAAAAGGTGATTACCGCGCTGGCGGCGCTGATTCAACTCGGCCCGGATTTC
CGTTTTACGACCACGCTGGAGACTAAAGGCAACGTCGATAACGGCATCCTGAAAGGTGAC
GTAATTGCGCGCTTTGGTGGCGATCCAACGCTAAAACGTCAGGATATTCGCAATATGGTC
GCGACGCTGAAAAAGTCCGGCGTCACGCAAATCGACGGCAATGTGCTTATCGACACGTCG
ATTTTCGCCAGCCACGATAAAGCGCCAGGCTGGCCGTGGAACGACCTGACGCAGTGTTTC
AGCGCGCCTCCCGCCGCCGCCATTGTTGACCGCAACTGTTTTTCTGTGTCGCTTTATAGC
GCGCAAAAACCAAACGATTTAGCCTTTATTCGCGTGGCTTCTTATTATCCGGTAACGATG
TTCAGCCAGGTACGGACTCTACCGCGCGGTTCGGCAGACGCGCAGTATTGCGAGCTGGAT
GTTGTGCCTGGCGATTTGAACCGTTATACGCTTACCGGCTGTTTACCACAGCGCGCCGAC
CCGCTGCCGCTGGCCTTTGCTATTCAGGACGGCGCCAGCTACGCGGGCGCGATCCTCAAG
CAGGAACTGAAAGAGGCCGGGATAACTTACCGCGGCACGCTGCTGCGCCAGACGCAGGTT
AACGAGCCCGGAACGATCGTCGCCAGTAAACAGTCGGCGCCACTGCACGATCTGCTTAAG
ATTATGCTGAAAAAGTCAGACAACATGATCGCCGATACTGTTTTTCGGATGATTGGTCAT
GTCAGGTTTAACGTGCCTGGCACCTGGCGGGCGGGATCGGATGCCGTTCGCCAGATCCTA
CGCCAACAGGCGGGAATTGATATCGGCAATACCATTATTGCCGACGGTTCCGGTCTTTCT
CGCCATAATCTGATCGCTCCCGCCACGATGATGCAGGTGCTGCAATATATCGCCCAACAC
GATAACGAGCTGAACTTTATCTCTATGCTGCCGCTGGCGGGCTATGATGGTTCATTACAA
TACCGCGCCGGGCTGCATCAGGCGGGCGTAGACGGTAAGGTTTCGGCGAAAACCGGATCA
CTGCAGGGCGTGTATAATCTGGCGGGCTTTATTACCACCGCAAGCGGACAACGGATGGCA

TTTGTACAGTATTTGTCCGGCTACGCGGTTCCACCTGCCGATCAGCGTAATCGCCGAATC
CCGCTGGTACGCTTCGAGAGTCGGTTGTATAAGGATATTTATCAGACTAACTGA
>EECEBICD_00153 hypothetical protein
ATGGTTATCAAAGAGGAAATTGTAGCTGACCTGGGACTGCGCGCCCGGGTTGGTGTTTTT
TTGCTTCTGACCCTGGTATGGCATATGCCGGGGCTGGCTTAG
>EECEBICD_00154 Transcription elongation factor GreA
ATGCAAGCTATTCCGATGACCTTACGCGGAGCCGAAAAACTGCGCGAAGAGCTGGATTTT
CTGAAATCTGTGCGCCGTCCTGAAATCATCGCCGCTATCGCCGAAGCGCGCGAACATGGC
GATTTAAAAGAAAACGCGGAGTATCATGCTGCACGCGAACAGCAGGGTTTCTGCGAAGGG
CGCATTAAAGATATCGAAGCAAAACTGTCGAATGCGCAGGTGATTGATGTCACTAAAATG
CCGAACAATGGCCGCGTGATTTTCGGCGCTACCGTCACGGTGCTGAACCTGGACACCGAC
GAAGAGCAGACCTACCGCATTGTTGGCGATGATGAGGCGGATTTTAAGCAAAATCTCATT
TCGGTAAACTCGCCAATCGCTCGCGGCCTGATCGGCAAAGAGCAGGATGACGTCGTGGTG
ATCAAAACGCCTGGCGGCGATGTGGAATACGAAGTGCTTAAAGTGGAATACCTTTAA
>EECEBICD_00155 RNA-binding protein YhbY
ATGACCCGTTTTCAATCCCAACGTAAGCAAAAATATACGATGAATCTGAGTACTAAACAA
AAACAGCACCTAAAAGGTCTGGCACATCCGCTCAAGCCTGTTGTTATGCTTGGCAATAAT
GGTTTGACCGAAGGGGTACTGGCCGAGATTGAACAAGCGTTAGAGCACCATGAACTTATC
AAGGTGAAAATCGCCTCGGAAGATCGCGAAACAAAAACCTTGATCGTGGACGCTATCGTG
CGCGAAACCGGCGCCTGTAACGTACAGGTCATCGGTAAAACGCTGGTGCTGTATCGCCCA
ACTAAAGAGCGTAAAATCTCGCTGCCGCGTTAA
>EECEBICD_00156 Ribosomal RNA large subunit methyltransferase E
GTGCCTGGTTTAAACTTGATGAAATACAGCAAAGTGACAAACTTTTTTAAGCCGGGAATG
ACGGTAGTCGATCTCGGCGCTGCGCCAGGAGGCTGGTCACAGTATGTGGTCACGCAGATT
GGCGGCAAAGGCCGCATCATCGCTTGTGATCTTTTACCTATGGATCCTATCGTTGGTGTG
GACTTTCTTCAGGGCGATTTTCGTGATGAACTTGTCATGAAAGCGTTACTGGAACGCGTG
GGTGACAGTAAAGTACAAGTTGTCATGTCGGATATGGCACCAAACATGAGCGGAACACCG
GCGGTGGATATCCCCCGCGCCATGTATCTGGTGGAGCTGGCGTTAGAGATGTGCCGCGAT
GTGCTGGCGCCAGGCGGAAGTTTTGTAGTGAAGGTGTTCCAGGGCGAAGGTTTCGATGAG
TATCTAAGGGAAATTCGCTCCCTGTTTACGAAGGTTAAAGTTCGTAAGCCGGACTCTTCG
CGTGCGCGTTCGCGTGAAGTGTACATTGTAGCGACCGGGCGAAAGTGA
>EECEBICD_00157 ATP-dependent zinc metalloprotease FtsH
TTGAGTGACATGGCGAAAAACCTAATACTCTGGCTGGTCATTGCCGTTGTGCTGATGTCA
GTATTCCAGAGCTTTGGGCCCAGCGAGTCTAATGGCCGTAAGGTGGATTACTCTACCTTC
CTGCAAGAGGTCAATCAGGACCAGGTTCGCGAAGCGCGTATCAACGGACGTGAGATCAAC
GTTACCAAGAAAGATAGTAACCGTTACACGACTTACATTCCGATTAATGATCCGAAGCTG
CTTGATAACCTGCTGACTAAAAACGTCAAGGTTGTTGGCGAACCACCTGAAGAGCCAAGC
CTGCTGGCTTCTATCTTCATTTCCTGGTTCCCGATGCTGTTGCTGATCGGCGTCTGGATC
TTCTTCATGCGTCAGATGCAGGGCGGCGGTGGCAAAGGCGCCATGTCGTTCGGTAAGAGC
AAGGCGCGTATGCTGACGGAAGATCAGATCAAAACCACGTTTGCTGACGTCGCGGGTTGT
GACGAAGCGAAAGAAGAAGTGGCGGAACTGGTTGAATACCTGCGTGAACCGAGCCGTTTC
CAGAAGCTGGGCGGTAAAATTCCGAAAGGCGTCCTGATGGTCGGCCCGCCGGGTACCGGT
AAAACCCTGCTGGCAAAAGCCATTGCGGGTGAAGCGAAGGTGCCATTCTTTACGATTTCC
GGTTCTGACTTTGTGGAAATGTTCGTCGGTGTCGGCGCGTCTCGAGTGCGTGACATGTTC
GAACAGGCCAAGAAAGCGGCGCCGTGCATTATCTTCATCGATGAAATCGACGCCGTAGGC
CGCCAGCGTGGCGCAGGTCTGGGCGGTGGTCACGATGAACGTGAGCAGACGTTGAACCAG
ATGCTGGTTGAGATGGACGGCTTCGAAGGTAACGAAGGTATCATCGTTATCGCGGCAACC
AACCGTCCGGACGTTCTGGACCCGGCGCTGCTGCGTCCAGGTCGTTTTGACCGTCAGGTG
GTGGTAGGCCTGCCGGATGTTCGCGGTCGTGAGCAGATTCTGAAGGTGCATATGCGTCGC
GTACCGTTAGCGACGGATATTGATGCGGCGATCATTGCGCGCGGTACGCCGGGCTTCTCC
GGTGCTGACCTCGCGAACCTGGTGAACGAAGCGGCGCTGTTTGCGGCTCGTGGTAACAAG
CGCGTTGTGTCGATGGTTGAGTTCGAGAAAGCGAAAGACAAAATCATGATGGGCGCCGAA
CGTCGCTCCATGGTGATGACGGAAGCGCAGAAAGAGTCGACCGCGTACCACGAAGCGGGC
CACGCGATTATCGGTCGCCTGGTGCCGGAACACGATCCGGTGCACAAAGTGACGATTATC

CCGCGCGGTCGTGCGCTGGGCGTGACCTTCTTCCTGCCTGAAGGCGACGCGATCAGCGCC
AGCCGTCAGAAGCTGGAAAGCCAAATCAGCACGCTGTACGGTGGCCGTCTGGCGGAAGAG
ATTATCTACGGCGTTGAGCATGTTTCTACCGGCGCGTCGAACGACATTAAAGTCGCGACT
AACCTGGCGCGTAACATGGTCACCCAGTGGGGCTTCTCGGAGAAACTCGGTCCGTTGCTG
TATGCGGAAGAAGAGGGCGAAGTATTCCTCGGCCGTAGCGTCGCAAAAGCGAAACATATG
TCTGATGAAACTGCGCGTATCATCGACCAGGAAGTGAAAGCGCTGATTGAACGTAACTAC
AATCGCGCTCGTCAGATCCTGACTGACAATATGGATATTCTGCACGCGATGAAAGATGCG
CTGATGAAATATGAAACCATCGATGCGCCGCAGATTGATGACCTGATGGCGCGTCGTGAA
GTGCGTCCGCCTGCGGGCTGGGAAGATCCAAACGGCACCAATAACTCTGACAGCAATGGT
ACGCCTCAGGCGCCGCGTCCGGTTGATGAACCACGCACGCCGAACCCGGGCAACACGATG
TCAGAGCAGCTGGGCGACAAATAA
>EECEBICD_00158 Dihydropteroate synthase
ATGAAACTCTTCGCTCAGGGCGCCACGCTCGATCTCACTCATCCGCATGTTATGGGTATC
CTGAACGTAACGCCGGATTCGTTCTCCGACGGCGGCGCGCATAACACGCTGATTGAGGCG
GTGAAACATGCGAATTTAATGGTGAATGCCGGTGCGACAATTATTGTGATGGTGGGGGAA
TCAACGCGACCAGGCGCGGCGGAAGTGAGCGTGGAAGAAGAGCTGGATCGCGTTATTCCG
GTACTGGAAGCAATCGCGCAACGTTTTGAAGTGTGGATTTCTGTGGATACCTCTAAACCC
GAGGTGATCCGTGAAGCGACAAGGGCGGGCGCGCATATTATCAATGATGTCCGTTCGCTC
TCCGAGCCCGGCGCGTTGGAAGCGGCAGCGGAAACCGGCTTGCCGGTCAGTCTTATGCAC
ATGCAGGGCAACCCCAAAACCATGCAGGAGGCGCCGAAATATGACGATGTCTTTGCCGAG
GTGAATCGCTACTTTATTGAGCAAATAGCACGTTGTGAGAAGGCCGGCATCGCAAAAGAG
AAATTGTTGCTCGACCCCGGATTCGGTTTCGGTAAAAATCTCTCTCACAATTATACCTTA
CTGGCGCGACTGGGTGAGTTTCATCATTTTAACCTGCCGCTGCTGGTGGGCATGTCACGT
AAAACGATGGTTGGTCAGTTACTTAACGTGGGACCATCAGACCGTCTGAACGGCAGCCTT
GCGTGCGCGGTAATTGCCGCGATGCAGGGCGCGCAGATCATTCGCGTCCATGACGTCAAA
GAAACCGTAGAGGCGATGCGGGTGGTAGAAGCCACTCTGTCTGCAAAGGGAAACAAACGC
TATGAGTAA
>EECEBICD_00159 Phosphoglucosamine mutase
ATGAGTAATCGTAAATATTTCGGTACCGATGGGATTCGCGGACGTGTAGGCAATGCCCCC
ATTACACCTGATTTCGTGCTTAAACTGGGTTGGGCTGCCGGAAAAGTGCTGGCGCGTCAT
GGCTCGCGTAAAATCATTATCGGTAAAGATACGCGTATTTCCGGCTATATGCTGGAGTCC
GCGCTGGAGGCGGGCCTTGCCGCTGCGGGGCTGTCGGCCTCGTTTACCGGACCTATGCCA
ACGCCAGCCGTCGCCTATTTGACGCGGACTTTCCGCGCGGAGGCGGGTATCGTTATCTCG
GCTTCGCATAACCCGTTCTATGATAACGGCATTAAATTCTTCTCTATCGACGGTACGAAA
CTGCCTGACGATGTGGAAGAAGCCATCGAAGCCGAAATGGAAAAAGAGATCACCTGCGTA
GACTCCGCCGAGCTGGGGAAAGCGAGCCGCATCGTCGATGCCGCCGGACGCTATATTGAG
TTCTGTAAAGGCACCTTCCCGAACGAACTGAGCCTCAATGGCCTGAAAGTGGTTGTGGAT
TGCGCCAACGGCGCTACCTACCATATCGCACCGAATGTGCTGCGCGAGCTGGGCGCGACC
GTTATCGCCATCGGCTGTGAGCCAAACGGCGTCAACATTAACGAAGAAGTGGGCGCGACC
GACGTGCGCGCGTTGCAGGCGCGCGTGTTGGCGGAGAAGGCGGACCTCGGCATCGCGCTT
GACGGCGACGGCGACCGGGTCATCATGGTTGACCATGAAGGCAATAAAGTCGATGGCGAT
CAGATTATGTATATCATCGCGCGCGAAGGGCTGCGCCAGGGGCAACTGCGCGGCGGCGCG
GTGGGGACGCTGATGAGCAATATGGGGCTGGAGCTGGCGCTCAAGCAGCTTGGCATTCCG
TTTGCCCGGGCGAAAGTGGGCGACCGCTACGTGCTGGAAAAATTGCAGGAAAAAGGCTGG
CGCATCGGCGCGGAAAACTCTGGTCACGTTATTCTGTTGGATAAAACGACCACTGGCGAC
GGTATTGTAGCGGGGCTACAAGTTCTGGCGGCGATGGTGCGTAATCATATGAGTCTGCAC
GATCTGTGCAGCGGTATGAAAATGTTCCCGCAGATTCTGGTTAACGTGCGTTACACCGCC
GGTAGCGGCGATCCGCTGGAAAATGAAGCGGTGAAAGCGGTAACGGCGGACGTAGAAGCC
ACGCTCGGCAATCGTGGGCGGGTACTGCTACGTAAATCCGGTACCGAACCGTTGATTCGC
GTGATGGTGGAAGGCGAGGATGAAGCGCAGGTTACGGCATTCGCCCATCGTATCGCGGAT
GCGGTAAAAGCCGTTTAA
>EECEBICD_00160 Protein-export membrane protein SecG
ATGTACGAAGCTCTTTTAGTTGTTTTCCTTATTGTGGCAATTGGCCTTGTAGGTCTGATC
ATGCTGCAGCAAGGTAAAGGCGCTGATATGGGAGCCTCCTTCGGAGCAGGCGCTTCCGCT

ACGCTATTTGGTTCAAGTGGTTCTGGTAACTTCATGACCCGAATGACGGCGGTATTGGCG
ACGCTGTTCTTCATCATCAGTCTGGTGTTGGGTAATATCAATAGCAACAAAACCAATAAA
GGAAGCGAGTGGGAAAATCTGAGCGCGCCGGCGAAGACCGAGCAGACTCAACCTGCTGCA
CCGGCCCAGCCGACCAGCGATATCCCGCGCTAA
>EECEBICD_00161 tRNA-Leu(gag)
GCCGAGGTGGTGGAATTGGTAGACACGCTACCTTGAGGTGGTAGTGCCCAATAGGGCTTA
CGGGTTCAAGTCCCGTCCTCGGTACCA
>EECEBICD_00162 hypothetical protein
ATGAACAGTGAACACTTTGTACGCCTAGCCTTAGATATCCTTAAATGCAGCCAAAAAGAA
CTTGCTGGAAAGCTAGGGGTTTCCTCCACTCAAATTAGCAAGTGGAAAAAAGGTGAACAT
ATGTCTGATGATATGGAAAAGAAATTCAGAAAAATCACCAACATAGGCGAGTATTCTCCA
CTGCTTGTTGAGTGGGCAGGCTCCGTCAGTAATGCAGAAAAATGGGATCGTCTGATGCAT
TTTATTGCTGATAGAGTACATGACAGAGCAGAAACCGGCTACGTCACAACACCTCTACTC
GACGAAGAAGGGTTCTTATGTGAAGAAACGATTGATACATTGGAAAAAATGGGGTTATCC
GCTCCAAAATCGTTCCCTGTTGAATTAGATATCAACTATGAGAATACAGATGATGAAGAA
ACTGAAGATCTATGGGACTCCATCAGCAATAACCCTCACAGTTCAATTATTGAAAAAATC
TATAACTCGCTGAATGATGTCTATGGCTTTTATGCTGCTTATGTTGATGAATTAATACAA
GATGAAGGGTTGGATATCTATTCAACTGATGCCATCAATATTATGTATTCTTTGATGTCG
CTTGCAGCTTGCAAAATTGAGATTGATTCAGCAACAGCACCGAATTTTCGACAATTCCGT
TATGAAGTTGAGAAGGATTATGAAAACTGGTTAAGCCAACTCAAACTTCTGGCATTCCGT
GCAGGAATTCCATTACGAGCAGAGCTCCTCCAGATGGTTTACGATAGTGCAGATGATCTG
AGTGTTGCAGCTGAGGCTGAAAGCTTAGATTTAAATAAATCACGAATCCACCCAGATATC
TACATGAATGAAATACTGACTGGTATGCGAATCATTCACCAAGTTCTCCCTGTAATTATG
GAAAAACTTGAAATTACAGATTTCGAGTTGGATGAAAGCGCCTTACACATAGGAAGATAA
>EECEBICD_00163 Argininosuccinate synthase
ATGACGACGATTCTTAAGCATCTTCCAGCAGGTCAACGTATTGGTATCGCTTTTTCCGGC
GGTCTGGACACCAGCGCCGCGCTGCTGTGGATGCGACAAAAAGGAGCTGTCCCATATGCT
TATACTGCGAATCTGGGACAACCGGATGAGGATGACTATGACGCCATTCCTCGTCGCGCA
ATGGAGTATGGCGCAGAGAATGCTCGCCTGATTGATTGCCGCAAGCAGTTGGTTGCAGAA
GGGATCGCAGCGATTCAGTGCGGCGCTTTCCATAATACGACTGGCGGACTGACCTATTTC
AATACCACGCCTCTGGGCCGTGCCGTAACCGGCACGATGCTGGTCGCCGCGATGAAAGAA
GACGGCGTGAATATCTGGGGCGACGGCAGTACCTATAAAGGCAACGATATTGAACGTTTC
TATCGTTACGGCCTGCTGACGAATGCTGAATTACAGATTTACAAACCGTGGCTGGATACC
GACTTTATCGATGAGCTGGGTGGTCGCCATGAGATGTCTGAATTTATGATTGCCTGCGGT
TTCGACTATAAAATGTCCGTTGAAAAAGCCTACTCCACCGACTCCAACATGCTCGGCGCC
ACGCATGAAGCGAAAGATCTGGAGTTTCTTAACTCCAGCGTCAAGATCGTCAACCCGATT
ATGGGCGTGAAGTTCTGGGACGAGAGCGTGAAGATCCCGGCAGAAGAGGTGACCGTCCGC
TTCGAACAGGGCCACCCTGTTGCCCTGAACGGTAAAACCTTCAGCGATGATGTTGAGATG
ATGCTGGAAGCAAACCGCATCGGCGGCCGTCACGGCCTGGGCATGAGCGACCAAATTGAA
AACCGCATCATTGAAGCAAAAAGCCGCGGTATTTATGAAGCCCCCGGAATGGCGCTGCTG
CACATCGCTTACGAGCGTCTGTTGACCGGTATTCACAACGAAGACACCATTGAACAGTAT
CATTCTCATGGTCGTCAGCTGGGTAAACTGCTGTATCAGGGCCGCTGGTTCGACTCGCAG
GCGCTGATGCTGCGTGACGGCCTGCAACGTTGGGTCGCCAGCCAGATTACCGGTGAAGTG
ACGTTGGAGCTGCGTCGCGGCAACGACTACTCCATCCTGAACACCGTGTCCGACAACCTG
ACCTATAAGGCTGAGCGTCTGACCATGGAGAAAGGCGAGTCCGTATTCTCACCGGACGAT
CGTATCGGTCAGTTGACCATGCGTAATCTGGACATTACCGATACCCGCGAGAAACTGTTC
GGCTACGCGAAAGCGGGTCTGTTAACCGCCTCTTCCGCGACCGGTCTGCCGCAGGTTGAG
AATCTGGAAAACAAAGGCAAATAA
>EECEBICD_00164 tRNA-Met(cat)
CGCGGGGTGGAGCAGCCTGGTAGCTCGTCGGGCTCATAACCCGAAGGTCGTCGGTTCAAA
TCCGGCCCCCGCAACCA
>EECEBICD_00165 Ribosome maturation factor RimP
TTGTCCACATTAGAGCAAAAATTAACAGAGATGATTACAGCACCAGTTGAAGCCTTAGGC
TACGAGCTGGTCGGCATCGAATTTATTCGCGGTCGCACATCCACACTGCGCATCTATATT

GATAGTGAAGATGGCATCAATGTTGATGATTGTGCTGATGTGAGCCACCAGGTAAGCGCG
GTGCTGGACGTTGAAGATCCTATCTCCGTGGCTTACAACCTTGAAGTCTCGTCACCGGGT
CTCGACCGTCCTATGTTCACTGCCGATCATTATGCGCGTTTCCAGGGTGAAGAAGTCGCG
CTGGTTCTTCGCATGGCGGTACAGAACCGTCGCAAGTGGCAGGGCATTATCAAAGCGGTG
GACGGTGAAATGATTACCGTCACAGTCGAAGGCAAAGATGAAGTGTTCGCGCTGAGTAAT
ATCCAGAAGGCGAACCTGGTTCCCCACTTTTAA
>EECEBICD_00166 Transcription termination/antitermination protein NusA
ATGAACAAAGAAATTTTGGCTGTTGTTGAAGCCGTCTCCAACGAAAAAGCGCTGCCACGC
GAAAAAATTTTTGAAGCGCTGGAAAGTGCGCTGGCGACAGCAACAAAGAAAAAATATGAG
CAGGAGATCGATGTTCGTGTAGAAATCGATCGTAAAAGCGGTGATTTTGATACTTTCCGC
CGTTGGTTGATCGTTGAAGAAGTGACCATGCCGACGAAGGAAATTACGCTGGAAGCGGCG
CGTTTTGAAGACGAAAGCCTGAATGTCGGCGACTATGTTGAAGATCAGATTGAATCTGTC
ACCTTTGACCGTATCACCACGCAGACTGCGAAACAGGTTATCGTACAGAAGGTCCGTGAA
GCTGAACGCGCGATGGTTGTCGATCAGTTCCGCGACCAGGAAGGCGAAATTGTCACTGGC
GTGGTGAAGAAAGTGAACCGCGACAATATCTCTCTGGAAATTAAATCCGAAGGGATGGCC
GGTAACGCTGAAGCGGTGATTCTGCGTGAAGATATGCTGCCGCGTGAAAACTTCCGTCCG
GGCGACCGCATCCGCGGTGTTCTGTACGCTGTACGTCCAGAAGCGCGTGGCGCGCAGCTG
TTCGTCACCCGTTCCAAGCCGGAAATGCTGATCGAACTGTTCCGCATCGAAGTGCCGGAA
ATCGGCGAAGAAGTGATTGAAATTAAAGCGGCGGCTCGCGATCCGGGTTCTCGTGCGAAA
ATCGCAGTGAAAACCAACGATAAACGTATCGATCCGGTCGGCGCTTGCGTGGGGATGCGC
GGCGCGCGCGTTCAGGCGGTCTCTACCGAACTGGGCGGTGAGCGTATCGATATCGTGCTG
TGGGATGATAACCCGGCGCAGTTCGTCATTAATGCGATGGCGCCGGCAGACGTCGCGTCT
ATCGTGGTGGACGAAGATAAACATACCATGGATATCGCCGTTGAAGCCGGTAATCTGGCG
CAGGCGATCGGACGTAATGGTCAGAACGTCCGCCTGGCTTCGCAATTGAGCGGCTGGGAA
CTCAACGTAATGACCGTTGATGACTTGCAGGCTAAACATCAGGCTGAAGCACATGCCGCT
ATCGAGATCTTTACTAAATATCTTGATATTGATGAAGAGTTCGCGACCGTTCTGGTAGAA
GAAGGTTTCTCCACGCTCGAGGAACTGGCCTATGTGCCAATGAAAGAACTGCTGGAAATT
GACGGCCTTGATGAGCCGACCGTTGAAGCACTGCGCGAGCGTGCTAAAAACGCACTGGCC
ACTCTGGCGCAGGACCAGGAAGCAAGCCTCGGTGATAACAAACCGGCTGACGATCTGCTG
AATCTGGAAGGATTAGATCGCGATATGGCTTTCAAACTGGCGGCTCGTGGTGTTTGTACG
CTGGAAGATCTCGCCGACCAGGGCATTGATGATCTGGCTGATATCGAAGGGTTGACCGAC
GAAAAAGCCGGTGAGCTGATTATGGCTGCCCGTAATATTTGCTGGTTCGGCGACGAAGCG
TAA
>EECEBICD_00167 Translation initiation factor IF-2
ATGACAGATGTAACCCTAAAAGCGCTGGCCGCAGAGAGACAGGTTTCCGTGGATCGCCTG
GTACAGCAGTTTGCTGATGCAGGTATCCGGAAATCTGCTGACGACTCTGTGTCCGCACAA
GAAAAACAGACTTTACTGGCGCACCTGAACCGCGAAGCGGTTTCTGGTCCCGATAAACTG
ACGCTGCAGCGTAAGACGCGCAGCACCCTGAACATTCCAGGTACCGGTGGAAAAAGTAAA
TCGGTACAAATCGAAGTCCGCAAGAAGCGCACCTTTGTGAAACGCGATCCGCAAGAGGCT
GAACGCCTGGCCGCGGAAGAGCAGGCGCAGCGTGAAGCGGAAGAGCAAGCCCGTCGTGAG
GCAGAAGAACAGGCCAAACGCGAGGCGCAACAAAAAGCTGAACGCGAGGCCGCAGAACAA
GCTAAGCGTGAAGCCGCTGAAAAAGCGAAACGTGAAGCTGCGGAAAAAGACAAAGTGAGC
AATCAACAGACTGACGATATGACCAAAACCGCCCAGGCCGAAAAAGCCCGCCGTGAGAAT
GAAGCTGCAGAGCTGAAGCGTAAAGCGGAAGAAGAAGCACGTCGCAAACTCGAAGAAGAA
GCGCGTCGTGTAGCAGAAGAAGCTCGCCGCATGGCGGAAGAAAACAAATGGACCGCAACG
CCTGAGCCAGTAGAAGACACCAGTGACTATCATGTCACCACTTCTCAGCACGCTCGCCAG
GCCGAAGACGAAAACGATCGTGAAGTTGAAGGCGGTCGTGGTCGTGGCCGTAATGCAAAA
GCAGCGCGTCCGGCGAAAAAAGGCAAACATGCCGAATCCAAAGCCGATCGCGAAGAAGCG
CGTGCTGCGGTTCGCGGCGGTAAAGGCGGCAAGCGTAAAGGGTCTTCTTTACAGCAAGGC
TTCCAGAAGCCCGCTCAGGCCGTTAACCGTGACGTGGTGATCGGTGAAACCATCACCGTT
GGCGAACTGGCGAACAAGATGGCGGTTAAAGGCTCTCAGGTCATCAAAGCGATGATGAAG
CTGGGTGCTATGGCCACCATCAACCAGGTTATCGACCAGGAAACCGCACAACTGGTTGCC
GAAGAGATGGGCCACAAAGTTATCCTGCGTCGTGAAAACGAACTGGAAGAAGCGGTAATG
AGCGACCGTGATACCGGCGCTGCGGCTGAACCGCGCGCACCGGTTGTGACCATCATGGGC

CACGTTGACCACGGTAAAACCTCTCTGCTGGACTACATTCGTTCCACGAAAGTAGCCTCT
GGCGAAGCGGGCGGCATTACCCAGCACATCGGTGCTTACCACGTTGAAACTGACAACGGG
ATGATCACCTTCCTGGATACCCCGGGTCACGCCGCGTTTACTTCTATGCGTGCTCGCGGC
GCCCAGGCAACGGATATCGTGGTTCTGGTGGTAGCGGCAGACGATGGCGTGATGCCGCAG
ACTATCGAAGCTATCCAGCACGCGAAAGCGGCAGGTGTGCCGGTCGTGGTTGCGGTGAAC
AAAATCGATAAGCCGGAAGCCGATCCGGATCGCGTTAAGAACGAACTGTCCCAGTACGGC
ATTCTGCCGGAAGAGTGGGGCGGCGAGAGCCAGTTCGTTCACGTGTCTGCGAAAGCGGGC
ACCGGTATCGACGAACTGCTGGACGCTATCCTGCTGCAGGCCGAAGTTCTGGAACTGAAA
GCCGTTCGTAAAGGTATGGCGAGCGGTGCGGTGATCGAATCCTTCCTGGATAAAGGTCGT
GGTCCGGTGGCTACCGTTCTGGTACGCGAAGGTACGCTGCATAAGGGCGATATCGTGCTG
TGTGGCTTCGAATACGGCCGCGTGCGTGCGATGCGTAACGAACTGGGTCAGGAAGTGCTG
GAAGCGGGGCCGTCCATTCCGGTGGAAATCCTCGGCCTGTCCGGCGTTCCGGCTGCGGGT
GACGAAGTGACCGTCGTTCGCGACGAGAAGAAAGCGCGTGAAGTTGCTCTGTATCGTCAG
GGTAAATTCCGTGAAGTTAAACTGGCGCGTCAGCAGAAATCTAAACTTGAGAACATGTTC
GCCAACATGACCGAAGGCGAAGTTCACGAAGTGAACATCGTGCTGAAGGCCGACGTACAG
GGTTCTGTGGAAGCGATCTCCGACTCTTTGCTGAAACTGTCTACCGACGAAGTGAAAGTG
AAGATCATCGGTTCTGGCGTAGGTGGTATCACCGAAACCGACGCGACCCTGGCTGCGGCG
TCCAACGCCATTCTGGTTGGCTTCAACGTTCGTGCCGATGCCTCTGCGCGTAAAGTGATC
GAATCTGAAAGCCTGGATCTGCGTTACTACTCCGTCATCTATAACCTGATCGACGAAGTG
AAAGCGGCGATGAGCGGTATGCTGTCTCCGGAACTGAAACAGCAGATTATCGGTCTGGCT
GAAGTGCGCGATGTGTTCAAATCGCCGAAATTCGGCGCGATCGCGGGCTGTATGGTTACC
GAAGGTACGATTAAACGCCATAACCCAATCCGCGTACTGCGCGACAACGTGGTTATCTAT
GAAGGCGAGCTGGAATCCCTGCGCCGCTTCAAAGATGACGTTAACGAAGTCCGTAACGGC
ATGGAATGTGGTATCGGCGTGAAGAACTACAACGACGTTCGCGTTGGCGATATGATTGAA
GTATTCGAAATTATCGAGATCCAACGTACCATCGCTTAA
>EECEBICD_00168 30S ribosome-binding factor
ATGGCGAAAGAATTTGGTCGCCCTCAGCGCGTAGCGCAAGAGATGCAAAAAGAGATCGCA
ATCATCCTGCAGCGCGAAATTAAAGACCCGCGCCTGGGCATGATGACTACCGTCTCCGGT
GTCGAAATGTCCCGTGACCTGGCCTATGCCAAAGTGTTTGTCACCTTTTTGAACGACAAA
GATGAAGACGCAGTAAAAGCGGGCATCAAAGCGTTGCAGGAAGCGTCTGGTTTCATCCGC
AGCTTGCTGGGTAAAGCGATGCGTCTGCGCATCGTGCCGGAACTGACCTTCTTCTACGAC
AACTCGCTGGTGGAAGGGATGCGTATGTCCAACCTGGTAACTAACGTGGTGAAACATGAC
GAAGAACGTCGTGTGAACCCGGACGACAGCAAGGAGGACTGA
>EECEBICD_00169 tRNA pseudouridine synthase B
ATGAGTCGTCCTCGTCGTCGTGGCCGCGACATTCACGGCGTTCTGCTGCTGGATAAGCCG
CAGGGTATGTCCAGCAATGACGTGCTGCAGAAAGTTAAGCGCATCTACAATGCCAACCGC
GCCGGGCATACCGGCGCGCTGGACCCGCTGGCGACCGGCATGTTGCCGATTTGCCTCGGC
GAAGCGACAAAATTTTCGCAGTACCTGCTGGATTCTGACAAACGCTATCGTGTTATTGCC
CGTCTGGGGCAGCGCACGGATACCTCTGACGCCGATGGTCAAATCGTGCAGGAGCGTCCG
GTGACTTTCAGCGCTGAGCAGCTTGCGAGCGCGCTGGAGACCTTTCGTGGGGATATCGAA
CAGATTCCCTCGATGTATTCGGCGCTGAAGTATCAGGGCAAGAAGCTGTACGAATATGCC
CGCCAGGGTATCGAAGTGCCGCGCGAAGCCCGCCCGATTACCGTCTATGAGCTGCTGTTT
ATTCGCCATGAAGGTAACGAGCTGGAGCTGGAAGTTCATTGCTCCAAAGGCACCTATATT
CGTACCATTATTGATGATTTAGGTGAAAAACTGGGCTGTGGCGCGCACGTAACGTACCTG
CGTCGTCTGACGGTCAGCAAGTATCCGGTGGATCGTATGGTGACGCTGGAGCATTTGCAG
ACGCTGGTAGCACAGGCGGAGCAGCAGGGCGTTCCGGCGGCGCAGTTGCTCGATCCATTA
TTGATGCCAATGGACAGTCCAGCTTCGGACTACCCTGTTGTTAATCTGCCGTTAACATCT
TCGGTATACTTCAAAAATGGCAACCCGGTTCGTACCACGGGCGCGCCGTTGAAGGGACTG
GTTCGTGTGACGGAAGGTGAAGACGATAAATTTATCGGTATGGGCGAAATTGACGACGAA
GGCCGTGTCGCGCCTCGTCGGCTGGTAGTAGAGTACCCAGCGTAA
>EECEBICD_00170 30S ribosomal protein S15
ATGTCTCTAAGTACTGAAGCTACAGCTAAAATCGTTTCTGAGTTCGGTCGTGACGCAAAC
GACACCGGTTCTACCGATGTTCAGGTTGCTCTGTTGACTGCACAGATTAACCACCTGCAG
GGTCACTTTGCAGAGCATAAAAAAGATCACCACAGCCGTCGTGGTCTGCTGCGTATGGTT

TCTCAGCGTCGTAAACTGCTCGACTACCTGAAACGTAAAGATGTTGCACGCTACACCGCG
CTGATTGAGCGTCTGGGTCTGCGTCGCTAA
>EECEBICD_00171 Polyribonucleotide nucleotidyltransferase
ATGCCGTCAAAAATTGGAAAGGATATTATTTTGCTTAATCCGATCGTTCGTAAATTCCAG
TACGGTCAGCATACCGTTACGCTGGAAACTGGCATGATGGCGCGTCAGGCCACTGCCGCT
GTTATGGTAAGCATGGATGACACCGCCGTATTCGTTACCGTTGTCGGCCAGAAAAAAGCC
AAGCCAGGCCAGGACTTCTTCCCACTGACCGTTAACTATCAGGAGCGTACCTACGCTGCT
GGTCGTATCCCGGGCAGCTTCTTCCGTCGTGAAGGCCGTCCAAGCGAAGGCGAAACCCTG
ATTGCGCGTCTGATTGACCGCCCGGTTCGTCCGCTGTTCCCGGAAGGCTTCGTTAACGAA
GTGCAGGTTATTGCGACCGTCGTTTCCGTTAACCCGCAGGTTAACCCAGACATCGTTGCC
ATGATCGGCGCATCCGCTGCGCTGTCGCTGTCCGGCATTCCGTTCAACGGCCCAATCGGC
GCGGCGCGCGTAGGTTACATCAATGACCAGTACGTGCTGAACCCGACCCAGGACGAGCTG
AAAGAAAGCAAGCTGGACCTGGTGGTTGCCGGTACCGAAGCGGCTGTGCTGATGGTGGAA
TCCGAAGCTGAACTGCTGAGCGAAGACACTATGCTGGGCGCGGTGGTATTCGGTCACGAG
CAGCAGCAGGTTGTTATCCAGGCGATCAACGATCTGGTGAAAGAAGCCGGTAAACCGCGT
TGGGACTGGCAGCCGGAAGCCGTCAACGACGCGTTGAACGCGCGTGTTGCTGCGCTTGCT
GAATCTCGTCTGAGCGACGCGTACCGCATCACCGATAAACAAGAACGCTATGCTCAGGTT
GACGTAATCAAATCCGAAACCATCGAGCAGCTTATTGCAGAAGATGAAACTCTGGATGCT
AACGAGCTGGGCGAAATCCTGCACGCCATCGAGAAAAACGTGGTGCGTAGCCGCGTACTG
GCAGGCGAGCCGCGTATTGATGGCCGTGAAAAAGACATGATCCGTGGTCTGGACGTGCGC
ACTGGCGTACTGCCGCGTACTCACGGTTCCGCTCTGTTTACCCGTGGTGAAACGCAGGCG
CTGGTTACCGCGACTCTGGGTACTGCCCGTGACGCGCAGGTGCTTGATGAACTGATGGGC
GAGCGCACTGACAGCTTCCTGTTCCACTACAACTTCCCTCCGTACTCCGTAGGCGAAACC
GGCATGGTCGGTTCTCCGAAGCGTCGTGAAATTGGTCACGGTCGTCTGGCGAAGCGCGGC
GTGCTGGCAGTGATGCCGGACATGGATAAATTCCCGTACACCGTTCGCGTGGTGTCTGAA
ATCACCGAATCCAACGGTTCTTCCTCTATGGCTTCCGTGTGCGGCGCTTCTCTGGCGCTG
ATGGACGCTGGCGTGCCAATTAAAGCCGCCGTAGCAGGTATCGCTATGGGTCTGGTGAAA
GAAGGCGACAACTACGTTGTACTGTCTGACATTCTGGGCGACGAAGATCACCTGGGCGAT
ATGGACTTCAAAGTGGCGGGTTCCCGCGACGGTATCTCTGCGCTGCAGATGGATATCAAA
ATTGAAGGTATCACCAAAGAGATCATGCAGGTTGCGTTGAACCAGGCTAAAGGTGCGCGT
CTGCACATCCTGGGCGTGATGGAACAGGCGATCAACGCGCCGCGCGGCGATATCTCTGAA
TTTGCGCCGCGTATCCATACCATCAAGATCAGCACGGACAAGATCAAAGACGTGATCGGT
AAAGGCGGTTCTGTGATCCGCGCGCTGACTGAAGAGACCGGCACCACCATCGAAATCGAA
GATGACGGTACTGTGAAGATCGCGGCGACCGACGGTGAAAAAGCGAAATACGCTATCCGT
CGTATCGAAGAGATTACCGCAGAGATCGAAGTCGGCCGTATCTACAATGGTAAAGTGACC
CGTATCGTTGACTTTGGCGCGTTTGTTGCCATCGGCGGCGGTAAAGAAGGTCTGGTTCAC
ATCTCTCAAATCGCGGATAAGCGTGTAGAGAAAGTGACCGATTATCTGCAGATGGGTCAG
GAAGTCCCGGTTAAGGTTCTGGAAGTTGACCGCCAGGGCCGTGTACGTCTGAGCATTAAA
GAAGCAACCGAGCAGTCTCAACCTGCGGCTGCGCCGGAAGCTCCGGCGAGCGAACAGGCC
GAGTAA
>EECEBICD_00172 Lipoprotein NlpI
ATGAAGCCTTTTTTGCGCTGGTGTTTCGTTGCGACAGCACTTACGCTTGCAGGATGCAGT
AATTCCGCCTGGCGTAAAAGTGAAGTCCTCGCAGTACCATTGCAACCGACTTTACAGCAG
GAAGTGATTCTGGCGCGTATGGAACAGATTCTTGCCAGTCGGGCTTTAACCGATGACGAA
CGCGCACAGCTTTTATATGAGCGCGGAGTGTTGTATGATAGTCTTGGTTTGAGGGCATTA
GCGCGAAATGATTTTTCACAAGCGCTGGCAATCAGACCGGATATGCCTGAAGTATTCAAT
TACTTAGGCATTTATTTAACGCAGGCAGGCAATTTTGATGCTGCCTATGAAGCGTTTGAT
TCTGTACTAGAGCTTGATCCAACTTACAACTACGCGCACTTAAACCGCGGTATCGCATTG
TATTACGGCGGCCGTGATAAGTTAGCGCAAGATGATCTGCTGGCGTTTTATCAAGACGAT
CCCAATGATCCTTACCGCAGCCTGTGGCTGTATCTTGTTGAGCAGAAGCTTAATGAGAAG
CAGGCGAAAGAAGCGTTAAAAGCACGCTTTGAAAAATCGGATAAAGAGCAATGGGGATGG
AACATTGTCGAGTTCTACCTGGGCGACATTAGCGAAGCAACGCTGATGGAAAGGCTCAAG
GCGGACGCAACGGATAACACCTCGCTCGCTGAGCATCTCAGTGAAACCAACTTCTATTTA
GGTAAGTATTACCTAAGTCTGGGGGATTTGGACAGCGCCACGGCTCTGTTCAAACTGGCG

GTGGCCAACAACGTTCATAACTTTGTTGAGCACCGATACGCATTGTTGGAATTATCGCTC
CTGGGCCAGGACCAAGATGACCTGGCAGAATCGGACCAGCAATAG
>EECEBICD_00173 ATP-dependent RNA helicase DeaD
ATGGCTGAATTCGAAACCACTTTTGCAGATCTGGGGCTGAAGGCTCCTATCCTTGAAGCC
CTTACCGATCTGGGTTACGAAAAACCATCTCCAATCCAGGCAGAGTGCATTCCGCATCTG
CTGGGCGGTCGCGACGTGCTGGGCATGGCCCAGACCGGTAGCGGCAAAACCGCAGCGTTC
TCTTTACCGCTGCTCAACAATCTTGATCCTGAGCTGAAGGCACCTCAGATTCTGGTGCTG
GCGCCAACCCGCGAACTGGCGGTACAGGTTGCCGAAGCCATGACGGATTTCTCTAAACAC
ATGCGCGGCGTAAACGTGGTTGCCCTGTACGGCGGTCAGCGTTATGACGTGCAGTTACGC
GCCCTGCGTCAGGGGCCGCAGATCGTTGTCGGTACGCCGGGACGTCTGCTTGATCACTTA
AAACGTGGCACTCTCGACCTCTCTAAACTGAGTGGTCTGGTGCTGGATGAAGCCGACGAA
ATGCTGCGTATGGGCTTCATCGAAGACGTTGAAACTATTATGGCGCAGATCCCGGAAGGT
CATCAGACCGCTCTGTTCTCCGCCACCATGCCGGAAGCGATTCGTCGCATTACCCGCCGC
TTTATGAAAGAGCCGCAGGAAGTGCGCATTCAGTCCAGCGTGACTACCCGTCCTGACATC
AGCCAGAGCTACTGGACTGTCTGGGGCATGCGTAAAAACGAAGCGCTGGTGCGTTTCCTG
GAAGCGGAAGACTTTGATGCGGCGATTATCTTCGTGCGTACCAAAAACGCGACCCTGGAA
GTAGCAGAAGCGCTGGAACGTAGCGGCTACAACAGCGCCGCGTTGAATGGCGATATGAAC
CAGGCGCTGCGTGAGCAGACTCTGGAACGCCTGAAAGATGGTCGTCTGGACATCCTGATC
GCTACCGACGTTGCGGCACGTGGTCTGGACGTTGAACGTATCAGCCTGGTGGTGAACTAC
GATATTCCGATGGACTCCGAGTCCTACGTTCACCGTATCGGTCGTACCGGCCGTGCGGGT
CGCGCCGGTCGCGCGCTGCTGTTCGTTGAGAACCGCGAGCGTCGTCTGCTGCGCAATATC
GAACGCACCATGAAGCTGACCATTCCGGAAGTGGAACTGCCGAACGCAGAACTGCTGGGT
AAACGCCGTCTGGAAAAATTCGCCGCGAAAGTTCAACAGCAACTGGAAAGCAGCGATCTG
GATCAGTACCGTGCGCTGCTGGCGAAAATCCAGCCGTCTGCTGAAGGCGAAGAGCTGGAT
CTCGAAACGCTGGCCGCCGCACTGCTGAAAATGGCGCAAGGCGAGCGTCCGCTGATTCTG
CCGCCGGATGCGCCGATGCGTCCTAAGCGTGAATTCCGTGACCGTGACGACCGTGGTCCG
CGCGAGCGTAATGACCGTGGCCCGCGTGGCGACCGTGAAGAACGTCCGCGTCGTGAACGT
CGTGACGTTGGCGATATGCAGTTATACCGCATTGAAGTGGGCCGTGATGACGGCGTTGAA
GTTCGTCATATCGTGGGCGCTATCGCTAACGAAGGCGACATCAGCAGCCGTTACATTGGT
AACATTAAGCTGTTCGCGTCTCACTCCACCATCGAGCTGCCGAAAGGTATGCCGGGCGAA
GTGTTGCAGCACTTCACGCGTACCCGCATTCTGAACAAGCCGATGAATATGCAGCTGTTG
GGTGATGCGGTTCCGCACGCGGGCGGCGAACGTCGCGGCGGTGGTCGTAGCTTCAGTGGC
GAACGTCGTGAAGGCGGTCGTAATTTCAGCGGCGAACGTCGTGAAGGTGGTCGTGGTGAT
GGTCGTCGCTTCAGCGGCGAGCGTCGTGAAAGCCGTGGTCCGCGTCGTGATGACTCTACC
GGTCGCCGTCGTTTCGGCGGTGATGCGTAA
>EECEBICD_00174 Tryptophan-specific transport protein
ATGGCAACACTTACCACCACCCAAACGTCACCTTCGCTGCTTGGCGGCGTGGTGATTATC
GGCGGCACCATCATTGGCGCGGGGATGTTCTCCCTGCCGGTGGTCATGTCCGGGGCGTGG
TTCTTCTGGTCGATGGCGGCGCTGGTCTTTACCTGGTTTTGTATGCTGCACTCCGGGCTG
ATGATCCTCGAAGCCAACCTTAACTACCGCATTGGTTCGAGTTTCGACACCATCACCAAA
GATCTGTTGGGGAAAGGCTGGAACATTGTCAACGGTATTTCCATTGCCTTTGTGCTCTAC
ATCCTGACTTACGCCTATATCTCGGCGAGCGGCTCGATTCTGCATCATACCTTTGCTGAG
ATGTCGTTGAACGTTCCGGCGCGAGCGGCAGGTTTTGCCTTTGCGCTGCTGGTGGCGTTT
GTAGTGTGGCTGAGTACGAAGGCGGTCAGTCGGATGACGGCGATTGTGCTGGGCGCGAAA
GTGATTACTTTCTTCCTGACCTTCGGCAGCCTGTTGGGGCACGTTCAGCCTGCCACGTTG
TTTAACGTGGCTGAAAGCCACGCGTCATATACGCCGTACCTGCTGATGACGCTGCCATTT
TGTCTGGCCTCTTTTGGTTATCACGGTAACGTACCGAGCTTGATGAAATATTACGGCAAA
GATCCGCGTACCATCGTCAAATGCCTGATTTATGGGACGTTACTGGCGCTGGCGCTCTAT
TCCGTCTGGCTGCTGGGGACGATGGGCAACATCCCGCGTCCGGAATTTATCGGCATTGCG
CAGAAAGGCGGCAATATTGATGTGCTGGTACAGGCGTTGAGCGGCGTGCTGAACAGCCGC
AGCCTGGATCTGCTGCTGGTGGTGTTCTCTAACTTTGCGGTCGCCAGTTCCTTCCTTGGC
GTGACGCTGGGGCTGTTTGACTATCTGGCGGATCTGTTTGGTTTTGATGACTCGGCAATG
GGGCGTTTTAAAACGGCGCTGCTGACTTTCCTGCCGCCGATGATTGGTGGCCTGCTGTAC
CCGAACGGTTTTCTGTACGCCATTGGCTATGCAGGTTTAGCTGCAACTATCTGGGCGGCC

ATTGTGCCTGCGCTGTTGGCACGTAAATCTCGCGAACGTTTCGGCAGCCCGAAATTTCGC
GTCTGGGGCGGTAAACCGATGATTGCCCTGATTTTGGTCTTCGGTGTTGGCAACGCGGTT
ATCCATATTCTGTCGAGCTTTAATTTGCTGCCGGTGTACCAGTAA
>EECEBICD_00175 hypothetical protein
ATGAGTGAACCTGCTTCCTTTTTTCTTCATGCACATATTACCGAGAGTAACCTCAAGAAA
TTTTTCCATTCACCGGCAACAAATATTAAAGACTATGACGACTGGTTGCCATGGTTTACT
GAGGAGCAAAGGTTGTACGGCGATCCTGCAAAAATGCTTAACAATCTTGCCACATGTAAT
TCTGGTGAAAGTGAAAAGAATATTTATGCTGAACATATCAACTTTAATAAAGAAACACAG
ATAGTCACGATGGATCACATATTTCTCTCAGAGAGCTATGAAATCTTCATGCCACTCATG
GCCTGTGTGCGGGGAATCGAGAAATTTATTACGCTAGGGGAAAATAATTTTGCGCTAATT
TATTATTACTGGTGGGGTTCGGAGATCGCCATCGCGCTCGAATTTGGCGCCAATGGTAGC
AGAATAACGGCAAATCCTAAGGCAGAAAATTTAACTATCGCCGATGCTTTTTTCGATGAA
CACGGGGAAGCACTCGCCGAAGAACTTTATAATAAACAGGGTTTTATTTGA
>EECEBICD_00176 hypothetical protein
ATGGGGCGTAAAGGACTACTCGCTATTGTATTATTGAGCCTGTTTATCGCTTTTATTTTA
AAATTTTTCTGGCTCACTCCGTACGATGAAGACGTTTACCTGCCAGTGGAAAAACCTGTC
GCATCGTCTTTAAAAATCATTCACCCCGGCGACCAGTTATTTATCCGCATTCTGAAAGCG
GAAGATAAGCTGGAACTGTGGGCAAGCGCCAATAACAAGCCTTACAAACTCTACAAAACC
TGGACTATATGCGCATGGTCTGGTGGCTTAGGGCCAAAACATAAACAGGGAGACGGTAAA
AGCCCGGAAGGCTTTTATGCCACCAACAAAGGGTTGCTTAACCCAAACAGCCGCTATCAT
CTGGCATTTAATATTGGCTACCCGAATGCTTACGATCGGGCAAATGGCTACACCGGTGAT
TTCATTATGGTTCACGGGAACTGCGTTTCGGCCGGTTGCTATGCCATGACCGATGCCGGT
ATTGAAGAGATTTATCAACTGGTGGCGCAGGCGCTAAACAGCGGGCAGAAGCGCGTGCCT
GTCCATATCTTCCCATTTACGATGAATGACGAAAATATGCGCCAGGCGCAAGCATGGCCG
GAATATAATTTCTGGCGTATGTTGAAGCCCGGCTATGATTACTTCGAAAAAAATCGCCGA
TTGCCAACCATCACCGTTGAAAATCGGCGTTATAAAATCAGCCCAACAACTCTTCCTTAA
>EECEBICD_00177 hypothetical protein
ATGACTGACAAAACTATTCCGTTCTCGGTGCTGGATCTGGCGCCGATCCCTGAAGGATCT
TCGGCAAAAGAAGCCTTCACGCACTCGCTTGATCTCGCACGGCTGGCTGAAAAGCGCGGT
TATCACCGCTACTGGCTGGCGGAACATCACAACATGACCGGTATCGCCAGCGCGGCGACC
TCGGTATTGATTGGTTATCTGGCGGCCAATACCACAACATTACATCTTGGCTCCGGCGGC
GTGATGCTGCCTAACCATTCACCGCTGGTGATTGCCGAGCAGTTCGGCACGCTGAATACG
CTCTACCCGGGACGTATCGACTTAGGGTTGGGACGCGCGCCGGGTAGCGATCAGCCGACG
ATGCGCGCGCTGCGTCGCCATATGAGCGGCGATATCGATAACTTTCCACGCGATGTCGCA
GAGCTGGTGGACTGGTTCGACGCTCGCGCCCCTAATCCGCATGTGCGCCCGGTGCCCGGC
TACGGCGAGCAGATCCCGGTCTGGCTGTTGGGCTCCAGCCTGTATAGCGCACAGTTGGCG
GCCCAGCTTGGCCTGCCGTTCGCCTTTGCTTCGCATTTCGCGCCGGATATGTTGTTCCAG
GCGCTGCATCTGTACCGTACCCAGTTCAAACCTTCAGCGCGGCTGGAAAAACCGTACGCG
ATGGTGTGTATCAATATTATCGCCGCCGACAGCAACCGCGACGCCGAATTCCTGTTTACC
TCCATGCAGCAGGCTTTCGTGAAGCTACGCCGTGGGGAAACCGGCCAACTGCCGCCGCCG
ATTGAGAATATGGAGACCTTCTGGTCGCCATCCGAGCAGTATGGCGTGCAGCAGGCGCTG
AGTATGTCGCTGGTCGGTGATAAAGCGAAAGTCCGCCACGGGCTGGAATCCATCCTGCGG
GAGACCCAGGCGGATGAAATTATGGTAAACGGGCAGATTTTCGACCACCAGGCACGCCTG
CATTCGTTTGATTTAGCGATGGATGTTAAGGAAGAGTTGTTGGGCTGA
>EECEBICD_00178 hypothetical protein
ATGAAATATTCCTTAGGACCGGTGCTTTATTACTGGCCGAAAGAGACGCTGGAAGATTTT
TATCAGCAGGCCGCAAAAAGCAGCGCTGATGTCATTTATCTGGGCGAAGCGGTGTGCAGC
AAGCGTCGCGCGACCAAAGTCGGCGACTGGCTGGAAATGGCGAAATCCCTCGCCGCCAGC
GGTAAGCAGGTGGTGCTCTCCACCCTGGCGCTGGTACAGGCGTCATCTGAATTAAGCGAG
CTGAAACGCTATGTGGACAACGGCGATTTTCTGCTGGAAGCCAGCGATCTTGGCGTGGTG
AATCTGTGCGCCGAGCGCAAACTGCCGTTTGTCGCCGGCCATGCGCTGAACTGCTACAAC
GCGGTCACGCTGCGCCGGTTGCTGAAAGAAGGCATGGTACGCTGGTGTATGCCGGTGGAG
CTGTCCCGCGACTGGCTGGTGAATTTGCTTAATCAGTGCGATGAACTGGGCATCCGCAAT
CAGTTTGAAGTGGAAGTGTTGAGCTACGGGCATCTGCCGCTGGCCTACTCCGCTCGCTGC

TTTACCGCCCGTTCGGAAGACCGCCCGAAAGATGAGTGCGAAACCTGCTGTATCAAGTAT
CCAAACGGTCGTGACGTGCTCTCGCAAGAGAATCAGCAGGTGTTTGTGCTTAACGGTATT
CAGACCATGAGCGGCTATGTCTATAACCTTGGCAACGAACTGACGTCGATGCAGGGATTG
GTCGATATCGTGCGGCTGTCGCCGTTGGGTACGGAGACATTTGCCATGCTTGATGCCTTT
CGCGCTAACGAAAACGGCGGCGCGCCATTAGCGCTTGCCGCGCATAGCGACTGCAACGGC
TACTGGAGGCGGCTGGCCGGGCTGGAGCTACAGGCCTAG
>EECEBICD_00179 putative protease YhbU
ATGGAGCTGCTCTGCCCTGCCGGAAATCTCCCGGCGCTGAAGGCGGCCATCGAAAACGGC
GCTGACGCCGTTTATATCGGGTTAAAAGACGATACCAATGCCCGTCATTTTGCCGGCCTT
AACTTTACCGAAAAAAAATTGCAGGAAGCGGTGAGTTTTGTTCACCAGCATCGCCGCAAA
TTACACATCGCCATTAATACTTTTGCGCATCCGGACGGCTATGCCCGCTGGCAGCGCGCC
GTGGATATGGCGGCGCAGCTTGGCGCCGACGCGTTGATTCTCGCCGACCTCGCTATGCTG
GAATATGCCGCAGAGCGTTACCCGCATATTGAGCGCCATGTTTCCGTTCAGGCCTCGGCA
ACCAACGAAGAGGCGATTCGCTTTTATCACCGCAACTTTGATGTCCACCGTGTAGTACTG
CCGCGTGTACTGTCGATTCACCAGGTAAAACAACTGGCCCGCGTCACGCCGGTGCCGCTG
GAGGTATTCGCGTTTGGCAGCCTGTGCATTATGGCGGAAGGTCGCTGCTATCTTTCTTCC
TACCTGACGGGGGAGTCGCCCAATACCGTCGGCGCCTGCTCTCCCGCCCGCTTTGTCCGT
TGGCAGCAAACGCCGCAGGGGCTGGAATCGCGCCTGAATGATGTCCTGATTGACCGTTAC
CAGGATGGCGAAAACGCAGGCTACCCAACGCTGTGTAAAGGCCGCTATTTAGTGGACGGC
GAGCGCTATCACGCGCTGGAGGAGCCAACCAGCCTGAACACGCTGGAACTGCTGCCGGAG
CTCATGGCGGCGAACATCGCTTCGGTGAAGATCGAAGGCCGCCAGCGCAGCCCGGCCTAC
GTCAGCCAGGTGGCGAAAGTGTGGCGCCAGGCGATCGATCGCTGCAAAGCCGCCCCGCAA
AACTTCGTTCCACAGCGCGACTGGATGGAGACGCTCGGCGCGATGTCCGAAGGCACCCAA
ACCACGCTTGGCGCATATCACCGTAAATGGCAGTGA
>EECEBICD_00180 Ubiquinone biosynthesis accessory factor UbiJ
GTGTTAGATAAACTGCGTTCACGCTTAGTCCATGCTGGCCCGTCTCTGATGAGTGTACCG
GTTAAACTGACGCCCTTTGCGCTAAAGCGCCAGGTTCTGGAACAGGTTCTGAGCTGGCAG
TTTCGTCAGGCGCTGGCCGATGGAGAGCTGGAATTCCTCGAAGGTCGTTGGTTAAGCATT
CATGTTCGTGACATCGACTTAAAATGGTATACCACGGTTGAAAATGAAAAGCTGATCGTT
AGCCAGCAGGCGGATGCTGACGTCAGTTTTAGCGCCGACGCCAGCGATCTGCTAATGATC
GCCGCCCGTAAACAGGATCCGGATACGCTCTTTTTCCAGCGTCGACTGGTCATTGAAGGC
GATACTGAGTTAGGGTTGTATGTGAAGAATCTGATGGACGCCATCGAACTGGAGCAGATG
CCGAAAGCGTTACGCGTTATGCTGCTGCAACTGGCGGATTTTGTTGAGGCGGGAATGAAA
AACTCGCCGGAAACCAAACAGACCTCGGTAGGTGAACCATGCTAA
>EECEBICD_00181 hypothetical protein
ATGCTAATTCGAGTAGAAATTCCCATTGATGCGCCTGGTATTGATGCGCTGTTACGCCGT
TCATTCGAAAGCGATGCGGAAGCGAAGCTGGTTCACGATTTGCGTGAAGATGGTTTTCTG
ACGCTCGGACTGGTGGCCACGGATGATGAAGGTCAGGTGGTGGGCTATGTCGCCTTTAGC
CCGGTTGATGTGCAGGGCGAAGATTTACAGTGGGTCGGCATGGCGCCGCTGGCGGTCGAT
GAAAAGTATCGCGGGCAAGGGCTGGCGCGCCAGTTAGTGTATGAAGGGCTGGATTCGCTC
AACGAGTTCGGCTACGCGGCGGTCGTGACGCTGGGCGATCCGGCGTTATATAGCCGCTTC
GGTTTTGAACTGGCCGCACATTACGATCTTCACTGCCGTTGGCCCGGTACGGAAAGCGCT
TTCCAGGTACACCGTTTGGCTGAGGATGCGCTGGAGGGCGTGACGGGGCTGGTGGAGTAT
CACGACCACTTCAATCGTTTTTAA
>EECEBICD_00182 hypothetical protein
ATGCTGATGGCGACAATGACACCCTGGTATCTGTATCTGATTCGTACTGCAGACAATGCG
CTTTACACCGGAATAACCACCGATGTGGCGCGTCGTTACAGGCAGCATCAAACGGGAAAA
GGCGCAAAAGCATTACGGGGAAAAGGCGAGTTAACGCTGGCGTTTGCGGCGCAGGTCGGC
AATCGCTCGCTGGCCCTGCGCATAGAGTATCGCATCAAACAGCTCACAAAGCGCCAGAAG
GAGCGCCTTGTGACGGAACGCGAAGCGTTTGAGGCGCTGCTGTCCAGCCTGCAAACGCCG
GTGCTTAAAAACGATTGA
>EECEBICD_00183 hypothetical protein
ATGGACACACTTACCGCTATTGGCCGTTGGCTGGCGAAACAACACGTGGTGACCTGGTGC
GTACACCATGAGGGAGAGCTGTGGTGCGCCAATGCGTTTTATTTGTTTGATGCGCAAAAC

GTCGCGCTGTATCTGTTAACGGATGATAAAACCCGCCATGCGCAAATGTCTGGCGCGTGC
GCGCCAGTTGCGGGAACGGTCAATGGTCAGCCGAAAACGGTGGCGCGCATTCGCGGCGTA
CAGTTTAAAGGGGAAATTCGCCGTCTGGAGGGGCAGGAGAGCGACGCCGCGCGTAAAGCT
TACCTTCGTCGCTTCCCGGTCGCCAGGGTGCTGCCAGCGCCGGTATGGGAAATCCGGCTG
GATGAAATTAAATTCACCGACAACACGCTGGGGTTTGGTAAAAAACTGCACTGGTTACGC
GACTCACGCGCCCAGCAGGCGTAG
>EECEBICD_00184 Protein/nucleic acid deglycase 2
TTGCTATTGCCGGGAGGTCATTCGCCGGACTACCTGCGCGGCGACAGCCGTTTCGTGGAT
TTTACCCGCGATTTCGTTAACAGCGGTAAACCGGTTTTTGCCATTTGCCACGGCCCACAG
TTGTTGATCAGCGCCGATGTCATTCGTGGCCGCAAACTCACGGCGGTGAAACCGATTATT
ATCGATGTGAAGAATGCGGGCGCGGAATTTTACGATCAGGAAGTCGTCGTCGATAAAGAT
CAGCTGGTGACCAGCCGCACGCCTGACGACCTGCCGGCATTTAACCGCGAGGCGCTACGC
CTGCTGGGCGCGTGA
>EECEBICD_00185 hypothetical protein
ATGAGTCAGGTACTGATTACCGGCGCGACCGGACTGGTCGGCGGACATCTGCTGAGAATG
TTAATCAACACGCCGCAGGTGAGCGCTATCGCCGCGCCGACGCGTCGCCCGCTGACGGAT
ATTGTCGGCGTTTATAACCCTCACGATCCGCAGCTTACCGACGCGTTAGCTCAGGTGACC
GACCCGGTGGATATTGTTTTTTGCTGCCTGGGCACGACCCGGCGTGAAGCGGGAAGTAAA
GCGGCGTTTATTCATGCGGATTATACGCTGGTGGTTGATACCGCGCTGACCGGGCGTCGA
CTGGGCGCGCAGCACATGTTGGTGGTCAGCGCAATGGGAGCAAACGCGCACTCTCCGTTT
TTCTACAATCGGGTGAAAGGAGAAATGGAGGAGGCGTTAATCGCGCAAAATTGGCCTCGT
TTGACGATTGCCCGGCCTTCTATGCTGCTCGGCGACAGAACAACGCGGCGGGTGAATGAA
ACGCTTTTCGCTCCGCTATTCCGGCTGTTGCCAGGAAACTGGAAATCGATTGATGCGCGG
GATGTGGCGCGGGCCATGCTGGCGGAAGCGCTGGCGCCAGCACAAGAAGGCGTGACGATT
CTGACCTCATCGCAACTGCGGGAAAAAGCGGAATAG
>EECEBICD_00186 Osmotically-inducible protein Y
ATGAAGGCATTTTCGCCGCTCGCAGTCCTTATTTCTGCGTTGCTATTGCAAGGTTGCGTC
GCCGCCGCGGTAGTGGGTACGGCAGCCGTCGGCACCAAAGCCGCAACCGACCCGCGTAGC
GTGGGCACCCAGGTGGATGACGGAACGCTGGAACTGCGTGTCAGCAGCGCGCTGTCGAAA
GATGAGCAAATCAAAAAAGAGACGCGCATCAATGTCACGGCTTATCAGGGCAAAGTTTTG
TTAGTGGGCCAGTCGCCAAACAGCGAACTCTCCGCACGCGCTAAACAGATAGCGATGGGC
GTTGAAGGCACCACCGAGGTCTATAACGAGATTCGCCAGGGTCAGCCTATCGGCCTGGGG
ACCGCCTCTAACGATACCTGGATCACCACGAAAGTTCGCTCACAGCTACTGACCAGCGAT
CAGGTAAAATCGTCAAACGTGAAAGTCACCACCGAAAATGGCGAAGTGTTCCTGCTGGGC
CTGGTGACTGAACGTGAAGGTAAAGCGGCAGCGGACATCGCCAGCCGGGTGAGCGGCGTG
AAACGCGTGACCACCGCCTTTACGTATATTAAGTAA
>EECEBICD_00187 DnaA initiator-associating protein DiaA
GTGCTCGAAAGAATCAAAGTTTGCTTTACAGAAAGCATTCAAACTCAAATTGCTGCGGCG
GAAGCGCTTCCGGATGCCATTTCCCGCGCCGCCATGACGCTGGTTCATTCCCTGCTTAAC
GGCAACAAAATCCTCTGTTGTGGAAACGGGACGTCCGCGGCCAACGCACAGCATTTTGCT
GCCAGCATGATTAACCGCTTTGAAACGGAGCGACCCAGTTTACCCGCCATTGCACTAAAT
ACCGATAATGTGGTCTTAACTGCGATCGCTAACGATCGCCTGCACGATGAAGTTTATGCA
AAACAAGTCCGGGCGCTGGGACATGCAGGCGATGTTTTATTGGCGATCTCTACGCGTGGT
AATAGTCGCGATATCGTAAAAGCCGTTGAAGCAGCCGTCACACGTGACATGACCATTGTG
GCGCTAACCGGGTATGACGGGGGTGAGCTGGCCGGACTATTAGGGCCGCAGGATGTCGAA
ATTCGTATCCCTTCACACCACAGCGCGCGCATTCAGGAAATGCATATGCTGACGGTAAAC
TGCCTGTGCGATTTGATCGATAACACGCTTTTTCCTCACCAGGATGATTAA
>EECEBICD_00188 hypothetical protein
ATGGCTCAAATACCAGCAAGGGGAGATTGTTCCCGCCAGCTAACGCGTAAACAGGCTGGC
GACGCGTGGGAAGCTGCCGCGCGTCGCTGGCTGGAAAGCAAAGGACTGCGGTTTATCGCC
GCCAACGTGCGCGAGCGCGGCGGCGAAATCGATCTGATCATGCGCGACGGAAAAAACGACG
GTTTTTGTTGAGGTCCGCTATCGGCGCTCCGGGCTTTATGGCGGCGCGGCGGCTAGTGTG
ACCCGCAGCAAGCAACACAAATTATTACATACTGCCCGCTTGTGGCTCGCCCGCCAGAAT
GGGAGTTTTGATACTGTGGATTGTCGGTTCGATGTGTTAGCCTTCACCGGAAATGAGATT

GAGTGGTTTCGGGATGCGTTTAACGACCACTCATAA
>EECEBICD_00189 Penicillin-binding protein activator LpoA
ATGGTACCCTCAACATTTTCTCGTTTGAACGCCGCGCGCGCGCTGCCTGTCGTCCTGGCT
GCGCTACTTTTCGCCGGGTGCGGCACCCAGGCGCCAGATCAAAGCGCAGCCTATATGCAG
GGTTCAGCGCAAGCTGACTCCGCCTTTTACCTGCATCAGATGCAGCAAAGCGCAGATGAT
AGCAAGACCAACTGGCAATTACTCGCCATTCATGCACTGCTGAAAGAAGGAAAAAGCCAG
CAGGCCGTCGACCTGTTCAACCAACTCCCGCAAAATCTGAACGATGCCCAGCGTCGCGAA
CAGTCTTTATTAGCGGTAGAAATCAAACTGGCGCAAAAAGATGTCGCAGGCGCGCAGGCC
TTGCTGGATAAACTAAAACCTGCCGACTTTGCGCCAAATCAGCAAGCGCGTTACTGGCAG
GCGCAGATCGTTGCCAGCCAGGGACGCCCGTCGCTTACCCTGTTGCGGGCGTTAATCGCC
CAGGAACCGCTACTGGCGGCGAAAGATAAACAAAAAAATATCGACGCCACCTGGCAGGCG
CTCTCCGCCATGACGCCGGATCAGGCCAAGACGTTAGTTATCAACGCCGATGAAAACGTG
CTTCAGGGCTGGCTGGATCTGCAACGCGTCTGGTTTGACAACCGCAACGATCCGGACATG
CTGAAAGCCGGGATCGCCGACTGGCAAAAACGCTACCCGCAAAATCCGGGGGCGAAAATG
CTGCCGACGCAGCTCGTCAATGTGCAACGTTTCAAACCGGCTTCCACCAGCAAAATCGCT
CTGCTGCTGCCGCTGAACGGTCAGGCTGCCGTGTTTGGCCGTACCATCCAGCAAGGTTTC
GAAGCCGCGAAAAACCTCGGCACCCAGGCGGTGGAGATGCAGCCTGCCGCCGCTCCTGAC
GCGCCGGTCGAACCTGGCGTGGAGGAGACGCAACCACAAATGACCAACGGCGTCGCCAGT
CCGTCGCAGGCCTCGGTGAGCGATCTGACTGATGACGCTCCAGCCCAGTCCGCTACGCCA
GTCAGCGCGCCACAAACTCCCCCTGCTACAGCAAGCGCGCCGGCGGATCCCTCCGCTGAA
TTAAAAATCTACGATACCTCTTCCCAGCCGTTGGATCAGGTGCTCGCTCAGGTTCAGCAA
GACGGCGCCAGTATCGTGGTCGGCCCGCTGTTGAAAAACAATGTGGAAGCGCTGATGAAA
AGCAACACGCCGCTCAACGTGCTGGCGCTCAACCAGCCGGAAACGGTACGTAGCTTCCCT
AATATCTGCTATTTCGCGCTCTCTCCAGAAGATGAAGCCCGTGACGCGGCGCATCATATT
TATGACCAGGGCAAGCAGTCGCCGCTGCTGTTGATTCCACGCAGCGCGCTTGGCGATCGC
GTGGCGAACGCCTTCACCCAGGAGTGGCAAAAACTGGGCGGCGGCATCGTGTTACAGCAA
AAATTCGGCTCCGTAGCCGAGCTGAAAATGGGCGTGAACGGCGGCGCGGGTATCGCGTTG
ACGGGCAGTCCGGTCGCCGCCAGCGTGCCTGCGCAGCCTGGCGTCACCATTGGCGGTCTG
ACCATCCCTGCGCCGCCGACCGACGCGCAAATCACCGGCGGCGGACGCGTAGACGCGGTC
TATATTCTGGCTACGCCGGAAGAGATTGGCTTTATCAAACCGATGATCGCCATGCGTAAC
GGCACCCAGAGCGGCGCGACGCTGTATGCCAGCTCTCGCAGCGCGCAAGGCACCTCCGGC
CCCGACTTCCGTCTGGAAATGGAGGGTTTACAATACAGTGAAATTCCTATGCTGGCAGGC
GGCAATATGCCGTTGATGCAGCAGGCGCTGAGCGCTGTACATAACGACTATTCTCTGGCG
CGGATGTACGCCATGGGCGTGGATGCCTGGACGCTGGCGAACCACTTTTCGCAGATGCGT
CAGGTGCAGGGATTTGAGATCAATGGTAATACCGGCGCATTAACCGCCAGCCCGGATTGT
GTGATTAACAGGAAGTTATCATGGCTCAAATACCAGCAAGGGGAGATTGTTCCCGCCAGC
TAA
>EECEBICD_00190 Ribosomal RNA small subunit methyltransferase I
ATGAAACAAAACGAATCGGCGGATAATTCTCAGGGCCAACTCTTTATTGTACCTACTCCT
ATCGGTAATCTGGCGGATATTACCCAGCGTGCGTTGGAGGTGTTACAGGCTGTAGATCTG
ATTGCCGCCGAGGATACTCGTCACACCGGTTTATTGCTGCAACATTTTGGGATTAATGCC
CGATTGTTCGCGCTGCACGACCATAACGAACAACAAAAAGCGGAAACGCTGGTCGCGAAG
CTAAAAGAAGGGCAGAACATTGCGCTGGTTTCCGACGCCGGTACGCCGCTGATTAACGAT
CCGGGCTACCATCTGGTGCGTACTTGCCGTGAAGCGGGGATTCGCGTGGTGCCGCTACCA
GGGCCGTGTGCTGCTATCACTGCATTAAGCGCCGCGGGTTTACCATCAGATCGTTTTTGT
TATGAAGGCTTCCTGCCTGCCAAATCAAAAGGCCGCCGTGATGCGCTAAAAGCCATTGAA
ACGGAACCGCGAACGCTGATTTTTTATGAGTCAACCCACCGCCTGTTAGATAGCCTGGAA
GATATCGTCGCGGTATTGGGAGAATCCCGTTATGTGGTGCTGGCGCGTGAGCTGACCAAA
ACCTGGGAAACCATTCACGGCGCGCCCGTTGGCGAGCTGCTGGCGTGGGTCAAAGAAGAT
GAAAACCGTCGCAAAGGCGAAATGGTGCTGATTGTCGAAGGCCATAAAGCGCAAGAAGAC
GACCTGCCCGCCGACGCGCTGCGCACGCTGGCGCTGCTACAGGCAGAACTGCCGCTGAAA
AAAGCGGCGGCGCTGGCAGCAGAAATTCACGGCGTGAAGAAAAATGCGCTGTATAAGTAT
GCGCTGGAGCAGCAGGAAGAATAA
>EECEBICD_00191 Glucitol operon repressor

ATGAACTCATTTGAGCGAAGAAATAAAATTGTCGACCTGATTAATACGCAAGGCAGCGTG
CTGGTGATGGACCTTTCAAATACATTTGGTATTTCTGAAGTGACTATCCGTGCTGACCTG
CGTCTGCTGGAAGAGAAAGGCCTTGTCACCCGTTTTCATGGCGGCGCGGCAAAACCGGGA
AGTCATCTGGCGGAAGGCGACAATCAGGAAGTCATTCTGGAAGATCGGTATCAACTCGCC
AGCGATCCGAAAAAGCGGATTGCCCAGGCGGCTGCGGCAATGGTTGAAGAAGGAATGACC
ATCATTCTGGATAGCGGGAGCACAACATTACTGATTGCGGAAGCGCTCGCGCGAAAAAGC
AATATTACGGTGATCACTAACAGCTTGCCTGCCGCTTTTACGCTATCAGAAAATAAAGAT
CTCACGCTGGTGGTCTGCGGCGGTACGGTACGGCATAAAACCCACTCAATGCACGGCACG
ATTGCGGAACGATCGCTACACGGCATCAGCGCCGATTTAATGTTTGTCGGGGCGGATGGC
ATTGATGCGACAAACGGTATTACCACGTTTAACGAAGGTTATTCCATTAGCGGCGTCATG
GCGGCGGCGGCACACAAAGTTATCGCGGTACTGGACGCGACCAAATTTAACCGTCGCGGC
TTTAACCAGGTATTGCCGATGGACAAGATCGACTGTGTGATAACCGATGACACCATCAGC
AAACAGGATAAAGCGGCATTAGCGAAAACAGGCGTGGAATTAATGATCGTCTAA
>EECEBICD_00192 Galactitol 1-phosphate 5-dehydrogenase
ATGAAATCAGTGGTTATTCACGCTGAGGGAGACGTGCGCGTTGAAGAACGTCCCTTACCA
CAACTGCAAGCCGAGGACGATGTGTTGGTCAAAGTCGTTAGCTCAGGGCTTTGCGGGTCG
GATATCCCGCGTATTTTCGCGCAGGGCGCCCATTATTATCCTATTACGTTAGGCCATGAA
TTCAGCGGCTACGTTGAATCCTACGGCACAGGCGTAACGGATATGCAGCCTGGCGATGCC
GTCGCTTGTGTCCCGCTTCTTCCCTGCTTTCGCTGTCCGCAATGCGAACGCGGTTACTTT
TCTTTATGCAAACAGTATCAGTTTGTAGGATCGCGCAGCGAAGGCGGTAATGCGGAATAT
GTGGTGGTCAAACGGGCCAATCTTTTCCGCTTGCCGTCTGATATGCCGATTGAAGATGGC
GCGTTTATCGAACCAATTACCGTGGGCTTACACGCCTTTCATCTGGCGCAGGGCTGCGAG
GGGAAAAACGTAATTATCGTTGGTGCCGGGACAATTGGCCTGCTGGCCTTACAGTGCGCG
CGGGAACTGGGCGCGCGAAGCGTAACGGCGATTGATATCAATCCGCAGAAACTGGAACTT
GCGAAAGCGCTCGGCGCAACGCATACCTGTAATAGCCGGGAAATGACCGCCGACGACATC
CAGACCGCGTTGTCCGACATTCAGTTTGATCAACTGGTTCTCGAAACGGCAGGAACTCCC
CAGACCGTCTCGCTGGCTATCGATATCGCCGGGCCGCGCGCGCAACTGGCGCTGGTGGGG
ACGTTACATCACGATCTCACCCTGACAACGCGTACATTCGGGCTAATTTTGCGTAAAGAG
CTCACGCTCCTCGGCAGTTGGATGAACTACTCCGCGCCATGGCCAGGAGAAGAATGGGAA
ACCGCAGCGCGCCTGTTGGCTGAAAAACGTCTTCAACTGACCCCTCTCATTGCCCATCGC
GGCGATGCGGAAAGCTTTGCAGAAGCAGTCAAAGCGCTTAATGGCGCGCCCATGCAGGGC
AAAATCCTACTTCAACTTTCCTGA
>EECEBICD_00193 PTS system galactitol-specific EIIC component
ATGTTTAGCGAAATAATGCGTTATATCCTCGACTTAGGCCCAACGGTGATGTTGCCTCTG
GTGATCATCGTGTTCTCTAAACTGCTGGGAATGAAGCTTGGGGATTGTTTTAAATCGGGT
TTGCATATTGGTATCGGCTTCGTGGGTATTGGTCTGGTCATCGGCCTGATGCTGGATTCT
ATCGGCCCCGCGGCGAAAGCCATGGCGGAACATTTCCAAATCAATCTCCACGTTATTGAC
GTCGGCTGGCCAGGTTCATCGCCCATGACCTGGGCGTCACAAATCGCGCTGGTCGCGATC
CCTGTCGCCATCGGGGTTAACGTCCTGATGCTGGTGACCCGCATGACCCGCGTGGTGAAT
GTTGATATCTGGAATATCTGGCACATGACCTTCACGGGCGCCATGCTGCATCTGGCGACC
GGTTCATACTGGCTGGGGATTCTGGGCGTTGTGGTTCATGCCGCCTTTGTCTACAAACTG
GGGGACTGGTTTGCCAAAGATACCCGCGACTATTTTGGCCTCGAGGGGATTGCTATCCCA
CACGGTTCATCCGCGTACCTGGGCCCCGTGGCGGTACTCGTTGATACCATTATCGAGAAA
ATTCCTGGTCTCAATCGTATTCACTTTAGCGCAGACGATGTCCAGAAACGCTTCGGACCG
TTTGGCGAGCCGGTGACTGTCGGCTTCGTGATGGGGCTGGTGATTGGTGTACTGGCAGGC
TACGACGCCAAAGCCGTTCTGCAACTGGCGGTCAAAACCGCAGCGGTGATGCTGCTTATG
CCACGCGTCATTAAACCTATTATGGATGGCCTAACGCCTATCGCGAAACATGCGCGTAAA
CGTCTACAGGCTAAATTTGGCGGGCAGGAGTTCCTGATAGGCCTTGATCCAGCGCTACTG
CTCGGTCATACCTCTGTTGTCTCCGCGAGCCTGATATTCATTCCGCTGACCATCCTGATT
GCCGTCTTAGTACCGGGGAACCAGGTGCTGCCGTTCGGCGACCTCGCCACCATCGGTTTC
TTTATTGCGATGGCGGTCGCGGTACACCAGGGCAACCTGTTCCGCACGCTGATTTCAGGT
GTCATTATTATGGGTATCACCCTGTGGATAGCCACCCAGACGATTGGCCTGCATACCCAA
CTGGCTGCCAATGCCGGAGCGCTAAAAGCTGGCGGACAAGTCGCCTCGCTGGATCAGGGC
GGTTCTCCCATCACCTGGCTGCTGATTCAACTTTTTACCTGGCAGAATATCGTCGGCTTC

GCCGTCATTGCCATTATCTATCTGGCCGGCGTACTGCTGACCTGGCGTCGCGCCCGACAG
TTTGTCGCGGCTGAGAAAGCCACGGCGCTACAGCAAAGTCAAATCGCCTCTTAA
>EECEBICD_00194 PTS system galactitol-specific EIIB component
ATGAAACGTAAAGTAATTGTGGCCTGTGGCGGTGCGGTTGCAACCTCAACGATGGCTGCG
GAAGAAATTAAAGAGCTATGTGATGCCAACCATATTGAGCTCGACTTAGTGCAATGCCGG
GTCACCGAAATCGAAACCTATATGGACGGCGCCGATCTTATCTGTACCACTGCCAGGGTC
GATCGCGCCTTTGGCGATATTCCCGTCGTTCACGGTATGCCTTTTGTTTCCGGCGTGGGT
ATTGAAGCATTACAGCAAAAAATTCTGTCCATTCTCATGGGGTAA
>EECEBICD_00195 PTS system galactitol-specific EIIA component
ATGAGCCAACTATTTGTCCGAACCGGAATCACATTCGACTCGAGCCAACAGGCGCTGGTG
CACATTGGCAAAGAGATGCTGGCGAAAGGCGTGGTACATGATAGTTATCCGCAGGCGCTG
GTGGAACGTGAAGCGTCTTTTCCTACCGGCATTGCGCTGGAGCGTCACGCCGTCGCTATC
CCTCATTGCGAGGCGGTTCATGCGAAATCACCCGCTATCTATCTGATTCGTCCAGATAAG
CCGGTCATGTTTCAACAGGCGGATGATGACGAAGAGATTGCCGTTTCGTTAATCATCGCC
TTAATCGTTGAAAACCCGGCGGCACAGCTAAAACTTCTGCGCCGCCTGTTTAGTGAGCTA
CAGAACCCGAGCACCCTCGATGCCCTGTTAAGCGCGCCGGATGCCGAACTGGCGATACGC
TTTCAGGAAACCATCCTTGAGCCTGAGCAGTGCGTCCAGGTTTGA
>EECEBICD_00196 D-tagatose-1,6-bisphosphate aldolase subunit GatZ
GTGAAAGAAATAATTTCTCGTCACAAAGCAGGGGAACAGATAGGGATCTGTTCCGTATGT
TCAGCACACCCGCTGGTGATTGAATCCGCATTACGTTTTGACCTGAATAGCGGGAACAAA
GTTCTGATCGAGGCGACCTCTAACCAGGTTAATCAGTTTGGCGGTTATACCGGCATGAAA
CCGGCTGACTTTCGTGATTTTGTCTATGGCATCGCCCAGGAAGTGGGTTTTCCACGCGAA
CGGCTTATTTTAGGCGGCGATCACCTGGGACCTAACTGCTGGCAAAACGAACCTGCGGAT
ACGGCGATGGAAAAAATCCGTCGAACTCATTAAGGCTTACGTCGCTGCCGGGTTTAGCAAA
ATCCATCTTGATGCCTCTATGTCCTGCGCTGACGACCCGACGCCGCTGGACCCAATGGTT
GTCGCTAAACGCGCCGCCTTGCTTTGCCAGGCAGCGGAAACGACGGCGACCGACGAACAA
AAACGCCATTTAACCTATGTTATCGGCACTGAAGTTCCGGTTCCGGGCGGGGAAGCCAGC
GCCATTAACGCCGTGCATGTTACCCGCGAACAGGATGCGGCCCGCACGCTGCAAACGCAT
CAGGCTGCTTTTCGCGCCCTGGGGCTGGATGAGGCATTGAACCGGGTTATCGCGATTGTC
GTACAGCCGGGCGTTGAATTTGATCATACCCAGATTATTCATTATCAGCCGCAGGCGGCA
CAGGCGCTCTCCGCATGGATAAAAGAAACGCCGATGGTCTATGAAGCGCATTCGACTGAT
TATCAGACGCGACAGGCCTACCGTGCGCTGGTACGCGATCATTACGCCATTCTGAAGGTC
GGCCCGGCGCTCACTTTCGCGCTGCGCGAAGCCATTTTCGCACTTGCGCAAATGGAAAAC
GAGCTGATCTCCCCGGAACAACGTAGTCGGGTACTGGAGGTCATTGATGAAGTTATGCTA
AACGAACCGGGCTACTGGAAAAAATACTACCGTCCAACCTGGAGCCAGGCGATGGTCGAC
ATCCACTTTAGTTTGTCTGACCGTATTCGCTATTACTGGCCGCACCCGCGTATTCGTCAA
AGCGTAGAGAAGTTAATCGCCAATCTGAACAACGTCACATTGCCGCTGGGATTAATTAGC
CAGTTTATGCCCGTACAGTTTGAACGCCTGTCTGAGGGCGTGCTCACCCCTACGCCGCAT
AACCTGATTATTGACAAAATTCAGGATGTTCTGCGCGCTTATCGCTTCGGTTGCACGCCG
GATGTCGCCTGA
>EECEBICD_00197 D-tagatose-1,6-bisphosphate aldolase subunit GatY
ATGTTTATCATTTCAGGCAGAACAATGCTAAAGAAGGCGCAGCAGGAAGGCTATGCTGTG
CCGGCGTTTAACATCCACAACCTGGAGACATTGCAAGTGGTGGTGGAAACCGCCGCAGAA
TTGCGCTCTCCGCTGATTGTCGCCGGTACGCCAGGCACCTTTAGCTACGCAGGCGTCGGT
AATATCGTGGCCATCGCCGCAGAACTGGCGAAAAGCTGGAACCATCCTCTCGCGGTACAT
CTCGATCATCATGAAAAACTGGCCGACATCAAAATGAAAGTCGCCGCCGGGGTACGCTCG
GTCATGATCGACGGGTCGCATTTCCCCTTTGCCGACAATATTGCGCTGGTGAAAAGTGTG
GTTGATTACTGTCATCGCTACGATGTCAGCGTTGAAGCTGAACTTGGGCGTCTCGGCGGG
CAGGAAGACGATCTTATCGTTGACGGTAAAGATGCGCTTTATACCCATCCGGAACAGGCC
CGGGAATTTGTAGAAAAAACGGGTATCGACTCGTTAGCCATTGCTATCGGCACCGCTCAC
GGCCTCTACACCGCTGAACCAAAACTTGATTTTGAACGACTGACGGAAATTCGTCAGCGG
GTTGATGTCCCCTTAGTCCTTCACGGCGCCTCTGGCCTGCCGACCCGCGATATTACCCGC
GCTATTTCGCTGGGCATCTGCAAAGTTAACGTCGCGACCGAGCTTAAAATCGCCTTTTCC
GGAGCGCTTAAAAACTATTTAACGCAACACGCAGAGGCCAGCGATCCCCGCCATTACATG

ATCCCGGCGAAAGCGGCCATGAAAGAGGTTGTACGTAAAGTGATTGCCGACTGCGGTTGT
GAAGGGAAGCTCTAA
>EECEBICD_00198 Galactarate dehydratase (L-threo-forming)
ATGGCCAATATCGAAATTCGCCAGGAATCGCCATCGGCGTTTTATATAAAAGTACATGAA
ACCGATAATGTCGCAATTATTGTTAATGACCACGGTCTTAAAGCCGGAACACGTTTTCCG
GATGGCCTGGAACTGACTGAACATATTCCGCAAGGCCATAAAGTTGCTCTGACGGATATC
CCCGCTCACGGCGAAATCATCCGTTATGGCGAAGTCATCGGTTATGCGGTACGCGACATC
CCGCGCGGAAGCTGGATCGACGAGTCGCTGGTTGAACTGCCAAAAGCGCCGCCGCTGAAT
ACGCTACCGCTGGCGACCAAAGTACCGGAACCGCTGCCGCCGCTGGAAGGCTATACGTTT
GAGGGCTATCGTAACGCCGATGGCAGCGTCGGCACCAAAAATCTGCTTGGCATTACCACC
AGCGTGCACTGTGTGGCGGGCGTGGTCGATTATGTGGTGAAAGTGATTGAGCGCGATCTC
CTGCCCAAATACCCGAATGTCGATGGCGTTGTAGGGCTAAACCATCTGTACGGCTGCGGC
GTGGCAATCAACGCGCCGGCGGCGGTCGTGCCGATTCGCACGATTCATAATATCGCGCTC
AACCCCAATTTTGGCGGCGAAGTGATGGTGATTGGCCTCGGTTGCGAAAAACTGCAGCCG
GAACGGCTGCTGGAAGGCACCGAGGATGTTCCCGCCATCGCCGTTGAAAGCGCCAGTATT
GTGCGTTTACAGGACGAGCAGCATGTCGGCTTTAAATCAATGGTCGACGATATTTTGCGG
GTCGCCGAACGCCATCTGACGAAACTGAACCAGCGCCAGCGCGAAACCTGCCCGGCCTCC
GAGCTGGTCGTCGGGATGCAGTGTGGCGGCAGCGATGCGTTTTCTGGCGTCACGGCAAAC
CCGGCGGTGGGTTATGCTTCCGATCTGCTGGTACGCTGCGGCGCGACCGTCATGTTCTCG
GAGGTCACCGAAGTGCGCGATGCCATTCATCTTCTGACGCCGCGCGCCATCAACGAAGCC
GTGGGTAAACGCCTGCTGGATGAAATGGCCTGGTACGATAACTATCTTGATATGGGGAAA
ACCGATCGCAGCGCTAACCCGTCCCCTGGCAACAAAAAAGGCGGCCTGGCGAACGTCGTG
GAAAAAGCGTTGGGCTCTATCGCTAAATCGGGTAAGAGCGCCATTGTCGAAGTATTATCG
CCGGGCCAGCGCCCCACCAAACGCGGCCTGATTTACGCCGCGACGCCCGCCAGCGATTTC
GTCTGCGGCACGCAGCAGGTCGCTTCCGGCATTACCGTACAGGTCTTTACCACCGGACGC
GGCACGCCGTACGGGCTGATGGCGGTGCCGGTCATTAAAATGGCGACGCGTACCGAGCTG
GCGAACCGCTGGTATGACTTAATGGATATCAATGCAGGCACCATCGCCACGGGTGAAGAG
ACAATCGAAGATGTGGGCTGGAAGCTATTTCACTTTATTCTCGATGTCGCCAGCGGCCGG
AAAAAGACTTTCTCAGATCAATGGGGATTGCATAACCAGCTGGCAGTGTTTAACCCCGCA
CCTGTGACCTGA
>EECEBICD_00199 5-keto-4-deoxy-D-glucarate aldolase
ATGAATAACGCTATTTTCCCGAATAAGTTTAAAGCGGCGCTGGCGGCGCAACAGGTACAG
ATTGGCTGCTGGTCTGCACTGGCAAGCCCAATCACCACCGAAGTTCTCGGTCTGGCGGGA
TTTGACTGGCTGGTGCTGGATGGCGAACATGCGCCGAACGATGTGACCACCTTAATTCCG
CAGTTAATGGCATTGAAAGGCAGCGCCAGCGCGCCTGTGGTACGTGTGCCAACCAATGAA
CCGGTCATTATCAAACGGATGCTGGATATTGGGTTTTATAATTTCCTGATCCCCTTTGTT
GAAACGCAAGAGGAAGCGGCTCGCGCCGTCGCCTCGACGCGTTACCCGCCGGAAGGCATT
CGCGGCGTCTCCGTTTCTCACCGCGCGAATATGTTCGGTACGGTGCCTGATTACTTTGCG
CAGTCGAACAAAAACATCACCATTATTGTTCAAATCGAAAGCCAGTTGGGTGTCGACAAT
GTTGATGCCATCGCCGCAACGGAAGGCGTTGATGGCATTTTTGTCGGCCCAAGCGATTTG
GCCGCCGCGCTGGGTCATCTCGGCAATGCTTCTCACCCTGACGTGCAGCAGACGATTCAA
CATATTTTCGCCCGCGCTAAAGCGCACGGTAAACCGTGCGGCATTCTGGCGCCGGTAGAG
GCGGATGCCCGCCGTTATCTGGAATGGGGTGCGACCTTTGTCGCCGTTGGCAGCGATTTA
GGCGCATTCCGCGCCAGCACGCAGAAGCTTGCCGACACCTTTAAAAAATAA
>EECEBICD_00200 2-hydroxy-3-oxopropionate reductase
ATGACGATGAAAGTTGGCTTTATTGGCTTAGGCATCATGGGTAAGCCCATGAGCAAAAAC
TTACTGAAAGCAGGGTATTCGCTGGTGGTGTCCGATCGTAATCCTGAAGCGATTGCCGAC
GTCATTGCCGCAGGCGCGGAAACGGCCTCTACGGCGAAGGCGATCGCTGAACAGTGTGAC
GTCATCATCACGATGCTGCCTAACTCTCCGCATGTGAAAGAGGTTGCGCTGGGCGAGAAC
GGTATCATCGAAGGCGCGAAGCCGGGAACCGTATTGATTGATATGAGTTCTATCGCGCCG
CTGGCAAGCCGTGAAATCAGCGATGCGCTGAAGGCGAAGGGCGTTGAGATGTTGGATGCG
CCGGTCAGCGGCGGCGAGCCGAAGGCGATTGATGGCACGTTGTCGGTGATGGTGGGGGGC
GATAAAGCCATTTTCGACAAGTACTACGACCTGATGAAAGCGATGGCGGGTTCCGTGGTG
CATACTGGCGATATCGGCGCAGGCAACGTCACAAAACTGGCTAACCAGGTGATCGTGGCG

TTGAATATCGCCGCCATGTCTGAAGCGCTGACGCTGGCGACTAAAGCGGGCGTCAACCCG
GATCTGGTCTATCAGGCGATTCGCGGCGGTCTGGCTGGCAGCACGGTACTGGATGCGAAA
GCGCCGATGGTCATGGACAGAAACTTTAAGCCAGGTTTCCGTATCGATCTGCACATTAAA
GATCTGGCGAATGCGCTGGATACCTCCCACGGCGTTGGCGCGCAGCTCCCGCTGACCGCC
GCCGTTATGGAAATGATGCAGGCGTTACGCGCGGATGGACACGGTAATGACGACCATAGC
GCGCTGGCGTGCTACTACGAAAAACTGGCGAAAGTGGAAGTCACTCGCTAA
>EECEBICD_00201 Glycerate 2-kinase
ATGAAAATCGTAATTGCCCCCGACTCCTACAAAGAAAGCCTTTCTGCCGCCGAGGTAGCG
CAGGCGATAGAAAAAGGATTTCGGGAAATTTTCCCCGATGCGCAATATGTCTCTGTTCCG
GTTGCCGATGGCGGCGAAGGAACGGTCGAAGCCATGATTGCCGCGACGCAGGGCGTTGAA
CGCACCGCATGGGTGACCGGGCCGTTGGGTGAAAAAGTGAAAGCCTGCTGGGGAATGTCC
GGGGATGGTAAAACCGCGTTTATTGAAATGGCGGCGGCCAGCGGACTGGCGCTGGTTCCA
CCAGAAAAACGCAACCCTTTGATTACTACCTCGCGGGGAACGGGGGAGCTGATATTGCAG
GCGCTGGAAAGCGGCGCAAGCAATATCATTATCGGCATTGGCGGCAGCGCGACAAATGAC
GGCGGCGCAGGGATGATGCAGGCGTTAGGCGCAAAACTGCGCGATGCAAACGGCGCGGAT
ATCGGTTACGGTGGCGGAAGCCTTCATTGTCTAAGCGACATTGATATTTCAGAGCTCGAT
CCCCGTTTAAAACTCTGCGCGATTCGTGTCGCATGCGATGTATCTAATCCTCTGATCGGC
GACAACGGCGCATCACGAATTTTTGGGCCGCAGAAAGGCGCGACCGAAGAGAACATTGTT
GAGCTGGACAGAAATCTTGCTCACTATGCCGACATCATAAAAAAATCGCTGAATGTCGAC
GTAAAGGCGGCGCCCGGCGCAGGCGCGGCAGGCGGTATGGGGGCGGCGTTAATGGCTTTT
CTCGGTGCGGAATTACGCAGCGGGATCGAAATTGTCACGGCGGCACTGAACCTGGAGGAG
CATATCCACGACTGTACGCTGGTGGTGACGGGAGAAGGGCGGATTGATAGCCAAAGTATT
CGCGGTAAAGTGCCGATTGGCGTCGCGAATGTGGCGAAAAAATATCATAAGCCAGTGATT
GGCATTGCCGGCAGCCTGACGCACGATGTAGGGATAGTGCATCATTATGGCATCGACGCT
GTCTTTAGCGTACTCACGCGCATCGTGACGCTGGAGGAGGCTTTTCGGGGCGCTTTTGAC
AATATTTATCGGGCATCCAGAAATGTTGCTGCCGCTCTGGCGATAGGAATGCGCAGCGCA
GGGTGA
>EECEBICD_00202 hypothetical protein
ATGAAACTGACCGATAAGCCGCTTTCTTTTGGGTATAGCGTCGTGGACAGTCATTCATCC
TGCCGCCCTGTAAAAGCAAAAACCCGCCGAAGCGGGTTTTATGAAGAGGTGAAACTGACC
GATAAGCCGGGTTCTGTCGTGGACAGTCATTCATCTAGGCCAGCAATCGCTCACTGGCTC
AAGCAGCCTACCCGGGTTCAGTACGGGCCGTACCTTATGAACCCCTATTTGGCCTTGCTC
CGGGTGGAGTTTACCGTGCCACGGACTGTTACCAGCCGCGCGGTGCGCTCTTACCGCACC
CTTTCACCCTTACCTGATCCCACTTGCGTGGGCCATCGGCGGTTTGCTCTCTGTTGCACT
GGTCGTAGGTTTCCCCCCAGGCGTTACCTGGCACCCTGCCCTATGGAGCCCGGACTTTCC
TCCCCTCCGCCCGTCTCCCCGAAAGGACGACGACGAAGCGGCGACTGTCTGGTCAGCTTC
GGCGCGCAGTATAGAGGGTTCGCGCGACTTTGTCACCCTGCGCTGCGCATTCCTATCGCC
AGAGCGGCAGCAACATTTCTGGATGCCCGATAA
>EECEBICD_00203 HTH-type transcriptional regulator TsaR
ATGAATACTCTTGTTCTCCCTAAAACACAGCATTTGGTGGTCTTTCAGGAAGTCATTAGA
AGTGGTTCTATCGGTTCTGCCGCAAAATCGTTAGGGTTAACCCAACCCGCCGTCAGTAAA
ATCATTAGCGATGTTGAGGCCTATTTTGGCGTTGAGCTAATTGTACGCAAAAATACCGGC
GTTACGTTGACCGAAGCGGGACAGGTACTGCTTTCCTGGTCTGAATCTATCACCCGCGAA
ATGAAAAACATGATCAACGAGATGAACAGTATGACGTGCAATACCGTTGTCGACGTCTCG
TTTGGTTTTCCTTCGCTGATTGGCTTTACCTTCATGTCCGATATGATTCATAAGTTCAAA
GAGGTATTTCCAAAAGCGCAGGTATCCATGTACGAAGCCCAGCTCTCTTCATTCCTGCCG
GCCCTGCGCGACGGGCGGCTGGACTTTGCTATCGGTACGCTCAGTAATGAAATGCAACTA
CAGGATCTGCATGTCGAGCCGCTGTTTGAATCAGAGTTTGTCCTGGTAGCCAGCAAGTCC
CGAACATGCACCGGCACCATCACACTGGAATCGTTAAAGGACGAACAGTGGGTGTTGCCG
CAAACGAATATGGGCTACTACAGCGAATTACTCACCACTCTACAACGAAATGGCATCAGC
ATCGAAAACATTGTTAAAACAGATTCCGTTGTCACAATATATAATCTTGTTCTCAATGCT
GATTTCTTAACTGTTATTCCCTGCGATATGACCACGCCTTTTGGCTCTAATCAATTTATC
ACGATACCCATAAAAGACACGTTACCCGTCGCACGTTATGCGGCGGTATGGTCTAAAAAT
TATCGTATCAAAAAAGCCGCTTCAGTATTAGTAGAATTGGCTAAACAATATTCATCATAT

AATGGATGCAGACGCAGGCAGTTAATAGAAATTGAATGA
>EECEBICD_00204 L-threonine dehydratase catabolic TdcB
ATGCACATTACATACGATCTCCCGGTTGCAATTGAAGATATCCTCGAAGCGAAAAAAAGA
CTGGCGGGGAAGATATATAAAACGGGAATGCCTCGCTCTAACTATTTTAGTGAACGTTGC
AAAGGCGAAATATTTCTCAAGTTTGAAAATATGCAGCGTACGGGTTCATTTAAAATTCGT
GGCGCTTTTAACAAACTTAGCTCGTTAACGGAGGCGGAAAAACGTAAAGGCGTCGTGGCC
TGTTCCGCAGGTAATCATGCACAGGGCGTTTCTCTTTCTTGCGCAATGCTGGGTATTGAC
GGCAAAGTCGTGATGCCGAAGGGCGCGCCAAAGTCAAAAGTGGCGGCCACCTGTGATTAT
TCGGCGGAAGTGGTGCTCCACGGTGACAACTTCAATGACACCATCGCTAAGGTGAGTGAA
ATTGTCGAAACCGAAGGCCGCATTTTTATTCCGCCTTACGACGATCCAAAAGTCATTGCC
GGACAGGGCACTATTGGTCTGGAAATTATGGAAGATCTGTACGATGTCGATAATGTCATC
GTGCCTATTGGCGGCGGCGGTTTAATTGCAGGTATTGCCATTGCGATTAAATCGATTAAC
CCGACCATTAAAGTTATCGGCGTACAGGCCGAAAATGTTCACGGTATGGCGGCGTCTTAT
TACGCTGGCGAAATAACCACGCACCGAACGACCGGCACCCTGGCGGATGGTTGTGATGTC
TCCCGCCCGGGTAATTTAACCTATGAAATCGTTCGTGAATTAGTCGATGACATTGTCCTG
GTTAGCGAAGATGAAATTCGTAACAGCATGATCGCTTTAATTCAGCGCAATAAGGTTATT
ACTGAAGGCGCTGGCGCTCTGGCTTGCGCCGCACTATTAAGTGGGAAATTAGATAGCCAT
ATTCAGAACAGAAAAACGGTCAGTATTATTTCTGGCGGCAATATCGATCTTTCTCGGGTA
TCACAAATTACGGGTTTAGTTGACGCTTAA
>EECEBICD_00205 Threonine/serine transporter TdcC
ATGAGTACTACTGATAGCATTGTATCCAGCCAGGCAAAACAGTCGTCATGGCGTAAATCG
GACACGACGTGGACGCTGGGTTTATTTGGTACCGCCATCGGCGCCGGGGTGCTGTTTTTC
CCTATCCGCGCCGGGTTTGGTGGGCTTATCCCCATTCTGCTGATGCTGGTTTTAGCTTAC
CCCATTGCGTTCTATTGTCACCGGGCGCTGGCGCGTCTGTGTTTATCCGGATCTAATCCG
TCCGGCAATATTACGGAAACGGTCGAAGAGCACTTTGGTAAAACGGGCGGGGTAGTTATC
ACGTTCCTTTACTTCTTTGCGATTTGTCCGCTGCTGTGGATTTATGGCGTCACCATCACT
AACACGTTCATGACGTTTTGGGAAAACCAGTTGCAGATGCCAGCGTTGAATCGCGGTTTC
GTGGCGCTGTTCTTGCTGCTGCTGATGGCCTTTGTGATCTGGTTTGGTAAAGATCTGATG
GTTAAGGTGATGAGTTACCTGGTATGGCCGTTTATCGCCAGTCTGGTGCTAATCTCATTA
TCGCTGATTCCTTACTGGAACTCGGCGGTTATCGATCAGGTTGATCTCAGTAATATCGCG
CTGACTGGGCACGATGGCATACTGGTTACGGTGTGGCTGGGCATCTCTATCATGGTGTTC
TCCTTTAACTTTTCGCCCATCGTCTCTTCCTTCGTGGTGTCTAAGCGTGAAGAGTATGAG
AAAGAGTTTGGCCGCGAATTTACCGAGCGTAAATGTTCACAGATCATCTCGCGCGCCAGT
ATGCTGATGGTCGCCGTGGTGATGTTCTTCGCCTTTAGCTGTCTGTTTACGCTCTCCCCG
CAGAACATGGCGGATGCGAAAGCGCAGAACATTCCGGTACTTTCCTACCTGGCTAACCAT
TTCGCCTCTTTGTCGGGGACAAAATCTACTTTTGCAACGGTGCTGGAGTATGGCGCGTCT
ATCATCGCGCTGGTTGCTATTTTCAAATCTTTCTTCGGTCACTACCTGGGGACGCTGGAA
GGGCTGAACGGCCTGGTGCTGAAATTCGGTTATAAAGGCGATAAAACCAAAGTCTCTATG
GGCAAGCTTAACACCATCAGCATGATCTTCATTATGGGGTCTACCTGGGTCGTAGCTTAC
GCAAACCCGAACATTCTGGACCTGATTGAAGCGATGGGCGCGCCGATTATCGCCTCGCTG
TTATGCCTGCTGCCGATGTACGCCATTCGTAAAGCGCCGTCGCTGGCGAAGTACCGTGGT
CGTCTGGATAACGTGTTCGTCACCCTGATTGGTCTGCTGACCATCCTGAATATTGTCTAC
AAATTGTTCTAA
>EECEBICD_00206 Propionate kinase
ATGAATGAATTTCCGGTCGTTCTGGTCATTAATTGCGGATCATCTTCTATCAAATTCTCT
GTACTGGATTTCGCTACCTGTGATGTTTTAATGGCGGGAATAGCAGATGGAATGAATACT
GAAAACGCCTTTTTATCTATTAATGGCGATAAGCCGATAAACCTCTCGCACTCTAATTAT
GAGGACGCGTTAAAAGCCATTGCCTTTGAGCTGGAAAAACGTGATTTAACGGATAGCGTG
GCATTAATTGGTCACCGTATCGCGCACGGTGGAGAGCTATTTACGCAGTCCGTCATTATT
ACCGATGAGATTATCGATAATATTCGTCGGGTATCACCTTTAGCGCCGCTGCATAATTAC
GCTAATTTAAGCGGCATTGACGCGGCGCGGCACCTTTTCCCGGCCGTGCGTCAGGTGGCG
GTATTTGATACCAGTTTCCACCAGACGCTGGCGCCGGAAGCCTATTTGTACGGATTGCCG
TGGGAATATTTTTCCAGCCTGGGTGTGCGTCGCTACGGCTTTCACGGTACGTCGCATCGC
TATGTTTCGCGGCGCGCGTATGAACTGCTTGACCTCGATGAAAAAAACTCGGGTCTGATT

GTCGCGCATCTGGGCAATGGGGCGTCTATCTGCGCGGTACGCAATGGTCAGAGCGTGGAT
ACCTCAATGGGGATGACGCCGCTTGAGGGGCTGATGATGGGCACTCGCAGTGGCGACGTT
GATTTCGGCGCGATGGCGTGGATAGCCAAAGAAACCGGGCAGACGTTGAGCGATCTGGAG
CGAGTGGTGAATAAAGAGTCCGGTTTGCTCGGCATCTCCGGGTTGTCATCCGACCTTAGA
GTCCTGGAAAAAGCCTGGCACGAAGGACATGAGCGCGCCCGGTTAGCGATAAAAACGTTT
GTCCACCGTATCGCCCGTCATATTGCCGGACACGCGGCATCGTTACATCGGCTGGATGGC
ATTATTTTTACCGGCGGTATTGGTGAAAATTCAGTATTAATTCGCCAGTTGGTCATTGAA
CATTTGGGCGTACTGGGGCTTACGTTAGACGTTGAAATGAATAAGCAGCCGAATTCTCAC
GGCGAGCGCATTATTTCCGCTAACCCTTCTCAGGTTATTTGCGCTGTTATTCCCACGAAT
GAAGAGAAAATGATTGCCCTTGATGCCATTCATTTAGGCAACGTGGAAGCACCAGTGGAA
TTTGCATAA
>EECEBICD_00207 PFL-like enzyme TdcE
ATGAAGGTAAATATCGATACCAGCGATATGCTGTATGCCGAAGCATGGCGTGATTTTAAA
GGTACGGACTGGAAAGAAGAAATTAATGTCCGTGATTTTATTCAGCATAACTATACGCCC
TATGAGGGGGATGAATCTTTTCTCGCCGACGCGACGCCAGCGACCACGGCGCTGTGGGAA
AAGGTGATGGCGGGAATACGTATCGAGAACGCGACCCATGCGCCGGTGGATTTCGATACC
AACATCGCGACGACGATTACCGCGCACGATGCGGGGTATATTGAAAAAGAGCTGGAAAAA
ATCGTCGGTTTACAAACGGATAAGCCGCTTAAACGCGCTTTGCATCCGTTTGGCGGCGTT
AACATGATCAAAAGTTCATTCCACGCCTATGGCCGCGAAATGGACGCCGATTTTGAATAT
ACCTTTACCGACCTGCGGAAAACCCATAATCAGGGCGTATTCGATGTTTACTCGCCGGAT
ATGCTGCGCTGCCGTAAATCCGGCGTATTAACCGGTTTGCCGGATGGTTATGGTCGCGGG
CGCATTATTGGCGATTATCGTCGCGTCGCGCTGTATGGCATCCGTTATCTGGTACGCGAA
CGCGAGCTGCAATTCGCCGACCTGCAGTCGAATCTGGAGCAGGGACAGAATCTTGAGGCG
ACGATTCGCCTGCGTGAGGAGTTGGCGGAGCACCGTCGCGCGCTGCTGCAAATGCAGGAG
ATGGCGGCGAAATACGGCTATGATATCTCCCGCCCGGCGCGGAACGCGCAGGAAGCGGTA
CAGTGGCTCTACTTCGCCTACCTGGCAGCGGTAAAATCGCAAAATGGCGGTGCAATGTCG
CTGGGAAGAACCGCGTCGTTCCTTGATATCTACATTGAGCGCGATTTCAACGCGGGCCTT
CTGACAGAGCAGCAGGCGCAGGAGCTGATCGACCATTTCATCATGAAGATCCGCATGGTG
CGCTTCCTGCGCACGCCGGAATTTGATTCGCTCTTTTCCGGCGATCCGATCTGGGCCACC
GAAGTTATTGGAGGGATGGGGCTCGATGGCCGCACTCTGGTGACGAAAAACTCCTTCCGT
TATCTGCATACCCTGCACACGATGGGACCAGCGCCGGAGCCGAACCTGACCATACTGTGG
TCAGAAGCGTTGCCGGTGGCATTCAAGAAATATGCTGCGCAGGTCTCCATCGTGACCTCA
TCTTTGCAGTATGAAAACGACGATTTAATGCGTACCGACTTTAACAGCGACGATTACGCC
ATCGCTTGTTGCGTCAGCCCGATGGTCATCGGTAAGCAGATGCAGTTCTTCGGCGCGCGC
GCCAACCTTGCCAAAACGTTGCTGTACGCTATCAATGGCGGCGTTGATGAGAAACTGAAA
ATCCAGGTCGGGCCGAAAACCGCGCCATTAATGGATGAGGTGCTGGATTACGACGCGGTA
ATGGAGAGCCTCGACCACTTTATGGACTGGCTGGCGGTGCAGTACATTAGCGCGCTGAAC
ATCATTCACTACATGCACGATAAGTACAGCTATGAAGCTTCGCTGATGGCGCTGCACGAC
CGTGATGTGTATCGGACGATGGCGTGCGGCATCGCCGGACTGTCGGTGGCGGCGGATTCC
CTGTCCGCCATCAGGTACGCTCAGGTGAAGCCGATTCGCGACGAAAATGGCCTTGCCATC
GACTTTGCTATTGAAGGCGAGTATCCGCAGTACGGCAACAACGATGAGCGCGTGGACAGC
ATCGCCTGCGACCTGGTGAAACGCTTTATGCAAAAAATTAGCGTGTTGCCGACGTATCGC
AATGCGGTGCCGACGCAGTCCATTTTGACCATCACCTCGAACGTCGTGTACGGGCAGAAG
ACCGGCAATACGCCGGATGGACGCCGCGCCGGAACGCCGTTCGCGCCGGGGGCGAACCCG
ATGCATGGTCGTGACCGCAAAGGGGCAGTGGCCTCGCTGACGTCGGTGGCGAAACTGCCG
TTTACCTACGCCAAAGACGGTATTTCGTACACCTTCTCGATTGTGCCTGCGGCGCTCGGG
AAAGAAGATGCGGTGCGGAAAACCAATCTTGTCGGCCTGCTGGACGGCTATTTCCATCAT
GAAGCACAGGTAGAAGGCGGGCAGCATCTGAACGTCAATGTCATGAACCGTGAAATGTTG
CTGGATGCGATTGAACATCCGGAAAACTACCCGAACCTGACGATCCGCGTTTCCGGCTAT
GCCGTGCGCTTCAATGCGCTGACGCGTGAGCAGCAACAGGACGTTATTTCGCGCACCTTT
ACTCAGGCGATGTAA
>EECEBICD_00208 L-serine dehydratase TdcG
ATGATTAGCGCATTTGATATTTTTAAGATTGGCATTGGACCTTCAAGCTCCCATACCGTA
GGGCCAATGAATGCAGGAAAATGTTTTATCGATCGGCTGATCGACAGCGGCGACCTGCCG

CGAACGACGCGCATCACGGTTGATCTTTACGGATCGCTATCGCTGACGGGCAAAGGCCAC
GCGACCGATACCGCCATCATTATGGGCCTGGCGGGCAATACGCCGCAGGATGTCAATATT
GATAGCATTCCGGCGTTTATCCAGGAGGTTGCCCGCAGCAGCCGTTTATCGGTGGCGGGT
GGCGCGCATGTGGTGGATTTTCCCGTCGCCGACAGTATTCTCTTTCACGCTGAAACCCTG
GCGCGCCATGAAAACGGTATGCGTATTACCGCCTGGAACGGACAAGCGCCGCTTTTACAC
AAAACGTATTACTCTATTGGCGGCGGCTTTATTGTCGAAGAGGAGCGTTTTGGTCAGTCG
CACGACGTAGAAAAATCGGTGCCGTATGATTTTCATTCCGCCAGCGAACTGTTGACGCTG
TGTGAAAGACAGGGGCTCTCGGTTTCCGGCCTGATGATGCAAAATGAACTGGCGTTGCGG
AGCAAAGAGCAGATTGACGCTGGCTTTGCCCGCATCTGGCAGGTGATGGCGACCGGAATT
GAACGTGGCATGAATACTGAGGGCGTGCTGCCGGGGCCGCTGAACGTTCCACGCCGCGCG
GTAGCGTTACGGCGTCTGCTGGTCTCCAGCGATAATCTCTCCCGCGATCCGATGAACGTC
ATTGACTGGATTAACATGTTCGCCCTGGCCGTGAGCGAAGAGAACGCGGCGGGCGGGCGA
GTGGTCACCGCGCCGACCAACGGCGCGTGCGGCATTATTCCCGCGGTGCTGGCCTATTAC
GACAAATTCCGTCGCCCGGTCAATGCCAACTCTATCGCCCGCTATCTGCTGTCGTCGGGG
GCGATTGGCATGCTGTACAAGATGAACGCCTCGATATCCGGCGCGGAAGTGGGGTGTCAG
GGTGAGGTTGGCGTCGCGTGTTCGATGGCGGCGGCGGGGCTGACAGAACTGCTGGGCGGC
AGCCCTGCGCAGGTCTGTATTGCGGCGGAAATTGCGATGGAGCATAACCTTGGCCTCACC
TGTGACCCGGTGGCGGGTCAGGTACAAATCCCTTGTATTGAACGTAATGCGATTAACGCC
GTTAAAGCGGTGAATGCGGCGCGAATGGCGCTACGCCGTACCTCTGAGCCGCGGGTTTCG
CTCGATAAAGTGATCGAAACCATGTACGAAACGGGTAAAGACATGAACGACAAATATCGC
GAGACGTCGCGCGGCGGTCTGGCGATTAAAGTCGTTTGTGGATAG
>EECEBICD_00209 putative serine transporter
ATGGAATCGGCTAGTAATACCAGCGTAATCCTCGACGCATCAGCCCCGGCGCGTCGGGCA
GGGATGACAGAAAGCGAATGGCGTGAAGCCATCAAATTTGATAGTACCGACACCGGTTGG
GTCATTATGAGTATCGGGATGGCGATCGGCGCAGGGATCGTTTTTCTTCCTGTCCAGGTT
GGCCTGATGGGACTTTGGGTCTTTTTACTCTCTTCTATTATTGGCTATCCGGCCATGTAC
CTGTTCCAGCGGCTGTTTATTAATACGCTGGCGGAATCCCCGGAATGTAAAGACTACCCC
AGCGTGATCAGCGGCTATCTGGGCAAAAACTGGGGTATTTTATTAGGCGCGCTGTACTTT
GTAATGCTCGTCATCTGGATGTTCGTCTATTCTACGGCAATTACTAACGACAGCGCCTCT
TATCTGCATACTTTCGGCGTAACGGAAGGATTACTTTCCGATAGTCCGTTTTATGGTCTG
GTGCTGATCTGCATCCTGGTGGCGATTTCCTCACGCGGTGAAAAATTACTGTTCAAAATC
TCCACCGGCATGGTGCTGACCAAGCTACTGGTAGTGGCGGCGCTGGGCGTCTCTATGGTG
GGTATGTGGCATCTGTATAACATTGGCGCGCTGCCGCCGATGGCCATGCTGATTAAAAAC
GCCATTATTACCCTGCCATTTACGCTGACCTCGATTTTATTTATCCAGACGTTAAGCCCA
ATGGTGATCTCTTACCGTTCACGCGAAAAATCTATTGAGGTCGCACGCCATAAAGCGCTG
CGTGCCATGAATATTGCGTTTGGCATCCTGTTCGTTACCGTATTTTTCTATGCGGTTTCC
TTCACCCTGGCGATGGGGCATGACGAAGCGGTGAAAGCCTACGAGCAGAATATTTCCGCC
CTGGCGATCGCCGCGCAATTTATTAGCGGCGATGGCGCAGGCTGGGTCAAAGTGGTTAGC
GTAATTCTGAACATCTTTGCCGTCATGACCGCCTTCTTTGGCGTTTATTTAGGATTCCGC
GAAGCGACGCAAGGCATTGTGATGAATATCCTGCGCCGTAAAATGCCCGCCGAGAAGATT
AAGGAAAATCTGGTACAGCGCGGCATTATGATTTTCGCTATTTTGCTGGCCTGGAGCGCC
ATTGTGCTCAATGCGCCGGTACTGAGCTTCACCTCTATTTGTAGCCCTATCTTCGGTATG
GTGGGCTGTTTAATTCCGGCATGGTTGGTCTACAAGGTTCCCGCATTGCACAAATATAAA
GGCGCTTCGTTGTACCTGATTATTATTACCGGTCTGCTGCTATGCGTTTCTCCGTTCCTG
GCATTCTCTTGA
>EECEBICD_00210 hypothetical protein
ATGTTTGAATCTAAAATAAATCCATTGTGGCAAAGTTTTATCCTGGCCGTACAGGAAGAA
GTAAAACCGGCGCTGGGGTGTACTGAGCCAATTTCACTGGCGCTGGCGGCAGCGGCGGCG
GCGGCTGAACTTGACGGCACAGTTGAACGCATTGACGCGTGGGTTTCGCCTAATCTGATG
AAAAATGGTATGGGTGTTACCGTTCCAGGAACGGGAATGGTAGGGCTACCGATCGCCGCG
GCGTTGGGCGCGTTGGGCGGTGATGCGAAAGCCGGGTTGGAGGTGTTAAAAGACGCTTCC
GCTAAAGCCGTTGCCGATGCAAAAGCGATGCTGGCCGCTGGACATGTCGCGGTGATGTTG
CAGGAGCCATGTAACGATATCCTGTTTTCACGGGCGAAAGTGTATAGCGGCGATAGCTGG
GCATGTGTCACGATCGTCGGCGATCATACAAATATTGTGCGGATAGAAACCAACAAGGGT

GTGGTATTTACCCAGGCCGATAATGCGCAGGAAGAAGAAAAAAACTCGCCACTGGGAGTG
CTGTCTCATACCTCGCTGGAAGAGATCCTGGCGTTTGTGAATGCGGTGCCCTTTGACGCT
ATTCGCTTTATTCTCGATGCGGCCAGGCTAAACGGCGCGTTGTCGCAGGAGGGACTGCGT
GGTTCCTGGGGGCTGCATATCGGTTCGACGCTGGCCAAACAGTGCGATCGCGGTCTGCTG
GCGAAAGATCTCTCCACGGCGATTTTGATCCGTACCAGCGCGGCGTCAGACGCCAGAATG
GGCGGCGCCACGTTGCCCGCGATGAGCAACTCCGGTTCTGGCAACCAGGGGATTACCGCC
ACGGTGCCAGTCATGGTGGTTGCTGAACATGTCGGCGCCGATGACGAGCGCCTGGCGCGC
GCGCTAATGTTATCGCATTTGAGCGCTATCTATATTCACCATCAGCTTCCGCGTTTGTCG
GCTCTGTGCGCGGCAACCACGGCGGCGATGGGCGCGGCGGCGGGTATGGCGTGGCTGATA
GATGGTCGTTACGACACTATCGCAATGGCTATCAGCAGTATGATTGGTGATGTCAGCGGG
ATGATTTGCGATGGCGCGTCGAATAGCTGCGCGATGAAAGTCTCTACCAGCGCGTCGGCG
GCGTGGAAAGCCGTACTGATGGCGTTGGACGATACGGCGGTGACCGGAAACGAAGGGATT
GTGGCGCACAATGTCGAACAATCTATTGCGAATTTGTGTTCGCTGGCGTGTCGCTCAATG
CAGCAGACAGACAAGCAGATCATCGAGATTATGGCCAGTAAAGCGCATTAA
>EECEBICD_00211 hypothetical protein
ATGAGCAAAAAATCGGCGAAGAAACGTCAGCCCGTGGTAAAACCTGCCGTACAGGAGACG
ATGAGCGCCACTGTGCCGCTGGGCTATGAAGAGATGCTAACCGAACTGGAAGCCATCGTG
GCGGACGCCGAAGCCCGGCTGGCAGAAGAAGAGGCCGCAGCCTGA
>EECEBICD_00212 Pirin-like protein YhaK
ATGATTACGACCCGAACCGCCAAACAATGTGGACAGGCAGACTACGGATGGCTACAGGCC
CGCTATACCTTCTCCTTTGGACACTATTTCGACCCTACACTGCTGGGCTACGCGTCGTTG
CGCGTACTGAACCAGGAAGTGCTGGCGCCGGGCGCCTCTTTTCAGCCTCGAACTTATCCA
AAGGTGGATATTTTAAACCTGATTCTGGACGGCGAAGCGGAATACCGCGACAGCGACGGC
AACCATGTACAGGCTAAAGCGGGCGAAGCGTTGCTGCTCGCCGCGCAGCCGGGCATTAGC
TACAGCGAACATAATCTCAGCAAAGTTAAGCCGTTAACCCGAATGCAACTGTGGCTTGAC
GCCTGTCCTGAACGGGAAAACGCGCTGGTACAAAAAATCCCACTCTCGACGGCGCAACAA
CAGCTTCTTGCCTCGCCCGACGGTGAGCAAAACAGCTTACAACTGCGCCAACAGGTCTGG
GTACATCATATTACGCTGGAGAAAGGCGAATCGCTTAACTTCCAGCTTCACGGGCCGCGC
GCCTATTTGCAGTCGATTCACGGTACGTTCCACGCGATGACGCACAACGAAGAACGCGAG
GCGTTAACCTGCGGAGATGGCGCATTTATTCGTGATGAACCTAACATTACGCTGGTTGCC
GATACGCCGCTGCGCGCATTACTGGTAGATTTACCGGTGTAG
>EECEBICD_00213 HTH-type transcriptional regulator YhaJ
ATGGCTAAAGAGAGAGCGCTGACGCTTGAAGCATTGCGTGTGATGGACGCCATTGACCGC
CGGGGCAGTTTTGCCGCAGCGGCTGATGAGTTGGGGCGGGTGCCCTCCGCGCTGAGTTAC
ACCATGCAGAAGCTGGAAGAAGAGTTGGACGTTGTACTGTTCGATCGCTCCGGCCATCGT
ACAAAATTCACCAACGTCGGACGTATGCTGCTGGAACGCGGGCGGGTATTGCTGGAAGCG
GCAGACAAGTTAACGACGGATGCTGAAGCGTTGGCCCGCGGTTGGGAAACGCATCTGACG
CTGGTCACTGAAGCTCTGGTACCGACGCCTGCTTTTTTTCCGCTGATCGACCGGCTGGCC
GCTAAAGCCAATACGCAGCTTTCATTGATTACCGAAGTACTGGCTGGCGCATGGGAGCGG
CTTGAGCAGGGACGGGCGGATATCGTTATCGCCCCGGATATGCATTTTCGATCGTCGTCG
GAAATTAACTCCCGTAAGTTGTATTCGTTAATGAATGTGTATGTCGCCGCGCCTGACCAC
CCTATTCATCAGGAGCCGGAGCCGCTTTCGGAAGTGACGCGGGTTAAATACCGCGGCGTA
GCGGTAGCGGATACCGCGCGCGAGCGGCCGGTATTAACGGTGCAACTGCTGGATAAGCAG
CCACGTCTGACGGTAAGCACGATTGAAGATAAACGTCAGGCGCTGCTGGCCGGGCTTGGC
GTCGCGACTATGCCATATTCGATGGTGGAACAGGATATTGCCGAAGGGCGGCTACGCGTG
GTCAGCCCGGAATCGACCAGCGAAATCGATATTATTATGGCCTGGCGGCGCGACAGTATG
GGGGAGGCAAAAGCCTGGTGCCTGCGGGAAATACCTAAGCTATTCGCCGGAAAATAA
>EECEBICD_00214 Inner membrane protein YhaH
ATGGATTGGTATTTAAAGGTTTTAAAAAATTATCTGGGATTTGGCGGCCGCGCGCGACGC
AAAGAGTACTGGATGTTTATTCTGGTCAATATCATCTTTACCTTTGTGTTGGGCCTGCTG
GACGCCATGCTTGGCTGGCAACGCGCCGGGGGTGAAGGCGTTCTGACCACCATTTACGGC
GTTCTGATCTTTTTACCGTGGTGGGCCGTGCAGTTCCGTCGTCTGCATGACACTGACCGC
TCCGCATGGTGGCTACTGTTGCTACTTATCCCCATTATCGGCTGGCTTATCATCATCGCG
TTCAACTGCCAGAATGGTACGCCGGGCGACAACCGCTTCGGCCCCGATCCGAAACGTTTT

TCCTGA
>EECEBICD_00215 Glutathionyl-hydroquinone reductase YqjG
ATGTTTAACTCCGCCTTCGATGGGCTGGGCGCTAAAGCCGGGGATTACTATCCCCCCGCG
CTGCAAAGCAAAATTGACGAGTTAAACGGCTGGATTTATGACAACGTTAATAACGGCGTT
TATAAAGCAGGCTTCGCCACCAGCCAGCAAGCGTATGATGAAGCGGTAGAAAAGGTGTTT
ACTGCGCTGGCGCGCCTGGAGCAGATTCTCGGACAGCACCGCTATCTGACGGGCGACCAG
TTGACCGAGGCGGATATCCGCTTATGGACGACCCTGGTACGCTTTGATCCGGTTTACGTG
ACTCATTTCAAATGCGACAAATACCGAATCAGCGACTACCTGAATCTGTACGGTTTCCTG
CGCGATATCTATCAAATACCGGGCATTGCTGAAACCGTTAATCTTGACCATATTCGTCAC
CACTATTTCCGTAGCCATAAAACGATTAACCCCACGGGTATCATTTCCATCGGACCGTGG
CAGGATCTGCTCGAACCTCACGGGCGCGACGTACGCTTTGGCTAA
>EECEBICD_00216 Inner membrane protein YqjF
ATGATTCTCTCCAGCGATAACAATGACGCGCCAAACCGCGTCATTGCTCATGAAAATAGC
AGCAGCCGCCGAGTCGGCCCCCTGGAGAATAAAATGAAAAAATTAGAAGATGTTGGTGTA
CTGATAGCGCGTATTTTAATGCCAGTTCTGTTTATTACCGCAGGATGGGGAAAAATTAGC
GGCTATGCGGGCACACAGCAGTATATGGAAGCGATGGGCGTACCAGGATTCCTGCTGCCG
CTCACCATACTGCTTGAGTTCGGCGGCGGTCTGGCGATTCTGCTGGGATTCCTCACCCGC
ACCACTGCGCTTTTTACCGCTGGTTTTACGTTGCTGACGGCATTGATCTTCCATAGCAAT
TTTGCAGAAGGTGTAAACTCGCTGATGTTTATGAAAAACCTGACCATTGCCGGCGGATTC
TTATTACTGGCGTTAACCGGGCCTGGCGCGTTCAGCCTTGACCGTCTGCTGAATAAAAAG
TGGTAA
>EECEBICD_00217 hypothetical protein
GTGAGCAGCAAAGGCGAACGCGAAAAACGTAAAGCGCTGCTTCTCAGTCAGATCCAGCAG
CAACGGCTGGATCTGTCCGCCAGCCGTCGCGACTGGCTGGAGACCACCGGCGCGTACGAT
CGCGGCTGGAATACGGTGTTAAGCCTGCGCTCCTGGGCGCTGGTCGGTAGCAGCGTAATG
GCCATCTGGACCATCCGTCATCCTAATATGTTGGTACGCTGGGCGAAACGCGGCCTGGGT
ATCTGGAGCGCCTGGCGCCTGGTAAAAACTACCCTCCGTCAACAACAGCTCCGCGGTTAA
>EECEBICD_00218 Inner membrane protein YqjE
ATGGCGGACTCTCGACAGGCACAAGGACCAGGCAAAAGCGTACTGGGTATCGGGCAGCGG
ATTGTCACCATTATCGTTGAGATGGTGGAAACCCGGCTGCGACTGGCGGTAGTAGAACTG
GAAGAAGAGAAGGCGAACCTCTTTCAGCTTTTGCTGATGGTAGGTCTTACCATGCTCTTC
GCGGCGTTTGGCTTAATGAGCCTGATGGTTCTGGTCATCTGGGCTATCGATCCTCAGTAC
CGTTTGAACGCGATGATCGCTACCACTGTTGTGCTGTTAGTGCTTGCGCTAATCGGCGGC
ATCTGGACTTTGCGCAAAGCGCGTCAGTCCACGCTATTGCGCCATACGCGGCACGAACTG
GCTAACGATCGGCAAATTCTGGAGGACGATCAGTCGTGA
>EECEBICD_00219 putative protein YqjD
ATGTCGAAAGATAACACTACGGAACATCTGCGCGCTGAGTTGAAATCCCTGACCGATACG
CTTGAAGAAGTGCTGAGCTCTTCGGGCGAAAAGTCGAAAGAAGAGTTGAGTAAGATTCGT
AGCAAAGCCGAGCGCGCGCTGAAAGAGAGCCGTTACCGCCTGGGCGAAACGGGTGATGTG
ATTGCCAAACAGACTCGCGTCGCCGCCGCCCGCGCCGATGATTATGTGCGCGAAAATCCC
TGGACAGGCGTAGGCATTGGCGCCGCTGTCGGTCTGGTGTTGGGCGTACTGCTGACGCGT
CGTTAA
>EECEBICD_00220 Protein YqjC
ATGAAATACCGCATCGCTTTGGCTATCACGCTTTTTACACTTAGCGCCGGTAGCTACGCT
AATTCGCTTTGTCAGGAAAAAGAGCAGGATATACAAAAGGAAATTAGCTACGCTGAAAAG
CACAACAATCAGCGCCGTATTGAGGGTCTGAACAAGGCGCTCAGCGAAGTACGCGCCAAT
TGTACTGACAGCAAGCTGCGCGCCGAGCATCAGAAAAAGATCGCTGAGCAAAAAGAAGAG
GTAGCCGAACGCCAACGTGATTTAGCCGAGGCAAAAGCAAAAGGCGATGCGGATAAAATA
GACAAACGTGAACGTAAGCTGGCCGAGGCTCAGGATGAGCTAAAAAAACTTGAGGCCAGA
GACTACTAA
>EECEBICD_00221 Modulator protein MzrA
ATGCTTAAACCACGCATAACTGCCCGACAACTCATTTGGATCAGCGCTTTTCTACTGATG
CTGACCATACTCATGATGACGTGGTCAACGCTGCGTCAGCAAGAGTCGACGCTGGCCATT
CGCGCGGTTAACCAGGGCGCCAGTATGCCAGACGGCTTCTCAGTTCTGCATCATCTGGAT

GCGAACGGCATTCATTTTAAAAGCATTACGCCTAAAAATGACATGTTGCTCATTACCTTC
GATTCCCCTGCGCAAAGCGCCGCAGCAAAAACAGTGCTTGATCAAACGTTACCACACGGT
TATGTCGTCGCTCAGCAGGACGATGACAACGAAACGGTACAGTGGCTGTCCCGCTTACGG
GAAAGCTCACACCGCTTTGGCTAA
>EECEBICD_00222 Inner membrane protein YqjA
ATGGAACTACTGACTCAATTGCTGAATGCCCTGTGGGCTCAGGACTTTGAAACACTGGCC
AATCCTTCCATGATTGGCATGTTATATTTTGTGTTATTTATGATTTTGTTTCTGGAAAAT
GGGCTGCTCCCTGCCGCCTTTTTGCCTGGCGACAGCCTGTTGATCCTGGTCGGCGTGCTG
ATCGCCAAAGGCGCGATGGGTTTTCCACAAACCATTTTGCTGCTTACCGTCGCGGCCAGC
CTGGGTTGCTGGGTGAGCTATATTCAGGGCCGCTGGCTGGGGAATACCCGCACCGTGCAA
AACTGGCTCTCTCATCTCCCCGCACATTATCATCAACGCGCCCATCACCTTTTCCATAAA
CACGGCCTGTCGGCATTATTGATAGGCCGTTTTATCGCGTTTGTCAGAACACTGCTGCCC
ACCATTGCCGGCATATCCGGCCTGAATAACGCGCGCTTTCAGTTCTTCAACTGGATGAGC
GGCTTGTTGTGGGTGCTTATCCTGACGTCGCTGGGCTATCTGCTGGGCAAAACGCCCGTC
TTTATGAAATATGAAGATCAGTTAATGTCATGCCTGATGCTGCTGCCCGTGGTGCTGCTC
TTTTTTGGCCTGGCGGGTTCACTGGTCATGCTGTGGAAAAAGAAGTATGGGAGCCGGGGA
TAA
>EECEBICD_00223 Serine/threonine transporter SstT
ATGGCTACGCAACGAGCATCAGGGCTACTGCAGCGTTTAGCGCAAGGCAGTCTGGTTAAA
CAAATTTTAGTGGGTCTGGTACTGGGGATTTTACTGGCATGGATTTCTAAGCCTGCGGCA
GAAGCGGTTGGCTTGCTTGGCACCCTGTTCGTCGGCGCCTTAAAAGCGGTCGCCCCCGTT
CTGGTGCTGATGCTGGTAATGGCCTCGATCGCCAACCACCAGCACGGCCAAAAAACCAAT
ATTCGCCCTATTCTCTTTCTTTACCTGCTAGGCACCTTTTCAGCCGCGCTGGCGGCCGTT
GTGTTCAGCTTCGCCTTCCCTTCAACGCTGCATCTGTCCAGTAGCGCCCAGGATATCGTG
CCGCCGTCCGGCATTGTAGAAGTCTTGCGCGGTCTGCTGATGAGCATGGTGTCGAACCCC
ATCGACGCGCTGCTGAACGCTAACTATATCGGGATTCTGGTGTGGGCCGTCGGTCTGGGA
TTTGCCCTGCGTCATGGCAATGAAACCACCAAAAACCTCGTGAACGATATGTCTAACGCC
GTGACCTTTATGGTGAAACTGGTGATCCGTTTCGCGCCAGTCGGTATTTTTGGTCTGGTT
TCATCAACGCTTGCAACCACCGGCTTCTCTACCCTGTGGGGTTATGCGCACCTGCTGGTC
GTCTTGATTGGCTGTATGCTGCTGGTCGCGCTGGTGGTCAACCCGCTGTTGGTATTCTGG
AAAATTCGTCGCAACCCTTATCCGCTGGTGTTCGCCTGCCTGCGTGAAAGCGGCGTTTAT
GCCTTCTTTACCCGCAGCTCTGCCGCCAATATTCCAGTGAATATGGCGCTGTGCGAAAAG
CTGAATCTGGACAGGGATACTTACTCCGTTTCCATTCCGCTGGGCGCCACTATCAATATG
GCCGGCGCGGCAATTACCATTACGGTTCTGACGTTGGCGGCAGTCCATACGTTAGGCGTC
CCGGTGGATCTGCCTACAGCGCTGCTGCTGAGCGTGGTGGCCTCGCTGTGTGCTTGCGGC
GCCTCCGGCGTGGCAGGCGGTTCATTGCTGCTGATCCCGCTGGCGTGCAACATGTTTGGC
ATCCCGAACGATATCGCGATGCAGGTAGTAGCGGTCGGCTTTATTATCGGCGTATTGCAG
GATTCGTGTGAAACGGCGCTGAACTCGTCTACCGATGTTCTGTTCACTGCGGCAGCCTGC
CAGGCAGAGGACGAACGTCTGGCGAATAACGCCTTGCGTAGCTAA
>EECEBICD_00224 Putative membrane-bound redox modulator Alx
ATGAATACTGTTGGTACGCCATTATTATGGGGTGGATTCGCGGTTGTTGTGGTGATAATG
CTATCCATCGACCTGTTGTTGCAGGGACGCCGTGGCGCACATGCGATGTCGATGAAACAA
GCGGCAGGATGGTCAATCCTGTGGGTAACGCTCTCGCTACTGTTTAACGCCGCTTTCTGG
TGGTATCTGGCTGAAACGCAAGGGCGCGAAGTTGCTGACCCGCAGGCATTGGCGTTCCTT
ACCGGCTATCTGATTGAGAAGTCGCTGGCCGTAGATAACGTCTTTGTCTGGCTGATGCTG
TTTAGCTATTTCTCCGTCCCTCCGGCTCTGCAACGCCGGGTGCTGGTGTATGGGGTTCTT
GGCGCGATCGTGTTGCGTACTATCATGATTTTCGCCGGTACCTGGCTTATCACTCAGTTC
GAATGGCTGCTGTATGTGTTCGGCGCCTTCCTGCTGTTTACCGGCGTAAAAATGGCGCTG
GCGAAGGAAGATGAATCCGGCATTGGCGAAAAACCGATGGTGCGTTGGCTGCGCGGGCAT
CTGCGCATGACCGATACGATCGAGAACGAGCACTTTTTCGTACGTAAAAACGGCCTGCTG
TATGCCACGCCGCTGCTGCTGGTGCTGATTATGGTTGAGTTCAGCGATGTGATCTTCGCC
GTTGACAGTATTCCGGCGATCTTTGCGGTCACCACCGACCCGTTCATCGTACTGACCTCT
AACCTGTTTGCCATCTTAGGACTGCGCGCGATGTACTTCCTGCTGTCCGGCGTGGCGGAA
CGCTTCTCGATGCTGAAATATGGTCTGGCGGTCATTCTGGTCTTTATCGGCATTAAGATG

CTGATCGTCGACTTCTACCATATTCCTATCGCGATTTCGCTGGGCGTAGTGTTTGGCATT
CTGACCATTACGCTGGTGATTAACGCCTGGGTTAACCATCAGCGCGATAAGAAACTACGA
GCGCAATAA
>EECEBICD_00225 scyllo-inositol 2-dehydrogenase (NADP(+)) IolU
ATGATACGTTTCGCTGTAATTGGGACTAACTGGATCACGCGCCAGTTTGTCGACGCCGCC
CATGAAACCGGCAAATTCAGGCTCGCCGCCGTTTACTCCCGTAGTCTTGAACAGGCGCAG
TCTTTCGCAAATGATTATCCCGTCGAGCATCTGTTTACCTCGCTGGAAGCGATGGCGCAA
AGCGACGCGATTGAGGCGGTTTATATCGCCAGCCCGAATTCCCTGCATTTCTCCCAGACG
CAGCGCTTTTTGCAGCATAAAAAACATGTCATGTGCGAAAAGCCGCTGGCTTCTAACCTG
GCGGAGGTTGATGCCGCTATCGCCTGCGCGCGCGATAATCAGCGGGTACTGTTTGAAGCC
TTTAAAACCGCCTGCCTGCCCAACTTCCTGCTGCTGCGGGAATCGTTGCCGAAAATCGGC
AGAATGCACAAAGCATTCCTAAATTACTGCCAGTACTCTTCACGTTATCAGCGCTATCTG
AACGGTGAAAATCCGAATACGTTTAATCCGGCCTTCTCCAATGGTTCGATTATGGATATC
GGGTATTACTGTCTGGCGTCGGCGATCGCGCTATGGGGCGAGCCGCGCAGCGTTCAGGCG
TCGGCGAATTTATTGGAAAGCGGAGTAGATGCACACGGCGTCGTGGTTATGGATTACGGC
GATTTTAGCGTCACGCTACAGCATTCCAAAGTCAGCGACTCGGTACTGGCCAGTGAAATT
CAGGGCGAGAGGGGATCGCTGGTCATTGAAAAGCTGTCGGAATGCCAGAAAGTGTGTTTT
GCGCCGCGCGGCGCGCTGATGCAGGATCTGACCCAGCCTCAGCATATTAATACCATGCTG
TATGAAGCCGGGGCGTTTGCCCAGCTTATTGAAAACCATGCCGTGGAACATCCGGGGTTG
TCGCTAAGTCGCGCCACGGCAAAATGGCTGACAGAAATACGCCGTCAAACCGGGGTCATC
TTCCCGGCGGACGATCTGACTCATCCCCTTACTGCGTAA
>EECEBICD_00226 hypothetical protein
ATGCTCTCACAATGTGCCCGTTTTATCCGCCGCCTCTGTTTTACACGCCGGGCGTTAACA
GTGGCGTGTTTTCTGCTTGTCGCAGCAGGCGTCGCGCTTTTCTATTCCAACTGGCTTATC
GTCAACGCCTCACAGCATCTGACATGGAATGGCATTCAGACCGTTCCCGCCCGCAACGTC
GGGCTGGTGTTAGGCGCTAAACCCGGCAATCGGTATTTTACCCGGCGGATTAATACTGCC
GCCGCGTTGTATCACGCCGGTAAAGTGAAGTGGCTGTTAGTCAGCGGCGACAATGGCAAA
AAAGAGTACGATGAACCTTCGGCGATGCAGCAGGCGCTGATAGCGAAAGGTGTGCCGGAA
GCGGCCATTTTTTGTGATTATGCCGGATTCTCCACGCTGGACTCCGTAGTTCGCGCCCGG
AAAGTGTTTGGCGAAAGCCGCATTACGATTATCTCGCAAGCATTCCATAATCAACGGGCG
ATCTGGCTGGCGCAACAATATGGCATAGACGCTATCGGCGTTAACGCGCCGGATCTGAAT
AAAAGGCACGGAACCTACACACGACTTCGGGAAAAGCTGGCGCGGGTTAGCGCGGTGCTG
GATGCCAAAATTTTGCACCGTCAGCCTAAATATCTGGGCGCTGGCGTCACTATTGGGGCT
GATAGCGCACACGGGTGCCCTTCTCGCCAGTAA
>EECEBICD_00227 UTP pyrophosphatase
ATGACCTCACTAACTTACCTTCAGGGGTATCCGGAACATCTCCTTGCCCAGGTGCGCGCC
TTAATCGCCGAGCAGCGCCTGGGAGCAGTGCTGGAAAAACGTTATCCCGGCGCGCATGAT
TACGCTACTGACAAAGCGCTATATCACTATACTCAGGAACTGAAAAGTCAGTTTCTGCGC
AATGCGCCGCCCATCAATAAAGTGATGTATGACAGCAAGATCCATGTGCTGAAAAATGCG
CTGGGACTGCATACCGCTGTCTCGCGCGTTCAGGGCGGCAAGCTCAAGGCAAAGGCGGAG
ATCCGCGTCGCCACCGTGTTTCGCAACGCCCCGGAGCCATTCTTACGGATGATTGTGGTT
CACGAACTGGCACACCTGAAAGAGAAAGATCATAACAAAGCGTTTTATCAGCTCTGCTGC
CATATGGAACCGCAGTACCATCAACTGGAGTTTGATACGCGACTGTGGCTGACGCATCAG
GCGCTGTCGGCACAATAA
>EECEBICD_00228 Ribosomal RNA large subunit methyltransferase G
ATGAGCCACGTAGACGACGGTTTCCGTTCACTGACGCTGAAACGTTTTCCGCAAACGGAT
GATGTTAACCCGCTTCTGGCGTGGGAGGCGGCCGATGAGTATCTGCTGCAACAGTTGGAT
GAGACGGAAATTCGCGGCCCGGTGCTGATCCTGAATGATACGTTTGGCGCGCTAAGCTGT
GCGCTGGCGGAGCATTCGCCATACAGTATTGGCGATTCGTATTTAAGCGAACTGGGTACG
CGAGAGAATTTACGCCATAACGGCATCGCGGAGTCCAGCGTAACGTTTCTCGACAGTACC
GCTGACTACCCGCAGGCACCGGGCGTGGTATTGATTAAAGTACCGAAAACACTGGCGTTG
CTGGAACAGCAGTTACGCGCTTTGCGTAAGGTCGTTACGGCGCAAACGCGTATTATCGCC
GGCGCGAAAGCGCGTGATATTCATACCTCTACGCTGGAACTGTTTGAAAAAGTGCTGGGC
CCAACCACGACGACGCTGGCGTGGAAGAAAGCGCGCCTGATTAACTGTACCTTCAGTCAT

CCGCAACTGGCCGACGCGCCGCAAACGCTAAGCTGGAAACTGGAAGATACAGGCTGGACG
ATTCACAACCACGCGAATGTGTTTTCTCGTACCGGATTGGATATCGGCGCGCGCTTCTTT
ATGCAGCATTTGCCGGAAAACCTCGACGGGGAGATTGTCGATCTCGGCTGCGGCAATGGG
GTGATTGGCCTGAGCTTACTGGCGAAAAATCCGCAGGCGAACGTAGTATTTGTCGATGAA
TCGCCGATGGCGGTAGATTCCAGCCGTTTGAATGTAGAAACGAACCTGCCGGAAGCATTC
GAACGCTGCGAATTTATGATCAACAACGCGCTCTCCGGCGTGGAGCCGTTCCGTTTTAAC
GCTGTGTTCTGCAACCCGCCGTTCCACCAGAAACATGCGCTGACGGATAATATCGCCTGG
GAGATGTTCCATCATGCCCGCCGCTGCCTGAAAATCAACGGCGAACTGTATATTGTGGCG
AACCGCCATCTGGACTATTTCCACAAGCTGAAGAAGATTTTCGGTAATTGCGCGACGATA
GCAACCAACAATAAGTTTGTTATTTTGAAAGCGGTGAAGCAGGGACACCGTCGATAA
>EECEBICD_00229 2,4-dienoyl-CoA reductase
ATGAGCTACCCATCGCTGTTCGCCCCGCTCGATCTGGGCTTTACTACTCTCAGAAACCGC
GTGCTGATGGGATCGATGCACACGGGTCTGGAAGAACATCCTGATGGCGCCGAGCGCCTG
GCGGCATTTTATGCCGAGCGCGCGCGACACGGCGTAGCGTTAATCGTCACCGGCGGTATT
GCGCCCGTTCCCTCCGGGGTCGTGATGACCGGCGGCGCGATGCTGAACGACGCCAGCCAA
TTAACGCCTCACCGCGTGGTGACCGATGCCGTACATGCGCAAGGCGGTAAAATCGCGCTG
CAAATTCTGCACACCGGACGTTACAGCTATCAGCCGCACCTGGTCGCGCCGTCGGCAATT
CAGGCGCCAATCAACCGCTTTATGCCGCATGAACTGACGCATGATGAAATCTTGCAGTTA
ATCGACGACTTCGCGCATTGCGCGCAACTGGCGCGGGAAGCAGGCTACGACGGCGTAGAG
GTGATGGGATCAGAGGGCTATCTGATTAATGAATTTCTCACCCGGCGAACTAACCATCGC
GATGACGAGTGGGGCGGCGACTATACCAGCCGTATGCGCTTCGCGGTTGAGGTAGTACGC
GCGGTACGCCAGCGGGTAGGGAACGACTTTATTATTATCTATCGCCTGTCGATGCTCGAT
CTGGTGGAAAATGGCGGCACGTTCGACGAAACGGTACAACTGGCGCAAGCCATTGAAGCC
GCAGGCGCAAGCCTAATTAATACCGGCATTGGCTGGCATGAGGCGCGAATCCCGACCATC
GCCACGCCAGTACCGCGTGGCGCCTTTAGCTGGGTGACGCGGAAACTCAAAGGTCATGTG
TCGGTTCCGCTGATCGCGACCAATCGCATTAACGATCCGCAGGTCGCCGAGACGATCCTG
ACGCGCGGCGATGCCGATATGGTGTCGATGGCGCGTCCCTTCCTTGCCGATGCCGAATTC
CTGACTAAAGCGCAATCCGGACGCGCAGATGAGATCAACACCTGTATCGGCTGTAATCAG
GCCTGTCTGGATCGGATCTTCATCGGCAAAGTGACCTCCTGTCTGGTGAACCCACGCGCC
TGTCACGAAACCCACATGCCGATAGCGCCGGCCATACGCAAAAAGAATCTGGCCGTTGTC
GGCGCAGGCCCGGCGGGGCTGGCTTTCGCGATTAACGCCGCGTCGCGCGGCCATCATGTC
ACGCTGTTTGACGCCCAGAGTGAAATTGGCGGCCAGTTTACTATCGCCAGGCAGATCCCG
GGTAAAGAGGAGTTTTACGAAACGCTGCGCTACTACCGTCGAATGATCGATGTTACTGGC
GTTACGCTGAAACTGAGCCAACGGGTGAACGCAGAGGATCTTCAGCCGTTTGACGAAGCG
ATCCTCGCCTGCGGAATAGTACCGCGCCGCCCGCCGATAGACGGTATCGATCATCCAAAA
GTATTGACCTACCTGGAGGTATTGCGCGATAAAGCGCCGGTTGGTAAGCGAGTGGCGATC
ATTGGCTGCGGCGGGATCGGTTTTGACACGGCCATGTATCTGAGCCAGCATGGCGAATCC
ACCAGCCAAAATATTGCTGAATTTTGTACGGAATGGGGAATTGATACCAGCCTGCAACAG
GCAGGCGGTCTGCGCCCGGAAGGCCCGCGCCTGGCACGTAGTCCACGGCAAATCGTCATG
CTGCAACGCAAAGCCAGCAAGCCGGGCGAAGGGTTAGGTAAAACCACCGGCTGGATCCAT
CGCGCGACGTTACTGGCGCGCGGCGTGAAGATGATCCCGGCGGTCAGTTACCAGAAGATC
GACGACGACGGATTACATCTCTTGATCGGCGGCGAACCGCAGTTGCTGGAAGTGGATCAT
GTGGTGATCTGCGCCGGACAGGAACCTCGCCGCGAGCTGGCGGACCCGTTACGCGCCGCA
GGTAAAACGGTACATCTTATCGGCGGATGCGATGTCGCGATGGAGCTGGATGCCCGACGG
GCGATTGCCCAGGGCATCCGACTGGCGCTGGAGATTTAA
>EECEBICD_00230 Putrescine aminotransferase
TTGAACAGGTTACCTTCCAGCGCATCGGCTTTGGCCTGTAGCGCGCACGCACTGAATCTC
ATCGAGAAGCGAACGCTAAACCATGAGGAAATGAAAGCACTAAACCGAGAGGTCATTGAC
TACTTTAAAGAGCATGTAAATCCAGGATTTCTGGAATACCGGAAATCTGTTACCGCCGGC
GGGGATTACGGAGCCGTAGAGTGGCAAGCGGGCAGTTTGAATACGCTTGTCGACACCCAG
GGGCAGGAGTTTATCGACTGTCTTGGGGGGTTTGGCATCTTTAACGTGGGGCACCGTAAT
CCTGTTGTGGTATCCGCCGTCCAGAATCAACTCGCAAAACAACCTCTCCACAGCCAGGAG
TTACTTGACCCCTTGCGGGCGATGCTGGCAAAAACGCTGGCGGCGCTGACACCGGGGAAA
CTCAAGTACAGCTTTTTCTGTAACAGCGGCACCGAATCCGTCGAAGCGGCGCTAAAACTG

GCAAAAGCGTATCAGTCGCCACGCGGCAAGTTTACCTTTATCGCCACCAGCGGCGCATTT
CACGGCAAATCATTGGGCGCGCTCTCCGCCACGGCCAAATCCACCTTCCGCAGACCCTTT
ATGCCTTTACTGCCGGGCTTCCGCCATGTGCCGTTTGGCAATATCGACGCGATGAGCATG
GCATTTAGCGAAGGCAAAAAAACGGGCGACGAGATAGCAGCCGTGATTCTGGAGCCTATT
CAGGGTGAAGGCGGCGTGATCCTGCCGCCGCAGGGATACCTGACAGAGGTACGTAAGCTC
TGTGACGAATTCGGCGCGCTGATGATTCTGGATGAAGTACAAACCGGCATGGGGCGTACC
GGCAAGATGTTTGCCTGTGAGCACGAAAATGTGCAGCCTGATATTCTGTGTCTGGCGAAA
GCCCTGGGCGGCGGCGTGATGCCGATTGGCGCCACCATCGCGACTGAAGAGGTGTTTTCG
GTTCTGTTCGACAATCCCTTCCTGCATACCACCACTTTTGGCGGTAATCCGCTGGCTTGT
GCTGCCGCGCTGGCGACCATTAACGTCCTGCTGGAGCAAAATCTCCCGGCCCAGGCGGAA
CAAAAAGGCGATACGCTCCTGGATGGTTTTCGACAACTTGCGCGTGAATATCCCAATCTG
GTACACGATGCGCGCGGCAAAGGTATGTTGATAGCCATTGAATTTGTCGATAACGAAACG
GGCTACCGTTTTGCCAGTGAGATGTTCCGTCAACGCGTGCTGGTCGCTGGTACGCTGAAT
AATGCGAAAACAATTCGTATCGAGCCACCGCTCACGCTAACCATTGAACTGTGCGAACAG
GTATTGAAATCAGCGCGTAATGCGCTGGCGGCAATGCAGGTGAGCGTTGAAGAGGTTTAA
>EECEBICD_00231 Aerotaxis receptor
ATGTCTTCTCATCCCTACGTCAGCCAGCTAAATACCCCGCTGGATGATGATACCACTCTG
ATGTCTACGACCGACCTGGAAAGCTATATCACTCACGCCAATGACACTTTTGTCCAGGTG
AGCGGCTATCAGTTAAACGAGTTACTGGCGCAGCCACATAATCTGGTGCGTCATCCGGAT
ATGCCGAAAGCTGCCTTCGCAGATATGTGGTACACCCTAAAACAGGGCGAACCGTGGAGC
GGCATTGTGAAAAACCGGCGTAAAAACGGCGACCATTATTGGGTGAGGGCCAACGCGGTA
CCGATGATACGTGAAGGGCGTGTGACGGGATATATGTCGATCCGTACCCGCGCCACGGAT
GATGAGATTGCCGCCGTCGAGCCTTTATATCAGGCGCTAAATGAAGGGCGGTGTAGTAAA
CGTATTCATAAAGGCCTGGTGGTTCGTCAGGGCTTGCTGGGCAAACTGCCCGCTATGCCT
GTTCGCTGGCGAGTGCGTAGTATTATGGGGCTAATGGCCGTAATGCTGGCGTTGGCGCTG
TTCGGTACGGATGCCTCATGGCAGGCGTTGTTGTTGGGCGCGTTGGCGATGCTGGCAGGT
ACGGCGCTATTTGAATGGCAAATTGTGCGTCCCATTGAAAATGTGGCGACGCAGGCGCTG
AAAGTGGCGACCGGCGAACGCAACAGCGTACAACACCTTAATCGTAGCGATGAGTTGGGG
CTGATGCTGAGGGCCGTGGGGCAGCTTGGCTTGATGTGCCGCTGGCTGATCAATGACGTA
TCAAGTCAGGTTTCCAGCGTCAGAAACGGCAGTGAAAGGCTGGCGAAGGGTAACAATGAT
CTGAACGAACACACCCGTCAGACCGTGGAGAATGTTCAGGAAACGGTAACGACCATGAAC
CAGATGGCGGAGTCCGTGAAGCTCAATTCCGAGACGGCTTCCGCTGCGGATAAGCTTTCC
ATGGCGGCCAGTAGCGCGGCGACTCAGGGAGGTGAGGCGATGGATACGGTGATTAAAACG
ATGGATGATATCGCTCACAGTACGCAACGTATCGGGACGATCACCACGCTAATTAACGAT
ATCGCTTTTCAGACGAATATCCTGGCGCTGAATGCGGCGGTAGAAGCGGCGAGAGCGGGC
GAGCAGGGGAAAGGGTTTGCCGTGGTTGCTGGCGAGGTACGTCATCTTGCCAGCCGTAGC
GCTAATGCGGCGAACGATATTCGTAAATTAATTGACGCCAGCGCAACAAAGGTGCAGTCA
GGCTCCGAGCAGGTTCACGCCGCAGGCCGTACCATGGATGACATTGTAGCCCAGGTGCAA
AATGTCACCCTGCTTATCGCACGGATCAGCCAGTCGACGCAGGAACAGACAGATGGGCTT
TCCAGCCTGACTCGCGCCGTGGACGAGTTGAACCGCATAACCCAGAAGAATGCGGCGCTG
GTGGAAGAGAGCGCACAAGTCTCCGCAATGGTAAAACACCGTGCCAGCCGGCTGGAGGAT
GCGGTCACGGTACTGCATTAA
>EECEBICD_00232 Methyl-accepting chemotaxis protein III
ATGTTTTTGCATAACATTAAAATACGTTCAAAATTATTTATGGCCTTTGGCTTATTCATT
GTTCTCATGGTGGTGAGTTCCGCTCTGTCTTTGTTTAGCCTTGATCGGGCTAATACGGGT
ATGCAGGACATTATTACCAATGATTATCCCACCACGGTAAAAGCCAATCTGTTAATCGAT
AATTTTAATGATTTCATCATCGCGCAGCAGCTCATGTTACTGGATGAAGAGGGGCGCTGG
AGCCAGAGCTCGCAGAAAGAACTCAGTGAGATAAGCCAGCGCATTTCGGCGCTACTGGAT
GAGCTTTCCAGGGAAAATAGTCACGATGCGGATTCACAGAAAATCATTAATGAGATCCGT
GAAGCGCGCCAGCAATACCTGGAGTCCAGATTCCGTATTTTGAAAGATATTCAAAGCAAT
AATCGTCAGGCGGCCATTCAGGAGATGATGACCAGAACGGTGCAGGTGCAAAAAGTCTAT
AAGGACAAAGTCCAGGAACTTATCGCTGTTCAGGACGCGCAGATGCATGAAGCGAGCGTG
CAGGTCAAAGAGGATTTTAAAAATAATCGGACGCTGTTAATCACTTTGGCGCTGATAAGC
ATCGCCGCCGGAGGCGTAATGGGATGGTATATTGTGCGTTCTATTACCCGGCCGCTTGAT

GACGCAGTACGCTTTGCCGAGGCGATTGCCGATGGCGATCTGACTCGCCATATCACCACC
GACTATAAAGATGAAACAGGCGTACTACTGCAAGCGTTAATGGCGATGAAAACGCGTCTA
CTGGATATCGTACAGGAAGTGCAAAACGGTTCGGAAAGTATCTCCACAGCGGCGGCGCAA
ATTGTCGCCGGTAACCAGGATTTGGCGGCGCGTACGGAAGAGCAGGCCAGCTCGGTTGAA
GAAACGGCGGCGTCGATGGAACAGATTACCGCCACGGTTAAAAATACGGCTGACCATACC
AGTGAAGCGACCAAACTCTCTGCCGGCGCCGCCAGCGTAGTGAAAAACAATGGGGAGATG
ATGAATCAAGTGACGCAGAAAATGCGCGTCATTAACGATACGGCAAATCGTATGTCGGAT
ATCATCAATATCATTGATTCCATTGCCTTTCAGACCAATATTCTGGCGCTGAACGCGGCG
GTTGAAGCGGCGCGCGCGGGCGAACATGGACGTGGTTTTGCCGTTGTCGCCGGAGAGGTT
CGCCAGTTGGCGCAAAAGAGCGCCTCGTCAGCCAGTGAAATCCGTAATTTGATTGAAGAT
TCAACCAGTCAGACTCAGGAAGGGATGCACCTGGTGGAGAAAGCCAGCGCCCTGATTAAT
GGCATGGTGGATAACGTCGAAGAGATGGATGTGATATTACGTGAGATTGGGCAGGCCAGC
CGCGAGCAAACTGACGGTATTTCGCAGATTAACAGCGCGATTGGCCTGATTGACGCCGCC
ACGCAACAAAACTCCTGCCTTGTGGAAGAGTCTGTTGCCGCCGCGGCGTCGCTGAACGAA
CAGGCGTTACATTTAAAAGAGCTGGTTAACGTGTTCCGCGTCCGCGAAGAGGACACGCAG
CCCGCTTAA
>EECEBICD_00233 Transcriptional regulator YqjI
ATGCAAAATCAACATGAAGGGTGTTGCAAAAACCGGGATCATATGCACGATGGTTGCTGC
AAAGATCGAGAACATCAGCATGAAGGCTGCCATAGCGCGCATCAGCATGAGAACGCCAGT
TGCGGCGGCGAGCACCGTCACGGACACGGCTGTGGTCGTCATGGACAGGGCGGTGGTCGA
CGTCAGCGATTTTTTGGCCACGGGGAACTTCGGCTGGTCATTCTTGATATCCTGACCCGG
GACGCCAGCCACGGCTATGAACTGATTAAAGCGATAGAAAACCTGACGGGGGGCGGCTAT
ACGCCAAGCGCTGGCGTGATCTACCCTACGCTGGATTTTTTACAGGATCAGCAATTTATC
ACGATTAGCGACGAAGAAGGCGGGCGGAAAAAAATCGCGATTACCGCGAACGGCGCGCAA
TGGCTTGATGAAAATCGCGAACACCTGACGCATATTCAGGCGCGGCTAAAAGCCCGTTGT
GTGGGCATGGAGTTACGTAAAAATCCGCAGATGAAGCGGGCGCTGGATAACTTCAAGGCC
GTGCTGGATCTGCGGATAAACCACAGCGATATCAACGACGCGCAAATCAAACGCATTATC
GGCGTGATTGATCGCGCGGCGCTGGAAATCGCCGAACTGGATTAA
>EECEBICD_00234 NADPH-dependent ferric-chelate reductase
ATGACGACATCATCCGTACGCTACCCACAGCGTGTTCGTAATGAGCTGCGTTTTCGCGAG
CTGATCGTTCTGCGGGTAGAACGTATTAGCGCAGGATTTCAGCGGATCGTACTTGGCGGC
GAGGCGCTGGACGGTTTCATCTCCCTGGGCTTTGACGATCACACGAAAGTTTTTTTCCCT
GAGCCAGGCTGTCGCTTTACGCCTCCGACAGTCACTGAGGAAGGCATTATCTGGGGGGAG
GGCGTTCGCCCGGTCTCCAGGGATTACACGCCGCTGTATGACGAGGCGCGTCGCGAGCTG
GCGCTGGATTTCTTTATCCACGATGGCGGCGTAGCCAGCCGTTGGGCAATGGAAGCCCGT
GAAGGCGATACACTGACGATCGGCGGGCCGCGCGGTTCGCTGGTGGTGCCGGAAGAGTAT
GCCTGTCAGGTTTATGTCTGCGATGAGTCCGGTATGCCTGCGCTACGTCGTCGTCTGGAA
TCGCTTAGCCGTCTGCCTGTGCGCCCTGCGGTAACCGCGCTGGTGAGCATTCAGGATGCG
GCATACCGCGATTATCTCGCCCATTTGACGGATATTACTGTGGAATATGTTGTTGGCGGC
GATGAACAGGCAATGCAAACGCGCCTGTCGCAGCTGACAATACCAGAATCAGACTATTTC
ATCTGGATTACTGGCGAAGGCAAAACGGTAAAACGTTTAAGTCAGTGCTTTGAAAAGGGG
TTTGATCCACACCTGGTACGTGCGGCAGCCTATTGGCATCGTAAATAG
>EECEBICD_00235 tRNA-Met(cat)
GGCCCCTTAGCTCAGTGGTTAGAGCAGGCGACTCATAATCGCTTGGTCGCTGGTTCAAGT
CCAGCAGGGGCCACCA
>EECEBICD_00236 G/U mismatch-specific DNA glycosylase
ATGGTTAAGGACATACTGGCGCCAGGGCTACGGGTGGTTTTTTGCGGCATCAATCCGGGA
CTCTCCTCGGCGAATACCGGCTTTCCGTTTGCGCATCCCGCGAATCGTTTTTGGAAAGTT
ATTCATTTGGCCGGTTTCACCGATCGACAGTTGAAACCGGAAGAGGCGGAAAAATTACTG
GATTTTCGCTGCGGGGTAACGAAACTCGTCGATCGACCGACGGTACAAGCGACTGAGGTG
AAGTTGCATGAATTACGTAGCGGCGGACGCAACCTGATTGAAAAAATAGAGGACTACCAG
CCCGCAGCGCTGGCGGTGCTTGGTAAACAGGCTTTTGAACAGGGATTCAGCCAGCGTGGT
ATCGCCTGGGGTAAACAAAAGATCGCCATTGGCGCGACGATGGTGTGGGTGTTGCCGAAT
CCCAGTGGCTTAAACCGAATTAAAACGGAAAAACTGGTCGAGGCCTATCGCGAACTGGAT

CAAGCGCTCATTATGCGTGGGCTATAG
>EECEBICD_00237 RNA polymerase sigma factor RpoD
ATGCCGCATATCGATCGGGAAGCCCCCGACAGCCGCAGTGAGAGGCAGCGGCCAAAATAT
AAGTATGCCCTCGCTTTAAATGTCGACAGCTATACCGTCGACACCAATCAAACGAATAAG
TGTGGATACCGTCTTATGGAGCAAAACCCGCAGTCACAGCTGAAACTTCTTGTCACCCGT
GGTAAGGAGCAAGGCTATCTGACCTATGCTGAGGTCAATGACCATCTGCCGGAAGATATC
GTCGATTCAGATCAAATTGAAGATATCATCCAAATGATCAACGACATGGGTATTCAGGTA
ATGGAAGAAGCGCCTGATGCCGATGATCTGCTGCTGGCTGAAAATACCACCAGCACCGAT
GAAGATGCGGAAGAAGCTGCTGCACAAGTTCTGTCCAGTGTTGAGTCTGAAATCGGTCGT
ACGACTGACCCGGTACGCATGTATATGCGTGAAATGGGCACTGTTGAACTGTTGACCCGG
GAAGGCGAAATCGACATCGCTAAACGTATCGAAGACGGGATCAACCAGGTTCAATGCTCC
GTTGCCGAATACCCGGAAGCCATTACCTATCTGCTGGAACAGTACGATCGCGTTGAGGCT
GAAGAGGCTCGTTTGTCCGATCTTATCACCGGCTTTGTCGATCCGAACGCGGAAGAAGAG
ATGGCGCCGACCGCAACTCACGTCGGTTCTGAACTCTCCCAGGAAGACCTGGATGATGAC
GAAGACGAAGATGAAGAAGACGGCGACGATGACGCCGCCGATGACGACAACAGCATTGAC
CCTGAACTGGCACGCGAAAAATTCGCTGAGCTGCGCGCGCAATACGTCGTTACGCGCGAC
ACCATCAAAGCGAAAGGCCGCAGCCATGCTGCCGCGCAGGAAGAGATTCTGAAGCTGTCT
GAAGTGTTCAAACAGTTCCGTCTGGTACCGAAGCAATTCGACTATCTGGTCAACAGTATG
CGCGTGATGATGGATCGCGTGCGTACCCAGGAACGTCTGATCATGAAGCTCTGCGTCGAG
CAGTGCAAAATGCCGAAGAAGAACTTTATCACGCTGTTTACCGGTAACGAAACCAGCGAA
ACCTGGTTCAACGCCGCTATCGCGATGAACAAACCGTGGTCGGAAAAACTGCACGATGTC
GCGGAAGAAGTGCAACGCTGTCTGCAAAAACTGCGGCAGATTGAAGAAGAGACCGGTCTG
ACCATCGAACAGGTGAAAGACATCAACCGTCGAATGTCCATCGGGGAAGCGAAAGCCCGC
CGTGCGAAGAAAGAGATGGTTGAAGCGAACTTACGTCTGGTTATTTCTATCGCTAAGAAA
TACACCAACCGTGGCTTGCAGTTCCTTGATCTGATTCAGGAAGGCAACATCGGTCTGATG
AAAGCGGTAGATAAGTTTGAATACCGTCGCGGCTACAAGTTCTCCACCTATGCAACCTGG
TGGATCCGTCAGGCGATCACCCGTTCTATCGCCGATCAGGCGCGCACCATCCGTATTCCG
GTGCATATGATTGAGACCATCAACAAGCTCAACCGTATTTCTCGCCAGATGCTGCAAGAG
ATGGGCCGCGAGCCAACGCCGGAAGAGCTGGCTGAACGGATGCTGATGCCGGAAGATAAA
ATTCGTAAGGTGCTGAAGATTGCCAAAGAGCCAATCTCCATGGAAACGCCGATCGGCGAC
GATGAAGATTCGCATCTGGGTGATTTCATCGAGGATACCACCCTCGAGCTGCCGCTGGAC
TCTGCCACTACCGAGAGCCTGCGTGCCGCCACTCACGACGTTTTGGCTGGCCTGACCGCT
CGTGAAGCGAAAGTGCTGCGTATGCGTTTCGGTATCGATATGAACACCGACCACACGCTG
GAAGAAGTGGGTAAACAGTTCGATGTTACCCGCGAACGTATCCGTCAGATCGAAGCGAAG
GCGCTGCGTAAACTGCGCCACCCGAGCCGTTCTGAAGTGCTGCGCAGCTTCCTCGACGAT
TAA
>EECEBICD_00238 DNA primase
ATGGCTGGACGAATCCCACGCGTATTCATTAATGACCTGCTGGCGAGAACCGATATCGTC
GATCTGATCGACGTGCGCGTAAAACTCAAGAAACAGGGCAAGAACTATCACGCGTGTTGT
CCATTCCATAATGAGAAAACCCCTTCTTTCACCGTAAACGGTGAAAAACAGTTTTATCAC
TGCTTTGGATGCGGCGCGCATGGCAATGCGATCGACTTTTTAATGAATTACGACAAACTC
GAATTTGTCGAAACCGTCGAAGAATTAGCGGCCATGCATAACCTGGAAATTCCCTATGAA
GCAGGCACCGGGCTTAGCCAGATAGAGCGCCATCAACGGCAAAACCTCTATCAGTTGATG
AATGGTCTGAATGACTTTTATCAGCAGTCGCTTACCCATCCTGCGGCAAAACCTGCGCGT
GACTATCTGCAAAAGCGCGGTCTGAGCGCTGAAATTATTCAGCGTTTCGCCATTGGCTTC
GCGCCGCCAGGGTGGGATAACGCGTTAAAGCGCTTCGGTAATAACAGCGATAACAAGGCA
TTGTTGCTGGATGCCGGGATGCTGGTCAATAACGAGCAGGGCAGCACTTACGATCGTTTC
CGTAATCGGGTCATGTTCCCTATTCGGGACAAACGCGGCAGGGTGATTGGTTTTGGCGGT
CGAGTACTGGGGAACGACACGCCGAAATACCTTAACTCTCCGGAAACGGACATTTTCCAT
AAAGGCCGCCAGCTATATGGTCTTTATGAGGCTCAGCAATATAGTGCTGAACCGCAACGC
TTGCTTGTGGTCGAAGGTTATATGGATGTCGTCGCACTAGCGCAATACGACATTAACTAT
GCGGTCGCTTCGCTGGGTACCTCAACCACGGCAGACCATATGCATATGTTATTCCGGGCG
ACGAACAACGTAATTTGCTGTTACGACGGCGACCGGGCTGGCCGTGATGCCGCCTGGAGA
GCGCTGGAAACAGCAATGCCTTATATGACCGACGGTCGGCAGGTACGCTTTATGTTTTTA

CCTGACGGCGAAGACCCGGATACGCTGGTGCGTAAAGAAGGCAAGGCGGCTTTTGAAGCG
CGGATGGAGCAGGCGCAGCCGCTCTCTACGTTTCTGTTTAATAGCCTCCTGCCGCAGGTG
GATTTAAGCTCGCCGGATGGCAGCACGCAGTTAGCCGCACTGGCGCTGCCGCTCATTAAT
CAGGTGCCTGGCGACGCACACCGTATTCAGTTGCGCCAGACGTTGGGGCTGAAGTTAGGC
ATTTTCGATGATAGTCAGCTTGATCGTTTAGTGCCAAAACAGGCGGAAAGCGGCGTTTCC
CGCCCGGCTCCGCAGCTAAAACGCACGACCATGCGTATACTGATAGGATTACTGGTGCAA
AATCCGGATCTCGCGCCGTTAGTGCCGCCCTTAGACGCGCTGGATCAGAATAAGCTGCCT
GGACTTGGCTTATTTAAAGAATTGGTCAAAACTTGTTTGGCTCAGCCAGGTCTTACTACC
GGACAACTTCTGGAACTCTACCGTGGCACAAATGATGCTGCGACCCTTGAAAAATTATCG
ATGTGGGACGATATAGCAGATAAGGCGATTGCCGAAAAGACCTTTACCGACTCACTCAAC
CATATGTTTGATTCGCTGCTGCAACTGCGACAAGAAGAGCTGATCGCTCGCGATCGCACT
CACGGTTTAAGCAGCGAAGAACGCCGAGAGCTCTGGACGTTAAACCAGGAGCTGGCCAGA
AAATGA
>EECEBICD_00239 30S ribosomal protein S21
ATGCCGGTAATTAAAGTACGTGAAAACGAGCCGTTCGACGTAGCTCTGCGTCGCTTCAAG
CGTTCCTGCGAAAAAGCAGGTGTTCTGGCGGAAGTTCGTCGTCGTGAGTTCTATGAAAAA
CCGACGACCGAACGTAAGCGCGCTAAAGCTTCTGCAGTGAAACGTCACGCGAAGAAACTG
GCTCGCGAAAACGCACGCCGTACTCGTCTGTACTAA
>EECEBICD_00240 tRNA N6-adenosine threonylcarbamoyltransferase
ATGCGTGTACTGGGTATTGAAACATCCTGCGATGAAACCGGCATCGCTATTTACGACGAC
AAAAAAGGTCTGTTAGCCAACCAATTGTATAGTCAGGTGAAATTACATGCTGACTACGGC
GGCGTAGTGCCTGAACTGGCTTCCCGCGATCATGTGCGTAAAACCGTGCCGCTGATTCAG
GCGGCATTAAAAGAAGCCGGTCTGACGGCGAGCGATATCGACGCGGTGGCCTATACCGCA
GGCCCGGGCCTGGTCGGCGCGCTGCTGGTCGGCGCAACCGTCGGGCGTTCGCTGGCATTT
GCCTGGAATGTGCCGGCCATTCCTGTACACCATATGGAAGGTCATCTGCTGGCGCCAATG
CTGGAAGATAATCCCCCGGAATTCCCGTTTGTGGCGCTACTGGTCTCCGGCGGACATACG
CAGCTCATTAGCGTGACCGGAATCGGTCAGTACGAACTGCTGGGAGAGTCGATTGACGAT
GCCGCCGGTGAAGCGTTTGATAAAACCGCCAAATTGTTGGGGCTGGATTATCCTGGCGGC
CCGATGCTGTCGAAAATGGCGTCGCAGGGGACGGCGGGACGTTTTGTCTTTCCGCGCCCG
ATGACCGATCGCCCGGGGCTGGATTTTAGTTTTTCCGGTCTGAAAACCTTTGCCGCTAAC
ACCATTCGTAGTAATGGCGGCGACGAACAAACTCGCGCTGATATCGCGCGCGCTTTTGAA
GATGCGGTCGTGGATACGCTGATGATCAAGTGCAAGCGCGCGCTGGAAAGCACCGGTTTT
AAGCGTCTGGTCATGGCGGGCGGCGTCAGCGCTAACCGCACGCTGCGCGCGAAGCTTGCC
GAAATGATGCAAAAACGCCGCGGCGAAGTGTTCTATGCGCGTCCGGAGTTTTGTACTGAC
AACGGGGCGATGATCGCCTATGCCGGAATGGTGCGGTTTAAGGCGGGCGTTACGGCGGAT
CTTGGCGTAACGGTACGTCCGCGCTGGCCGCTGGCCGAGCTGCCGGCGGCGTAA
>EECEBICD_00241 putative glycerol-3-phosphate acyltransferase
ATGAGTGCAATCGCGCCTGGAATGATCCTCTTCGCGTACCTCTGCGGCTCAATTTCCAGT
GCCATTCTGGTTTGCCGCATCGCCGGGCTGCCCGATCCGCGAGAAAGCGGCTCCGGCAAC
CCGGGCGCGACGAACGTATTACGAATCGGTGGCAAGGGAGCGGCTGTCGCGGTACTTATT
TTTGACATCCTTAAAGGCATGTTGCCCGTCTGGGGCGCGTATGCGTTAGGCGTTACCCCT
TTCTGGCTGGGGCTTATCGCTATCGCCGCCTGTCTGGGGCATATCTGGCCGGTCTTTTTT
GGCTTTAAAGGCGGGAAAGGCGTAGCAACCGCGTTTGGCGCCATTGCGCCCATCGGCTGG
GATTTAACCGGCGTCATGGCTGGCACCTGGCTGCTGACGGTCCTGTTAAGCGGTTATTCA
TCGTTAGGGGCGATTGTCAGCGCCTTGATTGCGCCGTTTTACGTCTGGTGGTTTAAACCG
CAGTTCACCTTCCCGGTATCGATGCTGTCATGTCTCATTTTGCTGCGCCATCATGACAAC
ATCCAGCGTCTGTGGCGTCGCCAGGAGACGAAAATCTGGACGAAGCTGAAAAAGAAACGC
CAGAAAGATTGA
>EECEBICD_00242 Dihydroneopterin aldolase
ATGGATATTGTATTTATAGAGCAACTTTCGGTAATCACCACTATCGGTGTTTACGACTGG
GAACAGACTATCGAGCAAAAACTGGTGTTCGATATCGAAATGGCGTGGGATAACCGTAAG
TCGGCGAAAAGCGATGATGTCGCAGATTGTCTAAGTTATGCGGATATCGCCGACACGGTG
ATAAATCATGTCGAAGGCGGGCGTTTTGCTCTGGTCGAGCGCGTGGCGGAAGAGGTGGCG
GATCTGCTGCTTAGCCGATTCAATTCTCCGTGGGTTCGCATCAAACTGAGCAAGCCTGGC

GCCGTCGCGCGCGCGGCGAACGTAGGGGTAATTATTGAGCGTGGCAATAATCTGAAATAA
>EECEBICD_00243 Undecaprenyl-diphosphatase
ATGAGCGATATGCACTCGCTGCTGATAGCGGCAATTTTGGGTGTGGTCGAAGGATTGACG
GAGTTTTTGCCGGTATCCAGCACGGGCCATATGATTATTGTGGGTCATCTGCTGGGGTTT
GAAGGCGATACGGCCAAGACATTCGAAGTGGTGATTCAACTTGGATCTATTCTGGCGGTC
GTGGTGATGTTCTGGCGGCGGTTGTTTGGCCTCATCGGTATTCACTTTGGCCGCCCGCTA
CAGCGTGAAGGTGAAAGTAAAGGTCGATTAACGTTGATTCACATCCTGCTGGGCATGATT
CCGGCGGTGGTGCTGGGGTTAGTCTTTCATGACACGATCAAGTCGCTATTTAACCCAATT
AACGTGATGTACGCGCTGGTGGTAGGCGGTCTGTTGCTGATTGCCGCCGAGTGCCTGAAG
CCGAAAGAACCGCGCGCGCCGGGGCTGGATGATATGACGTATCGTCAGGCGTTTATGATT
GGCTGTTTCCAGTGTCTGGCGCTGTGGCCGGGCTTCTCGCGTTCCGGAGCGACCATTTCC
GGTGGGATGCTGATGGGCGTCAGCCGTTACGCCGCCTCTGAGTTCTCGTTCTTACTGGCT
GTGCCGATGATGATGGGCGCAACGGTTCTGGATCTCTACAAAAGTTGGTCATTCCTGACC
GCAGCGGATATTCCGATGTTCGCAGTGGGCTTTGTGACGGCGTTTGTGGTGGCGCTGATC
GCTATCAAGACCTTCCTGCAACTGATTAAGCGTATCTCGTTTATTCCGTTTGCGATTTAC
CGCTTTGTGGTGGCGGCTGCCGTTTACGTTGTTTTCTTCTGA
>EECEBICD_00244 Multifunctional CCA protein
GTGAAGATTTATCTGGTCGGTGGTGCGGTTCGGGATGCGTTGTTAGGGCTACCGGTTAAA
GATAAAGATTGGGTCGTTGTCGGCGCCACGCCGCAGGAGATGCTTGATGCGGGCTACCAG
CAGGTAGGCCGTGATTTTCCTGTGTTTCTCCACCCGCAAACTCACGAAGAATATGCGCTG
GCGCGTACCGAGCGTAAATCCGGTTCCGGCTACACGGGCTTCACCTGTTATGCCGCGCCG
GACGTTACCCTGGAAGCCGACCTCCAGCGTCGCGATCTGACCATTAATGCTCTGGCGCGC
GACGATGACGGCCAGATTATCGACCCTTACCACGGGCGCCGGGATCTGGAGGCGCGTCTA
CTGCGACATGTCTCTCCCGCGTTTGGCGAAGATCCCTTGCGCGTATTGCGCGTGGCGCGT
TTTGCCGCCCGTTACGCTCATCTTAGTTTCCGTATCGCCGATGAAACACTGGCGCTAATG
CGCGAAATGACCGCGGCAGGCGAGCTGGAACATCTGACGCCGGAACGGGTATGGAAAGAG
ACGGAAAATGCGCTTACCACCCGTAATCCGCAGGTTTACTTCCAGGTATTGCGCGACTGC
GGCGCCCTGCGCGTCCTGTTTCCGGAAATCGACGCCCTGTTTGGCGTACCCGCGCCGGCA
AAGTGGCATCCGGAAATTGATACCGGAGTTCATACGTTGATGACGCTGTCGATGGCCGCC
ATGCTCAGTCCGCAGCTTGATGTTCGTTTCGCCACGCTTTGTCATGACCTGGGCAAAGGC
TTAACGCCGAAAAACCTCTGGCCGCGCCATCATGGCCACGGCCCGGCGGGCGTGAAACTG
GTCGAACAGCTATGCCAGCGTTTGCGCGTCCCCAATGACCTACGTGATTTAGCTAAACTG
GTCGCCGAGTACCATGATCTGATCCACACCTTTCCGATACTCCAGCCGAAAACGATCGTT
AAGCTGTTTGACGCCATTGATGCATGGCGTAAACCGCAGCGCGTCGAGCAGATTGCGCTG
ACCAGCGAAGCCGATGTCCGTGGACGTACCGGCTTTGAAGCGTCGGATTACCCGCAGGGG
CGCTGGCTGCGCGAAGCCTGGCAGGTGGCCCAAGCGGTACCGACGAAAGAGGTGGTGGAG
GCCGGATTTAAGGGCATTGAGATTCGCGAGGAGCTGACGAAGCGACGAATTGCAGCGGTC
GCCAACTGGAAAGAGAAGCGCTGCCCGAACCCCGCCAGCTAA
>EECEBICD_00245 hypothetical protein
ATGCCAAAATTACGCCTGATTGGACTAACTTTACTTGCGCTTAGCGCCACCGCCGTCTCT
CATGCTGAAGAGACGCGTTACGTTTCTGATGAACTGAATACCTGGGTCCGCAGCGGTCCG
GGAGATAATTATCGCCTTGTGGGTACCGTCAACGCCGGCGAGGAAGTCACGCTCTTACAG
AGCGATGCCAACTATGGTCAAATTAAAGACAGTTCTGGTCGTACTGCCTGGATACCGTTA
AAAGAACTGAATACCACACCAAGCCTGCGTACCCGCGTGCCGGATCTGGAAAATCAGGTC
AAAACGTTGACCGATAAACTCAACAATATTGATACCACGTGGAATCAGCGCACCGCCGAT
ATGCAGCAGAAAGTGGCGCAAAGCGACAGCATCATCAACGGGCTAAAAGAAGAGAACCAG
AAGCTGAAAAACGAGCTGATTGTCGCGCAGAAAAAAGTTAGCGCCGCGAATCTGCAACTG
GATGACAAACAGCGCACCATTATTATGCAGTGGTTTATGTATGGCGGCGGCGTATTGGGT
ATTGGCTTGCTGTTAGGGCTGGTGCTGCCGCATATGATCCCAAGCCGTAAACGCAAAGAC
CGCTGGATGAACTAA
>EECEBICD_00246 Inorganic triphosphatase
ATGGCTCAGGAAATCGAATTAAAGTTTATCGTCAATCACGACGCCGTAGATGCGCTGCGA
AATCATCTTCATACGCTCGGCGGCGAGCATCATGCGCCCAGCCAGTTGCTGAATATCTAC
TTCGAAACGCCTGATAACTGGCTACGCCGCCATGATATGGGGCTGCGTATTCGTGGTGAA

AATGGGCGCTATGAAATGACCATGAAAATTGCCGGCCGGGTAACGGGCGGGCTGCACCAG
CGACCCGAATACAATGTCGCGCTAAGCGAACCCGTGCTGGATCTGACGCAACTTCCGGCG
GAGGTCTGGCCGGATGGTAACCTGCCCGCCGGCCTGGCATCAAGCGTACAGCCGCTGTTC
AGTACCGATTTTTACCGGGAAAAATGGTGTCTGGACGTTGATGGCAGCAGGATTGAAATT
GCGCTCGATCTCGGCGACGTAAAAGCGGGCGAGTTTGCCGAACCGATTTGTGAGCTGGAG
CTTGAGTTGCTTCGCGGCGATACGCGCGCCGTGTTAAAACTGGCGAAGCAACTGCTGTCG
CAAACCGGTTTGCGTCAGGGCAGTCTGAGTAAGGCGGCGCGAGGTTATCACCTGGCGCAG
GGGAATGCGCCGCGAGAGAATACACCAACGGCAATTCTGCGGACTGCCGCGAAAGCCACG
GTAGAGCAAGGGCTGGAGGTCTCGCTGGATCTGGCGTTGTCACAGTGGCAGTACCATGAA
GAGCTCTGGCTGCGCGGCGACGAGTCGGCGGAAGAACATGTGCTGGATGCTATGGGATTA
GTGCGCCATGCCTTAATGCTTTTTGGTGGCATTGTTCCACGTAAAGCGAGCGCTCACTTA
CGTGATCTACTTACCCAGGCGGAAGCGACGATGACATCCGCGGTGTCCGCCGTCACAGCC
GTTTATAGTACGCAAACGGCGATGGCGAAACTGGCATTGACGGAATGGCTGGTGACGAAA
GCCTGGCAGCCATTTCTGGACGCGAAGGCGCAGGCCAAAATGGCCGATTCTTTTAAGCGT
TTCGCCGACATTCATCTTTCTCGTCATGCTGCTGAATTAAAAAAAGTTTTTGGACAACCG
CTGGGTGATAAGTATCGCGACCAGTTGCCGCGTTTGACTCGCGATATCGACAGCGTTTTA
CTGCTGGCCGGGTATTATGATGCGATGGTCGCGCAGGCATGGCTGGAAAACTGGCAGGGA
TTGCGTCACGCCATTCTTACCGGACAACGTATTGAAATTGAACATTTCCGTAATGAGGCC
ATTAACCAGCAGCCGTTCTGGTTGCACAGCGGAAAACGATAA
>EECEBICD_00247 Bifunctional glutamine synthetase adenylyltransferase/adenylyl-removing enzyme
ATGACGCCGCTCTCTTCACCCTTATCGCAGTACTGGCAGACCATTGTTGAGCGCCTACCA
GAAGGATTTACTGAGACATCGCTTAGCGTGCAGGCGAAGTCAGTACTTACATTTAGCGAT
TTTGCGCTGGATAGCGTGATAGCGCATCCGGAGTGGCTTGCCGAACTGGAATCCGCATCG
CCGCAGGCTGATGAATGGCGACATTATGCTGGCTGGTTACAGGAGGCGTTGGCAGGTGTC
TGTGACGACGCCTCGCTGATGCGTGAACTGAGGTTTTTTCGTCGTCGCATCATGGTACGC
ATCGCCTGGGCGCAGACGCTGTCGCTGGTGGACGATGAGACTATTTTACAACAGTTGAGC
CATCTGGCGGAGACGCTCATTGTGGGGGCGCGCGACTGGCTGTATGCCGCCTGTTGCCGC
GAATGGGGAACCCCCTGTAACCCGCAGGGCGTTCCGCAGCCGCTGTTGATTTTAGGCATG
GGCAAGCTGGGCGGCGGCGAACTGAATTTTTCGTCTGATATCGATCTGATTTTCGCCTGG
CCGGAACATGGCGAGACTCGGGGCGGGCGTCGTGAACTCGATAACGCCCAGTTTTTTACG
CGTCTCGGGCAGCGGCTGATCAAAGCGCTGGATCAGCCCACAATGGATGGTTTTGTCTAT
CGGGTGGACATGCGCCTGCGACCGTTTGGCGACAGCGGCCCGCTGGTACTGAGTTTTGCC
GCGCTGGAGGATTACTATCAGGAGCAAGGACGCGACTGGGAACGCTACGCTATGGTGAAA
GCGCGGCTAATGGGCGATAACGACGACGCCTGGTCTCGTGAATTACGCGCCATGCTGCGG
CCTTTCGTTTTCCGCCGCTATATCGATTTCAGTGTCATCCAGTCGCTACGTAATATGAAA
GGGATGATCGCCCGCGAAGTGCGACGCCGCGGTTTGAAAGACAACATTAAACTTGGCGCG
GGCGGTATTCGGGAAATTGAGTTTATCGTGCAGGTATTCCAGCTCATCCGCGGCGGGCGC
GAGCCGTCGCTACAGTCGCGTTCGTTACTGCCAACGCTGGATGCTATCGCTGCGCTGCAT
TTGCTGCCTGAGAATGACGTGGCGCAACTGCGCGTGGCCTATCTTTTTCTACGTCGTCTG
GAGAATCTGCTGCAAAGCATCAATGACGAACAGACGCAAACGTTGCCTGCCGACGATCTG
AATCGGGCGCGGCTGGCCTGGGGAATGAAGGCGGAGAACTGGCCGCAGCTTGTTGGCGAA
CTGACGGATCATATGGCTAATGTACGCCGCGTATTCAACGAATTAATTGGCGATGATGAA
GCCGATACCCCGCAGGAGGAAGAACGTTCAGAGCCGTGGCGTGAAGTATGGCAGGATGCG
CTACAGGAGGATGATTCGACGCCAGTGCTGGCGCATTTAGCCGATGAGGATCGGCGACAA
GTTCTGACCCTCATCGCTGACTTCCGTAAGGAACTGGATAAACGCCCTATCGGCCCGCGC
GGGCGGCAAGTGCTGGACCAACTGATGCCGCACCTGCTTGCTGATGTCTGTTCTCGCGAA
GACGCCGCTGTTACGCTATCGCGGATAACGCCGTTGCTGGCGGGGATTGTCACTCGCACC
ACCTATCTGGAGCTGCTGAGCGAATTTCCCGGCGCGCTGAAACACCTGATTATGCTCTGC
GCCGCTTCGCCAATGATAGCGAGCCAACTGGCGCGTTATCCGTTGTTGCTTGATGAACTG
CTCGACCCCGGCACGCTTTATCAGCCGACGGCTACCGACGCTTACCGCGACGAATTACGC
CAATATTTGCTGCGCGTGCCGGAAGAGGACGAAGAACAACAACTGGAGGCATTACGCCAG
TTTAAACAGGCACAGCTTTTGCGGATTGCGGCGGCGGATATTGCCGGTACGCTGCCGGTG
ATGAAAGTAAGCGATCACCTCACATGGCTGGCGGAGGCGATGATTGATGCTGTGGTGCAG

CAGGCGTGGACGCAAATGGTTGCGCGTTATGGTCAGCCAGCGCATCTGGACGAGCGCCAG
GGGCGGGGGTTTGCGGTTGTCGGTTATGGCAAGTTGGGCGGATGGGAGCTGGGCTACAGT
TCCGATTTGGATCTCATCTTCCTGCATGACTGTCCGATGGACGTCATGACCAACGGCGAA
CGCGAAATCGACGGGCGGCAGTTTTATCTGCGCCTGGCGCAGCGCATCATGCATCTGTTC
AGTACGCGCACTTCCTCCGGCATTTTGTATGAGGTGGATGCGCGTCTACGCCCCTCTGGC
GCGGCGGGAATGCTGGTGACTTCAGCGGACGCGTTTGCCGATTATCAGCAGCATGAAGCC
TGGACCTGGGAACATCAGGCATTGGTACGCGCTCGCGTGGTATATGGCGACCCGCAGCTT
ACCTCACAGTTTGATACGGTGCGCCGCACTATCATGACGACGGCGCGCGACGGCAAGACA
TTGCAGACTGAAGTGCGTGAAATGCGCGAGAAAATGCGGGCGCATCTCGGCAATAAGCAT
CGTGATCGTTTTGATATTAAAGCTGATGAAGGCGGTATTACGGATATTGAGTTTATCGCG
CAATATCTGGTACTGCGCTACGCTCATGAAAAACCTAAATTAACGCGCTGGTCTGATAAC
GTGCGCATCCTGGAGCTTCTGGCGCAAAACGGCATTATGGATGAACACGAGGCGCAGGCG
CTGACGGTAGCCTACACCACACTGCGTGATGAACTGCACCACCTGGCGTTGCAGGAGCTT
CCCGGTCATGTGGCGCAGACGTGTTTTAGCAAAGAGCGCGCGCTGGTGCAGGCAAGCTGG
CGGAAGTGGCTGGTAGCCGTTTGA
>EECEBICD_00248 Bifunctional protein HldE
ATGAAAGTAAATCTGCCAGCGTTTGAACGTGCAGGCGTCATGGTTGTGGGTGATGTAATG
CTGGATCGCTATTGGTATGGCCCCACTTGCCGTATTTCACCGGAAGCGCCGGTGCCCGTG
GTTAAGGTAAATACCGTTGAGGAACGTCCGGGCGGCGCGGCGAACGTGGCGATGAACATT
GCGTCTCTGGGAGCGAACGCCCGTCTGGTCGGCCTGACGGGTATTGATGACGCCGCGCGC
GCGCTGAGCAAAACGTTGGCGGAGGTCAATGTGAAGTGCGACTTCGTTTCTGTGCCGACG
CATCCGACAATTACTAAACTGCGCGTGCTATCACGTAATCAGCAGCTCATTCGTCTTGAT
TTTGAAGAAGGCTTTGAGGGGGTGGACCCGCAGCCGTTGCATGAGCGTATCAACCAGGCG
CTGGGATCGATCGGCGCGCTGGTGTTGTCCGATTATGCCAAAGGCGCTCTGACCAGCGTG
CAGACTATGATTTCCCTGGCGCGCCAGGCGGGCGTGCCGGTGCTCATCGATCCGAAAGGA
ACGGATTTTGAACGTTACCGCGGCGCCACGCTGCTGACGCCAAACCTTTCTGAATTCGAG
GCGGTCGCGGGGAAATGTAAAAGCGAAGACGAACTGGTTGAACGCGGCATGAAGCTCATT
GCCGATTACGACCTTTCCGCGCTGTTGGTCACGCGTTCCGAACAGGGAATGACGCTGCTG
CAACCGAATAAAGCGCCGCTACATATGCCGACGCAGGCGCAGGAAGTTTATGATGTTACC
GGTGCGGGCGATACGGTGATCGGCGTGCTGGCGGCGACGCTGGCGGCGGGAAATACCCTG
GAAGAGGCGTGTTATTTCGCCAATGCGGCGGCGGGCGTAGTGGTAGGTAAACTCGGGACG
TCAACGGTTTCCCCTATTGAGCTGGAAAACGCAGTGCGCGGACGCGCGGATACCGGCTTC
GGCGTTATGACCGAAGAGGAGTTGAGACAGGCCGTCGCCAGCGCGCGTAAGCGTGGCGAG
AAAGTGGTCATGACCAACGGCGTTTTCGATATTCTGCACGCGGGCCACGTCTCTTATCTG
GCGAACGCGCGCAAACTGGGCGACCGCCTGATTGTTGCGGTCAATAGTGACGCCTCGACT
AAACGTCTGAAAGGCGACAGCCGTCCGGTTAATCCGCTCGAACAGCGTATGATCGTGCTG
GGCGCGCTGGAGTCGGTCGACTGGGTTGTCTCTTTTGAAGAGGATACGCCGCAACGACTG
ATTGCCGGTATTCTGCCGGATCTGCTGGTAAAAGGCGGCGACTATAAGCCGGAAGAGATC
GCGGGCAGCGAAGAGGTCTGGGCCAACGGCGGCGAAGTCATGGTGCTGAACTTCGAAGAT
GGTTGTTCCACGACCAATATCATCAAAAAGATCCAGACCGAGAGCGAGAAGTAA
>EECEBICD_00249 Inner membrane protein YqiK
ATGGATGATGTTTTTGGCATATTGCCATCATGGATGTTTACCGCCATCGTTGCGGTTATC
GTCTTACTAATTATCGGGATCATTTTTGCTCGCCTCTATCGCCGCGCATCTGCGGAACAG
GCGTTTGTCCGTACCGGGCTTGGCGGGCAAAAAGTGGTGATGAGCGGCGGCGCTATTGTG
ATGCCAATCTTTCACGAAATTATTCCGATCAATATGAATACCCTAAAACTTGAAGTCAGC
CGTGCTACCGTCGACAGCCTGATCACCAAAGATCGTATGCGCGTTGACGTGGTCGTTGCT
TTCTTCGTACGTGTAAAACCCTCTGTTGAAGGTATTGCCACCGCCGCACAAACGCTTGGC
CAGCGAACGCTGTCGCCGGAAGATCTTCGTATGCTGGTTGAGGATAAATTCGTTGACGCC
TTACGCGCTACCGCGGCCCAGATGACGATGCATGAGCTACAGGATACGCGTGAAAACTTC
GTTCAGGGCGTACAAAACACCGTTGCGGAAGATTTGTCGAAAAACGGCCTGGAGCTGGAA
AGTGTCTCATTAACCAACTTTAACCAAACGTCCAAAGAGCATTTCAATCCAAACAATGCC
TTTGATGCCGAAGGTCTGACGAAACTGACGCAGGAAACCGAACGCCGCCGTCGTGAACGT
AATGAAGTGGAACAGGATGTGGAAGTGGCGGTGCGCGAAAAGAACCGCGACGCGCTTGAG
CGCAAGCTGGAAATCGAGCAACAAGAAGCCTTTATGACTCTGGAGCAGGAGCAACAGGTT

AAAACCCGCACCGCCGAACAAAACGCTAAAATTGCCGCTTTTGAGGCCGAGCGCCATCGT
GAAGCAGAACAGACGCGCATTCTTGCTGAGCGGCAGATCCAGGAGACGGAGATCGAACGT
GAGCAAGCAGTACGGTCCAGGAAAGTTGAGGCCGAGCGTGAAGTACGTATTAAAGAGATC
GAACAGCAACAGGTGACAGAAATCGCCAACCAGACGAAATCTATCGCCATTGCCGCCAAA
TCCGAGCAGCAATCACAAGCAGAAGCGCGCGCCAACGACGCGCTGGCCGATGCGGTTCGC
GCCCAGCAAAACGTAGAAACCACCCGCCAGACCGCCGAAGCGGATCGCGCCAAGCAGGTC
GCTCTGATCGCCGCGGCGCAGGATGCGGAAACCAAAGCGGTCGAACTGACCGTTCGGGCA
AAAGCAGAGAAAGAAGCGGCAGAATTGCAAGCCGCCGCCATCATCGAACTGGCGGAAGCA
ACGCGTAAAAAAGGTCTGGCGGAAGCCGAAGCGCAACGGGCGCTGAATGACGCTATCAAC
GTACTATCTGACGAACAAACCAGCCTCAAATTTAAACTAGCGCTCTTACAATCGTTGCCT
GCTGTTATTGAGAAGTCTGTTGAACCGATGAAATCCATCGACGGCATTAAGATTATTCAG
GTCGATGGCCTCAATCGCGGCGCGACGGCGGGCGACGTCGCCGCTGGCGGCGCTAACGGG
GGAAACCTGGCGGAACAAGCGTTATCTGCCGCGCTCACCTATCGTACCCAGGCACCGCTG
ATCGATTCGCTGTTAAACGAGATTGGCATCGCTGGCGGCTCGCTAAAGGCGCTGACCACG
CCGTTGGTTTCATCGGCAACGGATGAGATTAATCGCGAAGCAACGGTAAAAGAGCAATAA
>EECEBICD_00250 Inner membrane protein YqiJ
ATGACCTTATTTGCGGAGTATAACAGCCCCTATCTGTTTGCCATCGCGTTCGTTTTCTTT
ATCGGCGTGCTGGAGATGATTTCGCTGATTTTTGGCCACTTTCTTTCAGGCGCGCTCGAC
GCCCATCTCGATCATTATGACGCCCTGTCATCCGGCCCTGCGGGCCAGGCGCTTCATTAT
CTGAATATTGGACGCGTTCCCGCCCTGGTCGTCCTCTGTCTATTGGCCGGTTATTTCGGC
CTCTTCGGTATCCTCATCCAGCATGGCGGGATCATGCTCTGGCAAGCGCCGCTCAGCAAT
CTGCTGCTGGTCCCGTTGAGCATTGTCCTGTCGGTCTTTGCCGTGCATTACAGCGGAAAA
ATCCTCGCGCCCTGGCTACCCAGAGATGAAAGCTCCGCCCTCCGGGAAGAGGAATTTATC
GGCGGCATGGCGATTATTACCGGCCACGCCGCCGTCGCCGGTACACCTTGCGAAGGAAAA
TTTACCGATAAGTTCGGCCAGATTCACTATTTACTGCTGGAACCCGAAAAGGGAAAAGAA
TTTAAAAAAGGAGACAAGGTGCTGATTGTCTGCCGACTTTCCGCAACGCGTTATTTGGCT
GAGCGTACCTTCTATGTTTAA
>EECEBICD_00251 Surface composition regulator
ATGAACAACAATAACGTCTATTCACTAAATAATTTTGATTTTCTGGCGCGCAGTTTTGCC
AGAATGCAGGCGGAGGGGCGTCCTGTTGATATCCAGGCGGTAACCGGCAATATGGACGAG
GAGCATCGCGACTGGTTTTGCAAGCGTTACGCGCTTTACTGCCAGCAGGCGACTCAGGCG
AAAAGGTTAGAATTAGAACACTAA
>EECEBICD_00252 Ubiquinone biosynthesis accessory factor UbiK
ATGATTGACCCGAAAAAAATTGAGCAAATCGCACGCCAGGTTCATGAGTCAATGCCGAAA
GGTATTCGCGAGTTCGGGGAAGATATCGAGAAAAAAATCCGTCAAACGCTGCAATCTCAG
CTGACGCGTCTCGATCTGGTGAGCCGCGAAGAGTTTGATGTCCAGACTCAGGTCTTGCTG
CGCACCCGGGAAAAACTGGCGCTGCTGGAGCAACGCCTCAGCGAACTGGAAGCTCGCGAC
AAGCCTGAAGAAGTAAAACCTGCGCCCGCCATCCCGCCAGTCGATCCACAAGAGTAA
>EECEBICD_00253 3,4-dihydroxy-2-butanone 4-phosphate synthase
ATGAATCAGACGCTACTTTCCTCTTTTGGTACGCCTTTTGAACGTGTTGAACTTGCACTG
GATGCGCTGCGTGAAGGACGCGGTGTGATGGTGCTTGATGATGAAGATCGTGAAAACGAA
GGCGATATGATTTTCCCCGCAGAGACGATGACCGTAGAACAGATGGCGTTAACCATTCGT
CACGGTAGCGGTATTGTTTGCCTGTGTATTACTGAAGATCGTCGTAAGCAGCTCGACTTG
CCAATGATGGTGGAAAACAACACCAGCGCCTACGGCACCGGTTTTACCGTGACCATTGAA
GCCGCAGAAGGCGTGACCACCGGCGTTTCCGCCGCTGACCGTGTCACTACGGTGCGTGCG
GCAATTAAGGATGGCGCAAAACCGTCCGATCTTAACCGTCCGGGGCACGTTTTCCCACTG
CGCGCTCAGGCGGGCGGCGTGCTGACGCGCGGGGGTCATACGGAGGCGACCATCGATCTG
ATGACGCTGGCCGGTTTTAAACCTGCAGGTGTACTGTGCGAGTTGACTAACGACGATGGG
ACGATGGCGCGCGCGCCGGAGTGTATCGCGTTTGCAGGCCAGCATAATATGGCGGTAGTC
ACGATTGAAGATCTGGTGGCTTACCGCCAGGCGCATGAGCGCAAAGCCAGCTGA
>EECEBICD_00254 Protein-disulfide oxidoreductase DsbI
ATGGATTTTATTAAGGGATTATGGCGAGACCTACGCGCAAGACCAGTAGATACGCTGGTG
AGATGGCAGGAACAGCGCTTTCTGTGGCTGTTAATGGCCATCGCGATGGGAGGGTTAATT
ATCCTGGCGCACTCGTTCTTCCAAATCTATCTCTACATGGCGCCGTGCGAACAGTGTGTT

TACATTCGTTACGCGATGTTTGTGATGGTAATTGGCGGGGTGATTGCGGCGATAAATCCC
AAAAATATCGTGCTGAAGCTGATTGGCTGCATCGCGGCGTTTTATGGCAGCATTATGGGC
ATTAAGTTTTCTATTAAGCTTAATGGTATCCATCATGCGGTACATAATGCGGACCCCGAT
TCACTTTTTGGCGTGCAAGGATGTTCGACTGACCCGACCTTTCCTTTCAACTTACCGTTG
GCGGAATGGGCACCGGAGTGGTTTAAACCTACCGGAGACTGCGGTTATGACGCGCCTATC
GTCCCTGATGGCGTAACGCTCAGTAGCGTACAGCAGTGGTTTGTCGATCTCTACCAGCAG
TCCGAAGGCTGGTATCTGCTTCCGCCCTGGCACTTTATGAACATGGCGCAGGCATGTATG
TTGGCCTTTGGGTTGTGCCTGATTCTGCTGCTGGTGATGAGCGGCGCCTGGGCATTGAAA
CTGGCCAGAGGCAAATAA
>EECEBICD_00255 Thiol:disulfide interchange protein DsbL
ATGTCATCTAAATGGATTACCTCTTTATTTAAAAGCGTAGTATTAACCGCGGCATTAGTC
ACTCCTTTTGCGGCATCGGCTTTTACCGAGGGCACAGATTATATGGTTCTGGAGAAACCG
ATTCCTAATGCGGATAAAACGCTGATTAAGGTATTTAGCTACGCCTGCCCTTTCTGCTAC
AAATATGACAAAGCCGTAACAGGTCCGGTTTCTGATAAAGTAGCCGATCTCGTTACGTTC
ACTCCGTTCCACCTGGAAACAAAAGGCGAATATGGCAAACAGGCCAGCGAAGTATTTGCA
GTCCTGATCGCCAAAGATAAAGCTGCCGGGATTTCGTTATTTGATGCTAAATCACAATTC
AAAAAAGCGAAGTTCGCCTGGTATGCGGCTTACCATGATAAGAAAGAGCGCTGGTCAGAT
GGGAAAGATCCGGCGGCATTTATTAAAACCGGGCTGGACGCCGCAGGCATGAGCCAGGCT
GATTTTGAAGCGGCGCTGAAAGATCCTGCCGTTCAGGAGACTCTGGAAAAATGGAAAGCC
GCCTATGACGTTGCCAAAATTCAGGGTGTTCCGGCTTACGTCGTCAACGGCAAGTACCTT
ATCTATACCAAAAACATCAAATCCATCGATTCTATGGCTGAACTCGTTCGGGAACTGGCC
ACTAAAAAATAA
>EECEBICD_00256 Arylsulfate sulfotransferase AssT
ATGTTTGACCAATACCGGAAAACAATACTTGCCGGCGCCGTCGCACTGACATGCGGACTC
ACCGCAGCCAGTACGTTTGCCGCAGGTTTTCAACCGGCACAGCCCGCAGGGAAATTAGGC
GCAGTCGTTGTCGATCCTTACGGAAATGCCCCTCTCACCGCGCTGGTGGAATTAGATAGC
CATATTATTTCAGACGTTAAAGTTACTGTACATGGCAAAGGGGAAAAAGGCGTTCCTGTT
ACTTATACCGTTGGGAAAGAGTCTTTAGAAACCTATGACGGTATTCCTATTTTTGGCCTT
TATCAGAAATTTGCCAACAACGTCACGGTAGAATATAAAGAAAACGGCAAAGCCATGAAG
GATGACTATGTGGTGCAGACGTCCGCCATCGTCAACCATTATATGGATAACCGTTCTATT
TCAGATCTTCAGCAAACGAAAGTTATTAAAGTCGCGCCAGGATTTGAAGATCGCCTTTAT
CTGGTAAATACCCATACCTTTACGCCGCAGGGCGCTGAATTTCACTGGCACGGCGAAAAA
GATAAAAATGCGGGTATTCTTGATGCCGGTCCGGCGGGCGGTGCTTTGCCTTTCGATATC
GCCCCTTATACGTTTGTGGTTGACACCCAGGGTGAATACCGCTGGTGGCTGGATCAAGAT
ACCTTCTACGACGGCCACGATATGAATATCAACAAACGCGGCTACCTGATGGGTATTCGT
GAAACGCCTCGCGGCACCTTTACCGCGGTGCAGGGCCAACACTGGTACGAGTTTGACATG
ATGGGGCAAATTCTTGCCGATCACAAACTGCCGCGCGGGTTCCTGGATGCGTCTCATGAA
TCCATCGAAACCGTGAACGGCACCGTACTGCTGCGCGTCGGCAAACGCGATTACCGCAAA
GAAGACGGCATACATGTTCATACGATTCGTGACCAAATCATTGAGGTTGATAAGTCTGGC
CGCGTAGTAGACGTTTGGGATTTAACCAAAATCCTCGACCCTATGCGTGATGCGCTGCTC
GGCGCGCTGGATGCGGGCGCAGTATGCGTGAACGTCGATCTGGCCCATGCCGGACAGCAG
GCGAAACTCGAACCGGATACGCCGTATGGCGATGCGCTTGGCGTTGGTGCCGGTCGTAAC
TGGGCGCACGTCAACTCTATCGCTTATGACGCGAAAGACGACTCCATCATCCTTTCTTCC
CGCCATCAGGGTATTGTAAAAATTGGTCGCGATAAGCAGGTGAAATGGATACTGGCACCG
TCTAAAGGCTGGAATAAGCAGCTAGCCAGTAAATTGCTGAAACCGGTAGACGATCATGGT
AAGCCGTTGACCTGTGACGAAAACGGCAAGTGTAAGGACACCGATTTCGATTTCACCTAT
ACCCAACATACGGCATGGCTTTCCAGCAAAGGCACGTTAACGGTCTTTGATAACGGCGAT
GGTCGCGGCCTGGAGCAACCGGCTCTACCGACCATGAAATATTCCCGTTTTGTCGAATAT
AAGATCGATGAGAAGAAAGGCACCGTACAACAAGTTTGGGAATACGGTAAAGAACGTGGA
TATGATTTCTATAGTCCTATTACCTCGGTTGTTGAATATCAAAAAGACCGCGACACCATG
TTCGGCTTTGGCGGTTCTATTAACCTGTTCGACGTTGGTAAACCCACAGTCGGCAAACTG
AATGAGATTGACTATAAAACGAAAGAAGTGAAAGTTGAAATTGATGTGCTGTCGGATAAA
CCCAACCAGACTCACTATCGTGCATTACTGGTTCATCCAACGCAAATGTTTAAATAA
>EECEBICD_00257 Protein YqiD

ATGTTTATTGCCTGGTATTGGATAATATTAATCGTTCTGGTGGTAGTAGGGTATATTTGC
CATATGAAGCGTTATTGCAGAGCGTTTCGGCAGGACAGAGATGCATTACTTGAAGCGCGA
ACGAAACGTTTTCGCCAGACGAGCGAAGGCGACAGCGTGATGAATGAGCAGAAATAG
>EECEBICD_00258 Zinc transporter ZupT
ATGTCAGTACCACTTATTCTGACCTTACTGGCGGGCGCCGCCACCTTTATTGGCGCGTTT
CTTGGCGTTCTCGGCCAAAAACCGTCTAACCGCGTGCTGGCTTTTTCGCTGGGCTTCGCC
GCAGGGATCATGCTGCTTATCTCGCTGATGGAGATGCTGCCCGCCGCGCTTGATACCGAG
GGGATGTCGCCTGTACTGGGCTACGGAATGTTTATTATCGGCCTGTTGGGCTACTTCGGG
CTGGACCGCCTGCTTCCTCACGCTCATCCGCAGGATCTGGTGCAAAAAAGACAGCAGCCG
CTTCCCGGCTCGATAAAACGCACTGCGATTTTATTGACGCTCGGCATTAGCCTGCACAAC
TTTCCGGAAGGGATCGCCACCTTTGTCACTGCCAGCAGCAATCTTGAACTGGGTTTCGGC
ATCGCACTGGCGGTGGCGTTGCACAATATTCCTGAAGGTCTGGCGGTTGCCGGCCCGGTT
TATGCCGCGACGGGCTCAAAACGTACCGCGATTTTTTGGGCCGGTATCTCCGGCATGGCG
GAAATTCTTGGCGGCGTACTGGCGTGGCTGATTTTGGGCAGCCTGGTTTCACCGATCGTT
ATGGCGGCTATCATGGCGGCAGTCGCCGGCATTATGGTGGCGCTCTCCGTCGATGAACTG
ATGCCGCTGGCAAAAGAGATCGATCCTAACAATAACCCCAGCTATGGCGTGCTTTGCGGT
ATGTCCATCATGGGGCTCAGTCTCGTCATTTTGCAGACGATAGGTATCGGTTAA
>EECEBICD_00259 4,5-DOPA dioxygenase extradiol
ATGTCTTTAACGTGTATGCCAGCATTGTTTTTGGGGCATGGCAGCCCGATGAACGTGCTG
GACGATAATGATTATACCCGCGCCTGGCGGCGATTGGGGGAAGCCCTGCCGCGACCACAG
GCGATAGTGGTGGTGTCGGCGCATTGGTATACCCGCGGGACGGGCGTCACAGCGATGGAA
AGGCCGCAAACTCTCCATGATTTTGGCGGTTTTCCGCAGGCGCTGTATGACACGCATTAT
CCGGCCCCTGGATCGCCTGCGCTGGCGCAGCGTCTGGTTGAACTGTTGGCTCCCGTTCCC
GTCGCGCTCGATAAAGAAGCGTGGGGATTTGATCACGGCTCCTGGGGCGTATTGATCAAG
ATGTATCCGAATGCGGATATCCCGATGGTGCAGCTTAGTGTTGATAGTACCAAACCGGCG
GCATGGCATTTTGAGGTGGGCCGTAAACTGGCGACGCTGCGTGACGAAGGCGTGATGCTG
GTGGCCAGCGGGAATGTGGTTCATAACCTGCGGACTGTGCGTTGGCACGGTGACAATATA
CCGTATCCCTGGGCGGCGTCATTTAATGACTTTGTGAAAGCCAATCTGACGTGGCAGGGG
CCGGTAGAACAGCACCCGCTGGTGAATTATCTGCAACATGAAGGGGGGGCCTTATCGAAC
CCGACGCCGGAGCACTTTTTACCTTTGCTGTATGTACTTGGCGCCTGGGACGGCAAGGAA
CCTATCACCATCCCGGTCGACGGTATTGAGATGGGCAGTATAAGTATGTTGTCGGTGCAG
GTGGGGTAG
>EECEBICD_00260 Putative acid--amine ligase YgiC
ATGGAAAGAGTCAGTATTACCGAGCGCCCGGACTGGCGCGATAAAGCGACTGAATACGGG
TTTAATTTTCACACTATGTATGGTGAACCGTACTGGTGTGAAGACGCTTATTACAAGTTA
ACGCTCGCCCAGGTGGAAAAACTGGAAGACGTTACCGCCGAGCTGCACCAGATGTGCCTC
AAGGTAGTAGAACGCGTCATCGCCAGCGATGAGCTGATGACGAAGTTTCGTATTCCAAAA
CATACCTGGGGTTTTGTTCGCCAGTCCTGGCAAACGCAACAACCGTCGCTCTATTCCCGC
CTCGATTTGGCCTGGGACGGCATCGGCGAGCCTAAGCTGCTCGAAAATAATGCCGACACG
CCAACATCGCTGTATGAAGCGGCGTTTTTTCAGTGGATTTGGCTGGAAGATCAGATCAAC
GCGGGCAATCTGCCGGAAGGCAGCGATCAGTTTAATAGCCTGCAGGAAAAACTGATTGAA
CGCTTTGCTGAACTTCGCGAGCAGTACGGTTTTCAGTTGCTGCATCTCACCTGCTGTCGC
GATACGGTTGAGGATCGCGGCACCATTCAGTATTTACAGGACTGCGCGGCCGAAGCGGAA
ATCGCCACCGAATTTCTCTATATCGACGATATTGGTCTGGGGGAAAAAGGCCAATTTACG
GATCTGCAGGATCAGGTCATTGCGAACCTGTTCAAGCTCTATCCGTGGGAATTTATGCTG
CGCGAGATGTTTTCCACCAAGCTGGAAGATGCCGGCGTGCGCTGGCTGGAACCGGCCTGG
AAAAGCATTATCTCCAATAAAGCGTTGTTGCCACTGTTGTGGGAGATGTTCCCTGACCAT
CCGAATCTGTTGCCTGCGTATTTCGCCGAAGACGAACATCCGCCAATGGATAAATATGTC
GTGAAACCGATCTTCTCCCGCGAAGGGGCGAACGTATCGATTATTGAGAACGGTAAAACG
ATCGAATCCGTGGAAGGCCCGTATGGCGAAGAGGGTATGATCGTGCAACAGTTCTATCCG
CTGCCAAAATTCGGCGACAGTTACACCCTGATTGGTAGCTGGCTGATTAACGATCAGCCC
GCCGGAATTGGTATCCGTGAAGATCGGGCGCTGATCACCCAGGATCTCTCCCGCTTCTAT
CCGCATATTTTTGTCGAAGGATAG
>EECEBICD_00261 hypothetical protein

ATGAAACGGACAAAATCCATCCATCACGCATCATTTCGCAAAAGCTGGAGCGCGCGGCAT
TTAACGCCGGTCGCCCTGGCGGTTACGGCTGTTTTTATGCTGGCTGGCTGTGAAAAAAGC
GATGACACCGTATCGCTGTATCAAAACGCTGATGACTGTTCAGCGGCGAATCCGGGCAAA
AGCGCGGAGTGTACAACCGCGTATAACAATGCGCTGAAAGAGGCCGAACGTACTGCGCCT
AAGTACGCTACACGCGAAGATTGCGTCGCTGAGTTTGGCGAAGGTCAGTGCCAGCAAGCG
CCCGCACAGGCTGGCATGGCGCCGGAAAATCAGGCGCAGGCCCAACAATCCAGCGGCAGT
TTCTGGATGCCGCTTATGGCAGGTTACATGATGGGGCGTCTGATGGGCGGCGGCGCAGGC
TTTGCGCAACAGCCGCTGTTTAGCTCGAAAAACCCGGCCAGCCCTGCATACGGCAAATAT
ACCGATGCGGCAGGTAAAAACTACGGCGCGGCGCAACCGGGCCGGACAATGACCGTACCG
AAAACCGCGATGGCGCCGAAACCTGCCACCACAACAACCGTTACCCGCGGCGGTTTTGGC
GAATCCGTCGCCAAACAGAGCACCATGCAGCGTAGCGCTGCCGGTACTTCAACACGTTCG
ATGGGCGGCTGA
>EECEBICD_00262 Outer membrane protein TolC
ATGAAGAAATTGCTCCCCATCCTTATCGGCCTGAGCCTGTCGGGGTTCAGCACACTAAGC
CAGGCAGAGAACCTGATGCAAGTTTATCAGCAAGCACGCCTGAGCAACCCGGAATTGCGT
AAATCCGCTGCCGATCGCGATGCTGCATTCGAAAAAATTAACGAAGCGCGTAGTCCTTTA
CTGCCGCAACTGGGTTTAGGTGCCGACTACACCTACAGCAACGGTTATCGCGATGCGAAC
GGTATCAACTCCAATGAAACCAGCGCTTCCCTGCAATTAACGCAGACACTGTTTGATATG
TCGAAATGGCGTGGGCTCACCCTGCAAGAAAAAGCAGCAGGCATTCAGGATGTCACCTAT
CAGACCGATCAGCAGACGCTGATCCTCAATACCGCGAACGCGTATTTTAAGGTATTGAAC
GCTATTGATGTGCTTTCCTATACCCAGGCGCAAAAAGAGGCTATCTACCGTCAGTTAGAT
CAAACGACGCAACGTTTTAACGTGGGTCTGGTCGCCATTACCGACGTGCAAAACGCCCGT
GCGCAATATGATACCGTACTGGCGAATGAAGTGACCGCCCGCAACAACCTGGATAACGCG
GTAGAAGAGCTGCGCCAGGTAACCGGCAATTATTACCCGGAGCTGGCGTCGCTTAACGTC
GAGCATTTTAAAACCGACAAGCCTAAAGCTGTTAATGCGCTGCTGAAGGAAGCGGAAAAC
CGTAACCTGTCGCTGTTGCAGGCGCGTTTAAGTCAGGATCTGGCGCGCGAGCAAATCCGT
CAGGCGCAGGATGGTCATCTGCCGACGCTGAATTTAACGGCCTCAACCGGCATTTCTGAT
ACCTCTTATAGCGGTTCTAAAACCAACGCCGCCCAGTACGACGATAGCAACATGGGGCAG
AATAAAATCGGCCTGAACTTCTCCCTGCCGCTGTATCAAGGCGGGATGGTTAACTCGCAG
GTAAAACAGGCGCAGTATAACTTCGTCGGCGCAAGCGAACAGCTGGAAAGCGCGCACCGT
AGCGTGGTGCAGACCGTACGTTCTTCCTTTAACAATATTAACGCCTCCATCAGCAGCATC
AACGCGTATAAACAGGCAGTCGTTTCCGCGCAAAGTTCTTTGGATGCAATGGAAGCCGGT
TACTCGGTCGGTACACGTACCATTGTTGACGTACTGGATGCCACCACCACTCTGTATGAT
GCCAAGCAGCAACTGGCCAACGCGCGTTATACCTATTTGATTAATCAGTTAAATATCAAA
TATGCGCTCGGTACGCTGAACGAGCAGGATCTGCTCGCGCTTAACAGTACGTTGGGTAAA
CCTATCCCGACGTCGCCGGAAAGCGTAGCGCCGGAAACGCCAGATCAGGATGCTGCCGCA
GACGGTTATAATGCCCATAGCGCCGCGCCGGCAGTACAGCCGACCGCCGCTCGCGCCAAC
AGCAATAACGGCAATCCATTCCGGCATTGA
>EECEBICD_00263 ADP-ribose pyrophosphatase
ATGCGTAAATCAGACAACTTGCCAGTTACTTTCACCAAAAGCGATGTAGAAATTATTGCA
CGGGAAACGCGCTATCGCGGTTTTTTTTCATTGGATGTGTATCGATTCCGCCATCGTTTA
TTCAACGGAGAAATGAGTGGCGAGGTAAAGCGCGAAATTTTTGAGCGCGGGCACGCCGCC
GTCTTGCTACCCTTTGACCCTGAACGCGACGAGGTGGTGCTGGTCGAACAGATTCGTATT
GCCGCGTACGACACAAGCGTTACGCCGTGGCTGCTGGAGATGGTCGCCGGCATGATAGAA
GAAGGCGAAACCATTGAGGCGGTCGCCCGGCGGGAGGCGATGGAAGAAGCGGGGCTGACC
GTAGGCCGCACCCGACCGGTCATCAGTTACCTGGCGAGTCCAGGAGGAACCAGCGAGCGC
TCATCTATTCTGGTGGGTGAAGTGGACGCCACGACCGCGGTAGGTATTCATGGTCTGGCG
GATGAAAACGAAGATATTCGGGTTCATGTGGTAAGCCGGGAACAGGCATACCAGTGGGTA
GAAGAGGGGAAAATCGACAACGCAGCCGCAGTCATCGCTTTGCAATGGCTTCAGTTGCAT
CATCAAGAGTTAAAAAACGAGTGGAAAAAATGA
>EECEBICD_00264 hypothetical protein
ATGAAGCGTTATACACCTGACTTCCCAGAAATGATGCGTTTGTGCGAAACCAATTTTTCG
CAATTGCGCCGCCTGCTGCCGCGCAATGACGCCCCCGGTGAAACGGTGAGCTATCAGGTG
GGCAACGTACAATATCGGTTAACGATTGTGGAATCGACCCGATACACTACGCTTGTAACG

ATCGAGCAAACGGCGCCAGCCATCACCTACTGGAGCCTGCCGTCGCTGACGGTGCGCCTC
TACCATGATGCGATGGTGGCTGAAGTGTGTTCAAGCCAGCAGATCTTTCGCTTCAAAGCG
CGGTATGATTATCCAAATAAAAAGTTGCATCAACGCGACGAAAAGCATCAAATTAATCAG
TTTCTGGCCGACTGGCTACGATTCTGTTTAGCACATGGAGCGATGGCGATTCCGGTTTAT
TAG
>EECEBICD_00265 3',5'-cyclic adenosine monophosphate phosphodiesterase CpdA
TTGGAAAGCCTGTTAACCCTACCTCTGGCTGGTGAGGCCCGAGTCAGGATTTTACAAATT
ACAGATACTCACCTGTTTGCCGAAAAGCATGAAACGCTGTTAGGCGTCAATACCTGGGAA
AGTTACCAGGCCGTGCTGGAGGCTATTCGGGCGCAACAGTATGAATATGATCTGATCGTG
GCCACCGGCGATCTGGCGCAGGATCAATCCGCTGCGGCTTATCAGCATTTTGCTGAGGGT
ATCGCAAGCTTTCGCGCGCCTTGCGTTTGGCTACCGGGCAATCATGATTTTCAGCCGGCA
ATGTACAGCGCGCTGCAAGAGGCGGGGATTTCCCCGGCTAAACGCGTGTTAATCGGTGAA
CAATGGCAGATTTTGTTGCTGGATAGCCAGGTGTTTGGCGTCCCGCATGGCGAACTGAGC
GAGTTCCAACTGGAATGGCTTGAGCGTAAGCTCGCCGATGCGCCAGAGAGACAGACATTG
CTCCTGTTGCACCACCATCCGTTGCCGGCAGGGTGCAGTTGGCTGGATCAGCACAGCTTG
CGTAATGCTGGTGAGCTGGATAGCGTGCTGGCGAATTTCCCGCGTGTAAAGTATCTGCTA
TGCGGACATATTCACCAGGAGCTGGATCTCGACTGGAACGGACGGCGTCTGCTGGCGTCG
CCGTCAACCTGCGTACAGTTCAAACCGCACTGCGCGAACTTTACGCTGGATACTATCGCG
CCGGGCTGGCGTACGCTGGAGCTCCAGGCGAACGGCGTCCTGGAGACGGAAGTGCATCGC
CTGCAGGATACCCGGTTCCGACCCGATACCGCTTCGGAAGGATACTGA
>EECEBICD_00266 hypothetical protein
ATGTCTACGCTTCTCTATTTGCACGGATTCAACAGTTCCCCTCGCTCGGCAAAAGCGTGC
CAGCTAAAAAACTGGCTGGCGGAGCGTCATCCGCATGTCGAGATGATCGTCCCTCAACTA
CCGCCGTATCCTGCCGATGCGGCGGAGTTGCTGGAATCTCTCGTGCTTGAGCATGGCGGT
GCGCCATTAGGGCTGGTAGGATCGTCGCTGGGTGGTTATTACGCCACCTGGCTGTCGCAA
TGTTTTATGCTGCCGGCTGTGGTGGTGAATCCCGCCGTGCGGCCCTTTGAATTACTGACC
GACTATCTCGGTCAGAACGAGAACCCCTACACCGGGCAGCAATATGTGCTAGAGTCTCGC
CATATTTATGATCTTAAAGTCATGCAGATTGACCCGCTGGAAGCGCCGGACCTGATCTGG
CTACTGCAACAGACGGGCGATGAAGTGCTGGATTACCGCCAGGCGGTGGCATATTACGCC
TCCTGCCGTCAGACAGTGACCGAGGGTGGTAATCACGCATTCACGGGCTTCGAAGATTAT
TTCAACCAGATTGTCGATTTTCTTGGACTGCACAGTTGCTGA
>EECEBICD_00267 DNA topoisomerase 4 subunit B
ATGACGCAAACTTATAACGCTGATGCCATTGAGGTACTCACTGGGCTTGAGCCGGTACGC
CGCCGCCCGGGGATGTACACCGATACGACCCGTCCCAACCATTTGGGTCAGGAAGTAATT
GATAATAGTGTGGATGAAGCACTGGCAGGTCACGCCAAACGCGTGGATGTCATTTTACAT
GCCGATCAATCGCTGGAAGTGATTGACGACGGACGCGGGATGCCGGTGGATATCCATCCG
GAAGAGGGGGTTCCGGCGGTAGAACTGATCCTCTGTCGGCTTCATGCCGGCGGTAAATTC
TCCAATAAGAACTATCAGTTCTCTGGTGGTCTGCATGGGGTGGGGATTTCGGTCGTGAAT
GCCCTGTCAAAGCGCGTGGAAGTGACCGTGCGCCGCGACGGTCAGGTCTATAACATCGCG
TTTGAAAACGGCGAGAAAGTGCAGGATTTGCAGGTTGTCGGCACCTGCGGTAAACGTAAT
ACTGGCACCAGCGTCCATTTCTGGCCGGACGAAAGTTTCTTCGACAGCCCGCGTTTTTCT
GTCTCTCGCTTAATGCACGTTCTGAAAGCAAAAGCGGTGCTGTGTCCCGGCGTGGAAATC
ACTTTTAAAGATGAAGTGAATAACAGCGAGCAGCGCTGGTGCTACCAGGATGGTCTGAAC
GACTATCTGGGCGAAGCGGTAAACGGCCTGCCGACGCTGCCGGAAAAGCCGTTTATCGGT
AATTTTAACGGTGAAACGGAAGCTGTTGACTGGGCGCTATTGTGGCTGCCGGAAGGCGGC
GAATTACTGACGGAAAGCTACGTCAACCTGATCCCGACCATGCAGGGGGGACGCACGTCA
ACGGTCTGCGCCAGGGCCTGCTCGACGCGATGCGCGAATTTTGCGAATACCGCAATATTC
TGCCGCGCGGCGTCAAACTGTCGGCGGAAGATATCTGGGATCGCTGCGCTTATGTGCTCT
CCGTGA
>EECEBICD_00268 DNA topoisomerase 4 subunit B
ATGCAGGACCCGCAATTTGCCGGGCAGACCAAAGAGCGTCTGTCGTCGCGTCAATGTGCG
GCATTTGTTTCCGGCGTGGTGAAAGATGCCTTCAGCCTGTGGCTGAACCAGAACGTGCAG
GCGGCGGAACAACTGGCAGAGATGGCGATTGCCAGCGCGCAGCGGCGACTGCGCGCCGCA

AAAAAAGTGGTACGCAAAAAGCTCACCAGCGGTCCGGCGTTGCCGGGGAAACTGGCGGAC
TGTACCGCGCAGGATCTTAATCGGACCGAGCTGTTCCTTGTGGAAGGGGATTCGGCGGGC
GGTTCCGCCAAGCAGGCGCGCGATCGCGAATATCAGGCGATCATGCCGCTCAAAGGTAAG
ATCCTTAACACTTGGGAAGTCTCTTCCGATGAAGTGCTGGCCTCGCAAGAGGTGCATGAT
ATTTCCGTGGCGATCGGTATCGATCCGGACAGCGACGATCTGAGTCAGCTGCGCTACGGC
AAGATCTGTATCCTGGCGGATGCGGACTCCGATGGTTTGCATATCGCTACTCTGCTTTGC
GCGCTGTTTGTCAGACATTTCCGCGCGCTGGTGAAGAATGGTCATGTCTACGTCGCGCTG
CCGCCGCTATACCGTATCGATTTGGGTAAAGAGGTCTATTACGCGCTGACGGAAGAAGAG
AAGGCGGGCGTACTGGAACAACTGAAGCGTAAGAAAGGCAAGCCGAACGTACAGCGTTTC
AAAGGCCTGGGTGAAATGAACCCGATGCAGTTACGCGAAACCACGCTTGATCCAAATACT
CGCCGCCTGGTGCAGCTCACCATTAGCGATGAAGACGATCAGCGTACTAACGCGATGATG
GATATGCTGCTGGCGAAGAAACGTTCTGAAGATCGACGCAACTGGCTACAGGAAAAAGGC
GATCTCGCGGATCTCGACGTGTAA
>EECEBICD_00269 putative quinol monooxygenase YgiN
ATGCTTACCGTCATTGCCGAAATTCGCACGCGTCCGGGCCAACATCACCGTCAGGCCGTC
CTCGACCAGTTTGCGAAAATTATTCCAACCGTTCTTAAAGAAGAGGGGTGCCACGGTTAC
GCACCGATGGTGGATCATGCCGCGGGCGTGAGTTTTCAGACTCTGGCGCCTGACTCGATC
GTGATGATTGAACAGTGGGAAAGCGTTGCGCACCTTGAGGCGCATCTGCAAACCCCGCAT
ATGAAAGCCTACAGCGACGCGGTAAAAGACGATGTGCTGGAAATGAATATTCGTATTCTG
GAATCCGGCGTTTAA
>EECEBICD_00270 NADPH:quinone oxidoreductase MdaB
ATGAGTAACATCCTGATTATCAACGGGGCGAAAAAATTCGCGCACTCCAACGGCCAGTTA
AATGACACCCTGACCGAAGTTGCCGATGGGCTGCTGCGCGATCTGGGTCATCAGGTTAAG
ATCGTCCGCGCCGACAGCGACTATGACATTAAAGAAGAGGTGCAGAATTTCGTCTGGGCT
GACGTGGTGATTTGGCAGATGCCAGGCTGGTGGATGGGCGCGCCATGGACAGTAAAAAAA
TATATGGATGATGTCTTTACCGAAGGTCACGGCACGCTCTATGCCAGCGATGGCCGCACG
CGTTCCGATGCCGCTAAAAAGTACGGTTCCGGCGGTCTGGTGCAGGGGAAAAAATATATG
CTGTCACTGACGTGGAACGCGCCGATGGAAGCATTTACCGAAAAAGATCAATTTTTCCAC
GGCGTAGGCGTAGATGGCGTCTATCTGCCATTCCATAAAGCCAACCAGTTCCTGGGCATG
GACGCGCTGCCAACGTTTATTGCCAACGACGTGATTAAAATGCCGGATGTTCCGCGCTAT
ACCGCAGAATATCGCAAGCATCTGAGCGAAATTTTTGCTTAA
>EECEBICD_00271 Sensor protein QseC
ATGAAATTGACGCAACGTCTCAGCCTGCGCGTCAGGCTGACGCTTATTTTCCTGATTCTG
GTCTCCATCACCTGGGCAATCTCCAGTTTTGTGGCCTGGAGAAAGACGACGGATAATGTC
GACGAGCTGTTTGACACACAGTTGATGTTGTTCGCCAGACGGCTTAGCACCCTCGATCTT
AACGAGCTCAACGCCCCGCAGCGCATGGCGCATACGCCAAAGAAATTAAAACATGGGCAT
ATCGATGACGATGCGCTGGCGTTCGCCATCTTTTCCGCTGATGGCAAAATGTTGCTGCAT
GATGGCGACAACGGCCAGGATATTCCGTATCGCTATCGCCGGGAAGGTTTCGACAATGGC
TATCTGAAGGATGACAACGACCTCTGGCGTTTTTTGTGGTTGAACTCGGCGGACGGCAAG
TATCGTATCGTCGTCGGTCAGGAGTGGGATTACCGCGAAGATATGGCGCTGGCGATCGTA
GCGGCGCAATTAACGCCGTGGCTGATCGCCCTTCCCTTCATGCTGCTGATTTTATTGCTC
CTGCTTCACCGCGAGCTCAGACCGTTGAAAAAACTGGCGCAGGCGCTACGCTTCCGGTCG
CCGGAATCGGAAACGCCGCTCGACGCCAAAGGCGTACCGAGCGAGGTTCGCCCACTGGTC
GAAGCGCTTAATCAGCTTTTTAGCCGCATCCACTCCATGATGGTGCGCGAACGGCGATTT
ACCTCCGACGCCGCCCATGAATTGCGCAGCCCGCTGGCCGCGCTGAAAGTACAAACCGAG
GTGGCGCAGCTCTCGGGCGACGATCCGCTATCGCGTGATAAAGCGCTGACCCAGCTCCAT
GCGGGGATCGATCGCGCCACGCGGCTGGTCGATCAGCTACTGACGTTATCGCGTCTCGAC
TCGCTCAATAATTTGCAGGATGTAGCGGAGATTTCGCTGGAAGAGCTGCTGCAGTCCGCG
GTAATGGATATTTATCATCCGGCGCAGCAGGCGAATATCGACGTGCGTTTACAGCTTAAC
GCCCATGACGTCATACGTACCGGCCAGCCGCTACTCTTAAGCCTGTTAGTGCGTAACCTG
CTGGATAATGCGATTCGCTACAGTCCGCAAGGCAGCGTCGTCGACGTTACCTTACATGCC
CGCAGCTTCACCGTCAGAGATAATGGCCCTGGTGTAGCGCCAGAGATTCTGACGCATATC
GGCGAGCGTTTTTACCGCCCGCCTGGCCAAAGTGTGACCGGCAGCGGTCTGGGATTATCA
ATCGTCCGTCGCATCGCTACGCTGCACGGCATGACGGTGTCGTTTGGCAACGCCGCGGAA

GGTGGATTTGAGGCCGTAGTAAGTTGGTAA
>EECEBICD_00272 Transcriptional regulatory protein QseB
ATGCGAATTTTACTGGTAGAAGATGACACACTTATTGGCGATGGCATAAAAGCGGGCCTG
AGTAAAATGGGTTTCAGCGTCGACTGGTTTACCGAGGGACGTCCGGGCAAAGAGGCGCTT
TACAGCGCGCCTTATGATGCGGTTATTTTAGATTTGACGCTGCCAGGCATGGACGGACGG
GATATTTTGCGCGAATGGCGCGAAAAGGGTAAACAGGAACCGGTACTGATCCTGACGGCG
CGCGACGCGTTAGCAGAGCGCGTCGAGGGGCTACGTCTGGGCGCCGATGACTATTTATGT
AAACCTTTCGCGCTCATTGAGGTCGCCGCCCGTCTGGAGGCGCTGGTACGCCGCGCCAGC
GGTCAGGCCAGTAGCGAATTGCGCCACGGCCAGGTGACGCTCAATCCAGGCAATCTCGTC
GCGACGCTTGCCGGCGAACCGCTGGCCCTAAAACCAAAAGAGTTCGCGTTGCTGGAACTC
TTACTGCGCAACAAAGGTCGCGTGCTGCCGCGCAAGCTTATTGAAGAGAAGCTGTATAAC
TGGGATGACGACGTGTCCAGCAATGCAGTGGAAGTCCATGTTCATCATTTACGCCGCAAG
CTCGGCAGCGAATTTATTCGCACCGTGCACGGCATCGGCTACACCCTGGGTGACGCATGA
>EECEBICD_00273 Protein YgiW
ATGAAAAAATTAGCTGCAATCGTTGCCGTTATGGCACTGTGTTCTACCCCGGTTCTGGCG
GCTCAGCAGGGCGGTTTCTCCGGCCCGTCGGCGACGCAAACGCAAGGCGGCGGATTTGTC
GGCCCGAACGGCAGCAGCACGACGGTAGAGAGCGCTAAATCGCTGCGTGACGATGCGTGG
GTAACGTTGCGCGGGAATATCGTCGAGCGCATTTCCGACGACCTGTATGTGTTTAAAGAT
GCGACCGGTACTATCAATGTGGATATTGACCACAAGCGCTGGAACGGCCTGACCGTAACC
CCGCAGGATACCGTTGAAATTCAGGGTGAAGTGGATAAAGACTGGAACTCTGTCGAAATC
GACGTAAAACAGATTCGTAAAGTGAATCCGTAA
>EECEBICD_00274 HTH-type transcriptional activator RhaS
ATGAATGACCTGATCAGCGCGGCTTATTCCGAACGTCTGCGGCGCGTTTGCGACCACATT
GAGCGTCATCTGGATGAGCCTCTGTCGCTTGAGGCGTTAAGCCGGATGGCGCACAGCTCG
CCGTTTCATTTTCACCGGCAGTTTACCGTCTGGAGCGGGCTTCCGCTCTATCGCTATATC
CAGTGGCTGCGTTTGCGCCGGGCGTCATGGCGGCTGGCGTTTAATCCGCAAGATAAAGTT
ATCGATATCGCGCTGGATGCCGGATTTCAGAATCCGGAGTCGTTCACACGTGCTTTCAAA
ACCGCGTTTGGTCAAAGCCCGTGCCGGTTTCGGCAATCGCCGGACTGGCTGGCCTGGCAC
CAACGCGTTCCTAAACTTGCGTTACAGGAGCAGCATGTCATGGACGTAAAAATCGTTGAA
TTTCCACCTACCCGCGTCGCGATGCTGACGCATCTGGGGCATCCGGATAAGGTCAACGCC
AGCGCGGCGAAATTTATTGCATGGCGACGCGAAACGGGCCAGTCGCCGATTGCCAGCAGT
CAAACTTTTGGTATTGCCTGGCACGACCCGCAGACGACGCCGCCGGCTCAATTCCGCTTT
GATATCTGCGGCAGCGTCCGCCAGCCGATAGCCGAAAATGACGTTGGCGTGGTGAATAGC
GAGATTCCTGGCGGGCGCTGCGCGGTGGTGCGCCATCAGGGATCGCTCGACAGCCTCCCG
GAGAGCGTGTGGTATTTATTCCGCGAATGGCTACCCGCCAGCGGTGAGACGCCGCGCGAT
TTTCCGGTCTTCTTCCAGTACCTGAATTTCGTCCATGAGGTCGCGGAACATGAGCTGCTG
ACGGATATCTATCTGCCGCTTCGTTAA
>EECEBICD_00275 DNA topoisomerase 4 subunit A
ATGAGCGATATGGCAGAGCGCCTTGCGCTACATGAATTTACGGAAAACGCCTACTTAAAC
TACTCCATGTACGTGATCATGGATCGTGCGTTGCCGTTTATTGGCGACGGCCTGAAGCCG
GTACAGCGCCGCATCGTCTATGCGATGTCAGAGCTGGGGCTGAACGCCACCGCTAAATTT
AAAAAATCCGCCCGTACCGTTGGTGACGTACTGGGTAAGTATCACCCGCATGGCGACAGC
GCCTGCTATGAAGCCATGGTGCTGATGGCGCAGCCGTTCTCTTACCGTTACCCGCTGGTC
GATGGCCAGGGGAACTGGGGCGCGCCGGATGATCCGAAGTCATTCGCGGCGATGCGTTAT
ACCGAATCCCGCCTGTCCAAATACGCCGAGCTGCTGTTAAGCGAACTCGGTCAGGGGACG
GCGGACTGGGTGCCAAACTTCGACGGCACCATGCAGGAACCGAAAATGTTACCGGCGCGT
CTGCCGAACATCCTGCTGAACGGCACCACCGGTATTGCGGTGGGCATGGCAACAGATATT
CCGCCGCATAACCTGCGCGAAGTGGCGAAAGCGGCGATTACGCTGATTGAGCAGCCGAAA
ACGACGCTGGATCAGTTGCTGGATATCGTCCAGGGGCCGGATTACCCGACCGAAGCGGAG
ATCATTACCCCTCGTGCGGAAATTCGTAAAATTTACGAAAATGGGCGTGGCTCCGTGCGT
ATGCGCGCGGTATGGACCAAAGAAGACGGCGCCGTGGTGATCACCGCGTTGCCGCATCAG
GTATCTGGCGCAAAAGTGCTGGAGCAGATTGCTGCGCAGATGCGCAATAAAAAACTGCCG
ATGGTGGACGACCTGCGCGATGAATCGGATCACGAAAACCCGACGCGTTTAGTGATTGTC
CCGCGCTCCAACCGTGTGGATATGGAGCAGGTGATGAACCATCTGTTCGCCACCACCGAT

CTGGAAAAAAGCTACCGTATTAACCTGAACATGATCGGTCTGGATGGTCGTCCGGCGGTG
AAAAACCTGCTGGAGATCCTCACCGAGTGGCTGGCGTTCCGCCGCGACACGGTGCGCCGT
CGTCTGAACTATCGTCTGGAGAAAGTGCTTAAGCGCCTGCATATCCTCGAAGGTTTGCTG
GTGGCGTTTCTCAACATCGATGAAGTGATCGAGATTATCCGTAACGAGGATGAGCCGAAA
CCCGCGCTGATGTCGCGTTTCGGCATCAGCGAAACCCAGGCGGAAGCGATTCTCGAACTG
AAACTGCGTCATCTCGCCAAACTGGAAGAGATGAAAATTCGCGGCGAGCAGGACGAGCTG
GAAAAAGAGCGTGACCAGTTGCAGGGCATTCTCGCGTCCGAACGCAAAATGAATACCTTG
CTGAAAAAAGAGCTACAGGCAGATTCCGATGCCTATGGCGACGATCGCCGTTCTCCGCTG
CGTGAGCGCGAAGAAGCTAAAGCGATGAGCGAACACGACATGCTGCCGTCCGAACCGGTG
ACTATCGTGCTGTCGCAGATGGGCTGGGTGCGCAGCGCCAAAGGTCATGATATTGATGCG
CCGGGGCTTAACTATAAAGCGGGCGACAGCTTTAAAGCCGCGGTGAAAGGTAAGAGTAAT
CAACCGGTGGTGTTTATTGATACCACCGGGCGCAGCTATGCTATTGATCCCATTACGCTT
CCGTCGGCGCGTGGGCAGGGCGAGCCGCTGACCGGCAAACTCACGCTGCCGCCGGGGGCG
ACCGTAGAGCATATGCTGATGGAAGGCGATGACCAGAAACTGCTGATGGCGTCGGATGCG
GGCTACGGCTTCGTTTGTACGTTTAACGATCTGGTTGCCCGTAACCGTGCCGGTAAGGCG
TTGATTACACTGCCGGAAAATGCGCACGTCATGCCGCCGCTGGTGATTGAAGACGAGCAC
GATATGCTGCTGGCGATTACCCAGGCCGGACGGATGTTGATGTTCCCGGTAGACTCTCTG
CCGCAGCTGTCGAAAGGCAAAGGGAATAAGATCATTAATATCCCCTCTGCAGAAGCGGCG
AAAGGCGATGATGGACTGGCGCACCTGTACGTGCTGCCGCCACAAAGCACTCTGACTATC
CATGTCGGGAAGCGCAAAATCAAACTGCGCCCTGAAGAGTTACAAAAGGTAGTCGGTGAA
CGCGGACGCCGTGGCACATTAATGCGCGGCCTGCAGCGTATCGATCGCATTGAGATTGAT
TCGCCGCATCGCGTAAGTCATGGCGACAGCGAAGAGTAA
>EECEBICD_00276 1-acyl-sn-glycerol-3-phosphate acyltransferase
ATGCTATATATTTTTCGTCTTATTGTTACCGTTATTTACTCCATTCTGGTCTGTGTTTTC
GGTTCTATTTACTGCCTTTTCAGCCCACGTAACCCAAAACACGTGGCGACGTTTGGACAT
ATGTTCGGTCGTCTGGCCCCGCTGTTTGGCCTGAAAGTGGAGTGCCGCAAACCCGCCGAC
GCGGAAAACTATGGCAATGCTATCTATATTGCTAACCACCAGAATAATTATGATATGGTC
ACGGCGGCGAATATTGTGCAGCCTCCAACGGTGACGGTGGGGAAAAAAGCCTGCTTTGGA
TCCCGTTTTTTGGGCAACTGTACTGGCTGA
>EECEBICD_00277 1-acyl-sn-glycerol-3-phosphate acyltransferase
TTGATTGACCGTAATAACCGCGCCAAAGCGCATAGCACCATTGCGGCAGTGGTTAACCAT
TTTAAAAAGCGCCGTATTTCTATCTGGATGTTCCCTGAAGGTACGCGTAGCCGGGGACGC
GGTCTGCTGCCGTTTAAAACCGGCGCGTTTCATGCGGCTATCGCGGCTGGTGTGCCGATC
ATTCCTGTCTGCGTTTCCAACACCTCTAACAAAGTCAATCTAAACCGCCTGAACAACGGA
CTGGTTATTGTGGAAATGCTGCCGCCAGTCGATGTCAGCGAATATGGTAAAGATCAGGTT
CGCGAGCTGGCGGCCCACTGCCGTGCGTTAATGGAGCAAAAAATCGCGGAACTTGATAAA
GAGGTTGCGGAACGTGAAGCGACCGGTAAGGTTTAA
>EECEBICD_00278 Cell division protein FtsP
ATGTCATTCAGTCGGCGTCAGTTTCTTCAGGCATCGGGAATCGCACTATGTGCAGGCGCG
ATTCCCCTCAGGGCGAATGCTGCCGGTCAGCAACAGCCGTTGCCTGTTCCGCCGTTGCTG
GAGTCCCGTCGCGGGCAGCCGTTGTTTATGACGCTACAGCGAGCGCACTGGTCCTTTACG
CAAGGCACGCGCGCGCCGGTCTGGGGAGTGAACGGTCGTTATCTGGGGCCGACAATCCGC
GTCTGGAAAGGCGATGACGTTAAACTCATCTATAGCAACCGCCTGGCGGAAAATGTCTCC
ATGACGGTTGCTGGTTTACTGGTGCCGGGGCCGCTGATGGGTGGCCCGGCGCGTATGATG
TCGCCGAACGCGGACTGGGCGCCGGTGTTGCCGATTCGTCAGAGCGCCGCGACGCTGTGG
TATCACGCTAACACGCCTAACCGCACTGCGCAGCAGGTGTATAACGGCCTGGCCGGAATG
TGGCTGGTCGAAGACGACATTAGTAAAACGCTGCCCATTCCGAACCATTACGGCGTGGAT
GATTTTCCCGTCATTATCCAGGATAAACGGCTGGATAACTTTGGGACGCCGGAGTACAGC
GAACCGGGAAGCGGTGGTTTTGTCGGCGATACGCTGCTGGTGAACGGCGCGCAAAGCCCC
TATGTCGAAGTGTCCCGCGGCTGGGTGCGGTTACGGTTGTTGAACGCCTCGAACTCGCGG
CGCTATCAGCTTCAGATGAGCGATGGCCGGGCGCTGCATGTGATTTCAGGCGATCAGGGC
TTTTTACCTGCGCCGGTTTCCGTCAAACAGTTGTCACTGGCGCCCGGCGAGCGACGTGAA
ATCCTGGTTGATATGACCAACGGCGACGAAGTCTCGATCACCTGCGGGGAAGCGGCCAGC
ATAGTCGATCGGATTCGCGGTTTCTTTGAGCCTTCCAGTATTCTGGTTTCGACCCTGGTG

CTGACGTTACGGCCAACCGGATTGCTGCCGCTGGTGACCGATAATCTGCCGATGCGTCTG
TTGCCGACGGAGATCATGAGCGGCGCGCCTGTCCGCAGTCGGGACATCAGTCTTGGCGAC
GATCCGGGGATTAACGGCCAACTGTGGGATGTGAATCGCATTGATATTACCGCCCAACAA
GGCACCTGGGAGCGCTGGACGGTACGCGCCGATATGCCGCAGTCGTTCCATATTGAGGGC
GTCTCTTTCCTGATTCGTAACGTCAACGGCGCTATGCCGTTCCCGGAAGATAGAGGCTGG
AAAGATACCGTCTGGGTGGATGGGCAAGTCGAATTGCTGGTCTATTACGGTCAGCCGTCC
TGGCCGCACTTCCCGTTCTATTTCAACAGCCAGACGCTGGAAATGGCTGACCGCGGCTCG
ATTGGGCAAATGCTGGTGAATCCGGCGTCGTAA
>EECEBICD_00279 C4-dicarboxylate TRAP transporter large permease protein
DctM
ATGGACTTTGAATATATTTACCCAATAATTGTTTTATTTGGCAGCTTTGCCATTATGCTG
GCGGTCGGCGTTCCTATTACATTCGCTATCGGGCTGTCGTCCTTATTCTCAATCCTGATA
GCCCTTCCCCCGGATGCGGCAATCTCGGTTATTTCGCAAAAAATGACCGTCGGTCTCGAT
GGCTTTACGCTGCTGGCGATCCCCTTTTTTGTGTTAGCCGGCAATATCATGAATACCGGG
GGAATCGCCCGGCGCCTGGTGAATCTGGCTCAGGCGTTAGTTGGCCGCCTTCCCGGTTCG
CTGGCGCACTGCAATATTCTGGCGAACACCCTGTTTGGCGCGATATCCGGCTCGGCTGTC
GCTTCTGCGGCCGCGGTGGGCGGCATCATGTCGCCATTACAGGAAAAAGAGGGCTACTCT
CCGGCGTTCTCTACGGCGATCAACGTCGCTTCGGCGCCGATTGGCCTGATGATCCCGCCC
AGCAATGTGCTAATTGTCTATTCGCTGGCTAGCGGCGGCACCTCTGTCGCCGCCCTGTTT
TTGGCGGGCTACCTGCCCGGCATCTTAACCGCGCTGGCTTTAATGACGGTAGCGGCGCTG
TATGCCCATCGCCACCATTACCCGGTTGCCGAACGCATCAACCTGCGGCAGTTTCTGTCG
GTATTCCGCGATTCATTGCCCAGCCTGTTGCTGATATTTATTATTATCGGCGGCATTATC
GGCGGCGTATTTACGCCAACAGAGGCCTCTGCGATCGCGGTAATCTATAGCCTGGCGCTG
GCGGCCATTTATCGAGAAATTAATGTGAGTAAGTTGCGCGATATTCTGCTGGATTCGGTA
GTAACCAGCTCTATTGTTCTCTTACTGGTCGGCTGCTCAATGGGAATGTCATGGGCGATG
ACCAATGCTGATGTTCCTGAACTGATTAATGAAATGATTACCAGCGTGTCCGAAAATAAA
TGGGTCATCTTGCTGATTATCAATCTCATTTTGTTACTCGTCGGGACGTTTATGGATATT
ACCCCGGCTATTCTGATCTTTACGCCTATTTTCCTTCCCATTGCTCAACATCTGGGAATC
GACCCTGTCCATTTTGGCATTATTATGGTTTTCAATTTAACCATCGGACTGTGTACGCCC
CCGGTAGGCACTATTTTGTTCGTCGGTTGCAGTATCGGCAAAATCAGTATTGATAAAGTC
GTCAAACCGCTATTACCGATGTTTCTGGCACTCTTTGTGGTCATGGCGATGATTTGCTAC
TTCCCGCAGTTGAGTCTGCTGCTTCCCACGCTGTTTGCTCCTTCCTGA
>EECEBICD_00280 hypothetical protein
ATGCGAATACTCACCAATGCACTGAACAAAATACTGGCCGGGTGCTGCTGTATTATTCTG
GCGATCATGGTTGCCTGCGTATCCTGGCAGGTGGCGGCGCGCTTTATATTCAACGCGCCA
AGCAGCACGCTCGACGAATTCACCCAGATTCTTTTTATGTGGATGATTCTGTTAGGCGGC
GTCTATACCGCCGGATTAAAAAAACATTTAGCTATCGATTTGTTAGCGCAAAAACTCTCC
CGAACGCCCGCCCTCGTGCTCGACAGTATCATTCAGGTGATTATTACCGTGTTCGCGCTG
ATTTTTATGGTGTACGGCGGTGATATTATTGTTGAAAAAGCGGCGCACGTCAGCCAAATG
TCCCCGGTATTAAAATGGCCTATGGATAAAGTCTATTGGGTCATGCCGATTAGCGGCGTC
ATCCTGGTGTACTACACCATTGTGAACGTTATTGATAATTATCATCAGCGCCATCTTCGC
TAA
>EECEBICD_00281 Solute-binding protein
ATGAAAAACACACGTTCATTCACAACATCAGCGGTATTACTGGCCGGCTGTTTGCTACTG
GCATTTCCAGCGCTCGCCAAAACCACGCTGAAACTGAGCCACAATCAGGATAAAAGCCAC
GCCGTTCACAAAGCGATGAGCTATCTGGCCGATAAAGCGAAAGCCTATTCGGACGGCGAA
TTAAATATTCGTATTTACCCCAACGCCACGCTGGGCAACGAACGTGAATCGCTGGAATTG
ATGAACTCCGGCGCTCTGCAAATGGTGAAAGTCAATGCGGCATCGCTGGAGTCTTTTGCG
CCGGAATATAGCGTGTTTAGCCTGCCGTTTTTATTCCGCGACCGCGATCACTACTACAAC
GTACTGAAAAGCGACTTAGGGAAACGCATTCTCGCGTCCTCCGAAAGCAAAGGCTTCGTC
GGCTTAACCTGGTACGACGGCGGCGCCCGCAGTTTTTACGCTGGTAAGCCCATCACTCAA
CCCGACGATTTAGCCGGTATGAAAATCAGAGTGCAGCAAAGCCCCAGCGCTATCGCGATG
GTGAAAGCGCTCGGCGGCGTGCCGACGCCGATGGCGCAAGGCGAACTCTATACCGCGCTC
CAGCAAGGCGTGGTCGATGGCGGCGAAAACAACCCCGTGGTTTATGCCGATATGCGTCAT

GCGGAGGTGGCGAAATTCTATTCCCGCGACGAGCACACGATGGTGCCGGATGTCCTGGTC
ATCAGTACCAAAGTACTTAACAAATTGAGCGATAAAGAGCGGAAAGCGTTATATAAAGCC
GCAGATGAATCCATGCAGCAAATGAAAGACGTCATCTGGCCCGCCGCGGAAAAAGAGGCT
TATGAGAGCATGAAGGCCATGAATGCGACCGTTGTTGATATTGATAAATCCGCGTTCAAA
CAGCGTGTTAAGCCCTTGTTTGATGAGTTCCGCGCGAAAGATGCTCAATCAGCGAAGGAT
CTGGAATACATCGAGAATATGTAA
>EECEBICD_00282 hypothetical protein
ATGAGCGCTATCTCCCTGATCCAACCGGATCGCGACCTTTTCTCCTGGCCGCAGTACTGG
GCCGCCTGCTTTGGGCCGGCGCCGTTTTTGCCGATGTCACGTGACGAGATGGATCAACTT
GGCTGGGATAGCTGCGACATTATTCTGGTAACCGGCGACGCCTACGTCGACCATCCGAGC
TTCGGCATGGCGATTTGCGGTCGTATGCTGGAAGCGCAGGGCTTCCGCGTGGGGATCATC
GCCCAGCCTGACTGGAACAGCAAAGACGACTTTATGCGTCTGGGAAAACCGAACCTGTTC
TTCGGCGTCACCGCCGGCAACATGGACTCGATGATCAACCGTTACACCGCCGACCGTAAG
CTGCGCCATGACGACGCCTACACCCCGGATAACGTCGCGGGCAAACGCCCGGACCGCGCC
ACGCTGGTCTATACCCAGCGTTGCAAAGAAGCGTGGAAAGATGTCCCGGTGATCCTTGGC
GGCATTGAGGCCAGTCTGCGCCGTACCGCGCATTACGACTACTGGTCTGATACTGTGCGT
CGTTCGGTGTTAGTCGATTCCAAAGCCGACATGCTGATGTTTGGCAACGGCGAGCGTCCG
CTGGTGGAAGTGGCGCACCGTCTGGCAATGGGCGAAACCATCGATCAGATTCGCGACGTG
CGTAACACTGCGATTATGGTTAAAGAAGCGCTGCCTGGCTGGAGCGGCGTCGACTCCACT
CGTCTGGACACGCCGGGTAAAATCGATCCAATTCCCCATCCCTATGGTGAGGATCTGCCG
TGCGCGGATAACAAGCCGGTCGCGCCGAAAAAGCAGGAAGCTAAAGCGATTACCGTGCAG
CCGCCGCGTCCGAAGCCGTGGGAAAAAACCTATATCCTGCTGCCATCCTTTGAAAAGGTG
AAAGGCGACAAGGTGCTGTACGCCCATGCGTCGCGTATTCTGCATCACGAAACTAACCCT
GGCTGCGCGCGGGCGCTGATGCAAAAACACGGCGACCGCTATGTCTGGATCAACCCGCCC
GCCATACCGCTCTCCACTGAAGAGATGGATAGCGTGTTTGCGCTGCCGTATCAGCGTGTG
CCGCACCCGGCTTACGGCAATGCCCGTATTCCGGCCTATGAGATGATCCGCTTTTCGATC
AATATCATGCGCGGCTGCTTTGGCGGCTGCTCTTTCTGCTCGATCACTGAACATGAAGGG
CGCATCATTCAGAGCCGCTCGGAAGACTCTATTATCAACGAGATCGAAGCGATCCGCGAC
ACGGTGCCGGGGTTTACCGGCGTGATCTCCGATCTGGGCGGTCCTACCGCGAACATGTAT
ATGCTGCGCTGTAAGTCGCCGCGTGCGGAGCAGACCTGTCGTCGCCTGTCATGCGTCTAT
CCGGATATCTGTCCGCATATGGATACTGACCATACGCCGACGATCAATCTTTATCGCCGC
GCCCGTGAGCTGAAAGGCATTAAGAAAATCTTAATTGCCTCCGGCGTGCGCTACGACATC
GCCGTAGAAGATCCTCGCTACATTAAAGAGCTGGCGAGCCATCATGTGGGCGGCTATCTG
AAGATTGCGCCGGAGCATACTGAAGAAGGTCCGCTGTCGAAAATGATGAAGCCGGGTATG
GGCAGCTACGATCGCTTTAAAGAGTTATTTGACCTTTACTCTAAACAGGCCGGAAAAGAG
CAGTATCTGATCCCGTATTTTATCTCCGCGCATCCGGGCACGCGGGACGAGGATATGGTG
AATCTGGCGCTGTGGCTCAAGCGCCACCGCTTCCGTTTGGATCAGGTGCAGAACTTCTAC
CCGTCGCCGTTGGCGAATTCGACCACCATGTATTACACCGGCAAAAACCCGCTGGGTAAA
ATTGGTTATAAGAGTGAAGATGTGGTGGTGCCGAAGGGAGACAGGCAACGTCGCTTGCAT
AAGGCGCTGCTGCGTTATCACGATCCGGCGAACTGGCCGCTGATTCGCCAGGCGCTGGAA
GCGATGGGGAAAAAGCATTTGATTGGCGGTCGCCGTGAGTGTCTGGTGCCTGCGCCGACC
ATTGAAGAGATGCGCGAAGCGCGTCGCCAGAACCGCAATACCCGCCCGGCGTTGACCAAG
CATACGCCGGTGGAACATCAGCGGCAGGGGCTTGCCGCTAATAAGAAGCGCGGGAAAGGC
GCAGGGCGTTAA
>EECEBICD_00283 hypothetical protein
ATGGGTATGATTATTATTCCCTGTTTTATCGTAATTGCTATAATAGCTGTTGTTATATTC
ATAATGGCTATTAAAGCATGTAGTATGGAAAAACGTGAAGATAGGCAAATGACTCTTGCA
GGGATAATTTTAATGGGTGTTATTGCCGCTATTCCTGGATGGTTTTTGTACGAAATATTT
AGCCACGCGCCGTAA
>EECEBICD_00284 hypothetical protein
ATGACCTGCATTTTCTGTCAAATAGTTGAAGGTAAAGCACCTTGTCATAAAGTTTGGGAA
GATGAGCATCACCTGGCTTTTTTGTCGATTTTTCCGAATACCGATGGGTTTACCGTAGTG
ATCCCGAAAAAACATTACCCCAGCTACGCGTTCGATCTACCGCCGCAGGCGTTAGCGGAC
CTGATGCTGGCGACCCAAAAAGTGGCTAAGAAACTGGATAAGGCTTTCCCGGATGTCAGT

CGTACGGGAATGTTCTTCGAAGGTTTCGGCGTAGACCATGTGCACAGTAAATTAAGCCCC
ATGCACGGTACTGGCGATTTAACCCACTGGAAACCGATTGAATCGCGGCAGAATAAATTT
TTCGAACAGTATGAAGGATATCTGTCGTCACACGATCATGAACGGGCTGATGATGAAAAA
CTGGCCGCGCTGGCGGCCAGAATTCGTGAGGCATAA
>EECEBICD_00285 Putative malate transporter YflS
ATGTGTAATTGTGGCGGAAAAAAATCTCAGGGCGTCAAATGGCTCCCATTTATTCTTATT
CTGGTTATCGCCGCCGGACTGTGGCAGCTAACGCCCCCTTCCGGGTTAAGCGCGCCGGCC
TGGCATTCCGCGATTATTTTTGTCGCGACGATCGCCTCTATCGTGGCGAAAGTGCTGCCT
ATTGGCGCAGTCGGTATTATCGGCATCACCGTCTTTGCGCTCGCCTATGCTGCCGGCGAC
AAAACGGCCAGCGGCGCGATTACCACGGCCTTGAGCGAGCTGAACAGTTCGCTGATCTGG
CTTATCGTCGTCGCCTTTATGATTGCCCGTGGGTTTATCAAAACCGGGCTGGGTCGGCGT
ATCGCCCTACAAATGATCCGTCTGTTAGGTAAACGAACGCTGGGTCTGGCTTATGGTCTG
GCGTTTGCCGATCTGATCCTCTCCCCCGCGATGCCCAGTAACACCGCGCGCTGCGGCGGC
GTGATTTACCCGATCGCCGACTCGCTGGCGCGCAGCTTTGATTCACATCCGGAAGATGAG
TCTCGCAGCAAAATCGGCACCTTTCTGATCACCTGTATTGGCAATGTTAACGACGTCACC
GCCGCACTGTTTATGACCGGTTATACGGGTAACCTGCTGGCGGTAAAACTGGCGGCGAAC
GCCGGCGTTACGTTGAGCTGGGGAAGTTGGTTTATCGCCGCGCTCCTGCCTTGCCTGGTC
TCTTTTCTTATCGTCCCGCTGCTGGTCTACTGGCTGACGCGTCCGGAAATTAAACATACG
CCGGATGCGCCGGATTTGGCGCGCAAAGAGCTGGCGCAAATGGGCAGTATGACGCGCGGC
GAGTGGCTCATGCTGGCGACCGTCGGCGTGTTGCTGGTGCTATGGATTTTTGGCAGCTCG
CTCGGCGTGGATGCCACGACGGCCTCTTTTGTCGGTCTCTCTATTTTATTACTGAGCGGC
GTGCTCACCTGGGAAGACGTTAAAAGCGAAAAAGGCGCCTGGGATACGTTGATTTGGTTT
GCCGCGCTGCTGATGATGGCTAACCAGTTGAAGAAACTAGGATTCACCAGCTGGTTTGGC
AACCTGATTGGCGACAGCATCGGCAGCACGATGCATGGAACCAGTTGGATAATCATCCTG
CTGCTGCTTAACGCAGCCTATTTTTATACCCACTATTTTTTCGCCAGCGGCAACGCGCAA
ATCGCCGCGCTGTATGCGGTATTTCTGGGCGTCGGTCTGCATCTGAATATTCCGGCGGCG
CCGATGGCGCTCATGCTGGCGTTTACCAGCAGCCTGTACTGCTCTCTTACGCAGTATACC
CACGCACGCGGCCCCATTCTTTTTGGCGCCGGCTATGTACCGACGGGCGTCTGGTGGCGA
ACGGGATTTATTATCAGTCTCTTTAATCAGGCGGTCTTTTTGACAGTCGGCCTCGCATGG
TGGAAAGTGTTAGGGTTGTATTAA
>EECEBICD_00286 2,5-diketo-D-gluconic acid reductase A
ATGGCTAATCCAACCATTATCAGGCTACAGGATGGCAACGTAATGCCACAACTTGGGCTG
GGAGTCTGGAAAGCAAGCAATGAAGAGGTGATCGCGGCGATTCATAAAGCGCTGGAGGTG
GGCTATCGATCGATTGATACCGCCACAGCATATCAGAATGAAGAAGGCGTCGGCAAAGCG
CTAAAAGCAGCGAGCGTGGCGCGGGAAGAGCTGTTTATCACTACCAAGTTGTGGAATGAC
GATCAAAAACGTCCCCGCGAAGCCCTGCAGGAGAGTCTGAAAAAACTGCAGCTTGATTAT
CTCGATCTCTATCTGATGCACTGGCCCGTACCGGCTATCGATCACTATGTCGACGCCTGG
AAAGGCATGATCGCGCTGCAAAAAGAGGGGCTGGTAAAGAGTATCGGGGTCTGTAACTTT
CAGATCCATCACCTCCAGCGTCTGATCGATGAAACCGGCGTTACCCCTGTGATCAACCAG
ATCGAGCTTCACCCCTTGATGCAACAGCGCCAGCTACATGCGTGGAATGCGACGCATAAA
ATCCAGACCGAGTCCTGGAGCCCGCTGGCGCAGGGTGGCGAAGGCGTATTCGATCAGAAA
GTGATTCGCGAACTGGCGGATAAATACGGCAAAACGCCCGCCCAGATTGTTATTCGCTGG
CATCTGGACTGCGGGCTGGTCGTCATTCCGAAATCCGTCACCCCTTCCCGTATCGCGGAA
AACTTTGCTGTCTGGGATTTCCGTCTCGATAAAGATGAATTAGGCGAGATCGCAAAACTG
GATCAAGGAAAACGCCTCGGTCCCGATCCGGATCAGTTCGGCGGTTAA
>EECEBICD_00287 Alcohol dehydrogenase YqhD
ATGAACAATTTCAAATTACATACCCCTACCCGCATTCTGTTTGGTAAAGGCGCTATCGTC
GATCTTCGCGATCAAATCCCGCAGGACGCCCGCGTGCTGATTACCTATGGCGGCGGTAGC
GTGAAAAAGACCGGCGTACTGGCGCAAGTTCAGGAAGCGTTAAAAGGACTGGATGTTCGG
GAGTTTGGCGGTATTGAACCGAACCCGTCCTATGAAACGCTGATGAAGGCCGTGCAGCTT
GTGCGCGACGAAAATATTACCTTCCTGCTGGCCGTTGGCGGCGGTTCCGTACTGGACGGC
ACCAAATTTATCGCCGCAGCGGCGCAGTATACGGACGGCGTCGATCCGTGGCACATTCTG
GAAACCGGCGGTACTGAGATTCGTAGCGCAATCCCGATGGGGTCTGTGCTGACCCTACCG
GCAACCGGCTCAGAATCGAACGCCGGCGCAGTCATTTCCCGTAAAACCACTGGCGACAAA

CAGGCCTTCCACTCCTCGTTCGTGCAACCGGTGTTTGCCGTACTGGACCCGGTCTACACC
TATACATTGCCGCCGCGCCAGGTTGCGAACGGCGTCGTGGATGCTTTCGTCCATACCGTT
GAGCAGTACGTCACTTACCCGGTTAACGGCAAAATTCAGGATCGTTTCGCCGAAGGTATT
TTACTCACACTGATCGAAGAAGGTCCCAAAGCGCTGCAAGAGCCTGAAAATTATGACGTC
CGCGCTAACGTGATGTGGGCCGCTACTCAGGCGTTGAATGGCCTGATTGGCGCTGGTGTT
CCGCAGGACTGGGCGACACATATGCTGGGCCATGAGCTTACCGCGATGCACGGTCTCGAT
CATGCCCAGACGCTGGCTATCATTCTGCCTGCGCTATGGAACGAAAAACGAGACGTTAAA
CGTGCAAAACTCCTGCAATATGCTGAACGTGTATGGAATATCACCGACGGTTCCGACGAC
GAGCGTATTGATGCCGCCATTGCCGCCACCCGCCGTTTCTTTGAACAGATGGGCGTGCCT
ACCCGTCTGTCCGATTACGGTCTGGATGGCAGCACTATTCCGGCGCTACTGGCTAAACTT
GAGGCGCATGGATGCACAAATTTAGGTGAAAATCAGGATATTACGCTGGATGTCAGCCGT
CGGATTTATGAAGCGGCGCGCTAA
>EECEBICD_00288 HTH-type transcriptional activator RhaS
ATGAAAAAATGGCGCGTTATGAATCGTGAAGCAATTTGCTTACAGTTGGCGGATAAAATT
AATCATTTAAAAAATAATGATAAAATAATCAGCGAGCGGCTGGCGGGCATTCGTCTGTTG
TATGGTGTTGAACCGGGGCCGCGCACGCCGGTCATGTATCAGCCTGGCATCATATTTCTC
TTTTCCGGCCATAAAATTGGCTATATTAACAAGCGCAAGTTTCGTTATGACGCTAATGAA
TATCTCTTACTGACCGTGCCGCTCCCTTTTGAATGCGAGACCTGGGCAACGCCGGAGGTA
CCACTGGCCGGTATTCGGCTCGACATTGATGTGCTGCAACTGCAAGAGTTGCTCATGGAC
ATTGGCGAAGATGAACGTTTCCAGTTGCCGATGGCGGCCAGTGGGATTAACTCCGCCACG
CTGTCGGACGAGATTTTATGCGCGGTGGAAAGATTGCTGGATGTGATGGAGCGCCCGCTG
GATGCGCGGATTCTGGGAAAACAAATTATTCGCGAAATTCTTTATCATGTGCTGATGGGA
CCGCGCGGCGGGGCGCTGCTTGCACTGGTTAGTCGCCAGACGCACTTTAGCCTGATCAGT
CGCGTACTTAAACAGATCGAAATGAAATACACCGAGAACCTGAATGTTGAGCAACTGGCG
GCAGAAGCGAACATGAGCGTGTCGGCGTTTCACCATAATTTTAAGGCGGTCACCAGCACC
TCGCCGTTGCAGTATTTAAAAAGCTACCGACTGCATAAAGCGCGGATGATGATGATTCAC
GACGGTATGAAGGCCAGCGCCGCGGCGATGCGGGTCGGCTATGAGAGCGCATCGCAGTTT
AGCCGGGAGTTTAAACGTTATTTTGGCGTTACGCCGGGCGAAGATGCGGCAAGAATGCGG
ACAATGCAAGGGAGCTAA
>EECEBICD_00289 Inner membrane protein YghB
ATGGCTGTCATTCAAGATATTATCGCTGCGCTCTGGCAACATGATTTTGCCGCGCTGGCG
AATCCACACGTTGTTAGCGTCGTCTACTTTGTCATGTTCGCCACGCTATTTTTAGAAAAC
GGTCTGCTGCCAGCATCATTCTTACCTGGCGATAGTCTGCTGCTACTGGCAGGCGCGTTG
ATCGCCCAGGATGTGATGCATTTTTTGCCGACGATTGGCATTCTCACCACCGCGGCCAGT
CTCGGCTGCTGGCTAAGTTATATCCAGGGACGCTGGCTCGGTAATACGCGTACCGTCAAA
GGCTGGCTGGCGCAGCTTCCGGCAAAATATCATCAGCGCGCCACCTGCATGTTTGACCGC
CACGGTTTGTTAGCGTTGCTTGCCGGGCGCTTTCTGGCGTTTGTCCGCACTCTGCTGCCA
ACAATGGCGGGTATTTCCGGCCTGTCCAACCGCCGGTTTCAGTTTTTTAACTGGTTAAGC
GGCCTGCTGTGGGTGACTGTCGTGACCAGCTTCGGCTACGCGCTCAGTATGATCCCGTTT
GTTAAGCGCCATGAAGACCAGGTAATGACATTTTTAATGATCCTTCCCGTCGCGCTACTG
GTCGCCGGACTGTTAGGTACGCTGGTCGTGGTGATTAAAAAGAAATACTGTAACGCCTGA
>EECEBICD_00290 Cystathionine beta-lyase MetC
ATGACGGATAAACAGTTGGATACCAAACTGGTAAACGCAGGACGCAGCAAAAAATACACG
CTTGGCTCAGTGAATAGTGTGATTCAACGCGCTTCTTCACTGGTGTTTGACACCGTCGAG
GCCAAAAAACACGCTACCCGCAATCGCGCCAACGGCGAATTATTTTACGGACGCCGGGGA
ACGCTGACCCACTTTTCGCTCCAGGAAGCCATGTGTGAACTGGAAGGCGGCGCCGGATGC
GCGCTCTTCCCGTGCGGCACGGCGGCGGTCGCCAACACCATTCTGGCATTCGTTGAGCAG
GGCGACCACATCCTGATGACCAACACAGCCTATGAACCCAGCCAGGATTTCTGCAGCAAA
ATTCTCGGCAAACTGGGCGTGACGACAAGCTGGTTCGACCCGCTCATCGGCGCTGATATC
ACGCAACATATTCAACCGAACACGAAAGTCGTATTTCTGGAATCCCCCGGTTCTATTACC
ATGGAAGTACATGATATTCCGAGCATTGTTAGTGCGGTCAGACGCGTCGCGCCAGAAGCC
GTCATCATGATTGATAATACCTGGGCGGCTGGCGTTCTGTTCAAAGCTCTGGAATTCGAT
ATTGATATCTCTATCCAGGCGGCACTAAGTATCTGATTGGTCATTCTGATGCGATGGTC
GGTACTGCCGTTGCAAATGCGCGCTGCTGGGAACAATTGCGGGAAAACGCCTATCTGATG

GGGCAAATGCTCGACGCCGACACGGCTTATATGACCAGCCGCGGTCTGCGCACACTCGGC
GTGCGACTGCGCCAGCATCAAGAAAGCAGCCTCAAAATCGCCGCATGGCTGGCGAACCAT
CCTCAGGTCGCGCGCGTTAATCATCCGGCCCTGCCGGGTAGCAAAGGTCATGCGTTCTGG
AAACGAGACTTTACAGGCAGCAGCGGCCTGTTCTCCTTTGTGCTCAATAAAAAGCTGACC
GAGGCAGAATTATCAGCCTATCTGGATAACTTTTCGCTGTTCAGTATGGCCTACTCCTGG
GGAGGCTACGAATCGCTGATTATTGCTAATCAGCCGGAACAGATCGCCGCGATTCGTCCC
GCAGGCGGCGTAGATTTTACCGGTACGCTGGTCCGGGTGCATATTGGTTTAGAGAGTGTT
GATGATTTGATCGCTGATTTAGCCGCCGGCTTCGCCAGAATTGTGTAA
>EECEBICD_00291 hypothetical protein
ATGCGTAAGCGCAAAGAAAAAGCAGTAAAGGTGCGGCAGTATGTAAATAGTAATGAGAAC
GACTATCAATTCGACGTTGTTTTGATATTATTATGCTCAGATTTTGTGATTTGCGTCCTG
GAGATACAGAGTGGGTAA
>EECEBICD_00292 Biopolymer transport protein ExbB
GTGGGTAATAATTTGATGCAGACGGATCTTTCCGTCTGGGGCATGTATCAGCACGCCGAT
ATTGTCGTGAAGTGCGTGATGATTGGTTTGATTCTGGCGTCAGTCGTCACCTGGGCTATC
TTCTTCAGTAAAAGCGTTGAATTTTTCACGCAAAAGCGCCGCTTAAAGCGCGAACAACTG
CAATTAGCCGATGCGCGCTCTTTAGATCAGGCTTCCGACATTGCGGCAGGTTTCAGCGCA
AAAAGCCTCAGCGCACAGCTCATCAATGAAGCGCAGAATGAACTGGAACTCTCTCAGGGC
AGCGAAGATAACGAAGGGATTAAAGAACGTACCGGCTTCCGTCTGGAACGTCGCGTCGCA
GCGGTTGGCCGCTACATGGGACGAGGCAACGGTTATCTGGCGACGATCGGCGCCATTTCC
CCCTTCGTCGGGTTGTTTGGCACCGTATGGGGCATTATGAATAGCTTCATCGGTATTGCG
CAAACGCAGACCACCAACCTGGCGGTGGTCGCTCCTGGTATTGCGGAAGCGCTGCTGGCG
ACGGCGATTGGGCTGGTAGCCGCGATCCCGGCGGTCGTCATTTACAACATCTTCGCACGT
CAGATAGGCAGTTACAAAGCGACGCTGGGTGACGTGGCGGCGCAGGTATTGCTGCTGCAA
AGCCGCGATCTTGATCTGAATGCGAGCGCCAGTGCGCAGCCGGTACGCGCCGCGCAGAAA
TTACGGGTAGGGTAA
>EECEBICD_00293 Biopolymer transport protein ExbD
ATGGCAATGCGTCTTAACGAGAACCTGGATGACAACGGCGAAATGCATGAAATCAACGTG
ACGCCGTTTATCGATGTCATGTTGGTTCTGCTGATTATCTTTATGGTGGCTGCGCCGCTG
GCGACGGTGGACGTCAAGGTGAATCTGCCCGCTTCTACCAGTACGCCACAGCCGCGTCCG
GAAAAACCGGTTTATCTGTCGGTGAAAGCGGATAACACCATGTTTATCGGTAACGATCCG
GTGACGGATGATACGATGATTGCCGAGCTAACGGCCCTGACGGAAGGGAAAAAGGATACT
ACGATTTTCTTCCGTGCGGATAAAACCGTAGAGTATGAAACCCTGATGAAAGTGATGGAT
ATGCTGCATCAGGCGGGCTATCTCAAGATTGGTCTGGTCGGCGAAGAGACCGCGAAAGCG
AAATAA
>EECEBICD_00294 putative oxidoreductase YghA
ATGTCTCATCTATACGACCCTACCACGCAGTATTACACCGGCGAATATCCTAAGCAAAAA
CAGCCGGCGCCCGGCGTCCAGGCGAAAATGACGCCTGTTCCTGACTGTGGCGAAAAAAGT
TATGTCGGCAGTGGTCGCCTTAAAGATCGTAAGGCGCTGGTGACAGGGGGCGACTCCGGG
ATTGGGCGCGCTGCCGCTATCGCTTACGCGCGTGAAGGCGCAGATGTCGCGATCAACTAT
CTGCCAGCGGAAGAAGAGGACGCCCAGCAGGTTAAAGCGCTGATTGAGGAGTGCGGTCGT
AAAGCGGTGTTATTGCCAGGCGATCTTAGCGACGAATCCTTCGCCCGTTCGCTGGTCCAT
AAGGCGCGTGAAGCGCTGGGCGGGCTGGACATTCTGGCGCTGGTAGCCGGTAAACAGACG
GCAATCCCGGAGATAAAAGATCTGACCAGCGAGCAATTTCAGCAAACCTTCGCCGTTAAC
GTTTTTGCCCTGTTCTGGATAACCCAGGAAGCGATTCCGCTGCTGCCAAAAGGCGCCAGT
ATTATCACGACGTCGTCTATCCAGGCGTATCAGCCCAGTCCGCACCTGCTGGATTATGCG
GCAACCAAGGCGGCAATCCTCAATTACAGCCGTGGCCTGGCGAAACAGGTGGCGGAAAAA
GGAATTCGTGTGAATATTGTCGCGCCTGGCCCTATCTGGACAGCGCTGCAAATTTCCGGC
GGTCAAACCCAGGATAAAATTCCGCAGTTTGGTCAGCAGACGCCAATGAAGCGGGCCGGC
CAACCGGCAGAGCTGGCGCCGGTGTATGTCTATCTGGCAAGCCAGGAATCCAGTTACGTT
ACCGCAGAAGTGCACGGCGTGTGCGGCGGGGAGCATTTAGGCTAA
>EECEBICD_00295 hypothetical protein
ATGAAAGAAGTTGAAGACAATAACGTTTATTTGGCTTTAGACAACCATAAAAGTGATGAA
TTTATCTTAAAGCAAAATCTTGCAGCGCTAATCGCCAGTAAAAATGCCACCCTGGAAAAG

GTAACGCAGGAGGTGGTATCCATACCGGCGGCGTTAGTGCGCCTGAAGTGGCAAAACCGA
CGAGAGATGTATACGTTACAGGTCAAAGAGGAGATTTATGGCGCGACCATCAATGCTATT
ATCGAGCAGCATCCTGAATTACGAGACAAAATTATGTCTCGTCTTGAAAGTGACTGGCAG
CATCTGCTGGCTCGCGAGACGGCAACCTTACGTCTGACGCGTAAGCTTTCCGATGGCGAC
TACCGGACGCGCAACGTCACCACCGTCGCACGGCAAGAAAAATAA
>EECEBICD_00296 hypothetical protein
ATGCTTCCTGAGCATCTGGTTTCCTCTCATTTTCGCCTGTCATCGCCGCCCCCTGAAGCC
ACAGAAAACAGTAAAAATTTGCCTCAGGGAAAGACAAAACTCAGCGATTACGAGCCCGAT
ATTTTAATTTGCGCTAACGCAACCGGGCAACACAGAAACGTATTATTTGAAGAACGCGAC
CGTCATATTAAAGAACGTCTGTATTGTGTGGCTAAAATTGAAACGTTTGCCCGCCTGATA
AACGCACTACAGGCAGAAGGCAATATTGATGCCCAAACACTAAGTAAAATACTGGCGGAT
AAAACCGCAATGATAAATGAAAAAGGTAATGCCATTTGGCTTAATTTAATCACTCGCGAA
ACCAATATGCCGCTATTTTATTCACTGGAAGATAAAGATGAAAGAAGTTGA
>EECEBICD_00297 hypothetical protein
ATGAACAGTATTTTTTATTCTGTTATTATATTGCTATTACTGACCGGCGCGATACTCTTC
CTGATGTGGGAGGTTAATAAAAAACGTCCTGGTGGAAAAATCGTAAATTTAAATCAAACT
GAACCCATGACAAAGGAAGAGGGCGAAGATCATTTCTCAGTATTAATGAATTCGATAACG
CCGGTATGGTACTGGCGCGTTAACCATGAATATATCGATTTCCTTCATGCAACGATAAAA
CGAATGACAATGACTGAGTTGAATGAAACGCCTGGATTATTTGACGCACAGCGTCGTTGC
AGCGACCTTAACTCTGCCGTTTATAAGTATTACGACAATATTAAGAAGCGTTGTCTGAAC
GGAGAAAAGGTGCCGTATTCGGATTTGGACGTCCTTAATTTACGCCAGTGTTTTCGCGAG
TTCAGTCTTGAAGCCTACCCGGCATTGGTCGCGCTGGTATGGCCGGAATATCAGCGCCCG
CAGGTTAACCCGGATGAGATTTAG
>EECEBICD_00298 hypothetical protein
ATGGAACGCTTTCTTGAAAATGTAATGTATGCGTCTCGCTGGCTGCTCGCGCCGGTGTAT
TTTGGCCTCTCTTTGGCGCTGATCGCTTTAGCGCTAAAATTTTTCCAGGAAATTCTACAC
GTTCTGCCGAACGTTTTTGCCCTGGCGGAGGCCGATCTGATTCTGGTGCTGTTATCGCTG
GTTGATATGACGCTGGTCGGCGGATTGCTGGTGATGGTCATGTTCTCCGGGTATGAGAAT
TTTGTCTCTCAGCTGGATATCTCTGCGGGCAAGGAGAAGCTGAACTGGCTGGGGAAAATG
GATGCGACGTCGCTAAAAAATAAGGTGGCGGCATCGATAGTCGCTATCTCTTCCATTCAC
CTGTTACGCGTGTTTATGGACGCGAAAAACGTCCCGGATAACAAATTAATGTGGTATGTC
ATCATCCATCTGACCTTTGTCCTGTCGGCATTCGTTATGGGCTATCTGGACAGATTGACG
CGGCATAACCATTGA
>EECEBICD_00299 hypothetical protein
ATGCGTTTGCTGCAAAACTTTACTATTCGTATGGTCATGCTGACGATACTGGGACTTTTT
TGTCTACTCTGGTCGGGGGTGGGGTTATACAGCGTCCATGCTTTATCCGAAGTTTCGGAA
GGAAATGATATTGACCGGCATCTTGTGCGGCAAATGACGGTGCTAAGCCAGGGTAACGAT
CAATATTTCCGCTTTGTTACCCGCCTTAGCCGCGCGATGGATGTAAAAATCGGCGGCGGC
ACGCCTGATTTTGCGCCCGCCCGGCAGTCGCTCGAGAATATGCGTCAGAAGCTGGAGGAG
ATGAAAGCGCTATCGCCAGGGCCGATGAACCCTGATATCTCAAGGGAGGTCTTGTCCAAC
TGGCAGGCCTTGCTGGAGAAAGGCGTCGTTCCTCAAATGCAACTGGTGCAGCAAGGATCG
CTAACCGCCTGGAGCGAACATGCCAGTACGGTCACGCCCGCACTCAGTCGCGCGTTCGGC
GCCAGCGCGGAACGGTTTAGTCATGAAGCTGGCGCCATGCTGGATAACACCCGTGTGATG
GTCGATGGTAAAACCTATACCATCCGCATCCTGCTTATCACGGCGGTGATTCTGGGCATT
GCTATCCTGATTTTTACCGATCGCTATCTGGTTGCAATGATGGTGAAACCGCTGGAGCGT
ATCCGTCAGCAGTTTCAGCGTATCGCGCAAGGCGATCTTAGCCAGCCGATTGAAGCGTTG
GGGCGTAACTGCGTAGGCCGGTTGGTGCCGCTACTGCGCGCGATGCAGGACAGTTTACGC
GAAGCCGTGAGTACGATCCGCGCCGGAAGCGACAATATCTGGCGCGGCGCGACGGAAATT
TCTACCGGTAATAACGATCTCTCTTCCCGAACGGAAGAACAGGCCGCCGCGCTGGAAGAA
ACTGCCGCCAGCATGGAACAGCTTACCGCAACGGTGAAAATGAACGCCGAACACGCTCGC
CAGGCGAGTCAGCTTGCGGATGCCGCCTCGCTAACGGCAGGAAAAGGCGGTGAACTGGTT
TCCGATGTGGTAGAAACAATGAACGGTATTTCCGCCAGTTCGCAGCAGATCGCGGAAATT
ACTACCGTAATTAATAGTATTGCTTTCCAGACCAATATTCTGGCCTTGAACGCAGCGGTA
GAAGCGGCCAGAGCGGGCGAGCAGGGACGCGGCTTCGCGGTGGTCGCCGGTGAGGTACGT

AACCTGGCCAGCCGCAGCGCGGGGGCGGCGAAAGAAATTGAAGCCTTAATTGGCGAATCG
GTACGTCGCGTAGCGCAGGGCGCGCAGTTGGTCCAGGAGACCGGCGCGACGATGGACGCC
ATCCTGCGCGGCGTCACGGAAGTGACGACGATTATGAAACAGATCGCGTCCGCCTCCGAA
GAACAGAGTAAAGGTATTTCTCAGGTTGGCGTGGCGATCACGCAAATGGATAGCGTTACG
CAACAAAACGCCGCGCTGGTTGAGCAGGTTTCCGCGGCGGCGGCGGCGCTGGAACGGCAG
ACGGAGGATTTGCAACGCTCCGTACAGCAGTTCCGGCTTTCCGCTAGTGAGCCGCAACAA
CGCGTAACGGCGAAAGCGGCGCCTGGCGTTCAGAGAATGGCGTCGGCTCCCGCACAGTCT
ACTGATGAATGGGTTTCCTTCTGA
>EECEBICD_00300 hypothetical protein
ATGAATAATCATTTTGGGAAAGGGTTAATGGCTGGGTTGCACGCGCCATATGCATATAGC
GCGCATCATGCGGTGAATTTCTGTTCTGAGTATAAACGTGGCTTTGTATTGGGTTTTACA
CACCGTATGTTCGAAAAGACCGGCGATCGTCAACTTAGCGCGTGGGAGGCCGGAATTCTG
ACGCGTCGCTATGGTCTGGATAAAGAAATGGTGATGGATTTCTTTAAAGAGAATCATTCC
GGGATGGCGGTTCGCTTCTTTATGGTCGGTTATCGACTCGAAGGTTGA
>EECEBICD_00301 Hydrogenase-2 small chain
ATGACTGGAGATAATACTCTCATCACTTCTCACGGCATTAACCGTCGTGATTTCATGAAG
CTTTGTGCAGCACTGGCCGCTACTATGGGGCTCAGTAGCAAAGCCGCCGCAGAAATGGCA
GAATCGGTATCCAATCCACAGCGTCCGCCCGTTATCTGGATTGGCGCTCAGGAGTGTACC
GGTTGTACCGAATCACTGCTTCGTGCTACACACCCAACCGTTGAAAACCTCGTTCTGGAG
ACTATCTCTCTGGAATACCACGAGGTACTTTCCGCCGCATTCGGTCACCAGGTCGAAGAA
AACAAACATAACGCTCTGGAGAAGTATAAAGGGCAATATGTTCTGGTGGTGGATGGTTCT
ATCCCACTAAAAGATAACGGTATCTACTGCATGGTTGCCGGCGAGCCGATCGTGGATCAC
ATCCGCAAAGCCGCTGACGGCGCAGCCGCGATTATCGCTATCGGTTCCTGCTCGGCATGG
GGCGGCGTTGCTGCGGCTGGCGTAAACCCAACCGGCGCTGTCAGTCTGCAGGAAGTCTTA
CCGGGCAAAACGGTTATCAATATTCCAGGTTGTCCGCCAAACCCGCATAACTTCCTGGCG
ACCGTCGCGCATATCATCACTTACGGCACGCCGCCGAAGCTGGATGCGAAAAATCGTCCA
ACCTTTGCCTATGGCCGTCTGATTCATGAGCATTGCGAACGTCGTCCACACTTCGACGCA
GGCCGTTTTGCCAAAGAATTTGGCGACGAAGGCCACCGTCAGGGCTGGTGTCTCTACCAT
CTTGGCTGTAAAGGGCCGGAAACCTGGGGCAACTGTTCTACGTTACAGTTCTGTGACGTT
GGCGGCGTCTGGCCAGTGGCGATCGGTCATCCTTGCTATGGCTGTAACGAAGAAGGTATC
GGCTTCCATAAGGGCATTCACCAGCTTGCTCATGTCGAAAACCAAACTCCGCGTTCAGAG
AAACCTGACGTCAATATGAAAGAAGGCGGCAATATCTCTGCGGGCGCTGTCGGTCTGCTT
GGCGGCGTAGTCGGTCTGGTTGCCGGCGTCAGCGTGATGGCGGTACGTGAACTGGGGCGT
CAGCAAAAGAAAGATAACGCTGACTCACGGGGAGAATAA
>EECEBICD_00302 Formate dehydrogenase, nitrate-inducible, iron-sulfur subunit
GTGAACAGACGTGACTTTATTAAAGCAGCCTCCGGTGGAGCATTGCTGTTGGGCGCCGCG
CCGTCTGTCAGCCATGCGGCTGCAGAAAACCGTCCGCCGATTCCAGGTTCGCTGGGTATG
CTGTATGACTCCACTCTGTGCGTGGGCTGTCAGGCCTGCGTCACAAAGTGTCAGGATATC
AACTTCCCGGCGCGTAACCCGGAAGGGGAGCAGACCTGGTCGAACAACGACAAGCTGTCG
CCGTATACCAATAACATTATTCAGGTATGGCGTAGCGGCACGGGCGTGAACAAAGATCAG
GAAGAGAACGGCTATGCCTACATCAAGAAACAGTGTATGCACTGCGTTGATCCTAACTGC
GTTTCCGTTTGCCCGGTGTCGGCGCTGAAAAAAGATCCGAAAACCGGCATCGTCCACTAT
GACAAAGATGTCTGCACCGGCTGCCGTTACTGTATGGTCGCCTGTCCGTACAACGTACCT
AAGTACGACTACAACAACCCGTTTGGCGCGCTTCACAAGTGTGAGTTGTGTAACCAGAAA
GGCGTTGAGCGTCTGGATAAAGGCGGTTTGCCTGGGTGTGTGGAAGTTTGTCCGGCAGGG
GCCGTCATTTTCGGTACTCGTGAAGAGCTGATGGCGGAAGCGAAAAAACGTCTGGCCCTC
AAACCAGGCAGTGAATATCACTATCCGCGCCAGACGTTGAAAACGGACGACACCTATCTG
CATACGGTGCCTAAATACTACCCACACCTCTACGGTGAAAAAGAGGGCGGTGGTACTCAG
GTGCTGGTGCTGACAGGCGTTCCTTATGAAGATCTGGATCTGCCGAAGCTGGATGACCTT
TCAACCGGAGCGCGCTCCGAGCATGTTCAACACACGCTTTATAAAGGCATGATACTACCC
CTGGCTGCGCTGGCGGGCTTAACCGTGCTGGTTCGTCGTAATAGCAAAAACGACCATCAC
GATGGAGGAGACGATCATGAGTCATGA
>EECEBICD_00303 putative Ni/Fe-hydrogenase 2 b-type cytochrome subunit

ATGAGTCATGATCCAAAACCGCTGGGCGGAAAAATCATCAGTAAACCGGTCATTATTTTT
GGACCGTTAATCGTCCTGTGTATGCTCCTTATCGTGAAGCGTCTGGTCTTCGGGTTGGGC
TCCGTTTCCGACCTGAACGGCGGTTTCCCATGGGGCGTCTGGATTGCCTTTGACCTGTTG
ATCGGCACTGGCTTTGCCTGCGGCGGTTGGGCGTTGGCATGGGCGGTATATGTCTTTAAC
CGTGGGCAATACCATCCGTTGGTGCGCCCGGCGTTGCTGGCAAGCTTGTTTGGTTACTCG
CTGGGCGGCCTGTCGATCACTATCGACGTCGGTCGTTACTGGAACCTGCCGTACTTCTAC
ATTCCAGGTCACTTCAACGTGAACTCGGTACTGTTTGAGACGGCGGTCTGTATGACCATC
TACATCGGCGTGATGGCGCTGGAGTTTGCGCCTGCCTTGTTTGAACGTCTGGGCTGGAAA
GTGTCGCTCAAGCGTCTGAATAAGGTGATGTTCTTTATTATCGCGCTGGGCGCGCTGTTG
CCGACGATGCACCAGTCCTCAATGGGGTCATTGATGATCTCGGCGGGCTATAAAGTGCAT
CCGCTATGGCAAAGCTATGAAATGCTGCCGCTGTTCTCGGTTCTGACCGCGTTTATCATG
GGCTTCTCCATTGTCATATTTGAGGGTTCGCTGGTTCAGGCAGGCCTGAAAGGAAACGGT
CCGGATGAGAAAAATTTGTTTGTTAAGCTGACGAATACCATCAGCGTGCTGCTGGCGATT
TTCGTCGTCCTGCGCTTTGGCGAACTGATTTATCGCGACAAGCTGTCCTATGCGTTTGCC
GGCGATTTTTACTCCGCTATGTTCTGGATTGAAGTCGTCCTGATGGTCTTCCCGTTAGTG
GTGCTGCGTGTGGCGAAACTGCGTAATGACTCTCGTATGCTGTACCTGTCGGCGCTGAGC
GCGCTGTTGGGCTGCGCGACGTGGCGTCTGACCTATTCGCTGGTGGCATTCAACCCGGGT
GGCGGCTACCACTACTTCCCAACTTGGGAAGAATTGTTGATTTCTATTGGTTTTGTGGCT
ATTGAGATTTGTGCATACATCGTACTCATTCGTCTACTGCCGATACTTCCTCCTTTAAAA
CAAAACGATCATAATCGTCATGAGGCGAGCAAAGCATGA
>EECEBICD_00304 Hydrogenase-2 large chain
ATGAGCCAGAGAATTACTATTGATCCGGTAACCCGTATTGAGGGTCATTTACGCATTGAC
TGCGAAATTGAGAACGGCGTCGTATCCAAAGCATGGGCGTCCGGCACCATGTGGCGCGGT
ATGGAAGAGATCGTGAAGAACCGCGATCCGCGCGATGCGTGGATGATTGTGCAACGTATT
TGCGGCGTTTGTACGACAACTCACGCGATCTCCTCCGTTCGTGCGGCGGAAAGCGCGCTG
AATATTGATGTTCCCGTCAACGCGCAGTATATCCGTAATATTATTCTGGCGGCGCATACC
ACCCATGACCACATTGTCCATTTCTACCAGCTCTCTGCGCTGGACTGGGTGGACATTACC
TCTGCGCTGAAAGCCGATCCGGAAAAAGCCTCCGCGATGCTGGATGGCGTGTCGAGCTGG
CACCTGAACAGCGCGCAAGAGTTCACGAAAGTTCAGAACAAGATCAAGGATCTTGTCGCC
AGCGGTCAGCTAGGTATTTTCGCCAATGGCTACTGGGGGCACCCGGCAATGCAGTTACCG
CCGGAGGTCAACCTGATTGCGGTCGCCCACTATCTGCAGGCGCTGGAATGTCAGCGTGAC
GCTAACCGCGTGGTGGCGTTGCTGGGCGGTAAATCGCCGCATATCCAGAACCTGGCGGTC
GGTGGGGTGGCGAACCCGATCAATCTCGACAGCATCGGTACGTTGAACCTTGAACGCCTG
ATGTACATCAAATCGTTTATCGATAAGCTGAGCGATTTTGTCGAGCAGGTGTATAAAGTG
GATACCGCAGTCATCGCGGCCTACTATCCGGAATGGCTGGAGCGCGGCAAAGGCGCGGTG
AACTACCTGAGCGCGCCGGAATTCCCGACCGACGGTAAAAACGGCAGTTTCCTGTTCCCG
GGCGGTTACATTACCAATGCCGATCTGTCGACATACCGTCCGATCACCTCTCACTCCGAC
GAATTCCTGATTAAAGGTATTCAGGAGAGCGCGAAGCACGCCTGGTACAAAGATGAAGCG
CCGCAGGCGCCGTGGGAAGGCACTACGATCCCGGCTTACGACGGTTGGTCGGATGACGGT
AAATACTCATGGGTCAAGTCGCCGACATTCTACGGCAAAACCGTCGAAGTCGGGCCGCTG
GCGAACATGCTGTGTAAACTGGCTGCAGGTCGTGAATCCACGCAGACCAAGCTCAATGAA
ATCATTGCGCTTTATCAGAAGCTGACCGGCAAAACGTTGGAAATGGCGCAACTTCACTCT
ACGCTGGGTCGTATCATCGGGCGTACCGTTCACTGTTGTGAACTGCAAAACATATTGCAG
GATCAATACAACGCGCTGATTGTAAATATCGGCAAAGGCGATTACACCACGTTTGTGAAG
CCGAACATTCCGGCGACGGGCGAGTTCAAAGGCGTGGGCTTCCTGGAGGCGCCGCGCGGT
ATGCTGTCTCACTGGATGGTGATTAAAGACGGTATTATCAGTAACTATCAGGCGGTGGTG
CCATCGACCTGGAACTCAGGTCCGCGTAACTTCAACGATGACGTGGGGCCGTACGAACAG
TCGCTGGTCGGTACGCCGATCGCCGATCCGGCGAAGCCGCTGGAAGTGGTACGTACCATC
CACTCCTTCGACCCTTGCATGGCGTGCGCGGTACACGTGGTGGACGCCGATGGCAATGAA
GTGGTCTCGGTGAAGGTTTTGTAA
>EECEBICD_00305 Hydrogenase 2 maturation protease
ATGCGGATATTAGTCTTAGGGGTCGGCAATATTTTGCTGACCGATGAAGCCATCGGGGTG
CGTATTGTTGAAGCGTTAGAGCAACGATACACCCTGCCGGATTTCGTTGAGATTCTGGAT
GGTGGTACGGCGGGAATGGAACTTCTTGGCGATATGGCGAGTCGGGATCATTTGATCATT

GCCGACGCCATCGTGTCGAAAAAGAACGCGCCGGGAACCATTATGGTACTGCGCGACGAT
GAAGTTCCGGCGCTGTTCACCAACAAAATCTCCCCCCATCAGCTTGGACTGGCCGACGTA
TTGTCGGCCCTCCGCTTTACCGGCGAGTTTCCGAAGAAACTGACCCTGGTCGGCGTTATT
CCACAGTCGCTGGAACCGCACATCGGCTTAACGCCAACCGTCGAGGCGATGATTGAACCT
GCGCTTGAGCAGGTTCTTGCTGCGCTGCGAGAATCCGGCGTCGAAGCAATTCCTAAGGAG
ACGGCGCATGTCTGA
>EECEBICD_00306 Hydrogenase-2 operon protein HybE
ATGTCTGAAACGTTTTCCGGATTCGACACGGCGCCCGTCGCCCGGGTTCAGGCGGCGTTT
GAAGAGATAGCCCATCGTTCGATGCACGATCTTTCGTTTCTGCATCCGACTATGCCGGTG
CATGTCTCTGATTTTACGTTGTTTGAAGGACAATGGACGGGCACGGTAATTACGCCGTGG
ATGTTAAGCGCATTAATCTTCCCTGGCCCGGACCAGATCTGGCCCGGGCGTACCATCGGT
GAAAAACTCGGTCTGCAACTGCCTTATGGCACGATGACCTTTACCGTTGGCGAACTGGAA
GGCGTTTCACAATATCTTGCCTGCTCGTTGATGTCGCCCGCTTTCCCGTAGCCTGTCGCCG
GAAGAGGGCGTCCGGCTGGCGGATGACTGCGCGCGGATGCTGTTATCCCTGCCGGTAAGC
AATCCTGATGCGCCGCAGACAAGCCGGCGTGCGTTGCTGTTTGGCCGTCGGAGCTGTGAG
AATGCATGA
>EECEBICD_00307 Hydrogenase maturation factor HybF
ATGCATGAGCTGTCTCTTTGCCAAAGCGCCGTTGAAATTATTCAGCAGCAGGCAGAACAG
CACGGCGTGGCGCGCGTCACCGGCGTATGGCTGGAGATAGGCGCGCTTTCGTGCGTTGAA
GAGCGCGCGGTTCGTTTCAGCTTTGACATCGCCTGCCAGGGAACTTTAGCGCAAGGGTGC
GAATTGCACATCGACTACAGACCGGCCCAGGCCTGGTGCTGGGATTGCAGTCAGGTGGTG
GAAATTCTCAGGCATGACGCGCAATGTCCGCGTTGTCATGGCGATCGCCTGCGGGTGGAT
ACCGGCGATTCGTTGAAAGTAAAGAGTATTGAGGTCGAATAA
>EECEBICD_00308 Hydrogenase maturation factor HybG
ATGTGTATTGGCGTCCCCGGTCAGGTGCTGGCCGTCGGAGATGATATTCACCAGCTTGCG
CAGGTGGAAGTGTGTGGCATCAAACGTGATGTCAATATTGCTCTGATTTGTGAAGGTAAG
CCAGCCGATCTGGTAGGGCAATGGGTATTGGTACACGTTGGTTTTGCGATGAGCATCATT
GACGAAGACGAAGCGAAAGCCACGTTAGATGCGCTGCGCCGCATGGAGTACGACATCACC
AGCGCGTGA
>EECEBICD_00309 putative protein
ATGAATAAATTTTTGCGCCAGCTCTCTCTGCTGGCGTTGCTTTTTTGCTGGCCGTTAATG
TCACAGGCGGCGCGTACTTTTACCGATCAACTTGGCCGCCAGGTCACGGTTCCCGACACC
GTCGACAGAGTGGTGGTGCTCCAGCATCAAACGCTCAATCTCCTTGTACAGATGAATGCC
ACTGACAAAATCGTTGGGGTCATGGCGAACTGGAAACAGCAATTAGGCGACGGCTATGCG
CGTCTGGCGCCGGAACTGGCGCAAAAAGCCTCGCTGGGCGATCTAACGCACGTTGATCCT
GAAAAACTGGTGGCGCTGCGCCCGCAGGTGGTGTTTGTCACGAATTACGCTCCGCAGGAG
ATGATCGACAAAATCAGCCGTCTTGGCATTCCGGTGGTGGCGATTTCGCTGCGCCATGAT
GTTGCTGGCGAGCGGGCTAAAATGAACCCGACCCTGGCCGATGAAGAGCAGGCTTATAAT
CGCGGGCTGCGTGAAGGAATAACGCTGATTGGCGATATCGTCAATAAACCGCAGGAGGCG
AAAGCGCTGATTGAGGCGATGGATAAAGGTCGCAAGATGGTCAGCGACCGTTTGCAGTCG
GTTCCTGAAAATGAGCGGGTTCGCGCTTATATGGCGAATCCGGAGCTGACCACCTACGGT
TCCGGTAAATACACCGGTCTGATGATGGCCCATGCGGGGGCGCTGAATGTAGCGGCATCC
TCGGTGAAAGGCTTTAAGCAGGTGACAATGGAGCAGGTGATCGCCTGGGATCCACAGGTA
ATCTTTGTCCAGAATCGCTATCCAAAGGTCGTCAATGAGATCCTGCACAATCCCCAGTGG
CAGGTCATTGACGCGGTGAAGCATCATCGGGTTTACCTGATGCCGGATTATGCCAAAGCC
TGGGGATATCCGATGCCGGAGGCGGTGGGAATCGGCGAACTGTGGATGGCGAAAAAACTC
TATCCGGAAAAATTCCACGATATTGACATGCAAAAGGCCGCTAACGACTGGTATCAACGT
TTTTACCGTACTGACTATCAGGGCGTACAGTGA
>EECEBICD_00310 hypothetical protein
ATGCGAATACTGGCTGCGGGGAGCTTGCGCGTCGTCTGGCCGCAGCTAATGGCGGCATTT
CAGGCGGATGCGGTATGTGATTTCGGTCCGGCGGGGCTGTTACGTGAACGCATCGAAGCG
GGCGAAGCGTGCGACTTTTTTGCTTCGGCGAACCTGGCGCATCCGCAGGCGCTGCTGGAA
AGCGGGCGGGCGCTGCGGGTCGCGCCTTTTACGACGAACCGTTTGTGCCTGAGCGTGCGG
GCACAGGCGATGCGTGAAGGCGAGGACTGGCTGTCGCTATTAACCCGGCGGGATCTGCGG

ATCGGCACCTCAACAGCAGGTTGCGATCCTTCCGGCGATTATACGCAGCAGCTTTTTTCG
CGGATGGGCAAGGAGGGCGAAGCGGTCCGAAAACGCGCGGTAGCGCTGGTTGGCGGCAGG
CAGACTTTGCCGTTACCGGCGGGCCGGCTGGCTGCGGAATGGCTCATCAATCATGATTAC
ACCGATATCTTTATCGGCTATGCCAGCTATGCTCCCAGGCTACGGCAGGTTAATTCGCTG
CGAGTGGTTGATATCCCGGAACCTTATAATCCCGTGGCTGAATATGGCTTTGCCTGCCTG
AGCGAGCAGGGAAAAACGCTTGCCGACTTTTTACTGTCAGCACGGGCGAGGTTGATTCTC
ATGCAACATGGGTTTAGTGAAGCGCCGAATATGACACACTCGCAGAACTGA
>EECEBICD_00311 Disulfide-bond oxidoreductase YghU
ATGGCAGATAATACTTATCAACCCGCAAAAGTCTGGACGTGGGATAAATCCGGCGGCGGC
GCGTTCGCTAACATTAATCGCCCGGTTTCCGGCCCGACGCACGACAAAACGCTGCCCGTC
GGCAAACATCCGTTACAGCTTTATTCGCTGGGGACGCCTAACGGCCAGAAAGTGACTATT
ATGCTGGAAGAGCTCCTGGCGCTGGGCGTGACCGGCGCCGAATATGACGCCTGGTTGATT
CGCATCGGCGAGGGCGATCAGTTCTCCAGCGGCTTTGTTGACATCAATCCGAACTCAAAA
ATCCCGGCGCTGCGCGATAACTCACACAACCCGCCGATTCGAGTTTTTGAGTCGGGCGCA
ATTCTTGTTTATCTGGCGGATAAGTTTGGCCATTTCCTGCCGCAGGATCTGGCTAAGCGC
ACAGAAACCCTTAACTGGTTGTTCTGGCTACAGGGCGCGGCGCCGTTCCTCGGTGGCGGT
TTCGGTCATTTCTATCATTATGCGCCGGTAAAAATTGAGTACGCCATTAATCGCTTCACC
ATGGAAGCCAAGCGTCTGCTGGACGTGCTGGATAAGCAGTTAGCTCACCACCCGTATGTC
GCGGGAGAAGAGTACACTATTGCCGATATGGCCATCTGGCCGTGGTTCGGCAACGTGGTG
CTGGGGAATGTCTATGATGCAGCGGAGTTTCTGGATGCCGGAAGTTATCAAAACGTACAA
CGCTGGGCGAAGCAGGTAGCCGAACGTCCGGCGGTAAAGCGGGGGCGCATTGTCAACCGG
ACCAACGGCCCATTGAACGAACAGCTTCACGAACGCCATGACGCCAGCGACTTCGAAAAC
AATACCGAAGATAAACGTCAAAGTTAA
>EECEBICD_00312 Bifunctional glutathionylspermidine synthetase/amidase
ATGAGCAAAGGAACCACCAGTCAGGATGCCCCCTTCGGGACATTATTGGGTTACGCCCCC
GGCGGCGTAGCGATCTACTCTTCAGATTACAATTCCCTCGATCCGTGGGACGACGACGAC
GCAGCTTTTCGTAGCTACATCGACGATGAATACATGGGGCATAAATGGCAGTGCGTGGAA
TTTGCCCGCCGTTTTCTCTTTCTGAATTATGGCGTTGTTTTTACTGACGTCGGCATGGCC
TGGGAAATTTTCTCGCTGCGTTTTCTGCGCGAGGTCGTGAATGACAATATTTTGCCTTTG
CAGGCCTTCCCGAACGGTTCGCCGCGCGCGCCGGAGGCCGGGGCGTTGCTCATCTGGCAA
AAGGGCGGCGAGTTTAACGAAACCGGGCACGTTGCGATTATCACCCAACTCCTGGATAAC
AAAATTCGTATTGCTGAGCAAAACGTTATCCACACGCCATTGCCGCCAGGGCAGCAGTGG
ACTCGCGAACTGGAAATGGTCGTAGAAAACGGGTGTTACACCCTGCGCGACACCTTTGAT
GACACGACGATTTTAGGCTGGATGATCCAGACCGACGACACGCAATACAGTTTGTCACAG
CCTGACATCGCGAATCAGTCGCTGGCGATTCGCGGCGCAAGACTGCCAGAGAAAGGTCAG
TTTGACGGTCCGTGGCTGGATGAGCGCGATCCGTTACAAAAAGCCTATGTACAGGCGAAC
GGACACGTTATCAATCAGGACCCGTACCAGTATTTTACCATCACCGAGAGCGCTGAACAG
GAGCTTATCAAAGCGACCAATGAGTTGCATTTGATGTACCTGCACGCCACGGACAAAGTT
TTAAAAGACGATAATCTGCTGGCGCTATTCGATATCCCCAAAATTCTTTGGCCGCGCCTG
CGTCTCTCCTGGCAGCGCCGTCGTCACCATATGATCACCGGTCGTATGGATTTCTGCATG
GATGAACGCGGGCTGAAAGTCTATGAATACAATGCCGATTCCGCATCATGCCATACCGAA
GCGGGATTAATTCTCGAAAAATGGGCTGAGCAGGGCTATACCGGTAAAGGGCATAACCCG
GCGGAGGGCTTAATTAACGAGCTGGCAGGCGCCTGGAAACACAGTAAAGCGCGTCCTTTC
GTCCATATCATGCAGGATGATGATATCGAGGAGGACTATCACGCGCAGTTTATGCAGCAG
GCGCTGCATCAGGCCGGTTTCGCGAGCAAAATTCTCCGCGGTCTGGGCGAGTTGCGCTGG
GATGACGCCGGGCAGTTGATCGATGGCGACGGACGGCTGGTGAATTGTGTCTGGAAAACC
TGGGCGTGGGAAACGGCGATGGAGCAGATACGCGAAGTCAGCGAGACGGAGTATGCCGCA
GTGCCTATTCGTACCGGGCACCCGGAAAATGAAGTCCGGCTGATTGATGTATTACTGCGC
CCGGAAGTGTTGGTGTTTGAACCGCTGTGGACGGTGATCCCGGGTAACAAAGCGATCCTG
CCCATCCTGTGGTCGCTCTTCCCGCATCATCGCTATTTGCTCGACACGGATTTTACCGTT
AACGATGAGCTGGTGCAGACCGGCTATGCGGTAAAACCGATTGCCGGACGTTGCGGTAGT
AATATTGATTTGGTGAGCCATCAGGAAGAGCTCCTGGATAAAACCAGCGGCAAGTTCGCC
ACGCAGAAAAATATCTATCAGCAGTTGTGGTGCCTGCCGAAGGTCGCCGGGAAGTATATT
CAGGTCTGTACGTTTACCGTGGGCGGTAACTACGGCGGTACGTGCCTGCGCGGCGATGAT

TCATTAGTCATTAAAAAAGAGAGCGATATTGAACCGTTAATCGTCATCAAAGCGTAA
>EECEBICD_00313 hypothetical protein
ATGTTTAAAAAAGTATTAGGCTTGCGCCACTGGAATAATAATGTTGTAAAAATACCGCCG
CCAGCAGAATCTGGCGCTAATGCATCTGACGTGGTGGTGGATACTCCTGAGCCTTATGAG
CGGATACTGTGTCAAAAAATTCTGATGGGGATCTCGACGATCGATATCATTCGTAATGCC
ATCATTAAGTCCTGTGAGCAGCTCAACATTGAAAAAGAACGCATTAATGAACTTAACGAA
CAGAATGATAAAGCCAGAAGTTCATTAAAATCGCTCGTGGAATTTATCACCGAAATTGGC
ACGACTTCATCGGATATTGGCTGTCGTATGGGCGACTTAAATACCTCCTTAACGCAAATT
AATGCGTGTATTAAAGAGATACAGAAGATCGCTAACCAGACCAATTTGATAGCGATTAAC
TCCGCGATCGAGGCCGCGCGCGTAGGGGATGCCGGGCGTGGTTTTAGCGTTATTTCGAAA
GAGGTGAAAAACCTGTCCGAAGATGTTAAACACAGTTCGAAAAGCGTCAGTACGTTAACC
TCTGTAATCAAAGATAATACGGCCAGAGTGAGTGAGGTACTGGATAACCAACAGCCGGTT
ATTGATAATATTACGACTAACATAAATCAAATTGTGGAGTCTATTGGCATCGTCATCGAT
AAATCGCTGAGCATGAAATCGGTCATGCAGTACATCTCTACGGTGCAGTTTTTGAATATT
GTCAAAGTTGACCATGTTATCTGGAAAATGGAAGTCTACAAATTACTCTTAAATAAAGAC
ATAAACAGTAAAATAACGATGCACGATCAGTGTCGGCTGGGGAAATGGTACTACGGTTTT
GAAGGACAGCAGTTTAGCAATTACTACAGTTTCCGTAGTCTGGAAGCGCCTCATAAAGAG
GTGCATACCGCTGGCCACTCTGCGCTGAACTATTTTGCCGCAGGCGATATGAATGCGATG
TCGCAAGAACTCGACAGAATGGAGCGATCCAGTAATGAGGTGGTTAACCAGCTGGAAATG
CTGGCTGTCGATTTGTTAAAAGAAACCACACTGTGA
>EECEBICD_00314 Uronate isomerase
ATGGCTACGTTTATGACTGAAGATTTTCTACTTAAAAACGACATTGCCCGCACGCTGTAC
CATAAATACGCCGCGCCCATGCCGATTTATGACTTCCACTGCCATTTAAGTCCGCAGGAA
ATCGCCGACGATCGCCGTTTCGATAACCTCGGTCAGATCTGGCTGGAAGGCGACCACTAT
AAATGGCGAGCGCTACGAAGCGCAGGCGTGGATGAGTCGCTGATCACCGGCAAAGAGACC
AGCGATTATGAAAAATATATGGCCTGGGCCAATACCGTACCAAAAACGCTGGGCAATCCG
CTGTATCACTGGACGCACCTTGAACTACGCCGTCCATTTGGCATTACAGGTACGCTGTTC
GGACCGGATACCGCGGAAAGTATCTGGACGCAGTGTAATGAGAAACTGGCGACGCCGGCC
TTTTCCGCGCGCGGTATTATGCAGCAGATGAATGTGCGGATGGTCGGAACCACCGACGAC
CCGATAGATTCTCTGGAATATCACCGCCAGATAGCCGCCGACGACAGCATTGATATTGAA
GTCGCGCCAAGCTGGCGCCCCGACAAAGTTTTCAAAATCGAACTGGACGGCTTTGTCGAT
TACCTGAGGAAACTGGAAGCGGCGGCAGATGTCAGCATTACCCGTTTCGACGATTTACGT
CAGGCGCTCACTCGCCGCCTCGACCATTTCGCCGCCTGCGGCTGCCGCGCGTCGGATCAT
GGCATTGAAACGCTGCGATTTGCGCCGGTGCCCGACGACGCGCAGCTTGACGCCATTCTG
GGCAAACGTCTGGCTGGCGAAACGCTGAGCGAACTTGAGATCGCCCAGTTTACCACGGCG
GTGCTGGTCTGGCTGGGCCGTCAGTACGCCCGCGCGCGGCTGGGTGATGCAGCTACATATT
GGCGCGATCCGTAATAATAATACCCGGATGTTCCGCCTGCTGGGGCCGGATACCGGCTTT
GACTCCATTGGCGATAATAACATTAGCTGGGCGCTCTCCCGTTTGCTCGACAGTATGGAT
GTGACCAATGAACTGCCCAAGACTATCCTCTATTGCCTGAACCCACGTGATAACGAAGTC
CTGGCGACCATGATCGGTAACTTCCAGGGGCCGGGAATTGCCGGAAAAGTGCAGTTTGGT
TCCGGCTGGTGGTTTAACGATCAGAAAGACGGTATGCTGCGCCAACTGGAGCAACTGTCG
CAAATGGGACTGTTAAGTCAGTTTGTCGGGATGCTGACCGACTCCCGCAGTTTCCTTTCT
TATACGCGACATGAATATTTCCGTCGTATTCTCTGTAACCTGCTGGGACAGTGGGCGCAG
GACGGAGAGATTCCTGATGATGAAGCGATGCTAAGCCGTATGGTTCAGGATATCTGCTTC
AATAATGCCCAGCGTTATTTCACGATTAAATAA
>EECEBICD_00315 Polyol:NADP oxidoreductase
ATGGAACAGAATATCGCCACCGCCCAGGTTTCCGTCGCCCGCCCAAACTGGGACAAATCA
CGTCTGGTATCCCGTATTGTGCATCTGGGCTGCGGGGCGTTTCACCGCGCGCACCAGGCG
CTCTTTACCCATCATTTACTGGAAAAGAGCGACAGCGACTGGGGCATTTGTGAAGTGAAC
CTGATGCCGGGTAACGACGCGCGGCTGATCGCGAACCTGAAAGCGCAAAATCTGCTGTAC
ACCGTTGCAGAACGCGGCGCAGAAAGCACCGAACTGAAAATTATCGGTTCAATGAAAGAA
GCGCTGCACCCTGAATTCGACGGCCATGCAGGGATTCTGGCGGCAATGGCGCGCCCGGAA
ACCGCCATCGTCTCCTTAACCGTGACCGAAAAAGGCTACTGCACCGACCCCGCCAGCGGC
GAGCTTGATGTCAATAACCCGCTGATCCAAAACGATCTTGCCCATCCACAGCAGCCTAAA

TCCGCCATTGGCTATATTGTCGAAGCGCTAAACATGCGCCGGGAGCAAGGACTGAAAGCT
TTTACCGTGTTGTCATGCGATAACGTACGCGAGAATGGTCACGTCGCGAAGGCTGCCGTC
CTCGGCCTGGCAAAAGCTCGCGATGCCGCCCTCGCCGCATGGATTGCCGACAATGTGACC
TTCCCCTGCACCATGGTTGACCGCATTGTTCCGGCGGCGACCGAAGAGACGCTACAACTG
GTTGCCGACCAGTTAGGGGTTTATGATCCTTGCGCTATCGCCTGCGAACCCTTCCGCCAG
TGGGTCATCGAAGATAACTTTGTGAATGGTCGCCCGGACTGGGACACCGTCGGCGCGCAA
TTCGTTGCAGATGTCGTACCGTTTGAAATGATGAAGCTGCGTATGCTTAACGGCAGTCAC
TCTTTCCTCGCCTATCTGGGTTATCTCGGCGGGTATGACACTATCGCCGATACCATGACC
AACCCGGCTTACCGTCGCGCGGCGCTGGCGCTGATGCTTGATGAACAAGCGCCGACGTTA
TCAATGCCGGAAGGCACCGATCTGGAAGGGTATGCGAATTTGTTGATCGCGCGTTTCACT
AACCCCTCGTTGAAACACCGCACCTGGCAGATCGCGATGGATGGCAGTCAGAAACTGCCG
CAGCGTTTGTTGGACCCGGTACGTCTGCACTTGCAACAGGGCGATGACTATCGCCGCCTG
ACGCTGGGCGTCGCCGGATGGATGCGTTACGTCGGCGGTATCGATGAACAAGGTAAAACC
ATTGATGTCGTCGATCCGCTGCTCGCGCAGTATCAGGCGATTCATCAGCAATATCAGACG
CCGGAAGAACGCGTTCGCGGGCTACTGGCCATCGAGTCTATCTTTGGCAGCGACCTGCCG
AAGAACCACGAATTTGTGCAAGCCGTTACCGACGCTTACCAACAGCTATTGCAGAACGGC
GCGAAAGCCACGGTAGAAGCGCTGGCTAAGTAA
>EECEBICD_00316 Mannonate dehydratase
ATGAAACAAACCTGGCGCTGGTACGGACCTAACGACCCGGTAACGCTGTCAGATGTACGC
CAGGCTGGCGCAACCGGCGTGGTAACGGCGTTACACCATATCCCGAATGGAGAAATTTGG
TCGATAGACGAGATCCAGAAACGTAAAGCTATCGTTGAAGAGGCGGGTCTGGAGTGGTCT
GTGGTAGAGAGCGTACCTATCCACGAAGATATCAAAACCCACACCGGTCAGTACGATTTA
TGGATCAAAAACTACCAGCAAACGCTGCGTAACCTGGCGCAATGCGGTATCTATACGGTT
TGCTATAACTTTATGCCGGTGCTGGACTGGACACGTACGGATCTGGAATACGTATTGCCG
GATGGTTCAAAAGCGTTGCGTTTTGACCAGATTGAATTCGCCGCGTTCGAACTGCATATC
CTGAAGCGTCCGGGAGCAGAAGCCGACTATACGGCAGAAGAAATTGCTCAGGCAGAGCGA
CGTTTCGCCACCATGAGCGAGGAAGACAAAGCACGTCTGACCCGCAACATTATTGCCGGT
TTACCTGGCGCGGAAGAAGGCTATACGCTGGATCAGTTCCGTCAACACCTGGCGACGTAT
AAAGATATCGATAAAGCAAAACTGCGTGAACATTTTGCCTATTTCCTGAAAGCCATTATT
CCGGTTGCCGACGAGGTTGGCGTGCGTATGGCCGTTCACCCTGACGATCCGCCGCGCCCT
ATTCTCGGCCTGCCGCGCATTGTCTCTACCATTGAAGACATGCAATGGATGGTGGAAACC
GTTAATAGCATGGCGAACGGCTTCACCATGTGTACCGGATCTTACGGCGTGCGCGCCGAC
AACGATCTGGTTGATATGATCAAACAGTTTGGTCCGCGCATCTACTTTACGCATCTGCGC
TCTACGCTGCGCGAAGAGAATCCGAAGACCTTCCACGAGGCCGCCCATTTGCACGGCGAT
GTGGATATGTATGAAGTCGTTAAAGCGATTGTGGAAGAAGAGCACCGTCGTAAAGCCGAA
GGTAGCGACGATCTGATCCCAATGCGCCCGGACCACGGTCATCAGATGCTGGACGATCTG
AAGAAGAAAACGAATCCGGGTTATTCCGCCATTGGCCGTCTGAAAGGGCTTGCGGAAGTC
CGCGGCGTCGAACTGGCTATCCAGCGCGCTTTCTTTAGCAAATAA
>EECEBICD_00317 Hexuronate transporter
ATGAAGATGACAAAATTAAGATGGTGGATTATCGGCCTGGTCTGCGTAGGGACCATCGTA
AACTACCTGTCCCGTAGCTCGCTTAGCGTTGCGGCGCCAGCGATGATGAAAGAACTCCAT
TTTGATGAGCAACAATATTCCTGGGTGGTGAGCGCCTTCCAGCTTTGTTACACCATTGCG
CAGCCGATCACCGGCTATTTGATGGATGTCATCGGACTGAAAATCGGCTTCTTTATCTTT
GCCTTGTTATGGTCGCTGATCAACATGGCGCACGCGCTGGCCGGCGGCTGGATAAGTCTG
GCATTTCTGCGTGGTCTGATGGGGCTAACCGAAGCGTCAGCGATTCCGGCCGGGATTAAA
GCCAGCGCCGAATGGTTCCCGACGAAAGAACGCGGTATTGCGGGGGGACTGTTTAATATC
GGTACTTCGATTGGCGCGATGCTGGCGCCGCCGCTGGTGGTCTGGGCGATGCTGACCTTT
GCTGATAGCGGCATCGGTACTGAAATGGCGTTTGTGATTACCGGTGGGATCGGCGTGCTG
TTCGCCATCACCTGGTTCCTGATTTATAACTCGCCAAATAAACACCCGTGGATCACCCAC
AAAGAGCTGCGTTATATCGAAGACGGCCAGGAAAGCTATCTCCAGGACGATAATAAAAAA
CCGGCCGTTAAAGAGATCGTTAAAAAGCGTAACTTCTGGGCGTTGGCGATTACCCGTTTT
CTGGCTGACCCGGCATGGGGAACGCTGAGTTTCTGGATGCCGCTGTATCTGATTAACGTG
ATGCATCTGCCGTTAAAAGAGATCGCCATGTTTGCCTGGCTGCCGTTCCTGGCGGCGGAC
TTTGGCTGCGTTGCAGGCGGTTTCCTGGCGAAGTTCTTCATGGAAAAGATGCACATGACC

ACGATCAATGCGCGTCGTTGTAGCTTTACCATCGGCGCTGTTTTGATGATTTCGATCGGT
TTTGTCAGCATTACGACTAACCCGTATGTCGCCATTGCATTGATGAGTATTGGCGGTTTT
GCGCACCAGACGCTGTCTACCGTCGTGATTACCATGAGCGCTGACCTGTTCAAGAAAAAT
GAGGTCGCGACGGTTGCCGGTCTGGCAGGATCTGCGGCATGGATGGGACAGTTAAGCTTC
AACCTGTTTATGGGGGCATTGGTCGCTATTATCGGTTACGGCCCGTTCTTCATTGCACTC
AGTCTGTTCGATATTATCGGCGCCATCATCCTGTGGGTACTGATTAAAGATCCAGAAAAG
CACCACCCGCCAATGACGGAACAGCCGCTGGCGTCTCACCGCTAA
>EECEBICD_00318 hypothetical protein
ATGTCACGTAATTTTACTGTTTCAGCCTGTCAGTATATTGTTACCGAAATAAACACTTTT
GAAGATTTCATAACAAAAGTAAGGATACTTCTTAATAAAAGTCAGGGTGCTGATGTCGTT
ATCTTCCCGGAGCTATTTACCATTGAGTTATTTACATTATTGAAAAAATGGCAAGAAAGA
CCCATTTCACACCTTACTTTAATTGATCAGTTCACGGATGCCTATAAGCAGCTTTTTCAA
CAGGAAGCGAAAGAAAGAGGACAGTTCATTATTGCTGGCTCTCATCTGGAGCAGACTGGA
GCAGATCGCTATGAGAATGTTGCGCATATATGGGGACCCGATGGCGAACATTATGCTCAC
AGTAAGACGCATATCTTCCCGGCTGAACGGGGTTGGTATACTCAGGAAGGCGATAAAATG
GCTGTTTTTCAACTGCCATTTGCAAAGGTGGGTTTTAATATTTGTTATGAAGCAGAAATA
CCGGAGTGTGCCGCGACGCTGGCGGAACAGGGTGCGGAGTTAATTTTAACGCCTTCCGCT
ACGTTTACTGAACAGGGATTTTGGCGAGTCAGGCATTGTTGCCACGCACGTTGTATTGAA
AATCAAATTTATCTTGTTCACTGTTGCCTGGGCGGAAACCCGGGCGGGCCACTGCCAGGC
TGCTGGGCCCGAAGTTCAATTTTAAGCCCCTGTGATGTCGTGTGGAAAAATCCACAAGGT
ATTATCGCAGAGGCGCACGTCAATCAAGAGGATGTCATTTCAGGAGAAATTAACCTCGAT
GTTCTATATGAAAATAGACTTGGGGGGGCAGCAACAACGTTCAAGGACAGACGCAGAAAA
GCAGGCATATATAATATCTGGCCATCACACATTAAATAA
>EECEBICD_00319 Peptidoglycan deacetylase
ATGGGATATTTAAAATTACCTGAGGGTAAAAGGATTGCGGTCAATCTTGGTGTTGATGTA
GATGCTCAGTCTTTATGGCTGGGCGGATTTAATCGACCGTCGCCATCCTTTATGTCACGA
GGAGAGTTTGGTGCTCAAGTTGGCGTACCAAGGCTTCTGAAATTGTTCAAAGAAAATAAT
ATCAAAACCACCTTTTTTATTCCCGGACACACTGTAGACACCTTCCCGGAAATCATTAAA
GCAATTTTTGATGCCGGGCATGAAATCGCTCACCATGGCTACTATCATGAAAATCCGACG
CTGGTGAATCGTGATACAGAACGCCGACTTATGGATCTGGCGTTCGCCTGTTATAAAAGA
CATTTCGGCATACGCCCTGTGGGATACCGTTCTCCCTATTGGGATTATAGCGAGAATACG
CTGGATCTTCTGGAGGAAGCCGGTTTTCTCTATGACAGTAGCCTGATGGCGAGAGATCTT
GTGCCTTACCATCCTCAACGTTGGCAGGTGAATTGGGAGACGGGAAATATCGCAGGCCCG
GCCAGCGCGATTCTGGAAATTCCTGTTAGCTGGTATCTGGATGATTTTCCCGCACTGGCG
TATACCGGCAACCAGGAGGGGATGAGCGATACTGACGGTGTTCTGCGACGCTGGAAGGAT
ATTTTTACCTATGCTTATCACCATCAGGAAAACGGTTTATATGCGATGGCCCTTCATCCG
CAGATAATCGGTCATGGGCATCATATGATGATGTTGTCACGTTTGATTGAACATATTAAA
GGGCATGATGGCGTATGGTTTGCAACCTGTGAAGAGATAGCCAGTGTCTGGGTGGATGAC
GAAGAGGATCGACAAAAAATGCTCCGTCCTGATGTTCGTGGCGTAGCGCCGGTTCCGGAT
GATTATGGCTGGCCGGGCAAATGA
>EECEBICD_00320 Protein hcp1
ATGGATGCGATTTTTTTAAAGCTCGACAATATCAAAGGCGAAAGCCAGGTTGAAGGCTTT
GAAGATCAGATAGAGATCATGTCTTATAGCCACAATGTGGCAATGCAGGTAAATAACGAT
GTTAGCCTCACTGAGCGGACTTCCGGGCGCGCGCATGTTGGTGAAATGTCGCTCACCAAA
TTTGTCGACCTCGCCACGCCTGTGCTCAATGAGTACTGCTGTAGCGGGAGGCTAATTAGA
ACCGCGGTGCTGACGTTGTGCCGCAATGACGACGGCAAAATGCGGTCGCTTATCGTTTAT
ACCCTGACAAACGTGCTGATATCACAACTCAGTGTTAGCGGCGGCGCCGGCGGTAAGCCG
GTGGAAACGATGTCGCTGAACTTCACGAAAATCGAATGGCAAATCACTGCCGAGAAATCA
GACGGCGCTCAGCAGGAGTCCAGAACGTCAGTATGGGATCTGGCGATGGACCAGATAGGG
AAATGA
>EECEBICD_00321 Anti-adapter protein IraD
ATGATGACGCCAACCATTCCGGTCGCGCTATTCGACAGGCTCTTGGTTGAGGGGATAAGC
CCACATGAATTGGTACGACGCAAGTTAATGTGTTTATTCAACTCTTGCGCCGTGCCAGGT
GGCGAGACATTACCGCCGCTGCTCACTCGGGGTATGCCGGAATGGCACGAGGTGAATGTG

GGTGACAAACGGGTTCTCAACTGGTTTTGCCGTGAGCTTCGCGCTGCTATTTTACGTTAT
GAACCGAGCATCAACATGCTGAAAGTCAGTGTAAAAGATGCCCATCATCAGACGCTGGCG
TTGTCTTTGGAAGCGATGTTACAGGATGAGTCTGAACCGTTAAGGCTGGAGATAGCCTAC
TCTAACGGTAGATGGCGCTAG
>EECEBICD_00322 Phenylacetaldehyde dehydrogenase
ATGCACGAGGAGACCTTTGGCCCTGTCTGTAGCTTTATCGGTTATCAGAATGAAAAAGAG
GCGCTTTCGCATATCAACGCTTCGCCATTCGGCCTTGCTGCAAGTGTGTGGTCGGAAAAT
ATGTCTAAGGCATTACGCTACGCTGAAGATATTGATGCTGGCATGGTGTGGGTCAATATG
CATACCTTCCTCGATCCCGCGGTACCCTTTGGAGGGATGAAAGGATCGGGTAGTGGTCGT
GAATTTGGCAGCGCGTTTATTGATGACTATACCGAACTTAAATCTGTCATGGTCCGTTAT
TAA
>EECEBICD_00323 Phenylacetaldehyde dehydrogenase
ATGAGCGACGTGACATTACTTGCCGAAGTAACGACTTTTTTACGCCAACGACACGGACAA
TTTATTGCAGGTGAACGTCAGGCCGGAAACGGCACGAACTTCTCGGTCACTAACCCAGCC
ACCGGCAAAATCATCGCCGACGTTGTGTCGGCAACCCCTGCGCAGGCAGAAGAGGCCATG
CAGAGCGCCAGACGGGCGTTTGATGTCTGGCGTAAAATGCCAACGTTACAACGCGGCGCA
TTACTGCTGAAACTGGCTGATACTCTTGCCGCTCATCGTGAAGAGTTAGCTCAACTGGAA
AGCGTCTGTTCAGGTAAAACGATTACGCTGTCGCGCGGTCTTGAACTCGATCAGTCAGTG
GCCTTCCTGCGTTACTTTGCCGGTTGGGCAGGAAAAATAACCGGTGAAACGCTGAATGTC
TCCCTGCCATCAATGGGAGAAGAGAGATACACAGCGTTTACCCAACGCCAACCCATTGGC
GTGGTCGTCGGTATTGTGCCGTGGAATTTCTCAATTATGATTGCTATCTGGAAGCTGGCC
GCAGCGCTGGTATGTGGCTGCACCATCGTCATTAAACCAAGTGAATATACCCCGCTGACA
CTGCTGCGAGTCGCTGAACTGGCTAAAGAGGCAGGTTTCCCTGATGGCGTAATTAACGTG
GTAAACGGTGCTGGCGGTGAGATAGCGCAACAGCTGATCGCGCATCCAGATTGCGCCAAA
GTGAGTTTCACCGGGTCAGTTGAGACAGGTGAGAAAGTCCGGCGTTCGGCAACATCGTCA
GGAAAACGCGTTACCCTCGAACTGGGAGGGAAAAATGCGGCGCTGTTTCTCAATGATCTC
TCGGCACAAGCCATGGTCAACGGTATTCTTGAAGCCGGTTATCTGAATCAAGGACAGATT
TGTGCTGCCGCAGAGCGTTTTTATCTGCCCCAGGAAAAACTGGATACGGTCATGACGCTC
CTCAGGCAACGGTTATCGGAGATCGTGCCCGGCTCGCCTTTAGATGAAAAAACGGTGATG
GGCCCGCTGGCGAATCAGGTTCAGCTTGAAAAAGTGCTGCATCTGATTCAACGCGCACGG
GAAGAAGGGGATACTATTGTTTACGGCGGTGAAACTTTACCCGGCGGAGGGTTCTTTTAC
AGCCGACAGCGGTAA
>EECEBICD_00324 Gamma-glutamylputrescine oxidoreductase
ATGAATACAAAAATCGATGCGTTGAATTACTACGGCGCGACGAAAAAATACCCGTTGCAT
TTTCCTGCTCTGCAAGACGATATTGATGCCGATGTGGTGATCATTGGCGGTGGCTTTTCA
GGCATCAATACCGCGCTGGAACTGGCGGAGCAGGGGATCACTAATGTTGTCGTTCTGGAA
GCCCGCCATCTTGGGTATGGCGGCACCGGGCGAAACGGCGGTCAGGTGATGGCCGGTATA
GGTCACGACATCGAAGCCGTTAAAAAATATGTAGGCGCAGCGGGAATGGAGACGCTGTTT
AAAATCTCGAACCTCGGCGCAGGTATTATTCGGGAGCGAATTCGTAAATACCATATTGAG
GCTGATTTTGTCCCCGGATATGGTTATCTGGCCTACAACCAGCGGCAGTTAAAAACGCTG
CAACAATGGGAAAAAGAATTCAAAGCTGCTACGCCGGAAGATGATATTGAACTCTATACC
GGTAAAGACGTGCAGCAGGTTATTGGTTCCGACGTCTACTGCGGTGTTCTCAAGCATATG
GGGGGAGGGCATGTCCACTCGCTGAATTTATTGCTGGGCTCCGCTCAGGCTGCGAATTCG
CTGGGGGTGAAGATATTCGAATATTCACCTGTGGTTGAGGTCAGTTACGGTAAAACTGTG
CGGGTACGTACTGCGATGGGCAGCGTAAAAGCGGCCAAATTGCTGTGGGCCTGTGATAGT
TTTCTCAATAATCTGGAACCAGAAATTTATAAGAAAACGCTGGTTACTTATTCTTATCAG
GTCTCTACAGAACCTCTCTCCAGCGAGCTTGTTGAGCGGATCAGTCCCTTGCGCGGGGCT
TTTAGTGATATCCGCCCGGTGATAAATTACTACCGTCTCACAAAAGAAAACCGTCTGCTG
TTTGGCAGCGCCACAAGGTTTCTTGAATATACCCCGCACGATTTTGCTGCCTGGAACCGC
ACTCTGTTGACGGAGGTCTTTCCTTATTTGAAAGACGTTAAAATCGATTTTGCCTGGGGC
GGACCGATGGCCTGTAGCGCGAATCTGTTTCCGCAGATTGGCACGCTTCGTGAGCACAAT
AATGTTTTTTATGTTCAGGGTTATTCCGGATTTGGCGTCACGCCTTCCCATATTGTATGC
AAAATCCTGGCCGAAGGAATAAATGGCGGTTCCGAGCGGTATCGTCTGATGAGTAGCATT
CCCCACGCGACAATTTATGGTCGCGACAGTCTACGTCTATTGCTGGTCAGTGCGGGAAAA

CTGATACATCAAACAGCCGGTTTCTGGAAAGGCCGGAATTAA
>EECEBICD_00325 hypothetical protein
ATGCACGCTTTTAAACTCAATCAACCTGTTCCTGAATTACAGCCAATCGGGAGTGTCAGC
CTGTTGGGGGCGTTGCCAACAGAAGGCGATCCCCAGGTCGCCGTTGCGATGATTTACGGT
AAACCTGAGGACGTATTTACCTGTGGACTGTTTTCATCGACCCGCGGCGGTTTCACTATG
ATTTATCCCTTTACCGAGCACGCAACCGTGCTGGAAGGGGAAGTGGAACTCACTGTTGCG
GGTCAGGAACCGATACGTTTTTCACCAGGCGATAGTTGGTTTGTTGAACAGGGAACCGAA
GTGGCATGGAAAGTTTTAACGCCCCGTTTTGTAAAACATTATCTGGCAAAAGTTGAAAGC
CATAAGCAGGGTTGA
>EECEBICD_00326 Serine/threonine exchanger SteT
ATGTTTAAAAACAGACTTGCCAGCCATCTTGAACGTGGCGTTATTGGTTTCCCCACGACG
CTTGCCAGCTCCGTCGGGCTGATTATGGCAAGCCCGGTGATCCTGACCGTGACCAGTGGT
TTTGGCTCAGGGGGAGATACCTTTGCTCTTGCGGTATTACTGGCATTTATTATGATGCAA
GCGCAGATTACCACCTTTGCCGAAGCGGCAACGCTTATTCCCACGACTGGCTCCGTCTAC
GACTATATTTCCTGCGGGATGGGGCGTTTTTTCGCTATCACCGGTGCGCTTTGCGCTTAC
CTGATCGTACACATTTTTGCCGGCACGGCAGAAACGATTCTCGCTGGCGTCATGGCGCTG
GTGAACTTTGAGTCTGTTAGCGAACACGTGGCCATGCAGCACAATACCTGGATGGTTGGC
GTCGGCATGGTAGTGGTTTTCGGGTTACTGAATGCCATTGGTGTTGAAGTATTTGGCAAG
GTGGAAGTGTTTCTTACATTCGGTATGTGGACTACCCTGACCATCTTCGGTTTATGCGGG
ATCCTGTTATCCCCGGTCACTCATCATTCCGGGTGGTTTGGCGCACCGCTTGATGTCAGT
AATCTTTCTGGTCTGTTTGGTTATATTGGCATGGCGATGTTTATGTTCGTCGGTTGTGAA
CTGGTCACGCCAATGGCGCCGGAAATTAAAAAAGCGCATCGTACGATTCCCAGAGCGATG
GCGCTTGGCCTTTTAGCGGTGGCAGGTTGTATGTTCCTCTATGGCGCTGCGATTAACCGG
CAGGTAAATAATGTGGTGGTCGATGCGGCAAACCATATCTTTTTACTGGATACGCCGATG
GCTATTCCCGCCTTTGCCAATCGTGTCATGGGACATGTCGGGCAATACTGGCTGGGCGTG
GGGTTGTTGTTAGCGGGATGTGCGACGATTAATACGCTAATGGCGGCAGTACCGCGTATT
ATATACGGCATGGCGCTGGACGGCGCTTTGCCGCGGTTTCTGACCTGGTTGCACCCCCGC
TTTAAAACACCGGTCATCGCCATCGCATTCGGTGTTGCGATTCCCTGTTTACATGCCTGG
TATCTGAACGGTGATATCGACCACATTGTTCCGCTGATCCTCGCTGCGGTCTGCGCCTGG
GGAGTAGCTTATCTGCTGGTAACGTTATCGGTCGTTATCTTACGTATTCGGCGCCCCGAT
TTACCACGCGCTTACCGGGCCCCCTGGTTTCCCTTACCTCAGATTATTTCGAGCGTGGGT
ATTATTATCGCCATTATTAATATCGCGCCTCCAGGAATGAACAGTCGGGATGTACTTATA
CCATTCGGTATTATGCTTGGATTGACTGCCGCCTATGCCTTATTCTGGACATTATGTGTG
CAACGGGTTAATCCTTTCCTCCCGCTTCCGGTAGAAGAGGTCGTGGAACGGGCTATGGGC
AATTATGAAAAGCGAAACAATGAAGAGATGACGCCAGATGTCCTCCGTCCCCTGGTTTAA
>EECEBICD_00327 hypothetical protein
ATGTCCTCCGTCCCCTGGTTTAAGAACGCATTAATGAATATGGTGTTACGTGATTTGAGC
GGCTGGCGTTGTGAAAAAATTGACCGAACATAGCGCCGTGCTGCATCTGAATGCATTTACC
CAGGTTATCTGCCATGTTCAGCAAAAACGGTTATTCATGGCCAGTATTCACAGTTGTGAA
TTCAGGGTGAAAGGCACAATCAATTATCCTTTGCAGGGCAAAATTCGCGTTCATCAGCCC
GGATGGCTGAAACGTTATCCCGTCATTTTTACTGGCAGCAAAAGTACCGCCGGGCTGATT
AACTATCTTAATTGTTTCCCCAATTTACAACAGGCACTGAGTGAACTGGATTATCGTCGG
TTTACGCTGGTTTTACATCATAAGGAATGGTATTGCGGCATTGAGCTATGGGCTGCTAGC
GAAGTGGTTTGTAAAATGCCGCCGCTGCGCCGTTATCTGCGGCTTGAGCGGCACCAGCGT
GTTCTTCTGCTTAGTGTGATAAATATGATCAATCAGGCCATGAATCAATGGCTGCAACAG
GATACCGACGCCAGATAA
>EECEBICD_00328 HTH-type transcriptional regulator MalT
ATGTCAGCCTTATCAACTACTGGCCATAATGACGGGATCACTCTCCATTGCTTACAGAGC
ATTGCGCAACTCATTCCTCTGTCGTCGGCGGTTTTTTATCGTGTTAATGCTTATTTAAAG
CCGGAAGCCTATGTTTTACATAATATTTCTAATAGCACACACCAACAATATCTGGAGCAT
TTTCAACCATTAGACCCGCTATCGCCTTCCCGATTCGGCCAACAGGTTATTACAGTAGCG
ACGATGACGCCCGGCATCTGTGCTCGTCATCGCCATTATTATCATGAATTTATGCTACCT
AATCATGTGTGCGACATGATTGAGATCTTTATTCGTCGGGGCCATCGTATTATCGCGGGC
ATTTCATTAATGCGCGATATACCTTTTTCCAGCGAAGAGCGTTTGCGAGCCCAGGCAGTA

CAACCATTACTGGGACTGGCAATTCATGATTCTCTTCAGGAAGACAACGATCTGGCGTCA
ATATTGACCGCCAAAGAGCGTGAAATTGTGGGAATGGTCTGCGAAGGGGCCAGCAACAAA
TTGATTGCCCGTCAGTTGAATATTTCTCTCTCAACAGTGAAAACGCATTTACGTAATATT
TTTGCTAAAACAGAAGTGATAAATCGCACGGAACTGGTTTCCCGAACCAGAATGTCTTCG
GTACAACATTCACAGCATATGTGA
>EECEBICD_00329 Anaerobic sulfatase-maturating enzyme
ATGTTGAATCTCAACACATTACGGCAACAACAAATCCCCGTAATGACGGAATATCGCGCG
CAGATACCCTTTCATATTCTGGCAAAACCCATAGGCCCTGCATGTAATCTGGCCTGCCGC
TATTGTTATTACCCACAGGGCGAAACGCCCGTAGAAAAAATGAATGAATCAACGCTGGAG
GTTTTTATTTGTCGCTATATTGCGGCGCAACCTGCCAGTGCGCGTGAAATTAATTTTGTC
TGGCAAGGCGGTGAACCGCTTTTAGCCGGAATCGGTTTTTATAAAAAGGTAATAGCGCTT
CAACAACGATATGCGCCTGACGGCGTGACGATCAGTAATAGTCTGCAAACGAATGCGACG
TTGTTAAACGATGCCTGGTGCCGTCTGTTTCGCGACAATAATTTTACTATTGGCATCAGT
CTTGAGGGCAGTGAAGACTTGCAAAATCATCATCGTCCGGGCAAACGCGGCGAGGCCAGC
TATCCGGCGGTGTTGCGGGGAATCACATTGTTACAACACTATCGAGTCGATTTTAATGTA
CTGATTGTCGTGCATGATGACATGGCTCGCCATGCGGCAGCCATCTACGATCATGTTGTT
AGCCTTGGCGCTCGTTATCTGCAATTTCAGCCACTGATGAACGAAGGCAACGCCCTACAG
CAACGTTACCAATTGAGTGCGGATAACTGGGGACGTTTCATGATTGATATCTGGCGTCAA
TGGCGCAAACGCGGCGATATGGGACGGGTTTTTGTGATCAACATTGAACAGGCATGGGCA
CAATATTTTACGCATATCAGCGCCACCTGTGTCCATTCCGCCCGCTGCGGAACGAATCTG
GTCATGGAGCCGGACGGCAAACTCTATGCCTGCGATCATCTGATTAATAGCCAGCATTAC
CTGGGACAGCTTGCTAATAATACGTTAGCGCCAGCCGTAGATTCCGCAACCCGGCTTCCC
TTTGGTATTAAGAAAAGCCAGCGCCGGGAGTGTCAACGGTGTTCTGTGAAAATAGTCTGC
CAGGGAGGCTGCCCCGCACATATCAACAGTGCCGGCTACAACCGACTTTGTAGCGGCTAT
TACTCTTTTTTCACGGAGATTCTGGCTCCGCTACGCGCCTGGCCCCGGGATCTGAATGGA
CTGAAAGCCTGGCGTGCTGACGTTATGGGTAGATTTTCGGGCTGA
>EECEBICD_00330 Arylsulfatase
GTGAAAAAAGAAGTAACACTTGCCACACTCAGCATTATCTTCTCCGGTACGGCGCACAGT
ACGCAAAACGAACGTCCTGATATTATCGTGATTATCGCTGATGATATGGGATATTCTGAT
ATCACTCCCTTCGGTGGGGAAATCCCAACGCCTAATTTGCAGGCGATGGCTGAGAACGGC
GTGCGGATGAGTCAATATTACACGTCTCCCATGTCTGCTCCCGCCCGTGCGATGCTATTA
ACCGGGAACACCAGTCAGCAAGCGGGTATAGGCGGTATGTGGTGGTATGAAAATACCATA
GGTAAGGAAGGCTATGAATTGCGCCTGACTGATCGGGTGACGACCATGGCTGAACGCTTT
AAAGATGCTGGTTACAATACGCTGATGGCGGGTAAATGGCATCTTGGTTTTACGCCAGGC
TCGACGCCAAAAGATCGGGGCTTTCGTCATTCTTTCGCCTTGATGGGGGGAGGCGCCAGT
CACTTTGATGATGCCGTGCCGCTGGGAACCGTGGAGATATTTCATACCTATTATACCCGT
GACAATCAGCGCATTTCACTGCCCTCTAGTTTTTACTCCAGCGAAGCCTATGCCAGCCAG
ATTAATCGCTGGATCAGCGAGACGCCACGGGAACAACCTATCTTCGCGTGGTTGGCCTTT
ACTGCGCCACATGATCCTCTGCAGGCGCCGGATGAATGGATTAGTCGTTTTAAACGTCAG
TATGAACAGGGCTATGCAAACGTCTATCGTCAGCGTATTGCTCGTTTGAAGAAACTGGGT
TTCCTGCGTGATGACATACCTCTGCCAGGACTGGAACTTGATAAAGAATGGCAGGCAATG
ACCCCGGAACAGCAGAAATATACGGCGAAGGTGATGCAGGTTTACGCTGCTATGATCGCC
AATATGGATGCACAGATTGGCACCGTTATTGAGACATTAAAAAAGACCGGGCGCGATAAA
AACACGATTCTGGTCTTCTTAAGTGATAATGGGGTGAATCCGGCAGAGGGCTTTCACTAT
GAATCTGAACCAGATTTTTGGAAGCAATTCGATAATCGTTACGAAAATATTGGTCGTAAA
AATTCATTTATCTCTTATGGTCCCCACTGGGCTGATGTCAGCAATGCGCCTTATGGTCGC
TATCACAAAACGACCAGCGGTCAGGGGGGAATTAATACCAGTTTTATGATTTCCGGTCCT
GGTATCATCCATCATGGCGCCATAGATAACGCCACGATGGCGGCGTATGATGTTGCGCCC
ACGCTCTATGAATTTGCAGGTATTGATGCCAGTAAATCATTATCTGAAAGACCGACACTG
CCAATGATCGGCGTGAGTTTTAAACGCTATCTGACCGGTGAAAGTCTGCACGCGCCTCGC
ACACAATATGGTGTTGAACTCCATAATCAGGCGGCCTGGATAGATGGGGAATGGAAATTG
CGTCGTCTTGTCACAGTATTCCCACAGGCGGGTAATGCTCCATGGGAATTATTCAACCTG
CAACGTGACCCCCTGGAAACGCATAATCTCGCAGCAGATTATGTGGATAAAGTGAAAATA
CTGAGCAGTGCATATGAGGCATTTGCAAAACAGACAATGGTGCTTTATGCCAAAGGCAAG

CTTATCGATTATGTGGGTATCGACAGTAAAACCGGGCGTTATCTGGCTGTCGATCCACAG
ACATTGCAGCCAGTTCCTGCTCCGTTAGCGATTCCTTTAGACACAAAATCGGACCAATAA
>EECEBICD_00331 Hca operon transcriptional activator HcaR
GTGATCAAGGCCTGCGGTAAAGCGGGCTTTGAACCGAAAATTGGTCAACTCGTGCCGCAA
ATTTCGTCAGTCATTAACCTTGTGTCGGCAGAGATGGGCGTGTCGATGGTTCCAGATTCA
ATGAGACAGGTTAATGTCAAGGGAGTAGTATACCGCCCCGTTGCAGACCAGATGCCCGTC
GCAAAGCTGGCTCTGGCATACCGGCGCGGCGATACATCGCCAACGCTGAGAAACTTCATC
CTGAAAGTCACTGGCTGA
>EECEBICD_00332 HTH-type transcriptional regulator BenM
ATGGAACTGCGCCATATTCGCTATTTTCTCGCCGTCGCTGAAGAGCGGCATTTCACGCGG
GCTGCGACAAAATTAGGCATAGGTCAGCCGCCGCTAAGCCAACAGATTAAGGATCTGGAA
AGAGAGCTGGGAGCGCAGCTATTCCGGAGAGTGCCTCATGGCGCGGAATTAACCGAGGCT
GGAAAAGCATTTTATGATGTTGTTAAAGGGATGCCTGCTACCGCGACAAGAGCCGTGCTG
GCGGCGCAGCGTGTTGCCAGGGGGGAATCGGGCGTGTTAAGGGTGGGTTTTACTGCCTCT
GCGGCATTTAACAGTGTGGTGCCCGGCGCAATCCGTACTTTCAAGCGGGCTTATCCTGAC
GTTCGTTTGCAGCTTGAGGAAGGAAATACAACTCAACTGGCTGATGAATTGAATGAAGGA
TCGCTGGATGTTGCCTTTCTGAGACCCGGTTTTACAGGCAATGAACGGTTCCAGCTCAGG
TTACTGTCTGAAGAGCCAATGGTTATTGTGCTGGCAGAAACGCATCCTGCTGCGGCGTGT
AAACAGATTGCACTCTCGATTCTCAAAGATGAGTTTTTTACTCTTCCCACGTGAAATCG
GCCTGTCGCTTTATGACGCAGTGA
>EECEBICD_00333 L-malyl-CoA/beta-methylmalyl-CoA lyase
ATGCCCGATATTTCTCATACACCGACACGTAGCTGGCTTTTTACCCCAGCAATTCGACCA
GAACGTTTCATAAAAGCAGTTGAAAGTGCGGCGGATATTTCTATTATCGATCTTGAAGAT
TCCGTCACCCCGAATGACAAGGCGCAGGCCCGGAAAATTGCGATGCAGTTCCTTTCATCC
CGACCAAACTCATCGCTGAAAATAGCATTAAGAATTAATGGAATGAATACTCATGCTGGC
ATTGAAGACCTGCATATGTTACTGGAATGCCGTTTTTTTCCTGATTACATTATTCTTCCG
AAAACAGAGTCAGCCGCGCACCTGCAAATCGTGGATAGTCTTATCATGATGGCTGGTTCT
GATACTCGCCTTATTGGCATTATTGAGTCAGTTGTTGGGCTTAATGCCGTAGAGTCTATT
GCGGACGCCACCCCCAGACTCTGCGGGTTGATGTTTGGCGCCGCAGATATGGCGGCTGAC
ATTGGCGCGACACCCGCCTGGGAGCCTCTTGCGCTGGCGCGGGCCAGAATAGTGGCTGCC
TGCGCTATGAAAGGGCTTCTTGCAATAGATGCTCCCTTCTTTGATATAGGCGATTTTTCA
GGTCTGAAAGAGGAAACGCTGCAGGCGCTCAGTTTTGGTTTTTCTGCAAAATCGGCTATT
CACCCCGCTCAGATTAGTGTAATCAACGCCGCATTTACTCCTACAACGGCAGAAATTAAT
CATGCTCGTGCAGTACTGACAGAGAATGCCAAAGGCGTCGGGATCGTTAGCGGCATGATG
ATAGATGCAGCCGTTGCCCGTCAGGCCCGGAGATTACTCGCCCGGGCAGGGATCTTTTCC
TGA
>EECEBICD_00334 Mesaconyl-CoA hydratase
ATGAACACGTTATTACCCGCTTATAAAACCATTAGCGAGGGGCGCTACCGAGAAATTTCG
GGTCTTTACTGGGAGGATTTTCATCCCGGTGATGTTTTTGAACATCGTCCCGGCCGGACG
GTTCTGGACGCCGATAATGTGTGGTTTACCCTGCTCACTCTCAATGTGCAGCCGGTCCAT
TTCGATGCGGCTTACGCCAGCAAAACGGAATGGAAAAAGTTACTGGTTGATTCAACGTTC
ACTCTGGCGCTTTTAACCGGTATGAGCGTGCGTACTGTCAGCGCCAAGGTCGTGGCCAAT
CTTGGCTGGGATAAGGTACAAGCCGTCCATCCGGTTTTCGCAGGGGATACACTTTATGCT
GAAAGCACTGTACTCAGCAAACGCCTGTCAAATAGTCGCCCGGGACAAGGCATCGTTACC
GTTCGGACATGTGGTATTAACCAGAATGGTGTAGAGGTCATGCGCTTCGAACGAACAATG
TTAGTTTATTGCTGTGGATGTTCGCCTGAAGAAGACGCCGCTTATTGA
>EECEBICD_00335 Butyryl-CoA:acetate CoA-transferase
ATGATGAATGTTAATGCAGTGTATGCCGAAAAATGCGTGACGCCTGACGAGGCGGTTACT
TTAATTACCTCCGGCAGTCACCTCTCTATGGGAATGTTTGCAGCAGAGCCTCCAGCTTTG
CTGAATGCGCTGGCAAAGCGAGCGAAAAGGGGAGAAATCAACGATCTGCGGGTTTACTGT
TATGAAACAGCCAGCATTGCCGGGAACACTATTTTCCGTTATGAGCTAAGTGACTATATT
CATCTCTACAGTATGTTTATTACCGGTATTGAACGGGCTTTAATACGTCAGGGGATTGAG
AGTTCCGGGCGAAAAATAGTTAACTATGTACCTTCAAATTTTCATCAGGCGACGCGATTA
CTAGCTGATGATATTGGTATTGATACCTTTATTCACACCGTGTCCCCTATGGATAAGTAT

GGCTATTTTAATTTTGGAACTGGAAACGATTATTCCACCCGGATTGCGAGGACAGCTAAA
AAGCTTATTGTCGAAGTTAACAAATATATGCCGCGCGTTCATGGAGAAGGTGCTGCAATC
CATATTTCAGAAATTGACGCCATTGTTGAAAACCATGTCCCTTTAATTGAACTACCAATA
CGTACTGCCGTAGCCGAAGATATTGCTATCAGTCAGATTATCGCTTCTCTTGTGCCAGAT
GGCGCTTGTCTCCAGATGGGGGTCGGCGCATTACCGGAGCTCATCTGTAATGCGCTCAAG
GAACATAATGATCTGGGGGTCCATACAGAGGCGCTGAATCCGGGGTTGGTAAGTTTGATT
CAACAAGGCGTCGTTACCAATCAGCGTAAAAATATCGACCGGGGAATGTCTGTATTTACT
TTTGCTATGGGACAAAAGGATATGTATGACTATCTCAACGACAACCCCTCTTTTTTTAGT
CGGCCTGTCGATTATGTTAACGATCCGGGGATCATTGCGCAAAATGAGAATGTTGTCTCC
ATTAACGCTACCTTACAAATCGATCTCACTGGTGCCTGTAACTCTGAATATTTGCTTGGA
CACCAGTACAGCGCTTCAGGAGGGCAATTGGATTTTGTCAGAGGCGCATATGCATCTAAA
GGAGGTCGTTCCATTATTACGACCCGCTCAACGGCCGCGAATGATACGATTTCTCGTATT
GTTCCCCGCCTTGAGGGGCCTGTAACAACCCCACGAACGGATACGCACTGGATAGTCACT
GAATTTGGCTCCGTAAACCTGAAGGGGCTCTCTTCAACCGAGCGTGCGCTCAGCATCATT
GAATTGGCACATCCCAATTTTCGGCAGCAACTTAGAGTAGATGCAAAAAAAATGCATCTG
ATTTAA
>EECEBICD_00336 Virulence protein
ATGATAATTGACAGAATTGATCACCTTGTATTAACGGTATCTGATATATCAACCACAATA
AGATTCTACGAAGAGGTACTTGGTTTTTCTGCGGTAACATTTAAACAAAACAGAAAAGCG
TTAATATTTGGAGCGCAAAAAATTAACCTCCATCAGCAAGAAATGGAGTTCGAACCAAAA
GCGAGTAGACCAACGCCGGGATCTGCCGATTTATGTTTTATTACATCGACACCGATAAAT
GATGTTGTGTCTGAGATCTTACAGGCAGGCATTCCCATTGTTGAAGGCCCGGTTGAGAGA
ACTGGAGCCACAGGCGAGATAATGTCTATCTACATCAGGGACCCTGATGGAAACTTAATT
GAAATTTCGCAGTATGTTTAA
>EECEBICD_00337 tRNA-Phe(gaa)
GCCCGGATAGCTCAGTCGGTAGAGCAGGGGATTGAAAATCCCCGTGTCCTTGGTTCGATT
CCGAGTCCGGGCACCA
>EECEBICD_00338 hypothetical protein
ATGACCGGCGATCCAAACATTTTGATCGCTAAATCTTTTCTGGATTTTTTCACTGCCATT
ATTTTCGCCTGTTCGCTTGGCATCGCCGTTTCGGCGATTTGCGCGCCGATGTTGATTATC
CAGTTAACGCTTGCCGCCTGCGCCACCCTCATTCTTCCACTGACCACGCCGGCAATGATG
GGCGACTTTAGCGCCGTGGGTGGACTGCTGCTGGTGGCTACCGGCTTGCGTGTATGTGGA
ATCAAAATGTTTCCGGTAGTGAATATGCTCCCGGCGCTGTTGCTGGCGATGCCGATCTCC
GCCGCATGGGCGATGTTTTTTGCCTGA
>EECEBICD_00339 hypothetical protein
GTGGTAATTGGACCTTTTATTAACGCTGGCGCAATTTTATTCGGCGGCGTTATTGGCGCG
CTGTTAAGTCAGCGTCTGCCAGAACGGATTCGCGTCTCAATGACGTCTATTTTTGGTCTG
TGCTCTCTGGGAATTGGCATTCTGCTGGTGATGAAGTGCGCTAATCTTCCGGTGATGGTG
CTGGCGACGCTGGTCGGCGCGCTTATCGGCGAGTTTTGTTTGCTGGAAAAAGGCATTAAT
GGCGCAGTCGCGAAAATCCAACAATTATTTATGGCATCGGGTAAGAAGCCGACACACGAC
TCATTTATTCAAAGTTACGTCGCTATCATTGTACTTTTTGCGCCAGCGGCACCGGTATTT
TCGGCGCGATGCATGAAGGGATGA
>EECEBICD_00340 Constitutive ornithine decarboxylase
ATGAAATCAATGAATATTGCCGCCAGCGGTGAACTCATCCCCCGTCTATCCACCCACCGA
AACGTAGTGGCGCTGGACAGTACCGACTTTACCGATGTGGCCGCAGTCGTCATTACCACT
GCCGACAGCCGTAGCGGTATTCTGGCGTTGCTTAAACGCACCGGTTTTCACCTGCCGGTG
TTTATGCTTGCGGATGAACCCGTCAGCGCGCCTGTCGGCGTTACGGCGGTAATCGGCGGG
AACGCGCAGGAATGGCTGGAGTTGGAAAACGCCGCCAGTCGCTACGAAGCGGAACTTCTG
CCGCCCTTTTATGACACCCTGACCCAATATGTCGACATGGGGAATAGTACCTTTGCTTGT
CCGGGGCACCAGCATGGCGAGTTTTTTAGAAAACATCCCGCAGGCCGCCATTTTTATGAC
TTTTTTGGTGAAAACCTGTTTCGTGCGGATATGTGTAACGCCGATGTGAAACTTGGCGAT
CTGCTAATACATGAAGGTTCTGCCAAACACGCGCAGAAATTTGCGGCAAAAGTGTTTAAT
GCGGATAAAACCTACTTTGTGCTGAATGGGACTTCGGCGGCGAATAAAGTGGTCACCAAC
GCCCTGCTGACGCGCGGCGATCTGGTGCTGTTTGATCGTAATAATCACAAATCCAACCAC

CACGGCGCCCTGATTCAGGCCGGGGCGACGCCGGTCTATCTTGAAGCGGCGCGTAACCCG
TTTGGTTTTATCGGTGGTATTGATGCGCACTGTTTTGATGAAACGTACCTGCGCGACCAG
ATTCGTGACGTGATGCCGGAAAGCGCCGATGCGCTGCGTCCGTTCCGCCTGGCGATCATT
CAGCTGGGTACCTATGACGGCACTATTTACAACGCCCGTCAGGTGGTGGATAAAATCGGG
CATCTCTGCGATTACATTCTGTTTGACTCCGCGTGGGTAGGCTATGAGCAGTTTATCAAT
ATGATGGCGGATACCTCGCCGTTGCGGCTGGAGCTTAATGAAAACGACCCAGGGATCTTT
GTGACCCAATCGGTACATAAGCAACAGGCGGGGTTCTCACAAACCTCGCAGATCCACAAA
AAAGATAACCATATTCGCGGCCAGGCGCGTTTTTGCCCGCACAAACGCCTGAACAACGCC
TTCATGTTGCATGCTTCAACCAGTCCGTTCTACCCGCTGTTTGCGGCGCTGGATGTCAAT
GCCAAAATCCACGAAGGGGAGAGCGGTCGCCGCCTGTGGGCGGAGTGCGTTGCGCTGGGC
ATTGACGCGCGTAAAGCCATTCTCGCCCGTTGCAAACTGCTTCAGCCCTTTATCCCACTG
GTGGTTGACGGCAAGCCGTGGCAGGCATATCCGACGGAGACCATTGCCAGCAATCGTCGT
TTCTTCAGTTTTGAGCCCGGGGCGAAATGGCATGGCTTTGAGGGTTATGCGGATGACCAG
TATTTTGTCGACCCCTGTAAACTGTTACTGACCACGCCAGGTATTGATGCCGATAGCGGG
CAGTACACCGAATTCGGCATCCCGGCGACCATTCTCGCCCACTATCTGCGTGAGAACGGT
ATTGTGCCTGAGAAGTGCGATCTCAACTCGATTCTGTTCTTGCTGACACCCGCCGAGAGC
GAAGAGAAACTGGCGCGGCTGGTCGCGATGCTGGCGCAATTTGAACGGCACATTGAAGAC
GATACGCCGCTTGCGGATGTATTGCCGACGGTTTTTCAGAAATACCCGGTGCGCTACCGC
GATTACACCCTGCGTGAGCTATGTCAGGAGATGCACAATCTGTACGTTAGCTTTGACGTT
AAGGATCTGCAAAAAGCGATGTTCCGCAAAGAAAGCCTGCCGCATGTGGCGATGAACCCA
CAGGACGCCAACAGCGCCTTTATTCGCGGCGATGTAGAACTGGTGCGTATCAGCGAGGCG
GGCGGGCGTATTGCTGCCGAGGGGGCATTGCCATACCCGCCGGGCGTACTGTGCGTGGTG
CCCGGGGAGATTTGGGGCGGCGCGGCGCAGCGTTATTTTTGGCGCTGGAAGAGGGCATC
AATCTGTTGCCCGGTTTTTCGCCGGAATTGCAGGGGGTATATAGCGAGACCGATGCCGAT
GGAATTCAACGCTTGTACGGATATGTTTTAAAGTAA
>EECEBICD_00341 Nucleoside permease NupG
ATGGCCGCGGAAGTCACTACGCCTGGGGCGATGTTCTTTGTGATCCTGCTTAACTCGTTG
GCCTATATGCCGACGTTGGGCTTGATCAATACCATATCGTATTACCGCCTGCAGTCTGCC
GGCATGGATATCGTGACTGACTTCCCGCCTATCCGTATCTGGGGCACCATTGGCTTTATT
CTGGCGATGTGGGGCGTGAGTTTCTCCGGTTTCGAGCTGAGCCATATGCAGCTTTATATC
GGCGCGACGCTTTCCGTTCTGCTGGTACTGTTTACCTTTACCCTGCCGCACATTCCGGTG
GCGAACCAACAGAAAAACCAGAGCTGGACATCAATGCTGGGCCTTGACGCTTTTGCGCTG
TTTAAAAATAAGCGGATGGCGATTTTCTTCATCTTCTCCATGATGCTGGGCGCGGAACTG
CAGATCACCAACATGTTTGGCAACACCTTCCTGCATAGCTTTGATAAAGATCCGCTATTC
GCCAGTAGCTTTATCGTGCAGCACGCCTCGGTGATGATGTCGATTTCGCAGATTTCTGAG
ACGTTATTCATCCTGACCATTCCGTTCTTCCTGAGCCGTTATGGTATTAAGAACGTTATG
CTTATCAGTATTGTGGCGTGGATGCTGCGTTTCGGCCTGTTCGCTTATGGCGACCCGACG
CCGTTCGGTACCGTTCTGCTGGTACTGTCGATGATTGTGTACGGCTGCGCCTTCGACTTC
TTCAACATTTCTGGCTCGGTGTTTGTCGAAAAAGAAGTACGCCCGGAAATCCGCGCCAGC
GCGCAGGGGATGTTCCTGATGATGACCAATGGCTTCGGCTGTATCCTGGGCGGCATTGTG
AGCGGTAAAGTGGTGGAGTATTACACTCAAAACGGCATTACCGACTGGCAGACCGTGTGG
CTCATCTTCGCAGGCTACTCGCTGGTGCTGGCCTTCGCGTTCGTAGCCTTGTTCAAATAC
AAACACGTTCGCGTTCCGGCAAGTTCGCAACCCGTTGCACATTAA
>EECEBICD_00342 Nucleoside permease NupG
ATGAATCTTAAGCTGCAGCTTAAAATACTCTCTTTTCTGCAGTTCTGTCTGTGGGGAAGC
TGGCTCACTACCCTGGGCTCGTATATGTTCGTCACCTTAAAATTTGACGGCGCATCTATT
GGCGCAGTTTATAGTTCACTGGGGATTGCCGCCGTCTTTATGCCGACCTTGTTAGGCATT
GTGGCTGACAAATGGCTGAGCGCGAAATGGGTCTATGCCCTGTGTCATGTCGTCGCGCCA
TCACGCTATTCATGGCCGCGGAAGTCACTACGCCTGGGGCGATGTTCTTTGTGA
>EECEBICD_00343 Membrane-bound lytic murein transglycosylase C
ATGATGAAAAAATTACTCGCGCTTGCTGTTATTGCGCCGTTGCTCATCTCCTGCTCCAGT
TCGACCAAAAAAGGCGAGACGTATAATGAAGCCTGGGTCAAGGATACCAACGGCTTTGAT
ATTTTGATGGGGCAATTTGCCAACAATATTGAAAATCTGTGGGGATATAAGGAAGTCCTG
ATTGCCGGGCCGAAAGACTACGTCAAATATACCGATCAGTTCCAGACCCGTAGCCACATC

AACTTTGATGATGGGACCATTACCGTTGAAACCATCGCCGGGACGGAACCTACCGCACAT
CTGCGCCGGGCTATTATTAAAACGTTGCTCATGGGTGACGACCCAACCTCCGTCGATCTT
TACTCCGATGTCGATGACATAAAAATTTCTAAAGAACCCTTCCTGTATGGTCAGGTCCTC
GACAATACCGGCCAGCCTATTCGCTGGGAAGGCCGCGCCACGACATTCGCGGATTACTTG
CTGAAAACGCGGCTCAAAAGCCGCAGTAACGGCCTGCGTATTATTTACAGCGTCACCATT
AATCTGGTGCCGAACCACCTTGATAAACGCGCGCATAAATACATTGGCATGGTACGCCAG
GCCTCCCGTAAATACGGCGTTGACGAGTCGCTGATTCTGGCGATTATGCAAACGGAGTCC
TCGTTTAACCCGTATGCGGTCAGCCACGCGGATGCGCTGGGCCTGATGCAGGTGGTGCAA
CACAGCGCCGGGAAAGATGTTTTCCGTTCTCAGGGTAAGTCCGGCACGCCAAGCCGTAAC
TTCCTGTTCGACCCCGCCAGCAATATCGATACCGGCACAGCGTATCTGGCGATGTTGAAT
AATGTCTATCTGAGCGGTATCGAGAATCCGACGTCGCGGCGCTATGCGGTCATTACTGCC
TATAATGGCGGCGCAGGCAGCGTATTACGCGTATTCTCGAACGATAAGATTCAGGCCGCG
AATATGATTAACCGCATGTCGCCGGGGGATGTGTACCAGATATTAACCACCCGACATCCA
TCTGCGGAGTCGCGCCGCTATCTGTATAAGGTGAATTCCGCCCAGCGATCGTATCGCCGC
CGATAA
>EECEBICD_00344 putative Fe(2+)-trafficking protein
ATGAGCAGAACGATTTTTTGTACTTACCTGCAACGCGACGCTGAAGGGCAAGATTTTCAG
CTCTACCCTGGCGAACTGGGAAAACGTATCTATAACGAGATCTCTAAGGACGCGTGGGCG
CAGTGGCAGCATAAGCAGACCATGTTGATTAACGAGAAAAAGCTCAACATGATGAACGCC
GAGCACCGTAAGCTGCTGGAACAGGAGATGGTGAGCTTCCTGTTCGAAGGCAAAGACGTT
CATATCGAAGGTTATACGCCGGAAGATAAAAAATAG
>EECEBICD_00345 Adenine DNA glycosylase
GTGGAGAATACGCTGTGGACGTTGAGCGAGCAAGTGACGCCCGCACGCGGCGTGGAGCGT
TTTAATCAGGCGATGATGGATCTGGGCGCGATGGTTTGTACGCGTTCAAAGCCAAAGTGC
ACCCTGTGTCCGCTGCAAAACGGTTGTATCGCCGCTGCGCATGAAAGCTGGTCATGCTAT
CCGGGTAAGAAACCGAAACAGACGTTGCCGGAGCGGACGGGTTACTTTTTATTGTTACAG
CATAATCAGGAGATTTTCCTGGCGCAGCGCCCTCCCAGCGGTTTATGGGGCGGACTCTAC
TGCTTTCCGCAGTTCGCCAGCGAAGATGAATTACGTGAATGGCTGGCGCAACGGCATGTT
AACGCTGATAATTTGACCCAGCTTAACGCGTTTCGCCACACATTTAGCCATTTCCACCTG
GATATTGTGCCTATGTGGCTTCCCGTGTCGTCACTGGACGCCTGCATGGATGAAGGCAGC
GCGCTCTGGTATAACTTAGCGCAACCGCCGTCAGTCGGACTGGCGGCCCCCGTGGAGCGC
TTGTTACAGCAGTTACGTACCGGAGCGCCAGTTTAA
>EECEBICD_00346 Adenine DNA glycosylase
ATGCAAGCGTCTCAATTTTCAGCCCAGGTTCTGGACTGGTACGACAAATACGGGCGGAAA
ACGCTGCCCTGGCAAATTAACAAGACGCCTTACAAAGTATGGCTCTCGGAAGTCATGTTG
CAACAAACGCAGGTGACGACGGTGATTCCTTACTTTGAGCGATTTATGGCGCGCTTTCCG
ACGGTAACGGATTTAGCGAATGCGCCGCTGGATGAAGTACTCCATTTATGGACCGGGCTC
GGCTATTACGCCCGCGCACGTAATTTGCATAAAGCGGCGCAACAGGTGGCGACGCTTCAC
GGTGGAGAATTCCCGCAAACTTTTGCCGAAATCGCCGCGCTCCCCGGCGTCGGACGTTCA
ACCGCCGGCGCAATTCTCTCCCTCGCGTTAGGTAAACATTATCCGATTCTTGATGGAAAC
GTTAAACGTGTGCTGGCTCGCTGTTATGCTGTTAGCGGCTGGCCTGGAAAAAAGAGGTGG
AGAATACGCTGTGGACGTTGA
>EECEBICD_00347 tRNA (guanine-N(7)-)-methyltransferase
ATGAAAAACGACGTCATTTCACCGGAATTTGATGAAAACGGTCGCCCGCTGCGCCGTATT
CGTAGTTTTGTTCGTCGCCAGGGGCGACTGACAAAGGGGCAAGAACACGCGCTGGAAAAC
TACTGGCCGGTGATGGGCGTCGAGTTCAGCGAAGCGCCGGTAGATTTCGCCACGTTGTTT
GGTCGCGAAGCGCCGGTGACGCTGGAGATTGGTTTTGGCATGGGGGCGTCTCTCGTGGCG
ATGGCGAAAGCGCGCCCGGAGCAGAATTTCCTCGGCATTGAAGTGCATTCACCCGGTGTC
GGCGCGTGTCTGGCATCCGCTCATGAAGAGGGCGTTGAAAACCTGCGCGTCATGTGCCAC
GACGCGGTAGAAGTGTTGCATAAAATGATTCCTGACAATTCTTTATCGATGGTTCAGCTC
TTTTTCCCTGACCCGTGGCATAAAGCGCGTCATAATAAACGCCGTATCGTTCAGGTGCCG
TTTGCTGAACTGGTATTGAGCAAGCTGAAATTGGGCGGCGTTTTCCATATGGCGACCGAT
TGGGAAGCGTATGCGGAACACATGCTTGAAGTCATGTCTTCTATCGACGGGTACAAAAAT
CTGTCAGAGAGCAATGATTATGTACCGCGCCCGGAATCGCGCCCGGTAACGAAATTTGAA

CAACGTGGCCATCGTCTTGGTCATGGCGTATGGGACTTAATGTTCGAGAGGGTGAAATAA
>EECEBICD_00348 putative protein
ATGGCAAAGAACCGTAGTCGTCGTCTGCGTAAAAAAATGCACATTGACGAATTCCAGGAA
TTAGGATTTTCGGTGGCATGGCGTTTTCCGGAAGGTACATCTGAAGAGCAGGTCGATAAG
ACTGTCGATGACTTTATTAATGACGTTATTGAGCCTAATAAACTGGCGTTTGACGGCAGC
GGCTACCTGGCCTGGGAAGGGCTGATTTGTATGCAGGAAATCGGTAAATGTACCGAAGAA
CATCAGGCGATTGTGCGTAAGTGGCTGGAAGCGCGCAACCTTGAAGAAGTCCGAACCAGC
GAACTTTTCGACGTTTGGTGGGACTAA
>EECEBICD_00349 hypothetical protein
ATGATGCGCAAAACGCTGCTGGCGGCAGTGCTAACGTTTACGGCGATGGCCGCTCATGCA
GATTACAAATGCAGCGTCACGCCGCGTGACGATGTGATCCTGAGTCCACAAACGGTGCAG
GTGAAAGGTGAAAACGGCAACCTGGTGATCACGCCGGACGGCAATGTGATGTACAACGGT
AAGCAGTACACCCTTAGCGCCGCGCAACGCGAGCAAGCGAAAGATTACCAGGCGGAACTG
CGAAGCGCGCTGCCGTGGATTGATGAGGGCGCCCGATCGCGCGTCGAAAAAGGCCGCGTG
GCGCTGGATAAAATCATCGCTAAAGAGGTGGGCGAAAGCAGCAACATGCGTAGCCGTCTG
ACGAAGCTTGATGCGCAATTAAAAGCGCAAATGAACCGCATTATTGAACATCGTACGGAC
GGCCTGACGTTCCACTATAAGGCTATCGACCAGGTGCGTGCCGACGGTCAGCAGTTGGTG
AATCAGGCGATGGGCGGTATTTTGCAGGACAGCATTAACGAAATGGGCGCCAAAGTAGTG
CTTAAAGGCGGCGGCAATCCTTTACAGGGTGTGATGGGCAGCCTCGGTGGTTTGCAAACC
GCTATCCAGAACGAATGGAAAAACCAGGAGAAAGATTTCCAGCAGTTTGGCAAAGATGTC
TGTAGCCGCGTAGTGACCTTAGAAGATAGCCGCAAAGCGCTGGTTGGCAGCCTGAAGTAA
>EECEBICD_00350 L-asparaginase 2
ATGGAGTTTTTCAGGAAAACGGCATTAGCTGCTCTGGTAATGGGTTTCAGCGGCGCAGCG
TTCGCTTTACCGAATATCACCATCTTAGCGACCGGCGGAACGATCGCTGGCGGCGGTGAT
TCAGCAACAAAATCAAATTATACGGCAGGTAAAGTCGGTGTCGAAAATCTGGTGGATGCC
GTTCCTCAATTGAAGGACATTGCGATAGTGAAAGGCGAGCAGGTCGTTAACATTGGCTCG
CAGGATATGAACGATGAAGTCTGGCTCACGCTGGCGAAAAAAATCAATACCGAGTGCGAC
AGCACCGACGGTTTCGTGATCACCCACGGTACTGATACGATGGAAGAGACCGCTTATTTC
CTCGATCTGACCGTGAAATGCAATAAACCGGTGGTACTGGTCGGCGCGATGCGTCCCTCC
ACGTCAATGAGCGCAGATGGCCCGTTCAACCTCTATAATGCGGTAGTAACAGCGGCTGAT
AAACAGTCTGCCAACCGCGGCGTGCTGGTGGTAATGAATGATACCGTCATGGACGGACGC
GACGTCACTAAGACCAATACGACTGACGTGGCGACGTTTAAAGCGGTTAACTACGGCCCG
CTGGGTTATATCCACAACGGTAAAATTGACTACCAGCGTACGCCAGAGCGTAAACATACC
ACCTCCACCCCGTTCGACGTGTCGAAGCTGACCGCGTTGCCGAAAGTCGGTATCGTTTAT
AACTATGCGAATGCGTCCGATCTGCCGGCAAAAGCGCTGGTAGACGCGGGTTATGACGGG
ATCGTGAGCGCAGGCGTGGGTAACGGCAACCTGTATAAAACGGTCTTTGATACCCTGGCG
ACCGCCGCGCACAACGGTACGGTAGTGGTGCGTTCTTCACGTGTGCCGACTGGCGCAACC
ACGCAGGATGCGGAAGTTGACGATGCAAAATACGGCTTTGTCGCTTCCGGCACGCTGAAC
CCGCAAAAAGCGCGTGTCTTACTGCAACTGGCGTTAACCCAGACTAAAGATCCAAAACAG
ATCCAGACGATGTTCAATCAGTATTAA
>EECEBICD_00351 hypothetical protein
GTGGTTCGACAATGGATTGCTGGCGCAGCGCTGTTTGCCCTGATTTCAGGCTATAGTTGG
GCAGAGGTTGCTCAACCCTCCGATAACATCTTAAAAGAACAGTTTAGTAAGCAGTACCAC
GGCATACTTAAACTGGATTCCATTACCTTAAAAAATCTTGATTCGACGGGCAATCAGGCC
ACCTGGTCTGCCGAAGGGGATATTTCTTCCCGTGAAGATATGTATACCGGCGTTGGTATG
GCGGCGGATTATTATTTTGTTGAAAAAACCTGGACCAAAGATCGTCCCGTTAAATTTTCT
GCCATGTTAACCAGTAAAGGGACGCCAGCCTCTGGCTGGACGGTGAACTATTACTCATTA
CAAATGGCAGCCAGCGATCAGGGCCGGGCTATTGACGACATTAAAACGAATGATAAATAC
CTGATCGTCAACAGCGATGATTTTAACTATCGGTTTGGCAACATTGAAGCCTCCTGGCGT
GCGCAGAAAGCGTCGATCCCCGGTCTTGAAGAACAACTCTCTGCCCTGGATAAGAAAATA
GCGGTAGCTAAAAAAGAAGCGGACGCGTATTGGGGAAAAGGCGCTGATGGGAAACCGTTG
ACCCGCGCTGAGGCGTTTAAAAAGACGTTAAAAGAGCGCGATGATTATGTAAAAGCCAAC
GATAGCAGCGTATATGCCGAGAAATACGAAAAAGAGGTCTATCAACCGGCATTAGACGCG
TGCCGTAAGCAGAGTGAGCCGTGCAATGAAGCGGCTATTCAGCAGAAAAGAGATTTAGAC

ATACACGAGCAACGGCGGCAGGTATTTTTGAAATCAGAAGAGCTGCGTCGAAAAGCGCAG
AATGACTGGATAACGTTAGAGAAAGGACAATATCCGCTGAATATAGCGGTACAGAAACTT
CAGATGCAACAGAGTGATATCCGTGTGAAAATTATGGATATTAATGACGGTTACGAGCGC
TGGAAAAAAGATACGGACGATTTGCGTCGTAAAGGAGTTATTAAATAA
>EECEBICD_00352 Heme chaperone HemW
ATGGCTAAACTACCGCCGCTCAGTCTTTATATTCATATTCCCTGGTGTGTACAAAAATGT
CCATATTGCGACTTCAATTCCCATGCGTTGAAGGGCGAGGTGCCACATGACGACTACGTC
CAGCATCTGTTAAACGATCTGGATGCCGACGTCGCTTGGGCGCAAGGGCGTGAAGTAAAG
ACCATTTTTATTGGCGGCGGTACGCCAAGCCTGCTTTCCGGGCCGGCGATGCAAACGCTG
CTGGACGGCGTGCGTGCGCGCCTGAATCTGGCGGCGGATGCGGAAATTACTATGGAAGCG
AACCCCGGCACGGTCGAAGCCGACCGCTTCATCGACTATCAGCGCGCCGGCGTAAACCGG
ATCTCCATTGGCGTGCAGAGCTTTAGTGAGCCTAAGCTGAAACGTCTTGGCCGTATTCAC
GGTCCACAAGAGGCGATGCGGGCAGCAAGACTGGCAAATGGGCTTGGGCTACGCAGCTTT
AACCTCGACTTGATGCATGGATTGCCGGACCAAACGCTGGAAGAGGCGCTGAATGATTTG
CGACAGGCAATTGCGCTTAATCCGCCGCATCTCTCATGGTATCAATTGACGATTGAACCC
AACACTCTGTTCGGTTCGCGTCCGCCGGTTTTACCGGACGATGACGCTCTGTGGGATATC
TTTGAGCAGGGCCACCAGTTATTAACCGCCGCGGGCTATCAGCAATACGAAACGTCGGCC
TATGCCAAACCCGGTTATCAGTGCCAGCATAATCTGAACTACTGGCGCTTTGGCGACTAT
CTGGGGATTGGCTGCGGCGCCCACGGTAAAGTCACCTTCCCGGGCGGCAGGATCCTGCGC
ACCACCAAAACCCGTCACCCACGCGGTTATATGCAGGGACGTTACCTGGAAAGCCAGCGT
GACGTGAGTGATGACGATAAACCCTTTGAGTTCTTTATGAACCGTTTTCGGTTGCTGGAG
CGCGCGCCTCGCGCCGAATTTGTCGACTATACCGGGCTTACGGAAGCGGTTATTCGTCAG
CCAATCGACGAGGCTATTGCCCAGGGCTACCTGACCGAATGCGAGCAATACTGGCAGATT
ACCCGGCACGGTAAACTGTTTTTAAACTCTCTTCTTGAGTTGTTTCTCGCGGAATAA
>EECEBICD_00353 dITP/XTP pyrophosphatase
ATGCAAAAAGTTGTTCTCGCAACCGGCAACGCCGGTAAAGTGCGCGAGCTCGCGTCGCTG
TTAAGCGATTTTGGGCTTGATGTGGTAGCGCAAACGGAACTGGGCGTCGATTCCGCGGAA
GAAACCGGCCTGACGTTTATTGAAAACGCGATTCTCAAAGCGCGCCATGCCGCCAAAATG
ACCGGCCTGCCCGCCATTGCCGATGACTCCGGTCTGGCCGTTGACGTTTTAGGCGGCGCG
CCGGGTATCTATTCCGCGCGCTATTCTGGCGAAAACGCGACAGATCAGCAAAATCTGGAA
AAACTGCTGCATACTCTACGGGATGTTCCGGACGACAAACGCCAGGCGCGATTCCATTGT
GTACTGGTTTATTTGCGTCATGCGGAAGATCCCACACCGATAGTCTGTCACGGTAGCTGG
CCTGGCGTCATTACTCGCCAGGCGGCCGGAAATGGCGGATTTGGCTATGATCCCATTTTC
TTCGTACCGTCTGAAGGCAAAACCGCCGCAGAACTGACCCGTGAAGAAAAAAGCGCGATT
TCCCACCGTGGACAAGCGCTCAAGCTGCTGCTGGATGCGTTACGCAATGGCTAA
>EECEBICD_00354 hypothetical protein
ATGAGTGCTGTGACTCGCTGCGAAGACGGTCTGGTGTTACGGCTTTATATTCAGCCGAAA
GCCAGCCGCGACAGTATTGTGGGCTTACATGGCGACGAGGTGAAAGTCGCCATTACTGCC
CCGCCGGTTGACGGTCAGGCAAACAGCCATCTGACCAAGTTTCTTGGTAAACAGTTTCGC
GTGGCGAAAAGTCAGATCGTCATCGAAAAAGGCGAGCTGGGCCGTCACAAACAGGTAAAA
ATTATTCACCCGCAACAGATCCCGCCGGAAATTGCGGCGTTAACTGAGTAG
>EECEBICD_00355 hypothetical protein
ATGAATACGTTGACCTTCCTGCTCTCAACGGTAATTGAGCTGTATACCATGGTGCTATTA
TTGCGCGTGTGGATGCAGTGGGCCCGCTGTGACTTTTACAACCCTTTCTCCCAGTTCGTG
GTGAAAATTACGCAGCCCATCATCGGACCGCTGCGTCGTATTATCCCGCCAATGGGGCCG
ATTGACAGCGCTTCCCTGCTAGTGGCGTTTATTCTTAGCGTTATTAAGGCGATAGTGCTG
TTTAAGGTCATTACGTTTCAGGCGATTATCTGGATTGCCGCAGTGCTTATCTTGCTTAAA
ACAATCGGCCTGCTGATCTTCTGGGTGCTGCTGGTCATGGCTATCATGAGCTGGGTGAGC
CAGGGTCGTAGCCCGATTGAATATGTATTGATTCAGCTTGCCGAGCCGCTGCTCAGCCCG
ATTCGCCGTATACTGCCTGCGATGGGCGGCATCGACTTCTCGCCGATGGTTCTGGTGCTG
CTGCTGTATGTCGTTAATATGGGGATCGCTGAAGTCTTACAAGCGACAGGCAACATGTTG
CTGCCGGGGCTGTGGATGGCGCTATGA
>EECEBICD_00356 Pyridoxal phosphate homeostasis protein
ATGAAAGATATCGCGCATAACCTGGCATACATCCGGGACAAAATCTCCGCCGCGGCGACG

CGTTGCGGCCGCTCTTCAGAAGAAGTTACGTTGCTTGCAGTGAGTAAAACCAAACCTGCG
AGCGACATCGCAGAAGCCATTGCCGCCGGACAACGGGCATTTGGTGAAAATTATGTACAA
GAGGGGGTGGAAAAAATCCGCCACTTTCAGGAAGCGAAAGTGGAAGGCCTGCACTGGCAC
TTTATTGGCCCTTTACAGTCCAACAAAAGTCGGCTGGTGGCCGAGCATTTTGACTGGTGT
CATACCATCGATCGCCTGCGTATCGCCTCGCGGCTGAGCGAACAGCGTCCGGATAATCTG
CCGGCGCTAAATGTTCTGATCCAAATTAATATCAGCGATGAAAACAGCAAATCAGGCATT
CCGCTGGCGGAACTGGATGAGCTGGCCGCTGCGGTGGCTACACTGCCGCGTCTGCGGCTT
CGCGGACTGATGGCGATCCCGGCGCCAGAGTCAGATTATGTAAGGCAGTTTGAAGTCGCA
CGTCAAATGGCTGTAGCATTTGCCGGGCTGAAAGCGCGCTACCCTGACGTTGATACCCTA
TCTCTGGGTATGAGCGACGATATGGAAGCCGCGATTGCGGCAGGTAGCACAATGGTGCGC
ATCGGCACCGCTATCTTTGGTGCTCGTGATTACACAAAAAATTAA
>EECEBICD_00357 putative protein YggR
ATGAATATGGAAGAAATTGTGACGCTTAGTGTAAAGCATAACGTCTCGGATCTACACCTG
TGTAACGCCTGGCCTGCGCGATGGCGCAAACAAGGACGCATGGAGAACGCGCCTTTTACC
GCGCCTGATGTCGACAGGCTTCTGCTTGATTGGCTTAATGATGCGCAACAATATCAGTGG
CGAACGCACGGCCAGCTTGATTTCGCCGTCTCGCTGTCTGGCACGCGGCGCCTGCGCGCC
AGTGCGTTCACACATCAACAGGGAACGTCGCTGGCGCTACGGCTATTGCCCGAGCGTTGT
CCTGATCTGGCGGAAATCCAGACGCCGCCGATAGTACCCGCACTGCTTGCCAGCGAGAAC
GGACTGATTCTGGTCACTGGCGCTACTGGCTGCGGGAAATCCACTACGCTGGCGGCGATG
GTGGGACATCTTAATCAACATGCCGATAAACACATCCTGACGCTGGAAGATCCTATTGAG
TACCGTTACACCAGCAAGCGTTGCCTGATACAGCAGCGGGAAATCGGCCAGCATTGCGCC
ACTTTTGCCGCCGGGCTGCGCGCGGCGTTACGTGAAGATCCCGATGTCATTTTACTGGGA
GAGCTGCGTGACAGCGAGACCATTCGCCTGGCGCTGACGGCGGCGGAAACGGGGCATCTG
GTACTGGCAACCCTGCATACTCGCGGTGCGGCGCAGGCGGTGGAAAGATTAGTGGATAGT
TTTCCGGCGCAGGAAAAAGAACCGGTACGCAGCCAACTGGCGGGTAGTCTGCGCGCCGTA
CTATCACAAAAACTGGAGGTCGACAGACAGGATGGGCGCGTGGCGTTGTTTGAATTACTG
ATTAATACGCCAGCCACAGGGAATTTGATCCGTGAAGGCAAACTCCATCAGTTGGCCCAC
GTGATACAAACCGGGCAGCAGCAGGGGATGATGACATTCGCCCAGAGCGCGCAATGGCGT
CAGGCGCAGGGACGACTGTAG
>EECEBICD_00358 hypothetical protein
ATGTCATCACAACCGAATCAAAGCCTGATAGATGGCATTCGCTGTCTGCAATATCTGGTC
TCCAGCGACCGTGCTATCGGATGTCGCGAGCTTGCCCGGCTGATGGATATTAATACCACG
CGCGTTAATCGCCTGTTAATGACGATGGCCTCTATTGGACTCACGATGCAGGATGAGCAC
CGACGCTATTTACCCGGGCCAGGCATTCATGCTTTGGCGGCGCAGGCGATTCGTGGTTCA
GCGCTGTTCTCCCATGCGTTGCCTATCCTTGAACGCCATGCGCCCAAAGACATCGTGGTG
GCATTAGGCGTGCTATGGGAAGACCAGATTATCTATATTTATCACTCTACGCCGGGTTCG
CAGGGAAGTCAGGCGCTGGCGGGGTTTCGCATGTGCCCGGCCTGGCAGTCGGTAACAGGA
GTGGCGTTACTGGCGGCAGAAAGCGATGAGGCGCTAATGCAGCGTTTTACTCCGGAGCAA
TGGCGTAATCTGGCGCCGCATGTGGCGCAGCAGCGCCAGCGGGGATACGTGCTGTGGCAT
CACGCTGATGGTGAAGTTTCTATGGCGCAACCGCTAGGCAAGCACGCCGCAGCGCTGGCC
TTTGCGGGAATGTGGCGGATTGATGAGGCGGAGGCGGCTGCGCGTTTACAGGCGTTGAAG
GCATTAAACCAGCTTATTGCCCAGTAA
>EECEBICD_00359 Putative pre-16S rRNA nuclease
ATGAGCGACACCTTACTGGCCTTTGACTTCGGCACAAAAAGCATCGGCGTTGCAATAGGA
CAACGCATTACCGGCACCGCCCGTCCGCTTCCGGCAATCAAAGCACAGGACGGCACGCCC
GACTGGATGCTCATTGAACGTTTGCTGAAAGAGTGGCAGCCGGACGAAATTATTGTCGGG
CTACCGCTCAATATGGATGGCACTGAACAGCCGCTGACGGCGCGCGCACGTAAATTCGCC
AACCGTATTCATGGGCGTTTCGGGGTAACTGTCACGCTGCACGATGAACGACTCAGCACC
GTTGAAGCACGCTCCGGTCTGTTTGAACGAGGTGGGTATCGCGCACTGAACAAAGGCAAA
GTGGACTCGGCCTCCGCCGTGATTATCCTCGAAAGCTATTTTGAACAGGGGTATTAA
>EECEBICD_00360 hypothetical protein
ATGAATTTACAGCATCACTTTCTTATTGCCATGCCTGCGCTCCAGGACCCGATTTTCCGC
CGTTCCGTGGTGTATATTTGCGAGCACAACCAGGACGGCGCTATGGGGATTATTGTTAAT
AAGCCTTTGGAAAATCTGCAAATTGAAGGGATTCTGGAAAAACTAAAAATTACGCCGGAA

CCGCGCGATTCGTCAATTCGTCTTGATAAAGCCGTGATGCTCGGCGGCCCGCTGGCGGAA
GATCGCGGATTTATTCTCCATACTCCGCCGTCACGTTTCGCCTCCAGCATTCGTATCTCA
GACAATACCGTGATTACCACCTCCCGCGATGTGCTGGAAACGTTAGGTACCCAACAACAG
CCTTCCGATGTACTGGTCGCGCTGGGCTACGCCTCCTGGGATAAAGGCCAGCTTGAACAA
GAGCTGCTTGATAACGCCTGGCTTACCGCGCCCGCCGATCTCAATATTTTGTTCAAAACA
CCCATTGCCGAGCGCTGGCGCGAAGCGGCAAAACTTATTGGCATTGATATTCTGACCATG
CCTGGCGTTGCGGGGCACGCCTGA
>EECEBICD_00361 Glutathione synthetase
ATGATCAAGCTCGGCATCGTGATGGACCCCATCGCACACATCAACATCAAGAAAGACACC
AGTTTCGCTATGCTGCTGGAAGCCCAACGTCGTGGTTATGAACTTCATTATATGGAGATG
GCCGACCTTTATCTGATTAACGGCGAAGCCCGCGCCCGCACGCGAACGTTGAGCGTGGAG
CAAAATTACGATAAATGGTACGAGTTCGGCAGCGAGCAGGAGATTAAACTCGCCGATCTG
GACGTCATTTTGATGCGCAAAGATCCGCCGTTTGATACCGAGTTTATTTATGCGACCTAT
ATTCTGGAACGCGCTGAAGAAGAAGGGACGCTGATCGTCAACAAACCGCAAAGCCTGCGC
GACTGTAATGAAAAACTGTATACTGCCTGGTTTGCCGATCTGACGCCGGAAACGCTGGTA
ACGCGCAATAAAGCGCAGCTCAAAGCGTTTTGGGAAAAGCACGGCGATATCATTATGAAA
CCGCTGGACGGTATGGGCGGCGCGTCCATTTTCCGCGTTAAGGAAGGCGATCCTAACATA
GGCGTTATTGCCGAAACGCTGACCGAGCTGGGCAACCGCTACTGCATGGCACAGAACTAT
TTGCCCGCGATCAAAGATGGCGATAAGCGCGTCCTGGTCGTCGACGGCGAGCCGGTGCCT
TACTGCCTGGCGCGTATTCCGCAGGGCGGCGAAACCCGCGGCAACCTGGCGGCTGGTGGT
CGCGGCGAACCCCGTCCGTTGAGCGAAAGCGACTGGGAAATCGCCCGCCGCGTTGGGCCA
ACGCTTAAAGCCAAAGGGCTTATTTTCGTCGGTCTGGATATTATCGGCGACCGCTTAACG
GAAATTAACGTGACCAGCCCAACCTGCGTGCGTGAAATTGAAGCGGAATACCCGATCTCT
ATCACCGGGATGTTGATGGACGCCATCGAAGCGCGTCTGGCGAAGTAA
>EECEBICD_00362 Ribosomal RNA small subunit methyltransferase E
ATGCGTATTCCCCGCATTTATCACCCTGAATTGTTGACGTCCGGTACGCAGATTTCGTTA
TGCGAAGATGCGGCCAACCATATTGGTCGTGTACTGCGCATGGGAGCGGGACAAGCGTTA
CAGCTGTTTGACGGCAGTAATCAGGTATTCGATGCTGAAATCATTAGCGCCAGTAAGAAA
AGCGTTGAAGTGCAAGTGATGAAAGGCGAAATCGACGATCGTGAATCGCCGCTACATATC
CATCTGGGCCAGGTGATGTCGCGCGGTGAAAAAATGGAATTTACTATCCAGAAATCGATC
GAACTAGGTGTAAGCCTCATTACGCCACTGTTCTCTGAGCGCTGCGGCGTTAAACTGGAT
AATGAACGTCTGAACAAAAAGCGCCAGCAGTGGCAAAAGATCGCCATCGCCGCCTGCGAA
CAATGCGGGCGTAACCGGGTGCCGGAAATTCGCCCGCCGATGGCGCTGGAAGCCTGGTGC
GCCGAGCAAGATTCCGGGCTGAAGCTCAATCTTCATCCGCGCGCCCATGCCAGCATCAAC
ACTCTGCCGCTGCCGGTTGAACGCGTCCGACTGCTGATCGGCCCGGAAGGCGGGCTGTCG
GCGGACGAAATCGCGATGACCGCGCGCTATCAGTTTACTGATATTCTGTTAGGACCTCGC
GTTCTGCGTACTGAGACAACCGCGCTCACCGCCATTACCGCGCTACAGGTGCGTTTTGGC
GACTTAGGATGA
>EECEBICD_00363 Endonuclease-1
ATGTACCGTAATTTTTCTTTTGCCGCTGCGTTGCTGGCCGCAGCGTTTTCAGGCCAGGCC
CTGGCCGATGGCATTAACAATTTTTCTCAGGCCAAAGCGGCGAGCGTCAAAGTCAATGCC
GACGCGCCCGGCAGCTTTTACTGCGGGTGCCAAATCCGCTGGCAGGGTAAAAAAGGCGTC
GTAGACCTGGAGTCCTGCGGCTATAAGGTGCGTAAAAACGAGAATCGCGCCAGACGCATT
GAGTGGGAGCACGTTGTCCCCGCCTGGCAATTCGGTCATCAGCGCCAGTGCTGGCAGGAC
GGCGGGCGAAAAAACTGCGCTAAAGACCCGGTCTACCGCAAAATGGAAAGCGATATGCAT
AACCTGCAACCCGCGATTGGCGAAGTGAATGGCGATCGCGGCAACTTTATGTATAGCCAG
TGGAACGGCGGCGAAGGTCAGTACGGGCAGTGCGCCATGAAAGTAGATTTCAAAGCGAAG
CTCGCCGAGCCGCCCGCCCGCGCCCGTGGCGCAATCGCCCGCACTTATTTTTATATGCGC
GACCAATACCAACTGAAACTTTCCCGCCAACAAACGCAGCTTTTTAACGTCTGGGATAAG
CAGTACCCCGTTACCGCCTGGGAGTGCGAGCGCGATGCGCGTATCGCGAAGGTCCAGGGT
AATCATAATCCCTATGTGCAACGCGCTTGCCAGGCGCGAAAGAGCTAA
>EECEBICD_00364 Protein SprT
ATGCGCCGTCTGCGGGAAAATCTCGCCCAGGCGAACCTCAAGCTCGATAGACATTATCCG
GAGCCGAAACTGGTGTATACGCAACGCGGCACCTCGGCGGGCACCGCCTGGCTGGAGAGC

TACGAAATCCGCCTCAACCCGGTGTTACTGCTGGAAAACATCGACACCTTTATAGCAGAG
GTCGTGCCGCATGAACTGGCGCATCTGTTGGTGTGGAAGCACTTCGGACGCAAGGCTCCG
CATGGCAAGGAATGGAAGTGGATGATGGAAAGCGTGCTGGGCGTTCCGGCCAGACGTACT
CATCAATTTGCGCTGCAATCCGTACGGCGCAACACCTTTCCCTACCATTGCCAATGCCAG
CAACATCAACTCACCGTCCGCCGTCATAATCGGGTAGTACGCGGCGAAGCGGTTTATCGT
TGCGTTCACTGCGGCGAACCACTGGTCGCCGGGTAG
>EECEBICD_00365 Galactose-proton symporter
ATGCCTGACAATAAAAAACAAGGGCGTTCCAACAAGGCCATGACATTTTTTGTCTGCTTT
CTTGCAGCCCTGGCAGGATTACTTTTTGGCCTGGATATCGGCGTTATCGCCGGTGCTTTA
CCCTTCATTACCGACGAGTTTCAGATTACCGCACACACTCAGGAATGGGTGGTCAGCTCC
ATGATGTTTGGCGCGGCCGTCGGCGCTGTCGGCAGCGGCTGGCTCTCCTTTAAGCTGGGT
CGTAAAAAAAGCCTGATGATCGGCGCTATCCTGTTCGTCGCCGGTTCGCTGTTCTCCGCT
GCGGCGCCTAACGTTGAAGTCCTGATTATTTCTCGCGTGCTGCTGGGTCTGGCCGTCGGC
GTCGCTTCCTACACTGCACCGTTGTACCTGTCCGAGATCGCGCCGGAAAAAATTCGCGGC
AGTATGATCTCAATGTACCAACTCATGATCACCATCGGGATTCTGGGCGCTTATCTGTCA
GATACCGCGTTCAGCTACAGCGGCGCATGGCGCTGGATGCTCGGCGTGATCATCATCCCG
GCCATTCTGCTGCTGATCGGCGTCTTCTTCCTGCCGGACAGCCCGCGCTGGTTTGCGGCA
AAACGCCGCTTCCATGATGCTGAACGCGTCCTGTTGCGCCTGCGTGACACCAGTGCGGAA
GCAAAACGTGAGCTCGATGAAATTCGCGAAAGTTTGCAGGTTAAACAGAGCGGTTGGGCG
CTGTTCAAAGAGAACAGCAACTTCCGCCGCGCCGTGTTTCTTGGCATTTTGTTGCAGGTC
ATGCAGCAATTTACCGGGATGAACGTCATCATGTATTACGCGCCGAAAATCTTCGAACTG
GCGGGGTACACGAATACCACGGAGCAGATGTGGGGCACCGTTATTGTTGGTCTGACTAAC
GTGCTGGCAACCTTCATCGCTATCGGCCTGGTTGACCGCTGGGGCCGCAAGCCGACCCTG
ACGCTGGGCTTCCTGGTCATGGCGATTGGTATGGGGATTCTGGGTACGATGATGCACATC
GGCATCCATTCGCCGTCTGCGCAATACTTCGCTATCGCTATGCTGCTGATGTTCATCATT
GGATTTGCGATGAGCGCTGGTCCGCTGATTTGGGTACTGTGCTCCGAAATCCAGCCGCTG
AAAGGCCGCGATTTCGGCATCACCTGCTCCACCGCGACCAACTGGATCGCCAATATGATC
GTCGGCGCAACGTTCCTGACCATGCTGAACAATCTGGGTAACGCCAACACCTTCTGGGTC
TACGCTGGCCTGAACGTGCTGTTTATCCTGCTGACCCTGTGGCTGGTGCCGGAAACCAAA
CACGTTTCGCTGGAACACATTGAACGTAACCTGATGAAAGGTCGTAAACTGCGCGAAATC
GGCGCCCACGACTAA
>EECEBICD_00366 S-adenosylmethionine synthase
ATGGAAGAGATCATCAAACCCATTCTGCCGTCTGAATGGTTAAATACGTCCACTAAGTTT
TTTATCAACCCAACCGGTCGTTTTGTTATCGGCGGCCCGATGGGCGATTGTGGTCTGACC
GGTCGTAAGATCATTGTTGATACCTACGGCGGTATGGCGCGTCACGGCGGCGGCGCCTTC
TCCGGTAAAGATCCGTCTAAAGTTGACCGTTCTGCAGCTTACGCTGCGCGTTATGTCGCG
AAAAACATCGTGGCGGCAGGTCTGGCGGATCGCTGTGAAATCCAGGTCTCCTACGCTATC
GGCGTAGCGGAGCCGACGTCCATCATGGTGGAAACGTTCGGTACTGAAAAAGTGCCTGCT
GAACAGTTGATTCTGCTGGTGCGCGAATTCTTCGACCTGCGCCCGTACGGCTTGATTCAG
ATGCTGGATCTGCTGCACCCGATCTACAAAGAAACTGCGGCTTACGGCCACTTCGGTCGC
GAAAACTTCCCATGGGAAAAAACCGACAAAGCGCAACTGCTGCGCGATGCTGCCGGTCTG
AAATAA
>EECEBICD_00367 S-adenosylmethionine synthase
ATGGCAAAACACCTTTTTACGTCTGAGTCCGTATCAGAAGGGCATCCTGACAAAATTGCA
GACCAAATCTCTGATGCCGTGTTGGACGCTATCCTGCAACAGGATCCGAAAGCACGCGTC
GCCTGTGAAACCTACGTTAAAACCGGCATGGTTTTAGTTGGCGGTGAGATCACCACCAGC
GCCTGGGTCGATATCGAAGAGATTACCCGTAATACGGTGCGCGAAATTGGCTATGTGCAT
TCCGACATGGGCTTTGACGCCAACTCTTGCGCGGTACTGAGCGCAATTGGCAAACAGTCT
CCGGATATCAACCAGGGCGTTGACCGCGCAGACCCGCTGGAACAAGGCGCGGGCGACCAG
GGCCTGATGTTTGGCTACGCGACAAATGAAACCGACGTACTGATGCCTGCGCCAATCACC
TACGCGCACCGTCTGGTACAGCGTCAGGCTGAAGTGCGTAAAAATGGCACCCTGCCATGG
TTGCGTCCGGATGCAAAAGCCAGGTCACTTTCCAGTATGACGACGGCAAAATCGTCGGTA
TCGACGCCGTGGTTCTCTCTACGCAGCACGCAGAAGATATCGACCAAAAATCGCTGCAAG
AAGCGGTGA

>EECEBICD_00368 hypothetical protein
ATGACAGAAGATTCTACCATGAGTCTGAAGGTCGCTGACACTGGCAGTCTGAGTGTTAAT
CAGTATGGATGGATTAACATCTGGACGGCTATTTTAGGTCACTTCTTCGCCCAATTTCCA
GCTTTTTTTGAAGGCCGTCGCAATGTCGGTCAAAATCAGGCGATAAACGTTTCTGATAAT
GCTGACATTATGCGCATTTATGCTTTGCTTTTTTTCTGCGTTCTGGGTATAAAACGCGAC
GCGCGGCTCATACAAAAGCACACGACGTTTTCTTCGTGTCGCCACTTCCAGCCGGGTTAA
>EECEBICD_00369 Biosynthetic arginine decarboxylase
ATGTCTGACGACATGTCTATGGGTTCGCCTTCGTCAGCAGGCGAACAGGGTGTACTACGC
TCCATGCAGGAGGTTGCAATGAGCTCCCAGGAAGCCAGCAAGATGCTGCGCACTTACAAT
ATTGCCTGGTGGGGCAATAACTACTACGACGTCAACGAGCTGGGCCATATTAGCGTATGC
CCCGATCCCGACGTACCGGAAGCGCGTGTCGATCTTGCCAAACTGGTGAAAGCGCGCGAA
GCGCAAGGTCAGCGTCTGCCGGCGCTGTTCTGCTTCCCGCAGATCCTGCAACACCGTTTG
CGTTCAATTAACGCGGCGTTTAAGCGTGCGCGTGAATCTTACGGTTATAACGGCGACTAC
TTCCTCGTTTATCCGATTAAGGTCAATCAGCATCGCCGCGTGATCGAGTCCCTTATCCAT
TCCGGTGAACCGTTGGGGCTGGAAGCGGGGTCAAAAGCGGAATTGATGGCGGTGCTGGCG
CATGCCGGCATGACCCGTAGCGTGATCGTCTGTAATGGTTATAAAGACCGTGAATACATT
CGGCTGGCGCTGATTGGCGAGAAGATGGGCCATAAGGTTTATCTGGTCATCGAAAAGATG
TCTGAAATCGCGATTGTGCTGGAAGAGGCCGAGCGCCTGAATGTGGTTCCTCGCCTGGGC
GTGCGTGCGCGTCTGGCCTCTCAGGGGTCCGGTAAGTGGCAATCCTCCGGCGGCGAAAAA
TCAAAATTCGGCCTGGCGGCGACCCAGGTGCTGCAACTGGTGGAGACCCTGCGTGACGCC
GGGCGTCTGGACAGTCTGCAACTGTTGCACTTCCACCTGGGATCGCAGATGGCGAACATT
CGCGATATCGCGACCGGCGTGCGCGAGTCCGCGCGTTTCTATGTTGAGCTGCATAAGCTG
GGCGTTAATATCCAGTGCTTCGACGTGGGCGGCGGTCTGGGCGTGGATTATGAAGGTACC
CGCTCGCAGTCCGACTGTTCGGTGAACTATGGCCTGAACGAATATGCCAATAACATCATC
TGGGCGATTGGCGATGCCTGCGAAGAGCATGGTTTACCGCATCCGACGGTGATTACCGAG
TCTGGCCGCGCCGTCACTGCGCACCATACGGTACTGGTCTCTAACATTATCGGTGTGGAG
CGTAACGAATACACGGATCCGACTGCCCCTGCTGAAGATGCGCCGCGCGCGCTGCAAAAT
CTGTGGGAAACCTGGCAGGAGATGCATAAACCTGGCACCCGCCGCTCGCTGCGTGAGTGG
CTGCATGATAGCCAAATGGATCTGCACGATATTCATATCGGCTACTCTTCCGGCGCGTTT
AGTTTGCAGGAGCGCGCCTGGGCGGAGCAACTTTATCTCAGCATGTGCCATGAAGTGCAG
AAGCAACTGGACCCGCAAAACCGTGCGCATCGCCCGATTATCGACGAACTGCAAGAGCGG
ATGGCGGACAAAATGTACGTCAACTTCTCGCTGTTCCAGTCGATGCCGGACGCGTGGGGA
ATCGATCAGCTCTTTCCGGTGCTGCCGCTGGAAGGGTTAGATCAGGTACCGGAACGTCGT
GCCGTGCTGCTGGATATAACCTGTGATTCCGATGGCGCTATTGACCACTATATCGATGGC
GACGGTATCGCCACGACGATGCCGATGCCGGAATACGATCCAGAGAATCCACCGATGCTC
GGCTTCTTTATGGTCGGCGCCTATCAGGAGATCCTCGGTAACATGCACAACCTGTTTGGC
GATACTGAAGCGGTTGACGTGTTTGTCTTCCCGGATGGTAGCGTCGAGGTTGAGCTGTCG
GACGAAGGCGATACCGTTGCGGATATGCTGCAATATGTGCAACTGGATCCAAAAACGCTG
TTAACGCATTTCCGCGACCAGGTAAAACAGACGGATCTGGACGATGCGTTGCAGCAGCAG
TTCCTTGAGGAATTTGAAGCGGGACTGTACGGTTATACTTATCTTGAAGATGAGTAA
>EECEBICD_00370 hypothetical protein
ATGAGCAAATTTAAATTTAATGCCGTCATTGTTGGCTTGTTTTTGCTGTTAGGCGGTTGT
TCCAGCGGAATGCAATCTAATAATGGCTCTTCATCCGACGTCGGCACCGCCTGGGGCGGC
GACGTGCATTCATCGGTACAAAGCGTTTCGGTAGAACGTGCCGATCGAGAACCTGCCGAA
ATGGTGATAATAAATTACTCCACTCAATATCCGTCAGGTTACGATAAAGTTTATAGTATA
CGTATCAGCGATCTTGAATATGCTGTGCGCGACGCTAATTTTAATTCGATTCCAATTACT
CGTCGCTACAACGCATCAATGGGACAATGGCAATATAGTATTCCTGCAAGATCAGGGATG
AACTATCAGTTATATATTCGTAATTACAGTCATGATACCAATTACGAAATCGTCGCAACG
GTTGATGGCCTGGACGTGTTGAACGGAAAAGCAGGCTCGCTGAATCATCATGGCTATATT
GTTAACGCCGGCGATTCACTGGCGATTAAAGGGTTTCGCAAAGATAAACACACTGAGGCG
GCATTCCAGTTCGCCGACGTAGCTGACGCTTATGCGGCCCACTCCGCGCAGGGCGATGTA
CGCAACATCGGCGTCATCGGCTTTGCCGCTTTTGCATTGCAGGGTAAAGCAACAAATACG
CTACCGCCTTGTTCCTCGCAGGCTTTCCCGGCGGACAACAACGGGTATGCGCCGCCGCCC
TGCCGCAAATAA

>EECEBICD_00371 Pyruvate dehydrogenase complex repressor
ATGGAACAAATTTTAACCAAGCGGCGCTATTTTGATATAGGGCTGCAAATAGAAGAACTA
CTCTATTCAGGGGTATTTAAAGCTGGCGAGCGACTTCCGGCGGAGAGAGAACTCAGCGAG
CGTTTTCAAACCAGCCGCACGACGATTCGTGAGGCGATTATTATGCTTGAGCTGAAGGGG
GTGGTAGAGGTTAAGCAAGGCGCAGGCACTTACTTTATTGATAGTCTGGAAAAAATCAAT
CAAAAAGCGCTCTTGCCTTACTCAGACATTGGCCCCTTTGAATTGCTACAGGCCCGGCAG
GTTATCGAAAGTAACATCACCGGTTTTGCCGCTACGCAAATAAGATTTAATGAGCTTAAG
CAGCTTAAACGTATTATTGAGCAGCAGGAAAAGCAGATTGGCGGCGACAGCGACAAATTT
GAAGAACTGGATCGTCAGTTCCACAATATCATCGCGGAATCCACGCAAAATCGTGTATTA
ATGAAACAATCTGCCGAGCTGTGGCGGGCTGTCAGGACCGAAAATCCGCGCTGGAAGCAG
TTAAACTATAAGTATTTACATAAGAAAGAGTTAAGAATGAAATGGGTGGAAGATCACCGT
AGTATCTTCCTTGCATTGCAAAAACGCGATGCTGAGCAGGCCAGACAGGCCTCGTGGACC
CATCTTGAAAACAGTAAAAATGAGCTGGTAAAAATCTTCCAACAGGATGATTCGCTGGAA
GATTTTGATGATTTTTTCTTCGCCACCTGA
>EECEBICD_00372 Mannitol 2-dehydrogenase
ATGGACACCATAGCTCGCCAGTTAGACGCCGCAAGGCACCAATTCAAAACGACTTATTCA
CGTCAGGGAATGGAAGCCAACATCGTACATATTGGATTTGGCGCCTTTCACCGTGGTCAT
CAGGCGGTTTATAACGATCTCACGAATGAGATAACCGGCAACCGCTGGGGGATTTTTGAA
CTGAATCTGTTCGGCGATGCGGAGCTGGTGGATGCGCTAAATGCGCAACAGGGGCTGTTT
TCAGTGGTGGAAACGTCAGCCAGCGCCATCAATAGTCGCCTGGTTCGTACCATCACCGGC
GCGTTGCATACGCCGAAGTCAGGTATTCAGGCGGCGATCGAGAAGCTGACTGAGCCGCAG
GTAAAAATTGTTTCGTTGACGATAACGGAAAAAGGTTACTGCACCGATCCACGGTCCAGA
ACGCTCGATCTCAGCCATCCGCTCATCAAACACGATCTTGCCGATCCTGAACATCCCCGC
TCAGCACTCGGACTGATCGTCGAAGCGCTACGGATACGCCGCCAGCAAGGGCTTCCCCCG
TTTAGTGTACTGTCGTGCGATAACATTCCGGAAAACGGTCATTTGACCAAAACGGCGATC
GTTACGTTTGCCGATAATCTCGATCCGCAACTGGCGGCCTGGATAGCGAAGCATGTCACC
TTTCCTGGCACCATGGTCGATCGCATCGTGCCGGCAATGACGCCAGAGCAATTTGACATG
ATTAAAGACCAGATCGGTTTTGCCGATCCCTGCGGCATCGTATGCGAGGATTTCCGTCAG
TGGGTTATCGAAGATAATTTCGTTGCCGGTCGTCCTGACTGGGATAAAGCAGGGGCGATG
TTCGTCTCGGACGTGCTTCCCTATGAAGAAATGAAACTTCGTATGCTGAATGGCAGCCAT
TCGTTTCTCGCCTATAACGGCAGCCTGGCTGGATATGAGTTTATCTATCAGTGCATGGAA
GACGACGCTTTTAAAACCGCCGTACATCATTTAATGACGGAAGAGCAGGCAAAATCGCTG
CGCCCGAATTTGGCGGTAGATGTACATCAATATGCGCAGTTGCTCATTGAGCGATTCAGC
AACCCGAACATTAAACATAAGACCGGACAGATTGCGATGGACGGTTCTCAGAAGCTGCCT
CAGCGGGCCGTCGATCCTTATCTGACGCTTCAGGCGCGCGGCGTTAAGGGCAGGGCGTTA
GCGACGCTGATTGCGGGCTGGCTCCATTACGTCATTAATACACTCTCTCAGGGGCAGAGT
GTGGCCGACCCGCTCAATGATACCTTCAATGCGGCTATTAAAAATAAAAACACGCGATGG
GAACAAGCGCTGTCTTTGCTGCAGATAAATAGCATCTTTGGAACGCTGGCCGGCGACAAC
GCAGACTTTTTAAACGACATTCAGCAGGCCTTTAACCATATTGAGTTACATGGCGTCACG
GCCACCATTCACCGTTTATCTTCAAAGGGTTAA
>EECEBICD_00373 L-galactonate-5-dehydrogenase
ATGAAAACGCTTATCTGTAATAATCCGGGAAATATCGAATACATTGAAAGAGATATTCCT
CATTTAAAAGACGATGAAGTACTGTTAAAAATTAAAGCGGTTGGTATTTGCGGCACCGAT
ATCCATGCGTTTGCCGGTCGCCAGCCCTTTTTTGCCTATCCGCGCGTGCTGGGTCATGAA
ATTTGCGGCGTGGCGGAAATATTGGGTAAATCGTGCAGCACGGCGAAGGTCGGCCAACGC
TATAGCGTTATTCCCTGTATTCCTTGCGGCGCTTGCGCAGCCTGCCGGGAAGGAAAAACC
AACTGCTGTGAGAACGTCTCGCTGTATGGCGTTCACCAGGACGGCGGCTTTAGCGAATAT
CTGGCCGTTCGTGAACAAAATCTGGTTGAACTGTCGGATAACCTCACCGACAGCGCGGGC
GCGCTGGTTGAGTGTTTCGCCATCAGCGCCCACGCAGTACGCCGCGCTGACGTGAAGCCG
CAGCAAAACATTGTGGTGGTCGGCGCCGGGCCGATTGGGCTGGCCGCCGCCGCGATAGCA
AAAGCCAAAGGCGCTCGCGTCGCGGTAGCTGATATTGACGCCGAACGCCGTCGTCTGGTC
GCCGAAAAGGTAGGCGTTGCAACGCTCGATCCGTCGTCAGACGACTATATTGATGTGCTG
AAAGCCTGTTTCTCCGGTGAATTAGCCGGCATTGTGCTTGATGCCACCGGCAATAAATCC
TCCATGAGCCGCGCTGTTGATCTGATTCTTCACGGCGGAAAAATCGTCTTCATCGGGCTA

TATATCGGCGAGCTCGTCATTGACGATCCGACCTTCCATAAAAAAGAGACCACGCTATTG
AGCAGTCGTAACGCGACGCGGGAAGACTTCGAATGTGTCATTGAGCTAATGGCTCAGGGC
GCTATTAGCGAAACAATGATGAAAAACCAGGAATTCGATTTCTATACGTTCGGCAACCAG
TACCAGAAGAACGTGGTAGAAAACAAAAAATTGGTTAAAGGCGTTATCAAATTTTAA
>EECEBICD_00374 putative oxidoreductase YjmC
ATGTCAACAATATTAGTCAAAGAAAATGAATTGAAAGCGCTGGCGTTTAATAAACTCACC
CAGGCGGGCCTTGACGCGCAAACGGCGCAGCAGGTAGCCGACGTTTTGGTTCATGCCGAT
ATTACCGGCGTTCACTCCCACGGCGTTATTCGCGTAGAGCACTATTGTACCCGCCTGAAC
GCAGGCGGGCTCAATCCAAAAGCAACATTCAGCATCGAACAGATTTCGCCTTCTGTCGCT
ATTCTGGACTCTGATGATGGAATGGGGCACTGCGCGCTGATAAAGGCGACAGACCACGCT
ATTAGTCTGGCGAGAGAGACGGGGCTGGGCTTCGTCAGCGTCAAAAATACGTCCCATTGC
GGCGCGCTCTCGTGGTTCATTGAGCAGGCGACAAGCCAGGGAATGGTGGCTATCGCCATG
ACGCAAACGGATACCTGCGTTGCGCCGTATGGCGGCGCGGAGCGCTTTCTGGGGACTAAC
CCAATCGCCTTCGGCTTCCCCGTTAAAGACAGCCATCCGATGATCGTCGATATGGCGACC
AGCGCCATTGCGTTTGGTAAAATTCTGCACGCCAAAGAAACCGGGAAACCAATAGGTCAT
GGTCTGGCGCTGGATAAAGAGGGACATATCACTACCGATCCGCATAAAATTGAAAATCTG
CTGCCCTTTGGCGGACACAAAGGTTCTGGTATTGCCCTGGCGATTGATGCGCTCACGGGC
GTCCTGATGGGGGCAAACTTTAGCAACCATATTGTTCGGATGTATGGCGACTATGACAAG
ATGCGCAAGTTAGCGAGTCTGGTTATCGTTATTGATCCGCAGATGTTGGGCAATCCGCTG
TTTTCCTCAATAATGAGCACAATGGTCAATGAGCTAAGGGCTGTAAAACCGATGCCCGGT
GTTGATAAAGTGCTGGCGCCAAACGATCCGCAAATCGCCTATAAAGAAAAATGTTTAAAA
GAAGGTATTCCTGTGGCTGAAGGTATTTACCAGTACCTGATTGGCTGA
>EECEBICD_00375 Toxin-antitoxin biofilm protein TabA
ATGCTATATGGAAATATAGAGCAGCTTACTTTATTACCTTACGTTAACCACATTATTAAA
AAACTTATCATCGAAGCGGTGAAGATAGCTGAAGATCAGCCTGCTGGTAGATATGAACTT
TCTTTCCCGGAGTCATTTCTTATGATAAGCGAAGGAGAAACACACTCTTCGTTAAACCGC
AAAGCTGAACTTCATAAAAAATATATCGATGTTCAGATTTTACTAAGCGGTTATGAGGAG
ATTGGCTATTCCAACAAAATTGATACCAGGATAAAGGAGCTTGAGCATCTTCCTGATGAC
ATCATCTTTCCTGAATGTGTCGCTAATGAGCAGTTCGTTACATTAAATCCTGGCGATTTC
GCGCTTTTTTATCCCAACCAGATCCACCGTCCGCTTTGTACGCGTGGTAAACCTGCGCCG
GTTAAAAAAGCGATTGTGAAAATTCCGGCCACGGCATTTAGCGAGTCAAGCTTGCCCTGT
CAGACGAAGGAATAA
>EECEBICD_00376 Non-heme chloroperoxidase
ATGAATCATATTTTTAAATTAGCATTTATTTTTATTATCAATATTATATTTTTAATAAAT
GCTTCAGCAACAACGATGGACAAAGATAAATTTGTGGAAGCGAATGGTATCAAAATTCAT
TACGTTGAAGAGGGCGAGGGGCCTCCGCTGCTGTTGATTCACGGTGGTGGTCTGACGGCG
AAAAGTTGGCAGGGATTAGCGAAAGAGGCATCTCGATATTTTCGCGTCATCATGCCGGAC
AGTCGTGGACATGGATTAACCAATAACCCGCAAGGAACGTTTAGCTATGATCTGATGACG
GAAGATATGGCGGCTTTTGTTAAAGCGCTGAAGCTGGAAAAACCGCTGGTGATGGGTTAC
AGCGATGGCGGCATGGTCGTTCTGAAGTTAACCAGCCGATACCCGGATCTAGCGCGCGCT
GCTATCGTCGGCGGCGCAACGCATCGCTTTGCTACGACCCATTACATGCAGGGGATGGAA
ATTTTTTATGGTAAAGGAATGCCTTAG
>EECEBICD_00377 hypothetical protein
GTGGACAACGCCCACATCGCTGACGGAAGAAGAGGTGAAAAAATCCACATTCCTGTGCTA
TTACTGGACGGCGACCGGGATGAATTTTTCACCGTTGAAGAAGTTACCGAGCTGTACCGG
CTGCTGCCTCAGGCGGAGATGACCCTCATTCCCGGTAGCGGTCACGCCATTTTCCAGACG
CCGGGGAAAACACCGCTGTTTTACGCATTAGTGCTTGAGTTTTTACAGCGGCAACTCCCT
AAGGCCTCTTAA
>EECEBICD_00378 Agmatinase
ATGAGCACCTTAGGTCATCAGTACGATAACTCACTGGTTTCTAATGCGTTTGGTTTTTTA
CGTCTGCCAATGAACTTCCAGCCGTATGACAGCGATGCCGACTGGGTGATCACTGGCGTA
CCGTTTGATATGGCAACGTCCGGTCGCGCTGGCGGCCGTCATGGCCCGGCGGCGATCCGT
CAGGTGTCGACCAACCTCGCCTGGGAACATCACCGTTTCCCGTGGAGTTTTGACATGCGC
GAGCGCCTGAACGTCGTGGACTGCGGCGATTTGGTGTATGCGTTTGGCGATGCCCGTGAG

ATGAGTGAAAAATTACAGGCGCACGCTGAAAAACTGCTGTCTGCAGGCAAGCGTATGCTC
TCTTTCGGCGGCGACCACTTCGTCACGCTGCCGCTGCTGCGCGCCCACGCGAAACATTTT
GGCAAAATGGCGCTGGTACATTTTGATGCGCATACCGATACCTACGCTAACGGCTGCGAA
TTCGATCACGGCACGATGTTCTACACCGCGCCGAAAGAAGGCCTGATCGATCCGCATCAT
TCGGTACAGATCGGTATTCGTACTGAGTTTGACAAAGACAATGGCTTTACCGTGCTGGAT
GCCTGCCAGGTCAACGATCGCGGCGTGGATGATATTCTCGCTCAGGTGAAACAGATCGTC
GGCGATATGCCGGTCTATCTGACCTTTGATATCGACTGTCTGGACCCGGCGTTTGCGCCT
GGCACCGGTACGCCGGTGATCGGCGGTTTGACCTCCGATCGCGCCATTAAACTGGTACGC
GGTCTGAAAGATCTGAACATTGTCGGTATGGATGTAGTGGAAGTCGCGCCGGCTTACGAT
CAGTCGGAGATCACCGCTCTGGCGGCCGCGACGCTGGCATTAGAAATGCTCTATATCCAG
GCGGCGAAGAAAGGCGAGTAA
>EECEBICD_00379 Metalloprotease LoiP
ATGAAAATTCGCGCTTTACTGCTTGCTTTGGGCATGGCGACGGTGCTGACCGGTTGCCAG
AATATGGACTCTAATGGACTGCTTTCTTCGGGCGCTGAAGCTTTTCAGGCCTATACGTTA
AGCGATGCGCAGGTAAAAGCGTTGAGCGATCAATCCTGTCAAGAGCTCGACAGCAAAGCG
AAAATCGCGCCGGCGAGCAGCGAATACGCTAAACGACTGGCAAAAATTGCCGCTGCGCTG
GGGGATAACATTAACGGCCAGCCGGTGAACTATAAGGTTTACGAGACGAAAGACGTCAAC
GCGTTTGCCATGGCGAACGGTTGTATTCGCGTCTACAGCGGACTGATGGATATGATGACC
GACAACGAAGTTGAAGCCGTGATCGGCCATGAAATGGGCCATGTCGCGCTGGGTCATGTA
AAGAAAGGGATGCAGGTCGCGCTGGGCACTAACGCCGTGCGCGTCGCGGCGGCATCGGCA
GGAGGCGTTGTCGGCAGCCTGTCGCAATCACAGCTTGGCGATCTTGGCGAAAAACTGGTG
AACTCGCAGTTCTCCCAGCGTCAGGAGTCCGAAGCGGATGATTATTCTTACGATCTGTTG
CGCAAACGCGGTATCAGTCCGGCAGGACTTGCCACCAGCTTTGAGAAACTGGCAAAACTG
GAAGCGGGTCGTCAAAGCTCGATGTTCGACGATCACCCGGCGTCCGCCGCACGCGCACAG
CATGTGCGCGATCGTATGAGCGCGGACGGGATCAAATAA
>EECEBICD_00380 Transketolase 1
ATGTCCTCACGTAAAGAGCTTGCCAATGCTATTCGTGCGCTGAGCATGGACGCAGTACAG
AAAGCCAAATCCGGTCACCCGGGTGCCCCGATGGGTATGGCTGACATTGCCGAAGTCCTG
TGGCGTGATTTCCTGAACCACAACCCGACCAACCCGTCCTGGGCCGACCGCGACCGCTTC
GTGCTGTCCAATGGCCACGGCTCAATGCTGATTTACAGCCTGCTGCACCTCACCGGTTAT
GACCTGCCGATGTCCGAGCTGCAGAATTTCCGTCAGCTACATTCAAAAACCCCTGGTCAC
CCGGAAGTGGGCTACACCGCTGGCGTTGAAACCACTACTGGCCCGCTGGGTCAGGGTATT
GCCAACGCCGTCGGGATGGCGATTGCCGAGAAAACTCTGGCGGCGCAGTTTAATCGTCCT
GGCCACGATATCGTTGACCACTTCACCTACGTCTTTATGGGCGACGGCTGCATGATGGAA
GGCATTTCTCACGAAGTGTGCTCTCTGGCTGGTACTCTGAAACTGGGCAAACTGATCGCG
TTCTATGATGACAACGGTATCTCTATCGATGGTCATGTTGAAGGCTGGTTCACCGATGAC
ACCGCGAAACGTTTTGAAGCCTACGGCTGGCACGTTATCCGTGGCATTGACGGTCACGAT
GCCGACGCCATTAAACGCGCCACGGAAGAAGCTCGCGCCGTGACTGACAAACCGTCCCTG
CTGATGTGCAAAACCATCATCGGTTTCGGTTCGCCGAACAAACAGGGTACCCACGATTCC
CACGGCGCGCCGCTGGGCGACGCGGAAATCGCGCTGACCCGCGAACAGTTGGGCTGGAAA
TACGCGCCGTTCGAAATCCCGTCTGAAATCTACGCCCAGTGGGATGCGAAAGAAGCCGGC
CAGGCGAAAGAATCCGCATGGAATGAGAAATTCGCGGCTTATGAGAAAGCCTTCCCGCAG
GAAGCTGCTGAATTCACTCGTCGTATGAAAGGCGACATGCCGGCTGACTTCGATGCGAAA
GCGAATGAGTTCATCGCTAAACTGCAGGCGAATCCATCCAAAATCGCCAGCCGTAAAGCG
TCCCAGAATGCCATCGAAGCCTTCGGTCCGCTGTTGCCGGAATTCCTCGGCGGCTCCGCT
GACCTGGCACCTTCTAACCTGACCCTGTGGTCCGGTTCGAAAGCGATTAACGAAGATGCC
GCAGGTAACTACATTCATTACGGTGTGCGCGAATTCGGTATGACCGCTATCGCCAACGGT
ATCGCCCTGCACGGCGGTTTCCTGCCGTACACCTCCACCTTCCTGATGTTCGTCGAATAT
GCCCGTAACGCAGTGCGTATGGCAGCGTTGATGAAACAGCGTCAGGTGATGGTCTATACC
CACGACTCCATCGGTCTGGGCGAAGATGGCCCGACGCACCAGCCGGTCGAGCAGGTGGCA
AGCCTGCGCGTAACGCCGAACATGTCCACATGGCGTCCGTGCGACCAGGTGGAATCCGCG
GTGGCGTGGAAATATGGCGTAGAGCGTCAGGATGGCCCAACCGCGCTGATCCTCTCCCGT
CAGAACCTGGCGCAGCAGGAACGTACTGAAGAGCAACTGGCGAATATCGCCCGCGGTGGT
TACGTACTGAAAGATTGTGCGGGCCAGCCGCAGATTATCTTCATTGCGACCGGTTCAGAG

GTTGAGCTGGCGGTTGCCGCTTACGAAAAACTAACTGCCGAAGGCGTGAAGGCGCGCGTG
GTTTCCATGCCGTCTACCGACGCATTCGACAAGCAGGATGCGGCTTACCGTGAATCCGTG
CTGCCGAAGGACGTCTCTGCGCGTGTAGCGATTGAAGCGGGTATCGCTGACTACTGGTTC
AAATACGTCGGCCTGAACGGCGCTATTGTCGGGATGACCACCTTCGGTGAATCCGCCCCG
GCAGAGCTGTTGTTTGAAGAGTTTGGCTTCACGGTGGACAACGTGATCGCCAAAGCGAAA
GCACTGCTGTAA
>EECEBICD_00381 Nickel import ATP-binding protein LarO
ATGTTGACGTTAAATCAGGTCGCTTATCGCTGGCCGGATGCCGCAGCCGACTGTTTGCAC
GCTATCTCGCTTGAACTTCGTGACGGCGAATGGCTGGCGTTGACCGGCGATAACGGCGCG
GGAAAATCAACACTGTTACGGATTATGGCCGGCCTGCTTTCCCCCACTTCCGGCTCGGTA
ACGCTTAATGGAGAGCCGATTGCGCAGCTTAAAAATCGTCAACGCGCCGCCGCTATCGGC
GTACTCTTTCAGGAAGCGGAAAACCAGATTTTCCACAGCAATGTCGCGCAAGAGGTCGCT
TTTGGGCTGAAACTACAACGCTTATCGGCAGAGGAAATTTCACGGCGCACGCAGGCCGCG
CTGCGGTTATGCCAGCTAACGGACGTCGCCGAGGCGCACCCGCTTGATCTGCACGCCGCA
CCGCGCCGGATGGTGGCGGTCGCCAGTCTGGAGGCGATGGCGCCGCCTGTGTTATTGCTG
GATGAACCCAGCCGGGATTTTGACGCCCACTGGCTAACCGTATTTGAACACTGGCTGGCG
ACATGCCGGGCACGGGGAACCAGCGTCGTGGCGATTAGCCATGACGCTGCGTTTACCCGG
CGTCATTTTTCACGGGTGGTACGGCTGGAAAACGGCAGGTTGTCAAAGGGAGAATAA
>EECEBICD_00382 Energy-coupling factor transporter ATP-binding protein EcfA1
ATGGTTACGTTAGAGCAGTTTCGTTATCTTCCGTCCGATGCGACACGCCCGCCGGCATGT
TTTGATTTCCACTATTCAACACCCGGAATAGTGGCGATAGTCGGCGATAACGGCAGCGGA
AAAAGTACGCTGGCGCAACTGATGGCTGGCTGGTATCCGGATTATCTTCCCGGTGATATT
GACGGCACAGGCTTACTGCTTGGCGTCCCTATCGGGCAGCTCCCGCTGGTAGAACAGTCG
CCCACCATCCAGTTGGTGCAGCAATCGCCTTATTTACAGCTTTCCGGCTGTACCTTTAGC
GTAGAGGAAGAGGTGGCGTTTGGCCCGGAGAATCTCGGCCTCCATGAAGCGGAAATTCTA
CGGCGCATTGACGAGGCGCTGACTCTGACAAACTGCCAGTCGTTACGCCATCGCCATCCC
GGCACGCTCTCCGGCGGCGAAACGCAGCGGGTAGTGATTGCCAGCGCGCTTGCCATGCAG
CCCAGGCTATTAGTTCTTGATGAAGCCTTCAGTCGGCTGACGTCAGCGGCCACCGGGATG
CTGCTGGAACGGCTACAACAGTGGGCGCTGGAACGTCATTCTCTCATTGTGCTTTTTGAG
CGTAACCATTTTCCTTTTCTGACGCGATGCCAGCGGGTATGGCAATTGCGCGACGGAGCG
CTAACCCCATTATGTTGA
>EECEBICD_00383 hypothetical protein
ATGCATCCGTTTACCTCGTTAACCTTATGGGCGTTGGCGGCCTGCACCACGCTGCTACTC
CCTGCGCAGACCGTGCTCCCGGTTTATAGCGCGGCGGCCTTCCTCTGTCTGCTGGCGCTG
AAATCAACGCGGCGGCGGGCGAAGTATGTCGCCTGGCTGATGCTGTCTTTAGGGTTTGGG
CTATGGCTGGTACATGGCGGTTGGCTAACGGAATGGATAAGCGGGCAGCCTTGCGATCCG
CAGCGCTGGATATATGCCGTTACGCTATGGCTACGCCTGCTGGCGATCGTGTCGACGTCG
CAGCTATGGATGCAGTATGTGCCGGTTCAGCGGTTTATTCGCGCCCTGTTCGCTTCACGT
TTGCCGCCCGGCATCGCTTACCTGTTCGCTGGCCCGCTGCTGGTCGTCGAACAGCTCAAA
CGACAACTGACGATCGTTCATGAAGCCCAGCGCGCGCGTGGCGTACCGCTAGATGAAGGC
TGGTATCAGCGGCTACGGGCAATGCCTGCGCTGATCGTGCCGCTTACGCAAAATGCGCTT
AACGATCTGACAATACGCGGCGCGGCGTTGGATATGCGGGGGTTTCGGCTGCATCGAGCC
CGAACCACGCTGTGGGCGCCGAAAGATAGCATGTTACAACGGGTTGCGCGGTATGGCATG
GTACTGCTAATACTGGCGGAAGCGGGAGTGTGGATATGGTTACGTTAG
>EECEBICD_00384 hypothetical protein
ATGGCGCGTCGCCCGTTTTCCAGCCAGTCTCTGGTGCTGATTGTCATCGCTATTGCCATC
AATATGATTGGCGGACAGCTTATTAGCATGTTGAAGCTGCCTATTTTTCTCGACTCGATC
GGCACCTTAATTAGCGCGGTACTGTTAGGGCCGTTCATCGGCATGTTGACTGGGCTTCTG
ACCAACTTGCTGTGGGGGCTGCTTACCGACCCTATCGCTGCCGCGTTTGCCCCGGTAGCG
ATGGTTATCGGTCTGGTCGCCGGGTGGCTGGCGCGCGCAGGCTGGTTTCGTACCCTGCCC
AAAGTGATCGTCAGCGGGGTCGTCATTACGTTGGCGGTGACGCTGGTCGCCGTACCGCTT
CGTACCGCGCTTTTTGGCGGCGTTACCGGTAGCGGCGCTGATTTGTTCGTCGCCTGGATG
CATTCAATGGGCCAAAACCTGGTGGAGTCAGTCGCGATTACCGTCATAGGCGCTAATCTG

GTCGATAAAATTCTGACGGCGATCATCGTTTGGGTGCTGCTGCGTCAGCTACCGTTACGT
ACGACACGGCATTTTCCTGCGATGTCAGCCGTACGCTAA
>EECEBICD_00385 hypothetical protein
ATGACTGTATCTCACCACAATGCCTCTACAGCGCGCTTTTACGCGCTTCGTCTGCTTCCC
GGCCAGGAAGTGTTTTCCCAATTACACGCCTTTGTGCAACAGAACCAATTACATGCCGCC
TGGATTGCAGGGTGTACCGGCAGTCTGACCGACGTTGCGCTGCGTTATGCCGGGCAAGAA
GCGACCACATCGCTCACCGGCACGTTTGAAGTTATCTCGCTAAACGGCACGCTTGAATTG
ACCGGCGAGCATCTGCATCTCGCGGTATCGGATCCCTACGGGGCCATGCTCGGCGGCCAT
ATGATGCCAGGGTGTACCGTGCGTACCACGCTGGAACTGGTTATCGGCGAGCTGCCAGCG
CTGACGTTCAGCCGTCAACCGTGCGCCATTTCCGGTTATGACGAACTGCATATTTCTTCC
CGTTAA
>EECEBICD_00386 D-erythrose-4-phosphate dehydrogenase
ATGACCGTACGCATAGCGATTAATGGCTTTGGTCGCATCGGACGTAACGTGGTTCGTGCT
TTGTATGAATCCGGACGTCGCGCGGAAATTACCGTGGTGGCCATCAACGAGCTGGCGGAT
GCCGCAGGCATGGCGCATTTGTTGAAATACGATACCAGCCACGGGCGTTTTGCATGGGAG
GTTCGCCACGAGCGCGAGCAGCTTTTTGTCGGCGACGACGTCATTCGTATTCTGCATGAA
CGAACGCTGGCGGATCTGCCGTGGCGCGAACTGGGCGTGGATGTCGTGTTGGATTGTACG
GGCGTATATGGCAACCGGGAGCATGGCGAGGCGCATATTGCCGCTGGCGCGAAGAAAGTG
CTCTTTTCTCATCCGGGCAGCAACGATCTTGACGCCACCGTCGTTTTTGGCGTGAACCAG
AATGAACTGCGTGCGGAACATCGTATTGTCTCAAACGCATCCTGTACCACGAATTGCATA
ATTCCCGTCATTAAATTGTTGGATGATGCTTACGGCATCGAGTCTGGTACCGTCACGACG
ATCCACTCCGCGATGAACGATCAGCAGGTGATCGACGCGTATCACTCCGATCTGCGGCGC
ACGCGCGCGGCCAGCCAGTCGATTATCCCGGTAGATACAAAACTGGCGGCAGGCATTACG
CGTATATTCCCGCAGTTTAACGACCGTTTTGAGGCGATTGCCGTGCGCGTTCCAACCATT
AATGTGACGGCGATCGATTTAAGCGTAACGGTGAAAAAACCAGTAAAAGCCAGTGAAGTC
AACCAGTTGCTGCAAAAAGCAGCACAAGGTGCATTTCATGGTATAGTTGACTATACGGAA
TCACCGTTGGTCTCGATAGATTTTAACCACGACCCGCACAGCGCCATTGTTGATGGCACG
CAAACCCGGGTTAGTGGCGCCCACCTGATCAAGACGCTGGTCTGGTGCGATAATGAATGG
GGCTTTGCTAACCGGATGCTCGACACCACGTTAGCGATGGCCGCAGTTGGTTTCAGGCTC
GACGCGTCAGCGTCGACAAAACTTTAA
>EECEBICD_00387 Phosphoglycerate kinase
ATGTCTGTAATTAAGATGACCGATCTGGATCTGGCCGGGAAACGCGTTTTTATCCGTGCG
GATCTGAACGTACCTGTTAAAGAAGGGAAAGTGACCAGCGACGCGCGTATCCGCGCCTCT
CTGCCGACCATCGAACTGGCTCTGAAACAGGGCGCGAAAGTGATGGTAACTTCCCACCTG
GGTCGTCCTACCGAAGGCGAGTACAACGAAGAATTCTCTCTGCTGCCGGTTGTTAACTAC
CTGAAAGACAAACTGTCTAACCCGGTTCGCCTGGTCAAAGATTACCTGGACGGCGTTGAC
GTGGCCGAAGGTGAGCTGGTGGTTCTGGAAAACGTTCGCTTTAACAAAGGCGAAAAGAAA
GACGACGAAGCGCTGTCTAAAAAATACGCCGCCCTGTGTGACGTATTCGTGATGGACGCC
TTTGGTACTGCGCACCGCGCGCAGGCTTCTACCCACGGTATCGGCAAATTTGCTGATGTT
GCGTGCGCAGGCCCGCTGCTGGCCGCTGAACTGGATGCGCTGGGTAAAGCGCTGAAAGAA
CCGGCTCGTCCGATGGTGGCTATCGTTGGTGGTTCTAAAGTATCTACCAAACTGACCGTT
CTGGATTCCCTGTCTAAAATCGCTGACCAGCTGATTGTCGGCGGCGGTATCGCCAACACC
TTCGTTGCCGCTCAGGGCCACAGCGTCGGTAAATCCCTGTACGAAGCTGACCTGGTTGAC
GAAGCAAAACGTCTGCTGACCACTTGCGATATCCCGGTTCCGACCGATGTTCGCGTTGCC
ACCGAGTTCTCCGAAACCGCACCGGCGACTCTGAAATCCGTTAACGACGTGAAAGAAGAC
GAGCAGATCCTGGATATCGGCGATGCTTCCGCTCAGCAGCTGGCTGAAATCCTCAAGAAT
GCCAAAACCATTCTGTGGAACGGCCCGGTTGGCGTGTTTGAGTTCCCGAACTTCCGTAAA
GGGACTGAAATCGTGGCGAATGCCATCGCTGATAGCGAAGCGTTTTCCATCGCTGGCGGC
GGCGACACCCTGGCGGCAATCGACCTGTTCGGCATTGCTGACAAAATTTCCTACATTTCT
ACTGGCGGCGGCGCATTCCTCGAATTCGTGGAAGGTAAAGTTCTGCCAGCCGTAGCGATG
CTCGAAGAGCGCGCTAAGAAGTAA
>EECEBICD_00388 Fructose-bisphosphate aldolase class 2
ATGTCTAAAATTTTTGATTTCGTAAAACCAGGCGTGATCACTGGTGATGACGTACAGAAA
GTGTTCCAGGTAGCAAAAGAAAACAATTTTGCTCTGCCGGCAGTTAACTGCGTGGGCACT

GATTCCATCAACGCTGTTCTGGAAACCGCTGCAAAAGTTAAAGCACCGGTCATCGTTCAG
TTCTCCAACGGCGGCGCGTCCTTCATCGCGGGTAAAGGCGTGAAAACTGACGTACCTCAG
GGCGCGGCAATCCTGGGCGCTATCTCTGGCGCGCATCACGTTCACCAGATGGCTGAACAC
TACGGCGTGCCGGTTATCCTGCACACTGACCACTGCGCGAAGAAACTGCTGCCGTGGATC
GACGGTCTGCTGGACGCGGGTGAAAAACACTTCGCGGCTACCGGTAAACCGCTGTTCTCT
TCTCACATGATTGACCTGTCTGAAGAGTCTCTGCACGAAAACATCGAAATCTGCTCCAAA
TACCTGGCGCGCATGTCCAAAATTGGCATGACTCTGGAAATCGAACTGGGTTGCACCGGT
GGTGAAGAAGACGGCGTGGACAACAGCCACATGGACGCTTCTGCTCTGTACACCCAGCCG
GAAGACGTTGATTACGCGTACACTGAACTGAGCAAAATCAGCCCGCGTTTCACCATTGCC
GCTTCCTTCGGTAACGTACACGGCGTTTACAAGCCGGGTAACGTGGTTCTGACCCCGACC
ATTCTGCGTGATTCTCAGGACTATGTTTCTAAGAAACACAACCTGCCGCACAACAGCCTG
AACTTCGTTTTCCACGGCGGTTCCGGTTCTACTGCTCAGGAAATCAAAGACTCCGTAAGC
TACGGCGTAGTGAAAATGAACATCGATACCGATACCCAATGGGCAACCTGGGAAGGTGTT
CTGAACTACTACAAAGAAAACGAAGCGTATCTGCAAGGTCAGCTGGGCAACCCGAAAGGC
GAAGATCAGCCGAACAAGAAATACTATGACCCGCGCGTATGGCTGCGTGCTGGCCAGACT
TCGATGATCGCTCGTCTGGAAAAAGCATTTAAAGAACTGAACGCGATTGACGTTCTGTAA
>EECEBICD_00389 Small-conductance mechanosensitive channel
ATGGAAGATTTGAATGTTGTCGACAGCATAAATGGTGCGGGCACCTGGCTGGTACGCAAC
CAGGCGTTATTGCTCAGCTATGCTGTGAATATTGTTGCGGCTATCGCTATTATCATCATC
GGCTTGATTGTAGCGCGCGTGATTTCCAATACGGTAAATCGTCTGATGCGTGCGCGTCAT
ATTGATGCGACAGTGGCCGACTTTCTTTCTGCGCTGGTGCGCTATGGGGTTATCGCTTTT
ACGCTGATTGCGGCTCTGGGTCGCGTGGGGGTTCAAACCGCCTCGGTTATTGCCGTACTG
GGCGCCGCTGGTTTAGCCGTTGGCCTGGCATTACAGGGATCGCTTTCTAACCTTGCGGCT
GGGGTATTGCTGGTTATGTTCCGTCCGTTCCGCGCCGGTGAGTATGTCGATTTAGGCGGC
GTTGCCGGTACGGTACTTAACGTGCAAATCTTTTCCACGACAATGCGCGCGGTAGACGGC
AAAATTATTGTGATTCCGAATGGCAAAATTATCGCGGGAAATATCATCAACTATTCTCGT
GAGCCGGTTCGCCGCAACGAATTCATTATTGGGGTGGCCTACGATTCGGATATCGATCAG
GTGAAACAGCTGCTCACGACGATTATCGAGTCCGACGATCGTATTTTAAAAGATCGCGAA
ATGACGGTGCGTTTGAATGAGCTGGGCGCCTCATCAATTAATTTTGTGGTGCGTGTCTGG
AGTAAAAGCAGCGACCTGCAAAATGTCTACTGGGACGTGCTGGAACGTATTAAACGTGAA
TTCGACGCCGCTGGTATTAGCTTCCCGTACCCGCAGATGGACGTCAATTTTAAACGCGTG
AAAGATAACGCCGCGGAGTAA
>EECEBICD_00390 Arginine exporter protein ArgO
GTGATATCTTATTATTTTCAAGGGTTTGCCCTTGGCGCCGCCATGATCCTTCCGCTTGGC
CCGCAAAATGCGTTTGTGATGAATCAGGGAATCCGTCGCCAGTACCACCTCATGATAGCG
CTGCTTTGCGCGTTAAGTGATTTAGTCCTGATTAGCGCCGGTATTTTTGGCGGTAGCGCG
TTGCTGATGCAGTCGCCGTGGCTGCTGGCGCTGGTCACATGGGGCGGGGTTGCGTTCTTG
TTATGGTACGGTTTAGGCGCGCTGAAAACGGCAATGAGCAGTAATCTGGAGCTGGCCAGC
GCCGAGGTTATGAAGCAGGGGCGCTGGAAAATTATTGCTACGATGCTGGCGGTAACGTGG
CTGAATCCGCACGTTTATCTGGATACGTTTGTCGTGTTGGGCAGTCTCGGCGGACAACTG
GCTATGGAGCCAAAGCGCTGGTTTGCTCTCGGCACGATTAGCGCGTCGTTCTTGTGGTTT
TTTGGGTTAGCGCTACTGGCGGCCTGGCTTGCGCCTCGACTGCGTACGGCGAAAGCTCAA
CGCATTATCAACATTCTGGTAGGGGTGGTGATGTGGCTTATCGCCTTCCAACTGGCGCGG
GAAGGCGTCGCGCATATGCACGCGCTTTTCAACTAA
>EECEBICD_00391 26 kDa periplasmic immunogenic protein
GTGAAGTTCAAAGTGATGGCCCTGGCGGCATTAGTGGGTTTAAGTGCAATGTCGGCGCAG
GCAAGCGAATTGCCGGAAGGCCCGCACATCGTGACTTCCGGTACGGCAAGCGTCGATGCT
GTGCCTGATATCGCCACCTTAGCGATTGAAGTGAACGTCGCGGCGAAGGACGCGGCGACG
GCGAAGAAGCAGGCCGATGAGCGCGTGGCGCAATATCTTTCCTTCCTTGAGCAAAATCAG
ATTGCGAAAAAAGACATTAGCGCGGCGAATCTGCGCACGCAGCCGGATTATGATTACCAA
AACGGTAAAAGCATTCTGAAAGGGTATCGCGCGGTGCGAACCGTTGAGGTGACGCTGCGT
CAGTTAGATAAACTCAATTCGCTGCTGGACGGCGCGCTGAAGGCGGGTCTGAACGAGATT
CGTTCGGTATCGCTGGGCGTGGCGCAACCGGACGCCTATAAAGACAAAGCCCGCAAAGCC
GCCATTGATGACGCGATTCATCAGGCGCAAGCGCTGGCAGCCGGTTTTAACAGCAAATTG

GGGCCGGTTTACAGCGTTCGTTACCATGTCTCTAATTATCAGCCGAGTCCGGTGGTGCGA
ATGATGAAAGCCGCAGATGCTGCGCCGGTATCCGCGCAGGAAACTTACGAGCAGCCGACC
ATTCAGTTCGACGATCAGGTCGATGTGGTATTCCAGCTTGAACCTGGAACAGGGCAGACG
TCGACCACGGCCGCCAGCACGCAGTAA
>EECEBICD_00392 HTH-type transcriptional regulator ArgP
ATGAAACGTCCGGACTACAGAACACTACAGGCGCTGGATGCCGTTATACGTGAACGAGGG
TTCGAGCGCGCCGCCCAGAAGCTGTGCATCACGCAATCCGCCGTCTCACAGCGCATAAAA
CAGCTTGAGAATATGTTCGGACAACCTTTATTGGTGCGTACCGTTCCTCCTCGCCCTACG
GAGCAAGGGCAAAAATTACTGGCTCTGCTGCGTCAGGTTGAACTGCTGGAAGAAGAGTGG
CTGGGCGATGAACAAACCGGCTCTACACCGCTGCTGCTGTCGCTGGCGGTTAACGCCGAT
AGTCTGGCGACCTGGCTGCTTCCGGCGCTGGCGCCGGTGCTGGCGGACTCCCCTATCCGT
CTCAATTTACAGGTGGAAGATGAAACGCGCACGCAAGAACGTCTGCGGCGCGGCGAGGTG
GTGGGCGCGGTGAGTATTCAGCATCAGGCGCTGCCCAGTTGCCTGGTAGACAAACTGGGC
GCGCTCGACTATCTGTTTGTCGCGTCAAGGCCGTTTGCCGAGCGCTATTTCCCCAATGGC
GTTACCCGGTCGTCGCTGCTGAAAGCGCCCGCCGTCGCGTTCGACCATCTGGATGATATG
CATCAGGCCTTTTTGCAACAGAATTTCGATCTACCGCCCGGCAGCGTGCCGTGCCATATC
GTTAATTCATCCGAAGCATTTGTACAGCTTGCGCGTCAGGGCACGACCTGCTGCATGATC
CCTCACCTGCAGATTGAGAAAGAGCTGGAAAGCGGCGAGTTGATCAACTTAACGCCTGGT
TTGCTGCAGCGCCGGATGCTTTACTGGCACCGCTTTGCCCCGGAAAGCCGGATGATGCGC
AAAGTGACCGACGCGCTGCTGGAGTACGGGCACAAGGTGCTGCGTCAGGATTAG
>EECEBICD_00393 Ribose-5-phosphate isomerase A
ATGACGCAGGATGAACTGAAAAAAGCGGTAGGATGGGCGGCACTTCAGTATGTACAGCCC
GGCACCATTGTGGGCGTTGGTACAGGCTCAACGGCCGCACACTTTATTGATGCGCTGGGC
ACAATGAAAGGCCAGATTGAAGGCGCGGTCTCCAGCTCGGACGCCTCGACTGAAAAACTC
AAAGGCCTCGGTATTCACGTGTTTGATCTCAACGAAGTGGATAGCCTGGGTATCTATGTC
GATGGCGCGGACGAAATCAACGGTCATATGCAGATGATCAAAGGCGGCGGCGCGGCATTA
ACCCGTGAGAAAATTATTGCTTCCGTGGCGGAGAAATTTATCTGTATTGCGGATGCCTCT
AAGCAGGTGGATATTCTTGGCAAATTCCCGTTGCCGGTCGAAGTGATTCCGATGGCGCGC
AGCGCGGTCGCTCGTCAACTGGTGAAGCTGGGCGGGCGTCCGGAGTACCGTCAGAACGTG
GTCACGGATAACGGCAACGTGATTCTGGATGTTTATGGTATGGAAATCCTTGATCCCATC
GCGCTGGAAAACGCCATTAACGCGATTCCGGGCGTCGTGACCGTGGGGCTGTTCGCCAAC
CGTGGCGCCGATGTCGCACTGATCGGCACGCCGGACGGGGTGAAAACCATCGTAAAATGA
>EECEBICD_00394 D-3-phosphoglycerate dehydrogenase
ATGGCAAAGGTATCGCTGGAGAAAGATAAAATTAAATTTCTGCTGGTTGAAGGGGTGCAT
CAAAAGGCGCTGGAAAGCCTTCGTGCGGCAGGTTACACCAACATTGAATATCACAAGGGC
GCGCTGGACGCTGAACAGTTGAAAGCGTCCATCCGCGATGCCCACTTCATCGGTCTGCGA
TCCCGTACTCATCTGACAGAAGAAGTGATTAACGCCGCAGAGAAGCTGGTGGCGATTGGC
TGTTTTTGCATCGGCACCAACCAGGTGGATTTAAATGCGGCGGCAAAACGCGGTATTCCG
GTCTTTAACGCGCCATTCTCGAATACCCGTTCCGTGGCGGAGCTGGTGATTGGCGAGCTG
TTGCTGCTGCTGCGCGGCGTGCCGGAAGCGAACGCCAAAGCGCACCGCGGCGTATGGAAT
AAACTGGCGGCGGGATCGTTCGAAGCGCGCGGTAAAAAGCTTGGCATTATCGGTTACGGC
CATATAGGAACGCAGTTGGGCATTCTGGCGGAATCGCTGGGGATGCATGTCTATTTCTAT
GATATTGAAAATAAACTGCCGCTCGGTAACGCAACTCAGGTGCAGCACCTGTCTGAATTG
CTGAATATGAGCGATGTGGTCAGTCTGCACGTACCTGAAAACGCCTCCACCAAAAACATG
ATGGGCGCAAAAGAGATTGCCCTGATGAAGCCGGGTTCGCTGCTGATTAACGCCGCGCGC
GGTACGGTGGTAGATATCCCGGCTTTGTGCGACGCGCTGGCGACGAAACATCTGGCGGGG
GCGGCTATCGACGTCTTCCCGACGGAACCGGCCACTAATAGCGATCCGTTTACCTCTCCG
CTGTGCGAATTCGATAACGTGATCCTGACGCCGCATATCGGCGGCTCGACGCAGGAGGCG
CAAGAAAACATCGGTCTGGAAGTCGCAGGAAAACTGATTAAGTACTCGGATAACGGCTCG
ACGCTGTCCGCCGTGAACTTCCCGGAAGTGTCGCTGCCGCTGCACGGCGGTCGTCGCTTG
ATGCACATCCACGAAAACCGCCCAGGCGTATTGACCGCGCTTAACCAGATTTTTGCCGAG
CAGGGCGTTAACATTGCCGCGCAGTATCTGCAAACCTCCGCCCGAATGGGGTATGTGGTA
ATTGATATAGAAGCCGATGGCGATGTGGCGGAGAAAGCGCTGCTGGCGATGAAGGCGATT
CCAGGTACCATTCGCGCCCGTCTGCTGTACTAA

>EECEBICD_00395 5-formyltetrahydrofolate cyclo-ligase
ATGATTCGGCAACGTCGTCGTGCGCTGACGCCAGAGCAGCAACGCCGCTTTGGTCAACAG
GCCGCCGCCCGAATGCTCAGCTTTCCTCCCGTCGTGATGGCGCACACTGTCGCCGTGTTT
CTCTCCTTTGATGGCGAACTCGACACCCAGCCGCTGATTGAACAGCTATGGCGAGCAGGC
AAGCGCGTCTACCTTCCGGTTCTTCATCCCTTCAGCCCTGGCAACCTCCTGTTCCTGCAC
TATCATCCGCAGAGTGCGCTGGTGACCAACCGCTTAAAGATCCGGGAGCCACGGCTTGAT
GTCCGCGACGTATTGCCGCTGGCAAAGCTGGATGTGCTGGTAACGCCATTAGTGGCGTTT
GACGAAGGCGGGCAGCGCCTGGGGATGGGCGGCGGATTTTATGACCGTACGTTGCAAAAC
TGGCAGCAGCATAAGATCCAGCCCGTGGGCTACGCGCATGATTGCCAGTTAGTAGAAAAA
CTGCCAGTTGAAGAGTGGGATATCCCGCTGCCTGCGGTGGTGACGCCGTCCAAAATTTGG
GAGTGGTAG
>EECEBICD_00396 Cell division protein ZapA
ATGTCTGCACAACCCGTCGATATCCAAATATTTGGCCGTTCACTGCGAGTGAATTGCCCG
CCTGACCAAAGGGATGCGTTGAATCAGGCTGCGGACGATCTGAATCAGCGGTTGCAAGAT
CTGAAAGTTCGCACTAGAGTCACAAATACTGAGCAGTTGGTTTTCATTGCCGCATTGAAT
ATCAGCTATGAGTTAACTCAGGAAAAAGCGAAGACCCGCGATTACGCGGCGAGCATGGAA
CAACGTATTCGGATGCTACAACAGACCATCGAACAGGCGTTGCTTGATCAAGGTCGCATA
ACCGAAAAAACGGGCCAAAACTTTGAATAA
>EECEBICD_00397 hypothetical protein
ATGTCTATACAGAACGAAATGCCTGGTTACAACGAAATGAACCGGTTTTTGAATCAACAA
GGGGCCGGTCTGACCCCGGCTGAAATGCATGGTTTAATCAGCGGGATGATTTGCGGCGGC
AACAACGACAGCTCATGGCAGCCGCTGCTGCACGACCTGACGAACGAAGGTCTGGCCTTC
GGGCATGAGCTTGCTCAGGCGCTGCGCAAAATGCACGCGGCGACCAGCGACGCGCTGGAA
GACGACGGCTTTCTTTTTCAGCTTTATCTGCCTGAAGGCGACGATGTCAGCGTATTCGAT
CGCGCAGATGCCTTAGCCGGGTGGGTTAACCACTTTTTGCTTGGCCTCGGCGTAACGCAG
CCTAAACTTGATAAAGTCACCGGCGAAACGGGTGAAGCGATTGACGACCTGCGTAATATT
GCGCAGCTTGGTTATGATGAGAGCGAAGATCAGGAAGAGCTGGAGATGTCGCTCGAAGAG
ATCATCGAATATGTCCGTGTTGCGGCGCTGTTATGTCATGACACTTTCACGCGCCAACAG
CCGACCGCGCCGGAAGTGCGTAAACCGACGCTACACTAA
>EECEBICD_00398 Xaa-Pro aminopeptidase
ATGACTCAGCAGGAATACCAACGCCGTCGCCAGGCATTACTGGCGCAAATGCAGCCCGGC
AGCGCCGCGCTGATCTTTGCCGCGCCGGAGGCGACGCGCAGCGCAGACAGTGAATATCCG
TATCGCCAGAGTAGCGACTTCTGGTATTTCACCGGTTTTAACGAACCGGAAGCCGTGCTG
GTACTGATTAAGAGTGATGACACCCACAACCACAGCGTTTTGTTCAACCGCGTTCGCGAC
CTGACTGCGGAAATCTGGTTTGGTCGCCGTTTAGGACAGGATGCCGCACCGGAAAAACTG
GGCGTTGACCGGGCGCTGGCGTTTAGCGAAATCAACCAGCAACTCTTTCAGTTGCTTAAT
GGTCTGGATGTGGTGTACCACGCGCAGGGCGAATATGCGTATGCCGACGAGATTGTTCTG
GCTGCGCTGGGGAAGCTGCGTAAAGGCTCCCGCCAGAATCTGACCGCGCCGGCCACCATG
ACTGACTGGCGACCGATCGTCCATGAGATGCGCCTGTTCAAATCGCCGGAAGAGATTGCG
GTCATGCGCCGCGCCGGGGAAATTAGCGCTCTGGCGCATATCCGCGCGATGGAAAAATGC
CGTCCGGGGATGTTTGAGTATCAGCTGGAGGGTGAAATTCACCACGAATTTAATCGCCAT
GGCGCGCGCTATCCCTCCTATAACACCATTGTCGGCAGCGGCGAAAATGGCTGTATCCTG
CATTACACTGAAAACGAAAGTGAAATGCGCGACGGCGATTTAGTGCTTATCGACGCGGGT
TGTGAATATAAAGGTTACGCGGGCGACATCACGCGTACTTTCCCGGTGAACGGGAAATTT
ACGCCAGCTCAGCGTGAAATTTATGACATCGTTCTGGAATCGCTGGAGACCAGCCTGCGA
CTGTTCCGTCCTGGTACCTCTATTCAGCAGGTGACCGGCGAAGTCGTGCGCATCATGATA
ACCGGGCTGGTGAAGTTGGGGATTTTGCAAGGAGAGGTTGATCAACTGATTGCCGAGAAT
GCGCATCGTCCTTTCTTTATGCATGGCTTGAGCCACTGGCTGGGGCTGGATGTTCATGAT
GTCGGCGTTTATGGGCCGGATCGCTCCCGCATCCTGGAGCCGGGCATGGTGCTGACCGTA
GAGCCAGGCCTCTATATCGCGCCGGATGCCGACGTGCCGGAAGCGTATCGCGGCATTGGC
GTTCGAATTGAAGATGACATTGTCATTACCGAAACCGGTAATGAAAACCTGACCGCTGGC
GTTGTGAAGAAGGCGGATGACATTGAGGCATTAATGGCGGCGGCGCGGCAGCAATGA
>EECEBICD_00399 2-octaprenyl-6-methoxyphenol hydroxylase
ATGAGCGTCATTATCGTTGGCGGCGGCATGGCAGGCGCAACGCTGGCGCTGGCCATCTCT

CAGTTCAGTCATGGGACGTTGCCGGTACACCTGATTGAAGCGAAAGCGCCTGAAGCCGAC
GGTCATCCCGGCTTTGACGCCAGAGCGATTGCGCTGGCGGCGGGCACTTGCCAGCAACTG
GCGCGAATTGGCGTCTGGCAGGCGATTTCCGATTGCGCAACGGCAATCAACACCGTACAC
GTCAGCGATCGCGGTCATGTCGGATTTGTGACTCTCGACGCGCAGGATTACAGCCTGGCA
GCGTTGGGTCACGTTGCGGAGCTGCATGATATCGGGCTGCGTCTGTTTGCGCTCTTGCGT
AAAGCGCCAGGCGTTACGCTGCACTGCCCGGAGCGCGTAGCCAGCGTGTCGCGTACCCAA
CAGCAAGTTAACGTCACTCTGGAAAACGGCAATGTGATAGCTGGCAGCGTGCTGGTGGCG
GCTAACGGTACCCACTCTGCGCTGGCGTCAGCCTGCGGCGTTGACTGGCATCAGGAGCCT
TACGAACAGTTAGCGGTGATAGCGAATGTCGCGACCGCTATCCCGCATCAGGGACGCGCC
TTTGAACGTTTTACTCCCAATGGCCCGCTGGCGATGTTGCCGATGTCGCATGGCCGATGT
TCGCTGGTTTGGTGTCACCCACTCGACCAGCGCGATGAGGTGCTGAGCTGGTCCGACGCG
CAGTTCTGCCATGCATTGCAGGCCGCGTTCGGCTGGCGGCTGGGGCGAATTACTCAGGCC
GGTAAGCGCAGCGTGTATCCGCTTGCCCTGACTACGGCGGCAAAAGTGTTTACTCACCGG
ACTGTCATGGTCGGCAATGCGGCGCAGACGTTGCATCCTATCGCCGGACAAGGGTTTAAT
CTCGGTCTGCGCGATGTCATGAGCCTGGCGGAAACGCTGATCGCGGCACAGGACGCGGGC
GAGGATATTGGCGCCTGGCACGTGTTATCGCGCTATCAGCAGCGTCGTCAGGTTGACCGG
GATACGACGATTGGCGTAACGAACGGTCTGGTCCATCTGTTTGCAAATCGTTGGGCGCCG
CTGGTTATTGGGCGCAATATCGGTTTAATGGCGATGGAATTATTACCGCCTGCGCGCGAT
GCGCTGGCACAGCGGACCTTAGGTTGGGTTGCGCGTTAA
>EECEBICD_00400 2-octaprenylphenol hydroxylase
GTGCAAAGTGTTGATGTAGCAATAGTTGGCGGAGGCATGGTGGGACTGGCTGTCGCCTGC
GGCTTGCAGGGGAGCGGTTTGCGCGTTGCGGTACTGGATCACGTTGTCCCCCAGCCGTTG
GCGAACGATGCGTCGCCTCAGCTTCGGGTGTCGGCGATTAATGCCGCCAGCGAAAAACTG
CTTACCCGTCTTGGCGTATGGTCAAACATTGTGGCGCGACGGGCAAGTTGCTACCACGGT
ATGGAAGTCTGGGATAAAGACAGCTTTGGCCGTATTACGTTTGATGACGCAAGCATGGGA
TATAGCCATCTGGGGCATATTGTTGAAAACAGCGTCATTCACTACGCATTGTGGCAAAAA
GCGCAGCAAGCGGCGGATATCACCTTAATGGCGCCAGCGGAGTTACAGCAGGTCGCCTGG
GGGAGAACGACGCCTTCCTGA
>EECEBICD_00401 2-octaprenylphenol hydroxylase
ATGTTAACCGCGCGTCTGGTGATTGCCGCGGATGGCGCCAACTCCTGGCTGCGTAACAAA
GCGGATATTCCCTTAACTTTCTGGGACTACCGTCACCATGCGCTGGTAGCGACTATCCGC
ACCGAAGAGCCGCACGGCGCGGTTGCTCGCCAGGTGTTTCATGGTGAAGGTATTCTGGCG
TTTTTGCCGTTAAGCGATCCGCATCTGTGTTCCATTGTCTGGTCGCTTTCTCCGCAGGAT
GCGGAGCGTATGCAACAGAGCGGCGACGAGGCGTTTAACCAGGCGTTGACTATCGCCTTC
GATAATCGGCTGGGCTTATGCCGCCTGGAAAGCGAACGGCAGGTTTTTCCGCTGACGGGC
CGCTATGCTCGCCAGTTTGCCGCGCATCGTCTGGCGTTGGTCGGCGATGCCGCGCACACC
ATCCATCCGCTGGCAGGGCAGGGCGTCAACCTCGGCTTTATGGACGCGGCCGAACTGGTG
GACGATCTGCGCCGGCTTCAGCGCCAGGGCAAAGACATTGGCCAACATCTTTATCTCCGT
CGTTATGAGCGTAGCCGTAAACATAGCGCGGCGATGATGCTTGCCGGGATGCAAGGTTTT
CGTGACCTGTTTGCCGGCGAAAACCCGGCAAAAAAATTGCTGCGCGACATCGGGCTGAAA
CTGGCGGATACGCTCCCCGGCGTCAAACCCCAACTGCTTCGCCAGGCAATGGGGCTAAAC
GACCTTCCGCAATGGCTTCGTTAA
>EECEBICD_00402 Aminomethyltransferase
ATGGCCCAACAGACGCCTTTGTACGAACAACACACGCTATGCGGCGCCCGCATGGTCGAT
TTTCATGGCTGGATGATGCCCCTGCATTACGGCTCTCAGCTTGATGAACACCACGCTGTG
CGCACCGATGCCGGGATGTTTGATGTATCGCATATGACCATTGTCGATCTTCATGGCAGC
CGCACACGAGAGTTTCTACGTTATCTGCTGGCCAACGACGTCGCGAAGCTAACGAAGACG
GGTAAAGCCCTCTACTCCGGTATGCTTAACGCATCGGGCGGCGTGATTGATGACCTTATC
ATCTACTACTTCACTGAAGATTTCTTCCGTCTCGTTGTTAACTCCGCCACCCGCGAAAAA
GACCTCTCCTGGATTACCCAACACGCTGAACCTTATGCCATCGACATTACCGTTCGTGAC
GACCTGTCGCTGATTGCGGTACAGGGGCCGAATGCGCAGGAAAAAGCGGCGACTCTGTTT
ACTGACGAACAGCGCCATGCAGTAGAAGGTATGAAGCCGTTCTTCGGCGTTCAGGTGGGC
GATCTGTTTATCGCCACCACCGGTTATACCGGCGAAGCGGGCTATGAAATCGCCATGCCG
AATGAAAAAGCGGCGGATTTCTGGCGTGCGCTGGTGGAGGCGGGCGTGAAGCCGTGCGGT

CTTGGCGCGCGCGACACGCTGCGTCTGGAAGCTGGCATGAACCTGTACGGTCAGGAGATG
GATGAAGGTATCTCTCCGCTGGCGGCTAATATGGGATGGACTATCGCCTGGGAACCTGCT
GACCGCGACTTTATTGGCCGTGAGGCGCTGGAGATGCAGCGTGAAAAAGGCCATGAGCAG
CTTGTCGGTCTGGTGATGACCGAGAAAGGCGTATTGCGTAATGAACTGCCGGTGCGTTTT
ACCGATGCGCAGGGCAATCAGCAAGAAGGCATTATCACCAGCGGGACGTTTTCCCCAACG
TTGGGCTACAGCATTGCGCTGGCGCGTGTTCCGGCGGGCATCGGCGAGACGGCGATTGTA
CAAATTCGTAACCGTGAAATGCCGGTAAAAGTGACTAAACCTGTTTTTGTGCGTAACGGC
AAAGCCGTCGCGTGA
>EECEBICD_00403 Glycine cleavage system H protein
ATGAGCAACGTACCCGCAGAACTGAAATACAGCAAAGAACACGAATGGCTGCGTAAAGAA
GCGGATGGCACTTACACTGTTGGCATTACTGAACACGCTCAGGAGCTGTTGGGCGATATG
GTTTTTGTTGATCTGCCGGAAGTCGGCGCGACCGTAAGCGCGGGCGACGACTGCGCCGTG
GCGGAGTCCGTAAAAGCGGCGTCCGACATTTATGCGCCGGTAAGCGGCGAGATCGTGGCG
GTAAACGATGCGCTGAGCGACTCCCCGGAGCTGGTGAACAGCGAACCGTATGCCGGTGGC
TGGATCTTTAAAATCAAAGCCAGCGATGAAAGCGAACTTGAATCGCTGCTGGATGCCACC
GCGTATGAGGCATTGTTAGAAGACGAATAA
>EECEBICD_00404 Glycine dehydrogenase (decarboxylating)
ATGACACAGACGTTAAGCCAGCTTGAAAACCGCGGCGCTTTCATTGAACGCCACATCGGA
CCGGACGCCGCACAGCAACAAGAGATGCTGAATGCGGTTGGCGCCGAGTCGTTAAACGCG
CTGACAGGTCAGATTGTGCCGAAAGACATTCAGCTCGCGACTCCGCCGCAGGTTGGCGAG
GCGGCAACCGAATATGCCGCACTGGCGGAATTAAAAGCCATCGCCGGGCGTAACAAGCGC
TTCACGTCGTACATTGGCATGGGCTACACCGCCGTGCAGCTACCGCCGGTTATCCTGCGC
AACATGCTGGAAAATCCAGGCTGGTACACCGCTTATACCCCGTATCAACCGGAAGTCTCT
CAGGGGCGTCTGGAAGCGCTGCTCAACTTCCAACAGGTGACGCTGGACCTGACGGGGCTG
GACATGGCTTCCGCCTCGCTGCTGGATGAAGCGACCGCCGCCGCTGAAGCGATGGCGATG
GCAAAGCGCGTCAGCAAACTGAAAAATGCCAACCGGTTCTTTGTCGCCTCTGATGTCCAT
CCGCAAACTCTGGACGTCGTGCGTACTCGCGCCGAAACCTTTGGCTTTGACGTCATTGTG
GATGACGCGGCGAAAGCACTGGATCACCAGGATGTGTTTGGCGTGCTGTTGCAGCAGGTT
GGCTCCACGGGCGAAATTCACGATTACAGCGCGCTGATTAGCGAACTCAAAGCCCGTAAA
GTGATTGTCAGCGTCGCCGCCGACTTTATGGCGCTGGTGCTGCTTACCGCGCCGGGTAAA
CAGGGCGCGGACATTGTTTTCGGCTCTGCGCAACGCTTCGGCGTACCGATGGGGTACGGT
GGCCCGCATGCGGCGTTCTTTGCCGCCAAAGACGAATTTAAACGCTCCATGCCAGGCCGT
ATTATCGGCGTCTCAAAAGATGCGGCGGGCAATACCGCACTGCGCATGGCGATGCAGACT
CGCGAGCAGCATATCCGTCGTGAGAAAGCGAACTCCAACATTTGTACCTCGCAGGTACTG
CTGGCTAACATCGCCAGTCTGTATGCCGTTTATCATGGTCCGGTTGGTCTGAAGCGCATC
GCTAATCGTATCCACCGCCTGACCGATATTCTGGCGGCAGGTCTGCAACAGAAAGGCTTA
AAGCTGCGTCATGCGCACTACTTCGACACCCTGTGTGTGGAAGTGGCTGATAAAGCTGCG
GTACTGGCGCGTGCGGAAGCGGCAGAGATCAATCTGCGCAGCGATATTCATAACGCGGTT
GGTATTACGCTCGATGAAACCACCACCCGTGAGAATGTGGCGCAGTTGTTCAATGTTTTG
CTGGGCGGCAGCCACGGTCTGAACATTGAAACACTGGATAAAGACGTGGCGCTTGATAGC
CGTTCCATTCAGCAAAGTATGCTGCGTGACGATGCGATTCTGACGCATCCGGTATTTAAC
CGTTACCACAGCGAAACCGAGATGATGCGCTATATGCACTCGCTGGAGCGTAAAGATCTG
GCGCTGAACCAGGCGATGATCCCGCTGGGTTCCTGCACCATGAAGCTGAACGCCGCCGCC
GAGATGATTCCTATCACCTGGCCGGAATTCGCTGAACTGCACCCGTTCTGCCCGCCGGAA
CAGGCCGAAGGATATCATCAGATGATTAGCCAGCTTTCTGACTGGCTGGTGAAACTCACC
GGGTACGACGCCGTCTGTATGCAGCCTAACTCCGGCGCACAGGGGGAATACGCGGGGCTG
CTGGCGATTCGTCACTATCACGAAAGCCGCAACGAAGGGCATCGCGATATCTGCCTGATC
CCGGCCTCTGCTCACGGCACTAACCCTGCTTCTGCACATATGGCAGGAATGCAGGTGGTG
GTGGTCGCCTGTGATAAGAACGGCAACATCGACCTGGACGATCTGCGGGCAAAAGCAGAA
CAACATGCTGCGAACCTCTCTTGCATCATGGTGACTTATCCTTCCACCCACGGCGTCTAT
GAAGAAACGATCCGTGAAGTGTGTGAAGTTGTGCACCAGTTCGGCGGTCAGGTTTACCTT
GACGGCGCGAACATGAACGCCCAGGTCGGCATTACCTCACCGGGCTTTATTGGCGCGGAC
GTTTCGCATCTTAACCTGCATAAAACTTTCTGCATTCCGCACGGCGGCGGTGGCCCAGGC
ATGGGACCGATCGGCGTGAAAGCGCATCTGGCACCGTTCGTACCGGGCCATAGCGTGGTG

CAAATCGAAGGCATGTTAACCCGTCAGGGCGCGGTTTCTGCGGCGCCGTTCGGTAGTGCC
TCTATCCTGCCAATCAGCTGGATGTACATCCGCATGATGGGCGCAGAAGGCCTGAAGCAG
GCAAGTCAGGTCGCGATTCTTAACGCTAACTACATTGCCAGCCGTCTGAAAGATGCCTAT
CCGGTACTGTATACTGGTCGCGATGGCCGTGTGGCGCACGAATGTATTCTTGATATTCGC
CCATTGAAAGAAGAAACCGGCATCAGCGAGCTGGATATTGCCAAGCGTCTGATCGACTAC
GGTTTCCATGCGCCAACCATGTCGTTCCCGGTGGCGGGCACGCTGATGGTAGAGCCGACG
GAATCTGAAGGTAAGGCTGAGCTGGATCGCTTTATCGATGCGATGCTGGCTATTCGCGCA
GAGATTGACCAGGTGAAAGCGGGTGTCTGGCCGCTGGAAGATAACCCGCTGGTGAACGCG
CCGCACATTCAGAGCGAACTGGTCGCCGAGTGGGCGCATCCGTACAGCCGTGAAGTTGCC
GTATTCCCGGCAGGCGTGGCAGACAAATACTGGCCGACGGTGAAACGTCTGGATGATGTT
TATGGCGACCGTAACCTGTTCTGCTCCTGCGTACCGATTAGCGACTACCAGTAA
>EECEBICD_00405 hypothetical protein
ATGAAACGATTTATATTTCTGCCATTAATGACATATTTTCTATTAATGAATGCAATGGCA
GCTAACGGAGAAAATAATCCCAGTAATCTATTAGGTGCACCAGTTAATACTGCTATTTCT
GGTGGTTCAGTATTGCCGGAAGGAAAGTTATTAACCGCCGTGAATAGCTCATTTCGCGAT
AAAGATCATCAAATCGAGGGGCACGGAAGTCCGGATGTTTACTCCCAGATATGGTTATTG
AAGATTCGTTACGGTTTGACCGACAGACTGGAATTATCAACTGTGGGTTCCTATATCAAT
AACAAACGAGACAATCTGTCTCCTGAGCACATAGAGGGAATGGGCGACCAATCAGTGGGG
GTCAATTATGCTTTAATGAGCCAGCGAAGAGGTGATCCGTTTTGGGTTACGGTAGGTGGG
GCAGTATTACTACCCACCGGGCAAGATGGAGATAATCATTTGCCTGGGAATAGCGCCTGG
GGAGGAAGAGTAACACTCACTTTAACAAAATTATTTACTCCTAACATTAAAGGGGATATG
GGTTTTGTCTATCAGGGACCATTTGAGCGCGGTAACCAGGACGTGAAACGAGGTAACGAA
TTCCAGTGGAATACCCAGGTGCGGTATATGTTTAGCGATTTACCGCTGGATCTTGGTCTG
GAAAGTGCCTACAGCAATAATGCCTCTGGAACGAAAAAACTGTCTAATGGCAGCGTAATT
AATAACCATTCTGGAACTACCGAATGGGTTGTTGGTCCGTCATTTAATATTGCCGTCGAT
TCACTGAAATTATGGTTTGGTGCCGGAGCCTTCTTCCCGGTAATGCAGGAAGCGAAGTCG
CCAACCAAAATGGAAGATGTACGCTGGGAATTTAAAATAGGTAAGACCTGGTAA
>EECEBICD_00406 6-phospho-beta-glucosidase BglA
ATGAGAAAACTCACCTTGCCAAAAGATTTTTTATGGGGCGGCGCGGTTGCTGCACACCAG
GTTGAAGGCGGCTGGAATAAAGACGGTAAAGGCCCCAGCATCTGCGACGTGCTAACCGGC
GGCGCACACGGCGTGCCACGCGAAATCACCCAGAATGTCGTTGCTGGCAAATACTATCCG
AACCACGAGGCGGTGGATTTTTACGGACATTACAAAGAAGACATCCGTCTTTTCGCCGAA
ATGGGGTTCAAATGCTTCCGTACCTCTATTGCCTGGACGCGTATCTTCCCGAATGGCGAC
GAATCCCAGCCAAACGAGGCCGGTCTGAAATTCTACGACGACATGTTTGATGAGTTACTC
AAATACAACATCGAACCGGTCATCACCCTTTCCCACTTTGAAATGCCATTACATCTGGTA
CAGCACTACGGCGGCTGGACCAATCGTAAGGTCGTTGATTTCTTTGTCCGTTTTGCTGAA
GTCGTGTTTGAACGCTACAAACATAAGGTCAAATACTGGATGACCTTCAACGAAATTAAC
AACCAGCGAAACTGGCGCGCGCCGCTGTTTGGCTACTGCTGTTCCGGCGTGGTGTATACC
GAGCATGAGAATCCAGAAGAAACCATGTATCAGGTCTTACATCATCAGTTTGTCGCCAGC
GCGCTGGCGGTAAAAGCGGCACGTCGCATTAACCCGCAGATGAAAGTGGGTTGTATGCTG
GCGATGGTCGCGCTGTATCCTTTCTCCTGTAAACCAGAAGATGTGATGTTTGCTCAGGAG
TCGATGCGTGAACGCTACGTCTTTACCGATGTGCAGCTGCGCGGCTATTACCCGTCCTAT
GTGTTGAACGAGTGGGAGCGCCGCGGATTTAACATCAAAATGGAAGATGGCGATCTTGAA
GTGCTGCGCGAAGGCACCTGCGATTATCTTGGTTTCAGTTATTACATGACCAACGCGGTC
AAAGCCGAAGGCGGTAGCGGCGATGCGATTTCCGGTTTTGAAGGCAGCGTACCGAACCCC
TATGTTAAAGCATCTGACTGGGGCTGGCAGATTGATCCGGTGGGCCTGCGTTATTCATTG
TGTGAACTGTACGAACGCTATCAAAAGCCGCTGTTTATTGTCGAAAACGGTTTTGGTGCT
TACGACAAAGTAGAAGAAGATGGCAGCATCAACGACGACTACCGAATTGACTACCTGCGC
GCCCATATTGAAGAGATGAAAAAAGCGGTGACTTACGATGGTGTCGACCTGATGGGCTAC
ACGCCGTGGGGCTGCATCGACTGCGTGTCGTTCACCACCGGTCAGTACAGCAAGCGCTAC
GGCTTCATCTACGTGAACAAGCACGATGACGGTACGGGCGATATGTCGCGTTCGCGTAAG
AAGAGCTTCAACTGGTACAAAGAGGTGATTGCCAGCAACGGCGAGAAGCTTTAA
>EECEBICD_00407 hypothetical protein
ATGCAGCCAAACGACATTACTTTCTTTCAACGTTTCCAGAATGACATTCTGGCAGGGCGT

AAAACCATCACTATTCGTGATGCCTCCGAGTCCCACTTTAAAGCAGGCGATGTGCTACGC
GTGGGGCGGTTCGAAGATGATGGCTATTTTTGCACAATTGAAGTGACAGGAACGTCAACT
GTGACGCTGGATACGCTGAACGAAAAACATGCGCAACAGGAAAATATGTCGCTTGATGAG
CTGAAACGGGTCATTGCTGAAATCTATCCCAACCAGACGCAATTTTATGTGATTGATTTT
AAATGCCTTTGA
>EECEBICD_00408 hypothetical protein
ATGGTGCAAAAACCATTAATGACGCAGGGATATTCGCTGGCTGAGGAAATCGCCAACAGT
ATTAGCCACGGCATTGGATTGGTATTTGGTATTGTCGGCCTGGTGCTGTTGTTGGTTCAG
GCGGTAGAGGCGAATGCGGGTATGACGGCTATCGCCAGCTACAGTTTATATGGCGGGAGC
ATGATCCTGCTTTTTCTTGCCTCAACGCTTTATCATGCCATTCCTCATCAGCGGGCAAAA
ATCTGGTTAAAGAAATTTGATCACTGCGCTATCTATCTGCTCATTGCCGGAACCTATACG
CCATTTCTGCTGGTAGGTCTGGATTCGCCGCTGGCGCGCGGACTAATGATTGTGATCTGG
AGCCTGGCGCTATTCGGTATCCTCTTCAAGTTGACGATTGCGCACCGATTTAAAGTGCTT
TCACTGGTTACGTATCTGGCAATGGGCTGGCTGTCGCTGATAGTGGTTTATCAGTTGGCG
ATTAAGCTGGCGATAGGCGGCGTGACGCTGCTGGCGGTAGGTGGCGTCGTTTACTCGCTA
GGCGTAATTTTCTATGTTTGCAAACGCATACCTTATAACCACGCCATCTGGCATGGCTTT
GTGCTGGGCGGCAGCGTGTGCCACTTCCTGGCCATCTATTTGTATGTGGGACAGGCATAA
>EECEBICD_00409 hypothetical protein
ATGCCCGTGCCGGATGGCGGCGCTGTCGTACCTTATCCGGCCTACGAGAACAGCACGTGC
GAGCCTGATGGCGTTGCGCTATCAGGCTATCCGGCTTTTAACGTGATATCACTCTTCCAG
CGAGTAGGGCAGCGGGACGATATGCAGGGTGTTGGCATCGTCGCGAACGCGGAAGACGCT
TTCGGCTTCTAA
>EECEBICD_00410 tRNA-modifying protein YgfZ
ATGGCTTTTATCTCCTTTCCACCACGTCATCCTTCATCGTCAGCCCGCCTGCCGCTGACG
CTGATCGCGCTTGACGACTGGGCGCTGTCGACCATTACCGGCGTAGACAGTGAAAAGTAT
ATTCAGGGCCAGGTCACCGCTGACGTAAGCCAGATGACTGAACAGCAACACCTTCTCGCC
GCCCACTGCGATGCTAAAGGTAAGATGTGGAGCACTCTGCGCCTGTTCCGTGAGCGCGAC
GGTTTCGCCTGGATTGAACGCCGTAGCGTGCGTGAAGCGCAATTAACGGAGCTGAAAAAG
TACGCCGTATTTTCCAAAGTAGTCATCGCGCCGGATGACGAACGCGTATTGCTTGGCGTG
GCAGGTTTTCAGGCCCGCGCGGCGCTGGCTAATGTCTTTAGCGAACTGCCCAATAGCGAA
AATCAGGTGGTCAGAGACGGCGCCTCCACGCTGCTATGGTTTGAACATCCTGCGGAACGA
TTCCTGCTTGTCACCGATGTCGCGACGGCTAACATGTTAACGGAGAAGTTGCATGGCGAA
GCCGAACTGAATAACAGTCAACAGTGGCTGGCGTTGGATATTGAAGCAGGCATTCCGGTG
ATTGACGCGGCGAACAGCGGTCAGTTTATCCCCCAGGCGACTAACCTACAGGCGCTGGGC
GGCATCAGCTTCAAGAAAGGCTGCTACACCGGACAGGAGATGGTCGCACGGGCAAAATTC
CGCGGCGCCAATAAACGCGCGCTTTGGTTGCTGGCGGGCAAAGCCAGCCGGGTACCGGAA
GCAGGAGAAGATCTGGAGCTACAGATGGGCGAGAACTGGCGTCGTACCGGCGCTATTCTG
GCGGCGACGCAGTTGGACGATGGTCAGTTACTGGTTCAGGCCGTCATGAATAACGATTTA
GAAGCCGAAAGCGTCTTCCGCGTTCGCGACGATGCCAACACCCTGCATATCGTCCCGCTG
CCCTACTCGCTGGAAGAGTGA
>EECEBICD_00411 FAD assembly factor SdhE
ATGGATATTCACAATAAAGCACGTATTCACTGGGCATGTCGTCGCGGTATGCGCGAACTT
GATATTTCCATCATGCCGTTTTTTGAACATGAGTACGATAGTTTAAGTGATGAAGAAAAG
CGTATTTTTGTCCGTTTGTTGCAGAGTGACGACCCGGATTTATTTAACTGGTTAATGAAT
CACGGTAAACCGGCTGACGCTGAACTGGAACAGATGGTCCGACTCATTCAGACACGGAAT
CGGGAACGTGGTCCTGTGGCAATCTGA
>EECEBICD_00412 Inner membrane protein YgfX
GTGGTCCTGTGGCAATCTGATTTACGCGTCTCATGGCGCGCTCAGTGGATCTCATTGCTC
ATTCATGGGCTGGTTGCCGCGGTGATTTTATTGATGCCCTGGCCGCTGAGTTATACCCCG
TTATGGATGATACTACTGTCGCTGGTCGTGTTTGACTGTGTACGTAGCCAGCGCAGGATT
AATACCTGCCAGGGAGAGATCAAGCTACTCATGGACGGGCGCTTACGCTGGCAGGGGCAG
GACTGGACGCTCTTGCGTCCGCCCTGGTTACTGAAGAGCGGGATGGTGTTGCGTTTACGC
GCAGAGTCTGGACGTCATCAGCATTTATGGCTGGCGGCGGATAGCATGGAAGAAGCGGAA
TGGCGTGAGTTGCGTCGGATACTGTTACAGCAGCCGATATCAGGCCAACACTAA

>EECEBICD_00413 Flavodoxin 2
ATGAACATGGGTCTTTTTTACGGTTCCAGCACGTGCTACACCGAGATGGCCGCAGAGAAA
ATTCGCGATATCCTCGGCCCGGAGCTGGTGACATTACATAACCTCAAGGATGACGCCCCC
GCCTTAATGGAGCAGTACGACGTGCTCATTTTAGGCATCCCCACCTGGGATTTTGGCGAA
ATTCAGGAGGACTGGGAAGCCGTCTGGGAGCAACTGGACGATCTCAATCTGGAAGGCAAA
ATCGTTGCGCTTTACGGTATGGGCGACCAGCTCGGCTACGGCGAGTGGTTCCTGGATGCG
CTGGGTATGTTGCATGACAAACTTGCCCTCAAAGGCGTTAAATTTGTCGGCTACTGGCCC
ACAGAAGGCTACGAATTTACCAGCAATAAACCGGTCATTGCGGATGGGCAACTGTTTGTC
GGCCTGGCGCTGGATGAAACGAACCAGTACGATCTCAGCGATGAGCGTATACAGACCTGG
TGTGAACAAATTCTCGGCGAAATGGCCGAACACTACGCCTGA
>EECEBICD_00414 Tyrosine recombinase XerD
ATGGAACAGGATTTAGCGCGTATTGAGCAGTTTCTGGATGCCCTGTGGCTGGAACGAAAT
CTGGCGGAAAATACATTAAGCGCGTATCGTCGCGATTTATCGATGGTCGTTGCGTGGTTG
CATCATCGCGGAAAAACGCTTGCGACGGCGCAGGCTGATGATTTACAAACGCTATTGGCT
GAACGGGTGGAGGGCGGATACAAAGCGACCAGCTCCGCACGCCTGTTAAGCGCTATGCGG
CGCTTTTTCCAGCATCTGTACCGTGAAAAGTATCGTGAGGATGATCCGAGCGCGCAGTTG
GCCTCGCCTAAGCTTCCGCAGCGTTTACCGAAAGATCTTAGCGAGGCGCAGGTTGAACGG
CTCTTGCAGGCGCCGCTTATTGATCAACCTTTGGAGCTGCGCGATAAAGCTATGTTGGAA
GTGCTGTATGCCACCGGTTTGCGCGTGTCGGAACTGGTGGGGCTGACAATGAGCGATATC
AGTCTGCGGCAGGGCGTGGTGCGGGTTATCGGTAAAGGCAATAAAGAGCGGTTGGTGCCG
TTAGGTGAAGAGGCGGTATATTGGCTGGAAACGTATCTGGAGCATGGGCGTCCCTGGTTA
CTCAATGGCGTATCGATTGACGTATTATTCCCCAGCCAGCGTGCGCAGCAGATGACGCGG
CAGACCTTCTGGCACCGTATTAAACATTATGCGGTACTGGCTGGAATCGATAGCGAAAAA
CTATCGCCGCATGTTTTACGTCACGCTTTCGCCACGCATTTGCTGAATCACGGTGCCGAC
TTGCGCGTTGTACAAATGCTACTTGGGCATAGCGATCTTTCGACGACACAAATTTATACG
CATGTCGCAACGGAGCGTTTGCGTCAACTTCATCAACAGCACCATCCGCGGGCATGA
>EECEBICD_00415 Thiol:disulfide interchange protein DsbC
ATGAAAAAGCGTTTTATGATGTTCACTTTACTGGCGGCGGCGTTTTCAGGCGTGGCCCAT
GCGGATGATGCGGCTATCCGTCAGTCGCTGGCTAAGCTGGGCGTTCAGAGTACGGAAATT
CAGGCCTCGCCAGTGGCAGGGATGAAAACCGTCCTCACTCACAGCGGCGTACTGTACGTC
ACTGATGACGGCAAACATATTATTCAAGGGCCGATGTATGACGTGAGCGGCGCGCATCCG
GTAAACGTGACGAACAAACTGCTGATGAGCCAGCTAAATGCGCTGGAAAAAGAGATGATC
GTCTATAAAGCGCCGGATGAGAAACATGTCATTACTGTTTTTACCGATATCACCTGCGGC
TATTGTCATAAGCTACATGAAGAGATGAAAGATTATAACGCGCTGGGGATCACGGTACGT
TATCTGGCCTTCCCGCGCCAGGGGCTGGAAAGCCAGGCGGAGCAGGATATGAAATCTATC
TGGTGCGCCAAAGACAAAAACAAAGCGTTTGATGACGCCATGGCAGGTAAGGGCGTGAAA
CCGGCAAGCTGTGACGTGAACATTGCTGATCATTATGCGTTGGGCGTGCAGTTGGGCGTC
AGCGGTACGCCAGCCATTGTATTGAGTAACGGCTATGTGGTGCCTGGCTATCAGGGACCG
AAAGAGATGAAAGCGTTTCTTGATGAGCATCAAAAACAGACCAGTGGTAAATAA
>EECEBICD_00416 Single-stranded-DNA-specific exonuclease RecJ
GTGAAAGGAATGCTGCCCTGGCAACAGCTTAGCGGCATAGATAACGCGGTGGAGATCCTC
TACAACGCCTTTCGCGAAGGTATCCGCATTATTGTTGTTGGCGATTTTGACGCCGACGGC
GCGACCAGTACTGCGCTAAGCGTATTGGGAATGCGTGCGTTGGGATGTGACAACATCAGT
TATCTGGTGCCTAATCGCTTTGAGGACGGCTACGGTTTAAGCCCGGAAGTGGTCGATCAG
GCGAAGGCGCGCGGCGCGCAGCTTATCGTCACCGTAGATAACGGCATCTCATCCCACGCC
GGCGTAGCGCATGCAAAGACGTTGGGGATTCCGGTGATCGTGACCGATCACCACCTGCCT
GGCGATACATTGCCGGAGGCCGAAGCGATTATTAATCCCAATCTGCGCGACTGCGAATTC
CCGTCTAAGTCGCTGGCGGGCGTCGGCGTGGCGTTTTACCTGATGCTGGCGTTGCGAACA
TTTTTGCGCGACAAAGGATGGTTCGACGAGCGCAACATTGCGCCGCCGAATTTGGCGGAG
CTGCTGGATTTAGTGGCGTTGGGAACGGTAGCAGACGTTGTGCCGCTGGACGCTAACAAC
CGTATTCTGACCTGGCAAGGGCTAAGTCGTATTCGTGCCGGGAAATGCCGTCCGGGAATT
AAAGCGTTGCTGGAGATATCGAATCGCGATCCGCAGCAGCTTGCCGCCAGTGATTTAGGC
TTCGCGTTGGGGCCTCGCCTGAATGCCGCCGGCAGGCTGGATGATATGTCCGTTGGCGTG
GCGTTACTGTTGTGCGACAACCTCGGCGAAGCGCGTGTTCTGGCCAGCGAGCTGGATGCG

CTTAACCAGACGCGCAAAGAAATAGAGCAGGGGATGCAGGCGGAAGCGCTTATCCTGTGC
GAAAAGCTTGAGCGCAGTAGTGAAACGCTTCCGGGCGGTCTGGCGATGTATCATCCTGAA
TGGCACCAGGGCGTGGTCGGAATTCTGGCGTCGCGCATTAAAGAGCGTTTTCACCGCCCG
GTGATCGCCTTTGCGCCTGCGGGCGACGGCACGCTAAAAGGCTCAGGCCGATCGATTCAG
GGGCTGCATATGCGCGATGCGCTGGAACGGCTGGATACCCTTTACCCTGATCTGATGATC
AAGTTCGGCGGCCATGCGATGGCGGCGGGATTGTCGCTGGAAGAGCATAAATTCGAGCAG
TTTCAGCAACGGTTTGGCGAGCTGGTGACGGAATGGCTCGATCCTGCCCTGTTGCAAGGC
GAGGTGATCTCCGATGGTCCATTAAGCGCGGCGGAGATGTCTATGGAAGTGGCGCAACTG
TTGCGGGATGCCGGACCGTGGGGGCAAATGTTCCCGGAACCGTTATTCGATGGTCGTTTC
CGTCTGCTACAGCAGCGTCTGGTGGGCGAGCGTCACCTCAAGGTGATGGTGGAGCCTGTC
GGCGGCGGCCCGCTGTTGGATGGCATCGCATTTAATATTGATACGACCTGTTGGCCGGAT
AACGGCGTGCGGGAGGTAGAACTGGCTTATAAACTGGACATTAACGAGTTTCGCGGCAAC
CGTAGTTTACAGATTATTATCGATGATATTTGGCCGCTATGA
>EECEBICD_00417 Peptide chain release factor RF2
ATGACTCAGGGGCTGGACGACGTTTCCGGGCTGCTGGAACTGGCGGTAGAAGCTGACGAC
GAAGAGACGTTTAACGAAGCCGTCGCGGAACTGAATACGCTGGAAGAGAAACTGGCGCAG
TTGGAATTCCGTCGGATGTTCTCCGGCGAGTACGATAGCGCCGATTGCTATCTCGATATT
CAGGCCGGTTCCGGTGGTACTGAAGCGCAGGACTGGGCCAGCATGTTGCTGCGTATGTAT
CTGCGTTGGGCGGAAGCGCGCGGCTTCAAGACGGAAATCATTGAAGAATCGGAAGGGGAA
GTCGCCGGTATTAAGTCCGCGACTATCAAAATCTCCGGCGAATATGCCTATGGCTGGCTG
CGCACTGAAACTGGCGTACATCGTCTGGTGCGTAAAAGCCCGTTCGACTCCGGCGGTCGC
CGTCATACCTCGTTTAGCTCCGCGTTTGTGTACCCGGAAGTGGACGACGATATCGATATC
GACATTAACCCGGCGGATCTGCGTATCGACGTCTATCGCGCGTCTGGCGCAGGCGGTCAG
CACGTTAACCGTACGGAATCCGCCGTGCGTATCACCCATATTCCCACCGGGATTGTGACG
CAGTGCCAGAACGACCGTTCGCAGCACAAAAACAAAGACCAGGCCATGAAGCAGATGAAA
GCGAAGCTTTATGAACTGGAGATGCAGAAAAAGAACGCTGAAAAACAGGCGATGGAAGAC
ACTAAGTCCGATATCGGCTGGGGAAGCCAGATTCGTTCCTATGTCCTGGACGATTCCCGC
ATTAAAGACCTGCGTACTGGGGTGGAAACCCGCAATACACAGGCGGTACTGGACGGTAGT
CTGGATCAATTTATCGAAGCAAGTTTGAAAGCAGGGCTATGA
>EECEBICD_00418 Lysine--tRNA ligase
ATGTCTGAACAAAACGCACAGGGCGCTGACGAGGTAGTCGATCTTAACAATGAAATGAAG
GCACGTCGCGAGAAGCTGGCTGCACTGCGTGAGCAGGGTATCCCGTTCCCGAACGATTTC
CGTCGTGACCGTACCTCAGACCAACTGCACGCAGAGTTTGACGCGAAAGAAGCTGAAGAA
CTGGAAGCGCTGAATATTGAAGTGTCCGTCGCGGGCCGTATGATGACTCGCCGTATTATG
GGTAAGGCCTCTTTTGTTACCCTGCAAGACGTAGGCGGCCGCATTCAGCTGTATGTGGCG
CGCGATGATCTGCCGGAAGGCGTTTACAACGAGCAGTTCAAGAAGTGGGATCTTGGCGAT
ATCCTCGGCGCGAAAGGTAAGCTGTTCAAAACCAAAACCGGCGAGCTGTCTATCCACTGC
ACCGAACTGCGCCTGCTGACGAAAGCGCTGCGTCCGTTGCCGGATAAATTCCACGGCTTG
CAGGACCAGGAAGCGCGCTATCGCCAGCGTTATCTGGATCTCATCTCTAACGATGAATCC
CGTAACACCTTCAAAACCCGTTCGAAGATCCTGGCCGGTATTCGCCAGTTTATGGTTGCT
CGCGGCTTTATGGAAGTCGAAACCCCGATGATGCAGGTGATCCCAGGCGGCGCGTCTGCC
CGTCCGTTTATCACCCATCACAACGCGCTGGATCTGGACATGTACCTGCGTATCGCGCCG
GAACTGTACCTCAAGCGTCTGGTGGTTGGCGGCTTCGAACGCGTATTCGAAATTAACCGT
AACTTCCGTAACGAAGGCATCTCCGTGCGCCATAATCCTGAGTTCACCATGATGGAACTC
TATATGGCGTATGCGGACTACAAAGACCTGATCGAGCTGACGGAGTCTCTGTTCCGTACG
CTGGCGCAGGATGTTCTGGGCACCACCCAGGTTCCGTACGGTGATGAAGTGTTTGACTTC
GGTAAGCCGTTCGAAAAACTGACCATGCGTGAAGCGATCAAGAAATATCGCCCGGAAACG
GATATGGCTGATCTGGACAACTTCGATTCCGCGAAGGCGATTGCTGAAAGCATCGGCATT
CACGTCGAGAAAAGCTGGGGTCTGGGCCGTATCGTCACCGAGATCTTTGACGAAGTCGCT
GAAGCGCATCTGATTCAGCCGACCTTCATCACCGAATATCCGGCTGAAGTGTCTCCGCTG
GCGCGTCGTAATGATGTGAACCCGGAAATCACTGACCGCTTCGAGTTCTTTATCGGCGGT
CGTGAAATCGGTAATGGCTTTAGCGAGTTGAACGATGCGGAAGACCAGGCGCAGCGTTTC
CTGGATCAGGTTAATGCAAAAGCGGCGGGCGACGACGAAGCCATGTTCTACGACGAAGAC
TACGTGACGGCGCTGGAACACGGGTTACCGCCGACAGCGGGTCTGGGAATTGGTATCGAC

CGTATGGTGATGCTGTTTACTAACAGCCACACGATTCGCGACGTTATTCTGTTCCCGGCG
ATGCGTCCGGTGAAATAA
>EECEBICD_00419 Isopentenyl-diphosphate Delta-isomerase
ATGACTGAGGAACATGTCGTTTTATTGGATGAGCAGGATAAGCCTTCCGGCACACTGGAA
AAGTATGCGGCTCATACGCTTAATACGCCTTTGCATCTGGCTTTCTCCTGCTGGCTATTT
AACGAAGATGGCCAGTTGCTGGTAACACGACGGTCGCTGAGCAAGAAAGCCTGGCCAGGC
GTGTGGACGAATTCGGTTTGCGGTCATCCGCAACAAGGCGAAACCACCGAAGAGGCGATC
ATCCGCCGTTGTCGGTTCGAACTCGGCGTGGAAATTACCGATCTCACCCCCGTCTATCCG
CACTTCAGCTATCGAGCGACAGATCCAAACGGTATTGTGGAAAATGAAGTCTGTCCGGTA
TTTGCCGCGCGGGCAACCAGTGTGTTACAGGTTAACAGCGAAGAGGTGATGGACTATCAG
TGGAGCGAATTTAAAAGCGTATGGAAAAGCCTTCTCGCTACGCCGTGGGCATTTAGCCCG
TGGATGGTCATGCAAGCGTCAGACGAACAAGCGCGTGAACGACTGCTCAATTACTGCCAG
CGTTAA
>EECEBICD_00420 Murein hydrolase activator NlpD
ATGGTTTTGAGCGCAGGACGCCTGAACAAAAATAGCCTGGGTATTGCGGTGCTGTTATGC
ACAGGGCTGCTGCTGGCGGGGTGTTCCAGTAATTCCGGCTCAGGAACGTATTCCGGCTCC
GTCTATACCGTTAAGCGCGGCGATACGTTGTATCGTATATCGCGCGCTACGGGAACCAGC
GTGAAAGAACTGGCCCGACTGAATGGCATTTCTCCGCCTTATACTATTGAGGTGGGGCAG
CGAATTAAAGTGCGCGGCAGCGCAAAGAGTAGCAGTTCAACACGCAAAACCAGCAATAAA
ACAGCAACAAAAACTGCTGCGGTCAGACCGTCATCTTCGGTGCCAAAATCCTCCTGGCCG
CCGGTGGGGCAGCGTTGTTGGGTATGGCCTGCTAACGGTAAAGTGATTTTACCTTATTCC
ACCGCGGAGGGCGGAAATAAGGGGATCGATATTGCGGGCGCGCGCGGAACGCCTGTTTAT
GCGGCGGGAGCGGGTAAAGTTGTCTATGTCGGGAACCAGCTGCGCGGTTACGGCAATCTC
ATCATGATTAAGCACAATGAAGATTACATTACGGCTTACGCTCATAATGACACCATGCTG
GTTAATAATGGACAAAGCGTTAAAGCGGGGCAAAAAATCGCTACGATGGGCAGCACGGAT
GCTGCATCCGTAAGACTACACTTCCAGATTCGCTACCGGGCGACGGCCATCGATCCGCTG
CGTTATCTGCCGCCGCAGGGGAGTAAACCGAAGTGCTGA
>EECEBICD_00421 tRNA-Gly(ccc)
GCGGGCGTAGTTCAATGGTAGAACGAGAGCTTCCCAAGCTCTATACGAGGGTTCGATTCC
CTTCGCCCGCTCCA
>EECEBICD_00422 hypothetical protein
ATGAAGGTAAATACATGGACAATTTTGCTTATGTCGGCGCATCTGACGGCCTGTGCCGTC
CCTGGCACAGAAAAAATATCAGACCAGCATGGACAGCGTTACGGCAGAAAAAATAAGCCGG
ATTATCCAGTCGGATGTCATTCCTTATAAAGGCGAAAACCATGGAGAAGTTATCAGCCGC
GTATCGTCTGCTTTTCTCGGAACACCTTATCAGGCGGATACGCTCATCGGTGGGCCCGGC
ATCCCCGAAGTCCTTGTAGCGAATTTCAACGGCGTGGACTGCTTTACACTGGCTGATTAC
GTTGAAGCCCTGGCCCGCAGCGATAATCAGAAATCGTTTCTGCATAATCTTGCCAGGACT
CGCTACGCCGCGGGCAAAGTTGCTTATCTCAGCCGCCGGCACTTCTTCTCTGACTGGTTC
GCCGCAACACCGCGTAATGCACGGGATGTAACGCCGGATATCAGCCCTGATTATGTCGTT
GTTGACAAGCAGCTTAACCATAAACCTGATGGCGGCGAGTACATTCCTGGTCTGGGCATC
CATCCTCGTAAAATAAATTACATTCCCGGAAGAGCCATCAACCAGCAGGTCATGAACCAC
CTAAAAAACGGGGATTATATCGGTGTCTACTCCCCGCTTGACGGGCTGGACGTTTCACAT
GTGGGTATCGTAGTTCGCCATGATGAACAGGTGTGGTTCAGGAACGCCTCCTCATTGGCC
GCAAACAGAAAAGTGGTGGATACACCGTTTATGGAATACATGCATTCCAGACCGGGCATC
GTTGTGTTGCGGGCTGAATGA
>EECEBICD_00423 hypothetical protein
ATGCAAACAATGCATAAAAAACATATGTGCCGATGGCTTCTGCCCGGTTTGCTCGGTCTG
GCTCTGTGTGCGCCAGTACCACATACTTACGCAGCCATCATTGAGGCAGGATTTTATCCG
GAAGGTACAGATCTCCAGCTTGTTCTTAAGATAATTGAGACCGCGCGACAGGAAATCCGT
CTGATGGACTATTCCTTCACCTCTTGGGAGGTGGTCCGGTTCGAACATTCAGTGAGTACA
AAATTCTCCATGTCAAAGTGA
>EECEBICD_00424 Antitoxin VapB
ATGCACACAACACTTTTTTTTAGTAACCGGACCCAGGCAGTCAGATTGCCTAAATCGATA
TCCTTTCCGGAGGATGTGAAACATGTCGAAATCATTGCGGTTGGTAGAAGCAGAATCATT

ACTCCAGTCGGAGAATCCTGGGACAGCTGGTTCGATGGCGAAGGCGCCAGTACTGATTTT
ATGAGTACCAGAGAACAACCCGCAGTACAGGAACGGGAAGGATTTTGA
>EECEBICD_00425 tRNA(fMet)-specific endonuclease VapC
ATGCTGAAATTCATGCTTGATACCAATACCTGTATTTTCACCATCAAAAATAAGCCCGAA
CACATCAGAGAACGCTTCAACCTCAATACATCCCGAATGTGTATCAGCTCCATCACCTTA
ATGGAGCTGATTTACGGTGCTGAAAAAAGCCTGGCGCCGGAGCGTAATCTTGCCGTCGTG
GAGGGATTTATCTCCCGCCTTGAGGTTCTGGATTACGATACACAGGCAGCGATACATACC
GGTCAAATCCGTGCCGAACTGGCCCGCAAGGGAACACCTGTCGGGCCTTATGACCAGATG
ATTGCTGGCCATGCCGGTAGCCGCGGACTGGTCGTCGTCACAAACAATCTCCGCGAATTT
GAACGCATTCCGGGTATCCGAATCGAAGACTGGTGCTAA
>EECEBICD_00426 hypothetical protein
ATGCCGTTACTGCCCGATAACTGCCGCCCCCCCCTCTCCATAGCCGTTTCCTGCAACTT
AAATCACAGACGTTGATTTACCAGGCCGATATGCCTTCTTTCCCGTTATATATCCTTCAT
ACTTCAAGTTGCTTATGTGTTGGCTACGGATTACTCGGTCCATCCATGGGCCTCGCCCTG
ACGGGCCGTCGCACGCGACGTTCAAATCTGCTCCCGGCAGAATTGTCGCTCACCCCAGTC
ACAGAGTTATCTATGCTCCCGGGGACTCACTCCCTTGCCACCTTTAAGCAACTCGAATTA
TTTTGGGTATATATCAGTGAACTTCTATTTTTGTCAGTTTTTATTTTTCAGTTTATTTAT
TATTAA
>EECEBICD_00427 Attachment invasion locus protein
ATGAACAAAATGCTATTAGCCGGAAGTGCCGGTATTGTTCTGCTGTCTGCTGCAGCCAGT
CCGGTATGGGCAGACGATAATGCCAGTACATTTTCACTTGGATACGCCCAGTCCCATACT
AATCACGCCGGGACGTTGCGTGGGGTACGGCTGGCAAACAACTATGAAATGTCACCGGAC
TGGGGGCTGACAACCTCATTTGCATGGCTAAATGGAAGCCAGCGCTATTCCGATGAAAGC
AGTAATGGTCGTGTAACAACCCGTTATTACTCCCTGCTTGCAGGCCCCCCTGGGAAAAAA
AAAAACCATTTAAGTCTGTTAAGCAAAGGGGGCCCCGTATTATTGCATCAGCGTGATCAC
GGGATAAATGAGTCTGACAGTAAGGTCGGATATGGTTATTCTGCTGGCGTGGCTTATACC
CCGGTCAGTAGTGTCGCTATAACTCTGGGTTATGAGGGAGCGGATTTTGATGCTACCCAT
AACAGCGGAAGCCTTAATAGCAATGGTTTTAATCTTGGCGTGGGTTACCGGTTCTGA
>EECEBICD_00428 hypothetical protein
ATGAAAAGCCATATTATGCCAGGCGTTGCGCTGATGGTTATCCTGTTTTCAGGAAATATT
ATGGCCGCAACAAACATGACTGATAACGTTACTCTGAATAATGACAAGATATCCGGACAG
GCCTGGCAGGCCATGCGTGATATCGGAATGTCGCGATTCGAGTTGTTTAATGGTCGCACC
CAAAAGGCGGAACAACTGGCTGCGCAAGCAGAAAAACTCCTGAACGATGACAGCACTGAC
TGGAATCTGTATGTCAAATCAGATAAAAAAGCCCCAGTTGAAGGAGACCACTATATTCGG
ATAAATTCATCGATTACGGTAGCAGAAGATTATCTCCCTGCGGGCCAGAAAAATGATGCT
ATTAATAAAGCAAACCAGAAAATGAAAGAAGGAGATAAAAAAGGGACTATAGAGGCCCTG
AAACTAGCAGGCGTCAGTGTTATTGAAAATCAGGAACTCATTCCTCTTCAGCAAACCCGT
AAGGATGTGACCACAGCGCTTTCCTTGATGAACGAAGGAAAATACTATCAGGCAGGTCTA
TTACTGAAATCAGCTCAGGATGGCATAGTTGTAGACTCTCAGTCAGTTCAGGAATCACCC
ACGCACTCCGTGCAGCACGACGCCGCGCATTAA
>EECEBICD_00429 putative fimbrial-like protein YbgD
ATGCGTAATAAAATAATACTTGCCATGGCGGCTGCCGGTATGATGTATGGCGCTTCTGTA
TATGCAGTCGATCCACCCACAGCAGGCCCGTTCGGCTCAGGTAAAATTACCTTCACTGGC
ACCATCACCAACTCCCCCTGTGATATCGCACCGGGCGACGATGCGATCACCGTTCCGTTT
GGACAGATTTCTTACCGTAAACTGAATACCGCCGACGCAACTACGGAATCAAAACCGTTC
ACCATTCATCTACAGAACTGTGCTTTCGATCCGAATACCCCGGACACAGCTGGCTCCGCA
GGGAAAATGTCCAAAGTAACCGTATCTTTCTCTGGCGCAGCGAATACCAGCAAAAAGGCT
TACACCACCAGCGGTATCGCACAGCATGTTGGTGTCCAGCTACTGAAGAGTGACAACGCC
ACAATTATCGATCCCAACACCCCGATGCCGGATGGCGACGCTCAGCAACTGCAGGCCGGA
AACAACGAACTCAACTTCTTTGCCCGACTAATAGCACTGGATAATGGCGTCACTCCCGGT
GACTTCAACGCATCCGTCACCTACACCCTGAAATACCTGTGA
>EECEBICD_00430 Outer membrane usher protein PapC
ATGAAAATTACCCGGCTTGCCATTCTGATTACCCTGACATTTTCCGTCCTGAAGTCGCAG
GCCACGGAGTTCAATGCCAGCCTGCTGGACAGCGGGAACCTGTCTAACGTGGACCTCACG

GCCTTCAGCCGCGAGGGGTATGTGGCGCCCGGTAACTATATTCTCGATATCTGGCTCAAT
GACCAGCCGGTACGGGAACAGTATCCGGTCAGGGTTGTACCGGTTGCCGGACGCGACGCG
GCCGTCATCTGTGTGACAACAGACATGGTGGCCATGCTGGGTCTTAAGGATAAGATTATT
CATGGCCTGAAACCCGTTACCGGTATCCCGGACGGGCAATGCCTTGAGCTGCGCTCCGCT
GACAGTCAGGTACGGTACAGCGCTGAAAATCAGCGTCTGACCTTCATTATTCCCCAGGCC
TGGATGCGTTATCAGGACCCGGACTGGGTGCCCCCTTCACGCTGGAGCGACGGCGTCACC
GCCGGCCTGCTTGATTACAGCCTGATGGTCAACCGCTATATGCCGCAGCAGGGGGAAACC
TCTACCAGCTACAGCCTCTACGGCACCGCCGGGTTTAATCTCGGCGCCTGGCGATTGCGG
AGCGATTACCAGTACAGCCGTTTTGACAGTGGGCAGGGCGCATCACAGTCCGATTTTTAT
CTGCCGCAGACGTATCTGTTCCGTGCCTTGCCAGCACTGCGCTCAAAACTGACGCTCGGA
CAGACGTACCTCAGCTCCGCTATTTTTGACTCCTTCCGCTTTGCCGGGCTCACTTTGGCC
AGCGATGAGCGCATGCTGCCGCCCTCTCTTCAGGGGTATGCCCCGAAAATTAGCGGCATT
GCGAACAGTAATGCACAGGTCACCGTCAGCCAGAATGGTCGCATTCTGTACCAGACCCGG
GTGTCGCCGGGGCCTTTTGAGCTTCCCGACCTGAGCCAGAATATCAGCGGCAACCTGGAT
GTCAGCGTCCGGGAAAGCGACGGTAGCGTCCGGACCTGGCAGGTCAACACGGCCAGCGTC
CCCTTTATGGCACGCCAGGGACAGGTTCGCTATAAGGTGGCCGCCGGACGTCCGCTGTAC
GGCGGGACGCATAACAACAGCACGGTCAGCCCTGACTTTCTGCTCGGGGAGGCCACCTGG
GGAGCATTTAACAACACGTCGCTGTATGGCGGCCTCATTGCCTCCACCGGCGACTACCAG
TCCGCGGCGCTCGGGATCGGCCAGAATATGGGGCTGTTGGGCGCTCTGTCTGCGGACGTC
ACCCGCTCAGATGCGCGGTTGCCGCATGGGCAGAAGCAGTCCGGTTACAGCTACCGTATC
AACTATGCCAAAACGTTTGATAAAACCGGCAGTACCCTGGCGTTCGTCGGATACCGCTTC
TCGGACCGCCATTTTCTGTCCATGCCGGAGTACCTGCAGCGTCGGACCACTGACGGCGGC
GATGCATGGCATGAAAAACAGAGCTACACCGTGACTTATAGCCAGTCCGTTCCGGTACTC
AACATGAGCGCGGCCCTCTCGGTAAGCCGGCTCAATTACTGGAACGCACAGTCCAACAAT
AACTACATGCTCAGCCTCAACAAGGTGTTCAGTCTCGGCGACCTTCAGGGACTCTCGGCC
TCGGTGTCGTTTGCCCGTAACCAGTACACCGGCGGGGGTAGCCAGAACCAGGTCTACGCC
ACCATCAGTATCCCGTGGGGAGACAGTCGCCAGGTGAGTTACAGCGTACAGAAAGATAAC
CGGGGCGGCCTGCAGCAGACCGTGAATTACAGCGATTTCCATAACCCGGACACTACATGG
AACATCAGCGCCGGTCATAACCGTTATGACACCGGCAGCAGCTTCAGCGGCAGCGTGCAG
AGCCGTCTGCCGTGGGGCCAGGCGGCAGCGGACGCCACCCTGCAGCCCGGACAGTACCGC
AGCCTGGGCCTGAGCTGGTACGGCTCGGTGACTGCCACCGCCCACGGTGCCGCTTTCAGC
CAGTCGATGGCCGGGAATGAGCCCCGCATGATGATAGATACCGGAGACGTGGCCGGTGTG
CCGGTGAACGGCAACAGCGGGGTGACCAACCGCTTCGGGGTGGGCGTGGTGAGCGCCGGC
AGCAGCTATCGCCGCAGCGATATTTCGGTTGATGTGGCCGCCCTGCCGGAGGACGTGGAC
GTCAGCAGCTCCGTCATCAGTCAGGTACTGACGGAGGGCGCGGTGGGCTACCGGAAGATT
GACGCCAGCCAGGGGGAGCAGGTGCTCGGTCATATCCGGCTGGCTGATGGCGCAAGTCCG
CCGTTTGGCGCCCTGGTGGTGTCCGGGAAAACCGGCAGAACCGCGGGGATGGTCGGAGAT
GGCGGTCTGGCCTACCTGACAGGTCTCAGCGGGGAAGACCGCCGGACCCTGAATGTGTCC
TGGGATGGCCGGGTGCAGTGCAGACTCACCCTGCCGGAAACGGTGACACTCAGCCGGGGG
CCGCTGTTACTGCCCTGCAGATAA
>EECEBICD_00431 Chaperone protein PapD
GTGAAAAACAGCAGAAGAAGCCGTGTGATTTTACTCGCACTGGCGGCAGCATGGAGCCAG
TACAGCCCGGCGGCCGTCAATGTTGACCGGACCCGTATTATCATGGATGCGCCGCAGAAA
ACGGTGGCCATCACGCTGAATAACGATGACAAAACCACGCCGTTCCTGGCGCAGTCCTGG
GTGACGGACGCTGACGGGGTGAGAACCGATGCGCTGATGGCGCTGCCGCCGCTGCAGCGT
ATTGATGCCGGACAGAAATCCCAGGTCCGCATCACCCAGGTTCGCGGGCTGACAGATAAG
CTCCCTCAGGACAGGGAAACACTGTTCTGGTTTAACGTGCGGGGCGTTCCCCCGAAGCCA
GAAGACGACAACGTTCTGCAACTGGCCATGCAGAGCCAGCTGAAACTGTTTTACCGTCCG
AAAGCCATTATCAGAAGCAGCAGTGACCAGCCGGAAAGAAAGCTGACTGCTGAGCGGAAT
GCGGGACATCTGACCCTCAGAAACCCGACGCCGTACTACATCACTGTGGCGTGGCTGGGA
GCAGACCGTAGCCACAGGCTCAGCGGTTTCCGCGAGGGCGTGATGGTACCGCCCCTGGGG
AACCTGCCTTTAAAGGCTGTTCTGCCGGCAGAAACCCGGACGCTGTGGGTGGGGTATATT
GATGATTACGGGGGACTGCAGATGAATCGCTACACCTGCGATGCCCTGAACTGCGCGTTT
AAGGACGCAGGGGCGACATCATGA

>EECEBICD_00432 hypothetical protein
ATGACAGAATGGATTTTTAATCTGAAAACAAAACTGACCGTGCTGGTGATGATGTTGTGC
TCTTTATGTGTGACTAAAGTGTATGCGGTTGAGCTAGGTATAAATGAATGTGCAGTCACT
TCCGGTCAGAATATTAATTTAAGAAGTATAAATTTGACAACCGATGATTTTAAACCTGGC
ACGGATTCGGTTATCTATACAATCAATCAGGATGCAGTTTTTAAATGTTACATGGGATAT
GACACGCAATTTCCACAGCTGGTATTTAATCAGGGCTACTTCAGTAAATTTACCAAGACA
CTAGATGCGATGGGACTTGGATTCAGAATGTCCATTAAAGAAACAGGAAATGCTTCAAGT
GCTGTTTCGTTCTCCTGGGATGAAATAAAGAGTACTCAGTCAGGAAATGAGTTACGAAAA
GAGTTTGGCACAAAACTACCTGTTGGGACTACAGAACGAAAAGTAAGGATAACACTGGAT
TTCTTATATACAAAAGCATATTCAGAGAGTAGTGCAGTTACAGCTTTTACGGGGATAAGT
AATGTTTTAAATATTGTACCTTTTTCTTATTCTTTGAGACAAAACGGATTTATTTTATCT
GGTTTTAATGTGAGAATTCTCAGAAACGGATTGGGTAAAGTTGACATTGTTCCATTACAG
GTCAACTTTGGGCATATTTATACGACTTATGAACCTTCACAGACCAGACAGGCAAATTTT
ACAGTGATCGCCACGCAGGTTTTACGACCGGCTATGGGACAGGAATTCACGATCCCTCTG
GCCATCACCTTCGGGAAAGGTGCACTGACACAGGATACCGGGCAAACTCTGAATCTTGTC
AGCCTGGATGGCCCCAACAAAGGTCAGCCTAATGGACTCCGGCTTTCAATAAAAGATGAT
AAAGGAAAAGAGATTACTTTTGATAAACAAGAAGTACTGGGGGATATTACTATTACAGGA
GCTGTCACCGGTAATGTGTCGAAGGTATATACTGCAGTAATAACCCCGACCCCGGGGGGA
AGTGTAAAGACAGGAACGTTTTCTGCTGCAATACCCGTTACTGTTACCTATAACTAA
>EECEBICD_00433 hypothetical protein
ATGTGCCCTGATAATACACACGCAAAAAAGCAGTACCTTACCCCTGGTAACGATATTCAT
TATCCCGGACAGACCAACCATGATGCCTGTTTTATTCCGGAAAGCGTCCGCCAGTATGCC
GACGAGCCCCTGTATATCATTGTGGCACACTGGTGCCTGTTGCAGCAGAACTGGGTTCAG
CGTAACCAGATAGCAGAGGCGTTTCACATCACGGCCAGACGGGCCAGCTACCTGATTGCC
TATCTGCGAAGCAAAACATCGCGGGTGGTCAGCATCTGCCGTCACCAGACCCTGCCTAAT
AAAGCACGACGCTATGAGATTTATGTTATCCGGGTTCTTGACAGTCCCACCCCGTCCACG
CGCCGTGAAAAAGCCGGACCGCCGCTGGTATCAAAACGCCGGGTCGGTAATGGTGACAGG
TCGATGGCAAATGAACTGTGGAACCGGCTGTGCAGTAACAGAAATGCGGGGAAAATCCTG
AAAAAGAAGGAGGATGAGGATGACGGAACATAA
>EECEBICD_00434 hypothetical protein
ATGACGGAACATAACCGGATGCCGGCCCGGCAGATTATCGTTTACGGTGACTGCTGGCCG
GTGACGATCGCCGTGGCACACCTGGTCCGGAGATTTCTGCCGGGCTGCAACTGTGAAACC
GCATACAGTCTGCCTGTTCTGTTGCAGCAATTACGTCGGAAACCGGAGGCCATACTGATA
CTGTGTCTGCGTCCCCGTGAACATCTCTTTTTGTTCTATTCCCTGAGACAGATACTGCCG
GACTACCCGGTTATGATTATCAGCGATGAGCTGTTTTTCAGCGACCGGGTGGTACTGAAG
GTATACGGCGGCATCCCGGCGCTGCTGGAGCAGGAACTGGCGGAGATACTTATTCGGTGG
CGGCGGGATGAACAGTGGGCCGGGGGCGCACGCCTGCGCAGGACAGGAGGGCTGGACGCC
TTTTTGTTATCTCCGGATCCGGTGACGGGCTTTCTGGAAGTGCCACCGATATTTAACAAT
CCGAAGCGGTTGATGAACTATATGGATCAGTTGATGCACCGGGAGATACTGGCATGCGGA
GTGAGCCTGGCCCAGTTACGCTTGCTTCAGGAAGTTTACCGTGGACGAGGAAGGTTGTCA
GCGTTGTGTGGAAGGCTGAATACACAGGAGAAGCAAATCTGGCAGGACAAATACCGGTTA
CTGGTGAAGCTGGGAATGCGGAACCGATTACGGGAACTGCTGTTTGGTACCCGGTTTTGT
AAATCACTGCAGCGAACTCCGTTTATCGCTCCGCAATAA
>EECEBICD_00435 Nickel/cobalt efflux system RcnA
ATGGGTGAATTTTCGACACTTCTTCAGCAAGGAAACGGCTGGTTCTTCATTCCCAGCGCC
ATTTTATTAGGTATTTTGCACGGGCTTGAACCAGGGCACTCCAAAACTATGATGGCGGCT
TTTATTATTGCCATTAAAGGTACGGTTAAACAGGCCGTCATGCTCGGTCTGGCAGCAACG
CTTTCTCATACCGCGATCGTCTGGTTAATCGCGCTGGGTGGGATGTATCTTAGCCGGGCA
TTCACCGCACAATCAGTGGAACCATGGCTGCAGTTAATTTCTGCGATTATTATTCTGAGC
ACCGCGTGCTGGATGTTCTGGCGGACATGGCGAGGCGAGCAGCAGTGGCTGGCGGGAAAC
CACCATCACGACCATGACCATGACCATGACCATGACCATGACCATGACCATGACCATGAC
CATGACCATCATGGTCACATACATCCGGAAGGTGCAACGTCAAAAGCGTATCAGGATGCC
CATGAACGCGCCCATGCTGCCGATATTCAACGCCGTTTTGATGGTCAAACAGTGACTAAT
GGACAGATCCTGCTGTTCGGCCTGACCGGAGGGCTTATCCCCTGTCCGGCTGCGATCACC

GTTTTACTGATTTGTATCCAGCTTAAAGCGTTTACGCTGGGCGCCACGATGGTACTGAGC
TTTAGTCTTGGCCTGGCATTAACGCTGGTGACGGTAGGCGTTGGCGCGGCGATAAGCGTT
CAACAGGCAGCAAAGCGCTGGAGTGGTTTTTCGACGCTTGCCCGGCGGGCGCCCTATTTT
TCGAGCATTCTGATTGGTCTGGTCGGCGTGTATATGGGAATTCATGGCTATACCGGGATC
ATGCAGTAA
>EECEBICD_00436 Transcriptional repressor RcnR
ATGTCACATACCATCCGGGACAAACAAAAGCTTAAAGCCAGAACCAGCAAGATTCAGGGG
CAGGTGATAGCGCTAAAAAAAATGCTGGATGAACCGCATGAATGCGCGGCCGTGTTACAA
CAGATCGCGGCGATTCGCGGCGCGGTAAATGGCTTAATGCGCGAGGTGATTAAAGGGCAT
CTGACCGAACATATCGTTCATCAGAGTGATGAAGCCAGACGTGAAGAAGATCTGGACGTA
ATATTAAAGGTGCTGGATTCTTATATAAAATAG
>EECEBICD_00437 Inner membrane transport protein YqeG
ATGTCTAAAATTTGGTCAAAAGATGAGACTCTCTGGAGTTTTGCATTATACGGAACGGCT
GTAGGCGCAGGCACTCTTTTCCTTCCCATCCAGTTGGGATCGGCAGGCGCAATCGTGCTA
TTTATTACCGCGCTGGTCGCCTGGCCTCTGACATACTGGCCGCATAAGGCGCTGTGTCAG
TTTATTCTTTCCTCAAAGACTTCTGCCGGTGAGGGAATTACTGGCGCCGTTACTCATTAT
TACGGCAAGAAAATCGGCAGTATTATTACCACGCTTTATTTTATTGCGTTTTTTGTCGTG
GTATTAATTTATGCGGTGGCGATTACCAATTCCCTGACAGAGCAATTAGCGAAGCATATT
CAGATAGATATTCGCATCCGGATGGCAGTTAGCTTCGGCGTAGTACTTATCCTGAATATG
ATTTTCTTAATGGGAAGACATGCCACTCTTCGCGTAATGGGCTTTTTAGTCTTTCCACTT
ATCGCTTACTTCTTGTTCCTTTCGCTCTACTTAACCGGAAGCTGGCAACCCTCGTTACTG
ACCGGGCAGATGTCTTTGGATAGTCACACGTTGCACCAGGTATGGATTTCTATTCCGGTG
ATGGTTTTTGCTTTTAGCCATACGCCCATTATTTCGACGTTTGCCATCGACAGACGGGAA
AATTTTGGCGATCAGGCCATGGATAAATGCAAAAAGATCATGAAGGTCGCCTACCTGATT
ATCTGCCTGAGCGTGCTATTCTTTGTCTTTAGCTGCCTGCTCTCCATTCCGCCGTCATAC
ATTGAAGACGCGCGCAACGAAGGCGTTACCATCCTTTCCGCGCTCTCGATGATGCCCAAT
GCGCCGGCCTGGCTCTCTATTTCCGGTATTATCGTTGCCGTCGTGGCCATGTCGAAATCT
TTCCTCGGCACCTATTTCGGCGTCATCGAGGGCGCAACCGAAATGGTAAGAACCACCCTA
CAGCAGGTTGGCGTGAAAAAAAGCCGGGCGTTCAATCGCGCGCTGTCTATCATGCTGGTT
TCCGGGATTACGTTTATTATTTGCTGTATTAACCCGAATGCCATCTCTATGATTTATGCC
ATTAGCGGCCCGCTCATTGCGATGATTCTGTTTATTATGCCCACGTTGTCAACGTACCTG
ATACCGGCATTGAACCCCTACCGTTCAGTAGGTAATTTCATTACGTTGGTCGTCGGTTTA
CTTTGCGTATCGGTGATGTTTTTCGGCTAA
>EECEBICD_00438 hypothetical protein
ATGCAACATGACGCCATACAACGTCGCTCGCTGCCGGAGCGTATTTTCCATGCTGTCTGT
TTTGAAGGTATTGCGACTGCGATACTGGCGCCAACGACCGCCTGGCTAATGCAGCGCTCG
GTGCTAGAGATGGGCGGCCTGACGATATTGCTGGCAACGACGGCCATGATCTGGAATATC
ATTTATAACGCTCTGTTTGATCGACTGTGGCCTGCGCATCAGGTGAGACGAACCGCCAAA
GTGCGCGCACTCCATGCGTTAGGATTTGAAAGTGGGTTTATTGTCATTGGCGTGAGTATT
GTCGCCTGGGTACTGAATGTCAGCCTGCTACAAGCATTCACCCTGGAAATTGGTTTCTTC
CTGTTCTTTTTGCCGTACACGATGCTGTACAACTGGGCATACGATGTTTTACGCCAACGA
ATAGTGACTCGCCGCCAACAGCGCGTTAGTGCATGA
>EECEBICD_00439 HTH-type transcriptional activator AllS
ATGCGTTACTCACCTGAAGCGTTAATGGCATTTGTTGAGACCGTCTCTTGCGGTTCGTTT
TCTGCGGCGGCGCGCCGCCTGCGTAAAAGCCAGTCCACCATTAGTACCGCCATTGCGCAT
CTGGAGGCGGATCTGGGATTTTCATTATTTGATCGATCTTCGCGCCAGCCCGTTCTGACC
GAAGAAGGAAAACGGGTGCTTAGCTATGTTCAGGCTATTTTATCGGCGAGCGATCGTTTG
GATGAGGTGGCGCTGACGTTATCCGGCGAGACGGAGGCAAGACTCACGTTCGTCCTCTCT
GATACCCTGCACCCTGACAGTCTGGAAGAGTTAATGGCGCAGTTTGATCGTCAATTTCCG
CATACCGAGTTTGAATGCTTGATCGGAGAAAATGATGATGTCATCGACCTGTTGCAAAAA
GGGCGCGCCCAGATAGGCCTGATTGAAGCGCGTGATGACTATCCCACAGACATTGGCGCG
ACGCGTCTGCCTGTTCAAAGCTGGATGGGGGTTTATGTGGCGACCACGCATCCGTTGGCG
TCGCAGACGAACCTGCAATGGGAGCAACTGCATACCTGGCGCGAGCTGCGTCTGAATACC
TACCTTGAATCTAATGCCAACATTACGCGCGGCCCGGCCTGGTCAGCGCCAAACTATTTA

TTACTGTTGAGCATGGCGGTTCAGGGATTTGGCTGGTGCGTATTGCCCTGCGCGCTGGTG
GAAGAGTTTGCGGCGGAAAAACCGCTGGCCCAGCTGGAAGTTCCTGGCTGGCCGAGGGCG
ATTGCTATTGATTTGCTGTGGAACAAGAAGACGCCGCCCGGAACGGCAGGGAACTGGCTT
CGCTACCATTTGCAGCAGAAATAA
>EECEBICD_00440 Acetyl-CoA acetyltransferase
ATGAAAGAGGTTGTGATAGTGGGGGCTTTACGGACACCCATCGGCTGTTTTCAGGGAACG
CTGGCCCGTCATTCCGCCGTCGAGCTGGGGAGCATGGTCGTAAAAGCGTTAATTGAGCGC
ACCGGCGTGGACGCTAACGCTATCGATGAGGTCATCCTCGGTCAGGTGCTGACGGCTGGC
GCCGGGCAGAATCCGGCGCGCCAGTCAGCCATCAAAGGAGGGCTGCCGACTACCGTTTCC
GCCATTACTATTAATGACGTTTGTGGTTCCGGTTTAAAAGCGTTGCACCTGGCGACGCAG
GCTATTCAGTGCGGCGAAGCGGACATTGTTATTGCGGGCGGGCAGGAGAATATGAGCCGT
GCGCCGCATGTCCTCAATGACAGCCGTACCGGCGCGTTGCCTGATGCCGACAATCTGGTG
GATAGTCTGGTACATGATGGCTTATGGGATGCCTTCAACGATTATCACATTGGCGTGACG
GCAGAAAACCTGGCGCGGGAATATGGCATCAGCCGCGAGTTACAGGATGCTTATGCGCTC
AGTTCGCAGCAAAAAGCCAGGGCCGCGATTGATACCGGGCGTTTTAAAGATGAGATTGTC
CCCATCGTCACGCAACGTAATGGGCAGACCGCAATTGTCGATACGGATGAACAACCGCGG
GCCGACGCCAGTGCGGAGGGGTTAGCCCTGCTGCATCCGGCGTTTGATAGTTTAGGTTCG
GTGACGGCGGGTAATGCCTCCTCAATCAACGATGGCGCCGCCGCGGTGATGATGATGAGC
GAGGCGAAAGCCCAGGCGCTGGGGCTGCCGGTGCTGGCGCGCATCCGCGCGTTTGCCAGC
GTCGGCGTCGATCCTGCGCTAATGGGGATTGCGCCGGTATATGCTACCCGGCGCTGTCTG
GAGCGTGCCGGCTGGCAACTGACGGAGGTCGACCTTATCGAAGCCAACGAAGCGTTTGCC
GCCCAGGCGTTGTCGGTAGGAAAAATGCTGGAATGGGATGAGCGACGGGTAAACGTTAAT
GGCGGCGCGATTGCGCTGGGGCATCCCATTGGCGCTTCCGGTTGTCGAATTCTGGTTTCA
CTGGTGCACGAAATGGTTAAACGCGACGCGCGAAAAGGTCTGGCGACGCTGTGTATCGGT
GGCGGCCAGGGCGTTGCTTTAACCATTGAACGCGACTGA
>EECEBICD_00441 4-deoxy-L-threo-5-hexosulose-uronate ketol-isomerase
ATGATGCCGTTAAATAAATCAAAACAGTGTTTTGAATTTTATGCCATTGCGATCGATTTA
TCATTGGAGGGAAAAGTGGACGTAAGGCAAAGCATCCATAGCGAGCACGCTAAAACGCTG
GACACCCAGGCGCTGCGACGCGAGTTTTTAATAGAAAATATTTTTGTCGCTGATGAATAT
ACGATGGTCTACAGCCATATCGACCGTATTATTGTCGGCGGCATTATGCCCGTCAGCCAT
TCTGTTGAAATCGGCGGAGAGGTGGGTAAACAGCTTGGCGTTTCCCGGTTACTGGACCGC
CGCGAGCTGGGCGTCATTAATATTGGCGGCGCGGGCGCCATTATCGTTGACGGGCAGCGC
CATGATATTGGTCATCGTGATGCTCTGTATATTGGTAAAGGGGCGAAAGAGCTGGTCTTC
GTCAGCAATGAGGCGAGCAGACCGGCGAAGTTTTATTACAACTGCGCGCCAGCGCATACC
GCTTATCCGACGAAAAAAGTGTCGCCTGCCGACGTTGCGCCCGTCACCTTAGGCGATAAC
CTCACCAGTAACCGTCGCACCATCAATAAATACTTCGTACCGGATGTACTGGAAACCTGT
CAGCTCAGTATGGGACTCACCGAACTGGCGCCCGGTAATCTATGGAACACCATGCCATGC
CATACCCATGAGCGCCGTATGGAAGTTTACCTCTATTTCAATATGGAAGAGGACAGTTGC
GTTTTCCATATGATGGGGCAGCCTCAGGAAACCCGTCATATTGTGATGCGTAATGAACAG
GCGGTTATTTCCCCAAGCTGGTCAATACATTCTGGCGTTGGCACCAAAGCCTATACGTTT
ATTTGGGGCATGGTGGGAGAAAATCAGGTCTTCGATGACATGGATCACGTTGCGGTACAA
GATCTGCGCCAGTCGCGACCGGTGATAACGCCTGTGCGTGACAGGCATGACAAAAATAAG
GATTAA
>EECEBICD_00442 2-dehydro-3-deoxy-D-gluconate 5-dehydrogenase
ATGATTTTGAATGCATTTTCTCTTGAAGGTAAAGTCGCGGTTGTGACGGGGTGCGATACC
GGATTGGGGCAGGGAATGGCGTTAGGGCTGGCGGAAGCGGGCTGTGACATTGTCGGTATT
AACATTGTTGAACCGACGGAAACTATTGCGCGCGTCACCGCTCTGGGACGTCGTTTTTTA
AGCCTGACAGCCGATCTGCGTCAAATCGATGGTATTCCGGCATTACTGGAACGGGCGGTG
GCAGAATACGGTCATATTGATATTCTGGTGAATAACGCGGGTTTAATCCGTCGTGAAGAT
GCGATTGATTTCACGGAAAAAGACTGGGATGACGTCATGAACCTGAACATCAAGAGCGTG
TTCTTTATGTCTCAGGCGGCGGCAAAACATTTTATTGCCCAGGGCAACGGCGGCAAAATT
ATTAATATCGCGTCTATGCTTTCCTTCCAGGGCGGCATTCGCGTTCCGTCTTATACCGCG
TCGAAAAGCGGCGTAATGGGAATAACGCGTCTGATGGCGAACGAGTGGGCGAAACACAAT
ATCAATGTAAACGCCATCGCGCCGGGCTATATGTCCACGAATAATACCCAGCAATTGCGC

GCTGATGCACAACGTAGCGCCGAAATTCTCGACCGTATTCCGGCTGGACGTTGGGGGCTA
CCGGGCGATTTAATGGGTCCGGTAGTCTTCCTGGCATCCAGCGCCGCTGATTATGTTAAC
GGCTATACCATAGCGGTAGATGGCGGTTGGCTGGCGCGTTAA
>EECEBICD_00443 Arabinose-proton symporter
ATGGTCTCTATTAATCATGACTCTGCTTTAACGCCGCGTTCGCTTCGCGACACACGACGT
ATGAATATGTTTGTTTCGGTTTCTGCAGCGGTAGCGGGACTGTTATTTGGTCTGGATATC
GGCGTTATCGCCGGGGCGCTGCCTTTTATTACCGACCATTTCGTACTGACCAGCCGGCTG
CAGGAATGGGTCGTCAGCAGCATGATGCTTGGCGCGGCAATTGGCGCATTATTTAACGGC
TGGCTTTCATTCCGGCTGGGGCGTAAGTATAGCCTGATGGCTGGCGCGATTTTGTTCGTG
CTCGGCTCGCTGGGGTCGGCGTTTGCTTCCAGCGTGGAAGTATTGATTGGCGCCCGCGTG
ATACTGGGCGTAGCAGTAGGGATTGCCTCCTATACCGCGCCGCTTTATCTCTCTGAAATG
GCTAGCGAAAATGTTCGCGGCAAAATGATCAGTATGTATCAACTGATGGTGACGTTAGGC
ATTGTGCTGGCTTTTTTATCCGATACGGCATTCAGCTACAGCGGCAACTGGCGCGCGATG
TTGGGCGTGCTGGCGCTGCCTGCGGTGTTGCTCATTATTCTCGTGGTATTCCTGCCGAAT
AGTCCGCGTTGGCTGGCGCAAAAAGGTCGCCATATTGAAGCGGAAGAGGTGCTGCGTATG
CTGCGCGATACCTCGGAAAAAGCCCGTGATGAACTGAATGAAATTCGGGAAAGCCTCAAA
CTCAAGCAGGGAGGGTGGGCATTATTTAAAGCTAACCGCAATGTTCGCCGCGCCGTGTTC
CTCGGTATGCTGCTACAGGCAATGCAGCAGTTCACCGGCATGAACATCATTATGTACTAT
GCGCCGCGCATTTTTAAAATGGCCGGCTTTACCACCACGGAACAGCAAATGATCGCCACG
CTGGTGGTCGGACTGACTTTTATGTTCGCGACGTTTATCGCCGTCTTTACGGTCGATAAG
GCCGGGCGTAAACCGGCGTTAAAAATCGGTTTCAGCGTAATGGCGTTAGGGACATTGGTG
TTGGGCTACTGCCTGATGCAGTTTGATAACGGTACGGCATCAAGCGGTCTCTCCTGGCTT
TCCGTTGGGATGACGATGATGTGTATCGCCGGTTACGCGATGAGCGCCGCTCCGGTGGTG
TGGATACTGTGTTCGGAAATCCAGCCGCTGAAATGCCGTGATTTTGGCATTACCTGTTCA
ACCACGACAAACTGGGTATCGAACATGATCATCGGCGCGACATTCCTGACACTGTTGGAC
AGCATTGGCGCGGCAGGTACATTCTGGCTCTACACCGCGCTGAATATCGCTTTTATCGGC
ATCACTTTCTGGCTGATTCCGGAAACCAAAAATGTCACCCTGGAGCACATCGAACGCAAG
CTGATGGCGGGCGAGAAGCTAAGAAATATTGGCGTGTAA
>EECEBICD_00444 L-aspartate/glutamate-specific racemase
GTGAAAACGATCGGACTGTTGGGGGGGATGAGCTGGGAATCGACTATCCCTTATTACCGT
TTAATCAATGAAGGCATTAAACAGCAGCTGGGAGGCCTGCACTCGGCGAGCTTACTGCTG
CATAGCGTAGATTTCCACGATATTGAAGTATGTCAACGCCGCGGCGAGTGGGATAAAGCG
GGCGATATCCTGGCGCAGGCCGCGCAGGGGTTACAGCAGGCGGGCGCAGAAGGCATTGTG
CTGTGTACCAACACCATGCATAAAATCGCGCACGTTATTGAATCTCGTTGTTCTCTGCCT
TTCCTACACATCGCGGATGCTACCGGGCGAGCTATCGCCCGTCAGGGGTTGCATCGCGTG
GCGCTATTAGGGACCCGCTATACGATGGAGCAGGATTTTTATCGCGGGCGGCTGGAACAG
CAATTTGCGATTGAGACCATCGTGCCGGAGGCGGACGATCGGGCGCGTATCAATCAGGTC
ATTTTTGACGAGCTCTGTCAGGGGACATTCACCGACGCGTCGCGCCACTATTATTTACAG
GTTATCGAACAACTGGCTGCGCAAGGCGCGCAGGGCGTTATTTTTGGCTGCACTGAAATA
GGTTTGCTTGTTCCCCAGACGCAAAGCGCGCTACCGGTTTTCGATACCACGGCAATTCAT
GCTGCAGATGCCGTGAGCTTTATGCTTTCGTCATCGGCCCCAGAATAA
>EECEBICD_00445 Octopine catabolism/uptake operon regulatory protein OccR
ATGGCTGCCGTTAATTTACGTCACATTGAAATTTTTCATGCCGTAATGACCGCCGGAAAT
CTCACCGAAGCGGCGCGGCTGCTGCATACCTCGCAGCCTACCGTCAGTCGCGAGCTGGCG
CGTTTTGAGAAAGTGCTGGGATTAACGCTGTTTGAGCGCACACGTGGGCGCTTACATCCG
ACGGTGCAAGGGCTACGCCTGTTCGAAGAGGTACAACGCTCCTGGTATGGGTTGGATCGC
ATCGTCAGCGCGGCGGAAAGTCTGCGCGAGTTCCGTCAGGGCGAGCTCTCTATCGCCTGC
CTGCCGGTCTTTTCACAATCCTTTTTGCCGCCGTTACTACCGCCTTTTCTGGCGCGTTAT
CCGGATGTCAGCCTGAATATTGTGCCGCAGGAGTCGCCGTTGCTGGAAGAGTGGCTCTCC
GCCCAGCGCCACGACTTAGGGCTGACGGAGACGCTTCATGCCCCGGCAGGAACAACCCGC
ACCGAGTTACTAACGCTAAATGAAGTGTGCGTACTGCCCTGCGACCATCCGCTTGCCGCC
AAAACGGTACTGACGCCAGACGATTTTCAGGGGGAAAACTTTATCAGCCTCTCCCGCCTG
GACAGCTACCGACAGTTGTTGGATACGTTATTTGCCGAACATCAGGTGAAGCGCAGAATG
GTGGTGGAAACGCATAGCGCGGCCTCCGTCTGCGCGATGGTGCGCGCCGGGGCGGGCGTT

TCGATCGTTAACCCGCTGACCGCGCTGGGCTATGCCGCCAGCGGCGTCACCGTCCGCCGC
TTCAGCATTGACGTGCCCTTTACCGTCAGCCTGATTCGCCCGCTACACCGCCCCGCCTCC
GCGCTGGTTGACGCCTTCAGTAAGCATTTACAAACGCATCTTTCGCGCCTGGTCGAACCG
CTGGAGGTTATTCTGGGGCCGATGACGAAAGCATAA
>EECEBICD_00446 Diaminopimelate decarboxylase
ATGTCATTGCCGCATTATTACGCTGAAACTGACCTTAACGCTGAAAATTTGCTCAGATTG
CCTGCTGAATTCGGCTGTCCGGTCTGGGTCTATGACGCGCACATTATTCGCCGTCAGATA
GCCGCCCTGCAGCAGTTTGACGTCGTGCGTTTTGCGCAAAAAGCCTGTTCGAACATTCAT
ATCCTGCGCTTAATGCGCGCGCAGGGCGTCAAGGTGGATTCTGTTTCATTGGGGGAGATC
GAACGTGCGCTGGCGGCGGGCTACGATCCGCAAACGTGCCCTGATGATATCGTTTTTACC
GCCGATGTGATTGACGCCGCCACGCTGGCGCGCGTCAGCGAACTGCATATTCCGGTCAAT
GCCGGCTCGGTAGATATGCTGACGCAGCTTGGTCAGGTTTCGCCTGGGCATCGCGTCTGG
TTGCGCGTGAATCCTGGCTTTGGTCACGGCCACAGCCAGAAAACCAACACCGGGGGCGAA
AACAGCAAACACGGCATCTGGCATAGCGATCTTCCGGCGGCGCTGGCGGTAATGCAGAAA
TACCGACTGAAACTTGTCGGTATCCATATGCACATTGGTTCCGGCGTCGATTATGGCCAT
CTGGAGCAGGTTTGCGGCGCGATGGTACGCCAGGTGCTGGAATGTGGTCAGGATGTGGAG
GCGATTTCGGCAGGCGGCGGACTCTCGATACCTTATCGTGAGGGAGAAGAGCCCGTTGAT
ACGCGCCATTACTATGGTCTGTGGAACGCGGCGCGCGAGCAAATCGCCCGCCATCTGGGA
CATGCGGTCAAACTTGAGATCGAACCCGGACGTTTTCTGGTGGCGCAATCGGGCGTGCTG
GTAACGCAGGTGCGTAGCGTAAAACAGATGGGAAGCCGCCATTTCGTGCTGGTAGATGCG
GGTTTTAACGATCTGATGCGCCCGGCGATGTACGGTAGCTATCACCGTATTTCGGCGCTG
GCGGCGGATGGTCGCGCGCTTGAAAATGGCCCGTGGGTCGAGACGGTGGTCGCCGGGCCG
CTATGCGAATCAGGCGATGTTTTTACGCAGCAGGAAGGGGGAATGGTTGAAACACGCGCC
TTACCTGCGGTCATCCCCGGCGATTACCTCGTATTGCACGATACCGGCGCGTACGGCGCG
TCTATGTCATCGAATTACAACAGCCGCCCGCTCCTGCCGGAAGTACTGTTTGACAATGGA
CAGGCGCGGCTGATCCGCCGTCGTCAGACCATTGAAGAGCTGTTAGCGCTGGAGATGCTC
TGA
>EECEBICD_00447 HTH-type transcriptional regulator AscG
ATGGCGACAATGCTGGATGTTTCCCGCCATGCGGGCGTATCAAAGGCCACCGTCTCACGA
GTGCTGAATGGGACGGGGCAGGTAAAAGAAAGCACGCGCCAGAAAGTGTTTACGGCGATG
CAGGCTCTGGGCTATCGCCCCAACCTGCTGGCACGCTCGCTGGCGAATCGCACCAGCAAC
AGCATCGGTCTGGTCGTCTCTACGTTTGACGGCTTCTATTTCGGCAGTTTGTTGCGCCGG
GCGTCGCGCCAGGCGGAGTTTCATAACAAGCAGTTGATCGTCACCGATGGTCACGATACG
CCGGAACGAGAGCAGAAAGCCGTACAAATGTTGGCCGACAGACAGTGCGACGCTATTATT
CTTTACACTCGCTATATGGATGAGCCGGCGATTTTGTCGTTGATTGACGCCACGGAAATG
CCGCTGGTGATTATTAATCGCAACGTCACTCAGGCCCGCGATCGCGCTATTTTCTTCGAG
CAGGAGACGGCGGCATTCCAGGCGGTGGAATACCTGATTACGCAGGGCCATCGCGATATC
GCCTGTATTACGCTGCCTGTTCATACTCCCACTGGCACATCACGCGTAGCGGGTTATCGC
AAGGCGCTGGAAAAGTATGGTATTCCCTGGCAACCGGCAAAAGTGAAATACGGCGATTAC
ACGCTGACGCGCGGCTATGACGCCTGCCGGGAATTACTGGAGGAAGGCGTCACGTTTAGC
GCGCTATTCGCCTGTAATGATGACACGGCGCTGGGCGCGGCAAAAGCGCTGCGCCAGGCC
GGATTACGCATCCCGCAGGATGTGTCGCTGTTTGGTTTTGACGATGCGCCGGGCGCAACC
TGGCTTGAACCGGGGCTTTCAACAGTCTATTTACCCATCGAGGATATGATAGCCACCGCG
ATCGATCAGGCCGTTCGCCTGGCGAACAGCGAGCCGGTCGCCCCGATCCCGCCCTTTACC
GGCACGCTGATTCTGCGCGAGTCCGTCGCCGCGGGCCCGTTTTTTCAACGTCCGGCCTAA
>EECEBICD_00448 HTH-type transcriptional regulator GalR
ATGGCGACCATAAAAGATGTAGCCCGACTGGCCGGTGTTTCAGTCGCCACCGTTTCTCGC
GTTATTAACGATTCGCCAAAAGCCAGCGAAGCGTCCCGGCTGGCGGTGACCAGCGCAATG
GAGTCCCTGAGCTATCACCCTAACGCCAACGCGCGCGCGCTGGCACAGCAGGCAACGGAA
ACCCTCGGTCTGGTGGTCGGCGACGTTTCCGATCCTTTTTTCGGCGCGATGGTGAAAGCC
GTTGAACAGGTGGCGTATCACACCGGCAATTTTTTACTGATTGGCAACGGGTATCATAAC
GAACAAAAAGAGCGTCAGGCTATTGAACAGTTGATTCGTCATCGTTGCGCAGCGTTAGTG
GTACACGCCAAAATGATTCCGGATGCGGACCTGGCCTCATTAATGAAGCAAATCCCCGGC
ATGGTGCTGATTAACCGCATTTTACCGGGGTTAGAACACCGCTGTGTCGCGCTGGATGAC

CGTTACGGGGCATGGCTGGCGACCCGGCATCTGATCCAGCAAGGTCATACGCGTATTGGG
TATATCTGTTCCAATCACACTATCTCTGATGCCGAAGATCGCCTGAGGGGCTATTACGAT
GCGCTGGCGGAAAGCCATATCCCGGCTAACGATCGGCTGGTGACGTTCGGCGAACCGGAT
GAAAGCGGCGGCGAGCAGGCGATGACTGAGTTATTAGGCCGCGGCAGACATTTTACCGCG
GTGGCCTGCTATAACGACTCGATGGCGGCCGGCGCGATGGGAGTATTAAATGATAATGGC
GTGGGGGTGCCGGGCGAAGTATCGCTCATCGGTTTTGATGATGTACTGGTCTCACGCTAT
GTGCGTCCCCGACTGACCACCATTCGGTATCCGATCGTCACCATGGCGACACAGGCGGCG
GAGCTGGCGTTAGCGTTGGCGGGGAAATGCCCTACGCCAGAAGTAACTCATGTATTTAGT
CCGACACTGGTACGCCGACATTCGGTATCCACGCCGACGGATACCGGGCACCTGTCGACA
ACCGATTAA
>EECEBICD_00449 Bifunctional protein Aas
ATGCTGTTTGGATTTTTCCGTAACCTGTTTCGCGTGCTTTATCGTGTGCGCGTCACTGGC
GATGTCCGGGCGCTACAGGGGAACCGGGTCTTAATTACCCCCAATCACGTTTCATTTATC
GATGGTATGCTACTGGCGCTGTTTTTGCCGGTACGGCCTGTCTTTGCGGTATATACCTCC
ATCAGCCAGCAATGGTATATGCGCTGGTTAACGCCGCTGATCGATTTTGTGCTGCTCGAT
CCCACTAAACCCATGTCCATTAAACACCTGGTGCGTCTGGTGGAACAGGGGCGTCCAGTG
GTGATTTTCCCGGAAGGGCGGATTTCCGTGACCGGTTCGCTGATGAAAATTTATGACGGC
GCAGGCTTTGTGGCGGCAAAATCGGGCGCAACCGTCATTCCGCTCCGCATTGACGGCGCG
GAGCTTACCCCTTTCAGTCGCCTGAAAGGTCTGGTTAAACGGCGCTTATTCCCCCGCATT
CAGCTTCATATTTTACCGCCGACGCAAATACCGATGCCGGAAGCGCCGCGCGCGCGCGAC
AGACGTAAGATAGCGGGTGAGATGCTGCACCAGATCATGATGGAAGCGCGTATGGCGGTG
CGCCCGCGCGAGACGTTATACGAATCGTTGCTGGCGGCGCAATATCGTTACGGCGCGGGT
AAAAACTGTATTGAGGACATTAACTTTACGCCGGATACGTATCGTAAGTTACTGACCAAG
ACGCTCTTTGTTGGCCGGATTCTGGAAAAGTACAGCGTTGAGGGCGAGAAAATTGGTTTA
ATGCTGCCGAATGCCGCCATCAGCGCGGCGGTGATTTTCGGCGCGGTTTCTCGTCGCCGC
ATTCCGGCCATGATGAACTACACAGCGGGGGTAAAGGGCTGACCAGCGCGATTACCGCG
GCGGAAATTAAAACGATTTTTACCTCCCGCCAGTTCCTCGATAAAGGTAAATTGTGGCAC
CTGCCGGAGCAGCTAACGCAGGTTCGCTGGGTTTACCTGGAAGATTTAAAAGCGGACGTC
ACACCCGCCGACAAGCTGTGGATTTTTGCCCACCTCCTGGCGCCGCGACTGGCGCAGGTA
AAACAGCAGCCGGAAGACGCGGCGATCATTCTTTTTACCTCCGGCTCGGAAGGGCACCCG
AAAGGGGTGGTGCACAGCCATAAAAGCATTCTGGCGAACGTCGAGCAGATAAAAACGATT
GCGGATTTCACCGCCAATGACCGTTTTATGTCGGCGCTGCCGCTCTTTCACTCGTTCGGT
TTGACGGTGGGCCTGTTTACGCCGTTGTTGACCGGCGCGGAAGTGTTTCTTTATCCAAGT
CCGCTGCACTACCGCATTGTGCCGGAACTGGTCTATGACCGTAACTGTACCGTGTTGTTT
GGTACGTCAACCTTCCTCGGCAACTATGCGCGCTTTGCCAATCCGTATGATTTTTATCGC
CTGCGCTATGTGGTGGCCGGTGCGGAAAAACTTCAGGAGAGCACCAAACAACTGTGGCAG
GATAAATTCGGTCTGCGCATTCTTGAGGGCTACGGGGTAACGGAATGCGCGCCAGTAGTC
TCGATTAACGTGCCGATGGCGGCTAAGCCTGGCACTGTTGGCCGCATTCTGCCTGGCATG
GACGCGCGTCTGCTGGCGGTGCCCGGTATTGAAAACGGCGGTCGGCTACAGCTGAAAGGG
CCGAATATCATGAATGGCTATCTGCGCGTGGAAAAACCGGGCGTGCTGGAGGTGCCGTCG
GCGGAAAACGCCAGAGGCGAAACCGAGCGCGGGTGGTACGACACGGGCGATATTGTACGC
TTCGATGAAAATGGTTTTGTGCAGATTCAGGGGCGCGCGAAACGCTTTGCGAAAATTGCT
GGCGAAATGGTCTCGCTGGAGATGGTCGAGCAACTGGCGCTTGGCGTTTCGGCGGACAAA
ATGCACGCTACCGCCATTAAAAGTGATGCCAGCAAAGGTGAAGCGCTGGTACTGTTTACG
ACTGACAGCGAACTCACCCGAGAAAAGTTACAGCACTATGCTCGTGAACACGGTATTCCG
GAACTTGCCGTCCCGCGTGATATCCGTTATCTGAAGCAGCTTCCCTTGCTTGGCAGCGGG
AAACCCGATTTTGTGACGCTGAAAAGTTGGGTGGATGCGCCGGAACAACATCATGAGTGA
>EECEBICD_00450 Lysophospholipid transporter LplT
ATGAGTGAGTCAGTACGTACTAACACCTCCATCTGGTCGAAAGGGATGCTGTCGGTGATT
GTCGCGCAGTTTCTCTCGGCTTTTGGCGATAATGCGCTGCTGTTTGCGACGCTGGCACTG
CTGAAAGCTCAGTTTTACCCGGACTGGAGCCAGCCTGTTTTGCAAATGGTGTTTGTGGGC
GCTTACATTCTTTTTGCGCCGTTTGTCGGCCAGATGGCGGACAGCTTTGCCAAAGGCCGG
GTGATGATGGTTGCCAATGGTCTTAAACTGGCGGGAGCGGCAGATATCTGCCTCGGCGTA
AATCCTTTTGTCGGTTATACGTTGGTCGGGATTGGCGCGGCGGCTTATTCTCCGGCGAAA

TACGGTATTCTGGGCGAACTGACGACGGGTGATAAGCTGGTGAAAGCCAATGGCCTGATG
GAAGCCTCTACGATTGCGGCGATTTTGCTCGGTTCCGTCGCGGGCGGCGTATTGGCCGAC
TGGCATGTCATCGCCGCGCTGGTTGCCTGCGCGTTGGCCTACGCTGGCGCAGTGGCGGCT
AACCTGTTTATCCCGAAGCTTGTGGCGGCGCGTCCAGGACAATCCTGGCGGTTGTCGGCG
ATGACGCGTAGTTTCTTCAGCGCTTGCGTTGTCCTTTGGCGTAACGGTGAAACGCGTTTT
TCGCTGGTGGGGACCGGGCTATTCTGGGGCGCAGGCGTGACGCTGCGTTTTCTGCTGGTG
CTGTGGGTGCCAGTGGCGCTGGGCATTACGGATAACGCCACGCCGACTTACCTGAACGCC
ATGGTGGCGGTAGGCATTGTGGTCGGCGCGGGCGCGGCGGCAAAACTGGTGACGCTGGAG
ACCGTATCGCGCTGTATGCCCGCCGGGATTTTGATTGGCGTTGTGGTTGCGATCTTCTCA
TTACAGCACGCATTGCTGCCTGCTTATGCCTTGTTGCTGTTGATAGGTATGCTGGGCGGC
TTTTTTGTGGTGCCGCTCAACGCCTTGCTCCAGGAGCGCGGTAAGAAGAGCGTTGGTGCC
GGGAATGCCATTGCCGTACAAAATCTGGGCGAGAATAGCGCCATGCTATTAATGCTGGGA
CTGTACTCGCTGGCGGTGCTGGTTGGCGTTCCGGCAGTGGCGATAGGTATCGGCTTTGGC
GTGCTGTTCGCGCTGGCGATCGCGGCGCTGTGGATCTGGCAGCGCCGGCAGGCATCATAC
TGA
>EECEBICD_00451 Protein tas
ATGCACTATCACCGTATACCCCACAGCTCGCTCGAAGTAAGTACGCTAGGGCTGGGCACA
ATGACATTTGGTGAACAAAACAGCGAAGCCGACGCTCACGCACAGCTCGACTATGCCATC
GCAAATGGCATTAACCTTATCGATGCCGCCGAGATGTATCCGGTACCGCCGCGCCCGGAA
ACACAAGGGCTGACGGAGTCCTATATCGGTAACTGGCTCGCCAAACGCGGCAACCGTGAA
AAACTGATCATCGCCAGCAAAGTTAGCGGACCGGCACGTAATAATGATCAAGGCATCCGG
CCTCATCAGGCGCTGGATCGTAAAAATATTCGCGAAGCGCTACACGACAGTTTGACCCGA
TTGCAGACCGACTATCTGGATTTGTATCAGGTGCACTGGCCGCAGCGCCCCACTAACTGC
TTCGGTAAACTCGGCTATAACTGGACGGACTCCACGCCAGTAGTAAGTCTACTGGAGACG
TTAGACGCGCTAAGTGAGTTTCAGCGAGCGGGTAAGATTCGTTATATTGGCGTTTCAAAT
GAGACAGCGTTTGGCGTTATGCGCTATCTGCATCTGGCGGAAAAACATGACCTGCCGCGT
ATTGTCACGATCCAGAACCCCTATAGCCTGTTAAACCGCAGCTACGAGGTCGGGCTGGCG
GAAGTCAGCCAGTATGAAGGTGTTGAATTGCTCGCCTACTCCTGTCTGGCGTTTGGCACA
TTAACCGGTAAATACCTGAACGGGGCCAAACCCGCAGGCGCGCGGAATACCTTGTTCAGC
CGCTTCACACGTTACAGCGGCGAACAGGCGCAAAAAGCCGTTGCCGCCTACGTCGATATC
GCGAAGCGTCACAATCTTGATCCGGCGCAGATGGCGCTGGCCTTTGTTCGCCGCCAGCCG
TTTGTCGCCAGTACGTTACTGGGCGCAACCACGATGGCGCAGCTCAAGACGAATGTGGAA
AGTCTGCATCTGACGTTAAGCGAAGAGGTCCTGGCGGAGATAGAGGCGGCGCATCAGGTT
TATACCTACCCGGCGCCGTGA
>EECEBICD_00452 putative lipoprotein YgdR
ATGAAAAAGTGGGCAGTTGTAATTTCCGCAGTAGGGTTGGCTTTTGCGGTATCCGGATGC
AGCAGCGATTATGTAATGGCGACGAAAGATGGTCGTATGATCCTGACCGATGGAAAACCG
CAAGTTGACGATGATACCGGTCTGGTCAGCTATACCGATGCACAGGGAAACGAGATGCAA
ATCAACCGTGACGAAGTGTCTCAAATCATCGAACGTTAA
>EECEBICD_00453 hypothetical protein
ATGTTATTTGCATGGATAACCGATCCTAATGCCTGGCTGGCGCTCGGTACGCTGACGCTG
CTGGAGATTGTACTTGGGATCGACAACATTATTTTTCTTTCTCTGGTGGTAGCAAAACTT
CCTACGGCGCAACGGAACCACGCACGGCGGCTTGGGTTGGCGGCGGCGATGGTTATGCGC
CTGGCGCTGCTGGCGTCAATCGCCTGGGTCACTCGGCTGACTAATCCGCTGTTTGAGCTT
TTCGGCGAAGCGATTTCCGCCCGCGATCTCATTCTGTTACTGGGCGGGTTATTTTTAATC
TGGAAAGCCAGCAAGGAGATTCATGAGTCCATCGAAGGCGAAGAAGAAGGGTTAAAAACC
CGGGTTTCTTCTTTCCTGGGCGCAATAGTGCAGATTATGCTGCTGGATATTATTTTCAGC
CTTGATTCAGTTATCACAGCGGTTGGCCTCTCCGATCATCTGTTTATTATGATGGCGGCA
GTCGTTATTGCCGTGGGCGTCATGATGTTTGCGGCACGGCCCATCGGCGAGTTTGTCGAT
CGTCATCCGTCGGTAAAAATGCTGGCGCTCTCCTTTCTGATCCTGGTTGGCTTTACCCTG
ATTCTGGAAAGTTTCGACGTTCACGTTCCGAAAGGGTATATCTATTTCGCCATGTTCTTC
TCTATCGCAGTAGAGAGCCTGAACCTGTTGCGTAATAAAAAGAATCCGCTGTAA
>EECEBICD_00454 DNA mismatch repair protein MutH
ATGTCCGCTCTTTGTCCGCTACTGACTCCGCCTGCTTCGGAAGCATTGCTGTTAGCCCAG

GCGCGGCAGCTTTCCGGCTATACGTTAGGCGAGCTGGCGGCGATGGCCGGCATTACCACG
CCTAAAGATTTGAAGCGAGACAAAGGCTGGATCGGCGTCTTGCTGGAGATCTGGCTCGGC
GCCAGCGCCGGGAGCAAGCCGGAGCAGGATTTCGCCGCATTAGGCGTAGAACTGAAAACC
ATCCCGGTGGATAGTCTGGGTCGTCCTCTGGAAACAACCTTTGTTTGCGTGGCGCCGTTA
ACCGGTAACAGCGGCGTGACCTGGGAGACAAGTCACGTACGGCATAAATTGAAGCGCGTG
CTATGGGTGCCGGTCGAAGGCGATCGCAGCATACCGTTAGCCGAACGCCGCGTAGGTTCG
CCGCTACTCTGGAGTCCGAGTGAAGAAGAGGACAGGCAGCTACGCCTCGACTGGGAAGAG
CTGATGGACATGATAGTGCTGGGCCAGGTGGAACGCATTACCGCCCGCCATGGCGAAGTC
TTGCAACTGCGGCCCAAAGCCGCCAATGCCAGGGCGCTCACCGAGGCTATCGGTGCGCGC
GGGGAACCTATTTTGACGCTACCGCGTGGATTTTATCTGAAAAAATTTTACGCAGGCGCT
GCTGGCTCGCCATTTTTTATTGCAGAATCCGTAACGTCCATTACACGCAAATGCGCCTCC
GCTATGGCTACGCGTCGGACCGACGCCCGATAA
>EECEBICD_00455 RNA pyrophosphohydrolase
GTGATTGATGACGATGGCTACCGCCCAAATGTAGGTATCGTAATTTGTAATCGTCAGGGG
CAGGTGATGTGGGCCCGGCGATTTGGTCAGCACTCCTGGCAGTTCCCGCAGGGCGGGATA
AATCCAGGGGAGTCGGCAGAACAGGCGATGTACCGGGAACTGTTTGAAGAAGTTGGGTTA
AGCCGTAAAGATGTGCGGATCCTTGCTTCTACTCGCAACTGGTTGCGTTACAAGTTACCA
AAACGTTTGGTGCGTTGGGACACAAAGCCGGTATGTATCGGCCAGAAACAAAAGTGGTTT
CTTTTGCAGTTAATGAGCGCCGACGCCGAAATCAATATGCAAACCAGCAGTACGCCGGAG
TTTGACGGCTGGCGCTGGGTAAGTTACTGGTATCCGGTTCGACAAGTGGTGTCATTTAAA
CGCGATGTCTACCGCAGGGTAATGAAAGAGTTTGCAAGTGTTGTCATGGCGCTTCAGGAT
AATCCCCCTAAGCTACAAAGCGCGCCTGCTTATCGACGTAAAAGAGGTTAA
>EECEBICD_00456 Phosphoenolpyruvate-dependent phosphotransferase system
ATGCTCACTCGCCTGCGCGAAATAGTCGAAAAAGTGGCCAGTGCGCCGCGTCTGAACGAG
GCGCTGAATATTCTGGTCACGGATATCTGTCTTGCGATGGATACTGAGGTTTGCTCGGTT
TACCTGGCCGATCATGACCGACGCTGTTATTACCTGATGGCGACGCGGGGGTTGAAAAAA
CCGCGTGGTCGAACCGTTGCGCTTGCGTTTGATGAGGGGATCGTCGGTCTGGTGGGCAGA
CTGGCGGAACCCATTAACCTTGCCGATGCGCAAAAACATCCCAGCTTTAAATACATTCCT
TCGGTAAAAGAGGAGCGTTTTCGCGCGTTTCTCGGCGTGCCGATCATTCAGCGTCGCCAA
CTTCTCGGCGTGCTGGTCGTACAGCAGCGCGAATTGCGCCAGTACGATGAAAGCGAAGAG
TCTTTCCTTGTCACGCTCGCTACGCAGATGGCGGCTATCCTGTCACAGTCGCAGGTCACA
GCGCTGTTCGGGCAATACCGGCAGACGCGAATCCGCGCGCTTCCTGCGGCGCCGGGCGTG
GCTATCGCAACGGGCTGGCAAGACGCGACCATGCCGCTGATGGAACAGGTTTACGAAGCG
TCTACGCTGGATACCTCTCTTGAACGTGAACGACTGACCGGCGCGCTGGAAGAGGCGGCG
AACGAATTCCGACGATACAGCAAACGCTTTGCCGCCGGCGCGCAAAAAGAGACGGCGGCC
ATTTTTGATCTGTACTCACACCTGCTTTCAGACGCCAGACTGCGTCGCGAACTCTTCGCC
GAGGTCGACAAAGGCGCTGTTGCGGAGTGGGCCGTTAAAAAAATCATCGAAAAATTTGCC
GAGCAATTTGCGGCGCTAACCGATAACTACCTCAAAGAACGTGCGGGAGACTTACGCACG
CTGGGGCAGCGGCTGTTATTCCACCTTGATGATTCAGTTCAGGGGCCAAACGCCTGGCCG
GAACGTTTTATTCTGGTCGCGGATGAGCTGTCGGCGACCACCCTGGCGGAGCTGCCGCAG
GACAGGCTGGCGGGCGTTGTGGTGCGCGACGGCGCGGCAAACTCGCATGCGGCGATTATG
GTGCGCGCGCTCGGTATCCCGACCGTCATGGGTGCGGATATCCAGCCTTCGGTGCTACAT
CGCCGTACGTTGGTGGTGGATGGCTATCGCGGTGAATTACTGGTCGATCCTGAACCCGTA
CTTATTCAGGAATATCAGCGGCTCATTAGTGAAGAGATAGAACTCAGTCGTCTGGCGGAG
GACGACGTCAATCTTCCCGCCCAACTGAAAAGCGGCGAGCGTATCAAAGTGATGCTGAAT
GCCGGGCTCAGTCCCGAGCATGAGGAAAAGTTAGGCAGTCGGATCGACGGTATCGGCCTG
TACCGTACTGAAATTCCCTTTATGCTGCAAAGCGGGTTTCCCTCGGAAGAAGAGCAGGTG
GCGCAGTATCAAGGGATGCTGCAAATGTTTAACGATAAACCGGTGACGCTGCGGACCCTG
GATGTGGGAGCGGATAAGCAACTGCCCTATATGCCGATCAGCGAGGAGAACCCGTGTCTG
GGCTGGCGCGGGATCCGCATTACGCTGGATCAGCCGGAGATCTTTTTGATCCAGGTCCGC
GCGATGCTGCACGCCAATGCGGCGACTGGCAACCTCAGCATTTTGTTGCCGATGGTCACC
AGTATTGATGAAGTCGATGAAGCGCGGCGTCTGATCGAACGCGCCGGGCGCGAGGTCGAG
GAGATGATCGGCTATGCGATCCCGAAACCGCGTATCGGCATTATGCTGGAAGTACCGTCA
ATGGTATTTATGCTGCCGCATCTGGCTAACCGGATCGACTTTATCTCGGTCGGCACCAAC

GATTTAACGCAATATATCCTGGCGGTAGATCGCAACAATACCCGCGTGGCCAGTATTTAC
GACAGTCTGCATCCGGCTATGCTGCGCGCACTGAGCATGATTGCGCAGGAAGCCGAAAAA
AATGGCCTCGATCTTCGCTTGTGCGGCGAGATGGCGGGCGATCCGATGTGCGTGGCGATT
CTTATCGGTCTGGGATATCGCCATCTTTCGATGAATGGCCGTTCGGTAGCGCGTGTGAAA
TATCTGCTGCGGCATATCGATTTTGAAGACGCGCAAACCCTTGCCAGACGCAGCCTTGAG
GCGCAGATGGCGACCGAGGTGCGTCATCAGGTGGCGGCGTTTATGGAGCGCCGCGGGATG
GGGGGATTGATTCGCGGAGGGTTGTAA
>EECEBICD_00457 Phosphatidylglycerol--prolipoprotein diacylglyceryl transferase
ATGACCAGTAGCTATCTGCATTTTCCGGACTTTGATCCGGTCATTTTCTCAATTGGGCCC
GTCGCGCTTCACTGGTATGGCTTGATGTATCTGGTGGGGTTCGTTTTCGCGATGTGGTTG
GCGGTGCGTCGCGCTAACCGTCCGGGAAGCGGTTGGACCAAAAACGAAGTTGAAAATTTA
CTCTATGCAGGTTTCCTGGGGGTCTTCCTGGGGGGACGTATTGGCTATGTCCTGTTTTAT
AACTTCCCTCTGTTTCTGGATAACCCGCTCTATTTATTCCGCGTCTGGGACGGCGGCATG
TCCTTTCACGGCGGGCTTATCGGCGTGATACTGGTGATGATTATCTTCGCCAGGCGCACG
AAGCGCTCGTTCTTTCAGGTGTCTGATTTTATTGCGCCGTTAATTCCGTTCGGCCTGGGC
GCCGGGCGTCTGGGCAACTTTATCAACGGTGAATTGTGGGGGCGCGTCGATCCTGACTTC
CGGTTTGCCATGCTCTTTCCTGGCTCACGCGCGGAAGATATTGCGCTGCTGCCGTCACAT
CCGCAATGGCAGCCTATTTTTGATACCTACGGCGTATTGCCGCGCCACCCTTCCCAGTTG
TATGAGCTGGCATTAGAAGGCGTGGTGCTGTTTATCATCCTTAATCTCTTTATTCGCAAA
CCGCGTCCGATGGGCGCAGTCTCCGGATTATTCCTGATTGGCTATGGCGCGTTTCGTATC
ATTGTTGAATTCTTCCGCCAGCCGGACGCGCAATTTACCGGCGCGTGGGTACAGTACATC
AGCATGGGGCAGATTCTCTCTATCCCGATGATTATCGCTGGCGCGATCATGATGGTTTGG
GCATATCGCCGCCGCCCGCAGCAACACGTTTCCTGA
>EECEBICD_00458 Thymidylate synthase
ATGAAACAGTATTTAGAACTGATGCAAAAAGTGCTGGATGAAGGCACACAGAAAAACGAC
CGTACCGGCACCGGCACGCTTTCCATTTTTGGCCATCAGATGCGTTTTAACCTGCAGGAA
GGCTTTCCTCTGGTGACGACAAAACGCTGCCATTTACGTTCAATTATTCACGAACTGCTA
TGGTTTTTGCAGGGCGATACCAACATCGCTTACCTGCACGAAAATAATGTCACCATCTGG
GACGAATGGGCCGATGAGAACGGCGATTTAGGCCCGGTTTATGGTAAGCAGTGGCGTGCA
TGGCCAACACCGGATGGCCGCCATATCGATCAGATCGCCACGGTCTTAAGCCAGCTTAAA
AACGACCCGGACTCTCGTCGTATTATCGTTTCTGCGTGGAACGTCGGCGAGCTGGATAAG
ATGGCGCTGGCGCCGTGTCACGCCTTTTTCCAGTTTTATGTCGCAGATGGAAAACTCTCC
TGTCAGCTTTATCAGCGCTCCTGCGATGTGTTCCTCGGTCTGCCGTTCAACATTGCCAGC
TACGCGTTACTGGTGCATATGATGGCGCAACAGTGCGACCTGGACGTCGGCGATTTTGTC
TGGACCGGCGGCGACACGCACCTGTACAGCAACCATATGGAGCAGACGCACCTGCAATTA
AGCCGTGAACCTCGCGCGCTGCCGAAATTGGTGATTAAGCGTAAACCCGATTCGCTTTTT
GACTACCGGTTCGATGATTTCGAAATTGAAGGCTATGATCCGCACCCCGGTATTAAAGCG
CCGGTCGCTATCTAA
>EECEBICD_00459 hypothetical protein
ATGAAAAAACAACAGGGCTATACGCTCATTGAAACGGTGGTCGCGATGTCGCTCATTATC
ATTCTCAGCGCCACAGGACTTTATGGCTGGCAACGCTGGCAGCAGCAACAGCGCCTGTGG
CAAACCGCATGTCAGGTGCGGGATTACCTGCTGCAACTTCGTGAGGATGCTCACTGGCAT
AACCGCGATCACATTCTTCGCGTGGTCAGCGAAGGCGGTTCATGGTGCTTTGTCAGCTCT
GTTGCTACGCAAACATCGTGTACGCCATCTTCCCCCTTTGTGTTTACGCCCGACTGGCGT
GATGTCGTCCTTGCCGATATCACGCCGTCGTTAGCCTTCTTTGGCTTGCGCAATACGGCA
TGGGCAGGGCACATCGTACTGAAAAACGCTGCCGGAGAGTGGCGTCTGGTCGTCTCCTCC
TGGGGGCGAATCAGGCTATGCGAGCGTAACGAGGCCGCCACGTGTCAATAA
>EECEBICD_00460 hypothetical protein
GTGTCAATAACCACGCGGGGGTTCTCGCTACTGGAGGTCGTGCTGGCGATGGCGATAGGT
TCCATTTTACTGCTTGGGTCCGCACGATTTTTACCGGCGCTACAGCGTGAGATCTGGCAC
AACACGCGACAGTTATCGCTGGAAGATGAAATCTGGCAGCGAACCTATACCGTAGCGAAG
CATCTTCAACGTGCGGGTTACTGTCATGGCCACTGTATTGGCGAAGGGTTACATCTTGCC
GAACAGGGGCAATGCGTCATCGTTCAGTGGGACGGGAATAATAACGGTGTCTGGGATACC

ATACCGGCGAAAGAGGCCGACCAGACCGGGTTTCGTGTGAAGGATAACGTTCTGGAGACA
TTACGCGGAGCAACATCTTGCCAGGGTAAGGGATGGAAAAAATGACCGATCCCAATACG
GTACTCATCACGGCGTTTACCGTTGAGCGCCGGGATATAACGGGATTCTCGCCAGTGCTC
ATGCTGCATCTGCGCGGCGCCAGCAAAGCCGAGCCTCAGACGGTTATTGACGCGCAATAC
AGCGTAACGGGATTTAATCTGTGA
>EECEBICD_00461 hypothetical protein
GTGAATCGGGAACGTGGCGCTTCTTCGCTAATACTGGCGCTTTTAATACTGATTCTGGGC
AGTCTGCTACTGCAAGGCGTAAATCAGCAGCAGGCCAGCTATGCCACCCGCGTGACGACG
CAGAGCCTGGCGATACAACGCCAGGCGCTCGTACAGTCGGCGCTGGAATGGGGGCGGGGG
CAGCTATGGTCAGGCGTAACCGAAATGGAATGTCGGCGCTATTCCTCTTCAGGAGCGAGA
GTCTGTTTGCGGAGGTTACCGGGGGATGAGGTGGTGATGGCGGCGCAAGATGACAGCATG
ACGTTATGGCGTTTGGGCAACGTCATTCAGGGAAGCATTGTCTTTTCTCCCCACGGCTGG
AGCGATTTTTGTCCGTTAAAAGAGGTGGCGTTATGTCGCATCCCCTGA
>EECEBICD_00462 hypothetical protein
ATGTCGCATCCCCTGAATTTACAACGCGGGTTTAGCCTCCCGGAAGTTTTAGTGGCGATG
GTTCTAATGGTAATGATCGTCACCGCGCTGTCAGGCTATCAGCGGGTATTAATGCACAGT
TTTGCATTACGACACCAGTATCTTCAGATCTGGCGGCAGGCGTGGCAGCAAACGGCGCTG
TATCCATTTTCGCCTGCTGAAGGCTGGAAGGCCAACCGGATGCAGACAACGCAATCGGGA
TGTGTCAGCATCAGCGTCACGATGGTTTCACCCTCTGGCAGGCAGGGGCAGATGACGCGC
TTGCATTGTCCAAATCGTTAA
>EECEBICD_00463 RecBCD enzyme subunit RecC
ATGTTAAGGGTCTACCACTCCAATCGTCTGGATGTGCTGGAAGCGTTGATGGAATTCATC
GTCGAACGCGAGCGGCTGGATGATCCCTTTGAACCAGAAATGATTCTGGTACAAAGCACG
GGTATGGCGCAGTGGCTGCAGATGACCCTTTCACAAAAGTTTGGTATTGCGGCGAATATC
GCTTTTCCGTTACCGGCGAGTTTTATCTGGGAGATGTTTGTCCGCGTGTTGCCGGATATC
CCCAAAGAAAGCGCTTTCAGCAAACAAAGCATGAGCTGGAAACTGATGACCTTGCTGCCG
CAGTTGCTGGATAAGGATGAGTTTGTACTGCTGCGCCATTATCTGACCGACGACACCGAT
AAACGCAAACTCTTTCAGCTTTCCGCGCGCGCCGCCGACCTGTTCGATCAGTATCTGGTT
TATCGGCCTGACTGGCTTACGCAGTGGGAAGCGGGAAAAACCGTGGAAGGTCTGGGAGAG
GCGCAAAACTGGCAGGCGCCGTTGTGGAAAGCATTGGTGGAATATACCGCCGCGCTGGGG
CAGCCGCGCTGGCATCGCGCCAATCTCTACCAACGCTTTATTCAAACGCTGGAAAGCGCG
ACCGCCTGCCCGCCAGGGTTGCCGTCCCGCGTTTTTATTTGCGGCATTTCCGCGTTACCG
CCAGTGTATCTCAGAGCATTGCAGGCGCTGGGTAAACATATTGAAATCCATCTCCTTTTT
ACTAATCCGTGTCGGTATTACTGGGGGGATATTAAAGACCCAGCCTGGCTGGCAAAACTC
ATGGCTCGCCAGCGTCGTCACAGTTTTGAAGACCGTCATTTACCGTTGTTCCGTGAAAAC
CAGAATCCGGAAGCCTTATTTAACAGCGACGGTGAACAGGATATCGGTAATCCGCTACTG
GCCTCATGGGGAAAACTGGGACGGGATTATATCTACCTGCTATCTGAACTGGAAAACAGC
CAGGAGCTGGATGCGTTTGTTGATATTACGCCAGATAATCTTTTGCATCGGATTCAGGCG
GACATCCTTGAACTGGAAAGCCATGCGGTGGCGGGCGTAAATCTTGAGGAGTATTCGCGC
AGCGACAATAAGCGTCTGCTCGATCCCGAGGATAACAGCCTCTCTTTTCACGTTTGTCAC
AGCCCGCAGCGCGAGGTGGAGATACTGCACGATCGCCTGCTGGCGATGCTGGAAGCCGAT
CCGACACTAACGCCGCGCGACATTATCGTTATGGTGGCTGATATTGACAGCTATAGCCCG
TTTATCCAGGCGGTGTTTGGTAGCGCGCCAACGGAGCGCTATCTGCCTTATGCCATTTCC
GATCGGCGGGCGCGTCAGTCACATCCGGTGTTACAGGCATTTATCAGCCTACTTTCGTTA
CCGGACAGCCGCTTTGTCTCCGAGGATGTTCTGGCGTTGTTAGACGTACCGGTATTGGCT
GCCCGCTTTACGATCAACGAAGAAGGGTTGCGTTATTTGCGCTTGTGGGTCAATGAGTCA
GGCATTCGCTGGGGAATTGACGATGACAACGTACGCGAGCTGGAACTTCCTGCCACGGGG
CAGCATACGTGGCAATTTGGCCTGACGCGTATGCTGCTGGGGTATGCGATGGAAAGCGCC
CAGGGCGAGTGGCAGTCGGTTCTGCCTTATGACGAATCCAGCGGTCTGATTGCTGAGCTG
GTCGGGCATCTGGCTTCGCTGCTGATGCAGCTTAATATCTGGCGGCGCGGTCTGGCGCAG
GAGCGACCGCTGGAAGAGTGGCTGCCCGTTTGTCGCGATATGCTCAATGATTTCTTCCTG
CCCGATGCGGACACGGAAGCGGCAATGACATTGATTGAACAGCAGTGGCAGGCCATTATC
GCCGAAGGCGTCGCGGCGGAATATGGCGACGCGGTACCGATTTCGCTACTGCGGGATGAA
CTGGCGCAGCGTCTGGATCAGGAACGTATTAGCCAGCGCTTCCTGGCGGGCCCCATCAAT

ATTTGTACGCTGATGCCGATGCGTTCCATTCCGTTTAGAGTCGTTTGCCTGCTGGGAATG
AACGACGGCGTATACCCGCGCCAGCTTGCGCCGTTGGGCTTTGACCTGATGAGCCAAAAA
CCCATGCGCGGGGATCGTAGCCGTCGCGACGATGACCGCTACCTGTTTCTGGAAGCGTTA
ATCTCGGCGCAGCAGACGCTGTATATCAGCTACATCGGTCGCTCCATCCAGGATAATAGC
GAACGCTTTCCCTCTGTACTGGTGCAAGAATTGGTGGATTACATCGGACAAAGCCACTAT
TTGCCGGGCGACGAAACGCTGACCTGTGATGAGAGCGAAGCACGAGTCAAAGCGCATATT
ACGCGTCTGCATACCCGTATGCCTTTTGATGCGCAAAATTACCAACCCGGCGAACAGCAA
AGTTATGCCAGAGAGTGGTTGCCCGCGGCGAGTCAGTCGGGAAAAGCGCATTCTGACTTT
GTGCAGCCGCTTCCTTTTACGATGCCGGAAACATTGACTCTGGAAAGTCTGCAGCGTTTT
TGGGCCCATCCGGTACGCGCATTTTTTCAGATGCGGCTTCAGGTTAATTTCCGCTCGGAA
GAGAGTGAGATTCCTGATGCGGAACCGTTTGAATTAGAGGGATTGACGCGATATCAACTT
AACCAACAGTTGCTCAATACGCTGGTTGAAGAGGACGACGCCGAACGCTTGTTCCGCCGT
TTCCGGGCTGCGGGAGAGTTGCCTTACGGCGCGTTTGGCGAAATATTCTGGGATGCGCAA
TGTCAGGAGATGCAGCAACTGGCAAGCCGGGTCATCGCCTGCCGTAAGCCGAGCCAAAGC
CTGGAAGTGGATCTGCTTTGCAATGGCGTTCAGCTAACCGGCTGGCTGCCGCAGGTACAG
GAGGATGGATTATTGCGCTGGCGTCCCGCTTTAATCAGCGTGGCGCAAGGTGTGCAACTT
TGGCTGGAACACCTTGTCTATTGTGCCAGCGGCGGGAGTGGCGAAAGTCGCCTGTTTTTG
CGCAAAGAGGGAGAGTGGCGATTTCCGCCTCTTGATAAAACGCAGGCAATGGCTTACCTG
GCGCAACTGATTGAAGGCTACCGGGAAGGCATGTCTTCTCCGCTATTGGTACTGCCGGAA
AGCGGCGGCGCATGGATAAAAGCCTGTTATGACGCTGAAAATGATGTCATGTTAGACGAC
GATGACACCTTACAGAAGGCCAGAACAAAGTTTCTACAGGCTTATGAAGGCAACATGATG
GTGAGCGGCGAAGGCGAAGATATCTGGTATCAACGTCTTTGGCGTCAGCTGGAATCTGAA
ACGTTGCAGGCTATCATTGCGCAGTCACGGCATTACCTGTTACCGCTGTTTCGTTTTAAT
CAGTCTCGTTGA
>EECEBICD_00464 Protease 3
ATGCCCCGCAGCACCTGGTTCAAAGCGTTATTGTTATTGGTCGCCCTCTGGGGGCCTGCA
GTTCAGGCGGATATCGGTTGGCAACCGCTGCAAGAAACTATCCGTAAAAGCGATAAAGAT
ACCCGGCAGTATCAGGCGATACGTCTTGATAATGACATGGTGGTGTTGCTCGTATCCGAT
CCGCAGGCTGTAAAGTCGCTTTCAGCGTTAGTGGTGCCCGTTGGATCGCTTGAAGATCCT
GAGGCTCATCAGGGGCTTGCTCATTATCTTGAGCATATGTGCCTGATGGGGTCAAAAAAA
TATCCGCAGGCGGATAGCCTTGCCGAATACCTTAAAAGACATGGCGGTAGCCACAACGCC
AGCACCGCGCCTTATCGAACAGCCTTCTACCTGGAGGTTGAAAACGACGCGCTTCCAGGT
GCCGTAGATCGACTGGCCGACGCCATTGCCGCGCCATTGCTCAATAAAAAGTATGCCGAA
CGCGAACGAAACGCGGTGAATGCCGAGCTGACGATGGCGCGCACCCGCGACGGTATGCGT
ATGGCGCAGGTAAGCGCCGAAACCATTAACCCGGCGCATCCAGGCTCGCACTTTTCTGGC
GGCAATCTGGAAACGCTAAGCGATAAGCCGGGAAATCCGGTGCAACAGGCGTTGATCGCT
TTTCATGAAAAATACTATTCATCTAATCTGATGAAGGCGGTGATTTACAGTAATAAACCC
TTGCCGGAACTGGCGAGTATTGCCGCCGCAACCTATGGTCGCGTGCCGAATAAACAGATT
AAAAAACCGGAAATTACCGTACCTGTCATCACCGAGGCGCAGAAGGGCATCATTATTCAT
TACGTGCCGGCGCTACCGCGTAAAGTGCTGCGCGTGGAGTTTCGTATTGATAACAATAGC
GCGCAGTTCCGTAGCAAGACCGATGAACTGGTCTCTTATCTGATTGGCAATCGTAGCCCG
GGGACGCTCTCTGACTGGCTGCAAAAACAAGGACTGGTCGAAGGTATTAGCGCGGATTCC
GATCCGATTGTTAATGGTAACAGCGGCGTATTTGCCATTTCGGCAACGCTGACTGATAAA
GGTTTGGCCAATCGTGATGAAGTCGTCGCAGCTATCTTTAGCTATCTCAATACGTTACGT
GAAAAAGGGATAGATAAACGTTACTTTGACGAGCTGGCGCATGTGCTGGATCTCGACTTC
CGCTATCCGTCGATTACCCGCGATATGGACTATGTCGAGTGGCTGGCGGACACTATGATC
CGCGTCCCGGTAGCGCATACGCTGGATGCGGCGAATATCGCCGATCGTTACGATCCTGCC
GCTATCAAAAATCGCCTGGCGATGATGACGCCGCAGAATGCGCGCATCTGGTACATCAGT
CCCCAGGAACCCCATAATAAGATTGCGTATTTTGTGGATGCGCCTTATCAGGTCGATAAG
ATTAGCGAACAGACGTTTAAAAACTGGCAGCAAAAAGCACAGGGCATTGCGTTGTCGCTG
CCGGAGTTAAACCCCTATATTCCTGATGATTTTTCGCTTGTTAAGAATGACAAAAACTAC
GTGCGGCCAGAACTGATTGTCGATAAAGCGGATTTGCGCGTGGTTTATGCGCCGAGTCGT
TATTTCGCCAGCGAGCCAAAAGCTGACGTGAGCGTGGTATTGCGTAACCCGCAGGCGATG
GACAGCGCCCGCAATCAGGTACTGTTCGCCCTTAATGATTATCTGGCGGGAATGGCGCTC

GATCAGCTCAGCAACCAGGCGGCAGTGGGCGGCATTAGCTTTTCCACTAACGCCAACAAT
GGTCTGATGGTCACTGCGAACGGTTATACCCAGCGTTTGCCGCAGCTTTTCCTGGCGCTG
CTGGAAGGATATTTTAGCTACGACGCCACGGAGGAACAGCTGGCGCAGGCGAAATCCTGG
TATACGCAGATGATGGATTCTGCGGAGAAGGGAAAAGCCTACGAACAGGCGATTATGCCG
GTGCAGATGATTTCGCAGGTGCCTTATTTTTCCCGTGATGAACGTCGCGCTTTGCTGCCG
TCCATTACGTTAAAAGAGGTGATGGCCTATCGTAATGCGTTAAAAACGGGCGCTCGTCCG
GAATTTCTGGTTATAGGGAATATGAGCGAAGCCCAGGCGACCTCTCTGGCGCAAGATGTC
CAAAAACAGCTCGCGGCGAACGGATCGGCGTGGTGTCGTAACAAAGACGTGGTGGTTGAG
AAAAAGCAGTCCGTAATATTTGAAAAAGCGGGCAGTAGCACCGACTCCGCGTTAGCAGCG
GTCTTTGTCCCGGTCGGCTACGACGAGTACGTCAGCGCCGCCTACAGCGCGATGTTAGGT
CAGATTGTTCAACCGTGGTTTTACAATCAGCTACGAACCGAAGAGCAACTGGGATACGCT
GTTTTCGCCTTTCCGATGAGCGTTGGCCGTCAGTGGGGAATGGGATTCCTGCTACAGAGC
AACGATAAACAGCCCTCTTACCTGTGGCAACGCTATCAGGCATTTTTCCCTGACGCCGAG
GCGAAGCTGAGGGCGATGAAGCCGGAAGAGTTCGCCCAAATTCAGCAGGCGATCATTACG
CAAATGCGCCAGGCGCCGCAAACGTTGGGCGAAGAAGCATCCCGTTTAAGCAAGGATTTC
GATCGGGGTAATATGCGCTTTGACTCGCGTGATAAAATCATCGCTCAGATAAAATTGCTG
ACGCCACAAAAGCTTGCCGATTTCTTCCACCAGGCGGTGGTGGAACCACAAGGTATGGCA
ATATTGTCACAGATTGCCGGTAGCCAGAATGGAAAAGCAGAATACGTGCATCCGACAGGC
TGGAAAGTGTGGGATAACGTCAGCGCTTTGCAGCAAACGTTACCTCTAATGAGCGAAAAG
AATGAATGA
>EECEBICD_00465 RecBCD enzyme subunit RecB
ATGAATGATGTCGCCGAGACTCTTGATCCTCTGCGCTTGCCCCTTACAGGCGAGCGTTTG
ATTGAAGCATCAGCGGGCACAGGCAAAACGTTTACTATCGCGGCGCTGTATCTGCGGCTA
CTGCTCGGTTTAGGCGGCAGCACCGCATTTCCCCGTCCGTTGACCGTTGAAGAACTGCTG
GTGGTAACCTTTACGGAGGCCGCCACTGAAGAGCTGCGTGGGCGCATCCGCAGTAATATC
CATGAGTTGCGCATCGCCTGTCTGCGGGAAAGCACGGATAATCCGCTGTATGCCCGCTTG
CTGGAAGAAATTAGCGACAAAAAGCAGGCCGCGCAGTGGTTATTACTGGCAGAACGACAA
ATGGACGAGGCCGCGGTCTTTACCATCCACGGTTTTTGCCAGCGTATGCTCAGCCTGAAC
GCTTTCGAATCCGGTATGTTGTTTGAGCAACAGCTTATTGAAGACGAATCTTTGTTGCGT
TATCAGGCCTGCGCCGATTTCTGGCGGCGTCATTGCTATCCGCTGCCGCGCGACATCGCG
CAGGTGGTGTTTGACGTGTGGAAGGGACCGAAGGCGCTCTTAAAGGATATCGATCGTTAT
CTGCAGGGCGAAGCGCCGGTCATTAAAGCGCCGCCATCGCAGGAGGAGACGCTGGCATCG
CGTCATGAACAGATCCTGGCGCGCATCAACCAGGTGAAGCAGCAGTGGTGTGAGGCGGTT
AGCGAGCTGGACGCGCTGATCGAATCATCCGGCATCGATCGCAGAAAGTTCAACCGCGGC
AATCAGGCGAAATGGATCGAAAAGATCACCGCGTGGGCGCAAGAAGAGACGAAAAACTAT
CAATTACCCGAGGCGTTGGGAAAATTTTCACAGCGTTTTTTAGCTGAGCGCACCAAAGCT
GGCGGCGTGACGCCCCAGCACCCATTGTTTGTCGCCATTGATAACCTGTTGGGCGAACCG
CTATCGATTAAAGATCTGGTTCTTACCCGAGCGCTATCAGAGATCCGTGAAACGGTGGCT
CAGGAAAAACGCCGGCGCGGCGAGCTGGGGTTTGACGATATGCTCAGCCGTTTAGATACC
GCGCTGCGTAGTGAAAGCGGCGAGGCGCTGGCTGCGGCGATTCGTACCCGTTTTCCGGTG
GCGATGATCGATGAATTTCAGGATACCGACCCACAACAGTACCGTATTTTTCGCCGTATC
TGGCGACATCAACCCGATACGGCGCTATTGCTGATTGGCGATCCTAAACAGGCGATTTAT
GCGTTTCGTGGCGCGGATATCTTCACCTACATGAAGGCTCGCAGCGAAGTGAGCGCGCAC
TATACGCTTGACACTAACTGGCGCTCCGCGCCGGGGATGGTGAATAGCGTTAACAAGCTG
TTCAGTCAGATGAACGACGCCTTTATGTTCCGCGATATTCCCTTCAGTCCGGTAAAGTTT
GCGCCCAGAAACCAGTCGCTGCAATTTAAAGTGAATGACGCGCCTCAACCGGCGATGACC
CTGTGGCTAATGGAGGGGGAAAGCTGCGGTTCGGGCGATTATCAAAGTTATATGGCGCAG
GTGTGCGCCACGCAAATTCGCGACTGGCTGCGTGCCGGGCAAACGGGCGACGCGCTGCTG
ACGAACGGCGATTCGTCGCGGCCCGTTCGTGCCTCGGATATCAGCGTGTTGGTGCGCAGC
AGGCGGGAAGCCGCGCTGATACGGGATGCGCTGACCTTACTGGCGATCCCCTCCGTCTAT
CTTTCTAACCGCGACAGCGTATTCGAAACGCTCGAAGCGCAGGAGATGCTGTGGGTGTTG
CAGGCGGTGATGGCGCCCGAACGTGAGAATACGCTGCGTAGCGCTTTGGCGACATCAATG
ATGGGCCTTACCGCGCTGGATATCGAAACGCTTAACAATGATGAAAATGCGTGGGATGCG
GTTGTCGAAGAGTTTGATGGCTACCGACAGATTTGGCATAAACGCGGCGTGATGCCTATG

CTGCGCGCGCTAATGTCGGCGCGCAATATTGCTGAAAACCTGCTGGCGACGGCGGGAGGC
GAACGTCGGCTGACGGATATTTTGCATATTAGCGAGCTGTTACAGGAAGCCGGTTCGCAG
CTGGAAAGCGAACATGCGCTGGTGCGTTGGCTGGCGCAGCATATCCTTGAACCCGATAGC
AATGCTTCCAGCCAGCAACTACGTCTGGAAAGCGATAAGCATCTGGTGCAAATCGTCACC
ATTCATAAATCGAAAGGTCTGGAATACCCGCTGGTCTGGTTACCTTTTATCACCCATTTC
CGCGTGCAGGACCAGGCTTTTTATCACGACAGGCACTCCTACGAGGCGGTGCTGGACCTT
AGTCACGCCGAAGAGAGCATAGCACTGGCGGAAGCCGAACGCCTGGCGGAAGACCTGCGC
CTACTTTATGTCGCGCTGACCCGTGCAGTGTGGCATTGCAGCCTCGGCGTCGCGCCGCTG
GTGCGGCGTCGTAGCGATAAGAAAGGAGAGACGGATGTGCACCAAAGCGCGCTTGGGCGA
CTCCTGCAAAAAGGCGAACCGATGGATGCCGCAGGCCTTCGCGCCTGTATTGAGGCGCTA
TGCGACGAGGATATCGTGTGTCGAACGCCAGGCAATACGGATAACGACCGCTGGCAAATT
GCCGCTGCATCGCACGCGGAACTCAGTGCCAGAACGCTGCAACGCCTGCTTTATGATAGC
TGGCGCGTCACCAGTTATTCCGGTTTGCAGCAGCGGGGACATAGCGTGGCGCAAGATCTT
ATTCCTCGTCTCGACATTGACGCCGCAGGCGTTGGCGAAGCGGCGGAAGCGCCGGCAATG
ACGCCGCATCACTTTCCTCGCGGGGCATCGCCGGGAACATTTTTGCACAACCTGTTTGAA
GAGCTTGATTTTACTCAACCGATAAACCCCCAATGGGTACAGGAAAAACTGGAGCTGAGT
GGTTTTGAGACGCGATGGGAGCCCGTACTTACCCGCTGGCTGGACACCGTGTTGCACGTT
CCCCTTAACGAGACGGGCGTTAGTCTGAGCGTGCTGACCGAGCGTGAAAAACAGGTCGAA
ATGGAGTTTTATCTGCCGATTGCCCAGCCGCTTACCGCCGGAGAGCTGGATGCGTTGATT
CGTCGATACGACCCGCTTTCAGCAGGTTGCCCTGCGCTGGATTTCATGCAGGTTCGCGGC
ATGTTAAAGGGCTTTATCGACCTGGTTTTTCGTTATGAGGGGCGCTATTACCTGCTCGAC
TATAAATCTAACTGGCTGGGCGAGGATAGCGCCGCTTATACCCAGGCGGCGATGGCTACG
GCGATGCAGGCGCATCGCTATGATTTGCAGTACCAGCTTTATACGCTGGCATTGCATCGT
TACCTTCGTCATCGCATGGCGAATTACGACTATGAACGCCATTTCGGCGGCGTCATCTAT
CTCTTTTTACGCGGGGTGGATAGCGAACGTCCGCAGCAGGGTATTTTTACCACTCGCCCT
GCGGCGGCGTTAATTAACCAACTGGATGATATGTTTGCAGGTGAAATGAGTGAGGAGGCG
CAATGA
>EECEBICD_00466 RecBCD enzyme subunit RecD
ATGACAATCCAGGAGCGGTTGCTGGAGGCCGTTGAACAAAAACTCTTACGGCCCATTGAT
GCCCAGTTTGCTTTAACCGTCGCCGGTAACGACGATCCCGCCGTGACGCTGGCGGCAGCG
CTGCTTAGTCATGACGCAGGTGAAGGTCACGTGTGTCTGCCGTTGTCGCGTTTAACGTTA
ACGGAGGAGGCGCATCCCTTACTGGTCGCCTGTATAAGCGAAACGGCTACGCCAATCGAC
TGGAAAAAAGCGGTTACTGGCGTCTGCGGCGGTTAGCTGCGGCGATAGCCCCGCGCCGTTA
ATTCTGTGCGGCGAGCGTCTCTACCTGAATCGCATGTGGTGCAACGAACGTACGGTTGCG
CGCTTTTTTAACGAGGTTAACCAGGCCATCGCCGTAGATGAAGATCAGTTATCCCGTATT
CTGGACGCGCTATTCCCTCCGACAGACGAGGTGAACTGGCAAAAGGTGGCCGCCGCCGTA
GCGCTAACTCGCCGTATCTCCGTGATTTCAGGCGGTCCCGGCACCGGTAAAACCACCACC
GTCGCGAAGCTGCTGGCGGCATTAATTCAAATGGCGGATGGCGAACGTTGCCGTATCCGG
CTGGCAGCGCCGACCGGGAAAGCGGCCGCACGCCTGACGGAGTCGCTCGGCGCGGCGTTG
CGTCAGCTTCCACTTACCGATGCGCAGAAAAAGCGTATACCGGAGGACGCCAGTACGCTG
CACCGACTGCTAGGCGCACAGCCCGGCAGCCAGCGATTACGCCATCATGCGGGCAACCCG
CTACATCTGGACGTGCTGGTGGTCGATGAAGCATCAATGATTGATTTGCCGATGATGTCA
CGTTTGATTGACGCTCTGCCGCCGCACGGGCGGGTCATTTTTCTTGGCGATCGCGATCAG
TTGGCATCCGTTGAGGCGGGCGCTGTGTTGGGCGATATTTGCGCCTATGTCAACGCAGGG
TTTACGGCGGAACGCGCCCGACAGCTAAGTCGACTCACTGGTAGCGCCATTCCGGCGGGG
GCCGGAACGCAAGCCGCATCTTTACGCGATAGCCTCTGTTTGTTGCAAAAGAGCTATCGT
TTCGGCAGCGATTCTGGCATCGGCAAGCTGGCGGCGGCAATTAACTGCGGCGACAGGTCG
GCAATCCAGGCTGTTTTTCAGCAGGGGTTTAGCGATATAGAGAAGCGTACATTGCAAAGC
AGCGACGATTATGCCGGGATGCTGGATGAAGCGCTGGCGGGCTATGGTCGCTACCTGCGG
TTGCTGCATGAAAAAGCGACGCCGGAGGCAATCCTTCAGGCTTTCAATGAGTATCAACTG
CTTTGTGCGCTGCGCGAAGGCCCATTTGGCGTGAGGGGATTGAATGACCGCATTGAGCAG
GCGATGGTGCAGCAACGAAAAATTCAGCGGCACCCGCATTCTCGCTGGTACGAAGGGCGT
CCGGTAATGATTGCGCGCAACGATAGCGCGTTGGGGCTATTTAACGGCGATATTGGTATT
GCGCTCGATCGCGGGCAGGGGTTACGCGTCTGGTTTGTGATGCCGGACGGCACGATCAAG

TCCGTTCAGCCCAGCCGTCTGCCGGAACATGATACAACCTGGGCGATGACGGTACATAAA
TCACAGGGGTCTGAATTTGATCACGCCGCGCTGATTTTACCCAGCCAGCGTTCGCCGGTC
GTGACGCGGGAGCTGGTGTATACCGCTGTTACGCGAGCGCGGCGGCGGTTATCGCTGTAT
GCCGATGAACGGATCCTGGCAGGCGCGATTGTGACGCGTACGGAGCGGCGCAGCGGGCTA
GCCACCTTATTTGATGAAGTCAGTCGTACCGGATAA
>EECEBICD_00467 Amino-acid acetyltransferase
TTGCGCGGCGCGGTAAAAGCCTGTCGTAGCGGCGTTCGTCGTTGCCATCTGATCAGCTAT
CAGGAAGATGGCTCGCTATTACAGGAATTGTTCTCGCGTGACGGTATCGGTACGCAGATC
GTCATGGAGAGCGCCGAGCAGATCCGCCGCGCTACCATCAACGATATTGGCGGTATTCTG
GAGCTTATCCGCCCACTGGAACAGCAGGGCATTCTGGTACGCCGTTCCCGTGAACAACTG
GAGATGGAGATCGATAAATTTACCATTATTCAGCGCGATAATATGACTATCGCCTGCGCG
GCGCTGTATCCTTTTGTGGAAGAGAAAATAGGCGAAATGGCCTGCGTAGCGGTACATCCG
GATTACCGCAGCTCATCGCGTGGAGAAGTGCTTCTGGAGCGGGTTGCCGCCCAGGCGCGG
CAAATGGGGCTGCGTAAATTGTTTGTGCTGACGACGCGCAGTATTCACTGGTTCCAGGAG
CGCGGTTTCACGCCTGTCGATATTGAGTTACTGCCTGAGAGCAAGAAAAAAATGTATAAC
TATCAGCGACGTTCAAAGGTGCTGATGGCGGATTTAGGGTAA
>EECEBICD_00468 Amino-acid acetyltransferase
ATGATAAAGGAACGTAAAACCGAACTGGTAGAAGGGTTTCGCCATTCAGTTCCCTATATC
AACACCCATCGCGGAAAAACCTTTGTCATTATGCTCGGCGGCGAAGCCATTGAGCATGAC
AATTTTTCCAGCATCGTTAGCGATATCGGGCTACTGCATAGCCTCGGTATTCGCCTTGTC
GTGGTGTATGGCGCGCGACCGCAAATCGACGCCAATCTGGCCGCGCATCATCATGAACCG
ATTTACCACAAAAACACGCGGGTGACGGACGCCAAAGCACTGGAGCTGGTGAAACAGGCC
GCCGGTTTACTCCAGCTTGATATTACCGCCCGTCTGTCGATGAGTCTGAACAATACGCCG
TTGCAGGGCGCGCACATTAATGTCGTCAGCGGCAATTTCACCATTGCGCAGCCGCTGGGC
GTTGATGATGGCGTGGACTACTGTCATAGCGGCCGTATCCGGCGCATTGATGAAGATGCG
ATCAACCGCCAGTTGGATAACGGCGCGATCGTTCTGATGGGGCCGGTCGCCGTCTCAGTG
ACCGGCGAAAGTTTTAATCTGACCTCAGAAGAGATTGCCACTCAACTCGCCGTTAAACTG
AAAGCGGAAAAAATGATCGGTTTCTGTTCTTCTCAGGGCGTTACCAATAGCGAGGGCGGC
ATCATTTCTGAACTCTTCCCGAATGAAGCACAGGCGCGGGTGGAAGAGTTGGAAGCGCAG
GGCGATTACAACTCCGGAACCGTACGTTTTTGCGCGGCGCGGTAA
>EECEBICD_00469 N-acetylmuramoyl-L-alanine amidase AmiC
ATGTCGGGAGCCAATTCAGCAATCAGCCGTCGCCGTTTACTACAAGGCGCGGGCGCCATG
TGGCTATTAAGCGTGAGCCAGGTCGGGCTGGCTGCCGTAAGCCAGGTCGTTGCGGTGCGT
ATCTGGCCTGCGTCCAGCTATACCCGCGTCACGGTAGAGTCGAATCGTCTGCTGAAATAT
AAACAATTTGCCTTAAGTAACCCGGACCGCGTGGTAGTAGATATTGAGGATGTGAATCTG
AACTCGGTGCTTAAAGGCATTGGCGCGCAGATTCGCAGCGACGATCCGTATATCAAATCC
GCAAGGGTAGGGCAGTTTGATCCTAAGACTGTGCGTATGGTCTTTGAGCTAAAACAAAAC
GTGAAGCCGCAGCTATTTGCGCTGGCGCCTGTGGCAGGCTTTAAAGAACGTCTGGTGATG
GATCTTTATCCTGCCAATGCGCAGGATATGCAGGATCCGCTGCTGGCGCTGCTGGAAGAC
TACAATAAAGGCGATCTGGATAAACAGGTGCCGCCTTCGCAAAGCGGGCCGCAGCCCGGT
AAAGCGGGGCGCGATCGTCCCATTGTCATTATGCTTGATCCGGGACACGGCGGCGAAGAC
CCCGGCGCGATAGGCAAATACAAAACGCGTGAAAAAGACGTGGTGCTCCAGATAGCCCGC
CGCCTGCGCGCGTTGATCGAAAAAGAAGGCAACATGAAGGTCTATATGACGCGCAATGAA
GACATCTTCATTCCGTTAAAAGTCCGGGTGGCGAAAGCGCAGAAACAGCGCGCCGACCTG
TTTGTCTCTATTCATGCGGATGCCTTCACCAGCCGGCAGCCTAGCGGTTCATCCGTATTC
GCGCTATCGACCAAAGGCGCCACCAGTACCGCGGCGAAATATCTGGCGCAAACGCAGAAC
GCCTCGGACTTAATTGGCGGCGTGAGCAAAAGCGGTGACCGTTATGTCGACCATACGATG
TTCGATATGGTGCAATCGCTGACCATTGCCGATAGTTTGAAATTCGGTAAAGCGGTGTTG
AAGCAGCTTGGTAAAATTAATGACCTGCATAAAAACAAGGTCGAGCAGGCGGGGTTTGCG
GTGTTAAAGGCGCCCGATATTCCGTCTATTCTGGTCGAGACGGCGTTTATCAGTAACATT
GAAGAAGAACGCAAGCTCAAGACCGCGACGTTCCAGCAACAGGTCGCAGAGTCGATTCTG
GCGGGCATTAAAGCGTATTTTGCCGATGGGGCGACGCTGGCGAGAAGAAGTTAA
>EECEBICD_00470 tRNA-Met(cat)
CGCGGGGTGGAGCAGCCTGGTAGCTCGTCGGGCTCATAACCCGAAGGTCGTCGGTTCAAA

TCCGGCCCCCGCAACCA
>EECEBICD_00471 tRNA-Met(cat)
CGCGGGGTGGAGCAGCCTGGTAGCTCGTCGGGCTCATAACCCGAAGGTCGTCGGTTCAAA
TCCGGCCCCCGCAACCA
>EECEBICD_00472 Membrane-bound lytic murein transglycosylase A
ATGAAAGGACGTTGGGCAAAATATGTGGCGACAGGCGTAATGCTGGCAATGCTGGCTGCC
TGTTCATCAAAACCGACCGATCGCGGTCAGCAATATAAGGACGGGAAATTTACCCAGCCC
TTCTCCCTGGTAAACCAGCCGGACGCAGTGGGCGCGCCGATTAATGCTGGAGACTTTGCC
GAGCAGGTCAACCAGATCCGTTCCGCGTCGCCGCGTTTATACACCAATCAAAGCAACGTC
TATAACGCTGTCCAGAACTGGTTACGTTCCGGAGGCGATACGCGCACTATGCGTCAGTTT
GGTATCGATGCCTGGCAGATGGAAGGCACCGACAACTACGGTAACGTCCAGTTCACGGGC
TACTATACGCCCGTCGTTCAGGCTCGCCATACGCGCCAGGGCGCGTTCCAGTATCCTATC
TATAGTATGCCGCCAAAACGCGGACGCTTACCGTCCCGCGCTCAGATCTACGCAGGCGCG
CTGAGCGATAAGTACATCCTGGCCTGGAGTAATTCGCTGATGGATAACTTTATTATGGAT
GTCCAGGGCAGCGGTTATATTGATTTCGGTGACGGTAGTCCGCTGAACTTCTTTAGTTAT
GCCGGTAAAAACGGCTGGCCTTATCGCAGTATTGGTAAAGTGCTTATCGATCGCGGTGAA
GTGAAAAAAGAAGATATGTCGATGCAGGCTATACGCGAATGGGGAGAGAAGCACAGCGAA
GCGGAAGTGCGTGAATTACTGGAGCAGAACCCGTCGTTCGTCTTCTTTAAGCCGCAATCT
TTTGCGCCGGTCAAAGGCGCCAGCGCTGTACCGCTGATTGGGCGAGCATCCGTCGCCTCC
GACCGCAGCATCATTCCGCCTGGCACGACCTTGTTGGCGGAAGTACCGTTGCTGGATAAC
AACGGTAAATTTAGCGGTCAGTATGAGCTACGTTTGATGGTCGCACTGGATGTCGGCGGA
GCGATTAAAGGCCAGCACTTTGATATTTACCAGGGAATTGGCCCGGATGCCGGGCATCGT
GCCGGATGGTATAATCACTATGGTCGCGTCTGGGTGTTAAAAAGCGCGCCGGGCGCCGGT
AACGTGTTTAGCGGCTGA
>EECEBICD_00473 tRNA threonylcarbamoyladenosine dehydratase
ATGTCAGTGGTGATAAGTGATGCCTGGCGCCAACGCTTTGGCGGCACCGCGCGTCTGTAC
GGTGAAAAAGCGCTGCAGCGGTTTGCTGAGGCGCATATCTGCGTCGTGGGAATCGGCGGG
GTGGGATCATGGGCGGCAGAGGCGCTGGCGCGTACGGGCATCGGCGCGATTACGCTGATT
GATATGGACGATGTCTGCGTGACCAATACGAACCGTCAGATCCATGCGTTGCGTGATAAC
GTCGGGTTGGCGAAAGCGGAAGTGATGGCCGAACGGATTCGGCAAATTAACCCGGAGTGC
CGGGTAACGGTTATCGATGATTTCATCACGCCGGATAATGTGGCGGGTTATATGAACGCG
GGTTTTACCTATGTCATTGACGCCATTGACAGCGTTCGTCCAAAAGCCGCGCTGATTGCG
TACTGTCGCCGATATAAGGTTCCTTTAGTGACCACAGGCGGCGCGGGCGGGCAGATCGAT
CCGACGCAAATCCAGGTCGCTGATTTGGCGAAAACTATCCAGGATCCGCTGGCGGCAAAA
CTGCGTGAGAGATTGAAACATAATTTTGGCGTAGTGAAGAACAGTAAAGGTAAACTGGGC
GTGGACTGTGTTTTTTCCACCGAAGCGCTGGTTTACCCACAGGCGGACGGTTCCGTATGC
GCGATGAAAGCTACCGCAGAAGGGCCAAAACGGATGGATTGCGCCTCTGGCTTCGGCGCG
GCGACGATGGTGACGGCAACGTTCGGTTTTGTCGCCGTTTCCCATGCGCTGAAGAAGATC
ATGGCTAAAGCGGCGCGACAAGAATAA
>EECEBICD_00474 Protein RarD
GTGGAAAAAATATCTGCGTAACGGTACCATGTTTGTGGTGCTGGCATTTATATTATGGGGG
TTAACACCTTTATACTATCAATATTTATCAGGGGGGAACCTCGCACAAATATTGATATAC
CGGGTATTCTGGTCAATTCCATTATTACTGGCAGTAAGGTTCTTATTTCGGCAGCGAACG
CGATTTCATGATGTCTGGAAAGATAAAAAATCCTTTTTCTTCTGCATGATCGCCGGACTT
TTGATGATTGTATCATGGTCATCTTTTATTTATGCGCTAACCCATCATCTGGTGCTTGAT
GCCAGCCTCGGCTATTTTATCAACCCGCTATTTGTTATTGCGCTGGGATGCATTTTTCTT
AAAGAAAAACTGTCGTTGTTTCAGGCCATCGCCGTTTTTTCCGGTGTCTGCGGCCTGACT
TTTCAAATTATCATGCTACGGCATTTCCCGGCGCTGGCGCTAACCATGGGATTATCATTT
GCGCTATATGGCCTGGCGCGAAAGTTTATACATTATGATGTGATGACATCGATAACGATT
GAAACATTATGGGCATTGCCTGTCTCACTATTAATTTTTCTCTTTAGCGATACTGGACCA
ATTATATCCGCTAATACTCCTTTCTTTTTGTATGTCATGACGGCGCCAGTGACGATCATT
CCACTGGTATTATTTGCCATCGCGTTAAATCACACCTCGTTGATTGTCACCGGACTGGCG
CAATATATAGAGCCGTCTTTACAGTTCTTACTTGCCATTATGATATTTGGCGAGCACATT
AATTACGCGGAACTGCTCTGCTTTTGTGCAGTATGGTTCGGGTTGTTTCTATGCATATCT

GAAAATTTATATTCCCATTATCTCCGCGCCCGTCTGAAACCGGTGTTCGGCAGGGTACAG
CGCTTCTTTCGCTAA
>EECEBICD_00475 Sulfur acceptor protein CsdE
ATGACAAACCCTCTTTATACCGGACACCCGTTCGGCACAACGGTAACGGAAGAGACGTTA
CGCGCTATCTTTCTCCCGCTGACCCAGTGGGAGGATAAATATCGTCAGTTGATTCTGTTA
GGAAAACAGCTCCCCGCTCTGCCGGATGAATGCAAAGCGCAGGCGAAAGAGATAGCCGGC
TGCGAGAACCGCGTCTGGCTGGGCTTTACCCAGTCAGATAACGGAACGATGCACTTTTTC
GGCGACAGTGAGGGGCGGATTGCCCGCGGCCTGCTGGCGGTATTGCTCACCGCAGTGGAA
GGAAAAAACGCCGCCGAATTACAGGCTCGTTCACCGCTGGCGCTGTTTGATGAGCTGGGT
TTACGCGCCCAGCTTAGCGCCTCACGCAGTCAGGGGCTGAACGCGCTGAGCGAAGCGATT
GTTGCCGCAACGCATAAGGGTTGA
>EECEBICD_00476 Cysteine desulfurase CsdA
ATGAACGCTTTTAATCCCACGCAGTTTCGCGCGCAGTTTCCCGCGCTAGCCGATGCGGGT
GTTTATCTCGATAGCGCCGCCACGGCATTAAAGCCACAGGCAGTCATTGACGCCACGCAC
CAGTTTTATTGTTTGAGCGCCGGTAACGTTCATCGTAGCCAGTTTGCGCAGGCGCAGCGC
CTGACGGCGCAATATGAAGCGGCCAGAGCAAAAGCAGCGCGGCTGTTAAACGCGCCCGAT
GAAAAAAGTATCGTCTGGACACGCGGCACCACCGAAGCGATCAACATGGTGGCGCAGTGT
TACGCCCGTCCTCGTCTGCGCCCCGGCGATGAAATTATCGTTAGCGTCGCCGAGCATCAC
GCCAACCTTGTGCCCTGGCTGATGGTGGCGCAACAAACCGGCGCGCAGGTCATAAAACTG
CCGCTTAATGACCGACGTCTTCCTGATGTTGAGCGTCTGCCGGAACTGATCACGTCGCGC
AGCCGGATTCTGGCGCTGGGGCAAATGTCGAACGTAACGGGCGGCTGCCCGGATCTCGCA
AGCGCTATCAGCACCGCTCACGCAGCGGGAATGGTCGTGATGGTAGATGGCGCGCAAGGC
GCGGTACACTTCCCGGCGGATGTCCAGCAGCTTGATATCGATTTTTATGCTTTTTCCGCT
CACAAACTGTATGGCCCGACCGGTATCGGCGTGCTGTACGGTAAGCCGGAGCTTCTTGAG
GCGATGTCGCCCTGGCTCGGCGGCGGCAAGATGATCCGTGACGTTAGCTTTGAAGGCTTC
ACCACTCAAAGCGCTCCCTGGAAGCTGGAAGCGGGGACGCCGAACGTCGCCGGGGTCATC
GGCCTGAGCGCTGCGCTGGAATGGCTGTCCGATATCGATATTGAACAGGCCGAAAACTGG
AGCCGGGGGTTGGCGACGCTGGCGGAAGACGCACTGGCGAAACGTCCGGGCTTTCGTTCG
TTCCGCTGCCAGGACTCCAGCCTGCTGGCCTTTGATTTTGTCGGCGTGCACCACGGCGAT
ATGGTGACGCTGCTGGCGGAATACGGTATTGCGCTCCGGGCCGGGCAACATTGCGCCCAG
CCATTGCTGGCGGAACTTGGCGTCACAGGGACTCTGCGCGCCTCTTTTGCGCCGTATAAT
ACCCAACATGATGTGGATGCGTTGGTTAACGCCGTTGACCGCGCGCTGGAACTGCTGGTG
GATTAA
>EECEBICD_00477 putative lipoprotein YgdR
ATGAAAAAGACTGCCGCAATTATCTCTGCATGTATGCTGACTTTTGCCCTGAGCGCCTGT
TCCGGTCCTAATTACGTTATGCACACCAATGATGGACGTAGCATTGTGACCGACGGTAAA
CCCCAGACCGATAACGATACCGGTATGATTTCGTATAAAGACGCTAATGGCAACAAACAG
CAGATCAATCGTACTGACGTGAAAGAGATGGTGGCTCTGGAAAACTAA
>EECEBICD_00478 hypothetical protein
ATGTCTCAGATTAAAAAAACTAATAGGTTACATATTGTTACCTATTTGTTAAATTCATTC
GACATTAAGTCCAGAGGCCATGTCAAAACGATTACCTCCTTTAAATGCGTTACGCGTTTT
TGA
>EECEBICD_00479 Glycine cleavage system transcriptional activator
ATGTCAAAACGATTACCTCCTTTAAATGCGTTACGCGTTTTTGATGCTGCCGCGCGCCAT
TTGAGCTTTACGCGAGCAGCGGAAGAACTTTTTGTGACGCAGGCCGCAGTAAGCCACCAA
ATTAAGTCACTTGAGGATTTTTTGGGGCTAAAACTGTTCCGCCGCCGCAATCGTTCGCTG
CTACTGACGGAAGAAGGCCAAAGCTATTTTCTCGACATTAAAGAGATATTTTCCCAGCTC
ACGGAAGCAACGCGTAAGCTCCAGGCGCGCAGTGCCAAAGGGGCGCTGACGGTGAGTTTA
CCGCCCAGTTTTGCCATCCAATGGCTGGTGCCCAGGCTCTCCAGCTTTAACTCAGCTTAT
CCGGGAATTGACGTTCGGATACAAGCCGTTGACCGTCAGGAAGATAAACTGGCAGACGAT
GTCGATGTCGCGATTTTTTATGGCCGGGGAAACTGGCCGGGCTTGCGCGTCGAAAAATTA
TACGCAGAATATTTACTGCCCGTCTGTTCTCCTTTATTACTCACAGGCGAAAAACCGTTA
AAAACGCCGGAAGATCTGGCGAAACACACGTTATTGCACGATGCCTCCCGGCGTGACTGG
CAGGCCTATACTCGTCAGTTAGGGCTCAACCATATTAACGTTCAGCAAGGGCCGATATTT

AGCCACAGCGCGATGGTGCTACAGGCCGCTATTCACGGACAGGGGATCGCGCTGGCGAAT
AATGTTATGGCTCAGTCGGAAATTGAAGCCGGGCGACTGGTGTGTCCATTTAACGATGTT
CTGGTGAGTAAGAATGCTTTTTATCTGGTTTGTCATGACAGCCAGGCAGAACTGGGTAAA
ATAGCCGCCTTCCGTCAGTGGATTTTGGCGAAGGCGGCGACGGAGCAAGAAAAATTCCGT
TTTCGTTACGAACAATAA
>EECEBICD_00480 hypothetical protein
ATGACCAGCCGTTTTATGCTGATTGTCGCCGCTATCAGCGGTTTTATTTACGTTGCTCTG
GGCGCGTTCGGGGCGCATGTTTTGAGTAAAACCCTGGGCGTGGTTGAAATGGGCTGGATT
CAGACCGGCCTGCAATATCAGGCGTTCCATACGCTGGCGATCTTTGGCCTGGCGGTGGCG
ATGCAGCGCCGTATCAGCATCTGGTTTTACTGGAGCAGCGTTTTTCTGGCGCTGGGCACG
GTGCTGTTTAGCGGCAGTCTGTACTGCCTTGCGCTTTCGCATCTGCGCCTGTGGGCGTAT
ATCACGCCTGTCGGCGGCGTCAGTTTCCTGGTGGGCTGGGCGCTATTGCTGATTGGCGCC
ATTCGTATGAAACGTAAGGGCGCGAGCCATGAATAA
>EECEBICD_00481 Ribosomal RNA large subunit methyltransferase M
ATGAATAAAGTTGTATTGCTCTGCCGTCCGGGGTTTGAAAAAGAGTGCGCTGCAGAAATT
ACCGATAAAGCGGGAAAACGGGAAATATTTGGTTTTGCACGCGTAAAAGAGAACGCAGGC
TATGTCATTTATGAATGCTACCAGCCGGAAGATGGCGAAAAGCTGATTTCAGAGCTGCCG
TTCAGTTCGCTGATTTTCGCCCGCCAGTGGTTTGTGGTGGGTGAACTGCTGCAACATTTA
CCGCCGGAAGACAGAATTACGCCGATTGTCGGCATGTTGCAGGGCGTAGTGGAAAAGGGC
GGCGAGCTGCGAGTTGAAGTCGCCGACACCAATGAAAGTAAAGAACTGATGAAGTTCTGC
CGCAAATTTACGGTGCCGCTGCGCGCGGCTTTGCGTGATGCGGGCGTATTGACGAATTAT
GAAACGCCGAAGCGTCCGGTCGTTCATGTCTTTTTTATCGCGCCAGGATGCTGCTATACC
GGTTATTCGTTCGCGCATAACAATTCACCGTTTTATATGGGCATCCCGCGTTTGAAGTTT
CCGTCTGATGCGCCGAGCCGCTCAACGCTGAAACTGGAAGAGGCGCTCCACGTTTTCATC
CCGGAAGATGAGTGGGATGAACGTCTGGCGAACGGCATGTATGCGGTGGATTTGGGCGCC
TGTCCCGGCGGATGGACGTACCAGTTAGTGAAGCGCAATATGTGGGTTTACTCCGTAGAT
AACGGCCCGATGGCGCAAAGCCTGATGGACACCGGACAGGTGACCTGGTTGCGTGAGGAT
GGCTTCAGGTACCGTCCGAACCGGAATAATATTTCATGGATGGTCTGCGATATGGTGGAG
AAGCCGGCCAAAGTCACCGCGTTAATGGCGCAGTGGCTGGTGAACGGCTGGTGCCGGGAG
ACGATTTTTAACCTTAAACTGCCGATGAAAAAACGCTATGAAGAGGTATCGCATAACCTG
GCGTATCTTCAGGCGCAGTTGGATGAGCATGGCGTTAATGCGCAGATCCAGGCGCGTCAG
CTTTATCACGATCGCGAAGAAGTGACGGTACACGTGCGTCGGTTGTGGGCGGCTGTTGGC
GGGCGTCGCGACGAGCGATAA
>EECEBICD_00482 putative HTH-type transcriptional regulator YdjF
ATGAAAGCGGCACGCCAACAAGCGATAGTCGATCTGCTGATCAATCATAAAAGTCTGACC
ACCGAAGCGCTGGCCACCCGGCTTAACGTCAGTAAAGAGACCATCCGCCGCGATCTCAGT
GAACTACAGACGCAGGGAAAAGTGCTGCGTAATCACGGACGAGCTAAATATATTCATCGT
GAAAATCAGGACAGCGGCGATCCATTCCATATCCGCCTGAAAAGCCATTATGCGCACAAA
GCCGATATTGCCCGTGAAGCGTTAGCGTGGATAGAAGAAGGAATGACGATTGCGCTGGAT
GCCAGTTCAACCTGCTGGTATCTGGCCCGCCAGTTGCCGGATATTCCCATTCAGGTTTTT
ACCAATAGCCATCCTATCTGTCAGGAGCTGGGAAAACGCGAACGCATTACGCTGATAAGT
TCCGGCGGCCAGCTTGAACGTAAATATGGCTGTTATGTTAATCCGTCGCTTATCTCACAG
CTAAAATCGCTTGATATCGATCTGTTCATCTTTTCCTGTGAGGGGATCGACGGCGGCGGC
GATCTGTGGGACTCCAATGCGATCAACGCCGATTTTAAATCTATCCTGCTCAGGCGTGCC
TCGCAGGCTTTATTATTGATTGATAAAAGTAAATTTAATCGTTCAGGAGAGGCGCGCATC
GGCCATCTGGATGACGTGACGCATATTGTTTCAGATGCGCCGCAGTCATAG
>EECEBICD_00483 L-fucose mutarotase
ATGCTAAAAACCATTTCACCGTTAATTTCCCCGACACTGCTCAAAGTGCTGGCCGAGATG
GGCCACGGGGACGAAATCATTTTTTCCGACGCTCACTTTCCCGCGCATAGCCTGGGCCCA
CAGGTGATTCGCGCCGATGGTCTTAGCGTCAGCGATCTACTGCGGGCAATTATTCCGCTC
TTTGAGCTGGATAGCTACGCCCCGCCGCTGGTAATGATGGCCGCCGTGGAAGGCGACACG
CTCGACCCCAGCGTTGAAGCGCGCTATCGCGATGCTTTGTCATTAGAAGCGCCGTGCCCG
GATATCGTTCGTATCGACCGTTATGCGTTTTATGAGCGGGCGCAAAAAGCGTTTGCGATC
GTTATCACAGGTGAGTGCGCGAAGTACGGAAATATTCTTTTAAAAAAAGGGGTAACGCCG

TAA
>EECEBICD_00484 L-fuculokinase
ATGAAACAAGACGTTATCCTGGTTCTCGACTGCGGTGCGACTAACGTTCGGGCGATCGCC
GTTGATCGCCAGGGGAAAATTGTCGCCCGCGCCTCAACCGCTAACGCCAGCGACATTGCC
GCAGAAAATAGCGCCTGGCATCAGTGGTCGCTGGACGCCATCCTGCAACGTTTCGCCGAC
TGCTGCAGGTCACTCTCCAGCGCCCTGTCGGAGTGTGTTGTTCGCGGTATTACGGTGACT
ACGTTCGGCGTCGATGGCGCGCTGGTCGATGCGCAGGGTAAGCTGCTCTACCCGGTCATC
AGTTGGAAATGCCCGCGAACCGCCGCCGTGATGGAAACGATTGAGCGCTTTATCTCGCCC
CGGCAATTGCAGACGCTCTCCGGCGTCGGCGCGTTCAGCTTTAACACGCTGTATAAACTG
GTCTGGCTTAAAGAGAATCATCCGCGCCTGCTGGAACAAGCTCACTGCTGGCTGTTTATC
TCGTCGCTGATTAACCACCGTCTGACCGGCGAGTTTACAACCGATATCACTATGGCAGGC
ACCAGCCAGCTACTGGATATCCATCAGCGCGACTTTAGTCCGGAAATCCTGCAGGCGACG
GGCCTGGCGCGCCGCCTGTTCCCGCGTATCGTGGAAGCAGGCGCCCAGATAGGCACGCTA
CAGACAGACGCGGCCAGGCTACTCGGTCTCCCGGCGGGTGTGCCGGTGATCTCTGCCGGA
CACGATACTCAGTTCGCGTTATTCGGCGCTGGCGCGCAGCAAGGTGAACCGGTGCTCTCC
TCCGGAACATGGGAGATTCTGATGGTACGCAGCGGTCAGGTGGATACCTCGCTGCTCAGT
CAGTATCCGGGCTCGACCTGTGAACTGGACAGTCAGTCGGGGCTTTATAACCCCGGAATG
CAGTGGCTGGCCTCCGGTGTACTCGAATGGGTACGAAAACTGCTGTGGACGCCGGAAACA
CCATGGCAAACGCTGATCGACGAAGCGCGCGCTATTTCCGCTGGCGCGGAAGGCGTACGG
ATGCAGTGCGATCTGCTCGCCTGCCAAAATGCAGGCTGGCAGGGCGTGACGCTCAATACC
ACCCGCGGCCATTTCTATCGCGCCGCGCTGGAAGGCCTGACGGCGCAACTGCAGCGTAAT
CTGCGCACGCTGGAGAAAATCGGTCATTTTAACGCCACAGAACTGTTACTGGTTGGCGGC
GGCAGTCGCAACGCTTTGTGGAATCAAATTAAAGCGAATCAGCTTGATATCCCTATCAAA
GTGCTGGACGACGCGGAAACAACCGTGGCCGGCGCTGCCATGTTTGGCTGGTACGGCGTA
GGCGAATTTAGTAGCCCGGAACAGGCGCGCGCGCAGGTCAATTATCAGTATCGCTATTTC
TGGCCGCAAACCGAACCTGAAATTATTGAGGGAGTATGA
>EECEBICD_00485 L-fucose isomerase
ATGAAAAAAATCAGCTTACCGAAAATCGGTATCCGCCCGGTGATTGATGGACGTCGTATG
GGCGTACGCGAGTCGCTCGAAGAGCAAACCATGAACATGGCGAAAGCGACGGCGGCGCTC
ATCACCGAGAAAATGCGCCACGCCTGTGGGGCACAAGTGGAGTGCGTCATTGCGGATACC
TGTATTGCCGGAATGGCGGAATCCGCCGCCTGTGAAGAAAAATTCAGTAGCCAGAACGTG
GGCGTGACCATTACCGTTACGCCGTGCTGGTGCTATGGCAGCGAAACCATTGATATGGAC
CCGATGCGTCCAAAAGCCATCTGGGGCTTTAACGGCACAGAGCGTCCTGGGGCGGTTTAC
CTTGCCGCGGCGCTGGCGGCGCACAGCCAGAAAGGCATTCCAGCGTTCTCCATTTATGGC
CATGACGTGCAGGATGCCGACGATACCTCCATTCCTGCCGACGTCGAAGAAAAACTGCTG
CGTTTTGCGCGGGCAGGGTTAGCGGTAGCCAGCATGAAAGGCAAAAGCTACCTCTCCGTC
GGCGGCGTGTCGATGGGGATCGCGGGTTCCATTGTCGATCATAACTTCTTTGAATCCTGG
CTGGGCATGAAGGTGCAAGCGGTAGATATGACCGAACTGCGCCGCCGTATCGATCAAAAA
ATTTATGATGAGGCCGAACTGGAGATGGCGCTGGCGTGGGCGGATAAAAACTTCCGCTAC
GGCGAAGATCAGAACGCCTCGCAGTATAAGCGCAATGAAGCCCAGAACCGCGCGGTGTTA
AAAGAGAGCCTGCTGATGGCGATGTGTATTCGCGATATGATGCAGGGCAACAAGACGCTG
GCGGACAAAGGGTTAGTTGAAGAATCGCTGGGCTACAACGCCATTGCCGCCGGCTTCCAG
GGCCAGCGCCACTGGACCGATCAATACCCGAACGGCGATACCGCCGAAGCGCTGCTCAAT
AGTTCTTTTGACTGGAACGGCGTACGCGAACCGTTTGTCGTCGCCACCGAAAATGACAGT
CTGAACGGCGTAGCGATGCTGTTCGGTCATCAACTCACTGGTACCGCGCAGATATTCGCC
GACGTGCGAACCTACTGGTCGCCGGAAGCCGTAGAACGCGTCACCGGCCAGGCGCTCAGC
GGCCTGGCGGAACACGGCATTATTCACCTGATTAATTCCGGTTCTGCCGCTCTGGACGGT
GCCTGTAAACAGCGCGACAGCGAAGGCAAGCCGACCATGAAACCGCACTGGGAAATCAGC
CAACAAGAGGCGGATGCGTGTCTGGCGGCCACCGAATGGTGCCCGGCGATTCACGAATAC
TTCCGCGGCGGCGGCTACTCATCCCGTTTTCTGACCGAAGGCGGCGTGCCATTCACCATG
ACTCGCGTGAATATCATCAAAGGCCTGGGACCAGTACTGCAAATCGCCGAGGGCTGGAGC
GTCGAACTGCCGAAAGCGATGCACGATCAGCTTGATGCCCGCACCAACTCTACCTGGCCT
ACCACCTGGTTTGCGCCGCGTCTTACCGGCAAAGGCCCGTTTACCGATGTCTATTCGGTG
ATGGCGAACTGGGGCGCTAACCACGGCGTACTCACCATCGGCCATGTAGGGGCAGACTTT

ATTACTCTCGCCGCGATGCTGCGTATCCCGGTATGTATGCACAACGTGGAGGAGGCGAAG
ATCTACCGTCCTTCCGCCTGGGCGGCTCACGGTATGGATATCGAAGGCCAGGATTATCGC
GCCTGCCAAAATTATGGCCCGCTGTATAAACGTTAA
>EECEBICD_00486 L-fucose-proton symporter
ATGGGAAACACAACAATACAAACACAGAGTTTTCGTGCTGTGGATGCAGAGCAAAGCAAA
AGCAAGCGCTACATTATTCCATTCGCCTTACTTTGCTCGCTATTTTTTCTGTGGGCCGTC
GCCAATAATCTGAATGACATTTTATTACCGCAGTTTCAACAAGCTTTTACGCTAACTAAC
TTCCAGGCCGGACTTATTCAGTCAGCCTTTTATTTCGGTTATTTCGTCATTCCAATTCCC
GCTGGGATTTTGATGAAAAAGCTCAGTTATAAAGCAGGGATTATCACCGGGCTTTTTTTG
TATGCTGTTGGCGCCGCATTATTCTGGCCTGCCGCCGAGATAATGAATTACACATTATTT
TTAATTGGCCTGTTTATCATCGCCGCCGGTTTAGGCTGCCTTGAAACTGCGGCCAATCCT
TTTGTTACGGTATTAGGTCCAGAAAGCGGCGGACATTTCCGGCTTAATCTGGCGCAAACT
TTTAACTCCTTTGGCGCTATTATCGCCGTTGTGTTTGGGCAAAGCCTTATTTTGTCTAAC
GTGCCGCATCAATCACAAGAAGCGCTTGATAAAATGACGCCGGATCAGCTTAGCGCCTAT
AAACACAGCCTGGTGTTATCGGTACAAACGCCATACATGATTATCGTCGCCATCGTATTA
GTAGTTGCGCTGCTGATTATGCTGACCAAATTTCCGGCCCTGCAAAGTGACGATCATAGC
GATGCTAAACAAAGCTCTTTCTTATCTTCTCTCTCCCGACTCATCCGTATTCGCCACTGG
CGCTGGGCGGTGCTGGCGCAGTTCTGCTACGTGGGGGCGCAAACCGCCTGCTGGAGCTAT
CTGATCCGCTACGCCATTGAGGAGATTCCTGGAATGACGCCCGGTTTCGCCGCCAACTAC
CTGACCGGCACGATGGTGTGCTTCTTTATCGGCCGTTTCACCGGGACCTGGCTTATCAGC
CGCTTCGCGCCGCATAAAGTGCTGGCCGCCTACGCCCTGTTTGCCATGCTCCTGTGTCTG
ATTTCCGCCTTTAGCGGCGGACATATCGGCCTGCTGGCGCTGACGTTGTGTAGCGCATTT
ATGTCAATCCAGTACCCGACCATCTTCTCGTTGGGTATCAAAAATCTGGGACAGGACACT
AAGTACGGCTCGTCTTTTATCGTCATGACCATTATTGGCGGCGGTATTGTCACGCCAGTA
ATGGGCTTCGTTAGCGACGCCGCAGGCAAAATCCCGACCGCCGAACTGGTTCCGGCATTG
TGCTTTGCCGTCATCTTCATTTTTGCCCGTTTCCGTTCACAAGCGGCGACAAACTGA
>EECEBICD_00487 L-fuculose phosphate aldolase
ATGGAACGAAATAGACTGGCGCGGCAGATTATTGATACCTGTCTGGAAATGACGCGGTTA
GGGTTAAATCAGGGGACGGCAGGTAACGTGAGCGTACGTTATCAGGGCGGATTGCTGATT
ACGCCGACGGGGATCCCCTACGAGAAGCTGACAGAGTCGCACATTGTCTTTATTGATGCT
GACGGTCAGCATGAGCAGGGCAAGCTGCCTTCCAGCGAGTGGCGTTTTCATATGGCGGCA
TACCAGACGCGCCCTGACGCCAATGCCGTGGTACATAACCACGCTGTCCATTGTACGGCA
GTCTCGATCCTGAATCGCCCGATTCCGGCGATTCACTACATGATTGCCGCCGCAGGAGGA
AACTCTATTCCCTGCGCGCCCTACGCCACTTTTGGCACCCGCGAACTTTCTGACCATGTT
GCCGTGGCGCTTAAAAATCGTAAAGCGACGCTGTTACAGCATCACGGGCTTATCGCTTGT
GAAGAAAATTTAGACAAAGCGTTGTGGCTGGCGCATGAAGTGGAAGTGCTGGCGCAGCTC
TATTTAAGTACGCTGGCCATTGTCGATCCGGTACCGGTACTGGATGACGAGGCGATCGCT
ATCGTACTGGAGAAATTTAAAACTTACGGATTACGTATTGAAGAGTGA
>EECEBICD_00488 Lactaldehyde reductase
ATGGCGAACAGAATGATTCTGAATGAAACGGCATGGTTTGGCCGGGGAGCGGTGGACGCA
TTAACCGATGAGGTGACGCGCCCGCGGTTATCATAAAGCGCTGATTGTTACCGATAAAACG
CTGGTGCAATGCGGTGTGGTCGATAAAGTGACAAGCAGAATGGATGCCGCAGGGCTGGCG
TGGGAGATCTATGCTGGCGTGATCCCTAATCCTACGATTTCCGTTGTCCAGGAAGGGCTT
AAGGTCTTTACCCAAAGCGGCGCGGACTATCTCATCGCCATTGGCGGCGGTTCTCCGCAG
GATACCTGCAAAGCGATCGGTATTATCAGTAACAATCCGGAATTTGCCGATGTGCGCAGT
CTGGAAGGGCTATCGCCGACCCGTAAACCCAGCGTGCCGATTATGGCGATCCCGACAACG
GCGGGAACCGCGGCGGAAGTCACTATCAATTATGTGATTACCGATGAAGAGAAACGGCGC
AAATTCGTTTGTGTCGATCCGCATGATATTCCGCAGGCGGCATTTATCGATGCCGATATG
ATGGACGGTATGCCGCCCGCGCTGAAAGCGGCAACGGGCGTGGATGCGCTAACCCATGCT
ATTGAAGGCTATATTACCCGGGCCGCATGGGCGCTGACGGATGCATTACATATCAAAGCC
ATTGAGATTATTGCTGGCGCGCTGCGCGGCGCCGTGGCCGGAGAAAAAGAGGCCGGTGAG
GCGATGGCGCTGGGGCAATATGTTGCCGGGATGGGGTTTTCCAATGTGGGGCTGGGGCTG
GTGCATGGGATGGCGCATCCGCTGGGCGCGTTTTACAACACGCCGCATGGCGTCGCGAAT
GCTATTTTGCTTCCGCATGTGATGCGTTTTAATGCCGGGTCTACCAACGAGAAATTCCGT

GATATCGCCCGGGCGATGGGGGTGAAGGTAGAAGGATTGAGTCTTGAAGAGGCACGTAAT
GCCGCAGTGGAAGCCGTATTTACGCTAAATCGCGATGTGGGAATTCCGCTGCATTTGCGT
GATGTCGGCGTTCGAAAAGAGGATATCCCGGCGCTGGCGCAGGCGGCGTTCGATGATGTC
TGTACGGGCGGCAATCCGCGTGAAGCGAGTCTGGCGGACATTGTCGAACTGTATCATATC
GCCTGGTAA
>EECEBICD_00489 Flap endonuclease Xni
GTGGCTGCTCATCTGCTTATCGTCGACGCGCTCAATTTGATTCGCCGTATTCACGCCGTT
CAGGGATCGCCCTGTGTGGAAACCTGCCAACACGCGCTCGATCAATTGATTATCCACAGC
CAGCCAACCCATGCCGTAGCGGTATTTGATGACGACGCGCGCAGCAGCGGCTGGCGTCAT
CAGCGTTTACCGGACTACAAAGCAGGACGTCCGCCGATGCCTGACGATTTACATAATGAA
ATGCCCGCCCTACGCGCCGCATTTGAACAGCGCGGCATACGCTGTTGGGCATCAGACGGT
AACGAAGCGGACGATCTGGCGGCGACGCTGGCGCTAAAAGTTACCGAAGCCGGACACCAG
GCAACCATTGTGTCAACCGATAAAGGCTATTGCCAGTTGCTCTCTCCGGGATTGCGCATT
CGCGACTACTTCCAGAAACGCTGGCTGGACGCGCCGTTTATTGAAAAAGAGTTTGGCGTA
CTTCCCCGGCAGTTGCCGGATTACTGGGGTCTGGCAGGAATCAGCAGTTCGAAAGTCCCC
GGCGTTGCCGGTATCGGCCCCAAAAGCGCGACCCAGCTGTTGATTCAGTTTCAAAATCTG
GAGGGAATTTACGCCCATCTGGACGAGGTGCCGGAAAAGTGGCGCAAAAAGCTTGAGACG
CATAAAGAAATGGCATTTCTTTGCCGCGATATTGCACGTCTGCAAACCGATTTACATATC
GACGGAAACTTACAGCAGCTACGGCTGGCGCGGTAG
>EECEBICD_00490 L-serine dehydratase 2
ATGATTAGCGTATTCGATATTTTTAAAATCGGCATTGGCCCTTCCAGTTCTCACACCGTC
GGACCAATGAAAGCAGGCAAGCAGTTCACGGACGATCTGATTGCCCGCCATATCCTTACA
GATGTCACCCGCGTCGTGGTGGATGTCTACGGCTCTCTGTCATTGACGGGTAAAGGCCAC
CACACTGATATCGCCATTATTATGGGCCTGGCGGGGAATCTGCCGGACACCGTCGATATC
GACGCCATTCCGTCCTTTATCCAGGATGTGAACACCCATGGGCGTTTACTACTGGCTAAT
GGGCAGCATGAAGTCGAATTTCCGGTCGATAAGTGCATGAATTTCCACGCCGACAACCTG
TCTCTGCATGAGAACGGGATGCGTATTACCGCGCTGGCGGGCGATAAGGTGCTCTACAGC
CAAACCTACTACTCCATCGGCGGCGGCTTTATCGTCGATGAAGAGCACTTTGGTTTGACG
AATAGCGAACCCGTTAACGTACCGTACCCTTACAAAACCGCCGCGGATTTACAGCGCCAC
TGCCAGGAGACGGGTTTGTCGCTCTCCGGTCTGATGATGCAAAACGAACTGGCTCTGCAC
AGTAAAGAAGCGCTGGAGCAGCACTTCGCCAGGGTCTGGGAGGTAATGCGCAGCGGTATT
GAGCGCGGTATCACCACCGAGGGCGTATTGCCGGGCAAACTGCGCGTTCCGCGTCGCGCG
GCGGCGCTGCGTCGGGTGCTGGTCAGTCAGGATAAGACCACCACCGATCCGATGGCGGTC
GTCGACTGGATCAATATGTTCGCGCTGGCGGTGAACGAAGAGAATGCCGCAGGCGGACGA
GTAGTCACTGCGCCGACTAACGGCGCATGTGGTATTGTGCCGGCGGTGCTGGCGTACTAT
GACAAGTTTATCCGCGAAGTAAATGCTAACTCACTTGCTCGCTATATGCTGGTCGCCAGC
GCGATTGGCTCGCTGTATAAGATGAACGCCTCTATTTCCGGCGCCGAAGTGGGTTGTCAG
GGAGAGGTCGGCGTGGCCTGTTCCATGGCGGCAGCGGGTCTGGCCGAGCTGCTGGGCGGT
AGTCCGGCGCAGGTGTGTATCGCCGCAGAAATCGCCATGGAGCATAATCTGGGGTTAACT
TGCGATCCGGTTGCCGGTCAGGTACAGGTTCCCTGCATTGAGCGTAACGCGATTGCTTCA
GTCAAAGCGGTCAACGCCGCGCGTATGGCGCTGCGCCGTACCAGCGAACCGCGTGTATGC
CTCGATAAAGTGATTGAGACCATGTACGAAACCGGTAAAGACATGAACGCTAAGTACCGC
GAAACTTCGCGCGGCGGCCTGGCGATGAAGATCGTTACCTGCGATTAA
>EECEBICD_00491 Serine transporter
ATGGAAACCACTCAGACCAGCACTATTGCTTCGATTGACTCTCGAAGCGCATGGCGCAAA
ACGGATACCATGTGGATGCTGGGCCTTTACGGCACGGCTATCGGCGCGGGCGTTCTGTTC
CTGCCGATCAACGCAGGCGTCGGCGGCATGATTCCGCTCATCATCATGGCGATTCTCGCT
TTCCCGATGACCTTTTTCGCACACCGTGGCTTAACACGCTTCGTGCTGTCCGGTAAAAAC
CCGGGTGAAGACATCACTGAAGTCGTTGAGGAACACTTCGGTATCGGCGCAGGTAAACTG
ATTACCCTGCTCTACTTCTTCGCGATTTACCCCATCCTGCTGGTTTACAGCGTGGCGATT
ACTAATACCGTGGAAAGCTTCCTGACCCACCAGTTAGCGATTAATCCGCCGCCGCGGGCG
ATTCTTTCCCTGATCCTGATTGTTGGCATGATGACCATCGTGCGCTTCGGCGAGCAAATG
ATCGTTAAGGCGATGAGTATCCTGGTATTCCCGTTCGTCGCTGCGCTGATGCTGCTGGCC
TTGTACCTGATCCCGCAGTGGAACGGCGCGGCGCTGGAAACTCTGTCCTTTGATTCCGCC

GCGTCTACCGGTAACGGTCTGTGGATGACGCTGTGGCTGGCGATTCCGGTGATGGTTTTC
TCTTTCAACCACTCCCCGATCATCTCCTCCTTCGCGGTTGCGAAGCGTGAAGAGTACGGT
GAAGGCGCAGAGAAGAAATGTTCTAAAATTCTGGCCTTCGCCCACATCATGATGGTGCTG
ACCGTGATGTTCTTCGTCTTCAGCTGCGTGCTGAGCCTGACGCCGGCAGATCTGGCAGCG
GCAAAAGAGCAGAACATCTCTATTCTGTCTTACCTGGCGAACCATTTTAACGCGCCGATC
ATCGCCTGGATGGCGCCGATCATTGCGATGATTGCTATCACCAAATCCTTCCTCGGCCAC
TACCTGGGCGCGCGTGAAGGCTTTAACGGGATGGTGATTAAATCGCTGCGTGGTAAAGGT
AAATCCATCGAAATCAACAAACTGAATAAAATTACCGCGCTGTTCATGCTGGTCACCACC
TGGATTGTGGCGACGCTGAACCCCAGCATCCTGGGCATGATTGAAACCCTGGGCGGCCCG
ATCATCGCGATGATTCTGTTCCTGATGCCGATGTATGCCATCCAGAAAGTACCGGCAATG
CGTAAGTACAGCGGTCATATCAGCAACGTATTCGTTGTGATTATGGGCCTGATTGCCATC
TCCGCTATTTTCTACTCACTGTTCAGCTAA
>EECEBICD_00492 Pyrimidine/purine nucleotide 5'-monophosphate nucleosidase
TTGATTACACATATTAGCCCGCTTGGCTCAATGGATATGTTGTCGCAACTGGAAGTGGAC
ATGCTGAAACGCACGGCCAGTAGCGACCTGTATCAACTGTTTCGTAACTGTTCACTTGCC
GTTCTTAACTCCGGCAGCCTGACCGACAACAGCAAAGAGCTGCTGTCCCGCTTTGAAAAT
TTCGATATTAACGTACTGCGCCGCGAGCGCGGCGTGAAACTGGAATTGATTAATCCGCCT
GAAGACGCCTTTGTTGACGGGCGGATTATTCGTGCGCTACAGGCTAACCTGTTCGCGGTG
CTGCGCGACATTCTCTTTGTGAATGGGCAGATCCATAATGCCGGCCGTTTCCAGCATCTG
GATCTTGAAAGCTCAACACATATCACCAATCTGGTGTTCTCCATTTTGCGCAACGCCCGG
GCGCTGCACGTCGGCGAAGCGCCAAACATGGTGGTATGCTGGGGCGGCCACTCAATTAAC
GAAAACGAGTACCTGTATGCCCGTCGCGTCGGCACGCAGTTGGGATTACGCGAGCTGAAC
ATCTGCACCGGCTGCGGCCCTGGGGCGATGGAAGCCCCCATGAAGGGCGCGGCGGTCGGT
CACGCACAGCAGCGCTATAAAGACAGCCGCTTTATCGGCATGACTGAACCTTCCATTATT
GCGGCTGAACCGCCTAACCCGCTGGTGAACGAACTGATCATCATGCCGGATATCGAAAAA
CGCCTGGAAGCGTTCGTCCGTATCGCACACGGTATCATCATCTTCCCGGGCGGCGTGGGT
ACGGCGGAAGAGCTGCTTTATTTGCTGGGGATTCTGATGAATCCAGCCAATAAAAATCAG
GTGCTGCCGCTGATCCTGACCGGACCAAAAGAGAGCGCCGATTACTTCCGCGTGCTGGAT
GAGTTTATTACCCATACGCTGGGCGAAGCGGCCCGCCGTCATTACCGCATCATCATTGAC
GATGCGGCGGAGGTAGCGCGGCTGATGAAGAAAGCGATGCCGCAAGTGAAAGAGAATCGC
CGCGATACTGGCGACGCCTATAGTTTTAACTGGTCGATGCGTATTGCACCGGATCTGCAG
GTGCCGTTTGAGCCGTCGCATGAGAATATGGCCAACCTGAAACTGTACCCGGATCAGCCG
GTTGAGATTCTGGCGGCGGATCTGCGGCGCGCCTTCTCCGGTATTGTCGCGGGGAACGTG
AAGGAGGTCGGGATACGCGCCATTGAAGCTAATGGTCCGTATAAAATTCACGGCGATCGA
GAGATGATGCGTCGCATGGACGATCTGCTGCAGGGATTCGTCGCGCAGCATCGTATGAAA
TTGCCGGGTTCCGCCTACATCCCCTGTTACGAGATCTGCGCTTAA
>EECEBICD_00493 NADPH-dependent 7-cyano-7-deazaguanine reductase
ATGTCTTCTTATGAAAACCATCAGGCTCTCGATGGCCTGACTCTCGGAAAATCAACGGAT
TACCGGGACAATTATGACGCCAGCCTGCTGCAGGGCGTACCGCGCAGTTTGAATCGCGAC
CCGCTCGGATTAACAGCCGATAATTTGCCGTTCCACGGCGCGGATATCTGGACGTTGTAT
GAACTTTCCTGGCTTAACAGCCAGGGTCTTCCGCAGGTCGCCGTCGGGCATGTCGAACTG
GATTACACCAGCGTTAATTTAATCGAATCCAAAAGCTTTAAACTGTATCTGAACAGCTTT
AACCAGACGCGCTTTGACACCTGGGAGACGGTGCGCCAGACGCTGGAGCGCGATCTGCGC
GCCTGCGCGCAGGGCAACGTTAGCGTGAGGCTTCATCGTCTTGATGAGCTGGAAGGACAG
CCTGTCGCCCATTTCCACGGCACCTGTATTGACGATCAGGATATCAGTATCGACAACTAC
CAGTTTACTACCGACTATCTGCAACATGCGGTCAGCGGCGAGAAGCAAGTAGAAGAAACG
CTGGTCAGTCATCTGTTGAAATCCAACTGCCTGATCACCCACCAGCCGGACTGGGGCTCG
ATACAAATTCAGTATCGCGGACGCAAAATCGACAGGGAAAAACTGCTGCGTTATCTGGTC
TCCTTCCGTCACCATAATGAATTTCACGAGCAGTGCGTAGAGCGTATTTTCAATGACATT
CTGCGCTTCTGCCAGCCGGAAACGTTAAGCGTTTATGCACGCTATACCCGCCGCGGCGGG
CTGGACATTAATCCCTGGCGCAGCAATACCGATTTCGTGCCCGCCACGGGCAGGCTGGCG
CGACAGTAA
>EECEBICD_00494 Protein Syd

GTGGACGAACTGACAGCGCAGGCATTGAAAGCCTTTACGACGCGTTACTGCGACGCGTGG
CAAGAAAAACACGGTAGCTGGCCGTTGAGCGAAGAACTGTATGGCGTACCTTCTCCGTGC
ATTATTTCTTCAACCCGCGATGCCGTTTACTGGCAGCCGCAGCCTTTTGAAGGCGAAGAA
AATGTAAACGCGGTTGAACGCGCCTTTGATATTATGGTACAACCTGCGCTCCACGCGTTT
TACACCACGCAGTTTGCTGGCGACATGCCCGCGCAGTTTGCGGATGAAAAGCTTACGCTA
CTGCAAACCTGGAGTCAGGATGACTTTCGGCGCGTGCAGGAAAATCTGATAGGCCACCTG
GTCACGCAAAAGCGGCTCAAGCTTCCTCCCACGCTTTTTATTGCGACGCAGGAAAATGAG
CTTGAGGTCATTTCGGTCTGTAATCTGTCCGGCGAGGTGATTAAAGAAACGCTGGGCACC
CGTAATCGTACCGTTCTTGCCGCAACGCTTGCGGAATTCCTTACGCAACTTAATCCTCTT
CTGTAA
>EECEBICD_00495 putative protein YqcC
ATGACGACTCATGACCGTGTGCGTCAGCAGTTACATGCGCTTGAAACGCTGCTGCGCGAG
CATCGCCACTGGCGGCAGGATGCGCCGCAGGCGCACCTGTTTACCAGCACCCAACCATTT
TTTATGGATACCATGGAACCGCTGGAATGGCTGCAATGGGTATTGATTCCACGTATGCAT
ACCCTACTTGATAATGCGCAGCCGCTGCCCGAGGCATTTGCCGTCGCCCCTTATTATGAA
ATGGCGCTGGCGGCGGATCATCCGCAGCGGGAGGCGATCCTGGCGGTTTTGCAGGATCTG
GATGCGCTATTCGTCCGCGATAAATCCTGA
>EECEBICD_00496 tRNA pseudouridine synthase C
ATGCTGGAGATCCTCTATCAAGACCCGTGGCTGGTTGCCGTGAATAAACCCGCAGGCTGG
CTTGTTCACCGGAGCTGGCTGGATCGCGACGAAAAAGTCGTGGTCATGCAAACGGTGCGC
GACCAAATCGGCCAGCATGTTTTTACCGCCCACCGTCTCGACAGACCCACGTCGGGCGTA
CTGTTAATGGGGCTGTCCAGCGAAGCGGGACGCCGTCTGGCGCAGCAGTTCGAGCAGCAC
CACATCCGTAAACGCTACCATGCCATTGTGCGCGGCTGGCTGATGGATGACGCGGTACTG
GATTATCCGCTGGTGGAAGAGCGCGATAAAATTGCCGATAAATTCGCCCGCGAAGATAAA
GCGCCCCAGCCGGCTGTAACGCAGTATCGCGGACTGGCGACGGTCGAAATGGCGGTACCG
ACCGGGCGTTATCCCACCACGCGTTATGGTCTGGTTGAGCTAGAACCAAAAACGGGACGT
AAGCACCAGCTCCGTCGTCATCTGGCGCATCTGCGTCATCCTATCATCGGCGACAGTAAA
CATGGCGATTTGCGACAAAACCGCAGCGCCGCAGAGCATTTTGCCTGTCGTCGCCTGATG
CTTCATGCCAGTCGGCTTGAGCTGACGCACCCCTTCACCGGACAGCCGTTGATGATTCAG
GCCGGGTTGGATGAAACCTGGATGCAGGCGCTGACACAGTTTGGCTGGCGTGGACTTCTC
CCTGACAATGAAAGGGTTGAGTTTACGGCGGCGTCCCGGCAGGATGAGACTCATCAGGCA
TAA
>EECEBICD_00497 putative protein
ATGGCAGAAATTGGTATTTTTGTCGGGACCATGTATGGCAACTCACTGCTGGTGGCGGAA
GAAGCGGAAGCGATCCTCGCCAGACAGGGGCACAGCGCGATCGTGTTTGAAGATCCTGAA
CTGTCCGACTGGCAGCAATATCAGGATAAGGTTGCGCTGGTCGTTACCTCGACGACCGGG
CAGGGCGATCTACCGGATAGCATTGCGCCGCTCTTTCACGGCATTAAAGATACGTTGGGT
TTTCAGCCAAACCTGCGTTACGGGGTGATTGCGCTAGGTGATAGCAGCTACCCCAATTTC
TGTAATGGCGGCAAACAGTTTGATGCCCTGTTGCAAGAGCAAAGCGCGCAGCGGGTGGGG
GAGATGTTATTCATTGACGCCAGCGAACATCCGGAGCCGGAGAGCCAGTCCAATCCCTGG
GTTGAAAACTGGGGAACTTTACTTTCCTGA
>EECEBICD_00498 putative glucarate transporter
ATGAGTTCATTAAGTCACGCGGCGAGCAGTGCGGAGAAACGCACGAACGCCCGCTACTGG
ATAGTGGTGATGCTGTTTATCGTCACATCCTTCAACTACGGCGACCGCGCCACATTGTCC
ATTGCCGGCTCAGAAATGGCCAAAGATATCGGCCTTGATCCGGTAGGCATGGGCTATGTT
TTTTCTGCTTTTTCATGGGCCTATGTTATCGGGCAGATCCCGGGCGGCTGGCTGCTGGAC
CGCTTTGGTTCCAAACGCGTCTATTTCTGGTCTATTTTCATTTGGTCAGTCTTCACCCTG
TTGCAGGGTTTTGTCGATATCTTTAGTGGTTTCGGCATTATTGTCGCCCTCTTTACGCTT
CGTTTCCTGGTCGGTCTGGCGGAATCGCCTTCTTTCCCGGGCAACAGTCGCATCGTTGCG
GCCTGGTTCCCGGCGCAGGAGAGGGGAACGGCGGTGGCCATCTTTAACTCCGCGCAATAC
TTTGCCACGGTTATTTTCGCGCCGATTATGGGCTGGCTGACGCACGAAGTGGGCTGGTCG
CATGTCTTCTTCTTTATGGGCGGGTTAGGGATCATCATCAGCTTTATCTGGTTGAAAGTT
ATCCATGAACCAAATAACCATCCGGGCGTAAATAAGAAAGAGTTGGAGTACATCGCAGAA
GGCGGCGCGCTGATCAATATGGATCAGAAAGACACCAAAGCGAAAGTACCGTTTAGCGTC

AAATGGGGCCAGATCAAACAGCTGCTGGGCTCTCGTATGATGATTGGCGTTTACATCGGT
CAGTACTGTATTAACGCATTGACCTATTTCTTTATTACCTGGTTCCCGGTCTATTTGGTC
CAGGCGCGCGGCATGTCGATTCTCAAAGCGGGCTTTGTCGCCTCGGTTCCTGCAGTCTGC
GGCTTTATCGGCGGCGTGTTGGGCGGCATCATTTCCGACTGGCTGATGCGTCGTACTGGC
TCCCTGAATATCGCGCGCAAGACGCCGATTGTGCTGGGAATGTTGCTGTCAATGGTTATG
GTGTTCTGTAACTATGTGAACGTGGAATGGATGATCATCGGCTTTATGGCGTTGGCCTTC
TTCGGTAAAGGTATCGGCGCGTTGGGCTGGGCAGTAATGGCGGATACCGCGCCGAAAGAG
ATCAGCGGTCTTTCCGGCGGCTTGTTTAATATGTTCGGCAATATTTCCGGTATTGTGACG
CCAATCGCGATTGGTTACATCGTTGGCACCACCGGTTCCTTCAACGGCGCGTTGATCTAT
GTCGGCGTACATGCCCTGGTGGCCATCATCAGCTATCTGGTGCTGGTAGGCGATATTAAG
CGTATTGAACTGAAACCTGTCGCAGGACAATTATCATGA
>EECEBICD_00499 Glucarate dehydratase-related protein
ATGACAACGCAATCCAGTCCCGTTATTACGGACATGAAGGTCATTCCGGTTGCCGGACAT
GACAGTATGCTACTCAACATCGGCGGCGCGCATAACGCGTACTTCACCCGTAACATCGTC
GTCCTCACCGATAACGCCGGGCATACTGGCGTCGGCGAGGCGCCGGGCGGAGAAGTGATT
TATCAGACGCTGGTCGATGCTATTCCGATGGTATTAGGCCAGGAAGTCGCCCGCCTGAAC
AAAGTGGTGCAGCAGGTGCATAAAGGCAACCAGGCTGCTGATTTTGACACCTTTGGCAAA
GGCGCATGGACGTTTGAACTGCGGGTTAACGCGGTGGCGGCTTTGGAAGCGGCGCTGCTT
GATCTACTGGGACAGGCGCTTAATGTGCCGGTCTGTGAACTGTTAGGGCCAGGTAAACAG
CGCGATGCCGTCACCGTGTTGGGCTATCTCTTCTATATTGGCGATCGCACTAAAACCGAT
CTGCCGTATCTGGAAAGCACGCCGGGCAGTCATGAATGGTATCGTCTGCGCCATCAGGAA
GCGCTGAACAGCGACGCGGTCGTGCGGCTGGCGGAAGCGTCTCAGGATCGCTACGGCTTT
AAAGATTTTAAACTCAAGGGCGGCGTGCTGCCGGGCGAACAAGAGATTGATACTGTTCGC
GCGCTAAAAAAACGCTTCCCGGACGCGCGTATTACCGTGGATCCGAACGGCGCCTGGCTA
CTGGATGAAGCCATTGCGTTGTGCAAAGGGCTGAAAGATGTACTGACCTACGCGGAATAT
CCGTGCGGCGCGGAGCAAGGATTCTCTGGTCGTGAAGTCATGGCGGAGTTCCGCCGCGCG
ACAGGACTGCCGGTGGCGACCAATATGATCGCCACCAACTGGCGCGAAATGGGGCATGCG
GTGATGCTCAACGCGGTAGACATTCCTCTTGCCGACCCGCATTTCTGGACTCTCACCGGC
GCCGTTTGCGTGGCGCAACTGTGCGATGACTGGGGGCTAACCTGGGGCTGCCATTCGAAT
AACCATTTTGATATTTCACTGGCGATGTTTACCCATGTCGGCGCGGCGGCGCCAGGCAAA
CCCACCGCTATTGATACGCACTGGATCTGGCAGGAGGGCGATTGCCGCCTGACTAAAAAT
CCGCTGGAAATTAAAAACGGCAAAATTGCCGTTCCGGATGCGCCAGGACTGGGCGTGGAG
CTTGACTGGGAACAGGTGCGAAAAGCGCATGACGCGTATAAAAAGCTGCCAGGCGGCGCG
CGTAATGATGCAGGCCCGATGCAATACCTGATCCCCGGCTGGACTTTTGACCGCAAACGC
CCTGTTTTTGGTCGTCACTAA
>EECEBICD_00500 Glucarate dehydratase
ATGAATACTCAATTTACGACGCCTGTAGTGACTGAAATGCAGGTTATCCCGGTTGCGGGT
CATGACAGTATGCTGATGAACCTGAGCGGCGCGCACGCCCCGTTCTTCACGCGCAACATC
GTCATTATTAAAGATAACTCCGGCCATACCGGGGTCGGCGAGATTCCGGGCGGCGAAAAA
ATTCGCAAAACGCTGGAAGATGCGATCCCACTGGTGGTGGGAAAAACGCTGGGTGAATAT
AAAAATGTCCTGACCGCCGTTCGCAACCAGTTTGCCGATCGCGATGCGGGCGGACGCGGT
TTACAAACATTCGATCTCCGTACCACTATCCATGTGGTGACGGGTATTGAAGCGGCAATG
CTTGACCTTTTGGGCCAACACTTGGGCGTCAACGTCGCTTCGCTGTTAGGCGACGGTCAG
CAGCGCAGCGAAGTCGAAATGCTGGGTTATCTGTTCTTTGTCGGTAATCGCAAGGCTACG
CCGCTGCCGTATCAGAGCCAGCCGGATGAGCAATGCGACTGGTATCGTCTGCGCCATGAA
GAGGCGATGACGCCGGAAACGGTAGTGCGTCTGGCGGAAGCCGCCTATGAAAAATACGGC
TTCAACGACTTCAAACTGAAAGGCGGCGTGCTGGCGGGCGAAGAAGAGGCCGAGTCAATC
GTGGCGCTGGCGAAACGTTTCCCACAAGCGCGCGTCACGCTCGATCCAAACGGTGCCTGG
TCGCTGAACGAAGCGATCAGCATTGGTAAATACCTGAAAGGTTCTCTGGCCTATGCAGAA
GATCCGTGCGGCGCGGAGCAGGGTTTTTCCGGACGTGAAGTGATGGCGGAATTCCGTCGC
GCGACCGGATTACCGACGGCCACCAATATGATAGCGACCGACTGGCGTCAAATGGGGCAT
ACGCTGTCGCTGCAATCCGTCGATATCCCGCTGGCGGACCCGCACTTCTGGACTATGCAA
GGCTCTGTACGCGTGGCGCAAATGTGTCATGAGTTCGGTCTGACCTGGGGCTCGCACTCT
AACAACCACTTTGATATTTCGTTGGCGATGTTTACCCATGTTGCCGCGGCGGCGCCGGGC

AAGATCACCGCGATCGATACCCACTGGATCTGGCAGGAAGGCAACCAACGTCTGACTAAA
GAACCGTTTGAAATTAAAGGCGGCATGGTGCAAGTACCGACCAAACCGGGTCTGGGCGTT
GAGCTCGATATGGATCAGGTGATGAAAGCGCATGAGCTCTATCAAAAACATGGCTTAGGC
GCGCGTGACGACGCGATGGGAATGCAGTACTTAATTCCTGGCTGGACGTTTGATAATAAG
CGTCCTTGCATGGTGCGTTAA
>EECEBICD_00501 Glycerate 2-kinase
ATGAAAATAGTGATCGCACCAGACTCGTATAAGGAAAGTTTGAGTGCTCTTGAGGTAGCG
ACCGCCATTGAACAAGGATTTCGCGAGATCTGGCCTGACGCCGATTACCTGAAATTGCCG
CTGGCTGATGGCGGAGAGGGTACTGTCGAAGCGATGGTTGAAGCGACGGCGGGTCGAATC
GTGCATGTTGAGGTCACCGGGCCGTTGGGGCATCGCGTCAATGCGTTTTATGGGCTTTCC
GGCGATGCGCGTTCCGCATTCATTGAAATGGCGGCCGCCAGCGGGCTGGAGCAGGTGCCG
CCCGCGCAACGCGATCCGTTGAAGACCACGTCATGGGGAACCGGCGAGCTGATCCGTCAT
GCGCTGGATGCGGGCGTTGAGCATATCATTATTGGTATCGGCGGTAGCGCCACCAACGAT
GGCGGCGCGGGGATGGTACAGGCGCTGGGGGCCAGGCTACGGGATGCGCAGGGCAATGAT
ATTGCGCAAGGCGGCATTGGGCTGGAAACACTCGCCAGCATTGATATCAGCGGCCTGGAT
AAGCGTTTGTCGGCGTGCCATATAGAGGTCGCCTGCGATGTCACGAATCCCCTTACCGGC
AAAGAGGGCGCGTCTGCGGTTTTTGGTCCGCAAAAAGGGGCGACGCCGGAAATGATTGAA
CGCCTGGAGACGGCATTGACTCGCTATGCGCATTTGATTGCTCGCGATCTGCATGTCGAT
GTGTTGGATCTGGCAGGCGGCGGCGCGGCAGGCGGCATGGGGGCGGCGTTATACGCGTTT
TGCGGCGCGCAGCTACGGCGTGGCATTGAGATTGTCACCGATGCGCTTCATCTGGAAGCG
TGTCTTGCGGATGCTGATTTAGTCATCACCGGCGAGGGACGCATCGACAGCCAGACCATT
CACGGTAAAGTCCCTATTGGCGTGGCAAACATCGCCAAACGGTATAACAAGCCGGTAATT
GGTATTGCGGGTAGCCTGACGGAGGACGTTGGCGTGGTTCATGAACATGGGCTTGATGCC
GTTTTCAGCGTGATTTATACCATCTGCACGCTGGAGGACGCGCTGAAGAATGCGAGTGAA
AACGTACGAATGACCGCGCGCAACGTGGCTGCAACGCTGAAAGCGGGGCAACAATTACGC
TGA
>EECEBICD_00502 Signal transduction histidine-protein kinase BarA
ATGACCAACTACAGCCTGCGCGCACGCATGATGATTCTGATCCTGGCCCCGACCGTCCTG
ATAGGTTTGCTGCTCAGTATCTTTTTTGTAGTGCACCGCTATAACGACCTGCAGCGTCAA
CTGGAAGATGCCGGCGCCAGTATTATTGAACCGCTCGCCGTCTCCAGCGAATATGGTATG
AACTTACAAAACCGAGAGTCTATCGGCCAACTTATCAGCGTCCTGCACCGCAGACACTCT
GATATTGTGCGGGCAATTTCCGTTTATGACGATCATAACCGCCTGTTTGTAACGTCTAAT
TTCCATCTGGCCCCCTCACAGATGCAGCTTCCCGCCGGAGCGCCGTTTCCACGTCGTCTG
AGCGTCGATCGCCACGGCGATATTATGATTCTGCGCACCCCGATTATCTCGGAGAGCTAT
TCGCCGGACGAGTCAGCGATTGCCGACGCAAAAAATACCAAAAATATGCTGGGGTATGTG
GCGCTTGAACTGGATCTCAAGTCGGTCAGGCTACAGCAATACAAAGAGATTTTTATCTCC
AGCGTGATGATGCTTTTTTGTATTGGCATTGCGCTGATCTTTGGCTGGCGGCTTATGCGC
GATGTCACCGGGCCTATCCGTAATATGGTGAATACCGTTGACCGCATTCGCCGCGGACAA
CTGGATAGCCGGGTGGAAGGATTTATGCTGGGCGAGCTGGACATGCTGAAAAACGGCATT
AATTCCATGGCGATGTCGCTTGCCGCCTATCACGAAGAGATGCAGCATAATATCGATCAG
GCCACCTCGGACCTGCGTGAAACCCTTGAGCAGATGGAAATCCAAAACGTTGAGCTGGAT
CTGGCGAAAAAGCGCGCCCAGGAAGCGGCGCGTATTAAGTCGGAGTTCCTGGCAAACATG
TCGCACGAACTGCGAACGCCGCTGAACGGCGTCATTGGCTTTACCCGCCTGACATTAAAA
ACGGAGCTGAACCCCACCCAGCGCGACCATCTGAACACCATTGAGCGTTCCGCGAATAAT
CTGCTGGCGATCATTAATGACGTGCTTGATTTCTCCAAGCTGGAAGCCGGTAAGCTCATT
CTGGAAAGTATCCCTTTTCCACTGCGCAACACGCTGGATGAAGTGGTTACGCTGCTGGCT
CACTCGTCGCATGATAAAGGGCTGGAGTTGACGTTAAATATTAAAAACGACGTCCCGGAT
AATGTGATTGGCGACCCGCTGCGCCTGCAACAGGTCATTACTAATCTGGTGGGCAATGCC
ATTAAGTTCACCGAGAGTGGCAATATCGATATTCTGGTAGAAAAGCGGGCGCTCAGTAAC
ACCAAAGTACAGATTGAAGTGCAGATCCGCGATACGGGGATCGGCATTCCGGAACGCGAC
CAGTCTCGACTGTTTCAGGCGTTTCGCCAGGCCGATGCCAGTATTTCTCGCCGTCACGGC
GGCACCGGGCTTGGGCTGGTGATTACGCAAAAGCTGGTCAACGAAATGGGCGGGGATATC
TCTTTCCACAGCCAGCCTAATCGCGGCTCGACCTTCTGGTTTCATATTCATCTTGATCTT
AACCCAAATGTCATTATTGACGGGCCGTCGACCGCGTGTCTGGCCGGGAAACGACTGGCT

TATATCGAACCGAATGCTACCGCCGCGCAATGTACCCTGGATCTGCTGAGCGACACGCCG
GTGGAAGTGGTTTACAGCCCGACCTTCTCCGCGCTGCCGTTAGCGCACTACGATATTATG
ATTTTGAGCGTTCCGGTGACCTTCCGCGAGCCGCTCACCATGCAGCATGAACGTCTGGCG
AAAGCAGCGTCAATGACGGACTTTCTACTGCTGGCGCTACCTTGCCATGCGCAAATTAAC
GCCGAAAAGCTGAAACAAGGAGGCGCGGCGGCCTGTCTGTTAAAACCATTGACGTCAACG
CGCCTGTTGCCAGCGCTGACGGAATATTGCCAGTTGAATCACCATCCTGAACCGCTGCTA
ATGGATACCAGTAAAATCACCATGACGGTTATGGCGGTTGATGATAATCCCGCTAATCTG
AAGCTTATCGGCGCGTTACTGGAAGATAAAGTCCAGCACGTAGAACTTTGTGATAGCGGA
CATCAGGCGGTGGATCGGGCGAAACAAATGCAGTTTGATCTGATTTTGATGGATATTCAG
ATGCCGGATATGGACGGCATACGCGCCTGCGAATTGATTCACCAGCTTCCTCATCAGCAG
CAAACACCGGTTATTGCCGTTACGGCACATGCGATGGCCGGGCAAAAAGAGAAATTGCTC
AGCGCGGGCATGAACGACTATCTGGCTAAACCGATAGAAGAAGAGAAGTTGCATAATCTG
TTGCTGCGCTATAAACCTGGCGCCAACGTAGCAGCGCGCCTGATGGCGCCGGAACCGGCT
GAGTTTATCTTCAATCCGAATGCAACGCTCGACTGGCAGCTTGCGCTCCGCCAGGCTGCC
GGTAAGCCCGATCTGGCACGGGATATGCTGCAAATGCTGATTGATTTTCTGCCGGAAGTG
CGCAACAAAATTGAAGAACAACTGGTGGGAGAAAATCCCAACGGTTTGGTCGATCTGGTC
CATAAGCTACACGGGAGCTGCGGCTATAGCGGCGTACCGCGGATGAAGAACCTTTGCCAG
CTTATTGAGCAACAGCTTCGCAGCGGCGTCCACGAAGAGGAGCTGGAGCCTGAGTTTCTG
GAGCTGCTGGATGAGATGGATAATGTCGCGCGTGAAGCGAAGAAGATATTAGGCTGA
>EECEBICD_00503 23S rRNA (uracil(1939)-C(5))-methyltransferase RlmD
ATGGCGCAATTCTACTCTGCAAAACGGCGCGTGACGACGCGTCAGATCATAACCGTTAAA
GTCAATGACCTCGATTCATTTGGTCAGGGCGTAGCCCGTCATAACGGAAAAGCGCTGTTT
ATCCCCGGTTTATTGCCTGAGGAAAGCGCAGAAGTCATTATTACAGAAGATAAAAAGCAA
TTTGCCCGCGCACGCGTTTCGCGCCGTTTGAATGATAGCCCGGAACGAGAAACGCCGCGC
TGCCCGCATTTTGGCGTCTGCGGCGGTTGTCAGCAACAGCATGTCAGCATCGCCCTGCAA
CAACGCAGCAAAAGCGCGGCGCTGGCGCGTTTGATGAAACACGAGGTGAATGACATCATC
GCCGGCGCGCCCTGGGGTTATCGGCGTCGTGCGCGGTTAAGTCTGAATTGCCCGCCGGAT
AAACCGCTGCAAATGGGATTTCGCAAAGCGGGTTCCAGCGATATCGTCAATGTCGAACAG
TGCCCTGTTTTGGCGCCCCAGCTTGCAGCATTGCTGCCCCGAATCAGGGCGTGTCTGGCG
AGTTTACACGGCACCCGACATCTTGGGCATGTTGAGCTGGTACAGGCGGGAAGCGGTACG
CTGATGATTCTGCGCCATACCGCGCCATTAAGTGCGGCGGATAAAGAAAAACTGGAACGC
TTTTCGCATTCTGAGGGATTGTCTCTGTTTCTGGCGCCGTTTAGCGAGATACTGGAAACG
GTATCTGGCGAAGCGCCCTGGTATGATTCGCACGGCTTACGTTTAGCCTTCAGTCCGCGA
GACTTTATTCAGGTTAATGAGGCGGTAAACCAGCAAATGGTCGCCCGCGCGCTGGAGTGG
CTTGATGTGCGCGCCGAGGATCGTGTCCTGGATCTGTTCTGCGGAATGGGGAATTTTACG
CTGCCGCTGGCAACGCGCGCGGCAAGCGTGATTGGGGTTGAGGGCGTTCCGGCGCTGGTA
GAAAAAGGCCGTGAAAACGCCATCCGCAATGGTTTACATAATGTGACATTCTTCCATGAG
AACCTGGAGGAAGATGTCACGAAGCAGCCGTGGGCGAAAAACGGCTTTGACAAAGTCTTA
CTCGATCCTGCGCGTGCGGGGGCTACAGGAGTGATGCGACATATTATAAAATTAAAACCT
ATTCGCATTGTTTATGTATCCTGTAACCCGGCGACGCTGGCGCGCGATAGTGAAGCGCTG
GTCAATGCGGGATATGAGGTTACGCGTTTAGCGATGCTCGACATGTTCCCGCACACAGGA
CATCTGGAATCAATGGTTCTGTTCGAGCGCATGTAA
>EECEBICD_00504 GTP pyrophosphokinase
ATGGTCGCGGTAAGAAGTGCACATATTAATAAAGCTGGTGAATTTGATCCGAAGAAGTGG
ATCGCAAGCCTGGGAATTTCCAGCCAGCAGTCGTGTGAGCGCTTAGCCGAAACCTGGGCG
TATTGCCTGCAACAGACACAAGGACATCCGGATGCGGATCTGTTGCTGTGGCGTGGCGTG
GAGATGGTAGAAATTCTTTCCACGCTGAGTATGGATATCGACACGCTGCGGGCGGCGCTA
CTGTTCCCTCTGGCCGACGCCAACGTAGTCAGCGAAGATGTACTGCGCGAAAGCGTCGGC
AAATCTATCGTTACCCTGATTCATGGCGTGCGCGATATGGCGGCGATCCGTCAGCTAAAC
GCCACTCATAACGACTCTGTTTCTTCGGAGCAGGTTGATAACGTCCGTCGAATGTTATTG
GCGATGGTGGATGATTTCCGCTGCGTGGCGATCAAACTGGCCGAGCGAATCGCTCATTTG
CGCGAAGTGAAAGAGGCGCCGGAAGATGAGCGCGTGCTGGCGGCGAAAGAATGTACCAAC
ATCTATGCGCCGCTCGCTAATCGTCTGGGCATCGGGCAACTGAAGTGGGAACTGGAAGAC
TACTGTTTCCGCTACCTGCATCCGGCGGAATACAAACGCATCGCCAAACTGCTGCATGAG

CGCCGTCTCGATCGCGAACATTACATCGAAGAGTTTGTTGGACATCTGCGCGCCGAAATG
AAAAACGAAGGCGTGCAGGCGGAGGTCTACGGACGACCAAAACATATTTATAGCATCTGG
CGCAAAATGCAGAAAAAGCATCTGGCGTTTGATGAACTCTTTGACGTGCGCGCCGTGCGT
ATTGTCGCTGAACGTCTGCAGGACTGCTACGCCGCGTTGGGGATAGTGCATACGCACTAT
CGTCACCTGCCGGATGAATTCGATGATTATGTCGCTAACCCGAAACCGAACGGTTACCAG
TCTATCCACACCGTGGTCCTGGGACCGGGCGGTAAAACCGTTGAGATCCAGATCCGTACC
AAACAGATGCATGAAGACGCCGAACTGGGCGTGGCGGCACACTGGAAGTATAAAGAAGGC
GCCGCGTCCGGCGGCGTGCGCTCCGGTCATGAAGACAGAATTGCGTGGCTGCGTAAGCTG
ATCGCCTGGCAGGAAGAGATGGCCGATTCCGGCGAAATGCTGGATGAAGTGCGCAGCCAG
GTGTTTGACGATCGGGTCTACGTTTTTACGCCAAAAGGCGACGTGGTTGACTTGCCTGCC
GGATCTACGCCGCTCGATTTTGCTTACCACATCCACAGCGATGTTGGGCACCGCTGCATT
GGCGCTAAAATCGGCGGCCGTATTGTGCCATTCACCTATCAGTTGCAGATGGGTGATCAA
ATTGAAATTATCACTCAGAAGCAGCCGAATCCCAGCCGCGACTGGCTGAATCCAAACCTG
GGCTATGTGACGACCAGCCGCGGACGCTCGAAAATTCACGCCTGGTTCCGCAAGCAGGAT
CGTGACAAAAATATCCAGGCTGGACGGCAGATCCTCGACGATGAGCTGGCGCATTTGGGG
ATTAGCCTGAAAGAGGCAGAAAAACATCTGCTGCCGCGCTACAACTTTAATGAGCTGGAA
GAGTTGCTGGCGGCGATAGGCGGCGGCGATATCCGTCTTAATCAGATGGTGAATTTCCTG
CAATCGCAGTTCAATAAGCCGAGTGCGGAGGAGCAGGATGCAGCGGCGCTGAAACAGCTT
CAGCAAAAAAACATACGCGCCGCAAAATCGTCGTAAAGACGACGGGCGCGTGGTGGTGGAA
GGGGTTGGTAACCTGATGCACCACATTGCCCGCTGCTGCCAGCCGATTCCTGGCGATGAA
ATTGTCGGCTTCATTACTCAGGGGCGGGGAATTTCCGTGCACCGGGCCGACTGCGAACAG
CTGGCGGAACTGCGCTCCCATGCGCCGGAGCGGATCGTAGAGGCGGTATGGGGCGAAAGC
TACTCGGCGGGATATTCGCTGGTGGTGCGCGTCCAGGCCAACGATCGCAGCGGCTTGCTG
CGCGATATCACCACTATTCTGGCTAACGAAAAAGTCAACGTGCTGGGCGTCGCCAGCCGC
AGCGACATTAAACAGCAGATCGCCACCATTGATATGACCATCGAGATCTACAACCTGCAG
GTGCTGGGCCGCGTGCTCGGTAAGCTGAACCAGGTGCCGGATGTGATTGATGCACGGCGA
CTGCACGGGGGGTAA
>EECEBICD_00505 hypothetical protein
ATGAATCAAAAAGTATTATTTTTTCTAAGTATAATTGCATATCCTGGGATAACTCAGGCC
ATTAACCAAAATGTAATGATTGAACCATTAGTAATACGTGACTCAACAAATTATTTAGTA
CTACCTCAGGCTCAACAGGCTGAATTCTCACAATGCAAAAGCTGGCCGCATGATAGCACT
GTTATTCCAGCAAAATATGAGGCAACCCTGCTTAATGGAAGTCTCATATCTATGTTTATT
GGTGGCATAACAGTTAATTTATATACAACAGCAGATGTCAAAGTTAATACAGATGCCCAG
TGTACATTACTCAATGGTAATTTTCCTGGTTATTACGGCATAACATGCTTTGTTACTGGT
TATGACACAGACAAAGCTGTACCTGTAAGGACAGATAATGGCATGTCAATTATCGAAAGT
AAATTATATGCTACAGGAACAATGAGATATATAACAGAGATCCCGATGGATGGCAGTGGC
TTTCAGGGTATGGCGATTGGTGGTGCAGTAGTAATTAATACAGGCGCAATAACACCAACC
CCTAATACGGGTGTTATTTCTAACATAACAATGCCACCACTATATGATCCCGGGTTTATT
TTTACGGTACCTGGATGCGGTCAATACAAATATCATTCTGGGACAGGTTCTGTTTTTACG
AATAACAATTCAACTTATACGGTTGCATCGCCGATTGGTTTCATAACTGCGTCTTTGGAT
AAGAACTCCGTGTTAAATTATAATGAACTTCATTATAACGATAAGGCAGAAAATATAGAA
CTAGGGAAAATTTATTTGATGTATAAGGAGAAAAATGTCACGTGGGGTGAAGGATTCGAT
TATACATTGGAAAATAGCACGATCAATGTAGTTTGTGCTGATAGCAGGATTAAAACAAAT
GTAGATTATCAATGTAGGAATGGTGATATGGGGGCATGCAACAATGGCGAACTGGGAAGA
ATAATCGGCAACTGGGAACGAATAAACGTTGATACTAATTGCAGCGTGACAGTTATTTTG
CCCTGGCAATAG
>EECEBICD_00506 putative fimbrial-like protein YfcP
ATGTTATGGGCGGTTTGCAATACGCTACCTGCCGCCACGCCTAATGTGCACTACAGCGGT
AAGCTGGTTGCCGGCGCCTGTAACCTGGTAGTGGATAATGACACGATGGCGACGGTGGAT
TTTCATACTATTGGCAGTGACAATTTCGACGCCAGCGGGCAAACGACTCCTGTTCCATTC
ACACTTAGCCTCCAGGATTGCAAAACAGCGCTAGCCAACGGCGTGCTGGTGACCTTTCAG
GGCGTTGAAGACAGTACCCTGCCCGGACTGCTGGCGCTGGAGCCCTCGTCGGAAGCCAGT
GGTTTTGCGATTGGCGTGGAAACGGCTGCACAGCAACCAGTGAGTATCAATGCAACAGTG
GGTACAGCTTTTGTGCTGAAAGAAGGGATTACCACCATCAACTTGCAGGCCAGGCTGCAA

AAATACGCTGGTGAAGAGGTTATGCCGGGGGAATTTTCGGGTAGCGCAACAGTGAGTTTT
GAATATCAGTAA
>EECEBICD_00507 putative fimbrial-like protein YfcQ
ATGGAGAGATTCATAGTGAAAAGGGTGCTTATCCTGACGCTTTTAATCACCCAGTTTGCC
TGCGCCGACAATTTGACCTTTCACGGCAAACTGATAAACCCGCCCGCCTGTACTATCAAT
AACGGTGAAATGCTGGAGGTCAGTTTTGGTAGCGTCATTATTGATAATATTGACGGCGTC
AATTACCTGACCGAGATCCCGTGGACCCTTACCTGCGACAGCAGCTTTCGCGATGACGCA
TTAACTTTTACGTTGAGCTATCTGGGCACGGCGACGCCTTACTCCGCAAAAGCCTTAACC
ACCAGCGTCCCTGAGCTGGGCATTGAACTTCAACAGAACGGCACGGTGTTTCCTCCCGGC
ACGTCGCTAACCATCAATGAGTCATCGCTTCCCACACTAAAAGCCGTGCCGGTAAAACAG
CCCGGTAAAGAGCCTGCCGAAGGCGATTTTGAGGCTTTTGCCACGTTACAGGTGGATTAC
CAATGA
>EECEBICD_00508 putative fimbrial-like protein YfcR
ATGTTGACTCGCTGGAAAATGCTGGTGCTGCTGTGCGGTGGATTCGTTACCGGCACCGAG
GCTGCCGGCACAAAAACCGTGCAACTGGAGCTGCATCTGGTCGTCACTCAACCACCGCCC
TGTACGGTAGGCGGCGCTTCCGTAGAGTTTGGCGATGTCCTGACCACCAAAGTTGGCGAT
GCTTCTCAAACGAAACCTGTGGGCTACAGCCTGAACTGCGATGGCAGAGCGAGTGACTAT
CTGAAGCTACAAATTCAGGGAACCACCACCACCATCAGCGGGGAGCAGGTTCTGCAAACT
TCGGTGCAGGGGTTAGGCATCCGTATTCAGCAGGCGGGCAATAAGCAACTGGTTCCGGTT
GGCATAACGGATTGGCTGAACTTCACCCTGAGCGGCAGCAATGGGCCAGAGCTGGAAGCC
GTACCGGTTAAAGAGCCCACCACGCAGTTAGCGGGTGGCGATTTTAACGCCAGTGCGACG
TTAGTCGTGGATTACCAGTGA
>EECEBICD_00509 putative fimbrial chaperone YfcS
ATGAACAAGACCAACCATTTTAAGCGCCAGGCGCTTATCGCTTCGGTATTACTGGCTGCG
CCGCTGGTATCGCACAGCGCGATTGTGCCGGATCGTACCCGCGTTATTTTTAACGGCAAT
GAAAATTCAATCACCGTCACGCTGAAAAACGGCAACGCCACTCTGCCCTACCTCGCACAG
GCGTGGCTGGAAGACGATAAGTTCGCCAAAGATACCCGCTATTTTACCGCCCTGCCGCCG
CTGCAGCGTATTGAGCCAAAATCCGACGGGCAGGTTAAAGTCCAGCCGTTACCAGCCGCG
GCTAGCCTACCGCAGGACCGGGAAAGTCTGTTTTATTTCAACGTGCGGGAAATTCCGCCG
AAAAGCGATAAGCCTAATACGCTCCAGCTGGCGCTGCAAACGCGTATTAAGTTTTTCTAC
CGCCCGGTAGCCGTCGCCCGGCAGGTGGATAAGACGCACCCGTGGCAGACGAAGCTCACG
CTGACCTACCAGGGCGATGGTGTGATCTTCGACAACCCCACACCGTTTTACCTCGTCATC
AGCAATGCCGGTAGCAAAGAGAACGAAACCGCCTCCGGCTTTAAAAACCTGCTGATTGCC
CCGCGTGAAAAAGTGACCTCTCCCATCAAAGGTGCCTCGCTCGGCAGCTCGCCGGTGGTG
GGCTACGTCGATGATTACGGCGGCCATCGCCTGCTGGTTTTTACCTGCTCAGGGAATACC
TGCAAGGTCAATGAGGAAAAAACCCGTGACGCTGAGAAGAAAGCGAATAAGTAA
>EECEBICD_00510 Outer membrane usher protein PapC
ATGCTTCTAAGCCTCTCCCCTTATAGCGCGTCAGGCAAAGACATCGAATTTAATACCGAT
TTCCTCGATGTAAAAAATCGCGATAACGTTAACATTGCACAGTTTTCTCGTAAGGGTTTT
ATTCTGCCAGGCGTCTACCTTTTACAAATTAAAATTAACGGACAGACTCTGCCGCAGGAA
TTTCCTGTTAACTGGGTTATTCCAGAACATGATCCACAAGGAAGTGAGGTTTGCGCAGAA
CCAGAATTAGTTACGCAATTGGGTATAAAGCCGGAACTCGCGGAAAAACTCGTCTGGATA
ACGCACGGTGAACGACAATGTCTGGCGCCAGATTCACTGAAAGGCATGGATTTTCAGGCT
GACCTGGGACACTCCACGCTGCTGGTGAATTTACCCCAGGCGTATATGGAATACAGCGAT
GTCGACTGGGACCCGCCCGCCCGCTGGGATAATGGTATTCCCGGCATCATTCTGGATTAC
AACATTAATAATCAGCTCCGCCACGATCAAGAAAGCGGCAGCGAAGAGCAAAGCATCAGC
GGCAACGGGACGTTAGGCGCGAACCTGGGCGCATGGCGACTGCGGGCCGACTGGCAGGCC
AGCTACGACCATCGTGACGATGACGAGAACACTTCCACCCTCCACGATCAGAGCTGGAGC
CGCTACTACGCCTATCGCGCACTACCGACGCTCGGGGCCAAACTTACGCTGGGCGAAAGC
TATCTCCAGTCCGATGTTTTCGACAGCTTTAACTATATCGGTGCCAGCGTCGTTTCTGAC
GATCAGATGCTGCCGCCGAAACTGCGCGGCTATGCGCCGGAGATCGTGGGCATTGCGCGC
TCTAATGCCAAAGTCAAAGTCTCCTGGCAGGGGCGCGTACTGTATGAAACGCAGGTGCCC
GCAGGACCGTTCCGTATTCAGGATCTCAACCAGTCCGTTTCCGGTACGTTGCACGTCACC
GTGGAAGAGCAGAACGGTCAGACCCAGGAGTTTGACGTTAACACCGCATCGGTTCCCTTC

CTGACGCGCCCCGGCATGGTGCGCTACAAGATGGCGCTGGGCCGCCCGCAGGACTGGGAT
CATCACCCCATTACCGGCACATTCGCCTCGGCGGAAGCTTCGTGGGGGGTCACCAACGGC
TGGTCGCTCTATGGCGGCGCAATTGGAGAAAGCAACTATCAGGCCGTGGCGCTGGGAAGC
GGTAAGGATCTTGGCGTGGTGGGCGCGGTGGCGGTCGACATTACGCACTCCATCGCCCAC
ATGCCGCAAGACGACGGGTTTGACGGCGAAACGCTGCAGGGTAACTCATATCGCATCAGC
TACTCCCGTGACTTTGATGAAATCGACAGCCGACTAACCTTTGCCGGATACCGCTTCTCA
GAAAAGAACTTTATGAGCATGAGCGACTATCTGGATGCGAAAACCTATCATCATCTCAAT
GCCGGTCACGAAAAAGAACGCTATACGGTCACCTATAACCAGAACTTCCGTGAACAGGGC
ATGAGCGCCTATTTCAGCTACTCACGCAGTACCTTCTGGGACAGCCCGGATCAGAGTAAC
TATAACCTGTCTCTTTCCTGGTACTTCGACTTAGGGTCGATAAAAAATCTCAGTGCGTCG
CTGAACGGCTATCGCAGCGAATATAACGGTGATAAAGATGATGGCGTTTATATCTCGCTG
TCTGTTCCCTGGGGCAATGATTCCATCAGCTACAACGGTACGTTTAACGGTAGTCAACAC
CGTAATCAGCTCGGCTATTCCGGCCACAGCCAGAACGGCGATAACTGGCAGCTTCACGTC
GGGCAGGATGAACAAGGCGCACAGGCAGACGGTTATTACAGCCATCAGGGCGCGCTGACG
GACATCGATCTGAGCGCGGATTATGAAGAAGGATCGTACCGTTCGCTGGGCATGTCGCTG
CGCGGCGGCATGACGCTGACCACCCAGGGCGGCGCGCTACACCGGGGAAGTTTAGCGGGC
AGCACACGTTTGCTGGTTGATACCGACGGCATTGCGGACGTCCCCGTTAGCGGTAACGGC
TCGCCAACCTCAACCAACATTTTCGGCAAGGCCGTGATTGCGGATGTCGGAAGCTATTCG
CGCAGCCTGGCGCGTATCGATCTGAACAAATTGCCGGAGAAGGCGGAAGCTACTAAGTCG
GTTGTGCAGATCACGCTCACCGAAGGCGCCATCGGCTACCGTCACTTTGACGTGGTCAGC
GGCGAGAAAATGATGGCGGTTTTCCGGCTGGCAGACGGCGACTTCCCACCGTTCGGCGCC
GAAGTGAAAAACGAGCGCCAGCAGCAGTTGGGCCTGGTGGCCGATGACGGCAACGCCTGG
CTGGCGGGCGTAAAAGCCGGGGAAACATTGAAAGTCTTCTGGGATGGCGCGGCGCAGTGT
GAAGCATCACTCCCGTCCACGTTTACACCGGAGCTATTGGCCAACGCGCTATTGCTGCCG
TGCAAAATGCTGGAAGGTCAGCCCCCCACCGCACCGCAGAAAAGTTCTCCGCTGCCTGCG
CAACCGCTAATCCAGGAACATACGCAAACCGATGGCCAACCGGCCGCGCCGGTGGCGACA
ACCACTCAAACCCCGCCCATACCGCTGGCTGACAACCATGCGGTGAATCGCAAGGATATG
GAATAA
>EECEBICD_00511 Major MR/P fimbria protein
ATGAAGTCATCTCATTTTTGTAAACTGGCAGTAACTGCATCTTTAGTTATGGGAATTGTC
TCCGGCGCTCAAGCCGCGGGTAGCAACACAGCGAAGGTCACTTTCCTTGGTAATATTGTT
GACTCCCCCTGCTCCGTCACATTGGATACGGAAGATCAAACAGTCAATATGGGTTCAAGT
ATCGGTAATGGCACGCTGAGTAATGGTAAAACGACCATCAACAATGCCCGTACCTTTCAT
ATCGATCTTGAGGGTTGTACCTGGGCTACCGAGAAAAATATGAATGTGGTATTCACTACA
GGTAGTGGAACCACAGCGGCTACAGGCGCCACGGATAATCTCGCGCTGATGAAGACTGAC
GGCACTGGCGCTATTAGCAACGTAAGCCTGGCAATCGGCGATGCTGGCAAAAACAATATC
AAACTGGGCGATACCTATACACAGGCCATTGCGGACCTGGACGGAGATACCATTCTTGAT
GAGAAGCAAAGCCTGAATTTCACCGCCTGGCTGGTTGGCGCAGCAACCGGTACCGTGGGC
ACAGGTGAATTCAGCAGCGCCGCCAACGTCACTATCTCTTACCTGTAA
>EECEBICD_00512 Nucleoside triphosphate pyrophosphohydrolase
ATGACAACTAACCATCAAATTGACCGTCTTCTCACCCTCATGCAGCGCCTGCGCGACCCG
GAAAACGGCTGCCCGTGGGATAAAGAGCAGACCTTCGCCAGCATCGCGCCTTATACGCTG
GAAGAAACCTATGAAGTACTGGATGCGATTGCGCGTGAGGATTTTGACGATTTGCGCGGC
GAACTGGGCGACCTGTTGTTCCAGGTGGTGTTTTACGCGCAGATGGCGCAGGAAGAAGGC
CGCTTCGATTTTAATGATATCTGCGCTGCCATCAGCGACAAGCTGGAGCGCCGCCATCCG
CATGTTTTCGGCGAGCTTTCCGCGGATAATAGCGAAGAAGCATTAGTTCGCTGGGAGCAA
ATCAAAACGGAAGAGCGCGCGCAAAAAGCGCAGCATTCCGCGCTGGACGATATTCCGCGC
AGCTTACCGGCCTTGATGCGCGCGCAAAAAATCCAGAAACGTTGCTCAAACGTGGGCTTT
GACTGGACGACGCTGGGACCGGTCGTCGATAAGGTCTATGAAGAGATCGATGAGGTGATG
TTCGAGGCGCGGCAGGCGGTTGTTGATCAGGCTAAACTGGAAGAGGAAATGGGCGATTTG
CTCTTTGCCACGGTCAACATGGCGCGTCATTTAGGTACGAAAGCGGAGCTCGCTTTGCAA
AAAGCGAACGATAAATTTGAACGTCGTTTTCGCGAAGTTGAACGTATTGTCGCAGCCAGA
GGCCTGGAAATGACCGGGGTAGACCTGGAAACAATGGAAGAAGTTTGGCAGGAAGTAAAA
CGCCAGGAAATTGATCTCTAA

>EECEBICD_00513 CTP synthase
ATGACAACGAACTATATTTTTGTGACCGGCGGGGTCGTATCCTCTCTGGGTAAAGGCATT
GCCGCAGCCTCCCTCGCAGCCATTCTGGAAGCCCGTGGTCTCAACGTGACCATCATGAAA
CTGGATCCGTACATCAACGTCGATCCGGGTACTATGAGCCCAATCCAACACGGGGAAGTG
TTCGTTACTGAAGACGGCGCTGAAACCGACCTGGACTTGGGGCACTACGAGCGTTTCATC
CGCACCAAGATGTCTCGCCGCAACAACTTCACGACTGGCCGCATCTACTCCGACGTTCTG
CGTAAAGAACGCCGTGGCGACTATCTGGGCGCAACCGTACAGGTAATCCCTCACATCACT
AACGCGATTAAAGAGCGCGTGCTGGAAGGTGGCGAAGGCCACGATGTGGTACTGGTGGAA
ATCGGCGGTACCGTCGGTGATATCGAATCGCTGCCGTTTCTTGAGGCGATTCGTCAATTG
GCGGTAGATATCGGTCGTGAACACGCGCTGTTTATGCACCTGACGCTGGTACCTTACCTG
GCGGCTGCGGGCGAAGTGAAAACTAAACCGACTCAGCACTCCGTGAAAGAGCTGCTGTCT
ATCGGTATTCAGCCCGATATTCTGATTTGTCGTTCCGACCGTGCGGTTCCTGCCAACGAG
CGTGCAAAAATTGCATTGTTCTGTAATGTGCCGGAAAAAGCCGTTATTTCAATGAAAGAT
GTCGATTCCATTTATAAAATTCCAGGCCTGTTGAAATCTCAGGGGCTTGATGATTATATT
TGTAAACGATTCAGCTTGAACTGTCCGGAAGCTAACCTGTCTGAATGGGAACAGGTCATT
TACGAAGAAGCGAACCCGGCAGGCGAAGTGACTATCGGCATGGTCGGCAAATATATTGAA
CTGCCGGATGCCTATAAGTCGGTGATCGAAGCGCTGAAACACGGTGGTCTGAAAAACCGC
GTTACCGTCAACATCAAATTGATCGATTCGCAAGATGTTGAAACGCGCGGCGTCGAAATT
CTGAAAGATTTGGACGCTATCCTGATCCCCGGCGGCTTCGGCTATCGTGGCGTTGAAGGT
AAAATCGCCACTGCGCGCTATGCGCGTGAAAACAATATTCCTTATCTGGGCATTTGCCTG
GGGATGCAGGTTGCGTTGATTGAGTTCGCTCGTAACGTGGCCGGCATGGACAACGCCAAC
TCAACGGAATTTGTGCCAGACTGTAAGTACCCGGTTGTGGCGTTAATTACCGAGTGGCGC
GATGAAGACGGTAACGTTGAAGTCCGTAGCGAGAAAAGCGATCTGGGCGGCACCATGCGC
CTGGGCGCGCAGCAGTGCCAGCTTAGCGATGACAGTCTGGTACGTCAGTTGTACGGCGCG
TCAACGATTGTTGAACGTCATCGCCATCGTTACGAAGTCAACAATATGTTGTTGAAACAA
ATTGAAGCAGCGGGTCTGCGTGTCGCAGGCCGTTCCGGTGATGATCAGTTAGTCGAGATC
ATTGAGGTACCGAATCATCCGTGGTTCGTGGCCTGTCAGTTCCATCCGGAATTTACTTCC
ACGCCGCGTGATGGACATCCGTTGTTCGCTGGCTTTGTTAAAGCCGCCAACGAGCATCAG
AAACGTCAGGCGAAGTAA
>EECEBICD_00514 Enolase
ATGTCCAAAATCGTTAAAGTCATCGGTCGTGAAATCATCGACTCCCGTGGTAACCCGACT
GTTGAAGCTGAAGTACACCTGGAAGGTGGTTTCGTAGGTATGGCGGCGGCTCCGTCAGGT
GCTTCTACTGGTTCCCGCGAAGCGCTGGAACTGCGCGATGGCGACAAATCCCGTTTCCTG
GGTAAAGGCGTAACCAAAGCTGTTGGCGCGGTTAACGGCCCGATCGCTCAGGCTATTCTT
GGCAAAGACGCTAAAGACCAGGCTGGCATCGACAAAATCATGATCGACCTGGACGGTACT
GAAAATAAATCTAACTTCGGTGCAAACGCCATTCTGGCTGTCTCTCTGGCTAACGCCAAA
GCTGCTGCTGCCGCTAAAGGTATGCCGCTGTACGAGCACATTGCTGAACTGAATGGCACG
CCGGGCAAATACTCCATGCCGGTTCCGATGATGAACATCATCAACGGCGGCGAGCACGCT
GACAACAACGTCGACATCCAGGAATTCATGATCCAGCCGGTTGGCGCGAAAACGGTTAAA
GAAGCCATCCGTATGGGTTCTGAAGTTTTCCATCACCTGGCAAAAGTGCTGAAAGGCAAA
GGCATGAACACCGCTGTGGGTGACGAAGGCGGCTATGCGCCGAACCTGGGTTCCAACGCT
GAAGCGCTGGCTGTTATCGCTGAAGCGGTTAAAGCGGCTGGTTACGAGCTGGGTAAAGAC
ATCACCCTGGCGATGGACTGCGCAGCATCTGAATTCTACAAAGACGGTAAATACGTTCTG
GCTGGCGAAGGCAACAAAGCGTTCACCTCCGAAGAATTCACCCACTTCCTGGAAGAACTG
ACCAAACAGTACCCGATCGTTTCCATCGAAGATGGTCTGGACGAGTCTGACTGGGACGGT
TTTGCATACCAGACCAAAGTACTGGGCGACAAAATCCAGCTGGTTGGTGACGACCTGTTT
GTAACCAACACCAAAATCCTGAAAGAAGGCATCGAGAAAGGCATCGCTAACTCCATCCTG
ATCAAATTCAACCAGATCGGTTCTCTGACCGAAACGCTGGCTGCAATCAAGATGGCGAAA
GATGCTGGCTACACTGCTGTCATCTCTCACCGTTCTGGTGAAACTGAAGACGCTACCATT
GCTGACCTGGCTGTTGGTACCGCTGCAGGCCAGATCAAAACCGGTTCTATGAGCCGTTCT
GACCGCGTTGCTAAATACAACCAACTGATTCGTATCGAAGAAGCTCTGGGCGAAAAAGCA
CCGTACAACGGTCGTAAAGAGATCAAAGGCCAGGCGTAA
>EECEBICD_00515 7-carboxy-7-deazaguanine synthase
ATGCAGTACCCGATTAACGAGATGTTCCAGACTCTACAAGGCGAGGGTTATTTTACCGGC

GTCCCCGCTATTTTTATTCGTTTACAGGGATGTCCGGTCGGCTGTGCCTGGTGTGATACT
AAGCATACCTGGGATAAACTGAGCGATCGGGAAGTGTCTCTGTTCAGCATTCTGGCAAAA
ACCAAAGAGAGCGACAAATGGGGTGCTGCGAGCAGTGAAGACTTGCTGGCAGTAATTAAC
CGTCAGGGCTATACCGCGCGTCACGTTGTCATCACCGGTGGCGAACCCTGTATTCACGAC
CTCATGCCGCTCACCGATCTGCTTGAAAAGAGTGGTTTTAGTTGCCAGATTGAAACCAGC
GGGACGCATGAAGTGCGCTGTACGCCTAACACCTGGGTCACGGTTTCACCAAAAGTGAAT
ATGCGCGGGGGATATGACGTGCTTTCCCAGGCGCTGGAGCGCGCCAATGAAATCAAGCAT
CCCGTTGGGCGAGTTCGTGATATTGAGGCGCTGGATGAACTGTTAGCCACGTTGAGCGAT
GATAAACCGCGCGTGATTGCTTTGCAGCCCATTAGCCAGAAAGAAGACGCGACGCGTCTG
TGTATTGAAACGTGTATTGCACGTAACTGGCGGCTGTCTATGCAAACGCATAAATATTTA
AATATCGCCTGA
>EECEBICD_00516 hypothetical protein
ATGGCGTTAAGGATCAGGGTATTGCTCGAAAATCATAAGGGAGCTGGAGCGGATAAATCG
TTGAAGGCCCGGCCAGGATTAAGCCTGTTAGTCGAGGATGAGTCTACGTCGATCTTGTTC
GATACCGGCCCTGATGGCAGTTTTATGCAAAACGCGTTGGCGATGGGGATCGACCTGTCC
GATGTGTCTGCTGTGGTGCTTTCGCATGGTCATTACGATCATTGCGGCGGCGTGCCGTGG
CTTCCTGATAACAGTCGAATCATCTGCCATCCCGATATTGCGCGCGAACGTTATGCGGCA
ATGACTTTTCTCGGTATTACCCGAAAAATAAAAAAATTGTCGTGTGAGGTGGACTATTCA
CGCTACCGAATGATGTACACGCGTGACCCCCTGCCGATTGGCAAAAACTTTATCTGGTCA
GGGGAAATACCGGTGGTTGCGCCTGAAGCCTACGGCATTTTTGGCGGCCACGATGCGGAG
CCGGACTCCATTTTGGATGAGGGCGTACTGATTTACCAATCGACAAAAGGATTAGTCATC
ATTACGGGATGCGGGCATCGGGGGATCGCAAATATTGTCCGGCACTGTCAGAACATTACC
GGTATAAAGCGGATTTACGCGCTTGTCGGCGGATTTCATCTCCGCTGCGCATCACCGTTC
ACGCTGTGGCGAGTCAGGCGCTTTCTGCAAGAACAAAAACCTGAAAAACTATGCGGTTGT
CACTGTACAGGGGCCTGGGGGCGGTTGTGGCTACCGGAAATAACCGCACCTGCAACCGGT
GATGTGCTGCGCTTTTAA
>EECEBICD_00517 6-carboxy-5,6,7,8-tetrahydropterin synthase
ATGTCCACCACGTTGTATAAAGATTTCACCTTTGAAGCCGCTCACCGCCTGCCTCACGTG
CCTGAAGGGCATAAGTGCGGCCGCCTGCACGGACACTCGTTTATGGTGCGTCTCGAAATT
ACCGGTGAAGTCGATCCGCATACCGGTTGGATTATGGATTTCGCGGATCTCAAAGCGGCG
TTTAAACCGACTTACGATCGTCTTGATCACTACTATCTGAATGATATTCCAGGGCTGTCC
AATCCCACCAGCGAAGTGCTGGCAAAATGGATTTGGGATCAGGTTAAGCCCGTCGTGCCG
CTACTGAGCGCCGTGATGGTGAAAGAGACCTGTACAGCCGGCTGCGTCTATCGCGGCGAA
TAA
>EECEBICD_00518 Sulfite reductase [NADPH] flavoprotein alpha-component
ATGACGACACCGGCTCCACTGACTGGGTTGCTTCCGCTTAATCCGGAGCAACTGGCGCGC
CTTCAGGCCGCGACAACCGACCTTACTCCCGAACAGCTTGCCTGGGTCTCCGGCTATTTT
TGGGGCGTACTGAACCCGCGCTCAGGCGTTGTTGCCGTCACGCCTGTTCCTGAGCGGAAG
ATGCCGGGGGTTACGCTTATCTCCGCTTCGCAAACCGGCAATGCGCGTCGTGTGGCGGAA
GCGCTGCGTGATGATCTGCTGGCGGCCAACCTTAATGTGACACTGGTGAACGCCGGCGAC
TACAAATTCAAACAGATTGCCAGTGAAAAACTGTTGGTGATAGTGACGTCCACCCAGGGA
GAAGGCGAGCCGCCGGAAGAGGCCGTCGCGCTGCATAAATTCCTGTTCTCCAAAAAAGCG
CCGAAACTGGAAAACACCGCTTTTGCCGTGTTTAGCCTCGGCGACACCTCTTATGAATTT
TTCTGCCAGTCCGGAAAAGATTTCGACAGCAAACTGGCGGAACTGGGCGGTGAACGTCTG
CTCGACCGTGTCGATGCTGACGTTGAATATCAGGCAGCTGCCAGCGAATGGCGCGCCCGC
GTGGTTGATGTTCTGAAGTCTCGCGCACCTGTCGCGGCACCTTCGCAATCCGTCGCTACT
GGCGCGGTAAACGACATCCACACCAGCCCCTATACTAAAGACGCGCCGCTGATCGCGACC
CTCTCCGTGAATCAGAAAATCACCGGTCGCAACTCGGAAAAAGATGTGCGTCATATTGAG
ATTGATTTAGGCGACTCCGGCCTGCGCTATCAGCCGGGCGATGCGCTGGGCGTCTGGTAT
CAGAACGATCCGGCTCTGGTGAAGGAGCTGGTTGAGTTGCTGTGGCTGAAAGGCGATGAG
CCGGTGACGGTTGATGGTAAAACCCTGCCGCTCGCCGAAGCGCTGGAGTGGCACTTCGAG
CTGACGGTGAATACCGCCAACATTGTTGAGAACTACGCGACCCTGACGCGCAGTGAGTCG
CTGTTGCCATTAGTGGGCGACAAAGCGCAGCTCCAGCACTACGCCGCGACCACGCCCATT
GTTGATATGGTGCGTTTCTCTCCGGCGCAGCTCGACGCTGAAGCATTAATCGGCCTGCTG

CGTCCGCTGACGCCGCGTCTGTACTCCATCGCCTCCGCGCAGGCGGAAGTCGAAAGCGAA
GTACACGTAACCGTCGGCGTCGTGCGCTACGATATTGAAGGCCGCGCCCGCGCGGGTGGT
GCTTCCAGCTTCCTCGCCGACCGCGTGGAAGAAGAGGGCGAAGTGCGCGTCTTTATTGAA
CACAACGATAATTTCCGCCTGCCAGCGAATCCGCAGACTCCGGTGATTATGATTGGCCCA
GGTACGGGTATCGCGCCGTTCCGCGCCTTTATGCAGCAACGCGCCGCCGACGGTGCGGAA
GGCAAGAACTGGCTGTTCTTCGGCAACCCGCACTTTACCGAAGATTTCCTCTATCAGGTG
GAGTGGCAGCGCTACGTGAAAGAAGGCGTGCTGAGCCGGATCGATCTGGCCTGGTCCCGC
GATCAAAAAGAAAAAATTTACGTACAAGACAAGCTGCGTGAACAGGGTGCCGAGCTGTGG
TGCTGGATCAATGATGGCGCGCATATTTATGTCTGCGGCGATGCCAGGCGCATGGCCGCA
GATGTCGAAAAAGCATTGCTGGAAGTGATCGCTGAATTCGGCGGTATGGACCTCGAATCG
GCAGATGAATATTTAAGTGAGCTGCGCGTCGAGCGCCGTTATCAGCGAGATGTCTACTAA
>EECEBICD_00519 Sulfite reductase [NADPH] hemoprotein beta-component
ATGAGCGAAAAACATCCAGGCCCACTGGTGGTCGAAGGTAAACTGTCCGATGCCGAGCGC
ATGAAGCTGGAAAGCAACTATTTGCGCGGTACCATTGCGGAAGATCTGAACGACGGTCTC
ACCGGTGGTTTTAAAGGCGATAACTTCCTGCTGATCCGCTTCCACGGCATGTACCAGCAG
GATGACCGTGACATTCGTGCCGAGCGCGCCGAGCAGAAGCTGGAGCCGCGTCACGCGATG
CTGCTGCGCTGCCGTCTGCCGGGCGGGGTGATTACTACGACGCAATGGCAAGCGATCGAC
AAATTCGCGGCGGATAACACGATTTATGGCAGTATCCGTCTGACCAACCGCCAGACCTTC
CAGTTTCACGGCATTCTGAAGAAGAACGTCAAGCCGGTGCACCAGATGCTGCACTCGGTG
GGGCTGGACGCGCTGGCGACCGCTAACGATATGAACCGTAACGTGCTGTGCACCTCCAAT
CCCTACGAGTCTCAGCTGCACGCTGAAGCCTACGAGTGGGCGAAGAAGATCTCCGAACAT
CTGCTGCCGCGCACCCGTGCGTATGCGGAGATCTGGCTCGATCAGGAGAAGGTCGCGATC
ACCGATGAAGAACCGATCCTCGGGCAGACCTATCTGCCGCGTAAGTTCAAAACCACGGTA
GTGATCCCGCCGCAAAACGATATCGATCTGCACGCCAACGACATGAACTTCGTGGCGATT
GCCGAAAACGGCAAGTTGGTCGGCTTTAACCTGCTGGTGGGCGGCGGTCTGTCTATTGAG
CATGGTAATAAGAAAACCTACGCTCGTACGGCCAGCGAGTTTGGCTATCTGCCGCTGGAG
CACACCCTGGCGGTGGCCGAAGCGGTAGTGACGACGCAGCGCGACTGGGGTAACCGTACC
GACAGGAAAAATGCCAAAACCAAATACACCCTTGAGCGCGTGGGACTGGAGACATTTAAA
GCGGAAGTCGAGCGTCGCGCGGGGATCAAATTTGAGCCGATCCGCCCGTATGAGTTTACC
GGTCGCGGCGATCGTATCGGTTGGGTGAAAGGGATCGACAACAACTGGCACCTGACGCTG
TTTATCGAAAACGGTCGTATCCTGGATTATCCGGGGCGCCCGCTGAAAACCGGCCTGCTG
GAGATCGCGAAGATTCATCAGGGCGAGTTCCGTATTACCGCTAACCAGAACCTGATTATT
GCCAGCGTGCCGGAAAGCCAGAAAGCGAAGATCGAGACGCTGGCGCGCGACCACGGGTTG
ATGAATGCGGTGAGCGCGCAGCGTGAAAACTCAATGGCCTGTGTATCGTTCCCGACCTGC
CCGCTGGCGATGGCGGAAGCCGAGCGTTTCCTGCCGTCGTTCACGGACAAAGTGGAAGCG
ATTCTGGAAAAACACGGCATTCCTGATGAGCATATCGTGATGCGCGTGACCGGCTGCCCG
AACGGCTGTGGTCGCGCGATGCTGGCGGAAATTGGCTTAGTAGGGAAAGCGCCTGGTCGT
TATAACCTGCATCTTGGTGGAAACCGTATTGGGACACGTATCCCACGGATGTATCAAGAA
AATATCACCGAGCCGGATATTCTGGCCTCGCTGGATGAACTGATTGGGCGCTGGGCGAAA
GAGCGTGAAGCGGGTGAAGGCTTCGGCGACTTTACGGTGCGTGCGGGCATCATTCGCCCG
GTGCTCGATCCCGCGCGTGATTTCTGGGAATAA
>EECEBICD_00520 Phosphoadenosine phosphosulfate reductase
ATGTCCAAACTCGATCTAAATGCCCTGAATGAACTGCCAAAAGTCGATCGCGTACTGGCG
TTGGCGGAAACCAACGCTCAACTGGAAACGCTGACTGCCGAAGAACGCGTGGCTTGGGCG
CTGGAAAATCTGCCCGGCGAATACGTGCTTTCTTCAAGCTTTGGCATCCAGGCGGCGGTG
AGCCTGCACCTGGTGAATCAGATTCGCCCGGATATCCCGGTGATCCTCACCGATACCGGC
TATTTGTTCCCGGAAACCTATCAGTTTATTGATGAGCTAACGGACAAACTCAAACTCAAC
CTGAAAGTTTACCGCGCGGGCGAAAGTCCGGCCTGGCAGGAAGCGCGCTACGGCAAGCTG
TGGGAGCAGGGCGTTGAGGGCATTGAGAAATACAATGAGATCAACAAAGTCGAGCCGATG
AACCGGGCGCTAAAAGAGCTGAAAGCGCAGACGTGGTTTGCGGGTCTGCGTCGCGAGCAG
TCCGGCAGCCGCGCGCATTTACCTGTTCTCGCTATCCAACGCGGCGTGTTTAAAGTGCTA
CCGATTATCGACTGGGATAACCGCACGGTTTACCAGTATCTGCAAAAGCACGGTCTGAAA
TACCATCCGCTGTGGGATCAGGGCTATCTGTCGGTGGGCGATACGCACACCACCCGCAAA
TGGGAGCCGGGTATGGCGGAAGAAGAGACACGCTTCTTCGGACTGAAGCGCGAGTGCGGG

CTGCATGAAGGGTGA
>EECEBICD_00521 Secreted effector protein SopD
ATGCCAGTCACTTTAAGCTTCGGTAATCATCAAAATTATACGCTTAATGAAAGTCGGCTT
GCTCATCTGTTAAGCGCAGATAAAGAAAAAGCAATCCATATGGGGGGTTGGGATAAAGTC
CAGGATCATTTTAGAGCGGAAAAAAAGGACCATGCGCTGGAAGTGTTACACTCCATCATT
CACGGGCAGGGCCGTGGTGAACCTGGAGAGATGGAAGTTAACGTAGAGGATATAAATAAA
ATTTACGCCTTTAAACGGCTACAACATCTGGCCTGCCCGGCTCATCAAGATCTGTTTACT
ATCAAGATGGACGCTTCTCAGACACAATTTTTACTTATGGTCGGCGACACGGTGATCAGC
CAAAGCAATATTAAGGACATCCTCAATATATCAGATGATGCAGTGATAGAATCTATGAGT
AGAGAGGAGAGACAGTTATTTTTACAAATATGCGAGGTGATTGGTGCGAAGATGACCTGG
CACCCGGAATTACTTCAGGAGTCGATTTCTACACTACGAAAAGAAGTGACGGGCAATGCA
CAAATCAAAGCGGCGGTTTATGAGATGATGCGTCCCGCAGAGGCTCCAGACCACCCGCTT
GTCGAATGGCAGGACTCGCTTACTGAAGATGAAAAATCGATGCTGGCCTGTATTAATGCA
GGTAACTTTGAGCCTACAACTCAGTTTTGCAAAATAGGTTATCAGGAGGTACAAGGTGAA
GTCGCTTTTAGCATGATGCATCCGTGTATCTCCTATTTACTACATACCTATTCGCCGTTT
GCAGAGTTTAAACCAACAAATTCTGGTTTTTTAAAAAAACTTAATCAGGATTATAACGAC
TATCATGCAAAAAAAAATGTTTATTGATGTAATTCTTGAAAAGCTATATCTAACCCATGAA
CGTTCTTTGCACATTGGTAAAGATGGGTGCAGTCGTAATATATTACTGACATAA
>EECEBICD_00522 CRISPR-associated endonuclease/helicase Cas3
GTGTCGATATATCACTATTGGGGAAAGTCTCGAAGAGGAGAAACTGACGGCGGTGATGAT
TACCATTTGCTTTGCTGGCATTCTTTAGATGTTGCGGCTGTGGGTTACTGGATGGTGATA
AATAATATTTATTTTATTGACCACTATCTAAAAAAATTAGGCATCCAGGATAAGGAGCAG
GCGGCGCAATTTTTTGCCTGGATTTTATGTTGGCATGATATTGGAAAGTTTGCTCATTCC
TTCCAGCAACTATACCGTCATGAGGCTTTAAATATCTTTAATGAGCCTACACGGCATTAT
GAAAAAATCGCGCATACCACGCTGGGATACATGTTGTGGAACTCCTGGCTAAGTGAATGC
CCTGAATTGTTTCCTCCTTCTTCGCTTTCAGTTCGTAAAAGTAAGCGCGTTATGGCGCTT
TGGATGCCAGTCACTACAGGTCATCATGGACGCCCTCCAGAGGCAATCCAGGAGCTGGAC
CATTTTCGCCAGCAGGATAAAGACGCGGCAAGAGATTTTCTTCTGAGAATAAAAGCGCTC
TTTCCTTTAATTACTTTGCCTGAAGCCTGGGATGAAGATGAGGGTATCGACCAATTTCAG
CAACTTTCCTGGTTTATTTCCGCTGCGGTTGTACTGGCTGACTGGACTGGTTCTGCCAGC
CGTTATTTTCCGCGTACTGCGGAAAAAATGCCTGTTGATACCTACTGGCAGCAAGCTCTC
GCTAAAGCACAAACTGCCATCACGCTATTTCCCTCAGCGGCGAATGTGTCTGCCTTTACG
GGCATAGAAACGCTTTTCCCTTTTATTCAGCATCCCACACCGTTACAACAAAAGGCGCTT
GAGCTGGATATCAACGTGGATGGCGCCCAACTCTTTATTCTTGAAGATGTCACCGGGGCC
GGAAAAACAGAGGCGGCGCTCATATTAGCTCATCGACTGATGGCGGCAGGTAAAGCGCAG
GGACTCTATTTTGGACTGCCGACAATGGCGACAGCCAACGCGATGTTTGAACGTATGGCG
AACACCTGGCTGGCGCTGTATCAGCCGGACTCCCGTCCCAGCCTGATTCTGGCGCATAGC
GCGCGTCGCTTAATGGATCGTTTCAATCAGTCAATATGGTCGGTCACTCTTTCTGGTACG
GAAGAACCCGATGAAGCGCAGCCTTATAGTCAGGGATGCGCCGCCTGGTTTGCCGACAGC
AATAAAAAAGCGTTGTTGGCGGAGGTTGGCGTAGGCACGTTGGATCAGGCGATGATGGCG
GTAATGCCATTTAAACATAACAACCTGCGGTTACTGGGTCTTAGCAACAAGATCTTACTG
GCTGATGAGATCCATGCCTGTGATGCCTGGATGTCCCGAATACTTGAAGGTTTGATCGAA
CGGCAGGCCAGTAATGGCAACGCCACTATTCTGTTATCTGCGACGCTATCGCAGCAGCAG
CGAGATAAGCTGGTGGCGGCATTTTCCCGTGGGGTGAGGCGTAGTGTGCAGGCGCCGTTG
CTAGGCCATGACGATTATCCCTGGCTGACTCAGGTCACACAAACAGAGCTGATTTCTCAG
CGGGTTGATACACGCAAAGAGGTTGAGCGTTGCGTAGATATTGGCTGGCTACATAGTGAA
GAGGCGTGTCTTGAACGTATAGGTGAAGCAGTGGAAAAAGGAAACTGTATCGCCTGGATA
CGTAACTCCGTTGATGATGCGATTCGTATCTATCGCCAGCTTCAACTGAGTAAGGTCGTC
GCCACGGAAAACCTTTTACTCTTCCATAGTCGCTTTGCTTTTCACGATCGTCAGCGGATT
GAGTCGCAGACGCTGAATCTCTTTGGCAAACAGAGCGGCGCGCAACGTGCCGGTAAGGTC
ATTATCGCCACGCAGGTAATCGAACAAAGTCTGGATATTGACTGCGATGAGATGATCTCT
GATTTAGCGCCGGTGGATTTATTAATTCAGCGGGCCGGTCGACTACAGCGTCATATTCGC
GATCGTAACGGTCTGGTGAAAAAGAGTGGGCAGGATGAGCGAGAGACGCCAGTGCTGCGC
ATTCTTGCTCCGGAGTGGGATGACGCGCCGCGAGAGAACTGGTTATCCAGCGCCATGCGT

AACAGCGCCTATGTCTATCCCGATCATGGGCGCATGTGGCTGACGCAGCGCATATTACGT
GAGCAGGGGACGATTCGGATGCCGCAATCTGCCCGCTTGTTGATTGAGTCGGTCTACGGT
GAGGATGTCAACATGCCGGTTGGATTTGCAAAAACCGAGCAATTGCAGGAAGGCAAATTT
TATTGCGACCGGGCATTTGCCGGCCAGATGCTGCTTAACTTTGCGCCGGGCTACTGTGCT
GAAATTAGCGATTCTTTACCGGAGAAAATGTCAACGCGGCTGGCGGAAGAGTCTGTCACG
CTGTGGCTGGCGAAAATCGTGGATAGCGTCGTAACCCCTTATGCCAGCGGTGAACACGCC
TGGGAGATGAGCGTGCTGCGAGTACGTCAGAGCTGGTGGAATAAACATAAAGACGAGTTT
GAAAAATTAGACGGCGAACCCTTGCGTAAGTGGTGTGCGCAACAGCATCAGGATAAGGAT
TTTGCCACGGTGATTGTGGTGACGGACTTTGCCGCTTGTGGTTATTCGGCGAATGAGGGA
TTGATTGGCATGATGGGGGAATAA
>EECEBICD_00523 hypothetical protein
ATGGACAATTTTTCACTTTTAACAACGCCCTGGCTCCCCGTCCGTTTCAAAGACGGTTCC
ACGGGCAAGCTGGCCCCCGTCGATCTGGCGGATGAAAACGTGGTGGACATCGCCGCAACG
CGAGCAGATTTACAGGGAGCGGCCTGGCAGTTTCTGTTGGGATTGCTGCAATGCAGTATC
GCGCCGAAAAGATACAAAAATTGGGAGGATATCTGGTTTGATGGATTGCATGCCGATGTG
CTCCATAAGGCATTAGCACCGTTAGAACACGCTTTTCAGTTTGGCGCGGAATCCCCCTCG
TTTATGCAGGATTTTGAACCGTTAAGCGGCGAAAAAGTCTCTATTGCCTCATTGTTGCCG
GAAATACCTGGCGCGCAAACCACGAAGTTCAATAAAGATCATTTTGTCAAACGCGGCGTA
ACGGAACGTTTTTGTCCGCACTGCGCGGCGCTGGCGCTGTTCTCGTTGCAGCTTAACGCG
CCTGCGGGCGGCAAAGGCTATCGTACCGGGCTGCGCGGCGGCGGGCCACTGACCACGCTG
GTTGAATTGCAGGAATATCAGGGCGAGCGGCAAACGCCGATCTGGCGCAAGCTGTGGCTC
AACGTGATGCCGCAGGATACTGCGGATCTGCCTTTACCAGACCAGTGTGATGCGACCGTT
TTCCCGTGGCTTGCCGCGACGCGGACCAGCGAGCAGGCGAATGCCGTTACCACGCCGGAG
CAGGTCAATAAACTCCAGGCGTACTGGGGGATGCCGCGTCGTATCCGCCTGGATTTTGCC
ACCTTACAGTCAGGTTGCTGCGATATTTGCGGCGCTGAAAGCGATGAGCTTCTTGGCTTT
ATGACCGTCAAGAACTACGGCGTTAACTACGATGGCTGGCGGCACCCGCTGACGCCTTAT
CGCGCCCCGGTAAAAGATCAAAACGCCTTCTTTTCCGTTAAACCGCAGCCCGGCGGCCTT
ATCTGGCGCGACTGGCTGGGATTAAGTCAGAACAACCAGACGGAAGCGAATTACGAATCT
CCCGCGCAGGTAGTCAAGGTGTTTAACGCCCGCTCGCTGACTGACGTTAAAGCGGGGATC
CGGGGCTTTGGCGCGGATTTCGACAATATGAAAATCCGCTGCTGGTATGAGCATCACTTC
CCGTTGCTGATGACGGAAGGTCTGATCCCTGATTTACGTAAGGCCGTGCAAACTGCGGCC
CGCCTGTTGAGCCTGCTTCGCAGTGCGCTAAAAGAAGCGTGGTTCACCAATGCGAAGGAT
GCGCGGGGTGATTTCAGTTTTATCGACATTGATTTCTGGAACCTGACGCAGGGGCGCTTT
CTCAATCTGATCCACGATCTGGAAAACGGACACAAGCCGGACGAAAGGCTGAATAAATGG
CAAAGAGAACTTTGGCTGTTTACCCGTTGTTACTTCGATGATCACGTCTTTACCAACCCC
TACGAGAGCAGCGATCTGGAGCGCATCATGAAGGCGCGCAAAAAATATTTTACTTCATCG
GCGGAAAAGCAAAGCGCAAAAGCCGCCAAAGCAAAGAAACAGGAGGCTGCTGAATGA
>EECEBICD_00524 hypothetical protein
ATGAGTGTTGTGACGAAAGATGATAAAGCCACTTTGCGTCAGTGGCATGATGAACTACAA
GAAAAACGTGGATTACGTGCCAGCTTACGACGTAGTAAAACGGTGAATGATGCCTGTTTG
GCTGAGGGGCTTCATTCATTGTTAATGCAAACACATTCTTTATGGAAAAACAAAGCACCC
TGGAATGTTACTGCATTGGCAATCACAGCGGCACTGGCTGCACATATCAAATTTATTGAT
GAGCAAAAGAGTTTTGCTGCGCAGCTTGGACAGAAGAAAGGTGGCGACACTCCGGTGATG
TCAAAATTACGCTTCTCCCACCTATTAGCGGTAAAAACGCCGGATGAACTGCTGCGCCAG
TTACGGCGTGCGGTGAAGCTGTTAGATGGTTCGGTCAACCTGTTTTCTCTTGCAGACGAT
ATTTTCTGCTGGTGCCAGGAGCAGAATGACTTGCTGAACCACCATCGCCGCCAGCAGCGG
CCAACCGAATTTCTTCGTATCCGCTGGGCGCTGGAATACTACCAGGCTGGCGATACCGAT
AACGAACAAGATTAA
>EECEBICD_00525 CRISPR system Cascade subunit CasC
ATGACAACGTTTATTCAGCTCCACTTACTGACCGCTTACCCCGCCGCTAACCTGAACCGT
GATGATACCGGTGCGCCAAAAACCGTGGTGCTTGGCGGAGCAACACGTCTGCGTATCTCC
TCTCAGAGCCTGAAACGCGCCTGGCGTACATCTGAGTTATTTGAACAGGCATTAGCTGGC
CATATTGGTATTCGTACTGGTCGCATTGCTCGTGAGGCGGCGCAAATCCTCGTTGATAGC
GGCATTGACGCCAAAAAAGCGGTTGAGTACGTCAAGAACATCGCCAACTGCTTTGGCAAG

GTAAAAGAGGATAAGAAACCCAAAGATGAGTTGACGAATGCTGAAACCGAGCAACTGGTG
CATATCAGCCCTGCTGAGTTTGAGGCCGTGAAAGCGCTGGCGCGCCGTCTGGCAGAAGAA
AAACGTCCGGCAACAGAAGAGGAAGCGGAACTGTTACGTCACGATCGCATGGCCGTCGAT
ATTGCCATGTTTGGCCGGATGTTAGCGAAGAAAACTGATTTTAACGTGGAAGCCGCCTGC
CAGGTCGCCCACGCCTTCGGCGTCAGCGAAACGATCATCGAAGACGATTTCTTTACCGCT
GTGGATGACCTACGCCAGGCATCGGCAGAAGATGCAGGCGCAGGCCATCTCGGCGAAACC
GGCTTTGGCTCCGCGCTGTTTTACACCTATATCTGCATCGACAAAGATCTGCTGGTGAAA
AACCTGAACGGCAATGAAGAACTGGCAAACAAAACGCTGCGCGCCTTTACTGAAGCGGCG
CTGAAAGTGTCGCCGACCGGCAAACAGAACAGCTTTGCCAGCCGTGCCTATGCCTCGTGG
GCGCTGGCCGAAAAAGGCACCGACCAACCACGTTCACTGGCGGCCGCGTTTTATGAACCG
ATCAACGGTACAGACCAATTGAACGTTGCGGTTAAGCGTATTACCGCGCTGCATGAAAAT
ATGAATGAGGTCTATGCACAGGAGACGGCGTTCAAAAACTTTAACGTGATGAATCAGCAG
GGAAGCATGAAAGACGTGCTTGACTTCATCTGCGCGTAA
>EECEBICD_00526 hypothetical protein
ATGAGCCAATATCTGGTTTTTCAGCTTCATGGGCCGATGGCCTCCTGGGGCGTGGATGCG
CCAGGCGAAGTGCGCCACAGCCATGAACTTCCTTCGCGTTCTGCGCTGTTGGGGTTATTA
GCTGCGGCGCTGGGTATTCGCCGCGATGAAGAGGAACGGCTAAACACCTTTAATCGTCAT
TACCAATTTTTGCTGTGCGCCAGCGGCAACCCGCGCTGGGCGCGGGATTATCACACGGTG
CAGATGCCGAAAGAGGTACGTAAGGCGCGTTACTTCAGCCGCCGTGAAGAGTTGCAGGAT
CCTGAGTTACTGAGCGCGCTGATTTCCCGACGCGACTACTACACCGACGCCTGGTGGATG
ATCGCTGTCAGCGCGACGCCGGACGCGCCTTACACGTTGGCGCAGCTTCAGGCGGCGTTA
CAACATCCTGTGTTCCCGCTGTATCTGGGGCGCAAAAGTCACCCTCTGGCGTTGCCGCTT
GCGCCGCAACTGCTGGAAGGGAATGCGGCGGATGTCCTGCGTGAAGCATATCGTTGGTAT
CAGGATCAATTCAATGCGCTGAAACTGACTCTCCCCGGATTACAGAATGAATGCTGGTGG
GAAGGTGAACACGATGGCTTAACGGCGAACAAAATTTTACGTCGTCGCGACATGCCGTTG
AGCCGCCAGCAATGGCTGTTCGGTGAACGTAGCGTGAATCAGGGGCCGTGGCTCCGCAAG
GAGGACGCGTGTATCTCTCAAGAATAA
>EECEBICD_00527 CRISPR system Cascade subunit CasE
GTGTATCTCTCAAGAATAACGCTGCACACCAGCGAGCTTTCGCCGGCACAGCTCTTGCAT
CTGGTGGAGCGCGGCGAGTACGTGATGCACCAGTGGTTATGGGATCTCTTTCCCGGCGGC
AAGGAACGGCAGTTTCTTTATCGCCGCGAAGAGTTACAGGGCGCGTTTCGCTTTTTTGTT
CTCTCACAGGAACAGCCTGCCGCCAGCACGATTTTTGACGTACAGACTCGCCCGTTTGCG
CCAATGCTGAGCGCGGGACAGACGCTGCGGTTTAACTTGCGAGCCAATCCGACAATTTGC
AAAAACGGTAAGCGTCACGATTTGCTGATGGAGGCGAAACGCCAGCGTAAAACGCAGGGC
GATAGCCAGGATATTTGGTCTTATCAGCAGCAGGCGGCATTGGAATGGCTGGCGCGCCAG
GGGGAGCAAAATGGCTTTACTCTGCGCGAGGCGAGCGTTGATGCTTACCGGCAGCAGCAG
ATACGTCGAGAGAAATCCCGGCAGATGATTCAGTTTAGCAGCGTGGACTATACCGGCGTG
CTGGTGATTAACGAGCCCGCGCTTTTCCTGCAACGTCTGGCGCAGGGGTACGGTAAAAGC
CGGGCGTTTGGCTGCGGAATGATGATGATCAAACCCGGAGACGACGCGTGA
>EECEBICD_00528 CRISPR-associated endonuclease Cas1
GTGACGTTCGTACCGCTCAACCCGATCCCGTTAAAAGATCGAACCTCGATGATCTTCCTC
CAGTACGGTCAAATTGACGTGCTGGATGGGGCATTCGTGCTGATCGATAAAACGGGAATC
CGTACGCATATTCCCGTCGGTTCGGTCGCTTGTATCATGCTGGAACCGGGAACGCGGGTT
TCCCATGCGGCAGTGCATTTGGCATCAACGGTCGGCACCCTGTTGGTATGGGTGGGCGAG
GCGGGAGTGCGGGTCTATTCCTCCGGACAACCCGGCGGCGCACGAGCCGATAAGTTGCTT
TACCAGGCAAAGCTGGCGTTAGATGATGACCTGCGGCTTAAAGTAGTCCGCAAAATGTAT
GAACTGCGTTTTCGTGAGCCGCCTCCCGCCCGTCGTTCCGTTGAGCAACTGCGCGGTATT
GAAGGATCCCGTGTGCGGGCGACCTATGCATTACTGGCGAAGCAGTATGGCGTGAAATGG
CATGGTCGTAACTATGATCCGAAAGACTGGGAGAAGGGGGATGTCGTCAACCGATGTATT
AGCGCGGCGACATCGTGCCTGTACGGGATTTCAGAAGCGGCTATCCTGGCGGCGGGATAT
GCGCCGGCTATCGGTTTTATCCATAGCGGTAAGCCGCTTTCTTTTGTTTATGACATTGCC
GATATCATCAAATTTGAATCGGTGGTGCCCAAAGCATTTGAGATCGCCGCTCGTCACCCG
GCGGAACCTGATAAAGAAGTGCGCCTGGCCTGCCGGGATATTTTTCGCAGTTCGAAGCTG
ACCGGAAAATTGATCCCGCTGATCGAAGAGGTGCTCGCTGCCGGTGAAATTGAACCACCT

CAGCCTGCGCCGGATATGCTGCCGCCAGCAATACCGGAACCTGAATCACTGGGTGATAGC
GGCCATCGGGGGCATGGTTGA
>EECEBICD_00529 CRISPR-associated endoribonuclease Cas2
ATGAGTATGGTGGTTGTGGTCACGGAAAACGTACCGCCGCGCCTGCGGGGACGTCTTGCC
GTCTGGTTACTTGAGGTTCGTGCAGGTGTTTATGTTGGCGATACCTCGAAGCGTATTCGG
GAAATGATTTGGCAACAGATCACACAGCTTGGCGGAGTCGGAAACGTAGTAATGGCCTGG
GCAACAAATACTGAGTCTGGTTTTGAGTTCCAGACCTGGGGTGAAAACAGACGTATTCCG
GTAGATTTGGATGGACTGCGTTTGGTTTCTTTTCTTCCTGTTGAAAATCAATAA
>EECEBICD_00530 hypothetical protein
ATGTTTTCCGCAACGCGCCGCTTTGCCGTCATTCTGGCGCTCGGCGTAGGCTTTATCCTT
CCTGCACAAGCAGCATCACCAGGGCCTGGTGAAATAGCGAATACTCAGGCACGACATATC
GCCACCTTTTTTCCCGGGAGAATGACTGGCTCCCCCGCCGAGATGTTGTCTGCCGATTAT
TTACGCCAACAATTTACCCAGATGGGATATCAAAGCGATATTCGAACGTTTAATAGCCGA
TTTATTTATACCACGAAGGATAATCGCAAAAACTGGCATAACGTGACGGGCAGTACGGTC
ATCGCCGCCCATGAAGGGCGCGTGCCGCAACAGATCATCATTATGGCGCATCTGGATACG
TACGCTCCGCAGAGCGACGCTGATGTCGATGCCAATCTGGGCGGTTTAACGTTACAGGGA
ATGGATGATAATGCCGCGGGATTAGGCGTTATGCTGGAACTGGCGGCGCGTCTGAAAGAT
ATACCGACCCATTACGGGATTCGTTTTATCGCCACCAGCGGGGAAGAAGAGGGAAAGCTA
GGCGCGGAAAATTTACTCAAACGAATGAGTGACGCTGAGAAGAAAAATACGCTGCTGGTG
ATTAATCTCGATAACCTGATTGTTGGCGACAAGCTCTATTTTAATAGCGGGAAAAATACG
CCGGAAGCGGTGCGTACACTGACCCGCGATCGGGCATTAGCGATTGCGCGCCGTTATGGT
ATCGCCGCCAACACCAATCCGGGACGCAATCCATCCTACCCCAAAGGAACGGGTTGCTGT
AATGATGCGGAGGTTTTCGATAAAGCGGGAATATCGGTGCTTTCTGTTGAGGCGACGAAC
TGGAATCTGGGTAAAAAAGACGGATACCAGCAACGCGTGAAAAATGCCTCCTTCCCGAAC
GGCAATAGCTGGCACGACGTACGGCTTGATAATCAACAGCATATTGACAAGGCGCTGCCA
GGGCGAATTGAGCGCCGTAGCCGCGATGTAGTACGGATAATGCTGCCGTTGGTAAAAGAG
CTGGCGAAGGCGGAAAAAACGTCCTGA
>EECEBICD_00531 Sulfate adenylyltransferase subunit 2
ATGGATCAAAAACGACTCACCCACCTGCGGCAACTGGAGGCGGAAAGTATCCACATTATC
CGTGAAGTGGCAGCGGAATTCGCTAATCCGGTCATGCTGTACTCCATTGGTAAAGACTCC
AGCGTCATGCTGCATCTGGCGCGTAAGGCGTTTTATCCGGGTACGTTGCCGTTCCCGCTA
TTGCACGTTGATACCGGTTGGAAATTCCGTGAGATGTACGCCTTTCGCGATCGTACCGCC
AACGCATATGGCTGCGAGTTGCTGGTACATAAAAATCCGGAAGGCGTGGCGATGGGCATC
AATCCGTTCGTTCACGGTAGCGCTAAACACACCGACATTATGAAAACCGAAGGGTTAAAG
CAGGCGCTGAATAAGTACGGTTTTGATGCCGCGTTCGGCGGCGCACGGCGCGATGAGGAA
AAATCCCGTGCGAAAGAACGTATTTACTCCTTTCGTGACCGTTTCCATCGCTGGGACCCG
AAAAATCAGCGTCCAGAGTTGTGGCGTAACTATAACGGTCAGATTAATAAAGGTGAAAGT
ATACGCGTCTTCCCGCTCTCCAACTGGACAGAGCAGGATATCTGGCAATACATCTGGCTG
GAAAACATCGACATCGTGCCGCTGTATTTGGCTGCTGAACGCCCGGTTCTGGAACGTGAC
GGCATGTTGATGATGGTGGATGACGATCGTATCGATCTCCAGCCTGGCGAAGTCATCAAA
AAAAGGATGGTGCGCTTCCGTACACTTGGCTGTTGGCCGCTCACCGGGGCGGTGGAATCC
CACGCGCAAACGCTGCCGGAAATTATCGAAGAGATGCTGGTTTCTACCACCAGCGAGCGC
CAGGGGAGGATGATTGACCGCGATCAGGCGGGGTCGATGGAGCTTAAAAAACGTCAGGGG
TATTTCTAA
>EECEBICD_00532 Sulfate adenylyltransferase subunit 1
ATGAACACCATACTTGCACAACAAATCGCCAATGAAGGCGGCGTCGAAGCCTGGATGATT
GCCCAACAGCACAAAAGCCTGCTGCGTTTTTTAACCTGTGGCAGCGTTGATGACGGTAAA
AGTACGCTGATAGGTCGTTTGCTGCACGATACTCTGCAAATTTATGAAGATCAGCTCTCT
TCTCTGCATAATGACAGTAAACGTCATGGCACGCAGGGAGAAAAACTCGATCTGGCGCTG
CTGGTAGATGGGCTGCAGGCCGAGCGTGAGCAGGGCATTACTATTGACGTCGCGTACCGC
TATTTCTCCACGGAAAAACGAAAATTTATTATCGCCGACACGCCGGGCCATGAACAATAT
ACCCGTAATATGGCGACCGGGGCGTCCACCTGCGATCTGGCGATCCTACTGATCGACGCG
CGTAAAGGCGTGCTGGATCAGACGCGTCGCCATAGCTTTATCTCCACTCTGTTGGGGATC
AAACACCTGGTGGTGGCAATCAACAAAATGGATCTCGTTGACTACTGCGAAGAGACCTTC

GCCCGTATTCGCGAAGATTACCTGACCTTTGCTGAACAGTTGCCGGGCGATTTGGACATT
CGCTTTGTCCCGCTTTCGGCGCTGGAAGGGGATAATGTCGCCGCGCAGAGCGCGAATATG
CGCTGGTACAGCGGTCCGACGCTGCTGGAAGTCCTGGAAACGGTAGACATACAGCGTGCG
GTAGACCGTCAGCCGATGCGCTTTCCGGTACAATACGTTAATCGCCCGAATCTTGATTTT
CGGGGGTATGCCGGCACTCTGGCGTCTGGTAGCGTCAAAGTCGGAGAGCGCATTAAAGTG
CTGCCGTCCGGCGTGGAATCCAGCGTGGCGCGTATCGTCACTTTTGATGGCGATAAAGAA
GAAGCCTGTGCGGGCGAAGCCATTACCCTGGTACTCAACGACGACATTGATATTAGCCGC
GGCGATCTGCTGCTGGCGGCAAACGAGACGCTGGCACCTGCGCGGCACGCCGCGATCGAC
GTGGTATGGATGGCGGAACAGCCGCTGGCGCCGGGCCAAAGCTACGACGTTAAACTTGCG
GGTAAGAAAACCCGCGCGCGTATCGAGGCTATTCGCTATCAGATTGATATCAACAATCTG
ACCCAACGAGACGTCGAGAGCCTGCCGTTAAACGGTATTGGTCTGGTGGAGATGACATTC
GATGAGCCGCTCGCGCTGGATATTTATCAGCAAAATCCGGTGACGGGCGGGCTGATTTTT
ATCGACCGACTTTCTAACGTTACCGTGGGCGCTGGCATGGTGCGGGAACTGGACGAACGC
GGAGCGACGCCGCCCGTGGAATACAGTGCGTTTGAGCTGGAGCTGAATGCGCTGGTACGC
CGTCATTTTCCGCACTGGGACGCCCGAGATTTGCTGGGAGATAAACATGGCGCTGCATGA
>EECEBICD_00533 Adenylyl-sulfate kinase
ATGGCGCTGCATGATGAGAACGTGGTCTGGCACTCTCATCCCGTTACTGTCGCCGCCCGT
GAACAGCTCCACGGCCACCGTGGCGTGGTGCTGTGGTTTACCGGACTCTCCGGTTCGGGA
AAATCAACGGTGGCCGGCGCGCTGGAAGAGGCGTTACATCAGCGTGGCGTCAGTACCTAC
CTGTTGGATGGCGATAATGTGCGCCACGGTTTGTGCCGTGATTTAGGCTTTAGCGATGCC
GATCGCCAGGAGAATATCCGGCGGGTAGGCGAAGTGGCCAGCCTGATGGCGGACGCCGGC
CTGATTGTTTTGACGGCATTTATTTCTCCGCACCGCGCCGAGCGGCAACTGGTAAAAGAG
CGCGTCGGCCACGATCGTTTTATCGAGATTTATGTCAATACGCCGCTGGCTATCTGTGAA
CAGCGCGATCCTAAAGGGTTGTATAAAAAAAGCGCGTGCGGGCGAGTTGCGTAA
>EECEBICD_00534 Inner membrane protein YgbE
ATGCGTAATAGCCATAACATTACTTTCACAAGGTCAGACGCCTTCATGGTCGACGATGAC
GCCACCAGTGCATTTCCGGGGGCGGTGGTCGGCTTTGTGTCATGGCTGCTGGCGTTGGGG
ATCCCTTTTCTGCTTTATGGCCCTAACACGCTGTTTTTTTTCCTCTATACCTGGCCCTTT
TTCCTCGCGTTAATGCCGGTATCGGTGATTATCGGCATTGCGTTGCATCTGCTGGTAAAG
GGCAAAATACTTTTTAGCATTATGTTTACTCTACTGGCCGTAGGCGCGCTGTTCGGCGCG
TTATTTATATGGCTATTAGGGTAG
>EECEBICD_00535 Cell division protein FtsB
ATGGGTAAACTAACGCTGCTGTTGCTGGCTTTACTGGTCTGGCTACAGTACTCGCTGTGG
TTCGGTAAGAACGGCATACATGACTATAGCCGCGTCAACGATGATGTGGTCGCGCAGCAG
GCGACAAACGCCAAACTTAAAGCGCGTAACGATCAGCTTTTTGCCGAAATTGACGATCTC
AATGGCGGCCAGGAAGCGATTGAGGAGCGCGCGCGCAACGAACTTAGCATGACCAAACCG
GGCGAAACCTTCTATCGTCTGGTGCCTGACGCCTCAAAACGCGCGGCAACGGCGGGGCAA
ACTCATCGATAA
>EECEBICD_00536 2-C-methyl-D-erythritol 4-phosphate cytidylyltransferase
ATGGCAGCCACTTTATTGGACGTTTGCGCCGTGGTGCCGGCGGCCGGATTTGGCCGCCGA
ATGCAAACAGAATGTCCTAAGCAGTATCTCTCAATCGGTAATAAAACCATTCTGGAGCAC
TCAGTTCATGCGCTGCTGGCGCATCCTCGGGTGACACGCGTTGTTATTGCTATCAGCCCT
GGCGATCATCGCTTTGCCCAACTTCCGCTGGCGAATCATCCGCAAATCACCGTTGTGGAT
GGCGGCAACGAACGCGCTGATTCTGTTTTGGCCGGGTTGCAGGCCGTTGCAAAGGCGCAG
TGGGTGCTGGTGCACGACGCGGCAAGACCCTGTTTGCATCAGGATGACCTTGCGCGGTTG
CTGACCATCAGCGAAAACAGCCGGGTCGGCGGTATCCTCGCCAGTCCGGTGCGCGACACC
ATGAAACGCGGCGAACCGGGGAAGAACGCTATTGCCCATACCGTAGAGCGCGCCGATTTA
TGGCACGCGCTCACCCCGCAGTTTTTCCCCCGCGAACTCTTGCACGATTGTCTGACGCGT
GCGCTGAATGAAGGCGCGACCATCACCGACGAAGCGTCAGCGCTGGAATATTGTGGTTTT
CATCCGGCGCTGGTAGAAGGTCGCGCGGATAACATCAAGGTCACCCGTCCGGAAGATCTG
GCGCTGGCGGAATTTTATCTGACCCGAACCATCCACCAGGAGAAAGCATAA
>EECEBICD_00537 2-C-methyl-D-erythritol 2,4-cyclodiphosphate synthase
ATGCGAATTGGACACGGTTTTGACGTACACGCGTTTGGCGGAGAAGGGCCGATTATTATT
GGCGGCGTGCGTATCTCTTATGAAAAAGGGCTATTGGCGCATTCCGATGGCGATGTGGCG

TTACATGCGCTGACCGATGCGCTGCTGGGTGCGGCGGCGCTTGGCGATATCGGCAAGCTC
TTCCCGGATACCGATCCGGCATTTAAGGGAGCGGATAGCCGCGAACTACTGCGTGAAGCC
TGGCGTCGTATTCAGGCGAAGGGGTACACCCTCGGCAACGTAGATGTCACCATTATTGCG
CAGGCGCCGAAAATGCTGCCGCATATTCCGCAAATGCGCGTCTTTATCGCCGAAGATCTC
GGCTGCCATATGGATGATGTTAACGTGAAGGCGACGACGACGGAAAAGCTCGGATTTACC
GGACGCGGAGAAGGCATTGCCTGCGAAGCGGTCGCGTTGCTGATGAAGGCGGCGAAATGA
>EECEBICD_00538 tRNA pseudouridine synthase D
ATGACAGAGTTTGACAATCTCACCTGGCTGCACGGAAAACCACAAGGTAGCGGATTGCTG
AAAGCGAATCCGGAAGATTTTGTGGTGGTTGAAGATTTAGGCTTTACGCCAGATGGTGAA
GGCGAGCACATTCTGCTGCGTATTTTGAAAAACGGTTGCAATACCCGTTTTGTCGCTGAC
GCGCTGGCGAAATTCCTGAAAATTCACGCTCGCGAAGTGAGCTTTGCCGGGCAAAAAGAT
AAACACGCCGTTACCGAGCAGTGGCTGTGCGCGCGCGTGCCGGGGAAAGAGATGCCCGAT
TTCAGCGCCTTTCAGCTGGAAGGTTGTAAAGTGCTGGAATACGCGCGTCACAAGCGTAAG
CTGCGTTTAGGCGCGCTGAAAGGCAATGCCTTTACGCTGGTGTTGCGTGAAATAAGCGAT
CGCCGCGATGTCGAAACACGATTGCAGGCGATTCGCGATGGCGGCGTGCCGAACTATTTC
GGCGCACAGCGATTCGGCATTGGCGGCAGCAACTTGCAGGGCGCGCTGCGCTGGGCGCAA
AGTAACGCGCCGGTGCGCGATCGCAATAAACGCAGTTTTTGGTTGTCGGCGGCGCGTAGC
GCGTTGTTTAATCAAATTGTTCACCAGCGGCTGAAGAAACCAGACTTTAATCAAGTTGTT
GACGGCGATGCGCTACAATTAGCGGGGCGTGGAAGCTGGTTCGTCGCGACATCTGAAGAA
CTGCCTGAATTGCAGCGCCGGGTAGACGAAAAAGAGCTGATGATTACAGCGTCGTTGCCG
GGCAGCGGAGAGTGGGGAACACAGCGTGCGGCGCTGGCGTTTGAGCAAGATGCCATTGCC
CAGGAAACGGTGTTGCAATCGCTGTTGCTGCGCGAAAAAGTAGAGGCGTCGCGCAGAGCG
ATGCTGCTTTATCCGCAACAATTAAGCTGGAACTGGTGGGATGACGTTACCGTCGAGTTA
CGTTTCTGGCTGCCCGCAGGCAGTTTTGCCACCAGCGTAGTAAGGGAACTGATCAACACA
ATGGGTGATTATGCGCATATTGCTGAGTAA
>EECEBICD_00539 5'/3'-nucleotidase SurE
ATGCGCATATTGCTGAGTAACGATGACGGGGTTCACGCGCCCGGTATACAAACGCTGGCG
AAAGCGCTGCGTGAGTTTGCTGATGTACAGGTCGTAGCCCCGGATCGTAACCGCAGCGGC
GCGTCTAATTCCCTCACGCTGGAATCTTCGCTTCGTACTTTTACCTTTGATAATGGCGAT
ATCGCCGTACAGATGGGGACGCCGACCGATTGCGTCTATCTGGGCGTTAATGCCTTAATG
CGTCCGCGTCCGGATATTGTCGTTTCCGGTATTAACGCGGGTCCGAATCTGGGCGATGAT
GTGATCTATTCCGGTACTGTCGCCGCGGCGATGGAAGGTCGTCATCTCGGCTTTCCGGCA
TTAGCGGTCTCCCTTAACGGCTATCAACATTATGATACGGCTGCGGCCGTGACTTGCGCG
CTTTTGCGAGGGTTAAGCCGGGAGCCGTTGCGTACCGGGCGTATTCTCAACGTGAATGTC
CCGGATCTACCGTTAGCGCAGGTTAAAGGCATCCGCGTGACTCGCTGTGGTAGCCGCCAT
CCAGCGGATAAAGTAATCCCGCAGGAAGATCCGCGCGGTAATACATTGTACTGGATTGGC
CCGCCGGGCGATAAATACGATGCCGGGCCGGATACCGATTTCGCGGCGGTGGATGAAGGC
TACGTCTCCGTCACGCCGTTGCATGTGGATTTAACCGCGCACAGCGCGCATGATGTGGTT
TCAGACTGGTTAGACAGCGTGGGAGTTGGCACGCAATGGTAA
>EECEBICD_00540 Protein-L-isoaspartate O-methyltransferase
ATGGTAAGTGGACGTGTACAGGCTCTTCTTGAACAATTGCGCGCGCAGGGCATCAGAGAT
GAGCAGGTGCTTAATGCGCTTGCCGCAGTGCCGCGCGAGAAATTTATTGATGAAGCGTTT
GAACATAAGGCCTGGGAAAATATCGCTTTGCCGATAGGCCAGGGTCAGACGATTTCGCAG
CCCTACATGGTGGCGCGAATGACGGAACTGCTCGAACTGACGCCGCAATCCAGGGTGTTG
GAAATTGGCACCGGTTCCGGCTATCAGACGGCGATTCTGGCGCATCTGGTACATCACGTT
TGCTCCGTTGAGCGGATTAAGGGGCTGCAATGGCAGGCGCGCCGCCGCCTGAAGCAGCTC
GATTTACATAATGTTTCTACCCGTCATGGCGATGGCTGGCAAGGCTGGCAGGCGCGTGCG
CCGTTTGACGCTATCATTGTGACGGCCGCGCCACCGGAAATTCCTACCGCGCTCATGGCA
CAGTTGGATGAAGGCGGCATTCTTGTTCTGCCCGTGGGCGATGAGCAGCAGTTTTTGAAA
CGCGTGCGTCGCCGGGGCGGCGAATTTATTATCGATACCGTGGAGGCCGTTCGCTTCGTC
CCGTTAGTCAAGGGAGAGCTGGCCTAG
>EECEBICD_00541 Murein hydrolase activator NlpD
ATGAGCGCGGGAAGCCCAAAATTCACCGTTAGTCGCATTGCGGCACTATCACTGGTTTCA
CTATGGCTGGCAGGTTGTACCAGTTCGTCAAACCCTCCGGCGCCGGTCACGTCTGTGGAC

AGTGGGTCGTCATCAAATACCAATTCCGGGATGTTGATCACACCGCCGCCAAAAATGGGC
GCGACGACGCAACAAACGCCGCAACAAGCGCCGCAAATTCAACCCGTTCAGCGTCCTGTA
ACGCAGCCCATGCAAACTCAACCGGTCACGGAACAACCCGTGCAGATGGAAAATGGGCGA
ATTGTATACAATCGCCAGTATGGGAATATTCCGAAAGGTAGCTATACGGGTGGCAGTACT
TACACCGTGAAAAAGGGTGATACGCTTTTTTACATCGCCTGGATTACCGGGAACGATTTC
CGCGATCTGGCCCAGAGAAACAGTATTTCTGCCCCGTATAGCCTGAATGTAGGGCAAACT
TTGCAAGTTGGTAACGCATCCGGTACGCCAATTACTGGCGGAAATGCGATCACCCAGGCG
GATGCAGCACAGCAAGGAGTTGTGACCAGGTCTGCACAAAATTCCACCGTTGCAGTTGCG
TCTCAACCAACAATTACGTATTCTGAGGGCTCAGGTGAACAAAGTGCTAACAAAATGTTG
CCAAACAACAAGCCTGCTGGGACGGTTGTCACAGCGCCTGTAACGGCACCAACGGTAAGC
ACGACCGAACCAAATGCAAGCAGTACGTCAACCAGCGCGCCGATTTCCGCATGGCGCTGG
CCGACGGATGGCAAAGTGATCGAAAACTTTGGCGCTTCCGAAGGGGGCAATAAAGGGATC
GACATTGCAGGCAGTAAGGGACAGGCTATCGTCGCAACCGCTGATGGGCGCGTCGTATAT
GCCGGTAACGCACTGCGTGGTTACGGTAATCTTATTATCATCAAACATAACGATGATTAC
CTGAGTGCCTACGCCCATAATGATACGATGCTGGTCCGGGAACAACAGGAAGTTAAGGCG
GGGCAAAAAATCGCTACTATGGGTAGCACCGGCACCAGCTCTACACGCTTGCATTTTGAA
ATTCGTTACAAGGGGAAATCCGTAAACCCGCTGCGTTATTTACCGCAGCGATAA
>EECEBICD_00542 RNA polymerase sigma factor RpoS
GTGATAACGACCTGGCTGAAGAAGAGCTGTTATCGCAAAGGGGCCACACAGCGTGTGTTG
GACGCGACTCAGCTTTACCTTGGTGAGATTGGGTATTCACCACTGTTAACAGCCGAAGAA
GAAGTCTATTTTGCGCGTCGCGCACTGCGTGGAGATGTCGCTTCTCGCCGTCGCATGATT
GAGAGTAACCTGCGTCTGGTGGTAAAAATTGCCCGCCGTTATGGCAATCGTGGACTGGCG
TTGCTGGACCTGATTGAAGAGGGCAATCTGGGGCTTATCCGTGCAGTCGAGAAGTTTGAC
CCGGAACGCGGGTTCCGCTTCTCAACATACGCAACCTGGTGGATTCGCCAGACAATCGAA
CGGGCGATTATGAACCAAACCCGTACGATTCGCTTGCCGATTCACATTGTTAAAGAGCTG
AACGTATACCTGCGCACCGCACGTGAGTTGTCGCATAAACTGGACCACGAACCGAGTGCG
GAAGAAATTGCAGAGCAACTGGATAAACCGGTTGATGACGTCAGCCGTATGCTTCGTCTC
AACGAGCGCATTACCTCGGTAGACACCCCGCTGGGCGGTGATTCCGAAAAAGCGTTGCTG
GACATCCTGGCCGATGAAAAAGAGAACGGTCCGGAAGACACCACGCAAGATGACGATATG
AAACAGAGCATCGTCAAATGGTTGTTCGAACTGAACGCCAAACAGCGTGAAGTGCTGGCG
CGCCGTTTCGGTCTGCTGGGATATGAAGCTGCGACACTGGAAGATGTAGGCCGTGAAATC
GGTCTTACGCGTGAGCGTGTTCGTCAGATTCAGGTTGAAGGCCTGCGCCGTCTGCGCGAA
ATTCTGCAGACGCAGGGGCTGAATATCGAAGCGCTGTTCCGCGAGTAA
>EECEBICD_00543 Protein VdcD
ATGATTTGTCCACGCTGTGCCGATGCACATATTGAACTCATGGCGACTTCTCCGGTAAAA
GGAGTCTGGACGGTATATCAGTGTCAGCATTGTCTGTATACCTGGCGCGATACCGAACCG
TTACGCCGTACCAGCCGCGAACACTATCCACAAGCGTTTCGCATGACGCAAAAAGATATT
GATGACGCGCCAATGGTGCCGAGTATCCCGCCGCTGCTGGCAGAAGACAAACGGTAA
>EECEBICD_00544 Phenolic acid decarboxylase
ATGGCATTTGATGATTTGCGCAGTTTTTTACATGCGCTGGATCAGCAGGGACAACTGCTG
AAAATCAGCGAGGAAGTGAATGCGGAGCCGGATCTCGCCGCCGCTGCCAACGCAACGGGA
CGCATCGGCGATGGCGCGCCCGCGCTGTGGTTTGACAATATTCGTGGTTTTACCGACGCC
CGCGTGGCGATGAATACCATCGGTTCCTGGCAGAACCATGCCATCTCACTGGGTTTGCCG
CCTAACACGCCGGTTAAAAAACAGATCGATGAATTTATTCGCCGCTGGGACAATTTTCCG
GTAGCGCCGGAACGACGGGCGAATCCGGGCTGGGCGGAAAATACCGTCGACGGCGACGCA
ATCAACCTGTTTGATATCCTGCCGCTGTTTCGTCTGAACGACGGCGACGGCGGATTCTAT
CTGGATAAAGCCTGTGTGGTATCACGCGATCCGCTCGATCCCGATAACTTCGGCAAGCAG
AATGTCGGCATCTACCGCATGGAAGTTAAAGGCAAGCGTAAGCTGGGTCTGCAACCAGTG
CCGATGCATGATATCGCATTGCACCTGCACAAAGCTGAAGAGCGCGGGGAAGATCTGCCG
ATTGCGATCACGTTGGGTAACGATCCGATCATTACCCTGATGGGCGCAACGCCGCTGAAA
TACGATCAGTCAGAGTATGAAATGGCAGGCGCGCTGCGTGAAAGCCCCTATCCTATCGCC
ACCGCGCCGCTGACCGGTTTTGATGTGCCGTGGGGATCTGAAGTCATTCTTGAAGGGGTC
ATCGAAAGCCGTAAACGTGAAATTGAAGGACCGTTCGGCGAATTTACCGGCCACTATTCC
GGCGGTCGCAACATGACCGTGGTGCGTATCGATAAGGTCTCTTACCGCAGCAAACCCATT

TTTGAATCGCTCTATTTGGGGATGCCGTGGACGGAAATCGACTACCTGATGGGGCCGGCG
ACCTGCGTGCCACTGTATCAACAGCTGAAAGCCGAATTTCCGGAAGTGCAGGCGGTAAAC
GCCATGTACACCCACGGCTTGCTGGCCATCATCTCGACCAAAAAACGCTACGGCGGCTTT
GCCCGTGCGGTGGGCCTGCGAGCGATGACGACGCCGCACGGTCTGGGATATGTGAAGATG
GTGATCATGGTCGATGAAGATGTCGATCCATTCAATCTGCCACAGGTGATGTGGGCGTTG
TCGTCGAAAGTGAATCCGGCAGGCGATCTGGTACAGCTACCGAATATGTCCGTCCTGGAA
CTGGACCCGGGCTCAAGCCCGGCGGGGATTACCGACAAGCTGATCATTGACGCCACCACG
CCGGTTGCGCCAGATAACCGTGGTCACTATAGCCAGCCGGTTGTTGATTTACCGGAAACT
AAAGCCTGGGCTGAAAAGCTGACCGCCATGCTGGCCAACCGTAAATAA
>EECEBICD_00545 putative UbiX-like flavin prenyltransferase
ATGAGATTGATTGTGGGGATGACCGGTGCGACCGGCGCGCCGCTTGGCGTCGAGCTACTC
CAGGCATTGCGGGCGATACCTGATGTTGAAACGCATCTGGTAATGTCAAAGTGGGCGAAA
ACCACTATTGAACTGGAGACGCCCTACACGCCCGCTGAGGTAGCCGCCCTGGCAGATTAT
TGTCATAGCCCGGCCGATCAGGCGGCGACCATTTCCTCCGGCTCATTTCGTACCGACGGC
ATGATTATTATCCCCTGCAGTATGAAAACACTGGCGGGGGTTCGCGCCGGCTATGCCGAG
GGGCTGGTGGGACGCGCTGCCGATGTCGTGTTGAAAGAGGGTCGCAAGCTGGTGCTTGTG
CCGCGGGAAATGCCGCTCAGCACGATTCATCTGGAAAACATGCTAGCGCTTTCTCGTATG
GGGGTGGCGATAGTGCCGCCCATGCCCGCCTTCTACAACCTGCCGCAAACCGTTGACGAT
ATTATCCAGCACATCGTAGCGCGCGTTCTCGACCAGTTTGGTCTGGAGCATACGCGCACG
CGCCGCTGGCAAGGACTGCGACAGGCAGCAAATTTTTCACAGGAGAATGGATAA
>EECEBICD_00546 Transcriptional regulator HosA
ATGGAGTTACGAAACACCGCGTTTCATCTGTTACGCCAACTTTTTCAGCAACATACCGCA
CGCTGGCAGCATGAACTACCGGAGCTCACCAAACCTCAATATGCGGTGATGCGCGTTATT
GCTGAACATCCGGGTATTGAACAGGTCGATCTGACCGAAGCGGCGGTCAGCACTAAAGCG
ACGCTGGCGGAGATGTTAAGCCGGATGGAAAATCGTGGGCTGGTGAAGCGAGAAAACGAT
CCGCTGGATAAACGTCGGCGTTTTGTCTATCTCACCGTTCAGGGCCAAACGTTGCTCGCC
GCCGCGATACCACTGGGGGATCGCGTTGACGATGAGTTTTTGGGGCGACTGAGCGCTGAA
GAACGCGAACAGTTTACGCAACTGGTACGCAAGATGATGACGTAA
>EECEBICD_00547 HTH-type transcriptional repressor GlcR
TTGATACCTACTGAGCGTCGACAAATCATTCTTGATATGGTGGCGGAGAAAGGAATCGTC
AGTATCGCTGAACTGACCGAGCGCATGCATGTATCCCACATGACTATCCGCCGTGATTTA
CAAAAACTGGAGCAGCAAGGCGCGGTAATCCAGGTCTCTGGCGGCGTGCAATCATCGACT
CGCGTGGCGCACGAACCCTCGCATCAGATCAAAACCGAACTGGCGACGCCGCAGAAAGCG
GCGATTGGCAAACTGGCCGCCAGCCTGGTACAGCCAGAAAGCTGTATCTACCTTGATGCA
GGTACTACCACGCTGGCGATCGCCAGGCAGCTGGTTACGATGAATAAACTTACGGTGGTG
ACCAATGATTTCGTTATCGCCGATTACCTGATGGACAATAGCGATTGCACAATTATTCAC
ACTGGCGGGGCGGTATGCCGGGAAAACCGGTCGTGCGTCGGTGAAGCCGCGGCGACGCTT
TTGCGTGGGTTGATGATTGACCAGGCCTTTATTTCCGCCTCTTCCTGGAGCGTGCGCGGT
ATTTCAACGCCGGCAGAAGATAAAGTCACGGTGAAACGGGCCGTCGCCAGCGCCAGCCGC
CAAAAAATTCTGGTGTGCGACGCGACCAAATACGGCCAGGTCGCTACATGGCTGGCGTTA
CCGCTGGCGGAATTCAACCAAATCGTCACCGATGACGGCCTGCCGGAAAGCGCAATTCGG
GCGCTGGCCAAAGTGGATATCTCACTGCTGATGGCCAAACAATAG
>EECEBICD_00548 L-threonate dehydrogenase
ATGACAACAGGAACTGATTTTCATGTCGGCATTGTGGGGCTTGGCTCAATGGGAATGGGC
GCTGCGCGTTCATGCCTGCGCGCCGGGCTTTCCACATGGGGGGCCGATCTTAACCCGCAA
GCGTGCGCGAATCTGCTCGCTGAAGGGGCTTGTGGGGCTGCCGCCAGCGCCAGGGAGTTC
GCCGGCGTCGTGGATGCGTTAGTCATTCTGGTCGTCAATGCCGCCCAGGTCAGACAGGTG
TTATTCGGTGAGGATGGCGTCGCCCATCTGATGAAACCCGGAAGCGCGGTGATGGTTTCG
TCTACGATCTCAAGCGCCGACGCGCAAGAGATCGCCGCGGCGTTGACGGCATTGAATCTT
AACATGCTGGATGCGCCGGTTTCCGGCGGCGCGGTAAAAGCGGCGCAGGGCGAAATGACG
GTGATGGCCTCCGGGAGCGAGGCGGCGTTTACCCGCCTGAAGCCTGTGCTGGACGCCGTT
GCCAGCAACGTTTATCGCATCAGCGATACGCCGGGCGCAGGCTCAACCGTCAAAATCATT
CATCAGCTGCTGGCTGGCGTGCATATTGCGGCGGCGGCCGAAGCGATGGCGCTGGCCGCG
CGGGCTGGTATTCCTCTGGACGTGATGTATGACGTCGTGACCCATGCCGCCGGCAACTCC

TGGATGTTCGAAAACCGTATGCAGCATGTCGTTGACGGCGACTATACCCCGCGTTCGGCC
GTCGACATTTTTGTCAAAGATTTGGGGCTGGTTGCCGACACGGCGAAAGCGCTGCGCTTC
CCCTTACCGCTGGCCTCTACGGCGTTAAATATGTTCACCAGCGCCAGTAACGCCGGTTAT
GGCAAAGAGGATGACAGCGCTGTGATCAAAATTTTCTCAGGGATTACTCTGCCTGGCGTC
ACGCCTGAGGAGCCGTCATGTTAA
>EECEBICD_00549 3-oxo-tetronate kinase
ATGTTAAAAATTGGCGTCATTGCCGACGATTTTACCGGCGCGACTGACATCGCCAGTTTT
CTGGTCGAAAACGGGATGCCGACAGTGCAGATCAATGATGTCCCAACCGGGACGCAACCG
GAAGGGTGCGACGCGGTGGTTATCAGCCTGAAAACCCGCTCATGCCCGGCGCAAGAGGCG
ATAAAACAATCGCTGGCGGCGCTGGTATGGCTGAAAAAACAGGGCTGCCAGCAAGTCTAT
TTCAAATATTGCTCGACTTTCGATAGTACCGCCGAAGGCAATATCGGCCCGGTCACCGAT
GCGCTGATGGTGGCGCTGGATACCTCATTTACCGTGATTTCTCCCGCGCTGCCGGTTAAC
GGACGCACGGTTTATCAGGGCTATCTGTTTGTCATGAACCACTTGCTGGCGGAGTCCGGT
ATGCGCCACCACCCAATCAATCCGATGACCGACAGCTACCTGCCGCGTCTGATGGAAGCG
CAGGCGCAAGGGCGCTGCGGCGTTATTCCGGCTCAGACGCTTGATGAAGGCGTTGCCGCG
ACCCGTGCGGCGCTGTCGCGTTTACAGCAGGAAGGATATCGCTACGCAGTACTTGACGCG
CTCAATGAGCGGCACCTGGAAATCCAGGGCGAGGTTTTGCGTGATGCTCCGCTGGTGACC
GGCGGTTCTGGGCTGGCAATGGGGCTGGCGCGTCAGTGGGCGAAGCACGGCGTTTCTCAG
GCCCGTTCCGCAGGCTATCCGCTGAGCGGCCGCGCGGTGGTGCTTTCCGGTTCCTGTTCG
CAAATGACGAATCAGCAGGTGGCTTTCTATCGACAACATGCTCCCACACGCGACGTTGAT
GTGGCGCGCTGCCTGTCATCCGAGACGCGCGAGGCCTACGCTGAAGCGCTGGCGCAGTGG
GTGCTCAGTCAGGACAGCGAACTGGCGCCAATGATTAGCGCCACCGCCTCCACGCAGGCG
CTGGCCGCCATCCAGCAGCAATATGGCGCTACCGAAGCCAGCCATGCGGTAGAGGCGCTC
TTTTCCCTGCTGGCTGCTCGCTTAGCGGAAGGCGGTATCACCCGGTTTATTGTGGCGGGC
GGCGAAACCTCGGGCGTGGTGACGCAAAGCCTCGGTATTACCGGTTTTCACATCGGACCG
TGCATTTCACCCGGCGTGCCGTGGGTCAACGCGCTCCATGCGCCAGTCTCGCTGGCGCTA
AAGTCAGGTAATTTTGGCGATGAATCCTTTTTCATCCGCGCTCAAAGGGAGTTTCAGGTA
TGA
>EECEBICD_00550 3-oxo-tetronate 4-phosphate decarboxylase
ATGACGACACTGGCAAAAGAAGAGCACGCTTTGCGAGAAGAGATGGTCAGGATAGCCGCC
TCGTTTTTCCAGCGCGGCTATGCCACCGGCTCGGCAGGCAACCTCTCGCTGTTACTGCCG
GACGGCAATATTCTGGCGACGCCCACCGGTTCGTGTCTGGGTAATCTCGACCCGCAGCGG
CTATCCAAAGTGGACCCGCAGGGCGAATGGCTCAGCGGCGATAAACCGTCTAAAGAGGTG
CGTTTTCATCTGGCGTTGTATCGCAATAACCCTTGCTGCAAGGCCGTGGTACATCTGCAC
AGCACCTGGTCAACGGCGCTTTCCTGTCTCGAAGGGCTGGACCCGCAAAATGTGATTCGC
CCGTTTACCCCCTATGTGGTGATGCGGATGGGCGACATTCCGCTGGTTCCCTACTATCGC
CCGGGGGATGACCGAATTGCCCGCGATTTGGCTGCGCTGGCGGCCCGGCATCAGGCCTTT
TTACTGGCTAATCATGGGCCGGTCGTGTGCGGGGAGAATTTGCAGGAAGCCGCTAATAAT
ACCGAGGAGCTGGAAGAGACGGCAAAGCTGATTTTTATCCTTGGCGAACGCCCGATTCGT
TATTTAACCACCGAAGAAATTACGCAGTTAAGGAGATAA
>EECEBICD_00551 2-oxo-tetronate isomerase
ATGCCGCGTTTTGCCGCCAATTTGTCGATGATGTTTACCGAAGTGCCGTTTATCGAGCGT
TTTGCCGCCGCTGCCGAAGCCGGTTTTCAGGCCGTTGAGTTTCTGTTCCCCTACGGCTTT
GCCGCCAGCGAGATTAAAGCCCAGCTATCGCGTCATGATCTGACGCTGGCGCTGTTTAAT
ACTTCGGCGGGCGATACCGCGGCGGGCGAGTGGGGACGTGCCGCGCTACCGGGGCGCGAA
CACGACGCGCGCGCCGATATTGAACTGGCTCTGGAATACGCTCTGGCGCTGGAATGCGAG
CAGGTTCACATCATGGCGGGCGTAGTGCAGGATGGCGCTGATGGCGCACGTTATCGCGCC
ACGTTTATCGATAATTTGCGTTATGCCGCCGACCGGTTTGCCGCCCACGATAAACGTATT
TTGATCGAAGCGCTGAGTCCCGGCGTTAAACCCGGCTACCTCTTTTCCAGCCAATATCAG
GCGCTGGGCATCGCAGAAGAGGTTGACCGCCCGAATGTGTTCATCCAGCTCGACACTTTC
CACGCGCAAAAAGTGGATGGCAATTTGAGTCACCTGATTCGCGAGTACGCGGGCCGCTAT
GCCCATGTACAAATCGCCTCGCTACCGGACAGACATGAACCGGATGATGGCGAGATCAAC
TACCCGTGGCTGTTCCGGCTGTTCGATGACGTAGGTTATCGCGGCTGGATAGGCTGCGAA
TACCAACCACGGAATACCACTCAGGACGGGCTTGGCTGGTTTAACGCCTGGCGCTAA

>EECEBICD_00552 D-erythronate dehydrogenase
ATGCAGATTATCATTACCGGCGGGGGCGGCTTTTTAGGCCAGAAACTCGCAAGCGCCTTA
TTAAACTCATCGCTGGCGTTTAACGAACTGCTTCTTGTTGATTTAAAAATGCCTGCACGG
TTATCAGATTCCCCTCGTTTACGCTGCCTGGAAGCTGACTTAACCCAGCCGGGCGTGCTG
GAGAATGTGATTACCGCTAATACCTCTGTTGTTTATCATCTCGCTGCGATTGTCAGCAGC
CATGCGGAAGACGATTTCGATCTGGGATGGAAAGTTAACCTGGATCTTACACGCCAGTTA
CTTGAGACGTGTCGTCGACAACCGCAGAAAATTCGTTTTGTCTTCTCCAGCTCGCTTGCC
GTTTATGGCGGTACGCTGCCGGAATGCGTCACCGATACCACCGCGCTCACGCCGCGCTCG
TCTTATGGCGCGCAGAAAGCCGCCTGTGAACTGTTGGTCAACGATTATACCCGCAAAGGC
TATGTGGATGGGCTGGCGCTGCGTTTGCCGACGATCTGTGTTCGCCCGGGTAAACCAAAC
CGCGCCGCTTCTTCTTTTGTCAGCGCGATTATTCGTGAACCGTTGCAGGGCGAGACGACC
GTCTGCCCGGTGTCGGAAAGTTTGCGGCTGTGGATTTCCAGCCCGGCGACGGTGATCCAT
AACCTGTCGCTGGCCGCAACGTTACCCGCGCCTGGCGAGGCGAGCAGCATCAACTTACCC
GGGATCAGCGTAACCGTGGGCGAGATGCTGGAAACGTTGCGTCAGGCGGGCGGCCAGGCG
GCGCGCGATCGGGTTACGCATCAGCGCGATGAAGGCGTCGAGAAAATTGTCGCCTCCTGG
CCGGGACGTATCGATAACCAGCGTGCGCTGGCGTTAGGTTTTGTCGCCGATAAACGCTTC
GATGACATTATTGAACGCTTTCGACAAGATGATATGGAGGGGAGGTCATGA
>EECEBICD_00553 High-affinity gluconate transporter
ATGACAAGTTTACCGACCCCAATTATTGGCCTGATCGTTGCCGTTTTCGTGCTGGTATGG
CTGGTGTTACGCACCCGTGTTCATGCGCTGATCGCGATGCTGGCGGCGGCCTGCATCGCC
GGTCTGTTGGGGGGAATGGGCATTGATAAAACGCTCTCTGTCATCACCAGCGGATTCGGT
ACGACGCTGGGCAGCATTGGCCTGGTGATTGGTCTCGGCGTAATGATGGGGCGCTTGCTG
GAGGTTTCCGGCGCGGCGGAGCGTATCGCCTGGAGCTTTATCAAATGGCTGGGGAAACGC
CGTGAAGAGTGGGCGCTGGCGATCACGGGTTATATTGTCAGCATCCCCATTTTCGTCGAT
TCCGCTTTTGTCATTCTTTATCCGGTGGCGAAAGCGCTGGCGAAAAGCGGTAAACGTTCG
CTACTGACGCTGGGCGTCGCGCTGGCGGGCGGGCTGGTGGTCACTCACCATACGGTACCT
CCGACGCCCGGTCCGCTGGGGGTAGCCGGGATTTTTAACGTCGACATCGGCGCAATGTTA
TTGACCGGGATGGCGCTGGCGGTGCCCTGTGTGATCGGCATTGTGTTCTATGCGCAGTGG
CTGGATAAACGCTACCCTGATTTCGTGCCGCGCACGCTGAACGCCGATGAAGTCAATGCC
GCGCTGGAGCAGTACAATAAAGAGAAAGAGCAAAAGGATCTGCCGAGCCTGACGCTCTCT
CTGCTGCCTATTGTGGTGCCGATCGTGCTGATCTTCCTGAAGGCTATCTGTTCCACCCTC
GCCACTGTAGAAGGATGGTCCGGCCTGGCGACGCATCCGGTGGTACAGGCAATCAACTTT
GTGGGAAGTCCAGTGATCGCGTTGGCCATCAGCGTTCTGCTGGCGGTGTATACGCTGGTG
CCGCGCATGGATAAACATACCACCGCAGAGCGTCTGGAAGAGGGCTTGCAAAGCGCCGGG
ATTATCTTACTGGTCACGGGCGCTGGCGGCGCGTTGGGCGCGATCCTGCGCGACAGCGGC
GCGGGGCAACAGCTGGCGGAGCAGGTTGCCAACCTGCCGATCTCGCCGATCCTGATCCCG
TTTATCGTGGCGACGTTGGTACGCCTGATTCAGGGATCGGGGACGGTGGCGATGATCACT
GCCGCCTCGATTTCCGCGCCGATCCTGGCGCAGATCCCCGGCATCAATATGTTGCTTGCC
GCTCAGGCCGCAACGATGGGGTCGCTGTTCTTCGGCTACTTCAACGATAGCCTGTTCTGG
GTGGTTAACCGCATGATGGGCGTCTCTGATGTCAAACAGCAGATGGTGGTTTGGTCTGTA
CCGACGACTATTGCCTGGGCGATTGGCGGCACCGGCGTGGCGCTGATTAACCTGCTGTTC
GGTTCCGGCGGCAGTTGGCTCGATCCGTTACTGCCGATTGTCGTTCTGGCGGCAATTATG
CTGTGGGTGCGTTGGCAGGCGCAGGGGATCAAAGACAAGCTGGTAGTTAAGGATTAA
>EECEBICD_00554 HTH-type transcriptional regulator DmlR
ATGCTCAATTTGCAGCGTATGTCGCTGTTTATCGCCGTGGTAGATAGCGGCAGCTTCACG
GCGGCGGCGGCCGCATCGGGCCAAACAAAAGCGGTAGTGAGTTTTAATATTCGGCAGCTT
GAAAAAGAGCTTGGCGTAACGTTACTGCTACGTTCCACCCGACGACTGACGCTCACAGAC
GCCGGCGTCCTCTTTTACCAAAAAGGCGTGAATTTACTCAACGCAGCGAAAAATTTGCAG
GATGAAGTGCGTGCCAGCCATAGTGGGCTGGGCGGCGAGTTACGTATTACCACTACGCCC
GAGTTTGGCGAACAGGTGATCATTCCGTTACTGGCGCAGTTTAGTCAGCGCCATCCCGAT
CTCCGTATTCGGCATATGTCCTCATCACATCATGCCGATTTAATTGCCGAGCGCTTTGAT
GTGGCGATTCGGCTTGGCTCGCTGGCCGACTCCCGCTATCGCGCCGCGCTGATATCCCGC
TTTACCATTCTGCCGGTCGCCGCGCCGCAGTGGCTCGCCCGCCACCCCGTGTCGTCGCTG
GAGTCGTTGGCTCAGGCGGAATGGATTATTCATGAGCGATTACCTACGCCGCTACGGTGG

TCAGTAACGAATAATCATGGCCAACACTCACGCCTGGAGATAAGCAAAGCCGGCAAAATT
TCTGTCGACAGCGCGCGCTCGCTGATGGCCTTCGCTCTCGCGGGAAGCGGCGTCGCATTA
CTGCCGCAATGGTTAGTCAACACCGCACTGGAGGATGGAACGTTAATTCACGTGTTACCC
GACTATCATTTTCCCCGTCAGGGCATTTATGCCGTTTACCCGGACGCCCGCCATGTAACG
ACAAAAGTACGCGCATTTATTGATTTTTTACGTTCGCAATGGGATTGCGGGGAGCACGCC
CCATCATTGTAG
>EECEBICD_00555 Drug efflux pump JefA
ATGACGTATCGCAGTAAAGTGGCAGTGGTTTACCTGCTCGGCTTCTTTCTTGATCTGATT
AATCTGTTTATTGCCAGCGTGGCTTTTCCTGCGATGTCCGTGGAGCTGCATACGTCCATA
TCGGCGCTGGCATGGGTGAGTAACGGTTATATCGCAGGATTAACGCTGATCGTTCCGTTT
AGCGCGTTTCTTTCGCGTTATCTCGGCGCACGACGGCTGATTATATTTTCGCTCATTCTG
TTTAGCGTTGCCGCCGCTGCCGCAGGTTTTGCCGATTCCTTACACAGCCTTGTTTTCTGG
CGTATTGTGCAGGGGGCGGGCGGTGGCTTATTGATCCCGGTTGGTCAGGCGTTAACCTGG
CAACAGTTTAAACCGCATGAACGCGCTGGCGTGTCATCGGTTGTCATGATGGTGGCGCTA
CTGGCGCCGGCATGTTCGCCTGCGATTGGCGGGCTGCTGGTGGAAACGTGCGGCTGGCGC
TGGATATTCTTTGCCACGCTGCCGGTGGCCGTTCTCACTTTATTGCTCGCTTATCGTTGG
CTGAACGTCGCTTCGACGACAATGGTGTCAGCAAGATTGCTCCATCTGCCGTTGTTGACG
GACAAGCTGCTGCGTTTTGCCATGATCGTCTATCTGTGTGTGCCGGGTATGTTCATTGGT
ATCAGCGTGGTCGGGATGTTTTATCTACAGAACGTCGCGCAGCTATCGCCTGCGGCGGCG
GGGTCGCTGATGCTCCCCTGGTCGATCGCTTCATTTGTCGCCATTATGCTTACCGGACGT
TATTTCAATCGGCTTGGGCCGCGTCCGCTGATTATCGTCGGCTGTCTTCTTCAGGCCGCA
GGGATATTGCTTTTAACTAACGTTACGCCTGCGACATCGCACCGTGTACTGATGATGATA
TTCGCCTTAATGGGCGCTGGCGGCAGTTTGTGCAGCAGTACCGCGCAGAGTGGCGCTTTC
CTGACTATTGCTCGCCGGGACATGCCTGACGCCAGCGCGCTATGGAATCTTAATCGTCAG
ATCAGCTTTTTCCTTGGCGCGACGCTGTTGACGCTGCTGTTAAATGCGTTCCAGCGCGTT
ATGTCGCTTGAGGTTGCTTATCGCTGGACCTTTATTGCCGCCGCCGGTATCACCCTGCTG
CCCCTTATTTACGCTGTCTGTCTGAACAATAGACAGGCGCTTCTGTGCCTGAAAAAGGAA
AGACCATGA
>EECEBICD_00556 hypothetical protein
ATGAATCCATATAAAGAAGAAATTATTCACGCCCATGCGGCGATTGAGAACTGGCTTAGT
AAGGGGATGGGTAGCCTGGAGGCGTTGATAGCGCGTTTCGCTGTTGATTTTACGATGATA
ACGCCAGGAGGTATTTGTCTGGATTATCCGGCGCTGGGCGCTTTTTTCCAGGCGCAGCGC
GCGTGTCGCCCGGGGATGGTTATCGTGGTTGAGCACATTGACCTTGTGGCGGAGTGGCCG
GAAGGCGCCGCGCTGCGTTATCGCGAGCGACAGCAACTGCCGGGCCAGGCTGAGACGGTA
CGCTGGTCGACGGTCATTTTAAAAAGGGAAAGAGGACGGATAGTCTGGCGTCATTTACAT
GAAACGACAGCCACAGCCTGA
>EECEBICD_00557 DNA mismatch repair protein MutS
ATGAATGAGTCATTTGATAAGGACTTCTCCAACCACACCCCCATGATGCAGCAGTATCTC
AAGCTGAAAGCCCAGCATCCGGAGATCCTGCTCTTTTATCGTATGGGAGACTTTTACGAG
CTGTTTTATGACGACGCAAAGCGTGCGTCGCAGTTGCTCGATATTTCGCTGACCAAACGC
GGCGCATCGGCTGGCGAACCTATCCCTATGGCGGGTATCCCGCACCACGCCGTAGAAAAC
TACCTCGCGAAACTGGTCAATCAGGGCGAATCCGTCGCTATTTGCGAACAGATTGGCGAT
CCGGCCACCAGCAAAGGTCCCGTTGAACGTAAGGTCGTGCGTATCGTTACGCCTGGCACT
ATCAGCGATGAAGCCCTGTTACAGGAGCGTCAGGATAACCTGCTGGCGGCTATCTGGCAG
GATGGTAAAGGCTACGGTTACGCCACGCTGGATATCAGCTCCGGGCGCTTTCGTCTGAGC
GAACCCGCCGACCGTGAAACGATGGCCGCAGAGCTGCAGCGCACCAATCCCGCCGAACTG
TTGTATGCCGAAGATTTTGCTGAAATGGCGTTAATAGAGGGACGCCGGGGTCTGCGCCGT
CGCCCGTTGTGGGAGTTTGAGATTGATACCGCCCGCCAGCAGTTAAATCTGCAATTCGGT
ACGCGGGATCTGGTCGGTTTCGGCGTCGAAAATGCCTCGCGTGGATTATGTGCGGCAGGC
TGCCTGTTACAGTACGTAAAAGATACCCAGCGCACCTCCCTGCCGCATATTCGTTCCATT
ACGATGGAACGCCAGCAGGACAGCATCATTATGGATGCCGCGACCCGCCGCAATCTGGAA
ATTACCCAGAACCTGGCCGGCGGTGTCGAAAATACCCTCGCCGCGGTGCTTGACTGTACC
GTGACGCCTATGGGTAGCCGAATGCTTAAACGCTGGCTGCATATGCCGGTACGAAATACC
GACATATTACGTGAACGCCAGCAGACCATCGGCGCCTTGCAGGACACCGTCAGCGAACTG

CAACCGGTGCTGCGTCAGGTCGGCGATCTGGAGCGTATCCTTGCGCGTCTGGCGCTGCGC
ACCGCGCGTCCGCGCGATCTCGCCCGAATGCGTCACGCCTTCCAGCAGCTGCCGGAATTG
CACGCTCAGCTTGAAACCGTTGACAGCGCGCCGGTACAGGCGTTGCGTAAAAAAATGGGC
GATTTCGCCGAGCTGCGCGACCTCCTGGAACGCGCCATTATTGACGCGCCGCCGGTACTG
GTCCGCGACGGCGGCGTTATTGCGCCCGGCTACCATGAAGAGCTGGACGAATGGCGCGCG
CTGGCGGATGGCGCCACCGATTATCTCGATCGTCTGGAAATTCGCGAACGCGAGCGTACC
GGGCTGGATACGCTGAAAGTCGGCTATAACGCGGTACATGGTTATTACATTCAGATTAGC
CGTGGTCAAAGCCATCTGGCGCCCATCAATTATGTGCGTCGCCAGACGCTGAAAAATGCC
GAACGCTACATTATTCCTGAGCTAAAAGAGTATGAAGATAAAGTTCTGACCTCGAAAGGC
AAAGCGCTGGCGCTGGAAAAACAGCTATATGACGAATTGTTTGATCTGCTGTTGCCGCAT
CTGGCCGATTTACAGCAGAGCGCCAACGCGCTGGCGGAACTGGATGTGCTGGTTAACCTG
GCCGAACGCGCCTGGACGCTGAATTACACCTGCCCGACATTTACCGATAAACCCGGTATC
CGTATTACCGAAGGTCGCCACCCGGTGGTTGAACAGGTACTGAATGAGCCGTTTATCGCT
AACCCGCTCAATCTGTCGCCGCAGCGCCGGATGTTGATCATTACCGGCCCCAATATGGGC
GGTAAAAGTACCTATATGCGACAAACGGCATTGATCGCCCTGCTGGCCTATATTGGCAGT
TACGTTCCGGCGCAAAACGTGGAAATCGGCCCCATCGACCGTATTTTTACCCGCGTCGGC
GCAGCGGACGACCTGGCTTCCGGGCGTTCGACCTTTATGGTGGAGATGACCGAAACCGCG
AACATTCTGCATAATGCCACGGAAAACAGTCTGGTATTGATGGATGAAATCGGGCGCGGT
ACCTCTACGTATGACGGGCTGTCGTTGGCCTGGGCCTGCGCGGAGAATCTCGCGAATAAG
ATTAAAGCGTTAACGCTGTTCGCTACCCACTACTTCGAGCTGACCCAATTACCGGAGAAA
ATGGAAGGCGTGGCGAACGTCCACCTGGATGCGCTGGAACACGGCGATACTATCGCGTTT
ATGCACAGCGTGCAGGACGGCGCGGCAAGTAAGAGCTATGGCCTGGCGGTCGCGGCGCTT
GCGGGCGTCCCTAAAGAAGTCATCAAACGCGCGCGCCAAAAACTCCGCGAGCTGGAGAGC
ATCTCGCCCAATGCGGCGGCAACGCAGGTGGACGGCACGCAAATGTCGTTGCTTGCCGCC
CCGGAGGAGACATCGCCTGCCGTTGAAGCGCTGGAGAATCTCGATCCGGACTCCCTGACG
CCACGCCAGGCGCTGGAGTGGATCTATCGGCTGAAAAGTCTGGTGTAA
>EECEBICD_00558 hypothetical protein
ATGGAAAGTCGTCAATACACCCGGCATCTTTCGCTTTCGGAGCTGAAATGGTTTGCTATC
GGGATCGGTTTTTTTATTCTCAGCATAGCAACAGCCACCGTGAATTATCGGCTTTCCGGT
ATATCGCTACTGGTGGGCCTGTTGTTTATCATCTGGAAATTCAGCGTCACAGTTCTGTTT
CTGTTTACGCCGCGCAGGATGACGTTAACCGAAACCGCGTTGCAGGCCGGGCATCGTGTT
ATCCATTACGATGCGCTTGAATCAATGCGTCTGCTCCACCAATCCGACAAACTCATCTTG
CGCCACAGTGGCGGCAAGAAGTACGTCATCTACCTTGATTTCTGGAACGATGGCAACGGA
ATTTACGACCGACTGGCTGCTGAACTTGTCCGGCGACACGGTTCTGCGCTGGGGGCGCGC
CTCGCCGCCGATGGACGTCTTAAATTCGGCAAAGTGACCGCGCTGGCGGATCGGCTTGAA
CACAAAAACCGGGCGGTGCCGTATGCGCAGATCGCCAGTATTCGCACCCAGCGCGAGGAG
GGGGCTGGCAGTTCAATGAGCTATCTGATGATTAGCACCGCTACGGGCCGGATCTGCAAA
ATCGACCGCTCCACTATCGTCAATGAGCCGCTGCTGCTCAACTTTCTTTCCCAGCGTTTA
CCCGCCTGA
>EECEBICD_00559 hypothetical protein
ATGGAACTGTACGTATCAAAAACCAGCCAGCAAAACATGCCTGTGGAGTTCAATATCTAT
TATTCCAGATCTAAAACGTTGGTTGGTATTGTGTTCTGGATTTGTATGATGGGAGTTTTT
ATTGCGCTGGCGGTGAGTATGGCCACCGATCCCGAGGAGCCCAAAACTGGCGGTGCCGTC
TTTATGGGGCTTATTTGTTGCGGCAGTCTGTTATTTATTGTGCGCCTGGCTCGCTCATTT
TTAAGCGCGGCAAAGCCGGCAGTCACCATCAGTCGTGAAGGTATTCGCTTCTGTAATGGC
AAATTCGCGGCCTGGACAAATATCGCCGAAAACACCTGGCATTCACAGAGCATTAACTTC
ATCCCGGCCGCCGCCGGTATTAAGATTGTGTTGCATGAAGGCAAGCCGATCCATTTCGCC
ACCACGGTTATTGCGCTTTCTTCCGATAGGTATCTTGAGATGTGCGATCTTTACTCGTCT
CAGGCGCTCGGGTAA
>EECEBICD_00560 Serine/threonine-protein phosphatase 2
ATGGAATTAATCCGTTATGCTGACATCAACAGCGATCTCTATCGCCACATTTGGGTCGTT
GGTGATATTCATGGATGTTACTCCTTATTGTTAACCAGACTGGCTCAATTAAATTTCTCT
CCTGATACCGATCTCTTGATCTCCACTGGAGATAACATTGATCGAGGAAAAGAAAACCTT
GAAACACTGAGACTGTTAAATACCCCATGGTTTATTTCTGTAGTGGGTAATCATGAAGCT

ATGGCGCTTGACGCTTTTGAAACACAGGATGGCAATTTCTGGTATGTAAACGGTGGTTAC
TGGTACGACTCGGTCACCGAAAAAGACAGGCAAGAAGCCACTGAGCTATTACTAACTTTT
AAGCAGCGTCCTCACATCATAGAGGTTGAAACGTCCTCAAAGAAGTACGTGATTGCCCAT
GCCGATTATCCAGATGACAGCTACGATTATGGGAAGCAAGTTGATATTGATAGCGTTCTA
TGGTCACGCGATCGTCTGCTTGGTTCGTTACAGGGGAATATCCATCCAATACGCGGTGCG
GATACTTTCATCTTCGGCCATATGATTGTTGACTACACAACAACATTCGCCAATCAAATC
TACATTGACACAGGTTCTTTCTGTTCAGGCAATCTGTCTTTTTTTAAAATAAAGTAA
>EECEBICD_00561 hypothetical protein
GTGGGCTATCACCGTCGTTCAGTAGTGGAAACAGCGATGTTCCGTATCAAAACCTTGCTG
GGCGGCCATCTGAGCCTGCGGAACTATGACGCGCAGGTGGGTGAAGCGATGGCGATGGTC
AAAGCGCTGAACCGGATGACGTTGTTGGCAATGCCGTCCAGCGTCAGGCTCGTATAA
>EECEBICD_00562 hypothetical protein
ATGATGTTTACAGACTGGCATGAGGCCGCGATAGGGAAAACCCACAATCGAATGAATTTT
GATTGTGGGGATGCCGATCTGAACCAATTCCTGCAACGCCATGCACGACAAAATCATGAG
AAAGGGACAACGAAAACCTATGTTGCGCTTGATAATTCGGATGTTACACGTATCCACGGC
TTTTACTCAGTCAGTCCTGCATCACTGATATATGCACAGGTTCCCGGCGCAATCAGCAAA
GGATTAGGGAGATACGATGTGCCAGTGTTTCGTCTGGGTCGTTTAGCCGTAGACAAATCT
ATGCAGGGGCAAGGGCTGGGAGCACAACTTTTGTTATCTGCCGGAAAGCGTTGCATACAG
GCGGCTTTGCAGGTCGGTGGCGTAGCCCTACTTATTGATGCCAAAAATAAACAGGTCTGC
GACTGGTACAAAGGATTTGGCGCAGTACCATTAAACGATCAACCCCTTTCCTTGTTGCTG
TCGTTTAAAACGCTTTATGCTGCTTTATCTGCATCTGGTAGGTTATGA
>EECEBICD_00563 hypothetical protein
ATGCCTCAGATAGCTATAGAATCTAACGAAAGGCTGTCGTTGCGTGTATCGACGGATGCC
AAAAAGTTGATAGTCCGCGCTGCGGCAATCCAGCAAACCAACCTGACGGATTTTGTGGTC
AGTAACGTACTACCTGTCGCACAGAAGATTGTTGATGCCGCCGAACGGGTATATCTGACG
GAGCGCGATACTCAAATGATCATGGAAATTTTAGATAACCCGCCAGCGCCGAATGAAAAG
CTTCTTGCCGCTGCTTTCGCCCTGCCGGACATGAAGAAATGA
>EECEBICD_00564 Type 3 secretion system pilotin
ATGAAAAAATTTTATAGCTGTCTTCCTGTCTTTTTACTGATCGGCTGTGCCCAGGTGCCC
CTCCCTTCCTCCGTGAGCAAACCGGTACAGCAACCTGGCGCTCAGAAAGAGCAACTGGCC
AACGCAAATAGTATTGATGAGTGTCAGTCTCTTCCGTATGTGCCGTCAGACCTTGCGAAG
AATAAATCATTATCAAACCAGAACGCTGATAATTCCGCATCAAAAAATAGCGCAATCAGC
TCAAGCATTTTTTGCGAAAAATATAAACAAACCAAAGAGCAGGCGCTCACCTTCTTCCAG
GAACATCCACAATACATGCGTTCGAAAGAAGATGAAGAGCAACTCATGACCGAATTTAAA
AAAGTTCTTCTTGAACCCGGAAGTAAGAATTTAAGCATATATCAGACGTTACTTGCTGCC
CATGAAAGACTGCAATCCTTATAA
>EECEBICD_00565 Invasion protein InvF
ATGCTAAATACGCAGGAAGTACTTAAAGAAGGAGAGAAGCGGAAAATCCGCAGCCCGGAA
GCATGGTTTATACAGACGTGTTCCGCGCAAAAGCTGCATATGTCATTTTCTGAAAGCCGA
CACAATGAAAATTGCCTGATTCAGGAAGGCGCGCTGCTTTTTTGCGAGCAGGCCGTTGTC
GCACCAGTATCAGGAGACCTGGTTTTTCGACCGTTAAAAATTGAAGTACTCAGCAAATTA
CTGGCATTTATCGATGGCGCAGGATTAGTGGACACGACATATGCTGAATCCGATAAATGG
GTTTTGCTGAGTCCTGAGTTTCGCGCTATTTGGCAAGATCGTAAACGCTGCGAGTACTGG
TTTTTGCAGCAAATTATTACGCCTTCTCCGGCCTTCAATAAGGTACTGGCGCTGTTACGA
AAAAGCGAGAGTTACTGGTTGGTTGGCTATTTACTCGCTCAGTCAACCAGCGGCAACACG
ATGAGAATGCTGGGAGAAGACTATGGCGTTTCTTATACCCATTTTCGTCGTTTGTGCAGC
AGAGCGTTGGGCGGAAAAGCGAAGAGTGAATTACGAAACTGGCGTATGGCGCAATCGCTG
CTGAATAGTGTAGAAGGCCACGAGAACATCACCCAATTAGCCGTTAATCATGGTTACTCA
TCGCCTTCACATTTTTCTAGTGAGATCAAAGAGCTGATCGGCGTTTCGCCGCGGAAATTA
TCAAATATTATTCAATTGGCAGACAAATGA
>EECEBICD_00566 Type 3 secretion system secretin
ATGAAGACACATATTCTTTTGGCCAGAGTGCTGGCATGTGCCGCGCTTGTTCTGGTTACA
CCTGGTTATTCTAGTGAAAAAATACCTGTAACGGGAAGTGGGTTTGTTGCGAAAGACGAT
AGCCTGCGGACATTTTTCGATGCCATGGCGCTACAGCTAAAGGAGCCTGTCATTGTTAGC

AAAATGGCGGCACGAAAAAAAATTACGGGCAACTTTGAGTTTCACGATCCTAACGCATTA
CTGGAGAAGCTTTCCCTACAACTGGGGCTGATTTGGTATTTCGATGGGCAGGCTATCTAT
ATTTATGACGCCAGTGAAATGCGCAATGCCGTGGTTTCTTTACGCAACGTCTCACTCAAT
GAGTTCAACAATTTTCTAAAACGCTCAGGTTTATATAACAAAAATTACCCGCTACGTGGC
GATAACCGTAAAGGAACATTCTATGTTTCAGGGCCACCCGTCTATGTTGATATGGTGGTC
AACGCCGCCACCATGATGGACAAGCAAAACGATGGTATTGAGCTGGGACGTCAGAAAATA
GGGGTGATGCGTCTGAACAATACCTTCGTGGGCGATCGTACCTATAATCTGCGCGATCAG
AAAATGGTTATCCCCGGTATTGCCACGGCCATTGAAAGGTTGTTGCAGGGAGAAGAGCAA
CCTTTAGGTAATATTGTCAGTAGCGAACCCCCGGCGATGCCAGCGTTTTCAGCGAATGGA
GAAAAAGGTAAAGCAGCAAATTATGCCGGTGGCATGAGTCTGCAGGAAGCTTTAAAGCAA
AATGCCGCGGCGGGCAATATTAAAATCGTGGCCTATCCGGATACCAACAGTTTGTTAGTA
AAGGGAACGGCTGAGCAGGTGCATTTTATCGAAATGCTGGTTAAAGCGCTGGATGTCGCC
AAACGTCACGTAGAATTATCCCTGTGGATTGTCGATCTTAATAAAAGCGATCTGGAGCGT
TTGGGCACTTCATGGAGCGGCAGCATTACTATTGGGGACAAACTTGGCGTGTCATTAAAC
CAGTCTTCAATAAGTACCCTCGATGGCAGTCGATTCATCGCCGCGGTCAATGCGTTAGAA
GAGAAGAAACAGGCGACGGTGGTTTCACGCCCGGTATTACTGACCCAGGAAAATGTTCCC
GCTATTTTTGATAACAACAGAACGTTTTACACCAAGCTGATTGGGGAACGTAATGTGGCG
CTTGAGCATGTAACATACGGAACAATGATCCGAGTGCTGCCCCGTTTTTCCGCAGATGGT
CAGATAGAAATGTCGCTGGACATTGAAGATGGCAACGATAAGACGCCGCAATCCGATACT
ACCACCTCCGTAGATGCGTTACCCGAAGTCGGGCGAACGTTAATTAGCACTATTGCGAGA
GTGCCGCACGGAAAAAGTTTGCTGGTCGGTGGTTATACACGGGATGCAAATACCGATACT
GTCCAAAGTATTCCGTTTTTAGGCAAATTACCGCTTATTGGTAGCCTGTTCCGCTATTCC
AGTAAGAATAAAAGTAATGTTGTTCGTGTGTTCATGATTGAACCAAAAGAAATTGTCGAC
CCGTTAACGCCGGATGCCAGCGAATCGGTAAACAATATTCTGAAGCAAAGCGGTGCCTGG
AGTGGGGACGATAAGTTACAGAAATGGGTTCGTGTTTATCTGGATAGAGGTCAGGAGGCA
ATTAAATGA
>EECEBICD_00567 Protein MxiC
ATGATTCCTGGCTCAACCTCCGGTATTTCATTTTCCAGAATATTGTCCCGGCAGACATCT
CATCAGGATGCGACCCAGCATACTGATGCGCAACAGGCGGAAATACAACAGGCCGCAGAG
GATTCGTCTCCAGGGGCGGAAGTACAAAAATTTGTCCAGTCGACGGACGAAATGTCAGCG
GCGCTGGCGCAATTTCGTAACCGTCGCGATTATGAAAAAAAATCCAGTAATTTATCTAAC
AGTTTTGAACGCGTGCTGGAAGATGAGGCTTTACCGAAGGCGAAGCAAATCTTAAAGCTA
ATTAGCGTACATGGCGGCGCGTTAGAAGATTTTTTACGTCAGGCGCGTAGCTTATTTCCT
GACCCCAGTGATTTAGTCCTTGTGTTACGCGAATTGCTTCGTCGTAAAGACCTGGAAGAG
ATCGTGCGGAAAAAGCTGGAGTCGTTACTTAAGCACGTTGAAGAGCAAACCGATCCGAAG
ACCCTCAAGGCAGGGATTAATTGTGCGTTGAAGGCCCGGCTTTTTGGGAAAACATTATCG
TTAAAACCAGGCTTATTGCGCGCCAGCTATCGGCAATTTATCCAGAGTGAATCACATGAA
GTGGAGATTTACTCTGACTGGATAGCCAGTTATGGCTATCAACGTCGACTGGTGGTACTG
GATTTTATTGAGGGTTCGCTATTAACCGATATTGACGCGAATGACGCCAGTTGTTCGCGC
CTGGAGTTTGGCCAGCTTTTACGACGCCTGACGCAACTTAAAATGTTGCGCTCCGCTGAC
CTACTGTTTGTGAGTACATTGTTGTCGTATTCGTTTACCAAAGCGTTTAATGCGGAGGAG
TCGTCGTGGCTACTACTGATGCTTTCGCTATTGCAACAGCCACATGAAGTGGATTCGCTG
TTAGCCGATATTATAGGTTTGAATGCGTTATTGCTTAGTCATAAAGAACATGCATCCTTT
TTGCAGATATTTTATCAAGTATGTAAAGCCATACCCTCTTCACTCTTTTATGAAGAATAT
TGGCAGGAAGAATTGTTAATGGCGTTACGTAGTATGACCGATATTGCCTACAAGCATGAA
ATGGCAGAACAGCGTCGTACTATTGAAAAGCTGTCTTAA
>EECEBICD_00568 Invasion protein InvA
GTGCTGCTTTCTCTACTTAACAGTGCTCGTTTACGACCTGAATTACTGATTCTGGTACTA
ATGGTGATGATCATTTCTATGTTCGTCATTCCATTACCTACCTATCTGGTTGATTTCCTG
ATCGCACTGAATATCGTACTGGCGATATTGGTGTTTATGGGGTCGTTCTACATTGACAGA
ATCCTCAGTTTTTCAACGTTTCCTGCGGTACTGTTAATTACCACGCTCTTTCGTCTGGCA
TTATCGATCAGTACCAGCCGTCTTATCTTGATTGAAGCCGATGCCGGTGAAATTATCGCC
ACGTTCGGGCAATTCGTTATTGGCGATAGCCTGGCGGTGGGTTTTGTTGTCTTCTCTATT
GTCACCGTGGTCCAGTTTATCGTTATTACCAAAGGTTCAGAACGCGTCGCGGAAGTCGCG

GCCCGATTTTCTCTGGATGGTATGCCCGGTAAACAGATGAGTATTGATGCCGATTTGAAG
GCCGGTATTATTGATGCGGATGCTGCGCGCGAACGGCGAAGCGTACTGGAAAGGGAAAGC
CAGCTTTACGGTTCCTTTGACGGTGCGATGAAGTTTATCAAAGGTGACGCTATTGCCGGC
ATCATTATTATCTTTGTGAACTTTATTGGCGGTATTTCGGTGGGGATGACCCGCCATGGT
ATGGATTTGTCCTCCGCTCTGTCTACTTATACCATGCTGACCATTGGTGATGGTCTTGTC
GCCCAGATCCCCGCATTGTTGATTGCGATTAGTGCCGGTTTTATCGTGACTCGCGTAAAT
GGCGATAGCGATAATATGGGGCGGAATATCATGACGCAGCTGTTGAACAACCCATTTGTA
TTGGTTGTTACGGCTATTTTGACCATTTCAATGGGAACTCTGCCGGGATTCCCGCTGCCG
GTATTTGTTATTTTATCGGTGGTTTTAAGCGTACTCTTCTATTTTAAATTCCGTGAAGCA
AAACGTAGCGCCGCCAAACCTAAAACCAGCAAAGGCGAGCAGCCGCTTAGTATTGAGGAA
AAAGAAGGGTCGTCGTTGGGACTGATTGGCGATCTCGATAAAGTCTCTACAGAGACCGTA
CCGTTGATATTACTTGTGCCGAAGAGCCGGCGTGAAGATCTGGAAAAAGCTCAACTTGCG
GAGCGTCTACGTAGTCAGTTCTTTATTGATTATGGCGTGCGCCTGCCGGAAGTATTGTTA
CGCGATGGCGAGGGCCTGGACGATAACAGCATCGTATTGTTGATTAATGAGATCCGTGTT
GAACAATTTACGGTCTATTTTGATTTGATGCGAGTGGTAAATTATTCCGATGAAGTCGTG
TCCTTTGGTATTAATCCAACAATCCATCAGCAAGGTAGCAGTCAGTATTTCTGGGTAACG
CATGAAGAGGGGGAGAAACTCCGGGAGCTTGGCTATGTGTTGCGGAACGCGCTTGATGAG
CTTTACCACTGTCTGGCGGTGACGCTGGCGCGCAACGTCAATGAATATTTCGGTATTCAG
GAAACAAAACATATGCTGGACCAACTGGAAGCGAAATTTCCTGATTTACTTAAAGAAGTG
CTCAGACATGCCACGGTACAACGTATATCTGAAGTTTTGCAGCGTTTATTAAGCGAACGT
GTTTCCGTGCGTAATATGAAATTAATTATGGAAGCGCTCGCATTGTGGGCGCCAAGAGAA
AAAGATGTCATTAACCTTGTAGAGCATATTCGTGGAGCAATGGCGCGTTATATTTGTCAT
AAATTCGCCAATGGCGGCGAATTACGAGCAGTAATGGTATCTGCTGAAGTTGAGGATGTT
ATTCGCAAAGGGATCCGTCAGACCTCTGGCAGTACCTTCCTCAGCCTTGACCCGGAAGCC
TCCGCTAATTTGATGGATCTCATTACACTTAAGTTGGATGATTTATTGATTGCACATAAA
GATCTTGTCCTCCTTACGTCTGTCGATGTCCGTCGATTTATTAAGAAAATGATTGAAGGT
CGTTTTCCGGATCTGGAGGTTTTATCTTTCGGTGAGATAGCAGATAGCAAGTCAGTGAAT
GTTATAAAAACAATATAA
>EECEBICD_00569 Surface presentation of antigens protein SpaK
ATGCAACATTTGGATATCGCTGAATTAGTTCGTTCCGCACTGGAAGTAAGTGGTTGCGAT
CCTTCACTCATCGGAGGAATAGATAGCCATTCAACAATTGTTCTGGATTTATTTGCATTG
CCAAGTATCTGTATCAGCGTCAAGGACGATGATGTATGGATCTGGGCGCAATTGGGTGCT
GACAGCATGGTGGTATTACAACAGCGGGCTTATGAAATCTTAATGACCATCATGGAAGGA
TGCCATTTTGCCCGCGGCGGGCAATTACTACTGGGGGAGCAGAATGGGGAGCTAACGCTT
AAAGCCTTAGTGCATCCGGATTTTTTATCTGACGGTGAAAAGTTCTCTACTGCCTTGAAT
GGGTTTTACAACTATCTGGAAGTTTTTAGTCGGTCGCTAATGAGATGA
>EECEBICD_00570 putative ATP synthase SpaL
ATGAAAACACCTCGTTTACTGCAATATCTGGCCTACCCACAAAAAATAACCGGCCCAATT
ATTGAGGCGGAATTGCGCGATGTGGCCATTGGCGAACTGTGTGAAATACGCCGTGGCTGG
CACCAAAAACAGGTTGTTGCACGTGCGCAGGTGGTTGGCTTACAGCGGGAACGCACCGTG
TTGAGCCTTATCGGCAATGCCCAGGGGCTGAGCCGCGATGTCGTGCTTTATCCCACTGGA
CGTGCGTTATCGGCGTGGGTGGGATACTCGGTATTAGGCGCGGTGTTGGATCCGACAGGG
AAAATCGTTGAGCGTTTTACCCCTGAAGTGGCGCCGATTAGCGAAGAACGCGTTATTGAT
GTCGCACCGCCGTCTTACGCTTCACGCGTTGGCGTCCGTGAACCGCTGATTACCGGTGTG
CGCGCGATTGACGGGTTATTGACCTGTGGCGTAGGCCAGCGAATGGGCATTTTTGCCTCC
GCAGGATGCGGTAAGACCATGCTGATGCATATGCTGATCGAGCAAACGGAGGCGGATGTC
TTTGTTATCGGTCTTATCGGTGAACGAGGCCGTGAGGTCACTGAATTCGTGGATATGTTG
CGCGCTTCGCATAAGAAAGAAAAATGCGTGCTGGTTTTTGCCACTTCCGATTTCCCCTCG
GTCGATCGCTGCAATGCGGCGCAACTGGCGACAACCGTAGCGGAATATTTTCGCGACCAG
GGAAAACGGGTCGTGCTTTTTATCGATTCCATGACCCGTTATGCGCGTGCTTTGCGAGAC
GTGGCACTGGCGTCGGGAGAGCGTCCGGCCCGTCGAGGTTATCCCGCCTCCGTATTCGAT
AATTTGCCCCGCTTGCTGGAACGCCCAGGGGCGACCAGCGAGGGAAGCATTACTGCCTTT
TATACGGTACTGCTGGAAAGCGAGGAAGAGGCGGACCCGATGGCGGATGAAATTCGCTCT
ATCCTTGACGGTCACCTGTATCTGAGCAGAAAGCTGGCCGGGCAGGGACATTACCCGGCA

ATCGATGTACTGAAAAGCGTAAGCCGCGTTTTTGGACAAGTCACGACGCCGACACATGCT
GAACAGGCATCTGCCGTGCGTAAATTAATGACGCGTTTGGAAGAGCTCCAGCTTTTCATT
GACTTGGGAGAATATCGTCCTGGCGAAAATATCGATAACGATCGGGCGATGCAGATGCGG
GATAGCCTGAAAGCCTGGTTATGCCAGCCGGTAGCGCAGTATTCATCCTTTGATGACACG
TTGAGCGGTATGAATGCATTCGCTGACCAGAATTAA
>EECEBICD_00571 hypothetical protein
ATGCATTCGCTGACCAGAATTAAAGTATTGCAGCGGCGCTGTACGGTATTTCATTCACAG
TGTGAGTCGATATTACTTCGCTATCAGGATGAGGACCGCGGGCTGCAGGCCGAGGAGGAG
GCGATCCTTGAACAAATAGCGGGTCTGAAATTGTTATTAGATACGCTGCGTGCAGAAAAC
AGACAGCTCAGTCGTGAGGAAATTTATACGTTATTACGTAAGCAGTCTATTGTTCGCCGG
CAGATAAAAGATTTAGAACTCCAGATTATACAAATTCAGGAAAAACGGAGCGAGCTGGAA
AAGAAAAGGGAAGAGTTTCAGAAAAAAAGTAAATATTGGTTGCGCAAAGAAGGGAACTAT
CAACGCTGGATAATCCGTCAGAAAAGATTCTATATCCAGCGAGAGATACAGCAGGAAGAG
GCCGAGTCAGAGGAGATAATTTAA
>EECEBICD_00572 hypothetical protein
ATGGGCGATGTGTCAGCTGTCAGTTCATCCGGGAACATTTTACTGCCGCAGCAGGATGAG
GTTGGCGGTTTATCAGAAGCATTAAAAAAAGCGGTGGAAAAACATAAGACAGAATATTCC
GGTGATAAAAAAGATCGCGACTATGGCGATGCTTTCGTAATGCATAAAGAAACGGCTTTA
CCGGTATTACTGGCGGCATGGCGACATGGCGCGCCAGCGAAATCAGAACATCACAATGGC
AACGTTTCTGGTCTGCATCATAACGGAAAAGGCGAACTCAGGATTGCTGAAAAACTGTTG
AAAGTCACTGCTGAAAAATCTGTCGGTTTGATTTCTGCGGAGGCCAAAGTAGATAAATCC
GCAGCGTTGCTATCGTCTAAAAATAGGCCGTTAGAAAGCGTAAGCGGTAAAAAATTATCT
GCTGATTTAAAAGCCGTGGAGTCCGTTAGTGAAGTAGCCGATAACGCCACGGGAATCTCT
GACGATAATATCAAGGCATTGCCTGGGGATAATAAAGCCATCGCGGGCGAAGGCGTTCGT
AAAGAGGGCGCGCCGCTGGCGCGGGATGTCGCACCTGCCCGAATGGCCGCAGCCAATACC
GGTAAGCCTGACGATAAAGATCATAAAAAGGTTAAAGATGTTTCTCAGCTTCCGCTGCAA
CCAACCACTATCGCCGATCTTAGCCAATTAACCGGCGGCGATGAAAAAATGCCTTTAGCG
GCGCAATCAAAGCCGATGATGACTATTTTTCCGACTGCCGATGGCGTGAAAGGAGAGGAT
AGCTCGCTGACTTACCGTTTTCAGCGCTGGGGAAATGACTATTCCGTCAATATTCAGGCG
CGGCAAGCAGGGGAGTTTTCGTTAATACCGTCAAATACGCAGGTTGAACATCGTTTGCAT
GATCAATGGCAAAACGGTAATCCCCAGCGCTGGCACCTGACGCGAGACGATCAACAAAAT
CCGCAGCAGCAACAGCACAGACAGCAATCTGGCGAGGAGGATGACGCCTGA
>EECEBICD_00573 Surface presentation of antigens protein SpaO
ATGTCATTGCGTGTGAGACAGATTGATCGTCGCGAATGGCTATTGGCGCAAACCGCGACA
GAATGCCAGCGCCATGGCCAGGAAGCTACGCTGGAATATCCGACGCGACAAGGAATGTGG
GTTCGGTTGAGCGATGCAGAAAAACGGTGGTCGGCGTGGATTCAACCTGGGGACTGGCTT
GAGCATGTCTCTCCCGCTCTGGCGGGGGCGGCGGTTTCTGCTGGCGCTGAGCACCTGGTC
GTTCCCTGGCTTGCTGCGACAGAGCGACCGTTTGAGTTGCCCGTGCCGCATTTGTCCTGT
CGGCGTTTATGCGTAGAGAACCCCGTGCCGGGAAGCGCGCTGCCGGAAGGGAAATTGTTG
CACATTATGAGCGATCGGGGCGGCCTGTGGTTTGAATATCTTCCTGAACTGCCTGCAGTC
GGGGGCGGCAGGCCGAAAATGCTGCGTTGGCCGTTGCGCTTTGTAATCGGTAGCAGTGAT
ACGCAGCGTTCGTTGCTGGGCCGAATCGGGATCGGAGATGTACTCCTGATTCGTACTTCC
CGTGCGGAAGTTTATTGCTACGCGAAAAAGTTAGGTCATTTCAACCGTGTTGAAGGGGGA
ATTATTGTGGAAACGTTAGATATTCAACATATCGAAGAAGAAAATAATACAACTGAAACT
GCAGAAACTCTGCCTGGCTTGAATCAATTGCCCGTCAAACTGGAATTTGTTTTGTATCGT
AAGAACGTTACCCTCGCCGAACTCGAAGCCATGGGGCAGCAACAGCTATTATCACTGCCG
ACCAATGCTGAACTTAACGTTGAAATTATGGCGAATGGTGTTTTGCTGGGTAATGGCGAA
CTGGTACAGATGAATGACACCTTAGGCGTTGAGATCCATGAATGGCTGAGCGAGTCTGGT
AATGGGGAATGA
>EECEBICD_00574 Surface presentation of antigens protein SpaP
ATGGGGAATGATATCTCATTAATTGCCTTACTGGCATTTTCCACCCTGTTGCCATTTATT
ATTGCGTCAGGAACCTGTTTCGTTAAATTTTCTATTGTATTTGTCATGGTGCGTAACGCC
CTGGGATTGCAGCAGATACCTTCAAATATGACGCTTAACGGCGTCGCATTGCTGCTTTCT
ATGTTTGTTATGTGGCCAATAATGCATGATGCCTACGTCTATTTTGAGGACGAAGATGTC

ACCTTTAATGATATTTCGTCATTAAGTAAACACGTTGATGAAGGTCTGGATGGTTATCGC
GATTATCTGATCAAATATTCAGATCGCGAGTTAGTTCAGTTTTTTGAAAACGCGCAACTG
AAGCGTCAGTATGGAGAAGAGACCGAGACGGTAAAGCGTGACAAAGATGAAATTGAAAAA
CCATCAATATTTGCGTTATTACCTGCTTATGCGCTGAGCGAAATAAAAAGCGCGTTTAAA
ATTGGTTTTTATCTCTATTTGCCCTTTGTTGTCGTCGACCTGGTGGTATCCAGCGTGCTA
CTGGCGCTGGGGATGATGATGATGAGTCCGGTGACGATATCTACACCTATTAAGTTGGTG
CTTTTTGTCGCGCTTGATGGCTGGACCTTACTGTCTAAGGGATTGATATTACAGTATATG
GACATTGCAACATGA
>EECEBICD_00575 Surface presentation of antigens protein SpaQ
ATGGATGATTTAGTGTTTGCAGGTAATAAGGCGCTCTATCTTGTTTTGATCCTGTCAGGG
TGGCCGACGATTGTCGCAACGATTATCGGCCTCCTGGTAGGGTTATTCCAGACGGTAACG
CAATTACAGGAACAGACGCTGCCTTTTGGCATTAAATTACTTGGCGTGTGTTTATGCTTG
TTTTTACTGTCTGGCTGGTATGGCGAAGTTTTACTCTCTTACGGGCGTCAGGTGATATTC
CTGGCGTTGGCTAAGGGGTAA
>EECEBICD_00576 Surface presentation of antigens protein SpaR
ATGCTTTACGCGTTGTACTTTGAAATTCATCACCTGGTTGCGTCTGCGGCACTAGGGTTT
GCTCGCGTAGCGCCGATTTTTTTCTTCCTGCCGTTTTTGAATAGCGGGGTATTAAGCGGC
GCGCCGAGAAACGCCATTATCATCCTGGTGGCATTGGGAGTATGGCCGCATGCATTGAAC
GAGGCGCCGCCGTTTTTATCGGTGGCGATGATCCCGTTAGTTCTGCAAGAAGCGGCGGTA
GGCGTCATGCTGGGCTGTCTGCTGTCATGGCCTTTTTGGGTTATGCATGCGCTGGGTTGT
ATTATCGATAACCAGCGAGGGGCAACGCTAAGTAGCAGTATCGATCCGGCAAACGGTATT
GATACCTCGGAAATGGCTAATTTCCTGAATATGTTTGCCGCTGTCGTTTATTTACAAAAC
GGCGGTCTGGTCACGATGGTTGACGTGTTAAATAAAAGCTATCAGCTATGCGATCCGATG
AACGAGTGCACGCCTTCATTACCGCCGCTATTAACGTTTATTAATCAGGTGGCTCAAAAC
GCCTTGGTTCTGGCCAGTCCGGTGGTATTAGTGCTGTTGCTGTCAGAAGTATTCCTGGGT
TTATTGTCGCGCTTTGCTCCGCAAATGAACGCTTTTGCGATTTCACTGACGGTAAAAAGC
GGTATTGCCGTTTTAATTATGCTGCTTTATTTCTCTCCGGTACTACCGGACAATGTACTG
CGACTCTCTTTCCAGGCCACAGGGTTAAGCAGTTGGTTTTACGAGCGAGGGGCGACGCAT
GTCCTCGAATAA
>EECEBICD_00577 Surface presentation of antigens protein SpaS
ATGTCCTCGAATAAAACAGAAAAACCGACTAAAAAACGGCTGGAAGACTCCGCTAAAAAA
GGCCAGTCATTTAAAAGTAAAGATCTCATTATCGCCTGCCTGACGCTGGGAGGAATTGCC
TATCTGGTGTCGTATGGCTCATTTAATGAGTTTATGGGGATAATTAAGATCATTATTGCG
GATAATTTTGATCAGAGCATGGCTGACTACAGTTTGGCCGTTTTTGGGATAGGGTTAAAA
TATCTGATTCCATTTATGCTGCTCTGCTTAGTGTGTTCCGCATTACCGGCGTTATTACAG
GCCGGTTTTGTGCTGGCGACAGAAGCATTAAAGCCTAATTTATCGGCGTTAAACCCGGTA
GAAGGGGCAAAAAAACTTTTTAGTATGCGCACGGTTAAAGATACGGTCAAAACCCTACTG
TATCTCTCATCCTTTGTGGTGGCCGCCATCATTTGTTGGAAGAAATATAAGGTTGAAATC
TTTTCTCAGCTAAATGGCAATATTGTAGGTATTGCCGTCATTTGGCGTGAACTTCTCCTC
GCATTGGTATTAACTTGCCTTGCTTGCGCATTGATTGTCTTATTATTGGATGCTATTGCG
GAATATTTCCTGACCATGAAAGATATGAAAATGGATAAGGAAGAAGTGAAGCGTGAAATG
AAGGAGCAGGAAGGGAACCCAGAGGTTAAATCTAAAAGACGTGAAGTTCATATGGAAATT
CTGTCTGAACAGGTGAAATCTGATATTGAAAACTCACGCCTGATTGTTGCCAACCCCACG
CATATTACGATCGGGATTTATTTTAAACCCGAATTGATGCCGATTCCGATGATCTCGGTG
TATGAAACGAATCAGCGCGCACTGGCCGTCCGCGCCTATGCGGAGAAGGTTGGCGTACCT
GTGATCGTCGATATCAAACTGGCGCGCAGTCTTTTCAAAACCCATCGCCGTTATGATCTG
GTGAGTCTGGAAGAAATTGATGAAGTTTTACGTCTTCTGGTTTGGCTGGAAGAGGTAGAA
AACGCGGGCAAAGACGTTATTCAGCCACAAGAAAACGAGGTACGGCATTGA
>EECEBICD_00578 Chaperone protein SicA
ATGGATTATCAAAATAATGTCAGCGAAGAACGTGTTGCGGAAATGATTTGGGATGCCGTT
AGTGAAGGCGCCACGCTAAAAGACGTTCATGGGATCCCTCAAGATATGATGGACGGTTTA
TATGCTCATGCTTATGAGTTTTATAACCAGGGACGACTGGATGAAGCTGAGACATTCTTT
CGTTTCTTATGCATTTATGATTTTTACAATCCCGATTACACCATGGGACTGGCGGCGGTA
TGCCAACTGAAAAAACAATTTCAGAAAGCATGTGACCTTTATGCAGTAGCGTTTACGTTA

CTTAAAAATGATTATCGCCCCGTTTTTTTTACCGGGCAGTGTCAATTATTAATGCGTAAG
GCAGCAAAAGCCAGACAGTGTTTTGAACTTGTCAATGAACGTACTGAAGATGAGTCTCTG
CGGGCAAAAGCGTTGGTCTATCTGGAGGCGCTAAAAACGGCGGAGACAGAGCAGCACAGT
GAACAAGAAAAGGAATAA
>EECEBICD_00579 Cell invasion protein SipB
ATGGTAAATGACGCAAGTAGCATTAGCCGTAGCGGATATACCCAAAATCCGCGCCTCGCT
GAGGCGGCTTTTGAAGGCGTTCGTAAGAACACGGACTTTTTAAAAGCGGCGGATAAAGCT
TTTAAAGATGTGGTGGCAACGAAAGCGGGCGACCTTAAAGCCGGAACAAAGTCCGGCGAG
AGCGCTATTAATACGGTGGGTCTGAAGCCGCCTACGGACGCCGCCCGGGAAAAACTCTCC
AGCGAAGGGCAATTGACATTACTGCTTGGCAAGTTAATGACGCTACTGGGTGATGTTTCG
CTGTCTCAACTGGAGTCTCGTCTGGCGGTATGGCAGGCGATGATTGAGTCACAAAAAGAG
ATGGGGATTCAGGTATCGAAAGAATTCCAGACGGCTCTGGGAGAGGCTCAGGAGGCGACG
GATCTCTATGAAGCCAGTATCAAAAAGACGGATACCGCCAAGAGTGTTTATGACGCTGCG
ACCAAAAAACTGACGCAGGCGCAAAATAAATTGCAATCGCTGGACCCGGCTGACCCCGGC
TATGCACAAGCTGAAGCCGCGGTAGAACAGGCCGGAAAAGAAGCGACAGAGGCGAAAGAG
GCCTTAGATAAGGCCACGGATGCGACGGTTAAAGCAGGCACAGACGCCAAAGCGAAAGCC
GAGAAAGCGGATAACATTCTGACCAAATTCCAGGGAACGGCTAATGCCGCCTCTCAGAAT
CAGGTTTCCCAGGGTGAGCAGGATAATCTGTCAAATGTCGCCCGCCTCACTATGCTCATG
GCCATGTTTATTGAGATTGTGGGCAAAAATACGGAAGAAAGCCTGCAAAACGATCTTGCG
CTTTTCAACGCCTTGCAGGAAGGGCGTCAGGCGGAGATGGAAAAGAAATCGGCTGAATTC
CAGGAAGAGACGCGCAAAGCCGAGGAAACGAACCGCATTATGGGATGTATCGGGAAAGTC
CTCGGCGCGCTGCTAACCATTGTCAGCGTTGTGGCCGCTGTTTTTACCGGTGGGGCGAGT
CTGGCGCTGGCTGCGGTGGGACTTGCGGTAATGGTGGCCGATGAAATTGTGAAGGCGGCG
ACGGGGGTGTCGTTTATTCAGCAGGCGCTAAACCCGATTATGGAGCATGTGCTGAAGCCG
TTAATGGAGCTGATTGGCAAGGCGATTACCAAAGCGCTGGAAGGATTAGGCGTCGATAAG
AAAACGGCAGAGATGGCCGGCAGCATTGTTGGTGCGATTGTCGCCGCTATTGCCATGGTG
GCGGTCATTGTGGTGGTCGCAGTTGTCGGGAAAGGCGCGGCGGCGAAACTGGGTAACGCG
CTGAGCAAAATGATGGGCGAAACGATTAAGAAGTTGGTGCCTAACGTGCTGAAACAGTTG
GCGCAAAACGGCAGCAAACTCTTTACCCAGGGGATGCAACGTATTACTAGCGGTCTGGGT
AATGTAGGTAGCAAGATGGGCCTGCAAACGAATGCCTTAAGTAAAGAGCTGGTAGGTAAT
ACCCTAAATAAAGTGGCGTTGGGCATGGAAGTCACGAATACCGCAGCCCAGTCAGCCGGT
GGTGTTGCCGAGGGCGTATTTATTAAAAATGCCAGCGAGGCGCTTGCTGATTTTATGCTC
GCCCGTTTTGCCATGGATCAGATTCAGCAGTGGCTTAAACAATCCGTAGAAATATTTGGT
GAAAACCAGAAGGTAACGGCGGAACTGCAAAAAGCCATGTCTTCTGCGGTACAGCAAAAT
GCGGATGCTTCGCGTTTTATTCTGCGCCAGAGTCGCGCATAA
>EECEBICD_00580 Cell invasion protein SipC
ATGTTAATTAGTAATGTGGGAATAAATCCCGCCGCTTATTTAAATAATCATTCTGTTGAG
AATAGTTCACAGACAGCTTCGCAATCCGTTAGCGCTAAAGATATTCTGAATAGTATTGGT
ATTAGCAGCAGTAAAGTCAGTGACCTGGGGTTGAGTCCTACACTGAGCGCGCCTGCGCCA
GGGGTATTAACGCAAACCCCCGGAACGATCACGTCCTTTTTAAAAGCCAGTATTCAAAAT
ACCGACATGAATCAGGATTTGAATGCTCTGGCAAATAATGTCACGACTAAAGCGAATGAG
GTTGTGCAAACCCAGTTACGCGAGCAGCAGGCAGAAGTCGGAAAGTTTTTTGATATTAGC
GGAATGTCTTCCAGTGCCGTTGCGCTGTTGGCTGCCGCGAATACGTTAATGCTGACGTTG
AACCAGGCTGATAGCAAACTGTCTGGTAAGTTGTCATTAGTCAGCTTTGATGCAGCTAAA
ACGACAGCAAGCTCCATGATGCGCGAAGGGATGAATGCGTTGTCCGGTAGTATTTCCCAG
AGCGCGCTTCAGTTGGGGATCACTGGCGTGGGCGCCAAACTGGAATATAAGGGGCTGCAG
AATGAAAGAGGCGCGCTTAAACATAATGCCGCGAAGATCGATAAACTGACCACTGAAAGC
CACAGTATTAAAAACGTGCTGAACGGGCAGAATAGCGTCAAACTCGGTGCTGAAGGCGTC
GATTCTCTGAAATCGTTAAATATGAAGAAAACCGGTACCGATGCGACAAAAAATCTTAAT
GATGCGACGCTTAAATCTAATGCCGGAACCAGCGCCACGGAAAGTCTGGGTATTAAAGAC
AGTAATAAACAAATCTCCCCTGAACATCAGGCTATTCTGTCGAAACGTCTTGAGTCTGTC
GAATCCGATATTCGTCTTGAGCAGAATACCATGGATATGACCCGAATTGATGCGCGCAAG
ATGCAGATGACGGGCGATCTGATTATGAAGAACTCGGTCACGGTCGGTGGTATTGCAGGG
GCGTCCGGGCAGTACGCCGCTACTCAGGAACGTTCCGAGCAGCAAATTAGCCAGGTGAAT

AACCGTGTTGCCAGCACCGCATCGGACGAAGCCCGTGAAAGTTCACGTAAATCGACCAGC
CTGATTCAGGAAATGCTGAAAACAATGGAGAGCATTAACCAGTCGAAAGCATCCGCACTT
GCTGCTATCGCAGGCAATATTCGCGCTTAA
>EECEBICD_00581 Cell invasion protein SipD
ATGCTTAATATTCAAAATTATTCCGCTTCTCCTCATCCGGGGATCGTTGCCGAACGGCCG
CAGACTCCCTCGGCGAGCGAGCACGTCGAGACTGCCGTGGTACCGTCTACCACAGAACAT
CGCGGTACAGATATCATTTCATTATCGCAGGCGGCTACTAAAATCCAGCAGGCACAGCAG
ACGCTGCAGTCAACGCCACCGATCTCTGAAGAGAATAATGACGAGCGCACGCTGGCGCGC
CAGCAGTTGACCAGCAGCCTGAATGCGCTGGCGAAGTCTGGCGTGTCATTATCCGCAGAA
CAAAATGAGAACCTGCGGAGCGCGTTTTCTGCGCCGACGTCGGCCTTATTTAGCGCTTCG
CCTATGGCGCAGCCGAGAACAACCATTTCTGATGCTGAGATTTGGGATATGGTTTCCCAA
AATATATCGGCGATAGGTGACAGCTATCTGGGCGTTTATGAAAACGTTGTCGCAGTCTAT
ACCGATTTTTATCAGGCCTTCAGTGATATTCTTTCCAAAATGGGAGGCTGGTTATTACCA
GGTAAGGACGGTAATACCGTTAAGCTAGATGTTACCTCACTCAAAAATGATTTAAACAGT
TTAGTCAATAAATATAATCAAATAAACAGTAATACCGTTTTATTTCCAGCGCAGTCAGGC
AGCGGCGTTAAAGTAGCCACTGAAGCGGAAGCGAGACAGTGGCTCAGTGAATTGAATTTA
CCGAATAGCTGCCTGAAATCTTATGGATCCGGTTATGTCGTCACCGTTGATCTGACGCCA
TTACAAAAAATGGTTCAGGATATTGATGGTTTAGGCGCGCCGGGAAAAGACTCAAAACTC
GAAATGGATAACGCCAAATATCAAGCCTGGCAGTCGGGTTTTAAAGCGCAGGAAGAAAAT
ATGAAAACCACATTACAGACGCTGACGCAAAAATATAGCAATGCCAATTCATTGTACGAC
AACCTGGTAAAAGTGCTGAGCAGTACGATAAGTAGCAGCCTGGAAACCGCCAAAAGCTTC
CTGCAAGGATAA
>EECEBICD_00582 Cell invasion protein SipA
ATGGTTACAAGTGTAAGGACTCAGCCCCCCGTCATAATGCCAGGTATGCAGACCGAGATC
AAAACGCAGGCCACGAATCTTGCGGCGAATCTTTCCGCAGTCAGAGAAAGTGCCACAACG
ACGCTGTCAGGGGAAATTAAAGGCCCGCAACTGGAAGATTTTCCCGCGCTGATCAAACAG
GCGAGTCTGGATGCATTGTTTAAATGCGGGAAAGACGCTGAGGCGTTAAAAGAAGTTTTT
ACCAATTCAAATAATGTCGCCGGTAAGAAAGCGATAATGGAGTTTGCCGGGCTCTTTCGT
TCAGCGCTCAACGCCACCAGTGATTCTCCTGAGGCGAAGACGCTACTGATGAAGGTGGGG
GCAGAGTATACCGCGCAAATCATAAAAGATGGCCTGAAAGAAAAGTCAGCTTTTGGGCCA
TGGCTGCCAGAAACAAAGAAAGCGGAAGCGAAGCTGGAAAACCTGGAAAAGCAGCTGTTA
GATATTATCAAAAATAACACTGGTGGTGAATTAAGTAAATTATCGACGAATCTTGTTATG
CAGGAGGTGATGCCCTATATTGCCAGCTGCATTGAACATAACTTTGGCTGTACGTTAGAT
CCGTTAACCCGCAGCAATCTTACGCACCTTGTTGACAAAGCGGCGGCGAAGGCGGTTGAG
GCGCTTGATATGTGCCACCAAAAATTAACGCAAGAGCAGGGTACCAGCGTAGGACGGGAA
GCCCGGCACCTTGAAATGCAAACGTTGATACCCCTGCTGCTACGTAATGTTTTTGCACAA
ATTCCTGCAGATAAACTGCCTGACCCTAAAATTCCGGAGCCTGCGGCTGGACCAGTACCT
GATGGTGGGAAAAAAGCAGAACCTACGGGTATTAATATCAATATTAATATTGATAGCAGT
AACCATAGCGTGGATAACAGTAAGCATATTAACAATAGCCGAAGCCATGTCGATAATAGC
CAGCGCCATATCGATAATAGCAACCATGATAATAGCCGGAAGACGATTGATAATAGCCGA
ACATTTATTGATAACAGCCAACGCAATGGCGAGTCACACCATTCGACTAACAGCAGCAAC
GTAAGTCATAGTCATTCGCGTGTGGATTCGACTACGCATCAAACGGAGACGGCACACAGC
GCCAGCACAGGGGCAATTGACCATGGCATCGCGGGTAAAATTGACGTCACGGCTCATGCG
ACGGCAGAGGCTGTGACCAACGCTTCATCCGAAAGTAAAGATGGAAAGGTGGTCACGTCA
GAAAAGGGCACTACGGGTGAAACAACCTCTTTTGATGAAGTCGATGGCGTAACCAGCAAG
AGCATTATCGGCAAGCCGGTACAGGCCACGGTGCACGGCGTTGATGATAATAAACAGCAA
AGCCAGACGGCAGAGATTGTGAATGTGAAGCCGCTTGCCAGTCAACTGGCTGGCGTCGAG
AATGTTAAAACCGATACCTTACAATCAGACACGACGGTAATTACAGGTAATAAAGCCGGT
ACGACCGATAATGATAATAGTCAGACAGATAAGACTGGCCCATTTTCTGGTTTGAAGTTT
AAGCAAAATAGTTTCCTCTCAACAGTACCGAGCGTTACGAATATGCATTCAATGCATTTC
GACGCCCGTGAGACGTTTTTGGGTGTGATACGTAAAGCCCTGGAGCCAGATACCTCCACA
CCGTTCCCTGTACGCAGAGCGTTTGACGGCTTGCGTGCGGAAATATTACCCAATGACACG
ATAAAGAGTGCAGCGTTGAAAGCGCAATGTAGCGATATTGACAAGCACCCAGAATTGAAA
GCGAAAATGGAGACTCTCAAAGAGGTCATTACTCATCATCCACAAAAAGAAAAACTGGCT

GAGATTGCACTGCAGTTTGCCAGAGAGGCGGGGCTGACCAGGCTAAAAGGGGAAACTGAC
TATGTGTTAAGTAATGTGCTGGACGGCCTTATCGGAGACGGTAGCTGGCGAGCTGGCCCG
GCTTACGAGTCATACCTGAATAAACCTGGCGTGGATCGGGTTATTACTACCGTTGATGGC
TTGCACATGCAGCGTTAA
>EECEBICD_00583 Acyl carrier protein
ATGAATATGGATATTGAAGCAAGAGTCAAAAAAGTGATCACCTCCTGTATTGCCGTTGAT
GTTGATAGTATCAATGGTCAGACCCATCTGGTTGAGGATCTTTACGCTGACTCATTGGAT
TTAATTGATATTGTATTTGGTCTTAGTGAGGAGTTTGACATTAGTTGCAATGAAAACGAT
CTTCCTGATATGACGACCTTTGCGGATATATGCCGTGTTGTTAAAAAAAGTCTTGAGTCC
AGGGTGTAG
>EECEBICD_00584 hypothetical protein
ATGAAAATCATCACACATGTAGTACCCGGTTCAGGTATGGCTGCAATATATGATGATATT
GCTGACAGTTCGCGTTTTGTTATTAAAGGCAAGTTACGTCATGTTGAAAATGATCCGAAG
GAGCTGCTGATATGTGTTCCGATGCGTAGTGAATGGCTATTCTACTGGATTAAAGGAGAG
AAATATTGCGCCCGGCGATGGGCGCGAAAAAGTATCAAGACATTGTGTAACCAGATTAAA
TTTGAAGAAGCAATCTTATAA
>EECEBICD_00585 Chaperone protein sicP
TTGCAAGCACACCAGGATATTATCGCTAATATTGGTGAGAAATTGGGTTTACCGCTCACT
TTTGACGACAACAATCAGTGCTTATTATTACTCGATAGCGATATTTTTACGTCTATTGAA
GCTAAAGATGATATCTGGTTATTGAACGGTATGATTATACCGTTATCGCCTGTTTGTGGC
GATTCTATCTGGCGGCAGATTATGGTGATTAATGGTGAACTGGCTGCGAATAATGAAGGT
ACGTTAGCGTATATTGATGCCGCAGAGACGTTGTTGCTTATACATGCAATTACCGATCTG
ACAAATACTTACCATATTATATCGCAGCTTGAGTCATTTGTGAATCAGCAGGAAGTGCTC
AAAAACATACTGCAGGAATATGCTAAAGTATGA
>EECEBICD_00586 Secreted effector protein SptP
TTGAATAATTTAACGTTGTCTTCGTTTTCAAAAGTTGGTGTGTCGAATGATGCCCGACTT
TATATTGCTAAGGAAAATACTGATAAGGCATATGTTGCGCCTGAAAAATTTTCGTCAAAA
GTATTAACCTGGCTTGGAAAAATGCCGTTATTTAAAAACACTGAAGTGGTGCAAAAACAT
ACGGAAAATATCAGAGTACAGGACCAAAAGATTTTACAGACATTTCTCCATGCACTAACG
GAAAAATATGGGGAAACAGCGGTTAATGACGCACTGTTAATGTCCCGTATAAATATGAAC
AAACCCCTTACCCAACGTTTAGCAGTGCAGATCACGGAGTGTGTAAAAGCTGCTGACGAA
GGGTTTATAAACCTTATTAAGAGCAAGGATAATGTTGGTGTCAGGAATGCCGCTTTAGTC
ATAAAAGGCGGCGATACAAAAGTGGCAGAAAAAAATAACGATGTTGGAGCAGAAAGTAAG
CAACCTTTACTCGATATCGCGCTAAAGGGACTTAAAAGAACATTACCGCAACTGGAACAA
ATGGATGGAAATAGCCTGCGAGAAAACTTCCAGGAGATGGCTTCAGGTAACGGCCCGCTG
CGTTCATTAATGACGAATTTACAGAATTTAAATAAAATTCCAGAGGCTAAACAACTTAAT
GATTATGTTACAACGTTAACAAATATTCAGGTCGGTGTAGCCCGGTTCTCACAATGGGGA
ACCTGTGGAGGAGAGGTTGAGAGGTGGGTTGATAAAGCCTCTACTCATGAATTAACCCAG
GCTGTAAAAAAAATTCACGTTATTGCTAAAGAACTGA
>EECEBICD_00587 Secreted effector protein SptP
ATGCCACAGACGATGAGCGGACCGACACTGGGCCTGGCACGGTTTGCGGTCAGCAGCATT
CCAATTAATCAGCAAACCCAGGTGAAACTGAGCGATGGAATGCCTGTGCCAGTGAATACG
TTAACTTTTGACGGTAAGCCTGTGGCATTAGCCGGTTCGTACCCAAAAAATACGCCGGAT
GCGCTGGCAGCGCATATGAAAATGCTTCTTGAAAAAGAATGCTCATGCCTGGTGGTGTTA
ACGTCGGAAGATCAGATGCAGGCAAAACAATTACCACCCTATTTCAGAGGGAGCTACACC
TTTGGCGAGGTGCATACCAACAGCCAAAAAGTGAGCTCAGCCAGTCAGGGAGAAGCGATA
GACCAATACAATATGCAACTGTCCTGCGGGGAAAAGCGGTATACAATCCCGGTATTGCAT
GTGAAAAATTGGCCAGATCACCAGCCGTTACCGTCTACGGATCAGTTAGAATACCTGGCG
GATAGGGTGAAAAATAGTAACCAAAATGGCGCGCCGGGGCGTAGTTCATCAGATAAGCAT
TTACCGATGATTCATTGTCTGGGCGGAGTGGGGAGAACCGGAACGATGGCGGCGGCCCTT
GTACTTAAGGATAATCCTCATAGTAATCTGGAGCAGGTACGTGCAGATTTCCGGAATTCA
CGGAACAATAGAATGCTGGAAGACGCCTCACAGTTTGTACAACTAAAAGCAATGCAAGCC
CAGTTGCTTATGACGACGGCACGCTGA
>EECEBICD_00588 hypothetical protein

ATGCATTATTTTTTTATCATCGTAATCTGGTTGCTTAGCATAAATACGGCATGGGCTGAT
TGCTGGCTTCAGGCTGAAAAAATGTTCAATATTGAATCCGAACTACTTTACGCTATCGCC
CAGCAGGAATCGGCGATGAAACCTGGCGCCATTGGTCATAACCGAGATGGTTCAACCGAT
CTTGGCCTGATGCAAATTAACAGCTTCCATATGAAAAGGCTGAAAAAAATGGGGATTAGT
GAAAAACAGTTGTTACAGGACCCCTGCATTTCTGTCATTGTGGGCGCTTCCATTTTATCA
GATATGATGAAAATCTACGGTTATAGCTGGGAGGCCGTTGGCGCTTATAATGCCGGGACG
TCGCCGAAACGATCGGATATAAGGAAACGTTATGCTAAAAAAATTTGGGAGAATTACAGA
AAATTAAAAGGAATGTCAGCAGAAGAGAAAAACAAAAGACTTTCTATCGCGTCAAACAAA
TAA
>EECEBICD_00589 Transcriptional regulator HilA
ATGCAGGATCAGGTTCAATCCGAGAGTCTGCATTACTCTATCGTGAAGGGATTATCGCAG
TATGCGCCCTTTGGCCTGAGCGTGCTGCCGGTGACCATTACGAAGAACTGCCGCAGTGTT
AAGGATATTCTTGAGCTCATGGATCAATTACGCCCCGATTATTATATCTCCGGGCAGATG
ATACCCGATGGTAATGATAATATTGTACAGATTGAGATAGTTCGGGTTAAAGGTTATCAC
CTGCTGCACCAGGAAAGCATTAAGTTGATAGAACACCAACCCGCTTCTCTCTTGCAAAAC
AAAATTGCGAATCTTTTGCTCAGATGTATTCCCGGACTTCGCTGGGACACAAAGCAGATT
AGCGAGCTAAATTCGATTGACAGTACTATGGTTTACTTACGCGGTAAGCATGAGTTAAAT
CAATACACCCCCTATAGCTTACAGCAAGCGCTTAAATTGCTGACTCAATGCGTTAACATG
TCGCCAAACAGCATTGCGCCTTACTGTGCGCTGGCAGAATGCTACCTCAGCATGGCGCAA
ATGGGGATTTTTGATAAACAAAACGCTATGATCAAAGCTAAAGAACATGCGATTAAGGCG
ACAGAGCTGGACCACAATAATCCACAAGCTTTAGGATTACTGGGGCTAATTAATACGATT
CACTCAGAATACATCGTCGGGAGTTTGCTATTCAAACAAGCTAACTTACTTTCGCCCATT
TCTGCAGATATTAAATATTATTATGGCTGGAATCTTTTCATGGCTGGTCAGTTGGAGGAG
GCCTTACAAACGATTAACGAGTGTTTAAAATTGGACCCAACGCGCGCAGCCGCAGGGATC
ACTAAGCTGTGGATTACCTATTATCATACCGGTATTGATGATGCTATACGTTTAGGCGAT
GAATTACGCTCACAACACCTGCAGGATAATCCAATATTATTAAGTATGCAGGTTATGTTT
CTTTCGCTTAAAGGTAAACATGAACTGGCACGAAAATTAACTAAAGAAATATCCACGCAG
GAAATAACAGGACTTATTGCTGTTAATCTTCTTTACGCTGAATATTGTCAGAATAGTGAG
CGTGCCTTACCGACGATAAGAGAATTTCTGGAAAGTGAACAGCGTATAGATAATAATCCG
GGATTATTACCGTTAGTGCTGGTTGCCCACGGCGAAGCTATTGCCGAGAAAATGTGGAAT
AAATTTAAAAACGAAGACAATATTTGGTTCAAAAGATGGAAACAGGATCCCCGCTTGATT
AAATTACGGTAA
>EECEBICD_00590 Transcriptional regulator HilA
ATGCCACATTTTAATCCTGTTCCTGTATCGAATAAAAAATTCGTCTTTGATGATTTCATA
CTCAACATGGACGGCTCCCTGCTACGCTCAGAAAAGAAAGTCAATATTCCGCCAAAAGAA
TATGCCGTTCTGGTCATCCTGCTCGAAGCCGCCGGCGAGATTGTGAGTAAAAAACACCTT
ACTGGACCAGGTATGGGGCGACGCGGAAGTTAA
>EECEBICD_00591 HTH-type transcriptional activator RhaR
ATGGAAAATGTAACCTTTGTAAGTAATAGTCATCAGCGTCCTGCCGCAGATAACTTACAG
AAATTAAAATCACTTTTGACAAATACCCGGCAGCAAATTAAAAGTCAGACTCAGCAGGTT
ACCATCAAAAATCTTTATGTAAGCAGTTTCACTTTAGTTTGCTTTCGGAGCGGTAAACTG
ACGATTAGCAATAATCACGATACGATTTACTGTGACGAACCTGGGATGTTGGTGCTCAAA
AAAGAGCAGGTAGTTAACGTGACGCTTGAAGAGGTCAATGGCCACATGGATTTCGATATA
CTCGAGATACCGACGCAACGACTTGGCGCTCTCTATGCACTTATCCCAAACGAGCAGCAA
ACCAAAATGGCGGTACCCACAGAGAAAGCGCAGAAAATCTTCTATACGCCTGACTTTCCT
GCCAGAAGAGAGGTATTTGAACATCTGAAAACGGCGTTCTCCTGTACGAAGGATACAAGC
AAAGGTTGCAGTAACTGTAACAACAAAAGTTGTATTGAAAATGAAGAGTTAATTCCTTAT
TTTCTGCTGTTCCTGCTTACTGCTTTTCTCCGACTCCCGGAGAGTTATGAGATCATCCTT
AGCTCGGCTCAGATAACGTTAAAGGAGCGCGTTTACAACATTATATCTTCGTCACCCAGT
AGACAGTGGAAGCTTACGGATGTTGCCGATCATATATTTATGAGTACGTCAACGCTCAAA
CGGAAACTTGCAGAAGAAGGTACCAGCTTTAGCGACATCTACTTATCGGCAAGAATGAAT
CAGGCAGCAAAACTTTTACGCATAGGCAACCATAATGTTAATGCTGTAGCATTAAAATGT
GGTTATGATAGCACGTCCTACTTCATTCAATGTTTCAAAAAATATTTTAAAACTACGCCA
TCGACATTCATAAAAATGGCGAACCATTAA

>EECEBICD_00592 Protein PrgH
GTGTCCTGTGCGGTAATCTGCTGCTATCGAGAACGACAGACATCGCTAACAGTATATATG
GAAACATCAAAAGAGAAGACGATAACAAGCCCTGGGCCATACATAGTTCGATTACTTAAC
AGCTCACTGAACGGCTGTGAGTTTCCATTGCTGACAGGCCGAACACTCTTTGTGGTAGGT
CAGAGTGATGCGCTCACTGCTTCAGGCCAACTCCCTGATATACCTGCCGATAGCTTTTTT
ATCCCGCTGGACCATGGCGGAGTAAATTTTGAAATCCAGGTGGATACGGATACGACCGAA
ATTATACTCCATGAGCTGAAAGAAGGAAATTCTGAATCTCGTTCGGTGCAATTAAATACG
CCAATACAGGTCGGTGAATTGCTTATCCTGATTCGCCCGGAAAGCGAGCCGTGGGTGCCC
GAGCAGCCTGAGAAGTTAGAAACGTCTGCAAAAAAGAACGAGCCGCGTTTTAAAAACGGA
ATTGTAGCAGCACTGGCCGGGTTTTTTATATTGGGAATTGGGACTGTGGGGACGTTATGG
ATACTTAACTCGCCGCAGCGGCAGGCCGCAGAGCTCGATTCGTTATTGGGGCAGGAGAAG
GAGCGTTTTCAGGTGTTGCCAGGCCGGGACAAAATGCTCTATGTCGCTGCGCAAAATGAA
AGAGATACGCTGTGGGCTCGTCAGGTTTTAGCGAGGGGCGATTATGATAAAAATGCGCGA
GTGATTAACGAAAACGAAGAAAATAAGCGTATCTCTATCTGGCTGGATACCTATTATCCG
CAGCTGGCTTATTATCGGATTCATTTCGATGAGCCGCGTAAACCCGTTTTCTGGCTAAGC
CGCCAGCGAAACACGATGAGCAAGAAAGAGCTCGAGATGTTAAGTCAAAAGCTGAGAGCG
CTAATGCCTTACGCGGATTCGGTTAACATCACGTTGATGGACGATGTTACCGCAGCAGGC
CAGGCGGAAGCGGGGCTAAAACAGCAGGCGTTACCTTATTCCCGCAGGAATCATAAGGGG
GGCGTAACGTTTGTTATTCAGGGGGCGCTCGATGATGTAGAAATACTCAGAGCCCGTCAA
TTTGTCGATAGCTATTACCGCACATGGGGAGGACGCTATGTGCAGTTTGCGATCGAATTA
AAAGATGACTGGCTCAAGGGGCGCTCATTTCAGTACGGGGCGGAAGGTTATATCAAAATG
AGCCCAGGCCATTGGTATTTCCCAAGCCCACTTTAA
>EECEBICD_00593 Protein PrgI
ATGGCAACACCTTGGTCAGGCTATCTGGATGACGTCTCAGCAAAATTTGATACGGGCGTT
GATAATCTACAAACGCAGGTAACAGAGGCGCTGGATAAATTAGCAGCAAAACCCTCCGAT
CCGGCGCTACTGGCGGCGTATCAGAGTAAGCTCTCGGAATATAACTTGTACCGTAACGCG
CAATCGAACACGGTAAAAGTCTTTAAGGATATTGATGCTGCCATTATTCAGAACTTCCGT
TAA
>EECEBICD_00594 hypothetical protein
ATGTCGATTGCAACTATTGTCCCAGAGAATGCCGTTATAGGGCAGGCGGTCAATATCAGG
TCTATGGAGACGGACATTGTCTCGCTGGATGACCGGCTACTCCAGGCTTTTTCTGGTTCG
GCGATTGCCACGGCTGTGGATAAACAGACGATTACCAACAGGATTGAGGACCCTAATCTG
GTGACGGATCCTAAAGAGCTGGCTATTTCGCAAGAGATGATTTCAGATTATAACCTGTAT
GTTTCTATGGTAAGTACCCTTACTCGTAAAGGAGTCGGGGCTGTTGAAACGCTATTACGC
TCATGA
>EECEBICD_00595 Lipoprotein PrgK
ATGATTCGTCGATATCTATATACCTTTCTGCTGGTAATGACCCTTGCCGGCTGTAAGGAT
AAGGATCTTTTAAAAGGACTGGACCAGGAACAGGCTAATGAGGTCATTGCCGTTCTGCAA
ATGCACAATATAGAGGCGAATAAAATTGATAGCGGAAAATTGGGCTATAGCATTACCGTT
GCTGAGCCTGATTTTACCGCTGCGGTGTACTGGATTAAAACTTATCAGCTTCCTCCCCGG
CCACGGGTGGAAATAGCGCAGATGTTCCCGGCGGATTCGCTGGTATCGTCTCCGCGAGCT
GAAAAGGCCAGGTTATATTCGGCTATTGAACAGCGACTGGAACAGTCATTACAGACGATG
GAGGGCGTGCTCTCCGCCAGGGTCCATATTAGTTATGATATTGATGCTGGTGAAAATGGC
CGTCCGCCAAAACCTGTTCATCTGTCGGCATTAGCCGTATATGAACGAGGTTCGCCGCTT
GCGCATCAGATCAGCGATATCAAGCGTTTCTTAAAGAATAGTTTTGCCGATGTGGATTAT
GACAACATTTCTGTTGTGTTGTCAGAACGTTCTGATGCCCAATTACAGGCTCCCGGCACA
CCAGTAAAACGTAATTCTTTTGCAACCAGTTGGATTGTGTTGATTATTTTGTTATCCGTG
ATGTCAGCAGGCTTTGGCGTCTGGTATTACAAAAACCATTATGCCCGCAATAAGAAAGGC
ATAACGGCTGATGATAAGGCGAAATCGTCAAATGAATAG
>EECEBICD_00596 Oxygen-regulated invasion protein OrgA
ATGAATAGGCAGCCATTACCCATTATCTGGCAAAGAATCATTTTTGATCCGTTATCGTAT
ATCCATCCTCAGCGGTTGCAGATAGCGCCGGAAATGATTGTCAGACCCGCCGCCAGGGCG
GCGGCAAATGAGTTAATACTGGCGGCATGGCGGCTTAAGAACGGAGAAAAGGAGTGTATT
CAAAACTCACTGACGCAGCTGTGGCTGCGTCAGTGGCGCCGACTGCCGCAGGTAGCGTAT

TTACTCGGTTGCCATAAACTGAGAGCCGATCTGGCAAGGCAGGGAGCCTTGCTTGGCCTG
CCGGATTGGGCGCAAGCATTTTTGGCAATGCATCAGGGAACAAGTTTATCTGTCTGCAAT
AAGGCGCCGAATCACCGGTTTTTACTTAGCGTCGGGTATGCACAGTTAAATGCCCTAAAT
GAATTTTTACCTGAATCTTTAGCACAGCGTTTTCCTTTGCTTTTTCCTCCATTTATTGAG
GAGGCATTGAAGCAGGATGCTGTAGAAATGTCAATTTTGCTACTGGCCTTACAATATGCT
CAAAAATATCCCAATACCGTCCCCGCTTTCGCCTGTTGA
>EECEBICD_00597 Oxygen-regulated invasion protein OrgB
ATGCTCAAAAATATCCCAATACCGTCCCCGCTTTCGCCTGTTGAGGGGATACTGATAAAG
CGTAAAACGCTGGAACGTTATTTCTCTATTGAAAGATTAGAACAGCAGGCGCATCAACGG
GCTAAGCGTATTTTGCGAGAGGCGGAAGAAGAGGCAAAGACCTTGCGGATGTACGCCTAT
CAGGAAGGTTACGAGCAGGGAATGATAGACGCGTTACAGCAGGTCGCCGCTTATCTTACT
GATAATCAGACAATGGCCTGGAAGTGGATGGAAAAAATACAGATTTATGCCCGCGAGTTA
TTTTCAGCTGCGGTCGACCATCCCGAAACGCTTTTAACCGTCCTGGATGAGTGGCTAAGG
GATTTCGATAAGCCTGAGGGGCAACTTTTTTTAACACTGCCAGTTAATGCGAAAAAAGAT
CACCAAAAACTGATGGTGTTGCTTATGGAGAACTGGCCAGGCACTTTTAATCTTAAATAT
CATCAGGAACAGCGCTTTATCATGAGTTGCGGCGATCAGATCGCAGAATTTTCACCTGAA
CAATTTGTTGAAACGGCAGTAGGCGTCATTAAGCATCATCTTGATGAACTTCCACAAGAC
TGCCGGACAATTTCTGATAACGCCATTAACGCACTTATTGATGAATGGAAGACAAAAACG
CAAGCGGAGGTTATAAGGTGA
>EECEBICD_00598 hypothetical protein
GTGATACCGGGTACGATTCCGACTTCTTATTTGGTCCCGACAGCAGATACTGAAGCGACG
GGTGTGGTTTCGTTGAGTGCCAGGGCCGCTATGCTGAATAATGTGGATAGCGCGCCTTTA
AGCAATGGCGGCGACGTTGATCTTTATGATGCATTCTACCAACGACTTCTGGCGCTTCCT
GAGTCAGCCTCTTCTGAAACGCTTAAAGATAGCATTTATCAAGAGATGAATGCCTTCAAA
GATCCTAACAGTGGTGATTCGGCTTTTGTTTCTTTTGAGCAGCAAACGGCTATGCTACAA
AACATGTTAGCAAAAGTTGAACCGGGCACCCATTTATATGAGGCTTTAAACGGGGTATTA
GTTGGTACAATGAACGCACAGTCACAAATGACCTCCTGGATGCAGGAGATTATTTTATCG
GGCGGGGAAAACAAAGAGGCAATTGACTGGTGA
>EECEBICD_00599 HTH-type transcriptional activator RhaR
ATGGTATTGCCTTCAATGAATAAATCAGTTGAGGCCATTAGCAATAATCACCTTCAACAG
CCGAACAAATTTCCATTAATAAATGGATTAGCTGACGTAAGAGACTATTATGTCGCAAAC
TGCTTATTGTTTAAACTTAATAAAGGCAGTTTGCGAATTGAAAACGAATTTGGGGAGTTC
ATCGAACAATCTGCGCCGTGTTTATTTTTATTGGAAAAAGATCAAACAATCACGCTTAGT
ATGAGCGAAATAGAAGGGCATATTGATTTTTCTTCACTGGAAGTTTCCTATGACTTAATG
CAAAAATTCTACAAAGTTTTTTACAGTACAAGAAACTATAATGATCGAGAGCTATCATTA
AAAACGAAACCAAAGTATTTTTTTCATGCGGACTTGTTGCCAGGGATGAGTGATACTTTT
GACTCTATTTTGCATGGTGTGGCATGTCCACGGGTTTGTAGTAATGTGAGTATTGATGAT
CATGATTATTCATATTTCTCATTGATGTATCTTATATCGGCATTTGTACGTAAGCCCGGT
GGGTTTGATTTCCTTGAGCGAGCAATAAAAATTACGACAAAAGAGAAAGTTTATAACATT
ATTATCAGCGATCTCACCCGCAAATGGTCACAGGCTGAGGTGGCGGGAAAGCTGTTTATG
AGCGTATCAAGTCTGAAGCGAAAACTGGCCGCTGAAGAGGTGAGTTTTAGCAAAATATAC
CTAGATGCTCGTATGAATCAGGCTATAAAATTATTACGTATGGGGGCTGGAAATATTTCA
CAAGTCGCGACGATGTGTGGCTATGATACGCCTTCTTATTTTATCGCTATTTTTAAACGG
CATTTTAAGATTACACCGCTTAGCTTTATGCGTACGATGAACCATTGA
>EECEBICD_00600 hypothetical protein
ATGAGAAATGTAATTATATACGGTATTAACTGGACTAATTGTTATGCCCTTCAGTCTATT
TTTAAGCAAAAATATCCGGAAAAGTGCGTTAAAACGTGTAACTCATTAACCGCACTTTTG
CATTCTCTATCCGATATGCCGGACGCCGGGCTGATTTTAGCTTTGAACCCGCATGAGCAT
GTTTATCTTTTTCATGCGTTGCTGACGCGGTTGCAGAACCGTAAAGTTTTGGTAGTGGCA
GATCGCTTGTATTATATCGATCGCTGTGTGCTGCAATATTTTGGCGTTATGGATTATGTT
TTAAAAGACGAACTATCCTGCGCTATCCGCTCGGAGCGAGAGAAGCTCCGTCTTCCGGAA
GCCTGGCTGCGTTTCTGCCACAGGCCACAAAAGAAAACCGTGGCTGCTACGTATGCTTTT
AATGCCGGCGAGACGCCGGAAGAGGTATTGTTTAATATCAATCAATACGCCTGGTGGAAT
CTACCTCCCGGTGTCACGCAGGCGAAATATGCGCTGCTGATATTATTATCCTCCGGACAT

CCGGCAATTGAACTGGCGAAAAAATTTGGTCTGGGAACAAAAACCGTCAGTATTTATCGT
AAGAAGGTTATGTATCGTTTGGGGATGGACTCTTCACCGCTATCGCTGTTCCGGGGACTG
AAACTAGACGCGCATTTACAGCGCACTGCTTTTGCACATAACCCTGCTGTACCTGACGAT
AATTGCGCGTTACCGATTGCGGTAGGAATGAATTAA
>EECEBICD_00601 Effector protein YopJ
ATGCTAAGTCCTACGACTCGTAATATGGGGGCGAGTTTATCGCCTCAGCCTGACGTCAGC
GGGGAGCTAAACACCGAAGCATTGACCTGTATTGTTGAGCGTCTGGAAAGTGAAATTATA
GATGGCAGCTGGATTCATATCAGTTACGAGGAAACCGATCTCGAAATGATGCCTTTTCTT
GTTGCACAGGCCAATAAGAAGTATCCAGAGTTAAATCTTAAATTTGTTATGTCAGTCCAT
GAGCTTGTTTCCTCTATAAAGGAGACCAGAATGGAAGGCGTTGAATCTGCCCGATTTCTC
GTAAATATGGGAAGTTCAGGTATCCATATTTCAGTCGTCGATTTTAGAGTTATGGACGGA
AAGACATCGGTGATTTTGTTCGAACCAGCAGCGTGTAGCGCTTTTGGACCTGCTTTACTG
GCGTTGAGGACCAAAGCAGCTCTTGAACGTGAACAACTGCCTGATTGTTATTTTGCTATG
GTCGAGCTGGACATTCAACGAAGCTCTTCTGAATGCGGTATTTTTAGCCTGGCGCTCGCC
AAAAAACTTCAGCTTGAATTTATGAACTTAGTAAAAATTCATGAAGATAATATTTGTGAA
CGTCTGTGTGGTGAAGAACCTTTTCTCCCGTCCGATAAAGCAGACCGTTATCTGCCGGTG
AGTTTTTACAAACATACTCAAGGCGTACAACGATTAAATGAATATGTGGAGGCCAATCCG
GCGGCGGGAAGCAGTATAGTAAACAAAAAGAATGAAACGCTTTATGAGCGATTCGATAAC
AATGCCGTTATGCTAAACGATAAAAAACTCTCTATATCCGCTCATAAAAAAAGGATAGCT
GAATATAAGTCTTTACTTAAATCGTAA
>EECEBICD_00602 Manganese transport system membrane protein MntB
ATGGGCATCGTCTTTTCCGGGATGTTTGGCGCAGGTCTGGTGCTCTATGTTTCTATTCAA
TCGGAAGTGCATCTGGATCACATCCTGTTTGGGGATATGCTGGGCGTATCGCTGGGGGAT
ATCGTACAAACGTCAGTTATTGCACTGGGTATTGCGTTAATCATTGGGCTGAAATGGAAA
GATCTCCTGCTACACGCTTTTGACCCGCACCAGGCGAAAGCCAGCGGGCTGAATATCACG
CTACTGCATTATGGCCTGCTATGTATGATTGCGCTGACTATCGTCGCCACGCTGAAATCG
GTGGGTATTATTCTGTCGATTTCATTGCTTATCGCCCCCGGCGCAATTGCCATTTTGCTG
ACGCGACGCTTTGCGCGCGCGCTGGGATTAGCGGTGAGTCTGTCAGTTATTACCGCTTTT
GCGGGCGTCTATCTGTCTTTTTATCTTGATAGCGCGCCAGCGCCGACCATCGTGGTATTA
TTTGCCATCGTGTTTATTGCGGTATTTATTTACGCAACATGGCGCGACAGGCGTAATGAA
ATCGTACCAGAGGCGCAAGGTTAA
>EECEBICD_00603 hypothetical protein
ATGTTCTTAACAACGTTACTGGAACCCTTTCAGTTTGATTTTATGGTGAACGCCCTTATG
GTTTCGGTCATTGTGGCGATTCCCTGCGCCTTACTGTCCGTGTTTTTAGTGTTAAAAGGC
TGGGCGTTAATGGGCGATGCAATGAGTCATGCGGTATTCCCCGGGGTCGTGCTGGCATAT
ATTGTGGGGATCCCCTGGCGATTGGCGCTTTCATTGCCGGATTATTTTGCGCTATCGCGA
CCGGCTATCTGGACGATAACAGCCGCATCAAACGTGATACGGTGA
>EECEBICD_00604 Manganese transport system membrane protein MntB
ATGAACTGGCTTGTTGAGCCGTTTGGCTACCAGTATATGCTCAACGCGATGTGGGTCTCC
GCGATGGTGGGCGGTCTGTGCGCGTTTCTCTCCTGCTATTTGATGCTCAAAGGCTGGTCG
CTCATTGGCGATGCGCTATCTCACTCTATTGTGCCTGGCGTCGCTGGCGCCTGGATGTTA
GGGCTGCCCTTCTCGCTCGGCGCGTTTCTTTCCGGCGGACTGGCGGCAGGCAGTATGCTC
TTTCTTAACCAACGCTCACGCCTGAAAGAAGACGCGATTATCGGGCTTATCTTCTCCTCT
TTTTTTGGCGTCGGCCTTTTTATGGTGTCGCTCAATCCGATGTCGGTGAATATCCAGACG
ATTATTCTCGGCAACGTGCTGGCGATCGCGCCAGCGGATATTGCGCAACTGGCGATTATC
GGCGCAGTCTCGCTGACGATTCTGCTGTTAAAGTGGAAAGATCTGATGGTCGTCTTTTTC
GATGAAACCCATGCGCGTTCTATCGGGCTTAATCCGGGTCGACTAAAGCTGTTGTTTTTC
ACTCTATTGTCCGTCTCTACCGTGGCGGCCCTGCAAACCGTTGGGGCGTTCCTGGTGATC
TGTCTCGTCGTCACGCCGGGCGCGACGGCCTGGTTACTCACCGACCGTTTTCCACGTCTG
CTCATGATTGCCGTCGTGATTGGCAGCCTGACCAGTTTTCTGGGCGCATGGCTTAGCTAC
TGGCTGGATGGCGCCACGGGTGGAATTATTGTCGTCATGCAAACCTTACTGTTCATCACA
GCCTTTATTTTCGCCCCGAAACACGGTCTGTTAGCCAACCGTCGACGCGCCCGCCTGCAG
AAGGAGCCGACATGTTCTTAA
>EECEBICD_00605 Manganese transport system ATP-binding protein MntB

ATGAGTCAATCTGCGATTACCGTTGATCAAGTCACGGTGACGTATCGCAACGGTCATACC
GCCCTACGGGACGCCACTTTTCAGGTGCCTGGCGGTTCAATCGCCGCGCTGGTAGGCGTA
AATGGTTCCGGTAAGTCTACGTTATTTAAAGCGTTAATGGGCTTTGTGCATCTTGCGCAG
GGAGATATCACTATCCTGCAACAGTCAGTCAACAAGGCGCTGAAAAAAAACCTCATTGCT
TATGTTCCGCAATCGGAAGAAGTGGACTGGTCATTTCCGGTGCTGGTAGAAGATGTGGTG
ATGATGGGACGCTACGGCCATATGGGCTGGCTACGCCGTCCCACTGCGCATGATCACGCC
TGTGTTGACGCCGCGCTGGCGCGGGTGGATATGCAGGAATATCGTCACAGGCAGATTGGC
GAGCTGTCCGGCGGGCAGAAAAAACGTGTGTTTCTTGCCAGGGCCATCGCTCAGGATGGA
CAGGTAATCCTGCTGGATGAACCTTTTACCGGCGTGGATGTAAAAACCGAAGCGCGAATC
ATCGATCTACTACGGGAACTGCGCGATGAAGGCCGCACGATGCTGGTCTCAACGCATAAT
CTCGGCTCCGTCACCGAGTTTTGCGACTATACGGTAATGATTAAAGGAACCGTACTGGCC
AGCGGCCCTACCGAAACCACGTTTACCGCCGCGAATCTGGAACAGGCGTTTAGCGGCGTT
CTACGTCATATCGCGCTAAGCGGAGGTGAAGAGCACATCATCACCGATGACGAGCGCCCC
TTTATTTCCCGGCGCGTGGCCAGCGGAGGAAAATCGTCATGA
>EECEBICD_00606 putative periplasmic iron-binding protein
ATGACGAATCTACATCGTCTGAAAACACTCCTGATTGCCGGTATTGTCGCGATACTCGCG
CTATCGCCAGCCTATGCAAAAGAGAAATTTAAAGTCATCACCACGTTTACCGTTATTGCC
GACATGGCGAAAAACGTGGCGGGCGACGCAGCGGAAGTCAGCTCAATTACCAAGCCCGGC
GCTGAAATCCATGAGTATCAGCCAACGCCCGGCGATATTAAACGAGCGCAGGGGGCGCAG
CTTATCCTCGCGAATGGTCTGAACCTGGAGCGATGGTTCGCCCGCTTTTATCAGCACCTT
TCCGGCGTACCGGAAGTCATCGTCTCCACCGGCGTCAAACCGATGGGCATTACCGAAGGC
CCGTATAACGGTAAACCGAACCCGCACGCCTGGATGTCGGCAGAAAACGCGCTGATTTAT
GTCGATAACATTCGCGACGCCCTGGTGAAGTACGATCCGGATAATGCGCAGATCTATAAG
CAAAATGCCGAACGCTATAAAGCGAAAATTCGCCAGATGGCCGATCCGTTGCGTGCCGAA
CTGGAAAAAATTCCCGCCGATCAGCGCTGGCTGGTCACCAGTGAAGGCGCGTTCTCTTAC
CTGGCGCGCGATAACGACATGAAAGAGCTTTATCTCTGGCCAATTAACGCCGATCAACAG
GGTACGCCAAAACAGGTGCGTAAAGTGATTGATACCATTAAAAAGCACCATATTCCCGCC
ATCTTTAGCGAGAGTACGGTTTCCGATAAACCCGCCCGCCAGGTCGCGCGTGAATCCGGC
GCGCATTATGGCGGCGTACTGTATGTCGATTCTCTGAGCGCCGCTGACGGCCCTGTGCCA
ACCTATCTGGATCTGCTGCGCGTCACGACCGAAACCATCGTCAACGGCATTAACGACGGA
CTGAGGAGTCAACAATGA
>EECEBICD_00607 hypothetical protein
ATGCCTGGTAAACGTATCGCTCGTGAAAAACTGACGATCAAAAAAATGATCGCGCTGTAT
GAAAGCCAGTGTCCACAGGCGTCAGCGGTGCAGGGGCATTACGACGCGCTGTACGCCTAC
GCGCAAAAACGTCTTGATAAATGCGTATTTGGCGAGGAAAAACCCGCCTGTAAGCAGTGC
CCGGTGCACTGCTATCAGCCGGCTAAACGCGAGGAGATGAAGCAGATTATGCGTTGGGCA
GGTCCGCGAATGCTTTGGCGACACCCTGTCCTGACCGTGCGGCACCTTATCGACGATAAG
CGCCCCGTTCCGGAATTGCCGGAAAAATATCAGCGCAAGAAGTGA
>EECEBICD_00608 Formate hydrogenlyase transcriptional activator
ATGTCGTATACACCCATGAGCGATCTTGGACAGCAAGGCCTGTTCGATATCACTCGTACC
TTATTGCAGCAGCCCGATTTGGCGTCGCTCAGTGAAGCGCTTTCGCAACTGGTAAAGCGT
TCAGCGCTCGCCGACAGCGCCGGCATTGTGCTATGGCAGGCGCTGTCGCAACGCGCACAA
TATTATGCGACGCGAGAAAATGGCCGTCCTGTTGAATATGAAGATGAAACCGTACTGGCG
CACGGCCCGGTACGTCGCATCCTTTCTCGCCCCGATGCATTGCACTGTAATTTCCATGAG
TTTACCGAAACCTGGCCGCAGCTTGCCGCGAGCGGCCTCTACCCGGAATTTGGTCATTAC
TGTCTGCTTCCTTTAGCCGCGGAGGGCCGTATTTTCGGCGGCTGCGAATTCATCCGTCAG
GAAGATCGCCCCTGGAGTGAAAAGGAATATGACCGTCTGCATACGTTTACCCAAATTGTC
GGCGTGGTCGCCGAGCAAATTCAAAACCGGGTAAACAATAATGTCGATTATGATCTGTTG
TGCCGCGAACGCGATAACTTCCGTATTCTGGTTGCGATCACCAACGCCGTCCTGTCGCGT
CTGGATATTGATGAGCTGGTAAGCGAAGTCGCAAAAGAGATCCACCACTATTTCAATATC
GACGCAATCAGCATCGTGTTGCGCAGCCATCGCAAAAATAAGCTGAATATCTACTCCACC
CACTACCTTGATGAGCATCATCCGGCGCACGAACAGAGCGAAGTCGACGAAGCCGGCACG
CTCACGGAGCGCGTCTTTAAAAGTAAAGAGATGCTGCTTATCAACCTCAACGAACGCGAT
CCGCTTGCTCCCTACGAACGCATGCTGTTCGATACCTGGGGCAATCAGATCCAGACGCTT

TGCCTGCTGCCGTTGATGTCCGGCAAGACGATGCTTGGCGTGCTCAAACTGGCCCAGTGC
GAAGAAAAAGTGTTTACCACCGCCAACCTCAAGCTGCTGCGTCAGATTGCCGAACGAGTA
GCCATTGCCGTGGATAATGCGCTGGCCTATCAGGAAATCCACCGACTGAAGGAACGCCTG
GTCGACGAAAACCTGGCGCTCACGGAGCAGCTTAATAACGTCGACAGCGAGTTTGGCGAG
ATCATCGGGCGCAGCGAAGCGATGTACAACGTGCTAAAGCAGGTTGAGATGGTGGCGCAG
AGCGACAGTACCGTGCTGATTCTGGGCGAAACGGGAACCGGTAAAGAGTTGATTGCCCGC
GCCATTCACAACCTGAGCGGACGTAGCGGGCGCAGGATGGTAAAGATGAACTGCGCCGCG
ATGCCGGCGGGGCTGCTGGAAAGCGATTTGTTTGGTCATGAGCGCGGCGCGTTTACCGGC
GCCAGCGCGCAGCGGATTGGCCGTTTCGAGCTGGCGGATAAAAGCTCGCTGTTTCTGGAT
GAAGTGGGCGACATGCCGCTGGAGCTACAGCCTAAGCTGCTGCGCGTCCTTCAGGAGCAA
GAGTTTGAACGTCTCGGCAGCAATAAACTGATCCAGACCGACGTGCGGCTGATCGCCGCG
ACTAACCGCGATCTGAAAAAAATGGTCGCCGATCGCGAATTCCGTAACGATCTCTACTAT
CGGCTGAATGTTTTTCCGATCCAGCTTCCTCCGTTGCGCGAACGTCCGGAAGATATTCCA
CTACTGGTCAAAGCGTTCACTTTCAAAATCGCCCGCCGAATGGGGCGTAATATCGACAGC
ATTCCCGCCGAAACGCTGCGCACGCTCAGCAGCATGGAATGGCCGGGGAACGTTCGCGAG
CTGGAGAATGTCGTCGAGCGCGCCGTGCTGCTGACGCGCGGCAATGTGCTACAACTGTCG
CTGCCGGATATTACCGCCGTAACGCCCGATACCTCGCCTGTTGCGACTGAAAGCGCCAAA
GAAGGCGAAGATGAATATCAGCTTATTATACGCGTGCTGAAAGAGACCAATGGCGTAGTG
GCTGGACCAAAAGGCGCGGCGCAGCGTCTGGGGCTTAAACGAACCACCCTGCTGTCGCGT
ATGAAGCGATTGGGTATTGATAAAGACGCGTTAGCGTAA
>EECEBICD_00609 Carbamoyl dehydratase HypE
TTGAACAATATACAACTCGCCCACGGCAGCGGCGGCCAGGCTATGCAACAGTTGATTAAT
AGCCTGTTTATGGAGGCCTTTGCCAACCCGTGGCTGGCGGAACAAGAAGATCAGGCGCGT
CTGGAGCTGGCGCAACTGACGGCGGAGGGCGACCGTCTGGCGTTTTCTACCGATAGCTAT
GTGATCGATCCGCTCTTTTTCCCCGGCGGCAATATCGGCAAACTGGCGATTTGCGGTACG
GCGAACGACGTTGCGGTGAGCGGCGCGATCCCCCGCTATCTCTCCTGCGGCTTTATCCTT
GAAGAAGGGCTGCCGATGGAGACGTTAAAAAGCGTGGTCAATAGCATGGCGGCGACCGCG
CGGGAAGCGGATATCGCCATTGTAACCGGCGACACCAAAGTTGTGCAGCGCGGCGCGGCG
GATAAATTATTTATCAACACTGCCGGCATGGGGGCGATTCCCGCCGATATTCGCTGGGGC
GCGCAAACGCTGAGCGTTGGCGATGTGCTGTTAGTCAGCGGTACGCTTGGCGATCACGGT
GCCACTATCCTCAACCTGCGTGAGCAACTGGGACTGGATGGCGAGCTGGCGAGCGACTGC
GCAGTATTAACGCCGCTTATTCAGACGCTGCGTCATATTGACGGAGTGAAGGCATTGCGT
GACGCCACGCGCGGCGGCGTGAATGCGGTCGCCCATGAGTTTGCGACGTCCTGCGGTTAC
GGTATTGAATTGTCCGAATCCGCACTGCCGCTCAAACCTGCCGTGCGCGGCGTCTGCGAG
CTGTTGGGGCTGGATGCCCTGAACTTTGCCAACGAAGGAAAACTGGTGATTGCCGTTGAA
CGGCAGGCCGCAGATCGGGCGTTGGCGGCATTACGCGCGCATCCGCTGGGACGTGATGCA
GCGCTGATTGGCGAAGTCGTGGAACGCAAAGGCGTTCGCTTAGCCGGACTCTATGGCGTG
AAGCGAACCCTTGATTTGCCACACGCCGAACCATTACCTCGTATATGCTAG
>EECEBICD_00610 Hydrogenase maturation factor HypD
ATGCGTTTTGTTGACGAGTACCGTGCGCCGGAACAGGTAATGCAGCTCATTGAACACCTG
CGTGAACGCGCCGCCCTTCTTCCTTACACTGCCGAACGTCCGTTGCGCATTATGGAAGTC
TGCGGGGGTCATACCCACGCAATTTTTAAATTTGGTCTCGATCAGTTACTGCCCGAAAAC
GTGGAATTTATTCACGGCCCCGGTTGTCCGGTCTGCGTGCTGCCGATGGGACGCATCGAC
AGTTGCGTGGAAATCGCCAGCCATCCGGAGGTGATTTTCTGCACCTTTGGCGATGCCATG
CGCGTGCCGGGGAAACAGGGTTCTCTGTTGCAGGCTAAAGCGCGCGGCGCGGATGTCCGT
ATTGTTTACTCTCCGATGGACGCGCTGAAGCTGGCGCAGGATAACCCGACCCGCAAAGTG
GTATTTTTCGGGCTGGGCTTTGAAACCACCATGCCGACCACCGCCATCACGCTGCAGCAG
GCAAAACAGCGTGACGTGCGAAACTTCTACTTTTTCTGCCAGCACATTACGCTGATCCCG
ACGCTGCGTAGCCTACTGGAGCAGCCGGACAACGGCATTGACGCCTTTCTTGCGCCAGGC
CACGTCAGCATGGTCATCGGCACCGAGGCGTACCAGTTTATCGCCGCCGATTTTAATCGC
CCGCTGGTGGTGGCTGGATTCGAACCGCTTGATCTACTGCAAGGCGTGGTCATGCTGGTT
GAGCAGAAAATAGCGGCCCTAAGCCAGGTTGAAAACCAATACCGTCGCGTGGTGCCGGAT
GCCGGAAACATGCTGGCGCAGCAGGCCATCGCCGATGTGTTCTGCGTTAATGGCGACAGC
GAGTGGCGCGGTCTGGGCGTGATTGAATCCTCCGGCGTCCACCTGACGCCAGAGTATCAG

CGCTTTGACGCCGAAGCGCATTTCCGCCCAGCGCCACAACAGGTCTATGACGATCCGCGC
GCCCGCTGCGGCGAAGTGCTGACCGGCAGATGCAAGCCGCATCAGTGCCCGCTATTTGGT
AAAACATGTAATCCAGAGACCGCGTTCGGCGCGCTGATGGTCTCTTCCGAAGGCGCATGC
GCCGCCCGGTATCAGTATCGTCAGCAGGAGTGTGAAGTTTGA
>EECEBICD_00611 Hydrogenase maturation factor HypC
ATGTGCATAGGCGTGCCGGGTCAAATCCGCGCCATTGACGGCAATCAGGCAAAAGTTGAC
GTCTGCGGCATTCAGCGCGACGTCGATCTCACATTAGTCGGCAGTTGTGATGAAAACGGT
CAGCCGCGTCTGGGTCAGTGGGTGCTGGTTCATGTCGGATTCGCCATGAGCGTCATTAAC
GAAGCGGAAGCGCGCGACACGCTCGACGCGCTACAAAATATGTTTGATGTTGAGCCGGAC
GTCGGCGCACTGCTGTATGGTGAGGAGAGATAA
>EECEBICD_00612 Hydrogenase maturation factor HypB
ATGTGTACGACTTGCGGTTGCGCTGAAGGCAACCTTTATATAGAAGGCGATGAACATAAT
CCGCACTCAGCGTTTCGTAGCGCGCCCTTTGCCCCAGCGGCCCGCCCGGCGCTGAATATT
ACCGGCATTAAAACGCCTGACTTTGCCCCAAGTCAGACCGCAGAAGGCGATCTGCATTAC
GGCCACGGCGAAGCCGGAACCCACGCGCCTGGTATGAGTCAGCGGCGGATGCTTGAGGTT
GAAATCGACGTGCTGGACAAAAATAATCGTCTGGCTGAGCGCAACCGCGCCCGTTTCGCC
GCCCGCCAGCAACTGGTGCTCAACCTGGTGTCCAGCCCCGGGTCCGGTAAAACCACCCTG
CTGACCGAAACGCTGATGAAATTGAAAGACCGTGTACCGTGCGCGGTGATTGAAGGCGAC
CAGCAGACAGTGAACGACGCCGCCCGCATCCGCGCTACCGGCACGCCCGCCATTCAGGTC
AACACCGGTAAAGGCTGCCATCTTGACGCGCAAATGATCGCCGACGCCGCGCCGCGCCTG
CCGCTGGATGACCACGGTATTCTGTTTATCGAAAACGTCGGTAATCTCGTCTGCCCGGCC
AGCTTCGATCTGGGCGAAAAGCATAAAGTCGCAGTGCTTTCCGTGACCGAAGGTGAAGAT
AAGCCGCTGAAATATCCGCATATGTTTGCCGCTGCGTCGCTCATGCTGCTCAATAAAGTC
GATCTGCTGCCCTACCTCAATTTTGACGTTGAGAAGTGCATCGCCAGCGCGCGTGAAGTG
AACCCGGAGATTGAAATTATCCTGATTTCCGCCACCAGCGGCGAAGGCATGGATCAGTGG
CTGGCCTGGCTGGAGGCGCAGCGATGTGCATAG
>EECEBICD_00613 Hydrogenase maturation factor HypA
ATGCACGAAATCACCCTCTGCCAACGGGCACTGGAATTGATTGAACAACAGGCTTCAGCA
TACGGTGCAAAACGTGTGACAGCCGTCTGGATCAAAATTGGCGCATTTTCTTGCGTGGAA
ACCAGCGCTCTTTCCTTTTGTTTTGATCTGGTATGCCGCGGCACGATAGCAGAAGGTTGT
AAACTGCACCTCGAAGAACAGGAAGCCGAATGCTGGTGTGAGCATTGTCAACAATATGTC
ACGTTGTTAACCCACCGTGTACGTCGTTGTCCGCAATGTCATAGCGATACGCTGCGAATT
GTGGCCGATGACGGCTTACAAATTCGGCGCATAGAGATTGATGAAACTGAAGATTAA
>EECEBICD_00614 Formate hydrogenlyase regulatory protein HycA
ATGACTATTTGGGAAATAAGCGAAAAGGCCGATTACATCGCGCAACGGCATCGTCGCCTA
CAGGACCAGTGGCATATTTACTGCAACTCGCTGGTTCAGGGGATCACCCTCTCGAAAGCG
CGCCTGCATCACGCAATGAGCTGCGCGCCGGAAAGGGATCTTTGCTTTGTCCTGTTTGAG
CACTTTCGTATTTACGTCGCGTTGGCGGATGGTTTTAACAGCCACACCATTGAGTACTAC
GTTGAAACCAAAGATGGCGAAGATAAGCAATTGATTGCACAGGCGCAGCTCGATATCGAC
GGCAAGGTTGATGAACGGGTTAACAACCGCGATCGCGAGCAGGTGCTGGAGCACTATCTG
GAAAAAATCGCCAGCGTTTACGACAGCCTCTATACCGCTGTAGAAACCAACTCGCCGGTT
AATTTGCGTCAATTGGTAAAGGGAAACAGCCCGGCGGTATAA
>EECEBICD_00615 Hydrogenase-4 component A
GTGAATCGTTTTGTAATTGCTGACTCCACTCTCTGTATCGGCTGCCACACCTGTGAGGCC
GCCTGTTCAGAGACGCATCGCCAGCATGGCCTGCAGTCAATGCCGCGCCTGAAAGTGATG
CTGAATGAAAAAGAATCTGCGCCGCAGCTTTGTCACCACTGTGAAGATGCGCCATGCGCC
ACGGTATGCCCGGTTAACGCGATTAATCGCGTTGACGGCGCTGTCCAGCTTAATGAAAGC
CTGTGCGTGAGCTGCAAACTGTGTGGTATTGCCTGTCCGTTTGGCGCTATTGAGTTTTCC
GGCAGCCGTCCGCTGCATATTCCGGCGAACGCCAATACGCCGAAAGCGCCGCCCGCGCCG
CCAGCGCCTGCGCGCGTCAGTACGTTGCTCGACTGGGTGCCGGGCGTACGCGCTATCGCG
GTGAAATGCGACCTGTGCAGCTTTGACGAGCAAGGCCCGGCCTGTGTGCGGATGTGCCCG
ACTAAAGCGCTACACCTGGTGGAGAACACGGATATCGCCCGCGCCAGCAAACGTAAACGT
GAGCTGACGTTTAATACCGATTTTGGCGATCTCACCTTGTTTCAGCAGGCTCAGAGTGGG
GATGCATAA

>EECEBICD_00616 Hydrogenase-4 component B
ATGAGTTCACTTTCACTGATTACTAGCGGCGTCGCGTGGTTTGCCGCCGCCGCCGTTCTG
GCGTTTCTGTTTTCGTTTCATAAAGCGCTGAGCGGCTGGATTGCCGGTATCGGCGGCGCG
GTCGGCAGCCTGTGTACGGCTGGCGCGGGGTTCACCGCACTGACCAGCGCCGTTACGGTG
AGCGGCGTGATGCCGTTTACCGGTCACATGCTGCAAATAACGCCGCTGAATGCTATCTGG
CTGATTACGCTGGGGCTGTGCGGCCTGTTCGTCAGCCTGTTCAATATCGACTGGCATCGC
CATCCACAGGTAAAAGCCAACGGACTGTTGATCAACCTGCTGATGGCGGCAGCCGTCTGC
GCCGTGGTCGCCAGTAACCTGGGGACGATGGTGGTGATGGCGGAAATCATGGCGCTCTGC
GCCGTGTTCCTCACCGGCGGTAGCAAAGAGGGCAAACTGTGGTTTGCGCTGGGCCGTCTC
GGTACGCTGCTGCTGGCAATCGCCTGCTGGCTGGTGTGGCAGCGCTACGGCACGCTGGAT
CTGGGGCTGCTGGATCAACGCGCTCAGCAACTGCCGCTGGGCTCCGATATCTGGCTGCTC
GGCGTTATCGGCTTTGGTCTGCTGGCCGGGATTATCCCGCTACACGGTTGGGTGCCGCAG
GCGCACGCCAACGCCAGCGCTCCCGCCGCCGCGCTGTTCTCGACCGTGGTGATGAAAATC
GGGCTGCTGGGCATTCTCACGCTATCTCTGCTTGGCGGCAATGCGCCGCTGTGGTGGGGC
GTGGCGCTGTTGGTGCTTGGGATGATCACCGCCTTTGTCGGCGGTCTGTACGCGCTGATG
GAGCATAACATTCAGCGCCTGCTGGCCTATCACACGCTGGAAAATATCGGCATCATTTTG
CTCGGTCTGGGCGCAGGCGTTACCGGGATTGCTCTCAATCAGCCGGTACTGATCGCGCTG
GGTCTGACCGGCGGGCTCTATCACCTGCTGAACCATAGCCTGTTTAAAGCGTGCTGTTT
CTGGGCGCGGGCAGCATCTGGTTCCGTACCGGTCATCGCGATATCGAAAAACTGGGCGGT
ATTGGTAAACGGATGCCGGTTATTTCTATCGCCATGCTGGTCGGTCTGATGGCGATGGCT
GCGTTGCCGCCGCTGAACGGCTTTGCCGGCGAGTGGGTGATTTACCAGTCCTTCTTCAAA
CTGGGCAACAGCGGCGCGTTTGTTGGTCGTCTGTTAGGACCGCTGCTGGCGGTTGGCCTG
GCGATTACCGGCGCGCTGGCGGTGATGTGTATGGCGAAAGTTTACGGCGTGACGTTCCTT
GGCGCGCCGCGCACGAAGGAAGCTGAAAACGCCAGCTGCGCGCCTATCCTGATGGGCGTC
AGCGTGGTTGCGCTGGCTATCTGCTGTGTGATTGGTGGGGTTGCCGCGCCGTGGCTGCTG
CCGATGATTTCCACTGCGGTACCGTTGCCGCTGGAGACCGCGCACACCACCGTATCGCAG
CCGATGATCACGCTGCTGTTGGTCGCCTGTCCGCTACTGCCGTTCATCATTATGGCGATG
TTCAAAGGCAACCGTCTGCCGTCGCGTTCTCGCGGCGCGGCCTGGGTGTGCGGCTACGAC
CATGAGCAGTCGATGGTGATTACCGCGTACGGTTTCGCCATGCCGGTGAAAGAGGCCTTT
GCCCCGGTACTCAAATTGCGTAAATGGCTGAACCCGGTATCGCTGGTGCCGGGCTGGCAG
AACGCGGCGGCCGCGGTGCTGTTCCGTCGTCTGGCGCTGATTGAGCTGGCGGTGCTGGTG
GTGATTGTGGTTTCACGAGGAGCCTGA
>EECEBICD_00617 Formate hydrogenlyase subunit 4
ATGAGTGTGTTTTATCCGTTAATTCAGGCGCTGGTGCTATTTGCCGTTGCGCCATTGCTG
TCCGGTATTACTCGCGTGGCGCGCGCCCGTCTACACAATCGCCGTGGGCCGGGCGTGTTG
CAGGAGTACCGCGACATTATCAAACTGCTCGGTCGTCAGAGTATTGCGCCCTCTGATTCC
GGCTGGGTTTTTCGCCTGACGCCGTTTGTGATGGTCGGCGTGATGCTGACTATCGCCACC
GCGCTGCCGGTCGTCACCGTTGGATCTCCGTTGCCGCAACTGGGTGATTTAATCACCCTG
ATTTACCTGTTCGCCATCGCCCGTTTCTTCTTCTCTATTGCAGGCCTGGATACCGGCAGT
CCGTTCACCGCGATCGGCGCCAGCCGTGAAGCGATGCTCGGCGTGCTGGTGGAGCCGATT
CTGCTGCTGGGTCTGTGGGTCGCCGCGCAGGTGGCGGGTTCAACGCATATCAGCAATATC
GCCGATACCATTTATCACTGGCCTGTCGCGCGCAGTATTCCGCTGATTCTGGCGCTGTGC
GCCTGCGCCTTCGCCACCTTTATCGAAATGGGCAAGCTGCCGTTCGACCTGGCGGAAGCG
GAGCAGGAGTTGCAGGAAGGGCCGTTGACCGAATACAGCGGTAGCGGCTTTGCGGTGCTG
AAATGGGGCATCAGCCTTAAGCAACTGGTGGTGCTGCAAATGTTCGTCGGCGTGTTTCTG
CCGTGGGGACAGATGGAAACCTTCAGCGCAGGCGGACTGTTGCTGGCGCTGGTGATTGCC
ATCGTCAAGCTGATTGTCGGCGTACTGGTGATCGCCCTGTTTGAAAACAGCATGGCGCGA
CTGCGTTTTTGCGCCACTTCACGCGTGACCTGGGCCGGTTTTGGGTTTGCGTTTTTAGCC
TTCGTCTCCTTGCTGGCGGCGTGA
>EECEBICD_00618 Formate hydrogenlyase subunit 5
ATGTCTGAAGAAAAATTAGGTCAACAATACCTTGCGGCGCTGCACCAGGCGTTTCCGGGC
GTCGTGCTGGACGAAGCCTGGCAGACCAAAGATCAGCTGACTATTACGGTGAAAGTAAAC
TATCTGCCGGAAGTGGTGGAGTTTCTTTACTACCAGCAGGGTGGGTGGCTGTCGGTACTG
TTCGGTAATGACGAACGCCAGTTGTGCGGCCACTATGCCGTTTATTACGTGCTGTCGATG

GAGCAGGGCACGAAGTGCTGGATTACCGTCCGCGTTGAAGTGGATGCCAATAAGCTGGAA
TTCCCATCCGTTACGCCGCGCGTGCCGGCTGCCGTGTGGGGTGAGCGCGAAGTACGCGAC
ATGTACGGTTTAATCCCGGTCGGTCTGCCGGACGAGCGCCGTCTGGTGCTGCCGGACGAC
TGGCCGGATGAACTCTATCCGCTGCGTAAAGACAGTATGGATTATCGTCAGCGCCCGGCG
CCGACCACCGATGCGGAAACCTACGAGTTCATTAACGAGCTGGGTGACAAGAAAAATAAC
GTGGTGCCGATTGGCCCGCTGCATGTTACTTCCGATGAACCGGGCCACTTCCGTCTGTTC
GTCGATGGCGAAAACATTATCGACGCCGACTACCGCCTGTTCTACGTCCACCGTGGTATG
GAAAAACTGGCGGAAACCCGCATGGGTTATAACGAAGTCACCTTCCTGTCGGATCGCGTG
TGTGGTATCTGCGGCTTCGCCCACAGCACCGCCTACACCACTTCCGTGGAAAACGCGATG
GGCATTCAGGTGCCGGAGCGTGCGCAAATGATCCGCGCTATTCTGCTGGAAGTGGAACGC
CTGCACTCGCATCTGCTCAACCTTGGCCTGGCCTGCCACTTTACCGGCTTTGACTCCGGC
TTTATGCAGTTCTTCCGCGTGCGTGAAACCTCCATGAAGATGGCGGAAATACTGACCGGG
GCGCGCAAAACTTACGGTCTGAACCTGATCGGCGGGATTCGCCGCGATCTGCTCAAAGAG
GACATGATCCAGACCCGTCAACTGGCGCAGCAGATGCGTCGTGACGTGCAGGAGCTGGTG
GACATGCTGCTGAGCACGCCGAATATGGAACAGCGTACCGTGGGTATCGGTCGTCTGGAC
CCGGAAATTGCCCGCGACTTCAGTAATGTCGGCCCGATGGTGCGTGCCAGCGGTCACGCC
CGCGACACCCGCGCCGACCACCCGTTTGTCGGCTACGGTCTGCTGCCGATGGAAGTACAT
AGCGAGCAGGGTTGCGACGTGATTTCTCGTCTGAAAGTCCGTATCAACGAAGTCTACACC
TCGCTGAATATGATCGATTTCGGTCTGGATAATCTGCCGGGCGGCCCGCTGATGGTGGAA
GGCTTTACCTATATTCCGCACCGTTTTGCGCTCGGCTTCGCTGAAGCGCCGCGTGGTGAC
GATATCCACTGGAGCATGACTGGCGACAACCAGAAGCTGTACCGCTGGCGCTGCCGTGCG
GCGACCTACGCCAACTGGCCAACGCTGCGCTATATGCTGCGCGGCAACACCGTTTCTGAT
GCGCCGCTGATCATCGGCAGCCTCGACCCGTGCTACTCCTGTACCGACCGGATGACCGTG
GTCGATGTGCGTAAGAAGAAGAGCAAAGTCGTGCCGTACAAAGAACTTGAGCGCTACAGC
ATTGAGCGTAAAAACTCGCCGCTGAAATAA
>EECEBICD_00619 NAD(P)H-quinone oxidoreductase subunit I, chloroplastic
ATGTTTACCTTTATCAAAAAAGTTATCAAAACCGGCACGGCGACCTCGTCTTATCCGCTG
GAGCCGATTGCGGTTGATAAAAACTTCCGCGGCAAGCCGGAACATAATCCGCAGCAGTGC
ATCGGTTGCGCCGCCTGCGTTAATGCCTGCCCGTCGAACGCGCTGACGGTGGAAACCGAT
CTTGCGACCAATGAGCTGGCGTGGCAGTTCAATCTTGGCCGCTGCATCTTCTGCGGTCGC
TGCGAAGAAGTTTGCCCGACGGCGGCGATCAAACTGTCGCAAGAATACGAACTGGCAGTG
TGGAAGAAAGAAGACTTCCTGCAACAGTCCCGCTTCGCGCTGTGCAACTGCCGCGTATGT
AATCGTCCTTTCGCGGTACAGAAAGAGATCGACTACGCCATTGCGCTGCTTGCGCACAAC
GGCGACAGCCGCGCGGAAAACCACCGCGAAAGCTTTGAGACCTGCCCGGACTGTAAGCGT
CAGAAATGCCTGGTGCCGTCCGACCGTATTGAACTGACTCGCCATATGAAAGAGGTCAGC
TAA
>EECEBICD_00620 Formate hydrogenlyase subunit 7
ATGAGCAATTTATTAGGCCCGCGTGACGCCAACGGCATTCCGGCACCGATGACGGTGGAT
GAATCCATCGCCAGCATGAAGGCGTCTCTACTGAAAAATATTAAGCGTTCGGCATACGTT
TACCGCGTGGACTGCGGCGGCTGTAACGGCTGTGAAATCGAAATCTTTGCGACGCTTTCG
CCGCTGTTTGATGCCGAGCGTTTCGGCATCAAAGTGGTACCGTCGCCGCGTCATGCCGAT
ATTCTGCTGTTTACCGGCGCGGTGACCCGCGCCATGCGCTCTCCGGCGCTGCGCGCCTGG
CAGTCGGCGCCGGACCCGAAAATCTGTATCTCTTATGGGGCCTGCGGCAACAGCGGCGGC
ATCTTCCATGATCTGTACTGCGTCTGGGGCGGTACGGATAAAATCGTACCGGTGGATGTG
TATATTCCAGGCTGCCCGCCAACGCCTGCGGCGACGCTGTATGGCTTTGCGATGGCGCTT
GGCCTGCTGGAGCAGAAAATCCACGCCCGCGCGCCGGGTGAGCTGGACGATCAGCCCGCA
GAGATCCTGCATCCGGATATGGTGCAGCCGCTGCGCGTGAAGGTGGATCGCGCGGCGCGC
CGGCTGGCGGGTTATCGCTATGGTCGTCAGATCGCCGATGATTACCTGACACAGTTGGGG
CAGGGCGAGCAGCAAGTGGCGCGTTGGCTGGAAGCGGAAAACGATCCGCGTTTAACCGAA
ATTGTCACCCATCTCAATCATGTTGTTGAAGAGGCGCGTATCCGATGA
>EECEBICD_00621 hypothetical protein
ATGAGCGAACAGGTGGTGTTCAGTCAACTGAGCCGTAAGTTTATTGATGAGAACGATGCG
ACGCCTGCCGAGGCGCAGCAGGTTGTCTATTACAGCCTGGCGATTGGTCACCACTTGGGG
GTTATCGATTGTCTGGAGGCCGCGCTCACCTGCCCATGGGATGAGTATCTGGCGTGGATC

GCCACCCTGGAAGCCGGTAGCGATGCCCGGCGTAAAATGGAAGGCGTGCCGAAGTATGGC
GAGATCGTTATCGATTTCAACCATGTGCAAATGCTGGCGCGCGCGTTTGATGAGGCGCGC
GCCGCGCAGACCCCGCAGCAGCAAGAATGGAGCAAACTGATGCTCAGTATGCTGCATGAC
ATTCATCAGGAAAGCGCCATCTACCTGATGGTGAGGAGGCTTCGTGACTGA
>EECEBICD_00622 Hydrogenase 3 maturation protease
GTGACTGACGTTTTACTCTGTGTCGGCAACAGCATGATGGGCGACGACGGCGCAGGCCCA
TTACTGGCGGAAATGTGCGCGGCGCAGCCGAAGGGGAATTGGGTGGTGATTGATGGCGGC
AGCGCGCCGGAAAACGACATTGTGGCGATTCGCGAACTGCGCCCGCAGCGTCTGTTGATT
GTCGACGCCACCGACATGGGACTTAATCCGGGCGAGATCCGCATCATCGATCCGGATGAT
ATCGCCGAAATGTTTATGATGACCACCCACAATATGCCGCTCAACTACCTTATCGATCAG
CTGAAAGAGGATGTCGGCGAGGTGATTTTTGTTGGTATCCAGCCGGATATCGTCGGGTTT
TATTATCCGATGACTCAACCGATTAAGGACGCGGTAAATATCGTCTATCAGCGCCTGGAA
GGCTGGCAGGGAAACGGCGGATTTGCCGCGCTGGAAGCGCCGGAGGCGTAA
>EECEBICD_00623 hypothetical protein
GTGTTACACGGCAAAGGTTGTAAGAAGGACGTGAAGATTATCGTATGGAGCGGACTGCTA
TTTCTCTGCCTCGCCGCACGCGCACTGGCCACCGGTGTTGTCGGTTACCTGCCGATGAGC
GACGGTGAATATGCGCAAAAGCGTGCGCTGAAGCCGCTGTTGACCCTGCCTTATTCTGTA
TCGCCCGACCAGACCTGGCATTTTCGCCAGGTCGGAGTCAGTGGCGTGACGCTACTGCCG
GAGCCGAAAAAAGATAACGAATGGCGGATTAGCGGAAAAGACAGAGCCGGTAATTCGTGG
GTTGTCCCGGTGGGAAGGCTGATTAATTTGGCGGGTAATGCGCAATTTTATCGCGCTGAC
CTCGATCGTAACGGCATTCAGGATCTGGTTATCTGGTTGGGCAATCCTGGGTTGGGCCTG
GCGCCGAGCGCGCAATACATCATTTTCACCATTTCTCAAGAATGGCCGCCCCTGCGTCTT
TGA
>EECEBICD_00624 Hydrogenase-4 component A
ATGAACCGTTTCATCATTGCAGACGCGAGTAAGTGCATTGGTTGCCGTACCTGTGAAGTG
GCTTGCGTCGTATCCCATCAGGAAAATCAGGATTGCGCATCGCTGACCCCTGAAACCTTT
TTACCTCGTATCCATGTCATCAAAGGCGTGAACGTTTCCACGGCAACGCTGTGTCGCCAG
TGTGAAGACGCGCCGTGCGCCAACGTTTGCCCGAATGGCGCCATCAGCCGTGACAAAGGC
TTTGTTCATGTGATGCAGGAACGCTGCATTGGCTGTAAAACTTGCGTAGTGGCATGTCCT
TACGGCGCCATGGAAGTGGTGGTACGTCCGGTGGTTCGCAATAGCGGCGCGGGGCTGAAC
GTGCGTGCCGAAAAAGCCGAAGCCAACAAGTGCGATCTGTGTCACCACCGTGAAGCCGGT
CCGGCCTGTATGGCCGCTTGCCCAACGCACGCCCTAATCTGCGTGGACCGCAATAAGCTT
GAACAGCTGAGCGCGGAAAAACGCCGTCGCGCGGCGCTGGATTCAACGGCATCGCTGCTT
TTCTGA
>EECEBICD_00625 Carbamoyltransferase HypF
ATGGCGATAGACACACCTTCCGGCGTACAGCTACGCATTCGGGGCAAAGTACAGGGCGTC
GGTTTTCGTCCTTTTGTCTGGCAGCTGGCGCAGCAGTTGCGATTACACGGCGACGTGTGT
AATGACGGCGACGGCGTCGTCGTTCGGCTGCTGGAAGAGCCGTCGCAATTTATTGCCGCG
CTCTATCAGGATTGCCCGCCGCTGGCGCGCATTGACAGCGTTGAACACGCGTCGTTGGTA
TGGGAGCGCGCGCCGACGGATTTCACCATTCGTCAGAGCGCAGGCGGTTCGATGAACACG
CAAATCGTGCCGGATGCGGCGACCTGCCCGGCATGTCTTGCCGAGATGAATACCCCAGGC
GAGCGGCGCTACCGTTATCCTTTCATCAATTGCACCCACTGCGGACCACGCTTCACCATT
ATTCGCGCTATGCCCTATGACCGGCCATTTACGGTGATGGCGGCGTTTCCCTTGTGTCCG
GAATGCGACAGCGAATACCGCGATCCGTACGATCGCCGTTTTCATGCCCAGCCCGTTGCC
TGTCCGTCATGCGGGCCGCATCTTGAGTGGCGGAGCCAACATGAGCGAGCGGAAAAAGAG
GCGGCTTTGCAGGCGGCGGTCGCCCAACTGAACGCCGGAGGCATTATTGCCGTTAAAGGG
CTGGGGGGTTTCATCTGGCCTGCGATGCGCGCAACGATAACGCAGTGGCGATGCTGCGGG
CGCGTAAACATCGCCCGGCGAAACCATTGGCGGTGA
>EECEBICD_00626 Carbamoyltransferase HypF
ATGTTGCCCACGGCGCAAACGCTGCCGAGCGCGGCGCGTTCGCTGCTGACCACGCCAGCG
GCCCCGATTGTACTGGTGGATAAGCAGTATGTACCTTCGCTGAGTGAGGGCATCGCGCCA
GGACTTACGGAAGTGGGCGTGATGCTGCCAGCCAACCCATTGCAACACCTCTTGTTGCAG
GCGCTCAATTACCCGCTGGTGATGACATCCGGCAACCTGAGCGGCAAACCGCCCGCCATC
ACCAACGAACAGGCGCTGGACGATTTACACGATATTGCCGATGGTTTTCTGTTGCATAAT

CGCGACATTGTACAGCGCATGGACGACTCTGTCGTGCGCGACAGCGGCGAAATGCTGCGT
CGTTCGCGGGGATACGTGCCGGACGCGATTGCGTTGCCGCCCGGATTTCGCGATGTGCCG
CCGATACTTTGTCTGGGCGCGGATCTGAAAAACACGTTCTGTCTGGTACGCGGCGAACAG
GCGGTTGTCAGCCAGCATTTAGGCGATCTCAGCGATGACGGTATCCAGGCGCAGTGGCGC
GAGGCATTGCGTCTGATCCAGTCAATCTACGATTTTACGCCAGAGCGTATCGTCTGTGAT
GCGCATCCGGGCTATGTTTCCAGTCAGTGGGCCAGTGAGATGCGTCTGCCGACAGAGACG
GTGTTACACCATCATGCCCATGCGGCGGCTTGCCTGGCCGAGCATGGTTGGCCGCTGGAC
GGTGGAGAGGTGATTGCCCTGACGGTAGACGGTATCGGCATGGGTGAGAATGGCGCGCTA
TGGGGCGGAGAATGTTTGCGGGTCAATTATCGCGAGTGCGAACATTTAGGCGGTTTACCC
GCCGTGGCGCTGCCGGGAGGCGATCTGGCTGCCAAACATCCGTGGCGTAATCTGTTGGCG
CAGTGCCTGCGCTTTGTGCCGGACTGGCAGGATTACCCGGAGACTGCGGGGCTGCAACAG
CAAAACTGGAATGTCCTGGCGCGTGCCATTGAGCGCGGCGTCAATGCCCCGTTGGCCTCT
TCCTGCGGGAGGTTGTTCGACGCGGTGGCTGCCGCGCTTCGCTGCACGCCAGCATCGCTT
AGCTATGAGGGCGAGGCCGCCTGCGCGCTGGAGGCGCTGGCCTCTCAATGCGCTAACGTT
GAGCATCCGGTAACGATGCCGCTTAACGGCGCTCAACTGGACGTCGCCGTTTTCTGGCGG
CAATGGTTGAACTGGCAGGCCACGCCCGCGCAACGCGCCTGGGCTTTTCATGATGCGCTG
GCGTGCGGGTTTGCCACGCTAATGCGCCAGCAGGCTACGGCGCGGGGGATAACCACTCTG
GTCTTCAGCGGCGGGGTGATACACAACCGCTTACTTCGCGCGCGTCTTGCCTTTTATCTT
TCTGATTTTAAATTGTTATTTCCGCAGCGGTTACCGGCGGGCGACGGCGGACTGTCGTTT
GGACAGGGCGTGATTGCCGCAGCGCGAGCGTTAAGGGAAGTGTAG
>EECEBICD_00627 Nitric oxide reductase FlRd-NAD(+) reductase
ATGAGTCGGGGAATCATCATTATTGGGTCGGGCTTCGCCGCCCGCCAGCTCGTCAAAAAT
ATCCGTAAACAAGATGCGCATGTGCCGTTAACCCTGATTGCGGCGGACAGCATGGATGAG
TACAACAAGCCCGATCTCAGCCACGTTATCAGCCAGTCGCAGCGCGCCGATGATCTCACC
CGCCAGTTAGCGGGGGAATTTGCTGAACAGTTTAATCTGCGACTGTTCCCGCATACCTGG
GTTGCTGACATTGATGCCGATGCCCATGTGGTGAAAAGTCAGGATAAGCAGTGGCAGTAC
GACAAGCTGGTACTGGCGACCGGGGCGGCAGCCTTTGTGCCGCCGATTGCAGGACGTGAG
TTAATGCTTACTTTGAATAGCCAGCAGGAGTACCGCGCCTGTGAGACACAACTGCGCGAC
GCGCAGCGAGTGCTGATTGTCGGCGGCGGTCTCATTGGCAGTGAATTAGCGATGGATTTC
TGCCGCGCCGGGAAAACGGTAACGCTGATGGATAACGCCGCCAGCCTTCTCGCCTCACTG
ATGCCACCAGAAGTGAGCAGTCGCTTACAGCATCACCTGACCGATATGGGGGTGCATCTG
CTGCTGAAATCGCAGTTGCAAAAACTGGAGAAAACCGAAGCCGGTATCCGCGCTACGCTG
GTAAGCCAGCATAGTATTGAGGTCGATGCGGTGATTGCCGCCACCGGCTTACGCCCGGAA
ACCGCTCTGGCGCGCCCGCGCCGGGGTAGCCGTCAACCGCGGCGTCTGCGTGGATAGCTAT
CTACAGACCAGTCACCCGGATATTTATGCGATCGGCGACTGCGCGGAGATCAACGGTCAG
GTGCTGCCCTTCCTGCAACCGATCCAGTTAAGCGCGATGTACCTGGCGAAAAATCTGCTT
GGCGGCAACGCGCCGCTGAAGCTACCGGCGATGCTGGTCAAAGTCAAAACGCCGGAACTG
CCGCTGCATCTGGCGGGAGAAACTCAGCGCAGCGATTTGAGCTGGCAGATTACCGCAGAA
TCTGACGGTATGATTGCCAAAGGCATGAGCGGAGAAGGCCAACTACGCGCCTTTGTGGTG
AGTGAAGACCGCATGAAAGAGGCCTTTGCACTGCTGAAAACGTTGTCCGTATAA
>EECEBICD_00628 Anaerobic nitric oxide reductase flavorubredoxin
ATGTCTATTCTGGTTAAAAATAATATTCATTGGGTTGGCCAGCGCGACTGGGAAGTACGT
GATTTTCACGGCACTGAATACAAAACGCTGCGCGGCAGCAGCTATAACAGCTATCTCATT
CGTGAAGAAAAGAATGTGCTGATCGATACCGTCGATCATAAGTTTAGCCGTGAGTTTGTG
CAAAATCTGCGCAGTGAAATCGACCTCGCGGATATCGACTACATCATCATTAACCATGCC
GAAGAAGACCATGCAGGCGCGCTGACCGAACTGATGGCGCAGATCCCGGACACGCCTATC
TACTGTACCGCCAACGCCATTGACTCTATCAACGGCCATCACCATCACCCGGAATGGAAT
TTTAAGGTGGTGAAAACCGGCGATACGCTGGATATCGGCAACGGCAAGCAGTTGATCTTC
GTGGAAACGCCGATGCTGCACTGGCCGGACAGCATGATGACCTATATGACCGGCGACGCG
GTGTTATTCAGCAACGACGCCTTTGGTCAGCACTACTGCGACGAGCGCCTGTTCAATGAT
GAAGTGGACCAAACCGAACTGTTTGAACAATGCCAGCGCTACTACGCCAATATCCTGACG
CCGTTCAGCCGTCTGGTAACGCCAAAAATTACCGAAATCCTCGGCTTCAACCTGCCGGTG
GATATGATTGCCACCTCACACGGCGTGGTATGGCGCGACAACCCAACGCAAATCGTGGAA
CTGTATCTGAAATGGGCGGCAGATTATCAGGAAGATCGTATCACTATTTTCTACGATACC

ATGTCGAACAATACTCGCATGATGGCGGATGCCATCGCGCAGGGTATCAATGAAGTGGAC
CCGAACGTGGCGGTCAAAATCTTTAACGTCGCCCGCAGCGATAAAAATGAAATACTGACC
AATGTCTTCCGTTCGAAAGGCGTGCTGGTCGGCACTTCTACCATGAACAACGTGATGATG
CCGAAAATCGCTGGTCTGGTGGAAGAGATGACCGGCCTGCGCTTTCGCAACAAACGCGCC
AGCGCCTTCGGTTCTCATGGCTGGAGCGGCGGCGCGGTGGATCGACTGTCCACGCGCTTA
CAGGATGCCGGTTTTGAAATGTCCCTGAGTCTGAAAGCGAAATGGCGTCCGGATCTGGAT
GCGCTGGAGTTGTGCCGTCAGCACGGTCGCGACATCGCGCGTCAGTGGGCGCTTGCTCCG
CTGCCGGAAACCACACAGAAAACCGCGCCAGTCGAAGAAACCACAACCTGCGCCGCCGCA
GACTTCGGCCCCAAAATGCAATGCAGCGTATGCCAGTGGATTTACGACCCTGCGCTGGGC
GAGCCGTTGCAGGATGTCGCGCCGGGTACGCCGTGGAACGACGTGCCGGATAACTTCCTG
TGCCCGGAATGTTCATTAGGTAAAGACGTGTTCGACGTACTGGCGACGGAGGCAAAATGA
>EECEBICD_00629 Anaerobic nitric oxide reductase transcription regulator NorR
ATGAGTTTTTCCGTGGAGGTATTGGCGGGCATCGCCATTGAGTTACAACGCGGCATTGGG
CATCAGGATCGTTTTCAGCGGCTGATTACTACGCTGCGTCAGGTTCTGGCGTGCGACGCC
TCGGCGCTGTTGCGTTATGAATCACGACAATTTATTCCACTGGCGATTGATGGCCTGGCG
CAGGACGTTCTCGGCAGACGTTTTACCCTGGAAGGGCATCCGCGCCTGGAAGCCATCGCT
CGCGCGGGGGACGTGGTGCGTTTTCCGGCTGATAGCGATTTACCCGATCCGTATGACGGG
TTAATTCCCGGACAGGAAAGCCTGAAAGTCCACGCCTGTGTTGGTCTGCCGCTGTTTGCC
GGACAGAACCTCATCGGTGCGCTGACCCTTGATGCTATGACGCCGGAGCAGTTCGAGGTA
TTTAGCGATGAAGAGCTGCGGCTTGTCGCCGCGCTGGCCGCCGGGGCATTGAGCAATGCG
CTGTTAATTGAACAACTGGAAAGCCAGAACATGCTGCCGGGGTCGTCAGGCGTGTTTGAA
CCGATAAAAGAGACGCACATGATCGGCCTGTCGCCGGCGATGACGCAGTTGAAAAAAGAG
ATCGAGATTGTGGCGGGCTCCGATCTTAACGTGCTGATCGGCGGTGAAACGGGGACTGGT
AAAGAGCTGGTGGCGAAAGCCATTCACCAGGGCTCCCCCAGAGCGGTCAATCCGCTGGTC
TATCTCAACTGTGCCGCGCTGCCGGAAAGCGTGGCGGAGAGCGAGTTGTTTGGTCATGTA
AAAGGGGCTTTTACCGGGGCTATCAGTAATCGTAGCGGTAAGTTTGAAATGGCGGATAAC
GGCACGCTGTTTCTGGATGAGATTGGCGAACTGTCGCTGGCGCTACAGGCCAAGCTACTT
CGCGTGTTGCAGTATGGCGATATTCAGCGCGTCGGCGACGATCGTAGCCTGCGCGTGGAT
GTGCGGGTGTTGGCGGCGACCAACCGTGACCTGCGTGAAGAGGTGCTGGCGGGGCGTTTT
CGCGCCGACCTGTTCCATCGCCTGAGCGTTTTCCCGCTCTTTGTGCCGCCGCTGCGCGAG
CGCGGCGATGATGTGGTGTTGCTGGCGGGCTATTTTTGCGAGCAGTGCCGACTGCGGCTG
GGGCTGTCCCGCGTGGTACTGAGTCCCGGCGCGCGCCGCCATTTGCTCAACTATGGCTGG
CCGGGCAACGTCCGGGAGCTGGAACATGCCATTCACCGTGCGGTTGTACTGGCGAGAGCG
ACCCGGGCAGGGGATGAGGTGGTACTGGAGGAGCAGCATTTTGCCTTGTCAGAGGACGTT
TTACCCGCGCCGTCTGCGGAAAGCTTTCTGGCGCTGCCTGCCTGCCGCAATTTGCGCGAA
TCGACGGAGAACTTCCAGCGAGAGATGATTCGCCAGGCGCTGGCGCAAAATAATCACAAC
TGGGCGGCCAGTGCGCGGGCGCTGGAAACCGACGTCGCCAACCTGCATCGGCTGGCGAAG
CGTCTGGGACTGAAGGATTAG
>EECEBICD_00630 Arabinose 5-phosphate isomerase GutQ
ATGAGTGATGCACTACTAAACGCGGGCCGTCAGACCTTAATGCTGGAGCTACAAGAAGCC
AGCCGTCTGCCGGAGCGTCTGGGCGATGATTTTGTCCGCGCCGCCAATATCATTATTCAC
TGTGAAGGCAAAGTGATCGTTTCCGGTATTGGTAAATCAGGTCATATTGGTAAAAAAATC
GCCGCGACGCTTGCCAGTACCGGTACTCCCGCTTTTTTTGTTCATCCGGCGGAAGCACTG
CATGGCGATCTGGGGATGATTGAAAGCCGCGATGTGATGTTATTTATCTCCTATTCCGGC
GGCGCAAAAGAACTCGACCTCATCATCCCGCGTCTGGAAGATAAATCCGTCGCGCTGCTG
GCGATGACCGGTAAACCTCTCTCTCCGCTGGGGCGAGCGGCAAAAGCCGTTCTGGATATT
TCCGTCGAGCGTGAAGCCTGTCCGATGCATCTGGCGCCGACATCCAGTACCGTCAATACG
CTGATGATGGGCGATGCGCTGGCGATGGCGGTCATGCAGGCGCGCGGTTTTAACGAAGAA
GATTTCGCCCGTTCGCATCCGGCTGGCGCACTGGGCGCGCGTTTGCTCAATAATGTGCAT
CACCTGATGCGCCAGGGCGATGCAATACCGCAGGTAATGCTCGCCACCAGCGTGATGGAT
GCCATGCTGGAACTTAGCCGTACCGGGCTGGGGCTGGTGGCGGTTTGCGATGAGCAACAT
GTTGTGAAAGGCGTCTTTACCGACGGCGACCTGCGTCGCTGGCTGGTGGGCGGCGGCGCG
CTCACCACGCCGGTAAGCGAAGCCATGACGCCCAACGGTATTACGCTCCAGGCGCAAAGC

CGCGCCATTGACGCCAAAGAGCTCCTGATGAAACGCAAAATTACCGCCGCGCCGGTGGTC
GATGAAAACGGCAAACTCACCGGCGCCATTAACCTGCAGGATTTCTACCAGGCGGGGATT
ATCTAA
>EECEBICD_00631 Glucitol operon repressor
ATGAAACCTCGTCAGCGTCAGGCCGCGATTCTGGAGCACCTGCAAAAACAGGGAAAATGC
TCGGTTGAAGAACTGGCCCAGTACTTTGACACGACAGGCACCACTATCCGTAAAGATCTG
GTCATTCTGGAAAATGCCGGAACCGTCATCCGCACCTATGGCGGCGTGGTGTTGAATAAA
GAAGAGTCCGATCCGCCTATTGATCACAAAACGCTTATCAACACCCACAAAAAAGCGCTG
ATAGCCGCAGCCGCTGTGAAATATATCCATGATGGCGATTCCATTATTCTGGACGCCGGA
AGTACCGTGTTACAGATGGTGCCGTTGCTTTCGCGTTTTAGCAACATCACGGTGATGACC
AACAGCCTCCATATTGTCAACGCGCTATCGGAACTGGACAACGAACAAACTATCCTGATG
CCCGGCGGCACCTTCCGTAAAAAATCGGCGTCGTTTCACGGTCAACTGGCGGAAAACGCC
TTTGAGCAATTCAGCTTTGATAAACTTTTCATGGGAACTGACGGTATTGACCTGAACGTC
GGTGTGACCACGTTCAATGAGGTTTACACCGTCAGTAACGCAATGTGCAATGCAGCACGC
GAGGTGATTCTGATGGCGGACTCCTCAAAATTTGGTCGTAAAAGCCCCAATGTGGTGTGC
AGTCTGGAAACCGTCGACAAACTGATCACGGACGCGGGTATCGATCCGGCATTTCGTCAG
GCGCTGGAAGCAAAAGGGATTGAAGTAATCATAACCGGAGAAAGCAATGAGTGA
>EECEBICD_00632 hypothetical protein
ATGGTTTCCACTCTGATTACCGTCGCCGTTATCGCCTGGTGTGCGCAACTGGCGCTGGGC
GGCTGGCAGATTTCCCGCTTTAATCGGGCCTTCGATAAACTTAGCCAACAGGGGCGGGTC
GGCGTCGGGCGCTCCGGCGGGCGTTTTAAGCCCCGGGTGGTGGTTGCCGTGGCGCTGGAC
GAACAGCAGCGGGTGACGGACACTTTATTGATGAAAGGCCTTACGGTATTCGCCAGACCG
GTTAAAATTGCCGCAATGCAAGGAAAACATTTGCATGAATTACAGCCTGATGTGATCTTT
CCCCATGATTCGCTCGCGCAGAATGCACTATCATTGGCGCTTAAACTGAAACATGGGTAA
>EECEBICD_00633 Sorbitol-6-phosphate 2-dehydrogenase
ATGAATCAAGTTGCCGTTGTCATTGGCGGAGGCCAGACCCTGGGGGCATTCCTTTGCCGT
GGGCTTGCAGAAGAAGGATACCGCGTGGCGGTCGTTGATATTCAAAGCGATAAAGCCGCG
AATGTCGCCCAGGAAATTAACGCCGATTTTGGCGAAGGTATGGCGTACGGTTTTGGCGCC
GACGCCACCAGCGAGCAGAGCGTGCTGGCGCTTTCCCGCGGCGTAGACGAGATTTTCGGG
CGCGTCGATCTGCTGGTTTACAGCGCGGGTATCGCCAAAGCGGCGTTTATCAGCGATTTC
CAGTTGGGCGATTTTGACCGCTCGCTACAGGTGAATCTGGTGGGCTATTTCCTCTGCGCC
CGTGAGTTTTCCCGCCTGATGATCCGCGACGGCATTCAGGGGCGCATTATTCAGATTAAC
TCTAAATCCGGCAAAGTGGGCAGTAAACACAACTCCGGCTACAGCGCGGCGAAATTCGGC
GGCGTGGGGTTAACCCAGTCTCTGGCGCTGGATCTGGCGGAATACGGCATTACCGTACAC
TCGTTGATGCTCGGCAACCTGTTGAAATCGCCGATGTTCCAGTCACTGCTGCCGCAATAC
GCCACTAAACTGGGCATTAAGCCCGACGAGGTGGAACAGTATTACATTGATAAAGTACCG
CTCAAACGCGGGTGTGATTATCAGGATGTGCTGAACATGCTGCTATTTTATGCCAGCCCG
AAAGCGTCTTATTGCACCGGGCAGTCGATCAACGTAACCGGCGGTCAAGTGATGTTCTGA
>EECEBICD_00634 PTS system glucitol/sorbitol-specific EIIA component
ATGAGCGTGATTTATCAAACCACCATCACCCGTATCGGCCAGAGCGCGAAGGAGGCGCTC
GGCGAACAGATGCTGATTACGTTTCGGGAAGGCGCGCCGGCGGATATCGAAGAATTTTGC
TTCATCCATTGTCATGGCGAACTGACCGGCGCGCTGCAGCCTGGCGCGCGGTGTGAATTG
GGTCAACATTGCTATCCGGTCACGGCGGTCGGCAGCGTAGCCGAGCAAAACCTGCGTGAA
CTGGGGCATATCACTTTGCGCTTCGATGGTCTGCGCGAAGCAGAATTTCCCGGCACCGTA
CACGTTGCGGGGCCTGTACCGGACGATATCGCGCCGGGCTGTATTTTGACGTTTGTCGCT
TAA
>EECEBICD_00635 PTS system glucitol/sorbitol-specific EIIB component
GTGGACGGCTTTAAAGAAGGCGAACCGCCGGAAGCGGAGATCGGCGCGGCCATTATCGAC
TGTGGCGGTACGCTGCGCTGCGGTATCTATCCGAAACGCCGGATTCCAACCATTAATATT
CACTCGACGGGTAAGTCCGGCCCACTGGCGCAGTATATTGTTGAGGATATTTATGTCTCC
GGCGTAAAAGAAGAAAACATTACTCTTGTCGGCGAAACGCCTGCCAGTCCTCAGCCTGCC
AAAACGACATTAGGACGTGACTACGACACCAGCAAAAAAATCACCGAGCAGAGCGACGGG
CTGCTGGCAAAAGTCGGTATGGGAATGGGCTCCGCCGTGGCGGTACTGTTCCAGTCCGGT
CGCGACACCATTGATACGGTCCTGAAAACAATCCTGCCGTTTATGGCGTTCGTTTCGGCG

CTGATCGGCATCATTATGGCCTCAGGTCTTGGCGACTGGATCGCCCACGGCCTGGCGCCA
TTAGCCAGCCATCCACTGGGGCTGGTGACGCTGGCATTGATCTGCTCTTTCCCGCTGCTG
TCGCCCTTTCTCGGCCCTGGCGCGGTTATCGCTCAGGTCATTGGCGTCCTGATCGGCGTT
CAGATAGGCCTGGGCAATATCCCCCCGCATCTGGCGCTTCCCGCCCTGTTCGCGATTAAC
GCGCAGGCGGCCTGCGACTTTATCCCGGTCGGCCTGTCACTGGCGGAAGCGAAACAAGAC
ACCGTTCGCGTCGGCGTACCTTCTGTGCTGGTCGGACGCTTCCTGACTGGCGCGCCCACG
GTACTTATCGCCTGGTTTGTTTCCGGCTTTATCTATCAATAA
>EECEBICD_00636 hypothetical protein
ATGACACGGGTTCGTATTGAGAAAGGCGCCGGCGGCTGGGGCGGCCCGCTTGAACTGGAC
GTTACGCCAGGCAAAAAAGATCGTCTATATCACAGCCGGTACGCGCCCGGCGATCGTCGA
CAAACTGGCGCAACTAACAGGCTGGCAAGCGGTGGACGGCTTTAA
>EECEBICD_00637 PTS system glucitol/sorbitol-specific EIIC component
ATGATCGAAACCATTACACATGGTGCGGAGTGGTTCATCGGGTTATTCCAGAAAGGCGGA
GAGGTGTTTACCGGTATGGTGACAGGTATTCTTCCGCTACTGATAAGCCTGCTGGTTATC
ATGAATGCGCTCATCAATTTTATCGGCCAGCAGCGTATTGAGCGTTTTGCCCAACGCTGC
GCCGGAAATCCGCTATCCCGCTACCTGCTTTTACCGTTTATCGGAACGTTTGTTTTTTGT
AATCCGATGACCCTGAGTCTGGGACGCTTTATGCCGGAGAAGTACAAGCCGAGCTATTAC
GCGGCGGCCTCCTATAGCTGTCACTCCATGAACGGTCTGTTTCCGCATATCAATCCCGGC
GAACTGTTTGTTTATCTGGGGATCGCCAGCGGACTCACCACGTTAGGGCTTCCTCTCGGC
CCGCTCGCGGTGAGCTATCTGCTGGTAGGTCTGGTGACGAACTTCTTCCGCGGTTGGGTC
ACCGATCTCACCACCGCTATTTTTGAGAAAAAATGGGGATTCAACTTGAGCAGAAAGTTC
ATCTGA
>EECEBICD_00638 Membrane-bound lytic murein transglycosylase B
ATGTTCAAGCGTCGTTATGTAGCGTTACTTCCACTTTGCGTATTGCTTGCTGCCTGTAGC
AGCACGCCAAAATCTTCCGAGACTCAAGCGACGACGGGCACGCCTTCCGGCGGCTTTCTG
CTTGAGCCGCAGCATAACGTCATGCAAATGGGCGGCGACTTCGCCAACAACCCTAATGCG
CAGCAGTTCATTGATAAAATGGTCAATAAACACGGTTTCGACCGTCAGCAGCTGCAGGAG
ATCCTCTCGCAGTCTAAAAGACTGGATTACGTCCTGCGTCTGATGGACAGACAAGCGCCG
ACCACTCAGCCGCCCGCCGGACCAAATGGCGCCTGGTTGCGCTACCGCAAACAATTTATT
ACGCCGGATAACGTGCAAAACGGCGTCGCGTTCTGGAATCAGTATGAAGACGCGCTGAAC
CGCGCCTGGCAGGTATACGGCGTACCGCCGGAAATCATCGTGGGGATTATCGGCGTGGAA
ACCCGCTGGGGACGTATCATGGGGAAAACGCGGATTCTGGACGCGCTGGCGACGCTGTCA
TTTAACTATCCGCGCCGGGCGGAATATTTCTCCGGCGAGCTGGAAACCTTCCTGCTCATG
GCGCGCGATGAGCAAGACGATCCGCTCAATCTGAAAGGTTCCTTTGCGGGCGCGATGGGC
TACGGCCAGTTTATGCCATCTTCCTATAAAGAATATGCGGTCGATTTCAATGGCGATGGG
CACATTAACTTGTGGGATCCGGTAGATGCCATCGGCAGTGTGGCGAACTACTTCAAGGCG
CATGGTTGGGTGAAAGGCGATACGATTGCGGTGCCAGCGAATGGTCAGGCGCCGGGACTG
GCGAATGGTTTCAAAACAAAATACAGCCTCTCTCAGCTTGCCGCCGCAGGCTTAACGCCG
CAGCAATCGTTGGGCAATCATCAGGAAGCCAGCCTGCTTCGTTTGGATATCGGTACGGGC
TACCAGTACTGGTACGGCCTGCCGAACTTCTACACTATTACCCGATATAACCACAGCACG
CATTATGCGATGGCGGTTTGGCAACTGGGCCAGGCGGTAGCGCTGGCGCGGGTGCGCTAA
>EECEBICD_00639 hypothetical protein
ATGTCACTCAAAAAAACGGAAAGCACACAGCACAAACTGAAACAAACCTGTCAACTACGG
CTGTGGTTAATTGTGGGTGGAGTATGTTCATTAGGAATGGGAGTACTGAATATATTGTCG
TCAGGCTATTTTGAACCGTATGATGGCGGTCAAATTATCTTAGGCCTGGGATGTCTGGGA
TATAGTCTGGCACTATCAAAGAAGATATCCCACTTGCAGGCAGAGAATCAGCCCAAAAAG
CCATAA
>EECEBICD_00640 Nicotinamide-nucleotide amidohydrolase PncC
ATGACTGACAGTGAACTGATGCGGCTTAGCGAACAGGTCGGGCTGGCGTTAAAAGCGCGC
GGGGCGACCGTAACGACAGCAGAGTCCTGTACCGGCGGCTGGCTGGCGAAAGCCATTACC
GACATCGCCGGAAGCTCCGCCTGGTTTGAACGCGGCTTTGTCACCTATAGTAATGAAGCC
AAAGCGCAGATGATTGGCGTGCGTGAAGAGACTCTGGCGCAGCATGGCGCGGTCAGCGAA
CCCGTAGTGGTCGAAATGGCGATCGGGGCGCTGAAGGCCGCTCGCGCCGATTTTGCTGTC
GCTATTAGCGGAATTGCCGGGCCGGATGGCGGCAGTGAAGAAAAGCCGGTTGGTACGGTG

TGGTTTGCTTTCGCCAGCGTCAGCGGAGAAGGGATTACGCGTCGGGAATGCTTCAGCGGC
GACCGTGAATCGGTGCGTCGACAGGCGACGACGTACGCGCTACAAACCCTGTGGCAACAA
TTTCTACAAAACACTTGA
>EECEBICD_00641 Protein RecA
ATGGCTATCGACGAAAACAAACAGAAAGCGTTGGCGGCAGCACTGGGCCAAATTGAAAAG
CAATTTGGTAAAGGCTCCATCATGCGTCTGGGTGAAGACCGTTCTATGGATGTGGAAACT
ATCTCCACCGGTTCGCTTTCACTGGACATCGCACTCGGTGCGGGCGGTCTGCCGATGGGG
CGTATCGTCGAAATTTACGGCCGGAATCTTCCGGTAAAACGACCCTGACGCTGCAGGTG
ATTGCCGCTGCGCAGCGTGAAGGTAAAACCTGTGCGTTTATCGATGCGGAACACGCGCTT
GACCCTGTTTACGCACGCAAGCTGGGCGTCGATATCGATAACCTGCTCTGCTCTCAGCCG
GATACCGGCGAGCAGGCGCTGGAAATCTGTGACGCGCTGGCGCGTTCAGGCGCGGTGGAC
GTCATTGTGGTCGACTCCGTAGCGGCGCTAACGCCGAAAGCGGAAATCGAAGGTGAAATT
GGCGACTCTCACATGGGCCTCGCGGCGCGTATGATGAGCCAGGCGATGCGTAAGCTGGCG
GGGAACCTGAAACAGTCCAACACGCTGTTGATTTTCATCAACCAGATCCGTATGAAGATT
GGCGTGATGTTCGGTAACCCGGAAACCACCACCGGCGGTAACGCGCTGAAATTCTACGCC
TCTGTTCGTCTTGATATCCGTCGTATTGGCGCGGTGAAAGAGGGCGATAATGTCGTGGGT
AGCGAAACGCGTGTGAAAGTGGTGAAAAACAAAATCGCCGCGCCGTTTAAGCAGGCCGAG
TTCCAGATCCTCTACGGTGAAGGCATCAACTTCTATGGCGAACTGGTTGACCTGGGCGTG
AAAGAGAAGCTGATCGAGAAAGCGGGCGCATGGTACAGCTACAACGGCGAGAAGATTGGC
CAGGGTAAAGCGAACGCGACTACCTGGCTGAAAGAGAACCCGGCGACAGCGAAAGAGATC
GAGAAAAGGGTTCGTGAATTACTGTTGAGTAATCAGAATGCCACGCCCGATTTCGCCGTT
GACGATAGCGAAGGCGTTGCAGAAACCAACGAAGATTTTTAA
>EECEBICD_00642 Regulatory protein RecX
ATGCGGCCCTTTTGGCATTTCTCCCTGTTAAGGTTTTTTATGAGTGAACCCACATCGCGC
CGTCCCGCTTATGCTCGTCTCCTTGATCGTGCGGTACGTATTCTTGCTGTCCGCGATCAC
AGCGAGCAGGAATTACGGCGTAAACTCTCCGCGCCGGTGATGGGCAAAAATGGGCCGGAA
GAGATTGATGCGACGGCAGACGATTATGAACGCGTGATCGCCTGGTGCCACGAACATCAC
TATCTTGATGACGAGCGCTTCGTGATGCGCTTTATCGCCAGTCGTAGCCGCAAAGGCTAT
GGCCCGGCGCGTATTCGCCAGGAGTTGAATCAGAAAGGCATTTCCCGTGAATCGACTGAA
AAAGCGATGCGAGAATGTGAAATTGACTGGAGTGAAATGGCACGTGAACAGGCCGTTCGC
AAATATGGCGAGCCGCTCCCTTCAAACTTTTCAGAAAAGGTTAAGGTACAGCGCTTTTTG
CTCTATCGCGGGTATCTGATGGACGATATCCAGCAAATATGGCGAAATTTTGCAGATTGA
>EECEBICD_00643 Alanine--tRNA ligase
ATGAGCAAGAGCACCGCTGAGATCCGTCAGGCGTTTCTCGACTTTTTCCATAGTAAAGGG
CATCAGGTAGTTGCCAGCAGTTCCCTGGTGCCTAACAATGACCCCACTCTGTTGTTTACC
AATGCCGGGATGAACCAGTTCAAGGATGTTTTCCTTGGGCTCGATAAACGTAATTATTCC
CGTGCAACCACTTCCCAGCGTTGCGTACGCGCGGGGGGGAAACACAACGATCTGGAAAAC
GTCGGATACACTGCACGTCACCATACTTTCTTCGAAATGCTGGGCAACTTCAGCTTCGGC
GATTACTTCAAACACGACGCCATTCAGTTCGCATGGGAATTACTGACCGGTGAAAACTGG
TTTGCTCTGCCAAAAGAGCGTTTATGGGTCACCGTCTATGAAACCGACGACGAAGCCTAT
GAGATCTGGGAAAAAGAAGTCGGCATCCCGCGCGAGCGTATTATCCGTATTGGCGACAAT
AAAGGCGCGCCATACGCATCGGATAACTTCTGGCAGATGGGCGATACCGGTCCCTGCGGT
CCGTGCACCGAAATCTTCTACGATCATGGCGACCACATCTGGGGCGGCCCTCCGGGAAGC
CCGGAAGAAGACGGCGATCGCTATATTGAGATCTGGAACATCGTCTTTATGCAGTTCAAC
CGTCAGGCTGAAGGGACGATGGAACCATTGCCGAAACCGTCTGTAGATACCGGTATGGGG
CTGGAGCGTATTGCCGCGGTACTGCAGCACGTTAACTCTAACTATGACATCGACCTGTTC
CGCACGTTGATTGAAGCGGTAGCGAAAGTCACCGGCGCTACCGATCTGGGTAATAAATCG
CTGCGCGTTATCGCCGACCACATCCGTTCTTGCGCATTCCTGGTTGCCGATGGCGTGCTG
CCGTCGAATGAAAACCGTGGTTATGTGCTGCGTCGCATTATTCGTCGCGCGGTTCGTCAC
GGCAACATGCTGGGCGCGAAAGAGACCTTCTTCTACAAGCTGGTTGGGCCGCTGATTGAG
GTAATGGGCTCCGCAGGCGAAGAGTTGAAACGTCAGCAGGCGCAGGTCGAGCAGGTTCTG
AAAACGGAAGAAGAGCAGTTCGCTCGTACGCTGGAGCGCGGTCTGGCGCTGCTGGATGAA
GAACTGGCGAAATTGCAGGGCGACACCCTGGACGGCGAAACCGCTTTCCGTCTGTATGAT
ACTTATGGTTTCCCGGTCGATCTAACGGCGGACGTCTGCCGTGAGCGCAACATCAAAGTC

GACGAAGCCGGTTTTGAAGCCGCCATGGAAGAGCAACGCCGTCGCGCGCGCGAAGCTAGC
GGTTTTGGCGCGGACTACAACGCGATGATCCGCGTTGATAGCGCTTCTGAATTTAAAGGT
TATGACCATCTGGAACTGAACGGCAAAGTGACTGCGCTGTTTGTCGATGGTAAAGCGGTT
GAAGCGATTAACGCGGGACAGGAAGCTGTTGTCGTGCTGGATCAGACCCCATTCTATGCG
GAATCAGGCGGTCAGGTTGGCGATAAAGGCGAGCTGAAAGGCGCTGGATTTACTTTCGCT
GTGGACGACACGCAAAAATATGGTCAGGCGATTGGTCACCTCGGCAAATTGTCTGCGGGC
GCTCTGAAAGTGGGCGATGCGGTACAGGCTGACGTAGATGAAGCGCGCCGCGCACGTATC
CGTCTGAACCACTCCGCCACGCACCTGATGCACGCGGCGTTGCGCCAGGTGCTTGGGACA
CACGTTGCGCAGAAAGGTTCGCTGGTTAGCGATAAAGTGCTGCGCTTCGACTTCTCGCAC
AATGAAGCGATGAAGCCGTCGGAGATTCGTGAGGTTGAAGATTTGGTGAATGCGCAAATT
CGCCGCAACCTGCCGATTGAAACGAACATCATGGATCTCGACGCGGCGAAGGCGAAAGGG
GCCATGGCGCTGTTTGGCGAGAAATACGACGAACGTGTACGCGTTCTGAGCATGGGCGAT
TTTTCTACCGAACTGTGCGGCGGTACTCACGCCAGTCGTACCGGTGATATCGGTCTATTC
CGTATTATCTCTGAGTCCGGCACCGCAGCGGGCATTCGTCGTATTGAGGCGGTAACCGGT
GAAGGCGCTATGGCCACCGTTCATGCGCAAAGCGATCGCTTAAACGATATTGCGCATCTG
CTGAAGGGCGACAGCCAGAATCTGGGCGACAAAGTGCGTGCCGTGCTGGAACGTACACGT
CAGCTGGAAAAAGAGTTGCAGCAGTTGAAGGACCAGGCCGCAGCGCAGGAGAGTGCAAAT
CTTTCCAGTAAAGCGGTCGATCTCAACGGCGTGAAGCTCCTGGTCAGCGAGCTTGCTGGT
ATTGAGCCGAAAATGCTGCGAACCATGGTTGATGATCTGAAAAATCAACTGGGGTCTACC
GTTATCGTACTGGCAACGGTTGTTGAAGGTAAGGTTTCTCTGATTGCGGGCGTGTCGAAG
GATGTGACCGACCGGGTTAAAGCAGGGGAATTGATTGGTATGGTCGCTCAGCAGGTGGGC
GGTAAGGGTGGCGGTCGTCCGGACATGGCGCAAGCCGGTGGTACGGATGCGGCAGCTCTG
CCTGCAGCGTTAGCCAGTGTACAAGGCTGGGTCAGCGCAAAATTGCAATAA
>EECEBICD_00644 Carbon storage regulator
ATGCTGATTCTGACTCGTCGAGTTGGTGAGACCCTCATGATTGGCGATGAGGTCACCGTG
ACAGTTTTAGGGGTGAAGGGCAACCAGGTGCGTATTGGCGTGAACGCCCCGAAAGAAGTT
TCTGTCCATCGTGAAGAGATCTACCAGCGTATCCAGGCTGAAAAATCCCAGCAGTCCAGT
TACTAA
>EECEBICD_00645 tRNA-Ser(gct)
GGTGAGGTGGCCGAGAGGCTGAAGGCGCTCCCCTGCTAAGGGAGTATGCGGTCAAAAGCT
GCATCCGGGGTTCGAATCCCCGCCTCACCGCCA
>EECEBICD_00646 tRNA-Arg(acg)
GCATCCGTAGCTCAGCTGGATAGAGTACTCGGCTACGAACCGAGCGGTCGGAGGTTCGAA
TCCTCCCGGATGCACCA
>EECEBICD_00647 tRNA-Arg(acg)
GCATCCGTAGCTCAGCTGGATAGAGTACTCGGCTACGAACCGAGCGGTCGGAGGTTCGAA
TCCTCCCGGATGCACCA
>EECEBICD_00648 tRNA-Arg(acg)
GCATCCGTAGCTCAGCTGGATAGAGTACTCGGCTACGAACCGAGCGGTCGGAGGTTCGAA
TCCTCCCGGATGCACCA
>EECEBICD_00649 tRNA-Arg(acg)
GCATCCGTAGCTCAGCTGGATAGAGTACTCGGCTACGAACCGAGCGGTCGGAGGTTCGAA
TCCTCCCGGATGCACCA
>EECEBICD_00650 Fructose-1-phosphate phosphatase YqaB
ATGTACGCACGTTATGCTGGTTTGATTTTTGATATGGACGGTACTCTCCTGGATACCGAA
CCCACGCATCGCAAAGCGTGGCGTGAGGTTTTGGGGCGCTATGGTCTTCGTTTTGATGAA
CAGGCAATGGTCGCGCTTAACGGTTCGCCAACCTGGCTTATCGCCCAGTCAATCATTGAG
CTTAACCATGCCGATCTTGATCCCCTGTCACTGGCGCGTGAGAAAACCGACGCGGTAAAA
AGCATCCTGCTGGACTGCGTAGAACCGCTGCCGCTGGTTGAGGTGGTCAAAGCGTGGCAT
GGGCGGCGTCCCATGTCGGTCGGTACCGGTAGCGAAAGCGCTATCGCGGAAGCCTTGTTA
GCGCATCTGGGGCTGCGTCGTTATTTTGATGCGGTTGTGGCGGCAGATCACGTACAGCAT
CACAAACCCGCGCCTGACACCTTCCTACTGTGCGCCCAGCGGATGGGCATCATGCCGACG
CAGTGCGTGGTATTTGAAGATGCCGATTTTGGTTTACAGGCGGCGCGGGCGGCGGGAATG
GATGCCGTGGATGTCCGACTATTGTGA

>EECEBICD_00651 Inner membrane protein YqaA
GTGAGTGACGGACTGTCGCTCCTGTCTCTGTTTGCCAGTAGTTTTCTCAGCGCGACGCTG
TTACCCGGTAATTCTGAGGTGGTTTTGGTTGCTATGCTGGTCTCCGGCGTCAGTCATCCC
TGGGTCTTAGTCTTAACAGCAACAATGGGTAATAGCCTTGGAGGGGTAACTAACGTTATT
CTTGGGCGTTTCTTTCCGCTGCGCAAAACGTCGCGCTGGCAGGAAAAAGCGACTGGCTGG
CTAAAGCGTTATGGCGCAGCCACACTATTATTAAGCTGGATGCCGGTCATCGGTGATTTG
TTGTGCCTGTTAGCGGGATGGATGCGCATCCCGTGGGGGCGGACGATCTTTTTTTGTGTC
TTGGTAAGGCGCTGCGCTATGTTGCGCTCGCAGCGGCGACGGTTCAGGGCATGA
>EECEBICD_00652 Glutamate--cysteine ligase
TTGATCCCGGACGTATCACAGGCTCTGGCCTGGCTGGAAAAACATCCTCAGGCGTTAAAG
GGGATACAGCGCGGGTTAGAGCGCGAAACGCTGCGTGTCAATGCTGATGGCACGCTGGCG
ACAACAGGTCATCCTGAAGCGTTAGGTTCAGCGCTGACCCATAAATGGATTACCACGGAT
TTTGCGGAAGCCCTACTGGAGTTTATTACGCCAGTCGATGGCGATATCCAGCATATGCTC
ACCTTTATGCGCGATCTCCATCGGTATACGGCCAGGAAACTGGGCGATGAGCGGATGTGG
CCATTAAGTATGCCCTGCTATATTGCGGAAGGGCAGGACATAGAGCTGGCGCAGTACGGC
ACATCCAATACCGGGCGCTTTAAGACGCTCTATCGCGAAGGGCTGAAAAATCGTTATGGC
GCCTTGATGCAAACCATCTCCGGCGTACATTACAATTTTTCATTACCGATGGCGTTCTGG
CAGGCGAAATGCGGCGTTACGGAAGGCGAAGCGGCAAAAGAAAAAATTTCTGCCGGCTAT
TTTCGTCTGATTCGCAACTATTACCGTTTTGGCTGGGTCATCCCCTATCTGTTTGGCGCG
TCTCCGGCCATTTGTTCCTCTTTCCTGCAAGGGAAGCCAACCACATTACCGTTTGAAAAA
ACGGACTGCGGCATGTACTACCTGCCGTATGCGACGTCGCTGCGTTTGAGTGATTTGGGC
TATACCAATAAGTCGCAAAGCAATCTCGGAATTACGTTTAACGATTTGCATGAATACGTT
GCAGGTCTGAAGCGGGCGATTAAAACCCCTTCCGAGGAGTACGCGCGGATTGGTGTGGAA
AAAGACGGTAAGCGGCTACAGATTAACAGCAACGTTCTGCAAATTGAAAATGAGCTGTAC
GCGCCCATTCGTCCCAAGCGCGTGACGCGCAGCGGCGAATCGCCTTCTGACGCGCTTCTG
CGTGGTGGTATTGAGTATATTGAAGTTCGTTCTCTCGATATTAACCCGTTCTCACCGATC
GGCGTAGACGAGCAACAGGTGCGCTTCCTCGATCTGTTTATGGTCTGGTGCGTATTGGCC
GATGCGCCGGAAATGAGTAGCGATGAGCTGTTATGTACGCGTACTAACTGGAATCGCGTT
ATTCTGGAAGGGCGTAAGCCGGGCCTGACGTTAGGCATTGGCTGTGAAACTGCGCAGTTC
CCGCTGCCCAAAGTAGGGAAAGATCTCTTCCGCGATCTGAAACGCGTGGCGCAAACGCTG
GACAGTATCCACGGGGGAGAGGAGTATCAAAAGGTGTGTGACGAGTTGGTCGCCTGCTTT
GATAATCCTGAACTGACATTCTCCGCCCGAATCTTACGGTCTATGATTGATGAAGGCATT
GGCGGCACCGGGAAAGCGTTCGGTGAGGCTTACCGTAATCTGTTACGCGAAGAGCCGCTG
GAGATTTTACAGGAAGAAGAGTTTATTGCTGAACGTGACGCTTCAGTACGTCGTCAGCAG
GAGATTGAGGCGGCGGATACCGAGCCGTTTGCCGCGTGGCTTGCAAAACACGCCTGA
>EECEBICD_00653 S-ribosylhomocysteine lyase
ATGCCATTATTAGATAGCTTCGCAGTCGATCATACCCGGATGCAAGCGCCGGCGGTCCGG
GTTGCAAAAACGATGAACACCCCGCATGGCGACGCAATCACCGTGTTTGATCTGCGTTTT
TGCATTCCGAACAAAGAAGTGATGCCGGAAAAAGGGATTCATACGCTTGAGCATCTGTTT
GCTGGCTTTATGCGCGACCACCTCAACGGTAACGGCGTTGAGATTATCGATATCTCGCCG
ATGGGCTGCCGTACCGGTTTCTACATGAGCCTGATTGGCACGCCGGACGAGCAGCGTGTT
GCCGACGCGTGGAAAGCGGCGATGGCGGATGTGCTGAAAGTGCAGGATCAAAACCAGATT
CCGGAGCTGAACGTTTACCAGTGCGGTACGTATCAGATGCACTCGCTCAGTGAAGCGCAG
GACATTGCCCGTCATATTCTGGAGCGTGATGTGCGCGTGAACAGCAATAAAGAGCTGGCG
CTGCCGAAAGAAAAACTGCAGGAATTGCATATTTAG
>EECEBICD_00654 hypothetical protein
ATGAAGGCTAAATATTTGGCGCTGATGATCGGTGCTTGCTTTTCTCATAACCTTTGGGCA
GCGAATAATATCACTATTGAGCAGCGTCTGGCTGAACTGGAGCAACGTGTTGTTAATGCT
GAAAAACGGGCATCCGATGCCGAGGCGCAAATTCGCTCGTTGAAACAGCAGCAGGTCGCC
GCTACGCCGACGGTGAATGTCCAGTCCGCCGAGCCCATTGCAGCAGATAAAACGCCGCCG
AAGCTGACCTTATCCGGATTCAGCGATATTAAGTTCTATGGCGATGTCGAATTTAATATG
GATGCGGCAAGCCGTTCCGGTAGCCTGACCTCGATGAGAACGTCAGCGAATAAAGATTGG
GCATCAGGAACCAATGAACGCTGGGATATTAACGGGCGCTTGTTGCTGGGCTTTGATGGC
TACCAGCGGCTGGACAACGGTAATTTTGCCGGATTCTCTGTACAGCCTCTGGCGGACCTG

ACCGGAAAAATGAACCTTGATGATGCCGTTTTCTTCTTTGGTCGTGAGAATGACTGGAAA
ATTAAGGTTGGTCGTTTTGAAGCCTACGATATGTTCCCGCTGAATCAGGATACGTTTATT
GAATATTCGGGGAATACAGCGAACGATCTTTACAGTGACGGTTACGGCTATATCTATATG
ATGAAAGAAGGACGGGGACGTAGCGACAGTGGGGGTAACTTCCTGCTGAGCAAAACCATC
GACAACTGGTATTTCGAAGTTAACACATTGCTGGAAAATGGCAGTACGTTATATACAGAG
AAGCAGTACCACGGAATGGATTTAACCAACGATAAAAACGTGGCTTACGTCCGTCCGGTT
ATCGCCTGGCAAAACGGGCGTTTTTCAACGGCGATAGCGATGGAAAGTAACGTCGTTAAC
AACGCCTATGGCTATTATGAGAATGGGAAGTGGATCGATCAGTCAGATCGTACGGGCTAT
GGTTTTACCATGACCTGGAATGGTCAAAAAACTGACCCGGAAGATGGCGCAGTGATTAAC
CTGAATACCGCCTATATGGATGCGACCGATGAGACAGATTTTACCGCTGGGGTGAATGCG
CTGTGGCATCGATTTGAACTGGGTTATATCTATGCGCATAACAAAATCGAAGCCTTCAAT
GCTACCAATATCGATGCCGTTTGTGAGGACGATTGTTGGATCACCGATCCCGGCAATTAT
GATATTCACACTATTCATGCCTCATATTTATTCCCCAACGTGATGGATATGAAAAACTTT
AACATCTACCTCGGTGCCTATGCTTCATGGGTAGAGGCCAATCCGAATAATGGCGATAAC
AGTGAAGATGCGCGTTACGGCGGGCGTCTGAGATTCAAATATTTCTTTTGA
>EECEBICD_00655 Multidrug export protein EmrB
ATGCAACAGCAAAAACCGCTGGAGGGCGCGCAGCTCGTCATCATGACGATCGCGCTGTCG
CTGGCGACATTCATGCAGGTGCTGGACTCCACCATTGCCAACGTGGCGATTCCGACCATC
GCCGGGAACCTGGGTTCTTCCCTAAGCCAGGGGACATGGGTTATCACCTCTTTCGGGGTG
GCGAACGCTATCTCGATTCCCTTAACCGGCTGGCTGGCGAAACGTTTTGGCGAAGTCAAA
CTGTTCATGTGGTCGACGGTCGCCTTTGCCGCGGCCTCCTGGGCCTGTGGCGTATCCAGC
AGCCTGAATATGCTTATCTTCTTCCGCGTGGTGCAGGGCGTGGTCGCCGGGCCGCTGATT
CCGCTCTCGCAAAGCCTGTTGCTGAATAACTATCCGCCTGCAAAGCGCTCCATTGCGCTG
GCGCTCTGGTCAATGACCGTCATTGTCGCGCCGATTTGCGGCCCGATTCTGGGGGGCTAC
ATCAGTGATAATTACCACTGGGGATGGATTTTCTTCATCAACGTGCCGATTGGCATCGCC
GTCGTCCTGATGACGTTACACACGCTGCGCGGACGTGAAACGCATACCGAACGGCGACGT
ATTGACGCCGTCGGGCTGGCGTTACTGGTGATTGGCATCGGCAGCCTGCAAATTATGCTC
GATCGCGGTAAAGAGCTGGACTGGTTCTCCTCACAGGAAATTATCATCCTGACCGTGGTC
GCCGTCATCGCCATCAGTTTCCTGATCGTCTGGGAACTGACCGACGATCACCCTATCGTC
GATCTTTCTCTTTTTAAATCGCGTAACTTCACGATTGGCTGCTTGTGCATCAGCCTTGCC
TATATGCTCTATTTCGGCGCCATTGTTCTACTGCCGCAGCTTTTGCAAGAGGTGTACGGC
TATACCGCAACGTGGGCAGGTCTGGCGTCGGCGCCAGTCGGCATTATTCCGGTAATTTTG
TCGCCGATTATCGGACGCTTCGCGCATAAACTGGATATGCGCAGGCTGGTCACGTTCAGC
TTCATTATGTACGCCGTCTGTTTTTACTGGCGCGCCTGGACGTTCGAACCGGGTATGGAT
TTCGGCGCTTCCGCATGGCCGCAGTTTATTCAGGGATTTGCCGTCGCCTGCTTCTTTATG
CCGCTGACCACCATTACGCTCTCCGGGTTACCGCCAGAACGGCTGGCGGCGGCATCAAGC
CTGTCGAACTTCACGCGAACGCTGGCGGGCTCCATCGGCACCTCGATTACCACCACTATG
TGGACCGATCGTGAGTCGCTACACCATGCTCAGCTCACGGAGTCGGTTACGGCGTATAAT
CCAAACGCTCAGGCTATGTACGATAAACTGGAAGGCTTAGGGATGACGCACCAACAGGCG
TCAGGCTGGATAGCCCAGCAAATCACGAATCAGGGGCTGATTATTTCCGCCAACGAAATC
TTCTGGATGTCGGCGGGGATCTTCCTGGTGCTGCTCGGCCTGGTGTGGTTCGCCAAACCG
CCCTTCGGTGCGGGCGGCGGCGGTGGCGGCGCGCACTAA
>EECEBICD_00656 Multidrug export protein EmrA
ATGAGCGCAAATGCGGAGATCCAAACCCCGCAGCAACCGGCTAAGAAGAAAGGCAAACGC
AAAACAGCGCTGCTACTTCTTACCTTGCTCTTTGTTATTATTGCCGTGGCATATGGAATT
TATTGGTTTTTAGTATTGCGTCATATTGAAGAGACAGATGATGCTTACGTGGCAGGGAAC
CAGGTTCAAATCATGGCGCAGGTGTCAGGCAGCGTGACGAAAGTCTGGGCTGATAACACC
GACTTTGTAAAAGAGGGCGATGTTCTGGTCACGCTCGATCAGACTGACGCCAAACAAGCG
TTTGAAAGAGCCAAAACGGCGCTGGCCTCCAGCGTGCGCCAAACGCACCAGTTGATGATT
AACAGTAAGCAGTTGCAGGCGAATATCGACGTGCAAAAAACCGCCCTGGCGCAAGCGCAG
AGCGACCTTAACCGTCGTGTGCCGCTGGGTAATGCCAATCTTATTGGCCGGGAAGAGCTG
CAACACGCCCGCGATGCCGTCGCCAGCGCGCAGGCACAGCTGGATGTCGCCATTCAACAG
TACAATGCCAACCAGGCAATGATACTCAACAGTAATCTGGAAGATCAGCCTGCGGTTCAA
CAAGCGGCGACCGAAGTGCGTAACGCCTGGCTGGCGCTGGAGCGTACTCGCATCGTCAGC

CCAATGACTGGTTATGTCTCCCGCCGCGCCGTCCAGCCTGGCGCGCAAATCAGCCCCACC
ACGCCGCTGATGGCCGTGGTGCCTGCAACCGATCTGTGGGTGGACGCTAACTTTAAAGAA
ACCCAATTAGCGAATATGCGCATTGGGCAGCCAGTGACGGTGATTACTGATATTTATGGC
GACGACGTAAAATACACCGGTAAAGTCGTCGGTCTGGATATGGGGACAGGCAGCGCCTTC
TCCCTGCTGCCCGCGCAAAATGCGACGGGTAACTGGATTAAAGTGGTTCAACGTCTGCCG
GTACGCGTCGAACTGGACGCCCGCCAGTTAGAACAACATCCGCTGCGCATTGGTTTATCG
ACGCTGGTCACCGTGGATACCGCTAATCGCGACGGTCAGGTACTGGCCAGCCAGGTACGA
ACGACGCCGGTTGCCGAAAGTAACGCACGCGAAATTAATCTCGCGCCGGTCAATAAACTG
ATCGACGACATCGTACAGGCTAACGCGGGTTAA
>EECEBICD_00657 Transcriptional repressor MprA
ATGGATAGTTCGTTTACGCCCATTGAACAAATGCTAAAATTTCGCGCCAGCCGTCACGAA
GACTTTCCTTATCAGGAAATTCTTTTGACTCGTCTTTGTATGCACATGCAAGGCAAGCTC
CTGGAAAACCGCAATAAAATGCTGAAGGCTCAAGGGATTAACGAGACGTTGTTTATGGCG
TTGATTACGCTGGAGTCTCAGGAAAACCACAGTATTCAGCCGTCTGAATTAAGCTGCGCG
CTGGGTTCATCTCGCACCAATGCGACACGCATTGCAGATGAGCTGGAAAAACGCGGCTGG
ATTGAGCGTCGTGAGAGCGATAACGACCGCCGTTGCCTGCATCTGCAATTAACGGAGAAA
GGCCAGGCATTTTTGCAAGAAGTGCTTCCGCCTCAGCATCATTGTCTGCATCAACTCTGG
TCATCACTTAGCACAGCCGAAAAAGATCAACTTGAGCACATCACTCGTAAACTCCTGACG
CGTCTTGATCAAATGGAGCAGGAAGGCACTGTTCTTGAGGCGCTGCGCTAA
>EECEBICD_00658 putative transporter
ATGACGAAACCCACTCATGAGCTTAGCCCGGCGCTGATCGTTTTAATGTCTGTGGCCACG
GGTCTGGCGGTCGCCAGCAACTACTATGCCCAGCCGCTGCTTGATACCATCGCGCATCAC
TTTTCGCTTTCCGCCAGCTCCGCAGGGTTTATCGTTACCGCCGCGCAGTTGGGCTATGCC
GCTGGCCTGTTGTTTCTGGTGCCGCTCGGCGACATGTTTGAACGCCGAACGCTGATTGTC
TCCATGACGTTGCTGGCGGCTGGCGGAATGCTGATCACCGCCAGCAGTCAGTCGCTTAGC
ATGATGATACTCGGAACGGCCTTAACCGGACTGTTCTCCGTGGTGGCGCAGATTCTGGTT
CCGCTGGCCGCCACGCTTGCGACGCCCGCCACCCGCGGTAAAGTGGTCGGCACCATTATG
AGCGGCCTGTTGCTGGGGATCCTGCTGGCGCGAACGGTCGCCGGACTGCTGGCAAACCTC
GGCGGTTGGCGCACCGTATTTTGGGTAGCGTCGGCGCTGATGGCGCTGATGGCCGTCGCG
TTGTGGCGCGGACTGCCAAAGCTCAAATCCGACACCCATCTTAACTACCCGCAGCTGTTG
GGTTCTGTATTCAGCCTGTTTATTCACGATAAGCTGCTGCGTACCCGCGCGCTGCTGGGC
TGTCTGACCTTTGCTAATTTCAGCATCCTCTGGACGTCAATGGCCTTTTTGCTCGCCGCG
CCGCCGTTTAGCTATTCCGAGGGGATGATTGGCCTGTTTGGCCTGGCGGGGGCCGCCGGC
GCTTTAGGCGCGCGTCCGGCTGGCGGATTTGCCGATAAAGGTAAATCTCACCTCACCACC
ACGTTCGGCTTACTGCTGCTGTTACTCTCCTGGCTGGCTATCTGGCTTGGGCATACCTCG
GTACTGGCGCTGATTATTGGCATTCTGGTACTGGACCTCACCGTTCAGGGGGTACATATC
ACCAATCAGACGGTCATCTATCGTTTGCATCCGGATGCGCGTAACCGGCTCACCGCCGGC
TATATGACCAGCTACTTTATCGGTGGCGCCGCGGGGTCGCTGATTTCCGCCTCCGCCTGG
CAACATGCCGGCTGGGCCGGCGTTTGTCTGGCGGGTGTCACGGTAGCCTTACTTAATTTA
CTGGTCTGGTGGCGAGGTTTTCACCGACAGGAAGCCGTAAATTAA
>EECEBICD_00659 Glycine betaine/proline betaine-binding periplasmic protein
ATGCGACATACCGTGATATTTGCCTCAGCGTTTGCCACCCTTGTCACCGCCAGCGCTTTC
GCTGCCGACCTGCCTGGCAAAGGCATTACCGTCCAACCTATCCAAAGCACGATTTCTGAA
GAGACCTTCCAGACGTTACTGGTCAGTCGGGCGCTGGAAAAGTTGGGTTATACCGTAAAT
AAGCCGAGTGAAGTCGATTACAACGTAGGCTATACCTCCATCGCCTCTGGCGATGCGACG
TTTACCGCCGTGAACTGGCAGCCGCTGCATGATGATATGTATGCCGCAGCAGGCGGAGAC
AACAAGTTTTACCGCGAGGGCGTCTTCGTCTCCGGCGCTGCGCAGGGCTATCTGATCGAT
AAAAAAACCGCCGAGCAGTACAACATCACCAATATCGCTCAGCTAAAAGATCCGAAAATC
GCCAAAATCTTCGATACCAATGGCGACGGAAAAGCGGACATGATGGGCTGCTCGCCGGGC
TGGGGCTGCGAAGCCGTGATCAATCATCAGAATAAAGCGTTTGATCTGCAAAAAACCGTT
GAGGTCAGCCACGGTAACTATGCGGCGATGATGGCTGATACCATTACCCGTTTTAAAGAA
GGCAAGCCGGTGCTGTATTACACCTGGACCCCGTACTGGGTGAGCGACGTAATGAAGCCG
GGTAAAGATGTCGTCTGGCTACAGGTGCCGTTCTCCTCTCTGCCGGGCGAACAGAAAAAT

ATTGATACTAAACTGCCGAACGGCGCGAACTATGGGTTCCCGGTTAATACCATGCATATT
GTCGCCAATAAGGCATGGGCGGAGAAAAACCCGGCGGCGGCGAAACTGTTCGCCATCATG
AAGCTGCCGCTGGCGGATATCAACGCGCAGAACGCCATGATGCATGCCGGTAAATCGTCT
GAAGCCGATGTTCAGGGCCACGTGGACGGCTGGATCAACGCCCACCAGCAGCAGTTTGAC
GGCTGGGTGAAAGAAGCGCTGGCCGCGCAGAAATAA
>EECEBICD_00660 Vitamin B12 import ATP-binding protein BtuD
ATGGCAATTAAATTAGAAGTGAAGAATCTGTATAAAATATTTGGAGAGCATCCGCAGCGT
GCCTTCAAATATATTGAAAAGGGACTATCGAAAGAGCAAATACTGGAAAAAACGGGGCTA
TCGCTTGGCGTTAAAGACGCCAGTCTGGCCATTGAAGAAGGCGAGATATTTGTCATCATG
GGATTATCCGGCTCGGGTAAATCCACAATGGTACGCCTTCTCAATCGCCTGATTGAACCC
ACCCGCGGACAGGTACTGATTGACGGCGTTGATATTGCCAAAATATCAGACGCTGAGCTT
CGCGAGGTGCGCAGGAAAAAGATTGCGATGGTCTTCCAGTCATTTGCGCTCATGCCGCAT
ATGACCGTGCTGGATAATACGGCATTCGGTATGGAATTAGCGGGCATCGCGGCGCAAGAG
CGTCGCGAAAAAGCGCTGGACGCCTTGCGTCAGGTGGGGCTTGAAAATTACGCTCATGCC
TATCCGGATGAACTTTCCGGTGGGATGCGTCAGCGTGTTGGACTTGCCCGCGCGCTGGCA
ATCAACCCTGATATCTTATTAATGGACGAAGCGTTTTCCGCCCTCGATCCATTAATTCGT
ACCGAAATGCAGGATGAACTGGTGAAATTACAGGCGAAACATCAGCGCACCATTGTCTTT
ATTTCCCACGATCTTGATGAGGCTATGCGTATTGGCGACAGGATTGCCATTATGCAAAAT
GGCGAGGTCGTACAGGTTGGTACGCCGGATGAGATCCTGAATAATCCGGCAAATGATTAT
GTCCGCACGTTCTTCCGTGGCGTGGATATTAGTCAGGTCTTTAGCGCCAAAGATATTGCC
CGTCGCAGTCCGGTCGGCTTAATTCGTAAAACGCCAGGTTTCGGTCCCCGTTCGGCACTG
AAATTATTACAGGACGAAGACCGTGAATATGGTTACGTCATTGAGCGTGGCAATAAATTC
GTGGGCGTCGTGTCCATCGACTCATTAAAAGCGGCATTAAGCCAGGCGCAAGGGATTGAA
GCGGCGCTTATCGACGACCCTTTAGTTGTTGATGCGCAAACCCCACTCAGCGAGTTGCTC
TCTCACGTCGGCCAGGCGCCCTGCGCGGTGCCGGTTGTCGATGAAGAACACCAGTATGTT
GGCATTATTTCAAAACGTATGTTGCTACAGGCTTTAGATCGCGAGGGGGTAACAATGGCT
GATCAAACGAATCCGTGGGATACCGCACAGGTGGCCGATACTACGACGCAAACGGCTGAT
GCCTGGGGAACACCGGCAGGCGTAGCCACGGACGGCGGCAGTACCGACTGGTTGAACAGC
GCGCCCGCGCCAGCCCCTGAACACTTTTCTCTTCTGGACCCGTTCCATAAGACGCTGATC
CCGCTGGATAGCTGGGTCACAGAGGGAATCGACTGGGTCGTCACCCATTTCCGTCCCCTT
TTTCAGGGGATTCGTGTGCCGGTGGATTACATCCTCAACGGCTTTCAGCAACTGCTGCTG
GGAATGCCCGCCCCTGTGGCGATTATTCTCTTTGCGCTGATTGCCTGGCAGGTTTCCGGC
GTGGGCATGGGGATCGCGACGCTGATATCGCTGATCGCGATCGGCGCGATCGGTGCCTGG
TCGCAGGCGATGATTACCCTGGCGCTGGTGTTGACCGCCCTGTTGTTCTGCGTCGTGATC
GGATTACCGATGGGAATCTGGCTGGCGCGCAGCCCGCGTGCGGCCAAAATAGTTCGTCCG
CTGCTGGATGCGATGCAGACCACGCCCGCGTTTGTCTATCTGGTGCCGATTGTCATGTTA
TTCGGCATCGGTAACGTGCCGGGCGTGGTGGTGACGATTATTTTTGCTCTACCGCCGATT
GTACGCCTGACGATCCTGGGCATTAACCAGGTGCCTGCCGACTTAATTGAAGCGTCGCGC
TCGTTCGGCGCCAGCCCGCGCCAAATGTTGTTCAAAGTGCAACTACCGCTGGCGATGCCC
ACCATTATGGCAGGCGTTAATCAGACGCTGATGCTGGCTCTCTCGATGGTCGTCATCGCC
TCGATGATTGCGGTCGGCGGACTTGGCCAGATGGTACTACGCGGCATTGGTCGTCTTGAT
ATGGGGCTGGCAACCGTCGGCGGCGTCGGCATTGTGATTCTCGCCATCATTCTGGACCGT
CTGACGCAGGCCGTCGGGCGCGACTCGCGTAGCCGCGGTAACCGTCGCTGGTATACCACC
GGTCCTGTTGGGCTAATCACCCGCCCTTTCGTTAAGTAA
>EECEBICD_00661 Ribonucleoside-diphosphate reductase 2 subunit beta
ATGAAATTATCTCGTATTAGCGCCATCAACTGGAACAAGATCCAGGACGACAAAGATCTG
GAGGTGTGGAACCGGCTGACCAGTAACTTCTGGCTGCCGGAAAAAGTGCCGTTATCGAAT
GATATTCCGGCCTGGCAGACGCTGAGCGCCGCCGAACAGCAGCTCACCATTCGCGTGTTT
ACGGGACTTACGCTGCTCGACACTATCCAGAACATCGCAGGCGCGCCGTCGTTAATGGCA
GATGCCATCACGCCGCATGAAGAGGCAGTGCTGTCGAACATCAGCTTTATGGAAGCGGTA
CACGCCCGCTCTTACAGTTCTATTTTTTCCACGCTGTGCCAGACGAAAGAGGTTGATGCC
GCCTACGCCTGGAGCGAAGAAAACCCGCCGCTTCAGCGTAAGGCGCAGATTATTTTGGCT
CATTACGTCAGCGATGAACCGCTAAAGAAAAAGATTGCCAGCGTCTTTTTAGAGTCTTTT
CTGTTCTATTCCGGCTTCTGGCTGCCGATGTATTTCTCCAGCCGCGGTAAGCTCACGAAC

ACTGCCGACCTGATTCGTTTAATCATTCGCGATGAAGCGGTTCACGGTTATTATATTGGT
TATAAGTATCAGATAGCGCTACAAAAACTATCGGCAATCGAGCGTGAAGAGTTAAAGCTT
TTCGCGCTGGATTTGTTGATGGAACTGTACGACAACGAAATCTGCTACACAGAAGCGTTA
TATGCGGAAACCGGCTGGGTTAACGACGTCAAAGCCTTCTTGTGCTACAACGCCAATAAA
GCCTTAATGAACCTGGGTTATGAGGCGTTATTTCCGCCAGAGATGGCTGACGTGAATCCC
GCAATCCTTGCCGCGCTCTCGCCGAATGCCGACGAAAATCATGATTTCTTTTCCGGATCA
GGTTCATCTTATGTGATGGGGAAAACAGTCGAAACCGAAGACGAAGACTGGAATTTTTAA
>EECEBICD_00662 Ribonucleoside-diphosphate reductase 2 subunit alpha
ATGCAGGAAACCATGGATTACCACGCCCTGAACGCGATGCTGAATCTTTACGATAAAGCA
GGCCATATTCAGTTCGACAAGGACCAGCAGGCGATCGACGCCTTCTTTGCCACCCACGTC
CGCCCGCATTCCGTGACGTTTGCCAGCCAGCATGAACGTCTGGAGACGCTGGTTCGGGAA
GGGTATTACGATGACGCTGTCCTCGCGCGTTACAACCGCGCCTTCGTCCTCCGCCTGTTC
GAGCACGCCCACGCCAGCGGCTTTCGCTTCCAGACGTTTCTTGGCGCCTGGAAGTTCTAT
ACCAGTTACACGCTGAAAACCTTCGACGGCAAACGTTATCTGGAACACTTTGAAGATCGG
GTGACAATGGTGGCGTTGACGCTGGCGCAGGGTGACGAAACGCTGGCCACCCAACTGACC
GATGAAATGCTTTCTGGTCGCTTTCAGCCCGCTACCCCGACTTTTTTAAATTGCGGTAAA
CAGCAGCGTGGGGAACTGGTCTCCTGCTTCCTGCTCCGTATCGAAGACAACATGGAGTCG
ATCGGGCGGGCGGTGAATTCGGCGCTGCAACTCTCCAAACGCGGCGGCGGCGTCGCGTTT
TTACTCTCCAATCTGCGCGAGGCGGGCGCGCCGATCAAACGCATTGAGAATCAGTCTTCC
GGCGTGATCCCGGTGATGAAAATGCTGGAAGACGCGTTTTCGTATGCCAACCAGCTTGGC
GCGCGCCAGGGGGCCGGCGCGGTTTATCTCCATGCGCACCACCCGGATATTCTGCGTTTT
CTGGATACCAAACGGGAAAATGCCGACGAAAAAATCCGGATCAAAACGCTCTCTCTCGGC
GTGGTGATCCCGGATATCACCTTCCGGCTGGCGAAAGAAAACGCGCAAATGGCGCTCTTT
TCGCCCTATGACGTACAACGACGCTACGGCAAACCGTTTGGCGATATCGCCATTAGCGAA
CGGTACGATGAATTAATTGCCGATCCGCACGTGCGTAAAACCTATATTAACGCCCGTGAC
TTTTTTCAAACACTGGCGGAGATTCAGTTCGAATCCGGGTATCCCTACATCATGTTTGAA
GATACGGTAAACCGCGCGAATCCCATTGCTGGGCGCATTAATATGAGCAACCTGTGCTCG
GAAATTTTACAGGTCAATAGCGCCTCCCGTTACGACGATAACCTTGACTATACCCACATC
GGGCATGACATTTCCTGCAATCTCGGCTCGCTGAATATCGCTCACGTCATGGATTCACCG
GACATTGGTCGTACCGTAGAAACCGCTATTCGCGGCCTGACGGCGGTGTCGGACATGAGC
CATATACGCAGCGTACCCTCAATAGCCGCCGGTAATGCCGCCTCTCATGCCATCGGTCTG
GGCCAGATGAATCTGCATGGCTATCTGGCGAGAGAAGGCATTGCCTACGGTTCGCCGGAG
GCGTTGGATTTCACCAATCTCTATTTTTACACCATTACCTGGCATGCCGTGCATACTTCA
ATGCGGCTGGCCCGCGAACGCGGCAAAACCTTCGCCGGATTTGCGCAGTCGCGCTATGCC
AGCGGCGACTATTTTACGCAGTATTTACAGGACGACTGGCAACCGAAAACGGCGAAAGTC
AGAACGCTATTTGCCCGCAGCGGCATTACGCTGCCCACACGAGAAATGTGGCTAAAGCTG
CGCGACGATGTGATGCGCTATGGCATCTATAACCAAAATTTGCAGGCGGTGCCGCCGACC
GGTTCGATTTCTTACATTAATCATGCGACCTCCAGCATTCATCCGATTGTGGCCAAAATT
GAGATTCGCAAAGAGGGCAAAACCGGGCGTGTGTATTACCCCGCGCCGTTTATGACCAAT
GAAAATCTGGACATGTATCAGGATGCTTACGATATCGGTCCGGAAAAAATTATTGATACC
TATGCCGAGGCCACGCGCCACGTCGATCAAGGACTGTCGCTCACCCTGTTTTTCCCCGAT
ACCGCCACGACCCGCGATATCAACAAAGCGCAGATCTATGCCTGGCGAAAAGGTATTAAG
TCACTGTATTACATCCGGCTTCGCCAGTTGGCGCTGGAAGGTACTGAAATTGAAGGCTGC
GTATCCTGCGCGCTATAA
>EECEBICD_00663 Protein NrdI
ATGAGCGCGCTCGTCTACTTCTCCAGCAGCTCTGAAAATACGCACCGTTTTATGCAGCGT
CTGGGGCTGCCCGCCACGCGTATTCCGCTCAATGAGCGGGAGCGAATTCAGGTAGACGAA
CCGTACATTCTGGTTGTGCCGTCATACGGCGGCGGCGGGATGGCCGGTGCGGTGCCGCGA
CAGGTGATCCGCTTTTTAAATGATGAACACAACCGGGCGCGCATTCGCGGCGTTATCGCC
TCCGGTAATCGCAATTTCGGCGATGCCTGGGGATGCGCTGGCGATGTGATAGCACAAAAA
TGCGGCGTCCCCTGGCTGTACCGCTTTGAGCTCATGGGCACACAACGCGACATCGATAAC
GTCCGAAAAGGAGTAAATGAATTTTGGCAACAACTATCCCGGAGCGCGTAA
>EECEBICD_00664 Glutaredoxin-like protein NrdH
ATGAGCATTACTATTTACACTCGCAATAACTGTGTTCAGTGCCACGCCACAAAGCGGGCG

ATGGAAAGCCGTGGATTTGAATTTGAGATGGTGAACGTCGATCTGGTGCCGGATGCAGCG
GATACGCTCCGGGCGCAAGGGTTTCGTCAATTACCGGTGGTGATAGCGGGCGATTTGAGC
TGGTCTGGCTTCCGTCCGGACATGATTAACCGTCTGCACCCGACACCCCACGCGGCAAAC
GCATGA
>EECEBICD_00665 Alkyl hydroperoxide reductase AhpD
ATGACGACGTTACGCCAACCCTATTATGAACTGAGCCCGGAGGTTTACAGCGCTCTGGTG
CAGGCGAAAAACGCGCTGGAAAAGAGCACGATTGACCCCACTCTGATGGAGCTGATTTAC
CTGCGTGTTTCGCAAATAAATGGTTGTGCATTTTGCCTGGAGATGCACAGTAAAGCGCTG
CGTAAGGCTGGCGTGAATCAAGCTAAATTAGATGCGCTGGCAGGATGGCGGGTAAGCCAC
CATTTCAACGAGCAAGAGTGCGCGGCGTTAGCCTGGGCGGAATCGGTGACGCACATTGCT
GAAACCCACGCCGAAGATAATGTTTACCTGCCGCTGCTCGATCATTTCAGCGCGCGTGAA
ATCAGCGATCTGACCTTCGCTATTGGTTTAATGAACTGCTTTAACCGTCTGGCGATCGGT
ATGCGAATGTGA
>EECEBICD_00666 HTH-type transcriptional regulatory protein GabR
ATGCCGCGCTATCAGCACATCGCTCGTCAGTTAAAAACGGCCATTGAGCAAGGAGAACTC
GCGCCCGGAACGCGCTTGCCTTCCAGCCGGACGTGGGCGCAGGAACTGGGCGTTTCTCGC
GCCACGGTGGAAAATGCCTATGGCGAGCTGGTGGCGCAGGGCTGGCTGGAGCGACGTGGT
CAGGCAGGCACGTTTGTGAGCAACGCTCTACGGTTTGAGACGGCGCCGCCGATACCCGCT
GTTTTTGCCGGAGAAAGTCCGGAACCGAAACCCTTTCAGATGGGGTTACCGGCGCTGGAT
CTCTTTCCACGCGAGAAGTGGGCGCGAGTGATGGGGCGTCGGTTGCGCACGCAGACGCGC
TTCGATCTGGCATTAGGCGACGTCTGCGGCGAGGCGATTTTGCGCCAGGCGATAGTCGAT
TACCTGCGGGTTTCGCGTAGCATTGAATGCCTGCCGGAACAGGTATTTATTACCTCCGGA
TATGCGGATTCTATGCGGCTAATCCTGCGTACATTGTCTGTGCCGGGAGACAGCATGTGG
GTGGAAGATCCCGGTTTTCCGTTAATTCGCCCGGTGATAACGCAGGAGGGGATTACGCTG
GCGCCGATTCCGGTCGATGCGGATGGGCTGAATGTCGCGGCGGGGATGCGGGATTGCCCG
CAGGGGCGCTTTGCATTGGTGACGCCCGCCCATCAAAGTCCGTTGGGGGTGGCGCTGTCG
TTAACTCGCCGACGGCAACTTCTGGCATGGGCGGCGAATGTGCAGGCCTGGATTATTGAA
GATGACTACGACAGCGAATTTCGTTATCACGGTAAACCGCTTCCGCCGCTCAAGAGTCTG
GATGCCCCGCAGCGAGTGATTTACGCCGGAACGTTCAGTAAGTCGCTCTTTCCGGCATTA
CGTACCGCCTGGCTGGTGGTGCCGATAAAGCAGATTGAGCATTTCCGCCAGCAGGCGTCG
CTGATGCCCTGTAGCGTACCGTTGTTATGGCAGCACACGCTGGCTGATTTTATCCGTGAT
GGCCATTTCTGGCGGCATCTGAAAAAGATGCGTCAACATTATGCTCAGCGACGGTTATGG
ATTGAAGAGGCGCTGGCAGAACAGGGATTTGTCGTGACATTACAGAAAGGCGGTATTCAA
CTGGTTATTGAGGTTGAAGGCGATGATAAAGCGCAGGTAGCGAAAGCGAATCAGGCCGGA
CTGGCGGTACAGGCGCTAAGCCGTTGGCGAGTGGTTTCGTCAGGAAAGGGGGGCATTTTA
CTGTCGTTTACCAATATTACTTCCGCCGCCATGGCGAAACAGGTCGCATGGCAGCTTCGA
CAGGCGATACAGTAA
>EECEBICD_00667 putative protein YgaM
ATGCTTAACAAACCGAACCGAAACGACGTCGATGATGGTGTTCAGGATATTCAGAATGAT
GTCAATCGATTAGCCGACAGTCTGGAAGATGTCCTTAAATCCTGGGGAAGCGATGCGAAA
GACGAAGCCGAGGCTGCGCGACGTAAAGCACAGGCGTTGCTGAAAGAGACCCGGGCGCGG
ATGCATGGACGTACTCGCGTAAAACAAGCCGCCTGTGATGCGATGGGGTGCGCGGATACG
TTTGTGCGTGAAAAACCCTGGTGTAGCGTAGGAACCGCCGCAGCGGTAGGAATTTTTATC
GGGGCGTTGCTTAGCCTGCGTCGCTAA
>EECEBICD_00668 hypothetical protein
ATGTATTTACGACCTGACGAGGTGGCGCGGGTACTGGAAAAGGCAGGGTTTACCGTCGAT
GTGGTGACGAATAAGACGTATGGTTATCGACGCGGCGAGAATTATGTTTATGTTAACCGC
GAGGCGCGTATGGGGCGTACCGCGCTGATCATTCACCCCAGATTAAAAGACCGTAGCTCA
TCACTGGCTGACCCGGCTTCGGATATTAAAACCTGCGATCACTATCAAAATTTTCCGCTC
TATTTAGGCGGCGAGACCCACGAACATTACGGTATTCCCCACGGCTTTAGCTCCCGAATA
GCGTTAGAACGTTATCTGAATGGGCTTTTTGGCGACGAAAAAACCGACTAA
>EECEBICD_00669 L-alanine exporter AlaE
ATGTTCTCTCCACAGTCACGCTTGCGTCATGCCGTAGCAGACACGTTTGCGATGGTTGTT
TACTGTTCTGTTGTGAACATGTTGATCGAGATATTCCTCTCCGGAATGAGCTTTGAGCAG

TCGCTTTCTTCCAGGCTGGTGGCGATACCTGTCAACATTTTGATTGCATGGCCATACGGC
GTTTACCGCGATCTGATTATGCGCGTCGCGCGTAAGGCAAGCCCGGCGGGATGGGCCAAA
AACCTGGCGGATGTATTGGCTTATGTCACCTTTCAGTCACCCGTTTATATTATCATCTTG
CTGACCGTCGGTGCCGGCTGGCATCAGATAGTGGCCGCCGTGAGTTCAAACATTGTTGTT
TCAATGTTAATGGGCGCGGTTTATGGCTACTTCCTGGATTATTGTCGCCGCCTGTTTAAA
GTGAGCAGCTATCATCAGGCTAAGGCGTAA
>EECEBICD_00670 DNA-binding protein StpA
ATGAATTTGATGTTACAGAACTTAAATAATATCCGCACGCTGCGCGCTATGGCTCGCGAA
TTCTCCATTGACGTTCTTGAAGAAATGCTCGAAAAATTCAGGGTTGTCACTAAAGAAAGA
CGCGAAGAAGAAGAATTGCAGCAACGCCAGCTTGCCGAGAAGCAGGAGAAAATTAATGCC
TTTCTGGAGCTGATGAAAGCAGACGGTATTAACCCGGAAGAGTTATTTGCCATGGATTCA
GCAATGCCGCGTTCTGCTAAAAAGCGTCAGCCGCGTCCGGCAAAATATCGTTTTACTGAT
TTCAATGGCGAAGAAAAAACCTGGACCGGACAAGGCCGTACGCCTAAACCGATTGCCCAG
GCGCTGGCGGCGGGGAAATCTCTGGATGATTTCTTAATCTAA
>EECEBICD_00671 hypothetical protein
ATGTTCACACCAGGCGATATCGTACAGCCCCGAATGGGTGGGCCGAAACTGAAAGTGATT
GAAGTCAACGAGGATCATATTGTCGCGGTACAGGTCGGTAATGAGCAGGGCGAAAAATTG
ATCCTCAAAGCGGCAGATGTCACCCCCTATTGCGAAGAGGGCGATTTTGGCGTGTGCTGA
>EECEBICD_00672 Inner membrane protein YgaP
ATGTCCATTGGGATAGTTTCCCCCCGTGAGGCGCAAGCGCTGATAGCGCAAGGCGCGAAA
CTGATTGATGTACGCGACGCCGACGAATATCTGCGCGAGCATATTCCACATGCGCAACTG
GCGCCGCTTTCCCGGCTTGAACAGGGCGATTTGCCCGCCAACCTGCGAGCGGAACAGATT
ATTTTTCACTGTCAGTCAGGTAAACGAACCAGCAGCAATGCAGCAAAACTACAGGCCATT
GCCGCCCCTGCTCAGGTCAGTCTACTGGAAGGCGGCATCGACGGATGGAAAGCGGCAGGA
TTGCCCGTCACAGAAGACAAATCGCAACCGTTGCCACTGATGCGTCAGGTGCAAATCGCC
GCCGGCGGGTTGACGTTGCTTGGGGTGATTCTGGGCTATACGGTCCACGGCGGCTTTTTC
CTCATTAGCGGTTTTGTCGGGGCCGGGTTAATGCTGGCAGGCATGACCGGCTTCTGCGGA
ATGGCGCGACTACTGGAAAAAATGCCCTGGAATACCCGTACTCATTAA
>EECEBICD_00673 putative HTH-type transcriptional regulator YgaV
ATGACAGAGCTTGAACAACTTCAGGCCAGCGCAGAACAGGCGGCGGCCCTGCTCAAAGCG
ATGAGCCACCCAAAAAGATTACTGATTTTATGTATGCTTTGCGGTTCGCCAAAAACCAGC
GCAGGCGAACTGGCGCGCATTACCGGGCTAAGCCCCTCCGCGACGTCGCAACATCTGGCG
CGGATGCGGGAAGAAGGGCTGATTGATAGTCAACGCGATGCCCAGCGCATTCACTATTTC
ATTAAAAATGAGGCGGTTAATACGATTATCGCCACGTTAAAGAATCTCTATTGTCCATAA
>EECEBICD_00674 hypothetical protein
ATGGGTTTCTGGAGAATTGTATTTACGATTATTTTGCCTCCGCTCGGCGTACTTTTAGGA
AAAGGGTTCGGTTGGGCATTTATCCTTAATATCCTGCTGACATTACTCGGATATATTCCG
GGGCTTATCCACGCATTTTGGGTTCAGATGCGTCATTAA
>EECEBICD_00675 Potassium binding protein Kbp
ATGGGACTTTTCAATTTTGTAAAAGATGCAGGTGAAAAACTCTGGGACGCGGTAACCGCT
AATCATGATAAGGACGATCAGGCGAAAAAAGTCCAGGAACATTTAAATAAAACGGGAATA
CCCGACGCTGACAAAGTGAATGTGCAAATAGCTGATGGAAAAGCGACAGTGACGGGGGAT
GGGCTGAGTCAGGAGGCAAAAGAAAAAATTCTGGTTGCCGTGGGGAATATCGCCGGGATT
AGCAGCGTTGACGATCAGGTTAAAACCACGACGCCAGCGGCTGAAAGTCAGTTTTATACC
GTAAAATCAGGCGACACCCTGAGCGCTATTTCAAAGCAAGTGTATGGTAACGCCAATCTG
TATAATAAAATCTTCGAGGCAAATAAACCGATGCTGAAAAGCCCGGAAAAAAATATATCCG
GGACAAGTGTTGCGTATTCCTGAAGAATAA
>EECEBICD_00676 HTH-type transcriptional repressor GlaR
ATGACCGCCCTTTCCCAACCGACGGCCATAGATGGCTATCGCTGGCTTAAAAATGACATT
ATTCGCGGCACTTATCAGCCGGATGAGAAACTGCGGATGAGTTTATTAACCGCCCGCTAT
GCGCTCGGCGTAGGGCCGCTGCGTGAGGCGCTGTCGCAACTGGTGGCGGAACGGCTGGTC
ACCGTGGTGAATCAAAAAGGCTATCGCGTGGCATCCATGTCAGAGGCGGAACTCCTCGAT
ATTTTTGACGCGCGCGCCAATATGGAAGCGATGCTGGTACGCCTGGCGATTGAACGCGGC
GATGATGCGTGGGAGGCGGAGATCCTGGCGCGAGCGCATATGCTCAGTAAACTGGAAGCC

AGCGATGCCAGTGAACATATGCTCGATGAATGGGATCAGCGCCACCAGGCGTTCCATTCC
GCCATTGTCGCCGGGTGCGGCTCCCACTATCTGCTACAGATGCGCGAACGCTTATTTGAC
CTGGCGGCCCGCTACCGCTTTATTTGGTTACGTGAAACCGTTCTGTCCGTCGAGATGCTG
GAAGATAAACATATCCAGCATCACACGCTAACCGAAGCGATACTGGCGCGCGAGGCGGCA
CGCGCCAGCGAATTAATGCGTCAGCATTTGCTGACGCCGATTCCCATTATTCGCCAGGCG
ATGGCAGGGAAAATGTAG
>EECEBICD_00677 GABA permease
ATGGGGCAATTGTCAGAATCACATGCATTAGGGGGCGGGCTGAAATCACGCCACGTCACC
ATGTTATCGATCGCCGGCGTCATCGGCGCCAGCCTGTTTGTCGGTTCCAGCGTGGCGATT
GCGGAGGCCGGCCCTGCGGTACTGCTGGCCTATCTGTTCGCAGGACTTTTAGTCGTCATG
ATTATGCGAATGCTGGCTGAAATGGCCGTCGCCACACCGGATACCGGTTCTTTTTCCACC
TATGCGGATAAAGCGATCGGCCCCTGGGCCGGGTATACCATCGGCTGGTTATACTGGTGG
TTTTGGGTGCTGGTTATTCCGCTGGAGGCCAATATTGCCGCCATTATTCTGAATTCATGG
ATACCGGGTATTCCGGTATGGCTATTTTCATTAGTGATTACGCTTGCCTTAACCGGAAGC
AATTTATTGAGCGTCAAAAATTACGGCGAGTTCGAATTCTGGCTGGCGCTATGTAAAGTA
ATTGCCATTCTGGCGTTTATCGCGCTGGGCGCGACAGCGATTAGCGGTTTTTATCCGTAT
GCTGAGGTAAGCGGTATCTCCCGGCTATGGGATCACGGTGGTTTTATGCCTAACGGCTTC
GGCGCGGTGTTAAGCGCCATGTTGATCACCATGTTCTCTTTTATGGGCGCGGAAATCGTC
ACTATTGCTGCCGCGGAATCCGATACGCCGGATAAACATATCGTACGCGCCACCAATTCT
GTTATCTGGCGAATTTCCATCTTTTACCTGTGTTCCATTTTTGTGGTGGTGGCGCTGATC
CCGTGGAATATGCCGGGTCTGAAAAGCGTAGGTTCGTACCGTTCGGTACTGGAGCTGCTT
CATATTCCGCACGCAAAATTCATTATGGACTGCGTGATTTTGTTGTCGGTGACCAGTTGT
CTGAACTCCGCATTATATACCGCCTCCCGGATGCTCTATTCCCTTAGCCGCCGCGGCGAT
GCGCCTGCGATAATGGGAAAAACTAACCGTAGCAAAACGCCCTGGGTGGCGGTTCTCCTC
TCTACCGGCGCGGCCTTTTTAACCGTTATCGTCAATTATTACGCCCCGGCAAAAGTGTTT
AAATTTCTTATCGACAGTTCCGGCGCCATCGCGCTGCTGGTCTATCTGGTGATTGCGATT
TCGCAATTGCGGATGCGTAAAATTCTGCTGGCGCAGGGCGGAGAAATTAAGTTGAAAATG
TGGCTTTATCCGTGGCTCACCTGGCTGGTTATCGGCTTTATCTGCTTTGTTCTGGTGGTG
ATGCTATTTCGACCGGCACAGCAACTAGAAGTCATTTCAACGGGGCTGTTAGGGCTGGGC
ATAATAGGTACTGTCCCCATTATGTCTCGCTGGAAAAAACTGATAAGGTGGCAAAAAGCC
CCCCTGCAAAACCTACGTTAG
>EECEBICD_00678 4-aminobutyrate aminotransferase GabT
ATGAATACCAATAACGCTTTAATGCAGCGCCGTCATAACGCCGTTCCTCGCGGCGTGGGC
CAGATTCACCCAATTTTCGCCGAGCGGGCGGAAAATTGTCGGGTCTGGGATGTCGAAGGC
CGTGAGTATCTCGATTTCGCGGGCGGCATTGCCGTCCTGAACACCGGTCATTTACATCCG
GGCATTGTTTCAGCGGTCGAAGCGCAGTTGAAGAAATTGTCCCATACCTGTTTTCAGGTG
CTGGCCTATGAACCGTATCTGGCGTTGTGCGAACGCATGAACCAGAAGGTACCGGGCGAT
TTCGCCAAAAAAACGTTGCTGGTCACGACCGGTTCAGAAGCGGTTGAAAACGCCGTGAAA
ATCGCCCGCGCGGCAACAAAACGCAGCGGCGCTATCGCGTTTAGCGGCGCTTACCACGGT
CGTACCCACTACACGCTCTCTCTGACCGGGAAAGTACACCCGTACTCTGCTGGTATGGGA
TTGATGCCAGGCCATGTCTACCGCGCGCTTTATCCTTGCCCGCTACACAACGTCAGCGAC
GACGACGCGATCGCCAGCATTGAACGCATCTTTAAAAATGATGCCGCGCCGGAAGATATC
GCCGCCATTATTATTGAGCCGGTACAGGGCGAAGGCGGATTTTACGCCGCCTCTCCCGCG
TTTATGCAGCGACTGCGCGCGCTATGCGACCAACACGGCATTATGTTGATTGCCGACGAA
GTGCAAAGCGGCGCGGGCCGGACCGGTACGCTGTTCGCCATGGAACAGATGGGCGTTGCG
GCAGATATTACCACGTTTGCGAAATCGATCGCCGGCGGCTTTCCGCTGGCAGGCGTTACC
GGCCGGGCAGACGTGATGGACGCTATCGCGCCGGGCGGGCTGGGCGGCACCTATGCCGGA
AACCCGATTGCCTGCGCCGCCGCGCTGGCGGTACTGGACATTTTCGAGCAGGAAAATCTA
CTGCAAAAGGCGAATACGCTTGGCAAAACGCTGCGCGATGGCCTGATGGAGATAGCGGAA
ACGCACCGTGAGATTGGTGACGTGCGCGGACTGGGCGCCATGATCGCCATTGAGTTGTTC
GAAAATGGCGACCCCGGCAAACCGAACGCGGCTCTGACCGCCGACATTGTGGCCCGCGCC
CGCGAGAAAGGGTTAATTCTGCTCTCCTGCGGTCCTTACTACAACATCTTGCGCATCCTC
GTTCCACTCACTATTGAGGCCTCGCAGATTCGGCAAGGCCTGGAGATTATCGCCCAGTGC
TTTGATGAGGCGAAACAAGCGTAA

>EECEBICD_00679 Succinate-semialdehyde dehydrogenase [NADP(+)] GabD
ATGCAACTTAACGACTCTACCCTGTTCCGCCAGCAGGCCTTTATCGATGGCGACTGGCGC
GACGCGCGCGGCGGCGATGTCATCCCCGTGAGCAACCCCGCCAACGGGAAACCGCTCGGC
AATGTGCCCAAAATGGGGGCGGAAGAGACGCGCGACGCCATCAACGCCGCCAATCGCGCC
CTGCCCGCCTGGCGCGCGCTCACCGCCAAAGAGCGAGCCAATATCCTGCGTCGCTGGTTC
AATCTGATGATGGAACATCAGGACGATTTGGCGCATCTGATGACGCTGGAACAGGGTAAA
CCGCTGGCGGAAGCGAAAGGCGAAATTAGCTACGCCGCCTCGTTTATTGAATGGTTCGCC
GAAGAGGGGAAGCGTATCTATGGCGACACGATTCCAGGTCATCAGGCCGATAAACGGCTG
CTGGTGATTAAACAGCCCATCGGCGTCACCGCCGCGATTACCCCGTGGAACTTTCCCTCA
GCAATGATCACCCGTAAAGCCGGCCCCGCGCTGGCCGCAGGCTGCACGATGGTACTCAAA
CCCGCCAGCCAGACGCCGTTTTCCGCGCTGGCGCTGGCGGAACTGGCCCGGCGCGCCGGA
ATTCCCGCAGGTGTATTTAACGTCGTTACCGGTTCGGCAGGCGATATCGGCGGCGAGCTG
ACCAGCAACCCGCTGGTACGCAAACTGTCGTTTACCGGCTCGACGGAGATTGGCCGCCAG
CTAATGGAACAATGCGCCAAAGACATTAAAAAAGTGTCGCTGGAATTGGGCGGCAATGCG
CCGTTTATCGTCTTTGACGATGCCGATCTGGATAAAGCCGTCGAAGGCGCGCTGGCTTCG
AAGTTTCGCAACGCCGGGCAGACCTGTGTCTGCGCCAATAGATTGTATGTTCAGGACGGC
GTTTACGATCGTTTTGCGGAAAAACTCAACCAGGCGGTAAATAAGCTCGCGGTCGGCGAC
GGCCTGCAAGCCGACGTCGCTATCGGGCCGCTGATTGATGAAAAAGCCGTGGCGAAAGTC
CAGGAGCATATCGCCGACGCGCTGGAAAAAGGCGCCCGCGTCATTACGGGCGGTGAAGCG
CACAAATTGGGCGGCAACTTCTTTCAACCGACCATTCTGGCGGATGTTCCTGATAACGCG
AAAGTCGCCAAAGAAGAGACATTCGGGCCGCTCGCGCCTCTGTTCCGCTTCAGCGACGAG
GCGGATGTCATCAGACAGGCTAACGATACCGAGTTCGGCCTGGCAGCCTATTTTTATGCA
CGCGATTTAAGCCGCGTTTTCCGCGTCGGCGAAGCGCTGGAATACGGCATCGTCGGTATT
AATACCGGCATTATCTCCAACGAAGTCGCGCCTTTCGGCGGTATTAAGGCCTCCGGATTA
GGCCGTGAAGGTTCCAAATACGGCATCGAAGATTATTTAGAAATCAAATATATGTGCATC
GGCCTTTAA
>EECEBICD_00680 L-2-hydroxyglutarate dehydrogenase
ATGTATGATTTTGTGATTATCGGCGGCGGCATTATCGGCATGTCGACCGCCATGCAACTT
ATTGATGTCTACCCGGATGCGCGCATCGCATTGCTGGAAAAAGAGTCCGCGCCAGCCTGC
CACCAGACCGGGCATAACAGCGGCGTGAGCCATGCCGGGGTGTACTACACGCCCGGCAGC
CTCAAAGCGCGGTTTTGTCTGGCGGGCAATCAGGCGACCAAAACCTTTTGCGATCAAAAC
AACATCCGCTACGACACCTGCGGCAAAATGTTGGTCGCCACCTCGGAGCTGGAAATGGCG
CGGATGCGCGCGCTATGGGAGCGGACGGCGGCCAACGGCTTAGAGCGCGAGTGGTTAAGC
GCGGCGGAGCTGCGTGAACGCGAACCCAATATCATTGGCCTGGGCGGGATTTTTGTCCCT
TCCAGCGGGATTGTCAGCTATCGCGATGTCGCGACGGCCATGGCGAACCGCTTTCAGGCC
AAAGGCGGCGAGATCATCTACCATGCCGAGGTCAGCGCGCTAACAGAACATGCCGCTGGC
ATTGTTATTCGCACCTCGCAAGGTCGGGAAATAGAAACCGCTACGCTTATCGGCTGTGCA
GGGCTGATGGCGGATCGGCTGGTCAAAATGCTGGGCGTGGAGCCCGGTTTTATTATCTGC
CCTTTCCGCGGCGAGTACTTTCGTCTGGCCCCGCGACACAACCGGATCGTTAACCATTTG
ATCTACCCGATCCCCGATCCGGCAATGCCGTTTCTCGGCGTCCATCTTACCCGCATGATC
GATGGCAGCGTCACGGTCGGGCCGAATGCGGTGCTGGCGCTTAAACGCGAAGGCTACCGT
AAGCGCGACGTTTCCTTCACCGACACCCTGGAGATTTTCCGCTCCGCCGGCATTCGCCGC
GTACTGCAAAACCATCTGCTTTCCGGGCTGGGCGAGATGAAAAACTCGCTGTGCAAAAGC
GGCTATTTGCGGCGAGTACAAAAGTATTGCCCCAGCCTGACCGTCAACGATCTCCAGCCC
TGGCCGGCAGGCGTGCGGGCGCAGGCGGTTTCACCGGACGGCAAACTGATTGATGATTTT
CTGTTTGTCACGACGCCGCGTTCCATTCACACCTGTAACGCCCCCTCCCCGGCGGCGACA
TCCGCGATACCTATCGGCGCGCATATCGTCAGTAAAGTTCAGGCGCTACGCGCAAGCCAG
AGCAACCCCGGACGTACGCTGCGTGCGGCACGCAGCGTGGACGCATTACACGCCGCATTT
ACCCGTTAA
>EECEBICD_00681 Glutarate 2-hydroxylase
ATGAATGCACTGACTGCCGTAAAAGCGAACACAGATGATTTAGCCCAACGCCATACTGGT
TTCACCCTTGCCCCTTCGGCGCAGTCGCCGCGTCTGCTGGCGTTAACGTTTACGGCGGAT
ACCACAAGACAATTTCTCCATCAGGTTGCACAGTGGCCGGTGCAGGCGCTGGAATACAAA
TCTTTCTTACGTTTCAAAATCGGCAAGATCCTCGACGATCTGTGCGGCAATCAGCTACAA

CCGCTGTTGATCAAAACGCTACTCAATCGTGCACAAGGCGCGCTGTTGATCAGCGCCGAA
GGGCTCGATGATGTCGCACAGGCAGAAGAGATGGTCAAACTGGCGACGGCGGTGGCGCAT
CTTATTGGTCGCTCCAACTACGATGCGATGAGCGGCCAGTATTACGCGCGTTTCGTGGTC
AAAAACGTCGACAACTCCGACAGTTATCTGCGCCAGCCCCACCGCGTAATGGAACTGCAC
AATGACGGCACCTATGTTGAGGAGGTGACCGACTACGTGCTGATGATGAAAATCGACGAA
CAAAATATGGAAGGCGGTAATTCGCTGCTGCTGCACCTTGATGACTGGGAACATCTGGAG
TCGTTTTTTACCCATCCGCTGGCGCGTCGCGTCATGCGCTGGGCGGCACCGCCAAGCAAA
AATGTTAGCCATGACGTCTGGCACCCGGTGTTTGATGTCGACCAACAAGGCCGCCCGGTC
ATGCGTTATATCGACCAGTTCGTCCAGCCAAAAGACTTTGAAGAAGGCGTCTGGCTCAGC
GAGCTTTCAGACGCGCTGGAAACCAGTCAAAACATCCTCTCCGTGCCGGTACCGGTCGGC
AAATTCCTGCTGATTAACAATCTGTTCTGGCTACATGGACGCGATCGTTTTACGCCGCAT
CCTGACTTGCGTCGCGAACTGATGCGCCAGCGCGGTTACTTCGCCTACGCCGCCAGCCAT
TATCAAACTCACCAATAA
>EECEBICD_00682 hypothetical protein
ATGGGGAAAGTGATGCTGGCGGGGTTGCCCGGCCTGACACATGAGCGCGTAGGCCGGACA
AGTACAACGTATTTCAGCAGTCAATTAACCGGCGTCAACCTGCGGTTTACGGCTGTGTTT
ACGGAGCAGACGTAA
>EECEBICD_00683 hypothetical protein
ATGGATACCTGGATATATCTTTCTCAGGGCTTTGCGGTGGCGATGACGCCGGAAAACCTG
GTTATCGCGTTGATTGGCTGCTTCGTGGGCACGATCGTCGGTCTGCTGCCGGGCCTGGGA
CCGATCAACGGCGTGGCAATCTTACTGCCGCTGGCCTTTGCGTTGCATCTGCCTGCGGAG
TCGGCGCTAATTCTGCTGGCGACGGTGTACATTGGCTGTGAGTATGGCGGCAGGATCTCC
TCCATATTGCTCAACGTCCCCGGCGATGCGGCGGCGATCATGACGGCGCTGGACGGTTAC
CCAATGGCGCAGCAAGGGAAAGGCGGCGTAGCGCTGTCGATTTCCGCAGTCAGCTCATTT
TTCGGTTCATTAATCGCTATCGGCGGCATCATTCTGTTCGCCCCTTTACTGGCGCAATGG
TCGCTGGCCTTTGGTCCTGCGGAATATTTCGCCTTAATGGTTTTTGCCATCGCCTGTCTT
GGCAGCATGATGGCGCAAAACCCGCTTAAATCATTTCTGGCAGCGCTGATCGGTCTTGGC
CTTGCGACCGTCGGCGTGGACGCCAACACCGGGGTTTATCGCTTTACCTTTGACAGCGTT
CATCTTTCCGACGGCGTACAGTTTATCGTCGTGGTGATCGGCCTGTTCTCGGTATCAGAA
ATACTTTTAATGCTGGAACATACCAGCAGCGGGCAAACAATGGTCCGCAAAACGGGTCGA
ATGTTGTTCAACCTGAAAGAAGGCGCGCAGTGTATCGGCACCACCCTGCGTTCTTCGGTA
ATCGGCTTTTTTGTCGGCGTATTGCCCGGCGCCGGGGCGACCATTGCCAGCGCCATTACC
TATATGACCGAGAAAAAACTCAGCGGCAACAGCGATAGCTTCGGCAAAGGGGATATTCGC
GGCGTCGCGGCGCCGGAGGCGGCAAACAACGCCTCAGCCTGCGGCTCCTTCATCCCGATG
CTGACGCTGGGCGTTCCCGGTTCCGGTACTACGGCAGTGATGATGGGGGCGCTGACGCTG
TACAACATCACGCCAGGCCCGGCGATGTTCACCGAACAGCCGGATATCGTCTGGGGACTC
ATCGCTGCGCTGCTGATTGCGAACGTGATGCTGCTGATCATGAATATCCCGTTGATCGGT
CTGTTCACCCGTATGCTCACCATTCCGCTGTGGTTCCTGGTACCCGCCATCGCTGCCGTA
TCGGCGGTAGGGGTGTATGCGGTACACAGCACCACCTTCGATCTGGTGCTGATGGTCGCG
CTCGGCGTGTTAGGGTACATTTTACGTAAAATGCACTTCCCCATGTCGCCGCTGATCCTG
GGATTCGTACTGGGGGAAATGCTGGAGCAGAACCTGCGTCGCGCACTCTCCATCAGTAAC
GGCAATATGGCGATTTTGTGGCAAAGCGGCGTTGCCAAAGCCCTGCTGATCATGGCGATC
ATGGTCATTGTCGTACCGCCAGTGTTACGTCTGCTCCGTAAACACAGCCGTAAACCGCAG
GTTGACGCCGGTTAA
>EECEBICD_00684 hypothetical protein
ATGAGCGATCGTATCTTTGCGGGTATCTGGCTGTTGCTCTGTATCGCCGGGCTATTCATC
GCCTGGCAGATCCAAAGCGAATACAGCTATGAACCCGTCGGCCCCCGCCCTTTTCCGCTG
GGGATTATCGGCCTGATGGCGCTCTGCGCGCTGGCGCTGTTATTGCGCCATCCCGACACG
GTGAGCTGGCCGCGCCGCCATGTTCTGCAAAAGCTAATTACCATGATCATCATCCTGCTG
ATGTATGCCTGGGGATTCGAATGGCTGGGTTTTCCCATCGCCACCGCGCTTCTCACCATG
GTTATCGGGATGCTGTTCGGCGCCACGATTCCGGCGGCGGGTATCTCCGGCGCGGTATTG
GGTATTCTGCTGTGGTATGCCTTTGACCGGCTGCTTGATGTCACCTTACCTCTCGGCGCC
TGGCTGAGTTAA
>EECEBICD_00685 hypothetical protein

ATGAAAAAACAATTACTTCGTACCCTTACTGCAAGCATTTTATTAATGAGTACCTCTGTT
CTGGCGCAGGAGGCGCCGTCGCGAACGGAATGTATCGCGCCAGCCAAACCTGGCGGCGGT
TTCGACCTCACCTGTAAGCTGATTCAGGTGAGTTTGCTGGAGACTGGCGCTATCGAGAAA
CCCATGCGGGTAACGTATATGCCCGGCGGCGTCGGCGCTGTGGCCTATAACGCGATAGTC
GCTCAACGCCCTGGCGAACCCGGGACTGTGGTCGCCTTTTCCGGCGGTTCGCTGCTCAAC
CTGTCGCAGGGGAAATTTGGTCGCTACGGCGTGGATGATGTGCGCTGGCTGGCAAGCGTG
GGCACTGATTACGGCATGATCGCCGTGCGTGCGGATTCCCCGTGGAAAACGCTGAAAGAT
CTGATGACGGCGATGGAAAAAGATCCCAACAGCGTGGTCATTGGCGCTGGCGCCTCTATT
GGCAGCCAGGACTGGATGAAGTCGGCGTTGCTGGCGCAAAAGGCGAACGTCGACCCGCAC
AAGATGCGCTACGTTGCCTTTGAGGGCGGCGGCGAGCCGGTGACGGCATTAATGGGCAAC
CATGTTCAGGTTGTCTCCGGCGATCTCAGTGAAATGGTGCCTTATCTGGGCGGCGACAAA
ATCCGCGTGCTTGCCGTCTTTTCAGAAAATCGTCTGCCGGGCCAGCTTGCCAATGTCCCT
ACCGCTAAAGAACAGGGTTACGACCTGGTGTGGCCGATTATTCGCGGCTTCTACGTCGGG
CCCAAAGTCAGCGATGCCGATTACCAGTGGTGGGTGGATACCTTCAAGAAGCTCCAGCAA
ACCGACGAGTTTAAAAAGCAGCGCGATCTGCGCGGCCTGTTTGAGTTCGACATGACCGGC
CAGCAGCTCGATGACTACGTGAAAAAACAGGTTACTGATTACCGTGAACAGGCGAAAGCC
TTCGGACTCGCGAAATAA
>EECEBICD_00686 Transcriptional regulatory protein tctD
ATGCGTCTCTTATTGGCGGAAGATAACCGTGAGCTGGCTCACTGGCTGGAGAAAGCGCTG
GTGCAAAACGGCTTTGCCGTGGACTGCGTATTTGACGGCCTGGCGGCTGACCATCTTCTG
CACAGTGAAATGTACGCGCTGGCGGTGCTGGATATCAATATGCCGGGAATGGATGGACTG
GAGGTGGTGCAACGGCTGCGTAAACGCGGTCAGACCTTGCCCGTTTTGCTGCTCACGGCG
CGAAGCGCGGTGGCGGATCGTGTAAAAGGTCTCAACGTCGGCGCCGATGACTATCTGCCG
AAACCCTTTGAACTGGAAGAGCTGGATGCCCGTTTACGGGCCTTGCTGCGACGCAGCGCT
GGGCAGGTTCATGAAGTTCAGCAACTGGGGGAATTGATCTTTCATGATGAAGGGTATTTT
CTGTTACAGGGACAGCCGTTAGCGCTGACGCCGCGTGAGCAGGCGCTCTTAACCGTATTG
ATGTACCGACGAACGCGGCCGGTTTCGCGTCAGCAGCTTTTTGAACAGGTGTTCAGCCTG
AACGATGAGGTAAGCCCTGAAAGCATCGAACTTTATATTCATCGACTGCGTAAAAAACTC
CAGGGAAGCGATGTACGAATTACGACGCTGCGCGGCCTGGGTTATGTGCTGGAGCGCGGC
GATGAAGTGGGTTAA
>EECEBICD_00687 Sensor protein QseC
ATGAAGTGGGTTAAGCCGCAATCGTTGTACCTGCAACTCTTACTGTTCCTGGGGTTGCCG
CTGATTCTGCTATGGGGGCTGTCGGCGTTTAACAGCTATGTCAATGCGCTACAGGCCGCC
ACTCAGGCTTATGATCGTACGTTGCTCTCCTCGGCGCGAACGGTATCGGAACGGCTGGTG
GTGCGCAATAACCATCTGGAAGTTAACGTGCCCTGGGTGGTGCTGGATAGCTTCGAGCTT
AATATGAATGATCGACTCTATTACAAAGTCGTCGACCCTTCCGGAAAGGTCATTTCCGGT
TATGACGATCTGCCCGTCATGCCGCCCGCTACGCCCCGTACCCGGCTTTATCCGGCGCTG
GCGTGGTTCTATCATACCGAGTATCGCGGCGAGGCGATCCGCGTGGCGCGTTTGCTACAG
CCGGTCAACGAAGGCGGCATTATCGGCATGGCGGAGATTTATGTCGCAGAGACGCTGCAG
TCCCGTCGTTATCTGGCGGGCCAACTGCTGTTCTCTTCCTGGATCTCACAGGGGCTGCTG
GTGTTGCTGACCCTGGTGCTGGTTGGCTGGTTATTACGCCGGATCCTGCGCCCTATGCGT
CAACTTTCGTCATTAATGGTACGCCGCGAACCGGGTCTGTTAACGCCGCTGCCGGAACTG
CTGCCGTGGTCGGAAACACGCTTGTTAATCGTGGCGTTTAATCGCTACATTGACAGGCTC
CGCGGCATTCTCTCCCGGCAGGAACGCTTTAGTGCCGATGCTTCTCACCAGTTAAAAACG
CCGCTGGCGGTGTTAAAAACGCAGGCGGCGGTGGCGTTAGCCAGCCAGCACCCTCATCAT
TGGTACGAAAGTCTGCAGGCGATGAGCGTAACGCTGGATAGTACCATTCAGCTCACGGAA
AGATTGTTGCAACTTTCTGCGGTAAAGCATAAAGAGCAGGGCGAGCGGCGCTTTTCCCCG
GTGAATCTGTACGATATCGTACAGAGCGGCTGCTTTACCCGCCTGGCGCAGGCGCGGAGT
AAACATATCGACCTGGGCTACGAAGGCGAACAGGAAGCGATGTGGATCGAGGGGGATGAG
GTACTGCTTAGCGAGCTATGCGGAAACTTGCTGGATAATGCGCTGAAATACACGCCTGAG
CAAGGTATCGTCACCGCGCGGCTTGAACGGGATGGCGATGCCGTGACGCTGGTTGTCGAA
GATAGCGGCCCCGGCATTGATGATGAACACATCCATCTGGCGCTACAGCCGTTCCACCGG
CTCGATAACGTAGGAAATGTGGCCGGGGCGGGCATTGGTCTGGCTTTGGTCAATGATATT
GCACGCCTGCACCGTACTCATCCCCATTTTTCCCGTAGTGAAGCGTTGGGAGGGTTATAC

GTTCGGATACGCTTTTTAAGCCTTGTTCCGCAGTAA
>EECEBICD_00688 High-affinity nickel transport protein
ATGAATAATCATACTAAACGACGGGGAATAGCATTAACCGTCTTTCTTGTCGGGGTTAAC
ATCCTGGCCTGGATTTGGGCGTTCTGTGTTTTTCATCACCATGCAGTCATGCTAAGTGCA
GCCATACTGGCGTATAGCTTTGGGCTGCGTCATGCGGTTGATGCCGATCATATCGCAGCC
ATCGATACCGTCACCCGTAAACTGATGCAGCAGGGTAAAACGCCTTTAGGCGTGGGGGCG
TTTTTTTCATTGGGTCATTCGACCATTGTCGTTCTGGCATGTCTGGCGATTGTTGTGACG
TCGATGGCATTTCGCGATCGTATTGATGTTTTGCATCAATATGGTTCGTTGATTGGTACG
GCTGTTTCCGCTTTTTTTCTGCTGGCGATGGCCCTGCTTAACCTGTTTATTTTGTTCAAC
GTCTGGCGGCAATTTCGCTCGGTTACCCGCGGCGAGTCAGTGAGGGCGCATGACGAGGCC
ATACCTGGCGGGCTGATGACGCGTATATTTCAGCGTACGTTTCGTTTGGTCACTTCCAGT
TGGCATATGTATTTTGTCGGTTTTCTGTTTGGTCTGGGATTTGATACCGCAACCGAGGTT
GGGTTGCTGGGCATTTCCGCTTCAGCAGCCAACCAGGGGCTATCTCTCTGGTCAATGATG
ATTTTTCCTGTGCTGTTTACCGCCGGGATGGCGCTGGTAGATTCCCTGGATAACTTTGTC
ATGGTCGGCGCTTACGGTTGGGCTTTCTCGCATCCATTACGCAAGCTTTATTATAATATG
ACCATTACCGCCGCATCCGTCATTGTCGCGCTGGCGATTGGCGGGCTTGAGGCGCTGGGG
TTGATTGATGATGCGTTACAGTTAAGCGGAACATTTTGGCAGACCGTCAGCACGCTTAAC
GATCATATGGGGAATGTGGGCTTCTGGGTAGTGGGTGCTTTTGTGCTATTCTGGCTGCTC
TCGTTGTTGAATTATCGCTGGCGCGGCTATGATAAAATCACCCTCAATACCTGA
>EECEBICD_00689 hypothetical protein
ATGACACATGCAGACTGGCTGATTCTTGGGTATACAACTGTTATTATTATTTTTGGTATT
GTATGTTATATCGGATTGCTTAAATTGATAACGAAAGGCAAGCATGAAAAATAA
>EECEBICD_00690 hypothetical protein
GTGAAAATACAAGAAGTTAAGCGTATATTAACCCGCTGGCAGCCGTCTTCCTTTACCCTA
TACCGGGAGGTGTTTACGCAATACGGCGGTAGTATCAATATGCACCCAGATATTGTTGAT
TATTTTATGAAGCGCCATAACTGGCATTTTAAATTCTTCCACTATAAAGAAGATGATAAG
ATTAAAGGCGCCTACTTTATCTGTAATGATCAGAATATTGGCATCCTGACGCGCAGAACC
TTCCCGCTATCGTCAGATGAGATACTTATTCCAATGGCACCAGATTTACGCTGCTTCTTA
CCCGATCGTACTAACCGACTTTCGGCGTTGCATCAGCCACAAATACGCAATGCCATATGG
AAACTCGCCAGAAAGAAACAAAATTGTCTGGTCAAAGAAACGTTCTCTTCTAAGTTTGAA
AAAACACGCCGTAATGAATATCAGCGATTTCTTAAAAAAGGCGGAAGTGTAAAAAGCGTT
GCTGATTGTTCTTCTGATGAACTGACTCATATTTTTATCGAGTTATTTCGGTCTCGTTTC
GGCAATACTTCATCCTGCTATCCCGCTGATAACCTTGCCAACTTTTTTAGTCAACTGCAT
CACCTTCTGTTTGGGCATATTCTTTATATTGAAGGGATACCCTGTGCATTTGATATCGTA
TTGAAATCTGAAAGCCAGATGAACGTCTACTTTGACGTATCTAATGGTGCAATTAAAAAT
GAGTTCAGACCACTCAGTCCCGGCAGCATACTAATGTGGCTAAATATTAACCGTGCCAGA
CATTACTGCCAGGAAAGACAAAAAAAATTGCTTTTTTCCATCGGCATACTAAAGCCTGAA
TGGGAATATAAGCGCATGTGGTCGACCCCCTATTTTACCGGAAAATCGATTTGCTAA
>EECEBICD_00691 hypothetical protein
ATGACGATGCAGCAAAGTGATATGGAAAGATATAATCCATTATTAATGTTAAAAGAAGTC
ATGGCGCAGACGCCTTATCGCCATAAACGCTGGGGAGAGCGTAAGTTTCGCTATAAATTT
GTATTACGTTGCCTTATTAACCCCGTAACGACAATTAAATACTTCAATGAATTATGCCAT
CTGTCTCAGCCCAGAACGCTGATTATTCATCGTCCACTGTTGCCGGCAAAAATCCAGCGG
CCCTATTTGTATACCGGGCTTAGCATACGTTGCCGGGCAAAAGCTATTCTGGAACACTAC
CAGTTCGTTCAGTCCTTTCCGGAAAGTAAAATTAAAAAGATCCTCCTGTCCGAGGAACAA
ATCTTGCTCGCTCACCTGGAAGGAAAAAACGACGCTCTGGTAGATATCTACTGCGGTCCC
TGTGGCTATGACAGAGAAGGTGAGCTGACATTAACGCTGTGTTTCAATGATACGCCGTTG
GCACGTTTATCATTTTCCTTTATCCGCCATGAAGGGAAGCAAATAGCGCTGGTTGCCGGG
TTACAAGGGCCCAGTAAACATGTCGGCCCACAAGTCATACGTAACGCCACCAAAGATTGT
TACGGTCTCTTTCCTAAACGGATGCTGTATGAAGCTTTTGCAACATTGATGCAGGCCTGT
AATGTCGACGAGATTTATGCGGTAAGTGAAAACAACCATGTTTATCGTCAACTCAGGTAC
TTATTCCAGAAGAAAAAAACTTTTGTGGCCAGCTATAGTGAATTCTGGGAGTCGCTAAAC
GGCGTTAAGAAAGGTGCCTTATATCATCTGCCATCACAGGTGATGCGTAAAGCGCCTGAA
AGTATCCCCAGCAAAAAACGCGCAGAGTATCGGAAACGCTATCATATTCTGGATACAATA

ATACAAGAGGTTAACAGCCTCTCCCGGTAG
>EECEBICD_00692 Secreted effector protein PipB2
ATGGAGCGTTCACTCGATAGTCTGGCTGGTATGGCTAAATCTGCTTTTGGCGCGGGGACT
TCTGCTGCTATGCGGCAAGCTACCTCGCCCAAAACCATTCTGGAATATATCATTAACTTT
TTTACCTGTGGTGGGATACGTCGGAGAAATGAAACACAATATCAGGAATTGATAGAGACT
ATGGCTGAGACATTGAAAAGTACAATGCCTGACAGAGGTGCTCCGTTGCCAGAAAACATC
ATCCTGGATGATATGGATGGGTGTCGTGTCGAATTTAATCTTCCTGGTGAGAATAACGAA
GCTGGACAAGTTATTGTACGAGTCAGTAAAGGCGACCATTCTGAGACAAGAGAAATTCCG
CTTGCCTCTTTTGAAAAAATATGCCGAGCTTTACTATTCAGATGCGAATTTTCTCTCCCT
CAGGATTCTGTAATATTAACTGCCCAGGGAGGCATGAATCTTAAAGGCGCTGTCCTTACC
GGAGCAAATCTGACGTCAGAAAATTTATGTGACGCAGACTTAAGCGGCGCAAATTTAGAG
GGGGCAGTGCTGTTTATGGCGGATTGTGAAGGTGCAAATTTTAAGGGCGCAAATCTATCG
GGAACATCACTAGGCGACAGTAATTTCAAGAACGCCTGTCTGGAAGATAGCATTATGTGT
GGCGCTACCCTCGATCACGCTAATCTTACTGGCGCCAATTTACAACACGCGAGTCTGTTA
GGCTGTAGCATGATAGAATGTAATTGCTCCGGTGCAAATATGGATCACACTAATCTTTCA
GGCGCAACCCTTATACGTGCTGATATGAGCGGTGCGACATTACAGGGTGCTACTATAATG
GCTGCAATTATGGAAGACGCTGTCTTAACCCGGGCAAATCTGCGAAAGGCGAGTTTCATT
TCTACGAACCTGGACGGGGCTGATTTGGCTGAGGCTAATTTAAACAATACCTGTTTTAAA
GATTGTACACTAACCCATTTGCGTACCGAAGACGCCACAATGTCTACAAGTACACAAACA
CTCTTTAACGAATTTTATAGTGAAAATATTTAG
>EECEBICD_00693 Ferric enterobactin receptor
ATGAAAGTTAATAAGTTCCTCTGGTTAATAACCGTGGTTTCTACAGGGGTTAATAGTCCA
TTATCAGCAGCAGAATCTACAGACGACAACGGCGAAACGATGGTGGTTGAATCCACTGCC
GAGCAAGTATTAAAGCAACAGCCGGGCGTTTCGATTATTACTCGTGACGATATTCAGAAG
AATCCTCCCGTTAACGATCTTGCCGATATTATTCGTAAGATGCCTGGGGTTAATCTTACC
GGCAATAGCGCCTCGGGGACGCGCGGAAATAACCGACAAATTGATATTCGTGGTATGGGG
CCGGAAAATACCTTAGTGCTGATTGACGGCGTGCCGGTGACGTCACGAAATTCGGTTCGC
TATAGCTGGCGCGGAGAACGCGACACCCGTGGCGATACGAACTGGGTACCGCCGGAGATG
GTAGAACGTATTGAAGTGATCCGTGGGCCCGCCGCCGCGCGCTATGGCTCCGGTGCCGCT
GGCGGGGTGGTGAATATCATCACCAAACGTCCGACTAACGACTGGCATGGTTCGCTGTCG
CTCTATACCAACCAGCCGGAAAGCAGCAAAGAAGGTGACACCCGGCGGGGTAACTTTAGC
CTTAGCGGCCCGTTGGCTGGAGATACGTTGACGATGCGCCTGTACGGTAATCTCAACAGA
ACCGATGCCGACAGCTGGGATATTAACTCCTCCGCCGGTACCAAGAATGCCGCCGGACGG
GAGGGGGTCACCAATAAAGATATTAATAGCGTCTTCTCATGGAAAATGACCCCGCAGCAA
ATCCTTGATTTTGAAGCGGGATACAGTCGGCAGGGGAATATCTATGCCGGCGATACGCAA
AACAGTACTTCGAACGCGGTTACTAAAAGTCTGGCGCAGTCCGGGCGGGAGACCAATCGC
CTGTACCGGCAGAATTACGGTCTAACTCATAACGGCATCTGGGACTGGGGGCAAAGCCGC
CTGGGGTTCTACTATGAAAAAACCGATAATACCCGCATGAATGAGGGACTCTCCGGCGGC
GGCGAGGGGCGCATTACCAACGACCAGACGTTCACCACTAACCGCCTGATCTCGTATCGC
ACCAGCGGCGAGGTGAATGTCCCGGTGATATGGCTATTTGAGCAAACGCTCACCGTCGGC
GCGGAGTGGAACCGCGATGAGCTGAACGATCCTTCCTCTACCAGTCTGACGGTTAAGGAT
AGCAATATCGCCGGGATTCCCGGTTCGGCGGCTAACCGTAGCAGCAAAAATAAATCGGAA
ATCAGTGCGCTGTACGTTGAAGATAATATCGAACCCATGGCCGGTACCAATATTATTCCG
GGTCTGCGTTTTGACTATTTGAGCGAATCCGGGAGTAATTTTAGTCCCAGTCTTAACCTT
TCCCAGGAGTTAGGTGAATACGTCAAGGTGAAAGCCGGTATTGCCCGGGCCTTTAAGGCA
CCGAACTTGTATCAAACCAGTGAGGGGTATTTACTCTATTCGAAAGGCAACGGTTGTCCA
AAAGATATTACGTCAGGCGGCTGTTACCTGGTCGGTAATAAGAACCTCGATCCTGAAATT
AGTATCAATAAAGAGATTGGGCTGGAATTTACCGTAGACGATTATCACGCAAGCGTAACG
TATTTCCGTAACGATTATCAGAATAAGATTGTCGCAGGTGATCAGATTATTGGCAGAAGC
GCATCTGGCGCTTATGTATTGCAGTGGCAGAACGGCGGTAAAGCGCTGATTGAAGGTATT
GAAGCCAGCATGGCTGTTCCGCTGATGCCGGACCGTCTGAACTGGAATACCAATGCGACT
TATATGATCACTTCTGAGCAAAAAGATACCGGTAATCCCTTATCGATAATCCCGAAATAT
ACGGTGAATACCTTCCTCGACTGGACTATCACCAGCGCGCTCTCCGCTAACGTCAACTGG
ACGTTATACGGCAAGCAAAAACCGCGAACACATGCGGAGTCGCGTAGTGAAGAGACCAAA

GGATTGTCCGGGAAAGCGCTGGGGGCTTACTCACTGGTCGGCGCTAACGTTAACTACGAT
ATCAATAAGAACCTGCGTCTTAATGTCGGTATCAGCAATATCTTCGATAAACAGATCTAT
CGCTCCGCCGAAGGGGCAAATACCTATAACGAACCGGGTCGGGCTTATTATGCCGGTGTG
ACGGCCTCGTTTTGA
>EECEBICD_00694 hypothetical protein
ATGTATGGTCGCCAGTACCACAACAAGTGCTATCGCATGGCGCTATTTTTAGTGAGTTTT
TGTTTTAGCGCGCTGAGTTATGCGCAGCCGGATATGCAGCCATTAGGACCGAATATTGCT
GATAAAGGTTCGGGATATTACCATTTTAGGGTCAATGACTTTCAATCTGCCGATGGCGCA
AGACATTACCGGGTATGGACCGCTATTCCCAATAAAGCCGCCCCGCCGTCGGGTTATCCT
GTCTTATATATGCTGGACGGCAACGCAGTGATGGACAGACTGCCGGAGACGCTGCTCAAA
CAGCTGGCAGACCACTCGCCGCCGGTGATTGTGGCGATTGGCTATCAGACTAACCTGCCG
TTTGATCTTAACGGCAGAGCTTATGACTATACGCCGGCGCCGGGAATCGAAGGCAGTGAA
AATAACCCGCGTTTTCATCGTAAGACCGGCGGCGGGCCAGCGTTCCGCCAGCTACTGGAG
AGACATATCGCGCCGCAGGTGGAACAAGGAATAACCATCAACCCCGAACGGCGCGGGGTA
TGGGGACATTCTTATGGCGGCCTGTTCGTTTTAGACTCCTGGCTGTCATCATCCTTCTTT
CATATCTATTACAGCGCCAGCCCGTCACTCAGCAGGGATAACTTTGCCCTGTTGAATCGC
CTTACGGCAGTGAAGCCGTCGCCGTTCTGCCATAAAAAACTCATCATAATGGAAGGTTCA
GCGTCGAATGGGGATAGCAGGCAACGCCAGATGGCCGAGCTCCTGCAAAAAGTTCAGGAG
ACCGTGAGAACGCTTGAGAACAACGGCGTTAACGCGGCCCTGCAGCATTACCCGGGCCTG
GGCCACGGGCCCATGTTTAACGCCTCTTTTCGCAGTGCATTGCTGGATATCAGTCGCGAA
CCGGCAAGCCAAAAACCACGCTGTCATTAA
>EECEBICD_00695 Enterochelin esterase
ATGAACGGGAAGTCACCTTCCTGTGGCGTGCCGCCTCTCCTCTGCGGGGGGGTTTATGTT
CGCCTGAACCGTGTCACCGATAAAGATAACGTGGCGAAAGGTATGATGACGCAACTGCCC
ACAACGGATATCTGGCATCTGACGCTTCGCTTACCGGCGTCCTACTGCGGTTCCTATACG
ATGGTCGAAATACCACCGGAAACCCCGGACGAGACGGTGTTGCAGCTTGGAAGCCGTTTC
GCCTCCCTTGTCGGGAAGGCGGACCCGCTGAACAGCACGCCGGGAATAAATGTTCGGGGC
AATGCGCAGGAGTCCGTTCTGGCGCTGGATCATGCCCCGGCACAGGAGGAGTGGTCAGGC
TGTCGCGCTTACGCCGGGCAGCTTTTCACCTCAGAGCATCGGCTCGCCGGACAATGTCGC
CGTGTGCGGCTGTATCTTCCTGACGTTCCTGTCGTACAACCGCTGGGCTTGCTGGTACTC
ACGGATGGCGAGATCTGGTTCGACCATCTTGGGGTAAGCGCCGCCATTGACGCCGCCATC
CGCAGCGGACGCATCGCGCCGGTGGCCGTACTCGGCATTGATAACATCAACGCGCGTGAA
CGCGTCGCAATCCTCGGCGGTCGCCGCGAGCTGGTGCTCGACATTGCCGAACGCCTGCTG
CCAACTCTTCGCGCCAACTACCCGGAACGGCGGTGGGCGGACCGTACACAAACGGTGCTG
GCAGGGCAAAGCCTCGGCGGCGTGACCGCGCTGATGGCGGCCCGCCACACGCCAGAGAGC
TTCGGCCTGGTGCTGAGCCACTCCCCCTCAATGTGGTGGACGCCGGACAACCGCAATCGC
CCGCCCCATTTCAGCGCAGAAGAGCGCTCATGGGTCAGCGAACATGTGTTATCCGCGCCC
TCGCCAGCCGTACGTACGCATCTGTGCGTGGGAGCGCTGGAAGGTTCGACGGTCCCGCAG
GTCAAACAGTTGCATGAAAAATTGCGCGCCGCCGGCGTTGAAAGCCATTGCAGCGTGTAC
ACCGGCGGTCACGATTATGCCTGGTGGCGCGGCGCGCTGATTGACGGATTACGTCTACTG
CCCCGATAA
>EECEBICD_00696 Multidrug efflux ATP-binding/permease protein
ATGCCCGCGACTCATTCCCCCATGCCCGCTCGTGCCTGGATAGTTCGCCTTGCCCGCGTG
TGTTGGGAACGTAAAACACTGAGCATCATTGTCATCGTAGCATCAGTATCGACCATTTTA
CTGGCTGCGCTGACGCCGCTAATAACGCGTCAGGCCGTCAATGACGCGATAGCAGGCGAT
ACGACCCACCTGCCGTTACTTGCCTGCGGCCTGCTGTTAATTGCCCTTTTTGATTTTATC
GGGAATTACGTGCGCCGCGGCTATGCCGGGGAACTCTCGCTGTGGGTTCAGCATACGTTA
CGTAGCCGTGCGTTCGACAGTATTCAAAAACTGGACGGCGCAGGCCAGGACGCCTTGCGT
ACCGGGCAGGTCATTTCGCGTACCAACAGCGATCTTCAACAGGTACACACCTTGCTACAG
ATGTGTCCGGTGCCGCTGGCGGTGCTCACTTACTATGTGGCCGGTATCGCGGTGATGCTA
TGGATGTCGCCATCCATGACGCTTATCGTGATTTGCGTACTGGCCGCCCTTGCTATTACC
GCCCTGCGCGCCCGTCGCCGTGTTTTCGCACAAACGGGGCTTGCCTCAGACCGGCTGGCG
CACATGACGGAACATATGCGCGAAGTATTGGAACAGATCTCAGTGGTGAAGTCCTGCGTG
GCGGAGTTACGCGAAACGCGTTGGCTCGATGGTCAGTCGCGGCAGATGGTGCGGGTACGC

ATCGGCGCCGCTATCTCGCAGGCGATGCCGGGCGCAACCATGCTGGCGCTACCGGTGATA
GGGCAAATCGTCCTGCTGTGCTATGGCGGCTGGTCGGTAATGAATGGGCGCATCGATCTG
GGAACCTTCGTTGCGTTCGCGAGTTTTCTCGCTATGCTGACCGGCCCTACCCGCGTACTG
GCATCGTTTTTGGTTATCGCACAGCGCACACAGGCGTCGGTGGAGCGCGTCTTCGCGCTT
ATCGACACGCGTTCTCGCATGGAAGACGGTACTGAGTCGGTTGAAGGTCAGATTATCGGG
CTGGACGTGGAGAAGATGAGTTTCCACTACGACAACGGTAACCGTATCCTCAATGAGATC
TCGTTTTCCATTCACGCCGGTGAAACCGTGGCAGTGGTTGGCGCCTCCGGCTCCGGGAAA
TCAACGTTGCTGATGTTGCTGGCGCGCTTTTACGATCCCACCTCCGGCGGGGTGTGGCTC
AACACCACTACGGGTCAACAGAATATTCGCGACCTGAAACTGACGGCGCTTCGTCGTCGC
GTTGGCGTAGTGTTTGAAGACGCGTTTCTGTTTGCCGGTACGGTGGCGGAAAACATCGCC
TATGGGCACCCGCAAGCGACTCAGGACGACATTCGACGCGCCGCCGATGCCGCAGGCGCC
AGCGGGTTCATCAATGCGCTACCGCAGGGGTTCAACACCCGACTGGCCGAACGCGGAAGC
AACCTCTCCGGCGGCCAACGCCAGCGTATTGCGCTAGCCCGGGCACTGATTACCGCGCCG
GAACTGCTGATTCTCGATGACACAACCTCGGCGGTCGATGCCGGTACTGAAGCGGAAATT
AACACGGCTCTTGGTCGTTATGCCGATAATGAGCATATGCTGCTGGTGATTGCACGCCGC
CGTTCAACGCTGCAGTTAGCCGATCGGATCGTCGTGCTGGATAAAGGCCGCGTCGTGGAT
ATCGGTACCCAGGCGGAGCTGGATGCAAGGTGTCCGACGTTTCGCTCGCTGATGAGCGGC
GAGGGTGATTTTCTCGCCCTTGCCCCGGCAGAACAACGGACGCTATGGCCAACAACGCAG
GCGGCAAAATCCGACGACGCGCATGAGCGCCAGACACCCGCCGGAAAAGGTTTTGTCGAC
CGGATGACGCGCGTTCCGGAACGCGCCGTACAGATGGCGCTGGCAGGCCACGGGCGTCAA
GTCTCATCGCTGCTGACGCCAGTAGCCTGGATGTTCGTCATCGCCGCCCTGCTTATCGCG
CTTGATTCCGCCGCAGGCGTTGGCGTGCTGGTGCTTTTGCAGCGCGGTATTGACTCAGGC
GTTGCCGCAGGGGATATGTCGACTATTGGCATATGCGCTCTGCTCGCGCTGTGTCTGGTA
GCGATCGGTTGGTGCTGCTATGCGCTGCAAACGATCTTCGCCGCCCGCGCGGCAGAGTCG
GTACAGCATACGGTACGCTTACGCAGCTTCAGCCACCTGCTACGCCTGAGTCTTCCCTGG
CACGAGAAGCACATCGACTCGCGTCTTACTCGCATGACCGTCGACGTTGATTCACTCGCC
CGATTTCTGCAAAACGGTCTTGCCAGCGCGGCCACCAGCATCGTGACGATGGTCGCTATC
GCCGCGGCAATGTTCTGGCTTGACCCCATTCTTGCGTTAACGGCATTAAGCGCCGTACCT
GTAGTGATACTGGCGACGTGGATTTACCGCCGTTTGAGCTCGCCTGCCTACGCCCAGGCA
CGGCTGGAAATTGGTAAGGTGAACAGTACGCTTCAGGAAAAGGTCTCCGGTATGCGGGTA
GTGCAGTCACACGGTCAACAGAAGCAGGAAGCCGCCAGGCTACGGGCGTTATCAGACAAC
TTCCGCGCCACCCGCGTGCGGGCGCAAAAATACCTTGCTGTCTATTTTCCGTTCCTGACC
CTCTGCACCGAGGCCGCCTATGCCGCCGTGCTTTTAATCGGGGCTACCCGGGTCGCCGGA
GGCGAAATGACGCCCGGGATACTGGCGGCGTTCTTCCTGCTACTGGGACAATTTTATGGC
CCGGTACAGCAGTTGTCAGGCATTGTTGATTCCTGGCAGCAGGCGACCGCCAGCGGCAAA
CATATCAATGCGCTATTGGCGACTGAGGAAACGGAGAATATTGAACCGTCCTCCATAACG
CCTGGCACTGGCGGCGCGCTACGTCTGGAGGCATTGACATTCCGCTATCCCGAAAAAACG
CAACCTGTGCTTGATAATCTCTCGCTCACCATTCCGCCAGGAACGGTAGTCGCGGTGGTC
GGACGTAGCGGCGCCGGCAAATCGACGCTGATCAAGCTGCTGGCCGGGCTCTACTCTCCC
GGCAGCGGGCAAATCCGTGTCGGTGAGCGCCTAATTGATGCCGCGTCGCTTAGTGATTAT
CGTCGCCAGACGGGGCTGGTCACTCAGGATGTCGCATTATTTAGCGGCGATATTGCCGAA
AATATCCGCTATTCGCTGCCAGACAGCAGCGACACGGAGGTGGAGATCGCGGCGCGACAG
GCGGGACTCTTTGAAACTGTGCAACATCTGCCGCTGGGGTTCCGTACTCCGGTCAATAAC
GGCGGCACGGATCTGTCCGCGGGCCAGCGTCAGTTGATTGCCCTCGCCCGCGCCCACCTG
GCGCAGGCGCATATTCTGCTGCTCGACGAGGCGACAGCGCGTATCGACCGTAGCGCCGAG
GAGCGCTTAATGACCTCGCTTACCAGGGTGACGCATACCGAGAAACGCATCGCGCTTATC
GTCGCGCACCGGCTGACCACCGCTCGCCGTTGCGATGTTATTGTCGTAATCGATAAAGGA
TGTATCGCTGAATATGGCAGCCATGAGCAGTTGATAGCGACTCATGGCCTGTATGCTCGT
CTGTGGCGGGACAGCATCGGCCAGACACGCGATACGCAAGGAGAGGTCATAGGATAG
>EECEBICD_00697 Elloramycin glycosyltransferase ElmGT
ATGCGTATTCTGTTTGTCGGTCCACCACTGTATGGACTGCTATACCCTGTGCTGTCCCTG
GCGCAAGCGTTTCGTGTTAATGGCCATGAAGTGCTGATTGCAAGCGGTGGAAAATTTGCA
CAGAAAGCAGCCGAAGCTGGGTTGGTGGTATTTGACGCTGCGCCTGGTTTCGATTCGGAA
GCGGGTTATCGCCGTCAGGAGGCATTACGAAAAGAAAATAACATTGGAACAAAAATGGGG

AACTTCTCATTCTTCAGCGAAGAGATGACTGACCCGCTGGTCGCGTTCGTCGGGCAGTGG
CGACCAGATCTCATCGTCTACCCTCCCCTTGGGGTCGTTGGACCACTGATTGCCGCTAAG
TATGACATTCCGGTAGTGATGCAAACCGTCGGCTTCGGTCATACGCCCTGGCACATCAAA
GGCGTGACGAAATCACTTTCTAACGCCTACCGCCGCCATGGGGTCAGCGCGCCACCAAGA
GATCTGGCGTGGATAGACGTCACACCGCCCAGCATGAGCATACTGCAAAATGACGGAGAG
CCGGTTATCTCCATGCAATACGTCCCGTATAACGGCGGCGCCGTCTGGGAAGAATGGTGG
GAACGTACACCTGATCGCAAGCGTCTTCTGGTTAGCCTCGGCACCGTCAAACCGATGGTG
GATGGCCTGGATCTGATTTCCTGGGTAATGGATTCTGCCGGCGAAGTAGATGCCGAAATC
ATCCTGCATCTTCCGGCAAACGCCCGCTCGGATTTACGTTCGCTGCCGTCGAATGTCCGT
CTGGTCGACTGGCTTCCGATGGGCGTTTTCCTTAACGGCGCCGACGGTTTTATCCACCAT
GGCGGCGCAGGCAACACCCTGACGGCGCTACATGCTGGCATTCCGCAGATAGTCTTTGGC
CAGGGTGCCGACAGACCCGTCAATGCCCGCGCCGTGGTCGAGCGCGGATGCGGCATTATT
CCCGGTAAGAGCGGGCTTACGAGCAGCATGATCAATACCTTCCTCGGTAATCGCGCGCTT
CGCGAGGCGTCGCAGGAGGTCGCGGCGGAAATGGCGGCCCAGCCTTGCCCGACCGAGGTG
GCAAAAAAACTGATCGCCATGCTGCAACACGGCTAA
>EECEBICD_00698 IS200/IS605 family transposase IS200F
ATGCGGGATACTTACAAAGCGTTCTATGGAGAGAAGCGTAGGGCAGTAGGCAGCATATTA
AGAAAATTGTGTGAATGGAAAAACGTACGAATTCTGGAAGCAGAATGTTGTGCAGATCAT
ATTCACATGCTTCTGGAGATCCCGCCGAAGATGAGTGTGTCGAGTTTCATGGGATATCTG
AAGGGTAAAAGTAGTCTGATGCTTTACGAGCAGTTTGGGGATCTAAAATTCAAATACAGG
AACAGGGAGTTCTGGTGCAGAGGGTACTATGTCGATACGGTGGGTAAGAACACGGCGAAG
ATACAGGACTACATAAAGCACCAGCTTGAAGAGGATAAAATGGGTGAGCAATTATCGATC
CCTTATCCGGGCAGCCCGTTTACGGGCCGTAAGTAA
>EECEBICD_00699 IS3 family transposase IS1351
ATGCGTAAAGCCCGTTTTACTGAGCATCAGATCATCACTGTGATTAAGTCGGTTGAAGCT
GGACGAACCGTTAAAGATGTCTGCCGGGAGGCCGGTATCTCTGAGGCCACCTACTATAAC
TGGAAGTCCAGATACGGCGGCATGGAACCTTCTGATATTAAAAAGATAAAAGATCTTGAG
GACGAGAACCGACGCCTCAAACAGATGTTTGCCGACCTGAGCCTTGAGAACCGGGCGCTG
AAAGACGTTATCGAAAAAAAGCTTTAA
>EECEBICD_00700 IS3 family transposase IS1351
ATGTGGAATCACAAAAGGATCCACCGTATTTATTGTCTGCTGAAGCTGAATTTTCGCCGT
AAGGGCAAACAACGGCTGCCGGTGCGTAACCCTTCGCCACTGGCCACGCCGGAAGCGCTG
AACCAGAGCTGGTCTGTTGATTTTATGCATGATGCCCTGGTCTGTGGGCGTCGTTTTCGC
ACGTTCAATGTCGTTGATGACTTTAACCGTGAGGCGTTGTCGATTGAAATAGATCGGAAT
CTGCCAGCCCCACGAGTGGTCCGGGTGCTTGACAGGATCGCGGCAAACCGTGGCTATCCG
GCCATGCTTCGCATGGATAACGGCCCAGAATTTATCTCTCTGGCACTGACTGAATGGGCA
GAGAAACATGCCGTAAAACTGGTGTTTATCCAGCCGGGTAAGCCGACACAAAATGCTTTC
ATTGAGCGTTTTAACAGGACATATCGCACAGAAATACTCGATTTTTATCTGTTCAGAACG
CTGAATGAAGTACGTGAAATCACGGAAAAATGGTTATCAGAATATAACTGTGAACGTCCG
TATGAATCGCTGAACAATATGACACCGGAGGAATACCGACAGCACCATTATTTGGCCGGG
AACTCAAAAAATGTATGGAACTAA
>EECEBICD_00701 hypothetical protein
ATGGAATCGAACGATCCTGGAAAGCTGATTTGGCATGTCGCCTGCGATGAATCAGGGATA
GATGGTCAGCGATTTTATGGATTTGGTAGCTTATGGATGAAATACCAGAGGCGTGGCGAT
TTTGCGCGTATTGTCCGCGAACTTCGTGAGAAACATAACTGTAGCGATGAAATTAAATGG
CAGAAAGCTCATTCCAAGCGTAACGCTGCATTCTATCAGGATTTAATCGAGACCTTCTTC
AAACACCCTTGGCTAGCTTTTCATTGCATAGTCGTGGAAAAGTCGAAGGTAGAAAAAATCT
TTCCACGGTGGCGACTATGATCTTGCAATGCGTAAGCATTTTGGCAAGCTAATAGAGACT
AAAATTGGCAATGTCATCAAAGCCCACCCCGACAGAGAGTGTGAGTTTCGTGTTGAAGTA
GATCCATTACCATCCCGCTACAAAAAAGCTGATGAGGAATTCCATGTAATCACCAACCAC
ACTTTGGCACGTAGGTTTGGGCGCAAAGATATTATCAAAAGCGTAGTAAGTAAAGACTCA
AAGGCGTCAGAACATATTCAGATAGCAGATTTTTTGCTAGGTGCGGTCATGTGCGCTTAT
CAAGGTAAAGCAACTTCTGAGGCAAAGCTAGCCGTCGCAAACAATGTTGCATCCTACTTG
GGATGGGACTCACTGATGCATGATACTTGGCCAACAGAACGTAAATTCAATATTTGGTTT

TTCTTCGACAGGTCAAAAGGACCAAGGGATATTGTCACCCAAGAAGTTAAGCTCACTTAC
GCCCTTCCCAACACACGAAAATAG
>EECEBICD_00702 hypothetical protein
ATGCCAACACCTGAAAGTTTGTGGAAAATTCTGGCTTCACCCATTTCGACAAAGACGGCT
GTTAGGTATGGATTCCTCTCTGTAGCAATTGTCCTTTCTATCCGCTTTATACTCCCAATA
CTTAAGGACATTGCGCCTCTAACTGAACAAGACTTGCCGGGGTATTCCTATGCAGCGCAT
TTTGCTCTAACGATTTTGTTTTCACTTATTGCGGAAACTCTAACCTTTGAACTAGTAGCT
TGGATGTATCTCAAGCTCGAAAAATTCATAGAAAGGAGGAAATTAAGAAAAGAACTCGCC
CGCAAACAAGAGATTGAGAGCAAGGAAATCAAGAGAATCCAAGAAAAGTTTATTGAAAAT
TTCATACTGGCATGGCCCCTCATAGCCCCCCGCTATAAATATTTTGTCTATGAGCTACGA
AGGGGCCACAAGGGCTACTCAGATACGAATTCGGCCATCAATTACCTTAGACAACAAGGT
TGGATTCACCCAATAACTGAACTACCTTATAGCCATTGCCTTTATCGGTTAGACGAACTC
ATTTTTGAAGCCCTAAATAGGATAGAGTCAGCCGCAACAGCAACAGACTCCTAG
>EECEBICD_00703 transfer-messenger RNA, SsrA
GGGGCTGATTCTGGATTCGACGGGATTTGCGAAACCCAAGGTGCATGCCGAGGGGCGGTT
GGCCTCGTAAAAAGCCGCAAAAAAATAGTCGCAAACGACGAAACCTACGCTTTAGCAGCT
TAATAACCTGCTTAGAGCCCTCTCTCCCTAGCCTCCGCTCTTAGGACGGGGATCAAGAGA
GGTCAAACCCAAAAGAGATCGCGTGGATGCCCTGCCTGGGGTTGAAGCGTTAAAACGAAT
CAGGCTAGTCTGGTAGTGGCGTGTCCGTCCGCAGGTGCCAGGCGAATGTAAAGACTGACT
AAGCATGTAGTACCGAGGATGTAGGAATTTCGGACGCGGGTTCAACTCCCGCCAGCTCCA
CCA
>EECEBICD_00704 Type I secretion system membrane fusion protein PrsE
ATGAAAATCAATCAGCATGATGCCGCGATGGACGATCCCGATATTCAGCGTGAACGGGCG
TTTTCCGGCGCGGGTCGTATTGTTCTGATCTGCTCACTGTTATTTCTCATTCTCGGCATC
TGGGCGTGGTTTGGCCGACTGGATGAGGTTTCCACCGGCAACGGGAAAGTGATCCCCAGT
TCACGCGAACAGGTTCTGCAGTCGCTGGATGGCGGCATTCTGGCGCAGTTGACGGTGCGG
GAAGGCGACAGAGTTCAGGCTAACCAGATTGTCGCCCGGCTTGATCCGACGCGTCTGGCG
TCCAATGTGGGTGAAAGTGCGGCAAAATATCGCGCTTCACTCGCCTCCAGCGCACGGTTA
ACCGCGGAAGTCAACGACTTACCTCTCGCCTTCCCCGCTGAGCTGAACGGCTGGCCGGAT
CTGATTGCCGCAGAGACGCGTCTCTATAAAAGCCGCCGCGCGCAGCTGGCCGATACCGAA
GCCGAGCTACGGGATGCGCTGGCGTCGGTTAATAAAGAGCTGGCCATTACCCAGCGTCTG
GAGAAAAGCGGCGCGGCCAGTCATGTTGAAGTGCTGCGCCTGCAACGACAAAAAAGCGAT
TTAGGCTTAAAAATTACCGATCTGCGCTCACAATATTATGTGCAGGCACGCGAAGCGTTA
TCAAAAGCGAACGCTGAGGTCGATATGCTCTCCGCCATTTTAAAAGGACGCGAGGATTCC
GTCACCCGCCTTACCATACGTTCGCCGGTACGCGGCATTGTTAAAAATATCCAGGTCACG
ACGATTGGCGGCGTGATCCCGCCTAACGGTGAGATGATGGAGATAGTGCCGGTAGACGAT
CGTCTGTTGATTGAAACCCGCCTTTCGCCGCGTGATATCGCCTTTATTCATCCCGGCCAA
CGCGCATTGGTTAAAATTACTGCTTACGATTACGCCATTTACGGCGGGCTTGACGGCGTG
GTGGAGACCATTTCACCGGATACCATTCAGGATAAAGTGAAACCGGAAATTTTCTACTAT
CGCGTGTTTATCCGCACCCACCAGGACTATCTACAAAATAAATCAGGACGCCGTTTTTCG
ATTGTTCCAGGCATGATCGCCACGGTGGATATCAAAACCGGTGAAAAAACCATTGTCGAC
TATTTAATCAAACCGTTTAATCGCGCGAAAGAAGCGCTGCGCGAGCGGTAA
>EECEBICD_00705 Toxin RTX-I translocation ATP-binding protein
ATGCAGGCGGAGAACCGCTTTCTTCAGCAGTGGAATAGCTATATCCGTATTACCGGCGAG
TCGGGTCTACGCACCCGCAAACTCACGCAAGGGCTTATCAGTTGGGGGATGTCGGTACAA
AGTCTGGTGTATGCCGCAGTGATTATGTTCGGCGCGCCGATGGTCATTGAAGGCAGTATG
ACTACCGGCGCCGTGGTCGCCGCGTCGATGCTCGGCTCACGGATGATCGCGCCAATGGCT
AACCTGTGCGGCGTGCTGGCGCGCTGGCAGCAGGTGAAAGCGGCGAAGATGGGTCTCGAC
AACATTATGCAACTGCCCACCGAGACACAGCATGACGATAGCCTGATACACCGCGACATC
CTCCACGGGCATTACCTCTTTGAAAATGCTCAGTTTCGTTACCATAACGACGATCAACGC
ATACCGCTGCGCCTCGTGCGTCTGGAGATCATGCCAGGCGAGCGGATTGCGATACTGGGG
CGTAACGGCGCGGGTAAATCCACACTTTTACAGGCGATGGCGGGCGGCCTGGAGATGATT
CAGGGAGATGCCCGGCTTGATAATCTCAGCTTGTCGCACATCGATATGGCGGATTTACGC
CGCAACATCGGCTTTCTTAGCCAGAACGCGCGGCTTTTCTTCGGCACCCTACGAGAGAAC

CTGACGCTCGGCGCGCCGCACGCCAACGATGAACAGATTTTTGACGCGCTCGAAGTCAGC
GGCGGCGCCGTCTTCGTCAGGCGGCTGGCAAAAGGGTTGGATCATCCCATTATGGAGGGC
GGTAACGGCCTGTCCGGTGGGCAGCGCCAGTCCCTGTTGCTGGCGAGAATGCTGCTGCGC
TCCCCCAATATTGTGCTACTTGATGAACCCAGCGCCTCGCTGGACGAGCATACGGAACGA
GAGTTTATTCAGCGGTTACATCAGTGGCTTGGCAACCGTACCCTGGTCGTCGCGACGCAC
CGGGTACCAATACTGGAGCTGGTTGAGCGTGTCGTTGTCCTGAAAGAAGGACAACTGGTG
ATGGATGCGCCAAAAGCGCAGGCGCTTAACGCGGATCGGATGCAAAGTCACCGTCGGGAG
TGGAAAAATGAAAATCAATCAGCATGA
>EECEBICD_00706 Toxin RTX-I translocation ATP-binding protein
ATGACCCGCGCCGCCCCCGATGTAGAAGAGGTACTCAGTGAGCGCGCGCTAAGCCAATGG
GCGCAGGCCATCAGCTATGTCGCCGGTCATTATCGCGTTGCCTGTTCTCCCGGTTCAATT
CAGGCCAACGCCCCGTGGTTTAGGGGTAAAAGCAGAACGACCGCCTTAACGCAGCTCGCG
CGGCAGGCGGGTTTATCCTTTCATGCGCCGGACATAGACAAAACGGCATTTAGTCAGTGG
CGATTGCCGTTGGTTGTCGAGCTCCGGGACGGGCAGTTATTGGTCATCGAGCATGTTAAT
GGCGAAGATGCGGTAGACGTTTTTGTGATTGAAGAAGAGGGTCAGCGTAACCGCCTGACG
CTCAGCGAGTTATTGCCGGAGATCCTCTATGTTGCCGCGCTACGCCCGCTATCGGCGCTC
AAGGATAGCCGCGTCGATCGCTATATCTCCCGCTTTAAACCCGACTGGATGCGCGAGCTG
GTTCTGCAGGATATTCGCCCTTATTTACCGGTAATGGTCGCCGCCTTTCTGATTAACGTG
CTGTCGCTGGCCGGGATTGTGTTCTCTATGCAGGTTTACGATCGGGTGATCCCCGCTCAG
TCATATCCTACCCTGTACGTGCTCTCCTTCGGCGTGCTGGTAGCGGTGCTGTTCGGTTTT
CTGCTGCGTGAAGCGCGTACGCACATTATGGACGTGCTCGGTAAACGCGCCGATATGCGC
ATTTCCGATCGGGTATTCGGTCATGCGTTAAGGCTGCGCAACAGCGCTATCCCCCGTTCC
ACCGGCAGTTTTATCTCGCAGTTACGCGAACTAGAGCAGATCCGCGAGATGATTACCTCT
TCAACGCTGGCGACCATTGTCGATCTGCCTTTCTTTTTCCTGTTTATGATAGTCCTGGCG
ATTATTGCCCCACCGCTGGCATGGATAGCGCCCGTGGCGGCTCTGCTGATGATCCTGCCG
GGCGTCGCACTGCAAAAAAACTGGCTGTCCTCGCCAACCAGGCCGCCCACGAAGCGACGC
TACGCAATGCAGTCTTGGTCGAAAGCGTTCAGGGGCTGGAGGATATTAAACTGA
>EECEBICD_00707 Outer membrane efflux protein BepC
ATGGGAAGAGTCGCGCCTGTCGCCATCGTACTGGCATTTGCTTTGTTTCATCACCAGCCC
CGGGGCGCAGAAGCGCCGCCAATGATTACATCCGAAGGATTAGCAACGGACCAGATGCTT
CCTTCGCTGGATGGCTCCGCCGCTGAGTTGCCGCTCAGCGCCGCCGCGCCGGGCAACCTG
ACGCTCAATGACGCGGTCAATCGCGCCGTTAACTGGCATCCTTCTATTCGCGAAGCCGTC
GGCAAACTGCTCGCACAGAATGAACAAATAGAGGTCGCCAAATCGAAATATTATCCGCAA
GTCAGCGCTGGCGTGAACAATGGGTACAGCAATACCTACACCGATCACGGGTATAGCCCC
TCGCTGGTGCTGTCGGTATCGCAAATGCTTTATGACTTCGGCAAAGTGGCAAGCCAGGTT
CGCGCCGAAACCGCAGGCGCAGCGCAGCAACAGGCCAATGTGTTGCTCAGTATTGATACC
GTCGCGCACGAAACCGCCAACGCCATTGTACAAACGCAGAGCTGGCAGCAAATGGTAGAC
GCGGCGGAAGAACAGCTCGTCGCGCTGGACAGCATCGGTAAACTTATCCGGCAGCGCAGC
GATGAAGGCGCTACGTCGCTATCTGACGTGGTGCAAACCGAAGCCAGAATCGAATCCGCC
CGCTCGCAACTGGCGCAGTATCAGGCCAATCTCGACAGTGCGAAAGCCTCACTGATGAGC
TGGCTGGGCTGGAATTCGCTTAACGGCATCAATAATGACTTCCCGGCGAAACTTGCTCGC
AGCTGTGAGACGGCGACTCCCGACGATCGACTGGTGCCCGCGGTACTGGCCGCCTGGGCG
CAGGCTAACGTTGCGCGGGCGAATCTGGACTATGCCAGCGCGCAAATGACGCCGACGATT
TCGCTCGAACCTTCTGTACAACATTATCTTAATGATAAATATCCCAGTCATGAAGTGCTG
GATAAAACCCAGTATTCCACCTGGGTGAAAGTTGAGATGCCGCTTTACCAGGGCGGAGGG
CTGACTGCCCGACGCAACGCCGCCAGCCATGCGGTAGACGCGGCTCAGTCGACCATTCAG
CGCACCCGGCTTGATGTCCGCCAGAAACTGATGGAGGCGCGCAGCCAGGCAATGAGTCTT
GCCAGCGCGTTACAAATCCTTCGTCGGCAACAGCAGCTTAGCGAACGCACGCGCGAACTG
TATCAGCAGCAATACCTTAACCTCGGTTCCCGCCCGCTGCTCGACGTGCTTAATGCGGAG
CAGGAAGTTTACCAGGCGCGTTTTGCCGAACTGCAAACGGAAAGCCAGTTGCATCAGTTG
CAACTGAACTGTCTGTATAACACCGGCGCGCTTCGTCAGGCGTTCGCGTTAAATCATCGC
AGCATTCAATCCGTGGAGATCCAGCCATGA
>EECEBICD_00708 hypothetical protein
ATGCGTCTACTCGCCGTGGTTTCGAAATTGACTGGCGTCTCCACCACTGTGGAATCCTCA

GCGGTCACTCTTAACGCCCCGTCAATTGTTAAATTATCAGTGGCCCGGGAGGAAATTAGT
CAACTTACGCGCATTAATCAGGATCTGGTGGTGACGCTCCATTCCGGCGAAACGATCACG
ATTAAAAACTTTTACGTTACCAACGATCTGGGCGCAAGCCAGCTGGTACTGGCGGAAAAC
GATGGCACGTTATGGTGGGTAGAAAATCCGCAAGCCGGGCTACATTTTGAACAAATCGCT
GATATTAATGAACTGCTGGTCACTTCCGGCGCTTCCCATGAAGCAGGCGGCGCCGTTTGG
CCGTGGGTACTGGCTGGCGCGGTGGCGGCTGGCGGCATTGCCGCTATCGCGTCTTCCGGC
GGCGGCGATTCCCACCATCATTCGGATGGCGATAATCCGCCCCCCGATAACACCAATCCT
GACGGTAATCCCCCTGATAACAGCAATCCCGGCGGCAGTACCCCCAACGGCAATACTCCA
GGTAGCAGTAATCCTGTAGATACTACCCCGCCTCTCGCTCCCGGCGAATTATTGATTTCA
GCGGACGGAAAAACGGTAAGCGGGCAAGCCGAAGCGGGCAGTACCATTACCATCAAAGAT
CCCTCAGGCAACGTCGTTGGCGAGGGCAAAGCGGATAGCGACGGTAAATTTAGTATTGAT
CTGACAGCACCACAGATTAGCGGCGAACAACTTACCGTGACCGCGACTGACGATGCCGGC
AATACCGGCCCATCCGCAACCATCGATGCGCCCAACATTCCTCTCCCCGATACACCAGCT
ATCACCGCCGCTATCGATGATGCCGCTCCCCTCACCAGCACGCTGAGCAATAACCAGTTT
ACGAACGACAATACCCCCACTCTGGAGGGCACCGGCAGCGCAGGCACAGTCATCCATATT
TACGCCAATGGTCAGGAAATAGGCTCAACAACGGTTGATACCAGCGGAAACTGGCATTTT
GCCATTACCAGCGCGCTAGCGGATGGGGAAAATCATTTCACCGCCATTGCGACTAACGTG
AAAGGCGAAAGTAGCGAATCAGCCCGCTTTACGCTGACTATCGACACACTCATCCCCGAT
GCCCCACGTGTTGAACTGATTGCCGATAACACCGGTTTGCTCACCGGGCCGCTACAGAAT
AATGACCGAACTGACGAGGCAAAACCGCTATTTTCCGGGCAGGGAGAGGCAGGCAATACC
ATCACGATTAAAGAGGGTTCAACCGTTATCGGCAGCGCTACCGTAGACGAAAATGGACGC
TGGACCTTTACGCCGACTACGCCGTTAAGCGATGGCGAACATACCTTTACCGTCGAACAA
AGCGACAAAGCCGGAAACACGAGCCGCGTGACGACAACGCCTACTATCATTGTGGACACC
ACGCCGCCTGACGCCGCTATCATTGATAATGTTGCGAAAGACGGCACAACCGTTAGCGGC
ACCGCTGAAGCTGGCAGTACTGTGTCGATCTATGACCCGGCGGGAAATTACCTGGGCTCC
ACGATTACCGGAGAAAATAACCACTTCAGCATCACGCTGAATCCGGCTCAGACCCACGGC
GAGCGTCTGGAAGCGCGTATTCAGGACGCCGTCGGTAACATCGGCCCCGCCACGGAGTTT
ACCGCTTCTGACTCACAGTATCCTGCCCAGCCGACTATCCTTACCGTGACGGATGACGCT
GGCGCCGTTACCGGGCTGCTGAAAAATGGCGATGCCACAGATGATAACCGCCCAACCCTC
AGCGGTACTGCTGAACCAGGCAGTACGATATCGATTAACGATAATGGCTTTCCTGTAGCG
ACCTTTCCGCCCATTGTCGCTGACGCTGACGGCAAATGGAGCTTTACCCCCTCGCTGGCG
CTTGCCGATGGCGACCATGTCTTTACCGCTACCGCGACCAACGATCGCGGCACCAGCGGG
CAGTCCGTCTCCTTTACCATTGATATCGACACGCAGCCGCCGGTGCTGGAAGGCCTGGCG
GTTAGCGACGTCGGCGACAGACTCACCGGCACTACGGAAGCTGGCAGTACTGTGGTTATC
AAAGACAGCCTGGGAAATACGCTCGGGAGTGGAACGGCAGGCGACGACGGTACCTTCTCA
ATAGGTATTAGCCCGGCGAAAATTAACGGCGAAACATTAAGCATTAGCGTTACCGATAAA
GCCGCGAATAGCGGTCCGGTAGAAACGCTGAACGCGCCGGATAAAACTGCGCCTGCGGCA
CCGGACGGTCTTACCGTGGCGACCGACGGTCTGTCCGTAAGCGGTCAGGCGGAAGCCGGG
GCAACGGTCACTATCCGCGACAGTAGCAACACCGTACTTGGCAGCGCCGTCGCTAATGGC
AACGGACAATTTATCGTTCCGCTGAATACGGCGCAGACTAACGGCCAGGCGCTTATCGCT
ACCGCCACCGATGTCGCGAAAAACGAAAGCGCCGCCGCGACGGTTATCGCGCCGGACAGT
ACCGCGCCGGAAATGCCGAAAAACGTGGTAATTAGTGAGGATGGCACCAGTATCAGCGGC
ACCGCCGAACCGGGTAGCGCCATCACGATCGCCACGCCGGACGGCAAGCCGCTTGGCAGC
GGCAAAGCAGATGGCGAAGGTCATTTTACCCTTCCCCTCGTCCCCGCACAGACCAACGGC
GAACAGGTTACCGTCACCGCCACCGACAGCGCCAACAACGTCAGCCCGCCAACCACAGCG
CAAGCGCCCGATATCACCGCCCCGGATAAGCCCATTATCACTCAGGTACTGGACGATGTT
GAAAGCTTCACCGGGCCGCTGGTTAACGGACAAACCACCAACGACAACCGTCCCACCCTT
AGCGGTACGGCGGAGGCCGGCGCGCGTGTCGAAGTCTTTGATAACGGCGTTTCACTGGGA
CTCGCCACGCTACAGCCCAACGGTGCCTGGACGTTTACGCCGTCGCAAAATTTAGGTGAA
GGCGCGCATCGACTGACCGTAATCGCAACCGACGCTAAAGGCAATGCCAGTCAGGCCGCG
TCATTCGACCTGGTGGTCGATACGCAATCGCCGCAGCAACCGGTAATCACCTTCATTACA
GATGATGCGCCGGGTATTCTCGGTAGCGTCGCGCATCTGGGGCTCACTAACGACAGCACG
CCAACGATTAATGGTACAGGTGAACCGGGTTCCACAGTACACCTGTATCAGAATGGCGCC
CGGATAGCAGATATTATCGTCGGTAATTCCGGCGTCTGGAGCTACGCTTACACCACGGCC

TCGCCACTGGCGGACGACACCTACACCTTTACCGTGACGGCCAGCGACAGTAACGGCAAC
ACCACGCCTTTTTCGACCGATTTTACGATTACCATTGATACCCAGGCCCCTGCCGCCCCC
GGCGTTATCGGCGTAGCTGACGGCGACGGAAATACGATTGATACCAATCAGATTACCCAG
GAATCCCAGCCCCGGTTGAGCGGTAGCGGCACCGCAGGCGACACAATCATCCTTTACGAT
AATGGCAATGCCATAGGTCAGGCGCTGGTCGGCACGGACGGGCGCTGGCAGTTTACGCCG
CCTGCCGCGCTGGGCGACGGCGACCACCATCTGACCGCTCGCGCCAACGATCCGGCGGGG
AACGAAAGTCCCGAATCCATCAGCTTTACCCTACGCATCGATACCCAGGCGCCGGATGCG
CCGCAGATCGTGTCAGCCGCCATCACCGGCGGAGAAGGCGAGGTGCTACTGGCAAACGGC
AGTATTACCAATCAGCGTATGCCGACCCTCAGCGGCACCGGCGAACCCGGCACCATCATC
ACCCTGTACAATAACGGCGTAGAACTGGCTACCGTCCAGGTCAATCCACAGGGTAGCTGG
ACCTATCCGCTAACCCGTAATCTGAGCGAAGGGTTAAACATCCTGACGGCCACCGCCACG
GATGCCGCAGGCAATAGTAGCCCGACCTCCGGCGTTTTCTCCGTTACCCTTGATACCCAG
CCTCCAGCGCAGCCTGACGCGCCGCTAATCAGCGATAACGTCGCGCCGGTTATTGGCAAC
ATCGGCAATAATGGCGCAACGAACGATACCACGCCGACCTTCAGCGGCACGGGAGAGATC
GGCAGCACGATAATTCTCTACAATAATGGCAGTGAAATTGGTCGCACAACGGTAGGCGAT
AACGGTAGCTGGAACTTTACGCCTGCGGCACTGACGCCAGAAACCTATACCATTACCGTC
ACGGAAACCGATAGAGCGGGCAATATCAGTCCACCTTCCGCCTCAGTCACTTTTACGCTA
GACACCACTGCGCCCGCCAATCCGGTTATCACTTTTGCCGAAGATAACGTCGGCGAAGTC
CAGGATACTATTGTCAGCGGCGCAACCACTGACGACAATACACCGGTCATTCACGGCACT
GGCGACATCGGCAGCATTATTACGCTCTATAATGGCAGCAGCGTTTTAGGCGTAGTCACC
GTCGATGAGACCGGCACCTGGACGCTAGCGGTGACCAGCGCGCTGCCGGATGGCGTCTAC
ACCCTGACTGCCATTGCCGCCGATGCCGCCGGAAACAGCAGCGGCGTATCGAACAGCTTT
ACCTTCACCGTCGACACCGTTCCGTTGCAGCCGCCCGTCGTCAATGAAATCCTTGACGAT
GTTGCGCCAGTGACCGGGCCATTAACCGATGGCGCCTTTACTAACGATCGGACGCTGACT
ATTAACGGCAGCGGCGAAAACGGCAGCACCGTCACGATTTACGACAATGGCGTGGCAATC
GGGACGGCGCTCGTCACCGACGGGGTCTGGACATTCAATACGCCCGAATTATCAGAAGCC
AGCCATGCGCTAACCTTCAGCGCGACTGACGATGCTGGAAATACCACGGCGCAAACCCAG
CCGATCACTATTACCGTGGATATCACTGCCCCGCCCGCGCCAACGATCCAGACGGTGGAC
GACGATGGCACGCGCGTCGCCGGACTTGCCGATCCTTACGCTACCGTTGAAATTCACCAT
GCCGATGGCACCCTGGTCGGCAGCGCTGTCGCTAATGGCACCGGTGAATTCGTCGTTACG
CTCAGTCCGGCGCAAACCGATGGCGGTACGCTGACGGCAATTGCTATCGATCGCGCGGGG
AATAACGGCCCGGCTACGAATTTTCCCGCTTCCGACAGCGGTCTGCCCGCCGTCCCGGCC
ATCACGGCGATTGAAGATGATGTCGGGAGCGTACAGGGGAATATTGCGGCGGGCGGCGCC
ACGGACGACACCATGCCGACGCTGCGCGGCACCACGGATATCGGCTCTACCGTTGAAGTT
TTCATTGATGGCGATTCGGCAGGCTTTGCCACCGTTGACGCCAGCGGGAACTGGATCTTT
GAGATCGCGACGCCATTAAGCGAAAGCACACATTACTTCACCGTCCAGGCAACCAATGCG
AATGGCCCGGGCGGCCTGTCCGCACCGGTCGGGATCACTGTCGATCTTAGCGCGCCGGCG
CAACCGGTTATTACCAGCGCAACGGATGATGTCCCCGGCATGACCGGTACGCTGGATAAC
GGCGCGCTCACCAATGATTCACGCCCGACGCTCAACGGAACGGGAGAAGCTGGCGCCACG
ATCCGCATTCTGGATAACGGCGTAGAAATCGGTTCCGCCACGGTAGATCAAAGCGGCAAC
TGGCGCTTCACCCCGAACGCGCCGCTAGAGAGCAACGCGCACATCTTTACCGCCGTGGCG
ACCGATCCCGCTGGCAATAGCGGCCAGCCTTCGGACGGCTTTACGCTGAACATTGACGCG
CAGGCGCCAGATGTGCCGGTTATCACGTCCGTGATTGACGATAACAATCAACCGACCGTT
CCGGTGTTACCGGGGCAATCCACCGACGATCGGCAGCCAATACTGAACGGAACTGGCGAA
CCTGGCGCGACAATCACCATTTTTGATAACGGTACGCCGCTTGGCACGGCTCAGGTAGGC
GAAAACGGTAGCTGGACCTTCCCGGTGCCCCGCAATTTGTCAGAGGGAAGCCATAATCTG
ACGGTTAGCGCTACCGATCCGGCGGGCAATACCAGCGCGGTCTCCGCGCCGTGGACGATC
GTGGTCGATATTACGCCTCCGGCGATCCCGGTTCTCACCTCCGTCGTGGATGACCAGCCC
GGAATTACCGGCAACCTGGTTAGCGGGCAGCTAACGAACGATGCGACGCCCACCCTGAAC
GGGCGCGGAGAGGCAGGCGCGACGATTAATGTCTATCTTGACGGTAATCCCGCGTCCATC
GGTACCACGACGGTGAATAGCGACGGCACGTGGAGTTTCACGCCGCAGACGCCGCTTGCA
AACGGTAGCCACACGTTCACCCTTAGCGCCACCGATCCGGCGGGTAATAGCAGCTCGGTG
TCCAGCGGATTTGTGCTGACGATTGACGCCACACCGCCCGCCGCGCCGGTTATCGCCAGC
GTGGCAGATAATACGGCGCCGGTAACGGGCATCGTCCCCAACGGCGGCTCGACGAACGAA

ACCCGACCAACACTCTCGGGTACCGGTGAGGCGGGTACAACCATCTCGATTTATAATGGC
AGCGCGCTGGTCGGTACGGCGCAAGTTCAGGCCAACGGTAGCTGGAGCTTTACACCGTCT
ACCTCGCTGGGCGCGGGCGTCTGGAACCTGACGGCGACAGCAACCGATGCGGCAGGCAAT
ACCAGCGCCGCGTCCGAAATACGCTCGTTTACTATTGATACCACGGCTCCCGCCGCGCCT
GTTATTGATACGGTCTACGACGGTACGGGCCCCATTACCGGCAATCTGAGTTCAGGACAG
ATCACAGACGAGGCGCGCCCTGTCATTAGCGGCACCCGTGAAGCCAACACAACTATTCGT
CTCTACGATAACGGCACGCTGCTGGCTGAAATTCCCGCCGACAATAGCAGTAGCTGGCGC
TACACGCCCGACGCCTCGCTGGCGACGGGCAACCATGTAATTACCGTCATTGCCGTTGAT
GCCGCAGGCAACGCCAGCCCCGTTTCGGACAGCGTTAATTTCGTCGTCGATACTACGCCG
CCGCTGACGCCGGTAATCACATCAGTCAGTGACGATCAGGCGCCAGGCCTCGGCACGATC
GCGAACGGCCAAAATACCAACGATCCTACGCCAACCTTCAGCGGCACCGCAGAAGCCGGC
GCCACGATCACGCTCTATGAAAATGGTACGGTCATTGGCACGACAACGGCTCAGCCTGAC
GGCGCGTGGAGCGTCTCCACCTCAACGCTGGCAAGCGGAACGCACGTCATCACCGCCGTC
GCCACCGATGCCGCAGGAAACAGCAGCCCGAACAGTACGGCTTTCACCCTGACGGTCGAT
ACCACCGCGCCGCAAACGCCAATCCTGACGTCCGTGGTGGATGACGTCGCGGGTGGGGTC
ACAGGAAATCTCGCTAATGGTCAGATAACCAATGATAACCGCCCCACGCTGAACGGCACT
GCCGAAGCGGGCAGCGTGGTCAGTATCTATGATGGCGACACTCTGCTTGGCGTCACCTCG
GCTAACGCGAGCGGCGCGTGGAGCTTCACGCCGACGACAGGGTTAAACGACGGCACGCGC
ACATTAACAGTGACCGCCACCGACCCGGCAGGCAACGTTAGCCCGGCCACCAGCGGTTTT
ACTATCGTGGTCGATACCCTTGCGCCAACGGTTCCGCTTATAACCAGCATCGTTGATGAT
GTCCCGAACAATACCGGCGCCATTGGCAATGGACAATCGACCAACGACACACAGCCGACG
CTCAACGGTACTGCGGAAGCCAACAGCGCGGTAAGCATCTTCGATAATAGCGCGCTGGTC
GCGACCGTGAACGCCAATGCCAGCGGCAACTGGAGCTGGACGCCAACCGCCGCGCTCGGC
CAGGGAAGTCACGCCTATAGCGTTAGCGCCGCCGATGCGGCTGGCAACGTTAGCGCCGCT
TCGCCATCGATAACGATTATCGTGGATACCATTGCGCCCGGCGCGCCCGGCAACCTGGTC
ATCAATGCTACCGGTAATCGGGTGACGGGCACCGCGGAAGCAGGCAGTACAGTGACGATT
ACCTCTGATACTGGTGTGGTACTGGGAACCGCCACCGCCGACGGTACAGGCAGCTTCACC
GCCACACTCACGCCCGCGCAGACCAATGGTCAGCCGCTACTGGCATTTGCCCAGGATAAA
GCAGGCAACACAGGCATTGCCGCCGGATTTACCGCGCCCGATACGCGTGTGCCGGAAGCA
CCGATCATCACCAACGTAGTAGATGATGTGGGTATTTATACCGGCGCTATCGCCAACGGC
CAGGTCACTAATGACGCACAACCCACATTGAATGGTACCGCTCAGGCGGGCGCCACGGTG
AGCATTTATAACAACGGGGCGCTGCTCGGCACCACCACGGCGAACGCCAGCGGAAACTGG
AGCTTTACCCCGACAGGCAATTTGACCGAAGGCAGCCACGCCTTCACCGCCACCGCGACT
AACGCCAACGGTACAGGCAGCGTCTCCACCGCCGCGACGGTGATTGTCGATACGCTGGCG
CCCGGTACGCCGTCAGGTACGCTCAGCGCCGATGGCGGTTCACTTTCCGGGCTGGCAGAG
GCAAACAGCACCGTAACCGTCACGCTGACGGGGGGCGTGACGCTCACCACCACCGCTGGC
AGCAACGGCGCATGGTCTCTCACCTTGCCGACAAAACAAATTGAAGGTCAGCTCATTAAC
GTGACGGCCACTGACGCTGCGGGTAACGCCTCCGGCACGTTAGGCATTACCGCGCCGGTT
CTGCCGCTGGCGGCAAGGGATAACATCACCAGCCTTGATCTGACCTCTACCGCCGTCACC
AGCACGCAAAGCTATTCGGATTACGGCCTGCTGCTGGTTGGCGCGCTTGGCAATGTCGCC
TCGGTTTTGGGTAACGATACCGCTCAGGTTGAGTTCACCATTGCTGAAGGTGGTACGGGC
GACGTCACCATCGATGCCGCCGCAACGGGAATCGTGCTTTCGCTGCTCAGTACTCAGGAG
ATTGTGGTACAGCGCTACGACACCAGCCTCGGCGCCTGGACGACGATCGTCAACACCGCC
GTTGGCGACTTCGCGAATTTGCTTACCCTGACCGGGAGCGGCGTTACCCTGAACCTGAGC
GGCCTGGGCGAAGGCCAGTACCGGGTACTCACTTATAACACCAGTCTGCTCGCCACCGGG
TCATATACCAGCCTGGATGTCGATGTACACCAGACCAGCGCAGGTATTATTAGCGGGCCA
ACCATCAGTACCGGCAACGTCATGGCTGATGATACCGCGCCGACGGGCACCACGGTCACC
GCCATCACCAACGCCAACGGCGTCAGTACGCCGGTCGGCGCGGGCGGCGTGGATATCCAG
GGACAATACGGCACGCTGCACATTAATCAGGACGGCAGTTACACCTACACGCTGGCTAAG
CCCACGGCAGGATACGGACATAAAGAGAGCTTCACCTACACCATTACCCAGAATGGCGTC
GGTAGCAGCGCCGCGCAACTGGTTATCAATCTGGGTCCCGCGCCTGTACCGGGCAGCGTG
ATAGCGACAGACAATAACGCCTCGCTGGTCTTTGATACTCACGTTAGCTACGTCAACAAC
GGTCCCTCGACACAAAGCGGCGTCACGGTATTAAGCGTCGGACTTGGTAATGTACTGAAC
GCGAATCTGCTTGATGATATGACTAATCCGATCATCTTTAACGTTGAAGAAGGCGCTACG

CGAACCATGACGTTACAGGGAACCGTCGGCGGCGTCTCACTGGTTTCCACGTTCGATCTG
TACGTTTATCGCTTCAACGATGCCATTCAGCAATATGAGCAGTTCCGGGTGGAAAAGGGC
TGGATTAACACCCTGCTGTTAGCCGGACAGTCCCAGCCGCTGACCCTGACGTTGCCTGGC
GGCGAATACCTGTTCGTGCTGAATACCGCCAGCGGCATTAGCGTCCTCACTGGCTATACG
TTGGCGATTTCCCAGGACCACACCTATGCCGTTGACAGTATCACCGCCAACACCACCGGC
AACGTACTGACCAATGATGTCGTCCCTACGGACGCCCTCCTCACTGAAGTAAACGGCGTG
GCGATTGCGGCGACCGGCACAACGGAGGTAAACGGGCTGTATGGCTCGCTCATCATTGAC
GCAAGAGGCAACTATACCTACACGCTGAAGAACGGCGTCGGCGCCGACAGCATTAAAACG
CCGGACAGCTTTATCTATACGGTCAAAGCGCCAAACGGCGATACCGATACGGCCTCGCTC
AATATCACGCCAACCGCCAGGGCGCTGGATGCGATTAATGATGTCAGCGATACCCTCAGC
GTCGCCACGCTTCAGGATACCGCTGCCTGGCTGGACTCCAGCGTCGGCAGCGCCAGTTGG
GGGCTACTCGGCAAATCGGGCAGCGGGAGCGGCACCTTTGACGTTGCAACGGGCACCGTA
CTTAAAGGCGCGTCACTGGTCTTTGATGTCTCCACGCTCATTACGCTGGGCAATCTGAAT
ATTAGCTGGGCCATTCAGGAGAACGGGACCGTCATACGCAACGGAACCGTTCCGGTGGCG
AATATCACGCTGGGCAGCGCGACGGTGACCGTCAACCTGAGCGGTCTGGAGCTGGATGCC
GGAACGTACACGCTTAACTTTACCGGCACCAATACCCTGGCCGGGGCGGCGACGATCACG
CCACGCGTCATCGGCACCACCGTCGATCTGGATAATTTTGAAACGTCCGGAACGCATACC
GTTCTCGGCAATATTTTTGACGGCAGCGACGCGGCGGGGGCGATGGATCAGCTTAATACG
GTGAATACCCGCCTGAGCATTAGCGGGTATAACGGCAGCGCCGCCACGCTGGACGCCGCG
GCGAATACCACCAGCGCCACGATTCAGGGACATTACGGCACATTGCAAATTAACCTCGAT
GGCGCTTACACCTACACCCTGAATAATGGCGTCGCGATGTCGTCCATCACCAGTAAAGAG
GTCTTTACCTATCAACTGGATGACAAGATGGGTCATACGGATAGCGCCACATTGACCATT
GATATGGCGCCACAAATCGTCAGTACCAACCAAAACGATGTTCTCATCGGCTCCGCCTAT
GGCGATACGCTGATTTACCATCTGTTAAACGGCGCGGACGCGACCGGCGGCAACGGCGTC
GATCGCTGGCAAAACTTCTCTACCGCGCAGGGCGACAAGATCGATATCCACGAACTGCTG
ACCGGCTGGGATCACCAGGCGGCGACGCTGGGTAACTTTGTGCAGGTTCATACCAGCGGC
GCCAATACGGTGATATCCGTCGATCGCGACGGCGCCGGCAGTGCGTTTAAATCCACTGAC
CTTGTCACTCTGGAGAATGTGCAGCTCACGCTAAATGATCTGTTGCAGAACAACCACCTG
ATAACCGGCGGTTGA
>EECEBICD_00709 SsrA-binding protein
ATGGGGTGTTTTCGATATCAGATTACCTATGAATTCACGACACTTATGACGAAGAAAAAA
GCACATAAACCGGGCTCAGCCACCATCGCGCTGAATAAGCGCGCCCGGCACGAATACTTT
ATCGAAGAAGAGTTCGAGGCTGGTCTCGCCCTGCAAGGCTGGGAAGTAAAATCTCTGCGC
GCAGGAAAAGCCAATATCGGCGACAGTTACGTTATCCTCAAAGATGGCGAGGCCTGGCTG
TTTGGCGCAAACTTTACGCCGATGGCGGTCGCCTCGACACATGTCGTATGCGACCCAACC
CGAACCCGCAAACTGTTGTTAAATCAGCGTGAGCTTGATTCACTGTATGGCCGTATTAAT
CGTGAAGGTTATACTGTCGTCGCGCTTTCTCTGTACTGGAAAAATGCCTGGTGCAAAGTG
AAGATCGGCGTGGCGAAAGGTAAAAAACAACATGATAAACGTTCCGATCTTAAAGAACGT
GAGTGGCAACTGGATAAAGCACGCATTATGAAAAATGCAGGCCGATAA
>EECEBICD_00710 Ribosome association toxin RatA
ATGCCTCAGATTAGTCGGACTGCTTTAGTCCCCTACAGTGCGGAACAGATGTATCAGTTA
GTGAATGACGTGCAATCGTATCCCCAGTTTTTGCCTGGGTGTGTTGGTAGTCGCGTCCTG
GAGTCATCGCCAGCACAGATGACGGCGGCTGTTGACGTGTCGAAGGCGGGTATCAGCAAG
ACGTTTACGACGCGTAATCAGCTAACGCGTAACCAGAGTATTCTGATGCATCTGGTGGAT
GGCCCCTTTAAAAAGCTGATCGGCGGCTGGAAATTTACCCCGCTCAGCCCCGAGGCGTGC
CGCATTGAGTTTCAGCTTGATTTTGAGTTTACCAATAAACTCATTGAGCTGGCGTTTGGT
CGTATATTCAAAGAGTTAGCGTCCAACATGGTGCAGGCGTTTACGGTTCGCGCCAAAGAG
GTTTACCGTGCCGGATAA
>EECEBICD_00711 hypothetical protein
GTGCCGGATAAACTTGTGGTTGAAGTGGCTTATGCGCTGCCGGAAAAGCAGTATTTGCAG
CGCGTAACGCTGGAGGAGGGCGCGACGGTCGAAGAGGCGATTCGCGCTTCCGGCTTGCTG
GAATTGCGCACGGATATCGATCTGGCGAAAAATAAAGTAGGTATCTACAGTCGTCCGGTA
AAACTCACCGATACGGTGCAGGATGGCGATCGGGTTGAAATTTACCGTCCCTTAATCGCC
GATCCAAAAGCGTTACGCCGCCAGCGCGCCGAAAAATCGGCTGGCAGATAA

>EECEBICD_00712 Outer membrane protein assembly factor BamE
ATGCGCTGTAAAACGCTGACTGCTGCCGCAGCAGTACTCCTGATGTTGACCGCAGGCTGT
TCCACTCTGGAGCGAGTGGTTTACCGACCTGACATCAATCAGGGGAACTATCTTACACCT
ACGGATGTCGCCAAAGTGCGTGTTGGTATGACGCAACAACAGGTTGCGTATGCTTTAGGC
ACGCCGATGATGACCGATCCTTTTGGTACGAACACCTGGTTTTATGTCTTCCGCCAGCAG
CCAGGACATGAGAACGTGACGCAGCAGACTCTGACGCTCACCTTTAACAGCAGCGGCGTG
TTAACCAATATTGATAACAAACCGGCGTTGACGAAGTAA
>EECEBICD_00713 DNA repair protein RecN
ATGTTGGCGCAACTGACCATCAGCAATTTTGCTATCGTTCGCGAGCTTGAGATCGATTTT
CAGAGCGGAATGACCGTCATTACTGGTGAAACCGGCGCGGGTAAATCTATTGCGATTGAT
GCCCTGGGTCTGTGTCTGGGCGGTCGCGCTGAGGCCGATATGGTCCGTACCGGAGCGACC
CGCGCCGATTTGTGTGCCCGCTTTGCGCTGAAAGACACGCCTGCGGCATTGCGCTGGCTG
GAAGAAAACCAGCTTGAAGAAGGTCGCGAATGCTTACTTCGCCGCGTTATCAGCAGCGAT
GGCCGCTCACGGGGCTTTATTAACGGCACCGCTGTGCCCCTGTCTCAATTGCGCGAGCTC
GGTCAGTTGCTGATTCAAATTCATGGTCAACATGCGCACCAGCAGCTTACCAAACCCGAA
CAGCAAAAATCGCTGCTGGATAGCTATGCCAACGAAGCCGCTTTAGCGCAGCAGATGGCT
GCCCGCTACCAATTATGGCATCAAAGCTGCCGCGATCTTGCCCATCACCAGCAACAAAGC
CAGGAACGCGCCGCGCGCGCAGAACTGCTGCAATATCAGTTAAAAGAGTTGAACGACTTT
AATCCGCAGGCCGGTGAATTCGAGCAAATTGACGAAGAGTACAAACGGCTGGCCAACAGC
GGGCAGTTGCTTACGACCAGCCAGAACGCTCTGGCGCTACTCGCGGACGGCGAAGACGTT
AACCTGCAAAGCCAGTTATATAGCGCAAAACAACTGGTTAGCGAGCTGGTAGGTATGGAC
AGCAAGCTTTCCGGCATCCTGGATATGCTGGAAGAAGCCACTATCCAACTCACCGAAGCC
AGCGATGAATTACGCCACTATTGCGAGCGTCTGGATTTAGACCCCAATCGCCTGTTTGAG
CTTGAACAGCGCATCGCTAAACAAATTTCGCTGGCGCGAAAACATCACGTCAGCCCGGAA
GCGCTGCCGCAGCTTTATCAGTCACTGCTCGAAGAACAGCAGCAGCTCGACGATCAGGCC
GATTCGCTGGAAACGCTAACGCTGGCGGTTAATAAGCATCACCAACAAGCGCTGGAAACG
GCGCAAGCGCTGCATCAACAGCGTCAGTTTTACGCCCAGGAGCTGGGGCAGTTGATCACT
GAAAGTATGCATTTACTTTCGATGCCGCACGGTCTGTTTACTATCGACGTGAAATTTGAT
GAGCATCATCTGAGCAACGACGGCGCCGATCGCGTGGAGTTTAAAGTGACGACAAACCCG
GGTCAGCCGCTGCAGCCTATCGCTAAAGTCGCGTCTGGCGGTGAACTGTCGCGCATTGCG
CTGGCTATTCAGGTGATTACCGCGCGCAAAATGGAAACGCCGGCGCTAATTTTTGACGAG
GTCGATGTTGGCATCAGCGGCCCAACCGCCGCCGTGGTCGGTAAACTCCTGCGTCAGCTT
GGCGAATCCACTCAGGTGATGTGCGTCACTCACCTGCCACAGGTAGCCGGGTGCGGTCAC
CAGCACTTCTTTGTCAGTAAAGAAACAGACGGGGCAATGACCGAAACCCATATGCAACCG
TTGGATAAACGTGCGCGTTTACAGGAACTGGCCCGCCTGTTGGGAGGAAGTGAAGTTACG
CGTAATACGCTGGCAAACGCAAAAGAACTGCTGGCGGCGTAA
>EECEBICD_00714 NAD kinase
GTGGAGCAACAAATCGCCCACGAATTACAGCTAAAAAATGTGCCAACCGGCACGCTGGCG
GAAATTGGTCAACAGGCGGATCTGGCGGTAGTCGTGGGCGGCGACGGCAATATGCTTGGC
GCAGCGCGTACGCTGGCGCGTTATAACATCAATGTGATTGGCATTAACCGCGGTAATCTT
GGCTTCCTGACCGATCTCGATCCGGACAACGCCCTGCAACAATTATCTGATGTGCTGGAA
GGACGCTATATCTCCGAAAAACGTTTTCTGCTGGAAGCGCAGGTCTGCCAACAGGACCGC
CAGAAGCGTATCAGCACAGCCATTAATGAAGTGGTGCTGCATCCGGGAAAGGTGGCGCAT
ATGATCGAATTCGAGGTTTATATTGATGAAACCTTCGCTTTTTCACAGCGATCTGATGGT
CTGATCATTTCTACGCCGACCGGCTCCACCGCCTATTCGCTCTCCGCAGGCGGCCCTATT
CTGACCCCTTCACTGGATGCCATCACCCTGGTGCCGATGTTCCCGCATACCTTGTCGGCG
CGCCCGTTAGTCATTAACAGCAGTAGCACGATTCGTTTGCGTTTTTCGCATCGCCGTAGC
GATCTGGAAATTAGCTGCGACAGCCAGATAGCCCTGCCTATTCAGGAAGGAGAAGATGTG
CTGATTCGCCGCTGCGACTACCATCTCAACCTGATACATCCCAAAGACTACAGCTATTTC
AATACATTAAGCACAAAACTCGGCTGGTCAAAAAAATTATTCTAA
>EECEBICD_00715 Protein GrpE
ATGTGTCGTGAGTGCGGTGGGATGACGCGGATGCCCCACAATGCCGATACACTTGAAATG
ATTATTCATTTTTCTGAGGTCCTTGTAGCGAAGATTGATGACAATGTGAGTACTTCCCTT
GAAACCCTGAATCTGATCCCCATAATAAGCGAAGTTAGCGAGATGAATGCGAAAAAAAAC

GCGGAGAAATTCATGAGTAGTAAAGAACAGAAAACGCCTGAGGGGCAAGCCCCGGAAGAA
ATTATCATGGATCAGCACGAAGAGGTTGAGGCAGTTGAACCAAACGATTCTGCTGAGCAG
GTGGATCCGCGCGATGAAAAAATTGCGAATCTGGAAGTTCAGCTTGCAGAAGCCCAGACT
CGCGAACGCGACACCGTGTTGCGCATCAAAGCGGAAATGGAAAACCTGCGCCGTCGTACT
GAACAGGATATCGAAAAAGCGCATAAATTCGCTCTCGAGAAGTTCGTCAACGAATTGCTG
CCGGTCATCGATAGCCTGGATCGCGCGCTGGAAGTCGCAGACAAAGCCAATCCGGATATG
GCGGCAATGGTCGAAGGGATTGAGCTGACGCTGAAGTCTATGCTGGATGTGGTGCGTAAG
TTCGGCGTGGAAGTGATTGCTGAGACCAACGTGCCGCTGGACCCGAACGTGCATCAGGCG
ATTGCGATGGTGGAGTCGGAAGAGGTTCCGGCAGGCAATGTGCTGGGTATTATGCAGAAA
GGCTATACGCTGAACGGTCGTACCATTCGCGCGGCGATGGTGACTGTAGCGAAGGCGAAG
TAA
>EECEBICD_00716 hypothetical protein
ATGTCAGAAGATTATGTAATTGAATGGGATAAGAATTTTGCAGACGACCTCAACGTCGTC
GCCAACGTTTTTTTATCACACAACCCAACCTTATGGCCCACTATTTTCTCTCAACTTTCG
ACGCAACCTGAAATCTTTGAAGACGAAGATGAAGACGAATATGGGTTGCAGGACGTCCTG
GATTGTAGCGGTGGCGATCTCGGAAATAACGAGCTGGCACAGGCATTTTTACAAGTTCTA
CGCGGCGAAAGATTTATTCACCTCGTTGACTGGAAAGGTGAAGATGAAGAAGGCGAACTG
GCTAATTTTGCCGCAGATCGCTTTTATGAACTCACAAAAAACCTGACGAATTCTGAGGAA
TTAAGAAATCTTCTTGTCGAAATAACCCAAGAGGATGAAATCTCCGATGTCTGTGAAGCC
GGAGACCGTTACCTTGACGAGATCTTTGAACGCATCCAGACCGAACTCAATAAGAGGGGC
TTCCAGATTTTTGATCTGAATGAAGGATCTGACACCTATAATGTGGTCGTTCTGCCAATG
AGCGAATATAAAAAAATAGAGGACTTCAACACGCCGTGGCTGGAAGTGCAGGATTTTTTA
AGCTAA
>EECEBICD_00717 hypothetical protein
ATGGTGGTCATCTCGGCCTATTTTTCCGGTTCCGAAACTGGAATGATGACGCTAAACCGT
TACCGTTTACGCCATATGGCGAAGCAAGGTAATCGCTCGGCAAAACGGGTAGAAAAATTA
CTGCGTAAACCCGACCGACTGATAAGCCTGGTGCTGATCGGCAATAACCTGGTCAACATT
CTTGCCTCGGCGCTCGGCACGATTGTGGGTATGCGGCTTTACGGCGACGCTGGCGTGGCT
ATCGCCACCGGGGTACTGACGTTTGTCGTGTTAGTTTTTGCCGAAGTCCTGCCCAAAACT
ATCGCCGCGCTCTATCCGGAAAAAGTGGCCTATCCCAGCAGCTTCCTGCTGGCGCCGTTA
CAGATACTGATGATGCCGCTGGTGTGGTTACTTAACACCATCACCCGCCTGCTGATGCGT
CTGATGGGAATTAAAACGGACATCGTGGTTAGCGGGTCGTTAAGCAAAGAGGAATTACGC
ACTATCGTGCATGAATCCCGTTCGCAAATCTCTCGCCGCAATCAGGATATGCTGCTGTCG
GTTCTCGACCTGGAAAAGGTCAGCGTGGATGACATCATGGTGCCGCGTAATGAAATTATC
GGGATCGATATCAATGACGACTGGAAGTCTATCGAGCGGCAGCTCACCCACTCGCCACAC
GGACGCATTGTGCTCTATCGCGATTCGCTGGATGACGCCATCAGTATGCTGCGCGTGCGT
GAAGCCTGGCGGTTAATGGCCGAGAAAAAAGAGTTCACCAAAGAGATGATGCTGCGCGCC
GCCGATGAAATCTATTACGTCCCGGAAGGTACGCCGCTCAGTACACAACTGATTAAATTT
CAGCGCAATAAAAAGAAAGTCGGACTGGTCGTCAACGAATATGGCGATATCCAGGGGCTG
GTCACCGTGGAAGATATTCTCGAGGAGATCGTTGGCGACTTCACCACATCGATGTCGCCG
ACCCTGGCGGAAGAGGTCACGCCGCAAAACGACGGTTCGGTCATTATTGACGGTACCGCC
AACGTCCGGGAAATCAATAAAGCGTTTAACTGGCACCTGCCGGAAGACGACGCGCGCACC
GTCAACGGGGTGATTCTGGAGGCTCTGGAGGAAATTCCGGTTGCTGGCACGCGCGTGCGC
ATTGAGCAGTACGATATCGATATTCTCGATGTACAGGAAAATATGATTAAGCAGGTAAAG
GTTGTACCGGTAAAGCCGCTGCGCGAGAGTGTGGCGGAGTAA
>EECEBICD_00718 Inner membrane protein YpjD
ATGCCCGTTTTTGCTTTACTTGCCCTTGTCGCCTACTCCTTCAGCCTCGCGCTGATCGTT
CCCGGACTGTTGCAAAAAAACAGCGGCTGGCGGCGTATGGCTATTCTTTCTGCGGTCATC
GCGCTGGTGTGCCATGCTGTCGCGCTGGAATCGCGTATTCTGCCCGGCGGCGACAGTGGA
CAAAACCTGAGCCTGCTGAATGTCGGTTCGCTGGTCAGTCTGATGATCTGCACGGTGATG
ACCATTGTCGCGTCGCGTAATCGCGGCTGGCTACTTCTGCCCATTGTCTATGCTTTTGCG
CTGATTAACCTGGCCTTCGCCACGTTCATGCCTAATGAATATATTACGCATCTGGAAGCG
ACCCCAGGCATGATGGTGCACATCGGTCTGTCGCTTTTCTCTTACGCGACGCTGATAATC
GCCGCGCTGTATGCGCTACAACTGGCGTGGATCGACTATCAGTTGAAGAACAAAAAGCTG

GCGTTTAGTAATGAAATGCCGCCATTGATGAGCATTGAGCGCAAAATGTTTCATATCACG
CAGATTGGCGTCGTACTGCTGACCCTTACTCTGTGTACCGGCCTGTTTTACATGCATAAC
CTGTTCAGTACTGAAAATATTGATAAAGCCGTGCTCTCTATTGTGGCATGGTTTGTCTAT
ATCGTTCTGCTGTGGGGGCATTACCATGAGGGCTGGCGGGGTCGGCGGGTCGTGTGGTTT
AACGTCGCAGGCGCGGGTATTCTGACGCTGGCCTATTTTGGTAGCCGCATATTACAGCAA
TTTGTAAGCTAA
>EECEBICD_00719 Signal recognition particle protein
ATGTTTGATAATTTAACCGATCGTTTGTCGCGCACGCTGCGCAATATCAGTGGCCGTGGG
CGCCTTACTGAAGACAACGTTAAAGAGACGCTGCGCGAAGTGCGCATGGCGCTGCTGGAG
GCTGACGTTGCGCTGCCGGTAGTGCGTGAGTTCATCAATCGCGTAAAAGAGAAAGCGGTT
GGTCATGAAGTTAACAAGAGCCTGACCCCAGGGCAGGAGTTCGTCAAGATTGTCCGTAGC
GAACTGGTTGCGGCGATGGGCGAAGAGAACCAGACGCTGAATTTAGCTGCGCAGCCGCCA
GCCGTAGTATTGATGGCGGGTCTGCAGGGGGCGGGTAAAACCACCAGCGTCGGTAAGTTG
GGTAAATTCTTGCGCGAGAAGCACAAGAAGAAAGTGCTGGTCGTCTCTGCCGACGTCTAT
CGCCCGGCGGCGATCAAACAGCTCGAAACGCTGGCTGAACAGGTTGGCGTGGATTTCTTC
CCGTCTGATGTCGGCCAGAAACCGGTTGATATCGTCAACGCCGCGCTGAAAGAAGCGAAG
CTCAAATTCTACGACGTGCTGCTGGTGGATACCGCCGGTCGTCTGCACGTTGACGAAGCG
ATGATGGACGAAATCAAACAGGTCCACGCTTCTATCAAGCCAGTAGAAACGCTGTTTGTC
GTCGATGCGATGACCGGTCAGGATGCGGCGAATACCGCAAAAGCCTTTAACGAAGCGCTG
CCGTTAACCGGCGTGGTGCTGACCAAAGTTGATGGTGATGCTCGTGGCGGTGCGGCGCTC
TCTATTCGTCATATCACCGGCAAGCCGATTAAATTCCTCGGTGTCGGCGAGAAAACCGAC
GCGCTGGAACCGTTCCACCCGGATCGTATCGCCTCGCGTATTCTCGGTATGGGCGACGTA
CTGTCTCTTATCGAAGATATCGAAAGCAAAGTTGACCGCGCACAGGCTGAAAAGCTGGCG
ACTAAACTGAAGAAAGGCGACGGTTTCGACCTGAACGACTTCCTGGAACAGCTCAAACAG
ATGAAAAACATGGGCGGTATGGCCAGTCTGATGGGCAAATTACCGGGCATGGGGCAGATT
CCGGACAACGTTAAATCGCAGATGGATGACAAGGTGCTGGTGCGTATGGAAGCGATCATC
AATTCGATGACGCTGAAAGAGCGCGCGAAGCCGGAAATCATCAAAGGTTCCCGCAAGCGT
CGTATCGCGCAGGGCTGTGGGATGCAGGTGCAGGACGTTAACCGCCTTCTGAAACAGTTC
GACGACATGCAGCGCATGATGAAGAAGATGAAGAAGGGCGGGATGGCGAAGATGATGCGC
AGTATGAAGGGGATGATGCCGCCAGGCTTCCCGGGTCGCTAA
>EECEBICD_00720 30S ribosomal protein S16
ATGGTAACTATTCGTTTAGCTCGTCACGGCGCTAAAAAGCGTCCGTTCTACCAGGTTGTT
GTCACCGACAGCCGTAATGCACGCAACGGTCGCTTCATTGAGCGCGTTGGTTTCTTCAAC
CCAATCGCTAGCGAAAAAGAAGAAGGCACCCGCCTGGATCTGGATCGCATCGCTCACTGG
GTTGGCCAGGGCGCAACCATTTCCGATCGCGTTGCGGCGCTGATCAAAGAAGTAAAAAAA
GCAGCTTAA
>EECEBICD_00721 Ribosome maturation factor RimM
ATGAGCAAGCAACTCGCCGCACAAGTACCCGCTGAACCGGTTGTACTGGGGAAAATGGGT
TCTTCATACGGTATCCGTGGTTGGCTCAGAGTGTTTTCCTCCACTGAAGACGCCGAAAGC
ATTTTTGACTATCAGCCCTGGTTTATCCAGAAGGCGGGTCAGTGGCAGCAGGTACAGCTG
GAAAGCTGGAAGCACCACAATCAGGATCTGATCATCAAGCTGAAAGGCGTTGACGATCGA
GATGCCGCGAATCTTCTGACCAATTGCGAAATTGTCGTGGATTCCTCGCAACTTCCTGCG
CTTGAGGAGGGAGACTATTACTGGAAAGACCTGATGGGTTGCCAGGTAGTCACCGCTGAA
GGCTACGATCTCGGTAAAGTTATCGACATGATGGAAACCGGGTCGAATGACGTTCTCGTC
ATTAAGGCGAACCTGAAAGATGCATTTGGTATCAAGGAACGACTAGTACCGTTCCTCGAT
GGGCAGGTTATCAAGAAAGTCGATCTCGCTACTCGTACTATCGAAGTAGACTGGGATCCT
GGTTTTTAA
>EECEBICD_00722 tRNA (guanine-N(1)-)-methyltransferase
GTGTTTATTGGCATCGTTAGCCTGTTTCCTGAAATGTTCCGCGCAATTACCGATTACGGG
GTAACTGGCCGGGCAGTAAAAAAAGGCCTGCTGAACATCCAAAGCTGGAGTCCTCGCGAC
TTCGCGCATGACCGGCACCGTACCGTGGACGACCGTCCTTACGGCGGCGGACCGGGGATG
TTAATGATGGTGCAACCCTTGCGGGACGCCATTCACGCAGCAAAAGCCGCGGCAGGTGAA
GGCGCTAAAGTGATTTATCTGTCGCCTCAGGGACGCAAGCTTGATCAAGCGGGCGTTAGC
GAGCTGGCCACGAATCAGAAGCTTATTCTGGTGTGTGGTCGCTACGAAGGCGTAGATGAG

CGCGTAATTCAGACCGAAATTGACGAAGAATGGTCAATTGGCGATTACGTTCTCAGCGGT
GGCGAACTACCGGCAATGACGCTGATTGACTCCGTCGCCCGGTTTATACCGGGGGTTCTG
GGGCATGAGGCATCAGCAATCGAAGATTCGTTTGCTGATGGGTTGCTGGATTGTCCGCAC
TATACGCGCCCTGAAGTGTTAGAGGGGATGGAAGTACCGCCAGTATTGCTGTCGGGAAAC
CATGCCGAGATACGTCGCTGGCGCTTGAAACAGTCGCTGGGCCGAACCTGGCTTAGAAGA
CCTGAACTTCTGGAAAACCTGGCTCTGACTGAAGAGCAAGCAAGGTTGCTGGCGGAGTTC
AAAACAGAACACGCACAACAGCAGCATAAACATGATGGGATGGCATAG
>EECEBICD_00723 50S ribosomal protein L19
ATGAGCAACATTATTAAGCAACTTGAACAAGAGCAGATGAAGCAGAACGTACCTTCCTTC
CGTCCGGGCGATACCGTGGAAGTGAAAGTATGGGTTGTTGAAGGTACCAAGAAACGTCTG
CAGGCATTCGAGGGCGTGGTTATCGCTATTCGTAACCGCGGTCTGCACTCTGCATTCACT
GTTCGTAAAATTTCCAACGGCGAAGGCGTTGAGCGTGTCTTCCAGACTCACTCTCCGGTA
GTTGACAGCATTGCTGTTAAACGTCGTGGTGCTGTTCGTAAAGCTAAACTGTACTACCTG
CGTGAGCGTACTGGTAAAGCGGCTCGTATCAAAGAGCGTCTTAACTAA
>EECEBICD_00724 Diguanylate cyclase DgcN
ATGAATAAGGAATTTTCTCTGTCCAGACCAACATTTAAACGCACACTACGGCGGATTAGT
ATAATCAGCGTGCTGCTTACAATGACATTGATCTGGCTATTAATTTGCGTTGCGTCTGTC
CTTACGCTCAAACAGTATGCGCAAAAAAATCTCGATTTGACCGCCGCCACAATGGCCCAT
AGCCTTGAAGCGGCACTGGTATTTTCCGATAACGCGGCCGCAGCGGAAACGCTCGCCACA
CTGGGACGCCAGGGACAATTTTCAGCGGCGGAGGTCCGCGATAAAAATGGCCGTACTATC
GCCTCATGGCGCTATGATGCGCGAGCCGCAGACGATAAGCTCATCGGCTTAATTAGCCAC
TGGCTTTTTCCATTGCCGGTATCACAACCCGTCTGGCACAACGGCAGGGCCATCGGCGAA
GTACGGCTTGTCGCCCGCGACAGCCTTATTGGTCATTTTATCTGGCTATCGCTGGCAGTG
CTGACAGGATGTATTCTGCTGGCATCCGGCATTGCCCTGCTGCTCACGCGTTATTTGCAC
AATGGCGTTGTGGATGCGCTGCAAAATATTACTGAAGTTGTACACGACGTTCGCACTAAC
CGAAATTTTTCACGCCGGGTACCTGATGAGCGTATTGCGGAATTTCACCTGTTTGCGCAG
GATTTCAATAGCCTTCTGGATGAGATGGAAGAATGGCAGCTACGGCTTCAGGCTAAAAAT
GCCCAGTTACTACGTACCGCGCTGCACGATCCGCTGACGGGGCTTGCCAATCGCGCGGCA
TTTCGCAGCTGTATTAACGCGCTGATGAAGGACAATTCCGCTCGTAGCAGTTCGGCATTG
TTATTTCTGGATGGCGATAACTTTAAATATATTAATGATACCTGGGGACATGCGGCAGGC
GACCGCGTACTTATAGAGGTTGCCAAAAGATTAGCGGAATTCGGTGGTAGCCGTTATCAA
ACTTACCGACTCGGCGGCGATGAATTTGCGATGGTGCTTTACGATGTACATTCGGAGTAT
GAAGTACAACGTATTTGCGCAGCGCTATCCCAGGCGTTTAATCGACCTTTTGAACTGCAT
AACGGCCAGCGGATAACGATGACCCTGAGTATTGGCTTTGCGCTGACATGGGAACATGCC
ACTGCCGAAAAACTACAAGAACTGGCCGATCGAAATATGTATCAGGCTAAACACCGGCGT
GCGGAACGCTCGCTAAATTAA
>EECEBICD_00725 hypothetical protein
ATGCGTTTTTCTCACCGATTTATCCTTCTGTTATCGCTATTATTAGCAAGCTTGCCTCTG
TATGCTCAACGCGTCACGGAAGAAGAGAAATCCGTACGCGCCATCGTCTCCGGTATCGTC
AGTTATACACACTGGCCGGCGTTATCCGGGCCGCCAAGGCTATGCATATTTTCATCTGCG
CGTTTTGTCAGGGTACTTAGCGAAGAGGCTGATTGGGCTTTCCCTTATCAACCGCTTGTC
ATCCGTACCACACAAGAAGCCCTGAGCGCCCGCTGTGATGGTTTTTACTTTGGCAATGAA
TCGCCAGCTTATCAGGTGGAGTTAACGCGTCATTATCCAGTTAATGCCTTGTTATTAATT
GCCGAGCAAAATACCGAATGTATTATTGGCAGCGCATTTTGTCTGATCATTAACAATGAT
GAAGTCAAATTTTCCGTCAACCTGGACTCCCTCTCGCATAGCGGCGTGAGAGTAAATCCA
GAAGTATTAATGCTTGCACGGAATCAAAAGCATGAATAA
>EECEBICD_00726 Phospho-2-dehydro-3-deoxyheptonate aldolase, Tyr-sensitive
ATGCAAAAAGACGCGCTGAATAACGTACGTATCACCGATGAACAGGTATTAATGACGCCG
GAGCAGCTTAAAGCGGCCTTTCCGTTGAGCCTGGCGCAGGAAGCGCAGATAGCGCAGTCC
CGGGGAATCATTTCTGACATTATTGCCGGGCGCGATCCGCGTCTGTTGGTGGTATGCGGT
CCTTGTTCTATTCACGATCCTGAAACCGCTCTGGAATATGCCCGTCGATTTAAAGCCCTT
GCCGCAGAGGTCAGCGATAGCCTCTATCTGGTAATGCGCGTCTATTTTGAAAAGCCGCGA
ACTACCGTCGGCTGGAAAGGGCTGATTAACGATCCTCACATGGATGGCTCATTTGATGTG

GAAGCCGGGTTGAAAATAGCGCGTCAGCTACTGGTGGAACTGGTGAATATGGGGTTGCCA
TTGGCGACCGAAGCGTTGGATCCGAACAGCCCGCAATACCTGGGCGATCTGTTTAGCTGG
TCGGCGATAGGCGCGCGCACAACCGAATCGCAAACCCACCGCGAAATGGCGTCTGGTCTT
TCTATGCCGGTCGGCTTTAAAAACGGCACGGATGGCAGCCTGGCGACAGCGATTAACGCC
ATGCGCGCCGCTGCGCAACCTCATCGTTTTGTTGGCATTAACCAGGCCGGTCAGGTTGCG
TTATTGCAAACCCAGGGAAATCCGCATGGCCATGTGATTTTGCGTGGCGGCAAAGCGCCA
AACTATAGCCCGGCAGATGTCGCTCAGTGTGAAAAAGAGATGGAACAGGCGGGACTACGT
CCTTCGCTGATGGTAGATTGCAGTCATGGTAACTCCAATAAAGATTATCGCCGCCAGCCA
GCCGTTGCCGAATCTGTGGTTGCGCAGATTAAAGATGGCAATCGTTCAATCATTGGCTTA
ATGATTGAAAGTAATATTCATGAGGGTAATCAGTCTTCCGAACAGCCGCGCAGCGAAATG
AAGTATGGCGTTTCCGTCACCGATGCTTGTATTAGCTGGGAGATGACCGATGCCCTGTTA
CGTGAAATTCATAAAGATTTGAGCGGCCAGCTGGCGGTGCGCGTCGCATAA
>EECEBICD_00727 T-protein
ATGGTTGCTGAATTGACCGCGTTACGCGATCAAATAGATGATGTCGATAAAGCGTTGTTG
AATTTACTGGCTAAGCGCCTGGAACTGGTTGCCAAAGTCGGCGAGGTGAAAAGCCGTTTT
GGCCTGCCTATTTACGTGCCGGAGCGTGAGGCCTCTATGCTGGCTTCACGACGGGCGGAA
GCAGAAGCGATCGGTGTCCCGCCCGATCTCATTGAAGATGTCCTGCGCCGGGTAATGCGT
GAATCTTACTCCAGCGAAAATGATAAGGGGTTCAAAACGCTTTGTCCTTCTCTGCGTCCG
GTCGTCATTGTGGGCGGCGGCGGACAGATGGGGCGTCTGTTTGAAAAAATGCTCACGCTG
TCGGGCTATCAGGTCCGTATTCTGGAACAGCAGGACTGGCCGCGCGCCAGGGACATTGTC
GCCGATGCCGGAATGGTGATCGTCAGCGTGCCGATTCATGTTACTGAACAGGTCATAGCG
CAACTGCCGCCCCTGCCGTCCGACTGTATTCTGGTCGATCTGGCATCGGTGAAAAGCGAT
CCGTTACAGGCAATGTTGGCGGCCCATGATGGCCCCGTGTTGGGCTTGCATCCGATGTTT
GGCCCGGACAGCGGGAGCCTGGCGAAGCAGGTGGTGGTCTGGTGTGACGGGCGTCAACCG
GAAGCGTATCAGTGGTTCCTTGAGCAAATCCAGGTGTGGGGCGCTCGGTTGCACCGAATT
AGCGCTGTCGAGCACGATCAGAACATGGCTTTTATCCAGGCGTTGCGCCATTTTGCTACC
TTCGCTTATGGGCTGCATCTGGCGGAAGAGAACGTCCAGCTTGAGCAGCTTCTGGCGCTA
TCATCGCCGATTTATCGACTGGAGCTGGCGATGGTCGGGCGTCTGTTCGCCCAGGACCCG
CAGCTGTATGCGGACATTATTATGTCGTCGGAGCGCAATCTGGCGCTTATCAAGCGTTAC
TATAAACGTTTTGGCGATGCGATCGGGTTACTGGAACAAGGTGATAAGCAGGCTTTTATC
GACAGTTTTCGCAAAGTTGAACACTGGTTTGGCGATTATGCCAGACGCTTCCAGAATGAA
AGCCGTGTGTTATTGCGTCAGGCGAATGACAGCCGACCATAA
>EECEBICD_00728 6-deoxy-6-sulfogluconolactonase
ATGACTACGCCGCACGTTTTATTTGATTATGCCGGACATTTACCAGAATGCCCCACATGG
AGCGAGGATGAAAGCGCGCTGTATTGGACCGATATCCTGGAACAGGAGATCCATCGTTAC
CATCCGGCGAGCGGGACGCATAGCGTGCTGGCGTTTCCGGAAGAGGTGGGCTGTTTTGCC
TTGCGTGAGCAGGGCGGATTTATTGTGGCCATGCGTCATGCTATCTGGCTGGCCGATAAA
AATGGATTATTGCAGCGTAAGGTTTGCGACAATCCCTCTAATACGAAACTGGCGCGTTTC
AACGATGGCGGCACTGATGGCGATGGACGTTTTTATGCCGGAACGTTCTGGGCGCCAGGT
GACTATAATGGCGCGTTGCTGATGCGGATCGATCATGACCTGACGGCAAAAGTGATCCAG
TGCGATATCCAGGGACACAACGGCCTGGCGTTCAGCCCGGATAACCAGTGGATGTATACC
TCCGATACGCCGAATGGCGTGATATACCGCACGTTACTTGATAAGCATGGCGATCCGGGC
AAGCGTGAACTTTTTCGCCATTTTGGCGAAGGAGAAGGACTTCCCGACGGCGCGGCGATG
GATAGCGAAGGGTGCTACTGGAGCGCGATGTTTGACGGCTGGTGCGTGGCGCGTTTCTCA
CCGCAAGGAGAGCAGCTGGAAGAGTACCGGTTGCCGGTACGTTGTCCGACGATGGTTTGC
TTTGGCGGCGCAGATATGAAAACGCTGTTTATTACAACTACCAGAGAAAATATGTCTGCT
CAGGAAGTGGCGGATTATCCGCTCTCCGGCGCCATCTTCACCCTGCAGGTAGCCGTCGCA
GGGATGAAGAAAAGCCGTTTTATTGAACGTCAGGCAGGTTCTACCGGCACGACGTTTTCG
CTGGGATAG
>EECEBICD_00729 Bifunctional chorismate mutase/prephenate dehydratase
ATGACATCGGAAAACCCATTACTGGCGCTGCGAGATAAAATCAGCGCTTTAGACGAAGAG
TTACTGGCCTTACTGGCAAAACGACGCGCGCTGGCGATTGAAGTGGGACAAGCAAAACTA
CTGTCGCATCGTCCGGTTCGGGATATCGATCGTGAACGCGCGCTGCTGGACAGACTCATC
CATCTCGGTAAAGCCCACCATCTCGACGCACACTACATTACCCGTCTGTTCCAGCTTATC

ATTGAAGACTCCGTGCTTACTCAGCAGGCGCTGCTGCAACAACATCTGAATAATACTCAC
CCTCATTCGGCACGTATTGCGTTTCTTGGGCCGAAAGGCTCCTATTCTCATCTCGCGGCG
CGCCAGTATGCTGCACGCCATTTTGAGCAATTTATTGAGAGCGGCTGCGCAAAATTCGCC
GATATTTTTCATCAGGTCGAAACCGGCCAGGCCGATTACGCCGTGGTTCCGATAGAGAAC
ACCAGCTCCGGCGCTATCAACGATGTGTACGACTTATTGCAACACACCAGTCTGTCGATT
GTCGGTGAGATGACTGTCACTATCGATCACTGCGTGCTGGTTTCCGGCGCTACAGATCTG
AATACCATCGAAACGGTGTACAGCCATCCGCAGCCGTTTCAGCAGTGCAGTAAATTTTTG
AGCCGCTATCCGCACTGGAAAATCGACTATACCGAGAGTACGTCGGCAGCGATGGAAAAA
GTCGCGCAGGCAAACTCTCCGCGCGTCGCGGCGCTCGGCAGCGAGGCAGGCGGCATGTTG
CACGGTTTACAGGTGCTGGAACGCATTGCCGCAAACCAGACGCAGAATATCACCCGCTTT
CTGGTACTGGCGCGCAAAGCCATCAACGTTTCCGATCAGGTTCCGGCAAAAACCACTCTG
TTAATCGCCACCGGGCAGCAAGCTGGCGCGCTGGTCGAAGCGCTGCTGGTGCTGCGTAAC
CACAATCTCATCATGACGAAACTGGAGTCGCGCCCCATTCACGGCAATCCGTGGGAAGAG
ATGTTTTATCTCGATATTCAGGCGAACCTGGAGTCGCAGGTAATGCAAAGCGCGCTAAAA
GAGCTGGGCGAGATCACGCGCTCAATGAAAGTGCTTGGCTGCTATCCCAGCGAAAACGTC
GTGCCGGTAGAACCTGCCTGA
>EECEBICD_00730 Ribosome-associated inhibitor A
ATGACAATGAACATTACCAGTAAACAAATGGAAATTACTCCGGCAATCCGCCAACATGTC
GCAGACCGTCTCGCCAAACTGGAAAAATGGCAAACTCACCTGATTAATCCTCATATCATT
CTGTCTAAAGAGCCACAGGGCTTTATTGCTGACGCCACCATTAATACACCGAACGGACAT
CTGGTCGCCAGCGCAAAACACGAAGATATGTACACCGCTATTAATGAATTGATCAACAAG
CTGGAACGGCAGCTCAATAAAGTGCAGCATAAAGGCGAAGCCCGTCGTGCCACCGCATCG
GTGAAAGATGCCAGCTTCGTCGAAGCAGAAGAAGAGTAG
>EECEBICD_00731 Outer membrane protein assembly factor BamD
ATGACGCGCATGAAATATCTGGTGGCAGCAGCCACGTTGAGCCTGTTTCTGGCGGGTTGC
TCGGGTTCAAAGGAAGAGGTGCCCGATAATCCGCCGAATGAAATCTACGCGACTGCTCAG
CAAAAGCTGCAGGACGGTAACTGGAAACAGGCAATAACGCAATTGGAAGCGTTAGATAAT
CGTTATCCATTCGGACCGTATTCTCAGCAGGTGCAGTTGGATCTTATCTACGCCTATTAC
AAAAACGCCGATTTGCCGCTAGCGCAGGCCGCCATCGATCGTTTTATGCGTCTTAATCCA
ACGCACCCTAATATTGACTATGTCATGTACATGCGCGGCCTGACGAACATGGCGCTGGAT
GATAGCGCGCTGCAAGGTTTCTTTGGCGTCGATCGCAGCGATCGCGATCCGCAACATGCG
CGGGCGGCGTTCAATGACTTTTCGAAACTGGTACGCAGCTATCCTAATAGCCAGTACACC
ACCGACGCCACTAAGCGTCTGGTATTCCTGAAAGACCGTCTGGCGAAATATGAATATTCC
GTCGCAGAGTACTATACCGCGCGCGGCGCATGGGTTGCGGTCGTAAACCGGGTAGAAGGT
ATGCTACGCAATTATCCTGATACGCAGGCCACGCGCGATGCGTTGCCGCTGATGGAAAAC
GCCTATCGTCAGATGCAGCTGAATGCGCAGGCTGACAAAGTCGCGAAAATTATTGCCGCT
AACAGCAAAAACACCTGA
>EECEBICD_00732 Ribosomal large subunit pseudouridine synthase D
ATGGCACAACGAGTACAACTCACCGCAACGGTCTCCGAAAACCAACTCGGTCAACGCTTA
GATCAGGCTTTGGCCGAAATGTTCCCGGATTATTCACGTTCGCGTATAAAAGAATGGATT
TTGAATCAGCGCGTGTTGGTCAATGGTCAACTTTGCGATAAGCCAAAAGAAAAAGTGTTA
GGCGGCGAGCGGGTGGCTATTGATGCGGAGATCGACGAAGAGATTCGTTTTGAAGCGCAG
GATATCCCGTTAGATATTGTTTATGAAGATGACGATATTCTGGTTATCAACAAACCACGC
GATCTGGTGGTACATCCTGGCGCGGGTAACCCGGACGGTACGGTACTGAACGCGTTGCTA
CATTATTATCCGCCGATTGCGGATGTCCCGCGCGCGGGCATTGTGCATCGTCTGGATAAG
GACACGACGGGGCTGATGGTGGTGGCCAAAACCGTTCCGGCGCAAACGCGCCTGGTGGAG
TCTTTGCAGCTGCGCGAGATCACCCGCGAATATGAAGCGGTCGCGATCGGACATATGACG
GCGGGAGGAACGGTGAATGAACCCATCAGTCGTCACCCGACCAAACGTACCCATATGTCG
GTGCATCCCATGGGCAAACCGGCGGTGACCCACTACCGCATTATGGAACATTTCCGTGTG
CACACGCGTCTTAGGTTGCGTCTGGAGACCGGGCGTACGCACCAGATCCGCGTGCATATG
GCGCATATTACGCACCCGTTAGTGGGCGATCAGGTCTACGGTGGTCGTCCACGTCCACCG
AAAGGCGCATCGGAAGAATTTATTTCCACGCTGCGTAAGTTTGACCGCCAGGCGCTGCAT
GCAACAATGCTGCGTCTCTACCATCCTGTATCGGGTATCGAAATGGAGTGGCACGCGCCG
ATTCCACAAGATATGGTGGACCTTATCGATGCGATGCGCGCCGATTTTGAAGATCATAAA

GATGATGTGGACTGGTTATGA
>EECEBICD_00733 Polyphenol oxidase
ATGAATGCACTGATTGTCCCGCAGTGGCCGCTGCCGAAAGGCGTCGCAGCCTGTAGTTCT
ACCCGTATTGGCGGGGTGAGTTTACCGCCTTACGACTCGCTGAATCTGGGCGCGCATTGC
GGCGATAACCCGGAGCATGTTGAAGAGAACCGCAAGCGTCTGTTTGCGGCGGGCAATCTG
CCTTCGAAACCTGTCTGGCTTGAACAGGTTCACGGTAAAAATGTGTTGAGGCTTACCGGA
GAACCTTATGCCTCCAAACGTGCGGATGCGTCTTACAGCAATACGCCAGGAACCGTCTGC
GCGGTAATGACGGCAGACTGCCTGCCTGTGCTATTCTGTAACCGTGAGGGAACAGAAGTG
GCGGCGGCTCATGCGGGCTGGCGCGGATTATGTGAAGGCGTACTGGAGGAAACTGTCACC
TGCTTTGCTGACAAACCAGAAAATATCATCGCCTGGTTAGGTCCGGCGATCGGCCCGACC
GCGTTTGAAGTGGGGCCGGAAGTGCGTGACGCATTTTTAGCAAAAGATGCGCAAGCCGAT
AGCGCATTTTTGCCGCACGGGGAAAAATTTCTGGCGGATATTTATCAGCTTGCATGCCAA
CGTCTGGCGAATACCGGCGTTGAACATGTCTATGGCGGGGATCGCTGCACCTTTAGCGAA
AGTGAGACTTTCTTCTCTTATCGTCGCGACAAGACGACAGGTCGTATGGCAAGTTTTATT
TGGCTGATATAA
>EECEBICD_00734 Chaperone protein ClpB
ATGCGTCTGGATCGTCTTACTAACAAATTCCAGCTTGCTCTTGCCGATGCCCAGTCGCTC
GCGCTGGGGCACGACAACCAATTCATCGAACCTCTTCATTTAATGAGCGCCTTGCTGAAC
CAGGAAGGGGGATCGATACGTCCTTTATTAACCTCCGCCGGCATTAATGCTGGCCAGTTG
CGCACCGCTATCGATCAGGCGCTGAGCCGTTTACCGCAGGTGGAAGGCACCGGCGGCGAC
GTACAGCCTTCTTCGGAACTGGTACGCGTACTGAACCTTTGCGACAAGCTGGCGCAAAAA
CGGGGAGACAATTTTATTTCGTCAGAGCTGTTTGTTCTGGCGGCGCTTGAGTCTCGCGGC
ACGTTAACCGATCTGCTGAAATCAGCCGGTGCGACCACGGCCAATATCACTCAGGCAATT
GAACAGATGCGCGGAGGTGAAAGCGTGAACGATCAAGGGGCTGAAGACCAACGTCAGGCC
TTGAAAAAATATACCGTCGATCTGACCGAGCGGGCTGAACAAGGCAAGCTTGACCCGGTA
ATTGGTCGTGATGAAGAAATTCGCCGTACCATTCAGGTACTGCAACGTCGTACCAAAAAC
AACCCGGTGTTAATCGGTGAGCCAGGGGTCGGTAAAACGGCAATTGTTGAAGGACTGGCG
CAGCGCATTATTAACGGTGAAGTCCCTGAAGGCTTAAAAGGTCGCCGCGTACTGGCGCTG
GATATGGGCGCGCTGGTGGCGGGGGCGAAATATCGCGGCGAATTTGAAGAGCGTCTGAAA
GGCGTGCTGAACGATCTGGCGAAACAGGAAGGCAACGTCATCCTGTTTATTGACGAGCTG
CACACGATGGTTGGCGCAGGTAAGGCTGATGGCGCCATGGACGCCGGGAATATGCTGAAA
CCGGCGCTGGCGCGTGGTGAACTGCACTGTGTGGGCGCCACCACGCTGGATGAATACCGT
CAGTACATTGAAAAAGACGCGGCGCTGGAACGTCGTTTTCAAAAAGTGTTTGTCGCAGAA
CCTTCAGTGGAAGACACCATCGCTATTCTGCGTGGCCTGAAAGAACGTTACGAGCTGCAC
CACCATGTGCAGATCACTGACCCGGCGATTGTGGCGGCGGCAACATTATCTCACCGCTAT
ATCGCCGACCGTCAGTTGCCGGATAAAGCTATCGACCTCATCGATGAAGCGGCATCCAGC
ATTCGTATGCAGATTGACTCTAAGCCGGAGGAGCTGGACAGACTCGACCGCCGGATTATT
CAGCTCAAACTGGAACAGCAGGCGTTGATGAAAGAGTCTGACGAGGCGAGTAAAAAACGT
CTCGATATGCTCAACGAAGAACTGGACGATAAAGAGCGTCAGTACTCTGAGCTGGAAGAA
GAGTGGAAAGCGGAAAAAGCATCGCTTTCTGGGACGCAAACTATTAAAGCGGAGCTGGAG
CAGGCGAAGATCGCCATCGAGCAGGCGCGTCGCGTTGGCGACCTGGCGCGAATGTCTGAA
CTGCAGTACGGCAAAATTCCGGAGCTGGAAAAACAGCTGGAAGCCGCGACCCAGTCGGAA
GGTAAAACCATGCGTCTGTTACGTAACAAAGTAACGGATGCGGAAATTGCCGAAGTGCTG
GCGCGCTGGACCGGTATTCCGGTTGCCAGAATGCTGGAAGGCGAACGTGAAAAACTGTTG
CGTATGGAACAAGAGTTACACAGCCGTGTGATCGGGCAGAATGAAGCCGTTGAAGCAGTA
TCGAACGCCATACGTCGTAGCCGTGCTGGGCTGTCCGATCCGAACCGTCCGATTGGTTCC
TTCCTGTTCCTGGGGCCGACGGGGGTCGGTAAAACCGAACTGTGTAAAGCGCTGGCTAAC
TTTATGTTCGACAGCGACGACGCGATGGTGCGTATCGATATGTCCGAGTTTATGGAGAAA
CACTCCGTGTCTCGTTTGGTCGGGGCGCCTCCGGGATATGTCGGCTATGAAGAAGGCGGT
TATTTGACGGAAGCGGTACGCCGTCGTCCTTATTCCGTCATCTTGTTGGATGAAGTAGAA
AAAGCGCATCCGGATGTATTCAACATTCTGTTGCAGGTGCTGGACGACGGTCGGCTGACT
GACGGGCAGGGGAGAACGGTCGACTTCCGTAATACGGTGGTCATTATGACTTCTAACCTC
GGTTCCGATCTCATTCAGGAGCGCTTTGGCGAACTGGATTACGGTCGAATGAAAGAGATG
GTGCTGGGTGTGGTTAGCCAAAACTTCCGTCCGGAATTTATCAACCGTATTGATGAAGTT

GTGGTATTCCATCCGTTAGGTGAGCAACACATCGCTTCTATTGCTCAGATCCAGCTGCAG
CGTCTGTACAAACGTCTGGAAGAACGTGGTTATGAAATCCATATCTCCGACGAGGCGCTG
AAACTGTTGAGCGCCAACGGTTACGATCCGGTCTACGGGGCGCGTCCGTTAAAACGTGCT
ATTCAACAGCAGATCGAAAACCCACTGGCGCAGCAAATCCTGTCTGGTGAACTGGTTCCT
GGCAAAGTGATTCGCCTTGAAGCCAGTGATGATCGTATTGTGGCAGTGCAGTAA
>EECEBICD_00735 16S ribosomal RNA
TTGAAGAGTTTGATCATGGCTCAGATTGAACGCTGGCGGCAGGCCTAACACATGCAAGTC
GAACGGTAACAGGAAGCAGCTTGCTGCTTCGCTGACGAGTGGCGGACGGGTGAGTAATGT
CTGGGAAACTGCCTGATGGAGGGGGATAACTACTGGAAACGGTGGCTAATACCGCATAAC
GTCGCAAGACCAAAGAGGGGGACCTTCGGGCCTCTTGCCATCAGATGTGCCCAGATGGGA
TTAGCTTGTTGGTGAGGTAACGGCTCACCAAGGCGACGATCCCTAGCTGGTCTGAGAGGA
TGACCAGCCACACTGGAACTGAGACACGGTCCAGACTCCTACGGGAGGCAGCAGTGGGGA
ATATTGCACAATGGGCGCAAGCCTGATGCAGCCATGCCGCGTGTATGAAGAAGGCCTTCG
GGTTGTAAAGTACTTTCAGCGGGGAGGAAGGTGTTGTGGTTAATAACCGCAGCAATTGAC
GTTACCCGCAGAAGAAGCACCGGCTAACTCCGTGCCAGCAGCCGCGGTAATACGGAGGGT
GCAAGCGTTAATCGGAATTACTGGGCGTAAAGCGCACGCAGGCGGTCTGTCAAGTCGGAT
GTGAAATCCCCGGGCTCAACCTGGGAACTGCATTCGAAACTGGCAGGCTTGAGTCTTGTA
GAGGGGGGTAGAATTCCAGGTGTAGCGGTGAAATGCGTAGAGATCTGGAGGAATACCGGT
GGCGAAGGCGGCCCCCTGGACAAAGACTGACGCTCAGGTGCGAAAGCGTGGGGAGCAAAC
AGGATTAGATACCCTGGTAGTCCACGCCGTAAACGATGTCTACTTGGAGGTTGTGCCCTT
GAGGCGTGGCTTCCGGAGCTAACGCGTTAAGTAGACCGCCTGGGGAGTACGGCCGCAAGG
TTAAAACTCAAATGAATTGACGGGGGCCCGCACAAGCGGTGGAGCATGTGGTTTAATTCG
ATGCAACGCGAAGAACCTTACCTGGTCTTGACATCCACAGAAGAATCCAGAGATGGATTT
GTGCCTTCGGGAACTGTGAGACAGGTGCTGCATGGCTGTCGTCAGCTCGTGTTGTGAAAT
GTTGGGTTAAGTCCCGCAACGAGCGCAACCCTTATCCTTTGTTGCCAGCGGTTAGGCCGG
GAACTCAAAGGAGACTGCCAGTGATAAACTGGAGGAAGGTGGGGATGACGTCAAGTCATC
ATGGCCCTTACGACCAGGGCTACACACGTGCTACAATGGCGCATACAAAGAGAAGCGACC
TCGCGAGAGCAAGCGGACCTCATAAAGTGCGTCGTAGTCCGGATTGAGTCTGCAACTCGA
CTCCATGAAGTCGGAATCGCTAGTAATCGTGGATCAGAATGCCACGGTGAATACGTTCCC
GGGCCTTGTACACACCGCCCGTCACACCATGGGAGTGGGTTGCAAAAGAAGTAGGTAGCT
TAACCTTCGGGAGGGCGCTTACCACTTTGTGATTCATGACTGGGGTGAAGTCGTAACAAG
GTAACCGTAGGGGAACCTGCGGTTGGATCACCTCCTT
>EECEBICD_00736 tRNA-Glu(ttc)
GTCCCCTTCGTCTAGAGGCCCAGGACACCGCCCTTTCACGGCGGTAACAGGGGTTCGAAT
CCCCTAGGGGACGCCA
>EECEBICD_00737 23S ribosomal RNA
TTAAGCGACTAAGCGTACACGGTGGATGCCCTGGCAGTCAGAGGCGATGAAGGGCGTACT
AATCTGCGATAAGCGCCGGTAAGGTGATATGAACCGTTATAACCGGCGATACCCGAATGG
GGAAACCCAGTGTGATTCGTCACACTATCATTAACTGAATCCATAGGTTAATGAGGCGAA
CCGGGGGAACTGAAACATCTAAGTACCCCGAGGAAAAGAAATCAACCGAGATTCCCCCAG
TAGCGGCGAGCGAACGGGGAGGAGCCCAGAGCCTGAATCAGCATGTGTGTTAGTGGAAGC
GTCTGGAAAGGCGCGCGATACAGGGTGACAGCCCCGTACACAAAAGCGCATGTGCTGTGA
GCTCGATGAGTAGGGCGGGACACGTGGTATCCTGTCTGAATATGGGGGGACCATCCTCCA
AGGCTAAATACTCCTGACTGACCGATAGTGAACCAGTACCGTGAGGGAAAGGCGAAAAGA
ACCCCGGCGAGGGGAGTGAAAAAGAACCTGAAACCGTGTACGTACAAGCAGTGGGAGCAC
AGGTTTACCTGTGTGACTGCGTACCTTTTGTATAATGGGTCAGCGACTTATATTCTGTAG
CAAGGTTAACCGAATAGGGGAGCCGGAGGGAAACCGAGTCTTAATTGGGCGTTAAGTTGC
AGGGTATAGACCCGAAACCCGGTGATCTAGCCATGGGCAGGTTGAAGGTTGGGTAACACT
AACTGGAGGACCGAACCGACTAATGTTGAAAAATTAGCGGATGACCTGTGGCTGGGGGTG
AAAGGCCAATCAAACCGGGAGATAGCTGGTTCTCCCCGAAAGCTATTTAGGTAGCGCCTC
GTGAATTCATCTCCGGGGGTAGAGCACTGTTTCGGCTAGGGGGCCATCCCGGCTTACCAA
CCCGATGCAAACTGCGAATACCGGAGAATGTTATCACGGGAGACACACGGCGGGTGCTAA
CGTCCGTCGTGAAGAGGGAAACAACCCAGACCGCCAGCTAAGGTCCCAAAGTCATGGTTA
AGTGGGAAACGATGTGGGAAGGCCCAGACAGCCAGGATGTTGGCTTAGAAGCAGCCATCA

TTTAAAGAAAGCGTAATAGCTCACTGGTCGAGTCGGCCTGCGCGGAAGATGTAACGGGGC
TAAACCATGCACCGAAGCTGCGGCAGCGACGCTTATGCGTTGTTGGGTAGGGGAGCGTTC
TGTAAGCCTGCGAAGGTGGCCTGTGAGGGTTGCTGGAGGTATCAGAAGTGCGAATGCTGA
CATAAGTAACGATAAAGCGGGTGAAAAGCCCGCTCGCCGGAAGACCAAGGGTTCCTGTCC
AACGTTAATCGGGGCAGGGTGAGTCGACCCCTAAGGCGAGGCCGAAAGGCGTAGTCGATG
GGAAACGGGTTAATATTCCCGTACTTGGTGTTACTGCGAAGGGGGGACGGAGAAGGCTAT
GTTGGCCGGGCGACGGTTGTCCCGGTTTAAGCGTGTAGGTGTGTGTTCCAGGTAAATCCG
GTTCACTTTAACACTGAGGCGTGACGACGAGGCACTACGGTGCTGAAGCAACAAATGCCC
TGCTTCCAGGAAAAGCCTCTAAGCATCAGGTAACATCAAATCGTACCCCAAACCGACACA
GGTGGTCAGGTAGAGAATACCAAGGCGCTTGAGAGAACTCGGGTGAAGGAACTAGGCAAA
ATGGTGCCGTAACTTCGGGAGAAGGCACGCTGACACGTAGGTGAAGTGATTTACTCATGG
AGCTGAAGTCAGTCGAAGATACCAGCTGGCTGCAACTGTTTATTAAAAACACAGCACTGT
GCAAACACGAAAGTGGACGTATACGGTGTGACGCCTGCCCGGTGCCGGAAGGTTAATTGA
TGGGGTCAGCGCAAGCGAAGCTCCTGATCGAAGCCCCGGTAAACGGCGGCCGTAACTATA
ACGGTCCTAAGGTAGCGAAATTCCTTGTCGGGTAAGTTCCGACCTGCACGAATGGCGTAA
TGATGGCCAGGCTGTCTCCACCCGAGACTCAGTGAAATTGAACTCGCTGTGAAGATGCAG
TGTACCCGCGGCAAGACGGAAAGACCCCGTGAACCTTTACTATAGCTTGACACTGAACAT
TGAGCCTTGATGTGTAGGATAGGTGGGAGGCTTTGAAGTGTGGACGCCAGTCTGCATGGA
GCCGACCTTGAAATACCACCCTTTAATGTTTGATGTTCTAACGTGGACCCGTTACCCGGG
TTGCGGACAGTGTCTGGTGGGTAGTTTGACTGGGGCGGTCTCCTCCTAAAGAGTAACGGA
GGAGCACGAAGGTTGGCTAATCCTGGTCGGACATCAGGAGGTTAGTGCAATGGCATAAGC
CAGCTTGACTGCGAGCGTGACGGCGCGAGCAGGTGCGAAAGCAGGTCATAGTGATCCGGT
GGTTCTGAATGGAAGGGCCATCGCTCAACGGATAAAAGGTACTCCGGGGATAACAGGCTG
ATACCGCCCAAGAGTTCATATCGACGGCGGTGTTTGGCACCTCGATGTCGGCTCATCACA
TCCTGGGGCTGAAGTAGGTCCCAAGGGTATGGCTGTTCGCCATTTAAAGTGGTACGCGAG
CTGGGTTTAGAACGTCGTGAGACAGTTCGGTCCCTATCTGCCGTGGGCGCTGGAGAACTG
AGGGGGGCTGCTCCTAGTACGAGAGGACCGGAGTGGACGCATCACTGGTGTTCGGGTTGT
CATGCCAATGGCACTGCCCGGTAGCTAAATGCGGAAGAGATAAGTGCTGAAAGCATCTAA
GCACGAAACTTGCCCCGAGATGAGTTCTCCCTGAGACTTAAAGTCTCCTGAAGGAACGTT
GAAGACGACGACGTTGATAGGCCGGGTGTGTAAGCGCAGCGATGCGTTGAGCTAACCGGT
ACTAATGAACCGTGAGGCTTAACCT
>EECEBICD_00738 5S ribosomal RNA
TGGCGGCACTAGCGCGGTGGTCCCACCTGACCCCATGCCGAACTCAGAAGTGAAACGCCG
TAGCGCCGATGGTAGTGTGGGGTCTCCCCATGCGAGAGTAGGGAACTGCCA
>EECEBICD_00739 hypothetical protein
TTGGCTATCAAACCTTTTAACTACCAGCAGGACTTTTCCAGCATTGACTTCCGCCAGCAG
CCTGAATTGTATCAGGTTGGACGAGGCGAGCAGGGGGTGCTACTGGTTGAACCCTACAAA
AGCGAAATTCTTCCTTTCTGGCGCTATAAAGATGAAGCATCGGCGATGAAATCCGCAGAA
CAGGTCTACCAGTTGTTCGAAGCTTACCGGCAGCAGGATGATTTCGTTGGTATGGACATG
GCGCGTAAATTTATTCAGATGGGATACACCCGCGCCAGGCGATACGCTAACTATAAAGGC
GGTAAAAAGTATGCAGAAGACGGCAGCCTGAATACACGGGGTAATGATCCTATTAAGGCC
GCGGCGGCAACGGTTTTCAAAGGTTGGTGGGATAAAATCCGTCAGGACGAAGACTATCTG
AAACGGAAACGCCAGCATCAGGCACGGTGGGGATAA
>EECEBICD_00740 Alpha-ketoglutarate permease
ATGGCAGAAATTATTCAGCGTATAGATAAAAATAACGCTGAGGCTCCGGAGATGCGGCGA
CGCATATGGGCTATCGTGGGGGCCTCTTCCGGAAACTTAGTGGAATGGTTTGATTTCTAC
GTCTACTCTTTCTGCTCGCTCTATTTTGCACATATCTTCTTTCCTTCTGGTAATACCACT
ACTCAGCTTTTGCAAACGGCAGGCGTCTTTGCCGCCGGTTTTTTAATGCGGCCTATCGGT
GGTTGGCTGTTTGGACGCATTGCCGATCGACGCGGGCGCAAAGCCTCAATGTTGATTTCG
GTGTGCATGATGTGCATGGGGTCGCTGGTGATTGCCTGCTTACCTGGTTACGATACCATC
GGCACCTGGGCACCGGCGCTGCTACTTATTGCAAGGTTATTTCAGGGGCTCTCGGTAGGG
GGAGAGTATGGGACCAGCGCGACATATATGAGTGAAGTTGCGCTTGAAGGTCGTAAGGGA
TTTTTCGCGTCGTTTCAGTACGTTACGCTTATCGGTGGCCAACTACTGGCATTGCTGGTC
GTTGTGATTTTGCAGCAGGTTCTGGAGGATGCTGAGCTTCGTGCCTGGGGATGGCGTATT

CCCTTTGCGCTGGGCGCCGTGCTGGCGGTTGTGGCGTTATGGCTACGTCGCCAGCTGGAT
GAAACCTCGAAGCATGAGACTCGCGCATTAAAAGAAGCCGGTTCGTTGAAAGGTTTGTGG
CGTAATCGCAAAGCCTTTTTGATGGTGCTTGGTTTCACCGCGGCAGGCTCGCTGTGCTTT
TATACCTTCACCACCTATATACAGAAATATCTGGTAAATACAGCAGGTATGCATGCCAAT
GTCGCCAGCGGCATTATGACGGTCGCGCTGTGTGTTTTCATGTTTGTGCAGCCGTTATTC
GGCGCGTTATCCGATAAGATTGGCAGGCGCACCTCTATGCTATGTTTTGGCGCACTCGCG
ACGCTTTTCACCGTTCCAATTCTCTCCGCGTTGCAGAATGTGACGTCGCCTTATGCCGCA
TTTGCTTTAGTGATGTGCGCGCTATTGATTGTGAGTTTCTATACCTCGATTAGCGGGATC
CTGAAAGCAGAGATGTTTCCGGCGCAGGTTCGCGCCCTGGGCGTCGGTTTATCTTACGCG
GTAGCAAACGCGTTATTTGGCGGCTCTGCGGAATATGTCGCGCTGTCGCTAAAATCTGTC
GGTATGGAGAGTTCGTTCTTCTGGTATGTTACCGCGATGGCCGTTCTGGCTTTCCTGGTT
TCGTTGATGTTGCACCGTAAAGGAAAGGGCTTACGCCTTTAG
>EECEBICD_00741 hypothetical protein
GTGCGCGTGTTGATTCCTTTTACGGTGTTGTTTCTCTCCGGGTGCAGCCATCTGGCTAAC
GACCACTGGAGCGGTCAGGATAAAGCCCAGCATTTTATGGCATCAGCGATGCTATCGGCA
GCCGGTAATGAGTATGCGCGTCACCAGGGAGTAAGCCCTGACCGCAGCGCAGCGATAGGG
CTGATGTTTTCTTTGAGTCTCGGAGCCTCAAAAGAACTCTGGGACAGCCGCCCGGAAGGC
AGCGGCTGGAGCTGGAAAGATTTTGTCTGGGATGTCGCTGGCGCGACAACCGGTTACGCT
ATCTGGCAAATGGCGCGATACTAA
>EECEBICD_00742 CDP-diacylglycerol--serine O-phosphatidyltransferase
ATGTTGTCAAAATTTAAGCGTAATAAACATCAACAACACCTTGCCCAACTCCCTAAGATT
TCTCAGTCAGTTGATGATGTAGATTTCTTTTATACTCCTGCTACTTTTCGGGAGACACTG
CTGGAGAAAATCGCCAGCGCGACGCAGCGTATTTGTATCGTTGCCCTGTATCTGGAACAG
GACGATGGGGGTAAAGGTATACTCGACGCGCTCTATGCGGCTAAACGGCAGCGTCCTGAA
CTGGACGTGAGGGTGCTGGTCGACTGGCATCGCGCGCAACGCGGGCGTATTGGCGCAGCG
GCCTCGAATACCAATGCAGACTGGTATTGCCGACTGGCGCAGGAAAACCCCGGCATTGAT
GTCCCGGTTTACGGCGTACCGATTAATACGCGAGAAGCGCTTGGCGTACTGCATTTTAAA
GGCTTTATCATTGATGATAGCGTCCTGTACAGCGGCGCCAGCCTGAACGATGTCTATCTC
CATCAGCATGACAAATATCGCTACGATCGCTACCAGCTGATTCGCAATCGGCAAATGGCA
GACATCATGTTTGACTGGGTGACGCAAAATCTGATGAATGGCCGTGGTGTGAATCGTCTG
GATAATACCCAGCGGCCGAAAAGCCCGGAGATTAAAAACGATATCCGCTTATACCGTCAG
GAGCTGCGTGATGCGTCATATCATTTTCAGGGTGACGCTAATGACGAGCAACTCTCCGTT
ACGCCGCTGGTCGGGCTTGGCAAATCCAGTCTGCTGAACAAAACCATTTTCCATCTCATG
CCCTGTGCGGAGCATAAGCTCACCATTTGTACCCCTTATTTCAATCTACCAGCGGTGCTG
GTGCGGAATATTATTCAGCTACTGCGCGACGGAAAGAAAGTCGAAATCATTGTTGGCGAT
AAAACCGCGAATGATTTTTACATTCCGGAAGATGAACCGTTCAAGATCATTGGCGCGTTG
CCCTATCTCTATGAGATTAACCTGCGCCGCTTCCTGAGCCGTTTACAGTATTACGTCAAT
ACCGATCAGCTTGTGGTGCGTCTGTGGAAAGATGACGACAATACCTATCATTTGAAAGGG
ATGTGGGTAGATGATAAATGGATGCTACTCACCGGCAACAATCTGAATCCCCGCGCCTGG
CGTCTGGATCTGGAAAACGCCATTCTGATCCACGATCCTAAGCAGGAGCTTGCCCCTCAG
CGGGAAAAAGAGCTGGAACTTATCCGAACGCACACAACCATCGTAAAACATTATCGTGAC
CTGCAGAGCATTGCCGACTATCCGATAAAAGTGCGTAAATTGATTCGCCGTCTGCGCCGG
ATCCGCATCGACAGATTAATTAGTCGTATCCTGTAA
>EECEBICD_00743 Peptidyl-lysine N-acetyltransferase Pat
ATGAGCCAGCAAGGACTGGAAGCGCTACTGCGGCCAAAATCGATCGCGGTGATTGGCGCA
TCAATGAAGCCCCACCGCGCGGGTTACCTGATGATGCGTAACTTGTTGGCGGGCGGATTC
AATGGCCCCGTCCTTCCCGTGACGCCCGCCTGGAAAGCCGTTTTAGGCGTCATGGCCTGG
CCGGATATCGCCAGTCTCCCTTTCACCCCCGATCTGGCTATTTTATGCACTAACGCCAGC
CGTAACCTGGCATTACTGGACGCGCTTGGCGCGAAAGGGTGTAAAACGTGCATTATCCTT
TCTGCTCCCACGTCGCAACATGAAGAACTTCTTGCCTGTGCCCGGCATTATAAAATGCGT
CTGCTGGGTCCAAACAGTCTTGGGCTCCTCGCGCCGTGGCAAGGGCTGAATGCCAGCTTT
TCTCCCGTCCCGATTAAACAGGGCAAGCTCGCTTTTATTTCCCAGTCTGCCGCCGTTTCC
AATACTATTCTTGACTGGGCGCAACAGCGTGAAATGGGCTTTTCCTACTTTATCGCGCTG
GGCGATAGCCTGGATATTGATGTCGATGAACTACTGGACTATCTGGCGCGCGACAGCAAG

ACCAGCGCGATTTTGCTCTATCTGGAACAGTTAAGCGACGCCCGCCGTTTTGTTTCCGCC
GCCCGTAGCGCTTCACGTAACAAACCGATTCTGGTGATTAAAAGCGGCCGAAGCCCGGCA
GCCCAGCGTTTACTTAATACCAGCGCGGGAATGGACCCTGCGTGGGATGCGGCCATCCAG
CGCGCAGGCCTGCTGCGAGTCCAGGATACGCACGAGCTTTTTTCCGCCGTCGAAACACTG
AGCCATATGCGTCCGCTACGCGGCGACAGACTGATGATCATCAGCAATGGCGCCGCGCCT
GCCGCGCTGGCGTTAGATGAGTTGTGGTCGCGTAACGGCAAGCTGGCGACGTTGAGCGAA
GAGACCTGCCTGCAACTACGGCAGGCGCTTCCCGCGCACATTGATATTGCCAATCCGCTG
GATCTGTGTGATGACGCCAGCAGCGAACATTACGTCAAAACGCTGGATATCCTGCTCGCC
AGTCAGGATTTTGACGCGCTTATGGTTATCCACTCTCCCAGCGCTGCCGCGCCGGGTACG
GAAAGCGCCCATGCCCTGATCGAGACGATTAAGCGCCACCCCAGAGGCAAGCTTGTTACG
CTGCTGACAAACTGGTGCGGCGAGTTCTCGTCTCAGGAGGCAAGACGGCTATTCAGCGAA
GCCGGATTACCAACCTACCGTACGCCGGAAGGCACGATTACCGCGTTTATGCATATGGTG
GAATACCGGCGTAACCAGAAGCAACTGCGAGAAACGCCAGCGTTGCCGAGTAACCTGACG
TCCAATACCGCTGAGGCGCATAATCTGTTACAGCGGGCGATTGCGGAAGGCGCCGCCTCA
CTGGATACCCATGAAGTACAGCCGATTTTACACGCCTATGGGCTGCACACGCTCCCAACC
TGGATTGCCAGCGACAGCGCTGAAGCGGTGCATATCGCCGAACAGATAGGCTATCCGGTA
GCTCTCAAGCTGCGCTCGCCCGACATTCCGCATAAATCTGAAGTTCAGGGGGTCATGCTT
TACCTGCGGACCGCAAGCGAGGTACAACAGGCCGCGAACGCCATTTTTGATCGTGTAAAG
ATGGCCTGGCCGCAAGCGCGGATTCACGGTTTGCTGGTACAAAGCATGGCTAACCGCGCC
GGCGCGCAGGAGCTTCGTGTGGTGGTCGAGCACGATCCGGTGTTTGGTCCTTTGATTATG
TTGGGTGAAGGCGGCGTAGAGTGGCGTCCGGAAGAGCAGGCTGTCGTCGCGCTGCCGCCG
CTCAACATGAACCTGGCGCGCTATCTGGTGATTCAGGGCATTAAACAGCGGAAAATTCGC
GCCCGTAGCGCGCTGCGTCCGCTGGATATTGTCGGTTTAAGCCAATTGCTGGTCCAGGTT
TCAAACCTGATTGTCGACTGCCCGGAAATTCAGCGTCTGGATATCCATCCGCTGCTGGCT
TCCGCCAGTGAGTTTACCGCGCTGGATGTGACGCTGGATATTGCCCCGTTTGATGGCGAT
AACGAAAGTCGACTTGCGGTACGCTCCTATCCCCACCAGCTTGAAGAGTGGGTGGAGATG
AAAAATGGCGATCGCTGCCTGTTCCGTCCTATCCTGCCGGAAGATGAGCCCCAACTGCGA
CAATTCATCGCACAGGTCACCAAAGAGGATCTTTACTACCGTTATTTCAGCGAGATCAAC
GAATTCACCCATGAAGATTTAGCCAACATGACGCAGATCGACTACGATCGAGAAATGGCC
TTTGTGGCCGTGAGGCGGATGGACAATGCTGAAGAGATCCTCGGCGTAACGCGCGCGATC
TCCGATCCTGACAACGTAGATGCCGAATTTGCCGTATTGGTACGTTCAGATCTCAAAGGG
TTGGGTTTAGGACGCCGTTTAATGGAGAAATTGATTGCCTATACTCGCGATCACGGATTG
AAGCGGCTGAACGGTATTACGATGCCAAACAATCGCGGCATGGTCGCGCTGGCCAGAAAA
CTGGGATTTCAGGTCGATATTCAGCTCGAAGAGGGCATCGTGGGATTGACGCTGAATCTG
GCCAAATGTGATGAATCGTGA
>EECEBICD_00744 hypothetical protein
ATGACCAATAACGCTGTCCTTCAGTTACGCGCCGAACGGCTTGCGCGCGCTACTCGCCCT
TTTCTTGCCAGAGGCAATCGGGTTCGCCGCTGTCAACGCTGCCTGCTGCCGCTAAAATCT
TGCCTGTGTGATACGTTAACGCCTTCTCAGGCGAAAAGCCGTTTTTGTCTGGTCATGTTT
GACACTGAACCGATGAAACCCAGCAATACCGGCCGGCTGATAGCCGATATTTTGCCGGAT
ACCGCGGCGTTTCAGTGGTCGCGTACCGAGCCGCCACAGGCGTTGCTGGAGTTGGTACAG
CATCCGGATTATCAACCGATAGTGGTCTTTCCTGCGTCCTACGCCGGCGAAGCGCGCGAG
GTCATTTCCACCCCGCCCGCCGGGAAACCGCCGCTGTTTATTATGCTGGACGGCACCTGG
CCCGAAGCACGTAAAATGTTTCGTAAAAGCCCTTATCTGGATCATCTGCCGGTCATTTCC
GTCGATTTATCGCGTCTTTCAGCTTATCGTCTGCGTGAAATCCATGCCGAAGGGCAATAT
TGTACCGCCGAAGTCGCGATAGCGTTGTTAGATCTGGCGGGAGATACCGAGGCGGCAACC
AGTTTAGGTGAACATTTCACCCGTTTTAAAACGCGCTATCTGGCAGGAAAAACGCAACAT
CCGGGAAACGTCACAGCATAA
>EECEBICD_00745 Thioredoxin 2
ATGAACACCGTTTGTACCCATTGTCAGGCCATTAACCGCATCCCCGACGATCGGCTTCAG
GATGCGGCAAAATGTGGACGCTGCGGTCATGAGTTGTTTGACGGAGAAGTCATTAATGCG
ACCGGCGAAACGCTGGACAAACTGTTGAAGGACGATCTTCCAGTGGTGATCGACTTTTGG
GCCCCCTGGTGCAGCCCTTGCCGTAACTTTGCGCCAATCTTCGAGGATGTCGCAGAAGAG
CGCAGCGGTAAAGTCCGTTTCGTCAAAGTGAATACCGAGGCGGAACGGGAACTCAGCGCC

CGTTTCGGTATCCGCAGCATCCCGACGATTATGATTTTTAAACATGGACAGGTGGTCGAT
ATGCTTAATGGCGCAGTGCCTAAAGCGCCTTTCGACAGCTGGCTGAACGAAGCGCTGTAA
>EECEBICD_00746 putative tRNA/rRNA methyltransferase YfiF
ATGAACGATGAATTAAAAAACAAAAGCGGCAAAGTCAAAGTGATGTATGTCCGTAGTGAT
GATGACTCTGATAAACGCACCCATAACCCGCGTACCGGGAAAGGGGGAGGCCGCCCAGCA
AAGTCTCGCACTGACGGTGGACGTCGCCCCGCCCGTGATGAGCGAAATAATCAGAGCCGT
GACCGTAAACATGAAACTTCGCCGTGGCGCACCGTGTCCCGCGCGCCAGGCGATGAAACG
CCTGAGAAAGTCGATCATGGTGGTATTAGCGGTAAAAGTTTTATTGACCCTGAAGTTTTG
CGTCGCCAGCGTGCGGAAGAGACGCGCGTCTACGGTGAAAACGCCTGCCAGGCGCTATTC
CAGAGCCGTCCTGACGCCATCGTGCGCGCATGGTTTATCCAAAGCGTAACGCCGCGTTTT
AAAGAGGCGCTGCGCTGGATGGCGGCGAATCGTAAAGCCTACCATGTGGTGGATGAAGCC
GAACTGGCAAAAGCTTCCGGGACTGAGCACCACGGTGGCGTGTGTTTCCTGATTAAAAAG
CGTAACGGTACGACCGTTAAGCAATGGGTAAAGCAGGCGGCGGATCAAGACTGCGTGCTG
GCGTTGGAAGATGTCGCTAACCCGCATAATCTGGGCGGCATGATGCGTAGCTGCGCGCAC
TTCGGCGTGAAAGGCGTGGTGGTACAGGATGCGGCGTTGCTGGAGTCCGGTGCGGCGATT
CGTACTGCGGAAGGCGGGGCGGAGCATGTCCAGCCGATTACTGGTGAAAGCATTGTTGAC
GTACTGGATGATTTTCGTCAGGCGGGGTACACCGTGGTGACGACGTCCAGCGATCGCGGT
CAGGCGCTCTTTAGTACGACCCTGCCGGAAAAGATGGTGCTGGTCTTAGGCCGTGAATAT
GACTACCTGCCGGAAGCGGCGCGCGAACCCGACGATCTGTGCGTGAAAATTAACGGCACC
GGGAACGTGGAAAGCCTGAATGTGTCGGTTGCGACAGGCGTGCTGCTCGCCGAGTGGTGG
CGACAGAATAAAGCGTAA
>EECEBICD_00747 Uracil-DNA glycosylase
ATGGCGACTGAGTTAACCTGGCACGATGTGCTGGCTGATGAGAAACAGCAACCTTACTTT
ATCAATACGCTTCACACTGTTGCTGGCGAGCGTCAGTCGGGTATCACGGTCTACCCGCCG
CAAAAAGATGTGTTTAACGCCTTTCGCTTCACGGAACTGGGCGACGTTAAGGTGGTGATC
CTGGGACAAGATCCTTACCACGGCCCCGGCCAGGCACATGGTCTGGCGTTTTCCGTGCGC
CCTGGGATCGCTCCCCCGCCGTCATTAGTGAATATGTACAAAGAGCTGGAAGCCTCCATT
CCCGGCTTTGTTCGCCCAGCGCACGGTTATCTGGAAAGCTGGGCGCGCCAGGGCGTACTG
TTACTGAATACGGTGCTCACGGTACGGGCAGGCCAGGCGCACTCTCATGCCAGCCTGGGG
TGGGAAACCTTTACGGATAAAGTGATAAGCCTGATTAACCAACACCGCGAAGGGGTCGTC
TTCTTGCTGTGGGGCTCGCACGCCCAGAAAAAAGGCGCGATTATTGATCCACAGCGCCAC
CATATTCTGAAAGCTCCGCATCCCTCGCCACTCTCTGCGCACCGCGGATTTTTTGGCTGT
AACCATTTTGCGCTAACGAACCAATGGCTGGAACAACACGGCGAGAAAACTATCGACTGG
ACGCCAGTATTACCGGCTGAGAGTGAATAA
>EECEBICD_00748 Autonomous glycyl radical cofactor
ATGATTACAGGTATCCAGATTACTAAAGCCGCTAACGACGACCTGCTGAATTCCTTCTGG
CTGCTGGACAGCGAAAAAGGCGAAGCGCGCTGCATCGTTGCAAAATCCGGCTTTGCTGAA
GATGAAGTTGTGGCTGTCAGCAAACTGGGCGAGATTGAATACCGGGAAATTCCGATGGAA
GTTAAACCGGAAGTACGCGTTGAAGGCGGTCAGCATCTGAACGTTAACGTTCTGCGTCGT
GAAACTCTGGAAGACGCGGTTAAGCATCCGGAAAAATACCCGCAGCTGACTATTCGTGTT
TCTGGTTATGCGGTGCGTTTTAACTCCCTGACGCCGGAACAGCAGCGCGACGTTATCGCT
CGTACCTTTACCGAAAGCCTGTAA
>EECEBICD_00749 Cysteine/O-acetylserine efflux protein
GTGACACCGATGCTTTTAAGCGCTTTCTGGACTTATACATTAATCACCGCCCTTACCCCC
GGCCCAAACAATATTCTCGCGCTTAGCGCAGCAACTGCACATGGTTTTCGGCAGAGTATT
CGTGTACTGGCAGGTATGAGTCTGGGATTTTTGGTCGTCATGCTGCTGTGCGCCGGGATC
GCGTTTTCCCTTGCGGTTATCGATCCGGCAATCATTCATTTGCTCAGTTGGGTAGGGGCT
GCGTATATCCTGTGGCTGGCGTGGAAAATTGCCACCAGTCCAGCGGCGGATGAGAAGGTC
AGACCAAAACCGGTTGGCTTTTGGGTAAGCTTTGGCCTGCAGTTTGTGAACGTCAAAATT
ATTCTGTACGGCATTACCGCTCTGTCTACATTTGTTTTACCGCAAACGCAGGCGCTGAAC
TGGGTCATCGGCGTCAGTATATTGCTGGCATTGATCGGCACGTTCGGTAATGTGTGCTGG
GCGCTGGCGGGACATCTGTTTCAGCGGGCGTTTCGCCATTATGGTCGCCAGTTAAATATC
ATTCTGGCGTTACTGCTGGTGTACTGCGCGGTGCGGATTTTTTACTGA
>EECEBICD_00750 HTH-type transcriptional regulator GltR

ATGGATCTGCGTCGGTTTATTACGCTGAAAACGGTAGTGGAAGAGGGCTCGTTTCTGCGG
GCTTCGCAAAAGCTGTGCTGCACTCAGTCGACGGTAACTTTTCATATTCAACAGCTTGAA
CGTGAGTTTTCCCTGCAACTGTTCGAGAAAATAGGGCGCCGAATGTGCCTGACCACTGAG
GGGAAAAAGCTGATGCCGCATATCCATGAACTGACGCGTGTGATGGAGTTGATTCGTGAA
GCCGCAAGGCAGGATGCAGAACCCGGCGGAGAGCTGCGCGTGGCGACAGGTGAAACCTTA
CTGGCGTACAAAATGCCGCAGGTTCTACAACGTTTTAAGCTGCGAGCGCCGAATGTCAAA
TTGTCGCTACAGTCGCTGAATTGCTATGTGATACGTGATGCGTTGCTGAATGACGAGGTC
GATCTGGGCGTTTTTTATCGCGTAGGTAATGATGACGCGCTCACGATGCAGCAACTGGGG
GAGCAGTCGCTGGCGCTGGTGGCCTCTCCACTGCTACAGGATGCTGATTTTACGCAGCCA
GATCAGCATATCCCCTGCAGCTTCATCATCAATGAACCGCAATGTGTCTTCCGCCAGCTA
TTTGAAAGTACGCTACGGCAGAGGCGGATTACGCTGGAAAACACCATTGAACTGTGGAGT
ATTGAAAGTATTAAACAGTGTGTGGCTGCTAACCTGGGCATCAGCTTTTTACCACGTTTT
ACCGTTGAACGAGAACTTAGTACCGGTCAGTTGAAAGAGCTGCCGTTTGGCGCGCCGTCG
CTATCGATCATGGCGCTGTGCGCCCATCATGCCGGGAAAGCGGTCAGCCCGGCAATGCAG
ATATTTATACAGTGTATGGAGGCGTGTTTTACCGTGGAAGATAAAAAAATGCCGGGTTAA
>EECEBICD_00751 ATP-dependent RNA helicase SrmB
ATGACTGTAACGACTTTTTCCGAACTTGAACTCGACGAAAGCCTGCTGGATGCCCTCCAG
GATAAAGGTTTCACTCGCCCGACCGCCATTCAGGCTGCCGCCATTCCGCCTGCGCTCGAT
GGCCGTGATGTACTCGGTTCTGCGCCGACAGGCACCGGTAAAACGGCGGCATATCTGCTG
CCGGCGTTGCAGCACCTGCTCGACTTTCCGCGTAAAAAATCGGGGCCGCCGCGCATTCTG
ATCCTGACGCCAACCCGCGAACTGGCAATGCAGGTTGCCGATCACGCTCGTGAACTGGCT
AAGCATACTCATCTGGATATCGCCACGATTACCGGCGGCGTGGCCTATATGAACCATGCT
GAAGTGTTTAGCGAAAACCAGGATATCGTGGTCGCCACGACGGGCCGTCTGCTGCAATAT
ATAAAAGAAGAGAACTTTGACTGCCGGGCGGTTGAAACACTGATCCTTGACGAAGCTGAC
CGGATGCTCGACATGGGCTTCGCTCAGGACATTGAACATATCGCTGGTGAGACCCGCTGG
CGTAAACAGACGATGCTGTTCTCCGCCACGCTGGAAGGCGATGCGATTAAAGATTTTGCC
GAGCGCCTGCTGGAAGACCCGGTTGAGGTTTCCGCCAATCCGTCAACCCGCGAGCGTAAG
AAAATTCACCAGTGGTACTACCGTGCCGACAATTTCGAACATAAAGTTGCGCTGTTGAAG
CACCTGCTAAAACAAGACGATGCAACCCGTTCTATCGTGTTTGTACGTAAACGTGAACGC
GTCCATGAACTGGCCGAAACGCTGCGTCTGGCAGGGATCAACAATTGCTATCTTGAAGGT
GAGATGGCGCAGATCAAACGTAACGAAGGTATTAAGCGTCTCACCGACGGTCGCGTTAAC
GTGCTGGTCGCGACCGACGTCGCGGCACGTGGGATTGATATTCCTGACGTCAGCCACGTC
ATTAACTTCGACATGCCGCGCAGTGGCGATACCTATCTGCATCGCATTGGTCGTACAGGC
CGTGCTGGCCGCAAAGGCACTGCAATCTCGCTGGTAGAAGCCCACGACCATCTGTTGCTG
CTGAAAATCGGCCGCTACATCGAAGAGCCGCTAAAAGCGCGCGTCATTGATGAGCTTCGC
CCGACCACGCGTGCGCCCAGCGAAAAGCTGACCGGCAAACCGTCGAAAAAAGTGCTGGCG
AAACGCGCCGAGAAGAAAAAAGAGAAAGAAAAAGAGAAGCCGCGCGTGAAGAAACGTCAC
CGCGATACAAAAAATATCGGTAAGCGCCGCAAGCCAAGCGGTACAAAAATGCAGGAGCAA
TCCAGCGAAGAGTGA
>EECEBICD_00752 tRNA1(Val) (adenine(37)-N6)-methyltransferase
ATGTCTCAGTCTGGCTCCGTTCTTCGTCGTAACGGATTTACGTTTAAACAGTTTTTTGTC
GCACACGATCGGTGTGCGATGAAAGTGGGAACTGACGGTATTTTACTCGGGGCGTGGGCG
CCGGTCGCGGATGTAAAGCGAATTCTGGATATCGGTACTGGAAGCGGTCTGCTGGCGTTA
ATGCTGGCGCAGCGGACGGATGACAATGTGCCTATTGACGCCGTGGAACTGGATGCCGGG
GCTGCCATGCAAGCGCAGGAAAACGTGGCTCACTCTCCCTGGCCGCATCGAATAACGGTG
CATACTGACGATATTCAGAGTTGGGCGCCTCGCCAGACGGTACGCTTTGATCTTATTATC
AGCAACCCGCCTTATTACGAACCCGGTGTGGAATGCGCCACGCCGCAGCGGGAGCAGGCG
CGTTACACCGCTACGCTGGATCACCAGACGTTATTGGCTATTGCGGCGGATTGTATTACC
GAAGATGGCTTTTTTTGCGTAGTCTTGCCGGAACAAATAGGTAACGCTTTTACGCAGCAA
GCGCTCAACATGGGATGGCATTTGCGGCTACGTACTGACGTTGCGGAAAACGAAGCGCGC
TTGCCGCACCGGGTTTTGCTCGCATTCTCCCCGCAAGCCGGGGAGTGCTTTAGCGACAGA
TTAGTAATTCGCGGGTCAGACCAGCATTATTCTGAAAGTTATACGGCGCTGACCCAGGCA
TTTTATCTGTTTATGTGA
>EECEBICD_00753 L-aspartate oxidase

ATGATGACAACTCCTGAACTGTCCTGTGATGTGTTAATTATCGGCAGCGGCGCGGCCGGG
CTCTCGCTGGCGCTGCGTCTGGCTGAAAAGCATAAGGTTATCGTCCTGAGTAAAGGGCCG
GTGAGCGAGGGCTCCACGTTTTATGCGCAGGGCGGTATTGCCGCCGTGTTTGATGAAACC
GACAGTATTGCGTCGCATGTTGAAGATACGCTAATTGCTGGCGCCGGAATTTGCGATCGC
CACGCGGTGGAGTTTGTCGCCAGCAACGCCCGTACCTGTGTGCAGTGGCTTATCGATCAG
GGCGTCCTGTTTGATACGCATGTCCAGCCAAACGGCAAGGAGAGCTATCATCTCACGCGT
GAAGGCGGCCATAGCCATCGCCGTATCCTTCATGCCGCCGACGCTACCGGAAAGGAAGTC
GAAACCACGTTGGTCAGTCGGGCGCAAAATCATCCTAATATTCAGGTGCTGGAACGCAGC
AACGCCGTTGATTTAATTATCTCCGATAAAATGGGTCTGCCGGGGCCTCGCCGGGTCGTC
GGCGCCTGGATCTGGAATCGCAATAAAGAGTGGGTTGAAACCTGTCATGCGAAGTCGGTG
GTGCTGGCGACCGGCGGCGCCTCAAAGGTTTATCAATATACCACTAACCCGGATATTTCC
TCCGGCGACGGTATCGCGATGGCCTGGCGCGCAGGCTGTCGGGTCGCTAATCTGGAATTT
AACCAGTTTCATCCCACCGCCCTTTATCACCCGCAGGCGCGTAATTTTCTCCTTACCGAA
GCGCTGCGCGGCGAAGGCGCATACCTTAAACGTCCGGACGGTTCACGGTTTATGCCGGAC
GTTGACGAACGCGGGGAACTGGCGCCGCGCGATATTGTCGCCCGCGCTATTGACCATGAG
ATGAAACGACTCGGCGCAGACTGTATGTTTCTCGATATCAGCCACAAGCCTGACGATTTT
GTCCGCCAGCACTTCCCGATGATCTATGCGAAACTGCTCGATTTGGGGATGGATTTAACC
AAAGAACCGATTCCCATTGTTCCTGCCGCGCACTATACCTGCGGCGGCGTGGTCGTCGAT
GATTATGGTCGTACCGATGTAGACGGCTTATATGCCATTGGCGAAGTCAGTTACACCGGC
CTGCACGGCGCTAACCGTATGGCGTCCAACTCGTTGCTGGAATGCTTAGTGTACGGCTGG
TCCGCAGCAATGGATATCGACAGGCGAATGCCTTCTGTCCACAGCGTTGACGTCCTTCCC
GCCTGGGATGAAAGCCGCGTGGAGAACGCCGATGAGCGGGTGGTGATTCAGCATAACTGG
CACGAACTGCGTCTGCTGATGTGGGACTATGTGGGGATTGTGCGCACGACGAAACGGCTG
GAGCGCGCCCTACGGCGTATTACTATGTTGCAACAAGAGATTGACGAATATTACGCCAAC
TTTCGCGTCTCGAATAATTTACTGGAGCTGCGCAATCTGGTGCAAGTGGCCGAACTGATT
GTGCGTTGCGCGATGATGCGTAAAGAGAGCCGGGGGCTGCACTTTACGCTGGATTATCCG
CAACAACTGGCTGAATCAGGCCCATCGATCCTTTCGCCATTAACGCCTCACATAAACAGA
TAA
>EECEBICD_00754 ECF RNA polymerase sigma-E factor
ATGAGCGAGCAGTTAACGGACCAGGTCCTGGTTGAACGGGTCCAGAAGGGAGATCAGAAA
GCCTTTAACTTACTGGTAGTGCGCTACCAGCATAAAGTGGCGAGTCTGGTTTCCCGCTAT
GTGCCATCGGGCGACGTTCCCGATGTCGTACAGGAATCATTTATTAAGGCCTATCGCGCG
CTGGATTCTTTCCGGGGGGATAGTGCTTTTTATACCTGGTTGTATCGTATTGCGGTCAAT
ACTGCGAAGAACTACCTGGTTGCGCAGGGGCGTCGTCCGCCTTCCAGTGATGTAGACGCG
ATTGAAGCAGAAAACTTTGAAAGCGGCGGCGCGCTGAAAGAAATTTCGAACCCTGAGAAC
TTAATGTTGTCAGAAGAACTGAGACAGATAGTTTTCCGAACTATTGAGTCCCTCCCGGAA
GATTTACGTATGGCAATCACCTTACGGGAGCTGGATGGCCTGAGCTATGAAGAGATAGCG
GCTATCATGGATTGTCCGGTGGGGACGGTGCGTTCACGTATCTTCCGGGCGCGGGAAGCT
ATTGATAATAAAGTTCAACCGCTTATCAGGCGTTGA
>EECEBICD_00755 Anti-sigma-E factor RseA
ATGCAGAAAGAAAAACTTTCCGCTTTGATGGATGGCGAAACGTTGGATAGTGAGCTGCTC
AAAGCGCTTACGCACGACCCGGAAATGCAGAAAACCTGGGAGAGTTATCACCTGATCCGC
GATTCAATGCGGGGGGATACGCCTGACGTTCTCCATTTCGATATATCCGCTCGCGTGATG
GCCGCTATTGAAAACGAGCCAGTACGTCAGGTGTCGCCATTAATCCCTGAGGCCCAACCC
GCTCCGCAGCAATGGCAGAAAATGCCGTTCTGGAAAAAAGTGCGTCCGTGGGCGGCGCAG
CTTACCCAAATGGGCGTGGCGGCGTGCGTTTCACTTGCAGTTATCGTCGGTGTCCAGCAC
TATAATGGGCAATCTGAAACATCCCAACAACCTGAAACGCCGGTTTTCAATACCCTGCCT
ATGATGGGGAAAGCCAGCCCGGTAAGTCTGGGAGTGCCTTCTGAAGCAGCGCCTGTCGGC
AGTCAGCAACAGCAGGTACAGGAGCAGCGTCGTCGCATTAATGCCATGTTGCAGGACTAT
GAACTGCAGCGCCGACTGCACTCCGAACAGCTTCAGTTTGAGCAGGCGCAAACCCAGCAA
GCCGCGGTACAGGTGCCAGGAATCCAAACTTTAGGAACGCAATCGCAGTAA
>EECEBICD_00756 Sigma-E factor regulatory protein RseB
ATGAAGCAACTTTGGTTTGCCATGTCCCTTGTGGCGGCTAGCCTGTTCTTCTCAGCAAAC
GCCTCGGCCGACCCTGCGTCCGGGGCGTTATTGCAGCAGATGAACATCGCCAGTCAGTCA

CTGAATTACGAGCTGTCATTCGTCAGCATCACGAAACAAGGCGTTGAATCTCTGCGTTAT
CGACACGCACGCCTGGATGGTCGCCCGCTTGCGCAATTGCTACAGTTGGACGGTCCCCGC
CGGGAGGTCGTACAGCGTGGTAATGAAATCAGTTATTTTGAACCGGGCCTTGAGCCGTTC
ACCCTGAACGGTGATTATATTGTCGATTCTCTGCCTTCCCTGATTTACACCGACTTCAAG
CGCCTGGCGCCATACTACGACTTTATTTCTGTCGGACGCACCCGTATCGCAGACAGATTG
TGCGAAGTTATCCGCGTTGTGGCGCGCGATGGTACGCGTTACAGCTATATTGTCTGGATG
GACATGGACACCAAACTACCGATGCGCGTTGATCTGCTGGATCGCGATGGCGAGACGCTG
GAGCAGTTCCGCGTTATTGCGTTCACGGTGAGTCAGGATATCGGCAGCAACATGCAGGCG
CTGGCGAAGGCGAATCTGCCGCCGCTGCTTTCTGTTCCCGGTGGCGAAAAAACGAAATTT
AACTGGAGTCCATCCTGGGTGCCGCAAGGCTTTAGCGAAGTTTCCAGCAGCCGTCGTCCG
TTGCCGACGATGGATAACCTGCCTATCGAATCGCGGCTTTATTCTGACGGTCTGTTTAGC
TTTTCCGTCAATGTGAACCGGGCGACGCAAAACAGCAGCGATCAGATGCTGCGCACCGGG
CGCCGAACGGTTTATAGCAGCGTTCGCGACAACGCGGAAATCACCATTGTCGGCGAGCTT
CCGCCGCAAACGGCGAAGCGCATTGCCGACAGTATCAAATTCAGGGCAGTACAATGA
>EECEBICD_00757 Protein RseC
ATGATTAAAGAGTGGGCAACCGTTATCTCCTGGCAGAATGGTCAGGCGGTGGTGAGCTGC
GACGTTAAAGCGTCATGCAGCAGCTGCGCCTCCCGTGCGGGATGCGGCAGTCGCGTGCTG
AATAAATTAGGGCCGCAAACCACCCATACCATTGTTGTGCCAAGCGCGGAGCCGCTGGCG
CCGGGGCAGAAAGTCGAGTTGGGCATCGCCGAAAAAAGCCTTTTAGGTTCGGCGTTGCTG
GTCTATATGTCGCCTTTAGCGGGGCTATTTCTTTGTGCGGCGCTTTTTCAGATGCTGTTT
GGATCTGACCTTGCCGCATTAAGCGGGGCCGTGCTGGGCGGCGTGGGCGGTTTTCTGGTT
GCCCGCGGCTATTCCCGAAAACTGGCGGAGCGCGACGCCTGGCAGCCGGTGATTTTAAAC
GTTGCCCTCCCGCCGGACCTTGTTCGCGTCGAAACTACCTCAATCGAAACGCGCCAGTAA
>EECEBICD_00758 Elongation factor 4
ATGAAGAACATACGTAACTTTTCGATCATTGCTCACATTGACCACGGTAAATCGACGCTG
TCTGACCGTATTATCCAAATCTGCGGTGGCCTGTCTGACCGTGAAATGGAAGCTCAGGTA
CTTGATTCGATGGATCTTGAGCGTGAGCGCGGTATTACTATTAAAGCCCAGAGTGTGACG
CTGGATTTTAAAGCGTCTGATGGTGAAACTTATCAACTGAACTTTATCGACACGCCGGGA
CACGTTGACTTTTCCTATGAAGTTTCCCGTTCGTTAGCCGCCTGCGAGGGCGCGCTGCTG
GTGGTGGATGCCGGCCAGGGCGTAGAAGCGCAAACGTTGGCGAACTGCTACACCGCGATG
GAAATGGATCTTGAAGTGGTGCCGGTGCTTAACAAGATTGACCTGCCGGCCGCCGATCCG
GAGCGTGTGGCGGAAGAAATCGAAGACATTGTCGGTATCGATGCGACGGACGCGGTACGC
TGCTCCGCCAAAACGGGTGTCGGCGTGACGGATGTTCTGGAACGCCTGGTGCGCGATATC
CCGCCGCCGCAAGGCGATCCGGACGGCCCGCTGCAGGCGCTGATTATTGACTCCTGGTTC
GATAACTACCTGGGCGTGGTATCGCTGGTGCGTATTAAAAACGGCACCATGCGTAAAGGC
GACAAAATTAAAGTGATGAGCACCGGGCAGACCTACAACGCTGACCGCCTGGGGATCTTC
ACGCCAAAACAGGTTGATCGTACCGAGCTGAAGTGCGGCGAAGTAGGCTGGCTGGTCTGC
GCCATTAAAGATATCCTCGGCGCGCCGGTTGGCGATACCTTAACCTCAGCGCGTAACCCA
GCGGAAAAAGCGTTGCCGGGCTTTAAAAAGGTGAAACCGCAGGTCTATGCAGGTCTGTTC
CCGGTCAGCTCCGACGATTATGAAAGTTTCCGCGACGCGCTCGGCAAGCTGAGCCTGAAC
GATGCCTCACTGTTTTATGAACCGGAAAGCTCCTCGGCGCTGGGCTTTGGTTTCCGCTGC
GGCTTCCTCGGCCTGTTGCACATGGAGATCATTCAGGAGCGTCTGGAACGCGAATACGAT
CTGGATCTGATCACCACTGCGCCGACCGTGGTTTATGAAGTAGAAACAACGGCGAAAGAG
ACTATCTATGTTGATAGCCCCTCCAAGCTGCCGCCGTTGAATAACATTTATGAACTGCGC
GAGCCTATCGCCGAGTGTCATATGCTGTTACCACAAGCCTATTTAGGTAACGTTATTACG
CTGTGTATTGAGAAACGCGGCGTACAAACTAACATGGTGTATCACGGTAACCAGGTCGCG
TTGACCTATGAAATCCCGATGGCGGAAGTGGTGCTCGACTTCTTTGACCGTCTGAAATCA
ACGTCGCGCGGCTATGCGTCTCTGGATTATAACTTCAAGCGCTTCCAGGCTTCCGATATG
GTGCGTGTTGATGTGTTAATCAACAACGAGCGTGTCGATGCGCTGGCGCTGATCACGCAC
CGTGATAACTCGCAAAGCCGTGGTCGCGAGCTGGTGGAGAAGATGAAAGATTTGATTCCA
CGCCAGCAGTTTGATATCGCGATTCAGGCGGCGATTGGTACGCATATTATTGCCCGTTCG
ACGGTAAAACAGTTACGTAAAAACGTGCTGGCGAAGTGCTACGGCGGTGATATCAGTCGT
AAGAAAAAACTGCTGCAGAAACAGAAAGAAGGTAAGAAACGCATGAAGCAGATCGGTAAC
GTCGAGCTGCCTCAGGAGGCGTTCCTCGCCATTCTGCATGTCGGTAAAGACAATAAATAA

>EECEBICD_00759 Signal peptidase I
ATGGCGAGCATGTTTGCCCTGATTCTGGTGATAGCCACACTGGTGACGGGCATTTTATGG
TGCGTTGATAAGTTTGTTTTCGCGCCAAAACGTCGGGCGCGCCAGGCTGCCGCGCAAACG
GCGTCGGGAGATGCGCTGGATAACGCTACGCTCAATAAAGTGGCGCCTAAGCCGGGCTGG
CTGGAGACCGGGGCGTCGGTTTTCCCGGTTCTGGCGATCGTTCTGATCGTTCGTTCATTT
CTTTATGAACCCTTTCAGATCCCGTCAGGCTCAATGATGCCGACACTGCTTATCGGCGAT
TTTATTCTGGTGGAAAAATTTGCCTACGGCATTAAAGATCCGATCTACCAGAAAACCCTG
ATTGAAACCGGTCATCCAAAGCGCGGGGATATTGTGGTATTTAAATATCCGGAAGATCCT
AAGTTAGATTACATCAAACGCGCAGTCGGTTTGCCGGGCGATAAAATCACTTATGATCCG
GTTGCGAAAGAGGTGACGATTCAGCCTGGCTGTAGCTCCGGTCAGGCGTGCGAAAATGCG
CTGCCGGTTACCTACTCTAACGTTGAGCCGAGCGATTTTGTACAGACCTTTGCCCGCCGT
AACGGCGGAGAAGCGACCAGCGGTTTCTTTGAGGTTCCGCTAAACGAGACGAAAGAAAAC
GGCATTCGCCTGACCGAACGTAAAGAGACGTTAGGCGATGTGACGCACCGCATTCTGATG
GTGCCGATAGCCCAGGATCAGTTGGGCATGTATTACCAACAGCCAGGACAACCGCTGGCG
ACCTGGGTTGTACCACCGGGACAATATTTCATGATGGGCGACAACCGCGATAACAGCGCG
GATAGCCGTTACTGGGGATTTGTTCCGGAAGCGAATCTGGTCGGTAAAGCGGTCGCTATC
TGGATGAGCTTTGACAAGCAGGAAGGGGAGTGGCCGACAGGCGTACGCCTGAGTCGTATC
GGCGGTATTCACTAA
>EECEBICD_00760 Ribonuclease 3
GTGTATCAGGTCTGTTTCGTGTGCTGGATTGTTGACGCATTCATTTATTGGTATCGCATG
AACCCCATCGTAATTAATCGGCTTCAACGGAAGCTGGGCTACACTTTTAATCATCAGGAG
CTGTTGCAGCAGGCATTAACTCACCGCAGCGCCAGCAGTAAACATAACGAACGCCTGGAA
TTTTTAGGCGATTCAATCCTGAGCTTCGTTATCGCTAACGCGCTTTATCACCGTTTTCCA
CGGGTGGATGAGGGAGATATGAGCCGTATGCGCGCCACGTTGGTACGTGGTAATACGCTG
GCGGAATTAGCCCGTGAATTTGATTTAGGCGAGTGCCTGCGTTTAGGGCCGGGCGAATTA
AAAAGCGGCGGATTCCGTCGAGAGTCCATTCTGGCGGACACCGTGGAAGCGTTAATCGGC
GGAGTATTTCTCGATAGCAATATCCAGACCGTCGAGCAGTTAATCCTTAACTGGTATAAA
ACCCGTCTGGATGAGATAAGCCCGGGCGATAAACAAAAAGATCCTAAAACGCGCTTGCAG
GAGTATTTGCAGGGTCGCCATCTGCCGCTGCCATCTTACCTGGTTGTGCAGGTACGCGGC
GAGGCGCACGATCAGGAATTTACTATCCACTGCCAGGTCAGCGGCCTGAGCGAACCGGTG
GTTGGCACGGGTTCCAGCCGTCGTAAGGCGGAGCAGGCTGCCGCCGAACAGGCGCTGAAA
AAACTGGAGTTGGAATGA
>EECEBICD_00761 GTPase Era
ATGAGCACTGACAAAACTTACTGCGGATTTATTGCCATCGTCGGACGTCCGAACGTTGGC
AAATCCACCTTGCTGAACAAACTGCTTGGGCAGAAGATTTCGATCACTTCCCGTAAAGCG
CAGACCACGCGCCACCGCATTGTCGGTATTCATACCGAAGGCCCGTATCAGGCAATTTAT
GTCGATACCCCGGGACTGCATATGGAAGAAAAGCGCGCCATTAACCGTTTGATGAATAAG
GCGGCGAGCAGTTCGATTGGCGACGTTGAGCTGGTGATTTTCGTTGTGGAAGGCACCCGT
TGGACGCCGGACGACGAAATGGTCCTGAACAAACTGCGCGATGGCAAAGCGCCGGTTATT
CTCGCCGTTAACAAGGTGGATAACGTGCAGGAAAAAGCCGATTTGCTGCCGCACCTCCAG
TTCCTGGCAAGCCAGATGAACTTTCTTGATATCGTACCCATCTCTGCTGAAACGGGTATG
AACGTCGATACCATTGCCGGTATCGTGCGTAAACATTTACCGGAAGCGATTCATCACTTC
CCGGAAGATTACATCACCGATCGCTCTCAGCGCTTTATGGCGTCTGAAATCATTCGTGAA
AAGCTGATGCGTTTTCTCGGCGCCGAGCTGCCGTATTCCGTTACCGTTGAGATTGAACGT
TTTGTTACCAACGAACGCGGCGGCTATGATATCAACGGGCTGATCCTCGTCGAGCGCGAA
GGGCAGAAGAAGATGGTGATTGGCAACAAAGGCGCCAAAATCAAAACCATTGGTATTGAA
GCGCGTAAAGACATGCAGGAGATGTTTGAGGCGCCGGTACACCTGGAACTGTGGGTGAAA
GTGAAATCCGGCTGGGCCGATGACGAACGCGCTCTGCGCAGTCTCGGTTACGTAGACGAT
CTGTGA
>EECEBICD_00762 DNA repair protein RecO
GTGGAAGGGTGGCAGCGCGCATTTGTCCTGCACAGTCGCCCCTGGAGCGAAACCAGCCTG
ATGCTGGACGTCTTCACGGAAGAATCGGGGCGCGTGCGCCTTGTCGCCAAAGGCGCGCGA
TCTAAACGTTCCAATCTGAAAGGCGCGTTGCAGCCTTTTACGCCGTTATTGCTACGCTAC
AGCGGACGCGGCGAGGTGAAAACCCTGCGCAGTGCCGAGGCAGTTTCTTTGGCGCTGCCG

TTAAGCGGTATTACGCTCTATAGCGGCCTGTACATCAACGAACTCCTCTCTCGCGTACTG
GAATATGAAACGCGCTTCTCCGAACTCTTTTTGATTATCTGAACTGTATTCAGGCGCTG
GCGGGAACCACCGGCTCGCCTGAACCGGCGTTGCGACGTTTCGAACTGGCGTTGCTGGGT
CATCTGGGGTATGGCGTCAATTTCACCCACTGTGCAGGCAGCGGCGAACGGGTGGATGAC
ACCATGACCTACCGTTACCGCGAAGAAAAAGGCTTTTTCGCCAGCGTCGTCATCGATAAC
AACACCTTTACCGGACGGCACCTGAAAGCGTTGGAGGAGCGGGAATTTCCGGATGTAGAT
ACCCTGCGTGCCGCTAAACGCTTTACCCGTATGGCATTAAAGCCGTATCTTGGGGGAAAA
CCGTTAAAAAGCCGGGAGCTGTTCCGGCAATTTATGCCCAAACGCACAGTAAAAATGAAG
AAAGATTAA
>EECEBICD_00763 Pyridoxine 5'-phosphate synthase
ATGGCTGAATTACTGTTAGGCGTCAATATTGACCACATTGCCACGTTACGTAATGCGCGC
GGCACCGACTATCCGGATCCGGTACAGGCGGCGTTTATTGCTGAACAGGCAGGCGCGGAC
GGCATTACCGTACACCTGCGCGAAGATCGTCGCCACATTACCGATCGCGATGTGCGCATT
CTGCGTCAGACGCTGCATACGCGTATGAATCTGGAGATGGCGGTGACCGAAGAGATGCTG
GCGATCGCCGTAGAAACCAGGCCGCATTTCTGTTGTCTGGTGCCGGAAAAACGCCAGGAA
GTCACCACCGAAGGCGGTCTGGATGTGGCCGGACAGCGCGATAAAATGCGTGATGCCTGT
GCGCGCCTGGCGGCGGCTGGCATCCAGGTTTCGCTCTTTATCGATGCCGATGAAACGCAA
ATCAACGCGGCGGCGGAAGTCGGCGCGCCGTTTATCGAAATCCATACCGGCTGCTACGCC
AACGCAGAAACCGATGCGGAACAGGCAAAAGAGCTGGCGCGTATCGCCAGCGCCGCGACC
CTGGCGGCCCGTCTGGGGCTAAAAGTAAATGCGGGTCATGGTCTGACCTACCATAACGTC
AAAGCTATTGCCGCACTGCCGGAAATGCACGAGCTCAATATCGGTCATGCCATTATTGGT
CGGGCGGTGATGACGGGGCTGAAAGAGGCGGTGGCTGAAATGAAACGTTTGATGCTGGAA
GCGCGCGGCTAA
>EECEBICD_00764 Holo-[acyl-carrier-protein] synthase
ATGGCGATTCTCGGCCTGGGAACGGATATTGTAGAGATTGCCCGCATTGAGGCAGTGATC
TCCCGTTCCGGCGAACGTCTGGCAAGGCGTGTGCTCAGTGACAACGAGTGGGCTATCTGG
GAGACGCATCAGCAGCCGGTGCGTTTTCTCGCCAAGCGTTTTGCGGTCAAAGAGGCGGCA
GCGAAAGCTTTTGGCACCGGCATCCGCAACGGTCTGGCGTTCAATCAGTTCGAAGTGTTT
AACGATGAGCTGGGAAAACCACGTTTGCGGCTGTGGGGCGAGGCGTTAACGCTGGCGGAA
AAACTCGGTGTGGCGCATATGCATGTCACGTTGGCCGATGAACGCCATTACGCCTGCGCT
ACGGTCATTCTGGAAAGTTAG
>EECEBICD_00765 Ferredoxin YfhL
ATGGCGCTGTTAATCACCAAAAAATGTATCAATTGCGATATGTGCGAGCCGGAGTGCCCG
AATGAGGCGATTTCAATGGGCGACAGCATTTATGAGATTAACAGCGACAAATGCACCGAG
TGTGTAGGCCATTACGAAACGCCGACCTGTCAGAAAGTCTGCCCGATCCCCAATACGATT
TTGCAGGATCCCGCCCATGTCGAAACCGAAGAACAGTTGTGGGATAAGTTTGTTCTGATG
CATCACGCAGATAAACTGTAA
>EECEBICD_00766 HTH-type transcriptional regulator HdfR
ATGAATATCAAACAACTACACTCCTTCCTGATATTGTGTGATGAATTACATTATGGTCGG
GCAGCCAGTCGCCTGTTTATCACTCAGCCATCATTAAGTCAGCAAATCAAACAACTTGAG
TCATCACTTAAAACAATATTATTTAAGCGAAAAGGTAGAGGAATTAAATTAACAAAAGCA
GGCATTATTTTGCAACGACACGCTAACAAAATAATGCTGGATTTAAAAAATGCCGAAAAT
GAACTTCTTCCCTATCAGGACCAACAGCGCGATACTATTTCAATCGGGGTGTCAGGCAGT
TACCTGGTCCTGCCCGCTTTTCGCAACTTTATGGCGCACCATCCGGAAATATCTTTAAAT
GTTAAAGAGTTCTCAACTGAGCAAACCATTAAAAAGCTGACGGATAGCGCCGTTGATATT
GGCATTGTTTATCGAACAGCGCTCCCTGCCCAGCTAAGCTCGACCATGCTGTTTGAAGAT
GAAATTATCGCTGCAATCCCACTTTCTCATCCTCTGGCGACACGCGAACGTCTGCATTTA
AAAGATTTAAACGACCAGCCCATTATCGTGCTTAATGACTCTCTGCTATTAAGAGGGATC
ATCACCACAGAATTTAATAACCGCAAAGTCGTTCCTAACGTCATCTGCGAACTGGATAAC
CACTACTCATGCCTTGAGTATGCCGAAGCGCAGATTGGCATCGCATTTATTACCCGCTCG
CTAACCCATTTATCGACACCCAAAAATGTTCGCCTGGTTTCACTGGGCATCCCCGCATTC
TTTATTCCTGTCATGCTGGTGCACAGTAATGACCTTTCACTGGATAACGCAACCATCTGC
CTGCTCAAGCAGATCAAAAATTTTTACACGGTAGATAACAAATCAGCGCCAGAAAAGAGC
TGGCCCGAGCAGAAAATAGGCGTATAA

>EECEBICD_00767 L-lactate transporter
ATGTCGGCATCAATCAACCGCTGGGGAATGCTTGCCGCTCACGTGTGCATCAATTTTGTG
CTCGGGGGCGTCTACGCATTTAGCTATTTCAAAACACCACTCATGGCGCAATATCACTGG
GATCCGGCTACGCTGGCGTTAGCATTCTCTATCAATATGGGGATCATTCCTTTACCGATG
ATTTGGGGCGGGAGAATGATCGACAATGGTAAAGGAAAGCAGGCGATAGTTATCGGTGGT
ATCCTGTTTTCTTTAGGTTTTATCTTGTCCGGGTTTGTGGATAATTTGCCCATGCTGTTT
TTAACCTACGGCGTCATTGCCGGGTTGGGATCGGGCCTGGCTTTTACGGGTAATCTTAAT
AATATTCTGAAATTTTTCCCTGACCGTCGCGGTCTTGCCAGCGGTATCGTACTGGCGGGT
GTTGGCGTCGGGACGCTACTTTGCACCCGCCTGGCCGAATATTTTATGGCGCAAACTCAC
GATGTTAGTCGGGCGTTGTTATATCTGGGTATTGTTTATCTGGTGGTTATTTTTATCGTC
CAGTTCTTTATTCGTAGCGCGCCAGCAAAAGATAGCGGAGGAATTAAAGCGTCGCCACTG
GATAAAGACTATCGGCACATGCTGAAAGATCTGCGCTTCTGGCTGCTGTTTATGATTCTG
GCGCTGGGCGTGTTCTCTGGGATGGTAATTAGCTCAAGCTCTGCGCAAATTGGTATGACG
CAGTACGGTTTACTGTCCGGTGCATTAGTCGTTAGCCTGGTCTCGATATTTAACTCGATC
GGTCGCCTGTTCTGGGGAGGGTTAACCGATAAATTAGGCGGCTATAATACGCTGGTTATT
GTTTACCTCTTTACCTGCTTGTGTATGCTGCTGTTATTTTTCTTCAATGGCAATACTTCG
GTATTTTATTTCAGCGCTCTGGGCGTGGGCTTTGCTTATGCCGGTATATTAGTTATCTTC
CCTGGTTTGACCAGCCAGAATTTTGGTATGCGTAACCAGGGGCTAAACTACGGCTTTATG
TATTTTGGTTTTGCCGTCGGTGCGGTTATTGCTCCTTATGTAACGTCCGCTATTGCAAAA
TATACCGGAAGCTACAATACAGTATTTATTTTGACAACGGTGCTATTGCTTATTGGAGTC
GTGTTGACCCTGATAACGAAAAAATATGTCGCAACGGTTTTAGCCAAAATTCATTAA
>EECEBICD_00768 2-dehydropantoate 2-reductase
ATGAAAATTGCAATCGCAGGTGCAGGCGCTATGGGGTGTCGTTTTGGCTATATGCTGCTG
GAGGCCGGGCACGACGTGACGCTTATCGATAGCTGGCAGGAGCATGTCGACGCTATTCGT
AGCAAGGGGTTGTTTGTCGAAACGGAAACGACTCAGAAGTATTACCCCATCCCTGCTATG
TTGGCTGATGAATCCCAGGGGGAGTTTGAACTGGTTATTCTGTTTACCAAAGCCATGCAG
CTGGATAGCATGTTACTGCGTATTAAGCCGCTGCTGCCAGCCGCGAAGGTCGTGATGATT
TTATCTAACGGCCTGGGAAATATTGAAACGCTGGAGAAATATGTCGATCGGCAGAAAATC
TATGCGGGTGTGACGTTATGGTCCAGCGAACTGGAGGGGGCTGGGCATATTATGGCCACC
GGTACCGGAACGATTGAACTGCAGCCGATTGCCAGCCAGGATTCGGCTCAAGAGGCTAAG
GTCATTGCCACTCTTAATAGCGCGGGATTGAATGCTGAAATAAGCCCTGACGTATTATTA
TCGATCTGGAAGAAAGCAGCCTTTAATAGCGTAATGAACACCTATTGCGCGCTACTGGAT
TGTAATGTCGGCGGATTTGGTCAGCGGCCTGGTGCTTTAGATTTAGCGCAAGCGGTAGTT
GATGAGTTTGTATTGGTTGCTGCCAGCCAGAATATTCCGTTGACTGAGCAAATGGTGATG
AATACGGTGAAGAAAGTGTTCGATCCGCGTGAGAGCGGCCACCACTATCCTTCTATGCAT
CAGGATTTACATAAAGGCCGACTGACTGAAATCGACTATTTAAATGGTGCGATTGCGCGA
ATCGGCGCTCAGAACAATATTGCCGTACCGGTTAACACACTCCTGACGCAATTGATTCAC
GCGAAAGAAGCGCAATAA
>EECEBICD_00769 HTH-type transcriptional regulator MurR
ATGAACTGTTTGATTCGTATTCGCCAGCGTTATCCGGATCTCGCGCAAAGCGACAGGAAA
CTGGCGGATTATCTGCTCGCGCAACCGGATACAGCGCGACATCTGAGCTCGCAGCAACTG
GCGGCCGAAGCGGGCGTCAGCCAATCCAGCGTAGTCAAATTCGCGCAAAAACTGGGGTTT
AAAGGGTTTCCTGCATTGAAACTGGCTATCAGCGAAGCGTTGGCCAGCAATCCTAATCCG
CATTCCGTTCCCGTTCATAACCAGATTCGCGGCGACGATCCGATGCGCCTGGTCGGGGAA
AAATTAATTAAAGAGAATGTGGCGGCGATGCACGCCACGCTGGACGTTAACAGTGAAGAA
AAGCTGTTAGAAAGCGTCACGATGCTGCGCCACGCCCGCCGTATTGTGATCACCGGGATT
GGCGCCTCTGGGCTGGTGGCGCAAAATTTTGCCTGGAAGCTACTGAAGATTGGCATTAAC
GCGGTGGTTGAGCGCGATATGCACGCCCTGCTGGCAACAGTGCAGGCGCTGGCGCCGGAG
GATCTGCTGCTGGCGATCTCTTATTCCGGGGAACGGCGCGAGCTGAACCTGGCGGCGGAC
GAAACATTACGCGCAGGCGCGAAGATCCTGGCCATTACCGGTTTTTCTCCCAACGCCTTG
CAGCAGCGCGCCACCCGCTGTCTGTATACGATTGCCGAAGAGCAGGCGACGCGCAGCGCC
GCTATCTCCTCGACACATGCGCAAATGATGTTGACCGACTTACTGTTTATGGCGCTGGTA
CAACAGGATTTAGAGCGGGCGCCGGAGCGTATTCGCCATAGCGAGGCGCTGGTAAAAAAA
CTGGTTTGA

>EECEBICD_00770 N-acetylmuramic acid 6-phosphate etherase
ATGAATCTTGGTACCTTAGTGTCTGAAACCCGTAACCCGCAAACGATGGATCTGGATGCG
TTGCCTACTCCTGAATTAGTTAAGCGTTTTAATGAACAGGATACGCGGGTAGCGGAAGCG
GTAAAAGCCACTCTGCCTGACGTTGCGCGTGCGGTGGATGCCGCTGTGGCGGCGCTGAAG
TCTGGCGGACGTATCATCTATATGGGCGCGGGCACCAGTGGTCGGCTTGGCGTACTGGAT
GCCTCAGAATGCCCCCCGACGTTTGGCGTTCCGCACGGTCTGGTCGTCGGGCTGATTGCC
GGTGGGCCGGGCGCGTTGCTGAAGGCGGTCGAAGGCGCGGAAGACAGCCAACAGGCAGGC
GAAGACGATCTTGTCGCGCTAAATCTGCAAGAACAGGATCTGGTCGTGGGGCTCGCGGCG
TCGGGTCGTACACCCTATGTGATTGGCGGCCTGCGCTATGCGCGACAGTCCGGTTGTACC
ACTGTTGCCGTCTCCTGTAATCCAGATTCGCCTATTGCGCGGGAGGCTAATATCGCCATT
TCGCCCGTTGTCGGGCCGGAAGCGCTTACCGGCTCCACGCGGCTGAAATCCGGCACCGCG
CAGAAAATGGTACTCAATATGATTTCTACCGGCGCGATGGTGAAGTTCGGCAAGGTCTAT
CAAAACCTGATGGTGGATATGAAAGCCACCAACGTCAAACTGGTCGATCGCGCGTGTCGG
ATGGTCGTTGAAGCGACCGGGATAGGTCGTGAAGAGGCGGAAACGTTGCTGAAACAGACC
GATTTTGAGGTTAAGCCCGCCATTTTGATGGCGCTTACGGGGCTGGATGCTGCAGCCGCC
AGGGAAAAACTCGCCGCTCATCAGGGCTTTTTAAGGGCGGCGTTAGAACACTAA
>EECEBICD_00771 PTS system EIIBC component
ATGGATAAGACGGCAGCGCTCGCCAGCGACATTTTACGTGGTATCGGCGGAGAGCAAAAT
ATTCTGCGCCTGGAAAACTGTATGACGCGAGTCAGGGTGGAGGTACAGGACGACAGCCAA
CTGGATATTCCGCGCTTAAAAGCGTTGCCTGGCATCAGTGGATACGTAAAGCAAGGGGAG
CAACATCAGCTGATCGTTGGACCGGGGAAGGCTGCACAGGTGGTTGATGCGATGCGCGTG
CAGATTGCCGTAGGTGGTGTAAAGCCGGATGACGCTATGGCGCGTACCAAATCCGAGGCG
AAAGCGAAGTATAAAGCGCCGATGAGCGATGCGTTGCGTAAGCTGGCGAATGTTTTTATA
CCGCTTATCCCCGCCTTTATCGCATCCGGCTTGATTACCGGCATTATCAACATTCTTAAA
CGTCCGGATATCGTCGGCGATGTTGCCGTCCACTATCCTAACCTGCTGGGCCTGATGGGG
ATATTTGGTAGCGCGGTGTTCGCTATCATGAACATTCTGGTTGGCGTCAATACCGCGAAA
GTCTTTGGCGGATCGCAGGCGTTAGGCGGCGTGATGGCGGGGATCCTTTCCAGCCCGCAA
CTGGCGCAGATTACGCTCTTTGGCGAGGCGCTTCAGCCGGGACGGGGCGGCGTTATCGCC
GTCCTGCTGGTGGTGGCGTTAATGTGCTGGATTGAGCGCCAGTTCCGTAAGCTGCTGCCG
GGGTCGCTGGAGTTAATTCTTAACCCTTTATTGACGACTGTTATCACCGGCGCGGTAGCG
ATTGTCGCTCTGCAACCGCTTGGCGGCTGGATTTCAGACGCTATCGCTCACGGCGCGTCC
TGGGCTATCGATCGCGGCGGTTTTCTGGTGGGCGCGGTGCTAGCCGGAACTTTCCTGCCG
TTGGTACTGACCGGGCTGCATCAGGGGCTGGTGCCTATTCATGTGGAGCTGGTGCAAGCG
CACGGCTATAACGCGCTGTTTCCCATCCTGGCGATGGCGGGCGTTGGTCAGATAGGCGCG
GCCATTGCCGTACTGATGAAAACCCGCAATGCGCGGCTCAAAAAGGTGATTAAAGGCGCG
CTTCCGGTCGGACTGCTGGGGATTGGCGAGCCGCTGATTTTTGGCGTCACGCTGCCATTG
GGTAAGCCATTTATTGGCGCTTGCCTCGGCGGCGCGGTAGGCGGGGCGTTGATCAGCTAC
TGGAAAGTGGCGACGGTAATTACCTTTGGTATTTCCGGTTTGCCGCTGGCATTAACAATT
GTTGCCGGAAAAGTCCTGTTCTATCTGTTAGGCTATTTAATAGCGGTAATTGCCGGGTTT
ATTTTTACCTGGCTGCTGGGGTTCAATGATCCAGAGGAGTAA
>EECEBICD_00772 Phosphatidylglycerophosphatase C
TTGGTCAGCCACGAGCGTCGTGTCGTCTTTTTTGATTTGGATGGAACGTTACATCAACAG
GATATGTTCGGCAGTTTTCTGCGTTATTTGCTGCGCCGTCAGCCGCTAAATGCGCTGCTC
GTACTGCCGCTGTTGCCCATTATCGGTATCGGTTTACTGGTAAAAGGGCGTGCGGCCCGC
TGGCCGATGAGTCTGTTATTGTGGGGCTGTACTTTTGGCCATAGCGAAACGCGTTTGCAG
GCGCATCAGGCGGATTTTGTCCGTTGGTTTCGCGCTAACGTCACGGCGTTTCCGGTAGTA
CAGGAGCGTCTGACCACCTATCTATTGAGCTCTGACGCGGATATCTGGCTTATTACCGGT
TCTCCGCAATCATTGGTAGAGCAAGTCTATTTTGATACGCCGTGGCTGCCGCGCGTTAAC
CTCATCGCCAGCCAGATGGCGCGACGGTATGGCGGCTGGGTATTAACCGTGCGCTGTCTG
GGCCATGAAAAAGTCGCGCAACTGGAACGTAAAATCGGTACGCCGCTGCGTCTTTATAGC
GGCTACAGCGACAGCAAACAGGATAACCCGCTGCTTTATTTTTGCCAGCATCGCTGGCGG
GTGACGCCGCACGGCGAGTTGCAGCAGCTCGAATAG
>EECEBICD_00773 tRNA-specific adenosine deaminase
TTGTCTGATGTCGAATTAGATCACGAATACTGGATGCGCCACGCGCTGACGCTGGCGAAA

CGCGCCTGGGACGAACGCGAGGTTCCGGTTGGCGCGGTATTGGTGCATAACCATCGCGTC
ATTGGCGAAGGCTGGAACCGACCCATTGGCCGCCACGATCCTACTGCGCACGCTGAAATA
ATGGCGCTGCGTCAGGGCGGTCTGGTATTGCAGAATTATCGGTTACTGGATACCACGCTG
TATGTCACACTGGAACCCTGTGTGATGTGCGCAGGCGCTATGGTGCATAGCCGCATTGGA
CGCGTTGTCTTTGGCGCGCGCGATGCAAAAACCGGCGCGGCCGGATCGCTAATTGATGTG
CTGCACCATCCGGGAATGAATCACCGGGTCGACATTATTGAAGGGGTGCTGCGCGATGAA
TGTGCGACGCTGCTCAGTGATTTTTTCCGTATGCGTCGTCAGGAAATTAAAGCCCTGAAG
AAAGCCGACCGTGCGGAAGGCGCGGGGCCGGCAGTATAG
>EECEBICD_00774 Membrane-bound lytic murein transglycosylase F
TTGAAAAAATTAAAGATTAATTATCTGTTCATCGGCATATTGACGCTGCTGCTGGCAGCA
GCCCTTTGGCCCTCAATCCCCTGGTTCGGTAAAACCGAAAACCATATCGCCGCGATTCAA
GCCCGGGGGATACTGCGCGTCAGTACGATCGACTCGCCATTAACCTATTCCGTTATCAAC
GGTAAAAAATATGGTCTGGACTATGAACTGGCGCAGCAGTTCGCCAACTATCTCGGCGTG
AAGCTGAAAGTGACGGTGCGCCAGAATATCAGCCAGTTGTTTGACGATCTGGATAACGGC
AATGCCGACCTGCTCGCGGCAGGGCTGGTCTACGACAGCGCACGCGTTAAAAACTATCAG
CCCGGTCCCATGTATTACTCGGTTTCGCAACAGCTCGTCTACCGGGTCGGACAATATCGC
CCACGTTCGCTGGCGACCGTTAACGAGAATCAGCTTACCATTGCGCCAGGGCACGTCGTG
GTCAATGACTTACAGCGTTTAAAAGAGACAAAGTTTCCCGACCTGAGCTGGAAAGTGGAT
GATAAAAAAGGCTCGACGACGCTGCTGGAAGAGGTCATCAGCGGCAAACTGGATTACACC
ATTGCCGATTCAGTGGCGATCAGTCTGTTTCAGCGCGTTCATCCCGAGCTGGCCGTGGCG
CTGGATGTCACGGATGAACAGCCCGTGACCTGGTTTAGCCGACTGGACGACGATAATACG
CTCTCCGCCGCGCTGCTCGATTTTTTCAATTCGATCAATGAAGACGGGTCGTTGGCGCGG
ATCGAAGAGAAATATCTGGGACATGGCGATGATTTCGACTACGTTGATACCCGCTCTTTC
CTGCGAGCGGTAGATAACGTCCTGCCGGAGCTGGAACCGTTATTTAAAAAATATGCCAAA
GAGATAGACTGGCGCCTGCTGGCGGCGATTTCTTATCAGGAATCACACTGGGACCCGCTG
GCGACCTCGCCAACCGGCGTGCGCGGCCTGATGATGCTGACTAAAAATACCGCCCAAAGT
CTGGGGCTGACCGACCGTACCGATGCGGAGCAAAGCATCAGCGGCGGCGCTCGCTATCTT
GAAGATATGATGGCTAAAGTTCCCGAAACCGTTCCGGAAGACGAGCGTATCTGGTTTGCC
TTAGCCGCCTACAATATGGGTTATGCGCACATGCTCGATGCCAGATCGCTGACGGTAAAA
ACCAAAGGCAATCCGGATAGCTGGACCGATGTGAAGCAGCGTTTGCCGCTATTAAGCCAA
AAACCGTATTACAGTAAGTTAACCTACGGTTACGCGCGTGGACATGAGGCTTATGCTTAT
GTGGAAAATATCCGTAAATACCAGATAAGTTTGGTGGGGTATTTGCAGGAAAAAGAAAAG
CAGGAAGCGGAAGCTATGAAACTGGCGCAGGATTACCCGGCGGTGTCGCCGGAGGAGCTG
AATAAAGCACCCTTCCCCTTCCTGTCGTTTTTATCGCAGTCATCAGGCTACCTGACGCAT
TCGCCGTCTTTACTGTTTACGCCGCAGAAGAAAGAAGAAAAATAG
>EECEBICD_00775 Phosphoribosylformylglycinamidine synthase
ATGATGGAAATTCTGCGTGGTTCGCCTGCACTGTCTGCATTCCGTATCAATAAACTGCTG
GCGCGCTTTCAGGCTGCCAACCTCCAGGTCCACAATATATACGCTGAGTATGTCCATTTT
GCTGACCTGAACGCCCCGTTAAATGATAGCGAGCAGGCGCAGCTTACCCGTCTGTTGCAA
TACGGCCCGGCGCTTAACAGCCATACGCCGGCGGGTAAACTCCTGTTGGTCACACCGCGT
CCTGGTACCATCTCCCCTGGTCTTCAAAAGCAACGGATATCGCCCACAACTGCGGCCTGC
AACAGGTTGATCGGCTGGAGCGCGGCGTAG
>EECEBICD_00776 Phosphoribosylformylglycinamidine synthase
TTGACGGCGGAACAGTGGCGACAAGTGGCCGCAGAGCTGCACGACCGTATGATGGAGACG
GTGTTCTCTTCTTTAACCGATGCGGAAAAACTGTTTATCCACCATCAGCCTGCGCCGGTT
TCCAGTGTCGATCTGCTGGGAGAAGGCCGTCAGGCGTTAATTGACGCCAACCTGCGTCTT
GGTCTGGCGCTGGCGGAAGATGAAATCGACTATTTGCAGGAGGCGTTTACCAAACTGGGG
CGCAACCCGAACGATATCGAATTGTACATGTTCGCCCAGGCGAACTCCGAGCACTGCCGC
CACAAGATTTTTAACGCCGACTGGATTATCGACGGTAAACCGCAGCCGAAGTCGCTGTTT
AAAATGATTAAAAACACCTTCGAAACCACGCCGGACTATGTGCTCTCTGCCTATAAAGAC
AATGCCGCGGTGATGGAAGGATCTGCGGTGGGGCGCTACTTCGCCGACCACAATACTGGC
CGTTACGATTTTCATCAGGAGCCCGCGCACATTCTGATGAAGGTCGAAACGCATAACCAC
CCAACCGCCATCTCTCCGTGGCCGGGGGCGGCGACCGGTTCCGGCGGCGAAATTCGTGAT
GAAGGCGCCACCGGGCGTGGCGCGAAACCAAAAGCCGGGCTGGTAGGCTTCTCCGTTTCC

AACCTGCGTATTCCGGGCTTTGAACAGCCGTGGGAAGAGGATTTTGGCAAGCCGGAACGT
ATTGTCACCGCGCTGGATATCATGACCGAAGGTCCGCTGGGCGGCGCGGCGTTTAACAAC
GAATTTGGTCGCCCGGCGCTGACCGGTTACTTCCGTACTTATGAAGAGAAAGTGAACAGC
CACAACGGCGAAGAGCTGCGCGGCTACCATAAGCCGATCATGCTGGCGGGCGGGATCGGT
AATATTCGCGCCGATCACGTGCAGAAAGGCGAGATCGTCGTGGGCGCGAAGCTGATCGTC
CTTGGCGGCCCGGCGATGAATATCGGCCTCGGCGGCGGCGCGGCATCGTCAATGGCTTCC
GGCCAGTCTGATGCGGATCTCGATTTTGCCTCTGTTCAGCGCGACAACCCGGAAATGGAA
CGCCGCTGCCAGGAAGTGATCGACCGCTGCTGGCAGTTGGGCGACGCTAACCCAATCTTG
TTTATCCATGATGTGGGCGCGGGCGGTCTCTCCAATGCGATGCCGGAGCTGGTGAGCGAC
GGCGGGCGCGGCGGGAAATTCGAACTGCGCGACATTCTTAGCGACGAGCCGGGCATGAGC
CCGCTGGAAATCTGGTGTAACGAATCCCAGGAGCGCTACGTACTGGCGGTCGCGGCTGAC
CAGTTGCCGCTGTTCGACGAACTGTGTAAGCGCGAACGCGCGCCGTATGCGGTCATTGGC
GATGCTACCGAAGAACAGCATCTCTCGTTGCATGATAACCATTTCGACAATCAGCCGATC
GACCTGCCGCTGGATGTCCTGCTGGGCAAAACGCCGAAAATGACCCGCGATGTGCAAACA
TTGAAGGCGAAAGGCGATGCGCTGAACCGCGCGGATATCACGATTGCCGACGCGGTAAAT
CGTGTTCTGCATCTGCCGACCGTGGCGGAAAAAACCTTCCTTGTCACTATTGGCGACCGT
ACCGTCACCGGTATGGTGGCGCGTGACCAGATGGTTGGCCCGTGGCAGGTCCCGGTGGCT
GACTGCGCGGTCACTACCGCCAGCCTCGACAGCTACTACGGCGAAGCGATGTCGATTGGC
GAGCGCGCGCCGGTGGCGCTGCTGGACTTTGCCGCCTCCGCCCGTCTGGCCGTCGGTGAA
GCGTTGACTAACATTGCCGCGACGCAGATTGGCGATATCAAGCGCATTAAGCTTTCCGCT
AACTGGATGGCGGCAGCCGGTCATCCTGGCGAAGATGCCGGTTTATATGACGCGGTTAAA
GCGGTGGGAGAGGAACTCTGTCCGCAGCTTGGCCTGACGATTCCGGTGGGCAAAGATTCG
ATGTCGATGAAAACCCGCTGGCAGGAAGGCAACGAACAGCGCGAAATGACCTCGCCGCTG
TCGCTGGTGATTTCCGCGTTTGCCCGCGTGGAAGACGTGCGTCACACCCTTACGCCGCAG
CTGTCAACGGAAGATAACGCCTTGTTGCTGATCGATCTTGGCAAGGGCCACAACGCGCTG
GGCGCAACGGCGCTGGCGCAGGTCTATCGTCAGCTTGGCGACAAACCCGCGGACGTGCGT
GACGTCGCGCAACTGAAAGGCTTCTATGACGCCATGCAGGCGCTGGTCGCTGCGCGTAAA
TTACTGGCCTGGCACGACCGTTCCGACGGCGGTCTGCTGGTGACGCTGGCCGAGATGGCC
TTTGCCGGCCACTGCGGCGTGCAGGTCGATATCGCCGCGCTGGGCGATGACCATCTGGCC
GCACTGTTCAACGAGGAGCTGGGCGGCGTGATTCAGGTACGCGCTGAAGACAGAGACGCG
GTCGAAGCGCTGTTGGCGCAGTACGGTCTTGCTGACTGCGTCCACTACCTTGGTCAGGCC
CTGGCTGGCGATCGCTTTGTGATTACCGCTAATGACCAGACGGTATTCAGCGAAAGCCGC
ACTACGCTGCGTGTCTGGTGGGCGGAAACCACCTGGCAGATGCAGCGTCTGCGCGACAAT
CCGCAATGTGCTGATCAGGAACATGAGGCGAAAGCGAATGATGCCGATCCGGGCCTCAAC
GTGAAGCTGTCGTTTGATATTAATGAAGATATCGCCGCGCCGTACATTGCGACAGGCGCA
CGACCGAAAGTCGCTGTACTGCGCGAGCAGGGGGTGAACTCTCACGTGGAGATGGCGGCG
GCGTTCCATCGTGCGGGCTTTGACGCGATTGACGTCCATATGAGCGACCTGCTGGGCGGT
CGTATCGGTCTGGGCAACTTCCATGCGCTGGTGGCTTGCGGCGGCTTTTCCTACGGCGAC
GTGCTGGGGGCGGGCGAAGGCTGGGCGAAATCCATTCTGTTTAACCATCGGGTGCGCGAC
GAATTCGAAACCTTCTTCCATCGTCCGCAGACACTGGCGCTGGGCGTGTGCAACGGTTGC
CAGATGATGTCGAATCTGCGCGAGCTGATCCCGGGCAGCGAACTGTGGCCGCGGTTTGTA
CGTAACCATTCTGACCGCTTCGAAGCCCGCTTCAGCCTGGTGGAAGTGACGCAAAGCCCA
TCGCTGCTGCTGCAAGGAATGGTAGGCTCACAGATGCCGATTGCGGTTTCCCATGGGGAA
GGGCGCGTTGAGGTACGTGACGATGCGCATCTTGCCGCGCTTGAGAGCAAAGGCCTGGTC
GCTCTGCGCTACGTTGATAACTTTGGTAAGGTGACTGAAACCTATCCGGCTAACCCGAAC
GGTTCGCCGAACGGCATCACGGCGGTGACGACCGAAAACGGTCGGGTGACGATTATGATG
CCGCATCCGGAACGCGTATTCCGCACCGTCGCCAACTCCTGGCACCCGGAAAACTGGGGC
GAGGATAGCCCGTGGATGCGTATCTTCCGTAATGCGCGTAAGCAGCTTGGTTAA
>EECEBICD_00777 Sensor histidine kinase QseE
ATGCTGGCCTTTTTGCTGATCCTGCTGCCGTTGCTGGTTCTGGCCTGGCAGGCGTGGCAA
AGCCTTAACGCGTTAAGCGATCAGGCTGCGGTGACTAACCGCAGCACGCTTATTGACGCC
CGGCGCAGCGAGGCGATGACCAACGTCGCGCTGGAGATGGAGCGAAGCTACCGACAGTAT
TGTGTGCTTGACGATCCGACGCTGGCAAAAGTGTATCAAAGCCAGCGTAAGCGCTATAGC
GATATGCTGGACGCCCATGCCGGCGTCTTGCCGGATGATAAGTTGTATCAGGCGTTACGC

CAGGATTTAAACGCGCTGGCGCAATTGCAGTGTAAAGATAGCGGCCCGGAAGCGGCAGCG
GCCGCGCGTCTGGAGGCCTTTGCCAACGCGAATACGGAAATGGTGCAGGCGACGCGTACT
GTGGTTTATTCGCGTGGGCAACAGTTACAACAAGAAATCGCCGAGCGCGGACAATTTTTC
GGCTGGCAGGCGCTGGTGCTATTTCTGGTGAGCCTGGCGATGGTGCTACTTTTTACCCGC
ATGATTATCGGGCCGGTGAAAGGAATTGAACGGATGATTAATCGCCTGGGCGAAGGCCGT
TCTTTAGGGAATACCGTCACCTTTACCGGTCCGCGTGAATTACGATCGGTGGGGCAGCGC
ATTATCTGGCTGAGTGAGCGTCTGGCGTGGCTGGAATCCCAGCGCCATCAGTTTCTACGT
CACTTGTCCCATGAGCTGAAAACCCCGCTGGCCAGTATGCGCGAGGGGACAGAGCTGCTG
GCCGATCAGGTGGTTGGGCCGCTGACGCCGGAGCAAAAAGAGGTGGTCGATATTCTGGAT
GACAGCAGTCGTAATTTACAAAAATTGATTGAGCAATTGCTTGATTACAACCGGAAACTG
GTAGACAGTGCCACAGAGCTGGAAGCGGTCGATATCGCGCCGCTTGTGGATATGGTGGTT
TCCGCCCACAGTTTGCCTGCCCGCGCTAAAATGATGCATACCGATGTCGACCTGGAGGCC
GAACGCTGCATAGCAGAGCCCATGTTGCTGATGAGCGTGCTGGATAATCTCTATTCCAAC
GCGGTGCACTATGGCGCTGAATCCGGTAACATTTGTATCCGTAGTCGCTCTCAGGGCTCC
ACGGTTTATATCGATGTGGTTAATAGCGGCGAGCCGATTCCGCAAACCGAAAGAGAAATG
ATTTTTGAGCCTTTTTTCCAGGGAAGCCACCAGCGAAAAGGGGCGGTAAAAGGCAGTGGG
CTAGGGCTGAGTATCGCCAGAGATTGTATTCGTCGAATGCAGGGAGAGATCCAACTGGTG
GATGACAAAGCGCAGGAAGTCTGTTTTCGCATCTCGCTGCCGCTGCCTGCATCTGACAAA
CACTAA
>EECEBICD_00778 Quorum-sensing regulator protein G
ATGAATTTAAGCCTGGTGCGTATGTCACACGTTTTCGTCCAAACTATTAAACGATGCCTT
TTGCGGTGGGGAATACCGGTGGGCATATCGTGTCTGGCGCTGACCGCATGTGTTCCCCAT
GCTTCACAGCAACTGCCCGGTAGCGCAGCGCAGGACACGCTCCCCCATTATCAACTGGCG
GATTATCTCCCGACGGCGTGCGCGGATATCTGGTCGCTACGCGGGCAGGCGGTGGAGACG
AATCCGCTGTACTGGCTACGCACGATTGACTGCGCCGATCGTCTGATGCCGGTTCAGTCG
CGCGCAGAGGCGCGCGCGTTGACGGATGATAACTGGCAAAATGCCTTCAGGCGCGGGATT
TTACTGGCGGATGCAAAAATTACGCCGCCGGAGCGGCGCGCCATAGTGACCCGTCTGGAG
GCGTTAAGCGCGCAAATACCGGCTCAGGTACGGCCCGTCTACCAAATCTGGCATGACGGT
CAGGCGCTGCAATTAGCACTTTCTGCCGAACGTCAGCGTTATAGCAAGCTCCAGCAAACG
TCAGATAGTGAGCTTGATGCGCTGCGCCAGCAGCAACAGGCCTTGCAAACGCAGCTCGAT
CTTACCACCCGTAAGCTGGAGAGTTTAACCGATATTGAGCGCCAGCTTTCGACACGAAAA
CCGGCAGGGAATTACAACGCCGATACGCCGCATACGAATGACAAACCCGCGACGTCTGAA
GATGGCGCGGCGCCGTCGCCGTCACAAGATGAGGTAACGCCATGA
>EECEBICD_00779 Transcriptional regulatory protein QseF
ATGATAAGCCGTAAACCGGCGCACCTGCTGCTGGTCGATGACGATCCCGGATTACTGAAA
TTGCTCGGTATGCGCCTGACCAGTGAAGGATACAGTGTAGTGACCGCAGAGAGCGGCCAG
GAAGGACTGCGGGTACTGCATCGGGAAAAAGTGGATCTGGTAATAAGCGACCTGCGCATG
GATGAAATGGATGGTATGCAACTGTTCACAGAAATTCAGAAAGTACAGCCGGGAATGCCG
GTTATCATTCTGACGGCGCATGGTTCTATTCCGGATGCGGTGGCCGCTACCCAGAAAGGC
GTATTCAGCTTTTTGACGAAGCCGATCGACAGAGACGCGTTATATAAAGCGATAGATGAA
GCGCTGGAGCAGTCCGCTCCCGCGACGGACGATAGCTGGCGCAACGCTATCGTTACCCGT
AGCCCGCTGATGCTGCGACTGCTTGAACAGGCGCGCATGGTCGCGCAGTCGGATGTCAGC
GTATTGATTAACGGACAAAGCGGCACCGGGAAAGAGATTTTCGCACAGGCGATTCATAAT
GCCAGCCCGCGCAGTAACAAGCCGTTTGTCGCCATCAACTGCGGCGCGTTGCCGGAACAG
TTGCTGGAGTCCGAATTATTCGGCCATGCGCGCGGCGCCTTTACCGGCGCAGTGAGCAAT
CGTGAAGGGTTGTTCCAGGCGGCGGAAGGCGGCACGCTGTTTCTGGATGAAATTGGCGAT
ATGCCCGCGCCGTTACAGGTGAAGCTGCTGCGGGTGTTACAGGAGCGTAAAGTCAGGCCG
CTGGGCAGCAATCGCGATATTGATATCGACGTGCGAATTATTTCCGCGACGCACCGCGAT
CTGCCGAAGGCGATGGCGCGGGGCGAATTTCGTGAAGATTTGTACTACCGGCTGAATGTC
GTGAGCCTGAAAATTCCGGCGCTGGCGGAGCGTACCGAGGATATTCCGCTGCTGGCTAAT
CATCTGCTCAGACAGTCGGCACAGCGGCATAAACCGTTTGTTCGCGCCTTTTCTACTGAT
GCAATGAAACGGCTGATGACGGCAAGTTGGCCGGGCAACGTGCGCCAACTGGTCAACGTC
ATTGAACAATGCGTGGCGTTAACCTCTTCGCCGGTGATCAGCGATGCGTTGGTCGAGCAG
GCGCTGGAAGGCGAAAACACCGCGTTGCCAACTTTTGCCGAAGCGCGAAACCAATTTGAA

CTGAACTATCTGCGCAAGTTACTGCAAATTACCAAAGGTAACGTGACGCACGCGGCAAGG
ATGGCGGGGCGCAACCGCACGGAATTCTATAAGCTACTCTCCCGCCACGAACTGGATGCG
AATGATTTCAAAGAGTAG
>EECEBICD_00780 Nitrogen regulatory protein P-II 1
ATGAAAAAGATTGATGCGATTATTAAACCCTTCAAGCTGGACGATGTCCGTGAAGCGCTG
GCCGAGGTAGGTATTACCGGGATGACGGTCACTGAAGTCAAAGGCTTTGGCCGTCAAAAA
GGCCACACCGAGCTGTATCGCGGCGCGGAATATATGGTGGATTTTCTGCCGAAGGTAAAA
ATTGAGATCGTTGTGCCTGACGACATCGTGGATACCTGTGTTGACACCATCATTCGTACC
GCGCAGACGGGGAAAATCGGCGACGGTAAGATCTTTGTCTTTGATGTCGCCCGCGTTATT
CGTATCCGTACTGGCGAAGAAGACGACGCGGCTATCTGA
>EECEBICD_00781 Dipeptide and tripeptide permease C
ATGAAAACACCCTCACAACCGCGCGCGATTTTTTACATCGTGGCGATTCAAATTTGGGAA
TACTTCAGCTTTTACGGCATGCGTGCCTTACTCATCCTTTATCTTACCCACCAGCTTGGT
TTTAATGACAGCCACGCGATCAACCTCTTCAGCGCCTACGCCTCTTTAGTTTACGTTACC
CCTATTCTTGGCGGCTGGCTTGCCGACCGCCTGCTTGGCAACCGCGTGGCGGTTATCACC
GGCGCCTTATTGATGACGCTGGGTCATGTGGTATTGGGCTTAGAATCTGATTCAACGCTG
AGTTTATATGCGGCGCTGGCCATTATTATCTGCGGCTACGGCTTGTTTAAATCCAATATT
AGCTGTCTGCTGGGCGAACTTTACGCCCCTGATGACAACCGCCGCGACGGGGGATTCTCG
CTGCTCTACGCCGCCGGGAATATTGGATCGATAGCCGCGCCGATCGCCTGTGGTCTGGCG
GCGCAGTGGTATGGCTGGCATGTCGGCTTTGCGCTGGCGGGCGTGGGAATGTTCATCGGC
CTGCTCATCTTTTTAAGCGGTCATCGCCACTTTCAGCAGACGCGTGGGGTTAATCGCCCC
GCGCTGCGGGCGGTTAAATTCGCCCTTCCAACCTGGGGTTGGCTGGTATTAATGCTGTGT
ATCGCGCCGGTATTCTTCACCCTCCTGCTGGAAAATAACTGGTCTGGCTATGTGCTGGCC
ATTGTCTGCGTCTTCGCGGCGCAACTTATCGCCCGTATTATGGTTAAATTTCCCGAGCAC
CGTCGCGCCTTGTGGCAAATCGTTCTGTTAATGATCACCGGCACTCTCTTTTGGGTTCTG
GCGCAGCAGGGCGGAAGTTCGATCAGTTTATTCATCGACCGTTTTGTCAACCGCCACTGG
CTGAATATGACCGTCCCCACCGCATTGTTCCAGTCGGTCAATGCGATTGCGGTGATGGCG
GCGGGCGTGGTGCTGGCGTGGCTGAGCAGCCCGAAAGAGAGCGCCCGCTCGGTGCTGCGC
GTCTGGCTTAAATTTGCCGTGGGGCTGGTATTAATGGGCGGCGGTTTTATGCTGCTGGCG
CTAAACGCGCATCAGGCCCGGTTAGACGGGCAGGCATCAATGGGAATGATGATCGCAGGG
CTGGCACTGATGGGCTTTGCAGAGCTGTTTATTGATCCGGTCGCGATGGCGCAAATTACC
CGCCTCAACCTGCCTGGCGTCACGGGCGTGCTGACCGGTATTTATATGCTGGCGACCGGC
GCGGTCGCTAACTGGCTGGCTGGCGTCGTCGCGCAACAAACCACGGAATCGCAAATTAGT
GATACGGCGGTAGCCGCCTACGGACACTTTTTCTCGCAAATGGGCGAGTGGACGCTAAGT
TGCGTGGCGCTGATCGTCGCCATTGTCGGCCTGAGATGGGTTTGCACCCGGACTACATCG
GCGCTACCGCAGGGCGATTAA
>EECEBICD_00782 Inducible lysine decarboxylase
ATGAACGTTATTGCTATCATGAACCACATGGGCGTCTACTTTAAAGAAGAGCCTATTCGT
GAACTGCATCGTGCACTGGAAGGTTTAAATTTCCGTATCGTCTATCCAAACGACCGAGAA
GACCTGCTGAAGCTGATTGAAAATAACTCCCGCCTTTGCGGCGTCATTTTCGACTGGGAT
AAATATAACCTTGAGCTTTGCGAAGAGATTAGCAAGCTGAACGAGTATATGCCGCTGTAC
GCCTTCGCCAACAGCTACTCTACGCTGGACGTCAGCCTCAACGATCTGAGAATGCAGGTT
CGCTTCTTTGAATATGCTTTAGGCGCCGCAGCCGATATCGCGGCGAAAATTCGCCAGAAT
ACCGATGAATACATCGACAATATTCTACCGCCGCTGACAAAAGCGCTGTTCAAATATGTG
CGCGAAGGGAAATATACTTTCTGTACGCCTGGCCACATGGGCGGGACCGCTTTCCAGAAA
AGCCCCGTCGGCAGCATCTTTTATGATTTCTTCGGCCCGAACACGATGAAATCCGATATT
TCGATTTCCGTTTCTGAACTGGGTTCGCTGCTGGACCACTCCGGCCCGCATAAAGAAGCG
GAAGAGTATATCGCCCGCGTCTTTAACGCCGAACGTAGCTACATGGTGACGAACGGCACC
TCTACGGCGAACAAAATCGTCGGTATGTACTCCGCGCCGGCAGGCAGCACGGTACTGATT
GACCGTAACTGCCATAAATCGCTGACCCATCTGATGATGATGAGCGACATTACGCCGATT
TATTTCCGCCCAACCCGTAACGCCTACGGTATTCTTGGCGGTATTCCGCAAAGCGAGTTC
CAGCACGCCACTATCGCTAAACGGGTAAAAGAGACGCCTAACGCGACCTGGCCGGTCCAT
GCTGTTATTACTAACTCAACCTATGACGGTCTGTTGTATAACACTGACTACATCAAGAAA
ACTCTGGATGTGAAGTCCATTCATTTTGACTCCGCCTGGGTGCCTTATACCAATTTCTCC

CCTATTTATCAGGGTAAATGCGGTATGAGCGGCGACCGTGTAGAAGGCAAAATCATTTAT
GAAACCCAGTCCACGCATAAACTGCTGGCGGCGTTTTCACAGGCATCCATGATTCATGTT
AAAGGCGACATTAACGAAGAAACCTTTAACGAAGCCTACATGATGCATACCACCACTTCT
CCGCACTACGGTATCGTAGCGTCTACTGAAACCGCAGCGGCGATGATGAAAGGTAATGCG
GGTAAACGCCTGATTAACGGCTCTATTGAACGTGCGATTAAATTCCGTAAAGAAATTAAA
CGCCTGAAAAGCGAATCCGACGGCTGGTTCTTTGACGTATGGCAGCCGGAACATATCGAT
GGAGCTGAGTGCTGGCCGCTGCGTTCAGATAGCGCATGGCATGGCTTCAAAAATATCGAT
AACGAACACATGTACCTCGACCCGATCAAAGTCACTATCCTGACGCCGGGGATGAAGAAA
GACGGTACGATGGATGAATTCGGTATTCCGGCAAGTCTGGTCGCCAAATATCTCGATGAA
CGCGGCATCATCGTAGAAAAAACCGGTCCATACAACCTGCTGTTCCTGTTCAGCATTGGT
ATCGACAAAACCAAAGCTCTGAGCCTGCTGCGCGCGCTCACTGAATTTAAACGCGCCTTT
GACCTGAACCTGCGCGTCAAAAACATTCTGCCGGCACTGTACCGCGAAGCGCCTGAGTTT
TATGAAAATATGCGCATCCAGGAACTGGCGCAGAATATTCATAAACTGGTCGAACATCAT
AATCTGCCGGACTTAATGTACCGTGCGTTTGAAGTGCTGCCGAAAATGGTGATGACGCCG
TATACTGCGTTCCAGAAAGAGCTGCATGGCGAAACGGAAGAGGTGTATCTTGAAGAGATG
GTTGGACGCGTCAACGCCAACATGATCCTTCCTTACCCTCCGGGAGTGCCGCTGGTGATG
CCTGGTGAAATGATCACCGAGGAAAGCCGTCCGGTACTGGAATTCCTGCAAATGCTGTGC
GAAATCGGCGCCCACTATCCGGGCTTCGAAACCGATATCCACGGCGCCTATCGTCAGGCA
GACGGACGTTACACCGTTAAAGTGCTGAAAGAAAATACGAAATAA
>EECEBICD_00783 putative cadaverine/lysine antiporter
ATGAGTTCTGTCAAAAAGATCGGGCTATTTGCCTGTACTGGCGTCGTTGCCGGTAATATG
ATGGGGAGCGGGATTGCATTATTACCCGCCAACCTTGCCAGTATTGGTGGCATTGCCATC
TGGGGCTGGGTAATTTCTATTATTGGGGCAATGTCGCTGGCTTATGTTTATGCGAGGCTG
GCAACCAAAAACCCTCAACAGGGTGGCCCTATCGCTTATGCTGGGGAAATATCTCCGGCG
TTTGGTTTCCAGACCGGCGTTCTTTATTATCACGCAAACTGGATTGGTAACTTAGCCATC
GGCATTACCGCTGTTTCTTATCTTTCTACGTTCTTCCCGGCATTAAATAATCCAATACCG
GCAGGTATTGCCTGTATTGCTATCGTCTGGCTGTTCACCTTTATTAATATGCTGGGTGGG
GCCTGGGTCAGCCGCTTGACAACGATTGGCCTATTCCTGGTGCTGATCCCGGTAGTCTTG
ACCGCAGTCGCCGGCTGGCATTGGTTCGATATCGCGACCTATCACGCGAACTGGAACACG
TCCACGACGACCGACTCTCACGCCATTGTTAAAAGTATTCTGCTCTGCCTGTGGGCGTTC
GTTGGCGTTGAGTCTGCCGCCGTCAGCACCGGCATGGTGAAAAACCCGAAACGCACCGTA
CCGCTGGCAACCATGCTGGGTACCGCGCTGGCAGGCATCATCTATATCGCCGCGACTCAG
GTTATCGCCGGGATGTTCCCAGCTTCCGTTATGGCCTCTTCCGGCGCGCCGTTCGCTATC
AGTACCTCCACGATTCTGGGTGGCTGGGCTGCGCCGCTGGTTTCCGCCTTTACCGCTTTC
GCCTGCCTGACGTCTCTGGGCTCCTGGATGATGCTGGTCGGCCAGGCTGGGGTTCGCGCC
GCCAACGACGGCAACTTCCCGAAAATTTACGGTGAAATGGATAAAAACGGTATTCCGAAA
AAAGGTCTGCTGTTGGCTGCGGTGAAAATGACGGCGCTGATGATTCTGATCACCATCATG
AACTCCAGCGGCGGTAAAGCGTCCGACCTGTTCGGCGAACTGACCGGGATTGCCGTACTG
CTTACCATGCTGCCGTACTTCTACTCCTGTGTTGACCTGATTCGCTTCGAAGGGTTCAAC
ATCCGTAACTCGGTAAGCCTGATTTGTTCTGTACTGGGCTGCGCATTTTGCTTCATCGCA
CTGATGGGCGCCAGCTCCTTCGAACTCTCCGGCACCTTTATCGTCAGTCTGATCATCCTG
ATGTTCTACGGTCGCAAAATGCACCAGCGCCAGAACAACGACAGCGACAACAATACGGCT
GAAGCGCTTTAA
>EECEBICD_00784 Transcriptional activator CadC
ATGCAGCAACCTGTTGTACGCATTGGAGAGTGGCTGGTTACACCTTCTGTTAATCAGATC
AGTCGTCAGGGACGCCAGATTACTCTTGAACCACGGCTGATCGATCTTCTGATGTATTTT
GCGCATCACCCGGATGAAGTGTTAAGCCGGGATAACATTATTGATCATGTCTGGATGCGC
ACCATCGTGACCAACCATGTTGTCACCCAAAGCATTTCTGAACTACGCAAATCATTAAGA
GATGGCGGAGACAGCAATGCTGAATACATCGTTACCGTACCTAAACGCGGCTATAAGCTG
ACGGCGCCGGTTATCTGGTGCGAAGAAAATAGCGATGAGATCGATAATTCATCAACGTCG
CCGCCGCCGCCGATCGCAGCGACGAATGCAGAACCGACAGAGGGGGTTACTGCCGCTACG
CCTGTGCCGCCCGCCTCTTTGCAAACGCCGACTAAAAAGGCGAAAAAGCCGCGTATCGCC
GCTTTCTGGACGTGGGTGATGTTCCTGCTCTCGCTGGCGACGCTGGTGGTGTTTATCGTG
ATGTCGGTGGTAGATCATAATGCCGCGGTAACAAAAACGCGGTTATTACTTAACCCACGA

GATATAGATGTTCGTTTTGAAGGCGGCAATTCCTGTAATAACTGGGTCTCGCAGGAGTCC
TACGCTATCGGTCTGGGCGGCTTAATCACCGACTTGCTGAACACCTACTCGACCTTTATG
GTACATGATAAAACCAATTACCGGGTCAATGAGCCAAGCAGCTCCGGTAAGACGTTAACC
ATTCAGTTTGTCAATCAGCGCCACTATCGGGCGCAGCAGTGCTTTATGTCTGTGGTGCTC
ATTGATAACGCCGATGGTTCAACCATGCTGGATAAGCGTTATTTCGTCACCAATACTAAC
CAGTTGTCCATCCAGGATGATCTGTTTAATAGCCTGTCTTTCGTTTTAACACAGCCCTGG
CCTAATCGTATGCGAGAACAACTGGCCCTGTTCAGAACGCCGCAAAATACCGCGCTGATG
CGTTTTTATGAGGCGCGCCAGCTTATTCTGAGCGGCGATGCGCAGGCGCTAGGTAAAGCC
AGCGATATTCTGAATGGCATTATTAAGGAAACGCCTGATTTCAACTATGCCTATGAATAT
AAAGTGCTGGTAGATGTATTACGGCAATCACAACAGCCGTTTGATAAAGAACAAGTGGCT
GCGCTGAACGAAGAGTTTAAAAAAATAGATCAAATTCCGGGCGTTGAGAAAACGTCTGTT
TATTATAAAATCAAGACCGTTGATCTTTTGGGCAAAGGTGATATCGATGCAGCGTATGAG
GAAATCAATAAAAGCATCGAGCTTGAAATGTCGTGGTTTAACTATGTCCTGTTGGGGAAA
GTTTACGAAATGAAAGGTGAGAATCGTCTGGCCGCTGATGCCTATCTGACCGCCTTTAAT
TTACGTCCAGGTGAAAACACGCTGTACTGGATCGAGAACGGCGTATTTCAGACATCCGTT
CAGAAAATTGTCCCTTATCTGAATAGTTTTCTGGCAGAAGATTAA
>EECEBICD_00785 Flavohemoprotein
ATGCTTGACGCACAAACCATCGCTACAGTAAAGGCCACCATTCCCCTGCTGGTTGAAACA
GGACCGAAACTGACCGCCCACTTCTACGACCGTATGTTTACGCATAACCCGGAGCTCAAA
GAAATCTTCAATATGAGCAACCAGCGTAACGGCGATCAGCGTGAAGCCCTGTTTAATGCT
ATCGCGGCCTACGCCAGCAATATCGAAAATTTACCGGCGTTGCTGCCGGCGGTAGAAAAA
ATCGCGCAGAAGCACACCAGCTTCCAGATTAAGCCGGAGCAGTACAACATCGTCGGGACG
CATCTGCTGGCGACGCTGGACGAAATGTTCAACCCGGGCCAGGAAGTGCTGGACGCGTGG
GGCAAAGCCTATGGCGTACTGGCTAACGTCTTTATTCATCGGGAAGCCGAGATTTATCAC
GAGAACGCCAGCAAAGACGGCGGCTGGGAAGGCACGCGCCCCTTCCGCATCGTTGCGAAA
ACCCCGCGTAGCGCACTGATCACCAGCTTTGAGTTTGAACCGGTCGACGGCGGTACGGTG
GCGGAATACCGTCCCGGGCAGTATCTGGGGGTCTGGCTGAAGCCGGAAGGTTTTGCGCAT
CAGGAGATCCGCCAATATTCACTGACCCGTAAACCCGATGGCAAAGGGTACCGTATTGCC
GTGAAGCGTGAAGACGGCGGCCAGGTATCAAACTGGTTACACCATCACGCCAGCGTGGGC
GATGTGGTGCATCTGGCTGCGCCGGCAGGTGACTTCTTTATGAATGTCGCCGCTGATACC
CCCGTTTCGCTGATTTCCGCTGGCGTCGGCCAGACGCCGATGTTAGCGATGCTCGATACG
CTGGCGAAAGAACAGCATACCGCGCAGGTGAACTGGTTCCACGCGGCGGAGAACGGCGAC
GTCCATGCGTTTGCCGACGAAGTGAGCGAGCTGGGCCGTACACTGCCGCGTTTCACTGCC
CATACCTGGTACCGCGAGCCGACTGAGGCCGATCGCGCCCAACGCCTCTTCGACAGCGAA
GGGCTGATGGATTTAAGCAAACTGGAAGCTGCGATCAGCGATCCGGCAATGCAGTTCTAT
CTTTGCGGCCCGGTAGGTTTTATGCAGTTTGCCGCAAAACAACTGGTTTCACTTGGCGTG
AATCACGAAAACATTCATTACGAATGCTTCGGCCCGCATAAAGTGCTGTAA
>EECEBICD_00786 Serine hydroxymethyltransferase
ATGTTAAAGCGTGAAATGAACATTGCCGATTATGATGCCGAATTGTGGCAGGCTATGGAG
CAGGAAAAAGTACGTCAGGAAGAGCACATCGAACTGATCGCCTCCGAAAACTACACCAGC
CCGCGCGTGATGCAGGCGCAGGGGTCTCAGCTGACCAACAAATACGCTGAAGGATATCCG
GGCAAGCGCTACTACGGCGGTTGCGAATACGTTGATGTCGTAGAGCAGCTGGCTATCGAC
CGCGCGAAAGAACTGTTCGGCGCCGACTACGCTAACGTGCAGCCGCACTCCGGTTCTCAG
GCTAACTTCGCTGTTTACACCGCGCTGCTGCAGCCGGGCGATACCGTTCTGGGTATGAAC
CTGGCGCAGGGCGGCCACCTGACTCACGGCTCCCCGGTTAACTTCTCCGGTAAACTGTAC
AACATCGTACCTTACGGTATCGATGAGTCCGGTAAAATTGACTATGACGAGATGGCGAAG
CTGGCCAAAGAGCACAAGCCGAAGATGATTATCGGTGGCTTCTCTGCCTACTCCGGCGTG
GTTGACTGGGCAAAAATGCGTGAAATCGCTGACAGCATCGGCGCATACCTGTTTGTCGAC
ATGGCGCACGTGGCGGGCCTGATTGCCGCAGGCGTTTACCCGAACCCGGTTCCGCACGCT
CACGTTGTCACCACCACCACCCACAAAACCCTGGCGGGTCCGCGCGGCGGCCTGATCCTG
GCGAAAGGCGGCGATGAAGAGCTGTACAAAAAACTGAACTCCGCCGTCTTCCCAAGCGCG
CAGGGCGGCCCGCTGATGCACGTGATCGCGGGTAAAGCGGTGGCACTGAAAGAAGCGATG
GAGCCGGAGTTCAAAGTTTACCAGCAGCAGGTGGCGAAAAACGCCAAAGCGATGGTGGAA
GTGTTCCTGAACCGCGGCTACAAAGTGGTGTCTGGCGGCACTGAGAACCACCTGTTCCTG

CTGGACCTGGTGGATAAAAACCTGACCGGTAAAGAAGCTGACGCCGCGCTGGGCCGCGCT
AACATCACCGTGAACAAAAACAGCGTGCCGAACGATCCTAAGAGCCCGTTCGTGACTTCC
GGTATCCGTATCGGTTCTCCGGCGGTCACTCGTCGCGGCTTCAAAGAAGCGGAAGTGAAA
GAGCTGGCTGGCTGGATGTGTGACGTGCTGGACAACATCAATGATGAAGCCACCATTGAA
CGCGTGAAAGCGAAAGTGCTGGATATCTGCGCCCGCTTCCCGGTTTACGCGTAA
>EECEBICD_00787 putative 3-phenylpropionic acid transporter
ATGGCATTGCATTCCACGCGCTGGCTGGCGCTCAGTTATTTCACCTACTTCTTTAGTTAC
GGTATTTTTCTGCCCTTCTGGAGCGTCTGGCTCAAAGGTCTTGGGCTAACGCCGGAAACC
ATCGGTCTTCTGCTGGGCGTGGGTCTGGTCGCGCGTTTTCTCGGTAGTCTGCTCATTGCG
CCTCGCGTAAGCGATCCTTCGCGGTTGATCTCCGCGCTGCGCGTACTGGCATTGCTGACG
CTGGTATTTGCGCTGGCATTTTGGGCAGGAACGCATGTCGCGTGGCTAATGGTGGTGATG
GTTGGGTTTAACCTCTTCTTTTCACCACTGGTGCCGCTGACCGATGCGCTGGCCAATACC
TGGCAAAAGCAAATTACCCTGGACTATGGTCGGGTGCGGCTGTGGGGGTCCATCGCCTTC
GTGATAGGGTCGGCGCTGACCGGTAAGCTGGTGAGTTTATACGATTACCAGGCGATTCTG
GCGCTACTGACGCTCGGCGTCGCCTCGATGTTGCTGGGTATGTTGTTACGTCCCAGCGTG
CCGCCGCAAGGGGAAAGCCGTCAGCAGGAGAGCGCAGGCTGGCCCGCCTGGCGTACGCTG
GTGGCACAAAGTTGGCGTTTTCTCGCCTGCGTCTGTTTGCTGCAAGGGGCGCACGCCGCC
TACTACGGTTTTAGCGCCATCTACTGGCAGGGGGCTGGCTATTCAGCATCGGCGGTCGGC
TATTTATGGTCGTTGGGCGTGGTGGCGGAAGTGATCATTTTCGCCCTGAGTAAAAAGCTA
TTTCGCCGGTTTAGCGCCCGCGACTTGCTGTTGCTTTCCGCTGTGTGCGGCGTGGTACGC
TGGGGACTGATGGGCTGGAGTACGGCGTTGCCGTGGCTGATTGTGATACAAATCCTGCAC
TGTGGCACGTTTACCGTTTGCCATCTGGCCGCCATGCGCTATATCGCTGCGCGTCAGGGA
AGCGAGGTGATTCGTTTGCAGGCCGTCTATTCCGCTGTGGCGATGGGCGGCAGCATCGCG
ATTATGACGGTATTTGCCGGTTTTCTGTATCAACATCTGGGCGGCGGCGTGTTCTGGATT
ATGGCGCTGGTGGCATTGCCCGCTATCTTCCTTCGGCCCAAAGTGGTTGCCGCGTCATGA
>EECEBICD_00788 hypothetical protein
ATGATGCCGACAATTGCCCCACCATCTGTACTTTCCGCTCCCCAGCGCCGCTGTCAGGTA
TTGCTGACGCTTTTCCAGCCGGAGCCAATTGCCACGGTGGAAATCTTCAGCGCGCTTAAT
GGCGTTGATGATGATACTGCCCGTGAGGATATCACTGAGACAAGCCTGGAGATCCAGCGC
TATCATCGCCTTGCCATCACAACATGCCAGAACGGTTGCTATCGGATCGAAGGTACAGCA
CTCGATCAGCGCCTTTGCCTTTTACACTGGCTCAGGCGCGGCCTGCGTTTATGCCCGACC
TTCGTCACGCAACAGTTTACCCCGGCCTTAAAAAACGCGCTAAAGCAGCGCGGTATCGCG
CGTCCGCTGTATGACGATATTAATCTGCACGCGCTCATCAATCTGTGCGCCCGCCGGTTG
CAAAAACCATTTGAACATCGCGACGTGCAGTTCCTGCGTCTCTTTTTACAGTATTGTCTG
CTACAACACCATGCCGGCATCACCCCGGAATTCAACCCTGTGCAGCAGATTTGGGCGCAG
TCGTGCGCGGAATATCCACTCGCCCAGGAAATTGGTCGTCACTGGCAGCGCCACGTGATG
CAGGCCGCACCGCTCAATGAAGCGCTGTTTATGGCGTTACTGTTTTCAATGATTCGCCTC
CCCGACCCGATTCGCGACACGCATCAACGGGCGCAGCAACTGCGACTGGAGGTGGCGCGG
CTGGTGCTGCGCTTTCGAGAAAAAGGGAACGTACGCTTCAGCGACGAACAAGGGCTTAAC
GATCAGCTGTATGTCCATCTCGCCCAGGCGCTGAACCGAAGTTTATTCACGATTGGCATC
GATAATACGCTTCCGGAAGAGTTCAACCGTCTTTATCCGCGTCTGGTTCGCACCACACGT
GAGGCGCTCGCCGGGTTTGAAGCCGAATATGGTATCCACTTTTCCGAGGAAGAGACGGGG
CTGGTGGCAGTGATTTTCGGCGCCTGGCTAATGCAGGATAACGATTTGCATGAGAGACAG
ATCGTATTGTTAGCGGATAAAAATGATGCGCTGGAGACGCATATTGAGCAGCAACTGCGT
GAACTGACCCTGTTGCCGCTGAATATCAGACGGCTATCGCTCCAGGCATTTCAAAAAGAG
GGATGCCCGCGCGGCGTAGCGCTGATTGTTACCCCTTACGCCACGCCGCTACCGCTCTTC
TCACCGCCATTAATTCATGCGGATCGCACCCTCACAGAGCACCAACAGCAGCAGATCCGC
AAGATCCTGGAATCATGA
>EECEBICD_00789 hypothetical protein
ATGAAGCCAGTGAAACGCAGCGCCTTAACACTATTTCTTGCCGTTTTATCTTTCGTGGCG
GCGGCGCATCCGCACAGCTTCATCCGTCTGCAAACGCAGGTGGTCAGTGAAAATGAGCAA
TTTGTGGCGTTAAAAATGCGCTGGACAATGGACGCGCTGACGTCCGCAGATCTGCTGTAT
GACGCCGGAAACGCGGCACCGGGGTCAGAAATATGGAAAAAGTTGGCGGCGGAAGTGATG
GCTAACGTACTTGGGCAGCACTACTTTACAGAAGTGTGGCGCAATGGCGCGAAAGTGAAG

TTTAAAAACAGGCCGACAGAATACGGTATGACGCGTGACGCGCATCAGGCGGTATTGACC
TTTACGCTACCGCTGGCTGAACCGCAGCCGTTAAGCGGACAGACTTATACCTTTTCAACG
TTTGATCCCTCTTATTATGTCGATATGCATTATGACCAGGACAGCGATATCACGATGCCG
GAACCGCTGCGCGAAAAGTGTCGGATTCAGGTTTATACTCCTGCGCCCGGCGAAGAGACG
CTACGTTTCGCCCAGTCGCTGGATAAAGAGGACGCGCCGCCGGAGGATATGGATTTAGGT
AAACAGTTTGCCCAAACGGTGACCTTGCAATGTCAGTAA
>EECEBICD_00790 hypothetical protein
ATGTCAGTAATTTTTTCTCAACGCACTCCGGGGCGACGCTGGCTCTCGCTGTGGCCATTG
GCGTTATTCCTGCTGCTGATGCTGGTTGGCGGTCTGTGGATATGGCAGGCCTGGCCGCAG
GTGATGTTGAAAAGCGCTCTTTGGCAGCGGGACGTAAATCAGCAGTTGAGCGCGCTGCTT
AATGCGGTGGCGACGCATCCTGAGCGGGCGGGCGGATCGTTGCTTCTGCTGAGTTTTATG
TATGGCGTGCTTCATGCGCTGGGGCCGGGACACGGCAAAGTGGTGATCGCGACATGGCTG
GCGACGCATCCTTCGAAGTTGAAATCGAGTATCGTGTTAACCCTCGCCGCCGCGTTATTA
CAGGGGCTCGTGGCTATCGGGTTGGTGGTGGGCGTGCTGACCGTATTGCAACTTCCGGCC
AGGCAGCTCCATCTGAGCGGCTTCTGGCTGGAAAAGGGGAGCTATGCGTTAGTTGGCGGG
CTGGGCATTTTGCTGTGCTGGCGGGCCATAAAAAGGTTACGCGCGCTGCTACGTAAACCT
GTCTTTATCGCTTTTACGCCGCGCCATGTTCACCATGAAAAATGCGGCTGCGGACATCAG
CATTTACCGACGCAGGAACAATTGCATAGCGGCGATGACTGGCGCGCGCGACTCATGATC
GTACTGTCTATGGGGATGCGGCCCTGTTCCGGCGCGATAATGGTGCTGTTATTCAGTAAA
GTGATTGGCGTTTTTAGCTGGGGAATGGCGTCGGTACTGGCGATGGCGGCGGGAACCTCG
CTAACGATTACCTCGCTGGCGCTGCTGGTACACACTTTTCGTGATCTGGCGGTGAAACTC
AGTGGAAACAAAGCGCCGGCGTTATGGCGGCAGGTAGGCTGGTCAACGCTGGCGCTGGCC
GGAGGGGGCATACTGCTTGTTGCGGCGCTGGTCATGTGGTTTAGCGTGCCGCAGCCTGTG
GGCGGGCTGCGGCCATGGCGTGGTTGA
>EECEBICD_00791 Anaerobic sulfite reductase subunit C
ATGAGCATTGATATTGATATCATTAAAGCCCGTGCGAAAAACGAATATCGCCTGTCAAAA
GTGCGCGGCGAAGCCATGATCAGCGTCCGCATTCCCGGCGGTATTTTGCCCGCGCATTTG
CTAACGGTGGCGCGTGACATCGCCGAAACCTGGGGCAACGGACAAATTCACCTCACTACC
CGCCAGAAGCTGGCGATGCCGGGTATCCGTTACGAAGATATCGACAACGTGAACGCTGCG
CTGGAGCCGTTTCTTCGCGAGATTGAAATAGAACTGTGCGACGTCCAGGTTGAGGATACC
AAAGCGGGCTATCTCGCTATTGGCGGGCGAAATATTGTCGCCTGTCAGGGGAACCGCATT
TGCCAGAAAGCCAACACTGACACGACCGGTCTGTCCCGTCGTCTTGAAAAACTGGTCTAT
CCCAGCCCTTATCATCTCAAAACGGTCATCGTTGGCTGCCCGAATGATTGCGCGAAGGCG
TCAATGGCCGATCTGGGAATTATTGGCGTGGCGAAAATGCGCTTCACTGCCGATCGCTGT
ATCGGCTGCGGCGCCTGCGTGAAGGCCTGTAGTCACCACGCGGTAGGCTGCCTGGCGCTG
AAGAACGGCAAAGCGGTCAAAGAAGAGTCCGCCTGTATCGGCTGCGGCGAGTGCGTGCTG
GCCTGTCCGACGCTGGCCTGGCAACGTAAGCCGGATCAGCTCTGGCAAGTCCGTCTGGGC
GGACGCACCAGTAAAAAGACGCCGCGCGTCGGCAAGCTCTTCCTCAACTGGGTGACTGAA
GATGTGATAAAGCAGGTGATCGTTAACCTGTATGAATTTGAAAAAGAGATGCTCGGCGGA
AAACCGATCTATCTACATATGGGCCATCTGATCGACAAAGGCGGCTATCTGCGTTTTAAA
GAACGGGTACTGCGCGGCGTTCAGCTCAATCCGGAAGCGATGGTCGCCGAACGCATTTAC
TGGGCCGAAGACGAATCCGTCGCGCGGATGCATCTCAAACCCGCCGGACACTAA
>EECEBICD_00792 Anaerobic sulfite reductase subunit B
ATGTCACATTGTTCCTGTCATGATAAACCGCAGCACAGTTTGCTGCCTGCGGCGTACCGC
ATCCTCAGTATTACTCGCCACACGCCGCTGGAGTGGAATTTCCGCGTGGCCGTCGATTTT
CCCGCGCACTGGGGGCAATTTGTTGAGGTGTCGCTGCCGCGCGTCGGCGAAGCGCCCATC
TCCGTTTCCGACTACGGCGACGGCTGGATAGATCTGCTGATTCGTAATGTCGGAAAAGTG
ACCAGCGCCCTCTTTACGCTGAAAGAAGGCGACAACGTGTGGCTACGCGGCTGCTATGGC
AATGGCTATCCGGTCGATACGCTGCGCCATAAGCCCTTACTGGTTGTCGCAGGCGGTACG
GGCGTCGCGCCGGTGAAAGGCTTAATGCGCTATTTCGTTGAGAATCCCCAGGAAATTGGT
CAACTGGATATGATTCTCGGCTATAAAAATCGCGATTGCGTGCTGTACAAAGAAGAAATG
GCGACGTGGCGCGGCAAACATAATCTGGTACTCACGCTGGATGAAGGCGAGGCTGATGAC
CGTTACCAGATTGGCCGGGTGACCGATCGGCTTGCCGACATGACGCTTAGCGATATCGAC
ACTATGCAGGCCATCGTCGTTGGGCCGCCAATAATGATTACCTTTACCGTAAAAATGCTG

CTGCAAAAAGGGCTCAAGCCGGAGCAAATCTGGGTGGACTACGAACGCCGGATGGCCTGC
TCCGTCGGGAAGTGCGGCCACTGCCGTATGGGCGAAGTGTATGTCTGCACCGACGGTCCG
ATATTTAACTACGCCGTCGCGCAACGTTTTGCCGATTAA
>EECEBICD_00793 Anaerobic sulfite reductase subunit A
ATGGCTATCAAAATTACGCCTGACGAGTTTAGTTTGCTTATCCAGCGATTAAACAAAAAA
TGGCGCGTGTTTGCCCCGTCCGCAGAGTTTCGCGGCGGACGTTTTTCCGACACTGACAAC
ATTATTTATCAACGGATTAGCGGCTGGCGCGATCTCATCTGGCACGAGAAATCGCACATG
TCGCCGAATACCATTATCGCCCCTATCACCGAAACGCTCTTTTATTTCGATAAAGACACC
ATTCAAATTGCCGAGACAGACATTTCCCCGATAATCATCTTTGCCCGCGCCTGCGACATT
AACGCCATGTCGCGGCTGGATTATATGTATTTATCAAATGGGAATAATTCCGATTACAGT
TATCAACTGCTACGGGAGCATATTCGTTTCGTCCTTATTGAGTGTGAAGAAAGCTTCGAA
AATTGTTTCTGCGTCTCAATGGGTACCAATAAAACTGACTGTTATAGCGCCGCCATGCGT
TTTAGCGATGAGGGCGCGCTTGTCAGCATCCGCGATCCCTTTATCGAGGCGGCGATACAA
GGGCTGGGGCAGGAGGCTGACTATACCCCCTCTTTCGTCAGCGAAAACCGGGAAACCGTC
GTCACGCCGGACAGCGTTTGCCGCGATCCGCAAAAAATTCGCGACATTCTCACCCGCCAC
CCGCTATGGGACGCCTACGACAGCCGCTGCATCAGTTGTGGCCGCTGCACTACCGGGTGC
CCAACCTGTACCTGCTATAGCGTCTTTGACGTCGCCTATGATGAAAATCCGCAGCGCGGC
GAACGCCGTCGCCAGTGGGCAAGCTGCATGGTGCCGGGCTTTAGCGACATGGCCGGCGGT
CATGGTTTTCGCGAAAAACCCGGCGAGCGTCTGCGCTACCGCGCCCTGCATAAGGTCAAT
GACTACAAAGCGCGCAACGGCATTGAACATATGTGCGTCGGCTGCGGCCGCTGCGACGAT
CGCTGCCCGCAATACATCAAATTTTCTTTGATTATCAATAAAATGACCGCTGCCGTTCGG
CAGGCGCTGGCAGAGGAGGCATAA
>EECEBICD_00794 putative protein
ATGACCCTTCAACATACCCGACGCATCGTCAAATCCCTTTTTATTCTGTTTATCATTGTG
GTCTGTATTTATCTGCTACCCCGCGTAGCGATCAATGCCTTTTATTACCCGGACAATAAA
GTCTATGGGCCAACCCCTGCCGAAGCTGAATCCATTACCTTTACCGCCAAAGATGGCACG
CATCTGCACGGCTGGTTTATTCCCACTGCTTTCGGCAGGCCGGAAAACGCCGTCGCGACG
GTGATTCATGTCCATGGCAACGCCGGAAATATGTCGGCGCACTGGCCGTTAGTCAGTTGG
CTGCCAGAACGCAACGTAAACCTGTTTATGTTTGATTACCGTGGTTTTGGCGAATCGGAG
GGTACCCCATCACAAGAGGGGCTACTGGACGATACGAAAAGCGCGATAGATTATGTGCGG
CATCGCGCCGACGTTAACCCCGAGCGCCTGGTACTGCTGGGGCAGAGCCTGGGCGGCAAT
AATGTACTGGCCGCCGTAGGCCACTGCGTAGGCTGCGCAAATATGCGCTACGCCGACCAG
GCGGGCATACGTGCGATTATCCTTGACTCAACGTTTTTATCCTATTCCTCCATCGCCAAC
CAGATGATCCCGGGCAGCGGCTATTTGCTTGATGACCGCTATAGCGCGGATCGCAATATT
GCCAGCGTCAGCCCCATTCCGGTATTGATTCTTCATGGCACGGCAGACCATGTTATTCCC
TGGCAGGACAGTGAGAAATTGTATGCCCTCGCCCGGGAGCCCAAACAAAAAATCTTCATT
CCTGACGGCGATCACATTGATGCTTTTTCCGGACGTTATGCAAACCTTTACCGTGATGCA
ATGATTAAATTTATTCAGACAGCGCTATCCGCTAAATAG
>EECEBICD_00795 Inositol-1-monophosphatase
ATGCATCCGATGCTGACCATCGCCGTGCGCGCAGCGCGCAAGGCGGGTAATGTAATTGCC
AAAAACTATGAAACTCCGGACGCTGTAGAAGCGAGCCAAAAAGGCAGTAACGATTTTGTG
ACCAATGTAGATAAGGCCGCTGAAGCGGTGATTATCGACACCATTCGCAAATCTTACCCG
CAACACACTATTATCACCGAAGAAAGCGGTGAGCACGTTGGCACAGATCAGGATGTTCAA
TGGGTTATCGATCCGCTGGATGGCACCACCAACTTCATCAAACGTCTGCCACACTTCTCT
GTTTCCATTGCTGTACGCATTAAAGGCCGTACTGAAGTGGCTGTCGTTTACGATCCGATG
CGTAACGAACTGTTCACCGCCACCCGCGGTCAGGGCGCACAGCTGAACGGCTACCGTCTG
CGCGGCAGTACCGCGCGCGATCTGGACGGCACTATCCTCGCTACTGGCTTCCCGTTCAAA
GCCAAACAATACGCGACCACCTACATTAATATTATCGGCAAGCTGTTCAGCGAATGCGCC
GATTTCCGCCGCACCGGTTCCGCCGCGCTGGATCTGGCCTATGTGGCTGCAGGTCGTGTC
GATGGTTTCTTTGAAATTGGCCTTCGTCCGTGGGATTTCGCCGCGGGCGAACTGCTGGTT
CGTGAAGCGGGCGGTATCGTCAGCGATTTCACCGGAGGTCATAACTACATGATGACCGGT
AATATCGTGGCGGGGAATCCGCGCGTCGTTAAAGCCATGCTGGCGAATATGCGCGACGAA
CTGAGCGATGCGCTGAAGCGCTAA
>EECEBICD_00796 tRNA (cytidine/uridine-2'-O-)-methyltransferase TrmJ

ATGCTGCAAAACATTCGAATTGTGCTGGTGGAAACCTCCCACACCGGCAATATGGGCTCC
GTGGCTCGCGCTATGAAAACCATGGGCTTAACTAACCTGTGGCTGGTAAACCCGTTGGTG
AAACCGGACTCCCAGGCCATCGCCCTGGCGGCAGGCGCCAGCGATGTGATCGGTAATGCG
CAGATCGTCGATACGCTGGATGAAGCGTTGGCTGGCTGTAGCCTGGTGGTCGGCACCAGC
GCCCGTTCCCGCACTTTGCCGTGGCCGATGCTTGATCCGCGCGAGTGCGGCTTAAAAAGC
GTGGCGGAAGCGGCAAATACGCCGGTTGCGCTGGTTTTTGGTCGTGAGCGTGTCGGGCTG
ACTAACGATGAGTTGCAAAAATGCCATTATCACGTCGCTATTGCCGCTAACCCGGAGTAC
AGCTCGTTGAATCTGGCGATGGCGGTACAGGTCATCGCGTATGAAGTCCGCATGGCCTGG
CTGGCGACGCAGGAAAACGGCGACGCAGCAGATCATGAAGAGACGCCGTATCCGCTGGTG
GACGATCTTGAACGTTTCTACGGCCATCTGGAGCAGACGTTGCTCTCCACTGGCTTTATT
CGCGAAAACCATCCGGGGCAGGTGATGAATAAATTGCGCCGTCTGTTTACCCGCGCCCGA
CCGGAAAGCCAGGAGTTGAATATTCTGCGTGGGATTCTGGCGTCTATTGAGCAGCAGAAT
AAAGGCAAGTAA
>EECEBICD_00797 HTH-type transcriptional regulator IscR
ATGAGACTGACATCTAAAGGGCGTTATGCCGTGACCGCAATGTTAGACGTTGCGCTCAAC
TCCGAAGCGGGCCCGGTACCGTTGGCTGATATTTCTGAACGTCAGGGGATTTCCCTTTCT
TATCTGGAACAGCTATTTTCCCGTTTACGTAAAAACGGTCTGGTTTCCAGCGTACGCGGG
CCGGGCGGCGGTTATCTGCTGGGTAAAGATGCTGGCAGCATTGCGGTCGGCGAAGTGATC
AGCGCGGTTGATGAATCCGTTGACGCGACCCGTTGCCAGGGCAAAGGCGGCTGCCAGGGC
GGCGATAAATGCCTGACCCATGCGCTGTGGCGTGATTTAAGCGATCGCCTGACCGGTTTT
CTCAACAATATTACCTTAGGCGAGCTGGTGAATAACCAGGAAGTTCTGGATGTGTCTGGT
CGCCAGCACACGCATGACGCGCCGCGCGCCAGTGGTCGTGCGCAGGACGCTATCGACGTT
AAATTACGCGCTTAA
>EECEBICD_00798 Cysteine desulfurase IscS
ATGAAATTACCGATTTATCTCGACTACTCCGCAACCACGCCGGTGGACCCGCGTGTTGCC
GAGAAAATGATGCAGTTTCTGACCCTGGACGGAACCTTTGGGAACCCGGCGTCTCGTTCA
CACCGTTTCGGCTGGCAGGCTGAAGAAGCCGTCGATATCGCCCGCAACCAGATTGCTGAA
CTGGTCGGCGCCGACCCGCGTGAAATCGTCTTTACCTCAGGGGCGACGGAGTCTGATAAC
CTGGCGATTAAAGGCGCTGCCAACTTTTATCAGAAAAAAGGCAAGCACATCATCACCAGC
AAGACCGAGCACAAAGCGGTGCTGGACACCTGCCGTCAGCTTGAGCGCGAGGGGTTTGAA
GTAACCTACCTGGCGCCGCAGCGCAACGGCATCATCGATCTCAACGAGCTCGAAGCAGCG
ATGCGTGACGACACCATTCTGGTTTCCATCATGCACGTGAACAACGAAATCGGCGTGGTG
CAGGATATCGCGACCATCGGCGAAATGTGCCGCGCGCGCGGTATCATCTACCACGTTGAC
GCCACCCAGAGCGTGGGCAAACTGCCTATCGACCTGAGCCAACTGAAAGTGGATCTGATG
TCCTTCTCCGGTCATAAAATTTATGGTCCGAAAGGCATTGGCGCGCTGTATGTGCGTCGT
AAGCCGCGTATTCGCATTGAAGCGCAGATGCATGGCGGCGGGCACGAACGCGGTATGCGC
TCTGGTACTCTGCCTGTCCACCAGATCGTCGGCATGGGCGAAGCTTACCGTATCGCGAAA
GAAGAGATGGAGACCGAAATGGCCCGTCTGCGCGGTCTGCGTAACCGTCTGTGGAACGGC
ATCAAAGATATTGAAGAAGTTTACCTGAACGGCGACCTTGAGCAGGGCGCGCCAAACATT
CTCAACGTGAGCTTTAACTACGTTGAAGGCGAGTCGCTGATCATGGCGCTGAAAGACCTG
GCGGTCTCTTCCGGTTCCGCCTGCACCTCCGCCAGTCTGGAACCGTCTTACGTGCTGCGC
GCGTTGGGCATGAATGACGAGCTGGCGCATAGCTCTATCCGTTTCTCTTTAGGTCGTTTT
ACCACTGAAGAAGAGATCGACTACACCATTGATCTGGTTCGTAAATCCATTGGCCGTCTG
CGTGACCTTTCTCCACTGTGGGAAATGTACAAGCAGGGTGTGGATCTGAACAGCATCGAA
TGGGCACATCATTAA
>EECEBICD_00799 Iron-sulfur cluster assembly scaffold protein IscU
ATGGCTTACAGCGAAAAAGTAATCGATCACTATGAGAACCCGCGTAACGTAGGGTCGTTT
GACAACAACGACGATAACGTGGGAAGCGGCATGGTTGGCGCGCCGGCCTGCGGCGACGTC
ATGAAGTTGCAGATTAAAGTTAACGATGAAGGTATCATTGAAGACGCGCGCTTCAAGACT
TACGGCTGCGGTTCCGCTATCGCCTCCAGTTCTCTGGTAACGGAGTGGGTGAAAGGGAAA
TCCCTGGACGAAGCGCAGGCGATTAAAAACACCGACATTGCAGACGAACTGGAACTGCCG
CCGGTGAAAATTCACTGCTCTATTCTGGCGGAAGACGCGATTAAAGCCGCTATTGCGGAT
TACAAAAGCAAACGCGAAGCGAAATAA
>EECEBICD_00800 Iron-binding protein IscA

ATGTCGATTACACTTAGCGACAGTGCAGCAGCGCGAGTTAATACCTTCCTGGCCAACCGT
GGTAAAGGGTTTGGTCTGCGTCTGGGCGTGAGAACCTCCGGCTGCTCAGGTATGGCTTAT
GTACTGGAATTTGTTGACGAGCCAACGGCGGAAGACACCGTGTTTGAAGACAAAGGCGTT
AAGGTAGTGGTTGACGGCAAGAGCCTGCAATTTCTGGACGGTACACAGCTGGACTTCGTA
AAAGAAGGTCTGAACGAAGGGTTTAAATTCTCCAACCCGAATGTGAAAGATGAGTGTGGT
TGCGGCGAAAGCTTCCACGTGTAA
>EECEBICD_00801 Co-chaperone protein HscB
ATGGATTACTTCACCCTCTTTGGCTTACCAGCCCGTTATCAGATTGACACCCAGGCGCTG
AGCCTTCGTTTTCAGGATCTGCAACGCCAGTATCACCCTGACAAATTTGCCAACGGCACT
CAGGCGCAGCAGCTTGCCGCCGTCCAGCAATCCGCCACGATCAACCAGGCCTGGCAAACG
CTGCGTCACCCTTTGACGCGCGCGGAGTATCTGCTCTCCTTACACGGGTTCGATCTGGCC
AGCGAGCAACATACGGTACGCGACACCGCGTTTCTGATGGAACAACTCACGCTTCGCGAA
GAGCTGGACGATATCGAACAATCAAAAGACGACGTGCGGCTGGAAAGCTTCATCAAACGC
GTACAAAAGATGTTTGATGCTCGCCTTCAGCAAATGGTGGAACAGTTGGACAACGCCGCG
TGGGACGCGGCGGCGGATACGGTGCGCAAACTGCGGTTTCTCGATAAACTGCGAAGCAGC
GCAGAACAACTCGAAGAAAAGCTGCTCGATTTTTAA
>EECEBICD_00802 Chaperone protein HscA
ATGGCCTTATTACAAATTAGTGAGCCTGGTCTGAGCGCCGCGCCGCATCAGCGTCGTCTG
GCGGCGGGCATCGACTTAGGCACTACCAACTCTCTGGTTGCGACGGTTCGCAGCGGCCAG
GCGGAAACTCTGCCTGACCACGAAGGGCGCCACCTGTTGCCTTCAGTGGTTCACTATCAG
CAGCAGGGCCATACGGTCGGTTACGCTGCGCGCGACAATGCCGCGCAGGATACGGCCAAC
ACTATCAGCTCTGTGAAACGCATGATGGGACGCTCGCTGGCGGATATCCAGGCGCGCTAT
CCGCATCTGCCTTACCGGTTTAAGGCAAGCGTTAATGGACTGCCGATGATTGACACGGCG
GCTGGATTGCTCAATCCGGTACGCGTATCGGCTGATATTCTCAAAGCTCTGGCCGCGCGG
GCTAGCGAATCGCTCTCCGGCGAGCTGGATGGTGTGGTGATTACCGTTCCCGCCTATTTC
GATGATGCCCAACGTCAGGGAACGAAAGACGCCGCGCGGCTGGCAGGCCTGCATGTACTG
CGCCTGCTCAACGAACCGACGGCGGCGGCGATTGCCTACGGTCTGGACTCAGGCAAAGAA
GGCGTTATTGCTGTTTACGATCTCGGCGGCGGTACCTTCGATATCTCTATCTTACGCCTG
AGCCGCGGGGTGTTTGAAGTGCTGGCGACGGGCGGTGATTCTGCGCTCGGCGGCGACGAC
TTTGACCATCTGCTGGCGGACTATATTCGCGAGCAGGCGGGAATTGCCGATCGTAGCGAC
AATCGCGTTCAGCGCGAATTGCTGGATGCCGCGATTGCCGCGAAAATCGCCTTAAGCGAT
GCCGATACTGTTCGAGTTAACGTTGCGGGCTGGCAGGGGGAGATCACTCGCGAGCAGTTT
AACGATCTCATTTCCGCGCTGGTTAAGCGCACGCTATTGGCCTGCCGTCGCGCATTGAAA
GATGCCGGCGTTGATCCCCAGGACGTTCTGGAAGTGGTCATGGTCGGCGGTTCGACCCGT
GTACCGCTGGTGCGTGAGCGGGTGGGCGAATTTTTTGGCCGCACGCCGTTAACCGCTATT
GACCCGGATAAAGTGGTCGCTATCGGCGCAGCGATTCAGGCCGATATTCTGGTCGGCAAC
AAGCCGGACAGCGAAATGTTGCTGTTGGACGTGATTCCACTCTCTCTTGGGCTGGAAACG
ATGGGCGGCCTGGTGGAAAAAGTCATTCCGCGCAACACCACTATCCCGGTGGCGCGCGCG
CAGGATTTCACCACCTTCAAAGACGGCCAGACCGCGATGTCTATCCATGTGATGCAGGGC
GAACGCGAACTGGTGCAGGACTGCCGCTCGCTGGCGCGCTTTGCGCTGCGCGGGATTCCG
CCGCTGCCGGCGGGCGGGGCGCATATTCGCGTGACCTTCCAGGTAGACGCCGATGGCCTG
TTGAGCGTCACCGCAATGGAAAAATCCACCGGCGTCGAGGCCTCCATTCAGGTCAAACCT
TCCTATGGCCTGACCGATGGCGAAATCGCCTCGATGATCAAGGATTCCATGAGTTTTGCC
GAGCAGGACGTGAAAGCGCGTATGCTGGCGGAGCAAAAAGTCGAAGCCGCGCGCGTGCTG
GAAAGTTTGACCGGCGCGCTAACTGCCGACGCCGCGCTGTTAAGCGCCGCAGAACGTCAA
TGCATTGATGATGCCGCCGCACATTTAAGCGCGGTCGCACAAGGCGATGATGTTGACGCC
ATAGAACAAGCCATTAAAAACGTAGATAAACAAACCCAGGAATTTGCCGCTCGCCGCATG
GACCAGTCGGTTCGTCGCGCGTTGAAAGGCCATTCCGTAGACGAGGTTTAA
>EECEBICD_00803 2Fe-2S ferredoxin
ATGCCGAAGATTGTTATTCTGCCTCATCAGGATCTTTGTCCGGATGGCGCAGTTCTGGAA
GCTGAGACCGGTGAAACCATTCTTGACGTTGCGCTGCGCAACGGCATCGAGATTGAACAC
GCCTGTGAGAAATCGTGCGCCTGCACCACCTGTCACTGTATTGTACGTGAAGGCTTCGAC
TCCTTGCCGGAAAGTTCGGAAGAAGAAGACGATATGCTGGATAAAGCGTGGGGGCTGGAG
CCGGAAAGTCGACTGAGCTGCCAGGCCCGCGTTACCGACGACGATCTGGTTATCGAAATT

CCACGTTACACAATCAACCATGCGCGTGAGCATTAA
>EECEBICD_00804 Protein IscX
ATGGGACTCAAGTGGACCGATAGCCGCGAGATCGGCGAAGCGTTATACGACGCATATCCT
GATGTCGATCCTAAAACCGTTCGTTTCACCGATCTGCATCAGTGGATCTGCGAACTGGAC
GATTTTGACGACGACCCTAACGCATCCAATGAAAAAATTCTGGAGGCGATTCTGCTAGTC
TGGTTGGATGAAGCAGAGTAA
>EECEBICD_00805 Peptidase B
ATGACAGAAGCCATGAAAATTACGCTTTCCACGCAGCCTGCCGATGCGCGCTGGGGCGAT
AAAGCCACTTACAGTATTAATAACGACGGTATTACCTTGCATCTTAACGGTAAAGACGAT
CTGGGGTTGATCCAGCGTGCGGCGCGTAAAATCGACGGCCTGGGAATAAAACAGGTCGCG
CTGACAGGCGAAGGATGGGATATCGAGCGTTGTTGGGCATTCTGGGCGGGTTATAAAGGA
CCAAAAGGCGTCCGTACCGTAATGTGGCCGGATTTAGACGATGCCCAACGTCAGGAGCTG
GATAACCGCCTGACCATTATTGACTGGGTGCGCGACACCATTAATGCTCCGGCGGAAGAG
CTTGGACCGGAGCAACTGGCGCAGCGCGCGGTGGATCTGCTGTGCAGCGTCGCGTGCGAT
AGCGTCACTTACCGTATCACCAAAGGCGAAGATCTGCGCGAGCAGAACTATATGGGGTTG
CATACCGTAGGCCGTGGTTCTGAGCGTCCGCCGGTGCTGCTGGCGCTGGATTACAACCCG
ACGGGAGACAAAGATGCGCCGGTTTATGCCTGCCTGGTCGGTAAAGGTATCACCTTTGAC
TCCGGCGGCTACAGTATCAAACAAAGCGCTTTTATGGACTCAATGAAATCCGATATGGGC
GGCGCGGCGACAGTAACGGGCGCGCTGGCGTTCGCCATTACCCGTGGGTTGAATAAACGC
GTAAAACTGTTCCTGTGCTGCGCCGATAATCTGATCAGCGGCAATGCGTTTAAACTGGGC
GATATTATTCGCTACCGTAATGGTAAGAATGTCGAAGTCATGAATACCGATGCGGAAGGG
CGACTGGTACTGGCGGATGGCCTGATTGACGCCAGCGCGCAACACCCGGAGCTAATCATT
GATATGGCGACGTTAACCGGCGCGGCGAAAACGGCGCTGGGTAACGACTATCATGCGCTA
TTCAGTTTTGACGATACGCTGGCGGGGCGCTTGCTGACCAGCGCGGCGCAAGAGAATGAA
CCGTTCTGGCGTCTGCCGCTGGCGGAGTTCCACCGTAACCAGCTGCCGTCTAACTTTGCG
GAACTGAACAACACCGGCAGCGCGGCGTATCCCGCTGGGGCAAGCACCGCCGCGGGTTTT
CTGTCGCACTTTGTTGAAAATTACCGCGAAGGCTGGCTGCACATTGACTGTTCCGCCACT
TATCGCAAAGCGCCGGTTGAGCAGTGGGCTGCCGGGGCAACCGGTCTGGGTGTGCGCACC
ATCGCGAATTTGCTGACCGCCTGA
>EECEBICD_00806 hypothetical protein
ATGTATATGGGTTGTAGGCCTGATAAGACGCACGGCGTCGCCATCAGGCAAAATGCCGGG
TGGCGCTTCGCTTACCCGGCCTACAAAAACACCGCTCAGGCGGTCAGCAAATTCGCGATG
GTGCGCACACCCAGACCGGTTGCCCCGGCAGCCCACTGCTCAACCGGCGCTTTGCGATAA
>EECEBICD_00807 hypothetical protein
ATGTCCGAAACTAAAAATGAATTAGAAATCTTGCTGGAGAAAGCGGCGACTGAGCCCGCG
CATCGTCCGGCATTTTTCCGTACCTTACTGGAGTCCACCGTCTGGGTGCCGGGAAGCGCT
GCCGAAGGCGAGGCGATAGTGGAAGACAGCGCTCTGGATTTGCAGCACTGGGAAAAAGAA
GACGGCACGACCGTCATTCCCTTCTTTACTTCTCTGGAGGCGCTGCAGCAGGCCGTGGAA
GACGAACAGGCATTTGTGGTGATGCCGGCGCGCACGCTGTTTGAAATGACGCTTGGCGAA
ACGCTATTCCTCAACGCTAAATTGCCGACCGGCAAAGAGTTTATGCCGCGTGAAATCAGC
CTGCTTCTCGCTGAGGAAGGCAGTCCGTTAAGTACGCAGGAAGTGCTGGAAGGCGGCGAG
TCGCTTATTTTATCCGAAGTGGCCGAGCCGCCGTCGCAGATGATTGATTCCCTCACTACG
CTGTTTAAAACCATCAAACCGGTGAAACGCGCATTTCTCTGCGCGATTAAAGAACATGCG
GACGCCCAACCCAATCTGCTGATTGGCATTGAGGCTGACGGCGAAATTGAAGAGATCATT
CACGCAGCGGGCAATGTGGCGACCGACACGTTACCGGGTGACGAACCGATCGATATTTGT
CAGGTGAGGAAGGGCGAGCAGGGTATCAGCCACTTTATCACTGAGCATATCGCGCCGTTC
TACGAGCGCCGCTGGGGCGGGTTCCTGCGCGATTTTAAACAGAACCGCATCATCTGA
>EECEBICD_00808 hypothetical protein
ATGGATATTTCACGCGCTAATTTAATTGAGTTAGTTAAAAAGGTTAATCGCAATAAGGTG
CCTAATCCTATGCCTGCCGAGGAAATAAGTCGTCTGCGAGTGCGTAAGTATCGCGATCCG
CAAAATACGGAAACCACTGAGTTACCTGAAAGCCTGAAGGCGCTACTGGCCTACGACCGC
GATCTACTTAGCAATTATAATATGCCTGTGATTGAAACATTACAAAGATCTATTGATAAA
GAGGGAGTAATTCATTCTTACTCCCCTGATGAAGAGGCGTATTACGGCGCTGGCATGGAT
AGCTCAGGTATTGATATTGAAGATCTGATGCCTGTCTGGAGTAACGATCCCCGTCTTCCG

GCATTAATTCGTATCGATCATGTCGGCGATCAGGCCATTTTTATCTATATCACAGAACGG
GATGCCAACGGCGAATACCCTATTGCCCGCATGGAACGAAATGAATTCTGGCTGGCGGAG
TCTTCTCTGGTGGAATATCTCTATAATATTATTTCGGGCGCTAAAGACATTGGCTTTACC
GAAGAGGATTTACATCTTTCGCAATGGAAGGCACAGCAAAAAATGAATGAAAAACGCGAT
GCCGCTTTACTTGACCTGGAGGATTATCATGAGGCGTTTTGGGCAAAACTGGACGCGCTC
GTCGATTAA
>EECEBICD_00809 hypothetical protein
GTGAAAACCAAAACAGCGGTGATGATACTCCTTATACCCGGCGTAAAGTATTTACCGATA
AAAAATAGGATTTTATTAAGAGAAAATAATGCCCGTTTGCAGGACAGTGGAAAAGAGCTG
AATAAAAACAATGTCTGTAGATCGTTTTACATCAGCCTGAACGGTAGGCCGGATAAGCGT
GAGCGCCATCCGGCATGGCAGATATTAAGCGGGTTCAACCGGTAA
>EECEBICD_00810 3-mercaptopyruvate sulfurtransferase
ATGACCACCGCCTTTTTTGTCGCCGCCGACTGGCTTGCCGAACATATTGATGATCCGGAA
ATACAGATTCTTGACGCCCGTATGGCGCCGCCAGGACAGGAACATCGTGATATGGCGGGC
GAATATCGCGCCGGACATATTCCCGGCGCGCTGTTTTTTGATATTGAAGCGCTTTCCGAT
CGCGCATCGCCGCTGCCGCATATGATGCCGCGCCCGGAAGCGTTTGCGGTGGCCATGCGT
GAACTGGGTGTTCGCCAGGATAAACACCTGGTCATATACGATGAAGGCAATCTGTTCTCC
GCTCCGCGGGCATGGTGGATGCTGCGCACGTTCGGTGCCGAGAAAGTGTCCATTCTGGCG
GGCGGACTGGCGGGCTGGCAGCGCGACGAATGGCTATTGCGCGAAGGCGAAGAAGCGCAT
GAAGGAGGCGAGTTTGAGGCGAAATTCGCGCCGCAAGCCGTGGTGCGTCTCACCGACGTA
TTGCTGGCAAGCCATGAAAAAACCGCGCAAATCGTTGACGCGCGTCCGGCGGCGCGTTTT
AACGCTCAGGCTGATGAACCGCGTCCGGGACTGAGGCGCGGACACATTCCTGGCGCGCTA
AACGTCCCGTGGACGGAACTGGTGTATGAGGGCGAATTAAAGACCACTGACGAACTGAAT
GAGGTTTTTTTCAGCCACGGCGTGAGTTTCGACAGACCGATTATCGCCAGCTGCGGTTCC
GGTGTGACGGCCGCGGTCGTGGTGCTGGCGCTCGCCACGCTGGATGTGCCTGACGTCACG
CTTTACGATGGCGCGTGGAGCGAATGGGGCGCGCGTACCGATTTACCGGTTGAACCCGCT
TAA
>EECEBICD_00811 Alpha-2-macroglobulin
ATGAAACATTTACGCGTGGTGGCCTGCATGATCATGCTGGCGCTGGCGGGATGCGATAAC
AACGATAAAACCGCCCCGACGACTAAAAGCGAAGCGCCAGCCGTAGCGCAGCCCTCGCCC
GCGCAAGACCCGGCACAGCTACAAAAGCTGGCGCAGCAAAGCCAGGGCAAAGCGCTCACG
CTATTAGACGCCTCCGAAGCGCAGCTTGACGGCGCGGCGACGCTGGTGCTGACGTTTTCA
ATTCCTTTAGATCCTGAACAGGATTTCTCCCGTGTGGTTCACGTGGTTGATAAGAAAAGC
GGCAGCGTTGACGGCGCATGGGAGCTGGCGCCAAATTTAAAAGAGCTAAGGTTACGTCAT
CTGGAACCTGAACGCGTGCTGGTGGTCACGGTTGATCCCGCCGTTAAGGCGCTGAATAAC
GCCACCTTCGGCAAGTCTTATGAAAAAACGATTACCACGCGTGATGTCCAGCCCAGCGTC
GGCTTTGCCAGCCGGGGATCGCTGCTACCGGGGAAAATAGCGGAAGGACTGCCGGTCATG
GCGCTTAACGTCAACCACGTTGATGTGAACTTTTTCCGCGTTAAGCCCGGATCGCTGGCG
TCATTTGTCAGCCAGTGGGAGTACCGTAGTTCTCTCTCTAACTGGGAGTCCGACCATCTG
CTGAAAATGGCGGATCTGGTTTATACCGGTCGTTTTGATCTTAATCCGGCGCGCAATACG
CGTGAGAAACTGCTGCTGCCATTAAGCGATATTAAGCCGCTGCAACAGGCGGGCGTATAT
GTGGCGGTAATGAATCAGGCTGGACACTACAACTATAGTAATGCCGCTACGTTGTTTACC
CTTAGCGATATCGGCGTGTCCGCGCACCGTTACCATAATCGGCTGGATATCTTTACGCAA
AGCCTGGAAAACGGCGCGGCGCAGTCGGGAATTGAGATCGTTCTTCTGAATGATAAAGGT
CAGACGCTGGCGCAGGCGACGAGCGACGCGCAGGGACATGTGCAACTGGAGGCTGATAAA
GCGGCGGCGCTATTACTGGCGCGTAAAGAGGAGCAGACCACGCTGCTCGATCTCACGCTT
CCGGCTCTGGATCTGTCGGAATTTAATGTCGCTGGCGCGCCCGGCTACAGCAAGCAGTTC
TTTATGTTCGGCCCGCGCGATCTCTACCGCCCGGGCGAAACGGTTATCCTCAACGGATTA
CTGCGCGATAGCGACGGTAAAACGCTGCCCGATCAACCCGTTAAGCTGGAAGTGGTAAAA
CCAGACGGACAGGTGATGCGTACCGTCGTCAGCCAGGCGGAAAACGGGCTATACCGTTTG
AATTATCCGCTGGATATCAACGCGCCGACCGGCTTGTGGCATGTCCGCGCCAACACCGGC
GATAATTTGCTGCGGAGTTGGGATTTCCACGTGGAAGACTTTATGCCGGAGCGGATGGCG
CTCAACCTGACGGCGCAAAAAACGCCGCTGGCGCCTGCGGATGAGGTGAAATTCTCCGTT
GTCGGCTATTACTTGTATGGCGCGCCTGCTAACGGCAATACCCTGCAAGGGCAACTTTTC

CTGCGTCCGCTGCGCGACGCTGTCGCGGCGTTGCCTGGCTTCCAGTTCGGCAATATTGCT
GAAGAGAATCTTTCGCGCAGCCTGGACGAAGTTCAACTGACGTTGGATAAAGGCGGACGT
GGTGAAGTGAGTGCTGCCAGCCAGTGGCAAGAAGCGCATTCGCCGCTGCAGGTAATTCTA
CAGGCCAGCCTGCTGGAGTCTGGCGGTCGTCCGGTCACTCGTCGCGTAGAGCAGGCGATT
TGGCCTGCCGATACGTTACCGGGAATTCGTCCACAGTTCGCCGCCAAAGCGGTATACGAC
TACCGTACGGATACCACCGTTAATCAACCGATTGTCGACGAAGACAGCAACGCCGCATTC
GATATTGTTTACGCCAACGCGCAGGGCGAGAAAAAAGCGGTGTCCGGTTTACAGGTGCGG
CTCATCCGCGAGCGTCGCGACTATTACTGGAACTGGTCGGAAAGCGAAGGCTGGCAGTCG
CAGTTTGATCAAAAAGATCTGGTGGAGGGCGAGCAGACGCTGGATCTGAACGCGGATGAA
ACCGGAAAAGTCAGCTTCCCGGTGGAATGGGGCGCGTACCGTCTGGAGGTCAAAGCGCCG
AATGAGACGGTCAGCAGCGTTCGTTTCTGGGCCGGCTATAGCTGGCAGGATAACAGCGAC
GGTAGCGGCGCGGCGCGTCCGGATCGCGTCACCCTCAAACTGGATAAAGCGAGTTATCGT
CCAGGCGATACCATGAAATTGCATATCGCCGCGCCGGTCGCTGGTAAAGGTTATGCGATG
GTGGAGTCCAGCGACGGTCCGCTGTGGTGGCAGGCGATCGATGTGCCGGCACAAGGTCTG
GAGCTCACGATTCCGGTGGATAAAACCTGGAATCGCCACGATCTCTATCTCAGTACGCTG
GTGGTGCGTCCCGGCGATAAATCTCGTTCCGCGACGCCAAAACGCGCCGTGGGGTTACTA
CACCTACCGCTGGGGGATGAAAACCGCCGCCTCGATCTGGCGCTGGAAAGCCCGGCTAAA
ATGCGCCCGAATCAGCCGCTCACCGTCAGGGTGAAAGCCAGCGTTAAACACGGCGAAATG
CCAAAACAGATCAACGTGCTGGTCTCCGCGGTCGATAGCGGCGTATTGAATATCACCGAT
TACGCGACGCCGGACCCGTGGCAGGCGTTCTTCGGTCAAAAACGCTACGGTGCGGATATC
TACGATATTTACGGCCAGGTCATTGAAGGGCAGGGGCGGCTGGCGGCGTTGCGTTTTGGC
GGCGATGGCGACGACCTTAAGCGCGGCGGAAAACCGCCGGTAAACCATGTCAATATCATC
GCGCAGCAGGCGCAGCCGATCACGCTCAATGAGCAGGGCGAAGGGGTCGTAACGCTGCCG
ATTGGCGACTTTAACGGCGAACTGCGGGTTATGGCGCAGGCATGGACAGCGGACGATTTT
GGTCGCGGCGAAAGCAAAGTCGTTGTCGCCCGCGCCAGTGATTGCCGAGCTGAATATGCCG
CGTTTTCTGGCGGGAGGGGATGTTTCGCGACTGGTGCTGGACGTCACCAATCTGACCGAC
CGCCCGCAGACGCTGAATATTGCGCTCGCCGCCAGTGGGTTACTGGAACTGCTTAGTCAA
CAGCCGCAACCGGTCAACCTGGCGCCGGGCGTGCGCACCACCTTATTCGTTCCGGTACGT
GCGCTGGAAGGTTTTGGCGAAGGCGAAATCCAGGCGACCATTAGCGGTCTGAATCTGCCG
GGAGAAACCCTCGGCGCGCAGCATAAGCAGTGGCAAATCGGCGTGCGTCCGGCCTGGCCT
GCCCAAACGGTAAATAGCGGCATTGCGCTGGCGCCGGGAGAGAGCTGGCATGTACCAGAG
CAGCATCTGGCAAACGTCTCGCCAGCCACGTTACAGGGACAACTGCTGTTAAGCGGAAAA
CCGCCGCTCAATCTGGCGCGCTACATTCGCGAGCTGAAAGCATATCCGTACGGGTGCCTG
GAACAAACCACCAGCGGGTTATTCCCGGCGCTGTATACCAATGCCGCTCAATTGCAGTCG
CTCGGTATTACCGGCGATAGCGATGAAAAACGGCGCGCCGCGGTGGATATCGGCATCTCC
CGCATACTACAGATGCAGCGTGATAACGGCGGTTTTGCGCTATGGGATGAAAATGGGGCG
GAAGAGCCCTGGCTAACGGCCTACGCGATGGATTTCCTCATTCGCGCGGGCGAGCAGGGA
TATAGCGTCCCGCCGGAGGCCATTAACCGGGGCAATGAGCGACTGCTGCGCTATCTGCAG
GATCCCGGTACGATGCTGATTCGTTATAGCGATAATACCCAGGCCAGTACTTTTGCCGCT
CAGGCTTACGCCGCGCTGGTACTGGCGCGTCAGCAGAAAGCGCCGCTCGGCGCGCTGCGC
GAAATCTGGGAGCGCCGTAGTCAGGCGGCTTCAGGACTGCCGCTGATGCAATTGGGCATC
GCGTTAAACACGATGGGTGATGCCAGACGCGGCGAAGAGGCCATTACGCTGGCTCTGAAT
ACGCCGCGTCAGGACGAACGGCAATGGATAGCGGATTACGGCAGTTCTCTGCGCGATAAC
GCTCTGATGTTGTCGTTACTGGAAGAGAACAACCTCAGACCGGACGCGCAAAACGCGCTA
TTAAGCTCGCTTTCTGAGCAGGCCTTCGGTCAGCGCTGGCTCTCTACCCAGGAGAACAAT
GCCTTGTTCCTCGCCGCGCATTCGCGACAGGCCAGCGCGGGCGCCTGGCAGGCGCAGACC
TCGTTAGAGGCGCAGCCGCTGTCGGGCGACAAGGCGCTGACCCGTAATCTGGATGCTGAC
CAGCTGGCCGCCCTTGAGGTGACGAACACCGGTAGCCAGCCGCTATGGCTGCGTCTGGAT
AGCAGCGGCTATCCCTCATCTGCGCCTGAGCCTGCCAGCAACGTTTTGCAGATTGAACGA
CAAATACTGGGGACCGATGGTCAGCGTAAATCGCTGTCCTCGTTGCGTAGCGGCGAACTG
GTGCTGGTCTGGTTAACGGTAGTGGCCGATCGCAATGTGCCGGATGCGCTGGTGGTGGAC
CTGCTCCCGGCCGGGCTGGAGCTGGAAAACCAGAATCTGGCTGACAGCAGCGCCAGCCTG
CCGGAGAGCGGTAGCGAAGTGCAAAATCTGCTTAATCAGATGCAGCAGGCGGATATTCAG
TATATGGAATTCCGCGACGATCGGTTTGTGGCTGCCGTCGTTGTCAATGAGGGCCAGCCC

GTGACGCTGGTCTACCTGGCGCGCGCGGTAACGCCGGGGACGTACCAGCTTGCGCAACCG
CAGGTGGAATCGATGTACGCGCCTCAGTGGCGGGCAACCGGCGCGAGCGAGGGGCTGCTG
ATTGTGACGCCTTAA
>EECEBICD_00812 Penicillin-binding protein 1C
ATGAACGGTTGGCGTGGTAAACGTGGCCGCTGGCTGTGGCTGGCGGGCGCGCCGCTTTTT
ATTTTCCTGGCGTTATGGGCAGCGGATAAACTCTGGCCGCTGCCGCTCAATGAGGTCCAT
CCTGCGCGGGTCGTGGTAGCCCATGACGGTACGCCGCTGTGGCGCTTTGCCGACGCCGGG
GGGATCTGGCGCTATCCGGTGACGATTGAAGAGGTCTCTCCCCGCTACCTGGAGGCCTTA
ATCAATTACGAAGATCGCTGGTTCTGGCGGCACCCTGGCGTTAACCCTTTCTCCGTCGCG
CGCGCGGCATGGCAGGATCTTACCGCCGGGCGCGTTATTTCCGGCGGCAGTACGCTGACT
ATGCAGGTGGCGAGACTGCTGGACCCGCATTCGCGCACGTTCGGCGGTAAAATCCGCCAG
CTTTGGCGCGCCCTCCAGCTTGAATGGCATTTGTCCAAGCGCGATATCCTGACGCTGTAC
CTGAACCGCGCGCCGTTCGGCGGCACGCTACAGGGTATTGGCGCTGCAAGCTGGGCCTAC
TTCGGCAAGCCGCCCGCGCGCCTGAGCTACGCTGACGCGGCGCTGCTGGCCGTATTGCCG
CAGGCGCCGAGCCGGTTACGCCCCGACCGCTGGCCTGCGAGAGCCGAAGCGGCACGAAAT
AAAGTGCTGGATCGGATGGCGGCGCAGGGCGTCTGGCCTGCCGAAACGGTACGCGAGTCG
CGTGAAGAGCCGGTCTGGCTGGCGCCGCGTCAAATGCCGCAACTGGCGCCGCTTTTTGCC
CGAATGATGTTGAGTAAAAGCCGGAGCGACAAAATTGTGACCACGCTGGACGCCGGGTTG
CAGCGGCAACTGGAAGATCTGGCGCGGGCATGGAAAGGGCGGCTGCCTGCACGCAGTTCA
TTGGCGATGATCGTCGTCGATCATACCGATATGAGCGTGCGCGGCTGGGTAGGATCGGTA
GACCTTAATGACGACAGCCGTTTTGGTCATGTCGACATGGTAACGGCGATCCGATCGCCG
GGATCGGTACTCAAACCCTTTGTGTACGGTCTGGCGCTGGATGACGGCTTGATCCATCCC
GCATCATTATTACAGGACGTTCCGCGCCGCACCGGAGATTACCGCCCGGGAAACTTCGAC
AGCGGATTTCACGGCCCGGTCAGCATGAGCGACGCGCTGGTACGCTCGCTGAATCTGCCT
GCCGTTCAGGTGCTTGAAGCCTATGGGCCGAAACGATTTGCGGCAAAACTGCGTAACGTC
GGCCTGCCGCTATATTTGCCTGCTGGCGCGGCGCCCAATCTTTCGCTGATTCTGGGCGGC
GCGGGCGCGCGGCTTGACGAGATGGCGGCGGCATACAGCGCGTTCGCTCGTCATGGGAAA
GCGGCGAAACTACGGTTACAGCCGGACGATCCGCTGTCGGAAAGACCATTAATGTCGCCG
GGAGCGGCGTGGATCATTCGGCGGATTATGGCGGATGAAGCGCAACCCTTGCCGGATAAC
GCGCTGCCGCGCATTGTGCCGTTGGCGTGGAAAACCGGCACCAGCTACGGCTATCGCGAT
GCGTGGGCTATTGGCGTTAACGCGCGCTATATCATCGGCATCTGGACAGGCAGACCCGAC
GGCACACCTGTGGTGGGGCAATTTGGTTTTGCCAGCGCGGTGCCATTGCTTAACCAGGTC
AATAATCTGTTACTGGCGCATACGGGACGCCTGCCGGAAGATCCGCGTCCGCAGACGGTA
AGCCGCGGCGTCATTTGCTGGCCGGGCGGACAGACTCTGCCTGCCGGAGACAGCAACTGT
CGCCGCCGACTGGCGACATGGCTGCTCGATGACAGCCAGCCGCCCACGCTGCTGTTACCT
GAGCAGGAAGACATCAACGGTATTCGTTTCCCGGTCTGGCTGGATGATACGGGGCGGCGT
GTCGCTGCCGACTGTCCGCAGGCGTGTGCGCATACGTTTATCGTCTGGCCGCGGCCGCTG
GAGCCGTGGCTGCCGCCTGCGGAAAGACGTAGCGCCCGCTTGCCCGCGGCGTCCGACCAC
TGTCCGCCGCTACAGGGCAGTGATGCCGCGCCGTTAATGCTGTCAGGAGTGAGGGATGGC
GCGGTGATCAGGCAATTGCCAGGCCAGGAAAACGTTACGCTGCCCGTCTCGACTACCGGC
GGTAAAGGGCGTCGCTGGTGGTTTTTAAATGGCGAACCTGTTAATGGCGAAAATAATCGC
CTTTCTTTATTACTCAATATCGCTGGACGTTATCAACTTGTCGTAATGGATGAGTCCGGT
CAGGTTGCTGCGGTTAATTTTGAATTAATACGTTAA
>EECEBICD_00813 Dimethyl sulfoxide reductase DmsA
ATGAAAAATATAAAAAGCCAGGGAAATGGCGAACAGCCCGCTATTTCTCGCCGCCACTTT
ATCCAGGCCAGCTCCGCGCTTATTGCGTTACCGTTTGTCTCCTCGCCAGCTACCGCTCAG
GCGCGCGCTGTGACGGCCACTGAAAACAGGCCTGCGGAAAAAGTCGTTCAGACATGCAGC
ACTTTTGACTGCGGGGGCAAATGCGATATTCGGGCGCATGTTAGTGACGGTATCGTGACG
CGAATTTCAACGCGACCGGATAATGCGCTGGATGCGCAAATGCCGGTGATGCGTGCCTGT
GTTCGTGGACGCGCATACCGTAAATTTGTTTATCACCCCGATAGACTTAAATATCCCATG
AAGCGTGTAGGAAAACGCGGTGAAGGAAAGTTCGAGCGAATTTCCTGGGATGAAGCGACA
ACCTTAATCGCCGATAATTTAAAATCGATTACTGAAAAATATGGCCCGGCTTCGCGCTAT
GTTCATGTTGGCACCGCCGTTTCCGGCGGCACCTTTTCCGGCGATAAAATGGCGCGTCGC
CTGCTGAATCTGACCGGCGGCTATCTCGAAAGCTACCATTCTGTGAGTATGGGCAATACG

GCGGCCGCGACGCCTTATACTTACGGCATTGCCGCCAGCGGCAGTTCGCTGGATACGCTT
TTGGACACCAAACTGGTGATTCTGTGGGGCCATAACCCGACGGAGACCATTTTTGGTCAT
ACCAACTATTTCTTGCAGAAGATGAAGCAGAACGGTACGCGGTTTATCGTGGTCGATCCG
CGCTATTCCGACACCGTTTCCTCGCTGGCGGATCAGTGGATCCCGCTGCTGCCCACCACC
GACAATGCGCTGATGGACGCGATGATGTACGTCATCATCAGCGAGAATCTGCACGATCGC
GCATTTATTGCGCGCTACGCTATCGGTTTTGACGAAGACTCAATGCCGGAAGGCGTACCG
GCTAACGAATCGCTGGTGGCGTATCTGACCGGCGCCAAAGACGGTGTCGTTAAGTCCCCC
GAGTGGGCGGAAAAGATCACCCATGTGCCGGCGCAAACCATACGCCAGTTAGCGTGTGAT
TACGCGAATACCAAACCCGCCGCGTTGATTCAGGGGTGGGGACCACAGCGCCACAACTGC
GGCGAGCGCACCGCGCGCGGCTCGACCCTACTGGCGACCATTACCGGTAATGTCGGCATC
AAAGGCGGTTGGGCTGCGGGTTATGGCGGCTGCGCTAACCGTAAATTTGCCGCGGGTCCG
GAGATGCCGGATAACCCGGTAAAAGCCAAAATTTCGGTGATGAACTGGGTGCAGGCCGCA
GACGACGCCAGCAAAGTGACGCCGGATATGGGGCTGAAAGATGCCGATAAGCTGGACAGT
AACATCCGTATTCTGTTCTCGCTGGCCGGGAACTATCTGGCGAATCAGAACCCCGATCTG
CACCAGGCGGTACGCGTACTGGAGGATGAATCAAAGATCCAGTTTATTGTCGCCAGCGAT
CTGTTTATGACGCCGAGCGCGAAATACGCTGATCTACTGCTGCCGGAAACCAGCTTTATG
GAACGCTGGAACATCGGTGAAACCTGGGGCACGGCAAGCTACCTGATACTCTCTGAAAAA
CTGATCGAGCCTGAATTTGAGCGGCGCTCCGACTACGACTGGCTACGTGAGGTGGCGGCG
AAATTAGGCATTGAAAACGAATTTAGCCAGGGGCGCGATGAGAAAGCGTGGATTGAACAC
ATCTGGGAACAGACGCGCCTGGCGATGCCGGACGAAAATCTACCGGATTTCGCCACGCTG
CAAAAGACCCGTCAGCATCTGTTCAAAAGCGCGCCATTTATCGCCTTTGAAGACAATATT
CGCGATCCGGACAATCATCCGTTCCCGACGCCGTCAGGGAAAATAGAGATCTTCTCGAAG
CGCCTGTACGACATGCAGCATCCGGAAATCCCGGCGTTGTCGCACTACGTTCCCGCGCAT
GAAGGCCCGGAGGATGCGCTGGTGAAAGATTTTCCGCTCCAGTTAATTACGTGGAAAGGA
AAAAACCGCGCCAACTCAACGCAATACGCTAACCCGTGGCTGATTGAAGTGCAGCAGCAG
ACATTGTGGATCAACCCGCAGGATGCGCAAAAGCGCGGCATTACGCATGGCGACATGGTG
CGTATTCATAACTCACGCGGGATTTGCGAAATCCCGGCGGAAGTGACGCCGCGCATTATT
CCCGGCGTTGTCGCAATGCAGGCTGGCGCCTGGTGGCAGCCGGATGAAAACGGCGTTGAT
AAAGGCGGCTGTGCGAACGTCCTCAGCTCGGCCCGTATTACCGCGCTGGCGAAGGGAAAT
TCACATCAAACCATGCTGGTGGAGGTGGCTAAAGCATGA
>EECEBICD_00814 Anaerobic dimethyl sulfoxide reductase chain B
ATGAGTCAGTTTACACATTACCCGCCGGTGAGCGATAAACAACTGGGGTTTTTCATCGAC
TCCTCACGCTGCTCCGGTTGTAAAGCCTGCCAGGTGGCCTGCAAAGACAAAAACAATCTG
GAAGTAGGCCGTCGTTTCCGCCGCGTCTATGAAGTCAAGGGCGGAAGTTTCATCCCGACC
GGGCAGGGCGGGGTCAGCAATAACGTATTTGCTTATACGCTTTCTATTTCCTGCAATCAC
TGTGCGGACCCTGTCTGCACCAAGAATTGCCCGACTACCGCTATGCATAAACGCCCGGGC
GACGGCATCGTGCGCGTTGATACCGACAAATGCGTCGGCTGTGGCTATTGCGCTTGGTCC
TGCCCTTACGGCGCGCCGCAGCTTAACGAGCAGACCGGCCAAATGTCCAAGTGCGACATG
TGCGTTGATTTACTGGCGAAAGGTGAACCGCCCGTCTGTGTTGCGACCTGTCCGCTGGAG
GCCATCAAGTTTGCTCCGATTGATGAACTGCGCGCGAAGTACGGTAGCGTATGCGACGTT
AACGGGCTGCCAGATTCATCCATAACCAAACCTAATCTGGTTGTCAAAGCGCATCAGGGC
GCAGAAAAAGAGGGTAAACGCCATGCATGA
>EECEBICD_00815 Anaerobic dimethyl sulfoxide reductase chain C
ATGCATGAGTTACCGCTGCTGATTTTTACGCTCTGTTTGCAAGGGTCTGTGGGTGTGACA
GTGTGGCTGGCGTTGGGCCGCCAGTACGCCGTAGAGGGACGCGTGCCTGCACGCGGCGCG
CTTCCCGCGATGGCGGGGGCGTTTGTACTGGCCTGCGTAGGGCTGCTTGCTTCAGCGCTG
CACATGGGCTATCCCCTGAATGCGCTAAATGCGTTACGTCATGTCGCCAGTTCGTGGCTG
AGTCGCGAAATCGTCTTTGCCAGCCTTTATCTGGCGGCGCTGGGACTGGGGGTTGTGCTG
CTGTTTTTCCGAAAGCCAGGCTGGCAGCCGCTGCTGGCGTTGGCGGCAGCGTTCGGGCTG
GTGGATGTATTCTGCATGGCGCAGGTTTATATCCATGCCTCGGTGGCGACCTGGCAGCAT
AGCAACACGCTGGCGCTGTTCTTTGGCACATCAGGCATTATCGGCTCGGTAGTCATTGCG
CTGGCTTATCTGCGCAACGCTGGCGCCGCAATGCGCTGCGCTGTGGTGGTGGTCGCACTG
ATGGTGCTGATTCGCCTGATCATGCAGCCGCTATGGCTGGCGGATATTAATGCAGTGGAT
ACGACGGTCGTCACTTTCCCGCATCATCCGCTACAGGCGCTGGCGCAGTTGCGCGACGTC

TATCTTCTCGGCTGGTGTGTCTCAGCGGCGGGAATGCTCTGCTTTGCCGCAGGCGGTCTG
CGAAACGCCAGAGGAACTCTGGTGGCGGGCAGCGTATTGCTGCTTATCGGCGAGATTATG
CTGCGCTACGTCTTCTTTAGTATTGGCTGA
>EECEBICD_00816 Ferredoxin-type protein NapF
ATGGTTAACCTGACGATCGATCCGGCGGTAGACGTGACGCAGGCTTGCGTCCGTCGTCGC
TTTCGTTTTTCTTCCTGCCGCGCTTGCGCCGATGTTTGTCCGGCGCAGGCGTTTTCGCTG
GCGCAGGGGCAGGTCAGCATAGATACGACACGCTGCATAGCCTGTGGCGACTGTCTGTTT
GTCTGTCCTGTTGACGCCATTACCGGTATCAAACCGGTTAAACGCTTTGTGCAGGGGGAT
ACGTTGGTGGGGCCGTTTTCGCTACAGGCGCCAACGGTCGATGAACTGCTTCTCTGGCAC
AGCCAGTACGGTATTCGCTTTATTGATATTGCGGTGGAGCGATCGGCGCAGTGGCTGATG
GCGCTGGCGGGGCTAAATTTAGCGCTGCGCCGCTACGGCGAGCCGGGCTGGTCTTTCAAA
CACGTTGTCGGCGCGGAGATTAACGCCTCGCGGCGCACTCTGTTTCATGTTCCGCGCGAC
GCCATTACCCCGTGTGCCGTCGAGCCGGGGAAACGGCGTCTCAGACAGGCGTTTTCCGCG
TTCAGCGAATGCGTACCCGAAATCAGTCCGCAAGAGTGCAGGATGTGCGGCGCCTGCTGG
CGAAGCTGCCCGGAGAACGTTATCCAGTTTGACGACAATACCCTGACGATAGCGGCGGCG
CGTTGTACGGGATGCGGCGGCTGTGCGGCGGTATGTCCGCATCAGGCGCTGCGGCTGCGG
TTTGACGTGGAACCCGCCTCAACGCGGCATAGTGCGGCGCATACGCTCACCTGCGACATC
TGCAAACGTACTTTCCATGCTCTCACGCCTGAACACACGCACTGCGTGCTGTGTCAGTCC
CCTGAGTTTGCTGTGCGTCTTTAA
>EECEBICD_00817 Nucleoside diphosphate kinase
ATGGCTATTGAACGGACTTTTTCCATCATTAAACCCAACGCGGTGGCAAAAAACGTTATT
GGCAGCATCTTTGCGCGCTTTGAAGCTGCAGGGTTCAAAATTGTTGGCACTAAAATGCTG
CACCTGACCGTTGAGCAGGCGCGCGGCTTCTATGCTGAGCACGACGGCAAACCGTTCTTT
GACGGTCTGGTGGAGTTCATGACCTCTGGCCCTATCGTGGTATCCGTGCTGGAAAGTGAA
AATGCGGTACAGCGTCACCGCGATCTGCTTGGCGCCACCAATCCGGCGAACGCGTTGGCG
GGTACGCTGCGCGCCGACTACGCTGATAGCTTAACTGAAAACGGCACCCACGGTTCGGAT
TCGCTGGAATCCGCGCAGCGCGAAATCGCCTTTTTCTTCGGTGAAGGCGAAGTGTGCCCG
CGCACCCGTTAA
>EECEBICD_00818 Dual-specificity RNA methyltransferase RlmN
ATGTCTGAACAAATTGTCACACCTGAAAGCAGTACCCCGGTAGTTCTTAACAACGAAACA
AAAATCAACCTGCTGGATCTCAACCGTCAGCAGATGCGCGAGTTTTTTAAAAACTTAGGT
GAAAAGCCTTTCCGCGCCGATCAGGTGATGAAATGGATGTATCACTATTGCTGCGATAAT
TTTGATGAGATGACCGACATCAACAAAGTGTTGCGCGGCAAATTAAAAGAGGTCGCGGAA
ATCCGCGCGCCGGAGGTCGTTGAAGAGCAGCGTTCCTCAGACGGCACCATTAAGTGGGCG
ATTGCTGTCGGCGATCAGCGCGTTGAAACGGTCTATATCCCGGAAGACGACCGCGCTACG
CTGTGTGTCTCTTCACAGGTAGGATGCGCGCTGGAGTGCAAGTTTTGTTCAACTGCGCAG
CAGGGCTTTAACCGCAACCTGCGCGTATCGGAAATTATCGGTCAGGTCTGGCGTGCGGCG
AAAATCGTCGGCGCGGCCAAAGTCACCGGCCAGCGTCCGATCACCAACGTGGTCATGATG
GGGATGGGCGAGCCATTGCTTAACCTGACCAATGTCGTACCGGCGATGGAAATTATGCTC
GATGACTTTGGTTTCGGCCTGTCCAAACGTCGTGTCACGCTCTCAACGTCAGGCGTGGTG
CCTGCGCTGGATAAACTGGGCGATATGATAGATGTCGCGCTGGCTATCTCCTTACATGCG
CCGAATGACACTATTCGCGACGAAATCGTCCCGATCAACAAAAAATACAATATCGAAACG
TTCCTGGGCGCGGTTCGCCGCTATCTGGAGAAATCCAACGCCAATCAGGGGCGCGTGACG
ATTGAATACGTGATGCTTGACCACGTCAACGACGGCACTGAACACGCGCATCAACTGGCG
GAGCTACTAAAAGAGACGCCTTGTAAGATTAATCTGATTCCGTGGAACCCGTTCCCTGGC
GCGCCGTATGGCCGCAGCTCTAACAGCCGTATCGATCGCTTTTCTAAAGTTTTGATGAGT
TATGGTTTCACGACCATCGTACGCAAGACGCGTGGCGATGACATTGATGCGGCATGCGGA
CAGTTGGCAGGCGATGTGATTGACCGTACTAAACGTACCCTGCGTAAGCGTATGCAGGGA
GAGGTGATTGACATCAAGGCTATTTGA
>EECEBICD_00819 Cytoskeleton protein RodZ
ATGAATACTGAAGCCACGCACGACCAAAATGAAGCACAAACCACCGGCGTTCGTCTGCGC
AATGCCCGTGAACAACTCGGACTCAGCCAGCAGGCTGTCGCTGAGCGACTTTGCCTGAAG
GTTTCTACGGTACGCGATATTGAAGAAGATAAGGCGCCGTCCGATCTTGCTTCAACCTTC
CTGCGCGGATATATCCGCTCATATGCGCGTCTGGTGCATGTTCCAGAAGAGGAACTGCTG

CCGGGGCTGGAAAAACAAGCTCCGCTGCGCGCCGCTAAAGTCGCCCCGATGCAGAGTTTC
TCATTAGGAAAGCGTCGTAAAAAACGCGACGGCTGGTTAATGTCTTTTACCTGGCTGGTA
CTGTTTGTGGTCGTAGGGCTGACCGGCGCCTGGTGGTGGCAGAACCACAAGGCGCAGCAG
GAAGAGATCACGACGATGGCCGATCAAAGCACGGCTGAACTGAACGCAGATAAAGACAGC
GGCCAAAGCGTACCGCTGGACACCGGTGCCGTGACAAGCCAGGATACGACGCCAGCGCAA
ACGGCTCCGGCGCCTGCGACGCCGGTTGATTCGACGGCGGCGACGCAAACGCCAGCGCCA
ACGGCGGCGGCAACGCAAAATACGGTAGTCGCGCCTTCGCAGGCGAATGTCGATACGGCT
GCGACATCTGCCGCGCCAGCGGCAACGGAGACGCCGTCCGCGCTGCCGACCAGTCAGGCT
GGCGTAGCGGCGCCTGCTGCCGATCCGAACGCGCTGGTGATGAATTTTACCGCTGACTGC
TGGCTCGAAGTCACTGACGCCACGGGTAAGAAGCTGTTTAGTGGTATGCAGCGTAAAGAT
GGTAATTTAAACCTAACCGGTCAGGCGCCTTATAAACTTAAAATTGGCGCGCCGGCCGCG
GTGCAGATCCAGTATCAAGGAAAACCTGTCGATCTGAGCCGCTTTATCAGAACTAATCAG
GTTGCGCGTCTGACCCTTAATGCCGAACCGACACCGGCGCAGTAA
>EECEBICD_00820 4-hydroxy-3-methylbut-2-en-1-yl diphosphate synthase (flavodoxin)
ATGCATAACCAGGCTCCGATTCAACGTAGAAAATCGACACGTATTTACGTTGGGAATGTG
CCGATTGGCGATGGCGCCCCCATCGCCGTCCAGTCGATGACTAACACGCGTACCACTGAC
GTGGAAGCGACGGTTAATCAGATCAAAGCGCTGGAACGCGTTGGCGCAGATATTGTTCGT
GTTTCTGTACCGACCATGGACGCCGCCGAAGCGTTCAAGCTTATCAAACAGCAGGTTAAC
GTCCCGCTGGTTGCCGATATTCACTTCGACTACCGCATTGCGCTGAAAGTAGCGGAATAC
GGCGTTGACTGCTTGCGTATCAACCCCGGCAATATCGGTAACGAAGAACGCATTCGCATG
GTGGTGGACTGCGCGCGCGACAAAAACATCCCTATTCGTATAGGCGTAAATGCCGGATCG
CTGGAAAAAGATCTGCAGGAAAAATACGGTGAGCCGACGCCGCAGGCGCTGCTGGAATCA
GCCATGCGCCATGTCGATCATCTCGATCGTCTCAACTTTGATCAGTTCAAAGTGAGCGTA
AAAGCGTCCGATGTATTCCTTGCCGTTGAGTCTTATCGTTTGTTGGCTAAACAGATCGAT
CAGCCGCTGCATCTGGGGATCACCGAAGCGGGCGGCGCGCGTAGCGGGGCGGTCAAGTCG
GCGATCGGTTTAGGTCTGCTGTTATCGGAAGGTATCGGCGACACGCTGCGCGTATCGTTG
GCGGCCGACCCGGTGGAAGAGATCAAAGTCGGTTTCGATATTCTGAAGTCGCTGCGCATT
CGCGCGCGCGGCATCAATTTCATCGCCTGCCCGACCTGTTCTCGCCAGGAGTTCGACGTT
ATCGGCACGGTGAACGCGCTGGAGCAGCGTCTGGAAGATATCATCACCCCGATGGACGTC
TCGATCATCGGTTGCGTAGTGAACGGGCCGGGCGAAGCGCTGGTATCGACGCTGGGCGTG
ACGGGCGGCAATAAGAAAAGCGGCCTGTATGAAGACGGCGTGCGTAAGGACAGACTCGAT
AACGACGATATGATCGCGCAGCTTGAATCCCGTATTCGCGCGAAAGCAAGTCAACTTGAT
GAAGCGCGTCGGATTGACGTGCTGCAGGTTGAAAAATAA
>EECEBICD_00821 Histidine--tRNA ligase
GTGGCAAAAAACATTCAAGCCATTCGCGGCATGAACGATTATCTGCCTGGCGAAACCGCC
ATCTGGCAGCGCATTGAAGGCACACTCAAAAACGTGCTCGGCAGCTACGGTTACAGTGAA
ATCCGCTTGCCGATTGTAGAGCAGACCCCGTTATTCAAACGTGCGATCGGTGAAGTTACT
GACGTGGTTGAAAAAGAGATGTACACCTTTGAGGATCGCAACGGCGATAGCCTGACATTG
CGCCCTGAAGGTACGGCGGGCTGTGTACGCGCCGGCATCGAACATGGTCTCCTGTACAAT
CAGGAACAGCGTTTGTGGTATATCGGGCCGATGTTCCGTCATGAGCGTCCGCAAAAAGGC
CGCTACCGTCAGTTCCACCAGCTAGGTGCCGAAGTCTTTGGCTTACAAGGCCCGGATATC
GACGCGGAACTGATTATGCTGACCGCACGCTGGTGGCGCGCGCTGGGCATCTCTGAACAC
GTTAGCCTGGAGCTGAACTCTATTGGTTCCTTAGAGGCGCGTGCGAACTATCGCGATGCG
CTGGTCGCGTTCCTCGAACAGCATCAAGAGACGCTGGACGAAGACTGCAAACGCCGTATG
TATACCAATCCGCTGCGCGTGCTGGATTCAAAAAATCCGGACGTGCAGGCGCTGCTCAAC
GACGCGCCCGCTCTCGGCGACTATCTCGATGACGATTCACGCGAGCACTTTGCCGGCCTG
TGTAAATTGCTGGACGCGGCGGGGATTGCCTACACCGTCAACCAGCGTCTGGTACGCGGT
CTGGATTACTACAACCGCACCGTATTTGAATGGGTAACAAACAGTCTGGGGTCACAAGGC
ACCGTCTGTGCGGGTGGTCGTTATGACGGTCTGGTGGAACAACTGGGCGGTCGCGCTACC
CCGGCAGTGGGCTTTGCGATGGGCCTGGAACGACTTGTTTTGTTAGTTCAGGCAGTTAAT
CCGGAATTTATTGCCTCTCCTGTTGTCGATATATACCTGGTAGCTGCCGGCGCACAAACG
CAGTCTGCGGCAATGACGCTGGCGGAGCGGCTGCGCGATGAAATGCCAGGCGTGAAGCTA
ATGACAAACCACGGCGGCGGCAACTTTAAGAAACAGTTTGCCCGCGCCGATAAGTGGGGC

GCCCGTATTGCACTGGTTCTTGGCGAATCTGAAGTCGCCGATGGGACTGTTGTAGTGAAG
GATTTGCGCTCCGGTGAGCAAACGGCAGTGGCGCAGGACAGCGTCGCCGCGCATTTGCGC
ACTTTATTGGGCTAA
>EECEBICD_00822 hypothetical protein
GTGGAAATTTACGAGAACGAACACGACCAGGTGGATGCGATTAAACGCTTCTTTGCCGAA
AACGGCAAAGCGCTGGCTGTCGGAGTGATTTTAGGGGTTGGCGCGCTGGTTGGCTGGCGT
TACTGGAGCAGCCATCAGACGGAGTCCGCGCGTGCTGCTTCTCTGGCGTATCAAAATGCG
GTGACGGCGGTAAGCGCCGATAAGCCGGATAGCATTCCCGCCGCTGAAAAATTCGCGGCT
GAGAATAAAAACACCTATGGTGCGCTGGCGTCTATGGAACTGGCGCAGCAGTTTGTGGAT
AAGAATGAACTTAAAAAAGCCGAAGCCCAGTTGCAGCAGGGGTTGGCGGCCACCAGCGAT
GAAAATCTCAAAGCGGTGATTAATCTGCGTCTCGCTCGCGTTCAGTTGCAGCTTAAGCAA
GCCGACGCCGCGCTGAAAACTCTGGATGCCGTTAAAGGCGAAGGATGGACAGCCATTGTC
GCTGATTTGCGCGGCGAAGCGTTGCTGAGTAAGGGTGATAAAAAAGGCGCGCGCAGCGCA
TGGGAAGCGGGCGTCAATAGCGATGCTTCTCCGGCGTTGAGCGAAATGATGCAGATGAAA
ATCAATAATTTGTCCATCTGA
>EECEBICD_00823 Outer membrane protein assembly factor BamB
ATGCAATTGCGTAAATTACTTCTGCCAGGGTTGCTTTCCGTTACCCTGCTCAGCGGCTGT
TCACTGTTTAGCGGCGAAGAAGATGTCGTCAAGATGTCTCCATTACCGCAGGTTGAAAAC
CAGTTTACCCCGACCACTGTCTGGAGCACCTCTGTCGGTAACGGAATTGGCGAATTTTAT
TCTAACCTTCATCCGGTAATGGCAGATAACGTGGTCTATGCAGCCGATCGCGCGGGCGTG
GTAAAAGCGCTCAATGCGGATGATGGCAAAGAGATCTGGTCTGTGAATCTGGGCGAGAAA
GACGGCTGGTTCTCTCGTTCGTCCGCGTTGTTGTCCGGCGGCGTCACTGTCGCGGGCGGT
CACGTCTATATCGGCAGTGAAAAGGCCGAGGTTTATGCGCTGAATACCAGCGACGGTACC
ACAGCATGGCAGACGAAGGTCGCAGGCGAAGCGTTGTCTCGTCCGGTTGTCAGTGATGGA
ATCGTTCTTATCCATACCAGCAACGGTCAGTTGCAGGCGCTAAATCAGGCGGATGGCGCA
ATTAAATGGACGGTGAACCTGGATATGCCTTCGCTGTCGCTGCGCGGCGAATCTGCGCCG
GCAACCGCGTTCGGCGCGGCTATCGTTGGTGGTGATAATGGCCGCGTCAGCGCCGTGCTG
ATGCAACAAGGGCAGATGATTTGGCAGCAGCGTATCTCCCAGGCCACTGGCCCAACGGAA
ATTGACCGTCTGAGCGACGTCGATACCACGCCGGTTGTGGTAAACGGCGTCGTTTACGCG
TTGGCGTATAATGGTAACTTAACGGCGCTGGATCTGCGCAGTGGTCAGATTATGTGGAAA
CGCGAGCTGGGTTCGGTCAATGACTTTATCGTCGACGGAGACCGTATCTACCTGGTCGAT
CAGAACGATCGCGTGCTGGCGCTGACCACTGACGGCGGCGTAACGCTGTGGACGCAAAGC
GATTTGCTGCACCGTTTGCTGACCTCCCCGGTGCTGTATAATGGTGATTTAGTCGTCGGC
GATAGCGAAGGTTATCTGCACTGGATTAATGTTGATGACGGGCGTTTTGTGGCCCAACAA
AAAGTAGACAGTTCCGGTTTTCTGACCGAACCGACGGTGGCGGATGGTAAACTGCTCATC
CAGGCCAAAGACGGTACGGTCTACGCGATTACGCGTTAA
>EECEBICD_00824 GTPase Der
ATGGTACCTGTGGTCGCGCTTGTCGGGCGCCCTAACGTCGGAAAATCCACGCTATTTAAC
CGTCTGACCCGCACCCGAGATGCGCTGGTTGCGGATTTCCCGGGTCTGACTCGTGACCGT
AAGTACGGTCGTGCGGAGGTTGAAGGCCGCGAGTTTATCTGTATCGACACTGGCGGTATT
GACGGCACCGAAGACGGCGTCGAAACGCGGATGGCGGAACAGTCGCTGCTGGCGATTGAA
GAAGCGGATGTGGTGTTATTCATGGTAGATGCGCGCGCTGGCCTGATGCCGGCGGATGAA
GCGATTGCCAAACATCTGCGTTCCCGTGAAAAACCGACCTTCCTGGTGGCGAATAAAACG
GATGGTCTCGATCCCGATCAGGCGGTTGTCGATTTTTACTCGCTGGGATTAGGCGAAATT
TACCCTATCGCCGCCTCACATGGTCGCGGCGTATTGAGCCTGCTGGAACATGTGCTTCTG
CCGTGGATGGATGATGTCGCGCCGCAGGAAGAGGTGGATGAAGACGCGGAATACTGGGCG
CAGTTCGAGGTGGAACAAAATGGTGAAGAAGCGCCGGAAGATGATTTTGACCCGCAAAGC
CTGCCGATAAAGCTGGCGATTGTCGGTCGCCCGAACGTAGGTAAGTCCACACTCACTAAC
CGTATTCTCGGCGAAGAGCGCGTGGTGGTATACGACATGCCGGGCACCACTCGCGACAGC
ATTTATATCCCGATGGAACGCGACGAACGGGAATATGTGCTGATTGACACGGCAGGGGTG
CGTAAGCGCGGAAAAATCACCGATGCGGTAGAAAAGTTCTCGGTGATTAAAACGTTGCAG
GCGATTGAAGACGCTAACGTCGTACTGTTGGTTATCGATGCGCGCGAGGGGATCTCCGAC
CAGGATTTGTCTCTGCTGGGCTTTATCCTCAATAGTGGGCGCTCACTCGTTATCGTAGTC
AACAAATGGGATGGCCTGAGCCAGGAAGTCAAAGAGCAGGTGAAAGAGACGCTGGATTTC

CGTCTGGGCTTTATTGATTTTGCCCGCGTTCATTTTATCTCTGCGCTACACGGCAGCGGT
GTCGGCAACCTGTTTGAGTCCGTACGCGAGGCTTACGACAGTTCCACTCGCCGGGTGAGC
ACTGCGATGCTGACCCGTATCATGACGATGGCGGTGGAAGATCATCAGCCGCCGCTGGTG
CGCGGTCGCCGTGTGAAACTGAAATATGCCCACGCTGGCGGCTATAACCCGCCGATTGTG
GTTATTCACGGCAACCAGGTAAAAGATCTGCCAGACTCCTATAAGCGCTATCTGATGAAC
TACTTCCGCAAGTCGTTGGAAGTGATGGGGACGCCGATTCGCATTCAGTTTAAAGAAGGG
GAGAACCCGTATGCGAACAAGCGTAACACACTGACGCCAACGCAAATGCGCAAACGTAAG
CGTCTGATGAAACATATCAAGAAAAGCAAGTAA
>EECEBICD_00825 putative protein YfgJ
ATGGAAATAACCTGCCCCGTTTGCCATCACGCGCTGGAGCGCAATGGCGATACTGCGCAT
TGCGAGACATGCGCTAAAGATTTCTCGTTACAGGCACTTTGTCCTGACTGCCGCCAGCCG
TTACAGGTTTTGAAAGCCTGCGGCGCAGTGGACTATTTTTGCCAGAACGGTCATGGTTTA
ATTTCAAAAAAACGGGTTAATTTCGTCATTTCCGATCAATAA
>EECEBICD_00826 hypothetical protein
ATGTCACCTTATGGCAAGATCCGTACCAGCCTGTCCGTGGGCGAAGGCGGCGATCTGGAC
GGCAGTTCGCTGGATTATTTTATTCCCTGGTATGACAACCAGAGCACGCTTTTCTTTAGC
CAGATTTCGGCTCAGCGTAAGGAAGACCGCACCATTGGTAACTTTGGGCTTGGCGTTCGC
CAGAACGTCGGCAACTGGCTGCTTGGCGGCAACGCATTCTATGACTACGATTTTACGCGC
GGTCACCGTCGATTAGGTCTGGGAACCGAAGCCTGGACGGATTACCTGAAATTCTCCGGC
AACTATTACCATCCTCTTTCTGACTGGAAAGACTCCGAAGATTTCGACTTTTATGAAGAA
CGACCCGCGCGCGGCTGGGATATCCGCATGGAAAGCTGGCTGCCGTTTTATCCGCAGCTT
GGCGCTAAATTAGTTTACGAACAGTACTACGGCGATGAAGTAGCGCTGTTTGGCACCGAT
AATCTGCAAAAAGATCCTCATGCCGTCACTTTGGGGCTGGAATATACGCCGGTACCGCTG
GTCACGGTTGGTACGGATTACAAAGCCGGTACTGGCGATAGTAACGACTTTAGCGTTAAC
GCGACCGTGAACTACCAGATAGGCACGCCGCTGGCGGCGCAGCTCGATCCGGAAAACGTT
AAAATTCAGCACTCCTTAATGGGCAGCCGCACCGATTTTGTCGATCGTAACAATTTCATC
ATTCTCGAATATCGTGAGAAAGATCCGCTGGACGTCACGTTGTGGCTGAAGGCTGACGCC
ACCAACGAACACCCGGAATGCGTGATTGAAGATACGCCTGAGGCCGCGGTGGGGCTGGAG
AAATGTAAGTGGACGGTCAACGCGCTGATTAACCACCATTACAAAATTATTTCTGCCTCC
TGGCAGGCGAAAAACAACGCCGCCCGTACGCTGGTGATGCCGGTGGTAAAAGCTAATGCG
TTAACCGAAGGCAACAATAACAGCTGGAATCTGGTGCTTCCTGCCTGGGTTAATGCCGAT
ACCGAAGAGCAGCGCACCGCCCTGAACACCTGGAAAGTACGCATGACGCTGGAAGATGAA
AAGGGCAATAAGCAGAACTCCGGCGTGGTGGAAATCACCGTCCAGCAGGATCGCAAAATT
GAGCTGATCGTCGACAACATTGCCGATACCGATCGCAGCGATCACAGCCATGAAGCCAGC
GCGCTGGCCGATGGCGAAGACGGCGTGGTGATGGATCTGCTGATCACCGATTCCTTTGGC
GACTCCACGGATCGCAATGGTAACGAACTGGTGGACGATGCCATGACGCCCGTGTTGTAC
GACAGCAATGATAAAAAAGTCACCCTGGCCCAGACGCCCTGCACCACGGAAACCCCCTGC
GTGTTTATTGCCAGTCGTGACAAAGAAGCGGGGACGGTGACGCTTTCCAGCACCCTGCCG
GGGACTTTCCGCTGGAAGGCGAAAGCAGACGCCTATGGCGACAGTAACTACGTTGATGTC
ACTTTTATCGGCGACAACCTGAGCGCGCTGAATGCGGTGATTTATCAGGTCAAAGCCGCC
AACCCTGTCAACCTTATCGGCAAAGAAGATAAGCACCCTACGGTCAATAATGCTTACCGT
TTCCTGCTGTGGCGCGACAAAAATAAAGACGGCGTATTCCAGATGTCCGAGCAGCTCACG
GAAGAAGAGATGGCGCTGTACGACTACCAGTGGGAGTTTACCGGGCAGAGTACAAACGGA
CATACCGGCGCGCTGGCCAACACCATGAATGAAGATCTTGTCCTGCCAGTAACCAATAAA
GAGGCGGCGCAGAAGTTTGCCGCCAATGTGGAAGATGGCGTTCAGGGTTACGGCATACGC
GTTACGTATAGTCAGAAATAG
>EECEBICD_00827 hypothetical protein
ATGCAAGCAACAGTAAAACGGCGGCTGACGAAGGTCGCTTTAGCATTAGTAGTTGCCGGG
TATTGCGCCCGCGCCAGCGGTGGCGGCAAACGGTAATCTGAAATCGGGGCAATGGCAGATT
GTCTCTGAACAGACGGGCACCATTCAGGGTACGGTGCCGTGGATTACCCGTGCGGCGGAT
AAAACTGCGGATACCGACAAAGATCACGTAACGGTAACTATCGATCGCGGCGATCGTAAA
ATTGTGACCGAAGGCGATAAGCAGTTCCACGTTGGCGATAAGGTCACGGTGAACTGGGCG
ATTGGCGATACGGAAGGCGACCTGGACACCGACAACACGGCAACTAAAGCCACCGTGAAA
TGGGTGAGCTTTAGCGATCAGAATGGCAGCGACCCCAAAGATCTTGGTACTGGTGACAGT

TATGAAATCCAGGCCGCTGATGCGGATCGTTATATCGGTATCAAAATCACGCCAACCACT
ACCACCGGTGACCCTGCCGTGGCAACCGAACTGCTGTTGAAAGACTTGTCGACCGATGCG
GGCGGTGGTTCGGATGATGACGAAATTCCGGAAGGTCCGGTGGTGGATGAAAACGTCCAT
GTGGTTATTCATGAGAAAGATTCGAATACCAACCTGTTGAAGAATTCAGGGACGACACTC
AAAACGAATACTACCTATCAGGTATTGCTGTGGAGCGATAAAAACGGCAACGGCACCTAT
GATGCCGGTGAAAACGTTACCGACCAGTATGACTACCGCTGGAAATTCGTCGGCACCAGT
AAAATTGCAGGTACCGGTACTGGTGGTATCGTCAACGAAAACTGGAACGATAAAGACCTT
GTCATCCCGCTTACCAACGCCGAAGCGAAAGAGGCGTTCGAGGGTGCGGAAGGCGGCGTT
ACCGTGGGTAGCGACGGTGTCCAGGGCTTTGGTCTGTCCATCGACTACAAACGCAAATAA
>EECEBICD_00828 hypothetical protein
GTGAATAAAGTTGTACATTTCTTACTGACATTACTGATCATGTTTGCAGTGTCGATCGCA
CCTGCGCAGGCGCTATTGAAGGGCGGAACCTGGCAAGAGCTGAACTCAGTCACCGGTGCG
GTTAACGGCACCGCGCCGCTGGCCGACGGCGCTATTATCCCCCTTTACCAGGGAAGTACG
CTGCTTGACCCGAGCAAAAACCACGATATTGAATTCAGCGCCATGCCGCGCGATTTTAGC
GCGGATGCTACCTCAACCTCGATGCGGGCGGTCAATTCCACCGATACCGAAGGCGATCTC
TTTAGCGATCCGCCCACCATCGCCTGGGAAAACCGCCAGCCGCCCGCCATGGGCCTGGTA
TGGGCCGATGCCGCCACGCCCGACACGCCGCTCTCGCCGCAGCCGGTTCCGAACCTGACC
TTTTGCGCCCAAAACCTCGCCGGGCGACAGCTGGTGGCCTGGGCGCAGGTGGAAGATGAA
ACCAACGTTCCCGCGCTGTGGCTCTTTACGCGTACCGGGGTGCCGAATTATGCGACCATT
CCGCTGTTAAGCCCGAAAGTGGCGCTGAATATCAAACCAGCGGTGAGCGACCCTGTTTCT
GTGAGCGGCGATCATGTCGATGCCAGTTTTGAAGCATCGAAAGTTAAGGTCGGAGAAAGT
ATTACTCTTACTGTCACCACCAAAGCGTGTGATGGCGAACCTGCGATCAATGCGCCGTTT
GTGATCCGTCGCGAGGATGCAATAAATCGCCAGGGTGTGGTCAATAACGCTAACCCGGTG
CGCGTTGGCAATACCGAGTTAACGACTGCGCAAACAGAATATCATGGTGTAACGGACGGG
CAGGGCAACGCCACGGTGGTGGTAACGCAAGAGAACGGTCCGGGGGTAAAAACGCGACTT
ATCGTCTCTTCGCAAAACTATCCCACGCTGACCGACAATGTCGACGTCATTTTCACCACC
ATCACCAGTCCAGACAGCGATAAGGCCAGTATGTACGGCCACATGATAGAGAGCGCTACC
GCGACGCTGAACGGTATAACCTACACCTTCACCCGCCCGAAGCTGGCGGCGGAAGCGAGC
GGCGCTGACAAAAGCGTCGTCGATACCAACGAAACCTGGGCGCTGTTCGACTGGAACGGC
GCCGATAACCATTGCAATATTCTGCCGGATGCCGAACAACTGGTGCAGATGCGACATGAG
CACAGCACGCTGGCAACGTACACCGGCTGGCCGGCGACGGACGATGCCGAATACTGGTCG
TCGACCAAAGATCAGATGAGCGGCTACCACGCTGCCGTACATATGAACAGCGCATCGGTC
GTCAGGGCCGCCGACAGCGACACGCTGCTGGTCAGTTGTGTGGATAAAGCCCAGCCTGCG
GCGCACCCGCAGATTACTCTCTCCCCACAAGGACCGTATAAAGCGCAGGTGGGGGAGTCG
ATTGATCTGACGATGACCGTAGTTGACCGGGACACGCAAAAACCGCTGCCTTACCGCTAC
ATGGAGCTGTTTATCGACCCGGCGAAAAACCGCAAAGGGGAACATCAGGACGCATGGGAT
AACCTGCGGGTCACCGTTGATTCCGAAGGTATGAGCGCCTCGTCGCCGGAGCACTATACC
GGCGTGACTGACGTCAACGGCCAGGCGCACCTGACGTTAAAACATAACAGCGGCCTGGGC
GTCGAAACGCCCATCCGCATCGTGATGCCGGACGACGAAGGCGGCAACGTCGAGCTGTCC
TTTAGCGTAATTTTCACCGTGGTCACCAGCCCGGACGTGGACGGCGCCAACATGTGGGGC
CATATGCGCGGCGTGGTGGACGCGGGCAACCTGTACAAACGTCCGCTGCTGGCGGTGGAG
GCCAGCCATAAAGACGGCCAATTCAGCGAAAATAACGAAGAGTGGGCGACGTTTAACTCC
GTGGCGTCGGCCACCGCGCAGTGCGGTGTCGGCCAGGTGCCGGATCAGTCGTCGCTGGCG
CATTTGTACAGCGAACATGCGGGCGGACAGATGGAAAGCGAGCACGGCTGGCCGACTGAA
GATTATTTCATCGCCGCCGACAGCGACGCCTCCGGGACGGTACACGTCAACCTCGAAAAC
GGCGATAGCGGCAAATTTACCGATACGCCCAACTACCTGACCTGCTCGGCCAATGAAATG
GTGGCGGTGCTGGACGTCTACTTCAACGACGACCCGGTGATCAAAAACGCCGACGCCAAA
GTGGGTGAACAGGTTAAGATGAACATCCATTCGCGCAACGCCCTTAACGGCATAGCGATC
GGCAATACCGACTTCACCATCACCATGGCGAACGGCAGGCGGCGTGACGGGCTGACGACC
GGCTTTACCGATACCAGCAACGGCGAGATGCAGTTTGACGACGTGGGCTACGTTGCCGGG
CAGGTTTATCACGGGATCACCGACGCTAACGGCGATGCGACCATTATCCTCACCCAGAAA
AAGGGCGTGGGGCTGCTGACGCCGTTAAGCATCGTGCCCGTCAATTCATACATCAATACG
CCGGTTTCCCGCAGCGTGAAGTTTACCGTCGCGACCAGCCCGGATACGGCAAAAGCCAAA
ATGTGGGGCCATATGGCCGACACGATTACCGTGGGCGACTGGACTTTTGAGCGGCCCAAA

CTGGCGGGTGAAGTGAGCAACCCGCTGCGTACCCAGGACGAGAGCAATGAGACCTGGGCG
CGCGTGGCCCATAGCGACGCGGTGGGCAACCCGGATGCGGGCGGCTGCGCGGCGAACCGC
CTGCCGCGCATCGACCAGCTTGAGGCGCTGTATAACGCCAACAGCGGCGGCAAGATTCAC
AGCATTCAGGGCTGGCCGACCTATCTTAACTACTGGTCGTCCACCTACCAGAGCGCCACC
ACCTGGAAACTGATCGCGCTGACGAACGGCAGTGAATTTGCCAACAGCAACGTCTCGATT
TACGCCAGCTGCCTGGCCAGCGATAACCCGGTGGCGGCGTCCATCACCATTGAACCGGTC
AATCCGTCGCAGTGGTACGACGGCAGCGACGTCCACGCGGTCAAAGTGAAAAAGGGCGAG
ACGATGCAGCTCAAGGTGACGGTGAAGGACGCCAGCGGCAACCCGATCCCGGAAGCGCCG
TTTGTGCTCACCCGCGGCGACGGTTACGATCGCCGGGGCGAAAAGTATACCGCTCAGGAC
GGTGACGATCTGCAAGGCATCGTGACGCCCGTGGTGATCGACGGCGAATCGCTGGCATGG
ACCACCACCAAAATGGGGTCGCAGACCGGCACTGACGGCACCCGTATCATCAGCGTCACC
CGCCCGGACACCCACGGCACCCGCACCGCGATTAACGCGACGCTGTATGAAAATGCCGCC
GTGAGTGCCAGCATCGACACTATCTTTACCGTCGTCACCAGCCCGGATGTCAGCGTCGCC
CGCATGTGGGGGCATATGGCGCCATCGCTGACGGCGGCGGATGGCGCGGTGTACCAGCGC
CCGCTGCTGTACGCTGAGCTGTCCAGTACGGACAATACTGCGTCTAAGCAAGAAACCAAC
GAAACCTGGGCGGTTTTTCATGGGCCAGCCAGTGAAGGGGCCAATCCGGCCCGTTGCGCG
GCGGGCTATTACCCTGCCGTTGAGGCGCTGGATTCGCTCTACAGCAAATACCCGAGCCGA
ACGATAAATACCGCCCAGGGCTGGCCGGTCTACTATTCTTACTGGAGCGGATCGAACTCG
ACGTCATTTTCCAGCGGGGCAAAACTTGACTTCTATTACGCAGTCGATCTGGCTGATGGT
TCGCGTCGGTCAGAAAACAGCGCGACCAGCACTGCCTGGCAGTATCAAATTTGCGCCACG
ACGCCGCTGCCGCAGGCCACGCAGATTACCCTCACCTCCCCGCAGGCGATGGATGACGCT
ATTCAGGCGGTGAAGGCGAAAAACAGCGAGTCGATCCCGCTACTCATCACCACTACCGAT
GCGATGGGCAACCCGGTGCCTTATGCGACTTTTTCGCTTAAACGCGACGCCGGGAAGGCG
CGCAACACCGACTATAATAAATTTGTAGCCACTAACGGCACCAACATGACGGTGACGCCG
CTCACCGGTGCGCAGCAACAGTTTTACTATGCCACATCGGTTCTGACCGGCGCCACCGGC
GCGGATGGTACGCTGGCCCTGACGCTTGCCGAACCCGGCGGCATAGGGTTGAAAAACCAG
CTGACCGCGAATCTGAACGATACGCCGACGGCGACCTCATCGCTGCCGGTGGTGTTCACC
GTCCTCACCAGCCCGGACAGCGACAAGGCCAACATGTACGGCCATATGCCGGAGACCTTC
ACCGCCAGCAACGGTGCGGAGTTTAAGCGCCCGCTGGTGGCAGGCGAGCCCTCATCGGAG
GCGCACACGGATACATATTTCGAAACCAATGAAAACTGGATCATGGTGAATAGTTTTAAT
ACCGGCAATTACGGCGGGTGTCCGATGAACCAGATGGCTGCGATAGATGATTTCACTGCG
CTGTACAACGATCACCCAAGCGGCAAGGTGGCGACCGATATTGGATTGCCCGTCGGGAAG
CGGTGGTGGGCGGGGGATAGCCTGCTGAAAGGCTCAACCCTTTACTGGCAGTATAAAGAT
CTCAAGACGGGGAAAAACTACAGCATGTCGGAAAACCCGGGTAACTACTACTTACAACTC
TGCCTGACAACATCGCGCAGCGGGCTGAATATCGCGCTCTCTTCCGACGCATGGAACGCC
GATAAATCGGCGGCCGTGGCGAAAAAGGCGAAACGATACCGATGACGGTAACGGTGACT
AACGACGCCGGGCAGCCGCAGGCCGGGGTGGCCGTACTGTTGACGCGTGATTACGCTTAT
TCGCGCGGTGCGGTGGATAAGCAATACATTGAACCAGGCGTGATTGGCGAGCCGGTTCCG
TTTACAACTTCGCCTGCGAATATGATGCTGACGCCGGTTGCACCTGCGGGTACGGCAGTG
GCGTTTAATAACCAGAATGGCCTCAGCACAAAATGGTCTGGCTTCACCGGCGATGACGGT
AAGTTGCGGTTTACGTTAACTCAGGATAAATCGCTGGGGTTAAAAACGTCTGTCACGGCG
GCGCTTGCGAATCAATTTGATGAGGCGGCGAGCGTAGATGCAATCTTTACGGTACAAACC
AGCCCGGACACGCCGTATGCGTCATACTGGGGTCATATGCCGGATACCGTGCAGGTGAAC
GGCGTTACGCTGCGCCGCCCGTACCTGAAAGCCGAGCTCTCTGCCGCGCCCCGGGATACC
TGGCCGTTTAATAATGAAATCTGGGGGACGAACTATTATTATCAGTCCGAACACGTGGAA
ACGTCGTTGACGCATCTTTGCGGATCGCAGGAAAATATAGCCTCGCTGGACGATCTGAAA
GCGTTGCAGAGCGTTATCGGCACGTTGCAATGGCCGACGACATCATCCTGGGACTATGTG
TCGCAAGATGAAGGTCAAAGCAATAAGTACTACTGTTCATTTAACGAAACGACCGGCCAG
ACAACCTGTACTCGTGAAAAAGCGACGACCTCAGGCTTAGGCTCGTGCCGGGTGCCGTAG
>EECEBICD_00829 hypothetical protein
GTGAAATCGAAAAAGTGTGTCGGTTTTTTACCCTGGCTGCTGCTATGCGCCCTCAGTTTA
ACGCCTTTCTTCGCGCAGGCCGTGCGAAGCAGCGGCGCATGGCAGGAACTCACTGAGCAA
ACTGCGGCTATTAATGGCACTCCGCCTCAGGCCGACAGTGTAACGATCCCGGTATATCAG
GGCAGCGTGCAGTTGCGCTCTGATGCGGCGAATCCTGTTGACTACTCCGCGAAGCCCAGC

CAGTTCAGCACCAGTGATGCTGGCAGCGCGCTGACCTTAACCAATCCGCACGACACCGAA
GGCGATATCTTCGCCGTGCCGCCGCTGGTCTGGCAGGCTGAACGCGCGCCCACCGTCACG
CTGGTGTGGGCCGACGCCGCCACGCCGGACACGCCGCTTGATCCACAACCCGTCAGCAAT
ATAAGTTTCTGCGCGCAGAATCTGGCGGGCCGCCATCTGGTGGTCTGGTCGCAACTGGAT
ACCTCAACCGCCATACCGCCGCTGTGGTTGCTTACCCGCACCGGCGTGCCTTATAACACG
GCGGTCGAAGTGCTTGATCAAAAGTTTGCCGTGGATATCGCGCCCGCCGTGGGCGACCCG
GTAACGCTCGCCGCCGATCATCTGGACGAGTCGCTCAACGCCGCGAAGGTCAAAGCGGGC
GAAAGCATCACGCTGACGGTAACGACCAAAGACTGCGGCGGCGAACCGGAGGGCAACGTT
GATTTCGTGATTACCCGCGGCGACGCGCAAAACCGCCAGGGCGTGGTAAACAATGCCGCG
CCGGTGCGCGTCGGCAACACCGAACTGACCACCACCGCCACGGAATATCACGGTACGACC
AACGCCGAGGGCGTGGCGACCGTGGTCGTCACCCAGGCGAACGGCCCGGGCGTTAAAACG
CCGCTGATAGCGCACCCGTCGAGCGCGCCAGCGCTGAAAGCCAGCGTGGACGTGATTTTT
ACCACCCTCACCAGCCCGGACAGCAGCAGCGCCAACATGTACGGTCATATGGCAGAGACG
TCCACCGCCACGGTGGACGGCGCAAGCTACACCTTTTACCGCCCCAAACTGGCGGCGGAA
ACCGACGGTGAAGACCGCACCGCCAGCATCAACAATGAAACCTGGGCGCAGTTCAACTGG
GGCCACGCCGACGCGCACTGCGATATTCTGCCGGATGCACGCCAACTGGAAGGGTTAAAA
ATTGCGCGCGGCGATCTGGCCACCACCCTCGGCTGGCCGGTAGGGCTGACATCCGGTGAT
GAAGAGTACTGGTCCTCCTCCCAGGGATCGACTGCCACTGACCATATCAGCATTGATATG
CGCAGCCGCAGCCTCACGCAGATGCCGGACGCCACGCAATCGCTGGTTAGCTGCGTGGAT
AAAGCCCCGCCTGCGGTCACGCCGAAATTAGTGATTAGCGCCGACAATTTTGATTCAACG
GTCAACGCGGCGAAAGTAAAGGTGGGCGAAGAGATCAACATGAAGATCACCGTCACCGAC
AGCGCCACCAATAAACCGCTGCCTTATCGCTACCTTAACGTCTACCTGGGCGACGAGCAA
AATCGCCAGAATCAAAAGAATGCCGATATTGATAGCGGGCATCAGTGGACGGATGATCCG
GTAATTATCACCAATCTGGAAGGCAGCGACGGTCACTATCACGGCGTCACCGACGCCAAC
GGTCAGTTCTCGCTGGTGTTGACTCAGGACAAGGGGGCGGGCGTATTAACCCCGGTGCGC
GTGGTGCTGTTCGACGGCACGGAAGCGACGCAGAACGTGATTTTTACCGTCGTCACCAGC
CCGGACGTGGCGCAGGCGCGCATGTGGGGCCATATGCAGGGCGTGGTGGAAGCGGGCAAT
ATCTACAAACGCCCGCTGCTGGCGGACGAAGCCGCGCAGGATACCGGTTCCGAATTTGAG
AATAACGAATACTGGGCGACGTTTAACTCGGTAACGGCGGCGACGAACCAGTGCGGTACC
GGCCAGGTGCCGGGGCAACTGTTGCTGGATACGCTGTATCAGGCCCATTCCGGCAACGCG
ATGGAAACGACCTACGGCTGGCCGACGCAGAAACACAGCTATATCGCGGCGGATACTGAT
GGTTCCACCACCGCGCACGTCAACCTGGCGACCGGTGCGGACAGCACGTTCAGCGGCACC
GAACCGAACTATCTCTCCTGCTCCGGCAATGAGTTGGTGACCAGTCTGGACGTTTACTTT
AATGGCGATGAGTCGTTACGCAATGCGGTAGCCAAAGTCGGCGAACAAATCACGATGAAC
GTCCATTCCGTCAATGCGCTGAATGGGTTAAGCGTGCCCAACGCCAGCTTTATCGTCACC
ATGGCGCACGGCAAAAATCGCGACAACGCCACGACGGGCTTTACCGACTCCAGCGACGGA
ACGCTGGTGATGGGCGGAACGCCTTTCGGCTCTTCCCAGGCGTCGATGACGTATCAGGGC
ATGACCGACAATGAGGGCAACGCCACGCTGGTGATTGAACAGCCGCAGGGCGTGGGCTTG
CTGACGCCGCTGAGCGTACTGCCGGTCAACTCACTGATTACCACGCCCGTCAACCGCAGC
GTGAAATTTACCGTTCCCACCAGTCCCGATACGCCGGAGGCGCAGATGTGGGGTCATATG
GCGGACACGCTCACCGTGGGTGATATGGAATTCCAGCGGCCAAAGCTGGCGGCGGAAGCG
ACTGCCGCCACCCGTACCCAGGAGCAGGATAACGAAACCTGGGCGCGTGTCAGCCACGCC
GACGCGCTGAATAACCCCAACGCCGGGGGCTGCGAGGCCGGCCATCTGCCGCGCGCCGAT
CAGCTTGCCGCACTGTATGCCTCCAGCGACGGCAATAAAATCCACACAGTATCCGGCTGG
CCGACGACGTATGACTACTGGTCTTCCACCTTCGCCAGCGCCGCTACATGGCAGGCGGTA
TCACTGGCTGCGGGCGGCTATACCGCTTCCGGCGATGCGTCGGACTACGTGAGCTGTCTG
GTCAGCAAAAATCCGACGGCGGCGTCTATCACCATTGAGCCGGTGGATGCGGCATTGTGG
TATAACGCAAACAGCGAACACGCGGTGAAGGTGAAAAAGGGCGATACGCTCCAGCTTAAA
GTGACGGTAAAAGACGCCAGCGGCAATCCGCTGCCGCAGGCGCCGTTCGTGTTAAGCCGC
GGCGACGGCTATACCCGCCAGGGGGAGAAACATATCGCCGGTAGCGGCGACGGCATCGTC
TCGGCGGTGGTGATTGACGGCGATTCGCTCAACGACAGCGCCACCCAAATCGGCGGTATG
ACCGGTGAAAACGGCAGCAAAATCATCAACGTCACCCGCCCGGATGCCCACGGGACGAAA
GTCGCGATTACGGCGGCGCTGTACAATAACGCCAGCGCGACGGCCAGCATTGATACTATT
TTTACCGTGGTCACCAGCCCGGACAGCGACAAAGCCAAAATGTGGGGCCATATGCCGGAA

ACGACGACGGCGGCCAACGGCGAGGTGTTTAAGCGCCCGCTGTTATCGGCTGAAATCGCC
AGCGGCGTTACCCACGGCGACAATACGGAAAACAACGAAGCGTGGGGAATCGTCGATTTT
GAAATGGCGAACGACGCCTGCGGCGCGGGATACGTGCCGACCCTTGCCGATATGCAATCG
CTGTACGACGCCAGGCCCGGCGGCGCCATGAATACCCAGCAGGGCTGGCCGCTGGATGGT
AAAAACTACCAGGACAGCACCGCCGATTTGAGCCGGAGTACGCAAAACCGCTACGTTAAG
TCGATTAATCTGCGCGACGGCGGCGTCGGTTCGCTATTGTGGAATGAACAACTCTATTTT
GTCTGCCTGCAAAACGCCCATCCGGCGGCGACGCAGATAACGCTCACTTCGCCTGTGTAT
AACGACAGCGACGGATTCGCCAAAGCGAAAGTCGGCGAGACCATCCCGGTCATCATCACC
ACGCGGGATGCCCAGGGCAACCCGGCCGCCGACACGCCGGTTATCTTTACGCGCGGCGAC
AGCGTCGGGCGCGCGAATCAGGAGGTGAATAGCTCGGCGGCGGCGGATATACAGATAAAC
CACAGCGACGGGCGTAGCAGCGGCGCTAACTATTACACCGCCACGGGCGCCGACGGCACG
CTGACGCTGAATATCAGCCAGGACAGCGGCGCGGGCTTTAAAACGCCGCTGACGGCGGCA
ATAGAGCACAACGGCGTAACGAGCGCGCCGCTGCCGGTCATCTTTACCGTGGTCACCAGT
CCGGACACGCCGAAGGCCAACTACTGGGGCCATATGGCGGAAACGCTGACCGACAGCAGC
GGCGTGGCGTACAGGCGTCCGCTGCTGGCCAGCGAGTTTAGCGCTACGCCCGGCAAAACG
CTGACCATCGCCAACGGCTACTACGACAAAGGCGAAACCTGGGGCATGATAACCGTCGAT
AAAGCCTGGAACGGCACCGGTGGCGGCTGTGGGCGCGACACGCTACCGACCGTAGCCAAC
CTCCAGACGCTGTACGGGACATACCCGGATAACGCCATGCGCAGCCGTAACGGCTGGCCG
ATGACCAGTTCAGGCAACAACAACGTCAGCCGCTACTGGTGGGCGGGGGATTACGTGATA
TCGTCAGACGGCACGAAGTCGCTCTACGCGGCGGTCAACCTGTTTAACGACGGCAATGAT
GTCAAAACGACGACCAGCACCTCGATGTACTACATGCAGACCTGTCTTGCCTCGCCGCGC
AGCGCCGCCGCCAGCCTGACGCTGACGCTGGCAGGCCAGGATGAAACCACCGGAAGCGCA
AAGGCGAAGAAGGGCGAGCAGATGGCCGCCACCATCGACGTGAAAGACGCCGCAGGTCAG
CCGATGAAAAACGTGATGGTGAAAATCAGCCGCGGATCTTCGTACAATCGCGCCAACAGC
GCCACATCGTCCTCAAGCGTCACAGACGATATTACGCTGCGTAATGTGATGCCGTCCGGC
CTGGCGACATACCTGCTGGATACCTCGGCGAAATATCTGTATGCGCAAACTGATGCCCAG
GGACAGGTGACATTTACCCTTGCGCAGGACAGTACCGCCGGTTTAAAAACCACTATCAGC
GCCGCCACGATGGACGGCAGCAACCTGACAGACAGCAAAGACGCAATCTTTACCGTAGTG
ACCAGCCCGGACAGCGATAAGGCCAAATACTGGGGCCATATGCCGGAAACCTTTACCAAC
AGTAAGGGGGTTTAA
>EECEBICD_00830 hypothetical protein
ATGTATCAGGACACCGCCAGCCCGTGCGATCGGCTGGGGTTGCCGACGGTAAATGATTTA
CAGACGCTATATACCGATTATCCCAATGGCGCACTCACGACAACGCTTGGCCTACCGGTG
GCATCAGGAAAATATTGGGGGGCGGGTAACTCAGTACCGGATGCCACGCACTCAGACAGT
CAGTTCCAGTATGTCAGACTCAGCGATAATAATACGCTGACAACCAAAGCCAATACCGCC
ACGGCGCAGCTTTGTCTGGCAAAGCGCCGGGACTTGTCGATAGAACTCACCTCCAGCGCC
ATGGATGCGGATAAAGGCGCTCCAGTGGCGAAGAAGGGCGAGAGCCTGCCGTTGACCGTC
ACTGTGAGGGATGGTTCCGGCACGCCGCAGCCCAATACCACTATCCGTTTGGGACGAACG
CTTTCGATAGACCGGGCGGGTGTCGTTGATGGCAGTTCCGGCGGAGGCATGGTACTCACA
TCGGTTGCGCCGTCTACGGGCTCAATGACGTTTAACTGCACGGTATCTTCGTGCACAAGT
TATTGGTATGGCATTACGGATGAGGATGGTAAAGCGCAACTCGAAGTGACGCAGGATGAT
TCCCGTGGCCTGAGAACACCGCTGCAGGCGATGCTTGTCGACGATCCGCTGACAGTTAGC
GATATGGACGTCATTTTCACCGTCATTACCAGCCCGGACAGCGATAAAGCCAAATACTGG
GGACACATGCCGGAAACCGTGACCAACAGCGCGGGCGTGAAGTTCCGGCGTCCGCTGCTG
GCGGCGGAGATGACGTCGAATAGCGGTACTTACCTGGTTAACAATGAAACATGGCCCTTA
GTCACCGCAGCGAACACTGAAAAAGCTGGCGCAACCGGCTGTGATGCGGAATACCAGCCG
TTGTCGGGCGATCTGCAAACGTTGTATAGCGACAATCCTAACGGCGCAATCGGTACAAAT
TACGGCTGGCCGGTGGCGGGGAACAAATCCTGGTGGGCGGCGGATCGTGCGCCGAATACC
GGATATTACCAATTTATAAACCTGAATAGCGGCGGTAAGGGCACCGCCAGTTCATCGACT
GCCACCGGTGCTCAGGTGTGTCTGGTCGAGCCGCGTACTTCTACTCCTGCCAGTATTACG
CTGACCTCGACGGCGATGGACAGCGCGAAAAATGCGGCGGTAGTGCCGAAGGGAAGCGCG
ATGCCGTTAACGGTGACGGTAAAAGATAGCAGCGGTAACCCGGTCGCCAACGTTGGTTTT
ACGTTGTCACGTGGCGATTCGAAAAATCGGCAGGTATGGTGATAACGGATGGCGACGTG
GCAGCGGATGCCGGAGCAGACGATCTCATGCTTAAAGAGCTGACTCCCGCAAGCGCCAGC

CAAAGTATGACCACCACCGGGATTGTCTTTACCGGCACTACCGGCAGCGACGGTACGGCA
ACGTTTACGCTTAATCAGGATAAAAGCCTGGGGTTGAAAACGCCACTCACGGTGAAAGTG
ACGGACAATACGACTCTTCACGCATCGCTGGATGTGATTTTTATGGTACTTACCAGCCCG
GATACGGATAAAGCGTTGTTCTGGGGAAATATGTCGGATACGACATCCGTCAACGGAAAA
ACGCTGCATCGTCCGTGGCTTCAGGCTGAGATGCTGTCAGGCGTGACGCCAGTATTTACC
AACGGAGTGCATGCAAATAACGAGTACTGGGCGATGGCGCATACGGTGGACAACACGAAA
TGGGATATTGCCAAACAGTGCGGTAGCCTTAGCAAAGCACCGGATAATAACGACCTGTTG
ACGCTGTATCATTCAATTAGCTCGTTAGGCTGGCCGACGCTGGGCTATCCCTATTTATCT
AAATCAACCTCGAGCAGCGGCATGTATTGCGGCGTAGATGAGAATACCAAGAGTCAAAAC
TGCGCCATTAAGCCGGCAGGCACAGCGGGTTATGCGACGTGCGTGGAGTAA
>EECEBICD_00831 hypothetical protein
TTGAACAGGACTTACAGTATTGTCTGGAGCGCCGTGCGAAATATGTATGTCGTGGCTTCA
GAACTGGCGCGAGGCCACAGCAAAGTTAAAGCGCAAGTATGCGCGAGTGAAATCCATTCT
CCAAACCAAAAGTCCGAATATGGACAAATAATAAAAGCAACGCGCGCGGCGCTGGCTTGC
GCCGTGGCTGCAGCGCTCGGATTTTTCAGCCCGCTGGCCATGGCCGACAATCAGGTATCA
TATGCTGATGCGCAGACCCACGTTCTGGATGCGTCAACGCCGCCGATGAGCTATTCGGGC
ACCGATGAAGGCGCGGCGCTATATGTCTCGGGTGTCGCCACCGTCGGCTGGCAGCCCACC
ACGGTCACAGGGACGGGGCTCGTCATTGAAACAAGCGGAGGCGGAGCTGATGATCCGGAT
GGCGGTAAGTATGTTTCCAACGCAATTTCGCTGGATCATTACGCTATCCTTGAGCTTACC
GACGCGAAGATAACCACTACGGGCATTTATACCCAGGGTATTTCCGCAGCGGATGGCAGC
ACACTGACGTTAACAGACAGTACGCTAACGATTGACGGCAATTTCGGCGTAATGACGTTG
TATACGGGTTCAGAAGCAACGCTGGACGGTACCATCGTTGAGGCAGCTAACAGCAGCAGC
GCGCAGGTGCAACAAGGCAGTACGCTGAACGTGCTGGACGGTAGCACAATCACCCTGGCG
CAAGGCCAGATAAACGTTGTTGCCGGAAACACCGCGACAGACGAGGGTTCCACCCTGAAC
CTGTCTGATAGCTCGGTCAGCTCTGCAGGTACGATGAGCACCATCCAGGGTACCAATAAG
GCCGCTCTTAATCTCACCAACGCGACCATAACACATACCAATGCCAGCGGCGCGGCGGTA
CAGGCCAATAACGCCACTACCCTGGATATTACAGGCGGCAACATTACCTCTGCCGGTACG
GGCGTGTATATTCTGGCGTCGGACGCCAGGATCGACGGCGCCACCATCAACGCCGACGGC
GACGGTATCTTTATAACCAGCAAAAGAAAACTCGACGGGTATGAAGATCTCAACGCGCTG
ACGGTCAACAAAGCACAGGTTAATTCAGACACGATTGCGCTGCATGTGGACACTGGCACG
ACGATTAACGCTCCTATCGTGCTGACCGACAGCACATTTGAAGCGCCGGAAGTGATCAAG
CTTGGCAGCAAGGCGGTCATTCAGGCCAACAATACGACATTGATCGGTGACGTTGCTCAG
AGTGACATGTCATCGTCATCGCTCTCTTTAAGCCAGGGGCCACGTTAA
>EECEBICD_00832 hypothetical protein
ATGTTTACTACGCTGTCGCTCGACGACACCAGCCAGTGGAACATGACCGACCCGTCAACC
GTCGGCAATCTGACCAACGACGGCGATATTACGCTGGGCAACGCGAGCGGTTCGACAGGT
ACGCTGCTGACGGTGGACAATACCCTGACCTTACAGGATAACAGCCAGATAAACGCGACG
CTGGATACCGCCAACAGCGCGCCAATTATCAAAGCCGCCAACGTCACGCTGGACGGGACG
CTGAACCTTAGCTCAACGGCCACCTTTGTCGCGCCCGAAACCGACGAACACTTTGGATCG
ATCACGCTGATTGATTCACAAAGCGCAATCACTACCGATTTTGACAGCGTAACACTGGAT
GCTGACACCTCCGCTATGCCGGATTATCTGACGATCAACGCGGGCGTTGATGCGAATGAT
AATACCAACTACGAGTTGTCTACGGGCTTGAGTTGGTACGCGGGCGCAAATTCTGCGCGC
GCTGCGCATGGCACTTTTACCGTTGATGCAGGCAGTACCTTTACCGTCACCAGCGAACTG
GACGAAACCACGGCGACCTCCAACTGGAACGGCAGCAAGCTAACCAAACAAGGCGACGGC
ACGCTGATCCTCAGCAACACCGGCAACGACTACGGCGATACTGAAATTGACGGCGGCATT
CTGGCCGCCAAAGACGCCGCCGCGCTCGGCACTGGCGATGTGACGATTGCAGAAAGCGCA
ACGCTTGCGCTGAGCCAGGGCACGCTGGATAACAACGTGACCGGCGAGGGCCAAATTGTC
AAAAGCGGTAGCGATGAGCTGATCGTGACCGGCGATAACACTTACAGCGGCGGCACCACT
ATTTCCGGCGGTACGCTGACGGCTGACCATGCGGATTCGCTGGGTACCGGGGCTGTTGCT
AACAGCGGCGTGCTTCAGGTCGGCGAAGGCGAGCTGGAAAATACGCTTTCCGGCAGCGGC
TCGCTGGTGAAAACCGGCACAGGCGAGCTGACGCTAAGCGGCGACAACACTTACTCCGGC
GGCACCACCATTACCGGCGGTACGCTGACGGCTGACCATGCGGATTCGCTGGGCAGCGGC
GATATCGATAACAGCGGCGTGCTTAAGGTGGGCGAAGGCGATCTGGAAAATACGCTTTCC
GGCAGCGGTTCGCTGGTGAAAACCGGTACCGGCGAGCTGACGTTAAGCGGCGGCAACGAT

TACTCCGGCGGCACCACCATTATCGGCGGTACGCTGACGGCTGACCATGCGGATTCGCTG
GGTACCGGAGCTGTTGCCAACAGCGGCGTGCTTCAGGTCGGCGAGGGCGAGCTGGAAAAT
ACGCTTTCCGGCAGCGGTTCGCTGGTCAAAACCGGTACGGGCGAACTGACGTTAAGCGGC
GATAACAGTTACTCCGGCGGCACCACCATTATCGGCGGTACGCTGACGGCTGACCATGCG
GATTTGCTGGGTACCGGAGCTGTTGCCAACAGCGGTGTGCTTCAGGTCGGCGAGGGCGAG
CTGGAAAATACGCTTTCCGGCAGCGGTTCGCTGGTCAAAACCGGTACGGGCGAACTGACG
TTAAGCGGCGATAACAGTTACTCCGGCGGCACCACCATTATCGGCGGTACGCTGACGGCT
GACCATGCGGATTCGCTGGGTACCGGAGCTGTTGCCAACAGCGGTGTGCTTCAGGTCGGC
GAGGGCGAGCTGGAAAATACGCTTTCCGGCAGCGGTTCGCTGGTGAAAACCGGTACGGGC
GAGCTGACGTTAAGCGGCGATAACAGTTACTCCGGCGATACCACCATCGCCGACGGCACG
CTGATTGCCGCCAACGTCAACGCGCTGGGCAGCGGCAATATCGACAACAGCGGTACGCTG
ATGCTGGATGCCAACGGCGCATTTGAGCTTGCGAACATCACCACCCATACCGGCGCCACG
ACCGCGCTGGCGGCGGGCAGCACCCTTGACGCGGGCCAGCTCACCCAGGAAGACGGCAGT
ACGCTAAGTATTGACCTCGGCGCGGCCACCGACGACGCCGTGATTACCGCCGACAGCGTC
ACGCTGGGCGGCACTCTGAATGTTACCGGCATCGGCAGCGTTACAGACAGCTGGACGCCT
GAGGCATACACCTATACGCTGATTGACAGCGACAGCGCCATTACGAGCGATTTCGACGAT
CTGACCATTGCAGGGATGAACCGCGAAGATGTTGACTTCCTGACTATCGACGGAAAAGTC
GATGAAGCAGACAACACCCACTATGATCTGACGGCCTCCCTCTCCTGGTATGCGGACAGA
GACAACGCCACTACCGACGCCCACGGCACCTTTACCCTGAGCGACCCTGACGGCAGCTTT
AACGTTGCGGCTACACTGACAGACGTTGATGACACGCTGGATCCGGGTAGCAGATGGGAC
GGTAAATCGCTGACCAAAGAAGGCGCAGGCACTCTGATCCTCTCCGGCGACAACGACTAC
AGCGGCGGCACCACCATCAATGAAGGTACGCTGGTCGCCGCCAGTACGACGGCGCTGGGC
ACCGGTCTGGTAGACAATAACGCCACGCTGGTGCTGGACGTAGACGGTGAAGTCAGCGCG
GTGGGCGGCATCACCACTCATAGCGGCGCGACGACCCAACTGGCGCTCGGCACCTCGTTG
GATCTCGGCGACAGCGCGTTAATTCAGCAAGATGGCAGTACGCTGAACGTTGAGCTGAAT
AGTGACTCAGTGCAACCGCTGATCACCGGCGGCAGCGCCACGCTTGGCGGCGATTTGGTC
GTCAGCGACGCCAGCCTGCAAGCGCGCGCCTCCGACGCGGAGTTCCAGTCCTTTAAACTG
ATGGATATGGACAGCGATATCAGCGGCGACTTTACCAGCCTGACGATGAACCTCACCGAC
CAGCCGGACTATCTGACCGTAACCGGGACAATCAATCCTGCAGATGCGTCAGAATATTTG
CTTACCGAAGGGCTGAGCTGGAATGCGACCGCCACGTCAGCGACGCCAGCACACGGCACG
TTTACCCTGGGCGCAGGCGACAGTTTTGAAGTCACCAGCGTACTGGGCGATAAAACCGGC
AACGGCGACTGGGACGGTAAATCGCTGACCAAACTGGGCGCGGGTAAACTGACGCTGAGC
GGCGCGAACACCTACACCGGCGATACTAACGTACAGGAAGGCACGCTATGGCTGTCCGGA
GACGGCTCGATTGGCGAGATGGGCAGCCAGCAGGCGGTGAACGTCGCCTCCGGGGCGACC
TTCGGCGGCAGCAATGGTACGACCGTTAACGGTAAGGTGACCAACGAAGGGACGCTGGTC
TTCGGCGACAGCGAGGAGACAGGCGCTATCTTCACCCTCAATGGCGATCTGATCAACATG
GGGACGATCGCCAGCGGCAGCACCTCCAGCACGCCGGGCAATACGCTTTATGTTGATGGC
AATTACACCGGCAACGGCGGCTCGCTTTATCTGAATACCGTCCTGGGCGACGATGATTCG
GCGACCGATAAGCTGGTGATTACCGGCGATGCCTCCGGCACCACCGATCTGTACATCAAC
GGCATCGGCGATGGAGCACAGACCACTAACGGTATTGAAGTGGTGGATGTTGGCGGCGTA
TCGACCAGCGATGCGTTTGAGCTAAAAAATGAGGTTAACGCCAGTCTGTATACCTACCGT
CTGTACTGGAATGAAAGTGACAACGACTGGTATCTGGCGTCAAAAGCACAGTCTGATGAT
GACGACAGCGGTGGTGATGACAGTGATGTCACGCCTTCGGATGGCGGTGACGATGGCGGT
AATGTCACGCCTCCGGACGACGGCGGCGATGGCGGCGATGTCGCACCACAGTACCGTGCG
GATATCGGCGCGTACATGGGCAACCAGTGGATGGCGCGCAACCTGCAAATGCAGACCCTC
TATGACCGCGAGGGCAGCCAGTATCGTAATGCCGATGGCAGCGTATGGGCGCGCTTCAAA
GCGGGTAAAGCAGAATCAGAGGCTGTCAGCGGCAATATCGATATGGACAGCAACTACTCC
CAGTTCCAGTTAGGCGGCGACATTCTGGCCTGGGGCAACGGCCAGCAGAGCGTTACCGTT
GGCGTCATGGCGAGCTACATCAACGCCGATACCGACAGCACCGGTAACCGTGGCGCGGAC
GGTAGCCAGTTCACCAGTAGCGGCAACGTAGACGGCTATAACCTTGGCGTCTATGCCACC
TGGTTTGCCGATGCCCAAACGCATAGCGGCGCGTATGTCGACAGCTGGTACCAATATGGT
TTCTACAACAACAGCGTAGAGAGCGGTGATGCGGGATCTGAATCTTATGATTCAACCGCT
AACGCCGTCTCGCTGGAAACTGGTTATCGCTACGATATTGCGCTTAGCAACGGTAATACT
GTCAGTCTGATGCCGCAGGCGCAGGTTGTCTGGCAGAATTACTCAGCGGATAGCGTGAAG

GATAACTACGGCACCCGGATCGATGGTCAGGATGGCGACAGTTGGACAACGCGTCTGGGT
CTGCGTGTTGACGGCAAGCTGTACAAAGGCAGCCGTACGGTTATCCAGCCGTTTGCTGAA
GCTAACTGGCTGCACACCAGCGATGATGTGTCGGTATCGTTTGATGATGCTACGGTGAAA
CAGGATCTTCCGGCTAACCGTGCGGAGCTGAAAGTGGGTCTGCAGGCAGATATCGATAAG
CAGTGGAGCGTTCGCGCTCAGGTTGCCGGGCAGACTGGCAGCAATGACTTTGGCGATCTG
AACGGTAGCCTCAATCTGCGCTATAACTGGTAA
>EECEBICD_00833 Exodeoxyribonuclease 7 large subunit
ATGTTATCCTCTCAAACTTCCTCAATTTTTACCGTAAGCCGCCTTAACCAGACGGTTCGT
CTGCTGTTAGAACAGGAGATGGGACAGGTATGGATCAGCGGCGAGATTTCTAATTTCACG
CAGCCTGCGTCGGGCCACTGGTACTTTACGCTCAAAGACGATACCGCTCAGGTGCGCTGT
GCGATGTTCCGTAACAGCAATCGCCGGGTGACGTTCCGCCCGCAGCACGGGCAGCAGGTG
CTGGTACGCGCCAACATTACGCTGTACGAGCCGCGCGGCGATTATCAAATCATCGCCGAA
AGTATGCAGCCCGCGGGCGAAGGCTTGTTGCAGCAGAAATATGAGCAGCTAAAAGCCAAA
CTGCAGGCCGAAGGCTTGTTCGATCAGCAGCATAAACAGCCGTTGCCCTCCCCGGCCCAC
TGCGTTGGGGTGATCACTTCGAAAACCGGCGCGGCGCTGCACGATATTCTCCACGTGCTA
AAGCGTCGTGACCCATCCCTGCCCGTTATTATCTATCCAACCGCAGTCCAGGGCGACGAC
GCGCCGGGGCAGATCGTTCGTGCTATCGAACTGGCGAACGCGCGCGGCGAATGTGATGTA
CTGATCGTCGGACGCGGCGGCGGTTCGCTGGAAGATTTGTGGAGTTTTAACGACGAACGC
GTGGCGCGGGCGATTTTTGCCAGCCGCATTCCGGTGGTCAGCGCCGTCGGTCACGAAACG
GACGTCACGATAGCTGATTTTGTCGCCGACCTGCGCGCGCCTACGCCATCAGCAGCGGCA
GAAATTGTCAGCCGTAACCAGCAGGAGTTGTTGCGTCAGATCCAGTCCGCCCAGCAGCGT
CTGGGCATGGCGATGGACTACTACCTCGCCAATCGCAGCCGCCGTTTCACGCAGATTTTT
CACCGTCTGCAACAGCAACATCCGCAACTGCGTCTGGCCCGTCAGCAAACCGCGCTGGAA
CGTCTACGCCAACGGATGGGATTTGCGCTTGAGGCCAGGATTAAGCAGGCCAATCAACGC
CAACAGCGCGTAAGTCAACGCCTGAGCCAGCAAAATCCGCAGCCGCGCATTCACCGCGCG
CAGTCGCGCATCCAACAGCTGGAATATCGTCTGACGGAGAATATCCGCTCACGGTTAAGC
GAACAGCGCGAGCGTTTTGGCAACGCGGTCACGCATCTGGAAGCGGTCAGCCCGTTAGCC
ACGCTGGCGCGCGGCTATACGGTTTCGACGACAACGGACGGCAAGGTGCTGAAAAAGATC
AAACAGGTCAAAGCAGGCGATATCATGACCACGAGACTGGAGGACGGCTGGCTTGAAAGT
GAAGTCAAATCCGTCACGCCGGGCACGTAA
>EECEBICD_00834 Inosine-5'-monophosphate dehydrogenase
ATGCTACGTATCGCTAAAGAAGCTCTGACGTTTGACGACGTCCTCCTTGTTCCCGCTCAC
TCCACCGTTTTGCCGAATACTGCCGATCTCAGCACGCAGTTGACGAAAACTATTCGTCTG
AATATTCCTATGCTTTCTGCGGCGATGGACACCGTGACGGAAGCGCGCCTGGCAATTGCC
CTGGCCCAGGAAGGCGGCATTGGTTTTATCCACAAAAACATGTCTATTGAGCGCCAGGCG
GAAGAAGTTCGCCGCGTGAAGAAACACGAGTCCGGCGTAGTGACCGACCCGCAGACCGTC
CTGCCAACCACCACGTTGCATGAAGTGAAAGCCCTGACCGAGCGTAACGGTTTTGCGGGC
TATCCGGTGGTGACTGAAGATAACGAGCTGGTGGGTATCATCACCGGTCGTGACGTGCGT
TTTGTGACTGACCTGAACCAGCCGGTGAGTGTCTACATGACACCGAAAGAGCGTCTGGTG
ACCGTTCGTGAAGGCGAAGCCCGTGAAGTCGTGCTGGCAAAAATGCACGAAAAACGCGTA
GAAAAAGCGCTGGTCGTTGATGATAACTTCCATCTGCTTGGCATGATTACCGTAAAAGAT
TTCCAGAAAGCGGAACGTAAACCAAACTCCTGTAAAGATGAGCAGGGCCGTTTACGTGTC
GGCGCGGCGGTCGGCGCAGGCGCGGGCAACGAAGAGCGCGTTGACGCGCTGGTGGCGGCA
GGCGTTGACGTACTGCTGATCGACTCCTCTCACGGTCACTCTGAAGGCGTGTTGCAACGT
ATCCGTGAGACGCGTGCTAAATATCCTGACCTGCAAATCATCGGCGGCAACGTTGCGACG
GGCGCAGGCGCTCGCGCACTGGCGGAAGCCGGTTGCAGCGCGGTGAAAGTGGGTATCGGC
CCGGGTTCCATCTGTACGACTCGTATCGTGACTGGTGTGGGCGTTCCGCAGATCACCGCT
GTTTCTGACGCGGTGGAAGCGCTGGAAGGCACCGGGATTCCGGTTATCGCTGACGGCGGT
ATCCGTTTCTCCGGCGACATCGCCAAAGCCATCGCCGCAGGCGCGAGCGCGGTAATGGTG
GGTTCTATGCTGGCCGGTACCGAAGAATCCCCGGGCGAAATCGAACTCTACCAGGGCCGT
TCTTACAAATCTTATCGCGGTATGGGTTCTCTGGGCGCGATGTCCAAAGGTTCCTCCGAC
CGTTACTTCCAGAGCGACAACGCCGCCGACAAACTGGTGCCGGAAGGTATCGAAGGCCGC
GTAGCCTATAAAGGTCGCCTGAAAGAGATCATTCACCAGCAGATGGGCGGCCTGCGCTCC
TGTATGGGGCTGACCGGTTGTGCTACCATCGACGAACTGCGTACTAAAGCGGAGTTTGTG

CGTATCAGCGGTGCGGGTATCCAGGAAAGCCACGTTCACGACGTGACCATCACCAAAGAG
TCCCCGAACTACCGTCTGGGCTCCTGA
>EECEBICD_00835 GMP synthase [glutamine-hydrolyzing]
ATGACGGAAAACATTCATAAGCATCGCATCCTCATTCTGGACTTCGGTTCTCAGTACACT
CAACTGGTTGCGCGCCGCGTGCGTGAGCTGGGTGTTTACTGTGAACTGTGGGCGTGGGAT
GTGACAGAAGCACAAATTCGTGACTTCAACCCAAGCGGCATTATTCTTTCCGGCGGCCCG
GAAAGCACCACCGAAGAAAACAGCCCGCGCGCGCCGCAGTATGTCTTTGAAGCAGGCGTG
CCGGTATTTGGCGTTTGCTATGGTATGCAGACCATGGCGATGCAGCTTGGCGGTCATGTA
GAAGGTTCTAATGAGCGTGAATTTGGTTATGCGCAGGTCGAAGTGTTGACCGACAGCGCG
CTGGTTCGCGGTATTGAAGATTCCCTGACCGCAGACGGCAAACCGCTGCTGGACGTGTGG
ATGAGCCACGGCGATAAAGTGACGGCGATTCCGTCCGACTTCGTGACCGTCGCCAGCACC
GAGAGCTGCCCGTTCGCCATTATGGCCAACGAAGAAAAACGCTTCTACGGCGTACAGTTC
CACCCGGAAGTGACTCACACCCGCCAGGGTATGCGCATGCTGGAGCGTTTTGTACGCGAT
ATCTGCCAGTGTGAAGCGTTGTGGACGCCGGCGAAGATCATCGATGACGCCGTGGCGCGC
ATTCGCGAGCAGGTAGGCGACGATAAAGTGATCCTCGGTCTCTCCGGCGGCGTGGATTCT
TCCGTCACCGCCATGCTGCTGCACCGCGCGATCGGTAAAAATCTGACCTGTGTTTTCGTC
GACAACGGCCTGTTGCGCCTGAACGAAGCCGAGCAGGTGATGGACATGTTTGGCGACCAT
TTTGGTCTGAACATTGTTCACGTACCGGCAGAAGATCGCTTCCTGTCCGCGCTGGCTGGT
GAGAACGATCCGGAAGCCAAGCGTAAGATCATCGGCCGCGTTTTCGTTGAAGTGTTCGAC
GAAGAAGCGCTGAAACTGGAAGACGTGAAATGGCTGGCGCAGGGCACCATCTACCCTGAC
GTTATCGAATCTGCGGCGTCTGCAACCGGTAAAGCGCACGTCATCAAATCTCACCACAAT
GTCGGCGGCCTGCCGAAAGAGATGAAGATGGGGCTGGTTGAACCGCTGAAAGAGCTGTTC
AAAGACGAAGTGCGTAAGATTGGTCTGGAGCTGGGCCTGCCGTACGACATGCTGTATCGC
CATCCGTTCCCGGGGCCGGGCCTCGGCGTGCGCGTACTGGGCGAAGTGAAGAAAGAGTAC
TGCGACCTGCTGCGTCGCGCGGACGCTATCTTCATTGAAGAGCTGCGCAAAGCGGATCTG
TACGACAAAGTCAGTCAGGCGTTTACCGTCTTCCTGCCGGTCCGTTCCGTAGGCGTGATG
GGCGATGGTCGTAAGTACGATTGGGTTGTCTCTCTGCGTGCTGTCGAAACCATCGACTTT
ATGACCGCACACTGGGCACATCTGCCGTATGATTTCCTGGGTCGTGTTTCCAACCGCATC
ATCAATGAAGTCAACGGGATTTCCCGTGTGGTGTATGACATCAGCGGTAAACCACCAGCT
ACCATTGAGTGGGAATAA
>EECEBICD_00836 hypothetical protein
ATGACAAAAGACATTGTCCTGAATGCGCTTCTGATGGCCGTGTGGCGACGTCATCCCCAA
AAACAGGTGCTGGTTCAGTCTGATCAGGGTAGTCAGTACACCCGCTATGAATGGCTGAAA
TCGCACGGACTGGAGGGCAGTATGAGCCGTCGTGGCAACTGTCATAATGCGGTTGCAGAA
AGCTTTTTCCAGCTACTGAAGCGTGAGCGGATAAAGAAAAAGATCTACCAACGACTCAGA
AGTGTCCAGGTTATCCGTGGCGATTCAGCCCAAATAAGGGGTATTTAA
>EECEBICD_00837 hypothetical protein
ATGAGAGATATTGAGAAAGAAATTATCGATTTTATCGATCAGGAATATAACACTAAAAAG
TACTTTCTCTGTGGGCCAAAGCGAACCATCACGCTTGATATCAGTATTAGGGATGATCTA
AAACTCGTTTTTGAAGATAGTGAAGAACTTCTACAAGAGTACTTCAAGCGTTGGAATGTA
GATTCAGAAGGATTTGACATACTCAACTATCTGAATCCAGAGTACTTTGGAAGTAAAGAG
CCTGATCCGCGTAAGCCACTAACAGTTGGTATGCTCGTTGAAAGTGCTAAAACTGGACGT
TGGTTATATAGTTGA
>EECEBICD_00838 hypothetical protein
GTGACCTTTGAGTATACGTTTACGGTAAAGCACTGCATCGACACAATAGCGGGTTTCCTG
AAGTGCGTTGAGGTTGAGCCGCATCCGGTTATCGCAGAGATACTCACACAGTTTGAGAGT
ACCGTACGTCAGTGCCCGCTGGGATTCCAGATTTGCCCTGAACTTGTGCCAGCTACCGGG
AGTTTAACCACGCTGAAGGCTATCGGGTTTTGTATTCGGTCGAGGGAATCTTAG
>EECEBICD_00839 Protein YfgG
GTGAGTCAGGCCAACAGTATGCGAAAACGACATCGATTTAACACTCGCATGACCCGTATC
GTACTGCTTATCAGTTTTCTCTTCTTCTTTGGACGTTTCGTCTACTCTTCTATCGGCGCC
TGGCAGCACCATCAGGATAAAAAAGAAGCGCAACAGTCATCGCTTTCTGTCACCACGCCG
GGACAACGTTAA
>EECEBICD_00840 Cyclic di-GMP phosphodiesterase PdeF

ATGAAACTAAATAAAACTTATATAAATATCAGAGATAAATGGTGGGGACTACCGCTCATT
CTCCCTTCGATTTTACTGCCTGTTTTAAGTAGCGCAAATACTTATGCGCTTACCAGCACT
GGAAATGTGGTTCTTTTTTACCTGCCGTTAGCTTTTATGCTTAGCCTGATGCTCTTTTTC
GGCTGGGCAGCACTGCCGGGCATTGTGCTTGCTATCTTCTGGCGCAGATACCCGCAGACA
GGGCTGTATGAAACCCTTTCTGTGACGATGCACTTTATCATTACTATCGTGCTAAGCTGG
GGCGGCTATCGTGTCTTTTCCCCCCGGCGAAACAATGTTTCCCACGGCGACGCCCACCTG
CTGTTTCAACGAATATTCTGGCAGGTATTTTGTTCAGCAACATTATTTCTGGTCATCTAC
CAGTTTGCCGCTTTTGTTGGCATGTACGAAAGTAAAGCCAGCCTGATGGGGGTAATGCCC
TTTAATATCAACACGCTGATTAATTACCAGGCGTTACTGGTCGGTAACCTTGTCGGCGTG
CCATTGTGCTATTTTATTATTCGTACTCTCCGTAATCCGCTGCACCTGCGGGGGTATTAT
CAGCAATTAAAGCTACAGATTGATAGCAAGGCCACTAAAAAAGAGATCGTTATTTGGCTG
GCCGTATTGACTACACTAATGTTTATACTGTGTATGCCATTGACTGATAATAGTTCAATA
TTTAGTACCAACTATACGCTTTCGTTATTATTGCCGGTCATGCTGTGGGGTGCGATGCGC
TATGGCTATAAGTTTATTTCAATTATCTGGGCCGTCGTGCTAATTACGTCGATTCATTAT
TATCAGCGTTATATGCCCTGGTATTCAGGTTATGATACGCAATTAGCGATTACCTCTTCG
AGCTATCTGGTTTTTTCTTTTATTGTTAATTATATGTCTGTACTGGCCACGCGTCAGCGC
GTAGTGAGTGGTCGCGCACGGCGGCTGGCCTATCTTGACCCGGTTGTACATCTTCCCAAT
TTACGTGCGTTAAACCGCGCGCTCCAAAATGCGCCCTGGTCTACGATCTGTTTTTACAC
GTGCCGGGCCTGGAGCTATTGGGTAAAAATTATGGCGTCATGCTACGCATCCAGTATAAG
CAAAAGCTCTCTCACTGGATTACGCCGATGCTGGCCTCAAATGAGTGCGTCTACCAGATG
TCAGGGCACGACCTGGTGCTGCGTCTTAATACAGAGGCGCATCAGCAGCGTATTGAAGCG
CTGGATAAACATATTAAGCAGTTTCGGTTTATCTGGGATGGACTGCCGCTACAGCCGCCT
GTCGGCGTGAGCTACTGCTGCGTGCGTTCTCCGGTTAGCCACCTTTATCTTCTGCTGGGC
GAATTAAGCACCAGTTCCGATCTTTCGTTGACGACTAACGCGCCGGAGGATTTACAGCGG
CGCGGCGCAATGCACCTGCAGCGCGATTTGAAGGGCAGAATTGCGATGATGAATCGCCTG
CAACAGGCGTTGGAGCACGATCATTTTTTCCTGATGGCGCAGCCTATTTTCGGTGTTCGT
GGGGACGTTTACCATGAAATTTTACTGCGACTCAGGGATGACAACGGCGACGTTATCAAC
CCGGACAATTTTTTACCCGTGGCGCATGAGTTTGGTTTGTCTTCCGCTATCGATCTCTGG
GTGATTGAAAACACGCTGCGGTTTATGGCGGCCAGCCGGGAATATATGCCCGCGCATCGT
TTTGCCATTAATCTGTCGCCAACATCGGTATGCCGCGCTCGTTTCCCTGCAGAAGTTAAG
CAGTTGCTTACGCAATATCAGGTTGAACCCTGGCAACTGGTTTTTGAGGTAACGGAAAGC
AACTCTCTCACCAATGCTGAGCAGGCGCAGTTGACGCTGGGACAGCTACAGCAGTTTGGC
TGTCAGATAGCGATTGATGATTTCGGTACTGGCTACGCCAGCTATGCGCGGCTGAAAAAC
GTTGACGCCGATATTCTTAAAATTGATGGCAGTTTTATTCGCAATATAGTGTCAAACAGT
CTCGATTATCAGATTGTGGCTTCTATTTGTCATTTAGCGCGAATGAAAAATATGCAGGTC
GTCGCGGAGTATGTCGAGAGCGAAGAAATACGAAGCGTCGTGCTCTCGTTGGGGATCGAC
TATCTTCAGGGATATTTAATTGGCGAGCCGCAGCTATTATCTGAGATTCAGTAA
>EECEBICD_00841 Exopolyphosphatase
ATGCCAATTTACGATAAATCCCCTCGTCCGCAGGAGTTCGCTGCGGTCGATCTCGGCTCA
AACAGCTTTCATATGGTCATTGCCCGCGTGGTTGACGGCGCAATGCAGATTATCGGGCGT
TTAAAACAGCGCGTCCATCTGGCGGACGGGCTGGGCGCAGATAATAAACTCAGCGAAGAA
GCCATGGAACGGGGGCTTAGCTGTCTGTCGCTGTTTGCTGAACGCTTACAAGGTTTCTCC
CCTTCCAGCGTCTGTATCGTAGGCACCCATACGTTACGTCAGGCGCAAAATGCCGCTGAT
TTTCTCAAACGCGCGGAAAAGGTTATTCCCTACCCGATAGAGATTATTTCCGGTAACGAA
GAAGCGCGCCTGATTTTTATGGGCGTAGAACATACGCAGCCGGAAAAAGGCCGCAAGCTG
GTGATCGATATCGGCGGCGGGTCAACAGAGCTGGTCATTGGCGAAAACTTCGAACCCAGG
CTGGTTGAAAGCCGTCGTATGGGCTGCGTGAGCTTCGCGCAGCTCTACTTTCCCGGCGGC
GTTATCAATAAAGAAAACTTCCAGCGCGCCCGAATGGCGGCGGCGCAAAAACTGGAAACC
TTAACCTGGCAGTATCGTATTCAGGGTTGGAACGTAGCGATGGGCGCTTCCGGTACGATT
AAGGCCGCTCATGAAGTTCTCCTGGCGCTGGGTGAGAAAGATGGCTTCATTACGCCGGAT
CGCCTCGATAAACTGAAGTCAGAAGTGTTGAAGCACCGCTCCTTTAATGCGCTCAGCCTG
CCGGGTCTGTCTGAAGAACGAAAAGCGGTCTTTGTGCCGGGCCTGGCGATTCTGTGCGGC
GTTTTTGATGCTCTGGCTATCCGCGAGCTTCGCCTTTCCGACGGCGCGTTGCGCGAAGGC
GTGCTGTATGAAATGGAAGGCCGCTTCCGCCATCAGGATGTTCGCAGCCGTACCGCAAAA

AGTCTGGCCAATCAATACAACATTGACAGAGAACAGGCCAGACGCGTGCTGGAAACCACC
ATGCAGATGTACGAGCAGTGGCAGGCCCAGCAGCCAAAACTGGCGCATCCGCAGCTTGAA
GCGTTGCTCCGCTGGGCGGCAATGCTGCATGAGGTTGGACTGAATATTAATCACAGCGGT
TTACATCGCCATTCGGCTTATATTCTGCAACACAGCGATTTGCCCGGCTTTAATCAGGAG
CAGCAAATGATGATGGCGACGCTGGTGCGTTACCATCGTAAAGCCATAAAACTGGATGAT
ATGCCCCGCTTTACGCTGTTTAAGAAAAAACAGTATCTGCCGTTAATTCAGCTACTTCGG
CTGGGCGTATTACTGAACAACCAGCGGCAGGCGACCACTACGCCGCCGACGCTGCGACTA
ACGACCGATGACAGCCACTGGACGTTATGTTTTCCGCATGACTGGTTCAGCCAGAATGCG
CTGGTACTGCTTGATCTGGAAAAAGAACAGCAGTACTGGGAAGCTGTAACTGGCTGGCGT
CTCAATATTGAGGAAGAAAGCTCGCCGGAGATCGCCGCGTAA
>EECEBICD_00842 Polyphosphate kinase
ATGGGTCAGGAAAAGCTATATATCGAGAAAGAACTAAGCTGGTTAGCATTTAACGAACGC
GTGTTACAGGAAGCGGCGGACAAATCCAACCCGTTGATTGAGCGGATGCGGTTCCTGGGA
ATCTATTCTAATAACCTCGACGAGTTCTACAAAGTGCGTTTCGCTGAACTGAAACGACGC
ATTATTATCAGCGAAGAACAGGGTTCAAATTCTCATTCACGCCATTTGTTAGGCAAAATT
CAGTCCCGCGTGTTGAAAGCAGACCAGGAGTTCGACGGCCTGTACAACGAATTACTGCTG
GAAATGGCGCGTAATCAAATCTTTCTGATCAACGAGCGTCAGCTCTCGGTGAACCAACAA
AGCTGGTTGCGCCACTATTTTAAGCACTATTTGCGCCAGCATATTACCCCGATTCTTATT
AACCGGGAGACGGATCTTGTTCAGTTTTTAAAAGATGATTACACCTATCTGGCGGTGGAG
ATTATCCGCGGCGACACCATCAACTATGCGCTGCTGGAGATACCGTCCGACAAAGTCCCA
CGCTTCGTTAATCTGCCGCCGGAGACACCGCGCAGACGCAAGCCGATGATCCTGCTGGAT
AATATTCTGCGCTACTGCCTGGATGACATTTTTAAAGGGTTCTTCGACTATGACGCGCTT
AATGCCTATTCGATGAAAATGACCCGCGATGCCGAATATGACCTGGTGCATGAGATGGAA
TCCAGTCTGATGGAGCTGATGTCTTCCAGCCTGAAGCAGCGCCTGACCGCTGAGCCTGTG
CGCTTTGTCTATCAACGCGATATGCCCGCCGCGCTGGTCGACGTACTGCGGGAAAAACTG
ACGATCTCCCGGTATGACTCAATCGTACCCGGCGGGCGCTACCACAATTTTAAAGACTTT
ATTAATTTTCCGAATGTCGGTAAAGCCAACCTGGTCAATAAACCGTTACCGCGGTTACGC
CATCTTTGGTTTGATAAAGAGAAGTTTCGTAACGGTTTCGACGCGATTCGTGAACGTGAT
GTGCTGCTCTATTATCCTTATCACACCTTTGAACATGTGCTGGAACTGCTGCGTCAGGCG
TCGTTTGACCCAAGCGTGCTGGCGATCAAAATCAATATTTATCGCGTAGCGAAAGACTCG
CGCATTATTGACTCGATGATCCATGCCGCCCATAACGGCAAAAAAGTTACCGTGGTGGTG
GAGTTGCAGGCGCGCTTCGATGAAGAGGCGAATATTCACTGGGCCAAGCGGTTAACCGAA
GCGGGCGTCCATGTTATTTTCTCTGCGCCCGGGCTGAAAATTCACGCCAAACTCTTTCTC
ATCTCACGAAAAGAGGGTGATGACGTGGTGCGTTATGCGCATATCGGCACCGGTAACTTC
AACGAAAAAACAGCGCGGCTGTATACCGATTATTCGCTGCTGACCGCCGATGCGCGCATT
ACCAATGAAGTGCGTCGGGTCTTTAATTTTATTGAAAACCCCTATCGCCCGGTCACTTTC
GATTATTTGATGGTTTCTCCACAAAACTCACGGCGTCTGCTGTATGAGATGATCGATCGT
GAAATCGCCAATGCCCAGCAAGGCTTACCGTCAGGGATTACCTTAAAGCTTAACAACCTG
GTTGATAAAGGGTTGGTCGACAGGCTATACGCCGCGTCGGGTTCGGGCGTCCAGGTCAAT
CTGCTGGTACGCGGTATGTGTTCTCTTATTCCGCAACTGGAAGGCATTAGCGATAATATT
CGCGCGATTAGCATCGTGGATCGCTATCTGGAACACGATCGAGTTTATATTTTCGAAAAC
GGCGGTGATAAGCAAGTTTGGCTCTCTTCAGCTGACTGGATGACGCGTAATATTGACTAC
CGTATCGAAGTCGCTACGCCTATCCTCGACCCGCGGCTTAAACAGCGCGTCCTGGATATC
ATTGACATTCTGTTTAGCGACACGGTAAAAGCGCGTTTTATCGATAAAGAACTCAGCAAC
CGCTACGTACCGCGCGGCAATCGCCGTAAAGTCCAGGCGCAGCTGGCAATTTATGACTAC
ATCAAATCACTCGAGCAACCAGACTAA
>EECEBICD_00843 Phosphoribosylglycinamide formyltransferase
ATGAATATTGTGGTGCTGATTTCCGGCAATGGAAGCAATTTACAGGCGATTATCGATGCC
TGCGAAGCGAAGAAAATTAAAGGCACCCTCAGGGCAGTATTCAGCAATAAGGCCGACGCG
TTCGGCCTTGAACGCGCCAGAGAAGCCGGCATTCCGGCGCAAGCGCTGACTGCCGATCGG
TTTGACAGCCGCGACGCCTTCGATCGCGAGTTGATTCGCAAGATAGACGCGTATGCGCCC
GATGTGGTCGTGCTGGCCGGTTTTATGCGTATTCTGAGTCCGATGTTTGTCGCGCATTAC
TACGGGCGTCTGCTGAACATTCACCCTTCCCTGCTGCCAAAATATCCGGGGTTGCATACC
CATCGCCAGGCGCTGGAAAACGGCGATGAGGAGCACGGTACCTCGGTACATTTCGTGACA

GACGAACTCGACGGCGGCCCGGTCATTCTCCAGGCGAAGGTGCCGGTTTTTGCCGGCGAC
AGCGAAGATGATATCACCGCACGCGTACAGACTCAGGAACATGCGATTTATCCGCTGGTG
ATTAGCTGGTTTGCGCAGGGGCGTCTAAAGATGCGCGATAACGCCGCCTGGCTGGACGGG
CGTCGTCTGCCGCCGCAAGGTTATGCGTCTGACGAATAA
>EECEBICD_00844 Phosphoribosylformylglycinamidine cyclo-ligase
GTGACCGATAAGACCTCTCTTAGCTATAAAGATGCCGGCGTCGATATTGATGCGGGTAAC
GCTCTGGTTGATCGAATCAAAGGCGTAGTGAAGAAAACTCGCCGCCCGGAGGTTATGGGC
GGTCTGGGCGGTTTCGGTGCGCTGTGCGCGTTGCCGCAAAAATATCGTGAACCGGTACTG
GTTTCCGGCACTGACGGCGTAGGCACCAAACTTCGCCTGGCGATGGACTTAAAGCGCCAC
GACGCTATCGGTATTGATCTGGTGGCAATGTGCGTAAACGATCTGGTCGTTCAGGGCGCG
GAACCGCTGTTTTTCCTCGATTACTATGCCACGGGTAAACTGGATGTCGATACCGCCGCC
AGCGTGATCAACGGTATTGCCGAAGGCTGCCTGCAATCCGGCTGCGCGCTGGTCGGCGGC
GAGACGGCGGAAATGCCGGGCATGTATCACGGCGAAGATTACGATGTGGCGGGTTTCTGC
GTCGGCGTAGTCGAAAAATCAGAAATCATCGACGGCTCCCGGGTTGCCGAAGGCGACGTG
CTGATTGCGCTCGGCTCCAGCGGCCCGCACTCGAATGGATATTCGCTGGTGCGGAAAATT
ATTGACGTTAGCGGCTGCGACCCACAAACCACTCTGCTGGAAGGGAAGCCGCTGGCCGAT
CATCTGCTTGAACCGACCCGTATCTACGTAAAATCGGTTCTGGAACTGATTGAAAACGTC
GATGTGCACGCTATCGCCCACCTCACCGGCGGCGGCTTTTGGGAAAATATTCCGCGCGTT
CTGCCGGAGAATACCCAGGCGGTAATTAATGAGTCGTCATGGCAGTGGCCCGCCATCTTT
ACCTGGCTGCAAACCGCCGGTAATGTCAGCCGACATGAAATGTACCGTACCTTTAACTGC
GGCGTCGGCATGGTGATTGCGCTCTCCGCTCCGGAAGCGGACAAAGCGCTTGCTCTGCTA
AACGAGAAAGGTGAAAACGCATGGAAAATCGGTATCATCAAAGCCTTTGATTCCGAACAG
CGTGTGGTTATTGAATAA
>EECEBICD_00845 Uracil phosphoribosyltransferase
ATGAAGATCGTGGAAGTCAAACACCCACTCGTCAAACACAAGCTGGGTCTGATGCGTGAA
AACGACATTAGCACTAAACGCTTTCGTGAACTCGCCTCAGAAGTAGGCAGCCTGCTGACG
TATGAAGCGACAGCCGACCTGGAAACGGAAAAAGTCACCATCGAAGGCTGGAATGGCCCG
GTGGAAATCGACCAGATCAAAGGTAAAAAAATTACCGTTGTGCCGATTCTGCGCGCGGGT
CTGGGCATGATGGAAGGCGTTCTGGAAAATGTACCGAGCGCGCGTATCAGCGTAGTCGGG
ATGTACCGTAACGAAGAGACGCTTGAGCCAGTACCTTATTTCCAGAAACTGGTATCGAAC
ATTGATGAGCGCATGGCGCTGATCGTCGACCCGATGCTGGCGACCGGCGGTTCTGTCATC
GCGACCATCGACCTGCTGAAAAAAGCAGGCTGTAGCAGCATTAAGGTGCTGGTGCTGGTC
GCCGCGCCGGAAGGCATTGCGGCGCTGGAAAAAGCGCACCCGGACGTTGAACTGTACACC
GCCTCTATCGATCAGGGGCTTAACGAGCACGGATACATTATTCCGGGGCTTGGCGATGCC
GGCGATAAGATTTTTGGTACCAAATAA
>EECEBICD_00846 Uracil permease
ATGACGCGCCGTGCTATCGGGGTGAGTGAAAGACCGCCGCTTTTACAGACAATCCCGCTT
AGTTTACAGCACCTTTTCGCCATGTTTGGCGCGACCGTGCTGGTGCCAGTTCTGTTTCAT
ATCAATCCCGCGACGGTGCTGCTGTTTAACGGTATCGGAACGTTGCTGTATCTCTTTATC
TGCAAAGGTAAAATTCCTGCCTACCTCGGATCGAGCTTTGCCTTTATTTCCCCGGTACTA
CTGTTGTTGCCGCTGGGTTATGAAGTGGCGCTGGGCGGTTTTATTATGTGCGGCGTGTTG
TTCTGTCTGGTCTCTTTCATCGTTAAAAAAGCGGGCACCGGCTGGCTGGATGTGATGTTC
CCGCCTGCGGCAATGGGCGCAATCGTTGCCGTCATCGGTCTGGAGCTGGCTGGCGTCGCG
GCGGGGATGGCCGGATTGCTCCCTGCGCAAGGGCAGTCGCCGGACACGAAAACAATTATT
ATCTCCATGGTCACGCTGGCGGTGACGGTGTTCGGCTCCGTACTGTTTCGCGGTTTCCTG
GCGATCATTCCGATTTTGATCGGCGTGCTGGCGGGCTATGCGCTGTCATTCGCGCTGGGG
GTGGTCGATACCACGCCGATTGCCCAGGCGCACTGGTTTGCGCTGCCGACCTTCTATACG
CCGCGTTTTGAATGGTTCGCTATCCTGACGATTCTGCCCGCGGCGTTGGTCGTGATTGCC
GAGCATGTCGGTCATCTGGTGGTGACGGCGAATATCGTCAAAAAAGATTTAGTGCGCGAT
CCCGGTTTGCACCGCTCGATGTTCGCTAACGGATTGTCGACGATCATTTCCGGTTTCTTC
GGCTCCACGCCGAATACCACCTATGGGGAAAATATTGGCGTCATGGCGATCACCCGCGTT
TACAGTACCTGGGTTATCGGCGGCGCGGCGATTTTCGCCATTCTGCTTTCCTGCGTTGGC
AAACTGGCGGCGGCGATTCAGATTATCCCGTTACCCGTGATGGGCGGCGTCTCGCTGCTG
TTGTACGGCGTTATCGGCGCGTCGGGGATTCGCGTCTTGATCGAATCGAAAGTCGACTAC

AACAAAGCGCAAAACCTGATCCTCACCTCGGTGATTTTGATCATCGGCGTGAGCGGCGCG
AAAGTGCATATCGGCGCGGCAGAATTGAAAGGGATGGCGCTGGCGACCATCGTCGGGATT
GGCCTGAGCCTGATTTTTAAACTGATTAGCCTGTTGCGTCCGGAAGAAGTGGTACTGGAG
GCAAATGATGCGGAGCCTCCGCATCAGTAA
>EECEBICD_00847 DnaA regulatory inactivator Hda
ATGTCATCCTGGGTTGAGGTATCTCTGAACACACCGGCACAGCTCTCTTTGCCACTTTAT
CTTCCTGACGACGAAACTTTCGCAAGTTTCTGGCCGGGGGATAACGCCTCTCTACTGGCC
GCGTTACAAAACGTGTTGCGCCAGGAACATAGTGGATATATCTACCTTTGGGCGCGTGAA
GGCGCGGGCCGCAGCCATTTGCTGCACGCCGCCTGCGCTGAATTGTCGCAGCGTGGAGAT
GCGGTAGGCTACGTCCCGCTCGATAAACGTACCTGGTTCGTGCCGGAAGTGCTCGACGGT
ATGGAACACCTCTCGCTGGTGTGTATTGATAACATTGAGTGTGTCGCTGGCGATGAACTG
TGGGAGATGGCGATCTTTGATCTCTATAACCGCATTCTGGAGTCCGGTAAGACGCGGTTA
TTGATCACCGGCGATCGTCCGCCAAGACAGTTAAATCTGGGCTTGCCCGATCTCGCTTCC
CGTCTGGATTGGGGGCAAATCTATAAACTCCAGCCTCTTTCCGATGAAGACAAACTTCAG
GCGCTACAGTTGCGTGCCCGGTTACGGGGGTTTGAACTGCCGGAAGATGTTGGGCGGTTT
TTACTCAAACGTCTTGATCGCGAAATGCGCACGCTGTTTATGACGCTGGATCAGCTCGAT
CATGCGTCGATCACTGCCCAGCGAAAACTGACGATCCCGTTTGTCAAAGAGATTCTGAAA
CTGTAA
>EECEBICD_00848 putative protein YfgD
ATGACCGACACGATTAAAATCTACCATAACCCGCGCTGCTCGAAGAGCCGCGACACGCTG
AACCTACTGAAATCTAACGGCGTGGAACCGGAAGTGGTGCTTTATCTGGATACGCCCGCC
GACGCCGCCACCGTGCGGGAACTGCTGCGTATGTTGGGCATGTCCAGCGCGCGCGAACTG
ATGCGCCAGAAAGAAGATCTCTATAAAACGCTTCACCTGGCGGATAGCCAGCTGAGTGAA
GAGGCGTTAATCCAGGCGCTGGTTGAACACCCTAAACTGATGGAGCGCCCCATTGTGGTG
GCAAATGGTCAGGCGCGCATTGGCCGACCGCCTGAGCAGGTGCTGGACATCCTCGGTTAA
>EECEBICD_00849 Beta-barrel assembly-enhancing protease
ATGTTCAGGCAGTTGAAAAAAAAACCTGGTGGCAACCCTCATTGCAGCATTGGCTCTCGGT
CAGGTCGCGCCCGCATTTGCCGACCCTGCCGACACGCTGCCCGATATGGGAACCTCGGCA
GGAAGCACGCTTTCTATCGGACAAGAGATGCAAATGGGCGACTTTTATGTGCGCCAGCTA
CGCGGCAGCGCGCCGTTAATCAACGATCCGCTGCTGGTGCAATACATTAACGCGCTGGGT
ATGCGTCTGGTCTCGCACGCCGACTCCGTCAAAACGCCCTTCCATTTTTTCTTGATCAAT
AATGACGAAATCAACGCCTTCGCGTTCTTTGGCGGCAATGTGGTGCTGCACTCGGCGCTT
TTTCGCTACGCGGATAACGAAAGCCAGCTAGCTTCAGTCATGGCGCATGAAATCTCCCAC
GTGACGCAGCGCCACCTGGCGCGCGCGATGGAAGATCAAAAGCGCAGCGCGCCGCTTACC
TGGGTGGGCGCGCTGGGTTCCATTTTGCTGGCCATGGCCAGCCCACAGGCCGGTATGGCG
GCGCTAACCGGTACTCTGGCGGGAACGCGCCAGGGAATGATAAGTTTCACCCAGCAAAAT
GAGCAAGAAGCCGACCGTATTGGTATTCAGGTACTGCAACGCGCCGGATTTGACCCACAG
GCGATGCCCTCTTTCCTCGAAAAACTGCTCGACCAGGCGCGTTACTCCACGCGTCCGCCT
GAAATTTTGCTCACTCACCCCTTACCGGAAAGCCGCCTTGCGGATGCCCGCAACCGTGCC
AACCAGATGCGCCCGGTCGTGGTGCAATCTTCCGCCGACTTTTATTTCGCCAAAGCGCGC
GCCCTGGGAATGTACAATTCCGGACGTAACCAGCTCACCAGCGACCTGCTGGATCAGTGG
TCTAAAGGCAACGTGCGTCAGCAACATGCGGCGCAATATGGCCGGGCGTTGCAGGCGATG
GAAGCGAGCAAGTACGATGAAGCGCGCAAAACGTTGCAGCCGCTGTTAAGCGCAGAACCG
AACAATGCCTGGTATCTTGACCTCGCCACCGATATTGACCTCGGGCAGAAAAGAGCCAAC
GACGCGATTAATCGCCTGAAAAATGCCCGCGATCTGCGCGTTAATCCTGTGCTGCAGTTA
AACCTCGCCAATGCGTACCTCCAGGGAGGCCAGCCAAAAGCGGCGGAAACCATTCTGAAT
CGCTACACCTTTAGCCATAAAGATGACGGTAACGGCTGGGATCTGCTTGCTCAGGCCGAA
GCCGCGCTGAACAACCGCGATCAGGAGCTGGCGGCGCGCGCTGAAAGTTATGCGCTGGCG
GGACGACTGGATCAGGCAATTTCACTGCTCAGTAGCGCCAGCGCCCAGGCAAAACTGGGT
AGCCAGCAACAGGCGCGTTACGATGCGCGTATCGACCAGCTGCGCCAGTTACAGGAACGC
TTCAAGCCATACACGAAAATGTAA
>EECEBICD_00850 hypothetical protein
ATGCTCGAAATGTTAATGCAATGGTATCGCCGTCGCTTCAGCGACCCGGAGGCGATTGCC
TTGCTGGTCATTCTGGTGGCTGGTTTTAGCATCCTCTTTTTCTTTAGCGGCCTGTTAGCG

CCGCTGCTGGTCGCAATCGTACTGGCCTACCTGCTGGAGTGGCCGACGGCGCGCCTGCAG
GCGATTGGCTGTTCCCGGCGCTGGGCAGCCTCCATTGTCCTTATCCTCTTTGTCGGCATT
CTGTTATTAATGGCGTTTGTGGTGATGCCGATTGCCTGGCAGCAGGGTATTTACCTGATT
CGTGATATGCCCGGTATGCTGAATAAACTGTCCGATTTCGCCGCGACCTTGCCGCGACGC
TATCCGGCGCTGATGGATGCGGGCATTATTGATGCTATGGCGGAAAACATGCGTACCCGG
ATGCTTAACATGGGCGATTCGGTGGTGAAGTATTCGCTGGCGTCGTTAGTCGGGCTACTG
ACGCTGGCGGTATATCTGGTGCTGGTGCCGCTGATGGTCTTTTTCCTGGTAAAAGATAAA
GAGCAGATGCTGAATGCCGTGCGCCGCGTGCTGCCGCGTAATCGCGGGCTGGCGGGGCAG
GTGTGGAATGAGATGAATCAGCAGATCACCAACTATATCCGCGGAAAAGTGCTGGAGATG
GTAGTGGTGGGCGTGGCGACCTGGCTGGGCTTTTTGCTGTTTGGCCTCAACTATTCGCTA
CTGTTGGCGGTACTGGTGGGCTTTTCGGTCCTGATCCCCTATATCGGCGCCTTTGTGGTG
ACAATTCCGGTGGTGGGCGTCGCGCTGTTTCAGTTTGGCCTGGGGACGGAATTCTGGAGC
TGTTTTGCCGTTTATCTGATTATTCAGGCGCTGGATGGCAATTTGCTGGTACCGGTACTT
TTCTCCGAAGCGGTGAATTTGCATCCGCTGGTGATTATTCTGTCAGTGGTGATTTTCGGC
GGTCTGTGGGGATTCTGGGGGGTGTTTTTCGCCATTCCATTGGCGACGCTGATTAAAGCC
GTTGTCCATGCCTGGCCGGATGGTCAGGTGACGGATGCCTCATCCTGA
>EECEBICD_00851 Carbohydrate diacid regulator
GTGTTGAGAAAACATTACTTGAAAGGGTATACCGCGCGGCAAATTGTACAGCGAGCCATG
AAAATTATTCCTTACTCGGTAAACGTCATGGATGAGCATGGCGTCATTATCGCGTCTGGC
GAACCTTCGCGGCTTCGCCAGCGTCACGAAGGGGCCATTCTGGCGCTGAAGGAAAATCGT
TTTGTAGAAATTGATTCCGCTACCGCCAATCAGCTTAAAGGCGTGCGATCCGGCATTAAT
CTTCCCATCTCTTTTCATGAACAGCTTATCGGCGTGGTCGGCATTACCGGCGAGCCGGAG
GAGGTTCGCGCGTATGCCGAGCTGGTTAAAATGGCGGCGGAGCTGGTGATCGAGCATATG
GTGCTGATCGAACAGCGACAATGGGATAAACGCTATCGCGAAGAGCTGATCAACCAATTG
ATTTTGCGAGAAAATTCAACAGAGTCGTTGCGCTCCATGGCGGCCTATCTGGGTATTGAT
CTGGCGGTTCCCAGAGTGGTGCTGATTATTGAACTTTCCCAGCCGGATCGCGAAGCGCTG
CGCAATGTAATGGATTATTTCGAGAATCACGCGCGTAACCATTTGGTGACGTTTACCGAA
TTTAATGAATTAATCATTATTAAGCCTATCACGTTAAAAGAGGGAAAGTGGAATACCCGC
CAGGAAATGGGCGAATTGCAGATTTTTAAATCATGGGCTGCATCATCGGGCTTTAGCCGT
ATTTTGGTTGGGGGCTATTTTGCCGGCGAGACGGGATTGCACCGATCTTTGCTCACAGCG
AGAGCCACGCAGGCGATGGCGAAAAGACAAAAGCTGCGCAGCCAGTACATTTTTTATCAT
GACCACGCGCTTCCCGCGCTGCTAAGCGGGCTGTCTGAAAGTTGGCAAGTGCAGGAGTTA
TCGCGTCTGTGGCTGCAACTGGCGCAACATGACGCGAAAGGCGTATTGCAACAGACGCTG
CGGACTTGGTTTGAACATAATTGTGACCTGACGCAAACGGCCAAAGCATTGCATATTCAT
GTGAATACATTGCGCTATCGCTTACAGCGCTGTGAGGATATTACGCACATAAAAATCAAC
GAGTTAAAAAGTACGCTTTGGCTTTATATCGGTATGGAGCTTCAGGCCGAATCTGTACCG
TCCGACAAGTTACCGCTGCCTGGTCGAATCGAAATTTGTTGA
>EECEBICD_00852 High-affinity gluconate transporter
ATGACGTCGATATCCACCCTGGGAGCCATTGCCGCATTGGTTGTGGCCATCGTATTAATT
TTAAGAAAAGTGTCGCCAGCCTACGGCATGATGGCGGGCGCGCTGGTCGGCGGTTTGATT
GGCGGCGCCGATCTTCTGCAAACCGTGTCGCTGATGGTGTCAGGCGCGCAGGGCATCGTT
AATGCCGTATTGCGTATTCTGGCCGCAGGCGTACTGGCGGGCGTATTAATTGAGTCCGGG
GCGGCGAATACGATTGCTGAGACGATTGTCCGCAAAGTGGGGGAAACCCGGGCGTTATTG
GCATTAGCCATTGCCACACTATGTCTGACCGCCGTCGGCGTATTTATTGATGTGGCGGTG
ATTACCGTCGCGCCGATTGCGCTGTCGATTGCGCGTAATGCAGGCCTGTCAAAAAGCGCT
ATTCTGCTGGCGATGGTCGGCGGCGGCAAAGCGGGTAACGTGATGTCACCCAACCCGAAC
GCTATTGCCGCCTCCGATGCGTTTCATGTTCCTCTTACCTCTATTATGCTGGCGGGCGTG
GTTCCGGACATCGTTGGGCTTATTATCGCCTATCTGTTAGCGAAACGGCTGAATAATAAA
GGCGCGGGCGTCGCCGATCATGAAGTGACTCACCATGATGACTCCGTCGCCAGACCCGGT
TTTCTGGTGGCGATTAGCGCGCCGCTGGTCGCTATTTTCCTGCTTTCTCTCCGTCCGTTT
GCCGGCATTTCCATTGACCCGTTAATTGCCCTGCCCGTAGGGGGATTGGTCGGTCTGCTG
TTGATGGGACGCGCGCGCCACTGTAACCAGTACATGGTCGCTGGTCTGATGCGTATGGCG
CCTGTCGCTATTATGTTGCTGGGAACCGGTACGCTGGCCGGCATTATCGCTAATTCCGAA
CTTAAAGATGTCCTGATTCATGGCCTCACCGCTTCGGGATTGCCATCCTGGCTGCTGGCG

CCCGTGTCTGGCGCCATGATGTCAATGGCGACGGCCTCAACCACGGCGGGAACGGCGGTG
GCGTCTGGCGTTTTCTCTCCCACGTTACTTGAACTTGGCGTTTCCGCCCTGGCGGGAGCC
GCTATGATCCATGCGGGCGCCACGGTGCTGGACCATCTGCCGCACGGCAGCTTTTTCCAC
GCGACGGGCGGCAGCGTCAATATGCAAATTCATGAACGTTTAAAGCTAATGCCGTATGAA
ACGCTGGTGGGGTTAGCCATTACCTTTATTTCTACTCTGATGTTCGGTTTCTTTGGTTTT
GCAGGATAA
>EECEBICD_00853 Glycerate 2-kinase
ATGAAAATTGTTATTGCTCCTGATTCATTTAAAGAAAGTTTGTCCGCAATGGCGGTCGCT
GAGGCGATAGAAAAGGGGTTCCGCGAAATTTATCCTGACGCCGACTACGTTAAAGTGCCC
ATGGCGGATGGCGGGGAAGGAACCGTACAATCAATGGTGGAGGCCAGCGGCGGGCGTTAT
ATTGACCAGTGGGTACAAGGCCCGCTAGGGCAACCGGTGGCCGCGCGCTGGGGAATGCTG
GGCGATAGCGATACCGCCGTGATTGAAATGGCGGCGGCATCCGGGCTGCATCACGTTTCG
CCTGAATTGCGCAACCCGCTTAATACCACCAGCTACGGCACCGGCGAGTTAATCGTCGCG
GCGCTGGAGCGTGGCGTAAAGCGTATTATTCTGGGAATTGGCGGCAGCGCGACGAATGAC
GGCGGCGCGGGGATGATGCAGGCGCTTGGCGTTATATTGCGCGATAAACAGGGGCGCTCG
CTTTCTCCGGGCGGCGAGGCGCTGGCGGCGCTGGCCTCTATCGATCTGTCCGGTTGTCAC
CCATTGCTGCGTAAGGTATCGATAACCGTGGCGTGCGATGTCAACAATCCTCTGTGTGGT
CCCCAGGGCGCATCGGCCATTTTTGGCCCACAAAAAGGGGCGACAGCGGAAATGGTGAAC
ACCCTGGACGCGGCGCTGGAAAACTGGGGCCGACATATTTATCAGGCTACGGGGCGTGAG
GTGATTAATGCTCCCGGCGCGGGCGCGGCAGGTGGAATGGGCGCCGCGCTTTTAGGATTG
CTCAATGCGGAATTACGCGCCGGCGTAGAGATTGTCGTGGAAACGCTACAGCTTGAGCAG
GCGGTTAAAGATGCCGATCTGGTGATTACCGGAGAAGGGCGGCTGGACAGCCAAAGTATT
TGCGGCAAGACGCCGATTGGCGTCGCCAGAGTGGCGAAGCGGTATCATAAACCGGTTATC
GCGCTCGCCGGTGGGCTGCAACACGATCATCACGTTGTGTATCAACAGGGTATCGATGCA
GCGCTGTCGATTTTGTCGCATATCGTTACGCTGCCGGAAGCCTTACACGAGGCGGAATAT
AACCTGAGCCTGTCGGCGCGCAATGTGGCGGCGATATGGCGTCTGGCGCGGCAAGCCTGA
>EECEBICD_00854 Peroxiredoxin Bcp
ATGAATCCACTGAAAGCCGGTGATATCGCACCGAAATTTAGCTTGCCGGATCAAGACGGG
GAACAAGTAAATTTGACCGACTTCCAGGGACAGCGCGTTCTGGTTTATTTCTACCCAAAA
GCCATGACACCCGGCTGTACCGTGCAGGCCTGCGGCTTACGCGATAACATGGACGAGCTC
AAAAAAGCAGGCGTGGACGTGCTGGGCATCAGCACCGATAAACCTGAAAAACTCTCCCGT
TTCGCTGAAAAAGAGCTGCTGAACTTTACCCTTTTGTCCGATGAAAACCATCAGGTGTGT
GAACAGTTCGGCGTCTGGGGCGAAAAGTCGTTTATGGGCAAAACGTATGACGGCATTCAC
CGCATCAGCTTTTTGATTGACGCTGATGGTAAGATTGAACACGTCTTTAATGATTTTAAA
ACCAGCAATCACCATGATGTGGTGCTGAACTGGCTGAAAGAAAACGCGTAA
>EECEBICD_00855 Glycine cleavage system transcriptional repressor
TTGACACCGTCATCACAACACTATTTGGTTATCACTGCGCTGGGCGCCGACAGGCCCGGT
ATTGTGAATACCATTACTCGCCATGTCAGCAGTTGCGGCTGTAATATCGAAGACAGCCGA
CTGGCGATGCTGGGCGATGAATTCACGTTTATTATGCTGTTGTCCGGCACGTGGAACGCG
ATTACCTTGATTGAATCGACATTGCCGTTAAAAGGCGCGGAGCTGGATCTGCTGATTGTG
ATGAAACGGACGTCCGACCGCCCCCGCCCGGCAATGCCCGCGACGGTATGGGTTCAGGTT
GAGGTCGCCGACTCCCCGCATTTAATTGAGCGATTCACCGCGCTGTTTAATAGTCATGAA
ATGAATATTGCGGAGCTGGTCTCCCGCACGCAGCCGGCTGAAGGTGATAAAGCTGCGCAA
TTATTTATTCAAATCACCGCACATAGCCCGGCGTCGCAAAATTCCGCAAATATTGAGCAA
GCCTTTAAAGCACTATGTACAGAACTCAATGCGCAAGGCAGTATAAACGTCGTCAATTAC
TCACAACATGATGAACAGGATGGAGTTAAGTAA
>EECEBICD_00856 4-hydroxy-tetrahydrodipicolinate synthase
ATGTTCACGGGAAGTATTGTCGCGCTTGTTACGCCGATGGATGAGAAAGGTAACGTCAGT
AGGTCTTGCCTGAAAAAACTCATTGATTATCATGTCGCCAACGGTACCTCGGCGATTGTT
TCGGTTGGCACTACCGGCGAGTCTGCCACGCTAAGCCATGATGAACATGGCGATGTCGTG
ATGATGACGCTGGAACTGGCTGACGGACGTATTCCGGTTATCGCCGGCACGGGCGCAAAC
GCGACCGCGGAAGCGATTAGCCTGACGCAGCGTTTTAACGATAGCGGCATTGTTGGCTGC
CTGACGGTAACGCCGTACTACAATCGCCCCACGCAGGAAGGTTTGTTCCAGCATTTCAAA
GCCATCGCGGAACACACTGACTTGCCGCAAATTCTGTATAATGTGCCGTCCCGTACCGGT

TGCGACATGTTGCCGGAAACCGTGGGTCGTCTGGCGGAAATAAAAAATATTATCGCTATC
AAAGAGGCGACAGGGAACTTAACCCGCGTTCACCAGATCAAAGAGCTGGTTTCAGACGAT
TTTATTCTGCTTAGCGGTGATGACGCGTCTGCGCTGGACTTTATGCAACTGGGTGGTCAT
GGCGTGATTTCCGTTACGGCTAACGTAGCGGCGCGCGATATGGCTGACATGTGCAAACTG
GCGGCGGAAGGGCAATTTGCCGAGGCGCGCGCTATCAACCAGCGTCTGATGCCGTTACAC
AACAAACTATTTGTCGAACCCAATCCTATCCCGGTGAAATGGGCATGTAAGGCATTGGGT
CTTGTGGCGACCGACACGCTGCGCCTGCCAATGACGCCTATCACAGACCATGGTCGTGAC
ATCGTCAAAGCAGCGCTTCAGCATGCTGGCCTGCTGTAA
>EECEBICD_00857 Outer membrane protein assembly factor BamC
ATGGCTTACTCAGTACAAAAGTCGCGCCTGGCGAAGGTTGCGGGTGTTTCGCTTGTTTTG
CTGCTCGCTGCCTGTAGTTCCGATTCGCGCTACAAGCGCCAGGTAAGCGGCGATGAATCC
TATCTGGATGCCGCGCCGCTTGCTGAACTTCACGCCCCGGCAGGAATGATCCTGCCGATA
ATGACCGGCGACTACGTTATTCCGGTCACGAAGGGGAGCGGCGCGGTTGGTAAGGCGTTG
GATATCCGTCCGCCAGCGCAGCCGTTAGCGTTAGTGAGCGGCGCGCGTACCCAGTTCTCT
GGCGATACCGCTACGCTATTGGTGGAAAATGGTCGTAGCAGCACGCTGTGGCCGCAAGTC
GTCAGCGTGATTCAGGCGAAAAATTATCCGATTGAAAAACGTGACGATGCCAGCCAGACC
CTGACGACCGATTGGGTGAACTGGAACCGCCTGGATGAAGACGAACAGTATCGTGGACGT
TATCAAATCTCGGTGAAGCCGCAGGGCTATCAGCAGGCGGTAACGGTAAAACTGGTTAAT
CTGGAACAGGCTGGCAAGCCGGTCGCTGACGCGGCGTCGCTGCAGCGCTATAGCACGGAA
ATGATGAACGTCATTTCCGCCGGTCTGGATAAGACCGCTACGGATGCGGCAAATGCCGCG
CAGAACCGTTCCGCCGCAACGATGGATGTGCAGAGCGCCGCTGATGATACCGGTTTACCT
ATGCTGGTCGTTCGCGGTCCGTTCAACCTCGTGTGGCAACGCCTCCCGGCCGCGCTGGAA
AAAGTGGGCATGAAAGTCACCGACAGCACTCGCTCTCAGGGCAGCATGGCGGTAACGTAC
AAACCGTTGTCTGACAGCGACTGGCGGGATCTGGGCGCTAGCGACCCGGGCCTGGCATCC
GGAGACTATAAATTGCAGGTCGGCGATTTAGATAACCGCAGCAGCTTGCAGTTTATCGAT
CCTAAGGGGCATACTCTGACGCAAAGCCAGAACGACGCGCTGGTTGCCGTCTTCCAGGCA
GCGTTCAACAAGTAA
>EECEBICD_00858 Phosphoribosylaminoimidazole-succinocarboxamide synthase
ATGCAAAAGCAAGCTGAGTTGTATCGTGGTAAAGCGAAAACCGTATACAGCACGGAAAAC
CCGGACCTGTTGGTGCTCGAATTCCGCAATGATACGTCAGCAGGGGATGGCGCGCGCATT
GAACAGTTTGATCGCAAGGGCATGGTAAATAACAAATTCAACCATTTCATTATGACCAAA
CTGGCCGAGGCGGGCATCCCGACCCAGATGGAACGTTTGCTGTCTGATACCGAGTGTCTG
GTGAAGAAGCTGGAGATGGTGCCGGTTGAATGTGTGGTGCGCAACCGTGCGGCAGGGTCG
CTGGTCAAGCGTCTGGGCGTGGAAGAAGGCATGGAGCTTAACCCGCCGATCTTCGATCTG
TTCCTGAAAAACGACGCGCTGCACGACCCGATGGTGAATAGTTCCTACTGTGAAACGTTC
GGTTGGGTAAGTCAGGAAAATCTGGCGCGTATGAAAGAGCTAACCTACAAAGCCAACGAC
GTACTGAAAAAACTGTTTGATGACGCGGGTCTTATCCTGGTGGATTTCAAACTGGAGTTT
GGCCTGTATAAAGGCGAAGTGGTGCTGGGCGATGAGTTTTCGCCGGACGGCAGCCGCCTG
TGGGATAAAGAGACGCTGGACAAAATGGACAAAGACCGCTTCCGCCAGAGTCTGGGCGGT
CTGATTGAAGCCTATGAAGCCGTCGCGCATCGCCTGGGCGTGAAATTAGATTAA
>EECEBICD_00859 putative protein
ATGCGCTGGCAAGGGCGTCGTGAAAGTAACAATGTAGAGGACAGACGTAATCGGCCAGGC
GGCCCCTCGCTGGGTGGTCCTGGCTTTCGTCTTCCGCGCGGAAAAGGCGGCATCATTCTG
TTGGTGGTGGTGTTGGTCGCAGGGTACTATGGGGTGGATTTAACCGGTCTGTTGACCGGT
CAGCCGGTTTCGCAGCAGCAGTCGACGCGTTCGATAAGCCCCAATGATGATGAAGCGGCA
AAATTTACCTCGGTGATTCTGGCGACGACCGAAGATACCTGGGGGCAACTCTTTCAAAAA
ATGGGGCGCGGCTATCAGCAGCCAAAACTGGTGATGTACCGTGGTATGACGCGCACCGGC
TGTGGTGCGGGTCAGTCCGTGATGGGGCCGTTTTACTGCCCGGCGGACGGCACCGTCTAT
ATCGATCTCTCATTTTACGATGATATGAAAAATAAGCTGGGCGCGGACGGTGATTTTGCT
CAGGGTTATGTCATCGCCCACGAAGTCGGCCACCACGTACAAAAACTGTTAGGTATTGAG
CCTAAAGTACGCCAGTTACAGCAAAACGCGTCGCAAACGGAGGTGAATCGCCTTTCGGTG
CGCATGGAGCTACAGGCGGATTGCTTTGCCGGCGTGTGGGGACACAGTATGCAGCAGCAA
GGTGTACTGGAGGCGGGCGATCTGGAAGAGGCGCTCAATGCCGCACAGGCTATCGGCGAC
GATCGCTTACAGCAACAGGGGCAGGGGCGCGTCGTGCCGGATAGTTTTACCCACGGCACC

TCAGAACAGCGCTACAGCTGGTTTAAGCGTGGATTTGACAGCGGTGACCCGGCGCAATGT
AATACGTTTGGCAAAAACTTTTGA
>EECEBICD_00860 tRNA(Met) cytidine acetyltransferase TmcA
ATGTCTGATATCGATGCGCTACAGGCGTTAACGTCGCAAATGACACAGGAAGGCATTCGC
CGTTTACTGGTCATCAGCGGCGATGCGGCGTGGTGCCGGGAGCGGGCCGAGGCGATACGC
GCTGCATTGCCCGGTGACTGGCTGTGGGTCGCGCCTGACGCGCCCGCCCAGCCGTGCTGT
ACGCCACAGGCGTTACAAACGCTGCTGGGCCGTGAATTCCGTCATGCGATATTTGATGCC
TGGCAGGGCTTTGACGCCGCAGCGTTTGCCGCCCTGAGCGGACCTTGCAGGCCGGAAGC
TGGCTGCTTCTGCTGATGCCGCCGTACGAGACGTGGGAAAGCCGGCCTGATACCGATTCA
CTGCGCTGGAGCGACTGCGCGCAGCCTATCCCCACACCGCAGTTTGCACAGCATCTTAAA
CGGACTCTCTCCCGCGATCCGCAAACGCTGCTTTGGCGACAGCACCAGCCGTTTTGCTGG
CCATCTTATCCTTTCCGTGGACGCTGGCGACCCGCCACCGGCGAGCCGCAGCCGGAGCAG
GCGGCTATCTTATCGCGTTTACGTGAAATGCCGCCCGGCGTGGCGACGGTGATTGCCCCC
CGTGGGCGCGGCAAATCGGCGCTGGCCGGGCAATTCATCTCCCGGATGGCGGGCACGGCT
ATCGTGACCGCACCGGCGAAAACGGCAACGGATATCCTGGCAGCGTTTGCCGGTGAGCGG
TTCTGCTTTATGGCGCCGGATGCGTTGCTGGCGAGCGGGGCAAGAGCCGACTGGCTGGTA
GTTGACGAGGCGGCGGCAATCCCCGCGCCGCTGCTGCTGCAACTGGTTTCGCGCTTTCCC
CGCATACTACTCACCACCACCGTTCAGGGCTACGAAGGCACCGGACGCGGTTTTTTACTT
AAGTTTTGCGCCCGTTTTCCGCAGCTTCACCGCTTCACGCTTCGTCAGCCTGTCCGCTGG
GCGCCAGAATGCCCGCTGGAAAATATCGTCAGCGAGGCGCTGATATTCGACGATGAAGCG
TTTGCGCAGGCGCCACATGGCGCGATAGCAATCTCGGCGTTTTACCAACAGGCCTGGGTG
AATACGCCCGCGTTGCCGAGGGCTGTGTATCAGTTACTGTCGGGGGCGCACTATCGTACT
TCGCCGCTGGATCTGCGCCGAATGATGGATGCGCCAGGACAGCATTTCTTGCAGGCTACG
GCGAATAACCGTGTGGCGGGCGCCCTCTGGCTGGTCGAAGAGGGCGGGTTATCCGCAGAA
CTCAGTCAGGCTGTATGGTGCGGTTTCCGGCGGCCGCGTGGGAATCTGGTGGCGCAGTCG
CTGGCGGCGCACGGTAGCGACCCTTTAGCCGCGACGCTGGTTGGGCGTCGCGTAAGCCGT
ATCGCAGTGCACCCCGCTCGCCAGCGTGAAGGCATCGGTCAGCAGTTGATTGCCTGCGCG
TGCATGCAGGCGGCGCAGTGTGATTATCTTTCCGTCAGCTTCGGCTATACGCCGGAGCTG
TGGCGTTTCTGGCAACGCTGCGGCTTTGTACTGGTACGGATGGGCAACCATCGGGAGGCG
AGCAGCGGTTGCTATACGGCGATGGCGTTATTGCCGCTGAGCGACGCGGGAAAACGGCTG
GCGCAGCAGGAACACCAACGTTTGCGCCGCGATGCGGATATCCTGACGCAGTGGAACGGC
GAGGCGATACCGCTGGCGGCGTTAGATGAACAGGCGCTTAATGATGAAGACTGGCGTGAG
CTGGTCGGTTTTGCGTTCGCGCACCGTCCGCTACTGACATCGTTAGGTTGCCTGCATCGT
TTACTGCAATACAGCGCATTGCCATTACCGGCGCTGAGAGGGCGGCTGGAGGAAAAAGCC
AGCGATGCCGAACTGTGCGCCCGGCTACGCATTTCAGGCCGTAAAGCTCTGCTGGCGTTG
CAACGTGCGCAGGCGGCGCAGGCGCTGATTGCGTTGGATGCCGGACGAACGCAGCGTTTA
CGCGACGTAATGCCCGGCGGTGGGGAGCATGCCGGATAG
>EECEBICD_00861 hypothetical protein
ATGGACTGGCTGGCAAAATATTGGTGGATTCTGGTATTGGTGTTTCTGGTAGGCGTACTG
CTGAATGTGATCAAAGATCTCAAACGCATCGACCATAAGAAATTCCTTGCCAATAAACCT
GAGCTGCCCCCGCATCGCGATTTTAACGATAAGTGGGACGATGAAGAAGACTGGCCGAAG
AAAGATCAGCCGAAAAAGTAA
>EECEBICD_00862 Succinyl-diaminopimelate desuccinylase
ATGTCGTGCCCGGTTATTGAGCTGACACAGCAACTTATTCGTCGCCCCTCCCTGAGTCCG
GATGACGCAGGCTGTCAGGCATTAATGATTGAACGCCTGCGTAAAATCGGTTTTACCATT
GAGCATATGGATTTTGGCGACACGCAAAATTTTTGGGCATGGCGTGGGCGTGGCGAAACG
CTGGCGTTCGCCGGGCATACTGACGTCGTACCGGCTGGCGATGTCGATCGCTGGATCAAT
CCGCCGTTTGAACCTACGATTCGCGACGGAATGTTGTTCGGTCGCGGCGCGGCGGATATG
AAAGGTTCACTGGCGGCCATGGTGGTGGCTGCTGAACGTTTTGTTGCGCAGCATCCTCAT
CACCGGGGCCGCCTCGCATTTTTGATTACCTCTGATGAAGAAGCCAGCGCAAAAAATGGC
ACCGTAAAGGTGGTTGAAGCGCTGATGGCCCGTAACGAGCGGCTGGATTACTGTCTGGTC
GGCGAACCTTCCAGTACTGAAATTGTGGGCGATGTCGTCAAAAACGGGCGTCGCGGTTCT
CTCACCTGTAACCTCACTATCCATGGCGTTCAGGGGCACGTTGCCTATCCGCATCTGGCG
GATAACCCTGTTCATCGCGCCGCGCCTTTTCTTAATGAACTGGTCGCTATCGAGTGGGAT

CGGGGCAATGACTTTTTCCCGGCCACCAGCATGCAGGTAGCTAATATCCAGGCAGGCACC
GGCAGCAATAATGTCATTCCCGGTGAACTGTTCGTGCAGTTTAACTTCCGTTTCAGCACG
GAACTGACCGATGAGATGATCAAAGAGCGGGTACATGCGCTGCTTGAGAAGCATCAGCTA
CGCTATACCGTAGACTGGTGGCTTTCCGGCCAGCCGTTCCTGACCGCACGCGGCAAGCTG
GTGGACGCGGTGGTAAACGCCATTGAGCACTATAATGAAATTAAACCGCAATTACTGACG
ACGGGCGGTACGTCCGACGGACGTTTTATCGCCCGTATGGGAGCGCAGGTGGTAGAACTG
GGGCCGGTGAACGCCACCATTCATAAAATTAATGAATGTGTGAATGCCGCCGATCTCCAG
CTACTGGCCCGTATGTATCAACGCATTATGGAACAGCTCGTCGCCTGA
>EECEBICD_00863 Protein YffB
ATGATTACCCTCTATGGCATTAAGAATTGCGACACGATCAAAAAAGCGCGCCGTTGGCTG
GAGGAGCATGGCATTGATTATCGTTTTCACGATTACCGTGTGGATGGCATAGACCTCCCT
CTTCTTAATACCTTTATTGCCGAACTCGGCTGGCAGCCGTTACTGAATACGCGCGGCACA
ACATGGCGCAAACTGGATGAAGGCGACCGCAGCGGGATTACCGATGCCGACTCCGCCGCT
GCTCTCATGGTTGAACAGCCGGCAATCATCAAACGCCCATTGCTCTGCGCGCCCGGGAAG
CCTATGCTGCTTGGTTTCAGTGAATCCCGTTATCAGCAGTTTTTTGATGAGGTGTAG
>EECEBICD_00864 Multidrug efflux pump subunit AcrB
ATGGCGAATTTTTTTATCGATCGCCCCATTTTTGCCTGGGTGCTGGCTATCCTGTTGTGT
CTGACAGGGGCGTTAGCCATTTTTTCTTTACCTGTTGAACAATATCCCGATCTGGCACCG
CCCAACGTACGTATTACCGCGAATTATCCGGGAGCGTCGGCGCAAACGCTGGAAAATACC
GTAACCCAGGTTATTGAGCAGAATATGACGGGCCTCGATAATCTGATGTACATGTCATCA
CAAAGTAGCGGCACCGGACAGGCGACCATCACCCTGAGCTTTATTGCGGGAACCGATCCT
GATGAGGCGGTTCAGCAGGTGCAAAACCAGTTACAGTCCGCGATGCGTAAACTGCCGCAG
GCGGTACAGGATCAAGGCGTCACGGTACGCAAAACGGGCGATACCAATATTTTGACCATC
GCTTTCGTCTCTACCGACGGTTCTATGGACAAGCAGGATATCGCCGACTACGTCGCCAGT
AATATTCAGGACCCGCTCAGCCGCGTCAACGGCGTCGGCGATATTGACGCTTATGGTTCA
CAGTACTCTATGCGTATCTGGCTCGATCCGGCCAAATTGAATAGTTTTCAGATGACCACG
AAAGACGTGACCGATGCGATTGAGTCGCAGAATGCGCAAATCGCCGTCGGGCAGCTTGGC
GGTACGCCTTCGGTCGACAAGCAGGCGCTGAACGCCACCATTAATGCGCAGTCACTGCTG
CAAACGCCGCAACAATTTCGCGATATCACCCTGCGCGTTAATCAGGACGGTTCCGAGGTC
AAACTGGGCGATGTCGCCACCGTGGAGCTGGGGGCGGAAAAGTATGACTACCTCAGCCGT
TTTAACGGCAATCCGGCTTCCGGTCTTGGCGTTAAGCTGGCTTCCGGCGCGAACGAAATG
GCGACCGCGAAGCTGGTGCTGGATCGCCTCAACGAGCTGGCGCAGTACTTCCCTCACGGC
CTGGAATACAAGATCGCGTATGAAACCACCTCCTTTGTCAAAGCCTCGATTATCGATGTG
GTCAAAACGTTGCTGGAGGCTATCGCGCTGGTTTTCCTGGTGATGTACCTGTTCCTGCAA
AACTTTCGCGCCACGCTGATTCCGACGATCGCCGTGCCGGTAGTATTAATGGGCACCTTC
TCCGTGCTTTACGCGTTCGGCTACAGTATTAACACATTAACCATGTTCGCGATGGTGCTG
GCAATCGGGCTCCTGGTCGACGATGCCATCGTGGTGGTGGAAAACGTCGAACGTATCATG
AGCGAAGAAGGGCTCACGCCGCGTGAAGCGACGCGCAAATCCATGGGACAAATCCAGGGG
GCGCTGGTCGGTATCGCGATGGTGCTGTCTGCGGTATTCGTGCCGATGGCGTTCTTTGGC
GGTACCACCGGGGCAATTTATCGTCAGTTTTCTATTACCATTGTCTCGGCAATGGTGCTG
TCGGTGCTGGTCGCCATGATCCTGACACCGGCGCTGTGCGCAACGTTATTAAAACCGCTG
CACAAAGGCGAGCAGCACGGGCAACGCGGATTTTTCGGCTGGTTTAACCGTACCTTCAAT
CGTAATGCCGAACGTTATGAGAAAGGCGTAGCGAAAATTTTGCATCGCAGCCTGCGCTGG
ATTCTGATTTATGTTCTGTTGCTTGGCGGAATGGTGTTCCTGTTTTTGCGCCTCCCCACC
TCCTTTCTGCCGCAAGAAGATCGGGGCATGTTCACTACGTCTATCCAGCTACCGAGCGGC
TCTACGCAACAGCAGACCCTGAAAGTTGTTGAAAAGGTTGAAAACTATTACTTCACCCAT
GAGAAAAACAACATTATGTCGGTCTTCTCGACGGTCGGTTCCGGCCCTGGCGGGAACGGG
CAAAACGTCGCGCGCATGTTTGTTCGCTTGAAAGACTGGGACGCGCGCGATCCCACCACC
GGCTCCTCGTTCGCCATTATTGAGCGGGCGACAAAAGCATTTAACCAGATTAAAGAAGCT
CGCGTCTTCGCCAGCAGCCCGCCGGCAATTAGCGGTCTGGGCAGCTCCGCCGGTTTTGAT
ATGGAATTACAGGATCACGCCGGAGCAGGCCATGACGCGCTGATGGCCGCACGAGATCAA
CTCATTGAGCTGGCCGGGAAAAACAGTTCCTTGACCCGCGTGCGCCACAACGGCCTGGAC
GACAGCCCGCAACTGCAAATTGATATTGACCAACGAAAAGCGCAGGCGCTGGGCGTATCG
ATTGACGATATCAACGACACCCTGCAAACAGCCTGGGGATCGAGCTACGTCAACGACTTT

ATGGACCGGGGCCGCGTGAAGAAGGTCTATGTTCAGGCCGCAGCGAAATATCGTATGTTG
CCGGATGATATTAATCTTTGGTATGTCCGTAACAAAGATGGCGGCATGGTCCCCTTCTCC
GCCTTCGCCACCTCGCGCTGGGAAACCGGCTCGCCGCGTCTGGAACGCTATAACGGCTAT
TCGGCGGTAGAAATTGTCGGAGAGGCCGCGCCGGGAGTCAGTACCGGGACTGCGATGGAT
GTCATGGAGTCGTTGGTGCATCAGCTACCGGGCGGTTTTGGCCTGGAATGGACAGCCATG
TCTTACCAGGAACGGCTCTCCGGCGCGCAGGCGCCCGCGCTGTACGCTATTTCGCTATTA
GTCGTCTTCCTGTGTCTGGCGGCATTGTATGAAAGCTGGTCGGTGCCCTTCTCGGTGATG
CTGGTTGTGCCGCTCGGCGTCATTGGCGCGCTACTCGCTACCTGGATGCGCGGGCTGGAA
AACGATGTTTACTTCCAGGTGGGGCTGTTGACCGTTATCGGCCTCTCGGCGAAAAACGCG
ATTCTGATTGTGGAATTCGCCAACGAAATGAATCAGAAGGGACACGCGCTGTTAGACGCC
ACGCTGTACGCCAGCCGCCAGCGCCTGCGACCGATACTGATGACTTCGCTGGCGTTTATC
TTTGGCGTATTGCCGATGGCCACCAGCACCGGGGCAGGCTCGGGTAGCCAACATGCTGTC
GGAACCGGCGTGATGGGGGGAATGATCTCAGCAACCATTCTGGCTATCTTCTTTGTACCC
CTGTTTTTCGTGCTGATACGCCGCCGCTTCCCGCTGAAGCCGCGCCCGGAATAA
>EECEBICD_00865 Nitrate/nitrite sensor protein NarQ
GTGACGGTTAAACGCCCGGTCTCAGCCAGTCTGGCTAAGGCTTTTTTCTATATTGTGCTG
TTGTCTATTCTGTCCACCGGCAGCGCATTACTGACGCTGACCAGCAGTTTACGGGATGCC
GAAGCGATCAATATCGCCGGTTCGCTACGAATGCAGAGCTATCGGCTGGGTTACGATCTG
CAAAGCCGTAGCCCGCAGATTAATGCGCATCGTCAACTTTTCCAGCACGCGCTCAACTCG
CCGGTACTGCAAAATCTCAACGCCTGGTATGTGCCGCAGGCGGTGAAAACTCGCTACGCG
CGCCTGCACGCCAACTGGCTGGAGATGAATAGCCGCCTACAGGACGGCGACATTGCCTGG
TATCAGACCAATATCAACAATTACGTCGATCAAATTGATCTCTTCGTTCTGGCATTACAG
CATTACGCCGAGCGTAAAGTCATGCTGGTCGTCGCCATATCGCTGGCGGGCGGTATCGGC
ATATTTACGCTGGTCTTTTTTACCCTACGACGTATTCGCCAGCAGGTCGTTCGCCCGCTG
AATCAACTGGTCACCGCCAGTCAGCGAATTGAACATGGTCAGTTCGCCCCGCTGCCGCTG
GATACCAGCTTACCCAATGAGCTGGGATTGTTGGCGAAAACGTTTAGTCAGATGTCGAGC
GAGCTGCATAAACTTTATCGTTCGCTGGAAGCCTCGGTGGAAGAAAAAACGCACGATCTG
CACGAAGCGCACCGCCGCCTGGAGGTGTTATACCAGTGCTCCCAGGCGCTGAATACCAGC
CAAATCGACGTCCACTGTTTTCGCCATATTTTGCAAATTGTCCGTGAACATGACGCCGCC
TGGTATCTGGAGTTAACCGTGGGCGATAACTGGCGGATTAGCGAAGGCATGCAAAGCCCC
GACCTGCCGATGCAAATGCTGCCAGTGACGATGCAGGACACGGTCTATGGCGAACTCCAC
TGGCAAAGCCCCAACGTTAATGCCTCTACGCCGCTGCTGAACAGCGTCTCCACGATGCTG
GGGCGTGGTTTGTACTTTAATCAGGCGCAAAAACACTTCCAGCAACTTTTGTTAATGGAA
GAGCGCGCGACGATTGCCCGTGAGCTACACGATTCGCTGGCGCAGGTACTTTCTTATTTA
CGTATTCAGTTAACGCTTTTGAAGCGCGCTATTCCTGAGGATAACGCCGGCGCGCAAAGC
ATCATGGCGGATTTTTCCAGAGCGCTGAACGACGCCTACCGTCAGCTTCGCGAGCTACTC
ACCACGTTCCGTCTGACGTTACAGCAGGCCGATTTACCCTCCGCATTGCATGAGATGCTG
GAAGATTTACAGAGTCAGACTCCGGCCAAACTCACGTTGGATTGCCGTTTACCCACGCTG
GCGCTGGATGCGCAAATGCAGGTGCATTTATTGCAAATTGTGCGTGAAGCGGTCCTGAAC
GCCATTAAACACGCCAACGCGAGCGAAATCGCCGTGAGCTGCGTCACCGCGCCTGACGGC
GATCATACCGTTTATATTCGCGATAATGGCATTGGTATCGGCGAACCGCATGAGCCTGCC
GGACACTATGGTCTGAATATAATGCGTGAGCGCGCGGAGCGCCTTGGCGGCACGCTGAAT
TTTTCGCAACCCTCCGGCGGCGGCACACTGGTCAGTATCAGTTTTCGCTCCTCCAACAAG
GAAGAGAGTCAGTTAACGTAA
>EECEBICD_00866 Ferredoxin--NADP reductase
ATGAATCGTTTTATTATGGCCAATAGCCAGCAGTGTCTGGGCTGCCATGCCTGCGAGGTC
GCCTGCGTAATGGCTCACAATGATGAGCGACATGTCCTGACGCCACAGCGCTATCAGCCC
CGAATTACTGTCATCAAACATCAGCGCCAGCGCAGCGCGGTTACATGTCACCATTGCGAA
GATGCGCCCTGCGCCCGTAGCTGTCCGAACGGCGCAATCGCGCATATTAACGACAGCGTG
CAGGTCAATGCGCAGAAGTGTATCGGCTGTAAATCCTGCGTCGTCGCCTGCCCATTCGGC
ACCATGCAGATGGTATTGACGCCCGTCGCGCCAAACCAGTTCAAAGCCAGCGCGCATAAA
TGCGATCTGTGTCAGGGACGTGAACAGGGTCCGGCCTGTGTGGAAAATTGCCCGGCGGAT
GCGCTGCAACTGGTCACCGAAGACTCGTTGACCCGTCTGGCTAAAACGCGACGGCTGCGT
ACCGCCCCGGCAGGAGATTCGCCCCTGGCATACCGTGGATACACAACACAGTGGGACAGCA

AGCAGCAAAGTCGAGCGTATGCAGGCGACGCCGCCGCGCGGCGAACCGGATAAACTGGCG
ATTGAAGCGCGTAAAACCACCTTTGAGGAAATTTATCTGCCGTTTCGCGCCGCGCAGGCG
GAGCGTGAAGCGGCCCGCTGCCTCACATGCGGGGAACACAGTATCTGCGAATGGACCTGT
CCGCTGCATAACCATATTCCCCAGTGGATCGAACTGGTGAAAGCCGGCGATATTGATGCG
GCGGTAGAGCTTTCCCACCAGACCAACTGTCTGCCGGAAATTACCGGGCGCGTCTGTCCG
CAGGACCGGCTATGCGAAGGCGCGTGTACACTCCGCGATGAGTATGGCGCGGTTACCATC
GGCAACATTGAGCGCTATATCTCCGATCGCGCTCTGAGCAAAGGCTGGCGTCCCGACCTG
TCGGACGTACAGAAGAGCGATAAGCGCGTGGCGATTATCGGCGCGGGGCCGGCAGGTCTT
GCCTGCGCTGACGTGCTGGCGCGCCATGGCGTAAGCGCCACCGTCTACGATCGCCATCCG
GAAATCGGCGGTTTACTCACCTTTGGTATTCCGGCTTTCAAACTGGATAAATCTCTGCTG
GCGCGCCGTCGGGAAATCTTCAGCGCGATGGGGATTCGTTTCGAGCTTAACTGCGAAGTA
GGGAAAGATATTTCTCTGGAGACGCTGCTGGAGAGTTATGACGCCGTCTTTGTTGGGGTT
GGAACCTATCGTTCCATGAAGGCTGATTTACCGAATGAAGATGCGCCGGGGGTATATGAT
GCGTTGCCATTCCTCATCGCCAATACCAAACAGGTGATGGGGCTTCCGGCGTTGCCTGAC
GAGCCGTTTATTGATACTGCGGGCCTTAACGTGGTTGTGCTGGGCGGCGGTGACACCGCA
ATGGACTGCGTACGTACGGCGCTGCGTCATGGCGCTGCTAACGTCACCTGCGCTTATCGC
CGCGATGAGGCCAATATGCCGGGGTCGAAAAAAGAGGTGAAAAACGCGCGGGAAGAGGGC
GCGAACTTTGAATTTAACGTTCAGCCGGTTGAGTTGGTGCTGGATACGCACGGGCGCGCC
AGCGGTATTCGCTTTTTGCGTACGCGGCTTGGCGAACCGGATGGTCAGGGGCGACGCCGC
CCGGTACCTGTACCGGACAGCGAGTTCGTGATGCCGGCGGATGCGGTCATTATGGCGTTT
GGTTTTCATCCGCATGGTATGTCGTGGCTCGAATCGCATGGCGTGAAGGTGGATAACTGG
GGACGTATTGCCGCCAGCGTCGAGAGCGAGTTTCGTTACCAGACGTCCAACCCGAAAATT
TTCGCCGGCGGCGATGCGGTGCGCGGCGCCGATCTGGTGGTGACGGCGATGGCAGAAGGC
CGACATGCTGCGCAGGGGATACTCGACTGGTTAGCTAAATAA
>EECEBICD_00867 Putative D-alanyl-D-alanine carboxypeptidase
ATGAAATTTACTCTCGTTGCGACGGTTCTACTGACGTTTTCCCTTTCAGCTTTTGCCGTT
GAGTATCCGGTATTGACCATCGCGAGCCCTGATCAGGTGGGGTTTGATAGCCAAAAACTT
CACCGACTTGATGGCTGGATTCAAAATCAAATTGATGCCGGATATCCCAGCATCAATCTT
CTGGTGATAAAAGATAATCATATTGTTCTACAAAAAGCCTGGGGCTATGCCAAAAAATAT
GACGGTTCCACGCTGTTAGCCCATCCCATTCGGGCGACCACTAACACGATGTATGATCTG
GCTTCAAACACAAAAATGTATGCCACTAATTTCGCATTGCAAAAACTAGTGTATGAAGGA
AAAATAGATGTCAATGATTTGGTATCAAAATATATCCCCGGCTTTAAGGATATGCCTGGG
GATAAAATAAAAGGCAAAGATAAACTCCGCATTATTGATATTCTTCATCACGTCGCGGGC
TTTCCTGCCGACCCACAATATCCCAATAAAAACGTCGCCGGTAAATTATTTTCACAGAGT
AAAAGCACTACTCTGGAGATGATTAAAAAGACACCGCTGGAATATCAACCTGGGTCGAAG
CATATCTACAGCGACGTGGATTATATGATTCTTGGTTTTATCATTGAATCGATAACGGCA
ATGCCATTAGATCGCTACGTCGAAACCACCATTTATAAACCGCTCGGATTAAAACATACC
GTCTTTAACCCGCTGATGAAAGGCTTCACGCCGCCGCAAATCGCCGCAACGGAACTACAC
GGCAATACGCGTGACGGCGTGATTCATTTCCCCAATATTCGCACCAATACACTATGGGGC
CAGGTGCATGACGAAAAAGCCTGGTACTCAATGGGCGGCGTTTCAGGACACGCCGGACTG
TTTTCTGACACCCATGATATGGCTGTGTTAATGCAAGTCATGCTCAATGGGGGCGGCTAC
GGTAACCTGAAGCTATTCGACGACAAAACGGTTGCGCAGTTTACTCGCCACTCGCCAGAA
GATGCTACCTTTGGTCTGGGGTGGCGCGTTAACGGCAACGCCTCAATGACCCCAACGTTC
GGGGTATTAGCCAGTCCGCAAACGTATGGACATACCGGATGGACCGGAACGCTGACGTCT
ATCGATCCGGTTAATCATATGGCGATTGTTATTTTAGGTAACAGGCCGCACTCGCCGGTA
GCAAATCCTAAAGTGAACCCGAATGTTTTTGTCAGTGGATTATTACCTGCGGCGACGTAT
GGCTGGATTGTTGACCAGATATATGGTTCCTTAAAATAA
>EECEBICD_00868 GDP-mannose pyrophosphatase NudK
ATGTCGCAAACCATTACGCTTATTAAGGATAAAATTCTTTCTGATAATTATTTTACTCTG
CGCAACATTACCTATGACCTGACCCGTCGTAATGGCGAGGTTATCCGTCACAAGCGTGAG
GTTTACGATCGCGGGAATGGCGCAACCATTCTGCTTTACAACTCAACTAAAAAAACCGTG
GTTCTGGTTCGCCAGTTTCGCGTGGCGACATGGGTCAATGGTAATCAAGACGGTATGTTA
ATTGAAACCTGCGCTGGCCTGCTTGATAACGACGAGCCGGAGGTCTGTATCCGTAAAGAG
GCCATTGAAGAGACAGGGTATGACGTTGGTGAGGTACGCAAGATATTTGAGCTGTATATG

TCGCCAGGCGGCGTCACGGAGCTGATTCATTTCTTCATTGCCGAATATCATGATAGCGAG
CGTGCCAGCATCGGCGGCGGCGTGGAGGACGAAGAGATTGAGGTGCTTGAACTGCCGTTT
TCCCGGGCGCTGGAAATGGTGCGCTCAGGCGAGATTCGGGACGGTAAGACCGTGTTATTG
CTTAACTATTTACAGACGTCTCATCTAATGGACTGA
>EECEBICD_00869 hypothetical protein
ATGACATTAAGCCGCAGCGCCGGGGCGCGTACCGATGCGGTACTGCGTATCGATCGTGGC
GGTTTGGCGCCGCCCGACGCTAAAGAAGCCGCAATTGCGCCTCGCTTATTGCTGGATGGC
AAACCGCTTTCGTTCAACAGTCCACACTGGCGCGTGTCGCCCTGGCATCTCATGACCGGC
GATCCTGCCACCATCACCGCATTTTTACAGACGATTCAGGATGCGCAGGCCATCACCTTA
AAAAACGGCGTTCAGACGCTTTCGCTGGCGGGATTAAAAGCGGCATTACTGTTTATCGAC
GCGCAACAAAAACGTGTGGGCAGCGAAACCGCCTGGATTGAAAAAGGAAACGAACCGCCT
CTCAGCGTGCCCCCGGCACCGGCATTAAAGGGGATCGCCGTGATTAATCCAACGCCCGTA
CCGCTGAGTGAGGAAGAACGCGACGATCTGCTGGACTACGCAGCATGGCGGGTAAACGGC
ATTCGTTGTTCGCTTGACCCGCTGCGTCGCGAGACGCAGGTCAGCGCGTTGACGGATGAT
AAAGCGTTATTGATTGTTAACTGCGAAGCCGGCGCCTATAACACTATTGATCTCGCCTGG
GTAGTTTCACGCAAAAAAACGCTCGTCTCGCGCGCCGTACGTTTGCGGCTGCCGTTTAAT
CGCGGCGTCGAAAGTAATGACATGGAGTTGATGAACGCCTTCTTTGACGAGAAAACGCGT
GAACTGGTGACGTTGGCGAAAGGGCGGGGGTTAACCGACTGCGGTATCCAGACGCGCTGG
CGTTATGATGGCGATCGTTTTCGTCTGGTGCGTTATGCCGAAGAACCGAGCTGCGATAGC
TGGCACGGGCCGGATGCCTGGCCTACGCTGTGGATCACGCGTTGA
>EECEBICD_00870 hypothetical protein
ATGAAAATAAATAATGGCCCCGTATTATGCCCCCATTGTGGCTGCCTGAGCGCTTATTAT
GAAATAGATCGGCTGGCGGCGATAAGAGAAAAAGTGAATAAAGAAGGTGGATCAGCGGCG
TGGGATTCAACATTGCAGGCGCATAAAAAAAAGGCCTTTTGCCTGATGTGCCATAAATCC
ATTGATGAGGTCGTCATCGGGCAGTCAGATGCGCCTGAGTCTACGAAGTAA
>EECEBICD_00871 Transketolase 2
ATGTCCCGTAAAGACCTTGCCAATGCGATTCGTGCGCTGAGCATGGATGCCGTTCAAAAA
GCCAATTCCGGGCACCCCGGCGCGCCGATGGGGATGGCGGATATCGCTGAAGTGCTATGG
AACGATTTTCTTAAACATAACCCCACCGATCCAACCTGGTATGACCGTGACCGTTTTATC
CTTTCCAACGGTCACGCCTCCATGCTGCTCTATAGTCTGCTGCATTTAACCGGTTATGAC
CTGCCGCTGGAGGAGTTAAAAAACTTCCGTCAACTGCACTCTAAAACGCCAGGTCACCCG
GAGATTGGCTATACGCCCGGCGTTGAAACCACCACCGGACCGCTGGGCCAGGGGCTGGCG
AATGCCGTCGGGCTGGCGATTGCCGAGCGTACGTTAGGCGCACAGTTCAACCGGCCGGAC
CATGAGATTGTCGACCACTACACCTATGTCTTTATGGGCGACGGCTGTCTGATGGAGGGG
ATCTCGCATGAGGTGTGTTCGCTGGCTGGTACGCTGGGACTGGGCAAACTGATCGGCTTT
TACGATCACAACGGCATCTCCATTGATGGCGAAACCGAAGGCTGGTTTACCGACGATACC
GCCAAACGTTTTGAAGCCTACCACTGGCATGTCGTATATGACATTGACGGGCACGATCCG
GAGGCGGTGAAAAAAGCCATTCTCGAAGCCCAGAGCGTAAAAGATAAACCTTCGCTAATT
ATCTGTCGGACGGTGATAGGTTTTGGCTCGCCGAATAAAGCCGGGAAGGAGGAGTCGCAC
GGCGCGGCGCTGGGCGAAGAAGAGGTGGCGCTGACTCGCCAGAAGCTGGGATGGCATCAT
CCGGCGTTTGAGATCCCAAAAGAAATTTACCGCGCGTGGGATGGCCGTGAAAAAGGCGAG
AAAGCGCAGCAGCAGTGGCAGGAAAAGTTTGCTGCTTATGAGAAGGCTTATCCTGAGCTG
GCCGCGGAGTTTACCCGCCGGATGAGCGGCGGACTGCCGGAGGCATGGGAATCCGCCACG
CAAAAATTTATTAACGACTTGCAGGCTAATCCGGCCAAAATCGCCACCCGTAAAGCATCG
CAAAATACGCTCAATGCCTACGGTCCCCTGTTGCCGGAACTGTTGGGCGGATCGGCGGAT
CTGGCGCCGAGCAACCTGACTATCTGGAAAGGTTCGACCTCGTTGAAAGAGGATCCGGCA
GGTAATTATATTCACTATGGCGTGCGCGAATTCGGCATGACCGCCATTGCCAACGGCATC
GCCCATCACGGCGGCTTTGTACCGTATACCGCCACGTTTCTGATGTTTGTCGAATATGCG
CGTAACGCGGCGCGTATGGCGGCCTTAATGAAAGCCCGGCAGATTATGGTCTATACCCAC
GACTCCATCGGTTTAGGCGAAGATGGCCCGACGCACCAGGCCGTGGAACAACTGGCGAGC
CTGCGCTTAACGCCAAACTTCAGCACCTGGCGTCCCTGCGATCAGGTTGAGGCGGCGGTC
GGCTGGAAGCTGGCCATTGAACGTCAGCACGGGCCGACGGCGTTGATTCTCTCGCGGCAA
AATCTGGCGCAGGTGGAACGCACGCCGGAACAGGTGAAAGCGATTGCCCGCGGCGGTTAT
ATTCTGAAAGATAGCGGCGGCAAGCCGGATATCATTCTGATCGCTACCGGATCGGAAATG

GAAATTACCCTCCAGGCGGCGGAAAAAATTGACTGGCGAGGGGCATAACGTTCGCGTGGTG
TCGTTGCCTTCAACGGATATTTTCGATGCGCAGGACGAGGCATATCGCGAATCTGTGCTA
CCGGCGCACGTGACCGCCCGCGTCGCGGTGGAAGCCGGTATAGCCGACTACTGGTATAAA
TATGTTGGCCTAAAAGGCGCAATTATCGGCATGACGGGATACGGTGAATCCGCCCCGGCT
GACAAACTGTTCCCGTATTTTGGCTTTACCGTGGAAAATATTGTAGAAAAAGCCCGCCGG
GTGCTGAACATCAAAGGCTAA
>EECEBICD_00872 Transaldolase
ATGAACCAGCTAGACGGCATCAAACAATTCACTACCGTCGTCGCCGATAGCGGCGACATC
GAGTCCATCCGCCACTACCAGCCGCAGGATGCCACGACAAATCCCTCTTTACTGTTAAAA
GCCGCCGGGCTTGAGCAGTATGGTCATTTGATTGAGGATGCGATTGCCTGGGGGAAAAAA
CACGGCGGTACGCAGGAACAGCAGGTTGCGGCAGCCAGCGATAAACTGGCGGTTAATTTT
GGCGCGGAAATACTGAAAAGTATTCCCGGTCGCGTCTCCACTGAAGTCGACGCGCGGTTA
TCGTTCGATAAAGAAAAAAGTATCGAGAAAGCGCGCCATCTGGTGGATCTCTACCAGCAG
CAGGGTGTTGATAAATCACGCATTCTGATCAAGCTCGCCGCGACCTGGGAAGGTATCCGC
GCCGCCGGGCAGCTCGAAAAAGAGGGTATTAATTGCAACCTGACGTTGTTGTTCTCTTTT
GCCCAGGCGCGCGCCTGCGCCGAAGCGGGCGTCTATTTGATCTCTCCGTTCGTGGGCCGA
ATTTACGACTGGTATCAGGCGCGTAGTCCGCTGGAGCCGTATGTCGTGGAGGAAGATCCC
GGCGTGAAATCGGTACGCAATATCTACGATTACTTTAAACAGCATCGCTATGAAACCATC
GTGATGGGGGCAAGTTTCCGCCGTACTGAGCAAATCCTCGCGCTAACCGGCTGCGATCGA
CTGACTATCTCCCCTAACCTGCTTAAAGAGCTGAAAGAAAAAGAAGAACCGGTGATCCGT
AAACTGGTGCCTTCCTCGCAGATGTTCCACCGTCCTACGCCAATGACGGAAGCGGAGTTC
CGCTGGGAACATAATCAGGACGCGATGGCCGTAGAGAAGCTGTCGGAAGGTATCCGTCTT
TTTGCCATTGACCAGCGCAAACTGGAAGACCTGCTTGCCGCCAAACTATAA
>EECEBICD_00873 NADP-dependent malic enzyme
ATGGATGAGCAGTTAAAACAAAGCGCCCTTGATTTCCACGAATTTCCGGTACCCGGTAAA
ATTCAGGTTTCTCCGACTAAGCCCCTTGCCACCCAACGCGATCTGGCGTTGGCTTACTCG
CCAGGCGTGGCGGCGCCCTGCCTGGAAATCGAAAAAGATCCGCTGGCAGCCTACAAATAC
ACCGCGCGCGGCAACCTTGTCGCGGTCATTTCCAACGGTACAGCGGTGCTGGGGTTAGGC
AACATTGGCGCGCTGGCCGGCAAGCCGGTCATGGAAGGCAAAGGCGTTCTGTTTAAAAAA
TTCGCCGGCATTGATGTGTTTGACATTGAAGTCGATGAGCTTGACCCGGACAAATTTATC
AATGTGGTCGCAGCGCTGGAGCCGACTTTTGGCGGCATTAACCTCGAAGACATTAAAGCG
CCGGAATGTTTCTATATTGAACAGAAACTGCGCGAGCGCATGAACATCCCGGTTTTCCAT
GACGATCAGCACGGAACGGCGATCATTAGTACGGCCGCCATTCTTAATGGTCTGCGAGTC
GTAGAGAAAAATATTTCCGACGTACGGATGGTCGTCTCCGGCGCAGGGGCGGCGGCAATC
GCTTGTATGAACCTGCTGGTCGCGTTGGGGATGCAAAAGCACAACATCGTGGTGTGCGAC
TCTAAGGGCGTTATTTACAAAGGCCGTGAGCCGAATATGGCGGAGACCAAAGCGGCCTAT
GCGGTAGACGACAGCGGCAAGCGCACGCTGGATGAGGTCATTGACGGCGCGGATATTTTC
CTCGGCTGTTCAGGCCCGAAAGTGCTGACTCAGGAGATGGTGAAGAAAATGGCCCGCGCG
CCAATGATTCTGGCGCTGGCTAACCCGGAACCGGAAATTCTGCCGCCGCTGGCGAAAGAA
GTGCGTCCGGACGCCATCATCTGTACCGGTCGTTCCGACTATCCGAACCAGGTGAACAAC
GTATTGTGCTTCCCGTTTATCTTCCGCGGCGCACTGGACGTCGGCGCGACGGCGATCAAC
GAAGAGATGAAACTGGCGGCGGTCCGTGCGATTGCCGAGCTGGCGCACGCCGAACAGAGC
GAAGTGGTGGCCTCGGCCTATGGCGATCAGGACCTGAGCTTTGGGCCGGAATACATTATT
CCGAAACCGTTTGACCCGCGTCTGATTGTCAAAATCGCTCCGGCGGTCGCGAAAGCGGCA
ATGGATTCCGGCGTGGCGACGCGCCCGATTGCTGACTTTGATGCCTATATCGATAAACTG
ACCGAGTTTGTCTACAAAACCAACCTGTTCATGAAGCCGATCTTCTCGCAGGCACGCAAA
GATCCGAAGCGCGTAGTGCTGCCGGAAGGGGAAGAGGCGCGCGTGCTGCACGCAACCCAG
GAGTTGATAACCCTGGGACTGGCGAAGCCCATCCTGATCGGACGTCCGAGCGTGATCGAA
ATGCGTATCCAGAAACTGGGGCTACAGATCAAAGCGGGCGTTGATTTCGAGATCGTGAAT
AATGAATCCGATCCGCGCTTCAAAGAGTACTGGAGCGAGTATTACCAGATCATGAAGCGT
CGCGGGGTAACTCAGGAACAGGCGCAGCGTGCGATGATCGGCAACCATACGGCCATTGGC
GCGATCATGGTGCAGCGTGGGGAAGCGGATGCGATGATCTGCGGCACCATCGGCGACTAC
CATGAGCATTTCAGCGTCGTGAAAGCCGTCTTTGGCTACCGTGACGGCGTGCATACGGCA
GGGGCGATGAACGCCTTACTGCTGCCAAGCGGCAACACTTTTATCGCCGATACTTATGTT

AACGAAGATCCCACGCCGGAACAGTTGGCGGAGATCGCCGTAATGGCGGCGGAAACCGTT
CGCCGCTTTGGTATTGAGCCGAAAGTCGCGCTGTTGTCGCACTCTAACTTTGGTTCCTCA
AATTCGCTGTCCGCCAGCAAAATGCGCGAAACCCTTGAGCGGGTACGCGAACGAGCGCCC
GATCTGATGATTGACGGTGAGATGCACGGTGATGCGGCGTTGGTGGAAAGTATTCGTAAT
GACCGGATGCCGGATAGCCCGCTGAAGGGAGCCGCCAATATTCTGGTGATGCCGAATATG
GAAGCCGCCCGCATTAGTTACAACTTACTACGCGTCTCCAGTTCCGAAGGCGTCACGGTC
GGCCCGGTCCTGATGGGCGTGTCTAAACCGGTACATGTATTAACGCCGATCGCCTCTGTA
CGCCGTATCGTGAATATGGTGGCGTTAGCGGTAGTTGAAGCGCAAACCACGCCGCTTTAA
>EECEBICD_00874 Ethanolamine utilization protein EutS
ATGAATAAAGAACGCATTATTCAGGAATTTGTGCCGGGCAAACAGGTCACTCTGGCGCAT
CTCATTGCGCACCCTGGTGAAGAACTGGCGAAAAAGATCGGCGTTCCCGATGCTGGCGCG
ATTGGCATTATGACCTTAACGCCGGGGGAAACGGCGATGATCGCTGGCGATTTAGCGATG
AAGGCTGCGGATGTACACATCGGTTTTCTCGACAGATTTAGCGGCGCGCTGGTGATTTAT
GGCACCGTCGGCGCAGTAGAAGAAGCGCTATTACAGACGGTCAGCGGGCTGGGGCGATTA
TTAAACTTTACCCTGTGTGAGTTAACAAAAAGTTAA
>EECEBICD_00875 Propanediol utilization protein PduV
ATGAAACGTATTGCTTTTGTCGGCGCGGTCGGTGCCGGAAAAACAACGCTTTTTAACGCG
TTACGGGGAAATTATTCCCTCGCCAGAAAAACGCAGGCCGTGGAATTTAATGATCATGGC
GATATCGATACGCCGGGTGAATATTTTAGCCATCCCCGTTGGTATCACGCCTTAATTACC
ACGTTGCAGGATGTGGACACGCTGATTTATGTCCACGCGGCAAACGATAAAGAAAGTCGC
TTACCTGCCGGGTTATTGGATGTTGGAACCCGTAAACGACATATCGCCGTTATCAGCAAA
ACGGACATGCCGGATGCCGACGTCGCCGCTACGCGGCAGTTGCTTTGCGAGATTGGATTC
CGGGAGCCGATTTTCGAGCTCAACGGCCACGACCCGCAAAGCGTGCGGCAGTTGGTGGAT
TATCTGGCGGCTCTCAGTGAACAGGAGGAAGAGGCAGGTGAAAAAACTTATCACAGCTAA
>EECEBICD_00876 Ethanolamine utilization protein EutQ
GTGAAAAAACTTATCACAGCTAACGATATTCGTGCGGCCCACGCCCGCGGCGAACAGGCG
ATGTCGGTAGTGCTGCGCGCCAGCATTATTACCCCGGAGGCTCGCGAAGTGGCGGAACTA
CTGGGCTTCACGATTACCGAATGCGACGAGTCCGTTCCGGCATCCACATCGGCGCAGGCG
TGTAAAAACGAAAGCCAGCGCATTCGGGAAGCCATCATCGCGCAGCTCCCGGAAGGGCAG
TTTACTGAAAGCCTGGTGGCGCAGTTGATGGAAAAAGTGCTGAAGGAAAAGCAGTCGTTG
GAACTGGGGACGATGCAGCCGAGCTTTACCTCCGTCACGGGTAAAGGCGGCGTGAAAGTC
ATTGACGGCAGCAGCGTGAAATTTGGCCGCTTCGACGGCGCCGAACCGCACTGCGTCGGC
TTAACCGATCTGGTTACAGAGCAGGACGGCAGCAGCATGGCCGCCGGGTTTATGCAGTGG
GACAACGCCTTTTTTCCGTGGACGCTGAACTACGACGAAATCGACATGGTGCTGGAGGGC
GAACTGCACGTACGCCATGAAGGCGAAACCATGATTGCCAAAGCCGGAGATGTCATGTTC
ATCCCTAAAGGCTCCAGCATTGAATTTGGCACGCCAACGTCCGTGCGCTTTCTGTACGTC
GCCTGGCCTGCGAACTGGCAATCCGTATGA
>EECEBICD_00877 hypothetical protein
ATGAACGATTTCATCACCGAAACGTGGCTCAGAGCGAATCATACGCTCAGCGAAGGATCG
GAGATCCATCTGCCCGCTGATGCACGACTGACGCCCTCGGCCCGGGAACTGCTGGAAAGT
CGCCGCCTGCGCATTAAGTTTCTGGATCCGCAAGGCAGGCTGTTTGTCGATGATGACGAA
CAGCAGCCGCAGCCGGTACATGGCTTAACCAGTAGCGATACGCACCCACAGGCCTGCTGT
GAACTGTGCCGCCAGCCGGTGGTGAAAAAGCCCGATACGCTGACCCATCTGACGGCGGAC
AAAATGGTCGCCAAAAGCGACCCGCGCCTCGGCTTTCGCGCCGCGCTCGACAGCGCCATC
GCGCTGACCGTGTGGCTACAAATCGAACTGGCGGAACCCTGGCAGCCGTGGCTTTTCGAT
ATCCGCTCGCGGCTGGGCAACATTATGCGCGCCGACGCTATAGATGAACCGCTGGCGGCG
CAGGCGATCGTCGGGCTCAACGAGGATGAGCTGCATCGGCTTTCCCACCAGCCGCTGCGC
TACCTCGACCACGATCACCTGGTGCCGGAGGCCAGCCACGGTCGCGATGCGGCGCTGCTT
AATCTGCTGCGAACCAAAGTCCGCGAAACGGAAACCCTCGCCGCACAAGTGTTTATTACC
CGTAGTTTTGAGGTCTTGCGCCCGGACATTTTACAGGCGCTGAACCGTCTCTCCAGCACG
GTTTACGTGATGATGATTTTGAGCGTGGCGAAACATCCGCTGACGGTCGCCCAAATTCAA
CAACGGTTGGGAGAGAAGCCATGA
>EECEBICD_00878 Ethanolamine utilization protein EutD
ATGATCATTGAACGCGCCCGCGAACTGGCGGTACGCGCCCCCGCCAGAGTCGTCTTTCCT

GATGCGCTGGACGAACGCGTGCTGAAAGCGGCGCATTACCTGCAACAGTACGGTCTGGCT
CGCCCCGTTCTGGTCGCCAGCCCGTTTGCGCTGCGTCAGTTTGCCCTGAGTCATCGTATG
CCGATGGATGGCATTCAGGTCATCGACCCACACAGCAACCTGAGTATGCGCCAGCGTTTT
GCGCAACGCTGGCTTGCCCGCGCCGGAGAAAAAATGCCGCCGGACGCAGTGGAAAAGCTT
AGCGACCCGCTGATGTTCGCTGCCGCCATGGTGAGCGCCGGTGAGGCTGATGTGTGTATC
GCAGGCAACCTGTCGTCAACGGCGAATGTGCTGCGCGCAGGGCTGCGGGTGATTGGCCTG
CAACCTGGGTGCAAAACATTGTCGTCTATTTTCCTGATGCTGCCGCAGTATGCCGGTCCG
GCATTAGGGTTCGCCGATTGCAGCGTAGTACCGCAGCCGACGGCGGCGCAGCTGGCGGAT
ATCGCTATCGCCAGCGCCGACACCTGGCGGGCCATTACCGGCGAAGAGCCGCGCGTGGCG
ATGCTGTCATTTTCAAGCAACGGCAGCGCCCGTCACCCCAATGTCGCCAACGTGCAGCAG
GCGACAGAGCTCGTTCGCGAACGTGCGCCGCAACTGCTTGTGGACGGTGAATTGCAGTTT
GACGCCGCTTTCGTCCCGGAGGTCGCCGCGCAAAAAGCGCCTGACAGCCCGCTGCAAGGC
CGCGCCAACGTGATGATTTTCCCGTCGCTGGAGGCGGGCAATATTGGCTACAAAATCGCT
CAGCGTCTGGGAGGCTATCGCGCTGTTGGGCCGCTAATTCAGGGGCTTGCCGCGCCGCTT
CACGACCTCTCCCGAGGCTGTAGCGTGCAGGAAATTATCGAACTGGCGTTGGTGGCAGCC
GTGCCGCGCCAGGCTGACGTGAGCCGCGAACGCAGCTTACACACACTGGTTGAATGA
>EECEBICD_00879 Ethanolamine utilization protein EutM
ATGGAAGCATTAGGAATGATTGAAACCCGGGGCCTGGTTGCGCTGATTGAGGCCTCCGAT
GCGATGGTAAAAGCCGCGCGCGTGAAGCTGGTCGGCGTGAAGCAGATTGGCGGTGGCCTG
TGTACGGCCATGGTGCGTGGCGACGTGGCGGCGTGCAAAGCCGCAACCGATGCTGGCGCC
GCTGCGGCGCAGCGCATTGGCGAGCTGGTCTCCGTACACGTGATTCCACGCCCGCACGGC
GATCTGGAAGAAGTGTTCCCGATCAGCTTCAAAGGCGACAGCAACATTTGA
>EECEBICD_00880 Ethanolamine utilization protein EutN
ATGAAACTGGCAGTCGTCACAGGACAAATCGTATGTACCGTCCGCCATCAGGGACTGGCG
CATGACAAATTGCTGATGGTGGAAATGATCGATGCCCAGGGCAACCCCGACGGGCAGTGT
GCCGTCGCTATCGACAGTATCGGGGCGGGAACCGGAGAGTGGGTACTGCTGGTCAGCGGC
AGTTCCGCGCGCCAGGCGCATCGCAGCGAATTATCACCGGTCGATCTGTGCGTCATTGGC
ATCGTCGATGAAGTGGTGGCTGGCGGGAAAGTGGTTTTCCATAAATAG
>EECEBICD_00881 Aldehyde-alcohol dehydrogenase
ATGAATCAACAGGATATTGAACAGGTGGTGAAAGCGGTGCTGCTGAAAATGAAAGACAGC
AGCCAGCCGGCCAGTACTGTTCATGAGATGGGCGTTTTTGCCTCCCTGGATGACGCAGTG
GCGGCAGCAAAACGGGCCCAGCAGGGGCTGAAGAGCGTGGCGATGCGCCAGCTTGCCATT
CATGCCATTCGCGAAGCGGGCGAAAAACACGCCCGAGAATTAGCGGAACTTGCCGTCAGC
GAAACCGGCATGGGACGCGTTGACGATAAATTTGCCAAAAACGTGGCGCAGGCGCGCGGC
ACGCCGGGCGTCGAGTGTTTATCTCCGCAGGTGCTGACCGGCGATAACGGCCTGACGCTG
ATTGAAAATGCGCCGTGGGGCGTGGTGGCCTCGGTCACGCCGTCCACCAATCCGGCGGCG
ACGGTGATTAATAACGCCATCAGCCTGATTGCCGCAGGCAACAGCGTCGTGTTTGCCCCG
CATCCGGCGGCGAAAAAGGTCTCTCAGCGGGCAATCACTCTCCTCAATCAGGCGGTGGTC
GCCGCCGGTGGCCCGGAAAATTTGCTGGTCACCGTGGCGAACCCGGATATCGAAACCGCC
CAGCGGTTGTTCAAATATCCCGGTATTGGCCTGCTGGTGGTGACTGGCGGCGAAGCGGTG
GTGGACGCGGCGCGCAAACATACTAATAAACGCCTGATTGCCGCCGGTGCCGGCAATCCC
CCGGTGGTGGTGGATGAGACCGCCGACCTGCCGCGCGCCGCACAATCCATCGTCAAAGGC
GCATCGTTTGATAACAACATCATCTGCGCCGACGAAAAAGTGCTGATTGTGGTGGATAGT
GTCGCCGACGAGCTGATGCGTCTGATGGAAGGTCAGCACGCGGTGAAACTGACCGCCGCT
CAGGCCGAACAGCTCCAGCCGGTGCTGCTGAAGAATATCGATGAACGCGGCAAAGGTACC
GTCAGCCGTGACTGGGTCGGTCGTGACGCGGGCAAGATTGCCGCAGCCATCGGCCTGAAC
GTCCCGGATCAAACGCGCCTGCTGTTTGTGGAAACGCCAGCTAACCATCCGTTTGCCGTC
ACTGAAATGATGATGCCGGTACTGCCGGTGGTGCGGGTCGCCAACGTCGAAGAAGCCATC
GCCCTGGCGGTTCAGCTTGAAGGCGGTTGCCACCATACGGCGGCGATGCACTCGCGCAAT
ATCGACAACATGAACCAGATGGCGAACGCCATCGACACCAGCATTTTCGTCAAAAACGGG
CCGTGCATTGCCGGGCTTGGATTGGGCGGAGAAGGCTGGACCACCATGACTATCACTACG
CCAACCGGCGAAGGCGTGACCAGCGCACGTACCTTTGTGCGGCTGCGTCGATGCGTGCTG
GTGGATGCGTTTCGCATTGTATAA
>EECEBICD_00882 Cell division protein FtsA

ATGGCGCACGACGAACAACTCTGGCTCACCCCAAGACTGCAAAAAGCGGCGGCCCTGTGT
AACCAGACGCCCGCCGCCAGCGACGCGCCGCTGTGGCTGGGCGTCGATTTGGGCACCTGC
GACGTGGTGTCGATGGTGGTCGACGGCAACGCCCAGCCGGTGGCGGTGTGCCTTGACTGG
GCCGACGTTGTGCGTGACGGCATCGTCTGGGATTTCTTCGGTGCCGTCACCCTCGTGCGC
CGCCATCTCGACACCCTTGAACAGCAGCTCGGCTGCCGCTTCACCCACGCGGCGACCTCG
TTCCCACCGGGTACTGACCCGCGTATTTCGATCAACGTGCTGGAGTCTGCTGGGTTAGAA
GTCAGCCACGTACTGGATGAACCGACGGCGGTGGCGGATCTGCTGGCGCTTGATAATGCA
GGCGTGGTGGATATTGGCGGCGGCACTACCGGCATTGCCATCGTCAAACAGGGCAAGGTG
ACTTACTCCGCCGATGAAGCCACTGGCGGGCACCATATCTCCCTGACGCTGGCGGGCAAC
CGACGTATTCCCCTGGAAGAGGCGGAACAGTACAAGCGCAGCAACGCGCAGGAGATTTGG
CCGGTAGTGAAGCCGGTGTACGAAAAGATGGCGGAAATCGTCGCTTGCCATATTGCCGGG
CAGGGAGTGACAGATTTATGGCTGGCGGGCGGTTCCTGTATGCAGCCGGGCGTGGAGGCG
TTATTTCGCCAGCGTTTCCCGGAGTTACAGGTGCATTTACCGCAGCACAGCCTGTTTATG
ACCCCGCTGGCGATAGCCAACAGCGGGAGAGCGAAAGCGGAGGGACTCTATGCAAGCTGA
>EECEBICD_00883 NAD-dependent methanol dehydrogenase
ATGCAAGCTGAACTACAGACGGCGCTGTTTCAGGCATTCGACACCCTGAATCTGCAACGG
GTGAAAACGTTCAGCGTACCGCCGGTCACGCTGTGCGGACTTGGGGCGCTCGGCGCCTGC
GGCCAGGAAGCGCAAGCGCGAGGCGTAAGCCATCTGTTTGTGATGGTCGACAGCTTCCTG
CATCAGGCGGGAATGACCGCGCCGCTGGCACGCAGCCTGGCGATGAAAGGCGTGGCGATG
ACAGTCTGGCCGTGTCCGCCAGGCGAGCCGTGCATCACCGACGTTTGCGCGGCGGTGGCG
CAACTGCGTGAGGCGGCGTGCGACGGCGTAGTGGCCTTTGGCGGCGGTTCGGTGCTGGAC
GCGGCGAAAGCGGTCGCCCTGCTGGTGACTAACCCTGACCAGACGCTGAGCGCCATGACC
GAGCGCAGTACATTACGCCCGCGTCTGCCGCTGATTGCGGTGCCGACCACCGCCGGAACC
GGTTCTGAAACCACCAACGTGACGGTGATTATCGACGCGGTCAGCGGGCGCAAGCAGGTG
CTGGCACACGCGTCACTAATGCCGGACGTGGCGATTCTTGATGCTGCCGTGACCGAAGGC
GTTCCGCCAAACGTGACGGCGATGACCGGTATCGATGCGTTGACGCATGCGATTGAGGCC
TACAGCGCGCTCAGCGCCACGCCGTTTACCGACAGCCTGGCGATTGGCGCGATAGCGATG
ATTGGCAAATCGCTGCCGAAAGCCGTGGGTTACGGCCACGATCTGGCGGCGCGTGAAAAT
ATGTTGTTGGCCTCCTGTATGGCGGGAATGGCCTTTTCCAGCGCCGGTCTGGGGCTGTGT
CACGCGATGGCGCACCAGCCAGGGGCGGCGCTGCATATTCCGCACGGCCAGGCCAACGCC
ATGCTGCTACCGACGGTCATGGGCTTTAACCGGATGGTTTGCCGCGAACGCTTCAGTCAA
ATCGGTCGGGCGTTAACCAATAAGAAATCGGACGATCGCGATGCGATTGCGGCGGTGAGC
GAGCTGATTGCCGAAGTGGGCCAGAGCAAACGGCTGGCTGACGCTGGCGCCAAACCCGAA
CACTACAGCGCGTGGGCGCAAGCCGCGCTGGAGGATATTTGTCTGCGCAGTAACCCACGC
ACCGCCACACAGGCACAGATTATCGACCTGTACGCGGCTGCCGGGTAA
>EECEBICD_00884 hypothetical protein
ATGGGAATTAACGAAATCATCATGTACATCATGATGTTCTTTATGCTGATTGCCGCCGTG
GACAGGATCCTGTCGCAGTTCGGCGGGTCGGCGCGCTTCCTCGGTAAATTTGGCAAGAGT
ATCGAGGGGGCTGGCGGCCAGTTTGAAGAGGGCTTTATGGCGATGGGCGCGCTGGGACTG
GCGATGGTCGGTATGACCGCGCTGGCGCCGGTGCTGGCGCATGTACTGGGGCCGGTTATT
ATCCCGGTATACGAAATGCTGGGCGCGAACCCATCCATGTTCGCGGGTACGCTGCTGGCC
TGTGATATGGGCGGATTCTTCCTCGCCAAAGAGCTGGCCGGCGGTGATGTCGCGGCGTGG
CTATACTCAGGGTTAATACTTGGGTCGATGATGGGGCCGACCATTGTGTTCTCCATTCCG
GTGGCGCTCGGCATTATCGAACCGTCTGACCGCCGCTACCTGGCGCTTGGCGTACTGGCG
GGTATCGTCACCATTCCCATTGGCTGCATTGCGGGGGGATTGATCGCCATGTACTCAGGC
GTGCAGATCAATGGTCAGCCAGTGGAGTTTACCTTCGCGCTGATCCTGATGAACATGATC
CCGGTATTGATTGTCGCGGTGCTGGTAGCGCTGGGGCTGAAGTTCATCCCGGAAAAAATG
ATCAACGGTTTCCAGATTTTCGCCAAATTTCTGGTGGCGCTGATCACCATCGGTCTGGCG
GCTGCGGTGATTAAATTCCTGTTGGGCTGGGAGTTGATTCCGGGGCTCGACCCGATCTTT
ATGGCGCCAGGCGACAAACCTGGCGAAGTGATGCGCGCCATTGAAGTGATCGGCTCTATC
TCCTGCGTGCTGCTCGGCGCGTATCCGATGGTGCTGTTACTGACCCGCTGGTTTGAAAAA
CCGTTGATGAATGTCGGTAAGCTGCTGAACGTAAATAATATTGCGGCGGCAGGCATGGTG
GCGACCCTGGCGAACAATATCCCGATGTTCGGCATGATGAAGCAGATGGATACCCGCGGC
AAAGTGATTAACTGCGCATTTGCCGTCTCTGCGGCATTCGCGCTGGGCGACCATTTAGGC

TTCGCCGCTGCCAACATGAACGCCATGATCTTCCCGATGATTGTCGGCAAGCTGATCGGC
GGCGTGACGGCGATTGGCGTGGCGATGATGCTGGTGCCGAAAGATGACGCCGCCCAGGTG
AAAACCGAAGCGGAGGCGCAATCGTGA
>EECEBICD_00885 hypothetical protein
GTGAACACTCGCCAGCTACTGAGCGTCGGTATCGATATCGGCACCACCACCACGCAGGTG
ATCTTCTCGCGCCTGGAGTTGGTTAACCGTGCGGCGGTGTCGCAGGTGCCGCGCTACGAA
TTCATCAAACGCGACATTAGCTGGCAAAGCCCGGTCTTCTTTACGCCCGTGGATAAGCAG
GGCGGTCTGAAAGAGGCCGAACTTAAAGCGCTGATTCTGGCCCAGTATCAGGCCGCAGGC
ATCGCGCCTGAATCGGTGGACTCAGGGGCTATCATTATCACCGGCGAAAGCGCCAAAACC
CGCAACGCCCGTCCGGCGGTCATGACGCTCTCGCAGTCGCTGGGCGACTTTGTGGTCGCC
AGCGCCGGGCCGCATCTGGAATCGGTCATTGCCGGTTACGGCGCCGGAGCGCAAAGCTTG
TCTGAGCAGCGGATGTGCCGGGTGCTGAATATTGATATCGGCGGCGGCACGTCTAACTAC
GCGCTGTTTGACGCGGGAAAAGTCAGCGGCACCGCCTGCCTTAACGTCGGTGGTCGCCTG
CTGGAAACCGATGCTCAGGGGCGCGTGGTTTATGCCCATCAGCCGGGGCAGATGATTATC
GATGAGGTGTTCGGTTCGGGTACTGATGCTCGCGCCTTAGCTGCCGCACAGTTGGGACAG
GTGGCGCGGCGGATGGCGGATCTCATCGTCGAGGTTATCACTGGCGCGCTCTCGCCGCTG
GCGCAATCGCTGATGCAAACCGGGCTGCTGCCCGCCGATATTACGCCAGAGGTGATCACC
CTTTCCGGTGGGGTAGGTGAATGCTACCGCAACCAACCTGCCGATCCGTTCTGCTTTTCT
GATATCGGGCCGCTGCTGGCGACCGCGCTGCATGAGCACCCGCGTCTGCGCGAGATGAAC
GTGCAGTTTCCGGCGCAAACCGTGCGCGCCACGGTGATTGGCGCGGGGGCCCATACGCTG
TCGCTTTCCGGCAGTACTATCTGGCTGGAAGACGTCCAACTGCCGCTGCGCAACCTGCCG
GTGGCGATCCCGCAGGATGACGCCGATCTGGTGAACGCCTGGCGGCAGGCGCTTCTCCAG
CTTGATCTCGACCCGCAAACCGACGCTTACGTGCTGGCGCTTCCCGCCACGCTTCCGGTG
CGTTACGCCGCATTACTCACGGTCATCAACGCGCTGACGGCTTTTGTCGCGCGTTATCCC
AATCCACATCCCCTGCTGGTGGTGGCCGAGCAGGATTTTGGTAAAGCGCTGGGCATGTTA
TTGCGCCCACAGTTACCGCAACTTCCGCTGGCGGTCATCGATGAGGTGGTCGTGCGGGCG
GGAGACTATATCGACATTGGTACGCCTCTTTTTGGCGGATCGGTTGTGCCGGTGACGGTG
AAATCACTCGCATTTCCTTCCTGA
>EECEBICD_00886 Ethanolamine ammonia-lyase heavy chain
ATGAAACTAAAGACCACATTGTTCGGCAATGTTTATCAGTTTAAGGATGTAAAAGAGGTA
CTGGCTAAAGCCAACGAACTGCGTTCGGGGGATGTGCTGGCCGGGGTTGCCGCGGCAAGT
TCGCAGGAGCGCGTAGCGGCAAAACAGGTACTGTCGGAAATGACGGTGGCGGATATCCGC
AACAATCCGGTGATTGCCTATGAAGAGGACTGCGTGACGCGCCTGATTCAGGATGACGTC
AACGAAACGGCCTATAACCGGATTAAAAACTGGAGCATCAGCGAACTGCGCGAATACGTA
CTGAGCGATGAAACCTCCGTGGACGACATCGCGTTTACCCGCAAAGGGCTGACCTCCGAA
GTGGTGGCGGCAGTAGCGAAAATCTGCTCCAACGCTGACCTGATCTACGGCGGCAAGAAA
ATGCCGGTGATCAAAAAAGCCAATACCACCATCGGTATTCCGGGCACCTTTAGCTGCCGT
TTGCAGCCGAACGATACCCGTGACGATGTACAGAGTATCGCCGCGCAAATCTACGAAGGG
CTTTCTTTCGGCGCAGGCGATGCGGTGATCGGCGTTAACCCGGTGACCGATGACGTGGAG
AACCTGACCCGCGTGCTCGACACCGTTTACGGCGTTATCGATAAATTCAATATTCCGACC
CAGGGCTGCGTGCTGGCGCATGTCACCACCCAGATTGAAGCGATTCGCCGCGGCGCGCCG
GGCGGACTGATTTTCCAGAGCATTTGCGGCAGCGAGAAGGGCTTAAAAGAGTTCGGCGTC
GAGCTGGCTATGCTCGATGAAGCGCGGGCCGTGGGGGCGGAGTTCAACCGCATCGCCGGG
GAAAACTGCCTGTACTTTGAAACCGGACAGGGCTCGGCGCTGTCGGCTGGCGCGAACTTT
GGTGCCGACCAGGTGACAATGGAAGCGCGTAACTACGGGCTGGCGCGCCACTACGATCCG
TTCCTGGTGAACACCGTGGTGGGCTTTATCGGGCCGGAATATCTCTACAACGACAGGCAG
ATTATCCGCGCCGGTCTCGAAGATCACTTTATGGGCAAGCTGAGCGGCATCTCGATGGGC
TGCGACTGCTGCTATACCAACCATGCCGACGCCGACCAGAACCTTAACGAAAACCTGATG
ATTCTGCTCGCCACTGCCGGCTGTAACTACATCATGGGGATGCCGCTCGGCGACGACATC
ATGCTCAACTACCAGACCACCGCTTTCCACGATACCGCCACCGTCCGTCAGTTGCTGAAT
TTACGGCCGTCGCCGGAGTTTGAACGCTGGCTGGAAACGATGGGCATTATGGCAAACGGT
CGTCTGACCAAACGGGCGGGCGATCCGTCACTGTTCTTCTGA
>EECEBICD_00887 Ethanolamine ammonia-lyase light chain
ATGGATCAAAAACAGATTGAAGAAATTGTACGTAGCGTGATGGCGTCAATGGGACAGGAC

GTACCGCAGCCCGTCGCGCCGTCAACGCAGGAAGGCGCAAAGCCGCAGTGCGCCGCGCCG
ACGGTGACCGAAAGCTGCGCGCTGGATTTAGGTTCCGCAGAGGCAAAAGCCTGGATTGGC
GTCGAGAATCCGCATCGTGCGGACGTGCTGACCGAACTGCGTCGCAGTACTGCGGCACGC
GTCTGTACGGGGCGTGCCGGGCCGCGTCCGCGCACCCAGGCGCTGTTGCGCTTCCTGGCG
GATCACTCCCGTTCGAAAGATACCGTGCTCAAAGAAGTGCCGGAAGAGTGGGTAAAAGCG
CAAGGGCTGCTGGAAGTGCGTTCGGAGATCAGCGACAAAAACCTGTACCTGACGCGCCCG
GATATGGGGCGTCGTCTGAGCCCGGAAGCCATTGACGCGCTGAAGTCGCAGTGCGTGATG
AACCCGGATGTGCAGGTAGTGGTCTCCGATGGCCTCTCTACGGATGCGATCACCGCCAAC
TATGAAGAGATCCTGCCGCCGCTGCTTGCTGGTCTGAAGCAGGCCGGGCTGAACGTCGGC
ACGCCGTTCTTTGTGCGCTATGGCCGTGTGAAGATTGAAGATCAGATTGGCGAAATTCTC
GGCGCGAAGGTCGTCATCCTGCTGGTGGGCGAACGTCCGGGGCTGGGGCAGTCGGAAAGC
CTCTCCTGCTACGCGGTCTATTCCCCGCGCGTGGCGACCACCGTCGAGGCCGACAGAACC
TGTATTTCAAACATTCATCAGGGGGGACGCCGCCAGTAG
>EECEBICD_00888 Ethanolamine utilization protein EutL
ATGCCTGCATTAGATTTGATTCGACCTTCAGTGACTGCCATGCGCGTGATTGCCTCCGTG
AATGACGGCTTTGCGCGGGAACTTAAATTACCGCCACATATACGTAGCCTCGGACTCATC
ACGGCAGATTCTGATGATGTGACCTATATTGCCGCTGACGAAGCGACCAAACAGGCGATG
GTGGAAGTAGTCTATGGCCGCTCCCTCTATGCCGGGGCGGCTCACGGGCCGTCGCCTACC
GCTGGTGAGGTGTTGATTATGCTGGGCGGACCGAACCCGGCGGAAGTGCGCGCCGGTCTG
GATGCAATGGTCGCCAGCATTGAAAACGGCGCGGCGTTCCAGTGGGCGAACGATGCGGAA
AATACCGCATTCCTGGCGCATGTGGTTTCGCGTACCGGCTCGTACCTCTCGTCTACCGCA
GGTATCGCGCTGGGCGATCCGATGGCCTATCTGGTGGCGCCGCCGCTGGAAGCGACATTC
GGCATTGACGCGGCGATGAAATCCGCTGACGTTCAACTGGTGACTTACGTGCCGCCGCCG
TCGGAAACCAACTATTCGGCCGCGTTCTTAACGGGAAGCCAGGCCGCGTGTAAAGCCGCC
TGCAACGCCTTTACCGACGCCGTGCTGGATATTGCCCGTAACCCTGTCCAGCGTGCGTAA
>EECEBICD_00889 Ethanolamine utilization protein EutK
ATGATCAATGCCCTGGGATTACTGGAAGTGGACGGAATGGTCGCCGCCGTCGATGCGGCG
GATGCCATGCTGAAAGCGGCCAACGTGCGTTTGCTCAGTCATCAAGTGCTCGACCCTGGC
AGGCTCACGCTGGTGGTGGAGGGCGATCTGGCGGCGTGTCGTGCGGCGCTGGATGCCGGA
AGCGCCGCCGCGCAGCGCACAGGCCGTGTGATTAGTCGCAAAGAAATTGGTCGTCCGGAA
GAGGACACCCAGTGGCTGATTGGCGGGTTTGCGCGTGCGACAACGCCGACTGAGAAAGCG
CCCCAGGCACCCGCAACGCCTGAGTTCGCTGAGGCGTTACTGGCGCTGCTGGCATCAGTA
CGCCAGGGAATGACGGCGGGAGAGGTTGCCGCGCATTTTGGCTGGCCGCTGGAGCAGGCC
AGAAACGTGCTGGAACAACTCTTTTCTGACGGAGCGTTGCGTAAACGCAGTAGTCGCTAT
CGCATCAAAAATTAA
>EECEBICD_00890 hypothetical protein
ATGAAAAAGACCCGTACAGCGAATTTGCACCATCTTTATCATGAAGCGTTACCCGAAGAC
GTTAAACTTACGCCCAGAGTCGAGGTGGACAATGTTCATCAGCGACGGACGACGGATGTC
TATGAACATGCTCTGACTATCACCGCCTGGCAGCAGATCTACGATCAGTTACACCCGGGT
AAATTTCATGGCGAGTTCACGGAAATCCTGCTTGATGAGATTCAGGTGTTCCGTGAATAC
ACCGGCCTGGCGCTGCGTCAGTCGTGTCTGGTTTGGCCAAACTCTTTCTGGTTTGGTATT
CCGGCGACGCGCGGCGAACAGGGATTTATCGGCGCGCAGGGGCTCGGCAGCGCCGAGATT
GCCACCCGACCGGGAGGCACCGAGTTTGAACTGAGTACGCCGGATGATTACACCATTCTG
GGCGTCGTTATCTCGGAAGATGTTATTTCCCGTCAGGCCACGTTTTTGCATAACCCGGAA
AGGGTGCTGCATATGCTACGTAACCAGTTAGCGCTGGAGGTAAAAGAGCAGCATAAAGCG
GCGCTGTGGGGCTTTGTGCAGCAGGCGCTGGCCACCTTTAGCGAGAGTCCTGAAACCCTG
CATCAACCTGCGGTGCGTAAGGTCTTGAGTGATAATTTGTTGCTGGCGATGGGCACGATG
CTGGAAGAGGCGAAGCCGATTCATAGCGCCGAAAGCATCAGCCATCAGGGATATCGTAGG
CTACTGTCGCGGGCGCGGGAATATGTGCTGGAAAATATGTCGGAGCCGCTGACGGTGCTC
GACTTATGTAACCAGCTACACGTAAGCCGTCGCACGCTGCAAAATGCGTTTCACGCCATT
TTGGGCATTGGCCCCAATGCGTGGCTGAAACGTATTCGCTTAAACGCGGTACGCCGGGAA
CTGATTAGCCCGTGGTCGCAGAGCGCCACGGTGAAAGATGCCGCGATGCAGTGGGGATTC
TGGCATTTGGGGCAATTTGCCACCGATTATCAGCAATTATTTGCCGAAAAACCGTCGTTG
ACGCTGCATCAACGGATGCGGCAATGGGCTTGA

>EECEBICD_00891 hypothetical protein
ATGCAAACAAATACACGGAAAGTAGTAACCAGTGACGCCAAATGGCATCCCCATCTGGGT
ATAAGTTTTATTGGCTGCCTGCTTGCCATTACGCTAGAAATATATTTTGAAGAAAGAATA
TTCATCCCTCATAGCGGAGGGGTGAGCTTCGGTCTGATCGTGCTTCTGGTTATCAATATG
GTGACCATCCCTGTGGTCATGGCCCTGATAGCATTACTCTGCTTTATTATTCATATTCCG
CGTAAAAGCGTTAACTGTATCTTGCTATGCCTACTGGCATGTATTTTGACGATAGCCGGT
TTGTTTATCGCCTATCCGGTTGGTCGTTGA
>EECEBICD_00892 Oxygen-dependent coproporphyrinogen-III oxidase
ATGAAACCCGATGCACATCACGTAAAACAGTTTCTGCTACGCCTGCAGGATGATATTTGC
CAAAAGTTATCCGCTGTGGATGGCGCAAACTTTGTCGAAGATAGCTGGCGACGCGAAGCG
GGCGGCGGCGGACGCAGCCGGGTATTACGCAACGGCGGGATTTTCGAACAGGCGGGCGTT
AATTTCTCTCACGTTCACGGCGATGCAATGCCCGCGTCCGCCACCGCGCATCGGCCAGAA
CTCGCCGGGCGTAGCTTTGAAGCGATGGGCGTATCGCTGGTGGTGCATCCGCACAATCCG
TATATTCCCACCAGCCATGCTAACGTGCGCTTTTTTATTGCCGAAAAACCGGGCGCCGAT
CCGGTATGGTGGTTTGGCGGCGGGCTCGATTTAACGCCCTACTACGGCTTTGAAGAGGAT
GCCGTTCACTGGCACCGCACCGCCCGCGACCTGTGCCAGCCGTTTGGTGACAATGTGTAT
CCGCGTTATAAAAAGTGGTGCGATGACTACTTTTTCCTCAAGCATCGTAACGAGCAGCGC
GGTATCGGCGGCCTGTTTTTTGACGATTTGAATACGCCGGATTTCGACCACTGTTTTGCT
TTTATGCAGGCGGTCGGTAACGGCTACACCGAGGCGTATTTACCGATTGTCGAACGACGC
AAAGCGATGGTCTGGGGAGAGCGGGAGCGTAATTTTCAACTTTATCGCCGCGGTCGCTAC
GTGGAGTTCAATCTGGTATGGGACAGAGGCACCCTGTTTGGTCTGCAAACCGGCGGGCGC
ACTGAATCTATCCTGATGTCGATGCCGCCGCTGGTGCGCTGGGAGTACGACTGGCAGCCG
GAAGCGGGTAGCCCGGAAGCGGCGCTGAGCGAGTTTATTCAGGTACGCGACTGGATTTAA
>EECEBICD_00893 N-acetylmuramoyl-L-alanine amidase AmiA
ATGAGCACTTTTAAACTCCTAAAAACTCTCACATCGCGCCGCCAGGTACTCAAAACAGGT
CTGGCGGCCCTGACGCTGTCAGGTATGTCGCACGCAATAGCCAAAGAAGAAACGCTAAAA
ACCAGTAATGGTCACAGCAAACCCAAAACCAAAAAAACCGGCAGTAAACGGCTGGTGATG
CTCGACCCCGGACACGGCGGCATCGATACCGGCGCGATTGGCCGCAACGGTTCTCAGGAA
AAACACGTCGTACTGGCAATTGCCAAAAACGTGCGCGCTATTTTGCGAAATCACGGAATC
GATGCGCGTTTAACCCGGACAGGGGATACCTTTATTCCGCTGTACGATCGGGTGGAAATC
GCCCATAAGCACGGTGCGGATCTGTTTATGTCTATTCATGCCGATGGTTTTACTAACCCG
AAAGCAGCGGGCGCTTCCGTTTTTGCGCTTTCCAACCGCGGGGCCAGTAGCGCCATGGCG
AAGTATCTCTCCGAGCGTGAAAACCGCGCGGACGAAGTGGCGGGTAAAAAAGCCACTGAC
CGGGATCATCTATTACAACAAGTTTTGTTTGATCTGGTGCAAACCGATACGATTAAAAAC
AGTCTGACGCTGGGTTCGCATATTCTCAAGAAAATCAAACCTATACATAAATTACACAGC
CGCACTACCGAACAAGCGGCTTTCGTGGTGCTAAAATCACCGTCAATTCCGTCGGTGCTG
GTCGAAACCTCGTTTATTACCAATCCGGAAGAAGAACGCCTGCTGGGCACGACGGCGTTT
CGCCAGAAAATCGCTACGGCGATCGCCAACGGCATCATCAGTTATTTTCACTGGTTTGAT
AATCAGAAAGCACACACGAAGAAACGGTAA
>EECEBICD_00894 Acetyltransferase YpeA
ATGGAAATACGCGTTTTTCGCCAGGAAGATTTCGAAGAGGTGATTACCCTCTGGGAGCGT
TGCGACCTGCTGCGTCCATGGAACGATCCTGAAATGGATATTGAACGCAAGGTGAATCAC
GATGTCAGCTTGTTCCTCGTTGCGGAAGTTAGCGGCGAAGTCGTCGGTACGGTAATGGGC
GGTTATGACGGGCATCGCGGCTCGGCGTATTACCTGGGCGTTCACCCGGAGTTCCGCGGG
CGCGGCATTGCGAATGCGTTGCTTAACCGGCTGGAAAAAAAACTCATCGCCCGCGGGTGT
CCGAAAATCCAAATTATGGTGCGCGATGATAACGACGTTGTGCTGGGCATGTATGAACGT
CTGGGGTATGAGCATTCTGACGCCCTAAGTTTGGGTAAACGCCTGATTGAAGATGAAGAG
TACTGA
>EECEBICD_00895 Inner membrane protein YfeZ
ATGAAGAGTACTGAGTTTCATCCAGCCGATTATGATGTACACGGACGTTTACGCCTGCCG
TTTCTGTTCTGGTGCGTACTGTTACTTCAGGCGCGCGCCTGGGTACTCTTTGTCATTGCC
GGTTCATCCCGTGGACAGGGTAATACTTTGTTAAACTTTTTTTATCCCGACCATGACAAT
TTTTGGCTGGGGCTGTTGCCCGGCGTTCCGGCGGTAGTGGCATTTTTACTCAGCGGGCGG
CGTGAAGCGGTTCCTGGTGTGTGGCGCTGGCTGCGCGGACTCCTGATCCTTGCTCAACTG

GTGTCGTTGTGCTGGCTGCCGGTGATGTGGCTGGGTGGCGACCCGGTCAACGGCGTCGGG
CTGGCGCTGCTGCTGGCGGACATCGTCGCGCTGATCTGGCTGTTAACCAATCAACGTTTG
CGTGCCTGTTTTTCCCTTGAGAAAGAATAA
>EECEBICD_00896 putative protein YfeY
ATGAAATCGCTGCGTTTGACTTTACTCGCACTGCCGCTGGCGCTGACCGGCTGCTCAACG
CTCTCCTCGGTCAACTGGTCTGCCGCAAATCCATGGAACTGGTTTGGTTCCTCGACAGAA
GTCACTGAGCAAGGCGTGGGAGCGCTGACCGCCGCCACGCCGCTGGAAGAGCCGGCTATT
GCAGAGGCATTAGATGGCGATTATCGCCTGCGTAGCGGTATGAAAACCGATAACGGCAAC
GTGGTGCGCTTTTTCGAAGCGATGAAGGGCGACAGCGTCGCGATGGTGATCAACGGCGAG
CAGGGAACCGTGAGCCGTATTGATGTGCTGGACAGCGATATCCCAACCGCCGCGGGGGGC
AAAATCGGTACGCCGTTTAGCGACCTTTACAGCAAAGCGTTTGGTCACTGCGAGCCGGTT
TCCAGCGATAGCCATACCAGCGTCGAGTGTAAAGCCGAGGGAAGTCAGCATATTAGCTAT
GTTTTCTCCGGCGAGTGGAGCGGACCGGAAGGCTTAATGCCTCCTGATGACGTCTTAAAA
AACTGGGACGTGCGCAAAATTATCTGGCGTCGTTAA
>EECEBICD_00897 Dye-decolorizing peroxidase YfeX
ATGTCTCAGGTTCAAAGTGGCATTTTGCCGGAACATTGCCGTGCGGCAATTTGGATTGAA
GCCAATGTAAAAGGCGACCTGGACGCCCTGCGCGCGGCCAGCAGAACATTTGCCGATAAA
CTGGCGACCTTCGAAGTCAAATTTCCGGACGCCCATCTTGGGGCCGTTGTGGCGTTTGGC
AACAATACCTGGCGTGCCCTGAGTGGCGGCGTCGGGGCGGAAGAACTGAAAGATTTTATT
CCTTATGGTAAAGGGCTGGCGCCGGCCACGCAGTATGACGTGCTGATCCATATCCTTTCC
CTGCGCCACGATGTGAATTTTTCTGTTGCTCAGGCGGCGATGGAAGCCTTCGGCGACTGT
ATCGACGTGAAAGAAGAAGTTCACGGCTTCCGTTGGGTGGAAGAACGCGATCTGAGCGGC
TTTGTCGACGGGACAGAGAACCCGGCAGGCGAAGAGACGCGTCGTGAAGTGGCGGTGATC
AAAGATGGCGTGGATGCCGGCGGCAGTTATGTGTTCGTGCAGCGTTGGGAACACAATCTC
AAGCAACTTAACCGGATGAGTATTCACGATCAGGAGATGATGATCGGGCGTACCAAAGAA
GCCAATGAAGAGATTGACGGCGATGCGCGTCCGGCAACATCTCACCTGAGCCGCGTCGAT
CTGAAAGAAGATGGCAAAGGGCTGAAGATTGTGCGTCAGAGCCTGCCATACGGCACCGCC
AGCGGTACTCATGGTCTCTATTTCTGCGCTTACTGCGCACGCCTGCACAATATCGAACAG
CAGCTTCTGAGTATGTTCGGCGATACCGACGGTAAACGCGACGCAATGCTGCGCTTCACC
AAACCGGTGACTGGCGGCTACTATTTTGCGCCGTCGCTGGATCGCCTGTTAGCGCTATAA
>EECEBICD_00898 Oxidoreductase UcpA
ATGGGTAAACTCACGGGCAAGACAGCATTGATTACGGGCGCATCGCAGGGCATTGGCGAA
GGGATCGCTCGCGTATTCGCACGCCACGGCGCGAACTTAATCTTGCTGGATATCTCCGAT
GAGATTGAAAAACTGGCGGATGAGCTGGGCGGGCGCGGGCATCGCTGTACTGCCGTTAAG
GCTGACGTCAGAGATTTTGCTTCGGTGCAGGCGGCGGTTGCGCGCGCCAAAGAGACTGAA
GGTAGAATTGATATTTTGGTGAATAACGCTGGCGTGTGCCGTCTGGGCAACTTCCTCGAT
ATGAGTGAAGAAGATCGCGATTTCCACATTGATATTAATATTAAAGGTGTCTGGAACGTC
ACCAAAGCCGTCCTGCCGGAGATGATTAAACGTAAAGATGGCCGCATTGTGATGATGTCT
TCCGTCACGGGAGATATGGTGGCGGACCCGGGTGAAACGGCCTATGCGCTGTCAAAAGCC
GCCATTGTCGGTTTAACCAAATCGCTGGCGGTAGAGTATGCGCAGTCCGGTATTCGCGTG
AATGCCATTTGCCCCGGTTATGTAAGAACGCCGATGGCGGAAAGCATTGCCCGTCAGTCT
AATCCTGACGATCCGGAGTCGGTATTAACGGAAATGGCAAAAGCCATTCCGCTACGCCGC
CTTGCCGATCCACTGGAAGTAGGGGAACTGGCGGCATTTCTGGCTTCCGATGAGTCCAGC
TATCTTACCGGAACGCAAAACGTCATTGATGGCGGCAGTACCCTGCCTGAAAGCGTAAGC
GTAGGCGTCTGA
>EECEBICD_00899 Thiosulfate-binding protein
ATGGCCGTTAATTTACTGAAAAAGAGACCCCTGACGCTGGCAGCAATGCTGTTACTGGCA
GGGCAGGCGCAGGCAACGGAGCTGCTGAACAGCTCATACGATGTCTCCCGCGAGCTGTTT
GCCGCCCTTAACCCGCCGTTTGAGCAACAATGGGCGAAGGATAACGGCGGCGATAAGCTG
ACGATTAAGCAGTCTCATGCCGGGTCATCAAAACAGGCGCTGGCGATTTTGCAGGGACTG
AAGGCAGACGTCGTCACCTACAATCAGGTGACCGACGTACAGATTCTCCATGATAAAGGC
AAACTGATCCCTGCCGACTGGCAAAGCCGTCTGCCGAACAACAGTTCGCCATTCTATTCC
ACGATGGGCTTCCTGGTGCGCAAGGGGAACCCGAAAAATATTCACGACTGGAGCGATCTT
GTACGTTCCGACGTGAAGCTGATTTTCCCTAACCCGAAAACCTCCGGCAACGCCCGTTAC

ACGTATCTGGCGGCATGGGGCGCGGCGGATAACGCGGACGGCGGCGATAAAGCCAAAACC
GAACAGTTTATGACCCAGTTCCTGAAAAACGTCGAAGTGTTTGATACCGGCGGCCGCGGC
GCTACGACTACCTTTGCCGAGCGTGGTCTGGGCGATGTGCTGATTAGTTTTGAATCGGAA
GTGAACAACATCCGCAAACAATATGAAGCCCAGGGATTTGAAGTGGTGATCCCGAAAACG
AACATTCTTGCTGAATTCCCGGTCGCCTGGGTGGATAAAAACGTGCAGGCCAACGGCACA
GAAAAAGCCGCCAAAGCTTACCTGAACTGGCTGTATAGCCCGCAGGCGCAGACCATCATT
ACCGATTTCTACTACCGCGTGAATAACCCGGAAATCATGGGCAAGCAAGCAGATAAATTC
CCGCAGACCGAACTGTTCCGCGTGGAAGACAAGTTTGGTTCCTGGCCGGAAGTGATGAAA
ACGCACTTTGCCAGCGGCGGCGAGCTGGACAAACTATTGGCGGCGGGGCGTAAGTAA
>EECEBICD_00900 Sulfate transport system permease protein CysT
ATGCTTGCCGTTTCTTCCCGACGCGTGCTGCCCGGCTTTACGTTAAGCCTCGGCACCAGT
TTGCTGTTTGTCTGCCTGATCCTACTGTTGCCGTTGAGCGCGCTGGTGATGCAGCTCTCA
CAGATGAGCTGGGCGCAATACTGGGATGTCGTGACCAACCCACAGGTGGTGGCTGCCTAT
AAAGTGACGCTACTGGCGGCATTTGTAGCGTCGATTTTTAACGGCGTGTTTGGCCTGCTG
ATGGCGTGGATTTTAACCCGCTACCGTTTTCCAGGGCGCACGTTGCTGGATGCGCTGATG
GATCTGCCGTTTGCGCTGCCGACAGCGGTGGCGGGGTTAACGCTGGCCTCGCTGTTCTCA
GTGAACGGTTTCTACGGTCAGTTTCTTGCGCAGTTTGATATCAAAATGACCTATACCTGG
CTCGGTATTGCGGTGGCGATGGCCTTTACCAGCATTCCATTTGTGGTGCGCACCGTCCAG
CCGGTACTGGAAGAGTTGGGGCCGGAATATGAAGAAGCGGCACAAACGCTGGGGGCCACC
CGGCTGCAAAGTTTTCGCAAAGTCGTGCTGCCTGAGCTGTCGCCTGCGCTGATTGCCGGC
GTGGCGCTCTCTTTTACCCGTAGCCTGGGCGAGTTTGGCGCGGTGATTTTTATCGCCGGA
AATATCGCCTGGAAAACAGAAGTGACCTCGCTGATGATTTTTGTGCGTTTGCAGGAGTTT
GATTACCCCGCCGCCAGCGCCATTGCCTCGGTGATCCTTGCGGCATCCTTGCTGCTGCTG
TTCTCAATTAACACTCTGCAAAGTCGCTTTGGTCGACGTGTGGTAGGTCATTAA
>EECEBICD_00901 Sulfate transport system permease protein CysW
ATGGCGGAAGTGACTCAATTGAAGCGATACGACGTCCCCCGCATTAACTGGGGAAAATGG
TTTTTGATTGGCGTCGGGATGCTGGTTTCGGCGTTTATCCTGCTGGTGCCGATGATTTAC
ATCTTCGTGCAGGCGTTCAGCAAAGGGTTAATGCCGGTACTGCAAAATCTGGCCGATCCG
GACATGCTGCACGCCATCTGGCTGACGGTGTTGATTGCCTTGATCGCAGTGCCGGTGAAT
CTGGTGTTCGGCATTCTGCTGGCGTGGCTGGTGACGCGCTTTAACTTCCCCGGACGTCAG
CTACTGCTGACCCTGCTGGATATTCCCTTTGCCGTCTCGCCGGTGGTGGCCGGTCTGGTT
TATCTGCTGTTTTATGGTTCCAACGGCCCGTTGGGCGGCTGGCTGGATGAGCATAATTTA
CAAATGATGTTCTCCTGGCCGGGGATGGTACTGGTCACTATTTTTGTCACTTGTCCGTTT
GTGGTGCGCGAACTGGTGCCAGTAATGCTCAGCCAGGGCAGTCAGGAAGATGAGGCGGCT
ATTTTGCTGGGCGCATCCGGCTGGCAAATGTTTCGCCGCGTGACGTTGCCGAATATTCGC
TGGGCGCTACTCTACGGCGTGGTATTAACCAACGCCCGCGCGATTGGCGAGTTTGGCGCG
GTGTCGGTGGTGTCCGGCTCGATTCGCGGCGAAACGTTATCGCTACCGTTACAGATTGAA
TTACTGGAGCAGGACTACAACACCGTCGGCTCCTTTACCGCCGCCGCATTGTTGACGCTA
ATGGCGATTATTACCCTGTTTTTGAAGAGCATGTTGCAATGGCGTCTGGAGAATCAGGAA
AAACGCGCGCAACAGGAGGAAAATCATGAGCATTGA
>EECEBICD_00902 Sulfate/thiosulfate import ATP-binding protein CysA
ATGAGCATTGAGATCGCCAGAATTAAGAAATCGTTTGGTCGTACTCAGGTACTAAATGAT
ATCTCGCTGGATATTCCTTCCGGCCAGATGGTTGCCTTGTTGGGGCCGTCAGGTTCCGGC
AAAACGACGCTGCTGCGCATTATTGCCGGGCTGGAGCATCAGTCCAGCGGCCATATTCGT
TTCCACGGTACGGACGTTAGCCGCCTGCACGCGCGTGAGCGTAAAGTCGGTTTTGTGTTT
CAGCACTATGCGCTGTTTCGCCATATGACGGTGTTTGACAACATTGCTTTTGGCCTGACG
GTGCTGCCGCGACGCGACCGTCCAACTGCGGCGGCGATTAAAACGAAAGTGATGCAATTA
CTGGAGATGGTGCAACTGGCGCACCTCGCGGATCGCTTCCCCGCCCAGCTTTCCGGCGGA
CAAAAACAGCGTGTGGCGCTGGCGCGCGCGCTTGCCGTAGAACCGCAAATTCTACTGCTG
GATGAACCCTTTGGCGCGCTGGATGCCCAGGTACGTAAAGAGTTACGTCGCTGGTTGCGC
CAGCTACATGAAGAACTGAAATTCACCAGCGTCTTTGTCACCCACGATCAGGAAGAAGCG
ACGGAAGTGGCGGATCGGGTGGTGGTGATGAGTCAGGGCAATATCGAACAGGCTGATGCG
CCGGATCGGGTGTGGCGTGAACCGGCAACCCGCTTCGTACTGGAGTTTATGGGCGAGGTG
AACCGCCTGACAGGCACCGTCCGCGGCGGGCAGTTTCACGTTGGCGCGCATCGCTGGCCG

CTGGGTTATACGCCCGCGTACCAGGGGCCGGTCGATCTGTTCCTGCGTCCGTGGGAGGTG
GATATTAGCCGCCGCACCAGCCTGGATTCTCCGCTACCAGTACAGGTCATTGAGGCCAGC
CCGAAAGGTCACTATACACAGCTAGTGGTGCAGCCGCTGGGGTGGTATCACGATCCGCTG
ACGGTGGTGATGGCGGGTGAGGATGTTCCGGTTCGCGGCGAGCGTTTGTTTGTTGGACTG
CAAAAAGCGCGTCTGTATAACGGCGACCAGCGTATTGAAACGCGTGAAGAGGAACTTGCT
CTGGCGCAATCGGCCTGA
>EECEBICD_00903 Cysteine synthase B
GTGAATACATTAGAACAAACCATCGGCAATACGCCGCTGGTCAAATTACAACGTCTGGGG
CCGGATAACGGCAGTGAAATCTGGGTCAAGCTGGAAGGCAATAATCCGGCAGGATCGGTG
AAAGATCGCGCGGCGCTATCGATGATTGTCGAGGCGGAAAAGCGCGGGGAAATTAAACCG
GGCGATGTCCTGATTGAAGCGACCAGCGGCAACACTGGCATTGCGCTGGCGATGATCGCC
GCGCTGAAAGGTTATCGCATGAAGCTGTTGATGCCAGACAACATGAGCCAGGAGCGTCGG
GCGGCAATGCGGGCCTATGGCGCGGAACTGATTCTGGTCACTAAAGAGCAGGGAATGGAA
GGGGCGCGCGATTTAGCGCTCGCTATGTCTGAGCGAGGCGAGGGCAAACTGCTCGATCAG
TTTAATAATCCTGATAATCCCTACGCCCACTACACGACCACCGGCCCGGAAATCTGGCGG
CAAACGTCAGGTCGCATCACCCATTTTGTCTCCAGCATGGGCACGACCGGCACCATTACC
GGCGTGTCGCGTTTTCTGCGCGAGCAGGAAAAACCGGTCACCATCGTTGGCCTACAGCCG
GAAGAGGGCAGCAGTATTCCGGGCATTCGCCGCTGGCCTGCGGAATATATGCCGGGCATT
TTTAACGCCTCGCTGGTAGATGAGGTGCTGGATATTCATCAGAATGATGCCGAAAACACC
ATGCGTGAACTGGCCGTTCGTGAAGGCATTTTCTGCGGCGTGAGTTCCGGCGGCGCGGTA
GCGGGCGCGTTACGCGTCGCACGAGCCACGCCCGGCGCGATAGTGGTGGCGATCATCTGC
GATCGCGGCGACCGTTACCTTTCGACCGGCGTGTTTGGTGAAGAGCATTTCAGCCAGGGG
GCAGGGATTTAG
>EECEBICD_00904 hypothetical protein
ATGAAATTTGCTTTCCGGACACTGATTTTTGTCGGTTTGTTTTACGCCATTGCCCCTCTT
GCCTATGCAGAAACCTCGTTGGATGAGTTACAGCAACAGGCAGATCGAGGGGATATGAAT
GCGCAGTTTCAACTGGGCCGTAAATATCATATTGGCGATGGTGTGGAGCGTGATGTGGAG
AAAGCCGTTTTTTGGTATCAAAAAGCTGCAGCTCAAGGGAGCGCAAAAGCGACAAATAAT
CTGGGCGTGTTATATGAGCATGGACATGTTGCCCCCGAGGATGAGCACCGTTCAGTAGAG
TGGATAATGGAAAAATCATTTGCCTATTTTACGGATGCCGCTAAAAAGGGTCAGTGTACC
GCCCAGCAAAATATTGGTGCGACTTATGCTTATCGACACGAATATGTTCAGGCGTGGGCA
TGGCTCACGGTAGCAGCGACGCGAGGTTCTAAATGGGGATTGATAGACAGGGGACGTTTT
GAGAACAGTATGAGTTCCGATGAACGAAAACAGGCGGAGAAACTGGCGAAGGAATACCTG
CAAAAATACGACACGAAAGAAGGGCGGTGTAACGAGCAGACAATTGATTTGAGTACTTAT
AAGCGTTAA
>EECEBICD_00905 hypothetical protein
ATGAAGAAAATTGTCTGCGCCGTCGTTGCGCTCCTGCTGACCCTACCCGCCTGGGCGAAG
CTAAACGCCCACGAAGAAGCGCGCATTAACGCCATGCTGAATGCGCTGGCGCAGAAAAAG
GATCTGACCTTCGTTCGTAACGGTGATGCGCATAACTGCGAGGAAGCCGTATCGCACCTG
CGCTTAAAACTGGGTAACACGCGCAATCGTATCGACACCGCCGAACAGTTTATCGATAAA
GTCGCGTCGTCGTCGTCCATCACCGGAAAGCCCTATATCGTTAAAATTCCCGGTAAAAGC
GATGAAAACGCGCAGCCTTATTTACATGCGCTTATTGCGGAAACGGATAAAACGGTTGCG
CCGTAA
>EECEBICD_00906 hypothetical protein
GTGCGAGTTCATTTTGTTGTCCATGAGTCGTTTGAATCCGCTGGCGCTTATCTGAAATGG
GCTGAAGATCGCGGTTATACCATTTCATGGTCGCGCGTCTATGCCGGAGAAGCGCTACCG
CCCAATGCCGATGAGTTCGATATGCTCGTGGTATTTGGCGGTCCGCAGTCGCCGCGCACC
ACCCGCGAAGAGTGCCCGTACTTTGACTCTCGCGCCGAACAGCACTTAATTAATCAGGCG
ATTACGGCCCGGCGAATGGTTATCGGCATTTGTCTTGGTTCGCAGCTCATTGGCGAGGCG
CTGGGCGCCGCGGTGTGCCAAAGTCCGGAAAAAGAGATTGGCCACTACCCTATTACGCTC
ACTGAAGCCGGTCTGCGGCATCCGCTTATCGCCCACTTTGGCTCACCGTTAACCGTCGGC
CACTGGCACAACGATATGCCAGGACTCACCGATCAGGCGACCGTGCTGGCCGAAAGCGAA
GGCTGTCCACGTCAGATAGTGCAGTACGGTAATTTTGTCTACGGTTTCCAGTGCCATATG
GAATTTACCGTCGAGGCGGTTGAAGGATTAATCCAACATTCGCAGCAGGAGCTGGCCGAC

GCGCAAGGGAAGCGTTTTATCCGCTCGGTTGCCGAAATGCGCGCATGGAATTACCAGCAA
ATGAATGAAAAGCTGTGGCGTTTTCTGGATGAACTCACGCTGGCGCACTCACAAAAATAA
>EECEBICD_00907 Vitamin B6 salvage pathway transcriptional repressor PtsJ
ATGATCGACGGAAAAACCGCTAACGAAATTTTTGACAGTATCCGTCAACACATTATCGCC
GGCACGTTACGGGCGGAGGATTCGCTCCCGCCGGTACGGGAGCTGGCAAGCGAATTAAAG
GTGAACCGCAATACCGTCGCAGCGGCGTATAAACGGCTGATAACGGCGGGACTGGCGCAA
AGTTTAGGCCGCAATGGGACGGTGATTAAAGGCAGTCCGTCTCCCGTAGCGCTGGAAGGC
GGCGATCCGCATACGCCTCTGCACGACCTTTCCGGCGGCAATCCCGATCCGCAGCGTCTG
CCTGACCTTAGCCGTTATTTTGCCCGTCTCAGCCGCACGCCGCATCTCTACGGCGATGCG
CCCGTTTCCCCTGAACTACACGCCTGGGCGGCGCGCTGGCTACGGGATGCCACCCCCGTC
GCAGGCGAAATTGATATCACCAGCGGAGCTATCGACGCCATTGAAAGACTGCTTTGCGCG
CATCTGCTGCCGGGCGACAGCGTCGCGGTGGAAGATCCCTGTTTTTTAAGCAGTATCAAT
ATGCTGCGCTATGCCGGTTTTAGCGCCAGCCCGGTCAGTGTGGATAGCGAAGGAATGCAG
CCTGAAAAGCTGGAACAGGCGCTAAACCAGGGCGCGCGGGCAGTGATCTTAACGCCGCGC
GCGCACAACCCTACCGGCTGTAGCCTGAGCGCTCGCCGTGCCGCTGCGCTGCAAAATATA
CTGGCGCGTTACCCTCAGGTAGTGGTCATTATCGACGATCACTTCGCCCTGCTTTCTTCG
TCGCCCTGGCAGCCGGTTATTGCGCAGACAACGCAACACTGGGCGGTGATACGCTCCGTT
TCCAAAACCCTCGGCCCCGATTTACGTCTTGCTATTGTCGCCAGCGACAGCGCGACCTCG
GCAAAACTTCGGCTGCGGCTAAATGCCGGTAGTCAGTGGGTCAGCCATCTGTTACAGGAT
CTGGTCTACGCCTGCCTGACCGACCCTGAGTATCAGCACAGGCTGGCGCAAACGCGGCTG
TTTTACGCCACCCGGCAACAAAAACTGGCCCGCGCCCTACAGCAATATGGCATCGCCATT
TCTCCCGGCGACGGCGTGAATGCCTGGCTCCCGCTGGACACCCATAGCCAGGCTACCGCG
TTTACGCTGGCAAAATCCGGCTGGCTGGTACGTGAGGGAGAAGCCTTTGGCGTAAGCGCG
CCGTCTCACGGTCTACGTATCACGCTGTCGACATTAAATGACAGCGAGATCAACACGCTG
GCGGCTGATATCCATCAGGCGCTAAATCGCTAA
>EECEBICD_00908 Pyridoxine/pyridoxal/pyridoxamine kinase
ATGGGACAAGAGAGTGATATTCAGTCAGTGCTCTTCGACGACAACCATCGGGCATTACAG
ACCGATATCGTCGCCGTCCAGTCACAGGTCGTGTACGGCAGCGTAGGCAACAGTATCGCT
GTTCCGGCCATTAAAGCGCAGGGACTACGCGTGACGGCGGTCCCGACGGTGCTATTCAGC
AATACGCCGCACTATAAAACCTTTTATGGCGGCATTATTCCCGCCGAGTGGTTTGCAGGC
TACCTTACGGCGTTGAATGAGCGCGATGCGCTACGTGAATTAAAAGCGATTACCACCGGT
TACATGGGCAGCGCAGACCAAATTGTCTTGCTTTCGAAGTGGCTAATGGCGATCCGGGCT
TCGCATCCGGAGGTGTGTATTCTTGTTGATCCGGTCATTGGCGATACCGACAGTGGAATG
TATGTGCAGGCGGAAATTCCGCAGGCTTATCGCACGCATTTGTTGCCGCAGGCGCAGGGG
CTAACGCCGAACGTATTTGAGCTGGAGATGCTGAGTGGTAAACCGTGCCGCACGCTGGAA
GAGGCGGTGGCGGCGGCGCAATCGCTGCTTTCCGATACGTTGAAATGGGTGGTTATCACC
AGCGCGCCGGGCGAGTCTCTGGAGACCATAACGGTTGCGGTCGTTACTGCGCAGGTCGTG
GAGGTCTTTGCCCATCCGCGGGTGGCGACGGAGTTAAAAGGGACGGGAGATCTCTTCTGC
GCCGAGCTGGTCAGCGGTATTGTGCAGGGGAAAAAACTGACGACCGCGGCAAAAGATGCG
GCGCAGCGCGTGCTGGAGGTGATGACCTGGACACAGCAGTGTGGCTGTGATGAGTTAATT
CTGCCGCCGGCAGGGGAGGCGCGATGA
>EECEBICD_00909 hypothetical protein
ATGAAGAGTTATCGGCTGGTGGTTCGCCAGCAGGGACGTATCGTGGGGCATTTTGAAACG
AGCGGGCTGGACGCGCTGGAAGATATCTGCGTGGCGCGGGCGATGTTTGGCATCACTGGC
GGCTATCAGTGCGAGTTGCAAGTGTCCGATTCCGAACGCAGGATGCTGGAGAGTGGCCCG
GAGGGGATGAAGATCCTGATGCGCGAGAAGTGCTTTCGCCCGGTGACCTCGCAGTTGTAA
>EECEBICD_00910 PTS system glucose-specific EIIA component
ATGGGTTTGTTCGATAAACTAAAATCTCTGGTTTCTGATGATAAGAAAGACACCGGAACT
ATTGAGATTGTTGCCCCGCTCTCTGGCGAGATCGTCAACATCGAAGACGTGCCGGATGTA
GTTTTTGCTGAAAAAATCGTTGGTGATGGCATCGCTATCAAACCAACCGGTAACAAAATG
GTCGCCCCTGTTGACGGTACCATCGGCAAAATCTTTGAAACCAACCATGCGTTCTCTATC
GAATCCGATAGCGGCATTGAGCTGTTCGTTCACTTCGGTATCGACACCGTTGAGCTGAAA
GGCGAAGGCTTCAAGCGTATTGCTGAAGAAGGTCAGCGCGTGAAAGTTGGCGATCCGGTC
ATCGAGTTCGATCTGCCGTTGCTGGAAGAAAAAGCCAAGTCTACCCTGACTCCGGTTGTT

ATCTCCAACATGGATGAAATCAAAGAACTGATCAAACTGTCCGGTAGCGTGACCGTGGGT
GAAACTCCGGTTATCCGCATTAAGAAGTAA
>EECEBICD_00911 Phosphoenolpyruvate-protein phosphotransferase
ATGATTTCAGGCATTTTAGCATCCCCGGGTATCGCTTTCGGCAAAGCACTGCTGCTGAAA
GAAGACGAAATCGTCATTGACCGGAAAAAAATTTCTGCCGACAAGGTTGATCAGGAAGTT
GAACGTTTTCTGAGCGGTCGTGCCAAGGCATCTGCGCAACTGGAAGCGATCAAGACAAAA
GCTGGTGAAACGTTCGGTGAAGAAAAAGAAGCCATCTTTGAAGGGCATATTATGCTGCTC
GAAGATGAGGAGCTGGAGCAGGAAATCATAGCCCTGATTAAAGATAAGCACATGACGGCT
GACGCAGCCGCACATGAAGTTATCGAAGGTCAGGCCACTGCCCTGGAAGAACTGGATGAT
GAATACCTGAAAGAGCGCGCGGCTGACGTACGTGATATCGGTAAGCGCCTGCTGCGCAAC
ATCCTGGGTCTGGCCATTATCGATCTGAGCGCGATTCAGGAAGAAGTTATCCTGGTTGCC
TCTGACTTGACGCCGTCAGAAACCGCGCAGCTGAACCTGCAGAAAGTGCTGGGCTTCATC
ACCGACGCGGGCGGACGCACGTCTCATACCTCCATCATGGCGCGTTCTCTGGAACTGCCA
GCGATCGTAGGTACCGGTAGCGTCACCGCTCAGGTGAAAAACGGCGACTATCTGATTCTG
GATGCCGTAAACAACCAGGTTTACGTCAACCCGACCAACGATGTTATTGAGCAACTGCGC
GCCGTCCAGGAGCAGGTTGCGACCGAGAAAGCGGAACTCGCAAAACTGAAAGATCTGCCG
GCAATCACGCTGGATGGACATCAGGTTGAAGTTTGCGCCAACATCGGTACCGTTCGTGAC
GTTGAAGGCGCTGAGCGTAACGGCGCGGAAGGCGTTGGTCTGTATCGTACTGAATTCCTG
TTCATGGATCGCGACGCGCTGCCGACGGAAGAAGAGCAGTTTGCCGCCTATAAAGCGGTC
GCTGAAGCGTGCGGCTCGCAGGCGGTTATCGTCCGTACCATGGACATTGGCGGCGACAAA
GAGCTGCCGTACATGAACTTCCCGAAAGAAGAGAACCCGTTCCTGGGCTGGCGCGCCGTG
CGTATCGCCATGGATCGCAAAGAGATCCTGCGTGACCAGGTTCGCGCGATTCTGCGTGCC
TCCGCTTTCGGTAAATTGCGCATTATGTTCCCGATGATCATCTCTGTTGAAGAAGTTCGC
GCGCTGCGCAAAGAGATTGAAATCTACAAACAGGAACTGCGTGACGAAGGTAAAGCATTT
GACGAAAGCATTGAGATTGGCGTGATGGTGGAAACACCGGCTGCGGCGACAATTGCGCGT
CATTTAGCCAAAGAAGTTGATTTCTTTAGTATCGGCACCAATGATTTAACGCAGTACACC
CTGGCAGTTGACCGTGGTAATGATATGATTTCACACCTTTACCAGCCAATGTCACCGTCC
GTACTGAACTTGATCAAGCAAGTTATTGATGCTTCTCATGCAGAAGGTAAATGGACTGGC
ATGTGTGGTGAGCTTGCAGGCGACGAACGTGCTACACTTCTGTTGCTGGGGATGGGTCTG
GACGAATTCTCTATGAGCGCCATTTCTATCCCGCGCATTAAGAAGATTATCCGTAACACG
AACTTCGAAGATGCGAAGGTGTTAGCAGAGCAGGCTCTTGCTCAACCGACAACGGACGAG
TTAATGACGCTGGTTAACAAGTTCATTGAAGAAAAAACAATCTGCTAA
>EECEBICD_00912 Phosphocarrier protein HPr
ATGTTCCAGCAAGAAGTTACCATTACCGCTCCGAACGGTCTGCACACCCGCCCTGCTGCT
CAGTTTGTTAAAGAAGCAAAAGGCTTCACTTCTGAGATTACTGTGACTTCCAACGGCAAA
AGCGCCAGCGCGAAAAGCCTGTTTAAACTGCAGACTCTGGGCCTGACTCAAGGCACCGTC
GTCACCATCTCCGCAGAAGGTGAAGATGAGCAGAAAGCGGTTGAACATCTGGTCAAACTG
ATGGCGGAACTCGAGTAA
>EECEBICD_00913 Cysteine synthase A
ATGAGTAAGATTTATGAAGATAACTCGCTGACTATCGGTCATACGCCGCTGGTTCGACTG
AACCGTATCGGTAATGGACGCATTCTGGCGAAGGTGGAGTCGCGCAACCCGAGCTTCAGC
GTCAAGTGCCGTATCGGCGCCAATATGATTTGGGATGCCGAAAAGCGTGGCGTACTGAAA
CCTGGCGTGGAACTGGTGGAGCCGACCAGCGGCAACACCGGTATTGCGCTGGCGTATGTC
GCCGCCGCGCGCGGTTACAAACTCACCCTGGCCATGCCGGAAACCATGAGCATTGAACGC
CGCAAGCTACTCAAAGCATTAGGCGCGAATCTGGTGCTGACCGAAGGGGCGAAAGGGATG
AAAGGCGCTATTCAGAAAGCCGAAGAAATTGTCGCCAGCGATCCGCAAAAATATCTCCTG
CTCCAGCAGTTCAGCAACCCTGCCAACCCGGAAATCCATGAAAAAACCACCGGCCCGGAA
ATCTGGGAAGACACCGATGGTCAGGTGGATGTGTTTATCTCCGGCGTCGGCACTGGCGGT
ACGTTAACCGGCGTCACGCGTTATATCAAAGGGACGAAGGGTAAAACCGATCTTATCACC
GTTGCGGTAGAACCCACTGACTCCCCGGTTATTGCCCAGGCGCTGGCTGGTGAAGAGATC
AAACCAGGCCCGCATAAAATTCAGGGCATCGGCGCAGGCTTCATCCCGGGCAACCTGGAT
CTGAAACTGATTGATAAAGTGGTTGGCATCACCAATGAAGAAGCCATTTCTACCGCGCGC
CGTCTGATGGAAGAGGAAGGTATTCTGGCAGGAATTTCTTCCGGTGCCGCGGTTGCTGCA
GCGCTTAAGCTGCAGGAAGATGAAAGCTTTACCAATAAGAATATTGTGGTTATCCTACCT

TCATCAGGTGAGCGTTATCTGAGCACCGCGTTGTTCGCCGATCTCTTTACTGAGAAAGAA
CTGCAACAGTGA
>EECEBICD_00914 Sulfate transporter CysZ
ATGGTTTCATCATCCACAACCGTACCACGTAGCGGCGTCTATTATTTCTCGCAAGGATGG
AAGTTAGTCACCCTACCGGGAATCCGCCGCTTTGTTATTTTACCTTTGCTGGTCAACATT
GTGCTGATGGGCGGCGCATTTTGGTGGCTGTTTACCCAGCTTGACGCCTGGATACCTTCG
TTGATGAGTCATGTGCCGGACTGGCTGCAATGGCTGAGCTATCTGCTGTGGCCGATTGCG
GTTATCTCTGTTCTGCTGGTCTTTGGCTATTTTTTCTCTACCCTCGCCAACTGGATCGCC
GCGCCGTTTAACGGCCTTTTAGCCGAACAGCTTGAGGCGCGGTTAACCGGCGCCACGCCG
CCAGATACCGGCATACTCGGCATCATGAAAGATGTACCGCGCATTATGAAACGTGAATGG
CAAAAGCTGGCGTGGTATCTGCCGCGCGCTATTGTGCTGCTGGTTCTCTATTTTATTCCT
GGCATTGGCCAAACCATCGCGCCGGTGCTGTGGTTTTTGTTCAGCGCCTGGATGCTGGCG
ATCCAGTATTGCGACTACCCGTTTGATAACCACAAAGTGCCGTTTAAAACGATGCGCGCC
GCGTTGCGCACGCAGAAGGTGGCGAATATGCAATTTGGCGCGCTCACCAGCCTCTTTACG
ATGATTCCGGTGCTTAACCTTTTCATTATGCCTGTCGCCGTCTGTGGCGCGACGGCAATG
TGGGTTGATTGCTGGCGTGCTAAGCACGCGTTATGGAAATAA
>EECEBICD_00915 Cell division protein ZipA
ATGATGCAGGATTTGCGTCTGATATTAATCATTGTTGGCGCGATCGCCATAATCGCTTTA
CTGGTACATGGTTTCTGGACCAGCCGTAAAGAACGATCTTCTATGTTCCGCGATCGTCCA
CTAAAACGCATGAAATCGAAACGCGACGACGACTCGTATGACGACGATGTCGAAGAAGAC
GAAGGCGTGGGTGAAGTTCGTGTTCACCGCGTAAATCATGCCCCCGGCCAGTCGCAGGAG
CATGATGCCCCTCGTCAGTCGCCGCAACACCAGTACCAGCCGCCTTATGCGTCTGCGCAA
CCGCGTCCAGCGGCGCCGCCGCAGCCGCAAGCGCCGATGCAACAGCCGGTTCAGCAACCG
GTCCAACCTGCGCCGCAGCCTCAGCAGGTTCAGCCATCTGCGCCGCCGGTACAGCCGCCG
CAACAGCAGCCTGCACCGCCGTCGCAAGCGCCGCAGCCTGTTGCGCAGCCCGCGCCGCCG
CCATCAGCGCAAACCTTCCAGCCTGCGGAACCTGTGGTTGAGGCGGAACCGGTAGTGGAA
GAAGCGCCGGTTGTGGAAAAACCGCAGCGTAAAGAGGCGGTCATCATTATGAACGTCGCG
GCCCATCATGGCAGCGAGCTTAATGGTGAAGTGTTACTGAACAGTATTCAGCAGTCAGGC
TTTAAATTTGGCGATATGAATATTTTCCATCGTCATTTGAGCCCGGACGGCAGCGGCCCG
GCCTTGTTCAGTCTCGCGAATATGGTCAATCCGGGAACGTTCGATCCTGAAATGACGGAC
TTCACTACGCCTGGCGTCACTATCTTTATGCAGGTACCGTCTTACGGCGATGCGCTGCAG
AATTTCAAGCTGATGCTGCAATCCGCGCAGCATATCGCTGATGAGGTCGGCGGCGTCGTG
CTGGATGACCAGCGTCGGATGATGACGCCGCAGAAACTGCGCGAATATCAGGACCGCATC
CGCGAGGTGATGGACGCTAACGCCTGA
>EECEBICD_00916 DNA ligase
ATGGAACCAATCGAACAACAACTGACAGAACTGCGAACCACGCTTCGCCATCATGAATAT
CTGTATCATGTGATGGATGCGCCCGAGATTCCCGACGCCGAATATGACCGCCTGATGCGG
GAACTGCGAGAGCTGGAGGCGCAACGTCCGGACCTTATCACGCCGGATTCGCCCACGCAG
CGCGTTGGCGCGGCGCCGCTGACGGCATTCAACCAGATTCGCCATGAAGTACCGATGCTG
TCGCTCGATAACGTATTTGATGAAGAAAGCTTCCTGGCGTTTAACAAGCGAGTGCAGGAT
CGTCTAAAAAGCACGGAAAATGTCATCTGGTGCTGCGAGCTAAAGCTGGATGGTCTGGCG
GTCAGCATTCTGTATGAAAACGGCGTGCTGGTGAGCGCTGCGACGCGTGGCGACGGCACC
ACCGGTGAAGATATTACTTCAAACGTGCGCACGATTCGCGCCATTCCGTTAAAGCTGCAC
GGCGATAACATTCCGGCGCGTCTTGAAGTTCGCGGAGAGGTATTTTTACCGCAGGCCGGG
TTTGAAAAGATCAATGAAGACGCGCGCCGCACTGGCGGCAAAGTGTTTGCTAATCCGCGT
AACGCGGCGGCGGGCTCGTTGCGTCAGCTCGATCCGCGTATTACGGCGAAGCGACCGCTA
ACATTTTTCTGCTACGGCGTCGGTATTCTGGAAGGCGGCGAGTTGCCGGATACGCATCTG
GGACGTTTGCTGCAATTTAAAGCGTGGGGGTTACCGGTCAGCGATCGCGTGACGCTGTGC
GATTCTCCCCAGGCGGTACTGGATTTTTATCGCAACGTCGAAAAAGATCGCCCCACGCTC
GGTTTTGATATCGACGGCGTGGTGATTAAGGTCAACTCGCTGGCTTTGCAGGAACAGCTG
GGCTTTGTGGCCCGCGCGCCGCGCTGGGCGGTGGCGTTTAAATTTCCTGCTCAGGAGCAG
ATGACTTTTGTGCGCGATGTTGAGTTTCAGGTCGGGCGCACCGGGGCGATTACGCCGGTG
GCGCGTCTGGAACCGGTTCAGGTGGCTGGTGTGCTGGTCAGCAATGCCACGTTGCATAAT
GCCGATGAAATTGAGCGACTTGGCTTGCGCATTGGCGATAAGGTGGTTATCCGTCGGGCA

GGCGATGTGATCCCTCAGGTGGTCAACGTGGTTCTGTCCGAACGCCCTGAAGAGACGCGT
CCGATTGTGTTTCCAACGCACTGTCCGGTGTGCGGATCGGACGTAGAGCGCGTTGAAGGC
GAAGCCGTTACCCGTTGTACCGGCGGGCTGATTTGCGGCGCGCAGCGTAAAGAGTCGCTG
AAACATTTTGTGTCGCGCCGGGCGATGGATGTAGACGGGATGGGCGATAAGATCATCGAC
CAACTGGTGGAAAGGGAGTATGTCCATACGCCAGCGGATCTGTTCCGTCTGACGGCGGGT
AAACTGACCGGCCTGGACCGAATGGGGCCAAAGTCCGCGCAAAATGTTGTAAACGCGCTG
GAAAAAGCCAAAGCGACGACTTTTGCGCGTTTTCTCTATGCGCTGGGCATCCGTGAAGTG
GGTGAAGCGACGGCGGCGGGGCTGGCGGCTTATTTCGGCACGCTGGAGGCGCTGCAGACC
GCTACCATTGACGAGTTGCAGAAAGTACCCGACGTGGGGATTGTGGTCGCTACGCACGTC
TTTAACTTTTTTGCCGAAGAGAGCAATCGTGACGTGATTGGGCAGCTACTGGCGGAAGGG
GTTCACTGGCCCGCGCCGGTAGTCATTAATGTGCAAGAGGTCGATAGCCCGTTTGCCGGT
AAAACCGTGGTACTAACCGGTAGCCTGAGCCAGATGTCCCGCGATGACGCTAAAGCGCGT
TTAGCGGCGCTGGGCGCGAAAGTGGCAGGCAGCGTATCGAAGAAAACCGATCTGGTGATT
GCCGGTGAAGCGGCGGGCTCCAAACTGGCCAAAGCGCAGGAACTGGGTATTACCGTGATT
GATGAAGCGGAAATGATCCGCTTACTGGGTGCCTGA
>EECEBICD_00917 hypothetical protein
ATGGAAAAAGAGCAGCTGGTTGAAATCGCCAACACCGTGATGCCCTTTGGCAAATATCAG
GGACGTCGATTGATCGACCTTCCGGAAGAGTATTTGCTGTGGTTTGCCCGTAAAGATCAA
TTTCCGGCGGGTAAACTGGGGGAGTTGATGCAAATCACGCTGCTGATTAAAACCGAGGGG
CTGACGCAACTGGTACAGCCCCTGAAACGCCCGCTTTAA
>EECEBICD_00918 hypothetical protein
ATGGCCAATATTCTGTTTCCGACGTCAGTGCTGGGTATGATGGTATTACCGCTGATGATC
TTCCACCAGATCCAACTGATGGTCTGCGCAGGGCTGGCGCGGCGCTACAAACGCCAGACC
GAGAAGTTACAGGCGCAGCAGGAAAGCCGCGCCGCGAAAGCTTAA
>EECEBICD_00919 hypothetical protein
ATGAAACTTTTTCGTATCCTTGACCCTTTCACGCTGACACTTATCACCGTGGTACTACTG
GCCTCTTTTTTCCCGGCGGAAGGCGGCTTTGTGCCTGTCGTTGAAGGCATTACGACAGCG
GCCATCGCTCTGCTGTTTTTTATGCATGGCGCGAAGCTTTCACGTGAAGCGATCATCGCT
GGCGGCAGCCACTGGCGGTTGCATCTGTGGGTCATGTGCAGCACTTTTGTGCTCTTTCCT
GTCCTCGGCGTACTGTTTGCCTGGTGGGCGCCTGTCAATGTCGACCCTATGCTCTACAGT
GGCTTTCTGTATCTGTGCATTTTGCCCGCCACCGTGCAGTCGGCGATTGCATTTACTTCA
CTCGCGGGCGGTAACGTCGCGGCCGCGGTGTGCTCAGCGTCTGCGTCCAGTCTGCTGGGC
ATTTTCCTGTCGCCGCTGCTGGTCGGTCTGGTAATGAATATTCATGGCGCGCAGGGCAGT
CTGGAAGAGGTGGGCAAAATTATGCTGCAACTGCTGCTGCCTTTTGTGCTGGGACACCTG
TCTCGCCCGTGGATCGGCAACTGGGTCGCGCGCAACAAAAAGTGGATCGCGAAAACAGAT
CAGACCTCTATTTTGCTGGTGGTCTACTCGGCGTTCAGCGAAGCGGTGGTCAACGGCATC
TGGCACAAAGTCGGCTGGGGATCGCTGCTGTTTATCGTCGTGGTCAGTCTCATACTGTTG
GCGATCGTCATCACCATTAACGTCTTTGTCGCCCGTAAATGCGGCTTCAATAAAGCCGAC
GAAATCACTATTGTTTTCTGCGGTTCGAAAAAAGCCTGGCCAACGGGATCCCGATGGCCA
ATATTCTGTTTCCGACGTCAGTGCTGGGTATGA
>EECEBICD_00920 Hydrogen peroxide-inducible genes activator
ATGAACTATTCACTGCGTCAACTGCGTATTTTTATTACCGTCGCCCAGGCCAAAAGTTTC
AGTCGGGCAGGGGACAGGATTGGACTGAGCCAGTCTGCGGTCAGTCATAGCGTAAAAGAG
CTGGAGCGGCAAACCGGCGTCAGACTGCTGGATCGCACCACGCGTGAGGTGGTGCTGACC
GAGGCGGGGCAACAACTGGCGACGCGTCTGGAGCGGGTGCTTGATGAACTGAACAGCATA
CTGCGCGATGCAGGCAGAGTAGGGACACAATTAACCGGCACCGTGCGCGTTGCCGCGAGC
CAAACCATTTCTGCGCATCTTATTCCGCAATGCATTGCCCAAAGTAATTCACTTTATCCC
GCTATTGATTTTGTATTGCACGATCGTCCGCAGCAGTGGGTGCTGGAGAGCATCCGCCAG
GGTGAAGTGGATTTTGGCATCGTTATCGATCCCGGCGCAGCGGTGGATTTACAGTGTGAA
GCCATCTTATCAGAGCCTTTTTTACTGTTGTGTCGCCAGGATCATCCGCTGGCGCATCAG
GAATGGGTGAGCTGGCAGGATCTTAAACAGGCCTCGCTGGTCTTACAGGATTATGCTTCC
GGCAGTCGACCGCTGATTGATGCGGCGCTGGCCCATTTTGCTATTGAGGCGGATATCGTG
CAGGAGATTGGTCACCCGGCCACGCTGTTTCCAATGGTGGAAGCGGGAATCGGCATCAGC
GTGCTGCCCGCGCTGGCGTTACCGCTCCCGCAGGGCAGCCACTTGCAGGTTAAACGGCTG

ACGCCCGTAGTTGAAAGGCAGCTCATGCTGGCGCGACGAAAAAAACCGATCGCTCTCCACG
GCGGCGCAGGCGTTGTGGGACGTGGTGCGTACCCAGGCCAGCGAATTAACCGCCGCGAGG
GCGAAAGATCCGCTGTATCAGCTATAA
>EECEBICD_00921 hypothetical protein
ATGAAAAAAATTCTTACTCTGACGCTTTCTTCTTTACTCATTATTCCCGCACTCACCCAT
GCGGAATTTAAAGGCGGGTTCGCCGATATCGGCCTCCATTACCTCGACTGGACATCAGAC
ACCACGGAAAAAACCAGTAAAAAATCCCATAAAGATGATTTTGGGTACCTGGAACTGGAA
GGCGGAGCGAATTTTAGCTGGGGAGAAATGTACGGGTTCTTCGACTGGGAAAATTTTTAC
AATGGCCGCCACGCTAAACCAGGCAGCGAACAGCGTTACACCTTTAAAAATACCAACCGT
ATTTATCTGGGCGATACAGGATTAAACCTCTATCTCCATGCCTACGGGACGTACGGTTCA
CCTCATCGCGCTAATTTCCATGATGACATGTTTCTCTATGGTTTAGGCTATAACTTCACA
GGTAACGGTTACTGGTTTAAACCCTTCTTTGCCAAACGTTATACCGATCAGACCTACTAC
ACAGGCGATAATGGTTATGTATTAGGTTGGGTGGCCGGCTATAGCTTCTCGTTGGGTAGT
GAGAAATTCAGCGTAACGAACTGGAATGAGTATGAGTTTGACCGCGATGCGTCCTACGCT
GCGGGCAATGGCGGGAAAGATGGCATTAATGGCGCAGTAGCGCTGTGGTGGAATGCGACT
CCTCACCTGACCGCAGGAGTACAATATCGTTACGCTGATAATAAGTTAGGAGAATCCTTC
CTGCAGGATGGCATTATTTATTCCATCAAATACCTGTTTTAA
>EECEBICD_00922 Purine nucleoside phosphorylase 2
ATGCCTCACGCTCTTTTTTCTCATAATCCTGAGTATTGTATTGATATTATTCATGCACAT
AAACCGGCATTTACCCCGCGAGTCGCGTTTATTTTAGGCTCCGGGTTGGGTGCTCTGGCG
GATCAAATCGAAGATGCGGTAGCCATTTCTTACGAGAAATTGCCGGGATTTCCCGTCAGC
ACCGTACACGGCCATGCTGGTGAACTGGTGCTCGGCCATCTGGCGGGTGTTCCGGTGGCC
TGTATGAAAGGTCGCGGACATTTTTATGAAGGGCGTGGAATGGCGGTGATGACCGATGCC
ATTCGCACGTTGAAATTACTGGGCTGTGAAGTGTTGTTCTGCACCAATGCGGCAGGATCT
CTGCGTCCGGAAGTGGGGCCGGGTAGCCTGGTTGCGCTTAGCGATCACATTAATACGATG
CCCGGTACGCCGATGGTGGGGCCAAACGATGATCGTTATGGCGATCGCTTCTTCTCGCTG
GCTAATGCTTATGACGCAGATTATCGCGCCATGCTTCAGTCCGTTGCGGCAGAGGAGGGA
TTCCCCCTCACTGAAGGCGTATTTGTTTCTTATCCGGGTCCGAACTTCGAAACGGCGGCA
GAAATCCGCATGATGCAGATTATTGGCGGCGATGTGGTCGGAATGTCCGTTGTCCCTGAG
GTGATTAGCGCACGTCATTGTGGCCTCAAAGTTGTTGCCGTCTCAGCGATCACCAATCTG
GCGGAAGGGCTGGGCGATGTAAAACTCTCCCATGCGCAAACGCTGGCGGCGGCTGAACTT
TCCCGGCAGAACTTTATAAATCTGATTTGCGGCTTTTTACGTAAACTGGCCTAA
>EECEBICD_00923 Xanthosine permease
ATGGGTATTACGTCCCGCTTAAAAGTCATGTCGTTCTTGCAATATTTTATCTGGGGGAGC
TGGCTGGTTACCCTGGGCTCTTACATGATCAACACTCTGGATTTTACCGGCGCGAATGTC
GGTATGGTCTACAGCTCAAAAGGACTGGCAGCGATTATCATGCCGGGCATTATGGGGATC
ATTGCTGATAAATGGCTGCGCGCTGAGCGAGCCTATATGCTTTGCCATCTGGTTTGCGCG
GGGGCGTTATTGTACGCCACCACCGTTACCGATCCCCAGACGATGTTCTGGGTGATGTTG
GTTAATGCGATGGCGTATATGCCAACGATTGCATTATCCAATAGCGTTTCGTACTCCTGT
CTGGCGAAAGCAGGTCAGGATCCGGTAACGTCATTTCCGCCTGTGCGCGTTTTCGGCACA
ATAGGTTTTATTGTTGCGATGTGGACGGTGAGCCTGATGGGGCTGGAACTGAGCAGTGCG
CAATTATACATCGCTTCTGGCGCATCGTTATTGCTGGCCCTGTATGCGCTGACGTTACCG
AAAATTCCGGTAGCCGAGAAGAAGGCGAACACCACGCTTGCCAGTAAGCTCGGACTGGAT
GCTTTTGTTCTGTTTAAAAATCCACGCATGGCAATCTTCTTTTTGTTTGCGATGATGTTG
GGGGCGGTGCTGCAAATTACCAATGTCTTCGGTAATCCGTTCCTGCATGATTTTGCCCGT
AATCCTGAGTTTGCCGACAGCTTTGTGGTGAAGTATCCCTCTATCTTGCTTTCAGTTTCG
CAGATGGCGGAAGTGGGCTTTATCCTCACCATTCCGTTCTTCCTTAAACGCTTTGGTATT
AAAACGGTAATGCTGATGAGTATGCTGGCGTGGACGCTGCGTTTCGGCTTCTTTGCCTTT
GGCGATCCATCCCCGTTTGGCTTTGTGCTACTGCTGCTGTCGATGATTGTTTATGGCTGC
GCATTTGATTTCTTCAACATCTCAGGGTCAGTATTTGTAGAGCAGGAGGTGGACTCAAGT
ATTCGCGCCAGCGCGCAGGGGTTGTTTATGACCATGGTTAACGGCGTGGGGGCGTGGATT
GGGTCTCTTTTAAGCGGTATGGCCGTGGATTATTTTTCTATTGATGGCGTAAAAGATTGG
CAAACCATCTGGCTGGTCTTTGCCGCCTACGCTCTGGCATTGGCCGTTATTTTTGCATTG
TTCTTTAAATATCAGCACCATCCAGAAAAACTGTCGACCAAATCATTAGCACATTAA

>EECEBICD_00924 hypothetical protein
ATGAAAGAAATTATTCTCTCCTCCGGTATTGGATTTGGTATTGGCGCATTTTTTACTTTA
TGCCGTATACCTATTCCCGCACCAAATGTTTTACCCGGGGTATTATCTATTGTATTTATG
TATATTGGGTATCTTGTCGTTAAATCTATTTTTTACTAG
>EECEBICD_00925 Hca operon transcriptional activator HcaR
ATGGAACGAGCCCATCGTATTGATCTAAAGCTATTACGTTATTTCCTCGCTGTTGCAGAA
GAATTGCATTTTGGCCGGGCTGCTGCCCGGTTAAATATGTCACAACCTCCCTTGAGTATC
CATATCAAAGAACTTGAACAGCAACTGGGTACATTGCTGTTTATTCGTCATTCCCGCAGC
GTTGCATTAACCCATGCCGGGAAAGTTTTAATGGAGGAGTCACGTCGCTTGTTAGCCAGT
GCAAATCAGGCGCTGGCGCGAGTGGAGCAGATTGGTCGCGGTGAAGCGGGGCGCATTGAA
CTGGGTGTGGTTGGTACGGCGCTGTGGGGAAGAATGCGTCCTGCGATGCGTCACTTTTTG
AAAGCAAATCCCAATGTTGAAGTACTTTTTCGCGAAAAGTCTCCCGGTATGCAAATGGCG
TTGTTAGAGCGTCGGGAACTTGATGCAGGGATCTGGCGTATGGCTATCGAACCGGCAGTC
GGTTTTACCAGCATACGTTTGCATGAATCTGCATTCATGGTTGCAGTTCCGGAAGATCAC
GATCTGGCATCCAGAGATTCTGTGCCGCTGTCGGCGTTGCGCAATGAATATTTTGTTACG
TTGCCTTCCGTACATTCTGATTGGGGATTTTTACAACGTGTATGCCAACAGGCGGGTTTC
TCGCCAATGATTATTCGGGAAGTGGTTGAGCCGCAAACGGTGTTGGCGATGATCAGTATG
GGGATCGGGATTACGTTGATGGCTGATGGCTATGCGCAAATGAGCTGGCCGGGTGTGGTG
TTCCGTCCGTTAGAGGAACGTATTCCGGCGGATTTGTATATTGTGTATGACCAGCAGCAG
GCAACGCCAGCACTGGAGAAACTGGTGGCGGCGTTGACGGTATGA
>EECEBICD_00926 tRNA-Lys(ttt)
GGGTCGTTAGCTCAGTTGGTAGAGCAGTTGACTTTTAATCAATTGGTCGCAGGTTCGAAT
CCTGCACGACCCACCA
>EECEBICD_00927 tRNA-Val(tac)
GGGTGATTAGCTCAGCTGGGAGAGCACCTCCCTTACAAGGAGGGGGTCGGCGGTTCGATC
CCGTCATCACCCACCA
>EECEBICD_00928 tRNA-Val(tac)
GGGTGATTAGCTCAGCTGGGAGAGCACCTCCCTTACAAGGAGGGGGTCGGCGGTTCGATC
CCGTCATCACCCACCA
>EECEBICD_00929 tRNA-Val(tac)
GGGTGATTAGCTCAGCTGGGAGAGCACCTCCCTTACAAGGAGGGGGTCGGCGGTTCGATC
CCGTCATCACCCACCA
>EECEBICD_00930 Glutamate--tRNA ligase
ATGAAAATCAAAACTCGCTTCGCGCCTAGCCCGACAGGTTATCTGCACGTCGGCGGCGCG
CGTACTGCTCTTTATTCCTGGCTTTTCGCACGTCACCACGGCGGTGAATTCGTGCTGCGT
ATTGAAGACACCGATCTTGAGCGTTCCACGCCGGAAGCCATTGAAGCCATCATGGATGGT
ATGAACTGGCTGAACCTGGAATGGGACGAAGGCCCATATTTCCAGACCAAACGTTTTGAT
CGCTATAACGCGGTGATTGACGAGATGCTGGAGGCGGGAACAGCCTATAAATGCTACTGC
TCTAAAGAGCGTCTGGAGCAACTGCGTGAAGAGCAGATGGCGAAAGGCGAGAAGCCGCGT
TATGACGGGCGTTGCCGTCACAGTTACGAACATCATGCCGACGATGAGCCATGCGTGGTG
CGCTTTGCTAACCCGCAGGACGGTTCCGTTATTTTTGATGATCAGATCCGTGGCCCGATC
GAATTCAGCAATCAGGAACTGGACGATCTGATCATTCGTCGTACCGACGGTTCGCCGACC
TACAACTTCTGTGTGGTGGTGGACGACTGGGATATGGAAATTACCCATGTGATTCGTGGC
GAAGACCATATCAACAATACTCCGCGCCAGATTAACATTTTGAAAGCGTTAAACGCGCCG
GTGCCGATGTACGCGCATGTGTCGATGATTAACGGCGACGACGGTAAAAAGCTGTCTAAA
CGTCACGGTGCGGTGAGCGTGATGCAGTATCGCGATGACGGTTATCTGCCGGAAGCGTTG
CTGAACTATCTGGTGCGTCTGGGCTGGTCCAGCGGCGATCAGGAAATCTTCACTCGCGAA
GAGATGATCAAGCTGTTCTCCCTCGGTGCGGTGAGCAAGTCTGCGAGCGCGTTCAACACC
GATAAACTGCTGTGGCTGAACCACCACTATATCAATACGCTGGCGCCGGAATATGTGGCG
ACGCATTTGCAGTGGCATATTGAGCAGGAAAATATCGACACCCGTAACGGCCCGCAGCTT
GCTGAACTGGTGAAGTTGCTGGGCGAGCGCTGCAAGACTCTGAAAGAGATGGCGCAGAGC
TGCCGCTACTTCTATGAAGACTTCAGCGAGTTTGACGCCGATGCGGCGAAAAAACACCTC
CGCCCGGTAGCGCGTCAGCCGCTGGAAGTGGTGCGCGACAAGCTGTCGGCGATTACGGAC
TGGTCAGCAGAAAACGTCCACCACGCGATTCAGGCCACGGCGGATGAGCTGGAAGTGGGG

ATGGGTAAAGTGGGGATGCCGCTGCGTGTGGCTGTGACCGGTGCCGGGCAGTCGCCTGCG
CTGGATGTCACCGTACATGCGATTGGTAAAACCCGCAGCATTGAGCGCATCAACAAAGCG
CTGGGCTTTATCGCCGAGCGTGAAAGCCAGCAGTAA
>EECEBICD_00931 hypothetical protein
ATGACCACTGAAGAGCTGGCTGAATGCCTCGGCGTGGCCAGACAAACCGTCAATCGCTGG
ATCAGAGAACAACACTGGAAAACCGAAAAATTTCCCGGCGTCAAAGGCGGACGCGCAAGA
CTCATCCATATTGATGCCAGCGTGCGGGAGTTTATTCTCAATATTCCGGCTTTTCGCAAA
CTGCCTGCGTTTTATCAGGCGGAAGAGGCTTTCGCAGAATATGCGAACGCGGCGCACAGT
CACGCTTATCGACAAATTATTGATGCCGTTGAAAATATGTCTGCTCAGGAACAGGAAAAA
CTGGCGCTGTTTTTATCGCGGGAAGGTATTCGCGGATTTCTGACGCGTTTAGGTATCAAT
GAGGCGGACTAA
>EECEBICD_00932 hypothetical protein
ATGTTCAAGGAACGGATGACGCCAGAAGAACTCGCTAATCTCACCGGTTACAGCCGACAA
ACCATCAATAAATGGGTACGGAAAGAAGGCTGGGCAACATCGCCCAAACCTGGCGTCCAG
GGCGGTAAAGCCCGTCTGGTTCATGTAAACGAACAGGTTCGTGAATATATCCGCAGCGCT
GAACGTTCAGTTGACCATCACGCAGACACATTCACGCCTGCCAGCAACGCATCGCTTGAA
GCGCTGCTCATGACGCTCGCCAAAGAGATGACCTCATCCGAGCAGAAACAGTTTACCTCT
CTCCTCGTTCGTGAAGGGATTACCGGTTTGTTACAACGCTTAGGGATACGCGATAGCAAA
TAA
>EECEBICD_00933 tRNA-Ala(ggc)
GGGGCTATAGCTCAGCTGGGAGAGCGCTTGCATGGCATGCAAGAGGTCAGCGGTTCGATC
CCGCTTAGCTCCACCA
>EECEBICD_00934 tRNA-Ala(ggc)
GGGGCTATAGCTCAGCTGGGAGAGCGCTTGCATGGCATGCAAGAGGTCAGCGGTTCGATC
CCGCTTAGCTCCACCA
>EECEBICD_00935 putative cyclic di-GMP phosphodiesterase PdeA
ATGCCGGATAAGTGTAACGTATTAAAAAATATAAAAATATTTTTACTGGCCTTCTGCCTG
ACCGTTCCGGCTATTCTGCTATCTCGTCTCATTTCTCCCCGCGCCACAATTGATTCCAGC
TATATCTTTCTGGCCTGGCTACCGCTCTGCGTCATGTTTTCCGTGCTGTTTTTATTTGGC
CGCCGTGGCGTCGCGCCGATGGTAGGCGGAATGATGTTGACTAATGAATGGAATTTTCAT
TTACCTCTGCCACAGGCGATGGTGCTGCTATTTTGCCAGACGTTTCCCGTGTTGCTGGTT
TGCGCTATCGTACGCTGGCAGCTGGGCGCGCGCTGGCGCTACGGAATACCCAACCAAGGC
ATCTGGCTACGCGTTTTCTGGTTAGGGTTAATGACGCCGTTTGGCATAAAAATCAGTATG
CATCTGGCGGGGCACTACCTGGCTTTCCCGGTGACAATCTCTACCTTCTTTGGCACCGGC
ACCGCTATTTTCTCGATTGTTGATTTATTGAGTCTGATTTCTGCCGCGCTTATTTTTACC
CTCTTTTTCTATTATCCGCTGCGGATGATAGTCAGCCCGCACTATATTCGTATTTTCTGG
CGGAGGGATGTCGCTCCTTATCTCGCCAAAGAAAAACGCCTGTTTACGCTTCTCTGGTTT
GCCACTCTGGCGACGCTATTAGCGGTGCTGTGTACGCCTTTCGAGACAAAGTATATTGCT
GGCTATCTGGTGCCAGTTCTGTTCATTGGATTTACCATCGGCGTGGGGAAAATCAGCTAT
CCGCTGTTGAATTTTAGTTGGGCTCTCACTGCGCTATTCCTGCTTAGCTATAACCGCAAT
TTTCTGCAGGGCGTGGGCTCAGAATATTCGCTGGCATTTATCCTTTCAGTTCTCATTTCT
TTTAGTATTTGTTTGTTGTACATGGCGCGGATTAATCAGCGTAGCGAATGGTTGAATCGA
CAGTGGCACTCGCAGGCATTAACCGATCCGTTGACGCAACTGCCTAACCTGCGTGCGCTG
GAGCAGTTTCTTTTACAGGATGCCGGGCAAAGCGTGTGTTATCTACGCATGGAAAACCTG
GAATTTCTGAGCCGTCATTACGGTATGCAGATGCGCGTGCATTGCGAACGCGAGGTGTTT
CGCGTGTTGCAGCCGCTGCTGCTGGAAAAGGAGAAAATTTTCCATCTGCCGGGCAGTGAG
TTATTGCTGGTATTGACGGGACCGGAGACGGAAGCCCGCCTGCAATATATGCTCAATGTG
CTTAATAACCGCAAAATTTACTGGAACAATACCGGTCTGGACATGGAATACGGCGCATCG
TGGGGAACATTCGATGGTCGCCAGGAAACCTTGCAACCGCTGTTGGGGCAATTAAGCTGG
CTGGCGGAGCAATCCTGTTCGCACCGTCGGGTGCTTGCACTGACCCAGAGTATTGAAGCC
GCTTCCGGGCAGACGACGGAACGGGTGCTGCGGTTGCAGAAAATCCGTCAGGCGCTGGAG
CGCGGCGCGCTGGTTTTGTATGCGCAACCCATTCGCGACTCGCAGGGAAAAGGGTATGAC
GAGATTCTGACGCGGCTTCGGTGCGACGACGGTATCATGATGCCCAATCAGTTCATTCCG
CTTATCGCCCAGTTTAACCTGAGCGTACGGTTTGATATGCAGGTGATGGAAGCGCTCCTG

CAATGGTTGTCCGCGCATCCTTCGGCGGAGCAGGGGGCGCGTTTCTCGGTTAATTTGATG
CCGCTGACGCTGCTGCAAAAAGAGACGGCGTCGCGCATTATGCAGCTTTTTAAACGCTAT
GGCGTCCCGCCTGCTTCGGTCATCATCGAAATTACTGAAGAACAGGCCTTCTCTCATTCC
GAAATCAGTATGCACAATATCAACCAGCTGCGTAAGTTTGGCCTAAAAATCGCCATTGAT
GATTTCGGCACCGGCTATGCAAACTATGAACGGCTAAAACGCCTCAAGGCGGACATCATT
AAAATCGACGGTTGTTTCGTAAAAGATATTCTGACGGACTCCCTGGATGCGATGATCGTG
AAATCCATCACTGATTTAGCGAAAGCGAAATCGTTGAGCGTCGTGGCTGAGTTTGTTGAA
ACGCCTGCGCAGCGCGATCTGCTATTGCAGCTTGGCGTACACAGTTTGCAGGGATATCTG
ATTGGCCGCCCGCGCCCGTTAGGTAAATAA
>EECEBICD_00936 Nucleoside permease NupC
ATGGACCGCGTCCTTCATTTTGTCCTGGCGCTTGCCGTTGTTGCGGTACTCGCACTGCTG
GTTAGCAGCGACCGTAAAAAAATTCGCATTCGTTACGTCATTCAGTTACTTGTTATCGAA
GTATTACTGGCCTGGTTCTTCCTGAACTCGGATGTTGGCCTTGGCTTCGTAAAAGGTTTC
TCCGAGATGTTTGAAAAACTGCTCGGATTTGCCAACGAAGGGACAAACTTCGTCTTCGGC
AGTATGAACGATCAAGGTTTGGCGTTCTTCTTCCTGAAAGTATTGTGCCCGATCGTCTTT
ATTTCCGCGTTGATCGGTATTCTCCAGCATATCCGCGTTCTGCCAGTGGTCATCCGCGCT
ATTGGTTTCCTGTTGTCCAAAGTAAACGGTATGGGCAAACTGGAGTCTTTTAACGCCGTC
AGCTCGCTGATCCTCGGCCAGTCTGAGAACTTTATCGCTTATAAAGATATCCTCGGCAAA
ATGTCCCGTAACCGCATGTACACCATGGCGGCGACCGCAATGTCTACCGTTTCCATGTCT
ATCGTGGGCGCGTATATGACCATGCTGGACCCTAAATATGTGGTCGCAGCGCTGGTTCTG
AACATGTTCAGCACCTTTATCGTTCTGTCGCTGATCAACCCCTATGTCGTTGATGCCAGC
GAAGAAAATATTCAGATGTCGAACCTGCATGAAGGCCAAAGTTTCTTCGAAATGCTGGGC
GAGTACATTCTGGCAGGTTTTAAAGTGGCGATTATCGTCGCGGCGATGCTGATCGGCTTC
ATCGCGCTGATCGCGGCGCTGAACGCGCTGTTCGCAACAGTTACCGGTTGGTTCGGCTAC
AGCATCTCTTTCCAGGGCATTCTGGGTTACATCTTCTACCCGGTCGCATGGGTAATGGGC
GTACCGTCCAGTGAAGCGCTGCAGGTAGGAAGTATCATGGCGACCAAACTGGTTTCCAAC
GAATTCGTGGCGATGATGGATCTGCAGAAAATCGCCGCTACCCTCTCTCCACGCGCTGAA
GGCATTATCTCCGTCTTCCTGGTGTCCTTCGCGAACTTCTCTTCCATCGGTATTATTGCG
GGCGCGATTAAAGGCCTGAACGAAGAGCAAGGCAACGTGGTTTCCCGTTTTGGTCTGAAA
CTGGTTTACGGTTCTACGCTGGTGAGCGTACTGTCCGCCTCTATCGCGGCGCTGGTACTG
TAA
>EECEBICD_00937 Divalent metal cation transporter MntH
ATGACTGACAATCGCGTAGAGAATAGCAGCGGACGGGCGGCGCGCAAGCTAAGGCTCGCA
TTAATGGGACCTGCGTTCATTGCCGCGATTGGTTATATCGATCCTGGTAACTTCGCGACC
AATATTCAGGCCGGCGCCAGCTTTGGCTACCAGCTACTCTGGGTGGTGGTCTGGGCGAAC
CTGATGGCGATGCTGATTCAAATCCTCTCGGCAAAACTCGGAATCGCCACGGGCAAGAAC
CTGGCGGAGCAAATTCGCGACCATTATCCGCGCCCGGTAGTCTGGTTTTACTGGGTACAG
GCGGAAATCATCGCGATGGCCACCGATTTAGCTGAATTTATCGGCGCGGCAATAGGTTTT
AAGCTGATCCTGGGCGTCTCTTTATTACAGGGCGCGGTATTGACCGGTATCGCGACGTTT
CTGATTTTAATGTTACAACGCCGCGGTCAAAAACCGCTTGAAAAAGTCATCGGCGGTTTA
CTGCTTTTTGTCGCCGCTGCTTACATTGTGGAGCTGTTTTTCTCTCAGCCTGATATGGCG
CAGCTTGGCAAAGGAATGGTTATCCCGGCGCTACCGAACCCGGAAGCCGTATTCTTAGCC
GCCGGCGTGCTTGGCGCGACGATTATGCCGCACGTGATTTATCTGCACTCGTCATTAACA
CAGCATCTGCACGGCGGAACGCGGCAACAACGGTACTCAGCAACGAAATGGGATGTCGCT
ATTGCCATGACGATTGCCGGTTTTGTCAATCTGGCAATGATGGCGACCGCCGCGGCGGCG
TTTCATTTTAGCGGCCACACTGGCATCGCCGACCTCGATCAGGCATACCTGACGCTGGAA
CCGTTACTAAGCCATGCGGCGGCAACGGTGTTTGGTCTTAGTCTGGTAGCGGCAGGGCTT
TCCTCCACCGTGGTAGGGACTCTGGCGGGGCAGGTGGTGATGCAAGGGTTTGTCCGTTTT
CACATTCCGCTGTGGGTACGGCGCAGCATTACCATGCTGCCCTCATTTATTGTGATTTTG
ATGGGGCTGGACCCTACCCGAATTCTGGTGATGAGCCAGGTGCTGCTGAGTTTTGGTATT
GCGCTGGCGCTGGTGCCGCTCCTGATTTTCACCAGTAACGCTACGCTAATGGGCGAGCTG
GTGAACACGCGGCGAGTAAAACAGATTGGCTGGATAATTGTCGTGCTGGTCGTCGCCCTG
AATATCTGGTTGCTGGTTGGAACGGTGATGGGATTGTCATAA
>EECEBICD_00938 hypothetical protein

ATGTTCAGGTCACTGATTCTGGCGGCAGCCTTGCTGGCTTTTACACCGCTTGCCGCGAAT
GCGGGCGAAATCACCCTGTTGCCCTCAATAAAATTACAGATTGGCGATCGCGATGATTAC
GGTAACTACTGGGACGGTGGTCGCTGGCGCGACCGGGATTACTGGCACAACCATTATGAA
TGGCGTAAAAATCGCTGGTGGCGCCATGACAACGGCTATCATCGCGGCTGGGATAAGCGT
AAAGCGTATGAACGCGGTTATCGGGAAGGCTGGCGCGATCGCGACGATCATCGCGGGCGA
GGCCGGGGTCACAAACATCATCACTAA
>EECEBICD_00939 L-glyceraldehyde 3-phosphate reductase
ATGATTTATCAGCCCGATGAGAATCGTTATCACACAATGGAGTATCGTCGCTGCGGACGC
AGCGGCGTCAAACTTCCCGCTATTTCGCTGGGGTTATGGCACAACTTCGGCGATACGACA
CGGGTAGAAAATAGCCGGGCGCTGCTGCAACGCGCGTTCGATCTGGGCATTACCCACTTT
GATCTCGCCAATAATTATGGGCCGCCGCCGGGCTCCGCAGAATGCAATTTTGGCCGCATT
CTGCAGGAAGATTTTTTACCGTGGCGTGATGAATTGATCATCTCCACCAAAGCGGGCTAC
ACCATGTGGGATGGCCCTTATGGCGACTGGGGTTCACGGAAATACCTTATCGCCAGCCTC
GATCAAAGCCTGAAACGTATGGGGCTGGAGTATGTCGATATCTTTTATCACCATCGTCCC
GATCCCGAAACGCCGCTTAAAGAGACGATGAAAGCGTTAGATCACCTTGTGCGCCACGGC
AAAGCGTTGTACGTGGGGATCTCTAACTATCCTGCCGATCTGGCCAGACAAGCTATCGAT
ATTCTGGAGGATCTCGGCACGCCTTGCCTGATTCATCAGCCTAAATATTCGCTTTTTGAG
CGTTGGGTAGAGGACGGGCTGCTGGCGCTGTTGCAGGAAAAAGGCGTCGGCAGTATTGCC
TTCTCGCCGCTGGCGGGCGGGCAGCTCACTGACCGTTATCTGAATGGCATTCCGGAAGAT
TCCCGCGCGGCGAGCGGAAGCCGTTTCCTTAAACCAGAACAGATTACCGCCGACAAACTG
GAAAAAGTTCGCCGGTTGAATGAACTGGCCGCGCGACGGGGCCAAAAATTGTCTCAGATG
GCGCTCGCCTGGGTGCTGCGTAACGATAACGTTACCTCGGTACTGATTGGCGCCAGCAAA
CCGTCGCAAATCGAAGACGCCGTAGGAATGCTGGCCAATCGCCGTTTTTCGGCGGCGGAA
TGTGCAGAAATTGACGCTATTCTGGAGGGCCGGTTTTAG
>EECEBICD_00940 Indole-3-pyruvate decarboxylase
ATGCAAAACCCCTATACCGTGGCCGACTATTTGCTGGACAGACTGGCAGGATGCGGCATT
GGCCATCTTTTTGGCGTACCGGGCGATTATAACTTGCAGTTTCTTGACCATGTGATTGAC
CACCCGACCCTGCGTTGGGTGGGATGCGCCAATGAGCTGAACGCCGCTTATGCCGCGGAC
GGCTATGCGCGCATGTCGGGCGCTGGAGCGCTACTCACCACCTTTGGCGTGGGAGAACTT
AGCGCTATTAACGGTATCGCGGGCAGTTACGCGGAATATGTCCCGGTCTTGCATATCGTC
GGCGCGCCCTGTAGCGCTGCGCAGCAGCGTGGCGAATTGATGCACCATACCCTCGGTGAC
GGCGATTTTCGTCATTTTTATCGCATGAGTCAGGCGATATCCGCTGCCAGCGCAATATTA
GATGAACAGAACGCCTGTTTCGAGATTGACCGCGTGTTGGGTGAAATGCTTGCCGCACGC
AGGCCAGGATACATCATGTTGCCCGCCGATGTGGCGAAAAAAACGGCCATCCCACCTACG
CAGGCGCTGGCGTTGCCCGTGCATGAAGCGCAAAGCGGCGTGGAGACGGCTTTTCGTTAC
CACGCCCGTCAGTGCCTGATGAACAGTCGGCGTATTGCGCTATTGGCCGACTTTCTTGCC
GGGCGTTTTGGTTTACGACCACTGTTGCAGCGCTGGATGGCGGAAACGCCCATCGCTCAT
GCGACACTACTGATGGGGAAGGGGCTTTTTGATGAACAGCACCCGAACTTCGTTGGCACC
TATAGCGCTGGCGCCAGCAGCAAAGAAGTGCGTCAGGCCATAGAGGACGCCGATAGGGTT
ATCTGCGTCGGCACCCGTTTTGTCGATACCCTTACGGCCGGATTTACCCAACAATTACCG
ACGGAACGCACGCTGGAGATTCAGCCTTACGCGTCGCGCATCGGCGAAACCTGGTTCAAC
CTCCCGATGGCGCAGGCGGTGTCTACGCTGCGCGAACTGTGCCTGGAATGCGCTTTTGCG
CCGCCGCCGACGCGTTCCGCCGGACAGCCAGTGCGGATTGATAAGGGAGAACTGACCCAG
GAAAGCTTCTGGCAAACCTTACAGCAGTATCTCAAACCCGGCGATATTATCCTTGTCGAC
CAGGGGACCGCCGCCTTTGGCGCTGCCGCGCTGTCGCTTCCTGACGGCGCGGAAGTTGTG
GTACAGCCGCTGTGGGGGTCTATCGGCTATTCCTTGCCCGCCGCGTTTGGCGCGCAAACC
GCCTGTCCCGATCGGCGGGTGATTCTGATTATCGGCGATGGCGCGGCGCAGCTCACGATT
CAGGAGATGGGCTCGATGTTACGCGACGGGCAGGCGCCGGTCATCCTGCTGCTCAACAAT
GACGGCTATACCGTAGAGCGCGCCATTCACGGCGCGGCCCAGCGGTATAACGACATCGCG
AGCTGGAACTGGACGCAGATACCACCGGCGCTAAACGCGGCGCAACAGGCGGAGTGCTGG
CGGGTGACGCAGGCTATCCAACTGGCAGAGGTCCTCGAACGTCTGGCGCGCCCACAACGT
CTGTCATTTATTGAAGTGATGTTGCCAAAAGCCGATCTGCCGGAATTACTGCGTACCGTG
ACCCGGGCGCTGGAAGCCCGCAACGGGGATAA
>EECEBICD_00941 Putative ion-transport protein YfeO

ATGTTCCACCCGCGTGCCCGAACGATGCTGCTGCTGTCGCTCCCCGCGCTGATTATTGGC
GTAGCGTCAAGCCTGGTTTTAATCGCTGCGATGAAAGTCGCGTCGGTTTTTCAGCAATTT
CTTTGGCAACGACTGCCCACCAGCATCGGCATCGCTTATGATTCGCCGTTCTGGATAGTG
GGTATGCTCACGCTAACCGGGATCGTGGTGGGTTTGATTATCCGTTACAGTCCCGGACAC
GCTGGCCCCGATCCGGCCATCGAACCGTTAATCAGTATGCCGGTCTCGCCATCTGCGCTA
CCCGGGCTGCTTCTCGCCTTAATCATCGGCCTTGCTGGTGGGGTAAGTTTGGGGCCAGAG
CATCCGATAATGACAATAAATATCGCGCTGGCGGCGGCTTTCGGCTCCCGCCTGTTTCCG
CGTATCACGGCGTTAGACTGGACGATTCTGGCCTCAGCGGGCACCATCGGCGCGCTGTTC
GGCACGCCCGTCGCCGCAGCGCTGATTTTTTCGCAAACACTTAGCGGCTCTAACGATATT
CCCATGTGGGATCGCCTGTTTGCGCCGTTAATGGCGGCAGCGGCGGGGTCACTCACCACC
AGTCTGTTCTTTCATCCGCATTTTTCGTTACCCATCGCCCATTACACGCAGATGCGGCTG
GTGGACATTGCCAGCGGAGCGATCGTCGCGGCGATCGCCATTGCCGCCGGGATGGTCGCC
GTCTGGTGCCTTCCGCGTCTGCATGAGTTGCTGCACCGCCTGAAAAATCCGGTCCTGATT
CTCGGCATTGGCGGTTTTATACTGGGCATCCTGGGCGTCATTGGCGGGCCGCTTACCCTG
TTTAAAGGCCTGGACGAAATGCAACAAATGGCGTTTAGCCAGACGTTGGGCGCGGGAGAT
TATTTTACCTTAGCCGTCGTCAAACTCGCCGCGCTGGTCATCGCGGCGGCGAGCGGCTTT
CGCGGCGGGCGCATTTTTCCGGCTGTGTTTATCGGCGCGGCGCTGGGGCTCATGCTCCAT
GCCCATGTTGAGGCCGTACCCGCGGCGATTACCGTCTCCTGCGCGATTCTTGGCTTAGTG
CTGGTAGTGACGCGCGACGGCTGGCTCAGTCTGTTTATGGCGGCAGTCGTCGTACCGGAT
ACCAATCTGCTGCCGCTGCTCTGCATTGTGATGCTCCCCGCCTGGCTATTGCTGGCGGGA
AAACCGTTGCTGGCCGCCAACCGTCACGAACCCTAA
>EECEBICD_00942 Glucokinase
ATGACAAAGTATGCTTTAGTAGGAGATGTAGGCGGCACGAATGCGCGTCTTGCCCTGTGT
GATATCGCCAGTGGAGAAATCTCGCAGGCCAAAACGTATTCCGGCCTGGATTATCCCAGT
CTTGAGGCCGTGGTGCGCGTTTATCTCGATGAGCATAGCGTCAGCGTGGAAGATGGTTGT
ATCGCCATAGCCTGCCCGATTACCGGTGACTGGGTAGCGATGACTAACCATACCTGGGCT
TTTTCTATTGCGGAAATGAAAAAAATCTCGGCTTTAGCCATCTGGAAATCATCAACGATT
TCACCGCCGTGTCGATGGCGATCCCGATGCTGA
>EECEBICD_00943 Glucokinase
ATGGCGATCCCGATGCTGAAAAAAGAGCATTTAATTCAGTTCGGCGGCGGCGAACCGGTA
GACGGCAAACCTATTGCGGTGTATGGCGCGGGAACCGGCTTGGGCGTGGCACATCTGGTG
CATGTGGATAAGCGTTGGATTAGCCTTCCGGGCGAAGGCGGGCATGTCGATTTTGCGCCT
AATAGCGAAGAAGAAGCTATGATTCTGGAAATATTGCGCGCCGAAATTGGCCACGTTTCC
GCCGAGCGCGTGCTGTCTGGCCCGGGGCTGGTGAATCTTTACCGGGCGATTGTTAAGTCC
GACAACCGTCTGCCGGAAAATCTGCGCCCGAAAGATATTACCGAACGCGCCCTGGCGGAT
AGCTGTATCGATTGTCGCCGCGCGTTGTCGCTCTTTTGCGTCATCATGGGGCGATTTGGC
GGCGATCTGGCGTTAACCATGGGGACATTTGGCGGCGTTTATATCGCTGGCGGTATTGTC
CCGCGCTTCCTGGAATTTTTTAAAGCGTCCGGCTTCCGTGGTGGCTTTGAAGATAAAGGC
CGCTTTAAAGATTATGTACACGGTATTCCGGTCTATCTGATTGTCCATGATAACCCTGGC
TTGTTAGGCTCCGGCGCGCATCTGCGCCAGACATTAGGCCATATTCTATAA
>EECEBICD_00944 Glutamate-pyruvate aminotransferase AlaC
ATGGCTGACTTCCGCCCTGAACGTCGCTTTACGCGTATCGATCGCCTTCCGCCCTATGTT
TTTAACATCACCGCTGAACTCAAAATGGCTGCGCGTCGGCGCGGTGAAGATATTATTGAT
TTCAGTATGGGTAATCCTGATGGCGCAACCCCTCCCCACATTGTCGAAAAACTTTGCACC
GTCGCGCAGCGTCCGGATACGCACGGTTATTCCACGTCCCGCGGCATACCGCGTTTACGC
CGCGCGATTTCTCACTGGTATCAGGAACGTTATGATGTCGATATCGATCCGGAAACCGAA
GCGATTGTGACCATCGGTTCCAAAGAGGGACTGGCGCATTTAATGCTGGCGACGCTGGAT
CATGGCGATACGGTACTGGTGCCGAACCCCAGTTACCCGATTCATATCTATGGCGCCGTT
ATCGCCGGGGCGCAGGTGCGTTCGGTTCCATTGGTAGAGGGAGTGGATTTCTTCAACGAG
CTGGAGCGCGCTATTCGCGAAAGTTATCCGAAACCGAAAATGATGATTTTGGGTTTTCCG
TCTAACCCTACGGCCCAGTGCGTCGAACTGGAATTTTTTGAAAAAGTGGTCGCTCTGGCT
AAACGTTATGATGTGCTGGTGGTGCACGATCTGGCGTATGCCGATATCGTGTACGACGGC
TGGAAAGCGCCATCCATTATGCAGGTGCCCGGCGCGCGCGACGTGGCGGTTGAATTTTTC
ACGCTGTCGAAAAGCTATAATATGGCGGGCTGGCGTATTGGGTTTATGGTGGGCAATAAA

ACCCTGGTCAGCGCGCTGGCGCGCATTAAAAGTTATCACGATTATGGCACCTTTACCCCG
CTTCAGGTGGCGGCTATCGCCGCTCTGGAAGGCGATCAGCAGTGCGTGCGCGATATCGCC
GAGCAGTATAAACGTCGTCGCGATGTGCTGGTGAAGGGGCTGCATGAGGCGGGCTGGATG
GTTGAAATGCCGAAAGCCTCAATGTACGTCTGGGCGAAAATCCCGGAGCAATATGCGGCG
ATGGGATCGCTTGAGTTTGCCAAGAAGCTGCTGAATGAGGCGAAGGTTTGCGTTTCTCCA
GGGATAGGCTTTGGTGATTATGGCGACACCCATGTACGCTTTGCATTAATAGAGAATCGC
GACCGCATTCGCCAGGCCGTCAGAGGGATTAAAGCGATGTTCCGCGCCGATGGCGTGCTG
CCGTCACATCCAAAACCCGTTGAAGCGAGCACGGAGTAG
>EECEBICD_00945 Lipid A biosynthesis palmitoleoyltransferase
ATGTTTCCTCAAAGCAAATTTTCACGCGCGTTTTTGCACCCGCGCTACTGGCTGACGTGG
TTTGGCGTTGGCGTCCTCTGGTTACTGGTGCAGCTTCCCTACCCGGTTTTGCGTTTTCTG
GGCACCCGCACCGGTAAACTGGCGCGCCCCTTCCTGAAGCGACGTGAGTCAATCGCCCAG
AAAAATATTGAACTCTGTTTTCCGACCCTCTCCCGGGAAGAAAGGGAAAAACTGATCGCC
GAGAATTTCCACTCTCTTGGCATGGCGCTGCTTGAAACGGGCATGGCCTGGTTCTGGCCT
GATAACCGGGTTCGCAAATGGTTTGATGTGGACGGGCTGGACAACCTTACCCGTGCGCAG
GAGCAAAATCGCGGCGTAATGGTCGTTGGCGTGCATTTTATGTCGCTGGAATTGGGAGGC
CGGGTAATGGGTCTCTGCCAGCCGATGATGGCGACCTATCGTCCGCATAACAATCCGCTC
ATGGAGTGGGTGCAGACGCGCGGTCGTATGCGCTCAAATAAAGCGATGATCGGCAGAAAC
AATCTGCGCGGCATTGTGGGCGCGCTAAAAAAAGGGGAAGCCGTTTGGTTTGCTCCCGAC
CAGGACTACGGCCCTAAAGGCAGCTCCTTTGCGCCATTTTTCGCGGTTGAAAACGTCGCC
ACCACCAACGGCACCTATGTACTGTCCAGGCTGTCTGGCGCCGCCATGTTGACCGTTACA
ATGGTAAGGAAATCCGATAACTCGGGTTATCGTCTGTATATTACCCCTGAAATGGAAGGT
TACCCGGCAGATGAAAATCAAGCCGCTGCCTATATGAATAAAATTATTGAGAAAGAGATC
ATGCGCGCCCCGGAGCAATATCTCTGGATCCATCGCCGTTTCAAGACTCGCCCGTTGGGG
GAAGCGTCACTCTACATCTGA
>EECEBICD_00946 hypothetical protein
ATGTACCACCAGTTATTTACTCGCGCCCAGAAATTCTTTATCAAGAAGCGTAAATATTTC
TCCCGTAGCAAAAATAAGCTAATCATTATTTTTAATGCATTGTTTTTACAGTGA
>EECEBICD_00947 hypothetical protein
ATGATCTATTTATGGACATTTCTTGCAATAAGTATACTTGCCGTGAGTGGCTATATCGGT
CAGGTGATGGGGGCTTTTTCTGCGGTTTCTTCTTTTACGGGAATGGTTATCCTTGCCGCG
CTGATTTATTTACTCAATGTATGGTTGCAGGATGGCGATGACATCGTTAGCGGATTATTG
CTTTTTCTCGCACCGGCATGTGGGTTAATTATTCGCTTTATGGTGGGATACGGTAAGCGT
TAG
>EECEBICD_00948 Phosphoglycerate transporter protein
ATGTTAACAATATTAAAAACAGGGCAATCGGCGCATAAAGTCCCACCGGAAAAAGTCCAG
GCCACATATGGTCGATATCGTATACAGGCATTGTTAAGCGTATTTCTGGGATATCTCGCT
TACTATATAGTCAGAAACAATTTCACATTATCGACGCCTTATTTAAAAGAGCAATTGGAT
CTCAGCGCTACGCAAATCGGCCTGCTGAGTAGCTGTATGCTTATTGCTTACGGAATCAGT
AAAGGCGTAATGAGCAGTCTGGCGGATAAAGCCAGCCCAAAAGTCTTCATGGCGTGCGGT
CTGGTACTCTGCGCGATTGTTAACGTTGGGCTGGGATTCAGTAGCGCATTCTGGATATTT
GCCGCTCTGGTGGTCTTCAATGGCCTTTTTCAGGGCATGGGCGTCGGCCCCTCGTTTATT
ACTATTGCAAACTGGTTCCCTCGTCGGGAACGCGGACGCGTAGGCGCCTTCTGGAATATC
TCGCATAACGTTGGCGGCGGGATCGTCGCGCCTATCGTCGGTGCGGCTTTTGCGATATTG
GGCAACGAACACTGGCAAAGCGCCAGTTATATCGTTCCGGCCTGCGTCGCCGTCATTTTT
GCCTTAATTGTTCTGGTCTTGGGAAAAGGTTCGCCACGCGAAGAAGGTCTTCCCTCACTG
GAACAGATGATGCCGGAAGAAAAAGTCGTACTGAAAACGAAGAATACGGCAAAAGCGCCG
GAAAATATGAGCGCATGGCAAATCTTCTGTACTTATGTGCTGCGCAATAAAAATGCCTGG
TATATTTCGCTGGTGGATGTCTTCGTCTATATGGTGCGGTTTGGCATGATTAGCTGGTTG
CCTATCTATCTGTTGACAGTAAAACACTTTTCAAAAGAACAGATGAGCGTCGCGTTTCTC
TTTTTCGAGTGGGCGGCGATTCCCTCCACGCTACTGGCAGGCTGGCTGTCAGATAAACTG
TTTAAGGGCCGCAGAATGCCGTTAGCCATGATTTGCATGGCGCTGATTTTTGTCTGTCTG
ATCGGATACTGGAAAAGTGAATCTTTGCTGATGGTAACGATTTTCGCCGCCATTGTAGGT
TGTCTGATTTACGTCCCGCAGTTCCTCGCGTCCGTACAGACAATGGAGATAGTCCCCAGC

TTTGCCGTAGGTTCCGCCGTCGGTTTACGTGGGTTCATGAGCTATATTTTCGGCGCCTCG
TTGGGCACCAGCCTGTTTGGTGTAATGGTGGATAAACTTGGCTGGTACGGCGGATTTTAT
CTTCTGATGGGCGGCATCGTGTGCTGCATTCTATTCTGTTACCTCTCCCATCGCGGCGCG
CTGGAACTGGAACGCCAGCGCCAGAATGCGTTACATAATCAGGACTCACTGCAGCTTGCG
GACGCGCAATAA
>EECEBICD_00949 Phosphoglycerate transport regulatory protein PgtC
ATGAACGGGATCACAAATCGACTTTTCTCATGCTGTAGTCGTTTCGCCAGAGTGGGCGCT
TTTACGCTATGGCTGGCGGCAATGTTCGGTTCTTGCCAGGCGTACAGCCGCGAGCTGGTG
ATGGCAACGACCTTTTCACCCAGCGCGACCGCCTGGATTATCCAGCGCTGGCAGACGGAG
CCGGGTTCCGTCATGATTCGTACGCTCAACCGTACCAGCGGTTCTTTAGAGCAATTACTT
GATACGGCGAATGCGGAAAATGTCGATCTGATCCTGACGTCATCCCCCATGCTGTTGCAA
CACCTTCAGGAGCACCAAAAGCTGGCGCTATTAGATAGCGCGCCTGCCGCCAGCCAGAAG
CTGGTGCCGCGTTCTATCCGCTCAACTTCAGTGGCGGTAGCGGTATCCGGATTTGGGCTA
TTGATCAACCGTTCAGCGCTCGCTGCGCGCCATCTTCCGCCGCCTGCTGACTGGCAGGAT
ATGGGCCTGCCCAGTTACCAGGGCGCTCTATTAATGAGCAGTCCGTCACGTTCAGACACG
AATCATTTGATGGTGGAGTCGCTGTTGCAGCAAAAAGGCTGGACGGCAGGATGGGCGACG
CTGCTGGCAATCTCGGGTAATTTGGTCACGATTTCCTCTCGCAGTTTCGGCGTAGCGGAT
AAAATCAAAAGCGGGCTTGGCGTTGCCGGGCCGGTCATTGATAACTATGCTAATTTGCTA
CTTAACGATCCTAATCTTGCTTTTACCTATTTCCCGTATTCTGCGGTGTCGCCCACTTAC
GTCGCGGTGTTGAAAAACAGCCGTCATGCAGACGAAGCGCGTGCTTTTATTCATTACTTA
TTAAGTCCGAAAGGGCAAAGGATACTGGCTGACGCGAATACCGGTAAGTATCCCGTCGCA
CCGCTTAGCGCCGATAATCCTCGCGCCGCGCAACAACAGCGTTTGATGGCCCAGCCGCCG
TTGAACTATCGTCTGATCCTGAAGCGTCAGCAACTGGTACAACGCATGTTTGATACCGCG
ATCAGTTTTCGTCTCGCGCAGCTTAAAGATGCATGGCGAGCGTTGCATAGCGCAGAAACG
CGGCTTAAACGCCCCTTACCTGAAATCAGAGCCTTATTGACCAGCGTCCCGGTGGATGCG
GCAAGCAGCGAGGATGAAACCTGGCTGGCGCAGTTTGATAATAAAAGCTTTGCCGAACAA
AAAATGATGGAGTGGCAGATCTGGTTTCTGAACAATCAGCGGCTAGCGATTCACAAACTG
GAAGAGCTGAAATGA
>EECEBICD_00950 Phosphoglycerate transport system sensor protein PgtB
ATGAAGGGGAGATTATTGCAACGGCTTCGTCAGCTCAGTATCAGCAACAGTTTACGGGGT
GCATTTTTAACGGGCGCATTACTGACGCTGATTGTCAGTATGGTCAGCCTTTATTCCTGG
CATGAGCAAAGTTCGCAGGTTCGCTATTCGCTTGATGAATATTTTCCTCGCATCCACTCC
GCTTTTTTAATTGAGGGGAACCTGAATCTGGCTGTGGATCAACTCAATGAATTTTTGCTG
GCGCCGAACACGACGGTACGTTTGCAGTTACGTACGCAGATTATTCAGCATCTGGATAAG
ATTGAGCGTCTCAGCCAGGGGCTTCAGCTTGCTGAGCGCCGGCAACTGGCCGTCATTTTA
CAGGATAGCCGGACCTTGCTGGCGGAGCTGGACAACGCCCTCTACAACATGTTTTTGGTG
CGGGAGAAAGTCAGTGAGCTTTCCGCTCGCATCGACTGGCTCCATGATGATTTCACCACG
GAGCTAAATTCGCTGGTACAGGATTTTACCTGGCAGCAAGGGACGCTACTCGATCAAATT
GAGGCGAATCAGGGCGATGCAGCGCAGTATTTGCAACGTTCGCGCGAGGTGCAGAACGAG
CAGCAGCAGGTATATACCCTGGCGCGAATTGAAAATCAGATTGTTGATGACCTGCGCGAC
AGATTGAATGAGCTCAAGTCCGGCAATAATGACGGGATGCTGGTGGAGACCCATATTCGC
TATCTGGAAAACCTAAAAAAAACGGCGGATGAGAATATTCGTGCGCTGGATGACTGGCCC
AGTACGATCACGCTGCGGCAAACCATCGACGAACTATTGGAAATCGGGATGGTAAAAAAT
AAGATGCCCGATACCATGCGCGATTATGTTGCGGCGCAAAAAGCCTTACTGGACGCCAGC
CGCGCCAGAGAGGCTACGCTAGGGCGCTTTCGCACGCTGCTTGAGGCGCAGTTAGGTAGT
AGTCACCAGCAAATGCAGATGTTCAATCAACGCCTGGAACAGATTGTTCGCGTTAGCGGC
GGGCTTATTTTAGTGGCGACGCTCCTGGCATTGCTGCTGGCCTGGGGACTCAACCACTAT
TTTATCCGTTCCCGGCTGGTAAAGCGTTTTACCGCGCTTAATCAGGCTGTGGTACAGATA
GGGCTGGGGAGAACCGACTCGACTATCCCGGTTTACGGGCGCGATGAGCTAGGACGGATA
GCCCGTTTGCTACGTCATACGCTGGGCCAGCTCAATATGCAACGCAGGCAGCTTGAACAA
GAGGTCGCCGAGCGCAAAGAGATAGAAGCCGACCTGCGCGCCATGCAGGATGAGTTGATT
CAGACGGCGAAACTGGCGGTAGTCGGCCAGACCATGACCACGCTGGCGCATGAAATTAAC
CAGCCGCTGAATGCGCTTTCAATGTATTTATTTACGGCGGGACGCGCGATTGAACAAGGT
CAGAGCGGACAGGCGAGAAATACGCTCACCAAAGCGGAAGGGCTCATTAACCGGATTGAC

GCCATTATCCGTTCGCTGCGGCAGTTTACCCGCCGGGCGGAGCTGGAAACGCCATTGTAT
CCCGTTGATCTGCGCCAAACCTTCGTGGCGGCATGGGAGTTATTAGCCATGCGTCACCAA
TCCCGGCAGGGAGCGCTTTCACTGCCAACCGACACCGTATGGGTTAGCGGCGATGAAGTG
CGTATTCAGCAGGTTCTGGTGAATGTGCTGGCGAATGCGCTTGACGCCTGTTCGCATGAT
GCGGCGATCGCGGTGACATGGCAGACGCAGGGAGAGGCGCTGGAGGTTTATATTGCAGAT
AACGGCCCTGGCTGGCCTGTGGCGCTGTTGCCCTCTTTACTCAAGCCGTTTACTACCAGT
AAAGCTGTCGGGTTGGGTATTGGGCTGTCGATTAGCGTGTCGCTGATGGCGCAAATGAAG
GGCGACCTGCGTCTGGCGTCGACGCTGACGCGTAACGCTTGCGTGGTGCTGCAATTCTCT
GTAACGGATGTCGATGATGTTGAATGA
>EECEBICD_00951 C4-dicarboxylate transport transcriptional regulatory protein DctD
ATGATGTTGAATGATGAATGTTCTATTTTGCTGATTGATGATGACGTTGATGTACTTGAC
GCCTACACCCAAATGCTTGAGCAGGCAGGCTACCGCGTGCGTGGCTTTACCCATCCTTTC
GAGGCCAAAGAGTGGGTAGAAGCGGATTGGGAGGGGATCGTTTTGAGCGATGTGTGTATG
CCGGGGTGTTCCGGTATCGATCTTATGACGCTGTTTCATCAGGATGACGATCAACTGCCA
ATCTTATTGATTACCGGACATGGCGATGTACCGATGGCGGTTGATGCGGTGAAAAAAGGG
CGCCTGGGATTTTCTGCAAAAACCCGTCGATCCGGGCAAGCTGTTGATATTAATTGA
>EECEBICD_00952 C4-dicarboxylate transport transcriptional regulatory protein DctD
ATGAATCAGTTTCGACAACGGTTACAACAACTGGCGGAAACGGACATTGCCGTATGGTTT
TACGGTGAGCATGGCACTGGACGTATGACTGGCGCCCGTTATCTCCATCAACTGGGGCGT
AACGCGAAAGGGCCGTTTGTACGCTATGAACTCACGCCGGAGAATGCCGGGCAGTTGGAG
ACATTCATCGACCAGGCGCAAGGAGGTACGCTGGTGTTGAGTCATCCCGAATATCTGACA
CGCGAACAGCAGCACCATCTGGCGCGTTTACAAAGCCTGGAGCATCGGCCTTTTCGTTTG
GTGGGCGTTGGCAGCGCTTCGCTGGTGGAGCAGGCGGCAGCTAACCAGATTGCGGCCGAG
CTTTACTACTGTTTCGCCATGACCCAAATCGCCTGCCAGTCTCTTTCTCAGCGACCGGAT
GATATCGAACCGTTATTTCGCCATTATCTTCGAAAAGCCTGCCTGCGGCTCAATCATCCA
GTGCCGGAAATAGCGGGGGAATTACTGAAAGGAATAATGCGACGCGCCTGGCCAAGCAAT
GTGCGCGAACTGGCTAATGCGGCAGAGCTTTTTGCTGTTGGCGTGCTGCCGCTGGCGGAA
ACGGTCAACCCGCAGTTGCTTCTTCAGGAGCCGACCCCGCTTGACCGGCGCGTTGAAGAG
TATGAGCGACAAATTATTACCGAAGCATTAAATATTCATCAGGGACGAATTAATGAAGTG
GCGGAGTATCTGCAAATTCCCCGTAAAAAACTTTATCTGCGCATGAAAAAATATGGTCTA
AGTAAAGAGCATTATAAATTCTGA
>EECEBICD_00953 Coagulase/fibrinolysin
ATGAAAAAACATGCTATTGCAGTAATGATGATCGCCGTATTTTCTGAGTCGGTTTATGCG
GAGTCTACCTTATTTATTCCGGACGTCTCTCCTGATAGCGTCACGACATCCCTTTCTGTG
GGGGTGTTAAATGGTAAATCCAGGGAGCTGGTTTATGATACCGACACCGGGCGGAAGCTG
AGTCAACTGGACTGGAAAATAAAAAATGTCGCCACGTTGCAGGGGGATTTATCATGGGAA
CCCTATTCGTTCATGACGCTGGACGCCCGCGGCTGGACGTCTTTGGCGTCGGGATCGGGT
CATATGGTTGACCATGACTGGATGAGCAGTGAGCAGCCTGGCTGGACCGATCGTTCAATT
CATCCGGACACCAGCGCCAACTATGCTAATGAATACGATTTGAATGTGAAAGGTTGGTTA
TTGCAGGGCGATAACTACAAGGCGGGCGTGACAGCGGGCTATCAGGAAACCCGTTTTAGC
TGGACGGCAAGAGGCGGGTCTTATATTTATGATAATGGTCGGTATATTGGTAACTTTCCT
CATGGCGTGCGCGGTATAGGTTATAGCCAGCGTTTCGAAATGCCCTATATCGGGCTGGCG
GGTGATTATCGTATTAATGACTTTGAGTGTAATGTACTGTTTAAATACAGCGACTGGGTA
AATGCGCATGATAATGACGAACACTACATGCGCAAACTTACCTTCCGTGAAAAAAACGGAA
AATTCACGATATTATGGCGCGTCTATTGACGCCGGATATTATATTACCAGTAATGCAAAA
ATCTTTGCTGAGTTCGCTTACAGTAAATATGAAGAAGGTAAGGGCGGTACGCAAATCATA
GATAAAACCAGCGGTGATACGGCGTATTTTGGTGGCGATGCCGCAGGTATAGCTAATAAT
AACTATACGGTTACCGCGGGGTTGCAGTACCGCTTCTAG
>EECEBICD_00954 IS256 family transposase ISEc39
ATGGACGAAAAGAAACTCACAGCTCTTGCGGCTGAACTGGCTAAAGGTCTTAAAACTGAA
GCCGACCTCAGTCAGTTTTCCCATATGCTGACGACATTAACCGTCGAAACGGCGCTCAAT
GCTGAGCTGGCTGACCATCTCGGGCATGAGAAAAATGCTCCTAAAACGAGCTCAAACTCC

CACAATGGCTACTCATTAAAAACGGTGCTGTATGATGGCGGCGAGATAGTGCTGAACATG
CTGCGTGACCGTGAAAATACCTTTGAACTGCAGCTGATTAAGGAGCATCAGACGCGTATT
ACGAGATGGACAGTCAGATTTATCCCTTTATGCCAAAGGCATGACATGACTACCCGCGAA
ATCATCGCCACCTTCAAAGAGATGTACAACGCTGATGTGTCGCCCATGCTGATACTTAAG
ATCACCGCCGCAGTAAAAGAGTTGGTCAAAGGGGCGTCAAAAAAATGGAGTATGCCGATCC
AGGACTGGTCGCTGGCAATGA
>EECEBICD_00955 hypothetical protein
TTGGAACACTTAAAATACAGACCTGATATAGATGGATTACGCGCAATAGCGGTTTTATCT
GTGGTAATATTCCATTATTTCCCATCATTATTGCCGGGTGGGTTTGTTGGAGTAGATATA
TTCTTTGTGATATCTGGATACCTTATAACATCAATAATATTAAAATCTGCATCAAACAAA
TCATTTTCATACCTTGATTTCTATAAAAGGAGAGTGCTTAGAATATTTCCAGCATTATCC
ATAGTTCTTGTATCATGTCTTATTGTTGGCTGGGTTTATTTATTCCAGGATGATTACAAA
TTACTTGGTAAGCATGTTTTTAGCGGCTCATTCTTTATATCAAACTTTACTCTTTGGAGT
GAGTCTGGCTATTTTGATTCAAAGTCATACCTTAAACCTTTACTACATTTGTGGTCACTG
GGAATTGAAGAGCAATTTTATATAATATGGCCAGTAGTTATATTGCTATGCTTTAGAAGC
AAAAACCATAACAGAAACATAGTATTATCATGCGCAACTATATTTATAATTAGCTATGCG
ATTAGCATTTTTACAATGGCATCTGATGGCGGAGCTAATTACTACTCTCCCGCATCAAGA
TTTTGGGAGTTAATGGCTGGAGCGATTATATCCACATTGAGATTTATAGGAATAAACACT
TCGTTATCAAAATTAATGTCCCTGTTAGGAATTATACTAATCGCATTATCAATAACCATG
ATAGATGAAAAGATGTCATTTCCTGGATATATAGCAATAATCCCAGTACTTGGCGCCTCT
CTTATAATAGCATCTAATGGTAATGATTTAGTTGTGTCGAAATTGCTTAGTGTTAGGCCT
GTTGTTTTCTTTGGTCTTATTAGCTATCCTCTTTATTTGTGGCATTGGCCTATTTATTCA
TTCTATCGTTCAATATTTGCTGGTTCACCAGACTACCATGAATTAATTCTTCTTTTATTA
TCATCGTTCTTTTTGGCGATATTAACTTATTATTTAATTGAAAAACCACTGAGAAATGCC
AGAAATAAATATATCACAGCAATATTATTAGCATTATCAGTATTTGGGACAGGTTTAATT
GGCGCATTTATTTTTCATATAAATGGAGTTAAAGACAGGGAAATCAATAAATCAGCAGGT
GAATATGCTTCTGTTACTGACGTGTACAATTATTATAAATATGGAGAACTACTCCGTGGA
GGGATATGCCACTCAGTACAACTTACTGCTGCCATATCCAATGGATGTATAAAAAAATGGC
AAGCATAATATATTTATCATTGGTGATTCTTATGCGGCGGCTCTTTTCAATGGACTTTCT
CATTATATAGATAATAAAGGTTCTGATTATATAATAAGCCAAATGACAGATGGTAACGCT
CCTCCTCTATTTGTTGACGGTAAAGATGATTTACAGAGAAGTGTCATCACTCTAAACAAT
AATAGAATTAATGAAATTAAACGTGTTCAGCCTGAGGTGGTTCTGCTGACATGGTCAGTT
CGAGGAACAAATGGAGTACATGATAAAAAGTTAGCAATTGATACGTTATCATTAACCATA
AAAAAAATTAAAGAGGCATCCCCTGACTCAAGGATTATTTTCATTGGACCAGTCCCGGAA
TGGAATGCAAATTTAGTTAAAATAATATCTAACTACCTGAGTGAGTTTAAAAAAACCCCA
CCATTGTATATGACATATGGATTAAATAGTGAAATAAGCGAGTGGGACTCTTACTTTAGT
AACAATGTTCCAAAAATGGGAATTGAATATATATCAGCATACAAAGCATTATGTAACGAA
AGTGGATGTCTTACAAGAGTTGGTAATGGTCCTGATTTTATCACTGCCGTTGATTGGGGA
CATTTAACAAAGCCTGGTTCTGATTTCCTTTTTAATAAAATCGGAAATAAAATAATCAAA
TAG
>EECEBICD_00956 Prophage bactoprenol glucosyl transferase
ATGAAAATCTCTCTTGTCGTCCTAGTTTTTAACGAAGAAGACACGATACCGATTTTCTAT
AGAACGGTACATGAGTTTAATGAACTTGAAAAATATAAAGTTGAGATTATTTTTATTAAT
GACGGAAGTAAAGATGTGACAGAGTCAATAATTAAAATAATAGCCGTATCTGATCCACTC
GTCATTCCGTTTTCGTTTACACGAAACTTCGGGAATGATGCAAGATGCACAACCATTTTA
TTAATCTTTTTTTAA
>EECEBICD_00957 Prophage bactoprenol-linked glucose translocase
ATGTATGGGATGCATACGCATCAGGCGCTGACGAACTTTTCCGATTTTGTTATCGCTGTA
TCGTTCAGCTTCTATGCTAATGCGCGCTTCACCTTTAATGCCAGCACTACTGCAATTCGC
TACATGATGTATATGGGATTCATGGGAGCACTGAGCGCTGTTGTTGGATGGATGGCTGAC
CAATGTTCTTTGCCACCATTGGTTACCCTCATCACTTTCTCGGCAATTAGCCTGGTATGC
GGCTTTATCTATTCCAGATTCTTTATCTTCAGGGATAAAAATGAAAATCTCTCTTGTCGT
CCTAGTTTTTAA
>EECEBICD_00958 tRNA-Arg(cct)

GTCCCCTTAGTTAAATGGATATAACGAGCCCCTCCTAAGGGCTAGTTGCAGGTTCGATTC
CTGCAGGGGACACCA
>EECEBICD_00959 Inner membrane protein YfdC
ATGGATAGCCTTAACGACGATAAAATTAACCGGCAAAGCAGTGACCTGGAAGTTGAAAGC
GAAGAAAAACAAAGCGGTAAAGAGATTGAAGTGGATGAAGATCGTCTTCCTTCCCGCGCC
ATGGCGATTCATGAACATATTCGCCAGGATGGTGAAAAAGAGATGGAACGCGATGCGATG
GCTTTGCTCTGGTCAGCCATTGCCGCAGGACTTTCTATGGGGGCATCACTCCTGGCGAAA
GGGATTTTCCACGTGCAGCTTGAAGGCGTTCCCGGCGGCTTTTTACTGGAAAATCTCGGC
TATACCTTTGGTTTTATCATTGTCATCATGGCCCGCCAGCAATTATTTACTGAAAATACC
GTTACCGCCGTGTTGCCGGTAATGCAAAATCCCACTCTGAGTAACGTTGGCCTGCTGATG
CGCTTGTGGGGCGTAGTCTTATTGGGCAACCTTATTGGCACCGGGGTTGCGGCGTGGGCA
TTTGAATATATGCCTATATTTGATGAAGAGACCCGCGACGCCTTTGTCAAAATTGGTATG
GAGGTCATGAAAAATAGCCCAACGGAGATGTTTGCCAACGCGATTATCTCTGGCTGGATC
ATCGCCACAATGGTATGGATGTTTCCTGCAGCAGGCGGGGCAAAGATTGTGGTCATTATT
TTGATGACCTGGCTTATCGCGCTGGGCGATACCACCCATATTGTCGTCGGTTCCGTTGAA
ATTTTGTATTTGGTTTTCAACGGCACGCTGCCCTGGAGCGACTTTCTCTGGCCCTTCGCC
CTTCCCACACTTGCCGGAAATATCTGCGGCGGCACTTTTATTTTCGCATTGATGAGCCAC
GCGCAGATCCGCAACGATATGAGCAATAAGCGTAAGGAAGAAGCGAGGCTACGCGGCGAG
CGCCTGGAACGGGAGCGGAAAAAAGCGGAAAAACAGCGCTGA
>EECEBICD_00960 Intermembrane phospholipid transport system lipoprotein MlaA
ATGAAGCTTCGCCTGTCGGCGCTGGCGCTGGGAACCACACTGCTGGTCGGGTGTGCGAGT
TCCGGTACAGAACAGCAGGGGCGTTCAGACCCATTTGAAGGGTTTAACCGCACCATGTAC
AACTTCAACTTTAATGTGCTGGACCCGTATGTTGTTCGGCCGGTCGCGGTCGCCTGGCGT
GATTATGTTCCACAGCCTGCGCGTAACGGTTTAAGTAATTTCACCGGCAACCTCGAAGAA
CCGGCGATCATGGTGAACTATTTCCTGCAGGGCGATCCTTATCAGGGTATGGTGCATTTC
ACCCGTTTCTTCCTGAACACCTTATTAGGGATGGGCGGATTTATTGACGTCGCGGGAATG
GCGAATCCTAAGCTGCAGCGCGTTGAACCGCATCGCTTTGGCAGTACGCTGGGCCATTAT
GGCGTGGGGTATGGGCCTTATATGCAACTTCCGTTCTACGGCAGCTTCACGCTGCGTGAA
GATGGCGGGGATATGGCGGATACCTTGTATCCGGTGCTCTCGTGGTTGACCTGGCCAATG
TCCATAGGAAAATGGACTATCGAAGGGATAGAAACCCGCGCGCAGTTGCTGGACTCCGAT
GGTTTGCTGCGCCAGTCTTCCGATCCTTATATTATGGTGCGTGAGGCGTACTTCCAGCGT
CATGACTTTATCGCCAATGGCGGAAAACTCAAGCCGCAGGAAAACCCGAACGCGCAGGCG
ATTCAGGACGAACTCAAAGAGATCGACTCGGAATAA
>EECEBICD_00961 Long-chain fatty acid transport protein
ATGAGCCAGAAAACCCTGTTTACAAAGTCTGCTCTCGCAGTCGCAGTGGCAATCATCTCC
ACCCAGGCCTGGTCTGCAGGCTTTCAGTTAAACGAATTTTCTTCCTCTGGCCTTGGCCGG
GCCTATTCGGGTGAAGGCGCAATCGCAGATGATGCGGGTAACGTTAGCCGCAACCCGGCG
TTAATTACCATGTTTGACCGCCCGACGTTTTCAGCGGGCGCGGTTTATATTGATCCTGAT
GTTAATATTAGCGGGACATCGCCATCTCGTCGGACGTTAGATGCGGATAACATCGCGCCG
ACGGCCTGGGTGCCGAACGTCCATTTTGTCGCCCCGATTAACGATCAGTTTGGCTGGGGG
GCTTCTATTACCTCTAACTATGGTCTGGCGACAGAGTTTAACGACACATACGCCGGCGGC
TCGGTTGGCGGTACGACCGATCTTGAAACCATGAACCTGAATTTAAGCGGCGCTTATCGC
TTAAATGAGGCCTGGAGCTTCGGCCTTGGCTTTGATGCGGTATACGCGCGTGCGAAAATC
GAACGTTTTGCGGGCGATTTAGGCCAGTTGGTCGCGGCACAGAATCCGGCGCTGGCGCCG
GTCGCGGGTCAGATTCCCAGCGACACCAAGATTGCCCACCTGAACGGTAATCAGTGGGGC
TTTGGCTGGAATGCCGGTATCCTTTATGAACTGGATAAAAATAACCGCTACGCGCTGACC
TACCGCTCGGAAGTCAAAATTGACTTTAAAGGCAACTACAGCAGCGATCTTCCCATCGCT
ATAAACCGATTTAACCTGCCTATCCCAACGGCAACCGGCGGCGCGACGCAGTCAGGCTAC
CTGACGCTGAATCTGCCGGAAATGTGGGAAGTGTCCGGTTATAACCGTGTGGCGCCACAG
TGGGCCATCCACTATAGCCTGGCCTATACCAGCTGGAGCCAGTTCCAGGAATTGAAGGCA
AAATCGACCGCCGGCGATACGCTCTTTGAGAAGCATGAAGGCTTTAAAGACGCTTATCGC
ATTGCGTTGGGTACCACCTATTACTACGATGACAACTGGACGTTCCGTACCGGTATCGCC
TTCGATGACAGCCCGGTACCAGCGCAAAACCGTTCTATCTCTATTCCGGACCAGGACCGT

TTCTGGCTGAGCGCCGGTACGACTTACGCCTTTAATAAAGATGCGTCTGTCGACGTTGGC
GTCTCTTATATGCATGGTCAGAGCGTGAAAATTAATGAAGGCCCTTATCAGTTTGAATCT
GAAGGGAAAGCCTGGCTGTTCGGTACTAACTTTAACTACGCGTTCTAA
>EECEBICD_00962 hypothetical protein
ATGAGTAAATGCAGTGCTGATGAAACCCCGGTTTGCTGCTGTATGGATGTTGGAACCATC
ATGGACAACTCCGATTGCACCGCGTCATACAGCCGCGTATTCGCTACCCGCGCAGAGGCT
GAAGGGACGCTGGCGGCGTTAACCGAAAAAGCGCGTAGCGTGGAGTCTGAGCCTTGCCAA
ATTACGCCAACCTTTACCGAGGAATCCGAAGGCGTTCGTCTGGATATTGATTTTGTTTTC
GCCTGCGAAGCAGAAACGCTGATCTTCCAGCTCGGCCTGCGTTAA
>EECEBICD_00963 3-ketoacyl-CoA thiolase FadI
ATGCGTCAGGCTTTACCGTTGGTCACCCGTCAGGGCGATCGCATTGCTATTGTCAGTGGG
TTACGAACCCCCTTCGCTCGTCAGGCCACTGCATTTCATGGTATTCCCGCTGTTGATCTC
GGCAAGATGGTGGTAGGCGAGCTGCTGGCGCGCAGTGAAATTCCAGCCGATGCCATTGAG
CAACTGGTTTTTGGTCAGGTAGTACAAATGCCGGAAGCCCCCAATATTGCGCGCGAAATT
GTGCTGGGTACGGGAATGAATGTTCATACCGATGCCTATAGCGTAAGCCGCGCCTGCGCC
ACCAGTTTTCAGGCGGTAGCGAATGTGGCGGAAAGCCTGATGGCCGGCACGATACGCGCG
GGTATCGCTGGCGGCGCAGATTCGTCGTCGGTACTGCCAATAGGCGTGAGTAAAGCCCTG
GCGCGGGTACTGGTTGATGTCAATAAAGCCCGGACGACCCGCCAACGGCTGACGCTTTTT
TCTCGTCTGCGCTTACGCGACCTGCTGCCCGTGCCTCCCGCCGTTGCTGAGTATTCCACC
GGTCTGCGCATGGGCGATACCGCCGAACAGATGGCGAAAACGTATGGTATTACGCGTGAA
CAGCAGGACGCACTGGCGCATCGTTCACATCAGCGCGCCGCGCAGGCATGGGCGGAGGGT
AAACTGGCGGAAGAAGTGATGACGACCTATGTGCCGCCGTATAAAAACCCCTTTGCAGAA
GACAACAACATTCGCGGCGCCTCCACGCTGGCGGACTACGCTAAACTGCGTCCCGCTTTT
GACCGAAAACATGGCTCCGTAACCGCCGCCAACAGTACACCGCTGACCGACGGCGCTGCC
GCCGTTATTATGATGACCGAATCCCGCGCTAAAGAGCTGGGGCTGCGCCCGCTTGGTTAT
CTGCGCAGCTACGCCTTTACCGCTATTGATGTCTGGCAGGACATGCTATTGGGGCCGGCC
TGGTCAACGCCGCTGGCGCTGGAGCGGGCCGGGTTGACGATGGCCGATCTGACGCTTTTT
GACATGCATGAAGCGTTTGCCGCCCAGACTCTGGCGAACTTACAACTGCTGGGCAGCGAA
CGATTTGCCCGTGAGGTGCTTGGTCGTGCGCAGGCGACAGGGGAGGTGGATGACGCTAAA
TTCAACGTGCTCGGCGGCTCAATCGCCTACGGGCATCCTTTTGCCGCAACGGGCGCGCGG
ATGATAACCCAAACGTTGCATGAATTACGGCGTCGGGGCGGAGGTTTTGGCCTGGTCACG
GCCTGCGCGGCTGGCGGTCTTGGCGCGGCGATGGTTCTGGAGGCGGAATAA
>EECEBICD_00964 Fatty acid oxidation complex subunit alpha
ATGACTACCACATCAGCTTTTATGCTTAACGTGCGTCTGGATAATGTCGCGGTTGTCGCT
ATTGATGTCCCTGGAGAAAAGGTGAACACCCTGAAAGCGGAGTTTGCCGCGCAGGTACGC
GCCATCCTGAAGCAGATCCGGGAAAATAAAGCGTTGCAGGGCGTTGTTTTTATCTCAGCC
AAAGCAGATAACTTTATTGCTGGCGCTGATATCAACATGATAGGTCACTGCCAAAACGCG
CAGGAAGCGGAAACGCTGGCGCGTCAGGGGCAACAACTGATGGCGGAGATCCAGGCGTTG
CCTGTTCCGGTTATCGCCGCCATTCATGGCGCCTGTCTGGGGGGCGGCCTTGAAATGGCG
CTGGCCTGCCATCGACGTATCTGTACCGACGACGTTAAAACCGTTCTCGGACTGCCGGAG
GTACAGTTAGGGCTTTTACCGGGATCGGGCGGAACGCAGCGACTTCCACGGCTGGTGGGG
GTCAGTACCGCGCTGGATATGATCCTGACAGGTAAACAGCTTCGCGCCAGGCAGGCGCTT
AAAGCCGGGCTGGTTGATGATGTGGTGCCGCAAACTATTTTACTGGAAGCGGCTGTCGAG
CTGGCTAAAAAAGAACGCCTGGCGCAGCGGACATTACCGGTCCGTGAACGTATCCTGGCG
GGGCCGCTGGGGCGCGCGCTGTTATTTCGCCTGGTGCGTAAAAAGACAGCGCAAAAAACG
CAGGGTAATTATCCGGCGACGGAGCGGATCATTGATGTGATTGAAACCGGACTGGCGCAG
GGCAGCAGTAGCGGCTATGACGCGGAAGCGCGCGCTTTCGGCGAGCTGGCGATGACGCCT
CAATCGCAGGCGCTGCGCGCTGTTTTCTTTGCCAGTACCGAGGTGAAAAAAGATCCCGGT
AGCGATGCGCCGCCCGGCCCGCTGAACAGCGTGGGCATCTTAGGCGGCGGGTTAATGGGC
GGCGGTATCGCCTGGGTTACGGCCTGTAAAGGCGGTTTGCCAGTACGAATTAAGGATATA
AATACTCAGGGGATCAATCATGCTCTGAAATACAGCTGGGATCTGCTTGAAACAAAAGTA
CGCCGCCGCCATATCAAAGCCAACGAACGCGATAAACAGTTGGCATTGATATCAGGTTCG
ACCGATTACCGTGGTTTCAGTCATCGTGATCTGGTCATTGAAGCGGTGTTTGAAGATCTG
CCGTTAAAACAACAGATGGTGGCGGAAGTGGAACAAAATTGCGCTGCTCACACCATTTTT

GCCTCCAATACTTCTTCCCTGCCGATTGGCGATATTGCGGCGAATGCGGCCAGACCGGAA
CAGGTGATTGGATTGCACTTTTTTAGCCCGGTCGAAAAAATGCCGCTGGTTGAGGTTATT
CCTCATGCGTCTACTTCCGCGCAGACCATCGCCACGACCGTTAAGCTGGCGAAAAAACAG
GGTAAAACCCCGATAGTCGTCAGTGATAAAGCCGGGTTTTATGTTAACCGAATTCTTGCG
CCATATATCAATGAGGCGATTCGGATGCTGACTGAAGGCGAACGCGTCGAACATATTGAC
GCCGCCCTGGTAAAATTTGGTTTTCCGGTGGGGCCAATCCAACTTTTGGATGAGGTAGGA
ATCGACACGGGCACTAAAATTATTCCTGTGCTTGAGGCGGCGTATGGAGAACGTTTTAGC
GCGCCTGCAAATGTTGTCGCTTCAATTTTGAATGACGATCGCAAAGGTAGAAAAAATGGT
CGCGGTTTCTATCTTTATGGCGAAAAAGGGCGTAAAAGCAAAAAACAGGTTGACCCTGCT
ATTTATAAGCTTATTGGCGTGCAAGGGCAGAGTCGATTGTCTGCGCAGCAGGTGGCAGAG
CGCTGTGTCATGTTAATGCTTAACGAAGCGGCGCGCTGTTTCGACGAAAAAGTGATTCGC
AGCGCGCGCGATGGCGATATCGGCGCCGTGTTTGGTATCGGTTTTCCGCCGTTTCTCGGT
GGCCCGTTCCGCTATATGGATGCTCTCGGGCCGGGTGAAATGGTGGCGACATTACAGCGC
CTGGCCGCACTTTACGGTCCTCGTTACGCGCCTTGTGAACAATTAGTGCGCATGGCGGAG
CGCAGGGAGCATTTCTGGACGAATGGGGAAACTGACCAGGGAAATTGA
>EECEBICD_00965 Phosphohistidine phosphatase SixA
ATGCAAGTTTTTATCATGCGTCACGGCGACGCAGCCCTCGATGCCGCCAGTGATTCGGTT
CGTCCCTTGACCCCTTGTGGGTGTGACGAGTCTCGCCTGATGGCGAACTGGCTGAAAGGT
CAAAAAGTGGATATCGAACGTGTTCTGGTGAGTCCGTTTCTTCGGGCCGAACAGACGTTA
GATGTGGTCGGCGATTGTATGAATCTGCCGGCTCAGGTGGATGTCCTGCCGGAGCTAACG
CCCTGTGGCGATGTCGGTCTGGTCAGCGCCTACCTCCAGGCGCTGGCCAATGAAGAGATC
GGATCGGTGCTGGTGATTTCTCACTTACCTCTGGTCGGATACCTGGTATCTGAGCTTTGT
CCGGGCGAAACGCCGCCGATGTTCACGACGTCCGCAATTGCCAACGTTACGCTTGATGAA
AGCGGCAAGGGGACATTCAACTGGCAAATGAGTCCCTGCAATCTGAAAATGGCGAAAGCG
ATTTAA
>EECEBICD_00966 Endonuclease MutS2
ATGCGTCGCGGCGACTATTCGCCAGAACTGTTTCTGGATTTACACGGCTTAACGCAGCTT
CAGGCCAAGCAGGAACTGGGGGCGCTGATTGCCGCCTGCCGTCGGGAACATATTTTTTGC
GCCTGCGTCATGCATGGACACGGTAAACATATTCTGAAACAGCAAACGCCGTTATGGCTG
GCGCAGCATCCGCATGTGATGGCCTTTCATCAGGCGCCGAAAGAGTACGGCGGCGACGCC
GCGCTGCTGGTGTTGATTGAAGTGGAAGAGTGGCAACCGCCCGAATTACCCTGA
>EECEBICD_00967 hypothetical protein
ATGAAAAAGAAAACATCGCTCAGCGAGGAGGACCAGGCGCTGTTCCGTCAGCTGATGGTC
GGCACCCGAAAGATTAAACAGGATACTATCGTCCATCGGCCGCTGCGCAAGAAAATCACG
GAAGTCCCTACCCGAAGGTTAATTCAGGAACAGGCGGACGCCAGCCACTATTTTTCCGAT
GAGTTCCAGCCGCTGCTCAATACGGAAGGGCCGGTAAAGTATGTCCGTGAAGATGTCAGC
CATTTCGAGCTAAAAAAATGCGTCGCGGCGACTATTCGCCAGAACTGTTTCTGGATTTAC
ACGGCTTAA
>EECEBICD_00968 50S ribosomal protein L3 glutamine methyltransferase
GTGGATAAAATTTTCGTCGATGAAGCAGTAAGTGAACTGCATACCATTCAGGACATGTTG
CGCTGGGCGGTAAGCCGCTTTAGCGCGGCGAATATCTGGTATGGACACGGTACCGATAAC
CCGTGGGATGAAGCGGTACAACTGGTGTTGCCGTCTCTTTATCTGCCGCTGGATATTCCG
GAAGATATGCGGACCGCGCGGCTGACGTCCAGCGAAAAACACCGCATTGTCGAGCGAGTG
ATTCGTCGCATTAACGAGCGTATCCCGGTCGCCTACCTGACCAATAAAGCCTGGTTCTGC
GGCCACGAATTTTATGTCGATGAGCGCGTGCTGGTGCCGCGTTCGCCCATTGGCGAGCTG
ATTAATAACCACTTCGCCGGCCTTATTAGCCAACAGCCGAAATATATTCTGGATATGTGC
ACCGGCAGCGGCTGCATCGCCATCGCCTGTGCTTATGCTTTCCCGGACGCAGAGGTTGAT
GCGGTCGATATTTCGCCGGATGCGCTGGCTGTCGCCGAGCATAACATTGAAGAACACGGT
CTTATCCATCACGTGACGCCAATCCGTTCCGATCTGTTCCGCGATCTGCCGAAAGTTCAG
TACGATCTGATTGTCACTAACCCGCCTTATGTCGATGCGGAGGATATGTCCGATCTGCCG
AACGAATATCGCCACGAACCTGAGCTGGGGCTGGCGTCCGGCACTGACGGCCTCAAATTG
ACCCGTCGTATCCTGGGGAATGCGCCGGATTATCTGTCCGATGACGGCGTTCTGATTTGT
GAAGTCGGAAACAGCATGGTACATCTGATGGAGCAGTATCCGGATGTGCCGTTCACCTGG
CTGGAGTTTGACAACGGCGGCGATGGCGTCTTTATGTTGACCAAAGCGCAGTTGCTCGCG

GCCCGTGAACATTTCAACATTTATAAAGATTAA
>EECEBICD_00969 Chorismate synthase
ATGGCAGGAAACACAATTGGACAACTCTTTCGCGTAACCACTTTCGGCGAATCACACGGG
CTGGCGCTTGGGTGTATCGTCGATGGCGTGCCGCCCGGCATCCCGTTGACGGAGGCCGAT
CTGCAGCACGATCTCGACAGACGCCGCCCCGGCACCTCGCGCTATACTACCCAGCGCCGC
GAACCGGACCAGGTAAAAATTCTCTCCGGCGTGTTTGATGGCGTGACGACCGGCACCAGC
ATTGGCCTGCTGATTGAAAACACCGATCAGCGCTCGCAGGACTACAGCGCGATTAAAGAT
GTTTTTCGTCCGGGACACGCGGATTACACCTATGAGCAGAAATACGGCCTGCGCGATTAC
CGTGGCGGTGGACGTTCTTCCGCGCGTGAAACCGCGATGCGCGTAGCGGCAGGGGCGATC
GCCAAGAAATACCTGGCGGAAAAGTTCGGCATCGAAATCCGCGGCTGCCTGACCCAGATG
GGCGATATTCCGCTGGAGATTAAAGACTGGCGTCAGGTTGAGCTTAATCCGTTCTTTTGT
CCCGATGCGGACAAACTTGACGCGCTGGACGAACTGATGCGCGCGCTGAAAAAAGAGGGC
GACTCCATCGGCGCGAAAGTGACGGTGATGGCGAGCGGCGTGCCGGCAGGGCTTGGCGAA
CCGGTTTTTGACCGACTGGATGCGGACATCGCCCATGCGCTGATGAGCATCAATGCGGTG
AAAGGCGTGGAGATCGGCGAAGGATTTAACGTGGTGGCGCTGCGCGGCAGCCAGAATCGC
GATGAAATCACGGCGCAGGGTTTTCAGAGCAACCACGCTGGCGGCATCCTCGGTGGTATC
AGTAGCGGGCAACACATTGTGGCGCATATGGCGCTGAAACCTACCTCCAGCATTACCGTG
CCGGGACGTACGATCAACCGGATGGGTGAAGAAGTCGAAATGATCACCAAAGGGCGCCAC
GATCCGTGTGTGGGGATTCGCGCAGTGCCGATCGCAGAAGCCATGCTGGCGATCGTGCTG
ATGGATCACCTGCTGCGCCATCGGGCACAGAATGCGGATGTAAAGACAGAGATTCCACGC
TGGTAA
>EECEBICD_00970 Penicillin-insensitive murein endopeptidase
ATGAAAAAAACCGCGATTGCGCTGCTGGCATGGTTTGTCAGTAGCGCCAGCCTGGCGGCG
ACGCCGTGGCAGAAAATAACCCATCCTGTCCCCGGCGCCGCCCAGTCTATCGGTAGCTTT
GCCAACGGATGCATCATCGGCGCCGACACGTTGCCGGTACAGTCCGATAATTATCAGGTG
ATGCGCACCGATCAGCGCCGTTATTTCGGCCACCCGGACTTGGTCATGTTTATCCAGCGG
TTGAGTCATCAGGCGCAGCAACGGGGGCTCGGAACCGTCCTGATAGGCGACATGGGGATG
CCTGCCGGAGGCCGCTTTAATGGCGGACACGCCAGTCATCAGACCGGGCTTGATGTGGAT
ATTTTCTTGCAGTTGCCGAAAACGCGCTGGAGCCAGGCGCAGCTATTGCGCCCGCAGGCG
TTAGATCTGGTGTCCCGCGACGGTAAACACGTCGTGCCGTCGCGCTGGTCGTCGGATATC
GCCAGTCTGATCAAACTGGCGGCACAAGACAATGACGTCACCCGTATTTTCGTCAATCCG
GCTATTAAACAACAGCTTTGCCTCGATGCCGGAAACGATCGTGACTGGCTACGTAAAGTA
CGCCCCTGGTTCCAGCATCGCGCGCATATGCACGTGCGTTTACGCTGCCCTGCCGACAGC
CTGGAGTGCGAAGATCAACCTTTACCCCCGCCGGGCGATGGATGCGGCGCTGAACTGCAA
AGCTGGTTCGAACCGCCAAAACCTGGCACCACAAAGCCTGAGAAGAAGACACCGCCGCCG
CTGCCGCCTTCCTGCCAGGCGCTACTGGATGAGCATGTACTCTGA
>EECEBICD_00971 putative membrane transporter protein YfcA
ATGGACAATTTTTATGATCTGTTTATGGTCTCCCCGCTGCTGCTGGTGGTGCTGTTTTTT
GTCGCCGTACTGGCAGGATTTATCGATTCTATCGCCGGGGGCGGAGGGCTGCTCACTATC
CCTGCGCTGATGGCCGCCGGGATGTCGCCGGCAAACGCGCTGGCGACCAATAAATTACAG
GCGTGCGGCGGCTCCCTCTCGTCTTCGCTCTATTTTATTCGCCGTAAAGTGGTAAACCTG
GCCGAGCAAAAGCTCAATATTCTGATGACGTTCATTGGCTCGATGAGCGGCGCGCTGCTG
GTGCAGCACGTGCAGGCGGATATTTTGCGCCAGATCTTGCCTATCCTGGTGATTTTCATC
GGCCTCTATTTTTATTGA
>EECEBICD_00972 putative membrane transporter protein YfcA
ATGCCGAAGCTGGGCGAGGAAGATCGCCAGCGCCGCCTGTATGGATTACCGTTCGCGCTG
ATAGCCGGGGGATGCGTCGGGTTTTACGACGGCTTTTTCGGGCCTGCCGCCGGGTCGTTT
TACGCTCTGGCGTTTGTCACCTTATGTGGCTATAACCTGGCGAAATCCACGGCACATGCC
AAAGTGCTTAACGCTACCTCCAACGTTGGCGGCCTGCTGTTATTTATCATCGGCGGCAAA
GTGATCTGGGCGACGGGCTTCGTGATGCTGGTTGGTCAGTTTTTAGGGGCGCGAATGGGG
TCGCGTCTGGTATTGAGCAAAGGCCAAAAGCTTATCAGACCGATGATCGTTATTGTCTCG
GCGGTAATGAGCGCCAGGTTACTTTATGATAGCCACGGACAGGAGATCCTCCACTGGTTG
GGGATGAATTAA
>EECEBICD_00973 Elongation factor P hydroxylase

ATGAACAATACGCACCATTACGAACAGTTAATTGAGATTTTTAACGGCTGCTTTGCCGAA
GAATTTAATACCCGTCTGATTAAAGGCGACGACGAACCGATCTATCTTCCTGCTGACGCG
CAGGTACCGTACCACCGCATCGTCTTTGCGCATGGGTTTTACGCCAGCGCGCTGCATGAG
ATTTCTCACTGGTGTATCGCCGGTAAGGCGCGCCGCGAACTGGTTGACTTTGGTTACTGG
TACTGCCCGGATGGACGCGACGCGCAGACCCAAAGTCAATTTGAAGACGTCGAGGTGAAG
CCACAGGCTTTCGACTGGTTGTTTTGCGTGGCGGCGGGGTATCCCTTTAACGTCAGTTGC
GACAATCTGGAAGGCGATATTGAGCCGGATCGCGTGGCGTTTCAGCGCCGGGTTCATGCG
CAGGTAATGGCGTATCTGGAGCAGGGCATTCCTGAACGTCCGGCCCGCTTCATCAAAGCG
CTACAGAATTACTATCACACGCCCGAACTTAAGGCGGAACAGTTCCCGTGGCCGGAAGCG
CTTAATTAA
>EECEBICD_00974 hypothetical protein
ATGATCGCGGAGTTTGAATCACGCATTCTGGCATTAATTGACGATATGGTAGAGCACGCC
AGCGACGATGAGCTTTTTGCCAGTGGATACCTGCGTGGCCACCTGACGTTAGCCATTGCC
GAGCTGGAGAGCGGTGATGACCACTCCGTTGAGGCGGTCTATGCCAACGTTAGCCAAAGT
CTGGAGAAAGCGATCGGCGCCGGCGAACTGTCGCCGCGCGATCAGGCGTTAGTCAAAGCG
ATGTGGGACAATTTGTTCGACAAGGCAAAACAATAA
>EECEBICD_00975 tRNA 5-methylaminomethyl-2-thiouridine biosynthesis bifunctional protein MnmC
GTGAAACAATACGCCATACAACCCGCCACACTCGAATTTAACGCTGAGGGTACACCTGTT
TCCCGAGATTTTGATGATGTCTATTTTTCTAATGACAATGGACTCGAAGAGACACGCTAC
GTTTTTCTTGGCGGCAATCGTCTTGCGGAGCGATTTCCCGTACATTCACATCCATTATTT
ATAGTCGCGGAAAGCGGTTTTGGCACCGGACTCAACTTCCTGACGCTGTGGCAGGCGTTT
GACAGCTTTCGTTCTGCGCACCCACAGGCGACGCTGCAAAGGTTACATTTCATCAGTTTT
GAGAAATTCCCGTTAACGCGCGACGATCTGGCGCTGGCGCACCAACACTGGCCGGAGCTC
GCGCCCTGGGCAGAGCAGCTGCAGGCGCAATGGCCGCTGCCGCTCCCGGGGTGCCACCGC
CTATTGCTGGATCGGGGTCGGGTGACGCTGGATTTGTGGTTTGGCGATATTAATGAGCTG
ACCGACCAACTGGACGCCACGCTGAATCAAACAGTCGACGCCTGGTTCCTCGACGGTTTT
GCCCCGGCGAAAAATCCGGATATGTGGACGCCAAACCTGTTTAACGCGATGGCGCGGCTG
GCGCGGCCCGGCGCAACGCTGGCGACGTTTACCTCGGCGGGCTTCGTTCGTCGGGGATTA
CAGGAAGCCGGTTTTACCATGCAAAAACGCAAAGGCTTCGGACGTAAACGCGAGATGCTT
TGTGGAGTGATGGAACAACACCTCATGCCGACCCTTTCCGCGCCCTGGTTTTACCGCAGC
GGAAGCGAAAAACGTGAAACGGCGATCATTGGCGGCGGCATCGCCAGCGCGCTGTTATCA
CTGGCGTTACTGCGTCGCGGCTGGCAGGTGACGCTCTATTGCGCGGACGACCAGCCCGCC
CAGGGCGCCTCCGGTAATCGACAGGGTGCGCTTTATCCACTCCTTAGCAAACATGACGCC
GCCATTAATCGCTTTTTCCCGACCGCATTCACCTTTGCCCGTCGCCTGTATGATGCCCTG
CCCGTCTCTTTTGATCATGACTGGTGCGGCGTGACACAGCTCGGATGGGATGAGAAAAGC
CAGCAGAAAATTGCCCAGATGCTGTCCCTGGCGCTGCCCGCCGAACTGGCCTCGGCGCTC
AATGCCGAAGAGGCGGAACAGGCGGTTGGCGTGACGACACGCTGCGGAGGAATTACCTAT
CCGGCAGGCGGCTGGCTATGCCCGGAACAATTAACTCGCGCAGTCATCGCACTGGCGACC
GAACAGGGTTTACAAACGCGCTTTCGCCATACGCTCACGTCGCTCGTTGCGCAGGAGTCG
CGCTGGCAACTGCGCTTTATGTCAGGTGAAACCGCCAGCCATGAGACCGTCGTCCTGGCG
AACGGCCATCAGATTAACCGTTTTGATCAGACCCGGCCGCTGCCGGTGTATGCCGTCGGG
GGGCAGGTCAGTCATATTCCCACCACCCCCGCCCTCTCGGCGTTGCGTCAAGTGTTATGC
TACGACGGCTATCTGACGCCGCAAAATCCGCATAATCAGCAGCACTGTATTGGCGCCAGC
TATCACCGGGGCGACGAAAGCACCGTCTGGCGCGAAGAAGATCAGCGTCAGAACCGACAG
CGTCTGCTTGACTGTTTTCCGGATGCGAAATGGGCCACAGAGGTTGACGTTAGCGGCAAC
AGCGCCCGTTGCGGCGTGCGTTGCGCCACCCGCGACCATCTGCCGATGGTGGGCAACGTC
CCGGATTACCACGCCACGCTGACGCATTACGCCGATTTAGCCGATAACAAAACGTCGGCG
GCGCCTGCGCCTGTGTATCCGGGTTTATTTGTGCTTGGCGCACTGGGATCGCGCGGTTTG
TGCTCAGCGCCATTATGCGCAGAAATTCTGGCGGCGCAGATGAGTAACGAGCCGATTCCA
CTGGATGCCGGTACGCTGGCGGCGTTAAATCCAAATCGGTTATGGGTGAGAAAGTTATTG
AAGGGAAAAGCGGTGAAGTAA
>EECEBICD_00976 3-oxoacyl-[acyl-carrier-protein] synthase 1
ATGAAACGTGCAGTGATTACTGGCCTGGGCATCGTTTCCAGCATCGGTAATAACCAGCAG

GAAGTCCTGGCATCTCTGCGTGAAGGACGTTCAGGGATCACTTTCTCTCAGGAGCTGAAG
GACGCCGGCATGCGTAGCCAGGTATGGGGCAACGTAAAACTGGATACCACTGGCCTCATT
GACCGCAAAGTCGTGCGCTTTATGAGCGACGCCTCCATCTATGCTTATCTGTCTATGGAG
CAGGCAGTGGCCGACGCCGGTCTGGCGCCGGAAGTCTATCAGAATAACCCGCGTGTCGGC
CTGATTGCAGGTTCCGGCGGCGGCTCCCCGAAATTCCAGGTCTTTGGCGCAGATGCCATG
CGTAGCCCGCGTGGTCTGAAAGCCGTGGGCCCGTATGTGGTGACGAAAGCAATGGCATCC
GGCGTTTCCGCCTGCCTCGCTACGCCGTTTAAAATTTATGGCGTGAACTACTCCATTAGT
TCCGCTTGCGCGACCTCCGCACACTGTATCGGCAATGCGGTTGAACAGATCCAACTGGGC
AAACAGGACATCGTTTTTGCTGGCGGCGGCGAAGAGCTGTGCTGGGAAATGGCCTGTGAG
TTCGATGCGATGGGCGCACTGTCCACCAAATATAACGATACGCCTGAAAAAGCGTCCCGT
ACCTATGATGCGCACCGTGATGGGTTTGTCATCGCGGGCGGTGGCGGTATGGTTGTTGTC
GAAGAGCTGGAACACGCTCTGGCGCGCGGCGCGCATATCTATGCGGAAATCGTCGGTTAC
GGCGCAACATCCGACGGCGCAGACATGGTTGCTCCGTCTGGTGAAGGCGCAGTACGTTGC
ATGCAGATGGCGATGCACGGCGTAGACACGCCGATCGACTACCTGAACTCTCACGGTACG
TCCACGCCGGTAGGCGACGTGAAAGAACTCGGTGCAATCCGTGAAGTGTTTGGCGATAAC
AGCCCGGCTATCTCCGCGACCAAAGCGATGACCGGCCACTCGCTGGGTGCGGCTGGCGTG
CAGGAAGCCATCTACTCTCTGCTGATGCTGGAGCACGGTTTTATCGCGCCGAGTATCAAC
ATCGAAGAGCTGGATGAGCAGGCTGCGGGTCTGAACATCGTGACCGAAACGACCGAGCGT
GAACTGACTACCGTAATGTCTAACAGCTTCGGTTTTGGCGGCACCAACGCCACGCTGGTG
ATGCGTAAGTTGTAA
>EECEBICD_00977 hypothetical protein
ATGCCAACTAATACATTAGATAAAATTAGGCATAGTTTGAGTTGTGTGGCTGTTTTATTT
GGTTTGTTCGGCATTTTTGTTTTTGCCTCATTTTCGCCTTCCTATGCCTGGCTTTATTTG
GGAGGTCTGGCTGCGCCTTTTATATATAGTATTGTTTTTGTCTATGCGATTGCCGCCTGG
AGTATTTATTCAAAGTATTACCCGTTTTTATCATTGGGTCGCTTATCATTCGTGGAGTGT
TTTGTTCCGGCGCTGGCCCTGGTGTGCCTTACCGTTCTCTACAATGTTTTTAGTGGCCCG
GAACCCTGGATGGCCGAGTTATCAAGGCAATTTTTTCTTCATAAATTTCTTAACACGCTG
GCGATGTGCTTTCTGGCTCCTGTGGCGGAGGAAATTATATTCAGAGGGTTTTTATTAAAC
AGCAGTATCGGTTGGGGTCGATACTCAAGAGCTTCCGGTATTATTATTACTTCACTCGCT
TTTGCATTTATGCATACACAATATCTCTTTGCGGTGACTTTTGTTTATCTGTTTGTTTTT
TCATCGATATTATGCGTTGTTCGAATGAGAAGTCGAGGGTTAATGATACCGATAATACTT
CATATTTTAAATAATGCCTGGGTTATTTTCGGCTTACTGTTTTCAGCGACGGAATAA
>EECEBICD_00978 hypothetical protein
ATGAAAACGTTCTCCCTTGTCGCGCTTATCCTGTTGCTTTGTAGTTGCTCCACGCCTCAT
CACGACAGTACGCAGGCCGTGAAGCAGTTCTATACCTCATGGATGACGACGTTCACGAAC
GATGTGAATCCCCCTGACGACACCACCGCCTTAATGCAACGTTACGTTGCCAAAGAGGTT
ATTCATCGCCTGGCGCTAATACAGTCCCTTTATGAACAGGAGATTGTGGGGGCAGATTAT
TTTATGTATGCGCAGGACTATGCGCCAGAGTGGATTCCACAGTTACGGGTTGGGAAGGCA
CACCCTTTCCTCGGCGGAGAAAAAGTGGATGTTCTGTTGGCTACAGAGTCAACGCCCATC
CACCTGGAAGTTTATACCCGTTGGGAAGAGGGACGCTGGAAAATTTACCGCGTTCGGGAT
GCTGACAGGGGTTATGAACAGCCGATTTATGACGCAGGCGCCATCACCCAGGCTGAGGCC
TGGTCAGCGAAAGTCGCGCCGGAATATAAGAAGCATTAA
>EECEBICD_00979 hypothetical protein
ATGAGTTGGGATAAAAGAGTGGCGGTTAATTATGCCAAAACGCATGCAGGTTCTCATTCT
CAAGGCCGGTGTGCGGAATTTACCCGAAAGGCAATTCAGGCGGGCGGTATCACGCTGGGG
CATACGTATCATGCGAAAGATTATGGCCCAATGTTAAGAAGCGCCGGATTCACCGCTATT
GGTACCTATGAAATGCCGCGCGAGGGTGACGTCATCATTATTCAACCTTACGCTGGCGGA
AACCCGAGCGGTCACATGGCGATATACGATGGCACAGAGTGGTATTCAGATTTTAAACAA
CGCGATATGTGGGCCGGCCCGGGCTACCGGGCAGCCAGGCCCTCATATACCATCTACAGG
AAGAATTAA
>EECEBICD_00980 hypothetical protein
ATGGAACCAAAAAGCCAAGACTGGCATCGTGCAGATATTAAAAGTGCACTTGAAAAAAGA
GGTATTACGCTCAGAGATCTTTCTCGTCAGGCCGGTTTATCACCTGATTCACTGCGCAAT
GTCTTTACCCGTTCCTGGCCGAGAGCTGAACGGATTATTGCGGACGCGCTGGGCATCACG

CCGCAGGAGATCTGGCCGTCGCGCTATGATGATATGCAAATAAAAAATGATGCCGATATC
GCAGAATAA
>EECEBICD_00981 hypothetical protein
ATGGCAAAGGAATGGTTCACGGTTAAGGAATGTCTTGGCTTACCGGGGTTTCCGGGAAGC
GAGCCTGCGGTTCGCGAACGTTTATATAAGTACAGTGAAGGGAAAGCGGGGGTCAGGCGT
AAGCGTGTTAAATCAAAGGCCGAGGAGTTTCATATATCTGTTTTCCCTCTCTATGTTCAT
CGTTATCTGGACGACAGCGGAGAGGAACCGACGCCAGAACCCATTTCCTTACAGGAAGCG
GAGCCTGAAGATATCTGGGAGATGATGTTCAGGTTACTGACGCCAGAACAGCGCAAGCAG
GTGACCGGACGGTTTAAGGTGAGAGGGATGAAAGCGGTCTTTCCCTTTCTGTTTGATGAC
ACGCCGCCACGTTGA
>EECEBICD_00982 putative MFS-type transporter YfcJ
ATGACAGCAGTTAGCCAAAAAACAACGACCCCGTCTGCGAATTTTTCGCTGTTCCGCATC
GCCTTTGCGGTTTTCCTGACCTATATGACGGTCGGCCTGCCGTTACCCGTCATCCCGCTT
TTTGTTCATCACGAACTGGGCTATAGCAATACGATGGTTGGGATCGCCGTGGGCATTCAG
TTTTTTGCGACGGTACTGACGCGCGGCTATGCCGGACGACTGGCGGATCAATATGGCGCT
AAACGATCGGCGTTACAGGGGATGTTTGCCTGCGGGCTGGCGGGCGCCGCCTGGCTCCTG
GCCGCGCTGTTGCCGGTTTCCGCGCCGATAAAATTCGCGCTGCTTATTGTCGGGCGTTTG
ATCCTCGGATTTGGCGAAAGCCAGCTGCTGACCGGAACCCTGACCTGGGGACTGGGGCTG
GTTGGCCCAACGCGTTCCGGTAAGGTGATGTCGTGGAACGGGATGGCGATTTACGGCGCG
CTGGCGGCCGGTGCGCCGTTGGGCTTGCTGATCCATAGCCACGGCTTCGCGGCGCTGGCG
GGGACGACGATGGTTTTACCGCTGCTGGCCTGGGCATTCAACGGCACCGTGCGTAAAGTG
CCTGCGTATACGGGGGAGCGACCTTCACTGTGGAGCGTGGTCGGGCTTATCTGGAAACCG
GGCCTGGGCCTGGCATTGCAGGGCGTGGGCTTTGCCGTCATCGGTACGTTTATTTCACTT
TATTTTGTCAGCAATGGCTGGACGATGGCGGGATTCACCCTGACCGCGTTTGGCGGTGCG
TTTGTACTGATGCGCATATTGTTCGGTTGGATGCCGGACCGCTTTGGCGGCGTGAAGGTC
GCCGTTGTCTCATTGCTGGTTGAGACGGCAGGGTTGCTGCTCTTGTGGCTGGCGCCGACG
GCCTGGATAGCGCTGGTAGGCGCGGCGCTCACCGGGGCAGGGTGTTCACTGATTTTCCCG
GCGCTGGGCGTGGAGGTTGTGAAACGCGTACCTGCACAGGTACGCGGTACGGCGCTCGGC
GGCTACGCCGCGTTTCAGGATATCTCCTACGGCGTTACCGGCCCGCTGGCGGGCATGCTG
GCCACGTCATGCGGCTATCCTTCCGTATTCCTCGCGGGAGCGATCTCCGCTGTGGTTGGC
ATCCTGGTGACGATATTATCGTTCCGGCGTGGTTAA
>EECEBICD_00983 Flagellar regulator flk
ATGCATCCCATCTCCGGCGCCCCTGCACAGCCTCCAGGCGAAGGGCGTAATCCTCTTTCC
GCTGCGAGCGAGCAGCCGCTTTCCATGCAACAGCGTACGGTACTGGAAAGGCTGATCACG
CGCCTGATTTCACTGACCCAGCAGCAAAGCGCGGAAGTATGGGCCGGAATGAAACACGAT
CTGGGGATAAAAAATGACGCGCCGCTACTGTCGCGCCACTTCCCCGCCGCTGAACAGAAT
CTTACCCAGCGTCTGGGCGTAGCGCAGCAAAATCATGCCAATCGTCAGGTGCTTTCACAG
CTAACAGAGCTGCTTGGCGTCGGGAATAATCGTCAGGCCGTCAGCGATTTTATCCGTCAA
CAATACGGTCAAACCGCGCTTAGCCAGCTTACGCCCGATCAACTCAAAAACGTGCTGACG
CTCCTGCAACAGGGGCAGCTTTCCATTCCGCAGCCGCAGCAACGTCCGGCAACGGATCGG
CCATTATTGCCTGCTGAACATAATACGTTGAATCAGTTGGTGACGAAGCTGGCAGCCGCG
ACCGGCGAGTCAAACAAACTTATCTGGCAATCAATGCTGGAACTCTCCGGCGTGAAAAGC
GGCGAGCTCATTCCGGCAAAGCAGTTTACCCATCTGGCGACATGGCTACAGGCGCGGCAG
ACGCTGTCACTACAACACGCGCCTACGCTACATACGCTTCAGGCTGCGTTAAAACAACCG
CTTGAGCCGGACGAACTCACAGCGATCAAAGAGTATGCCCAGCACACTTATCAAATTCAG
CCGCAAACGGTGCTGACCACCGCCCAGGTGCAGGATTTACTTAACCATATTTTCCTGCGT
CGCGTAGAGCGGGAGACTGATGAGCTGGAACCGCTCTCTATTCAACCCATCTACCGCCCG
TTTGCGCCGATGATTGAAACCGTGAAAAATCTGTCTGCGCGGCCAGGCCTGTTGTTTATC
GCGCTAATAATTGTACTGGCGCTTTTCTGGCTGGTTTCTTAA
>EECEBICD_00984 Erythronate-4-phosphate dehydrogenase
GTGAAAATTCTCGTTGATGAAAATATGCCTTACGCCCGCGAATTATTTAGCCGCCTGGGT
GAGGTAAAGGCAGTGCCGGGTCGCCCGATCCCCGTTGAAGAATTGAACCATGCCGATGCG
TTAATGGTGCGTTCGGTCACGAAAGTGAATGAATCGCTGCTGAGCGGCACGCCGATTAAC
TTCGTTGGTACTGCTACGGCAGGCACCGATCATGTGGATGAGGCATGGCTGAAGCAGGCG

GGCATCGGTTTTTCAGCCGCGCCCGGCTGTAATGCTATCGCCGTGGTGGAGTATGTGTTT
TCCGCGCTGTTGATGCTGGCCGAACGTGACGGCTTTTCGTTACGCGATCGCACTATAGGG
ATTGTCGGCGTGGGTAACGTCGGAAGCCGGCTACAGACCAGGCTTGAAGCGTTGGGTATC
CGCACGTTGCTGTGCGATCCGCCACGCGCCGCACGCGGGGACGAGGGTGATTTTCGTACG
CTGGATGAGCTGGTGCAGGAAGCGGATGTCCTGACTTTCCATACGCCGCTCTATAAAGAC
GGACCGTACAAAACGCTGCATCTGGCCGATGAGACGCTGATCCGTCGACTGAAACCCGGC
GCCATTCTCATTAACGCCTGCCGCGGTCCGGTGGTGGATAACGCCGCGTTGCTGGCGCGA
CTTAATGCCGGGCAGCCGCTGAGCGTGGTGCTTGATGTGTGGGAGGGCGAACCGGATCTT
AACGTCGCGCTACTGGAAGCCGTGGATATCGGCACGTCGCATATTGCCGGTTATACGCTG
GAAGGCAAAGCGCGCGGCACCACTCAGGTCTTTGAGGCGTATAGCGCGTTTATTGGCCGC
GAGCAGCATGTCGCGCTGGAGACATTACTGCCTGCGCCTGAGTTTGGTCGTATTACGCTT
CATGGCCCGCTGGATCAACCGACGCTGAAAAAGACTGGCGCATTTGGTGTATGA
>EECEBICD_00985 USG-1 protein
ATGTCTGAAGGCTGGAACATTGCCATTCTGGGCGCAACGGGCGCCGTAGGCGAAGCCCTG
CTTGAAACACTGGCTGAACGCCAGTTCCCGGTCGGTGAAATTTATGCGTTGGCGCGTCAT
GAAAGCGCGGGCGAGCACCTCCGTTTTGGTGGCAAGTCGGTCATTGTCCAGGATGCGGCG
GACTTTGACTGGACGCAGGCGCAGTTGGCGTTTTTCGTTGCCGGCGCAGAGGCATCGGCT
GCCTGGATAGACGACGCGACTAATGCAGGTTGCCTGGTGATAGATTCCAGCGGTCTGTTC
GCGCTGGAGCCTGATGTGCCGCTGGTCGTGCCGGAAGTTAACCCGTATGTACTGGCGGAT
TACCGTAACCGTAACGTGATTGCGGTCGCCGATAGTCTGACCAGTCAACTGCTGGCGGCG
CTCAAGCCGTTAATCGATCAGGGGGGACTGTCGCGCATCGCCGTCACCAGTATGTTATCA
GCGTCCGCGCAGGGGAAAAAAGCGGTCGATGCGCTGGCTGGACAGAGTGCGAAGCTGCTT
AACGGCATACCGATTGATGAAGATGATTTCTTTGGCCGCCAGTTGGCGTTCAATATGCTG
CCGCTGCTGCCGGATCGGGAAGGCAGCGTGCGCCAGGAACGTCGTATCGTCGATGAAGTG
CGTAAAATTTTGCAGGACGATGGCGTGATGATCTCCGCCAGTGTCGTGCAGTCACCGGTG
TTCTATGGCCATGCGCAGATGGTGAGCTTTGAAGCGCTCCGTCCGCTGGCGGCGGAAGAG
GCGCGCGAGGCATTTTCCCGCGGTGAAGATATCGTACTGTCAGAAGAGACGGATTACCCG
ACGCAGGTGGGCGACGCTTCCGGCAATCCACAGCTCTCTATTGGCTGTGTGCATAATGAT
TACGGTATGCCGGAACAAATTCAGTTCTGGTCAGTGGCGGACAACGTGCGTTTCGGCGGC
GCGCTGATGGCCGTTAAAATTGCGGAGAAGCTGGTGCAGGAGTATCTGTACTGA
>EECEBICD_00986 tRNA pseudouridine synthase A
ATGTCCGGACAGCAATCATCGCCGGTCTATAAAATTGCGCTGGGTATTGAGTACGACGGC
AGCAAGTATTATGGCTGGCAGCGTCAGAATGAAGTGCGCAGCGTGCAGGAAAAGCTGGAA
AAAGCGCTCTCGCAGGTGGCAAATGAACCTATTAACGTGTTCTGCGCCGGGCGCACCGAT
GCCGGCGTTCACGGCACCGGGCAGGTTGTCCATTTCGAAACGACCGCATTGCGCAAAGAT
GCGGCATGGACGCTGGGGGTAAATGCGAATTTGCCTGGTGACATTGCCGTGCGCTGGGTG
AAAACTGTACCGGATGATTTTCACGCGCGATTTAGCGCTACGGCTCGCCGTTATCGCTAT
ATCATCTACAATCATCGACTGCGTCCTGCGGTATTAGCAAAAGGCGTGACGCATTACTAT
GAGCCGCTGGATGCAGAGCGGATGCATCGCGCGGCGCAGTGTCTGCTTGGCGAAAATGAT
TTTACCTCGTTTCGCGCGGTGCAGTGCCAGTCGCGCACGCCGTGGCGTAATGTGATGCAC
ATCAACGTTACCCGCCACGGCCCTTATGTGGTGGTCGATATCAAAGCGAATGCCTTTGTT
CATCATATGGTCAGGAATATTGTCGGCAGCCTGCTGGAAGTAGGCGCTCACAACCAGCCG
GAGAGCTGGATCGCAGAGCTGTTAGCGGCCAGGGACAGAACGCTGGCGGCGGCAACGGCG
AAGGCGGAGGGGCTATATTTGGTTGCGGTGGATTACCCTGACCGGTTTGACCTTCCAAAA
CCGCCAATGGGCCCGCTATTTCTGGCGGACTAA
>EECEBICD_00987 Protein DedA
ATGGACCTGATCTACTTTATTATTGATTTTATTCTGCACATCGATGTGCATCTGGCGGAG
CTGGTCGCGGAGTACGGCGTCTGGGTGTACGCTATTTTGTTTTTGATTCTGTTTTGTGAA
ACCGGCCTGGTCGTCACGCCGTTCCTGCCGGGTGACTCGCTGCTGTTTGTCGCCGGGGCG
CTGGCTTCGCTGGAAACCAACGATCTCAATGTGCATGTCATGGTGGCGTTAATGCTGATC
GCCGCAATTGTCGGCGATGCGGTAAATTACACGATTGGTCGACTGTTCGGCGAAAAACTG
TTCAGTAATCCGAATTCGAAAATTTTCCGCCGCAGTTATCTGGATAAAACCCACCAGTTT
TATGAAAAACACGGCGGTAAAACTATCATTCTCGCTCGCTTCGTGCCGATCGTCAGAACC
TTTGCGCCGTTTGTGGCAGGGATGGGGCACATGTCCTATCGGCATTTTGCCGCCTATAAC

GTCATCGGCGCGCTGCTGTGGGTGCTGCTCTTTACCTACGCCGGTTATTTCTTCGGTACG
ATACCGTTGATTCAGGATAACCTTAAGCTGCTGATTGTGGGGATTATCGTGGTTTCGATA
TTACCTGGCGTCATTGAGATTGTCCGGCATAAGCGCGCTGCGTCGCGTGCCGCAAAATAG
>EECEBICD_00988 Acetyl-coenzyme A carboxylase carboxyl transferase subunit beta
ATGAGCTGGATTGAACGAATTAAAAGCAACATTACTCCCACCCGCAAGGCTAGCATTCCT
GAAGGGGTGTGGACCAAGTGTGATAGCTGCGGTCAGGTTTTATACCGCGCTGAGCTGGAA
CGTAATCTTGAGGTCTGTCCTAAGTGTGACCATCATATGCGCATGTCGGCGCGCAATCGC
CTGCATAGCCTGTTAGATGAAGGTTCCCTTGTGGAGTTGGGTAGCGAGCTGGAGCCAAAA
GACGTGCTCAAGTTCCGCGACTCCAAAAAATATAAAGACAGACTGGCGTCCGCGCAGAAA
GAAACCGGCGAGAAAGACGCGCTGGTGGTCATGAAAGGGACGCTTCACGGTATGCCGGTT
GTCGCCGCCGCGTTTGAATTCGCGTTCATGGGCGGCTCAATGGGGTCTGTCGTTGGCGCA
CGCTTCGTTCGCGCCGTTGAACAGGCGCTGGAAGACAACTGTCCGTTAGTGTGTTTCTCT
GCTTCCGGCGGCGCGCGTATGCAAGAAGCGCTGATGTCGCTGATGCAGATGGCGAAAACC
TCGGCGGCGCTGGCTAAAATGCAGGAACGTGGTCTGCCCTACATCTCGGTATTGACCGAT
CCGACAATGGGCGGCGTTTCCGCCAGTTTTGCGATGTTGGGCGATCTCAACATCGCCGAG
CCAAAAGCCCTCATTGGCTTCGCTGGCCCGCGCGTTATCGAACAAACCGTTCGTGAGAAG
CTCCCGCCGGGATTCCAGCGCAGTGAGTTCCTGATCGAAAAAGGCGCTATTGATATGATA
GTTCGCCGCCCGGAAATGCGCCTGAAGCTGGCGAGCATTCTGGCGAAGCTGATGAATCTG
CCCGCGCCGAACCCGGACGCCCCGCGTGAAGGCGTGGTGGTGCCTCCGGCGCCGGATCAG
GAGTCTGAGGCCTGA
>EECEBICD_00989 Dihydrofolate synthase/folylpolyglutamate synthase
ATGAAAAACAAATCTATACCTCAAGCTGCGTCGCCCCTGGCCTCGTGGCTTTCTTATCTG
GAAAACCTGCACAGCAAGAGTATCGATTTAGGCCTTGAGCGCGTAAGCCAGGTGGCAGCG
CGTCTGGACATCCTCAAGCCTGCGCCGTTCGTTTTTACCGTGGCGGGAACCAACGGTAAA
GGTACGACCTGTCGAACGCTGGAGTCGGTGTTAATCGCGGCGGGATACCGTGTGGGCGTC
TATAGTTCGCCTCATCTGGTACGTTACACCGAGCGCGTGCGCGTGCAGGGGAAAGAATTA
GCGGAATCCGCGCATACCGCGTCCTTTGCGGAAATAGAAGCGGCGCGCGGCGATATTTCA
TTGACCTATTTTGAATATGGTACGTTGTCGGCATTGTGGCTGTTTAAACAGGCTAACCTT
GACGTGGTGATTCTGGAGGTCGGGTTGGGCGGCCGCCTGGACGCCACGAACATTGTGGAT
GCCGATGTAGCGGTAATTACCAGCATCGCGCTTGACCATACTGACTGGCTGGGGCCGGAT
CGCGAAAGTATCGGCCGCGAGAAGGCCGGGATTTTTCGCGCGGAAAAACCCGCTATCGTC
GGCGAACCGGAAATGCCGACAACCATCGCGGATGTCGCGCAGGAGACCGGCGCGCTGTTG
CGGCGTCGCGGCGTTGACTGGCGTTATGAGGTGACAGCGACCCACTGGGCGTTTACCGAT
GGTGACGGCACGCTTGCCGGACTGCCGTTGCCGCAGGTTCCTCAGCCGAATGCCGCCACC
GCTCTGGCCGCGCTGCGCGCCAGCAGGTTGAACATTGACGAGCAGGCGATTCGCGACGGT
ATTGCGCAGGCTACCTTGCCGGGACGTTTTCAAATCGTGAGTGAGTCGCCGCGCGTTATC
TTTGATGTCGCCCATAATCCCCACGCGGCGGAATACCTGACGGGTCGTCTGAAAATGTTG
CCGAAACGCGGGCGCGTGCTTGCGGTGATTGGTATGCTGCATGATAAAGATATCGCCGGC
ACGTTAGCCTGGTTGAAAAGCGTGGTTGACGACTGGTATTGTGCTCCGTTAGAGGGGCCG
CGCGGCGCGACGGCGGAACAACTTCTGGAACATCTGGGGAAAGGGAATGTCTATGATAGC
GTCGCGCAGGCATGGCAGGCCGCTATCGACGCGGCGCAGCCAGAAGATACCGTGCTGGTG
TGCGGATCATTCCACACCGTCGCACATGTTATGGAAGTGATAGACGCGGGGAGAATCGGT
GGCGAGTAA
>EECEBICD_00990 Cell division protein DedD
GTGGCGAGTAAGTTTCAGAATCGTTTAGTCGGCACGATTGTGTTAGTCGCGCTGGGGGTT
ATTGTGCTTCCCGGTCTGCTGGACGGACAGAAAAAGCATTATCAGGATGAATTTGCTGCG
ATTCCACTGGTACCGAAGCCTGGCGACCGTGATGAGCCGGATATGATGCCTGCCGCAACG
CAGGCGCTGCCGACACAGCCGCCGGAAGGGGCGGCTGAAGAGGTGCGGGCGGGAGATGCG
GCAGCGCCATCGCTGGACCCGTCGCGCATGGCGTCTAACAATGTTGAGCTTGATCCGATA
CCGGCGGAGACGCCTAAACCGAAGCCGGTTGAAAAGCCGAAACCGCAGCCTAAACCGCAA
CAGCCGGTTGTCGCCGCTTCCACGCCGACGCCCGCGCCGCAACCAGTCGCCGACGATAAA
CCTGCGCCGACCGGCAAAGCCTATGTCGTACAGTTGGGCGCGCTGAAAAACGCGGATAAA
GTCAACGAGATAGTCGGCAAGCTTCGTTCCGCCGGGTTCCGGGTTTATACTTCCCCCTCA

ACGCCGGTACAGGGTAAAATAACCCGTATTCTCGTCGGGCCGGATGCGTCGAAAGATAAG
ATGAAAGGATCGCTTGGCGAGCTCAAGCAGATTTCCGGGTTAAGCGGCGTGGTGATGGGA
TATAGCCCGAACTAA
>EECEBICD_00991 Colicin V production protein
ATGGTCTGGATTGATTACGCCATTATCGCGGTGATTGCTTTTTCCTGTCTGGTTAGCCTG
ATCCGCGGCTTTGTTCGTGAAGCGTTATCGCTGGTGACGTGGGGTTGTGCTTTCTTTGTC
GCCAGTCACTACTACACTTACCTGTCTGTCTGGTTTACGGGCTTTGAAGACGAACTGGTT
CGTAATGGGATTGCTATCGCGGTACTGTTTATCGCGACGCTTATCGTCGGCGCTATCGTG
AATTTTGTGATAGGTCAGCTAGTAGAGAAGACCGGCCTGTCGGGAACTGACCGGGTGTTG
GGGATCTGTTTCGGCGCACTGCGAGGCGCGCTGATTGTCGCCGCCATTCTGTTCTTCCTT
GATACCTTTACCGGCTTGTCGAAAAGCGAAGACTGGAGCAAATCGCAGCTCATCCCGCAG
TTCAGCTTCATCATCAGATGGTTCTTTGACTATCTGCAAAGCTCGTCGAGTTTCTTGCCC
AGGACGTAA
>EECEBICD_00992 Amidophosphoribosyltransferase
ATGTGCGGTATTGTCGGTATCGCCGGTGTTATGCCGGTCAACCAGTCGATTTATGACGCG
TTAACAGTGCTCCAGCATCGTGGTCAGGATGCTGCCGGTATCATCACCATTGATGCGAAT
AACTGCTTCCGCTTGCGTAAGGCAAACGGGCTGGTGAACGATATTTTTGAAGCTCGCCAC
ATGCAGCGTTTGCAGGGCAACATGGGGATCGGCCATGTGCGCTACCCTACCGCAGGCAGC
TCCAGCGCTTCCGAAGCGCAACCTTTTTATGTTAACTCGCCGTATGGCATCACGTTGGCC
CATAATGGCAATCTGACTAACGCGCATGAGCTACGTAAAAAGCTGTTTGAAGAAAAGCGC
CGCCATATCAACACCACCTCCGATTCCGAAATCCTGCTCAATATTTTCGCCAGCGAGCTC
GACAACTTCCGTCACTATCCGCTGGAAGCCGACAATATTTTTGCCGCGATTGCCGCGACT
AACCGTCAAATCCGCGGCGCCTATGCCTGCGTGGCGATGATTATCGGCCACGGCATGGTG
GCTTTTCGCGATCCGCACGGCATTCGCCCGTTAGTGCTGGGCAAGCGTGACGTTGGCGAT
GGTCGTACGGAGTATATGGTCGCTTCTGAAAGCGTGGCGCTGGATACGCTGGGCTTTGAG
TTTCTGCGCGATGTCGCGCCGGGCGAAGCTATCTATATTACCGAAAAGGGGCAACTGTTC
ACGCGCCAGTGCGCGGATAACCCGGTCAGTAATCCCTGCCTGTTCGAGTACGTTTATTTT
GCTCGCCCGGACTCCTTTATCGACAAAATCTCCGTCTACAGCGCTCGTGTGAATATGGGC
ACCAAACTTGGCGAAAAAATCGCTCGTGAATGGGAAGATCTGGACATCGACGTCGTGATC
CCTATCCCGGAAACCTCCTGCGATATCGCACTGGAAATTGCCCGTATTCTCGGCAAGCCG
TACCGCCAGGGATTTGTCAAAAATCGTTACGTGGGACGTACCTTTATCATGCCGGGCCAA
CAGCTACGCCGTAAATCCGTGCGCCGTAAGCTCAATGCTAATCGCGCGGAATTCCGCGAT
AAGAACGTCCTGCTGGTGGATGATTCCATCGTACGTGGCACCACATCAGAGCAGATCATC
GAGATGGCCCGGGAAGCCGGCGCGAAGAAGGTTTATCTGGCGTCCGCCGCGCCGGAAATT
CGTTTCCCCAACGTCTATGGCATTGATATGCCGACCGCCAATGAGCTGATCGCGCATGGT
CGCGAGGTGGATGAGATTCGGCAGATCATCGGCGCAGACGGGCTTATCTTCCAGGATCTG
AACGATCTGATCGAAGCCGTACGCGCGGAAAACCCGGATATCCAGCAGTTTGAATGTTCG
GTATTTAACGGGGTATATGTCACCAAAGACGTAGACCAGCAATACCTCGACTTCCTGGAT
TCATTACGTAATGACGACGCCAAAGCGGTGCTGTTCCAGAACGAAATGGAAAACCTGGAA
ATGCATAACGAAGGCTAA
>EECEBICD_00993 Arginine utilization regulatory protein RocR
ATGGCATCCACCAATCAGGAACTGGCCTCGGCGCTCAGGATGTTTTCCCGCTTTTTCGAT
TTGATTCATCAGCCGTTGGCCGTCATTAATGAGCGTGGTGAATACGTTTACTACAATCAG
GAAAGCGCGGATCTGGACGGCTACAGCATTGAACGGGCAATGGGAAAACATATGCTGGAT
GTCTATCCGGGCATGAAAGAGACGCAAAGTACGATGCTTTCATCGTTAAAAAAAGGCGTG
GAATACATTGGCCATTATCAAATTTATTACAATGCGCGAGGCCAGGCCGTTGACTATCAG
CACACCACCGCACCGCTCTATGCAAGCGATGGCGGCATGGTCGGCGTTATCGAAATCGGC
AGGAATATGTCCGGCGTCAGGCGGCTCCAGGAGCAGGTGGTAGAACTGAACCAACTGCTG
TATGCCGATCGCCATGAGAAGCACCATGCCATTATTACCGAAAACCCGGAAATGCTCAGT
AATATCGCCAAAGCCAAACGGCTGGCCGCCAGTAATATTCCGGTGACGATTGTCGGGGAG
ACGGGTACGGGTAAGGAGCTATTTTCCCGCCTGATACATCAATGCAGCAAGCGGGCGAAT
AAGCCATTTATCGCCCTGAACTGCGGCGCTCTACCGCCTACGCTTATCGAAAGTACGCTT
TTCGGCACCGTGCGCGGCGCCTATACCGGCGCGGAAAATAGCCAGGGCTATCTGGAACTG
GCAAACGGGGGAACGCTCTTTCTTGATGAGCTGAACGCCATGCCGATAGAAATGCAAAGT

AAGCTGCTGCGATTCTTGCAGGATAAAACCTTCTGGCGGCTCGGCGGACAACAGCAACTC
CACTCTGATGTCAGGATCGTTGCCGCCATGAACGAAGCGCCCGTCAAATTAATTCAACAA
GAACGCCTGCGGGCGGATCTTTTTTATCGGTTGAGCGTCGGAATGTTGACGTTACCGCCG
CTGCGCGCCCGCCCGGAAGATATTCCCTTACTGGCAAATTATTTTATTGATAAATACCGT
AATGATGTGCCGCAGGACATTCACGGACTAAGCGAGACGGCGCGTGCTGATCTGCTCAAT
CACGCCTGGCCGGGTAATGTCAGGATGCTGGAAAACGCGATTGTACGCAGCATGATCATG
CAGGAAAAAGACGGGCTGCTGAAACACATCATTTTTGAACAGGACGAGTTAAATTTAGGC
GTACCGGAAACCGCGCCGGAAAATCCCCTTCCCTCGTCACCCGATCCGCAGTATGAAGGG
TCGCTGGAGGTACGGGTTGCTAACTACGAAAGGCATTTAATAGAAACCGCGCTGGATACG
CATCAGGGGAATATTGCCGCCGCGGCCCGCAGCCTTAACGTATCGCGCACCACGTTACAG
TACAAAGTACAAAAATACGCTATTCGCTTCGGCGTGGTGCGTAATTGA
>EECEBICD_00994 Diaminopimelate decarboxylase
ATGACTGACTCGATTATGCAGAATTACAACCAGCTACGTGAGCAGGTAATCAACGGCGAT
CGCCGTTTCCAGCACAAAGACGGTCATTTGTGTTTTGAAGGGGTCGATCTGGACGCACTG
GCGCGCCAGTACCCCACGCCGTTCTATGTGTTTTCTGAGCCGGAGATTATTCGTAACATT
CATGAAATCCAGCAGGCATTTGCGGCGCATAAAAATACCAAAACCTTCTTTGCGTCAAAA
ACCTGCTCGGTCATGGGGGTATTGAAAGCCATTCGTGACGCCGGAATTTGCGCGGAGGCA
AACTCACAATATGAGGTTCGCAAGTGTCTGGAGATAGGCTTTCGCGGAGATCAGATCGTC
TTCAACGGCGTGGTGAAAAAGCCTGCCGATCTGGAATATGCCATCGCTAACGATCTGTAT
CTTATTAATGTTGATAGCCTGTATGAGCTGGAACATATTGATGCCATCTCTCGCAAGCTG
AAGAAAGTCGCCAATGTTTGCGTCCGCGTAGAACCGAATGTTCCCTCGGCGACGCATGCG
GAACTGGTTACCGCTTTCCATGCGAAGTCGGGTCTCGACCTGGAACAGGCGGAAGAAACC
TGCCGCCGCATTCTGGCGATGCCTTATGTGCATTTACGCGGCTTGCATATGCATGTAGGC
GATCAGGTGCCGGAGTCAGAGCCTTTCGCAAAAGCGACTAAAGTGCTGGTGGATGAATCC
CGTCGTCTGGAAGAGGTGCTCGGTATTAAATTTGATTTGATTAACGTGGGCGGCGGAATT
CCGGTTCCCTATAAATATGACGACGAGAATGGCGACCCGTTAAAAGATAATATGTATGCC
GGTATTACCGCCCAGGATTTTGCCGATGCGGTCATTCGCGAAGTGCATAAATGGCGTACC
GATGTGGAAATATGTATTGAACCGGGACGTAAAGTGACCGGGTCGGCGGCGGTTTTACTG
ACGGAAGTCTCCTGCGAAAAACGTAAAACAAATTACGATCTTAACGGCAATGTGGAATGC
CACGTTGAATGGAAATTTGTCGACGCTGGTTACAGCGTACTCTCTGACAGCCAGCATTTT
GACTGGTTCTTTTATGTCTATAACGCCTCTCGCATGACGGCAGCGCACGACGCCTGGGTA
AAATTAGCCGGGCCGCTGTGTGATGGCGGAGATTATTTCCACATGGGGGTGAAAGGCGAA
GAGTTTTTACTGCCGAAGGAGACTCATGTTGGCGATATTGTCGCGTTTCTTGATGCCGGG
GCATACACCATTGAAAGTCAAACCGTTTATAACAACCGTCCGCGCACGGGGGTGGTGATG
ATCGATAAAAATGGTGAGACGCGTCTGATTCGTCGGGAAGACAGTTACGAAGATATGGTC
AAGTACGACATTTATTAA
>EECEBICD_00995 Glutamate/gamma-aminobutyrate antiporter
ATGAGTAACACGAATTCTGGCTTACTTGGCATCAAAGACATTGTCTTTATGAATGTCATC
GCTATTCTGAGTTTGCGGCAAATCCCTAACGTGGCGCCTTACGGGGCATCGGCCATGTTG
TTATGGGTGATCGCCGCATTCTGCTTGTTTTTTCCTTTAGCGATGGTATGCGGCGAGCTG
TCAACCGGCTGGCCGAAAGATGGCGGGATATTTGTCTGGATTAAAGAGGCATTTGGTAAA
CGTATTGCATGGATTGTCGTGGTTTGTTTTCTCTTTTCCTGCGTACTGTTTTTCCCATTA
ATGCTGCAATTCGGCTTTACCGCGTTGGGCTATATGATCGGCGGCGGCCTGGCGGAAAAT
AAAGCGTTTATCGGTATTGGTTCCGCGGTAATTTTCTGGCTATTAACGCTGATGAATATC
CGCGGGATGGAATGGACAAAAATTATTAATAGCATCAGCGCCTGGTGCGGCGTATTTATT
CCCTCCGCCATCCTCATTTTGCTGGCGGTAGTATGGCTATGTACCGGACACCAGATGCAG
ACGGACTACACTACAGCGAAAAACTGGATTCCGGATCTCGGACACTGGGATACGATCGTT
TTCTTATCCAGCATGATGTTTGCGTTTGCCGGACTGGAGGTCGCCCCGATGATTGCCGGG
CGTACCCGTAATCCACAGCGTGATTTTCCGCGAGCGATGGCCGTCTCCGCCGCCGTTATC
GTCGGGATCTATATGGTCGGCACCTGGGCGCTAAATACGCTTTTACCCGCCGGGAAAACC
GACATTGTGGCAGGCGTCATGCAGGCTATGCATGCTGCTGCGGATACGCTGCATATGCCG
TGGTTAATTCCTGTTATGGCCATCTGTATGTTTTTTGGCGCTCTCGGGCAGATTAACTCC
TGGCTGGTGGGGCCGATTTATATGCTCCAGGAAGCCTCGCGTGAAGATAATCTGCTGGGG
GACCGAATCGGCAAATTACATCCGGTGTGGAAAACGCCCGCCTTTGCCTTGACTGTACAG

GCGATTATTGTCACGGTCCTGTGTTTTTCGACTTTTATTTCGCCAAGCGTTGCGGCGGCT
TACTGGATGTTAACGGCGCTAACGACCATCACCTATTTTATTCCTTACCTGGTGATGTTC
CCGGCGTTCTGGCGTTTACGTAAGACTCAGCCGGATACGCCAAGAAGTTTTAAAATTCCG
GGCAAGGTTTTACCGGCTATTTTACCCGCGCTGGGCTTTCTTTCTATCGCCTTTGCCGTT
GCGCTGTTATTTATTCCGCCTTCTCAAATTGATATGGGCGGATATTTCCAGTACGCCGGG
AAAATTATTGGCGGAGCCGTGCTGGCGGTTGTCGTCGCGGAGTACATTTACCATCGGGCG
CAGAAACGTAATGCCCGTTTAAGCATGGCGGGAGGAAAATAA
>EECEBICD_00996 Ornithine racemase
ATGTATATGCCAGTTCTGGAAATAAATCTACGCAAGCTGGAAGAAAACGCCCGCACGGAA
AAAGCTCTGCTGGCAAGCAGCGGTATTGACGTGATGGCTGTCAATAAAGTTTTTGACGGC
TGCGTGGAAACCGCGCAGGCGGTGTTCAACGGCGGAATTACCGTTATCGCTGAATCACGA
ACCTATAATTTGAAAAAAATACGCGAGACAGGATGTACGACCTGCCTGTTACGCAGCCCG
TGTCTGAGTGAAATCGAAGATGTGGTGCGCTACGCGGATATTAGTTTAAATAGCGAGCCG
GTGGTATTACGGGCATTATCGCAAGAAGCCCAACGCCAGGGGAAAACGCATCAGGTATTA
CTGATGGTGGATATGGGCGATTTACGCGAGGGTATCTGGTTTGGTGAATATCAACGCATT
CTTGAGACCGTGACTCTGATTACTGGTCTGCCCGGTCTGGAATTATATGGACTGGGCACC
AACTTTAATTGTTACGGCACCGTGTTGCCAACGGTAAAAAATGGCGAGGATTTTCTGGCG
CTGGCCGCCCGGCTGGAAGCTGACAGCGGTATTCCTGTCCGGCGGCTTTCTGCGGGAAAC
TGCACCAGCTATCACCTGCTGGATAAAGGTATCTGGCCGCAGGGACTGAATCATCTGCGC
ATCGGCGGACTGCATGAATTCGGCATTGAATATGTGGACATGAAATACCTGAATGAATTT
CACCACTCCGCAAAACCGGTCGATAAAGCCTGTTCCGATATGTATATCCTGGAAGCGGAA
ATCATCGAACTGAACAGTAAACCTACCGTCCCGGTCGGGGAGCTGGGCGTCGATGCCTTT
TTGCAAAGTAAAACCTTTGTCGATCGTGGCATACGCCGACGCGCATTGCTGGCGTTTGGT
CGCCAGGATGTGCCGTCGGATAACTGTGTGCCATGCGACGACGCTATCACTATCTTAGGA
CAAACCAGCGATCATACGCTGGTGGATATTGAGGATTGTCGTCAGTCGCTAAAAGTAGGC
GATGTGGTTCGCTTTGAGCTGGATTATACGGGCCTGTTAATGGCCTGCCAGACCAAAGGC
GTCGCCTGGAGGTTTACGCGTTAA
>EECEBICD_00997 Amino-acid permease RocC
ATGGATACCCTGCAACAATCTCCGCCAGCAGAGCCTGAAGGCGGTCTGCGGCGTAACCTG
AAGAAGCGGCACCTGCTGATGATGTCGCTGGGCGGCACGATTGGCACCGGCTTATTTATC
GGCATTGCCGAACCGTTAAGCAGCGTCGGGCCGGCGGGAACCTTACTGGCCTATCTGTTT
GCCGGAACCATTATGCTGGCGACGATGATGTGTCTTGGCGAATTATCATGCGCTTTTCCT
CACTCCGGTTCATTTCAACATTACGCGCTGATGTTTATGCCGTCACCTTGCTGGAGCTAT
ACCATTGGCTGGCTTTACTGGTTTAGCTGGGTCTTTTCCTTGGCAGCCGACCTCACTGCC
GCAGGGTTTATTGCTCACCAGTTTTTCCCCGCGGTTCCGGTATATATGTTCTGCCTGGCG
ATTCTGCTCATCCTGACGGCTATCAATCTCACCTCCGCAAAAAGTTTTGGCGAATGCGAA
TACTGGCTTTCGGCCATTAAGGTCTTCGCGATCGTGCTGTTTATCTGCGCGGGCGGGGTC
ATGATCTACTCGCTAATGGGCCATAGTGACTGGCACCCGACGTTAAAAATAGACGGAATG
TGGTTTCCCCACGGTTGGGAGCAAATTGTGGTCTGTATGACGATCGTGATTTACTCCTTC
CAGGGGTGGAACTGGTCGGAAATGCGGCAGGGGAGACCGAATCGCCGCACATTATTTTAC
CGAAAGTGA
>EECEBICD_00998 Amino-acid permease RocE
GTGATCCTGGGGATCGGTCTCAGAATCATTTTGTTTTACGGCCTGGCGATTGCGGTGCTG
GCGCTGGTCTATCCGCATGAACTGACGCCAAATGGACAAAGTCCCTTTGTATGGGTATTC
TCCCACGCGGGCATTCCGGGGGCGGATACGCTGATGACGCTGGTGATCTTTTCCGCCGCG
GTTTCTGCCGCTAACTCGGCCATTTACGCCAGTTCGCGTATGCTGTGGTCTATGGCGGGC
GATCGCTTTGCGCCAGCCTGCTTTGGTAAAACGAACGGCGGCGGCGTGCCGGTGTATGCT
ATTTTAATCACGGCCTTGTTGGCGTTAGTGTCGTTGCTGACCCGCTATATTCCCGCACAG
CAATTTTATTTATATCTGATCGCCAGTACCGGGCAGGTGGGTTGCCTGGCATGGATAACC
ATCGGATGGTGCCAGTATCGTTTCCGACAATCGGTGCGTAATGGAACGTACGCCAGCGAT
TTGCTACGCTATCGATCGCCGCTTTTCCCCTGGACGGCCCGATTTGTCATTATCACCAAT
TTTGCCATTATGGTCGGCACCTGGTTTAGTGAGCAAGGGGTAGTGATAATGTTGGTTGAG
CTGGCTTTTATGATAGGGATTCTTTTGTCATGGTATCTCTTCAGACCCACTTTGAGCCGA
TTACGTAACACAGTGGGATGA

>EECEBICD_00999 Flavin prenyltransferase UbiX
ATGAAACGACTCATTGTAGGAATTAGCGGCGCCAGCGGAGCGATTTATGGCGTGCGCTTA
TTACAAATTTTGCGCGATGTCGATAGCGTGGAAACGCATCTGGTGATGAGCCAGGCGGCC
CGGCAAACGCTGGCGCTGGAGACCCATTTTTCGCTGCGCGAGGTGCAGGCGCTGGCCGAT
GTGACGCACGATGCGCGCGACATTGCCGCCAGCATTTCCTCGGGCTCTTACCCGACGGCG
GGAATGGTTATCTTGCCCTGTTCGATTAAAACGCTGTCGGGGATTGTCCACAGTTATACT
GACGGACTGCTTACCCGCGCGGCGGATGTGATATTAAAAGAGCGGCGACCTCTGGTGCTG
TGTGTGCGCGAAACGCCGCTGCATATCGGGCATTTGCGTTTAATGACCCAGGCCGCTGAG
ATAGGCGCGGTGATTATGCCGCCGGTTCCGGCTTTTTATCATCTTCCTCAGACGTTAGAT
GATGTTATAAATCAAACGGTTAACCGAGTACTGGATCAGTTTGACATCCCCCTTCCGCAC
GATCTTTTTGTTCGCTGGCAGGGCGCATAG
>EECEBICD_01000 Lysine/arginine/ornithine-binding periplasmic protein
ATGAAGAAGACCGTTCTCGCTTTGTCTTTGCTGATAGGTCTGGGCGCGACGGCGGCCAGT
TACGCCGCGCTCCCGCAAACGGTTCGTATTGGAACAGATACCACCTACGCGCCTTTCTCA
TCAAAAGATGCCAAAGGCGAGTTTATTGGCTTTGATATCGATCTCGGTAATGAAATGTGT
AAGCGTATGCAGGTGAAATGTACCTGGGTCGCCAGCGACTTTGATGCGCTTATTCCCTCA
CTAAAAGCGAAAAAAATTGATGCCATTATTTCATCGCTCTCTATTACCGATAAACGCCAG
CAAGAAATTGCGTTTTCCGACAAACTTTACGCGGCGGATTCACGCCTGATTGCGGCAAAA
GGGTCTCCCATTCAGCCGACGCTGGAATCGCTGAAAGGAAAGCATGTCGGCGTGCTGCAA
GGGTCCACGCAAGAGGCTTACGCCAATGATAACTGGCGCACAAAAGGTGTGGATGTGGTG
GCTTATGCCAACCAGGATCTTATCTATTCCGATTTAACCGCCGGTCGTCTGGATGCCGCA
TTACAGGATGAAGTCGCCGCCAGCGAAGGTTTCCTGAAGCAGCCAGCGGGCAAAGAGTAT
GCGTTTGCCGGTCCTTCCGTGAAGGATAAAAAATATTTTGGCGACGGGACGGGGGTTGGG
CTGCGCAAAGACGATACCGAGCTAAAAGCCGCGTTTGATAAAGCGCTGATCGAACTGCGC
CAGGACGGGACTTACGACAAAATGGCCAAAAAGTACTTCGATTTTAATGTCTACGGCGAT
TGA
>EECEBICD_01001 Histidine-binding periplasmic protein
ATGAAAAAACTGGCGTTATCCCTTTCTCTGGTGCTGGCATTTTCCAGCGCTACCGCAGCA
TTTGCCGCTATTCCACAAAAGATTCGTATCGGTACCGATCCTACATACGCACCGTTTGAA
TCCAAAAATGCACAAGGTGAATTGGTCGGCTTTGATATCGATCTGGCCAAAGAGCTGTGC
AAACGTATCAACACACAGTGTACGTTCGTGGAAAACCCGCTGGATGCGCTGATTCCGTCT
TTAAAAGCGAAGAAAATTGATGCCATCATGTCCTCGCTGTCCATCACTGAAAAGCGCCAG
CAGGAAATCGCGTTTACCGACAAGCTTTACGCCGCTGATTCCCGTCTGGTGGTGGCGAAG
AACTCTGATATTCAGCCGACCGTCGCGTCGCTGAAAGGTAAGCGCGTCGGCGTGCTACAG
GGGACGACGCAGGAGACCTTCGGCAATGAGCACTGGGCGCCGAAAGGGATTGAGATCGTC
TCTTACCAGGGGCAGGACAACATTTATTCCGATCTGACGGCTGGCCGTATTGATGCGGCG
TTCCAGGACGAGGTCGCGGCCAGCGAAGGTTTCCTTAAACAGCCCGTCGGAAAAGACTAT
AAATTCGGCGGCCCGGCGGTGAAAGATGAGAAGTTATTCGGCGTAGGAACCGGTATGGGG
CTGCGCAAAGAGGATAACGAGCTGCGCGAAGCGCTGAACAAAGCCTTTGCTGAAATGCGT
GCTGACGGTACTTACGAAAAGCTGGCGAAAAAGTACTTTGATTTTGATGTTTATGGCGGT
TAA
>EECEBICD_01002 Histidine transport system permease protein HisQ
ATGTTGTACGGGTTTTCAGGCGTAATTTTACAGGGCGCGATCGTCACGCTGGAACTGGCG
CTCAGTTCCGTGGTGCTGGCCGTGCTTATCGGTCTCGTCGGCGCGGGGGCGAAGCTTTCG
CAAAACCGGGTAACGGGGCTTATTTTTGAAGGGTACACCACCCTGATTCGCGGGGTGCCG
GATCTGGTATTAATGCTGCTGATATTTTACGGATTGCAAATCGCGCTTAACGTGGTGACC
GATTCGCTGGGTATCGACCAGATAGATATTGATCCGATGGTGGCCGGTATTATCACCCTT
GGTTTCATCTACGGCGCCTATTTTACCGAAACCTTTCGCGGCGCGTTTATGGCGGTGCCG
AAAGGCCATATCGAGGCGGCGACGGCTTTTGGTTTTACCCACGGGCAAACCTTCCGGCGG
ATTATGTTCCCGGCGATGATGCGTTACGCCCTGCCGGGGATCGGCAATAACTGGCAGGTG
ATACTCAAAGCGACGGCGCTGGTGTCGCTGCTGGGCCTGGAAGATGTCGTCAAAGCCACG
CAGCTTGCCGGTAAAAGCACCTGGGAGCCTTTCTATTTTGCCGTGGTCTGTGGACTTATC
TACCTGGTATTTACCACCGTTTCCAATGGTGTGCTGCTCTTGCTTGAGCGTCGCTATTCT
GTGGGCGTGAAGAGGGCCGATTTGTGA

>EECEBICD_01003 Histidine transport system permease protein HisM
GTGATTGAGATCATTCAGGAGTACTGGAAATCCCTGCTGTGGACGGATGGCTATCGTTTT
ACCGGCGTGGCGATTACGCTGTGGTTGTTAATTTCATCGGTGGTGATGGGCGGTCTTCTG
GCGGTGATTCTGGCGGTAGGTCGCGTTTCCAGTAATAAATTCATCCGTTTTCCCATCTGG
CTGTTTACGTATATTTTTCGCGGCACGCCGCTGTACGTACAGCTACTGGTGTTCTATTCG
GGGATGTATACGCTGGAGATAGTCAAAGGCACCGATCTTCTTAACGCGTTTTTCCGCAGC
GGACTGAACTGTACCGTACTGGCGCTGACGCTGAATACCTGCGCGTACACCACGGAAATT
TTCGCGGGGGCGATTCGTTCGGTACCGCACGGTGAAATTGAAGCGGCACGGGCCTATGGC
TTCTCGTCATTTAAAATGTATCGCTGCATTATTTTGCCGTCGGCATTACGTATCGCCTTG
CCTGCCTACAGCAACGAAGTGATTTTAATGCTGCACTCTACGGCGCTGGCGTTTACCGCG
ACGGTGCCGGATTTGTTGAAAATCGCCCGCGATATTAACTCAGCGACTTACCAGCCGTTT
ACCGCGTTTGGCATTGCCGCCGTGTTGTATTTACTCATTTCGTATGTCCTGATAAGCCTG
TTCCGTCGGGCGGAAAGACGCTGGTTGCAGCACGTGTCCTCTAAATAA
>EECEBICD_01004 Histidine transport ATP-binding protein HisP
ATGTCAGAAAATAAATTACACGTTATCGATTTGCACAAACGCTACGGCGGTCATGAAGTG
CTGAAAGGGGTATCGTTGCAGGCCCGCGCCGGAGATGTGATTAGCATCATCGGCTCGTCC
GGCTCCGGTAAAAGCACTTTTTTGCGCTGTATTAACTTCCTCGAAAAACCGAGCGAAGGC
GCGATTATCGTGAACGGTCAGAACATTAATCTGGTGCGCGACAAAGACGGGCAGCTCAAA
GTGGCGGATAAAAATCAGCTACGCTTGTTGCGTACCCGCCTGACGATGGTGTTTCAGCAC
TTTAACCTCTGGAGCCACATGACGGTGCTGGAAAATGTGATGGAAGCGCCGATTCAGGTA
CTGGGATTAAGCAAGCACGACGCGCGCGAGCGGGCGTTGAAATATCTGGCGAAGGTGGGG
ATTGATGAGCGCGCTCAGGGCAAATATCCCGTCCATCTCTCCGGCGGCCAACAGCAGCGC
GTTTCTATTGCGCGCGCGCTGGCGATGGAACCTGACGTTTTACTGTTCGATGAACCCACA
TCGGCGCTCGATCCTGAACTGGTCGGCGAAGTGTTGCGCATCATGCAACAACTGGCGGAA
GAAGGCAAAACGATGGTGGTGGTCACGCATGAAATGGGCTTCGCCCGCCATGTCTCTTCG
CACGTTATTTTTCTGCATCAGGGGAAAATTGAAGAAGAGGGCGATCCGGAGCAGGTGTTC
GGCAATCCGCAAAGCCCGCGTTTACAGCAATTCCTGAAAGGCTCGCTGAAATAA
>EECEBICD_01005 Epimerase family protein
ATGCAGATTCTGATTACCGGCGGTACAGGCCTGATAGGGCGTCATCTCATTCCCCGGCTG
TTAACACTGGGGCATCAGGTGACGGTCGTTACGCGCAATCCCGATAACGCGCGTCAGATT
CTCGATTCCAGAGTGACATTATGGAAAGGGCTGGCGGAACGTGAGCACCTCAATGAGATT
GATGCCATCATCAATCTGGCGGGCGAACCGATTGCCGACAAGCGCTGGACCTCGCAACAA
AAAGAACGGTTGTGCCAAAGCCGCTGGGCTATCACGCAAAAGTTGGTGGATTTAATTCAT
GCCAGCGCTACGCCGCCCTCGGTGTTGATATCCGGCTCCGCCACGGGCTATTACGGCGAT
CTGGGCGACGTTGTCGTCACTGAAGATGAGCCGCCGCACAACGAATTTACGCATAAACTT
TGCGCCCGCTGGGAACAGATTGCCTGTCGTGCGCAGAGCGACCAGACCCGCGTCTGCCTG
CTGCGTACCGGCGTTGTCCTGGCGCCGCAAGGCGGGATTCTGGGGAAAATGGTTCCGCCG
TTTCGCCTCGGACTCGGCGGTCCGGTGGGTAATGGTCGACAATATCTGGCCTGGATTCAC
ATCGACGATATGGTCAACGGCATTCTGTGGCTGCTGGATAACGATCTTCGCGGTCCCTTC
AATATGGTCTCGCCGTATCCCGTCCATAATGAGCAGTTTGCTCATGCGCTGGGTCGCGCG
CTGCGCCGCCCGGCGATTATTCGCATTCCGGCGACGGCAATTCGCTTATTGATGGGAGAG
TCGTCAGTGCTGGTGCTGGGCGGACAGCGGGCGCTGCCAAAACGGCTTGAAGCCGCCGGA
TTCGCATTTCGCTGGTATGATTTAGAAGAGGCGCTGGCGGATGTGATTCGCTAG
>EECEBICD_01006 Disulfide-bond oxidoreductase YfcG
ATGATTGACCTTTACTACGCCCCCACGCCCAACGGGCATAAAATAACGCTGTTTCTGGAA
GAGGCGGAGCTGGCGTATCGTTTGCTAAAAGTAGATATCAGCAAGGGCAATCAGTTTCGC
CCTGATTTTTTGGCTATCTCTCCCAATAATAAAATTCCTGCCATCGTCGATCACGCCCCG
GCAGACGGCGGTCAACCGCTTAGTCTTTTTGAATCCGGCGAAATTCTTCTCTACCTGGCG
GAAAAAAGCGGCAAACTGCTTAGCGGGGAGCTGCGTGAACGCCATACCACGCTGCAATGG
CTGTTCTGGCAGGTCGGCGGGTTAGGGCCGATGCTGGGCCAGAATCATCACTTTAACCAC
TTTGCGCCGCAGGCCATCCCCTATGCAATTGAACGCTATCAGGTTGAAACGCAACGCTTA
TACAACGTCCTGAATAAACGCCTGGAAACCTCCCCGTGGCTGGGCGGCGACCACTACAGT
ATTGCGGATATCGCCAGTTGGCCGTGGGTTAACGCACATCAGCGTCAGCGAATCGATCTT
GATACCTACCCGGCGGTGTATAACTGGTTTGAACGCATTCGCACGCGTCCTGCGACAGCG

CGCGCACTGTTACAAGCGCAACTGCACTGTAACAGTACGAAAGCGTAA
>EECEBICD_01007 Glutathione S-transferase YfcF
ATGAGTAAGCCTGTTATTGTGCTGTGGTCGGATGCGAACTTTTTCTCTCCCTACGTCTTG
TCCGCCTGGGTGGCATTACAAGAAAAAGGGCTTTCCTTTACTCTGAAAACTCGCGATCTG
GACCAGGGAGAACATCTGCAACCTGGCTGGCGAGGATATGCGCTGACGCAGCGCGTACCG
GTGCTTGAGGCCGACAATTTTGAGCTGAGCGAGTCGTCCGCTATTGCGGAATACCTGGAG
GAGCGTTTCGCGCCGCCGCAGTGGGAGCGTATCTACCCGCACGATTTACAAAAACGCGCC
CGCGCGCGACAGATTCAGGCGTGGCTACGCAGCGATTTACTGCCGCTTCGCGAAGAACGG
CCTACCGACGTGGTGTTTGCCGGGGCGAAAAAAGCGCCGTTAAGCGAGGCTGGAAAAGCC
AGCGCGGCAAAACTGTTTGCGACGGCTGAAGCGCTATTGGGGCAGGGGACGCAAAATTTA
TTTGGTGAATGGTGCATTGCCGATACCGATCTGGCATTGATGATCAATCGTCTGGCGCTG
CATGGCGATGACGTGCCGACATCGCTGGCGGCATATGCGACATTCCAGTGGCAGCGTGCT
TCCGTACAGCGTTTTATTGCGCTTTCCTCGAAGCGTTCAGGCTGA
>EECEBICD_01008 Phosphodiesterase YfcE
ATGAAACTGATGTTCGCATCGGACATTCATGGTTCTTTACCCGCCACGGAACGCGTGCTT
GAGCGGTTCGCGCAAAGCGGCGCGCGTTGGCTGGTGATATTAGGCGATGTCCTTAACCAC
GGGCCGCGAAATGCGCTGCCTGAAGGCTATGCGCCCGCCCAGGTCGCCGAACGCCTGAAC
GCGGTGGCGACGCAAATCATTGCCGTGCGCGGCAACTGCGATAGCGAAGTGGATCAGATG
CTGCTGCATTTCCCGATCACTGCGCCGTGGCAGCAAATATTGACGCAAGAGAGGCGGCTA
TTTTTAACGCATGGGCACCTCTTCGGGCCGACGAATCTGCCCGCTCTGCATACTGGCGAT
GTGTTGGTTTATGGTCATACGCATCTGCCGGTGGCGCAACAGCAGGAAGGACTGTATCAC
TTTAATCCTGGGTCAGTGAGTATCCCGAAAGGCGGGTATGCGGCAAGCTACGGTATTCTG
GATGATAATGTGTTGAGCGTAATCGCACTCAATGATCAAAGTATCATTGCGCAGGTTGCA
ATTAACTCGTAA
>EECEBICD_01009 putative Nudix hydrolase YfcD
ATGGTGGAACAGCGTCGTTTGGCAAGTACGGAATGGGTGGATATTGTGAATGAAGACAAT
GAAGTCATTGCACAGTCCAGCCGGGAACAGATGCGTGCGCAACGCTTACGGCATCGTGCG
ACGTATATCGTCGTTCATGATGGTATGGGTAAAATTCTGGTGCAGCGTCGTACCGAAACA
AAAGATTTTTTGCCCGGTATGTTAGATGCTACCGCAGGCGGCGTGGTTCAGGCTGATGAG
CAGCTACTGGAATCTGCCCGCCGCGAAGCGGAAGAAGAGTTGGGTATTGCAGGCGTGCCG
TTTGCCGAACACGGTCTGTTTTACTTTGAAGACCAACATTGCCGGGTTTGGGGCGCGCTG
TTTAGCTGCGTTTCCCACGGGCCTTTCGCGCTTCAGGAAGATGAAGTCAGCGAAGTGTGT
TGGCTGACCCCGGAGGAGATCACCGCGCGCTGTGATGAGTTTACGCCAGATTCGCTCAAA
GCGCTGGCGCTGTGGATGACCCGCAACGCCAAAAACGAAGCCGCGCTCCAGGAGAAACCG
GAAGAGACGGAATAA
>EECEBICD_01010 HTH-type transcriptional regulator GntR
ATGTCGATACCCCGTAAACGGCGCAGTACCGGTAAAGTGACTATCGCCGATGTAGCCCAA
CTTGCCGGTGTAGGCACGATGACCGTATCACGAGCCTTACGCACGCCGGAACAGGTCTCT
GATAAACTCCGGGAAAAAATCGAAGCGGCAGTGCATGAGTTGGGCTATATGCCTAATCTC
GCCGCCAGCGCGCTGGCGTCTGCCTCCTCGCACACCATCGCGATGGTCGTACCGAACCTT
GCGGAAGCGGGGTGTTCTGAAATGTTCGCCGGGCTGCAACAAATTTTACAGCCCGCAGGC
TACCAGATTATGCTGGCGGAATCGCAGCATCGCGTGGAACAAGAGGAGAAGCTGCTCGAA
ACGCTGCTGGCCTCCAACATTGCCGCAGCGATTCTGCTTAGCGTCGAACATAGCACTACC
GTGCGCCAGTGGCTAAAAAATGCCTCTATCCCGGTGATGGAGATGGGCGCGATTCGCAGC
GATCCTATTGATATGAATATCGGTATTGATAACGTTGCGGCAATGTATGAGCTGACGGAA
ATGCTGATTCAGCGCGGTTATCAGAATATCGGGCTGCTATGCGCCAATCAGGAGCAGTGG
ATTTTTCAGCAACATCTGCACGGCTGGTACAAAGCGATGCTTCGCCACCATATGTCGCCA
AACCGGGTGATTAATGCCGCCTTGCCGCCCAATTTTTCTACCGGCGCGTCGCAACTGCCG
GAGTTTTTACTGGCGTGGCCGGAGCTGGACGCGCTGGTATGCGTATCGGATGAGCTGGCC
TGCGGCGCGTTATACGAGTGCCAACGACGGCGGATCAAAGTTCCGGACGATTTAGCGGTG
GTCGGGTTTGGCGATAGCGATGTCAGCCGGGTCTGCCAGCCGCCGTTAACCACCATGGCC
GTACCGCACCGTAAGATTGGCAGTGAAGCCGGACGGGCGCTGTTGGAAAGGTTAAATCAG
GGAAACTGGAGCGATCGAAAATCCATCGCCTCCAGTTTATGTATGCGGGAAAGCTGCTAA
>EECEBICD_01011 Ascorbate-specific PTS system EIIA component

ATGCTGGGAACCTGGTTATCTGACGCAACCATCACGCTACGGGAGAGCGTGGAAACGTGG
TCGCAGGCGCTGGAAATTTGCGGCAAACCCCTGCTGGACGCAGGTGTTATCGCACCGGAA
TACATTACGGCGATTGTTCAGCAGCACCAAAAGCTTGGGCCGTATTATGTATTAGCGCCA
GGGTTAGCGATGCCGCATGCGAGGCCGGAAGAGGGGGCGAAAGGGTTAGGTTTATCGTTA
CTGAAACTACAGCACGGCGTTTCATTTGGCGCAGACGAATTCGATCCGGTTGACATCATT
ATTATGCTGGCGGCGCCTGATAAGCACAGTCATATCGAAATGATATCGGCGCTGGCGGAA
TTATTTTCCAGTGATGTAGATATGGAAAAATTACATCAGGCAAAAAACCTGGAGGACATT
AAAACGATTATTGACCGTTTCTGA
>EECEBICD_01012 hypothetical protein
ATGAAAATCATGGCAATTTGCGGTTCCGGCCTGGGTAGTAGTTTTATGGTAGAAATGAAC
ATTAAAAAAGTATTAAAGAAACTCAATATTGACGCCGAGGTGGAACACTCCGATCTTTCA
TCGGCCACGCCCGGGGCTGCCGATCTTTTTGTAATGGCCAAAGATATCGCGGCCAGCGCC
AGCGTACCGGAAAGCCAACTGGTGGTGATCAATAACATCATCGACATCAACGAACTGGAA
ACGCAGTTGCGCGCATGGTTCGCTAAACAATAA
>EECEBICD_01013 Ascorbate-specific PTS system EIIC component
ATGTTTATCCTTGAAACGCTGAATTTTGTTGTTGATATTTTAAAAGTACCCTCGGTGCTT
GTAGGGTTAATTGCGCTTATTGGTCTGGTGGCGCAGAAAAAAGCGTTTTCCGATGTGGTA
AAAGGCACGATTAAAACCATTTTAGGTTTTATTGTGCTTGGTGGCGGCGCAACCGTACTG
GTGGGTTCATTAAACCCGCTAGGGGGGATGTTTGAACACGCTTTTAATATTCAGGGCATT
ATTCCTAATAATGAAGCGATAGTCTCAATCGCACTGGAAAAATATGGCGTCCACGGCG
TTAATTATGGCATTTGGTATGGTGGCCAATATTGTCGTGGCGCGCTTTACGCGTCTTAAA
TATATCTTTTTGACCGGGCATCACACCTTTTACATGGCCTGTATGATTGGCGTAATCCTG
ACCGTGGCGGGGTTTGAGGGCGTAGGGCTGGTCTTCACCGGATCGCTAATCCTGGGGCTG
GTGATGGCCTTCTTCCCGGCGTTGGCCCAACGTTATATGAGGCGGATTACCGGTACCGAT
GACATTGCGTTTGGTCACTTCGGTACCCTGGGATATGTGCTTTCCGGCTGGATTGGCAGC
CTGTGTGGTAAAGGCTCCCGCTCCACGGAAGAGATGAACCTGCCGAAAAACCTCAGTTTT
TTACGCGACAGTTCTATCTCGATTTCGCTGACCATGATGATTATCTATCTGATCATGGCA
GTCAGCGCTGGGCGTGAGTATGTCGAAGCGACGTTTAGCGGCGGGCAAAACTATCTGGTG
TACGCCATCATCATGGCAATCACCTTCGCCGCCGGCGTGTTCATCATTCTGCAAGGCGTC
CGTCTGATTCTGGCTGAAATTGTTCCGGCCTTTACCGGCTTTTCAGAAAAACTGGTGCCA
AACGCGCGTCCGGCGCTGGACTGCCCGGTGGTGTATCCCTATGCCCCTAACGCGGTACTG
ATTGGCTTCCTGTTCAGTTTTCTCGGCGGGCTGGTGGGGCTGTTCCTGCTGGGACAGATG
AAGCTTGTGTTAATCCTGCCTGGCGTTGTGCCGCACTTCTTTACCGGCGCGACCGCAGGC
GTCTTTGGCAATGCCACCGGCGGTCGCCGCGGGGCGATGATCGGCGCATTTGCTAATGGT
CTATTAATCACTTTCCTGCCAGTGCTGTTACTGCCGGTTCTCGGCGCGATCGGCTTCGCC
AATACCACGTTCTCGGATGCTGACTTTGGCGTAATCGGTATTTTGTTGGGCAACCTGGCG
CGTTATCTGTCGCCGATGGCTATCACGGGGCTGGTGGTGGCGCTATTCGCGCTACTGGTG
GCGTACAACGTGTTGGCCAAAAACAAAAAAGCGACTGCCGAAGTGCAGGAAAATAGCGGA
GCGAAAGAATGA
>EECEBICD_01014 Apulose-4-phosphate transketolase subunit A
ATGAACGTAACCGAAATCACCCAACTGGCGCGCGATATTCGCGTCGCCACGCTCAAATCC
TTAAACCATCTTGGCTTTGGTCACTATGGCGGCAGCATGTCGGTAGTGGAGACTCTGGCG
GTTCTGTACGGCGCGGTGATGAAAATCGACCCTGCCGACCCGGACTGGCCGGAACGCGAT
TACTTCGTACTGTCGAAAGGCCATGCGGGCCCCGCGTTGTACAGTACCCTGGCGATTAAA
GGCTATTTCCCGCGCGAAGAGCTGAATACGCTTAACCAGAACGGCACCCGTCTTCCCAGC
CATCCGGATCGGCTAAAAACGCGCGGCGTGGACGCCACGACCGGCTCACTGGGACAGGGC
ATCTCTATTGCGGGCGGAATGGCGTTGTCGCATAAACTGGCGCGGCGACCCAACCGGGTA
TTTTGCATTGTCGGCGATGGCGAGCTCAATGAAGGGCAGTGCTGGGAGGCGTTTCAGTTT
ATTGCCCACCACCGCCTGAATAATCTGACCGTTTTCATTGACTGGAATAAACAGCAGCTT
GACGGCGAGCTGGAGGAGATTATCAATCCATTCGATCTGGAAGGGAAATTCCGTGCGTTT
GGTTTCGATGTCGTCACCGTCAAAGGCGATGATATCGCCGGACTACTGGCGGTGGTTCAG
CCTGTGCTGCCCGCTGATGCCCGGCCACGGGTTGTGATCCTTGACAGCATTAAAGGGCAG
GGGGTGCCATACCTGGAGCAGTTAACGAATTCTCACCACTTACGCCTGACGGATGGCATG
AAGCAGACGCTCAATGAGGCCATTCATCAACTGGAGGTCATGCATGATTAA

>EECEBICD_01015 Apulose-4-phosphate transketolase subunit B
ATGATTAAGCTTGCGCCCGCAGGGCTTAAAGACGATATCGAAATGCGCAAAGTTTACGCC
GGTTTTGTCGCCGGGCAGATTGAGGCCGGAAGCCCAATTATTGCGCTTGAGGCTGATTTA
ATGAGCTCAATGGCGATGGACAGCGTGGCGCGTGACTACCCGCAGCATGTCATTAACTGC
GGTATTATGGAGGCGAATGTGATTGGCACTGCGGCGGGGCTGGCGCTGACCGGTCGTAAA
CCGTTTGTGCATACCTTTACCGCTTTTGCCAGCCGTCGCTGTTTTGATCAGCTCTTCATG
GCGCTGGATTATCAGCGTAACAACGTCAAAGTGATCGCCTCCGATGCCGGAGTGACGGCG
TGCCATAACGGCGGCACCCATATGTCTTTTGAAGATATGGGGATTGTTCGCGGGCTGGCG
CACTCCGTAGTACTGGAGGTGACGGATGCGGTGATGTTCGCGGATATTTTACGCCAGCTA
ATGGACCTGGACGGTTTTTACTGGCTGCGTACCATCCGTAAGCAGGCGACGAGTATCTAT
GCGCCAGGCTCTACCTTTACCATTGGCAAGGGCAACGTACTGCGCGAGGGCGATGATATT
ACGCTTATCGCCAACGGCATTATGGTGGCGGAGGCGCTGGAGGCGGCGCGCCAACTGGAG
CAGGAAGGCGTGAGCGCCGCGGTCATTGATATGTTCACCTTAAAGCCTATCGATCGCATG
TTGGTGAAAAACTACGCGGAAAAAACGCGGCGTATCGTCACGTGTGAAAACCACAGCATC
CATAATGGACTGGGGTCAGCGGTGGCGGAAGTGCTGGTGGAAAACTGCCCGGTGCCGATG
CGCCGTGTTGGCGTCAAAGAGCGTTATGGGCAGGTAGGAACGCAGGATTTCTTACAACAA
GAGTACGGACTCACCGCCGAAGCGATTGTAGAAGCCGCGAAATCACTGCTTTAA
>EECEBICD_01016 hypothetical protein
ATGTCTGTCGTTACAGAATCAAAAACAGCCAGAAAATGGGCAATGCCCGATACGCTGGTC
ATCATCTTTTTTGTCGCCATTTTAACCAGTATCGCCACCTGGGTCGTACCCGTTGGGATG
TTTGATAGCCAGGAAGTGCAGTATCAGGTCGATGGTCAGACAAAAACCCGCAAAGTCGTC
GACCCGCACTCTTTTCGCATTGTGACCAATGAGGCCGGGGAAGCGCAGTATCATCGCGTA
CAATTTTTCACTACCGGCGATGAAAGACCAGGGCTGATGAATTTTCCGTTTGAAGGGTTA
ACATCCGGATCGAAATTCGGCACTGCCGTCGGCATCATTATGTTTATGTTGGTGATTGGC
GGCGCATTCGGCATCGTGATGCGTACCGGCACCGTGGATAACGGCATTCTGGCGCTTATT
CGCCACACGCGGGGCAACGAAGTCCTGTTTATTCCGGTGCTATTTGTCCTCTTTTCGTTA
GGGGGCGCCGTATTTGGCATGGGCGAAGAGGCGGTCGCCTTTGCAATTATCATAGCCCCG
CTGATGGTCAGGCTGGGCTATGACAGCATTACCACCGTCCTGGTGACCTATATCGCCACC
CAAATTGGCTTTGCCAGTTCGTGGATGAACCCTTTCTGCGTAGTCGTCGCCCAGGGGATT
GCCGGCGTGCCGGTACTTTCCGGCTCCGGGCTGCGTATTGTGGTCTGGATTGTCGCCACG
CTGATTGGGCTGGTTTTTACACTGGTCTACGCCTCACGCGTGAAAAAGAATCCGCTGCTG
TCCCGCGTCCACGAATCCGATCGCTATTTCCGCGAACAGCAGGATGAAGTCGTCCAGCGC
CCTTTCACCTTCGGTGACTGGCTGGTACTGCTGGTCCTGACCGGCGTAATGATTTGGGTC
GTCTGGGGCGTGATTGTCCACGCCTGGTTTATTCCGGAGATCGCCAGCCAGTTTTTCACC
ATGGGCGTGGTGATCGGCCTCATCGGCGTTATCTTCCGCCTCAACGGTATGACGGTCAAC
GTGATGGCGTCCTCATTCACCGAAGGGGCGAGGATGATGATCGCGCCCGCCTTGCTGGTG
GGTTTTGCTAAAGGCATTCTGCTGCTGGTCGGCAACGGTGAAGCCGGGGAACCCAGCGTG
CTCAATACGCTGCTTAACAGTATCGCGCATGGCATCAGCGGGCTTAATAATGCTATCGCC
GCCTGGTTTATGCTGCTCTTCCAGGCGGTATTTAATTTCTTTGTCACGTCCGGTTCGGGT
CAGGCGGCGTTAACCATGCCATTACTGGCGCCGCTCGGCGATCTGGTCGGTGTTAACCGT
CAGGTGACTGTTCTGGCCTTCCAGTTTGGCGATGGCTTCAGCCATATTATCTATCCGACA
TCAGCCTCGCTGATGGCCACGCTGGGCGTGTGCCGGGTCGATTTTCGCAACTGGCTGAAA
GTGGGAGCGTCGCTGCTGGGACTACTGTTTATTATGTCCAGCGTGGTCGTCATTGGCGCG
CAGATGATGGGATATCATTAA
>EECEBICD_01017 Phosphate acetyltransferase
GTGTCCCGTATTATTATGCTGATCCCTACCGGAACCAGCGTCGGCCTGACCAGCGTCAGC
CTCGGCGTCATCCGTGCTATGGAACGCAAAGGCGTTCGTCTGAGCGTCTTTAAGCCTATC
GCCCAGCCTCGCGCTGGCGGCGATGCGCCTGACCAGACCACCACTATCGTTCGCGCGAAC
TCTACCCTGCCGGCGGCTGAACCGCTGAAGATGAGCCACGTTGAATCTCTGCTCTCCAGC
AACCAGAAAGACGTGCTGATGGAAGAGATCATCGCGAACTACCATGCGAATACCAAAGAC
GCGGAGGTGGTGCTGGTTGAAGGTCTGGTTCCGACCCGTAAACATCAGTTCGCTCAGTCT
CTGAACTATGAAATCGCGAAAACGCTGAATGCGGAAATCGTTTTTGTCATGTCTCAGGGT
ACCGACACGCCAGAACAGCTGAACGAGCGTATCGAACTGACGCGCAGCAGCTTCGGCGGC
GCGAAAAACACCAACATCACCGGCGTTATCATCAACAAACTGAATGCACCGGTTGATGAA

CAAGGTCGTACTCGCCCGGATCTGTCGGAGATTTTTGACGACTCTTCCAAAGCGCAGGTG
ATCAAAATCGATCCTGCTAAACTGCAGGAATCCAGCCCGCTGCCGGTTCTGGGCGCGGTG
CCGTGGAGCTTCGACCTGATTGCGACCCGCGCTATCGATATGGCGCGTCACCTGAACGCC
ACCATCATTAACGAAGGCGATATCAAAACCCGCCGAGTTAAATCCGTCACTTTCTGCGCG
CGTAGTATTCCGCATATGCTGGAACATTTCCGCGCAGGCTCGTTGTTAGTGACCTCCGCT
GACCGTCCGGACGTACTGGTCGCAGCTTGCCTGGCCGCGATGAACGGCGTAGAAATCGGC
GCCCTGTTGCTGACCGGCGGCTATGAAATGGACGCACGCATTTCTAAACTGTGCGAACGC
GCATTCGCCACCGGTCTGCCGGTCTTTATGGTGAACACCAACACCTGGCAGACTTCGCTG
AGCCTGCAAAGCTTCAATCTGGAAGTCCCGGTTGATGACCATGAGCGCATCGAGAAAGTT
CAGGAATACGTCGCGAACTACGTTAACGCTGAGTGGATCGAGTCGCTGACCGCCACTTCC
GAGCGTAGCCGTCGTCTCTCTCCGCCGGCGTTCCGCTACCAACTGACCGAGCTGGCGCGT
AAAGCCGGTAAACGCGTAGTGCTGCCGGAAGGCGACGAACCGCGTACCGTCAAAGCGGCG
GCAATCTGCGCTGAACGCGGCATCGCCACTTGCGTTCTGCTGGGCAACCCGGATGAAATC
AACCGCGTCGCGGCATCTCAGGGCGTTGAGCTGGGCGCAGGTATTGAAATCGTCGATCCG
GAAGTGGTGCGTGAAAGCTATGTCGCTCGCCTGGTTGAGCTGCGTAAGAGCAAAGGCATG
ACCGAACCGGTTGCCCGCGAGCAGCTGGAAGACAACGTGGTGCTCGGCACGCTGATGCTG
GAGCAAGACGAAGTCGACGGCCTGGTTTCCGGCGCGGTTCATACCACCGCCAACACCATC
CGTCCGCCGCTGCAGCTTATTAAAACGGCGCCGGGTAGCTCCCTGGTCTCTTCTGTGTTC
TTTATGCTACTGCCGGAACAGGTTTACGTTTACGGCGACTGCGCAATCAACCCAGACCCG
ACTGCAGAGCAGCTGGCAGAAATCGCGATTCAGTCTGCGGATTCCGCCATTGCCTTCGGC
ATCGAACCGCGTGTGGCAATGCTCTCCTACTCCACCGGCACCTCTGGCGCGGGCAGCGAC
GTAGAGAAAGTACGTGAAGCGACGCGTCTGGCGCAGGAAAAACGTCCTGACCTGATGATC
GACGGTCCGTTGCAGTACGACGCCGCGGTCATGGCTGACGTAGCGAAATCCAAAGCGCCG
AACTCGCCGGTTGCGGGTCGCGCTACCGTGTTCATTTTCCCGGATCTGAACACCGGTAAC
ACCACCTACAAAGCGGTACAGCGTTCTGCCGACCTGATCTCCATCGGGCCGATGCTGCAG
GGTATGCGCAAGCCGGTGAACGACCTGTCCCGTGGCGCACTGGTTGACGATATCGTCTAT
ACCATCGCCCTGACGGCGATCCAGGCTTCCCAACAGCAGCAGTAA
>EECEBICD_01018 Acetate kinase
ATGTCGAGTAAGTTAGTACTGGTTCTGAACTGCGGTAGTTCTTCACTGAAATTTGCAATT
ATTGATGCAGTTAATGGTGATGAATACCTTTCTGGTTTAGCCGAATGTTTCCATCTGCCA
GAAGCACGCATCAAATGGAAAATGGACGGCAGTAAACAAGAAGCGGCTTTAGGTGCAGGC
GCCGCTCACAGTGAAGCGCTGAACTTTATCGTTAACACTATTCTGGCACAAAAACCAGAA
CTGTCTGCGCAGTTAACTGCAATTGGTCACCGTATCGTACACGGCGGCGAGAAGTATACC
AGTTCCGTAGTCATTGATGAGTCCGTCATTCAGGGCATCAAAGATTCCGCCTCTTTCGCA
CCGCTGCATAACCCGGCTCACCTGATTGGTATCGCCGAAGCGCTGAAATCTTTCCCGCAG
CTGAAAGATAAAAACGTTGCAGTATTCGACACCGCCTTCCATCAGACCATGCCGGAAGAA
TCTTACCTGTATGCCCTGCCGTACAGCCTGTATAAAGAACACGGCGTTCGTCGCTATGGC
GCACACGGCACCAGCCACTTCTATGTAACTCAGGAAGCGGCAAAAATGCTGAACAAGCCG
GTAGAAGAACTGAACATTATCACCTGCCACTTGGGCAACGGCGGTTCTGTTTCCGCTATC
CGTAACGGTAAATGCGTGGATACCTCTATGGGTCTGACCCCGCTGGAAGGCCTGGTCATG
GGTACCCGTTCCGGCGATATCGACCCTGCGATTATCTTCCACCTGCACGACACTCTGGGT
ATGAGCGTGGATCAGATCAACAAAATGCTGACCAAAGAGTCCGGTCTGCTGGGTCTGACT
GAAGTGACCAGCGACTGCCGTTATGTTGAAGACAACTACGCCACCAAAGAAGACGCAAAA
CGTGCGATGGACGTTTACTGCCACCGCCTGGCGAAGTACATCGGTTCTTACACCGCTCTG
ATGGACGGTCGTCTGGATGCAGTCGTGTTCACCGGCGGTATCGGTGAAAACGCCGCGATG
GTTCGCGAACTGTCTCTGGGTAAACTGGGCGTGCTGGGCTTTGAAGTCGACCACGAACGT
AACCTGGCGGCCCGCTTCGGCAAATCTGGTTTCATCAACAAAGAAGGTACCCGTCCGGCG
GTGGTGATCCCAACCAACGAAGAACTGGTTATCGCGCAGGACGCGAGCCGTCTGACTGCC
TGA
>EECEBICD_01019 hypothetical protein
ATGTCGACACCGGATAATCGCTCCGTAAATTTTTTTAGCTTATTTCGCCGGGGGCAGCAC
TACGCGAAGACGTGGCCGATGGAAAAGCGGCTGGCGCCCGTTTTCGTCGAAAACCGCGTC
ATTCGTATGACGCGCTATGCCATACGATTTATGCCGCCTGTCGCCGTCTTTACATTATGC
TGGCAGATAGCGCTGGGCGGTCAGTTAGGTCCGGCCGTGGCGACCGCCCTGTTCGCCCTG

AGCTTGCCTATGCAGGGATTATGGTGGTTAGGGAAACGTTCCGTGACGCCGTTGCCGCCT
TCAATCCTTAACTGGTTTTACGAAGTTCGCGGTAAATTGCAGGAGGCTGGACAAGCGCTG
GCGCCGGTAGAAGGAAAGCCGGATTACCAGGCATTAGCTGACACGCTTAAGCGCGCCTTC
AAACAACTGGATAAAACTTTCCTGGATGATTTGTAA
>EECEBICD_01020 hypothetical protein
ATGGAAATGACCAACGCGCAACGTCTGATTTTGTCTAACCAGTACAAAATGATGACGATG
CTCGATCCCACCAACGCCGAGCGTTACCGTCGCTTACAAACAATTATTGAACGCGGCTAC
GGCCTGCAGATGCGCGAGCTGGATCGCGAGTTTGGCGAGCTGACGGAAGAGACCTGCCGT
ACCATCATCGATATTATGGAGATGTACCATGCGCTGCACGTCTCCTGGACCAACCTCAAA
GATACGCAAGCCATCGACGAACGTCGCGTCACCTTCCTGGGGTTTGACGCCGCGACGGAA
GCGCGCTACCTCGGCTATGTCCGTTTTATGGTGAATATAGAAGGACGCTACACTCACTTT
GACGCCGGGACACACGGCTTTAATGCCCAGACGCCGATGTGGGAAAAATACCAGCGGATG
TTGAATGTCTGGCATGCCTGCCCGCGTCAGTACCACCTGAGCGCGAATGAAATCAATCAA
ATCATTAATGCCTGA
>EECEBICD_01021 Hexitol phosphatase A
GTGCAGTGTAAAGGTTTTCTGTTTGATCTGGATGGAACGTTGGTTGACTCATTACCTGCC
GTCGAACGCGCGTGGTGTAGCTGGGCCGATCGTTTTAACCTTGCTCATGATGAGGTGCTG
GGCTTTATTCATGGCAAACAGGCGATCACCTCGTTGCGGCATTTTATGGCGGGTAAATCG
GAGGCGGAAATTGCCGCTGAGTTTACGCGCCTGGAGCAAATAGAAGCCACTGAAACCGCA
GGGATCACCGCGTTGCCGGGGGCAGTCGATTTACTCAATCACCTGAATAAAGCGGGCATT
CCGTGGGCTATCGTCACCTCCGGTTCAATGCCGGTCGCGCGTGCGCGCCATCAGGTCGCG
GGCCTTCCAGCGCCAGAGGTGTTTGTCACCGCCGAACGAGTCAAACGCGGCAAACCGGAG
CCGGATGCGTATTTGCTGGGCGCACAACTGCTTGGCCTGGCCCCTCAGGAGTGCGTCGTA
GTAGAAGATGCCCCGGCTGGCGTGCTGTCCGGGCTGGCTGCGGGCTGCCACGTCATTGCG
GTCAATGCGCCGGCCGACACGCCGCGTCTGGCGGATGTTGATTTTGCGTTGGATAGCCTG
ACGCAATTATCGGTCGCTAAACAACCAAACGGCGATGTGGTGGTGTTAAGAAAGACGTGA
>EECEBICD_01022 hypothetical protein
GTGAACGGTGAATTGATTTGGGTTCTCTCTTTACTGGCGATTGCCGTCGTGTTGTTTGCG
ACGGGCAAAGTACGTATGGATGCGGTGGCTTTATTCGTCATTGTTGCTTTTGTCCTTAGC
GGTACCTTGACCCTTCCGGAAGCCTTCTCCGGCTTTAGCGATCCGAATGTCATTCTTATC
GCCGCCTTATTTATTATTGGCGACGGCCTCGTGCGCACCGGCGTTGCGACGGTGGTCGGT
ACCTGGCTGGTTAAAATGGCGGGCAGCAGTGAAATCAAAATGCTGGTTTTACTGATGATC
ACCGTCGCCGGACTTGGCGCTTTCATGAGTTCAACCGGCGTGGTGGCGATCTTTATCCCC
GTTGTTCTTAGCGTTTCTATGCATATGCAGACCTCGCCTTCGCGCCTGATGATGCCGCTG
AGTTTTGCCGGGCTTATCAGCGGAATGATGACGCTGGTGGCGACGCCGCCGAACCTGGTG
GTTAACAGTGAACTGCTGCGTGAAGGCCTGCACGGGTTCAACTTTTTTAGCGTGACGCCG
CTGGGGATAGTGGTGTTGGCGCTCGGCATTGTTTATATGCTGGTGATGCGCTTCATGCTA
AAAGGCGATGCGCCCGGACAACAGGCCGGTAAGCGGCGCACTTTCCGCGATCTTATCCGC
GAGTACCGTCTGACGGGGCGCGCTCGCCGTCTGGCGATTCGCCCCGGTTCGCCAATGGTG
GGACAGCGGCTGGACGACCTTAAACTGCGCGAACGTTACGGCGCCAATGTCATCGGCGTT
GAACGCTGGCGGCGCTTTCGGCGGGTTATCGTCAATGTGAACGGCGTATCGGAGTTTCGC
GCTCGCGACGTTCTGCTGATTGATATGTCGGCGGCGGAGGTTGACCTCAGAGAGTTCTGC
GCCGAGCAACTGCTGGAACCGATGGTATTGCGTGGAGAATATTTTTCCGATCAGGCGTTG
GATGTTGGCATGGCGGAAATTTCGCTTATTCCGGAGTCTGAACTGATAGGCAAGTCGGTG
CGTGAAATCGCCTTCCGTACACGTTACGGGCTGAATATGGTCGGCCTGAAACGCGACGGC
GTCGCGCTGGAAGGGTCGCTGGCCGATGAACCGTTATTGATGGGCGATATCATTCTGGTC
GTGGGGAACTGGAAGTTAATCAGCCTGCTGGGGCAAAAAGGCCGCGACTTTGTGGTGCTG
AATATGCCGGTCGAGGTCAGCGAAGCCTCTCCTGCGCACAGCCAGGCGCCGCACGCCATT
TTCTGCCTGGTACTGATGGTGGCGCTGATGTTGACCGATGAAATTCCCAACCCGATCGCG
GCGATTATCGCCTGCCTGCTGATGGGGAAATTTCGCTGTATTGATGCTGAAAGCGCGTAT
AAAGCGATCCACTGGCCGAGTATTATCTTGATTGTGGGTATGATGCCGTTTGCGCTGGCG
TTGCAAAAAACCGGCGGCGTCTCTCTGGTCGTGCAAGGATTAATGGATATCGGCGGCGGC
TATGGGCCGTATATGATGCTGGGATGCCTGTTCGTGCTGTGCGCCGTCATAGGACTGTTC
ATTTCTAACACGGCAACGGCGGTGCTGATGGCGCCGATTGCGCTGGCGGCGGCGAAATCG

ATGGGGGTGTCGCCCTATCCGTTTGCGATGGCGGTGGCGATGGCGGCATCCGCCGCCTTT
ATGACGCCGGTTTCATCGCCGGTCAATACTCTGGTTCTGGGGCCGGGAAACTATAGCTTT
AGCGATTTTGTGAAGCTTGGCGTGCCGTTCACGCTTATTGTGATGGCGGTTTGTATTGTG
ATGATTCCGATGTTGTTTCCGTTTTAA
>EECEBICD_01023 5'-deoxynucleotidase YfbR
ATGAAGCAGAGCCATTTTTTCGCCCATCTCTCCCGCATGAAGTTGATTAACCGCTGGCCG
CTGATGCGTAACGTGCGTACGGAAAACGTCTCCGAACATAGTCTGCAGGTGGCGATGGTC
GCCCATGCGTTGGCGGCGATAAAAAACCGTAAATTTGGCGGTCAGCTCAATGCTGAACGC
ATCGCGCTTCTGGCGATGTATCATGATGCCTCGGAAGTACTGACCGGCGATCTGCCGACG
CCGGTAAAATATTTTAATTCCCAGATAGCCCAGGAATATAAAGCGATCGAAAAAATCGCC
CAGCAAAAGCTGGTTGATATGGCGCCGGATGAATTACGCGATATTTTCGCACCGCTTATT
GATGAGAATGCCTGGAGCGAAGAAGAACAGGCAATCGTAAAACAGGCCGATGCGCTTTGC
GCCTACCTGAAATGCCTGGAGGAGCTCTCAGCGGGAAACAATGAGTTTGGACTGGCAAAA
ACGCGGCTGGAAAAAACGCTGGAATTACGTCGCAGCCAGGAGATGGACTATTTTATGGCG
GTGTTCGTACCAAGTTTCCATCTCTCTCTTGACGAGATAAGCCAGGATTCACCGCTTTAA
>EECEBICD_01024 Glutamate-pyruvate aminotransferase AlaA
ATGTCCCCCATTGAAAAATCCAGCAAACTCGAAAATGTCTGTTATGACATTCGTGGCCCC
GTTCTGAAAGAAGCGAAACGCCTTGAGGAAGAAGGTAATAAAGTTCTTAAACTGAATATC
GGCAACCCCGCCCCCTTCGGATTCGAAGCGCCGGACGAAATTCTGGTCGATGTCATTCGT
AATTTGCCGACGGCGCAAGGATATTGCGACTCGAAAGGGCTTTATTCTGCCCGTAAAGCG
ATTATGCAACATTATCAGGCCCGTGGAATGCGCGATGTAACCGTGGAAGATATCTATATC
GGTAACGGCGTTTCCGAGCTGATCGTTCAGGCGATGCAGGCGTTGCTTAACAGTGGCGAT
GAAATGCTGGTTCCCGCGCCCGATTATCCGTTATGGACAGCGGCGGTATCGCTCTCCAGC
GGCAAGGCGGTACATTATCTTTGCGATGAATCTTCCGACTGGTTCCCGGACCTTGACGAT
ATCCGCGCCAAAATCACCCCACGTACGCGTGGTATTGTCATTATTAATCCAAACAACCCT
ACTGGCGCCGTCTATTCTAAAGAATTGTTGATGGAAATTGTCAACATTGCGCGCGAGCAT
AATCTCATCATCTTCGCCGATGAAATTTATGACAAGATTCTCTATGACGATGCCGAGCAT
CACTCTATCGCCGCACTGGCACCTGATCTGCTGACCATCACCTTTAATGGGTTGTCTAAA
ACCTATCGCGTCGCCGGCTTCCGCCAGGGCTGGATGGTGCTGAACGGGCCGAAAAAACAT
GCGAAGGGCTATATTGAAGGGCTGGAGATGTTAGCCTCAATGCGCCTGTGCGCCAACGTC
CCGGCGCAACATGCTATTCAGACCGCGCTCGGCGGCTATCAAAGCATTAGCGAATTTATT
CTGCCGGGCGGTCGACTGTATGAACAGCGTAACCGCGCCTGGGAGTTGATTAACGATATC
CCCGGCGTTTCCTGCGTAAAACCGCGCGGCGCGTTGTACATGTTCCCCAGGATCGACGCT
AAACGTTTTAATATTCACGACGACCAGAAGATGGTGCTGGACTTCCTGCTACAGGAAAAG
GTGCTGCTGGTGCAAGGTACCGCCTTTAACTGGCCGTGGCCGGATCACTTCCGCATTGTG
ACGCTACCGCGTGAAGATGACCTGGAGATGGCCATCAACCGTTTTGGGCGTTTCCTCTCA
GGTTACCATCAATAA
>EECEBICD_01025 HTH-type transcriptional regulator HdfR
ATGATAAATGCAAATCGTCCGATAATTAACCTCGATCTCGATCTGCTGAGAACGTTCGTG
GCCGTTGCCGATCTGAACACCTTCGCCGCGGCGGCGGCGGCGGTATGTCGTACTCAGTCT
GCGGTCAGTCAGCAAATGCAGCGTCTTGAGCAACTCGTGGGAAAGGAATTGTTCGCCCGC
CATGGTCGTAACAAACTTTTGACCGAACACGGTATCCAGCTTCTGGGGTACGCCAGAAAA
ATCCTGCGTTTTAATGATGAGGCGTGTTCATCATTAATGTTCAGTAATCTTCAGGGAGTG
TTAACAATCGGCGCTTCTGATGAATCAGCTGATACTATCCTGCCTTTTTTGCTTAGCCGA
ATCAGCTCGGTTTATCCGAAACTCGCGCTTGATGTACGGGTAAAACGCAACGCCTATATG
GTGGACATGGTTAAATCGCAGGAAGTAGATTTGGTGGTGACGACTAACCAACCTCATTCG
CTGGACTGTCTGAATTTGCGGACCTCGCCCACCCATTGGTATTGTGCGGCTGAGTATGTT
TTGCAAAGGGGAGAGCCCGTACCACTAGTCCTGCTGGACGATCCCAGTCCATTCCGCGAT
ATGGTGCTGGAGACGCTAAATGCAGCGGGTATTCCCTGGCGGCTGGCCTATGTGGCGTCT
ACATTACCGGCGGTACGCGCGGCCGTTAAAGCCGGTCTTGGCGTAACGGCTCGACCTGTC
GAAATGATGAGTCCCGACCTTCGCGTGCTGGGCGCCGCAGACGGATTACCGCCTTTGCCG
GATACGGAATATCTGTTGTGCCGCGATCCCAATAGCCAGAACGAGCTGGCGATGGTGATT
TTCCATGCGATGGAAAGCTATCAAAACCCATGGCATTACAACCAGTTTTCTGCAGAGGGC
GGAGATGATCCGCTGATGGTGGAAGGGGGTTTTGAGTAG

>EECEBICD_01026 NAD(P)H-quinone oxidoreductase subunit 3
ATGAGTATGTCAACATCCACTGAAGTCATCGCTCATCACTGGGCATTCGCTATCTTTCTT
ATCGTTGCCATTGGCCTGTGCTGCCTGATGCTGGTAGGCGGTTGGTTTTTGGGCGGTCGC
GCACGCGCGAGGCACAAGAACGTTCCGTTTGAATCAGGTATCGACTCGGTCGGCACCGCC
CGCTTACGCCTGTCTGCCAAGTTTTACCTGGTAGCCATGTTCTTCGTTATCTTCGACGTT
GAAGCGCTGTATCTGTTCGCATGGTCGACTTCTATCCGCGAAAGCGGCTGGGTTGGCTTT
GTGGAAGCTGCAATTTTTATTTTTGTGTTACTGGCTGGTCTGGTTTATCTGGCGCGTATT
GGCGCGTTGGACTGGACGCCCGCGCGTTCACGCCGCGAGCGTATGAACCCGGAAACGAAC
AGTATCGCTAATCGTCAACGCTAA
>EECEBICD_01027 NADH-quinone oxidoreductase subunit B
ATGGATTATACGCTCACCCGCATAGATCCTAACGGTGAGAATGACCGTTACCCCCTGCAA
AAACAGGAGATCGTAACCGACCCCCTGGAGCAGGAAGTTAACAAAAACGTGTTCATGGGC
AAACTGCATGACATGGTTAACTGGGGCCGTAAAAACTCAATTTGGCCCTACAACTTCGGC
CTTTCTTGCTGCTATGTAGAGATGGTGACCTCTTTTACCGCAGTGCATGACGTTGCCCGT
TTCGGCGCGGAAGTGCTGCGTGCGTCGCCACGTCAGGCTGACCTGATGGTGGTGGCCGGG
ACCTGCTTTACCAAAATGGCCCCGGTTATTCAGCGTCTGTATGACCAAATGCTGGAGCCA
AAATGGGTCATTTCTATGGGCGCCTGCGCCAACTCCGGCGGCATGTACGATATTTATTCG
GTAGTGCAGGGCGTTGATAAGTTCATCCCGGTGGATGTGTATATCCCTGGCTGTCCGCCG
CGTCCGGAAGCGTATATGCAGGCGTTGATGCTGCTGCAAGAATCGATTGGCAAAGAGCGC
CGTCCGCTCTCATGGGTGGTGGGCGATCAGGGCGTGTATCGCGCCAATATGCAGCCAGAA
CGCGAACGTAAACGCGGCGAGCGTATTGCCGTCACGAACCTGCGTACCCCGGACGAGATT
TAA
>EECEBICD_01028 NADH-quinone oxidoreductase subunit C/D
ATGGTGAACAATATGACCGACTTAACCGCGCAAGACGCTGCATGGAGTACCCGCGATCAT
CTTGATGATCCGGTTATCGGCGAACTGCGCAACCGTTTTGGGCCGGATGCCTTTACCGTT
CAGGCGACCCGCACCGGTATCCCCGTTGTTTGGGTCAAGCGTGAACAACTACTGGAGGTT
GGCGATTTCTTAAAGAAACTGCCGAAACCTTACGTCATGCTGTTTGACTTACACGGCATG
GATGAACGCCTGCGTACGCACCGTGACGGGTTACCCGCCGCGGATTTTTCCGTTTTCTAC
CACCTGATTTCCATTGAGCGTAATCGCGATATCATGCTCAAGGTGGCGTTGTCTGAAAAC
GACCTGCGCGTGCCGACCTTCACTAAACTTTTCCCTAACGCCAACTGGTACGAGCGTGAG
ACCTGGGAGATGTTTGGTATTGATATCGAAGGCCACCCGCACCTGACGCGCATCATGATG
CCGCAAACCTGGGAAGGCCATCCGCTGCGTAAAGATTATCCGGCGCGCGCGACCGAATTC
GACCCGTTTGAGCTGACCAAAGCCAAGCAGGATCTGGAAATGGAAGCGCTGACCTTTAAG
CCGGAAGACTGGGGGATGAAGCGCGGGACTGACAACGAGGACTTTATGTTCCTCAACCTC
GGCCCGAACCACCCGTCTGCGCACGGTGCGTTTCGTATTATCCTGCAACTTGACGGTGAA
GAAATCGTCGACTGCGTGCCGGATATCGGCTATCACCACCGCGGCGCCGAAAAAATGGGC
GAGCGACAGTCCTGGCATAGCTACATTCCCTATACCGACCGTATCGAATACCTCGGTGGT
TGCGTCAATGAAATGCCGTACGTGCTGGCGGTAGAAAAGTTGGCAGGGATCACCGTCCCG
GATCGTGTGAACGTCATCCGCGTGATGCTCTCCGAACTGTTCCGCATTAACAGTCATCTG
CTGTACATCTCGACCTTTATTCAGGACGTCGGCGCGATGACGCCGGTCTTCTTTGCCTTT
ACCGACCGGCAGAAAATTTATGACCTGGTGGAAGCCATTACCGGTTTCCGTATGCACCCG
GCCTGGTTCCGTATCGGCGGCGTCGCGCACGACTTGCCGCGCGGTTGGGATCGCCTGCTG
CGAGAGTTCCTTGAGTGGATGCCGAAGCGTCTGGATTCTTACGAGAAAGCCGCGCTGCGT
AACACTATTCTGAAAGGGCGTTCGCAGGGCGTTGCCGCTTACGGCGCGAAAGAGGCGCTG
GAGTGGGGCACCACCGGCGCAGGGCTGCGCGCGACCGGGATCGACTTCGACGTACGTAAA
TGGCGTCCATACTCTGGCTACGAAAACTTTGACTTTGAAGTGCCGGTGGGCGGCGGCGTT
TCCGACTGCTATACCCGCGTGATGCTGAAAGTGGAAGAGCTGCGCCAGAGTCTGCGCATT
CTTCAGCAGTGCCTCGATAACATGCCGGAAGGGCCGTTCAAAGCGGATCACCCGCTGACC
ACGCCGCCGCCGAAAGAGCGCACGCTGCAACATATCGAAACCCTGATCACCCACTTCCTG
CAGGTCTCCTGGGGCCCGGTGATGCCGGCGCAGGAGTCCTTCCAGATGGTGGAAGCGACG
AAGGGGATTAACAGTTACTACCTGACCAGCGACGGCAGCACTATGAGCTACCGTACCCGC
GTGCGCACGCCGAGCTTTGCGCACCTGCAGCAGATTCCCTCGGCGATCCGCGGTAGCCTG
GTCTCCGACCTGATTGTCTATCTGGGTAGTATCGATTTTGTTATGTCAGATGTGGACCGC
TAA

>EECEBICD_01029 NADH-quinone oxidoreductase subunit E
ATGCACGAGAATCAACAACCACAAACCGAGGCTTTTGAGCTGAGTGCGGCAGAGCGTGAA
GCGATAGAGCACGAGAAGCACCACTACGAAGACCCGCGTGCGGCGTCCATTGAAGCGCTG
AAAATCGTTCAGAAGCAGCGTGGCTGGGTGCCGGACGGCGCGATCTACGCTATTGCCGAT
GTGCTGGGTATTCCGGCGAGCGACGTCGAAGGCGTCGCCACATTCTACAGCCAGATTTTC
CGTCAGCCGGTCGGTCGCCACGTGATTCGCTACTGCGACAGCGTGGTGTGCCATATCACC
GGCTATCAGGGGATTCAGGCCGCGCTGGAAAAAAATCTCAACATCAAACCGGGGCAGACG
ACCTTTGATGGTCGCTTTACGCTGCTGCCGACCTGCTGCCTGGGGAATTGCGATAAAGGG
CCGAACATGATGATTGATGAGGATACTCACAGCCATCTGACCCCGGAAGCCATTCCTGAA
CTACTGGAGCGGTATAAATGA
>EECEBICD_01030 NADH-quinone oxidoreductase subunit F
ATGAAAAACGTTATCCGTACTCCCGAAACCCATCCGCTGACCTGGCGTCTGCGCGATGAC
AAACAGCCGGTCTGGCTGGATGAATATCGTAGCAAAAACGGCTACGAAGGCGCGCGTAAG
GCGCTGACCGGCCTGTCTCCGGATGAAATCGTCAGCCAGGTAAAAGACGCCGGTCTGAAA
GGGCGCGGCGGCGCGGGCTTTTCCACCGGTCTGAAGTGGAGCCTGATGCCGAAAGATGAA
TCCATGAACATCCGTTACCTGCTGTGTAACGCCGATGAAATGGAGCCGGGCACCTATAAA
GACCGTCTGCTGATGGAACAACTGCCGCACCTGCTGGTGGAAGGCATGCTGATCTCCGCG
TTCGCGCTGAAAGCGTATCGCGGCTACATCTTCCTGCGCGGCGAATATATCGAAGCGGCT
GTACATTTGCGCCGTGCGATTGCTGAAGCCACCGAGGCCGGGCTGCTTGGCAAAAATATC
ATGGGCACCGGGTTCGACTTTGAACTGTTTGTCCACACCGGGGCAGGGCGTTACATCTGT
GGTGAAGAAACGGCGCTGATTAACTCCCTCGAAGGCCGCCGCGCGAACCCACGCTCCAAG
CCGCCCTTCCCGGCGACCTCCGGCGTATGGGGCAAACCGACCTGTGTGAATAACGTCGAA
ACGTTGTGCAACGTCCCGGCAATCCTCGCCAACGGCGTGGAGTGGTACCAGAACATCTCG
AAAAGCAAAGATGCGGGTACCAAGCTGATGGGCTTCTCAGGCCGCGTGAAGAATCCGGGC
CTGTGGGAATTACCGTTCGGCACCACCGCGCGCGAGATCCTCGAAGACTACGCGGGCGGT
ATGCGCGATGGCCTGAAATTCAAAGCCTGGCAGCCTGGCGGGGCGGGAACCGACTTCCTG
ACCGAGGCGCATCTTGACCTGCCGATGGAGTTCGAAAGCATCGGTAAAGCAGGCAGCCGT
CTGGGCACCGCGCTGGCGATGGCGGTAGACCACGAAATCGGCATGGTGTCGCTGGTGCGT
AACCTGGAAGAGTTCTTTGCCCGCGAATCCTGCGGCTGGTGTACGCCATGCCGCGACGGG
CTGCCGTGGAGCGTGAAAATTCTGCGCGCGCTGGAGCGTGGCGAAGGCCAGCCGGGGGAT
ATCGAGACACTTGAGCAACTGTGTCGTTTCCTCGGACCGGGTAAAACCTTCTGCGCGCAT
GCGCCGGGCGCGGTTGAGCCACTGCAAAGCGCAATTAAGTATTTCCGCGAAGAATTCGAA
GCTGGCATCAAGCAGCCGTTCAGCAATACCCATTTAATCAATGGTATTCAGCCGAACCTG
CTCAAAGAGCGCTGGTGA
>EECEBICD_01031 NADH-quinone oxidoreductase subunit G
ATGGCTACGATTCATGTAGACGGCAAAGAATACGAGGTCAACGGCGCGGACAACCTGCTA
CAGGCATGTCTGTCCCTCGGCCTTGATATTCCGTATTTTTGCTGGCACCCGGCGCTGGGA
AGCGTCGGTGCTTGCCGCCAGTGTGCGGTGAAGCAATACCAAAACGCGGAAGATACGCGT
GGTCGCCTGGTGATGTCCTGTATGACGCCAGCCACCGACGGTACCTTTATCTCTATTGAC
GACGAAGAAGCGAAACAGTTCCGTGAAAGCGTGGTGGAGTGGCTGATGACTAACCACCCG
CACGACTGTCCGGTCTGTGAAGAGGGCGGTAACTGCCATCTTCAGGATATGACCGTGATG
ACGGGTCACAGTTTCCGTCGCTACCGTTTCACCAAACGTACCCACCGCAACCAGGATCTG
GGGCCGTTCATCTCTCACGAAATGAACCGCTGCATCGCCTGCTACCGTTGTGTGCGTTAC
TACAAAGATTATGCCGACGGCACCGATCTGGGCGTCTATGGCGCGCATGACAACGTCTAC
TTCGGGCGTCCGGAAGACGGTACGCTGGAAAGCGAATTCTCCGGTAACCTGGTCGAGATT
TGCCCGACCGGCGTCTTCACCGATAAAACGCACTCCGAGCGTTACAACCGTAAATGGGAC
ATGCAGTTTGCGCCGAGCATCTGCCAGCAGTGTTCCATCGGCTGTAACATCAGCCCGGGC
GAGCGCTATGGCGAATTACGCCGTATCGAAAACCGTTACAACGGTACCGTTAACCACTAC
TTCCTCTGCGACCGCGGTCGTTTCGGCTATGGCTATGTGAACCTGAAAGACCGTCCGCGT
CAGCCGGTTCAGCGCCGTGGCGACGACTTCATCACCCTCAACGCCGAGCAGGCGATGCAG
GGCGCGGCGGATATTCTGCGCCAGTCGAAGAAAGTGATCGGTATCGGTTCGCCACGCGCC
AGCATTGAGAGCAACTTTGCGCTGCGTGAACTGGTTGGTGCGGAAAACTTCTACACCGGT
ATCGCGCGCGGCGAGCAGGAACGTCTGCAACTGGCGCTGAAAGTGCTGCGTGAAGGCGGC
ATTTACACCCCGGCGCTGCGGGAAATCGAATCTTATGACGCGGTACTGGTCTTGGGCGAA

GACGTTACCCAGACCGGCGCGCGCGTTGCGCTGGCGGTACGTCAGGCGGTGAAAGGTAAA
GCGCGTGAAATGGCGGCGGCGCAGAAAGTGGCCGACTGGCAGATTGCGGCCATCCTTAAC
ATCGGCCAGCGTGCCAAACATCCGCTGTTCGTGACCAACGTGGACGACACCCGTCTGGAT
GATATCGCCGCCTGGACCTATCGCGCGCCGGTGGAAGATCAGGCGCGCCTGGGCTTTGCG
ATTGCCCATGCGCTGGACAATACAGCGCCTGCGGTTGACGGTATCGACAGCGACTTGCAA
AACAAAATTGATGTCATTGTGCAGGCGTTGGCCGGCGCGAAAAAACCGCTGATTATCTCC
GGCACTAACGCCGGTAGCAGTGAGGTGATTCAGGCGGCCGCCAACGTGGCGAAAGCGCTG
AAAGGCCGTGGCGCCGACGTCGGTATTACTATGATTGCCCGCTCCGTGAACAGCATGGGG
CTGGGTATGATGGGAGGCGGTTCGCTTGACGATGCGTTGGGCGAGCTGGAAACCGGCAGC
GCGGACGCGGTGGTGGTGCTGGAAAACGATCTCCACCGTCATGCAAGCGCTACGCGCGTG
AACGCCGCGCTCGCCAAAGCGCCGCTGGTGATGGTGGTTGACCATCAGCGCACGGCGATT
ATGGAAAACGCGCATCTGGTGCTTTCCGCGGCAAGCTTCGCCGAAAGCGATGGTACGGTC
ATCAACAACGAAGGCCGCGCGCAGCGCTTCTTCCAGGTTTATGATCCGGCCTACTACGAT
AACAAGACGATTATGCTGGAAAGCTGGCGCTGGCTGCATTCGCTGCACAGCACCGTCGAA
AACCGCGAAGTGGACTGGACACAGCTTGACCATGTGATCGACGCGGTCATTGCCGCCATG
CCGCAATTTGCCGGCATTAAAGACGCCGCGCCGGATGCGACCTTCCGCATTCGTGGGCAG
AAGCTGGCGCGCGAACCGCATCGTTACAGCGGTCGTACGGCGATGCGCGCCAATATCAGC
GTGCATGAACCACGTCAGCCGCAGGATAAAGACACCATGTTCGCCTTCTCGATGGAAGGG
AATAACCAACCGACTGCCCCGCGATCTGAAATTCCGTTCGCCTGGGCGCCGGGCTGGAAC
TCCCCGCAGGCGTGGAACAAATTCCAGGATGAAGTGGGCGGCAAACTGCGTCACGGCGAT
CCGGGCGTGCGTTTGATTGAAGCGACTGAGGGCGGTCTGGATTATTTCACTACCGTGCCG
GCAAGCTTCCAGGCGCAGGACGGTCAGTGGCGTATTGCGCCGTATTACCACCTGTTTGGC
AGCGACGAATTGTCTCAGCGTTCTCCGGTCTTCCAGAGCCGTATGCCGCAGCCGTATATC
AAACTTAACCCGGCGGATGCCGCGAAGTTGGGCGTCAATGCCGGGACGCGCGTCTCCTTT
AGCTACGATGGCAATACGGTGACGCTGCCGGTTGAAATCTCTGAAGGGTTAGCGGCAGGG
CAGGTAGGGCTGCCGATGGGTATGCCTGGCATCGCGCCGGTTCTGGCTGGCGCGCGTCTT
GAGGATCTGCGGGAGGCGCAACAATGA
>EECEBICD_01032 NADH-quinone oxidoreductase subunit H
ATGAGTTGGATTACACCGGATCTGATTGAGATCCTGCTGAGCATTCTCAAAGCGGTGGTG
ATTCTGCTGGTGGTCGTCACCTGCGGGGCCTTCATGAGCTTTGGCGAACGTCGTCTGCTG
GGTCTGTTCCAGAACCGTTATGGACCAAACCGCGTTGGCTGGGGCGGCTCGCTCCAGCTG
GTCGCGGATATGATCAAGATGTTCTTTAAAGAAGACTGGATCCCGAAATTCTCGGATCGC
GTCATCTTTACCCTGGCGCCGATGATCGCATTTACTTCGCTGCTGCTCTCCTTCGCCATT
GTGCCGGTTAGCCCTAATTGGGTGGTGGCCGATTTAAACATCGGGATTTTGTTCTTCCTG
ATGATGGCCGGTCTGGCGGTTTACGCGGTGCTGTTCGCCGGTTGGTCGAGCAACAACAAA
TACTCGCTGCTGGGCGCGATGCGCGCGTCTGCCCAAACGGTGAGCTATGAAGTGTTTCTT
GGTCTTTCCCTGATGGGCGTGGTGGCGCAGGCCGGTTCATTTAATATGACCGATATCGTC
AATAACCAGGCGCATCTGTGGAACGTGATTCCGCAATTCTTTGGGTTTGTTACTTTCGCC
ATCGCGGGCGTAGCGGTCTGTCACCGTCACCCGTTTGACCAGCCGGAAGCCGAGCAGGAA
CTGGCGGACGGTTACCACATCGAATATTCCGGTATGAAATTCGGTCTGTTCTTCGTGGGG
GAGTACATCGGTATCGTCACCGTTTCCGCGCTGATGGTAACGCTGTTCTTCGGTGGCTGG
CATGGCCCGTTCTTACCGCCATTCGTCTGGTTCGCGCTGAAAACCGCGTTCTTCATGATG
ATGTTCATTTTGATTCGTGCGTCGTTACCGCGTCCGCGTTATGACCAGGTAATGTCCTTC
GGCTGGAAAGTTTGCCTGCCGCTGACGCTCATCAACTTGCTGGTAACGGCGGCTGTCATT
CTGTGGCAGGCGCAATAA
>EECEBICD_01033 NAD(P)H-quinone oxidoreductase subunit I, chloroplastic
ATGACCTTAAAAGAATTATTAGTAGGTTTCGGCACTCAGGTACGCAGTATCTGGATGATC
GGCCTGCATGCGTTCGCCAAACGCGAAACGCGGATGTATCCGGAAGAGCCGGTCTATTTA
CCGCCCCGTTACCGTGGCCGTATCGTGCTCACGCGCGACCCGGACGGCGAAGAGCGCTGC
GTTGCCTGCAACCTGTGCGCGGTAGCCTGTCCGGTAGGCTGTATCTCTCTGCAAAAAGCG
GAGACCAAAGATGGCCGCTGGTATCCGGAATTTTCCGCATTAACTTCTCACGCTGCATCT
TCTGCGGCCTGTGCGAAGAAGCGTGCCCGACCACGGCGATTCAGTTGA
>EECEBICD_01034 NADH-quinone oxidoreductase subunit I
TTGACCCCGGACTTCGAACTGGGCGAATACAAACGTCAGGATCTGGTGTACGAAAAAGAG

GATCTGCTGATTTCCGGTCCGGGCAAATACCCGGAATATAACTTCTACCGGATGGCGGGT
ATGGCAATCGACGGCAAAGATAAAGGCGAAGCAGAGAACGAAGCCAAGCCTATCGACGTC
AAGAGCCTGTTACCGTAA
>EECEBICD_01035 NADH-quinone oxidoreductase subunit J
ATGGAATTCGCTTTTTATATCTGTGGCCTGATAGCCATCCTCGCGACCCTGCGAGTGATC
ACCCATACCAATCCGGTACACGCGCTGTTGTATCTGGTGATATCGCTACTGGCGATTTCC
GGGGTGTTCTTTTCGCTGGGCGCTTACTTTGCCGGTGCGCTGGAAATTATCGTCTACGCA
GGCGCCATCATGGTGCTGTTTGTGTTCGTGGTGATGATGCTCAACCTCGGCGGCAGCGAA
ATCGAACAGGAACGCCAGTGGCTGAAGCCGCAGGTGTGGATTGGTCCGGCGGTTTTGTCG
GCCATTATGCTGGCGGTCATTGTGTACGCCATTCTGGGCGTTAACGACCAGGGCATCGAC
GGCACGCCGATTAGCGCGAAAGCGGTGGGCATTACGCTGTTCGGGCCTTATGTTCTGGCG
GTCGAACTGGCGTCTATGCTGCTGCTGGCAGGCCTGGTGGTGGCGTTCCACGTCGGTCGT
GAAGAGCGTGCGGGCGAAGTGCTAAGCAACCGCGCCGATGACCGCGCGAAAAGAAAAACG
GAGGAGCGCGCATGA
>EECEBICD_01036 NADH-quinone oxidoreductase subunit K
ATGATCCCCTTAACACACGGACTGATCCTCGCTGCGATTTTATTCGTCCTGGGTTTAACC
GGTCTGGTTATCCGCCGCAATCTGCTGTTTATGCTGATCGGGCTGGAAATCATGATTAAC
GCCTCCGCGCTGGCCTTTGTGGTCGCCGGGAGCTACTGGGGCCAGACCGATGGTCAGGTG
ATGTACATTCTCGCCATCAGCCTTGCGGCTGCGGAAGCGAGTATTGGACTTGCGCTGTTG
CTGCAACTCCATCGCCGTCGCCAGAACCTGAACATCGATTCAGTAAGTGAGATGCGTGGA
TGA
>EECEBICD_01037 NADH-quinone oxidoreductase subunit L
ATGAACATGCTTGCCTTAACCATTATTCTGCCATTGATTGGCTTTGTACTGCTGGCGTTC
TCTCGCGGACGCTGGTCGGAAAATCTCTCCGCGACTATTGGCGTGGGCTCCGTTGGTCTG
GCGGCGTTGGTGACAGCGTTTGTCGGGATGGATTTCTTCGCCAACGGCAAACAGGCGTTC
AGCCAGCCGCTGTGGACGTGGATGTCGGTCGGTAACTTCAACATCGGTTTTAACCTGGTG
CTGGACGGCCTGTCGCTGACTATGCTCTCTGTGGTCACCGGCGTCGGCTTCCTGATCCAC
ATGTTCGCGTCCTGGTACATGCGTGGGGAAGAGGGCTACTCCCGCTTCTTCGCTTATACC
AACCTGTTTATCGCCAGCATGGTTGTTCTGGTGCTGTCCGATAACCTGCTGTTGATGTAC
CTCGGCTGGGAAGGCGTGGGCCTGTGCTCTTATCTGTTGATCGGCTTCTATTACAGCGAT
CCGAAGAATGGCGCAGCGGCAATGAAAGCCTTCGTGGTGACCCGTGTCGGTGACGTGTTC
CTCGCCTTCGCGCTGTTCATTCTCTACAACGAACTGGGCACGCTGAACTTCCGCGAAATG
GTCGAGCTGGCGCCAGCGCACTTTGCTGACGGCAACAACATGCTGATGTGGGCGACGCTG
ATGCTGCTGGGCGGCGCGGTCGGTAAATCTGCGCAGCTACCGTTGCAGACCTGGCTTGCC
GACGCGATGGCCGGCCCGACGCCGGTCTCCGCGTTGATCCACGCCGCGACGATGGTTACC
GCCGGTGTCTACCTGATTGCACGCACGCATGGCCTGTTCCTGATGACGCCGGAAATTCTG
CATCTGGTGGGTATTATCGGGGCGATTACGCTGGCCATGGCGGGTTTCGCCGCGCTGGTG
CAGACCGACATCAAACGCGTGCTGGCCTATTCCACCATGAGCCAGATTGGCTACATGTTC
CTGGCGCTGGGCGTGCAGGCGTGGGATGCGGCGATTTTCCATCTGATGACCCATGCGTTC
TTCAAAGCGCTGCTGTTCCTGGCGTCCGGTTCCGTCATTCTGGCCTGCCATCACGAACAG
AACATCTTTAAGATGGGCGGTCTGCGTAAGTCTATTCCGCTGGTGTATGCCTGCTTCCTG
GTGGGCGGCGCGGCGCTGTCGGCGCTGCCGCTGGTCACTGCGGGCTTCTTCAGTAAGGAT
GAGATCCTGGCTGGCGCGATGGCGAACGGTCATATCAATCTGATGGTGGCAGGTCTGGTC
GGCGCGTTCATGACCTCCTTGTACACCTTCCGGATGATTTTCATCGTATTCCACGGTAAA
GAACAAATTCATGCTCATGCAGGGAAGGGGATTACCCACCATCTGCCGCTGATTGTCCTG
ATGATTCTGTCTACCTTTGTTGGCGCGCTGATTGTGCCGCCTCTGCAGGGGGCTGCCGCA
GACAACTGA
>EECEBICD_01038 NADH-quinone oxidoreductase subunit L
TTGTGCCGCCTCTGCAGGGGGCTGCCGCAGACAACTGAACTGGCGCATGGTCGTGTGTTG
ACCCTGGAAATTACCTCTGGCGTAGTGGCGATTGCCGGCATCCTGATTGCCGCATGGCTG
TGGCTGGGTAAACGTACGCTGGTGACGTCGATCGCCAACAGCGCGCCGGGCCGTCTGCTG
GGCACCTGGTGGTATAACGCCTGGGGCTTTGACTGGCTGTACGACAAAGCATTCGTCAAG
CCATTCCTCGGTATCGCCTGGCTGCTGAAGCGCGACCCGCTTAATGCGTTGATGAACATT
CCGGCTATCCTTTCCCGTTTTGCAGGCAAAGGCCTGGTACTGAGCGAGAACGGTTATTTG

CGCTGGTATGTGGCATCCATGAGCATCGGCGCGGTTGTAGTGCTGGCGCTGTTGATGGTA
CTGCGTTGA
>EECEBICD_01039 NADH-quinone oxidoreductase subunit M
ATGTTATTACCCTGGTTGATATTAATCCCCTTTATCGGCGGCTTTCTGTGCTGGCAGACC
GAACGCTTTGGCGTGAAGGTGCCGCGCTGGATAGCGCTGATTACCATGGGGCTGACGCTG
GCGCTCGGTCTGCAATTGTGGTTGCAGGGCGGCTACTCACTGACGCAATCCGCCGGTATT
CCACAGTGGCAATCTGAATTTGTTCTGCCGTGGATCCCGCGTTTTGGCATCTCTATCCAT
CTGGCGCTCGACGGACTGTCGCTGCTAATGGTGGTGCTGACCGGCCTGCTCGGCGTTCTG
GCGGTGCTTTGCTCCTGGCGAGAAATCGAAAAATATCAGGGCTTCTTCCACCTGAACCTG
ATGTGGATCTTAGGCGGCGTTATCGGCGTGTTCCTGGCCATCGACATGTTCCTGTTCTTC
TTCTTCTGGGAAATGATGCTGGTGCCGATGTACTTCCTGATAGCGCTGTGGGGCCACAAA
GCTTCTGACGGGAAAACGCGTATCACGGCGGCGACCAAGTTCTTCATCTACACCCAGGCG
AGTGGTCTGGTGATGCTGATTGCCATCCTGGCGCTGGTCTTTGTGCACTACAACGCGACC
GGCGTGTGGACCTTCAACTATGAAGAGCTGCTGAATACGCCGATGTCCCACGGTGTGGAA
TATCTGCTGATGCTGGGCTTCTTCATCGCCTTCGCGGTGAAAATGCCGGTGGTTCCGCTG
CATGGCTGGCTGCCGGATGCGCACTCCCAGGCGCCGACCGCCGGTTCCGTCGACCTGGCC
GGTATCTTGCTGAAAACCGCCGCTTACGGTCTGCTGCGTTTCTCCCTGCCATTGTTCCCG
AACGCGTCGGCGGAGTTTGCGCCTATCGCCATGTGGCTGGGCGTTATCGGCATTTTCTAC
GGCGCGTGGATGGCCTTTACCCAATACGACATCAAGCGCCTGATTGCCTATACCTCCGTT
TCCCACATGGGCTTCGTGCTGATCGCTATCTACACCGGCAGTCAGCTGGCGTACCAGGGC
GCGGTGATTCAGATGATTGCACACGGTTTGTCCGCAGCCGGTCTGTTCATTCTGTGCGGC
CAGTTGTACGAACGTCTGCATACGCGTGACATGCGTATGATGGGCGGCCTGTGGGGCAAG
ATGAAGTGGTTGCCTGCGCTGTCCATGTTCTTTGCGGTGGCGACGCTCGGTATGCCGGGC
ACCGGTAACTTTGTCGGCGAATTTATGATTCTGTTCGGCAGCTACCAGGTGGTGCCGATG
ATTACCGTGATCTCCACCTTTGGGTTGGTATTCGCCTCTGTATACTCGCTGGCGATGCTG
CATCGCGCTTACTTTGGTAAAGCGAAGAGCCAGATTGCCAGCCAGGAACTGCCAGGGATG
TCGCTGCGCGAGCTGTTTATCATTCTGTTGCTGGTGGTGCTTCTGGTACTGCTTGGCTTC
TATCCGCAGCCGATTCTGGATACCTCGCATTCTGCGATGAGCAACATCCAGCAGTGGTTT
GTTAATTCTGTTACTACTACAAGGCCGTAA
>EECEBICD_01040 NADH-quinone oxidoreductase subunit N
ATGACAATAACTCCACAACACCTGATTGCGCTGCTACCGCTGCTGATCGTCGGCTTGACG
GTGGTGGTTGTGATGCTCTCCATTGCGTGGCGACGCAATCATTTCCTCAATGCCACGCTG
TCGGTCATTGGGCTTAACGCTGCTCTGGTTTCGCTTTGGTTTGTCGGCCTGGCTGGGGCA
ATGGACGTCACGCCGCTAATGCGCGTTGACGGCTTTGCGATGCTCTACACCGGGCTGGTG
CTGCTGGCGAGCCTCGCCACCTGCACCTTTGCCTACCCGTGGCTTGAAGGCTATAACGAT
AACCAGGAAGAGTTCTACCTGCTGGTGTTAATCGCCAGCCTGGGCGGAATTCTGCTGGCG
AACGCCAACCATCTGGCGGCGCTGTTCCTCGGTATTGAACTGATCTCACTGCCGCTGTTC
GGCCTGATTGGTTATGCTTTCCGCCAGAAACGCTCGCTGGAAGCCAGTATCAAATACACC
ATCCTCTCCGCCGCCGCCTCGTCTTTCCTGCTGTTCGGGATGGCGTTGGTGTACGCGCAG
TCCGGCAACCTGTCTTTCGAAGCGCTCGGTAAGAGCCTCGGCGATGGTATGCTGCACGAG
CCGTTGCTGCTGGCGGGCTTTGGCCTGATGATTGTCGGTCTGGGCTTTAAACTCTCTCTG
GTGCCGTTCCACCTGTGGACGCCAGACGTTTACCAGGGCGCGCCTGCGCCGGTTTCCACC
TTCCTGGCGACGGCGAGCAAAATCGCTATCTTCGGCGTGGTGATGCGTCTGTTCCTGTAT
GCGCCGGTAGGCGACAGCGAAGCGGTACGCGTGGTGCTGGGCATTATCGCCTTTGCCTCT
ATCATCTTCGGTAACCTGATGGCGCTGAGCCAGACCAACATCAAACGTCTGCTCGGTTAC
TCTTCTATCTCTCACCTCGGCTATCTGCTGGTGGCGCTGATTGCCCTGCAGAGCGGCGAG
ATGTCGATGGAAGCGGTAGGCGTTTATCTGGCCGGTTATCTGTTCAGCAGCCTCGGCGCG
TTTGGCGTGGTCAGCCTGATGTCCAGCCCGTTCCGCGGCCCGGATGCCGACTCCCTGTAC
TCTTACCGTGGTCTGTTCTGGCACCGTCCGGTTCTTGCCGCGGTGATGACGGTGATGATG
CTGTCGCTGGCAGGTATCCCAATGACGCTGGGCTTTATCGGCAAATTCTACGTGCTGGCG
GTCGGCGTTCAGGCCAGCCTGTGGTGGCTGGTCGCCGCCGTGGTGGTGGGTTCCGCGATT
GGTTTGTACTACTACCTGCGCGTTGCGGTGAGTCTCTACCTGCACGCGCCGCAGCAACCG
GGACGCGACGCGCCGACAAACTGGCAGTACAGCGCAGGCGGTATCGTGGTGCTGATCTCC
GCGTTGCTGGTGTTGGTACTGGGCGTCTGGCCGCAGCCGCTGATTAGCCTTGTGCAACTG

GCGATGCCGCTGATGTAA
>EECEBICD_01041 hypothetical protein
ATGCTTAATGGAAAAACATTAATGCTGTTGTTGGGTGGGGTTGTTCTCTCGGGCTGTGGG
CCGGAGCCCTCCGACCCACAGGGAAATAATCCGGCTGAGTTAAAACAGGAGCAGGCTATC
CAGAAAGAAAATAGCGCTCAGGCCGGGGATGACACTGTCCAGAAAAGACAAGCCGAGGCC
GCCCAACAGGCTGCGAAAAAAGCAGCCGAATATAAAGCGAATACTGAAGCCAAAGCCCTG
GCAGATGCCAAAGCGGCATCTCTGGCAACAGCCGAAGCACCGCAATATGAAATGCGGACA
CGTGCCGCGGCATCAAAAGCGTTCGCTGCACAAGGCGGTAATGTAATGGGGACCGCGCGT
TACGAACACTACGATGAAAATCCGATTAAACAGGTAAGTCAGGCGCCGCTCGCCACGTTT
AGTCTGGATGTAGACACTGGCAGCTACGCTAACGTGCGGCGCTTTCTGAATCAGGGACAA
CTGCCGCCGCCGGAAGCCGTGCGGGTAGAAGAGATGCTCAACTATTTTCCCGCGCCTCAG
CCCGTTGCGGATAAGCAGGATAACACTAAACCCATTGCGGCCTGTATACCGATGCCATTT
GCGGTTAAATACGAACTGGCTCCCTCGCCGTGGAACGCGCAGCGTACGCTATTAAAAGTT
GATGTTCAGGCCCGGGACATGCAGGTCAGGGATCTGCCACCTGCCAACCTGGTTTTTCTC
ATTGATACTTCTGGTTCTATGCAGCCAGCGGAACGCCTGCCGCTGATCCAGTCGGCGCTA
AAACTGCTGGTGAACGATCTGCGTGCGCAGGATAACATCACGATTGTGACCTACGCGGGC
GGCACTCACGTCGCGTTGGCGTCTACAGCGGGAAATAACACAACCGCGATTAAAGCGGCA
ATTGATAATCTGGATGCTTACGGGAGTACCGGTGGTGAAGCGGGATTACGGCTAGCCTAC
GAGCAGGCCGAAAAAGGGTTTATCAAAGGCGGCGTTAACCGCATTTTGTTGACCACCGAC
GGTGATTTTAACCTCGGTATTACCGATCCCAAAGACATCGAAGCGCTGGTAAAAAAAGAG
CGTGAGAAAGGTATTACCTTATCTACGCTGGGCGTCGGCGATGACAATTTCAACGAAGCC
ATGATGGTGAGGATTGCTGATGTGGGTAACGGCAATTACAGCTACATCGACTCCCTCTCC
GAGGCGCAAAAAGTCCTCAAGGATGAGATGCATCAAACGCTGGTCACCGTTGCCAAAGAT
GTAAAATCGCAAATCGAATTTAATCCGCAGTGGGTGACTGAGTACCGGCAGATTGGTTAT
GAAAAACGCCAACTGCGCGACGAGGATTTCAATAACGATAAGGTTGATGCCGGTGATATC
GGCGCGGGTAAACACGTCACGCTATTCTTTGAACTGACGCTTAACGGGCAGAAAGCCTCG
GTGGATAAACTGCGCTACGCTCAGGACAAAGCCGCCTCAAAGCCAACAAAATCAAGCGAG
CTGGCGTGGATCAAATTGCGCTGGAAAGCGCCGCAGGGCAGCGAAAGTACATTAGCCGAG
TTCCCGGTCGTTATGGGAAAAATGCCGATCTTTGCTGACGCCTCTGAAGATTTTCGTTTC
CGCGCGGCGGTAGCGGCTTTCGGGCAAAAACTGCGTGGCTCAGAAACGCTGGCAGATACG
ACCTGGCCGCAAATTATTAAATGGGGTGAACAGGCGCGCGGGGAAGATAGACAAGGCTAT
CGCGCGGAGTTTATTAAACTGGTAAAACTGGCGGAAGGCTTGTCTCACTAA
>EECEBICD_01042 Chemotaxis protein CheV
ATGGACAATTTTCAGAAAGATATTGATGACAGGGCCAATTTAACCCTGTCCAACCGTTTT
GAACTGCTGTTATTCCGTCTTGGTACATCGCTGCATGAACAGAAGTCAGAGCTGTTCGGC
ATCAATGTATTTAAGCTGCGCGAAATCGTACCAATGCCCGCCTTTACGCGCCCGGCAGGC
ATGAAAGCGCCGCTATTGGGCATGGTCAATATTCGGGATCAGGTCATTCCAGTGATTGAT
TTGCCAGCGGTAGCGGGCTGCAAGCCGGAAACCGGGCTGAATATTTTGCTGATCACCGAA
TATGCGCGCAGCGTACAGGCATTTGCCGTCGAGTCGGTGGAAAATATTATGCGTCTTGAC
TGGCAACAGGTGCATACCGCCGAGAAAGCCGTCAACGGTCGCTACATCACCAGTATTGCC
TGCCTGGATGATAACAAAGAAACCAACAACCTGGCGCTGGTGCTCGACGTTGAGCAGATC
CTTTATGATATTGTCCCCTCCAGCCACGACCTGCGCGCCACCAATCTGAAGACCAATAAA
TTTTATATTACCCCTGGCGCGGTAGCCATTGTGGCGGAAGACTCCAAAGTCGCCCGCGCG
ATGCTGGAAAAAGGGCTGAACGCGATGGGAATTCCGCACCAGATGCATGTGACCGGCAAG
GATGCATGGGAGAGAATCCAGCAACTGGCGCAGGAAGCGGAGGCCGAAGGTAAGCCAATT
AGCGAGAAAATCGCGCTGGTCCTGACAGATCTTGAGATGCCGGAGATGGATGGCTTTACG
TTAACGCGTAAAATTAAAACCGATGAACGGCTGAAAAAGATCCCGGTCGTTATTCACTCT
TCCCTTTCCGGCAGCGCCAACGAAGATCATATACGTAAGGTGAAGGCCGACGGCTACGTG
GCCAAGTTTGAGATTAACGAACTCTCTTCGGTCATTCAGGAAGTGCTGGAGCGCGCAGCA
ACAAACAGCCAGGGACCACTGATCAGCCGCAAAAGCGCCTGA
>EECEBICD_01043 Ribonuclease BN
ATGGAATTAATTTTTTTAGGTACGTCCGCAGGCGTTCCCACGCGCTCACGCAATGTAACG
GCAATTTTATTACATCTGCAACATCCAACGCAGCCGGGAGTATGGCTTTTTGATTGCGGC
GAAGGGACACAGCATCAAATGCTTAATACCGCGTTTCATCCCGGTAAGCTTGAGCGCATT

TTTATCAGCCATCTTCACGGCGATCATCTGTTTGGCCTGCCGGGTCTGCTATGCAGCCGG
TCGATGGCGGGGAACCCCCATCCGCTAACCGTATACGGGCCTCAAGGCGTGCGTGAGTTT
ATTGCAACCACACTGCGTCTTAGCGGTTCATGGACCGATTTTCCGTTGCAAATTGAAGAA
GTTAGCGCAGGCGATATTCTTGACGACGGTCTGCGTAAAGTGACCGCATTCAGGCTGGAG
CATCCACTGGAGTGCTACGGATATCGCGTTGTTGAACATGACAAGCCTGGCGCGCTGAAT
GCCAGGGCGTTAAAAGCCGCAGGCGTTACGCCCGGCCCGCTGTTTCAGGCGTTGAAGGCA
GGAAAAACCGTCACACTTGCCGATGGAAGGCAGATCAATGGCGCCGACTACCTTGCTCCC
GCCGTGGCCGGGAAATCAGTGGCGATTTTCGGCGATACCGCGCCCTGCGAGGCCGCCCTC
GCGCTGGCGCAAGGCGTGGATGTGATGGTCCATGAAACCACGCTGGATGCGTCAATGGAG
GAGAAAGCCAACGCTCGCGGACACAGCTCCACCCGTCAAACCGCGACGCTGGCGCGGGAA
GCTGCCGTCGGTCGGCTGATCATGACCCATATTAGCTCACGCTATGACGATAAAGGCTGT
CAGCGCTTACTGGCGGAATGCCGCGCTATTTTCCCGGCGACGGAGCTGGCTTACGATTTT
TCTGTTTTCCCGGTTTAG
>EECEBICD_01044 Protein ElaA
ATGATTGACTGGCAGGATCTCCACCATAGTGAACTTACCGTTCCTCAACTTTACGCGTTA
CTCAAATTGCGCTGCGCGGTCTTTGTGGTAGAACAGCGCTGTCCTTACCTGGATGTGGAT
GGCGATGATTTAGTCGGCGACAATCGGCATATTCTGGGCTGGCATCAGGATGAGCTGGTG
GCGTATGCGAGGATTCTGAAAAGCGATAACGAATCAGACCCGGTTGTCATTGGCCGGGTG
ATCGTCAGCGATGCCTGGCGAGGGGCGAAACTGGGCCAGCAATTAATGGCGAAAACGCTG
GAATCTTGTGGGCGGCACTGGCCGGATAAGCCGCTATATCTGGGAGCGCAGGCGCATCTG
CAACCTTTTTATGCGCGGTTTGGTTTTATTCCCGTCACCGATGTTTATGACGAAGACGGC
ATTCCGCACCGGGGAATGGCGCGGGAGGTTCATCAGGCATAA
>EECEBICD_01045 Protein ElaB
ATGTCATATCAATTTGGTGAATCACGTGTTGATGATGATCTGACTTTGCTCAGCGAAACG
CTGGAAGAGGTTTTACGTTCCTCAGGCGATCCCGCCGATCAAAAATACATTGAGCTGAAA
GCCCGCGCGGAACAAGCGCTGGAAGAGGTGAAAAACCGCGTAAGCCATGCGTCGGATAGC
TATTATTACCGGGCAAAGCAGGCCGTTTATAAAGCGGATGACTACGTTCATGAAAAACCC
TGGCAGGGTATTGGCGTAGGCGCGGCGGTAGGACTGGTGCTTGGACTGCTGCTGGCGCGG
CGCTAA
>EECEBICD_01046 Isochorismate synthase MenF
GTGCATTCTATTACTACTGCGCTGGAAAATCTGACGCGTCAGCTATCGCAAGAGATACCG
GCGACGCCAGGGCTTTGCGTTTTCGATGCGCCTTTCCCTTTAAACGATGCCTTCGATGCA
TTGAGCTGGCTGGCCAGCCAGTCATCCTTTCCGCAATTCTACTGGCAACAGCGCAATGGC
GACGAAGAAGCTGCCGTTCTCGGGGCCATCACCGTATTTTCATCGCTCGATCTGGCGCAA
CGGTTTTTACGTCAGCACGCGCAGCCTGACCTGCGTATTTGGGGGCTGAATGCGTTCGAG
CCGAAAGAGGGCTACTTATTGCTGCCGCGGCTGGAGTGGCGACGTAGCGGTGGTACGGCG
ACCTTACGCTTACATCTGCACAGTGACGTTTCGCTACGCGATGATGCGCGCCAGGCGAAA
GCGTTTCTCGCTTCTCTGGTCGGCATTAAGCCTGTTCCGGCGCTACGCCTTTCGCTCACG
AGCGAACAACACTGGCCGGACAAAGACGGTTGGGTGAAGCTGATTCAGCAGGCCACCCAC
ACCATTGCGCAAGAGGCGTTTGATAAAGTTGTGCTGGCGCGGGCCACCGACTTACAATTC
TCCCGTCCGGTCAATGCCGCCGCGATGATGGCGTCCAGTCGTCGTTTAAATCTCAACTGC
TACCACTTTTTTATGGCCTTTTCCGCCGATACCGCCTTTCTGGGATCATCGCCGGAGCGG
CTCTGGCGGCGGCGTGAAACGGCGCTGCGTACCGAAGCGCTGGCTGGGACGGTGGCGAAC
CATCCGGATAACCATAAAGCCTGGCAGCTTGGCGAATGGTTAATGAAAGACGATAAAAAC
CAGCGAGAAAATATGCTGGTTGTCGAAGACATCTGCCAGCGACTGCAAAACTGCACCCAC
TCGCTGGATGTGCTGCCGCCGCAGGTGCTCAGACTGCGCAAGGTTCAGCATTTGCGCCGC
TGTATCTGGACGGCGCTTAATCAGGCGGACGACACGCTCTGCCTGCACCAGTTACAGCCT
ACCGCAGCGGTCGCCGGGATCCCTCGTGAACCCGCGCGTTGTTTTATAGAACAACATGAG
CCGTTCGAACGTGAATGGTATGCCGGGTCGGCAGGCTATCTCTCCACGCGGCAAAGTGAG
TTTTGCGTGACGTTACGTTCAGCCAAAGTGACGGCCAATGTGGTGCGATTGTATGCCGGC
GCGGGAATCGTCCGCGGCTCCGATCCGGAACAGGAATGGCAGGAAATAGATAATAAAGCG
GCCGGGTTACGTACCTTATTGCAGATGGATGCGTAA
>EECEBICD_01047 2-succinyl-5-enolpyruvyl-6-hydroxy-3-cyclohexene-1-carboxylate synthase

ATGTCAGTAAGTGCATTTAACCGGCGCTGGGCGGCGGTCATCCTGGAAGCCTTAACCCGT
CATGGCGTCAGGCATGTTTGTATCGCCCCTGGTTCCCGTTCCACGCCGCTCACGTTGGCG
GCGGCAGAGAATCCTGCTTTTATTCACCATACGCACTTTGATGAGCGTGGGCTTGGTCAT
CTGGCGTTAGGGCTGGCGAAAGTCAGCCAACAGCCGGTTGCCGTTATTGTCACTTCAGGT
ACCGCCGTGGCGAACCTCTATCCGGCGCTGATTGAGGCGGGGCTGACGGGCGAGAAGCTC
ATTTTACTTACCGCCGATCGCCCGCCGGAGCTGATTGACTGCGGCGCCAACCAGGCGATC
CGTCAGGCGGGGATGTTTGCCTCGCACCCCTCCCAAACTCTGTCGTTGCCGCGTCCCACG
CAGGATATTCCGGCGCGCTGGCTGGTGTCGACCATCGACAATGCGCTCGCCATGCTTCAT
GCGGGCGCGCTGCACATTAACTGTCCTTTTGCCGAACCGCTGTATGGCGATATGAATGAC
ACCGGTCTGGTCTGGCAGCAGCGTCTCGGCGACTGGTGGCAGGATGAAAAACCCTGGCTG
CGCGAAGCGCGTCGACTGGAAAGCGACAAACAGCGCGACTGGTTTTTCTGGCGACAAAAA
CGCGGCGTCGTTGTCGCCGGGCGTATGAGCGCGGAAGAAGGCAAAAAAGTGGCGCAGTGG
GCGCAAACTCTGGGATGGCCATTAATTGGCGACGTATTGTCGCAAACCGGGCAGCCGCTG
CCGTGCGCCGACCTGTGGCTCGGCAACGCCAAAGCGGTGACCGAACTGCAGCAGGCGCAG
ATTGTCGTCCAGCTTGGCAGTAGCCTTACCGGAAAACGCTTACTGCAATGGCAGGCAACC
TGCGAGCCGGAAGAGTATTGGGTAATCGACAATATTGAAGGTCGTCTGGACCCTGCGCAC
CATCGTGGACGCAGACTGGTAGCGAAAATCGCCGACTGGCTGGAACTACATCCGGCGGAA
AAACGTAAACCGTGGTGTGTAGAGATCCCGCGTCTGGCGGAACTGGCGTGGCAACGGGTG
GTCGCGCAGCGGACACCTTTGGCGAAGCGCAGCTGGCGCACCGTATCCGCGACTATCTGC
CGGAGCAGGGGCAATTGTTCGTGGGCAATAGTCTGGTGGTGCGTCTGA
>EECEBICD_01048 2-succinyl-5-enolpyruvyl-6-hydroxy-3-cyclohexene-1-carboxylate synthase
TTGTTCGTGGGCAATAGTCTGGTGGTGCGTCTGATCGACGCGCTGTCACAGTTGCCGGCT
GGCTATCCGGTATACAGCAATCGCGGGGCGAGCGGCATTGACGGCCTGCTTTCCACGGCG
GCAGGCGTGCAGCGCGCCAGCGCCAAATCGACGCTGGCTATCGTCGGCGATTTATCCGCG
CTGTACGATCTTAACGCGCTGGCGTTACTACGTCAGGTATCCGCGCCGTTTGTGTTGATT
GTGGTCAACAATAACGGCGGGCAGATCTTTTCCCTGCTGCCGACGCCGCAAAGCAAACGC
GAGCGCTTTTATTTGATGCCGCAAAACGTCCATTTTGACCATGCGGCCGCCATGTTTAAC
CTGCGCTATCATCGCCCGGAAAACTGGGAAGAGCTGGAGTCTGCGCTGGCTGGCGCGTGG
CGAACCCCGGCGACAACGGTGATTGAGCTGGTGGTAAACGATACGGACGGCGCGCAGACG
TTGCAGCAACTTTTGGCTCAGGTAAGCCATCTATGA
>EECEBICD_01049 2-succinyl-6-hydroxy-2,4-cyclohexadiene-1-carboxylate synthase
ATGATGTTGCACGCGCAGCACATGCCCGGACAGCCTGGGGCGCCCTCACTGGTTTTCCTG
CACGGTTTTTCTGGCGATTGCCGTGAATGGCAGCCGGTTGGCGAACAGTTTCACGGCTGT
TCGCGGCTTTATATTGACTTACCGGGACATGGCGGATCTGCGGCTATTCCGGTCGGCGGC
TTTGCCGATGTCATCAGATTACTGCGCGCTACGTTAATTAGTTACAACATACTAAAGTTT
TGGTTGGTGGGATACTCCCTTGGCGGCCGGGTGGCGATGATGGCAGCCTGCCAGGGAATA
CCAGGCCTGTGCGGCCTGGTGGTTGAAGGCGGTCATCCGGGGCTGCAAAATGAACAGGCG
CGCGCTGAAAGACGATTGTCCGACGGGCGCTGGGCAGAGCGTTTTCGTCATGAACCATTG
ACTGAGGTTTTTCACGACTGGTATCAGCAGCCGGTGTTCGCCTCGCTGACGGCGCAGCAA
AGACAGGCGCTAACGGCGCTGCGTAGCCAGAATAACGGCGAAACGCTGGCTGCGATGCTG
GAGGCGACATCGCTTGCCGTCCAGCCGGATTTACGCGAGGCGCTCAACGCGCTGGCATTT
CCGTTTTATTATTTATGTGGCGAACGCGACAGCAAATTCCGCGCCCTGGCGCAGGAAGTG
GCGGCGACATGCCATGTCATTCGTAACGCCGGACACAACGCGCATCGGGAAAACCCCGCT
GGCGTGGTAGACAGTCTGGCTCAGATTCTGCGTCTCTGA
>EECEBICD_01050 1,4-dihydroxy-2-naphthoyl-CoA synthase
ATGATCTATCCTGATGAAACAATGCTTTACGCACCGGTAGAATGGCACGACTGCTCGGAG
GGTTACACCGACATTCGTTATGAAAAATCCACCGACGGTATTGCAAAAATCACTATTAAT
CGTCCGCAGGTGCGCAATGCATTTCGTCCGTTGACCGTCAAAGAGATGATTCAGGCGCTG
GCGGATGCCCGTTACGACGACAACGTGGGCGTGATTATTCTGACCGGCGAAGGCGATAAA
GCGTTTTGCGCGGGCGGCGACCAGAAGGTGCGCGGCGATTACGGCGGTTATCAGGATGAC
TCCGGCGTGCATCACCTGAACGTGCTGGACTTCCAGCGCCAGATTCGCACCTGTCCAAAA
CCGGTTGTCGCGATGGTCGCGGGTTACTCCATCGGCGGTGGTCATGTGCTGCACATGATG

TGCGACCTGACCATCGCGGCGGAAAACGCCATCTTCGGCCAGACCGGTCCGAAAGTCGGC
TCCTTTGACGGCGGTTGGGGCGCTTCGTATATGGCGCGTATCGTTGGTCAGAAGAAAGCG
CGTGAAATCTGGTTCCTGTGCCGCCAGTACGATGCGCAACAGGCGCTGGACATGGGGCTG
GTTAACACCGTCGTTCCGCTGGCGGATCTGGAAAAAAGAGACCGTGCGCTGGTGCCGCGA
AATGTTGCAAAACAGTCCGATGGCGCTGCGTTGCCTGAAAGCGGCGCTGAATGCCGATTG
TGA
>EECEBICD_01051 1,4-dihydroxy-2-naphthoyl-CoA synthase
ATGTTGCAAAACAGTCCGATGGCGCTGCGTTGCCTGAAAGCGGCGCTGAATGCCGATTGT
GACGGTCAGGCCGGGCTGCAGGAGCTGGCGGGCAACGCTACCATGCTGTTCTACATGACG
GAAGAAGGGCAGGAAGGCCGTAACGCCTTTAACCAGAAGCGTCAGCCTGACTTCAGCAAA
TTCAAACGGAACCCATAA
>EECEBICD_01052 o-succinylbenzoate synthase
ATGCGTAGCGCGCAGGTATACCGCTGGCAGATCCCCATGGACGCGGGGGTGGTTCTGCGC
GACAGGCGGTTAAAAACTCGCGACGGGCTGTATGTTTGTCTGCGTGACGGCGAGCGTGAG
GGATGGGGAGAGATCTCTCCGCTGCCGGGCTTCAGTCAGGAAACGTGGGAAGAGGCGCAG
ACGGCGCTCCTGACGTGGGTGAACGACTGGCTTCAGGGGAGCGAGGGATTACCGGAGATG
CCTTCGGTCGCGTTTGGCGCAAGCTGCGCGCTGGCGGAACTGACTGGCGTCTTGCCGGAG
GCGGCGGACTATCGCGCTGCGCCGTTATGCACTGGCGATCCTGACGATTTGGTGCTGCGG
CTTGCCGATATGCCCGGCGAGAAAATCGCTAAGGTCAAAGTGGGTCTTTATGAAGCGGTA
CGCGACGGCATGGTGGTTAATTTGCTGCTGGAGGCGATCCCGGATCTGCATCTGCGTCTG
GATGCGAATCGCGCCTGGACGCCGCTAAAAGCCCAACAGTTCGCAAAGTATGTTAATCCG
GATTACCGCGCTCGTATCGCCTTTCTCGAAGAACCGTGTAAGACGCGGGATGATTCCCGC
GCCTTTGCCCGTGAAACCGGCATCGCGATTGCCTGGGACGAAAGTCTGCGCGAAGCGGAT
TTCACCTTTGAAGCCGAAGAGGGCGTCAGGGCTGTGGTTATCAAACCTACGCTGACCGGA
TCGCTTGATAAAGTGCGTGAGCAAGTCGCTGCCGCCCATGCGTTGGGACTGACGGCGGTC
ATCAGCTCTTCGATCGAGTCCAGCCTCGGCCTGACGCAACTGGCGCGGATTGCCGCCTGG
TTGACGCCGGGAACGCTGCCCGGACTGGATACCTTGCATCTGATGCAGGCGCAACAGGTT
CGCCCCTGGCCCGGTAGCGCGTTGCCTTGTCTGAAGCGTGATGAGCTGGAACGACTGTTA
TGA
>EECEBICD_01053 2-succinylbenzoate--CoA ligase
ATGACATTCACGGATTGGCCGTGGCGCCACTGGCGTCAGGTTCGCAGCCAGGCGCCCGCT
CTGCGCCTGAACGATGAAGTGCTAAGCTGGCGCGCCCTCTGTGAGCGTATTGATGCGCTG
GCGGGCGGGTTTGCCGCGCAGGGCGTACGCGAGGGCGACGGTGTCCTGCTGCGCGCCGGG
AATCAGCCGCGCACTCTGTTGGCATGGCTTGCGCTGATGCAGTGCGGCGCGCGGGTTCTG
CCCGTCAATCCGCAGTTGCCCCAGACGTTGCTGGAGGCGCTGGTGCCGAAGTTAACGCTG
CGTTTTGCGCTGACACTGGAAGGCGAAAACGCGTTTTCCGGATTAACCGCGTTGCAGATA
CAAAAGAGCACAGCGGCGTATGCTGTGGACTGGCAACCGCAGCGTCTGGTTTCCATGACG
CTGACATCGGGTTCCACAGGATTGCCAAAAGCGGCTGTACATACGTGCCAGGCGCATCTT
GCCAGCGCGCAGGGGGTGCTTTCGCTAATGCCGTTTGGCCCTCAGGATGACTGGCTGCTA
TCGCTGCCGCTGTTTCATGTATCCGGGCAGGGTATTATGTGGCGTTGGCTCTTCGCCGGG
GCGCGCATGACGGTACGCGATAAGCAACCGCTGGAGCAGATGCTGGCAGGGTGTACTCAT
GCTTCGCTGGTGCCGACGCAGCTGTGGCGGCTGCTGGCGAATCGGGCGGCGGTGACGCTG
AAAGCCGTTTTGCTCGGCGGCGCGGTGATTCCGGTTGAACTCACCGACCAGGCCAGCAAA
CAAGGGATTCGCTGCTGGTGCGGGTATGGGCTCACGGAGTTCGCGTCTACAGTATGCGCC
AAGGAGGCCGATGGTTCTGATGATGTGGGCGCGCCGCTCCCCGGCAGAGAGATCAGAATC
GTCGATAATGAAGTCTGGCTGCGCGCCGCCAGTATGGCGGAAGGTTACTGGCGTGATGGC
AAACTGATCCCGCTGGTTAATGACGAGGGCTGGTTTGCCACGCGCGATCGCGGCGATCTG
AACCACGGCAGGCTAACCATCGCCGGGCGGCTGGACAACCTGTTCTTTAGCGGCGGCGAA
GGCATTCAGCCGGAGGAAGTCGAACGCGTTATTAATGCGCATCCGCTAGTGCAACAAGCC
TTCGTCGTGCCGGTAGAAGATAAAGAGTTTGGCCATCGTCCCGTTGCGGTTGTGGAGTAT
GCATCGCAGGCCGGAGACGTGAACCTCGCTGAATGGGTCCGCGATAAGTTGGCGCGTTTT
CAGCAGCCGGTTCGCTGGTTGACGCTGCCGTCAGAACTGAAAAACGGAGGGATTAAAATA
TCTCGCCGTGCGCTCCAGCAGTGGGTGTGTGAGAACTGTAAAAATTAA
>EECEBICD_01054 Signal transduction protein PmrD

ATGGAATGGTTGGTTAAGAAATCGCATTATGTCAAAAAGAGGGCGTGCCATGTTCTGGTG
CTGTGCGATAGCGGCGGTTCGCTAAAAATGATCGCCGAGGCGAATTCCATGATATTACTG
AGTCCCGGCGATATCCTGTCGCCTTTACAGGATGCGCAGTATTGTATTAATCGGGAAAAA
CACCAGACCTTAAAAATCGTTGATGCACGCTGTTATTCCTGCGACGAATGGCAGCGGTTG
ACGCGCAAGCCATCATGA
>EECEBICD_01055 putative 4-amino-4-deoxy-L-arabinose-phosphoundecaprenol flippase subunit ArnF
ATGGGCGTAATGTGGGGACTGATAAGCGTTGCGATTGCCTCCCTCGCCCAGTTGAGTTTA
GGATTCGCCATGATGCGACTGCCATCGATAGCGCATCCACTGGCGTTTATTTCCGGGCTG
GGCGCTCTTAATGCCGCGACCCTGGCGCTATTCGCCGGTTTGGCGGGGTATCTGGTTTCC
GTCTTCTGCTGGCATAAAACATTACACACGCTCGCGCTCAGCAAAGCCTATGCGCTGCTG
AGTCTCAGCTATGTGCTTGTCTGGGTAGCCTCAATGTTGTTACCCGGTTTGCAAGGCGCT
TTCAGCCTAAAAGCGATGCTCGGCGTATTGTGCATTATGGCGGGGGTAATGCTGATTTTT
CTGCCCGCCAGATCATGA
>EECEBICD_01056 putative 4-amino-4-deoxy-L-arabinose-phosphoundecaprenol flippase subunit ArnE
ATGATCGGCGTCATTCTGGTGCTTGCCAGTCTGCTCAGCGTTGGCGGTCAGCTCTGCCAG
AAGCAAGCGACACGGCCATTGACGGTGGGCGGACGCCGCCGTCACTTGATGCTGTGGCTG
GGCCTGGCGCTGATATGCATGGGTGCAGCGATGGTGCTGTGGCTGCTGGTGCTACAAACC
CTGCCGGTGGGAATTGCTTACCCGATGTTGAGTCTGAATTTTGTCTGGGTCACGCTGGCG
GCCTGGAAAATCTGGCATGAACAGGTTCCGCCCCGCCACTGGTTTGGCGTCGCATTAATT
ATCAGCGGCATTATCATTCTGGGGAGTGCGGCATAA
>EECEBICD_01057 Undecaprenyl phosphate-alpha-4-amino-4-deoxy-L-arabinose arabinosyl transferase
ATGATGAAATCGATACGCTATTATCTGGCTTTCGCCGCATTTATCGCGCTCTACTATGTT
ATTCCTGTCAATAGCCGCCTGCTCTGGCAGCCGGATGAAACGCGCTATGCCGAAATAAGT
CGGGAGATGCTGGCGTCTGGCGACTGGATCGTGCCGCATTTTCTGGGATTACGTTATTTT
GAAAAACCGATAGCCGGTTACTGGATAAACAGTCTGGGTCAGTGGCTTTTTGGCGCTACC
AATTTTGGCGTGCGGGCTGGCGCTATTTTAACGACATTGCTGGCCGCAGCGCTGGTGGCG
TGGCTGACGTTCCGCTTATGGCGGGATAAACGTACCGCGTTGCTCGCCTCCGTTATCTTT
CTCTCCCTCTTTGCCGTTTACAGTATCGGGACGTATGCGGTACTCGACCCGATGATCGCG
CTTTGGCTTACGGCCGGGATGTGTTGTTTCTGGCAGGGGATGCAGGCCACGACCCGAACG
GGCAAGATTGGGATGTTTTTGCTACTGGGCGCAACCTGCGGTCTGGGTGTATTAACTAAA
GGGTTCCTCGCCCTGGCCGTACCGGTGGTGAGCGTGCTGCCGTGGGTTATTGTTCAGAAA
CGCTGGAAGGACTTTTTGCTATACGGCTGGCTGGCGGTCTTGAGCTGCTTTGTGGTTGTC
CTTCCCTGGGCGATCGCCATTGCGCGACGCGAAGCCGATTTCTGGCATTACTTTTTCTGG
GTGGAGCATATCCAGCGATTCGCCATGAGCGATGCCCAGCATAAAGCCCCTTTCTGGTAC
TATCTGCCGGTACTGCTCGCGGGAAGTTTACCGTGGCTGGGGTTACTGCCTGGCGCGCTT
AAACTGGGCTGGCGCGAGCGAAACGGCGCATTCTATTTGCTCGGATGGACGATCATGCCG
CTTCTCTTTTTCAGCATTGCGAAAGGGAAACTGCCCACCTATGTTCTTTCCTGTTTCGCA
CCGATAGCGATACTCATGGCGCGCTTCGTCCTGCATAACGTAAAAGAAGGCGTCGCCGCG
CTACGTGTCAACGGCGGGATCAACCTGGTGTTCGGGATAATCGGGATCGTCGCGGCGTTT
GTCGTCTCCTCATGGGGCCCGCTGAAATCGCCGGTGTGGACGCATATCGAAACCTATAAA
GTGTTTTGCGTCTGGGGCGTCTTTACTGTTTGGGCGTTTGTGGGCTGGTATAGCCTCTGT
CATAGCCAACAATACCTATTACCCGCGTTTTGTCCGCTGGGACTGGCGCTGCTGTTCGGC
TTTTCCATCCCCGACAGGGTGATGGAGTCCAAACAGCCACAGTTTTTTGTCGAAATGACC
CAGGCGCCTTTGGCATCAAGCCGCTATATTCTTGCCGACAACGTCGGCGTCGCCGCCGGT
CTGGCCTGGAGTCTGAAACGAGACGATATTATGCTCTACGGACACGCGGGCGAACTCAGG
TATGGTCTTAGCTATCCTGATGTGCAGGATAAATTCGTTAAAGCCGACGATTTTAACGCC
TGGCTCAACCAGCATCGTCAGGAGGGCATTATTACGCTAGTACTTTCAATAGCCAAAGAC
GAAGACATCAGCGCTCTTTCCCTTCCCCCTGCGGACAATGTTGATTATCAGGGGCGTCTG
GTGTTAATTCAGTATCGGCCTAAATGA
>EECEBICD_01058 putative 4-deoxy-4-formamido-L-arabinose-phosphoundecaprenol deformylase ArnD

ATGACGAAAGTCGGTTTACGCATTGATGTTGATACCTTGCGAGGAACGCGTGAAGGTGTT
CCCCGGCTGCTGGCGACATTGCATCGCCACGGCGTTCAGGCCAGCTTTTTCTTCAGCGTC
GGGCCGGACAATATGGGACGTCATCTTTGGCGTCTGATAAGACCGCGATTTTTATGGAAA
ATGCTACGTTCAAACGCCGCTTCGCTCTACGGCTGGGATATCCTGCTGGCCGGTACCGCC
TGGCCGGGAAAAAATATCGGCAACGCCAATGCCGGGATTATTCGTGAAACGGCAACATAC
CATGAGACGGGACTACATGCCTGGGATCACCATGCGTGGCAGACTCACAGCGGCCACTGG
AGTATCCGCCAGCTTGAAGAAGATATCGCGCGCGGCATAACGGCGCTTGAGGCCATTATC
GGAAAACCCGTCACCTGCTCAGCCGCGGCCGGCTGGCGAGCGGATGGGCGCGTTGTTCGT
GCGAAAGAGCCGTTCAACCTGCGGTATAACAGCGATTGCCGTGGAACCACCTTATTCCGT
CCGCTACTGATGCCGGGCCAGACCGGGACGCCACAAATCCCGGTCACCTTACCCACGTGG
GATGAGGTTATCGGCCCCGCCGTTCAGGCGCAGTCGTTCAATACCTGGATTATCTCCCGT
ATGTTGCAGGACAAAGGCACGCCGGTATATACCATCCATGCGGAAGTCGAAGGTATTGTC
CATCAGCCGCTGTTTGAAGATTTGCTCGTTCGTGCACGTGACGCGGGGATCACCTTTTGT
CCTCTGGGCGAACTGCTGCCGACATCGCCTGAGTCTCTGCCGTTGGGGCAAATTGTACGT
GGGCACATCCCTGGCCGTGAAGGTTGGCTGGGGTGCCAACAGGCAGCGAGCGCCTCATGA
>EECEBICD_01059 Bifunctional polymyxin resistance protein ArnA
ATGAAAGCCGTTATTTTTGCCTATCACGATATGGGATGTCAGGGGGTGCAGGCCGTACTG
GATGCCGGGTATGAGATTGCCGCGATTTTCACTCATGCCGACAATCCTGCGGAAAACACC
TTTTTTGGCTCCGTCTCCCGGCTGGCGGCAGAATTAGGGATTCCGGTTTATGCACCGGAT
AACGTAAACCATCCTATCTGGGTCGACCGCATCGCTGAGCTTGCGCCGGATATTATTTTT
TCGTTCTATTACCGCAACCTGTTAAGCGAAGAAATTCTGCACCTCGCCCCGGCTGGCGCG
TTCAATCTGCACGGTTCTCTATTGCCTGCCTATCGTGGCCGTGCGCCGCTAAACTGGGTT
CTGGTTAACGGCGAAAGCGAAACGGGCGTAACGCTGCATCGTATGGTTAAACGTGCCGAC
GCCGGAGAGATTGTCGCCAGTCAACGCGTTGCTATTGCGCAGGACGATGTTGCGCTTACC
CTGCATCATAAGCTATGTCAGGCCGCGCGCCAGTTGCTTAATAGCATCCTGCCCACCATG
AAATGCGGCGATATACCTTCTGTTCCCCAGCGTGAGTCTGACGCTACATATTATGGTCGC
CGCAGGCCGGAAGATGGCCTTATCGACTGGCACAAACCGGTTTCCACGGTGCATAACCTG
GTGCGGGCGGTAGCCGCCCCCTGGCCCGGCGCGTTCAGCTATAACGGCTCGCAAAAATTC
ACTATCTGGTCTTCCCGAATTTGTCCGGATGCGCAGGGCGCGCTTCCCGGCTCGGTAATT
TCGGTCTCTCCGCTGCGGGTCGCCTGCGCTGATGGCGCGCTGGAGATTATTACCGGCCAG
GCCGGAGACGGCATTACTGTACAGGGCTCGCAGCTTGCACAGACGCTTGGTCTGGTGGCG
GGCGCGCGTTTGAACCGTCCACCAGCGACCAGCGGTAAACGTCGCATTCGCGTTCTCATC
CTCGGCGTCAACGGTTTTATCGGCAATCATTTGACCGAACGGTTACTGAATGAAGAGAAT
TACGAGGTCTACGGTATGGATATCGGCAGCAACGCCATTAGCCGATTCCTGCTCCACCCT
CGATTTCATTTCGTGGAGGGAGATATCAGCATCCACTCAGAATGGATCGAATATCATGTC
AAAAAATGCGATGTCGTACTGCCGCTGGTAGCAATTGCAACCCCGATTGAATACACCCGC
AACCCGCTGCGCGTCTTTGAACTTGATTTTGAAGAAAACCTGCGGATTATCCGTTATTGC
GTGAAATACCGTAAACGCGTGGTTTTCCCGTCTACCTCTGAAGTGTACGGCATGTGTACG
GACGCCTCTTTTGACGAGGACAAATCGAATCTGATCGTCGGGCCGGTGAATAAGCCACGC
TGGATTTACTCCGTTTCCAAACAGCTTCTCGACCGGGTGATTTGGGCTTATGGCGAAAAA
GAGGGATTGCGCTTCACGCTTTTCCGTCCCTTTAACTGGATGGGGCCGCGGCTGGATAGC
CTGAACGCGGCGCGTATCGGCAGTTCACGCGCGATAACGCAGTTGATTCTGAACCTGGTA
GAAGGGACGCCGATAAAGCTTATCGACGGCGGTCAGCAGAAACGCTGTTTCACCGATATT
CGCGATGGGATCGAAGCATTATTCCGCATTATTGTGAATGATGGCGATCGGTGCGATGGC
AAAATCATTAATATCGGCAACCCTGACAACGAAGCCAGTATTCAGGAACTGGCGACGCTG
TTGCTCGACAGTTTTGATAAGCATCCGCTGCGCTGCCATTTCCCACCGTTTGCCGGTTTC
CAGGTCGTCGAAAGCCGTAGTTATTACGGCAAGGGTTATCAGGATGTCGCACACCGGAAA
CCCAGTATCGACAATGCCAGGCGCTGTCTGGGCTGGGAACCGTCTATTGCGATGCGTGAT
ACGGTTGAAGAAACGCTGGACTTCTTCCTGCGCAGCGTAGATGTCGCGGAACGCGCATCA
TGA
>EECEBICD_01060 Undecaprenyl-phosphate 4-deoxy-4-formamido-L-arabinose transferase
ATGTTTGACGCCGCCCCCATAAAAAAAGTCTCGGTGGTTATTCCTGTCTATAACGAACAG
GAAAGCCTGCCGGAATTGATAAGACGCACCACCGCCGCCTGCGAGAGTCTGGGCAAAGCG

TGGGAGATTTTATTGATAGATGATGGTAGCAGCGATAGCTCGGCGGAGCTGATGGTTAAG
GCGTCCCAGGAAGCCGATAGCCATATCATCTCTATTTTACTCAACCGTAACTACGGTCAA
CATGCAGCGATCATGGCGGGGTTTAGCCATGTAAGCGGCGATCTCATCATTACGCTGGAT
GCCGATCTGCAAAACCCGCCGGAAGAGATCCCGCGCCTGGTGGCGAAAGCCGATGAAGGG
TTCGATGTGGTAGGCACCGTGCGCCAGAACCGACAGGACAGCCTGTTTCGCAAATCGGCG
TCAAAAATCATTAATCTACTGATCCAGCGCACAACGGGCAAAGCGATGGGCGATTACGGC
TGCATGTTACGTGCCTATCGCCGCCCTATTATCGATACTATGTTGCGCTGCCATGAACGC
AGCACCTTTATCCCTATTCTGGCCAACATTTTCGCTCGTCGGGCTACCGAGATCCCGGTG
CATCACGCTGAACGTGAATTTGGCGACTCTAAATACAGTTTTATGCGTCTCATTAACCTG
ATGTATGACCTGGTGACCTGCCTGACGACGACGCCGCTGCGCCTGTTGAGTCTGCTGGGC
AGCGTTATCGCCATCGGCGGGTTTAGCCTGTCGGTATTGCTTATTGTACTTCGCCTGGCG
CTGGGGCCGCAGTGGGCGGCGGAAGGCGTATTTATGCTCTTTGCCGTTCTGTTTACCTTC
ATCGGCGCGCAATTCATCGGTATGGGACTGCTGGGCGAATACATCGGTCGCATTTATAAC
GATGTCCGCGCCCGCCCCCGCTATTTTGTTCAACAAGTTATCTACCCGGAAAGCACGCCG
TTTACTGAGGAAAGCCACCAATGA
>EECEBICD_01061 UDP-4-amino-4-deoxy-L-arabinose--oxoglutarate aminotransferase
ATGTCGGACTTTTTGCCTTTCTCCCGTCCGGCGATGGGCGCGGAAGAGCTCGCTGCCGTT
AAGACCGTACTGGACTCAGGTTGGATAACAACCGGCCCGAAAAATCAGGAACTGGAAGCG
GCGTTTTGTCGGCTAACTGGCAATCAATATGCCGTTGCCGTCAGTTCAGCCACTGCCGGT
ATGCACATCGCGTTGATGGCGCTGGGCATTGGTGAAGGCGACGAGGTCATTACACCGTCA
ATGACGTGGGTTTCTACGCTAAATATGATCGTTTTACTCGGCGCGACCCCCGTCATGGTC
GATGTCGATCGCGATACGCTGATGGTCACGCCTGAACATATCGAAGCCGCGATTACGCCA
CAAACCAAAGCAATTATCCCCGTCCACTACGCTGGCGCGCCCGCCGATCTGGACGCTATT
TACGCTCTTGGCGAGCGTTATGGCATCCCGGTGATTGAAGACGCGGCGCACGCGACAGGA
ACCAGCTACAAAGGTCGCCACATTGGCGCCCGGGGCACCGCGATTTTCTCCTTCCACGCC
ATTAAGAACATCACCTGCGCTGAAGGCGGTATCGTCGTCACGGATAACCCGCAATTCGCC
GATAAACTGCGCAGTCTCAAGTTTCACGGGCTTGGCGTTGATGCCTGGGATCGACAGAGC
GGAGGACGCGCGCCACAAGCGGAAGTCCTGGCGCCGGGCTACAAATATAATTTGCCCGAC
CTCAACGCCGCTATCGCGCTTGCTCAGTTACAGAAACTGGATGCGCTTAACGCTCGCCGC
GCCGCCATCGCAGCGCAGTACCACCAGGCAATGGCAGACCTGCCTTTTCAGCCTCTGAGC
CTTCCGTCGTGGGAACATATTCACGCCTGGCATTTATTCATTATCCGGGTCGATGAAGCC
CGTTGCGGTATTACCCGGGATGCTCTGATGGCATCGCTGAAAACGAAAGGCATCGGCACA
GGGCTGCATTTCCGCGCCGCGCATACGCAAAAATATTATCGCGAACGTTTCCCAACGCTC
ACGCTGCCTGACACCGAGTGGAACAGCGAGCGGATCTGCTCACTGCCGCTCTTCCCGGAC
ATGACCGAGAGTGACTTTGATCGGGTCATTACCGCCCTTCATCAGATAGCAGGACAATAA
>EECEBICD_01062 Lipopolysaccharide core heptose(II)-phosphate phosphatase
GTGCTGGCATTTACCCTACGCTTTATTAAAAACAAACGCTATTTCGCGATTCTGGCGGGG
GCGTTGGTTATTATCGCCGGGTTGACCTCACAACATGCCTGGAGTGGTAACGGTCTACCG
CAGATTAACGGTAAAGCGCTGGCGGCGCTGGCGAAGCAGCATCCGGTAGTCGTGTTATTC
CGCCATGCCGAGCGGTGCGATCGTTCCGATAATACCTGCCTGTCAGACAGCACGGGGATT
ACGGTCAAGGGTGCGCAAGATGCCAGAGCGCTGGGTAAAGCCTTCAGTGCCGATATACAA
AATTACAATCTCTATTCCAGTAATACGGTGCGCACCATCCAGTCGGCAACCTGGTTTTCT
GCGGGCAGGTCGCTTACGGTGGATAAAAAAATGATGGATTGCGGCAGCGGTATTTATGCT
TCAATCAATACGTTACTGAAAAAGTCGCAGAATAAAAATATAGTCATTTTTACCCATAAT
CATTGTTTGACGTATATTGCAAAAAATAAGCGCGGCGTAAAGTTTGATCCGGACTATCTT
AATGCGCTCGTTATGCATGCGGAAAACGGAAAACTCTTTTTAGATGGTGAATTTGTTCCC
GGCTAG
>EECEBICD_01063 Nucleoside triphosphatase NudI
ATGCGTCAACGAACTATAGTTTGCCCATTAATTCAAAATGATGGCTGTTATCTGCTGTGC
AAAATGGCCGACAACCGCGGCGTCTTTCCCGGTCAGTGGGCGCTGTCGGGCGGCGGGGTC
GAGCCAGGCGAACGCATTGAAGAGGCGCTGCGTCGGGAGATTCGGGAAGAACTCGGCGAA
CAACTGATTCTGAGCGATATTACGCCGTGGACCTTCCGCGACGATATCCGCGTTAAAACC
TACGCTGATGGCAGACAAGAAGAGATTTATATGATTTATCTCATCTTCGACTGTGTTTCC

GCTAATCGCGATATATGTATTAACGACGAATTTCAGGACTACGCCTGGGTCAAGCCAGAA
GAACTGGCGCTGTATGATTTGAATGTAGCGACACGCCACACATTAGCATTGAAAGGGTTA
TTATAA
>EECEBICD_01064 hypothetical protein
ATGTGCTTGCTGGAAAGGGTAAAAATGAAAAAAAGCATATTATTAGGTTTTGCCGGAATG
CTGTTCGTTTCTGCATCGGCGCAGGCCATCAGTATTAGCGGCCAGGCGGGCGAAGATTAC
ACCAACATTGGCGTGGGTTTTGGTACGGAATCGACGGGACTGGCGCTAAGCGGCAACTGG
ATGCACAACGATGATGATGGCGATGCTGCGGGCGTGGGTCTGGGGCTGAACATTCCGGTG
GGACCGCTGCTGGCGACGGTAGGCGGTAAAGGTATTTATACCAATCCGAAAGACAGCGAT
GAAGGCTATGCGGCGGCTGTCGGCGGTGGGCTACAATGGAAAATCGGCAACAGCTTCCGT
CTGTTCGGCGAGTATTATTATTCACCAGATTCGCTGTCCAGCGGTATCGACAGTTATGAA
GAAGCGAATGCAGGCGCGCGTTTCACCATTATGCGCCCGCTGAGCATTGAAGCCGGTTAT
CGTTACCTGAACCTGGCGGGTAAAGACGGCAACCGCGATAATGCGATTGCTGACGGCCCT
TATGTTGGCGTAAACGCCAGCTTTTGA
>EECEBICD_01065 Nicotinamide-nucleotide amidohydrolase PncC
ATGCTAAATGTGGAAATGCTATCCACCGGGGATGAGGTGTTGCACGGACAAATCGTTGAC
ACCAATGCCGCCTGGCTGGCTGATTTTTTCTTTCATCAGGGCGTTCCGTTATCACGGCGA
AATACGGTAGGCGATAATCTGGACAGTCTGGTCGCTATCTTGCGTGAACGCAGTCAACAT
GCTGATGTTTTGATTGTTAATGGCGGGTTAGGGCCGACCAGCGATGATTTAAGCGCGCTC
GCCGCGGCAACGGCAAAAGGCGAAGGATTAGTACTGCACCAGGCGTGGCTGACCGAAATG
GAACGCTACTTTCAGCAGCGTGGCCGCGTGATGGCGCCGAGCAACCGTAAACAGGCGGAA
CTGCCTGCCAGCGCGGAGTTTATTAATAATCCGGTCGGCACTGCCTGTGGATTTGCGCTC
CAGCTCAACCGCTGCCTGATGTTTTTCACCCCCGGCGTACCGTCGGAATTTAAGGTTATG
GTGGAGAAGGAAATCTTACCGCGGTTACGCGCGCGTTTTTCGCTGCCGGAGCCGCCGCTT
TGCCTGCGGTTAACGACTTTTGGACGTTCAGAAAGCGACCTCGCGCAGCGTCTCGATTCA
CTGCCGTTGCCGCCCGGGGTGACGATGGGGTATCGCTCATCAATGCCGATCATTGAACTG
AAATTGACCGGCCCGGCTACGCAACGGGAGGCGATGCTGGCGTTATGGCCAGAGGTCAAA
CGGGTTGCCGGGCAGAACCTGATTTTTGAAGGTACGGAAAACCTGCCGGCGCAAATAGCC
CGCCGCCTGCAGGAAAGGCAGTTGAGTCTGACGTTGAGCGAGCAATACACCAGCGGCTTA
CTGGCGCTACAGCTTTCCCGCGCCGGGGCGCCTGTTCTTGCCAGCGAAGTCGTTCCTTCT
CAGGAAGAGACGCTGGCGCAAACGGCGCACTGGACGACAGAACGGCGCAGCAATCATTAT
GCCGGTCTGGCGCTGGCCGTGTCGGGGCTGGAAAACGAACATCTTAATTTCGCGCTGGCG
ACGCCGGATGGCACGTATGCCTTACGCGTGCGTTTTAGCGCCAATCGGTATAGCCTGGCA
ATACGTCAGGAGGTCTGCGCGATGATGGCGCTAAATATGCTGCGCCGCTGGCTTAATGGC
GAGGACATTACCAGCGAGCATGGCTGGATAGACGTGGTTGAATCATTGACATCATAA
>EECEBICD_01066 putative HTH-type transcriptional regulator RhmR
ATGTTGGAATCAAGTAAAGTCCCGGCTCTTACTCGCGCAATCGACATCCTCAATTTGATC
GCGCGTATTGGCCCATGCAGCGCGGCAATCATTATTGAAACGCTGCGTATTCCAAAAAGC
ACCGCGTACCTGCTGTTGAATGAATTAAAACGTCAGCGATTTATTAGCCTCGATCATCAG
GATAACTACTGCCTGTGGACTAAGCTCGTTGAATTGTCGGGTCATGCGCTCAGCAAGATG
GATCTGCGGGAGCTGGCGCGTCCTCGTCTAACACAACTGATGGATGAAACCGGGCTGCTG
TGTCACTTGGGGATCATCGACCACGAAAATGCGTATTACATACTGAAAGTGGAATCCTCA
TCCACTATCAGCGTGCGCTCGCATGAGGGGAAAAGCCTCTCTCTTTATCGTTCCGGCATC
GGCAAATGCCTGCTGGCATGGCAGCCGGCCGCCGTACGCAAAGCCATTATTGAACAACTG
GTATGGGAGCGAGCCACGCTGACCACCATCACCGAGCCGCAACAATTGAATGATGAGCTG
GAACGCATTCGCCAGCGTGGATGGAGCTTTGATAACGGGGAGGATTATCCGGATGTCCGC
TGCGTTGCCGCACCGGTATTTAATGCCAGAAACGAACTGACCGCGGCAATTTCAGTGGTC
GGCACGCGCCTGCAAATCAACGAAGAGAATCTTGATTATTTGGCCGGTAAAGCTATTGCC
TGTGCCAAAGATATTTCACGCCTGTTGGGATGGAAAAGTCCCTTCGACTCACTCGCATCT
TAA
>EECEBICD_01067 L-rhamnonate dehydratase
ATGACTCTTCCTAAAATTAAACACGTTCGAGCCTGGTTTATTGGGGGCGCTACCGCAGAG
AAAGGGGCTGGCGGCGGGGATTATCACGACCAGGGAGGAAATCACTGGATTGATGACCAT
ATCGCCACTCCCATGAGCAAATATCGCGACTATGAGCAGTCTCGTCAGTCTTTTGGAATC

AACGTGTTAGGGACGCTGATTGTCGAAGTAGAGGCGGAAAATGGGCAGACGGGGTTCGCC
GTTTCGACAGCAGGGGAGATGGGCTGTTTTATCGTAGAAAAACATCTTAACCGGTTTATT
GAAGGCAAATGCGTAAGCGATATCAAACTTATTCACGATCAGATGCTCGGCGCGACCATG
TACTACTCCGGTTCCGGCGGTCTGGTGATGAACACGATTTCCTGTGTGGATCTGGCGCTG
TGGGATCTGTTTGGCAAGGTGGTTGGGCTGCCGGTTTATAAATTGCTGGGCGGAGCGGTG
CGCGATGAAATTCAGTTTTACGCCACCGGCGCACGCCCGGATCTGGCTAAAGAAATGGGC
TTTATCGGCGGTAAAATGCCGACTCACTGGGGGCCGCACGATGGTGATGCGGGGATCCGT
AAAGATGCCGCGATGGTGGCGGATATGCGTGAAAAGTGCGGGCCGGATTTCTGGCTGATG
CTCGACTGCTGGATGAGTCAGGATGTGAACTATGCGACTAAACTGGCTCACGCCTGTGCG
CCATTTAATCTCAAATGGATTGAAGAGTGTCTGCCGCCGCAGCAGTATGAAGGCTATCGC
GAATTAAAACGCAACGCGCCTGCAGGGATGATGGTTACCAGCGGCGAACACCATGGTACG
CTGCAATCCTTCCGCACTCTGGCAGAAACCGGCATTGATATTATGCAGCCGGATGTTGGT
TGGTGCGGCGGATTAACCACGCTGGTTGAGATCGCCGCGCTTGCCAAATCGCGCGGGCAA
CTGGTGGTGCCGCACGGTTCGTCTGTCTATTCGCACCATGCAGTTATTACTTTCACTAAT
ACCCCTTTCAGCGAGTTCCTGATGACCAGCCCGGACTGTTCCACCCTGCGTCCGCAATTT
GACCCGATTCTCCTGGATGAGCCGGTGCCGGTGAATGGACGCATCCATAAATCAGTGCTG
GATAAGCCGGGCTTCGGCGTCGAGCTTAACCGTGACTGTCACTTGAAACGCCCTTATAGC
CACTAA
>EECEBICD_01068 Inner membrane transport protein RhmT
ATGAGCATCACTTTACTTGACGGCGTAGTGAAGAAAAACCGCGCACGTTTAATTCCTTTT
ATGTTGGCACTGTATGTACTGGCGTTTCTGGATCGTTCAAATATTGGTTTTGCCAAAGAG
ACCTATCAGATAGATACCGGGCTAAGTAATGAAGCCTACGCGCTAGGGGCCGGTATTTTC
TTTGTGGTTTACGCTTTCCTTGGCGTACCGGCTAACCTGCTGATGCGCAAATTTGGCGCA
AGAACCTGGATCGGCACGACTACCCTGCTGTGGGGCTTTCTCTCTGCTGCGATGGCCTGG
GCGGATAGTGAAGCAAAATTCCTGATAATCCGCACGCTATTGGGCGCGGCGGAGGCTGGA
TTTTTCCCCGGCATGATCTATCTAACTTCACAATGGTTCCCGCAGCGTAACCGTGCCAGC
ATTATGGGGCTATTTTATATGGGCGCGCCGCTGGCGTTAACGTTGGGTTCGCCGCTCTCC
GGCGCTCTGCTGGAGATGCATGGTTTTATGGGGCACCCGGGATGGTTCTGGATGTTCGTT
ATCGAAGGCCTACTGGCGATTGGCGCAGGGATATTCACCTTTTTCTGGCTTGATGACACT
CCGCAGCAGGCGCGTTTTCTTAGCCTGGAAGAAAAGAACGCGCTTATCAGGCAACTGGCG
AGCGAAGAAGAGAAAAAGGTCACGTCAAGGCTTGCGGATGCGCTGCGTAATGGACGCGTC
TGGCAACTGGCTATTATTTATCTGACGATTCAGGTGGCGGTATACGGACTCATCTTTTTC
CTGCCGACCTAG
>EECEBICD_01069 Inner membrane transport protein RhmT
TTGGGCACTAAAGTGGGCTTTACCGCTTCGGTGGTAACAGCGGTTCCCTGGGTGGCGGCT
TTGCTTGGCACATGGCTTATTCCTCGCTACTCCGACCGTACCGGCGATCGCCGTAACGTT
GCGGCAGTGACGTTACTGGCTGCGGGTATTGGCATTGGCCTGTCAGGTCTGGTTTCTCCG
GTGCTCGCTATTCTGGCGCTCTGTGTTGCAGCAGTAGGATTTATTGCCGTTCAGCCCGTA
TTCTGGACCATGCCGACGCAGCTACTTTCCGGTACGGCTCTGGCGGCAGGAATTGGTTTC
GTTAATCTGTTTGGCGCTGTGGGCGGTTTTATTGCCCCAATTCTGCGCGTAAAAGCGGAA
ACGTTGTTTGCCAGCGATGCGGCGGGCCTGCTGACGCTGGCCGGAGTCGCCATCATTGGC
TCATTAATTATCTTCACGCTGAGCGTTAATCGTCCGGTGGCGCAATCCGGCGCTGCGCAT
CATTAA
>EECEBICD_01070 2-keto-3-deoxy-L-rhamnonate aldolase
ATGAACGCATTGTTATCAAATCCTTTTAAAGAAGGATTACGTAAAGGGGATACGCAAATT
GGTTTGTGGCTCAGTTCAACCACCTCTTATATGGCGGAGATTGCGGCGACGTCGGGTTAT
GACTGGTTGTTAATAGATGGCGAGCACGCGCCTAATACGGTGCAGGACTTGTATCATCAG
TTACAGGCCATCGCCCCATACGCCAGCCAGCCGGTGATTCGCCCGATCGAGGGAAGTAAA
GCGCTCATTAAACAGGTACTGGACATCGGGGCGCAAACGCTACTGATCCCGATGGTTGAC
ACCGCAGAACAGGCGCGCCAGGTCGTTTCCGCTACCCGATACCCGCCGTTGGGGCAACGA
GGCGTTGGCGCGAGCGTGGCGCGTGCGGCGCGCTGGGGGAGAATCGACAACTATATGGCA
CAGGCCAATGAGTCGCTCTGCCTGTTGGTGCAGGTTGAAAGCAAAGTGGCGCTGGAAAAC
CTTGACGCGATTCTGGAGGTTGAAGGCATTGATGGCGTCTTTATCGGTCCGGCAGATCTC
TCTGCTTCATTGGGTTACCCCGATAATGCCGGACACCCGGAAGTGCAGCGAATCATTGAA

TCGTGTATTTATCGTATTCGCGCTGCCGGAAAAGCGGCGGGTTTTTTGGCGGTCGATCCG
GCAATGGCGCAGAAATGTCTGGCGTGGGGCGCGAATTTTGTCGCGGTCGGGGTAGATACG
ATGCTCTACACCGAGGCGCTGGACAGCAGGTTGGCGATGTTTAAATCTGTTCAGTCAGTA
AGCACGGCAAAACGTAGTTATTGA
>EECEBICD_01071 hypothetical protein
ATGCCGGATGGCACGTATGCCTTACGCATGCGTTTTAGCGCCTATCGGTATAGTCTGGCA
ATACGTCAGGAGGTTTGTGCGGTGATGGCGCTAAATATGCTGCGCCGTTGGCTTAATGGC
GAGGACATTACCAGCGAGCATGGCTGGATAGACGTGGTTGAATCATTGACGGCATAG
>EECEBICD_01072 Deubiquitinase SseL
ATGAATATATGTGTAAATTCACTTTACCGATTGAGCACACCGCAATTTCACAGTTTATAT
TCAGAAGATGTGAGCGATGAGGCGCTTGCGTTGTTGATTGGCGAAGTAGAGAACGGTAAT
CAGAATTGTATTGATCTGTTATGCAATCTTGCGTTACGCAACGATGACCTGGGACATAAA
GTTGAGAAATTACTTTTTGATCTCTTTAGCGGAAAAAGATCGGGATCACCAGATATAGAC
AAAAAAATCAATCAGGCTTGCCTTGTATTACATCAAATCGCCAATAACGATATAACAAAA
AATAATACTGAGTGGAAAAAGCTACATGCCCCTTCCAGATTACTTTATATGGCCGGTTCA
GCGACAACCGACCTTTCTAAAAAAATAGGAATAGCACATAAAATTATGGGCGACCAGTTC
GCTCAGACAGATCAAGAACAGGTAGGAGTTGAAAATCTTTGGTGTGGTGCGCGAATGTTG
TCGTCAGATGAGCTGGCAGCTGCAACGCAAGGTCTGGTTCAAGAATCACCTCTTCTCTCG
GTAAACTATCCTATTGGGCTGATTCATCCTACCACCAAAGAAAATATATTAAGCACTCAG
CTACTTGAAAAGATTGCTCAATCAGGATTATCTCACAATGAAGTCTTCCTGGTAAATACA
GGAGATCACTGGCTTCTCTGTTTATTTTATAAACTTGCAGAAAAAATAAAATGCCTTATA
TTTAATACTTATTATGATTTAAATGAAAATACTAAGCAAGAGATTATAGAAGCAGCAAAA
ATTGCAGGTATATCAGAAAACGAAAATATTGATTTTATTGAAACTAATTTACAAAATAAT
GTACCCAACGGCTGTGGTCTATTTTGTTACCATGCAATTCAACTCTTATCGAATGCAGGA
CAAAACGATCCTGCTACCACACTACGAGAGTTTGCGGAAAATTTCTTAACGCTTTCAGTA
GAGGAACAAACACTATTTAACACCCAAACCCGGCGACAAATATATGAATACAGTCTCCAG
TAA
>EECEBICD_01073 Anaerobic glycerol-3-phosphate dehydrogenase subunit C
ATGAGCGACACACGTTTTGAAAGTTGTATTAAATGTACGGTGTGCACCACCGCATGCCCG
GTCAGCCGCGTTAATCCTGGCTACCCTGGCCCTAAACAGGCCGGACCGGATGGCGAGCGC
CTGCGTCTGAAAGACGGCGCACTGTATGATGAGGCGCTGAAATATTGCATTAACTGCAAA
CGGTGTGAGGTCGCTTGTCCGTCGGACGTGAAGATTGGCGATATCATCCAGCGCGCCCGC
GCAAAATACGATACGACGCGCCCGTCATTGCGTAATTTTATTCTCAGCCATACCGATCTG
ATGGGCAGCGTTTCAACGCCGTTCGCGCCGGTGGTCAATACCGCCACCGCATTAAAGCCA
GTGCGCCAGTTGCTTGATTACGCGCTCAAAATCGATCATCGCCGCACCTTACCAAAATAC
TCTTTCGGCACCTTCCGCCGCTGGTATCGTAGCGTGGCGGCGCAGCAGGCTAAATACAAA
GATCAGGTCGCCTTTTTCCATGGCTGTTTTGTGAATTACAACCATCCGCAGCTCGGAAAA
GATCTGATTAAGGTGCTGAACGCAATGGGTACTGGCGTACAGTTACTGAGTAAAGAGAAG
TGTTGCGGCGTACCGCTGATCGCTAACGGTTTTACGGATAAAGCTCGCAAACAGGCTATC
AGCAATGTGGAGTCGCTACGCGAGGCGATTGCCGTCAAAGGTATTCCGGTCATCGCCACG
TCATCCACCTGTACGTTTGCGTTGCGCGATGAATATCCTGAAGTGCTGGACGTGGACAAC
GCCGGGCTGCGCGAACATATCGAGCTGGCCACCCGCTGGCTATGGCGCAAACTGGATGCC
GGGAAAACGCTGCCGCTTAATCCTTTACCGCTGAAAGTGGTCTACCATACGCCGTGTCAT
ATGGAAAAAATGGGCTGGACGCTTTACACGCTTGAACTGTTGCGGCAAATTCCGGGGCTT
GAATTGACGGTACTTGATTCACAGTGCTGCGGCATTGCCGGTACGTATGGATTTAAAAAG
GAAAACTATCCGACATCACAGTCCATCGGCGCGCCACTGTTCCGCCAGATTGAAGAGAGC
GGCGCCGACATTGTCGTCACCGATTGTGAAACCTGTAAGTGGCAAATTGAGATGTCTACA
AGCAAACGCTGCGAACACCCCATTACGCTACTGGCCCAGGCGCTCGGCTAA
>EECEBICD_01074 Anaerobic glycerol-3-phosphate dehydrogenase subunit B
ATGAAATTTGATACGGTTATTATGGGCGGCGGTCTCGCCGGTTTGCTATGCGGACTGCAA
CTCCAGCAGCATGGATTACGCTGCGCCATCGTCACCCGCGGACAAAGCGCGCTGCATTTT
TCCTCCGGCTCGCTGGATCTACTCAGCGCCCTGCCCGACGGCCAACCGGTGACCGACATT
ACCGCCGGGCTTGATGCGCTGTGCCGCCAGGCGCCTGAACATCCTTATTCGCGTTTAGGC
GCGCAAAAAGTCCTTACGCTGGCGCAACAGGCGCAAACGCTACTTAACGCCAGCGGCGCG

CAGTTATACGGTGACGTACAGCAGGCGCACCAGCGTGTCACGCCGCTTGGCACATTGCGT
TCAACCTGGCTTAGCTCGCCGGAAGTACCGGTCTGGCCGTTATCCGCACAGCGAATTTGC
GTCGTCGGCGTTAGCGGATTACTGGATTTCCAGGCGCACCTTGCGGCAGCGTCGCTGCGC
CAACGCGACCTCAACGTGGAAACCGCAGAAATCGACCTTCCGGAACTGGACGTACTGCGT
GATAACCCGACGGAATTTCGCGCGGTCAATATTGCGCGTCTGCTTGATAACGAAGAAAAA
TGGCCGCTGCTGTACGACGCGCTGTCGCCAATAGCGACAAACTGCGACATGATCATCATG
CCCGCCTGTTTCGGGCTGGCGAACGATACGCTGTGGCGCTGGCTTAACGAACGTCTGCCC
TGCGCGCTCACGCTGCTGCCAACGCTGCCGCCATCGGTGCTCGGTATTCGTCTGCACAAT
CAGCTCCAGCGCCAGTTTGTCCGTCAGGGCGGCATCTGGATGCCGGGCGATGAAGTGAAG
AAAGTCACCTGCCGTCGCGGTACCGTGAGCGAAATATGGACCCGCAACCATGCCGATATC
CCACTGCGCCCGCGTTTTGCCGTACTTGCCAGCGGTAGCTTCTTTAGTAGCGGTCTGGTT
GCCGAGCGCGAAGGGATCCGCGAACCTATTTTAGGGCTCGACGTACAGCAAACGGCCACC
CGCGCCGAATGGTATCAACAACATTTCTTTGACCCACAGCCCTGGCAGCAGTTTGGCGTC
GTGACGGATGACGCCTTCCGCCCCTCCCTTGCGGGCAACACGGTAGAAAATCTGTATGCA
ATAGGTTCTGTGCTGGCGGGATTCGATCCTATAGCTGAGGGATGCGGCGGCGGTGTCTGC
GCAGTCAGCGCCCTCCAGGCCGCGCACCATATCGCTGAGCGTGCAGGAGAACAGCAATGA
>EECEBICD_01075 Anaerobic glycerol-3-phosphate dehydrogenase subunit A
ATGAAAACTCGCGACTCGCAAGCAAGTGACGTGATTATCATTGGCGGTGGAGCAACAGGC
GCAGGGATCGCCCGCGACTGCGCGCTGCGCGGTTTACGTGTCATTCTGGTGGAACGGCAT
GATATAGCGACCGGCGCGACCGGGCGTAACCACGGACTGCTGCACAGCGGCGCTCGTTAT
GCGGTTACCGACGCGGAATCCGCGCGCGAGTGCATTAGTGAAAACCAGATTTTAAAACGG
ATCGCCCGCCATTGCGTCGAACCGACGGATGGTCTGTTTATTACGTTGCCGGAAGATGAT
CTTGCGTTTCAGGCCACCTTTATTCGCGCCTGTGAAGCGGCGGGCATTCGCGCTGAAGCC
ATCGATCCGCAGCAGGCTCGCATCATCGAACCTTCCGTTAACCCTGCGCTCATCGGTGCG
GTAAAAGTGCCGGATGGCACCGTGGACCCTTTCCGCCTTACCGCCGCCAATATGCTGGAT
GCCAGAGAACATGGCGCAATCGTGTTAACGGCGCATGAAGTGACCGGGCTCATTCGTGAA
GGCGCAACGGTCTGCGGCGTCCATGTGCGCAACCATCTCACTGGCGAAACCCAGACGCTT
CACGCGCCGGTGGTGGTCAATGCGGCTGGTATCTGGGGGCAACGGATCGCCGAATACGCC
GACCTGAGTATTCGCATGTTCCCGGCGAAAGGCTCGCTGTTGATCATGGACCATCGGATC
AACCAGCATGTCATTAACCGCTGCCGGAAACCATCGGATGCCGACATTCTGGTACCTGGC
GATACGATTTCATTGATCGGCACTACCTCTACCCATATTGATTACAATGAGATAGACAGC
AACCGCGTCACCGCAGATGAAGTCGATATCCTGCTGCGTGAAGGGGAAAAACTGGCCCCT
GTCATGGCGAAAACGCGTATTCTGCGCGCGTATTCCGGCGTCCGCCCGCTGGTGGCCAGC
GATGACGATCCCAGCGGTCGGAACGTCAGCCGCGGTATTGTGTTGTTCGACCACGCCGAA
CGCGACGGGCTTGATGGCTTCATTACCATTACCGGCGGGAAGCTAATGACTTACCGCCTG
ATGGCGGAATGGGCGACCGACGCCGTGTGCCGCAAGTTGGGCAATACTCGCCCCTGCATC
ACGGCGGATACGCCGTTGCCTGGCTCAAAAGAGTCCACGGAACATACGCTGAAACGCATT
ATCTCGCTTCCCGCGCCGCTACGCGGATCGGCGGTTTATCGGCATGGCGATCGCACGCCG
GGGTGGCTGAGTGAAGGCCGCCAACACCGTAGCCTGGTCTGTGAATGCGAGGCCGTCACG
GCGGGGGAAGTGCAGTATGCCGTCGAAAACTTAACCGTGAATAGCCTGCTGGATCTGCGC
CGTCGCACCCGCGTAGGGATGGGCACTTGCCAGGGCGAACTGTGCGCCTGCCGCGCGGCC
GGGCTATTGCAGCGTTTTAATGTGACGACCGCGGCGCAATCTATTACGCAACTCTCTGAA
TTTCTGAATGAGCGCTGGAAAGGCGTACAACCTGTCGCCTGGGGAGACGCGCTGCGCGAA
AGCGAATTCACGCGCTGGGTTTATCAGGGATTATGCGGTCTGGAGAAGGAGCACCAGGAT
GAAATTTGA
>EECEBICD_01076 Glycerol-3-phosphate transporter
ATGTTGAGTATTTTTAAACCAGCGCCGCATAAAGCGCGCTTGCCAGCGGCGGAGATTGAT
CCGACCTATCGCCGATTACGCTGGCAGATTTTCCTGGGGATATTCTTTGGCTATGCCGCG
TATTATCTGGTGCGCAAGAACTTTGCCCTCGCGATGCCCTACCTGGTAGAACAGGGTTTT
TCACGCGGCGATCTGGGCTTTGCGTTGTCCGGGATTTCCATCGCTTATGGTTTTTCGAAA
TTTATAATGGGTTCGGTGTCCGATCGCTCGAATCCGCGCGTTTTTCTGCCGGCAGGGTTG
ATTCTGGCCGCAGCAGTCATGTTGTTTATGGGCTTTGTGCCGTGGGCGACATCCAGCATC
GCCGTGATGTTTGTGCTGTTGTTCCTTTGCGGCTGGTTCCAGGGGATGGGGTGGCCGCCG
TGCGGACGTACGATGGTTCACTGGTGGTCGCAGAAAGAGCGCGGCGGCATTGTGTCGGTC

TGGAACTGTGCGCATAACGTCGGCGGCGGGATCCCGCCACTGCTGTTCCTGCTGGGGATG
GCGTGGTTTAACGACTGGAAAGCGGCGCTCTATATGCCAGCCTTTGGCGCGATTGTGGTG
GCGCTGTTCGCTTTCGCAATGATGCGCGATACGCCGCAATCCTGCGGTCTGCCGCCTATC
GAAGAGTATAAAAACGACTATCCGGACGACTACAACGAAAAAGCGGAAGAAGAGCTGACT
GCGAAGCAGATCTTCATGCAGTACGTACTGCCGAACAAATTACTATGGTATATCGCTATC
GCCAACGTGTTTGTTTATCTGCTGCGCTACGGCATCCTCGACTGGTCGCCGACCTACCTG
AAAGAAGTGAAGCATTTCGCACTGGATAAATCCTCCTGGGCCTACTTCCTGTATGAATAC
GCGGGTATTCCGGGCACGCTGCTGTGTGGCTGGATGTCGGATAAAGTCTTCCGCGGCAAC
CGCGGTGCGACCGGCGTGTTCTTTATGACCCTGGTGACTATCGCGACCATCGTTTACTGG
ATGAACCCGGCAGGGAACCCGAACGTTGATATGGCTTGTATGATTATCATCGGCTTCCTG
ATCTACGGCCCGGTAATGTTGATCGGTCTGCACGCGCTGGAACTGGCGCCGAAAAAAGCG
GCGGGTACGGCGGCGGGCTTTACCGGTCTGTTTGGTTACCTGGGCGGTTCTGTCGCTGCA
AGCGCCATTGTGGGTTATACCGTTGACTTCTTCGGTTGGGATGGCGGCTTTATGGTGATG
ATTGGCGGCAGCATTCTGGCGGTAATCCTGCTGGTGGTCGTGATGATTGGTGAAAAACGC
CATCATGATGAGCTGCAGTTAAAACGCAACGGAGGCTAA
>EECEBICD_01077 Glycerophosphodiester phosphodiesterase, periplasmic
ATGAAAACCACACTGAAAAACCTTAGCGTGGCGTTAATGTTGGCAGGAATGACCATCGGA
AGCGGCGCCGTGGCGGCTGAAAAAGTCGTTATCGCCCACCGTGGTGCCAGCGGTTATCTG
CCGGAGCATACGCTACCGGCGAAAGCGATGGCGTATGCGCAAGGGGCAGATTATCTGGAA
CAGGATTTAGTGATGACAAAGGACGACCATCTGGTCGTCCTCCATGACCACTACCTTGAC
CGGGTGACTGACGTCGCTGACCGTTTTCCGGACCGGGCGCGTAAAGATGGGCGCTATTAC
GCTATCGATTTTACGCTGGATGAAATTAAATCCCTGAAGTTTACCGAAGGGTTTGATATT
GAAAACGGCAAAAAAGTACAAACTTATCCGGGCCGTTTCCCGATGGGAAAATCTGATTTC
CGCATTCATACCTTTGAAGAGGAAATTGAATTCGTTCAGGGATTAAATCACTCTACCGGT
AAAAATATCGGTATTTATCCGGAAATTAAAGCGCCGTGGTTCCATCATCAGGAAGGGAAA
GATATTGCCGCGAAAACGCTGGAAGTGCTGAAGAAGTATGGTTACACCGGCAAGCAGGAC
AATGTCTATTTGCAGTGTTTTGATGTCGCTGAGCTGAAACGTATTAAGAATGAACTGGAA
CCCAAAATGGGGATGGATCTCAATCTGGTTCAACTTATTGCGTATACCGACTGGAATGAA
ACACAGCAGAAACAGCCGGACGGTCGTTGGGTAAATTACAACTACGACTGGATGTTTAAG
CCGGGCGCTATGAAGCAGGTGGCGGAATATGCGGACGGTATTGGTCCGGATTACCATATG
CTGGTTGCGGAAGGCTCAACGAAAGGGAATATCAAGCTGACTGGAATGGTGCAAGACGCG
CATCAGAATAAGATGGTAGTGCATCCTTACACTGTGCGTGCCGATCAATTGCCGGACTAT
GCCACAGACGTCAATCAGTTGTACGACATCCTGTATAACAAGGCGGGCGTCGACGGGCTG
TTCACTGACTTCCCGGATAAGGCGGTTATGTTCCTGCAAAAAAATGACTAA
>EECEBICD_01078 hypothetical protein
ATGTATTGCGTCCCCATTTACCGGGATAAATTCACCGTAGTGGTGCCTGAAAATCATCCC
CTGGCGGCCAATAGTACGGTAACGGTCGAAGAACTGATGGACGAACCACTTATTGTCAGT
AAAGGTCGTTATGAATTAAGCATTATGGCGTTGTTTAAAGAAAAGGGTATTGAGCCGATA
TTTAAATATGAATTTAACCATCCCGATACTGCGCTGAATTTTATTCGCCAGGGATTAGGT
ATCGCCTTATTGCCAGAATTAACCTTAAAAGCCACGACAGGAAAATTATGCTCCGTGGCG
CTGGAACCCACCTTTTATCGACAAATTTCCCTGTTAGCAAAAGAACCGCCGGTAGAAGGA
AGTCCGTTATTTTTATTACAAAAATGTATGGAAACACTGACCGATGAGGGGTTGCTATAA
>EECEBICD_01079 Hydrogen peroxide-inducible genes activator
ATGACACTAACTCAAATCCACGCCCTGCTTGCCGTACTGGAGTACGGCGGATTTACCGAG
GCCAGCAAACGGCTATACATGACGCAATCCGCCGTCAGCCAGGCCATCTCCGCGCTTGAG
GAGGAGCTCGGCGTTGACATATTGATTCGTGAGCGTCGTAAAGAGATTGAGCTTACCGCC
GCCGGTAGCCGCATCGTCAGGCACTTACGCGCGATCCAGCGCGACGTCAACGCGGTAAAA
GAGATTGCCGAACAAGAAAAAAGAACCCGGCCCGTACGCTACGGATTGGCTGTTTTCCCA
GCGCCTGCGCCTGCATTTTACCCGGCGTCATTCGCTATTTTGAAAGCCATCATCCAAATG
TAA
>EECEBICD_01080 Staphylopine export protein
GTGCGCCATCTGAGCGGGTCGTTAAGAATGAAAGAAAAATTATGGACGAAGGATTTTTGG
GCAATCACCATAGTCAGTTTTATTATCTTTTTTGTTTTTTATGTACTGCTAACTTTATTA
CCCATTTATATCGCTGACCGCCTGCATGCTTCTGCAGATAAGGCCGGGCTGTTAGTGACA

TGTTTCCTGGCCGCGGCAATTGTTATTCGGCCATTTGCCGGACAATGGGTGGGAAAATAT
TCGAATAAAAAGATTCTGGTGCTCTCTTCTCTGGCTTTTTGGTGATTACCGCGCTGTAT
CCGGTTTGCCATTCTATCGAGTCGCTGCTCTTTATCCGCGTACTTCATGGGATTACGTTT
GGTGTTATTACCACGGTAAAGGAACTATTTCAGCGCGGTTAATTCCGGCCTCCCGACGT
GGAGAGGGCATCAGCTTTTTCTCTTTAGCGATGGGCCTTGCGATGGTCGTAGGACCGTGG
ATCGGTTTGAATATGGCCCGCTGGAACGCGTTCACTGCCGCATTCTGGCTTTGTTCCGCT
GTAGCGGCGTTAGGGATTGTACTTTCCCTGATTGTGACGGTCCCTCCGGTAATTCGTCAT
GCCGATGGTTCGAAACCGAATCTGGGTTTTGCCGCGATGTTTGACCGCGCCGCCTTGCCT
TTTGCGCTGGTCACCTTTTTTATGACTTTCTCCTATGCCGGCGTATCGGCTTTCCTGGCG
CTGTATGCGCGTGAGCTGGATTTAATGGCGGCGGCCAGTAATTTCCTGCTGTGCTACGCG
ATTTTCCTGATGATATGCCGTACCTTCACCGGGAATATTTGTGATAAAAAAGGGCCGAAA
TATGTTGTTTTCCCATGCCTGTTGGCTTTTACCATCGGTCTGGTGGCGCTGGGCTACACC
AATGGCAGCATCATGATGATTATTTCCGGCGGTCTCATCGGTATCGGCTACGGTTCGGTC
ACGCCGGTTTTCCAGACGCAAATTATCAGCTCGGTTGAACCGCATAAAATCGGCGTGGCG
AATTCACTGTTCTTTAACGCGATGGATGCCGGAATGGCGATTGGCGCCTTTATCATGGGA
ATGATGGTTGAGTCGGTTGGCTATCGTATGATTTATGTCGCTGGCGCGGTGCTGGTGGTA
CTCGCTGGCGCACTGTATGCCGTGCAGATGAAAAAACGTGGCGTGATGCCTTTGGTTTCC
ACCAGCGAGTTGCACTGA
>EECEBICD_01081 hypothetical protein
ATGGGACGCGTCACGCTGCGTATCACTGGCACACAGCTGCTGTGCCAGGATGAACACCCT
TCTCTGCTGGCCGCGCTGGAATCGCATAACGTTGAGGTGGAGTATCAGTGCCGCGAAGGT
TATTGCGGCTCCTGCCGTACCCGACTGGTTGCCGGTCAGGTTGACTGGATTACGGAACCT
TTAGCGTTTATCCAGCCAGGAGAGATTTTGCCCTGCTGCTGCAGGGCAAAAGGCGATATC
GAAATCGAGATGTAA
>EECEBICD_01082 Ribonucleoside-diphosphate reductase 1 subunit beta
ATGGCATACACCACTTTTTCACAGACGAAAAACGATCAGCTCAAGGAACCCATGTTCTTT
GGCCAGCCGGTCAACGTTGCACGCTATGACCAGCAGAAATATGACATCTTCGAAAAACTG
ATCGAAAAACAGCTCTCTTTCTTCTGGCGTCCGGAAGAGGTCGACGTCTCCCGCGATCGT
ATCGATTATCAGGCGTTGCCGGAGCACGAAAAACATATCTTCATCAGCAACCTCAAATAC
CAGACGCTGCTGGATTCCATTCAGGGACGTAGCCCGAACGTCGCCCTGCTGCCGCTCATT
TCCATCCCGGAGCTGGAGACGTGGGTAGAGACCTGGGCGTTTTCTGAGACGATTCACTCA
CGATCCTACACCCACATTATTCGTAACATCGTCAACGATCCGGCGGTCGTGTTTGACGAT
ATCGTGACCAATGAGCAAATTCAGAAGCGCGCCGAAGGTATCTCCGCCTACTACGACGAA
CTTATTGAGATGACCAGTTACTGGCACCTGCTCGGTGAAGGCACCCATACCGTTAATGGC
AAAACGGTTGTCGTGAATCTGCGTGAGCTGAAGAAAAAACTGTATCTGTGCCTGATGAGC
GTGAACGCGCTGGAAGCCATCCGCTTCTACGTCAGCTTCGCCTGCTCTTTCGCATTTGCC
GAGCGTGAACTGATGGAAGGCAACGCCAAAATTATCCGTCTGATCGCCCGTGACGAAGCT
CTGCATCTGACCGGCACGCAACATATGCTGAACCTGTTGCGTAGCGGCGTGGACGACCCG
GAGATGGCGGAAATCGCCGAAGAGTGTAAACAGGAATGTTATGACCTGTTCGTGCAGGCG
GCGCAGCAGGAAAAAGAGTGGGCGGACTATCTGTTCCGCGACGGTTCGATGATCGGCCTG
AATAAAGATATCCTGTGTCAATACGTTGAATATATTACTAATATTCGTATGCAGGCGGTA
GGGCTGGATCTGCCGTTCCAGACCCGCTCCAACCCAATCCCGTGGATCAATACCTGGCTG
GTGTCCGATAACGTTCAGGTTGCGCCACAGGAAGTGGAAGTTAGTTCTTATCTGGTCGGA
CAAATCGATTCGGAAGTCGATACCGACGATCTGAGTAATTTCCAGCTCTGA
>EECEBICD_01083 Ribonucleoside-diphosphate reductase 1 subunit alpha
ATGAATCAGAGTCTGCTGGTGACAAAGCGTGACGGCCGTACTGAGCGCATCAATCTCGAC
AAAATTCATCGCGTGCTTGATTGGGCGGCAGAAGGACTGAATAACGTATCGGTTTCTCAG
GTCGAGCTACGCTCGCATATTCAGTTTTATGATGGGATTAAGACCTCCGATATCCATGAA
ACTATCATTAAGGCCGCCGCAGACCTGATCTCTCGCGATGCGCCGGATTATCAATACCTG
GCTGCGCGTCTGGCGATTTTCCACCTGCGTAAGAAAGCCTTCGGCCAGTTTGAGCCGCCC
GCACTTTACCACCATGTGGTAAAAATGGTTGAGCTTGGCAAATACGACAATCATCTGCTG
GAAGACTACACGGAAGAAGAGTTCAAGCAGATGGATTCGTTTATCGTTCACGATCGCGAT
ATGACCTTCTCCTACGCTGCCGTTAAGCAGCTGGAAGGGAAATATCTGGTGCAAAACCGC
GTGACCGGCGAAATCTATGAAAGCGCACAGTTCCTCTACATTCTGGTAGCGGCCTGCCTG

TTCTCGAACTACCCGCGTGAAACGCGTCTTGACTATGTAAAACGTTTTTACGATGCGGTC
TCTACGTTCAAAATCTCGCTGCCGACGCCCATCATGTCCGGTGTGCGTACCCCAACCCGT
CAGTTCAGCTCCTGCGTACTGATCGAGTGTGGCGACAGTCTGGATTCCATCAACGCCACC
TCCAGCGCTATCGTGAAATACGTTTCTCAGCGCGCCGGTATCGGCATCAACGCGGGACGC
ATTCGTGCGCTGGGCAGCCCGATTCGCGGCGGCGAAGCGTTCCACACCGGCTGTATTCCG
TTCTACAAGCACTTCCAGACGGCGGTGAAATCCTGTTCGCAGGGCGGCGTGCGCGGCGGC
GCGGCAACCCTCTTCTACCCGATGTGGCATCTGGAAGTGGAAAGCCTGCTGGTGCTGAAA
AACAACCGCGGCGTGGAAGGCAACCGCGTACGTCACATGGACTACGGCGTACAGATCAAC
AAACTGATGTATACCCGCCTGCTGAAGGGCGGCGACATTACGCTGTTCAGTCCGTCGGAT
GTACCCGGGCTATACGATGCGTTCTTCGCCGACCAGGACGAATTCGAACGCCTGTACGTG
AAATATGAACACGACGACAGCATCCGCAAACAGCGTGTGAAAGCGGTCGAACTGTTCTCG
CTGATGATGCAGGAACGCGCCTCTACCGGTCGTATCTATATCCAGAACGTTGACCACTGC
AATACTCACAGTCCGTTCGATCCGGTTGTCGCCCCGGTACGCCAGTCTAACCTGTGTCTG
GAGATTGCCCTGCCAACCAAACCGCTGAACGATGTCAACGATGAAAACGGCGAAATTGCG
TTGTGTACGCTGTCTGCATTCAACCTGGGCGCCATTAAAACTCTGGACGAGCTGGAAGAA
CTGGCTATTCTGGCAGTACGCGCTTTGGATGCTCTGCTCGATTATCAGGATTACCCGATT
CCGGCTGCCAAACGCGGCGCAATGGGCCGTCGTACGCTGGGCATTGGCGTGATTAACTTC
GCCTACTGGCTGGCGAAAAACGGTAAGCGTTATTCCGACGGCAGCGCCAATAATCTGACG
CATAAAACCTTCGAAGCCATTCAGTACTATCTGCTTAAAGCGTCCAACGAACTGGCGAAA
GAGCAAGGCGCCTGCCCATGGTTCAACGAAACCACCTATGCAAAAGGCATTTTGCCGATC
GACACCTACAAAAAAGATCTGGATGCGATCGTCAACGAACCGCTGCACTACGACTGGCAA
CAGCTGCGTGAGTCGATCAAAACCCACGGTCTGCGTAACTCCACGCTATCGGCGCTAATG
CCGTCGGAAACGTCGTCCCAGATCTCCAACGCCACCAACGGCATTGAGCCGCCGCGCGGC
TACGTCAGCATCAAGGCCTCGAAAGACGGTATTTTGCGTCAGGTCGTGCCGGATTATGAG
CATCTGAAAGACGCTTACGAACTGTTGTGGGAGATGCCGAACAACGACGGTTATCTGCAA
CTGGTGGGTATCATGCAGAAGTTTATCGATCAGTCGATTTCCGCCAATACCAACTACGAT
CCGTCACGTTTCCCGTCAGGAAAAGTGCCGATGCAGCAACTGCTGAAAGATTTGCTCACC
GCGTATAAGTTTGGCGTGAAAACTCTGTACTATCAAAATACCCGCGACGGCGCGGAAGAT
GCGCAGGACGATTTGGCGCCTTCTATTCAGGACGATGGCTGCGAAAGCGGCGCTTGTAAG
ATCTGA
>EECEBICD_01084 Ubiquinone biosynthesis O-methyltransferase
ATGAACACTGAAAAACCGTCGGTAGCTCACAACGTTGACCATAATGAAATCGCCAAATTC
GAAGCCGTCGCGTCGCGCTGGTGGGATCTGGAGGGCGAATTCAAGCCATTGCATCGTATC
AACCCCCTACGCCTTGGCTACATTACTGAGCGCTCAGGCGGCCTGTTTGGTAAAAAGGTG
CTCGACGTCGGCTGTGGCGGCGGTATTCTGGCGGAGAGCATGGCGCGCGAAGGCGCAACC
GTCACCGGGCTGGATATGGGCTTTGAGCCTTTGCAGGTCGCCAAACTGCATGCGCTGGAA
AGCGGCATCGAGGTAGAGTACATGCAGGAGACGGTTGAGGAACATGCGGCAAAACACGCC
CAGCAATACGACGTCGTCACCTGTATGGAAATGCTGGAGCACGTTCCCGATCCGCAATCG
GTCGTCCACGCCTGCGCCCAACTGGTTAAACCCGGCGGCGAGGTGTTCTTCTCAACGTTG
AACCGCAATGGGAAATCCTGGCTGATGGCGGTCGTCGGCGCTGAGTATATTCTGCGCATG
GTGCCAAAAGGCACGCACGACGTGAAGAAATTCATTAAGCCTGCTGAACTGTTAAGCTGG
GTAGATGAAACCGTCCTGAAAGAGCAACATATCACCGGGCTGCACTACAACCCGATAACC
AATACTTTCAAACTCGGTCCCGGAGTAGACGTTAACTACATGTTGCATACCCGCGCTAAA
AAAGCCTGA
>EECEBICD_01085 hypothetical protein
ATGTTACGTAAAAAACAATACAATTCAAAAGCTAATGTGTCATATCTGATATATCAGCAA
GCAAACTTAATGAAATATACAATAACAACAATAAAGTATGTAAATTTTCTTATATCTGAT
ATATTAGATCCGTTAATTTTGAATAAATTAATTACAATGGAGCCGGGTTTAACCATGGAG
GAGAAGAAAATGAAAAATAGATTAAAGCGTGACTTTAGTCACATATCAATTGAAATACCC
AAATCCATCACCCAGGTGATTAAAGAGAAAATAAGGGAGATGATTATCCATGGCGATTTC
GATCTTGGGCAAGCGATCAGTGAAAATGAACTATCAAATATTCTGATGGTAAGTAAAACA
CCAATTCGTGAAGCTTTTATTTGGCTGAGCTATAATGAAAACTTAGTCAACATCATTCCC
CGCAGTGGGACATTTGTTTTTTCTGTCACCGACGAGGATATCAATGATCTCATCAAAATG
AGGGTTATCCTTGAACAGGGCGCAATTCGTGAAGCGATGGAGAAAAATGCTAACAATGTC

ATAGTCGAATTAAGCAATATTTTATCGAAGTCAGCAAAAATTAATGCAGAGCGCGATACG
CAAGCTTACCTCAAACTGGATCATGATTTTCATTATGTTTTTGTGAAATATGCCGATAAT
AAATACATTTCACAAGCACACCTGTTGATCTCAGCACGTTTATTGGCAATTCGCTACAGA
CTGGATTTTACCACTGAATATATCACCAGCTCTAACCGTGGACATGCCACTATTTTGGAT
ATGTTAAAAAACAACAACGTCGAGGGCGTCTGTAATTTTATCACGCACCACATCGGTAGC
GGTTTTACAGAACGCGCAAGAAAACTTCTTGCGCTGAAAGCGTAA
>EECEBICD_01086 hypothetical protein
ATGAGTAGTCCACAAGAAAATTTATACGATGCAATTCGTATTGTAAAAAGGAAAATCATT
CCTTTGGCCTTTATTCTTTACTTTTTTAACTATATGGATCGTGTCAATATTGGGTTTGCT
GCATTACGCATGAATGAATCTCTCGGTATTACGCCTGAGGATTTCGCTAATATTTCTTCT
GTATTCTTCATATCATATTTAATCTTTCAAATTCCGAGTAGCATTGGCTTGCAAAAGCTT
GGCGCAAGAAAATGGATTAGTTCCATTATTATCGGCTGGGGAGCGGTAACAGGGCTGATT
TTTTTGCTAAAGATACTCAACATATTTTGCTGGCTCGCATTTTTTTGGGGGTATTTGAAG
CTGGCTTTTTCCCGGGCATGGTTTATTATCTTGCATGTTGGTTCCCGGCTCGTGAACGCG
GAAAAGTCAATAGTTTCTTCATGTTATCTATTGCTGTTGCATCGGTATTGGCTGCACCGA
TGTCTGGTTGGATCATCGAGCATTTGA
>EECEBICD_01087 Putative tartrate transporter
ATGTCTGGTTGGATCATCGAGCATTTGAATACCCCGGATTATGAAGGCTGGCGCTGGCTT
TTTGCTATTGAAGGTATCCCTACCGTATTCCTTGGCATCCTGACATTTTATTTACTGCCT
GACAGTCCAGAAAAGGCTAAATGGCTTACGCCACAGCAGATATCGGCCTTAGTTAATAAA
TTACGTACTGACAATGAAACCGCAGCTGCATTAAATAAAAATACCAACTCATCCTTTCTT
TCCGTAATCAAAAATCCAGTTTTGCTTCAGCTTTCTTTCGCCTACATGTTGATTCAGGCG
GCTGCTCTGGCGGCAAACTACTGGTTGCCGGGGTTGGTGAAGGGATTTTCAGCTGATTTT
ACGGATACTGACGTTGGTTTAATCATGAGTATTCCGTTTATTTTCGCTATGTTCAGCATG
CCCTGGTGGGGCTGGCATTCCGATAAAAAGAATGAACGAAAATGGCATGCTGCATTACCC
ATGTTTCTGGCAGGCTGTGGTTTTCTGATGATTGCTCTTGTCCCCTCAATGTCGCTGAGA
ATGCTGGGGCTAACGTTCTATGGTGTTGGGATCCTCAGCTATTACGGACCTTACTGGGCG
CTGCCTTCTGCATTATTGTCGCCGTCAGGGTTGGCTATCAGCATTGCGTTTATTAACTCA
TGTTCAAGTCTTGGCGGATTCCTGATTAATAAGTCACTGGGATTTGTTTCTACTCATTAT
GGTGCGACAGGAATATTTATTGTAGAGGCAATACTTTGCTTCGCTGCGGTAGCCGTTTTG
GCGTTAATGAAAATTGATGTGAAAAAAGAAAAAGCCAACAAACAAATGTTGTATCACGAA
CCTGATAAATGGAGAAATTATGAAAATTACATCTATTGA
>EECEBICD_01088 D-galactonate dehydratase family member RspA
ATGTCATCCTATAATCCAGTACTTATCCGGGTGAATACCGACTCAGGATTAAGTGGTATT
GGCGAAGTAGGACTGGCCTATGGCGCAGGCGCTAAAGCTGGCGTAGGGATCATCAGAGAT
CTGGCGCCATTGGTTGTGGGTGAAGATCCATTAAATATCGAAAAAATTTGGGAGTTCTTT
TTCAGAAAGACTTTTTGGGGGATGGGGGGCGGAAACGTTTTTTATGCCGGAATGAGCGCT
ATTGATATTGCTCTTTGGGATATCAAAGGTAAATATTTAGGTGTACCTGTTTATCAGCTC
CTTGGCGGGAAAACTAATGAAAAATTAAGAACCTATGCTAGTCAGTTACAATTTGGTTGG
GGGGATAAACGCCAGATATTAGTGACACCTGAGGAATATGCTGAGGCGGCGCGTGCGGCT
CTTGACGATGGATATGATGCGATTAAAGTGGATCCACTCGAAATCGATCGTAATGGCGAT
GACTGTGTTTTTCAGAATAAAAATCGTAACTATTCAGGATTGTTACTGGCCGATCAATTA
AAAATGGGGGAAGCGCGAATTGCCGCTATGCGTGAAGCAATGGGGGATGATGCTGATATT
ATCGTGGAAATCCATTCTCTTCTCGGTACAAACTCAGCTATTCAATTTGCGAAGGCGATA
GAAAAGTATCGTATCTTTCTTTATGAAGAACCGATTCATCCATTAAATTCTGATAATATG
CAGAAAGTTTCCCGTTCAACAACGATTCCAATCGCCACTGGCGAGCGTTCCTACACAAGA
TGGGGATACAGGGAATTACTGGAGAAGCAGTCTATTGCCGTAGCGCAACCTGATTTGTGT
CTCTGCGGCGGAATTACCGAAGGAAAGAAAATCTGTGACTATGCTAATATTTATGACACC
ACTGTACAGGTGCATGTTTGCGGCGGTCCTGTTTCCACGGTAGCCGCGCTGCATATGGAA
ACAGCAATTCCCAACTTTATTATTCATGAGCATCATACCAATGCGATGAAAGCATCTATT
CGGGAACTTTGTACCCACGATTATCAACCGGAAAATGGCTATTATGTCGCGCCGGAACAG
CCCGGATTGGGTCAGGAATTAAACGATGAGGTAGTGAAAGAATACCTGGCCTATGTGATT
AAATAG
>EECEBICD_01089 DNA gyrase subunit A

ATGAGCGACCTTGCGAGAGAAATTACACCGGTCAACATTGAGGAAGAGCTGAAAAGCTCC
TATCTGGATTATGCGATGTCGGTCATTGTTGGCCGTGCGCTGCCGGATGTCCGAGATGGC
CTGAAGCCGGTACACCGTCGCGTACTTTACGCCATGAACGTATTGGGCAATGACTGGAAC
AAAGCCTATAAAAAATCTGCCCGTGTCGTTGGTGACGTAATCGGTAAATACCATCCCCAC
GGCGATTCCGCAGTGTATGACACCATCGTTCGTATGGCGCAGCCATTCTCGCTGCGTTAC
ATGCTGGTGGATGGTCAGGGTAACTTCGGTTCTATTGACGGCGACTCCGCGGCGGCAATG
CGTTATACGGAGATCCGTCTGGCGAAAATCGCCCACGAACTGATGGCCGATCTCGAAAAA
GAGACGGTGGATTTCGTGGATAACTATGACGGTACGGAAAAAATTCCGGACGTCATGCCG
ACCAAAATTCCGAATCTGCTGGTGAACGGTTCTTCCGGTATCGCAGTAGGTATGGCGACG
AATATCCCGCCGCACAACCTGACGGAAGTGATTAACGGCTGCCTGGCGTATATCGACAAC
GAAGACATCAGCATTGAAGGGCTGATGGAACATATTCCGGGGCCGGACTTCCCGACCGCC
GCGATCATCAACGGTCGTCGTGGTATCGAAGAAGCCTACCGTACCGGTCGTGGCAAAGTG
TACATTCGCGCCCGCGCGGAAGTTGAAGCTGACGCGAAAACGGGCCGTGAAACCATCATC
GTCCATGAAATTCCCTATCAGGTGAACAAAGCGCGCCTGATCGAGAAAATCGCCGAGCTG
GTGAAAGATAAACGCGTGGAAGGCATCAGCGCGCTGCGTGACGAATCCGACAAAGACGGG
ATGCGCATCGTGATTGAAGTGAAACGCGATGCGGTGGGCGAGGTGGTGCTTAATAATCTC
TACTCCCAGACCCAGCTACAGGTTTCCTTCGGTATTAACATGGTGGCGCTGCATCACGGC
CAGCCGAAGATCATGAACCTGAAAGACATCATTTCAGCGTTCGTGCGCCACCGCCGTGAA
GTGGTGACGCGTCGTACTATTTTTGAACTGCGTAAAGCCCGTGACCGCGCGCATATCCTT
GAAGCTCTGGCGATTGCGCTGGCTAACATCGACCCGATTATCGAACTGATTCGCCGCGCG
CCAACGCCGGCAGAAGCAAAAGCGGCGCTGATTTCGCGTCCGTGGGATCTGGGCAACGTT
GCTGCGATGCTGGAGCGCGCTGGTGATGACGCTGCGCGTCCGGAATGGCTGGAGCCAGAA
TTTGGCGTGCGTGACGGTCAGTATTACCTGACTGAGCAGCAGGCGCAGGCGATTCTGGAT
CTGCGTTTGCAGAAACTGACCGGCCTGGAGCATGAAAAACTGCTCGACGAATACAAAGAG
TTGCTGGAGCAGATTGCTGAACTGCTGCACATTCTGGGCAGCGCCGATCGCCTGATGGAA
GTGATCCGCGAAGAGATGGAGTTAATTCGCGATCAGTTCGGCGATGAGCGTCGTACCGAA
ATCACCGCCAACAGCGCCGATATTAATATCGAAGATCTGATTAGCCAGGAAGATGTTGTC
GTGACGCTGTCTCACCAGGGTTACGTCAAATATCAACCGCTGACGGATTACGAAGCGCAA
CGTCGTGGTGGGAAAGGTAAATCTGCCGCGCGTATTAAAGAAGAAGACTTTATCGACCGC
CTGCTGGTGGCCAACACCCATGACACCATCCTCTGCTTCTCCAGCCGGGGCCGTCTGTAC
TGGATGAAGGTCTATCAGTTGCCGGAAGCCAGCCGCGGCGCGCGCGGTCGTCCGATCGTT
AACCTGCTGCCGCTGGAAGCCAACGAACGTATCACCGCGATTCTGCCGGTTCGTGAATAC
GAAGAAGGCGTCAACGTCTTTATGGCGACCGCCAGCGGTACCGTGAAGAAAACGGCGCTG
ACCGAATTCAGCCGTCCGCGTTCCGCCGGTATTATCGCGGTGAACCTCAACGACGGCGAC
GAGCTGATTGGCGTTGACCTGACTTCTGGTTCTGACGAAGTCATGCTGTTCTCGGCCGCG
GGTAAAGTGGTGCGCTTCAAAGAAGACGCCGTCCGTGCGATGGGGCGTACCGCGACCGGT
GTGCGCGGTATTAAGCTGGCGGGAGACGATAAAGTCGTCTCTCTGATCATCCCACGCGGC
GAAGGCGCTATTCTGACCGTAACGCAAAACGGCTACGGGAAGCGTACCGCAGCGGACGAG
TACCCGACCAAGTCTCGTGCGACGCAGGGCGTTATCTCTATCAAAGTGACCGAGCGCAAC
GGTTCCGTTGTCGGTGCGGTACAGGTAGACGATTGCGACCAGATCATGATGATCACGGAT
GCCGGTACTCTGGTGCGTACCCGAGTGTCCGAGATCAGCGTCGTGGGACGTAATACCCAG
GGCGTTATCCTTATCCGCACGGCGGAAGATGAAAACGTGGTGGGTCTGCAACGCGTTGCT
GAACCGGTAGATGACGAAGAACTCGACGCTATCGACGGCAGCGTGGCGGAAGGGGATGAG
GATATCGCCCCGGAAGCGGAAAGCGATGACGACGTTGCGGATGACGCTGACGAGTAA
>EECEBICD_01090 Sensor histidine kinase RcsC
TTGAAATACCTTGCTTCCTTTCGAACTACGCTGAAAGTCTCGCGCTACTTATTCAGAGCG
TTAGCGTTACTCATTTGGCTCTTAATCGCCTTTGTTTCGGTGTTTTACATCGTCAATGCC
CTGCACCAGCGGGAATCTGAAATTCGTCAGGAGTTCAACCTTAGCTCCGATCAGGCGCAG
CGTTTTATCCAGCGCACGTCGGATGTGATGAAAGAGCTCAAATATATCGCCGAGAACCGT
TTAACGGCGGAAAACGGCGTCATGTCGTCGCGTGCGCGTGATGACAAAATGGTGGTGCCG
GACTTTGAACCGCTATTTGCCGACTCAGACTGTGCGGCGATGGGCTCCGCCTGGCGCGGA
TCGCTGGAGTCGCTGGCCTGGTTTATGCGTTACTGGCGCGACAACTTTTCAGCGGCGTAT
GATCTTAACCGCGTTTTCCTGATTGGCAGCGATAACCTCTGTATGGCGAATTTCGGCCTG
CGTGAAATGCCCGTGGAGCGCGATGATGCGTTAAAAGCGTTACATGAGCGGATTATGAAA

TACCGCAACGCGCCGCAGGAAGAAAGTGGGAATAACCTTTTCTGGATAAGCCAGGGCGCG
CGTCAGGGCGTGGGCTACTTTTATGCGCTGACGCCGGTGTATCTGGCCAACCGTCTGCAG
GCGCTGCTGGGCGTCGAGCAGTCCATCCGTATGGAAAATTTTTTCACGCCCGGCAGTTTG
CCGATGGGCGTCACTATTATTGATGAGAACGGCCATTCGCTGATTTCGTTGACCGGTCCT
GACGGCAATATTAAAGCGGAGCCGCGCTGGATGCAGGAGCGCTCCTGGTTTGGTTATACG
CCAGGTTTTCGCGAACTGGTACTCAAAAAAAGCCTGCCGCCATCCTCGCTCAGTATTGTC
TATTCTGTGCCTGTAGATTTAGTCCTGGAGCGTATTCGTATTCTTATCCTGAACGCTATC
CTGCTGAACGTGCTGGTGGGCGCAGGGCTGTTTACGCTTGCGCGGATGTATGAGCGGCGA
ATTTTCATTCCGGCGGAGAGCGATGCCCAGCGTCTGGAAGAGCATGAACAGTTCAACCGT
AAAATCGTCGCCTCCGCGCCGGTGGGGATCTGTATTTTGCGCACTATTGATGGTGTCAAT
ATTCTGAGTAACGAACTGGCGCACACCTACCTGAACATGCTCACGCATGAAGATAGACAG
CGCCTGACGCAAATCATTTGCGGCCAGCAGGTTAACTTCGTTGATGTATTGACCAGCAAT
AATACCAATTTGCAAATTAGCTTCGTCCATTCCCGCTATCGCAATGAAAACGTCGCGATT
TGCGTGTTGGTAGATGTGAGTACGCGCGTCAAAATGGAAGAGTCGCTGCAAGAGATGGCG
CAGGCGGCGGAGCAGGCAAGCCAGTCTAAATCCATGTTCCTTGCGACGGTCAGCCATGAA
TTGCGCACACCGCTGTACGGTATCATTGGCAATCTCGATCTGCTGCAAACCAAAGAGCTG
CCAAAAGGCGTGGATCGGCTGGTCACGGCGATGAATAACTCCTCCAGTCTGTTGCTGAAA
ATTATCAGCGACATTCTCGACTTCTCGAAAATTGAGTCGGAACAGCTAAAGATTGAGCCG
CGCGAATTTTCACCGCGAGAGGTGATGAACCATATCACCGCCAATTATCTGCCGCTGGTG
GTGCGTAAGCAGCTCGGCCTGTATTGTTTTATTGAGCCTGACGTGCCGGTATCGCTGAAC
GGTGACCCGATGCGTTTACAGCAGGTTATTTCAAACCTGTTGAGCAACGCCATTAAGTTT
ACCGATATTGGCTGCATCGTGCTGCATGTGCGCTGTGATGGGGATTATCTCAGCATTCGC
GTGCGGGATACCGGCGTCGGTATTCCGGCAAAAGAGGTGGTTCGCCTGTTCGATCCCTTC
TTCCAGGTGGGAACCGGCGTACAGCGTAACTTTCAGGGGACCGGTCTGGGGCTGGCGATT
TGCGAAAAATTGATCAGCATGATGGACGGCGATATTTCCGTCGATTCGGAGCCGGGCATG
GGGAGCCAGTTTACGCTGCGCATCCCGCTTTATGGCGCGCAATACCCTGTGAAAAAGAGC
GTGGAAGGACTCGCCGGAACCTGCTGTTGGCTGGCGGTGCGCAATACTTCTTTGTGCCAG
TTTATTGAGACCAGTCTGGCGCGTAGCGGCGTTCATACGCAGCGTTATGAAGGGCAAGAA
CCCGCCGCGGACGATATTTTGATTGTCGATGACGCGCTGGAGCATACCTGGCAGGGGAGG
GCTGCGGTCGTCTTTTGCCGCCGTCATATCGGGATTCCGTTGGAAAGAGCGCCGGGCGAG
TGGGTGCATAGTGTGGCGTCGGTGCATGAGTTGCCCGCGCTGTTGGCGCGTATCTATAGT
ATTGAGCTGGATAGTGAAGCGCTTTCCAGCGCATTGCCGACGACGGATAAAACCGCAGAT
TCAAATGACGACATGATGATTCTGGTCGTTGACGATCATCCGATCAATCGCCGCTTGCTG
GCCGATCAACTGGGATCGCTGGGGTATCAGTGTAAGACAGCCAATGATGGCGTGGACGCT
CTGAATGTGCTGAGTAAAAATGCTATCGATATTGTTCTGAGCGATGTGAACATGCCGAAC
ATGGACGGCTATCGCCTGACGCAGCGTATCCGTCAGCTTGGGCTGACGCTGCCGGTCGTC
GGCGTGACGGCGAACGCGCTGGCGGAAGAGAAACAACGTTGCCTGGAGTCCGGTATGGAC
AGTTGTTTGTCGAAGCCTGTGACGCTGGATGTGTTGAAACAGACGCTGGCGGTGTATGCC
GAGCGGGTACGTAAAACGCGGGCATAA
>EECEBICD_01091 Transcriptional regulatory protein RcsB
ATGAACAATATGAACGTAATTATTGCCGATGACCACCCGATTGTACTGTTCGGTATTCGC
AAATCACTTGAACAAATCGAGTGGGTGAATGTTGTCGGCGAATTTGAAGATTCCACAGCA
TTGATCAACAACCTGCCGAAATTAGATGCGCATGTGTTGATCACTGACCTCTCCATGCCG
GGAGATAAATACGGCGATGGGATCACCTTGATCAAGTACATCAAGCGTCATTTTCCGAGC
CTGTCTATTATCGTTCTGACCATGAACAACAATCCGGCGATCCTGAGCGCCGTGTTGGAT
CTTGATATTGAAGGGATCGTACTCAAGCAGGGTGCGCCAACCGATCTGCCTAAGGCGCTG
GCCGCGCTGCAAAAAGGCAAAAAATTCACCCCGGAAAGCGTTTCTCGTCTGCTGGAGAAA
ATCAGCGCAGGCGGTTACGGCGATAAGCGCCTGTCGCCAAAAGAGAGCGAAGTATTACGC
CTGTTCGCCGAGGGTTTCCTGGTCACCGAAATCGCCAAGAAGCTCAACCGCAGCATTAAG
ACCATCAGCAGCCAGAAGAAATCGGCGATGATGAAACTGGGCGTAGAGAATGATATCGCG
CTGCTCAACTATCTCTCTTCCGTCACCCTGAGTCCGACAGACAAAGAATAA
>EECEBICD_01092 Phosphotransferase RcsD
ATGAGTCAGTCTGACACAACGGTCTCTACCCGATTCTCCCTTCTACCGGGAAGCATTACC
CGTTTCTTCTTATTGCTGATCATTGTGCTGCTGGTAACGATGGGCGTTATGGTACAGAGC

GCCGTCAACGCCTGGTTGAAAGACAAAAGCTATCAAATTGTCGATATCACTCACGCTATT
CATAAACGCGTGGACACCTGGCGGTATGTTACCTGGCAAATTTATGACAACATCGCCGCC
ACCACTACGCCATCGACAGGTGAAGGACTTCAGGAAACGCGTCTGAAACAGGATGTGTAT
TATCTTGAAAAGCCGCGACGTAAAACTGAAGCGCTGATTTTCGGTTCCCATGACAGCGCC
ACGCTGGAAATGACGCAGCGTATGTCAACCTATCTGGATACGCTGTGGGGCGCGGAAAAC
GTGCCGTGGTCAATGTATTATCTCAACGGACAGGATAACAGCCTGATTCTTATCTCTACC
CTACCGTTGAAAGATCTCTCTTCCGGCTTTAAAGAGTCCACTATTGGCAATATCGTCGAT
TCCCGCCGGGCAGAGATGTTGCAGCAGGCGAATGCGCTGGATGAGCGCGAAAGTTTCTCC
TCATTGCGAAAACTGGCCTGGCAAAACGGTCACTATTTTACGCTGCGCACGACCTTCAAT
CAGCCAGGACATCTGGCGACCGTGGTGGCGTTTGATCTGCCCATTAATGATTTAATCCCG
CCGGGAATGCCGCTGGACAGTTTTCGGATAGAGCCTGACGCAACCCAGGCGACGGGGCGA
AGCAGTGAAAAGGAAGCGCCAGATAGCGTCACGATTAGCTTTAACGGTTCAAAAATTGAG
ATCTCCTCAGCGCTTAACTCTACCGGAATGCGCCTGATCTGGCAGGTACCGTTCGGTACG
CTTTTGCTTGATACGTTGCAAAATATTCTGCTGCCTTTACTGCTGAACATCGGGCTGCTG
GCGTTGGCGTTATTTGGCTACGCCACGTTCCGTCATCAGCCGGGACGGTCGACGGAATCG
ACATCCGGCAACGCGGCGAATAACGAGCTTCGCGTCTTACGCGCCATTAATGAAGAGATC
GTTTCTCTGCTGCCGCTGGGTCTGCTGGTCTACGATCAGGAAGGCAACCGCACCGTGATC
AGCAATAAAATCGCCGATCATCTGTTGCCGCATCTTAACCTGCAAAATATTACCAGCATG
GCGGAACAGCATCAGGGCGTGATTCAGGCGACAATTAATAACGAACTGTATGAGATCCGT
CTGTTTCGCAGCCAGATCGCTCCCCGCACGCAGATCTTTATTATTCGCGATCAGGACCGG
GAAGTGCTGGTGAATAAGAAGCTTAAACAGGCGCAGCGTTTGTATGAGAAGAATCAGCAG
GGTCGCGCCGCCTTTATGCAAAATATCAGCAACACGCTTAAAGAGCCCGTACGCCAACTG
GCGGTTAACGCCGCGGCAGTCACCACGCCGGAAAGCCTGAAACTGGCGGATCAGGCGGAT
GTATTGGTACGTATGATCGATGAAATCCAACTGGCGAATATGCTGGAAAATGACGCATGG
AAGAGCGAAGCCACGCTGTTCTCCCTGCAGGATCTTATTGACGAAGTCGTGCCGGAAGTG
CTGCCCGCCATTAAGCGTAAAGGGCTGCAACTGCTGATTAATAACCATCTTAGCGGCAAC
GATGAACGCTGGGGCGATCGCGACGCCCTGCGTCGGATTTTGCTGCTGTTGATTCAGTAT
GCCGTCACAACGACCGCTATGGGTAAAATTACGCTTGAAGTCGAGCAGGATGAATCTATC
GCGGAGCGTCTGACTTTCCGTATTCTGGATACGGGCGAAGGCGTAACGTTAAACGAGATT
GATAATCTGCATTTCCCGTATATGAATGAAACGCAGGGCGATCGTTACGGCAAAGCGAAT
CCGCTCACCTTCTGGCTGTGCAATCAACTGGCGCGCAAACTGGGCGGTCATCTCAACATT
AAAGCGCGAGAAACGCTGGGCACGCGCTATACCGTTCATGTCAAAATGCTTCCTCACGAT
CAGCATACTCAGGTCGAAGAGCGCCTGCTGGACGACGTGAGCGTGATGGTCGATGTGACG
TCCAACGAGGTTCGCGCGATTGTGCTGCGTCAGTTAGAAAACTGGGGCGCCACCTGCATC
ACGCCGGATGAAAGGCAGATAAGTCAAGAATATGATCTCTTTTTAACGGATAATCCGTCT
AATCTTACTGCCTCGGGCTTGCTTTTAAGCGATGATGAGTCAGGCGTGCGGAAAATTGGC
CCGGGTCAGTTGCGCGTCAACTTTAATATGAGCAATGCTATGCAGGAAGCTGTACTACAA
CTTATCGAAGAGCAGCTGGCGCAGGAAGAGATTCCCGCCTCCCCGCTCGGCGGAGATGAA
AATGCCGAGCTTCATGCCAGCGGCTATTATGCGCTATTTGTAGACACAGTACCGGATGAT
GTTAAGAGGTTGTATACTGAGGCGGCAACCAGCGATTTCGCTGCGCTGGCCCAAACGGCC
CACCGCCTGAAAGGGGTGTTTGCCATGCTTAATCTGGTACCCGGCAAGCAGTTATGTGAA
ACGCTGGAACATCTGATTCGTGAGAAAGATGCTCCAGGTATAGAAAAATATATCAGCGAC
ATTGACGCCTACGTCAAAAGCTTGCTGTAG
>EECEBICD_01093 Outer membrane porin C
ATGAAAGTTAAAGTACTGTCCCTCCTGGTACCAGCTCTGCTGGTGGCGGGCGCAGCGAAT
GCGGCTGAAATTTATAATAAAGACGGCAACAAATTAGACCTGTTTGGTAAAGTTGATGGC
CTGCACTACTTCTCTGACGACAAAGGCAGCGACGGCGACCAGACCTACATGCGTATCGGC
TTCAAAGGCGAAACGCAGGTTAACGATCAGCTGACCGGTTATGGCCAGTGGGAATATCAG
ATTCAGGGCAACCAGACTGAAGGCAGCAACGACTCCTGGACGCGTGTGGCGTTTGCGGGT
CTGAAATTCGCTGACGCAGGTTCCTTCGATTATGGTCGTAACTACGGCGTAACCTATGAT
GTGACCTCCTGGACCGATGTTCTGCCGGAGTTCGGCGGCGACACCTACGGCGCTGACAAC
TTTATGCAGCAGCGTGGTAACGGCTATGCCACTTACCGTAACACCGACTTCTTCGGCCTG
GTGGATGGCCTGGACTTCGCGTTGCAGTATCAGGGCAAAAATGGCAGCGTGAGCGGTGAA
AACACCAACGGTCGCAGCCTGCTGAACCAGAACGGTGACGGTTACGGCGGATCGCTGACT

TATGCAATCGGCGAAGGCTTCTCTGTCGGTGGCGCTATCACCACGTCTAAACGTACTGCC
GATCAGAACAACACCGCTGACGAACACCTGTATGGTAACGGCGATCGCGCTACGGTCTAC
ACCGGCGGCCTGAAATACGATGCGAACAACATCTATCTGGCAGCGCAGTATTCTCAGACC
TATAACGCAACCCGTTTTGGTACCTCTAACGGCAACAATAAGTCCACCTCTTACGGTTTT
GCCAACAAAGCGCAGAACTTTGAAGTGGTTGCTCAGTACCAGTTCGACTTTGGTCTGCGT
CCGTCTGTGGCTTACCTGCAGTCTAAAGGTAAGGACATCAGCAACGGCTACGGCGCCAGC
TATGGCGACCAGGACATCGTAAAATATGTTGATGTCGGCGCGACTTACTACTTCAACAAA
AACATGTCCACCTATGTTGATTACAAAATCAACCTGCTGGATAAAAACGACTTTACCCGC
GATGCGGGCATCAACACCGACGACATCGTAGCGCTGGGTCTGGTTTACCAGTTCTAA
>EECEBICD_01094 FAD:protein FMN transferase
ATGAAAATGACTTTTTGCCGGGCCGTGTGTCTGGCGGCGGCTTTTTTACTTATGGGCTGC
GATGAGGCTCCCGAAACGACAACAGCGTCACCTGCCGCTCAGGTGCTGGAAGGTAAAACG
ATGGGGACCCTCTGGCGGGTGAGCGTGGTTGGCATCGATGCGAAACGCGCCGCAGAGTTA
CAGACTAAAATCCAGACTCAGCTTGATGCTGATGATTGGTTGCTTTCTACCTATAAAAAT
GACTCCGCGCTAATGCGTTTTAACCATTCACGCAGCCTTGCGCCCTGGCCGGTCAGCGAA
GCCATGGCGGATATCGTGACCTCGGCGCTACGTATTGGCGCGAAGACGGATGGCGCGATG
GATATCACCGTGGGCCCGCTGGTCAATCTGTGGGGGTTTGGGCCGGATCGGCAGCCGCTG
CATATCCCAACACCAGCACAAATCGATGCGGCAAAAGCGAAAACAGGCCTGCAACATTTG
CAGGTTATCGACAGGGCTGGACATCAGTTTTTGCAAAAAGATCTGCCGGATCTTTATGTT
GATCTCTCCACGGTCGGGGAGGGCTATGCGGCGGATCATCTGGCGCGACTGATGGAGCAG
GAGGGCATTGCGCGTTATCTGGTCTCGGTGGGCGGCGCATTAAGCAGTCGCGGGATGAAT
GCGCAGGGGCAGCCGTGGCGCGTCGCGATTCAGAAGCCGACCGACCGGGAAAACGCGGTG
CAGGCGATTGTGGATATCAACGGGCATGGCATCAGCACCTCCGGCAGCTACCGTAACTAT
TACGAGCTGGATGGCAAGCGCGTATCGCACGTTATCGATCCACAAACGGGGCGCCCCATT
GAACACAATCTGGTATCGGTTACGGTCATCGCGCCAACGGCGCTGGAAGCGGACGGCTGG
GACACCGGCCTGATGGTGCTCGGTACGCAAAAGGCGCAAGAGGTCGTGCGGCGGGAAGGG
CTGGCGGTCTTTATGATCATGAAAGAAGGTGAAGGCTTTAAAACCTGGATGTCGCCGCAG
TTCAAAACGTTCCTGGTGAGCGATAAGAATTAA
>EECEBICD_01095 Bifunctional transcriptional activator/DNA repair enzyme Ada
ATGAAAAAAGCGTTACTTACCGATGATGAATGCTGGCTGCGGGTGCAGGCGCGCGATGCC
AGCGCGGATGGGCGTTTCGTTTTTGCAGTGCGAACCACTGGCGTTTTTTGCCGCCCTTCT
TGTCGCTCGAAGCGGGCGTTACGTAAAAATGTTCGCTTTTTTGCCAACGCGGAGCAGGCG
CTGGACGCCGGTTTTCGCCCCTGCAAGCGCTGTCAGCCGGATAATGCGCGCGCGCAGCAA
CGGCGGTTGGATAAGATTGCCTGCGCCTGCCGTTTACTTGAGCAGGAGACGCCGGTAACG
CTGGCGTCTCTGGCGCAGGCGGTGGCGATGAGCCCGTTTCATCTGCACCGTCTGTTTAAA
GTGAGCACCGGAATGACGCCGAAAGGGTGGCAGCAGGCGTGGCGCGCCCGGCGGCTGCGT
GAGGCGTTGGCGAAAGGAGAGCCGATCACGGCGGCTATTTATCGCGCTGGCTTCCCGGAT
AGCAGTAGCTACTACCGTCATGCCGACCAGACGCTGGGCATGACGGCAAAACAGTTTCGC
AAAGGCGGCGATAATGTCTCCGTTCGCTATGCGCTGACGGACTGGGTTTACGGACGGTGC
CTGGTGGCGGAGAGCGAGCGGGGGATTTGCGCGATTCTCCCCGGTGATAGCGACGACGCG
CTACTGGCTGAATTACACACCCTTTTCCCGACGGCCCGCCACGAACCTGCCGACGCGTCT
TTTCAGCAACGGGTGCGGCAGGTTGTCGCGGCTATCAACACACGCGATGTGCTGCTCTCG
TTGCCGCTGGATATCCAGGGAACCGCGTTTCAACAGCAGGTCTGGCAGGCGTTATGCGCA
ATTCCCTGCGGCGAAACCGTAAGCTATCAACAGCTTGCCGCGACTATCGGCAAACCCACG
GCGGTACGCGCGGTCGCCAGCGCGTGCGGCGCGAATAAACTGGCGATGGTGATCCCGTGT
CATCGGGTCGTGCGTCGCGATGGCGCGCTCTCCGGTTATCGTTGGGGCGTGCGTCGAAAA
GCGCAGCTATTAAAGCGAGAAGCGCAAAAAGAGGAGTAG
>EECEBICD_01096 Alpha-ketoglutarate-dependent dioxygenase AlkB
ATGCTGGATCTATTTGCTGATGAAGCGCCCTGGCAAGAGCCCCTGGCGCCTGGCGCGGTG
GTGTTGCGCCGCTTTGCGTTTCGCGCGGCGCAGTCGCTATTAGACGATATCGGGTTTGTC
GCCAGTCAGTCTCCGTTTCGTCAGATGGTGACGCCGGGCGGCTACACCATGTCGGTGGCG
ATGACCAACTGCAGCGCGTTAGGGTGGACCACCGATCGTCACGGTTATTGTTATGCCGTG
CGCGATCCGTTGACCGATAAACCCTGGCCTGCGCTGCCGTTATCGTTTGCCAGCGTATGT

CGTCAGGCGGCAATCGCGGCAGGGTATGCGAGCTTCCAACCGGACGCCTGCCTGATCAAT
CGCTACGCGCCCGGCGCAAAACTGTCGCTGCATCAGGATAAAGACGAGCCGGATCTGCGC
GCGCCTATTGTCTCAGTTTCGTTGGGGGTGCCGGCGGTTTTTCAGTTTGGCGGCCTGCGT
CGCAGCGATCCGCTCCAGCGGATCTTACTGGAGCATGGCGATATCGTGGTCTGGGGCGGC
GAGTCCCGTCTGTTTTATCATGGTATCCAGCCGCTTAAAGCGGGCTTTCATCCCATGACC
GGTGAATTTCGTTACAATCTCACCTTCCGTCAGGCGGCTGAAAAAGAATAA
>EECEBICD_01097 ABC transporter ATP-binding/permease protein YojI
ATGGAACTTCTTATTCTTGTCTGGCGGCAGTACCGCTGGCCATTTATTAGCGTGATGGCG
CTGAGCCTCGCCAGCGCGGCGCTGGGGATTGGCCTGATCGCGTTTATCAATCAGCGCCTG
ATTGAAACGGTGGACACCAACGTCATGGTGCTGCCGGAATTTCTCGGCCTGCTCCTGTTG
CTAATGGTCGTCACATTAGGTTCCCAACTGGCGCTCACCACTCTGGGACACCATTTCGTT
TACCGCCTGCGCAGCGAATTTATCAAGCGCATTCTGGATACGCACGTTGAACGCATTGAG
CAACTGGGCAGCGCCTCTTTACTGGCCGGTCTGACCAGCGATATTCGCAATATTACTATC
GCCTTTGTGCGTCTGCCTGAGCTGGTGCAGGGCATTATCCTCACCGTCGGCTCGGCGGCG
TATCTGGCGATGCTATCGACAAAAATGCTGCTGGTTACGGCTATCTGGATGGCGGTCACT
ATCTGGGGCGGATTCGTGCTGGTGGCGCGGGTTTACCAGCATATGGCGACCCTGCGCGAG
ACCGAAGATAAGCTGTACAACGACTATCAGACGGTGCTGGAAGGACGCAAAGAGCTGACG
CTAAACCGCGAGCGTGCGGAGCAGATTTTTCAGCAATGCTATACCCCGGACGCCAAAGAG
TACCGTCATCACATTATCCGTGCCGATACTTTTCATCTGAGCGCCGTCAACTGGTCGAAT
ATCATGATGCTGGGGGCGATAGGCCTGGTCTTCTGGATGGCGAATAGTCTCGGCTGGGCG
GATACCAACGTCGCGGCGACCTATTCACTGACGTTGCTGTTCCTGCGCACGCCGCTGCTG
TCTGCGGTGGGGGCGTTGCCAACCCTACTGACCGCCCAGGTGGCGTTTAATAAACTGAAT
AAATTCGCGCTCGCCCCGTATAAGCCTGATTTTCCACAGCCGAAAGCCTTTCCCGACTGG
AAGACGCTGGAGCTGCGCAACGTGGTTTTCCACTATCAGGATAACGCGTTTTCCGTGGGG
CCCATCAACCTGACGATTCACCGCGGCGAACTGCTGTTTTTAATTGGCGGCAACGGTAGC
GGGAAATCGACGCTGGCCATGTTATTGACCGGTTTATACCAGCCGCAATCGGGAACGATT
TTACTTGACGGTCAACCTATTGCCGCCGGGCAGCCGGAAGATTATCGCAAACTGTTTTCT
GCGGTGTTCACCGATGTGTGGTTATTCGACCGCCTGCTGGGGCCGCAGGGGAAACCCGCC
AATCCCGCGCTGGTCGAAAAGTGGCTTGAGCACCTGAAAATGACGCACAAGCTGGAGCTA
AACGACGGGCGCATTTTGAATCTTAAACTGTCGAAAGGGCAAAAAAAGCGCATTGCGCTG
CTGCTGGCGCTGGCGGAAGAGCGCGATATTATTTTGCTGGATGAGTGGGCCGCCGATCAG
GACCCGCATTTCCGCCGGGAGTTTTATCAGGTGCTGTTGCCGTTAATGCAGGAAATGGGC
AAAACCATTTTCGCCATCAGCCATGATGATCACTATTTTATTCACGCCGATCGTCTGCTT
GAAATGCGCAACGGACAGCTAACCGAACTCACCGGCGACGAACGTGATGCCGCCTCGCGT
GATGCGGTTGCGCGTACTGCGTAA
>EECEBICD_01098 Ecotin
GTGAACAATCAAATGAAGATGTTTGTCCCTGCCGTTGTCTTCGCCGCCCTCGCCAGCGCC
TCTGCCTGGGCTAACAATGGTGATACCGCCCAGCCGCTGGAAAAAATCGCCCCCTATCCG
CAGGCGGAAAAAGGAATGAAGCGGCAAGTGATAACCCTTACCCCTCAGCAGGATGAATCT
ACCCTCAAAGTGGAACTGTTGATTGGTCAAACGCTGAATGTGGATTGTAACCAGCATCGC
CTCGGCGGCACGCTGGAAACAAAAACGCTGGAAGGCTGGGGCTATGACTATTATGTCTTT
GATAACGTCACCTCTCCGGTATCAACCATGATGGCCTGCCCTGAAGGTAAGAAAGAGCAA
AAATTCGTCACCGCCTGGCTGGGTGAAGACGGGATGCTGCGCTACAACAGCAAGCTGCCG
ATCGTGGTGTATACCCCGGCGAATGTGGACGTGAAATACCGCATCTGGAAAGCGGACGCT
AACGTACAGAACGCCGTCGCGCGATAA
>EECEBICD_01099 hypothetical protein
ATGCATACCCCGATAGAGGTAAATGCTGTTGCCGGATCAAAAGAGTGCCAGGTAAAGACC
CGTAAACGTGCTTTTTCTCACATTTCAATGAGTTATATAGATGCTTATATAACGAAAATC
ACTAATAGTTGA
>EECEBICD_01100 Ferredoxin-type protein NapF
ATGGTTGATTTATCCCGTCGAAGCATGTTGACCGGCAGTTGGCGCAACGCCAGCAATGGG
ATTCTTCCGCCGTGGGCCAGAGAAACAACCTATTTTCTCGCACATTGTCTGCGTTGCGAC
GCCTGCATCCAGGCGTGTGAGGCCGACATCCTGCAGCGAGGGGCGGGGGGATATCCGAGC
GTAGATTTCAAACGGGGCGAGTGCAGCTTTTGTTATGCCTGTGCGCAGGCCTGTACTGAA

TCGCTTTTTCTTCCGCGCCACACCAGGGCCTGGGATCTGATTTTTACGCTCACGGAAAAC
TGCCTCGCCAGGCAATCGGTTGAATGCCATCGCTGCCAGGACAGTTGTGAACCTATGGCC
ATTACCTTTCGTCCCACGCTATCCGGCATTTACCAGCCGCAGCTTGACTCGCAGGCCTGT
AACGGATGCGGGGCGTGTGTGGCTATCTGTCCCGTGTCAGCCATCAAAGCGGAGAACCAC
CATGCGCACTAA
>EECEBICD_01101 Chaperone NapD
ATGCGCACTAACTGGCAGGTCTGTAGCCTGGTCGTGCAGGCCAAAAGTCAGAACATCGCC
GCCATTGGCACACAGTTGAACACGTTGCCGGGTTGCGAAGTCGCGTTAAGCGACGTTGAA
AGCGGGCAACTGATCGTGGTGGCGGAAGCAGATCAGAGTGAAACGCTGATGCAAACAATT
GAGTCAGTACGCAATGTCGAGGGCGTACTGGCGGTGTCGCTGGTTTATCACCAGCAGGAT
GAGCAAGGTGAGGAAACACCATGA
>EECEBICD_01102 Periplasmic nitrate reductase
ATGAAACTCAGTCGTCGTAGCTTTATGAAAGCTAACGCCGTTGCGGCCGCTGCGGCGGCT
GCCGGTCTGAGCGTGCCGGGCGTCGCCCGCGCCGTTGTCGGCAGCAGGAAGCCATCAAA
TGGGATAAAGCGCCGTGCCGTTTTTGCGGAACGGGTTGCGGTGTGTTGGTGGGAACGCAG
CAGGGCCGTGTGGTCGCCTGCCAGGGCGACCCCGACGCGCCGGTCAACCGTGGTCTGAAC
TGCATTAAAGGCTACTTCCTGCCCAAAATCATGTACGGTAAAGATCGTCTGACGCAGCCG
ATGCTGCGCATGAAAGACGGCAGCTATCACAAAGACGGCGAGTTTACGCCGGTGAGCTGG
GAACAGGCCTTCGATGTGATGGAGGAGAAGTTTAAAACCTCCCTGAAAGAGAAAGGACCA
GAAGCGATCGGCATGTTTGGATCGGGTCAATGGACCATCTGGGAAGGCTATGCCGCCGCG
AAACTGTTCAAGGCTGGTTTCCGCTCTAACAACATCGACCCGAACGCGCGTCACTGCATG
GCCTCGGCGGTGGTGGGCTTTATGCGCACCTTTGGCATGGATGAACCGATGGGCTGCTAC
GACGATATTGAGCAGGCCGATGCGTTTGTGCTGTGGGGTTCCAACATGGCGGAGATGCAC
CCGATTTTATGGTCGCGCATCACTAACCGCCGTTTGTCCGACCCGAACGTGAAGGTTGCC
GTCCTCTCCACCTTCCAGCACCGCAGCTTTGAACTGGCGGACAACGGTATTGTCTTTACC
CCGCAAAGCGATCTGGTGATCCTTAACTATATCGCGAATTACATTATTCAGAACAACGCG
GTAAATCAGGATTTCTTCACAAAGCATGTGAACCTGCGTAAAGGGGCGACGGATATCGGC
TACGGCTTACGCCCGACGCACCCGCTGGAAAAAGCGGCGAAGAATCCGGGCTCCGACGCT
TCCGAGCCGATGAGCTTCGACGAGTATAAAGCCTTCGTGGCAGAATATACGCTGGACAAG
ACCGCTGAAATGACCGGCGTGCCAAAAGATCAACTGGAACAGTTGGCACAGCTCTACGCC
GATCCGAATAAGCGCGTGATCTCTTACTGGACGATGGGTTTTAACCAGCATACCCGCGGC
GTGTGGGCGAATAACCTGGTTTACAACCTCCATCTGCTGACCGGAAAAATCTCCCAGCCT
GGCTGCGGTCCTTTCTCGCTGACAGGCCAGCCTTCCGCCTGCGGTACGGCGCGTGAAGTG
GGAACCTTCTCACACCGTCTACCCGCAGACATGGTCGTGACGAACGAAAAACACCGTGAT
ATCTGCGAAAAACACTGGCAGATCCCCGCTGGCACTATTCCGGCAAAAGTGGGTTTACAC
GCGGTGGCGCAGGATCGCGCGTTGAAAGACGGCAAGCTGAACGTCTACTGGGTAATGTGT
AATAACAATATGCAGGCCGGGCCGAACATCAATGAAGATCGGATGCCGGGCTGGCGCGAT
CCGCGTAACTTTATCATTGTTTCCGATCCTTACCCGACGGTGAGCGCGCTGTCTGCGGAT
CTGATCCTGCCGACCGCCATGTGGGTTGAGAAAGAAGGCGCTTACGGCAACGCCGAACGC
CGCACGCAGTTCTGGCGTCAGCAAATTAAAGCGCCGGGCGAAGCTAAATCCGACCTCTGG
CAACTGGTGCAGTTCTCCCGTCGTTTTAAAACCGAGGAGGTCTGGCCGGAGGCGTTGTTG
GCTCAGAAGCCGGAGCTGCGCGGCAAAACGCTGTATGACGTTCTGTTCGCCACGCCGGCG
GTCAGCAAATTCCCGCTGAGCGAGCTGAAAGAGGATCAGTTAAACGATGAGTCTCGTGAG
CTGGGCTTCTATCTGCAAAAAGGGCTGTTTGAAGAATACGCCTGGTTTGGACGTGGCCAC
GGGCACGATCTGGCGCCGTTCGACGATTACCACAACGCGCGCGGTCTGCGCTGGCCGGTC
GTCGAAGGCAAAGAGACGCAATGGCGTTACAGCGAAGGCAACGACCCGTATGTAAAAGCG
GGGGAAGGGTACAAATTCTACGGTAAACCGGATGGCAAAGCGGTGATCTTCGCGCTGCCA
TTTGAACCCGCGGCGGAGTCGCCGGACAACGAGTACGATCTGTGGCTTTCCACCGGTCGC
GTGCTGGAACACTGGCATACCGGCAGTATGACGCGCCGCGTGCCTGAATTGCATCGCGCC
TTCCCGGAAGCGGTGGTCTTTATTCACCCACTGGATGCGAAAGCGCGCGATCTGCGTCGC
GGGGATAAGGTTAAAGTGTCGTCCCGCCGCGGTGAAGTGATTTCGATTGTCGAAACGCGT
GGACGTAACCGTCCGCCGCAGGGACTGGTGTATATGCCGTTCTTCGATGCCGCTCAGCTA
GTGAACAACCTGACGCTGGATGCGACGGACCCGCTCTCTAAAGAGACGGACTTCAAGAAG
TGCGCCGTTAAGCTGGCGAAGGTGTAA

>EECEBICD_01103 Ferredoxin-type protein NapG
ATGTCCCGTACAGCGAAACCCCAAAATGGCCGCCGCCGCTTCCTGCGTGATGTTGTGCGC
ACTGCGGGCGGGCTGGCGGCGGTTGGCGTGGCGCTGGGCTTGCAACAGCAAACCGCGCGC
GCATCGGGCGTGCGGCTGCGTCCGCCTGGCGCGCTGAATGAAAATGTCTTCGCCAGCGCC
TGTGTGCGCTGCGGGCAGTGCGTTCAGGCGTGTCCATACGACACGCTAAAACTGGCGACG
CTGGCCTCCGGGCTATCGGCAGGCACGCCCTATTTCGTCGCTCGCGACATCCCCTGCGAA
ATGTGTGAAGACATTCCGTGCGCCAAAGTCTGCCCCAGCGGGGCGCTGAATAAAGATATC
GCCTCCATTGATGACTCGCGCATGGGGCTGGCGGTGCTGCTGGATCAGGAAAACTGCCTC
AACTTTCAGGGGCTGCGCTGCGACGTTTGTTACCGCGAATGCCCGAAAATCGATGAGGCC
ATCACGCTGGAGCTGGATCGCAACATGCGCACCGGCAAACATGCGCGCTTTCTGCCCACC
GTACACAGCGACGCCTGTACCGGCTGCGGCAAATGCGAAAAAGTCTGCGTACTGGAGCAA
CCAGCGATAAAAGTGTTGCCGTTGTCATTGGCGAAAGGGGAGTTAGGACACCATTACCGC
TTCGGCTGGCTGGAGGGGAAAGATGGCAAATCGTAA
>EECEBICD_01104 Ferredoxin-type protein NapH
ATGGCAAATCGTAAACGCGACGCCGGGCGAGAAGCGCGGGCGAAAAAAGGCTGGTGGCGT
AGCCACCGCTGGCTGGTGTTACGCCGTATCAGCCAGTTTATGGTGCTGGGGATGTTTCTC
AGCGGCCCGTGGCTGGGCTTTTGGGTTTTGCATGGCAACTACAGCAGTAGTTTGCTGTTC
GATACCCTCCCGTTGACCGATCCGCTGATTACCCTGGAAAGTCTCGCCAGTGGACATCTT
CCCGCTACCGTGGCGCTGACGGGAGCAGTGATTATCGCTGTGCTGTATGCGCTGGCGGGA
AAGCGACTATTTTGCAGTTGGGTTTGCCCGCTAAACCCCGTTACCGATTTGGCTAACTGG
ATGCGCAGGAAATTTGACCTGAACCAGTCTGCGACGATTCCACGCCATATACGGTACGTC
CTTCTGGTGGTGGTGTTAATCGGTTCGGCGTTGACCGGCACGCTGCTGTGGGAATGGATT
AATCCCGTGTCGCTGTTAGGCCGTAGTCTGGTTATGGGATTTAGTAGCGGCGCGTTGCTG
ATTATTGCTTTGTTTTTATTTGATTTATTAGTCGTTGAGCACGGTTGGTGCGGCCATATT
TGCCCGATGGGCGCGCTGTATGGCGTGCTGGGCAGTAAAGGCGTAGTGACCGTGACGGCC
AAAAAACGTGAAAAAATGTAATCGCTGTATGGATTGTTTTCATGTTTGCCCGGAACCGCAT
GTGCTACGTGCGCCGGTGCTGGATGAGCAAAGCCCGGCGCAGGTCACCCACCGCGACTGC
ATGACCTGCGGCCGCTGCGTGGATGTCTGTTCTGAGGATGTTTTTACAATAACGATACAT
AACATCATTCGGGATGCATCGCGGCGGTAA
>EECEBICD_01105 Periplasmic nitrate reductase, electron transfer subunit
ATGAAAAGCCATGACCTGATGAAAGCGCTGTGTCAATGGATGGCCACGCTTGCCCTGGTG
GTAAGTGGTGCGGTTTGGGCGGCAAACGGAGTGGATCTGAGCCAGTCGCCGGAAGTATCC
GGGACGCAGGAAGGGGCGATTCGTATGCCGAAAGAGCAGGAGCGTATGCCGCTAAATTAC
GTCAACCAGCCACCGATGATCCCGCACAGCGTCGAGGGCTACCAGGTGACGACCAATACC
AACCGCTGTCTGCAATGCCACGGCGTGGAAAGTTATCGTACGACGGGCGCGCCGCGTATC
AGCCCGACGCACTTTATGGACAGTGACGGTAAAGTGTCGGGCAACGTCGCGCCGCGTCGT
TATTTCTGCCTGCAGTGCCACGTACCGCAGTCTGATACCGCGCCGATTATCGACAATACC
TTTACCCCCTCGCAAGGTTACGGGAAATAA
>EECEBICD_01106 Cytochrome c-type protein NapC
ATGGAAAATTCTAACCGTAAACCCGGCTGGATAAAGCGCGTCTGGCGATGGTGGCGCAGC
CCCAGCCGCCTGGCGCTGGGGACGCTGCTGTTAATCGGTTTTATCGGCGGCATCATCTTC
TGGGGCGGCTTCAATACCGGAATGGAAAAGGCTAACACCGAAGAGTTTTGTATTAGCTGC
CATGAAATGCGCAACACGGTGTATGAGGAGTATATGGAAACCGTCCACTACAACAACCGT
AGTGGCGTTCGTGCCACCTGTCCTGACTGCCATGTGCCGCATGAGTGGGGGCCGAAGATG
ATCCGTAAGATCAAAGCCAGTAAAGAGCTGTATGCGAAGGTATTTGGGCTGATAGATACA
CCGCAGAAATTTGAAGCTCATCGCCTGACAATGGCACAAAATGAGTGGCGGCGCATGAAA
GACAACAACTCGCAGGAATGCCGTAACTGCCACAATTTCGACTTTATGGATCTGACCGCG
CAAAAAGGCGTCGCGGCGAAAATGCACGATCAGGCGGTAAAAGACGGACAGACCTGTATC
GATTGCCACAAAGGGATCGCGCATAAACTGCCGGATATGCGTGATGTTAAACCAGGTTTC
TGA
>EECEBICD_01107 Cytochrome c biogenesis ATP-binding export protein CcmA
ATGCTTGAAGCCAGAGATCTGCACTGCGAACGGGACGAGAGGACGCTGTTTCGCGGGCTG
TCGTTCACCGTGGACGCCGGGGAGTGGGTGCAGGTCACCGGCGGCAACGGCGCCGGAAAA
ACCACCCTGCTGCGCCTGCTGACCGGGCTGGCGCGCCCGGACGGCGGGGAGGTGTACTGG

CAGGGCGAGCCCCTGCGCCGCGTGCGCGACAGCTTCCATCGCAGCCTGCTGTGGATAGGG
CACCAGCCGGGGATTAAATCCCGCCTGACGGCGCGGGAGAACCTGCACTTTTTCCACCCC
GGCGACGGCGCGCGTCTCCCGGAGGCGCTGGCGCAGGCCGGGCTGGCGGGATTTGAGGAC
GTGCCGGTCGCTCAGCTCTCGGCCGGGCAGCAGCGCCGGGTGGCGCTGGCCCGCCTGTGG
CTGACCCGCGCCGCGCTGTGGGTGCTCGACGAGCCGTTCACCGCCATTGACGTTAACGGC
GTGGCGCGCCTCACCCGGCGGATGGCGGCGCACACGGCGCAGGGCGGGATGGTCATTCTC
ACCACCCACCAGCCGCTGCCGGGGGCCGCGGACACCGTCCGCCGCCTGGCGCTGACCGGC
GGGGGGGCCGGGCTGTGA
>EECEBICD_01108 Heme exporter protein B
ATGTGGCGTGTCTTCTGTCTCGAGCTGCGCGTGGCGTTCCGCCACGGCGCGGACATCGCC
GGCCCGCTGTGGTTCTTCCTGATGGTCATCACCCTGTTTCCGTTAAGCGTCGGGCCGCAG
CCGCAGCTGCTGGCGCGTATCGCGCCGGGCATCATCCAGGTGGCGGCGCTGCTGGCCTCG
CTGCTGGCGCTGGAGCGGCTGTTTCGCGACGACCTGCAGGACGGCAGCCTGGAGCAGCTG
ATGCTGCTGCCGGTGCCGCTGCCGGCGGTGGTGCTGGCGAAGGTGCTGGCCCACTGGGCG
GTGACCGGCCTGCCGCTGATGATGCTCTCCCCGCTGGTGGCGCTGCTGCTGGGGATGGAC
GTGTACGGCTGGAAAATCATGGCGCTGACGCTGCTGCTCGGCACGCCGGCGCTGGGGTTT
CTCGCCGCGCCGGGCGTCGCGCTGACGGCCGGCCTGCGGCGCGGCGGCGTCCTGCTGGGC
ATTCTGGTGCTGCCGCTGAGTGTCCCGGTGCTGATTTTCGCCACCGCGGCGATGGACGCG
GCATCGATGCATTTACCCGTTGACGGCTATCTGGCGGTGCTGGGGGCGCTGCTGGCGGGC
AGCGCGACGTTAAGCCCGTTCGCCACCGCGGCGGCGCTGCGCCTCAGCGTGCAGTAG
>EECEBICD_01109 Heme exporter protein C
ATGTACCTGCATGTCCCGGCGGCCATCTGGTCGATGGGTATCTATGCGGCGATGGCGGTG
GCGGCGTTCACCGGGCTGGTCTGGCAGATGAAAATGGCCAGCCTTGCCGTCGCGGCGATG
GCGCCGGTGGGGGCGGTGTACACCTTCATCGCGCTGGTCACCGGCGCGGCGTGGGGCAAA
CCGATGTGGGGCACCTGGTGGGTGTGGGACGCGCGCCTGACCTCGGAGCTGGTGCTGCTG
TTTCTCTACGCCGGGGTCATCGCCCTGTGGCACGCCTTTGACGACCGTAAAATGGCCGGG
CGCGCGGCGGGCATTCTGGTGCTGGTCGGCGTGGTGAACCTGCCGGTTATCCACTATTCC
GTCGAGTGGTGGAACACCCTGCACCAGGGCTCGACGCGGATGCAGCAGAGTATCGACCCG
GCGATGCGCTCGCCGCTGCGCTGGGCCATCGCCGGCTTCCTGCTGCTCTTTATGACGCTG
GCGCTGATGCGGATGCGCAACCTGATTTTACTGATGGAAAAACGCCGCCCGTGGGTGAGC
GAACTGATACTGAAAAGGGGGCACCGGTGA
>EECEBICD_01110 Heme exporter protein D
ATGACCGTGGCGCCGCTGGTGCTGCTGGCGCTGCACACGGTGCTGCAGCGCCGGGCCATT
CTGCGCGGCGTGGCGCAGCAGCGGGCGCGCGAGGCGCGGATGCGTGCCGCACAGGCGCAA
CAGGAGGCCGCGTGA
>EECEBICD_01111 Cytochrome c-type biogenesis protein CcmE
GTGAACCTGCGACGTAAAAACCGGCTATGGGTGGTCTGCGCGGTGCTGGCGGGCCTGGCG
CTGACCACCGCCCTGGTCCTGTACGCGCTGCGCGCGAATATCGACCTGTTCTATACCCCC
GGCGAAATCCTCTACGGCAAGCGCGAGACGCAGCAGCTGCCGGCGGTGGGCCAGCGCCTG
CGCGTCGGCGGGATGGTGATGCCCGGCAGCGTCAGGCGCGACCCGGACTCGCTGAAGGTG
AACTTCAGCCTCTACGACGCCGAAGGGTCGGTGACGGTGAGCTATGAGGGGATACTGCCG
GACCTGTTCCGCGAGGGACAGGGGGTGGTGGTGCAGGGCACCCTGGAGAAGGGCAGCCAC
GTCCTGGCGCAGGAGGTGCTGGCCAAGCATGACGAGAACTACACCCCGCCGGAAGTGGAA
AAGGCGATGCAGGAAAACCACCGCCGCCCGCAACGCGCTGATAAGGACACCTCATCATGA
>EECEBICD_01112 Cytochrome c-type biogenesis protein CcmF
ATGATGCCTGAATACGGCCACGCGCTGCTGTGCCTGGCGCTCGGCGTGGCGCTGCTGCTG
TCCGTTTACCCGCTGTGGGGCGTGGCGCGCGGCGACGCGCGGATGATGGCGTCGGCCGGG
GTGTTCGCCTGGCTGCTGTTCATCTGCGTGGCGGGCGCGTTTTTCGTGCTGGTGCACGCC
TTTGTGGTTAACGACTTCACCGTGGCCTATGTCGCCGGCAACTCGAACACGCAGCTGCCG
GTGTGGTACCGGGTGGCCGCCACCTGGGGGGCGCACGAGGGCTCGCTGCTGCTGTGGGTG
CTGCTGATGAGCGGCTGGACCCTGGCGGTGGCGGTGTTCAGCCGGCCGGTGCCGGCGGAT
ATCGTCGCCCGGGTGCTGGCGGTGATGGGGATGGTCTGCGCCGGTTTTCTGGCGTTCATC
CTGTTCACCTCCGGCCCGTTCGCCCGCACGCTGCCGGCCTTTCCGGTGGAGGGGCGCGAC
CTGAACCCGCTGCTGCAGGACCCGGGGCTGATTTTCCACCCGCCGCTGCTGTACATGGGC

TATGTCGGCTTCTCGGTGGCCTTCGCCTTCGCCATCGCCGCGCTGCTGAGCGGGCGTCTG
GACAGCGCGTTCACCCGTTTTGCCCGCCCGTGGACGCTGGCGGCGTGGGTGTTCCTGACG
CTGGGCATCGTGCTCGGCTCGGCGTGGGCCTACTACGAGCTGGGCTGGGGCGGCTGGTGG
TTCTGGGACCCGGTGGAGAACGCCTCCTTTATGCCGTGGCTGGCGGGCACCGCCCTGCTG
CACTCGCTGGCGGTCACCGAACAGCGCGCCGGCTTTAAGGCGTGGACGCTGCTGCTGTCC
ATCTGCGCCTTCTCGCTGTGCCTGCTGGGCACCTTCCTGGTGCGCTCCGGGGTGCTGGTG
TCGGTGCACGCCTTCGCCTCCGACCCGGCGCGCGGAATGTTTATCCTCGCCTTTATGGTG
CTGGTCACCGGCGGCTCGCTGCTGCTGTTCGCCGTGCGCGGGCACAGGGTGCGTTCGCGG
GTGAACAACGCGCTGTGGTCGCGCGAGTCGCTGCTGCTCGGCAACAACGTCCTGCTGATG
GCCGCCATGCTGGTGGTGCTGCTGGGTACCCTGCTGCCGCTGGTGCACAAACAGCTGGGG
CTGGGCAGCATTTCGGTGGGGGAGCCGTTCTTTAACACCATGTTCACCTGGCTGATGGTC
CCCTTTGCCCTGCTGCTGGGGGTGGGGCCGCTGGTGCGCTGGGGCCGGGACCGGCCACGT
AACATCAGGAAACTGCTGTGGGCTGCCGTTGTCACTACCCTGGTGCTGTCGGTACTTTTG
CCGTGGCTGCTGGAAGATAAAATCATCGCCATGACGGCGGTGGGGATGGCGATGGCCTGC
TGGATTGCGGTGCTGGCGGTGGCCGAAGCCGTACAGCGCGTGTCCCGCGGCACGAAAACC
TCTCTCAGCTACTGGGGAATGGTGGCGGCGCACCTCGGGCTGGCGGTGACGATTACCGGC
ATCGCCTTCAGCCAGAATTACAGCGTGGAGCGTGACGTGCGGATGCGGGCGGGCGACAGC
GTGACCATTCACGACTACCGCTTCACCTTCCGGGAGGTGCGGGACATCACCGGGCCCAAC
TACCGCGGCGGGGTGGCCCTCATCGGGGTGACGCGCCACGGCGAGCCGGAGGCGGTGCTG
CACGCGGAGAAACGGCTCTACAACACCAGCCGGATGGTGATGACCGAGGCGGCGATTGAC
GGCGGGCTGACCCGCGACCTGTACGCCGCGCTCGGGGAGGAGCTGGACAACGGCGCGTGG
GCCGTGCGCCTGTACTACAAACCGTTTGTCCGCTGGATATGGGCCGGGGGGCTGCTGATG
GCGCTGGGCGGGCTGCTGTGCCTGGTGGACCCGCGCTACCGCCGCCGTAAACCATTGCCG
GAGGCCGGATGA
>EECEBICD_01113 Thiol:disulfide interchange protein DsbE
ATGAAACGCAACGTACTGTTATTACCGTTACTGATTTTTCTGCTGATTGCCGCGGCGCTG
CTGTGGCAGCTGACGCGCAACGCGCAGGGGGATGACCCGACGAATCTCGAATCGGCGCTG
ACCGGGAAGCCGGTGCCGGCGTTCCGCCTGGAATCGCTGGAGACGCCGGGTCAGTACTAT
GAGGCGGAGGTGCTGACGCAGGGGAAACCGGTGCTGCTTAACGTCTGGGCCACCTGGTGC
CCGACCTGCCGCGCCGAGCATCAGTACCTGAACCAGCTTTCTGCGCAGGGTATCCGGGTG
GTCGGGCTGAACTATAAGGACGACCGGGCGAAGGCGGTGGCCTGGTTAAAGGAACTGGGC
AACCCGTATGCGCTGAGTTTATCGGACAGCGACGGGATGCTGGGGCTGGACCTGGGCGTG
TACGGCGCGCCGGAAACCTTCCTCATCGACGGCAGGGGGATTATCCGCTACCGCCATGCG
GGCGATTTGAATGCCCGGGTGTGGGAGAGTGAACTGAAACCGCTGTGGGACAGATACAGC
CGGGAGGCGGCGCAATGA
>EECEBICD_01114 Cytochrome c-type biogenesis protein CcmH
ATGAGACTGTTACCGGGCATGGTGATGCTGATGCTGGTGCTGGTTATCTCCGGGTCAGCG
CGGGCGACCACCGACGTGATGCCGTTTAAAGATGAAGCGCAGGAGCAGCAGTTCCGCCAG
CTCACGGAGCAGCTGCGCTGCCCGAAATGCCAGAACAACAGCATTGCGGACTCGAACGCG
ATGATAGCCACCGACATGCGCCGCAGGGTGTATGACCTGATGCAGGAGGGGAAGAGCCGC
CAGGAAATCATCGATTACATGGTGGCGCGCTACGGCAACTTCGTCACCTACGACCCGCCG
CTGACCCCGTTGACGGTGCTGCTGTGGGTGCTGCCGCTGGCCGCCATCGTAGCGGGCGGG
TGGATAATCGTTGCCCGCACGCGCCGGCGGGTGCGCCTGCGCCGGGAGCCGCTGCCGGCG
GACACCCCGGTTTGCGGCGCGCGCGCCGGGTGGGGCGTTTACGTGCCGGGGGCCGTCATT
GCGCTGGCGGTCGGCGCCGGCAGCTACGCCCTGACCGGCAGCTATCAGCAGGTCAGGGCC
TGGCAGCAGGCAACGGCGCAGACGCCCGGGCTGCTGGCGCGGGCGCTGGACCCGGCGGCG
CAGCCGCTGAATGAAGAGGAGATGGCGCGGCTGGCGCTGGGGCTGCGCACCCGCCTGCAG
AATGATGCCGGCAATGTTGAGGGCTGGCTCATGCTGGGGCGCACCGGTATGGTACTGGGT
AATGCCGGTACCGCCACCGGGGCGTATGCGAACGCCTACCGTCTGGCCCCGAAAAACAGC
GATGCGGCGCTGGGCTACGCGGAGGCGCTGACGCGCTCGTCCGACCCGGAGGATAACCGC
CGCGGCGGGGAGCTGCTGCGTCAGCTGGTGAGCCGCGACCACACGGATATCCGGGTGTTA
AGCCTGTATGCGTTTAACGCCTTTGAGCAGCGGCGCTTTGGCGAGGCGGTGGCGGCCTGG
GAGATGATGCTGAAACTGCTGCCGGCGGGTGACGCCCGGCGGGCGGTGATAGAGCGCAGT
ATCCGGCTGGCGCAGGAGAAATAA

>EECEBICD_01115 Nitrate/nitrite response regulator protein NarL
ATGCCTGAAGTAACACCTTTTCAGGTGCTTATTGTGGACGACCATCCACTTATGCGGCGA
GGTATTCGTCAATTACTGGAACTGGACCCGGCTTTTCATGTCGTTGCCGAAGCGGGTGAC
GGGGCGAGTGCGATCGATCTGGCGAATCGCATTGAGCCCGATCTGATCCTGCTGGATCTG
AATATGAAAGGTCTGAGCGGGTTAGATACCCTGAACGCGCTACGCCGGGATGGTGTGACG
GCGCAAATTATTATTCTTACCGTCTCGGATTCCGCCAGCGACATCTACGCGCTGATCGAC
GCCGGCGCCGACGGTTATCTGCTCAAAGATAGCGATCCGGAAGTGCTGCTGGAGGCCATT
CGCAAAGGCGCTAACGGCGGTAAAGTCTTTAGCGATCGGGTCAATGAATATCTGCGTGAG
CGCGAACGGTTTGGCGCGCAGGAAGATCCCTTTAGTATCCTGACCGAACGAGAGTTAGAT
GTCTTGCATGAGCTGGCGCAGGGGCTTTCTAACAAACAGATTGCGTCAGTACTGAATATT
TCTGAGCAGACGGTTAAGGTGCACATTCGTAATCTACTTCGCAAGCTAAACGTGCGCTCA
CGCGTCGCGGCGACCATTCTATTTTTACAAACACGCGGAATACAGTAA
>EECEBICD_01116 hypothetical protein
ATGAAGAGAATATTTATATATCTATTATTACCTTGTGCATTCGCATGTTCTGCTAATGAT
AATGTTTTTTTTGGCAAGGGCAACAAGCATCAGATCTCTTTTGCTGCGGGAGAAAGTATA
AGAAGAGGAGGGGTTGAGCACTTATATACGGCTTTTCTGACATACAGTGAACCCAGCGAT
TTTTTCTTTTTACAGGCAAGAAATAATCTGGAGTTAGGAGGATTTAAGGCTAAGGGTAGC
GATGATTGCAGTAAACATTCTGGCAGCGTTCCCTGCAATAAATATAACCAGGGCGTATTG
GGTATCTCGAAGGATGTGGCGCTGGTTCATTTCGCTGGTATCTATACCGGTATTGGTCTG
GGGGCTTATATAAAATCTAAGTCGCGAGATGATATGCGTGTCAATTCTGCATTTACCTTT
GGAGAAAAAGCGTTTCTTGGCTGGAACTTTGGGGCTTTTTCTACAGAAGCTTATATCCGG
CATTTCTCGAATGGATCACTTACGGATAAAAATTCAGGGCATAATTTTGTAGGTGCTTCA
ATTAGTTATAATTTCTGA
>EECEBICD_01117 hypothetical protein
TTGCATGAACTGATTTTGCATGAACTGATTGGACATCTTTCCTGTGCAGTGGTCAACGTA
AAGTCAATGCAGGCAAATAACGTAAACCATGTTTGTACTAAAACAGAAAAAGCGCATCTA
CACCCTATGCTGGAAGAAATGTTTGTAGTGGCAGGTAAATGA
>EECEBICD_01118 hypothetical protein
ATGCTGATGACGAGGCGGCTGCCGCCGTTCTGGCTGTGTCAGCCGACGCCGCGCTGGCTG
ATGCCTCCCCGGCTTTTGTGGTCGCCGTACCTGCGCTGCTCTCCGCAGATGCTGCGGATG
AAGCTGCCGACCAAAATTCCCCTTGAAAACCAGCCTGTCAGGGCTGGTTTTTTTTATGCC
TACAATTAA
>EECEBICD_01119 hypothetical protein
GTGATTTCCCTGGAAGATGCAAGCCTGACGAAAAAAGGTATCGTTAAATTAAGCAGTGCC
ACGGACAGTGACAGCGAAGCGCTGGCGGCCACACCAAAGGCGGTAAAAACCGTTATGGGT
GAGGTACGGACCAAAGCGCCGCTGGACAGCCCGGCATTCACTGGAACGCCGACCACACCG
ACGCCGCCAGGCGATGCTAAAGGGCTTCAGACAACAAACGCGGAGTTTGTCCGCAAACTG
ATTGCCGCGCTGGTTGGTTCCGTACTGGAGCCACTGGACACCCTGCAGGAACTGGCTGAC
GCGTTGGGAAATGATCCGAACTTTGCCACCACGGTACTGAATAAACTGGCGGGCAAGCAG
CCGCTGGACGAAACCCTGACGGCGCTGTCAGGAAAAAGCGTTGACGGTCTTATCGAATAC
GTTGGTTTGCGAGAAACCATAAGTCGTGCCGCCGATGCATTATAA
>EECEBICD_01120 hypothetical protein
GTGCGTCGTATCGGTGCCGCGCGAGCGTTTGATGGCGCAGTTACTATCGGCTGTGATGAT
AATCCGTGGACGACGGCGGAGTTTATCGTCTGGCTGGAGTCTCAGGGCGCATTCAATCAC
CCTTACTGGATGTGTCGTGGCTCCTGGTCTTACGCTTATAACAAAATCATCACGGATACT
GGCTGCGGTACTATCTGTCTCGCTGGCGCAGTGATTGAGGTAATGGGAGTGCGTGGCGCG
ATGACTATTCGGGTGACAACGTCCCATTCAGTATCTGGTTGGTGA
>EECEBICD_01121 E3 ubiquitin-protein ligase SspH2
ATGCCCTTTCATATTGGAAGCGGATGTCTTCCCGCCACCATCAGTAATCGCCGCATTTAT
CGTATTGCCTGGTCTGATACCCCCCCTGAAATGAGTTCCTGGGAAAAAAATGAAGGAATTT
TTTTGCTCAACGCACCAGACTGAAGCGCTGGAGTGCATCTGGACGATTTGTCACCCGCCG
GCCGGAACGACGCGGGAGGATGTGGTCAGCAGATTTGAACTGCTCAGGACGCTCGCGTAT
GCCGGATGGGAGGAAAGCATTCATTCCGGCCAGCACGGGGAAAATTACTTCTGTATTCTG
GATGAAGACAGTCAGGAGATATTGTCAGTCACCCTTGATGATGCCGGGAACTATACCGTA

AATTGCCAGGGGTACAGTGAAACACATCGCCTCACCCTGGACACAGCACAGGGTGAGGAG
GGCACAGGACACGCGGAAGGGGCATCCGGGACATTCAGGACATCCTTCCTCCCTGCCACA
ACGGCTCCACAGACGCCAGCAGAGTATGATGCTGTCTGGTCAGCGTGGAGAAGGGCTGCA
CCCGCAGAAGAGTCACGCGGCCGTGCAGCAGTGGTACAGAAAATGCGTGCCTGCCTGAAT
AATGGCAATGCAGTGCTTAACGTGGGAGAATCAGGTCTTACCACCTTGCCAGACTGTTTA
CCCGCGCATATTACCACACTGGTTATTCCTGATAATAATCTGACCAGCCTGCCGGCGCTG
CCGCCAGAACTGCGGACGCTGGAGGTCTCTGGTAACCAGCTGACTAGCCTGCCGGTGCTG
CCGCCAGGACTACTGGAACTGTCGATCTTTAGTAACCCGCTGACCCACCTGCCGGCGCTG
CCGTCAGGACTATGTAAGCTGTGGATCTTTGGTAATCAACTGACCAGCCTGCCGGTGTTG
CCGCCAGGGCTACAGGAGCTGTCGGTATCTGATAACCAACTGGCCAGCCTGCCGGCGCTG
CCGTCAGAATTATGTAAGCTGTGGGCCTATAATAACCAGCTGACCAGCCTGCCGACGTTG
CCGTCAGGGCTACAGGAGCTGTCGGTATCTGATAACCAACTGGCCAGCCTGCCGACGCTG
CCGTCAGAATTATATAAGCTGTGGGCCTATAATAATCGGCTGACCAGCCTGCCGGCGTTG
CCGTCAGGACTGAAGGAGCTGATTGTATCTGGTAACCGGCTGACCAGTCTGCCGGTGCTG
CCGTCAGAACTGAAGGAGCTGATGGTATCTGGTAACCGGCTGACCAGCCTGCCGATGCTG
CCGTCAGGACTACTGTCGCTGTCGGTCTATCGTAACCAGCTGACCCGCCTGCCGGAAAGT
CTCATTCATCTGTCTTCAGAGACAACCGTAAATCTGGAAGGGAACCCACTGTCTGAACGT
ACTTTGCAGGCGCTGCGGGAGATCACCAGCGCGCCTGGCTATTCAGGCCCCATAATACAA
TTCGATATGGCGGGAGCCTCCGCCCCCCGGGAAACTCGGGCACTGCACCTGGCGGCCGCT
GACTGGCTGGTGCCTGCCCGGGAGGGGGAACCGGCTCCTGCAGACAGATGGCATATGTTC
GGACAGGAAGATAACGCCGACGCCTTCAGCCTCTTCCTGGACAGACTGAGTGAGACGGAA
AACTTCATAAAGGACGCGGGGTTTAAGGCACAGATATCGTCCTGGCTGGCACAACTGGCT
GAAGATGAGGCGTTGAGAGCAAACACCTTTGCTATGGCAACAGAGGCAACCTCAAGCTGC
GAGGACCGGGTCACATTTTTTTTGCACCAGATGAAGAACGTACAGCTGGTACATAATGCA
GAAAAAGGGCAATACGATAACGATCTCGCGGCGCTGGTTGCCACGGGGCGTGAGATGTTC
CGTCTGGGAAAACTGGAACAGATTGCCCGGGAAAAGGTCAGAACGCTGGCTCTCGTTGAT
GAAATTGAGGTCTGGCTGGCGTATCAGAATAAGCTGAAGAAATCACTCGGGCTGACCAGC
GTGACGGCAGAAATGCGTTTCTTTGACGTATCCGGCGTGACGGTTACAGACCTTCAGGAC
GCGGAGCTTCAGGTGAAAGCCGCTGAAAAAAGCGAGTTCAGGGAGTGGATACTGCAGTGG
GGGCCGTTACACAGAGTGCTGGAGCGCAAAGCGCCGGAACGCGTTAACGCGCTTCGTGAA
AAGCAAATATCGGATTATGAGGAAACGTACCGGATGCTGTCTGACACAGAGCTGAGACCG
TCTGGGCTGGTCGGTAATACCGATGCAGAGCGCACTATCGGAGCAAGAGCGATGGAGAGC
GCGAAAAAGACATTTTTGGATGGCCTGCGACCTCTTGTAGAGGAGATGCTGGGGAGCTAT
CTGAACGTTCAGTGGCGTCGTAACTGA
>EECEBICD_01122 Protein UmuC
ATGGAAGTACTGGATCACCTGAATGCAAAGAAGGGCTGGGGAAAGCTGTACTTTGCCGGG
GAGGGTATCCAGCAACAGTGGGCTATGAAGAGAAACATACTGTCATCCCGATATACCACC
CGCTATGAAGACCTGCTTCAGGTTAAGTGA
>EECEBICD_01123 tRNA-Pro(ggg)
CGGCACGTAGCGCAGCCTGGTAGCGCACCGTCATGGGGTGTCGGGGGTCGGAGGTTCAAA
TCCTCTCGTGCCGACCA
>EECEBICD_01124 Inner membrane protein YejM
ATGGTAACTCATCGTCAGCGCTACCGTGAAAAAGTCTCCCAGATGGTTAGCTGGGGGCAC
TGGTTCGCTCTGTTCAATATACTGTTGGCTACGTTGCTCGGCAGCCGTTACCTGTTTGTC
GCCGACTGGCCGACAACGCTTGCGGGTCGCATATACTCCTACCTGAGCATTGTCGGGCAC
TTTAGCTTTCTGGTGTTCGCCACCTATTTGCTTATTCTTTTTCCGCTCACGTTTATCGTG
ATGTCCCAGCGGCTGATGCGGTTTATATCAGCCATTCTGGCGACCGCGGGTATGACGTTG
TTGCTTATCGACAGCGAAGTCTTTACCCGTTTTCACCTGCATCTTAATCCCATTGTCTGG
GAGCTGGTCATCAACCCTGACCAGAACGAAATGGCGCGCGACTGGCAGCTTATGTTTATT
AGCGTGCCGGTTATCTTATTGATTGAGATGCTATTTGCGACATGGAGTTGGCAAAAGCTG
CGCAGTCTCACGCGCCGTCGCCATTTCGCAAGGCCGCTTGCGGCATTCTTTTTCGTCTCC
TTTATCGCATCGCATCTTATCTACATCTGGGCAGATGCTAATTTTTACCGGCCGATTACC
ATGCAGCGGGCAAACCTGCCGCTCTCTTATCCGATGACGGCGCGACGTTTTCTTGAAAAA
CACGGTCTGCTGGATGCGCAGGAATATCAGCGCCGTCTGGTAGAGCAAGGCAATCCGGAA

GCGGTCTCCGTTCAGTACCCGCTCAGCAATTTGCACTATCGCGATATGGGCACCGGTCAG
AATGTGCTGCTCATTACCGTAGACGGCCTGAACTATTCTCGCTTTGAGAAGCAGATGCCG
GAGCTGGCTACATTTGCCGAGCAAAACATCGACTTCACCCGTCATATGAGTTCAGGGAAT
ACCACTGATAACGGTATTTTCGGCCTGTTCTATGGCATATCGCCAGGCTATATGGATGGC
GTGTTATCCACCAGAACGCCTGCGGCGCTCATTACCGCGTTGAATCAGCAAGGCTACCAA
CTGGGACTGTTTTCTTCGGATGGCTTCGCCAGCCCGCTTTATCGTCAGGCATTACTGTCC
GATTTCTCGATGCCGGCAGCGCAAACGCAGTCTGATGCGCAAACGGCCAGCCAGTGGATA
GACTGGCTTGGGCGCTACGCACAGGAAGATAACCGCTGGTTCTCATGGATATCGTTTAAC
GGTACTAATATTGATGACAGTAACCAGAAAAATTTTGTTAAGCGTTACGCCAGTGCCGCC
AGCGACGTGGATGCGCAAATCAATCGGGTTCTGAACGCGCTGCGAGAGGCCGGTAAATTC
GAGAATACTGTCGTGATCATTACCGCAGGACGCGGCATACCGTTGACGCCGGAAGAAAAT
CGCTTCGACTGGTCGCAAGGTCATCTGCAAGTACCGCTGGTGATCCACTGGCCGGGGACG
CCTGCGCAGCGTATTAATGTGCTTACCGATCATACCGATGTGATGACCACGTTGATGCAA
CGCCTGTTACATGTCAGCACGCCAGCGAACGAATATTCACAGGGCCAGGACATCTTTACC
GTTCCACGTCGTCATAACTGGGTAACGGCGGCAGACGGCAGTACGCTGGCAATCACGACG
CCGCAAATGACCCTGGTGCTCAATAATAACGGTCATTATCAGACCTATGACTTACACGGC
GAGAAGATCAAAGATCAGAAGCCGCAGCTCAGTCTGCTGCTGCAAGTGCTAACAGAAGAG
AAACGTTTTATCGCTAACTGA
>EECEBICD_01125 hypothetical protein
ATGCCACAACTCTCCCGCTATAGTGATGAACATGTTGAACAATTGCTGAGCGAACTGCTC
AGTGTACTGGAAAAACATAAGGCGCCGACCGATCTTTCCCTGATGGTGCTGGGAAATATG
GTCACCAACCTTATCAACACCAGCGTTGCGCCAGCTCAACGCCAGGCGATTGCGAACTCT
TTTGCCCGCGCCTTGCAGTCTTCGATTAGCGAAGACAACGCGCACTAA
>EECEBICD_01126 Nucleoid-associated protein YejK
ATGAGTCTGGATATCAACCAGATTGCCCTGCACCAGCTTATCAAGCGCGATGAGCAAAAT
CTTGAGCTGGTCTTGCGCGATTCATTGCTGGAGCCGACAACCACCGTTGTCGAGATGGTG
GCTGAACTGCATCGGGTCTATAGCGCCAAGAATAAGGCGTATGGCCTGTTTAATGAAGAG
AGTGAACTGGCGCAAGCGCTGCGGTTGCAACGTCAGGGGGAAGAAGATTTTCTTGCCTTT
AGCCGGGCGGCGACCGGACGCCTGCGTGACGAACTGGCGAAATATCCCTTTGCGGACGGC
GGCATTGTATTGTTCTGCCATTATCGTTACCTGGCGGTGGAGTATCTGCTGGTTACGGTA
CTGAACAACCTGAGCAGTATGCGGGTCAATGAAAATCTGGACATTAACCCGACGCATTAT
CTTGATATCAATCATGCGGATATCGTGGCGCGTATCGATCTTACCGAGTGGGAAACTAAT
CCGCAATCGACCCGCTACCTGACGTTTCTCAAAGGTCGGGTAGGGCGCAAGGTCGCTGAC
TTCTTTATGGATTTCCTCGGCGCCAGCGAAGGGTTGAACGCCAAAGCGCAGAATCGCGGC
CTGTTGCAGGCAGTGGATGATTTCACCGCAGAAGCGCAGTTGGATAAAGCAGAACGTCAG
AACGTGCGTCAGCAGGTGTACAGCTACTGCAACGAGCAGTTACAAGCCGGGGAAGAGATT
GAGCTGGAATCGCTGTCTAAAGAGCTTTCCGGCGTCAGTGAAGTCAGCTTCAGCGAATTT
ACTGCCGAAAAAGGCTATGAGCTGGAAGAGAGCTTCCCGGCAGATCGCAGTACGCTACGC
CAGTTAACCAAATATGCCGGCAGCGGCGGCGGGTTAACGATTAACTTTGATGCGATGCTA
CTGGGCGAGCGGATTTTCTGGGACCCGGCGACCGATACCCTGACTATCAAAGGGACGCCG
CCGAATCTGCGCGAGCAGTTGCAGCGACGCACGTCGGGCGAGAAATAA
>EECEBICD_01127 hypothetical protein
GTGAGTAAAGGAATACGACAGAAGCCGCCTGTTAATTTCAGTTTATTTCGGCAGGTTTTG
TATGCGCACATTGTCGCATTTTTGATGATGTTGGTGCTCGGCATGGTGTTCACCGTTCTC
TCGTTGGTACTGTTTTATACTTATGGTGCCAACTGGTTACTGAGCTTGTTCATATGCCCG
CTGTTCTTGTTAAGTGGTTTATTCATTACTGGATTTGCTTTTAAATCAACTTGGTCCAGC
ATACGTTACTATTACGACAAGGGGCAGTTGAAACGGTATGGACTCAATCTTGATGCAACT
TTGACGCATAAAGAGAAGGTGGAAATACGCATCGATAATGCAAAGCGTCAAGTGCGTGTA
GATGAACTGGAGCTCCATGTCTTATTTGATTTTCAGTTTGATAGCAAGACATGGAGCTGC
GGCGACTTACTGACCAATGAAAAGGTATTTGATGCGTTGAATGACGGGCAAACTATACCA
ATACGAATTTTGCCATGGAAACCGGAAAGTGCCAGCGTTCGTCAACGGGCATTATTCAAT
CGGCTTAAAGGTATGAATACCGCGGCGGAAACTACAGATTCTCGATTAGGGGAGGCGCTA
ATTGAATGTGGCGAGGTGTAA
>EECEBICD_01128 50S ribosomal protein L25

ATGTTTACTATCAACGCAGAAGTACGTAAAGAGCAGGGTAAGGGTGCGAGCCGCCGCCTG
CGCGCCGCTAACAAGTTCCCGGCAATCATCTACGGCGGTTCTGAAGCCCCGATTGCTATC
GAACTGGACCACGACCAGGTGATGAACATGCAAGCTAAAGCTGAATTCTACAGCGAAGTT
CTGACCCTCGTTGTTGACGGTAAAGAAGTAAAAGTTAAAGCTCAGGCTGTACAGCGTCAC
GCTTACAAACCGAAGCTGACTCACATCGACTTCGTTCGCGCGTAA
>EECEBICD_01129 Putative DNA repair helicase RadD
ATGATTTTTACACTCCGCCCCTACCAGCAAGAAGCCGTAGACGCCACGCTCAGCCACTTT
CGCCGCCACCGTACGCCCGCCGTGATTGTTCTGCCGACCGGCGCAGGTAAAAGCCTGGTG
ATCGCCGAACTGGCGCGCGTCGCCCGCGGACGGGTACTGGTGCTGGCGCATGTGAAAGAG
CTGGTCGCGCAGAACCACGCCAAATATTGCGCGCTGGGGCTGGAAGCAGATATTTTCGCC
GCCGGACTCAAACGTAAAGAGAGTCAGGGCAAAGTCGTGTTCGGCAGCGTACAGTCGGTG
GCGCGTAATCTTGACGCCTTCCAGGAGGAGTTTTCGCTGTTGATTGTCGATGAATGCCAC
CGCATCGGTGACGATGAAGACAGTCAGTATCAGCAAATCCTCACTCACCTGAGTAAAGTT
AATCCTCACTTACGTCTGCTTGGGCTAACCGCCACGCCCTTCCGCCTCGGAAAAGGCTGG
ATTTATCAATTTCATTACCACGGTATGGTGCGCGGCAACGACAACGCGCTGTTTCGCGAC
TGTATTTATGAACTGCCGCTGCGCTATATGATTAAACACGGCTATCTGACGCCGCCTGAG
CGGCTTGATATGCCAGTGGTCCAATACGATTTCAGCCGCCTGCAGGCCCAAAGCAATGGG
CTGTTCAGCGAAGCCGACCTGAACCGTGAGCTGAAAAAGCAGCAGCGGATTACGCCGCAC
ATCATCAGCCAGATTATGGAATTTGCGCAAACGCGCAAAGGCGTGATGATTTTCGCCGCC
ACGGTCGAACATGCGAAAGAGATTGTCGGTCTGCTTCCGGCGGACGACGCGGCGCTGATT
ACCGGCGATACGCCAGGGCCCGAGCGCGACGCGCTGATTGATAATTTCAAGGCGCAGCGT
TTTCGCTATCTGGTTAACGTCTCGGTGCTGACCACCGGCTTTGACGCCCCACACGTTGAT
CTCATCGCGATTCTACGTCCCACCGAGTCAGTTAGTCTTTACCAACAAATTGTCGGGCGT
GGTCTGCGCCTTGCGCCGGGAAAGACCGATTGCCTGATTCTTGATTACGCAGGCAACCCG
CACGACCTGTATGCCCCGGAGGTCGGTAGCCCGAAGGGAAAAAGCGATAACGTCCCTGTC
CAGGTATTTTGCCCGGCCTGCGGCTTTGCCAACACCTTCTGGGGGAAAACCACTGCCGAC
GGCACGCTGATTGAACACTTTGGCCGTCGCTGCCAGGGCTGGTTTGACGATGACGACGGC
CATCGCGAGCAGTGCGATTTTCGCTTTCGCTTCAAAAACTGCCCGCAGTGTAATGCCGAA
AACGATATTGCCGCCCGACGCTGCCGGGAATGTGACGCTATTCTGGTCGACCCGGACGAT
ATGTTAAAAGCGGCGCTCAGACTCAAGGATGCGTTAGTCCTGCGCTGTAGCGGAATGACG
ATGCAGCATGGGCAGGATGAGAAAGGCGAATGGCTGAAAATCACTTACTATGACGAGGAC
GGCGCGGATGTCAGTGAGCGCTTCCGCTTGCACACGCCCGCCCAGCGTACCGCTTTCGAA
CAGCTATTTATTCGCCCGCATACCCGCACGCCCGGCGTTCCTTTACGCTGGATCACGGCG
GCGGATATTGTCGCGCAGCAGGCGCTGTTGCGGCATCCCGATTTTGTGGTCGCGCGGATG
AAAGGCCAGTACTGGCAGGTGCGTGAAAAAGTGTTCGACTATGAAGGCCGCTTCCGCCGG
GCGCACGAATTACGTGGTTAA
>EECEBICD_01130 Ribosomal small subunit pseudouridine synthase A
ATGCGACTTGATAAATTTATCGCTCAGCAGCTTGGCGTCAGCCGCGCTATTGCCGGGCGT
GAAATTCGTGGTAACCGCGTTACCGTCGATGGCGACATCATTAAAAATGCGGCCTTCAAA
CTGCTCCCGGAACATGCGGTTGCGTATGACGGCAATCCCTTAGCGCAGCAACACGGGCCA
CGCTATTTTATGCTTAACAAGCCGCAGGGATACGTTTGTTCAACCGATGATCCCGATCAT
CCAACGGTACTGTATTTCCTGGATGAGCCGGTGGCGTATAAGCTGCATGCCGCAGGACGT
CTGGATATCGATACTACCGGTCTGGTGTTAATGACAGATGACGGTCAGTGGTCGCACCGC
ATTACGTCGCCGCGCCATCACTGTGAAAAAACCTATCTGGTGACCCTGGAGTCGCCGGTG
GCCGACGATACGGTAGCGCAATTTGCGAAAGGCGTGCAGTTGCATAATGAAAAAGATCTC
ACTAAACCCGCTACGCTGGAGGTGATAACGCCTGTGCAGGTCCGTCTGACCATCAGCGAA
GGCCGTTATCATCAGGTGAAGCGGATGTTTGCCGCAGTAGGCAATCGCGTTGTGGAACTG
CACCGCGAACGGATTGGCGCCATTACGCTGGATGAGAATCTGGCTCCCGGCGAGTACCGC
CCGTTGACTGAAGAAGAAATCGCCAGCGTCGGCTAA
>EECEBICD_01131 Bicyclomycin resistance protein
GTGACCACCCGGCAGCACTCTTCCTTTGCCATTGTCTTTATCCTTGGCCTGTTGGCCATG
TTAATGCCGCTGTCGATTGATATGTATCTTCCTGCGCTGCCGGTGATTTCTGCGCAATTC
GGCGTGCCTGCCGGTAGCACGCAGATGACGCTCAGTACCTATATTCTGGGGTTTGCGCTG
GGTCAGCTTATCTATGGACCGATGGCGGATAGCCTCGGGCGTAAGCCGGTCATCCTGGGC

GGGACGCTGGTATTTGCTGCTGCGGCGGTCGCCTGTGCGTTGGCGCAGACTATCGATCAA
CTGATCGTGATGCGTTTCTTTCACGGTTTGGCGGCCGCGGCGGCAAGCGTCGTCATCAAT
GCGCTGATGCGGGATATTTATCCAAAGGAAGAGTTTTCGCGCATGATGTCATTTGTCATG
CTGGTCACTACGATAGCGCCGTTAATGGCGCCCATTGTCGGCGGCTGGGTGTTGGTATGG
TTAAGCTGGCACTATATCTTCTGGATACTGGCCATTGCGGCGATTCTGGCGTCAGTCATG
ATCTTTGCTCTGATTAAAGAGACGCTGCCCGTTGAGCGGCGTCAGCCTTTTCATATTCGT
ACCACAATAGGTAACTTTGCCGCGTTGTTTCGCCACAAACGCGTACTGAGCTATATGCTG
GCGAGCGGGTTTAGTTTTGCCGGTATGTTCTCTTTTTTGAGCGCGGGGCCGTTTGTTTAT
ATCGAAATTAATCATGTTCCGCCGCAGGATTTCGGCTACTACTTCGCGTTGAACATCGTA
TTTCTGTTTGTGATGACGATTATCAACAGCCGTTTTGTCAGACGGGTAGGGGCGCTAAAC
ATGTTTCGGGCCGGGCTATGGATTCAGTTTGCGATGGCGGTGTGGATGGTTTTCAGCGCG
CTGATGGGCATTGGATTCTGGGCGCTGGTGGTCGGCGTTGCGGCGTTTGTCGGCTGTGTG
TCGATGGTTTCGTCCAATGCGATGGCGGTCATTTTGGATGAGTTTCCGCATATGGCCGGA
ACGGCGTCTTCGTTGGCGGGCACTTTCCGCTTTGGTATTGGCGCTATCGTCGGCGCGTTG
CTGTCGCTGGCTACCTTTACCAGTGCGTGGCCGATGATCTGGTCGATTGCGCTTTGCGCC
GCCTGTTCCATTCTGTTTTATCTCTACGCCAGCCGTCCCAAAAAACGGTGA
>EECEBICD_01132 putative protein YejG
GTGAATACATTACAGCTCTCAATTGTTCACCGTCTGCCGCAAAACTATCGCTGGTCGGCG
GGTTTTGCGGGGTCAAAGGTTGAACCGATTCCGCAAAACGGGCAGAGCACCGAGAATAGT
CTGGTGGCGCTTAAACTGTTGAGTCCGGCAGGCGATAGCGCGTGGTCAGTGATGCATAAG
CTCAGCCAGGCGCTAAGCGATATTGAAGTTCCTTGCTCCGTGCTGGAGTGCGAAGGGGAA
CCGTGCCTGTTCGTTAATCGTCAGGATGAGTTTGCCGCCACCTGTCGGCTGAAAAATTTC
GGCGTGGCGATAGCGGAACCTTTTTCTAACAATAATCCTTTTTAG
>EECEBICD_01133 putative ABC transporter ATP-binding protein YejF
ATGACATCTCCATTGCTTGCGATTGAAAATTTATCGGTAGGTTTCCGTCAGCAACAGCAC
GTGCGTCCTGTCGTTAACGCCATTTCGTTACAGGTGAACGCCGGGGAAACGCTGGCGCTG
GTCGGCGAGTCCGGGTCAGGGAAAAGCGTGACGGCGCTTTCCATTCTGCGTCTTTTGCCT
ACCCCTCCCGCCGTCTACCTCTCCGGCGATATTCGCTTTCACGGCGAATCATTGCTTCAT
GCCAGCGAGCAGACGCTACGCGGCGTGCGCGGCAATAAAATCGCCATGATTTTTCAGGAA
CCGATGGTTTCACTTAACCCGCTGCATACGCTGGAAAAACAGCTCTATGAAGTGCTGTCG
CTCCATCGGGGAATGCGTCGGGAGGCGGCAAGGGCGGAGATGATCGGTTGTCTGGATCGG
GTCGGTATCCGCCAGGCGAGCCAGCGTCTGCGCGATTATCCGCATCAGCTTTCCGGCGGC
GAACGCCAGCGCGTCATGATAGCCATGGCGCTGTTAACACGGCCGGAATTACTGATCGCC
GATGAGCCGACCACCGCCCTCGACGTCTCCGTACAGGCGCAGATCTTATCTCTACTGCGG
GAACTCCAGCGCGAGCTTAATATGGGATTGCTGTTTATCACCCATAACCTCAGCATTGTA
AAAAAACTGGCGGATTCAGTGGCGGTAATGCAACACGGCAAGTGCGTAGAGAACCAACGC
GCCGACACGCTGCTCTCCGCGCCGACCCATCCGTACACGCAAAAACTACTCAACAGCGAA
CCCACAGGCGATCCGGTTCCGCTCCCCGCCGGGCAGGCGCCGTTGCTGGAAGTGGACAAG
CTGCGCGTCGCCTTCCCAATCCGCAAAGGCATTCTGAAGCGCGTCGTGGATCATAATGTG
GTGGTTAACAATATCAGTTTCACCCTGCATCCAGGCGAAACGCTGGGTCTGGTCGGCGAG
TCAGGATCGGGAAAAAGCACCACCGGTCTGGCGCTGTTACGGCTTATCCGCTCCGAAGGC
CGCATCGTGTTTGACGGTCAATCGCTGGATACGTTAAACCGCCGCCAGCTTTTACCTGTT
CGCCACCGTATCCAGGTCGTATTCCAGGACCCAAACTCATCGCTAAACCCGCGTTTAAAC
GTGTTGCAAATTATCGAAGAAGGCCTGCGCGTCCACCAGCCTACGCTTTCAGGCGCGCAG
CGAGAACAGCAGGTGAAAGCGGTCATGATGGAAGTCGGCCTGGACCCGGAAACGCGGCAT
CGTTACCCCGCTGAGTTTTCCGGCGGCCAGCGTCAACGTATCGCCGTCGCCAGGGCGCTG
ATTTTAAAACCGTCGCTTATTATTCTGGATGAACCGACCTCATCGCTGGATAAAACCGTT
CAGGCGCAGATTCTTGCCCTCCTGAAATCGCTCCAGCAAAAGCACCGTCTGGCCTATATC
TTCATTAGCCACGATCTGCATGTAGTACACGCGCTGTGCCATCAGGTTATCGTGCTGCGG
CAGGGGGAGGTGGTTGAACAGGGGCAATGCGAGCGCGTGTTTACCGCACCGCAACAGGCC
TATACGCGTCAGCTACTCGCGTTAAGCTGA
>EECEBICD_01134 Inner membrane ABC transporter permease protein YejE
GTGGGCCCCCCGTACGCTTTGGCGCCAATACCATTAATTTTGCCACCACGCAGCCTTTCC
CCTCCCCGCCTTCGGCGAAAAAACTGGCTGGGCACCGATGCGAATGGCGGCGACGTTTTC

GCCCGCATCCTGTACGGCACCCGCATTTCTATTTATTCGGCCTGATGTTAACGATTTGC
TCCAGCGTCATGGGCGTACTGGCGGGCGCGCTACAGGGCTATTATGGCGGCAAAGTCGAT
TTATGGGGGCAACGTCTCATCGAAGTCTGGTCGGGGATGCCGACCCTGTTTCTGATTATT
TTACTTTCCAGCGTAGTGCAGCCTAACTTCTGGTGGCTGCTGGCCATAACCGTGTTGTTT
GGCTGGATGAGTCTGGTGGGCGTAGTGCGCGCCGAGTTTTTACGGACCCGCAATTTTGAC
TATATCCGCGCCGCGCAGGCGCTTGGCGTCAGCGATCGTGACATTATCCTGCGCCATATG
TTGCCTAATGCGATGGTCGCTACCCTGACATTTTTACCGTTCATTTTATGTAGCTCCATC
ACCACCCTGACGTCGCTGGATTTTCTGGGATTCGGTCTGCCGCTTGGCTCCCCTTCTCTC
GGCGAACTTCTTTTACAGGGGAAAAACAACTTACAGGCTCCCTGGCTGGGGATCGCCGCC
TTTCTGTCTGTCGCCATTCTGCTATCGCTGCTGATTTTTATCGGCGAAGCGGTACGCGAC
GCCTTTGATCCTGCTAAGGCGGTATAA
>EECEBICD_01135 Inner membrane ABC transporter permease protein YejB
ATGGGCGCCTATCTTATCCGCCGTCTGTTACTGGTGATCCCCACGCTATGGGCCATTATT
ACCATTAACTTTTTTATCGTGCAGATCGCTCCCGGCGGCCCTGTCGATCAGGCGATTGCC
GCGATTGAATTTGGCCAGGGCGGCGCGTTGCCCGGCGCCAGCGGCGAAGGCGTTCGCGCC
AGTCATGCGCAAACCGGCGTCGGCAATATCAGCGACAGCCATTATCGCGGCGGCCGGGGA
CTCGATCCGGAGGTGATCGCGGAGATCACCCATCGCTACGGCTTCGACAAACCGCTGCAT
GAGCGTTATTTCAAGATGCTACGGGACTATCTCTCTTTTGACTTCGGCGACAGTCTGTTT
CGCAGCGCATCCGTACTGACGCTAATCAAAGACAGCCTTCCCGTCTCCATTACCCTGGGA
TTATGGAGCACGCTGATTATTTATCTGGTGTCTATCCCGCTGGGGATTCGCAAAGCCGTC
CATAACGGCAGTCGCTTTGATGTGTGGAGCAGCGCGTTTATCATTATTGGTTATGCGATC
CCCGCCTTTCTGTTTGCTATTTTATTAATCGTCTTTTTTGCCGGCGGCAGCTACTACGAT
ATTTTTCCGCTACGTGGCCTGGTCTCCGCCAACTTTGACACATTACCGTGGTACCAGAAA
ATCACCGATTATTTGTGGCATATTACGCTGCCGGTGTTGGCGACCGTCATCGGCGGTTTT
GCCGCCCTGACGATGCTGACGAAAAATTCGTTTCTCGACGAGGTGCGTAAACAGTATGTC
GTTACCGCTCGCGCGAAAGGCGTGAGTGAAAAAAACATTCTCTGGAAACATGTTTTTCGT
AACGCCATGCTGCTGGTGATCGCCGGGTTTCCCGCCACTTTTATCAGTATGTTTTTCACC
GGCTCGTTGCTAATTGAAGTCATGTTCTCGCTCAACGGCCTGGGGCTGCTGGGTTATGAA
GCAACCGTTTCGCGCGACTATCCGGTGATGTTCGGTACGCTTTATATCTTTACCCTGATT
GGCTTATTACTGAATATCCTGAGCGATATCAGCTATACGCTGGTCGATCCACGCATTGAT
TTTGAGGGACGATAA
>EECEBICD_01136 hypothetical protein
ATGATTGCGCGCGTAATGCTTCTGCTTGTCGCACTGGTTAGCGCTGGCGCCCAGGCCCAG
GAAATCAAAGAAAGCTACGCTTTCGCCGTACTCGGCGAACCTAAGTATGCTTTTAACTTT
GATCACTTTGATTATGTGAATCCTGCTGCGCCGAAAGGCGGTCAGATGACGCTTTCCGCC
ATTGGTACGTTCGATAATTTCAATCGCTATTCGCTGCGCGGCAATCCCGGCGTACGTACC
GAAGCCCTTTACGATACGCTTTTTACCACCTCGGATGATGAACCCGGAAGCTATTACCCG
CTGATTGCCGACCATGCCCGCTATGCCGCCGACTATTCCTGGGTGGAAATCTCGATTAAT
CCCCGCGCTCGTTTTCACGATGGCACGCCCATTACCGCCCGCGATGTGGCCTTTACCTTT
CATAAGTTTATGACCGAAGGCGTACCGCAGTTCCGTCTGGTCTATAAAGGTACTACCGTG
AAGGCGATTGCGCCTTTAACCGTGCGAATTGAGCTGGCGAAGCCTGGTAAAGAAGACATG
CTTAGTCTGTTTTCATTACCGATCATGCCCGAAAAATTCTGGAAAAATCACAAGCTCAGC
GATCCGCTTTCAACGCCGCCCTTAGCCAGCGGCCCATACCGGATTACTCAGTGGAAAATG
GGCCAGTACATTGTCTATTCACGCGTCAAAAACTACTGGGCGGCTAATCTGCCGGTCAAT
CGTGGACGTTTTAACCTCGACACTATCCGCTACGATTACTACCTTGATGACAATGTCGCT
TTCGAGGCGTTTAAAGCGGGCGCATTTGATCTACGGCTGGAAAACGACGCTAAAAACTGG
GCAACGCGCTACATCGGTAAAAATTTCGATAATCATTACATCATTAAAGAAGAGCAGAAA
AACGAGTCGGCGCAGGACACACGCTGGCTGGCCTTTAACATTCAGCGCCCGGTATTTAAA
GACCGGCGGGTACGTGAAGCTGTCACCCTGGCCTTCGATTTTGAGTGGATGAATAAAGCG
CTGTTCTATAATGCCTGCAGCCGAACCAACAGTTACTTCCAGAATACCGAGTACGCCGCC
AGAAATTATCCTGACGCCGATGAGCTGGTATTACTCGCGCCGATGAAAAAAGATCTTCCT
CCTGAAGTCTTCACTCAGATCTATCAGCCGCCGGTCTCTAACGGCGACGGCTACGATCGC
GAAAATCTTCTTAAAGCTGACGCCTTGTTGACGCAGGCCGGATGGGTGATCAACGGACAG
CAACGGGTCAATAGCGTCACCGGTAAGCCTCTGACGTTTGAACTTCTCCTTCCTGCCAGC

AGTAATAGCCAGTGGGTTCTGCCCTTCCAGCATAATCTTCAGCGTCTGGGCATTACGATG
ACTATCCGTCAGGTTGATAATTCTCAGCTCACCAACCGGATGCGCAGCCGCGACTATGAC
ATGATGCCGAGGCTATGGCGGGCGATGCCCTGGCCCAGCTCCGATCTACAAATCTCATGG
GCGTCGGAATACATTGACTCCAGTTATAACGCTCCCGGCGTACAAAGCCCGGTGGTGGAT
AAACTGATCGCGCAAATTATCGCAGCGCAGGGTGATAAAGCGAAACTGGTGCCGCTGGGA
CGGGCGCTGGATCGCGTGCTGACCTGGAACTATTACATGCTGCCGATGTGGTATATGGCG
CAAGACAGGCTCGCCTGGTGGGATAAATTCTCCCATCCGGCGATTCGCCCGGTATATACC
ATCGGGTTAGATACCTGGTGGTATGATGTCAACAAAGCCGCCAAACTACCGGCAGCCAGG
AGGTAG
>EECEBICD_01137 putative cyclic di-GMP phosphodiesterase PdeN
ATGCTTACACGCGACTCCTCTTCCGGTCGGAAAATCCTGCTTACCTGTATCATTACGGGG
ATAGCGGCAGCATTACTTATTGGTAGCCTGCAATTTTTCCTGTCGTGGCATAAACGGGAT
GTTAAATACAATACGCTCCTTTCTGATGTACAACATTACCTGGCCAGCTATTTCGCCGAC
CTGAAAGCCACCACCGATATTCTTCAGCCGTTGACGATCAACACCTGTCAGCAGGTTGGC
GCAGAACTCACCTCAAGAGCCGCTTTTAGCCTTAATGTCCGCGCCTTCTTATTGATTAAA
GATAAAAAAGTGTTCTGCTCTTCCGCCACTGGCGCCATGAATATGCCGCTTCAGCAACTG
GTACCGGATATAGATATCCGCAAAGACGTTGCTATGGCGATTCTGCCCGGCACGCCAATG
ATGCCCAACAAACCGACAATGGTGATTTGGTATCGAAACCCATTATTAAATGACAGCGGC
GTATTCACATCGCTGAATATTAACCTGACGCCCTACTTACTCTATACCACGCGCCAGGAT
GATTTTAACGGCATCGCGCTGATTGTCGGCAATACCGCGCTGTCGACCTTTTCTTCGCGT
CTGCTGACCGTCGCGGAGCTGCCGGGCACGCCTTCACGACAGGCAACTATCAACGGCCTG
CCGCTTAAAATACAGCTTTATGCCGATAGCTGGACCTACAATGACTTGTGGTATGCGTTG
ATGCTGGGCTGCATTAGCGGCATCGTAGCGGGGTTTATCTGTTACTTTATCTATGCCCTC
CGGACGCGACCGGGCAAAGAGATTTTAACGGCGATCAAACACGAGCAGTTTTATGTGGTT
TATCAGCCCGTCGTCGATACTCGTACGCTGAGCGTCACCGGGCTTGAAGTCTTACTGCGC
TGGCGTCACCCTACGGCAGGAGAGATTCCGCCGGATGCCTTTATCCACTATGCCGAAGCG
CAGCAACTTATCGTACCGTTAACGCAGCATCTGTTCGAGCTGATCGCCCGCGACGCGCCC
ACGTTACAGAAGGTGATGCCCGTCGGTGCCAAATTTGGCATTAACATCGCGCCGGAACAT
CTGCACAGCGACAGTTTTAAAGAGGATATGCACCGGCTCAGGGACGCCCTGCCCGCGCAC
CATTTTCATATCGTGCTGGAAATCACTGAACGCGATATGCTCCAGCAGTATGAAGCGGCC
AAACTGTTCGAATGGCTGCATTCCGCCGGGTTTGAAATCGCTATAGATGATTTCGGTACA
GGCCATAGCGCCCTGATTTATCTGGAGCGATTTACTTTAGATTATCTGAAAATCGATCGC
GGTTTTATCCAGGCGATCGGCACGGAAACCGTCACGTCTCCGGTTCTGGATACCGTGCTG
ACGCTCTCCAGACGCCTGAATATGCTGACGGTGGCCGAGGGCGTTGAAACGCCGGAGCAG
GCCCGTTGGCTACGCGATCACGGTGTTAATTACTTGCAGGGGTACTGGATTAGTCGTCCT
TTACCCCTACAAGATTTCGTGCTGTGGATGTCGGCGCCTTCTGTGCCTGAATGGTAG
>EECEBICD_01138 Murein DD-endopeptidase MepS/Murein LD-carboxypeptidase
ATGGTCAAATCTCAACCGATTTTGAGATATATTTTGCGCGGGATTCCTGCGATTGCAGTT
GCGGTTCTGCTTTCTGCTTGTAGCACAACCACCAATACCGCAAAGAATATGCATTCTGAG
ACGCATGCTGTGGGCAATAGCGATAGCTCTTCACTGCAAGCCTCTCAGGATGAATTTGAA
AATATGGTGCGTAACCTCGACGTTAAGTCGCGGATTATGGATCAGTATGCTGACTGGAAA
GGCGTGCGTTACCGCCTGGGCGGCAGTACCAAGAAAGGCGTCGACTGTTCCAGCTTTGTA
CAGCGCACCTTCCGTGAACAGTTTGGTTTAGAGCTTCCGCGTTCAACCTATGAACAGCAG
GAAATGGGCAAAGCAGTTTCACGCAATAACCTGCGTACGGGCGATCTGGTTCTGTTCCGC
GCCGGTTCCACTGGTCGTCATGTCGGTATTTATATCGGCAATAACCAGTTTGTCCATGCG
TCTACCAGTAGCGGCGTCATTATCTCCAGTATGAACGAACCGTACTGGAAAAAACGCTAC
AATGAAGCGCGTCGAGTTCTGAGCCGCAGTTAA
>EECEBICD_01139 Lipid A 1-diphosphate synthase
ATGACGATGAAAACCCGCTATTCCTTGATAATTCTGCTTAATGCCGCCGGTCTGGCGCTG
TTTTTGTCATGGTATCTTCCCGTCAATCACGGTTTCTGGTTCACCATTGATTCCGGCATA
TTCCACTTCTTTAACCAGAAACTGGTGGAAAGCCACGCGTTCCTCTGGTGGGTCGCTATT
ACCAATAATCGGGCGTTTGACGGCTGTTCGCTGCTGGCGATGGGCGGACTGATGCTCAGT
TTCTGGCTAAAAGAAGACGCGTCGGGCCGGCGGCGCATTGTGATAATCGGATTGGTCATG
CTGCTAACAGCGGTGGTGCTTAACCAACTCGGTCAGGCGCTGATCCCCGTCAAGCGCGCC

AGCCCAACGCTCTCTTTTGAGCATATTTATCGCGTCAGCGAGTTGCTGCACATCCCGACA
AAAGACGCGTCAAAAGACAGTTTCCCAGGCGATCACGGTATGATGCTGCTGATATTTTCC
GCCTTTATGCTGCGTTATTTCGGCAAAACGGCGGGGATTATCGCCCTTATTATATTTGTG
GTTTTCGCGTTTCCGCGCGTTATGATTGGCGCTCACTGGTTCACCGATATTGTGGTCGGG
TCGCTCACGGTTATTTTGATTGGCCTCCCCTGGTGGCTGATGACCCCGCTCAGCGACCGG
GCGATAGCGCTTTTTGAGAATTATCTCCCGGGCGGAAATAAACAAATTTTAAACAAATAA
>EECEBICD_01140 Zinc-binding GTPase YeiR
GTGACCAAAACCAATCTTATTACTGGATTTCTCGGTAGCGGAAAAACCACCTCTATCCTT
CATTTATTAGCTCATAAAGATCCGGCTGAAAAGTGGGCCGTCCTGGTTAATGAATTTGGT
GAAGTGGGTATTGACGGCGCGCTGCTTGCCGACAGCGGCGCACTGCTAAAAGAGATCCCC
GGCGGCTGCATGTGCTGCGTCAATGGATTGCCTATGCAGGTGGGGCTCAACACGCTGCTG
CGCCAGGGCAAACCTGACCGGTTGCTGATTGAACCAACCGGACTGGGGCACCCAAAACAG
ATTCTGGATTTATTAACTGCGCCGGTTTATGAGCCGTGGATTGATTTACGCGCTACGCTC
TGCATCCTTGACCCTCGCCTGCTACTGGACCAACAGAACGTCGCCAATGAAAATTTCCGC
GATCAGCTCGCCTCAGCCGATATTATCATCGCCAATAAGACCGATCGCGCCACGGCGCAG
AGCGATGCCGCCCTGCAACAGTGGTGGCGACAGTACGGCGGCGATCGTCAACTGATTCAT
GCCGAACATGGACAGATAGACGGTAAGCTTCTGGATTTACCGCGACAAAATCTGGCGGAA
CTGCCGGCCAGCGCCGCGCATTCTCACACTCATGCCAGTAAAAAAGGACTCGCCGCGCTA
AATCTGCCCGCCCAGCAGCGCTGGCGACGCAGCCTTAATAGCGGACAGGGTCATCAGGCC
TGCGGCTGGATTTTCGATGCCGATACCGTGTTTGACACCATTGGCCTCCTCGAATGGGCT
CGTCTGGCGCCGGTGGGCCGGGTGAAAGGCGTTATGCGCATACAAGAGGGGCTGGTACGC
ATCAATCGCCAGGGCGATGACCTGCACATCGAAACACAGAGTGTCGCGCCGCCGGATAGC
CGGGTTGAACTTATCTCAAACACAGAAACCGACTGGAATACGTTACAGACGGCCTTGTTG
AAGCTTCGTTTAGCGACGCACGCGTAA
>EECEBICD_01141 Elongation factor P-like protein
ATGCCAAGAGCGAACGAAATCAAAAAAGGTATGGTACTGAACTACAACGGCAAATTGCTG
ATTGTGAAAGATATCGACATTCAGTCGCCTACCGCCCGTGGCGCCGCCACACTGTATAAA
ATGCGTTTTTCCGATGTCCGCACCGGTCTGAAAGTGGAAGAACGTTTCAAAGGCGACGAT
ATCGTTGATACCGTCACCCTGAGCCGTCGCGGCGTTGACTTTTCTTATGTCGATGGCAAC
GAATATGTGTTCATGGATAAAGAAGACTATACGCCGTATACCTTCACCAAAGATCAGATT
GAAGAAGAGTTGCTGTTTATGCCTGAAGGCGGAATGCCGGATATGCAGGTTCTGACCTGG
GACGGCCAACTGCTGGCGCTAGAGCTTCCGCAGACCGTGGATCTGGAAATCGTCGAAACC
GCGCCGGGTATTAAAGGAGCGTCCGCCAGCGCACGTAACAAACCCGCGACATTAAGCACC
GGTCTGGTGATTCAGGTTCCCGAATACCTGAGCGCAGGAGAGAAAATCCGCATTCATATC
GAAGAGCGCCGTTATATGGGGCGCGCGGATTAA
>EECEBICD_01142 hypothetical protein
ATGGACTGTCGTAGCGGTTGTGGTGCTTGCTGTATTGCGCCTTCAATTTCGTCGCCGATA
CCCGGAATGCCGAATGGCAAGCCGATGAATACCAGATGCGTGCAGCTTTCGGAAGATAAC
TTGTGCCTTATCTTTGGCTCGCCTTTACGCCCAAAAGTGTGCTCTGGTTTGCAACCGACA
GGGGACATGTGTTTAACGACCCGGGAAGAGGCCATTATTTATTTGCTGGAACTCGAACAG
AAAACCTTACCATGA
>EECEBICD_01143 hypothetical protein
ATGCGGGGAGAAAATAGTGAAATTGATTTAGTCGACGTTTCCTTATTCCTTATAAGAAAA
TGGCTAAGTATCGTGATCGTTACCTTTATTTTTGTCGCCATTGGCTATGTTTTTGTGAGC
GTGCAGCATGATACAAAGGTTGTGAGTATTACAATCAAACCTAATCTGGATACGCCTGCT
ACCTATGCGTTATGTGGCTATACTAATGATATTCAATGTAAAACAACGGTTATCCTTAAC
CAGTTTAGCCGTTTCTTGCCTGCGGAATATAAAAATAAAATAAGTTTTGACGCGAAAAAT
CAGCTTGTTCTTTTTAAGGCGCAGGGGAGGGAAGAAAGCGTCAATAATATTATTTCCACC
CTGAAAAATAGTGTCGATAAAATGGCGGCATGGTATATCGATGATGGTGACATTAAACGT
TATAGCGTGCATAAAAATATGTTACAAACGGAAACATATGCAAAGCTATCCTTGTTGTCG
GAGGCGGTTAAAAATAATGCTAACATTGAAGTGATAGATGTTGCTGTCACACCAAAATAT
AAGATGCGGCTGGTGTTGGTTCTTTGCGGACTGATGGGCGTAATTTTCTCAATAACCGGT
CTTTTATGTAGGAGGGCGCTTACAGAATATCGTTTTGAGAAAAAGACAATGAATGACCGC
GCATGA

>EECEBICD_01144 Sugar efflux transporter B
ATGTGGGGAACTAACAGCCTGTATATCATTAATATGCCGCTGTTTATCATCAATGAGCTG
CATCTTCCGGAAAAACTTGCAGGCGTGATGATGGGCACCGCCGCCGGGCTGGAGATTCCC
ACAATGCTGATTGCAGGTTACTTCGCCAAACGGTTGGGAAAACGCCTGTTAATGTGCATC
GCCGTTGTCGCCGGACTGTGCTTTTATGCGGGGATGTTGCTGGCGCACGCGCCGGCAACG
TTACTCGGCTTGCAGTTGCTGAACGCGATTTACATCGGCATTCTTGGCGGGATTGGCATG
CTCTATTTTCAGGATCTCATGCCAGGCCAGGCGGGTTCAGCCACCACGCTGTATACCAAT
ACCATTCGCGTCGGCTGGATCATTGCCGGTTCGCTGGCGGGAATCGCCGCAGAAATCTGG
AATTATCATGCGGTCTTCTGGTTTGCGTTAGTCATGATTGTCGCCACCATGTTCTGCCTG
GCGCGTATTAAAGATGTTTAA
>EECEBICD_01145 Sugar efflux transporter B
ATGCACAACAGTCCCGCCGCCGCTGCGCCTAAATCGTTTGACCTGACGTCTACCGCCTTT
TTGATCGTCGCTTTTCTCACCGGTATTGCCGGTGCGCTACAGACGCCGACGCTCAGTATT
TTTCTGACGGATGAGGTTCACGCCCGCCCCGGCATGGTGGGATTCTTTTTTACCGGCAGC
GCGGTTATCGGCATTATCGTGAGCCAGTTTCTGGCCGGTCGCTCAGATAAAAAAGGCGAT
CGCAAAAAACTGATCGTTTTCTGTTGCGTGCTGGGCATGTTGGCCTGCGTGCTGTTTGCC
TGGAACCGCAACTACTTTATTTTGCTGTTTATCGGCGTCTTTCTCAGTAGCTTCGGCTCT
ACCGCCAACCCGCAGATGTTTGCGCTCGCCCGTGAACATGCCGATCGTACAGGTCGTGAA
GCGGTGATGTTCAGCTCAATCCTCCGCGCCCAGGTCTCATTGGCCTGGGTCATTGGGCCG
CCGCTGGCCTATGCGCTGGCGATGGGTTTTAGCTTTACGGTGATGTATTTGAGCGCGGCG
GTAGCTTTTATCGTCTGCGGCGTCATGGTGTGGTTCTTTTTACCGTCAATGCGCAAAAGA
TGCGCCGCTGGCAACCGGCACGCTGGAAGCACCGCGTCGTAA
>EECEBICD_01146 hypothetical protein
ATGTGCTTCACCTCAAAAAATTGCTACAAAACCGGTAACTTTTCTTGCGTGCTGAAGCAG
AAAGATCCAGACTTCTTGAAACGTTTCAGCGCGATCTGGTTTTTTTGA
>EECEBICD_01147 Multiphosphoryl transfer protein
ATGTTCCAGTTATCGGTTCAGGACATTCATCCGGGCGAGCAGGCCGGAAATAAAGAAGAG
GCCATTCGGCAAATCGCCGCCGCGTTAGCGCAGGCGGGGAATGTGGCGGGCGGCTATGTT
GACGGTATGCTGGCGCGCGAACAGCAAACCTCGACCTTTCTTGGCAATGGTATTGCGATT
CCCCATGGCACAACAGACACGCGCGATCAGGTGCTGAAAACCGGCGTTCAGGTCTTTCAA
TTTCCGCAGGGCGTTACCTGGGGAGAAGGCCAGGTCGCCTACGTGGCGATTGGTATTGCC
GCCAGTTCGGACGAACACCTCGGCCTGCTGCGTCAGCTGACGCATGTGCTGAGCGATGAC
TCCGTGGCTGAACAGCTGAAATCCGCGATAACGGCGGAAGAGCTGCGCGCATTGCTGATG
GGCGAGAAGCAAAGTGAACAATTAAAACTTGATAACGAGACGATGACGCTGGATGTCATC
GCCAGCTCGCTGGTCACATTGCAGGCGCTAAATGCGGCGCGTCTGAAAGAGGCGGGAGCG
GTTGATGCGGCGTTTGTCGCAAAAACGATTAACGATAGTCCGATGAATCTGGGACAAGGC
ATCTGGCTGAACGACAGCGCGGAAGGCAATTTGCGCAGCGCTGTTGCGGTAAGCCGTGCG
ACCCAGGCGTTTGACGTAGAGGGTGAAAAAGCAGCCTTATTAGTCACTGTCGCGATGAAC
GACGAGCAGCCGATTGCCGTGTTAAAACGCCTCGGCGACCTGTTGCTGAACAATAAAGCC
GATCGTCTGCTGAGCGCCGACGCCGCGACGCTGCTGGCGTTGCTGACCAGTGATGATGCG
CTGACGGATGACGTACTGAGCGCGGAATTTGTAGTGCGTAATGAGCATGGTCTCCATGCC
CGTCCGGGCACCATGCTGGTGAATACGATTAAACAATTTAACAGTGAAATTACCGTGACA
AACCTTGATGGAACCGGCAAACCGGCAAACGGACGTAGTCTGATGAAAGTGGTGGCGTTA
GGCGTGAAGAAAGGCCATCGTCTGCGCTTTACCGCGCAGGGTGAAGATGCTGAACAGGCG
CTGAAAGCGATTGGCGATGCGATCGCCGCCGGTCTCGGGGAGGGCGCATAA
>EECEBICD_01148 Tagatose-6-phosphate kinase
ATGAGCAGGCGTGTCGCCACCATTACCCTTAATCCGGCTTACGATCTGGTTGGGTTTTGC
CCTGAAATCGAACGCGGCGAAGTGAACCTGGTGAAAACCACGGGTCTGCACGCGGCGGGC
AAAGGCATTAACGTTGCCAAGGTGCTAAAAGACTTAGGCATCGACGTCACCGTCGGCGGT
TTTCTCGGTAAAGATAACCAGGACGGTTTTCAGCAATTGTTCAGCGAACTGGGCATCGCT
AACCGCTTTCAGGTGGTTCAGGGCCGGACCCGCATCAACGTGAAGCTGACGGAAAAAGAT
GGCGAAGTGACCGACTTCAACTTTTCCGGCTTTGACGTGACCCCCGCAGACTGGGAACGC
TTTGTTAACGACTCCCTGAGCTGGCTGGGCCAGTTCGATATGGTCTGCGTCAGCGGTAGC
TTACCGGCTGGCGTGAGTCCGGAAGCGTTCACCGACTGGATGACGCGTCTGCGCAGCCAG

TGTCCATGCATTATCTTTGATAGTAGCCGTGAAGCGTTAGTCGCCGGTCTTAAAGCTGCG
CCGTGGCTGGTGAAACCGAATCGCCGCGAACTGGAAATTTGGGCGGGTCGTAAGCTCCCG
GAAATGAAAGATGTAATTGATGCGGCGCACGCGTTACGCGAACAGGGTATCGCTCATGTG
GTGATTTCGCTGGGGGCGGAAGGCGCGCTGTGGGTTAACGCCTCAGGAGAATGGATCGCT
AAACCACCGGCAGTTGACGTGGTAAGTACCGTCGGCGCAGGCGATTCCATGGTTGGCGGA
TTGATTTACGGCCTGCTGATGCGCGAGTCCAGCGAACATACGCTGCGTCTGGCGACGGCG
GTTGCCGCGCTGGCGGTCAGCCAGAGCAATGTGGGTATTACCGATCGTCCTCAGTTGGCC
GCAATGATGGCGCGTGTCGACTTACAACCGTTTAACTGA
>EECEBICD_01149 PTS system fructose-specific EIIB'BC component
ATGAAAACGCTGCTGATAATTGACGCTAATCTCGGCCAGGCTCGCGCTTACATGGCGAAA
ACCCTGCTTGGAGCGGCGGCGCATAAAGCAAATCTGGAAATTATCGACAATCCGAATGAT
GCTGAGCTGGCGATCGTGCTGGGCGAATCTCTGCCGAACGACAATGCTCTGAACGGCAAA
AAGGTTTGGCTGGGGGATATTGGTCGCGCCGTCGCGCATCCGGAACTGTTTCTGAGTGAA
GCCAAAAGCCATGCGACCCTTTACAGCGCCCCTGCTGCGGCTGCTCCTGCGGCGAGCGGC
GGTCCGAAACGCGTGGTCGCGGTAACGGCCTGCCCGACCGGCGTCGCCCATACTTTTATG
GCGGCTGAAGCCATTGAAACAGAAGCGAAAAAACGCGGCTGGTGGGTAAAAGTCGAAACG
CGCGGCTCCGTGGGGGCCGGCAATGCCATCACCCCGGAAGAGGTGGCGGAAGCGGATTTA
GTGATTGTGGCGGCGGATATCGAAGTGGATCTGGCGAAGTTTGCCGGTCTCCCGATGTAC
CGCACGTCGACCGGCCTGGCGCTGAAAAAGACGGCGCAGGAGCTGGATAAAGCCGTAGCG
GAAGCGACGCCGTATCAACCGGCGGGTAAGGCATCGCAAGCGGCGACCGAAGGGAAGAAA
GAGAGCGCTGGCGCATACCGGCATCTGTTGACGGGCGTTTCTTACATGCTGCCTATGGTG
GTTGCCGGGGGACTGTGTATTGCGCTTTCCTTCGCCTTTGGTATTGAAGCCTTTAAGGTG
CCGGGCACGCTGGCGGCGGCGCTGATGCAGATTGGCGGCGGTTCGGCGTTTGCGCTGATG
GTGCCGGTACTGGCGGGTTATATCGCTTTCTCCATCGCGGATCGCCCAGGCCTTACGCCA
GGTCTGATTGGCGGTATGCTGGCGGTTAGCACCGGTTCTGGTTTTATCGGCGGGATCATT
GCCGGCTTCCTTGCCGGGTATATGGCGAAGCTTATCAGTACCAAACTGAAACTTCCGCAA
AGTATGGAAGCGCTGAAGCCAATCCTGATCATCCCGTTAATTTCCAGTCTGGTGGTGGGG
CTGGCGATGATTTACCTGATCGGTAAACCGGTTGCCGGGATTCTGGAAGGGCTCACCCAC
TGGCTGCAAACCATGGGGACCGCAAACGCGGTGCTGCTGGGCGCGATTCTCGGCGGGATG
ATGTGTACCGACATGGGTGGCCCGGTGAACAAAGCGGCGTATGCGTTTGGCGTTGGTCTG
CTGAGTACGCAAACTTACGCGCCGATGGCGGCGATCATGGCCGCAGGTATGGTACCGCCG
TTAGCGCTGGGTCTGGCAACGATGGTGGCGCGTCGTAAGTTCGACAAAGCGCAGCAGGAA
GGCGGTAAAGCGGCGTTGGTACTGGGGCTGTGCTTTATCACTGAAGGCGCTATTCCGTTT
GCGGCGCGCGACCCGATGCGTGTACTGCCGTGCTGTATCGTTGGCGGCGCGTTGACCGGG
GCTATTTCTATGGCGGTAGGCGCAAAATTGATGGCGCCGCATGGCGGTCTGTTTGTTCTG
CTTATCCCAGGCGCAATTACGCCGGTATTGGGATACCTGCTGGCAATTGTGGCCGGTACG
CTGGTGGCAGGACTGGCTTATGCCGTCCTGAAACGTCCGGAGACGGAAGTCGCGGCAAAA
GCGGCATAA
>EECEBICD_01150 Endonuclease 4
ATGAAATACATCGGAGCGCACGTCAGCGCTGCTGGCGGTCTGGCTAACGCCCCTGCCCGC
GCGGCTGAAATTGGCGCAACGGCCTTTGCGCTTTTCACAAAAAACCAGCGTCAGTGGCGT
GCCGCCCCCCTTACTCCCCAGGTCATTGATGACTTTAAAATCGCCTGTGAAAAGTATCAT
TTCTCGGCGGCGCAAATTCTTCCCCACGATAGTTACCTGATTAATCTGGGCCATCCGGTC
AGTGAAGCGCTGGAAAAATCACGCGATGCCTTTCTCGATGAAATGCAGCGCTGTGAACAA
CTCGGCTTAACCTTGCTTAATTTTCATCCCGGTAGCCATCTGATGCAGATTGCACAGGAG
GATTGCCTGGCGCGGATCGCGGAATCCATCAATATTGCCCTCGCGCAGACCGAGGGCGTT
ACGGCGGTTATCGAAAATACAGCCGGTCAGGGCAGTAATCTGGGGTTTGAGTTTGAACAG
TTAGCCGCCATCATCGACGGCGTGGAAGATAAGTCGCGCGTTGGCGTCTGTATCGATACC
TGCCATGCCTTTGCCGCCGGATACGATCTGCGTACGCCAGAGGCGTGCGAAAAAACATTC
TCCGAATTCGGGAAAATTGTCGGATTTCAGTATTTGCGCGGAATGCACCTGAACGACGCC
AAAAGCGCCTTCGGTAGCCGCGTTGACCGCCATCACAGTCTGGGTGAAGGCAATATCGGC
CACGATGCGTTTCGTTGGATTATGCAGGATGGGCGTTTTGACGGTATTCCGCTGATACTG
GAGACCATCAATCCTGATATCTGGGCGGAAGAGATTGCGTGGTTAAAAGCCCAGCAAATT
GCCGAAGCGATGGCCTGA

>EECEBICD_01151 hypothetical protein
ATGACAGAACTCACCTTGCAGAATCATCGTCGTACAATGTGGCATTTTATACCGGGGCTC
GCTCTGAGCGCCGTCATTACAGGAGTCGCCCTGTGGGGCGGCGCTATCCCTGCCGTTGCA
GGCGCGGGGTTCAGCGCCCTGACCCTGGCTATCCTGCTGGGTATGGTTATCGGGAATACC
GTCTATCCACAAATATGGAAACAATGCGACGGCGGCGTGCTGTTTGCAAAACAACATCTG
CTGCGTTTAGGGATTATCCTTTACGGCTTCCGCCTTACCTTTTCACAAATCGCCGACGTG
GGTATTAGCGGTATTGTGATTGACGTATTAACGCTTTCCAGCACGTTCATGCTGGCCTGC
TTCTTAGGCCAAAAAGTCTTTGGTCTGGACAGGCATACCAGCTGGCTGATTGGCGCCGGT
AGCAGTATCTGCGGCGCGGCGGCGGTACTGGCGACAGAACCGGTGGTAAAAGCGGAAGCC
AGTAAGGTTACTGTAGCCGTCGCGACTGTCGTTATTTTCGGCACCATCGCTATTTTCCTT
TACCCGGCAATGTATCCGCTGCTGGCGCACTGGTTCAGCCCGGAAACTTACGGCATCTAT
ATCGGCTCAACCATGCATGAAGTCGCGCAAGTGGTCGCAGCGGGTCATGCCGTTAGCCCG
GATGCGGAAAATGCGGCAGTTATCGCTAAGATGCTGCGTGTCATGATGCTGGCGCCGTTC
CTGATCATTCTGGCGGCACGCGTTAAACAGCTTTCACCGGCGACCGGCGCGGAAAAAAGC
AAAATTACCATTCCGTGGTTTGCTATTTTCTTCATCGTAGTGGCGATCTTCAACTCTTTC
CACCTGCTGCCGAAAGCCGTGGTGGATATGCTGGTGACGCTGGACACAGTGCTGCTGGCG
ATGGCGATGGCGGCGTTGGGTCTGACCACTCACGTTAGCGCATTGAAAAAAGCGGGGGCG
AAACCGCTGCTGATGGCGCTGGCACTATTCGCCTGGCTGATTATTGGCGGTGGCGCCATT
AACGTCCTAATCCATAGCCTTATCGCATAA
>EECEBICD_01152 HTH-type transcriptional activator CmpR
ATGCATATTACGCTACGACAACTTGAAGTGTTTGCTGAAGTACTGAAAAGCGGTTCAACA
ACCCAGGCGTCGGTCATGCTGTCATTATCGCAGTCCGCCGTTAGCGCCGCGCTCACCGAT
CTGGAAGGTCAGCTCGGCGTACAGTTGTTTGATCGGGTGGGGAAACGGCTGGTGGTCAAC
GAACATGGGCGCTTGTTATACCCGCGCGCGCTGGCATTGCTGGAGCAAGCGGTAGAAATT
GAGCAGCTATTTCGTGAAGATAACGGCGCGATACGGGTTTATGCCAGTAGCACCATCGGC
AATTACATTTTACCCGCCATGATTGCCCGCTATCGCCAGCATTATCCGGCCTTGCCGCTG
GAACTCAGCGTGGGAAACAGCCAGGACGTGATTAACGCGGTGTTGGATTTTCGGGTTGAT
ATCGGCCTGATTGAAGGGCCGTGTCACAGTACAGAGATTATCTCTGAGCCGTGGCTGGAA
GATGAGCTGGTGGTCTTTGCCGCGCCGTCCTCGCCGCTGACGCAGGGGCCGGTGACGCTG
GAACAGCTTGCGGCATCCCCATGGATTTTACGCGAGCGCGGTTCCGGTACGCGGGAAATT
GTCGATTATTTGCTGTTGTCGCATTTGCCGCGCTTTCATATGGCGATGGAGTTGGGGAAT
TCAGAAGCGATCAAACATGCGGTGCGTCATGGACTGGGGATAAGCTGTTTATCGCGCCGG
GTGATTGCTGAACAACTCCAGGCGGGAACCCTGGGCGAAGTCGCGGTGCCGTTACCGCGC
CTGGTGCGCACGTTGTGGCGTGTTCATCATCGGCAGAAACATCTTTCTAACGCGTTGCAG
CGTTTTTTGAGTTACTGTGAATAA
>EECEBICD_01153 Lysine-specific permease
ATGGGTTCCAAAACTAAAACCACAGAAGCGCCCGCGCTACGTCGCGAATTAAAGGCGCGT
CACCTGACGATGATTGCCATTGGCGGCTCCATCGGGACAGGTCTTTTCGTTGCATCTGGT
GCGACAATTTCTCAGGCGGGGCCAGGCGGCGCGCTGTTTTCTTATATTCTGATTGGTTTG
ATGGTCTACTTCCTGATGACCAGTCTTGGCGAGCTGGCGGCCTATATGCCAGTATCTGGC
TCGTTTGCGACATATGGTCAGAACTATGTGGAAGAAGGCTTTGGTTTCGCGCTGGGCTGG
AACTACTGGTACAACTGGGCGGTGACTATCGCTGTTGACCTGGTTGCCGCGCAATTGGTG
ATGGGCTGGTGGTTCCCGGACACGCCGGGCTGGATCTGGAGCGCGCTATTCCTCTGCGTC
ATTTTCTTGCTGAACTACATTTCTGTACGCGGTTTTGGCGAGGCGGAGTACTGGTTCTCG
TTAATTAAAGTCGCGACCGTTATTATCTTCATCATCGTTGGCGTAGCGATGATTATCGGT
ATCTTTAAAGGTGTCGAGCCGGTCGGCTGGAGCAACTGGACGACGGGCGATGCGCCTTTC
GCTGGCGGTTTTGCGGCAATGATTGGCGTCGCGATGATCGTGGGCTTCTCTTTCCAGGGA
ACTGAGCTGATTGGTATTGCCGCCGGGGAATCTGAAAACCCGGAGAAGAATATTCCACGC
GCGGTGCGTCAGGTGTTCTGGCGTATTCTGCTGTTTTATGTTTTCGCGATTTTGATTATC
AGCCTGATCATTCCTTATACCGATCCAAACCTGCTGCGTAACGACGTCAAAGACATTAGC
GTCAGTCCGTTTACCTTAGTGTTCCAGCATGCGGGCTTGTTGTCTGCGGCGGCGATAATG
AATGCGGTTATCCTGACGGCGGTACTGTCTGCGGGTAACTCCGGGATGTATGCCTCGACC
CGTATGCTCTACACCCTGGCCTGTGACGGTAAGGCCCCGCGTATTTTTGCGAAATTGTCC
CGCGGCGGCGTACCGCGCAATGCGCTTTATGCCACCACCGTGATTGCCGGTCTTTGCTTT

TTGACCTCAATGTTTGGCAACCAGACGGTCTATCTGTGGCTGTTGAACACCTCCGGTATG
ACGGGCTTCATCGCCTGGCTGGGGATTGCCATTAGCCATTATCGCTTCCGTCGCGGTTAC
GTGTTACAAGGGTATGATGTGAATGATTTGCCGTACCGTTCCGGGTTCTTCCCGCTGGGG
CCGATTTTCGCCTTTGTACTGTGCCTGATTATTACGTTGGGGCAGAACTATGAAGCGTTC
CTGAAAGATACCATCGACTGGGGCGGCGTCGCGGCGACCTATATCGGTATTCCGCTGTTC
CTGCTTATCTGGTTCGGCTACAAGCTGATAAAAGGTACGCATTTCGTGCGCTATAGCGAA
ATGCACTTCCCGGAACGCGTTAAAAAGTAA
>EECEBICD_01154 Colicin I receptor
ATGTTTAGGTTTAACCCTTTCGTTCGGGTAGGGCTTTGTATGTCCGCAGTTACGTTGGCC
TGGCCGGTTGCCGCGGCAACGGATGATGGTGAAACCATGGTCGTTACCGCGTCCGCCATC
GAACAAAATCTGAAAGATGCGCCAGCAAGTATCAGCGTCATTACCCAGCAAGATTTACAG
CGCAGGCCCGTGCAAAACCTGAAAGATGTCCTGAAAGAGGTGCCGGGCGTACAGCTCACT
AACGAAGGGGATAACCGTAAAGGCGTGAGTATTCGTGGTCTGGACAGCAGTTATACGCTG
ATCCTGATCGACGGTAAGCGCGTTAACTCCCGTAACGCGGTCTTCCGCCATAATGATTTT
GATCTGAACTGGATACCGGTTGACGCTATCGAACGCATTGAAGTGGTGCGTGGCCCCATG
TCCTCGCTGTATGGTTCCGATGCGCTGGGCGGCGTGGTCAACATTATCACCAAAAAAATT
GGTCAGAAATGGCACGGCAGCGTCACGGTTGACTCCACTATTCAGGAACATCGCGACCGT
GGCGATACTTATAACGGTCAGTTCTTTACCAGCGGTCCGCTGATCGACGGCGTGCTGGGA
ATGAAAGCCTACGGTAGCCTGGCGAAACGCGAAAAAGACGAGCAGCAGAGTTCCGCCACC
ACCGCTACGGGTGAAACGCCGCGCATTGAAGGATTTACCAGCCGGGATGGAAACGTCGAG
TTTGCCTGGACACCAAACGAAAATCATGACGTTACCGCAGGCTACGGTTTTGATCGCCAG
GACCGGGACTCCGACTCGCTGGATAAAAACCGTCTGGAACGGCAAAACTATGCGTTGAGC
CATAACGGACGTTGGGATCTCGGCAATAGTGAGCTCAAATTCTACGGCGAAAAAGTGGAA
AACAAAAACCCCGGCAACAGTAGCCCTATCACGTCAGAAAGCAATTCGATTGACGGGAAA
TACGTTCTGCCGTTAGCGGCTGTCAACCAGTTCCTTACCTTCGGCGGCGAATGGCGTCAC
GATAAACTGAGCGATGCGGTCAACCTGACCGGCGGTTCCAGTACTAAAACCTCCGCCAGC
CAGTATGCTCTGTTCCTTGAAGATGAGTGGCGCATCTTCGAACCGCTGGCGCTGACCACC
GGTATTCGTATGGATGATCATGAAACCTATGGCGATCACTGGAGCCCGCGTGCTTATCTG
GTCTATAACGCAACGGATACCCTGACGGTGAAAGGTGGGTGGGCCACGGCCTTTAAAGCG
CCGTCGCTATTGCAATTAAGTCCGGACTGGGCGACCAACTCCTGCCGTGGCGGATGCCGC
ATTGTCGGTAGCCCGGATCTGAAACCTGAAACCAGTGAAAGCTGGGAGCTCGGCCTTTAT
TACCGGGGCGAAGAAGGCATACTGGAGGGCGTGGAAGCGAGCGTTACCACGTTCCGTAAC
GATGTGGACAATCGCATCAGCATTAGTCGTACCCCTGACGTTAACGCCGCGCCCGGCTAT
TCCAACTTTGTCGGTTTCGAAACCAACAGTCGGGGACAGCGCGTTCCGGTCTTCCGTTAT
TACAACGTGAATAAAGCGCGTATTCAGGGCGTGGAAACCGAGTTGAAAGTTCCGTTTAAC
GAGGCGTGGAAACTGTCGTTGAATTACACCTATAACGACGGACGCGACGTCAGTAATGGC
GGGAACAAGCCGCTTTCCGATCTGCCGTTCCACACCGCGAACGGTACGCTGGACTGGAAA
CCGGCGCAGCTCGAAGACTGGTCATTCTACGTATCAGGGAAGTATACCGGGCGTAAGCGG
GCCGACAGCGCAACAGCGAAAACGCCGGGCGGGTACGTGGTCTGGGATACCGGCGCGGCA
TGGCAGGCGACGAAAAACGTTAAACTGCGTGCGGGCGTTCTGAACGTGGGCGATAAAGAT
CTCAAACGCGACGACTACGGTTATACCGAAGACGGACGTCGTTACTTTATGGCGGTGGAT
TACCGTTTCTGA
>EECEBICD_01155 Membrane sensor protein UhpC
ATGAGTATAAAAACATTTATTTTCAGCCAGCCCGATAAGCCGATTGTAAAAGAGAAAAAA
GAGATAGACCAGACCTATAAAAAATTTCGTTTTGAGATTATCGCCTCGGTCTTTATCTCT
TATGCCGTCTTTTATCTTACCCGTAAAAACTTCTCCGCCGCGATGCCGGCCATGCTGACG
GAAACGTCGCTGACCGCAGAAGACTTCGCCATTATGTCTTCTATTTTTTACATTCTTTAT
GGCGCGATGAAGTTTGTCGGCGGGATGCTGGTCGATAAAATTAACCCGAAAGCCATGACC
GGCCCGGTGCTGATTGGCGTGGGGATTGTAAATATTCTGTTCGGCTTTTCCGATAGTGTG
GCGGCATTCTACGTACTGTACAGCCTCAACGCGATCTTACAGGGCACCAGCTTTCCGCCG
ATGGCGAAAATTATGGCCTCGTGGTTTTCGAAAAACGAACGGGGACGCTGGTGGGCTATC
GTTGAAGCGGCGCACAATATCGGCGGCAGCCTCGCGCCGCTGCTGACCAGCTTTGCTATC
GCCTTTAGCGGTAGCTGGAAAATGGGGTTTTATGTTCCCGGCGCCATTTCGCTGCTGATG
GGGATAGTGGCGCTATTTACCATTAAAGATCGTCCCGGTACGTTAGGTTTGCCCAACGTG

GGGCAGTGGCGTAACGACCCGACAGAACTGGCCCAGGTCAAGGCCAGCCCGGTCAACCTG
AGCTTCTGGCAGATTTTTGTAAAATATATCCTGACCAATCCACTGGTATGGATCATTATT
ATCGGCGATATGTCAGTTTATATTGCGCGCACCATCCTTAACGACTGGCCGCAGATTTAC
TATTCGCAGGTTCACGGCTGGAGCCTGATAAAAGCGAACTCGATTATTTCCTGGTTTGAG
GCGGGCGGACTGGCAGGTGGGTTGCTGGCAGGCTACTTGTCTGACTTTATGTTCAAAAGT
AACCGCTGGATGACCGGATTAATCTTCGCGTTAGCGCTGTGCATATGCATCGTGCTGGTG
CCGCTGGTTCAGGATACCTCTTACACCCTCACCGCGATTCTGTTCACCATCATGGGCTTC
GCCTTATACGGACCGCATATGCTTTTTGCCGTCGGCTGTCTGGATGTGACCCATAAGGAT
GCGGCGGGATCGATTACCGGCTTTCGAGGATTGTTCAGCTATGTCGGCGCGGCAATGGCC
GGTGTGCCGGTAATTATGGTGAAAAATAGCTGGGCGTGGTCGGGCGTTTATATCTATGCG
GTGATCGCCATTCTGCTAACGACTCTGTCGCTGGCGCTGCTCTCCAGGCTGCATCGGTTA
TAA
>EECEBICD_01156 Phosphoserine phosphatase SerB2
ATGAGCATTATTTATTTTATTACTACACAGGATATCGATACGTTTCAGAAAAAATTACAG
GAAACGTTATTTAATGCCGTCACCATGCCGTTTCCGCTATTATTTGATAAACGTTATGCC
GCGCTTATCAATACCGCTTATTTAAAGCTTACGCTGCCAGCGGAATGTCTGACGCCTGAA
TTTTATCGCTATTTACGCGAACTCTCATTGCAATGGCAGTTTGATTTTTTTATTAAGCCG
CAGCCACTGCCTGCTAACGGTATTATCGCGTTTGATATGGACTCGACATTTATTGCGGAA
GAAGGCGTGGATGAAATTGCCCGCGAGCTTGGCATGTCGACGCAAATCACCGCCATTACC
CAACAGGCGATGGAGGGCAAGCTTGATTTTAACGCCAGTTTTACCCGACGTATCGGAATG
TTGAAAGGCACACCGAAAGCAGTGCTGAACGCCGTATGCGATCGCATGACCCTTTCGCCA
GGCCTGTTAACTATTCTGCCAGTCATCAAGGCCAAAGGATTTAAAACCGCGATTATCTCC
GGGGGACTGGATATATTCACCCAGCGTCTAAAAGCGCGGTATCAACTGGATTATGCCTTT
TCGAATACGGTTGAAATACGCGATAACGTTCTGACGGATAACATTACCTTGCCGATTATG
AATGCCGCAAACAAAAAACAGACTCTGGTTGACCTGGCTGCCCGGTTAAATATTGCCACG
GAGAATATTATTGCCTGCGGTGATGGCGCCAACGATCTGCCGATGTTAGAACATGCTGGC
ACCGGTATTGCCTGGAAAGCGAAGCCGGTTGTACGGGAAAAAATCCACCATCAGATTAAT
TATCACGGTTTCGAATTGCTTCTTTTTCTTATTGAAGATGAATTATAA
>EECEBICD_01157 L-serine dehydratase 1
ATGGAGAGTTGCCTTGATATATTCAAAATAGTAATAGGTCCATCCAGCTCACGTACCGTA
GGGCCGATGCGCGCCGCCTGTCACTTTATTTCATTGCTCAGAGAACAAGAGACGTTGCCG
CTAATTCGGGAAATTGAAATCGAGCTGTATGGCGCGTTATCGTTAAGCCGCAAGTGCCAC
AATGTGGATACCGCCCTTTATCTTGGGCTGCTGGGTTGCCAGCCGGAGAATGTCGATCTC
CGTTCGCATATGGCGGTGATTAAGCGTGCGGAAAATGAAAACAAAATCGAACTCCCGCTC
TCCGACGCGGGCGGAATCACGATAAAAGTCAAGATTATCGCCAATCACCAGGCGCATCCA
GGCCATCCTTATGCCATGACGTTTCGCGCCAGAGATGATTATTTCACGGTGTATGAAGAG
ACCTGGTTTTCGACCGGCGCCGGACAGGTTCGCAAACATGGCGAGCCGCTGACGCCATCG
CTTCCTTTGCGTACAGTGTCGCCCTTTGAATTTAGTCATGCCGCGCAGTTGCTGGCGCTG
TGCCGACGTAATGGCCTGTCCGTGGCGGCGCTGATGATGAAAAATGAGCTGTGCCGCCAC
TCGCCACAGACGTTGCAGAATTATCTGGCGCAAATATGGGACGTGATGCAGCAGGCGGTT
TATCGCGGCCTGCATACTGAGGGGGTATTACCCGGTCCCTATCAGGTACCGCGGCGCGCC
TGTGCGTTACATAAGACGTTACAGGCTAACCGCTCCGCCAGCGATTTTTTAACCGCGCTG
AACTGGGTCAATGCCTTCGCGATTGCGGTAAGCGAAGAAAATGCCAGCGGCGGACAGATT
GTTACCGCGCCAACTAATGGCGCGTGCGGCATCATTCCGGCAGCGCTCTGCTGGTATGAT
AAATTTGTCACGCCGCTGGAGCCCGGTGCTCTGACCCGTTTCTTCCTGACCGCCGCCGCT
ATCGCGATATTGTTTAAACAAAACGCCTCTATTCTGGGATCTGAAGTTGGCTGTCAGGGG
GAAATCGGCGTGGCGTGCTCAATGGCGGCGGCGGGCCTGGCTGAACTGATGGGCGCATCC
GTGGAGCAAACGCTGAGCGCCGCTGAAATTGCCATGGAACATCATCTGGGATTGACCTGC
GATCCGCTGGGCGGGCAGGTACAAATTCCCTGCATAGAGAGAAATGCCATCTCTGCCGTG
AAAGCGATAAACGCGGCGACGATGGCGATGAGTCGCGTAAGCGAACCCTGTATTTCGCTC
GATGAGATCATTGCTGCGATGTACGAGACCGGTAAAGACATGAGCGCTAAATACCGTGAA
ACGTATCATGGTTCGCTGGGGAAAATTCAGCCGCGCAAGAGAGGGTAA
>EECEBICD_01158 hypothetical protein
GTGAAATACAATACGATGAATAACGACGAGATTATCCTGTCGTTGTGCGCCCGCTTAAAG

GAGACGCGTCTGTCTCTTTCGATGACCCAGCAGCAGCTTGCCGATCGCGCGCACGTTGGC
ATCGCCACCATCAAGCGTATCGAGAAGGGGGAGGGCTCAATCTTGATACGCTAA
>EECEBICD_01159 S-formylglutathione hydrolase YeiG
ATGGAAATGCTCGAAGAGCACCGATGTTTTGGCGGCTGGCAGCAGCGCTGGCGTCATCAT
GCCGCCACGCTAAACTGCGCCATGACATTCAGCATTTTTCTTCCGCCGACTCAGGATAAT
GAACCGCCTCCGGTACTGTACTGGTTGTCCGGGTTGACCTGCAATGATGAGAACTTTACC
ACCAAAGCAGGCGCGCAGCGTATCGCAGCAGAACTGGGCATTGTATTGGTGATGCCGGAT
ACCAGTCCACGCGGTGAACAGGTCGCCGACGATAGCGGATACGATTTAGGCCACGGCGCC
GGGTTTTATCTCAATGCCACACAGCCGCCCTGGGCCAGCCATTATCGCATGTACGATTAC
CTGCGCGATGAGCTTCCGGCGCTTATCCAGACACAGTTTAACGTCAGCGACCGTTGCGCC
ATCAGCGGGCACTCGATGGGCGGCCACGGCGCATTAATCATGGCGCTGAAAAATCCGGGC
AAATACACCAGCGTCTCTGCTTTTGCGCCTATCGTCAACCCCAGCCGCGTGCCGTGGGGA
ATCAAAGCGCTTACCGCTTATCTCGGCGAAGATGAATCAGCCTGGACTGAGTGGGATAGC
TGCGAATTAATGCTGGCCAGCGAGCCGCAGGACGCCATTCCTGTGCTCATCGATCAGGGC
GACAGCGATCAATTCCTCGCCGATCAGCTTCAGCCCGCCGTACTGGCGGAAGCCGCGCGG
CAAACCGCCTGGCCGATGACGCTACGAATCCAGCCTGGTTACGATCATAGCTATTATTTT
ATTGCGTCCTTCATCGAGGACCATCTGCGCTTTCATGCCAGGTATTTACGGGACGAGCGT
GAGACGTCACCAACATAA
>EECEBICD_01160 GTP cyclohydrolase 1
ATGCCATCACTCAGTAAAGAAGCGGCCCTGGTTCATGACGCGCTGGTTGCCCGGGGGCTG
GAAACGCCGCTGCGCCCGCCTATGGATGAACTGGATAATGAAACGCGAAAAAGCCTTATC
GCAGGGCATATGACTGAGATTATGCAGTTGCTGAATCTCGATTTGAGCGATGACAGTCTG
ATGGAAACGCCGCATCGTATCGCTAAGATGTACGTTGACGAGATTTTCGCCGGCCTCGAC
TATGCCAATTTCCCGAAAATTACCCTCATTGAAAATAAAATGAAGGTGGATGAAATGGTC
ACCGTCCGCGATATCACGCTGACCAGCACCTGTGAACATCACTTCGTGACCATTGATGGC
AAAGCGACCGTCGCGTATATCCCCAAAGATTCCGTGATCGGCCTGTCGAAAATTAACCGC
ATCGTGCAATTCTTCGCCCAGCGTCCACAGGTTCAGGAACGTTTGACCCAACAAATTCTG
ACGGCGCTGCAAACCTTGCTCGGCACCAATAATGTAGCGGTATCGATTGATGCGGTGCAT
TACTGCGTGAAAGCGCGCGGTATTCGTGACGCAACCAGCGCCACGACGACGACGTCGCTG
GGTGGATTGTTTAAGTCCAGCCAGAATACGCGCCAGGAGTTTCTGCGTGCCGTTCGCCAT
CATCCATGA
>EECEBICD_01161 hypothetical protein
ATGATTAAGGCGGAGACGATGGAGCGTAATGTCACGCTGGATTTTGTACGTGGCGTCGCT
ATCCTCGGTATCCTGCTTCTTAACATTAGCGCCTTTGGATTGCCGAAGGCGGCTTATCTC
AATCCCGCCTGGTACGGCGCGATCGTTCCCGAAGACGCCTGGTCCTGGGCGATTCTCGGT
ATTGTGGCGCAGGCTAAGTTTCTTACGCTGTTCGCGTTGTTGTTTGGCGCGGGGCTACAA
ATGCTGCTTCCGCGTGGTAAACAGTGGATTCAGTCGCGCTTAACGCTATTGGTACTGTTA
GGTTTTATCCATGCGTTATTCTTTTGGGACGGCGACATTCTGCTGGCCTACGGTCTGGTC
GGGTTAATTTGCTGGCGACTGATACGCGATGCGCCTTCGGTAAAAAGCCTGTTCAATACC
GGCATACTCCTTTATCTGGTAGGGATTGGCGTGCTACTGCTGCTGGGCGTGGTATCGAGT
AGCGAAACCAGCCGCGCCTGGACGCCGGATGCGTCGGCAATATTGTATGAAAAATACTGG
AAGCTGAACGGCGGCATGGAGGCTATCAGCAACCGGGCAGAGATGCTGTCGAATAGTTTA
CTGGCGCTCGGCGCGCAGTATGGCTGGCAACTGGCGGGTATGATGCTGCTGGGCGCCGCG
CTAATGCGTAGCGGCTGGCTGAAAGGTCAGTACAGTCTGCGGCACTATCGCCGTACCGGG
GCTCTGCTGGTGGCGTTAGGGCTACTGATCAACCTGCCTGCGGTCATTTTACAATGGCGG
CTGGACTGGGCGTACCGCTGGTGCGCCTTTTTATTGCAGGCGCCGCGTGAACTGAGCGCG
CCGTTGCAAACGCTGGGCTATGCCGCGCTGATGTTTGGCTTCTGGCCGCAGCTTAGCCGA
TGCAGACTGACGCTTGCCATTGCCTGCGTAGGGCGAATGGCGCTGACCAATTATCTGCTG
CAGACGATCATCTGTACCACGCTGTTTTATCAGTTTGGCTTATTTATGAAATTCAATCGA
CTGGAACTCTTGTTCTTTGTCGTTCCGGTATGGGCAATAAACCTTCTCTTTTCCGTCATC
TGGCTACGTTTCTGGCGACAGGGACCGGTCGAGTGGTTATGGCGTCAACTCACGTTGCGC
GCGTCAGGATCGCTCAGATAA
>EECEBICD_01162 HTH-type transcriptional regulator GalS
ATGATCACCATTCGTGATGTAGCGCGCCAGGCTGGCGTGTCTGTAGCGACCGTTTCCCGC

GTACTGAATAACAGCGCGTTGGTCAGTCCCGACACCCGTGACGCCGTTATGCAGGCCGTC
ACCCTGCTGGGATATCGGCCAAATGCGAATGCGCAAGCGCTGGCCACCCAGGTGAGCGAT
ACCATCGGCGTCGTGGTCATGGATGTTTCCGATGCCTTTTTCGGCGCTCTGGTGAAAGCC
GTAGATCTGGTCGCGCAGCAGCACCAGAAATATGTTCTCATTGGCAACAGTTATCATGAG
GCGGAAAAAGAGCGCCATGCGATTGAAGTCTTGATCCGTCAGCGTTGTAACGCATTGATT
GTTCACTCAAAAGCCTTAACCGATCGCGAGCTGAGCGACTTTATGGATCAGATCCCCGGT
ATGGTGCTGATTAACCGTATCGTGCCGGGTTATGCGCATCGTTGTGTTTGTCTCGACAAT
GTGAGCGGCGCCAGAATGGCGACCCGAATGTTGCTGAATAATGGACATCAACGCATCGGC
TACCTGGCCTCCAGCCACCGTATTGAAGATGACGCGATGCGCAGAGAAGGGTGGTTACAC
GCGCTGCAAGAGCAGGGGATTGCTGCGTCGGAGAGCTGGATAGGCACCGGCACGCCGGAC
ATGCAGGGCGGCGAGTCGGCAATGGTTGAGTTGCTGGGACGCAATCTGCAACTGACGGCG
GTATTTGCCTATAACGATAACATGGCGGCGGGTGCGCTGACGGCGTTAAAAGATAACGGC
ATCGCCATTCCCTTGCATCTGTCTGTCATCGGTTTCGACGATATCCCTATTGCTCGTTAT
ACCGATCCTCAGTTGACTACCGTGCGCTATCCTATTGCTTCTATGGCGAAAATCGCGACC
GAGCTGGCGTTACAGGGGGCTGCAGGCACGCTGGATATCACGGCGACGCACTGCTTCATG
CCGACCCTGGTACGGCGCCATTCGGTGGCATGGCGACAGAATGCGGTACTGATCACTAAC
TGA
>EECEBICD_01163 D-galactose-binding periplasmic protein
ATGAATAAGAAGGTACTGACCCTTTCTGCCGTGATGGCAAGTCTGTTATTCGGCGCGCAC
GCGCAGGCGGCTGATACTCGTATTGGCGTGACGATTTATAAATATGACGATAACTTTATG
TCCGTGGTGCGTAAGGCTATTGAAAAAGATGGCAAATCCGCGCCGGATGTTCAGCTACTG
ATGAATGACTCGCAAAACGATCAATCTAAACAGAATGATCAAATTGACGTTTTATTGGCG
AAAGGGGTTAAAGCTCTGGCGATTAACCTGGTCGACCCGGCAGCCGCCGGTACGGTGATT
GAGAAAGCGCGCGGGCAAAATGTGCCGGTGGTGTTCTTTAACAAAGAGCCTTCCCGCAAA
GCGCTGGACAGCTATGACAAGGCGTATTATGTCGGGACTGACTCTAAAGAATCCGGTGTG
ATTCAGGGCGACTTGATTGCCAAACACTGGCAGGCGAATCAGGGTTGGGATCTGAATAAA
GACGGTAAAATTCAGTATGTTCTGCTGAAAGGCGAGCCGGGGCACCCGGATGCTGAAGCT
CGTACGACGTATGTGGTGAAAGAGTTAAATGCTAAAGGTATTCAGACCGAGCAACTGGCG
TTAGACACCGCCATGTGGGATACCGCGCAGGCAAAAGATAAGATGGATGCCTGGCTATCT
GGCCCGAACGCTAACAAGATTGAAGTGGTTATCGCGAATAACGATGCGATGGCGATGGGC
GCGGTAGAGGCGCTGAAAGCGCATAATAAATCATCGATTCCGGTCTTTGGCGTCGATGCG
TTACCGGAAGCTCTGGCGCTGGTGAAATCGGGCGCGATGGCCGGTACGGTACTGAATGAC
GCCAACAATCAGGCGAAAGCGACATTCGATCTGGCGAAAAACCTCGCCGAAGGCAAGGGC
GCGGCTGACGGCACCAACTGGAAGATTGAGAACAAAATCGTGCGCGTGCCTTATGTCGGC
GTGGACAAAGACAATCTGAGCGAATTTACCCAAAAATAA
>EECEBICD_01164 Galactose/methyl galactoside import ATP-binding protein
MglA
ATGGGCAGCACGATCTCTCCGTCGTCGGGTGAATATTTGTTGGAAATGCGTGGCATTAAC
AAATCATTTCCCGGCGTGAAAGCGCTTGATAATGTCAACTTAAACGTTCGTCCGCACTCT
ATTCATGCATTAATGGGCGAAAACGGCGCAGGCAAATCAACATTATTAAAATGTCTTTTC
GGTATTTATCAAAAAGACTCCGGCAGCATTGTATTTCAGGGAAAAGAAGTGGACTTCCAT
TCGGCGAAAGAAGCGCTGGAGAATGGGATTTCGATGGTACACCAGGAGTTAAACCTGGTA
TTACAACGTTCGGTCATGGATAACATGTGGCTGGGGCGTTATCCTACCAAAGGTATGTTT
GTCGACCAGGATAAAATGTACCAGGATACCAAAGCGATATTTGATGAGCTGGATATCGAT
ATTGACCCGCGCGCGCGCGTAGGAACGTTATCCGTTTCGCAAATGCAGATGATCGAAATT
GCGAAGGCGTTTTCCTATAACGCTAAGATCGTTATTATGGATGAACCGACCTCATCGTTA
ACTGAGAAAGAGGTTAATCATCTGTTCACCATTATTCGCAAGCTGAAAGAACGCGGCTGC
GGCATTGTTTATATCTCGCATAAAATGGAAGAAATTTTTCAATTGTGCGATGAAATTACT
ATTTTGCGCGACGGTCAGTGGATTGCCACCCAGCCGCTGGAAGGGCTGGATATGGATAAG
ATCATTGCGATGATGGTCGGGCGTTCCCTGAACCAGCGCTTTCCGGATAAAGAAAATAAG
CCGGGCGACGTGATTCTGGAGGTTCGTCACCTGACCTCGCTGCGCCAGCCTTCCATTCGC
GATGTCTCCTTTGATTTGCACAAGGGCGAAATTCTGGGCATTGCCGGTCTGGTTGGGGCA
AAGCGTACCGATATCGTCGAAACGCTGTTTGGCATTCGTGAGAAGTCGTCCGGGACAATC
ACTTTACATGGCAAGAAAATTAATAATCACACTGCGAACGAAGCGATTAACCACGGTTTT

GCGCTGGTGACGGAAGAACGTCGTTCCACCGGAATTTACGCCTATCTGGATATCGGATTT
AACTCATTAATTTCGAATATCCGTAATTATAAAAATAAAGTGGGGTTACTGGATAATTCC
CGGATGAAAAGCGACACCCAATGGGTCATTGATTCCATGCGGGTAAAAACGCCGGGGCAT
CGCACGCAAATTGGTTCGCTTTCCGGCGGCAATCAGCAAAAGGTGATCATTGGTCGCTGG
CTATTAACGCAGCCGGAAATATTAATGCTGGACGAACCGACCCGCGGCATTGATGTCGGC
GCAAAGTTTGAAATTTATCAGCTTATTGCGGAACTGGCGAAAAAAGGCAAGGGGATCATC
ATTATCTCTTCTGAAATGCCGGAGCTGTTAGGGATTACCGATCGTATCCTGGTGATGAGT
AACGGTCTCGTTTCCGGCATTGTCGATACTAAAACAACAACGCAAAACGAAATTTTGCGT
CTTGCTTCTTTGCACCTTTAA
>EECEBICD_01165 Galactoside transport system permease protein MglC
ATGAGTGCGTTAAACAAGAAAAGCTTTTTGACCTGGCTGAAAGAGGGCGGAATTTACGTC
GTTCTTTTAGTGTTGCTGGCGATTATTATTTTCCAGGACCCGACTTTTTTAAGTTTGCTG
AATTTAAGTAATATTCTGACGCAATCTTCGGTACGTATTATTATCGCGCTGGGCGTGGCG
GGACTCATCGTCACCCAGGGGACAGACCTGTCGGCGGGGCGTCAGGTAGGGCTGGCGGCG
GTCGTGGCGGCAACATTACTGCAATCAATGGAAAACGCCAACAAAGTGTTTCCGGAAATG
GCGACCATGCCGATTGCGCTGGTCATCCTGATTGTCTGCGCGATTGGCGCGGTGATTGGC
CTGGTGAACGGCATCATCATTGCTTACCTGAACGTGACGCCGTTTATTACCACGCTTGGC
ACGATGATTATCGTTTATGGGATCAACTCCCTTTACTATGACTTTGTCGGCGCTTCGCCC
ATTTCCGGCTTTGACAGCGGCTTTTCCACCTTTGCGCAGGGCTTTGTGGCGATGGGCAGT
TTCCGGCTCTCATACATCACCTTTTACGCTCTGATTGCGGTAGCGTTTGTCTGGGTGCTG
TGGAATAAGACCCGTTTTGGTAAAAACATTTTTGCTATTGGCGGCAACCCGGAGGCGGCG
AAAGTTTCTGGCGTAAACGTGGCGCTGAACCTGCTGATGATTTATGCGCTCTCCGGCGTG
TTTTATGCCTTCGGCGGCTTACTGGAAGCAGGGCGTATTGGTTCTGCCACCAACAACCTC
GGCTTTATGTATGAACTGGATGCGATTGCCGCGTGCGTGGTCGGGGGCGTATCGTTTAGC
GGCGGAGTGGGGACGGTCTTCGGCGTGGTGACCGGCGTCATTATCTTTACCGTCATCAAC
TATGGCCTGACCTATATAGGGGTAAACCCGTACTGGCAGTACATTATCAAAGGCGGCATT
ATCATTTTCGCCGTGGCGCTGGACTCGCTGAAATACGCGCGTAAGAAGTAG
>EECEBICD_01166 NAD-dependent dihydropyrimidine dehydrogenase subunit
PreA
ATGTTAACAAAAGATTTGTCTGTTACCTTTTGCGGCGTTAAGTTTCCCAATCCGTTTTGT
CTTTCTTCTTCTCCGGTAGGCAATTGTTATGAGATGTGCGCCAAAGCCTATGATACCGGC
TGGGGCGGGATCGTTTTTAAAACCATTGGTTTTTTATTGCCAACGAAGTCTCTCCACGT
TTTGATCACCTGACGAAAGAAGATACCGGTTTTATTGGTTTCAAAAACATGGAGCAAATT
GCTGAGCATCCGCTGGAAGAGAATCTGGCCGCCATTCGACGGCTAAAACAGGATTATCCG
GATAAGGTGCTGATTGCTTCCATCATGGGGGAAAATGAGCAGCAGTGGCAGGAACTGGCG
CGTCTGGTTGAAGAAGCCGGCGCGGATATGATTGAGTGTAACTTCTCTTGCCCGCAGATG
ACCTCGCACGCCATGGGAAGCGATGTAGGGCAAAGCCCGGAGCTGGTTGAAAAATACTGC
CGCGCCGTAAAACGCGGTTCATCTTTGCCAATGCTGGCGAAAATGACGCCGAATATTGGC
GATATGTGCGAAGTCGCGCTGGCCGCCAAACGCGGCGGAGCCGACGGTATCGCCACCATC
AACACCGTGAAATCCATTACTAACATCGACCTGAACCGCAAAATCGGGATGCCGGTGGTT
AACGGTAAATCCAGTATCTCCGGTTATTCAGGAAAAGCGGTGAAACCCATTGCGCTGCGT
TTTATCCAGCAGTTGCGAATGCACCCTGAACTGCGCGATTTCCCGATTAGCGGTATCGGC
GGCATTGAAACCTGGGAAGACGCTGCCGAATTTCTGCTGCTGGGCGCGGCGACCCTACAG
GTGACGACAGGCATTATGCAATACGGTTACCGCATTGTAGAAGATATGGCGAGCGGCCTC
AGCCACTATCTGGCCGATCAGGGATTTGCCTCATTACAGGAGATGATCGGGCTGGCGAAT
GGCAATATTATCCCGGCGGAAGACCTGGATCGCAGCTATATCGTCTATCCGCGGATTAAT
CAGGAGAAATGCGTCGGCTGCGGACGTTGCTATATCTCCTGCTATGACGGCGGTCATCAG
GCGATGGAGTGGGATGAACACAGCCGCACGCCGCATTGCAATACGGAAAAATGCGTCGGT
TGTCTGCTGTGCGGTCATGTCTGCCCGGTCGCCTGTATTGATTTAGGCGAGGTCAAATTT
AAAAAAGGCGAAAAAGAACACGCGTTAACGCTGTAG
>EECEBICD_01167 NAD-dependent dihydropyrimidine dehydrogenase subunit
PreT
ATGCCGCAACAAAATTATCTGGATGAACTCACGCCTGGTTTTACGCCATTACTGGCAATA
AAAGAGGCTTCACGTTGTTTATTATGTCACGATGCGCCCTGTAGTCAGGATTGCCCGGCG

CAAACCGATCCGGGGAAATTTATTCGTTCTATTTACTTTCGCAATTTTAAAGGCGCAGCG
GAAACTATCCGGGAAAATAATGCGCTTGGTGCTGTCTGCGCCAGAGTCTGTCCGACGGAA
AAATTATGCCAGCGCGGATGTACACGTTCCGGAATAGATAAACCTATTGATATTGCACGC
CTGCAACGTTTTATTACCGATTTTGAACAGCAGACTGCCATGCAGATTTATCAGCCCGGG
AGTAAAACACGCGGGAAAGTCGCGATTATTGGCGCGGGTCCGGCAGGTTTGCAGGCAAGC
GTGACGCTCACACATTTAGGCTATGACGTCACCATTTACGAAAAACAGCCGCAGCCCGGC
GGATGGCTGCGTCACGGCATCCCGGCATTTCGTCTGCCGCAAAGCGTACTCGATCAGGAA
ATTGCCCGCATTGTAGAAATGGGCGTCAATATTAAATGTAATTGTGACGTGGGCGGGTCG
CTATCACTTGCGCAACTCAAAGCCGAATATCGCGCCGTGCTGATGACCGTCGGGATGTCC
TGTGGCTCTGATTTACCGTTATTTGAGCAAGCCAGTCACGTAGAGATTGCCGTTGATTTT
TTACAGCGCGCCCGTCAGGCCGACGGCGATATTAGCGTTCCGCGTAGTGCATTAATTATT
GGCGGCGGCGACGTAGCGATGGATGTCGCCAGTACGTTAAAAATTCTCGGCTGCCCTTCC
GTCACCTGCGTGGCGCGGGAAGAATTAGCCCAATTTCCCGCCAGCGAAAAAGAATTTACC
AGCACGCAGGCATTAGGCGTATCGATTATTGATGGCTTTACGCCTGTCGCCGTCAGCGGA
AATAAAGTGACCTTCCACCATGTACGCCACTCAGGAGAACTGACGCTGGAAGCGGAAAAT
ATTATTTTGGCCGTGGGGCAGCACGCGCGACTGGATACCTTTGCGGAGATAAAAGCGCAG
CATAATATTATCGACACGCATAATTATCAAACCGACGACCCGGCGATCTTTGCCGCTGGC
GATATTGTTAAAGGTGATAAGACCGTCGTTTATGCGGTAAAAACCGGAAAAGAAGCCGCT
CAGGCTATTCATCATTATTTAGAGGAGGCCTGCTCATGTTAA
>EECEBICD_01168 hypothetical protein
ATGTTAAAGCGCGTGTTTTACAGCCTGTTGGTCCTGGTAGGCTTGCTGCTGTTGACGGTG
CTTGGCCTCGATCGGTGGATGAGCTGGAAAACAGCGCCCTATATCTATGACGAGCTACAG
GATCTCCCCTACCGCCAGGTTGGCGTCGTGTTAGGGACAGCCAAATACTATCGCAAGGGT
GTCATTAATCAGTACTATCGTTATCGTATCCAGGGCGCGTTAAACGCTTACAATAGCGGT
AAAGTCAATTACCTGCTGCTCAGCGGCGACAATGCCCTGCAAAGCTATAACGAACCGATG
ACCATGCGCAAAGACCTTATCGCGGCGGGCGTCGATCCTGCGGACATTGTGCTTGATTAC
GCTGGCTTCCGTACGCTTGACTCCATTGTGCGTACCCGCAAAGTGTTTGATACCAACGAC
TTTATTATCATCACTCAGCGCTTCCACTGCGAACGCGCCTTGTTTATTGCGCTGCATATG
GGCATTCAGGCGCAGTGTTACGCGGTACCTTCGCCCAAAAATATGCTGACAGTGCGCCTG
CGCGAATTTGGCGCCCGCTTTAGCGCGCTGGCGGATCTCTATATTTTTAAACGCGAACCG
CGTTTTTTAGGCCCGCTGGTACCTATTCCCACGCAGCATCAGGTGCCGAATGACGCCCAG
GGGTATCCGGCAGTTACGCCGGAACAATTGCTTGAACTGGAAAAGAAAAAAGGGGAAATG
ATCATGGATGTACAGACGATATTTGTCATATTAGCCTTTCTGCTACTCCCGCTTTTTTGC
TTCCGTGAGGCCTGGAAAGGCTGGCGAACCGGCGCGGTAGATAAAGTTGTCAAAAATGCG
CGAAAACCGGTTTATGTCTATCGCCATGCCGATCCCGTACAATATTGGTCTTATCTTTTT
CTTTATACCGGTTGCGGTTTTTTATTTACTGGAATGATTATTTATCTTCTTTTTTATCGT
TAA
>EECEBICD_01169 Cytidine deaminase
ATGAACGAGCTGAACAGCGGTCTGGATCTGCGCATTCATCTGCCGGGCCGCGCGCCGCAT
ACGTTACGCGATTATCTGCCTGACGCTTTCGGGCCAAAAGATCTGGAGATCAAAACGCTG
CTGATGGACGAGCAGGATCATGGCTTTACGTTAACGGGCGATACGCTAACGCAAGCGGCG
ATAACGGCAGCCAATAAGAGCCATATGCCTTATAGCCACTCACCGAGCGGCGTGGCGCTG
GAATGTAAAGATGGCCGTATTTTCACCGGCAGCTATGCGGAAAACGCCGCCTTTAACCCT
ACGCTTCCCCCGCTGCAAGGCGCGCTAAACCTGCTGAGTCTCAATGGCTATGACTATGCG
GATATTCAGCGCGCGATTCTGGCGGAAAAAGGCGATGCAGCATTAATTCAATGGGATGCC
ACCGCCGCAACGCTGAAAGCGTTAGGTTGCCATAATATTGACCGCGTCCTTCTCGGCTAA
>EECEBICD_01170 Cytidine deaminase
ATGCATCCACGTTTTCAAACTGCTTTTGCCCAGCTTGCGGATAATTTGCAATCAGCGCTG
GCCCCTATCCTGGCGGACCACCATTTTCCCGCCATGCTGACCGCAGAGCAGGTCTCGACG
CTTAAAAATACGGCGGGACTGGACGAAGACGCGCTGGCTTTCGCACTGTTACCTCTGGCG
GCGGCCTGCGCCCGTACCGATTTGTCCCATTTTAACGTCGGCGCGATTGCGCGCGGCGTC
AGCGGCAACTGGTATTTCGGCGCGAATATGGAGTTCCTGGGCGCCACCATGCAACAAACT
GTGCATGCTGAACAAAGCGCGATTAGCCACGCCTGGCTACGCGGCGAAAAAAGGGCTCGC
CGCCGTCACCGTTAA

>EECEBICD_01171 Inner membrane protein YohK
ATGATGACATATATCTGGTGGTCGTTACCGCTGACGCTGGCGGTATTTTTCGCCGCCCGC
CGACTGGCGGCGCATTTTAAAATGCCGTTGCTGAATCCGCTGCTGGTGGCAATGGTAGTC
ATTATTCCTTTTCTGTTGCTGACCGGTATTCCCTACGAGCACTACTTCAAAGGCAGCGAA
GTACTTAACGATCTGCTGCAACCGGCGGTAGTGGCACTGGCCTATCCCCTTTATGAACAA
TTGCACCAGATTCGCGCGCGTTGGAAATCCATTATTAGCATCTGTTTTGTCGGCAGTCTG
GTAGCGATGATTACCGGGACATCCGTTGCCTTGCTGATGGGCGCAACGCCTGAAATCGCG
GCATCGGTACTGCCTAAATCCGTGACTACGCCTATCGCGATGGCGGTGGGCGGCAGCATC
GGCGGCATCCCCGCGATCAGCGCGGTATGCGTAATTTTTGTCGGTATTCTGGGCGCGGTG
TTTGGCCACACGCTGTTAAACGCGATGCACATCCGTACTAAGGCCGCACGCGGGCTGGCA
ATGGGGACCGCCTCACATGCGCTCGGTACTGCGCGCTGCGCCGAGCTGGATTACCAGGAA
GGCGCGTTTAGCTCCCTGGCGCTGGTGATCTGCGGCATTATTACCTCGCTGGTAGCGCCC
TTTTTGTTTCCGCTCATTCTGGCGGTAATGCGCTAA
>EECEBICD_01172 hypothetical protein
ATGAGTAAGTCACTGAATATTATCTGGCAATATATACGCGCTTTTGTATTGATTTATGCC
TGTTTGTACGCAGGCATTTTTCTTGCATCACTGCTTCCTATCACTATTCCCGGCAGCATT
ATCGGGATGTTAATTCTGTTTGTATTGCTGGCGCTGCAAATTCTGCCGGCAAAGTGGGTT
AACCCCGGCTGTTATGTGCTGATTCGTTATATGGCGTTGCTGTTTGTGCCGATTGGCGTT
GGGGTCATGCAATATTTTGACTTACTGCGCGCGCAATTCGGCCCGGTGGTGGTCTCTTGC
GCTATCAGTACGCTGGTCGTTTTTGTGGTCGTAAGCTGGAGTTCACATCTGATACACGGT
GAGCGTAAAGTGGTTGGGCAGAAGGGAACGAAAAAATGA
>EECEBICD_01173 HTH-type transcriptional regulator GbpR
ATGGCGAACTGGGCGCAGAAATTGAAATTACATCACCTGCAAACGCTGGTTGCGCTGGGC
GAACAGGGCAACCTGACGCACGTCGCCCGGATGATGAATATCTCCCAGCCGGCGCTATCG
AAGTGGCTATCTCAACTCGAAGATGAGATAGGCATCACGCTTTTTGAGCGCCACAGTAAA
GGACTACGCCCCTCGGAAGGCGGTAAATTACTGCTCCAGCACGCCCAGCGCCTAATTAAC
GATCTGGAGCGTTCACAGTATGAAATCGCCCGTTTCAAGCAGGGCGGCCTGGTTGGCAGC
CTGAAGATCGGCTGTTCGCCCGTGGCGACTGACTGCGTATCACAGGCGATTCTGAGCCTG
CTTAACGAAATGCCGACGCTGCATCTGAATATTGAAGAGAAGGTAATGACGCCGCTGCTG
CACGATTTGCTTGCCGGTCAGGTGGATGTGGTGGTAGGGCGTGTCGGCGGCCGCGCCCTG
CAACTGCCGCTTAATTATCAGGTACTTTATACCGAGCCGGTCTGCTTTGTGGCGCGTCCG
CACCATCCGCTGGCCGCGCGAGCGCAGATCGCCTGGAGCGATCTGGCGCACTGGCGGTGG
ATAGTCTGGCCAACCGGCACCCCTATTCGCATCAGTATTGATAACGCCCTCGTCGATAAT
GGCGTCATGCTCCCGGAAAACACCATTGAATCGGCGTCAATGAACGTCAGCACCAATTTG
CTGCAAAGCAGCGATATGATCTCTATTCTTTCTTTACGGCTGGCGCAACGCTATGCCAGC
CAGGGGCAACTGGCGATTTTAAACTTGCCAAAAATTGAGCAAAAAGGCAGCGTAGGGATG
TTCTGGCGTAAGAATGAGACGCCGTCTTTGGCCCTGAGTCGCTTTTTGTATTTTTTAGCC
CAGGTTTAA
>EECEBICD_01174 3-hydroxybenzoate transporter MhbT
ATGACTCAACGACGTGATCTACAAGCCCTTATTGATGCCGCGCCCGTCGGCAAAATGCAG
TGGCGCGTTATCATCTGCTGTTTTCTGGTGGTTATGCTCGACGGTTTTGACACCGCCGCG
ATTGGCTTCATCGCCCCGGATATTCGTACTCACTGGCAGCTAAGCGCCAGCGAACTTGCG
CCGCTGTTTGGCGCAGGGCTGCTGGGGCTTACGGCCGGCGCGCTGCTATGCGGGCCGCTG
GCGGATCGTTTTGGCCGCAAGCGGGTCATTGAGCTTTGCGTGGCGCTATTCGGCGCATTG
AGCCTGCTTTCCGCTTTCTCGCCGGATATAGAAACCCTGGTGTTGCTGCGCTTCTTAACC
GGTCTGGGACTGGGCGGAGCGATGCCGAATACCATCACCATGACGTCGGAATACCTTCCC
GCTCGTCGACGCGGAGCGCTGGTCACGCTGATGTTCTGCGGTTTTACCCTGGGGTCGGCG
ATGGGCGGGATTGTGAGCGCGCAACTGGTGCCGCTGATTGGCTGGCACGGAATTCTGGCG
CTAGGCGGCATCTTGCCTTTGATGCTGTTTTTCGGCCTGCTGTTCGCGCTGCCGGAATCT
CCCCGCTGGCAGGTACGCCGCCAACTACCGCAAGCCGTTGTCGCCCGGACGGTCAGCGCC
ATTACCGGCGAGCGCTATCACGATACGCAATTCTTTCTGCATGAGACGGCAGCCGTCGCC
AAAGGCAGTATTCGCCAGCTTTTTGCCGGGCGACAGCTTGTCATTACCCTGATGTTATGG
GTGGTGTTCTTTATGAGCCTGCTCATTATCTATCTGCTTTCCAGCTGGATGCCGACGTTA
CTTAACCATCGCGGTATTGATCTGCAACAGGCGTCGTGGGTGACTGCCGCATTCCAGGTT

GGCGGCACGCTTGGCGCGCTGTTACTCGGCGTGTTGATGGATCGGCTTAACCCGTTCCGG
GTACTGGCGGTGAGCTATGCGCTGGGCGCGGTTTGCATTGTCATGATAGGCCTGAGCGAA
AACGGCCTTTGGCTGATGGCGCTGGCGATTTTTGGTACCGGCATCGGTATTAGCGGTTCA
CAGGTCGGGCTTAATGCTCTGACGGCGACGCTGTACCCCACCCAAAGCCGGGCGACGGGC
GTGAGCTGGTCGAACGCCATTGGACGCTGCGGGGCGATTGTCGGTTCGCTCTCCGGCGGC
ATGATGATGGCCCTCAATTTCTCTTTCGATACGTTGTTTTTTGTCATTGCTATTCCGGCG
GCTATCAGCGCGGTAATGCTTACCCTGCTGACGGTGGTTGTCCGCCTTTCGATTTCTGTA
CCTGACGACCTGCCGCGTGCCAGCGTCGTAAACGAATAA
>EECEBICD_01175 Gentisate 1,2-dioxygenase
ATGTATGAAATAAATCAGAACGTAAAAGATAGCCGTCAGCAGTATTACCAGCATATTTCC
GGGCAGAATCTGACGCCGCTGTGGGAATCGTTACATCACCTGGTACCGCAGACGCCAAAC
GCCAACTGCGCGCCGGCCTACTGGAATTATCAGGAAATTCGTCCGCTACTGATGGAAAGC
GGCAATGTCATTGGCGCGAAAGAGGCGATCCGCCGGGTGCTGGTGCTGGAAAATCCGGCA
TTGCGCGGTCAGTCGTCGATCACGGCGACCTTATACGCTGGTTTACAGCTGATCCTGCCC
GGCGAAGTCGCGCCGAGTCATCGCCATAACCAGTCGGCGCTGCGTTTTATCGTCGAAGGT
AAAGGCGCATTTACCGCGGTGGACGGCGAGCGCACGCCAATGCATACCGGCGATTTTATC
CTGACGCCGCAGTGGCGCTGGCACGATCATGGTAATCCGGGGTCAGAGCCGGTGGTATGG
CTGGATGGTCTGGATCTGCCGTTAGTCAACCTCCTGGGCTGTGGGTTTGCGGAAGACTAT
CCCGAAGATCAGCAGCCGGTGACGCGAAAAGAGGGCGATTATCTGCCGCGCTATGCAGCG
AATATGCTGCCGCTGCGCCACCAGCGCGGGAACTCGTCGCCGATTTTCAACTACCGTTAC
GACCGCAGTCGCGAGGCGTTGCACGATCTGACCCGTATGGGCGATCCGGATGAGTGGGAA
GGCTACAAGCTGCGTTACGTTAATCCCGTCACCGGCGGTTATCCGATGCCGTCGATGGGC
GCGTTCCTGCAACTGCTGCCAAAAGGCTTTGCCTCGCGTGTGGCGCGGAGCACCGACAGC
ACTATCTACCACGTCGTTGAAGGGGCAGGGCAGGTCACTATCGGCAACGAAACTTTTCAT
TTTTCCGCAAAAGACATTTTTGTGGCGCCGACCTGGCATGAGGTGTCGTTTCGCAGCAGC
GAAGACACGGTGTTATTCAGTTTTTCGGACAAGCCGGTTCAGGAAGCCCTGGGGCTGTTC
CGCGAAGCACGTTATTAA
>EECEBICD_01176 Fumarylpyruvate hydrolase
ATGACCAAATATGTATTCCAGCCGCAGGCTCCGGTAACAGTACCCGTGGCGGGCAGTGAT
GAGCAGTTTCCGGTACGTCGCGTTTACTGCGTGGGTCGCAACTATGCCGCCCATGCCCGT
GAAATGGGCTTTGATCCCGACCGCGAACCGCCGTTCTTCTTTTGTAAACCAGCGGACGCT
GTCGTCCCGGTAGCGGCGGGGGATACCCTAGAACTGCCGTATCCGGCGCAGACTGATAAC
TACCATTATGAAATTGAGCTGGTGGTGGCGATTGGTAAAAAAGGGAGCGATATTCCGCTG
GAAAAAGCCCATGAATATGTCTGGGGATACGCCACCGGTCTGGACATGACGCGTCGCGAT
CGTCAGATGGAAATGCGTCAGATGGGACGCCCGTGGGAAATCGGCAAAGCGTTCGATCTC
TCCGCGCCCATTGCGCCATTACACAAAGCCGCTGAAACGCATAATGTGGATAACGCCCCT
ATTTGGCTACAGGTTAACGGCGAAGACCATCAGCGCAGCGATATTCGTCACCTGATTTGG
TCGGTGAATGAAACCATCAGCTATTTGTCCGGCTTTTTTGAATTGCAGCCAGGCGATTTG
ATTTTCACCGGTACGCCAGAGGGGGGTGGGCGCCGTGGTGAAAGGGGATGTCATCACCGGG
AACGTAGAAGGTCTGACGCCGATTGCTGTGAAAATTGTCTGA
>EECEBICD_01177 Maleylpyruvate isomerase
ATGAAGCTGTACAGTTTCTTTAATAGTTCGGCGTCTTATCGTGTACGTATTGCGCTGGCG
TTAAAGGGGATTGATTACCAGACGGTGGGCGTCAATATTCGTATCGGCCAGCAGAATGCG
CTGGCCTACCGACGGATGAATCCGGTAGGCCTGGTGCCGACGCTGGTCACTGATGACGGC
GAATCGTTGGGGCAATCGCTGGCCATCGTCGACTGGCTGGACCGACATTTTCCGCAAACG
CCGCTGCTGCCGACGAGCGACCCGGCGCGCAGCCAGGCGCTGGAAATTGTCTACGCTATC
GCCTGCGATATACACCCAATCAATAACATGCGCGTGTTGCGCTACCTGACCGATGAGCTG
AAGGTTAGCGAAGAGGATAAAAAACGCTGGTACGCCCACTGGATACAGCAAGGGTTAAGC
GCGGTAGAGCAGCTATTGCGTAAAAGTCAGTCCGGACGATTCTGCGTGGGCGACGCGCCA
GGACTTGCGGACTGCTGCCTGATTCCTCAGTGGGCGAATGCGCTCAGGATGGGCTGCGAT
TTATCAGGCTATCCGCGCTGTAAAGCCGTTTATGACGCCTGCGTACAGCTACCGGCGTTT
ATTGCCGCCGCGCCGGAAAATCAACAAGATAAAATCCCGGCCTGA
>EECEBICD_01178 3-hydroxybenzoate 6-hydroxylase
ATGACTCACGTGACAAGCGCAATTATTGTAGGTGGCGGAATTGGCGGCGCGGCGACCGCG

CTGTCCCTGGCGCGCCAGGGAATAAAAGTCATGCTGTTGGAAAAAGCGCACGAAATTGGC
GAGATTGGCGCGGGTATTCAGCTTGGGCCGAACGCTTTTTCGGCGCTGGATAGTCTCGGT
GTGGGCGATGTTGCCCGCCAGCGTGCGGTGTTTACCGATCACATTACCATGATGGATGCC
GTGAATGCCGAAGAAGTGGTATGCATTGAAACCGGACAGGCGTTCCGCGACCATTTTGGC
GGGCCGTATGCGGTAATTCACCGGGTAGATATTCATGCAACGGTATGGGAAGCGGTGCTG
ACGCACCCTGGCGTGGAGTATCGTACCTCGACCCATATTGTCGATATCCGCCAGACGCCG
GATGACGTTACGGTGTTTGATGAGCAGGGAAATAGCTGGACGGCGGATATTCTCGTGGGC
TGCGACGGCGTTAAATCGGTCGTGCGGCAAAGTTTACTCGGCGATGCGCCGCGCGTAACG
GGGCATGTAGTTTATCGGGCGGTGATTGATTGCGACGATATGCCGGACGATTTACGTATT
AACGCACCGGTGCTGTGGGCAGGCCCGCATTGTCACCTGGTCCATTATCCGCTCCGTGGA
GGTCAACAGTACAATCTGGTGGTGACATTCCACAGCCGCCAGCAAGAAGAGTGGGGCGTG
AAAGACGGCAGTAAAGAAGAGGTGCTCTCTTATTTGCCGGCATTCATCCCCGCCCGCGC
CAGATGCTGGATAAGCCGACCTCCTGGCGCCGCTGGTCGACCGCCGATCGCGAGCCGGTC
GCGAAATGGGGAACCGAGCGTATCACTCTGGTGGGCGACGCCGCGCATCCGGTGGCGCAA
TATATGGCCCAGGGCGCCTGTATGGCGCTGGAAGACGCCGTCACGCTGGGCAAAGCGCTG
GAACGCTGCGACGGCGACGCGCAGCAGGCATTTGCGCTGTATGAGTCGGTGCGTATTCCC
CGCACGGCGCGTATCGTCTGGTCTACCCGCGAGATGGGACGTCTCTATCACGCCGCCGGG
GTGGAGCGTCAGGTGCGTAATCTGCTGTGGAAAGGGAAATCGCAGGAGGCGTTTTATCGC
GGTATCGAATGGCTGTACGGCTGGAAAGAAGATAACTGTCTTGAGCCGCGTTGA
>EECEBICD_01179 tRNA-dihydrouridine(16) synthase
ATGCGTGTTTTACTGGCGCCGATGGAAGGCGTGCTCGACGCGTTAGTGCGCGAGCTGCTG
ACCGAAGTGAATGATTACGATCTCTGCATCACCGAATTTGTGCGCGTGGTGGATCAGCTG
CTGCCGGTAAAAGTGTTTCATCGCATCTGCCCGGAGTTGCATCACGCCAGCCGCACGCCG
TCCGGCACGCCGGTGCGTATTCAGCTTCTGGGCCAGCATCCGCAGTGGCTGGCGGAAAAC
GCCGCGCGGGCGACGGCGTTGGGATCGTATGGCGTGGACCTGAACTGCGGCTGTCCGTCA
AAAGTGGTGAACGGCAGCGGCGGCGGCGCGACATTGCTCAAAGATCCCGAACTCATCTAT
CAGGGCGCGAAAGCGATGCGGGCCGCGGTACCGTCGCATCTGCCGGTGACGGTAAAAGTG
CGTCTCGGCTGGGATAGCGGCGATAGAAAATTTGAAATCGCCGATGCGGTGCAGCAGGCC
GGCGCCAGTGAACTGGTGGTGCATGGTCGTACCAAAGCGCAGGGCTACCGCGCCGAGCAT
ATCGACTGGCAGGCGATCGGCGAAATACGCCAGCGTTTGACTATTCCGGTTATCGCGAAT
GGCGAAATCTGGGACTGGCAGAGCGCGCAGGCATGTATGGCGACCAGCGGCTGCGATGCG
GTGATGATTGGCCGTGGGGCGTTAAATATTCCTAACCTGAGTCGGGTGGTGAAGTATAAC
GAACCGCGTATGCCGTGGCCGGAAGTGGTGACGTTATTACAAAAATATACCCGACTGGAA
AAGCAGGGCGATACCGGTTTATACCATGTCGCGCGTATTAAACAGTGGTTGGGATATTTA
CGTAAGGAATATATTGAGGCGATAGAACTTTTTCAGTCGATTCGGGCGTTAAACCGTTCG
TCCGAGATTGCGCGGGCGATTCAGGCTATTAAAATCTAA
>EECEBICD_01180 Toluene efflux pump outer membrane protein TtgI
ATGAATCGTAATTCTTTCCTTGCCGCGACAGCGAGCTTACCGCTTTTTATTTTGCTGGCG
GGTTGCGCCCCCCATGCACGATACCCGCCAGTCGCTGACCCAGCAGACGCCCTCTTCTCAT
GTTGATTCCAGCCTGCCGGCGGCATTGAAAAACGGTTGGCCGGATAGCCAGTGGTGGAAG
GCGTATCACGATGCGCAGCTTGACGCGCTGATCGATAATGCCATACAGCATTCGCCGGAT
ATGCAGGTGGCTGAACAGCGTATTCAACTGGCGGAAGCGCAGGCGAAAGCGGTTGAGGCG
CAGGACGGCCCGCAGCTCGACTTTTCTGCCGACATTGAGCGCCAGCGAATGTCCGCGGAA
GGGCTGATGGGGCCATTCGCGATTACCGACCCCGCGGCGGGAACGACCGGGCCGTGGTAT
ACCAACGGTACATTCGGCCTGACGGCGGGCTGGGATCTGGATTTATGGGGGAAAAACCGC
GCTGAAGTTACCGCGCGGATCGGGGCGGTCAAAGCGCGCGAAGCGGAGCAGGAGCAGACC
CGACAATTGCTGGCAAGCGGCGTGGCGCGACTCTACTGGGAGTGGCAAACACAGGCAGCG
CTGAAAAATGTGCTGATGCAAATCGAGCATGAGCAGCAAAACGTGGTGGCGGTAAACCGG
GAGTTGTATCAACACGGTATTACTTCTTCTGTTGAAGGGGTCGAGACCGATATCGACGCC
AGCAAAACCCAGCAGCAGTTGAATGACGTTAACGGTAAAATGAAGGTTATTGAAGCGCGG
CTCAGCGCGTTAACCAATACGCAATCGGCGGCGCTCAAGCTGCGTCAGGTGAGTCTCCCC
GCCGTTGAAAGCCAGTTACCGTCGCAACTGGGGTATTCGTTGCTGGCGCGCCGGGCGGAT
CTACAGGCCGCGCACTGGTACATTGAATCCTCGTTGAGCAGCATTGACGCCGCGAAGGCC
GCGTTTTATCCCGATATCAATCTGATGGCTTTTTTACAGCAGGATGCGTTACATCTGAGC

GATCTGTTCCGCCATTCCGCACAGCAATATGGCATTACCGGCGGGCTGACGCTGCCGATT
TTCGACAGCGGCAGGCTGAATGCCAACCTCGATATCGCCAAAGCGCAAAGCAACCTTTCT
ATCGCCAACTACAACAAAGCGGTGGTAGATGCGGTTAATGATGTGGCGCGCGCCGCCAGT
CAGGTAGAGACGCTGGCGCAGAAAAATCAGCATCAGCAGCAGATTGAGCATGACGCGCAG
CGTGTGGTCGGTCTGGCGCAAGCGCGCTTTAATGCCGGCATTATTGCGGGTTCCCGCGTG
AGCGAGGCCAAAATCCCGGCACTGCGCGAGCAGTGTAACGGCTTGCTGTTGCAGGGCCAG
TGGCTGGATGCCTCTATTCAGCTTACCAGCGCGCTGGGCGGCGGTTATCATTCCTGA
>EECEBICD_01181 putative oxidoreductase YohF
ATGACCAAAGTCGCAATTGTAACGGCATCAGATTCGGGAATTGGCAAAGCGTGTGCGTTG
TTATTAGCACAAAACGGTTTTGATATTGGTATCACCTGGCATTCCGATGAACGCGGCGCG
CAGGAGACGGCGAAAAAAGCGGCGCAGTTTGGTGTGCGGGCAGAGACCATTCATCTTGAC
CTGAGCCAGTTACCCGAGGGCGCGCAGGTCATTGAACATCTTATCCAGCGCCTGGGGCGC
GTTGACGTGCTGGTCAATAATGCCGGGGCGATGACCAAATCCGCGTTTATTGATATGCCG
TTTACGCAGTGGCGGCAAATTTTTACCGTGGATGTCGATGGCGCGTTTCTCTGCGCGCAG
ATCGCCGCCCGGCATATGATTAAACAAGGAGAGGGAGGGCGCATTATCAACATCACCTCC
GTCCATGAGCATACGCCGCTTCCGCAAGCCAGCGCCTATACCGCGGCGAAACATGCGTTG
GGGGGATTGACTAAATCCATGGCGCTGGAACTGATTGAGCATCATATTTTGGTCAACGCC
GTCGCGCCAGGCGCTATCGCCACGCCGATGAATGATATGGATGACAGCGACATTGAGCCG
GGATCGGAACCCTCGATCCCTATCGCCCGACCGGGTTCTACGCATGAAATAGCCAGCCTC
GTCGCCTGGCTGTGTTCGGAAGGAGCAAGCTATACCACCGGTCAGTCGCTGATTGTCGAC
GGCGGGTTTATGCTGGCGAATCCGCAATTCAACGCCAAATAA
>EECEBICD_01182 Inner membrane protein YohD
ATGGATATCAACACGCTTATTACGCATTATGGCTATGCGGCGCTGGTTATCGGCAGTATG
GCCGAAGGTGAAACCGTTACCCTGTTGGGGGGCGTCGCGGCGCATCAGGGATTACTCAAG
TTTCCGCTGGTGGCGGCGGCCGTCGCGCTGGGAGGGATGATGGGCGATCAGCTACTTTAC
CTGTTAGGACGATGCTATGGCGGGAAGATCCTGCGTCGTTTCCCGCGTTATCATACGAAG
ATTCGTCGCGCGCAGAAAATGATCCAACGCCATCCGTATCTGTTTGTGATCGGCACGCGT
TTTATGTACGGCTTCCGGGTGGTTGGCCCGCTGCTGATTGGCGCCAGCCGCCTGCCGCCT
AAAATTTTTCTGCCGCTGAATATTGTAGGCGCGCTGATCTGGGCGCTGCTGTTTACCACG
CTGGGATATCTTGGCGGCGAGGTGATCGCGCCATGGCTGCACGACCTCGATCAGCATCTC
AGGCACGGGGTCTGGCTGATTCTGGCCATCGTCCTGGTGGTGGGCGTGCGCTGGTGGCTG
AAAAGACGCGGCAAAGCGGAGGCCCGTTAA
>EECEBICD_01183 Inner membrane protein YohC
GTGATCAAAAGCGAAAACGAAACCGTTTCGCATCACTATACGCACCATGTTTTGCTGATG
GCGGCGATTCCCGTCGTCTGCGCCTTTATCGGCACGACGCAAATCGGCTGGAATTTTGGC
GACGGCAACGTGTTACAACTCTCGCTGTTTACCGCTTTTGCGCTGGCGGTCTTGTTCTAC
GGCGTGATGCTGGCGGGCGTCGCGGTGATGGGACGCGTCATCTGGTGGATGGCGCGAAAC
TATCCGCAACGGCCATCGCTTGCGCGCTGTATGGTTTTTGCCGGCTATGTCGCGACGCCG
CTGTTTTTAAGTGGTCTGGTGGCGCTCTATCCCCTGGTATGGCTATGCGCACTGGTCGGC
GCTGTCGCGCTCTTTTACACCGGCTATTTGCTGTATTTGGGTATTCCGACTTTCCTTAAC
ATTAATAGAGAGGAAGGATTAAGCTTCTCCAGTTCGACGCTCGCTATCGGCGTACTGGTC
CTGGAGGTGCTACTGGCGATCACCGTGATCCTGTGGGGATACGGCTACCGCCTGTTCTGA
>EECEBICD_01184 D-alanyl-D-alanine endopeptidase
ATGTTGGCCGTTCCTTTTGCCCCGCAGGCTGTGGCGAAAACGGCAGCGACAACCGCGGCG
TCTCAACCTGAGATTGCCTCCGGTAGCGCAATGATTGTTGATCTCAACACCAATAAAGTG
ATCTATTCGAACCACCCGGATCTGGTGCGTCCGATTGCCTCGATAACCAAATTAATGACA
GCGATGGTCGTGCTCGACGCGCGATTGCCGCTGGATGAAATACTGAAGGTCGATATCAGC
CAGACGCCGGAAATGAAAGGGGTTTACTCCCGCGTGCGCCTGAACAGCGAAATTAGCCGC
AAAAATATGTTGCTGCTGGCGTTGATGTCATCCGAAAACCGTGCGGCGGCAAGCCTGGCG
CACTATTATCCTGGCGGTTATAACGCGTTTATTAAAGCGATGAATGCGAAGGCGAAAGCG
CTGGGCATGACGCATACCCGCTTCGTTGAGCCGACGGGGCTGTCGATTCATAACGTCTCG
ACCGCGCGGGATTTAACAAAATTGCTGATTGCCACGAAGCAATACCCGCTGATTGGCCAG
CTAAGCACCACGCGCGAAGACATGGCGACATTCTCGCATCCGGCGTATACGCTGCCGTTT
CGCAATACCAACCATTTGGTCTACCGTGATAACTGGAATATCCAGTTAACGAAAACCGGC

TTTACTAATGCGGCGGGGCATTGCCTGGTGATGCGTACCGTGATCAATAACAAACCGGTC
GCGCTGGTGGTCATGGACGCGTTCGGCAAATATACTCACTTTGCGGACGCCAGTCGCCTG
CGTACATGGATTGAAACCGGTAAAGTCATGCCCGTTCCGGCTGCCGCGCTGAGCTATAAA
AAGCAAAAAGCCGCGCAAATGGCGGCAGCCAGCGCCAGCGCCGGAGCGCAAACCGCACAG
AACGATTAA
>EECEBICD_01185 Quinone-dependent D-lactate dehydrogenase
ATGTCTTCCATAACAATGACTGATAATAAAACGTTCCTGAATGAGCTGGCCCGCCTGGTA
GGCCATTCTCACCTGCTGACAGACCCCGCCAAAACCGCCCGCTATCGCAAGGGTTTCCGT
TCCGGTCAGGGCGACGCGCTGGCCGTTGTTTTCCCGGGTTCGTTACTGGAACTGTGGCGC
GTGCTCAACGCCTGCGTTAACGCTGACAAAATTATTCTGATGCAGGCCGCCAACACCGGT
CTGACGGAAGGCTCCACGCCGAACGGCAACGACTACGATCGCGATATTGTGATTATCAGC
ACCCAGCGACTGGATAAGCTGCACTTACTGGATAACGGCCAACAGGTTCTGGCCTGGCCC
GGTACGACGCTCTATGCGCTGGAAAAAGCGCTTAAACCGCTGGGCCGTGAACCGCACTCG
GTGATTGGTTCGTCGTGCATCGGCGCGTCGGTCATCGGCGGTATCTGTAATAACTCCGGC
GGCTCGCTGGTGCAGCGCGGCCCGGCCTATACCGAGATGTCGCTTTTTGCCCGTATTGAC
GACCAAGGCAAACTGCAACTGGTCAATCATCTGGGTATCGATCTTGGGCATACGCCGGAG
CAGATCCTGAGCAAGCTGGATGACGAACGGATTAAAGATGAGGATGTGCGCCACGATGGT
CGTCATGCGCACGATCACGACTACGTGACGCGGGTACGAGATATCAACGCCGATACGCCC
GCCCGCTATAACGCCGACCCGGACCGTTTGTTTGAATCTTCCGGCTGCGCCGGAAAACTG
GCGGTTTTTGCCGTTCGCCTTGATACCTTTGCAGCGGAAAAAAATCAGCAGGTTTTTTAT
ATCGGCACCAATCAGCCCGCGGTACTGACTGAAATCCGTCGGCATATTCTGGCGAACTTC
GACAATCTGCCAGTGGCGGGAGAATATATGCATCGCGATATCTACGATATTGCGGAGCAG
TACGGTAAAGATACCTTTCTGATGATCGATAAACTCGGCACCGACAAGATGCCATTCTTT
TTCACGCTAAAAGGCCGAACCGATGCGATGCTGGAAAAAGTAAAATTTTTCCGTCCGCAT
TTCACCGACCGCGCGATGCAGAAATTCGGCCATCTGTTTCCCAGCCATCTGCCGCCGCGA
ATGAAAAACTGGCGCGATAAATATGAGCATCATCTGCTGTTAAAAATGGCGGGAGACGGC
GTCGCAGAAGCGCAACGCTGGCTGAATGAATTTTTTAAATCCGCTGAGGGCGGTTTCTTC
ACCTGTACGCCGGAAGAAGGCAGCAAAGCCTTCCTGCACCGCTTCGCCGCTGCTGGCGCG
GCGATTCGTTATCAGGCCGTACACGCTGATGAAGTGGAAGATATCCTGGCGCTGGATATC
GCTCTGCGCCGCAACGATACCGACTGGTTCGAGCATCTGCCGCCGGAAATCGACAGCCAA
CTGGTACATAAACTCTATTACGGCCACTTTATGTGCCACGTTTTTCATCAGGACTATATC
GTGAAGAAAGGCGTCGACGTCCACGCGCTGAAAGCGCAAATGCTGGAGCTGTTGCAGGCG
CGGGGGGCACAATACCCGGCGGAGCACAACGTTGGACATCTGTATAAAGCGCCGGAAACG
CTGACGCGTTTTTATCGGCAAAATGACCCAACCAACAGTATGAACCCCGGTATCGGCAAA
ACGAGTAAGCGGAAATTCTGGCAGGAAAATACGCCCGACGAAACGCATTAA
>EECEBICD_01186 Beta-xylosidase
ATGAAATGGTTATGTTCAGTAGGTGTTGCTGTGAGTCTGGCGATGCAGCCAGCATTGGCG
GAAAACCTGTTTGGCAATCACCCGCTGACGCCCGAAGCGCGCGACGCGTTCGTCACGGAT
TTACTCAAGAAAATGACTGTCGATGAAAAAATTGGTCAGTTACGTCTGATTAGCGTCGGC
CCTGATAACCCGAAAGAAGCGATTCGCGAGATGATCAAAGACGGGCAGGTAGGGGCGATT
TTCAATACCGTCACCCGCCAGGATATCCGTCAAATGCAGGATCAGGTGATGGCATTGAGC
CGCCTGAAAATTCCTCTCTTTTTTGCCTATGACGTGGTACACGGCCAGCGAACCGTCTTC
CCGATAAGCCTCGGTCTGGCTTCTTCTTTTAATCTGGATGCGGTAAGAACCGTAGGACGC
GTATCGGCGTATGAAGCGGCGGACGACGGTCTGAATATGACCTGGGCGCCGATGGTGGAT
GTCTCCCGCGATCCGCGTTGGGGACGCGCCTCCGAAGGCTTTGGCGAAGATACATATTTA
ACCTCTATCATGGGCGAAACCATGGTGAAAGCGATGCAGGGCAAAAGCCCGGCGGACCGC
TATTCGGTGATGACCAGCGTGAAACATTTTGCCGCCTATGGCGCCGTTGAAGGCGGTAAA
GAATACAACACCGTGGATATGAGTTCCCAGCGTCTGTTTAATGACTACATGCCGCCGTAC
AAAGCGGGGCTGGATGCGGGGAGCGGCGCGGTGATGGTGGCGCTGAACTCGCTGAACGGC
ACGCCAGCGACGTCAGATTCCTGGCTATTGAAAGATGTCCTGCGCGATGAGTGGGGCTTT
AAGGGAATTACCGTTTCCGACCACGGCGCTATTAAAGAACTGATTAAACACGGTACCGCG
GCCGACCCGGAAGATGCGGTGCGCGTGGCGCTAAAGGCAGGGGTGGATATGAGCATGGCG
GATGAGTATTACAGCAAATATCTGCCGGGTCTGATTAAGTCGGGCAAAGTGACGATGGCG
GAACTGGACGACGCGACGCGCCATGTGCTCAACGTAAAATATGACATGGGATTGTTTAAC

GACCCCTATAGCCATCTGGGACCGAAAGAGAGCGACCCGGTGGACACCAATGCCGAAAGC
CGCCTGCATCGTAAAGAGGCACGGGAAGTGGCGCGGGAAAGTGTGGTACTGCTGAAAAAC
CGGCTGGAAACGCTGCCGCTGAAAAAATCGGGCACCATTGCCGTTGTTGGGCCGCTGGCC
GACAGTCAACGTGATGTAATGGGAAGCTGGTCTGCTGCTGGGGTCGCGAATCAGTCAGTG
ACGGTGCTGGCGGGTATTCAAAATGCCGTCGACGACGGGGCGAAGATCCTCTATGCCAAA
GGGGCGAATATCACTAACGATAAAGATATCGTCGATTTCTTAAACCTGTATGAAGAGGCG
GTGAAAATCGATCCGCGCTCACCGCAGGCGATGATTGATGAGGCGGTGCAGGCGGCGAAA
CAGGCCGATGTAGTGGTCGCAGTGGTAGGCGAGTCGCAGGGGATGGCGCATGAAGCCTCC
AGTCGTACCAATATCACCATTCCGCAGAGTCAGCGCGACTTAATCACCGCTCTGAAAGCC
ACCGGTAAGCCGCTGGTACTGGTATTAATGAACGGCAGACCGCTGGCGCTGGTAAAAGAA
GATCAACAGGCCGATGCGATTCTGGAGACCTGGTTTGCCGGGACGGAAGGCGGCAACGCC
ATTGCCGATGTGCTGTTTGGCGATTACAACCCGTCAGGCAAACTGCCGATCTCCTTCCCG
CGTTCGGTGGGACAAATTCCGGTCTATTACAGCCATCTCAATACGGGACGCCCGTATAAT
CCTGAAAAGCCAAACAAATACACCTCACGCTATTTTGACGAAGCGAATGGCCCGCTCTAT
CCGTTTGGTTACGGTCTGAGCTACACCACATTTACGGTGTCTGACGTCACGCTCTCCTCA
CCGACAATGCAACGCGACGGCAAAGTCACGGCCAGCGTAGAGGTCACGAACACCGGTAAA
CGCGAAGGGGCCACGGTCATCCAGATGTACTTACAGGATGTCACGGCGTCGATGAGCCGC
CCGGTGAAGCAATTAAAAGGCTTTGAAAAAATCACCCTCAAGCCCGGCGAACGTAAAACC
GTGAGTTTCCCGATTGATATCGAGGCGTTGAAGTTCTGGAATCAGCAGATGAAATATGAC
GCGGAACCCGGCAAGTTCAACGTCTTTATCGGCGTGGATTCCGCTCGCGTTAAACAAGGA
TCGTTCGAGCTACTGTAA
>EECEBICD_01187 Glycine betaine-binding protein YehZ
ATGACGATCTCAAAGCTATGGGTAAGTTCGCTGGCGTTGCTGGCGACGGTGAGTTTACCT
CTACAGGCGGCATCGCCCGTGACGGTCGGGTCAAAGATTGATACCGAAGGCGCACTGCTC
GGCAACATGATTTTGCAGGTACTGGAAAGCCACGGCGTTAAAACGGTCAATAAAATTCAG
CTCGGGACGACGCCAGTCGTTCGTGGCGCGATTACCGCCGGAGAGCTGGATATCTATCCG
GAATACACCGGTAACGGGGCGTTCTTTTTTAAAGATGAAAACGATCCGGCATGGAAAAAT
GCAAAGCAAGGCTTTGAGAAAGTCAAAAAACTGGATGCGGAGCAGAATAAGCTGGTCTGG
TTGACGCCTGCGCCGGCAAATAACACCTGGACCATCGCGGTACGCCAGGACATCGCGGAA
AAAAATAAACTCTCCTCGCTTGCCGATCTCAGCCGTTATCTGAAAGAGGGCGGCACTTTT
AAACTGGCGGCGTCTGCGGAATTTATCGAACGGGCGGATGCGCTGCCGGCATTTGAAAAA
GCCTATGATTTTACCTTGAATCAGAACCAGCTCTTGTCGCTGGCGGGCGGCGATACGGCG
GTCACGATCAAAGCCGCCGCGCAACAAACATCCGGCGTTAACGCCGCGATGGCGTATGGC
ACCGACGGCCCTGTCGCCGCGCTGGGTCTGCAAACCCTGAGCGACCCGAAAGGCGTCCAG
CCTATCTATGCGCCCGCGCCGGTAGTGCGTGAATCGGTGTTACAGGCCTATCCGCAGATA
GCCGACTGGCTGCAGCCCGTTTTCGCCAGCCTTGATGAGAAAACGCTGCAACAGTTGAAC
GCCAGAATCGCCGTAGAAGGGCTGGATGCGAAAAAAGTCGCCGCAGATTACCTCCGACAA
AAAGGGTGGGTGAAGTAA
>EECEBICD_01188 Glycine betaine uptake system permease protein YehY
GTGAGTGATGCTGCCAAAAAAATCACGGTTAATCGTGTGCTGTTGCTGTTGGTTGTTCTG
ACCCTGCTTGCTGTCGCGCTGCCTTTCATTAATTACGCCCCGAATCGTCTGGTGAGCGGC
GAGGGGCGCCAGTTATGGGAGATCTGGCCGGCGACCATCTGGATGCTGACTGGCGCGGGA
TGCGCGCTGTTTACGTTGTGCTTTGTACCCGGTAAACGCGGTAGCGTACTGACGCTGATG
ATGGCGCAGACGCTTTTTATTGTCATGCTGTGGGGAGTCGGCAGGGTGGCGACCCAATTG
GCGCAGGAGGGCAGCCCGCTGGCGCGTACCAGTCTGGGAAGTGGATTGTGGCTGGGCCTC
GGTCTGATGCTGCTGGCCTGTAGCGATGCGATCCGCCGTATTACCACTGGGCCGCTGTGG
CGCTGGTTGCTGCACGCGCAAATTGTCATTGTCCCGCTGGTGCTGCTGTTTTCAGGCACG
TTCGACAACCTCTCGCTTCTGAAAGAGTATACCAACCGCCAGGATGTTTTCGATGCCGCG
CTGGTGCAACATTTGATGTTACTGGCGGGCACGGTGCTGCCGGCGTTGGCGATCGGACTA
CCGTTGGGCGTCTGGTGTTATTTTTCCGCCTCACGCCAGGGGCCGGTGTTTACCGTGCTG
AATGTGATCCAGACTATCCCTTCCGTCGCGCTGTTTGGCCTGCTGATTGCGCCGTTGGCC
GGGCTGGTGAAACAGTTTCCGTGGCTTGCCGAATTCGGCGTCGCGGGAACCGGCATGACG
CCTGCACTGATAGCGCTGGTGCTCTATGCGTTATTGCCGCTGGTGCGCGGCGTGGTCGCC
GGGCTCAATCAGGTGCCGCGCGATGTGCTGGAGAGCGCAAGAGCAATGGGAATGAGTAGC

GGCCAGCGCTTCAGGCATGTGCAACTGCCGCTGGCGCTGCCGGTCTTTTTACGTAGCCTG
CGGGTAGTAATGGTTCAGACAGTGGGAATGGCCGTCGTCGCCGCGCTGATTGGCGCGGGA
GGTTTCGGCGCGCTGGTGTTTCAGGGGCTATTGAGCAGCGCCATTGATTTAGTCCTGTTG
GGCGTCATTCCGGTGATTGCGCTGGCGGTAGTGATCGACGCGCTGTTTGATTTATGGATC
GCGTTTTTGAAGGGAGCAACCGATGATTGA
>EECEBICD_01189 Glycine betaine uptake system ATP-binding protein YehX
ATGATTGAATTTAACCATGTCAGTAAAACCTTCGGCGATCAACAGGCTGTTAGCGACCTC
AATTTGCACTTTAGCGAAGGCAGCTTTTCGGTGTTAATTGGCACCTCCGGTTCGGGAAAA
TCGACCACTCTGAAGATGATTAACCGGCTGGTAGAGCATGATAGCGGAACGATCCGTTTT
GCCGGGGAAGAGATCCGCAGCCTGCCGGTGCTTGAACTGCGCCGTCGCATGGGCTATGCC
ATTCAGTCTATCGGTCTTTTTCCCCACTGGACGGTGGCGCAAAATATCGCCACCGTACCG
CAACTACAAAAGTGGTCGCGTGCGCGGATTAACGATCGTATTGATGAACTGATGGCATTA
TTGGGTCTGGAAAGCGCGCTGCGCGATCGTTATCCGCATCAGCTTTCCGGCGGGCAACAG
CAGCGGGTCGGCGTTGCGCGGGCGCTGGCTGCCGATCCGCAGGTATTGCTGATGGACGAG
CCTTTCGGCGCGCTTGATCCGGTAACGCGCGGCGCATTGCAGCAGGAGATGACCCGCATT
CATCAGCTACTGGGGCGCACCATCGTACTGGTGACGCACGACATCGACGAGGCGCTACGC
CTCGCCGACCATCTGGTGCTGATGGACGGGGGCAATGTTATCCAACAGGGATCGCCACTT
TCTATGCTGACCTCGCCGGAAAATGATTTCGTGCAGGCGTTTTTTGGCCGCAGCGAGCTG
GGCGTAAGGCTGCTTTCGTTACGTAGCGTAGGCGATTATGTACGTCGGCATGAACAGCTC
AGCGGCGACGCGCTGGTGGAAGAGATGACGCTACGCGATGCGCTATCGATGTTTGTCGCC
CGTCGGTGCGACGTCCTGCCGGTGGCGAATCAGCAGGGCGAGCCCTGCGGTACGCTCCAT
TTCCGCGATCTACTTTCGGAGACGTCCCCCCGTGAAACGACTGTGTGA
>EECEBICD_01190 Glycine betaine uptake system permease protein YehW
GTGAAACGACTGTGTGATCCGCTTCTCTGGCTTATTGTTCTGTTCTTGCTTCTGCTGTTT
GGATTGCCTTATAGCCAGCCGTTCTTCGCCGCGCTGTTTCCCGATTTACCGCGCCCGGTC
TACCAACAGGAGAGTTTTGCCGCCCTCGCGCTCGCCCATTTCTGGTTGGTGGGCATCTCA
AGTCTGTTTGCCGTCGTGGTGGGCGTCGGCGCAGGGATTGCGGTCACGCGAGAAAGTGGG
AAGGAGTTTCGTCCCCTGGTGGAGACTATCGCCGCCGTCGGGCAGACCTTTCCCCCGGTC
GCGGTACTGGCGATCGCGGTACCCGTCATGGGTTTTGGTCAGCAACCAGCCATTATCGCC
TTGATCCTGTATGGAGTGTTGCCCATCCTGCAGGCGACCCTGGCCGGGCTGGGCGCGGTG
CCTGCCAGCGTGATGAGCGTTGCCAGCGGTATGGGAATGAGCCGTCGCCAACAGTTGTAT
CAGGTTGAACTGCCGCTGGCCGCGCCGGTGATTCTGGCGGGCATCCGAACCTCGGTGATT
ATCAATATTGGTACGGCGACCATCGCTTCAACAGTGGGGGCCAGTACGTTAGGCACGCCG
ATCATTATCGGGCTTAGCGGCTTTAATACGGCCTATGTTATCCAGGGGGCGCTGCTGGTG
GCGCTGGCGGCGATCATTATCGATCGCCTGTTTGAAAGGCTGACGCGCGCGCTTACCCGG
CACGCAAAATAA
>EECEBICD_01191 hypothetical protein
ATGCGCGTAGCGAAAATCGGGGTGATCGCCCTTTTCCTGCTGATGGCTATTGGCGGTATC
GGCGGCGTGATGCTGGCAGGTTACAGTTTTATTTTGCGTGCCGGGTAA
>EECEBICD_01192 HTH-type transcriptional regulator MlrA
ATGGCGCTTTACACAATTGGTGAAGTGGCTTTGCTTTGTGATATCAATCCTGTCACGTTG
CGCGCGTGGCAGAGACGTTATGGACTTTTAAAACCACAGCGAACGGATGGCGGTCATCGT
CTGTTTAACGATGCCGATATCGACAGAATCCGCGAAATCAAGCGCTGGATAAATAACGGC
GTCCAGGTCAGCAAAGTCAAAGTGCTGCTCAGTAGCGACAGTAGCGAACAACCTAACGGC
TGGCGCGAACAGCAGGAGATCCTGCTGCACTACCTGCAAAGCAGTAATCTGCACAGTTTA
CGGTTATGGGTCAAAGAACGCTGTCAGGATTATCCAGCCCAAACATTGACCACTAACCTG
TTCGTCCCGCTGCGGCGACGATTACAGTGCCAACAACCTGCCCTTCAGGCGCTGCTCGGC
ATTCTTGACGGTATCCTGATCAACTATATTGCGCTCTGCCTGGCGTCTGCGCGTAAGAAA
CAGGGAAAAGATGCGTTGGTGATCGGCTGGAATATCCATGATACCACCCGCCTGTGGCTG
GAAGGTTGGGTCGCCAGCCAACAGGGATGGCGAATCGACGTGCTGGCGCATTCGCTTAGC
CAGTTCCGCCCGGAACTGTTTGACGGCAAGACGTTACTGGTATGGTGCGGAGAAAACCAG
ACGCTGGCGCAGCAGCAGCAACTCCTGGCATGGCGCGCCCAGGGACGCGACATTCATCCC
CTTGGCGTTTAA
>EECEBICD_01193 Sensor histidine kinase BtsS

ATGTACGAGTTTAATCTGGTGTTGCTGCTGCTTCAGCAGATGTGCGTGTTTCTGGTCATT
GCGTGGCTAATGAGTAAAACGCGTCTGTTCATCCCGCTTATGCAGGTCACGGTTCGTCTG
CCGCACAAGCTTCTGTGTTACGTCACGTTTTCTATCTTCTGCATCATGGGCACTTATTTT
GGGCTGCATATCGAAGATTCGATTGCCAATACCCGCGCAATTGGCGCGGTGATGGGCGGC
CTACTCGGCGGGCCGGTCGTCGGCGGGCTGGTCGGTCTGACCGGTGGGTTACATCGGTAT
TCTATGGGCGGCATGACGGCGCTGAGCTGTATGATTTCCACCATCGTCGAAGGGCTGCTG
GGCGGGTTGGTACACAGCGTTCTCATACGTCGCGGACGCCCGGACAAAGTGTTTAGCCCG
CTGACGGCGGGAGCAATTACGTGTATTGCCGAACTGGTGCAGATGCTGATCATTTTACTG
ATAGCCAGGCCGTTTGACGATGCCTTGCATCTGGTCAGTAATATTGCCGCGCCGATGATG
GTGACGAATACCGTTGGCGCCGCGCTGTTCATGCGTATTTTGCTCGATAAGCGCGCCATG
TTCGAAAAATATACTTCGGCATTTTCTGCTACCGCGCTGAAGGTCGCCGCGTCAACGGAG
GGGATTCTGCGTCAGGGATTTAACGAAGTGAACAGTATGAAGGTGGCGCAGGTGTTATAT
CAGGAGCTGGATATTGGCGCCGTCGCCATCACCGATCGCGAAAAACTGCTGGCTTTTACT
GGTATTGGCGACGATCACCATCTACCGGGCAAACCCATTTCATCAGGTTATACGCTGAAA
GCAATTGAAACCGGAGAGGTGGTTTATGCCGATGGCAACGAAGTGCCGTATCGCTGTTCG
CTACACCCGCAGTGTAAACTCGGCTCGACGCTGGTGATCCCGCTGCGTGGCGAAAATCAG
CGAGTCATGGGCACCATTAAATTGTACGAAGCGAAAAACCGGCTGTTTAGCTCAATTAAC
CGCACCCTGGGAGAGGGTATTGCGCAGCTTTTATCCGCGCAGATCCTGGCCGGGCAGTAT
GAACGGCAGAAGGCGTTGCTGACGCAGTCAGAGATCAAGCTGTTGCACGCGCAGGTGAAC
CCGCATTTTCTGTTTAACGCGCTCAATACCATTAAAGCGGTGATTCGCCGCGACAGCGAA
CAGGCCAGCCAACTGGTGCAGTACTTGTCGACCTTTTTTCGCAAAAATTTAAAACGCCCG
TCGGAAATCGTCACGCTGGCGGATGAAATTGAACACGTAAACGCTTATCTGCAAATTGAA
AAAGCGCGTTTTCAGTCGCGTCTGCAGGTACAGCTTGATGTTCCATCGACGCTTTCACGT
CAGAAATTGCCTGCGTTTACATTACAGCCGATTGTTGAGAACGCCATTAAACATGGCACG
TCGCAACTGCTTGATACCGGCAACGTCGCTATTCGCGCCCGGCGCGAAGGGCAGCATTTG
ATGTTAGATATTGAGGATAATGCGGGACTGTATCAGCCTTCCGCCGGCAGTAGCGGGCTG
GGGATGAGTCTGGTTGATAAACGTCTGCGCGAACACTTTGGCGATGATTATGGTATTAGC
GTGGCCTGCGAGCCGGACTGTTTTACCCGAATTACATTACGACTTCCACTGGAGGAGGAC
GCATGA
>EECEBICD_01194 Transcriptional regulatory protein BtsR
ATGATTAAAGTGCTGATTGTGGATGATGAGCCGTTAGCGCGGGAAAATCTGCGGATTTTG
CTCCAGGGGCAGGATGACATTGAGATTGTGGGAGAGTGCGCGAACGCGGTAGAAGCGATT
GGCGCGGTACATAAGTTGCGACCTGATGTGCTGTTTCTGGATATTCAGATGCCGCGTATC
AGTGGACTGGAGATGGTAGGAATGCTTGATCCGGAACACCGCCCGTATATCGTTTTTTTA
ACCGCGTTTGACGAATACGCCATCAAAGCCTTTGAAGAACACGCTTTTGATTATCTGCTC
AAGCCGATAGAGGAGAAACGGCTGGAAAAAACGTTACATCGTCTGCGTCAGGAGCGCAGT
AAACAGGATGTTTCGTTGTTGCCGGAAAACCAGCAGGCGCTTAAATTCATTCCCTGTACC
GGACACAGCCGGATCTATTTGTTGCAAATGGATGATGTCGCCTTTGTCAGTAGCCGTATG
AGCGGCGTTTATGTGACCAGCAGTGAAGGGAAAGAGGGGTTTACCGAGCTGACGCTGCGC
ACGCTGGAAAGCCGGACGCCGCTACTGCGTTGTCATCGTCAGTTTCTGGTGAATATGGCC
CATTTGCAGGAAATTCGGCTGGAGGATAATGGGCAGGCAGAGCTGATTTTACGCAACGGC
CTGACGGTGCCGGTAAGCCGTCGCTATCTGAAAAGTTTAAAAGAGGCGATTGGCCTGTAA
>EECEBICD_01195 hypothetical protein
ATGCTGAGTAACGATATTCTGCGTAGCGTGCGCTACATTTTAAAAGCTAATAATACCGAT
CTGGCGCGTATCCTGGCGCTGGGTAACGTTGATGCTACGCCGGAGCAGATAGCAATCTGG
TTGCGCAAAGAAGAGGAAGAGGGGTTTCAGCGTTGCCCGGATATCGTGTTGTCCTCATTT
CTCAATGGCCTCATTTATGAAAAACGCGGCAAAGATGAGGCGGCGCCTGCATTGACGGCG
GAACGTCGTATCAACAACAATATTGTGCTGAAAAAGCTGCGTATTGCCTTTTCGCTAAAA
ACAGATGATATCCTGGCGATACTTACCGGTCAGTTGTTTCGTGTCTCAATGTCAGAGATC
ACCGCGATGATGCGCGCGCCGGACCATAAGAACTTCCGCGAATGCGGCGATCAGTTTATG
CGTTATTTTCTGCGCGGTCTGGCGGCCCGTGAACACGCGGCGAAGTAA
>EECEBICD_01196 hypothetical protein
ATGCAGGTTCTACGTCTTATGGCACTGCCACTATTCGCGCTCTCTCTATCGGTTAGCATA
ACTGGCTGCGATCAGAAAAACGATACTCTCCAGGGAAAGCAAAATAACATGACAGCGTTT

ATCAAGAAGATAGCCGCTAGCAAAGAGTCAGAGGAAACACAACGCTATGTAGGTAATCTC
AACGGTATTGAAATCAAGTTAACCTATTACTACAAAGGGGATATCGTTTTACGTCAAATA
TCTGAACATAAACTACTTTATAAGACCCTGAAAGCCAATAATAAAGAAGAAGCACAAAAA
ATGCTGAGTCAAGTCGGCGAAGCTTATCAGGGTATGCCGGGTTTGACTGAACGAATCGAC
TATTATGATAGCTATGCTACGGAATATGTGGATATTGATTTTACCCAGGCAAAAATAAGC
GACCTCTGTAAATTGCCAGGATCATCAATTGACAACTGTTCCGCGTACTATCTGTCAATG
ATTCGCTCGCAGAAACTGTTGGAAGAGAGCGGGTATCATAGAATCAATTAG
>EECEBICD_01197 putative lipoprotein YehR
ATGAAAATTTCTGGCAAGTTATTGTCCACGGCTTTGGCTTCCGTACTGGTGTTCTCTCTT
GCTGGCTGTGGCGATAAAGAAGAATCAAAGACCTTTAACGCAAACCTGGCGGGGACAGAA
ATTTCAATTACTTACACCTATAAAGGTGACAAAATCATTAAGCAGACGTCTGAAAGTAAA
ATCAGCTATGCCACTGTAGGCGCTAAAACGAAAGAAGATGCCGCCAAAATTCTCGATCCG
CTGAGCGCGAAATATAAAAATATCGCCGGAGTGGAAGAAAAATTAACCTATGAAGATACC
TATGCCCAGGAAAACGTCTCTGTGGATATGGAAAAAGTGGACTTTAAAGCGTTACAGCAA
ATCTCAGGGACGATGGTGTCCGGCGATACCAGCAAAGGTATCAGCATGAAACAAACCCAG
ACGCTGCTGGAAGCTGCTGGTTTTAAAGAAGCGAAATAA
>EECEBICD_01198 Methionine--tRNA ligase
ATGACTCAAGTCGCGAAGAAAATTCTGGTAACGTGCGCGCTGCCGTACGCCAACGGCTCT
ATCCACCTCGGCCATATGCTGGAGCACATCCAGGCTGATGTCTGGGTCCGTTACCAGCGA
ATGCGCGGCCATGAGGTTAACTTCATCTGTGCCGATGACGCTCATGGCACGCCGATCATG
CTGAAAGCGCAGCAGCTTGGTATTACGCCGGAGCAAATGATCGGTGAAATGAGCCAGGAG
CACCAGACCGATTTCGCCGGTTTTAATATTAGCTACGACAACTACCACTCAACGCACAGC
GACGAGAATCGCGAGCTGTCCGAGCTGATTTATACGCGCCTGAAAGAGAACGGTTTTATT
AAGAACCGCACTATCTCTCAACTCTACGATCCGGAAAAAGGCATGTTCCTGCCGGACCGA
TTTGTGAAAGGCACCTGCCCGAAATGTAAATCCGCGGACCAGTACGGCGATAACTGTGAA
GTCTGCGGCGCAACTTACAGCCCGACCGAACTTATCGAGCCGAAATCCGTGGTGTCCGGC
GCGACGCCGGTAATGCGTGACTCCGAGCACTTTTTCTTTGATCTGCCGTCATTCAGCGAA
ATGCTGCAGGCGTGGACCCGCAGCGGCGCGCTGCAGGAGCAGGTGGCGAACAAAATGCAG
GAGTGGTTTGAATCCGGCCTGCAACAGTGGGACATTTCCCGCGACGCGCCGTATTTTGGT
TTCGAAATCCCGAACGCGCCGGGCAAATATTTCTACGTCTGGCTGGACGCGCCGATTGGC
TATATGGGCTCCTTCAAAAATCTGTGCGATAAGCGCGGTGACACGACCAGTTTTGATGAG
TACTGGAAAAAAGACTCCGACGCCGAGCTGTATCACTTTATCGGCAAAGACATCGTCTAT
TTCCACAGCCTGTTCTGGCCTGCCATGCTGGAAGGCAGCCACTTCCGTAAGCCGACCAAC
CTGTTCGTTCACGGTTACGTGACGGTGAACGGCGCGAAGATGTCTAAGTCTCGCGGCACC
TTTATTAAGGCCAGCACCTGGCTGAAACACTTTGACGCCGACAGCCTGCGCTACTACTAC
ACCGCGAAGCTTTCTTCACGCATTGATGACATCGACCTGAACCTGGAAGACTTTGTCCAG
CGCGTCAATGCCGATATCGTCAATAAAGTAGTCAACCTGGCATCCCGTAACGCCGGTTTT
ATCAATAAGCGTTTCGACGGCGTGCTGGCGGCTGAACTGGCCGATCCGCAATTGTACAAA
ACCTTTACTGACGCCGCTGCGGTGATTGGCGAAGCATGGGAAAGCCGTGAATTCGGCAAA
GCTATCCGTGAGATTATGGCGCTGGCCGACGTCGCTAACCGTTATGTTGACGAGCAAGCG
CCGTGGGTGGTGGCTAAACAGGAAGGCCGCGACGCTGACCTGCAGGCCATTTGCTCGATG
GGCATCAACCTGTTCCGCGTGCTGATGACGTATCTGAAACCGGTACTGCCGACGCTTTCT
GAACGCGTTGAAGCCTTCCTGAACAGCGAACTGAACTGGGATGCCATCGAACAGCCGCTG
CTCAGTCACAAGGTCAACACCTTTAAGGCGCTCTACAATCGCATCGACATGAAGCAAGTT
GAAGCGCTGGTTGAAGCGTCTAAAGAAGAGGTGAAAGCCGCAGCCGCACCGGTTACCGGC
CCGTTAGCCGACTTCCCGATTCAGGAAACCATCACCTTTGACGATTTCGCCAAAATTGAC
CTGCGCGTAGCATTGATTGAAAACGCTGAGTTCGTGGAAGGCTCCGACAAATTGCTGCGT
CTGACGCTGGATCTGGGCGGCGAGAAGCGTAACGTCTTCTCCGGCATTCGTTCCGCCTAC
CCGGACCCGCAGGCGCTGATCGGCCGCCAGACGGTAATGGTCGCCAACCTCGCGCCGCGC
AAAATGCGCTTTGGCGTCTCCGAGGGAATGGTGATGGCCGCAGGCCCTGGCGGGAAAGAT
ATCTTCCTGTTAAGCCCTGATGACGGCGCGAAGCCTGGCCAACAGGTGAAATAA
>EECEBICD_01199 Iron-sulfur cluster carrier protein
ATGAACGAACAATCCCAGGCCAAATCACCAGATACCCTGCGAGCGATGGTCGCCGGGACG
CTGGCGAATTTTCAGCACCCAACCCTGAAGCATAACCTCACCACGCTTAAGGCGTTGCAC

CACGTCGCCTGGATGGATGATACGCTGCACGTTGAGCTGGTCATGCCGTTCGTCTGGAAC
AGCGCGTTTGAGGTATTAAAAGAGCAATGCAGCGCCGATCTGTTACGTATTACGGGCGCA
AAAGCGATCGACTGGAAACTGTCGTACAACATTGCGACGCTGAAACGCGTCAAAAATCAG
CCTGGCATTAATGGCGTGAAAAACATCATTGCCGTGAGCTCCGGTAAAGGCGGGGTGGGG
GAAATCGTCCACGGCGGTAAACCTGGCGCTGGCGTTAGCGGCTGA
>EECEBICD_01200 Iron-sulfur cluster carrier protein
ATGCTTGGCGCAGAAGATCAGCGCCCGACATCGCCAGACGGTACGCATATGGCGCCGATT
ATGTCTCACGGCCTGGCGACCAACTCGATCGGCTATCTGGTGACTGACGATAACGCGATG
GTATGGCGCGGCCCAATGGCCAGTAAGGCGCTGATGCAGATGTTGCAGGAGACGCTGTGG
CCGGATCTGGATTATCTGGTACTGGATATGCCGCCGGGAACCGGGGATATTCAGCTAACG
CTGGCGCAGAACATTCCTGTTACCGGCGCGGTGGTGGTGACTACGCCGCAGGATATCGCG
CTGATCGACGCCAAAAAAGGTATCGTGATGTTCGAGAAGGTGGAAGTGCCGGTGCTCGGC
ATTGTGGAAAATATGAGTATGCATATTTGTAGCAACTGCGGCCATCACGAACCGATCTTC
GGCACGGGCGGCGCGCAGAAGCTGGCGGAAAAATACCATACTCAGCTTCTGGGTCAGATG
CCGCTGCATATTAGTCTGCGCGAAGATCTCGATCGTGGTACGCCGACGGTGGTTAGCCGT
CCGGAGAGCGAATTTACCGCGATCTATCGCGAACTGGCCGATCGCGTTGCGGCACAGCTT
TACTGGCAGGGTGAAGTGATTCCGGGTGAAATCGCCTTCCGCGCGGTGTAA
>EECEBICD_01201 hypothetical protein
ATGAAAAAATATTTGCTGATGGGAATTATAGTTTCGGCTTATGGGATAAGCGTACCCGTT
TTTGCCTCCGACACCGCAACATTAACCATCAGTGGAAAAGTGACCGCGCCAACGTGCAGT
ACAGAAGTGGTAAACGCACAGTTGCAGCAGCGTTGCGGCAACACTATCCACGTTTCAACT
CTGCAAACACCCGCCGCTACGCCGATGAGAGGTGTGACAACGCAGCTATACACGGTGCCT
GGTGATAGCACCCGACAGATTGTTGTTAATCATTACGACTAA
>EECEBICD_01202 putative fimbrial-like protein YehD
ATGAAACGTTCACTTATTGCTGCTTCTGTATTGTCTGCTGTATTTATGAGCGCTGGGGTT
TTTGCTGCTGATGAAGATATGGGGGAATTAAAAATAAACGGAGAAGTGGTGGGAACATCC
TGTACTTTCGAAGGTGCAAATAGCGCGACTATTGAATTATCCCAGGTAGGTGTTGATAGA
TTGACTGACTTAAACCCTGGCGATATATATACAGGATACACTAGCCCAGAAGCGATTTTA
AAAGTAAGATGTACGAATACAGCTAATCCACGAATTAGTTTTAACCGTTCTCAATTTGTG
GATAACATGCAAATCACCAAAAATAATGCTACTAATAATGGTGCGGGCTTCGCTGTTTAT
CTTGATGGTATTCAGGTGAAACCGGATGAGGCGGGGAATTACACTCTGAATTCAAGTAAG
TTTGAAAATGGTGTATATACCCTGAACTTTTCTGCCCGCTATGCCGCCGTTGAAAATACT
GTAACACCAGGTTCTGTTGAATCTGTACTGACGATGACGGTATTAACTGATTAA
>EECEBICD_01203 putative fimbrial chaperone YehC
ATGAAATGGATGATTGCATTATGCCTGGCTTGTGCCGCTATGCCTGCGTGGAGCGGCATT
TATATATATGGTACACGTATTATTTATCCGGCGCAGAAAAAAGATATTACCGTACAATTA
ATGAATGACGGTAAACGAAGTTCACTCATCCAGGCGTGGATTGATAACGGTGATACCAGC
CTGCCGCCAGAAAAATTACAGGTTCCATTTATTATGACCCCCCTGTAA
>EECEBICD_01204 putative fimbrial chaperone YehC
TTGCCTGGCGATCGTGAAAGTCTTTTCTACCTCAATGTATTGGATATTCCACCAAATTCA
GACGAGAACAAAGATAAAAATATCATTAAATTTGCGCTACAAAACCGGATAAAGTTGATC
TATCGCCCACCGGGGGTACAGAAAGTCGATAAAGCAACATTTTCCCGCTTGCAGTTGCTT
CGTGCGCCAGGGAGTATTAGCGTGAAAAATAATTCCGCAAACTGGATCACTATTCCAGAA
ATTAAAGCAAAAAGTAAAGTAAATAAAGAAACCTTATTACTGGCTCCATGGTCATCGCAG
AGTATCACCACAACGGTAGTCGTAAATAGCTATACCGTGACGCTAATTGATGATTCTGGT
AATTATTTAAACGAGACTGTCAAAATAGAAAATTAA
>EECEBICD_01205 Outer membrane usher protein YehB
ATGGAAAAGTTAAACGCCATCACGCTGTCACTTTTTGCAGCATTACTTATTTCACCCGCC
TGGGCTGAAGAAGAAACATTCGATACTAACTTTATGTTCGGTGGGTTGAAAGGTGAAAAG
GTGTCTCGTTATCAGATTGATAGCACCAAACCCATGGCTGGCGTTTATGAAATGGACGTT
TATGTCAATAAAGAATGGCGTGGAACTTATGAGGTTAATATTCAGGATGACCCCGATAGC
ACCTGTATTTCTCCGGACCTGATCGCCAGCCTGGGAATTAAATTTACCCCGCAGAGTACA
ACAGTAGAAAATGAATGTATTGCGCTGAAAACGGTGGTTCATGGAGGTAGTGTTTCTTAT
GACACTGCCGCTTTTAACCTTTATCTGAGCGTCCCTCAGGCTTATGTGCTGGAATATGAA

GCGGGCTATGCGTCTCCAGAGACATGGGACCGTGGAATTAACGCTTTTTATACATCGTAT
TATGCCAGCGAATATTACAGCCATTATAAAAGCGGCGGTAGTGAGAAAAATACTTACGCA
AATTTTGTCAGCGGATTGAATCTGCTTGGATGGCAATTACATTCTAACGCTAATTTTTCC
AAAAGTGAAAATTCAGCTGGCAAATGGCAGAGCAATACTCTGTATCTGGAACGTGATTTC
CCGGCGGTACTGGGGACAATGCGTCTGGGGGAACAGTACACCAGCGGGGATATGTTTGAT
ACGGTGCGCTTTCGCGGGGTGCGCTTCTGGCGTGATATGCAAATGCTGCCGCACTCTAAG
CAAAATTTCGCTCCGGTTGTGCGTGGCGTGGCGCAAAGTAACGCCCTGGTGACGGTTGAA
CAGAATGGCTTCATTGTTTATCAAAAAGAAGTTCCGCCTGGCCCCTTTGTATTTGAAGAT
CTTCAGCTGGCAGGGGGGAGCCGATCTGGACGTGAGCGTTAA
>EECEBICD_01206 Outer membrane usher protein YehB
TTGTATTTGAAGATCTTCAGCTGGCAGGGGGGAGCCGATCTGGACGTGAGCGTTAAAGAG
GCTGACGGTACTGTTTCACGGTTTATTGTTCCGTATTCTTCCGTTCCCAATATGGTGCAG
CCCGGCGTAGCAAAATATGATTTCGCCGCCGGACGAAGCAGAATTGAAGGAGCCTCTCAG
CAAACTGATTTTCTCCAGGGAACCTATCAGTACGGGGTCAATAACCTGCTAACGTTGTAT
GGCGGAACGATGTTAGCCAGCGATTATCGCTCATTTACCCTTGGCACCGGATGGAACACG
CTGATTGGCGCGGTATCTGTCGACGGTACGTTGTCTCATAGCAAGCAGGATAATGGCGAT
GTTTTTGATGGGGAAAGCTACCAGGTTGCCTGGAATAAATATCTCCCGCAATCGGCCACC
CATTTTTCTCTGGCAGCGTATCGCTACTCATCACGTGATTATCGCACTTTTAACGATCAT
GTCTGGGCTAATAACCGGGATAACTATCGTCGCGATGATGACGATATTTATGATATTGCT
GATTATTATGAGAATGACTTTGGGCGTAAAAATACTTTCACTCTCAATATTAATCAGACG
TTACCCGATGGGTGGGGATATTTTACTGCCAGCGCTCTGTGGCGCGACTATTGGGGGCGT
AGCGGCACGGGAAAGGATTATCAGTTAAGCTACAGTAATACCTGGCAGCGGCTCAGTTTT
ACGCTATCGGCAACTCAGACGTATGACAGCAATAACCGTGAAGATAAGCGCTTTAATATC
TATTTATCGATTCCCTTAACATGGGGATTAAAGGAGAATGGGGGAAATCGTGATATCCAT
TTATCGAATTCCACCACCTTTGACGATCAAGGTTATGAGGCGAATAACACCAGCCTTAGC
GGCACGTTCGGTAATCGCGATCAGTTTAATTATACGACTAACCTCAGCCAGCAGCGACAG
GAACACCAAACGACATTCGGGGGATCGGTAACCTGGAACGCCCCGCTGGCGACAGTCGGC
GGCAGTTACAGCCAGTCCAATAAATACCATCAGGTGGGCGGAAATATTCAGGGAGGGCTG
GTTGCATGGGCCGATGGCGTGCATTTAGCCTCTCGCCTTAATGATACTATCGCGATTATT
AACGCGCCGTACCTGGAAGGCGCGGCGGTGCAGGGGCGTCCCTATTTACGTACCAATGCA
AAAGGTTATGCCGTATTTGAAGCATTGACGCCATACCGGCAAAATTTTATCTCTCTTGAT
GTCTCTGAGAGTGAAAGCGATGTCGCCCTGTTGGGAAATCGAAAAGTTACAGTGCCTTAT
CGCGGAGCCGTAGTATTGGTTCATTTTGAAACAGAGACCAGTAAACCTTTTTATTTCCTG
GCGCGGCGCGCAGATGGCGAGCCTTTAACATTTGGTTATGAAGTGGAAGATGACGAGGGA
AATAATGTAGGACTGGTAGGTCAGGGGAGCCGGGTATTTATTCGTACTGAAAAGGTTCCC
ATTTCGGTAAAAATCGCAACAGATAAACAACAGGGTCTTTTCTGTAAGATCACTTTTGAT
AAACAGATTGATGAAAATAACGTTTATATTTGTCGGTAA
>EECEBICD_01207 hypothetical protein
ATGAAAAGACTACTATTTTTATTAATGATGATGCAGTTTACGCAGTTGGGTTACGCGGCG
TGTAATGCGACCTTAACCAACACGAAGGATTATACGATTCAATCCGACAGCTTAATGCTG
GGGGGGAGGAGTCAGCCATTATTACAAATGGTTTCACAGATGCCAATTGTTCTAATAGTG
ATGTTGTGA
>EECEBICD_01208 putative fimbrial-like protein YehA
GTGACTAAATTAGAGACATCCGATCATATTATCGGTATGGGACCGAATGACAGTGTTAAG
TTAAAGTTAAAAGTTGAATGGCAGAGCAGTAGTGCGATAAATATGCGCAATCAAAACGGT
GTAATTGAAGCCCCCTATAAGATAACGATTTCAGAAATAAACTCACTGGAGGCGGTGACT
GTGTCTGCCAGGGGGGGGTACTCGGTTACGATAGATAATATAACGGTGATGTCGGGTGCA
AAGAATCAATGGAGCAGTAGCGATTGGGTGGTATTTATTTTAAGATATATTGGCTGTTGG
GGGAATAGAGAGTGTGTTGCAAACGTTATCACTGATCTGAATGGGAAAGGCGTTTATAAA
GCCAGTTTAACGATAAATTACAACCGTAAAGAAACTACCTGCGCCCCCCAGAATCTCACC
ATCACACTGGACCCGGTGCCTATGACTAAACTCCGTGCTAAAGGAGAGGTTAGTGAGTTT
TCTAAGCAGGGTGATATTATTCTGGATTGCAAAAATCAGGTGCGGAATGCGATGCTGACA
TCACGGCAAATAGCGGTAAATCTACGTAGTGATTTAGTCTGGGATGAGAATGAAGCAGTC
CTGAAACCGGATAACGATAATGGCGTTGGGTTTATCTTAAGAGATTCAAATCGGAGCCTG

CTTAAAATAAACAAAGGAGTAGCGATAAATAGTATTTTCAAAACCTATGCCAAAGGCAGC
GCAGTGAATTCGGTGGAAGCTATTCCTGTCTTTGCCACATATTATGTTCTCGATCCTGCA
AAAGTTAAAGCGGGGCCATTACAATCGAAAGCATTAATCGTGGTATCTTATAATTAA
>EECEBICD_01209 Nickel/cobalt homeostasis protein RcnB
ATGAAAAGTAAGTTGCTGCCTTGCGCGCTGTTGCTTGCAACCAGTTTTGCATGGGCCGCC
CCCGCGACAACGGGCATTGATCAATATGAGTTAAAAAGCTTTATCGCCGACTTCACCCAC
TTCAAGCCCGGCGATACCGTTCCGCAAATGTACCGCACCGATGAGTATAATATTAAACAG
TGGAAATTACGTAATCTGCCCGCGCCAGATGCCGGTACTCACTGGACCTATATGGGCGGC
GCGTATGTACTGATTAACGATACCGATGGAAAAATTATCAAAGCCTATGATGGCGAGATT
TTTTATCATCGATAA
>EECEBICD_01210 Hydroxyethylthiazole kinase
ATGCAGCCTGACCTGCACTGCCGCACGCTTGCGGCGCATACGTTAAAACACTTTCGCGCG
CTCTCCCCGCTTACGCACTGTATGACGAATGACGTCGTACAAACGTTTACCGCCAATACG
TTGCTGGCGCTGGGCGCTTCACCCGCCATGGTGATTGATCCTGTCGAGGCCAGACCGTTT
GCCGCCATCGCCAACGCCTTGCTGATTAATGTCGGAACATTAACTGCCTCACGCGCTGAC
GCGATGCGTGCGGCGGTAGAAAGCGCTTATGATGCCAAAACGCCGTGGACGCTTGATCCT
GTCGCGGTGGGCGCGCTGGAATTTCGTCGGCGATTTTGCCTGGATCTCTTGTCCCTGCGC
CCGGCGGCAATACGCGGCAACGCCTCGGAAATCCTGGCGTTATCCGGCATGGCGCTGGGC
GGACGTGGCGTAGATACCACCGAGGCGGCGTTGGCCGCACTGCCTGCGGCGCAGGCGCTG
GCGCGTCAGATAGACTGCATCGTTGTGGTTACCGGAGAGATAGATTACGTCACTAATGGT
CAGCGCACGCTGAGCATTCCCGGCGGCGATCCGTTAATGACTCGCATTGTAGGCACCGGC
TGCGCGTTGTCGGCGGTCGTCGCCGCCAGTTGCGCGTTACCGGGCGCCGCGCTGGACAAT
GTCGCGTCGGCCTGCTGCTGGATGAAACTGGCTGGACAGGCCGCGGCAGAGCGTAGCGAA
GGACCTGGTAGCTTCATCCCGGCTTTTCTTGATGCGCTCTATCATCTGGATGTGGAGGCG
GCCAATGCAACGAATTAA
>EECEBICD_01211 Hydroxymethylpyrimidine/phosphomethylpyrimidine kinase
ATGCAACGAATTAACGCGCTGACGATTGCCGGCACCGATCCCAGCGGCGGGGCCGGTATC
CAGGCGGATCTCAAGACCTTTTCCGCGCTGGGCGCATATGGCTGTTCGGTCATCACCGCG
CTGGTGGCGCAAAATACCTGTGGCGTACAGTCGGTGTACCGGATAGAGCCGGACTTTGTC
GCCGCTCAACTTGATTCCGTGTTCAGCGATGTGCGCATTGATACCACAAAAATCGGCATG
CTGGCGGAAACCGATATTGTCGAGGCCGTGGCGGAGCGACTACAGCGTCATCATGTACGT
AATGTGGTGCTGGATACGGTGATGCTGGCGAAAAGCGGCGATCCGCTGCTCTCGCCCTCT
GCGATAGAAACATTACGCGTCCGGCTGTTGCCACAGGTATCGCTGATTACGCCTAATTTG
CCGGAAGCCGCAGCGTTGCTGGATGCGCCCCATGCACGTACGGAACAGGAGATGCTGGCG
CAGGGGCGGGCGTTACTGGCGATGGGGTGTGAAGCGGTATTGATGAAAGGCGGGCATTTA
GAGGATGCGCAAAGCCCGGACTGGCTCTTTACCCGTGAGGGCGAGCAACGTTTTAGCGCG
CCGCGTGTGAACACCAAAAATACGCATGGGACGGGCTGTACGCTGTCGGCGGCGCTGGCG
GCGTTACGGCCCCGGCATCGCTGTTGGGGAGAGACGGTAAACGAGGCGAAGGCATGGCTT
TCGGCGGCGCTGGCGCAGGCGGACACGCTGGAAGTGGGGAAGGGCATTGGTCCGGTACAT
CATTTCCACGCGTGGTGGTAG
>EECEBICD_01212 putative fructoselysine utilization operon
transcriptional repressor
ATGGAACAAGCGCATACCCGGCTCATCGCGCAACTGAATGAACGAATTTCAGCGGCGGAT
AATACGCCGCTATACATGAAATTCGCCCAGACGGTAAAGGATGCCGTGCGCAGCGGTATT
CTTGAGCACGGTAATATTTTGCCTGGCGAACGCGATCTCAGCCAGCTTACCGGCGTCTCG
CGCATCACGGTACGAAAAGCGATGCAGGCGCTGGAAGAAGAGGGGGTCGTCACGCGCGCC
CGCGGCTACGGTACGCAAATCAATAATATCTTTGAATATTCCCTGAAAGAGGCGCGGGGA
TTTTCTCAGCAGGTTGTCTTGCGCGGCAAAAAACCGGATACGCTATGGGTTAACAAACGC
GTGGTAAGTTGTCCGGATGAGGTCGCGCAGCATCTGGCGATCCCCGCAGGCAGCGACGTC
TTTTTACTCAAACGCATTCGTTATGTTGATGAGGACGCGGTATCGATTGAAGAGTCCTGG
GTGCCCGCGCATCTGATTCATGATGTCGACGCGATTGGCATTTCGCTGTATGACTATTTT
CGTCGCCAGCACATTTACCCTCAGCGTACCCGATCCCGCGTCAGCGCCCGGATGCCGGAT
GATGAGTTTCAGTCGCATATTCAAATGGATGGTAAAGTGCCGGTGCTGGTGATTAAGCAG
GTGGCGCTCGATCAGCAGCAGCGGCCTATTGAATACAGCATCAGTTATTGTCGTAGCGAC

CTATACGTTTTTGTGTGCGAGGAGTAG
>EECEBICD_01213 putative sugar kinase YdjH
ATGAACGGCGCACGGTTGCTGACATTATTACCGACCCTGCATACCCGGCGTCCGCTCACC
GTTATCGGCGCGGCAGTGATCGATATCATTGCCGACGCCTGGACGTTGCCGCGTCGCGGA
GGCGATATTGAACTGCAACAACAGAGCGTCAATGTCGGCGGCTGTGCGCTGAATATTGCC
GTGGCGTTGAAACGACTGGGGATTGACGCCAGCAATGCGCTGCCGCTGGGCCAGGGCGTG
TGGGCGGAGAGGATTCGCCGCCGTCTGGCAAAAGAGGGATTATCCAGCGTGATTGACGCG
GTTGAAGGAGATAACGGCTGGTGTCTGGCGTTAGTCGAACCGGATGGCGAACGCACGTTT
ATGTCTTTTAGCGGCGTGGAAAACCAGTGGAATGCGGACTGGCTGTCGCAGCTCAGGACA
CCGCGGGGCAGTCTTGTCTACCTGTCCGGCTATCAACTGGCCTCGTCCTGTGGCGAGCCA
CTGATCCAGTGGCTGGAAACCGTCGACGATGTAACGCCTTTTATCGATTTTGGCCCGCGA
ATCGGTGATATTACAGACGATGCAATGGCGCGCATTATGGCCTGCCGGCCCATCGTATCC
CTCAATCGCCAGGAAGCCATCATCGCCGCGGAACGCGCCGGGCTTGCGACCGATACCCGC
ACTTTCGGCGAAGCATGGGTAAAACGCTTTTCCGCGCCGCTGGTGGTGCGCCACGATAAA
GACGGAGCGTGGTATTTTAGCGCGCAATCTTCCGGCGTTGTGCCAGCGTTTCCGGCAACC
GTGGTGGATACCATCGGCGCAGGCGACAGCCATGCTGGCGGCGTGCTGGCAGGACTGGCG
TCAGGCTGGTCGCTCGCGGATGCCGTACTGCTGGGAAATGCGGTGGCGAGCTGGGTGGTC
GGGCATCGCGGCGGCGACTGCGCGCCCACGCGCGAGGCGCTACTCCTCGCACACAAAAAC
GTATAG
>EECEBICD_01214 ADP-ribosyl-[dinitrogen reductase] glycohydrolase
ATGAAAGCAGAACGTATTCTCGGTGCTCTTTACGGGCAAGCGTTAGGGGATGCGATGGGA
ATGCCCTCTGAGCTGTGGCCGAGAACGCGCGTCAAAGCGCATTTCGGCTGGATTGACCGC
TTTCTGCCCGGGCCAAAAGAGAATAATGCCGCCTGCTATTTTAACCGCGCGGAGTTTACG
GATGATACCGCAATGGCGCTCTGCCTGGCCGACGCGCTACTCGAATGCGAAGGCAACATT
GATCCCGAGATTATTGGGCGTAACATCCTCGCCTGGGCGGAACGCTTTGATGCGTTCAAT
AAAAATGTACTCGGCCCGACGTCAAAAATTGCGCTGAATGCGATTCGCGACGGCAAACCC
ATCGCGGCGCTGGAAAATAACGGCGTGACTAACGGCGCGGCCATGCGCGTTTCGCCGTTG
GGCTGCCTGTTGCCGCCAACTAACCTGACAGCCTTTATAGCAGAAGTAGCGTTAGCCTCC
AGCCCGACGCATAAATCCGATCTGGCGGTGGCTGGCGCCGTGGTTGTCGCCTGGGCCGTC
TCGCGAGCGGTTAACGGCGACACGTGGCAAAGTATTGCCGACTCGCTCCCCGCCATCGCC
CATCAGGCGCAGACAGCGCGTATTACCACGTTTAGCGCCTCGCTATCGGCACGTCTGGAA
CTGGCGCTAAACATTGTCCGCCACGCCGACGGCGTCGAATCTGCCAGCGAACAGCTCTAT
CAGATCATCGGCGCGGGAACCAGCACCATTGAATCGGTTCCCTGCGCTATCGCCATGGTG
GAGCTGGCGAATACCGATCCTAACCGCTGTGCCGTGCTGTGCGCTAACCTCGGCGGCGAT
ACCGACACTATCGGCGCGATGGCTACCGCCATTTGCGGCGCGTTGCACGGGGTAAGCGCC
ATTAACCCCCAGTTAAAGCAAACGCTGGACAAGGTTAATCAGCTGGATTTTGCGCGCTAT
GCCGTTGCGCTGGCGAGCTATCGTCAACGGCGGGAGGCACTATGA
>EECEBICD_01215 Putative nucleoside transporter YegT
ATGAAAACGACCTTTAAACTGTCGTTCATGATGTTCGTGGAGTGGTTTATCTGGGGCGCC
TGGTTCGTCCCCCTGTGGCTATGGCTAAATAAAAGCGGGTTTTCCGCCGGTGAAATAGGC
TGGTCTTATGCCTGTACCGCCATTGCGGCGATCCTGTCGCCGATTCTGGTCGGCTCCGTT
ACCGACCGTTTTTTCTCAGCGCAAAAAGTCCTGGCGGTACTGATGTTTGCGGGTGCCGTA
CTGATGTATTTCGCGGCACAGCAAACCACCTTTGCCGGATTCTTTCCGCTGCTCCTGGCC
TATTCATTGACCTATATGCCGACCATCGCGCTGACAAACAGCATTGCCTTCGCTAACGTA
CCGGACGTTGAACGCGATTTTCCACGCATTCGCGTTATGGGAACCATTGGGTGGATTGCC
TCCGGCCTCGCCTGTGGATTCTTGCCGCAAATGTTAGGCTATAACGATATTTCCCCCACC
AACATTCCACTGCTCATAACCGCCGCAAGTTCAGCGTTGCTGGGCGTATTTGCGTTCTGT
CTGCCAGACACGCCGCCAAAAAGCACCGGCAAGATGGATATTAAAGTGATGCTGGGGCTG
GATGCGCTGGTTCTGCTGCGCGATAAAAACTTTCTCGTTTTCTTCTTCTGTTCCTTCCTG
TTTGCGATGCCGCTGGCCTTCTACTACATCTTCGCTAACGGCTATCTGACAGAAGTGGGA
ATGAAAAACGCCACCGGCTGGATGACGCTCGGCCAGTTCTCTGAAATCTTCTTTATGCTG
GCGCTACCCTTTTTCACTAAGCGCTTTGGTATTAAAAAGGTATTATTACTTGGTCTTATT
ACCGCCGCGATCCGCTACGGCTTTTTTGTCTATGGCGGCGCGGAGACGTACTTCACTTAC
GCCCTGCTGTTCCTCGGTATTTTGCTGCACGGCGTAAGTTACGATTTCTACTACGTCACC

GCCTACATTTATGTCGATAAAAAAGCGCCGGTTCATATGCGTACCGCTGCGCAAGGGCTT
ATCACCCTCTGCTGTCAGGGATTCGGCAGCCTGCTGGGGTATCGCCTCGGCGGCGTGATG
ATGGAAAAAATGTTTGCTTATCCGCAACCGGTTAACGGTCTGACATTCAACTGGGCGGGC
ATGTGGACGTTTGGCGCAGTGATGATTGCCGTCATCGCGCTACTGTTTATGATTTTCTTT
CGCGAATCGGACAAAGAGATCACTGCTATTGACGATCGCGATATTGCGTTGACACAAGGG
GAAGTAAAATGA
>EECEBICD_01216 Fructose-bisphosphate aldolase class 1
ATGACTGATATTGCGCAGTTGCTTGGCAAAGACGCCGACAGCCTTTTACAGCATCGTTGT
ATGACCATTCCTTCCGATCAGCTTTATCTACCCGGCAAGGATTACGTTGACCGGGTGATG
ATTGACAATAATCGTCCACCAGCCGTTCTGCGTAATATGCAGACGCTCTATAATACTGGA
CGCCTGGCGGGAACAGGATACCTTTCGATACTGCCCGTCGATCAGGGAGTGGAGCACTCC
GCCGGCGCTTCTTTTGCGGCCAACCCGCGCTACTTTGACCCAAAAAATATTGTTGAATTG
GCGATTGAGGCCGGCTGTAACTGCGTGGCCTCTACCTACGGCGTACTGGCCTCGGTTTCA
CGGCGCTATGCGCACCGTATTCCGTTTTTGGTCAAACTGAATCACAACGAAACATTGAGC
TATCCGACAGAGTACGATCAGACCCTGTACGCCAGCGTTGAGCAGGCGTTCAATATGGGC
GCCGTGGCGGTAGGCGCGACGATCTATTTCGGTTCGGAACAGTCCCGTCGCCAGATTGAA
GAGATCTCCGCCGCTTTTGAGCGTGCGCATGAGCTGGGTATGGTGACCGTATTGTGGGCT
TACCTGCGTAATTCTTCCTTTAAAAAAGACGGCGTCGATTACCATGTATCCGCTGACCTG
ACGGGGCAAGCCAACCATCTGGCCGCTACGATTGGTGCGGATATTGTGAAACAGAAAATG
GCCGAGAATAATGGCGGTTATAAAGCGGTCAACTTCGGCTATACCGACGATCGCGTGTAC
AGCAAACTGACCAGCGATAACCCGATTGATTTGGTTCGTTACCAGTTGGCGAACTGCTAT
ATGGGACGCGCCGGGCTTATCAACTCCGGCGGCGCGGACGGCGGTGACACTGATCTCGGC
GACGCGGTTCGTACCGCGGTGATCAACAAACGAGCCGGTGGAATGGGACTAATTCTGGGA
CGTAAAGCCTTTAAGAAATCGATGGCTGATGGCGTGAAGTTGATTAATGCCGTACAGGAT
GTCTATCTGGATAGCAAAGTCACCATCGCCTGA
>EECEBICD_01217 putative lipid kinase YegS
ATGGCTAACTTTCCCGCCAGCTTACTGATCCTCAATGGCAAAAGCGCTGATAACCAGCCG
CTACGTGAGGCCATTACTCTACTTCGCGACGAAGGTATACAGATTCACGTTCGGGTAACC
TGGGAAAAAGGCGACGCGCAGCGTTACGTTGATGAAGCCCGTAGACTGGGCGTTGAAACG
GTGATTGCCGGCGGCGGAGACGGTACCATTAATGAAGTGTCGACCGCGCTCATCCAGATC
CGCGACGGCGTAGCCCCTGCACTGGGCCTTTTGCCGCTCGGCACTGCCAACGATTTTGCC
ACCAGCGCCGGTATTCCTGAAGCGCTGGACAAAGCGCTGAAACTGGCGATTGCCGGAAAC
GCGATGGAAATTGATATGGCCAGGGTCAATGATAAAACCTGTTTTATCAATATGGCGACG
GGCGGTTTTGGCACCCGCATTACCACCGAAACGCCGGAAAAACTTAAAGCCGCGTTGGGC
GGCGTCTCCTATCTTATTCACGGTCTGATGAGAATGGATACGCTGACCCCTGACCGCTGC
GAAATACGTGGCGAAAACTTTCACTGGCAGGGAGATGCGCTGGTGATCGGCATCGGCAAC
GGTCGTCAGGGCCGGCGGCGGGCAGCAATTATGTCCTACCGCGCTGGTTAA
>EECEBICD_01218 hypothetical protein
ATGAATGTCACTTCAGGCGTGAATGCGCAAACGCCATTGCTCCCTCCTTCTGAACGGGGT
AATGACGAAAAACCGGTAGCTGAAATCGTTGATTTTAACGCTTATGGCAATAAACCCAGA
TGTTTAATGTGCCTGGGAACCACGGCATTGTTTGCAGGTGTTTTCAGTGGGGTATGCTCA
GGGGCTGTCGCCAGTGTCAGTAGTGGTACTGCATATACTACCGCCCTGACGGTTTTAGGC
GCATCGTTTGGCATGGGGGGAATTGGTATGATGGGAATATGCGCCGGACTATACCTTAGC
GCAAATGGCATCAGAACCCGGCCAGCCTGGCCATAA
>EECEBICD_01219 Tir chaperone
ATGTATTCAAGAGCCGATCGCTTATTAAGACAGTTTTCACTAAAATTAAATGCCGATTCA
ATTGTCTTTGACGAAAACAGATTATGCTCATTTATTATAGATAACCGTTACCGTATCTTA
CTAACAAGCACTAACAGTGAGTATATAATGATATACGGTTTTTGCGGAAGACCTCCGGAT
AATAATAATCTTGCTTTTGAATTTCTCAATGCTAATTTATGGTTTGCAGAGAATAATGGA
CCACATTTGTGTTATGATAATAACTCACAATCATTATTATTGGCTCTCAACTTCTCACTA
AATGAAAGTTCTGTAGAGAAACTGGAATGTGAAATTGAGGTAGTTATTAGATCAATGGAG
AAACTGTATCACATTTTACAGGATAAAGGAATAACTCTGGATACCGATTATACGTGA
>EECEBICD_01220 hypothetical protein
GTGAAGAATAAATTCTCCGTTGGCGATAGTCTGGAGCTGATGACTCCGCAGGGAAATATC

AATTTCACCCTGGAACAGATGGAGAACGCCAAAGGCGACGCTATGCCGGTGGCACCTGGC
GATGGCTATACCGTCTGGATGCCCGTCCCGCAGGACGTTACGCTGGATTACGCACTATTG
ATGCGTAATTTCTCAGGCGAATCAACGCGTAACCCCCATGCTAAGTAG
>EECEBICD_01221 23S rRNA 5-hydroxycytidine synthase
ATGTTTAAACCAGAACTTCTTTCGCCGGCGGGAACGCTGAAAAATATGCGTTACGCTTTC
GCTTACGGTGCCGATGCCGTCTATGCGGGCCAACCACGCTACTCTTTACGCGTGCGTAAT
AACGAATTCAATCACGAAAATTTGCAGCTTGGCATCAACGAAGCCCACGCGCTCGGAAAA
AAATTCTACGTGGTGGTGAACATCGCCCCGCATAACGCCAAGCTCAAAACCTTTATCCGT
GACCTGAAACCCGTCGTCGAGATGGGCCCGGATGCGCTGATCATGTCCGATCCAGGGTTG
ATTATGCTGGTACGCGAGCACTTCCCGGCAATGCCGATTCACCTGTCGGTACAGGCTAAC
GCCGTAAACTGGGCGACGGTAAAATTCTGGCAGCAGATGGGGCTGACCCGTGTGATTCTC
TCCCGCGAACTGTCGCTGGAAGAGATTGAGGAAATTCGCCAGCAGGTGCCGGATATGGAA
ATAGAAATTTTCGTCCACGGCGCGCTATGCATGGCCTATTCCGGCCGCTGCCTGCTTTCC
GGCTACATCAATAAACGCGATCCGAATCAGGGCACCTGCACCAATGCCTGCCGTTGGGAA
TATAACGTGCAGGAAGGAAAAGAAGACGTTGTCGGCAACATCGTGCATAAGCACGAACCG
ATTCCGGTACAGAACGTTGAGCCGACGCTCGGTATCGGCGCGCCGACGGATAAAGTGTTT
ATGATAGAAGAGGCCCAAAGACCGGGCGAATACATGACCGCGTTCGAAGACGAGCATGGC
ACCTATATCATGAACTCAAAAGATTTGCGCGCTATCGCCCACGTGGAGCGCCTGACGAAA
ATGGGCGTCCACTCGCTGAAAATCGAAGGCCGCACCAAATCCTTTTATTACTGCGCCCGT
ACCGCACAGGTCTACCGTAAGGCCATCGACGACGCCGCCGCGGGTAAACCCTTCTGA
>EECEBICD_01222 hypothetical protein
ATGGAACAGGATGATCGTCTGCTCAACGCTATGTTTGAGATGTGCAACCACAAAAATCCC
CTCAATGACGGGCAACGAGAGTGGCATATCGCCGATATTTCCGGCCTGCTGCGCGAAGAA
CGCTATGACGAACTGGACGAACGCTACAACCAGACGCTGACGGAGAGCTTTACCAGCCGC
GAAGCGGAAAAGCGCTATTTCTTCGCCTGGAATCAAATGGACAACCCTTTTTACGATATG
GACACGCTGGTTGAGGCTGGGCCGCAAGGATTAGCGCTGATCAAAAACTGGCAGCGCGCC
CGCCCCCGCTCCACTCACGCCTGGCTTGCCGAGGCGCAATACTGGAATCACCGTGCGTGG
CTGTATCGCAGCTATGGCTGGGCGAGAGAAACCACCCGAGCGATGTGGATCTGCGCCGCC
GCCTGCAACGAACGGATGGTCATCGCCGTGCTCAATGCTATCGACTGTGAACCGCGCCAG
TGGATGGCGGCGGCCCTGACCAGCACCAACTCAAAAGTCTTCGGCCAGCCCGACTGGCTG
GTTGAGTTCCTCGAGGGCGCGGATGTTGCCGGGCAGCCATTAATGGAGGATCTGGCTGAA
TATCATCGCCATTCACCGCAGGAGGTGGACGCGCTGATGGCCCATTCCGGGCTGTCGTTT
GCCGACGCGGTTTGCCCAAATTTGCCGCGCCCGTCCGTGCTGCCGGAGTGCAATGACGAT
GCCGGGCAAAAATACTGGCTCACCGTCTGCCTGGCTATTTTCCCGACGGCATTTTACGTG
CTGGATGAATATATCCCGTTTCGTATGCCGCGCTGGGGAGGGTCTCACGAGGAAATCCGC
GAATTTCTGGAATCAAGCGTATGCGATCACCTTTCTGCCGCAGAACGTGAACATCTGGAG
CTATTGATCTGGTGGGACGATCACCGCGATCTGCGCATCAAAGAGGTTGACTCCCCGGCA
GAACAGGAACGCATTATCGCCAAAGCCGAAGAGATTAGCCTGCGAGCGCATATCCAGGAA
TCTCGCCATAACACGCTGAAGTGGCTGCGGGTCTGCTATAGCGATCTGGACGATAATGAC
GCGCTCTGGCGAACCCTTCAGCGTTCGATCGTGGAAAAAGTTAAGTTCAACAATTACTTC
TTTGATGACACGATAAAGTTCGCCCTGCGCGATTTTCCTGACACCTGGTGGATGTATAAT
TTTCTCTGTCAGAATGCGCAGCAAACGGAATTCGCAGTGCCGAAAATCCGTCGTGGCTAC
TTTCAGTACGCCGGGTTATTGGGTTTCGAAAAGGATGAGGCGCAAGGCCTCGCCTGGCTC
GATAGCGTCGCCGACATTCAATATAACCATAACTGGCGGGCGGCGATTAAAAATTTCGAC
TGGTTCGGGCTCCCGGAACATTTCGTGCCGCTGGCAGAGCTGGGCGCACAAAGAAATATA
CCCGCCGCGCTAAACCTGCTGGGCCTTGAACACAATAATAAAGAGAACAACGGCCTACTA
CCTTACGATCCTGCCATCGCGTTAGGGTATTTTCAGCGCGCGGCGGAGATTTTGCACCGG
CAGTTAGCGCTGCGCGAGAGCCCCCCGTACAAATTAATAGATAACGGAGGCTATACCGAC
TATGAAAATGACCTGCAAAATATTCATTTCAGCATTGGCATCTGCAACCAGCGTCTGAGT
AAACAAGAACCTGATACCGAAAAACGTTCCGCTTATGAAAAAGAACTGCTCGATAATCTC
TGGCTGGCGCATCAGTACGGGCATAAAGAGGCCTGGGGCTTGTTCCTGCTGAATATTTTT
GAAGTGAAGGATATTACGCTGGCGCATAAGCATCTGGAACTGGTGCAACAAGAAGCCAAC
AAAGGCACGCTACACGCTATGGTGACCCTATCGCGCCTGCATGGTAATAAACACGATCGA
ACGCTGTTTAATATGAAGCTAAGCGCACGCTGGGCGCATTTTGCCTTCACGCTTTACCCG

GATAATGAAATCGTTATGGACTGCCTGGACCACCTGCATTTCGATTCTTTCTGGAAACGC
TTCCGTTTCGCGTGGTATACCGTCCGCATCCCGAATTCAGAGCTGCCGGGACAGGTCAAC
TCAATGGTGTAA
>EECEBICD_01223 hypothetical protein
ATGTTCTGGATTGTTCTTATTATTGCCGCCCTATTTTTTAGCTGGCGCAACTACAAAAAA
AACAAACCGCTGATTGCGGCCCGTATCGCCGAGGAGAAAGAAAAAGATCGCCTGAAAGAC
CTGCGTCGCGATACCCGACGGCGGCAATGGGCGCTGGCGCTAGCTGATATCCTCGCCCGG
CGCAACGGGCTACCGATCAAAGGCGTTGAGCTGGTATTTAAACTTGACGATGACAAACGC
CGCTATCTGGCGCAGCAGGTAAAAAAAGAGCTGGGCTTGAGCGAAAACCTCGACGGCGCG
GCGTTACGCCACAAGGTTGAGGATATCTTACGCCGCTGGCCTGCGGGCATCGGCAGCTCG
CCGCGCACGTTTTATCATCATCTGGCGGCCCAGGGCCAGGTCCGCGACGCGCTGGCCTTC
GACTGTATGCGCACCGCCTTTCTGACCCGCTGTATCGCCGGGCTCGGCTGGTGTAACGAA
AACGAGGCCTGGCTGGTGCTATTGCTCAACGCCCAGCGCGCTCAGGACTGTTTTGATTCC
TGGGAGGATTACGCCACCGCCTACGTTCGCGCTCGCCGGGTATGGCTCACGCTACGTGAC
ACGCCAACAGCGCTTGCCGGACGGGATTTACAGGAAGCCACCCATTATCTGCAAGACCCT
GTCAGCCGCTGGCGACAACTGCCGTGGAATGAATTCAAAATTTTCGAGCCGATTTAA
>EECEBICD_01224 hypothetical protein
ATGACAGAAACGTCATCTCACCGCTATAAACCACGCAATATAATAAATGCGCCAAATGTA
AAATCGTCTATTTTTTCCCGGAGCCAACAACGCGGCGACAGCGAAAATATCCAGCGCTGG
TTAAGCAACCATTTTTATCGTTGGATCATCGGTGACTTTCCTCATGTTTATCCTGTGCGT
TCCGTCGCCGATTATGCCGTTTATTTTAGCGCTGATGCAGAAATCCCCGCCTGGCTGGCG
CCAAAGCTGGGTGGCGATGAGCGCTTTTACTACCTTAACGTTCAGCACCCGCAACTTGTG
GCAATGGAAAGAGATTTAGTGGAATTTCTTTCGCGTCAGGAAGGAACGCGGCTGGAAACT
AAACTGCAGCGCATCAACTGCTTTACCGTGCTGGCAATGCGCGAAGCGGAGCATCAAAAA
ATGCAGCGCCTGCGTGAACAAGGTTGGTATCCCTCCAACAGCGAGGCGCTAAAACCGGTG
ATGACGGTGAATAACGGTGTGCTGGTTGAACTTGATGCAACAAATCCCGGCCTGCGCAGT
GAGATGGCGTATGAATCCTGGCATATGCAACACTGCGACGGAGATTTTGATAATAAAGGC
GCGCTTTCCGGCGGCTACGGCGATTACTACGCCCGGCAAATGGAACAACAAAAACTTCGC
CTGTTCAGCCTGCGCGACGGCAATAATATTCCCCACGTCACAATAAGCCTGGTGGTTGGT
AATAACGGCCTGAGCATTGATCAGATTAAAGGTAAGCAAAATCGCCATCCTATTAAGAAA
TATGCTAACGATGTTTTATCTCTGTTACGCCATCTGCAACCACTTCCTGAGCGCCACGCT
GATTGCGAAGGGATGGGAATAGTTTATGAATCGACGCCGGAATATTCAGGTTGGAAATTT
ATTACTCATATTCATGACCTCAATTTTCTGCTCAATGTGCTGCATGACAATTTTCATTTA
ATGGAGCACTTCCCTACGCCTCCGGTGGCTTTACAGTGGCTGCTGCTGCACAGCGCCCCG
GAGGCACTGCGCTACCTTCAGGTTGTTGATCCTAACGTGGCGACGGCTGCGGAAATGCTG
TTTCCGCAGCATGAATGGCATCCTACGCTTGCCGGGAAAAACACCAGTAGCGAACCTTTC
GAAATTGAAAGCCTGACGCTACAAACCACGCGTTACCTCCCTGTCATCAAGGAGGTGCAA
TAA
>EECEBICD_01225 Transcriptional regulatory protein BaeR
ATGACTGAATTACCCATTGATGAAAACACGCCGCGCATTTTGATTGTTGAAGATGAACCC
AAGCTGGGTCAGCTGCTTATTGACTACTTACGCGCGGCGAGTTACGCCCCCACGCTCATC
AATCACGGCGACAAGGTACTGCCGTATGTGCGTCAAACGCCGCCGGATCTGATTCTGCTG
GATCTGATGCTGCCGGGTACCGATGGTCTGACGCTATGCCGGGAGATTCGCCGTTTTTCC
GACATCCCCATTGTGATGGTCACAGCCAAAATTGAAGAGATCGACCGGCTGCTGGGGCTG
GAAATCGGCGCCGATGATTATATCTGTAAGCCTTACAGCCCGCGCGAAGTCGTGGCCCGC
GTGAAAACCATTTTGCGCCGCTGTAAACCGCAGCGCGAACTCCAGCAGCAGGATGCCGAA
AGCCCGTTAATGATTGATGAAAGCCGCTTTCAGGCCTCCTGGTGTGGTAAAGCGCTGGAT
TTAACCCCCGCTGAATTCCGCCTGCTAAAAACCTTATCGCTTGAACCGGGTAAAGTTTTT
TCCCGCGAGCAGTTGTTAAACCACCTCTACGACGACTACCGTGTGGTGACCGATCGTACT
ATCGACAGCCACATTAAAAACTTGCGCCGTAAACTGGAATCGCTGGATGCCGAACAGTCA
TTTATACGCGCGGTCTACGGGGTGGGATATCGCTGGGAAGCGGATGCGTGTCGCCTGGTA
TAA
>EECEBICD_01226 Signal transduction histidine-protein kinase BaeS
ATGAAAGTCTGGCGGCCCGGAATCACCGGCAAGCTGTTTCTGGCGATTTTCGCCACCTGC

ATCGTGCTGCTGATCAGTATGCATTGGGCCGTGCGCGTCAGTTTTGAACGCGGATTTATT
GACTATATCAAACACGGCAACGAGCAGCGGCTACAAATGCTGGGCGATGCGCTGGGCGAC
CAGTACCAGCAACACGGCAACTGGCGTTTTTTACGCAACAACGATCGCTTCGTTTTCCAG
ATTTTGCGTTCATTTGAGCATGACAACGACCGGGATAAACCCGGCCCCGGTATGCCGCCG
CACGGTTGGCGCACCCAATTCTGGGTCGTCGATCAAAATGGCCGGGTGCTGGTCGGCCCA
CGCGGTCCGGTGCCGCATGACGGTACTCGCCGCCCCATTCTGGTCAATGGCGCCGAAGTC
GGCGCGATTATCGCCTCTCCTGTCGAGCGGCTGACGCGCAATACGGATATTAATTTTGAT
ATGCAGCAGCGGCGCGCCAGTTGGATGATCGTGGCGTTATCAACGTTGCTGGCCGCGCTG
GCGACGTTTTTGCTGGCGCGTGGCCTGCTCGCCCCGGTCAAACGGTTGGTCGAGGGTACG
CACAGACTGGCGGCGGGGGATTTTACCACGCGCGTGACCCCAACCAGCGCCGATGAGCTG
GGAAAACTGGCGCAGGACTTTAACCAGCTTGCCAGTACGCTGGAAAAAAACCAGCAGATG
CGCCGCGACTTTATGGCGGATATTTCCCACGAACTGCGCACGCCGCTGGCGGTATTGCGC
GGCGAACTGGAAGCCATTCAGGATGGCGTTCGCCAGTTCACGCCAGAATCGGTTACCTCG
CTACAGGCGGAAGTCGGTACGCTTACCAAACTGGTTGACGACCTGCATCAGCTGTCGTTG
TCTGACGAGGGCGCGCTGGCTTACCAAAAAACGACCGTCGATCTTGTTCCCCTGTTGGAA
GTCGCTGGCGGCGCGTTCAGAGAGCGCTTTACCAGCCGGGGGCTAACGCTACATTACGCG
TTGCCGGATAGTATGACCGTCTTCGGCGACCCGGATCGCCTGATGCAACTGTTTAATAAT
CTACTGGAAAACAGTCTGCGCTACACCGACAGCGGCGGCGGCCTGCATATTTCCGCCGAA
CAACGCGACAAATCATTATTCCTGACCTTTGCCGATTCTGCACCGGGCGTCAGCGATGAA
CAGTTACAAAAGCTTTTTGACCGTTTTTACCGCACGGAAGTGTCCCGTAACCGCGCCAGC
GGCGGTTCTGGACTGGGGCTGGCAATATGCGTCAATATTGTACACGCGCATAACGGTCAT
CTTCACGCCGCTCATTCGCCTTTTGGCGGGGTTAGCATTACAGTAGAGTTACCGCTGGAT
CGCGATTTACAGAGAGAAGTATGA
>EECEBICD_01227 Putative multidrug resistance protein MdtD
ATGACGCCGGTCTTTTTACAAATTGGTCTTGGTTTTTCACCGTTTCATGCCGGTTTGATG
ATGATCCCCATGGTGCTAGGCAGCATGGGAATGAAGCGGATTGTGGTGCAGATAGTTAAC
CGTTTTGGCTATCGTCGGGTTCTGGTGGCGACTACGCTCGGACTGGCATTGGTTAGCCTG
TTGTTTATGTCGGTCGCGCTGCTCGGCTGGTATTATCTCCTGCCGCTGGTACTGCTTTTG
CAGGGAATGGTGAACTCGGCGCGCTTTTCTTCCATGAATACCTTAACGCTAAAAGACCTT
CCCGATACGCTGGCCAGCAGCGGCAATAGCTTATTATCGATGATTATGCAGCTCTCGATG
AGTATCGGCGTCACTATTGCCGGAATGCTGCTGGGTATGTTTGGCCAACAGCATATCGGG
ATAGACAGTAGCGCCACACATCACGTCTTTATGTACACCTGGTTATGTATGGCGGTTATT
ATCGCGCTACCGGCGATAATTTTTGCCCGCGTGCCGAACGATACACAGCAAAACATGGTC
ATTTCACGGCGTAAAAGGAGCCTGTAA
>EECEBICD_01228 Putative multidrug resistance protein MdtD
ATGACAGAACTTCCTGACAATACCCGCTGGCAACTCTGGATTGTGGCATTCGGCTTTTTT
ATGCAGTCGCTGGATACCACCATCGTCAACACTGCGCTGCCCTCCATGGCGAAAAGCCTG
GGGGAAAGCCCGCTGCATATGCACATGGTAGTGGTGTCATATGTTCTTACCGTCGCCGTA
ATGCTCCCCGCCAGCGGCTGGCTGGCAGATAAAATCGGCGTGCGTAATATCTTCTTTGCC
GCCATTGTGCTGTTCACGCTGGGTTCGCTGTTTTGCGCCCTTTCCGGCACGCTTAATCAA
CTGGTATTAGCGCGGGTTTTACAAGGCGTAGGCGGCGCGATGATGGTGCCGGTCGGACGC
CTGACGGTCATGAAAATCGTGCCGCGCGCGCAATATATGGCGGCGATGACCTTCGTCACC
CTACCGGGACAGATCGGGCCGTTACTCGGCCCCGCGCTGGGCGGGGTGCTGGTGGAATAT
GCTTCATGGCACTGGATCTTTCTGATCAATATCCCGGTGGGAATCGTCGGCGCTATGGCA
ACCTTTATGCTGATGCCAAACTACACCATAGAGACCCGGCGCTTTGATCTGCCGGGGTTC
CTGCTGTTAGCTATCGGCATGGCGGTACTCACGCTGGCGCTGGATGGTAGTAAAAGCATG
GGGATTTCGCCCTGGACATTAGCCGGACTTGCTGCGGGCGGCGCGGCAGCCATTCTGCTC
TATCTCTTCCATGCCAAAAAAACTCAGGCGCGTTATTCAGCCTGCGATTGTTCCGCACGC
CCACGTTTTCACTGGGGTTGCTGGGCAGTTTTGCCGGGCGTATCGGCAGCGGTATGCTGC
CGTTTATGA
>EECEBICD_01229 Multidrug resistance protein MdtC
ATGCGCTTTTTCGCCCTTTTCATCTACCGCCCGGTCGCCACCATTTTGATTGCCGCCGCC
ATTACGCTGTGCGGCATTCTGGGCTTTCGTCTGCTGCCGGTCGCCCCGCTGCCGCAGGTC
GATTTCCCGGTGATTATGGTTAGCGCCTCGCTGCCGGGCGCCTCGCCGGAAACCATGGCT

TCGTCGGTGGCGACGCCGTTGGAACGTTCTTTGGGACGCATTGCAGGCGTCAATGAAATG
ACCTCCAGCAGCTCGCTCGGCAGTACACGCATTATTCTCGAATTTAATTTCGATCGTGAT
ATTAACGGCGCGGCGCGCGACGTGCAGGCCGCCATTAACGCCGCGCAAAGCTTGTTGCCA
GGCGGAATGCCCAGCCGCCCGACTTATCGCAAGGCCAACCCGTCCGACGCGCCGATTATG
ATTTTAACGCTTACCTCGGAGAGCTGGTCACAGGGCAAACTGTATGATTTCGCCTCTACC
CAACTGGCGCAAACCATCGCGCAAATTGACGGCGTCGGCGATGTTGACGTCGGCGGCAGC
TCCCTGCCCGCAGTACGTGTAGGCTTAAACCCGCAGGCGCTCTTTAACCAGGGCGTCTCG
CTGGATGAGGTCCGCGAAGCGATCGACAGCGCCAACGTACGCCGACCACAAGGCGCAATT
GAAGATAGCGTCCACCGCTGGCAAATCCAGACCAACGACGAACTGAAAACCGCCGCCGAA
TATCAGCCGCTGATTATTCACTATAACAACGGCGCGGCGGTACGCCTGGGCGACGTCGCC
AGCGTCACTGACTCGGTGCAGGATGTCCGTAACGCCGGGATGACGAACGCTAAACCCGCT
ATTTTGTTGATGATCCGCAAGCTGCCGGAGGCCAACATTATTCAGACGGTCGACGGCATC
CGGGCAAAACTGCCGGAACTGCGGGCAATGATCCCCGCCGCTATCGATTTACAAATCGCC
CAGGATCGTTCGCCGACGATTCGCGCATCGCTGCAAGAGGTAGAAGAGACACTGGCTATC
TCTGTTGCGCTGGTGATCCTGGTGGTGTTTTTATTCCTGCGTTCCGGGCGCGCCACGCTA
ATCCCCGCCGTCGCCGTTCCCGTTTCGCTCATCGGCACCTTCGCCGCCATGTATCTGTGC
GGCTTCAGCCTCAACAATCTGTCGCTGATGGCGCTGACTATCGCGACCGGATTTGTCGTT
GATGATGCCATTGTGGTGCTGGAAAATATCGCCCGCCATCTGGAGGCGGGAATGAAACCT
TTGCAGGCGGCATTACAGGGTACGCGGGAAGTCGGGTTTACGGTCATCTCCATGAGTCTG
TCGCTGGTGGCGGTATTTCTGCCGCTGCTGTTAATGGGCGGCCTGCCAGGACGATTATTA
CGGGAATTCGCCGTTACCCTCTCGGTGGCGATTGGCATTTCGCTGGTGGTCTCGCTCACA
CTGACGCCGATGATGTGCGGCTGGATGCTTAAATCAAGCAAACCGCGCACCCAACCGCGT
AAACGGGGCGTTGGCCGTTTGCTGGTCGCCTTGCAACAGGGTTACGGCACGTCGTTAAAA
TGGGTGCTTAACCATACGCGTCTTGTCGGGGTGGTTTTTCTTGGCACCGTTGCGCTGAAC
ATCTGGCTTTATATCGCCATCCCTAAAACATTCTTTCCGGAGCAGGACACCGGCGTGTTG
ATGGGCGGTATTCAGGCTGACCAAAGCATCTCTTTCCAGGCCATGCGCGGCAAGCTGCAG
GATTTTATGAAAATTATTCGCGACGATCCGGCGGTGAATAATGTCACTGGTTTTACCGGC
GGATCGAGGGTGAATAGCGGCATGATGTTTATTACGCTGAAGCCGCGTGGCGAACGCAAA
GAGACGGCGCAGCAAATCATTGATCGACTGCGGGTCAAACTGGCAAAAGAACCTGGCGCC
AGGCTGTTTCTGATGGCGGTACAGGATATTCGCGTCGGCGGGCGGCAGGCTAACGCCAGT
TACCAATATACGTTGCTGTCTGACTCTCTGGCGGCGCTGCGCGAATGGGAGCCGAAAATA
CGCAAAGCGCTCTCGGCCCTGCCGCAACTGGCGGACGTAAACTCCGACCAGCAGGATAAC
GGCGCGGAGATGAACCTTATCTACGACCGCGACACCATGTCACGGCTGGGTATTGATGTT
CAGGCCGCAAACAGTCTGTTAAATAATGCTTTCGGCCAGCGGCAAATTTCCACCATTTAT
CAGCCGATGAACCAGTATAAAGTGGTGATGGAAGTCGATCCGCGCTATAGCCAGGATATC
AGCGCGCTGGAGAAAATGTTCGTTATCAACCGTGACGGAAAAGCGATTCCCCTCTCTTAT
TTCGCCCAATGGCGGCCCGCCAATGCGCCGCTGTCGGTGAACCATCAGGGACTTTCCGCG
GCGTCCACGATTGCCTTTAACCTGCCGACCGGCACATCGTTATCGCAGGCGACAGAGGCC
ATTAATCGCACCATGACGCAGCTTGGCGTCCCCTCGACGGTACGCGGCAGTTTTTCCGGA
ACGGCGCAAGTCTTCCAGCAGACCATGAATTCACAGCTTATTTTGATAGTGGCGGCGATC
GCTACCGTCTACATTGTGCTGGGGATACTGTACGAAAGCTACGTCCATCCGCTGACCATT
CTCTCTACTCTACCATCGGCGGGCGTTGGGGCGCTTCTGGCGCTGGAACTCTTCAATGCC
CCTTTCAGCCTAATCGCCCTGATAGGGATCATGCTATTAATTGGCATTGTGAAGAAAAAC
GCCATTATGATGGTCGATTTTGCGCTTGAAGCGCAACGAAGCGGCGGCCTGACGCCGGCA
CAAGCCATTTTCCAGGCCTGCTTGTTACGCTTCCGTCCAATAATGATGACCACGCTGGCG
GCGCTGTTCGGCGCACTGCCATTGGTGTTATCTGGCGGAGACGGTTCGGAATTACGGCAG
CCGTTGGGGATAACCATTGTCGGCGGTTTGGTCATGAGCCAGCTCCTGACGCTCTATACC
ACGCCGGTGGTGTACCTCTTTTTCGATCGTCTGCGGCTGCGTTTTTCGCGTAAAAATAGC
AAACCGGTAGTAGAAATATGA
>EECEBICD_01230 Multidrug resistance protein MdtB
ATGCAGGTATTACCTCCGGGCAGCACGGGCGGCCCCTCGCGTCTGTTTATTCTGCGCCCC
GTGGCCACCACTCTGCTGATGGCGGCGATTTTACTCGCCGGGATTATCGGCTATCGCTTC
CTGCCCGTCGCCGCTTTGCCGGAGGTCGACTACCCCACTATTCAGGTTGTTACGCTCTAC
CCTGGCGCCAGCCCGGATGTCATGACCTCCGCCGTCACCGCGCCGCTTGAGCGCCAGTTC

GGCCAGATGTCAGGACTGAAGCAGATGTCGTCGCAAAGCTCCGGCGGCGCGTCGGTGGTA
ACGCTACAGTTTCAGTTGACGCTGCCGCTGGACGTTGCCGAGCAGGAAGTACAGGCGGCG
ATTAACGCAGCCACCAATTTATTGCCTTCCGACCTGCCGAATCCGCCGATTTATAGCAAA
GTCAATCCGGCGGACCCGCCGATTATGACGCTTGCCGTCACCTCAAACGCGATGCCAATG
ACTCAGGTAGAGGACATGGTAGAAACCCGCGTGGCGCAGAAGATCTCGCAGGTCTCCGGC
GTCGGACTGGTGACGCTTGCCGGCGGGCAGCGCCCTGCAGTACGCGTAAAACTGAATGCT
CAGGCTATCGCCGCGCTCGGTCTGACCAGCGAAACGGTCCGTACCGCAATTACCGGCGCC
AACGTCAACTCGGCGAAAGGCAGTCTGGATGGCCCCGAGCGGGCGGTGACGCTTTCTGCT
AACGATCAGATGCAGTCTGCCGACGAATACCGCAAGCTTATCATCGCGTATCAAAACGGC
GCGCCGGTACGGTTGGGCGATGTCGCCACCGTCGAACAGGGGGCGGAAAATAGCTGGCTC
GGCGCATGGGCGAATCAAGCGCCGGCTATCGTGATGAACGTTCAACGCCAGCCCGGCGCC
AATATCATTGCGACAGCGGACAGCATTCGCCAGATGCTGCCCCAGCTTACCGAAAGCCTG
CCGAAATCGGTGAAGGTCACGGTCCTGTCCGATCGCACCACCAATATTCGCGCTTCCGTG
CGCGATACCCAGTTTGAACTGATGCTGGCGATCGCGCTGGTCGTCATGATTATCTATCTG
TTTTTACGTAATATTCCCGCCACAATTATTCCCGGCGTCGCCGTACCGCTGTCGCTTATC
GGCACCTTTGCGGTGATGGTGTTTTTGGATTTTTCCATTAATAACCTGACGCTGATGGCG
CTCACTATCGCCACGGGTTTCGTGGTGGACGATGCGATTGTGGTGATCGAGAACATCTCG
CGCTACATCGAAAAAGGAGAAAAACCGCTGGCGGCGGCGCTCAAAGGCGCGGGCGAAATC
GGCTTTACCATTATTTCCCTCACCTTTTCACTGATTGCGGTGCTGATCCCGTTGCTCTTT
ATGGGCGATATTGTTGGTCGACTGTTCCGCGAATTTGCGGTGACGTTGGCGGTAGCGATT
TTAATCTCCGCCGTCGTCTCTTTGACGCTCACGCCCATGATGTGCGCGCGTATGCTCAGC
CAGCAGTCTCTGCGTAAACAAAACCGCTTTTCCCGCGCCTGCGAGCGGATGTTTGACCGC
GTGATCGCCAGCTACGGACGCGGATTAGCGAAAGTGCTCAACCATCCGTGGCTTACATTG
AGCGTGGCATTCGCCACGCTCCTGCTCAGCGTTATGCTGTGGATAGTCATTCCGAAAGGG
TTCTTTCCGGTACAGGATAACGGCATTATCCAGGGGACGCTGCAGGCGCCGCAATCGTCA
TCGTATGCCAGTATGGCGCAACGTCAACGCCAGGTGGCGGAGCGGATATTACAGGACCCG
GCGGTGCAAAGCCTGACGACTTTTGTTGGCGTAGACGGCGCTAACCCCACGCTCAATAGC
GCGCGCCTGCAAATTAACCTCAAGCCGCTGGATGCGCGTGATGACCGCGTGCAGCAGGTG
ATCTCCCGGCTGCAAACCGCCGTGGCGACGATTCCCGGCGTGGCGCTGTATCTCCAGCCG
ACGCAGGATTTAACCATCGACACGCAGGTCAGCCGCACGCAGTATCAGTTTACCCTGCAG
GCCACGACGCTCGATGCGCTCAGCCACTGGGTGCCAAAACTGCAGAACGCGCTACAGTCG
TTACCACAGCTCTCTGAGGTAAGCAGTGACTGGCAAGATCGAGGATTAGCGGCCTGGGTG
AATGTCGACCGCGACAGCGCCAGCCGTCTGGGTATCAGCATGGCGGATGTGGATAACGCG
CTCTACAACGCGTTCGGACAACGCCTGATTTCAACGATTTATACCCAGGCGAACCAGTAC
CGCGTCGTGCTGGAACATAATACCGCCAGCACGCCGGGCCTGGCGGCGCTGGAGACGATT
CGCCTGACGAGCCGCGACGGCGGCACCGTACCGCTCAGCGCGATTGCCCGCATTGAGCAG
CGCTTCGCTCCGCTCTCCATCAATCATTTAGATCAGTTCCCGGTTACGACATTTTCGTTT
AACGTGCCGGAGGGCTATTCGCTCGGCGATGCGGTGCAGGCGATTCTCGATACGGAAAAA
ACGCTCGCCCTGCCAGCAGATATTACAACGCAGTTTCAGGGTAGTACGCTCGCCTTCCAG
GCGGCGCTAGGCAGCACCGTCTGGCTTATTGTCGCCGCCGTGGTGGCGATGTATATCGTG
CTCGGCGTGCTGTATGAGAGTTTTATCCATCCGATCACGATTCTCTCAACGCTGCCGACG
GCGGGCGTCGGCGCGCTGCTGGCGCTGATCATCGCTGGTAGCGAGCTCGATATTATCGCC
ATTATCGGCATTATTTTGCTGATCGGTATCGTGAAGAAAAACGCCATCATGATGATTGAC
TTCGCCCTCGCCGCTGAACGCGAACAGGGGATGAGTCCGCGCGACGCTATCTTCCAGGCC
TGTCTGCTGCGTTTTCGACCGATTCTGATGACCACGCTGGCGGCGTTGCTCGGGGCATTG
CCATTAATGTTGAGTACCGGCGTTGGCGCGGAATTACGTCGCCCGTTGGGGATCGCGATG
GTGGGCGGCCTACTGGTCAGCCAGGTATTAACTCTGTTTACCACACCGGTGATTTATCTC
CTGTTTGACCGCCTGTCGCTGTACGTGAAAAGTCGCTTTCCGCGCCATAAAGAGGAGGCG
TAG
>EECEBICD_01231 Multidrug resistance protein MdtA
ATGAAAGGCAGTAATACTTTCCGCTGGGCAATAGCGATTGGGGTTGTAGTGGCCGCCGCC
GCATTCTGGTTCTGGCATAGCCGTAGCGAAAGCCCGACCGCCGCGCCGGGCGTCGCCGCG
CAAGCGCCGCATACCGCCGCCGCAGGTCGCCGCGGTATGCGCGACGGCCCTCTGGCGCCG
GTACAGGCCGCGACCGCGACCACGCAGGCCGTACCGCGCTATCTGAGCGGGCTGGGTACC

GTGACCGCCGCGAATACCGTTACGGTGCGTAGCCGCGTGGATGGTCAACTCATCTCCCTG
CACTTTCAGGAAGGTCAGCAGGTCAACGCAGGCGATCTGCTGGCGCAAATCGATCCCAGC
CAGTTTAAGGTCGCCCTGGCGCAGGCTCAGGGACAGTTGGCGAAAGATAACGCTACGCTG
GCGAATGCGCGTCGTGATCTGGCGCGCTATCAGCAACTGGCAAAAACCAATCTGGTTTCC
CGTCAGGAACTGGATGCGCAACAGGCGCTGGTCAACGAAACCCAGGGAACCATTAAAGCG
GATGAAGCCAATGTCGCCAGCGCGCAGTTACAGCTCGACTGGAGTCGTATCACGGCCCCG
GTCTCGGGACGCGTGGGTCTGAAACAGGTGGATGTCGGCAACCAGATTTCCAGCAGCGAT
ACCGCAGGTATTGTCGTCATTACGCAAACGCACCCGATTGATCTCATTTTTACTCTGCCG
GAAAGCGATATCGCGACCGTGGTTCAGGCGCAGAAAGCGGGGAAAACGCTGGTCGTAGAA
GCCTGGGATCGGACTAACTCGCACAAATTGAGCGAAGGTGTGTTGCTCAGTCTGGACAAC
CAGATTGATCCCACAACGGGAACGATCAAAATTAAAGCGCGCTTTACCAATCAGGACGAT
ACGCTGTTCCCCAATCAATTTGTGAACGCCCGGATGCTGGTCGATACCGAACAAAATGCC
GTTGTGGTGCCTGCCGCGGCGGTGCAAATGGGCAATGAGGGCCACTTTGTGTGGGTGCTG
AACGACGAAAATAACGTCAGCAAGAAGCGGGTAAAAATCGGTATTCAGGATAACCGAAAC
GTGGTGATCAGCGCTGGCTTGTCGGCAGGCGATCGCGTCGTTACCGATGGTATTGATCGG
CTGACGGAAGGCGCAAAAGTCGAGGTCGTTGAGCCGCAAACCACCGTGGCGGATGAAAAA
TCCCCTTCCCGCCATGAAGGTCAAAAAGGAGCGCGCGCCTGA
>EECEBICD_01232 hypothetical protein
ATGATGACTACCTTCATCATAACCCTTTCCTTATTTTGGCCCCTTCCTCGGGAGGGGCTT
TCCCGTTTCAGCGTCCTGCTGAAATCGGTGGCTTACCTTCATTGCCGTGCCGCCACTTTA
CCGCACTCCGGCGCGTTAACGATTTTCACCGATGAGAGTTGCGTAGCGCCTCGCAAAGCG
AGTCGCCATTCAGAATAA
>EECEBICD_01233 Chaperone protein DnaK
GTGTTTATCGGATTTGATTACGGTACGGCAAACTGCTCGGTCGCCATTATGCGCGACGGG
CATCCACAGTTGCTGACAATGGAGAATAACAGCGCATTACTGCCGTCTATGCTGTGCGCG
CCGACGCGCGAAGCGGTGAGCGAGTGGCTGTATCGCCATCATGATGTGCCGGCAACGGAC
GAAGAAACCCAGGCGCTGCTACGCCGCGCGATACGCTACAACCGCGAGGAAGATATTGAG
GTTGGCGCGCAAAGCGTACAGTTTGGTCTGGCGTCGCTGGCGCACTATATTGACGATCCC
CAGGAGGTCTGGTTTGTCAAATCGCCGAAATCCTTTTTAGGCGCCAGCGGCCTGAAACCG
CAGCAGGTCGCGCTGTTTGAAGATTTAGTCTGCGCCATGATGGTACATATTCGTCATACG
GCGCACAGCCAATTGCCGGAGGCCATTACCCAGGCGGTGATCGGTCGCCCGATCAACTTT
CAGGGACTCGGCGGCGATGACGCGAACCGCCAGGCGCAGGGTATTCTGGAGCGGGCGGCA
AAACGCGCCGGTTTTCAGGAGGTGGTATTCCAGTATGAACCGGTTGCGGCGGGCCTGGAC
TACGAAGCTACGCTGCGGGAAGAGAAGCGTGTGCTGGTCGTGGATATTGGCGGCGGTACC
ACCGACTGTTCCATGCTGCTGATGGGGCCGCAGTGGCGTCAGCGCGCCGATCGTGAAAAC
AGCCTGTTGGGGCACAGCGGTTGCCGCGTGGGCGGAAACGATCTGGATATCGCGCTGGCC
TTTAAAAATCTGATGCCTTTGCTGGGCATGGGCGGCGAAACCGAAAAAGGCATCGCCCTA
CCGGTGCTGCCGTGGTGGAACGCCGTGGCGATTAACGACGTCCCGGCGCAAAGCGATTTT
TACAGTAGCGCGAATGGTCGTCTGTTGAACGATTTGGTTCGCAACGCGCGTGAAGCAGAC
AAGGTCGCGTTGCTGCTCAAGGTCTGGCGTCAGCGGCTTAGTTACCGCCTGGTACGCTGC
GCGGAAGAGAGCAAAATCGCCCTTTCCGGCCAGGCGGATGTTACGGCGAGGCTGCCATTT
ATCAGCGACGACCTTGCGGTCGCTATCAGCCAGCAAGGACTGGAAGCGGCGCTGGATCAA
CCGCTGGCGCGTATTCTGGAACAGGTGCAACTGGCGCTGGACAGCGCGCAGGAAAAACCG
GATGTCATCTATCTGACCGGCGGCAGCGCCCGTTCGCCATTGATTAAAAAAGCGCTTTCC
GAACAGTTACCGGGCATACCCGTCGCTGGCGGCGATGATTTCGGCTCCGTCACCGCAGGA
CTGGCTCGCTGGGCAGAGGTCGTTTTCCGCTAA
>EECEBICD_01234 DNA-3-methyladenine glycosylase 2
ATGTTTACCCTGAGCTGGCAGCCGCCTTACGACTGGTCGTGGATGTTGGGCTTTCTTGCC
GCGCGTGCGGTGGACGGCGTGGAAACGGTAGGCGAGGGCTTTTATGCGCGCAGCCTGGTG
GTGGGCGAGCATCGCGGACTGATCAGCGTGAGCCCCCATCTGCCCACCCACACTGTGCAG
GTCAGCGTGAGCGCCGGATTACTTCCCGTCGCGCCGGCGTGTCTGGCGAAGGTTTCGCGT
CTGTTTGATCTTGACTGCCAGCCCGCGCAGGTTGCCGCCGTGCTGGGGCCACTGGGCGAG
GACAGACCGGGGCTGCGTTTACCGGGATCGGTTGATACGTTTGAACAAGGCGTGCGGGCG
ATACTGGGGCAACTGGTGAGTGTCGCGATGGCGGCAAGGTTGACGGCGAAAGTGGCGCGC

CGCTATGGCGAGGCGTTGCCGGATGCGCCCGATTATGTCTGTTTTCCCGGTCCGGAAACG
CTGGCGTTGGCCGATCCGCTGGCGCTGAAAGCTCTGGGGATGCCGCTGCGGCGCGCCGAA
GCGCTGATTCATCTGGCGCAGGCGACGCTGGCGGGAAAACTGGCGCTCGCCGCGCCGCCG
GATATTGAGCAGAGCGTTAAAAATTTACAAACGTTTCCGGGAATTGGCCGCTGGACGGCG
AACTATTTCGCCCTGCGCGGTTGGCAGGCAAAAGATATCTTTTTACCCGATGATTACTTA
ATTAAGCAGCGCTTCGCCGGGATGACGCCCGCGCAGATCCGTCGCTACGCGGAGCGCTGG
AAACCGTGGCGTTCCTACGCGCTACTGCATATCTGGTATACCCACGGCTGGCAACCGTCA
ATGGATAGCGAAATAGCTGGTATTCAGTAA
>EECEBICD_01235 hypothetical protein
ATGAGTAAACCATCCCAACATGTTTTCGTTACCGTCCCCCACCCGCTATTGCGTCTGGTC
AGTTTAGGCCTGGTCGCGTTTGTCTTTACGCTCTTTTCGCTCGTATTATCGCGTGTCGGC
ACCCAACTCGCGCCGCTGTGGTTCCCCACCTCCATTATGATGGTCGCGTTTTACCGCCAT
GCGGGCCGTCTGTGGCCAGGGATCGCGGTCGCCTGCTCGCTCGGCAGCATCGGAGCCTCA
CTGACTTTATTTCCTGCCGCCTCGCTTAACTTTAGCTGGACGGCAATAAATATTATCGAG
GCGGCCACTGGCGCGATACTGCTGCGAAAACTGCTCCCCAGCTACAATCCCTTACAAAAT
TTGAATGACTGGTTTCGTCTGGCTATCGGCAGCGCCGTGATTCCGCCGCTACTCGGCGGC
CTGTTATTTTGGCTGATAGCGCCGGAAGCGGTCGCCTCGAAAGCGTTTCTGATTTGGGTA
TTATCGGAAGCCATCGGCGCGCTGACGCTGGTGCCGTTAGGTTTACTGTTTAAACCACAC
TATCTATTGCGTCATCGCGACCCGCATCTGTTGCTGGAAACGCTGTTGACGCTGGTCATT
ACGCTGGCGCTAAGCTGGCTCGCTATGCGCTATATTCCGTGGCCGTTTACGTGCGTTATC
GTCCTGTTGATGTGGAGTGCGGTGCGCCTGCCGCGAATGGAGGCGTTTTTAATTTTTTTA
GCTACCGTTATCGTGGTCTCGCTCATGCTGGCCAATGACCCCACGCTGCTCGCCACGCCG
AAAACCGACGTGATGGTCAATATGCCGTGGCTGCCTTTTTTAATGATCCTGCTGCCTGCC
AATATGATGACGATGGTGATGTACGCGTTTCGCACGGAGCGCAAACATATCACCGAGAGC
GAATCCCGCTTTCGCAACGCGATGGAGTATTCCGCCATTGGTATGGCGCTGGTCGGTACG
GAGGGTCAGTGGCTACAGGTCAATAAATCTCTGAGCCATTTTCTTGGCTATAGCCAGGAT
GAATTACGCACGATGACATTTCAGCAATTGACCTGGCCGGAAGACCTGAATAATGACCTT
GAACAGCTAAACATGCTGGTGCGCGGCGACATCAATAGCTATTCCATGGAAAAACGCTAC
TACACCCGCAACGGCGATGTCGTCTGGGCGCTACTGGCCGTCTCGCTGGTTCGTCATAAA
GACAATAAACCTCTCTATTTTATTGCTCAGATTGAAGATATTAACGATCTCAAGCAAAGC
GAGCAGGAAAACCAGCGGTTGATGGAACGTATTACACAAGCGAACGACGCGCTGTTTCAG
GAAAAAGAACGGTTGCACATCACGCTTGACTCTATCGGAGAAGCGGTGGTGTGTATTGAT
GTCGCGATGAATATCACCTTTATGAACCCGATCGCCGAGAAGATGAGCGGCTGGCGGCAA
GAGGATGCTTTAGGCGCGCCATTATTAACGGTTCTGCGCATTACGTCAGGTGATAAGGGG
CCGCTGCTGGAGGATATTTATCGCGCCGACAGGTCGCGATCGGACATGGAACAGGAGATA
GTGCTCCATTGTCATAACGGCGGCAGCTACGATATTCATTACAGTATCACGCCGCTCAGT
ACGCTTGACGGCGATAAGATTGGTTCGGTTCTGGTTATCCAGGACGTTACCGAATCACGA
AAAATGTTACGCCAGCTCAGCTACAGCGCAACGCATGACGCCCTCACCCATCTTGCCAAC
CGCGCTAGTTTTGAAAAACAGCTTCAACAGCGCCTGCAAACAATACAGGAATCGCCTCAA
CATCATGCGCTGGTGTTTATCGATCTGGACCGTTTTAAAGCGGTTAACGACAGCGCGGGA
CATGCCGCCGGCGATGCCTTATTGCGCGAACTCGCGTCACTGATGCTCAGTATGCTTCGA
TCCGGCGATCTTCTGGCCCGTCTGGGTGGCGATGAGTTTGGCCTGTTGCTGCCGGATTGT
AATAGTGACAGCGCACGCTTTATCGTGACGCGGCTTATCAACGCCGTTAATGAGTATCAC
TTTATGTGGGAAGGACGCCTGCATCGGATCGGCGCCAGCGCCGGGATCACAATGATAAAT
GAACATAACTGCCAGCTCACAGAGGTCGTGTCTCAGGCCGATATAGCCTGCTACGCCGCG
AAAAATAGCGGTCGCGGCCGTCTGACCGTGTACGAACCGCAGCACGCATTAACCTCCTCA
AAGGGAATGATGCCGCTGGAAGAACAGTGGCATATGATTAAAAATAACCACCTGCTGATG
CTTGCCAGAAACGTCGCGCCGCCGCGTACGCCGGAGGCCACCAGCTTTTGGCTGGTCTCA
CTCAGGCTGTGGACCAGCGAAGGGGAAGTGTTGGAAGAGCGCGCGTTCCGGGCAGGGTTA
GCCGACTCCGCGCTGCATCACGCGCTTGACCGACGGGTATTCCATGAGTTTTTCCACCAT
GCGGCGACCGCCGTCGCCAGCAAGGGATTAAGCGTTGCGCTGCCGCTGTCCGCCGCGGGA
TTATACAGCGCGACCCTTATTGATGAGCTGCTGGAACAACTGGAGCATAGCCCACTGCCG
CCACGGTTATTACATCTGATTATCCCGGCTGACGTTATCGTTAAGCAGGCGCAGACCGCC
GCTGCGACTCTGCGAAAACTGCGGCAACGCGGCTGCCAGGTGATTCTCAGCCATGTCGGG

CGCGATTTACAGCTGTTTAATTTACTGCCCCCGCATATCGTCGATTATTTACTGCTGGAT
AGCGACCTTATCGCCAACGTTCATGAAAGTTTAATGGATGAAATGCTGACCTCGATCATC
CAGGGACACGCCCAGCATCTGGACATCAAAACGCTCGCCGGGCCGGTACAAAACCCACAG
GTGCTGGATACGCTCTCCTGTATTGGCGTGGATCTGATTTATGGCGACACTATCGCCGAA
GCGCAACCGCTGGATTTATTACTGAATACCAGCTATTTCGCTATCCATTGA
>EECEBICD_01236 Uridine kinase
ATGACTGATCAGTCTCATCAGTGCGTCATTATCGGTATCGCTGGCGCATCGGCTTCCGGC
AAGAGTCTTATTGCCAGTACCCTTTACCGCGAATTGCGTGAACAAGTGGGTGACGAACAT
ATTGGCGTTATTCCCGAAGACAGTTATTACAAAGATCAAAGCCATCTGTCGATGGAAGAA
CGCGTAAAAACTAACTATGACCATCCGAATGCGATGGATCACAGCCTGCTATTTCAGCAT
CTTCAGGCGCTCAAGCGCGGTTCGGCGATTGAATTACCGGTATACAGTTATGTTGAGCAT
ACCCGTATGCAGGAAACGGTTCGCGTAGAACCGAAGAAAGTGATTATTCTTGAAGGTATT
CTGTTACTGACCGACGCGCGTTTGCGTGAAGAGATGAACTTCTCTATTTTTGTCGATACG
CCGCTGGATATCTGTTTGATGCGCCGTATCAAACGCGATGTGAACGAGCGTGGTCGCTCA
ATGGATTCGGTGATGGCGCAATATCAGAAAACGGTGCGTCCGATGTTCCTGCAATTTATT
GAGCCTTCCAAACAATATGCGGATATTATCGTGCCGCGCGGCGGTAAAAACCGCATCGCT
ATTGATATTCTGAAAGCGAAAATCAGCCAGTTCTTTGAATAA
>EECEBICD_01237 dCTP deaminase
ATGCGTTTATGCGACCGAGATATTGAAGCCTGGCTGGATGAAGGCCGTTTGTCTATCACC
CCGCGACCTCCCGTAGAGCGCATTAACGGCGCGACGGTCGATGTACGTCTTGGCAATAAA
TTTCGAACCTTCCGTGGCCATACGGCGGCGTTTATCGATCTGAGCGGGCCGAAAGACGAG
GTCAGCGCGGCGCTGGATCGCGTGATGAGCGATGAAATCGTTTTGCCGGACGGCGAGGCA
TTTTATCTGCATCCGGGCGAGTTGGCGCTGGCTGTCACCTTTGAATCCGTTACCCTGCCG
CCGGATTTAGTGGGCTGGCTGGATGGTCGCTCCTCGCTGGCGCGTCTGGGATTGATGGTG
CATGTGACGGCGCACCGTATCGATCCCGGCTGGTCGGGCTGCATTGTGCTTGAGTTTTAC
AACTCCGGGAAGCTGCCGCTGGCGTTGCGTCCGGATATGCTCATCGGCGCGCTGAGCTTT
GAGCCGCTGTCCGGCCCGGCGGCGCGGCCTTATAACCGCCGTCAGGATGCCAAATATCGC
GATCAGCAGGGCGCTGTCGCCAGCCGGATTGATAAAGACTGA
>EECEBICD_01238 hypothetical protein
ATGAGACGATTTCTGACGACGCTGATGATTCTCCTGGTCGTGCTGGTGGCCGGATTCTCT
GCGTTGGTATTGTTGGTCAATCCGAATGATTTCCGCGCCTATATGGTGCAGCAGGTTGCC
GCGCGTAGTGAATACCAGTTGCAACTGGACGGGCCGCTGCGCTGGCACGTATGGCCGCAA
CTCAGCATCCTTTCCGGGCGAATGACGCTAACGGCGCGAGGCGCCAGCGAACCGCTGGTG
CGGGCTGACAATATGCGCCTGGATGTCGCTTTATGGCCGCTGTTGAGTCATCAGCTTCAT
GTTAAGCAGGTGATGCTGAAAGGCGGGGTGATTCAACTGACGCCGCAAACGGAAGCGGTA
CGCAGTGATGATGCGCCGGTCGCGCCGAAAGATAATACCTTGCCGGACGTAGCGGAAGAT
CGCGGCTGGTCGTTTGATATTCGTAGCCTGCGCGTTGCCGACAGCGTGCTGGTTTTCCAG
CATGAAAATGATGATCAGGTCACGGTGCGCGATATTCGTCTGAATATGGAACAAGATGCC
GAGCATCGCGGCACGTTTGATTTTTCGGGACGCGTGAACCGCGATCAGCGGGATCTTGCT
TTATCGTTCAGCGGAACGGTGGATGCTTCCGATTATCCGCATAACTTAACCGCGGGTATT
GAGCAACTGCGCTGGCAATTGCAGGGCGCGGATTTACCGGCGCAGGGGATTGAGGGGCAG
GGACAGCTGCAGGCGCAGTGGCAGGAGGCGCAAAAACGTCTTTCATTTAACCACTTAAAT
TTAACGGCGAATGACAGTTCTCTGACCGGACACGTTCAGGTAACGCTGGCGGAGCAGCCG
GAATGGCAAATTGACCTCCAGTCCAGCAAGCTTAATCTGGACAACCTGTTGCCGCATCAT
AGCGCCGTGGCTCAGACGGGCGGCGCGGTGTCGCAGGGGCAAAATACGCTCCCCCTGACC
AGGCCAGTTATCGCGTCGCGTGTTGGTGCGCCGCCTTATCAGGGGCTGCAAAGCTTTACG
GCGGAGATTGCTTTACAGGCGGATTCAGTTCGCTGGCGGGGAATGGACTTTACCCAGGTT
TCGACGAAGATGTCTAATCAGGCCGGATTGCTGGATATTACCGAGTTGCAGGGGAAACTG
GCCGACGGAGAGATGTCGCTGCCCGGCACGCTTGACGCCCGCACCGCCAGTCCACGCATC
GAGTTCCACCCTCGCCTCAACCATGTTGAGATTGGGACTATCCTGAAAGCCTTCAATTAT
CCGATTAGCCTGACCGGTAAGATGTCGCTGGTTGGCGATTTCTCCGGGGCGGATATTGAC
GCGGAGGCATTTCGCCACAGCTGGAAAGGAAAAGCGCATGTTGACATGAGCAATACGCGC
CTCGAAGGAATGAATTTCCAGCAACTGGTGCAGCAGGCCGTAGAGCGAAGCGGCGGCGAC
GCGCAGCAGTCGCAGGAAAATATGGACAACGCGACCCGACTGGATCGCTTCACGACCGAT

CTGACGCTAAATAAGGGCACGCTGACGCTCGACGACATGGTCGGGCAATCTTCTATGCTG
GCGTTAACCGGCAGCGGAACGCTGGACCTGGTTAAGCAGAGCTGTGATACGCAATTTAAT
CTGCGTGTGCTGGGCGGCTGGAACGGCGATAGCAACCTGATCACCTTCCTGAAAGAGACG
CCGGTGCCGCTGCGCGTCTATGGCAAGTGGCAGGAGCTGAACTATACCCTGCAAGTGGAT
CAGTTATTGCGTAAGCATTTACAGGATGAGGCGAAGCGTCGGCTCAACGACTGGGCGGAG
CGCAATAAAGATACCCGCAATGGTAAAGATGTGAAGAAACTGCTGGATAAGCTGTAG
>EECEBICD_01239 hypothetical protein
ATGGAATGGATTGCCGATCCGTCAATCTGGGCCGGACTGGTAACGTTAATCGTTATCGAA
CTTGTTCTCGGCATTGATAACCTGGTCTTTATTGCCATCCTCGCTGAAAAACTACCGCCG
GGGCAGCGCGACCGCGCGCGCATTACCGGCCTGATACTGGCGATGATCATGCGCCTGCTG
CTGCTGGCGTCAATCTCGTGGCTGGTCACCTTGACTAAGCCGCTTTTTAGCGTACAGGCG
TTAAGCTTTAGCGCCCGCGATCTGATTATGCTGTTCGGCGGCTTCTTCCTGCTCTTTAAA
GCCACCATGGAGCTCAACGAACGGCTGGAAGGGAAAGATAGCGCCAACCCTACCCAGCGT
AAGGGGGCGAAATTCTGGGCGGTGGTCGCGCAAATCGTGGTGCTGGACGCTATCTTTTCT
CTCGACTCCGTGATTACCGCCGTGGGGATGGTCGATCACCTGGCGGTCATGATGGCGGCC
GTGATAATCGCCATTAGCCTGATGCTGCTCGCCAGCAAGTCACTGACCCGATTTGTGAAC
AACCATCCCACCATTGTCATTTTGTGTCTTAGCTTCCTGCTAATGATCGGCTTTAGCCTG
GTGGCGGAAGGGTTTGGTTTCCACATTCCGAAAGGGTATCTGTACGCGGCTATCGGCTTC
TCGGTGATGATCGAGGCGCTCAACCAACTGGCTATTTTTAACCGCCGCCGTTTTCTTTCC
GCTAACCATACGCTGCGCCAGCGCACAACGGAGGCCGTGATGCGGCTGATTAGCGGTAAA
AAAGAGGATGCCGAACTGGATGCGGAAACCGCCGCGATGCTGGCAGACCATGACGACAGC
CAGATTTTTAATCCGCAGGAGCGCAGGATGATTAAGCGGGTGCTGAATCTCAACCAGCGC
ACCGTAAGTAGCATCATGACGTCGCGTCACGATATCGAGCATATCGATCTCAACGCGCCG
GAAGCGGAAATCCGCGCTCTGCTGGAAAAGAACCAGCATACGCGTCTGGTCGTGACGGGT
GAGAATGAACAGGAAGATTTACTCGGCGTCGTCCACGTCATCGATCTGTTGCAACAATCG
CTGCGCGGCGAACCGCTCAACCTGCGGGCGCTGATTCGCCAACCGCTGGTTTTCCCGGAA
ACATTGCCGCTGCTGCCTGCGCTGGAGCAGTTCCGTAACGCCAGAACCCATTTTGCCTTT
GTCGTAGATGAGTTTGGCTCGGTTGAAGGCATTGTGACATTAAGCGATGTCATGGAGACT
ATCGCCGGTAATTTGCCGAATGAAGTCGAGGAAATTGACGCCCGCCACGATATTCAGAAG
AACCCGGACGGTTCCTGGACAGCAAACGGCCATATGCCGCTGGAGGATTTGGTGCAGTAC
GTGCCGCTACCGCTGGATGAAAAACGGGAATACCATACGATTGCCGGATTATTGATGGAA
TATTTGCAGCGCATACCGCAAACCGGTGAAGAGGTTCAGGTGGGTGATTATTTGCTGAAA
ACATTGCAGGTAGAAAGCCATCGAGTGCAGAAAGTGCAGTTAATTCCGCTGCATAACGGC
GAGCCAGACCAGGAGATGTAA
>EECEBICD_01240 hypothetical protein
ATGATGAAATCCAAAATGAAATTGATGCCATTATTGGCGTCGCTCAGCTTGATAAGTGGT
TGCACAGTACTTCCGGGCAGCAATATGTCTACGATGGGGAAAGATGTGATCAAACAGCAA
GACGCTGACTTTGATCTCGACCGGATGGTCAATGTGTATCCGCTGACGCCACGGCTGGTT
GAGCAATTACGCCCGCGGCCCAATGTCGCGCAACCGAATATGTCGCTGGACCAGGAGATC
GCCAGCTATCAGTATCGCGTCGGGCCTGGCGATGTGCTGAATGTCACCGTCTGGGATCAC
CCGGAATTGACCACGCCAGCGGGCCAGTACCGTAGCTCAAGCGATACCGGCAACTGGGTA
CAGCCGGACGGCACCATGTTTTATCCCTACATTGGCAAGGTTAGCGTCGTCGGTAAAACT
TTGTCAGAGATTCGCAGCGATATTACCGGGCGTTTAGCGAAGTACATCGCGGACCCGCAG
GTGGATGTCAATATCGCCGCTTTCCGCTCGCAAAAAGCGTATATCTCCGGCCAGGTGAAT
AAATCCGGTCAGCAGGCCATTACTAACGTACCGCTAACCGTCCTGGATGCGATTAACGCC
GCGGGCGGCCTGACCGATATGGCGGACTGGCGCAACGTCGTGTTGACGCACAACGGCAAA
GAACAGCGCATTTCGCTACAGGCGCTGATGCAAAATGGCGATCTTAGTCAGAACCGCTTG
CTCTACCCTGGCGACATTCTGTATGTGCCGCGCAATGACGATCTGAAAGTCTTTGTCATG
GGCGAAGTGAAAAAACAGAGCACCCTCAAAATGGATTTCAGCGGCATGACGCTCACCGAA
GCATTGGGCAATGCGGAAGGCATCGATCTGACCACCTCCAACGCCAGCGGCATTTTTGTG
ATTCGTCCGTTGAAAGGCGAGGGGGAACGCGGCGGCAAGATCGCCAATATCTACCAGCTT
GATATGTCTGACGCCACGTCATTGGTGATGGCGACGGAATTCCGACTTCAGCCTTACGAT
GTGGTGTACGTCACGACCGCGCCGGTTGCTCGCTGGAACCGTCTGATCAATCAGTTGCTG
CCAACCATTAGCGGTGTCCGTTATATGACGGATACGGCCAGCGACATTCATTCCTGGTAA

>EECEBICD_01241 Low molecular weight protein-tyrosine-phosphatase Wzb
ATGTTTAACAAAATCTTAGTGGTCTGTGTGGGGAATGTTTGCCGTTCCCCGACGGCGGAA
CGTTTGCTAAAACGCTTTCATCCCTCGCTGACGGTTGCGTCCGCAGGATTAGGCGCGCTG
GTCGGCAAAGGCGCCGATCCTGCGGCGGCGAGCGTGGCGAGCGCCCACAATCTGTCTTTG
GAAAATCACTGCGCAAGGCAAATTTCCGCGCGTCTGTGCCGGGAATATGACCTGATTCTG
ACGATGGAGAAACGTCACATCGCCGCCCTGTGCGACATTGCGCCGGAAATGCGCGGCAAG
GTAATGCTGTTTGGTCACTGGGACAGCGAGCGTGAAATACCGGACCCGTATCGCAAAAGC
CGCGATGCGTTTGAGGCGGTGTACACATTACTGGAAAGGTCTGCCCGCCAGTGGGCGCAG
GCACTGAATGCAGAGCAGGGAAAACCATGA
>EECEBICD_01242 Tyrosine-protein kinase wzc
ATGACAGAAAAAGTAAAACAATCTGCGGCGGTTACGGGTAGCGATGAAATCGATATCGGG
CGCCTGGTCGGAACGGTGATTGAAGCGCGTTGGTGGGTGTTGGGGACGACGGCCATATTT
GCGCTGTGCGCGGTAATTTATACGTTCTTCGCCACGCCCATCTATAGCGCCGATGCGCTG
GTGCAGATTGAACAAAACGCCGGTAATTCGCTGGTACAGGATATCAACAGTGCATTAGCG
AATAAGCCGCCGGCTTCCGATGCGGAAATTCAGCTCATTCGATCGCGCCTGGTGCTTGGG
AAGACCGTTGACGATCTGGATCTGGATATTGCCGTCACCAAAAACACCTTTCCGCTGTTC
GGCGCCGGGTGGGAACGGCTGATGGGGCGTCACAATGAAATGGTGAAAGTCACGACATTC
ACCCGACCAGAGACGATGAGCGGACAGATCTTCACCCTCAAAGTGCTGGGCGACAAACGT
TATCAGTTGGTTAGCGACGGCGGATTTAGCGCGCAGGGCGTTGTGGGCCAGCCACTCAAT
AAAGACGGCGTGACGATGCAGGTAGAGGCGATTGACGCCCGTCCTGATACAGAATTTACG
GTGAGTAAATTCTCAACGCTTGGCATGATTAATAACCTGCAAAATAACCTCACCGTAACG
GAAACCGGCAAAGATACCGGCGTTCTGAGCCTGACGTTCACGGGAGAGGATCGCGACCAG
ATCCGCGAAATTCTCAACAGTATTACCCGTAACTATCTGCAACAGGATATTGCGCGGAAA
TCTGAAGAGGCGGGGAAGAGCCTGGCGTTTCTGGCAAAACAACTGCCGGAAGTCCGCAGC
CGTCTGGATGTGGCGGAAAACAAGCTAAACGCTTTCCGCCAGGATAAAGACTCGGTGGAT
TTACCGCTGGAGGCGAAGGCGGTGCTCGATTCGATGGTTAATATCGACGCCCAGTTGAAT
GAGTTGACGTTTAAAGAGGCGGAAATTTCCAAGCTCTTTACCAAAGCGCATCCGGCCTAC
CGCACCCTGCTGGAAAAACGTAAAGCGCTGGAAGATGAAAAAGCCAAACTGAACGGGCGG
GTGACGGCAATGCCGAAAACCCAGCAGGAGATTGTGCGTTTGACCCGCGATGTGGAGTCC
GGCCAGCAGGTTTATATGCAACTGCTTAATAAACAGCAGGAGCTGAAAATCACCGAAGCC
AGCACCGTTGGCGATGTGCGCATCGTTGACCCGGCGATTACCCAGCCGGGCGTGCTGAAG
CCGAAAAAGGCGTTGATTATTCTCGGCAGCATTATTCTGGGATTAATGTTATCGATTGTC
GGCGTGCTGCTGCGCTCGCTGTTTAATCGCGGTATCGAAAGCCCGCAGGCGCTGGAGGAG
CACGGGATCAGCGTCTATGCCAGTATTCCGCTGTCGGAATGGCAAAAAGCGCGCGATAGC
GTCAAAACCATTAAAGGGATTAAGCGTTACAAGCAGAGCCAACTGCTGGCGGTGGGCAAT
CCGACCGATCTGGCGATTGAGGCGATTCGCAGCCTGCGCACCAGCCTCCATTTCGCCATG
ATGCAGGCGCAAAATAATGTACTCATGCTCACAGGCGTCAGCCCCTCTATCGGTAAAACC
TTTGTTTGCGCCAACCTGGCGGCCGTCATCAGCCAGACGCATAAACGGGTATTACTGATC
GACTGCGATATGCGCAAGGGCTACACCCATGAACTGCTCGGCACCAATAACGTGGACGGC
TTATCTGACATTCTGGCGGGCAAAGGCGAGATAGCCTCCTGCGCGAAACCTACGGCGATC
GCTAATTTTGATCTTATTCCGCGCGGTCAGGTGCCGCCTAATCCATCGGAACTGCTGATG
AGCGAGCGCTTCGGCGAGCTGATCGCCTGGGCAAGCAGCCGCTACGACCTGGTATTAATC
GACACGCCGCCGATTCTGGCCGTGACGGACGCCGCGATTGTGGGTCGCCACGTCGGGACG
ACGCTGATGGTGGCGCGTTATGCCGTCAACACCTTAAAAGAGGTGGAAACCAGTCTGAGC
CGTTTTGACCAGAATGGCATTCAGGTCAAAGGCGTCATTCTCAACTCCATTTTTCGCCGG
GCGACGGGCTACCAGGATTATGGTTATTACGAGTATGAATACCAGTCAGATTCCAAATAA
>EECEBICD_01243 hypothetical protein
ATGGCGCGGCCTGTGACTGTAGGCCGGACAAGCGTAGCGCCATGCGGCAAAACTATCTGG
GGAATAAGCATGACAACAGACAATCCGCTGATCTCAATTTATATGCCAACCTGGAACAGG
CAGCAGTTGGCTATCCGGGCGATTAAATCAGTACTACGTCAGGACTACCGCCACTGGGAG
ATGATCATCGTGGATGATTGCTCCTCGTCTTATCAGCAGCTACAACAATTTGTTGAGGAG
CTGAACGACCCGCGCGTCCGTTATACCCATAACGCCAGGAATACCGGCGCCTGCGCGGTT
CGCAACCAGGCCATTATGCAGGCGCAAGGACACTACATTACCGGTATTGATGATGACGAT
GAATGGACGCCAAACCGTCTGAGCGTGTTTCTGGCCCATAAACACCAGTTGACTACGCAC

GCCTTTTTGTATGCCAACGACTATGTGTGCGAGGGCGAGGTCTACTCTCAGCCTGCCAGC
CTGCCGCTGTATCCGAAATCCCCTTATTCGCGCCGCTTGTTCTACAAGCGCAATATTATT
GGCAATCAGGTTTTTACCTGGGCCTGGCGTTTTAAAGCGTGTTTGTTCGATACCGAACTC
AAGGCGGCGCAGGATTACGACATTTTTCTGCGCATGGTGGTGGAGTATGGCGAACCGTGG
AAAGTTGAAGAGGCGACGCAAATTTTACATATCAACCACGGCGAGATGCAGATTACCTCT
TCGCCGAAAAAGTTCTCCGGCTATTTCCATTTTTATCGTAAGCACAAAGATAAATTCGAT
CGCGCCAGCAAAAAATACCAGCTTTTTACGCTGTACCAAATTCGCAATAAGCGAATGACC
TGGCGCACGTTGCTGACGTTGCTCTCCGTGCGTAACAGCAAACGCCTGGCGGCCGGGCTG
CGGGGGCGATAA
>EECEBICD_01244 Serine acetyltransferase
ATGTTGGAAGACTTACGCGCGAATAGCTGGAGCTTACGCCCCTGTTGCATGGTGCTGGCC
TACCGTATCGCTCATTTTTGTTCCGTCTGGCGCAAAAAGAATGTGCTGAATAATCTGTGG
GCCGCGCCGGTGCTGGTGCTGTATCGCGTGATCACGGAATGCCTGTTCGGGTATGAAATC
CAGGCCGCCGCGACCATTGGTCGGCGGTTCACTATCCATCATGGTTACGCGGTAGTGATT
AACAAACATGTCGTCGCCGGCGACGATTTTACCATTCGCCACGGCGTCACCATTGGCAAT
CGCGGCGCGGACAGCCTGGCTTGTCCGGTGATTGGTCACGGCGTTGAGCTGGGGGCGAAT
GTCATCCTGCTTGGCGATATTACTATCGGTAATCACGTCACGATTGGCGCGGGTAGCGTG
GTACTTGACAGTATTCCGGACCATGCGCTGGTGGTGGGAGAAAAAGCACGGGTGAAGGTC
AGCACATGA
>EECEBICD_01245 hypothetical protein
ATGAATATTCTGCAATTTAATGTACGGCTGGCGGAAGGCGGCGCGGCGGGAGTGGCGTTG
GATCTCCATCTGCGGGCGCTGCAAAAAGGGCTGACGTCACGTTTTGTCTATGGTTATGGC
AAAGGCGGAAAAAAAGCGTCAGCCACCACCGTTATCCGCAGGTGA
>EECEBICD_01246 hypothetical protein
GTGATAAAACAGACGCCGCGCGGCACGGCAATCGCTAATATCGCGCTGTTTCGTTTCCTG
AATCGCGATCTGTTTGGCAATCTCGACAATCTTTACCGCACGGTTATCCAGACATCCGGC
CCGCTGGTGCTGCATTTTCATGTTCTCCACAGTTACTGGCTAAACCTGGCGGATATCGTG
ACGTTTTGCGAAAAAGTCAAAGCGCAAAAACCGGACGTCACGCTGGTCTGGACGCTGCAC
GATCACTGGAGCGTCACCGGGCGTTGCGCCTTCACCGACGGTTGCGAGGGCTGGAAAAGC
GGCTGCCAAAAAATGTCCGACCCTAAGCAATTATCCGCCGGTCAGGGTGGATCGGGCGCAC
CAGCTTATTGGCGGCAAACGTCAGCGCTTTCGGGACATGCTGCGGCTGGGCTGCCAGTTT
ATTTCGCCGAGCCAGCATGTGGCCGAGGCCTTTAACAGCGTTTATGGCGCGGGACTCTGC
CGGGTGATTAACAACGGTATCGATCTGGCGACCGAGGCGATTCTCGCGCAACTATCACCT
GTGCCGCTGAATCCGGGCAAACCGCGGATCGCCATTGTGGCGCATGACTTGCGTTACGAC
GGCAAAACTGACCAGCGTCTGGTACACGACATGATGGCGCTGGGCGAAAAAATTGAACTG
CATACCTTCGGTAAATTTTCGCCTTTTACCGGCCAAAACGTTGTTAATCACGGTTTTGAA
ACCGATAAGCGCAAATTAATGAGCGCGCTCAATGAGATGGATGCGCTGGTCTTTAGCTCG
CGGGTCGATAACTATCCGCTGATCTTGTGTGAAGCGCTCTCGATCGGCGTACCGGTGCTC
GCTACCCACAGCGAGGCGGCGCAGGAGGTGCTGGCGAAATCCGGCGGCCAGACCTTTGCC
GCTACAGATGTACTGCGCCTGGCGCAGCGGCGTAAGCCAGAGATTGCTCAGGCGGTATTT
GGCGCCACGCTGGACGCCTTTCGTATGCGTAGCCGCGTCGCGTACAGCGGTCAACAGATG
CTGGAGGAGTATGTCTCGTTCTATCAGAATCTGTAG
>EECEBICD_01247 hypothetical protein
ATGTCTCGTTCTATCAGAATCTGTAGCTATCTGCTGCTGCCGCTGATCTACCTACTGGTC
AATGTCAAGATTGCCCAACTGGGGGAAAGCTTTCCCATTACCATCGTCACTTTTTTACCG
CTGTTGCTGCTGTTATTTGTGGAACGCATTAGCGTAAAAAAATTGATGATCGCCTTAGGC
ATCGGCGCGGGGCTTACGGCATTTAACTTCCTGTTCGGCCAGTCGCTGAATGCCGGTAAA
TATGTCACATCCACGATGCTGTTTGTCTATATTGTGGTCATTATCGGGATGGTCTGGAGT
ATCCGATTCAAAACCATTTCCGCGCATAACCACCGAAAGATTTTGCGTTTTTTTTATCTG
GTGGTGGGTATAGTGGTCGCGCTCGCCGCGGTTGAGATGGCGCAAATTATCCTTACCGGC
GGCAGCAGTATTATGGAAGGAATTTCGAAATATCTCATTTACAGTAATAGCTACGTACTG
AACTTCATAAAATTTGGCGGTAAGCGTACCACTGCGCTTTATTTTGAACCGGCATTTTTC
GCTTTGGCACTAATCTCAATTTGGCTGAGCATCAAACAGTTTGGTATCAAAACACCGAAA
AGCGATGCTATGATTCTGGCAGGGATAATATTATCAGGATCATTTTCAGGGGTAATGACC

TTTATCCTGTTTTACCTTCTGGAGTGGGCGTTCCAATATTTGAATAAGGATGCGATAAAG
AAAAAACTTCCACTGGCGCTGGTCTCATTAACCCTGTTTTTGGTTGGGGTAATTATTGCA
TTTCCTTATATCGCGACACGACTTGGCGATTTAGGGACGGAAGGCTCATCTTCTTATTAT
CGTATTGTGGGCCCCTTAGTCATGGTCGGATATTCCTTGACCCATATTGATGGTGTAGTC
AGATTTGGCTCACTTTATGAATATGTCGCATCATTCGGAATCTTTAACGGTGCGGATGTC
GGGAAAACCATAGACAATGGATTGTATCTGCTGATTATTTATTTTTCCTGGTTCGCAGTG
CTAATGACGCTGTGGTATATGGGGAAAGTTTTAAAAATGGCGCTAAATGCGTTTGGCGAT
AATCGCAATTTTCGGGTACAGCTCTATCTTTTTACGCCGGTGTCACTGTTTTTTACCGGT
TCAATATTTAGCCCGGAATATGCTTTTTTAATCGTCTGTCCGTTCATTTTGCGCAAGGCG
TTAAAAATTTCATAA
>EECEBICD_01248 hypothetical protein
ATGTTTTTAAGCGTCGTTACCGTCGCCTTTCGCAATTACGAAGGGGTGGTAAAAACCTGG
CGCTCGCTGCGCAATCTGGCGCGCGATCCGGGTCTGTCATTTGAATGGATCGTAGTGGAT
GGCGGTTCGGAGGACGGCACGCGCGAATTTCTGCAAAAACGTCACGGTGAGTTTAACCTG
CGTTTTATCAGTGAAAAGGATCGCGGTATTTATGATGCCATGAACAAAGGCATTGCGCTG
GCGCAGGGGCGTTATACCCTGTTTCTTAATTCCGGTGATGCGTTCCATGATGATGCCGCG
CTGTTTGTCCGCCAACTGGCGCGGCAAAAGGGCGAGGCGATGTATATTGGCGACGCGCTG
CTGGATTTCGGCGGCGGGCATAAAATACGCCGGAAGGCTAAACCCGGGTATTATATCTAC
CATAGTTTGCCAGCCAGCCATCAGGCGATATTTTTCCCGACGGCAGCGTTAAAAAAGTAT
CCTTATGATCTGCAATATCATGTTTCTTCAGATTATGCGCTGGCGGCCCGTCTTTATAAA
GCAGGCTATCCTTTTCGGCGAATTAACGGACTGGTATCTGAATTTTCGATGGGCGGCGTG
TCAACCTCGAATAATCTGGAATTATGTCAGGATGCAAAAAATGTACAGCGAAAAATATTA
CGTGTGCCAGGATTTTGGGCGCAATTATCCTATTTATTACGTCTGAAAACGACGGGTAAA
ACAAAAGCGCGGTATAACAAAGTCTAA
>EECEBICD_01249 hypothetical protein
ATGCAGGATCTTAGCGGCTTTACGGTGCCGAAGGGGTTCCGCGGCGCTAATGTGCTAAAA
GTACAATTATGGTGGGCGGTACAGGCGACATTATTCGCCGGGTCGCCGCAAATATTGTAT
CGCTGGCGAGCCTTTTTGCTGCGTCTGTTTGGCGCCAAAATTGGAAAGAATGTGGTTATT
CGACCGTCAGTAAAAATTACCTATCCGTGGAAATTAACCGTCGGCGATTATGCCTGGGTA
GGCGACGACGCTGTGTTATATACGTTGGGTGAAATTAATATTGGCGCACATGCGGTCATT
TCACAAAAAGGGTATTTGTGCACCGGTAGCCATGATTATACCAGCGCCCATTTCGATATT
AACGCCGCGCCGATTGTTATTGGCGAAAAATGTTGGCTGGCGACCGATGTTTTTGTCGCG
CCCGGCGTGACGATAGGTCATGGCACCGTCGTCGGCGCGCGCAGCAGCGTATTTAAATCA
TTACCGGCAAATGCGATTTGTCGGGGCAATCCCGCAGTGGTAACGCGCCAGCGCGTTCAG
AAAGTTACTCCCTAA
>EECEBICD_01250 GDP-mannose 4,6-dehydratase
ATGTCAAAAGTCGCTCTCATTACTGGCGTAACCGGACAGGATGGGTCTTACCTGGCAGAA
TTTCTGCTGGAAAAAGGGTATGAGGTGCATGGTATCAAGCGCCGCGCGTCATCGTTTAAT
ACCGAGCGCGTGGACCATATTTATCAGGACCCGCACAGCTGCAACCCGAAATTTCATCTG
CATTATGGCGACCTGACCGACGCCTCCAACCTGACCCGCATTTTACAGGAAGTGCAGCCG
GATGAGGTCTACAACCTGGGCGCGATGAGCCATGTGGCGGTGTCGTTTGAGTCGCCGGAA
TATACCGCCGATGTGGATGCGATGGGCACGCTGCGCCTGCTGGAGGCGATCCGCTTCCTC
GGTCTTGAAAAGAAAACGCGGTTCTACCAGGCCTCCACCTCTGAACTGTACGGGCTGGTG
CAGGAGATCCCGCAGAAAGAGACCACGCCGTTCTACCCGCGTTCCCCCTATGCGGTGGCG
AAACTGTACGCCTACTGGATCACCGTTAACTACCGTGAATCCTACGGTATTTACGCCTGT
AACGGCATTCTGTTTAACCACGAGTCCCCGCGTCGCGGCGAAACCTTCGTCACCCGTAAG
ATCACCCGCGCCATCGCCAATATCGCCCAGGGACTGGAGTCCTGCCTGTATCTCGGCAAC
ATGGACTCGCTGCGCGACTGGGGTCATGCGAAAGATTACGTGCGGATGCAGTGGATGATG
TTACAGCAGGAGCAGCCGGAAGATTTCGTGATTGCCACCGGCGTGCAGTATTCCGTGCGC
CAGTTTGTGGAGCTGGCGGCGGCGCAACTGGGGATAAAACTGCGCTTTGAAGGCGAAGGC
ATTAATGAGAAAGGGATTGTGGTATCCGTTACCGGACACGATGCGCCGGGCGTGAAACCG
GGCGATGTGATTGTGGCCGTTGATCCGCGTTATTTCCGTCCGGCGGAAGTGGAAACCCTG
CTGGGCGACCCGTCCAAAGCGCATGAGAAACTGGGCTGGAAACCGGAAATCACCCTGTCG
GAGATGGTCTCCGAGATGGTGGCGAACGATCTGGAGGCCGCGAAAAAACACTCACTGCTG

AAATCTCACGGTTACGAGGTGGCCATCGCGCTGGAGTCCTGA
>EECEBICD_01251 GDP-L-fucose synthase
ATGAATAAGCAACGAATTTTTGTGGCGGGCCATCGCGGGATGGTGGGCTCCGCCATTGTA
CGGCAGCTTGCGCCGCGCGGCGACGTGGAACTGGTGCTGCGCACCCGCGATGAGCTGGAT
CTGCTCGACGGGCGCGCGGTACAGGCGTTCTTTGCCGGGGCGGGTATCGACCAGGTTTAT
CTGGCGGCGGCGAAAGTGGGCGGCATTGTCGCCAACAACACATATCCGGCGGATTTTATC
TATGAAAACATGATGATAGAGAGCAACATTATTCACGCCGCGCACCTGCACAACGTGAAC
AAACTGCTGTTTCTCGGTTCGTCCTGTATCTATCCGAAACTGGCAAGGCAGCCGATGGCG
GAAAGCGAGCTGCTGCAGGGGACGCTGGAGCCGACCAACGAGCCGTATGCCATCGCCAAG
ATCGCCGGGATTAAATTGTGCGAGTCCTACAACCGGCAGTACGGTCGCGACTACCGTTCG
GTGATGCCAACCAACCTGTACGGCCCGCATGACAATTTCCATCCGGACAATTCACATGTG
ATCCCGGCGCTGCTGCGCCGCTTTCATGAGGCCGCGCAGAGCCACGCGCCGGAGGTGGTG
GTGTGGGGCAGCGGCACGCCGATGCGCGAATTTCTGCACGTTGACGATATGGCGGCGGCC
AGCATTCACGTGATGGAGCTGGCGCGCGAAGTGTGGCAGGAGAACACCGCTCCGATGCTG
TCGCACATTAACGTCGGCACTGGCGTGGACTGCACCATCCGCGAGCTGGCGCAGACTATC
GCAAAGGTGGTGGGTTACCAGGGCCGGGTGGTGTTCGACGCCACGAAGCCGGACGGCACG
CCGCGTAAATTGCTCGACGTCACGCGCCTGCATCAGCTTGGCTGGTATCACGAAATTTCA
CTGGAGGCAGGGCTTGCCGGTACTTACCAGTGGTTCCTTGAGAATCAGCACCGGTTCCGG
GGGTGA
>EECEBICD_01252 GDP-mannose mannosyl hydrolase
TTGCCGGTACTTACCAGTGGTTCCTTGAGAATCAGCACCGGTTCCGGGGGTGACAATGTT
TTTACGTCAGGAAGATTTCGCCGCCGTGGTGCGGACCACGCCCCTCATCTCCCTCGATTT
CATCGTGGAAAACGGCCAGGGGAAATTTTACTGGGCCAGCGTCTCAACCGTCCGGCGCAG
GGCTACTGGTTTGTGCCGGGGGGGCGGGTGTGCAAAGACGAAACGCTGGAGGCCGCCTTT
GCACGCCTGACGCAGGCGGAACTGGGCGTGCGTCTGCCGCTGGCGGCAGGGACGTTTTAT
GGCGTCTGGCAGCACTTCTATGACGACAACTTTTCCGGTGAGGATTTTTCAACTCACTAC
ATCGTGCTCGGCTTTCGTCTGCGCGTGACGGAGAGCGATTTACGCCTGCCTGATGCCCAG
CATGGCAGTTACCGCTGGCTGACGCCGGAACAGCTTCTGGCAAGCGACAATGTTCATGAG
AACAGTCGGGCCTACTTCCTTCCTGACGCGCCGGCGGTGGGCCTATGA
>EECEBICD_01253 hypothetical protein
ATGAAGATCCTGGTCTATGGCATTAACTATTCGCCGGAACTGACCGGTATCGGCAAATAT
ACCGGCGAAATGGTGGCGTGGATGGCGCAGGAAGGCCATGAGGTGCGGGTCATTACTGCG
CCGCCGTACTACCCGCAGTGGAAGGTCGGTGAGCGCTATTCCGCCTGGCGCTATCGCCGG
GAAGAGGGCGAGGCTACCGTCTGGCGCTGCCCGCTGTATGTGCCGAAACAGCCCTCCACC
TTAAAACGACTGCTTCATCTGGGGAGCTTTGCATTGAGCAGTTTTTTCCCACTGATGGCG
CAGCGTCGCTGGAAGCCGGATCGGATTATCGGCGTTGTACCGACGCTCTTTTGTACGCCG
GGAATGCGCCTGCTGGCGACACTCTCCGGCGCGCGTACCGTTCTGCATATTCAGGATTAC
GAAGTGGATGCCATGCTCGGCTTAGGAATGGCGGGAAAAGGCAAGCGCGGCAGCGTAGCG
CGGTTAGCGACGGCCTTTGAACGCAGCGCGCTGCGTAACGTTGATAATGTTTCGACCATC
TCTCGCTCCATGATGAATAAAGCGCGGGAAAAGGGCGTCGCGGCGGAAAAAATCCTCTTT
TTTCCGAACTGGTCGGAAGTGGCGCGCTTTCAGGACGTTAATGACGCTGACGTGACGGCT
TTGCGTCAGCAGCTTGGCTTGCCAGAGGGCAAAAAAATTGTGCTTTATTCCGGCAATATC
GGCGAAAAGCAAGGGCTGGAAAAGGTGATTGACGCCGCGGAACGATTACGCGATCGACCG
TTGATTTTTGCGATTGTCGGCCAGGGGGGCGGCAAGGCGCGGCTGGAGAACATGGCCCGC
GAACGCGGCCTGGCAAATATTAAATTCCTCCCGCTGCAGCCTTACGACGCCTTACCCGCG
CTGCTGAAAATGGGCGACTGCCATCTGGTGGTGCAAAAGCGCGGCGCGGCGGATGCGGTA
CTGCCCTCCAAGCTGACTAACATTCTGGCGGTCGGCGGTAATGCGGTGATTACCGCTGAG
CCGCACACCGAATTAGGACAACTTTGCGCGCGCTATCCGGGCATCGCCGTTTGCGTGGAA
CCGGAGTCGACCGACGCGCTGGTCAACGGGATTAGCCAGGCGCTCGCCATGCCGAAAAAC
AACACGACGGCACGTGAATACGCCGAACGCACTCTCAATAAAGAGAACGTGCTACGCCAA
TTTATTGCAGATATTCGGGGATGA
>EECEBICD_01254 Mannose-1-phosphate guanylyltransferase 1
ATGCGTCAGACAAAACTTTACCCGGTTGTGATGGCCGGTGGCTCCGGTAGCCGATTGTGG
CCGCTTTCTCGCGTGCTTTATCCAAAACAGTTTCTGTGCCTGAAAGGCGATCTCACCATG

CTGCAAACCACCATTTGCCGTCTGAACGGCGTGGAGTGCGAAAGCCCGGTGGTGATCTGT
AACGAACAGCACCGTTTTATCGTTGCCGAACAGCTCCGCCAGCTCAATAAGCTGACGGAG
AACATTATTCTTGAGCCCGCCGGGCGCAATACCGCGCCGGCGATTGCGCTGGCGGCGCTG
GCGGCGACGCGCCAGCATACGGACTGCGATCCGCTGATGCTGGTACTGGCGGCGGATCAT
GCAATCGCCAATGAAGAGGCCTTCCGCGACGCGGTGCGCGGCGCCATGCCCTATGCCGAC
GCGGGCAAACTGGTGACCTTCGGTATCGTGCCGGATCTGCCGGAAACCGGCTATGGCTAT
ATCCGGCGCGGTGATGTCGTGCCGGGCGCGACGGATGCGGTGGCTTTTGAGGTGGCGCAG
TTCGTTGAGAAGCCGGGGCTGGAAACCGCTCAGGCCTACGTCGCCAGCGGCGATTACTAC
TGGAACAGCGGCATGTTTCTGTTCCGCGCCGGACGCTATCTGGAAGAGCTGAAAAAGTTC
CGCCCCGATATTCTGGCCGCCTGTGAACAGGCGATGCGCGGCGTCGATCCGGATCTTGAC
TTCATTCGTGTGGATGAAGAGGCCTTCCTCGCCTGTCCGGAAGAGTCCATTGACTACGCG
GTGATGGAGCGAACGGTGGACGCGGTGGTGATGCCGATGGACGCGGGCTGGAGCGATGTC
GGCTCGTGGTCCTCGCTGTGGGAAATCAGCGCCCACACCCCGGAGGGAAACGTCCACCAC
GGCGACGTCATCAGCCATAAAACGGAAAACAGCTACGTTTACGCCGAATCCGGCCTGGTG
ACCACCGTCGGCGTGAAGGATCTGGTGGTGGTGCAGACCAAAGACGCCGTGCTGATTGCC
GATCGCCACGCGGTGCAGGACGTGAAAAAGGTGGTGGAGAAGATCAAGGCCGACGGACGC
CATGAGCACCACATGCACCGTGAGGTTTACCGTCCGTGGGGCAAATACGACTCCATCGAC
GCGGGCGAGCGCTATCAGGTGAAGCGTATTACCGTCAAGCCGGGGGAGGGACTGTCGGTG
CAGATGCACCATCACCGCGCCGAGCACTGGGTGGTGGTGGCGGGGACCGCCAGAGTCACC
ATCAACGGCGAGGTTAAGCTGCTCGGTGAAAACGAGTCCATCTATATTCCGCTGGGGGCG
ACTCACTGCCTGGAAAATCCGGGAAAAATACCGCTCGATTTAATTGAAGTGCGTTCCGGC
TCGTATCTCGAAGAGGACGACGTGGTGCGGTTCGAGGACCGTTACGGGCGGGTCTGA
>EECEBICD_01255 Phosphomannomutase/phosphoglucomutase
ATGACAAAATTAACCTGTTTCAAGGCCTACGACATTCGCGGCAGGCTGGGCGAAGAGCTC
AATGAAGATATCGCGTGGCGGATTGGCCGCGCCTACGGCGAATATTTAAAACCGAAAACC
ATCGTGCTGGGCGGCGACGTGCGTCTGACCAGCGAGGCGCTGAAGCTGGCGCTGGCGAAA
GGGTTACAGGACGCGGGCGTTGACGTGCTGGATATCGGTATGTCCGGCACCGAAGAGATT
TACTTCGCCACCTTCCACCTTGGCGTGGACGGCGGTATTGAGGTGACCGCCAGCCATAAC
CCGATGGACTACAACGGTATGAAGCTGGTACGCGAGGGCGCCCGCCCGATAAGCGGCGAC
ACCGGTCTGCGCGACGTTCAGCGTCTGGTGGAAGCGGGCGACTTCCCGCCGGTGAACGAG
GCCGCGCGCGGCAGCTACAGACAAATCAGCCTGCGCGACGCCTACATCGGCCACCTGCTC
GGCTATATCAGTGTTAACAATCTCACCCCGCTGAAGCTGGTGTTTAACGCCGGCAACGGC
GCGGCGGGGCCGGTCATTGACGCCATCGAAGCGCGCCTGAAGGCGCTGGGCGCGCCGGTG
GAGTTCATCAAAATCCACAACACCCCGGACGGCACCTTCCCGAACGGCATTCCCAACCCG
TTGCTGCCGGAGTGTCGCGACGACACCCGCAAGGCCGTTATTGAACACGGGGCGGACATG
GGCATCGCCTTTGACGGCGACTTCGACCGCTGCTTCCTGTTCGATGAGAAAGGGCAGTTT
ATCGAGGGGTACTACATCGTCGGGCTGCTGGCGGAAGCGTTTCTGGAAAAACACCCGGGG
GCGAAGATTATCCACGACCCGCGCCTGACCTGGAACACGGAGGCAGTGGTGACGGCGGCG
GGCGGGACGCCGGTGATGTCAAAAACCGGGCACGCCTTTATCAAGGAGCGGATGCGTACG
GAGGATGCCATTTATGGCGGGGAAATGAGCGCGCATCACTATTTTCGTGATTTTGCCTAC
TGCGACAGCGGGATGATCCCGTGGCTGCTGGTGGCGGAGCTGGTGTGCCTGAAGAGACAG
TCGCTGGGTGAGCTGGTTCGCGACCGGATGGCGGCGTTCCCGGCGAGCGGGGAAATCAAC
AGCAGACTGGCGGAGCCGGCGGCGGCCATTGCGCGGGTGGAAGCGCACTTTGCGGAAGAG
GCGCAGGCGGTGGATCGCACGGACGGTCTCAGCATGTCGTTCGCCGACTGGCGTTTCAAC
CTGCGCTCGTCGAACACCGAACCGGTGGTGCGCCTGAATGTGGAATCACGCGGCGATATT
CCGCTGATGGAAGCGCGAACACGCACTCTGCTGGCGCTGCTGAATCAGTAA
>EECEBICD_01256 UDP-glucose:undecaprenyl-phosphate glucose-1-phosphate transferase
ATGACAAATCTAAAAAAGCGCGAACGAGCCAAAACCAATGCATCGTTAATCTCTATGGTG
CAACGCTTTTCAGATATCACCATCATGTTTGGCGGGCTGTGGTTCATCTGTGAAGTCAGT
GGACTGCCATTCCTGTACATGCACCTGTTGGTGGCGCTGATTACGCTGGTTGTTTTTCAA
ATGCTGGGCGGGATGACCGATTTTTATCGCTCCTGGCGCGGCGTCAAAATGTCGACAGAG
TTAACGCTGTTACTGCAAAACTGGACGCTGAGCCTGATCTTCAGCGCCGGTCTGGTGGCG
TTTAATGATGACTTTGACAACCGCCTCGCCACCTGGCTGGCTTGGTATGCGCTGTCCAGC

CTGGGCTTAGTGTTATGCCGTTCATTTATTCGTTTCGGCGCGGGCTGGCTACGCAATCGT
GGTTATAACACTCGCCGGGTCGCCGTCGCCGGAGATTTGCCTGCGGGTCAGGCGTTGCTG
GATAGCTTCCGTAATGAGCCGTGGCTGGGTTTTGAGGTGGTGGGCGTCTATCACGATCCT
AAACCGGGCGGCGTCTCTGCTGACTGGGCCGGGAACTTCGAGCAGTTAATTGACGATGCG
AAAGCCTCGCGAATTCACAATGTTTATATCGCCATGTCGATGAGCGACGGCGCCATGGTG
AAAAAACTGGTGCGCGAGCTGGCCGACACCACCTGCTCGGTGATCCTTATTCCCGATGTG
TTCACCTTTAATATTCTCCACTCGCGCGTGGAGGAGATGAGCGGCGTTCCGGTCGTTCCG
CTGTATGACACGCCGCTATCAGGGATTAACCGTCTGCTTAAACGGGCAGAAGATATCGTG
CTGGCGTCGCTGATTCTGCTGCTCATCTCACCGGTACTGTGCTGCATTGCGCTGGCGGTC
AAATTGAGCTCGCCCGGGCCGGTGATTTTCCGTCAGACGCGCTACGGGATGGACGGCAAG
CCGATCAAGGTCTGGAAGTTCCGCTCAATGAAGGTGATGGAGAACGATAAGGTCGTTACC
CAGGCGACGCAAAACGATCCGCGCGTCACCCGGGTAGGTAACTTTTTGCGTCGTACCTCG
CTGGATGAATTGCCGCAGTTTATCAACGTATTAACTGGCGGGATGTCCATCGTCGGTCCT
CGCCCGCACGCGGTGGCGCATAACGAACAGTATCGTCAGTTGATTGAAGGTTACATGCTA
CGCCACAAGATGAAGCCGGGCATCACCGGCTGGGCGCAAATTAATGGCTGGCGCGGTGAG
ACCGATACGCTGGAGAAAATGGAAAAACGCGTGGAATTCGACCTGGAATACATCCGCGAA
TGGAGTATTTGGTTCGATATCAAAATCGTTTTTCTGACGATTTTTAAAGGGTTTGTTAAT
AAAGCGGCGTATTAA
>EECEBICD_01257 Lipopolysaccharide biosynthesis protein WzxC
ATGAGTTTACGACAAAAAACGATCAGCGGCGCTAAATGGTCGGCTATCGCCACGATAGTG
ATTATCGGTCTGGGGTTAATTCAGATGACGGTGCTGGCGCGGATCATCGACAACCACCAG
TTTGGCCTGTTGACCGTCTCGCTGGTGATTATCGCGCTGGCCGACACGATCTCGGACTTT
GGCATCGCGAACTCGATTATCCAGCGTAAAACGATTGGGCATCTGGAGCTGACCACGCTC
TACTGGCTAAACGTTGGGCTTGGGATTGTGGTATTTGCGGTGGTCTTTTGGCTGAGCGAT
GCGATTGCCCATGTTTTGCATAACCCGGATCTCGCGCCGTTAATCAAAACGTTGTCGCTG
GCGTTCATCGTGATCCCTCACGGGCAGCAGTTCCGCGCTCTGATGCAAAAAGAGCTGGAG
TTCAATAAGATCGGCATGATCGAAACGACCTCCGTGCTGGCGGGGTTCACCTTTACGGTG
ATTAGCGCCCATTACTGGCCGCTGGCGTTAACCGCGATTCTCGGCTATCTGGTGAACAGT
GCGGTGCGAACGCTGCTGTTTGGTTACTTTGGCCGCAAGATTTACCGTCCGGGGCTGCAT
TTTTCGCTGGCATCGGTGTCCACGAACCTGCGTTTTGGCGCGTGGCTGACGGCGGACAGT
ATCGTCAATTACATCAACACGAATTTATCGACGCTGGTACTGGCGAGAATTCTCGGCGCA
AGCGTCGCCGGGGGATATAACCTCGCGTACAACGTGGCGGTTGTGCCGCCGGCGAAGCTT
AACCCCATCATTACTCGCGTGCTATTTCCGGCATTCGCCAAAATCCAGGACGATACCGAG
AAGCTGCGCGTCAACTTCTATAAGTTGCTTTCCGTGGTGGGAATTATCAATTTTCCCGCG
CTGTTGGGCCTGATGGTAGTGGCGAACAATTTTGTGCCGTTAGTGTTTGGCGAGAAGTGG
AACAGTATTATCCCGATCCTGCAATTGCTGTGCGTGGTGGGGCTGTTGCGCTCGGTCGGC
AACCCGATTGGTTCGCTGCTGATGGCGAAAGCGCGCGTGGATATCAGCTTTAAGTTCAAC
GTCTTTAAAACGTTTCTGTTTATCCCGGCAATTCTCATTGGCGGCCATCTGGCGGGGGCG
ATTGGCGTGACGCTGGGGTTCCTGGTGGTGCAAATCATCAACACCATTCTGAGCTATTTC
GTGATGATTAAGCCGGTACTCGGCTCCAGCTATCGTCAGTATATTCTCAGCCTGTGGCTA
CCGTTTTATCTCTCATTGCCAACATTTATTACCAGCTACGGCGCAGGAAAGCTGGCCGAC
GGCTATTTACCGTTGTCAGGGGTATTCGCACTACAGGTTATGGTCGGTATTTTGAGTTTT
ATTTTGATGATTATTTTTTCACGCAATGCGCTGGTGATGGAAATTAAAAATCAGCTTGTT
GGCAGTGCAAAAATGAAAAAGTTATTACGTGTCGGATGA
>EECEBICD_01258 hypothetical protein
ATGAAATTATTAATTTTAGGCAACCACACATGCGGCAACCGTGGTGATAGCGCCATTATG
CGCGGTTTGCTTGATGCCATCCGTCAACAGGCGCCGGAGGCGGAGATGGATGTGATGAGC
CGTTTCCCGGTGAGTTCCGCCTGGTTGCAGGGGCGCCCGATTATTGCCGACCCGCTGTAT
CAGTTAAGCCAGAAACAGCAGGCGGCGGCAGGGCTTAACGGTCGGGTGAAAAAAGTATTA
CGCCGTCGTTTTCAGCATAAAATTTTATTGTCTAAAGTCGCCCAGGAAGGATCGCTGCGT
AATTTTGCTATCGCGCCGGAATTTGCCGAATTCGCGCAATTTATTGCGCAGTATGACGCC
GTCATTCAGGTGGGCGGTTCAAACTATGTCGACTTATACGGTTTGACTCACTTTGAATAT
CCGCTGTGCGCGTTTATGGCGAACAAACCAATTTATATGGTGGGACATAGCGTCGGCCCT
TTCCAGAATCCTGACTTTAACCAGCTCGCCAATTATGTTTTTGGCCGCACCAGTGCGCTG

ATCCTGCGTGAAACCGTCAGCCGCGATATGATGACCGCCGCGCAGATCGATACCCGGAAA
GTGGAGCAGGGGGTGGATACCGCCTGGTTAGTCGAACGCCGTCACGACGAGTTTGTTGCC
AGTTATGCTGTGCAGCACTGGCTGGACGTCACCGCGCGCCGCAAAACCGTCGCGATTACG
TTGCGCGATCTGGAGCCGTTTGATAAACGCCTGGGCACCACTCAGCAGGCCTATGAGCAG
GCATTTGCCCAGGTCGTGAATCGCCTGATTGCGGACGGTTATCAGGTACTGGCGCTGTCA
ACCTGCACCAGTATCGATCGCTATAACCGCGATGACCGGATGGTTGCGTTACGGATGGCG
AAGTATGTAAACGACTCAGAGAATTTCCATATCGCGATGGACGAACTCAATGACGTGGAA
ATCGGAAAGATCCTCGCCTCCTGCGTACTGACCATCGGGACGCGCCTGCATTCCGCCATT
ATCTCCATGAATTTTGGCACACCCGCCATCGCCATTAATTATGAACATAAATCCGCCGGG
ATTATGCAGCAGCTAGGCCTGGCGGAATTGGCCATCGATATCCGTCACTTGCTGAACGGT
TCATTACAAAGCGTGGTGGCCGATACGCTCAATCAATTGCCTGCGCTAAAGGCAAAAGTG
GCTAACGCCGTCACCGAGGAGCGGGCGAAAGGGTTCACGATGATCAAATCTGTTCTCGAC
AGAATTCGGGAGGCGCAATGA
>EECEBICD_01259 UDP-N-acetylglucosamine--peptide N-acetylglucosaminyltransferase GtfA subunit
ATGAAAGTCAGCTTTTTTCTGCTGAAATTTCCACTCTCATCGGAAACCTTTGTGCTGAAT
CAGATTACTGCGTTTATTGATATGGGCCATGAGGTGGAGATTGTCGCGTTACAAAAAGGC
GATACCCAACATACTCACGCCGCCTGGGAGAAGTATGGCCTGGCGGCGAAAACCCGCTGG
TTACAGGATGAGCCCCAGGGACGGCTGGCGAAACTGCGCTACCGGGCATGTAAAACGCTG
CCGGGGCTGCATCGGGCGGCGACCTGGAAAGCGCTCAATTTTACCCGCTATGGCGATGAA
TCACGCAATTTGATCCTTTCCGCGATTTGCGCGCAGGTGAGCCAGCCTTTTGGGGCGGAT
GTGTTCATCGCACACTTTGGTCCGGCGGGCGTGACGGCGGCCAAACTACGCGAACTGGGC
GTGCTTCGCGGCAAAATCGCGACTATTTTCCACGGGATTGATATTTCCAGCCGCGAAGTG
CTCAGTCATTACACGCCGGAGTATCAGCAGTTGTTTCGTCGTGGCGATCTGATGCTGCCC
ATCAGCGAACTGTGGGCCGGTCGCCTGAAAAGTATGGGCTGTCCGCCGGAAAAGATTGCC
GTTTCGCGCATGGGCGTCGACATGACGCGTTTTACCTATCGTCCGGTGAAAGCGCCAGGG
ATGCCGCTGGAGATGATTTCCGTCGCGCGCCTGACTGAGAAAAAAGGCCTGCATGTGGCG
ATTGAAGCCTGTCGGCAACTGAAAGCGCAGGGCGTGGCGTTTCGCTACCGCATTCTGGGC
ATTGGCCCGTGGGAACGTCGGCTGCGCACGCTCATCGAGCAGTATCAGCTAGAGGATGTC
ATTGAGATGCCGGGGTTTAAACCGAGCCATGAAGTGAAGGCGATGCTGGATGACGCCGAT
GTTTTTTTGCTGCCGTCGATTACCGGTACGGATGGCGATATGGAAGGTATTCCGGTAGCG
CTGATGGAGGCGATGGCGGTAGGGATTCCCGTGGTATCTACCGTGCATAGCGGTATTCCG
GAACTGGTGGAGGCCGGCAAATCCGGCTGGCTGGTGCCGGAAAACGATGCGCAGGCGCTG
GCGGCCCGACTCGCTGAGTTCAGCCGGATTGACCACGACACGCTGGAGTCGGTGATCACG
CGCGCCCGTGAAAAAGTGGCGCAAGATTTTAATCAGCAGGCGATTAATCGCCAGCTAGCC
AGCCTGCTACAAACGATATAA
>EECEBICD_01260 hypothetical protein
ATGCCCGTGAATAAGTTCTCCCGACGTACCCTCCTGACGGCAGGTTCCGCGCTTGCTGTT
CTTCCTTTTCTGCGCGCCTTGCCGGTACAGGCGCGTGAACCTCGCGAGACCGTCGATATT
AAGGATTATCCGGCGGATGACGGTATCGCCTCGTTCAAACAGGCCTTCGCCGACGGACAG
ACCGTGGTCTTACCGCCAGGATGGGTGTGTGAAAATATCAATGCGGCGATAACGATTCCG
GCGGGAAAAACGCTGCGGGTACAGGGCGCGGTGCGTGGGAATGGCCGGGGACGGTTTATT
TTGCAGGACGGGTGTCAGGTGGTGGGGGAGCAGGGCGGCAGTCTGCACAATGTGACGCTG
GATGTTCGCGGGTCGGACTGTGTGATTAAAGGCGTGACGATGAGCGGCTTTGGCCCCGTC
GCGCAAATTTTCATCGGCGGTAAGGAACCGCAGGTGATGCGTAATCTCATTATCGATGAC
ATCACCGTTACCCACGCCAACTACGCCATTCTCCGCCAGGGATTTCATAACCAAATGGAC
GGCGCGCGGATTACGCATAGCCGCTTTAGCGATTTGCAGGGGGACGCCATTGAGTGGAAT
GTCGCGATTCACGACCGCGACATCCTGATTTCCGATCATGTCATCGAACGCATTGATTGT
ACCAATGGCAAAATCAACTGGGGGATCGGCATCGGGCTGGCGGGTAGCACCTATGACAAC
AGTTATCCTGAAGATCAGGCAGTAAAAAACTTTGTGGTGGCCAATATTACCGGATCTGAT
TGCCGACAGCTGGTGCACGTAGAAAATGGCAAACATTTCGTCATTCGCAATGTCAAAGCC
AAAAACATCACGCCCGATTTCAGTAAAAATGCGGGTATTGATAACGCAACGATCGCCATT
TATGGCTGTGATAATTTCGTCATTGATAATATTGATATGACGAATAGTGCTGGGATGCTC
ATCGGCTATGGCGTCGTTAAAGGAAAATACCTGTCAATTCCGCAAAACTTTAAATTAAAC

GCTATTCGGTTGGATAATCGCCAGGTTGCTTATAAATTACGCGGCATTCAAATTTCCTCC
GGCAACATCCCCTCTTTTGTCGCCATCACCAATGTACGGATGACGCGTGCTACGCTGGAA
CTGCATAATCAACCGCAGCACCTCTTTCTGCGTAATATCAACGTGATGCAAACTTCAGCG
ATTGGCCCGGCGTTAAAAATGCATTTCGATTTGCGTAAAGATGTCCGTGGTCAATTTATG
GCCCGCCAGGACACGCTGCTTTCCCTCGCTAATGTTCATGCCATCAATGAAAACGGGCAG
AGTTCCGTGGATATCGACAGGATTAATCACCAAACCGTGAATGTCGAAGCAGTGAATTTT
TCGCTGCCGAAGCGGGGAGGGTAA
>EECEBICD_01261 UTP--glucose-1-phosphate uridylyltransferase
ATGATGAATTTGAAAGCAGTTATACCGGTAGCGGGTTTGGGTATGCATATGTTGCCTGCC
ACCAAGGCAATCCCAAAAGAGATGCTACCGATCGTCGACAAGCCAATGATTCAGTACATT
GTCGATGAGATTGTGGCTGCAGGGATCAAAGAAATCGTACTGGTGACTCACGCGTCTAAA
AACGCCGTTGAGAACCACTTCGACACCTCTTATGAACTTGAATCACTTCTTGAGCAGCGC
GTTAAGCGCCAGCTTTTGGCGGAAGTGCAATCTATCTGCCCACCGGGCGTGACGATTATG
AACGTTCGCCAGGCGCAGCCGTTAGGGCTGGGGCACTCTATTCTGTGCGCGCGTCCGGTC
GTGGGCGATAACCCTTTCATTGTGGTACTCCCGGATATTATTATCGATGATGCTACCGCC
GATCCGCTGCGCTATAACCTTGCGGCGATGGTGGCGCGTTTCAATGAAACGGGTCGCAGC
CAGGTGCTGGCGAAGCGCATGAAAGGTGATTTATCGGAGTATTCCGTTATCCAGACGAAA
GAACCTCTGGATAATGAAGGCAAAGTCAGCCGGATTGTGGAGTTTATCGAAAAACCGGAT
CAGCCGCAGACGCTGGATTCCGATTTGATGGCGGTAGGCCGTTATGTGCTTTCAGCCGAC
ATCTGGGCGGAACTGGAAAGAACCGAACCGGGCGCCTGGGGCCGTATCCAGCTCACCGAT
GCCATTGCAGAACTGGCGAAAAAACAGTCGGTTGACGCGATGCTAATGACGGGTGACAGC
TATGACTGCGGTAAAAAAATGGGCTACATGCAGGCATTTGTGAAGTACGGGCTGCGCAAC
CTGAAAGAAGGAGCGAAGTTCCGTAAGAGCATAGAGCAGCTTTTGCATGAATAA
>EECEBICD_01262 GDP-mannose 4,6-dehydratase
GTGAAGATACTTATTACTGGCGGGGCAGGTTTTATTGGATCAGCTGTTGTCCGCCATATT
ATTAAGAATACACAGGACACTGTAGTTAATATTGATAAATTAACCTACGCCGGTAATCTT
GAATCCCTTTCTGATATTTCTGAGAGTAATCGCTACAATTTTGAACACGCGGATATTTGT
GATTCCGCTGAAATAACGCGTATTTTTGAGCAGTACCAGCCGGACGCGGTGATGCATTTG
GCTGCGGAAAGTCATGTGGACCGTTCGATTACCGGGCCAGCAGCATTTATTGAAACCAAT
ATCGTCGGCACCTATGTACTTCTTGAAGTTGCGCGTAAATACTGGTCTGCGCTTGGCGAA
GATAAAAAAATAATTTTCGTTTTCATCATATTTCCACTGATGAAGTTTACGGCGATTTAC
CGCATCCTGATGAAGTTGAAAACAGCGTTACGCTGCCGTTATTTACTGAAACGACGGCAT
ATGCGCCAAGTAGCCCCTATTCTGCGTCAAAAGCATCCAGCGATCATTTAG
>EECEBICD_01263 dTDP-glucose 4,6-dehydratase
TTGCCAATTTATGGCAAAGGGGATCAGATTCGCGATTGGCTATATGTAGAAGATCATGCT
CGCGCGCTTCATATGGTAGTGACTGAAGGCAAGGCGGGGGAGACTTATAACATTGGTGGC
CACAATGAGAAGAAAAATCTCGATGTGGTATTTACCATCTGTGATCTGCTGGACGAGATT
GTACCCAAAGCGACTTCTTATCGTGAACAAATCACTTATGTCGCGGATCGTCCGGGCCAT
GATCGTCGTTATGCCATTGATGCAGGTAAAATTAGCCGCGAATTAGGCTGGAAACCGCTG
GAGACCTTTGAAAGCGGTATTCGTAAAACAGTGGAATGGTACCTTGCAAATACTCAATGG
GTAAACAATGTTAAAAGTGGGGCGTATCAGAGTTGGATAGAACAGAACTATGAAGGACGC
CAGTAA
>EECEBICD_01264 dTDP-4-dehydrorhamnose reductase
ATGAATATCTTACTTTTTGGTAAGACAGGGCAAGTAGGCTGGGAGTTGCAACGTTCTCTG
GCACCAGTAGGGAATCTGATTGCCCTGGATGTCCATTCAAAAGAGTTTTGCGGTGATTTT
AGTAATCCGAAAGGCGTTGCCGAAACCGTTCGTAAGCTTCGTCCCGATGTGATTGTTAAC
GCAGCAGCACATACTGCAGTAGATAAAGCAGAGTCTGAACCAGAACTGGCGCAGTTACTT
AACGCCACCAGTGTGGAAGCCATCGCTAAAGCAGCCAACGAAACTGGCGCATGGGTAGTG
CATTATTCAACCGATTATGTATTTCCTGGTACCGGCGATATCCCATGGCAGGAAACGGAC
GCTACGTCGCCGCTGAATGTCTATGGCAAGACCAAACTGGCGGGAGAAAAGGCCCTGCAG
GATAACTGCCCTAAGCATCTTATCTTCCGCACCAGTTGGGTTTATGCAGGTAAGGGCAAT
AATTTCGCAAAGACAATGCTTCGTCTGGCGAAAGAGCGTCAGACACTTTCAGTCATCAAC
GATCAGTACGGTGCGCCAACCGGTGCAGAATTACTGGCTGACTGCACGGCTCATGCGATC
CGTGTGGCGTTAAAGAAACCAGAAGTCGCAGGTCTTTACCATCTGGTTGCCGGGGGAACC

ACAACCTGGCATGACTACGCGGCCTTAGTCTTTGACGAGGCGCGCAAAGCAGGGATAACG
CTTGCGCTGACTGAGCTTAATGCTGTGCCGACCAGCGCCTACCCGACGCCGGCGAGCAGA
CCAGGCAATTCGCGTCTCAATACTGAAAAGTTTCAGCGTAATTTTGACCTTATTCTGCCG
CAATGGGAATTAGGAGTTAAGCGCATGCTGACTGAAATGTTTACGACGACAACCATCTGA
>EECEBICD_01265 Glucose-1-phosphate thymidylyltransferase
ATGAAAACGCGTAAGGGCATTATTTTAGCGGGGGGCTCCGGCACCCGTCTTTATCCGGTG
ACCATGGCGGTAAGTAAGCAATTGCTACCAATTTATGATAAACCGATGATTTACTATCCC
CTTTCCACGCTTATGCTGGCAGGCATTCGGGATATCCTGATCATCAGTACGCCACAGGAC
ACGCCGCGTTTTCAACAACTGCTGGGAGACGGCAGCCAGTGGGGGCTGAATCTTCAATAT
AAAGTACAGCCAAGCCCGGATGGCTTAGCACAGGCGTTTATTATTGGTGAAGAGTTCATT
GGTAATGATGATTGTGCATTAGTACTGGGTGACAATATCTTCTATGGTCATGATTTACCA
AAGTTAATGGAAGCTGCCGTTAATAAAGAAAGTGGTGCTACCGTCTTTGCTTATCATGTA
AACGATCCGGAGCGCTACGGTGTGGTTGAGTTTGACCAAAGTGGCACAGCCGTTAGTCTG
GAGGAAAAACCGTTACAACCGAAGAGTAATTACGCGGTAACGGGGCTGTATTTTTATGAT
AATAGCGTGGTGGAGATGGCGAAAAATCTTAAGCCTTCCGCTCGCGGTGAGTTAGAAATC
ACGGATATTAACCGTATCTATATGGATCAGGGAAGATTGTCTGTCGCCATGATGGGGCGC
GGTTATGCCTGGCTGGATACAGGGACGCATCAGAGTTTGATAGAGGCCAGTAATTTTATT
GCAACCATCGAAGAACGCCAGGGGCTAAAAGTGTCCTGCCCGGAAGAGATCGCATTTCGT
AAAAATTTTATAAATGCACAACAGGTTATAGAACTGGCCGGGCCATTATCAAAAAATGAT
TATGGCAAATATTTGCTGAAGATGGTGAAAGGTTTATAA
>EECEBICD_01266 dTDP-4-dehydrorhamnose 3,5-epimerase
ATGATTGTGATTAAAACAGCAATACCAGATGTCTTGATCTTAGAGCCTAAAGTTTTTGGC
GATGAGAGGGGATTCTTTTTTGAAAGTTATAACCAGCAGACCTTTGAAGAGTTGATTGGA
CGTAAAGTTACATTTGTTCAAGATAATCATTCAAAATCCAAAAAGAACGTACTCAGAGGG
CTACATTTTCAGAGAGGAGAAAATGCACAGGGGAAGTTAGTTCGTTGTGCTGTCGGTGAG
GTTTTTGATGTTGCGGTCGATATCCGAAAAGAATCGCCTACTTTTGGTCAATGGGTTGGC
GTAAATCTATCTGCTGAGAATAAGCGACAGCTTTGGATTCCAGAAGGTTTTGCTCATGGT
TTTGTTACTCTTAGTGAGTATGCAGAGTTTCTGTACAAAGCAACTAATTATTACTCACCT
TCATCGGAAGGTAGCATTTTATGGAATGATGAGACAATAGGTATTGAATGGCCTTTTTCT
CAGCTGCCTGAGCTTTCAGCAAAAGATGCTGCAGCACCTTTACTGCATCAAGCCTTGTTA
ACAGAGTAA
>EECEBICD_01267 CDP-6-deoxy-L-threo-D-glycero-4-hexulose-3-dehydrase reductase
GTGTCTCATATTATTAAGATTTTTCCATCAAATATTGAATTTTCCGGTAGAGAGGATGAA
TCAATCCTCGATGCTGCGCTATCGGCTGGCATCCATCTTGAACATAGCTGCAAAGCGGGT
GATTGTGGTATCTGTGAGTCCGATTTGTTGGCGGGAGAAGTTGTTGACTCCAAAGGTAAT
ATTTTTGGACAGGGTGATAAAATACTAACCTGCTGCTGTAAACCTAAAACCGCCCCTTGAG
CTAAATGCGCATTTTTTTCCTGAACTAGCTGGACAGACAAAAAAAATTGTCCCATGCAAG
GTAAATAGTGCTGTACTGGTTTCAGGCGATGTTATGACTTTGAAGTTACGCACACCACCA
ACAGCAAAAATTGGCTTCCTTCCAGGGCAGTATATCAATTTACATTATAAAGGTGTAACT
CGCAGTTATTCTATCGCTAATAGTGATGAGTCGAATGGTATTGAGTTGCATGTAAGGAAT
GTTCCCAATGGTCAGATGAGCTCTCTCATTTTTGGGGAGTTACAAGAAAATACTCTTATG
CGCATTGAAGGACCTTGCGGAACATTTTTTATTCGTGAAAGTGACAGACCTATAATCTTC
CTTGCAGGCGGTACTGGATTCGCTCCAGTTAAATCAATGGTTGAGCATCTCATTCAGGGA
AAATGTCGTCGTGAGATCTACATCTACTGGGGAATGCAAGATAGTAAAGATTTTTACTCT
GCATTACCGCAGCAGTGGAGTGAACAGCACGACAACGTTCATTATATCCCTGTTGTTTCT
GGTGATGACGCCGAATGGGGGGAAGAAAGGGATTTGTCCATCATGCTGTGA
>EECEBICD_01268 hypothetical protein
ATGGATGATTTTGATTCTCTAGAGTTCTTCGATATATATGCATGTGGTTCACCTGTGATG
ATCGATGCCAGTAAAAAGGACTTTATGATGAAAAATCTCTCTGTAGAACATTTCTATTCT
GATGCATTTACCGCATCTAAATAA
>EECEBICD_01269 Glucose-1-phosphate cytidylyltransferase
ATGAAAGCGGTCATCCTGGCTGGTGGACTTGGTACCAGACTAAGTGAAGAAACAATTGTA
AAACCAAAACCGATGGTAGAAATTGGTGGCAAGCCTATTCTTTGGCACATTATGAAAATG

TATTCTGTGCATGGTATCAAGGATTTTATTATCTGCTGTGGTTATAAAGGATATGTGATT
AAAGAATATTTTGCGAACTACTTCCTTCACATGTCAGATGTAACATTCCATATGGCTGAA
AATCGTATGGAAGTTCACCATAAACGTGTTGAACCATGGAATGTCACATTGGTTGATACG
GGTGATTCTTCAATGACTGGTGGTCGTCTGAAACGTGTTGCTGAATACGTAAAAGATGAC
GAGGCTTTCCTGTTTACTTATGGTGATGGCGTTGCCGACCTTGATATCAAAGCGACTATC
GATTTCCATAAGGCTCACGGTAAGAAAGCGACTTTAACAGCTACTTTTCCACCAGGACGT
TTTGGCGCATTAGATATCCAAGCTGGTCAGGTCCGGTCATTCCAGGAAAAACCGAAAGGC
GATGGGGCAATGATCAATGGTGGTTTCTTTGTGTTGAATCCATCGGTTATCGATCTCATC
GATAACGATGCAACAACCTGGGAACAAGAGCCATTAATGACATTGGCACAACAGGGGGAG
TTAATGGCTTTTGAACACCCAGGTTTCTGGCAGCCGATGGATACCCTACGTGATAAAGTT
TACCTTGAAGGGCTGTGGGAAAAAGGTAAAGCTCCGTGGAAAACCTGGGAGTAA
>EECEBICD_01270 CDP-glucose 4,6-dehydratase
ATGATTGATAAAAATTTTTGGCAAGGTAAACGTGTATTCGTTACCGGCCATACTGGCTTT
AAAGGAAGCTGGCTTTCGCTATGGCTGACTGAAATGGGTGCAATTGTAAAAGGCTATGCA
CTTGATGCGCCAACTGTTCCAAGTTTATTTGAGATAGTGCGTCTTAGTGATCTTATGGAA
TCTCATATTGGCGACATTCGTGATTTTGAAAAGCTGCGCAATTCTATTGCAGAATTTAAG
CCAGAAATTGTTTTCCATATGGCAGCCCAGCCTTTAGTGCGCCTATCTTATGAACAGCCA
ATCGAAACATACTCAACAAATGTTATGGGTACTGTCCATTTGCTTGAAGCAGTTAAGCAA
GTAGGTAACATAAAGGCAGTCGTAAATATCACCAGTGATAAGTGCTACGACAATCGTGAG
TGGGTGTGGGGCTATCGTGAGAACGAACCCATGGGAGGGTACGATCCATACTCTAATAGT
AAAGGTTGTGCAGAATTAGTCGCGTCTGCATTCCGGAACTCATTCTTCAATCCTGCAAAT
TATGAGCAACATGGCGTTGGTTTGGCGTCTGTGAGGGCTGGTAATGTCATAGGCGGAGGC
GATTGGGCTAAAGACCGTTTAATTCCCGATATTCTGCGCTCATTTGAAAATAACCAGCAG
GTTATTATTCGAAACCCATATTCTATCCGTCCCTGGCAGCATGTACTGGAGCCTCTTTCT
GGTTACATTGTGGTGGCGCAACGCTTATATACAGAAGGTGCTAAGTTTTCTGAAGGATGG
AATTTCGGCCCGCGTGATGAAGATGCGAAGACGGTCGAATTTATTGTTGACAAGATGGTC
ACGCTTTGGGGTGATGATGCAAGCTGGTTACTGGATGGTGAGAATCATCCTCATGAGGCA
CATTACCTGAAACTGGATTGCTCTAAAGCAAATATGCAATTAGGATGGCATCCGCGTTGG
GGATTGACTGAAACACTTGGTCGCATCGTAAAATGGCATAAAGCATGGATTCGCGGCGAA
GATATGTTGATTTGTTCAAAGCGTGAAATCAGCGACTATATGTCTGCAACTACTCGTTAA
>EECEBICD_01271 dTDP-4-dehydro-2,6-dideoxy-D-glucose 3-dehydratase
ATGACAGCAAATAACCTGCGTGAGCAAATCTCTCAGCTTGTCGCTCAGTATGCGAATGAG
GCATTGAGCCCGAAACCTTTTGTTGCAGGTACAAGCGTTGTGCCTCCTTCCGGGAAGGTT
ATTGGTGCCAAAGAGTTACAATTGATGGTTGAGGCGTCTCTTGATGGATGGCTAACTACT
GGTCGTTTCAATGATGCCTTTGAGAAAAAACTTGGGGAATTTATTGGGGTTCCTCATGTT
TTAACGACTACATCTGGCTCTTCGGCAAACTTGCTGGCACTGACTGCGCTGACTTCCCCA
AAATTAGGCGAGCGTGCTCTCAAACCTGGTGATGAGGTTATTACTGTCGCTGCTGGCTTC
CCGACTACAGTTAACCCGGCGATCCAGAATGGTTTAATACCGGTATTCGTGGATGTTGAT
ATCCCGACATATAATATCGATGCCTCTCTCATTGAAGCTGCAGTTACTGAGAAATCAAAA
GCGATAATGATCGCTCATACACTCGGTAATGCATTTAACCTGAGTGAAGTTCGTCGGATT
GCCGATAAATATAACTTATGGTTGATTGAAGACTGCTGTGATGCCCTTGGGACGACTTAT
GAAGGCCAGATGGTAGGTACCTTTGGTGACATCGGAACCGTTAGTTTTTATCCGGCTCAC
CATATCACAATGGGTGAAGGCGGTGCTGTATTCACCAAGTCAGGTGAACTGAAGAAAATT
ATTGAGTCGTTCCGTGACTGGGGCCGGGATTGTTATTGTGCGCCAGGATGCGATAACACC
TGCGGTAAACGTTTTGGTCAGCAATTGGGATCACTTCCTCAAGGCTATGATCACAAATAT
ACTTATTCCCACCTCGGATATAATCTCAAAATCACGGACATGCAGGCAGCATGTGGTCTG
GCTCAGTTGGAGCGCGTAGAAGAGTTTGTAGAGCAGCGTAAAGCTAACTTTTCCTATCTG
AAACAGGGCTTGCAATCTTGCACTGAATTCCTCGAATTACCAGAAGCAACAGAGAAATCA
GACCCATCCTGGTTTGGCTTCCCTATCACCCTGAAAGAAACTAGCGGTGTTAACCGTGTC
GAACTGGTGAAATTCCTTGATGAAGCAAAAATCGGTACACGTTTACTGTTTGCTGGAAAT
CTGATTCGCCAACCGTATTTTGCTAATGTGAAATATCGTGTAGTGGGTGAGTTGACAAAT
ACCGACCGTATAATGAATCAAACGTTCTGGATTGGTATTTATCCTGGCTTGACTACAGAG
CATTTAGATTATGTAGTTAGCAAATTTGAAGAGTTTTTTGGTTTAAATTTCTAA
>EECEBICD_01272 CDP-abequose synthase

ATGGGAGCGTTTGGGTTCCTTGGATCACGACTTACATCCTACTTCGAAAGTCGACATACT
GTGATTGGCTTAGCAAGGAAGAGGAACAATGAAGCTACCATAAATAATATTATTTACACG
ACAGAAAATAATTGGATCGAAAAAATAGTAGAATTTGAACCGAATATTATTATTAACACT
ATTGCTTGCTATGGAAGACATAACGAACCTGCAACAGCTTTAATAGAAAGCAATATTCTT
ATGCCTATCAGAGTATTAGAATCTATCTCATCACTTGATGCAGTATTCATAAATTGTGGA
ACATCACTGCCACCAAATACGAGTTTATATGCATATACTAAACAAAAAGCAAATGAACTC
GCCGCCGCCATTATAGATAAAGTTTGTGGTAAATATATAGAGTTAAAATTGGAGCATTTC
TATGGAGCTTTTGATGGAGACGATAAGTTTACCAGTATGGTTATTAGACGTTGTTTAAGT
AACCAGCCAGTAAAGTTAACATCTGGTTTGCAACAGAGAGATTTCTTGTATATAAAAGAT
CTACTAACAGCGTTCGATTGTATTATAAATAATGTTAATAATTTCCCCAAATTTCATAGT
ATTGAAGTTGGTAGTGGAAAGGCTATATCAATTCGTGAATATGTAGAGACTGTTAAAAAT
ATCACAAAAAGCAATTCTATAATTGAATTTGGTGTGGTCAAAGAAAGAGCCAATGAATTG
ATGTATAGTTGTGCTGATATAGCAGAACTTGAAAAAATAGGATGGAAAAGAGAGTTCTCT
CTTGTTGATGCATTAACTGAAATAATTGAAGAGGAAGGGAAATGA
>EECEBICD_01273 CDP-paratose 2-epimerase
ATGAAGCTTTTAATTACCGGTGGATGTGGCTTTCTTGGGAGTAATCTTGCCTCCTTTGCT
TTAAGTCAAGGGATTGATTTAATTGTATTCGATAATCTATCACGTAAAGGTGCAACAGAT
AATTTACATTGGTTATCCTCCTTAGGAAACTTTGAGTTTGTACATGGTGATATTCGCAAC
AAAAATGATGTTACAAGATTAATAACTAAGTATATGCCTGATAGCTGTTTTCATCTTGCA
GGTCAAGTGGCAATGACTACATCTATTGACAATCCTTGTATGGATTTTGAAATTAATGTA
GGTGGAACTTTAAATTTACTTGAGGCAGTACGGCAGTATAATTCAAATTGTAATATAATT
TATTCATCAACAAATAAAGTATACGGCGATCTTGAGCAATATAAATACAATGAAACAGAA
ACTAGATATACTTGTATAGATAAGCCTAATGGATATGATGAGAGCACACAATTAGATTTC
CACTCACCATATGGTTGTTCAAAAGGTGCTGCAGATCAATACATGCTTGATTATGCAAGG
ATTTTTGGTTTGAATACAGTGGTGTTCAGGCATTCATCAATGTATGGTGGGAGACAGTTT
GCTACTTATGATCAAGGCTGGGTAGGTTGGTTTTGTCAAAAAGCGGTTGAAATTAAAAAT
GGTATTAACAAACCCTTCACTATTTCTGGTAATGGTAAGCAAGTTAGGGATGTTTTGCAT
GCTGAAGATATGATTTCGTTATATTTCACTGCCTTGGCAAATGTATCAAAAATTAGGGGG
AACGCTTTTAATATTGGTGGTACCATTGTCAACAGCCTATCATTACTTGAATTATTCAAA
TTGCTTGAAGATTATTGCAACATTGATATGAGGTTCACTAATTTACCAGTAAGGGAAAGG
GATCAGCGTGTTTTTGTTGCAGATATTAAAAAAATCACTAATGCAATTGACTGGAGCCCG
AAAGTCTCGGCAAAAGATGGTGTCCAGAAAATGTATGATTGGACTAGTTCTATATGA
>EECEBICD_01274 hypothetical protein
TTGGTTAGAATTCCAAGACATCTTATTATTGCCGCTTCCTCTTGGCTTTCAAAGATAATA
ATTGCCGGTGTTCAGTTAGTAAGTGTTAAATTTCTTTTGGAAATTCTTGGCGAAGAATCA
TACGCTGTATTTACTCTTTTAACTGGATTATTGGTCTGGTTTAGCATTGCAGATATTGGG
ATTGGTAGTAGTCTACAAAATTATATATCTGAGTTGAAAGCTGATAGAAAATCATATGAT
GCATATATCAAGGCCGCAATTCATATTCTATTCGCATCCTTAATCATTTTAAGCTCTACA
TTATTCTTCTTATCAGATAAATTATCGTCACTATATCTTACTTCATTTAGCGATGAATTG
AAAAACAACTCAGGAAGTTATTTTTTTATAGCAAGTATATTATTTATATTCATTGGCGTT
GGGAGTGTGGTCTATAAAATATTATTTGCGGAACTGTTAGGGTGGAAAGCTAATATAATT
AATGCATTATCTTATCTTTTAGGTTTTTTAGATGTAGTTGCGATCCATTATTTAATGCCA
GATTCGAGTATTACCTTCGCTTTAGTAGCATTGTATGCTCCGGTAGCAATACTGCCCATT
ATATATATATCGTTTCGGTACATATATGTTCTTAAAACGAAAGTAAACTTTAACACCTAT
AAATTATTACTATCACGTTCATCAGGGTTTCTGATTTTTTCGTCCTTATCGATAATAGTT
TTACAAACTGATTATATTGTGATGTCTCAGAAATTATCTGCTGCAGATATTATAAAATAT
ACTGTAACGATGAAAATATTTGGTTTAATGTTTTTTATTTATACTGCGGTATTACAAGCA
TTATGGCCAGTATGTGCTGAATTACGAGTGAAAATGCAGTGGAGAAAGTTGCATAGAATC
ATTTTCCTAAATATTATTGGTGGGGTATTTTTTGTTGGTCTTGGTACGTTATTCATTTAT
GTTTTAAAGGATTATATCTATAGCATAATTGCTAACGGTATAGATTATAATATTAGTGGG
GCTGTTTTTGTTTTACTGGCTGTGTATTTTAGTATAAGAGTTTGGTGTGATACATTTGCT
ATGTTACTTCAAAGTATGAACCAATTAAAAATTCTTTGGCTCATAGTTCCGTGTCAGGCA
TTAATTGGTGGTGTGACTCAATGGTATTTTGCAGAGCATTATGGAATAGTTGGTATTTTA
TACGGACTAATTTTATCGTTCTCGCTAACTGTTTTTTGGGGATTGCCAGTGTATTATATG

TATAAGAGTAAAAGGCTAGCATAA
>EECEBICD_01275 Abequosyltransferase RfbV
ATGAAGGTATCATTTTGTATCCCAACGTATAATCGAGTAAAATTCATTGAAGACCTTCTT
GAAAGTATTAATAATCAATCTTCTCACTCCTTAATTGTAGAAGTATGTATTTCAGATAAT
GCATCAACTGATGGAACGGAAGAGTCAATAAATATCTGGAGAGATAGATTTAATTTTCCA
ATTTTGTATCAAAGGCATAACGAAAATATTGGGCCAGATAGGAATTATTTATCTGCAGTT
AATATGGGGACTGGAGATTATTGTTGGATTTTCGGAAGTGATGATATTCTTACAAAAAAT
TCCCTTGCATTGATGGAAGATAAATTGGCTGCCGGAAGTGACATTTATTTATGTGACAGA
CGAGAATTAGATATTTCAATGACGAAAATATCTAATCCACATCGGCGATGGTTAAATGGT
GGCAGTAGATTATTTTCTTTTAGCAATGAAGCTGATTTGATAGAGTACTTTAGTAAATGT
AACTCAGTCGGCGGACTTTTTAGCTACTTAAGCTCTATTATAGTGAAAAGGAATAAATGG
TCTGACGTCATATTTGACGAGTCATACATTGGTACAGCTTATGCGCATGTATATATTTTA
TTAAGAATAATTAACAATATGAATTCGACCCTGCAGTACATATCACTGCCACTGGTTGAT
TGCAGAGGAGATAACGATACATTCGAAAGTAATGGGAAGGCTCGTCGAATAAAAATTGAT
TTCATCGGTTATTTAAAACTGAGGGAGGATTTTTATAATAACAATACAAAAATATATATC
TCATTTGGACGGGTTCTTACAAAAGAGCGTCCTTGGTTTTATACTTCTTTAGCTATGGCA
TGTTATGGTGATTCCACCGATAGGGCTGAACTTGCATCATTTTATAAGAAGTTGGGATAT
CCGAAAATAGCCACCAATTTAATATTTCGTCTTAAAGGGCTGGCTAGCTACACTAAAAAA
ATAAAATTAGCAAAAATGGTAATAAAAAAAATATTTTCATAG
>EECEBICD_01276 D-inositol-3-phosphate glycosyltransferase
ATGATCGTTAACCTATCACGTTTAGGTAAAAGTGGTACGGGAATGTGGCAATACTCGATT
AAATTTTTAACGGCACTGCGAGAAATAGCTGATGTTGACGCAATAATCTGTAGCAAGGTA
CACGCTGATTATTTTGAAAAGCTCGGTTATGCAGTAGTTACTGTTCCGAATATTGTTAGC
AACACATCAAAAACATCGCGACTTAGACCATTAGTATGGTATGTATATAGTTACTGGCTT
GCGCTGAGGGTTTTAATTAAGTTTGGTAATAAAAAATTGGTGTGTACTACACATCACACT
ATCCCCTTACTGAGAAACCAAACGATAACCGTACATGATATAAGACCTTTTTATTATCCA
GATAGTTTTATTCAGAAAGTGTATTTTCGCTTTTTATTAAAAATGTCCGTTAAGCGATGT
AAGCATGTTTTAACGGTATCTTATACCGTTAAAGATAGCATTGCTAAAACTTATAATGTA
GATAGTGAGAAAATATCAGTAATTTATAATAGTGTTAATAAATCTGATTTTATACAAAAA
AAAGAAAAAGAGAATTACTTTTTAGCTGTTGGTGCAAGTTGGCCACATAAAAATATTCAT
TCATTCATAAAAAATAAAAAAGTTTGGTCTGACTCTTATAATTTAATTATTGTATGTGGT
CGTACTGACTATGCAATGTCTCTCCAACAAATGGTCGTTGATCTGGAACTAAAAGATAAA
GTGACTTTTTTACATGAAGTCTCATTTAATGAATTAAAGATTTTATATTCTAAAGCCTAC
GCGCTTGTTTATCCATCTATTGATGAGGGTTTTGGTATACCTCCTATTGAAGCGATGGCA
TCAAATACTCCAGTTATAGTGTCCGATATACCAGTATTTCATGAAGTGTTAACCAATGGT
GCATTATATGTGAATCCGGATGATGAAAAAAGCTGGCAGAGTGCAATTAAAAATATAGAG
CAGTTGCCTGATGCAATTTCCCGATTTAACAACTATGTCGCACGGTATGACTTTGATAAT
ATGAAGCAGATGGTTGGCAATTGGTTGGCGGAATCAAAATAA
>EECEBICD_01277 hypothetical protein
ATGAAAATAACATTAATTATTCCCACATATAATGCAGGGTCGCTTTGGCCTAATGTTCTG
GATGCGATTAAGCAGCAAACTATATATCCGGATAAATTGATTGTTATAGACTCAGGTTCT
AAAGATGAAACGGTTCCGTTAGCCTCAGACCTGAAAAATATATCAATATTTAATATTGAC
TCTAAAGATTTTAATCATGGAGGAACCAGAAATTTAGCAGTTGCAAAAACTCTGGACGCT
GATGTTATAATTTTTCTAACGCAAGATGCAATTCTCGCGGATTCGGATGCAATTAAAAAT
TTGGTTTATTATTTTTCAGATCCATTGATAGCAGCGGTTTGTGGTAGACAACTTCCTCAT
AAAGATGCTAATCCCCTTGCAGTGCATGCCAGAAATTTTAATTATAGTTCAAAATCTATT
GTTAAAAGTAAGGCAGATATAGAAAAATTGGGTATTAAAACTGTATTTATGTCCAATTCT
TTTGCTGCCTATCGCCGTTCCGTTTTTGAAGAGTTAAGTGGGTTTCCTGAACATACAATT
CTTGCCGAGGATATGTTTATGGCGGCTAAGATGATTCAGGCGGGTTATAAGGTCGCCTAC
TGCGCTGAAGCGGTGGTAAGACACTCCCATAATTATACCCCGCGAGAAGAGTTTCAACGA
TATTTTGATACTGGTGTATTTCATGCTTGTTCTCCGTGGATTCAGCGTGACTTTGGCGGA
GCCGGTGGTGAGGGTTTCCGCTTCGTAAAATCAGAGATTCAATTCCTGCTTAAAAATGCA
CCGTTCTGGATTCCAAGAGCTTTATTAACAACCTTTGCTAAATTCTTGGGTTACAAATTA
GGCAAGCATTGGCAATCTTTACCGTTGTCTACATGTCGCTATTTTAGCATGTACAAGAGT

TATTGGAATAATATCCAATATTCTTCGTCAAAAGAGATAAAATAA
>EECEBICD_01278 Mannose-1-phosphate guanylyltransferase RfbM
ATGTCTTTTCTTCCCGTAATTATGGCTGGCGGCACAGGTAGCCGTTTATGGCCGCTTTCA
CGCGAATATCATCCGAAGCAGTTTCTAAGCGTTGAAGGTAAACTATCAATGCTGCAAAAT
ACTATAAAGCGATTAGCTTCACTTTCTACAGAAGAACCCGTTGTCATTTGCAATGACAGA
CACCGTTTCTTAGTCGCTGAACAACTCCGTGAAATTGACAAGTTAGCAAATAATATTATT
CTCGAACCGGTAGGCCGTAATACTGCACCAGCGATCGCTCTTGCCGCGTTTTGTGCGCTC
CAGAATGCTGATAATGCTGATCCTCTTTTGTTGGTTCTTGCTGCAGATCATGTGATTCAG
GATGAAATAGCTTTTACGAAAGCTGTCAGACATGCTGAAGAATACGCTGCAAATGGTAAG
CTTGTAACTTTTGGTATTGTTCCAACGCATGCTGAAACGGGTTATGGATATATTCGTCGT
GGTGAGTTGATAGGAAATGACGCTTATGCAGTGGCTGAATTTGTGGAGAAACCGGATATC
GATACCGCCGGTGACTATTTCAAATCAGGGAAATATTACTGGAATAGCGGTATGTTTTTA
TTTCGTGCAAGCTCTTATTTAAACGAATTAAAGTATTTATCACCTGAAATTTATAAAGCT
TGTGAAAAGGCGGTAGGACATATAAATCCCGATCTTGATTTTATTCGTATTGATAAAGAA
GAGTTTATGTCATGCCCGAGTGATTCTATCGATTATGCAGTTATGGAGCACACACAGCAT
GCGGTGGTGATACCAATGAGCGCTGGCTGGTCGGATGTGGGTTCCTGGTCCTCACTTTGG
GATATATCGAATAAAGATCATCAGAGAAATGTTTTAAAAGGAGATATCTTCGCACATGCT
TGTAATGATAATTACATTTATTCCGAAGATATGTTTATAAGTGCGATTGGTGTAAGCAAT
CTTGTCATTGTTCAAACAACAGACGCTTTACTGGTGGCTAATAAAGATACAGTACAAGAT
GTTAAAAAAATTGTCGATTATTTAAAACGGAATGATAGGAACGAATATAAACATCATCAA
GAAGTTTTCCGCCCCTGGGGAAAATATAATGTGATTGATAGCGGCAAAAATTACCTCGTT
CGATGTATCACTGTTAAGCCGGGTGAGAAATTTGTGGCGCAGATGCATCACCACCGGGCT
GAGCATTGGATAGTATTATCCGGGACTGCTCGTGTTACAAAGGGAGAGCAGATTTATATG
GTTTCTGAAAATGAATCAACATTTATTCCTCCGAATACTATTCACGCGCTGGAAAATCCT
GGAATGACCCCCCTGAAGTTAATTGAGATTCAATCAGGTACCTATCTTGGTGAGGATGAT
ATTATTCGTTTAGAACAACGTTCTGGATTTTCGAAGGAGTGGACTAATGAACGTAGTTAA
>EECEBICD_01279 Phosphoglucosamine mutase
ATGAACGTAGTTAATAATAGCCGTGATGTTATTTATTCATCAGGTATTGTGTTTGGAACG
AGTGGGGCTCGCGGTCTTGTAAAAGATTTTACACCTCAGGTATGTGCTGCTTTTACGGTT
TCATTTGTTGCCGTTATGCAGGAACATTTTTCCTTTGATACCGTAGCATTGGCAATAGAT
AATCGTCCAAGTAGTTATGGGATGGCTCAGGCGTGTGCTGCTGCATTGGCGGATAAAGGC
GTTAACTGTATTTTTTATGGAGTGGTACCAACCCCAGCTTTGGCCTTTCAGTCTATGTCT
GACAATATGCCTGCGATAATGGTTACGGGAAGTCATATTCCATTCGAGCGGAACGGCCTC
AAGTTTTATCGTCCTGATGGTGAAATCACGAAACATGATGAGGCTGCGATCCTTAGTGTT
GAAGATACGTGCAGCCATTTAGAGCTTAAAGAACTCATAGTTTCAGAAATGGCTGCTGTT
AATTATATATCTCGTTATACATCTTTATTTTCTACCCCCTTCTAA
>EECEBICD_01280 hypothetical protein
ATGGATAATATTGATAATAAGTATAATCCACAGCTATGTAAAATTTTTTTGGCTATATCG
GATTTGATTTTTTTTAATTTAGCCTTATGGTTTTCATTAGGATGTGTCTATTTTATTTTT
GATCAAGTACAGCGATTTATTCCTCAAGACCAATTAGATACAAGAGTTATTACGCATTTT
ATTTTGTCAGTAGTATGTGTCGGTTGGTTTTGGATTCGTTTGCGACATTATACATACCGC
AAGCCATTTTGGTATGAGTTAAAAGAAATTTTTCGTACGATCGTTATTTTTGCTATATTT
GATTTGGCTCTGATAGCGTTTACAAAATGGCAGTTTTCACGCTATGTCTGGGTGTTTTGT
TGGACTTTTGCTCTAATCCTGGTGCCTTTTTTTCGCGCACTTACAAAGCATTTATTGAAC
AAGCTAGGTATCTGGAAGAAAAAAACTATCATCCTGGGGAGCGGACAGAATGCTCGTGGT
GCATATTCTGCGCTGCAAAGTGAGGAGATGATGGGGTTTGATGTTATCGCTTTTTTTGAT
ACGGATGCGTCAGATGCTGAAATAAATATGTTGCCGGTGATAAAGGATACTGAGATTATT
TGGGATTTAAATCGTACAGGTGATGTCCATTATATCCTTGCTTATGAATACACCGAGTTG
GAGAAAACACATTTTTGGCTACGTGAACTTTCAAAACATCATTGTCGTTCTGTTACTGTA
GTCCCCTCGTTTAGAGGATTGCCATTATATAATACTGATATGTCTTTTATCTTTAGCCAT
GAAGTTATGTTATTAAGGATACAAAATAACTTGGCTAAAAGGTCGTCCCGTTTTCTCAAA
CGGACATTTGATATTGTTTGTTCAATAATGATTCTTATAATTGCATCACCACTTATGATT
TATCTGTGGTATAAAGTTACTCGAGATGGTGGTCCGGCTATTTATGGTCACCAGCGAGTA
GGTCGGCATGGAAAACTTTTTCCATGCTACAAATTTCGTTCTATGGTTATGAATTCTCAA

GAGGTACTAAAAGAACTTTTGGCTAACGATCCTATTGCCAGGGCTGAATGGGAGAAAGAT
TTTAAACTGAAAAATGATCCTCGAATCACAGCTGTAGGTCGATTTATACGTAAAACTAGC
CTTGATGAGTTGCCACAACTTTTTAATGTACTAAAAGGTGATATGAGCCTGGTTGGACCA
CGACCTATCGTTTCGGATGAACTGGAGCGTTATTGTGATGATGTTGATTATTATTTGATG
GCAAAGCCGGGCATGACAGGTCTATGGCAAGTGAGTGGGCGTAATGATGTTGATTATGAC
ACTCGTGTTTATTTTGATTCCTGGTATGTTAAAAACTGGACGCTTTGGAATGATATTGCC
ATTCTGTTTAAAACAGCGAAAGTTGTTTTGCGGCGAGATGGTGCGTATTAA
>EECEBICD_01281 hypothetical protein
ATGTCCAAGCAACAGATCGGCGTCGTCGGTATGGCAGTGATGGGGCGCAACCTCGCGCTC
AACATCGAAAGCCGTGGTTATACCGTCTCCGTTTTCAACCGCTCCCGTGAAAAAGACCGA
AGAAGTGATTGCCGAGAATCCCGGCAAAAAGCTGGTGCCTTATTACACGGTGAAAGAGTT
TGTTGA
>EECEBICD_01282 6-phosphogluconate dehydrogenase, decarboxylating
GTGATTGCCGAGAATCCCGGCAAAAAGCTGGTGCCTTATTACACGGTGAAAGAGTTTGTT
GAATCCCTCGAAACGCCTCGTCGTATCCTGTTAATGGTGAAAGCGGGTGCAGGTACTGAT
GCAGCTATCGATTCGCTGAAACCGTATCTGGAAAAAGGCGATATCATTATTGATGGCGGT
AACACCTTCTTCCAGGACACAATCCGTCGCAATCGCGAGCTGTCTGCGGAAGGTTTTAAC
TTTATCGGTACCGGTGTTTCCGGTGGTGAAGAGGGCGCGCTGAAAGGGCCATCTATCATG
CCTGGCGGTCAGAAAGATGCCTATGAACTGGTGGCGCCGATCCTGACGAAGATTGCTGCT
GTGGCGGAAGATGGCGAACCGTGCGTGACCTATATCGGCGCCGATGGTGCTGGTCACTAC
GTCAAGATGGTCCACAATGGTATTGAATATGGCGATATGCAGCTTATCGCTGAAGCTTAC
TCCCTGCTGAAAGGCGGCCTGAATCTCAGCAATGAAGAGTTGGCGAACACTTTTACTGAG
TGGAATAACGGCGAGCTGAGCAGCTATCTGATCGACATCACTAAAGACATCTTCACTAAA
AAAGATGAAGACGGAAACTACCTGGTGGATGTCATTCTTGATGAAGCCGCCAACAAAGGG
ACCGGTAAATGGACCAGCCAGAGCGCGCTGGATCTGGGTGAACCGCTGTCGCTGATTACC
GAATCCGTCTTTGCGCGTTACATCTCTTCGTTGAAAGCGCAACGTGTTGCCGCATCTAAA
GTGCTCTCCGGGCCGAAAGCGCAGCCGGCAGGCGACAAAGCTGAATTTATCGAGAAAGTG
CGTCGCGCGCTGTATCTTGGCAAGATCGTCTCCTATGCGCAGGGCTTCTCGCAGCTACGC
GCCGCGTCTGATGAATATCACTGGGATCTCAACTACGGCGAGATTGCTAAGATTTTCCGT
GCGGGCTGTATCATTCGCGCTCAGTTTCTGCAGAAGATCACTGATGCTTACGCAGAAAAC
GCTGGTATCGCTAACTTGCTGTTAGCGCCTTACTTCAAGAAAATTGCTGATGAGTACCAG
CAGGCGCTGCGTGATGTTGTGGCTTACGCGGTGCAGAACGGCATTCCGGTGCCGACATTC
TCTGCGGCAGTGGCCTACTATGACAGCTATCGCGCCGCAGTACTGCCAGCTAACTTAATT
CAGGCGCAGCGTGACTACTTTGGTGCGCACACCTATAAACGTACTGATAAAGAAGGCATT
TTCCATACCGAGTGGTTGGAATAA
>EECEBICD_01283 UDP-glucose 6-dehydrogenase
ATGAAAATCACGATATCCGGGACGGGTTATGTTGGCTTGTCTAATGGTTTGCTTATTGCA
CAACATCATGATGTGGTGGCGTTAGACATTGTTCCTTCCCGTGTTGAACTGTTAAATGAT
CGGATATCTCCGATTGTTGATAAAGAAATTCAGCAATTTTTAAAGGAAGATAACATCAGA
TTCAGGGCAACGCTGGATAAGTTTGATGCGTACCAGAATGCAGATTACGTCATTATTGCT
ACGCCTACGGATTACGATCCCAAAACCAACTATTTTAATACGTCCAGTGTGGAATCGGTC
ATTCAGGATGTGATCAGCATCAACCCCGCAGCCGTGATGATTATTAAGTCTACGGTGCCG
GTTGGCTTCACGGCGGCAATGCGTCAGAAGTTTGCGACAGAAAATATCATTTTCTCTCCG
GAGTTTTTACGTGAAGGTAAAGCCCTTTACGATAACCTTTATCCCTCCCGTATCGTGATT
GGGGAACAGTCTGAGCGCGCCAGAGAATTTGCCGCATTACTCCAGGAAGGCGCTATTAAA
CAGGAGATTCCAACGCTGTTTACGGACTCCACTGAGGCGGAAGCTATTAAACTTTTCGCC
AATACTTATCTCGCTATGCGAGTCGCTTATTTTAATGAATTAGACAGCTATGCTGAAACG
TTAGGATTAAATACCCGACAAATTATTGAAGGGGTGTGCCTCGACCCGCGTATTGGCAAT
CATTATAATAATCCCTCTTTTGGTTATGGCGGATATTGTTTGCCAAAAGATACCAAACAG
TTGCTGGCCAATTATCAGTCGGTGCCTAATAATATTATCTCCGCTATCGTTGAAGCAAAC
CGTACGCGTAAAGATTTTATTGCCGACGCTATCCTCGCCAGAAAGCCTAAAGTGGTGGGC
ATTTATCGCCTGATTATGAAGAGTGGCTCCGATAACTTCCGCGCCTCATCGATTCAGGGG
ATTATGAAGCGCATTAAAGCGAAAGGCGTAGAGGTCATCATCTATGAGCCGGTAATGGAA
GAAGATACTTTCTTTAACTCACGTCTTGAACGTGATTTGCACTGCTTTAAACAGCAGGCG

GATGTCATTATTTCCAACAGGATGGCGGCAGAGCTTTTGGATGTTGCTGAAAAAGTGTAT
ACCCGCGATCTTTTCGGTAGTGATTAA
>EECEBICD_01284 Chain length determinant protein
ATGACAGTGGATAGTAATACGTCTTCCGGGCGTGGGAACGATCCGGAACAGATTGATTTG
ATTGAGTTATTGCTACAGTTATGGCGTGGGAAGATGACCATTATTGTAGCCGTTATTATC
GCCATTTTGCTGGCCGTAGGCTACCTGATGATAGCCAAAGAAAAATGGACATCCACGGCG
ATTATTACCCAACCTGATGCCGCGCAGGTTGCCACCTATACCAATGCGCTCAACGTCTTG
TATGGCGGGAATGCCCCCAAAATCTCTGAAGTGCAGGCGAATTTTATAAGCCGCTTTAGC
TCTGCGTTTTCCGCATTATCGGAAGCGCTGGATAATCAGAAAGAGCGGGAAAAGCTCACC
ATTGAACAGTCGGTAAAGGGGCAGGCGCTGCCACTCTCGGTTTCTTATGTGAGTACTACC
GCTGAAGGGGCGCAGCGTCGGCTGGCGGAATATATCCAACAGGTGGATGAAGAGGTCGCT
AAGGAACTGGAAGTTGACCTGAAAGATAACATCACGCTGCAAACCAAAACGTTGCAGGAG
TCCCTTGAGACGCAGGAAGTTGTGGCGCAGGAGCAAAAAGATCTGCGTATTAAGCAAATC
GAAGAAGCGTTGCGCTATGCGGATGAGGCCAAAATCACGCAGCCGCAGATTCAGCAAACC
CAGGATGTTACCCAGGACACGATGTTCCTGCTGGGGAGCGATGCGCTAAAATCGATGATA
CAGAACGAAGCGACGCGTCCACTGGTCTTTTCTCCGGCCTATTACCAGACGAAGCAGACA
CTGCTGGACATTAAAAATCTGAAAGTGACTGCCGATACGGTGCACGTCTATCGTTATGTG
ATGAAGCCGACGCTGCCGGTCCGTCGCGATAGCCCGAAAAAAGCCATTACCCTTGTGCTG
GCTGTATTGCTGGGCGGGATGATCGGTGCCGGGATTGTGCTGGGGCGCAATGCGCTACGT
AGCTATAAGCCAAAAGCCTTGTAA
>EECEBICD_01285 Histidine biosynthesis bifunctional protein HisIE
ATGTTAACAGAGCAACAACGCCGCGAGCTGGACTGGGAAAAAACCGATGGCCTGATGCCA
GCCATCGTGCAACATGCGGTATCCGGCGAAGTATTGATGCTGGGCTATATGAACCCACAA
GCGCTGGACAAAACCATTGAATCCGGCCATGTCACGTTCTTCTCACGCACGAAACAGCGT
TTATGGACCAAAGGCGAAACCTCAGGCCATGTGCTGAATGTCGTCAGCATTGCGCCGGAT
TGCGACAACGATACACTGCTGGTGCTGGCAAATCCTGTCGGCCGACCTGCCACAAAGGT
ACCAGCTGCTGCTTTGGCTACGCCGGCCATCAGTGGTTATTCCTGTATCAACTGGAGCAG
CTACTGGCAGAGCGCAAAACCGCCGATCCGGCCAGCTCTTACACGGCGAAACTGTACGCC
AGCGGTACCAAACGTATTGCGCAAAAAGTTGGCGAAGAAGGTGTAGAAACCGCGCTGGCG
GCCACCGTCAATGATCGTTTTGAATTAACCAATGAAGCGTCTGATTTGATGTACCACCTG
CTGGTGCTGTTGCAGGATCAGGATCTCAACCTCACCACCGTGATCGACAATCTTCGTAAG
CGTCATCAATAA
>EECEBICD_01286 Imidazole glycerol phosphate synthase subunit HisF
ATGCTGGCAAAACGTATAATTCCGTGTCTGGATGTTCGTGATGGTCAGGTGGTGAAAGGC
GTACAGTTTCGCAACCATGAGATCATTGGCGATATCGTTCCGCTGGCCAAACGCTATGCC
GACGAAGGCGCGGACGAACTGGTGTTCTATGACATTACCGCCTCCAGCGATGGTCGCGTA
GTCGATAAAAGCTGGGTGGCGCGCGTTGCCGAGGTGATCGACATTCCGTTTTGTGTAGCG
GGCGGCATCCGGTCAATTGACGACGCCGCCAAAATTCTTTCTTTCGGGGCGGATAAGATC
TCTATCAATTCCCCTGCACTGGCTGACCCAACGCTGATTACCCGTCTGGCTGACCGTTTT
GGCGTACAGTGCATTGTCGTCGGGATTGATACCTGGTTTGACGACGCCACGGGGAAATAT
CATGTTAACCAGTATACCGGCGATGAAAACCGTACCCGCGTGACGCAGTGGGAGACGCTG
GATTGGGTGCAAGAGGTACAACAGCGCGGCGCGGGGGAAATCGTCCTGAATATGATGAAC
CAGGACGGCGTGCGTAACGGTTACGATCTGACGCAGTTGAAAAAAGTCCGTGACGTTTGC
CGCGTGCCGCTGATCGCCTCCGGCGGCGCGGGCACGATGGAACACTTTCTTGAGGCATTC
CGTGATGCCGATGTCGACGGCGCGCTTGCCGCCTCCGTTTTTCACAAGCAAATCATCAAT
ATTGGCGAATTAAAAGCGTACCTGGCAGGCCAGGGCGTGGAGATCAGGATATGTTAA
>EECEBICD_01287 1-(5-phosphoribosyl)-5-[(5-
phosphoribosylamino)methylideneamino] imidazole-4-carboxamide isomerase
ATGATTATTCCGGCATTAGATTTAATTGACGGCACCGTGGTGCGTCTCCACCAGGGCGAC
TACGCCCGGCAGCGGGATTACGGTAACGATCCCCTGCCCCGTTTGCAGGATTACGCCGCC
CAGGGCGCCGGGGTGCTGCATCTGGTAGATCTGACCGGCGCTAAAGATCCGGCTAAGCGA
CAGATACCGCTGATTAAAACCCTGGTCGCGGGCGTGAACGTGCCTGTTCAGGTCGGCGGC
GGCGTGCGTACCGAAGAAGACGTTGCGGCATTACTGAAAGCTGGCGTTGCCCGTGTGGTC
ATCGGTTCAACGGCGGTGAAATCTCCTGACGTGGTGAAAGGCTGGTTTGAACGTTTTGGC

GCGCAGGCGCTGGTACTGGCGCTGGACGTTCGCATAGACGAACACGGCAACAAGCAGGTG
GCGGTTAGCGGCTGGCAGGAAAATTCCGGCGTCTCGCTGGAACAACTGGTGGAGACCTAT
CTCCCCGTCGGCCTGAAACATGTACTGTGTACCGATATTTCTCGCGACGGCACGCTGGCG
GGCTCTAACGTTTCACTGTACGAAGAGATATGCGCCAGATATCCGCAGATCGCCTTTCAA
TCCTCCGGCGGTATTGGCGATATCGATGATATTGCCGCCCTGCGCGGCACCGGCGTGCGC
GGCGTGATTGTCGGACGCGCTCTGCTGGAAGGGAAATTTACCGTTAAGGAGGCCATCCAA
TGCTGGCAAAACGTATAA
>EECEBICD_01288 Imidazole glycerol phosphate synthase subunit HisH
ATGAACGTCGTCATCCTTGACACCGGCTGCGCCAATTTAAGCTCGGTAAAATCCGCCGTG
GCGCGCCACGGTTACACCCCGGTGGTCAGCCGCGAAGCGGAAATCGTATTACGCGCTGAC
AAACTCTTTCTGCCCGGCGTCGGCACGGCGCAGGCCGCTATGGATCAACTGCGCGAACGT
GAACTGATCGACTTAATTAAAGCGTGTACTCAGCCGGTACTGGGTATCTGCTTAGGGATG
CAACTGCTGGGTCGTCGCAGCGAAGAGACGCGCGGCGTGGATCTGCTGAACATTATCGAA
CAAGACGTGCCGAAAATGACCGACTTTGGTCTGCCGCTGCCGCACATGGGCTGGAATCGC
GTGTATCCGCAGGCGGGCAACCGGCTGTTTCAGGGCATTGAAGATGGCGCCTATTTTTAC
TTTGTTCACAGCTACGCGATGCCGGTCAACCCGTGGACTATCGCCCAGTGCAATTACGGC
GAGCCGTTTACCGCGGCGGTACAGAAAGATAATTTCTTCGGCGTACAGTTCCATCCGGAA
CGTTCGGGCGCGGCGGGCGCACAGTTGCTGAAAAACTTCCTGGAGATGTAA
>EECEBICD_01289 Histidine biosynthesis bifunctional protein HisB
ATGAGTCAGAAGTATCTTTTCATCGACCGGGACGGAACCTTAATTTCCGAACCGCCGAGC
GATTTTCAGGTAGACCGCTTTGATAAACTGGCCTTTGAGCCAGAGGTGATTCCCGTATTG
CTGAAGCTGCAAAAAGCCGGTTTTAAGCTGGTGATGATCACTAACCAGGATGGACTTGGC
ACGCAAAGCTTCCCGCAGGCGGACTTCGACGGACCGCACAACCTGATGATGCAGATTTTC
ACCTCTCAGGGCGTATGCTTTGATGAGGTGCTGATCTGCCCTCACCTGCCCGCAGACGAC
TGCGACTGCCGCAAGCCCAAAGTGAAGCTGGTGGAGCGTTATCTTGCGGAACAAGCGATG
GATAGCGCCAACAGCTATGTGATTGGCGATCGTGCGACCGATATCCAGCTCGCTGATAAC
ATGGGCATTACTGGTTTACGCTATCACCGTGAAACGCTGAACTGGACGATGATTGGCGAA
CAGCTAACGAAACGCGATCGCTATGCGCATGTGATCCGTAACACCAAAGAAACACAGATT
GATGTCAGTGTCTGGCTGGATCGCGAAGGCAACAGCAAGATTAATACCGGCGTCGGCTTC
TTTGACCATATGCTCGATCAAATCGCCACCCACGGCGGCTTTCGCATGGAGATTACCGTT
AAGGGTGATCTCTATATCGACGATCACCACACGGTAGAAGATACTGGACTGGCGCTCGGT
GAGGCATTAAAACTGGCACTCGGCGACAAGCGCGGTATCTGCCGTTTTGGCTTTGTACTA
CCGATGGATGAATGTCTGGCGCGCTGCGCGCTGGATATTTCCGGTCGTCCGCATCTGGAA
TATAAAGCTGAATTTACCTACCAGCGTGTGGGCGATTTGAGCACAGAGATGATTGAACAC
TTTTTCCGCTCACTCTCTTACACGATGGGCGTCACTCTGCATCTCAAGACTAAAGGTAAA
AACGATCACCACCGTGTCGAAAGTTTGTTTAAAGCCTTTGGTCGGACGCTACGCCAGGCT
ATTCGCGTGGAGGGCGATACATTACCGTCCTCGAAAGGAGTGCTGTGA
>EECEBICD_01290 Histidinol-phosphate aminotransferase
ATGAGCACTGAAAACACTCTCAGCGTCGCTGACTTAGCCCGTGAAAATGTCCGCAACCTG
GTACCGTATCAGTCTGCCCGCCGTCTGGGCGGTAACGGCGATGTCTGGCTGAACGCGAAT
GAATTCCCGACAGCGGTGGAGTTTCAGCTCACCCAACAAACGCTTAACCGCTACCCGGAA
TGCCAGCCAAAGGCCGTGATTGAAAACTACGCGCAATATGCTGGCGTAAAGCCGGAGCAG
GTGCTGGTCAGCCGCGGCGCGGATGAAGGGATCGAGCTGGTGATCCGCGCCTTCTGTGAA
CCGGGGAAAGACGCTATTCTCTACTGTCCGCCCACTTACGGTATGTACAGCGTCAGCGCC
GAAACCATTGGCGTAGAGCGCCGGACGGTTCCCGCGCTTGAAAACTGGCAGCTGGATCTA
CAGGGGATTTCCGACAACCTTGACGGCACAAAAGTGGTGTTCGTTTGTAGCCCCAATAAT
CCTACCGGACAACTTATCAACCCGCAGGATCTGCGCACGCTGCTGGAGTTGACACGCGGT
AAAGCGATAGTCGTCGCCGACGAAGCTTATATTGAGTTTTGCCCGCAGGCCACGCTGACA
GGCTGGCTGGTTGAATATCCTCATCTGGTTATCCTGCGCACATTGTCGAAAGCTTTTGCG
CTGGCGGGTCTGCGCTGCGGCTTTACACTGGCTAATGAAGAGGTGATCAACCTGCTGTTA
AAAGTGATCGCCCCTTATCCGCTTTCTACGCCAGTGGCGGATATCGCCGCCCAGGCGCTG
AGCCCGCAGGGAATAAACGCAATGCGCGATCGCGTGGCGCAGACAGTGCAGGAACGTCAG
TATCTGGTGAATGCCCTGCAACAGACCGCCTGCGTAGAACACGTCTTTGACTCTGAAACC
AACTATATTCTGGCGCGGTTTACCGCCTCCAGCAGCGTGTTTAAATCCTTATGGGATCAG

GGCATTATCTTACGCGATCAGAATAAACAACCTTCTTTAAGCGGCTGCCTGCGGATTACG
GTCGGCACCCGCCAGGAAAACCAGCGCGTCATTGACGCCTTACGTGCGGAGCCAGTATGA
>EECEBICD_01291 Histidinol dehydrogenase
ATGAGCTTCAATACCCTGATTGACTGGAACAGCTGTAGCCCTGAACAGCAGCGTGCGCTG
CTGACGCGTCCGGCGATTTCCGCCTCTGACAGTATTACCCGGACGGTCAGCGATATTCTG
GATAATGTAAAAACGCGCGGTGACGATGCCCTGCGTGAATACAGCGCTAAATTTGATAAA
ACAGAAGTGACAGCGCTACGCGTCACCCCTGAAGAGATCGCCGCCGCCGGCGCGCGTCTG
AGCGACGAATTAAAACAGGCGATGGCCGCTGCCGTCAAAAATATTGAAACGTTCCATTCC
GCGCAGACGCTACCGCCTGTAGATGTGGAAACCCAGCCAGGCGTGCGTTGCCAGCAGGTT
ACGCGTCCCGTCGCGTCTGTCGGTCTGTATATTCCCGGCGGCTCGGCTCCGCTCTTCTCA
ACGGTGCTGATGCTGGCGACGCCGGCGCGTATTGCGGGATGTCAGAACGTGGTTCTGTGC
TCGCCGCCGCCCATCGCTGATGAAATCCTCTATGCGGCGCAACTGTGTGGCGTGCAGGAA
ATCTTTAACGTCGGCGGCGCGCAGGCGATTGCCGCTCTGGCCTTCGGCAGCGAGTCCGTA
CCGAAAGTGGATAAAATTTTTGGCCCCGGCAACGCCTTTGTAACCGAAGCCAAACGTCAG
GTCAGCCAACGCCTCGACGGCGCGGCTATCGATATGCCAGCCGGGCCGTCTGAAGTACTG
GTGATCGCCGACAGCGGCGCAACACCGGATTTCGTCGCTTCTGACCTGCTCTCCCAGGCT
GAGCACGGTCCGGATTCGCAGGTGATTCTGCTGACGCCTGATGCTGACATTGCCTGCAAG
GTGGCGGAGGCGGTAGAACGTCAACTGGCAGAACTGCCGCGCGCGGACACCGCCAGGCAG
GCCCTGAGCGCCAGTCGTCTGATTGTGACCAAAGATTTAGCGCAGTGCGTCGCCATCTCT
AATCAGTATGGGCCGGAACACTTAATTATCCAGACGCGCAATGCGCGCGATTTGGTGGAT
GCGATTACCAGCGCAGGTTCGGTATTTCTCGGCGACTGGTCGCCGGAATCCGCCGGTGAT
TACGCTTCCGGAACCAACCATGTTTTACCGACCTATGGCTATACTGCTACCTGTTCCAGC
CTTGGGTTAGCGGATTTCCAGAAACGGATGACCGTTCAGGAACTGTCGAAAGCGGGCTTT
TCCGCTCTGGCATCAACCATTGAAACATTGGCGGCGGCAGAACGTCTGACCGCCCATAAA
AATGCCGTGACCCTGCGCGTAAACGCCCTCAAGGAGCAAGCATGA
>EECEBICD_01292 ATP phosphoribosyltransferase
ATGTTAGACAACACCCGCTTACGCATAGCTATTCAGAAATCAGGCCGTTTAAGCGATGAT
TCACGAGAATTGCTGGCCCGCTGCGGCATAAAAATTAATTTACACACTCAGCGCCTGATT
GCGATGGCGGAAAACATGCCGATTGATATCCTGCGCGTGCGTGATGATGACATTCCGGGT
CTGGTGATGGATGGCGTGGTCGATCTCGGCATTATCGGCGAGAACGTGCTGGAAGAAGAG
CTACTCAACCGCCGCGCGCAGGGTGAAGATCCGCGCTATTTTACCCTGCGCCGTCTTGAC
TTCGGCGGCTGCCGTTTATCGCTGGCAACGCCGGTTGACGAAGCCTGGGACGGCCCGGCC
GCGCTGGACGGTAAACGTATCGCTACCTCTTATCCGCACCTCCTCAAACGCTACCTCGAC
CAGAAAGGCGTCTCTTTTAAATCGTGTCTGTTAAATGGTTCTGTCGAAGTCGCGCCGCGC
GCGGGGCTGGCCGACGCTATCTGCGATTTGGTCTCTACCGGCGCGACGCTTGAAGCTAAC
GGCCTGCGTGAAGTCGAAGTTATCTACCGCTCTAAAGCCTGTCTGATTCAGCGCGACGGT
GAGATGGCACAGAGCAAGCAACAGCTGATCGATAAATTGCTGACCCGTATTCAGGGCGTG
ATTCAGGCGCGCGAATCGAAATACATCATGATGCACGCGCCAAGTGAACGCCTGGAAGAG
GTTATCGCCCTGCTGCCAGGCGCCGAAAGGCCGACAATTCTGCCGCTGGCAGGCGAGCAA
CAGCGCGTGGCGATGCACATGGTCAGCAGCGAAACGTTGTTCTGGGAAACCATGGAGAAA
CTGAAAGCGCTTGGCGCCAGCTCGATTCTGGTACTGCCGATCGAGAAGATGATGGAGTGA
>EECEBICD_01293 Protein YeeZ
ATGAAAAAGGTCGCAATTGTCGGTTTAGGTTGGTTAGGCATGCCGCTGGCAATGTCGCTG
GCAGCGAGAGGCTGGCAGGTTACCGGCAGCAAAACCACCCTTGACGGGGTGGAAGCGGCC
CGTATGAGTGGAATTGAAAGCTATCCGCTGCGTCTGGAGCCGGAGCTGGTCTGTGAGGCG
GACGATCTTGACGCTCTGCTGGATGTCGATGCGCTGGTGATAACACTGCCCGCGCGCCGT
AGCGGTCCGGGAGAGGATTTTTATCTGCAGGCGATGCAGGAGCTGGTCGACAGCGCGCTG
GCTTATCGTATCCCGCGTATTATTTTTACCAGTTCGACGTCTGTTTACGGCGATGCGCAA
GGCGTGGTGAAAGAGAATACGCCGCGTAATCCGGTCACGGCCAGCGGGCGTACCCTGAAG
GAACTGGAAGATTGGCTGCATAATCTGCCGGGCACGTCAGTAGATATTCTGCGGCTGGCG
GGGTTGGTCGGGCCTGGACGCCATCCCGGTCGTTTCTTTGCCGGAAAAACCGCGCCGGAT
GGTCAACACGGGGTCAATCTGGTGCATCTTGAAGACGTGGTTGGCGCAATTACCCTTTTG
CTACAGGCGCCAAAAGGGGGGCACATCTATAATATATGTGCGCCGGCGCATCCAGCCCGC
AACGTGTTTTATCCGCAAATGACGCGCTTGCTGGGAATGGCGCCGCCGCATTTTCGTAAC

GCACCGGACAACGGCAAAGGGAAGATTATCGACGGTAGCCGGATTTGTAATGAGCTGGGA
TTTGAATATCAATATCCCGATCCGCTGGTGATGCCGATGGAGTGA
>EECEBICD_01294 HTH-type transcriptional regulator YhaJ
ATGAAACCACTGCTGGACGTTCTGATTATTCTTGATGCGTTAGAAAAAGAAGGCAGCTTC
GCGGCGGCGTCGGCAAAATTATTTAAAACGCCGTCCGCCCTCAGTTATACCGTTCATAAG
CTGGAAAACGATCTTAATATCCAATTGCTCGACCGTAGCGGGCATCGCGCGCGATTTACG
CGAACCGGTAAGATGCTACTGGAAAAAGGCCGTGAAGTGCTGCACACCGTTCGCGAGCTG
GAAAAGCAGGCGATTAAACTGCATGAAGGCTGGGAGAACGAACTGGTCATCGGTGTGGAT
GATACCTTTCCCTTTTCTCTTCTGGCCCCGCTTATCGACGCTTTTTATCAACGCCACAGC
GTGACGCGCCTTAAATTTGTCAATGGCGTTCTCGGCGGTTCATGGGAGACGCTGACGCAG
GGAAGGGCGGATATTATTGTTGGCGCCATGCGCGCGCCGCCGCCTGACACCGGCTTTGGC
TTTGCAAGACTGGGCGATCTTGAACTGGTGTTTGCTGTCGCTCCGCATCATCCGCTGGTT
CATGAAGAGGAACCGCTTAGCCGCCAGACGATCAAACGTTTTCGCGCGATTGTGGTTGGC
GATAGCGTCCATTCCTCGCGATCAATAGGGTCTGAACTGCTCGACGCTCAGGAAGCGATT
ACCGTGTTTGATTTTAAAACCAAGCTGGAGCTGCAAATTAGCGGCCTGGGATGCGGCTAT
TTACCGCGTTATCTGGCGCAGCGATTTTTGGAAAGCGGCGCGCTTATAGAGAAGAAAGTT
GTGGCGCAAATCGTCTATGAACCGGTGTGGGTTGGCTGGAATGAACAGACCGCCGGGCTG
GCCAGCGGCTGGTGGCGGGATGAAATTTTAGCAAATAATGCTATTGCTGGGGTGTATGCA
AAATCGACGGTTTAA
>EECEBICD_01295 Low-affinity putrescine importer PlaP
ATGTCGCATAACGTTACTCCAAACACCTCTCGCGTGGAATTACGTAAAACGCTTACGTTA
GTTCCGGTTGTAATGATGGGTCTTGCCTATATGCAGCCGATGACGCTGTTCGATACATTT
GGTATCGTTTCAGGCCTCACTGACGGTCACGTACCGACGGCGTATGCATTCGCGTTAATC
GCGATTCTGTTTACGGCGTTGAGCTACGGGAAACTGGTGCGTCGCTATCCTTCCGCGGGT
TCGGCGTACACCTATGCCCAGAAATCCATCAGCCCGACCGTTGGCTTTATGGTGGGTTGG
TCTTCTCTGCTCGATTATCTGTTCGCGCCGATGATCAACATTCTGTTGGCGAAAATTTAT
TTTGAAGCGCTGGTGCCCTCCATTCCGTCATGGGTGTTTGTCGTGGCGCTGGTGGCCTTT
ATGACGGCCTTTAACCTGCGCAGCCTCAAATCGGTCGCTAACTTCAACACCGTGATTGTA
GTGCTACAGGTGGTGCTGATTGCGGTCATTCTGGGCATGGTGGTTTATGGCGTATTTGAA
GGCGAAGGCGCCGGTACGCTGGCGAGCACCCGTCCGTTCTGGTCCGGCGATGCGCACGTT
ATCCCGATGATTACGGGAGCGACGATCCTGTGCTTCTCCTTTACCGGCTTTGACGGCATC
AGTAACCTGTCGGAAGAGACCAAAGATGCTGAGCGCGTGATCCCGCGTGCGATTTTTCTG
ACCGCGCTGATTGGCGGCCTGATCTTCATCTTTGCAACCTATTTCCTGCAATTGTACTTC
CCGGATATCTCTCGCTTTAAAGATCCGGATGCGTCTCAGCCGGAAATCATGCTGTACGTG
GCGGGTAAAACCTTCCAGGTGGGCGCGCTGATCTTCTCTACTATCACCGTACTGGCTTCC
GGTATGGCGGCGCATGCAGGCGTAGCGCGCCTGATGTACGTAATGGGGCGCGACGGCGTA
TTCCCGAAAAGCTTCTTCGGTTACGTTCATCCGAAATGGCGTACGCCAGCGATGAACATC
ATCCTGGTCGGGGCGATCGCGCTGCTGGCGATTAATTTTGACCTGGTGATGGCGACGGCG
CTGATTAACTTTGGTGCGCTGGTCGCGTTTACCTTTGTTAACCTGTCGGTGATTTCGCAG
TTCTGGATCCGTGAGAAGCGTAATAAAACGTTGAAAGATCACTTCCAGTATCTGTTCCTG
CCCATGTGCGGCGCGCTCACGGTTGGCGCGTTGTGGGTTAACCTGGAAGAGAGCTCAATG
GTACTGGGCCTGATCTGGGCAGGTATCGGGCTGATTTATCTGGCGTGCGTAACTAAAAGC
TTCCGCAACCCGGTTCCACAGTACGAAGACGTCGCGTAA
>EECEBICD_01296 Exodeoxyribonuclease I
GTGACAGTTAAAAACGACAGCATACAGAGCACATTCCTCTTCCACGATTACGAAACCTTC
GGTACGCATCCGGCCCTCGACAGACCTGCGCAATTCGCCGCGCTCCGTACGGATAACGAC
TTCAACGTTATCGGCGAGCCGGAGGTGTTTTATTGCAAACCCGCCGATGATTATCTACCG
CAGCCCGGCGCGGTGCTGATTACCGGCATCACGCCGCAGGAAGCGCGTGAGAAAGGAGAA
AACGAAGCCGCTTTCGCCAGACGCATTCATGCGCTGTTTACCGTTCCTAAAACCTGCGTT
GTGGGCTACAACAATGTGCGTTTTGATGATGAAGTCACGCGCAATATTTTTTATCGCAAC
TTTTACGATCCCTATGCCTGGAGCTGGCAGCATGATAATTCACGTTGGGATCTATTGGAT
GTCATGCGCGCCTGTTATGCGCTGCGCCCGGAGGGAATTAACTGGCCGGAAAACGACGAC
GGCCTGCCCAGCTTTCGTCTGGAACATTTAACCCAGGCGAACGGGATCGAACACAGCAAC
GCGCATGATGCGATGGCGGATGTCTACGCCACTATTGCGATGGCGCAACTGGTGAAAACA

CGCCAGCCGCGGCTGTTTGATTATCTTTATAGCCACCGCAGTAAACATAAACTGGCGGCG
CTGATTGACGTCCCGCAAATGAAGCCGCTGGTGCATGTCTCCGGCATGTTTGGCGCGTGG
CGCGGTAATACAAGCTGGGTCGCGCCGCTGGCGTGGCATCCTGAAAACCGTAACGCGGTG
ATCATGGTCGATTTAGCAGGCGATATTTCTCCTCTTCTTGAGCTGGACAGCGACACCCTT
CGCGAGCGGCTTTATACGGCCAAAGCCGATCTTGGCGATCACGCCGCAGTGCCGGTAAAG
CTGGTGCATATCAATAAATGTCCGGTACTGGCGCAGGCGAATACCTTGCGCCCGGAGGAT
GCCGACCGGCTGGGAATTAACCGCCAGCATTGCCTGGATAACCTGAAAGTGTTGCGTGAA
AACCCGCAGGTTCGCGACAAAGTGGTGGCGATTTTCGCCGAAGCCGAACCTTTTGCCGCC
TCGGATAACGTTGATGCCCAGCTCTATGATGGTTTTTTCAGCGATGCCGATCGCGCAGCC
ATGAAAATCGTACTCGAAACCGAGCCACGTAACCTGCCCGCGCTGGACATTACCTTTGTC
GATAAGCGCATTGAGAAGCTGCTGTTTAATTACCGCGCACGCAATTTTCCCGGTACGCTG
GATGACGCAGAGCAGCAGCGCTGGCTAGAGCATCGCCGTCAGGTGCTGACGCCGGAGTTT
TTACAACAATATGCCAATGAATTGCAGATGCTTTCTCAGCAGTATGCGGAAGATAAAACG
AAGCTGGGGTTGCTGAAATCACTGTGGCAGTACGCAACTGAGATTGTGTAA
>EECEBICD_01297 E3 ubiquitin-protein ligase SopA
ATGAAGATATCATCAGGCGCAATTAATTTTTCTACTATTCCTAACCAGGTTAAAAAATTA
ATTACCTCTATTCGTGAACATACGAAAAACGGGCTCGCCTCAAAAATAACCAGTGTTAAA
AACACGCATGCTTCTTTAAATGAAAAACTTAAAACAGGAAAGAGCTCATCGATTGAGTTC
GCGTTACCACAAAAAATAAAAGACTTCTTTCAGCCGAAAGATAAAAACACCTTAAACAAA
ACATTGATTACTGTTAAAAATATTAAAGACACAAATAATGCAGGCAAGAAAAATATTTCA
GCAGAAGATGTCTCAAAAATGAATGCAGCATTCATGCGTAAGCATATTGCAAATCAAACA
TGTGATTATAATTACAGAATGACAGGTGCGGCCCCGCTCCCCGGTGGAGTCTCTGTATCA
GCCAATAACAGGCCCACGGTTTCTGAAGGTAGAACACCACCAGTATCCCCCTCCCTCTCA
CTTCAGGCTACGTCTTCCCCGTCATCACCTGCCGACTGGGCTAAGAAACTCACGGATGCA
GTTTTACGACAGAAAGCCGGAGAAACCCTTACGGCCGCAGATCGCGATTTTTCAAACGCA
GATTTCCGTAATATTACATTCAGCAAAATATTGCCCCCCAGCTTCATGGAGCGAGACGGC
GATATTATTAAGGGGTTCAACTTTTCAAATTCAAAATTTACTTATTCTGATATATCTCAT
TTACATTTTGACGAATGCCGATTCACTTATTCGACACTGAGTGATGTAGTCTGCAGTAAT
ACGAAATTTAGTAATTCAGACATGAATGAAGTGTTTTTACAGTATTCAATTACTACACAA
CAACAGCCCTCTTTTATTGATACAACATTAAAAAATACGCTTATACGTCACAAAGCCAAC
CTCTCTGGCGTTATTTTAAATGAACCGGATAATTCATCACCTCCGTCAGTGTCAGGGGGC
GGAAATTTTATTCGTCTAGGTGATATCTGGCTGCAAATGCCACTCCTTTGGACTGAGAAC
GCTGTGGATGGATTTTTAAATCATGAGCACAATAATGGTAAAAGTATTCTGATGACCATT
GACAGCCTGCCCGATAAATACAGTCAGGAAAAAGTCCAGGCAATGGAAGACCTGGTTAAG
TCATTGCGGGGTGGCCGCTTAACAGAGGCATGTATCCGGCCAGTTGAAAGTTCGCTGGTA
AGCGTACTGGCCCACCCTCCCTATACGCAAAGTGCGCTTATCCGCGAGTGGCTCGGGCCT
GTTCAGGAACGTTTTTTTGCCCACCAGTGCCAGACCTATAATGACGTTCCCCTGCCGACT
CCTGACACATATTATCAGCAGCGCATACTGCCTGTGTTGCTGGATTCGTTTGACAGGAAC
AGCGCCGCCATGACCACTCACAGCGGACTCTTTAATCAGGTGATTTTACACTGTATGACA
GGCGTGGACTGCACTGATGGCACCCGCCAGAAAGCTGCAGCGCTTTATGAACAGTATCTT
GCTCACCCGGCGGTGTCTCCCCACATCCATAATGGGCTCTTCGGCAATTATGACGGCAGC
CCGGACTGGACAACCCGCGCTGCAGATAATTTCCTGCTGCTTTCCTCCCAAGATTCAGAC
ACGGCGATGATGCTCTCCACTGACACGCTGTTAACAATGCTAAACCCTACTCCTGACACT
GCATGGGACAACTTTTACCTGCTGCGAGCCGGAGAGAACGTTTCCACCGCGCAAATCTCT
CCGGTAGAGTTATTCCGTCATGACTTTCCGGTGTTTCTCGCCGCATTTAATCAGCAGGCC
ACGCAGCGACGCTTTGGGGAGCTGATTGATATCATCCTCAGCACTGAAGAGCACGGGGAG
CTGAACCAGCAGTTTATTGCCGCCACGAACCAGAAACATTCCACCGTGAAGTTGATTGAT
GATGCCTCAGTGTCGCGTCTGGCCACCATTTTTGCCCCCTTGCTTCCTGAAGGCAAACTC
AGCCCGGCACACTACCAGCACATCCTCAGTGCTTATCACCTGACGGACGCCACCCCACAG
AAGCAGGCGGAAACCCTGTTCTGTCTCAGTACCGCATTCGCACGCTATTCCTCCAGCGCC
ATTTTCGGCACTGAGCATGACTCTCCGCCGGCCCTGAGAGGCTATGCGGAGGCGCTGATG
CAGAAAGCCTGGGAGCTGTCTCCGGCGATCTTCCCGTCCAGCGAACAGTTTACCGACTGG
TCCGACCGTTTTCACGGCCTCCATGGCGCCTTTACCTGTACCAGCGTTGTGGCGGATAGT
ATGCAACGTCATGCCAGAAAATATTTCCCGAGTGTTCTGTCATCCATCCTGCCACTGGCC

TGGGCCTAG
>EECEBICD_01298 Thiosulfate reductase molybdopterin-containing subunit PhsA
ATGAGCATTAGTCGTCGTTCTTTTTTGCAGGGGGTAGGCATAGGCTGCTCTGCCTGCGCG
CTGGGCGCTTTTCCGCCCGGGGCGCTGGCGCGCAACCCGATTGCCGGCATTAACGGTAAA
ACCACGCTAACGCCAAGTTTGTGCGAGATGTGTTCCTTTCGTTGCCCTATTCAGGCGCAG
GTAGTCAATAACAAGACCGTTTTTATCCAGGGCAATCCTTCTGCGCCGCAGCAGGGGACG
CGCATATGCGCCAGGGGCGGGAGCGGCGTCAGCCTGGTCAATGACCCGCAACGGATTGTA
AAACCTATGAAACGCACCGGGCCACGCGGCGACGGTGAATGGCAGGTGATTAGCTGGCAA
CAGGCTTACCAGGAAATCGCGGCGAAAATGAATGCCATCAAAGCGCAGCATGGCCCCGAG
AGCGTGGCTTTCTCTTCCAAATCGGGCTCGCTCTCCAGCCATCTTTTCCATCTGGCTACG
GCCTTTGGTTCGCCTAATACCTTTACTCACGCCTCAACATGCCCTGCCGGGAAAGCCATT
GCGGCAAAAGTGATGATGGGCGGCGATCTGGCGATGGATATCGCTAACACGCGCTATCTG
GTTTCGTTTGGCCACAATTTGTATGAAGGGATTGAAGTTGCCGATACCCATGAGTTAATG
ACCGCGCAGGAGAAGGGGGCCAAAATGGTGAGCTTCGATCCGCGTTTGTCTATATTTTCC
AGCAAGGCGGATGAGTGGCACGCTATTCGTCCCGGGGGGATTTAG
>EECEBICD_01299 Thiosulfate reductase molybdopterin-containing subunit PhsA
GTGGCACGCTATTCGTCCCGGGGGGATTTAGCGGTTCTGCTGGCGATGTGCCACGTCATG
ATTGATGAACAGCTCTACGATGCGTCTTTTGTTGAGCGTTATACCAGCGGATTTGAACAG
TTAGCACAGGCGGTAAAAGAGACGACGCCGGAATGGGCCGCCGCGCAGGCCGATGTCCCA
GCCGACGTTATTGTCCGGGTGACACGCGAACTGGCTGCCTGCGCGCCTCACGCTATTGTC
AGTCCTGGTCATCGCGCGACGTTCTCGCAGGAAGAGATCGATATGCGGCGTATGATTTTT
ACGCTTAATGTGCTGCTCGGTAATATTGAGCGCGAAGGCGGGCTATATCAGAAAAAAAAC
GCGTCTGTTTACAATAAACTGGCCGGAGAAAAGGTCGCGCCAACGCTGGCGAAACTCAAC
ATTAAAAATATGCCGAAACCGACGGCGCAACGCATCGATTTGGTCGCACCGCAGTTTAAA
TATATCGCCGCTGGCGGCGGCGTGGTGCAAAGCATTATTGACTCGGCGTTAACCCAGAAG
CCTTACCCGATAAAGGCGTGGATTATGTCGCGGCATAATCCTTTCCAGACCGTCACCTGT
CGTTCGGACCTGGTAAAAACCGTTGAGCAACTGGATCTGGTGGTCAGCTGCGATGTCTAT
TTGAGCGAGAGCGCGGCATATGCCGACTATCTGCTGCCGGAATGCACCTATCTCGAACGG
GACGAAGAGGTATCCGATATGTCGGGACTGCACCCGGCTTACGCTCTGCGCCAACAGGTC
GTAGAGCCGATTGGCGAGGCGCGTCCGAGTTGGCAAATCTGGAAAGAACTTGGCGAGCAG
TTGGGATTAGGGCAGTACTATCCGTGGCAGGATATGCAGACGCGCCAACTCTATCAGTTG
AACGGCGACCATGCCTTAGCGAAGGAACTGCGACAGAAAGGGTATCTCGAATGGGGCGTT
CCGCTGCTATTACGCGAACCAGAATCCGTTCGTCAGTTTACGGCGCGTTACCCCGGCGCT
ATCGCGACGGACAGTGACAACACCTATGGCGAACAGCTTCGCTTCAAATCGCCCTCCGGC
AAAATCGAACTTTATTCCGCAACCCTGGAGGAATTGCTCCCTGGCTACGGCGTTCCGCGC
GTTCGTGACTTTGCGCTGAAAAAAGAGAATGAGCTTTACTTCATTCAGGGCAAGGTGGCC
GTGCATACCAATGGCGCGACGCAGTACGTACCTTTACTCAGCGAGCTAATGTGGGATAAC
GCGGTCTGGGTTCATCCGCAAACGGCGCAAGAAAAAGGCATTAAGACCGGCGATGAGATC
TGGCTGGAAAATGCCACGGGTAAAGAGAAAGGTAAGGCGCTGGTGACGCCCGGTATCCGC
CCGGACACGCTTTTTGTCTATATGGGATTTGGCGCTAAAGCTGGGGCCAAAACGGCGGCG
ACGACACACGGTATCCACTGTGGCAATTTACTGCCGCACGTGACGAGTCCGGTATCCGGT
ACGGTAGTGCATACCGCAGGCGTGACGCTGAGCCGGGCATGA
>EECEBICD_01300 Thiosulfate reductase electron transfer subunit PhsB
ATGAATCATTTAACGAATCAGTACGTCATGCTGCATGATGAAAAACGTTGTATCGGCTGC
CAGGCGTGTACCGTTGCCTGCAAAGTGCTTAATGACGTACCGGAGGGATTTAGCCGCGTA
CAGGTACAAATTCGCGCCCCTGAACAGGCATCCAACGCACTAATCCATTTTCAATTTGTC
CGCGTCTCCTGTCAGCACTGTGAAAATGCGCCATGTGTCAGCGTTTGTCCTACCGGAGCG
TCATATCGTGATGAAAACGGGATCGTGCAGGTGGATAAATCGCGCTGTATTGGCTGCGAT
TATTGTGTTGCCGCGTGTCCTTTCCATGTGCGCTATTTGAATCCGCAAACCGGCGTCGCC
GACAAGTGTAACTTCTGTGCCGACACGCGGTTGGCGGCTGGCCAGTCTCCGGCGTGCGTA
TCCGTTTGCCCAACGGACGCGCTGAAATTCGGCAGACTGGATGAGAGCGAGATCCAGCGC
TGGGTCGGTCAGAAAGAGGTCTATCGCCAGCAGGAAGCGCGTAGCGGCGCGGTCAGTCTG

TACCGTCGTAAAGAAATCCATCAGGAGGGTAAAGCATGA
>EECEBICD_01301 Thiosulfate reductase cytochrome B subunit PhsC
ATGAATACTATCTGGGGAGCGGAACTACATTATGCGCCAGATTATTGGCCGCTGTGGTTA
ATTTATGCAGGCGTCGTGGTGCTGCTCATGCTTGTTGGGCTGGTTATCCATGCGTTATTG
CGCCGGATGCTGGCGCCAAAAACGGCGGGCGGCGAAGAGCATCGTGACTATCTCTACTCG
CTGGCGATTCGCCGTTGGCATTGGGGAAATGCGTTACTGTTTGTTTTATTACTGTTAAGC
GGTTTATTTGGTCATTTTTCTCTCGGCCCTGTAGCGCTAATGGTACAAGTGCATACCTGG
TGTGGTTTTGCCTTACTGGCTTTCTGGGTCGGGTTTGTGCTGATCAACCTCACCACAGGT
AACGGGCGTCACTATCGGGTAAATTTTTCCGGACTGGTAACGCGCTGCATACGCCAGACG
CGTTTTTACCTTTTTGGCATTATGAAAGGGGAAGCGCATCCGTTCGCGGCAACAGAGCAG
AATAAGTTCAATCCACTGCAACAACTGGCATATCTGGCGATTATGTACGCGCTGGTACCG
CTGTTAATCATCACCGGTTTGCTGTGTCTCTATCCGCAGGTTGCGGGTCTGGGCCCTGTG
ATGCTGGTGCTGCATATGGCGCTTGCTATCATCGGCTTACTGTTTATTTGCGCGCATCTC
TATCTGTGTACTCTTGGCGACACGCCGGGACAAATTTTCCGTAGCATGGTTGACGGCTAT
CATCGTCATCGTACCGCGCCGCGCGGGGATAAGTCCGCCGTCTGA
>EECEBICD_01302 D-alanyl-D-alanine carboxypeptidase DacD
TTGAAACGCCGTTTGTTTATTGCCGCTTCTTTATTCGCGATGCACCTTTCTCCTGCGCTT
GCGGCCGATGCCGTAAGTTTTGCTCCCCAACCGCCAGCCATTGATGCAGGCGCCTGGGTG
TTGATGGATTACACTACGGGGCAGGTTCTTACCGCCGGTAATGAACACCAGCAACGTAAT
CCCGCCAGCCTTACCAAGCTAATGACGGGCTACGTCGTCGATCGCGCCATCGATAGTCAT
CGTATCAGCCCTGACGATATCGTCACAGTTGGGCGCGACGCCTGGGCGAAGGATAATCCG
GTGTTTGTCGGCTCCTCGTTGATGTTTTTAAAAGAAGGGGATCGCGTGTCGGTACGCGAT
TTAAGCCGTGGCTTAATTGTGGATTCCGGTAACGATGCCTGTGTGGCGCTGGCGGATTAT
ATCGCGGGCGGGCAGCCGCAGTTTGTGGCGATGATGAACAGCTATGTGAAAAAACTCAAT
TTGCAGGATACCCATTTTGAAACCGTCCACGGTCTGGATGCGCCGGGACAACATAGCTCC
GCGTATGATCTGGCCGTACTCTCCCGGGCGATTATTCACGGCGAACCTGAATTTTATCAC
ATGTACAGCGAGAAAAGCCTGACCTGGAACGGCATCACGCAGCAGAACCGCAACGGCTTG
TTGTGGGATAAGACCATGCATATCGATGGGCTTAAAACCGGACACACATCGGGGGCGGGA
TTTAATCTTATCGCATCGGCGGTGGACGGGCAGCGTCGACTTATCGCCGTCGTGATGGGG
GCGAAAAGTTCGAAAGGCCGTGAGGAACAGGCGCGAAAACTGTTGCAGTGGGGCCAGCAA
AACTTTGCGACGGTGCAGATTTTGCACAGCGGTAAAAAAGTGGGCAGCGAGCGAATCTGG
TATGGAGATAAAGAAAAGATTGCGCTGGGTACGGAACAGGATTTCTGGATGGCGCTGCCT
AAAGCGGAAATTCCGCATATCAAAGCAAAATATGTGCTGGATAGAAAAGAGCTGGAGGCG
CCAATCGCCGCTCATCAACGGGTAGGTGAAATAGAGCTATACGATCGGGATAAACTGATC
GCTCAGTGGCCGCTGGTCACTCTGGAGTCGGTGGGTAAAGGCGGTATGTTCTCTCGTCTG
AGCGACTATTTTCAGCATAAAGCGTAA
>EECEBICD_01303 DNA gyrase inhibitor
ATGGATTACGAAATCAGGCAGGAACAAAAACGCAAGATAGCCGGTTTCCACATGGTCGGT
CCATGGGAACACACCGTGAAGCAGGGTTTTGAACAACTGATGACGTGGGTGGATCGACAG
CGGATCGTGCCAGTTGAATGGATTGCCGTCTATTACGACAATCCAGATGTCGTACCTGCA
GAAAAAACTCCGCTGCGATACCGTGGTGTCCGTGGCAGAGAATTTCATCCTGCCGGACAA
TAG
>EECEBICD_01304 DNA gyrase inhibitor
GTGGTGTCCGTGGCAGAGAATTTCATCCTGCCGGACAATAGCGAAGGCGTAATCGTGACC
GCCATTGAAGGCGGCGAATATGCCACTGCTGTCGCGCGAGTGGAAGACCGTGACTTTGCG
AAGCCGTGGGAACGGTTTTTTGATGTCCTGGAGCAGGATAGCGCTTATCAGATAGCCTCT
GCGCCCTGTTTTGAAACCTATCTGAATAACGGCATGGAAGATGGATACTGGGATATTGAA
ATGTATATTCCGGTACAGCGGAAGTGA
>EECEBICD_01305 Inner membrane protein YeeA
GTGCGTGCCGATAAGTCATTAAGCCCTTTTGAAATACGGTTGTACCGTCATTATCGCATC
GTGCATGGCATACGTATCGCCCTGGCGTTCATTCTTACCTTTCTGCTGGTTCGTCTGTTT
TCTATTCCCGAGGGCACATGGCCGCTAATCACGTTAGTGGTGATTATGGGGCCCATCTCA
TTCTGGGGAAATGTAGTGCCTCGTGCTTTTGAGCGCATAGGCGGTACGATTCTGGGCGCG
GCGTTAGGGCTGGTGGCGCTACGCCTGGAGCTCTTTTCCTTACCGCTGATGCTGGTGTGG

TGTGCGATAGCGATGTTTTTGTGCGGCTGGCTGGCCTTAGGGAAAAAACCGTATCAGGCG
CTACTGATAGGCATAACGCTTGCCGTTGTTGTCGGCGCTCCCGCTGGTGATATGGATACG
GCATTATGGCGTGGCGGGGATGTGATTTTGGGGTCGCTACTGGCAATGTTGTTTACCGGC
ATCTGGCCCCAGCGCGCCTTTTTTGCACTGGCGCATCCAACTGGCTCATTGCGTCACGGC
GTATAA
>EECEBICD_01306 Inner membrane protein YeeA
TTGGATAAACATTTGCAACGGTTGCTGAACGATGTCGTCAAAATGCGCGGGCTGATAACG
CCTGCCAGTAAAGAAACCCGGATACAAAAATCTATTTTCGAGGCCATTCAGACCATCAAC
CGCAACCTGGTTTGTATGCTTGAATTACAAATCAATGCCCACTGGGCGACGCGCGCCAGC
CATTTTGTGATGCTTAACGCGCACACATTGCGTGAAACCCAGCAGATGACGCAGCAAACC
TTGCTAACGATCGCCCACGCGTTGTTTGAAGGCAACCCGCAGCCGGTACTGGCAAACACC
GGTAAACTGAATGATATTGCTGCCGAGTTGCGGCAGCTAATGAACGAGCAGCAGGGCGAC
GCGGTGGCTGAAACCCCCATCCACGGCTATGTTTGGCTAAGTATGGAAACTGCCCGACAG
TTGGAACTGCTTTCACATTTGATCTGCCGGGCTTTGCGCAAATAA
>EECEBICD_01307 hypothetical protein
ATGGAAACAACCAAGCCTTCTTTCCAGGACGTGCTGGAGTTTGTTCGTCTGTTCCGTCGC
AAAAATAAACTGCAGCGTGAAATTCAGGACATTGAGAAGAAGATCCGTGACAACCAGAAG
CGTGTGCTGCTGCTGGACAACCTGAGCGACTACATCAAGCCAGGTATGAGCGTTGAGGCG
ATTCAGGGTATCATCGCCAGTATGAAAAGCGACTATGAAGATCGCGTTGATGACTACATC
ATTAAAAATGCCGAAATCTCCAAAGAGCGTCGCGACATTTCCAAAAAGCTGAAAGCGATG
GGCGAAATGAAACACGCCGATGTAAAAGCCGAATAA
>EECEBICD_01308 L-threonine kinase
ATGCGCGCACACTATTCGTACCTGAAAGGTGATAACGTGGCTGTTGCGCAATGCCCCGCC
TCATGCGGGGAACTTATCCAGGGATGGATTCTGGGCAGTGAGAAACTGGTCTCCTGCCCC
GTTGACTGGTACAGCACCGTAGCAGTCACGGCTGCGCCGCCGTTGGTAAACGAGCGTCCA
TTGTCGCGGGCGATGGTGGAGCGCGTTCTGGCGCACTGGCAGTATCCTGCGCACTGGAGT
AACGAGATTCGCGTCGATGTGCGTTCGTCAATTCCCGTTGCCAAAGGCATGGCCAGCAGC
ACCGCAGATATCGCCGCTACGGCGGTGGCAACGGCGCATCATCTTGGCCATTCGCTGGAT
GAAACTACCCTTGCACAGCTTTGCGTCTCAATCGAACCCACTGATAGCACCGTTTTTCAT
CAGTTAACGCTGTTTGATCATAATAATGCGGTCACGCAAATCGCCTGCGAGCCACCGCCG
CCAATCGATTTGCTGGTACTGGAAAGCCCGGTCACCCTGCGCACGCAAGATTACCACCGT
CTCCCTCGCCAGCAGAAATTAATAGCAAGTTCAGCAACCTTGCAGCAGGCCTGGAATCTG
GTGCAGGAAGCCTGTATAACGCAAAATCCGCTCCGACTGGGTGAGGCGGCTACGCTTAGC
GCTATCGCCAGCCAGACGCTGTTACCTAAGCCGGGCTTTACCGCCCTGCTGTCGCTGGTC
GAAGAGTGTGATTTATACGGATTGAACGTGGCGCATAGCGGTAGCGTGGTGGGTCTGATG
CTGGACCGAAAACGTCATGATATTGCGCGCCTGAAAGGTAAGCTGGCAGAGAAAAAAACT
TACCCGACACTGGCCAAAACAACATTTACTCAAGATGGTCACAGGCGGGGTCAAACTGCA
GTGAGAGCCTGA
>EECEBICD_01309 putative propionate kinase
ATGTCTTACAAAATAATGGCCATTAACGCCGGCAGCTCATCGTTAAAATTCCAGCTGCTG
GAGATGCCGCAGGGCGACATGCTCTGTCAGGGGCTGATTGAGCGTATCGGTATGGCGGAT
GCGCAGGTGACAATTAAGACGCACAGCCAAAAATGGCAGGAAACCGTCCCCGTTGCCGAT
CATCGCGATGCGGTAACGCTGCTTCTGGAAAAGCTGCTGGGTTATCAAATCATTAACAGT
CTGCGGGACATTGACGGGGTGGGCCATCGTGTGGCGCACGGCGGTGAGTTTTTTAAAGAC
TCGACGCTGGTCACGGATGAAACGTTAGCGCAAATCGAACGTCTTGCTGAACTGGCCCCG
CTTCACAACCCGGTAAACGCGTTGGGTATTCACGTTTTTCGTCAACTGTTGCCTGATGCC
CCTTCCGTAGCCGTGTTTGATACGGCATTTCACCAGACGCTGGATGAACCCGCCTATATT
TACCCGCTTCCCTGGCATTATTACGCGGAACTGGGGATTCGCCGTTATGGCTTTCATGGC
ACCAGCCATAAGTATGTCAGCGGCGTTCTGGCGGAAAAGCTGGGCGTACCACTCAGCGCA
TTACGGGTGATTTGTTGTCATCTTGGAAACGGTAGCAGCATCTGCGCCATCAAGAACGGG
CGTTCGGTCAATACGTCGATGGGCTTTACGCCGCAGTCAGGCGTGATGATGGGCACCCGT
AGCGGAGATATTGATCCCTCTATTCTGCCGTGGATAGCCCAGCGCGAAAGCAAAACGCCG
CAACAGTTGAATCAGTTATTAAATAATGAGTCAGGCTTGCTGGGCGTTTCTGGCGTGTCG
TCTGATTACCGCGATGTCGAACAGGCAGCCAATACCGGTAACCGGCAGGCTAAACTCGCT

CTCACTCTGTTTGCCGAACGCATTCGCGCCACCATTGGCAGCTATATTATGCAAATGGGA
GGACTGGATGCGCTGGTATTCACTGGGGGCATTGGCGAGAACTCAGCGCGCGCCAGATCG
GCGGTGTGCCATAATTTGCAATTTTTGGGCTTAGCCGTTGATGAAGAGAAAAATCAGCGT
AATGCGACATTTATCCAGACGGAGAATGCCCTGGTGAAAGTCGCCGTCATCAACACCAAC
GAAGAGTTAATGATCGCACAAGACGTTATGCGCATTGCGCTACCGGCCACGGAAGGGCTT
TGTGTACCAGCCTGA
>EECEBICD_01310 Propanediol utilization protein PduV
ATGAAGCGTTTGATGTTTATCGGCCCCAGCCAGTGCGGAAAAACCTCGCTGACCCAAAGC
CTGCGCGGCGAAGCGCTCCATTATAAGAAAACTCAGGCCATTGAATGGTCGCCGATGGCC
ATCGATACGCCGGGCGAATATCTGGAGAACCGCTGCCTGTACAGCGCCCTGCTGACCAGC
GCCTGCGAGGCCGACGTCATCGCCCTGGTGCTGAATGCCGACGCCCAGTGGTCGCCCTTT
TCTCCGGGTTTTACCGCCCCGATGAACCGCCCGACGATTGGCCTGGTCACCAAGGCCGAT
CTCGCCGAGCCGCAGCGTATTTCGCTGGTTGCGGAGTGGCTTACGCAGGCTGGCGCCCGG
CAGATTTTTATTACCAGCGCGTTGAATAATTCGGGTCTCGACGCCGTGCTGGATTTTCTG
AATTCGAAGGAACCTTTATGTCTTACAAAATAA
>EECEBICD_01311 Propanediol utilization protein PduU
ATGGAAAGACAACCGACAACGGATCGCATGATTCAGGAATACGTGCCGGGGAAACAGGTC
ACTCTCGCGCACCTGATTGCTAATCCAGGGAAAGATCTCTTTAAGAAGCTGGGCCTGCAG
GATGCGGTGTCCGCCATTGGCATCCTTACCATCACGCCGAGCGAAGCATCTATTATTGCT
TGCGATATTGCTACCAAATCCGGCGCGGTGGAGATTGGGTTTCTTGACCGCTTTACCGGC
GCGGTGGTGCTGACTGGCGATGTTTCTGCCGTCGAGTATGCCCTCAAGCAGGTAACGCGC
ACGCTGGGCGAAATGATGCAGTTCACCACTTGCTCGATCACCCGGACGTAA
>EECEBICD_01312 hypothetical protein
ATGTCTCAGGCTATAGGAATTTTAGAACTCACCAGCATCGCCAAAGGGATGGAGCTTGGT
GATGCGATGCTGAAAAGCGCCAACGTCGATCTACTGGTCAGCAAGACCATCTGCCCGGGG
AAGTTTTTACTGATGCTGGGCGGCGATATCGGCGCTATCCAGCAGGCCATTGAAACCGGT
ACGTCGCAGGCCGGCGAAATGCTCGTCGACAGCCTGGTGCTGGCGAATATTCATCCCAGC
GTACTTCCGGCCATCAGCGGCCTGAACAGCGTCGATAAGCGCCAGGCGGTAGGTATCGTC
GAAACCTGGAGCGTGGCGGCCTGCATTAGCGCCGCAGACCGGGCGGTGAAAGGCTCAAAC
GTCACCCTGGTGCGGGTGCATATGGCCTTCGGTATCGGCGGTAAATGCTACATGGTGGTG
GCGGGCGACGTTTCTGACGTCAATAACGCCGTGACGGTTGCCAGCGAAAGCGCGGGCGAG
AAAGGGTTGTTGGTTTACCGTTCGGTGATCCCACGCCCGCATGAAGCCATGTGGCGACAG
ATGGTGGAGGGATAA
>EECEBICD_01313 Ion-translocating oxidoreductase complex subunit C
ATGAGCACCGCCATCAACAGCGTTGAAATGTCCCTTAGCGCCGATGAAATTCGTGAGCGC
GTCCGCGCAGCGGGCGTTGTCGGCGCAGGCGGCGCAGGTTTTCCCGCTCACGTCAAACTA
CAGGCGCAGGTAGAGATTTTTCTGGTGAACGCCGCCGAGTGTGAACCGATGCTCAAAGTT
GACCAGCAACTGATGTGGCAGCAAGCTGCGCGCCTTGTGCGCGGCGTACAGTACGCCATG
ACGGCGACCGGCGCGCGCGAAGGGGTGATTGCCCTGAAAGAAAAATACCGCTGGGCCATC
GACGCCCTTACCCCGCTACTGCCCGCCGGTATCCGCCTGCATATTCTGCCGGATGTTTAT
CCGGCGGGCGATGAGGTGCTGACTATCTGGATGGCAACCGGCCGCCGGGTCGCCCCGGCT
GCGCTACCGGCCAGCGTCGGCGTGGTCGTCAATAACGTGCAAACGGTGCTGAATATTGCC
CGGGCTGTCGAGCAGCAGTTTCCGGTCACTCGTCGCACGTTGACCGTGAACGGCGCGGTC
GCCAGACCGTTGACCGTTACTGTTCCCATTGGCATGTCGCTGCGGGAAGTACTGGCGCTG
GCGGGCGGCGCAACGGTCGACGACCCTGGTTTTATTAACGGCGGCCCGATGATGGGTGGC
CTGATTACCTCTCTTGATAATCCGGTGACGAAAACTACCGGCGGCCTGCTGGTGCTCCCC
AAAAGCCATCCGCTTATCCAGCGGAGAATGCAGGACGAGCGCACGGTGCTTTCCGTCGCG
CGCACCGTCTGCGAACAGTGCCGACTGTGTACCGATCTCTGTCCGCGACACCTGATCGGC
CACGAACTCTCTCCGCACCTGCTGGTGCGGGCGGTGAACTTTCACCAGGCTGCCACGCCG
CAGCTGCTGCTGAGCGCCCTTACCTGCTCGGAATGCAACGTTTGCGAAAGCGTGGCCTGT
CCGGTGGGGATCTCGCCGATGCGCATCAACCGCATGTTAAAACGCGAGCTGCGGGCGCAA
AACCAGCGCTACGAAGGGCCGCTGAATCCGTCCGACGAAATGGCGAAATATCGCCTGGTG
CCGGTGAAGCGGCTGATCGCCAAACTGGGACTAAGCCCCTGGTACCAGGAAGCGCCGCTG
GTTGAAGAAGAGCCGTCAGTAGAAAAAGTCACTTTGCAGCTGCGCCAGCATATTGGTGCC

AGCGCGGTAGCGAACGTTGCGGTGGGAGAACGCGTGACGCGCGGGCAATGCGTTGCCGAT
GTCCCGCCTGGCGCGCTCGGCGCACCTATTCACGCCAGCATCGACGGCATTGTTTCGGCC
ATCAGCGAACAGGCCATCACGGTTGTAAGAGGTTAA
>EECEBICD_01314 Aldehyde-alcohol dehydrogenase
ATGAATACCTTCTCACTACAAACGCGGTTGTACAGCGGTCAGGGCAGCCTGGCGGTGCTC
AAGCGCTTTACCAATAAGCACATCTGGATAATCTGCGATGGCTTTCTGGCGCGCTCTCCG
CTGCTGGATACCCTGCGTAATGCGCTGCCCGCAGATAACCGCATCAGCGTCTTTAGCGAG
ATAACGCCGGACCCCACCATCCACACAGTGGTTCAGGGCATTGCGCAAATGCAGGCTCTG
CAACCGCAGGTGGTGATCGGTTTTGGCGGCGGCTCGGCAATGGACGCGGCGAAAGCGATT
GTCTGGTTTAGCCAGCAGAGCGGGATCAACATCGAAACCTGCGTGGCGATCCCGACCACC
AGCGGCACCGGCTCGGAAGTGACCAGCGCCTGCGTAATTAGCGACCCGGATAAAGGCATC
AAGTATCCGCTGTTCAACAATGCGCTGTATCCGGATATGGCGATCCTCGACCCGGAGCTG
GTGGTCAGCGTACCGCCGCAGATTACCGCCAACACCGGCATGGACGTGCTGACCCACGCC
CTGGAGGCCTGGGTGTCACCGCGCGCCAGCGACTTTACCGACGCGCTAGCGGAAAAAGCC
GCCAAACTGGTGTTCCAGTATCTGCCCACGGCGGTGGAAAAAGGCGACTGCGTGGCGACG
CGCGGGAAAATGCACAATGCCTCAACGCTCGCCGGGATGGCCTTCAGCCAGGCGGGGCTG
GGGCTTAACCACGCGATAGCCCACCAGCTTGGCGGGCAGTTTCATCTGCCGCATGGCCTG
GCCAACGCCCTGCTGTTAACCACGGTTATCCGTTTTAACGCCGGCGTTCCGCGCGCCGCC
AAACGCTACGCGCGGCTGGCCAAAGCCTGCGGTTTTTGCCCGGCAGAAGCCAATGATATC
GCGGCGATCAATGCGCTGATTCAGCAAATCGAACTGCTTAAGCAACGCTGCGCCCTTCCC
TCACTGGCCGTTGCGCTTAAAGAAGGAAGATCCAACTTTTCCGCACGCATTCCGGCGATG
GTGCAGGCCGCGCTGGCGGACGTTACGCTGCGCACCAACCCGCGCCCGGCCAGCGCCGAA
GAAATTCGTGAACTGCTGGAGGAACTGCTATGA
>EECEBICD_01315 Aldehyde-alcohol dehydrogenase
ATGAATACTTCTGAACTCGAAACCCTGATTCGCACCATTCTTAGCGAGCAATTAACCACG
CCGGCGCAAACGCCGGTCCAGCCTCAGGGCAAAGGGATTTTCCAGTCCGTGAGCGAGGCC
ATCGACGCCGCGCACCAGGCGTTCTTACGTTATCAGCAGTGCCCGCTAAAAACCCGCAGC
GCCATTATCAGCGCGATGCGTCAGGAGCTGACGCCGCTGCTGGCGACCCTGGCGGAAGAG
AGCGCCAATGAAACGGGGATGGGCAACAAAGAAGATAAATTCCTCAAAAACAAGGCTGCG
CTGGACAACACGCCGGGCGTAGAAGACCTCACCACCACCGCGCTGACCGGCGACGGCGGC
ATGGTGCTGTTTGAATACTCACCGTTTGGCGTTATCGGTTCGGTCGCACCGAGCACCAAC
CCGACGGAAACCATCATCAACAACAGCATCAGCATGTTGGCGGCGGGCAACAGCGTCTAC
TTTAGCCCGCATCCGGGGGCGAAAAAGGTCTCTCTGAAGCTCATCAGCCTGATTGAAGAG
ATTGCCTTCCGCTGCTGCGGCATCCGCAATCTGGTGGTGACTGTAGCTGAACCCACCTTC
GAAGCGACCCAGCAGATGATGGCCCACCCGCGAATCGCAGTACTGGCCATTACCGGCGGC
CCCGGCATTGTGGCAATGGGCATGAAGAGCGGTAAGAAGGTGATTGGCGCTGGCGCGGGT
AACCCGCCCTGCATCGTTGATGAAACGGCGGACCTGGTGAAAGCGGCGGAAGATATCATC
AACGGCGCGTCATTCGATTACAACCTGCCCTGCATTGCCGAGAAGAGCCTGATCGTAGTG
GAGAGTGTCGCCGAACGTCTGGTGCAGCAAATGCAAACCTTCGGCGCGCTGCTGCTAAGC
CCGACCGATACCGACAAACTCCGCGCCGTCTGCCTGCCTGAAGGCCAGGCGAATAAAAAA
CTGGTCGGCAAGAGCCCATCGGCCATGCTGGAAGCCGCCGGGATCGCTGTCCCGGCAAAA
GCGCCGCGTCTGCTGATTGCCGTTGTCAACGCCGACGATCCGTGGGTCACCAGCGAACAG
CTGATGCCGATGCTGCCGGTGGTAAAAGTCAGCGATTTCGATAGCGCACTGGCGCTGGCC
CTGAAGGTTGAAGAGGGGCTGCATCATACCGCCATTATGCACTCGCAGAACGTGTCACGC
CTGAACCTCGCGGCCCGCACCCTGCAAACCTCGATATTCGTCAAAAACGGCCCCTCTTAT
GCCGGGATCGGCGTCGGCGGCGAAGGCTTTACCACCTTCACTATCGCCACGCCAACCGGT
GAAGGGACCACGTCAGCGCGTACTTTTGCCCGTTCCCGGCGCTGCGTACTGACCAACGGC
TTTTCTATTCGCTAA
>EECEBICD_01316 hypothetical protein
ATGGCGATTTATACCCGAACAGGTGACGCAGGCACCACATCACTGTTTACCGGCCAGCGG
GTGAGCAAAACGCACCCGCGGGTTGAAGCCTACGGCACGCTGGATGAGCTGAATGCCGCG
CTGAGCCTGTGCGCCTGCGCCGCTGCGGATGAAAATCACCGCACCTTACTCGAGGCCATC
CAGCAGCAACTTTTTTGGTTTAGCGCAGAGCTGGCCAGCGACAGCGAGCAGCCATCGCCC
AAACAGCGCTACATCAGCAGCGAAGAGATTTCGGCCCTGGAAGCCGCTATCGATCGGGCA

ATGGCCCGCGTCGAACCGCTGCACAGCTTTATTTACCCGGACGCTGCGAAGCCGCGAGC
CGCTTACATTTTGCCCGCACGCTGGCGCGCCGCGCCGAACGCCGTCTGGTTGAACTGGCA
ACTGAAGTCAACGTACGCCAGGTGCTGATGCGCTACATCAACCGCTTATCGGACTGCCTG
TACGCCCTGGCCCGCGCGGAAGATAGCGATGCGCACCAGGCCAACATCATCCGCGAAGTT
AGCAAGCGCTATCTGGCTGCCAGCCAGCCGACCCGCAGCAAGGAGACAACGCCCGTGGCC
CTCTCATTCCACGATCTGCACCAGCTCACCCGCGCCGCCGTTGAACGCGCGCAGCAACTG
CAGGTTCCGGTAGTCGTCAGCATCGTTGACGCGCACGGCACGGAAACTGTGACCTGGCGG
ATGCCGGACGCCCTGCTGGTCAGCAGCGAACTGGCGCCGAAAAAGGCCTGGACCGCAGTG
GCAATGAAAACGGCGACCCATGAGCTGAGCGATGTTGTTCAGCCAGGCGCCGCGCTTTAT
GGCCTGGAAAGTCATTTACAGGGAAAAGTGGTCACCTTTGGCGGCGGTTACGCCCTATGG
CGCGACGGCATATTAATTGGGGGGTCTTGGCATCAGCGGCGGCAGCGTTGA
>EECEBICD_01317 Carbon dioxide concentrating mechanism protein CcmL
ATGCATCTGGCACGAGTCACGGGCGCGGTTGTCTCCACGCAAAAATCACCTTCTTTGATT
GGGAAAAAGCTGCTGCTGGTGCGTCGGGTCAGCGCCGATGGCGAACTCCCCGCCTCGCCA
ACCTCCGGCGATGAAGTGGCCGTGGATTCCGTCGGCGCAGGCATCGGCGAGCTGGTTCTG
CTCAGCGGCGGCTCCAGCGCCAGGCACGTTTTTTCCGGGCCAAATGAGGCCATCGACCTC
GCCGTTGTCGGCATTGTCGATACGCTTTCACGTTAA
>EECEBICD_01318 hypothetical protein
ATGAACGGCGAAACTCTGCAGCGCATTGTCGAGGAGATTGTCTCCCGACTGCAGCGCCGT
GCCCAGAGCACGGTGACGCTGAGCGTTGCGCAGCTTCGCGATGCCGACTGCCCAGCGCTG
TTCTGTCAGCACGCGTCGCTGCGCATCCTGCTCGTCGACCTGCCGCTGCTGGGTCAGCTT
GCGGATGCAGAAACCGACGATACCGCCGCCCGGAAAATTCACGACGCGCTGGCGTTTGGC
ATCCGCGTACAGCTCAGCCTGCACAGCCAGCTGCTGCCGGTTATCCCGGTAAAAAAACTG
GCTCGTCTGCCGCTGGTATTCACCGATGAGCGCGGACTGCCGCTGGTTCTGCACGCGGGT
TCGGTGCTGAGCTATCGCGATGTCGCGCTGCTAAGCCGGGGGCGTGTGGTTGTCCACCGC
AAATGCATCGTGACGGCCCTGGCGCGCGAAGCAGCTAATGCGCGGAATATTCAATTAATT
AAGCAGGAGTAA
>EECEBICD_01319 Phosphate propanoyltransferase
ATGGATAAAGAGCTTTTGCAATCAACGGTCCGTAAAGTTCTCGACGAGATGCGCCAGCGG
CCTATTCCGCTGGGCGTCTCAAACCGTCATATCCACCTGTCCGCGCAGGATTATGAGCGG
CTGTTTCCCGGCCATCCCATCAGCGAAAAAAAAGCGCTACTGCAGCCGGGACAATATGCT
GCGGAGCAAACCGTCACCCTGGTAGGGCCGAAAGGACAGCTCAACAATGTGCGCCTGCTC
GGCCCGCTGCGCTCGGTAAGCCAGGTGGAAATTTCGCGTACCGATGCGAGAACGCTGGGG
ATTGCCGCGCCGCTGAGAATGTCCGGCAACCTGAAGGGCACACCTGGCATCCGCCTGGTC
AGCCCCTTTGGCGAACTGGAGCTACCTTCCGGGGTTATCGTCGCGCAGCGGCATATTCAT
ATGTCGCCGCTTGATGCGCTGATCCTGAAGGTCTCCCACGGCGATAGGGTTTCGGTAGCG
ATTGAAGGCGACGAGCGCGGGCTGATTTTCAACAATGTGGCGATTCGCGTTTCACCGGAC
ATGCGCCTCGAAATGCACATTGATACCGATGAAGCCAACGCCGCTGGTGCCGATAACCCG
CAGGCCTTTGCCCGGCTGGTAGGCCCGCGATGA
>EECEBICD_01320 hypothetical protein
GTGAAGCAATCACTGGGATTACTTGAAGTTTGTGGTCTGGCACTGGCTATCAGCTGCGCC
GATATCATGGCGAAATCCGCTTCTATCACGCTGCTCGCCCTCGAAAAGACCAATGGTTCA
GGCTGGATGGTGATTAAAATTACCGGTGATGTGGCCTCCGTGCAGGCGGCCATCACCACC
GGTGCGCAATTTGCCGAACAGCGGAATGGTCTGGTGGCCCACAAGGTTATCGCCAGGCCC
GGAGAAGGGATCCTGCTCGCAGAGACACCCTCCCCCTCCGTCATTGAACCTGAGCCTGAA
GCGTCAGAGATAGCTGATGTCGTTTCTGAAGCGCCAGCCGAAGAGGCCCCGCAGGAATCA
GAACTGGTCAGCTGCAATCTGTGTCTGGATCCAAAATGTCCGCGCCAGAAGGGCGAACCT
CGTACGCTTTGCATTCATTCCGGCAAGCGAGGTGAAGCGTAA
>EECEBICD_01321 Propanediol utilization protein PduA
ATGAATAACGCACTGGGACTGGTTGAAACAAAAGGGCTGGTCGGCGCCATTGAAGCCGCC
GATGCAATGGTTAAATCCGCCAACGTACAGCTGGTGGGCTACGAAAAAATTGGTTCTGGC
CTGGTTACCGTCATGGTTCGCGGCGATGTCGGCGCGGTTAAAGCAGCCGTAGATGCCGGC
AGCGCGGCGGCAAGCGTCGTGGGTGAAGTGAAATCCTGCCACGTTATCCCGCGTCCGCAC
AGTGATGTTGAGGCCATTTTACCGAAATCAGCCTAA

>EECEBICD_01322 hypothetical protein
ATGGACAGTAATCACAGCGCCCCGGCCATCGTCATTACCGTTATCAACGACTGCGCCAGC
CTCTGGCACGAAGTGCTGCTGGGTATCGAAGAGGAAGGCATCCCTTTCCTGCTTCAGCAT
CACCCGGCTGGAGATGTCGTTGACAGCGCCTGGCAGGCGGCGCGCAGCTCGCCGCTGCTG
GTCGGCATTGCCTGCGATCGACATACGCTGGTCGTACATTACAAGAATTTACCCGTATCG
GCGCCGCTTTTTACGCTGATGCATCATCAGGACAGTCAGGCCCAACGCAACACCGGTAAT
AACGCGGCACGGCTGGTCAAAGGGATCCCCTTTCGGGATCTCCATGCTTAA
>EECEBICD_01323 Diol dehydratase-reactivating factor alpha subunit
ATGCGATATATAGCTGGCATTGACATCGGTAACTCATCAACGGAAGTCGCACTGGCGCGG
CAAGATGAGACTGGCGCACTGACGATTACGCACAGCGCGCTGGCGGAAACCACCGGGATC
AAAGGCACGTTGCGTAACGTGTTCGGCATTCAGGAAGCGCTCGCCCTCGTCGCAAAGCGC
GCGGGGATCAATGTCAGCGATATTTCGCTCATCCGCATTAACGAAGCCACGCCGGTGATT
GGCGATGTGGCGATGGAAACCATTACCGAAACCATCATCACCGAATCGACAATGATCGGC
CATAACCCAAAAACGCCGGGCGGAGCAGGCCTTGGTGTGGGTATCACGATTACGCCGGAG
GAGCTGTTAACCCGCCCTGCGGACTCGTCCTATATTCTGGTGGTATCGTCAGCATTCGAT
TTTGCCGACATCGCCAATGTTATCAACGCCTCAATGCGCGCCGGATACCAGATTACCGGC
GTCATTTTGCAGCGCGACGATGGCGTGCTGGTCAGCAACCGGCTGGAAAAATCGCTGCCG
ATCGTTGACGAAGTGCTCTACATCGACCGCATTCCGCTGGGGATGCTGGCGGCGATTGAA
GTCGCCGTGCCGGGGAAGGTTATCGAGACCCTCTCCAACCCTTACGGCATCGCCACCGTA
TTCAACCTCAACGCCGATGAGACGAAAAACATCGTCCCGATGGCGCGCGCGCTGATTGGC
AACCGTTCCGCCGTGGTGGTTAAAACGCCATCCGGCGACGTCAAAGCGCGCGCAATACCC
GCCGGTAACCTGGAGCTGCAGGCTCAGGGTCGTACCGTGCGCGTGGATGTTGCCGCCGGT
GCCGAAGCTATCATGAAAGCGGTGGACGGCTGCGGCAAGCTCGACAACGTCACCGGCGAG
GCCGGGACCAATATCGGCGGCATGCTGGAGCACGTACGCCAGACCATGGCCGAGCTAACC
AATAAGCCCAGCAGCGAGATTTTCATTCAGGATCTTCTGGCCGTTGACACCTCGGTTCCG
GTGAGCGTCACCGGCGGTCTGGCCGGGGAGTTCTCACTGGAGCAGGCCGTCGGCATCGCC
TCGATGGTGAAATCAGACCGTCTGCAGATGGCGATGATTGCCCGTGAAATTGAGCAGAAG
CTTAATATCGACGTGCAGATCGGCGGCGCTGAGGCTGAAGCCGCCATTCTGGGCGCGCTG
ACCACGCCGGGTACCACCCGACCGCTGGCGATCCTTGACCTCGGCGCGGGCTCCACCGAT
GCCTCCATCATCAACCCTAAAGGCGAAATCATCGCCACTCACCTCGCCGGTGCAGGCGAC
ATGGTGACGATGATTATTGCCCGCGAGCTGGGGCTGGAAGACCGCTATCTGGCGGAAGAG
ATCAAAAAATACCCGCTGGCGAAGGTCGAAAGCCTGTTCCACTTACGCCACGAGGACGGC
AGCGTCCAGTTCTTCCCGACGCCGCTGCCTCCCGCCGTGTTCGCCCGCGTCTGCGTGGTG
AAACCGGACGAACTGGTGCCGCTTCCCGGCGACTTAGCGCTGGAAAAAGTGCGCGCCATT
CGCCGCAGCGCTAAAGAACGCGTCTTTGTCACCAACGCCCTGCGCGCGCTGCGCCAGGTC
AGTCCAACCGGCAACATTCGCGATATTCCGTTCGTGGTGCTGGTCGGCGGCTCGTCGCTG
GATTTCGAAGTTCCGCAGCTGGTCACCGATGCGCTGGCGCACTACCGCCTGGTCGCCGGG
CGAGGAAATATTCGCGGCAGCGAAGGCCCAAGAAACGCGGTGGCCACCGGCCTGATTCTC
TCCTGGCACAAGGAGTTTGCACATGGACAGTAA
>EECEBICD_01324 Propanediol dehydratase small subunit
ATGAATACCGACGCAATTGAATCGATGGTTCGGGACGTATTGAGCCGCATGAACAGCCTG
CAGGGCGATGCGCCAGCAGCGGCTCCTGCGGCAGGCGGCACGTCCCGCAGCGCAAAGGTC
AGCGACTACCCGCTGGCGAACAAACACCCGGAATGGGTGAAAACCGCCACCAATAAAACG
CTGGACGACTTTACGCTGGAAAACGTGCTGAGCAATAAAGTCACCGCTCAGGATATGCGC
ATTACCCCGGAAACCCTGCGCTTACAGGCGTCTATCGCCAAAGATGCGGGCCGCGACCGG
CTGGCGATGAACTTCGAACGCGCCGCCGAACTGACCGCGGTACCGGACGATCGCATTCTT
GAAATCTACAACGCCCTTCGTCCGTATCGTTCAACGAAAGAAGAGCTGCTCGCTATCGCC
GACGATCTCGAAAACCGTTATCAGGCAAAGATTTGCGCAGCTTTCGTTCGTGAAGCGGCA
GGGCTGTACGTTGAGCGTAAAAAACTCAAAGGCGACGATTAA
>EECEBICD_01325 Propanediol dehydratase medium subunit
ATGGAAATTAATGAAAAATTGCTGCGCCAGATAATTGAAGACGTGCTTCGCGATATGAAG
GGCAGCGATAAACCCGTCTCGTTTAATGCGCCTGCGGTATCCACAGCACCACAGACCGCT
GCGCCTGCGGGCGACGGCTTTCTGACCGAAGTGGGCGAAGCGCGCCAGGGCACTCAGCAG
GACGAAGTCATTATCGCCGTCGGCCCGGCATTTGGCCTGGCGCAAACCGTCAATATCGTC

GGCTTACCGCATAAGAGCATTTTGCGCGAAGTCATTGCCGGTATTGAAGAAGAAGGTATC
AGGGCGCGCGTGATTCGCTGCTTTAAATCTTCCGACGTGGCGTTCGTCGCCGTGGAAGGT
AACCGCCTGAGCGGCTCCGGCATCTCCATCGGCATCTAG
>EECEBICD_01326 Propanediol dehydratase large subunit
ATGAGATCGAAAAGATTTGAAGCACTGGCGAAACGCCCTGTGAATCAGGACGGCTTTGTT
AAGGAGTGGATCGAAGAAGGCTTTATCGCGATGGAAAGCCCGAACGACCCAAAACCGTCG
ATAAAAATCGTTAACGGCGCGGTAACCGAGCTGGACGGAAAACCGGTTAGCGAATTCGAC
CTGATCGACCACTTTATCGCCCGCTACGGCATCAACCTGAACCGCGCCGAAGAAGTGATG
GCGATGGATTCGGTCAAGCTGGCTAACATGCTGTGCGATCCGAACGTCAAGCGCAGCGAA
ATCGTTCCGCTAACCACCGCGATGACCCCAGCGAAAATTGTCGAAGTGGTTTCGCATATG
AACGTGGTTGAGATGATGATGGCGATGCAGAAAATGCGCGCCCGCCGTACTCCATCTCAA
CAGGCGCACGTCACCAACGTTAAAGACAACCCGGTGCAAATTGCCGCCGATGCCGCCGAA
GGCGCATGGCGCGGGTTTGACGAACAAGAGACGACGGTTGCGGTAGCGCGCTATGCGCCG
TTCAACGCCATCGCGCTGCTGGTTGGTTCTCAGGTAGGTCGTCCGGGGGTACTGACTCAA
TGCTCGCTGGAAGAAGCCACCGAGCTGAAGCTCGGCATGCTGGGCCACACCTGCTACGCC
GAAACCATCTCCGTTTACGGCACCGAGCCGGTCTTCACCGACGGTGACGATACCCCATGG
TCGAAGGGCTTCTTAGCCTCTTCCTACGCCTCTCGCGGCCTGAAAATGCGCTTCACCTCC
GGCTCCGGCTCCGAAGTGCAGATGGGCTACGCCGAAGGCAAATCCATGCTGTATCTGGAA
GCGCGCTGCATCTATATCACCAAAGCCGCGGGCGTTCAGGGGCTGCAAAACGGCTCCGTA
AGCTGCATCGGCGTACCGTCTGCCGTGCCGTCAGGCATTCGTGCCGTGCTGGCGGAAAAC
CTGATCTGCTCTTCGCTGGATCTGGAATGCGCCTCCAGTAACGACCAGACCTTCACCCAC
TCCGATATGCGTCGTACCGCGCGCCTGCTGATGCAGTTCCTGCCGGGTACCGACTTTATC
TCCTCTGGTTATTCCGCGGTGCCGAACTACGACAACATGTTCGCCGGTTCCAACGAAGAT
GCGGAAGACTTTGACGACTACAACGTTATCCAGCGTGACCTGAAAGTGGACGGTGGTCTG
CGCCCGGTTCGCGAAGAGGACGTTATCGCCATCCGTAACAAAGCCGCCCGCGCGCTGCAG
GCCGTGTTTGCCGGAATGGGACTGCCGCCGATTACCGATGAAGAAGTTGAAGCTGCGACC
TATGCCCACGGTTCGAAAGATATGCCGGAGCGTAACATCGTCGAAGACATCAAGTTCGCC
CAAGAAATCATCAATAAAAACCGCAACGGTCTGGAAGTCGTTAAAGCGCTGGCGCAGGGC
GGGTTTACCGACGTGGCCCAGGACATGCTCAACATCCAGAAAGCCAAGCTAACCGGCGAC
TATTTGCACACCTCCGCCATTATCGTCGGCGACGGACAAGTGCTCTCTGCGGTTAATGAC
GTCAATGACTATGCCGGTCCGGCAACAGGTTATCGCCTGCAGGGAGAACGCTGGGAAGAG
ATTAAAAACATCCCTGGCGCTCTTGATCCCAACGAGATTGATTAA
>EECEBICD_01327 Propanediol utilization protein PduB
ATGAGCAGCAATGAGCTGGTGGAACAGATCATGGCGCAGGTGATTGCCCGTGTGGCAACG
CCGGAACAACAGGCCATCCCTGGTCAACCCCAACCTATACGAGAGACGGCTATGGCAGAA
AAAAGCTGCAGTTTAACGGAATTTGTCGGGACCGCCATTGGCGATACCCTGGGCCTGGTT
ATCGCCAACGTCGATACCGCCCTGCTGGACGCGATGAAGCTTGAGAAGCGCTATCGTTCC
ATCGGTATTCTCGGCGCCCGCACCGGCGCAGGCCCGCACATTATGGCGGCGGACGAAGCG
GTGAAAGCCACCAACACCGAAGTGGTCAGCATTGAGCTGCCGCGCGATACCAAAGGCGGA
GCGGGCCACGGTTCGCTGATTATTTTAGGCGGTAACGATGTATCCGACGTCAAGCGCGGT
ATCGAAGTCGCGCTGAAAGAGCTGGACCGCACCTTCGGTGATGTTTACGGCAACGAAGCC
GGACATATCGAGCTGCAGTACACCGCTCGCGCCAGCTATGCGCTGGAAAAAGCGTTCGGC
GCGCCGATTGGCCGCGCCTGCGGCATTATCGTCGGCGCTCCGGCTTCCGTCGGCGTACTG
ATGGCCGATACCGCGCTGAAGTCAGCCAACGTTGAAGTCGTGGCGTACAGCTCTCCGGCG
CACGGCACCAGCTTTAGTAACGAAGCTATTCTGGTGATTTCCGGCGACTCCGGCGCGGTC
CGTCAGGCGGTGACCTCCGCCCGCGAAATCGGCAAAACCGTCCTTGCGACCCTCGGTTCT
GAACCGAAAAACGATCGCCCGTCCTACATCTGA
>EECEBICD_01328 Propanediol utilization protein PduA
ATGCAACAAGAAGCACTAGGAATGGTAGAAACCAAAGGCTTAACCGCAGCCATAGAGGCC
GCTGATGCAATGGTTAAGTCAGCCAATGTGATGTTAGTGGGCTATGAAAAGATTGGCTCC
GGGCTGGTAACCGTCATCGTGCGCGGCGATGTTGGCGCGGTCAAAGCGGCCACCGATGCA
GGTGCCGCAGCCGCACGCAACGTGGGTGAAGTGAAAGCCGTACACGTCATCCCACGCCCT
CACACCGATGTAGAAAAAATCTTACCGAAGGGAATTAGCCAATGA
>EECEBICD_01329 Propanediol diffusion facilitator

ATGAATGATTCTCTAAAGGCGCAATGTGGTGCGGAATTTTTAGGCACCGGACTCTTTTTG
TTTTTTGGTATCGGTTGTTTGAGCGCGCTCAAAGTCGCAGGCGCCAGCCTCGGATTATGG
GAGATTTGCATTATTTGGGGGCTTGGAATTTCACTGGCCGTTTACCTTACTGCCGGAATC
TCCGGCGGACATCTTAACCCTGCGGTGACGATTGCGCTGTGGCTGTTTGCCTGCTTTCCC
AAACAGAAAGTATTGCCTTATATTATTGCCCAGTTTGCCGGCGCTTTTGGCGGCGCTCTG
CTGGCGTATGTGCTTTATAGTAGCCTTTTTACTGAATTTGAGACTGCGCATCATATGGTG
CGCGGTAGCGTCGAAAGTTTGCAATTAGCCAGCATTTTCAGTACCTATCCGGCCGCAGCG
CTAAATGTATGGCAGGCTGCGTTGGTGGAAGTGGTCATTACGTCGATTCTGATGGGCATG
ATCATGGCATTGACGGATGATGGCAATGGCATTCCTAAAGGGCCGCTTGCGCCTTTGCTT
ATTGGGATATTGGTTGCCGTTATCGGCGCGTCTACTGGACCCCTTACCGGTTTTGCAATG
AACCCGGCGCGTGATTTCGGTCCCAAACTGTTCACATGGTTAGCGGGGTGGGGGAACATG
GCGATGAGCGGTGGGCGAGAGATCCCTTATTTTATCGTGCCGATTGTCGCGCCTGTTATC
GGCGCCTGTGCCGGAGCAGCCATATATCGCTATTTTATTGGTAAGAATTTACCTTGTAAC
CGCTGTGAACTGTAA
>EECEBICD_01330 HTH-type transcriptional activator RhaS
ATGATTTCTGCGAGCGCTCTGAACTCAGAACTCATTAATAAAATCGCACAGGATTTTGCA
CAAGCCACCGGTCTGGCGGTTGTGGTTGTCAATATTCATGGTGATGAAATATCTGAGCTA
TTCAATTTCACGCCGTTTTGTCAGTTGATGCGGCAGCATCCCCAGCATAGTACACGTTGT
CGTATGAGCGATCGCTGCGGGGGACTGGAGGCATCGAAGTCCGATCAACCCTGTATTTAT
CGTTGTCATGCCGGTCTAACGGATTTTTCTATTCCGCTGGTAATCGCAGGGCATTTAGTT
GGCTTTGTTTTATGCGGGCAGGTTCGTTTAAGTAATGACGTGGAGCTGGTAAACATTCTT
AATGTCGACGATCGCTGGCAGGCTGATCCTGAGCTCTTGAATGAATTTCGGAATGTGCCA
GAGATGGACTATTCACGCGTTATCGCTTCCGCCGATCTTCTTAAACTGATAGTTGAGAAT
TGCCTGAAAAAACAGCTCAATTTCGTGGTTATCAAAGACAATCCGCAGCAATCGGAGGCA
AATAAAACCACTCGTGGTCCCACTCCGCATGACAGTAAAATGAAAAAAGCGCTTCGCTAT
ATTGATGCGCATTTGTCCGACGATCTTCGTCTTGAGGATGTCGCCTCACATGTCTATCTC
AGCCCCTATTACTTCAGTAAGCTATTCAAAAAATATCAGGGAATCGGATTTAATGCGTGG
GTAAACCGCCAGAGAATGGTTAGCGCCAGAGAATTGCTTTGCCATAGTGACTGGAGTATT
GCCAGTATTGCGCGTAATTTAGGCTTTTCGCAAACCAGCTATTTTTGCAAAGTCTTCCGC
CAGACCTATCAGGTTACGCCGCAGGCTTATCGCCAGCAGATAAATGAGAATTCTCATCCT
CCATCGTTATGA
>EECEBICD_01331 Cobyrinate a,c-diamide synthase
ATGGCGGCAAGGCATCACGCATTTATTCTTGCAGGCACAGGAAGCGGTTGCGGCAAAACC
ACGGTGACGCTTGGTTTGTTGAGGCTGCTGCAAAAACGCGCGCTGCGCGTACAGCCGTTT
AAAGTTGGGCCAGATTACCTTGATACCGGCTGGCATACCGCTATCTGTGGCGTGGCGTCC
CGTAATCTTGACAGTTTCATGCTTCCTCCTCCTGTTCTCAACGCCTTATTTTGCGAACAA
ATGCGGCAGGCGGATATCGCTGTCATTGAAGGCGTCATGGGGCTGTATGACGGCTATGGC
GTCGACCCCAATTACTGCAGCACAGCCGCGATGGCCAAACAGCTTGGTTGCCCGGTCATC
CTACTGGTGGACGGCAAGGCTGTTTCAACGTCGCTTGCGGCTACCGTTATGGGATTTCAG
CACTTCGACCCGACCCTCAACCTCGCGGGCGTGATTGTGAATCGCGTCACTAGCGACGCC
CACTATCAGCTCCTCAAAAATGCCATTGAACATTATTGCTCACTGCCGGTACTGGGGTAT
GTTCCTCCTTGCGACGGCGTCGCATTACCTGAACGCCACCTGGGGTTGATTACCGCCAGA
GAATCCCTCGTCAATCAGCAATCATGGCATGATTTTGCCGCCTCGCTGGAACAAACAGTG
GATGTGGATGCGCTACTCTCGCTGAGCGTGTTATCAGCGCTTCCGACGGGAATGTGGCCT
GAGCGCCCCGATAACACAGCGGGGGCAGGGCTGACGCTTGCTCTGGCGGATGATGAGGCC
TTTAACTTTTATTATCCCGATAACATCGATTTTCTGGAGCGTGCCGGCGTTAACATTGTG
CGTTTTAGCCCACTGCATGACCGCGCATTGCCTGATTGTCAGATGATCTGGCTGGGCGGT
GGATATCCTGAATTATACGCCGCGGATCTGGCGGCAAATACGGCGATGTTAAAACATTTG
CGGGCGGCGCATCAGCGCGGCGTGGCGATTTACGCGGAGTGTGGCGGTCTGATGTATCTG
GGGAGCACTCTGGAGGATAGCGGTGGGGAAATTCATCAGATGGCGAATATTATCCCCGGT
CACAGCAAAATGGGTAAGCGACTTACCCGCTTTGGCTACTGTGAGGCGCAGGCTATGCAG
CCAACGCTATTGGCGGCGCCTGGCGAGATAGTGCGCGGCCATGAATTTCACTACTCAGAT
TTTATCCCCGAAACACCCGCCGTTATGGCCTGTCGTAAGGTTCGCGATGGCCGCGTGTTA
CAGGAATGGACTGGCGGCTGGCAGACAGGCAACACGTTTGCCAGCTATCTGCACGTCCAT

TTCGCCCAGCGTCCGGAAATGTTGCAGCACTGGCTGGCGGCGGCGAGGCGCGTATTATGA
>EECEBICD_01332 Cobalamin biosynthesis protein CobD
ATGACGATCCTTGCCTGGTGTATCGCCTGGGTGCTGGATTTTATCATCGGCGACCCTCAA
CACTGGCCCCATCCGGTACGCTGGATAGGCCGATTAATTACGTTTGTGCAGCGTATTGTG
CGTCGCTATTGTCCCGGCGATAAAGCGCTGCGGATTGGCGGCGGCGTAATGTGGGTTGTG
GTTGTCGGGGCGACCTGGGGCGTGGCATGGGGCGTACTGGCGCTGGCGCAGCGGATTCAC
CCCTGGTTTGGCTGGAGCGTCGAAGTCTGGATGATCTTTACCACGCTGGCCGGACGCTCT
CTGGCCCGCGCCGCGCAGGAGGTTGAACGACCGTTGCGGGAGAACGACCTTGCGGAAAGC
CGAATAAAACTCTCCTGGATCGTCGGGCGCGATACGTCGCAACTTCAGCCAGCGCAGATC
AATCGCGGCGTGGTGGAAACGGTTGCAGAAAACACCGTTGACGGCATTATCGCGCCGCTC
TTTTTCCTCTTTCTCGGCGGCGCGCCGCTGGCGATGGCCTACAAAGCCGTCAATACCCTG
GATTCAATGGTGGGCTACAAACATGAAAAATACCGGGCGATTGGTATGGTCAGCGCCCGT
ATGGACGACGTAGCGAACTATCTTCCTGCCCGACTGAGCTGGCTGTTGCTGGGCATTGCG
GCAGGACTGTGTCGCCTGAGTGGTTGGCGTGCGCTGCGTATTGGCTGGCGTGACCGTTAT
AACCACAGTAGCCCAAATTGCGCCTGGTCGGAAGCGTGTGTGGCCGGCGCCTTAGGTATC
CAGCTCGGCGGCCCAAATAACTACTTTGGCGAGCGTGTGGACAAGCCCTGGATAGGCGAC
GCGCAGCGCGATATTTCCGTGGACGATATTTCCCGAACGATTCGATTGATGTGGGTCGCT
TCAACCCTGGCGCTGGCGCTGTTTATTGCGGCGCGGTGCGGGTTATCTGGCGTGGCCTGA
>EECEBICD_01333 Cobalt-precorrin-8 methylmutase
ATGCATTATATCCAGCAGCCCCAAACCATTGAGGCCAACAGTTTCACCATTATTAGCGAC
ATTATTCGCGAAACGCGTCCTGATTACCGTTTCGCCAGTCCGCTCCATGAGGCCATTATC
AAGCGGGTTATTCATACCACCGCCGATTTCGACTGGCTGGACATTTTGTGGTTCTCTGCC
GATGCGCTGGAGCAGCTTTGTGATGCGTTACGCCATCCGTGCATTATTTATACCGATACC
ACGATGGCGCTGTCCGGCATTAATAAAAGATTACTGGCGACGTTTGGCGGCGAGTGTCGC
TGTTATATCAGCGATCCGCGCGTGGTGCGCGCCGCACAAACGCAGGGGATTACCCGTTCA
ATGGCCGCCGTCGATATCGCTATCGCCGAGGAGGAGAAAAACAAGCTCTTTGTTTTCGGT
AATGCGCCGACGGCCCTGTTCCGTTTGCTTGAACATAACGTGACGGTGAGCGGCGTGGTG
GGCGTACCGGTAGGCTTTGTCGGCGCGGCGGAGTCCAAAGAGGCACTCACCCATAGCCAT
TTCCCCGCGGTTGCCGCGTTAGGGCGTAAAGGCGGCAGTAACGTGGCTGCCGCTATCGTC
AATGCGCTGCTCTATCATCTCCGGGAGGCCTGA
>EECEBICD_01334 Cobalt-precorrin-5B C(1)-methyltransferase
ATGAGCGAGCTTTCTTTTGACGCGCCGGTCTGGCACCACGGTAAAGCGTTACGTAAAGGA
TATACCACCGGTTCCTGCGCGACGGCGGCGGCAAAAGTCGCCGCGCTGATGGTATTACGT
CAACATCTGATTCATCAGGTCTCCATCGTCACGCCGTCGGGCGTCACGCTATGCCTGAAC
GTGGAGTCGCCGCACATTGAAGGCCAGCAGGCGATAGCCGCAATTCGTAAAGATGGCGGC
GATGACGTGGATGCCACGCACGGAATGCTGATTTTTGCCCGCGTTACGCTCAACGACAGC
GGTGAGATTACGCTCACTGGCGGCGAAGGTATTGGTACGGTAACGCGTAAAGGGATTGGA
CTGCCGCTCGGGAGCGCCGCCATCAATCGTACGCCGCGCCATACCATTGAGTCAGCGGTG
CGCGAAGCGATAGGCCCGGCGCGTGGGGCCGATGTGGAGATTTTTGCCCCGGAAGGCGAA
GTGCGGGCGCAAAAAACGTATAACTCGCGGCTTGGCATCCTTGGCGGCATTTCCATTATT
GGCACTACCGGCATTGTGACGCCGATGTCGGAAGAAAGCTGGAAACGCTCACTATCGCTG
GAACTGGAGATCAAACGGGCGTCAGGATTAACGCGGGTGATACTCGTGCCGGGCAACCAC
GGCGAACGGTTTGTTCGCGAACAAATGGGCGTCGACACACAGGCAGTCGTCACCATGAGC
AATTTTGTCGGCTACATGATTGAAGAGGCGGTACGGCTGGGATTTTGCCAGATAGTGCTG
GTGGGGCATCCTGGAAAACTGATCAAAATCGCCGCCGGGATCTTTCATACCCATAGCCAT
ATTGCCGATGCGCGCATGGAAACGCTGGTCGCGCATTTAGCTTTACTGGGCGCGCCGCTG
GAGTTACTCACCCTGGTCGGCGATTGCGATACCACTGAAGCGGCAATGGAGCACATTGAA
GCATATGGCTTCGGGCATATCTATAACCATCTTGCCAGGCGTATTTGTTTGCGAGTCATG
CAGATGCTGCGTTTTACCAAAACGCCGCCTGTCTGCGACGCCATCCTGTTTTCTTTTGAT
AACCATATTCTCGGCAGTAATCGTCCCGTCGACGAGATTGCTAAGGAGCTGCAATGCTAA
>EECEBICD_01335 Cobalt-precorrin-7 C(5)-methyltransferase
ATGCTAACGGTCGTGGGAATGGGGCCCGCCGGACGGCATTTGATGACCCCTGCGGCGCTG
GAAGCGATAGACCACGCCGATGCGCTGGCAGGCGGTAAACGGCATTTGGCGCAGTTTCCG
GCGTTTGGCGGCGAACGATTTACGCTGGGCGCGGATATTGGCGCGTTGTTGTCCTGGATT

GCCGCCCGCTGGGATAAAGGGATCGTGGTGCTGGCCTCCGGCGATCCGCTGTTTTATGGC
ATCGGTACACGCCTGGTGGCGCATTTTGGTATCGAACAGGTTCGCATTATCCCTGGCATC
AGCGCCGTACAGTATCTGTGCGCGCAGGCTGGAATTGATATGAATGACATGTGGTTGACC
AGTAGCCACGGTCGTTGCGTCAGCTTCGATCAGTTGGCGAGCCATCGCAAAGTGGCGATG
GTCACGGATGCCCGCTGCGGGCCGCGAGAAATCGCGCGGGAACTGGTCGCGCGGGGCAAA
GGGCATCGCTTAATGGTGATTGGTGAAAATCTGGCGATGGAAAATGAGCGGATCCACTGG
CTACCCGTCAGTGCGGTCAACGCCGACTATGAAATGAATACAGTGGTGATCCTGGATGAA
AGATGA
>EECEBICD_01336 Cobalt-precorrin-6B C(15)-methyltransferase
(decarboxylating)
ATGAAAGATGAGCTTTTTCTGCGCGGCGAGAACGTGCCAATGACCAAAGAGGCGGTACGG
GCGCTGGCGCTGTCGAAACTTGAGCTCCACCGCGCCAGTCACCTGATTGACGTCGGGGCG
GGAACGGGAAGCGTCTCAATTGAAGCGGCGCTGCAATTTCCTTCGTTGCAGGTGACGGCT
ATCGAGCGTAATCCTGCCGCGCTACGGCTGCTTGATGAGAATCGTCAGCGCTTTGCCTGC
GGCAATATCGATATTCTCCCCGGCGAAGCGCCAATGACCATAACCGGGAAAGCCGATGCG
GTGTTTATGGGCGGCAGCGGCGGACATCTGACGGCGCTGATTGACTGGGCGATGGGCCAT
CTGCATCCCGGTGGCCGTCTGGTGATGACCTTTATCTTGCAGGAAAACCTTCATAGCGCG
CTGGCGCATCTTGCGCACATCGGCGCATGTCGAATGGACTGTGTACAGCTTCAGCTTTCA
TCGTTAACGCCGCTGGGGGCGGGGCACTATTTCAAACCGAACAATCCTGTTTTTGTTATC
GCCTGTCAGAAGGAAGAAAACCATGTCAGAGACATTTGA
>EECEBICD_01337 Cobalt-precorrin-4 C(11)-methyltransferase
ATGTCAGAGACATTTGATCCACGCTGTGTATGGTTCGTCGGCGCCGGGCCGGGCGATCGC
GAGCTGATCACGCTTAAAGGCTACCGCCTGCTGCAACAGGCGCAGGTAGTTATCTATGCC
GGTTCGCTGATCAATACCGAACTGCTGGATTACTGCCCCGCGCAGGCAGAACGCTACGAC
AGCGCCGAACTGCATCTTGAGCAGATTATCGAGCTGATGGCGGCGGGCGTGAAAGCGGGA
AAAACGGTGGTGCGTTTGCAAACGGGCGATGTCTCGCTGTACGGCTCCGTACGTGAGCAA
GGCGAAGAGTTAACCCGCCGTGGTATCGACTGGCAGGTAGTGCCTGGCGTCAGCGCGTTT
CTCGGCGCTGCGGCGGAGCTGGGTGTGGAATATACCGTACCGGAGGTATCGCAGAGTCTC
ATTATTACCCGTCTGGAAGGGCGTACGCCAGTACCGGCGCGCGAGCAACTGGAGGCGTTC
GCCAGTCACCAGACGTCGATGGCGATTTATCTCTCCGTTCAGCGTATTCACCGAGTGGCG
GAACGTCTCATTGCCGGCGGTTATCCGGCAGCTACGCCTGTCGCGGTCATCTATAAGGCT
ACCTGGCCGGAAAGCCAGACCGTTCGCGGCACGTTAGCGGATATTAGCGACAAAGTACGT
GATGCTGGCATCCGTAAGACGGCGCTGATTCTGGTCGGGAATTTTCTGGGTAAGGAGTAT
CACTACTCAAGACTTTATGCAGCGGACTTTAGCCATGAATACCGTAAAGCCTGA
>EECEBICD_01338 Cobalt-precorrin-5A hydrolase
ATGAATACCGTAAAGCCTGAATCCATTGCGTTGTTTTGCCTGACGCCCGGCGGCGTGGCG
CTGGCAAAGAGACTCTCCGCGATGCTGCCGTTAACCTGCTTTACCAGTGAAAAACTGCGG
GAAGAGGGATTTATTCCCTTCGATGGCGGATTCGCTAATACCGCCCGGCAGGCTTTTACC
ACTTATACCGCGCTTATTTTTATCGGCGCGACCGGCATTGCCGTTCGTGTCCTGGCGCCG
TTAGTGAACGACAAGTTCAGCGATCCGGCGGTGGTCGTCATTGATGAACGAGGTCAGCAT
GTCATTAGCCTGCTTTCCGGTCATGCGGGCGGGGCCAATGCCTTGACGCGCTACCTGGCA
GGAATGTTAGGCGCCGATCCGGTGATTACCACGGCAACGGATGTCAATGAGATGTCCGCG
TTGGACACCTTAGCTTTCCAGCTTAACGCCCGCATGTCCGATCTTCGCACGGCGGTAAAA
ACCGTTAACCAGATGTTGGTCAGTCATCAACGTGTGGGGTTATGGTGGGATGCCGAACTA
ACGGAAGAGATCGGCCAGTGCGATATTCGCGGTTTTATCCCTGTTGATGATTTGCAGCGG
TTGCCTGAGCTGGATGCGCTTATCTGCGTCTCTTTGCGTAATGACCTCCCTGAGCTTCCC
GTACTGCACTGGAAACTGGTGCCCCAGCGTGTGGTGGCGGGAATTGGCTGTCGCCGCGAT
ACGCCATTTCCCCTGTTAGCGACATTACTGGCGCGTCAGCTTGAAGCGCAGAAACTCGAT
CCGCTGGCGTTAAAAGCGATTGGCAGCGTCACGCTCAAAAAAGGGGAGCCGGGGCTTATT
CAGCTCGCCTCCTGCTGCCGCGTGCCTTTTAAAACCTTTACCGCCGAAGCGTTGCGTGAA
TTCGAACACCATTTTCCCGGTTCTGGCTTCGTCAGAAAAACGGTGGGCGTTGGCAGCGTA
TCCGGCCCGGCAGCGTGGTTATTAAGCCAGGGACAATTGTTAGGCGAGACCCTGCGAGAA
CAGGGCGTCACTATTACTTTGGGAGTTTCACACTGA
>EECEBICD_01339 putative cobalt-factor III C(17)-methyltransferase

ATGTTAAGCGTAATCGGAATTGGCCCTGGCTCACAGTCAATGATGACCATGGAAGCGATT
GAGGCGTTACAGGCGGCGGAAATCGTCGTGGGCTACAAAACCTATACCCATCTGGTTAAA
GCATTCACCGGCGATAAGCAGGTGATCAAGACCGGCATGTGTAAGGAAATTGAACGCTGC
CAGGCGGCTATTGAACTGGCGCAGGCTGGGCATAACGTAGCGCTCATCAGCAGCGGGGAT
GCGGGAATTTACGGCATGGCGGGTCTGGTGCTGGAACTGGTCGGCAAGCAAAAACTGGAC
GTCGAGGTGCGGCTCATTCCTGGCATGACCGCCAGCATCGCCGCCGCCTCTTTACTGGGC
GCGCCGTTGATGCATGACTTCTGTCATATCAGCCTCAGCGACCTGCTGACCCCGTGGCCG
GTTATTGAAAAACGTATTGTTGCCGCCGGAGAGGCAGACTTTGTTATCTGTTTTTACAAC
CCGCGCAGCCGCGGGCGCGAAGGGCATCTGGCGCGCGCGTTTGACCTGCTTGCCGCCAGT
AAAAGCGCGCAAACGCCGGTTGGCGTGGTGAAATCCGCCGGACGTAAGAAAGAGGAAAAA
TGGCTGACTACGCTCGGCGATATGGATTTTGAACCGGTAGATATGACCAGTCTGGTCATC
GTTGGTAATAAAACGACCTATGTTCAGGATGGGCTGATGATAACGCCGCGAGGTTACACG
TTGTGA
>EECEBICD_01340 Cobalt-precorrin-6A reductase
GTGAATGAGGGGGAGGTGCTGGTGGTGGGCGGCACCAGCGATGCGCGCGCGTTATGCCGA
CAACTGGATGCGGCGAACGTCGCGTACACCTTATCCGTGGCGACGCCAGCCGGAAAAGCG
CTGGCTGGCGACATTAAAGGACAGGTGCGCTGTGGTCGTCTGGAGTACGGGCAGATGGTC
GCCTGGCTAAAAGAAAACCGTACTCGCTGGGTAATAGATGCATCGCATCCTTATGCAGAA
ATGGTCAGCCATAACCTCCTTCGCGCCTGTGAAACGGCGGGCGTGTTGTTAAGTCGTTAT
CAGCGTCCGGAGCAGCTTAGCAATCTGACGCATCCGCTTCTCTATACGGCGCGCAGTATT
GCCGATGCCTGCGAGATAGCGAGGCGTTTTGGTCCGCGCGTGTTACTGACGACCGGCAGT
AAAGATCTGGCCGTCTGGCGTGCCGGGCTGGCGGAAAAAACGTTGCTGGCGCGCGTACTG
CCCGTGGCGGAGGTGATTCAACGCTGCAGCGAACTGGGGTTCGGCGTTGGAGAAATTTTC
GCACTGTGTGGGCCGTTCAGCGCTGATTTTAACGCCGCGTTTTACCACCAGTGCCGGGCC
GACGTAGTCATTACCAAAGCATCCGGCGCGGAGGGGGGATATCAGGAAAAAGTTCAGCCC
TGTCTGGATGCGGGCATTCCCTGCATCGTTATTGCGCGCCCGACGCCGCTTGTGACGGGT
GATGAGTTACTGGAAAGCCAGGCCGCCTTTGCGCAGCGTTTATCGCGCTGGCTGGCCGCA
GCTAAGGAGTAA
>EECEBICD_01341 Sirohydrochlorin cobaltochelatase
ATGAAAAAAGCGCTTCTGGTGGTCAGTTTTGGCACCAGCTATCACGACACCTGTGAAAAA
AACATTGTGGCGTGCGAACGTGATCTGGCGGCGTCCTGTCCCGATCGCGACCTGTTTCGC
GCCTTCACTTCCGGAATGATCATTCGCAAGCTTCGCCAGCGTGACGGTATCGACATCGAT
ACGCCATTACAGGCTTTGCAAAAGCTTGCCGCACAGGGATATCAGGACGTGGCGATTCAG
TCGTTGCACATTATTAACGGCGACGAATATGAAAAAATTGTCCGTGAAGTACAACTCTTG
CGTCCGTTATTCACTCGCCTGACGCTGGGCGTGCCGTTGCTGAGCAGCCATAACGATTAT
GTGCAGCTAATGCAGGCGTTGCGACAACAGATGCCATCGCTCAGACAAACGGAGAAAGTC
GTTTTTATGGGCCACGGCGCCAGCCATCACGCATTCGCAGCTTATGCCTGTCTCGATCAT
ATGATGACCGCGCAGCGCTTTCCGGCGCGCGTCGGCGCCGTAGAAAGCTACCCGGAGGTC
GACATCCTGATCGACAGCCTGCGTGACGAAGGCGTCACCGGCGTACATTTGATGCCATTA
ATGCTGGTGGCGGGCGATCACGCCATTAATGATATGGCTTCAGACGACGGCGACTCATGG
AAAATGCGCTTTAACGCGGCGGGCATTCCGGCGACGCCGTGGCTGAGCGGGCTGGGAGAA
AACCCGGCTATCCGGGCGATGTTTGTCGCGCATTTGCGCCAGGCGCTGAACATGACGGTA
GAGGAGGCGGCATGA
>EECEBICD_01342 Cobalt-precorrin-2 C(20)-methyltransferase
ATGAACGGTAAATTATACGCATTAAGTACCGGGCCAGGCGCGCCCGACCTGATAACCGTC
CGCGCAGCGCGGATTCTTGGCTCGCTGGACATTCTGTATGCGCCCGCCGGTCGTAAGGGG
GGCGATAGCCTGGCGCTGTCGATTGTGCGCGACTATCTCGGCGAGCAGACGGAAGTACGT
TGTTGCCATTTCCCTATGAGCGCTGACGGCGCCGAGAAAGAAGCGGTCTGGAACGAGGTT
GCCGCCGCGCTTACCACAGAGGTGGAAGCGGGTAAACAGGTCGGTTTTATTACCCTGGGC
GATGCGATGCTATTCAGTACCTGGATTTTTTTACTCCAGAGAATCGGCTGCCCGGAGTGG
CTGGAGATTGTCCCCGGCGTCACGTCCTTCGCCGCGATAGCCGCCCGCGCGAAAATGCCG
CTCGCCATAGAACGGCAATCGCTGGCGGTTATTTCTTGTACCGCGCCGGAGGCGGAAATA
GCCCAGGCGCTACAACAGCATGATAGCCTGGTGTTGATGAAAGTGTATGGCCGATTCGCC
CGTATTAAAGCGTTGCTGGCGCAGGCTGGCCTGCTGGAATGCGCCTTAATGATGTCCGAG

GCCACGCTGCTCGGCGAGCAGTGCTGGCGCCACCTCCACGAGGTCAACGATGATCGAGCG
TTGCCCTATTTTTCGACCATTCTGGTCAATAAACAGTGGGAGTATGCAGAATGA
>EECEBICD_01343 Cobalt transport protein CbiM
ATGAAACTTGAACAGCAGCTTAGACAGTTGTCTTTCAGTGGACTGGCTGCAGCGCTATTG
CTGATGGTTGTCCCGCAACAGGCGTTTGCGATGCATATCATGGAGGGCTTTTTACCGCCA
GTGTGGGCGCTGGCCTGGTGGTTACTGTTTTTACCCTGCTTGTGGTATGGGCTGGTACGC
TTACGGCGTATTGTGCAGGAGGATAATCATCAGAAGGTACTGCTGGCGCTGTGCGGCGCG
TTTATTTTTGTCCTGTCCGCGCTCAAAATTCCGTCGGTAACGGGGAGTTGTTCACATCCG
ACCGGCGTTGGTCTGGCGGTTATCTTGTTCGGGCCTGGGGTGGTGGCGATTCTCGGCGCG
GTTGTGCTGTTGTTTCAGGCGCTATTGCTGGCGCATGGCGGCCTGACGACGCTTGGCGCA
AATGGAATGTCGATGGCGGTGATTGGCCCGGTTGTCGGTTATCTGGTGTGGAAAATGGCC
TGCCGTGCCGGACTGCGCCGCGATGTCGCCGTTTTTCTGTGCGCGATGCTGGCGGATTTG
GCGACCTATTTTGTGACTTCAGTCCAGCTTGGCGTTGCGTTTCCAGATCCCCATGCCGGA
GCGACAGGGTCTGTTGTGAAGTTTATGGGGATTTTCTGCCTCACACAGATTCCTGTCGCT
ATTGCTGAAGGTTTATTAACCGTCATGATTTATGACCAGTTAACCAAACGGCAAGTGATT
ACCGTACAAGGACATTAA
>EECEBICD_01344 Cobalt transport protein CbiN
ATGAAAAAAACGTTGATGTTGTTAGCGATGGTGGTGGCGTTGGTGATTCTGCCGTTTTTT
ATTAACCATGGCGGTGAATATGGCGGTTCAGACGGCGAAGCGGAAAGCCAGATTCAGGCT
ATTGCGCCGCAGTATAAACCCTGGTTTCAGCCGTTATATGAACCTGCCAGCGGTGAAATT
GAAAGCCTGCTGTTTACCCTTCAGGGCTCTCTTGGCGCGGCGGTGATTTTTTATATCCTG
GGGTATTGCAAAGGTAAACAACGCCGTGATGACCGGGCTTGA
>EECEBICD_01345 Cobalt transport protein CbiQ
ATGACCGGGCTTGATCGACTCAGCTATCAAAGCCGCTGGGCACACGTTGCGCCGCAGCGC
AAGTTTTTGCTGTGGCTGGCGATGATAATACTGGCGTTTGTTCTGCCGCCTGTAGGGCAG
GGCATTGAGCTGCTGATCATTGCCGGATTGAGCTGTTGGCTATTGCGCATATCCCTCTGG
CGCTGGTGTCGATGGATGGCGATACCTTTTGGATTTTTGTTGGTCGGCGTGATAACGATT
ATCTTCAGCATCAGCCGCGAGCCGCAAATGCTGCTGGCTGGCATATCTGTTGGGCCGTAC
TGGATCGGGATTACCCGTGCGGGCGTGGTTACGGCGAATGAAACGTTCTGGCGTAGCCTG
ACGGCGCTGTCCGCGACGTTGTGGCTGGTGATGAACCTACCGTTTCCGCAGTTAATTAGC
TTGCTTAAGCGCGCGCATATTCCACGCCTGCTTACCGAGCAAATCCTGTTAACGTGGCGC
TTTCTCTTTATTCTTCTGGATGAGGCGGCGGCAATTCGTCGCTCGCAAACGCTGCGTTTT
GGTTATTGCAGCCTGCCGAACGGCTATCGATCTCTGGCAATGCTGACTGGACTCCTGTTT
ACGCGGGTGTTAATGCGTTATCAGCAAATGACTACCACGCTGGATATCAAACTGTATCAG
GGTGATTTTCACCTGTAA
>EECEBICD_01346 Cobalt import ATP-binding protein CbiO
ATGCTTGCCACTTCAGACCTGTGGTTCCGATATCAGGATGAGCCGGTACTTAAAGGGTTA
AATCTGGATTTTTCGCTCTCGCCTGTTACCGGTTTGGTGGGCGCGAACGGCTGTGGGAAG
TCTACGCTTTTTATGAACCTGAGCGGCCTGTTGCGTCCGCAAAAAGGGGCCGTGTTGTGG
CAGGGAAAACCGCTGGATTACAGCAAGCGCGGACTACTGGCGCTGCGCCAGCAAGTTGCG
ACGGTTTTTCAGGACCCTGAACAGCAGATTTTTTATACTGATATCGATAGCGACATTGCG
TTTAGTTTACGTAATTTGGGGGTGCCGGAGGCGGAAATCACGCGCCGCGTCGACGAGGCG
CTAACGCTGGTTGACGCCCAACATTTTCGCCATCAGCCCATTCAGTGTTTGAGTCATGGG
CAAAAAAAACGTGTGGCTATCGCCGGAGCGCTGGTGCTACAGGCGCGCTATCTATTGCTG
GATGAACCCACTGCTGGTCTCGATCCCGCAGGGCGCACTCAGATGCTTGCTATCATCAGG
CGGATTGTGGCGCAGGGCAATCATGTCATTATCTCCAGCCATGATATCGATCTTATTTAT
GAAATTAGCGATGCCGTATACGTGTTACGCCAGGGCCAGATCCTGACGCATGGCGCGCCT
GGCGAGGTTTTCGCGTGTACGGAAGCGATGGAACAAGCGGGGCTAACCCAGCCCTGGCTG
GTCAAACTTCATACTCAACTGGGACTGCCGCTGTGTAAAACAGAAACGGAGTTTTTTCAT
CGAATGCAAAAGTGCGCATTCAGGGAGGCGTCATGA
>EECEBICD_01347 Cobyric acid synthase
ATGACGCAGGCAGTTATGTTGCAGGGGACGGCATCCGACGTCGGCAAAAGTGTGCTGGCG
GCGGGTTTATGCCGCATTTTTTATCAGGATGGTCTGCGTACCGCGCCGTTTAAATCGCAA
AATATGGCGCTTAATTCTGGTATTACGTCGGATGGTAAAGAGATGGGGCGGGCGCAGATT

TTTCAGGCGGAAGCCGCGGGGATCACGCCAGATGTGCGTATGAACCCGGTGCTGCTCAAA
CCGACCAGCGATCGCCAGGCGCAGGTCGTGCTGATGGGGAAAGTGGCGACCAATATGGAT
GCGGTGAGTTACCATGACTATAAACCGCGTTTGCGTGAGCAAATCCTTGCGGTCTATAAC
AGCCTGGCGCAGGAATATGACGTTATTGTGCTGGAAGGCGCCGGAAGCCCGGCGGAAATC
AATCTGCGCGATCGCGATATCGTCAATATGGGAATGGCGGAAATGGCCCAGTGTCCGGTT
ATTCTGGTGGCGGATATCGACCGAGGCGGCGTATTCGCCGCTATTTATGGCACGCTGGCG
CTGCTGCATAAACAGGAGCGTGACAGAGTAAAGGGCGTCATTATCAACAAATTCCGCGGC
GACGTGGCGCTACTCTATTCCGGTATCGAACAAATCGAATCGCTTACCGGCGTCCCGGTC
CTGGGAGTGATGCCGTGGCTGGATGTGGATCTGGAAGATGAAGATGGCGTCGCGCTGCAA
AACGATAAATATCGCGGTAACGCCCCCCGTGATATCACTATTGCCATTGTGCAACTGCCA
CATATTTCTAACTTTACGGATTTTAACGCTCTGGCGGCGCAGCCGGATGTGCGCATACGC
TATATTCGCCGCCCGGAAGCGTTGACTGACGCTGATCTGGTGATCCTGCCGGGCAGTAAG
AATACGCTGAGCGACCTCGCCTGGCTGCGCGAAAGCGGGATGGCGGATGCGCTATTGCAG
ACACATCGGCAGGGCGTGCCGGTGATGGGAATTTGCGGTGGCTACCAGATGCTGGGCGAC
ACTATTGTGGATGAGGTGGAGTCGGGGCTGGGTACGCAGCCGGGCCTGGGGCTGCTTAAT
ACGATCACTCGCTTTGCACAGGATAAAATCACGACGCAGGTTAACGCGACAATGTCAGGC
GAGCTGCCCAGCTGGCTGGCTGCCGCGGCGGGGCTACCGGTACGCGGTTATGAAATCCAC
ATGGGGGAAACGGTGCTGCAGGAAGGATGCTGTACGGCCATGACGTTACAGAAAAATGGA
TGCTCTGTGGCGGATGGCGCGGTCACCGCCGACGGACTGGCGTTTGGCACTTACCTTCAC
GGCCTGTTTGACAGCGATGCGTTTACCCGTGCGGTGGTCAATGGGTTACGGGCACGAAAA
GGGCTTGCGCCGTGGGAAACGACTTTTTGTTATGCGGAGCATAAAGCGCGGCAATTTGAT
CTGCTGGCGGAAGCGATGCGGCAACATATTGATATTGATAAAATTTATACCATCATGCAA
CAGCATCAGGAGCCGGTATGA
>EECEBICD_01348 Bifunctional adenosylcobalamin biosynthesis protein CobU
ATGATGATTCTGGTGACGGGCGGGGCGCGTAGTGGTAAAAGCCGTCATGCTGAAGCCTTA
ATTGGCGATGCGCCGCAGGTACTGTATATCGCCACCTCGCAGATTCTTGATGACGAGATG
GCGGCGAGAATTCAGCATCATAAAGATGGCAGGCCGGCACACTGGCGGACCGCAGAATGC
TGGCGGCATCTTGATACGTTGATTACCGCGGATCTTGCCCCTGACGACGCGATTTTGCTG
GAATGTATTACCACCATGGTGACGAATCTGCTGTTTGCGCTGGGAGGCGAAAACGATCCC
GAACAGTGGGATTACGCGGCGATGGAGCGCGCCATTGACGATGAAATTCAGATTTTAATT
GCAGCCTGCCAGCGCTGCCCGGCGAAAGTGGTACTGGTGACAAATGAGGTGGGAATGGGG
ATCGTCCCGGAAAACCGTCTGGCGCGCCATTTTCGTGATATTGCCGGTCGGGTCAACCAA
CGACTGTCGGCAGCGGCGGATGAGGTCTGGCTGGTGGTCTCAGGTATTGGAGTCAAAATT
AAATGA
>EECEBICD_01349 Adenosylcobinamide-GDP ribazoletransferase
ATGAGTAAGCTGTTTTGGGCCATGCTCGCTTTTATTAGCCGCTTGCCCGTGCCGTCACGC
TGGTCGCAGGGACTGGATTTCGAGCAGTATTCGCGTGGGATCGTGATGTTTCCCTTTATC
GGGTTGATTCTGGGAGGGGTAAGCGGCCTGATTTTCATCCTCCTGCAACCCTGGTGCGGT
ATTCCGCTAGCGGCGTTGTTCTGTATTCTGGCGCTGGCGTTGCTGACCGGTGGTTTTCAC
CTGGATGGGTTGGCCGATACCTGCGATGGTATTTTTTCCGCGCGCCGCCGTGAGCGAATG
CTGGAGATTATGCGTGATAGTCGTCTGGGAACCCATGGCGGGCTGGCGCTTATTTTTGTG
CTACTGGCAAAAATTCTGGTGGTCAGCGAACTGGCGTTACGTGGAACGCCAATGTTGGCG
GCGTTGGCGGCGGCCTGTGCGGCCGGACGCGGCAGCGCCGTTTTATTGATGTACCGTCAT
CGCTACGCCCGTGAAGAGGGGCTTGGCAATGTATTTATCGGTAAAGTCAGCGGACGGCAA
ACCTGCATTACTCTGGGATTGGCGGTAATCGTCGCCACGGTATTACTGCCTGGTATGCAA
GGGTTGGCCGCAATGGTGGTCACATGCGCGGCGATTTTCATCCTTGGCCAGTTGCTTAAA
CGTACGCTTGGCGGTCAAACCGGCGATACGCTGGGCGCGGCGATCGAACTTGGTGAATTG
ATCTTTCTGCTGGCTCTGTTATGA
>EECEBICD_01350 Nicotinate-nucleotide--dimethylbenzimidazole phosphoribosyltransferase
ATGCAGACACTACACGCTTTACTCCGTGACATTCCTGCGCCGGACGCCGAGGCGATGGCG
CGTGCGCAGCAACATATTGACGGCCTGCTCAAACCGCCGGGCAGCCTGGGCAGACTGGAA
ACCTTAGCCGTTCAGCTCGCGGGTATGCCGGGTCTTAACGGTACGCCGCAGGTAGGTGAA
AAGGCGGTGCTGGTGATGTGCGCCGACCATGGCGTCTGGGATGAAGGCGTAGCGGTTTCG

CCCAAAATCGTGACGGCGATTCAGGCGGCGAATATGACGCGGGGAACAACCGGCGTATGC
GTGCTTGCCGCTCAGGCCGGTGCGAAGGTGCATGTCATTGATGTCGGTATTGATGCCGAA
CCTATTCCTGGCGTAGTTAATATGCGCGTCGCGCGCGGCTGCGGAAATATTGCCGTTGGC
CCGGCGATGAGTCGCTTGCAGGCTGAGGCGCTTTTGCTGGAGGTTTCCCGCTACACCTGC
GACCTGGCGCAACGCGGCGTGACCTTATTTGGCGTAGGGGAGCTGGGAATGGCGAACACT
ACGCCAGCCGCCGCGATGGTCAGCGTTTTTACAGGAAGTGATGCAAAAGAGGTGGTGGGG
ATTGGCGCGAATCTTCCGCCTTCCCGCATCGATAATAAAGTGGACGTCGTGCGGCGGGCG
ATTGCGATTAATCAGCCCAATCCGCGCGACGGCATTGATGTGTTGTCGAAGGTGGGTGGT
TTTGATCTGGTCGGGATGACCGGCGTGATGCTTGGCGCGGCAAGGTGTGGCTTACCCGTA
TTGCTGGATGGCTTTCTTTCCTACTCGGCAGCGCTGGCGGCCTGTCAGATTGCGCCTGCG
GTGAGACCTTATCTGATCCCGTCGCACTTTTCGGCGGAAAAGGGCGCCCGCATCGCGCTC
GCGCATTTGTCTATGGAACCCTATTTGCATATGGCGATGCGGTTAGGTGAAGGAAGCGGC
GCGGCGCTGGCGATGCCGATCGTGGAAGCCGCGTGCGCCATGTTCCACAACATGGGGGAG
TTGGCGGCCAGTAATATTGTCCTGCCGGAGGGGAACGCAAACGCAACATAA
>EECEBICD_01351 putative L,D-transpeptidase ErfK/SrfK
ATGCGTCGTATCACACCTTTTTTTCCGTTTTTCGTCTTGTTGGTAAGCCATTTCTCGCTG
GCTATAAGCTATCCGTTGCCGCCCGAAGGCAGTCGTCTGGTAGGACAGCCTGTCACAATC
GCCGTACCGCAGAACAATACTCAGCCCTTAGAGTCATTTGCCGCCCGCTACGGTCAGGGG
CTGAGTAATATGCTTGAAGCGAATCCCGGCGTGGATGTGTTTTTACCTCAGTCTGGTTCC
ACACTGGTGGTGCCGCAGCAGTTGATTCTGCCTGACACCGTTCGTGAAGGTATTGTGGTA
AACGTCGCCGAGATGCGCCTTTATTATTATCCCGCAGGCACGAATACGGTTGAGGTGCTA
CCTATTGGTATTGGTCAGGCTGGCCGTGAAACGCCGCGCAACTGGGTAACTGCGGTTGAA
CGCAAACAAGATGGCCCGGTTTGGGTGCCAACGGCTAATACGCGTCGGGAATATGCTAAA
GAGGGAAAAACGCTGCCCGCGATGGTGCCTGCGGGGCCGGATAACCCTATGGGGCTATAC
GCAATATATATCGGCAGGTTATATGCCATCCATGGCACTAACGCTAACTTTGGCATTGGC
CTTCGCGTCAGTCAGGGCTGTATCCGTTTACGCAATGACGATATTAAATATTTATTCGAC
CATGTTCCTGTAGGAACCCGCGTTCAGATTATCGACAGGCCCGTTAAATTTTCGGTAGAG
CCGGATGGCAGTCGTTGGCTGGAGGTACACGAACCGCTTTCTCGCAACCGTGCTGAATTT
GAGTCAGACAAAAAAGTTCCATTGCCCGTTACGCCAGTGCTGCGCACGTTTATCAAAGGG
GATGACGTTGATACTTCGCGGGTTAATGAGGTGCTGGAGCGCCGTTCCGGAATGCCGGTC
AACATTAGCGCGGGCAGGCCTGGCCTTTAA
>EECEBICD_01352 tRNA-Asn(gtt)
TCCTCTGTAGTTCAGTCGGTAGAACGGCGGACTGTTAATCCGTATGTCACTGGTTCGAGT
CCAGTCAGAGGAGCCA
>EECEBICD_01353 putative FMN/FAD exporter YeeO
TTGTTGCATTTGCAGACATTTTTTCTGCAAAAAGCTTTGCAGCCTTACGATTTCTCGCTC
ATTATTGAAATGTGGTTTCAACTCTCTTCTTTAAGGTCCATTTTGAACGTCTCCACTGCC
TTACGCCAGGCTGTCGTCCGTACGCCCTGGTATGCCAAACGCAAGAGCTACAAAGTGTTG
TTCTGGCGTGAAATCACCCCACTTGCTATCCCTATTTTTCTGGAAAATACCTGTGTTTTG
CTAATGGGCGTGCTCAGTACTTTTCTCGTCAGTTGGCTGGGCAAGGAAGCAATGGCGGGC
GTGGGGCTTGCCGACAGTTTTAATATGGTAATTATGGCTTTTTTTGCGGCTATCGATCTT
GGTACTACGGTGGTGGTTGCCTTTAGCCTGGGCAAGCGCGACAGGCGACGGGCAAGGGCG
GCGGCGCGCCAGTCGCTGGTGATTATGACGCTATTTGCCGTTGTGCTGGCAGTGGTCATT
CATTATTTCGGCAGTGAAATTATTAATATTGTCGCAGGCGAGGCGACGTCAGAAGTAAAG
GGGCTGGCGTTAACGTACCTTGAACTGACAGTGCTGAGTTATCCGGCTGCGGCAATTGCG
CTAATCGGTAGCGGCGCGCTGCGTGGGGCAGGGAATACGAAAATCCCGTTGATGATTAAC
GGCGGGATGAACATTCTCAATATTATCATCAGCAGCATCCTGATTTACGGGGCTTTCTCC
TGGCAAGGGCTGGGTTTTGCCGGCGCGGGGCTGGGATTAACCATTTCGCGCTACATCGGC
GCAGTAGCGATTATTTGGGTGCTGATGATTGGTTTTAATCCGGCGCTGCGCATTCCGCTG
AAAAGCTATCTGAAGCCGCTGAATTTCGGCATTATCTGGGAAGTGATGGGTATCGGTATT
CCGGCGAGCATTGAATCAGTGCTGTTCAACGGTGGCAAGCTACTGACGCAAATGTTTGTC
GCCGGAATGGGCACTAACGTTATTGCGGGTAACTTTATTGCCTTTTCCGTGGCGGCGCTT
ATCAACCTGCCGGGTAACGCCCTTGGTTCGGCGTCGACTATTATCACCGGTAAGCGTCTT
GGTACCGGACAAATTGGTCAGGCAGAGCGTCAATTGCGCCATGTATTCTGGATGTCGACT

ATCGTACTTACGGCAATCGCCTGGGGGACAGCGCCGTTCGCGGGTCTGTTTGCCTCATTT
TATACCCAGGAGCAGGACGTAAAAGAGGTAGTGAAAGTTCTGCTCTGGCTTAATGCTGTC
TTTATGCCAATTTGGGCGGCCGCGTGGGTGTTGCCGTCGGGTTTTAAAGGCGCGCGCGAT
GTGCGATTTGCGATGTGGGTATCGATGCTGGGGATGTGGGGCTGTCGCGTTGTGGCAGGG
TATACGCTTGGTATTGTGCTGGGTATGGGGGTTGTAGGGGTTTGGCTGGGGATGTTTCTT
GACTGGGCCGTGCGCGGCGCGCTGTTTTACTGGCGTCTGGTAAGCGGGCGCTGGCTGTGG
AGATACCCGCGCGTAAAGAGGGAGTAA
>EECEBICD_01354 tRNA-Asn(gtt)
TCCTCTGTAGTTCAGTCGGTAGAACGGCGGACTGTTAATCCGTATGTCACTGGTTCGAGT
CCAGTCAGAGGAGCCA
>EECEBICD_01355 hypothetical protein
ATGGTAGACAATATTGAACAGCAAGTTTACGAGCTTGTACGGCCTTATGCTGGCACATAT
CTGTTCAACATTAAGCAAGTGGAATTAACACCAGAGATTGACCTTGATACTGATCTGAGC
ATTGATGAACTTGAAGCCGAAGATCTCATGAATAAATTCTTCGAGAAACTCAATGTCGAG
CGAGGTAACTTCAGAATAGAAACCTATTTTCCTAATCACCCTTTTTCATGGCATCCGTTC
AAGAAAACGGAACCTGTACCCGTCCCAGATTTCACTATCAGCATGCTGATCGAGTCAGCA
AAAGCCGGGAAATGGTTATATGACTAA
>EECEBICD_01356 AMP nucleosidase
ATGGAAAATAAAGGCACAAACCTAACGCCCGAGCAGGCGCTGGATCGCCTGGAGGAGCTA
TACGAGCAGTCGGTTAACGCGCTCCGTGAAGCCATCGCTGACTATGTTGATAACGGTACG
CTGCCCGATCCCCACGCCAGGCTTAACGGACTGTTTGTTTATCCTTCGCTGTCTGTAAGC
TGGGATGGCGCGACACCGAACCCGCCTAAAACACGCGCTTTTGGACGCTTTACTCATCCC
GGCTGTTATACCACTACCGTTACGCGGCCTGCGCTATTTCGCGCTTATCTTCTGGAACAG
CTTAACCTCGTTTACCATGATTATGGCGCGCATATTGCGGTAGAGGCCTCGCACCATGAG
ATCCCGTATCCTTATGTGATCGATGGCTCGGCTCTGACGCTGGATCGTTCTATGAGCGCC
GGGCTAACGCGCCATTTTCCTACCACAGAACTGGCGCAGATTGGCGATGAGACGGCAGAC
GGTCTGTTCCACCCCGGCGAATTCTATCCGCTATCGCACTTCGACGCGCGTCGTGTTGAC
TTCTCGTTGGCCCGGTTACGGCACTATACCGGTACGCCGGTTGAACATTTTCAGCCCTTC
GTTTTGTTTACCAACTACACCCGCTATGTCGATGAGTTTGTGCGCTGGGGATGCAGCCAA
ATTCTTGATCCTGACAGTCCCTATATCGCGCTTTCCTGTGCCGGCGGAATTTGGATCACG
GCAGAAACGGAAGCGCCGGAAGAGGCTATTTCCGATCTGGCCTGGAAGAAGCATCAGATG
CCCGCCTGGCACCTGGTAACGGCAGATGGGCAGGGGATTACGCTGGTAAATATCGGTGTC
GGCCCTTCAAATGCTAAAACGATTTGCGACCATTTGGCCGTGTTGCGGCCTGACGTCTGG
CTTATGATTGGGCACTGTGGAGGGTTACGAGAAAGTCAGGCGATTGGTGATTATGTGCTG
GCCCATGCCTACTTACGCGATGATCATGTGCTGGATGCCGTTCTGCCGCCTGATATTCCT
ATTCCAAGCATTGCGGAAGTACAGCGTGCGCTGTACGACGCTACCAAAGCGGTTAGCGGA
ATGCCCGGTGAAGAGGTGAAACAGCGACTGCGCACGGGAACGGTCGTCACCACGGATGAT
CGTAACTGGGAGCTTCGTTATTCCGCATCGGCGCTGCGATTCAACCTTAGCCGCGCGGTC
GCGATTGATATGGAGAGCGCGACGATTGCAGCCCAGGGCTATCGCTTCCGCGTGCCTTAC
GGCACGCTGCTTTGTGTTTCTGACAAACCTTTACACGGTGAAATTAAACTGCCCGGGCAG
GCAAACCGGTTTTATGAGGGGGCTATTTCAGAACATTTGCAAATTGGTATTCGTGCTATT
GATTTACTACGCGCAGAAGGTGATCGTTTGCACTCACGTAAATTACGCACGTTTAATGAG
CCACCGTTTCGCTAA
>EECEBICD_01357 hypothetical protein
ATGTTGACCCAACACCGCCTGCCGGAAGACCCGCGCTGGAACGCCCTGCACGAGGAGTTC
AGCTTTGACGACGACGACAACCTGCTGGGCATGAACATGCCGCCCGAAGGTGGCGAGCAT
CAGGCGAAAGTCAAAGGCGACCGCCTGCTGCTACAGGGCAGACCACCACTATGA
>EECEBICD_01358 hypothetical protein
ATGAAATTTAAATTCGCCACATTACTCACAGCCGCCACGGTTTTTTACTCTTATCAGAGC
GTTGCACAAAGTCAGCAATCGCCTGATGTCTGGTCAGTTGTCGAACAGAAACTGCAAAAT
GCCGTCCCTTTAAATGAAAACACCACTTTTAAATCGCAGGCAGCCTGGTTTGAATTACAC
AGGGAACTGTTTATGTACGGCCCGATGAGCCGTGCGGATGCATTACTGAAAAAGCTGGAC
ACACAATCGAATGATGCGAAGTTTTCGCTAAACCATGAGGCCTCGTGGATCGCTGAAAAT
AATAGCCCGGATGCCGCGATGGTTTTCCTGTCCAAAATTGGTCTCGATAAACCGTCGAGC

ATTACCGCCTACGAGAGCTACGTTGATGGCTGGGTTAATCGCAAACAGCCTGAAAAAGCC
TTAGCCCTTCTTAGCAAAAATGCCGACGCGAGGAATTTTTATTTAGCCACGGTTTTAAAA
TCCTGGCACTCAGATCCTGATAAAACCGCCGCTATTTATAATGAAAATTACGCTGATAAA
ATTGTTGTTCCTTATACTCAGCTAAAAATGCTGCTTATTATTGCGAAGCAGTACCATGCC
AAGGGCGACACCGCAAAGGCGTTGGTTTACGCAGATAGCGCGCTGAAAATGTTTGATACG
GCTATTGCTCAACAACCGAGTGCTGAAGCCTACCGTTATCAGGAGTATCTCGACCTGATG
GAGATCTATTACGCCACGGGTAATAAAGAGAAGGCGATGGCGTTGTCTGCGAGATTGCGC
AAAGCAACCGGCAATAAAGGAAGCTATTTCCAGTATTCATTACCGGGATTGCTCTCTTTT
TATAAGAAAAACGAGCTGACACAGAATTATCAGGAGACGCTTTCAACCTATGTGACGCAG
GTCGATAAAATATTCAATTTTGCCCCTTCGCCGCGCATTGAAATGGAGCTGATTGATTTA
TTAAGCAAGCTTGATGATGTTGCGCTGATGAATAAGCGTATCGATTTGCTGATGAGCGCG
CCGGAATATACCTGCTACGACGACAGGTATTGCTACGAGTACAAAATCAAAGCGCTGAAG
AATCTCTTTCAGCATAAGCAGAATGCTGTGGCGGAAAAGCATATCGCAACCCTCTCCGCC
GAGGCGCAAACCCTGACGTTCGCCGAATGGGAAGATGCAACAAATGAAATAGGTGAAGTA
CTCAAAGAAATTGGCCATCCTGAGGCAGCGCAAAAATTAGCCGTCAGCGCCGAGGCGATT
TACCTCAGCCAAGCAAAAGCCTGGCCTGATGAAGATATGTCGCGTTCCTTTCAAAGACTT
GCAGAGCTTTACGGCTATGGCAACGACACGGTGAATGCCAAACGGGTGTTACACCAGCAC
GTTCCCTCTCTCGAAGAGGAGGCGATGATCGACCACTACATGAATGCAAAACAGTGGAGT
CAGGCAAGGGAATTGATGATTAACGCAGATAGGGTCGATAATAAAAACCTGATGCTGTTA
CGCCAGATTTGTTCTGAAAACACGCCCGAGTGTCAGGAACATATTACCTTTACGCTGAAA
AAATTAACCACGCAGGCGTCGATTACCCGCCAGGACGACACCGGTAATCAGCAGCTTTAT
CAGATTGGGAATATTTTTCACAGATTGGGTATCATACCGGGAGCGGAACAGCAGGCGCTG
ATTCAGGCGTTGTATAACAAGGCCGCAGAGCCTAAAAAAGCCACGCCTTAA
>EECEBICD_01359 hypothetical protein
TTGAAGAAAATATTCATAACAATTATAGTTTCATTATATTGCGCGAATATATGTGCAAAA
CAATCCCCCAGTACAGAATCCAAACCCGTTAATTTCATCGCCCAAATTGAAAATATCGAT
TTTAATAAAACCGCCATTAGCAGTGACCTGAAACTTTTACTCGCCGACCGTTATTATTTT
AAAGATAAAACACCCTGTAATACTAATACCCTTTCTGCGCGCGCAGAATCAATGGGACTG
ACGACAGAAGAATATATTAATAAAATACGCAGTTTAAGACCGGCTATTCTCGATGACCGT
TATTTTTACCTGACGGTCGATCAGTGCGATGCTGGCGGCACGCCCATGCTGACTGGAATC
GAGCTATGTACCGAAGCGCTGTGCGGTGCCGAATACATGAAAAGATCGTCTGACTTGTGG
CTGGATGATGAACTGCAACCGACGGTTAAACGTCAGGCAACAACAGTAGTCCATATGCCC
CTCCCCTATGACAAAGAGAAAAAAATTATGGAAAGTCACCGGCTGGTATCTGGAATCCTCA
GAAGAAACAGGGGAGGTGATGCAGAGTAAACAGATAGCCTTTGAAGGCTATACCAACGAA
GAAAACTTCGCTAACCGACAACGTGTTTCTGTATTTAAATCTTTCTATGAAAGCGGCAAT
CTCAAAAATATTTATCATTATAATGCGCAAAATAAGCGTGACGGAAAAGCTGAAACTTAC
TTTGATGAAAAAGACAAAATAGCGGAAACGCTGACGTTTAAAGATGGTCAACCCGAAGGG
GAATACATCGTATACCATGAAAACGGAGCCGTTGAAAGTAAACGTTACTTTGCCCAAGGT
AAAATTAAAGATGGCGAATGCCCACATTTTTATGATAACGGCGTTTTAAAACAAAAACAC
AGCTATTTAAATCAGAAACTGGAAGGCCCGGCATTTGAATATTTCCCTGATGGGAAAATA
AAGGGGAAATATTCATACAGAAAAGGCACCATTGTCGGCACCAGTACGGAATATTATTCT
ACCGGTAAAATTCGTGGTGTTTACCACAGAAATAACCAAGGTGAAAACGACGGAACGTTC
GAACAATACAGTGAAGAGGGCAAACTCCTTTCTAAAGCGACTTATAAAAATGGCAAACAG
CTTTCTGCTCAGAGCTGGTATGGAAATGGGCATCCCAAAGAAGAATCCTCTTTTGATAGT
GAAGGCCGTAAACATGGTGCGGTCAAAGAATGGTTCAGCAACGGAAAGCCTGCCTCATCC
AAAATGTATAAACATGATGTGTTAGACGGGGATTCCGAAAAGTGGTACGAAAATGGTCAC
CGCGAATCAGTCTATCCCTATAAAAACGGTATGCTAAATGGGGATGCTAAGCACTGGAAT
GAACAGGGCAAACTCACCTATACCACCGAATATAAAGACGATAAAAAGCAGGGAGCCGAT
CGCCGCTGGAGCGAACGTACCGGTAAACTTGTTGAAGAGGTTATGTTTGCTAATGATGAA
AGAAACGGCCTGAAAAGAGAGTTTAACGATCGTACGGGTAAGGTCTTAAGTGCGCTGCCC
TATGTCGATGGCGACAAGGAAGGTACAGAAGAAGCCTATGATGAAGACGGCATAAAATAC
ATCCGTTGTTACCACAACGATGAAGAGCTTTCAGAGCTATATGCGCCGACTGACGTCACC
AATAAGGCAAAGCAGGGTGACAGCACTGCGCAATATCATTTAGGTAAGTATGAGTTTGAA
TGCACAAACTACGATGCGGCGATGAAATGGTTAACTCAGTCCGCAGAGCAAAATCATCCC

GGCGCGCTTCTCTTTTTGGCCTATGCGTACAACGACGGTGATGGCGTAACGCAAGACAGC
AAAAAATATCTCTCCTATTTATTCAAAGCGGCAGAGCTTGGCGAAAGTGATGCGCAGTTA
GAAGTGGGATATTTAAATCTTATCGGCGAAGGAATGCCGAAAAACCTGCCGGAAGCCTAT
AAGTGGATTAAAAAATCCGCTGACCAGGGAAATGCTCAGGCGCACTATAACCTTGGACTC
ATGTACAGGAATGGTGATGGCGTTGAGAAAGACCTCAACAAAGCCAAACTTCATCTTACA
GCGGCGGTAAAAGGCGGCGTCAAACCTGCACTGGCAGCCTTAAAGGAATTAACGCCTCAA
ACTAAGTAA
>EECEBICD_01360 hypothetical protein
ATGGGCAAGTTAGGTGGTGAAATGAAGGCACTGGCAAAGCACTGTGGCGGTAGCCATAAA
ACGGTGAACGATTGCATTCATATCGTGCAGCGTTTCGATCATCACTTACGGGCTTTGAAC
GTCCATATGCAGCGGGTTGCGCAGATTAAGGTGCGCCACATTGAAAGCTATATTCATGAA
AGGTTGGCGCAGGGGATCGGCAAACGCACGCTGCAAAATGAAATGGCCTCGCTGCGCGCT
GTTCTGCAACAGGCCGGGCGTAAACAGGTGGCAGAGCATGAGTGGCTGACTAATAAATCG
CTGGGTCTGGCTGGTGCATCGCGCAGCGGGACGCGACAGGCGATCACGCCGGAACATTAT
CATCATGTGCTGGAGACGGCCCGCATGAAAGATCCGGGACTGGCGGCGGCGCTGGAACTG
GCGAGGCTGATGGGCTTACGTTCACAGGAAGCGGTGCAGAGTGTGCAATGGATCTGGGGC
ATGGGGACGGGCGTGGGCGGTATGTGGCGCAGGTGTATGGGCAAATTTGACAGCTGGCTA
AGAGCGAGCAGCGGATGGAGCAGGTTACCACCGTTCACAGTTAGTGAAAGACTGGGCGGA
GGTGGTAAATATCAGGCTGCATTACCTTCCCCCTTACAGTCCAAACCTGAATCCGATAGA
ACGAATGTGGAAATTAATGAACGAACATGCCCGGAATAA
>EECEBICD_01361 hypothetical protein
ATGGCATTAACCGATACCGCTGGCGGCACGCAGACCGATCCGGGTACAGGAAAACCGTCA
GAAAATCCACCCGCCGATCCACCGTCAGATGGTCCCGCTTCTGAAAAACCACACGACCCA
CCCGCCGATCCCAATAAGCCCACTCCACCGAAAGAAGAAACGCCGCCCGGCGCGCCGGAG
AAATACGCGTTCACCGCCCCTGAAGGTCAGGAACTCGATACCAGCGCGCTGGCGCAGTTC
GAACCAGTGGCGCGTGAACTGAACCTGACGCAGGAGCAGGCGCAGAAGCTGGTGGACGTC
TATCCGAAAGTGCTGGCGGGAGTGCAACAACAGCAAGCGGAGTCATGGCAGAAGCAAACA
GAAGACTGGGCTGCGGCAGTTAAAGCCGATAAAGACATTGGCGGTGACAAGCTGGCGTCT
AATCTTGGCGCAGCTCAGCGGGCTATCGACACGTTCGGTACCAAAGAACTGAAAGAGTAT
CTCGATGGTACCTGCGCCAGATCTCTGGTCAATGCTGCACCGTGA
>EECEBICD_01362 hypothetical protein
ATGACAAAAAGATTGCCTGTCGCGGAGATAGTGGAAGCATTAAGCAAATGGTTTGATGTT
AGCCGCTATGATGCGCTGAAAAACCTCACTCTGGAACAAATTTATGCGGAACTGGAAAGG
CGCATGTTTGCCTACAAAGCCAGACAACAATGGGAAACACTGGATGATAAGCATCGTAAT
GCCGTTATTCACCATGATGCGATGATCCACTCTGGCAGGGTCTTGCTGGAGGATAAATGG
ATCTCTGATAGCCACATGCTGGCTCATAGCTATGCCGTCAGGCCAATGACAAGGGATTCA
CTGTTTAACTATGGACGGGCAATGTATCGACTGGAGAATACACCTCCAGAAGAAAATGTT
TCAGTGAGTTCGGATTACATATCTGAATACCTGAAACAGGGAGGGCTGAACCCTGCTAAC
AAGATGCTGATTGAAATTGATTTGGAAGAAGCCAGTAGCGACGATCTGGCTGAACACCTG
AAAGTGCTTATCAATCAATGGCAGAAGCACCTGAAAGTGCCTAAACCACCGGAAAAAGAT
TTTCGTTTTGGTCATAAAACATTCCAGAAGATACTCGATTACAAGATTATCCCTCTGATG
GACCTCATAGCTTGGGAACAGCTCAACAATCAGAAAATCAAATACCCTGTATTGGCTGGT
ATCCTGCACCCTGATATGCGTTATGCCAGAGGTAGCGAGCAAATCAAAGACACTGATTAT
CCGCTTGCCCACGGTTTTTTAAACAATGACAATTATTTCAAGTCACTGAATGATTTTTTC
ATAAAAAACAATCTGGTGAAAAACTCACCTATTCTTGATGTCATCGCAATGAATGACAAA
CCAGAAACAAAAAAGAAAACAAGGGACATCCACTAA
>EECEBICD_01363 hypothetical protein
ATGGTTAAAAGAGAAATAAAAGCAAAGACAACGTTTATCCGTTTGCCAGAAGTAATAAAA
CGCACCGGATTTGGTAAAACGTGGATTTACGAACTGATTAAGGCCGGGCGTTTTCCATCT
CAGGTGAAAACAGGGATAAGAGCAGTGGCATTCATTGAAAGTGAGATTGATGAATGGATT
GAAGCGACTATTCGAAACTCACGCCAGAACGCAGCATAA
>EECEBICD_01364 hypothetical protein
ATGAAGATAGAACTTATTACAACAAAACAGTTTATTGAACAAGCCGAATGTTATTTCAGG
AACTATATGGACGGATTGCGGAGGAATGCACCAGATGATTTTTATTATTTTCTCAACAAT

AAATACAATATGAATGACATTATGGAAAGTATCATTAAAAAAACGAGATATTATTTTTAT
GATGACACCGAAGAAGGCAAAAGGAATCGGATTTATGGAGAAGTAAGTCACTGTAAGGTA
AAGCAACATCTTCGTCAGCTTTGGATTATCTATAAGTGTGTATACCGCTAA
>EECEBICD_01365 hypothetical protein
ATGAATACATTTAAAAATAAAACTACTGAAATCTTTTATGTTGTGTCCTTGCATATCTAT
GCGGAGCTTTTTAACTCAAAGGATAAAACAACCAGTAATATGATTATGACGCATGTAATG
GATCATGAGTTTGTCTGTAGGCTAATCGATCTGGCAATGAGGAATGCTGAGAAGCATCTT
CTGAAAAAAGCATGGAAAAAAAACGCTGCTGAAAAACTGTCAGAGGTGGATTTTAAAGGG
GTTAAGCAGGCATTAGCCAAAATGCACTACACCGTTCTGGCAGAGTCAATATGTTGA
>EECEBICD_01366 hypothetical protein
ATGAGCACCGCAACTGACTTTAAGACACTCCTCGACAATATAAAAATAGATAATGCAGGC
CAGATTAGTAAAAGGTATGGTCGTATAACTAAGGCTTTGAACCAATACTTTTATAACTTA
GATTCTAAGACAGCCAATTCACTACAGGTTGGTTCCTATGGGCGCTTCACAGGGATTCGA
GGGATCTCTGATCTTGATATGCTTTACTTTCTACCTGCAACTGCATGGCCAAGATTCCGA
GATCGACAATCGTATTTATTACAGGTTGTGAAAACAGAAATCAAGAAAACTTTCAAAAAT
ACAGATATTCGCGGTGATGGGCAAGTTGTTGTTGTTAAATTTAAGAATCAAGAGGTTGAG
GTAGTTCCTGTATTCAGTAATGAAGATGGCACTTTTACATACCCGGATACACATGATGGT
GGATCGTGGAAGGTATGTAACCCTAGGGCCGAAATGTCGTCTTTTAGGGCACTGAATGAT
GATAGGAAGGGACATCTGAGACGTCTATCTAAAATGATTCGAGCATGGAAAGCTCGTCAT
GAAGTTGAGATAAGTGGATTCTTAATTGATACACTGTGTTATAATTTTTTCTCTAATCTA
ACTGAATATGATGATAAGAGTTTCAAAAGTTATGATCAACTTTCGCTTGATTTTTTCACT
TTCTTAGAGAATGAAGGTGACCGAGTATTTTATTATGCTCCCGGTAGTCGCTCAAAAGTG
AGCGTAAAAAAAATCATTTAATAAAGTAGCAAAATTAACAAAAGAATATTGTGAAGAAGCT
TTATCTGCTACAAGTGAAAACTCAAGAAACTTAGCTTGGAAAAAAGTTTTTGGCAGGCCT
TTTCCAAATTATACGACAAAAGCACTCAGTAATGTCAATGTATCTGAACAGTTTATTGAA
GATCAATATGAGATGAATTTATATGGTCATGTTTCGATAGAATGTGAGATTAGAAAGAAT
AATTTACTGGAAGCTCTTCTTTCAAATCTTCTTGGCGAAGGGCATGATATTAGCACGAAT
CGCAAGTTAAGATTCTATGTTGATGAGATAAATAATATATCTCACCCATATAAGATTAAA
TGGAAAATAAAAAATGTAGGTGATGAAGCTGAGCGCCGAGGAAATGTTAGAGGTGAAATT
TTAGACGATGAAGGCGGTTCTGAGCGTTTCGAGACCGCAGATTTCTCAGGACCTCATTTT
GTTGAATGTTATGTTATTTATGGTAATCAAGTTGTAGCAAGAGATAGAATCGACGTACCT
ATACATAATTAG
>EECEBICD_01367 hypothetical protein
ATGGATAATAATATATTAGAAGGGCAATTAAGAGAATGCTATGGAAGGGTCGTTTACACA
CATAAAACTCACGAGAAATGTGCAGATATCCTTCAAAAAAAACTAGGCTGCATGAAAAAT
TTACAGTTAGTTCTTTTAGCAATAACTACCGGTAGTTTTATAACTACAGTGGTGGGAGAT
GCTAAAACAGGTGCTATAATTGGGTCAGTACTTTCCGCTATCCTCCTATTTTTAAACTCA
TATTTAAAGGATTATGATTTAGGCTCTATAGCTCAAAAACACAGACAAGCTGCTGGGGAT
ATGTGGTTAATCAGGGAAAGATATTTATCTCTTCTCACAGACTTGAAAATGCAAACTAAA
AGTATCGAGGAAATTCTTAAAGAAAGAGACGCCTTGATGATTGAACTTTCTGCAATTTAT
ATCGGAGCACCATCCACGAATTACAAGGCATATTCAATGGCTCAAAAGGCACTGAAGGAA
CTTGAGGATATGACTTTCTCTGATGAGGAAATTGATAAATTTCTTCCCACTGAATTGAAA
CGGAAATGA
>EECEBICD_01368 DNA-binding protein H-NS
ATGAGTGAAGCTCTCAAGGCACTGAACAATATTCGTACACTTCGCGCACATGCTCGTGAA
ACAGATCTGGCAACTCTGGAAGAGATGCTGGAAAAACTCTCCGCTGTCGTTGAAGATCGT
CGTGAGGAAGAAAGTGCCGTCCGTAAGGAACAAGAAGAACGTCAGGCTAAGCTGGAAGCT
TTCCGTCAGAAACTGTTAGAAGATGGTATTGACCCGGCAGAACTCCTTGCTGCTGTTGGC
TCATCCCAACCTAAAACCAAATCGACCCGCGCTCCACGCCCTGCCAAATACAAATATACA
GATGAAAACGGCAACGAGCAGACTTGGACAGGTCAGGGTCGCACTCCTAAAGCGATCGCT
GCTGCTATCGAAGCAGGTAAAAAACTGGAAGACTTCGCACTCTGA
>EECEBICD_01369 hypothetical protein
ATGAGAAAAGTAAAAACAGACAACAGTGATTTAATCGAATACGTAAATACAGTCAAAGAA
CTAAAAAATCATATTTCGATAGATGAATACAGGAATGAATATCGTAGGCTACGCTCTGAT

GACATTCCATTGGTTAAGTCTCAAAAATTTAAGTCTGCACATACTGAGCTTCGCAGACTT
GAAAAGAAGAGAGAATCTCTTATTGAATACTTTATTGACGAGTTAAATCCTATTAGCAGT
TCAAAAGCTAACACATCAGCGAGATCAACTGGTAATCTCGATTTATTTAACGAACGCGTG
TTATACCGTAAAGCACTTTCAGAAAAAAGCGATGAAGAAATAATTGCCTTAGTCATAAAA
CAGCGTACAGAAGCCGCTGTTGAGTTCAAGCGTTCCATAGAACAAAGCCTGAATCAACTC
TCTCACATCTCGTCAGAGTTCGCCCCATCAAGCCAGAAGCGCAGGAAAATGTCTCTTTAA
>EECEBICD_01370 hypothetical protein
ATGAAAAAACTACCCGGTTCTCTTGAGATTAAGCTCCACGAAAAACTATCCAAGAGTGAT
ATACTAAATATATTAGCTGAGCAGATGACCATGCTTGAGGAAACATTTGGCATTCAGGAG
TTTAAGATTTTCTCTTATCTTGAATGCTATATTGGAGATAAGAAAAAAGCATTATATTAC
AGAAGTCGCAACAGTGCCGTAGCCACATTCAAATTAAAGGGTCTTGAATCCCCTGTGAAC
ACGGCTAAACTCATATCAAAAGAAAACGGACAACGTATTGTTTCCTTTGATAAAGATCTG
GATATAAACAGAATATCTGCTACAGTCCGAAACATACAAAACAATAATCCTTATCGTGGG
TGGTCTGAAGGTATCAGTGTTGTTCCTGCCTCAATCATTAGTAAAATAATTGAAGTGGAT
ATAATACGCGCGCAGGAAGAACAAGGCAGACTTTACCGTATTGAAGAACAGCGCAAGAAA
GAAGAACAAATACGCAAAGCTAAAGAAAGAGAGGAGTATGAACGACCTTTAAAGGCTTTT
ATTTCTTCAAAAATAAAAGAAAGTGATTTATCTGAGAAGGATTTCAAAAAGCAGGTTTGT
TCAAGCTGCGACTATCTGAAAGATCGCTCCACAAAATCAAGATATTTTACTGAACGACCA
GACCTGTTAGACAAATATCATAATGAAAGATTAATTCGTTTCTCGATAAAAGGCACTGAT
GGGAAAGTAGGAAAAATAGAAATTTATACAGATACAGGCGAGCTTATTTTTGAACGGTAT
AAAACCAAGTAA
>EECEBICD_01371 hypothetical protein
ATGAATACACCAGATGCTGATCTATTACACTTATTTGAATCATTAACTACCGAGCAAAAG
ATTGATATCGCAAGACGCTTACTACAAAACAAAGAAATAAAATCAACAACAGTCATTTCT
TCTGAGGAAGATAAAGCTATTTCGTTTTCACAACCTTATGTATTTGCTTTTACTTCCAAC
TTTCGTGTTTTCTCTGATCTTGATATATCCAAGAATGAATTCCGTGTATTAACCTATATC
TTGGAGTATATGGAGTTTGGAAATCTGATCAATCTTTCACAATCATCGCTGTGCAAAGCA
TTGAATATCGCTTCCGGTAATATGTCTAACATTTTTAAAAAACTCAGACAGAAAGGAATA
CTCGTCGAGCATCATGGTCATCTCTATATCAACTCTAACATTTTCGCTAAAGGAATGAGT
CATCAACTGGATCAGAAACGTAAAGAACAGTTGAGCAACGCTCGCAATCTTTGCGCAGAA
ATAGAACAGTATCCAGAAAGTTATTCAGCTATGTCAAAAAGTGAAAGAAGAACCCTATTC
TTAGAAACTGAAACAAAAGAAATCTATACTGACGCATTTGTTTTCAGTAAGAAGAAAAAG
AATAACGCTGTTCCTAATCCCAATAACAGCATACATGTTGATAAACCACGAAAAGTATAT
AAAAGAACAAAGGAAAATGAGGAAGCAAGAAGAAACGCTGTTGACGCAGACTCTCATATG
CAGAAAGTATCCTGA
>EECEBICD_01372 hypothetical protein
ATGCTATGTTTAAACATAGGATCCGAATTAAAATTTAAAAGGAAAATAGAACTGACTATG
ATCGAATACATTCGCTTATTACTTCATAGTAGTAAAATACAAAATGTCAGTTTTAGGGGC
GTAGCCGTGCGTAAGCACAGAGATAAAGATCTTTTCAAAAAGAAGTTTAATAATCAGTAG
>EECEBICD_01373 hypothetical protein
ATGAACAATATCAAAATGATCACACTCTTCCATCCACATGACAAAACACCATTTATGATC
TGTATTGTAAGCAAAGTAGAAGATACAGAACACGGATTGAAATTAACCCTTGAAAACGGC
AACAACATTTGTGTCAATAATTATAGTCATTATCTTTTATCAGATTCTGTAAGTCGTTGT
GATAAAGACAGGTTGAAAAATATTTATATTCGCTTGGTTTCAGAACTCACACAGATGAGT
GAAGAAACTATAAAATCACAGATGTTATAA
>EECEBICD_01374 hypothetical protein
ATGCGGAAAATAAAAACAAAGATCTATATACCTACAACTTTAACAGGAAACATGGGTAGA
CACTATATCCCTGTTTTAGAGGATTTAAGAAAAACAATATACAGCGACAGAATATTATCA
AGGCTTGCAGATAGCGGCAATATTGTTATCCACAGTTCAGTTGGGTATCCGGTTGCTAAA
TATAAAAACACGGGGATTAGCATAGGCATAGAACCATTAAATCCAATGATAAGACAAGAT
CTAACTCTTGGATATATCGTGGTAATCAGAAACGGAAAAGCATCACAGGAAGTTAATGGT
TTACTTAACAGGTCATTGCCAAAAGCGATAAGCACTTTTAAGGACCATATTAATGAATAT
GAAGCAGCAAAAAGTAAAATGCTATAA
>EECEBICD_01375 hypothetical protein

ATGGAAGGTCAGTTAATTTTTTGTTCCGATTCAATACTACGGTTTCAGTCCGATTACGAT
GAAACCGCTGCCGTTCCATTGCTATCCATCCAGAACGTCATAGCCGATACCGATCCGTTC
TTTCTTCTCCGTTTCTTCCACCATACCGTTCTGATTGAAGAAGGAACAACGCTGGCAAGT
ATCTTTCTGGCAATAGAACCGTGGAAGGCTTTGTTAGCGGCTTATCTGGACAGAGATGTT
GGTGCTTATATAGATGAGGTAAGAAAACCGTCCGGGCCAACGACATGGGATATTGAATGG
ATTGGGATTGATCGCCGTAGCATGGTTTACCGCGCTTATAAACGTCAGGAGATGCAGGAT
GGAGAGGATTTTTCTGACTATTTAAACCGGGAACGCGTTCTGACAGATGAATTTGAGATT
GAAAGTGGCTGTGAGGCTTCTGGCTTCATTAAAGGTGATAAAGAACGATGGAGCATCAGT
GGTGATGTTCACGAGATAAAGAATCTGCCTGTAATCCTCTATAGCAAACAGACATTGATG
ACTTCACCGAAAGACGGTTTGTTGAAGAAAAACATATCGGGTGTGAAGAGTTCGAAGCAC
AGTTGCTTCATTTATGGCGATACCTCGTTTTCTTTCAGTGAGGTTATGGAAGCCATCTTC
ATTAGCGGGCTGTTCTTCTATGCACCAAAAGACGCCGCCAGCAGTCTTGATGAGCTGAAA
GCGAGTCTGGCTGAACTGGAAGAAGAACGCGCTGAAAATCCAAATGCGGAGTCGACTGGC
AATGAAACAGATGAAGAACCTACCATCGTTGTAGCCGAAGGTGCATTTGATTCGCTCGCT
GCTCACATGGAGTCTGAAAAAGCGGAGTGGCAATCCATTAAGAAGTTATGCCAGCAAGAA
GGTGGGCTGCCCATACGCATTGGCAACATTAAAATGTCTGAGTCTATTCAGATTATACCT
TAG
>EECEBICD_01376 hypothetical protein
ATGTATAAAGAAGAAAATAAAAATATAGCTCGTAAAAGTGTTTTAAAAGCTGCGATTGAG
GCGCTTACTTTGTGTAGAAAAGACTCGACTTTAGCTCCTAAAGACTATATTCGAAAAGTT
AAAGCGTTTTATAGGAAAGACGAGAGTGATCCACGTGCGTTTATTGTTGATGAGTTATCG
GAGGAAACAATAATTCGGTGGGAGGAATTCTATGATTCAGTGATTCAGGATAGGACTGCA
CGTAGTATTAAAGTCGCATATTTATCTGGGCCTAACCCTGAAAATGATTTAACTGAAATG
ACTGATATGGGGCTTTTGCCAGAAAATATTTGGGCATTTGAAAGTGATGCTAAAATATAT
AATGAGGCTGTAATTTCAGCACTTTCTTCAAAATTTCCGTTTATAAAATTAATTAAAGCT
AATGTTGGGGATTTTTTTGAGGTGTCACCGCAGAAATTTGATCTTATATATCTTGATTTC
TGTGGGCCATTGCCGAGTAAGAAAGCTGGTCAGAAAACATTAAAGGCAATAACATCAATA
TTAAAATATCATGCGCTTTCACCTCTTGGTGTTATGATAACAAATGTAAGTCTACCGTCA
AAAGAACAAAACGCGAATGAACATAAAAATATCGTAAATTTAGTTGCGAGTTATCTCTAT
CCAAAATCAACACTGGAGAGTAATAATCCTGAATGGAACTGTACTGATGGTGCAATTTCT
GAAGGTTATAGCCTTGATGAGTGGCATAAAAAAGTTGAATGTGAAATTGAAGATTTCTAT
GGGCAGTATATAACGAGATTATTAGTTGATCTGATTTCGGTGATCTCTCCATATGATAAT
TTCACCTCATCGCATTCTTTATATAAAAATATGTTTAAAATTTCTAATTACAACGATCTA
ACTAAAAGCGTAAATGATTTATTTCATTTTGATAGTAATGGGAATGGTGGGGATATTATA
GTTGATTCAGGGCTATTTCCAATCCTTTGGACAATTGCATCAATAGATAAAAAAATACAAT
AATAAAGATAAGAATTACTATCAAGATATTTATTGCGATGATGATTTTAACGATTATGCT
CAGTCTTTTTTATCGCAAATGAGTGCTAATGGTAATGCTCATGATTTAATAAAAAACATT
TCTAATATGCACTTTCTATTAAATGAAGGGCGTACAGAGAATAATTTCTATAGTGATTCT
CTGCGAAATCTCAATAAAATAAATTGGTATCAAAAAGTTTATCCATTTTGTGATCTTTTT
TTGTTTCATCAAATTAAAGAGGTTTTATTCAGACAGTTATCCGTACCTTATCATGTAAAT
ATGGAAAAAAACACTGAGATGGAAATACAAAGCAAAAGACACCAATATGTATATGGATATG
TTGGTGCTTGATGAATGTCGTTACCTATACGATTGGATGCCTTCACTTGATATGTTTTAT
AGTGGGATGATGGATATCGAGAGGCAATTTAGTTTTAGGTTTATTCTTGATGCTGTAGCT
AAGCATCGAATGGTTTATAATAATGAATTCTTTTATGGGACTGCATCTGTTAGTAAATTC
GAAACTGATTATGTTGAAAAAGTATTGAGTGTTAGAAAGAATATAATCTAA
>EECEBICD_01377 hypothetical protein
ATGAAAAAATTATTATTTGCTATTTTTATCATACTTCCGCTTGGTGGCTGTACCGTTCAT
GGTAATAAATCTCTAACTGATGAAAATCATCAGACGGTTAAATTAAAAATCGTCAAGGGT
AAAACTACCCAACGAGAAATTCTTGCTGCCTTTGGAGAACCTCAGACACGCGCTACGAAT
GATGGACAGGAGATGTGGAACTACTCAAGTATGACGGGAGAAAGCCAATTATCTAACTAC
ATACCCGGCCTTGCCCTATTGACCAATAGCAGCACGGCACACATTAAATCACTGGATATC
TGGTTTAAAGGTGATGTTGTGGAACGTTATAATTTTAGTCAGACAGCCAGTAAAGTTACA
CGAGGTATGATTGATTAG
>EECEBICD_01378 hypothetical protein

GTGGAAGCAACAGGTATCGTATTTTTAGTGGTGCTCTTCGTCATTATCATGACCGCAGCA
GATATACAGAAGAAAAAGCACTACAACAGCTTTACGGAAGTTCTGGACGGCGACATTCTT
TCGTATGAATGCCAGCAGACAGGTATTGTTATTGATACTCAAAAACATACTGTCCGTATT
TTCAATAAAGATAAAGACAGCACATATACTTTTGATGAAATCCGGGAAATAAATTATACG
CTTTCAGAGGGTGGTAAGTTTTACGGCAATGGAACATTACGAGGTATGAATAATGCGGCT
ATTGCTAACTGGAGAGAACAACTTTCAGCTAATAAACGGTCTGGTCTAAATATTTTACCG
ATGATATTAAAAATCCCATGTGGAAAGTTAATGTTCCCCTGA
>EECEBICD_01379 hypothetical protein
ATGAAGAAGATTTTACTCGTTGCTGGCGCGGCTCTGGCGCTGGCGGGATGTGGTGAGAAA
GGTGAGTTTGAGAAAGCGATCAATGCCAAGATTGGGCAGGACAAATACTGCTTCTCGCTG
GATAACAATAACACCAGCTTCCCGATCCGTCTTGCCAAGCCACGGCTTGATTCGACTGGC
ACAGGGACGAATTCAGTGATCCTCGACGGTTTTATTGAGCAGGGCCTGATGGTTTTTGAA
CAGGGCTATGACTCGAACGTATTAGGGATAACAGAGGAAGGCAAAAAAGCCAAAGTCTGG
AGCACAACAGACGGTGCCTGTGTTGGTCGTCGTGCTGTTGACGAGATTAAGGAATGGACT
GAGCCGGGAAACGGTAATCAGAAAGTAGTTCGTGTGTCTTATACGTGGAAGCTGGTTGAC
GTTCCGAACTGGATTGACAAAAAGGCATTCGCCAGTGTTAAAGGAATGAATGAACCTGCT
GATGGTGCAATGAATCTCTTTAAGACCAGCAATGGCTGGAAAGCCAATTAA
>EECEBICD_01380 hypothetical protein
ATGGAAAACAATACCATCAAATGCCCGTTCTGTTTCAAAGAGAGCCAGCGTGGGGTTCAC
GTTTGCACAGGTTGCTTAGCGACTGTGTTATGTGGCACTTATCCCGGCTGGTATGCTGCT
GTTGTTTTGTTGTTGACTTTTGGTTTATCGACGTTGATCGGTATGGCAGGAGCTACTATC
AGCCTTCCGATAATTGCTATCGTAGGGTTTGTTGCTGGCAAAGTTATTTTTGCTGATAAT
GTAGTGTTTAGGCGAAGAATGTAA
>EECEBICD_01381 hypothetical protein
ATGAATACAATCTATCATTATTGCAGCCCTGAATCTTTCTTCAGTATTATTCAGAATCAG
CGATTATGGCTAAGCTCTATGGATCATATGAACGATTACATGGAGAAAAAGTGGTTTTAC
TCTACACTTAAAAAAATATTTATATAAAAACTTAGATGCTAACTGCGTTGACCAGTTTATA
GCACATTTGGATGACAATATATCTATTGGAACACCATTTGCTTGTTGTCTATCTAAATCT
GGGGATATTTTAAGTCAATGGCGTGCGTATGCAAAAGATGGCTTTGGTGTATCCATCGGA
TTCGACAGAGAAAAGCTCGATGTCTATGATGGGATTATAGGTAATAACCTTGATCCTAAA
CACCGACTAACTCTTAGCGATATATCTTATATGGATATTAACGTAATTGAATGCTTAGCT
GAAAGGATATTATCAAGATATTCATTCATAAAAAAATACTATATGAATGAAATTATATCG
ACATCAAAATTTAACCGCTACGACAAATGCATCCTTGAATTAATTTCCAATATTATTCAC
TTAAATACCACGACTAAAAACCCAGCATTTAAGGAGGAAAAAGAAGTACGATTAGTTTAT
CAAACTCTTGATACAGGAAGATATGAATACCCAGAATCATCATCAATTAAAGATCTGAAA
TATAGAATATCCAATAATCAAATTATATCATATTACGAATTAGGTTTCCCAAAAGATGCC
GTTTCAGAATTGATATTAGGTCCAAACAATAAATTTAAAGAAAGCGATATTGTTAACTTT
TTGCAGTATAATGGTTTTGAACACAGCATAAAAATATTGAAATCAAAGGCCAGTTATGGA
GCTTAA
>EECEBICD_01382 hypothetical protein
ATGGCGATTTTTCATCTGGATTTTAAAATTGTGAAACGAAGCGAAGGCATGACTTCCGTT
GCGAAGGCTGCCTACCATACCCGTACACGTATTACAGATGATCGCATCGGTGAAACATAC
GATTTCAGCCACAGAACAGATCTACATGGTCATATTATATTGGCACCTGTTTCCGCACCT
GCTCATATTGTTGAAAGCTCCTCAGCATTGTGGAATGAAGTTGAAAGAGTGGAACGCCAG
AACAACGGACAAACAGCGCGTTATTTTGATGTCGCTATCCCCGTAGAACTCAGCAATGAT
GACAAGAAAAAATTAGTTGCCGAATACTGCCAGAAAAACTTTGTCGATAAGGGGATGATT
GCTGATATCGCATTTCATGATCTGGACAGTAAAAACCCACATGCTCACGTCATGTTAACC
CTGAAAACCATTGGTCCAGAAGGCTTCGGCAAAAAAGACAGAAGCTGGAATGATAAGAAG
ATGGTTGTTCAGTGGCGTGAGTCGTGGGCCACGATGTCAAACAGCTACCTTGAGACCGCA
GGCCGTGAAGAACGTATCGATCACCGTTCTCTCAGAACTCAATGTGCTGATGCCCCTTGCC
CAAGCAGAAGAAGCATTCAGCGCTGAAGAAAAAGCCTTCTGGCTTGCCAAAGCAACCGAA
ACAAACCGTCCAGCAATGCAACGAGTTCATCGTGCCAAGTGGAATAACACCGAGTCACAG
GAACAGCGAGCTACAGAACAAGCTTTACGTGATCAGCAAATAGAGGAAGCGAAGAAGGTC
TACAACACATTCAGTGAGCTACCACTGGAGATCGTGGTTGATGTCAGAAGCTTTACGATT

ACACATCTTGCTGAACCCGAAGAAATCGTTATCCCTGACTTCCCGGCTACAACTAAACAG
CAACCTGTTATGACGACACACGCCACAAGCAGAAGACCAGCAACAAAATCTTACCGTAAT
CCCAATAAGGTCAGTAAAGTAAATGTATCAGGGAAGAAATCTCCCGTTCTGATTGTTCCT
GAACCAACTGCATCCACTAAATTGAAAACCCCATCTATTCAAAACACCAGAGCGAAGAAC
CGCGCTCCGATGAGGAGTAGGAAACAGACCAAGCCCCGCCAGAATGGTTTGTTTAAACGT
TTCACGCTACTGATAGTAGGATATATAAGAGAGAAATTTATATGGGCCAGAAGGAAACCC
GATACCACTGATGCTGACCATGATAAGCGTATCGCAGAGAACTATGTCTATGATGAAGTG
CTGGGTATCAATGTTCCACGCTCGGAGTTTGAAAAACGCGCTAAATTTAACAATGACCAG
ACATCACCAGAAGCAAGCATCTATGGCAATGAATCAGACCATGACAAAACCGTTCGTTTT
CCAAGTCGTCCGAAAGATGTACAAACAGAGATTGATAAATACATAGATCTCATACCATCA
AGCCCCTCAGAGTATTCAGGAAAGTCCCATGTTTCAAAGTTGAGACCACTGGCATATCGT
AATAATAATTATAATGAATAG
>EECEBICD_01383 hypothetical protein
ATGGATAATGAAACTAAAAGATCAAGAACAGAAAAAACATTAAAACAAAAGGTGGCATTT
GCTCAACTTGAACTTAACCGTTTAAAGTCAATGGAGAAATCAGAACAAAAGAAAGTTGAA
ACAAGGCTTAAGATTATTCTTGGGGCAGAAGTAGCCAAGGCTATGAATTGTGGTATCGAA
CAGGTGGATAAGGAACTTGTTATGGGGATTTTACTTTCAGCATCTGAGTTGAATGATATT
GAAAGAGTCAAATATATAAAAGCAGGGAGATGGTTTCTTGCTCAAATGGATGGCAGACAA
AAATAA
>EECEBICD_01384 hypothetical protein
ATGGTTTTATTAAATAGTAAAAGAAAAAGCAAAAAAGGTTTCTCATTACTCGAATTATTA
CTTGTTCTGGGGATTATTGCCGCATTAGTAGTAGCGGCTTTTATTGTGTATCCCAAGGTT
CAGGCTTCACAAAGAGCGCAAGCGGAAAGTAATAATATTGCCACCATACAGGCAGGTGTC
AAAGCACTCTATACATCAGCATCCAGCTTTACTGGGCTAACGAATACCGTAGCTGTTCAG
GCAAAAATCTTCCCTGATAATATGTTAAGTGGAACAGGCAATGCAGCTAAACCAATTAAC
GCATTCAAAGGGAACGTGACGCTGGCTGCGGCAGCTACAGGTCCGTCATCTGCGGCAGGT
TCTTCTTTCACTATCACATATGACAACGTGCCAGCAGCAGAATGTGTGAAAATAACCACA
GCGGCGGCAGGCAATTTTTACACTGCTAAAGTGGGTTCTAAAGTAGTAAAAGCTGCAGAT
GGTACCCTTGATGTAGCAGCTACCGCCGCAGCATGTAATAACGCTACAAGTAATACATTA
GTATTCACGTCTATTTAA
>EECEBICD_01385 hypothetical protein
ATGATAAAGAAAAGAGGATTTACTCTATTAGAGATCACCATCGTTTTAGGTATAGGTTCT
CTTATTGGTTTTATGAAGTTTCAGGACATGAGGAAAGAACAGGAAGCAGTAATGGCACAG
GCGGTAGGCTTTCAAATGAAACAGGTTGGCGAAGCAGTTAACCGTTATATTAGCATTCGC
TATAATAAGCTTTCCACACTGTCATCTTCACGCAGTCAAAGTAGCGATCCGGGACCAAGA
ACCTGTACGGCTAATGGCTGTGAAATCACTTACCAGACGCTGGTGAATGAAAGTCTCTTG
CCCTCTACATATGCGGGAGTTAATGCCCAAAAGTCTTCTTATAAAATCCTGTTAAAACGA
TCAGGCGTATCACCAAACTATGTTATCAATGGTTTGATTGCTACGACGATACCTTGGGTC
GAAGGTGGAAAAAACGCGATACGACCTGTTAGGAAAATCGATGCAATCAGCAGGTATTGAT
AGCGGTATGACGAAATCGCCTGCTCAGGCATCAGGATATAATGGAGTGTGGACAGAAAAG
TCTACTGATTACCCGGCAATTAATAAAGCAGGATTACTGGTTTACCGTGTGGGCTATGAT
AGTTCTATGTATTCTGTTTACTTGCGTCGCGATGGAACATTGCCTATGACTGGAAATCTC
AATATGGGCACTAACGATATTAATAATGCCAAGAACATTACCGCATCGGGAACGGGGATC
TTTGGTGGGGATGTTTCTTCCGCAGGGAAAATAATCGCAGCACAAGAAATAATAGCGCAT
AATGGTTATGGTGATGCCATACATTTTGGCGGCGATGCTGTTAATAACGACTATGAAATA
ACCATGTCTAAGGATAAGACCTTATCTATTCATATGGCAAGCAATCGAACAGATCTCACT
ACGCTGAAAATTAGTGGAGGGCTGTCTACGATTGGTAATGGAACGATTAGTGGAACATTA
GATACAGGTAAAAGTATCACATCCGGTGGACAATTTAATGGTCATAACGGTGGTGGTGAT
AGTTTCAGTATTGGTGGTGGAGATGCTAACGATTATGAGTTCAGATTAGATACTGTTAAG
CCGCTAACCATCTGGCGAAATGGTGGCACCAGTACTGAAACAAGGCTTCAGGTTTTTGGG
AAACAAACCAACCAAGGTGATTTTGCTATTACTCCGGGGACAGGAAGCACTGGTAGTATT
GCAGCATCTGGAAATATTCAGGGACATACTCTACAACCTACATCGACGAATACAACTGGT
GGGACATGTCCAAGTATAGGCCTGATATCTAAAGACAACATGGGAAATATATTATCCTGC
GTTAATGGAAAATGGTCTGTTGTCAGTAACCTGCCAGTTGGTTCACCTGTTCCTTGGCCT

TCAAATACAGCGCCTGTAGGTTGGTTAATATGCAGGGGACAATCTTTTAATACTGCCCTA
TATCCTCAGTTAGCAAAAGCCTATCCAAGAGGAAGACTTCCTGATTTAAGAGGAGTATTT
ATTCGAGGGCTGGATTCGGGAAGAGGACTTGACTCAGGAAGAGTTATTAATTCTTATCAG
GATGATCAGATTCAAAATATTACAGGTCATATGGCGGCTGATGTATCACAGAGTGGAAAC
ATCGGTAAATATGTGAGTGGCGCTTTCGCGGATAGTGGTGCGTTAGGTGAAGGTGATGAA
GGTCATAAAAGCAATGAAGTAAGGAAATATACGTTTGATGCTTCGAGAGTCGTTCGGGCA
GGTAATGAAACAAGACCTAAGAATGTTGCCATGAATTATATCGTTCAGGCACAATGA
>EECEBICD_01386 hypothetical protein
ATGTCTATAAGCACCACAATGTCAAATATCAACAGAATACAAAAAGACATTGCTAGTCTA
CAAAAACAACTTTCTGATGAGCAGCGTAAAGAGGCCCAACTTTCAGGAAAAATCAATCAA
AAAAATCGCGTCCCTACCCCATCTTCATTTTCTACACCTTATTTTTCTAAATTTTTATAT
CCATTCTATTATAATATAAAATTTTTCAAGTCTAAAGCACAAAATGTGATTTAA
>EECEBICD_01387 hypothetical protein
ATGTGTATAATCGATTATTGTTTTATGTATACGATATGGTGGGAAATGAGTAATACTACA
GATTGGATTAATACTATATCTAATGTGATGATCGCCGGTTCTGCGATTTATGGCGCTTTT
AAGGCTAAGAGCTATTTCAAAAGTAAAAATGATGAGGAAGCATATAATGATGCAAAAAAA
TTGATATTTGAACTGTATCCTCAATATAGAATTGATTTGCATAAATTGGTTATCGTTTTG
TTATCAATGACATCCAATAGAATTCCAAATGATATATTGGAAACTCGGTTGGAGAAAATT
ACCAATAGACTGTCTAATACACAAATTGAAATTGTAACTATCAGTTCCAATATTATTGAA
CGCCATAGATGGAAGATCAGAGATGATTTTAAAGAGTCTTTTGAAATAATTAAAAATATC
TATAGGAATAAATTAAGTTTTCCATTGATTAATGTAGTGGATGAAGTAAGTCAAAGCACA
GAAACTGAAAGGCAAAATATACTTAGAGAATTAATAGGGAAATGTCATGAAGGTATTGAT
GCCATTCATACATTCACAGATAATAAATTTAGAGTTGACGAGTATTTCGTTCGTCCCTTT
TTAAACTAA
>EECEBICD_01388 hypothetical protein
ATGAACTCTATTAGAGTGATTGAACGTCAACATGAAATAGATATGATTGAAGGTGAAGCA
AAACATAAAGCAGAACTTGAAAGAATAAAATCTGAATCTCAATACAATACGAGCGAGTTA
AAAGAAAAACTTGAACGGCTTGATATAATTGTCAAAAACCAAGAAGCGCGAAATAATGAA
CTATTAGAAGAGCGAAAAAATTAG
>EECEBICD_01389 hypothetical protein
ATGATATTATCCCCTATATATGAGATAATAAAGGAATCAAAATTTAAAGACATAGAGAAA
TATCTAGAAAAAGTCGGTGTTCTTGAAAATAGTTTAAAACGTCTCCGAGACTCCGAAAGA
AAAACATTTTCAATGGATGAACTGGAAAATAACGACTATAATGCAATAAATGCCGATTAA
>EECEBICD_01390 Prophage integrase IntA
ATGTCACTTACTGATACCAAAGTAAAAAATACCAGACCATCGGAAAAGGCCGTCAAGCTC
ACTGACGGGTTTGGCCTCTATCTGCTGGTGCATCCTAACGGTTCAAAATACTGGCAGTTA
GGCTATCGCTTTGATGGCAAACAGAAGGTGTTTTCCATTGGGGTTTACCCTGCGGTTTCA
CTTGCCGATGCCAGACAACGCCGGGACGAGGCCAAAAGGCTGCTGACTCAGGGGATTGAC
CCGAACGCTAAAAAACAGGCTGATGAAAAAGTCCTTCAGGAGAAGCGGGATAAAACCCGC
TCGTTCCGTGTCGTCGCCAAAAGCTGGTTTGCCACCAAAACAAAATGGTCAGAAGATTAC
GCCGATACTGTCTGGAAGCGCCTTGAAACCTATGTCTTCCCGGATATAGGCGACAGAAAC
GTTTCAGAACTGGATACGGGTGATCTGCTTGTCCCGGTCAAAAAAGCGGAAACACTCGGC
TACCTTGAAATTGCCATGCGGATTAAGCAATACATCACCGCGATCCTACGTCATGCCGTC
CAGCAAAAGCTTATGCGTCATAATCCGGCCTATGATATGGAAGGCGCTGTCCAGAAACCA
GAGACGGAACACCGCCCTGCACTGGAGCTGGAAGAGATCCCGCTACTGCTTGAACGTATT
GATGCCTACAAAGGTCGTGGACTGACTACGCTAGCGATTAAACTCAATCTGCTGATCTTC
ATTCGTTCCAGCGAACTTCGTTTCGCCCGGTGGTCGGAAATCGACTTCAAAAGTAAGTTA
TGGGTGATCCCCGAACAGCGGGAAGCGATTGAAAACGTCAAGCACTCGACTCGTGGGGCT
AAAATGAAACGTCAGCACTTCGTTCCCCTTTGCAGGCAGGCTCTCAAGATACTGAAAGAG
ATCCGCCAGCTTACCTATGAAGAAGGTAACGAAGCAGAATTAATTTTCACTGGCTGTTAT
GATTCATTCAAACCCATGAGTGAAAACACTATTAACAAGGCGCTACGTAAGATGGGCTAT
GACACCACACAGGACATCTGCGGTCATGGTTTCCGCACACTGGCGTGTAGTGCCTTAATT
GAGTCTGGTCTATGGTCAGAAGATGCTGTAGAGCTTCAAATGAGCCATAAGGAAAGTAAC
AGCGTTCGCGCAGCCTATACCCATAAGGCCAAGCATCTTGACCAACGCCGCCTGATGCTC

CAGTGGTGGGCTGATTTTCTTGATGAGAATCGGTATGAGATGGTCAGGCCGTTTGAGTTT
GCTCAGAAACAATAA
>EECEBICD_01391 tRNA-Asn(gtt)
TCCTCTGTAGTTCAGTCGGTAGAACGGCGGACTGTTAATCCGTATGTCACTGGTTCGAGT
CCAGTCAGAGGAGCCA
>EECEBICD_01392 tRNA-Asn(gtt)
TCCTCTGTAGTTCAGTCGGTAGAACGGCGGACTGTTAATCCGTATGTCACTGGTTCGAGT
CCAGTCAGAGGAGCCA
>EECEBICD_01393 Protein MtfA
ATGATTAAGTGGCCCTGGAAAGCACAAGAAATAACCCAGAACGAAGACTGGCCGTGGGAT
GATGCGCTGGCTATACCTCTTCTGGTAAACCTCACCGCGCAAGAACAGGCTCGGCTTATT
GCGCTAGCCGAACGTTTTTTGCAGCAGAAAAGACTGGTAGCGCTACAGGGATTTGAGCTC
GACTCGTTAAAAAGTGCACGTATTGCGTTAATTTTTTGCTTACCGATCCTGGAGCTCGGT
ATTGAGTGGCTTGATGGTTTTCATGAAGTGCTCATTTATCCCGCGCCCTTTGTGGTAGAT
GATGAATGGGAAGATGACATAGGTCTGGTGCATAGCCAGCGTGTCGTACAGTCGGGGCAA
AGCTGGCAACAAGGGCCCATCATTCTGAACTGGCTGGATATCCAGGACTCGTTCGATGCT
TCGGGTTTCAACCTCATTATTCATGAAGTCGCGCACAAACTGGATATGCGTAATGGCGAT
CGCGCCAGCGGCATCCCTTTCATCCCGTTACGCGATGTGGCTGGCTGGGAACACGATCTC
CACGCGGCAATGAATAATATTCAGGATGAAATCGATCTTGTTGGCGAAAGCGCTGCCAGT
ATAGATGCCTATGCCGCCACCGACCCTGCAGAATGTTTTGCCGTGTTGTCAGAGTATTTT
TTCAGCGCGCCAGAACTGTTTGCTCCACGTTTCCCGGCACTATGGCAGCGTTTTTGCCAG
TTCTATCGCCAGGATCCTTCTCAGCGCTTACGGGTAAGCGCTGCCGAAGGCGACTACGGC
GAGGAATCCGAACATTAA
>EECEBICD_01394 tRNA-Ser(cga)
GGAGAGATGCCGGAGCGGCTGAACGGACCGGTCTCGAAAACCGTTGCGGGGGTAACTCCG
CCGAGGGTTCGAATCCCTCTCTCTCCGCCA
>EECEBICD_01395 hypothetical protein
ATGGAAATAGCTTATTTATGTGCTGCCAGAGTTTCTGATGTATTGAAAATGAACTGGAAT
CAAATACTGGAGAAAGGAATTTTTATTCAGCAAGGAAAAACCGGAGTTAAACAAATTAAA
TCCTGGACAGATCGCTTACGTGATGCCGTTGAAATATGTCGTGAATGGGGAGAGGAAGGC
CCTGTTATCAGGACTATGTATGGCGAGCGTTATTCTTATAAAGGATTTAACGAGGCGTGG
AGAAAGGCGCGAAAGGCTGCGGGGGATGATCTGGGACGTCCTCTTGACTGCACTTTCCAC
GATCTAAAGGCAAAGGGGATTTCAGACTATGAGGGAACGGCAAAAGACAAGCAGAAGTAC
AGTGGCCACAAAACCGAATCCCAGGTTCTTGTTTACGATCGCAAGGTGAAAATGAGCCCA
ACCCTGGACAGGAAGCGTTGA
>EECEBICD_01396 hypothetical protein
ATGATTCGCCTGGACACGGCTTTTTTGCCCACGGGCATCCATATAACGCCGGATATGTTC
TGGCTTTATTGCTTTAGCTTCGGCATCACCAAATACGGCAAGTATATATTTCTCATGTGC
CAGATAATCTTTCTGCGTTCTTGGGGCCAGATCAGCATAATCGGCACTGGCAAGAAATTT
TCGCCATAA
>EECEBICD_01397 hypothetical protein
ATGAGCGATCGCTTCCTGACTGAGGAGGAACTGGAAGATGCTACAGGAGCAAGCCAGAAG
TCACTCCAGAAAGAAGTATTAACGCTGAACGGTATTTATTTTATAGAACGCCGGGACGGT
TCAATCAGAACAACCTGGTATCATATAAATCACCCAGTTTCGCGCCTTCTTCCACCAGCA
GGGTATCAGCCTGTACCAGGCATGAATTTTGACGCTATAGAGAGTTAA
>EECEBICD_01398 hypothetical protein
ATGAACAACTTAATGGTCGACCTTGAAACGATGGGTAAAAAACCTAACGCGCCTGTTGTC
TCCATCGGTGCTGTGTTCTTCGATCCGCAAAGTGGTGAAATTGGACCTGAGTTCTATACC
GCCGTTAGCCTTGAAAGCGCAATGGAACAAGGTGCCGTTCCTGATGGCGATACCATTCTA
TGGTGGTTAAGACAAAGCCCGGAAGCGCGAGCGGCTATTTGCGCTGATGCAGTATCTGTT
ACGACCGCGCTTATTGAGTTCAATGACTTTATCACCTGTCACGCCGACGATTTGAAATAC
CTGAAGGTATGGGGTAACGGTGCCAATTTCGATAACGTTATCCTGCGTGGCGCTTTCGAA
CGTGCCAGCCTCCCCTGCCTGTGGAATTACCGGAACGATCATGACGTCCGCACGATGGTT
ACTTTGGGTCGTGCAATCGGCTTCGATCCCAAACGTGACATGCCGTTCGAAGGCGATATG

CACAACGCGCTGGCTGATGCCAGGCATCAGGCGAAATACGTTTCAGCTATCTGGCAGAAA
CTGATCCCGCCCACCAGCAACAATATCTGA
>EECEBICD_01399 hypothetical protein
ATGACCACTATTACCAAAGAATGGCTACAGCAAACTATCGCTGAATTTGAAAACACTCGC
GACGATATTCCGTTTGGCCTGAGCGATGACGACGCCAAAGTTCTTATTGTGCTGAAGCGT
GCGCTGGCATCGCTGGAAGCTGAGCCAGCCGGATATCACGTAATCAAAGAGTGCGGAAAG
GTTGGCTGTAGTGTTGCAACGCTTGAGGAAGCCGAGAAAACGCGAGATTTCTGGAATAAA
AAGTGGACTATCAGACCGTATTTTTACACCGCCCAGCCAGTACAGGAAACTGGCGTTTAC
AATGATGTGCTAAATATCATCAGCCTGTTGGAAAACAACGAATGGGCTGAGCACTGCACG
AGTACAGTTTTAGGTTCACTTCTGGAATCAGAAATAACGCGTCTGGTTGGCAAAGAACAG
TCAGCGCCGGTAGTCACTTTCTATCGCGATGGCGTTGAAGCCGCCGCCAAATGGATAGAT
CAGCAGCGTGAGGCATACGACAGCGAGCATGGATGGTCTGATCCTGATACCGGAGCGTTC
GAGTTCGGCAATGATGCCCAACGCGGATATTCATCCACCCTGGAAGAATTGGCCGAAGGC
ATTCGCGCTCTGCATCCAAATGCTGGCAACTCTCCGGTAATTCCGGATGGTTGGATAAGC
TGTAGTGAGCGGATGCCAGAAGACGAGCAAGAAGTAATTGTTCATAACAAGTTGGGATAC
CGTTATGTTTCATATTTTGATGAGCATTCTGGACTATTTTTTGACATGCGAGGCGGCAAT
CAGATGAACTGTATTGAGCACATCTTTGTTACACACTGGATGCCAGTGCCAGCAGCACCA
AAACCGGAGATAAATAACGAATGA
>EECEBICD_01400 hypothetical protein
ATGTGGAGAGGATTAAATCGCGGCGGCAGCCAGATGATTCTAACTGCCTACGAATACGAT
CCGGAAACCGAAAAATCTCAGTCTGTTTACCTTCTACGGCATCACAGCAAGGTTAAGAAG
ACCACGCTTGAACAGAAGCTGACAGTTAAGAACGACGCCTTCGGACGGTTTAAGCCTTTC
GTTGAACTTGAAGATTTTCCGGAAGGGCTTAGCGAACGCGAAGCAATGCTGAAATTAGCT
GACTGGCTGCACCGACTTAGTGTGGCTATCGAAGATAACTGGAGTACACCATGA
>EECEBICD_01401 hypothetical protein
ATGTGTAACTTCCACGAACGCAAAGTTCGTCGAACTGAATATTACCAACGTTTCGTTTTT
GGCTGGAAGCTGCGTCCCTGCACGGCGTGTAACGGCAGTGGTTATTACGACCACAACGGT
AGTCCGAAATGCTCGTCCTGCAATGGCACAGGCAAAGAGCGGTACAAACCAAATTAG
>EECEBICD_01402 hypothetical protein
ATGAGCAACATCGACAAACTCAATGACCATGAACTGGTTGATCTGAAAAACGCTATCGAA
AGAGAGCTTAAACGACGCGCTGATGGGCCAAAAGTCACCACGTATTATGTCGTCTCCTGC
ATAACTGATGCTCAGCATTTTACTGATTTGGACTGCGCTTTACGTTGCTTAAAAAGTGTC
ACCGAAAACCTTATGGAGTGGGTAACGGAATCCCCAGAAAACCGGGATTACGTCAATCAA
TGCACAGGCATTGTTGGGGCAAAACTCCAGGTGAAGGAGATGAATCTCGATCACTTCAAC
ATGCGCGTAGCAGAAAAATATTTCGACGATATTTGTTATCCACAGGAGACAGCCCAATGA
>EECEBICD_01403 hypothetical protein
ATGAGCAACCGAATTCGCAATGCTCAGGTATTCGACGCGCGCACCGGTGAGTACCCGGTT
GATATGTACATTCGCTGGATCATTGGTGGTGAACTTGATTTTGATGCCAATTATCAGCGC
GGGTATGTCTGGGGGCATGAAGAGCAGCAGGCATTCTTAAACGCAGTTATTTCTGGTTTT
CCTATCGGCTCAGTGGCGCTGGCAAAGGCACCTGACTGGTGTTCGCGTGAACTTCCTTAC
ATAGAAGTTGTTGACGGCAAGCAGCGTCTCACAACCCTGAAAAAACTCATCACCAATGAG
ATTCCAATCATCTTGGCTGATGGTCCGCTTTACTGGCGAGATATGACTCGTGCGGAGCAA
TTGGCATTTGGGCGTCGTCCACTCCCAGCAGTTGTGCTGGATGAGGTGACGTACAAGGAT
CGCCTGGCTTATTTCATGGCGGTGAACTTTACAGGTGTTCCGCAAAGCGAAGAGCACAAG
CGACACGTAATGCAACTAATGGAGGCTGCCCAATGA
>EECEBICD_01404 hypothetical protein
ATGAAACTGGAAATGTACACCCTGGACGGATCGGTGATTGTTGATAGCAATCTGGTAACA
CAGTTCTATCCAGACTACAAAAGCGGCGGCGAGTTAACAGTCATCGAGACTATTTCAGCC
ACCGGAGAAACTTTCACCGTGAGGGTTAAACACTCGTTTCTTCAGGTAACAAGTGCGCTG
GCTACGGCGTGGAGCGTTGATGAAAAGAAAGCTGAAGGAGCAGCTCAATGA
>EECEBICD_01405 hypothetical protein
ATGAGTTTTATTCAAACACTTTCAGGTAAACAATTTGATTATCTCAGCGCAACTATTGAC
GACATTGATATTGAAGATATCGCCGTGGCGCTTTCCAATATTTGCCGCTTCTCCGGACAT
CTTCCTGAATTTTATAGCGTGGCGCAGCATTCCGTACTGTGCAGCCAGCTTGTATCACCG

GAGTTTGCCTTTGAAGCCCTGATGCACGACGCAGCCGAAGCGTATTGCCAGGATATCCCT
GCCCCATTAAAAGCGTTACTGCCTGATTATCGCGAGATTGAGAAACGTACCGATCAACTG
ATCCGCTTTAAGTTTGGCTTGCCACTGGAAGAAGCCAGCGTAGTGAAGTATGCAGATCTG
ACCATGCTGGCAACTGAACGCCGTGATCTGGATATTGATGACAGTATTCCCTGGGTAATA
CTGGAAGGTATCCCCCCGACAGATTTATTCGAAATCTACCCACTTCGCCCCGGTCAGGCT
TTCGGCCTGTTTATGGCCCGCTTTAATGAACTGATGGAGCTACGCCAATGTGCTGCATAA
>EECEBICD_01406 Recombination-associated protein RdgC
ATGCATAACCCATTTTTTAAAAATATGCTGATATATCGCTTCAGCCGCGACTTTAACATC
GACATAGACTCTCTTGATAAGAAACTTGAGCTGTTTCGCTTCTCACCATGCGGAAGCCAG
GATATGGCAAAAAGCGGATGGTTTTCACCATTAGTCCAGTATTCAGATGTGCTATATCAT
GCAGTCAATAACCAGTTACTTTTGGTTATTCGTCGTGAAGAAAAAATCATACCTAAACAA
ACGATCGCCGATGAGATTAATAAGAAGGTTTCCACGCTTGAGCGAGAGCAAGGCCGCCGT
CTTAAGAAAACTGAGAAAGACTCTATTCGTGATGAGGTTCTTCATTCCCTGTTACCCAGG
GCGTTTACTAAAAACAGTCTGGTTCGTATTTGGATAAACACTGCAGCCGGGTTTATCGTT
GTTGATACATCCAGCATCAAGCGCGCAGAAGATTCTCTCGCCCTGCTTCGTAAAACCCTA
GGTTCCTTGCCAGTCGTGCCGCTGACGATGGAAAACCCTATCGAGCTCACGCTAACTGAG
TGGGTTCGTAGCGAAGCGGCTCCTTCCGGGTTCTCCATCGGCGATGAAGCGGTCCTTAAA
GCTATTCTCGAAGATGGTGGCACAGGCCGGTTTAAAAAGCAGGATCTTGCCTGCGATGAA
ATTCTTACCCATATCGAAGCTGGGAAGGTAGTTACCCAGATTTCAATGGAATGGCAGCAG
CGTATCAGTTTCACCCTTTCTTGTGACGGTATATTGAAACGCATAAAATTTGCAGATCAG
TTAATCAGCCAGAACGACGATATCGACAGCGAAGATGTCGTGCAGCGATTTGATGCTGAC
ATTACGCTGATGACAGGCGAACTCAGTAATCTTATTTCTGATTTAACGGCTGCCCTCGGC
GGCGAAGCCAAGCGATAA
>EECEBICD_01407 hypothetical protein
ATGTCTCAGAAGGATGATATTCCTGTCTTTCCCGTAACCGGCTGGCAGGCTGGACCGCTT
CCTGGTTACGACGCTCTGGTAGTGAAATTCCAGTTTCTCTCATCACCGATGCAACCAATT
GAGTCTGCTCAGGAAACGCAATTTTTAGTACTTACTCCTGAGATGGCTGAGAGCCTGGCT
TCAGACTTGCAAAGGCATATTCAGGATTTGCGAAATTCCGACGTTCACAGCCCACAAGAA
GGCAAGCACTAA
>EECEBICD_01408 hypothetical protein
ATGAATAACTACTACACATGCTCCTTCTGCGGGGTCAGTGAGCTGGATGCAAAAAAGCTC
ATCGCTAAAGGAAGTAAGGACGAGCCAGCTATCTGTTCCGAGTGCGTTGTTTCATGCGTA
AACATTCTTATCAACTACGCAGCCGTGATAAAGCCAGTAAAACTGAACGTCACGAAGGGG
GAATGA
>EECEBICD_01409 putative HTH-type transcriptional regulator
ATGAATATAGGAAACCGTGTCAGACAACTTCGCCGCGCGAAGAACATGAAAATTGCTGAG
CTAGCAGAAGCGATCGGCGTGGATGCCGCAAACATCTCTCGTCTGGAGACTGGCAAGCAA
AAGCAATTTACCGAACAAACACTTTCTAGGCTGGCTGACTGCTTAAGTGTTGATATAGCA
GAACTCTTTACCTCAGACCCAAAAGGTAATACTGTATGTAAACACAGTGATATGAGGAAG
GATTCAGCTAACGTGAAGGATTTGTTCCGTATCGAGATACTGGATGTCAGTGCAAGCGCC
GGTAATGGACTCATTCAGGGCGGTGATGTTATCGATGTAATCCATGCTATCGAATATAAC
AAGGACAAAGCACTAGCTATGTTCGGCGGGCGTCCTGCCGCTGAGCTTAAAGTGATTAAC
GTGCGTGGTGACAGCATGGCACCAACAATTGAACCCGGAGATCTTATTTTTGTCGATATA
AGCATCAACCAGTTCGATGGTGATGGCATCTATGTCTTTGGCTTTGATGATAAAATATAC
GTAAAAAGGCTACAGATGATCCCCGATAAATTACTGGTGATATCTGATAACACTAACTAC
AGGGAATGGAGTATTACCAAAGACAACGAGTGCAGGTTCGGTGTTTTTGGCAAGGTTCTG
ATAAGCCAGACGCAGTCACTCAAACGACACAATTAA
>EECEBICD_01410 hypothetical protein
ATGCAATCACCATTGAGAAAATTGCGGAAATCGCATGGTTATACGTTACAGCACGTCGCT
AAAGGGGTTCAGGTTGATCCTGCAACATTAAGCCGGGTTGAAAGATGCGAGCAGGCTCCT
TCAACAGAGCTTGCTGAGCGCCTGGCTCAATTTTACGCCGGAGAAATTAGCGAGATGCAA
ATTTTGTATCCAAACAGATATCAGCTTAGTGATTCGGCGATTTGA
>EECEBICD_01411 hypothetical protein
GTGGGACATGAACCTGAATGGAAAGTTGAAAAGCAGCCCCGCTGGCTGGTGGCTGCGATT

AAAAAGACGATTTCCAGTCTGCATGGCGGTTATGAAGAAGCTGCGGAATGGCTGGATGTC
ACCAAAGATGCTCTGTTTAACCGCCTGCGTACTGGTGGTGATCAGATCTTCCCGATTGGG
TGGGCGCTGGTACTGCAACGTGCCGGAGGAACCTATCACCTGGCACATTCAGTAGCCAGG
GCATCAGGTGGCGTTTTTGTTCCGCTGGCAGATATGGAAGAAGTGGATAACGCAGATATT
AATCATCGCCTGCTGGAAGCGATTGAGCAGATCACCAGTTATTCCCAGCAAATCAGGGTG
GCTATCGAAGATGGCGTTATTGAGCCACATGAAAAAGCCGTGATTGATGAGGAGTTGTAT
CAGGCGATCGCAAAGCTGCAACAGCATTCGACACTGGTATACAGAGTTTTTTGCGTGCCA
GAAAAGGGTGACGCCCGCGAGTGTGCAGCTCCGGGCGCCGTGGCGTCAAATTTTATGGAG
AAAACCAACGCATGA
>EECEBICD_01412 hypothetical protein
ATGAACAGTTTAACGGTAAATAACCGTTTGTCGCAACAACCGGGGATGTATGAGTACCGG
CCGTTGCGTCATGAATGCAGATTATCAAATAGCCTGGTCGTGCGTAACCACAGGGAACAC
AGCCTGACCGTGGGGGATGAATCGTGCAGGAACTTAACCGCTGGTTTCGGGATGGAAGGG
GACTTTATGTCCATGTCATTCGCTGGGAACCAGAAACTGAGCGCGTTATCTATCTGCGCA
AGGGCTATCCGCATGAGTGTTTTAGCCCTTTGTGGAAATTCAGGCGTGATTTTGTTGAGT
GTGAAGCGCCAGGAACACATTGATTCTGCAATTCCGGGACGTTACACTGTTCAGGCACCT
CATAAAGCGGGTGCCGGGCGTGGAAACCCGGAATTCAATATAGAGCACAACCGCGCTCAT
GCGGTTTTTTCTTGTCATGAGCATTGTTACGCCCAAATTATGGTGGGGCGTGCAGGGCCA
GTTTCGGCTGGGCCGGGTTCTATGTTGACCGGTATTTCCACCCCTGTACGTCTCACCACC
TATAAGGTCGTGGAAAGCCTTGGTGGTGAGTTCATTGAATTCAACATAGAGGCTGCCACT
ATGGCTACTGTCCCAACCCTCGCTCAACCTGAAATTAGAATTATTAACGGCCAAGCCGTT
ACTTCCTCCCTGGCTGTTGCCGACTACTTCATCAAGCGTCACGCTGATGTTATCCGTAAA
ATAGAATCTCTCGAATGTTCCACTCTATTTCGTAAACGCAATTTTGCGTTTACATCGATT
TCAATAAATCAGCCCAACGGCGGTACTCGCAAACTCCCATGCTATCAAATCACACGCGAT
GGTTTTGCGTTTTTGGCAATGGGTTTCACTGGTAAACGTGCTGCTCAGTTTAAAGAGGCA
TACATCGATGCCTTTAACCAGATGGAGAAACAGCTTTCAACTCCATCGGTGCTGAGCGAT
GCAGCACATAATGCCAGCGTTCTTTATTCCTACATTTCATCCATTCATCAGGTTTGGTTA
CAGCAGCTTTATCCCATGCTGGAAAAAGCGGAATCTCCGCTGGCTGTAAGCCTGCACGAT
CGCATCAATGACGCTGCGGCGCTTGCGAGCCTTATCAATATGACACTGAACCGTTCAGAG
GTAAGGGGGCGCAAATGA
>EECEBICD_01413 hypothetical protein
ATGATCCGGAATATTTTTAAACGGTTCACCAGCCAACGTTTTCATTGCCCTCGTCCAGGA
CAGTGGTACAGCACACCAGAAGGGTACGTTCTGCGTATTAGCCTGGTCGATCGCGAATGT
CAGAAGGTTGTCTGTGAGCCTCTTGGGCGTAATTACCGCGTCAACATGCCGCTTATTGCC
TTTCGTTCCGGCAAAAACATGAAGCATCTCGGAGGTGCTGCATGA
>EECEBICD_01414 hypothetical protein
ATGAGTTCCCTTATTCAATTACTCGATCGCCCCATCGCCTACAACCCTGCTTTTGCAAAA
CTGAAAGCCGGGAAGGTAAAAGCTGGCCCGGTTGCGGCAGTATTCCTGTCCCAGCTTGTT
TACTGGCATAACCGGATGGATGGCGGCTGGATGTACAAAACACAGGCTGATATTGCCAGT
GAAACGGCGCTAACCCGCGACGAACAGGAAACAGCACGTAAACGTCTGGTAGCACTTGGT
GTACTGGAAGAAGCCCGTCGCGGTGTACCTGCCACCATGCACTACCGCATCAACACCGCA
CGGCTTGAAGCGCTGTTGCTGGAAACGGCGAAGCCAGTGAAAAAGGGCGCTCAGGAGAAA
ACCAGATTGCGGGACTTCCAGAATGTGGAAACCCCGCAATCTGGATTGGTGCAACCCCGC
AAACCAGATTGCGGTGATGCCGCAAACAAGAATGTGGAAACCCCGCAAACAAGTACGGGG
CAACCCAACGAACAAGCATGTGGCGATCCCACAATCTTTCCTACAGGAGATTACACAGAG
ACTACTCAGGAGATTACACAGGAGAGTAAAACCCCTTTTTGTCCGGTTGCTGAGCAACCC
GACCCCGAAGTGACGCTCACCGATCAGGCGATTGAGGTTTTAACCCACCTGAACCAGGTA
AGTGGCTCCCGGTATCAGAAGTCAAAAACCTCCCTGGAAAACATCCGTGCCCGACTGCGT
GAGGGGTACAGCGTTGCTGATCTGCAACTGGTTATCGACCTGAAGCATGAGCACTGGCAC
GAGAACGACGAGCAGTACCAGTACATGCGCCCGGAAACGCTGTTCGGTCCGAAGAAATTC
GAGAGCTATCTGCAAAGCGCTACCCGCTGGGATCAGAAGGGGCGGCCTAAACGCGCTGAC
TGGGGTGCGAAGAAGCGCGATGTGATGGCTTTTGGTCCGGTTGATACAACGATTCCGGAG
GGATTCAGAGGATGA
>EECEBICD_01415 hypothetical protein

ATGAGTCTGTTAGCAAAAGTGCAGGCGTTTATCGAGCTTAATCCGGGGCTGACATCAAAT
GAGATTGCCGATGCTTTTCCTGAATACGCACGCTTTGATGTGCAGCGTTCAGCGAGCAAG
TTGTATCGGTGTAAGCGTGTTAACCGCCGCCTGGATGGAGATGTATTTCGCTATTACGCG
GGTAAAGACGAGGCAGTGATTTTGACGTTACGACAGAAAAGGTCAGGTCATACAGGTTCG
GGTGATCCGATGGTGATTGCAAAGCTGGTAAGCCGCGCTGAAGAACTGGAATCCAGAGGG
TTATTTAATCGTGCATCGATAGTGTGGCTGGAGGCATTTAGCGAAAGCCAGTTTATCTAC
GAACGCGAGGAATTTTTACGCCGCCGTCAGAAGTGTCTGAACCGCATCAAAAAGAGAATC
AGACCCGTAGAGCAGGTTTATCTGGCAGGGCGATTTGTGGGGAACGTGGAATGA
>EECEBICD_01416 DNA adenine methylase
ATGACCAGTGAATCCGTTTGTATTGAAAGCAGTGATGTAACGATATCTGTTGATGAATCC
GCTTCGCGCACCTGGCGTCGCCCGTTCCTGAAATGGGCAGGCGGTAAATATTCCATGTTA
CCCGATCTTTACCAGGTCATTCCGGCAGGTATGCGCCTGATTGAACCGTTTGTCGGCGGT
GGTTCGGTGTTTCTCAACTCAGACAAACACGCCTGCTTCCTGCTGGCCGATGTGAATACC
GACCTTATCAATCTGTATCAGATGCTGGCTGTTGTACCTGGTGCGGTGATAAGACATGCT
AGGGTAATGTTTGACCGTCTCAATGACGCTGAAAGCTATATGGCGCTACGGGAAGAGTTC
AATGCTCAGGTGATGGACGCTCCGGAACGCGCCGCCGCTTTCCTTTTCCTTAATCGTCAC
TGCTTCAATGGCCTGATCCGGTACAACCGCAACAACCAGTTTAACGTTGGCTGGGGCAAA
TACCCGTCGCCTTATTTCCCGGAAGAAGAAATCAGGGCATTTACCGAAATGGCGCACAAC
TGCGTATTCATGGCGGCAGGATTTCGCCGGACGCTGGCACTTGCGGGAGAGGGTGACGTT
GTGTACTGCGATCCACCCTACGAACCGATGCCCGGCAAGGATGGTTTTACTCACTACGCC
GCTGGTGGCTTTACCTGGGATGATCATATCGCGCTGGCGGAATGTTGTGTTGCTGCTCAT
CAGCGAGGTGCCAGAGTCGTGATCGGCAATTCCACATCTCCGCGTGTTATCGACCTGTAC
TCGCAGCACGGCTTTGAAATCCGCTATATCAGCGCCCGCCGCTCAATATCAAGTAAGGGC
AGTACCCGCGAGAAAGCGAAAGATCTCGTGGCGATTCTGTAG
>EECEBICD_01417 Crossover junction endodeoxyribonuclease RusA
ATGAAACTGACATTGCCATTTCCACCCAGCGTTAACACCTACTGGCGGGCTCCGAATAAG
GGACCGCTTAAAGGTCGTCACATGGTCAGCGCCAGCGGCCGGAAGTATCAGAGCGAGGCG
TGCGCGGCAGTGATTGAGCAGTTACGCCGTCTGCCAAAACCTTCAACAGCCCCGGCAGCG
GTGGAAATCACCCTGTATCCGCCAGACAAGCGGATCAGGGATCTGGACAACTACAACAAG
GCGCTGTTTGACGCCCTGACCCACGCGGGTGTGTGGGAAGACGACAGCCAGGTGAAAAGA
ATGCTGGTGGAGTGGGGACCAGTTTTCCCGAAGGGGAAGGTAGAAATCACGATCACGAAA
TTTGAAACAGGGGCGGGTGCAGCTGCCTGA
>EECEBICD_01418 hypothetical protein
ATGAATAATTTAATGGTCATTGATGGTATCGAAGTTCGCCGCGACGTTCATGGGCGCTAT
TGTCTTAACGATTTGCACCGGGCTGCGGGTGGAGAGCAGAAATACCGTCCGAAGTACTGG
CTTGATAATAAGCAAACCCGTGAGCTGATTGAGCAACTTTTCACCGAGGGCGGAATTCCA
CCCTCGGAACAAAATCAATCTGTTAGCTTTTTTCAGGGCGGTAGTGATACCCGAAGTTTG
GCACGTGCTCCAGTAAATACTGTTCGCGGTGGTGCTGAACAAGGTACATACGTATGCAAA
GAACTGGTATTTGCTTATGCAATGTGGATCAGTCCGTCTTTCCATCTCAAGGTGATCCGC
ACGTTCGATCGGATTACCAGTGCGCCACAAATATCTTCTGGTATGGCTGCCGATAAGATG
CAGGCGGGGGTGATTCTGCTGGGTTTTATGCGCAAAGAGTTAAACCTGTCCAATTCATCG
GTACTGGGCGCGTGCCAGAAACTCCAGGAGGCAGTGGGACTACCTAACCTGGCGCCACAA
TATGCCATTGATGCTCCGGCTGGCGCGCCGGATGGTTCAAGCCGCCCGACGCTTGCACTG
AGCGCGCTGTTAAAACAGCATGGTATCCGGATGACGGCTAATCAGGCGTATCAGCAGTTA
GCAAAGCTGGGTGTTGTTGAACATCGTGAGCGTTACAGTCGCTCCGCGATTAACGGCATT
AAAAAATTTTGGTCGCTGACGGCGAAAGGCTGCATGTTCGGCAAAAACATCACCAGCCCG
GCAAACCCTCGCGAGACGCAGCCGCATTTCTTCGAATCCAAATTCCCTGAGCTGCTGAAG
CTGCTCGATACCGTTCATTGA
>EECEBICD_01419 hypothetical protein
GTGAGAGCGTTACTGACCCCTGAAATTGCTCCTCGTATGGGCGTTGTATTGTTCAGGCCG
GGATCGGAACTGATGCCCCTGTTTATGCAGGGGCGTGTTCTGCTTGAACCAGAGCCGGAA
CAATATTCATCTTTCGCCTGCGGCGCGGTCCCGGCGGTATCACAGCCGCTGGCGGATGAT
CCTGCTGTTCGTGATGTGTTCCGTAATGAGTCGGTTATCTATCGTGCTGGTGGTCTCGAT
AGTCTGGAAAGCTGGCTACTCCGGGGGAATGGCTGTCAGTGGCCGCATTCAGTCTGGCAC

AGCGAACAGATGACAACCATGCGCCACGCACCGGGGGCAATCCGACTGTGCTGGCACTGC
GATAACCTGCTGCGCGAACAGTTTACGGAACGGCTGGAATCAATAGCTGTGGAGAACACG
ACAAAATGGGTTTTATCGGTTGTTTGTCGTGATCTGGGTTTTGACGATATGCACGCAGTC
ACGCTCCCGGAACTGTGCTGGTGGATGGTACGCAATGACCTGGCAGAAGTCTTACCGGAG
AGCGCTGCGAGAAAAGCATTAAGGATGCCGAAGGCAATTGTCCAGTCAGCTACCCGTGAA
AGTGAAATTGTCCCCTCGGTGCCGGCCACCAGCATTGTACAGGATAAGGCGAAAAAGGTA
CTGGCACTCAGGGTTGATCCGGAATCGCCGGAAAGCTTCATGTTACGTCCGAAACGCCGT
CGATGGATCAATGAAAGATATACCCGCTGGGTTAAATCCCAGCCGTGCGCGTGCTGCGGG
AAGCAGGCGGATGATCCGCACCACCTGACAGGCCACGGTCAGGGAGGGATGGGAACAAAG
GCGCATGACCTCTTTGTGCTGCCGTTGTGCAGAACGCATCACAATGAGTTACATGCGGAC
ACCGTGGCATTCGAAGAGAAATACGGCTCTCAACTGGAGTTGATATTTCGTTTTATCGAT
CGCGCGCTGGCGATCGGCGTGCTGGCGTAA
>EECEBICD_01420 hypothetical protein
ATGAACCTTGAAGCCTTACCAAAATATTACTCACCAAAATCTCCAAAATTGAGCGATGAC
GCTCCGGCGACAACCTCCGAATCTTTGACGATTACGGATGTAATGGCGGCGCAGGGGATG
GTGCAATCGAAAGCACCACTGGGGTTTGCTTTATTCCTGGCAAAAGTTGGTATTCAGAAT
CCTGACTTCGCGATTGAAGGGCTGATTCATTACGCGGTGGCACTGGATAACCCGACACTG
AATAAATTGAGTGAAGAAACTCGGTTACAGATTGTTCCTTACCTCGTGAATTTTGCATTT
GCTGATTATTCCAGATCTGCTGCAAGCAAGGCTCGCTGTGAGCATTGTGCTGGTACGGGA
TTTCATCATGTATTACGTGAAGTGGTGAAACACTCCAGAAATGGTGAACCCGTCATCAAA
GAGGAGTGGGAGAAGGAACTATGTCAGCATTGTCATGGTAAGGGAGAAGTCAGCACGGTG
TGCAGAGGGTGTAAGGGTAAAGGTATTGTCTTGGATGAAAAAAGAACTCGGTTTCATGGC
GCGCCTGTTTATAAGATTTGTGGGCGTTGCAATGGAAACCGGTTTAGTCGTTTACCAACC
ACACTGGCGCGGCACCATGTCCAGAAACTGGTACCGGATCTGACGGATTATCAGTGGTAC
AAAGGATATGCAGACGTCATTGATAAACTGGTTACAAAATGCTGGCAGGAAGAAGCATAT
GCAGAAGCGCAATTAAGAAAGGTGACAAGATGA
>EECEBICD_01421 hypothetical protein
ATGGATAAGATTCTCCCAACGATGGGCTTTGTATGTCTATCGTTGATAAGTCTCAAGAAC
CCGCCTCCGAGTGGGTTTTTTATTTGTGATCACTTTATTTTTTGTCTTGCTAAGTTATTG
TATGGACAAGAACTAAAATTAAGTGGTGACATTGTGCTCTCTAATAACGAACGTTGGGTT
TCCTTTTTTGACTTTGCTTTTACGCCTACACACGCAGCGGCGCCGAGTATTCCCATTGAA
GACATACTCAAGAAATTGAAGGTACTGGTGAGCTCAGGGAGTGCTGTAAAGTTATACAAT
CATAGGTCTAGAGCGCTTAGGATTTCGGAGATGAAATATTCTATTGGGGATAGCCAGGCG
ACTCTACTTATCCAGCTTTGTGATAAAAATGGTTCTGACCCTGTTTTTGGTGAGTTAACA
ACAGGCAACCTTAGAGTAGAACCTAAGCTTGCCGGTGAAGGTATCGCAGTTTCTTGTCAC
ATTGTAATATCCACAGATGTTGTCAAAAACACTGCCGATCACCACAAAACTCTCGTTGAA
TCTGTCCCCGGTATCAGTAAGTCAGTTCTTGAGCCATTTTTAAATGCTATGCTCAGAGAA
GCCTTCGCTGGATGTGAGTTTAAAAATCCTGCAACTAAAGGTATGTGCCAGCACCGCCCA
AAGCTGGAAATCTATTCTCATGGTTCACAAACGCTGATGGATGCATTAAAAGGTGCAAAG
ATTCATAACGTTAAACTTGTGAGTACAAGAAGGAAAGGTGGATTGGACCAAACGGCGTAC
ACTGAGCTCTCAGAAAGGTCCGTAAAGTATAAAATCATTAGACAGCCGCCATTGAAAGAT
AAAGAAAGGTTGTTAGAGATTTTAAGAAAGAAAGGGCAGCAGTCTGGATATACCAAGGTT
TCAATTAGTTACTCAAAAGATGGCAAGCAAGCCAGTTTGGATCTTGACCGTAACGAAGAT
GCTGCCACAAAACTGTTCACTAAAAGTGAGAGGGTAATATTAGGTAACCTCATCAACCAA
TGTGAAAGCACAGTACATCTGCAGCTTGAAACAAAAATGATAGGGTTGCTCTAA
>EECEBICD_01422 hypothetical protein
ATGAAACTTTTTTCACCGCTGAGTTATCTCCGCATCAAGCATGAGGAAAAGGACTGGTAT
GATTACAAAATACCAGCTGCAGTGTCTCTAATCGTCACTATTGTTTATTATTTTCACGCT
AGCAAAATTTCTTTAATCGAGACTAACGGACTCCTGCTTCAGGTTAATGGGTTACTTCAA
GTCTTGATTGGTTTTTATATCGCAGCACTGGCTGCGGTTTCTACTTTTTCTAGCTCTTCG
ATCGACGAAGTAATGGCGGGCGTACCTCCGACTCTAGTAGAGAAATTCCGAGGGCAGAAG
CTTACTGTAGAACTGACGCGCAGGCGCTTTGTTTGTTACCTTTTTGGTTATCTAGCTCTT
GTGAGCTTTATGTTATTTTGCTTAGGGATGATTTCTATTCTGATTGGGAAGCCTTTCCAT
TTGTGGCTGCTCACATTCTGTTCTCCTGATGCAATCTTGTGGCTTAAAACGGTATTTGTT

GGCGTTTATATATTCATCTTAATGAATATCATAACAACAACTTTGCTGGGACTTTACTTC
CTTGCAGTTCGGTTCCACCAATCATCGCTGTAA
>EECEBICD_01423 hypothetical protein
ATGTCCGAACCTGTATCCAGTGCGACAGTGTTGGCTGGTGGATTAATGGGGGCCAGTGTA
TTCGGTCTGGCAACCGGAACTGATTATGGTGTGGTATTCGGCGCTTTTGCCGGCGCGGTG
TTTTATGTCGCCACGGCAACCAACATCGGACGCATCAGGCTGGTCGCTTATTTTATTACA
TCATTTATTGTGGGAGTGCTTGGTGCCGGGCTGATAGGTACTAAGCTTGCGGCAATAACG
CATTATGAAAAACCACTGGATGCACTTGGCGCAGTGATTATTTCTGCAATGTGTATAAAG
TTTCTCACTTTTCTCAACAGTCAGGATCTGAACACCCTGTTCAGTATTCTCTCTCGTATC
AGGGGAGGGGATCAGATGGTAGCAAATGACCCTTCTGCAGCTCTGAATGCCGTAATTTGT
GGGGTGATAGTCATCGTTCTGATGTTTTACCGACGCGGTGATGCGACACACCGCCCCCTG
ATTTCGTTACTGGCCTATGTCATGGTGCTGGTATATGCCAGCGTCCCTTTCCGGTTTGTT
TTTGGTTTATATGAATCATCCCACTGGCTGGTGGTGATGGTGAATATCCTTATCTGCGCC
GCTGTGCTGTGGGCTCGCGGTAATGTGGCGCGTCTGGTCGATGCACTGAGGCACTGA
>EECEBICD_01424 hypothetical protein
ATGAATCAACAACAATTTCAGCAGGCGGCTGGTATTAGCGCCGGGCTTTCTGCACGCTGG
TTTCCGCACATTGATGCGGCAATGAAAGAGTTTGGTATTACAGCAGTTAATGATCAGGCC
ATGTTTATTGCACAAACGGGACATGAATCAGCAGGATTTACTGTTCTGAAGGAAAGCTTC
AATTATTCGGTGGAGGCGCTGAAAAAGACGTTTGGTAAACGCCTGACGCCGTATCAGTGT
GAAATGCTGGGGCGTATTGATGGTCGCCAGGTTGCCCACCAGCCGCAAATAGCCAATCTG
GTTTACGGTGGCCGCATGGGTAACAAAGACGCCGGAGATGGCTGGAAGTATCGCGGGCGT
GGTCTGCTTCAAATCACCGGCCGCGAGAACTACGTCAAATGCGGAGCTGCGCTGAAGCTT
GATCTGATCAGCACACCAGAGTTGCTGGCACAGGAGAAGCATGCAGCCCGTTCTGCTGCA
TGGTTTTTCACATTACGTGGTTGCCTGATGTATTCAGGTGATGTTGTCCGTGTAACGCAG
ATCATCAACGGTGGCCAGAATGGACTGGCTGACAGAAATAGTCGTTATAACAAAGCGCGG
GCGGCGTTGCTGGTATGA
>EECEBICD_01425 hypothetical protein
ATGCACAAGCACAAGCCGCTAAATCTGCTGCCACTGCTGCTGGCCTGTCTGCCACTGTTA
GCCAGCTGCGTACCGAAGCAACAAAGCTTGCCGCCCGCCTGGACGCCGCAAAGCACACCT
CAGATCTTGCCGCTGCCGTCAGAAGCAAAACAGCCGGAGCCGACGCCGCAGTGCTCGCCG
ACATGCTCGGACGCCTTGCAGAAGAAGCTCGATATTATGCTGAGCGATCTGACGAAAGCT
ACCGCGCAGGAATGA
>EECEBICD_01426 hypothetical protein
ATGCCACCAAGAACACCTAAATCCTGTCGTGTTCGCGGCTGTCGCAGTACAACAACAGAT
CCATCCGGATATTGTGAAAGTCACAGAAGCGAGGGCTGGAAACAATACAAGCCAGGACAA
TCCCGTCATCAACGCGGTTATGGTTCGAAGTGGGACGTTATCCGTGAGCGCATACTGAAG
CGTGATAAAGGTTTATGCCAGTTATGTCTGCGTGCCGGTGTGGTGCGTGAGGCGAAAACT
GTTGACCACATTATCCCTAAAGCGCATGGCGGAACTGATGCCGACAGTAATCTGCAGAGT
CTGTGCTGGCCCTGCCATAAGGCGAAGACGGCTCGTGAACGGCTGAAATAA
>EECEBICD_01427 hypothetical protein
ATGGGTGCTGTTGTGAGATCTTCCGGTGGTGGCCGTAAGCGCAATTTGCCTTCGGGCCAG
AAAAGCAAGCTGACCAGGATCGCACCGCCGGAAGAGTTAATGAGTGATATCGCGATCCGC
ATCTGGAAAACGCAGAGCAAAATTTTAATTGAGCGGGGCGTTTTTGATCTTGAAGACGCG
CCGCTACTCCTGGCGTACTGCAATGCGTTTCACTTGATGATTGAGGCCGAAAAAGTCATC
GCGGAAGAAGGCCTGACCGTATCAAGTGAAATGGGTGGTGAGAAAAAACACCCTGCAGTC
AATGTCCGTAATGACTCCGTTTCGCAGCTCGCCCGTCTGGGTTCACTTCTCGGGTTAGAC
CCGCTCAGCCGCATAAGAATGACCAGCGGAAAAAATGATCCGGACGATGAAGGGAATGAA
TTTGATGAGTTTGACTGA
>EECEBICD_01428 hypothetical protein
ATGGCTACATATCCGAACGTCAATGCGGCGAACCAGTATGCGCGGGACGTCGTGAACGGG
AAGATACTGGCCTGCCGGTTAACCATGCTTGCCTGTCAGCGACATCTTGACGACCTGGAA
CGTGCCAAAGATCCGCATTGGCCTTACCGCTTCGATAAAAATAAAGCAGAACGTTTCCTT
CGCTTTTCCCAGAAAATGCCGCACACCTCCGGAGAGTGGGCTCGCCGGAAGTTGCGGATA
GAATTTGAACCCTGGCAAAAATTTGCGCTGGGCGTGCCGTTTGGCTGGGTGCGCAAGGAT

ACCGGTTTTCGCCGCTTCACTGAGATTTACATCGAGGTACCGCGTAAAAATGGGAAATCG
GCGATTGCGGCCGCCGTCGGTAACTATATGTTCTGTGCAGATGGCGAGTACGCAGCGGAA
GTTTACTGTGGTGCCACAACGGAAAAACAAGCCTGGAAAGTTTTTGCGCCTGCACTGGCG
ATGGTGAAAAAGCTGCCGGCGTTGCGTCAGAAGTTCTGTATCAAACCCTGGGCAAAGAAA
ATGACTCGCCCGGATGGTTCCCTGTTCGCGCCAATTATCGGTGACCCTGGAGATGGCGAC
TCACCATCATGTGCGATCATCGATGAGTACCACGAGCATGATACTGACGCGCTATACACC
ACAATGACTACCGGGATGGGGGCGAGGGAGCAGCCCATCACGCTGATCATCACCACGGCA
GGCTTTGATATTGCCTCGCCTTGCTATGAAAAACGTACTCAGGTGGTCGAGATACTGGAG
CGCATCCGGGAGGGTGGTGAAAACGAGGCAATTTTCGGGATCATCTATACCCTGGATGAT
GACGATGACTGGACACAGCCGGAAGCTCTGATCAAAGCCAACCCGAATTACAACATTTCG
GTGAAAGAGGGATTCCTCAAGGCTAAACAGTTGCTGGCGATGTCCACGCCAGGCCAGACC
AATAAAATACTCACCAAGCATTTCAACAAATGGGTGAGTTCTAAAGCAGCTTACTACAAC
CTGCAGAAGTGGATGACCGCAGCAGACAAAACGCTCAGACTGTCCGATTTTGCAGGTGAG
GAGTGTTATCCCGGCATCGACCTGGCATCAAAACTTGACCTTAATGCAGTGGTGCCGGTA
TTCCGCCGTGAAATAGACGGCCTGAGTCATTATTACTGCGTTTCGCCTATGTTCTGGGTA
CCGGAAGACACCGTCTACGCCACGGACCCGGCGTTGAAAACTATTGCAGACCGTTACCAG
TCTTTTGTTAATCAGGGCGTGCTGGTTCCGTCAGACGGTGCAGAAGTGGATTACCGCCTT
ATCCTGGAAGCGATCCTGAAATTACGGGAAACGGTGAAGATAGCCGCGAGTCCGATTGAC
CCCTACGGTGCAACAGGCTTATCTCATATGCTGCAGGATGAAGGGCTTGAACCTGTCACC
ATTACCCAGAACTACACGAACATGAGCGACCCGATGCGTGAGATTGAGGCTGCGATCGCT
GCTGGCCGATTCCATCATGACGGTAATCCCTTGATGACCTGGTGTATTTCGAACGTGGTT
GGCAAGTACCTGCCTGGTAGCGACGATGTTGTTCGCCCGGTGAAAGAAGGCGCAGGCAAC
AAAATTGATGGTGCAGTTGGCCTGATGATGGGGGTTGGCCGCGCAATGCTGAACGAGCCG
AAAGACTTTCTTTCTAACCTCGATCCTGATGAGGAACTGTTATTCCTGTGA
>EECEBICD_01429 hypothetical protein
ATGCTGATTGATGCCATTTTCAGAAGCAACTCGCTGGAAAACCCAGCTGTTCCGGTCACC
GTTGAAGCGGTCGAAAACGACGGGATCTTTAATGGTGATGTGATTGTTAACCCCCGGACG
GCAATGAAACTGGCGGCGGTGTATGCATGTATCTACGTTATTTCATCCAACGTTGCGCAG
ATGCCCCTGCACGTCATGCGGCGAACCGGGAAGAAGGTTGAAACTGCCCGCGACCATCCT
GCCTTTTACCTGGTTCATGACGAACCCAATTCCTGGCAGACCAGCTATAAATGGCGCGAG
CTCAAACAACGTCACATTCTGGGCTGGGGTAACGGATATACCAGAGTTCTCCGTCACCGC
CGAACCGGTGAAGTCACTGGCCTTGAAGCCTGTATGCCGTGGGAAACAACGCTGCTGAAC
ACCGGCGGGCGCTATACCTACGGCGTGTATAACGAAGAAGGTTCCTTTGCCATTAATCCT
GATGACATGATCCACGTCAGGGCGTTGGGTAACGATCAGAAAATGGGGCTCAGTCCGGTT
CTTCAGCACGCCGAAACCATCGGTATGGGTATGAGCGGGCAGAAATACACGGAAAGTTTT
TTCAGCGGTAACGCCAGACCAGCGGGCATAGTTTCAGTAAAAGGAGAATTGAATGACGGC
TCCTGGAAAAGGCTGAAAGAGATGTGGCAAAAAGCCACGGCGATGCTGCGCAGCCAGGAA
AACAGGACAATGTTGCTCCCGGCTGAACTGGATTATAAAGCGCTGACGGTTTCCCCAGTC
GATGCCCAGCTCATCGACATGATGAAGCTCAACCGTTCCATGATTGCCGGGATTTTCAAC
GTGCCGGCACACATGATCAACGACCTCGAAAAAGCCACCTTCTCCAATATTTCCGAACAG
GCGATTCAGTTTGTTCGCTACACAATGATGCCGTGGGTGACGAACTGGGAGCAGGAGCTT
AACCGTCGGTTGTTCACCCGCGCCGAACGGGAAGCCGGGTATTACGTGCGCTTTAACCTG
GCGGGTTTATTGCGCGGTACTGCCAAAGAGCGCGCGGAGTTCTATCACTTCGCTATCACC
GATGGCTGGATGAGCCGCAACGAAGCACGCGCGTTTGAGGATATGAATCCGAAAGACGGC
CTTGATGAAATGCTGGTCAGCGTTAACGCCTCCCGGCCAGCCAAATCCACAACCCAGGAG
AACACTCAAGATGAGTGA
>EECEBICD_01430 hypothetical protein
ATGAGTGAACGTGAAATTCGCTGTTACAGCGGCGAGGTGCGCGCAGAAACGCACGACAGC
GAGCCCAGCCGGATCATCGGGTATGGTTCGGTCTTTGACAGCCGTTCTGAACTGATTTTC
GGTTCGTTTCGCGAAATCATCCGGCCCGGTGCGTTTGATGAAGTGCTGAATGACGATGTA
CGGGCGTTATTCAACCATGACCCCAATTTTATCCTGGGTCGCAGAAGTGCGGGCACGCTG
GCACTGACGGTTGATGAGCGGGGTCTGCGTTATGACATCACCGCGCCAGAAACTCAGACA
ATCCGTGATCTGGTGCTGGCACCAATGCAGCGCGGGGATATCAACCAGTCCTCTTTTGCA
TTTCGCGTCGCCCGCGACGGAGAGGAATGGTACCAGGACGAGGATGGTGTGGTGATTCGT

GAGATTACCCGTTTTTCCCGTCTGCTGGATGTCAGCCCTGTGACATATCCGGCGTATCAG
GAGGCAGATTCCGCCGTCCGCTCTATGAAAGCCTGGCAGGAGGCGCGCGATAGTAGCGCA
CTGCAGAAAGCCATTAACCAACGAATGGCGCGTGAGCGCGTCCTGACCCTTCTTAACGCG
TAA
>EECEBICD_01431 hypothetical protein
ATGAAATTGCATGAACTGAAACAAAAACGTAACACCATCGCGACCGACATGCGCGCGCTG
AACGAAAAAATCGGCGATAACCCATGGACGGATGAGCAGCGTACCGAATGGAACAAGGCA
AAATCTGAACTGGAAGCACTCGACGAGCGCATCGCCCGCGAAGAAGAGCTGCGCCGCCAG
GACCAGACCTACGTTGATGAAAACGAGGAAGAGCAGCGCAATAATCAGGATCCTGATAAA
GACCCGCAGCAGGACGAAAAACGCGGCCAGATTTTTGATAAATGGATGCGTCACGGCGCC
AGCGAACTGAGTTCCGAAGAGCGCAAAGCCTTACGCGAACTGCGTGCGCAGGGTGTGGCG
CCGGATGAAAAGGGCGGCTATACCGTGCCTGATACCTTCCTGGCGAAAGTGGTCGAACAG
ATGAAATCCTACGGTGGTATTGCCAGCGTGGCGCAGATCCTCGCTACATCCGATGGGCGC
ACTATGGAATGGGCCACTGCTGATGGTACCGCTGAAGTGGGTGTGCTGCTGGGTGAAAAC
GAAGAAGCGGGTGAAGAAGATACCGAATTCGGTATGGATAGTCTGGGCGCGCTGAAAATG
ACATCCAAAATTATCCGCGTATCCAACGAGCTGCTACAGGACAGTGCGATCGACATGGAA
GCCTATCTCGCCCGCCGTATTGCGGAGCGCATTGGCCGCGGTGAAGCGCGTTACCTTATT
CAGGGGACCGGCACCGGTACGCCAAAACAGCCTAAGGGTCTGAAAGCATCCGTAACCGGC
ACTACGCAGACGGCCGCTGCCGGAGCTGTTAAATGGCAAGAGATTCTGGCGCTGAAACAC
AGTATTGATCCGGCGTACCGCCGCGGGCCGAAGTTCCGCCTGGCGTTCAATGACAATACG
CTGAAACTCATCAGCGAGATGGAAGACGGTCAGGGCCGTCCACTCTGGCTGCCTGATATC
GTCGGCGTGGCGCCAGCATCAGTGCTGAATGTTCCGTACGTTATTGATCAGGAGATCGAT
GATATTGGCGCGGGCAAAAAATTCATGTTCTGTGGCGACTTCGACCGCTTCATTATCCGC
CGTGTTCGCTACATGATCCTGAAGCGCCTGGTGGAGCGTTACGCGGAATTCGACCAGACC
GGCTTCCTGGCGTTCCATCGCTTTGACTGTATTCTCGAAGATACCTCTGCGATTAAAGCG
CTGGTGGGCAAAGGCTCGGCAAGCAGCTAA
>EECEBICD_01432 hypothetical protein
ATGTTGCTTTCTCCTGAGGAGATCAAGTTGCAGCTCAGGCTGGATGAGGATTACGCCGAT
GAAGATAAATTTCTTGAGCTGTTGGGGCGGGCGGTTCAGGCCAGGACAGAAAATTTTCTG
AACCGGAGACTTTATACGGCGGAGGCGGGGGTGCCAGCCGACGATCCGGAGGGGCTTATT
CTCTCGGATGACATCAGGATGGGGATGCTGCTTCTGGTGACGCACTTCTACGAGAATCGT
TCTACCGTCACCGAAGTGGAGAAAGTCGAACTGCCGATGAGCTTTAACTGGCTCGTCGGT
CCATACAGGTACATCCCGCTATGA
>EECEBICD_01433 hypothetical protein
ATGAAACTCAGGCAGGCGCAGGCCAGCGCCACATACCTTTTGCCCGACCCAGGCGAACTT
GACCAGCGCATTGTTATCCGGCGGCGTGTCGATGTTCCGGCTGATGACTTTGGCGTAACG
CCGACGTACCCGGAGCAGATCCGGGCGTGGGCCAAAAAAGCGCAACCCGGCGCGGCAGCT
TATCAGGGGGCTGTGCAGATAGAAAACAGGGTGACGCACTATTTCACCATCCGTTTTCGC
CGCGGTATCACCGCCGATCATGAAGTGCTCCACGACGATATTTCTTATCGGGTTAAACGG
GTCCGTGATCTGAACAGTAAACGCCGCTTTCTGTTGCTCGAGTGCGAAGAGCTGGGTACC
GATAACGGGAGTGACTATGCCGCAGAAAGCATATTTACACGTTGA
>EECEBICD_01434 hypothetical protein
ATGCCGCAGAAAGCATATTTACACGTTGATTTTGAACAGCCGGAAACGCTTGTTTTTAAC
CGGGCGCGTATGCGCCGGGCGTTTGTCAGTATCGGGCAGGTACATATGCGCGATGCCCGC
CGCCTGGTCATGAAGCGGGGGCGTTCCGGACCCGGCGATAATCCTTCATACAGAACGGGA
AAACTGGCACGCTCCATCGGGTATTACGTTCCGCGGGCATCCAGTCGCCGTCCTGGATTG
ATGGTGAAAATTGCCCCTAATCAGAAGAACGGGGAAGGGAACCGCCCGATCTCAGGCGCA
TTTTACCCTGCCTTTCTGTTCTACGGTGTTCGCCGTGGGGCGAAGCGTAAGAAAGGCCAT
CATCGAGGCGCATCAGGCGGCAGCGGCTGGCGTGTGGCACCACGTAACAACTACATGACT
GAGGTTCTGGATAAACGCCGCAGCTGGACACGTTATGTGCTCTCCCGCGAATTGCGAAAA
TCACTCCGTCCTCAGCGAAGGAAGAAAAAATGA
>EECEBICD_01435 hypothetical protein
ATGAAATTAACCCCGATTATTGCGGCACTTCGCAGCCGTTGCCCTCGGTTTGAAAACCGT
GTGGGTGGCGCAGCGCAGTTTAAAGCGATACCGGAGGCCGGAAAGCTCAGGCTACCAGCC

GCGTATGTTGTGCCAGCCGAAGACGTCACGGGTGAGCAGAAATCGCAGACCGACTACTGG
CAGGATTTGACGGAGGGTTTTTCCGTCATCGTGGTACTCAGCAACGAACGGGATGAAAAA
GGGCAGTGGGCTTCTTACGACGCAGTTCACGACGTCAGGCAGGAAATCTGGAAGGCGCTG
CTGGGGTGGGAACCGGACCCGCAGGCGCATGAAATTCAGTATGCGGGTGGGATGCTTCTC
GATCTGAACCGCCACGAACTGTATTACCAGTTCGACTTCACGGTGAAGTATGAAATTACC
GAAACAGACACCCGCCAGCAGGATGATCTGGACGGCCTGCCCGACCTTAAAACGCTCAGT
ATTGATGTTGATTTTATCGAACCCGGTACCGGGCCAGATGGCGACATCGAGCACCACACC
GAAATTACATTTCAGGAATAA
>EECEBICD_01436 hypothetical protein
ATGTTTGTGAAACCCGCAAAAGGGCGATCGGTTCCCGATCCGGCCCGTGGCGACCTTTTA
CCTGAAGGAGGTCGAAATGTTGATGAGAATAACTACTGGCTGCGCCGCGAGGCCGCTGGT
GATGTCCGGCGCACGAATAAAAAGGTGAAAACAAATGGCGATTAG
>EECEBICD_01437 hypothetical protein
ATGGCGATTAGTTTTAATTCCATCCCGTCAGATACGCGGGTTCCGCTGTTTTATGCCGAG
ATGGATAACTCGGCGGCAAATACCGCCCGGGACAGCGGGGCATCACTGCTGATTGGTCAC
GCCAGCAATGATGCGTCAATTGCCGTCAACAGTCTTGTTCTGGTGTCATCGGTTGATTAT
GCCCGTCAGATTTGCGGTGCAGGAAGCCAGCTGGCCCGTATGGTCGGGGCGTACCGTAAG
ACCGATCCATTTGGCGAACTGTATGTCATTGCCGTACCTGAATCCACAGGCGCGGCAGCA
ACCGTCGCTTTGACGGTAACTGGCGAAGCGACGGAAACCGGAACGGTGAATGTCTATACC
GGCCGAACCCGCGTTCAGGCTCCCGTGACCAGCGGTGATGACGCTGCGGCGGTGGCTGTG
AGCATTAAGGATGCGGTCAATGCAAACCCTGATCTTCCCTTTACGGCAACATCAGAAGCG
GGGGTGGTGACACTGACTGCGCGCCACAAGGGGTTATATGGAAATGAAATTCCGGTCACT
CTCAATTATTACGGCTTTGGCGGTGGGGAGGTGTTACCGGCAGGTGTGAATATTACGGTT
GCCAGCGGCGTGAAGGGGGCTGGTGCGCCAGCTCTTAACGACGCGGTGGCAGCGATGGGA
GATGAGCCGTTCGATTATATCGGCCTTCCGTTTAACGACACGGCATCGGTGAACACGATG
GCAACTGAAATGAATGATTCCAGCGGTCGCTGGAGTTATATCCGGCAGTTGTATGGTCAC
GTTTATACGGCGAAGACGGGGACGCTGTCGGAGCTTGTGGCCGCGGGTGACCAGTTTAAC
CTGCAGCACATCACCCTGGCGGGCTATGAGAAAGACACCCAGACGCCTGCTGATGAACTG
GCTGCAAGCCGTACTGCCCGTGCTGCGGTTTTTATCCGTAACGATCCGGCGCGCCCGACC
CAGACCGGGGAACTGGTGGACATGCTGCCGGCACCGAAAGGCAAACGCTTCACGACGACT
GAACAGCAGACGTTACTTTCCCACGGTGTGGCAACGGCGTATGTGGAAAGCGGCGTGCTG
CGTATTCAGCGGGATATCACGACGTACAGGAAAAATGCGTATGGTGTGGCGGATAACAGC
TACCTTGACAGCGAGACGCTGCATACCAGTGCTTATGTGTTGCGCCGTCTGAAATCTGTT
ATTACCAGTAAATACGGGCGCCATAAACTTGCTAATGATGGTACGCGTTTCGGGCCTGGT
CAGGCCATTGTCACGCCTGCCGTTATCCGTGGTGAGCTGGGATCAACATATCGCCAGCTG
GAGCGGGAAGGCATCGTGGAAAACTTCGATCTGTTCCAGCAACATCTGATAGTTGAGCGT
AACGCGAACGATTCGAACCGCCTTGATGTGCTGTTTCCGCCTGATTATGTCAATCAGTTA
CGTGTGTTTGCGGTGCTTAACCAGTTCCGTCTGCAGTACAGCGAGGAGGCTGCATAA
>EECEBICD_01438 hypothetical protein
ATGGGAAAAATTGCGGGAACAACATATTTCAAAATCGACGGACAGCAACTGTCGGTAACC
GGAGGGATTGAAGTCCCCATGAACACCAAAGTTCGTGACGACGTGATTGGCCTGGATGGT
TCCGTTGACTACAAGGAAACCAGCCGGGCACCGTATACGAAGGTGACCGCCAAAGTGCCG
AAAAACTTCCCGGTCGATAAAATTACGTCTTCTGATGTCATGACCATCACATCAGAGCTG
GCAAATGGTCAGGTGTATGTTCTCTCAAACGCCTGGCTGCACGGCGAAGCCAACCATAAC
CCGGAAGAGGGCACCGTGGATCTTGAGTTCCACGGTGAGGAGGGATTTTACCAGTGA
>EECEBICD_01439 hypothetical protein
GTGATAAAAGAACTTGTGCTCAAAAAGCCGATTATGGCGCATAACGAAAAGCTTCATGTG
CTGGAGCTGCGCGAACCGTCCTACGATGAAATCGAAGCCATTGGTTTTCCGTTCACCGTT
TCCGGTGATGGCGGCGTCCGGCTGGACAGTTCGGTTGCTCTGAAATATATCCCTGTGCTG
GCAGGTATTCCACGCTCCTCGGCAGCGCAACTGGCAAAACTGGATATTTTCAAAGCCTGT
ATGTTGATCCTCAATTTTTTTACCCGGTCGGAGACGGAGGAGGACTCAGAAAGCGGGTCT
ACAACACCGCATACTTCTGGCGAATAA
>EECEBICD_01440 hypothetical protein
ATGGCTGACAGTTTCCAGTTAAAGGCCATTATCACTGCCGTTGACCAGTTATCGGGTCCG

CTGAAAGGGATGCAGCGGGAACTGAAGGGATTTCAGAAAGAAATGGCCGGGCTGGCGATC
GGTGCTGCCGCTGCCGGGACCGCTGTTCTTGGGGCGCTGGCGCTGCCCGTGAATGCTGCG
ATCGGCTTTGAGTCAAAAATGGCTGACATCCGGAAGGTGGTTGACGGCCTGGATGATAAA
AAAGCATTCGCGCAGATGAGTGACGATATCCTGACGCTGTCCACACAGTTACCGATGGCG
GCGGAGGGAATTGCAGAGATCGTGGCGGCGGGCGGGCAGGCAGGCATTGCCCGCGGCGAT
TTGATGCAGTTTGCGAACGACGCAGTGAAAATGGGTGTGGCGTTTGATACCACTGCCGAA
GAGTCCGGTCAGATGATGGCGCAGTGGCGGACAGCGTTCAGACTGACGCAGGAAGACGTG
GTTGTCCTGGCCGATAAAATCAACTATCTGGGGAATACCGGCCCGGCAAATGCGAAGAAA
ATTTCTGATATCGTGACGCGGATTGGTCCGCTGGGCGGTGTTGCCGGAGTGGCATCCGGC
GAAATTGCCGCGATGGGCGCCACCATTGCCGGGATGGGGGTTGAATCAGAAATTGCCTCC
ACCGGCATCAAAAACTTCATGCTGTCGTTAACCGCAGGTAATTCGGCAACCAAAGCCCAG
AAACAGGCTATGGCTTTCCTGAAGCTGAATCCCCGGAAACTCGCTGAGGATATGCAAAAG
GATTCGCGCGGGGCCATGCTGAAGGTGCTGGACTCGCTCGCGAAAGTGCCAAAAGCTAAA
CAGGCCGCCGTCATGAATGCGCTGTTTGGCAAGGAGTCACTTAGCGCGATTGCCCCGCTG
CTGACCAACCTGGATTTGTTACGCACCAATTTTGATCGTGTGGCTGATGCCCAGGAATAT
GGCGGCTCGATGCAGAAGGAATACGCATCCCGCGCGTCCACAACAGAAAACCAGCTGGTT
CTGCTGAAAAACAGCGTCAATGCGATTTCGGTAACGCTGGGCGATACCTTCCTGCCCGCC
ATTAACGAAGCTGCAGAAGCGGTCATGCCTTACCTGGAGCAGCTCCGGACATTCGTTCGC
GCGAATCCTGAACTGGTTCAGTCTGCGGCGAAGTTCGGCGCGGCGCTGCTGGCTGTTGGC
GTATCCATTGGCAGCCTGTCCCGGGCTGTCAAAATCCTGAACAGTGTCATTAACCTCTCT
CCGGCGAAAGTCGCCATTGCGGCGCTGGTGGCCGGCGCTATGCTGATCATTGAGAACTGG
GACGATGTTGCTCCGGTGATTAAGGCGGTATGGCAGGAGGTCGATAACGTTGCGCAGGAG
ATGGGCGGATGGGAGACGGTGATTGAAGGGGTTGGTCTGGTTATGGCTGGTTCTTTTACC
GTCAGGACCATTGGTGCCCTGCAGCAGTCCGTCCTGCTGGCCGGACGGCTTTCCGGTCTG
CTGGGTAAAATTGGCCGGATGGGGGCCATGACGCTGACAATTGGCGTGGCGGTGTCACTC
TTTAAAGAGCTTAAGGATCTGGAGCAGGGGGCAAAGGATGCGGGTATGGATGCTGGCGCA
TTCGCTGTACAGAAGCTGCAAACGAAGGAGCGTGAACGCGGGTATAACGGTTTTATTCCC
AGACTCAAAGAGCTTCTTGGTATGGACACCCCGATTCCGCAGGGGCGTTATCAACCTTAT
GTGCCACTGACCCGGCGTTCTGGCGTACTCGGGCGAGCTGTCCCGCCATCAACGCAGCGC
AGCGAACTCAAAGTGACATTTGAGAATGCACCACAAGGTATGCGTGTGACTGATATACCG
AAATCCGGTAATCCATTGATGAACATCAGCCATGATGTGGGTTACTCACCCTTTCGTACA
TCACGATAA
>EECEBICD_01441 hypothetical protein
ATGGCTTTTTTCTCCTCAACTGGCTGGCGCGGGCGCCTGCGTGATGCATCATTTCGTGGA
GTGCCTTTCTCCGTTGAAGATGATGAAAGCACGTTTGGACGCCGCGTACAGGTACATGAA
TATCCGAACAGGGATAAGCCCTGGACGGAGGATTTAGGTCGCGCCACGCGCCGCCTGACG
ATAAATGCTTATCTTGTCGGTGATGATTACGCAGACAGGCGGGATCGTCTTATTGGTGCC
ATTGAAACCGCAGGCCCTGGTACGCTGGTCCATCCGCAGTATGGCGAAATGCAGGGCAGC
ATTGACGGACAGGTCAGGATCACTCACAGCAGTACAGAAGGGCGCATGTGTCGTGTCTCC
TTTCAGTTTGTGGAAAGTGGTGAACTTTCTTTTCCTGTGGCGGGAATGGCAACGGCGAAG
CGCCTGGAAACATCAGGCGGGCTTTTCGACGATGCGATTGACAGTATGTTTTCCACATTC
TCGTTGTCAGGTATTTCTGATTTTATCCAGAACGATGTCATTGCCGATGCTGCCTCCATG
CTGGGCGATGTTGCCGATGCTTTCAGGATGGTTGACTCCGGCGTGTCTGCCGCAATGCGG
CTGTTACAGGGGGATTTGTCTGTCATTCTGATGCCACCGGGCGCCGCAAGTGATTTCGTT
AACGCACTGCAAAAAGCCTGGCGCTCAGGTGACAGGCTCAGAGGCAGTACATCGGATCTG
GTCACGATGATAAAAACGATGTCAGGTATCACCCTTGATCCCGGTCTTTCCCCCCGTGGC
ACCTGGCCCACTGACTCCGGATCTGCTGCGAAACAGAAAATGCAACGCAATATGATCGCA
GCCGCCATCAGGACAACAGCCATCAGCACAGCCGTCCACGCCGTGACAACACTGAAGCAG
CCGCGTGATGTACCTGATGTCCGGGGCGTAAATCAGCCTGCAGGAACAGGCCGTGACTCA
GACATTATCACTGTCATGCACCCGGCGCTGGATGGTGTACAGACAGTCAGTAATGGCAGC
TTTCCACCGAATTATGAAGATCTGAAAGCTATCCGGACCGCGCTCAATGCTGCGATTGAC
CAGGAGCAGTTGCGTATCCGGGATGATGTGCTTTTCCAGCAAATTTCCGTTATGCGGACG
GATCTCAATCGCGATATTTCTGCACGACTGGCACAGGTTGAACGTACTGCATTGCGAACG
CCTGATGATGTTCTGCCTGCACTGGTACTGGCTGCAGCCTGGTATGACGACGCCGGGCGG

GAATCTGATATCCTCACTCGTAATCCCGTTCCCCATCCGGGATTTATCCCGGTTGAGCCG
CTGAGGGTTCCGGTACGATGA
>EECEBICD_01442 hypothetical protein
ATGAATAATACGGTTTTTTTACGCGTCAACGGGCGTGACTGGGGAGGATGGACGTCAGTA
CGGATAAGTGCGGGCATTGACCGTATTGCCCGGGACTTTAATGTCTCGATCACCCGGCAG
TGGCCTGGTGGAGAAGACGTACCGCCAGTAAAAAATGGTGACGCTGTAGAGGTACTCATT
GGCGATGATTTAGTTATTACCGGCTGGGTTGAGGCGTTACCGCTACGTTATGATGCGCAG
ACCATTATGACGGGCATTGTCGGGCGCAGCAAAACGGCAGATCTTATCGACTGTTCTGCA
TCGCCTGCACAGCATAACGGGAAAAATTTATTCCTGATCGCCAGCGCACTTGCCCGGCCA
TTCGGTGTGGACGTTGTTGATGCAGGCGCGCCGGCAGCCGCCGTTATTGAGGCTCAGCCG
GAACATGGTGAAACGGTTGTGGACTGTCTGAACAGGCTGCTTGGACAGGCTCAGGCGCTG
GCATATGACGACGAACGGGGACGGCTGGTTCTCGGCAGGCCGGGCAGTATGAAAGCAGCC
ACGGCACTGGTACTTGGCGAAAATATTCTTTCCTGTGATACCGAGCGTAGTGTTCGTGAG
CGTTTCTCCAGTTATCTGGTTACGGGGCAGCGTCCTGGTACGGATGACGATTTCGGCGAG
GCAACCATTGCTGCTATCCGGCAGAGTACTGGTGATGCAGGCGTCACGCGGTATCGTCCC
CACACCATTCAGCAGTCAGGAACTGCCACAACTGACAGCTGCAAATCCCGCTGTGAATTT
GAAGCCCGTCAGCGTGCGGCGAAAACGCTGGAAACCACCTATACCGTACAGGGATGGAGA
CAGGGGAATGGCGAATTGTGGAAACCGAATCAGGCCGTGGTGGTGTATGACCCGCTGAAC
GGTTTTGACAATGAAACGCTGGTGATCGCCGAAGTGACGTACAGCCAGGACAATAACGGC
ACCCTGACCGAAATCCGGGTGGGGCCTGCGGATGCCTATCTTCCTGAACCATTCAGGCCG
AAAGCGAAGAAAAAAGTCAGTGAGGAGGCAGATTTCTGA
>EECEBICD_01443 hypothetical protein
ATGGCTAACCATCCTCTTCAGAACATGATAACGCGCGCAGTCATTACCGCGATTGATACC
GTCAGAAAATGCCAGACTGCCGGACTGAAACTTATTGCCGGTGAAAAAAAGAGAATGTGG
AGCATCTTGAACCTTACGGTTTCACCTCTGCAGCACAGAATGGCGCAGAAGCGGTGGTAT
TGTTTCCCGGCGGTGGCCGTTCGCACGGAGTGGCTGTGGTTGTGGCTGACCGCCGCTTCA
GACTGA
>EECEBICD_01444 hypothetical protein
GTGGTTGTGGCTGACCGCCGCTTCAGACTGAAAGGGCTGGCGCGCGGGGAAGTCGCGCTA
TATGACGATCAGGGGCAGTCGGTCACATTAACCCGAGCCGGAATAGTGGTAAATGGCGGC
GGAAAGCCAGTTATTTTCACGAATGCCACTAAAGCACGTTTTGAAATGCCGATCGAATCC
ACTGGCGATATCAGGGACAACTGTGACAGCAGTGGAAAAACGATGGCTGAAATGCGCACG
ACCTATAACGGTCATACCCATAAAGAAAATGGCGATGGCGGCGGTATAACCGATAAGCCT
GGCCAACCCATGAGCTGA
>EECEBICD_01445 hypothetical protein
ATGATCCTTTATGTTAATGGAATCCGTAAGGATGCCACGGCTTCGCTCGACTTTCTGACG
CGGGCAGTGGTGATTTCTCTTTTTACCTGGCGCCGGGCGGAGCGGGATGACAGGACCCCA
CAGCCATACGGCTGGTGGGGGGACACCTGGCCTGCTGTTCAGAATGACCGCATCGGTTCC
CGCCTCTACCTGCTGAAACGCCGCAAACTCACCAATAAAACGCCGCAGGATGCCCGCGAA
TACATGCAGCAGGCGCTGGCGTGGATGACAGACGATGGCGTGGCGGCACGTATTGATGTG
ACATCTGAACGCACAGGAACAGATACCCTGGCAGCTGGCGTGACGATATATCAGCGGGAC
GGGGTAATTCACAATATTACATTCGATGATATATGGAGCAAACTTAATGGCTGA
>EECEBICD_01446 hypothetical protein
ATGGCTGACAGTCAATTTGCACGTCCTGAACTTCCTCAGTTGATTGCTACCATTCGCAGC
GATTTACTGACCCGTTTTCAGCAGGATGTTGTGTTACGTCGCATGGATGCCGAGGTTTAC
AGCCGGGTACAGGCTGCTGCCGTACATACGCTGTATGGTTATATCGATTATCTGGCCCGG
AATATGCTGCCTGATATGTGTGATGAGGACTGGCTTTACCGTCACGCGAGGATTAAGCGT
TGTCCCAGGAAAAATGCCGTATCTGCGAAGGGATTTGCACGCTGGGATGGTATTGCCGGA
ACGCCGGAGATCCCCGCGGGTACACAGATTCAGCGGGATGATCAGGTTACATTCACGACC
CTGCAGACGGTGAAAGCTTCCGGCGGCCTGTTACGTGTGCCGGTTATTGCTGATGTGGCG
GGAACTGCCGGTAATACTGACGATGGTACGGCGTTACGCCTTGGCACGCCGATTACTGGT
ATTCCTTCTACAGGTTACGCTGACACTCTGACCGGGGGGGCTGATACAGAGGAGCCTGAA
ACGTGGCGCGCGCGCGTCATGGAACGCTATTACTGGATACCACAGGGGGGCGCTGATCCT
GATTACGTCATCTGGGCAAAGGAAATCGCGGGAATAACCCGTGCGTGGACATTCCGCCAT

TATAAGGGGACCGGCACCGTTGGTGTGATGGTGGCTACCAGTAACCCGGTGAATCCGGCT
CCTGGCGACGATCTCGTTAAGGCTGTACGTGACCATATTTTGCCGCTGGCACCTGTTGCT
GGCGGCGGACTCTTTGTTTTCGCTGCCACTGAAAAAAGCATTCCGGTAACAGTCGCACTG
GCCAAAGATACCCCGGAAATTCGTACTGCCATTATTGCGGAGCTAAATGCGCTGATGCTG
CGTGATGGCGCGCCGTCCGGAAAAATTTATGTTTCGCGAATCAGCGAGGCGATAAGTCTG
GCGACCGGGGAAGTGGCACATCAGCTGCGTGTGCCGGCGGCAGATGTGGTGCTGGGAAAA
ACTGAACTTCCTGTCCTGGGGAATATAACCTGGGCCACCTATACCGGGGAGAACGGATAA
>EECEBICD_01447 hypothetical protein
ATGGCATTACAGGACGAATATACGCAGTTACTTTATCACCTTCTGCCGGAAGGGCCTGCC
TGGGACGGAGAAAACCCACTGATTGAAGGGCTGGCGCCGTCGCTGAACCGGGTACATCAG
AGAGCGGATGAACTGATGGCTGAAATTGATCCGGCCAGAACCACAGAACTGATAGACCGT
TATGAACAGCTGTATGGCCTGCCTGATTCCTGTGCACCGGAAGGCGTTCAGACATTACAG
CAGCGCCAGCAACGGCTGGATGCAAAGGCAAATGTTGCTGGCGGTATAAACGAGAGGTTT
TATCGGGAACAGCTTGATGCGTTGGGGTATACCGCTGCCACCATTGAGCAGTTTCAGAAT
CTCGACAGCACACCCGATCCTGAATGGGGGGAATTCTGGCGTTACTACTGGCGTGTGAAT
ATTCCGGCTGATGCGAACATCAGCTGGCAGACCTGTACAAGCACCTGCGACTCTGCGATC
AGAACGTGGGGCGATACTGTTGCTGAATGTGTGATTGATAAGCTTTGTCCGTCACATACG
GTTGTTGTTTTTGCTTATCCGGAAGGAAAAGAGAATGCACAGAATTGA
>EECEBICD_01448 hypothetical protein
ATGCACAGAATTGATACGCCCACCGCGCAAAAAGATAAATTTGGTCAGGGAAAAAACGGA
TTTACGAATGGTGATCCCGCCACGGGCCGCCGCGCAACGGATCTCAACAGTGATATGTGG
GATGCAGTCCAGGAAGAGGTCTGTACTGTTATTGAAGCCGCCGGCATACCACTCAGTAAA
GGCGAACATACGCAGCTTCACGCCGCCATTGGCAGGCTGATCGATGAACAGGTTAAAACC
CGTCTTGAAAAAAATCAGAATGGCGCGGACATCCCGAATAAGCCGCTGTTTCTCCAGAAC
GTCGGTTTAGGAGAAACGATAAATCTCGCTGCAGGGGCCCTGCAAAAATCGCAGAACGGC
GGCGATATTCCTGACAAAAAACAATTTGCGAGAACCATCGGTGCGGTAACGTCAACCACC
ATTACACTTGGCGAATCAGGCTGGTTCAAAATCGCCACGGTTGTAATGCCGCAGGCTACA
TCAACTGCGGTGATTAAACTGTACGGTGGGGCGGGGTTTAACGCTGGTTCACCTGAACAG
GCGGCAATCAGCGAACTGGTATTGCGTGCCGGTAATGGTTCACCTGTTGGAATAACCGCC
ACATTATGGAGGCGTTCACCTTCTGCTGCTAACGAGGTCGCATGGGTTAATACATCAGGC
GACACCTACGATATTTATATTAATATCGGCCAGTATGCGTACTGGTTAATTGCGCAATAT
GATTACACCGGTAATGCAAATGTCACGCTGCACAGTACGCCTGAATATTCATCAGTTCAG
CCGGGAAACTCAACCAGCGGTCAGACATATACACTGTTTAATAGTCTGATGAAACCCACA
GCCGGTGACGTTGAGGCACTGTCAGTTAATGGAGGGAGGCTAAACGGTCCGTTAGGCATT
GGTACTGATAATGCGCTGGGTGGTAATTCGATTGTATTCGGAGATAACGATACAGGGTTT
AAGTGGCACAGTGACGGCGTTCTGGGGATTTATGCCAATAATGCTCTGGTTGGTTATATC
GACAATTCCGGGCTGCACATGTCAGTAGATGTTCTCACTAATGGTGCCGTACGCGCAGGC
AACGCAAAAAAACTGTCACTGACGAGCAATAATAATTCGACAATGACAGCCACGTTTAAT
TTATGGGGCGACGCAAACAGGCCAACAGTTATTGAACTGGACGACGATCAGGGATGGCAT
CTGTACAGCCAGCGAAATCCTGACGGTTCGATTGTCTTTACGGTCAATGGAGATATCACC
GCTAACACGCTTCGTGCAAGCGGGGCTATCTATCAGAATAACGGCGACATCTTTGGTTCG
CTATGGGGAAATGGCTGGTTAAGTACCTGGATTAATAATAATCTCGTCTTAGATGTTCAG
TTAGGGGCTGGCACATCAGTGACTACCTGGAACAATGCAGGTTCCTGGCCTAACACTCCC
GGATATGTAGTTACCTCCGTCTGGAAAGATTATCAGGGCGAAAATATTGATGGTATTAAT
TATGCGCCTTTGCAAAAACGAGTCGGGAGTCAGTGGTATACCGTACAAGGGGGAACGGTA
TAA
>EECEBICD_01449 hypothetical protein
ATGAAAAAATATCAGAATATCAAAAATTTCAGACTTATTGACGCGCCCGTAAACAGGGAT
AAAACTCAGGCTGAAATAAATATAGGTGCATATTTTCTGGAGTCGGACGATGGACAGGAC
TGGTATGAGTGTCAGTCATTATTTTCTGATGATACTGCAAAAATAATGTACGACCATGAG
GGGGTTATCTGGGGTGTTGTTAATAAGCCAGTCCCGCAACGAGGAAACACATATTCTGTA
TCAATGTTGTGGCCGGTTAATATGTCTGTTGCGGAAATAGACGCTGCTGACTGCCCTGAT
GATTGCCGTGGTGATGGTACGTGGTTATATCAGGACGGTAAAGTCGTTCAACGGGGTTAT
TCTCCGGAAGAGCTGCGTAAAAAGGCGGAGGCTGAAAAAGTTCGCCGCCTTGCTGAGGCT

GAATCAGCCATCGCACCACTGGCACGGGCAGTAAAACTAAAAATTGCCACAGATGAAGAG
ATTAAACGGCTGGAAGCATGGGAACTTTATAGCGTAATGGTAAACAGGGTGGATACATCT
GCGCCTGACTGGCCGGATATACCACGCTAA
>EECEBICD_01450 hypothetical protein
ATGAGTGATTTTCACCACGGCACGCAGGTCATCCAATTTAATGGAGGTACGCGCGTCACA
TCCACGATATCGATCGAAATCGTCAGTATGGTCTTTACGTTTAGCTTTGCCGAAACATTG
CTCGCGTTTATATCATACGTTTGCAAATAA
>EECEBICD_01451 hypothetical protein
ATGTATGACAAGAAACTATCAGATATTTATTTAGAAAACATCGCTAAAATTGAAGCTCAA
CCAGCAAATGTCAGAGATGAATATCTGCTTGGTGAGATAAAAAAGAGTTTAAATGAAGTT
TTAAAGAACAATCCAGAGGAGTCCCTTGTTTCGTCCCATGATAAACGCTTGGGACATGTA
CGGTTTGATTTTTACAGAAATCTTTTTTTATTAAAAGGAAGTAATGCTTTTCTGGAGGCG
GGCAAACATGGCTGCCATCACCTGCAACCTGGAGGTGGCTGCATATATCTTGATGCTGAT
ATGTTACTTACAGGTAAACTCGGCACTTTGTATTTACCTGATGGTATTGCTGTTCATGTA
AGTCGTAAAGGTAATAGCATGAGTCTTGAAAATGGGATTATCGCCGTTAACCGCAGCGAG
CATCCGGCATTGAAAAAAGGACTTGAAATTATGCACAGTAAACCTTATGGCGATCCATAC
ATTGATGGTGTCTGCGGTGGGCTAAGGCATTATTTTAATTGTTCTATACGGCACAATTAT
GAAGAGTTTTGTAATTTTATAGAATTTAAGCATGAACATATTTTTATGGATACCAGCAGT
TTGACTATCAGCTCCTGGAGATAA
>EECEBICD_01452 hypothetical protein
ATGCAATTACCAGAACAGGATGAGTTTTCTGATTTTTTTGCTGCCAATGATGATGAACAA
GCCTCTTTAAGGCGTAAGTTTTTTTTGGAGAAACATAAAGAACCGTGTCTGTCTGAGTCT
GCATTAGAGGACTACCAGGCGCTGTTTATGAGTATCTACGGAATTAATATTGACTGGAAA
GAGGGGACTTTTAGCCTGCTTGAGGCACTTTCAGATAATCAGGAGGGAAGCCTGTCACGG
TCAAATTCGATTATGACAGTGAGATTGAAACAGCAACGATAA
>EECEBICD_01453 hypothetical protein
ATGGGAAGTGATGGTTTTGATACAGAACTGGTGCGCATTGAGCATATATTGGCTAATAGT
GGATATAGTTTGCGGGTATATCAGAACAGCACTTTTAGTGATACATTATCGTTCTTACTC
ATTCCGTCAGATGAGTGGAAACGTGTTGAACAGCATTATAGCCCAGAGCATATTTCTGAA
TACTTCGTTCCGTATGGAAAACAACTTGTTATTCCTGAGGTTACTGCTCCAGTCGTAAAT
TATGTGCCATCCGTTAAACAAGAAGCATCAAATGTCCCAGCGTTGTTTAATGCTCGGGGT
ATCCGTATTTGTTTTTTAAGTATAATGCTAATCGCATTTGCAATTTATATTTTGTGGAAT
ATCCTGACAAAAATAGAGCCTTTATCATCAGGCCAACCTGCTGGCTGTGAAAATCTACAA
AATTTATACTCAAAATTACGTCCAGAAGTAGCCGAGCCATTAAAAGAAAAAATGCGTAAG
AGTTTGGGCTGTAAATGA
>EECEBICD_01454 hypothetical protein
ATGACTCTTATTAATTTTGTTGCCGCAAATCAAAAATACTACGCTTTTAATTTGTTGTCA
GTGTTTATCATGGTGTTCTGGTCAGTTCAGTACCTCCTGCAGCATCTCCTTGTCCAGACT
CAGGTCAGCCACCAGCTTCTTCAGCCGCTGATTCTCATCCTCCAGTTGCCGCAGACGCCG
CAGTTCCGTCACGCCCGGCCCGGCAAATTTTTTCTTCCAGTTAATGGGATGGACTACTTC
CTCCCGCTGCGGTTAACTGTACGAAATGTGCTCACCAGGAGAATCACCATGAATATCGTA
TTTCTGGGTATTGATCTGGCTAAAAATGTTTTTCAGCTCTGCGGGTTAAACCAGGCCGGC
AAACCGGTTTATACGAAACGCACTGGCCGAAAAGAATTGCTCCAGACGCTGGCAAATATT
CCTGCATGTCTGATTGGGATCGAAGCGTCCACCGGGGCATTTTACTGGCAGCGTGAGTTT
GAGAAACTGGGGCACAAAGTAAAGGTCATCAGTCCTCAGTATGTAAGACCCTTTGTCCGC
GGGCAAAAAAATGATGGTAATGATGCACAGGCCATCGCAGTGGCTCTGATGCAACCGACA
ATGCAGTTCGTGCCGCCAAAAAGCCCCGAACAGCAGGATATCCAGGCTTTACACCGGGCA
AGGCAGCGTATTGTCAATCACCGCACTGCTACAGTCTGTCAAATAAGGGGGCTGTTACTT
GACCGGGGGGATCCCCATTGGCAGTGCTGTCTCCAGAGCTCGCCGTGCTATTCCTCTTATC
CTTGAAGATGCAGAAAACGGTCTAAGTTCCCGTATGCGCAGAACAATTGCCGAACTCTAT
GATCTCTTTAACGATCTCGGGCGTCGGATCCATTTTTTTGATAAGGAAATTGAAACAGTA
TTCAGGCAATCAGAAGCCTGTCCGCGTATCGCCAAAGTTAAAGGCATTGGTCCTAAAACG
GCCACGGCCGTTGTTGCTGCTATTGGCAAAGGAACTGAATTTAAGAATGGTCGCCACTTT
GCTGCATGGCTGGGTCTGGTTCCACGCCAGCATTCGAGTGGCGACAGGCAGGTGCTGATG

AATATGACGAAAAAAGGCGACAAGCATCTGCGGACACTTTTTATTCATGGTGCCCGCGCT
GTCGTCAGGGTTGCCACGAATAACAATGATGGTCATATGAATCAGTGGGTTAACCAGTTA
AAGGAACGGCGCGGATTTAATAAAACGACCGTGGCGGTCGCTAACAAAAACGCGAGAATA
ATCTGGTCGATGCTGAGAAATGATACCGGGTATCAGGTAGTGTGTAATTAA
>EECEBICD_01455 hypothetical protein
ATGAAAAAGACCCGTTATACCGAAGAACAGATTGCATTTGCCCTGAAACAGGCTGAAACC
GGCACCCGCGTCGGGGAAGTCTGCAGAAAGATGGGAATTTCTGAGGCCACATTTTACATC
TATGGACTACCTCCGTTTTGCAAGTACTGA
>EECEBICD_01456 tRNA-Ser(gga)
GGTGAGGTGTCCGAGTGGCTGAAGGAGCACGCCTGGAAAGTGTGTATACGGCAACGTATC
GGGGGTTCGAATCCCCCCCTCACCGCCA
>EECEBICD_01457 Glyoxylate/hydroxypyruvate reductase A
ATGGAGATAATTTTTTATCACCCGACATTTAACACCGCCTGGTGGGTAAATGCGCTGGAG
AAGGCTCTCCCACATGCGCGCGTTCGTGAATGGAAGGTCGGTGATAACAACCCCGCAGAC
TATGCGCTTGTATGGCAGCCCCCGGTTGAAATGCTGGCCGGAAGACGCTTAAAAGCCGTC
TTTGCGCTGGGCGCGGGGGTGGATGCAATTCTGAGTAAATTAAATGCGCATCCGGAAATG
CTGGACGCCTCCATTCCTCTATTCCGTCTGGAAGATACCGGAATGGGCCTGCAAATGCAG
GAATATGCCGTAAGTCAGGTATTACACTGGTTCCGTCGTTTCGATGATTATCAGGCGCTG
AAAAATCAGGCGCTATGGAAACCGTTGCCGGAATATACCCGCGAAGAGTTTAGCGTCGGT
ATCATGGGCGCAGGGGTACTGGGCGCAAAAGTGGCAGAAAGTCTACAGGCGTGGGGGTTC
CCGTTACGTTGCTGGAGTCGTAGCCGCAAATCCTGGCCTGGCGTGGAAAGTTATGTTGGG
CGTGAAGAGCTGCACGCTTTCCTGAACCAGACGCGGGTGCTGATTAATCTGCTGCCGAAT
ACGGCCCAAACGGTAGGAATTATTAATAGCGAATTGTTGGATCAATTGCCGGATGGCGCT
TACGTGTTGAATCTCGCGCGCGGCGTGCATGTTCAGGAGGCGGATCTGCTGGCTGCGCTT
GATAGCGGTAAGCTAAAAGGCGCGATGTTGGATGTCTTTAGCCAGGAACCGTTACCGCAG
GAAAGTCCATTATGGCGCCATCCGCGAGTCGCCATGACGCCGCACATTGCGGCAGTCACC
CGTCCAGCGGAAGCCATCGATTATATTAGCCGCACCATTACCCAGCTGGAGAAGGGAGAG
CCGGTGACGGGGCAGGTGGATCGGGCGAGAGGATATTGA
>EECEBICD_01458 putative phosphatase YcdX
ATGTATCCCGTTGACCTGCATATGCATACCGTCGCCAGCACTCATGCCTACAGTACTCTG
AGCGATTATATCGCGGAAGCCAAACGCAAAGGCATTAAACTTTTTGCGATTACCGATCAT
GGTCCGGACATGGAAGATGCGCCGCATCACTGGCATTTTATTAACATGCGCATCTGGCCG
CGTCTGGTTGACGGCGTGGGGATACTGCGTGGCATTGAGGCGAATATCAAGAACATTAAC
GGTGAAATTGATTGTTCCGGAAAGATGTTCGACTCGCTGGATCTGATTATCGCAGGCTTC
CATGAGCCCGTTTTTGCGCCACATGATAAAGAAACCAATACCCAGGCGATGATCGCGACC
ATCGCCAGCGGCAAGGTGCATATAATTAGTCACCCCGGAAATCCAAAGTATCCAGTGGAG
GTTAAAGCCATCGCGCAGGCGGCGGCGAAACACCATGTAGCGCTGGAAATCAACAACTCT
TCTTTTCTGCATTCGCGTAAAGGAAGCGAAGATAATTGCCGCGCGGTCGCTGCCGCCGTA
CGCGATGCGGGAGGCTGGCTGGGTAGCGTTAGGCTCTGA
>EECEBICD_01459 Chaperone protein YcdY
ATGAATGAGTTTTCAATCCTGTGCCGTGTGCTGGGATCGTTGTTTTACCGCCAACCGCAA
GATCCTTTACTGGTTCCGCTGTTTACGTTAATCCGTGAAGGTAAACTGGCGGCAAACTGG
CCGCTGGAGCAGGATGACATGCTGGCGCGTTTACAGAAAAGCTGCGATATCACGCAGATT
TCCACTGATTACAATGCGTTATTTGTTGGGGAAGAGTGCGCGGTAGCGCCATACCGCAGT
GCGTGGGTCGAAGGCGCGGAAGAGTCTGAGGTGCGCGCTTTTTTAACGTCGCGAGGGATG
CCGCTGGCCGATACGCCTGCCGATCACATTGGCACTTTATTGCTCGCGGCCTCCTGGCTG
GAAGATCAGTCTGCCGAAGATGAAAGTGAAGCGCTGGAAACCTTATTTGCCGATTATCTG
CTTCCCTGGTGCAATACCTTCCTCGGTAAAGTTGAAGCCCATGCCGTTACGCCATTCTGG
CGCACTCTGGCGCCGCTAACGCGTGATGCGATAGGGGCCATGTGGGATGAACTTCAGGAA
GAAGATGAAGAATAA
>EECEBICD_01460 Inner membrane protein YcdZ
ATGATGCGCGCCATGAACATACTTCTTTCTATTGCTATCACGACGGGCATCCTTTCTGGA
ATATGGGGATGGGTGGCCGTCTCCCTGGGGTTACTAAGCTGGGCCGGTTTTTTAGGTTGT
ACGGCTTATTTCGCCTGTCCGCAGGGCGGCTTTAAGGGATTGTTAATTTCCGTCTGTACG

CTGTTAAGCGGTATGGTGTGGGCGCTGGTCATTATTCACGGTAGCGCGTTGGCGCCGCAT
CTGGAAATTGTCAGTTACGTGTTGACGGGGATCGTGGCATTCCTGATGTGTATCCAGGCA
AAGCAGCTATTGCTTTCTTTTGTTCCGGGAACATTTATCGGCGCCTGCGCGACATTTGCA
GGGCAGGGTGACTGGCGGTTGGTATTACCGTCGCTGGCGCTGGGGCTAATCTTTGGCTAT
GCCATGAAAAATAGTGGGCTATGGCTGGCATCACGCCGCGAGCAACATTCAGCGAATACG
GCGGTCACAAAATAA
>EECEBICD_01461 Curli production assembly/transport component CsgG
ATGCCGCGCTTACTTATTTGGTTGCCGTTTTATTGTTGAGCGGATGCTTAACTGCCCCG
CCGAAACAAGCTGCGAAACCGACATTAATGCCCCGCGCACAAAGTTACAAAGATTTGACG
CACTTACCTGCTCCCACCGGTAAGATCTTTGTTTCGGTATATAACATTCAGGATGAAACG
GGCCAATTTAAACCTTACCCGGCAAGTAACTTTTCTACGGCTGTGCCGCAGAGCGCCACC
GCTATGTTGGTCACTGCGCTGAAAGATTCGCGCTGGTTTATCCCGCTGGAACGACAAGGC
TTACAGAATCTTTTGAATGAACGGAAAATTATTCGCGCAGCCCAGGAAAACGGCACCGTG
GCGATGAATAACCGTATCCCGCTTCAGTCGCTGACGGCGGCAAATATTATGGTGGAAGGT
TCTATTATTGGTTATGAAAGTAACGTCAAATCCGGCGGGGTCGGCGCAAGATATTTCGGT
ATTGGCGCCGATACGCAGTATCAGCTGGATCAGATTGCTGTCAACCTGCGCGTGGTTAAC
GTCAGTACAGGCGAGATCCTTTCTTCGGTGAACACCAGTAAAACAATCCTTTCCTATGAA
GTACAGGCAGGCGTGTTCCGTTTTATTGATTACCAGCGCTTACTGGAAGGCGAAATCGGC
TATACCTCGAACGAACCGGTGATGCTGTGTCTGATGTCAGCCATTGAAACCGGCGTTATC
TTCCTCATTAATGATGGTATCGATCGCGGACTGTGGGATTTGCAGAATAAAGCGGACAGG
CAAAATGATATTCTGGTGAAATACCGTGAGCTGTCAGTACCGCCAGAATCCTGA
>EECEBICD_01462 Curli production assembly/transport component CsgF
ATGCGTGTTAAACATGCAGTAGTGCTGCTCATGCTTTTTTCGCCATTAACCTGGGCTGGA
AATATGACGTTCCAGTTCCGTAATCCTAACTTTGGTGGAAACCCCAATAACGGTTCCTTT
TTATTGAATAGCGCCCAGGCGCAAAATTCATATAAAGACCCCGCTTATGATAACGATTTT
GGTATCGAAACCCCCTCAGCGTTGGATAACTTTACGCAGGCTATTCAATCGCAAATTCTG
GGCGGCTTGTTGACCAATATTAATACCGGGAAACCAGGACGTATGGTGACCAACGATTTT
ATTATCGATATCGCTAATCGCGACGGACAGCTCCAGCTCAACGTCACGGACAGAAAAACG
GGAAGAACCTCGACCATCGAAGTGTCAGGTTTACAAACTCAGTCAACCGATTTTTAA
>EECEBICD_01463 Curli production assembly/transport component CsgE
ATGAAACGCTATCTGACCTGGATTGTAGCAGCAGAGTTACTGTTCGCTACCGGAAACCTG
CATGCCAATGAAGTTGAAGTCGAGGTTCCCGGATTGTTAACCGACCATACCGTCTCTTCG
ATAGGACATGAATTCTATCGTGCATTCAGCGACAAATGGGAAAGCGAATACACCGGCAAT
CTGACCATTAATGAAAGACCCAGTGCGCGTTGGGGAAGCTGGATCACCATAACGGTAAAT
CAGGACGTTATTTTCCAGACCTTTTTATTTCCAATGAAAAGAGACTTCGAGAAAACCGTC
GTCTTCGCATTAGCGCAAACAGAGGAAGCATTAAATCGCCGACAAATAGATCAAACGCTA
TTAAGTACGAGTGATTTAGCGCGTGATGAATTCTAA
>EECEBICD_01464 putative csgAB operon transcriptional regulatory protein
ATGTTTAATGAAGTCCATAGTAGTCATGGTCACACACTATTGTTGATCACAAAGCCATCT
CTGCAAGCTACGGCATTATTGCAACATTTAAAGCAATCGCTGGCCATAACCGGAAAACTG
CATAATATTCAACGTTCTCTGGAAGATATCTCAGCCGGTTGCATTGTTTTAATGGATATG
ATGGAAGCGGATAAGAAGCTTATCCACTATTGGCAGGATAATTTAAGCCGCAAAAACAAT
AATATAAAAACATTATTGTTAAATACCCCTGACGATTATCCCTACCGTGAAATTGAAAAC
TGGCCTCATATTAACGGCGTGTTTTACGCCACTGAAGACCAGGAACACGTGGTCAGCGGA
TTACAGGGTATTCTGCGTGGCGAATGCTATTTTTCACAAAAATTAGCCAGTTACCTGATT
ACGCACTCAGGAAATTACCGCTACAACAGCACCGAGTCCGCATTACTCACTCATCGCGAA
AAAGAGATCCTCAATAAGTTACGTATTGGTGCCTCTAATAATGAAATCGCGAGGTCGCTA
TTTATCAGCGAGAATACGGTTAAGACACATCTTTATAATCTTTTCAAAAAGATAGCTGTC
AAAAATCGCACCCAGGCAGTTTCATGGGCAAACGATAATCTCAGGCGGTAA
>EECEBICD_01465 Minor curlin subunit
ATGAAAAACAAATTGTTATTTATGATGTTGACAATACTGGGTGCGCCTGGGATTGCAACC
GCGACAAATTATGATCTGGCTCGTTCAGAATATAATTTTGCGGTAAATGAATTAAGCAAG
TCTTCATTTAATCAGGCGGCCATTATTGGTCAAGTCGGCACGGATAATAGTGCCAGAGTA
CGCCAGGAAGGATCAAAACTATTGTCCGTTATTTCACAAGAAGGAGGAAATAATCGGGCG

AAAGTCGACCAGGCAGGGAATTATAACTTTGCGTATATTGAGCAAACGGGCAATGCCAAC
GATGCCAGTATATCGCAAAGCGCTTACGGTAATAGTGCAGCTATTATCCAGAAAGGTTCT
GGAAATAAGGCCAATATTACCCAGTACGGTACGCAGAAAACAGCAGTTGTAGTGCAGAAA
CAGTCGCATATGGCTATTCGCGTCACCCAACGCTAA
>EECEBICD_01466 Major curlin subunit
ATGAAACTTTTAAAAGTGGCAGCATTCGCAGCAATCGTAGTTTCTGGCAGTGCTCTGGCT
GGCGTCGTTCCACAATGGGGCGGCGGCGGTAATCATAACGGCGGCGGCAATAGTTCCGGC
CCGGACTCAACGTTGAGCATTTATCAGTACGGTTCCGCTAACGCTGCGCTTGCTCTGCAA
AGCGATGCCCGTAAATCTGAAACGACCATTACCCAGAGCGGTTATGGTAACGGCGCCGAT
GTAGGCCAGGGTGCGGATAATAGTACTATTGAACTGACTCAGAATGGTTTCAGAAATAAT
GCCACCATCGACCAGTGGAACGCTAAAAACTCCGATATTACTGTCGGCCAATACGGCGGT
AATAACGCCGCGCTGGTTAATCAGACCGCATCTGATTCCAGCGTAATGGTGCGTCAGGTT
GGTTTTGGCAACAACGCCACGGCTAACCAGTATTAA
>EECEBICD_01467 Curli assembly protein CsgC
ATGCATACTTTATTGCTCCTTGCCGCACTTTCAAATCAGATTACGTTTACCACGACTCAG
CAAGGCGATATTTACACGGTGATCCCTCAGGTCACATTAAACGAACCCTGCGTCTGTCAG
GTGCAAATTCTCTCTGTGCGCGACGGCGTCGGGGGACAAAGCCATACACAGCAAAAACAA
ACGCTATCTTTACCTGCTAATCAACCGATTGAGTTGTCTCGTCTTAGTGTAAATATATCT
TCAGAGGACTCGGTTAAAATTATTGTTACTGTTTCGGACGGACAATCACTGCATTTATCA
CAACAATGGCCGCCTTCTGCACAGTAG
>EECEBICD_01468 putative protein YmdA
ATGTCCGTAATCAAGAAAAATATCCCTGGCATAGGCCTGTGTATCTGCGCTTTTTTTATC
CATTCTGCGGTAGGGCAACAAACGGTACAGGGCGGCGTTATCCATTTTCGCGGCGCGATT
GTTGAGCCACTGTGCGATATTTCTACTCATGCCGAAAATATTGATTTAACCTGCCTGCGC
GAAGGTAAAAAGCAAATGCACCGGATTGACCTTCGGCAGGCATCTGGATTACCGCAGGAT
ATTCAGTCCATTGCGACGGTGAGGCTGCATTATCTCGATGCGCAAAAAAGCCTGGCGGTG
ATGAATATTGAGTACCGTTAA
>EECEBICD_01469 O-acetyl-ADP-ribose deacetylase
ATGACATCACGTCTTCAGGTCATACAGGGTGATATCACTCAACTTAGCGTCGATGCGATT
GTGAATGCCGCTAACGCATCATTAATGGGCGGCGGTGGCGTAGACGGCGCGATTCATCGC
GCGGCGGGGCCGGCATTGCTGGACGCCTGTAAACTCATCCGTCAGCAACAGGGCGAATGT
CAGACGGGACATGCGGTTATCACGCCTGCTGGCAAGCTTTCGGCAAAGGCGGTTATTCAC
ACAGTGGGGCCCGTCTGGCGAGGCGGCGAACACCAGGAAGCTGAGCTACTCGAAGAGGCA
TACCGGAATTGTTTGCTGCTTGCCGAGGCGAATCACTTTCGTTCCATCGCTTTTCCGGCA
ATCAGTACCGGCGTTTATGGCTATCCACGCGCCCAGGCCGCTGAAGTCGCCGTCAGGACG
GTTTCAGATTTTATTACCCGTTACGCTCTGCCTGAACAGGTATACTTTGTCTGTTATGAT
GAAGAAACTGCCCGGCTTTACGCAAGATTACTTACTCAGCAAGGCGACGACCCTGCCTGA
>EECEBICD_01470 Cardiolipin synthase C
ATGAAGAAACTGCCCGGCTTTACGCAAGATTACTTACTCAGCAAGGCGACGACCCTGCCT
GATAAAACACGCCTGGAGCGTGCCGTTGAACCGCTATGCGCGCGCCATCCCGGAGAGTGC
GGCATTCTCGCGCTGGATAACAGTCTGGACGCTTTTGCCGCCCGCTACCGCCTGACCGAA
ATGGCGGCGCGGACGCTGGATGTGCAGTATTATATTTGGGAAGACGATATGTCCGGGCGA
CTGCTCTTTTCGGTACTGCTGTCGGCGGCGAAGCGCGGCGTTCATGTTCGTCTGCTGCTG
GATGATAACAATACGCCTGGTCTGGATGATACGTTGCGCTTGCTGGATAGCCATCCTAAT
ATCGAAGTTCGTCTGTTTAATCCTTTCTCTTTTCGTACGCTACGCGCGCTGGGATATTTG
ACGGATTTTGCGCGGCTGAATCGGCGGATGCACAATAAAAGTTACACTGCCGACGGCGTA
GTGACGCTGGTCGGTGGGCGCAACATCGGCGATGCCTATTTCGGCGCTGGCGAGGAGCCG
CTATTTTCCGATCTGGACGTGATGGCCATTGGCCCGGTGGTCAATGATGTCGCCAATGAT
TTTGAACGTTACTGGCGCTGTAGTTCAGTGTCGACATTGCAGCAAGTATTATCCCTTTCT
GAGCAGGAACTGACGCAGCGTATCGAACTTCCCGAATCCTGGTATAACGATGAGATCACC
CGCCGTTATCTGCATAAGCTGGAAACCAGCCAGTTTATGGCGGATCTCGATCGCGGAACG
TTGCCGCTGATTTGGGCAAAAACACGCTTGCTTAGCGATGACCCTTCTAAAGGCGAGGGG
AAGGCGCAGCGCCATTCGCTTCTTCCGCAGCGATTATTTGACGTGATGGGGTCGCCGACG
GAGCGTATCGACATTATTTCCGCTTACTTTGTCCCTACGCGCGCAGGCGTGGCGCAGTTG

CTTAATCTGGTCAGGAAAGGCGTGAAGATCGCCATCTTAACTAACTCTCTGGCGGCCAAC
GATGTGGCGGTCGTTCACGCAGGGTACGCGCGCTGGCGCAAGAAATTACTGCGCTATGGC
GTGGAGCTCTACGAACTGAAACCGACCCGCGAACATGAAACCGCCGTACATGATCGCGGA
CTCACCGGGAACTCAGGTTCCAGCTTACATGCTAAAACGTTCAGTATTGATGGTAGTAAG
GTGTTTATCGGGTCGCTTAATTTTGATCCCCGTTCAACGCTTTTAAATACCGAAATGGGC
TTTGTCATTGAAAGTGAAACGCTGGCGACGCTTATTCATAAGCGTTTTACGCAGAGCCAA
CGCGATGCGGCCTGGCAACTGCGGCTGGATCGCTGGGGACGAATTAACTGGATCGATCGT
CAGCAAGAAGAGGAAAAGGTGTTAAAGAAAGAACCCGCTACGCGTTTCTGGCAGCGAGTT
CTGGTACGGTTGGCGGCAATTTTACCTGTGGAATGGTTGCTGTGA
>EECEBICD_01471 Glucans biosynthesis protein C
ATGAGCTCTGTACCCGCGCCGCGTGAATATTTCTTGACTCTATCCGCGCATGGCTGATG
TTGTTAGGGATTCCCTTTCATATCTCGTTGATCTATTCCACTCACAGTTGGCATGTCAAT
AGCGCCACGCCATCGTGGTGGCTAACTCTGTTTAACGATTTTATCCACGCTTTTCGTATG
CAGGTGTTTTTTGTTATTTCTGGTTATTTTTCGTACATGTTGTTTTTACGTTATCCGTTA
AAACGCTGGTGGAAAGTACGGGTAGAACGTGTGGGTATTCCCATGCTTACCGCAATCCCT
TTGCTTACCTTGCCGCAATTTATCCTGTTGCAATATGTCAAAGAGAAAACAGAGAACTGG
CCTACACTCTCTGCCTATGAAAAATATAATACGTTAGCGTGGGAACTCATTTCACATCTG
TGGTTTTTACTGGTGCTGGTGATATTAACCACCGTCAGCATCGGGATTTTTACCTGGTTC
CAAAAAAGGCAGGAAACAAGCAAGCCTCGTCCCGCCGCTATTTCACTGGTCAGGCTTTCG
CTTATTTTTTTCCTGCTGGGGATGGCTTACGCCGCTATCAGGCGCATTATTTTCATCGTA
TATCCAGCAATCCTCAGTGACGGCATGTTCAATTTTATTGTGATGCAAACGCTATTTTAT
GTGCCGTTTTTTATTCTCGGTGCGTTGGCCTTCATTCACCCCGATCTGAAAGCGCGCTTC
ACCACGCCCTCACGCGGATGCACTTTAGGCGCTGCCGTTGCTTTTATCGCGTATCTGCTG
AATCAACGTTATGGGAGCGGCGACGCCTGGATGTACGAAACCGAATCCGTGATTACGATG
GTAATGGGGCTGTGGATGGTGAACGTGGTATTCTCACTGGGGCATCGCTTGTTAAACTTC
CAGTCCGCGCGTGTCACCTATTTCGTGAATGCTTCGCTGTTTATCTATCTGGTGCATCAT
CCCTTAACGCTTTTCTTTGGCGCGTATATTACGCCGCATATCTCCTCCAACCTGATCGGG
TTCTTGTGTGGGCTGATATTTGTCATGGGTATTGCGTTAATTCTGTATGAAATTCATTTA
CGCATCCCGCTCCTGAAATTTCTCTTTTCAGGTAAACCGCCGGTAAAACAAGAAAGCCGC
GCCGCGATCGGGTAG
>EECEBICD_01472 Glucans biosynthesis protein G
ATGAAACATAAACGACAAATGATGAAAATGCGTTGGTTGGGCGCAGCTATTATGTTAACG
CTCTACGCATCATCGAGCTGGGCGTTCAGTATTGATGACGTGGCAAAACAAGCTCAATCT
TTAGCCGGGAAAGGCTATGAGGCGCCTAAAAGCAACTTGCCCTCCGTTTTCCGCGACATG
AAATATGCGGATTATCAGCAGATCCAGTTTAACAGCGATAAAGCCTACTGGAACAACTTA
AAGACCCCTTTTAAGCTCGAATTTTACCATCAGGGGATGTACTTCGATACGCCGGTCAAG
ATTAACGAAGTGACGGCGACGACGGTCAAAAGAATCAAATACAGCCCGGATTACTTCAAT
TTTGGCAATGTTCAGCACGATAAAGACACGGTAAAAGATTTAGGCTTCGCCGGGTTCAAA
GTTCTGTACCCCATCAACAGTAAAGATAAGAACGACGAAATCGTCAGTATGCTTGGCGCC
AGCTATTTCCGCGTTATCGGCGCAGGCCAGGTATATGGCTTATCTGCGCGCGGCCTGGCG
ATTGATACCGCCTTACCATCTGGTGAAGAGTTTCCCCGCTTTCGCGAGTTCTGGATTGAG
CGTCCAAAACCCACCGATAAGCGTTTGACCGTCTATGCATTACTGGATTCTCCGCGCGCG
ACCGGCGCTTACCGTTTTGTGATCATTCCTGGCCGCGATACCGTGGTGGACGTGCAGTCA
AAAGTCTATCTGCGCGATAAAGTGGGCAAGCTGGGCGTTGCGCCATTAACCAGTATGTTC
CTGTTTGGGCCAAACCAGCCGTCGCCGACGACCAACTATCGTCCGGAACTGCATGACTCG
AACGGCTTATCCATTCATGCGGGTAATGGCGAGTGGATTTGGCGTCCGCTGAACAATCCA
AAACACCTCGCTGTGAGCAGCTATGCAATGGAAAACCCTCAGGGATTCGGCCTGTTGCAG
CGTGGTCGCGAGTTCTCGCGCTTTGAAGATTTAGACGATCGCTACGACCTGCGTCCAAGC
GCCTGGATTACCCCGAAAGGCGACTGGGGCAAAGGTAAGGTTGAACTGGTTGAAATTCCG
ACCAATGATGAAACCAACGATAACATCGTCGCTTACTGGACTCCGGATCAACTGCCGGAA
CCGGGTAAAGAGATGAACTTCAAGTACACTCTGACCTTCAGCCGCGATGAAGATAAACTT
CATGCGCCGGATAATGCCTGGGTGCTGCAAACACGCCGCTCAACGGGCGACGTTAAACAG
TCGAATCTGATTCGCCAGCCCGACGGCACTATTGCCTTTGTGGTGGATTTCGTTGGCGCC
GACATGAAAAAACTGCCGCCGGATACGCCTGTCGCTGCGCAAACCAGCATTGGCGATAAC

GGTGAAATCGTTGACAGTAATGTACGCTATAACCCAGTCACTAAAGGCTGGCGTTTAATG
CTGCGCGTGAAAGTCAAAGACGCGAAGAAAACCACGGAAATGCGTGCCGCATTGGTGAAT
GCCGATCAGACGCTAAGTGAAACCTGGAGCTACCAGTTACCTGCCAATGAATAA
>EECEBICD_01473 Glucans biosynthesis glucosyltransferase H
ATGAATAAAACAACTGAGTATATTGACGCACTGCTGCTTTCTGAACGTGAGAAAGCGGCA
TTGCCGAAAACTGACATCCGCGCCGTGCATCAGGCGCTGGATGCCGAGCATCGGACTTAC
TCGCGAGAAGACGATTCACCGCAGGGTTCCGTAAAAGCCCGCCTTGAACACGCCTGGCCG
GATTCATTGGCGAAGGGGCAGTTAATTAAAGATGATGAAGGGCGCGATCAGTTGCAGGCT
ATGCCAAAAGCGACGCGCTCTTCGATGTTTCCCGATCCCTGGCGAACCAACCCGGTTGGC
CGTTTCTGGGATCGTCTGCGTGGGCGGGATGTAACGCCGCGCTATGTTTCTCGTCTGACA
AAAGAAGAGCAGGCGAGCGAGCAAAAATGGCGTACCGTCGGCACTATACGCCGCTATATT
TTGTTAATTTTGACTCTGGCGCAAACCGTCGTCGCGACCTGGTATATGAAGACCATTCTG
CCTTATCAGGGATGGGCGCTCATCAATCCTATGGATATGGTGGGGCAGGATATTTGGGTC
TCTTTTATGCAGCTCCTGCCCTACATGCTGCAAACCGGTATCCTGATTCTGTTTGCCGTG
CTGTTCTGCTGGGTGTCAGCCGGATTCTGGACGGCGCTGATGGGCTTCCTGCAACTGCTT
ATCGGGCGCGATAAGTACAGCATCTCCGCGTCTACGGTTGGCGATGAGCCCCTCAATCCG
GAACACCGGACGGCGCTGATCATGCCTATCTGTAATGAAGACGTTAGTCGCGTTTTCGCC
GGTCTGCGCGCGACCTGGGAGTCCGTTAAAGCCACAGGCAACGCCGCGCATTTCGACGTC
TATATCCTTAGCGACAGTTATAACCCGGATATCTGCGTGGCGGAGCAAAAGGCGTGGATG
GAGCTTATCGCGGAAGTGCAGGGCGAAGGCCAAATTTTTTACCGTCGCCGCCGCCGCCGT
ATGAAACGCAAAAGCGGCAATATTGACGATTTTTGCCGCCGCTGGGGCAATCAGTACAGC
TATATGGTGGTGCTGGACGCGGACTCAGTGATGAGCGGCGAGTGTCTGAGCGGCTTGGTG
CGCCTGATGGAAGCGAATCCAAACGCCGGGATTATCCAGTCTTCGCCGAAAGCGTCGGGG
ATGGATACTCTGTATGCCCGCTGCCAACAGTTCGCGACCCGCGTCTATGGGCCGCTGTTT
ACCGCCGGGCTGCACTTCTGGCAGTTGGGGGAGTCGCACTACTGGGGGCACAATGCCATT
ATCCGCGTGAAGCCGTTTATCGAGCACTGCGCTCTGGCGCCGCTGCCGGGAGAAGGTTCG
TTCGCCGGATCGATTCTTTCCCACGACTTTGTGGAGGCGGCGCTAATGCGTCGGGCAGGG
TGGGGCGTCTGGATTGCCTACGATCTCCCCGGCTCCTATGAAGAGCTGCCGCCAAACCTG
CTGGATGAGCTTAAACGCGACCGCCGCTGGTGTCACGGCAACCTGATGAACTTTCGTCTG
TTCCTGGTGAAAGGAATGCACCCGGTGCATCGCGCCGTGTTCCTGACGGGGGTAATGTCA
TATCTGTCCGCGCCGTTATGGTTTATGTTCCTCGCGCTTTCTACCGCGCTGCAGGTCGTT
CATGCGTTAACAGAGCCGCAATATTTCCTTCAGCCGCGCCAGCTTTTTCCGGTCTGGCCG
CAGTGGCGTCCGGAACTGGCAATCGCGCTGTTTGCATCAACGATGGTGCTGCTGTTCCTG
CCGAAGCTGCTCAGTATTATGCTGATCTGGTGTAAAGGCACCAAAGAGTATGGCGGTTTC
TGGCGCGTTACGTTGTCGCTGTTGCTGGAAGTGCTGTTCTCCGTGTTGCTGGCGCCGGTG
CGTATGCTGTTTCATACCGTGTTTGTGGTCAGCGCGTTCCTCGGCTGGGAAGTGGTCTGG
AACTCACCGCAACGCGACGATGATTCTACGCCGTGGGGAGAAGCCTTTATGCGTCACGGC
TCTCAACTGCTGCTGGGGCTGGTCTGGGCGGTGGGTATGGCGTGGCTGGATTTACGCTTT
CTGTTCTGGCTGGCGCCGATTGTCTTTTCGCTGATTCTGTCGCCATTTGTTTCGGTGATC
TCCAGTCGTTCAACGGTAGGATTACGCACCAAACGCTGGAAGCTGTTCCTGATCCCGGAA
GAGTATTCGCCGCCTCAGGTGTTGGTCGATACCGATAAATATCTGGAGATGAATCGCCGC
CGTATTCTGGACGATGGCTTTATGCATGCGGTATTTAACCCGTCGCTTAATGCGCTGGCG
ACCGCGATGGCCACTGCGCGTCACCGCGCCAGTAAGGTGCTGGAAATAGCCCGCGATCGT
CATGTGGAGCAGGCGCTAAACGAAACGCCGGAGAAACTGAACCGCGATCGGCGTCTGGTT
TTGCTCAGCGATCCGGTGACAATGGCGCGTTTACACTATCGGGTCTGGAATGCGCCAGAG
AGATACTCTTCCTGGGTAAACCATTATCAGTCTCTCGTCCTGAATCCGCAGGCGTTGCAG
GGACGAACATCGTCAGCGAGATAA
>EECEBICD_01474 hypothetical protein
GTGCGTATATTCGCGGTGAGCATAATGGTGATTACCCTGAGCGGCTGCGGCAGTATTATC
AGCAGAACGATCCCCGGACAAGGACACGGCAACCAGTATTACCCTGGCGTGCAGTGGGAT
ATGCGTGATTCCGCATGGCGCTATATCACTATCCTCGATCTGCCCTTCTCACTGATCTTC
GATACACTGCTACTGCCGCTCGATATTCACCACGGGCCTTATGAGTAA
>EECEBICD_01475 hypothetical protein
ATGACCATGTATGCCACGCTGGAAGAAGCTATCGATGCAGCCCGGGAAGAATTTCTGGCT

GACCATCCAGGCCTCGAACAAGATGAAGCGAATGTGCAGCAGTTCAACGTTCAGAAATAT
GTACTGCAGGATGGGGACATCATGTGGCAGGTCGAATTTTTCGCCGATGAAGGTGAAGAT
GGCGAATGTCTGCCGATGCTGAGTGGTGAAGCCGCGCAGAGCGTGTTTGACGGCGATTAT
GATGAGATAGAGATCCGCCAGGAATGGCAGGAAGAGAATACTTTGCATGAATGGGATGAA
GGGGAATTCCAGCTTGAACCCCCGCTTGATACCGAGGAAGGCCGTACTGCGGCAGACGAA
TGGGATGAGCGTTAA
>EECEBICD_01476 Staphyloferrin B transporter
ATGTCACCCTCTGATGTCCCCATAAACTGGAAACGCAATCTAACCGTTACCTGGCTGGGC
TGTTTTTTAACCGGCGCAGCATTCAGTCTGGTCATGCCTTTTCTCCCTCTCTACGTTGAG
CAACTCGGCGTAACAGGCCATAGCGCGCTCAACATGTGGTCCGGGCTGGTCTTTAGTATT
ACGTTCCTCTTTTCAGCTATCGCTTCCCCTTTTTGGGGCGGTCTGGCGGATCGTAAAGGG
CGTAAGATCATGCTGTTGCGTTCCGCTCTCGGCATGGCCATTGTGATGCTGTTAATGGGC
ATGGCGCAAAATATCTGGCAATTTCTGATCCTCCGCGCGTTATTAGGCCTGCTGGGCGGA
TTTATTCCCAACGCCAATGCGCTCATCGCCACTCAGGTGCCGCGCCACAAAAGCGGCTGG
GCGTTAGGGACGCTCTCTACTGGCGGCGTCAGCGGCGCTCTGCTTGGCCCACTTGCGGGC
GGTCTGCTCGCCGACCATTACGGCCTTCGTCCGGTCTTTTTTATTACCGCCAGCGTTTTG
TTTATCTGTTTTCTGCTCACCTTCTTTTTCATTCACGAGAATTTCCTGCCGGTGAGCAAA
AAAGAGATGCTTCACGTTCGGGAAGTGGTCGCCTCGTTAAAAAATCCCCGCCTGGTGTTA
AGCCTGTTTGTCACCACGCTGATTATTCAGGTGGCGACGGGGTCTATTGCGCCCATCCTG
ACGCTCTATGTGCGCGAACTCGCGGGAAATGTCAGTAATATTGCGTTCATTAGCGGGATG
ATAGCGTCCGTTCCCGGCGTGGCGGCATTACTTAGCGCCCCCCGGCTCGGCAAGCTTGGC
GACAGAATTGGCCCGGAAAAGATCCTTATCGTCGCGCTGATTATTTCCGTACTGCTGCTG
ATTCCAATGTCTTTTGTGCAAACGCCGTGGCAGCTCGCGCTATTGCGGTTTCTGCTTGGC
GCGGCGGATGGCGCGCTGCTGCCAGCCGTTCAAACTCTGCTGGTTTACAACTCTACCAAC
CAGATAGCCGGGCGCATATTCAGTTACAACCAATCTTTCCGCGATATCGGCAACGTCACC
GGCCCTCTCATGGGTGCCGCAATTTCCGCGAGCTATGGCTTCCGCGCCGTATTCTGCGTC
ACGGCAGGCGTGGTGTTGTTCAATGCTATCTATTCATGGAACAGCTTACGACGGCGCAGA
CTGGCAATAGAATAA
>EECEBICD_01477 Lipid A biosynthesis lauroyltransferase
ATGACGAAGTTGCCTAAATTCTCCGTGGCCTTACTGCACCCGCGCTATTGGTTAACCTGG
TTGGGTATAGGCGCGCTTTGGTTGGTCGTGCAACTCCCCTACCCGGTCATCTATAAATTG
GGTTGCGCATTGGGCCACCTGGCGCGACGCGTAATGAAACGCCGCGCAAAAATCGCCTAT
CGCAACCTTGAATTATGCTTTCCGGAAATGACCGCGCAGGAACGCCACACTATGGTGGTC
AAAAACTTTGAATCCGTCGGTATGGGCGTGATGGAAACCGGCATGGCCTGGTTCTGGCCC
GATCGGCGAGTGAACCGCTGGATGGAAGCGAGCGGTCTGGAGCATATCCGTGAAGTTAAG
GCGCAGGGGCTGGGGTTCATTCTGGTAGGAATACATTTTCTTACCCTTGAGTTTGGCGCC
CGCATGTTTGGTATGCATAACCCGGGGATCGGAGTTTATCGCCCGAATGATAATCCGTTG
CTCGACTGGTTACAGACCTGGGGACGCTTACGCTCCAATAAATCGATGTTGGATCGCAAA
GATCTGAAAGGCATGGTTAAGGCATTGAAAAGCGGCGAATTGATCTGGTATGCGCCGGAT
CACGATTACGGCCCGCGCGCCAGCGTCTTTGTACCGTTATTCGCCGTCGACCAGGCCGCT
ACGACCTCCGGTACCTGGATGCTCGCGCGCATGTCTAAAGCCTGTATTATCCCCTTCGTG
CCTCGCCGTAAACCCGATGGGAAAGGCTACGAACTGATCATTCTTCCTGCGGAATATTCG
CCTCCGCTGGAGAGCGCAGAAGCGACTGCCGCATGGATGAATAAGATTGTTGAGCAGTGC
ATTATGATGGCGCCAGAGCAGTATATGTGGCTGCATCGCCGCTTTAAAACTCGCCCTGAA
GGCGTACCGTCGCGCTATTGA
>EECEBICD_01478 hypothetical protein
ATGCCAGTGTTACACAACCGCATCTCTAATGACGAGCTGAAAGCCAAAATGCTGGCGGAA
AGCGAGCCGCGTACGACAATTTCTTTTTATAAATATTTCACTATCGCCTCGCCGCAGCAG
ACGCGGGACGCGTTGTATCAGGTGTTTACGGCGTTGGACGTTTTGGTCGTGTTTACCTG
GCGCATGAAGGCATCAATGCGCAAATCAGCGTGCCGCAAAGCAAGGTCGAGACCTTTCGT
CAACAGCTTTATACGTTCGACCCCGCGCTGGACGGGCTGCGTTTAAATATCGCGCTGGAG
GATGACGGAAAGTCATTTTGGGTGCTGCGTATGAAAGTTCGCGACCGTATCGTCGCTGAC
GGAATTGACGATCCGACTTTTGACGCCAGTAATGTCGGCGATTATCTGAAGGCGGCAGAT
GTGAATGCGATGCTGGACGATCCTGATGCGGTCTTTATTGATATGCGCAACCACTATGAG

TATGAAGTCGGCCATTTCGAAAATGCTCTGGAAATCCCGGCGGATACGTTTCGTGAACAG
TTGCCAAAAGCGGTTGAAATGCTGCGGGAACATGCAGATAAAAAGATAGTGATGTACTGT
ACCGGCGGTATTCGTTGTGAGAAAGCCAGCGCCTGGATGAAACACAACGGTTTCAATAAA
GTCTGGCATATTGAGGGTGGCATCATTGAGTACGCCCGTCGCGCGCGCGAGCAGGGGCTT
CCCGTTCGCTTTATCGGCAAAAACTTTGTATTTGATGAGCGAATGGGCGAACGAATCTCG
GATGAGGTTATCGCGCATTGCCATCAGTGCGGCGCGCCCTGCGATAGCCATACCAACTGC
AAAAATGACGGTTGCCATCTGCTGTTTATCCAGTGTCCGCAGTGCGCCAGTAAATTTAAC
GGCTGCTGTAGTGAACAATGCTGTGAAGAGTTGGCCTTGCCGGAGGAAGAACAGCGCCGA
CGTCGCGCGGGTCGCGAGAACGGCAATAAAATTTTTAATAAATCGCGTGGTCGGCTTAAT
AGCAAACTGAGCATTCCCGATCCGGCTGAGTAA
>EECEBICD_01479 Protein YceI
ATGAAAAAAAAACCTGCTGGGATTCACCCTCGCATCCTTGTTATTCACGACCGGTTCCGCC
GTGGCGGCGGAGTATAAAATTGATAAAGAAGGCCAACATGCGTTCGTCAATTTCCGCATT
CAGCATCTAGGCTACAGCTGGCTATACGGCACCTTTAAAGATTTCGACGGCACGTTCACT
TTTGACGAAAAAAAATCCGTCGGCAGACAAAGTGAACGTGACCATTAACACCAATAGCGTC
GACACTAACCATGCCGAACGTGACAAACACCTGCGTAGCGCGGAGTTTCTTAATGTTGCG
AAATTCCCGCAGGCAACCTTCACCTCTACCAGCGTGAAAAAAGAGGGCGATGAACTGGAT
ATTACCGGCAATCTGACGCTCAATGGCGTGACTAAACCGGTGACGCTGGAAGCGAAGCTG
ATGGGCCAGGGCGACGATCCGTGGGGCGGTAAGCGCGCGGGCTTTGAGGCCGAAGGAAAA
ATTAAGCTGAAGGATTTCAATATAACTACCGATCTCGGCCCGGCCTCACAAGAGGTTGAG
CTTATCATCTCAGTAGAAGGCGTTCAGCAGAAGTAA
>EECEBICD_01480 Cytochrome b561
ATGCAATTTAAAAATACTCCACAGCGTTATGGCGTGGTTTCCGCCGCGCTCCACTGGCTG
ACCGCCCTGGTGGTCTATGGCATGTTTGCGTTGGGTTTATGGATGGTCACGCTCAGTTAT
TACGACGGCTGGTATCACCAGGCACCGGAAATACATAAAAGTATTGGCATATTACTGATG
ATGGCGCTGATCGTGCGTATTATCTGGCGGCTTTATTCTCCGCCTCCCGTTGCGTTGACC
AGCTATTCCCGCTTAACGCGCGCTGGCGCCGCCGCAGGTCATCTCCTCCTGTATCTCCTG
CTCTTTGCGATAATCATTAGCGGCTACCTGATTTCCACCGCCGACGGTAAACCGATTAGC
GTCTTTGGCTGGTTTGAGATTCCGGCCACGCTTACGGACGCGGGCGCGCAGGCTGACATC
GCCGGAACGCTGCATCTGTGGTTTGCCTGGTCGCTGGTCATTATCTCGCTCTCGCATGGG
GTTATGGCGCTAAAACACCATTTCATCGATAAAGACGACACACTGAAACGTATGACAGGA
ATGTCGTCATCTGACTATGGAGCTCAAAAATGA
>EECEBICD_01481 putative protein YceO
ATGCGCCGTCTCTTTCATTTTCTGATGAATAATATTCGCGAACATTTCATGCTTTACGTT
ATTCTTTGGTCACTGCTAGCGGTGATGGATATTGTTTATCTCCTTTTCTTCTGA
>EECEBICD_01482 N-methyl-L-tryptophan oxidase
ATGAAATACGACCTTATTATTATCGGCAGCGGTTCGGTTGGCGCCGCCGCTGGTTATTAC
GCCACCCGCGCCGGGCTAAAGGTCCTGATGACCGATGCGCATATGCCGCCTCATCAACAG
GGCAGCCACCACGGCGATACCCGTCTTATCCGCCATGCTTATGGTGAAGGCGAAAAATAT
GTCCCGCTGATGCTTCGCGCCCAGACGCTTTGGGATGAACTCTCCACACACAATGAAGAG
CCTATTTTTGTCCGCTCCGGCGTCGTCAACCTCGGCCCGGCCGATTCCGCTTTCTTAGCC
AACGTCGCACGAAGCGCGCAACAGTGGCAATTGAACGTCGAGCGCCTGGACGCGACGGCC
CTCATGACGCGCTGGCCGGAAATTCGCGTGCCCGATAATTATATCGGGCTGTTTGAAGCT
GACTCCGGTTTTCTGCGCAGCGAATTAGCCATTACCACATGGCTTCGTCTGGCCCGAGAG
GCTGGCTGCGCACAGCTATTCAACAGCCAGGTAAGCCATATTCATCATGATGATAACGGT
GTGACGATAGAGACGAGTGAAGGCAGCTACCACGCCAGCAAGGCGCTAATTAGCGCGGGC
ACCTGGGTCAAAGCGCTGGTACCGGAGCTGCCCGTTCAGCCCGTACGTAAAGTTTTTGCC
TGGTTTAAGGCGGATGGACGTTACAGCACTAAAAACCGCTTTCCGGCCTTTACCGGCGAA
ATGCCCAACGGCGATCAATATTACGACTTCCCGGCGGAGAACGACGAGTTAAAAATCGGC
AAACACAATGGCGGACAGCTAATACAGGCTCCGGAAGAGCGCAAGCCCTTTGCCGCCGTT
GCCAGCGATGGCGCGGAAGCATTTCCTTTCCTGCGTAACGTACTGCCGGGTATCGGCGGT
TGTTTACATGGGGCGGCATGTACCTATGATAATTCGCCGGACGAGAATTTTATTATCGAT
ACGCTGCCTGGCCATGAGAACACGCTTGTCATCACTGGACTCAGCGGACATGGTTTTAAA
TTCGCCCCGGTGTTAGGAGAAATCGCTGCGGATTTTGCGTTGGGAAAAACATCCTCCTTT

GATCTGACGCCGTTCCGGCTTTCCCGTTTTAGCCAATAA
>EECEBICD_01483 Biofilm regulator BssS
ATGGAAAAGAATAATGAAGTCATTCAGACCCATCCGCTTGTAGGATGGGACATCAGCACC
GTCGATAGCTATGATGCGCTGATGCTGCGTTTGCACTACCAGACCCCAAATCGTCCGGAA
CCGGAAGGGACTGAAGTTGGTCAAACGCTCTGGTTAACGACAGATGTAGCCAGGCAATTT
ATTTCAATATTAGAAGCCGGCATCGCCAAAATAGAATCAGGTGATTACCAGGAAAACGAG
TATCGTCGCCATTAG
>EECEBICD_01484 DNA damage-inducible protein I
ATGCGTATTGAAGTCACTATAGCCAAAACGTCACCTCTGCCTGCCGGTGCGATTGATGCG
CTGGCGGGCGAACTCTCCCGTCGTATTAGCCATCATTTTCCTGAGAACTTGGGTAACGTC
ACCGTGCGTTACGCTACCGCCAACAACTTGTCCGTCATTGGCGCATCAAAAGAGGACAAA
GAACGCATTAGCGAGATTCTCCAGGAGACCTGGGAGAGCGCTGATGACTGGTTCATCAAT
GAATAA
>EECEBICD_01485 Dihydroorotase
ATGACTGCACCATCCCAGGTTTTAAAGATCCGCCGCCCGGACGACTGGCACGTTCACCTT
CGCGATGGCGACATGTTAAAAACGGTCGTACCCTATACCAGCGAAATTTATGGTCGCGCT
ATCGTGATGCCGAACCTGGCGTCCCCCATCACGACCGTTGATGCAGCGATCGCCTACCGC
CAGCGTATTCTCGATGCGGTGCCCGCCGGGCATGATTTCACGCCGTTAATGACCTGCTAT
TTAACGGATTCGCTCGATGCCGATGAACTGGAGCGTGGTTTCCATGAAGGCGTATTTACT
GCGGCTAAGCTTTACCCGGCCAATGCCACCACTAACTCCAGTCATGGCGTAACGTCAGTC
GACGCTATCATGCCGGTACTGGAGCGGATGGAAAAACTCGGAATGCCATTGCTGGTCCAC
GGTGAGGTGACCCATGCGGAGGTTGATATCTTCGATCGCGAAGCGCGTTTTATCGACACC
GTAATGGAACCGCTACGCCAGCGTCTGACCGCGCTTAAAGTGGTCTTTGAGCACATCACG
ACCAAAGATGCCGCGCAGTATGTACGTGACGGCAACGACTACCTGGCGGCTACCATTACG
CCTCAACATTTAATGTTTAACCGTAATGATATGCTGGTCGGCGGCATTCGTCCTCACCTG
TACTGTCTGCCGATTCTGAAACGCAATATTCACCAGCAGGCGTTACGCGAACTGGTTGCC
AGTGGTTTTACGCGCGCCTTCCTGGGGACGGATTCAGCGCCGCATTCACGTCATCGTAAA
GAGACTAGTTGCGGCTGCGCCGGTTGTTTCAACGCTCCCTCCGCCCTTGGCAGTTATGCC
GCCGTGTTTGAGGAAATGAACGCGCTGGCGCACTTTGAAGCGTTCTGTTCACTGAATGGC
CCGCAATTCTATGGCCTGCCGGTGAATGCGGGGTGGGTGGAACTGGTTCGCGATGAACAA
CAGGTACCGGAAAATATCGCGCTGGCTGATGATTCGCTGGTGCCTTTTTTAGCGGGTGAA
ACAGTACGCTGGTCAGTAAAAAAATAA
>EECEBICD_01486 putative lipoprotein YceB
GTGGGTTGTAATCAACTTACGCAATACACCATTAGCGAACAAGAAATCAATCAAGCGCTT
GAAAAACGGAATAATTTCTCAAAAGATATTGGTCTGCCCGGCATTGCCGACGCGCATATC
GTATTGACTAACCTTGTCAGCCAGATTGGTCGGGAAGAACCTAATAAAGTCACCCTTACT
GGCGATGCCAGGCTGGATATGAATTCCCTGTTTGGCAGTCAAAAAGCGACGATGAAACTC
AAGCTAAAAGCCCTGCCGGTCTTTGATAAAGAAAAAGGCGCCATCTATCTTCAGGAGATG
GAAGTAGTAGACGCCACCGTGACGCCGGAGAAAATGCAATCGGTGCTACAAACGCTCCTG
CCTTATTTAAATCAGTCTTTACGTAGCTACTTTAACCAGCGGCCCGCTTACGTGTTGCGT
GAGGACAGCAGTAAAGGCGAAGCGTTAGCAAAGAAACTCGCGAAAGGCATTGAAGTTAAA
CCTGGTGAAATCGTTATTCCTTTCACCAATTGA
>EECEBICD_01487 Glutaredoxin 2
GTGAAGCTTTATATTTACGATCATTGCCCTTTCTGCGTTAAAGCCCGCATGATTTTCGGC
CTGAAGAACATCCCCGTTGAACTTAACGTGTTACAAAATGACGACGAGGCGACGCCCACC
CGGATGATTGGTCAAAAGATGGTGCCCATCCTGCAAAAAGATGATAGTCGCTACCTTCCT
GAAAGTATGGATATTGTACATTATGTCGACAACCTCGACGGCAAACCGCTGTTAACCGGG
AAACGCAATCCAGCCATTGAAGAGTGGTTGCGCAAAGTTAACGGTTACGTTAATCAGCTA
CTGCTGCCGCGATTTGCAAAATCCGCTTTCGATGAATTTTCTACCCCTGCGGCGCGTCAG
TATTTTATCCGCAAAAAAGAAGCCTCATCTGGCAGTTTTGACAACCATCTTGCGCACTCT
GCAGGACTGATTAAAAAGATCGGCGATGATTTACGTTTGCTGGATAAACTCATCGTACAG
CCTAACGCCGTTAACGGCGAATTATCGGAAGATGATATTCATCTGTTTCCGCTTCTGCGT
AATCTGACGCTGGTCGCCGGTATCCACTGGCCGACAAAAGTCGCGGACTATCGCGATAAT
ATGGCGAAGCAGACGCAAATTAATTTACTCTCCTCAATGGCTATCTAA

>EECEBICD_01488 Multidrug resistance protein MdtH
ATGTCGCGCGTCTCGCAGGCGAGGAACCTGGGTAAATATTTTCTTCTCATCGATAACATG
TTAGTGGTGCTGGGTTTTTTCGTCGTCTTCCCGCTCATCTCTATTCGCTTTGTCGATCAA
ATGGGGTGGGCTGCCGTAATGGTAGGGATCGCGCTCGGCCTGCGCCAGTTTATTCAACAA
GGTCTGGGCATTTTTGGCGGCGCCATCGCCGATCGCTTTGGCGCGAAACCGATGATTGTC
ACCGGTATGCTGATGCGCGCCGCCGGCTTTGCCACCATGGGTATCGCGCATGAGCCCTGG
CTCTTGTGGTTTTCCTGCTTTCTTTCCGGTCTCGGCGGTACGCTTTTTGACCCGCCGCGT
TCAGCGCTGGTGGTCAAATTAATTCGTCCGGAGCAACGGGGCCGCTTCTTCTCTCTGTTG
ATGATGCAGGACAGCGCGGGCGCGGTGATTGGCGCGCTGCTGGGAAGCTGGTTGCTACAA
TACGATTTTCGCCTGGTCTGCGCGACAGGCGCTATTTTGTTCATATTATGCGCCCTTTTC
AACGCATGGCTGCTTCCGGCCTGGAAGCTATCAACGGTCAGAACGCCGGTGCGTGAAGGA
ATGCGCCGCGTCATGAGCGATAAAAGGTTTGTCACCTACGTGCTGACGCTGGCGGGCTAC
TATATGCTGGCGGTACAGGTCATGTTAATGCTGCCGATTATGGTAAACGATATCGCCGGT
TCGCCTGCTGCCGTGAAATGGATGTACGCTATTGAGGCGTGTCTCTCGCTGACGTTGCTC
TACCCGATTGCCCGCTGGAGCGAAAAGCGTTTTCGGCTGGAGCATCGGCTGATGGCCGGT
TTGCTCGTCATGTCGCTGAGCATGCTCCCCATCGGGATGGTGGGCAATTTACAGCAGCTT
TTTACGCTTATTTGCGCTTTCTACATCGGCTCGGTTATCGCCGAACCGGCGCGCGAAACG
CTCAGCGCGTCGCTCGCAGACGCGAGGGCGCGGGGAAGCTATATGGGCTTTAGCCGTCTG
GGATTAGCCATTGGCGGCGCGATTGGTTATATCGGCGGCGGCTGGTTGTTTGATATGGGG
AAAGCGCTTGCGCAGCCTGAACTACCGTGGATGATGCTCGGTATTATCGGCTTTATCACC
TTTTTGGCTTTAGGCTGGCAATTTAGTCATAAACGCACGCCGCGCCGGATGCTGGAACCC
GGCGCCTGA
>EECEBICD_01489 [Ribosomal protein S5]-alanine N-acetyltransferase
ATGTTTGGCTATCGTAGTAACGTGCCAAAAGTGCGCTTAACCACCGATCGTCTGGTGGTA
CGTTTAGTGCATGAGCGTGATGCCTGGCGTCTGGCCGATTATTACGCGGAAAATCGTCAT
TTTTTAAAACCCTGGGAACCGGTCCGTGATGAAAGTCATTGTTATCCTTCAGGATGGCAG
GCGCGTCTGGGAATGATCGGTGAATTTCACAAACAGGGCTCCGCCTTCTATTTCGCGCTA
CTTGATCCGGAAGAAAAAGAAATTATCGGCGTGGCGAATTTTTCCAATGTGGTGCGCGGT
TCTTTTCATGCCTGTTATCTGGGCTATTCCATTGCGCAGAAGTGGCAGGGGCAAGGGCTG
ATGTTTGAAGCCTTAACCGCTGCGATTCGCTATATGCAGCGCACTCAGCATATCCACCGT
ATCATGGCGAACTATATGCCGCACAACAAACGTAGCGGCGCGTTGCTGGCGCGGCTTGGC
TTTGAAAAAGAAGGCTATGCGAAAGATTACCTGTTGATTGATGGACAATGGCGCGACCAT
GTCCTGACGGCGTTAACCACGCCGTTATGGACGCCGGGGCGTTGA
>EECEBICD_01490 hypothetical protein
ATGAAATATGAATTAACCGCCACTGAAGCGCGAGTGATTGGCTGTCTGCTGGAAAAGCAG
GTGACAACGCCGGAACAGTATCCGCTTTCCGTCAACGGCGTGGTGACAGCCTGTAATCAG
AAAACCAACCGTGAACCGGTGATGAACCTGACGGAACAAGAGGTACAAGAACAGCTCGAT
AACCTGGTGAAACGCCACTTTTTGCGTACGGTCAGCGGGTTTGGCAATCGCGTCACCAAA
TATGAACAGCGCTTCTGTAATTCCGAATTTGGCGATCTGAAACTTAGCGCGGCGGAAGTG
GCGCTTGTCACTACGCTGCTGCTGCGCGGCGCGCAAACGCCCGGCGAGTTGCGTAGCCGG
GCGTCGCGGATGCATGAATTCAGCGATATGGCGGAAGTTGAATCCACGCTGGAACGGCTT
GCCAGTCGTGAGGACGGCCCGTATGTCGTCCGTCTGGCGCGTGAACCGGGTAAGCGCGAA
AGCCGTTATATGCACCTTTTTTGCGGCGACGTCGATGAACTGTCTCTCCAGACGTCTGCG
CCGGAAAGTGCGTCGGGCGATCTTCAGTCGCGCGTCGAAGCGCTGGAAAGCGAAGTGGCG
GAGTTAAAGCAGCGGCTGGATTCTTTGTTAGCTCACCTGGGAGAGTAA
>EECEBICD_01491 Putative oxidoreductase YceM
GTGAGAACATTACGGATTGGCATTGTCGGATTAGGCGGTATTGCGCAGAAGGCCTGGCTG
CCGGTATTAACCAACACCGCCGGATGGACGTTACAGGGCGCCTGGTCTCCTTCGCGGGAT
AAAGCCTTACGTATCTGCGAAAGCTGGAGCATACCGTATGTGGATTCGCTGGCGAATTTA
GCGTCCGGCTGCGATGCGGTCTTCGTCCACTCCAGTACCGCAAGCCATTATGCCGTGGTC
AGCGAACTTCTCAATGCCGGCGTCCATGTTTGCGTGGATAAACCGCTGGCGGAAAATCTA
CGTGATGCCGAACGGCTGGTGGCGCTGGCGGCGCAAAAAAATTGA
>EECEBICD_01492 Putative oxidoreductase YceM
ATGGTTGGCTTTAATCGCCGTTTCGCGCCGCTGTACCGCGAACTGAAGACGCGCCTCGGC

ACTGCGGCGTCACTGCGTATGGATAAACATCGTACCGATAGCGTCGGGCCGCACGACTTA
CGTTTTACTTTGCTCGATGACTATCTGCATGTCGTGGATACCGCTCTGTGGCTGGCGGGC
GGCGAGGCGCGCCTTGCCAGCGGCACGTTGCTCACCAGCGAGTCCGGCGAAATGTGCTAT
GCGGAACATCATTTTTCCGCCGACAAACTACAAATTACCACCAGTATGCACCGGCGCGCC
GGAAGTCAGCGTGAATCGGTCCAGGCCGTCACCGACGGCGGGCTATATGACGTGACGGAT
ATGCGTGAATGGCGCGAAGAGCGCGGGCAGGGTATTCTCATCAAACCCATTCCTAGTTGG
CAAACGACGCTTGAACAGCGTGGTTTTGTCGGATGCGCGCGGCATTTCATTGACTGCGTA
CAAAATCAGACGGTTCCGGAAACGGCGGGGGAGCAGGCGATTTTGGCCCAGCGCGTCGTG
GAGGCGCTGTGGCGGGACGCCATCAGCGAATAA
>EECEBICD_01493 putative lipid II flippase MurJ
ATGCAGGAGTTTTACGCCAGGGTCTGGAATACAAAAGAAATGAATTTATTGAAATCGCTG
GCTGCCGTCAGCTCGATGACTATGTTTTCACGCGTGTTGGGCTTTGCCCGTGATGCGATT
GTCGCCAGAATTTTTGGCGCAGGGATGGCGACCGACGCCTTTTTTGTGGCGTTTAAACTC
CCCAATCTACTACGCCGGATCTTTGCCGAAGGCGCTTTTTCTCAGGCCTTTGTGCCTATC
CTGGCGGAATATAAGAGCAAGCAGGGTGAAGAAGCGACGCGGATCTTTGTCGCTTACGTT
TCCGGCCTGTTGACGCTGGCATTGGCCGTCGTGACGGTGGCCGGTATGCTGGCCGCCCCG
TGGGTGATTATGGTAACCGCGCCGGGTTTTGCCGATACTGCGGATAAATTCGCGCTGACG
ACGCAACTGCTGCGGATTACGTTTCCCTATATTCTGCTGATCTCGCTGGCTTCACTGGTT
GGCGCCATTCTCAACACCTGGAATCGCTTCTCTATTCCCGCTTTTGCGCCGACATTTCTT
AATATCAGCATGATCGGTTTTGCATTATTCGCCGCGCCATACTTTAATCCGCCGGTGCTG
GCGTTAGCCTGGGCAGTCACCGTCGGCGGCGTGCTGCAACTGGTGTATCAACTTCCGTAT
TTGAAAAAGATCGGTATGCTGGTGCTGCCGCGCATTAACTTTCGCGACACCGGGGCGATG
CGGGTGGTCAAACAGATGGGGCCGGCGATTTTGGGCGTATCCGTCAGTCAGATCTCTCTT
ATCATCAATACCATTTTTGCCTCGTTTCTGGCCTCCGGCTCGGTCTCATGGATGTACTAT
GCCGATCGGTTGATGGAGTTCCCGTCCGGCGTGCTGGGCGTGGCGTTGGGGACCATCCTG
TTGCCGTCATTGTCGAAAAGCTTTGCCAGCGGCAATCATGATGAGTACTGCCGCCTGATG
GACTGGGGGCTGCGTTTGTGCTTTTTACTGGCGTTGCCGAGCGCGGTAGCGTTAGGCATT
CTGGCGAAGCCGCTGACGGTCTCGCTGTTTCAGTACGGTAAATTCACCGCCTTTGATGCG
GCGATGACGCAGCGGGCGTTAATCGCCTATTCGGTGGGGCTGATTGGCTTGATCGTCGTA
AAAGTGTTGGCCCCGGGCTTCTATTCTCGCCAGGATATTAAAACGCCGGTGAAAATCGCC
ATAGTGACGTTAATCATGACTCAGTTAATGAACCTGGCGTTTATTGGACCGCTGAAACAC
GCCGGGCTGTCGCTCTCTATTGGTCTGGCGGCATGTCTCAATGCGTCGCTGCTGTACTGG
CAACTGCGCAAACAGAATATCTTTACGCCACAACCGGGTTGGATGTGGTTCCTGATGCGT
CTGATCATTTCCGTACTGGTGATGGCCGCCGTATTGTTCGGCGTGTTGCATATTATGCCG
GAGTGGTCGCAAGGGTCGATGTTATGGCGTTTGCTACGTTTGATGGCGGTAGTGATCGCG
GGTATTGCGGCCTATTTCGCCGCGCTTGCCGTGCTGGGCTTTAAAGTGAAAGAGTTTGTT
CGCCGGACGGCGTAA
>EECEBICD_01494 Flagella synthesis protein FlgN
ATGACTCGTTTGTCAGAAATACTTGACCAGATGACCACCGTCCTGAATGACCTGAAGACG
GTGATGGACGCCGAGCAACAACAGCTTTCCGTAGGCCAGATTAACGGCAGCCAGCTACAG
CGTATTACAGAAGAAAAAAGCTCGTTGCTGGCGACGCTGGATTATCTGGAACAACAGCGC
CGTCTGGAGCAGAACGCGCAGCGTAGCGCAAACGATGACATTGCAGAGCGCTGGCAGGCG
ATTACCGAAAAAACGCAGCATCTTCGCGACCTCAACCAGCACAACGGCTGGCTGCTGGAA
GGGCAAATCGAGCGTAATCAACAGGCGCTGGAGGTGCTGAAACCTCACCAGGAGCCGACC
TTGTACGGGGCTGACGGTCAGACTTCCGTCTCTCACCGCGGCGGTAAAAAAATTTCTATC
TAA
>EECEBICD_01495 Negative regulator of flagellin synthesis
ATGAGCATTGACCGTACCTCACCTTTGAAACCCGTTAGCACTGTCCAGACGCGCGAAACC
AGCGACACGCCGGTACAAAAAACGCGTCAGGAAAAAACGTCCGCCGCGACGAGCGCCAGC
GTAACGTTAAGCGACGCGCAAGCGAAGCTCATGCAGCCAGGCGTCAGCGACATTAATATG
GAACGCGTCGAAGCATTAAAAACGGCTATCCGTAACGGTGAGTTAAAAATGGATACGGGA
AAAATAGCAGACTCGCTCATTCGCGAGGCGCAGAGCTACTTACAGAGTAAATAA
>EECEBICD_01496 Flagella basal body P-ring formation protein FlgA
ATGCAAACGTTAAAACGAGGATTCGCCGTGGCGGCTTTGCTGTTCAGCCCCCTGACAATG

GCGCAGGACATCAACGCCCAGCTGACCACGTGGTTTTCCCAGCGTCTGGCTGGCTTCAGC
GACGAGGTGGTTGTCACGCTCCGTTCATCGCCCAATCTGCTACCGAGCTGCGAACAGCCA
GCCTTCAGCATGACGGGCAGCGCGAAGCTGTGGGGCAACGTCAATGTGGTGGCGCGCTGC
GCCAATGAAAAACGTTATTTGCAGGTGAATGTGCAAGCGACCGGCAATTATGTCGCCGTT
GCCGCGCCCGTCGCGCGCGGCGGAAAATTGACGCCGGCCAACGTCACGCTAAAACGTGGC
AGGCTGGATCAGTTACCGCCGCGGACGGTACTGGATATCCGTCAGATTCAGGATGCCGTC
AGTTTGCGCGATCTCGCACCCGGGCAGCCGGTACAGCTTACGATGATACGTCAGGCCTGG
CGCGTCAAAGCGGGGCAACGAGTACAGGTCATCGCCAATGGCGAGGGGTTCAGCGTCAAT
GCGGAAGGTCAGGCGATGAATAATGCCGCCGTCGCGCAAAATGCGCGCGTGCGCATGACA
TCGGGTCAAATCGTGAGTGGAACCGTCGATTCTGATGGGAATATTCTTATTAACCTATAA
>EECEBICD_01497 Flagellar basal body rod protein FlgB
ATGCTCGACAGGCTCGATGCCGCCTTACGATTTCAGCAGGAAGCGCTTAATCTGCGCGCG
CAACGTCAGGAAATATTAGCGGCGAATATCGCCAATGCCGATACGCCGGGGTATCAGGCG
CGCGATATTGATTTTGCCAGTGAGTTAAAAAAGGTGATGGTGCGTGGACGGGAAGAAACC
GGCGGCGTCGCGTTAACGTTGACTTCCTCTCACCATATTCCCGCCCAGGCGGTCTCTTCT
CCCGCAGTGGATCTGCTTTACCGCGTACCCGATCAGCCTTCTTTGGATGGTAACACCGTA
GATATGGACAGGGAACGTACGCAGTTTGCGGATAACAGTCTCAAATATCAGATGGGGCTT
ACCGTTCTGGGTAGCCAACTCAAAGGCATGATGAATGTGCTACAGGGAGGAAACTAA
>EECEBICD_01498 Flagellar basal-body rod protein FlgC
GTGGCGCTGTTAAACATTTTTGATATTGCCGGATCGGCGCTTGCCGCGCAGTCCAAGCGG
TTGAACGTTGCGGCCAGTAACCTGGCGAATGCGGATAGCGTCACCGGCCCGGACGGACAG
CCTTATCGCGCCAAACAGGTGGTTTTTCAGGTGGACGCCGCGCCGGGTCAAGCCACTGGC
GGGGTAAAGGTCGCCAGCGTGATTGAAAGTCAGGCACCGGAAAAGCTGGTTTATGAGCCA
GGCAATCCGCTGGCGGACGCTAATGGTTACGTCAAAATGCCCAACGTCGATGTGGTCGGC
GAAATGGTCAACACGATGTCAGCCTCGCGCAGCTATCAGGCAAATATCGAAGTCCTGAAT
ACCGTAAAAAGCATGATGCTTAAAACGCTGACATTAGGCCAGTAA
>EECEBICD_01499 Basal-body rod modification protein FlgD
ATGTCTATTGCCGTAAATATGAATGACCCGACCAACACGGGCGTCAAAACGACGACCGGC
AGCGGGTCGATGACCGGAAGCAACGCTGCCGATCTGCAAAGCAGTTTCCTGACCTTACTG
GTCGCGCAATTGAAGAACCAGGACCCGACTAACCCATTACAAAATAATGAGTTAACGACA
CAGTTGGCGCAAATCAGTACCGTGAGCGGCATTGAAAAACTGAATACGACGCTGGGGGCT
ATTTCCGGGCAAATCGATAATAGTCAGTCCCTACAGGCGACCACGCTGATTGGACATGGC
GTTATGGTGCCTGGCACCACAATTCTGGCGGGTAAAGGCGCGGAAGAAGGGGCCGTGACG
TCCACGACGCCGTTTGGCGTGGAATTGCAACAGCCTGCGGACAAAGTGACGGCAACCATT
ACCGATAAAGATGGCCGGGTGGTACGGACGCTGGAGATCGGTGAGTTGCGAGCCGGGGTA
CACACCTTTACCTGGGATGGTAAGCAAACGGACGGAACAACGGTACCGAATGGTTCTTAC
AACATTGCGATTACCGCCAGCAATGGCGGAACGCAACTGGTGGCGCAGCCGCTGCAATTC
GCTCTGGTACAGGGCGTGACGAAGGGCAGTAACGGCAACCTGTTGGATCTGGGTACCTAC
GGCACCACCACACTCGACGAAGTTCGGCAAATAATCTAA
>EECEBICD_01500 Flagellar hook protein FlgE
ATGTCTTTTTCTCAAGCGGTTAGCGGCCTGAACGCTGCGGCCACCAACCTTGATGTTATC
GGTAATAACATCGCCAACTCCGCCACCTATGGCTTTAAGTCCGGTACGGCATCATTTGCC
GATATGTTCGCCGGTTCCAAAGTGGGGCTGGGCGTAAAAGTGGCGGGGATTACCCAGGAT
TTTACCGACGGTACGACAACGAACACCGGGCGCGGGCTGGATGTCGCGATTAGCCAGAAC
GGTTTTTTCCGCCTGGTAGACAGCAACGGTTCCGTGTTCTATAGCCGCAACGGCCAGTTC
AAACTGGACGAGAACCGTAACCTGGTCAATATGCAGGGGATGCAGTTGACCGGCTATCCG
GCCACCGGTACGCCGCCGACCATTCAGCAGGGGGCGAATCCTGCGCCGATCACCATTCCG
AACACGCTGATGGCGGCGAAATCGACCACCACCGCGTCAATGCAGATCAACCTGAACTCA
ACGGACCCTGTACCGTCTAAAACGCCCTTTAGCGTGAGTGATGCGGATTCGTATAACAAA
AAAGGCACCGTCACCGTTTATGACAGCCAGGGTAATGCCCATGACATGAACGTCTATTTT
GTGAAAACCAAAGATAATGAATGGGCTGTGTACACCCATGACAGCAGCGATCCTGCAGCC
ACTGCGCCAACAACGGCGTCCACTACGCTGAAATTCAATGAAAACGGGATTCTGGAGTCT
GGCGGTACGGTGAACATCACCACCGGTACGATTAATGGCGCGACAGCGGCCACCTTCTCC
CTCAGCTTCCTTAACTCCATGCAGCAGAACACCGGGGCTAATAACATCGTCGCCACCAAT

CAAAACGGCTATAAGCCGGGCGACCTGGTGAGCTACCAGATTAACAACGACGGCACCGTG
GTTGGCAACTACTCCAACGAGCAGGAGCAGGTGCTGGGGCAGATTGTGCTGGCTAACTTC
GCCAACAACGAAGGTCTGGCATCCCAGGGCGATAACGTCTGGGCGGCGACGCAGGCTTCC
GGGGTTGCGCTGCTGGGGACTGCCGGTTCCGGCAACTTCGGTAAGCTGACGAACGGCGCG
CTGGAAGCCTCTAACGTGGATTTGAGTAAAGAGCTGGTGAATATGATCGTCGCGCAGCGT
AACTACCAGTCGAATGCGCAGACCATCAAAACTCAGGACCAGATCCTCAATACGCTGGTT
AACCTGCGCTAA
>EECEBICD_01501 Flagellar basal-body rod protein FlgF
ATGGATCACGCAATTTATACCGCTATGGGGGCGGCCAGCCAGACGCTTAACCAGCAGGCG
GTAACGGCCAGCAACCTGGCTAATGCCTCAACGCCGGGCTTTCGCGCGCAGCTTAACGCG
CTACGCGCGGTGCCCGTTGATGGCCTCTCTTTAGCAACGCGCACGCTGGTTACGGCGTCG
ACGCCCGGGGCGGATATGACCCCGGGTCAGTTGGACTACACCTCCCGTCCGCTGGATGTC
GCGTTACAGCAGGACGGCTGGCTGGTAGTGCAAGCGGCGGATGGCGCTGAAGGATATACC
CGTAACGGGAATATCCAGGTGGGTCCGACCGGGCAGTTAACCATTCAGGGACATCCGGTT
ATCGGCGAAGGCGGCCCGATTACCGTTCCTGAAGGGTCGGAAATCACCATTGCGGCAGAC
GGCACGATCTCCGCGCTCAATCCCGGCGACCCGCCAAACACGGTGGCGCCCGTTGGGCGA
CTGAAGCTGGTCAAAGCGGAAGGCAATGAGGTGCAGCGGAGCGATGACGGTTTATTCCGC
CTTACCGCCGAGGCACAGGCTGAACGCGGGGCGGTACTGGCCGCCGACCCGTCAATTCGC
ATTATGTCGGGCGTGCTGGAGGGCAGTAACGTCAAGCCGGTTGAAGCCATGACCGACATG
ATCGCCAACGCACGTCGTTTTGAAATGCAGATGAAGGTTATCACCAGCGTAGATGAGAAC
GAAGGGCGAGCTAACCAACTGCTGTCGATGAGTTAA
>EECEBICD_01502 Flagellar basal-body rod protein FlgG
ATGATCAGTTCATTATGGATCGCCAAAACCGGTCTGGACGCGCAGCAAACCAATATGGAT
GTGATTGCCAATAACCTGGCAAACGTCAGCACCAATGGTTTTAAGCGTCAGCGCGCGGTA
TTTGAAGATCTGTTGTATCAGACCATCCGCCAGCCGGGCGCGCAGTCGTCCGAGCAGACG
ACGCTGCCTTCAGGGCTGCAAATCGGTACCGGCGTGCGTCCGGTCGCCACGGAGCGTCTG
CACAGTCAGGGGAACCTGTCGCAGACCAACAACAGTAAAGATGTGGCGATTAAAGGGCAG
GGCTTTTTCCAGGTCATGCTGCCGGACGGTACGTCTGCCTATACCCGCGACGGCTCTTTC
CAGGTGGATCAGAATGGTCAACTGGTGACGGCGGGCGGTTTTCAGGTGCAGCCGGCAATC
ACCATTCCGGCCAACGCGTTAAGCATCACGATTGGCCGCGACGGCGTGGTCAGCGTTACC
CAGCAGGGGCAGGCCGCGCCGGTTCAGGTCGGGCAGCTTAACCTGACCACCTTTATGAAC
GACACCGGGCTGGAAAGCATTGGCGAGAACCTCTATATCGAAACGCAATCGTCCGGCGCG
CCGAACGAAAGCACGCCGGGGCTCAACGGCGCGGGGTTGTTGTATCAAGGGTATGTCGAA
ACGTCGAACGTTAACGTGGCGGAAGAGCTGGTGAACATGATTCAGGTTCAACGCGCCTAT
GAAATTAACAGTAAAGCAGTATCGACGACCGATCAGATGCTGCAGAAACTGACGCAACTC
TAA
>EECEBICD_01503 Flagellar L-ring protein
GTGGCGAATGGCTCCATATTTCAGTCTGCGCAGCCGATTAATTATGGCTATCAGCCGCTT
TTTGAAGATCGTCGACCGCGTAATATCGGCGATACGCTCACGATTGTGTTACAGGAAAAC
GTCAGCGCCAGTAAAAGCTCGTCGGCAAATGCCAGTCGCGACGGCAAAACCAGTTTTGGT
TTTGATACGGTACCGCGTTATCTGCAGGGATTATTCGGTAATTCCCGCGCGGATATGGAG
GCCTCCGGCGGCAACTCTTTTAACGGCAAAGGCGGCGCGAATGCCAGCAATACCTTTAGC
GGCACGCTGACCGTGACCGTCGATCAGGTTCTGGCCAATGGCAATTTACACGTCGTGGGG
GAAAAACAGATCGCGATTAATCAGGGAACGGAATTCATCCGCTTCTCCGGCGTGGTAAAT
CCACGCACCATCAGCGGCAGCAACTCCGTTCCCTCGACACAGGTGGCGGATGCGCGGATT
GAATATGTCGGGAACGGCTATATTAACGAAGCGCAAAATATGGGCTGGCTGCAACGTTTC
TTCCTTAATTTGTCGCCGATGTAA
>EECEBICD_01504 Flagellar P-ring protein
GTGTTTAAAGCTCTTGCAGGAATCGTTCTGGCACTGGTTGCCACTCTGGCGCACGCCGAG
CGTATCCGGGATCTGACCAGTGTCCAGGGAGTACGGGAAAACTCGCTGATCGGCTACGGG
CTGGTGGTCGGGCTGGACGGTACGGGCGACCAGACGACCCAGACGCCATTTACCACCCAG
ACGCTGAATAACATGCTGTCACAACTGGGGATTACGGTCCCCACCGGCACCAACATGCAG
TTGAAAAACGTGGCGGCGGTGATGGTGACGGCGTCGTATCCGCCTTTTGCGCGACAGGGA
CAAACGATCGATGTCGTCGTTTCCTCAATGGGGAACGCTAAAAGTCTGCGTGGCGGGACG

TTATTAATGACGCCGTTAAAAGGGGTGGACAGCCAGGTGTATGCTCTGGCGCAGGGCAAT
ATTCTGGTCGGCGGCGCGGGCGCTTCCGCAGGCGGCAGTAGCGTGCAGGTTAACCAGCTT
AATGGCGGGCGCATCACTAATGGCGCGATTATCGAACGCGAGTTGCCGACTCAGTTCGGC
GCTGGCAACACCATTAATCTGCAATTGAACGACGAAGATTTTACGATGGCGCAGCAAATT
ACCGACGCCATCAACCGCGCCCGCGGTTACGGCAGCGCCACTGCGCTTGATGCGCGAACG
GTACAGGTACGCGTGCCCAGCGGCAACAGCTCGCAGGTGCGTTTTCTGGCGGACATTCAA
AATATGGAAGTCAACGTGACGCCGCAGGATGCAAAAGTTGTGATCAACTCGCGTACCGGT
TCGGTGGTCATGAATCGGGAAGTCACGCTGGATAGCTGCGCTGTGGCGCAGGGCAATTTG
TCAGTGACAGTCAATCGCCAGCTCAACGTCAACCAGCCGAATACGCCATTTGGCGGCGGG
CAGACCGTGGTGACGCCACAGACTCAAATAGATTTGCGTCAGAGCGGCGGATCGCTACAG
AGCGTGCGTTCCAGCGCCAATCTGAACAGCGTAGTGCGTGCGCTGAATGCGCTTGGCGCG
ACGCCGATGGACCTGATGTCGATTTTGCAGTCCATGCAGAGCGCGGGCTGTCTACGCGCC
AAACTGGAAATCATCTGA
>EECEBICD_01505 Peptidoglycan hydrolase FlgJ
ATGATCGGAGACGGTAAATTGCTGGCCAGCGCGGCCTGGGATGCGCAATCTCTGAACGAA
CTGAAAGCGAAAGCGGGCCAGGACCCGGCGGCGAATATCCGTCCTGTGGCCCGTCAGGTG
GAAGGGATGTTTGTGCAGATGATGCTGAAAAGTATGCGCGAGGCTTTACCCAAAGATGGT
TTATTCAGCAGCGATCAGACGCGTCTGTATACCAGCATGTATGACCAGCAGATCGCCCAG
CAGATGACCGCCGGTAAGGGATTGGGGCTGGCGGATATGATGGTTAAACAGATGACGGGC
GGGCAGACGATGCCTGCGGATGATGCGCCGCAAGTACCGCTTAAATTCTCCCTGGAGACG
GTAAACAGCTATCAAAATCAGGCGCTGACCCAACTGGTGCGCAAAGCCATACCGAAAACG
CCGGACAGCAGCGATGCGCCGCTCTCCGGCGACAGTAAAGACTTTCTGGCCCGGCTTTCG
CTCCCGGCGAGGCTGGCCAGCGAACAAAGCGGGGTGCCGCATCATCTGATTCTGGCGCAG
GCGGCGCTGGAGTCCGGCTGGGGGCAACGGCAAATCCTGCGGGAGAATGGCGAACCCAGC
TATAACGTATTTGGCGTGAAAGCGACCGCCAGTTGGAAAGGGCCGGTGACGGAAATCACC
ACCACTGAATACGAGAATGGCGAAGCGAAAAAAGTGAAAGCGAAATTCCGCGTCTATAGC
TCGTATCTGGAGGCATTATCGGATTATGTCGCGCTGTTAGCGCGTAACCCACGCTACGCT
GCCGTGACCACTGCCGCCACGGCAGAGCAGGGCGCAGTGGCTCTGCAAAACGCCGGATAC
GCCACTGACCCGAATTACGCGCGTAAATTGACCAGCATGATTCAGCAGTTGAAAGCGATG
AGTGAAAAGGTCAGCAAAACCTACAGCGCGAATCTCGACAATCTCTTTTAA
>EECEBICD_01506 Flagellar hook-associated protein 1
ATGTCCAGCTTGATTAATCACGCCATGAGCGGACTTAACGCCGCGCAGGCCGCGTTAAAT
ACGGTCAGTAATAACATCAACAATTATAACGTTGCGGGTTATACCCGGCAGACAACTATT
CTGGCGCAGGCAAACAGTACGTTAGGGGCTGGCGGCTGGATAGGTAATGGCGTTTACGTT
TCAGGCGTACAGCGCGAATATGATGCGTTTATCACTAATCAGCTACGCGGCGCGCAAAAC
CAGAGCAGCGGCTTGACCACGCGCTATGAACAGATGTCGAAAATCGACAACCTGCTGGCC
GATAAATCCAGCTCACTGTCTGGTTCGTTGCAGAGTTTTTTTACCAGCCTGCAAACGTTA
GTCAGTAATGCGGAAGATCCTGCGGCGCGTCAGGCGCTGATTGGTAAAGCGGAAGGGCTG
GTAAACCAGTTCAAAACCACCGATCAGTATCTGCGCGATCAGGATAAACAGGTCAATATC
GCGATTGGCTCCAGCGTGGCGCAAATCAACAATTACGCGAAGCAGATAGCTAACCTGAAC
GATCAAATCTCCCGTATGACGGGCGTAGGCGCGGGCGCATCGCCGAACGACCTGCTCGAT
CAACGTGATCAGTTGGTTAGCGAGCTTAACAAGATCGTTGGCGTCGAGGTGAGTGTACAG
GACGGCGGCACCTATAACCTGACGATGGCCAATGGCTATACGCTGGTGCAGGGGTCGACG
GCGCGTCAGTTGGCGGCGGTTCCCTCCAGCGCCGACCCGACGCGAACGACTGTCGCTTAT
GTCGATGAGGCCGCCGGTAACATCGAAATTCCGGAAAAGTTGCTGAACACCGGTTCGCTC
GGCGGGCTACTGACGTTCCGTTCCCAGGATCTGGATCAGACTCGTAATACGCTGGGCCAG
TTGGCGTTGGCGTTTGCCGATGCGTTTAACGCGCAGCATACCAAAGGTTATGACGCCGAC
GGCAATAAAGGGAAAGACTTCTTTAGCATTGGCTCGCCGGTGGTATATAGCAACAGTAAT
AATGCCGATAAAACGGTATCGCTAACCGCTAAGGTGGTCGATAGCACGAAGGTTCAGGCG
ACGGATTATAAGATTGTTTTTGACGGTACAGACTGGCAGGTTACTCGCACTGCGGATAAC
ACCACCTTCACGGCGACAAAAGATGCTGACGGAAAACTGGAGATTGACGGTCTGAAAGTG
ACGGTAGGGACCGGCGCACAGAAAAACGACAGTTTTCTTCTCAAGCCGGTTAGCAATGCT
ATCGTCGACATGAACGTTAAAGTGACGAATGAAGCCGAGATTGCGATGGCGTCTGAGTCA
AAACTCGATCCTGACGTGGATACCGGCGACAGCGATAACCGCAATGGTCAGGCATTGCTG

GACTTACAAAACAGCAATGTAGTGGGCGGCAACAAAACTTTTAACGATGCTTACGCCACG
TTGGTCAGCGATGTGGGTAACAAAACGTCAACGCTGAAAACCAGCAGCACCACGCAGGCG
AATGTGGTTAAACAGCTTTATAAACAGCAACAGTCGGTTTCCGGCGTTAACCTCGACGAA
GAGTACGGCAATTTGCAGCGTTATCAGCAGTATTATCTGGCGAATGCGCAAGTATTGCAG
ACCGCGAATGCGCTGTTTGATGCGTTATTGAATATTCGCTAA
>EECEBICD_01507 Flagellar hook-associated protein 3
ATGCGTATCAGTACCCAGATGATGTACGAACAAAATATGAGCGGCATCACTAATTCTCAG
GCCGAATGGATGAAGCTGGGCGAGCAGATGTCTACCGGTAAGCGCGTTACCAACCCATCT
GACGATCCAATCGCCGCGTCGCAGGCGGTAGTACTCTCTCAGGCGCAGGCGCAGAATAGC
CAGTATGCCCTGGCGCGTACGTTTGCCACCCAAAAAGTGTCGCTGGAAGAAAGCGTACTC
AGTCAGGTGACGACGGCGATTCAAACCGCGCAGGAAAAAATCGTCTATGCCGGAAACGGC
ACGTTAAGCGACGATGACCGCGCGTCGCTGGCGACGGATTTACAGGGGATCCGCGATCAG
CTGATGAACCTGGCAAACAGCACTGACGGCAATGGTCGCTATATCTTTGCCGGGTATAAA
ACGGAAGCGGCGCCATTCGACCAGGCGACAGGTGGTTATCATGGCGGCGAGAAAAGTGTT
ACCCAGCAGGTGGATTCCGCACGCACGATGGTAATTGGCCATACGGGAGCGCAAATTTTT
AATAGCATCACCAGCAATGCGGTGCCGGAACCGGATGGCTCAGACTCCGAAAAGAATCTG
TTTGTCATGCTCGATACGGCAATTGCCGCGCTCAAGACCCCGGTGGAAGGCAATGACGTG
GAAAAAGAAAAAGCCGCTGCCGCCATTGATAAAACCAATCGCGGCTTAAAAAATTCGCTT
AATAACGTCCTGACCGTTCGTGCGGAACTGGGAACGCAACTGAGCGAACTCAGTACGCTG
GATTCACTGGGAAGCGACCGTGCGCTGGGACAGAAACTGCAGATGAGCAACCTGGTAGAT
GTGGACTGGAACTCGGTCATTTCTTCCTACGTCATGCAACAGGCGGCATTACAGGCGTCC
TATAAAACGTTTACCGACATGCAGGGAATGTCGCTTTTCCAGTTGAACCGGTAA
>EECEBICD_01508 Ribonuclease E
ATGAAAAGAATGTTAATCAACGCGACTCAGCAGGAAGAGTTGCGCGTCGCCCTTGTAGAT
GGGCAGCGTCTGTACGATCTGGATATAGAAAGCCCCGGACACGAGCAGAAAAAAGCGAAC
ATCTATAAAGGCAAAATTACCCGTATTGAACCGAGCCTGGAAGCGGCTTTTGTTGATTAC
GGCGCAGAACGCCACGGTTTCCTCCCGTTAAAAGAAATTGCCCGCGAATATTTCCCCGCT
AACTACAGCGCTCATGGTCGCCCGAACATTAAAGACGTGTTACGCGAAGGCCAGGAAGTC
ATTGTTCAAATCGATAAAGAAGAGCGCGGCAACAAAGGCGCGGCGTTAACCACCTTTATC
AGTCTGGCGGGCAGCTACCTGGTACTGATGCCGAATAACCCGCGCGCGGGTGGTATTTCC
CGTCGCATTGAAGGCGACGATCGTACCGAGTTAAAAGAAGCGTTGGCCAGCCTGGAACTT
CCTGAAGGGATGGGGCTTATCGTGCGTACCGCGGGCGTGGGTAAATCCGCCGAAGCGTTG
CAGTGGGATTTAAGCTTCCGCCTGAAACACTGGGAGGCCATTCAGAAAGCCGCTGAAAGC
CGTCCCGCGCCGTTCCTGATCCATCAGGAAAGCAACGTCATCGTACGCGCGTTTCGCGAC
TATCTGCGTCAGGATATCGGTGAAATCCTGATCGACAATCCAAAAGTGATGGAAATGGCG
CGTCAGCATATCGCCGCGTTGGGCCGCCCGGATTTTAGCAGCAAAATTAAGCTGTACACC
GGTGAAATCCCGTTGTTCAGCCATTACCAGATCGAATCGCAGATCGAGTCAGCGTTCCAG
CGTGAAGTGCGCCTGCCCTCCGGCGGCTCCATCGTCATTGACAGTACCGAAGCGTTAACC
GCCATCGATATCAACTCCGCCCGCGCGACTCGCGGCGGCGATATCGAAGAAACGGCGTTT
AATACCAACCTGGAAGCGGCCGATGAGATTGCCCGCCAGTTGCGTCTGCGCGACCTGGGC
GGCCTGATCGTTATCGACTTTATCGATATGACGCCGGTACGTCACCAGCGCGCGGTAGAA
AACCGTCTGCGTGAAGCGGTGCGTCAGGACCGGGCGCGTATTCAGATTAGCCATATTTCC
CGCTTCGGCCTGCTCGAAATGTCGCGTCAGCGCCTGAGCCCGTCGCTGGGCGAATCCAGC
CATCACGTGTGTCCGCGCTGTAGCGGTACCGGCACCGTGCGCGATAACGAATCTCTGTCG
CTGTCCATTCTGCGCCTGATTGAAGAAGAAGCGCTGAAAGAGAATACGCAGGAAGTCCAT
GCCATTGTTCCGGTGCCGATCGCCTCTTATCTGCTTAACGAAAAACGGACGGCAGTTAAC
GCTATTGAGACGCGTCAGGATGGCGTCCGCTGTGTCATCGTCCCGAACGATCAGATGGAA
ACGCCGCACTATTCCGTGCTGCGCGTACGCAAAGGGGAAGAAACGCCGACCCTGAGCTAC
ATGCTGCCGAAGCTGCACGAAGAAGCGATGGCGCTGCCTTCTGAAGAAGAGTACGCCGAG
CGTAAACGCCCGGAACAGCCTGCGCTGGCGACATTCGCTATGCCAGATGTGCCTCCGGCG
CCGACGCCTGTCGAACCGGCGGTTAGCGTCGCCACGGCGAAAAAGGATAATGTTGCCGCC
GCGCAGCCTGCCCAGCCGGGTCTGTTTAGCCGCTTCCTTAACGCGCTGAAACAACTGTTC
AGCGGTGAAGAAACGAAAGCCGTCGAAACAGCCGCGCCGAAAGCGGAAGAAAAAGCGGAG
CGTCAGCAGGATCGTCGCAAGCCACGGCAGAACAACCGTCGCGATCGTAACGAACGCCGC

GACACCCGTGATAACCGTGCCGGACGTGACGGCGGCGAGAGCCGCGACGATAATCGTCGT
AACCGTCGCCAGACGCAGCAGCAAAATGCGGAAGCGCGCGATACCCGCCAGCAGGAAACG
GCGGAAAAAGTGAAAACCGGCGATGAACAGCAACAGACGCCGCGTCGCGAACGCAGCCGC
CGCCGCAATGATGACAAGCGCCAGGCGCAGCAGGAAGTAAAAGCGCTTAACCGCGAAGAA
CAGCCTGTCCAGGAGACCGAGCAGGAAGAACGCGTTCAGCAGGTTCAGCCGCGCCGCAAG
CAGCGTCAGCTTAATCAGAAAGTCCGCTTTACCAACAGCGCAGTGGTTGAAACGGTTGAC
ACGCCGGTCGTCGTCGACGAACCACGCCCGGTTGAAAACGTTGAACAGCCCGTTCCAGCG
CCGCGTACCGAGCTGGCGAAAGTTGATTTACCTGTCGTCGCCGATATCGCGCCGGAGCAG
GATGATAGCGTAGAACCTCGCGATAATACCGGTATGCCGCGTCGCTCCCGCCGTTCTCCG
CGCCACCTGCGCGTAAGCGGTCAGCGTCGCCGTCGCTATCGTGACGAGCGCTACCCGACA
CAATCGCCGATGCCGTTAACCGTCGCCTGCGCGTCGCCGGAAATGGCGTCCGGTAAAGTC
TGGATCCGCTACCCGATTGTCCGTCCTCAGGAAACGCAGGTTGTTGAAGAGCAGCGTGAA
GCAGATCTGGCGCTGCCACAACCGGTTGTCGCTGAACCGCAGGTTATCGCTGCGACCGTA
GCGTTAGAACCACAAGCGTCGGTACAGGCGGTAGAAAACGTTGCCGTTGAGCCGCAGACC
GTCGCCGAACCACAGGCGCCGGAAGTCGTCAAGGTCGAGACAACGCATCCGGAAGTGATT
GCCGCGCCGGTTGATGAACAGCCGCAACTGATTGCCGAAAGCGATACGCCGGAAGCGCAA
GAGGTTATTGCTGACGCCGAACCGGTAGCAGAAACCGCCGACGCGTCCATTACGGTGGCT
GAAAACGTGGCTGATGTTGTCGTTGTCGAGCCAGAAGAAGAGACAAAAGCGGAAGCGGCT
GTTGTGGAGCACACGGCAGAAGAAACCGTTATCGCACCGGCCCAGGTGGTGGAAAAGCCG
CAGGACGTTGTCTGCGTTGACGACGATCATAATCACGCCAGCGCGCCGATGACGCGTGCG
CCAGCGCCGGAATATGTGCCGGAAACCCCGCACCATAGCGACTGGCAGCGCCCTTCCTTC
CACTTTGAAGGTAAAGGCGCGGCAGGCGGACACAGCGCTACACGTCATGCCAGCGCCCCG
GCGACACGCCCACAGCCGGTAGAATGA
>EECEBICD_01509 Ribosomal large subunit pseudouridine synthase C
ATGTTCATCCACTGGCGTAGACGTCTCTATAAGCATAGAAAAATGTGTGGCGCGAATCTC
ACAGGCTATTTAGAATCGCCCCCCATGAAAACAGAAACGCCATCCGTAAAAATTGTTGCT
ATCGCCGCTGACGAAGCGGGGCAACGCATTGATAACTTTTTGCGCACCCAACTGAAAGGG
GTACCAAAAAGTATGATTTATCGCATCTTGCGTAAAGGCGAGGTGCGGGTGAACAAAAAA
CGCATTAAACCGGAATACAAACTGGAAGCGGGCGATGAAGTCCGCATTCCGCCGGTTCGC
GTTGCTGAACGCGAAGAAGAGGCCGTGTCGCCGCATTTGCAAAAAGTGGCGGCGTTGGCG
GACGTCATTTTGTATGAAGACGACCATATTCTGGTGCTGAATAAGCCTTCCGGCACCGCC
GTACACGGCGGGAGCGGGTTAAGTTTTGGCGTCATCGAAGGGCTGCGGGCGTTGCGTCCG
GAAGCGCGATTCCTGGAACTGGTGCATCGTCTTGACCGCGATACCTCCGGCGTGCTGTTG
GTGGCGAAAAAACGCTCCGCGCTGCGTTCGCTGCATGAGCAACTGCGCGAAAAGGGAATG
CAGAAAGATTATCTGGCGCTGGTGCGCGGCCAGTGGCAATCGCACGTTAAAACCGTACAG
GCGCCATTATTAAAGAACATTCTGCAAAGCGGCGAACGTATTGTTCGCGTGAGCCAGGAA
GGCAAACCGTCAGAAACGCGCTTCAAGGTGGAAGAGCGCTATGCCTTCGCCACGCTGGTG
CGCTGTAGCCCGGTAACGGGCCGTACGCATCAGATTCGCGTACATACCCAATATGCCGGT
CACCCTATTGCGTTCGACGATCGCTACGGCGACCGTGAGTTTGATCAACAGCTTACCGAA
GCGGGAACCGGTCTTAAACGTCTCTTTCTACACGCCGCGGCGCTGAAGTTCACGCATCCG
GGAACGGGCGAAGTCATGCGCATTGAAGCGCCGATGGATAACGCCCTGAAGCGCTGTCTG
CAGGTGCTGCGTAACGCAAAATAG
>EECEBICD_01510 hypothetical protein
ATGGCCAATACTAGCTGGATGCGCTCATTTACTCAGCGTTGGGGAAAATACGTTATCGTG
CCGCTCGTGATCGCGATGCTGACCGGTTGCGAAGGGAAGGGCGATAGTTCACGACGGGCT
TTCGTTGAGTATTTGAATACGCAAATCATTACGTCTCCCGTGGTGGGCGTCTGTGAGTTG
ACGCCAGCCCAACGTCAGGCTTTTGGTCATTACGCCGATGACTACGATGTACTATTAGCT
GCCTACGGGCAAATCTTTGCTGTGGTGTATGAGACCGGCCAGGAGGATAAGATCACCGGT
GTTATTCAGTCTGAGCGTGATAATCTGGTTTTCCTGAACGAGCTACGTGTGGCTCGTGAC
GTAAACAATCGATTTATCCTTCGGTTGAAAAATGTGAGTAGTGAAAACAGAAAAAAATAT
TCTGCTTTAAAGCTCCCCTCTGATGTGAAGCCTGTCTTTGACGCTGCGTGGAATAAAACC
GTAACACCGCTGGATGAGACGATGACTCGCTATTATTTTCTGCGCGGAAAGCGGCTCGAC
CAGCTGGTCGCAATGGGCGAGTTTCTGCAAAAACAGGGAAATAAAGTCCGCTTTGAAACG
GATGGAAAAGCGGTTTTTGTCGACCTGGCAGCAAAAGAACACTTTCAGGATCAACAATTT

AATTTTTTCATTACGCAGAATGATATAAACCATCTTACTCAGCAGATGGTTGAATTTATA
AATAGTCAACAAGATGCGGCGGATGCTGGCGGCGTTAATTAA
>EECEBICD_01511 hypothetical protein
ATGGGGAATTCATTAACGCGCACGTACACCGCTGTTGATACCCTATGGGATAAAACGCAC
CGTACATCGGTTCGTAATACCAATGCTTTTTTTCTGGTCGGTGAATGGATTGACCAGGAA
ACGGGCCCGCACGAAGAGAGTGAAGATCGGCAGGCGCTAAATTGTTCACAACAAGCGCTT
TCCAGTTGGTTAGAACTGGAAGCGGTCGAAACTGCTCCCCAATAA
>EECEBICD_01512 7-methyl-GTP pyrophosphatase
ATGCCCCGTCTCATCCTTGCGTCTACCTCTCCCTGGCGTCGCGCGCTGCTGGAAAAGCTG
ACGATTCCTTTCGAATGCGCCACGCCGGATGTTGATGAAACCCCCATGCCGGGCGAAGCG
CCGCGTCAGTTGGTGCTGCGGCTGGCGCAGGCGAAGGCGCAATCGCTCGCCCGCGCGTTTC
CCGAATCATTTGATTATTGGCTCCGACCAAATTTGTGTTCTGGACGGCGAAATCACCGGC
AAACCGCTGACGGAAGAAAAAGCGCGTCAGCAATTAGCCAAAGCCAGCGGAAATATTGTG
ACGTTTTATACCGGACTGGCACTGTATAACTCCGCCTCCGGGCACTTACAAACCGAAGTG
GAGCCTTTTGACGTTCATTTTCGCCATTTAAGCGAAGCGGAAATAGACGACTATGTCCGT
AAAGAGCATCCGCTGCACTGCGCGGGAAGTTTTAAAAGCGAAGGTTTAGGCATTGCATTA
TTTGAGCGCCTGGAGGGCCGCGATCCGAACACGCTGATCGGCTTGCCGCTTATTGCTCTG
TGCCAGATGCTGCGACGGGAAGGCTTCAACCCGCTACTGCAGTAA
>EECEBICD_01513 Large ribosomal RNA subunit accumulation protein YceD
ATGCAAAAGGTAAAATTACCCCTGACTCTTGATCCGGTTCGTACCGCTCAAAAACGCCTT
GATTACCAGGGTATCTATACCCCCGATCAGGTTGAGCGCGTTGCCGAATCTGTAGTCAGT
GTGGACAGTGATGTGGAATGCTCCATGTCGTTCGCTATCGATAACCAGCGTCTCGCCGTT
TTAACCGGCGATGCGGTCGTTACGGTGTCGCTCGAATGCCAGCGTTGCGGGAAACCGTTT
ACCCATCAGGTTCACACAACGTATTGTTTTAGCCCTGTCCGTTCCGACGAGCAGGCTGAA
GCACTGCCGGAAGCGTATGAGCCGATTGAGGTAAACGAATTCGGCGAAATCGATCTGCTG
GCGACGGTTGAAGATGAAATCATCCTCGCCTTGCCGGTAGTTCCGGTGCATGATTCTGAA
CACTGTGAAGTGTCCGAGGCGGACATGGTCTTTGGCGAACTGCCTGATGAAGCGCAAAAG
CCAAACCCATTTGCCGTATTAGCCAGCTTAAAGCGTAAGTAA
>EECEBICD_01514 50S ribosomal protein L32
ATGGCCGTACAACAGAATAAACCAACCCGTTCCAAACGTGGCATGCGTCGTTCCCATGAC
GCTCTGACCGCAGTCACCAGCCTGTCTGTAGACAAAACTTCTGGTGAAAAACACCTGCGT
CACCACATCACTGCCGACGGTTACTACCGCGGCCGTAAGGTCATCGCTAAGTAA
>EECEBICD_01515 Phosphate acyltransferase
ATGGGGGGCGATTTTGGTCCTTCCGTGACGGTGCCTGCAGCATTGCAGGCACTGAACGCT
AATTCGCAGCTCACTCTTCTTTTAGTCGGGAATCCCGATATTATCACGCCATTACTTGCC
AAAGCTGACTTTGAACAACGTTCGCGTCTGCAGATTATCCCTGCACAGTCAGTTATTGCC
AGTGATGCCCGGCCTTCGCAGGCGATCCGCGCCAGTCGCGGGACATCCATGCGTGTGGCC
CTGGAGCTTGTGAAAGAAGGCCGAGCCGAAGCTTGCGTTAGCGCCGGTAATACCGGGGCG
CTGATGGGGCTGGCGAAATTATTGCTCAAGCCTCTTGAGGGGATTGAGCGTCCGGCGTTG
GTGACGGTATTACCGCACCAGCAAAAGGGCAAAACGGTAGTGCTTGATTTGGGGGCCAAC
GTCGACTGTGACAGTACCATGCTGGTGCAGTTTGCCGTGATGGGCGCCGTTTTGGCGGAA
GAGGTCGTCGGGATCAAAAATCCCCGCGTGGCGCTACTCAATATCGGGGAAGAAGAAACC
AAAGGCCTCGACAGTATTCGCGAAGCCTCTTTAATGTTAAAAACAGTCCCAACCATCAAT
TACATCGGCTACCTTGAGGCGAATGAGTTACTGACGGGAAAAACCGATGTACTGGTGTGT
GACGGCTTTACAGGCAATGTCACACTTAAAACGATGGAGGGGGTTGTCAGAATGTTCCTT
TCACTGCTGAAATCACAGGGCGAGGGGAAAAAACGGTCGTGGTGGCTGCTGTTATTAAAA
CGTTGGTTACAAAAAAGCCTGACGAGGCGATTCAGTCACCTCAACCCCGACCAGTATAAT
GGCGCCTGTCTGTTAGGATTGCGCGGCACGGTGATAAAAAGTCATGGTGCGGCCAATCAG
CGAGCATTTGCAGTCGCGATTGAACAGGCAGTGCAGGCGGTGCAGCGACAAGTTCCTCAG
CGAATTGCCGCTCGCCTGGAATCTGTATACCCAGCCGGATTTGAACCGCTGGACGATGGC
AAAGGCGTAAACCTGCGGGCGCACAGATAA
>EECEBICD_01516 3-oxoacyl-[acyl-carrier-protein] synthase 3
ATGTATACGAAGATTATTGGTACTGGCAGCTATCTGCCCGAACAAGTGCGGACTAACGCC
GATCTGGAAAAAATGGTTGAGACCTCTGACGAGTGGATTGTCACTCGTACAGGTATTCGT

GAACGCCATATCGCCGCGCCTAATGAAACTGTCGCGACGATGGGCTTTACCGCTGCGAAT
CGCGCGATTGAGATGGCGGGGATCGATAAAGACCAAATTGGCTTGATTGTGGTGGCTACC
ACATCAGCAACGCATGCATTTCCAAGCGCGGCATGTCAGATTCAAAGTATGCTCGGTATT
AAAGGTTGCCCGGCGTTTGATGTCGCGGCAGCGTGCGCAGGTTTCACCTACGCGTTAAGC
ATCGCCGACCAGTACGTTAAATCCGGCGCGGTTAAACACGCGCTGGTGGTCGGTTCCGAT
GTATTAGCCCGCACTTGCGATCCTGGCGATCGCGGTACGATCATTATTTTCGGCGATGGC
GCAGGCGCGGCCGTACTGAGCGCTTCTGAAGAACCGGGTATTATCTCCACTCATCTTCAT
GCCGATGGCCGTTACGGTGAATTACTGACCCTGCCGAATGCCGATCGCGTAAATCCGGAT
AACCCGATTTACCTGACGATGGCGGGCAATGAAGTCTTTAAAGTGGCGGTCACTGAACTG
GCGCATATTGTCGATGAGACGCTGGCGGCTAATAACCTGGATCGCTCAGAACTCGATTGG
CTGGTGCCGCATCAGGCTAACCTGCGTATCATTAGCGCGACAGCGAAAAAACTCGGCATG
TCGATGGACAATGTCGTCGTCACGCTGGACAGGCACGGCAATACCTCCGCGGCTTCTGTG
CCGTGCGCGCTGGATGAAGCCGTGCGTGACGGGCGAATTAAAGCCGGTCAGCTGGTATTG
CTTGAAGCCTTCGGGGGTGGATTCACCTGGGGCTCCGCGCTGATTCGTTTCTAG
>EECEBICD_01517 Malonyl CoA-acyl carrier protein transacylase
ATGACGCAATTTGCATTTGTGTTCCCCGGTCAGGGTTCTCAGAGCGTTGGGATGTTGGCC
GAGATGGCGGCAAATTACCCTATCGTAGAAGAAACGTTTGCTGAAGCTTCTGCGGCTCTG
GGATATGATCTGTGGGCGCTCACCCAGCAAGGTCCAGCGGAAGAACTGAATAAAACCTGG
CAGACGCAGCCGGCGCTATTAACCGCTTCCGTCGCGCTTTGGCGCGTTTGGCAGCAGCAG
GGCGGTAAAATGCCTGCGTTAATGGCAGGTCACAGCCTGGGCGAATATTCCGCGCTGGTT
TGCGCTGGCGTCATCAACTTTGCTGATGCCGTTCGTCTGGTGGAAATGCGCGGTAAATTC
ATGCAGGAAGCGGTTCCGGAAGGTACTGGCGGCATGTCTGCGATCATAGGGCTGGATGAT
GCCTCTATTGCTAAAGCCTGTGAAGAATCTGCCGAAGGGCAGGTTGTTTCACCGGTTAAC
TTTAACTCGCCGGGACAGGTGGTTATCGCCGGGCATAAAGAGGCGGTAGAACGCGCAGGC
GCAGCCTGTAAAGCCGCTGGCGCGAAACGCGCGCTGCCGCTGCCGGTGAGCGTACCGTCG
CACTGCGCGCTGATGAAACCAGCGGCAGATAAGCTGGCGGTTGAATTAGCCAAAATTACC
TTTAACGTGCCAACGGTGCCGGTAGTAAACAACGTTGACGTGAAATGTGAAACCGATGCC
GCCGCTATCCGCGATGCGCTGGTTCGCCAGTTGTACAATCCGGTACAGTGGACGAAGAGC
GTGGAATTTATCGCGGCGCAGGGCGTTGAACATCTTTATGAAGTGGGTCCAGGTAAAGTC
CTCACTGGTCTGACGAAACGTATTGTCGACACCCTGACAGCGTCGGCGCTGAACGAGCCG
GCGGCGCTGTCTGCGGCACTTACGCAATAA
>EECEBICD_01518 3-oxoacyl-[acyl-carrier-protein] reductase FabG
ATGAGCTTTGAAGGAAAGATTGCGCTGGTGACTGGTGCAAGCCGTGGCATAGGCCGCGCA
ATTGCAGAGACTCTCGTTGCCCGCGGCGCGAAAGTTATCGGGACTGCGACCAGTGAAAAT
GGTGCGAAGAACATTAGCGACTATTTAGGTGCTAACGGGAAAGGTTTGATGCTGAATGTG
ACCGATCCTGCATCTATTGAATCTGTTCTGGAAAATATTCGCGCAGAATTTGGTGAAGTG
GATATCCTGGTTAATAATGCCGGTATCACTCGTGATAATCTGTTGATGCGAATGAAAGAT
GATGAGTGGAACGATATTATCGAAACCAATTTATCATCCGTTTTCCGCCTGTCAAAAGCG
GTAATGCGCGCTATGATGAAAAAGCGTTGTGGTCGCATTATCACTATTGGTTCTGTGGTT
GGTACCATGGGAAATGCAGGTCAGGCAAACTACGCTGCGGCGAAAGCGGGCCTGATCGGT
TTCAGTAAATCACTGGCGCGTGAAGTTGCGTCCCGTGGTATTACTGTCAATGTTGTGGCT
CCGGGTTTTATTGAAACGGACATGACGCGTGCGCTGTCTGACGATCAGCGTGCGGGTATC
CTGGCGCAGGTGCCTGCGGGTCGCCTCGGCGGCGCTCAGGAAATCGCCAGTGCGGTTGCA
TTTTTAGCCTCTGACGAAGCGAGTTACATCACTGGTGAGACTCTGCACGTCAACGGCGGA
ATGTACATGGTTTAA
>EECEBICD_01519 Acyl carrier protein
ATGAGCACTATCGAAGAACGCGTTAAGAAAATTATCGGCGAACAGCTGGGCGTTAAGCAG
GAAGAAGTAACCAACAATGCTTCTTTCGTTGAAGACCTGGGCGCAGATTCTCTTGACACC
GTTGAGCTGGTAATGGCTCTGGAAGAAGAGTTTGATACTGAGATTCCGGACGAAGAAGCT
GAGAAAATCACCACCGTTCAGGCTGCCATTGATTACATCAACGGCCACCAGGCGTAA
>EECEBICD_01520 3-oxoacyl-[acyl-carrier-protein] synthase 2
GTGTCTAAGCGTCGTGTAGTTGTGACCGGACTGGGCATGTTGTCTCCTGTCGGCAATACC
GTAGAGTCTACCTGGAAAGCTCTCCTTGCCGGTCAGAGTGGCATCAGCCTGATCGACCAT
TTCGATACTAGCGCCTATGCAACGAAATTTGCTGGCTTAGTAAAGGATTTTAACTGTGAT

GACATTATCTCGCGCAAAGAACAGCGCAAGATGGATGCCTTCATTCAATATGGAATTGTC
GCTGGCGTACAGGCCATGCAGGATTCTGGCCTTGAAGTGACGGAAGAGAACGCAAGCCGT
ATTGGCGCCGCTATCGGCTCCGGTATCGGTGGTCTCGGTCTGATCGAAGAAAACCACAGT
TCGCTGGTAAAAGGCGGACCGCGTAAGATTAGCCCGTTCTTTGTCCCGTCGACGATTGTT
AACATGGTAGCAGGCCATCTGACTATCATGTATGGCCTGCGTGGGCCAAGCATCTCTATT
GCGACTGCCTGTACTTCAGGCGTGCATAACATCGGCCATGCCGCGCGTATCATCGCCTAT
GGCGATGCGGACGCGATGGTGGCAGGTGGCGCAGAAAAAGCCAGTACGCCGCTGGGTGTA
GGCGGTTTTGGCGCAGCGCGCGCGCTGTCGACCCGCAACGATAACCCACAGGCGGCGAGC
CGTCCGTGGGATAAAGAACGCGATGGTTTTGTACTGGGCGACGGTGCCGGTATGTTGGTG
CTTGAAGAGTACGAACATGCGAAAGCGCGTGGTGCGAAAATCTATGCTGAAATCGTCGGC
TTTGGTATGAGCAGCGATGCTTACCATATGACGTCGCCGCCGGAAAATGGCGCAGGCGCT
GCGCTGGCGATGGTTAACGCGTTGCGTGATGCCGCGATTGAGCCTGCTCAGATTGGCTAC
GTCAACGCGCACGGCACGTCGACGCCTGCCGGCGACAAAGCTGAGACGCAGGCGGTTAAG
TCAGTCTTTGGCGATGCCGCAAGCCGAGTGATGGTGAGCTCGACCAAATCGATGACCGGC
CATCTGTTGGGCGCCGCAGGCGCAGTAGAGTCTATTTTCTCTATTCTGGCGTTACGCGAT
CAGGCCATTCCGCCAACCATTAATCTGGATAACCCGGATGAAGGTTGCGATCTGGACTTC
GTGCCGCATGAAGCGCGCCAGGTCAGCGATCTGGAGTACGCTCTGTGTAACTCCTTTGGC
TTTGGCGGAACTAACGGTTCCTTGATTTTTAAAAAGATCTAA
>EECEBICD_01521 Aminodeoxychorismate lyase
ATGTTTTTAATTAATGGCCATGCGCAGGATCAACTTGCTGTAAGCGACCGGGCGACGCAG
TTCGGCGATGGCAGTTTTACGACCGCACGTATTGTTGATGGCAACATTTGCCATCTGGAA
GCGCATCTTCAGCGCTTGCAGGTTGCCTGTGAAAAATTACGGATCGCTTTTAGCCATTGG
GCGACCCTTCGGCAAGAGATGACAATGTTGGCCACAGGGCATGATTCAGGCGTGTTGAAA
GTGATCATTAGCCGTGGTAGCGGTGGCCGGGGATACAGCGCCATGAATTGTCAGGCAGCT
ACCCGAATCCTCTCCGTTTCTGCTTATCCCGCTTATTATTCTCAGTGGCGTAAGCAAGGC
ATCACTCTTACCCTTAGTCCCATACCGCTTGGGCGCAATCCTTATCTTGCCGGATTAAAA
CATCTGAACCGCCTCGAACAGGTGTTGATTCGCTCTCATCTTGAGCAGACGGACGCCGAT
GAGGCGCTGGTTCTTGACAGCGAGGGATGGGTTACGGAATGCTGTGCGGCTAATTTGTTC
TGGCGTACAGGCGACATTGTTTTTACGCCGCGTCTGGATCAGGCCGGGGTGAACGGCATT
ATGCGACAATTTTGTTTACGCCAACTGGCGCAATCTCCTTTCCAGGTTCTTGAAGTACAG
GCGAGAGAGGAGGCAGTCAGGCAGGCGGATGAGATTATCATTTGCAACGCGCTAATGCCG
ATTATTCCCATACGCGCCTATCACGGGACGTCGTATTCTTCGCGAACACTGTTTCAATTT
TTAGCCCCATTTTGTGAGCATCCGAATTAG
>EECEBICD_01522 Endolytic murein transglycosylase
ATGAAAAAATTGTCAGGCGTTTTCCTTCTGCTGTTGGTTGTGCTGGGTATTGCCGCGGGC
GTGGGGATGTGGAAAGTTCGCCATCTGGCGAACAGCACGTTACTTATTAAAAACGAGACT
ATCTTTACGCTCAAGGCGGGAACGGGGCGGCTGGCGCTTGGCGACCAGCTTTATGATGAA
AAAATCATTAATCGCCCCCGGGTATTTCAGTGGCTGCTGCGCGTGGAGCCTGAGTTATCG
CACTTTAAAGCGGGAACTTACCGTTTTACGCCGGGGATGACCGTACGGGAGATGCTTGAG
TTGCTGGAGAGCGGCAAAGAAGCGCAATTCCCGTTGCGGTTTGTGGAAGGGATGCGCCTT
AGCGATTACCTGAAACAGTTACGAGAGGCGCCGTATATTCGCCATACATTGCCGGATGAT
GACTACGCCACTGTCGCTCAGGCATTAAAGCTTGCGCATCCGGAATGGGTAGAAGGGGGG
TTCTGGCCCGATACCTGGATGTATACCGCCAACACCAGCGATGTCGCTATTCTCAAGCGA
GCGCACCAAAAGATGGTGAAAGCTGTCGATACTGTCTGGAAAGGCCGGGCCGAGGGGCTG
CCTTATAAAGATCAGAACCAACTGGTGACAATGGCCTCGATTATTGAAAAAGAAACGGCT
GTCGCCAGCGAACGCGATCAGGTGGCCTCAGTCTTTATTAATCGCCTGAGAATCGGTATG
CGCCTTCAGACCGATCCCACCGTGATTTACGGAATGGGGACGAGTTATAATGGTAACTTG
TCGCGTGCGGATCTGGAAAAGCCGACGGCTTATAACACGTATACCATAACCGGGCTGCCG
CCAGGACCGATTGCATCGCCCAGCGAAGCGTCATTGCAGGCGGCGGCGCATCCGGCGAAA
ACGCCGTATCTCTATTTTGTGGCCGACGGTAAAGGCGGTCACACATTTAACACCAATCTT
GCCAGCCATAATCGGTCAGTGCAGGAGTACCTGAAAGTGCTTAAGGAAAAAAATGGGCAG
TAA
>EECEBICD_01523 Thymidylate kinase
ATGGGCAGTAATTATATCGTCATCGAGGGCCTGGAAGGCGCCGGAAAAACCACTGCGCGC

GACGTGGTGGTGGAGACCCTTGAGCAACTGGGTATTCGTAATATGATTTTTACCCGAGAG
CCGGGCGGGACGCAACTTGCCGAAAAACTAAGAAGTCTGGTGCTGGATATCCGATCGGTA
GGCGACGAAGTGATTACCGATAAAGCGGAAGTGCTGATGTTTTATGCCGCTCGCGTACAG
CTCGTCGAAACGGTAATCAAACCCGCGCTGGCGCAAGGCATATGGGTGATCGGCGATCGC
CACGATCTCTCGACCCAGGCGTATCAGGGGGAGGGCGCGGCATTGATCAAACCATGCTGG
CGACACTACGCGATGCCGTGCTGGGCGATTTTCGTCCTGACCTGA
>EECEBICD_01524 Thymidylate kinase
ATGCTGGCGACACTACGCGATGCCGTGCTGGGCGATTTTCGTCCTGACCTGACGCTGTAT
CTGGATGTCACGCCGGAAGTCGGGCTAAAACGCGCCAGAGCGCGCGGCGATTTGGATCGC
ATTGAGCAGGAATCTTTTGATTTCTTTAACCGGACTCGCGCGCGCTATCTGGAACTGGCG
GCGCAAGACTCGCGTATCCGCACCATTGACGCGACCCAGCCGCTGGATGCCGTCATGCGC
GACATTCGCGCCACGGTGACGAAGTGGGTGCAGGAGCAAGCGGCATGA
>EECEBICD_01525 DNA polymerase III subunit delta'
ATGAAATGGTATCCATGGTTACGGCCCGCCTATGAAAAACTGGTAGAAAGTTACCAGGCG
GGACGTGGGCACCATGCGCTACTGATTCAGGCCTTACCTGGCATGGGCGACGAGGCGCTG
TGCTATGCGCTGAGCCGTTATCTGTTATGCCAGCAGCCTGAGGGCCACAAAAGCTGTGGG
CATTGCCGTGGTTGTCAGCTTATGCAGGCTGGCACGCATCCGGACTATTACACGCTGACG
CCGGATAAAGGCAAAAGCAGCCTCGGCGTAGATGCGGTACGCGAGGTCAGCGAGAAATTG
TACGAGCACTCCCGTCTGGGCGGCGCGAAGGTGGTGTGGATTGCGGATGCGGCGCTATTA
ACCGACGCCGCCGCCAATGCGTTGCTCAAAACGCTGGAAGAGCCGCCGGAGCAAACCTGG
TTTTTCCTTGCCAGCCCGGAGCCTGCTCGTCTGTTGGCGACATTACGTAGCCGCTGTCGG
CTGCATCATCTGGCGGCGCCGTCCGAATCGTATGCGATGTCATGGCTGTCGCGAGAAGTG
ACGGCGTCACAAGAAGCATTGCTCACGGCGCTACGCCTGAATGCGGGGTCGCCCGGCGCC
GCGCTGGCGCTGTTGCAGTCTGAACGTTGGGCGCAGCGTGAGGCGCTGTGTCAGGCGTTG
ATGGACAGTTTACATACGGGCGACTGGTATGCGTTATTAACGGCGCTCAATCATGAGCAA
GCCCCGGCGCGCCTGCATTGGCTGGCGACGCTGCTGGTAGACGCGCTGAAACGTCAGCAC
GGCGCGTCGTACCTGACAAACGTTGATGCGGACGCGGTTGTTGCCGCGCTGGCGGGGCCG
CTATCCCCGGCGCGTATTCAGGCAATCCTGAACGATGTTTGCCATTGTCGTGATCAATTA
CTTCATGTGACCGGATTAAATCGCGAACTGGTATTAACCGATCTCATTCTCCGCATCGAG
CATTACCTGCAACCAGGCACCTTGTTGCCTGTTCCTCATCTTTGA
>EECEBICD_01526 putative metal-dependent hydrolase YcfH
ATGTTTTTAGTCGACTCACACTGCCATCTTGATGGTCTGGATTATCAATCTTTGCATAAG
GACGTGGACGATGTGCTGGCGAAAGCCGCCGCGCGCGATGTGAAGTTCTGTTTAGCGGTG
GCGACGACGCTGCCGGGGTATCGCCATATGCGCGAGCTGGTGGGCAAGCGCGATAATGTG
GTCTTTTCCTGCGGCGTGCATCCGTTAAATCAGGATGAAGCATATGACGTAGAAGAACTG
CGCCTCCTTGCTGCAGAAGACGATGTGGTGGCGATGGGCGAGACCGGGCTTGATTACTTT
TACACGCCTGAGACCAAAATCCGCCAGCAGGCGTCTTTTATTCATCATATCCAGATTGGC
CGCGAATTGCATAAACCGGTGATTGTCCATACTCGCGACGCGCGTGCCGATACGCTGGCT
ATCCTGCGCGAAGAAAAAGTGACGGATTGCGGCGGCGTACTACACTGTTTTACAGAAGAC
AGAGAAACGGCCGGCAAATTGCTGGATCTGGGGTTTTATATCTCTTTTTCCGGCATCGTA
ACGTTCCGTAACGCTGAACAGTTGCGTGATGCGGCGCGCTATGTGCCGCTGGATCGGTTA
TTAGTCGAGACCGATTCTCCTTATCTGGCGCCGGTGCCGCATCGCGGTAAAGAGAACCAG
CCTGCGATGGTACGCGACGTCGCAGAATATATGGCGGTATTGAAAGGGGTTGCCGTTGAA
GAACTGGCGCAGATCACCACCGATAACTTTGCTCGCCTGTTCCATATTGACGCGTCTCGC
CTGTCATCAATCCGTTAA
>EECEBICD_01527 PTS system glucose-specific EIICB component
ATGTTTAAGAATGCATTTGCTAACCTGCAAAAGGTCGGTAAATCGCTGATGCTGCCGGTA
TCCGTACTCCCTATCGCAGGTATCCTGCTGGGTGTCGGTTCCGCTAACTTCAGCTGGCTG
CCAGCCGTTGTATCGCACGTTATGGCAGAAGCGGGCGGATCTGTTTTTGCCAATATGCCG
CTGATTTTCGCTATCGGCGTGGCGTTGGGCTTCACCAACAACGACGGCGTTTCGGCTCTG
GCGGCGGTAGTAGCGTATGGCATCATGGTGAAAACCATGGCTGTGGTTGCGCCGCTGGTT
CTGCATTTACCTGCTGAAGAGATCGCCGCTAAACACCTTGCTGATACGGGTGTTCTCGGG
GGGATTATCTCCGGTGCGATCGCAGCGTATATGTTTAACCGCTTCTACCGTATCAAACTG
CCTGAGTATCTGGGCTTCTTTGCGGGCAAACGCTTCGTGCCGATCATTTCGGGTCTGGCC

GCTATTTTTACCGGTGTGGTGCTGTCTTTCGTATGGCCGCCAATCGGTACGGCTATCCAG
GCGTTCTCGCAGTGGGCGGCTTATCAGAATCCGGTTGTCGCGTTTGGTATTTACGGTTTT
ATCGAGCGTTGCCTGGTGCCGTTTGGTCTGCATCATATCTGGAACGTTCCTTTCCAGATG
CAGATCGGCGAATACACCAACGCAGCGGGTCAGGTATTCCACGGCGACATTCCGCGCTAT
ATGGCGGGCGACCGGACGGCGGGTATGTTGTCTGGCGGCTTCCTGTTCAAAATGTACGGT
CTGCCGGCTGCGGCCATTGCTATCTGGCACTCTGCTAAACCGGAAAACCGCGCAAAAGTG
GGCGGTATCATGATTTCCGCCGCGCTGACCTCGTTTTTGACGGGTATTACCGAGCCTATC
GAGTTCTCCTTCATGTTCGTGGCGCCGATCCTGTACATCATTCACGCGATTCTGGCAGGT
CTGGCATTCCCGATCTGTATCCTGCTGGGGATGCGTGACGGTACGTCGTTCTCCCACGGT
CTGATCGACTTTATCGTGCTGTCCGGTAACAGCAGCAAATTGTGGCTGTTCCCGATTGTT
GGTGCCGGTTATGCGATTGTGTACTACACCGTGTTCCGTGTGCTGATCAAAGCGCTGGAT
CTGAAAACGCCGGGTCGTGAAGACACTACTGATGACGCAAAAGCGGGCGCGACCAGCGAA
ATGGCGCCGGCTTTGGTTGCGGCGTTTGGCGGTAAAGAGAACATCACTAACCTGGATGCC
TGTATCACGCGTCTGCGCGTGAGCGTAGCCGATGTAGCGAAAGTGGATCAGGCTGGCCTG
AAGAAACTGGGTGCGGCAGGCGTTGTCGTCGCGGGTTCCGGCGTTCAGGCTATTTTCGGT
ACGAAATCCGATAACCTGAAAACGGAAATGGATGAGTACATCCGTAACAGCTAA
>EECEBICD_01528 FhuE receptor
ATGTCTTTCATTCAATACCGCCGGGACAAACACCTTCCGTCCACCGCCGCACCGTCGCTG
TTAGCTATGGGTATGGCGATGGCATTTATGCCTGCCGCCTTCGCGGCTGAGGATACCGTT
ATTGTCGAAGGCGCGACGACCGCTGACGCCGTAAACCGCGAAGAACAGGATTACAGCGTG
AAAACGACCGCAGCGGGCACCAAAATGCCGATGACTCAGCGCGATATCCCGCAGTCGGTC
AGTATTGTCAGCCAGCAGCGTATGGAAGACCAGCAGTTGCAAACCCTGGGCGAAGTGATG
ACCAATACGCTAGGGATCAGCGGAAGCCAGGCTGACTCCGATCGCATCAGCTATTACTCG
CGCGGGTTTGAAATTGACAACTATATGGTTGACGGTATTCCAACGTATTTTGAGTCCCGC
TGGAATCTGGGCGATGCGCTAACGGATACCGCGCTGTATGAGCGCGTGGAGGTGGTTCGC
GGCGCGAATGGGCTGATGACCGGGACCGGTAATCCTTCGGCCTCAATTAATATGATCCGT
AAACACGCCACCAGTCGGGAGTTCAAAGGTAACGTTTCCACAGAATACGGTAGCTGGAAT
AAGCAGCGTTACGTCATGGATCTGCAAAGTCCGCTCACCGCGGACGGCAATGTCCGTGGG
CGCATCGTGGCGGGTTATCAGAATAATGATTCCTGGCTGGACCGCTATAACAGTGAGAAA
GCGTTCTTTTCCGGAATTGTCGATGCCGATCTGGGTACAACCACCAACCTTTCAGCCGGT
TATGAATATCAAAAAATTGATGTCAACAGCCCAACCTGGGGCGGTCTGCCGCGCTGGAAC
ACCGATGGCAGCAAAAATAGCTATGACCGCGCGCGTAGTACCGCCCCGGACTGGGCTTAT
AACAACAAAGAGATAAATAAATTCTTCGTCACGCTTAAACAGCGTTTTGCCGAAAGCTGG
CAGGCGACCCTGAATGCGACCCATACTGAGGTCAAATTCGACAGTAAAATGATGTATATC
GATGCGTTAGTCGATAAAGAGACAGGTACGCTGGTGAGTCCTTATGGCGCAAGCTACCCC
GTGGTCGGCGGTACGGGCTGGAACAGCGGCAAGCGCAAAGTAGATGCCATAGATCTCTTT
GCCGATGGCGCTTATGAGCTGTTTGGCCGTCAGCACAATATGATGTTTGGCGGCAGTTAT
AGTAAACAGAATAACCGCTATTTTAGCGCCTGGGCGAACGTCTTTCCGGACGACATTGGC
AACTTCAGCGCCTTTAACGGTAATTTCCCCCAAACCCACTGGGCGCCACAAAATCTGGCG
CAGGACGATACCACGCATATGAAATCGCTGTATGCCGCTACGCGCATTTCGCTGGCCGAC
CCGCTGCATCTGATTCTCGGCGCTCGTTATACCAACTGGCGTGTTGATACACTGACCTAC
AGCATGGAGAAAAACCATACCACGCCGTATGCCGGGCTGATTTATGATATCAACGACAAC
TGGTCCGCTTATGCCAGTTATACCTCTATCTTTCAGCCGCAGAATAAACGCGACAAAGCG
GGTCAATATCTGGCTCCGATTACCGGTAATAACTATGAAGCGGGTCTGAAGTCCGACTGG
ATGAACAGTCGCCTCACCACCACGTTGTCCGTGTTCCGCATTGAGCAGAATAACGTCGCT
CAGGCAACCACTATTCCCATCCCTGGCAGTAATGGGGAGTTTGCCTGGAAGTCTACTGAC
GGAACGGTGAGCAAAGGCGTCGAATTTGAAGTGAACGGCGCAATTACCGATAACTGGCAG
ATGACTTTCGGGGCTACCCGGTATGTTGCCGAGGACAATGAAGGTAACGCCGTGAACCCT
AATCTCCCGCGCACCAGCGTGAAGTTATTCACCCGCTATCGTCTGCCGGCGATACCGGAG
CTGACGGTCGGCGGCGGCGTGAACTGGCAGAATCGGGTTTACAAAGATACCACGACGCCG
TACGGCACTTTCCGCGCCGAACAGGGCAGCTATGCGCTGGTCGATCTGTTTACCCGCTAT
CAGGTGACGAAAAATTTCTCCGTACAGGGAAACATCAACAACCTGTTTGATAAAACCTAC
GACACCAATATTGATGGTTCCATTGTTTACGGCGCGCCGCGTAACGTCAGCCTCACCGCC
AATTATCAGTTCTGA

>EECEBICD_01529 Purine nucleoside phosphoramidase
ATGAAAAAGGAAAACGTCGTGGCAGAAGAAACTATATTCAGCAAAATTATTCGTCGTGAA
ATCCCTTCGGATATCGTTTATCAGGATGAGCTGGTCACCGCCTTTCGTGATATTTCACCG
CAGGCGCCGACGCATATCTTGATTATTCCCAATATCCTGATCCCAACCGTCAATGACGTG
ACGGCGGAACATGAACAGGCGTTAGGTCGGATGATCACCGTGGCGGCGAAAATCGCCGAA
CAGGAAGGCATTGCCGAAGATGGGTATCGTTTAATCATGAATACCAACCGTCATGGGGGC
CAGGAGGTTTATCATATCCATATGCACCTACTGGGCGGCCGCCCGCTAGGCCCGATGCTG
GCGCATAAAGGCTTGTAA
>EECEBICD_01530 hypothetical protein
ATGATCAAAGGATGCATCGCGGTTTCTCTGGGCTTGCTGCTGCTTACCGGCTGCCGGTCA
CACCCTGAAATTCCGGTAAGCGACGCGCAGTCGCTGGTGATGGAGTCCACGGTTCTGGCG
GCGGGCGTCACCGCAGAGCCGCCTGAACTGACGGCGTCTGATATCCAGCCATCAGCCTCT
TCCCGCGTCTATAACGAAAGGCAAGAGCCCATAACCGTTCATTATCGGTTTTACTGGTAT
GACGCGCGGGGGCTGGAGATGCATCCGCTGGAGGCGCCGCGTAGCGTAACCATACCCGCG
CGTTCGTCGGTAACGCTATTTGGCAGCGCCAATTATCTGGGGGCGCATAAGGTCAGGCTT
TATCTTTATTTGTGA
>EECEBICD_01531 Penicillin-binding protein activator LpoB
ATGACAAAAATGCATCGCTACGCCGCCATTGCTGCGCTGGCGATATTTCTCTCCGGGTGT
ATGGCGCAACGTCAACCTGCGCCAGTTGAAGAGGTAAAACCAGCGCCAGAACAACCCGCG
CAGCCACCGCAACCTCCTGTTGTGCCGTCTGTGCCAACCATTCCACAGCAACCGGGGCCG
ATCGAACATGAAGATCAAACGGGCCAGCCAGCGCCTAAGGTGCGCCACTATGACTGGAAC
GGCGCGATGCAACCGCTGGTCAGCAAAATGTTGCAGGCTGATGGCGTGACGGCCGGAAGC
GTATTGCTTGTCGACAGCGTGAATAACCGGACGAATGGGTCGTTAAATGCGAACGAGGCA
ACGGAAACGTTACGTAATGCGCTGGCGAACAACGGTAAATTTACGCTGGTCTCCGTACAG
CAACTCTCGATGGCAAAACAGCAGTTAGGGTTGTCTCCGCAGGATAGTCTGGGAACGCGC
AGTAAAGCTATTGGCATTGCGCGCAATGTCGGGGCGCAATATGTACTTTACTCCAGCGCA
TCCGGTAACGTTAACGCGCCTGCATTACAGATGCAGTTAATGCTGGTGCAAACGGGCGAG
ATAATCTGGTCAGGTAAAGGTGCGGTCCAACAACAATAA
>EECEBICD_01532 Thiamine kinase
GTGCGGTCCAACAACAATAATCCCTTAACGCGCGACGAGATCCTGTCGCGCTATTTTCCC
CAGTATCGTCCCGCCGTCGCCGCATCGCAGGGGCTAAGCGGCGGGAGCTGTATTATTGCC
CACGATACTCACCGTATCGTGCTGCGCCGTCATCACGACCCCGACGCTCCACCAGCCCAT
TTTTTACGTCATTACCGCGCCTTATCCCAATTGCCGGCCAGTCTTGCGCCGCGAGCGCTT
TTTTATACGCCGGGCTGGATGGCGGTAGAATATCTGCATGGTGTGGTAAATTCCGCTCTG
CCAGATGCCGACGAACTGGCGGCCTTACTGTATCATTTGCATCAACAGCCGCGTTTTGGC
TGGCGTATCGCGCTATCGCCACTATTGGCGCAGTACTGGTCGTGTTGCGATCCGGCGAGG
CGTACGCCGTTTTGGTTGCGGCGGCTCAAACAGTTGCAAAAAAACGGTGAACCTCGCCCG
CTGCGGCTCGCGCCTTTGCATATGGATGTCCATGGCGACAATATAGTATTAACGTCCGCC
GGGTTGAGACTGATTGACTGGGAGTATGCCGGCGACGGCGATATTGCGTTGGAGCTGGCG
GCAGTATGGGTTGAGGATGAACGCCAGCACCGACAACTGGCAGACGCGTATGCAGCGCGC
GCGCGAATCGACGCCCGGCAGCTATGGCGACAGATACGATTATGGCACCCCTGGGTCATT
ATGCTAAAAGCAGGGTGGTTCGAATACCGCTGGCGACAAACCGGCGAGCAACAATTTATC
AGGCTGGCCGATGAAACCTGGCGCCAGCTACGTATGAAAGGATAA
>EECEBICD_01533 Beta-hexosaminidase
GTGGGCCCAGTAATGTTGAATGTCGAAGGATGTGAGCTGGATGCGGAGGAGCGCGAGATA
CTGGCGCATCCGCTGGTTGGCGGGCTTATTCTGTTCACGCGTAATTACCATGACCCGGAA
CAGCTGCGCGAATTAGTGCGCCAGATTCGGGCGGCATCGCGTAACCATCTGGTGGTGGCC
GTCGATCAGGAAGGCGGGCGCGTACAGCGTTTCCGTGAAGGGTTTACCCGCCTGCCCGCG
GCGCAATCTTTTTTGCGCTGCATGGGCTGGAAGAGGGGGGCGACTGGCGCAGGAGGCTGG
GTGGCTGATGGCCAGCGAAATGATCGCGATGGATATTGA
>EECEBICD_01534 Beta-hexosaminidase
ATGGCCAGCGAAATGATCGCGATGGATATTGATATCAGTTTTGCGCCAGTGCTGGACGTC
GGGCATATCAGCGCGGCAATTGGCGAGCGTTCTTATCATGCCGATCCCGCAAAAGCGTTG
GCAATGGCAACACGCTTTATTGACGGTATGCATGACGCCGGGATGAAAACGACCGGTAAG

CATTTCCCCGGCCACGGCGCGGTCACGGCGGACTCCCACAAAGAGACGCCGTGCGATCCA
CGTCCTGAAACGGACATCCGTGGCAAAGATATGTCGGTGTTCCGCACGCTGATTAGCGAG
AATAAACTGGATGCGATTATGCCTGCGCACGTGATCTACCGCGCTATCGATCCGCGTCCG
GCAAGCGGTTCGCCGTACTGGCTGAAAACGGTGCTGCGTCAGGAGTTAGGCTTTGATGGC
GTCATTTTCTCCGACGATCTGTCGATGGAGGGCGCGGCGATTATGGGCAGCTATGCTGAG
CGCGCTCAGGCGTCGCTGGATGCGGGTTGCGATATGATTCTGGTCTGCAATAATCGTAAA
GGCGCAGTCAGCGTGCTGGATAATCTGTCGCCGATCAAAGCAGAACGTGTTACACGTTTG
TATCATAAAGGTTCATTTTCACGCCGGGAGCTCATGGACTCAGCACGCTGGAAAACAGCC
AGCGCCCAGCTCAACCAGTTACATGAACGCTGGCAGGAAGAAAAAGCAGGTCATTAA
>EECEBICD_01535 hypothetical protein
ATGATTATTTATTTACACGGTTTTGATTCAAATAGTCCGGGCAACCATGAAAAAGTGTTG
CAATTACAGTTTATCGACCCGGATGTTCGGCTGGTAAGCTACAGTACGCGGCATCCTAAG
CACGATATGCAGCATCTGCTGAAAGAAGTGGACAAAATGTTGCAGTTGAATGTGGACGAG
CGCCCTTTAATCTGTGGCGTGGGGCTGGGCGGTTACTGGGCGGAGCGGATTGGCTTTTTG
TGCGATATCCGTCAGGTGGTTTTTAACCCGAACCTGTTCCCCTATGAAAACATGGAGGGG
AAAATCGATCGCCCGGAAGAATATGCGGACATCGCTACAAAGTGCGTGACCAACTTTCGC
GAAAAAAATCGCGATCGTTGTCTGGTGATCCTCTCGCGTCATGACGAAGCGCTGGACAGC
CAACGCTCAGCGCAAGCGCTTCACCCGTATTATGAAATTGTGTGGGATGAGGAACAGACT
CACAAATTCAAAAATATTTCGCCGCATTTGCAACGTATCAAAGCGTTTAAAACGCTGGGA
TAA
>EECEBICD_01536 NADH dehydrogenase
TTGACTACACCATTAAAAAAGATCGTGATTGTCGGCGGCGGCGCTGGCGGGCTGGAAATG
GCGACGCAGTTAGGCCATAAACTGGGGCGCAAGAAAAAAGCGAAAATCACGCTGGTAGAC
AGAAATCACAGCCATCTGTGGAAACCATTGCTGCACGAAGTGGCGACTGGCTCTCTGGAC
GAAGGCGTGGATGCGCTGAGCTATCTGGCTCATGCGCGTAATCATGGTTTCCAGTTCCAG
CTGGGGTCGGTGATGGATATCGATCGCGAAGCGAAGACCATCACCATTGCCGAGTTGCGT
GATGAAAAGGGCGAACTGCTGGTGCCGGAGCGCAAAATCGCGTATGACACGCTGGTGATG
GCGCTGGGCAGCACCTCTAATGATTTCAACACGCCGGGCGTGAAAGAGCACTGTATCTTC
CTCGATAACCCGCATCAGGCGCGCCGTTTTCACCAGGAGATGCTGAACCTGTTCCTCAAG
TATTCGGCTAATCTGGGCGCGAACGGTAAGGTCAATATCGCCATCGTTGGCGGCGGTGCG
ACGGGGGTTGAGCTGTCGGCTGAACTGCATAATGCGGTAAAACAGCTGCATAGCTATGGT
TATAAGGGGCTGACCAACGACGCGCTGAACGTGACGTTGGTTGAGGCTGGCGAGCGTATT
CTGCCTGCGTTGCCGCCGCGTATCTCCAGCGCCGCGCATAATGAACTGACCAAACTGGGC
GTTCGCGTGCTGACGCAAACTATGGTCACCAGCGCCGACGAAGGCGGTCTGCATACGAAA
GAAGGTGAATATATTCAGGCCGATCTGATGGTGTGGGCGGCGGGTATCAAAGCGCCAGAT
TTTATGAAAGAGATTGGTGGTCTGGAAACGAACCGCATTAACCAACTGGTGGTGGAACCG
ACGCTGCAAACCACGCGCGATCCTGATATTTATGCGATTGGCGACTGCGCTTCCTGCGCA
CGTCCGGAGGGAGGTTTTGTGCCGCCTCGCGCTCAGGCGGCGCATCAGATGGCTACCTGC
GCGATGAATAACATTCTGGCGCAGATGAATGGCAAACCGCTGAAAGCTTACCAGTATAAA
GATCATGGATCGCTGGTCTCTCTGTCCAACTTCTCTACCGTGGGTAGTCTGATGGGGAAC
CTGACCCGCGGTTCAATGATGATTGAAGGACGTATCGCCCGCTTCGTGTATATCTCGCTA
TACCGTATGCACCAGATAGCGCTGCATGGATACTTTAAAACCGGCCTGATGATGCTGGTG
GGCAGTATTAACCGCGTCATTCGTCCGCGTCTGAAACTGCATTAA
>EECEBICD_01537 hypothetical protein
GTGAATAAATCAATGTTGGCGGGTATAGGGATTGGCGTAGCGGCTGCGCTGGGTGTGGCG
GCAGTGGCCAGTCTCAACGTATTTGATCGTAGTCCGCAATATGCGCAGGTGATATCGGCT
AAACCGATTAAAGAGACGGTAAAAACGCCGCGTCAGGAGTGCCGTAATGTCACGGTCACG
CATCGCAGACCGGTTCAGAACGAAAACCGTATTGCCGGCTCGGTGCTTGGCGCAGTAGCA
GGCGGCGTGATTGGACATCAATTTGGCGGCGGTCGTGGTAAAGATGTGGCGACGGTCGTC
GGGGCGCTGGGCGGCGGTTATGCTGGCAATCAAATCCAGGGGTCAATGCAGGAAAGTGAT
ACTTACACTACCACGCAGCAACGCTGTAAGACGGTGTATGACAAATCTGAAAAGATGCTT
GGCTACGACGTGACCTACAAAATTGGCGATCAGCAGGGCAAAATCCGCATGGATAAAGAC
CCCGGAACGCAAATTCCCCTCGACGGCAATGGCCAACTGGTTCTGAATAACAAAGCATAA
>EECEBICD_01538 HTH-type transcriptional repressor ComR

ATGACAACGGATACCACGAGTTGCGCGAAGAAAAGCCGTGGCCGACCAAAAGTGTTCGAC
AGGGAAGCCGCGCTTGATAAGGCCATGACTCTCTTCTGGCAGCACGGCTATGAAGCAACT
TCGCTTGCCGATCTCGTAGAGGCTACCGGGGCGAAAGCGCCTACTCTCTATGCGGAATTC
ACTAATAAAGAAGGATTATTTCGCGCTGTCCTGGATCGCTACATCACTCGCTTTGCCTCA
AAGCACGAAGCCCAGCTCTTTTGTGAAGAGAAAAGCGTGGAATCGGCGCTGGAAGATTAC
TTCACAGAAATTGCCGCCTGCTTTACCAGTACAGACACGCCGGCTGGCTGCTTTATGATT
AATACGTCCGCCACCCTCGCCGCCTCCTCGCGCGATATCGCCCGCACGGTAAAATCACGC
CACGCCATGCAGGAACAGACGCTCATCCAGTTTCTGCGCCAACGTCAGGAGCGCGGTGAA
ATTCCGGCGCACTGTAACCCGCAGGCGCTCGCGGAATATATAAACTGTATTTTACAGGGA
ATGTCGATCAGCGCCCGTGAAGGAGCCACCTTCGAACAACTGATGCAAATTACGCGTACG
ACTCTGCGCATCTGGCCTGAACTACTTAAGTCCTGA
>EECEBICD_01539 Multiple stress resistance protein BhsA
ATGAAAAACGTAAAAAACCTCATCGCTGCTGCCGTACTGAGTTCGCTCTCATTCGCCAGC
TTCGCCGCCGTTGAAGTTCAGGCAACGCCAGAAGGTCAACAGAAGTTCGGTACCATTTCC
GCGAATGGCGGCACGAATCTGGGCTCGCTGGAAGATCAGCTGGCGCAAAAAGCGCAGGAG
ATGGGGGCGAAGTCTTTCCGTATTACCTCTGTTACCGGCCCGAATACCCTTCACGGTACG
GCGGTAATCTATAAGTAA
>EECEBICD_01540 putative L,D-transpeptidase YcfS
ATGTTCAACAATACGCGACTCTCTCGCTGGTGGGCGTTGTTTGCGCTTACCGCGACCATC
ATGCTTGCCCTTCCCGCGCAGGCGAATACGTGGCCTCTTCCGCCCCCCGGCAGTCGACTG
GTGGGAGAAAATAAATTCCATGTCGTTGAAGACGACGGCGGCTCGCTGGAGGCGATCGCC
AAAAAATATAACGTGGGCTTTTTAGCCCTGTTACAGGCCAACCCCGGCATTGATCCTTAC
GTGCCACGCGCAGGCAGCGTGCTGACGATTCCGCTGCAAACGCTGCTACCGGACGCGCCA
CGTGAAGGGATTGTCATTAACCTCGCGGAATTACGGCTTTACTATTATCAACCGGGGAAA
AATACGGTCACGGTCTACCCTATCGGGATTGGCCAACTGGGCGGCGATACCCTGACGCCA
ACGATGGTGACGACCATTTCGGATAAACGCGCCAATCCAACCTGGACGCCGACCGCCAAT
ATTCGCGCACGTTATAAAGCGCAAGGCATCGATCTCCCCGCCGTGGTGCCTGCCGGTCCC
GATAACCCAATGGGGCATCACGCGATTCGTTTAGCGGCCTACGGCGGCGTCTATCTGTTA
CATGGCACGAATGCCGATTTTGGCATTGGTATGCGCGTCAGTTCCGGCTGTATCCGTCTG
CGCGACGGTGATATAGAGACATTGTTCCGTCAGGTCACGCCGGGAACCAAAGTGAATATT
ATCAATACGCCGATTAAAGCCTCGGTGGAGCCAGGCGGCGTGCGACTGGTGGAAGTTCAT
CAGCCGCTGTCTAAAAATATAGGCGACGATCCGCAGGTGCTGCCGATTGTGCTGAATGGC
CCGATGCAGGCCTTTAAGGACGCGCCGCAGACCGATGCTGCGGTGATGGAACATGTTATG
GAAGTGCGTTCCGGAATGCCGGTGGATGTAACCCGTCAAGCAGAGGCGAAACCGCAGTCG
CTGTAA
>EECEBICD_01541 Transcription-repair-coupling factor
ATGCCTGAAAAACAACGTTACACGCTGCCGACAAAAGCGGGCGATCAGCGCCAGTTGGGC
GAACTTACCGGTGCCGCCTGCGCTACGCTGGTGGCGGAGATAGCCGAACGCCATGCCGGT
CCGGTGGTACTCATAGCGCCTGACATGCAAAATGCTCTGCGATTGCATGATGAAATCCGC
CAGTTTACCGATCAGATGGTGATGAACCTGGCGGACTGGGAAACGCTACCGTATGACAGC
TTCTCCCCGCATCAGGAAATTATCTCTTCGCGCCTGTCCACGCTCTACCAGTTGCCGTCG
ATGCAGCGCGGCGTCTTGATTGTACCGGTAAACACCCTGATGCAGCGCGTTTGTCCGCAT
AGCTATCTGCATGGTCATGCGCTGGTGATGAAAAAAGGCCAGCGGCTGTCCCGCGACGCT
CTGCGCGCGCAGCTTGACAGCGCCGGTTATCGGCATGTCGATCAGGTGATGGAGCATGGC
GAATACGCGACGCGCGGCGCGCTGTTGGATCTCTTTCCGATGGGCAGCGAACAGCCTTAT
CGCCTCGACTTTTTTGATGATGAAATCGACAGCCTGCGTCTGTTTGACGCGGATACCCAG
CGTACGCTGGAGGAGGTCGAGGCCATCAATTTGTTGCCCGCGCACGAATTTCCTACCGAC
AAAGCCGCTATTGAGCTTTTCCGTAGCCAGTGGCGCGATACCTTTGAGGTAAAACGCGAC
GCCGAACACATCTATCAGCAGGTCAGCAAAGGCACCTTGCCCGCCGGGATCGAATACTGG
CAGCCGCTCTTTTTCAGCGAACCGTTACCGCCATTATTTAGCTATTTCCCCGCCAATACT
CTGGTGGTGAATACTGGCTCGCTGGAAACCAGCGCCGAACGCTTTCAGGCCGATACGCTG
GCGCGTTTTGAAAATCGCGGCGTCGATCCTATGCGCCCGCTTTTGCCGCCGGAAGCCCTC
TGGCTACGGGTAGATGAGCTCTTTTCCGAGCTTAAACGCTGGCCTCGCCTCCAGCTTAAA
ACCGACCATCTGCCGGAAAAAGCCGCCAATACCAATCTTGGTTTCCAGAAATTACCGGAT

TTGGCTATCCAGGCACAGCAGAAAGCGCCGCTGGACGCGCTGCGTAAGTTCCTCGAATCT
TTCAGCGGCCCGGTGATTTTTTCTGTGGAAAGCGAAGGACGCCGCGAAGCGCTGGGAGAG
CTCCTGGCTCGCATAAAAATCGCGCCGAAGCGGATATTACGCCAGGACGAGGCGCAGGAT
GCGGGACGCTATCTGATGATTGGCGCCGCAGAACATGGCTTTATCGACACCCAACGCAAT
CTGGCGCTGATTTGTGAAAGCGATCTTTTGGGTGAGCGGGTGGCGCGTCGCCGCCTGGAT
TCCCGCCGCACGATCAACCCGGATACGTTGATTCGTAACCTGGCGGAGCTCCACGTCGGT
CAGCCGGTAGTGCATCTGGAGCACGGCGTCGGACGCTATGCCGGCATGACCACTCTGGAG
GCTGGCGGCATCAAGGGTGAGTATCTGATGCTCACCTACGCCAATGACGCCAAACTGTAT
GTGCCGGTCTCCTCATTGCATCTGATTAGCCGCTATGCCGGCGGCGCGGAAGAAAGTGCG
CCGCTGCATAAACTGGGCGGCGACGCCTGGTCGCGCGCCCGGCAGAAAGCGGCGGAAAAA
GTGCGCGATGTCGCAGCGGAACTGTTGGATATTTATGCGCAACGGGCGGCAAAAGAGGGG
TTTGCCTTTAAGCATGATCGTGAGCAGTATCAGTTATTCTGCGACAGCTTCCCGTTTGAA
ACGACGCCCGATCAGGCACAGGCGATTAACGCCGTATTGAGCGATATGTGCCAACCGCTG
GCGATGGACAGGCTGGTGTGCGGCGACGTCGGCTTCGGCAAAACCGAAGTGGCGATGCGC
GCCGCGTTCCTGGCAGTGGAAAACCATAAACAAGTAGCGGTACTGGTGCCAACCACGCTG
CTGGCGCAGCAACACTATGATAACTTCCGCGACCGTTTCGCCAACTGGCCGGTACGCATT
GAGATGCTGTCCCGCTTTCGCAGCGCCAAAGAGCAGACACAAATCCTGGCGGAAGCCGCC
GAGGGTAAGATCGATATTCTGATCGGTACGCATAAACTGCTACAAAGCGATGTGAAGCTG
CGCGACCTGGGCCTGCTGATTGTCGATGAAGAGCACCGTTTCGGCGTGCGCCACAAAGAG
CGAATTAAGGCGATGCGCGCCGATGTCGATATTCTGACGCTCACGGCGACGCCGATCCCG
CGGACGCTGAATATGGCGATGAGCGGTATGCGCGATCTCTCCATTATCGCCACTCCCCCT
GCCCGTCGTCTGGCGGTAAAAACCTTTGTTCGCGAGTATGATAGCCTGGTAGTAAGGGAA
GCGATTCTGCGTGAAATCCTGCGCGGCGGTCAGGTTTACTATTTGTACAATGACGTCGAA
AATATTCAGAAAGCCGCAGAGCGGCTGGCGGAACTGGTACCGGAAGCGCGAATCGCTATA
GGCCACGGGCAGATGCGTGAACGTGAGCTGGAACGGGTCATGAACGATTTCCACCATCAA
CGTTTTAACGTGCTGGTGTGTACTACCATTATCGAAACGGGGATCGACATTCCCACCGCT
AACACGATCATCATTGAACGCGCGGATCATTTCGGCCTCGCGCAGTTGCACCAGCTGCGT
GGCCGCGTTGGGCGCTCGCACCACCAGGCCTACGCCTGGCTGTTGACGCCACATCCTAAG
GCCATGACCACCGATGCGCAAAAACGGCTGGAGGCGATTGCGTCGCTGGAGGATTTAGGC
GCCGGGTTTGCGCTGGCGACCCACGACCTGGAGATCCGCGGCGCGGGCGAACTGCTTGGC
GAAGAGCAAAGCGGCTCAATGGAAACCATTGGCTTCTCACTCTACATGGAACTGCTGGAA
AACGCCGTCGATGCGCTGAAAGCAGGCCGCGAACCCTCGCTGGAAGATCTCACCAGTCAG
CAAACGGAAGTCGAGCTGCGAATGCCGTCGTTGCTGCCAGATGATTTTATTCCGGATGTT
AATACCCGGCTGTCATTCTATAAACGCATTGCCAGCGCGAAGAACGAAAACGAGCTGGAG
GAGATCAAAGTGGAATTGATCGACCGCTTCGGCCTGCTGCCCGATCCGGCGCGTAATCTG
CTGGATATCGCCCGCCTGCGCCAACAGGCGCAAAAGCTCGGCATCCGAAAACTGGAGGGT
AACGAGAAAGGCGGAACGATTGAGTTCGCCGAGAAAAACCACGTTGACCCCGCCTGGCTT
ATCGGGTTGCTGCAAAAACAGCCGCAGCATTTCCGTCTGGATGGCCCGACGCGCCTCAAG
TTTATTCAGGATTTAAGCGAACGTAAAACGCGTATAGACTGGGTACGCCAGTTTATGCAG
CAACTGGAAGAAAACGCTATTGCATAA
>EECEBICD_01542 Lipoprotein-releasing system transmembrane protein LolC
ATGTATCAACCTGTCGCTCTATTCATCGGCCTGCGTTACATGCGCGGGCGTGCAGCAGAT
CGCTTCGGTCGCTTCGTTTCCTGGCTTTCCACTATCGGCATTACCCTGGGGGTGATGGCG
CTGGTCACGGTATTGTCGGTGATGAACGGTTTTGAGCGCGAACTGCAAAATAACATTCTT
GGTTTGATGCCTCAGGCCATCCTCTCTGCTGAACATGGTTCTCTTAACCCGAGTCAGATG
CCGGAAAAAGCGGTGAATCTGCAAGGCGTTAACCGTATTGCGCCGCTGACGACGGGCGAT
GTCGTACTGCAAAGCGCGCGTAGCGTTGCGGTTGGCGTGATGTTGGGTATCGATCCGGCG
CAAAAAGATCCGTTAACGCCGTATCTGGTCAATGTGAAGCAAAGCGAATTACAGCCGGGT
AAGTATAATGTCATCCTCGGCGAGCAGCTCGCCGGACAGTTAGGGGTCAACCGTGGCGAT
CAGATTCGGCTAATGGTGCCATCTGCCAGCCAGTTTACGCCAATGGGACGCCTGCCCAGC
CAGCGTCTGTTTACGGTGATTGGCACATTTGCCGCCAATAGCGAGGTCGATGGCTATGAA
ATGCTGGTCAATATTCAGGACGCGTCGCGTTTAATGCGCTATCCGGCAGGCAATATTACC
GGCTGGCGTCTGTGGCTGGATGAACCGCTGCAAGTCGATACCCTCAGTCAACAGACGCTG
CCGCAAGGCACGAAGTGGCAGGACTGGCGCGAACGCAAAGGCGAGTTGTTCCAGGCTGTG

CGCATGGAAAAGAACATGATGGGATTGTTGCTGAGTCTCATCGTGGCAGTCGCGGCTTTT
AACATTATTACTTCGTTGGGCCTGATGGTCATGGAGAAGCAGGGCGAAGTGGCGATTTTA
CAAACTCAGGGGCTGACGCCGCGACAAATTATGATGGTCTTTATGGTGCAGGGCGCCAGC
GCCGGGATTATCGGCGCGCTGTTGGGCGCCGCGCTGGGCGCATTGCTTGCCAGCCAACTG
AATAACCTGATGCCGATCATCGGCGCGTTTCTGGATGGCGCTGCGTTGCCCGTGGCGATA
GAGCCGTTACAGGTGATCGTTATCGCTCTGGTGGCAATGGCCATCGCGCTGCTTTCTACG
CTTTATCCTTCCTGGCGCGCTGCCGCCACTCAACCCGCTGAGGCTTTACGTTATGAATAA
>EECEBICD_01543 Lipoprotein-releasing system ATP-binding protein LolD
ATGAATAAGATCCTGTTGCAATGCGACAACCTGTGCAAACGCTATCAGGAAGGCACCGTA
CAAACCGACGTGTTGCACGATGTCAGCTTCAGTATCGGCGAAGGCGAAATGATGGCGATC
GTCGGCAGTTCCGGTTCGGGTAAAAGTACGTTGCTGCATCTGTTAGGCGGGCTGGATACG
CCCACCTCCGGCGATGTGATTTTTAGCGGCCAGCCGATGAGCAAACTCTCTTCTGCGGCA
AAAGCGGAATTACGTAACCAGAAACTGGGGTTTATCTATCAGTTTCATCATCTCCTGCCG
GATTTTACGGCGCTGGAAAACGTGGCGATGCCATTATTGATTGGCAAAAAGAAACCGGCG
GAGATTGACGCTCGGGCGCGAGAAATGCTGCACGCCGTAGGGCTGGAGCATCGCGCAACC
CATCGACCTTCGGAACTTTCCGGCGGTGAGCGCCAGCGTGTCGCGATAGCGCGCGCGTTG
GTGAATAACCCGCGTCTGGTACTGGCGGATGAGCCGACCGGTAATCTGGATGCGCGTAAT
GCCGACAGTATTTTTGAGCTTCTGGGCGAGTTAAATCGTCTGCAGGGAACCGCGTTTCTG
GTGGTGACTCACGATTTGCAACTGGCGAAACGTATGAGCCGCCAGCTTGAGATGCGCGAT
GGCCGCCTGACGGCGGAACTGAGCCTGATGGGGGCGGAGTAA
>EECEBICD_01544 Lipoprotein-releasing system transmembrane protein LolE
ATGGCGTCGCCTTTATCATTGTTAATTGGCTTGCGCTTCAGCCGTGGGCGGCGGCGCGGC
GGTATGGTTTCGCTCATTTCCGTCATTTCTACCATCGGTATCGCGCTGGGCGTGGCGGTA
TTGATTGTCGGTTTAAGCGCGATGAACGGTTTCGAACGCGAGTTGAATAACCGTATTCTG
GCGGTGGTTCCGCACGGGGAGATTGAAGCGGTCAATCAGCCGTGGACCAACTGGCGTGAA
GCGCTGGCAAAAGTGCAGAAGGTGCCGGGCATTGCCGCCGCCGCGCCGTATATCAATTTT
ACCGGCCTGGTAGAGAGCGGGGCGAATTTGCGCGCCATTCAGGTTAAAGGCGTCGACCCT
AAGCAAGAGCAGCAGCTTAGCGCGTTACCGTCATTTGTGCAAAATCACGCCTGGGATCAT
TTCAAAGCGGGCGAACAGCAGATCATTATCGGCAAAGGCGTCGCCGATGCGCTGAACGTG
AAACAGGGCGACTGGGTGTCCATCATGATCCCCAATGCGAATGCCGACCATAAGCTGCTC
CAGCCCAAACGCGTGCGTTTGCATGTCATCGGGATTCTGCAACTGAGCGGCCAGCTCGAT
CACAGCTTCGCCATGATCCCGCTTGAGGATGCGCAGCAGTATCTGGATATGGGGTCCAGC
GTTTCCGGTATCGCGCTTAAAGTCCACGACGTGTTTAATGCGAACAAACTGGTGCGTGAC
GCGGGTGAAGTGACTAACAGCTATGTCTACATTAAGAGCTGGATCGGCACCTACGGCTAT
ATGTATCGCGATATCCAGATGATTCGCGCCATTATGTATCTGGCGATGATTCTGGTGATC
GGCGTGGCCTGTTTTAATATCGTTTCCACCTTAGTGATGGCGGTAAAAGACAAAAGCGGC
GACATTGCGGTATTAAGAACGCTGGGGGCGAAAGATGGTTTGATTCGTGCCATTTTCGTC
TGGTACGGGCTACTGGCGGGCTTGCTGGGCAGCTTGATTGGCGTGGCGATCGGCGTTGTC
GTTTCGCTTCAGCTTACCGCCATTATTAATGGGATTGAAAAGGAGATCGGGCATCAGTTT
TTGTCCGGCGATATCTATTTTATCGACTTCCTGCCATCCGAATTACATTGGCTGGACGTT
GTTTACGTACTGGTCACGGCGTTGTTGTTAAGCTTACTGGCGAGCTGGTATCCGGCGCGG
CGGGCGAGCAACATTGACCCTGCGCGAGTATTGAGCGGGCAGTAA
>EECEBICD_01545 N-acetyl-D-glucosamine kinase
ATGTATTACGGGTTTGACATTGGCGGAACAAAGATTGCGTTAGGCGTATTTGACTCAACG
CGGCGGCTGCAGTGGGAAAAACGGGTTCCCACGCCCCATACCAGCTATAGCGCCTTTTTA
GACGCCGTATGCGAACTGGTCGCGGAAGCCGACCAGCGATTTGGCGTAAAAGGGTCGGTA
GGGATTGGCATACCCGGTATGCCGGAAACCGAAGACGGCACGCTGTACGCCGCGAATGTC
CCGGCAGCCAGCGGTAAGCCGTTGCGCGCCGATCTCAGCGCCCGACTGGATCGCGATGTG
CGTCTGGACAATGACGCTAACTGTTTTGCCTTGTCCGAAGCCTGGGATGATGAATTCACG
CAATATCCTTTGGTTATGGGGCTTATCCTCGGCACCGGCGTCGGCGGTGGCCTGGTGCTA
AACGGGAAGCCGATTACCGGTCAGAGCTATATCACCGGCGAGTTTGGTCATATGCGTTTG
CCGGTTGACGCGCTAACGTTGATGGGGTTTGATTTCCTCTCCGCCGTTGTGGATGCGGT
CAGATGGGCTGCATTGAGAATTATCTGTCCGGGCGCGGGTTTGCGTGGCTATATCAGCAT
TATTATGATCAATCGCTGCAGGCGCCGGAGATTATCGCGTTGTGGGAGCAGGGCGATGAG

CAGGCGCACGCGCATGTTGAGCGCTATCTGGATTTACTGGCGGTTTGTCTGGGGAATATA
CTGACGATTGTCGATCCCGATTTATTGGTGATCGGCGGCGGACTATCGAACTTTACGGCA
ATAACAACGCAACTGGCGGAAAGACTGCCGCGCCATCTCCTCCCTGTTGCCCGCGCGCCG
CGCATTGAGCGTGCGCGGCATGGGGATGCAGGTGGGATGCGCGGTGCTGCTTTTTTACAT
CTTACCGACTAA
>EECEBICD_01546 NAD-dependent protein deacylase
ATGCAGTCGCGTCGGTTTCATCGATTAAGCCGCTTTCGTAAAAATAAACGTCTGTTGCGT
GAACGTCTGCGCCAGCGGATCTTTTTCAGAGACAGAGTGGTGCCGGAAATGATGGAAAAC
CCAAGAGTATTAGTCCTTACAGGTGCAGGAATTTCTGCAGAGTCTGGTATTCGTACGTTT
CGCGCGGCGGATGGCCTTTGGGAAGAGCATCGGGTTGAAGACGTGGCAACGCCGGAAGGA
TTCGCCCGTAATCCGGGGCTGGTGCAGACATTTTATAATGCCCGCCGTCAGCAGCTTCAA
CAGCCGGAAATACAACCCAATGCGGCGCATCTGGCGTTAGCGAAGCTTGAAGAAGCGCTT
GGCGATCGCTTTTTGCTGGTGACGCAAAACATCGACAATTTGCATGAACGCGCGGGCAAT
CGCAACATCATTCATATGCATGGCGAGCTGTTGAAGGTGCGCTGTTCGCAAAGCGGCCAA
ATTCTGGAATGGAACGGCGATGTGATGCCGGAAGATAAATGTCACTGCTGCCAGTTCCCG
GCGCCGCTACGTCCGCACGTGGTGTGGTTTGGCGAGATGCCGCTTGGCATGGATGAAATT
TATATGGCGCTGTCGATGGCGGATATTTTTATCGCCATTGGCACATCCGGTCATGTCTAT
CCGGCTGCGGGGTTTGTGCATGAAGCAAAACTGCACGGCGCACATACGGTGGAACTGAAT
CTTGAGCCAAGCCAGGTCGGCAGCGAGTTTGAAGAGAAGCACTACGGCCCGGCAAGCCAG
GTTGTGCCGGAATTTGTTGATAAATTCCTGAAAGGGCTGTAG
>EECEBICD_01547 Spermidine/putrescine-binding periplasmic protein
ATGAAAAAATGGTCACGCCACCTGCTCGCGGCGGGCGCCCTGGCGCTGGGTATGAGTGCC
GCTCATGCCTCCGACAACGACACGCTCTATTTCTATAACTGGACTGAGTATGTACCGCCG
GGCCTGCTGGAACAGTTCACTAAAGAGACAGGCATTAAGGTGATTTATTCGACCTACGAG
TCGAACGAAACCATGTATGCCAAACTCAAAACCTATAAAGATGGCGCGTATGACCTGGTT
GTTCCTTCTACATACTATGTCGACAAAATGCGCAAAGAAGGCATGATCCAGAAGATCGAT
AAGTCGAAATTAACCAATTTCCACAACCTCGATCCGGAAATGCTCAACAAACCGTTTGAC
CCCAACAACGATTATTCTGTTCCGTATATCTGGGGCGCAACGGCGATTGGCGTGAACAGC
GATGCTATCGATCCAAAAACCATTACCAGCTGGGCTGACTTGTGGAAACCGGAATATAAA
AACAGCCTGCTTTTAACCGACGATGCGCGAGAAGTCTTCCAGATGGCGCTGCGCAAGCTA
GGTTATTCCGGTAACACTACCGATCCGAAAGAGATCGAAGCGGCATATGAAGAGTTGAAA
AAACTGATGCCGAACGTAGCGGCCTTCAACTCCGATAATCCGGCGAATCCGTATATGGAA
GGGGAAGTCAATCTGGGGATGGTGTGGAACGGTTCGGCCTTCGTTGCTCGTCAGGCTGGC
ACGCCGCTGGAGGTCGTCTGGCCGAAAGAAGGCGGTATTTTCTGGATGGATAGCCTGGCT
ATTCCGGCAAACGCGAAAAATAAAGAGGGCGCGCTGAAGCTAATCAACTTCCTGCTGCGT
CCTGACGTCGCGAAAGAGGTGGCAGAAACCATCGGCTACCCTACCCCCAACCTGGCGGCG
CGTAAGCTGTTAAGCCCGGAGGTCGCCAATGATAAGTCGCTCTATCCGGATGCGCAAACC
ATCAGCAAAGGCGAGTGGCAAAACGATGTCGGCGACGCCAGCGCCATTTATGAAGAGTAT
TATCAGAAGCTAAAAGCAGGACGCTAA
>EECEBICD_01548 Inner membrane ABC transporter permease protein YdcV
ATGATCGGTCGGCTGCTCCGTGGCGGTTTCATGACTGCCATCTATGCGTATCTGTATATT
CCCATCATTATTCTGATCGTGAACTCCTTCAACAGTTCGCGGTTCGGCATTAACTGGCAG
GGCTTTACCACTAAATGGTACGAACTCCTGGTCAATAACGACAGTCTGTTGCAGGCCGCT
CAACATTCACTGACGATGGCGATTTTTTCAGCGACCTTCGCCACGTTGATTGGCTCGTTA
ACCGCGGTGGCGCTTTATCGCTACCGCTTTCGCGGGAAGCCATTCGTCAGCGGGATGCTG
TTTGTAGTAATGATGTCGCCGGATATCGTGATGGCGATCTCCCTGCTGGTGCTGTTTATG
TTACTCGGCATTCAACTGGGCTTTTGGTCGTTGCTGTTCTCGCACATCACCTTTAGCCTG
CCGTTTGTGGTCGTCACGGTCTATTCGCGCCTGAAAGGCTTTGATGTCAGAATGCTGGAA
GCCGCAAAAGACCTGGGGGCCAGTGAGGTAACGATTTTACGCAAAATCATTCTGCCGCTG
GCGATGCCGGCGGTGGCGGCGGGCTGGTTGCTGAGCTTTACGTTGTCGATGGACGATGTA
GTGGTCTCGTCGTTTGTCACCGGGCCGGGCTATGAAATTTTACCCTTAAAGATTTATTCC
ATGGTAAAAGTCGGCGTTTCACCAGAAGTGAACGCGCTGGCGACCGTTTTATTGGTGCTG
TCTCTGGTAATGGTCATTGCCAGCCAGCTTATTGCTCGTGATAAAACCAAGGGCCGATAA
>EECEBICD_01549 Secreted effector protein SifA

ATGGCGATGTGTGGATTAAAACAGCTTGAAATACCACCACCGCATACCCACATACCTATT
GAAAAAATGGTAAAAGAGGTTTTACTAGCGGATAAGACGTTTCAGGCGTTCCTCGTAACG
GATCCCAGCACCAGCCAAAGTATGTTAGCTGAGATAATCGAAACCATCTCTGATCAGGTT
TTTCACGCCATTTTTAGAATAGACCCCCAGGCTATACAAAAAATGGCGGAAGAACAGTTA
ACCACGCTACACGTTCGCTCAGAACAACAAAGCGGCTGTTTATGTTGTTTTTTATAA
>EECEBICD_01550 Secreted effector protein SifA
ATGTTGTTTGCCGAACGCGCCCCCACACGAGAGCGGCTTACAGAGATTTTTTTTGAGTTG
AAAGAGTTAGCCTGCGCATCGCAAAGAGATAGATTTCAGGTTCATAATCCTCATGAAAAT
GATGCCACCATTATTCTTCGCATCATGGATCAAAACGAAGAGAACGAATTGTTACGTATC
ACTCAAAATACCGATACCTTTAGCTGTGAAGTCATGGGGAATCTTTATTTTTTAATGAAA
GATCGCCCGGATATTTTAAAATCGCATCCACAAATGACGGCCATGATTAAGAGAAGATAT
AGCGAAATCGTAGACTACCCCTCCCTTCGACATTATGTCTCAATCCTGCTGGCGCGCCGA
TATTATCGGTTCCATTAG
>EECEBICD_01551 hypothetical protein
ATGCCGATTACTATAGGGAATGGTTTTTTAAAAAGTGAAATCCTTACCAACTCCCCAAGG
AATACGAAAGAAGCATGGTGGAAAGTTTTATGGGAAAAAATTAAAGACTTCTTTTTTCTA
CTGGCAAAGCAAAAGCGGACCGTTGTCTACATGAGATGTTGTTTGCCGAACGCGCCCCCA
CACGAGAGCGGCTTACAGAGATTTTTTTTGAGTTGA
>EECEBICD_01552 Spermidine/putrescine transport system permease protein
PotB
ATGAAGAACACAAGTAAATTCCAGAATGTAGTGATTGTCACTATCGTCGGTTGGCTTGTG
TTATTTGTTTTTCTGCCCAACCTGATGATCATTGGCACCAGCTTTCTGACCCGTGACGAC
GCCAGTTTCGTCAAGATGGTCTTTACGCTGGATAATTACGCGCGCCTGCTCGATCCGCTC
TATTTTGAAGTGCTCCTGCACTCTCTGAATATGGCGCTGATCGCCACCCTCTCCTGCCTG
GTGCTGGGCTATCCTTTTGCCTGGTTTCTGGCGAAGTTACCGGAAAAGATACGTCCGTTA
TTGCTGTTTTTGCTGATTGTTCCTTTCTGGACCAACTCACTGATCCGCATCTATGGGCTG
AAAATATTCCTGAGCACTAAAGGTTATCTCAATGAATTCCTGCTGTGGCTGGGCGTGATT
GATACACCGATTCGCATCATGTTCACCCCGAGCGCCGTTATTATCGGCCTGGTTTACATT
TTGCTGCCATTTATGGTCATGCCGCTCTACTCCAGCATTGAGAAGCTCGACAAACCACTA
CTGGAGGCCGCACGCGATCTCGGCGCCAGTAAAATGCAAACGTTCATCCGCATCATTATT
CCATTAACCATGCCGGGCATTGTCGCCGGTTGCTTGCTCGTGATGCTACCGGCAATGGGC
CTGTTCTACGTCTCCGATTTAATGGGTGGCGCGAAAAACCTGCTGATTGGTAACGTCATT
AAGGTACAGTTCCTGAATATTCGCGACTGGCCGTTTGGCGCGGCTACCAGCATTACGCTG
ACCATTGTGATGGGACTCATGCTATTGATTTACTGGCGGGCTTCCCGCTTATTGAATAAG
AAGGTGAGTGATATAAGCGATTAA
>EECEBICD_01553 Spermidine/putrescine import ATP-binding protein PotA
ATGGGACAGAGTAAGAAATTGAATAATCAACCGCGTTCGCTTTCACCGCTGGTGCTTTTG
TCTGGAATTAGCAAAAGTTTCGATGGCAAAGAGGTCATTTCGCAACTGGATTTGACCATT
AACAATGGCGAATTCCTCACGCTGCTTGGCCCTTCCGGCTGCGGTAAAACAACCGTTCTT
CGCCTGATTGCCGGCCTGGAAACCGTTGATGCCGGGCATATCATGTTGGATAACCAGGAT
ATCACCCACGTTCCCGCCGAAAATCGTTATGTAAATACCGTATTTCAAAGCTACGCTTTA
TTTCCGCATATGACGGTGTTTGAAAATGTCGCGTTTGGCTTACGAATGCAAAAAACGCCA
GCCGCCGAGATAGCGCCTCGCGTGACCGATGCGCTGCGTATGGTGCAGCTTGAAGAGTTT
GCACAACGTAAACCGCACCAGCTTTCCGGCGGACAGCAGCAGCGTGTGGCTATCGCTCGC
GCGGTGGTGAATAAACCTCGCCTGTTATTACTGGATGAATCGCTTTCCGCGCTGGACTAT
AAATTGCGCAAACAAATGCAGAACGAGCTTAAAGCATTACAGCGTAAACTCGGTATCACG
TTTGTTTTTGTCACTCACGATCAGGAAGAAGCGCTCACCATGTCCGATCGTATCGTGGTA
ATGCGTAATGGCGTGATTGAGCAAGACGGCACGCCGCGTGAAATCTATGAAGAGCCAAAA
AACCTGTTTGTCGCCGGTTTTATCGGCGAAATTAACCGCTTTGACGCAACCGTCATTGAA
CGGCTTGATGAACAGCGCGTCCGCGCCAGCGTAGAAGGTCGGGAGTGCAATATATACGTC
AATTTCGCTGTCGAACCGGGCCAAAAACTGAACGTCCTGCTGCGACCGGAAGATCTACGC
GTCGAAGAGATCAACGATGATAACCACATCGAAGGGCTTATCGGCTACGTGCGCGAGCGC
AATTATAAAGGTATGACGCTGGAGTCGGTGGTTGAACTGGAAAATGGCAAAATGGTGATG
GTCAGCGAATTCTTCAACGAAGACGATCCTGACTTCGACCATTCACTTGACCAAAAAATG

GCCATTAGTTGGGTAGAAAGCTGGGAGGTCGTACTGGCTGATGAAGAACACAAGTAA
>EECEBICD_01554 Peptidase T
ATGGATAAACTACTTGAGCGTTTTTTACACTACGTATCGCTGGATACCCAATCAAAGTCG
GGTGTTCGGCAGGTTCCCAGCACTGAGGGGCAGTGGAAGTTACTACGTTTGCTCAAACAG
CAGCTCGAAGAGATGGGGCTGGTTAACATTACATTAAGTGAAAAAGGGACGTTGATGGCG
ACGCTCCCGGCCAATGTTGAGGGGGATATTCCCGCCATTGGTTTTATCTCCCATGTGGAT
ACCTCTCCGGATTTCAGCGGTAAAAACGTTAACCCGCAGATTGTCGAGAATTATCGCGGC
GGCGATATAGCATTAGGGATTGGCGATGAGGTGTTGTCACCCGTGATGTTCCCGGTACTG
CATCAATTACTGGGACAGACGCTGATTACTACCGATGGTAAGACATTGCTGGGCGCGGAC
GATAAAGCCGGCGTTGCGGAGATCATGACCGCGCTGGCGGTGCTGAAAGGTAATCCTATT
CCCCACGGTGAAATTAAAGTGGCGTTTACGCCTGACGAAGAGGTAGGGAAAGGCGCGAAG
CACTTCGATGTTGAGGCCTTTGGCGCGCAGTGGGCCTATACGGTCGACGGCGGCGGCGTG
GGCGAACTGGAGTTTGAAAACTTCAATGCCGCCTCGGTGAATATCAAAATCGTCGGCAAT
AACGTGCATCCCGGTACGGCAAAAGGCGTGATGGTCAATGCGCTGTCGTTGGCGGCGAGG
ATTCACGCGGAAGTGCCGGCGGATGAAACGCCTGAAACCACTGAAGGTTACGAAGGGTTT
TATCATCTGGCCAGCATGAAAGGCACCGTTGACCGGGCCGAAATGCACTACATCATTCGC
GATTTCGACCGTAAGCAGTTTGAAGCGCGTAAACGCAAAATGATGGAGATTGCCAAAAAA
GTCGGTAAGGGGCTGCATCCGGACTGCTATATCGAACTGGTGATTGAAGACAGTTATTAC
AATATGCGCGAAAAAGTGGTTGAGCATCCGCATATTCTCGATATCGCCCAGCAGGCCATG
CGTGACTGTCATATTACGCCGGAGATGAAACCAATTCGCGGCGGTACAGACGGGGCGCAA
CTGTCGTTTATGGGCCTGCCGTGTCCTAATCTCTTTACCGGCGGATATAACTATCATGGT
AAACATGAGTTTGTGACGCTGGAGGGGATGGAAAAAGCGGTACAGGTGATTGTACGTATC
GCGGAGCTGACGGCGAAGCGCGGCCAGTAG
>EECEBICD_01555 hypothetical protein
ATGTTACGCATTGTGGTGAAGCGTTTTATTATTTATGGCGGGATATTTTCCGCCATCAAT
TTTTCTGCCTGGAGCGCCGAATATACGCCTTCCTGGTCCCAGCGCCAGCAACAATCTGCA
GCATGTTTTATGACCGGCGATGAAACCTGCATGACGTTTATTGATGATGCGGTGAGGCTG
GCCTCGCAGCAATATGGCAAACGCTCAATACAGCTTGTTCGTAGCCTGCTATTACAAAGC
GATATTTATCAATGGCTGGGTAAACCTGAATTAACGCCGCAAATGCTGCTGCGAGCGCGG
GCAATAATGAAAACATTTCCGGCGGGCACTTATCCGGGCGATCGGGCCGATATGTTTGAA
CACCTGGCGGCCTTTTATGTTTACAGTGACGATCGCCATATAGAGTATTCATCTACAGAG
CAGTGGCGATACGAGATTAAGGTTGATTACCGTCAGCGAATAGCCTGGCAAGAGCAGGCA
TTAACGTGGCGTCTTAAAGACAAAAAGGCATCGACGGAGGCATTGGTTTATACCTTAAAC
AGGATGCGCGACGCTTATTCTGATGCGCTGGAAGAGCGGGATGTCGAATGTGATAGTGCG
CGTAAAGCCTATTATTTGGCGAAGATAGACACAACGGAGCGGCAGTGGCTGAGCGTTATT
TTACGAGATAAAACCTGGGATAATCGTGAGCGCGTCGCCTCTTTTTTGCAGCAGAAAGCG
GATATTGCTTATAATGCAGGGCATATTAGCGAGGCGATTAATGCGCTAAGTCAGGCGCTA
AAAATTGAGCAAACGCTTTATGGCGCCGAATTCGGCGAAATGACGGTTGACAGCAATAAT
CTTGCTGGCTTTTATGCACAGGGCCATCATTATAAGGAAGCGAAAGATCTCTACCTTAAA
CTTATCGCGTATTACCAGAGCCGTTTAACGCCAATGGCGACGGTAATATCACGATTACGC
TTTTATTTGCCCGAAAACATCGACCTGGACAGTACCTCCCCCTATCTCCCATTGCTGGAA
GAATATAAACGTCGGCAGTCCGACGTATCCATGGTGTTGTATGGTATTTCGCTGCTTTAT
CAGAATAATCAACAGCTTGAACAAGCAAAAGACTTTGCGGAACGCGCCTTTACGCTTGAT
GCCGTAGCTTATCCAGCAAAGATGCAGTATGAACGATTACAGCGACTGGCTAATATTGCC
GAAGGTCTTGGCGATAACGTACTGGCGCGACGTTACCGACAATTGTCATTTCGCCATCGA
ATGGCCCACTCGATTTATCCGGGCGATCCGCAATATAACGATTTCGCCAAACCTGGCGGC
GATCGTTGTGGATAG
>EECEBICD_01556 50S ribosomal protein L16 3-hydroxylase
ATGGAATACCAATTAACTCTTAACTGGCCCGACTTTCTTGAACGCCACTGGCAAAAACGC
CCGGTGGTCTTAAAACGTGGGTTTAGCAACTTTATCGATCCGCTCTCGCCGGATGAGCTG
GCGGGGCTGGCGATGGAAAGCGAAATCGACAGCCGACTCGTTAGCCATCAGGATGGCAAA
TGGCAGGTCAGCCATGGCCCTTTCGAAAGCTACGATCATCTGGGTGAGAGCAATTGGTCG
CTGCTGGTACAGGCGGTAAACCACTGGCACGAACCCACCGCCGCGCTGATGCGCCCGTTC
CGCGCTCTGCCGGACTGGCGTATCGACGATCTGATGATCTCCTTTTCCGTTCCCGGCGGC

GGCGTTGGCCCGCATCTGGATCAGTATGATGTGTTTATTATTCAGGGAACCGGTCGCCGT
CGCTGGCGCGTGGGCGAAAAGCTGCAAATGCGTCAGCACTGCCCGCATCCCGACCTGTTA
CAAGTCGATCCGTTCGAGGCCATTATTGATGAAGAGCTGGAGCCGGGCGACATTCTGTAT
ATTCCGCCAGGATTCCCGCATGAAGGCTATGCGCTGGAAAACGCCATGAACTATTCGGTC
GGTTTTCGCGCGCCCAATAGCCGTGAGCTGATCAGCGGTTTTGCCGATTACGTCCTGCAA
CGGGAGTTGGGCAATACATACTACAGCGATCCGGATATGCCGTCCCGCAAGCATCCGGCG
GATATCCTGCCGCAGGAGATGGATAAACTGCGTAATATGATGCTCGATTTGATCAATCAA
CCCGCGCATTTCCAACAGTGGCTTGGCGAGTTTCTCTCTCAGTCACGCCACGAACTGGAT
ATTGCGCCGCCGGAGCCGCCGTATCAGCCTGATGAAATTTACGATGCCTTAAAGCAAGGC
GAGGTATTAGTACGTCTCGGCGGGCTTCGCGTGTTGCGTATCGGCGATGAGGTCTATGCG
AATGGCGAAAAAATAGACTCTCCGCATCGCCCCGCTCTGGAGGCGCTCGCCAGTCATATT
GCACTGACCGCTGAAAACTTCGGCGATGCGCTGGAAGACCCGTCCTTCCTCGCGATGCTG
GCGGCGCTGGTGAATAGCGGATACTGGTTTTTCGAAGGATAA
>EECEBICD_01557 Virulence sensor histidine kinase PhoQ
ATGAATAAATTTGCTCGCCATTTTCTGCCGCTGTCGCTGCGGGTTCGTTTTTTGCTGGCG
ACAGCCGGCGTCGTGCTGGTGCTTTCTCTGGCATATGGCATAGTGGCGCTGGTCGGCTAT
AGCGTAAGTTTTGATAAAACCACCTTTCGTTTGCTGCGCGGCGAAAGCAACCTGTTTTAT
ACCCTCGCCAAATGGGAAAATAATAAAATCAGCGTTGAGCTGCCTGAAAATCTGGACATG
CAAAGCCCGACCATGACGCTGATTTACGATGAAACGGGCAAATTATTATGGACGCAGCGC
AATATCCCCTGGCTGATTAAAAGCATTCAACCGGAATGGTTAAAAACGAACGGCTTCCAT
GAAATTGAAACCAACGTAGACGCCACCAGCACGCTGTTGAGCGAGGACCATTCCGCGCAG
GAAAAACTCAAAGAAGTACGTGAAGATGACGATGATGCGGAGATGACCCACTCGGTAGCG
GTAAATATTTATCCTGCCACGGCGCGGATGCCGCAGTTAACCATCGTGGTGGTCGATACC
ATTCCGATAGAACTAAAACGCTCCTATATGGTGTGGAGCTGGTTCGTATACGTGCTGGCC
GCCAATTTACTGTTAGTCATTCCTTTACTGTGGATCGCCGCCTGGTGGAGCTTACGCCCT
ATCGAGGCGCTGGCACGGGAAGTCCGCGAGCTTGAAGATCATCACCGCGAAATGCTCAAT
CCGGAGACGACGCGTGAACTGACCAGCCTTGTGCGCAACCTTAATCAACTGCTCAAAAGC
GAGCGTGAACGTTATAACAAATACCGCACGACCCTGACCGACCTGACACACAGTTTAAAA
ACGCCGCTCGCGGTTTTGCAGAGTACATTACGCTCTTTACGCAACGAAAAGATGAGCGTC
AGCAAAGCTGAACCGGTGATGCTGGAACAGATCAGCCGGATTTCCCAGCAGATCGGCTAT
TATCTGCATCGCGCCAGTATGCGCGGTAGCGGCGTGTTGTTAAGCCGCGAACTGCATCCC
GTCGCGCCGTTGTTAGATAACCTGATCTCCGCGCTAAATAAAGTTTATCAGCGTAAAGGG
GTGAATATCAGTATGGATATTTCACCAGAGATCAGTTTTGTCGGCGAGCAAAACGACTTT
GTCGAAGTAATGGGCAACGTACTGGACAACGCTTGTAAATATTGTCTGGAGTTTGTCGAG
ATTTCGGCTCGCCAGACCGACGATCATTTGCATATTTTCGTCGAAGATGATGGCCCAGGC
ATTCCCCACAGTAAACGTTCCCTGGTGTTTGATCGCGGTCAGCGCGCCGATACCCTACGA
CCAGGACAAGGCGTGGGGCTGGCTGTCGCGCGCGAGATTACGGAACAATACGCCGGGCAG
ATCATTGCCAGCGACAGTCTGCTCGGCGGTGCCCGTATGGAGGTCGTTTTTGGCCGACAG
CATCCCACACAGAAAGAGGAATAA
>EECEBICD_01558 Virulence transcriptional regulatory protein PhoP
ATGATGCGCGTACTGGTTGTAGAGGATAATGCATTATTACGCCACCACCTGAAGGTTCAG
CTCCAGGATTCAGGTCACCAGGTCGATGCCGCAGAAGATGCCAGGGAAGCTGATTACTAC
CTTAATGAACACCTTCCGGATATCGCTATTGTCGATTTAGGTCTGCCGGATGAAGACGGC
CTTTCCTTGATACGCCGCTGGCGCAGCAGTGATGTTTCACTGCCGGTTCTGGTGTTAACC
GCGCGCGAAGGCTGGCAGGATAAAGTCGAGGTTCTCAGCTCCGGGGCCGATGACTACGTG
ACGAAGCCATTCCACATCGAAGAGGTAATGGCGCGTATGCAGGCGTTAATGCGCCGTAAT
AGCGGTCTGGCCTCCCAAGTGATCAACATCCCGCCGTTCCAGGTGGATCTCTCACGCCGG
GAATTATCCGTCAATGAAGAGGTCATCAAACTCACGGCGTTCGAATACACCATTATGGAA
ACACTTATCCGTAACAACGGTAAAGTGGTCAGCAAGGATTCGCTGATGCTTCAGCTGTAT
CCGGATGCGGAACTGCGGGAAAGTCATACCATTGATGTTCTCATGGGGCGTCTGCGGAAA
AAAATACAGGCCCAGTATCCGCACGATGTCATTACCACCGTACGCGGACAAGGATATCTT
TTTGAATTGCGCTAA
>EECEBICD_01559 Adenylosuccinate lyase
ATGGAATTATCCTCACTGACCGCCGTTTCCCCTGTCGATGGACGCTACGGCGATAAAGTC

AGCGCGCTGCGCGGAATTTTTAGCGAATACGGTTTGCTGAAATTTCGTGTACAAGTCGAA
GTACGTTGGCTGCAGAAATTAGCCGCGCACGCAGCGATCAAGGAAGTTCCTGCTTTTGCT
GCCGACGCAAACGGTTACCTGGATACGCTTGTTGCAAACTTCAATGAAGAAGATGCCGCG
CGCATTAAAACCATTGAGCGCACCACGAATCACGATGTGAAAGCGGTTGAGTATTTTCTG
AAAGAAAAAGTCGCCGCGATCCCGGCGCTACATGACGTTTCCGAATTTATCCACTTTGCC
TGCACTTCTGAGGACATTAACAACCTGTCGCACGCGTTAATGCTCAAAACCGCGCGCGAT
GAAGTGATCCTGCCTTACTGGCGTCAGATGATTAACGCGGTTAAAGATCTCGCCACGCAG
TATCGCGACATTCCTCTGCTCTCCCGCACCCACGGCCAGCCGGCAACGCCTTCCACTCTG
GGTAAAGAGATGGCGAACGTGGCGTATCGGATGGAGCGTCAGTTCCGCCAGCTCAACCAG
GTGGAGATCCTCGGTAAAATTAACGGCGCCGTAGGCAACTATAACGCGCATATCGCCGCC
TATCCGGAAGTTGACTGGCATCAGTTCAGCGAAGAGTTCGTCACCTCGCTGGGCATCCAG
TGGAATCCTTACACCACCCAGATTGAACCGCATGATTATATTGCGGAACTGTTTGACTGT
ATCGCGCGCTTTAACACCATCCTGATCGATTTCGATCGCGATGTCTGGGGCTATATTGCG
TTGAACCATTTCAAACAGAAAACCATCGCCGGGGAGATCGGTTCTTCTACCATGCCGCAT
AAAGTTAACCCCATTGACTTTGAAAACTCAGAAGGCAACCTCGGTCTGTCTAACGCAGTG
TTGCACCATCTGGCAAACAAACTGCCGGTTTCCCGCTGGCAGCGCGATCTGACCGACTCA
ACCGTCCTGCGTAACCTGGGTGTCGGCATCGGCTATGCGCTTATCGCTTATCAGTCCACC
CTGAAGGGCGTCAGCAAGCTGGAAGTAAACCGCGATCATCTGCTTGACGAACTGGATCAC
AACTGGGAAGTATTAGCCGAACCGATCCAGACCGTCATGCGCCGCTATGGTATTGAAAAA
CCATATGAAAAACTGAAAGAGTTGACCCGTGGCAAGCGTGTTGATGCCGAAGGAATGAAA
CAGTTTATTGATAGTCTGGCCCTGCCGGAAGCAGAAAAAACGCGCCTTAAAGCCATGACG
CCGGCAAATTATATCGGTCGCGCTGTGACTCTGGTCGACGAACTTAAATAA
>EECEBICD_01560 High frequency lysogenization protein HflD
GTGGCAAAGAATTATTATGACATCACCCTCGCGCTGTCTGGCATTTGCCAGTCGGCACGC
CTGGTGCAACAACTGGCGCACCAGGGGCACTGTGATGCCGACGCGTTGCACGTTTCACTC
AACAGTGTCATTGATATGAATCCCAGTTCTACTCTGGGCGTGTTCGGCGGCAGCGAAGCT
AACCTGCGTCTTGGTCTGGAAACACTGCTCGGCGTGCTGAATGCCAGCAGCCGACAAGGA
CTTAACGCCGAACTGACGCGTTATACGCTTAGCCTGATGGTGCTGGAGCGCAAGCTGTCA
TCCGCCAAAGGCGCGTTGAATACGTTAGGCGATCGCATTAACGGCCTGCAGCGCCAGCTT
GACCATTTTGATTTACAGTCAGACACGTTAATGAGCGCCATGGCGGGCATCTACGTGGAT
GTGATTAGCCCATTGGGCCCGCGCATTCAGGTGACGGGCTCCCCGGCCGTTCTGCAAAGT
CCGCAGGTGCAGGCCAAAGTCCGCGCCTCGCTACTGGCAGGCATCCGTGCAGCCGTGCTC
TGGCATCAGGTAGGCGGTGGTCGCCTACAGCTTATGTTTTCTCGTCATCGCCTGACGACT
CAGGCAAAACAAATTCTTGCTCATTTAACCCCGGAGTTATGA
>EECEBICD_01561 tRNA-specific 2-thiouridylase MnmA
ATGTCAGAAAGCCCAAAAAAAGTGATCGTCGGCATGTCCGGCGGTGTCGACTCCTCCGTT
TCCGCCTGGCTGTTGCAACAACAGGGTTATCAGGTAGAAGGCCTGTTCATGAAGAACTGG
GAAGAGGACGATGGTGAGGAATACTGTACCGCGGCAGCCGATCTGGCTGACGCGCAGGCC
GTGTGCGACAAGCTTGGCATTGAACTGCATACCGTTAATTTTGCCGCAGAATATTGGGAT
AACGTCTTTGAACTGTTCCTTGAAGAGTACAAAGCGGGCCGTACGCCGAACCCGGATATT
CTGTGCAATAAAGAGATCAAATTTAAAGCCTTCCTTGAATTTGCCGCAGAAGATTTAGGC
GCCGACTATATCGCCACCGGTCACTACGTTCGCCGCGCGGATGTAAACGGCAAAAGCCGG
TTGTTGCGCGGGCTCGACGGCAACAAAGATCAGAGCTACTTCCTCTATACGCTTGGCCAT
GAGCAGATTGCGCAAAGTCTGTTCCCGGTTGGCGAGCTGGAAAAACCGCAGGTGCGTAAA
ATCGCCGAAGATCTGGGGCTGGTAACCGCCAAGAAAAAAGATTCTACCGGTATCTGCTTT
ATCGGCGAGCGCAAATTCCGCGACTTTTTAGGGCGCTATCTGCCTGCGCAGCCGGGGAAA
ATCATTACCGTTGACGGCGATGAAATCGGCGAGCATCAGGGTCTGATGTATCATACGCTC
GGTCAGCGTAAAGGGCTGGGAATCGGCGGCACCAAAGACGGTAGCGAAGACCCCTGGTAT
GTGGTGGATAAAGACGTTGAAAACAATGTGTTAATTGTCGCCCAGGGGCATGAGCACCCA
CGTCTGATGTCGGTAGGCCTCATCGCCCAGCAGCTACATTGGGTCGATCGCGAACCGTTC
ACCGGCACCCTGCGCTGTACGGTAAAAACCCGCTACCGCCAGACCGATATTCCGTGCACG
ATTAACGCGCTGGATGACGATCGTATCGAGGTCATTTTTGACGAGCCGGTTGCCGCCGTT
ACGCCGGGCCAGTCCGCTGTCTTTTACAGCGGCGAAGTCTGCCTGGGCGGCGGCATTATC
GAGCAGCGACTGCCGCTGACGGTCTGA

>EECEBICD_01562 Phosphatase NudJ
ATGTTCAAGCCGCACGTGACCGTTGCCTGCATCGTACATGCCGAAGATAAATTTCTGGTG
GTGGAAGAGACCATCAACGGTAAATCGTTATGGAATCAACCTGCCGGACACCTGGAGGCA
GATGAAACGCTGGCGCAGGCCGCCGCGCGTGAACTGTGGGAAGAGACTGGCATCACGGCG
CAACCACAGTATTTTATCCGTATGCATCAGTGGATTGCGCCGGATAAGACCCCGTTCCTG
CGCTTTTTATTCGCTATCAAGCTTGATCATATTTGCGCGACCGAACCCCATGACAATGAC
ATCGATTGCTGCCGTTGGGTGAGCGCCGACGAGATTCTGAACGCGCCGAATTTACGTTCT
CCGCTGGTGGCTGAAAGCATTCGCAGTTATCAAACGGGACAATGTTACCCCTTATCTATA
GTGGGCGAATTTAACTGGCCGTTTACAGAGGGTGTCAAGTAA
>EECEBICD_01563 hypothetical protein
ATGAAACGGCTTCTCATTGCTTTTGCTTTATTAACACCGTTATCCGTCGATGCGGCCAGC
TTCGACTGCCAGAAAGCGCAGGCTGCGGATGAAAAGGCTATCTGCGCGCACCTGACGCTG
AATGATAAAAACGTGGAAATGCACACCAAATATCAGTTTCTGAAAGGGTTGTTCGCGATG
GGCAGTCGCGGCGCGCTGCAGGACGCCCAGCAGAGCTGGCTTAAACAGCGTCAGCAGTGC
AAGGCCGATGTCACCTGCCTGACAAAAGCTTATAACGAGCGTCTGAAACAGCTTGACGCG
ATCTATGACCACATTGATAAACCGCTTTAA
>EECEBICD_01564 Ribosomal large subunit pseudouridine synthase E
ATGTTTTGTAATGAGCATGTCAATACTACCCTATTGTTTGCCGCCATGAAAGACACTCAT
TATTGCGCTATAATGCGGCAATTAATAAGTTCTGAAAACACCATGCAAAAAACTTCTTTT
AGAAATCACTACGTTAAGCGATTCAGCTCACGACAAGCATCTAAATCACGGAAAGAAAAC
CAGCCAAAACGCGTGGTTTTGTTCAATAAACCTTACGATGTGTTGCCGCAGTTCACGGAT
GAAGCAGGTCGCCGCACGTTAAAAGATTTTATTCCTGTGCAAGGCGTTTATGCCGCAGGC
CGCCTGGACCGCGACAGCGAAGGGCTTTTGGTGCTGACTAATGACGGCGCGCTGCAGGCG
CGCCTCACGCAACCGGGCAAACGCACCGGCAAGATTTACTATGTTCAGGTAGAAGGTATC
CCGGATAATGCAGCGCTGCAGGCGCTTCGTACTGGCGTCACGCTCAACGATGGCCCTACC
CTGCCCGCCGGTATTGAGATTGTCGCGGAGCCTGACTGGCTGTGGCCGCGCACCCCGCCG
ATTCGCGAACGGAAGAATATTCCCACCAGTTGGCTAAAAGTCACCTTATATGAAGGCCGC
AACCGCCAGGTAAGACGCATGACAGCGCATGTCGGTCATCCCACTCTACGATTAATTCGT
TACAGCATGGGCGACTACACGCTCAACGGCCTCGATAATGGACAGTGGCGCGAGATCGCA
CAGGAGAAAGACAGATGA
>EECEBICD_01565 Isocitrate dehydrogenase [NADP]
ATGGAAAGCAAAGTAGTTGTTCCGGTGGAAGGTAAGAAGATCACCCTGCAAAACGGCAAA
CTCAACGTTCCTGAGAATCCGATTATCCCGTTTATTGAAGGCGATGGTATCGGTGTTGAT
GTCACTCCAGCCATGCTTAAAGTAGTCGATGCCGCCGTCGAGAAAGCCTATAAAGGCGAG
CGTAAAATTTCCTGGATGGAAATTTACACCGGAGAAAAAATCCACACAGGTTTACGGCCAG
GATGTCTGGCTTCCCGCTGAAACCCTTGATTTAATTCGTGACTACCGCGTAGCGATTAAA
GGTCCATTGACCACGCCGGTTGGCGGCGGTATCCGCTCTCTGAACGTTGCCCTGCGTCAG
GAGCTTGACCTGTACGTTTGTCTGCGTCCCGTTCGTTACTATCAGGGCACGCCAAGTCCG
GTGAAACATCCGGAGCTGACCGATATGGTCATCTTCCGTGAAAACTCTGAAGACATCTAC
GCCGGAATTGAATGGAAAGCTGACTCTGCAGACGCTGAGAAAGTGATCAAGTTCCTGCGC
GAAGAGATGGGCGTGAAGAAAATTCGCTTCCCGGAGCACTGCGGTATCGGTATCAAACCG
TGTTCTGAAGAAGGCACTAAACGTCTGGTTCGCGCGGCGATTGAATACGCTATTACCAAC
GATCGCGATTCCGTCACGCTGGTACATAAAGGCAATATCATGAAGTTCACCGAAGGCGCG
TTTAAAGACTGGGGCTACCAGTTGGCGCGCGACGAGTTCGGCGGTGAGCTTATTGACGGC
GGTCCGTGGCTGAAAATTAAGAACCCGAACACGGGTAAAGAGATCGTGGTTAAAGACGTT
ATCGCCGATGCGTTCCTGCAACAAATCCTGTTGCGTCCGGCGGAATACGACGTTATCGCC
TGTATGAACCTGAACGGTGACTATATTTCCGATGCCCTGGCGGCTCAGGTTGGCGGTATC
GGTATTGCACCGGGTGCGAATATCGGCGACGAATGCGCGTTGTTTGAAGCGACTCACGGG
ACTGCGCCGAAATATGCGGGCCAGGATAAAGTCAACCCAGGCTCCATTATTCTGTCTGCG
GAGATGATGCTGCGCCACATGCAGTGGTTCGAAGCCGCAGACCTGATTGTTAAAGGTATG
GAAGGCGCGATTGCCGCGAAGACCGTGACCTATGACTTTGAACGCCTGATGGAAGGCGCT
AAGCTGCTGAAATGTAGTGAGTTTGGTGACGCGATTATCGCAAATATGTAA
>EECEBICD_01566 hypothetical protein
ATGCTCAAACCTATCTGCCATAGTGGAAGTATAAAGGTTCCGGAATATCTGGAAACAGAT

AAGGAAAAAAAATGCCGGACGTACTCCACTGTCTTCAGACATTCAGCAAGTTAGAAATGTT
GTTGAAGATGTTCCGCCATTTCCAGAAAGCAGAACGGCGAGAGGTTCTGTGAGCGCAGCG
TACAGGCTTTCTTTTGACGAAGTGTTTTGCGGTCTCAGCAATGAAGAGCGTAAAAAGGTG
TATGGTCGCCTTTTTGGAAAACAAGTACTTGCGCATATTCATTCCAGGTGTCAGCGAGAC
GCTGACATAATAAGAGAAAAAGCGCTCAGGCGGATAAGCCGTGAGTGTGGTACCGAAATA
GATTGCACTCTCTTGCTAAATAAGATGGTGGATATTTTACAAAATGCCCGGCTGACAATA
AATTTTAATGCGGCAAAAATTGACTTTGCCTCTCTCTTAAAAAATAAAGAATATCTGAAC
TCTTATGCATTAGGTTGCAGACCAGGAGATTTACCTGCTTATAATGTCGGACGTGATTCA
GTTGAAACTAAAGCATTTGAACTGGAGAGGCTTGCAGATTCACCTTATGCCCCATATGGT
CAGACAGGCGGTTTTTCCGTAGCATATACTCCCAACAGTAGAACTTTTAGCCCTACAAGC
AGACCAATTTATGCTGCACTGGACTTTCTGAACGGTGAAAATGGAGGTGCCAGCGCCTAT
GGGAAATCATTTTTTGAATTAAATGATAATGTAAAAACAAATTGTACATTATCACCTTTT
GATATCTACGGCCACAGATTTGGTCTGGATACGAGTAAACTATCAACATTTTGGCATATG
GAGAACCTGATTGCATCCTGTCAAAATGATTTTTTTGGTTATAATTGCTTTAAGAGTTTG
GTTAAAATGGCTAAGGGCGAAAAATTTTTAGCTCATTCTAATTATGGTAAAGGCTATGAA
GGGAATTATATAGAAGCTCATATTCATGGTGATGTATGTTTATTCAGAGATATAAAACAC
GTTTATTTGTCTTTGCAAGAAAACTCTTACTCGGAAAGTCAACTATATGATTATGCAAAA
CAAATAAACCTGGTGCTTAATAGAGACTGCATAATATTATATTGA
>EECEBICD_01567 hypothetical protein
ATGAAGAATTTTTTCAAAATAATTACTGATTTCATCGCGGATATTTCCCTTGATCTATTT
GCTATATTTTTATGCATGTTATTCGTATACAAAACAGGACCATCAATTGGTGTGATATCA
TTTTTTATTGCATTAATTATTTATATCATTCTTCATTTTGTTTTTTACTCATTTCGTGAA
AAAATCATAAAAAAAAATATTCAAATAA
>EECEBICD_01568 hypothetical protein
ATGAATAAAATACATGTTACATATAAAAATCTCTTACTTCCGATTACCTTCATTGCAGCA
ACTCTAATTAGCGCCTGTGATAACGATAAAGATGCCATGGCGGAAGCTGAAAAAAAATCAA
GAGAAATACATGCAAAAAATCCAGCAAAAAGAGCACCAGCAATCAATGTTCTTTTACGAC
AAAGCCGAAATGCAAAAAGCTATTGCCAATATCAACGCAAAAGGTGGAGCCAATCTTGCG
ATTATTGAAGTCCGTTTCTTCAAGGGCGGGTATTCATTCATTCGACAAAGCGTTAACACC
CCTGCTAAAGTAGAGATGTTTAAATTTAACAACGGCTACTGGGGGGACCTTCGCCTGTCA
ACTTAA
>EECEBICD_01569 hypothetical protein
ATGTTCGTCGAACTCGTTTATGACAAGCGAAATGTTGAAGGTTTGCCAGGCGCACGCGAA
ATCATCCTCAATGAACTCACAAAACGCGTACATCAACTTTTTCCCGATGCGCAAGTGAAA
GTTAAGCCAATGCAGGCGAACGCATTAAACAGTGACTGTACAAAAAACCGAGAAAGAACGG
CTGCACCGTATGCTGGAAGAGATGTTTGAAGAGGCTGATATGTGGCTGGTCGCCGAATAA
>EECEBICD_01570 hypothetical protein
ATGACTTTGCTTTCAGGAAAAACCACACTGGTTCTCTGCCTCTCCTCTATTTTATGTGGA
TGTACGACGAACGGCTTACCCACACCTTATAGTATTAATTTGTCGTTCCCGGTCATTACA
CAAAACCAGATTAATTCCGGTGGTTATTACATAAATGACGCGGAACAAATTCGGACAACT
GATGGTCTGTGCCTTGATACAGGCCCAGATCAACAGAATCGTTTGACGCTGCGGGAGTGT
AAGCATGTGCAATCTCAGCTTTTCTCATTTCACCGAGACAGAATCACGCAGGGTGAGAAA
TGTCTGGATGCCGCAGGACAAGGTACAAAAGAAGGCACACCAATCATTCTTTATTCATGC
ACGGGTAATGATAACCAGCGCTGGCTCACTGATGATAACAAAATTAAGGGGAAACAGAGC
CGAAAATGCCTGGGCACAAATAGCATTATTGTCAGAAAAGGCGACCCTGTTGTGTTGGCC
GATTGCGATTTTAGTCGCGCCCTGGAATTTACCATCAGGTAG
>EECEBICD_01571 hypothetical protein
ATGAAACATCATGCTTTTATGCTTTGGTCATTACTTATTTTTTCATTCCATGTTTTGGCC
AGTTCAGGCCATTGTTCTGGTTTACAACAGGCATCATGGGATATTTTTATCTACGATTTT
GGTAGTAAAACCCCGCAACCACCTACAAATACTGATAAAAAGCAAGCCAGGCAGATTAGT
TCACCGTCCTGCCCGACGACAAAACCCATGATGTCCGCACCAGTCAATGACGCCAGGAAA
GGGAATACTTTCTCCAGAACATAA
>EECEBICD_01572 Attachment invasion locus protein
ATGAAAAATATTATTTTATCCACTTTATTTATTACTACAAGCGTTTTGGTTGTAAATGTT

GCACAGGCCGATACTAACGCCTTTTCCGTGGGGTATGCACAAAGTAAAGTTCAGGATTTC
AAAAATATCCGAGGGGTAAATGTGAAATACCGTTATGAGGATGACTCTCCGGTAAGTTTT
ATTTCCTCGCTAAGTTACTTATATGGAGACAGACAGGCTTCCGGGTCTGTTGAGCCTGAA
GGTATTCATTACCATGACAAGTTTGAGGTGAAGTACGGTTCTTTAATGGTTGGGCCAGCC
TATCGATTGTCTGACAATTTTTCGTTATACGCGCTGGCGGGTGTCGGCACGGTAAAGGCG
ACATTTAAAGAACATTCCACTCAGGATGGCGATTCTTTTTCTAACAAAATTTCCTCAAGG
AAAACGGGATTTGCCTGGGGCGCGGGTGTACAGATGAATCCGCTGGAGAATATCGTCGTC
GATGTTGGGTATGAAGGAAGCAACATCTCCTCTACAAAAATAAACGGCTTCAACGTCGGG
GTTGGATACCGTTTCTGA
>EECEBICD_01573 tRNA-Arg(cct)
CCGCCACTAGCTCAGCTGGATAGAGCATCAACCTCCTAAGTTGATGGTGCGAGGTTCGAG
GCCTCGGTGGCGGTCCA
>EECEBICD_01574 hypothetical protein
ATGATGAAACGTAAATTGATCCCATTTACCCTCTTTCTCGCTGCGCTTAGCGCCAGTACC
ACCTCTATAGCAGCTTCGCAGGAGATTTCTAAATCTATATACACCTGTAATGATAACCAG
GTTATGGAGGTGATTTACGTCAACACCGAGGCTGGAAACGCCTATGCGATTATCAGTCAG
GTAAACGAGATGATCCCGATGCGGTTAATGAAAATGGCCTCCGGAGCAAATTACGAAGCC
ATAGACAAAAATTATACTTATAAACTTTATACCAAAGGCAAAACGGCTGAACTGGTGGAA
GGTGATGATAAACCTGTTCTCAGTAACTGTTCCTTAGCTAACTAA
>EECEBICD_01575 hypothetical protein
ATGGTCGCCAAAAATAAAATTCACGGTCGTCGTATTCGTCAGTCTCTGCAACAGTTACAT
CATCGTTGTCTTTTTCAGCAACTGGCGGTAAGCCCACCAGGAGCAAAATATGTTTCAGTC
ACCGCTGAAACAAAAAACTGGCTTATTCATCTTGTGACTTCAGAGGGCCGTTAG
>EECEBICD_01576 Small heat shock protein IbpB
TTGCTGATTCTCTTTTTCTCTGACCGTTTCAACCGTATTGATAGACTTTTCAGTCAATTA
ACAGGAGATACGCCAGTCACTGCGACGCCAGCTTACGATCTGCAAAAGCGCGATGCGAAT
AACTATCTGCTTACCGTGAGCGTTCCTGGCTGGAAAGAGGAAGAGCTTGAAATTGAAACG
GTTGGCGGCAACCTGAATATTACGGGTAAACACACTGAAGAGACGGTAGAGGATCAGACG
CACTGGATTTATCGTGGTATTCGTAAGGCGGATTTCCAGTTGAGTTTTTCTTTGCCTGAA
CATGCTAAGGTGAATAATGCGAAACTGGAACAGGGCCTCTTGTTGGTCGAGATTTACCAG
GAGATCCCTGAAAGCGAGAAACCGAAAAAAATTGCCATAGAAAGCAAACCAAAGGCGATT
GAACACAAATCATAA
>EECEBICD_01577 hypothetical protein
ATGAAATTAGTGCTCGTCAATCGGCAGGTCATTTTACCGGAAAGCGGAACCGAGTCCTTC
CAGTGCCATGCCTCTACGCTTGTTCGCTTACCGTGCGGTACGCTCGTGGCCGCATGGTTC
GCGGGGTTGCGCGAAGGAAGCGAAGATACAGCCATCTGGCTATCTCGCTATGAACATAAT
ATCTGGACAACACCGCAGCGAGTGGCGGCACGCGAGGGGGAAGCGCACTGGAATCCGGTG
TTATTTTACCCGTCGGATAAATTGTGGTTATTTTATAAAGTAGGCAGCGACGTACACGTG
TGGAAAACCTGGTTTATCACCTCCAGCGATCGAGGATTTACCTGGAGTACGCCAGCTCCG
TTGGTTAACGATGATATTTTGCCGCGCGGTCCCGTAAAAAATAAACTCCTGCTGGCATCA
AATGGCGCCTGGATTGCGCCAGGGTCGATTGAAAGTCCTGAACGGTGGAGGGCATTTGTG
GATCGATCCTCTGACGAAGGAAAACACTGGAATATCTCTTTTGTTCCGCTGGAGCCTGAT
AACGCTATTTCCGGGACAAACGTAGCGTTGTGGGATGGTGTAAAAAAGGGGATGTTGTGG
GAATGTTGTCTGGAAAACCTCCTGCGCTGGGATGGCGTTATTCAGCCTACGCTGTGGGAG
TCTTCTCCGGGGCATATCCATATGTTGTTACGAAGCACACGCGGGGCAATTTTTCGCAGT
GATTCAATCGACTATGGCGCTACCTGGTCTGTTGCCCGCGCGACATCTTTATCGAATAAT
AATAGTGGTATCGATCTGGTTAGTATGCAGGACGGTACGCTGATTCTTGCGTTAAATCTG
GTCAACGGCAACTGGGGAAAGCGCTATCCGCTTTCACTGATTGCTTCCCAGGATAATGGC
GAATCATGGCTACCGCTACTGGACCTGGAAAGTGACCACGGTGAGTATTCTTATCCCGCT
ATTATTAGCGAGGGCGGCGTCGTACATATCACCTACACGTGGAACCGGAAAAATATCGTA
TATTGTCGATTACAAACGGTGTAA
>EECEBICD_01578 Cytochrome b561
ATGGCACATTTCTCAAGACTGCAAATAACGCTTCACTGGTTAACACTGCTCCTGACAGGC
ATCGCATATGCTGCTATTGAGTTACGGGGGTGGGCGCCAAAAGGCTCCAGCGTTTACCTG

TTTATGAAAGACACGCACTATGATATGGGCGTTCTGGTCTGGGCGCTGATGTTTTTACGG
TTATATCTTAAACACAAATACCCGGATCCGGTCATTACGCCTCCCCCTCCTCACTGGCAG
CACGTAGCCGCTAAATTAATGCATATCGCGCTTTATCTCACTTTTCTCGCCTTACCGCTG
CTGGGCGTCGCGATGATGGCATCAGGCGGGAAAAGCTGGAGCTTTTTTGGTTTTACTGTA
CCGGTATTTTTGACGCCAGATAGCGCCCTAAAATCGGATATAAAGCGTATTCATGAAATG
CTGGCCAATATCGGCTATTTCCTGATTGCCATGCATGCAGCAGCGGCGTTGTTTCACCAT
TACATACAAAAAGATGATACATTTTCAAGGATGTTACCCGGTAAATCATGA
>EECEBICD_01579 Oligopeptide-binding protein AppA
ATGCTGGGCTGGAGCCACGGCAGCTACTTATTGCTTCATGCGCCGTTGCTTAAGCAAAAC
GCCGACATGAGTTGGGGTAATTTACTGACGGAAAAAGTGGATACCAGCCCCGTCGGTAAA
ATCTGGACGCTAACCTTAAAGCCCGGGCTGAAATTCTCCGATGGCTCGCCATTAACTGCC
GAAGATGTCGTTTTTACATACAATAAAGCGGCGAAGAGCGGCGGTAAAATTGACATGGGC
AATTTTAGCCATGCGCGAGCGCTGGACGCACGCCGAATTGAGATGACGCTGAGCCATCCG
CAGAGCACCTTTGTGAATGTACTCGGGTCGTTAGGGATTGTTCCGGCCAGCCGTTATGAT
GAAAAAACGTTCGCCCGCGAACCGATAGGCGCCGGTCCGTACCGACTGGTCAGCTTTCAG
CCGGGTCAGCAACTGATCGTTGAAGCTAATCCCTGGTATGCGGGTAAAAAGAATGACTTT
AACCGGCTGGTTTTTGTCTTTCTGGATGAAGATAATGCTTATGCCGCCGCGCGCAGCGGA
CAGTTGGGACTGGTGCGCATTGCCCCTTCTATGGCGGTGGCCCCGCAGCAGGATAATCTT
AAACTCTGGGTACGCGATAGCGTTGAAAACCGGGGCATTGTCTTCCCGATGGTGCCTGCC
GGTAAAAAGGATGCTAACGGTTATCCTGTCGGCAACGATGTGACCGCTGATGTCGCTATC
AGACGCGCAATTAACTATGCCATTAATCGTAAGCAACTGGCGGAACAGGTGATGGAAGGC
CATGCGATACCCGCCTATAGCGCGGTGCAGGGATTGCCGTGGCAAAATCCTTCAGTGATA
TTCAGCGATGGCGATATTGCAAAAGCGCGCGCCATCCTGGAAGAGGCTGGCTGGAAGATA
AACCGTGCGGGCGTGCGTGAAAAAGCGGGTAAAGAAGCGCGTCTGACCTTATGGTATGCC
AGTGGCGACAGCACCCGACGGGATCTGGCCGAGGCGGTGCGCGCCATGTTGCAGCCTTTA
GGCATTGTCGTCTCGTTGCAATCGGGAAGCTGGGAAACTGTAGAGCGCCATATGCACGCT
AACCCTACGCTGTTTGGCTGGGGAAGTCTGGACCCGATGGAACTCTTCCATCACTACAGT
GGGAAAGCCGCTGGTGTGGAATATTATAACCCGGGCTATTACAGCAACCCTGCGGTAGAA
GCACATCTGAAACAGGCTATAGATGCGCCTGACTGGCAAAAGGCGATTCCTTTCTGGCAG
CAGGTTGAGTGGGATGGGAAGCAGGGCGCGGGCGTCCAGGGCGATGCGGCATGGGCATGG
CTGCTTAATATTCAGCATACCTATCTGGCCAACCCCTGTATTGATCTGGGAAAAGGCGCG
CCAGAAATCCACGGTAGCTGGTCGGTGTTAAACAATCTTGATGACTGGACCTGGACCTGT
CGGTGA
>EECEBICD_01580 Dipeptide transport system permease protein DppB
ATGAAATTGTTCTTCAGTCATTTTTTGCGGCTGATTATTCTGTTGGTGCTGGTGGCTGCC
GGCACCTTCATTTTGCTTAGCTTTTCGCCCGTTGATCCTATCCGTGCTTATATTGGCAAC
GATCTGCTCCATGTTCCGCCAGAACAATATGCGCGGATTGCGGCGCGCTGGGGGCTCGAC
CAGCCGCTGTGGGAGCGTTTCGGTCACTGGTTTTGGCGTCTGCTCCAGGGTGACATGGGG
TATTCCATGCTGTTCAACATGCCTGTCGCCAGCGTGATCCGGGAACGTTTTGCGACGTCA
TTCGCGCTGCTGGCGGGCGCCTGGCTATTGTCTGGTGTGCTGGGCGTGACATTGGGTTTT
CTGGCCGGACGTTTTCTCCACCGTTGGCCGGATAAGATGATTTGCCGTATCAGCTACTTA
TTGTCATCGTTGCCCACTTTCTGGATAGCAATGCTGTTGCTGGCGCTATTTGCCGTCCGC
TGGCCGGTATTACCTGTTTGTTGCGCCTGGGACCCCGGAAATAATGCAGGAACAGCTCTT
TTATCGGAACGTTTACGCCATCTTGTGTTGCCGGTATGTGCGTTAAGTCTATTGGGCATG
GGGCAAATTGCGCTGCATACGCGAGAAAAAATAGCCAGCGTAATGAAGAGCGAATTTATT
CGTTTTGCGCGCGCTCAGGGGGATAAAGGATGGTCGCTGTTACGTCACCAGGTGTTACGT
CATGCGATTACCCCAGCACTCTGTTTACAGTTTGCTTCTCTGGGCGAACTGATGGGGGGA
GCGTTACTGGCAGAAAAAGTCTTTGCGTATCCGGGGCTGGGACAGGCCACTATCGATGCC
GGTCTGCGTGGCGATGTCCCGCTACTCATGGGGATCGTTTTATTCTGTACACTATTAGTG
TTTGCCGGAAATACGATTTCAGCCTGGCTTGTGGTGGTGCTTAATCGATCGCTGGAGCGT
CCCGATGCTCTATAA
>EECEBICD_01581 Dipeptide transport system permease protein DppC
ATGCTCTATAATCCTGCCCCGACGTTGTTTCGTCTGATCCTCTCCGTTACCTGTCTGCTG
TTTATTGCCGGATACGGTTATGCCACGTTGAGCCAGCCGCCAGAGGTTAATCTGCTTGCC

CGGCATCTGTCGCCTGATATCCAGCACTGGTTTGGCACCGATAATCTGGGGCGCGATGTA
TGGCTTCGTTGCTTTCAGGGGGCCTTCACCAGTTTACAAATTGGCGTTGGCGCCGCGCTG
TGCAGCGGCGTTATCGCGCTAGTGATGGCGGCAGTGGCGCGCATTCATCCCCGGCTGGAC
CTCCTGATGCGGTTGATAACCGATGCTATGCTCGCCATGCCGCATTTACTTTTGCTGATC
CTTATCTGTTTTACGCTCGGCGGCGGTAAAAGCGGCGTCATTGCGGCCGTGGCGTTAACG
CACTGGCCCCGTCTGGCGCTGATTCTGCGCGCCGACGCGGAGCGGGTAGCGCAGAGTGAC
TACCTGACATTGACCTATCGTCTGGGGCATGGACACCTTTATTGCTGGCGATATCACTAT
TTTCCGGCGCTATTGCCGCAATGGTTGACGGGAACGTTATTGATGTTTCCTCATGCGGTA
TTGCACAGCGCGGCATTAAGTTTTCTGGGGTTTGGGCTGGCGCCGCATGAGCCTTCGCTG
GGGCTGTTGCTGGCGGATGCGTTACGGTTTATTAGCCATGGTAACTGGTGGCTGGTGCTG
TTTCCCGGCCTAATGTTATTCACGCTGGTGATGCTCTTCGATCAGTTTGCGCGCGCCATT
CAGCGGCTGTGGCTAAGGAGTGACGTATGCTAA
>EECEBICD_01582 Vitamin B12 import ATP-binding protein BtuD
ATGCTAAGTTTACAACAGGTTACGCTGGAAAGCGCGCGTTATCGCTGGTATGGCACGAGA
CGCTGGTCGCCGTTATTACAGAATGTCTCTTTTGATATCGCACCCGGCGAAATGGTGGCA
TTGGTTGGCGGCAGCGGGGAGGGAAAAAGTCTGCTGCTGCAATGCCTGCTCGATCTGCTG
CCGGAAAACTTACGCTTTCGGGGGGAGATTACGCTTGATGGCAACCGGCTGGACAGACAT
ACCATCAGGCAGCTTAGGGGCAATACGTTTAGCTACGTGCCGCAGGGGGTACAGGCGCTT
AATCCCATGCTGAATATCAGAAAACATTTGAACAGAGCATGTCATCTGACCGGACGCGCC
TGGGATGAGACGCAGATGGTACAGCTATTACAGCAAAGCGATCTTGAACCGACAGTCCTG
GAACGCTTTCCCCGCCAGCTCTCTGGCGGGATGGCTAAACGAATTCTGGCGTGCCACGCC
TCGCTCAGCCAGGCTCGTTATATCCTCGCGGATGAGATCACCGCCTGGCTTGATACCGCA
CTGGCAAACCAGTTGCTTGAGCACTTGCGAGGTCTCTGCGAGCGGGGATGCGGCGTGCTG
TGGGTGACTCATGACCTGCTGCTGGCGGCGCGGTATGCCGATCGCATTGTGGCGCTGCAT
CAGGGTTATATCACGGACAATATCCGCTGCGAGCAGTTACAGCCGGAAGAGATGAGTGAG
CCGTTAAAACGGCAATGGCAAGCATTGCCTGAACTAAACCCGTTATTTATGCCGACCGGA
GAGGGAATAGAATGCTGA
>EECEBICD_01583 Oligopeptide transport ATP-binding protein OppF
ATGCTGAGCTGCCGCGACCTCGTTATTCGTCAGGGCGGGAAAGTCTTATGGCAGAACCTG
ACGTTTAGGATTTCTGCCGGTGAACGGGTAGGAATTCATGCGCCAAGCGGAACGGGGAAA
ACAACGTTGGGGCGCGTCCTGGCCGGATGGCAAAAGCCGACGGCGGGCGACGTTCTGCTG
GACGGCAGCCCGTTACCCCTGCATCAGTATTGTCCGGTACAGCTTGTCCCCCAGCATCCT
GAACTGACGTTTAATCCCTGGCGAAGCGCCGGAGACGCTGTACGTGATGCCTGGCAGCCT
GACCCGGAAACGATGAGGCGTTTGCATGTGCAGCCCGAGTGGCTGACGCGTCGGCCAATG
CAGCTCTCAGGCGGGGAGTTGGCGCGTATTGCGATACTGCGCGCGCTTGACCCACGAACG
CGTTTTCTGATTGCCGACGAAATGACGGCGCAACTCGATCCGTCTATTCAAAAAGCGATA
TGGGTATACGTGCTTGAGGTGTGCCGCAGCCGTTCATTAGGGATGTTGGTGATTAGTCAT
CAGTCTGCGCTACTTGATCAAGTCTGTACCCGCCATTTACAGGTGGAATAG
>EECEBICD_01584 hypothetical protein
ATGGAGTATTTAGCACGACTAAATATCGTTGCGATGCTGATGTCAACGACGTTCTGGATC
AATCTGGCGGTCGTTTTTTTCGTCACCCTCATTACTTACTGGCTCATCAACTGGCTACTT
AATGTGGTCTATAAAGCGCTACAACGCCCGGATAAAAAGAATGATGCTCATGGGCGGCTG
CGGCCTATTGTTTTCGAGATGCTGAAAAAGACCAGTAAGATGCTAATCTTTTTTGCCGCA
TTTCTCTTTAGCCTGCGCTTTGTCGCCCTGCCCGACCGGTTATTCTCTACCCTCTCTCAC
GCCTGGTTTTTAGTGGTTGCGATTCAAATGGCGATTTGGCTTGATCAGGGCGTACAGTCA
TGGATGCGTCATTTACTGTATGCCCCAGGCTCCAATAAAAACCCGGTGACGTTAGTCATT
CTTGGTATGATTTTGCGCGTGCTCGTCTGGTCTATGATGTTGCTCTCCATCCTGGCGAAC
GTCGGCGTGGATATTACCGCACTGGTAGCCAGCCTCGGCGTGGGCGGTATTGCTATTGCC
CTGGCGGTACAAACCGTTCTGAGCGATGTCTTCGCTTCGCTGTCGATTGGTTTCGATAAG
CCCTTCGAGATCGGCGATTTCGTCGTCTTTAATGATGTAGCCGGTACGATTGAACATATC
GGTTTGAAAACCACTCGCATCCGTAGCCTCAGCGGCGAACAGATCGTTTGTGCGAACGCG
CAATTGTTGCAGCAAACCATACACAACTATAAGCGGATGCAAACGCGTCGTATCGTGTTT
ACTTTTGGCGTCGCCACTGCGACACCGCCGGAAAAGTTACGCCTCATCGGCGATATGGTG
AAAAAGATTATTACCGATGTCGGCGAAACACAATTCGACCGCGCCCATCTACTGGCCTTT

GGTCAGGATCGGCTGACCTATGAGGTGGTGCATATCGTCAATACCGCGGATTACAATAAA
TACATGGATATTCAGCAAGAGATAAATATCCGTATTATTGAAAAACTCATTGAGAATGAT
ATCGAACTGGCGCTGCCTAGTCTGGTTGTCAGAGCCCCTGTACAGGTTGAAGAGACCAGG
GACTACAGTAATACCGCTGATGCAAAAAACGCAGATAAACGCGTCATGCAGTAA
>EECEBICD_01585 putative cysteine-rich protein YhjQ
ATGCAACAAGAACATCGTGAGTGTATTGAGCAGTGTTATGAATGCGCGGCGGCGTGTGAT
ATCTGCGCCTCTTCTTGTCTGCGTGAAGATAACGTCGAAATGATGAAGCACTGCATCCAG
TTAGATATGCAATGTGCGGCCATTTGTCGCCTGGCGGCGCAATTTATGGCATTGGAAAGC
GAGTACTCACAAAAATTATGCCGCCTGTGCGCGGATATCTGCAAAGCCTGCGCCGAAGAG
TGTGCAAGGCACGATCACGACCATTGCCAGAATTGCGCGCGGGCATGCAGCCAATGCGCA
GACGCCTGCCTTAAAATGGCCGCGTAA
>EECEBICD_01586 tRNA-Arg(tct)
CCGCCATTAGCTCAACCGGATAGAGCATAGAGCTTCTACCTCTAAGGTTCGGGGTTCAAT
TCCTCGATGGCGGACCA
>EECEBICD_01587 Metal-binding protein ZinT
TTGGTGATTCATTTAAAAAAACTGACAATGCTTCTGGGAATGCTGTTGGTCAATAGTCCT
GCCTTCGCGCATGGTCATCATGCTCATGGCGCGCCGATGACGGAGGTTGAGCAAAAAGCG
GCAGCGGGCGTGTTTGATGACGCGAATGTACGCGATCGCGCGCTCACCGACTGGGATGGT
ATGTGGCAATCCGTCTATCCTTATCTGGTCAGCGGCGAGCTGGACCCTGTCTTCAGACAA
AAGGCGAAGAAAGACCCGGAGAAAACGTTTGAGGATATTAAAGCGTATTATCGCAAAGGA
TACGTGACGAATGTTGAGACGATCGGCATTGAAAATGGCGTTATAGAATTCCATCGCGAC
AATAACGTTGCATCCTGTAAATACAATTATGCCGGCTATAAAATTCTGACTTACGCATCG
GGTAAAAAAGGGGTGCGCTACCTGTTCGAATGTAAAGATGCGAATAGCAAAGCGCCTAAA
TATGTTCAGTTTAGCGATCATATCATTGCCCCGCGCAAATCGGCGCACTTCCATATCTTT
ATGGGAAATACCTCGCAGCAGGCATTGTTGCAAGAGATGGAAAACTGGCCTACCTATTAT
CCTTATCAGTTAAAGGCGAATGAGGTCGTTGACGAAATGCTACATCATTAA
>EECEBICD_01588 Streptomycin 3''-adenylyltransferase
ATGACGCTGTCCATACCGCCCTCGATTCAATGCCAAACGGAAGCGGCGTGTCGCCTGATT
ACTCGTGTCACAGGCGATACGTTGCGTGCTATTCATTTATATGGTTCCGCTGTCGCGGGC
GGTCTGAAGCCAAACAGCGACATTGATCTACTGGTGACAATATGCCAGCTGCTGACGGAG
GCCCAACGCGCAACGCTGATGCAGGAATTGCTGGCGCTTTCATCGCCGCCGGGGGCGTCG
GCAGAAAAACGCGCTCTGGAGGTCACTGTCGTGCTTTATTCGCAGCTGGTTCCCTGGTGT
TTTCCACCTGCCCGGGAAATGCAATTTGGCGAGTGGCTAAGAGAGGACATTTGTCAGGGG
ATTTATGAACCAGCGCAGCAGGACTGGGACATTGTGTTACTGATAACCCAAATTCTGGAA
ACCAGTATACCGCTAAAGGGAGAACGCGCGGAACGGCTGTTCACGCCAGCCCCTGCGGCG
CAGTTGCTGAAGGCCTTGCGTTATCCCCTCGACCTTTGGCAATCCACGGCAGATGTGCAG
GGAGATGAGTATCATATCGTTTTAACCCTGGCGCGTATCTGGTATACCCTTTCTACCGGG
AGATTTACCTCTAAGGATGCGGCGGCTGACTGGCTGTTACCTCAGTTGCCAGAAGATTAT
GCTGCCACGTTGCGTGCGGCGCAGCGTGAATATTTAGGTCTGGAGCAACAGGACTGGCAT
ATTTTGCTGCCTGCGGTCGTACGCTTTGTGGATTTTGCCAAAGCGCACATCCCCACGCAG
TTCACATAA
>EECEBICD_01589 hypothetical protein
ATGACTGGTTCCGCATCATCTGTTTTAAAGACAGATACAAGACTATTGAGCGAAAACCTG
TTTCTTAATTATGGTCTGGAGTCCTTATTTAAGGACGTCGCAATAATAGCTGGCAACGTA
AATTATATTATTTTTGATATCGACGATTACTACACCGTACAGCAGTATATTACTTCCACA
TTTAAAGCGGTACTCATTGGAATTGTTACCCATAATGAATATAACTTTATTGATGAACAT
CATGCTATATATCGAATAAAGGTCGATGCCTCTATTGCTGAGTGGCGCGAATTACTAATT
TTGGCCGCTGCTGGTCAATTTTTTCCCAGGTCAGAGAAAGTCAGATTAACGGCGAATGAA
AGAAATGTTTTGGCGATGCTTGTTAAAGGAATGGATATCCGACAAATAAGCTGTGAGCTG
AATGTACATTTAAAAACTATTTACTCAGTACGCTACCATGTTCTGACTAAATTAGGTTGT
AGAACAGTTCTGGACTATCAAATACTGTCCGTTTCTAAAGCCTTTACGCACTGGCTTACA
ATAAATAATGTTGATAATGTAATTTCCCGTGTAAATAGTAAGGTCATTGCTTAA
>EECEBICD_01590 HTH-type transcriptional repressor BluR
ATGGCTACTTTTAGTATTAGCGCAATGGCGGCGCAGTGTGGTGTCACAACAGCAAATATC

AGGTCCTGGGAACGCTATGGCCTGCTTCAACCTACCAAAGATGAAGCTGGCAATCGTTTT
TATTGCATAGAAGATGCTATCAGAATACAGCATATTATCGACGCGTTAAGCAAAGGCTTT
TCATTAAAAGAGATTAAACCTGCCCTCTATGGTGAAGAAACGCATTGCCGCTCGGGATGG
TTATTCTACCAGGAAGAGATTTTAGCGCAGTGTAGAAAATTCGAACCCGCCAAATTGCGA
AAATTACTCTGGCGTTATGGTCGGGAAATTCCGCCGTGTATCGTAATTGAGTCAATACTG
CGTCCTTTACGGCTGTGGTTGTCCGCAGGCGATGATGATGCACGCCGATTTGAAAAGGCA
TTACTGGATACTGCGATTATTGAATACGCCACGTTCCAGCTAAGTAGCGTACGAAAGGCG
CCAGCAGGCACAATGCTTATTGCTGCGTTTTCGCTGAACGACCCCATTGAGCTATGGCTG
GAAACGATTAAATATTGTAGTGAAGGTATGCGAGTTGAAGTCATCCCCTGGCAGGCGCCG
ATGCCCGATCTTTTATCAACGTCTTTCGAGCATATTGTTCTGTGGCATGATGAGACATTA
ACTCCCGCGCAGGAAAATCTTATTCAGGCTTTAAAAGAATCGGGTAAATTTAGTCTTCAT
GTGAAAAATGGCTCAGGCTTAACATTACTGCCAGCCGTATACCGGCAGCATGAGATGCCC
GAAAGCATGCAGCATGTTGCACCGCAAATCCATGCAAACGGCTACGAAAAATCAGGCACC
GCACGTTGA
>EECEBICD_01591 hypothetical protein
ATGACTACCATTACACTAGTCAACGAACAAAATAGTACTGGCAATCCTTTTTCTGCACAT
ATGTTATGTGAACAGCGAGCTATTCAGGAAATTACTTACGAACTATTGCAATCGCAGCAG
CATGTCTCAAATAAGGATATCATCGCCAAACTTATTGAAAAACTGGAAACAGAAAAAGAC
GTAGTGCAGCTTGATATTTACCGTAATGCCTTAGAAGCCGTTTTGTTTCAGACACCTGAC
GACATCTAA
>EECEBICD_01592 Monofunctional chorismate mutase
ATGATTCGTCATATCGCCATTTTTCTTTGTTCTTTATTGATGTGCAGCACCACTTTTGCC
GATTCGGTAACGTCGGTATCGCTTGGCGCGCTCTTAACCGCGCTCAATGAACGCATGTTA
TTAATGAAAGATGTGGCTGCTTATAAAATGAAGCACCATCTGCCGATAGAGGATTTCACA
CGTGAACAAAATGTTTTTGCCGAGGCTGAAGAAGAAGCGAAAAATAACGGTCTGGACCCG
CATTCGATAACCCCTTTTATTCGTTCGCTAATGGATGCCAGTAAAGCGATACAGTACCGC
TATTTAGCGCAGTGGCGAACCGGCTCGGAACCCTCCTTTCCGATACAAACCTTGTCGGTC
ACCCGGCAACGTATTCGACAACTTGATAATCAAATGTTGATCATTATCAGCCAGCGCTTG
ATGGTCGGCGCTTTCAGCCACGACGATATGGTGTGGCTGAGAGCGCAGTTTAATGCGCCG
AATCTGAATGAGAGCGATATCAGTAATGTGTTGGCAGCCTTATCTTTGGTGCGACGCGCG
CGTTAA
>EECEBICD_01593 Leucine efflux protein
GTGTTCGCGGAGTATGGTGTTCTGAATTATTGGACGTACCTGGTCGGGGCGATTTTTATC
GTTCTGGTACCGGGGCCGAATACGCTTTTTGTGCTTAAAAATAGCGTGGGACGCGGGGTT
AAAGGAGGATATCTTGCCGCATGCGGCGTGTTTATCGGCGATGCGATATTAATGTTTCTG
GCGTATGCGGGCGTGGCGACGCTGATCAAAACAACGCCGGTTCTGTTTAACATTGTTCGC
TATCTCGGCGCGTTTTATCTGCTCTACCTTGGCGCAAAAATCCTTTACGCCACTCTGACC
TCAAAAGGGCGTGCGGCGACGGAAACGGTAGTTCCTTTTGGCGCTATTTTTAAGCGTGCT
TTAATTCTTAGCCTGACGAATCCTAAAGCCATACTTTTTTATGTTTCCTTTTTTGTGCAA
TTTATTGATGTTACTGCGCCGCATACTGGCGTGTCATTTTTTATTTTGGCCACCACCCTT
GAAATTGTCAGTTTTTGCTATCTGAGCTTTTTAATTCTTTCTGGCGCTTTTGTAACCCAC
TATATTGGTACCAAAAAGAAACTGGCAAAAGTGGGCAATTCTTTGATTGGCCTTCTTTTC
GTCGGGTTTGCCGCCCGCCTGGCAACGCTCCAGTCTTAA
>EECEBICD_01594 putative protein YeaR
ATGCGGCAAATTCCACAAAATCATATCCATACACGTTCCACGCCCTTCTGGAACAAAGAG
ACGGCGCCTGCCGGAATTTTCGAACGCCACCTTGATAAGGGAACGCGACCCGGCGTTTAT
CCTCGTCTCTCGGTGATGCAGGGGGCGGTCAAATATCTCGGATACGCTGATGAACATTGC
TCTGAGCCGGAAGAGATCATGGTGATCAATGCCGGCGAGTTTGGCGTGTTTCCTCCGGAA
AAGTGGCACAATATTGAGGTCATGACGGATGATACCTATTTTAACATCGACTTTTTTGTC
GCGCCGGAAGTACTGATGGAAGGGGCGACCTCACGAAAAGTCATTCATGCCGGGAGAGAA
TAA
>EECEBICD_01595 Protein YoaG
ATGGGAAAGGCCACTTATACCGTCACGGTCACGAATAATAGCAACGGCGTCTCTGTGGAC
TATGAAACGGAAGCACCGATGACGTTGCTGGTACCTGAAGTTGCAGCAGAAGTTGTGAAG

GATTTGGTCAATACCGTGCGTTCCTACGATACCGAAAACGAACACGACGTTTGCGGTTGG
TAA
>EECEBICD_01596 hypothetical protein
ATGAAACTCGATACCCGATTAACTTCGTCAGCACTTACTCTTGCGCTTGCCGCTGTGGTC
ATCCCGTTTACGGCGGACTGGCAATTGCCTTTGCTTAACGGCGTGGTGGTCCGATGGATT
GAAAACGGACAAGCGTTATGGTTACTGTTTGGGGCGCTATTTACCGCCTGGTATATACGG
CCACTTTCCCGTCCCGAAGGGGCAAAGCAGTTTTGGCTATGGGCTGTTGTCTGGTGGGTG
GTGCTATTGGGTAGAAGCACCAGTTGGGGGCGTGACTATTTCCCTGACGAGCCGCGTATG
CTGTTCAGAACGATTTCAGTGCTATTAATTGCTGCGTTAGTCTTGCCAGTCCTGTTTTCT
GCCGGGCTCCGTAAGGAGATTGTTCGTCGCCTACGGGACGCTCCTCTGCCATTGTGGCTG
TTTACCGTGACGGCATGTTCATATCTGATTTCCGACACTGTCGAGCATCATCGGTGGTTG
TCGCCGATATTCCTGCATAACGCTCGTTACACAGATTTGATTGAAGAGCTTTATGAAGTG
CCATTTATGATCGGTTTGTTTATGGTGACCGTGGTGTTTATGCAGCAGGACAAGCAGGAT
GAATGTACGGCGCTGGAGATGACCCCGTATCACGCAAAATAG
>EECEBICD_01597 hypothetical protein
ATGGGCATTCTTTCATGGATTATTTTTGGACTTATTGCGGGTATTTTGGCTAAGTGGATC
ATGCCAGGTAAAGATGGCGGCGGATTCTTTATGACGATCATTCTGGGGATCGTTGGTGCG
GTAGTCGGAGGATGGATCAGTACGTTCTTTGGCTTTGGCAAAGTGGATGGTTTCAACTTC
GGCAGCTTTGCAGTCGCGGTGATCGGTGCGATTGTCGTGCTGTTAATCTACAGAAAAATC
AAAAGCTAA
>EECEBICD_01598 hypothetical protein
ATGAAATTTACGTGGATTGCGTTGCCAGGCGTATTGCTGCTGAGCGCTTGTTCATCGTCG
CAACCCAACGCCCCCCGGCCGCCGCGCATTGGGATGCCTAATCCGGCAGCCGTCTACTGT
GAGCAACAGGGCGGTACGCTGATGCCGGTGCAGACCCCACAAGGCGTGCGTTCCGATTGC
AAATTGCCCAATGGCGAAATCATTGATGAGTGGGCATTATGGCGCAGAGAGCACCCTGAT
ACGAAAAAGTGA
>EECEBICD_01599 hypothetical protein
ATGTTTCAGTTAAAGCCGGGCGCGTTGGCAATAGTTATTGGCGCTAAGACACCCGCCGGA
CGGCGGAATATTGGTAAATCGGTTGAGCTATTTTGCCTGTGCCAACCCGGAGATGAATTC
ATTAATCCGGTAAATGGACACGTCACCTTACTGCCGAAAGAAGCGCCTCGGGCGCTGTGG
CTGGTTACGGGCGACGTAGCCTCCGCCGATGGTCAGCACGGTTTTGCCTGGGTGCGAGCA
GAACATTTGATGCCGTTGTCCCCGGACAGACAACCAGGCCAGGCGGCGGCACGACAATCA
CAGCTCTCGTAG
>EECEBICD_01600 hypothetical protein
ATGCGAATCCAGTGTAAGCGCGTCTATTTCCCGGCGGAAAAAGACGATGGTTATCGGGTA
TTGGTTGACAGGCTATGGCCACGCGGCATTAAAAAATCGGCGCTGGTCTATGATGAATGG
AATAAGGCCATTACGCCCTCCACCGAATTGCGCAAAGCCTTTCATAGCGAACTGATTGAT
TTTAACCACTTCGCGCAGCAATACCGGGCAGAACTTGCGCAACAGCATCACGAAGGCAAA
CGGCTGGCGGATATTGCCCGACACCAGCCGCTAACGTTGCTATACGCCGCTAAAGATACT
CGCCAGAACCATGCCATTGTGCTGGCAGAGTGGCTACGAGAGCTGTGA
>EECEBICD_01601 2-nitroimidazole transporter
ATGACGACAGCCCTTTCACCACGCGGCAAACAGGGCGCTTTACTGATTGCCGGTATTTTG
ATGATAGCCACTACGCTGCGCGTAACATTTACCGGCGCTGCGCCGTTGCTGGAGACAATC
CGTTCGGATTACGGTCTTTCCACCGCCCAGACGGGCCTGCTAACAACCTTGCCGCTTCTG
GCGTTTGCACTGGTTTCGCCGCTGGCGGCCGGTATTGCCCGCCGGTTTGGGATGGAACGC
AGTCTGTTTGCCGCAATGCTGCTGATTTGCGCGGGTATTGCTCTCCGCTCCCTGCCCTCC
GCTGCATTGTTGTTTGCCGGAACTGCCATTATTGGGTGCGGGATAGCGCTGGGTAATGTG
CTGCTACCAGGATTGATTAAGCGCGATTTTTCACAACATGTCGCCAGGCTGACGGGCGCG
TATTCATTGACGATGGGCGCCGCCGCCGCGTTGGGGTCCGCGCTGGTGGTTCCGCTGGCG
TTGCACGGTTTTGGCTGGCGCGGCGCGCTGTTAATGCTAATGCTGTTTCCGCTGTTGGCG
TTTCTTATCTGGTTGCCGCAGTGGCGCACTACCCGCTCAGCTAACCTGAGTAGCTCCCGG
GCATTACATGAACGCGGTATCTGGCGTTCGCCTTTAGCCAGGCAAGTTACGTTGTTTCTG
GGACTTAACTCACTGATTTATTATGTGATTATCGGCTGGTTACCAACAATACTCATCAGC
CACGGTTACAGCGAAGCACAGGCAGGATCGCTACATGGCCTGCTGCAACTGGCGACCGCG

GCGCCTGGGTTAGCCATTCCGCTTATTTTGCCTCGCTTTAACGATCAACGCTGGATTGCC
GCGCTGGTCTCACTCTTGTGCGCAGTGGGCGCGGCGGGGCTTTGGTTTGTGCCGGGCCAG
GCGATCATCTGGACGCTACTGTTCGGCTTCGGTTCAGGCGCGACGATGATCCTCGGCCTG
ACGTTTATCGGCCTGCGCGCCAGTTCGGCGCATCAGGCGGCGGCGCTTTCCGGCATGGCG
CAGTCGGTCGGGTATTTACTGGCGGCATGTGGGCCGCCCGTCATGGGAAAACTTCATGAT
GCCAGCGGTAGCTGGTATCTGCCGCTATCGGGCGTAACGGTTCTGGCTATCATCATGGCG
ATTTTAGGCCTCTACGCCGGACGCGATCGAGAGATAGCGTCATAA
>EECEBICD_01602 HTH-type transcriptional regulator NimR
ATGATTGGCCTGAGACTGGACGGTTACGATCCCGACCTTCACCACGATGCCGCTGTGGCC
TTTTGTATTCGCGCCAGGGATGATGAACTGTTTAGCCCTCGCCATCAGCATCGCAAGGGA
CAGTTGATCCTCGCTCTGCACGGCGCGATCACCTGTGAGGTGGAAAATGCGATGTGGATG
GTTCCGCCCCAGTATGCCGTCTGGCTCCCCGGCTCGCTGCCGCACAGTAATCATGTGACC
GCAGGGGCGGAGCTGTGCTTTTTATTTATTGAACCTGCGGCGGTCGTCATGCCGGAGCGT
TGCTGCACGCTGAAAATTTCGCCGTTATGCCGGGAGTTGATTTTGTCGCTGGCGCGCAGA
ACCGATCCCGAAAGAGCGCAAATGCCAACGCAGCGCCTTATTCAGGTGTTGTTTGATGAA
CTGCCGCAGCAGCCGCAGGAACAATTGCAATTACCGGTATCGGGGCATCCGAAAATCCGC
CAAATGGTTGAGACAATGGCGCAGGAGCCCGCGCGATGGAATACGTTAGGGCAGTGGGCC
AGCGTATTCGCGATGAGCGAGCGTAACCTTGCCCGGCTGGTCGTCAAAGAGACGGGGCTA
AGCTTTCGTCGCTGGCGTCATCAGTTGCAATTAATTCTGGCGCTACAAGCGCTGATCGCG
GGACGTAATGTACAGCAGACGGCGCAAATGCTGGGTTATGATTCCACCACGGCATTCATT
ACCATGTTTAAAAAAGGCCTGGGGCAGACGCCAGGCCAGTATCTGACGGGCGAGTTACTG
CTTCCCCACAATTAA
>EECEBICD_01603 hypothetical protein
ATGTTTGATGTCACTCTGCTGATCCTGCTCGGACTGGCAGCTCTGGGATTTATCAGCCAC
AACACCACTGTCGCCGTCTCAATTCTGGTGCTGATTATTGTCCGCGTAACCCCGTTGAAT
ACCTTTTTTCCCTGGATTGAAAAACAGGGGCTCACCGTCGGGATTATCATTCTGACCATC
GGCGTGATGGCCCCCATCGCCAGCGGAACCCTACCGCCCTCCACATTGATCCACTCTTTT
GTGAACTGGAAATCGCTGGTCGCGATTGCCGTCGGCGTTTTCGTCTCCTGGCTGGGCGGT
CGCGGCATTACGCTGATGGGAAACCAACCGCAGCTCGTCGCCGGGCTGCTGGTCGGTACG
GTACTTGGCGTCGCGCTGTTTCGCGGCGTGCCGGTCGGCCCACTGATTGCGGCGGGGCTG
GTCTCTTTAATTGTGGGGAAGCAGTAA
>EECEBICD_01604 hypothetical protein
ATGTACGATCCACCGTTTCTTGAAGCGCTGATGATAACGGCGTCGTTTTTCGCCATTTTT
ATCATTATCGTTGTGTCTGTATTGCTCCTTGAAGGGGAAGGGGACTAA
>EECEBICD_01605 hypothetical protein
ATGGCCGAAATGACTGAAGTCGCAAAGGGTGTCGCTACACACCAACGTTTAGTGGCTTTA
TTAACCCAGGAGAACGCGCGCTATCGTGTGGTGAACCATGAAGCGGTAGGGAAATGCGAA
GCGGTATCTGAAATACGCGGTACTGCGCTGGGCCAGGGCGCAAAAGCGCTGGTCTGTAAA
GTCAAAGGCAACGGCGTCAACCAGCACGTTCTTGCTATTCTGGCGGCCGATCAGCAAGCC
GACCTCTCTCAGCTTGCTTCACATCTGGGCGGCTTACGCGCTTCCCTTGCCAGCCCGGCG
GAAGTCGATATGCTGACCGGCTGCGTTTTTGGCGCGATCCCGCCTTTCAGTTTTCACCCC
AATCTGAAACTGGTCGCCGATCCGCTCCTGTTTGAGCGTTTCGATGAAATTGCCTTTAAT
GCCGGCCTGCTGGAAAAAATCAGTCATTATGAATACACAGGACTATCTGCGCATCGCCCGA
CCAGAACTGGCAGTCTTCCGCCGCTTTCAGTCATCGCCAACGGCTTAG
>EECEBICD_01606 putative diguanylate cyclase DgcJ
ATGAATTTGCATCATAAAGCGCTCAGGCACTTTATCTCGGCAAGCGTCATCGTTTTGACA
TCGTCCTTCCTGATTTACGAACTTATTGCCAGCGACAGGGCAATGAATGCCTATATGCGT
TATATCATGGAGAGAGCCGATTCGTCGTTTTTGTACGATAAGTACCAGAATCAGAGCATC
GCCGCACATTTGATGCGAACTTTTGAAGCGCCGGGAGACCCCGTCACTGCAGAAAAACGC
CGCGCGTTTTGCGACGCCTTTGAGGCTATTAACGGTACGCACGGCGTCAACCTGACCCGG
CATAATTATCCGGGGCTACATGGCACCCTGCAAACCGCCGCTACACAGTGTACCGATAAT
CTTGATGACGCCCTTTTACTGCCTGCATTTGATCAGGCGGTAAGCATCAACCGTTCGCAG
GACGACCACAGTCACGGGCTGGGCACACTGGAGCTTAAATTCCGTTATTACGTTGATTTA
AATAAACATTATGTCCATTTCTATGATTTAATCAACTCACGGCGCTTCGCCATGCATCGT

TGGACTTTTTTACAAAAAGGCACAATGGGTATTAACAGAAAAGATATAGATAAACTTTTT
ACCGGCCGTACGGTTATTTCAAGTATTTACATGGATGATATTACCCAGGAAAACGTCATG
AGCTTTTTAACGCCAGTCTATCTGGCGGGAACGTTAAAAGGTATCGTGATGGTGGATGTT
AACCAGGATAATTTAAAAAATATTTTTTATACCCAGGACCGTCCGCTGGTTTGGCGTTAT
CTTAACGTAACACTAAAGGACATGGACTCCGGAAAAGAAATTATTATTAATCAAAGCAAA
AATAATCTGTTTCAATATGTGAATTATAGCCATGATATCCCGGGTGGACTGCGCGTTTCG
TTGTCCCTTGATTTAACCTATTTCCTTGTCTCGTCCTGGAAAGCGCTGGCCTTTTACTTA
CTGGCAACGGCGCTCCTGCTTAATATGGTACGGATGCACTTTCGGCTTTATCGCAACGTC
ACACGCGAAAATATTAGCGATGCCATGACCGGGCTTTACAACCGTAAAATATTAACACCG
GAGCTGGAACAGCGACTGCAACGCCTGGTCAATGCCGGGACGCCGGTGACATTTGTCGCT
ATTGATTGCGATAGGTTAAAACTGATCAACGATACCCAGGGGCACCAGGAAGGCGACCGA
ATTATTACCCTGTTGGCGAAAGCGATTAAAACATCGATTCGTAAAAGCGATTACGCCATT
CGCCTCGGCGGCGATGAGTTCTGTATTATTCTTGTTGATTACGCGGCGGATTTGGCTATC
CATCTGCCGGAGCGTATTATTCGTAACCTGCAAATTATTGCACCGGATAAGACAGTCCAT
TTTTCCGCCGGGATTTATAATATGCAGCCCAATGATACGATTAATGATGCCTACCAGGCT
TCCGATGCGCAGCTCTATCTGAACAAACAGCAAAAACAACATCGTTCATCATAG
>EECEBICD_01607 hypothetical protein
ATGACCTGGTTCATAGACCGACGTCTTAACGGCAAAAATAAAAGCACGGTGAATCGCCAG
CGCTTTTTGCGCCGTTATAAAGCACAAATTAAGCAGTCAATTTCCGAAGCGATTAATAAA
CGCTCTGTGACCGATGTCGACAGCGGAGAGTCCGTCTCTATTCCAACCGATGATATTAGC
GAACCGATGTTTCATCAGGGGCGCGGCGGTCTGCGCCATCGCGTCCATCCGGGTAACGAT
CACTTTATCCAGAATGATCGCATTGAGCGTCCGCAAGGCGGTGGCGGCGGCGGTTCCGGC
AGCGGTCAAGGTCAGGCCAGCCAGGACGGCGAAGGCCAGGATGAGTTTGTTTTTCAGATT
TCAAAAGATGAATATCTGGATCTGCTCTTTGAAGATTTAGCGCTGCCTAATCTGAAGAAA
AACCAGCATCGCCAGCTTAACGAGTATAAAACTCACCGCGCCGGTTTCACCTCAAACGGC
GTACCGGCCAATATCAGCGTGGTACGTTCGCTACAAAACTCTCTGGCGCGCCGTACAGCA
ATGACGGCAGGAAAACGCCGCGAACTGCACGCGCTGGAAACGGAACTGGAGACCATCAGC
CATAGCGAACCAGCGCAACTGCTTGAAGAGGAGCGGTTACGTCGGGAAATTGCCGAACTA
CGGGCTAAAATCGAGCGAGTGCCGTTTATCGACACCTTTGATTTACGCTATAAAAATTAT
GAAAAACGGCCTGAGCCCTCCAGCCAGGCGGTGATGTTCTGTCTGATGGACGTCTCGGGT
TCGATGGACCAGGCAACCAAAGATATGGCCAAGCGTTTTTACATTCTGCTCTATCTGTTT
TTGAGCCGAACATATAAGAACGTAGAAGTGGTTTATATCCGCCACCATACCCAGGCGAAG
GAAGTGGACGAACATGAGTTCTTTTATTCGCAAGAGACCGGGGGGACGATTGTCTCCAGC
GCGCTTAAACTCATGGATGAAGTGGTTAAAGAGCGCTACGACCCGGGGCAGTGGAACATC
TATGCGGCGCAAGCGTCAGACGGTGATAACTGGGCCGACGATTCACCGCTGTGTCATGAG
ATTCTGGCGAAAAAGCTGCTGCCGGTAGTGCGCTATTACAGCTATATCGAGATTACCCGC
CGCGCCCACCAGACCTTATGGCGCGAGTATGAACATCTGCAGGCGACGTTCGATAACTTC
GCCATGCAGCATATTCGCGATCAGGAGGATATTTATCCGGTATTCCGCGAATTGTTTCAG
AAACAGAGCGCCAATCAAAGCGTATAA
>EECEBICD_01608 hypothetical protein
ATGAATATATTCGATCACTATCGCCAGCGTTACGAAGCTGCCAAGGACGAAGAGTTCACA
CTGCAGGATTTTCTTACCATTTGTCGGCAAGATCGCAGTGCTTATGCCAACGCGGCAGAG
CGGCTATTAATGGCTATCGGCGAGCCTAATATGGTCGACACTGCCCAGGAACCCCGACTT
TCCCGACTCTTCTCCAACCGGGTTATTGCTCGCTATCCTGCGTTTGAAGAGTTTTACGGC
ATGGAAGACGCGATTGAACAGATTGTCTCTTATCTGAAACATGCGGCGCAAGGGCTGGAA
GAGAAGAAGCAAATTCTCTATTTGCTGGGACCTGTGGGGGGTGGCAAATCATCGCTCGCT
GAACGGCTGAAATCGCTAATGCAGCGCGTGCCAATTTATGTTTTAAGCGCCAACGGCGAG
CGTAGTCCGGTCAACGATCATCCGTTATGCCTGTTTAATCCGCAGGAAGACGCGCAGATT
CTGGAAAAAGAGTACGGTATTCCGCGCCGCTATCTCGGTACGATTATGTCCCCGTGGGCG
GCCAAACGCCTGCATGAGTTTGGCGGCGATATTACTAAATTCCGCGTGGTGAAAGTCTGG
CCCTCCATTCTGGAGCAGATTGCCATCGCCAAAACGGAACCCGGCGATGAAAACAACCAG
GATATTTCGGCGCTGGTCGGCAAGGTGGATATTCGTAAACTCGAACATCACGCCCAGAAC
GATCCGGATGCGTATGGTTACTCTGGCGCGCTGTGCCGCGCTAACCAGGGGATCATGGAG
TTCGTCGAGATGTTTAAAGCGCCTATTAAGGTGCTGCACCCCTTGCTGACCGCAACTCAG

GAAGGTAACTACAATGGTACGGAAGGTATTTCCGCCCTGCCCTTTAATGGCATCATACTT
GCCCACTCAAACGAATCTGAATGGGTGACTTTCCGTAACAATAAAAATAACGAAGCGTTC
CTCGACCGTGTCTATATCGTGAAGGTGCCTTATTGCCTGCGAATCTCCGAAGAGATCAAA
ATCTACGAGAAACTGCTTAATCATAGCGAGCTGGCTCATGCGCCCTGCGCGCCGGGCACG
TTGGAAACGCTTTCGCGCTTCTCGATTCTTTCCCGCCTGAAAGAGCCGGAAAATTCCAGT
ATTTACTCGAAAATGCGCGTTTATGACGGTGAAAGCCTGAAAGATACCGACCCTAAAGCT
AAATCGTACCAGGAGTATCGCGACTATGCGGGCGTGGACGAAGGGATGAACGGTCTCTCC
ACCCGATTCGCCTTTAAGATCCTCTCCCGCGTATTCAACTTCGACCATGCGGAAGTGGCG
GCAAACCCGGTACATCTGTTCTACGTTCTGGAACAACAAATCGAGCGTGAACAGTTCCCG
CAGGAGCAAGCTGAGCGTTATCTGGAGTTCCTGAAAGGCTACCTGATCCCGAAATACGCC
GAATTTATCGGCAAGGAGATCCAGACGGCCTATCTCGAATCTTATTCTGAGTACGGGCAG
AACATTTTTGATCGTTACGTGACTTATGCGGATTTCTGGATTCAGGATCAGGAGTATCGC
GACCCGGATACCGGTCAACTGTTCGATCGCGAGTCGCTGAACGCCGAACTGGAAAAAATT
GAAAAACCAGCGGGTATCAGTAACCCGAAAGATTTCCGTAATGAGATAGTAAACTTTGTC
CTGCGCGCCAGAGCCAACAATAGCGGCCGTAATCCAAACTGGACCAGTTACGAGAAGCTG
CGCACGGTGATTGAGAAAAAAATGTTCTCTAATACTGAAGAGCTGTTGCCGGTCATTTCT
TTTAACGCCAAAACGTCGACTGACGAGCAGAAAAAGCATGACGACTTTGTCGATCGGATG
ATGGAGAAAGGATATACCCGTAAGCAGGTTCGTTTGCTGTGCGAGTGGTACCTGCGCGTG
CGTAAATCGTCGTGA
>EECEBICD_01609 MltA-interacting protein
GTGACCAAACTTAAACTTCTGGCTCTTGGCGTGTTTATTGCAACATCCGCCAGCGTCGCG
CACGCTGAAAGCAACCTGACGTTGGGCGCAGGCGTTGGTGTGGTTGAACACCCTTATAAA
GATTACGATAGCGATGTTTATCCGATACCGGTCATCGCTTATGAAAGCGAAAACTTCTGG
TTCCGTGGTCTGGGCGGCGGGTATTACTTGTGGAATGACAATGCCGATAAGTTGTCAATC
ATGGCATACTGGTCCCCGATGTACTTCAAACCGGGCGACAGCGATGACCATCAGTTGCGT
CGTCTGGACAGGCGTAAAAGTACTATGATGGCGGGCGTTTCTTACGCGCATCACACCCAG
TATGGATTCCTGCGTACCTCACTGGCAGGGGATACGTTGGATAACAGTAACGGTATTGTC
TGGGATCTGGCCTGGCTGTATCGGTATACCAATGGCGGGCTGACCTTAACGCCAGGTATC
GGGGTTGAATGGAATAGCGAGAACCAGAACGATTACTATTACGGCGTATCACGTAAAGAA
TCTTCGCGTAGCGGCCTGCGCGGCTATAACCCGAACGATAGCTGGAACCCGTATCTTGAA
CTTAGCGCTAACTATAATTTCGCCGGTAACTGGAGCGTATATGGTACTGCGCGCTACACC
CGCCTGTCGGATGAAATTACCGACAGCCCGATGGTGGATAAATCCTGGACCGGTATCCTT
TCTACTGGCGTGACCTATAGATTCTGA
>EECEBICD_01610 Anaerobic sulfatase-maturating enzyme
ATGAGCATAGTATTCAATACCGTAGCCAAGCCCAGCGGCAGTCTGTGTAACTTATCCTGC
AAGTACTGTTTCTATCTTGATAAACCCCGGGGGCAGCGCGTCATTTCTGACGATGTGCTG
GAGACGTATATCCGCCGGGTAATTGATGATACGCCATCCTCAGAGGTCTCGTTTTGCTGG
CAGGGGGGAGAGCCGACGCTATGCGGTCTTTCTTTTTACCAAAAAGTGGTGCGCTTGCAG
CAACGCTATGCCAACGGCAAAACTATCTACAACAGTCTGCAAACCAATGGCGTATTAATC
AATGAAGAGTGGGCGGCTTTCTTTGCGCAGCACCAGTTCCTGATTGGTATATCGATTGAT
GGGCCGCAAGTCGTTCATGATAATTACCGGAAAACGCCGTCAGGGCGGGCGTCTTTTTCC
CGAGTCGTTAATGCTATCCGCCTTCTGCAGGCAAATGATGTCGAGTTCAACACGCTCACT
GTCGTGAATGATGCGTCATGCCGTCATGGCAACGCTATTTATCATTTTTTGACGCAGGAA
CTGGAAAGTAAACACCTGCAATTTATTCCCATTGTTGAGCCGCTCGCGCAAAAAGCGCAG
CGTTCTTTGACGTTATCTGACAATGAGGATTCGCCTTCGCTGATGCCCTTTTCCGTCACG
CCTGAAGGGTGGGGCGCCTTTATGTGCGATGTTTTTGATCAATGGATACGTCACGATGTC
GGACGCATATTCGTACAGCTTTTTGATAACTTACTTGGCGTCTGGATGGGGGAGCCCGCC
ACGCTTTGTACGATGCAGTCGACCTGCGGGCAAAGTTTGCTGGTGGAGCAGAATGGCGAC
GTGTTTAGCTGTGACCATTTTGTTTTTCCCGCCTATAAACTGGGCAATCTGCAGCAACAC
TCTTTAGAAGAAATGGCGGCCTCTCCTTTTCAGCAGCAGTTTGGCGCGGCTAAAGCAAAC
CTTTCCTCACGCTGCCAGAACTGTACGTGGCGCTTTGCCTGTCACGGCGGTTGTCCGAAA
CATCGAATTTGTATGGACGGCGGCGAACGGCAAAATTATCTCTGTAAAGGATATCTGGAG
TTCTTTCAACATGTGACGCCCTATATGAATGTGATGCGTCAATTATTACTAAATCAGCGA
CCCGCCGCGCATATTACCCGCATCGTCGACATGATTGCGGATGACGTTCGTCAGTGA

>EECEBICD_01611 putative oxidoreductase
GTGAAAAACGTAACGTTTTGCGGGCAGGTGACGCTTCCTGCCATCGGACAGGGAACATGG
TATATGGGCGAGCGCGCGGATCAACGTCAGCGCGAGGTTTCGGCGCTGCGCGCGGGGCTG
GATTTGGGGTTACGCCTTATTGATACGGCGGAAATGTATGCCGACGGCGGGGCGGAAAAG
GTCGTGGGCGAGGCGCTGGCAGGGCGACGCGATGAGGCATTTCTGGTGTCAAAAGTGTAC
CCGTGGAACGCTGGCGGCCAGAAGATAGTTGCCGCCTGTGAGGAAAGCCTGCGTCGTCTG
AAAACAGATTATCTCGATCTCTATCTATTGCACTGGCCGGAAGCTTTTCATTCGAAGAG
ACCGTTGAAGGTATGGAGCGGCTAATCGCGCAGGGGAAAATTCGCCGTTGGGGAGTCTCT
AATCTTGATTATGACGATATGCAGGCGCTGTGGCGCATCCCTGGGGGACAGCAATGCACG
ACAAATCAGGTGCTCTATCATCTGGCGTCACGCGGTATTGAGTATGATTTATTGCCGTGG
TGTCAACAACAGCGAATGCCAGTGATGGCCTATAGCCCGCTGGCGCAGGCTGGCCGGTTA
CGTAGCGGTTTACTGAATCATCCGGTGGTGAATGAAATTGCGCAAGCGCATAACGCAACG
GCGGCGCAAATCTTACTGACGTGGGTGATTGCACATCCGGGCGTGATGGCGATCCCGAAA
GCCGGAAGTACAGAACACGTGAAGCAAAATGCGGCGGCGCTTGAGATCGCCTTGTCAGCA
AACGAACTTGCCTTACTGGACAACGCCTGGCCTGCGCCAAAAGGGAAAACCGCGTTGGAT
ATGGTGTGA
>EECEBICD_01612 Putative glucose-6-phosphate 1-epimerase
ATGATTAATAAAATTTTTGCCCTCCCGGTTATCGAACAGCTTACCCCTGTCCTTTCCCGT
CGTCAGCTTGATGACCTGGATCTGATCGTCGTTGACCATCCGCAGGTTAAAGCCTCTTTT
GCATTACAGGGCGCGCACCTTCTCTCCTGGAAACCCGTTGGCGAGGAAGAGGTATTGTGG
CTCAGTAATAATACGCCTTTCAAAACCGGCGTGGCGCTACGCGGCGGTGTACCGATCTGC
TGGCCCTGGTTTGGACCTGCGGCTCAACAGGGTCTACCGTCACACGGCTTTGCGCGTAAT
CTACCGTGGGCGCTAAAAGCGCATAATGAAGATGATAATGGCGTAATGCTGACATTTGAG
CTGCAAAGCAGTGAGGCCACCCGGAAATACTGGCCGCACGATTTCACGCTGCTGGCACGT
TTTAAAGTGGGCAAAACCTGTGAAATTGAACTGGAAGCGCATGGCGAGTTTGCAACCACA
TCCGCGCTCCACAGCTATTTTAACGTTGGCGACATCGCCAACGTTAAGGTCAGCGGACTC
GGCGATCGGTTTATTGATAAAGTTAACGACGCTAAAGAAGGTGTCCTGGCCGGCGGTATA
CAAACCTTTCCGGACCGTACCGATCGCGTTTACCTCAATCCTGAAGCATGTAGCGTGATC
CACGATGCGACATTAAACCGCACGATTGATGTAGTTCACCACCATCACCTTAACGTCGTA
GGCTGGAACCCTGGCCCGGCGTTGTCCGTCAGCATGGGCGACATGCCGGATGACGGCTAT
AAAACGTTTGTCTGCGTTGAAACGGTTTACGCTACTGCGCCGCAACAAGCCACAGAAGAA
AAACCCTCTCGTCTGGCGCAGACTATCTGCGTCGCTAAACGCTAA
>EECEBICD_01613 Glyceraldehyde-3-phosphate dehydrogenase A
ATGACTATCAAAGTAGGTATCAACGGTTTTGGCCGTATCGGTCGCATTGTTTTCCGTGCT
GCTCAGAAACGTTCTGACATCGAGATCGTTGCAATCAACGACCTGTTAGACGCAGAGTAC
ATGGCATACATGCTGAAATATGACTCCACTCACGGCCGTTTCGACGGTACTGTTGAAGTG
AAAGACGGTCATCTGATCGTTAACGGTAAAAAAATCCGTGTTACCGCTGAACGCGATCCG
GCTAACCTGAAATGGGACGAAGTTGGTGTTGACGTAGTGGCTGAAGCTACCGGTATCTTC
CTGACTGACGAAACCGCGCGTAAACACATCACCGCTGGCGCGAAAAAAGTGGTTCTGACG
GGTCCATCTAAAGACAACACCCCAATGTTTGTTAAAGGCGCTAACTTTGACAAATACGAA
GGCCAGGACATCGTTTCCAACGCTTCCTGCACCACCAACTGCCTGGCGCCGCTGGCTAAA
GTTATCAACGACAACTTCGGCATCATCGAAGGTCTGATGACTACTGTTCACGCGACTACC
GCAACCCAGAAAACCGTTGACGGCCCGTCTCACAAAGACTGGCGCGGCGGCCGCGGCGCA
TCTCAGAACATCATCCCGTCCTCTACTGGCGCTGCTAAAGCGGTAGGTAAAGTACTGCCA
GAACTGAATGGCAAACTGACTGGTATGGCGTTCCGCGTTCCGACTCCGAACGTATCCGTT
GTTGACCTGACCGTTCGTCTGGAAAAAGCGGCTACCTACGAGCAGATCAAAGCTGCTGTT
AAAGCTGCTGCCGAAGGCGAAATGAAAGGCGTTCTGGGTTACACCGAAGACGACGTTGTA
TCTACCGATTTCAACGGCGAAGTATGCACCTCCGTGTTCGATGCTAAAGCAGGCATCGCG
CTGAACGACAACTTCGTGAAACTGGTCTCCTGGTACGATAACGAAACCGGTTACTCCAAC
AAAGTACTGGACCTGATTGCTCACATCTCCAAATAA
>EECEBICD_01614 Peptide methionine sulfoxide reductase MsrB
ATGGCTAACCAACCTTCAGCAGAAGAACTCAAGAAAAAATTGTCCGAAATGCAATTCTAC
GTGACGCAGGATCGTGGCACAGAACCGCCATTTACCGGACGCCTGCTACATAATAAACGC
GATGGGGTTTATCACTGCCTGGTCTGTGATACGCCGCTTTTTCACTCCCACACCAAATAC

GATTCAGGCTGCGGCTGGCCTAGCTTTTACCAGCCTGTCAGCGAAGAGGCGATTCGTTAC
ATAGACGATTTTTCTCATGGCATGCAGCGTGTCGAAATTCGTTGCGGTAACTGCGATGCG
CATCTCGGACACGTTTTCCCGGACGGGCCGCAGCCAACCGGTGAGCGCTATTGCGTTAAC
TCGGCCTCGCTGGCCTTTTCTGACGAGAAAAACGGCGATCAACTGAAAGGCTGA
>EECEBICD_01615 hypothetical protein
ATGAATCTTGATGAGATCATTAACAGCATGACGCCGGAAGTGTATCAACGTTTATCCACC
GCCGTTGAGCTGGGTAAATGGCCGGATGGCGTGGCGTTGACGCCGGAACAGAAAGAAAAC
AGCCTGCAACTGGTGATGCTATGGCAGGCGCGGCACAATACCGAAGCGCAACATATGACG
ATCGATACTCACGGTCAGATGGTGATGAAAAGCAAGCAGCAGCTCAAAGCGGATTTCGGT
ATTACGTCAAAGCCGATTGCGACATTGAAAATGCAGTAA
>EECEBICD_01616 D-arabitol-phosphate dehydrogenase
ATGAAAGCACTGGCGAGATTTGGCAAGGCCTTTGGCGGCTACAAAATGATTGATGTCCCG
GAGCCGGTATGCGGTCCGGAAGACGTGATTATCGAAATCAAAGCGGCGGCGATCTGCGGC
GCGGATATGAAGCACTATAACGTTGACAGCGGGTCCGACGAATTTAACTCCGTTCGCGGT
CATGAATTCGCCGGACGTATCGCGCGGGTGGGCGAAAAAGTGAAAGACTGGAAAGTCGGC
CAGCGCGTGGTTTCTGACAACAGCGGACACGTTTGCGGCGTTTGCCCTGCCTGTGAACAA
GGCGATTTTCTGTGTTGTACAGAAAAAGTGAATCTGGGTCTGGATAACAACACCTGGGGC
GGTGGATTTTCCAAATACTGCCTGGTTCCGGGAGAAATCCTGAAAATCCATCGCCATGCC
CTGTGGGAGATCCCGGAAGGCGTGGATTACGAGGAAGCCGCAGTACTGGACCCTATCTGC
AACGCCTATAAATCTATCGCCCAGCAATCCAAATTTTTACCCGGCCAGGACGTGGTAGTC
ATCGGCACCGGTCCGTTAGGTCTGTTTTCGGTCCAAATGGCCCGCATTATGGGGGCGGTA
AATATCGTGGTGGTTGGCCTGGATGAGGATGTTCCTGTCCGTTTCCCTGTGGCCAAAGAA
CTCGGCGCGACGGCGTTTGTTAACGGCTCCACGGAAGATGTCGTCGCACGCTGCCGGGAG
ATTTGCGGCAAAGACAACCTCGGTCTGGTGATCGAATGCTCCGGTGCCAACATTGCGCTT
AAACAGGCCATCGACATGCTGCGGCCAAATGGTGAAGTGGTTCGCGTCGGTATGGGGTTT
AAGCCGCTCGATTTCTCCATTAACGACATTACTTCCTGGAACAAAAGCATCATTGGGCAT
ATGGCCTATGACTCCACATCCTGGCGCAATGCGATTCGCCTGCTTGCCAGCGGCGCTATC
AAAGTAAAACCGATGATCACCCATCGTATCGGTCTTTCTCAGTGGCGCGAAGGGTTTGAC
GCAATGGTCGATAAAACAGCCATTAAAGTGATCATGACCTACGACTTTGATGAATAA
>EECEBICD_01617 Inner membrane metabolite transport protein YdjE
ATGGAACAGATAACAAAACCGCACTGCGGCGCCCGGCTGGATCGCCTGCCGGATTGCCGG
TGGCATTCATCAATGTTTGCCGTCGTCGCCTTTGGTCTGCTTGTTTGCTGGAGTAATGCC
GTCGGCGGATTAATTCTTGCCCAGCTCAAGGAGTTAGGCTGGACCGATAATTCCACGACA
GCCACCTTCTCCGCCATTACCACGGCGGGGATGTTTCTCGGCGCGCTGGTGGGCGGCATT
ATCGGCGATAAAACCGGACGCAGGAACGCCTTTATTCTTTATGAAGCGATTCACATTGCC
TCGATGGTCGTGGGCGCATTTTCACCGAATATGATGTTCCTGATTGCCTGTCGTTTTGTG
ATGGGCGTGGGATTAGGGGCACTACTGGTCACGCTGTTCGCCGGATTTACCGAGTACATG
CCCGGCAGAAACCGGGGAACCTGGTCGAGTCGCGTTTCGTTTATTGGCAACTGGTCATAT
CCATTGTGTTCCCTGATTGCAATGGGGCTGACGCCGCTAATTAGCGCGGAATGGAACTGG
CGCGTGCAGTTACTGATCCCGGCTGTGCTGTCGCTGATTGCTACCGCGCTTGCCTGGCGA
TACTTCCCTGAATCTCCCCGCTGGCTGGAATCGCGCGGGCGTTATCAGGAAGCCGAAAAG
GTTATGCAGGCCATTGAAGCAGGCATCGAACGTCAGACAGGAAAACCGCTGCCGCCGGTT
GTTATTGAAAACAGCGGCAAGCCGCCGCGTTCCGTACCGTATTCAGCGTTGCTCACTGGC
GTATTACTGAAACGCGTTATTTTGGGTTCCTGCGTGCTGATTGCCATGAACGTTGTGCAA
TACACGTTAATTAACTGGCTGCCCACCATCTTTATGACACAGGGCATCAACCTCAAAGAT
TCGATCGTTTTAAATACCATGAGTATGTTCGGCGCACCGTTTGGCATATTTATCGCTATG
CTGGTAATGGATAAAATTCCGCGCAAAACCATGGGTGTCGGTTTATTAATTCTGATTGCC
GTGCTGGGATATATTTATTCGCTGCAAACCAGCATGTTGCTTATCACCTTAATTGGCTTT
TTCCTGATTACCTTTGTCTATATGTATGTCTGTTATGCCTCGGCGGTCTATGTGCCGGAG
ATCTGGCCAACAGAAGCGAAACTTCGCGGTTCCGGCCTGGCGAACGCTGTCGGCAGAATC
AGCGGTATCGCTGCCCCCTATGCGGTGGCGGTATTACTCAACGGTTATGGCGTGACGGGC
GTTTTCGTCCTGCTGGGCGCTGTCTCGATTATCGTCGCCGTCGCAATAGCGACTATCGGA
ATCGAGACCAAGGGTGTTTCGGTTGAAAGTCTGAGCATTGATAAGGTCACGAGTAAATAA

>EECEBICD_01618 putative zinc-type alcohol dehydrogenase-like protein YdjJ
ATGAAAAATTCAAAAGCGATACTAAAAACGCCGGGCACCATGACAATTATAGCGGCTGAT
ATTCCAGTACCAAAAGAAAACGAAGTATTGATCAAAGTGGAATATGTCGGTATTTGCGGT
TCAGATGTTCACGGTTTTGAATCCGGGCCATTCATTCCGCCGAAGGATCCAAATCAGGAA
ATTGGTCTCGGTCATGAGTGTGCTGGTACGGTCGTTGCGGTCGGCAATCGGGTAAGCAAA
TTTAAGCCAGGCGATCGGGTTAATATCGAGCCGGGCGTGCCGTGCGGCCACTGCCGCTAT
TGTCTGGAAGGAAAATACAATATTTGTCCGGATGTTGATTTTATGGCGACGCAGCCGAAT
TATCGCGGGGCCTTAACGCACTATCTGTGCCATCCGGAAAGTTTTACGTACAAGCTTCCG
GACAATATGGACACTATGGAAGGTGCGCTGGTGGAACCTGCTGCTGTTGGAATGCACGCG
GCAATGCTGGCGGATGTTAAACCGGGTAAGAAAATCGTCATTCTCGGCGCGGGCTGCATT
GGTTTAATGACCCTGCAAGCGTGTAAGTGTCTGGGGGCGACCAATATCGCGGTAGTGGAT
GTGCTGGAAAAACGGCTGGCAATGGCTGAACGACTGGGCGCGACAACCGTTATCAATGGG
GCGAAAGAAGATACTGTCGCGCTCTGCCAGCAGTTCACCGACGATATGGGCGCCGATATT
GTGTTTGAAACCGCCGGTTCCGCCGTCACAACTCAGCAAGCGCCGTATCTGGTCATGCGC
GGCGGGAAGATCATGATTGTTGGCACTGTCGCAGGAGATTCAGCGATTAATTTCCTCAAA
ATTAACCGTGAAGTCTCCATCCAGACGGTATTCCGCTATGCCAACCGCTATCCGGTGACT
ATTGATGCCATCTCCTCCGGGCGTTTCGATGTGAAATCAATGGTGACGCATATTTACGAT
TACAAAGACGTACAACGTGCATTTGAAGAGTCGGTGAATAACAAACGCGACATTATTAAA
GGCGTTATTAAAGTTTGCGATTAA
>EECEBICD_01619 putative protein YdjI
ATGTTGGCAGATATAAAATATTGGGAAAATGATGCACAAAATAAACATTACGCGATTGCG
CACTTTAATGTGTGGAATGCGGAAATGTTGATGGGAGTGATTGATGCGGCGGAAGAGGCA
AATTCGCCGGTTATTATCTCCTTTGGTACCGGATTTGTGGGCAATACTTCATTTGAAGAC
TTCTCTCATATGATGGTTTCCATGGCTAAAAAAGCCACTGTGCCGGTAATTACCCACTGG
GATCATGGACGTAGCATGGACATTATCCATAATGCCTGGACGCACGGCATGAACTCGCTG
ATGCGTGACGCTTCTTCCTTTGATTTTGAAGAAAATATTCGCCTGACCAAAGAAGCCGTG
GATTTCTTCCATCCGCTGGGTATTCCGGTAGAGGCGGAGTTAGGACACGTAGGGAACGAA
ACGGTGTATGAAGAGGCGTTGGCGGGGTATCACTATACCGACCCGGATCAGGCGGCGGAA
TTTGTAGCGCGCACCGGTTGTGACTCGCTGGCGGTCGCCATCGGCAACCAGCATGGCGTG
TATACCTCTAAACCAAAACTGAATTTTGAGGTCGTGGAGCGCGTGCGCCAGGCAGTTTCC
GTACCGCTAGTGTTACACGGCGCTTCCGGTATTAGCGACGCGGATATCAAAAAAGCCATT
TCGCTGGGCATCTCGAAAATCAATATCCACACCGAACTGTGTCAGGCCGCGATGGTAGCG
GTAAAAGAGAATCAGGATCAGCCGTTCCTGCATCTGGAACGCGAAGTACGCAAAGCGGTA
AAAGCCCGTGCGCTGGAAAAAATCAAACTGTTTGGTTCAGATGGTAAAGCGGAATGA
>EECEBICD_01620 putative sugar kinase YdjH
ATGGACGAAATTGAAGTTCTTTGCATAGGGGCGGCCATTGTCGACATTCCTTTGCAACCG
GTTAGTAAAAATATCTTTGATGTCGATTCGTATCCATTAGAACGAATTGCCATGACCACA
GGGGGAGATGCGATTAATGAAGCCACGATTATTTCGCGTCTCGGGCATCGCACTGCGCTA
ATGAGTCGCGTTGGCGACGATGCGGCAGGATATTTTATTCTCGACCACTGTCGGAAGGAG
AATATCGATATTCAGAGCCTGAAGCAGGATGCGGATATTGATACCTCAATTAATGTTGGG
CTGGTGACTGATGATGGTGAGCGCACCTTTGTCACTAACCGTAACGGCAGCCTGTGGAAG
CTAAATATTAACGATGTCGATTTTGACCGTTTTTCTCAGGTCAGGCTGTTATCACTGGCA
AGTATCTTTAACAGCCCGCTGCTGGATGGCAAAGCATTAACGGCTATTTTTACGCAGGCG
AAAGCGCATCAACTGGTTATCTGCGCTGACATGATCAAACCGCGGCTGAATGAAACTCTG
GACGATATTCGCGAAGCGCTAAGTTTTGTCGATTATCTGTTTCCTAATATTGATGAAGCC
AAAAAGCTGACCGGGAAAGAGACGCTGGATGATATTGCCGATTGTTTTTTGCAATGTGGC
GTAAAAACAGTGGTGATTAAAACTGGCAAGAAAGGCTGCTTTATCAAAAGCGCCGATATA
AAAATGGAGGTGCCTGCGGTGTCAGGCATTACCGCCATCGACACTATTGGCGCTGGTGAT
AATTTTGCATCGGGATTTATCGCCGCGCTACTGGAGGGAAAAAACGCTGCGTGAATGCGCA
CGGTTCGCCAATGCGACGGCCGCGATATCGGTACTCAGCGTGGGCGCCACAACGGGCGTG
AAAAACAGAATGCTAGTGGAACAACTCCTGAATGAATACGAAGGGTAA
>EECEBICD_01621 NADH-specific methylglyoxal reductase
ATGAAAATGATACCTTTAGGTAATACAGATATAGCGCTTTCCCGTATGGGACTCGGCACC

TGGGCTATCGGCGGCGGTCCGGCATGGAATGGCAACCTCGATATGCAGGTCTGCATCGAT
ACTATCGTTGAGGCGCACCGTTGTGGTATTAACCTGATTGATACGGCGCCGGGCTATAAC
TTTGGCAATAGCGAAGTGATTGTCGGCCAGGCGCTGAAAAAAATTACCGCGTAA
>EECEBICD_01622 NADH-specific methylglyoxal reductase
ATGGTGGTGGAGACCAAATGCGGGATCGTCTGGGAGCGGGAAGGGAGTCTGTTCAATAAA
GTCGGCGATCGCCAGCTCTATAAAAATCTTTCTCCGGACTCTATTCGTAAAGAAGTCGAT
GCCAGTCTCCAGCGACTAGGGATTGATTATATCGATATCTATATGACCCACTGGCAGTCG
GTACCGCCGTTTTTTACGCCGATTGCGGAAACCGTTGGCGTGCTCAATGCTCTGAAAGCA
GAGGGGAAAATCCGTTCTATCGGGGCGGCGAATGTAGATGTCAGCCATATCCGCGAATAC
CTTAAACATGGCGAGCTGGACATCATACAGGCGAAATACAGCATTCTTGACCGGGCGCTG
GAAACGGAGCTGCTGCCGCTATGCCGCGAGCACGGCATCGTGGTACAGGTTTATTCGCCA
CTGGAGCAGGGATTGTTAACCGGCACCATTACCCGTGACTATGTTCCCGGCGGGGCGCGC
GCGAATAAACTCTGGTTCCAGCGTGAGAATATGTTGCGGGTTATTGATATGCTGGAGCAG
TGGCAGCCATTGTGCGACAAATACCATTGCGCCGTCCCGACGCTGGCGCTGGCGTGGATA
TTGCGACAAAGCGAGCTGATTTCGATACTGAGCGGCGCCACGTCGCCGGAGCAGGTACGA
GAGAATATCGCTGCGCTGTCCATCACCTTAACGGATGACGATGCGTTATTGATGAGACAA
ATGGCGGAAGCGCTGGACAATCCATAA
>EECEBICD_01623 putative HTH-type transcriptional regulator YdjF
GTGGCGGCGAAAGACAGGATTCAGGCGATCAAACAAATGGTCGCTAATGATAAAAAAGTA
ACAGTCTCTAACTTGAGTTCAATTTTTCAGGTAACCGAAGAGACAATTCGACGCGATCTG
GAAAAACTCGAAGATGAAGGCTTTTTGACTCGAACTTATGGCGGTGCGGTATTAAATTCG
GCGGTGCTGGCTGACAATATCCATTTTTATAAGCGTGCAAAATCGTTCTATGAAGAGAAG
CAAATTATAGCCCGTAATACGCTTCCGTTTATTAAAAACAAAACAACGATGGCCGCCGAC
TCCAGCAGTACGGTAATGGAATTGCTAAAACTCCTGAAAGACCGAAACGATCTTACTTTG
TTAACTAACTCAGCGGAAGCTTTCCATGAACTGGCGCAATCGGACATTAATCTTGTTTCT
ACGGGCGGTGAGTTAAATAAAAATACGCTATCGCTGCAGGGGCGTATCACTAAAGAAATA
ATCAGCCGTTATCATGTTGATATTATGGTAATGAGTTGTAAAGGGCTGGATATGGCGAGC
GGGGCGCTTGATTCTAACGAGGCGGAAGCTGAAATCAAAAAAACGATGATTCGCCAGGCC
ACAGAGGTGGCGTTATTGGTGGACCACTCAAAATTTGATCGTAAAGCGTTCGTGCGGTTG
GTCGATTTTAGTCATATCCATTACCTGATAACGAATAAAACGCCAGGTGATGAATGGATC
GCATTTTGCGATAAAAATAATATCCAGCTTGTCTATTAA
>EECEBICD_01624 Inner membrane metabolite transport protein YdjE
ATGGAAGACAATGATCATATCGGCGCCAGACTAGACCGCTTACCGTTATCCCGGTTTCAT
TTTCGTATTTTTGGAATAATTAGTTTTGGCCTGCTATTAACGGGGTTTTTGAGTTATTCA
GGAAATGTGGTTCTGGCCAAATTGATAAACAATGGCTGGTCGAATAATTATCTTAATGCC
GCGTTCACATCGGCACTGATGCTTGGCTATTTTATTGGTTCGCTTACCGGCGGTTTTATT
GGGGATTACCTGGGGCGTCGAAAAGCGTTTCGCATTAATTTGTTGCTTGTGGGCATCTCC
GCAACGGCTGCCGCATTTGTTCCCAATATGTACTGGCTGATTTTCTTTCGTTGTCTGATG
GGGACAGGCATGGGGGCATTGATTATGGTTGGATATGCTTCTTTTACCGAGTTTATCCCT
CCCATTGTACGAGGAAAATGGTCCGCGCGTCTCTCTTTTGTAGGGAACTGGTCACCGATG
CTGTCTGCGGGAATTGGCGTAGTGGTCATTGCGTTTTTAAGCTGGCGGATGATGTTTCTT
CTTGGTGGGATAGGGATGCTGCTGGCGTGGTCTCTTTCGGGGAAGTACTTTATCGAATCG
CCCAGGTGGTTAGCAGGTAAAGGGCGGCAACGCGAGGCTGAACAGCAGCTTCTCATGATT
GAATCGCAAATAGAAAAAGAAAAAAGTATTGTTTTGCCGCCGCATCAGGAAAGCAATAAA
TCCACGGAATCGCGCTTTGAAAACGGTACATTCTGGCTCTTATTCAAAGGACAAATGTTG
CGGCGCACGTTAGTGGCGATCACTGTATTGATTGCGATGAACATTTCGCTTTATACCATT
ACGGTATGGATCCCGACAATTTTTGTTAATTCAGGAATTGATGTTACAAAATCCATTTTC
ATGACCGCCATCATTATGATTGGCGCGCCGGTGGGCATATTTATCGCCGCGCTCATTATT
GATCATTTCCCCAGACGGTTATTTGGTTCGCTGTTGCTTATCGTTATTGCGGTAGTAGGA
TATTTTTACTCAATACAGACTGAGGAATGGGCCATTCTGGTTTATGGACTAGTCATGATT
TCCTTTCTGTATATGTACGTCTGCTTCGCCTCGGCGGTTTACGTTCCCGAATTATGGCCT
ACGCACCTTAGACTACGTGGATCGGGTTTTGTGAATGCGGTAGGGCGGATCGTCGCTGTC
TTTACCCCCTACGGCGTGGCGGTTTTACTGACCCGGTATGGATCTGTCACCGTTTTTGTT
GTGCTGGGTATGCTGCTGGCGGCGTGCGCGCTGATATTATTTTGTTTTGGCATTGAAACG

AGAAGAGTTTCGTTAGAAGCGCTCTCCAGCCTGCGTTGA
>EECEBICD_01625 Nicotinamidase
ATGACAAACCGGGCCTTATTACTGGTCGACTTGCAAAATGACTTTTGCGCCGGCGGCGCG
CTGGCTGTAGCGGAAGGCGACAGTACCATCGACATCGCCAACGCTCTTATTGACTGGTGC
CAGCCCCGGCAGATTCCGGTGCTGGCCAGCCAGGACTGGCATCCCGCGCAGCATGGCAGC
TTCGCCAGCCAGCATCAGGCGGAACCCTATAGTCAGGGAGAGCTGGACGGGCTGCCGCAA
ACGCTCTGGCCGGATCACTGCGTTCAACATACCGACGGCGCGGCGTTACATCCCCTGCTT
AACCAACATGCGATTGACGCCTGCATCTATAAGGGTGAGAACCCGCTGATTGATAGCTAC
AGCGCGTTTTTTGACAACGAACATCGGCAGAAAACTACCCTTGATACCTGGCTTCGCGAG
CATGACGTTACCGAGCTTATCGTGATGGGGTTGGCGACCGATTACTGCGTAAAATTTACG
GTGCTGGATGCGCTAGAGTTGGGCTATGCCGTTAACGTTATCACCGATGGCTGTCGCGGC
GTGAATATTCACCCGCAGGATAGCGCACACGCGTTTATGGAGATGGCGTCGGCAGGCGCG
ACGCTGTATACGCTGGCAGACTGGGAAGAGACGCAGGGCCAGGCTGTCGCCAGGTAG
>EECEBICD_01626 L-asparaginase 1
ATGGCGGCGTCTGCTCATAGCCGCCATCCGATAATCATTAATACGTCCTTCGCGACACGA
CTGAACATTATCATGCAAAAGAAATCAATTTACGTTGCCTATACCGGCGGTACTATCGGT
ATGCAGCGCTCAGATAAAGGGTATATCCCGGTGTCCGGGCACCTTCAGCGCCAGCTGGCG
CTGATGCCGGAATTCCACCGCCCGGAGATGCCGGATTTTACCATCCATGAATATGCCCCG
CTGATGGATTCATCAGATATGACGCCGGAAGACTGGCAGCATATCGCTGCAGATATCAAA
GCGCACTACGATGAGTATGACGGTTTTGTTATTCTGCACGGCACCGACACGATGGCGTAT
ACCGCATCGGCGCTCTCTTTCATGCTGGAAAATCTGGGTAAACCGGTTATTGTGACAGGG
TCACAAATTCCGCTGGCTGAGCTGCGCTCCGACGGGCAAATTAATTTGCTGAATGCGCTT
TATGTCGCCGCCAACTACCCTGTTAATGAAGTAACGCTGTTTTTTAATAATCGTCTGTTC
CGCGGCAATCGCACGACCAAAGCACATGCGGATGGTTTTGACGCTTTTGCCTCTCCTAAT
CTGCCGCCCTTGCTGGAAGCAGGAATTCACATTCGCCGTTTAAATACGCCTCCCGCGCCC
TACGGTTCTGGTGAATTAATCGTTCACCCTATTACGCCGCAGCCGATAGGCGTAGTGACG
ATTTATCCGGGGATTTCCGCCGAGGTAGTACGTAACTTTCTGCGTCAGCCGGTAAAAGCG
CTGATTCTGCGCTCTTATGGCGTAGGCAACGCGCCGCAAAATAAAGCGTTTTTACAGGAG
CTGAAAGAGGCCAGTTCGCGCGGCATCGTGGTGGTTAACCTGACGCAATGCATGTCAGGG
AGAGTCAATATGGGCGGGTACGCTACCGGTAATGCGCTGGCGCACGCTGGCGTGATTGGC
GGTGCCGATATGACCGTCGAAGCTACCTTAACCAAACTGCATTATTTGTTAAGCCAGGGT
CTGGACACTCAGGCGATTCGTAGCGCGATGGCGCAAAACTTGCGTGGTGAATTGACGCCG
GATGATTAA
>EECEBICD_01627 Protease 4
ATGCGAACCCTGTGGCGATTTATTGCCGGATTTTTTAAATGGACGTGGCGAGTGTTGAAC
TTCGTCCGTGAAATGGTACTCAACCTGTTCTTTATTTTTCTGGTCCTGGTGGGCGTTGGG
ATCTGGATGCAGATCGGTAACGGCAGCAACAGTGAACAAACGGCGCGCGGCGCTCTGCTG
CTGGATATTTCCGGCGTCATTGTGGATAAACCCTCCACCAATCATCGTCTGGGCGCGCTG
GGTCGCCAGTTATTTGGCGCCAGCTCCGACCGTCTGCAGGAAAACTCTCTGTTTGACATC
GTCAACGCTATTCGTCAGGCGAAAGATGACCGTAACATCACGGGTATCGTTCTGGATCTG
AAAAACTTCACCGGTGCCGATCAGCCGTCAATGCGCTATATCGGTAAAGCGCTGCGCGAA
TTCCGCGATAGCGGCAAACCGGTTTTCGCCGTGGGTGAAAACTACAGCCAGGGTCAGTAT
TACCTCGCCAGTTTCGCCAATAAAATTTGGCTTTCCCCACAGGGTCAGGTAGATCTTCAC
GGCTTCGCTACGAATGGCCTGTACTACAAAACGCTGCTGGATAAACTGAAAGTCTCTACC
CACGTTTTCCGGGTCGGCACCTATAAATCCGCCGTCGAGCCGTTTATCCGCGACGATATG
TCGCCCGCCGCCCGCGAGGCCGACAGCCGCTGGATAGGCGAACTGTGGCAGAACTACCTG
CATACCGTTTCTGCCAATCGCCAGATTTCGCCGCAACAACTGTTCCCCGGCGCGCAGGCT
ATTATCGACGGGTTAACCAGCGTGGGCGGCGACACCGCCAAATATGCGCTCGACCATAAA
CTGGTGGACGCCCTCGCCTCCAGCGCAGATGTTGAAAAAGCGCTGACGAAGCAGTTTGGC
TGGAGCAAAACCGAAAATAACTATCGCGCGATCAGCTATTACGATTATTCGCTGAAAACG
CCTGCGGATACCGGCGGTACTATTGCGGTTATTTTCGCCAATGGCGCGATTATGGATGGC
GAAGAAACGCCAGGGAATGTCGGCGGCGACACTACGGCATCGCAGATCCGCGACGCACGC
CTTGATCCTAAAGTGAAAGCGATTGTGCTGCGCGTCAATAGCCCAGGCGGGAGCGTCAAC
GCCTCCGAAGTTATCCGCGCCGAACTGGCGGCAGCAAGAGCGGCTGGCAAACCGGTGGTG

GTCTCAATGGGCGGCATGGCGGCCTCCGGCGGTTACTGGATCTCTACGCCGGCAAACTAT
ATCGTGGCCAGCCCCAGCACGCTGACGGGTTCAATTGGCATCTTCGGCGTCATCAATACG
GTAGAAAACAGCCTGTCGTCGATTGGCGTACACAGCGACGGCGTTTCCACCTCGCCGCTG
GCGGATATTTCGATGACCAAAGCACTGTCACCGGAAGTGCAGCAGATGATGCAACTCAGT
ATTGAGTACGGCTACAAACGCTTTATCACGCTGGTGGCAGACGCGCGTAAGCGTACGCCG
GAGCAAATTGATAAAATCGCACAAGGCCATGTCTGGACCGGAGAAGACGCGAAAGCCAAT
GGTCTGGTGGACAGTCTCGGCGACTTTGACGACGCCGTCGCCAAAGCGGCGGAGCTGGCG
AAACTGAAACAGTGGCATCTTGATTACTATCAGGACGAACCGACGGTCCTTGATATGGTC
ATGGACAGTATGACCGGATCAGTACGCGCCATGCTGCCGGAGGCCATTCAGGCTATGCTC
CCGGCGCCGCTCGTTTCCGCCGCCAACACGGTGAAGGCCGAGGGGGATAAACTGGCGGCA
TTTAACGATCCCCAAAACCGTTATGCGTTCTGTTTGACTTGCGCGAACGTTCGCTAA
>EECEBICD_01628 Putative NAD(P)H nitroreductase YdjA
ATGGATGCACTAGAACTGCTGGTCAACCGTCGTAGCGCCTCTCGTCTCGCAGAACCGGCG
CCTGTCGGAGAGCAATTACAGAACATTCTGCGGGCGGGAATGCGTGTTCCCGATCATAAA
TCTCTACAGCCGTGGCGTTTTTTTGTGATTGAAGGAGAGGGGCGCGACCGCTTCAGCGCC
GTGCTTGAACGAGGCGCTGTCGCTGCCGGAGGCGATGAGAAAGCGATCGAAAAAGCGCGT
AATTCGCCGTTTCGCGCGCCGCTGATCATTACCGTGGTGGCGAAATGCGAAGAAAACCAT
AAGGTTCCCGTGTGGGAACAGGAGATGTCTGCCGGATGCGCGGTGATGGCGATGCAAATG
GCGGCGATTGCGCAAGGGTTTAATGGTATCTGGCGTAGCGGCGCATTAACGGAGAGCGCT
ATCGTGCGGGAAGCCTTTGAGTGCCGCCCGCAGGATAAAATTGTTGGTTTTCTCTATCTC
GGCACGCCGCAGCTCAAAGCGTCAACGACGATCAGTACCCCCGACCCCACGCCGTTTGTG
CGTTATTTCTGA
>EECEBICD_01629 Selenide, water dikinase
ATGAGCGAGCAAGCCATTCGTTTAACGCAATACAGCCACGGCGCTGGTTGCGGTTGTAAA
ATTTCCCCTAAAGTGCTGGAGACTATCCTGCATAGCGAGCAGGCGAAGTTCGTCGACCCG
AACCTGCTGGTGGGTAATGAAACCCGCGATGATGCGGCGGTTTACGATCTGGGTAATGGC
ACCAGTATTATCAGCACCACCGACTTCTTTATGCCGATAGTCGACAACCCGTTTGATTTT
GGCCGCATTGCGGCAACAAACGCCATCAGCGATATTTTTGCGATGGGCGGCAAACCGATT
ATGGCGATCGCGATCCTTGGCTGGCCGATTAATACCCTGTCGCCCGATATTGCGCGTGAA
GTGACCGAGGGGGGGGCGTTTTGCCTGCCGTCAGGCCGGTATCGCGCTGGCGGGCGGACA
CTCTATTGA
>EECEBICD_01630 Selenide, water dikinase
GTGCCGACCGAACGGGTGAAGAAAAACAGTACCGCGCAGGCGGGATGCAAACTCTTTCTG
ACCAAACCGTTGGGGATTGGCGTGTTGACCACCGCCGAGAAAAAATCGCTGCTTAAACCT
GAACATCAGGGGCTGGCGACGGAAGTCATGTGTCGGATGAACGTTGCTGGCGCGGCGTTT
GCCAATATCGACGGCGTAAAAGCCATGACTGACGTTACCGGTTTTGGCCTGCTGGGGCAC
CTGAGCGAGATGTGCCAGGGCGCAGGCGTGCAGGCGCTGCTTTGCTATCAGGACATCCCT
AAACTGCCGGGCGTGGAAGAGTATATTGCTCTGGGCGCCGTACCGGGCGGCACAGAGCGC
AACTTCGCCAGCTATGGTCATCTGATGGGCGACATGTCGCGTGAAGTTCGTAGCCTGCTG
TGCGATCCGCAAACGTCAGGCGGTCTGCTGTTGGCGGTAACGCCGGACGCCGAAGACGAT
GTTAAAGCCACCGCGGCGGAATTTGGTATCGATCTGACCGCGATTGGCGAACTGGTCGAG
GCCCGCGGCGGTCGCGCTATGGTTGAGATTCGTTAA
>EECEBICD_01631 DNA topoisomerase 3
ATGCGGTTGTTTATTGCCGAAAAACCGAGTCTGGGGCGCGCCATTGCGGATGTGCTGCCA
AAACCGCACCGTAAAGGCGATGGTTTTATTGAGTGCGGAAACGGGCAGGTCGTCACCTGG
TGTATTGGTCATTTGCTGGAACAGGCGCAGCCGGATGCGTATGACAGCCGTTATGCGCGC
TGGAATCTGGCTGACCTGCCTATCGTGCCGGAAAAATGGCAGCTTCAGCCTCGTCCTTCC
GTCACCAAACAGCTCAATGTGATTAAGCGCTTTTTGCATCAAGCCGGTGAAATTATTCAC
GCCGGCGACCCGGATCGCGAAGGACAGCTTCTGGTTGATGAAGTGCTGGATTATCTCCAG
CTTCCTGCCGAAAAGCGCCAGCAGGTGCGGCGTTGTCTGATAAACGACCTTAACCCGCAG
GCGGTCGAGCGTGCTATTGACAGGCTGCGGGCGAACAGCGACTTCGTGCCGCTGTGCGTC
TCCGCTCTGGCGCGAGCGCGAGCGGACTGGCTGTATGGCATTAATATGACCCGTGCTTAC
ACGATCCTGGGCCGTAATGCCGGCTATCAGGGCGTGTTATCCGTGGGACGCGTGCAGACG
CCTGTACTGGGGCTGGTGGTGCGGCGAGACGAAGAGATTGAGAACTTCGTCGCCAAAGAC

TTCTTTGAAGTAAAGGCGCACATCGTTACGCCTGCCGACGAGCGTTTTACCGCTATCTGG
CAGCCGAGCGAGGCGTGCGAACCTTATCAGGATGAAGAGGGGCGCTTGCTTCATCGTCCG
CTGGCGGAGCATGTAGTGAACCGAATCAACGGTCAGCCCGCGCTGGTAACCAGTTATAAT
GATAAACGGGAATCAGAATCCGCGCCGCTGCCGTTTTCGCTCTCGACGCTACAGATTGAA
GCCGCCAAACGCTTTGGCCTGAGCGCGCAAAACGTGCTTGATATTTGTCAGAAGCTATAT
GAAACCCACAAACTGATTACCTATCCGCGTTCCGACTGCCGTTATCTGCCGGAAGAACAC
TTTGCCGGACGGCAGGCGGTCATGAACGCGATTAGCGTCCACGCCCCGGATTTACTGCCG
CAGTCTGTGGTTAATCCTGACACGCGCAATCGCTGCTGGGATGACAAAAAAGTGGATGCG
CACCACGCGATTATCCCGACGGCGCGCAGTTCTTCTGTCCATTTGACGGAAAACGAAGCG
AAAGTGTACACCCTGATTGCGCGTCAGTATCTGATGCAGTTCTGCCCGGACGCGGTGTTT
CGTAAATGCGTTATTGAACTGGAAATCGCCAAAGGAAAATTTGTCGCCAAAGCGCGTTTT
CTGGCGGAGGCCGGTTGGCGGACGTTACTGGGCAGTAAAGAGCGCGACGAGGAAAACGAC
GGTACGCCGCTGCCGGTTGTCGCCAAAGGTGATGAGTTGCTGTGTGAAAAGGGGGAAGTG
GTCGAGCGCCAAACCCAGCCGCCGCGTCATTTCACCGATGCGACATTGCTTTCCGCGATG
ACCGGAATTGCCCGCTTCGTGCAGGATAAAGATCTGAAAAAGATCCTGCGCGCGACCGAT
GGGCTGGGGACGGAAGCCACGCGCGCCGGGATTATCGAGCTGCTGTTCAAACGTAGCTTT
CTGACCAAAAAAGGGCGCTACATTCATTCTACCGATGCTGGCAAAGCGTTAATACATTCG
CTGCCGGAAATGGCGGCCCGTCCAGATATGACCGCGCACTGGGAATCTGTTTTGACGCAA
ATCAGCGAAAAGCAGTGCCGTTACCAGGATTTTATGCAACCGCTGGTCGGCACGTTATAT
CAGCTGATCGATCAGGCTAAGCGCACGCCGGTGAAGCGTTTCAGAGGGATAGTCGCGCCA
GGCGGTGGAGAAAAGAAAAAGAGCGCGCCGCGTAAGCGAGCGGGCAAAAAAAGCCCGCCT
GCTGAGGAGACAGGCCGTCAGACCGAATAA
>EECEBICD_01632 NADP-specific glutamate dehydrogenase
ATGGATCAGACATGTTCTCTGGAATCGTTCCTCAACCATGTACAAAAGCGCGACCCGCAT
CAAACCGAGTTCGCGCAAGCCGTCCGTGAAGTCATGACCACCCTGTGGCCGTTTCTTGAA
CAAAACCCCCGTTACCGCCACATGTCTTTACTGGAACGTTTGGTTGAACCAGAGCGCGTA
ATTCAGTTTCGCGTAGTGTGGCTGGACGACAAAAACCAGGTGCAGGTAAATCGCGCCTGG
CGCGTGCAGTTCAACTCTGCTATCGGCCCCTATAAAGGCGGTATGCGCTTCCATCCTTCG
GTAAACCTTTCCATCCTTAAGTTCCTCGGCTTTGAGCAAACCTTTAAAAATGCCCTCACC
ACGTTGCCGATGGGCGGCGGTAAAGGCGGCAGCGATTTCGATCCGAAAGGGAAAAGCGAA
GGCGAAGTGATGCGTTTCTGCCAGGCGCTGATGACTGAACTGTATCGCCATTTAGGGCCG
GATACCGACGTGCCTGCCGGGGATATCGGCGTGGGCGGGCGCGAAGTGGGCTTTATGGCG
GGAATGATGCGCAAGCTTTCCAATAACAGCGCCTGCGTCTTCACCGGCAAAGGGCTTTCC
TTCGGCGGCAGCCTGATTCGTCCGGAGGCGACGGGCTATGGCCTGGTCTACTTCACCGAA
GCGATGCTTAAACGTCATGGTCTCGGTTTTGAAGGGATGCGCGTCGCCGTGTCCGGCTCC
GGCAACGTAGCGCAATACGCCATTGAGAAAGCGATGGCCTTTGGCGCGCGCGTGGTTACC
GCGTCGGATTCGAGCGGGACGGTGGTCGATGAAAGTGGCTTCACGCCAGAGAAGCTGGCG
CGTCTGTGTGAAATTAAAGCCAGCCGTGACGGTCGCGTCGCTGACTACGCCCGTGAGTTT
GGCTTAACCTATCTGGAAGGCCAGCAGCCCTGGAGCGTACCGGTGGATATCGCCCTGCCA
TGCGCGACGCAGAACGAACTGGACGTTGACGCCGCGCGCGTACTGATTGCCAATGGCGTG
AAAGCCGTGGCGGAAGGGGCAAATATGCCAACCACGATTGAAGCGACCGATCTCTTCCTT
GAAGCGGGCGTACTGTTTGCGCCGGGTAAAGCCGCCAACGCGGGCGGCGTGGCGACATCA
GGGCTGGAAATGGCGCAGAACGCCGCGCGGTTGAGCTGGAAAGCCGAGAAAGTGGACGCC
CGTCTGCATCACATTATGCTGGATATCCACCACGCCTGTGTCGAGTACGGCGGCGATAAT
AAGCATACCAACTACGTTCAGGGCGCCAACATCGCCGGTTTCGTGAAAGTCGCCGACGCC
ATGCTGGCGCAGGGCGTCATTTAA
>EECEBICD_01633 hypothetical protein
ATGTGCCGAAGCCTCGTGGTCGCCGTGATGGCGATAATGCCAATGGTCGGGCTGGCAAAC
CAGAACAGTCGTCCTGATATTCAGGTGAATGTCCCGCCGGAAGTTTTCAGCACGCGCGGT
CAGTCGTCCCAGCCCTGCATTCAGTGCTGCGTTTACCAGGATCAGAATTACTCCGAAGGC
GCGGTAATAAAAGTGGAAGGCGTCGTGCTCCAGTGCCAGCGTGACGAAAAAACCATCAGC
ACGAATCCGTTAGTCTGGCGTCGCGTAAAGCCATAA
>EECEBICD_01634 CTP pyrophosphohydrolase
ATGATAAAAAAACTCGATGTTGTTGCCGCCATCATTGAATGCGACGGCAAAATTCTGCTG

GCCCAGCGCCCAGCTCATGCGGATCAAGCCGGTTTATGGGAGTTTGCCGGCGGTAAAGTC
GAACCCGGCGAAACCCAGCCGCAGGCGCTTATCCGCGAGCTACGCGAAGAGTTAGGGATT
GACGCCACGCCCGGCGTCTATATCGCCAGCCATCAACGCGACGTGTCTGGCCGACGGATT
CATTTGCACGCCTGGCATGTGCCTGCGTTTAACGGTCTGATACGTGCGCTGGAACATCAG
GCATTAGCCTGGTGTACGCCGGAAGAGGCGCTGGAATACCCCCTCGCCCCGGCGGATATT
CCGCTATTGCAGGCGTTTATGGCTTTACGCGACGCCAGACTAACGGATTCGTGCTGA
>EECEBICD_01635 Exodeoxyribonuclease III
ATGAGTGGCAGTCACCCGGTAAAATTTCTGTTACCATCCCAACACGATTCACCTTGCAAA
GTGGCAGTGACTATGAAATTTGTCTCTTTTAATATCAACGGCCTGCGCGCCCGTCCCCAT
CAGCTTGAAGCGATTGTCGAAAAACACCAGCCCGATGTGATCGGTCTACAGGAGACAAAA
GTCCACGACGAAATGTTCCCGCTCGAAGAGGTCGCGAAGCTGGGCTATAACGTTTTCTAT
CATGGACAAAAGGGCCATTACGGCGTCGCGCTGCTAACAAAAGCCACGCCCATTTCCGTC
AGACGCGGTTTCCCGGATGATGGCGAAGAGGCGCAACGCCGCATTATCATGGCGGAAATC
CCCTCGCCGCTGGGTAATATCACCGTCATTAACGGCTATTTCCCGCAGGGGGAAAACCGG
GATCATCCGTTGAAGTTCCCCGCGAAAGCGCAGTTTTACCAGAACTTGCAAAACTATCTT
GAAACGGAACTCAAGTGCGATAATCCGGTATTAATTATGGGCGACATGAATATCAGCCCG
ACCGATCTGGATATTGGCATCGGCGAAGAGAACCGCAAACGCTGGCTGCGTACCGGTAAA
TGCTCCTTCCTGCCGGAGGAGCGTGAATGGATGTCACGGTTACTGAAGTGGGGATTAGTG
GACACGTTCCGTCAGGCGAATCCGCAAACGATGGATAAATTTTCGTGGTTTGATTACCGC
TCAAAAGGTTTCGTGGATAATCGCGGACTGCGCATTGACCTCTTGCTGGCAAGCGCTCCG
CTGGCGGAACGTTGCGCAGAAACCGGTATAGACTACGACATTCGCAGTATGGAAAAACCC
TCCGACCATGCGCCGGTCTGGGCGACGTTTCGTGTTTGA
>EECEBICD_01636 Succinylornithine transaminase
ATGTCTCTGTCAGTTACGCGCGAAAACTTTGATGAATGGATGGTGCCGGTATATGTTCCC
GCGCCTTTTATTCCTGTACGCGGAGAAGGCTCACGCCTGTGGGATCAGCAGGGGAAAGAG
TACATTGATTTTGCTGGCGGGATCGCGGTCAACGCATTGGGACATGCGCATCCGGCGCTG
CGCGAGGCGTTAAATGAACAGGCGAATCGCTTCTGGCATACCGGGAACGGTTACACCAAT
GAACCGGCGCTGCGACTGGCGAAAAAACTGATCGACGCGACCTTCGCCGAACGCGTTTTC
TTTTGTAATTCAGGCGCTGAAGCAAATGAAGCAGCATTAAAACTGGCGCGCAAATATGCC
CACGATCGTGTCGGCAATCATAAAAGCGGCATTGTAGCCTTTAAAAACGCGTTCCATGGT
CGCACGCTCTTTACCGTCAGCGCTGGCGGTCAACCCACCTATTCACAGGACTTTGCGCCG
CTGCCGCCGGATATTCGTCATGCAGCCTATAACGATCTGAACTCCGCCAGCGCGCTCATT
GATGACAATACCTGCGCCGTAATCGTAGAGCCTGTGCAGGGCGAGGGCGGCGTGATCCCC
GCCACGAAAGCTTTTTTGCAGGGGCTGCGTGAATTGTGCGACCGGCATCAGGCGTTACTG
ATTTTTGATGAGGTGCAGACGGGCGTCGGACGTACCGGCGAACTGTATGCATATATGCAT
TATGGCGTTACGCCCGATATCCTGACGACTGCTAAAGCGTTGGGTGGGGGCTTCCCGATC
GGCGCGATGCTGACCACGCAAGACTATGCCAGCGTTATGACGCCAGGCACGCATGGCACC
ACCTACGGCGGTAATCCGCTGGCGACGGCAGTTGCCGGAAAAGTACTGGATATCATCAAT
ACGCCGGAGATGCAAAACGGCGTCAGACAGCGCCACGATGCGTTTATTGAGCGCCTAAAT
ACCATTAATGCACGTTTTGGCATGTTCAGTGAAATTCGCGGTCTGGGGCTACTGCTTGGT
TGTGTGCTACAGACGGAGTTTGCCGGAAAAGCCAAACTCATCGCCCAGGAAGCGGCAAAA
GCAGGCGTGATGGTGCTCATCGCCGGCGGCGACGTGGTGCGTTTTGCGCCAGCGTTAAAT
GTTAGCGACGAGGAGATAGCCACCGGGCTTGATCGTTTTGCCCTGGCGTGCGAACGCCTT
CAGACGGGAGGCGCATCATGCGGGTGA
>EECEBICD_01637 Arginine N-succinyltransferase
ATGCGGGTGATTCGTCCTGTCGAACATGCGGATATCGCCGCGCTGATGCAGCTTGCAGGC
AAAACGGGCGGCGGCTTAACCTCGCTACCGGCAAATGAAGCGACGCTGGCGGCCCGCATT
GAACGCGCGCTAAAAACCTGGTCCGGCGAGCTGCCAAAAGGCGAACAAGGATATGTATTC
GTTCTCGAGGATAGTGAAACGGGTGAGGTCGGCGGGATCTGCGCCATTGAGGTCGCGGTC
GGCCTTAACGACCCCTGGTATAACTATCGTGTCGGTACGCTGGTTCATGCCTCCAAAGAG
CTGAATGTATATAACGCGTTGCCAACATTATTCCTGAGTAATGATCATACCGGCAGCAGT
GAGCTTTGCACGCTGTTTCTCGATCCCGAATGGCGTAAAGAGGGTAACGGCTACCTGTTG
TCAAAATCGCGCTTTATGTTTATGGCCGCGTTTCGCGATAAATTCAATGAGAAAGTGGTG
GCGGAAATGCGCGGCGTCATTGACGAGCACGGTTATTCTCCCTTCTGGCAAAGTCTCGGC

AAACGCTTTTTTTCTATGGATTTCAGCCGGGCGGATTTTTTATGCGGTACCGGGCAAAAA
GCGTTTATCGCCGAGCTGATGCCCAAACATCCTATTTATACCCACTTCTTATCTGAAGAG
GCACAGGCCGTGATTGGCGAAGTCCACCCGCAAACGGCGCCAGCCCGCGCGGTGCTGGAA
AAGGAAGGTTTTCGCTATCGCCACTATATCGACATCTTCGACGGTGGGCCGACGCTGGAG
TGTGATATTGACCGCGTGCGGGCCATTCGTAAAAGCCGACTGGTGGAAGTCGCGGAAGGG
CAACCCGCGCCTGGCGACTATCCGGCATGTCTGGTCGCCAATGAAAACTATCACCACTTT
CGGGCCGCGCTGGTGCGTGCCGATCCGCAGACTTCACGGCTTGTACTTACCGCCGCGCAG
TTGGATGCGCTGAAATGTCGCGCGGGCGACCACGTTCGGCTGGTACGCCTTTGCGCTGAG
GAGAAAACCGTATGA
>EECEBICD_01638 N-succinylglutamate 5-semialdehyde dehydrogenase
ATGACGCTATGGATAAACGGCGACTGGATAACCGGTCAGGGCGAACGCCGCCGCAAAACG
AACCCGGTGAGTGCGGAGATACTTTGGCAGGGGAATGACGCTAATGCGGCACAGGTCGCC
GAGGCCTGCCAGGTGGCGCGCGCGGCGTTTCCTCGTTGGGCCAGACAGCCTTTTGCGGCA
CGACAGGCTATCGTAGAGAAATTTGCCGCCCTGCTGGAGGCGCATAAAGCCGAGCTCACG
GAGGTCATCGCGCGTGAAACCGGTAAACCGCGCTGGGAGGCGGCAACGGAAGTGACGGCG
ATGATCAATAAGATTGCCATCTCGATTAAGGCTTACCATGCCAGAACAGGCGAACAAAAA
AGCGAACTTGTCGATGGCGCCGCGACGTTGCGCCATCGTCCTCACGGTGTGCTGGCGGTA
TTCGGCCCTTATAACTTTCCCGGCCATTTACCGAATGGCCATATTGTGCCCGCGTTGCTG
GCAGGCAATACGCTGATCTTCAAACCTAGCGAGCTAACGCCATGGACCGGGGAAACGGTA
ATAAAACTCTGGGAACGGGCGGGGCTACCGGCAGGCGTTCTTAATCTGGTGCAGGGCGGC
CGGGAGACCGGACAAGCGCTAAGTTCGCTCGACGATCTCGACGGACTGCTGTTTACCGGC
AGCGCCAGTACCGGATATCAGCTTCATCGCCAGCTATCCGGCCAGCCGGAAAAAAATACTG
GCCCTTGAAATGGGCGGAAACAATCCGCTCATTATTGAGGATGTGGCAAATACAGATGCG
GCGGTACATCTGACGCTGCAATCGGCGTTTATTACCGCCGGACAGCGCTGTACCTGCGCG
CGACGCCTTCTGGTAAAACAGGGTGCGCAGGGAGATGCATTTCTGGCGCGGCTGGTTGAC
GTCGCCGGACGTCTGCAGCCCGGCAGATGGGACGACGATCCGCAGCCGTTTATCGGGGGA
CTGATTTCAGCGCAGGCGGCACAGCATGTGATGGAGGCCTGGCGTCAACGAGAGGCATTA
GGCGGTCGCACGCTACTGGCGCCGCGGAAGGTCAAAGAGGGAACCTCTCTGCTGACGCCT
GGCATCATTGAGCTGACGGGCGTCGCGGATGTGCCGGATGAAGAGGTGTTTGGTCCGCTG
CTGAACGTCTGGCGTTATGCGCATTTCGATGAGGCGATTCGTCTGGCGAATAATACCCGT
TTTGGTCTGTCGTGTGGGTTGGTGTCGACGGATCGCGCGCAGTTCGAACAGCTCTTGCTG
GAGGCGCGGGCAGGGATCGTTAACTGGAATAAACCGCTCACCGGGGCAGCGAGTACCGCG
CCGTTTGGTGGTATCGGCGCGTCTGGCAACCATCGGCCCAGCGCCTGGTATGCCGCCGAT
TATTGCGCCTGGCCGATGGCCAGTCTGGAATCTCCCGAACTGACGTTGCCTGCGACATTA
AGCCCCGGCCTCGACTTTTCTCGCAGGGAGGCGGTATGA
>EECEBICD_01639 N-succinylarginine dihydrolase
ATGACGGCGCATGAAGTTAATTTTGACGGGCTGGTGGGGCTTACGCACCATTATGCCGGG
CTATCCTTCGGCAATGAGGCATCGACCCGCCACCGTTTTCAGGTGTCGAATCCTCGTCTG
GCGGTAAAGCAGGGGCTGCTAAAGATGAAAGCTCTGGCGGATGCCGGCTTTCTCCAGGCG
GTGATCCCGCCGCATGAGCGGCCTTTTATTCCGGCGTTGCGTCAGCTCGGCTTCACGGGT
AGCGATGAGCAGATTCTGGATAAGGTTGCGCGTCAGGCGCCACGCTGGCTTTCCAGCGTG
AGTTCCGCGTCGCCAATGTGGGTTGCGAATGCGGCGACGGTTTGCCCATCGGCAGACGCG
CTGGACGGGAAAGTCCACCTGACGGTGGCGAATTTAAACAATAAATTTCATCGCGCTCTT
GAGGCGCCTGTGACCGAAGCGCTGCTACGCGCCATATTTCGCGATGAAAACCAGTTTTCA
GTGCATAGCGCGTTACCGCAGGTCGCATTATTGGGAGATGAAGGCGCGGCGAATCATAAC
CGTCTTGGCGGCGAGTATGGTTCGGCAGGCGTGCAGCTTTTTGTCTATGGGCGCGAAGAG
GAGAATGAAATACGACCCGCTCGTTATCCGGCGCGCCAGAGCCGCGAAGCCAGCGAGGCC
GTGGCGCGTCTTAACCAGGTGAATCCGCAACAGGTTATCTTCGCTCAGCAGAACCCGGAG
GTCATCGATCAAGGCGTATTCCATAATGATGTCATCGCCGTTTCGAATCGACAGGTATTG
TTTTGTCACGAAGCGGCGTTTGCCCGGCAGAAAGTGCTCATTAATCAGTTGCGTACGCGC
GTTGACGGTTTTATGGCGATAGAGGTGCCCGCCGAAGAGGTTTCTGTATCAGATGCTGTG
GCGACCTACCTGTTTAATAGTCAGTTGCTAAGCCGTGACGACGGCTCAATGCTGTTAGTG
TTGCCGCGGGAATGTCAGGACCATGCCGGCGTCTGGCGCTATCTGAATAAGCTGGTGGCG
GAGGATAACCCCATCAGCGCGATACAGGTGTTTGATTTGCGAGAAAGTATGGCCAACGGC

GGCGGCCCGGCCTGTCTGCGATTACGCGTGGTGTTAACAGAAGAAGAACGACGGGCGGTG
AATCCAGCGGTAATGATGAATGACGCTCTGTTTACGGCCCTTAACGCGTGGGCGGATCGT
TATTATCGCGATCGCCTGACCGCCGCCGATCTGGCCGATCCGTTATTATTGCGAGAAGGC
CGGGAGGCGCTGGATGTGTTAACGCGTCTGCTGGATTTGGGGTCGGTTTATCCTTTCCAG
CAAACGGGGGCGGCTGATGGATAA
>EECEBICD_01640 Succinylglutamate desuccinylase
ATGGATAACTTTCTGGCGTTGACGCTAAGCGGCACGACACCACGGGTGAGGCAGGGGAAG
GGCGCGGGTTTTCGCTGGCGCTGGTTAAGCCATGGTCTGCTTGAACTCACGCCCGATGCG
CCGGTCGACCGCGCGTTAATTCTTTCTGCGGGGATACACGGCAATGAAACCGCGCCGGTC
GAGATGCTGGATAAGCTGCTGTCGGCGCTGTATTCAGGCAGCTTGACGTTAACGTGGCGA
GTGCTGGTGGTACTCGGCAATCCGCAGGCGCTGGCGGCGGGAATACGCTATTGTCACAGC
GATATGAACCGTATGTTTGGCGGGCGTTGGCAGTCCTTTGCCGAAAGCGATGAAACGCGG
CGGGCGCGTGAGCTGGAGCTAAGTCTGGATACCTTCTTTTCATCTGGCCAGGCGCGGGTA
CGCTGGCATCTGGATCTGCATACCGCCATTCGTGGCTCGCATCATTTGCGTTTTGGCGTA
TTGCCGCAGCGCGACCGCCCGTGGGAGACAGATTTTCTGGCGTGGCTGGGCGCGGCAGGA
CTAGAGGCGTTGGTATTTCATCAGGCGCCCGGCGGTACGTTTACGCACTTTAGCTCTGAA
CATTTCGGCGCGCTTTCCTGTACGCTGGAGTTGGGAAAGGCGTTGCCGTTTAGGCAAAAC
GATCTGACGCAGTTCAACGTAACCTCGCAGGCGTTGTCGGCGTTGCTGAGCGGTGTCGAA
ACGTCAACCTCGTTTTCGCCGCCGCTACGCTATCGGGTGGTGTCGCAAATCACGCGTCAC
AGCGACAAGTTCGCGCTTTATATGGATGCGCAAACGCTGAATTTTACTGCCTTTGCGAAG
GGAACGTTGCTGGCCGAGGAGGGGGATAAGCGCGTGACGGTGACGCATGACGTTGAATAT
GTTCTCTTTCCTAATCCCTCTGTCGCCTGCGGATTGCGGGCTGGATTAATGCTGGAAAGA
CTGCCCTGA
>EECEBICD_01641 hypothetical protein
ATGATTTGGGACACAGTTTCATGCTATTACGGATTATTCCTGGAGAATTGCTTTATTAAT
AAGCGTTGCTGCGTTATAGTTCTGCATAATTTCTGCTATATCATGCTGTTGTATATTTCT
CTTTCAACTCTGCGCAATTTATTCTTTTTTTCTTCACAATTGGCGAAAAAGTGTTTTTTA
TACTTTCATTGTTTTACCGTTGTTATGACTAATTGA
>EECEBICD_01642 Periplasmic chaperone Spy
ATGCGTAAACTGACTGCTCTATTTGTTGCCTCTACCCTGGCGATGGGGGCTGCGAATCTG
GCTCATGCCGCTGAGACTACCACCGCCGCGCCAGCGGATGCGAAGCCAATGATGCAGCAT
AAAGGTAAATTTGGCCCACATCACGATATGATGTTTAAAAACCTGAACCTGACAGACGCG
CAGAAGCAACAAATTCGCGACATTATAAAAGCGCAGCGCGAGCAAATGAAACGTCCGCTG
CTGGAAGAGCGTCGCGCCATGCATGATATTATTGCCAGCGATACCTTCGATAAAGCCAAA
GCCGAAGCGCAAATTACGAAAATGGAAGCGCAGCGCAAGGCCAATATGCTGGCGCATATG
GAAACCCAGAATAAAATTTATAACGTTCTGACGCCGGAGCAGAAAAAGCAATATAACGCT
AATTTTGAGAAGCGTCTGACAGAACGCCCGGCGCAAGAAGGTAAAATGCCTGCTGCGGCA
GAATAA
>EECEBICD_01643 Excinuclease cho
GTGGTACGGCGTCAATCAGCCCCCCGCCTTGAGTTTGAAGCGGCGGCCATTTACGAATAT
CCAGAACATTTACGTCCTTTCCTCAGCGAAGCGCCAGCCTTACCGGGCGTCTATGTATTT
CATAGCGAAAGCGATACGCTGCCGCTTTATATCGGTAAAAGCGTGAATATTCGCAGCCGG
GTGCTCTCACATCTGCGAACGCCTGACGAGGCCGCTATGCTGCGACAGGCGCGGCGGATA
AGCTGGATCTGCACCGCTGGGGAAATGGGCGCGTTGCTGCTGGAGGCACGGCTGATCAAA
GAACAACAGCCGCTGTTTAACAAGCGGTTACGTCGTAATCGCCAGCTTTGCTCGCTACAG
TTGAGCGAACAAAAAATTGAGGTCGTTTCCGCCCGCAGCGTCGATTTTTCCCATGAGCCA
AACCTGTTTGGCCTCTTCGCCAACCGTCGGGCGGCGCTGCAAAGCTTACAGAACCTCGCC
GATGAACAAAAATTGTGTTATGGCCTGCTGGGGCTTGAACCGGTAAGCCGTGGGCGCGCC
TGTTTTCGTTTCGCTCTGAAGCGCTGCGCTGGCGCGTGTTGTGGGCAAGAAACCCCGCAG
GCGCATTTTCTTCGTCTGCAGGCCTCGCTGGAACGGTTACGTGTGGTTTGCTGGCCCTGG
AAAGGCGCTATCGCGTTAAAAGAGAGCCGTCCACAAATGACCCAGTTCCACATTATCAAC
AACTGGTTATGGCTGGGGGCGGTCCCCTCGCTGGATGAGGCCGCCACGCTGGTGCGCACC
CCTGCGGGCTTTGATCAGGACGGCTATAAGATTCTCTGTAAACCGTTAATGTCAGGTCAA
TATGAGATCATTGAACTGCACACCGACTGTCGCCAGTCATAA

>EECEBICD_01644 NH(3)-dependent NAD(+) synthetase
ATGACTCTGCAGCAAGAGATAATCCAGGCGCTTGGCGCGAAACCGCATATCAACCCTGAA
GAAGAAATTCGCCGCAGCGTGGATTTTCTTAAAGCGTACCTGGAAACCTATCCCTTTTTG
AAATCGCTGGTGTTAGGCATCAGCGGCGGGCAGGATTCGACGCTGGCCGGGAAACTCAGC
CAGATGGCTATCGCCGAATTGCGAGAAGAGACCGGTGATAATGCGCTGCAGTTTATCGCC
GTTCGTCTGCCTTATGGCGTACAGGCCGATGAACAGGATTGCCAGGACGCCATTGCTTTT
ATTCAGCCGGACAGAGTACTCACCGTCAATATTAAAGGCGCCGTCCTCGCCAGCGAACAG
GCGCTGCGCGAAGCGGGAATTGAGCTGAGCGATTTTGTTCGCGGCAATGAAAAAGCGCGT
GAGCGAATGAAAGCGCAATATAGCATCGCCGGTATGACTCACGGCGTCGTGGTGGGCACC
GACCATGCGGCGGAAGCGATAACTGGGTTCTTCACCAAATATGGCGACGGCGGTACCGAT
ATTAACCCGCTTCATCGCCTTAACAAACGCCAGGGCAAACAGCTTCTCGCAGCGCTGGGC
TGTCCTGAGCATCTGTATAAAAAAGTCCCCACCGCCGATTTGGAAGACGACCGCCCTTCA
CTGCCTGATGAAGCCGCGCTGGGCGTAACGTATGACAATATTGACGATTACCTGGAAGGC
AAAACGCTGGACCCCGCCATCGCTAAAACTATCGAAGGCTGGTATGTAAAAACTGAACAT
AAGCGCCGTTTACCGATTACGGTATTCGACGACTTTTGGAAAAGGTAA
>EECEBICD_01645 Osmotically-inducible putative lipoprotein OsmE
ATGAACAAGAACGTAGCAGGAATTTTAAGCGCAGCGGCGGTAATGACTATGCTGGCAGGC
TGTACGGCGTATGATCGTACAAAAGACCAATTTGTTGAGCCTGTCGTTAAAGATGTTAAA
AAAGGAATGAGCAGGGCGCAAGTTGCGCAGATTGCGGGCAAACCGTCATCTGAAGTGAGT
ATGATTCATGCGCGCGGTACCTGTCAGACCTACATCCTGGGTCAACGTGATGGTAAAGCG
GAAACCTACTTTGTTGCGCTGGATGATACCGGGCACGTGATTAACTCCGGCTACCAGACC
TGTGCGGAGTACGACACTGACCCACAGGCGCCGAAGCAGTAA
>EECEBICD_01646 PTS system N,N'-diacetylchitobiose-specific EIIB component
ATGGAAAAGAAACACATTTATCTGTTTTGTTCTGCGGGCATGTCAACATCGTTACTGGTA
TCAAAAATGCGCGCTCAGGCTGAAAAATATGAAGTGCCGGTGATTATTGAAGCATTCCCT
GAAACCCTGGCCGGTGAAAAAGGCCCGGCAGCGGACGTCGTGTTATTAGGCCCGCAAATT
GCTTATATGCTCCCCGAAATTCAGCGTTTATTACCCGGTAAACCGGTAGAAGTCATTGAT
TCAATGTTGTACGGCAAGGTAGATGGCTTAGGCGTGCTTAAAGCTGCGGTAGCGGCAATT
AAAAAAGCCGCCGCGAATTAA
>EECEBICD_01647 PTS system N,N'-diacetylchitobiose-specific EIIC component
ATGAGTAACGTCATTGCTTCACTTGAAAAGGTACTCCTTCCTTTTGCTGTAAAAATAGGA
AAGCAGCCACACGTTAATGCAATCAAGAACGGCTTTATTCGTTTAATGCCATTAACCCTT
GCGGGGGCGATGTTTGTATTAATTAATAACGTTTTCCTGAGCTTTGGGGAGGGGTCGTTT
TTTTATTCCATGGGCATTCGGCTTGATGCCTCAACCATTGAAACACTTAATGGATTGAAA
GGCATCGGCGGGAACGTCTATAACGGCACATTGGGCATTATGTCGCTCATGGCGCCATTT
TTTATCGGCATGGCGCTGGCGGAAGAGCGCAAGGTTGATGCGCTGGCCGCCGGATTGCTT
TCCGTGGCGGCATTTATGACCGTAACGCCGTATAGTGTCGGCGAGGCCTATGCCGTAGGC
GCTAACTGGCTGGGCGGCGCGAATATTATCTCCGGTATTATTATTGGCCTGGTGGTCGCG
GAGATGTTTACATTTATTGTCCGGCGCAACTGGGTGATTAAACTACCGGACAGCGTACCG
GCTTCGGTGTCTCGTTCATTCTCAGCATTAATTCCCGGCTTTATTATTCTCTCTATTATG
GGGATTATTGCCTGGGCGCTGTCTAATTACGGTTCTAACTTCCATCAGATTATTATGGAC
ACTATCTCTACGCCGCTGGCATCGCTGGGTAGCGTGGTAGGGTGGGCATATGTCATTTTT
GTACCGCTGCTGTGGTTCTTTGGTATTCATGGTTCGCTGGCGCTGACCGCGCTGGACAGC
GGCATCATGACGCCCTGGGCGCTGGAAAACATCTCTATTTACCAGCAGTATGGCTCCGTC
GATGCGGCACTGGAAGCCGGTAAAACGTTCCATATCTGGGCGAAACCGATGCTGGATTCT
TATATCTTCCTCGGTGGTAGCGGCGCAACGCTGGGTCTGATCATCGCTATCTTCCTGGCA
TCCCGTCGCGCGGACTATCGCCAGGTGGCAAAACTGGCGCTGCCGTCAGGTATCTTCCAG
ATTAACGAACCCATCCTGTTTGGCCTGCCGATTATTATGAACCCGGTGATGTTTATCCCC
TTCATTCTGGTTCAGCCGATTCTGGCGGCGATTACGCTGGTGGCTTACTATTTGGGTATT
ATTCCGCCGATTACCAATATTGCGCCGTGGACCATGCCAACCGGGTTGGGGGCGTTCTTT
AACACCAACGGCAGTGTCGCCGCGTTGCTGGTTGCGCTATTTAACCTGGCGGTCGCTACC
CTGATTTATCTCCCCTTCGTGGTGGTGGCTAACAAAGCGCAGAACGCCATCGAGCAGGAA

GAAAGCGAAGAAGATATCGCTAACGCACTGAAATTCTAA
>EECEBICD_01648 PTS system N,N'-diacetylchitobiose-specific EIIA component
ATGTTTGATCTTGAAAATGTTGTAGATAACCAGACAGCGGCTGAGGAACTCGAAGAAGTC
GTTATGGGGCTTATCATCAATTCTGGTCAGGCGCGCAGCCTGGCATATGGCGCGCTCAGA
AAAGCGAAAGAAGGCGATTTTGAGGCGGCGAAAGCGATGATGGACCAGTCGCGCATAGCG
CTCAATGAAGCGCATCTTGTGCAGACAAAACTCATCGAAGGCGATCAGGGCGAAGGCAAA
ATGAAAGTCAGTCTGGTCCTGGTTCACGCTCAGGATCATTTGATGACCTCAATGCTGGCT
CGTGAACTGATCGCCGAGTTGATTGAGCTCCATGAGAAACTGAAATAA
>EECEBICD_01649 HTH-type transcriptional regulator ChbR
ATGCAGCTACAGGTTAACGCAACGGAAATCAAAACGGTTTATGAGCAGCAGCTCTTCAAT
GGCAAAAATTTCCATGTGTTTATCTATAACAAGACGGAAAGCGTCACCGGGCTGCATCAG
CACGACTACTATGAATTTACCCTGGTATTAACCGGACGTTATTACCAGGAGATTAACGGG
AAGCGTGTGCTGCTGGAACGTGGAGATTTTGTTTTTATCCCGGTGGGGTCGAATCACCAA
AGTTTCTATGAGTTTGGCGCAACGCGTATTCTGAACGTAGGAATTAGTAAACGTTTTTTT
GAACAGCATTATCTTCCGTTACTGCCGTTTTGTTTTGTGGCATCGCAGGTATACAGGGCG
AATAGCACCTTCCTTACCTATATTGAAACGGTGATTGCGTCGTTGAATTTTCGCGGCAAT
GGGCTCGACGAATTTATCGAAGTGGTGACATTTTATATTATAAACCGCTTACGCCATTAC
CGCGAAGAGCAGGTTTATGATGATATTCCACAATGGCTGAAAGCAACCGTGGAGATAATG
CATGATAAAACGCAGTTTGGCGAACATGCGCTGGAAAATATGGTGCGCCTGTCGGCAAAA
TCCCAGGAGTATCTGACGCGCGCCACCCGACGTTATTACAGCAAAACGCCGATGCAAATC
ATTAATGAGATTCGCATTAATTTTGCCAAGAAACAGTTGGAAATGACCAACTATTCGGTT
ACGGATATTGCTTATGAAGCCGGGTATAGCAGTCCAAGTCTGTTTATTAAAACGTTTAAG
AAGATGACCTCATTTACGCCGAATAGTTATCGGAAGCGATTGACGGAAATTAATGAGTAA
>EECEBICD_01650 6-phospho-beta-glucosidase
ATGAGCCAGAAATTAAAAGTCGTCACTATTGGTGGCGGGAGCAGCTACACCCCTGAATTA
CTTGAAGGCTTTATTAAGCGCTACCATGAATTACCTGTTACCGAATTATGGCTGGTTGAC
GTTGAAGACGGGAAAGAGAAGCTGGGCATTATTTATGATCTCTGCCAGCGAATGATTGAT
AAAGCAGGCGTTCCGCTAAAATTGTATAAAACGCTGGATCGCCGGGAAGCGCTGAAAGAC
GCTAATTTTGTCACTACCCAGCTACGCGTTGGTCAACTCAAAGCCCGTGAACTGGACGAG
CGTATCCCGCTTAGCCACGGCTATCTGGGGCAAGAAACCAACGGCGCTGGCGGTTTATTT
AAAGGGTTGCGTACCATTCCGGTTATTTTTGACATCATTAAGGATGTTGAAGAATTATGT
CCGAATGCGTGGGTCATTAACTTTACTAACCCGGCGGGGATGGTGACGGAAGCGGTTTAT
CGTCATACCAACTTTAAAAAGTTCATTGGCGTATGTAATATTCCTGTCGGCATGAAAATG
TTTATTCATGACGTGCTGGCGCTGAATGAGACTGACGATCTTTCCATTGACCTGTTTGGT
CTAAACCATATGGTCTTTATTAAAGATGTGCTGGTGAATGGCACCTCACGGTTCGCAGAA
TTACTGGATGGCGTGGCGTCCGGTCAGTTGAAAGCGTCAACCGTAAAAAATATCTTTGAT
CTGCCGTTTAGTGAAGGATTGATTCGCTCGCTGAACATGCTGCCGTGCTCTTATTTGTTG
TATTACTTCAAGCAAAAAGAGATGTTGGCGATTGAAATGGGCGAATATTACAAAGGCGGC
GCGCGCGCTCAGGTCGTACAAAAAGTGGAGAAACAACTCTTCGACTTGTACAAAAAATCCT
GAGCTAAACGTGAAGCCGAAAGAGCTTGAGCAACGCGGCGGCGCTTATTATTCCGATGCC
GCTTGTGAAGTCATTAACGCTATTTATAATGACAAGCAGACTGAGCATTACGTTAATATT
CCACATCATGGGCATGTCGAGAATATCCCGGCGGACTGGGCGGTGGAAATGACCTGCATT
CTGGGACGCAATGGCGCGACGCCGCACCCGCGTATCACCCGTTTTGACGAAAAAGTGCTG
GGGCTTATCCACACTATTAAAGGATTTGAGGTCGCGGCCAGCAATGCGGCGCTGAGCGGA
AACTTTAATGATGTGCTGCTGGCGCTTAACCTGAGTCCGCTGGTGCATTCCGACCGCGAC
GCAGAAGTCCTGGCGCGTGAGCTCATTCTGGCGCATGAAAAATGGCTGCCTAATTTTGCC
GCTTGCATCGAAGCGCTTAAAGGTAAGCACCACTGA
>EECEBICD_01651 Chitooligosaccharide deacetylase ChbG
ATGGAACGCGTGTTGATTGTGAATGCCGATGATTTTGGCCTGAGTAAAGGGCAAAATTAC
GGCATTGTTGAAGCATATCGCAACGGCGTTGTGACCTCCACGACGGCGCTGGTCAATGGC
GAGGCTATTGACCATGCGGCGCAGCTTAGCCGCGAGCTGCCTGCGCTTGGGGTGGGAATG
CATTTTGTTTTGACGTTAGGCAAACCGGTGTCAGAGATGCCGGGCCTGACGCGAGACGGC
CTGTTGGGGAAATGGATCTGGCAAATGGCGGAGGAGGATACGCTTCCGCTGGATGAAATT

GCGCATGAACTGGCGTGTCAGTATCAGCGTTTCATCGACGTATTTGGCCGCGAGCCAACG
CATCTCGACAGTCACCATCATGTGCATATGTTCCCGCAAATTTTCCCCATCGTCGCCCAC
TTTGCGGCGCAGCGCGGGATTGCGTTACGTATTGACCGCCAGACGGTGTTGAACGCCGAC
GATCTGCCGTCGGATTTGCGCAGCACTCAAGGATTTAGTAGTGAATTTTACGGCGAGGAG
ATCACCGAAGCGTGCTTTTTACGCATTCTGGATGCCTCGGCGCATCGAGGAGAAGCCTCT
CTGGAGGTGATGTGTCATCCGGCGTTTGTGGATAACATTATTCGCCAGAGCGCCTATTGT
TACCCCCGCTTAACTGAACTGGAGGTGTTGACGTCTGCGTCACTCAAGGCAGCGATTGCG
GAACGTGGATATCGTCCTGGCTGTTTTCTTGATATTTAG
>EECEBICD_01652 Catalase HPII
ATGTCGCATAATGAGAAATCCCCCCATCAGTCCCCCGTGCATGATACCCGTGAATCACAG
CCCGGCCTGGACTCCCTGGCGCCGTCAGACGGTTCACATCGCCCAACGCCAGAACCGACG
CCGCCGGGCGCGCAACCAACGGCCCCCGGCAGCTTAAAAGCGCCCGAGACCGCGAATGAT
AAGCTCACCGCCCTCGACGCCTTTCGTAAAGGCAGCGAAAATTATGCGCTGACCACCAAT
CAGGGCGTGCGCATCGCCGACGATCAAAATTCTCTTCGCGCCGGCAGCCGTGGCCCTACG
CTGTTGGAAGATTTTATCCTGCGCGAAAAAATTACCCACTTTGACCATGAGCGTATCCCG
GAGAGGATCGTTCACGCCCGCGGATCAGCAGCGCACGGCTATTTTCAGTCATATAAAGAC
CTGAGCGACATCACGAAAGCCGCGTTTCTGTGCGATCCACAGAAAATTACCCCGGTCTTT
GTTCGCTTTTCGACAGTACAGGGCGGCGCGGGGTCTGCTGATACCGTACGTGATATCCGC
GGTTTTGCGACCAAATTTTATACCGAAGAGGGAATTTTCGATCTCGTCGGTAATAACACG
CCGATCTTTTTTATCCAGGATGCGCATAAGTTTCCGGATTTTGTGCACGCCGTTAAGCCC
GAACCACATTGGGCCATTCCGCAAGGACAGAGCGCGCACGACACCTTCTGGGACTATGTT
TCCTTACAGCCTGAGACGCTGCACAACGTCATGTGGGCAATGTCGGATCGCGGCATTCCA
CGCAGTTATCGGACGATGGAAGGGTTCGGCATTCACACCTTCCGTCTGATCAATGCGCAA
GGGAAAGCGACCTTTGTCCGTTTCCACTGGAAACCGTTGGCGGGTAAAGCCTCGCTGGTA
TGGGATGAGTCACAAAAACTGACCGGTCGCGATCCGGATTTTCACCGCCGGGATCTGTGG
GAGGCCATCGAAGCGGGCGATTTCCCGGAATACGAGCTGGGCCTACAACTCATTGCCGAA
GAGGATGAATTTAAATTCGATTTTGATCTGCTCGATCCGACCAAACTGATTCCGGAAGAG
TTGGTGCCAGTACAACGCGTCGGCAAAATGGTGTTAAACCGTAATCCGGATAACTTTTTC
GCTGAAAACGAACAGGCGGCATTTCATCCAGGGCATATTGTTCCCGGCATTGATTTTACC
AACGATCCGCTGTTACAGGGGCGTCTGTTCTCTTATACCGACACGCAAATCAGTCGTCTG
GGCGGGCCAAACTTCCACGAAATTCCGATTAACCGCCCTACCTGCCCCTACCATAACTTC
CAGCGTGACGGTATGCACCGGATGGATATCGACACCAATCCGGCGAACTATGAACCGAAC
TCCATTAATGACAACTGGCCGCGCGAAACGCCGCCAGCGCCAAAACGCGGCGGCTTCGAA
TCATACCAGGAACGCGTGGATGGCAATAAAATACGCGAACGCAGCCCCTCTTTCGGTGAA
TATTACTCGCATCCTCGCCTGTTCTGGCTCAGTCAGACGCCGATAGAACAGCAGCATATT
ATTGACGCGTTCAGCTTTGAACTGGGTAAAGTCGCCCGCGCGTATATTCGGGAGCGGGTG
GTCGATCAGTTAGCGCATATTGATGTCACGCTGGCTCAGGGCGTCGCGCATAATCTCGGT
TTTGCACTCACCCATGAGCAAACGCAAATTGCCCCGCCTCCTGATGTCAACGGTCTGAAA
AAAGATCCGGCGCTCAGCCTGTATGCGGTGCCGGACGGCGATGTTAAAGGCCGAGTGGTC
GCTATTCTGCTGAACGACAAGGTTAACGCCGCCGAACTGTTGACCATTTTGCAGGCTTTA
AAAGCCAAAGGCGTCCACGCAAAACTGCTCTATTCACGGATGGGTGAAGTAACGGCTGAT
GACGGTTCAACATTAACTATTGCGGCAACCTTTGCCGGCGCGCCGTCGCTAACGGTCGAT
GCGGTAATAGTACCGTGCGGCAATATTGCCGATATCGAGAGTTGCGGCGATGCTCGTTAC
TACCTTCTGGAAGCCTACAAACATCTGAAGCCCATCGCACTGGCAGGCGACGCCCGACGG
TTTAAGGCGCTTTTAAACATTGATAGCCAGGGGGAAGAAGGACTTGTTGAGGCAGATAAC
GTTGACCACCACTTTATGGACACTCTGCTGACGCTGATGGCGGCACACCGTGTCTGGTCG
CGTGCGGGGAAAATTAACGCGATTCCTGCATAA
>EECEBICD_01653 Cell division activator CedA
ATGATGAAACCTCTTCGCCAGCAAAATCGCCAGATTATTAGCTATATTCCGCGTGTTGAA
CCCGCGCCGCCGGAACATGCGATAAAAATGGATACGTTTCGCGACGTCTGGATACTGCGC
GGTAAATATGTTGCCTTTGTTCTGACGGGGGAGTCATTTCAACGGTCGCCCGCCTTTAGC
GTGCCAGAATCGGCGCAACGTTGGGCGAATCAGGTTCGCCAGGAAAACGAAATCGCTGAC
TAA
>EECEBICD_01654 L-cystine uptake protein TcyP

ATGAATTTTCCATTAATTGCGAACATTGCGGTGTTCGTCATTCTGCTGTTTGTACTGGCG
CAGGCCCGTCATAAACAGTGGAGTCTGGCTAAAAAAGTGCTTGTCGGCCTTGTGATGGGC
GTGGTCTTTGGTCTGGCCCTGCACACCATTTATGGCGCTGACAGTCAGGTGTTAAAAGAC
TCCATTCAGTGGTTCAATATTGTCGGCAACGGCTATGTGCAATTGCTACAAATGATTGTG
ATGCCGCTGGTATTCGCCTCTATTTTAAGCGCCGTGGCACGACTGCATAACGCCTCTCAA
TTGGGGAAAATCAGTTTTCTGACTATCGGCACGCTGCTGTTTACTACCCTTATCGCAGCG
CTGGTGGGCGTGCTGGTCACCAATCTGTTCGGACTGACGGCGGAAGGTCTGGTTCAGGGC
GGCGCGGAAACCGCGCGTCTGAATGCCATTGAGACGAGCTATGTCGGCAAAGTGGCCGAT
CTCAGCGTGCCGCAACTGGTGCTGTCTTTTGTACCGAAAAATCCGTTTGCGGATCTGACT
GGCGCAAATCCTACGTCTATTATCAGCGTGGTGATTTTCGCCGCATTCCTGGGCGTGGCC
GCGCTTAAACTGCTGAAAGATGATGCGCCAAAAGGCGAGCGTGTGCTTGTTGCTATCGAT
ACCCTGCAAAGCTGGGTGATGAAGCTGGTGCGTCTGGTAATGCAACTTACGCCTTACGGC
GTACTGGCGCTGATGACGAAAGTGGTTGCCGGCTCTAATCTGCAGGACATCATTAAGCTT
GGAAGCTTCGTGGTAGCGTCATATCTTGGTCTGGCGATAATGTTTGTGGTTCACGGCATA
CTATTGGGCGTGAACGGGATTAGCCCGCTGAAATATTTCCGCAAGGTGTGGCCGGTGCTG
ACATTTGCCTTTACCAGCCGCTCCAGCGCCGCCTCCATTCCACTTAATGTCGAAGCGCAA
ACCCGCCGCCTGGGCGTGCCGGAGTCCATCGCCAGCTTTGCTGCCTCTTTTGGCGCGACC
ATCGGCCAAAACGGTTGTGCCGGTCTTTATCCTGCAATGTTAGCGGTAATGGTTGCGCCG
ACGGTCGGCATCAACCCCTTAGACCCCGTATGGATAGCCACGCTGGTGGGTATCGTTACC
GTAAGTTCGGCGGGTGTTGCCGGCGTTGGCGGCGGCGCGACCTTCGCCGCGCTGATTGTG
CTGCCTGCCATGGGCTTACCGGTCACGCTGGTCGCGCTGTTAATCTCCGTCGAACCGTTG
ATTGATATGGGCCGTACCGCACTGAACGTGAGCGGCTCAATGACCGCGGGTACGCTGACC
AGCCAGTGGCTGAAGCAGACCGATAAAACCATTCTCGATAGCGAAGAAGACGCTGAACTG
GCGCATCGATAA
>EECEBICD_01655 Inner membrane protein YdjM
ATGGGTTTTATCATGACGGCGGAAGGACATCTTCTTTTTTCTATAGCCAGCGCGGTTTTC
GCCAAGAATGCCGAGTTAACGCCCGTCCTTGCGCACGGCGACTGGTGGCATATTATTCCT
TCGGCTATTTTGACCTGTCTGCTGCCGGATATCGACCACCCCAAATCTTTTCTTGGTCAG
CGCCTGAAATGGGTATCGAAACCGATCGCCCGCGCGTTCGGTCACCGGGGGTTTACCCAT
AGTCTGCTGGCGGTCTTTGCCCTACTGGCGACCTTTTATCTCAAGGTACCGGATACGTGG
ATTATCCCGGCGGATGCGCTGCAAGGCATGGTACTTGGCTATTTAAGCCATATCGTCGCG
GATATGCTCACCCCCGCAGGCGTTCCCTTACTGTGGCCGTGCCGCTGGCGTTTCCGCCTG
CCTATTCTGGCGCCGCGCAAAGGCAATCAACTGGAGCGATTTTTATGCATGGCGTTGTTT
GCCTGGGCGGTGTGGATGCCACAGTCCATGCCGGAAAACAACGTCATACGCTGGTCTTCG
CAGATGATTACTATCCTGCAAATGCAGTTCAATCGTTTTATAAATCACCAGATTGATTAG
>EECEBICD_01656 Hexitol phosphatase B
ATGTCAACCCCACGTCAAATTCTTGCTGCAATTTTCGATATGGATGGACTACTGATCGAT
TCCGAACCGCTCTGGGATCGCGCTGAACTGGACGTGATGGCAAGTTTAGGCGTAGATATT
ACTCGGCGCCATGAACTGCCAGACACGCTTGGGCTACGCATTGATATGGTTGTCGATCTC
TGGTTTGCGCAGCAACCGTGGAACGGCCCCGATCGCCAGGAAGTGACAAACCGTGTTATT
GCCCGCGCGATTACGCTTATCGAAGAGACGCGCCCTCTTTTACCGGGTGTGCGCGAGGCC
GTGGCGCTGTGTAAAGCGCAGGGGCTATTAGTGGGGCTTGCCTCGGCATCGCCGCTGCAT
ATGCTGGAAAAAGTGCTCACCATGTTTGAACTGCGCGACAGTTTTGACGCGCTGGCCTCT
GCGGAAAAATTGCCCTACAGCAAACCGCATCCGCAGGTCTATCTGGATTGTGCGGCCAAA
TTAGGTGTTGATCCATTAACCTGCGTGGCGCTGGAGGATTCGGTGAATGGTCTAATCGCG
GCAAAGGCGGCGCGAATGCGCGCCATTGTGGTGCCAGCAGAAGAAAACCAGCCTGATCCG
CGCTTTGCGCTGGCGAATGTCAAACTTAACTCACTGACAGAGCTGACGGCGGCGCATCTA
CTCGGTTAA
>EECEBICD_01657 hypothetical protein
ATGACGTATCAACAAGCTGGACGCATTGCAATCCTAAAAAGAGTTGTTGGGTGGGTTATT
TTTATTCCGGCACTGCTTTCCACGCTCATCTCAGTCCTTAAATTTATGTATGCGCACAGT
GAAAAGCAGGAGGGGATAAATGCGGTATTGCTGGATTTTACCCACGTAATGATAGACATG
ATGCGCGTGAATACGCCGTTTCTGAATGTTTTTTGGTACAACTCGCCGACGCCGAATTTT
CAGGGAAGCCTGAATATCGGTTTCTGGCTCATTTTTATTCTGATTTTTGTCGGGTTGGCG

ATGCAGGATTCCGGCGCCAGAATGAGCCGCCAGTCACGTTTTTTGCGTGAAGGCGTGGAA
GATCAGTTGATCCTGGAAAAAGCAAAAGGCGCGGAAGGGCTAACGCGTGAGCAGATAGAG
TCACGTATCGTGGTGCCGCACCATACCATCTTTTTACAGTTTTTCCCGCTCTATATTTTG
CCGGTCATTATTATCGTGCTCGGCTACTTTTTCTTTTCCTTGTTAGGCTTCATGTAA
>EECEBICD_01658 putative ketoamine kinase
ATGTGGCAAGCTATCAGTCGTCTTTTAAGCGAGCAAGTCGGTGAAGGCGAAATCGAACTG
CGTAATGAACTGCCTGGCGGAGAAGTTCATGCCGCATGGCATTTACGTTATTCAGGCCAT
GATTTTTTCGTAAAGTGCGATGAACGAGAAATGCTTCGCGGTTTTACCGCTGAAGCCGAT
CAGCTGGAGCTGCTCTCCCGCAGTAAAACCGTTGTTGTGCCTAAGGTATGGGCGGTAGGT
TCGGATCGGGATTACAGCTTTTTAGTCATGGACTACCTCTCGCCCCGCCCGCTGGATGCC
CATAATGCGTTTATTCTTGGGCAACAGCTGGCGCGCCTGCACCAATGGAGCGACCAACCT
CAATTCGGCCTCGATTTTGACAACGCGCTCTCTACCACGCCGCAGCCGAATACCTGGCAA
CGCCGTTGGTCGACTTTTTTCGCCGAACAGCGCATTGGCTGGCAACTTGAACTCGCCGCG
GAAAAAGGCATTACTTTTGGTAATATTGACGCCATTGTTGAACATGTTCAGCAACGTCTG
GCGTCGCATCAACCGCAGCCTTCTCTTTTGCATGGCGATCTATGGTCGGCGAACTGCGCG
CTTGGCCCTGACGGCCCCTATATCTTCGATCCAGCCTGCTATTGGGGCGACAGAGAATGC
GATCTGGCGATGCTGCCGCTGCATACCGATCAGCCACCGCAGATTTATGACGGCTATCAG
TCCGTTTCGCCGCTACCGCTGGATTTTCTCGACAGGCAGCCGATTTACCAGCTCTACACC
CTGCTTAATCGGGCAAGGCTGTTTGGCGGTCAGCACCTGGCCACTGCGCAAAAAGCGATG
GATCGGCTGCTTGCGGTATAG
>EECEBICD_01659 hypothetical protein
ATGGCCAGTGGCGATATTACCCGTTATGTTATTACCGTAACCTTCCATGAAGATTCCCTG
ACCGAGATTAACGAACTCAATAATCATCTGACGCGCAGCGGTTTTTTGCTGACATTAACG
GACGATGAAGGGAATGTGCATGAGCTCGGCACCAATACGTTTGGCTTTGTTAGCGCTCAG
AGCGCTGATGAAATCAAAGCGCTTGTCGCCGGACTTGCGAAAAGCGCATTAGATAAGGAT
GTCGACATTACCGTGGCGACCTGGGAGGCGTGGAGTAAAAACGCACAATAA
>EECEBICD_01660 ATP-dependent 6-phosphofructokinase isozyme 2
ATGGTACGTATCTATACGTTGACGCTTGCGCCCTCTCTCGATAGCGCAACAATCACGCCG
CAAATTTATCCCGAAGGTAAACTGCGGTGCTCCGCGCCGGTATTCGAACCCGGCGGCGGG
GGTATTAATGTCGCTCGCGCCATTGCGCATCTTGGCGGCACCGCGACCGCAATTTTCCCG
GCTGGCGGCGCTACCGGCGAACATCTGGTCGCACTACTGGCTGACGAAAATGTCCCCGTG
TCTACCGTCGATGCGAAAGACTGGACGCGTCAGAACCTGCACGTCCACGTCGAGTCCAGC
GGCGAACAGTATCGTTTTGTTATGCCCGGCGCCACGCTGGACGATGACGAATTTCGCCAG
CTCGAAGAACAGGTACTGGAAATTGAATCCGGGGCAATTCTGGTTATCAGCGGCAGCCTT
CCGCCCGGCGTTAAGGTCGAGAAACTCACGCAACTGATTTCCGCCGCACAGAAGCAAGGC
ATTCGCTGCATTATTGATAGCTCTGGCGACGCGCTCACCGCCGCGCTGGCGCTTGGCGAT
ATCGAACTGGTGAAACCTAACCTGAAAGAATTGAGCGCGTTGGTTAATCGCGATCTGACG
CAGCCTGATGATGTGCGTAAAGCGGCCCAGGAATTAGTGCAAAGCGGCAAGGCTCGCCGG
GTAGTGGTCTCTCTGGGGCCGCAAGGGGCGCTGGGCATAGACAGTGAGAATTGCATCCAG
GTTGTTCCTCCCCCGGTGAAAAGTCAAAGTACTGTCGGCGCAGGCGACAGTATGGTGGGC
GCGATGACGTTAAAACTGGCGCAAGACGCGTCGCTGGAAGAGATGGTGCGCTTTGGTGTG
GCGGCAGGCAGCGCCGCTACGCTCAATCAAGGTACGCGTCTGTGTTCCCGTGACGATACC
CAAAAAATATACGCCTATCTTTCCGCGCAATAA
>EECEBICD_01661 hypothetical protein
ATGAAGCTTTTGAAGGCAGTACCCGCTGTAGTTATGCTGGCGGGTGGCGTGTTCGCGTCT
CTGTATGCCGCCGCCGATGATTCCGTTTTTACTGTCATGGATGATCCCTCCACCGCGAAA
AAACCTTTTGAAGGTAATCTGAATGCGGGCTACCTTGCTCAGTCAGGCAATACTAAAAGT
TCTTCCTTAACGGCTGACACGGCGATGACCTGGTATGGACAACGTACTGCCTGGTCATTA
TGGGGCAATGCCAGCAATACCTCCTCTAATGATGAACGCTCTTCAGAAAAAATATGCGGTA
GGCGCGCGTAACCGTTTTAATATGACGGATTATGATTATACCTTTGGCCAGGCCAGTTGG
CTGACAGACCGTTTCAACGGTTATCGTCAGCGCGACGTACTGACCGCCGGTTACGGGCGG
CAGTTCCTTAATGGTCCAGTGCACAGCTTCCGTTTTGAATTTGGTCCAGGGGTTCGCTAT
GACGAACATACGGACGATACCACTGAAACGCAGCCGTTGGGTTATGCTTCCGGCAGCTAT
GCCTGGCAGTTAACTGACAATGCTAAGTTTACACAGGGGGTGTCAGTATTTGGCGCAGAA

GACACCACACTAAATTCGGAAACAGCCCTCAATGTCGCCATTAACGAACATTTTGGTCTA
AAGGTGGGGTATAATTTAACGTGGAACTCGCAGCCGCCTGAATCAGCGCCTGAACATACC
GATCGTCGCACTACGGTAACGCTGGGCTATAAGATGTAA
>EECEBICD_01662 hypothetical protein
GTGAACAAATACAGCTATTGCGCAACGATGATTGCCGCCATACTCAGTACGACCACAATG
GCCAATGCCAGTAGCCTTGCTATTTCAGTGGCGAATGATGATGCAGGTATATTTCAACCA
TCCTTAAATGCCCTTTATGGGCACCCGGCTGCAGACCGGGGTGATTATACTGCCGGGCTC
TTTTTAGGCTACAGCCACGATCTCACTGACGCCAGTCAACTCTCTTTTCATATCGCGCAA
GATATTTATTCTCCATCAGGCGCCAATAAGAGAAAGCCTGAAGCCGTCAAAGGCGACCGC
GCGTTCAGCGCCTTTCTTCATACCGGACTGGAGTGGAATTCATTAGCCACCAATTGGTTA
CGTTATCGTTTAGGTACCGATATCGGCGTTATTGGTCCTGACGCAGGCGGTCAGGAGGTT
CAGAATCGGGCGCATCGAATAATAGGCGCAGAAAAATACCCTGCCTGGCAGGATCAAATT
GAAAACCGTTATGGTTACACGGCAAAAGGAATGGTCTCCCTTACGCCAGCTATTGATATT
CTGGGTGTCAATGTGGGCTTCTATCCAGAAGTCTCTGCTGTCGGCGGCAATCTCTTCCAG
TATTTGGGGTACGGCGCAACCGTTGCGCTGGGAAATGATAAGACCTTCAACTCTGATAAT
GGGTTTGGGTTATTGTCCCGCCGAGGGTTAATACACACTCAAAAAGAGGGATTGATCTAT
AAAGTATTTGCTGGCGTTGAGCGCAGGGAAGTTGATAAAAACTACACGTTACAGGGGAAA
ACGCTGCAAACAAAAATGGAAACGGTGGATATTAATAAAACCGTGGATGAATATCGCGTC
GGTGCCACCATCGGCTATTCTCCCGTCGCATTTTCTCTTTCTTTAAACAAAGTCACGTCG
GAATTTCGCACCGGAGACGACTATTCTTATATCAACGGAGACATCACCTTCTTCTTCTGA
>EECEBICD_01663 hypothetical protein
ATGTTGAAAAATGGTGTTAAAAAACACGCGCCAGATATTATTGAATATCCATTTTTCATT
CGCTATCTGAGTGCGAGAAATTATTGGCTTCACGATTATGCATATAATACGATGTTTTTT
GGTATCAATATGAATATCACGTTGTATTCTTTTGAGCTCATTTTCTATGATGTCTTCGAT
GTTTATCTGTTATTAATTTTTACCGTGATAGTGTTGTCTTTAATGATGAGCGCATCTAAC
GGCTGGTAG
>EECEBICD_01664 Nuclease
GTGAATAAAACCATTAATCTGCTAAAATTACTGCCCGTAGTATTATTAAGCGCATGTACT
ACATCGTATCCTCCCCAGGATATAATATCGGCACCCGAGTTACCCCATCGTAACGTACTC
GTTCAGCAACCTGATAACTGTAGCGTTGGCTGTCCTCAAGGAGGAAGTCAACAAACAATC
TATCGCCATGTCTATACGCTCAATAATAATAGCGCCACGAAATTTGCCAACTGGGTTGCC
TATAGCGTGACAAAGACCAGCCAGGCAAGCGGTCGCCCGCGGAACTGGGCGCAGGACCCC
GATTTACCGCCCTCGGATACGTTGGCCCCTTCGGCCTATAAAAATGCCCATACGCTATTA
AAAGTCGACAGGGGACACCAGGCGCCGTTGGCAGGATTGGGCGGCGTTTCGGACTGGCCG
TCGTTAAATTATTTATCGAATATTACGCCGCAGAAATCCGCCCTGAACCAGGGAGCATGG
GCTGCACTGGAAAACCGGGTGCGCGAACTTGCCAAACAGGCTGATGTATCTGTAGTGCAC
GTAGTGACCGGCCCCCTTTTTGAGCGGCATATCGCCACATTGCCAGAAGATGCGACGGTA
GAAATTCCCAGCGGGTACTGGAAGGTTTTATTCACCGGAACGGCGCCGTCAAAAAGTGAA
GGAAATTACGCTGCGTTTATTATGGATCAGAATACGCCCCGTTCGGCGAATTTTTGCGAC
TATCAGGTTACCGTGGAGGCTATCGAACATAAAACGAAGCCAGTGCTGACGCTGTGGTCT
GCCTTGCCTGAAGCGGTAGCCAGCGAGGTGAAAACGACAAAGGGGAGTCTGGTGCAGAAG
TTAGGTTGTCGATGA
>EECEBICD_01665 hypothetical protein
ATGCTTATAATTTCATACATTGTATTATGCTTATTATTTATAGTTTATCTCTATACTCTT
TCCGTAAGAATTGAGGGAAAAATAATAAATGTAATGGTCCCATACCTGATAATAACAGTG
CCTACACTGTACGTGTTTGAAGGTATATTTGTATACCTCTCAGAGGTGCGGAAGTATACA
GTGGAATATTTGTTTTTCTATACTTGCTATATAACATACATAGCATCATTTGTTATTTCT
TATCTTTACACACAAAGAAAACCCATATACAACAAATCAAACACAAAAAATAAACCAAGG
TATGTGTTTACTTCATTGTTATTCACCTTCCTTGCTTTTATCATTTACCTTCCAGTGTTG
ATGGAGTTCAGAGAGTACATACTTAGCCCAAGAAGAATATACGAATTAACCAGAACAGGG
TATGGTATATACTTCTATCCTTCATTAATGTTTTCTCTTGTCGCTTCTATTTGCGCGTTC
TTTACATACAAAAAAATCAAAGTTATTTTGTATTTCCATAGTTTTATTTAACTGTATACTT
ATTTTCTTGCATGGTAACAAAGGACCAATATTTAGTATATTTATAGCATTCATCCTTTAT
CTTTCATATATTGAAAATAAAAAAATTAAATTTATGTTCCTGGTGAAATCGTTTGCTGTT

ATAGCAGTCATTGTAACGGCATTCTTTGCATATACGTTTACTGATGGGAATCCGATAGAA
AATATGGCGAATTACTCGGATTATACCCGTAATGCTGTTCTTGTTGCTTCCTCAAACTTT
GACTTTATGTACGGAAAATTACTAATGGAGAGCGAGGTTTACTCGAGAATTCCGAGGGCT
ATTTGGCCTGATAAGCCTGAAGATTTTGGCGCATTGTATCTGGCAAAAGTATTTTTCCCT
GATGCATTCTACAGGAATCAGGGCGCTCCTGCTTTCGGGTATGGTGAACTATACGCAGAT
TTCGGGCTTTTTACACCAGTTTGGTTAGTTATATCTGGAGTATTTAAAGGCGTCCTGGCT
AAGTATTTCTCCAATAAAACTCAGGAAACAAAGTCAGCGCATTATTTCATAATGTTCCTA
TTTTGCATTGGAATAAGTGTGATTCCTGTTAGCATGGGATGGTTGTTCCCTGAGCATTTG
ATGATTGCCTTTATAGTATACATTGCATCTTCCTTTGTTTTTTCAGCGCATATAAGATTC
GTTTTACTAAGAAGCGATAAATAA
>EECEBICD_01666 Threonine--tRNA ligase
ATGCCTGTTATTACTCTTCCTGATGGCAGCCAACGCCATTATGACCACCCTGTAAGCCCG
ATGGATGTTGCTCTGGACATTGGTCCTGGCCTGGCGAAAGCCACCATTGCGGGCCGTGTG
AATGGCGAGCTGGTTGATGCTTCCGATCTGATTGAAAATGATGCGACGCTTGCCATCATC
ACCGCAAAAGATGAAGAGGGTCTGGAGATCATTCGTCACTCTTGCGCGCATCTGTTAGGT
CACGCAATCAAGCAACTTTGGCCGCACACGAAAATGGCGATCGGCCCGGTTGTCGACAAC
GGTTTTTACTATGACGTTGATCTTGACCGCACGCTAACTCAGGAAGATGTCGAAGCGCTC
GAAAAGCGGATGCATGAGCTCGCCGAGAAAAATTACGACGTTATCAAGAAAAAGGTGAGC
TGGCATGACGCGCGCGAAACCTTCGTGAAGCGCGGCGAGACTTACAAAGTCGCTATTCTT
GATGAAAATATCGCCCATGATGATAAGCCAGGCTTGTACCATCATGAAGAATATGTCGAC
ATGTGTCGTGGTCCGCACGTGCCGAATATGCGTTTCTGCCATCACTTTAAACTGATGAAA
ACTGCTGGCGCATACTGGCGCGGTGACAGCAATAATAAGATGCTGCAGCGTATTTACGGT
ACGGCATGGGCAGATAAAAAAGCCCTGAACGCTTATCTGCAGCGCCTGGAAGAGGCCGCA
AAACGCGACCACCGTAAAATTGGTAAGCAGCTCGACCTGTATCATATGCAGGAAGAAGCG
CCGGGCATGGTGTTCTGGCATAACGACGGCTGGACTATCTTCCGCGAGCTGGAAGTCTTT
GTTCGTTCTAAACTCAAAGAGTACCAGTATCAAGAAGTTAAAGGCCCGTTCATGATGGAC
CGTGTGCTGTGGGAAAAAACCGGGCACTGGGACAACTATAAAGATGCGATGTTCACCACA
TCCTCAGAAAACCGCGAATATTGCATCAAGCCGATGAACTGCCCGGGCCACGTTCAGATC
TTTAACCAGGGTCTGAAATCCTATCGTGATTTGCCGCTGCGTATGGCGGAATTCGGTAGC
TGCCACCGTAACGAGCCATCAGGCGCGCTGCATGGTCTGATGCGCGTACGCGGCTTTACG
CAGGATGATGCGCATATCTTCTGCACCGAAGAGCAGATTCGCGATGAAGTTAACGCTTGT
ATTCGTATGGTCTACGATATGTACAGCACCTTTGGCTTCGAGAAGATCGTCGTCAAGCTT
TCCACTCGTCCTGACAAGCGTATCGGCAGCGATGAGATGTGGGATCGTGCTGAGGCGGAT
CTGGCGGTTGCGCTGGAAGAAAATAATATCCCGTTTGAGTATCAACTGGGTGAAGGCGCA
TTCTACGGTCCGAAAATTGAATTTACCTTATATGACTGCCTCGATCGTGCATGGCAGTGC
GGTACAGTACAGCTGGACTTCTCCTTACCGTCTCGTCTGAGCGCCTCCTATGTAGGCGAA
GACAACGAGCGCAAGGTGCCGGTAATGATTCACCGTGCGATTCTTGGGTCGATGGAACGC
TTCATCGGTATCCTGACCGAAGAGTTCGCTGGTTTCTTCCCGACATGGCTCGCGCCTGTT
CAGGTAGTCGTGATGAATATTACCGATTCGCAGTCTGAATACGTTAACGAATTGACGCAG
AAACTACAAAATGCGGGCATTCGTGTAAAAGCAGACTTGAGAAATGAGAAGATTGGCTTT
AAAATCCGCGAGCACACTTTACGTCGTGTCCCTTATATGTTGGTCTGTGGTGATAAAGAG
GTGGAAGCAGGCAAAGTTGCCGTTCGCACCCGCCGCGGTAAAGACCTGGGCAGTCTGGAC
GTAAATGACGTGATTGAGAAGCTGCAACAAGAGATTCGCAGCCGCAGTCTTCAACAACTG
GAGGAATAA
>EECEBICD_01667 Translation initiation factor IF-3
GTGAGTCTGAGAGAAGCTCTGGAAAAAGCTGAAGAAGCCGGAGTAGACTTAGTCGAAATC
AGCCCTAACGCCGAGCCGCCGGTTTGTCGTATAATGGATTACGGCAAATTCCTCTATGAA
AAGAGTAAGTCTTCTAAGGAACAGAAGAAAAAGCAAAAAGTTATCCAGGTGAAGGAAATT
AAATTCCGTCCTGGGACAGATGAAGGCGACTATCAGGTAAAACTCCGCAGCCTGATTCGC
TTTCTCGAAGAGGGCGATAAGGCCAAAATCACGCTGCGTTTCCGCGGTCGTGAAATGGCC
CACCAGCAAATCGGTATGGAAGTGCTTAATCGCGTGAAAGACGATTTGCAAGAACTGGCA
GTGGTCGAATCCTTCCCAACGAAGATCGAAGGCCGTCAGATGATCATGGTGCTTGCTCCT
AAGAAGAAACAGTAA
>EECEBICD_01668 50S ribosomal protein L35

ATGCCAAAAATTAAGACCGTACGCGGTGCTGCTAAGCGCTTCAAAAAAACCGGTAAAGGT
GGTTTTAAGCACAAGCACGCTAACCTGCGTCACATTCTGACCAAAAAAGCGACTAAACGT
AAACGTCACCTGCGTCCGAAAGCCATGGTTTCTAAAGGCGATCTGGGCCTGGTTATCGCG
TGCCTGCCGTACGCATAA
>EECEBICD_01669 50S ribosomal protein L20
ATGGCTCGCGTAAAACGTGGTGTAATTGCACGCGCACGTCACAAGAAAATTTTGAAACAA
GCCAAAGGCTACTACGGTGCGCGTTCTCGCGTATACCGCGTTGCCTTCCAGGCAGTTATC
AAAGCTGGTCAGTATGCTTACCGTGACCGTCGTCAACGTAAGCGTCAGTTCCGTCAACTG
TGGATTGCGCGTATCAACGCAGCAGCACGTCAGAACGGTATTTCTTACAGCAAATTCATC
AACGGCCTGAAAAAAGCCTCTGTTGAAATCGACCGTAAGATCCTGGCTGATATCGCCGTA
TTCGACAAAGTCGCGTTCACCGCGCTGGTTGAAAAAGCGAAAGCAGCTCTGGCATAA
>EECEBICD_01670 Phenylalanine--tRNA ligase alpha subunit
ATGTCACATCTCGCAGAGCTGGTTGCCAATGCAGCGGCCGCCATTAACCAGGCGTCAGAT
GTTGCCGCGTTAGACAATGTACGCGTCGAATATTTGGGCAAGAAAGGGCATTTAACCCTT
CAGATGACGACCCTACGCGATCTGCCGCCGGAAGAGCGTCCGGCAGCCGGGGCAGTCATT
AACGCCGCGAAAGAGCAGGTACAACAGGCGCTTAACGCGCGTAAAGCCGAGCTGGAAAGC
GCAGCGCTTAACGCCCGTCTGGCCGCTGAAACCATCGATATCTCCCTGCCGGGGCGCCGT
ATTGAGAACGGCGGTCTGCATCCGGTCACGCGTACCATTGACCGTATTGAAAGTTTCTTC
GGTGAGCTTGGTTTTACCGTGGCGACTGGCCCGGAAATCGAAGATGACTACCATAACTTC
GATGCGTTAAACATTCCGGGTCATCATCCGGCCCGCGCCGATCATGATACCTTCTGGTTT
GACGCGACCCGTCTGCTGCGGACCCAGACGTCTGGCGTCCAGATCCGGACGATGAAAGCG
CAGCAACCGCCGATCCGTATTATTGCGCCGGGCCGTGTATACCGTAATGACTACGACCAG
ACGCATACTCCGATGTTCCATCAGATGGAAGGTCTGATTGTTGATACCAACATCAGCTTC
ACCAACCTCAAAGGTACGCTGCACGACTTCCTGCGTAATTTCTTTGAGGAGGATCTGCAG
ATTCGCTTCCGTCCTTCTTATTTTCCGTTTACCGAACCTTCTGCAGAAGTGGATGTGATG
GGGAAAAACGGCAAATGGCTGGAAGTGCTGGGCTGCGGGATGGTGCATCCGAATGTGCTG
CGCAATGTCGGTATCGATCCGGAAATCTATTCTGGCTTCGCTTTTGGTATGGGGATGGAA
CGTCTGACCATGTTGCGTTATGGCGTCACCGACCTGCGTTCATTCTTCGAAAACGATCTG
CGTTTCCTCAAACAGTTTAAATAA
>EECEBICD_01671 Phenylalanine--tRNA ligase beta subunit
ATGAAATTCAGCGAACTGTGGTTACGCGAGTGGGTTAACCCGGCAATTGATAGCGATGCG
TTGGCTAACCAGATTACGATGGCTGGCCTGGAAGTAGACGGCGTTGAGCCGGTCGCGGGA
AGCTTCAATGGCGTTGTGGTTGGGGAAGTGGTTGAGTGCGCCCAGCATCCGAACGCTGAC
AAATTGCGTGTAACGAAAGTCAACGTCGGCGGAGAACGCCTGTTGGATATCGTCTGCGGC
GCGCCAAACTGTCGTCAGGGGTTGAAAGTAGCGGTGGCCACCATTGGCGCTATTCTGCCG
GGCGATTTCAAAATTAAAGCGGCAAAACTGCGCGGCGAACCGTCTGAAGGGATGCTGTGC
TCCTTCTCCGAACTGGGGATTTCCGACGATCACAGCGGCATTATCGAACTGCCGGCGGAT
GCGCCGCTGGGTACCGATATCCGTGAATACCTGAAGCTTGACGACAATACCATCGAAATT
AGCGTCACGCCGAACCGTGCGGATTGCTTAGGTATCATCGGCGTTGCGCGTGATGTGGCG
GTACTGAACAAAGCGCCGCTCCAGGAGCCTGAAATGGTGCCGGTTACGGCAACCATCAGC
GATACGCTGCCGATTACGGTAGAGGCCGCTGATGCGTGTCCTCGCTATCTTGGCCGGGTG
GTAAAAGGCATCAATGTTAACGCGCCGACGCCGCTGTGGATGAAAGAGAAACTGCGTCGC
TGCGGTATTCGCTCCATCGATGCGGTGGTTGACGTCACGAACTATGTGCTGCTCGAACTG
GGTCAGCCAATGCATGCTTTCGATAAAGATCGCATTGATGGCGGTATTGTCGTGCGGATG
GCAAAAGAGGGTGAAACCGTCGTCCTGCTGGACGGTAGCGAAGCGACGTTAAATGCGGAC
ACGCTGGTGATCGCCGATCACCATAAGGCGCTGGGGATAGCCGGTATTTTTGGCGGCGAA
CATTCCGGCGTGAACGGCGAAACGCAAAATGTGCTGCTGGAGTGTGCGTACTTTAATCCG
CTGTCTATCACCGGTCGCGCTCGCCGCCACGGTCTGCATACCGATGCGTCTCACCGCTAC
GAGCGCGGGGTGGACCCAGCGCTGCAATATAAAGCGATAGAGCGCGCTACTCGTCTGCTT
CTTGATATCTGTGGCGGTGATGCGGGTCCGATAATTGATGTGAGCAACGAAGCAACGTTG
CCGAAACGCGCCACCATTACGCTGCGTCGCAGTAAACTGGATCGCCTGATCGGGCATCAT
ATTGCGGATGAGCAGGTCAGTGATATTCTGCGTCGTCTGGGCTGTGAAGTGACCGAAGGG
CAGGACGAGTGGAAAGCCGTAGCGCCGACCTGGCGTTTCGATATGGAGATTGAAGAAGAT
CTGGTGGAAGAGGTGGCCCGCGTTTACGGCTATAACAATATTCCGGACGAGCCAATTCAG

GCCGGTTTAATCATGGGGACGCACCGTGAAGCCGATCTGTCGTTGAAGCGGGTTAAAACC
ATGCTGAACGACAAAGGCTATCAGGAAGTGATTACCTATAGCTTTGTCGATCCTAAAGTA
CAACAGCTCATCCATCCAGGCGCAGAAGCGCTGCTGCTGCCAAACCCTATCTCCGTTGAG
ATGTCGGCGATGCGTCTGTCTCTGTGGAGCGGATTGTTGGCGACGGTGGTCTATAACCAA
AACCGTCAGCAGAATCGCGTGCGTATTTTTGAAACCGGTTTACGTTTCGTTCCGGATACA
CAAGCCAATCTGGGTATTCGACAGGATCTGATGCTGGCTGGCGTGATTTGCGGTAACCGC
TATGATGAGCACTGGAACCTGGCAAAAGAGACCGTTGATTTCTATGATTTGAAAGGCGAT
CTGGAAGCAGTATTGGATTTAACCGGCAAACTGGGTGATATCCAGTTTAAGGCGGAGATG
AATCCGGCTCTGCATCCGGGACAGTCTGCGGCGATTTATCTGAAAGATGAACGTATTGGT
TTTATTGGGGTTGTTCACCCTGAACTGGAACGTAAACTGGATCTGAATGGTCGTACGCTG
GTGTTTGAACTGGAATGGAATAAGCTCGCAGACCGTATCGTGCCGCAGGCGCGGGAGATT
TCACGCTTCCCGGCCAACCGTCGCGATATTGCGGTTGTTGTTGCAGAAAACGTTCCCGCA
GCGGATATTTTATCCGAATGTAAGAAAGTTGGCGTAAATCAGGTAGTTGGCGTAAACTTA
TTTGACGTGTACCGCGGTAAGGGTGTTGCGGAGGGTTATAAGAGCCTCGCTATCAGCCTG
ATCCTTCAGGATACCAACCGTACACTCGAAGAAGAGGAGATTGCCGCTACCGTCGCCAAA
TGTGTAGAGGCATTAAAAGAGCGATTCCAGGCATCATTGAGGGATTGA
>EECEBICD_01672 Integration host factor subunit alpha
ATGGCGCTTACAAAAGCTGAAATGTCAGAATATCTGTTTGATAAGCTTGGGCTTAGCAAG
CGGGATGCCAAAGAACTGGTTGAACTGTTTTTCGAAGAGATCCGTCGTGCTCTGGAAAAC
GGTGAGCAGGTGAAACTCTCTGGTTTTGGTAACTTCGATCTGCGTGATAAAAATCAACGT
CCGGGGCGTAACCCGAAAACGGGTGAAGATATTCCTATTACAGCACGGCGCGTGGTGACC
TTCAGACCCGGGCAGAAGTTAAAGAGCCGGGTCGAAAACGCTTCGCCCAAAGAAGAGTAA
>EECEBICD_01673 Vitamin B12 import system permease protein BtuC
ATGCTGACTTTTGCCCGCCAACAACAGCGACGAAACGTTCGCTGGCTTCTGAGCCTGTCA
CTGCTGGTGCTACTGGCTACACTTCTGAGCTTATGCGCAGGCGAACAGTGGATTGCCCCC
GGTGACTGGTTAAGCGCCCGGGGGGAACTGTTTGTCTGGCAAATTCGCCTTCCCCGCACG
CTTGCGGTATTGCTGGTTGGCGCTGCGCTGGCGCTATCTGGCGCCGTGATGCAGGCGCTG
TTTGAAAACCCACTTGCTGAACCGGGTCTGCTCGGCGTTTCGAATGGGGCCGGTGTTGGG
CTTATTGCCGCCGTCTTACTGGGGCAGGGGCAACTGCCAGGATGGGCGCTGGGACTGTGC
GCTATAGCCGGCGCGCTCATTATTACGTTAATCCTGCTGCGTTTTGCGCGTCGCCATCTC
TCTACCAGCCGCTTGTTGTTGGCGGGCGTCGCGCTGGGCATTATCTGTAGCGCGCTGATG
ACGTGGGCTATCTATTTTTCCACCTCTTTCGATCTGCGGCAATTAATGTACTGGATGATG
GGAGGATTTGGCGGCGTTGACTGGCAGCAGAGCTGGCTAATGATTGCGCTCATCCCGGTA
CTGATCTGGATATGTTGCCAGTCGCAACCGCTGAATATGCTGGCGCTAGGGGAAACCTCG
GCGCGGCAGCTTGGCCTGCCGCTGTGGTTCTGGCGCAATTTGTTGGTCATCGCCACCGGC
TGGATGGTGGGCGTCAGCGTGGCGATGGCGGGGGCGATTGGTTTTATCGGTCTGGTTATT
CCGCACATCCTGCGCTTATGTGGTTTAACCGATCACCGGGTTTTACTTCCCGGCTGCGCG
CTGGCCGGGGCTATCGCCCTGCTATTGGCTGATGTGGTCGCCCGACTGGCGCTGGCGTCG
GCTGAACTGCCTATCGGGGTGGTCACCGCCACATTGGGGGCACCAGTGTTTATCTGGCTG
CTACTCAAATCCGCGCGTTAG
>EECEBICD_01674 Thioredoxin/glutathione peroxidase BtuE
ATGCAAAACTCGCTTCTTAATACTCACGTTACGACGATTGATGGAGAGGTCACTACACTT
GAAAAATACGCCGGAAAAGTGCTGCTCATTGTAAACGTCGCCTCCAGATGTGGCCTGACG
CCGCAATATGAACAGTTGGAAAATATCCAGAAGGCGTGGGCAGATCAGGGGTTTGTCGTG
TTGGGTTTTCCCTGCAACCAGTTTATGGGGCAGGAGCCAGGCAGCGAAGAGGAGATTAAA
ACCTATTGTGCCAGCACCTGGGGCGTCACGTTCCCGATGTTCAGTAAAATTGATGTGAAT
GGCGACGCGCGCCATCCGCTGTATCAAAAACTGATAGCCGCTGCGCCAACGGCAGTCGCG
CCGGAGAAGAGCGGCTTTTACGAACGGATGGTAAGCAAAGGGCGTACGCCACTCTATCCG
GATGATATTCTGTGGAATTTTGAAAAATTTTTGGTAGGCCGGGATGGTCAGGTGGTACAG
CGTTTTTCGCCGGATATGACGCCTGAAGATCCTATTGTGATGGAAAGCATAAAAATCACG
TTGGCAAAATAA
>EECEBICD_01675 Vitamin B12 import ATP-binding protein BtuD
ATGTCTCAATTGATGCAATTAAAGGATGTCGCCGAGAGCACCCGCCTGGGGCCGCTTTCC
GGCGAAGTAAGCGCAGGAGAGATCCTTCATCTTGTCGGGCCGAACGGTGCCGGGAAAAGC

ACGCTACTGGCGCGTATGGCGGGGTTAACCTCCGGCGAGGGGAGCATCAGGTTTGGCGGA
GCGCCGCTGGAGGCATGGGCGACGGCAACGCTTGCCCAACACCGCGCTTATCTTGCGCAA
CAGCAAAATCCGCCGTTCGCCATGCCGGTCTGGCATTACCTGACATTGCATCAACCTGAT
AAAACGCGAACCGGGCAGCTTAACGAGGTGGCCGATATGCTTGGATTAGGCGACAAACTG
GGGCGAAGCGTGAATCAGCTTTCCGGTGGCGAGTGGCAGCGTGTTCGCCTTGCTGCCGTT
GTACTACAAATTCATCCCGACGCTAATCCGGTCGGTCAATTATTGCTGCTTGATGAGCCA
ATGAACAGTCTCGATGTTGCGCAACAAAACGCTCTGGATCGGGTATTACATCATTTATGT
CAGGCAGGAATTGCGATTGTGATGAGTAGCCACGATCTGAACCACACGCTGCGCCATGCG
CATAAAGCCTGGTTACTAAAGCGCGGTAAACTCATTGCCTGCGGCCGCCGGGAAGAGGTG
CTCACCCCCTCTTATCTGGCGCAAGCCTACGGTCTGCGCTTTCGACGACTCGACGTCGAG
GGGCATCCAACGCTCATTTCGGCCACTTAA
>EECEBICD_01676 Murein DD-endopeptidase MepS/Murein LD-carboxypeptidase
ATGCGTTTTTGGCTTCTTGTTATCACAGCATTGTTCCTGGCAGGATGCAGTACTCATCGG
GCACCGGCGCCAAATGCCAGATTATCAGACTCCATTACCGTCATTGCGGGTCTGAACGAT
CAGTTGCAGTCCTGGCGCGGTACGCCATACCGTTACGGCGGCATGAGTCGCAGAGGCGTG
GACTGTTCCGGCTTTGTGGTGGTCACTATGCGCGACAAGTTTGATTTGCAGTTGCCTCGT
GATACGCGTGAACAATCAAAAATCGGCACCAGAATCGATAAAGATGAATTGCTGCCCGGA
GATTTGGTTTTCTTTAAAACCGGTTCGGGCGAAAGTGGTTTACATGTTGGTATTTACGAT
ACAAATAATGAGTTTATTCATGCCTCTACCAGTCGTGGCGTGATGCGTTCATCGCTGGAT
AATGTGTACTGGCGAAAAAACTTCTGGCAGGCACGGCGAATTTAA
>EECEBICD_01677 Anti-FlhC(2)FlhD(4) factor YdiV
ATGATTGCTTCACTTGATGAGCTTTACCATTCCGAACTCTTTTTCCTTCCCGTCATGGAT
GAAAATGCTCGGCTTGTTGGTTTAGAGATTATTGCAACGTTTGCCGCTGAAGACGGCGCC
GTGCGGATGCCGACTGAGTTGGTAGCGCCTCGTCTTTCGGTGGAGGAACAATACTGTCTG
TTTGTTGAAAAACTGGCGTTGTTAGAAACCTGTCAGCACTTTTTTATTCAGCATAAACTT
ATTGCATGGCTTAATTTACCGCCCGCAATTAGTGACTTATTATTATTAGATAGCGAACTT
TTTTCCCGGGCAGCGCGCTTTCCGTTTCTTGAATTAGCTATAAATGAGAACTATCCTGGG
TTAAACCAGGGCAAAAACAATGAAACGTTAGCTAATCTGGCAATGCATTTCCCATTAATG
CTGGCAAATTTTGGGGCAGGCGAGGCGTCGACTAAAGCTATTTTCGATGGGCTTTTCAAA
CGAGTCATGTTGGATAAAAATTTTATTCAGCAGCGAGCTGAAATGATCTCATTCGAACCA
TTCATGCACGCTATTGTTGCCCAGATCTCCTCCAGCTGCGAGTCGCTGATGATTGCCGGT
ATTGATAATGAGGCGATGTTTTCGCGCGCAGCGCCATTGGGATTTAGCGCATTCCAGGGC
GGACTATGGCCTCCGGTGCCGGTAAGCCAGCTCATTAAACTCGTTCAGCGATAA
>EECEBICD_01678 Protein adenylyltransferase SelO
ATGACCCTGTCTTTTACTGCCCGTTGGCGCGATGAACTGCCAGCAACGTATACCGCGCTT
TTACCCACACCGTTAAAAAATGCGCGACTGATTTGGTATAACGACAAACTGGCGCAGCAG
TTAGCAATTCCCGCGTCGTTATTTGACGCTACCAATGGCGCTGGCGTTTGGGGCGGCGAA
ACCTTATTACCCGGTATGTCGCCTGTCGCGCAGGTATATAGCGGGCATCAGTTCGGCGTC
TGGGCGGGACAACTCGGCGACGGACGCGGTATTTTGCTGGGCGAACAGCTCCTGGCGTAT
GGCTCGACCCTCGACTGGCATCTGAAAGGCGCCGGATTAACACCCTATTCGCGAATGGGG
GATGGACGCGCCGTCCTGCGTTCAACCATACGAGAAAGCCTTGCCAGCGAAGCCATGCAT
TATCTGGGTATTCCCACTACGCGCGCGTTATCTATCGTAGCCAGCGATACGCCGGTTCAG
CGAGAGACGCAGGAAACGGGCGCGATGTTAATGCGTCTGGCGCAAAGTCATATGCGTTTT
GGTCATTTCGAACATTTCTACTACCGCCGTGAGCCGGAAAAAGTTCAGCAACTGGCTGAT
TTCGCTATTCGTCATTACTGGCCGCAATGGCAGGATGTCCCGGAAAAATATGCTTTGTGG
TTTGAAGAGGTGGCGGCGCGTACCGGAAGACTCATCGCCGAGTGGCAAACCGTCGGTTTT
TCGCACGGCGTTATGAATACGGATAACATGTCGATTCTGGGGCTGACCATCGACTATGGC
CCCTTTGGTTTTCTCGACGACTACGATCCCGGCTTCATTGGTAACCATTCCGATCATCAG
GGACGTTACCGCTTCGATAATCAACCGTTGGTGGCGCTGTGGAATTTACAACGTCTGGCG
CAGACGTTAACCCCTTTTATTGAGATCGACGCGTTAAACAGAGCGCTGGATCGTTACCAG
GACGCGTTGTTAACGCACTATGGGCAGCGAATGCGCCAGAAGCTGGGTTTCTTTACCGAG
CAAAAAGACGATAACGCTTTACTGAATGAATTGTTTAGTCTGATGGCGCGCGAAGGGAGT
GACTATACCCGTACATTTCGGATGTTAAGCCATACGGAGCAACAGAGCGCTTCCTCGCCG
TTGCGGGATACGTTTATCGATCGCGCGGCGTTCGATGCCTGGTTCGATCGCTACCGGGCG

CGATTGCGCACAGAGGCGGTGGATGACGCGCTGCGCCAGCAGCAGATGCAACGCGTGAAT
CCTGCGGTTGTGTTGCGCAACTGGCTGGCGCAACGCGCGATAGATGCGGCTGAACAAGGA
GACATGGCGGAACTGCATCGGTTACATGAAGTTTTACGTCAACCTTTTACCGATCGAGAT
GATGACTATGCCAGCCGACCGCCGGAGTGGGGAAAGCGGCTGGAGGTCAGTTGTTCAAGT
TAG
>EECEBICD_01679 putative protein YdiE
ATGTCGCGTATGGATAACACCGAGCTGCCTCACCCTAAGGAAATAGACAATGAAACGCTT
CTCCCTGCCGCTGAACGGCGCGTCAACAGTCAGGCGCTATTAGGTCCGGACGGCAAAGTC
ATCATCGATCACAACGGTCAGGAGTATCTGTTGCGCAAAACTCAGGCTGGTAAGCTACTG
TTAACCAAATAA
>EECEBICD_01680 Phospho-2-dehydro-3-deoxyheptonate aldolase, Trp-sensitive
ATGAACAGAACCGACGAACTCCGTACTGCGCGTATCGACAGCCTGGTAACACCGACCGAA
CTCGCGCAGCGGTATCCTGTATCGTCCTCCGTCGCCAGTCACGTTACCGACTCCCGACGC
CGGATAGAAAAGATTTTAAATGGTGAAGATCCACGGCTACTGGTCGTCATTGGCCCCTGT
TCGATTCACGATCTGAATGCTGCCATGGAATACGCGACGCAGCTCCAGGCACAACGCCAA
AAGCATCAGGCGCGTCTGGAAATCGTCATGCGCACCTATTTTGAAAAACCGCGCACCGTT
GTGGGATGGAAAGGCCTGATTTCCGATCCTGACTTGAATGGAAGTTACCGCGTCAATTAT
GGGCTTGAACTGGCGCGTCGCTTGCTATTGCAGGTGAACGAACTGGGAGTACCGACCGCC
ACAGAATTTCTTGATATGGTCACTGGCCAGTTTATTGCCGATCTGATCAGTTGGGGAGCG
ATTGGCGCGCGTACCACCGAAAGCCAAATCCATCGGGAAATGGCTTCTGCGCTCTCTTGT
CCGGTCGGCTTTAAAAATGGTACGGATGGCAATACTCGCATTGCCGTTGACGCTATTCGC
GCCTCCCGCGCCAGCCATATGTTTCTCTCGCCGGATAAAGACGGACAGATGACCATCTAC
CAGACGAGTGGCAACCCGTATGGGCACATCATCATGCGCGGCGGTAAAAAACCGAACTAC
CACGCTGAAGATATTGCCGCCGCCTGCGATACGTTGCATGAATTTGATCTGCCGGAACAT
CTGGTCGTCGACTTCAGCCACGGCAACTGTCAAAAACAGCATCGCCGCCAGCTGGAGGTA
TGTGATGATATTTGCCAGCAGATTCGTAATGGCTCCACGGCAATTGCCGGGATTATGGCC
GAGAGTTTTTTACGGGAAGGCACGCAAAAAATTATCAGCGGTCAACCATTAATCTATGGT
CAGTCCATTACCGATCCCTGCCTGAACTGGGAAGATACGGAAGTTTTGTTGGAAAAACTT
GCCGCAGCGGTAGACAGCCGCTTTTAA
>EECEBICD_01681 Phosphoenolpyruvate synthase regulatory protein
ATGGATAATGTTGTTGATCGCCATGTTTTCTATATTTCTGATGGTACCGCCATCACCGCC
GAGGTCCTGGGTCACGCGGTTATGTCGCAGTTTCCGGTGACGATCAGCAGCATCACGCTG
CCATTCGTGGAAAACGAAAGTCGCGCCCGCGCCGTCAAAGACCAAATTGACGCCATTTAT
CAGCAAACCGGCGTTCGCCCGCTGGTGTTTTACTCGATTGTTTTACCTGAAATTCGCGCC
ATCATCTTACAGAGCGAAGGTTTCTGCCAGGATATCGTCCAGGCGCTGGTGGCGCCGCTT
CAGCAGGAGATGAAACTGGATCCGACGCCGATCGCTCATCGTACTCATGGGCTTAATCCG
GGTAACCTGAACAAGTATGACGCCCGTATTGCCGCTATCGATTATACCCTGGCGCATGAT
GATGGCATTTCATTGCGCAACCTCGATCAGGCTCAGGTCATTTTACTTGGCGTTTCGCGC
TGTGGAAAAACGCCCACCAGCCTCTATCTGGCGATGCAATTCGGTATTCGTGCGGCTAAC
TACCCTTTCATCGCCGATGATATGGATAATTTGACGCTGCCCACATCGTTAAAACCGCTA
CAACATAAACTGTTCGGCCTGACAATTGACCCTGAACGCCTGGCGGCAATTCGCGAAGAA
CGCCGTGAAAACAGTCGCTATGCCTCGTTGCGTCAGTGCCGCATGGAAGTGGCGGAGGTC
GAAGCGCTGTATCGTAAGAATCAGATCCCCTGCCTGAACAGCACTAATTATTCCGTTGAA
GAGATAGCCACCAAGATTCTCGATATTATGGGGCTTAACCGCAGGATGTACTAG
>EECEBICD_01682 Phosphoenolpyruvate synthase
ATGTCCAACAATGGCTCGTCACCGCTGGTGCTTTGGTATAACCAACTCGGCATGAATGAT
GTAGACAGAGTTGGGGGCAAAAATGCCTCCCTGGGTGAAATGATTACTAACCTTTCCGGC
ATGGGCGTATCCGTTCCGAATGGTTTTGCAACCACTGCCGATGCGTTTAACCAGTTTTTG
GATCAAAGCGGCGTAAACCAGCGCATTTATGAGCTGCTGGATAAGACAGATATTGATGAT
GTCTCCCAGCTGGCGAAAGCGGGCGCGCAGATCCGCCAGTGGATTATCGACACTCCTTTC
CAGCCGGCGCTGGAAAACGCCATCCGCGATGCCTATGCGCAGCTTTCGTCGGATGATGAA
AATGCCTCTTTTGCCGTGCGTTCCTCCGCCACGGCGGAAGATATGCCAGATGCTTCGTTT
GCGGGTCAACAGGAAACGTTTCTTAACGTTCAGGGCTTCGACGCGGTGCTGGTGGCAGTG

AAACACGTATTTGCTTCTCTGTTTAACGATCGCGCCATCTCTTATCGCGTTCACCAGGGA
TATGATCATCGCGGCGTAGCGCTTTCCGCTGGCGTGCAGCGGATGGTTCGCTCCGATCTC
GCCTCGTCAGGGGTGATGTTCTCTATCGATACCGAATCCGGCTTTGACCAGGTGGTCTTT
ATCACCTCGGCATGGGGACTGGGTGAAATGGTGGTGCAGGGCGCGGTTAACCCGGATGAA
TTTTATGTCCATAAGCCAACCCTGGCGGCAGGCCGTCCGGCCATCGTACGCCGCACCATG
GGGTCGAAAAAAATCCGCATGGTTTACGCCGCCACCCAGGAGCACGGCAAGCAAGTGAAG
ATTGAGGATGTGCCGCAGCAACAGCGCGACATTTTCTCGCTCACCAACGAAGAAGTGCAG
GAACTGGCGAAACAAGCGGTGCAGATTGAGCAGCACTATGGTCGTCCGATGGATATCGAA
TGGGCGAAAGACGGTCATACCGGTAAGCTCTTTATTGTGCAGGCACGTCCGGAAACCGTG
CGCTCCCGTGGCCAGGTGATGGAGCGTTATACGCTACATGCGCAGGGGAAAATCATTGCC
GAAGGTCGCGCCATTGGTCATCGTATCGGCGCGGGTACGGTAAAAGTTATTCATGATATC
GGCGAAATGAACCGTATTGAGCCTGGCGATGTGCTTGTCACCGATATGACCGACCCGGAC
TGGGAACCGATCATGAAAAAAGCGGCGGCGATTGTCACTAACCGTGGCGGGCGCACCTGT
CACGCGGCGATTATCGCCCGCGAATTGGGCATTCCGGCGGTTGTCGGCTGCGGCGACGCC
ACCGAACGCATGAAAGATGGCGAAAAAGTAACCGTCTCCTGTGCGGAAGGTGATACCGGT
TATGTGTATGCCGACATGCTGGATTTCAGCGTAAAAAGCTCCAGCGTAGATACCATGCCA
GAGCTACCGCTGAAGGTCATGATGAACGTCGGCAACCCGGACAGGGCATTTGACTTTGCC
TGCCTGCCGAATGAAGGCGTAGGTCTGGCGCGACTGGAATTCATCATTAACCGTATGATT
GGCGTTCACCCACGCGCGCTGCTGGAGTTTGACGACCAGGATGCCGCGCTGCAAAACGAC
ATTCGTGAGATGATGAAAGGGTTTGACTCGCCGCGTGAATTTTACGTTGGTCGACTGACG
GAAGGGATCGCGACGTTAGGCGCCGCCTTCTACCCGAAACGCGTCATCGTTCGCCTGTCT
GACTTTAAGTCCAACGAATACGCCAACCTGGTCGGCGGCGAACGTTATGAGCCGCATGAA
GAAAACCCGATGCTGGGCTTCCGCGGCGCGGGCCGTTACGTCGCAGAAAGTTTCCGCGAT
TGTTTTGCGCTGGAATGCGAAGCGGTGAAACGCGTGCGCAACGATATGGGCCTTACCAAC
GTCGAAATCATGATCCCGTTCGTGCGGACGGTCGACCAGGCGAAAGCGGTCATCGAAGAG
CTGGCGCGCCAGGGGCTTAAACGCGGCGAGAACGGGCTGAAGATCATCATGATGTGCGAG
ATCCCGTCCAATGCCTTGCTGGCTGAACAGTTCCTCGAGTATTTTGATGGCTTCTCGATT
GGTTCTAACGACATGACGCAGCTGGCGTTGGGGCTGGATCGCGATTCCGGCGTGGTGTCT
GAACTGTTTGACGAGCGTAACGACGCGGTGAAAGCGCTGCTGTCGATGGCGATCCGCGCG
GCGAAAAAACAGGGCAAATATGTTGGAATTTGCGGTCAGGGTCCGTCCGACCATGAAGAC
TTTGCCGCCTGGCTGATGGAAGAGGGGATCGACAGCCTGTCGCTGAACCCGGACACCGTG
GTTCAGACCTGGCTGAGTCTGGCGGAACTGAAGAAGTAG
>EECEBICD_01683 Medium-chain fatty-acid--CoA ligase
ATGAGTGTTACATTAACGTTTGACGCCGCAAGGCGGAAAACCTATCGCGAGTCCGGCTAC
TGGGGCGATGCTTCACTGGGCGACTACTGGCGGCAAACCGCACGCGCCGTACCCGATAAA
ATCGCCGTGGTCGATAATCATGGCGCGTCCTGGACCTACGCCGCGCTCGACTACGCGGCA
AGCCGCCTGGCAAACTGGTTACTGTCTCAGGGGATTCAACCGGGCGATCGCGTGGCCTTT
CAGCTTCCGGGCTGGTGTGAGTTTACCCTCATTTATCTGGCCTGCCTGAAAACGGGCGCG
GTATCTGTCCCGCTACTTCCCGCCTGGCGAGAAGCAGAGCTGGTTTGGGTGCTGAACAAA
TGTCAGGCTAAAATATTCTTCGCCCCCACCGTATTCAAACAGAATCGTCCGGTCGATCTT
ATCCTTCCGCTACAAAATCAACTGCGCCATCTGACGCATATTGTCGGCGTGGATAAACTG
GCGCCTGCCACCACAGCGCTTGCGCTTAGTCAAATCATCGACCGCAGCGAACCGTTGCAG
TCAGACATTAACATTCACGGTGATGAACTGGCGGCAGTGCTGTTTACCTCCGGCACAGAA
GGAATGCCGAAAGGGGTGATGTTGACCCACAATAATATTCTTGCCAGTGAACGGGCGTAT
TGCGCGCGGTTGAATTTAACCTGGCAAGATGTGTTCCTGATGCCTGCGCCACTGGGCCAC
GCCACCGGATTTTTACACGGCGTCACCGCGCCCTTTTTAATCGGGGCGCGTAGCGTATTG
CTGGACATCTTTACCCCAGAAGCCTGCCTTACCTTATTAGCGCAGCAACGCTGTACCTGT
ATGTCAGGCGCGACGCCGTTTATTTACGATCTGCTTTGTGCCGTTGAGCAACAGCCTGCC
GACCTCTCCTCACTGCGATTCTTCTTATGCGGCGGCACGATTATTCCCAAAAAGGTTGCC
CGCGACTGCCAGCAGCGCGGTATCAAATTATTGAGTATTTACGGTTCTACAGAAAGTTCT
CCACACTCGATGGTTAATCTGGGTGATTCGACTTCACGCATGATGAATACCGATGGTTAT
GCCGCCACCGGTGTAGAAATTAAAATCGTGGATGAAGATCGCAATACGCTTCCAGCAGGC
CACGAAGGCGAAGAAGCCTCGCGCGGGCCGAACGTTTTTATGGGATACCTGGACGAGCCG
GAACTTACCGCCCGCGCACTGGATAACGAGGGCTGGTATTACAGCGGCGACCTCTGCCGC

ATGGATGAAGACGGTTATATCAAAATTACCGGGCGCAAAAAGGATATTATTATACGTGGA
GGTGAAAATATCAGCAGTCGCGAGGTGGAGGATATTTTATTGCAACACCCCCGCATACAC
GATGCTTGCGTCGTTGCTATGCCTGATGAACGCTTAGGCGAACGTTCATGCGCGTATGTA
GTCTTAAAACCGCCGCACCTTTCGCTGACGCTGGAAGAGGTGATCGCTTTTTTCAGTCGA
AAACGGGTAGCGAAGTATAAATATCCGGAACGGATCGTCATCGTAGAAAAACTGCCCAGA
ACCGCCTCTGGCAAAGTGCAGAAGTTTCTGTTGCGGCAGGATATTATTGAGCGGCTGCGC
CAGGAGCACACTGCGGTATAA
>EECEBICD_01684 Ferredoxin-like protein FixX
ATGAGTTCGGACAATAAGGTGAATGTTGACGTCAAATTGGGCGTAAATAAATTTTACGTA
GATGAAGGACACCCACATATCATTTTGCGCTCCTCGCCTGACATGCAGGAGTTTAATAAA
TTAATAAAAGCCTGCCCTGCCGGACTCTATAAGCTGGACGATGCGGGAAATATTCATTTT
GACTCCGCGGGCTGCCTGGAATGCGGGACCTGCAGGGTTCTCTGCGGCAATACTCTTCTC
GAAAAATGGGAATACCCGGCGGGAACGTTCGGCGTTGAGTTTCGCTACGGCTGA
>EECEBICD_01685 hypothetical protein
ATGATGCCGGAGCTGGCAAATGACGGCGTGATGATTGTCGGCGACGCCGCCGGTCTTTGC
CTGAATCTCGGCTATACCGTCCGCGGCATGGATTTAGCCATCGCGTCCGCGCAAGCGGCC
GCTCAAACCGCCATTGACGCTAAAGCGCGGCAAGATTTTTCCGCCGCGAGTCTTGCCGGT
TACAAACGCGCGCTGGAGAAAAACGGCGTGTTACGCGATATGCGGCATTTCCGCAAACTC
CCCGGATTAATGGAAAACCCACGGCTTTTTACCGAGTACCCGCGAATGGCGGCAAACATT
GTGGGCGATCTGTTCACCGTTGATGGTGGTCCAGTCCCACCGTTGCGTAAAACTATCCTG
TCACATGCGAAAAAAATTGGACTGGTGAATCTATTAAAAGACGGCATAAAAGGGGCTACG
GCATTATGA
>EECEBICD_01686 Electron transfer flavoprotein-ubiquinone oxidoreductase
ATGTCGGATGATAAATTTGACGCTATTGTGGTGGGCGCAGGCGTAGCAGGAACGGTCGCC
GCGTATGTGATGGCAAAAGCGGGACTGGATGTACTGGTTATTGAACGCGGAAACAGCGCG
GGCAGCAAAAATATGACCGGTGGCCGGCTCTACGCTCACAGTATCGAAAGTATTATGCCG
GGATTCGCAACGTCCGCCCCCATCGAACGCGTCGTTACCCGTGAAAAAATCTCCTTTTTA
ACCGAAGAAAGCGCCGTCACACTTGATTTTCACCGCGCGCCCCCTACTCCGCCCCTTACG
TCATATACCGTACTGCGCAACAAACTGGACCCGTGGTTAATGGCGCAGGCGGAGCAGGCT
GGCGCGCAGTTTATCCCAGGTGTACGGGTCGACGCTCTGGTACGTGAAGGCAATCGCGTC
ACTGGCGTTCAGGCCGGGGATGATATTCTTGATGCGAATATTGTGATTCTCGCGGACGGC
GTGAATTCCATGCTGGGCCGTTCACTGGACATGGTGCCTGCCTCGTCTGCGCATCACTAT
GCCGTCGGCGTAAAAGAGTTGATTGGGCTCTCTCCGGCACTTATTGAAGAGCGTTTTAAT
CTGGCGTCCCATGAAGGCGCCGCCTGGTTATTCGCTGGCGCGCCCTCCAACGGGCTCATG
GGCGGCGGGTTTCTTTATACCAACCGCGATTCGGTTTCTCTGGGGCTGGTATGTGGCCTG
GGCGACATTGCTCACGCGACCAAAAGCGTACCGCAAATGCTGGAAGATTTTAAACAGCAT
CCGGCGATACGCCCTTTGATTCAGGGGGAACGCTGCTCGAATACTCCGCTCATATGGTGC
CCGAAGGCGGCATAG
>EECEBICD_01687 Protein FixB
ATGAATCATTTAGCTAACGTATGGGTATTTAGCGATAACGTAGAGCGTTATGCCGAATTA
ATGACCGGCGCGCGGCAGTGGGGAGAAAAAGTTTACGCCATCGTGCAAGGAAACACGGAG
ATCGACTACGTGAAAGCGCTTGGCGCTGATGAGATAGTCGTTCTTGAATCGCATACGGAT
TTGCAGCGCGTGGAAAACTATGCCGAAACGCTGGCCTCATTATTAGGCGACCAAAATGGG
CTGCTGCTCATGGCGGCGACCAAGCGCTGTAAAGCGCTGGGCGCCCGGCTGAGCATCCAG
CTTGACGCCATGATGGTGAACGACGCGACGTCTATTGACCTCCTCGACGGGACGCTACAC
GCAGAGCATCGCATGTACGGCGGACTTGCTTTCGGCAAAGAGAAATTAAACAGTCCGCAG
GCGATTATTACGCTGGCACCCGGCGTGCTGGAACCCGCCGCCCCTCTCCCCGGTCATACT
TGCCCGCAGACCACGCTGGCATATATTGCCCCACGCCACGAGGTATTGTGCCAGCAGCGG
CGCGCGAAATCGCTAAGCAGCGTCGATTTGAGCAAAGCCAAACGGGTCATCGGCGTAGGG
CGAGGCCTGGCGGCGCAAAGCGATCTGGATATGGTGCATAAACTGGCGGCCGTTCTCGGC
GCAGAAGTGGGCTGTTCCCGCCCCATTGCGGAAGGTGAAAACTGGATGGAACGTGAGCGT
TATATTGGCGTATCCGGCGTTCTGTTGAAATCCGATCTCTATCTGACGCTGGGCATTTCC
GGCCAAATTCAACATATGGTGGGTGGAAACGGCGCAAAAATTATTGTCGCAATTAACAAA
GATAAAAACGCGCCCATTTTCAATTATGCCGATTATGGCCTGGTGGGCGATATCTATAAG

GTTGTTCCTGCCCTCATCGACCAGTTAAGCCGCTAA
>EECEBICD_01688 Protein FixA
ATGGTTCAGGATGCGCAACTGGAGCACGCCCTGCCCAAAGAAACCGCGCTGGCGCTAGCA
TCCGCGGCGGAGAAAATTGGCTTTGACCTGCTGCTCTTCGGGGAGGGCTCCGGCGATATT
TATGCCCAACAGGTGGGCCTGCTGACTGGCGAACTCCTGAAACTCCCCGTCATCAACGCA
GTAAGCCATATTCAACGCGACGGCGACAAAATCATCGTTGAACGCACACTGGAAGAAGAC
GTTGAAATTATCGAGCTCACGCTGCCCGCGGTGCTTTGCGTGACCTCAGATATTAACGTT
CCACGCATCCCGTCAATGAAAGCCATTCTCGGCGCCGGGAAAAAAGCCGTTACGTCGTGG
CTGGCGAGCGATATCCACTGGACCCCAACCACGCCACTGGCGGAACTGGTCGCCATCTCG
GTACCGCCGCAGACAGAACGCAAGCATATCATTCTCGATAACGATTCGCCGGAAGCCATT
ACCGCACTGGCCGACCATTTGAAAAAATCCCTGAACTAA
>EECEBICD_01689 HTH-type transcriptional activator RhaS
ATGTATCAGCGCTGTTTTGACTCCGCCGCGGAGACGATTTTTGAACAGGACAAGACCCCT
CGATTTTCACGTTTTGTCATTAGCGATGATCCAAACTGGGAGTCTGGCCATCATATGCAT
GATAATGAAACAGAGCTTATTTACGTCAAAAAAGGGATAGCCAGGTTAATTATTGACTCT
TCACTCTACGTCGCGCATGCGGACGATATTGTCGTCGTTGAGCGGGGGCGGCTACACGCT
GTAGCATCCGACAGCAATTCGCCGGCTACCACCTATACCTGTGCGCTGTATGGGTTTTGT
TTTCCAGGTTGGGAAGAAAACCAACTATTGCAGAGTCATTCCTGTCCGGTGGTTTCCGTG
GTTCAGGGGAAAGAGGTCATTAAAAGTATCTTTAATGAATTAAGCGTGTTACTTCCGCAG
GGGAAAAACGTATTGGCTTCCTCTGTATACGATGCATTTGCTTACACTCTGACCGCACTT
TACTATGAAAATTTTAAAAATGCCTATCGTTCTGAACAAGGCTACATTAAAAAAGATGTA
TTAATTAAGGATGTGCTTGTTTATCTCAATAATAATTATCGTGAAAAAATCACGCTTGAC
CAGCTATCGAAAAAATTTCGTGCCAGCGTCAGTTATATATGCCATGAATTTGCCAAAGAA
TATCATATATCCCCTATCAACTATGTTATTCAACGACGTATGACCGAGGCCAAGTTTGCG
CTGACAAACACTGAGTCGCCACTGGCAGAAATTTCATGGCGGGTAGGATATGAAAATGTG
GACCATTTTGCCAAATTGTTTATGCGGCATGTAGGATGTTCTCCCAACGATTATCGTAAG
CAATTTAAAAATAGTCTGGTTGAGCAGGCGTATTTGCTGCCAAATACCTGA
>EECEBICD_01690 Crotonobetainyl-CoA reductase
ATGGATTTTTCATTAACTGAAGAACAAGAACTATTACTGGCCAGTATTCGCGAGTTAATC
ACCAGCAATTTTCCGGAAGAGTATTTCCGTACCTGCGATCAAACGGGCACCTATCCCAAA
GAATTTATGCAGGCGCTGGCGGAAAACGGTATTTCGATGCTGGGCGTCCCGGAAGAATTC
GGCGGCATTCCGGCTGACTATGTAACGCAAATGCTGGCGCTGATGGAAATCTCAAAATGC
GGCGCTCCGGCCTTTTTGATTACCAACGGCCAATGCATTCACAGCATGCGCCGTTTTGGT
TCCGCAGAACAACTGCGTAAAACAGCAGAAAGTACTCTGGCCACCGGCGATCCAGCATAT
GCGCTAGCCTTAACAGAACCTGGCGCGGGCTCTGATAATAATAGCGCCACAACAACCTGG
ACGCGTAAAAACGGCAAGGTTTATCTTAACGGCCAAAAAACATTTATCACCGGTGCAAAA
GAATATCCTTACATGCTGGTTCTGGCGCGCGATACGGAGCCAAAAGATCCTAAAAAAGCG
TTCACGCTTTGGTGGGTGGACTCAAATAAACCGGGCATAAAGATTAATCCGCTGCACAAA
ATTGGCTGGCACATGTTGAGTACATGCGAAGTTTATCTCGATAATGTCGAGGTGGACGAA
AGCGACCGGGTCGGTGAAGAAGGCATGGGTTTCCTCAATGTCATGTACAATTTTGAGATG
GAGCGTTTGATTAATGCGGCGCGCAGTACAGGTTTCGCCGAATGCGCATTTGAAGATGCC
GCCCGTTACGCCAACCAGCGTATCGCCTTTGGTAAACCCATCGGCCATAACCAGATGATC
CAGGAAAAGCTGGCGTTAATGGCCATCAAAATCGAAAACATGCGCAATATGGTGTTGAAA
GTCGCCTGGCAGGCCGATCGGGAAGAGTCACTGCGTACCAGCGCCGCGCTGGCTAAGCTG
TATTGCGCACGCACAGCGATGGAAGTGATTGACGATGCGATTCAGATTATGGGTGGGCTT
GGTTATACTGACGAAGCCCGTGTCTCCCGCTTCTGGCGCGATGTTCGCTGCGAACGTATC
GGTGGCGGCACGGATGAAATTATGATCTATGTGGCAGGACGCCAGATTCTGAAAGACTAT
CAGAACAAGTAA
>EECEBICD_01691 Acetate CoA-transferase YdiF
ATGAAAATCAATAAGCCATCACGCATTAATGGCCGCGTTCCCGTGCTCTCTGCACAAGAA
GCAGTAAATTATATTCCAGATGAGGCCACTTTATGTATTCTGGGCGCTGGCGGCGGTATT
CTCGAAGCGACAACATTAATTACCGCACTGGCAGATAAATATCAAACAACCCAGTCACCA
CGAGATTTATCTATTATTAGCCCTACCGGATTAGGTGACCGCGCCGATCGCGGCATCAGC
CCGCTGGCGCAGGAGGGATTGGTAAAATGGGCGCTATGCGGACATTGGGGGCAATCTCCC

CGTATTTCTGAACTGGCTGAACAAAATAAAATCGCCGCCTATAACTACCCACAAGGCGTA
TTAACCCAGACGCTGCGCGCCAGCGCCGCACACCAGCCGGGTATTCTGAGCGATATCGGT
ATTGGTACCTTTGTCGACCCTCGCCAACAGGGCGGAAAATTAAACGACGTGACTAAAGAA
GATCTCATTAAGCTGGTAGAGATCGATAATAAAGAATATCTCTATTATAAAGCGATAGCG
CCTAATGTGGCTTTTATCCGCGCCACCACCTGCGACAGCGAAGGCTATGCATCATTTGAA
GATGAGGTGATGTATCTTGATGCACTGGTGATCGCCCAGGCCGTTCACAATAATGGCGGT
ATTGTAATGATGCAGGTGCAGAAAATGGTAAAAAAAGCCACGCTGCATCCAAAATCCGTG
CGTATTCCCGGCTATCTGGTGGATATTGTGGTTGTCGACGCCGACCAGACGCAACTCTAT
GGCGGCGCGCCGGTGAACCGATTTATCTCCGGGGATTTTACTCTTGATGATAGTACCCAG
CTTACCCTGCCCCTTAATCAGCGCAAGCTGGTAGCCCGACGCGCGCTGTTTGAAATGCGT
AAAGGCGCGGTAGGCAATGTGGGCGTAGGCATTGCCGACGGCATTGGTCTGGTCGCTCGC
GAGGAAGGCTGCGCCGATGACTTCGTGCTGACCGTAGAAACTGGCCCGGTGGGCGGTATT
ACCTCTCAGGGTGTGGCCTTCGGCGCCAATGTCAATACCCGCGCGATCCTCGATATGACC
TCACAATTTGATTTTTATCACGGCGGTGGGCTCGATGTCTGCTATTTAAGTTTTGCCGAA
GTGGATCAACACGGTAATGTCGGTGTACATAAATTCAACGGCAAAATTATGGGCACCGGC
GGGTTTATCGATATTAGCGCAACGTCGCAAAAAATTATTTTCTGCGGAACTCTGACGGCC
GGAAGTTTAAAAACTGAAATTACCGATGGCAAATTAAATATTCTGCAGGAAGGTCGCGTG
AAAAAAATTCGTCAGCGAACTGCCGGAAATTACGTTCAGTGGAAAAATTGCGCTTGA
>EECEBICD_01692 3-dehydroquinate dehydratase
ATGAAAACCGTAACTGTAAGAGATCTCGTGGTTGGCGAAGGCGCGCCAAAGATCATTGTG
TCGCTAATGGGAAAAACCATTACCGATGTGAAATCGGAAGCACTCGCCTACCGTGAAGCG
GATTTCGATATTCTGGAGTGGCGCGTTGACCATTTTGCCAACGTGACAACGGCGGAAAGC
GTACTTGAGGCCGCCGGCGCCATCCGGGAGATTATTACCGATAAACCCTTGCTATTTACC
TTCCGCAGCGCGAAAGAAGGCGGCGAACAGGCGCTAACTACCGGACAGTATATCGCTCTG
AATCGTGCAGCGGTTGACAGCGGTCTGGTCGATATGATCGATCTTGAGCTTTTTACCGGC
GACGACGAGGTGAAAGCCACCGTCGGCTATGCTCATCAACACAATGTTGCGGTGATCATG
TCTAACCATGATTTTCATAAAACGCCCGCAGCGGAAGAGATTGTTCAGCGTCTGCGTAAA
ATGCAGGAACTGGGCGCCGATATTCCGAAGATCGCCGTCATGCCACAGACTAAAGCCGAT
GTCCTGACCTTACTTACCGCCACTGTAGAAATGCAGGAGCGCTATGCGGATCGTCCGATT
ATTACCATGTCGATGTCGAAAACCGGGGTAATATCTCGTCTTGCCGGCGAAGTGTTCGGT
TCTGCAGCAACGTTTGGCGCGGTGAAAAAAGCATCTGCGCCGGGACAAATATCGGTAGCC
GATCTGCGTACCGTATTAACTATATTGCACCAGGCGTAA
>EECEBICD_01693 Quinate/shikimate dehydrogenase
ATGGATGTTACCGCAAAATATGAATTAATTGGTCTTATGGCGTATCCTATCCGTCATAGC
TTATCGCCCGAAATGCAAAACAAAGCGTTAGAAAAAGCGGGGCTACCGTATACCTATATG
GCTTTCGAAGTGGATAACACGACGTTTGCCAGCGCCATTGAAGGACTGAAAGCATTAAAA
ATGCGCGGTACGGGCGTCTCCATGCCAAACAAACAGCTCGCGTGTGAGTATGTCGATGAA
TTAACGCCAGCAGCAAAACTGGTTGGCGCTATCAACACCATTGTTAACGATGACGGCTAT
CTACGCGGCTACAACACGGATGGTACGGGCCATATTCGGGCAATTAAAGAAAGCGGTTTC
GATATTCGGGGAAAAACCATGGTGCTGCTCGGCGCGGGAGGCGCGGCTACCGCCATTGGC
GCCCAGGCAGCCATTGAAGGCATTAAGGAAATTAAACTGTTTAACCGTAAAGATGACTTT
TTCGAAAAAGCGGTTGCCTTTGCGAAGCGGGTGAATGAAAACACCGATTGCGTCGTTACC
GTCACCGATCTTGCCGATCAGCACGCTTTTACTGAAGCGCTTGCCAGCGCAGATATTCTG
ACGAACGGCACCAAAGTTGGCATGAAACCATTGGAGAACGAATCCCTGATCGGCGATGTT
TCCCTGCTCCGCCCTGAATTGCTGGTCACCGAATGTGTTTATAATCCGCATATGACAAAG
TTATTGCAGCAGGCGCAACAAGCTGGTTGCAAAACAATTGATGGTTACGGCATGTTGTTA
TGGCAAGGAGCCGAACAGTTTGAACTGTGGACAGGAAAAGCATTTCCATTGGACTATGTG
AAGCAGGTGATGGGATTCACAGCCTGA
>EECEBICD_01694 Inner membrane transport protein YdiN
ATGTCTCAGAACAAGGCTCGCAACATGCCATATTTGCTGGCTGTTATCTGCATTTATTTT
AGTTACTTTCTCCACGGCATGAGTGTTATTACACTAGCCCAGAACATGACCTCCCTTGCA
CAGAAATTCTCCACGGATAGTGCCGGTATCGCCTATTTAATCTCTGGCATTGGTCTTGGT
CGTCTGGTCAGTATTTTATTCTTTGGCGTACTGTCCGATAAATTTGGCCGTCGGGCAATA
ATACTGCTTGGCGCCGTACTATATATGCTATTTTTCTTCGGTATTCCCGCCAGTCCTAAC

CTGATGATCGCTTTCATATTAGCGGTGTGTGTCGGCGTGGCGAACTCCGCGCTGGATACC
GGCGGATACCCTGCATTAATGGAGTGTTTTCCCAAAGCATCGGGCTCGGCAGTTATTCTG
GTTAAAGCGATGGTCTCTTTTGGGCAAATGATTTATCCCCTTATTGTCAGCGCCTTGTTA
GTCAACCATATCTGGTACGGCTACGCGGTGGTAATCCCCGGTATCCTTTTCGTCCTCATC
ACGTTGATGCTGTTGAAAAGCCGTTTTCCCAGCCAACTTGTCGATGCCAGTATTGCGAAA
GAATTACCCCAGATGAACAGTACTCCCCTCGTCTGGCTGGAAGGCGTAGCTTCCGTTTTA
TTTGGCGTCGCCGCGTTCTCAACCTTCTATGTGATTGTGGTCTGGATGCCTAAATATGCG
ATGGCCTTCGCCGGAATGGCGGAATCCGACGCGCTGAAAACCATCTCTTATTACAGTATG
GGATCGTTGGTTTGCGTGTTTATTTTTGCCGCATTGCTGAAAAAAATGGTTCGCCCTATC
TGGGCCAATGTTTTCAATGCCGGGCTGGCGACACTCACCGCTGCGGCAATTTACCTGTAT
CCCTCTCCACTGATCTGTAATGCTGGCGCCTTCGTGATTGGTTTTTCCGCTGCTGGAGGT
ATTTTACAATTAGGTGTATCGGTAATGTCGGAATTTTTCCCTAAGAGTAAAGCTAAAGTC
ACCAGTATATATATGATGATGGGGGGCGTAGCTAACTTTATTATTCCACTGATCACCGGT
TATCTCTCTACTATTGGCCTGCAATATATCATTTTGTTAGATTTTGCCTTTGCACTACTG
ACATTTATCACCGCCATTATTGTATTTATTCGCTATTATCGCGTATTTAAGATCCCGCAA
AACGATGTCCGATTTGGCGAGCGTTATTTCCAGTAA
>EECEBICD_01695 Inner membrane transport protein YdiM
ATGAAAAATCGTTATTTCCCTACCGCTGTAGGGTTGTATTTTAATTACTTTGTACATGGC
ATGGGGGTTATCCTGATGAGCCTGAACATGTCATCGCTTGAACAGCAATGGCATACCAGT
GCCGCAGGCGTATCTATTGTGATCTCTTCGCTTGGTATTGGCCGGCTAAGCGTTTTGTTA
ATCGCCGGTATGCTTTCCGATCGCTTTGGTCGCCGTCCTTTTATCATTCTGGGGACCGCC
TGTTACCTGATTTTTTTCATTGGCATTCTTTATGCTCAGACGATTTTTGTCGCTTATGCC
TGTGGCTTTTTAGCGGGTATGGCCAACAGTTTTTTGGATGCAGGTACCTATCCCAGTTTG
ATGGAAGCCTTCCCTCGCTCCCCCAGTACGGCAAATATTCTGATCAAAGCATTTGTCTCT
GGCGGGCAATTCTTGTTGCCCATTATTATCAGTTTATTAGTCTGGGCTAATATGTGGTTT
GGCTGGTCCTTCCTGCTTGCCGGGGCGATCATGCTTATTAATGCTCTGTTTTTATTACGC
TGTCCTTTTCCACCCTATCCTGGTCGGATATTGAAGCCCAAAATCTCGCAAGCGCCTGTC
GCAGGCGTACATCATTGCTCCCTCATCGATCTTATCAGCTACACCCTTTATGGCTATATC
TCGATGGCAACCTTTTATCTTATCAGCCAGTGGCTGGCGCAGTATGGACAGTTCGTCGCA
GGAATGTCGTATACCCAATCCATCAAACTGTTAAGTATTTATACCTGCGGATCGCTACTC
TGCGTTTTTATAACCGCCCCGCTGGTCAGAAAAACAATTCGCTCTACCACATTATTGATG
TTTTATACCTTTATCTCTTTTATTGCGCTTCTGACGGTCTGCCTGCATCCTCAGGCCTAT
GTTGTTATGATCTTCGCATTCGTCATTGGATTCTCTTCCGCCGGTGGCGTAGTACAAATC
GGCCTGACGCTGATGGCGGCCCGTTTTCCACAGGAGAAAGGTAAAGCGACCGGCATTTAT
TACAGCGCCGGCAGTATCGCGACGTTCACTATTCCGTTGATCACAGCGCGGATTTCAGAA
ATGAGCATTGCACATATTATGTGGTTTGATACCGGTATCGCCGCAGTGGGATTTCTCCTC
GCACTGTTCATCGGTTACCGAAGTCGGGCTGAATCACAACATCACGCTGAAGCAGCGTCA
ACCGCGCAATAA
>EECEBICD_01696 hypothetical protein
ATGATGAACGCGTTAGAACTACAAGCACTACGGCGTATTTTTGATATGACGATTGAAGAA
TGCACAATCTATATCACCCAGGATAATAATTCGACAACCTGGCAACGATGGGAAGCTGGC
GACACACCCATCTCTCCAGAAATCATTGCCAGGCTGAAGGAAATGAAAGCCAGGAGACAG
CGACGAATTAACGCCATTGTTGACAAAATAAATAACCGCATCGGCAATAATACAATGCGT
TACTTCCCTGATTTATCTTCTTTTCAGTCCATTTATACAGAGGGTGATTTTATCGAGTGG
AAAATTTATCAATCTGTTGCTGCCGAACTTTTCGCACATGATCTGGAGCGGCTGTGTTAG
>EECEBICD_01697 Putative transport protein YdiK
ATGGTAAATGTTCGTCAGCCCAGGGATATTGCGCAAGTGCTGCTATCGGTGCTGTTTTTA
GCCATCATGATTGTGGCCTGTTTGTGGATAGTACAGCCTTTTATTTTGGGGTTTGCATGG
GCAGGTACGATTGTTATCGCGACCTGGCCCGTTTTGCTGAAATTACAAAAAATATTGTGG
GGCCGCCGCTCGCTTGCCGTATTGGTCATGACGCTGCTGTTAGTGCTGCTCTTCGTTATT
CCCGTCGCCTTGCTGGTGAACAGCATTGTCGATGGTAGCGGCCCGCTCATCCATGCAGTG
ACCGGCGGCGATATGACGCTACCCGATCTGGCATGGTTAAATAATATTCCGTTGGTTGGC
GCCAAACTGTATGCCGGATGGCATAGCCTGCTGGATATGGGCGGTAGCGCTATTATGGCG
AAAGTGCGGCCTTATATCGGCACAACCACGACCTGGTTTGTCGGTCAGGCGGCGCATATC

GGACGCTTTATGATGCACTGTGCCCTGATGCTGTTGTTTAGCGCCCTGCTTTACTGGCGC
GGCGAACAAGTGGCGATGGGTATACGCCATTTTGCCTGTCGTCTCGCGGCTAAACGCGGC
GATGCGGCGGTTCTGCTGGCTGCCCAGGCGATCCGCGCCGTGGCGCTTGGCGTGGTGGTT
ACCGCGCTGGTACAGGCGGTACTTGGCGGCGTCGGGCTGGCGATTTCCGGCGTGCCTTAC
GCTACCCTGTTGACGGTAGTGATGATCCTTTCGTGTCTGGTTCAGTTGGGGCCGTTGCCG
GTATTGATTCCAGCCATTATCTGGCTTTACTGGACAGGCGACACCACCTGGGGCACCGTG
CTGCTGGTTTGGAGCGCCGTCGTCGGCACTCTGGATAACGTGATTCGCCCGGTACTCATC
CGCATGGGTGCCGATTTACCGCTGCTTCTGATCCTTTCCGGGGTAATCGGCGGCCTGATT
GCATTCGGTATGATTGGTCTGTTTATTGGACCGGTGCTTCTGGCCGTGACCTGGCGTTTG
TTCTCCGCATGGGTACATGAAGTTCCGGCGCCGACGAATGAACCAGAGGAGATCCTTGAA
GAGCTGGATGACATCGAGGAAGCCAACAAGCAGTCATAA
>EECEBICD_01698 putative protein
ATGATTCCACAAATTTCTCAGGCACCCGGCGTCGTTCAGCTGGTGCTCAATTTTTTGCAG
GCGCTGGAGCAGCAAGGGTTTACGGGCGATACGGCAACCAGCTATGCCGACCGTCTGACA
ATGTCGACCGATAACAGTATCTACCAGCTTCTTCCCGATGCTGTTGTGTTTCCCCGTTCT
ACGGCGGATGTCGCGCTTCTTGCCCGTCTGGCGGCGGAGCCGCGCTTTACGTCGCTGATA
TTTACCCCTCGAGGCGGCGGTACTGGCACGAATGGCCAGGCGCTAAACCAGGGCATTATT
GTTGATATGTCCCGCTATATGAACCGCATTATTGAAATTAATCCTGAAGAGGGATGGGTG
CGGGTAGAGGCGGGAGTGATAAAAGATCAGCTTAATCAAGCGTTAAAGCCGTATGGCTAT
TTTTTTGCGCCGGAACTTTCCACCAGTAATCGCGCCACGCTTGGCGGTATGATTAATACC
GATGCCTCCGGTCAGGGTTCGCTGGTCTATGGAAAAACGTCAGATCACGTCTTAGGCATA
CGGGCCGTATTGTTGGGGGGCGATATCCTTGACACGCAATCGATGCCCGTAGAGCTTGCC
CGGACGCTGGGTGAAAACACGACGACGCCAGGCCGCATCTATCAGACCGTCTATCAGTCC
TGCCTGGAAAACCGCCAACTTATCCTCGATAGCTTTCCAAAGCTCAATCGCTTCCTGACA
GGCTACGATTTACGCCACGTCTTTAACGATGACATGACCCGCTTCGATCTCACCCGCATC
TTAACGGGGTCGGAAGGGACGCTGGCGTTTATTACTGAAGCCCGGCTGGATATTACCCCG
ATACCCAAAGTCCGCCGTCTGGTGAATGTAAAATACGACTCCTTTGATTCCGCGCTGCGT
AACGCCCCTTTTATGGTTGATGCGCGCGCCTTATCCGTCGAGACGGTCGATTCCAAAGTC
CTTAACCTCGCGCGGGAAGATATTGTCTGGCACTCCGTTAGCGAATTGATTACCGATGTG
CCGGATAAAGAGATGCTTGGCCTGAATATCGTGGAATTCGCGGGTGACGATGAAGCGCTT
ATTGACGGACAGGTCGAGGCGCTGTGCCACCGGCTGGATGGCCTGATGGCGCGAGCGGAA
GCGGGCGTGATCGGCTGGCAACTTTGTACCGATTTGACGGGTATTGAGCGTATTTACGCG
ATGCGTAAAAAAGCGGTAGGGCTGCTGGGTAATGCGAAAGGCGCTGCCAAACCGATTCCT
TTCGCGGAAGATACCTGCGTGCCGCCTGAACATCTGGCCGATTACATCGCCGAATTTCGC
GCTCTGCTGGATGGTCATGGCTTAAGTTACGGCATGTTCGGCCACGTGGATGCCGGGGTG
CTGCATGTGCGTCCGGCGCTGGATATGTGCGATCCGCAGCAGGAACTGTTGATGAAACAG
ATCTCCGATGAGGTCGTCGCGCTGACCGCCAGATACGGTGGGCTGTTGTGGGGCGAGCAT
GGAAAAGGTTTTCGTGCGGAATACAGCCCGGCCTTTTTCGGCGAAGTTCTGTATGGCGAG
CTGCGCAAAATAAAAGCGGCTTTTGATCCGCATAATCGACTTAACCCGGGTAAAATCTGT
CCGCCGCAGGGGATAGAGGCGCCGATGATGAAGGTGGATGCGGTAAAACGCGGCACCTGG
GATCGGCAAATTCCGCTGGCGGTACGGCAGACCTGGCGCGGCGCTATGGAGTGTAATGGT
AACGGCCTGTGCTTTAATTTCGATGCGAAAAGCCCGATGTGCCCGTCGATGAAAATTAGC
CTTAACCGGATCCACTCGCCGAAAGGGCGGGCGACGCTGGTGCGGGAATGGCTCCGTCTA
TTGGCCGATCGCGGCGTCGATCCGTTGAAGCTGGAAAAGGAGTTGCCAGAAAAACGCGCC
AGCTTACGCACGCTGATTGCCCGCACCCGGAATAGTTGGCACAAACGCAAAGGCGAGTAT
GACTTTTCCCATGAAGTTAAAGAGGCCATGTCCGGCTGTCTGGCGTGTAAAGCCTGTACG
ACCCAGTGTCCGATTAAAATCGATGTTCCGGAGTTTCGTTCACGTTTCTTACAGCTTTAC
CATACGCGTTATCTGCGCCCGGTACGCGATCATCTGGTGGCGACGGTCGAGACCTACGCG
CCGCTGATGGCGCGTGCGCCAAAGACCTTTAACTTTTTTATTAACCAACCGGTGGTTCGT
AATCTGGCGAAAAAACATATCGGTATGGTGGATTTACCTCTGCTTTCTGCCCCTTCATTG
CAACAGCAACTGGTGGGCCATCCGTCTGCCAATATGACGCTTGAGCAACTGGAACGCATG
AGCGCCGAGCAAAAAGCGAAAACGGTACTGGTGGTTCAGGATCCGTTTACCAGCTACTAC
GACGCCCGGGTCGTGGCGGACTTTATCCGCCTGGCGGAAAAACTGGGCAGGAGGCCGGTA
TTGTTACCGTTCTCGCCGAATGGTAAGGCGCAGCATATTAAAGGTTTTCTTAATCGCTTT

GCGAAAACGGCGAAAAAAACCTCTGAGTTCCTTAATCGGATCGCCGCGTTGGGGATGCCG
ATGGTGGGCGTCGATCCGGCGCTGGTTCTGTGTTATCGCGACGAATACAAACTGGCGCTG
GGTGAACAGCGCGGCGATTTTCATGTGTTGCTGGTGAATGAATGGCTGGCGCAGGAAATA
AATGCAAGACTGTCCGTCGAGGTGAGCGGCGAACCGTGGTACTTTTTTGGTCATTGTACG
GAAGTCACGGCTTTACCCGGCGCGCCTGCGCAGTGGGCCGCCATCTTTGCCCATTTTGGC
GCGAAGCTGGAAAATGTCAGCGTAGGCTGCTGCGGTATGGCGGGCACTTATGGACATGAA
CTCACCAATCATAAAAATTCGCTGGGAATTTATGAGCTATCCTGGCATCAGGCGATGCAA
CGTCTACCGCGAAACCGCTGCCTGGCAACCGGCTATTCCTGCCGAAGCCAGGTTAAACGC
ATTGAAGGTACGGGCGTGCGTCACCCGTTGCAGGCATTACTGGAGATTATAGGTTGA
>EECEBICD_01699 1,4-dihydroxy-2-naphthoyl-CoA hydrolase
TTGATCTGGAAACGTGAAGTGACCCTCGATGCGCTTAATGCGATGGGGGAAGGAAATATG
GTTGGCCTGCTTGATATCCGCTTTGAGCGTATTGGGGACGATACGCTGGAAGCGACAATG
CCCGTCGATCATCGCACCAAACAGCCGTTCGGCTTGCTGCATGGCGGAGCGTCTGTGGTG
CTGGCGGAAAGTATCGGCTCGGTCGCAGGTTATCTGTGTACGCAGGGGGAGCAAAAGGTG
GTGGGGCTGGAGGTGAATGCCAACCATGTACGGTCGGCGCGTCAGGGGCGCGTACGAGGC
GTCTGTAAAGCGTTGCATACCGGGGCTCGCCATCAGGTCTGGCAGATAGAGATCTTTGAT
GAACAGGGACGTCTGTGCTGTTCTTCGCGTCTTACTACGGCGATTGTGTGA
>EECEBICD_01700 hypothetical protein
ATGTCTACTGATTTAGACCCGACCCAACTGGCGATTGAATTTTTACGCCGTGATAAAACC
GAACTTTCTCCCGCGCAGTATTTGAAGCGTTTAAAACAGCTGGAGTTAGAGTTTGCCGAT
CTCCTCACCCTCTCTGCAACCGAACTGAAAGAAGAGATCTATTTCGCCTGGCGGTTGGGC
GTGCATTAA
>EECEBICD_01701 L-cystine uptake protein TcyP
ATGGTTGTTACGCTCGCGTATATCGCACTTTTTTTGGTTTTCTCGTGGGTCATCTTGAGA
ATTAATCAAAAAAGCGATTCCCTGTCGAAAAGCGTTTTTATCGCTATCTTTTTAGGGGCT
GTTATTGGTTTATCCCTGCATTTTATTTCAGCAAATCACACTAAAACTATTATCGAATGG
TACAGCATCGTCGGCAATGGTTACGTCCACCTGTTAAAACTGGTCGCTATACCGCTAATT
TTTATTTCTATTCTTTCCGCCATCAATAAACTGGAAAATAGCGCCGGCATCGGAAAAATG
TCGCTGACGATCGTCGGATGCATGTTATGCCTGGTGATGGTTGCCGGTTTTATCGGATTA
CTGACCGCTCATGTGCTGGGGCTTGACGCCAGCGCTTTTGTGCACATGCCGTCAATGTTA
ACTACAGAAGAAGTCAATAAGACTGCCGCGGTGTCGATTCCCCAGTTAGTGACATCGCTG
ATCCCGACTAATATTTTTCTTGATCTTACGGGAGCCAGAAGTGTTTCCGTTATCGGCATC
GTTATCTTCACGCTAATAGCGGGGATCGCTCTGTTAAAGGTCAAAAAAGAGGCGCCGGAA
GAAGGTCAGAAATTAAGCGCAGGCATTAACGCTATCCAGATCTGGGTCATGAAGATGGTA
CGTATCGTTATTGCATTAACGCCCTATGGCGTCATGGCATTAATGACTACCGTATTTTCA
TCATACCACTTTGAACAATTCGCCAGCTTACTTGGTTTTATCGGCGCCTGTTATATCGCG
ATTTTCATGATGTTTATCGTGCATGCCATCTTGCTGATCCTCAGCGGTAATAATCCAGCG
CGTTATTTCAGTATGGTCTGGCCCGTCTTAACCTTTGCGTTTGTTTCCCGCAGCAGCGCA
GCCTCTATCCCGCTGGCCATTTCCGCCCAGGAAAAAATTTGGCGTACAAAGCACTATTGCC
AATATTTCCGCCTCGTTTGGCTCCAGTATGGGGCAAAATGGCTGCGCCGGGATTTATCCG
GCTATTATGGTAGCGATGATTGCGCCCACCATCGGCATTGATCCGCTCTCGCTGCATTTT
CTGGCCGCGATGTTGCCTGCCATTGCGCTAGGGTCTATTGGCGTAGCCGGCGTCGGCGGC
GGCGGTACGTTCGCGGCGCTGATTGTCCTGTCGACGTTGAATTTTCCCGTTGCGCTGGTC
GGTATTTTTATTGCCATCGAACCTATCGTTGATATGGCCCGCACGGCTCTGAACGTTAAC
GGATCGATGATGTCAGGTGTGCTGGCTAACCGTATTTTGAATAATCATACGGCTGACGAC
ATGCCAGCGGTTATTGACAGACCTTAG
>EECEBICD_01702 Protein SufA
ATGGAATTGCATTCAGACACGTTTAACCCGGAGGACTTCCCCTGGCAAGGGCTGACGCTA
ACGCCAGCGGCGGCAGCGCACATACGTGAACTGGCAGAAAAGCAGCCGGGCATGTTGGGC
GTGCGGTTAAGCGTAAAACAGACGGGATGTGCCGGATTTGGCTATGTTCTGGATACCGTT
CGGGAGCCGGATAAAGACGATCTGGTTTTTGAAGCCGAAGGCGCAAAGCTTTTCGCACCG
TTACAGGCGATGCCTTTTATTGACGGAACAGAAGTGGATTACGTACAGGAAGGGCTGAAC
CAGTTATTTAAATTTCATAACCCCAAAGCCCAGAATGAATGCGGCTGCGGCGAAAGTTTT
GGGGTATAG

>EECEBICD_01703 FeS cluster assembly protein SufB
ATGTCTCGTAACACTGAAGCAACGGAAGAGGTCGGCATCTGGACAGGCGGACGCCTTAAT
TACAAAGAAGGATTTTTCACGCAACTACCCACTGATGAACTGGCAAAAGGCATCAGCGAA
GAGGTGGTGCGCGCGATTTCCGCAAAGCGTAACGAACCTGAGTGGATGCTGGAATTTCGT
CTCAATGCCTATAGAGCCTGGCTGGACATGGATGAACCGCACTGGCTGAAGGCGCACTAC
GATAAACTGAACTATCAGGACTACAGCTATTACTCTGCGCCATCATGCGGAAACTGCGAC
GATAGCTGCGTTTCCCAGCCGGGAGCCGTACAGCAAACGGGCGCGAATACCTTTTTGAGT
AAAGAGGTTGAAGACGCCTTCGAGCAACTTGGCGTTCCGGTTCGCGAAGGGAAAGAGGTC
GCCGTCGATGCGATTTTTGATTCGGTCTCCGTAGCGACCACATACCGTGAAAAACTGGCG
GAACAGGGCATTATTTTCTGTTCTTTTGGCGAAGCGATACACGATCACCCTGACCTGGTC
AGGAAATATCTTGGCACCGTCGTGCCGGGGAACGATAACTTTTTTGCCGCGCTGAATGCG
GCAGTCGCTTCCGATGGGACATTTATCTATGTTCCCAAAGGCGTGCGTTGCCCAATGGAG
TTATCCACCTATTTTCGCATTAACGCCGAGAAAACCGGTCAGTTTGAGCGCACGATTCTG
GTGGCGGATGAAGGCAGCTATGTGAGTTATATCGAAGGCTGCTCCGCGCCCGTACGCGAC
AGTTATCAGCTCCACGCCGCGGTAGTGGAAGTCATCATCCATAAAGATGCTGAAGTAAAA
TATTCCACCGTACAAAACTGGTTCCCGGGCGACAACAACACGGGCGGCATTCTGAACTTT
GTGACCAAGCGGGCGTTGTGCGAAGGGGAAAACAGCAAGATGTCGTGGACCCAGTCGGAA
ACCGGTTCGGCGATTACCTGGAAATACCCGAGCTGTATCCTGCGCGGTGATAATTCTATC
GGCGAGTTCTATTCCGTTGCGCTGACCAGTGGACATCAGCAGGCTGACACCGGCACCAAA
ATGATCCATATCGGTAAAAACACGCGCTCAACCATTATCTCAAAAGGCATTTCTGCCGGC
CACAGCCAGAACAGCTACCGTGGGCTGGTAAAAATTATGCCGACGGCAACCAACGCCCGA
AATTTCACCCAATGCGACTCCATGTTGATTGGAGCGGACTGCGGCGCGCATACGTTCCCG
TATGTTGAATGCCGCAATAACAGCGCGCAGCTGGAGCACGAGGCGACAACATCACGCATC
GGTGAAGATCAGCTTTTCTATTGTCTGCAACGCGGCATCAGCGAAGAAGACGCTATTTCG
ATGATCGTCAACGGTTTCTGCAAAGACGTTTTCTCGGAACTACCGCTGGAGTTCGCCGTT
GAAGCGCAAAAACTGCTGACGATTAGCCTTGAGCACAGCGTCGGTTAA
>EECEBICD_01704 putative ATP-dependent transporter SufC
ATGTTAAGCATTAAAGATTTACAGGTCAGTGTGGAAGAAAAAGCGATTTTGCGTGGCCTG
AATCTGGAGATTCGCCCCGGCGAAGTTCACGCCATTATGGGGCCGAACGGTTCCGGGAAA
AGCACGCTCTCCGCCACCCTGGCGGGACGTGAAGATTATGAGGTCACCGGCGGCTCGGTT
ACGTTCAAGGGTAAAGATCTGCTTGAACTGTCGCCGGAGGAACGGGCGGGCGAAGGGATT
TTTATGGCCTTCCAGTACCCGGTAGAAATCCCCGGCGTCAGTAACCAGTTTTTCCTGCAA
ACCGCGCTGAATGCCGTGCGCGCCTACCGCGGGCAGACGTCGTTGGATCGCTTTGATTTT
CAGGATCTGATGGAAGAAAAGATCGCATTGCTGAAAATGCCGGAAGATTTACTGACCCGT
TCGGTAAACGTCGGTTTTTCCGGCGGGGAGAAAAAGCGCAATGATATTTTGCAAATGGCG
GTGCTGGAGCCGGAACTGTGCATCCTTGATGAGTCGGACTCCGGACTGGATATCGATGCG
TTGAAAATTGTGGCGGAAGGCGTCAACGCGTTACGCGACGATAAACGCGCTTTTATTATC
GTGACCCACTATCAGCGCATACTGGACTATATCAAACCCGATTACGTCCATGTCCTCTAT
CAGGGGCGAATTGTCCGATCCGGCGATTTTACTCTGGTCAAACAACTGGAGGAGCAGGGT
TATGGCTGGCTTACCGAACAGCAGTAA
>EECEBICD_01705 FeS cluster assembly protein SufD
ATGGCTGGCTTACCGAACAGCAGTAAAGCGCTACAGCAATGGCAGCATCTCTTTGAGGAA
AAAAGAGAAAGTCGAACTGAGCAGGCAAGACAACATTTACAACAGATGTTACGTCTGGGA
TTGCCGACGCGTAAACATGAAGACTGGAAATACACGCCGCTGGAGGGCTTAACCCATAGC
CAGTTTATCCAGCAGTGTGCGACTATCAGCGCCGCACAGCGTGATGCGTTGGCGCTGCAA
ATCGATGCCGTGCGGCTGGTGTTTGTCGATGGTCGGTTTATGCCAGAGCTCAGTGATAGC
ACGCAAAACAGCGGATTTGACGTTAGCGTCAGGGATGAGCGCCAGACGCTTGCCGCGCCG
GTACAGCCTGAGGTTTTTCTGCATCTGACGGAAAGCCTGGCGCAATGTGTCACGTATATA
CAGGTACGGCGAAATCAGCGTCCGACCAGGCCGCTACTGTTAATGCATATCACGCAGGGC
GTGGATGGCGACGAGCTGAACACCGCGCATTATCGCCACCATCTGGCGCTGGCGGAAGGC
GCGGAAGCGACGGTTATTGAGCATTATGTCAGCCTTACAGCGGCAAAACATTTTACCGGC
GCGCGTCTGACGATGAACGTTGCAGACAATGCGCAACTGCATCATATCAAACTGGCGTTT
GAAAATGCGTCCAGCTATCACTTTGCGCATAACGATCTTCTGCTGGCGACAGACGCCTCG
GCGTTTAGCCACAGTTTTCTGCTGGGCGCCGCAGTACTACGTCACCACAGCAGCTCACAA

CTGAATGGTGAAAACGCGACGCTTCGACTGAATAGTCTGGCGATGCCGGTGAAAAATGAA
GTTTGCGATACGCGGACATGGCTGGAACATAACAAAGGCTACTGTAACAGCCGCCAGTCG
CACAAGACCATCGTCAGCGACAAAGGACGCGCGGTCTTTAATGGCCTGATTAACGTGGCG
CAACACGCCATTAAAACCGACGGCCAGATGACCAATAATAACCTGTTGTTGGGGAAACTG
GCTGAAGTCGATACCAAACCGCAATTAGAGATCTATGCCGATGACGTGAAGTGCAGCCAC
GGCGCGACGATCGGGCGTATTGACGATGAGCAGATGTTTTATCTGCAATCACGCGGCATC
AGACAGCAGGAGGCGCGGCACATGATTCTTTACGCGTTTGCCGCAGAGTTAACGGAAGCT
ATCCATGATAGCGCCCTTAAACAGCAGGTGCTGGCGCGAATCGGCCAGCGACTGCCCGGA
GGCCTGGTATGA
>EECEBICD_01706 Cysteine desulfurase
ATGACATTTCCTGTAGAAAAAGTACGGGCGGATTTTCCCATACTGCAGCGTGAAGTTAAC
GGCCTGCCGCTGGCTTACCTGGACAGCGCAGCCAGCGCTCAAAAACCTAATCAGGTGATT
GATGCTGAATCTGCCTTCTACCGTCACGGCTATGCTGCGGTACATCGAGGTATCCATACG
TTAAGCGCGCAGGCGACCGAAAGCATGGAGAATGTGCGTAAGCAGGCGTCGCGGTTTATT
AACGCCCGCTCCGCAGAAGAACTGGTGTTCGTGCGCGGTACGACGGAGGGCATTAACCTT
GTCGCCAACAGTTGGGGAACGGAAAATATTCGCGCCGGGGATAACATTATCATCAGCGAG
ATGGAGCATCACGCCAACATCGTTCCCTGGCAGATGCTGTGCGAGCGCAAAGGCGCTGAA
CTGCGCGTGATCCCGTTGCATCCTGACGGTACGCTGCGGCTGGAGACCTTAGCTGCGCTG
TTCGATGACCGGACCCGACTGCTGGCCATTACCCATGTTTCCAATGTGCTGGGGACGGAA
AACCCACTGCCGGACATGATTGCGCTGGCGCGCCAGCATGGGGCGAAAGTGCTGGTGGAT
GGCGCCCAGGCCGTGATGCACCATGCTGTTGACGTCCAGGCGCTGGACTGCGATTTTTAC
GTTTTCTCCGGCCATAAACTTTACGGGCCGACCGGCATCGGCATTCTGTATGTTAAAGAG
GCGTTGCTGCAAGAAATGCCGCCGTGGGAAGGGGGCGGGTCGATGATCTCGACCGTCAGC
CTGACGCAGGGAACGACATGGGCGAAAGCGCCCTGGCGTTTTGAGGCGGGAACGCCGAAT
ACTGGCGGCATCATCGGTCTCGGCGCGGCGATTGACTATGTGACGTCGCTGGGACTGGAT
AAGATTGGCGATTATGAGCAGATGCTGATGCGCTATGCGCTGGAGCAACTGGCGCAGGTG
CCTGATATCACGCTGTATGGTCCGGCGCAGCGATTGGGCGTCATCGCGTTTAATCTGGGT
AAACACCATGCTTACGACGTCGGCAGCTTTCTTGATAATTACGGTATCGCGGTACGAACG
GGACATCACTGCGCAATGCCGCTCATGGCCTGGTATGGCGTGCCGGCAATGTGCCGGGCT
TCGCTGGCGATGTATAACACCCATGAAGAAGTGGACCGACTGGTGGCAGGATTAACGCGT
ATCCACCGCTTATTGGGATAA
>EECEBICD_01707 Cysteine desulfuration protein SufE
ATGGCTGCGCTACCGGATAAAGAAAAACTGCTGCGTAACTTTACGCGTTGCGCTAATTGG
GAAGAGAAATATTTATATATTATTGAGTTGGGGCAGCGCCTGGCGGAGTTAAATCCGCAG
GATCGTAATCCGCAAAATACGATTCATGGCTGTCAGAGCCAGGTGTGGATCGTGATGCGG
CGAAACGCCAACGGCATTATCGAATTGCAGGGCGACAGCGACGCGGCGATCGTTAAGGGC
CTTATGGCGGTGGTATTTATTTTGTACCACCAAATGACGGCGCAGGATATTGTGCATTTT
GATGTCCGGCCATGGTTCGAAAAAATGGCGCTTGCGCAGCACCTCACACCTTCTCGTTCT
CAGGGGCTGGAAGCAATGATTCGCGCAATTCGCGCTAAGGCCGCCACTCTTAGCTAA
>EECEBICD_01708 putative L,D-transpeptidase YnhG
ATGAAACGTGCGTCTTTCATTACGCTAACTATTATCGGCGCGTATAGCGCGTTACAGGCA
GCCTGGGCGGTTGATTATCCATTACCGCCCGAAGGCAGCCGTCTTATTGGTCAGAATCAA
ACCTATACCGTACAGGAAGGTGATAAAAACCTGCAGGCTATCGCCCGGCGTTTTGATACG
GCGGCGATGCTCATTCTTGAAGCGAACAATACGATTGCGCCGGTGCCTAAGCCCGGTACG
CTAATTACCATTCCCTCGCAGATGCTATTGCCGGATGCGCCCAGGGAAGGCGTTATCGTC
AACCTCGCGGAGCTACGGCTGTATTATTACCCGCCGGGAGAAAACCGCGTTCAGGTGTAT
CCCATCGGCATCGGTTTGCAGGGACTGGAAACTCCCGTCATGGACACCCGGATAGGGCAA
AAGATCCCCAACCCGACGTGGACGCCGACGGCGGGCATACGCCAACGTTCGCTTGAGCGG
GGGATCACGCTGCCGCCGGTGATCCCTGCCGGGCCAAATAACCCGCTGGGACGCTATGCG
CTGCGTCTGGCGCACGGTAATGGGGAATATCTCATTCATGGCACCAGCGCGCCGGATAGC
GTAGGTCTGCGCGTGAGTTCCGGTTGCATTCGCATGAACGCGCCGGACATCAAAGCGTTA
TTTGCGCAGGTCAGAACGGGGACGCCGGTAAAAGTGATTAACCAGCCGGTGAAATTCTCT
GTCGAGCCGAATGGCATTCGTTATGTAGAGGTGCACAGGCCGCTATCGCCGGAAGAAGAG
CAAAACGTGCAGACGATGCCCTACGTGTTGCCTGCGGAATTTACCGCGTTCAGAAACGCG

CAAGGGGTGGATAGTCGCCTGGTCGATAAGGCGCTATACCGGCGAGCCGGGTATCCAGTC
AGCGTGAGCGCCGGGCAGACGCCAGCGGTAAATACGACCGCAGTCGAATCCGCTCAGAAC
GGTTTTGTCGGGGAAGAGGGGCAAACGCGCGCGACGCAGTAG
>EECEBICD_01709 Major outer membrane lipoprotein Lpp
ATGGCGCACATCGTGCGCCATTTTTTTACCCATATAACCACGCAAAGTATAATGTTATT
GTTATGAACCGTACTAACCAGCTGATCTTAGGTGCAGTAGTTCTGGGTTCCACGTTACTG
GCCGGTTGTTCAAACAACGCTAAAATCGATCAGTTGTCTTCCGATGTACAGACGCTGAGC
GCTAAAGTTGAGCAACTGAGTAATGACGTAAATGCAATGCGTTCCGACGTTCAGGCTGCT
AAAGACGACGCAGCTCGCGAGAACCAGCGTCTGGACAACAAAGTAGTATCCCACGTCCGT
AAGTAA
>EECEBICD_01710 Major outer membrane lipoprotein Lpp 1
ATGAATCGTACTAAACTGGTACTGGGCGCGGTAATCCTGGGTTCTACTCTGCTGGCTGGT
TGCTCCAGCAACGCTAAAATCGATCAGCTGTCTTCTGACGTTCAGACTCTGAACGCTAAA
GTTGACCAGCTGAGCAACGACGTGAACGCAATGCGTTCCGACGTTCAGGCTGCTAAAGAC
GACGCAGCTCGCGCTAACCAGCGTCTGGACAACCAGGCTACTAAATACCGTAAGTAA
>EECEBICD_01711 Pyruvate kinase I
ATGAAAAAGACGAAAATTGTTTGTACTATCGGTCCGAAAACCGAATCCGAAGAGATGTTA
AGCAAAATGCTGGACGCGGGCATGAACGTGATGCGTCTGAACTTCTCTCATGGCGATTAT
GCAGAACACGGTCAGCGTATCCAGAACTTGCGCAACGTGATGAGCAAAACCGGGAAAAAA
GCCGCTATTCTGCTTGATACTAAAGGACCAGAAATCCGCACCATTAAACTGGAAGGCGGT
AACGACGTTTCGCTGAAAGCGGGTCAAACCTTCACTTTTACCACCGACAAATCCGTGGTC
GGTAATAATGAAATCGTTGCCGTCACCTATGAAGGCTTCACCAGCGACCTGAGCGTTGGC
AACACCGTCCTGGTTGACGATGGTCTGATCGGTATGGAAGTGACCGCCATCGAAGGCAAT
AAAGTCATTTGTAAGGTGCTGAACAACGGCGATCTGGGCGAGAACAAAGGCGTTAACCTG
CCGGGCGTTTCTATTGCTCTGCCAGCGCTGGCTGAAAAAGACAAACAGGACCTGATCTTC
GGTTGCGAACAGGGCGTTGACTTTGTTGCTGCGTCCTTTATCCGTAAACGTTCTGACGTT
GTCGAAATTCGCGAGCATCTGAAAGCCCACGGCGGCGAAAACATCCAGATTATCTCTAAG
ATCGAAAACCAGGAAGGCCTGAACAACTTCGATGAAATTCTCGAAGCTTCCGACGGCATC
ATGGTTGCGCGTGGTGACCTGGGCGTAGAAATCCCGGTTGAAGAAGTGATTTTTGCGCAG
AAAATGATGATCGAAAAATGTATCCGCGCACGTAAAGTCGTGATCACCGCGACTCAGATG
CTGGATTCTATGATCAAAAACCCGCGTCCGACCCGCGCAGAAGCAGGCGACGTAGCGAAC
GCCATCCTCGACGGTACGGATGCGGTTATGCTGTCCGGCGAATCCGCCAAAGGGAAATAC
CCGCTGGAAGCCGTTTCTATCATGGCGACCATCTGCGAACGTACCGACCGCGTCATGAAC
AGCCGTCTGGACTACAACAACGACAGCCGTAAACTGCGCATTACGGAAGCAGTATGCCGC
GGCGCGGTAGAAACCGCCGAAAAACTGGAAGCGCCGCTGATCGTGGTAGCGACCCAGGGC
GGTAAATCCGCTCGCGCCGTGCGCAAATACTTCCCGGACGCCACCATCCTGGCATTGACC
ACCAATGAAGTGACCGCCCGTCAGTTAGTACTGAGCAAAGGCGTGGTATCGCAGCTGGTG
AAAGAAATCAATTCTACTGATGATTTCTACCGTCTGGGCAAAGATGTCGCCCTGCAAAGC
GGCCTCGCCCAGAAAGGCGACGTCGTAGTGATGGTCTCCGGCGCGCTGGTTCCAAGCGGC
ACCACTAACACCGCCTCTGTTCACGTACTGTAA
>EECEBICD_01712 Putrescine importer PuuP
ATGAAAAAAGTCAAAAAATTGTCTCTTACCGATTTAGTGCTTTATGGTCTGGTGTTTATG
GTGCCTATCGCGCCAGTCTCTCTTTATGGCGTCGTTTATAATCTTTCGCACGGTATGGTT
GCGCTGGTTTATATTATTGGCGCTATCGCGATGTTTTTTACCGCGTACAGCTACTCTACC
TTATCGCAGCATATCTCCTCGTCGGGTTCGGCCTATGCGTATGCTGGCGTATGCATTAAT
CCCGCCGTAGGGTTTCTGACCGGATGGATATTACTGCTGGATTATCTTTTGTTCCCTACG
CTCGTGGCAGTGCTGGGCGGCGTCGCGGTCCACGCTATTTTACCGCAAATCCCGGTCTGG
GTCTGGCCGTTGATTTATGTGGCTATCGGCACCGGAGTCAACTATCTCGGGATACAGCAA
ACGGCGAAATTCGATAAGTTACTGGTCTTTATTCAACTCGGTATTCTGGCGATTTTCGTG
CTACTCATTGTCCGTCTGATGCTGCTGGACAGTAGTCAGATAACGCTTTCCTTCCGGCCT
TTTTTCGATTCACAGTGGTTCACCCCAGGGCTCATCGCGACGGCGATTTCGGTGGCTGCG
CTGAACTTTCTCGGCTTTGACGCTATCAGTACATTAAGTGAAGAAAGCGAAGGCGGGGGA
CGCGCCGTCAGTAAAGCAACGCTGTTGGCGTTAGTTTTGGCGACGGTACTGTTTATTATC
GTTGTGGCGTTTGCGGCGTTCGCCACCGGTAATGTCGACCGCTTCGCGGAAGGCAATGCG

ACGAATGAAGCATTTTTCACCATTGCCGGCAACGTTGGCGGTATCTGGCTCAAAGTGGCA
TTTTCCATCATTGTGGCGTTTGTGTGCGCCGTCGGCAACATTATTACCGCGCAAACGGCG
GTGTCCAGAGTGCTATTTTCAATGGGGCGCGACCGAATGTTGCCCGCCTTCCTCGCTCAC
GTCCATACCACGCGTAAAACGCCGGATTATGCCATTCTGTTTACCGGCGGCGTCACCCTG
CTGCTCAGCTACCTGTTTTCCGGCAAGATTGAGTCTATCTCCACGCTGGTGAACTTTGGC
GCGCTTTTCGCTTTTTTTGTCGTGAACCTGTGTGTGTTTATCCTGTTCAATTTCCGGATG
AAAGCCCAGCGGCGCATTTTCGCCCATGTCATTTCGCCGATCATGGGGATGATCGTCATC
GGCTACGTCTGTTTAAACATGAACATTCACGCTCTGATACTCGGAATTAGCTGGGCGGCG
ATCGGGATAGCGATTCTGTGCTATCGAAAAGCACATAACCAAAACATCGCCATCGACCTG
GAAGGCAAAAAGTTGCTCGATTGA
>EECEBICD_01713 L-amino acid amidase
ATGAATGATACTTATCTCTATGGCGAAATGTTTTCTGAAAACATATTGACAAGCTATCGG
ATTTATGGAGACAGAAAATCATTACTCACGCCGCTCATTATCCTTCATGGCGGCCCTTCT
GGCGGCTTTGATTATTTATTAAATTATCGCCGTCTGGCTGATGACGGGCGCATGGTAATA
TTTTACGATCAATATGGCTGCGGTCGTTCAACGCATTTTCCTCATGCTGACGCGTCTTTC
TGGACTATTGCGCGTTATCTCAGTCAATTAACCCAATTAATTCATCACCTGGGTATTGGA
CACGGGTATTCGATACTGGGACATTCCTGGGGCGGAATGCTTGCCGCAGAGCACGCCTGC
TTACAGCCTGCCGGGCTTCGCGGGACGATACTGGCCAGTTCGCCGGCCAGTATCGCCCTG
TGGCAACAGGAAGCCATACGGCTCTTTAACGCTCTGACGCCAATGTCCGATGACGATATC
AAAAACGTCATTATGCCCGCCGTCATTTATCAAAACCCACCGGAACAGCTGGTCGCTTAT
TATGCCAGACACGTTTATACCCTCGCGGAAGAAGCCGTTCATGTTCAGCGCTCTAATGCG
CAGTTCGCCGCCGACCCTACGGGTTATCACATTTTATGGGGAACCAATGAACTGGCGGCA
AATGGCAAACTTGCCGACTGGGATATTACGCCCCATTTGTGCCAGATACGCTGTCCTGTT
CTGGTACTGCGCGGCGAAAACGATCAGGCAACCGAGCGCGTCGTCTCTCCGCTTCTCTCT
CACATTTCAGACTGCCGCGCGGTCACTATTCCTGGCAGCAGCCATAACCCGCATGAAGAA
AATATCGCGCCCTGCCTCGCAGCGGTAAGCGCGTTTTTACGCGATCTGGCATAA
>EECEBICD_01714 hypothetical protein
ATGAAAATTTTATTTATTGGCGAATCCTGGCACATTCATATGATTCACTCTAAAGGTTTT
GATAGCTTTACATCGAGCAAATATGAAGAAGGCGCAGACTATTTATTATCCTGCTTACGC
CAGGGTAATATCGATGTGGACTACATGCCTGCCCATATTGTGCAAACTCGTTTTCCACAT
ACCGCCGAAGCATTAGCCTGCTATGATGCGATCGTCATCAGTGATATCGGTAGCAATACG
TTCCTGCTACAAAACAGAACGTTTTATAATATGGACATTATTCCTGATGCATTACAACTT
ATCGCCGATTATGTTGCTGAGGGCGGCGGTCTGTTGATGATCGGCGGGTATTTATCCTTT
ACGGGAATAGAAGCAAAAGCCAATTATAAAAATACGGTTCTGGCGGAGGTATTGCCGGTT
GATATGCTGGATGTAGATGACCGCGTAGAACTACCGCAAGGATGTAAAGCAGTAAATACT
GCGGTAGAGCATGTTATTACCCAGCCATTTAGCGAATGGCCGCCGCTGTTGGGCTATAAC
AAGCTTATCGCCAAAGAAAATAGTCAAGTCCTTGCTGAAATTAATGGCGATCCACTGTTA
GTTATGGGTACTTACCATAAAGGGAAAGTATGTTGCTTTGCCAGTGATTGTTCGCCGCAC
TGGGGAAGCCCGCAGTTTCTGCAATGGGAACATTACGCCACCTTCTGGTGTAATGTTTTA
CACACAATAAAAAAATAA
>EECEBICD_01715 Ribokinase
ATGTTTAAAGAAGAAAGACGTCATGCCATCATTAATTTACTGATAAAGGATAATAGTGTT
AGCGTCAGTAAACTTTCAGACCTTTATAAGGTTAGCCAGGAGACTATTCGTTCCGATCTA
CGCTATTTCCAGAAATCAGGTATGCTTCAGCGTTGCTATGGCGGAGGGATTTTAAACCGT
GACGCGCTGAGTAAGCTTATCACTGAAAATAAGATTGATATCTCCAGCACTATCGCCACG
CCAATCCATCAGGATGCAAAACTGCGCCGGGAAAACCCAAAAAAAGCAGGCAAGGTGTGT
GTTTTAGGCTCATTCAATATTGATGTTTCAGCAACCGTGCCGTGGTTTCCACAAAGCGGA
GAATCCATTCTGGCCAGTCAATTTGGATTCTATCCTGGCGGTAAAGGAGCCAACCAGGCT
TTAGCGGCGAACAATGCCGGCGCTGCGGCACATTTTATTTTTAAAGTGGGCAAAGATCAG
TTCAGCGCATTTGCTATGAATCATATTATTCAATCAGGTATCGCCTCATACAGCGCGTAT
CAAACAGATAAAGCGCCCACCGGTAGCGCATTGATCTATGTCTCCGCCGTGGATGGCGAT
AATATTATCGCCATCTACCCTGGCGCCAATATGATGCTCACCACGCAAGAGATTAACGAG
CAACACCGTTATATCGCCGAGTCTGACGTTATGTTAATGCAGCTCGAAACGAACATTGAA
GCGTTGACTGAATTTATTCGTCTGGGCAAACAAGAAAATAAAATGATCATGCTGAATCCT

GCCCCCTATACGAAACAGGTGACGCATTTATTATCTGATATTGACATCATCACGCCGAAT
GAAACTGAAGCCTCTTTTTTATCCGGCGTAACCATTACTGATATTAATGATGCGAAAAAA
GCCGGAAATATTATTCTGCAATCCGGGGTGAAAAAAGTCATCATTACCCTTGGCGCCCGT
GGATCTCTGCTCTGTGAGCACGCCCGCACGTTGTATATTCCTGCGTGGAGCGCCGTGGTA
AAAGATGCCGCCGGGGCCGGTGACGCTTTTAATGGCGCCTTAGCCGCCGCGCTGGCGCGA
CAAGCAGACATGGTCGCAGCCATTCAATATGCCTCCGCTTTCGCTTCTCTGGCGGTGGAA
CAAGTCGGTGCGTCGAGTATGCCTCAGCACTTGCAGGTTTTACATCGAATGCGTACCCAA
TCTAATAAAGTCATTCACATTAATTAA
>EECEBICD_01716 Tetrathionate reductase subunit A
ATGGCTAATTTAACCCGTCGTCAGTGGCTAAAAGTCGGTCTCGCCGTCGGTGGGATGGTC
ACTTTTGGTCTGAGCTACCGTGATGTGGCGAAACGCGCAATTGATGGCCTGTTAAACGGG
ACATCCGGCAAGGTAACGCGCGACCGCATCTTTGGCAATGCGTTAATTCCGGAGGCGCAG
GCGCAAACGCACTGGCAGCAAAATCCACAACAAACCATCGCCATGACGCAATGCTTCGGC
TGTTGGACACAGTGCGGTATCCGCGCCCGGGTTAATGCCGATGGCAAAGTGATACGCATC
GCCGGCAATCCCTATCACCCCTTGTCGCAGGAACACCCGATTGACTCGTCCGTCCCTTTT
AGCGAAGCCATGGAGCAACTGGCGGGAGAAAGCGGTCTTGACGCCCGCTCAACCGCCTGC
GCGCGCGGCGCCACGCTGCTGGAAAGCCTGTACAGTCCGCTACGACTGCTTGAACCGATG
AAACGCGTGGGTAAACGCGGCGAAGGGAAATGGCAGCGCATCAGCTTTGAGCAACTTATT
GAAGAAGCCGTGGAAGGCGGCGATCTGTTTGGCGAAGGTCATGTGGACGGACTGCGCGCT
ATTCATGCGCCGGATACGCCAATTGACGCAAAGCACCCCAGTTTCGGGCCCAAAACCAAT
CAGTTACTGGTCACGAATACCAGCGACGAAGGCCGCGATGCGTTTCTGCGTCGTTTTGCG
CTAAATAGCTTCGGCAGCAAGAATTTCGGCGCGCATGGCGCCTACTGTGGACTGGCTTAC
CGGGCCGGCTCCGGGGCATTGATGGGCGATCTGGATAAAAACCCGCATGTCAAACCCGAC
TGGGAAAACGTGGAGTTTGCGCTCTTTATGGGCACCTCCCCGGCACAGTCCGGCAATCCG
TTTAAACGCCAGGCACGTCAGTTGGCGAGCGCCCGACTGCGTGAGAATTTTCAATACGTC
GTGGTTGCCCCCGCCCTCCCCTTATCAACGGTGCTCGCCGATCCTCGCGGTCGCTGGCAA
CCGGTCATGCCCGGCAGCGATTCGGCGCTGGCAATGGGGATGATCCGCTGGATCATGGAT
AATGAACGTTATAATGCTGATTATCTGGCGATTCCCGGCGTACAGGCTATGCAGCAGGCC
GGCGAGCAAAGTTGGACCAACGCCACGCACCTGGTCATTGCGGATGAACTGCCGACGCTT
GCCGGACAACACCTGACGCTGCGCCATCTTACGCCCGATGGCGAAGAGACCCCTGTCGTG
CTGAATACCGACGGCGAGTTGGTCGATGCGTCCACTTGCCGACAGGCACGGCTTTTCGTG
ACGCAGTACGTTACGCTCGCCGACGGCCAACGGGTCACGGTGAAGAGCGGGTTGCAACGC
CTGAAAGAGGCGGCAGAAAAGCTCTCGTTGGCGCAATACAGCGAACAGTGCGGCGTACCG
GAAGCGCAAATTATCGCGCTGGCGGAAACCTTTACCAGTCACGGACGTAAAGCCGCGGTC
ATCAGTCACGGCGGCATGATGGCCGGCAATGGGTTTTATAACGCCTGGTCGGTCATGATG
CTTAACGCGCTGATCGGCAACCTCAGCTTGTCCGGCGGCGTCTTTGTCGGCGGCGGCAAA
TTCAACGGCGTTAGCGACGGCCCCCGCTACAACATGAACAGTTTTGCCGGAAAAGTGAAA
CCGTCCGGGTTAAGTATTGCCCGTAGCAAAACCGCTTATGAAGCATCGGAAGAATACCGC
GACAAAATTGCCGGCGGGCAATCCCCTTATCCAGCCAAAGCGCCGTGGTATCCCTTTGTG
GCAGGCCAGCTTACCGAACTGTTGACCTCCGCGCTCGAAGGCTATCCTTATCCGCTTAAA
GCCTGGATTTCCAATATGAGCAACCCGTTTTACGGTGTTCCCGGTCTACGCGCCGTGGCG
GAAGAAAAGCTAAAAGACCCTCGCCGACTGCCGCTCTTTATCGCGATTGACGCCTTTATG
AATGAAACGACGGCGCTGGCGGATTACATTGTGCCGGATACGCACAATTTTGAGAGCTGG
GGCTTTACGGCGCCCTGGGGCGGCGTAGCCAGTAAAGCCACTACCGCCCGCTGGCCGGTT
GTCGCCCCCGCCACTCGCCGAACGGTGGACGGGCAACCTGTCTCAATGGAAGCATTTTGT
ATTGCGGTAGCAAAACGGCTCCATCTGCCCGGCTTCGGCGACCGGGCGATAACCGATCCG
CAGGGCAATACTTTTCCACTGAACCGGGCGGAAGACTTCTATCTGCGCGTAGCCGCTAAT
ATCGCCTTTATGGGCAAGACGCCGGTCGCGCTGGCAAATCAGGAAGATATTTCGCTTACC
GGCGTCAGCCGCATTCTGCCAGCAATTCAGCACACGCTTAAAGCTGATGAGGTCGGTCGC
GTGGCGTTTATCTACTCGCGTGGCGGCCGGTTTGCGCCCGAGGATAGCGGCTATACGGAG
CAACGGTTAGGTAACGCGTGGAAAAAACCCTTACAGATCTGGAATGCAGATGTCGCCGCC
CACCGTCACGCCATCACCGGGGAGCGCTTCAGCGGTTGCCCGGTCTGGTATCCGGCGCGT
TTGTCAGATGGTCGTGCGATTGACGACCAGTTTCCCATTGGGCAATGGCCGCTGAAACTG
ATTTCATTTAAATCAAATACCATGTCCAGCTCAACAGCCGTCATCCCGCGCTTACACCAT

GTGAAGCCAGCAAACCTGGTGGCGCTGAATCCGCAAGACGGCGAGCGTTATGGACTGCAA
CATGGCGATCGGGTACGGATCATTACGCCGGGCGGTCAGGTCGTGGCGCAAATCAGTTTG
TTAAATGGCGTGATGCCAGGCGTCATCGCCATCGAACACGGATATGGCCACCGCGAGATG
GGCGCAACGCAGCACTCTCTGGATGGCGTGCCTATGCCGTATGATCCACAAATCAGGGCA
GGCATAAATCTTAACGATCTGGGCTTTGCCGATCCGACAAGAACCATTACCAACACCTGG
CTCGACTGGGTTTCTGGCGCGGCAGTACGTCAGGGGCTGCCGGCAAAAATCGAGCGTATA
TAA
>EECEBICD_01717 Tetrathionate reductase subunit C
ATGACGCATTCACTCATCATTGAAGAAGTGCTGGCTCACCCGCAGGACATTAGTTGGCTG
CCGTGGGCGGTACAATATTTCTTTTTTATTGGCATTGCCGCCTGCGCCGCACTGTTTGCC
TGTTATCTTCACTGGCGGAAAAAAGACGCCGCAACAGAAGAAAATCGGGCATTACTGATT
GCCATTACCTGTGCGATTACCGCACCGCTGGCGCTGACGGCGGATCTGCACCAGACTGCC
CGCGTCTGGCATTTCTATGCCTGGCCGACGCCCTGGTCGTGGATGCCCTGGGGAGCGTTG
TTCCTGCCGCTGTTTACCGGATTTCTCGCTCTGTGGTTCCTGGCGCAGCAGATTAAACGA
TTATTCAATAAAAGTTACAACGTCACTAAATGGTTGGCGTTAGCCAGCGCGCTTTGCGCG
GTGGGCCTGTTGATTTATACCGGCCGCGAAGTCTCCGTTGTGCTGGCGCGCCCAATCTGG
TTTAGCTACGCCTTCCCCGTGGCGATGTTCCTTAGCGCCTTACAGGCGTTCTTCGCGCTG
ATGATTGTCGCCGCCCGACGCGACTCGGTAAGGCTGCCAAAAATATTGTGGGGACAAATC
TGGACGCTGGCGGCGCTGGGGCTGGTTGTGGCCATGTGGGTTAGCGGCGATACGCTTTCC
GGCACGGCAATCCGTCAGTGGATTACCGTCGCCCTGTCAGCCAAATATTACGCTGTCGGC
TGGCTAGCGCTGTGGGTATTCACGCTGCTGTTCTGTAGCCTGACGCTACGTCATCCGTTA
TCGCAGCTAAGACGCGTCCTGCTGGTTCTCAGCGCGCTGGCGCTATGTTGGCTGATGCGC
TGGACATTGTTGATTCAGGTACAAACCGTCCCCAAGTTCAACGCGCAATTTAACCCTTAC
TCGTTACCAGGCGGAACGGATGGCTGGCTGGCTATTCTCGGCACCTTCGGCCTGTGGATA
GCGCTACTGATTATTATTCGTGAAACGCTGAACGGACTCACCAGGAGATTACAACATGGC
TAA
>EECEBICD_01718 Tetrathionate reductase subunit B
ATGGACAGCAGTAAACGGCAATTTCTCCAGCAGCTCGGCGTCCTGACCGCTGGCGCCTCG
CTGGTTCCGCTGGCTGAAGCGAAATTTCCTTTTTCGCCGGAGCGGCATGAAGGCTCTCCC
CGACACCGTTACGCCATGCTTATCGACCTGCGGCGTTGTATCGGCTGTCAGTCCTGTACC
GTAAGTTGCACTATTGAAAACCAAACGCCGCAGGGCGCGTTTCGTACGACGGTGAACCAA
TACCAGGTCCAGCGTGAAGGTAGTCAGGAAGTCACGAATGTGCTATTGCCGCGTCTGTGC
AACCATTGCGATAACCCCCCCTGTGTGCCGGTCTGCCCGGTACAAGCCACCTTTCAGCGG
GAAGATGGCATTGTGGTGGTGGATAACAAACGCTGCGTCGGCTGCGCCTATTGTGTCCAG
GCGTGTCCTTACGACGCCCGATTTATCAATCATGAAACGCAAACTGCCGATAAATGCACG
TTTTGCGTCCATCGTCTGGAAGCCGGGCTGTTACCCGCTTGCGTAGAGTCCTGCGTCGGC
GGCGCGCGTATTATTGGCGATATCAAAGATCCCCATAGCCGCATCGCCACCATGCTTCAT
CAGCATCGCGACGCTATCAAGGTATTAAAGCCGGAAAACGGCACGTCGCCCCATGTTTTC
TACCTGGGTCTGGACGACGCCTTTGTCACCCCATTAATGGGCCGTGCGCAGCCCGCGCTT
TGGCAGGAGGTCTGA
>EECEBICD_01719 Tetrathionate sensor histidine kinase TtrS
GTGAGAGGTAAAACCGTAAGGCGCCTGGCGGTGTTGGCGGCAGTAGGGCTACTTTGTCAT
GGCGCGTGGGCAGGGACGTGGAATATCGGTATTTTGGCCATGCGCGGCGAGGCGTCTACG
CGTAGCCACTGGCAACCGTTGGCAAAGACATTAAGCCAACAGCTTCCAGGCGAAACCTTT
CATATCCAGCCGCTGGATCTGCATCAAATGCAGGAAGCCGTTAACCAGGGAACCGTGCAG
TTTGTGATAACCAACCCGGCGCAATTTGTCCAACTGAACAGCCATGCGCCGCTGCGCTGG
TTAGCTTCCCTGCGTTCCACGCGCGATGGGAAAGCGGTGAGTAATGTTATTGGCAGCGTG
ATTTTGACCCGGCGCGATAGCGGCATCACCACGGCGCATGATCTCATCGGTAAGACAGTC
GGCGCGATTGATGCTCAGGCGTTTGGCGGCTATTTATTAGGCTATAAAGCGCTCAGCGAC
GCGGGCTTACGCCCGGAGCGCGATTTTCATCTTCGTTTTACCGGATTTCCTGGCGATGCC
TTAGTCTATATGCTGCGCGAAAAAGCGGTGCAGGCGGCAATTGTGCCAGTGTGCCTGTTA
GAAAATATGGATCAGGAAGGATTGATTAATAAAAAGGACTTTATCGCGCTGCTTTCCCGA
CCGACGCCACTGCCTTGCTTAACCAGTACGCCGTTATATCCTGACTGGTCGTTCGCGGCG
CTACCTGCGGTAAGCGATGCGCTGGCGGATCGCGTAACGCGAGCGCTATTCAACGCGCCC

GCCGCCGCGTCATTTCACTGGGGCGCGCCAGCGTCGACCAGTCAGGTGGAAGCTTTGCTG
CGTGATGTTCGTCAGCACCCTCAGCAGCGCCGACTGTGGCTGGATGTCAAAAGTTGGTTA
ATCCAGCACCAGCTAATGGTCGGTGGCGTGATTCTGGCGTTCCTGTTGCTCACGCTCAAT
TATATTTGGGTCATGCTGCTGGTGCGTCGACGCGGAAAACAACTGGAACGTAATAATGTA
GTCCTTCATCAGCATGAGCGGGCGCTGGAAACCGCCCGGCAAATGAGCGTGTTGGGTGAA
ATGACCTCCGGGTTTGCCCATGAGCTTAATCAGCCGCTTTCCGCGATTCGACATTATGCC
CAGGGGGGCCTGATTCGACTGCGCGCTGCAGATGAACAGCATCCCTTGCTGCCGGCGCTG
GAGCAGATTGACCAGCAGGCGCAACGCGGTGCGGATACTCTGCGTAACCTGCGTCACTGG
GTCAGCCAGGCGCAGGGCAACCCGGTGCTAACCGAAGCGTGGAAGGCCATAGCCATTCGC
GAGGCGATTGATCATGTCTGGCAATTGTTGCGTATGGCGCAACAGTTTCCGACAGTGACT
CTGCATACCGAGGTTAGCGCTGCGCTGCGCGTAACGCTGCCGTCAGTGCTGCTGGAACAG
GTGCTGGCGAATATCATTCTTAATGCGGCTCAGGCGGGCGCCACCCATTTATGGGTCGTT
GCTGAACGCACTGAACACGGCATCAGTATCGCTTTACAGGATAACGCCGGGGGAATCGAT
GAGGCGCTATTACGTCAGGCGTTTCAGCCGTTTATGACCACCCGCAAAGAGGGGATGGGC
TTAGGGCTGGCGATTTGCCAGCGGCTGGTGCGGTATGGGCGGGGCGATATCAGTATCAGG
AACCAGACCGCGCCGGACGGTCTGTCGGGAACGGTGGTCACGATACATTTCTTACATGAA
AATGGGGGCAGGGATGGCGACAATTCATCTACTGGATGA
>EECEBICD_01720 Tetrathionate response regulatory protein TtrR
ATGGCGACAATTCATCTACTGGATGATGATACGGCGGTCACTAACGCGTGCGCGTTTTTA
CTGGAAAGTCTGGGATATGACGTAAAATGCTGGACGCAGGGGGCGGATTTTTTGGCGCAG
GCCAGTCTGTATCAGGCCGGGGTCGTATTACTGGATATGCGAATGCCGGTACTGGATGGG
CAGGGCGTTCATGATGCGTTGCGCCAGTGCGGAAGTACCCTGGCGGTTGTTTTTCTTACC
GGGCATGGCGATGTACCGATGGCCGTGGAGCAGATGAAACGCGGCGCCGTCGATTTTCTG
CAAAAACCGGTATCGGTAAAACCGCTACAGGCGGCGCTGGAGCGTGCGCTGACGGTTTCA
TCGGCAGCGGTGGCGCGTCGTGAGATTATACTGTGTTACCAGCAGTTGACGCCGAAAGAG
CGTGAGCTGGCCAGCCTGGTGGCAAAAGGATTTATGAACCGTGAAATTGCGGAAGCGATG
AATATCGCGGTGCGTACCGTAGAGGTGCACCGCGCCAGAGTCATGGAAAAAATGCAGGCC
GGTAGCCTGGCGGAACTGATTAGGCGTTTCGAAAAAATGGCCTCGCCAGAGACCAGAATA
CGAACAACGTATGAGCCATGA
>EECEBICD_01721 Fumarase D
ATGACAAAACCGACAAAAGATGACGAACTGTACCGCGAGATGTGCCGGGTGGTGGGCAAG
GTCGTGCTGGAAATGCGCGATCTGGGGCAGGAGCCTAAATATATTGTTATTGCGGGCGTG
TTAAGAACCGCGCTGGCGAATCAACGCATCCAACGTAGCGCGTTAGAAAAACAGGCTATG
GAAACCGTGATTAACGCTCTGGCGCGGTCATAG
>EECEBICD_01722 hypothetical protein
ATGAATAACAAACCCGTAGTCGGCATTAGCGGATGTCTGACTGGCGCCACGGTACGCTTT
GATGGCGGACATAAGCGTATGGATTTTGTCATGAACTCTCTGGCGCCGTGGGTGGACTAC
AAACCGATCTGCCCGGAGGTGGCGATCGGCCTGCCCGTGCCTCGCCCTGCGCTGCGTCTT
ATCCAGACGACCTGTGGCGACATTCGGATGCGCTATAGCCATGCCCCGCACGATGACGTC
ACCGAGCGAATGAATGCCTTTGCCGATCGGTTTCTGCCAACAATAGGCGAACTGGCGGGG
TTTATTGTGTGTGCAAAATCGCCCAGTTGCGGAATGGAGCGCGTTCGCCTGTATGATGAA
AAGGGTAACAGGGGCCGCAAAGCTGGCACGGGGCTGTTTACCGCCGCGATGATGGACAAA
TATCCCTGGCTGCCGATAGGGGAAGATGGTCGCCTGCACGATCCGGTACTACGCGAGAAT
TTCATCGCACGTATCTTCGCGCTACATGAATTAAATGCGCTACGCGCGCAGGGCTTGAGT
CGTCACAGTTTGCTCGCGTTTCATAGCCGATACAAACTTCAGCTTCTCGCCCACCACCAG
GCCGGCTACCGCGAAATTGGCCCTTTCGTCGCGCGTCTGCATGAGTGGGGCGATCTGGAT
GCCTTCTTTGTCCGATACAGAGAAAAACTGATGGCTATCCTGCGCCACCCCGCTTCACGT
AAAAATCACACCAATGTGCTGATGCATATCCAGGGCTATTTTCACCGCGCGTTGAACAGC
AGGCAACGCGCGGAATTACGCGAGGTGATTCTCGGCTACCGGGCGGGCCAACTACCTATC
CTGGCACCGTTAACGCTGCTGAAGCACTATCTGGCGGAACATCCAGATGACTATCTACGC
AGCCAGAATTATTTTGAGCCGTACCCTGATACGTTAGGGCTGCGCTTAGCCATCACCTGA
>EECEBICD_01723 HTH-type transcriptional regulator MlrA
ATGTCGTATTCTATAGGCGAATTTGCCAGACTATGCGGTATCAATGCCGCCACGCTAAGG
GCATGGCAGCGACGCTATGGTTTATTGAAACCGCAGCGTACTGATGGCGGGCATCGCTTA

TACAGCGATGACGATATTCGACAAGCGCTTAGCATCCTCGACTGGGTGAGAAAAGGCGTA
CCGATAAGCCAGGTCAAACCCTTACTGTCGCGTCCGGTCATTCGCCTGGGCGATAACTGG
ATAACGATCCAGGAGACGATGCTCCAACATCTCCACGAAGGGCGAATTGACGCGCTGCGG
CTGTTGATTTATGACTGTGGCCGGGAATATCCTCGCGCAGAACTGGTGACCCATTTATTG
CGTCCGTTGCGCAGCAAAGTGTCCGCGCACCTTCCCGCCGTGATGACGCTGCGCGAAATA
CTGGATGGCATCATTATTGCTTACACCTCTTTTTGCCTTGAAGGCGACAGAAAAGCGCCT
GGCAACAATGCCTTTATTAGCGGATGGCATCTCTCGGATCACTGTGAAATCTGGCTGGAA
GCGTTGACTCGTACCGGACAAGAGCTGCGGCTCAATGTGCTTCCCTCTCCACCAGCCGCG
CTGGCGCCCGAGCTGTTCGCCCAGAGAAAATGGTTCCTGGTGACCACCGGAAAGCTCACT
GCCGGGCAGAAAAAACAGCTTGCCCAGTGGCGCAACGTGGTCGCTTCGCTGGAGGTTATC
ACGCTATAA
>EECEBICD_01724 Oxygen regulatory protein NreC
ATGAAAGAATATAAGATCTTATTAGTAGACGATCATGAAATCATCATTAACGGCATTATG
AATGCCTTATTACCCTGGCCTCATTTTAAAATTGTAGAGCATGTTAAAAATGGTCTTGAG
GTTTATAATGCCTGTTGCGCATACGAGCCTGACATACTTATCCTTGATCTTAGCTTACCT
GGCATCAATGGCCTGGATATCATTCCTCAATTACATCAGCGTTGGCCAGCAATGAATATT
CTGGTTTACACAGCATACCAACAAGAGTATATGACCATTAAAACTTTAGCCGCAGGTGCT
AATGGCTATGTTTTAAAAAGCAGTAGTCAGCAAGTTCTGTTAGCGGCATTGCAAACAGTA
GCAGTAAACAAGCGTTACATTGACCCAACGTTGAATCGGGAAGCTATCCTGGCTGAATTA
AACGCTGACACGACCAATCATCAACTGCTTACTTTGCGCGAGCGTCAGGTTCTTAAACTT
ATTGACGAGGGGTATACCAATCATGGGATCAGCGAAAAGCTACATATCAGTATAAAAACC
GTCGAAACACACCGGATGAATATGATGAGAAAGCTACAGGTTCATAAAGTGACAGAGTTA
CTTAACTGTGCCCGAAGAATGAGGTTAATAGAGTATTAA
>EECEBICD_01725 hypothetical protein
GTGACATTGGCTACCTTAGCTCGCTATATCAGTATTGCCGCAGAATATCAACTTTTGCGA
AATATAGAGCTACAGGAGCAGGATCCGAGTCGCTGCTCAGCGTTACTGGCGACAGATGAT
ATGGTCATTAATAGCAAGATTTTCCAATCACTGGACCTCTTGCTGGCTGATATTGAAAAT
GCTGTATCGGCTGGACAAAAAATCGATCAGTTAATTCACACATTAAAAGGCTGTTTAGGT
CAAATAGGGCAGACTGAATTGGTATGCTATGTCATAGACATTGAGAATCGCGTAAAAATG
GGGAAAATCATCGCGCTGGAGGAACTAACCGACTTACGCCAGAAAATACGTATGATCTTC
AAAAACTACACCATTACTTAA
>EECEBICD_01726 Response regulator receiver protein
ATGCTTGTCAGCCTGGGACAACACGTCACTGTTGCCGCCAGTAGTAACGAGGCTCTGACT
TTATCACAACAGCAGCGATTCGATTTAGTACTGATTGACATTAGAATGCCAGAAATAGAT
GGTATTGAATGTGTACAATTATGGCACGATGAGCCGAATAATTTAGATCCTGACTGCATG
TTTGTGGCGCTATCCGCTAGCGTAGCGACAGAAGATATTCATCGTTGTAAAAAAATGGGA
TTCATCATTACATTACCAAACCAGTGA
>EECEBICD_01727 Sensor histidine kinase RcsC
ATGAATTTGCTCAATCTCAAGAATACGCTGCAAACATCTTTAGTAATCAGGCTAACTTTT
TTATTTTTATTAACAACAATAATTATTTGGCTGCTATCTGTGCTTACCGCAGCTTATATA
TCAATGGTTCAGAAACGGCAGCATATAATAGAGGATTTATCCGTTCTATCCGAGATGAAT
ATTGTACTAAGCAATCAACGGTTTGAAGAAGCTGAACGTGACGCTAAAAATTTAATGTAT
CAATGCTCATTAGCGACTGAGATTCATCATAACGATATTTTCCCTGAGGTGAGCCGGCAT
CTATCTGTCGGTCCTTCAAATTGCACGCCGACGCTAAACGGAGAGAAGCACCGTCTCTTT
CTGCAGTCCTCTGATATCGATGAAAATAGCTTTCGTCGCGATAGTTTTATTCTTAATCAT
AAAAATGAGATTTCGTTATTATCTACTGATAACCCTTCAGATTATTCAACTCTACAGCCT
TTAACGCGAAAAAGCTTTCCTTTATACCCAACCCATGCCGGGTTTTACTGGAGTGAACCA
GAATACATAAACGGCAAAGGATGGCACGCTTCCGTTGCGGTTGCCGATCAGCAAGGCGTA
TTTTTTGGGGTGACGGTTAAACTTCCCGATCTCATTACTAAGAGCCACCTGCCATTAGAT
GATAGTATTCGAGTATGGCTGGATCAAAACAACCACTTATTGCCGTTTTCATACATCCCG
CAAAAAATACGTACACAGTTAGAAAATGTAACGCTGCATGATGGATGGCAGCAAATTCCC
GGATTTCTGATATTACGCACAACCTTGCATGGCCCCGGATGGAGTCTGGTTACGCTGTAC
CCATACGGTAATCTACATAATCGCATCTTAAAAATTATCCTTCAACAAATCCCCTTTACA
TTAACAGCATTGGTGTTGATGACGTCGGCTTTTTGCTGGTTACTACATCGCTCACTGGCC

AAACCGTTATGGCGTTTTGTCGATGTCATTAATAAAACCGCAACTGCACCGCTGAGCACA
CGTTTACCAGCACAACGACTGGATGAATTAGATAGTATTGCCGGTGCTTTTAACCAACTG
CTTGATACTCTACAAGTCCAATACGACAATCTGGAAAACAAAGTCGCAGAGCGCACCCAG
GCGCTAAATGAAGCAAAAAAACGCGCTGAGCGAGCTAACAAACGTAAAAGCATTCATCTT
ACGGTAATAAGTCATGAGTTACGTACTCCGATGAATGGCGTACTCGGTGCGATTGAATTA
TTACAAACCACCCCTTTAAACATAGAGCAGCAAGGATTAGCTGATACCGCCAGAAATTGT
ACACTGTCTTTGTTAGCTATTATTAATAATCTGCTGGATTTTTCACGCATCGAGTCTGGT
CATTTCACATTACATATGGAAGAAACAGCGTTACTGCCGTTACTAGACCAGGCAATGCAA
ACCATCCAGGGGCCAGCGCAAAGCAAAAAACTGTCATTACGTACTTTTGTCGGTCAACAT
GTCCCTCTCTATTTTCATACCGACAGTATCCGTTTACGGCAAATTTTGGTTAATTTACTC
GGGAATGCGGTAAAATTTACCGAAACCGGAGGGATACGTCTGACGGTCAAGCGTCATGAG
GAACAATTAATATTTCTGGTTAGCGATAGCGGTAAAGGGATTGAAATACAGCAGCAGTCT
CAAATCTTTACTGCTTTTTATCAAGCAGACACAAATTCGCAAGGTACAGGAATTGGACTG
ACTATTGCGTCAAGCCTGGCTAAAATGATGGGCGGTAATCTGACACTAAAAAGTGTCCCC
GGGGTTGGAACCTGTGTCTCGCTAGTATTACCCTTACAAGAATACCAGCCGCCTCAACCA
ATTAAAGGGACACTATCAGCGCCGTTCTGCCTGCATCGCCAACTGGCTTGCTGGGGAATA
CGCGGCGAACCACCCCACCAGCAAAATGCGCTTCTCAACGCAGAGCTTTTGTATTTCCCC
GGAAAACTCTACGACCTGGCGCAACAGTTAATATTGTGTACACCAAATATACCAGTAATA
AATAATTTGTTACCCCCTGGCAGTTGCAGATTCTTTTGGTTGATGATGCCGATATTAATC
GGGATATCATCGGCAAAATGCTTGTCAGCCTGGGACAACACGTCACTGTTGCCGCCAGTA
GTAACGAGGCTCTGA
>EECEBICD_01728 Salmonella pathogenicity island 2 protein C
ATGCTGGCAGTTTTAAAAGGCATTGCATTAATTCAGGATATCAAGGCCGAAGGTAATAGC
CGATCCTGGATAATGACTATTGATGGGCATCCTGCCAGAGGAGAAATTTTCTCAGAAGCA
TTTTCTATTTCTTTGTTCTTAAATGACCTGGAAAGCTTACCTAAGCCTTGTCTTGCCTAT
GTGACACTACTGCTTGCAGCACACCCGGACGTCCATGATTATGCTATACAGCTCACAGCG
GATGGGGGATGGTTAAACGGTTATTATACCACAAGTAGTAGCTCTGAGCTTATTGCTATT
GAGATAGAAAAACACCTGGCTTTAACTTGCATTTTAAAAAATGTAATACGCAATCACCAT
AAACTTTATTCGGGTGGGGTATAA
>EECEBICD_01729 Type 3 secretion system secretin
ATGGTAGTAAATAAACGTTTAATCTTAATTTTACTATTTATACTCAATACAGCAAAGAGT
GATGAGTTATCATGGAAAGGTAATGACTTCACCCTTTATGCCAGACAAATGCCATTAGCA
GAGGTTTTACATCTGCTCTCAGAGAACTATGATACGGCTATTACTATTAGCCCATTGATA
ACAGCTACATTTAGTGGAAAAAATTCCGCCTGGACCACCGGTCGATATTTTGAATAACCTG
GCAGCACAATATGATTTGCTTACCTGGTTTGATGGCAGCATGTTATATGTATATCCTGCA
TCGTTATTAAAACATCAGGTTATCACTTTCAATATTTTATCTACTGGACGGTTCATTCAT
TACTTACGCAGCCAGAATATCCTTTCATCACCGGGATGCGAGGTTAAAGAAATTACCGGT
ACCAAAGCTGTGGAGGTGAGCGGTGTTCCCAGCTGCCTGACTCGTATTAGTCAATTAGCT
TCAGTGCTGGATAATGCGTTAATCAAACGAAAAGACAGTGCGGTGAGTGTAAGTATATAC
ACGCTTAAGTATGCCACTGCGATGGATACCCAATACCAATATCGCGATCAGTCCGTCGTG
GTTCCAGGGGTCGTGAGTGTATTGCGTGAGATGAGTAAAACCAGCGTACCGGCGTCATCG
ATGAACAATGGTTCACCCGCTACACAGGCATTGCCCATGTTTGCTGCCGACCCACGCCAG
AATGCAGTGATCGTTCGTGATTATGCGGCCAATATGGCCGGGTATCGGAAACTTATCACA
GAATTAGATCAACGCCAGCAGATGATAGAGATTTCGGTGAAAATTATCGATGTTAATGCT
GGAGATATTAACCAGTTAGGCATCGACTGGGGAACGGCAGTGTCGCTGGGTGGCAAGAAA
ATTGCGTTCAATACAGGTTTGAATGACGGTGGTGCTAGCGGTTTTTCAACGGTAATCAGC
GATACCTCAAACTTTATGGTGCGTTTGAATGCCCTAGAAAAAAGCTCTCAGGCTTATGTA
CTTTCCCAGCCATCTGTGGTGACTTTAAATAATATCCAGGCTGTGCTGGATAAAAATATT
ACTTTCTATACCAAACTGCAGGGAGAAAAAGTGGCTAAACTTGAATCCATCACTACGGGT
TCTTTGTTGCGCGTTACACCTCGCTTGTTAAATGATAATGGCACGCAAAAAATAATGCTT
AATCTTAATATTCAGGATGGACAACAAAGTGATACGCAAAGCGAAACAGACCCGCTGCCC
GAAGTGCAAAATTCTGAAATTGCTTCGCAGGCCACATTATTGGCCGGGCAAAGTCTATTG
CTGGGAGGATTTAAACAAGGTAAACAAATCCACTCGCAAAATAAAATTCCTTTATTGGGC
GATATTCCTGTCGTAGGCCATTTGTTTCGCAATGATACGACTCAAGTACATAGTGTAATC

AGGCTTTTTTTGATTAAAGCCTCAGTAGTAAATAATGGCATATCTCATGGTTAA
>EECEBICD_01730 hypothetical protein
GTGGCTATCGCGTTTGGTAAACAGGATTGTGAACTTGCTGATTATCAAATACCCGTTTCC
AGATCAGGGTACTGGTGGTTGGCTGGCGTATTCTTGATTTTCATCGGTGGAATGGGTGTC
CTGTTAAGTATTAGTGGTCAGCCTGAAACGGTAAATGACTTACCTTTGCGGGTTAAGTTT
TTATTAGACAAAAGCAATATTCATTATGTGCGGGCGCAATGGAAAGAAGATGGCAGCCTG
CAGTTGTCCGGTTATTGCTCGTCAAGCGAACAGATGCAAAAGGTGAGAGCGACTCTCGAA
TCATGGGGGGTCATGTATCGGGATGGTGTAATCTGTGATGACTTATTGGTACGAGAAGTG
CAGGATGTTTTGATAAAAATGGGTTACCCGCATGCTGAAGTATCCAGCGAAGGGCCGGGG
AGCGTGTTAATTCATGATGATATACAAATGGATCAGCAATGGCGTAAGGTTCAACCATTA
CTTGCAGATATTCCCGGGTTATTGCACTGGCAGATTAGTCACTCTCATCAGTCTCAGGGA
GATGATATTATTTCTGCGATAATAGAGAACGGTTTAGTGGGGCTTGTCAATGTTACGCCA
ATGCGGCGCTCTTTTGTTATCAGTGGTGTACTGGATGAATCTCATCAACGCATTTTGCAA
GAAACGTTAGCAGCATTAAAGAAAAAGGATCCCGCTCTTTCTTTAATTTATCAGGATATT
GCGCCTTCCCATGATGAAAGCAAGTATCTGCCTGCGCCAGTGGCTGGCTTTGTACAGAGT
CGCCATGGTAATTACTTATTACTGACGAATAAAGAGCGTTTACGTGTAGGGGCATTGTTA
CCCAATGGGGGAGAAATTGTCCATCTGAGTGCCGATGTGGTAACGATTAAACATTATGAT
ACTTTGATTAACTATCCATTAGATTTTAAGTGA
>EECEBICD_01731 hypothetical protein
ATGACAACTTTGACCCGGTTAGAAGATTTGCTGCTTCATTCGCGTGAAGAGGCCAAAGGC
ATAATTTTACAATTAAGGGCTGCCCGGAAACAGTTAGAAGAGAACAACGGCAAGTTACAG
GATCCGCAGCAATATCAGCAAAACACCTTATTGCTTGAAGCGATCGAGCAGGCCGAAAAT
ATCATCAACATTATTTATTATCGTTACCATAACAGCGCACTTGTAGTGAGTGAGCAAGAG
TAA
>EECEBICD_01732 Type III secretion system chaperone SseA
ATGATAAAGAAAAAGGCTGCGTTTAGTGAATATCGTGATTTAGAGCAAAGTTACATGCAG
CTAAATCACTGTCTTAAAAAATTTCACCAAATCCGGGCTAAGGTGAGTCAACAGCTTGCT
GAAAGGGCAGAGAGCCCCAAAAAGAGCAGAGAGACAGAGAGTATTCTTCATAACCTATTT
CCACAAGGCGTTGCCGGGGTTAACCAGGAGGCCGAGAAGGATTTAAAGAAAATAGTAAGT
TTGTTTAAACAACTTGAAGTACGACTGAAACAACTTAATGCTCAAGCCCCGGTGGAGATA
CCGTCAGGAAAAACAAAAAGGTAA
>EECEBICD_01733 Secreted effector protein SseB
ATGTCTTCAGGAAACATCTTATGGGGAAGTCAAAACCCTATTGTGTTTAAAAATAGCTTC
GGCGTCAGCAACGCTGATACCGGGAGCCAGGATGACTTATCCCAGCAAAATCCGTTTGCC
GAAGGGTATGGTGTTTTGCTTATTCTCCTTATGGTTATTCAGGCTATCGCAAATAATAAA
TTTATTGAAGTCCAGAAGAACGCTGAACGTGCCAGAAATACCCAGGAAAAGTCAAATGAG
ATGGATGAGGTGATTGCTAAAGCAGCCAAAGGGGATGCTAAAACCAAAGAGGAGGTGCCT
GAGGATGTAATTAAATACATGCGTGATAATGGTATTCTCATCGATGGTATGACCATTGAT
GATTATATGGCTAAATATGGCGATCATGGGAAGCTGGATAAAGGTGGCCTACAGGCGATC
AAAGCGGCTTTGGATAATGACGCCAACCGGAATACCGATCTTATGAGTCAGGGGCAGATA
ACAATTCAAAAAATGTCTCAGGAGCTTAACGCTGTCCTTACCCAACTGACAGGGCTTATC
AGTAAGTGGGGGGAAATTTCCAGTATGATAGCGCAGAAAACGTACTCATGA
>EECEBICD_01734 hypothetical protein
ATGAAAAAAGACCCGACCCTACAACAGGCACATGACACGATGCGGTTTTTCCGGCGTGGC
GGCTCGCTGCGTATGTTGTTGGATGACGATGTTACACAGCCGCTTAATACTCTGTATCGC
TATGCCATGCAGCTTATGGAGGTAAAAGAGTTCGCCGGCGCAGCGCGACTTTTTCAATTG
TTGACGATATATGATGCCTGGTCATTTGACTACTGGTTTAGGTTAGGGGAATGCTGCCAG
GCTCAAAAACATTGGGGGGAAGCGATATACGCTTATGGACGCGCGGCACAAATTAAGATT
GATGCGCCGCAGGCGCCATGGGCCGCAGCGGAATGCTATCTCGCGTGTGATAACGTCTGT
TATGCAATCAAAGCGTTAAAGGCCGTGGTGCGTATTTGCGGCGAGGTCAGTGAACATCAA
ATTCTCCGACAGCGTGCAGAAAAGATGTTACAGCAACTTTCTGACAGGAGCTAA
>EECEBICD_01735 Secreted effector protein SseC
ATGAATCGAATTCACAGTAATAGCGACAGCGCCGCAGGAGTAACCGCCTTAACACATCAT
CACTTAAGCAATGTCAGTTGCGTTTCCTCGGGTTCGCTGGGAAAGCGCCAGCATCGTGTG

AATTCTACTTTTGGCGATGGCAACGCCGCGTGCCTGTTATCAGGGAAAATTAGTCTTCAG
GAGGCAAGCAATGCGTTGAAGCAACTGCTTGATGCCGTACCCGGAAATCATAAGCGTCCA
TCATTGCCTGACTTTTTGCAGACCAATCCCGCGGTTTTATCAATGTTGATGACGACATTA
ATACTCAGCGTTTTTGGTAATAACGCTCAATCGTTATGCCAACAGCTTGAGCGGGCAACT
GAGGTGCAAAATGCATTACGTGATAAGCAGGTAAAGGAGTATCAGGAGCAGATCCAGAAA
GCGATAGAGCAGGAGGATAAAGCGCGTAAAGCGGGCATTTTTGGCGCTATTTTTGACTGG
ATTACCGGCATATTTGAAACTGTGATTGGCGCCTTAAAAGTTGTGGAGGGTTTTCTGTCC
GGTAATCCCGCAGAAATGGCTAGCGGCGTAGCTTATATAGCCGCAGGTTGTGCAGGAATG
GTTAAAGCCGGAGCCGAAACGGCAATGATGTGCGGTGCTGACCACGATACCTGTCAGGCA
ATTATTGACGTGACAAGTAAGATTCAATTTGGTTGTGAAGCCGTCGCGCTGGCACTGGAT
GTTTTCCAGATTGGCCGTGCTTTTATGGCGACGAGAGGTTTATCTGGCGCAGCTGCAAAA
GTGCTTGACTCCGGTTTTGGCGAGGAAGTGGTTGAGCGTATGGTAGGTGCAGGGGAAGCA
GAAATAGAGGAGTTGGCTGAAAAGTTTGGCGAAGAAGTGAGCGAAAGTTTTTCCAAACAA
TTTGAGCCGCTTGAACGTGAAATGGCTATGGCGAATGAGATGGCAGAGGAGGCTGCCGAG
TTTTCTCGTAACGTAGAAAATAATATGACGCGAAGCGCGGGAAAAAGCTTTACGAAAGAG
GGGTGA
>EECEBICD_01736 Secreted effector protein SseC
ATGGCAAAAGAAGCGGCAAAAGAAGCCCTGGAAAAATGTGTGCAAGAAGGTGGAAAGTTC
CTGTTAAAAAAATTCCGTAATAAAGTTCTCTTCAATATGTTCAAAAAAATCCTGTATGCC
TTACTGAGGGATTGTTCATTTAAAGGCTTACAGGCTATCAGATGTGCAACCGAGGGCGCC
AGTCAGATGAATACTGGCATGGTTAACACAGAAAAAGCGAAGATCGAAAAGAAAATAGAG
CAATTAATAACTCAGCAACGGTTTCTGGATTTCATAATGCAACAAACAGAAAACCAGAAA
AAGATAGAACAAAAACGCTTAGAGGAGCTTTATAAGGGGAGCGGTGCCGCGCTTAGAGAT
GTATTAGATACCATTGATCACTATAGTAGCGTTCAGGCGAGAATAGCTGGCTATCGCGCT
TAA
>EECEBICD_01737 Secreted effector protein SseD
ATGGAAGCGAGTAACGTAGCACTGGTATTACCAGCGCCTTCCTTGTTAACACCTTCTTCC
ACTCCATCTCCCTCCGGGGAGGGAATGGGTACTGAATCAATGCTTCTGTTATTTGATGAT
ATCTGGATGAAGCTAATGGAGCTTGCCAAAAAGCTGCGCGATATCATGCGCAGCTATAAC
GTAGAAAAACAACGGCTGGCCTGGGAACTGCAAGTCAATGTTTTACAGACGCAAATGAAA
ACAATTGATGAAGCGTTTAGAGCATCAATGATTACTGCGGGTGGCGCAATGTTGTCGGGT
GTACTGACGATAGGATTAGGGGCCGTAGGCGGGGAAACCGGTCTTATAGCGGGTCAAGCC
GTAGGCCACACAGCTGGGGGCGTCATGGGCCTGGGGGCTGGTGTAGCGCAACGTCAAAGT
GATCAAGATAAAGCGATTGCCGACCTGCAACAAAATGGGGCCCAATCTTATAATAAATCC
CTGACGGAAATTATGGAGAAAGCAACTGAAATTATGCAGCAAATCATCGGCGTGGGGTCG
TCACTGGTCACGGTTCTTGCTGAAATACTCCGGGCATTAACGAGGTAA
>EECEBICD_01738 hypothetical protein
ATGGTGCAAGAAATAGAGCAATGGTTGCGTCGGCATCAGGTGTTTACTGAGCCTGCATAT
TTAGGGGAGACCGCCATATTACTTGGGCAGCAGTTTATATTATCGCCTTACCTGGTGATC
TATCGTATTGAGGCAAAAGAAATGATTATTTGTGAGTTCAGGCGCCTGACGCCCGGGCAA
CCTCGACCACAGCAATTGTTTCACTTACTGGGACTTTTACGCGGGATATTTGTGCATCAC
CCGCAGTTAACATGTTTAAAGATGTTGATAATCACCGACGTTCTGGATGAAAAAAAGCCA
TGCTACGCAGGAAATTATTGCGCATCCTGA
>EECEBICD_01739 hypothetical protein
ATGAAAGAAGATCAGAAAAATAAAATACCCGAAGACATTCTGAAACAGCTATTATCCGTT
GATCCGGAAACCGTTTATGCCAGTGGTTACGCCTCATGGCAGGAGGGGGATTATTCGCGC
GCCGTAATCGATTTTAGTTGGCTGGTGATGGCCCAGCCATGGAGTTGGCGTGCCCATATT
GCATTGGCTGGCACCTGGATGATGCTTAAAGAATACACGACGGCCATTAATTTCTATGGA
CATGCCTTGATGCTGGATGCCAGCCATCCAGAACCGGTTTACCAAACGGGCGTCTGTCTC
AAAATGATGGGGGAACCCGGGTTGGCGAGAGAGGCTTTTCAAACCGCAATCAAGATGAGT
TATGCGGATGCCTCATGGAGTGAGATTCGCCAGAATGCGCAAATAATGGTTGATACTCTT
ATTGCTTAA
>EECEBICD_01740 hypothetical protein
ATGAAAATTCATATTCCGTCAGCGGCAAGTAATATAGTCGATGGTAATAGTCCTCCTTCC

GATATACAAGCGAAGGAGGTATCGTTTCCTCCCCCTGAAATTCCAGCGCCTGGTACCCCC
GCAGCCCCTGTGCTGCTTACGCCTGAACAAATAAGGCAGCAGAGGGATTATGCTATACAT
TTTATGCAATACACTATTCGTGCGCTGGGTGCGACAGTCGTGTTTGGGTTATCGGTTGCT
GCAGCGGTAATTTCTGGCGGGGCAGGATTACCCATTGCTATTCTTGCGGGGGCGGCGCTC
GTGATTGCTATTGGGGATGCTTGCTGTGCGTATCATAATTATCAATCGATATGTCAGCAA
AAGGAGCCATTACAAACCGCCAGTGATAGCGTTGCTCTTGTGGTCAGTGCGCTGGCCTTA
AAATGTGGGGCAAGTCTTAACTGCGCTAACACCCTTGCTAATTGTCTTTCTTTATTAATA
CGTTCAGGAATCGCTATTTCTATGTTGGTTTTACCCCTACAGTTTCCACTGCCCGCGGCT
GAAAATATTGCGGCCTCTTTGGACATGGGGAGTGTAATTACCTCCGTTAGCCTGACGGCG
ATAGGTGCGGTACTGGATTATTGCCTTGCCCGCCCCTCTGGCGACGATCAGGAAAATTCT
GTTGATGAACTTTATGCCGATCCCAGTGCGTTATTGGCGGAACAAATGGCAGCGCTCTGT
CAATCTGCTACTGCACCTGCATTAATGGACAGTTCTGGTCATACATCTCGGGGAGAACCA
TGA
>EECEBICD_01741 hypothetical protein
ATGAAACCTGTTAGCCCAAATGCTCAGGTAGGAGGGCAACGTCCTGTTAACGCGCCTGAG
GAATCACCTCCATGTCCCTCATTGCCACATCCGGAAACCAATATGGAGAGTGGTAGAATA
GGTCCTCAACAAGGAAAAGAGCGGTTATTGGCCGGACTTGCGAAACGAGTGATAGAGTGT
TTTCCAAAAGAAATTTTTAGTTGGCAAACGGTTATTTTGGGCGGACAGATTTTATGCTGT
TCCGCTGGAATAGCATTAACAGTGCTAAGTGGTGGAGGCGCGCCGCTCGTAGCCCTGGCA
GGGATTGGCCTTGCTATTGCCATCGCGGATGTCGCCTGTCTTATCTACCATCATAAACAT
CATTTGCCTATGGCTCACGACAGTATAGGCAATGCCGTTTTTTATATTGCTAATTGTTTC
GCCAATCAACGCAAAAGTATGGCGATTGCTAAAGCCGTCTCCCTGGGCGGTAGATTAGCC
TTAACCGCGACGGTAATGACGCATTCATACTGGAGTGGTAGTTTGGGACTACAGCCTCAT
TTATTAGAGCGTCTTAATGATATTACCTATGGACTAATGAGTTTTACTCGCTTCGGTATG
GATGGGATGGCAATGACCGGTATGCAGGTCAGCAGCCCATTATATCGTTTGCTGGCTCAG
GTAACGCCAGAACAACGTGCGCCGGAGTAA
>EECEBICD_01742 hypothetical protein
ATGGATATTGCACAATTAGTGGATATGCTCTCCCACATGGCGCATCAGGCAGGCCAGGCC
ATTAATGACAAAATGAATGGTAATGATTTGCTCAACCCAGAATCGATGATTAAAGCGCAA
TTTGCCTTACAGCAGTATTCTACATTTATTAATTACGAAAGTTCACTGATCAAAATGATC
AAGGATATGCTTAGTGGAATCATTGCTAAAATCTGA
>EECEBICD_01743 hypothetical protein
GTGGAATCATTGCTAAAATCTGAAGTTATTAGCGACGATGTTCGACGGTTGCTGCTGGAA
ATCATGTTTGCGGGCGTTAACCATAGCCTGATTTCCCAGGTACATGCGATGTTACCAGCG
CTAACGGTTATTGTTCCGGATAAAAAATTACAGTTGGTATGTCTGGCATTATTGTTGGCG
GGTTTAAATGAGCCGCTAAAAGCCGCGAAAATTTTATCGGATATAGATTTGCCAGAGGCT
ATGGCGCTGCGTCTGTTATTTCCTGCACCAAATGAGGGGTTTGAAAATTGA
>EECEBICD_01744 hypothetical protein
TTGAATATTTCTGATATGAGCGTAGTGCCTGTAAGCACTCAATCTTATGTAAAGTCCTCT
GCAGAACCGAGCCAGGAGCAAATTAATTTTTTTGAACAATTGCTGAAAGATGAAGCATCC
ACCAGTAACGCCAGTGCTTTATTACCGCAGGTTATGTTGACCAGACAAATGGATTATATG
CAGTTAACGGTAGGCGTCGATTATCTTGCCAGAATATCAGGCGCAGCATCGCAAGCGCTT
AATAAGCTGGATAACATGGCATGA
>EECEBICD_01745 Yop proteins translocation lipoprotein J
ATGAAGGTTCATCGTATAGTATTTCTTACTGTCCTTACGTTCTTTCTTACGGCATGTGAT
GTGGATCTTTATCGCTCATTGCCAGAGGATGAGGCGAATCAAATGCTGGCATTACTTATG
CAGCATCATATTGATGCGGAAAAAAAACAGGAAGAGGACGGTGTTACCTTACGTGTCGAG
CAGTCGCAGTTTATTAATGCGGTTGAGCTACTTAGACTTAACGGTTATCCGCATCGTCAG
TTTACAACGGCGGATAAGATGTTTCCGGCTAATCAGTTAGTGGTATCACCCAGGAAGAAC
AGCAGAAGATTAATTTTTTAA
>EECEBICD_01746 hypothetical protein
ATGGAGGGCGTGATTAATGCAAAAGTGACCATTGCGCTACCGACTTATGATGAGGGAAGT
AACGCTTCTCCGAGCTCAGTTGCCGTATTTATAAAATATTCACCACAGGTCAATATGGAG
GCCTTTCGGGTAAAAATTAAGGATTTAATAGAGATGTCAATCCCTGGGTTGCAATACAGT

AAGATTAGTATCTTGATGCAGCCTGCTGAATTCAGAATGGTAGCTGACGTACCCGCGAGA
CAAACATTCTGGATTATGGACGTTATCAACGCCAATAAAGGGAAGGTGGTGAAGTGGTTG
ATGAAATACCCTTATCAGTTGATGTTATCGTTGACAGGACTGTTATTAGGAGTGGGCATC
CTGATCGGCTATTTTTGCCTGAGACGCCGTTTTTGA
>EECEBICD_01747 hypothetical protein
TTGCTTCTACAGTGGTTTAGCGAGGATGAGATCTGGCAGCTATATGGTTGGTTGGGGCAA
AGAGATGGCAAATTACTTCCTCCGCAAGTGATGCAACAAACTGCATTGCAGATCGGTACC
GCCATTCTTAATCGGGAAGCGCATGACGATGCGGTTTTACATGCGCTATTAGTATTATTA
CCCCCTCCGCAGCGTATACTTTGGCCGAAGACTTCTCTTACCGAGATTATCTTCATGGAG
CATTTGCTATGA
>EECEBICD_01748 hypothetical protein
ATGAGTTTTACTTCACTTCCTCTGACGGAAATTAACCATAAGCTACCCGCTCGAAATATT
ATTGAGTCACAGTGGATAACATTACAATTAACTTTATTTGCGCAAGAGCAACAAGCTAAG
AGAGTTTCACATGCTATTGTGAGCTCCGCTTACCGTAAGGCTGAAAAAATCATCCGAGAC
GCCTATCGTTATCAGCGTGAACAGAAAGTTGAGCAGCAACAAGAACTAGCGTGCTTGCGT
AAAAATACGCTGGAAAAAATGGAAGTGGAATGGCTGGAACAGCATGTAAAACATTTACAA
GAAGATGAAAATCAATTTCGTTCATTGGTCGATCACGCAGCGCATCATATTAAAAATAGT
ATAGAACAGGTTCTGTTGGCCTGGTTCGACCAACAGTCGGTAGACAGTGTTATGTGCCAT
CGTCTGGCACGCCAGGCCACGGCTATGGCGGAAGAGGGAGCGCTTTATTTGCGTATTCAT
CCTGAAAAAGAGGCATTGATGCGTGAAACTTTTGGCAAGCGGTTTACGTTGATTATCGAG
CCTGGTTTCTCTCCCGATCAGGCTGAACTTTCCTCAACACGATATGCCGTTGAGTTTTCA
CTTTCTCGTCATTTCAACGCGTTACTGAAATGGTTACGTAATGGTGAAGATAAAAGAGGT
AGCGATGAATATTAA
>EECEBICD_01749 hypothetical protein
ATGAATATTAAAATTAATGAGATAAAAATGACGCCCCCTACAGCATTTACCCCTGGCCAG
GTTATAGAGGAACAAGAGGTTATTTCGCCTTCAATGTTAGCTCTCCAGGAGTTACAGGAA
ACGACGGGGGCAGCGCTCTATGAGACGATGGAAGAAATAGGAATGGCGCTGAGTGGTAAA
CTGCGCGAAAGTAATAAATTCACTGATGCTGAGAAACTGGAGCGCAGGCAGCAGGCTTTG
CTGCGTTTGATAAAACAAATACAGGAGGATAATGGGGCAGCGTTGCGTCCGCTTACCGAA
GAGAATAGTGATCCTGATTTACAGAATGCGTATCAAATTATCGCTCTTGCAATGGCGCTT
ACTGCCGGCGGGTTGTCAAAAAAGAAAAAACGCGATTTGCAATCGCAACTGGATACGCTT
ACAGCGGAGGAGGGATGGGAACTTGCCGTTTTTAGTTTACTGGAACTTGGCGAAGTGGAT
ACCGCTACGCTGTCCTCGCTGAAGCGTTTTATGCAACAGGCGATAGACAACGATGAAATG
CCCTTATCGCAGTGGTTCAGACGCGTGGCAGACTGGCCGGATCGCTGTGAACGGGTCCGT
ATTTTGCTAAGAGCAATAGCCTTTGAACTTAGCATATGCATCGAACCCTCGGAGCAAAGT
CGTTTGGCCGCAGCATTAGTACGTTTGCGTCGTTTGCTGTTATTCCTTGGCCTTGAAAAA
GAGTGCCAGCGTGAGGAGTGGATTTGCCAGTTGCCGCCTAATACATTACTGCCGCTACTA
CTCGATATTATTTGTGAGCGCTGGCTTTTCAGTGATTGGTTGCTTGATAGACTTACCGCT
ATAGTTTCTTCATCGAAGATGTTCAATCGGTTACTCCAACAACTTGATGCGCAGTTTATG
CTGATACCCGATAACTGTTTTAACGACGAAGATCAACGTGAACAAATTCTCGAAACGCTT
CGTGAAGTAAAGATAAATCAGGTTTTATTCTGA
>EECEBICD_01750 hypothetical protein
ATGGATTGGGATCTCATTACTGAACGTAATATTCAGCTTTTTATTCAATTAGCAGGATTA
GCTGAACGGCCTTTAGCGACCAATATGTTCTGGCGGCAAGGACAATATGAAACCTATCTA
AACTATCATAACGGTCGTATTCACTTATGTCAGATACTCAAGCAAACCTTCTTAGACGAA
GAACTGCTTTTTAAAGCGTTGGCTAACTGGAAACCCGCAGCGTTCCAGGGTATTCCTCAA
CGATTATTTTTGTTGCGCGATGGGCTTGCAATGAGTTGTTCTCCACCTCTTTCCAGCTCC
GCCGAGCTCTGGTTACGATTACATCATCGACAAATAAAATTTCTGGAGTCGCAATGCGTT
CATGGTTAG
>EECEBICD_01751 Secretion system apparatus protein SsaV
ATGCGTTCATGGTTAGGTGAGGGAGTCAGGGCGCAACAGTGGCTCAGTGTATGCGCGGGT
CGGCAGGATATGGTTCTGGCGACGGTGTTATTAATCGCTATTGTGATGATGCTGTTACCT
TTGCCGACCTGGATGGTTGATATCCTGATTACTATCAACCTTATGTTTTCAGTGATCCTG
CTCTTAATTGCTATTTATCTTAGTGACCCTCTCGATTTATCGGTATTTCCGTCTTTATTA

CTTATTACTACATTATATCGTTTGTCACTCACAATCAGCACATCACGGCTGGTACTGTTA
CAACATAATGCCGGTAATATTGTGGATGCTTTCGGTAAGTTTGTCGTAGGAGGAAATCTC
ACCGTTGGGTTGGTCGTATTTACCATCATTACTATCGTGCAATTTATTGTCATTACAAAA
GGTATCGAGAGGGTGGCGGAAGTTAGCGCACGTTTCTCGCTTGATGGGATGCCAGGCAAA
CAAATGAGTATCGATGGCGATTTGCGTGCCGGAGTTATCGATGCAGACCATGCCCGTACA
TTAAGACAGCATGTCCAGCAGGAAAGCCGCTTTCTCGGTGCGATGGACGGTGCGATGAAA
TTTGTTAAAGGCGATACGATTGCCGGTATTATTGTTGTTCTGGTGAACATTATCGGCGGT
ATCATTATCGCTATCGTACAATATGATATGTCGATGAGTGAGGCTGTTCACACTTATAGC
GTACTGTCAATCGGAGATGGTTTATGTGGGCAAATTCCATCGCTGCTGATTTCCCTTAGC
GCGGGAATTATTGTCACCCGTGTCCCGGGTGAGAAACGCCAGAACCTGGCGACAGAGTTG
AGTTCTCAAATTGCCAGACAACCTCAGTCGCTCATATTAACCGCTGTGGTTTTAATGCTC
CTCGCTTTAATTCCTGGCTTTCCTTTTATCACTCTCGCTTTCTTTTCAGCGTTGTTAGCA
TTGCCAATTATCCTCATTCGCCGCAAAAAGTCTGTGGTTTCCGCAAATGGCGTCGAAGCA
CCGGAAAAAGATAGTATGGTTCCCGGCGCATGTCCTCTAATCTTACGTCTTAGCCCGACG
TTACATTCTGCCGACCTGATTCGTGATATTGACGCCATGAGATGGTTTTTATTTGAGGAT
ACCGGCGTCCCTCTCCCTGAGGTGAATATTGAGGTTTTGCCTGAACCCACCGAAAAATTG
ACGGTACTGCTATATCAGGAACCCGTATTTAGTTTATCTATTCCCGCTCAGGCGGATTAT
TTATTGATAGGCGCGGACGCTAGTGTGGTGGGTGACAGCCAGACGTTACCGAACGGGATG
GGGCAGATCTGTTGGCTTACAAAAGACATGGCCCATAAGGCGCAAGGTTTTGGACTGGAC
GTTTTCGCGGGCAGCCAACGTATCTCTGCCTTATTAAAATGTGTCCTGCTTCGGCATATG
GGAGAGTTTATTGGTGTTCAGGAAACGCGTTATCTAATGAATGCGATGGAAAAAAAACTAC
TCTGAGCTGGTGAAAGAGCTTCAACGCCAGTTACCCATTAATAAAATCGCTGAAACTTTG
CAACGGCTTGTATCAGAACGGGTTTCTATTAGAGATTTACGTCTTATTTTCGGCACCTTA
ATTGACTGGGCGCCACGTGAAAAAGATGTCCTGATGTTGACAGAATATGTCCGTATCGCG
CTTCGCCGTCATATTCTGCGTCGTCTTAATCCGGAAGGAAAACCGCTGCCGATTTTGCGG
ATCGGCGAAGGTATTGAGAACCTCGTGCGTGAATCCATTCGCCAGACGGCAATGGGGACC
TATACTGCGCTGTCGTCTCGTCATAAGACGCAGATCCTGCAACTTATCGAGCAGGCGCTG
AAGCAGTCAGCCAAATTATTCATTGTCACTTCTGTCGACACCCGACGTTTCTTGCGAAAA
ATTACAGAAGCCACCTTGTTCGACGTACCGATTTTGTCATGGCAGGAATTAGGAGAGGAG
AGCCTTATACAAGTGGTAGAAAGTATTGACCTTAGCGAAGAGGAGTTGGCGGACAATGAA
GAATGA
>EECEBICD_01752 putative secretion system apparatus ATP synthase SsaN
ATGAAGAATGAATTGATGCAACGTCTGAGGCTGAAATATCCGCCCCCCGATGGTTATTGT
CGATGGGGCCGAATTCAGGATGTCAGCGCAACGTTGTTAAATGCGTGGTTGCCTGGGGTA
TTTATGGGTGAGTTGTGCTGTATAAAGCCTGGAGAAGAACTTGCTGAAGTCGTGGGGATT
AATGGCAGCAAAGCTTTGCTATCTCCTTTTACGAGTACAATCGGACTTCACTGCGGGCAG
CAAGTGATGGCCTTAAGGCGACGCCATCAGGTTCCCGTGGGCGAAGCGTTATTAGGGCGA
GTCATTGATGGTTTTGGTCGTCCCCTTGATGGCCGCGAACTGCCCGACGTCTGCTGGAAA
GACTATGATGCAATGCCTCCTCCCGCAATGGTTCGACAGCCTATCACTCAACCATTAATG
ACGGGGATTCGCGCTATTGATAGCGTTGCAACCTGTGGCGAAGGGCAACGAGTGGGTATT
TTTTCTGCTCCTGGCGTGGGGAAAAGCACGCTTCTGGCGATGCTGTGTAATGCGCCAGAC
GCAGACAGCAATGTTCTGGTGTTAATTGGTGAACGTGGACGAGAAGTCCGCGAATTCATC
GATTTTACACTGTCTGAAGAGACCCGAAAACGCTGTGTCATTGTTGTCGCAACCTCTGAC
AGACCCGCCTTAGAGCGCGTGAGGGCGCTGTTTGTGGCTACCACGATAGCAGAATTTTTT
CGCGATAATGGAAAGCGAGTCGTCTTGCTTGCCGACTCACTGACGCGTTATGCCAGGGCC
GCACGAGAAATCGCTCTGGCCGCCGGAGAGACCGCAGTTTCTGGAGAATATCCGCCAGGC
GTATTTAGTGCATTGCCACGACTTTTAGAACGTACAGGAATGGGGGAAAAAGGCAGTATT
ACCGCATTTTATACGGTACTGGTGGAAGGCGATGATATGAATGAGCCGTTGGCGGATGAA
GTCCGTTCACTACTTGATGGACATATTGTGCTATCCCGGCGGCTTGCAGAGAGGGGGCAT
TATCCTGCCATTGACGTGCTGGCAACGCTCAGCCGCGTTTTTCCAGTCGTTACCAGCCAT
GAGCATCGTCAACTGGCGGCGATATTGCGGCGGCGCCTGGCGCTTTACCAGGAGGTTGAA
CTGTTAATACGCATTGGGGAATACCAGCGAGGAGTTGATACTGATACTGACAAAGCCATT
GATACCTATCCGGATATTTGCACATTTTTGCGACAAAGTAAGGATGAAGTATGCGGACCC
GAGCTACTTATAGAAAAATTACACCAAATACTCACCGAGTGA

>EECEBICD_01753 hypothetical protein
ATGGAAACTTTGCTGGAGATAATCGCGCGGCGTGAAAAGCAATTACGCGGCAAGCTTACC
GTACTTGATCAGCAGCAACAGACGATTATTACGGAACAGCAGATTTGCCAGACGCGCGCT
TTAGCAGTGTCTACCAGACTGAAAGAATTAATGGGCTGGCAAGGTACGTTATCTTGTCAT
TTATTGTTGGATAAGAAACAACAAATGGCCGGGTTATTCACTCAGGCGCAGAGCTTTTTG
ACGCAACGGCAGCAGTTAGAGAATCAGTATCAGCAGCTTGTCTCCCGGCGAAGCGAATTA
CAGAAGAATTTTAATGCGCTTATGAAAAAGAAAGAAAAAATTACTATGGTATTAAGCGAT
GCGTATTACCAAAGTTGA
>EECEBICD_01754 hypothetical protein
ATGCGTATTACCAAAGTTGAGGGAAGTCTTGGGTTGCCATGCCAGTCTTATCAGGATGAT
AACGAGGCGGAGGCGGAACGTATGGACTTTGAACAACTCATGCACCAGGCATTACCCATT
GGTGAGAATAATCCTCCTGCAGCATTGAATAAGAACGTGGTTTTCACGCAACGTTATCGT
GTTAGTGGCGGTTATCTTGACGGTGTAGAGTGTGAAGTCTGTGAATCAGGGGGCTAATT
CAGTTAAGAATCAATGTCCCTCATCATGAAATTTACCGTTCGATGAAAGCGCTAAAGCAG
TGGCTGGAGTCTCAGTTGCTGCATATGGGGTATATAATTTCCCTGGAGATATTCTATGTT
AAGAATAGCGAATGA
>EECEBICD_01755 hypothetical protein
ATGTTAAGAATAGCGAATGAAGAGCGTCCGTGGATGGAGATGCTTCCAACGCAAGGCGCT
ACCATTGGTGAGCTGACATTGAGTATGCAACAATATCCAGTACAGCAAGGGACATTATTT
ACCATAAATTATCATAATGAGCTGGGTAGGGTGTGGATTGCAGAAGAATGCTGGCAGCGC
TGGTGTGAAGGGTTAATTGGCACCGCTAATCGATCGGCTATCGATCCTGAATTGCTATAT
GGAATAGCTGAATGGGGGCTGGCGCCGTTATTGCAAGCCAGTGATGCAACCCTCTGTCAG
AACGAACCGCCAACATCCTGCAGTAATCTACCACATCAGCTAGCGTTGCATATTAAATGG
ACAGTTGAAGAACATGAGTTCCATAGCATTATTTTTACATGGCCAACGGGTTTTTTGCGC
AATATAGTCGGAGAGCTTTCTGCTGCGCGACAACAGATTTATCCTGCCCCTCCTGTAGTA
GTCCCTGTATATTTAGGCTGGTGCCAGCTTACGTTAATTGAACTTGAGTCTATCGAAATC
GGCATGGGCGTTCGGATTCATTGCTTTGGCGACATCAGACTCGGTTTTTTTGCTATTCAA
CTACCTGGGGGGATCTACGCGAGGGTGTTGCTGACAGAGGATAACACGATGAAATTTGAT
GAATTAGTCCAGGATATCGAAACGCTACTTGCGTCAGGGAGTCCAATGTCAAAGAGTGAC
GGAACATCTTCAGTAGAACTTGAGCAGATACCACAACAGGTGCTCTTTGAGATCGGACGT
GCGAGTCTGGAAATTGGACAATTACGACAACTTAAAACGGGGGACGTTTTGCCTGTAGGT
GGATGTTTTGCGCCAGAGGTGACGATAAGAGTAAATGACCGTATTATTGGGCAAGGTGAG
TTGATTGCCTGTGGCAATGAATTTATGGTGCGTATTACACGTTGGTATCTTTGCAAAAAT
ACAGCGTAA
>EECEBICD_01756 Surface presentation of antigens protein SpaP
ATGTCTTTACCCGATTCGCCTTTGCAACTGATTGGTATATTGTTTCTGCTTTCAATACTG
CCTCTCATTATCGTCATGGGAACCTCTTTCCTTAAACTGGCGGTGGTATTTTCGATTTTA
CGAAATGCTCTGGGTATTCAACAAGTTCCCCCAAATATCGCACTGTATGGCCTCGCGCTT
GTACTTTCCTTATTCATTATGGGGCCGACGCTATTAGCTGTAAAAGAGCGCTGGCATCCG
GTTCAGGTCGCTGGCGCTCCTTTCTGGACGTCTGAGTGGGACAGTAAAGCATTAGCGCCT
TATCGACAGTTTTTGCAAAAAAACTCTGAAGAGAAGGAAGCCAATTATTTTCGGAGTTTG
ATAAAACGAACCTGGCCTGAAGACATAAAAAGAAAGATAAAACCTGATTCTTTGCTCATA
TTAATTCCGGCATTTACGGTGAGTCAGTTGACGCAGGCATTTCGGATTGGATTACTTATT
TATCTTCCCTTTCTGGCTATTGACCTGCTTATTTCAAATATACTGCTGGCTATGGGGATG
ATGATGGTATCGCCAATGACCATTTCATTACCGTTTAAGCTGCTAATATTTTTACTGGCA
GGCGGTTGGGATCTGACACTGGCGCAATTGGTACAGAGCTTTTCATGA
>EECEBICD_01757 Surface presentation of antigens protein SpaQ
ATGAATGATTCTGAATTGACGCAATTTGTAACGCAACTTTTATGGATCGTCCTTTTTACG
TCTATGCCGGTGGTGTTGGTGGCATCGGTAGTTGGTGTCATCGTAAGCCTTGTTCAGGCC
TTGACTCAAATACAGGACCAAACGCTACAGTTCATGATTAAATTATTGGCAATTGCAATA
ACCTTAATGGTCAGCTACCCATGGCTTAGCGGTATCCTGTTGAATTATACCCGGCAGATA
ATGTTACGAATTGGAGAGCATGGTTGA
>EECEBICD_01758 Surface presentation of antigens protein SpaR
ATGGCACAACAGGTAAATGAGTGGCTTATTGCATTGGCTGTGGCTTTTATTCGACCATTG

AGCCTCTCTTTATTACTTCCCCTATTAAAAAGTGGCAGTTTAGGGTCCGCGATTTTACGT
AATGGCGTGCTTATGTCACTTACCTTTCCCATATTACCAATCATTTACCAGCAGAAGATT
ATGATGCATATTGGTAAAGATTACAGCTGGTTAGGGTTAGTCACCGGAGAGGTGATTATT
GGTTTTTTAATTGGGTTTTGTGCGGCGGTTCCCTTTTGGGCCGTTGATATGGCGGGGTTT
CTGCTTGATACTTTACGTGGCGCGACAATGGGTACGATATTCAATTCTACAATAGAAGCT
GAAACCTCACTTTTTGGCTTGCTTTTCAGCCAGTTCTTGTGTGTTATTTTCTTTATAAGC
GGCGGCATGGAGTTTATATTAAACATTCTGTATGAGTCATATCAATATTTACCACCAGGG
CGTACTTTATTATTTGACCAGCAATTTTTAAAATATATCCAGGCAGAGTGGAGAACGCTT
TATCAATTATGTATCAGCTTCTCTCTTCCTGCCATAATATGTATGGTATTAGCCGATCTG
GCTTTAGGTCTTTTAAATCGGTCGGCACAACAATTGAATGTGTTTTTCTTCTCAATGCCG
CTCAAAAGTATATTGGTTCTACTGACGCTCCTGATCTCATTCCCTTATGCTCTTCATCAC
TATTTGGTTGAAAGCGATAAATTTTATATTTATCTAAAAGACTGGTTTCCATCTGTATGA
>EECEBICD_01759 Yop proteins translocation protein U
ATGAGCGAGAAAACAGAACAGCCTACAGAAAAGAAATTACGTGATGGCCGTAAGGAAGGG
CAGGTTGTCAAAAGTATTGAAATAACATCATTATTTCAGCTGATTGCGCTTTATTTGTAT
TTTCATTTCTTTACTGAAAAGATGATTTTGATACTGATTGAGTCAATAACTTTCACATTA
CAATTAGTAAATAAACCATTTTCTTATGCATTAACGCAATTGAGTCATGCTTTAATAGAG
TCACTGACTTCTGCACTGCTGTTTCTGGGCGCTGGGGTAATAGTTGCTACTGTGGGTAGC
GTGTTTCTTCAGGTGGGGGTGGTTATTGCCAGCAAAGCCATAGGTTTTAAAAGCGAGCAT
ATAAATCCGGTAAGTAATTTTAAGCAGATATTCTCTTTACATAGCGTAGTAGAATTATGT
AAATCCAGCCTGAAAGTTATCATGCTATCTCTTATCTTTGCTTTTTTCTTTTATTATTAT
GCCAGTACTTTTCGGGCGCTACCGTACTGTGGGTTAGCCTGTGGCCTGCTTGTGGTTTCT
TCTTTAATAAAATGGTTATGGGTTGGGGTGATGGCTTTTTATATCGTCGTTGGCATACTG
GACTATTCTTTTCAATATTATAAGATTAGAAAAGATCTAAAAATGAGTAAAGATGACGTA
AAACAGGAGCATAAAGATCTGGAGGGCGATCCTCAAATGAAGACACGGCGTCGGGAAATG
CAGAGTGAAATACAAAGTGGGAGTTTAGCTCAATCTGTTAAACAATCTGTTGCGGTAGTG
CGTAATCCAACGCATATTGCGGTTTGTCTTGGCTATCATCCCACCGATATGCCAATACCA
CGCGTCCTGGAAAAAGGCAGTGATGCTCAAGCTAACTATATTGTTAACATCGCTGAACGC
AACTGCATCCCCGTTGTTGAAAATGTTGAGCTGGCCCGCTCATTATTTTTGAAGTGGAA
CGCGGAGATAAAATTCCTGAAACGTTATTTGAACCCGTTGCAGCCTTGTTACGTATGGTG
ATGAAGATAGATTATGCGCATTCTACCGAAACACCATAA
>EECEBICD_01760 tRNA-Val(gac)
GCGTCTGTAGCTCAGTTGGTTAGAGCACCACCTTGACATGGTGGGGGTCGATGGTTCGAG
TCCATTCAGACGCACCA
>EECEBICD_01761 tRNA-Val(gac)
GCGTTCATAGCTCAGTTGGTTAGAGCACCACCTTGACATGGTGGGGGTCGTTGGTTCGAG
TCCAATTGAACGCACCA
>EECEBICD_01762 Multidrug resistance protein MdtK
GTGCAGAAGTATACCAGTGAAGCGCGTCAGTTATTAGCGTTAGCGATACCGGTGATCCTT
GCGCAAGTGGCACAGACCGCAATGGGATTCGTCGATACCGTCATGGCGGGAGGCTATAGC
GCTACGGACATGGCTGCCGTCGCTATCGGCACCTCTATCTGGCTTCCCGCCATTTTGTTT
GGTCACGGCTTACTGCTGGCCTTGACGCCTGTGATTGCGCAACTCAATGGCTCCGGCCGT
CGTGAGCGTATCGCGCATCAGGTTCGACAAGGGTTCTGGCTGGCGGGCTTTGTCTCCGTG
CTGGTGATGATCGTCCTGTGGAATGCCGGATACATTATCCGCTCTATGCATAATATTGAT
CCGGCGCTGGCCGATAAAGCCGTGGGTTATCTTCGCGCGCTGCTGTGGGGCGCGCCAGGA
TACCTTTTCTTTCAGGTAGCGCGAAACCAGTGCGAAGGTCTGGCCAAAACCAAACCGGGT
ATGGTGATGGGATTTTTGGGCCTGCTGGTGAATATTCCGGTGAACTATATTTTTATCTAT
GGCCATTTTGGTATGCCGGAACTCGGCGGTATCGGCTGCGGCGTTGCGACTGCGGCGGTC
TATTGGGTGATGTTTATCGCCATGCTTTCTTACATTAAACACGCGCGTTCAATGCGTGAT
ATTCGTAATGAAAAAGGCTTTGGCAAACCTGATAGCATCGTCATGAAGCGCCTGATTCAA
CTGGGGTTGCCGATTGCGCTGGCGCTGTTCTTTGAAGTGACGTTATTTGCGGTCGTTGCG
CTGCTGGTTTCGCCATTAGGTATTGTGGATGTCGCCGGTCATCAGATTGCGCTTAACTTT
AGCTCGCTGATGTTCGTACTGCCGATGTCGCTGGCTGCGGCGGTAACGATTCGAGTGGGT
TATCGTCTGGGCCAGGGCTCAACGCTGGATGCGCAAACCGCAGCGCGTACCGGTTTAGGC

GTCGGCATTTGTATGGCTGTGGTTACCGCTATTTTTACCGTTACTCTGCGTAAGCATATT
GCTTTGCTCTACAATGACAACCCTGAGGTCGTGGCGCTTGCCGCGCAATTAATGCTGCTC
GCTGCGGTTTACCAGATTTCCGACTCTATACAGGTTATCGGCAGCGGTATTCTGCGCGGT
TATAAAGATACGCGTTCCATCTTTTTTATCACCTTTACCGCCTACTGGGTGTTAGGTTTA
CCCAGCGGTTATATCCTGGCGTTAACGGATTTGGTGGTGGATCGTATGGGACCGGCAGGC
TTCTGGATGGGGTTCATCATCGGCCTGACCTCGGCGGCGGTGTTAATGATGCTACGGATG
CGCTATCTACAACGTCAGCCGTCCGCCATCATTTTGCAGCGCGCTGCGCGATAA
>EECEBICD_01763 Riboflavin synthase
ATGTTTACGGGGATCGTACAGGGTACCGCGAAACTGGTATCGATTGATGAAAAACCCAAC
TTTCGCACCCATGTGGTGACGTTACCGGACTACATGCTGGAGGGGCTGGAGACGGGGGCG
TCGGTAGCGCATAACGGATGCTGTTTGACGGTGACTGAAATAAACGGCAACCAGATAAGT
TTTGATCTGATGAAAGAAACGTTGCGCATCACCAATCTGGGCACGTTGAGGGTAGGTGAT
GAGGTCAACGTAGAGCGTGCCGCTAAATTCAGCGATGAAATCGGTGGTCATCTGATGTCG
GGGCATATTATTACGACGGCGGAGATCGCGAAAATCCTGACCTCCGAAAATAACCATCAG
GTGTGGTTTAAAGTGCAGGATGCGTCCCTGATGAAGTATATCCTGTACAAAGGCTTTATC
GGCGTCGACGGCATCAGTTTGACCGTCGGCGAGGTAACGCCAACGCGCTTCTGCGTGCAT
CTCATTCCGGAAACGCTGGAACGTACTACGCTTGGCAGAAAAAAACTGGGTGAGCGTGTG
AATATCGAGATCGATCCGCAAACGCAGGCGGTTGTCGATACCGTAGAACGCGTACTGGCT
GCGCGAGAAAATGCGGTCAGAAATCAGGCCGACATTGGCTAA
>EECEBICD_01764 Cyclopropane-fatty-acyl-phospholipid synthase
ATGAGTTCATCGTGTATAGAAGAAGTCAGCGTACCGGATGATAACTGGTACCGAATCGCC
AACGAATTGTTAAGTCGCGCGGATATTACAATTAATGGTTCCGCCCCGTCTGACATTCGT
GTTAAGAATCCCGATTTTTTTAAACGCGTCCTTCAGGAAGGATCGTTGGGGTTAGGTGAG
AGTTACATGGATGGCTGGTGGGAATGCGAGCGTCTGGATATTTTCTTCAGCAAAGTCTTA
CGTGCCGGTCTTGAAAATCAACTCCCCCACCATGTTAAAGATACGCTCCGTATCCTCGGC
GCGCGTCTGATTAATCTACAAAGTAAAAAACGCGCCTGGATTGTCGGCAAGGAACATTAC
GATCTTGGTAATGACCTTTTTTCCCGTATGCTCGATCCCTATATGCAATATTCTTGCGCC
TACTGGAAAGATGCCGATACGCTGGAAGCGGCACAGCAAGCCAAGCTGAAATTAATTTGC
GAAAAGCTACAGCTACAACCGGGGATGCGTGTGCTGGATATCGGCTGCGGCTGGGGCGGT
CTGTCGCAATATATGGCGACCCATTATGGCGTTAGCGTGGTGGGCGTAACGATCTCCGCT
GAACAACAAAAAATGGCGCAGACGCGTTGCGAAGGTCTGGATGTCTCCATTCTCCTGGAA
GATTATCGCGACCTTAACGATCAGTTTGACCGAATCGTCTCCGTCGGCATGTTCGAACAT
GTCGGGCCAAAAAACTACAACACCTATTTTGAGGTTGTTGACAGGAATTTAAAACCGGAT
GGTCTTTTCCTGCTCCATACTATTGGTTCTAAAAAAACCGATCATAATGTCGATCCGTGG
ATCAATAAGTATATTTTCCCCAACGGTTGCTTGCCGTCTGTTCGTCAAATCGCCGAGGCC
AGCGAATCACACTTTGTAATGGAAGACTGGCATAACTTTGGCGCCGACTATGACACGACT
CTGATGGCGTGGCATGAGCGCTTTATCAATGCCTGGCCAGAGATTGCGGGCAATTATAAC
GAGCGCTTTAAACGTATGTTCAGCTATTATCTCAACGCCTGCGCGGGTGCGTTTCGCGCG
CGTGATATCCAGCTTTGGCAGGTGGTATTTACGCGCGGCGTTGAAAACGGACTGCGCGTT
CCTCGCTAA
>EECEBICD_01765 Inner membrane transport protein YdhC
ATGCAACCTGGAAAAGGTTTTTTAGTCTGGTTAGCCGGTCTTAGCGTGCTGGGATTTCTG
GCTACGGACATGTACCTGCCCGCGTTTGCCGCCATACAGGCGGATTTACAAACGCCTGCC
GCCGCGGTCAGCGCCAGCCTAAGCCTGTTTTTAGCTGGCTTTGCCGTCGCGCAACTCCTC
TGGGGACCGCTATCGGATCGTTATGGCCGTAAACCGATTTTACTGCTAGGGTTATCTATC
TTTGCACTCGGAAGTCTGGGGATGCTGTGGGTAGAAAGCGCGGCAGCACTGCTGACGCTG
CGCTTCGTGCAGGCCGTCGGCGTTTGCGCGGCAACGGTAATCTGGCAAGCGCTGGTGACC
GATTATTACCCTTCGCAAAAAATTAACCGTATCTTCGCCACGATCATGCCACTGGTGGGA
TTATCCCCGGCGCTTGCGCCCTTACTGGGAAGCTGGATACTGACGCATTTCTCATGGCAG
GCGATTTTCGCCACTCTGTTCGTGATTACGTTGTTGCTGATGTTGCCGGCGCTGCGTTTA
AAACCATCGGTAAAAGCGCGTACCGAAGGACAGGATAAACTCACTTTCGCGACGTTGTTA
CGCTCTAAAACCTATCGCGGTAATGTGCTGATTTATGCCGCCTGTTCCGCCAGCTTTTTT
GCGTGGCTAACAGGGTCCCCGTTTATTCTCAGCGCGATGGGTTACAGCCCGGCAGTGATT
GGCCTGAGCTATGTTCCGCAAACCATCGCTTTTCTGATTGGCGGATATGGTTGCCGCGCG

GCGCTACAAAAATGGCAGGGCTACCAATTGCTTCCGTGGCTGTTAGGGCTGTACGCGCTG
AGCGTTATCGCCACCTGGGGAGCGGGCCTTCTGAATAATGCCTCACTGGTGGAGATCCTG
ATTCCTTTCTGCGTAATGGCGATTGCTAATGGCGCCATCTACCCTATCGTCGTCGCGCAA
GCGTTACGCCCTTTCCCTCAAGCCACGGGGCGCGCTGCGGCGCTACAAAATACGCTGCAA
CTGGGGTTATGTTTCCTGGCAAGCCTGGTGGTTTCATGGCTCATCAGCACGCCGCTGCTG
ACCACCACCAGCGTAATGCTCTCTACCGTTGTTCTGGCGGCGTTAGGCTACAAAATGCAG
TCTCATGCAGATTGCGCTGAGACCGGATTTCCCCATGCCAATGTCGCCCACGACAAGTCT
CACTAA
>EECEBICD_01766 putative HTH-type transcriptional regulator YahB
ATGTGGTCGGAATACTCGCTTGAAGTGGTTGACGCCGTAGCGCGCAATGGCAGTTTTAGC
GCGGCGGCGCAGGAACTGCACCGTGTGCCTTCAGCAGTCAGTTACACCGTTCGTCAGTTA
GAGGAGTGGCTGGCCGTACCGCTTTTTGTACGACGTCACCGTGATGTTGAACTGACGCCT
GCTGGCGTCTGGTTTTTAAAAGAAGGGCGTTCTGTTATCAAAAAAATGCAGATCACTCGC
CAACAGTGTCAGCAAATTGCTAATGGTTGGCGTGGGCAGTTAGCTATTGCGGTGGACAAT
ATCGTCAGACCAGAACGTACCCGGCAGATGATCGTCGATTTCTATCGCCATTTTGACGAT
GTGGAACTGCTGGTTTTTCAGGAGGTGTTTAACGGCGTCTGGGACGCGCTCTCCGACGGG
CGTGTCGAACTGGCGATTGGGGCGACGCAGGCGATTCCGGTAGGGGGGCGTTACGCTTTT
CGGGATATGGGGACCCTGAGCTGGAGCTGCGTAGTGGCAAGCGATCACCCGCTGGCGTCG
ATGCCTGGGCCGTTAAGCGATGATACCCTGCGCAACTGGCCGTCGCTGGTCAGGGAAGAC
ACTTCGCGAACCTTACCGAAACGGATTACCTGGCTGCTGGATAACCAAAAAAGGGTCGTC
GTACCGGACTGGGAGTCATCGGCAACCTGCCTGTCGGCAGGACTATGCGTGGGAATGGTG
CCGACGCATTTTGCCCGACAGTGGATAGACAGCGGAAAATGGGTGGCGCTGACGTTAGAG
AATCCGTTTCCCGATGCGGCCTGTTGCGTGACGTGGCAGCAAAACGAAGCCTCGCCCGCG
CTGGCATGGCTGCTGGACTATTTGGGCGATAGCGAAACGTTGAATCGGGAGTGGCTGCGG
GAGCCAGAAGAGGCTCCCGACAGCGGGGATTAA
>EECEBICD_01767 HTH-type transcriptional repressor PurR
ATGGCAACAATTAAAGATGTAGCGAAACGAGCAAACGTTTCCACTACAACTGTATCACAC
GTAATCAACAAAACGCGTTTTGTCGCTGAAGAAACGCGTAACGCGGTCTGGGCGGCAATT
AAAGAGCTGCACTACTCTCCCAGCGCCGTCGCGCGTAGCCTGAAGGTTAACCATACCAAG
TCGATAGGCTTACTGGCGACCAGCAGCGAAGCGGCCTATTTTGCCGAAATTATCGAGGCG
GTTGAAAAAAACTGTTTCCAGAAAGGCTATACGCTGATTTTAGGCAACGCCTGGAATAAC
CTGGAAAAACAGCGCGCCTACCTGTCCATGATGGCGCAAAAGCGCGTGGATGGCCTGCTG
GTGATGTGTTCTGAGTATCCAGAACCTCTGCTTTCCATGCTGGAAGAGTATCGCCATATT
CCGATGGTGGTGATGGACTGGGGTGAAGCGAAGGCCGATTTTACCGACACGGTGATTGAT
AACGCCTTTGCAGGCGGCTATATGGCGGGTCGTTATCTGGTTGAGCGCGGCCACCGGGAT
ATCGGCGTTATTCCCGGCCCGCTGGAGCGCAACACCGGCGCGGGCCGGCTGGCAGGCTTT
ATGAAAGCCATGGAGGAGGCGCTGATCAACGTGCCGGACAACTGGATTGTTCAGGGCGAC
TTCGAGCCGGAATCCGGTTACCACGCGATGCAGCAAATTTTATCGCAGTCACATCGCCCT
ACCGCCGTTTTCTGCGGCGGCGATATTATGGCGATGGGCGCGCTTTGCGCGGCTGACGAA
ATGGGGCTTCGCGTACCGCAGGACGTTTCGGTGATCGGTTATGACAATGTGCGTAACGCC
CGTTACTTTACCCCGGCGCTGACGACGATTCACCAGCCCAAAGACTCTTTAGGCGAAACC
GCATTTAATATGCTATTGGATCGCATCGTCAATAAGCGCGAAGAGTCACAGTCTATTGAA
GTTCATCCACGCCTGGTTGAGCGTCGCTCGGTCGCTGACGGCCCGTTCCGCGACTATCGG
CGTTAA
>EECEBICD_01768 putative protein YnhF
ATGAGTACCGACCTGAAATTTTCGTTGATTACTACGCTGATTGTTCTGGGCGTGATTGTC
GCAGGTGGATTAACGGCCGCTCTGCACTGA
>EECEBICD_01769 Superoxide dismutase [Fe]
ATGTCGTTCGAATTACCTGCATTACCGTATGCCAAAGATGCTCTGGCGCCGCATATTTCT
GCTGAGACGCTGGAATATCACTATGGCAAACATCATCAGACTTACGTGACCAATCTTAAC
AATTTGATCAAAGGCACGGCGTTTGAAGGCAAATCGCTGGAAGAGATCGTGCGCACTTCC
GAAGGCGGCATCTTTAATAACGCCGCTCAGGTGTGGAACCACACCTTCTACTGGAACTGC
CTGGCGCCAAACGCTGGCGGCGAACCGACCGGGAAACTGGCTGATGCTATCGCGGCATCT
TTTGGCAGCTTCGCGGAATTTAAAGCGCAGTTCACCGATGCCGCTATCAAAAACTTTGGT

TCCGGATGGACATGGCTGGTAAAAAGCGCTGATGGCAAACTGGCTATCGTTTCTACCTCA
AATGCCGGTACGCCGCTGACTACGGATGCAACGCCGTTGCTGACGGTTGATGTGTGGGAA
CACGCGTATTACATCGACTACCGCAACGCCCGTCCAAACTATCTGGAGCATTTCTGGGCG
CTGGTTAACTGGGAATTTGTTGCGAAAAATCTCGCCGCATAA
>EECEBICD_01770 Murein DD-endopeptidase MepH
GTGGCGCGGATAAACAAAATCTCGATCACGCTCTGTGCTTTACTTTTTACTACTCTCCCT
TTTACGCCAATGGCCCATGCTTCCAGGCAAGCCAAAGCGACGCCTGCTGTTTCTCATGTC
ACGAAAACGGCAGATAAAAAGAAAAGCACAACGAAAAAGATCAGTAAGACCAGCAAAAAA
AACGTAAAAAAAACCTCAAAAACCAGCCGTTCTTCTTCCGCTTCTACGCCAAAACGTACC
ACCGCCGCCACGAAATCTCGTCATACGACGCATCGTCGTAACAGAACCGCGCCCGCGTCA
ATCGCCGCCCTCGACGTAACGGAAAAATGCACGACGCGTAAAGGCCGTAAGCCGCACTGC
GTGAAGGGGAAAGGAACATTGCCGGTCTCTATTGCCGATGCGCATCGTGCAAAAGTACAA
AAAGCGACGAAAACCGCGATGTCCAAACTCATGAACCAGATTGGTAAACCGTATCATTGG
GGAGGCGCGTCGCCGCGTACCGGTTTTGATTGCAGCGGTCTGGTTTATTATGCCTATAAA
GATTTAGTGAAGATCCGTATCCCCCGAACGGCGAATGAGATGTATCACCTGCGTGACGCG
GCGCCCATTGAGCGTAGCGAGTTGAAAAACGGCGATCTGGTGTTCTTCCGTACGCAGGGG
CGCGGCACCGCCGATCACGTTGGCGTCTATGTCGGCAACGGTAAGTTTATCCAGTCTCCG
CGCAGCGGTCAGGAAATTAAGATCACCTCACTGAACGAAGAATACTGGCAACGTCATTAT
GTCGGCGCCCGCCGGGTGATGACGCCTAAAACAATTCGCTAA
>EECEBICD_01771 Glutaredoxin 4
ATGAGCACCACTATCGAAAAAATTCAGCGCCAGATCGCTGAAAACCCGATTCTCCTGTAT
ATGAAAGGTTCACCGAAACTGCCAAGCTGTGGTTTTTCTGCGCAGGCCGTTCAGGCGCTT
TCAGCCTGCGGGGAACGTTTTGCCTATGTTGATATCCTGCAAAACCCGGATATTCGCGCT
GAATTACCGAAATACGCCAACTGGCCGACATTCCCACAACTGTGGGTGGACGGCGAGCTG
GTCGGCGGGTGTGATATTGTGATTGAGATGTATCAGCGTGGTGAGTTGCAGCAGCTTATT
AAAGAAACTGCGGCAAAATATAAAACTCAGGAGCCGGACGCAGAGTAA
>EECEBICD_01772 Ribonuclease T
ATGTCCGATAACGCTCAACTTTCCGGTCTGTGCGACCGTTTTCGTGGTTTTTATCCTGTC
GTCATCGACGTTGAAACTGCAGGATTTAACGCTAAAACCGATGCGCTGCTCGAAATCGCC
GCCATCACGCTGAAAATGGATGAACAAGGCTGGCTGATGCCGGACATGACGCTGCATTTC
CATGTGGAGCCGTTCGCTGGAGCCAACTTACAGCCGGAAGCGCTTGCGTTTAACGGTATC
GATCCCTCTAACCCGCTACGCGGCGCGGTGAGTGAATATGAGGCGCTACACGCTATTTTC
AAAATGGTGCGTAAAGGTATCAAAGAAAGCGGTTGTAGCCGCGCCATTATGGTCGCGCAT
AACGCCACTTTTGATCACAGTTTTATGATGGCCGCCGCAGAGCGCGCCTCGCTGAAACGT
AACCCGTTTCATCCGTTCGTCACCTTTGATACCGCGGCATTGAGCGGACTGGCGCTGGGG
CAAACGGTGTTATCAAAAGCGTGCCTGGCGGCAGGTATGGAGTTTGACGGCGAAAAGGCC
CATTCCGCTTTGTATGATACCGAGCGGACAGCGGTGCTGTTTTGTGAAATCGTCAATCGC
TGGAAGCGCCTGGGCGGCTGGCCGTTACCTTTGCCGACGGATAAATAA
>EECEBICD_01773 Lactoylglutathione lyase
ATGCGTTTATTGCATACTATGCTGCGCGTCGGCGATTTGCAGCGTTCCATCGCTTTTTAC
ACCAACGTTCTGGGCATGAAATTGCTGCGTACCAGTGAGAATCCGGAATACAAATACTCG
CTGGCCTTTGTGGGATATGGTCCGGAAACGGAAGAAGCGGTCATCGAACTAACCTATAAC
TGGGGCGTTGAGAGCTATGACATGGGCAATGCGTACGGGCATATCGCGCTGAGCGTTGAC
AATGCCGCTGAAGCCTGTGAACGCATTCGTCAGAACGGCGGCAACGTGACCCGTGAAGCG
GGGCCGGTAAAAGGCGGTTCGACTATCATCGCCTTCGTTGAAGATCCGGATGGCTATAAA
ATCGAATTGATTGAAGCTAAAGACGCAGGCCGCGGTCTGGGTAACTGA
>EECEBICD_01774 N-ethylmaleimide reductase
ATGGCGCCTTTGACGCGTTTGCGGAGTATTGAACCGGGCGACATTCCTACGCCGTTAATG
GCGGAATATTACCGTCAACGCGCCAGCGCCGGATTGATCATCAGCGAAGCCACACAAATC
TCCGCACAGGCGAAAGGCTATGCCGGCGCACCGGGATTACACAGCGATGAGCAGATCGCC
GCCTGGAAAAAAATCACTCAGGGAGTTCATGCTCAGGGCGGCCATATGGCGGTGCAACTG
TGGCATACGGGCCGTATTTCACACGCCAGCCTGCAACCAGGCGGACAAGCGCCCGTTGCG
CCATCCGCTATCAATGCGGGAACTCGCACTTCTTTGCGTGACGAAAACGGCCAGGCCATC
CGCGTAGAAACTTCCACGCCGCGCGCGCTGGATACTCACGAGATCCCCGGGATCGTCAAC

GATTTCCGCCAGGCTATCGCCAATGCCCGAGAGGCAGGCTTCGACCTGGTGGAGCTGCAC
TCCGCGCACGGTTATCTGCTGCACCAGTTCCTCTCTCCGTCTTCTAATCACCGTACCGAT
CGGTACGGCGGCAGCGTAGAGAATCGCGCCCGTCTGGTGCTGGAGGTGGTTGATGCCGGC
ATTAAGGAATGGGGCGCCGACCGTATCGGCATCCGCCTTTCTCCTGTCGGCACTTTCCAG
AATGTCGATAACGGCCCGAATGAAGAAGCTGACGCCCTTTATCTGATTGAAGCGTTAGGC
AAACGCGGCATTGCTTATCTGCATATGTCTGAACCAGACTGGGCGGGCGGAGAACCGTAC
AGCGACGCATTCCGTGAAAAAGTGCGCGCGTGTTTCCACGGCCCCATCATCGGCGCTGGC
GCCTATACGCCAGAAAAAGCGGAAGATCTGATCGAAAAAGGGCTGATTGACGCCGTGGCG
TTTGGTCGCGATTACATCGCTAACCCGGATCTGGTGGCGCGTCTGCAACGTAAAGCGGAA
CTTAATCCACAGCGTCCCGAAAGTTTTTATGGCGGCGGCGCGGAAGGCTACACTGACTAC
CCTACTCTGTAA
>EECEBICD_01775 HTH-type transcriptional repressor NemR
ATGAATAAGCAAACCGAACATGATACCCGCGAACATCTGCTGGCCACCGGCGAGCAGCTT
TGTATGCAACGGGGTTTTACCGGCATGGGGCTTAGCGAATTGCTCAAAACCGCCCAGGTG
CCTAAAGGGTCGTTTTATCACTACTTTCGCTCGAAAGAAGCCTTCGGCGTCGCCATGCTT
GAGCGACATTACGCCTGCTATCATCAGCGTCTTACGGCGCACTTCACCTCCGGTCCCGGT
AATCATCGCGATCGCCTTCTCGCCTGGTATCAGGAAACGTTAAAGCAGTTTTGCCAGCAA
GGCGCCATCAGCGGTTGCCTGACGGTGAAACTGTCAGCGGAGGTCTGCGATCTGTCGGAG
GATATGCGCTCTACGATGGATAAAGGGGCGCAGGAGATCATGGCACTGCTTGCACGCGCG
CTGGAAGATGGCCGTAATAGCCACTGTTTACATTTCACCGGCCAGCCGTTGCCACAGGCG
CAGGTACTTTATGCCCTGTGGCTGGGCGCCAATTTGCAGGCCAAAATATCCCGTAGCGCC
GCGCCGCTGGAAAATGCGTTGGCGCATGTTAAGACCATCATTGCAACGCCTGAGCAGTAA
>EECEBICD_01776 hypothetical protein
GTGGCCGAGCAGTTGGAGTTTTTTCCTGTAGCAAGCCCATGTCGCGGTATCTGCCAGTCT
GATGAGCGTGGCTTTTGTCGGGGCTGTATGCGCAGCCGCGACGAACGGTTTAACTGGCAA
AAAATGAGCGATGCCGAGAAACAAAATGTACTGCGGCTTTGTCGCCAGCGTTTCTTGCGT
AAAATACGCGCAAACAAGCCTCTCCCATCGGAAGATCCTCAACAACCTTCACTCTTTTAA
>EECEBICD_01777 Oxidoreductase YdhF
ATGGTTCAGCGTGTCACCATCGCTCCACAAGGCCCGGAGTTTTCGCGTTTTGTCATGGGT
TACTGGCGTTTGATGGACTGGAATATGTCCGCGCGTCAGTTAGTGAGTTTTATCGAAGAA
CATCTGGATTTGGGCGTCACTACCGTTGACCATGCGGACATTTATGGCGGTTATCAGTGC
GAAGCGGCATTTGGCGAGGCGCTAACGCTGGCGCCGCATTTGCGGGAAAGACTGCAAATC
GTCACCAAATGCGGTATTGCCACCACTGCCAGAGCGGAAAATAAGCTCGGTCATTATATT
ACCGATCGCCGCCATATTATTCTGAGCGCCGAACAGTCATTAAAAAATCTGGCGACAGAT
TATCTGGATATGTTGTTAATTCACCGTCCCGATCCGTTAATGGATGCGGATGACGTCGCC
GAGGCGTTTCAACATCTGCACCAAAGCGGCAAAGTGCGCCATTTTGGCGTTTCTAATTTT
ACGCCAGCGCAATTTACGTTGCTGCAATCGCGCCTGCCTTTCACCCTGGCGACCAACCAG
GTGGAGATCTCGCCGGTTCACCAGCCTTTGTTGCTGGACGGCACATTAGATCAATTACAG
CAACTGCGTATTCGTCCGATGGCCTGGTCATGTCTTGGCGGCGGTCGGCTGTTTAATGAT
GAGGCTTATCAGCCGTTGCGCCAGGAACTTTCCGTTATCGCGCAAGAACTCAACGCCAGT
TCTATTGAACAGGTGGTCTATGCCTGGATTTTACGTTTGCCGTCGCAGCCGCTGCCGATT
ATTGGCTCGGGTAAAATCGAACGCGTACGTGCCGCACTAGAAGCGGAAACGCTGTCATTG
TCGCGTCAACAGTGGTTCCGAATCCGCAAGGCGGCACTGGGTTATGACGTGCCGTAA
>EECEBICD_01778 Superoxide dismutase [Cu-Zn]
ATGAAGCGATTAAGTTTAGCGATGGTGACGCTGCTGGCCTGTGCGGGTGCGCAGGCTGCC
AGCGAGAAAGTAGAGATGAATCTGGTGACGGCGCAAGGCGTAGGACAGTCTATCGGCACC
GTCGTCATCGATGAAACCGAAGGCGGCTTAAAATTTACCCCACACCTTAAAGCGTTGCCG
CCGGGCGAGCATGGTTTTCACATTCATGCCAACGGTAGCTGCCAGCCAGCGATTAAAGAC
GGCCAAGCGGTTGCCGCAGAAGCCGCTGGTGGTCATCTGGACCCACAAAATACCGGCAAG
CATGAAGGACCGGAAGGCCAGGGGCATCTGGGCGACCTCCCGGTGTTAGTCGTTAATAAT
GATGGTATCGCCACCGAACCGGTTACTGCGCCGCGTCTGAAGTCTCTTGATGAAGTGAAA
GATAAAGCGCTCATGATCCATGTGGGCGGCGATAACATGTCCGATCAGCCGAAACCGCTC
GGCGGCGGCGGAACGCGTTACGCCTGCGGCGTCATTAAATAA
>EECEBICD_01779 p-hydroxybenzoic acid efflux pump subunit AaeB

ATGAAACTCTCCCTGCCAGCCCTGCGAAATACGCCCTGGTTTAAAGCGACCTCCGGCCAG
TGGCGTTACGCGTTGCGCAATACCATTGCCATGTGTCTCGCGCTGACATTCGCTTATTAT
CTCAATCTGGATGAGCCCTACTGGGCGATGACTTCGGCCGCAGTGGTGAGCTTTCCGACC
GTAGGCGGCGTAATCAGTAAAAGTCTGGGGCGTATTGCCGGGAGTTTACTCGGCGCGACC
GCCGCGTTGATTATTGCCGGCCATACGCTGAATGAACCCTGGTTGTTCTTATTTAGCATG
GCCGCCTGGATCGGTTTTTGTACCTGGGCCTGCGCGCATTTTACCAATAATGCCGCTTAT
GCTTTCCAGTTGTCCGGCTACACGGCGGCGATCATCGCGTTCCCGATGGTTAATATTGTA
GAAATCACGCAGTTGTGGGATATCGCCCAGGCACGTGTATGTGAAGTTATCGTCGGCATC
CTCTGTGGCGGCATGATGATGATGATCCTACCCAGCACATCGGATGGTACCGCCCTGCTC
ACCGCGTTGAAAAACATGCATGCGCGCTTGCTGGAACATGCCAGTCTACTGTGGCAGCCG
GAGACTACCGACGCTATCCGCTCCGCGCATGAGGGGGTCATTGGGCAAATCCTGACCATG
AACCTGCTGCGTATTCAGGCCTTCTGGAGTCACTACCGTTTTCGCCGCCAGAATGCATTG
CTAAATGCATTGTTACACCAGCAATTACGGCTAACCAGCGTGATCTCCAGTCTGCGTAGA
ATGCTTTTAAATTGGCCGACGCCGCCGGAAAACAGCCGCGAAGTCATTGAACAACTCTTA
GCTGAGTTGGCAAAACCGCGCGCGGACAGCTACACCGTGGCGCGGATTATTGCGCCGCTA
CGCCCGCAGGATGAACAGGATTATCGTCACCTCGCGTTCTGGCAGCGATTACGCTATTTT
TGCCAGCTATATCTGCGCAGCAGCCGTCAGTTATATCTGATCGAAAGCGGCGCGCCGGTA
GATCAAATCCATATCCGCCGCACTCCCGGCCTTGCGCGGCATACGGATAACGCAGAGGCC
ATCTGGAGCGGCGTGCGTACCTTTTGCACGCTCACGGTAATTGGCGCCTGGAGTATTGGC
GCGCAATGGGAATCCGGGCCCGGCGCATTAACCCTGGCGGCGATCAGTTGCGTCCTCTAC
TCCATCGTCGCCACGCCGTTTAAGTCGCTTTCCTTATTGATGCGTACTTTAGTGCTGCTG
TCCTTATTCAGCTTCGTGGTGAAATTTGGCCTGATGGTGCAAATCACCGATCTGTGGCAA
TTCTTGCTTTTCCTTTTTCCGCTGTTCGTCACGATGCAACTGCTTAAACTGCAGATGCCT
AAGCTGGCGGGACTGTGGGGGCAGCTTATCGTTTTTATGGGCTCATTCATCGCCGTGACC
AATCCACCGGTGTACGACTTCGCCGACTTCCTTAACGATAATACCGCCAAGATCGTCGGC
GTCGCGATTTCCTGGCTGGCGTTTGCGATTTTGCGCCCCGGTTCCGATGCCGTAAAAAGC
CGCCGCCATATCCGCGCGCTACGCCGCGATTTCGTCGATCAGCTTAGCCGTCACCCTTCC
CATAACGAAAGCGAGTTTGAATCGCTGACCTATCATCACGTCAGTCAGCTCAGCAATAGC
CAGGATGCGCTGGCGCGCCGGTGGTTATTACGTTGGGGAGTGGTACTGCTGAACTGCTCG
CATGTGGTGTGGCAGCTGCGCGCCTGGGAGTCGCGTTCCGATCCGCTTTCCCGGGTGCGG
GATATTTGTATATCCCTGCTGCGCGATGTGATGAGCGAGCGCGGCGTACAGCAACGCCCG
CTTGCCGTTACGCTACAGGAACTACAGCGTATCTGCGACACGCTCGCACATCATCATCAA
CCCGCCGCGCATGAACTGGCGGCGATTATCTGGCGTTTACACTGTTCGCTGTCGCAGCTG
GAACAGGCTCCAGCGCAGGGAACACTGTCTCCGGGTTATTTAATGACGCCGCAGGCGTAA
>EECEBICD_01780 p-hydroxybenzoic acid efflux pump subunit AaeA
ATGTCGTTAAAAACAATAAAATATTTTTCTACTCTCATTGTCGCCGTCGTCGCCGTTCTG
GCCGCATGGTGGTTATGGAATTACTATATGCAGTCACCGTGGACGCGCGATGGTAAAATC
CGCGCGGAACAGGTCAGCGTAACGCCGCAGGTTTCTGGCAGCATTACCCAACTCAATATC
AAAGATAATCAGTTCGTCAACGCCGGGGACGTTCTTTTTGTCATTGATAAAACGCCCTTC
CATATTGCAGAACTTAACGCGCAGGCGCAGTTGGCCAAAGCGCAGTCCGATCTGGCAAAA
GCGAATAATGAAGCCGATCGACGTCGACATTTATCGCGTAACTACATCTCTGCTGAAGAT
CTGGATAGCGCCAATCTGAATGTTAAAGCGATGCAGGCCAGCGTAGATGTCGCACTGGCG
ACGTTAAAACAGGCGCAGTGGCAGCTTAGCCAGACGGAAGTAAAAGCGCCGGTATCCGGC
TGGGTGACCAACCTTTCCACGCGTACCGGTGATTATGCCAGTACCGGTAAGCCACTTTTC
GCCCTGGTAGATAGCCATTCATTTTATGTAATGGGATATTTCGAAGAAACCAAACTACGC
CATATCCGCGAAGGTGAGCCTGCATTAATAACACTGTATAGTGGCAATGTTAAGTTACAG
GGTCACGTTGGCAGTATCGGGCGCGCTATTTACGACCAAAGCGTGGAAAGTGACTCCGGT
TTAGTGCCCGATATTAAACCCAACGTACCGTGGGTACGTCTGGCGCAACGCGTACCGGTA
CGTATCGAATTTGACGCTTTGCCGCAGGATATCACGCTCGTCTCCGGCACTACCTGCAGC
GTAGCCATTGGGCAGCGGTAA
>EECEBICD_01781 hypothetical protein
GTGAAGTGTATACTCAACGCCACAGGATTGCCCTTACAAGATCTCATGTTGGGAGCATCT
GTTTACTTTCCGCCATTCTTTAAAGCCTTTGCGCTTGGGTTCGTTATCTGGTTGTTTATT
CACCGACTCCTGCGTGACCGGATATACTCTGATGAAATCTGGCATCCCCTGTTAATGGAT

CTCTCCCTCTTTACGCTTTGCGTATGTCTGGGCCTCGTTTTACTGATCGCGTGGTAA
>EECEBICD_01782 Transcriptional regulator SlyA
TTGGAATCGCCACTAGGTTCTGATCTGGCACGGTTGGTGCGCATTTGGCGTGCTCTGATT
GACCATCGCCTCAAGCCTCTGGAATTGACGCAGACACATTGGGTCACGTTGCACAATATT
CATCAATTGCCGCCTGACCAGTCGCAGATTCAATTGGCTAAAGCGATAGGCATTGAGCAG
CCATCGCTGGTACGCACGTTGGATCAACTTGAAGATAAGGGGCTAATTTCGCGGCAAACC
TGCGCCAGCGATCGTCGCGCTAAGCGCATTAAACTGACCGAGAAAGCGGAGCCGCTGATC
GCTGAGATGGAAGCGGTCATTCATAAAACGCGCGGTGAAATTTTGGCTGGGATTTCTTCA
GAGGAGATTGAGCTTCTGATTAAACTTATCGCCAAACTTGAACACAATATTATGGAATTG
CACTCTCACGATTGA
>EECEBICD_01783 Outer membrane lipoprotein SlyB
ATGATTAAACGTGTACTGGCCGTTTCACTGATGGGGTTATCCTTAGCTGGGTGTGTTAAC
AATGATAGCCTTTCGGGTGATGTTTATACCGCATCCGAGGCTAAACAAGTTCAGAATGTA
ACGTACGGTACTATCGTTAACGTTCGTCCGGTTCAAATTCAGGGCGGTGATGATTCTAAC
GTGATTGGCGCTATCGGCGGCGCGGTGTTGGGCGGATTCCTCGGCAACACTATCGGCGGC
GGTACGGGTCGTTCACTGGCGACCGCGGCAGGCGCGGTGGCTGGCGGCGTAGCGGGCCAG
GGCGTCCAGAGCGCAATGAATAAAACCCAGGGTGTGGAACTGGAAATTCGTAAAGACGAT
GGCAACACCATTATGGTTGTGCAGAAACAAGGAAATACGCGTTTCTCGGCAGGTCAGCGT
GTCGTGCTGGCCAGCAACGGCAGCCAGGTTACCGTCTCTCCACGCTAA
>EECEBICD_01784 Anhydro-N-acetylmuramic acid kinase
ATGAAATCGGGCCGTTTTATTGGCGTTATGTCCGGAACCAGCCTTGATGGCGTGGATGTT
GTACTGGCGGCAATTGATGAAACTATGGTTGCGCAACAGGCAAGTCTCACCTGGCCTATT
CCCGTTCATCTGAAAAAAGGCATTCTCGATATTTGCCAGGGACAGCCGCTTACGCTCTCG
CAACTCGGCCAGCTGGATACCCAGTTAGGCCGGCTTTTTGCACAGGCGGTTAATGCGCTG
TTAGCGCGACAACGTTTGCAGCCGCGGGATATCGTCGCCATTGGTTGTCATGGGCAAACG
GTCTGGCATGAACCGACCGGCGAGGCCCCGCATACGCTGCAAATTGGCGATAATAATCAC
ATCGTCGCCCATACGGGGATTACAGTGGTCGGCGATTTTCGGCGGCGGGATATTGCGCTG
GGCGGACAAGGCGCGCCGTTGGTGCCTGCTTTCCATCACGCGCTGTTGGGACATCCGACG
GAAAAACGTATGGTCCTGAATATCGGCGGCATTGCCAATTTATCTCTGCTTTTCCCCGGA
CAGGCGGTGCGCGGGTACGACACGGGGCCGGGTAATATGTTGATGGATGCGTGGATTTGG
CGACAATGCGCGCAGCCCTACGACAAAGACGCGGCATGGGCGAAAGAAGGTCAGGTTATT
CTTCCATTGCTGCAAAAGATGTTGCGCGATCCGTACTTTGCTGCGTCGGCGCCCAAAAGT
ACAGGACGCGAATATTTCAACTATGGCTGGTTAGAGCGTCATCTTACGGCTTTCCCTGGC
GCAGACGCGCGCGATGTACAGGCCACGCTGGCGGAGCTGACGGCGGTCTCCATCGCCCAA
CAGGTGCTGCTCAATGGCGGCTGCGAGCGGCTGATGGTCTGTGGCGGCGGAAGCCGGAAT
CCGTTAGTGATGGCCCGTCTGGCGGCGCTGTTGCCCGGTATCGAGGTTTCGACCACCGAT
AAGGCGGGGATCAGCGGCGATGATATGGAAGCGCTGGCCTTTGCATGGCTTGCCTGGCGA
ACGCTCGCGGGCCTGCCGGGAAATTTACCTTCCGTGACGGGGGCTACAGAAGCCAGTGTT
TTAGGCGCTATTTACCCGGCTAACCCAATAACTCAGAGTTAA
>EECEBICD_01785 Membrane-bound lysozyme inhibitor of C-type lysozyme
TTGCCCTTCCAGAAGCAGGAGAGCTTTCGGGATATGCCTATGAAAAAGATCGCAATAATG
TGTTTACCTGTGCTGCTGACCGGCTGTAGCGTTTATCAACAATTTGTTGAACGTATGCAA
ACGGATACGCTGGAATACCAGTGTGATGAGAAGCCGCTTACCGTTAAAGTCAATAACCCG
CGTGAAGAGGTCAGTTTTGTTTATGACAATAAGCTGCTGACCCTGAAGCAAGGAATCTCG
GCCTCGGGCGCGCGTTATACCGACGGGATTTATGTTTTCTGGTCGCAAGGCGAGAGCGCC
ACGGTCTATAAACGAGACAGAATTGTGCTTAATAACTGTCAGTTACAAAATCCGAAGCGT
TGA
>EECEBICD_01786 Pyridoxine/pyridoxamine 5'-phosphate oxidase
ATGTCTGATAACGACCAATTGCAGCAGATCGCGCATCTGCGCCGTGAATACACGAAAGGC
GGCTTGCGTCGCCGCGATTTACCCGCTGAACCGTTAACCTTATTTGAGCGCTGGCTTGGG
CAAGCGTGCGACGCCAGACTGGCCGATCCGACGGCAATGGTCGTGGCGACCGTAGATGAC
AAGGGGCAACCGTACCAGCGTATTGTATTGCTCAAGCATTATGATGAAAAAGGCCTGGTC
TTTTATACCAACCTGGGAAGCCGCAAGGCGCATCAAATTGAGCACAATCCGCGTATCAGC
CTGTTATTTCCCTGGCATATGCTTGAACGCCAGGTGATGGTGACAGGCAAAGCCGAGCGT

CTCTCCACACTTGAGGTGGTGAGGTATTTCCACAGCCGTCCGCGCGACAGCCAGATCGGC
GCATGGGTTTCTAAACAATCCAGCCGCATTTCCGCGCGCGGTATCCTCGAAAGCAAATTC
CTGGAGCTCAAGCAGAAGTTTCAACAGGGCGAAGTGCCGTTACCCAGCTTTTGGGGTGGT
TTTCGCGTGAGTATCGAACAGATGGAGTTCTGGCAGGGCGGCGAACATCGTCTGCACGAT
CGCTTTTTATACCAACGCGACGACGGCGCATGGAAAATCGACCGTCTTGCGCCCTAA
>EECEBICD_01787 Tyrosine--tRNA ligase
ATGGCAAGCAGTAACTTGATTAAACAATTGCAAGAGCGGGGACTGGTAGCCCAGGTGACG
GACGAAGACGCGTTGGCAGAGCGACTGGCGCAAGGCCCGGTCGCGCTCTATTGCGGCTTC
GATCCTACCGCTGACAGCTTGCATTTGGGGCATCTGGTTCCATTGTTATGCCTGAAACGC
TTCCAGCAGGCAGGGCACAAACCTGTTGCGCTGGTAGGCGGCGCGACCGGTCTGATTGGC
GACCCGAGCTTTAAAGCCGCCGAGCGTAAACTGAACACTGAAGAGACCGTTCAGGAGTGG
GTGGCGAAAATTCGTAAACAGGTTGCCCCATTCCTGGACTTTGATTGCGGCGAAAACTCC
GCCATCGCGGCCAATAACTATGACTGGTTCGGCAGCATGAATGTGCTGACTTTCCTGCGC
GATATCGGTAAACATTTTTCCGTTAATCAGATGATCAACAAAGAAGCGGTGAAGCAGCGT
CTGAACCGTGACGATCAGGGTATCTCTTTCACGGAGTTCTCTTATAACCTGCTACAGGGG
TACGACTTCGCTTGTCTGAATAAACTGCACGGCGTTGCCTTACAGATTGGCGGTTCTGAC
CAGTGGGGGAACATCACTTCCGGTATCGACCTGACCCGTCGCCTGCACCAGAATCAGGTA
TTTGGTTTGACCGTTCCGCTGATCACCAAAGCTGACGGCACGAAATTCGGTAAAACCGAA
GGCGGCGCTGTCTGGCTGGATCCGAAGAAAACCAGTCCGTACAAATTCTACCAGTTCTGG
ATTAACACCGCAGATGCCGATGTTTATCGCTTCCTGAAATTCTTCACTTTTATGGACATT
GAGGAGATCAATGCCCTGGAAGAAGAAGATAAAAACAGCGGTAAAGCGCCGCGCGCGCAG
TATGTACTGGCCGAGCAGGTGACGCGCCTGGTTCATGGTGAAGAGGGGCTGGTCGCGGCG
AAACGGATCACTGAATGCCTGTTCAGCGGTTCACTGAGCGCGCTGAGCGAAGCTGACTTT
GAGCAGTTGGCGCAGGACGGCGTACCGATGGTTGAAATGGAAAAAAGGCGCCGACCTGAT
GCAGGCGCTGGTAGACGCCGAACTCCAGCCGTCGCGTGGTCAGGCGCGTAA
>EECEBICD_01788 Tyrosine--tRNA ligase
ATGCAGGCGCTGGTAGACGCCGAACTCCAGCCGTCGCGTGGTCAGGCGCGTAAGACTATC
GCCTCCAACGCTGTCACTATCAATGGTGAAAAACAATCCGATCCTGAATACATCTTTAAC
GATGAGGACCGTCTGTTTGGCCGCTATACCTTATTACGTCGCGGCAAAAAGAACTATTGT
CTGATTTGCTGGAAATAA
>EECEBICD_01789 Pyridoxal kinase PdxY
ATGAAGAATATCCTCGCTATCCAGTCCCATGTTGTTTTCGGACATGCGGGCAACAGCGCC
GCCGAATTTCCCATGCGCCGTCTTGGCGCGAATGTTTGGCCGCTGAATACCGTTCAGTTT
TCTAACCACACGCAATATGGTAAATGGACCGGCTGTGTCATGCCGCCCAGCCATCTGACT
GAGATTGTCCAGGGGATTGCGGACATTGGCCAGTTAGCGCACTGCGACGCCGTACTTAGC
GGTTACCTGGGATCTGCCGAGCAGGGAGAACACATTCTCGGTATTGTGCGCCAGGTTAAA
GCAGCAAATCCGCAGGCAAAATATTTCTGCGATCCCGTGATGGGGCATCCGGAAAAGGGT
TGTATCGTGGCGCCTGGCGTCGCAGAGTTCCATGTGCGTTATGCGCTACCCGCCAGCGAT
ATTATCGCGCCTAACCTGATTGAACTGGAAATTCTCAGCAAACATAGCGTGAATAATGTG
AATGACGCCGTGCAGGCGGCGCGAGAACTGATTGCTCAGGGGCCAGAAATTGTACTGGTA
AAACATCTCGCCCGCGCCGGTTACAGTTCAGAACGCTTTGAAATGCTGCTGGTGACAGCG
CAGGAAGCCTGGCATATTAGCCGTCCGCTGGTGGATTTCGGTTCACGTCAGCCGGTTGGC
GTTGGCGATGTCACCAGTGGTTTATTGCTGGTCAAACTTTTGCAGGGCGCGACGTTGCAG
CAGGCGCTGGAGCACGTTACGGCGGCAGTCTATGAGATTATGATCGCCACCAAAACGATG
CAGGAGTACGAACTGCAGGTCGTGGCGGCCCAGGACAGGATCGCTAACCCTGAACATTAC
TTCAGCGCAACGCGGCTGTAA
>EECEBICD_01790 Glutathione S-transferase GstA
ATGAAACTGTTCTACAAACCGGGTGCCTGCTCTCTTGCTTCCCACATTACCCTGCGCGAA
ATTGGCAAAGACTTTACGCTTGATGGCGTCGATCTCGCAAAAAAACGCCTGGAAAACGGT
GATGATTACCTGGCAGTCAACCCTAAAGGACAGGTTCCGGCTCTGCTGCTGGATGACGGC
ACACTGTTGACTGAAGGCGTCGCCATTATGCAGTACCTCGCCGACAGCGTTCCCGACCGC
CAGCTTCTGGCGCCGGTAAGTAGTCTTGCTCGCTATCATACGCTGGAATGGCTCAACTAT
ATTGCAACGGAACTGCATAAAGGCTTTACCCCGCTGTTCCGCCCTGACACGCCGGAAACT
CTTAAACCCGCCGTTCGCGCCGCTCTGGAAAAGAAATTTCAGTATGTGGATGAATCGCTT

TCAGACGACCAGTGGATTTGCGGCCAGCGTTTTACCATCGCAGACGCTTATCTGTTTACC
GTATTGCGTTGGGCCTATGGCGTAAAACTGAATATGGACGGTTAACGCATATTGAAAGT
TATATGCAGCGTGTCGCAAAAAGACCGACCGTTGCGGCAGCGCTTAAAGCGGAAGGACTG
AATTAA
>EECEBICD_01791 Dipeptide and tripeptide permease A
GTGTCTACTGCAAACAAAAAACCAACTGAAAGCGTCAGTTTGAACGCTTTCAAACAACCG
AAGGCGTTCTATCTCATCTTCTCGATAGAGTTATGGGAGCGTTTTGGTTATTACGGCCTG
CAAGGCATTATGGCCGTCTACCTGGTAAAACAACTGGGTATGTCAGAAGCGGATTCCATC
ACCCTTTTCTCTTCCTTCAGTGCGCTGGTGTACGGCCTGGTCGCCATTGGCGGTTGGTTA
GGTGATAAAATTCTGGGCACCAAACGCGTTATTATGTTGGGTGCCGTGGTGCTGGCTATC
GGTTATGCGCTGGTCGCATGGTCTGGTCATGACGCTGGCATCGTCTATATGGGCATGGCG
GCTATCGCTGTGGGTAACGGTCTGTTTAAAGCTAACCCGTCCTCCCTGCTGTCTACCTGC
TACGCGAAGGATGATCCGCGTCTCGACGGCGCATTTACGATGTATTATATGTCCGTCAAC
ATCGGCTCCTTCTTCTCTATGCTGGCGACGCCGTGGCTGGCGGCGCGTTATGGCTGGAGC
ACCGCGTTTGCGCTTAGCGTCGTCGGTATGCTGATAACCGTCGTTAACTTTGCTTTCTGC
CAGCGCTGGGTGAAAAGCTACGGTTCCAAACCCGACTTTGAACCGATCAACTTCCGTAAT
CTGCTGCTGACAATCGTGGGTATTGTCGTTCTGATTGCGGTCGCCACCTGGCTGCTGCAC
AACCAGGATATCGCGCGTATGGTGCTGGGCGTAATCGCTCTTGGCATCGTCATTATCTTC
GGTAAAGAAGCGTTTTCCATGCACGGCGCGGCTCGCCGTAAAATGATCGTCGCCTTTATT
TTGATGCTGCAGGCGATTATCTTCTTCGTACTGTACAGCCAGATGCCGACGTCGCTGAAC
TTCTTTGCCATTCGTAACGTCGAGCACTCTATTCTCGGTATCGCGTTCGAACCGGAACAG
TATCAGGCGCTGAACCCATTCTGGATCATTATCGGCAGCCCGATTCTGGCCGCTATCTAT
AACAGGATGGGCGATACGCTGCCGATGCCGATGAAATTCGCCATTGGTATGGTGCTGTGT
TCCGGCGCGTTCCTGATTCTGCCGCTGGGCGCCAAATTTGCGAACGATGCCGGCATCGTA
TCCGTAAACTGGCTGATCGCAAGCTACGGCCTGCAAAGTATCGGTGAGCTGATGATCTCC
GGTCTGGGACTGGCAATGGTCGCACAGTTGGTTCCGCAGCGTCTGATGGGTTTCATCATG
GGTAGCTGGTTCCTGACAACGGCAGGCGCTAACATCATCGGCGGTTATGTCGCCAACCTG
ATGGCTGTGCCGAGCGACGTGACTGACCCACTGATGTCCCTGGAAGTCTATGGTCGCGTA
TTTATGCAGATTGGCATAGCAACCGCCGTTATCGCCGTCCTGATGCTGCTGACAGCGCCT
AAGCTGAACCGTATGACCCAGGACGATGATACCGCTGAAAAAGGCTCTAAAGCCGCTACA
GTTTAA
>EECEBICD_01792 Endonuclease III
ATGAACAAAGCAAAAAGGCTGGAAATTCTGACGCGCCTGCGCGACAACAATCCGCACCCC
ACAACGGAACTCAATTTCACGTCGCCGTTTGAGTTGCTGATTGCCGTGTTGCTTTCCGCG
CAGGCAACCGACGTCAGCGTGAACAAGGCTACCGCCAAACTTTACCCGGTCGCCAATACG
CCAGCCGCGATGCTGGAACTGGGCGTAGAAGGCGTGAAATCCTATATCAAAACCATCGGT
TTATTTAACAGTAAAGCGGAAAACGTGATTAAGACCTGCCGTATTTTACTGGACAAGCAT
AATGGCGAAGTGCCGGAGGATCGGGCGGCGCTGGAAGCGCTTCCCGGCGTGGGGCGAAAA
ACCGCTAACGTAGTACTAAATACCGCCTTCGGCTGGCCGACCATCGCCGTTGATACGCAT
ATATTTCGTGTATGCAACCGAACACAGTTCGCGCCGGGTAAAAATGTCGAACAGGTAGAA
GAGAAGTTGCTGAAAGTGGTGCCGAATGAGTTCAAAGTGGACTGCCATCACTGGTTAATT
TTGCATGGCCGCTATACCTGTATCGCCCGAAAACCCCGCTGCGGCTCCTGCCTCATTGAA
GACCTTTGCGAATACAAAGAAAAAGTCGATATTTGA
>EECEBICD_01793 Ion-translocating oxidoreductase complex subunit E
ATGAGCGAAATAAAAGACATTGTTGTTCAGGGGTTATGGAAAAATAACTCTGCTCTGGTG
CAGCTACTTGGCCTGTGCCCTTTACTGGCAGTGACGTCTACGGCAACCAACGCTCTGGGG
CTGGGTCTGGCCACAACGCTGGTATTAACGCTGACCAACCTTACGGTGTCCGCACTACGT
CGCTGGACGCCGGCAGAAATCCGTATCCCGATTTACGTTATGATCATTGCCTCGGTGGTA
AGCGCAGTACAGATGCTGATCAATGCGTATGCGTTCGGTTTGTATCAGTCTTTAGGGATT
TTTATTCCGCTGATTGTAACGAACTGCATTGTTGTTGGTCGCGCCGAAGCGTTTGCCGCG
AAAAAAGGACCGTGGCTTTCCGCGTTGGACGGTTTTTCTATCGGCATGGGCGCGACAGGC
GCGATGTTCGTGCTGGGTTCGCTGCGCGAGATCCTCGGGAATGGTACGCTTTTCGATGGC
GCGGATAGCCTGCTGGGAGGCTGGGCAAAAGTATTACGGGTCGAGATATTTCACACCGAC
TCGCCTTTCCTGCTGGCGATGCTGCCGCCAGGCGCGTTTATCGGCCTGGGGCTCATGCTG

GCGGTAAAATATCTTATTGATGAAAAAATGAAGAAACGCCGCGCCGAAACCGCGCCTTCC
GCTGTTCCGGCAGGTGAAACAGGGAAAGTCTGA
>EECEBICD_01794 Ion-translocating oxidoreductase complex subunit G
ATGCTGAAAACGATTCGTAAACACGGTATTACGCTGGCGCTTTTTGCCGCAGGCTCTACG
GGATTGACCGCGGTGATTAATCAGATGACAAAATCAACCATCCACGAACAAGCGCTGCAA
CAACAACATGCCCTGTTCGATCAGGTATTGCCGCCAGATCGTTACAATAATAATCTGCAA
GAGAGTTGTTATCTGGTCGATGCGCCCGCCCTCGGCAAAGGCACACACCGTGTTTTTATC
GCTCGCAAGGATGATAAACCCGTAGCGGCCATCATTGAGGCCACCGCGCCAGATGGCTAT
TCCGGCGCTATCCAGCTTATTGTTGGCGCAGATTTCAACGGTACCGTCCTGGGGACGCGT
GTGACGGAACATCATGAGACGCCAGGGTTAGGTGATAAAATTGAGCGCCGTTTGTCTGAC
TGGATTACCCACTTTAGCGGTAAAACCATCAGCGGTGAAAATGATACGCACTGGGCGGTG
AAAAAAGATGGCGGTGATTTTGATCAATTTACCGGGGCGACAATAACGCCGCGTGCCGTA
GTGAATGCGGTTAAACGCGCCGGGCTGTACGCCGAATCACTGCCCGCTCAGCTCCCCCAT
TTAACCGCCTGTGGAGAATAG
>EECEBICD_01795 Ion-translocating oxidoreductase complex subunit D
ATGGTATTCAGAATCGCAAGCTCGCCTTATACCCATAACCAGCGCCAGACATCGCGAATC
ATGTTGCTGGTAGTAATCGCCGCTCTGCCGGGGATTGCCGCGCAGACGTGGTTTTTTGGT
TGGGGAACGCTTTTCCAGATCGTTCTGGCTGCTATCACGGCGCTCGTCGCAGAAGCCATT
GTCCTCAGGCTGCGCAAACAATCGGTCGCCAGTCACCTGCAGGATTATTCCGCGTTATTG
ACGGGGCTTTTGCTGGCGGTCAGCATCCCTCCGCTTGCGCCCTGGTGGATGGTCGTGCTG
GGAACCGGATTCGCCATTATTATCGCTAAACAGTTATATGGCGGGCTGGGACAAAACCCC
TTTAATCCGGCAATGATCGGTTACGTGGTGCTATTAATTTCCTTTCCGGTACAAATGACA
AGCTGGTTGCCGCCTTATGAAATAGCCGCAACAACGCCCGATATGCTGGACACGTTACGG
ATGATTTTTAGCGGGCATACCGCCAGCGGCGGCGATATGACGCTGCTACGTATCGGTATT
GACGGTATCAGCCAGGCGACGCCGCTGGATACGTTTAAAACGTCCTTGCGCGCCGGACAT
TCCGTTGAGCAAATTATGCAGTACCCGATATACAGCGGCGCGCTGGCCGGCGTGGGCTGG
CAGTGGGTTAACCTCGCCTGGCTTGTCGGCGGCGTATTCCTGCTGTGGCAAAAAGCTATC
CGCTGGCATATCCCGGTCAGTTTTCTGCTGACGCTGGCGCTATGCGCCGCGTTAGGTTGG
CTTTTCTCGCCCGCTACGCTGGCCTCGCCGCAGCTTCATCTGCTGTCAGGCGCCACCATG
CTGGGTGCATTTTTTATCTTAACGGATCCGGTTACCGCCTCCACGACCAACCATGGACGT
TTAATTTTCGGCGCGCTGGCTGGGGTATTAGTGTGGCTGATTCGTAGCTTCGGCGGCTAT
CCGGACGGCGTTGCTTTTGCTGTCCTGTTGGCGAATATCACGGTTCCTCTGATCGACTAC
TACACGCGCCCCCGCGTCTATGGACATCGCAAAGGATAA
>EECEBICD_01796 Ion-translocating oxidoreductase complex subunit C
ATGCTTAAACTATTTTCCGCTTTCAGAAAAGATAAAATCTGGGATTTCGACGGTGGTATT
CATCCGCCCGAAATGAAGACCCAGTCAAACGGCACGCCGCTGCGTCAGGTGCCTTTAGCG
CCGCGCTTCGTTATCCCCTTAAAACAGCACATTGGCGCTGAGGGCGAGTTGTGCGTTAGC
GTCGGCGATCGCGTGCTGCGCGGCCAGGCGCTGACCCGTGGACGTGGGAGAATGCTGCCG
GTACACGCGCCCACCTCCGGGACGGTTATCGCTATTGCCCCCCACTCGACGGCACATCCT
TCGGCGCTGGCGGAGTTAAGCGTCATCATTGATGCCGACGGCGAGGACCGCTGGATTGAA
CGCGAGGGCTGGTCTGATTACCGCGCCCACAGCCGTGAAGCGCTCATTGAACGCATTCAT
CAGTATGGCGTTGCCGGGCTTGGCGGCGCAGGGTTTCCCACCGGCGTTAAATTGCAGGGC
GGCGGCGATAAAATCACCACGCTTATTATCAATGCCGCAGAGTGCGAACCTTACATTACG
GCGGACGATCGTCTGATGCAGGACTGCGCCGCGCAGATTGTGGAAGGTATTCGCATCCTG
GCGCATATTCTGCAACCGCGTGAGGTATTAATCGGCATTGAAGATAACAAACCGCAGGCT
ATTTCCATGCTGCGCGCCGTACTGGCTGACGCTCACGACATTTCGTTACGTGTGATCCCA
ACCAAATACCCTTCCGGCGGCGCAAAACAACTCACCCAAATACTGACGGGAAAACAGGTT
CCCCACGGCGGGCGCTCCTCTGATATTGGCGTGCTGATGCAAAACGTCGGTACGGCTTAT
GCCGTTAAACGCGCCGTGGTTGACGGCGAACCGATCACAGAACGCGTCGTCACCTTAACG
GGTGAAGCGGTAAGCCGTCCGGGTAACGTCTGGGCGCGGCTCGGCACTCCCGTTCGTCAT
TTGCTTAACGATGCCGGATTCTGCCCTTCTGCTGACCAGATGGTCATCATGGGCGGCCCG
TTGATGGGATTCCCCCTTCCCTGGCTTGACGTACCGGTCGTAAAAATCACCAACTGCCTG
CTGGCGCCATCCGTGACTGAAATGGGAGCGCCTCAGGAAGAGAAAAGCTGTATTCGTTGC
AGCGCTTGCGCTGACGCTTGCCCTGCCGACCTTTTACCGCAGCAGCTTTATTGGTTCAGC

AAAGGGCAGCAGCATGACAAGGCTACCGCTCACCATATTGCTGATTGTATAGAATGCGGC
GCCTGCGCCTGGGTCTGCCCCAGTAATATTCCGCTCGTCCAATATTTCCGTCAGGAAAAA
GCGGAGATTAATGCGATCAGGCTTGAAGAAAAACGCGCCGCCGAAGCCAAAGCGCGTTTT
GAGGCTCGTCAGGCAAGACTGGAGCGCGAAAAAGCCGCCCGCCTTGCACGTCATAAGAGC
GCAGCGGTACAACCAGCGGCAAAAGATCAGGATGCCATTGCCGCTGCGCTGGCGCGGGTA
AAAGAGAAACAGGCGCAGGCCACGCAACCGGTGGTTATTCAGGCGGGTAGTCTGCCAGAC
AATAGCGCCGTCATCGCGGCCCGTGAAGCGCGTAAAGCGCAAGCGCGCGCTAAACAAGCC
GCTCATCCGGTGGCTGACAGCGCGATATCCGGCGGCGACCCGCGTAAAGCCGCAGTGGAG
GCGGCTATCGCTCGCGCCAAAGCCCGCAAACAGGAGCAGCAGGCCGGAAGCGAACCTGCC
GAACCGGTCGATCCGCGCAAAGCCGCGGTGGAGGCGGCCATCGCCCGCGCCAAAGCCCGC
AAACAGGAGCAGCAGGCCGGAAGCGAACCTGCCGAACCGGTCGACCCGCGCAAAGCCGCG
GTGGAAGCGGCTATCGCCCGCGCCAAAGCCCGCAAGCAGGAGCAGCAGGCCGGAGGCGAA
CCTGCCGAACCGGTCGATCCGCGTAAAGCCGCGGTGGAAGCGGCCATCGCCCGCGCCAAA
GCCCGTAAGCAGGAGCAGCAGGCCGGAAGCGAACCTGCCGAACCGGCCGACCCGCGTAAA
GCCGCGGTTGCTGCGGCTATCGCCCGCGTTCAGGCAAAAAAAGCAGCACAGCAGCAGGTT
GTTAACGAGGATTAA
>EECEBICD_01797 Ion-translocating oxidoreductase complex subunit B
ATGAATACTATCTGGATTGCCGTTGGCGCCCTGACCCTTCTGGGCCTGGTGTTCGGCGCC
ATTCTGGGTTATGCCTCCCGCCGATTCGCGGTTGAAGATGATCCGGTTGTTGAAAAAATC
GATGCGATTTTGCCGCAGAGCCAGTGTGGTCAGTGCGGCTATCCCGGCTGTCGCCCCTAC
GCTGAAGCCGTGGGTCTCCAGGGCGAGAAAATCAATCGTTGCGCGCCCGGCGGCGAAGCC
GTCATGCTGAAAATGGCAGAGCTGCTAAACGTAGAGCCGCAGCCATGCGATGGTGAAGAA
CAGCAGGCCGCGCCAGTACGTATGCTGGCAGTTATTGATGAAAATAACTGCATTGGCTGC
ACAAAATGTATTCAGGCCTGCCCGGTTGACGCCATTGTTGGCGCAACGCGCGCCATGCAC
ACGGTCATGAGCGATCTCTGTACTGGCTGTAATCTGTGCGTCGATCCGTGTCCGACGCAC
TGCATCGAATTACGTCCGGTGAATGAGACGCCCGACAGTTGGAAATGGGATTTGAACACC
ATTCCCGTTCGCATCATTCCCGTGGAACAACATGCTTAA
>EECEBICD_01798 Ion-translocating oxidoreductase complex subunit A
ATGACTGATTACCTGCTGCTCTTTGTCGGAACTGTACTGGTCAATAACTTTGTACTGGTC
AAATTCCTGGGCCTTTGTCCCTTTATGGGCGTATCCAAAAAACTGGAAACCGCCATGGGG
ATGGGCCTCGCGACGACCTTTGTCATGACGCTGGCGTCGATATGCGCCTGGCTTATCGAC
ACCTGGATACTTATTCCGCTGGATCTGATTTATCTGCGGACGCTGGCCTTTATCCTCGTC
ATTGCTGTGGTCGTGCAATTTACCGAAATGGTTGTACGGAAAACCAGCCCGGCGTTATAC
CGCTTGTTAGGCATCTTCTTACCACTGATTACCACTAACTGTGCGGTTCTGGGCGTAGCG
TTACTGAATATTAATCTCGGTCATCACTTTCTTCAGTCGGCGCTGTACGGATTTTCCGCC
GCCGTCGGTTTTTCATTAGTGATGGTTCTGTTTGCCGCCATCCGCGAACGCCTTGCCGTG
GCGGATGTGCCCGCCCCTTTTCGTGGTAACGCGATAGCATTGATTACCGCAGGATTAATG
TCTTTGGCCTTTATGGGCTTTAGTGGTTTGGTGAAGTTGTAA
>EECEBICD_01799 Inner membrane protein YdgK
ATGACTACAACACCGCCACAACGAATTGGAGGCTGGCTTCTTGGCCCGTTAGCCTGGCTG
TTAGTCGCGTTGTTGAGCGCCTCGCTGGCGTTATTACTCTACGTTATGGCGCTTGCCACA
CCGCAAACGTTCAAGACGCTTAGCGGACAGGAAACCGGTAATTTACTGCTATGGGGCATT
TCATTTATTACCGCTATCGCCATGTGGTACTATACCCTGTGGTTGACGATCGCATTCTTC
AAGCGCAGACGCTGCGTGCCGAAGCATTACATTATCTGGCTGCTGGTGTCGGTGTTACTG
GCGGTTAAAGCATTTGCCTTTTCGCCCGTCTCAGATGCCTTTGCCGTGCGTCAGTTACTC
TTCCCACTGTTGGCGACGGCGCTCATTGTGCCTTATCTCAAACGTTCAGCGCGCGTTAAA
ACAACGTTTGTTAATCCGTAA
>EECEBICD_01800 Transcription modulator YdgT
ATGACTGTTCAGGATTATCTATTAAAATTCCGCAAAATTAGTTCGCTCGAAAGTCTGGAA
AAACTGTTCGACCACCTTAACTATACCCTTACTGATGACATGGACATTGTTAATATGTAT
CGCGCTGCAGATCACCGCCGTGCCGAACTGGTTTCCGGAGGACGCCTGTTTGATGTAGGT
CAGGTTCCGCAATCCGTCTGGCGCTATGTGCAATAA
>EECEBICD_01801 putative oxidoreductase YdgJ
ATGAATGACAAAATCCGCGTTGGGTTAATCGGTTATGGCTACGCCAGTAAAACCTTTCAT

GCGCCCTTAATTATGGGAACGCCAGGGCTAGAGCTTGCGGCGGTATCCAGCAGCGATGAA
ACAAAAGTGAAAGCAGATTGGCCGGCGGTTTCCGTGGTCTCTGAGCCCCGACATTTATTT
AACGATCCGCATATCGACCTTATCGTGATCCCGACGCCCAACGATACCCATTTCCCGCTG
GCGAAAGCTGCGCTGGAAGCCGGTAAACATGTGGTGGTTGATAAACCCTTTACCGTGACA
CTGTCACAGGCGCGAGAATTGGATGCGCTTGCGCGGAGCACTGGCCGGGTACTTTCCGTC
TTCCACAACCGACGCTGGGACAGCGATTTTTTGACGCTGAAAGCCTTGCTGGCGGATGGC
GTGCTCGGCGAGGTGTCCTTGTTTGAATCCCACTTTGATCGCTTCCGTCCCCAGGTGCGC
GACCGCTGGCGCGAGCAAGGCGGGCCGGGAAGTGGTATCTGGTACGATCTGGCGCCGCAC
TTGTTGGATCAGGCCATTCAGCTTTTTGGCCTGCCGGTCAGCATGACGGTGGATTTAGCG
CAACTGCGTCCGGGCGCGCAATCTACAGATTATTTCCACGCGATTTTATCCTACCCCCAG
CGCCGCGTGATTTTGCACGGTACGATGCTGGCCGCGGCGGAATCCGCGCGATACATCGTT
CATGGTTCGCGCGGTAGCTATGTTAAATATGGCCTCGATCCCCAGGAAGAACGGTTAAAA
AACGGCGAGCGTTTACCGCAGGAAGACTGGGGATACGACATGCGTGACGGCGTATTGACC
CTGGTTGAGGGAGAGACGCGCAAAGAAGAGAACTGGCTTACGTTGCCGGGTAACTACCCG
GCATATTATGCCGCCATCCGCGATGCGTTAAACGGTAACGGTGAAAATCCCGTGCCGGCA
AGCCAGGCGATTCAAATTATGGAGTTGATTGAACTGGGCATGGAATCGGCAAAACATCGC
GCGACGCTGTGTCTTGCCTGA
>EECEBICD_01802 Adenosine deaminase
TTGGGGGTAAAAGTCCTGGCATCGCTTGACGCCTGCCGCCGTGTGGCCTTTGAAAATATT
GAAGATGCCGCACGTAACGGCCTGCATTACGTCGAGCTTCGCTTTTCGCCCGGCTATATG
GCGATGGCGCATCAGTTACCGATAGCAGGCGTGGTTGAAGCCGTTATCGACGGCGTACGC
GACGGCTGTAACACGTTTGGCGTCGAGGCGCGACTCATTGGCATTATGAGCCGTACCTTC
GGCGAGGCGGCATGTTTACAGGAGCTTGATGCGCTGCTGGCGCACAGAGAGAACATCACC
GCGCTGGATTTAGCCGGTGATGAGCTGGGCTTTCCCGGCAGTCTGTTTTTGTCGCACTTT
AATCGGGCGCGCGATGCGGGCTGGCATATTACCGTTCACGCTGGCGAAGCCGCAGGGCCG
GAAAGTATCTGGCAGGCGATTCGCGAACTGGGGGCCGAGCGTATCGGACATGGCGTAAAA
GCTGTCGAAGATCGCGCGTTAATGGATTTTCTTGTACAACAGCGTATCGGTATTGAATCC
TGTCTGACCTCGAATATTCAGACCAGTACCGTCGCATCGCTGGCAGATCATCCGCTCAAA
ACCTTCCTTGAGCATGGCGTTCTCGCCAGTCTTAATACGGACGATCCGGCTGTTCAGGGC
GTAGATATTATCCATGAATATCACGTTGCCGCGCCAGCGGCAGGATTGAGCCGCGAACAG
ATTCGCCAGGCGCAGATTAACGGTCTGGAAATCGCCTTTTTGAGCGATGGCGAAAAACGT
GCGCTGCGCGAAAAAGTGGCCGCGGCGTAA
>EECEBICD_01803 Adenosine deaminase
ATGATTGATATTACGCTTCCTTTGACTGACATCCACCGCCACCTTGATGGCAACATTCGC
GCCCAAACAATTCTGGATCTTGGTCGGCAGTTCAACATCGCCCTTCCCGCCCAAACGCTG
GAAACGCTGATTCCGCATGTCCAGGTCACCTCAACCGAACCCGATTTAGTGAGCTTTCTC
ACGAAGCTCGATTGGGGGTAA
>EECEBICD_01804 Protein YdgA
ATGAAAAAAACACTGGTAGCTGCAGGTGTAGTGATTGCACTTGGCATCGTCTGGACAGGC
GGCGCCTGGTATACGGGGAAAAAGCTGGAGAACCATCTTTCAGAAATGGTGACTCAGGCC
AATGAACAGCTCAAGCGTACTGCGCCGGAGGCCGGTGTCGAATTAAGTTATCAAAACTAC
CAGCGTGGCGTGTTCAGTAGCCATCTGCAACTGGTTGTCAAACCGGTTGCCGGCGCAGAC
ACTACCTGGTTGAAGCCCGGGCAAAGCATTGTGCTGGACGAGTCCGTTTCACATGGCCCC
TTCCCGCTCGCCCAGCTCAAAACGCTCAATCTGATCCCTTCTATGGCGTCGGTAAAAACC
ACGCTGGTGAATAACGATGCGGCAAAACCCCTCTTTGATATCGCGAAAGGCGACACGCCC
TTTGTTATCAATACCCGCATTGGTTATGGCGGCGATACCCGCTCCGATATCTCTCTCAAG
CCGTTAAACTACGAGAATGCCGGTGAAAAGGTCGCCTTTAGCGGCGGCGAGTTCCAGCTT
AACGCCGATAAAGACGGTAATGTGGTTTCGCTGTCGGGCGAAGCGCAGAGCGGCCTGGTC
GATGCCGTTAACGAATATAACCAGAAGGTCCAACTCACCTTTAATAATTTGAAAACCGAC
GGTACCAGTAAACTGGCAAGCTTTGGCGAACGTGTGGGCGACCAAAAGTTAACGCTGGAT
AAACTGTCCATCGCCATTGAGGGTAAAGAGATGGCGGTGCTGGAAGGGATGGAAATTGCC
GGCAAGTCCGATCTCGTCAACGACGGAAAAACCATTAACAGCCAGTTGGATTACTCGCTT
AACAGCCTGAAAGTGCAGAATCAGGATTTGGGCAGCGGTAAATTAACGCTGAAAGTCGGT
CAAATCGACGGCGAAGCCTGGCACCAGTTTAGTCAGCAATATCATGCACAAACGCAGGCG

CTGCTGAATCAGCCGGACGTGGCGCAGAATCCTGAGCTTTATCAGCAAAAAGTCACTGAA
GCGTTCTTTAGCGCTCTCCCCGTGCTGTTAAAAGGCGATCCGGTACTGACCCTGGCGCCG
CTTAGCTGGAAAAACGCCAAAGGGGAAACCACGCTGAATCTGTCGCTGTTCCTCAAAGAT
CCGGCAACCACAACGGCACAACCGCAAACGCTGGCGCAGGAAGTCGACCGCTCCGTGAAA
TCGCTGGATGCGAAACTGGCAATTCCGATGGACATGGCGGTCGAGTTTATGACGCAGATA
GCAAAGCTGGAAGGCTATCAGCAGGATGATGCGGAAAAACTGGCAAAACAGCAGGTGCAG
GGGCTGTCCGCAATGGGTCAGATGTTCCGACTGACAACGCTGAAGGATAATACTATCGCC
TCCAGTTTGCAGTACGCCAATGGCCAGATCACGCTCAACGGACAAAAAATGCCGTTGGAA
GACTTTGTCGGCCTGTTTGGGATGCCGGCGCTTTCCGTACCGGACGTTCCGGCGCTGCCT
CAACAATAA
>EECEBICD_01805 Mannose-6-phosphate isomerase
ATGCAAAAACTCATTAACTCAGTGCAAAACTATGCCTGGGGAAGTAAAACTGCGTTAACG
GAACTTTATGGCATCGCCAATCCGCAGCAGCAGCCAATGGCTGAACTCTGGATGGGCGCG
CATCCCAAAAGCAGCTCGCGAATCACCACCGCCAACGGCGAAACCGTCTCCCTGCGTGAC
GCCATCGAAAAGAATAAAACCGCCATGCTGGGCGAAGCGGTAGCCAACCGTTTCGGCGAA
CTGCCGTTTCTGTTTAAAGTACTGTGCGCCGCGCAGCCGCTCTCTATTCAAGTGCACCCG
AATAAGCGCAATTCCGAAATCGGTTTCGCGAAAGAAAATGCGGCGGGTATCCCCATGGAT
GCCGCAGAGCGGAACTATAAAGATCCTAACCATAAACCAGAGCTGGTTTTTGCCCTGACG
CCTTTCCTGGCGATGAACGCGTTCCGCGAATTTTCTGACATTGTCTCTTTACTGCAACCT
GTCGCCGGCGCGCATTCCGCTATCGCCCACTTTTTGCAGGCGCCGAATGCTGAACGTCTG
AGCCAGCTTTTCGCCAGCCTGTTGAATATGCAAGGCGAAGAAAAATCCCGCGCGTTAGCC
GTACTCAAAGCGGCGCTTAACAGCCAGCAAGGCGAACCGTGGCAAACGATCCGCGTGATT
TCAGAGTATTATCCTGACGACAGCGGGCTTTTCTCTCCTTTGTTGCTGAATGTGGTCAAA
CTGAATCCCGGCGAGGCGATGTTCCTGTTTGCCGAAACGCCTCATGCTTATCTGCAGGGC
GTTGCGCTGGAAGTCATGGCGAACTCCGATAACGTTCTGCGCGCTGGCCTTACGCCAAAA
TATATCGACATCCCTGAGCTGGTCGCGAACGTGAAGTTCGAACCTAAACCTGCCGGCGAG
TTGCTGACTGCCCCGGTGAAAAGCGGCGCGGAGCTGGACTTCCCAATTCCGGTTGACGAT
TTTGCTTTTTCACTGCACGACCTGGCGCTTCAGGAGACGAGCATCGGCCAACACAGCGCC
GCGATTCTGTTCTGCGTTGAGGGTGAGGCGGTGTTACGTAAAGATGAACAGCGTCTGGTA
CTGAAGCCGGGTGAATCGGCCTTTATCGGCGCGGATGAGTCTCCGGTTAACGCCAGCGGC
ACAGGCCGTTTAGCGCGTGTTTATAACAAGCTGTAG
>EECEBICD_01806 Fumarate hydratase class I, aerobic
ATGTCAAACAAACCCTTCTTTTATCAAGACCCTTTTCCCCTCAAAAAGGACGATACCGAG
TACTACTTGTTGACCAGCGAACATGTTTCCGTCGCCGAATTCGAAGGACAAGAGATCCTG
AAAGTCGCGCCGGAAGCGCTAACGCTGTTGGCGCGTCAGGCCTTCCACGATGCGTCTTTT
ATGCTACGTCCCGCGCATCAGCAGCAGGTGGCGGATATTCTGCGCGATCCGCAGGCCAGT
GAAAACGACAAATATGTCGCGCTGCAATTCCTGCGAAACTCCGATATCGCGGCCAAGGGC
GTGTTGCCCACCTGCCAGGATACCGGTACGGCCATTATTGTCGGCAAAAAAGGCCAACGC
GTCTGGACTGGTGGAGGTGATGAAGCGGCGCTGGCTCGCGGCGTATATAACACCTATATC
GAAGATAACCTGCGCTATTCGCAGAACGCGGCGCTGGATATGTACAAAGAGGTCAATACC
GGTACTAACCTGCCGGCGCAGATCGATCTCTACAGCGTCGATGGCGATGAATATAAATTC
CTTTGTATTGCTAAAGGCGGCGGTTCGGCGAATAAAACCTACCTCTATCAGGAGACAAAA
GCGCTTCTGACGCCGGGTAAGCTCAAAAATTATCTCGTCGATAAGATGCGTACGCTGGGC
ACCGCCGCCTGTCCGCCGTATCACATTGCGTTTGTCATTGGCGGCACCTCGGCGGAAGCG
AACCTGAAGACCGTTAAGCTGGCCTCGGCAAAATACTATGATGCGCTACCAACCGAAGGC
AATGAACACGGGCAGGCCTTCCGCGATATTGAGCTGGAAAAAGAATTGTTGCTTGAGGCG
CAAAATCTTGGCTTAGGCGCGCAGTTCGGCGGCAAATATTTCGCTCACGATATTCGTGTC
ATCCGATTGCCGCGTCACGGGGCATCGTGTCCGGTAGGTATGGGCGTGTCCTGCTCCGCG
GATCGAAACATTAAAGCGAAGATCAACCGGGAAGGGATCTGGATCGAGAAGCTGGAGCGC
AATCCCGGTAAATATATTCCTGAGGCGCTGCGCCAGGCGGGAGAGGGCGAGGCGGTGCGC
GTTGATCTTAACCGTCCCATGAGCGAGATACTGCAACAATTGTCGCAGTATCCGGTATCA
ACGCGCCTGTCGCTGAACGGCACAATTATTGTGGGCCGCGACATCGCGCACGCTAAACTG
AAAGAACGGATGGACAGCGGCGAAGGTTTGCCGCAGTACATCAAAGATCATCCTATCTAT
TATGCGGGGCCGGCAAAAACGCCGGAAGGGTACGCCTCCGGGTCGCTTGGGCCGACTACC

GCAGGACGGATGGACTCTTATGTTGATCAGCTCCAGTCGCAGGGCGGCAGTATGATCATG
TTGGCGAAAGGCAACCGCAGCCAGCAGGTGACCGATGCCTGTAAGAAACATGGCGGCTTC
TACCTGGGCAGTATCGGCGGCCCGGCAGCCGTTCTGGCGCAGGGCAGTATCAAGCGCCTG
GAGTGCGTGGAATATCCTGAACTGGGTATGGAGGCTATCTGGAAAATTGAAGTGGAGGAT
TTTCCGGCCTTTATTCTGGTGGATGATAAAGGCAACGATTTCTTCCAGCAGATTCAGTCA
TCGCAGTGCGCACGCTGCGTTAAGTAA
>EECEBICD_01807 Fumarate hydratase class II
ATGGTAACGGTACGCCGCGAGAAAGATTCAATGGGCGCGATTGAAGTCCCGGCAGATAAA
CTGTGGGGGGCGCAGACTCAACGTTCGCTGGAGCATTTTCGGATTTCCACGGAGAAAATG
CCCGTCTCGCTCATTCACGCTCTGGCGTTGACCAAGCGCGCCGCTGCGAAGGTCAACCAG
GACTTAGGGTTGTTGGCGGCGGAAAAAGCCAGCGCGATTATCCAGGCGGCTGATGAAGTG
CTGGCGGGAAAACATGCTGATGAGTTTCCGCTGGCGATTTGGCAGACCGGGTCCGGTACG
CAAAGCAACATGAATATGAATGAGGTGTTAGCGAACCGCGCCAGTGAAATTTTGGGCGGC
GTTCGCGGTATGGAACGCAAGGTGCATCCCAATGATGACGTCAATAAGAGTCAAAGCTCA
AACGATGTGTTCCCAACCGCCATGCACGTGGCGGCGTTACTGGCGTTACGCGAACACCTT
ATCCCGCAATTATCCGCATTAACGGATACGCTTCGCGATAAATCCCACGCTTTCGCCGAT
ATTGTCAAAATTGGCCGTACTCACCTGCAGGACGCGACGCCGCTCACCTTAGGCCAGGAG
ATTTCCGGTTGGGTAGCCATGCTGGAACATAACCTCAGGCATATTGAGCACAGTTTACCG
CACGTCGCGGAACTGGCGCTCGGCGGAACCGCAGTAGGGACAGGGCTTAATACCCACCCG
GAATATGCCCGGCGCGTGGCCGAGGAACTGGCGACGATTACCGCGGCGCCGTTTGTTACC
GCCCCGAATAAATTCGAAGCGCTGGCGACCTGTGACGCGTTGGTACAGGCGCATGGCGCA
TTAAAAGGACTGGCGGCCTCGCTGATGAAAATCGCCAACGATGTTCGCTGGCTGGCGTCC
GGCCCGCGCTGCGGCATTGGCGAGATTGCTATCCCGGAGAATGAGCCAGGCAGTTCCATT
ATGCCTGGTAAAGTGAATCCGACCCAGTGTGAAGCGGTAACGATGCTATGTTGCCAGGTG
ATGGGTAACGATGTGGCCATCAATATGGGGGGCGCATCGGGCAACTTTGAGCTCAACGTC
TATCGTCCGATGGTTATTCATAATTTTCTGCAAACGGTGCGCCTGCTGGCCGATGGCATG
GCGAGTTTCAATCAACACTGTGCGTCAGGAATCGAGCCAAACCGCGAGCGTATTACGCAG
TTGCTGAATGAGTCGCTGATGCTGGTCACTGCGCTCAATACGCACATCGGTTACGATAAA
GCGGCGGAGATTGCGAAGAAGGCGCATAAAGAAGGGCTGACGCTCAAAGCCTCAGCCGTG
GCATTAGGATACCTTAGCGACGAGGAGTTCGACGCCTGGGTACGTCCGGAGTTGATGGTT
GGCAGTATGACGCCGGGACGTTAA
>EECEBICD_01808 DNA replication terminus site-binding protein
ATGTCCCGCTACGATCTCGTTGAACGCCTGAATGGCACTTTTCGCCAGATAGAACAACAC
CTGGCGGCGCTGACGGATAATCTACAGCAGCATTCGCTACTTATCGCCCGTGTCTTTTCA
CTGCCGCAGGTGACCAAAGAGGCGGAACATGCGCCGCTGGATACTATCGAGGTAACGCAA
CATCTGGGGAAAGAGGCCGAAACGCTGGCGCTACGTCACTATCGCCATTTGTTTATTCAA
CAGCAGTCCGAAAACCGCAGCAGTAAAGCCGCCGTCCGCCTGCCTGGCGTTTTATGCTAT
CAAGTGGATAATGCGACACAGCTAGATCTGGAAAATCAGGTACAGCGCATTAACCAGTTG
AAAACCACCTTCGAGCAGATGGTTACGGTAGAGTCCGGTCTTCCCTCAGCGGCACGTTTT
GAGTGGGTGCATCGGCATCTACCCGGCTTAATTACGCTGAACGCTTACCGCACGCTGACG
CTTATCAATAACCCCGCCACCATACGTTTCGGTTGGGCAAATAAACATATTATTAAAAAT
TTAAGCCGGGATGAGGTTCTGAGCCAGTTGAAAAAAAGCCTCGCGTCGCCGCGCAGCGTC
CCGCCGTGGACGCGCGAACAATGGCAATTCAAGCTGGAACGGGAATATCAGGATATCGCT
GCCTTACCGCAGCAGGCCCGACTCAAGATTAAGCGTCCGGTTAAAGTACAGCCGATCGCC
CGCATCTGGTATAAAGGCCAGCAAAAACAGGTGCAACACGCCTGCCCCACGCCGATTATC
GCATTAATCAACACGGACAACGGCGCTGGCGTGCCGGATATTGGCGGGCTGGAAAACTAT
GACGCCGACAATATTCAGCACCGTTTTAAACCACAGGCGCAGCCGCTGCGACTGATTATT
CCGCGACTGCACCTGTATGTGGCGGATTAA
>EECEBICD_01809 Sensor protein RstB
ATGAAAAAGCTGTTTGTGCAGTTTTACCTTTTGCTGTTCGTCTGTTTTCTGGTCATGACC
TTATTGGTCGGACTGGTGTACAAATTCACCGCCGAACGCGCGGGCAGGCAATCGCTCGAC
GATCTGATGAAAAGTTCGCTCTATCTGATGCGCAGCGAACTGCGGGAAATTCCGCCGCGT
GAATGGGGGAAAACGTTAAAAGAGATGGACCTGAACCTCTCTTTCGATTTACGCGTTGAA
CCGTTAAATCACTATAAGCTTGATGCCGCCACCACGCAGCGACTGCGTGAAGGCGATATT

GTCGCGCTTGACGATCAATACACCTTTATTCAGCGTATCCCGCGCAGCCATTACGTCCTG
GCGGTTGGCCCGGTTCCCTATCTCTATTTCCTGCATCAAATGCGACTGCTGGATATCGCC
TTAATGGCGCTCATTGCCTTTTCTCTCGCCTTTCCTGTGTTTATCTGGATGCGCCCGCAC
TGGCAAGAAATGCTCAGGCTCGAATCCGCCGCGCAGCGCTTTGGCGAGGGTCATTTGACT
GAACGCCTGCACTTCGATAATGGCTCCAGTTTCGAACGTCTGGGCGTCGCGTTCAACCAG
ATGGCGGATAACATTAATGCCCTGATCGCCAGTAAAAAACAGCTGATTGACGGTATCGCC
CATGAGCTTCGCACGCCGCTGGTACGTTTACGCTATCGGCTTGAAATGAGTGAAAATCTG
ACACCGCCGGAATCACAGGCGCTCAATCGCGATATTGGTCAACTGGAAGCGCTGATTGAA
GAACTGCTCACCTATGCGCGCCTTGACCGACCACAAAATGAACTGATGTTGACCGAACCC
GACCTCCCCGCATGGTTGCTCGCGCATTTGCAGGATGTGCAGAGCGTGACCCCGGAACGT
GCCGTAAACCTTCTCACATGTGTAATCGGCGATTATGGCGCGCTTGATATGCGGTTAATG
TCGCGAGTGCTGGATAACCTGCTGAATAATGCGTTGCGCTACAGTCGTACCACCGTTCAG
GTCAGCCTGTTACTCGACGGCAGTCAGGCAACGTTGATAGTCGAAGATGATGGCCCCGGC
ATTGAAGCGGATGCGCGTGAGCGCATATTTGAACCTTTTGTCAGACTCGACCCCAGTAGA
GACCGGGCGACAGGCGGCTGCGGGCTTGGACTCGCCATCGTACACTCTATCGCCCTGGCG
ATGGGCGGTAGCGTCGTCTGTGACGAGAGCGAACTGGGCGGCGCAAAATTTAGTTTTCGC
TGGCCGGTCTGGCACGCGATGCCCGACATGACCGCTGCCTGA
>EECEBICD_01810 hypothetical protein
GTGGGGTATCGCATTCCGGTACAGCCAGAAGCGAAAGGTCATGAAGCGCAGTGGTTCAGC
AACCTGATGGCCTTTCGCCCGGCGCGTGCCGATGACGGGCAACTGGCTTTTCTTGGCCTG
GGGGAAAGCCTGGTGGCCGGAATGAATGACGGCGTGCGGATGGCCCCAGGGTTTTCTTCC
TCAGAGCAGGACAAAACCGCGCTTCGCCAGGTCGCGACATTTGCGGCAAAACGGGGAATA
CCGCTTGAGATCCACGCCTATACCGATGACAGCGCCGACGCGATATTGACGATTTTTGAG
CAGGTAGCGCAGCAGTACGATCTGCGCCCTCTCCGCTGGTCTATTGCGCATCTGAATACC
GGTTCGCCACAGACGCTTGAGCGAATGCGTAAGCTGGGTCTGGCATACACTGTGCAAATG
GGGCCTTACTTTGAGGGGCTTGCCATCCGTGACGCCAATCCACCCGGCGCGACGGACAAT
TCGCCGCCGGTTCGACTGGCGCTGGATAAAGGGCTTGTCGTAGCTGGCGGTACCGATTCG
ACGCGTATTGGCATTGCCGGTGTCTGGCACGCTATCGAATATCATATCACCGGTATAGCG
TCAGGCGGTTCCGTGCGTAAACCCGCCAGCGAGCGGCTCACGCGTCTGGAAGCGCTAGCG
TTATATACACGTCATGCCGCCTGGCTCGCCTTTGCCGAACAACACCGGGGCCAGCTTCGC
GTCGGAAAACAGGCCGATCTGGCGGTACTCAATCAGCCATTTATGACGATGCCGGAAGAC
AGAATTGATACCATTCGCGCTGTTTTGACGCTTGTCGATGGACGCATTGTTCACGAAAGT
CCGGACCTTAACGCCGGACAATGA
>EECEBICD_01811 Adenine deaminase
ATGATCAGTCTCGTCCCGCCTCTGCTTTCCAGAACCGCGCTTCTCTTTCTGCTCACGGCC
ACAGGCGCAGCCACCGCCGCCCGCCCGGCAGCGGATATTATTTTGCATAACGGTAATATC
ATCACTCTGAATGATGCCCAGCCGCAGGCCAGCGCGCTGGTGATTTCCGGCTCGCGGATT
GTGGCGATTGGCGATGATACGGCGACAGATGAATGGCGCGGCGACCATACCCGTACCATC
GATTTACAGGGTAAAACCGTGATACCCGGCCTGACCGATACCCACATCCACGCCATTCGC
GGCGGACAAACCTGGACATTCGAAACCTACTGGTACGACAGCCCTTCGCTTAAAGACGCG
CTGGATAAATTACGCGCCGACGCTAACCGTCGTCCCCACGATCAATGGGTAGCCGTAGTG
GGATCGTGGATACCGGCGCAATTTGCAGAAAACCGGGCGCCGACGGTAGCCGAATTGAGC
CACGCCCTTCCCGATCATCCGGCTTATATTCAGTATCTTTACGACTATGCGTTAGTGAAT
CAGCGCGGTATAGACGTACTTGGCCTTAACGACACCCCTCCTCCTGATTTAGCGGGAATC
CGCGTAGAGCGCGACGCAAAAGGTAGCGCCACGGGGAAATTATTTGGTGACATCGCCGCG
TTTAACCAGCTTTTTGCCAGCATAAGTAGTAACGCCGATCGCGAGGGCGGTCTGCGACAA
TTTTTCGCTGATATGAACGCTCGCGGCGTGACCGGCATCATTGACCCCTCTGCCGGGCCT
GCCGCCGCTTATGAGCCTTTATTTGCAATGCGAAACCAGGGGGGATTTACCGCTGCGCGT
GGGGTATCGCATTCCGGTACAGCCAGAAGCGAAAGGTCATGA
>EECEBICD_01812 Outer membrane porin N
ATGAAAAGAAAAGTATTGGCACTTGTCATCCCGGCTCTGCTGGCTGCTGGCGCAGCACAC
GCCGCTGAAATTTATAACAAAGACGGCAACAAACTGGACCTTTACGGGAAAGTAGACGGC
CTGCACTATTTCTCTGATGACTCTTCTAAAGATGGCGATCAGACCTATATGCGTGTCGGC
TTTAAAGGCGAAACGCAGATTAACGACCAACTGACCGGTTACGGCCAGTGGGAATATAAC

GTTCAGGCTAACACTACCGAAGGCGAAGGCGCTAATTCCTGGACTCGTCTGGCATTCGCC
GGCCTGAAATTCGGCGACTACGGCTCATTCGACTATGGTCGTAACTACGGCGTCCTGTAC
GACGTAGAAGGCTGGACCGATATGCTGCCTGAGTTCGGCGGCGACTCCTATACTTACGCT
GACAACTACATGACCGGTCGTGCTAACGGCGTGGCCACCTACCGTAACACTGATTTCTTC
GGTCTGGTAGACGGTCTGAACTTCGCGTTGCAATATCAGGGTAAAAATGAAAGCCAAAGC
GCTGACGATGTAAATATCGGTACCAATAACCGTAATAATGGCGACGATATTCGTTACGAT
AACGGTGATGGCTTCGGTATTTCTACTACCTATGATATTGGCATGGGCTTCAGCGCAGGC
GCTGCGTATACCACTTCCGATCGTACTAATGAGCAAGTCAATGCTGGCGGCACGATTGCT
GGCGGCGACAAAGCGGATGCATGGACAGCTGGCTTGAAATATGATGCCAACAACATCTAT
TTAGCGACTATGTATTCTGAAACCCGTAATATGACGCCTTATGGTAAAACGGATGCGGGT
TATGCTGGCGGCGTAGCAAACAAAACGCAAAACTTCGAAGTTACAGCCCAATATCAATTT
GATTTCGGTCTGCGCCCGGCGGTTTCTTTCCTGATGTCTAAAGGTAAAGATCTGACCTAT
AACAACGTTAATGGCGATGATAAAGACCTGGTTAAATATGCTGATGTAGGTGCTACCTAC
TATTTCAACAAAAACTTCTCCACCTATGTTGATTATAAAATCAACCTGCTGGATGATGAC
GACCAGTTCTATAAAGATGCCGGTATCAGCACTGATGATATCGTCGCGCTGGGTATGGTT
TACCAGTTCTAA
>EECEBICD_01813 Transcriptional regulatory protein RstA
ATGAACCGCATTGTATTTGTTGAAGACGACGCTGAAGTGGGTTCTCTCATTGCCGCTTAT
CTGGCGAAACATGATATTGATGTCATTGTTGAGCCGCGTGGTGACCGGGCGGAGGATCTT
ATCCTCACCACTCAGCCCGATCTGGTTTTACTGGATATTATGCTTCCAGGTAAAGATGGC
ATGACGATATGTCGCGATTTACGCCACCGCTGGCAAGGCCCGATTGTCCTGCTCACCTCG
CTGGACAGTGATATGAATCATATTCTGGCGCTGGAGATGGGCGCCTGCGACTACATCCTC
AAAACCACGCCTCCGGCCGTTTTGCTGGCGCGTCTGCGCTTACACCTGCGGCAAAGCGAA
CAAACGCAACAGGCAAAAAGTCTTCAGGAGAGCGCGCTTACGCCGCATAAAGCGCTGCGT
TTTGGCGCGTTAACCATCGATCCCCTTAACCGGGCGGTCCAGCTCAATGGCGATTTCATA
TCGCTCTCCACCGCCGATTTTGAATTGCTCTGGGAACTGGCGACACACGCCGGACAAATT
ATGGATCGCGACGCGTTGCTAAAAACGCTGCGCGGCGTCAATTATGATGGTCTGGATCGC
AGCGTGGATGTGGCGATTTCCCGCCTGCGTAAGAAACTGCTGGATAGCGCTGCTGAGCCC
TATCGTATAAAGACCATTCGCAATAAAGGCTATCTTTTTGCCCCTCATGCCTGGGACGAA
ACGACGGGATAA
>EECEBICD_01814 Inner membrane protein YdgC
ATGGGACTCGTGATTAAAGCTGCCCTGGGGGCGCTGGTCGTCGTATTGATTGGTCTGCTG
TCAAAAACGAAAAATTATTATATCGCCGGATTAATTCCTCTGTTTCCAACGTTTGCGCTT
ATCGCGCACTATATCGTGGCCAGCGAGCGGGGCATCGACGCGATGCGTACCACCATCGTC
TTTAGCATGTGGTCGATCATTCCTTATTTCATTTATCTTGCAACGCTATGGTATTTCAGC
GGCGTGATGCGCCTGCCCGTCGCGCTTGGCGGTGCGGTGGTCTGCTGGGGGCTAAGCGCG
TGGCTGCTGATTTTTTTGCTGGATTAAATGGCATTAA
>EECEBICD_01815 Putative arginine/ornithine antiporter
ATGGAAAAAAAACTGGGACTGAGCGCGCTGACCGCGCTGGTTTTAAGCTCAATGCTTGGC
GCAGGTGTTTTCAGTCTGCCCCAGAATATGGCCGCCGTTGCCAGTCCGGCTGCGTTGTTA
ATTGGCTGGGCCATTACCGGCGTGGGGATACTCTTACTGGCATTCGCCATGTTAATCCTT
ACGCGCATTCGCTCCGAGCTTGACGGCGGCATCTTTACTTACGCCCGCGAAGGCTTCGGC
GAACTGATTGGCTTTTGTTCTGCCTGGGGCTATTGGCTGTGCGCGGTCATCGCCAACGTA
TCCTATTTGGTTATCGTTTTTTCAGCGCTGAGTTTCTTTACCGATACGCCTGCGCTGCGT
CTGTTTGGCGACGGTAATACCTGGCAGGCTATTGTCGGCGCTTCAGTCCTGCTCTGGATT
GTCCATTTCCTCATCCTGCGCGGCGTACAAACGGCAGCCAGCATCAATCTGGTCGCGACT
CTGGCGAAACTGTTGCCGCTCGGCGCGTTTATTGTGCTGGCTATCATGATGTTCAAACTG
GATACCTTCACACTGGACTTCACCGGTGTTGAACTCGGTATTCCCGTATGGAACAGGTC
AAGAACACCATGCTTATTACGCTGTGGGTATTTATTGGCGTTGAAGGCGCCGTCGTCGTC
TCGGCCCGAGCGAAAAATAAACGCGATGTAGGCCGCGCCACGCTACTGGCGGTTCTCGCG
GCGCTGGGAATATATCTCCTGGTCACGCTGCTTTCTCTTGGCGTTCTTGCGCGTCCGGAA
CTGGCGGAGATGCGTAACCCGTCGATGGCCGGACTGATGGTAAAAATGATGGGGCCGTGG
GGAGAAATTATTATTGCAGCGGGACTGATTGTGTCTGTCTGCGGGGCTTATCTAAGCTGG
ACTATCATGGCGGCAGAGGTGCCGTTTCTGGCGGCAGCCTATAAATCCTTCCCGGGCATC

TTCGCGCGACAAAATGCGCAAGGCGCGCCTTCTGCATCGTTATGGCTCACCAATATCTGT
GTGCAAATCTGTCTGGTGCTGATCTGGCTGACCGGTTCGGACTACAACACGCTATTGACG
ATTGCGTCAGAAATGATTCTGGTGCCCTACTTTCTGGTCGGCGCATTTCTGCTGAAGATT
GCAACACGTCCCCTCCATCGGGCGGTCGGTATCGGAGCCTGTATTTATGGCTTATGGTTG
TTATACGCGTCGGGTCCCATGCACCTGCTGCTGTCCGTCGTACTGTACGCGCCTGGATTA
CTGGTTTTCCTGTATGCGCGTCGGACGCACAAGCATGACAACGTGTTAAATCGTCAGGAA
GTCGTGCTGATTGGTTTATTGCTGGTCGCGGCGGTTCCGGCTACGTGGATGCTGGTGGGT
TGA
>EECEBICD_01816 Protein YdgH
ATGAAGCTTAAGAACACTCTCCTGGCGTCGGCTCTGCTTTCTGCGGCTGCTTTTTCTGTC
CATGCAGCAACAGAACTGACGCCGGAGCAAGCGGCTGCACTAAAACCTTATGACCGCATC
GTTATTACCGGTCGTTTTAATGCTATTGGCGATGCGGTCTCCGCCGTGTCCCGCCGCGCA
GATAAAGAAGGCGCAGCCTCCTTCTATGTTGTCGACACCTCGGAGTTCGGCAACAGTGGT
AACTGGCGTGTTGTGGCGGACGTGTATAAAGCCGACGCGCCGAAAGCCGACGCGCCCAAA
AATCGTGTCATTAATGGCATCGTGGAATTACCGAAAGACCAGGCCGTACAATTAGAACCG
TATGATACCGTCACCGTTCAGGGCTTCTACCGTAGCCAGCCGGAAGTTAACGATGCCATT
ACGAAAGCGGCGAAACAAAAAGGCGCATACGCTTTCTATATTGTTCGTCAGATAGATGCG
AATCAGGGCGGAAACCAGCGCATCACTGCATTTATCTATAAACAAGATGCGAAAAAACGC
ATCGTCCAGAGCCCGGACGCTATTCCGGCAGACTCTGAAGCTGGCCGCGCCGCGCTGGCT
CAGGGCGGCGAAGCTGCGAAGAAAGTCGAAATTCCAGGCGTCGCCACGTCAGCCTCGCCG
AGCGCCGAAGTAGGCCGTTTCTTCGAAACGCAATCCTCCAAAGGCGGACGTTACACTGTC
ACACTTCCGGATGGCACAAAAGTTGAGGAGCTGAATAAAGCGACGGCTGCGATGATGGTG
CCTTTCGACAGCGTTAAGTTCACCGGCAACTACGGCAATATGACAGAAATCTCCTATCAG
GTCGCCAAACGCGCGGCGAAGAAAGGCGCTAAGTATTATCATATTACGCGTCAGTGGCAG
GAGCGCGGCAATAACATCACCATCAGCGCCGACCTCTATAAATAA
>EECEBICD_01817 NAD(P) transhydrogenase subunit alpha
ATGCGAATTGGCATACCAAAAGAGCGGTTACCCAATGAAACCCGTGTCGCTGCGACGCCG
AAAACGGTAGAGCAGCTACTGAAGCTGGGGTTTAGCGTCGCTATCGAAAGCGGGGCGGGT
CAACTGGCAAGTTTTGACGACAAAGCGTTTGCGCAGGCTGGCGCGGATATTGTAGACGGC
AATGCTATCTGGCAGTCGGAAATTATTCTCAAGGTTAATGCGCCTGAAGAAGAGGAAATC
GCTTTACTGAACCCCGGGACTACGCTGGTCAGTTTTATCTGGCCTGCGCAAAATCCTGGG
TTAATGGAGAAACTTGCCGAACGTAAGGTTACGGTGATGGCGATGGACTCCGTGCCGCGT
ATTTCCCGCGCGCAATCGCTCGACGCGTTAAGCTCAATGGCGAATATCGCGGGTTATCGC
GCCATTGTAGAGGCCGCGCATGAATTTGGGCGCTTCTTTACCGGCCAAATCACTGCCGCC
GGGAAAGTGCCGCCGGCGAAGGTGATGGTGATTGGCGCGGGCGTTGCCGGTCTTGCGGCT
ATCGGCGCGGCAAACAGCCTGGGGGCGATTGTCCGCGCGTTTGACACTCGTCCTGAAGTC
AAAGAGCAGGTCCAGAGTATGGGGGCTGAGTTCCTCGAACTGGATTTTAAAGAGGAAGCC
GGTAGCGGCGATGGTTACGCCAAAGTGATGTCCGAGGCGTTTATCAAAGCGGAAATGGCG
CTCTTTGCCGCCCAGGCCAAAGAGGTCGATATCATTGTTACCACCGCGTTGATTCCGGGC
AAACCTGCGCCGAAGCTGATTACCCGCGATATGGTCGATTCTATGAAGGCCGGCAGCGTG
ATTGTCGACCTGGCCGCGCAGAACGGCGGTAACTGCGAATATACCGTCGCTAATCAGGTT
GTGACGACGGACAATGGCGTGAAAGTGATTGGCTACACCGATCTACCGGGGCGTTTGCCG
ACGCAGTCATCCCAACTTTATGGCACCAACCTGGTTAACCTGCTGAAGCTGCTGTGCAAA
GAGAAAGACGGCAATATTGACGTGGATTTTGACGATGTCGTGATCCGTGGCGTGACGGTG
ATTCGTGACGGCGACATTACCTGGCCTGCGCCGCCAATCCAGGTTTCCGCGCAGCCACAG
GCCGCGCCTAAAGCAGCACCTGCGCCAAAAGAGCCGGAAAAGCCCGCTTCACCCTGGCGG
AAGTATGCGTTAATGGCGCTGGCGATCATTCTTTTTGGCTGGCTGGCCGATGTCGCGCCA
AAAGAGTTCCTCGGCCATTTTACCGTGTTTGCGCTCGCCTGCGTGGTTGGTTATTACGTG
GTGTGGAACGTGTCTCATGCGCTTCATACGCCATTAATGTCGGTGACCAACGCAATTTCA
GGGATTATTGTTGTCGGGGCATTATTGCAAATTGGTCAGGGCGGCTGGGTCAGCTTCCTG
AGCTTTATCGCGGTGCTTATCGCCAGCATTAATATTTTCGGTGGCTTCACCGTGACTCAG
CGTATGCTGAAAATGTTCCGGAAAAACTAA
>EECEBICD_01818 NAD(P) transhydrogenase subunit beta
ATGTCTGGAGGATTAGTTACAGCTGCATACATTGTTGCCGCGATCCTGTTTATTTTCAGT

CTTGCCGGGCTTTCAAAGCATGAAACGTCCCGCCAGGGGAATAACTTCGGTATTGCCGGG
ATGGCGATCGCGTTGCTGGCGACCATTTTCGGGCCGGATACCGGCAACGTGGCATGGATT
CTGGTCGCCATGATTATTGGCGGCGCGATTGGCATCCGCATGGCGAAAAAAGTTGAGATG
ACCGAGATGCCGGAGCTGGTCGCGATTCTGCACAGTTTCGTGGGCCTGGCGGCGGTACTG
GTTGGCTTTAACAGCTATCTGTACCATGACGCCAGTCTGGCGCCGGTGCTGGTGAATATT
CATCTGACCGAAGTCTTCCTCGGTATCTTTATTGGCGCAGTCACTTTCACTGGATCGATC
GCAGCGTTTGGTAAACTGCGCGGCAAAATCTCTTCTAAGCCGTTGATGTTGCCTAACCGT
CACAAAATGAACCTGGCGGCGCTGGTTGTTTCTTTCCTGTTGCTGCTGGTTTTTGTCCGC
ACCGAAAGCGTGGGTCTACAGGTTCTGGCGCTGCTGCTGATGACCATTATCGCGCTGGCG
TTTGGTTGGCATCTGGTCGCCTCTATCGGCGGGGCGGACATGCCGGTGGTTGTTTCAATG
CTGAACTCCTATTCCGGTTGGGCTGCCGCGGCGGCTGGCTTCATGTTGAGCAACGATTTG
CTGATTGTGACCGGCGCGCTGGTCGGTTCGTCAGGCGCGATCCTGTCTTACATTATGTGT
AAAGCGATGAACCGCTCTTTCATCAGCGTTATTGCTGGTGGGTTCGGTACTGACGGCTCC
TCAACGGGCGACGATCAGGAAGCTGGCGAACATCGCGAAATCACGGCGGAAGAGACCGCT
GAGATGCTGAAAAACTCCCACTCCGTCATTATTACGCCGGGGTACGGGATGGCGGTGGCG
CAGGCGCAGTATCCGGTGGCGGAAATCACCGAAAAACTGCGTGCGCGCGGTATCAATGTG
CGTTTCGGCATTCATCCGGTGGCGGGTCGCCTGCCGGGGCATATGAACGTGCTGTTGGCA
GAGGCGAAAGTGCCTTATGACATCGTGCTGGAAATGGACGAAATCAATGATGATTTCGCC
GATACCGATACCGTGCTGGTGATTGGCGCGAACGATACGGTGAACCCGGCGGCGCAAGAC
GATCCCCGCAGCCCGATCGCCGGTATGCCGGTTCTCGAAGTGTGGAAAGCACAGAATGTC
GTGGTCTTTAAGCGTTCAATGAATACTGGTTATGCTGGTGTGCAGAACCCGCTGTTCTTT
AAAGAGAATACGCAAATGCTGTTTGGCGATGCCAAAGCCAGCGTGGATGCAATTCTGAAA
GCGCTTTAA
>EECEBICD_01819 AI-2 transport protein TqsA
ATGGCAAAACCGATCATCACGCTCAATGGTCTAAAAATCGTCATTATGCTGGGAATGCTG
GTGGTTATTCTGAGCGGTATCCGCTTCGCCGCAGATATCATTGTTCCCTTTATTTTGGCG
TTATTTATTGCGGTCGTTCTCAATCCCGTTGTGCAACGGATGACCCGATGTCGAATTCCG
CGCGTTATCGCCGTGTCATTGCTCATTGTGATTATTGTCATGCTAATGGTACTGCTACTG
GCCTATTTGGGGACATCATTAAATGAACTGGCCCGTACCCTACCGCAGTATCGCTCCTCA
TTGGTGATACCGTTGCAACGTATCGAACCGTGGTTGCAGCGAGCAGGCATTGGCGTGTCG
GTTGATGAGCTGGCGAAATATATCGATCCAAACGCCGCAATGACTCTGGTAACGAACCTG
CTCACCCAGCTGTCCAATGCCATGTCGTCCATCTTTTTATTGTTATTGACCGTGGTATTT
ATGCTGCTGGAGGTTCCACAATTGCCTGAAAAACTGCAGCAGATGATGAGCCGTCCGGTG
GAGGGCATGGCGGCAATTCAACGCGCGCTTGATAGCGTTTCCCATTATCTGGTGCTAAAA
ACAGCGATCAGTATCGTGACGGGACTGGTCGCATGGGGAATGCTGGCCGCGCTGGATGTC
CGCTTCGCCTTTGTCTGGGGACTACTGGCCTTCGCGCTGAACTATATTCCTAATATTGGA
TCGGTGCTCGCTGCCCTGCCGCCCATTATTCAGGTGCTGGTATTTAACGGGTTTTATGAT
GCGCTGCTGGTGCTGGCAGGCTATCTGCTGATTAATCTGGTCTTCGGCAATATCCTTGAG
CCACGAATCATGGGACGTGGTTTAGGCCTTTCAACCTTAGTGGTATTTTTGTCGTTAATT
TTCTGGGGCTGGCTATTAGGTCCGGTGGGCATGCTGCTCTCCGTTCCGTTAACCATCATT
GTCAAAATCGCCCTCGAACAGACCACCGGCGGGCAGAGTATCGCCGTTCTGCTTAGCGAT
TTAAACAAAAGATAA
>EECEBICD_01820 Spermidine export protein MdtJ
ATGTTTTACTGGATTTTATTAGCTCTGGCTATCGCGACTGAAATTACCGGCACGCTCTCA
ATGAAATGGGCTAGCGTGGGTAATGGCAATGCGGGTTTTATTTTAATGCTGGTGATGATC
ACGCTGTCATATATATTTCTTTCTTTTGCGGTAAAAAAGATTGCCCTGGGTGTAGCGTAT
GCCTTATGGGAAGGGATCGGCATTTTATTTATTACCATTTTTAGCGTGCTGCTATTTGAT
GAAGCATTATCAACCATGAAGATTGCCGGCTTGCTGACGCTGGTCGCCGGCATAGTATTG
ATCAAATCAGGGACGCGTAAACCCGGCAAACCGGTGAAGGAGGCGACTCGTGCAACAATT
TGA
>EECEBICD_01821 Spermidine export protein MdtI
GTGCAACAATTTGAATGGATACACGGCGCCTGGCTTGGGCTGGCAATAATGCTGGAAATC
GCGGCTAACGTACTGTTGAAATTTTCGGACGGCTTTCGCCGTAAATGCTATGGCATCTTA
TCGCTTGCCGCTGTTCTGGCCGCCTTCAGCGCGCTTTCCCAGGCGGTAAAAGGGATAGAC

CTGTCGGTGGCGTATGCGTTATGGGGCGGTTTTGGTATTGCCGCCACGCTGGCGGCGGGC
TGGGTGCTCTTCGGCCAGCGCTTAAACCCCAAAGGCTGGGTTGGCGTGATATTGCTGCTC
GCCGGAATGGTCATGATCAAATTTGCCTGA
>EECEBICD_01822 hypothetical protein
GTGTTTTTTACCGGTTATGGCGAAAGCCAGGGGGCTGAAACAGACGCCACGGATGCCAGC
GACGTCAAAACGCTTTTTTTTAACCATGATGATCGGGTGCCCGTCGCCGATCCTACCCAA
TCGCCGTGGGATGCCATCGGGCAACTAGAGACCGCCAGCGGTAATTTGTGTACGGCAACG
CTTATCTCCCCGCATATCGCCCTGACAGCAGGTCACTGCCTGCTAACGCCACCGAAAGGA
AAACCAGACAAGGCGGTTGCGCTGCGTTTTGTGGCGCGAAAAGGAGTATGGCGCTATGAA
ATCCACGGCATTGAAGGCCGCGTTGAACCGTCGCTGGGCCGACGCCTGAAAGCGGATGGC
GATGGCTGGATTGTGCCGCCTGCCGCGGCAAGCTGGGATTTTGGTCTGGTAATATTGCGC
TACCCACCTTCCGGTATCACGCCAGTGCCTTTATTTACCGGCGATAAAGCGGCGCTCACT
GCGGCGTTGAAAGCCGCAGACCGAAAAGTCACGCAGTCCGGTTATCCGGAAGATCATCTG
AATGCGCTCTATTCCCATCAGGATTGCGTCGTGACCGGCTGGGCGCAGAATGCCGTACTC
TCACACCAGTGCGACACGTTACCTGGCGACAGCGGCTCGCCGCTCTTATTGCACACGGAT
AGCGGATGGCAGCTCATTGGCGTACAAAGCTCCGCCCCTGCGGCTAAAGATCGCTGGCGC
GCCGATAACCGCGCTATATCCGTCACCGGCTTTCGCGATAAGCTGAAAGCTCTGGCGCAG
GATTAA
>EECEBICD_01823 Acid shock protein
ATGAAAAAAGTATTAGCTCTGGTTGTTGCCGCTGCAATGGGTCTCTCTTCCGCAGCATTC
GCGGCTGAGACTGCAACGCCTGCGAAAACCGCAGCGCCTGCCAAAACAACCCAGAACACC
CAGCACCACAAAAAACAGCATAAAAAAACCGTTGAGCAAAAAGCGCAGGCGGCTAAAAAG
CACCAGAAAAAAGGTGGTAAAGCGCCAGCCAAAACCCCGTCAAAACCAACTACTCAACCG
GCTGCGTAA
>EECEBICD_01824 Inner membrane transport protein YnfM
GTGAGTCGTACAACTATTCTCGATGACGCCACGGCGAGCGATATCGATGAACAACGCCAT
TCTCAGCCGGTTCAATTTATTAAACGCGGTACAGCGCCTTTCATGCGCGTCACGCTGGCC
CTTTTTTCCGCGGGCCTGGCGACTTTCGCGCTCCTTTATTGCGTTCAGCCTATACTACCG
GTACTTTCCCAGGAGTTTGGCGTCTCCCCCGCCAGCAGCAGCGTTTCACTTTCTATTTCT
ACCGCCATGCTGGCTATCGGCTTACTTTTTACCGGCCCGCTTTCTGATGCGATAGGCCGT
AAACCGGTGATGGTCACCGCCTTGTTATTAGCCTCCTGCTGCACATTGTTATCAACCATG
ATGACCAGCTGGCATGGTATTCTGATTATGCGCGCGCTGATCGGTCTGTCGTTAAGCGGC
GTCGCCGCGGTGGGAATGACCTACCTGAGCGAAGAAATCCATCCCAGCTTTGTCGCTTTC
TCTATGGGGCTTTATATTAGCGGTAATTCCATCGGCGGGATGAGCGGGCGTTTATTAAGC
GGCGTCATGACCGACTTTTTTAACTGGCGCATCGCGCTGGCGGCCATCGGATGCTTTGCG
CTGGCGTCCGCGCTGATGTTCTGGAAGATTTTACCCGCCTCGCAACATTTCCGTCCCACG
TCGCTACGGCCTAAAACGCTATTTATTAATTTCCGTCTCCACTGGCGCGATCGGGGGTTA
CCTCTGCTGTTTGCAGAAGGTTTTTTACTGATGGGCGCGTTCGTCACCCTGTTTAACTAC
ATTGGTTATCGCCTGATGCTCTCCCCCTGGGAATTAAGCCAGGCCGTGGTCGGACTGTTA
TCCGTAGCCTATCTCACCGGCACATGGAGCTCGCCGAAAGCTGGCGCCATGACCTCACGC
TATGGCCGGGGGCCGGTGATGCTGTTCTCTACCGCAGTAATGCTGGGTGGGCTTTTACTG
ACGCTTTTTACCTCGCTTTGGCTGATTTTTGCTGGGATGCTGCTTTTTTCCGCCGGCTTT
TTCGCCGCGCACTCCGTGGCCAGTAGCTGGATTGGCCCGCGTGCGCGTCGGGCGAAGGGA
CAGGCCTCGTCACTTTATCTTTTCAGCTATTATCTGGGGTCCAGTATTGCCGGGACGCTA
GGCGGTGTATTCTGGCATAGTTACGGCTGGAACGGCGTAGGTGGTTTTATCGCGCTAATG
CTGGTGCTGGCCATCCTGGTCGGGACGCGGTTACATCACCGTCTTCATGCCTGA
>EECEBICD_01825 HTH-type transcriptional regulator BenM
ATGAATATCGAATTACGTCATTTACGTTACTTTGTGGCGGTAGCAGAAGAGCTGCATTTT
GGTCGCGCCGCCGCGCGCCTCAATATCTCGCAGCCGCCGCTGAGTCAGCAGATACAAATT
CTGGAGCAGCAGGTCGGCGCGCGATTACTGGCGCGGACTAACCGCAGCGTAGCGTTAACC
GCCGCCGGGCGACAGTTTCTGGTGGATAGCCGACACATCCTGAGTCTGGTAAACGACGCC
GCCGCTCGCGCCGAGCGGTTGCATCAGGGCGAAGCGGGTGAAATCCGTATTGGCTTTACG
TCTTCAGCGCCTTTTATTCGGGCGGTATCGCATACCTTTTCGCTGTTCCGCCAGTCCTAT
CCGGGGGTGCATATGCAAACGCGTGAAATGAATACGCGTGAGCAAATCGCGCCGCTCAAC

GAAGGCGTACTGGATATTGGCTTACTGCGGAATACGCCGTTGCCCGACACACTTAATCGC
GAGGTGATTTTGCATGAGCCGCTTATGGCCATGATCCCGCGCGAACATCGGCTGGCGCAA
AAGCCGGTCGTCAGTCTGACAGAACTGGCGGAAGAACCTTTCGTCTTTTTCGATCCGCAT
GTCGGGACGGGCCTCTACGACGATATCCTCGGCCTGATGCGGCGCTATAACCTGTCGCCG
ACCATCACGCAGGAGGTTGGCGAGGCAATGACCATTATCGGTCTGGTAGCGGCGGGACTG
GGTGTCTCGATTCTGCCGGCATCCTTTAAGCGTGTACAGATGAGCGAGATGCGCTGGGTT
CCCCTGGCGGAAGAGGACGCCGTATCTGAAATGTGGCTGGTATGGCCAAAGCATCATGAA
CAGAGTCATGCCGTACAGCGGTTTTGCCAACTGCTATTACTGGCTGCCCGACGGGATTAA
>EECEBICD_01826 Protein mlc
GTGGTTGCTGATAGTCAGCCTGGGCATATCGATCAAATTAAGCAGACCAATGCTGGCGCA
GTGTATCGCCTGATTGATCAGCTCGGACCGGTATCGCGAATTGACCTGTCTCGTCTGGCG
CAGTTGGCGCCTGCCAGTATTACGAAAATTGTTCGCGAAATGCTGGAAGCGCATTTGGTT
CAGGAACTTGAAATTAAAGAGGCGGGCAGTCGCGGACGTCCCGCCGTAGGGCTGATGGTG
GAAACGGAAGCCTGGCACTATTTATCTATTCGTATTAGCCGTGGCGAAATTTTCCTTGCA
CTGCGCGATCTTAGCAGCAAACTGGTGGTAGAAGAGTGTCTGGCGCTGCCGTTAAACGAA
GCTACGCCGTTGCTTGAGCGAATTATTACGCACGTTGATCGGTTTTTACCCGCCATCAG
CAGAAACTGGAGCGTCTGACCTCCATTGCCATTACGTTACCGGGCATTATCGATACCGAA
AACGGCGTTGTGCACCGGATGCCGTATTACGAAGATGTCAAAGAGATGCCTTTGGGAGAT
GCGCTGGAGCGGCACACCGGCGTACCGGTTTACATTCAGCATGATATTAGCGCCTGGACG
ATGGCAGAGGCGCTTTTTGGCGCCTCACGCGGCGCGCGCGACGTTATCCAGGTGGTGATT
GATCATAATGTGGGGGCGGGCGTTATCACCGACGGTCATTTGCTTCATGCGGGTAGTAGC
AGTCTGGTAGAGATTGGGCATACCCAGGTCGATCCTTATGGTAAGCGCTGTTATTGCGGT
AATCATGGCTGTCTGGAGACCATCGCCAGCGTCGATAGCGTGCTGGAACTTACGCAGCTT
CGGCTTAATCAGTCGATGAGTTCAATGTTGCACGGCCAGCCGTTAACGGTAGATTCACTG
TGTCAGGCGGCGATGCAGGGAGATCTATTAGCAAAAGATATTATTAGCGGCGTTGGCGCG
CATGTCGGACGCATTCTGGCTATCATGGTGAATTTATTTAATCCGCAAAAAATTCTTATT
GGTTCGCCGCTAAGTAAAGCGGCTGATATCCTTTTTCCAGCCATTGCTGACAGTATCCGT
CAACAGGCGCTGCCCGCCTACAGCAGGAATACGGTTGTGGAAAGCACGCAGTTTACCAAC
CAGGGTACGATGGCCGGGGCGGCGTTGGTAAAAGACGCGATGTATAACGGCTCTTTGTTG
ATTCGTCTATTACAGGGTTAA
>EECEBICD_01827 ATP-dependent dethiobiotin synthetase BioD 1
ATGCTGAAGCGTTTCTTTATTACAGGTACAGACACTTCTGTTGGGAAGACGGTGGTTTCC
CGCGCATTACTACAAGCGTTAGCGTCGAGTGGTAAGAGCGTTGCAGGGTATAAACCTGTC
GCTAAAGGGAGCAAAGAGACTGCAGAAGGGATGCGCAACAAAGATGCGCTGGTATTGCAA
AGCGTCTCTTCGCTGGAGTTGCCGTATGAGGCGATTAATCCGATCGCGTTAAGCGAAGAA
GAAAGCAGCGTCGCGCATAGCTGTCCTATAAATTACACCTTGCTGTCCAACGGTCTGGCG
AGCCTGAGCGATAAAGTGGACCACGTTGTTGTGGAGGGCACTGGTGGCTGGCGTAGTTTG
ATGAATGATTTACGTCCATTGTCTGAATGGGTGGTACAGGAACAGTTACCGGTATTGATG
GTGGTAGGGATTCAGGAGGGGTGCATTAATCATGCGCTACTGACTGCGCAAGCTGTCGCT
AATGATGGACTGCCCCTTAATTGGCTGGGTGGCGAATCGTATCAATCCTGGCCTGGCGCAT
TATGCTGAAATTATTGATGTGCTTGGGAAAAAACTGCCAGCGCCGCTGATTGGCGAGCTA
CCGTACCTGCCGCGCGCCGAGCAGCGTGAGCTGGGGCAGTATATTCGTTTGTCAATGCTC
GGCAGCGTGCTGGCGGTAGATAGAATCATGGCGTAA
>EECEBICD_01828 Voltage-gated ClC-type chloride channel ClcB
ATGTTTCGTCGCCTGCTGATCGCTACGCTCATCGGTATTCTCGCGGCGCTGGCTGTCGCC
GCCTTCCGTCATGCGATGCAATTGCTGGAGTGGATTTTCCTGAGTAATGACACCGGCAGT
CTGGTGAATGCGGCTGAGGGGCTTTCGCCCTGGCGACGGCTGATTACGCCAGCGCTTGGC
GGCCTGGCCGCTGGTCTGTTGCTGTGGGGGTGGCAAAAAATGAACCAACAGCGCCCCCAT
GCGCCCACTGACTATATGGAAGCATTGCAAACCGACGGGCAATTTGACGTTGGCGCCAGT
CTGGTGAAATCGCTGGCCTCGCTGCTGGTCGTGGTCAGCGGCAGCGCAATTGGTCGCGAA
GGCGCAATGATCCTCCTCGCCGCGCTGGCGGCATCCAGCTTTGCCCGACGCTTCACGCCG
CGTGAGGAATGGAAATTATGGATCGCCAGCGGCGCCGCTGCGGGCATGGCGGGCGCTTAC
CATGCGCCGCTGGCGGGCAGCCTGTTTATCGCGGAAATCCTGTTCGGTACCTTAATGCTG
GCTTCCCTCGGCCCGGTTGTCGTTTCCGCCGTCGTCGCCCTGCTAACCACCCATCTATTA

AACGGTAGCGATTCACTACTCTATACCGTACATCTGACCGTTGATCTCCACGCGCGGGAG
TACGTCATGATAGTCAGTACCGGCCTGGTCGCCGGTCTGTGCGGTCCATTACTGATGTGG
CTGATGACGGCCAGCCATAATAGTTTCTTACGCTTAAAACTGTCCCCGCCCTGGCAACTG
GCGTTGGGCGGGCTTATTGTCGGATTACTGTCGTTACTCACGCCCACGGTATGGGGAAAC
GGCTATAGCGTCGTACAATCATTTTTGCTCTCTCCGCCGCTATTCTCGCTGATTGGCGGA
ATTTTTGCGTGCAAAATCCTGGCGGTGCTCGCCAGCAGCGGGTCTGGCGCGCCGGGCGGC
GTCTTTACGCCAACATTATTTGTCGGATTATCAATTGGAATGTTTCTGGGTCGGATATGG
GGATTCTGGCTGCCAGGCTCGGATGAAATAGCGATTTTATTGGGGCTGGCGGGTATGGCG
ACGCTACTGGCGGCGACAACCCATGCGCCGATAATGTCCACCCTGATGATTTGTGAAATG
ACCGGCGAGTATCAGTTACTCCCCGGTCTGCTGATTGCCTGTGTAGTCGCGTCAGTGCTC
TCGCGAACGTTACGCCATGATTCTATCTACCGCCAGCACGCTGCCGAGCATTGA
>EECEBICD_01829 Osmoprotectant import ATP-binding protein OsmV
ATGATAAAACTGGAAAATCTCACCAAACAATTTGTACAGAAAAAGGGTCAGCCGCTAAAA
GCCGTCGACAACGTCAACCTGAACGTGCCGGAAGGCGAAATGTGCGTCTTACTCGGTCCC
TCCGGCTGCGGCAAAACCACCACGTTAAAGATGATCAACCGGCTGATCGCGCCCAGCAGC
GGTAATATTCTTATCAACGGCGAGAATACCAACGATATGGATACGGTAACGCTACGCCGC
AATATTGGGTATGTTATCCAGCAGATCGGCCTGTTTCCTAATATGACGATTGAAGAAAAT
ATTACCGTTGTGCCGCGCATGTTGGGTTGGGACAAAGCGCGCTGTAAGCAACGCGCCGAA
GAGTTGATGGACATGGTGGCGCTGGATGCCAGAAAATTTCTGCATCGCTATCCAAAGGAG
ATGTCCGGGGGACAGCAACAGCGTATTGGCGTGATCCGCGCTCTGGCCGCCGATCCGCCG
GTACTGCTGATGGATGAGCCCTTCGGCGCGGTGGACCCCATTAACCGCGAAGTGATCCAG
AACCAGTTTCTGGATATGCAGCGTAAGCTAAAAAAGACCGTGATGCTGGTCAGCCATGAT
ATCGACGAAGCGCTAAAGCTCGGCGACCGTATCGCCGTGTTCCGTCAGGGGCGAATTGTA
CAGTGCGCCAGTCCTGATGAACTGCTGGCCAAACCGGCTAACGAGTTTGTCGGGTCGTTT
GTCGGTCAGGACAGAACGTTAAAGCGTTTGCTACTGGTCTCCGCGGGCGATGTTACCGAC
CAACAGCCAACCATTACCGCGCGGCCTTCCATGCCGTTAAGCGAGGCATTCGGCATTATG
GATGATCACGATATTCGCGCCATTACCGTCATTGACAACGACGGTAAGCCACTGGGATTT
GTGAAGCGCCGGGAAGCGCGGAACGCCAGCGGAATCTGCGCCGATATCACCCATCCTTTC
CGTATTACGGGCAAAGCGGAAGATAATCTGCGTATTGTGCTGTCCAGATTGTACGAAAGT
AATACTAGCTGGATGCCGATCGTTGATGAAGACGGACGCTATAACGGTGAAATCTCTCAG
GATTACATCGCCGATTATCTCAGCTCAGGCCGCACGCGTCGGGCGCTAAATATTCACGAA
AGCAGCTAA
>EECEBICD_01830 Osmoprotectant import permease protein OsmW
ATGGATACGATACATTATATGCTGGATAATGCAGGCTATCTCGCCAGCCTTACCTTTCAG
CATCTCTGGCTGGTCGCGCTGGCCGTCGGTCTGGCGATTATCATCGGCGTACCGTTAGGC
GTTTTGATTGTACGCCACAAATGGCTGGCAATGCCGGTATTGGGCGCCGCCACGCTACTT
CTTACCATTCCGTCTATCGCGCTGTTTGGATTAATGATCCCGCTCTTTTCGCTGATCGGG
CATGGTATTGGCGTTCTGCCCGCTGTGACGGCCGTTTTTCTCTACTCTTTACTGCCGATT
GTCCGTAATACCCATACTGCGCTTGATAGTTTGCCGCCCGGTCTGCGTGAAGCGGGACGC
GGCATCGGTATGACTTTCTGGCAACGGCTGCGCTGGGTCGAAATCCCAATGGCCCTACCG
GTTATTTTCGGGGGTATCCGCACCGCGGTAGTGATGAATATCGGCGTGATGGCGATTGCC
GCGGTGATCGGCGCTGGTGGTCTGGGGCTGCTACTGTTAAACGGCATTAGCGGCAGCGAT
ATTCGCATGTTAATTGCCGGCGCGCTAATGATCTGTCTACTGGCAATTGTACTTGACTGG
CTGTTGCATCGTCTGCAAGTCGTACTGACACCTAAGGGGATACGATAA
>EECEBICD_01831 Osmoprotectant-binding protein OsmX
ATGAGATTCAAAAAACACCTGTTGGGATGGCTTGCCGCAACGCTGTTGTTCAGTAGCCAG
ACGCAGGCCGCGCCGCTGGTTCTCGCCACCAAAAGCTTTACCGAGCAGCATATTCTTTCC
GCTATGACCGTTCAGTATTTGCAGAAGAAAGGCTTTCAGGTTCAGCCGCAAACCAATATC
GCCGCGGTGATTTCACGTAATGCGATGGTGAATAAACAAATTGATATTACCTGGGAATAC
ACCGGTACATCGCTGATTATTTTCAACCGTATCGATAAGCGCATGAGTCCACAGGAAACC
TACGACACGGTAAAACGCCTGGATGCGAAGCTGGGCCTGGTATGGCTCAAACCGGCTGAC
ATGAACAATACTTACGCGTTCGCGATGCAGCGCAAACGCGCCGAGTCGGAAAATATCACC
ACCATCTCGCAAATGGTGGCAAAAATCGAACAGGTCCGGCAGAACGATCCTGACCACAAC
TGGATGCTCGGTCTCGATCTGGAATTTGCCGGGCGCAGCGATGGGATGAAGCCCCTTCAG

CAAGCCTACCAAATGCAGCTTGATCGCCCGCAAATACGACAGATGGACCCAGGGCTGGTC
TATAACGCCGTTCGGGATGGGCTGGTTGACGCCGGGCTGGTCTATACCACCGACGGACGG
GTGAAAGGGTTTGATCTGAAAGTGCTGGAAGATGATAAAGGCTTCTTTCCAAGTTACGCT
GTCACGCCCGTGGTGCGTAAAGAGGTGCTGGAAGCCAATCCTGGCCTTGATGACGCCTTA
AACACCCTTTCCGGCCTGCTCAATAACGATGTGATATCGACCCTAAACGCCCAGGTCGAT
ATCGAGCATCGCACGCCGCAACAGGTAGCCCATCAATTTTTGCAGGACAAAGGTCTGCTG
TAA
>EECEBICD_01832 Osmoprotectant import permease protein OsmY
ATGCACACCCTCACCCTAAAACGTGTACTTGGGTTCACTATCGTCATTTTGCTGTTACTG
GCGCTGTTTATCTGGGGAATCGGTCTGGAGACGCTGAAAGCGCGTCAGGTCGATCTGCTG
TATCTCGGGCAACGGCATTTGATGCTGGTCTTTACGTCCATGTTTTTTGCTTTACTGGTC
GGTATCCCGAGCGGGATTTTACTCAGCCGACCTGCGGCAAAAGGATTTGCTGAATATGTC
ATGCAAATCTTTAATGTTGGTAATACCTTGCCGCCGCTGGCCGTTCTGGCCTTAGCGATG
GTGATTATCGGGATCGGCGATACGCCCGCCATTGTCGCGCTATTTCTGGCCTCCCTTCTG
CCTATCGTCCGCAATACCTATGCGGGCCTCTGTTCCGTTCCCGCCTCACTGATTGAAGCG
GCGAACGGGATTGGGATGACAAAATGGCAGCGGCTTCGTCAGGTAGAGCTACCTAATGCG
TGGCCAGTGATGCTGTCCGGTATTCGCATCGCGACCGCCATCAATGTCGGCACCGCGCCG
CTGGCCTTTCTGATCGGCGCCAGCAGCTACGGCGAGCTGATTTTCCCGGGAATTTATCTC
AACGATTTTCCGACGTTGATCCTCGGCGCAACGGCCACGGCGTTATTCGCGCTGATTCTT
GACACTCTGCTTGCCTGGTTTGGGCGACGCCTTAGCCCTCATACCGTCTGA
>EECEBICD_01833 Tat proofreading chaperone DmsD
ATGACCACTTTTTTACAACGTGATGATTTTTCAGTAACGGCGCGTGTTCTTGGCGCGTTG
TTTTATTATTCGCCGGAAAGCCACGAAACCGCTCCTCTGGTTCAGGCGCTATTAACCGAC
GACTGGCAGGCGCAGTGGCCGCTTGATGCCGAGGCGCTTGCGCCTGTGGCGGCTATGTTT
AAGACCCACAGCGAAGAGTCGTTGCCACAGGCCTGGCAGCGTCTGTTTATCGGCCCTTAC
GCTCTGCCGTCTCCGCCGTGGGGTTCCGTCTGGCTGGATCGTGAGAGCGTATTATTTGGC
GATTCTACGCTGGCGCTACGTCAGTGGATGCGCGAAAACGGAATTCAGTTTGAAATGCAA
CAAAACGAGCCAGAAGATCATTTTGGGTCGCTGTTGCTGCTGGCGGCCTGGCTTGCCGAG
AATGATCGTCATCATGAATGCGAACAACTCCTGGCCTGGCATCTGTTTCCGTGGTCGTCC
CGCTTTCTGGACGTATTTATCGATCATGCCGGGCATCCGTTTTATCAGGCGTTGGGGCAA
CTGGCGCGTCTGACTCTGGCGCAATGGCAAGCTCAACTTATTATTCCCGTCGCTGTAAAA
CCGTTATTCCGTTAA
>EECEBICD_01834 Anaerobic dimethyl sulfoxide reductase chain C
ATGGGAAGTGGATGGCATGAATGGCCGTTGATGATCTTCACGGTCTTTGGTCAGTGCGTG
GTCGGCGCGTTAATCGTTAGCGGACTGGGCTGGCTGACGGCAAAAGACGATACCATCGCC
CGTCAGCGCATCGTGCGTAGCATGTTTTTTCTGTGGCTGGTGATGGGACTAGGATTCCTC
GCGTCGATTATGCATCTCGGCTCGCCGATGCGCGCGTTTAACTCGCTTAACCGCGTAGGC
GCTTCCGCACTGAGTAATGAAATCGCCGCAGGATCGGTGTTCTTTGCCGTAGGCGGCATC
TGGTGGCTGGTGGCGGTACTCGGTAAAATGCCCCCGGCGCTGGGTAAAGTGTGGCTGCTG
GTCAGTATGGCGCTCGGCGTTGCATTCATCTGGGCAATGACGCTCGTTTATCAGATAGAT
ACCGTGCCAACCTGGTATAATGGCTACACCACGCTGGCCTTTTTCCTCACGGCATTCCTG
TGCGGCCCGGTGTTTGCGGCGCTACTGCTACGCATCGCGCGCGTCCCATTTTGCAGCGTG
ACGTTTGCCAGTATTAGCGGCCTAGCATTGGTGGTGTGCGTGGCGGTCATCGTACTACAA
GGACTGTCTCTCTCAACCATTCACAGTTCCGTGCAACAGGCCAGCCATCTCGCGCCGGAT
TACGGTATGCTACAGGTCTGGCGCATTGTCTTGCTTGCCGCCGGATTAGGCTGCTGGCTA
TGTCCGCTAATCCGTCGCCCGCGAACCGCATACCGTTGGTTTGCTGCTCGGTGTCGTGCTG
GTGCTGGCGGGCGAAATTATTGGCCGCGGACTTTTTTATGGCCTGCATATGACCGTAGGT
ATGGCCATCGCAGGTTAA
>EECEBICD_01835 Anaerobic dimethyl sulfoxide reductase chain B
ATGACAACCCAGTATGGATTTTTTATTGATTCCAGTCGTTGCACCGGTTGCAAAACCTGC
GAACTGGCCTGCAAGGACTACAAAGATCTCACTCCCGACGTCAGTTTCCGGCGGATATAT
GAATACGCAGGTGGCGACTGGCAGGAAGATAACGGCGTCTGGCACCAGAACGTCTTTGCC
TATTATCTCTCCATTTCATGCAACCACTGCGAAGACCCGGCCTGTACCAAAGTCTGCCCG
AGCGGCGCGATGCACAAGCGCGATGACGGTTTTGTGGTGGTGAACGAAGAGGTCTGTATC

GGCTGCCGCTACTGCCACATGGCCTGCCCGTACGGCGCGCCGCAGTACAACGCCGCCAAA
GGTCATATGACTAAGTGCGACGGCTGTCATGATCGCGTCGCCGACGGCAAAAAGCCGATT
TGCGTGGAGTCCTGCCCGCTGCGGGCGCTGGACTTTGGCCCGATAGACGAACTGCGTAAA
AAACACGGAGAGCTGGCTGCCGTCGCGCCGTTGCCGCGCGCGCATTTCACGAAGCCGAAT
ATTGTGATTAAACCGAACGCCAACAGCCGCCCGACCGGGGACACTACCGGTTATCTGGCC
AATCCGAAGGAGGTATAA
>EECEBICD_01836 putative dimethyl sulfoxide reductase chain YnfF
ATGGCGGCCAGTATCAGCCGACGAAGCCTGGTGAAAACCTCCGCTATCGGTAGCCTTGCG
CTTGCCAGTAGCGCTTTCACCCTTCCGTTCTCCCGCATCGCACACGCCGCAGCAGACCTC
GCATCCGGCAATGTGGCAGAAAAAGCGGTCTGGAGTTCATGTACCGTTAACTGCGGCAGC
CGCTGCCTGCTGCGCCTACACGTAAAAGATGACACCGTATATTGGGTTGAATCAGATACC
ACGGGCAACGATGAATATGGCAACCATCAGGTACGCGCCTGCCTGCGCGGCCGTTCTATT
CGTCGCCGGATGAATCATCCCGATCGCCTGAAATATCCGATGAAACGCGTTGGTAAACGA
GGAGAAGGCAAATTCGAACGTATCAGTTGGGATGAAGCGTTGGATACCATCGGCGATAAC
CTGAAACGTATCCTCAAGGACTACGGCAACGAAGCGGTACATGTCCTTTACGGTACCGGC
GTGGACGGCGGCAATATTACCAATTCTAACGTACCGTATCGCCTGATGAACGCCTGCGGC
GGCTATCTCAGCCGTTACGGTAGCTACAGCACCGCCCAAATCAGCGCGGCCATGAGCTAT
ATGTTCGGCGGCAACGACGGCAACAGTCCGGATGATATCGCCAATACAAAACTGGTGGTG
ATGTTTGGCAATAACCCCGCGGAAACACGTATGAGCGGCGGCGGCGTGACATACTATGTC
GAACAAGCGCGCGAGCGCTCCAACGCCCGGATGATCGTGATTGACCCGCGTTATAACGAC
ACTGCCGCCGGCCGCGAAGACGAGTGGCTGCCGATTCGCCCTGGTACCGATGGCGCGCTG
GCGGCGGCAATCGCCTGGGTGCTGATTACCGAAGATCTTATTGATAAACCGTTTCTGGAT
AAATACTGCATCGGCTACGACGAAACCACTCTGCCCGCCTCGGCCCCGCGTAACGCGCAC
TATAAAGCCTATATTTTGGGTCAGGGCGACGACGGTATTGCTAAAACACCGCAATGGGCC
GCGCAGATTACCAGTATTCCGGCGGAAAAAATCATTCAACTGGCGCGGGAAATCGGCTCG
GCGAAACCGGCTTACATTTGCCAGGGCTGGGGACCGCAACGCCACTCTAACGGGGAGCAG
ACCGCACGCGCAATCGCCATGCTGTCTGTACTCACCGGTAACGTCGGCATAAATGGCGGT
AATTCCGGCGTGCGCGAAGGCACCTGGGATCTGGGCGTCGAATGGTTTTCTATGCTGGAG
AACCCGGTAAAAACCCAGATTTCGGTCTTTACCTGGACCGACGCTATCGATCATGGCGCG
GAAATGACCGCCACACGCGACGGCGTGCGGGGAAAAGACAAGCTCGACGTGCCGATCAAA
TTTTTGTGGTGCTACGCCAGTAATACGCTGATCAATCAGCATGGCGACATCGCCCACACC
CACGAGGTGCTTCAGGACGACAGTAAATGTGAAATGATCGTCGGCATCGAGCACTTTATG
ACCGCGTCGGCAAAATATTGCGACATTCTGCTGCCTGATTTAATGCCGACCGAGCAGGAA
GATCTGATTTCCCACGAATCCGCAGGCAATATGGGCTATGTCATTCTTGGTCAGCCCGCC
ACCTCGCCGAAATTTGAAAGAAAGCCCATTTACTGGACGCTCAGTGAAGTGGCTAAACGG
CTTGGTCCGGACGTATACCAGACCTTCACAGAAGGCCGCACGCAGCATGAGTGGGTCAAA
TACCTGCACGCAAAAACCAAAGCGCGTAACCCGGAAATGCCGGATTACGAAGAGATGAAA
CAGACAGGGATTTTCAAGAAAAAATGCCCGGAGGAGCACTACGTCGCTTTCCGCTCATTC
CGCGAAGACCCTGCCGCCAACCCACTGAAAACGCCGTCCGGTAAAATCGAAATCTATTCC
GAGCGTCTGGCAACCCTCGCCAACACGTGGGAGCTGAAGAAAGATGAGATTATCCATCCT
TTACCCGCCTATACGCCAGGCTTTGACGGTTGGGACGATCCGCTTCGTCAGCGCTACCCG
CTACAGCTAACCGGCTTCCACTATAAAGCGCGTACCCACTCCAGCTACGGCAATATCGAC
ATCCTGCAACAGGCGTGTCCGCAGGAGATCTGGATCAACCCGATTGATGCGCAAGCGCGC
GGCATTCAGCATGGCGACACCGTGCGGGTCTTTAACCAGAATGGAGAGATGCTCATTCTG
GCAAAAGTCACGCCGCGCATATTGCCTGGCGTCACCGCTATCGGCCAGGGCGCCTGGTTA
AATGCCGACATGTTTGGCGACAAAGTCGATCGCGGCGGCTCCATTAACATTCTGACGTCG
CACCGGCCTTCCCCACTGGCGAAAGGTAATCCGTCACACAGTAATCTTGTTCAGGTTGAA
AAGGCGTAA
>EECEBICD_01837 Putative dimethyl sulfoxide reductase chain YnfE
ATGCCCGGAGAAAAGCAACAAACAGGCGTTAGCCGCAGGACGTTAGTGAAATCAGCGGCG
CTTGGCTCACTGGCGCTGGCTGCCGGAGGCGTATCTTTACCGTTTGGGATGCGTACCGCG
GCCGCGGCGGTGCAGCAGGCTATGCGCAACGAAGAAGATAAAATCGTCTGGGGCGCCTGT
TCGGTCAACTGCGGCAGTCGCTGCGCCCTGCGTCTGCATGTAAAAGATAATGAAGTCTGG
TGGGTTGAAACTGATAACACTGGCGACGATGTCTACGGCAATCATCAGGTTCGCGCCTGC

CTGCGGGGCCGTTCAATCCGTCGGCGTATTAACCATCCGGATCGCCTTAACTATCCCATG
AAACGCGTTGGCCGCCGCGGCGAAGGCAAATTTGAACGCATTAGCTGGCAGGAAGCACTG
GACACCATTAGCGCCAGCCTGAAAAAGACCGTAGAGACCTACGGCAATGAAGCCGTTTAT
ATCCACTATTCATCCGGCATCGTAGGCGGCAACATAACCCGCTCGTCGCCTGCCGCCTCG
CCGGTGAAGCGTTTGATGAACTGCTACGGCGGTTCGTTGAATCAATACGGCTCATACAGT
ACCGCGCAAATTTCTTGCGCCATGCCCTACACCTATGGTTCCAACGACGGCAACAGCACA
TCTGATATCGAAAACAGTAAACTGGTGGTCATGTTCGGCAATAATCCGGCTGAAACACGC
ATGAGCGGCGGCGGCATTACCTGGTTTCTGGAGCAAGCGCGTGAACGCTCAAATGCGCGC
ATGATCGTAATAGATCCGCGCTATACCGATACCGCTGCCGGACGTGAAGACGAGTGGATA
CCGATTCGCCCCGGCACCGATGCCGCGCTGGTGGCGGGAATTGCCTGGGTATTGATTAAC
GAAAATCTCGTTGACCAGCCTTTTCTCGATAACTATTGCATCGGCTACGATGAAAAGACC
CTTCCGGCTGACGCGCCCCCCAATGGCCATTATAAGGCTTATATTTTGGGCCAGGGCGAG
GACGGTATCGCCAAAACGCCGCAGTGGGCATCGCATATTACCGGCATCCCCGCCGATCGG
ATTATCAAACTGGCGCGCGAGATAGGCAGCGTCAAGCCGGCCTACATCTGTCAGGGTTGG
GGCCCGCAGCGTCAGGCGAACGGCGAACAAACCGCTCGCGCCATCGCCATGTTACCGATT
CTGACCGGCAACGTCGGCATTCATGGCGGTAATAGCGGCGCACGCGAATCCACTTACACC
ATTACCATTGAACGTCTGCCGGTCCTGGAGAATCCCGTTAAAACCACCATCTCCTGCTTT
AGCTGGACGGACGCCATTGCGCGCGGCCCGGAAATGACCGCCCTGCGCGACGGCGTACGC
GGCAAAGATAAGCTGGATGTCCCGATCAAATTTCTATGGAACTACGCTGGCAACACCCTG
ATTAATCAGCATTCGGATATTAATAAAACCCATGAAATTCTTCAGGATGAAACGAAATGC
GAAATGATTGTGGTCATCGACAATTTTATGACCTCTTCCGCGAAGTACGCCGATATTTTA
CTGCCGGATCTCATGACCGTCGAACAGGAAGATATCATCCCCAATGATTACGCCGGCAAT
ATGGGATATCTGATTTTTATCCAACCGGCGACGACGCCTAAGTTTGAACGCAAGCCCATC
TACTGGGTACTCAGCGAAATCGCCAGACGCCTCGGCGACGATGTGTATCAGCGGTTTACC
GAAGGCCGTACACAGGCGCAATGGCTGCAATATCTGTACGCCAAAATGCAGGCAAGAGAT
CCGGCATTGCCCGCGTATGACGATCTGAAAAAAATGGGCATCTATAAACGCAAAGATCCC
AACGGCCACTTTGTCGCCTATAAAAAATTTCGTGAAGATCCGCAGGCCAATCCGTTAAAA
ACGCCGTCCGGTAAAATCGAAATCTACTCAAGCAAACTCGCTCACATCGCCAGTACCTGG
GAGCTGGCGGAAGGCGACGTGATAAGCCCACTGCCGATTTATACCCCTACTTTTGAAGGA
TGGGACGATCCGAAGCGGAGCACCTTCCCGCTACAGCTGTTTGGCTTTCACTATAAATCC
CGTACCCATTCAAGCTACGGCAATATTGATGTTCTGCAGGCCGCCTGTCGCCAGGAGGTG
TGGATAAATCCGCTCGATGCGCAAAAACGTGGTATCGCGAATGGCGATAAGGTTCGCGTC
TTTAACGATCGTGGAGAGGTGCGTCTACCCGCCAAAGTCACGCCGCGTATTCTGCCGGGC
GTCAGCGCGATGGGTCAGGGCGCATGGCATAATGCGGATATGGCTGGCGATAAAATTGAC
CACGGCGCCTGCATCAATACGCTCACGACGCTGCGTCCTTCCCCCTGGCAAAAGGGAATC
CTCAACATACAAATCTGGTGGAAATCGAAAAAATCTAAGCCGCCTGCTCCGGGACGTTTT
TACGCGCCCGGAGCTCCCCGTTTTGCGAACACAACGACATTTAAAACTCTTTTTTGGATG
AGCATAGTGAGCTGTTATGAAAATCACCAACCCTGA
>EECEBICD_01838 putative protein YnfD
ATGAAAATCGCAACTGCAATAGCCGCTCTGGCGTTAGTTAGCGCCCCCTCGCTGGTGATG
GCGGCACCGGGATCGTGCGAGAGAGTAAAAAGCGATATTGAACAACGTATCATCAATAAC
GGCGTGCCGGCGGATAATTTTACGCTCACTATCGTTCCTAACGACCAGGCCGATCAGCCG
GACTCACAGGTGGTGGGCCATTGCGCGAACGACACGCATAAAATTCTTTATACCCGCACC
AGCAGCGGCAATGCGCCAGCCAATACCTCTCCCGCTCAGGACGGTTCATCCGCCGAACCG
CAATAA
>EECEBICD_01839 hypothetical protein
GTGATACGTGTGAAGAAACCCCTTTTACTGACGTTATTGTGCATGATACTGGCAGGTTGT
GATAACCCGAAATCCCTTGAGTCTTTTACGCCGGAAATGGCCAGTTTTTCGAACGAATTT
GATTTTGATCCATTACGTGGTCCGGTAAAGGATTTCAGCCAGACATTGATGAGTGAGAAT
GGCGAAGTGGCGAAGCAGGTCACCGGTACGCTTTCACAGGAAGGCTGTTTTGATACGCTT
GAGTTACACGATCTGGAGAATAATACGGGGCTGGCACTCGTGCTGGATGCCAATTATTAC
CGGGATGCTCAGACGCTGGAAAAAAAGGTGCAGTTACAGGGGAAGTGCCAGCTGGCGGCG
TTGCCTTCGGCGGGCGTGACCTGGGAAACGGACGACAACGGTTTTGTGGTATCGGCGACG
GGCAAAGAGATGAAGGTCGAGTACCGCTATGATAGCGAAGGCTATCCGTTAGGTAAGACA

ACCATAAACTCGCAGAATACGCTCTCTGTGACGGCAAAACCCTCCGCGGACCCGCGTAAA
AAGCTGGATTATACGGCAGTCAGCCGGGTGGATGATCGACAGGTCGGCAACGTCACACAG
AGCTGCGAATATGATGCGTACGCGAACCCTGTTGACTGCCGGCTTGTTATCGTCGATGAG
AGCGTTAAGCCGGCAGTGTTCCACCATTACACCATTAAAAATCGTATCGATTACTACTAA
>EECEBICD_01840 Spermidine N(1)-acetyltransferase
ATGACAAGTGACTACAAGGTCAGACTGCGCCCGCTTGAGCGTGAAGATTTACGATTTGTC
CACCAGCTTGATAATAATGCCAGCGTTATGCGTTACTGGTTTGAAGAGCCGTATGAGGCT
TTTGTCGAACTGTCGGATCTCTACGACAAACACATCCACGACCAAAGCGAACGGCGCTTT
GTCGTCGAATGCGACGGTGAAAACGCCGGGCTGGTAGAACTGGTGGAAATTAACCATGTG
CATCGGCGGGCGGAATTTCAGATTATTATCTCGCCGGAGTACCAGGGAAAAGGCCTGGCG
TCACGCGCGGCCAAACTGGCGATGGATTACGGATTTACCGTACTCAATCTCTATAAATTG
TATTTGATCGTCGATAAAGAGAATGAAAAAGCGATTCACATCTACCGTAAACTCGGGTTT
AGAGTAGAAGGCGAGCTGATTCATGAGTTCTTCATTAACGGCGAATACCGTAACACCATC
CGCATGTGTATCTTCCAGCAGCAGTATCTGGATGAACATAAAACGTCCGGTTCTACGCTC
CTGAAACCCACCGCGCAGTAA
>EECEBICD_01841 hypothetical protein
ATGAACAATACGCTGAGCAAACGCCTGTGCCTGACGGCAATGCTTACGTTGGCGGCCGTT
GTATATACTACTTCCGCATTCGCTGAAACCAGTAAACTGGTGATTGAATCGGGAGATAGC
GCACAAAGTCGTCAGGAAGCGGCTATGGAAAAGGAACAATGGAACGACACTCGCAGTCTG
CGCCAGAAAGTGAATACCCGCGCAGAAAAAGAGTGGGACAAAGCCGATGCCGCCTTTGAT
AACCGCGATAAATGCGAGCAAAGCGCCAACATCAACGCCTATTGGGAGCCCAATACGCTG
CGGTGTCTGGATCGTCGTACAGGTCGCGTTATTACGCCCTAA
>EECEBICD_01842 hypothetical protein
ATGAATCTCATGCTAAAGACGACTCTGCTTTTCTTTGTCACCGCCCTCTGTGAAATCATT
GGATGTTTTCTTCCCTGGCTATGGCTTAAGCGCGGCGCCAGCGTGTGGTGGCTTCTGCCC
GCCGCTGCGTCACTTGCGTTATTCGTCTGGCTTTTGACCTTGCATCCGGCGGCGAGTGGG
CGGGTTTATGCGGCTTACGGTGGGGTATATGTCTGTACTGCGCTCCTGTGGTTACGTGTT
GTCGATGGCGTCCGGCTCACGGTTTATGACTGGTGCGGCGCGCTGATCGCGCTGTGTGGA
ATGCTGATTATCGTCGTCGGATGGGGACGTACGTAA
>EECEBICD_01843 D-galactonate dehydratase family member RspA
ATGAAGATTGTAGGGGCTGAAGTTTTTGTTACGTGTCCAGGGCGTAACTTCGTCACCTTA
AAGATAACCACGGAAGACGGGATCACCGGTCTGGGGGATGCCACCCTGAACGGACGCGAG
CTCCCGGTGGCCTCGTACCTTACCGATCACCTGTGCCCACAGCTCATCGGGCGGGATGCT
CGCCGCATCGAAGATATTTGGCAGTTCTTCTACAAAGGCGCGTACTGGCGCCGTGGCCCT
GTCACCATGTCCGCCATTTCCGCGGTTGATATGGCGCTATGGGATATTAAAGCCAAAGCC
GCCAATATGCCGTTGTATCAGTTACTCGGCGGCGCTTCGCGTGAAGGCGTCATGGTGTAT
TGCCATACCACAGGCCACACCATCGACGATGTGCTGGAAGACTACGCGCGTCATAAAGAG
CAAGGGTTCAAAGCAATCCGCGTGCAGTGCGGCGTTCCGGGGATGAAAACCACCTACGGC
ATGGCGAAAGGCAAGGGGCTGGCCTATGAGCCCGCGACAAAAGGCCAGTGGCCGGAAGAA
CAATTGTGGTCAACGGAAAAGTATCTCGATTTTACGCCAAAGCTGTTTGACGCCGTGCGT
AATACGTTCGGGTTTAATGAACATCTTCTGCACGATATGCATCACCGACTGACGCCTATT
GAGGCTGCCCGCTTTGGCAAAGGTATTGAAGACTATCGTTTGTTCTGGATGGAAGATCCG
ACGCCGGCTGAAAATCAGGCATGCTTCCGCCTCATCCGCCAGCATACGGTCACGCCGATT
GCGGTAGGCGAAGTCTTTAACAGTATCTGGGATTGCAAACAGCTCATTGAAGAGCAATTG
ATTGACTATATCCGCACCACGCTTACCCATGCCGGTGGTATTACCGGGATGCGCCGCATT
GCCGATTTTGCCTCGCTCTACCAGGTGCGTACCGGTTCGCATGGGCCGTCCGATCTGTCG
CCGGTTTGTATGGCCGCTGCATTACATTTCGACCTGTGGGTGCCTAACTTTGGCGTGCAG
GAGTTTATGGGCTACTCCGAACAGATGCTGGAGGTTTTCCCGCATAACTGGACGTTCGAG
GGCGGCTATATGCATCCGGGCGACAAGCCAGGACTGGGCATTGAGTTCGATGAAAAACTG
GCGGCGAAATATCCGTACGATCCAGCCTATTTGCCGGTGGCCCGCCTGGAAGACGGCACA
CTGTGGAACTGGTAA
>EECEBICD_01844 Starvation-sensing protein RspB
ATGAAAAGCATCGTAATCGAAAAACCGAATACATTAACTATCGAAACGCGCGCGCTTCCG
CAGCCTGCGCCGGGCGAAGTTCGCATTAAAGTGAAACTGGCGGGTATCTGCGGTTCCGAT

AGCCATATTTATCGGGGACATAATCCCTTTGCAAAATATCCCCGTGTCATAGGCCATGAA
TTTTTCGGTGTCATCGACGCGGTAGGCGACAACATCAATCGCGACCGGATAGGAGAACGG
GTATCCGTCGATCCGGTGATCAGTTGCGGGCATTGTTACCCTTGTTCAGTCGGTAAACCC
AATGTGTGTACCTCGCTGGTGGTGCTGGGCGTGCATCGTGACGGCGGTTTCAGCGAGTAT
GCGGTGGCGCCGGCCCGTAATGCTCATCGCATACCGGACAACATCGCAGATCATCATGCC
GTCATGGTTGAGCCGTTTACCATTGCGGCAAATGTGACGGGACAAGTGAATCCGACCGAG
CAGGACGTGGCGCTTATCTATGGCGCAGGCCCGATGGGACTGACGACCGTACAGGCGCTG
AAGGGGGTTTATCAGGTTAAAACGGTCATAGTCGTTGATCGCATTGAAGAGCGTTTAGCG
ATGGCGAAGCAGAGCGGCGCTGACTGGACCCTCAATAATGGTCAACATTCACTGGCGGAA
TTTCTACAGCAGAAGGCGCTAAAGCCGACCTTGATTGTCGATGCCGCCTGTCATCCCGCT
ATCCTGCAAGAGGCGATTACACTTGCATCGTCTGCGGCACGCATTGTCCTGATGGGATTT
TCCAGCGACCCGTGTGAGATTGTACAGCAGGGTATTACCGGAAAAGAGCTGGCTATTTAT
TCATCGCGTCTTAATGCCAATAAATTCCCGGTCGTGATTGACTGGCTGAATAAAGGGCTT
ATTGATCCGGATAAGCTGATAACGCATACATTTGATTATCAACATGTCACCGACGCGATT
GAGCTTTTCGAGAAAGACCAGCGTCAATGCTGCAAAGTCTTACTCACGTTTGCGAAATAA
>EECEBICD_01845 Putative transporter YdfJ
ATGACACAAATAAAGCATGAACGATCGACTCAGGATTTAGTGCGCGCTGCCGTATCTGGA
TGGTTAGGCACCGCGCTTGAATTTATGGATTTTCAGTTATATTCACTAGGCGCGGCTCTG
GTCTTCCATGAGATATTTTTTCCGGAACAGTCTGCCGCGATGGCGCTTATTCTGGCAATG
GGCACCTATGGTGCAGGTTATATAGCCAGAATTATCGGCGCCTTTGTTTTTGGTAAAATG
GGCGACCGAATCGGACGAAAAAAAGTATTGTTTATTACCATCACCATGATGGGGATCTGC
ACCACGCTGATTGGCGTACTGCCGACGTATGCGCAAATCGGCATTTTTGCGCCAGTATTA
CTGGTGACATTGCGAATTATACAGGGGCTGGGCGCCGGGGCGGAAATTTCCGGCGCGGGT
ACTATGCTGGCCGAGTATGCGCCGAAAGGAAAGCGTGGTATCATTTCCTCGCTGGTCGCG
ATGGGCACCAACTGCGGTACGTTGAGCGCGACGGCGATCTGGGCGGTGATGTTCTTTGCG
CTTGAACGCGAACAACTGATCGCCTGGGGATGGCGTATTCCTTTCCTGGCGAGCGTCGTT
GTTATGATATTCGCGATCTGGCTGCGAATGAATCTGAAAGAAAGTCCGGTATTCGAAAAA
GTAAGCGAAGGCGAAAAAATCGCCAGCGTTAACGCCTGCGTCAGAAAATACATTGGGCGCG
ATGTTTACCAGCAAATCTTTCTGGCTGGCTACCGGTTTACGTTTTGGGCAGGCGGGCAAT
TCCGGGCTTATTCAAACCTTCCTTGCCGGATATTTAGTTCAGACGTTATTATTTAACAAA
TCTATCCCAACCGATGCGTTAATGATAAGTTCCGTAATTGGTTTTATCACTATCCCACTC
TTGGGGTGGTTGTCCGATAAATACGGTCGTCGGCTGCCGTATATAATATTGAATATCTCC
GCGATTATTCTCGCCTGGCCGATGCTGTCTATTGTGGTAGATAAAACGTATAGCCCTGGC
GTAATTATGGCGGCGCTCATTGTTATTCATAACTTTGCCGTGCTGGGATTATTTGCGCTG
GAAAACATCACCATGGCGGAAATTTTCGGATCGCGTAATCGCTTCACCCGGATGGCGATT
TCAAAAGAGGCAGGGGGCTGGTGGCGGTCGGTTTTGGCCCCGTCCTCGCAGGGATCTTTT
GTAACATGA
>EECEBICD_01846 hypothetical protein
GTGGCGGTCGGTTTTGGCCCCGTCCTCGCAGGGATCTTTTGTAACATGACCGATTCCTGG
CTGCCCATCTTAATTATGCTGGTGCTCTACTCGTGTATTGGTCTGATTTCTGCGTTGTTG
ATGCCGGAGGTCAGAGATCGCGATCTGAGCTTGCCTGAGGATGCGGCGGAAGCGACAGCG
GCAGAGAAGTTACGCCACTCCGCAACCCAGACGAGTTAA
>EECEBICD_01847 Polyol:NADP oxidoreductase
ATGCAAAACAGGCTTTTATCGGCAAAGGCGACGCTTCCTGATTACGATCGTGCGGCGCTG
GTGGCGCGGATGGTCCATCTCGGTTTTGGCGCGTTTCATCGCGCGCATCAGGGGGTTTAT
ACAGATATTCTGGCTGCGGAGCAGCATAGCGACTGGGGCTATTATGAGGTTAATCTTATT
GGCGGCGAACAGCAGATTGCCGATCTAAAGCAGCAGGATAACCTCTATACCGTGGCGGAG
ATGTCGGCGGAAGCCTGGACGGCGCGTGTGGTGGGCGTCGTCAAAGCGGCGTTGCATGTT
CAGGTTGATGGTCTGGAACGTGTGCTGGCGGCGATGTGCGAACCGCAAATAGCGATTGTG
TCTCTGACCATTACCGAGAAAGGGTATTGTCATTCGCCAGCCACGGGCCAACTTCTGCTG
GAACATCCCATGATTGCCGCCGACCTGCAAAATCCGCATCAACCGCTAACGGCACCGGGT
ATTATTGTCGAAGCGTTAGCGCGTCGTAAAGCCGCAGGCTTACCGGCATTTACCGTGATG
TCCTGCGACAACATGCCGGAAAATGGGCATGTGACGCGTCAGGTTGTGACGGCTTACGCC
CGGGAGGTGGATGCGGAACTGGCGATATGGATCGAGCAAAACGTGACATTTCCGTCAACC

ATGGTTGATCGGATTGTCCCTGCGGTCACGCCGGAGACGCTGGATAAAATCGAACAACTG
ACCGGCGTGCGCGATCCGGCGGGCGTTGCCTGTGAACCGTTTCGCCAGTGGGTGATAGAG
GATACGTTTGTGGCGGGTCGTCCGCTGTGGGAAAACGCGGGCGCGACGTTGGTCGCCGAC
GTGGTTCCGTTTGAAGAGATGAAACTGCGAATGCTTAACGGCAGTCATTCATTCCTGGCT
TATCTGGGGTATCTTGCCGGCTATCAGCATATTAATGACTGCATGGCTGACGACAACTAT
CGTCTGACCGCGCAGGCGTTAATGTTACGCGAGCAGGCGCCCACGCTGAAAGTCCAGGGG
GTAGATTTACAGCGTTATGCCGATCAGTTGATTGCGCGTTACCGTAATCCGGCGCTGCGT
CATCGTACCTGGCAGATTGCGATGGATGGCAGCCAGAAACTTCCGCAGCGTATGCTGGAC
TCCGTTCGCTGGCATCTTGCTAATCATAGCGATTTTGATTTGCTGGCGCTGGGCGTGGCG
GGGTGGATGCGTTACGTTGGCGGCGTGGATGAGCAGGGTAAGGCGATCGACGTCAGCGAT
CCGCTATTGCCGGTTATTCAGCGCGCCGTGGCAAACAGTGAAGAAGGCGCCAGTCGTGTT
AAGGCGCTGCTGGGAATGGCTGAAATTTTCGGCAACGATTTGCCGCAGGCGGAGCGGTTT
ACGCAAAAAGTGCAAGAAGCCTATGACAGTTTACTGACATATGGCGCGAAAGCGAGCGTG
GCGAAATATGCCGAGCGCTTGAAATAG
>EECEBICD_01848 Putative selenoprotein YdfZ
ATGATGACATACGATCGTAACCGTAACGCAATCACTACCGGTAGCCGGGTCATGATAAGC
GGCACGGGTCATACCGGGATTATCAAAGCCATTGAGAGCGAAGGTCTGGATGCAGGTCAA
ATTCGCCGCGGCAAAACGGTGATTGTCGAAGGTTGTGAAGGTAAATTCGCGCCGGTTGAA
TTGATTCGTCTTGGCATGAACTGA
>EECEBICD_01849 HTH-type transcriptional repressor RspR
ATGACCGTCGAAACGCAGCTTAACCCTACACAGCCTGTCAACCAGCAAATTTACCGTATT
CTTCGGCGTGATATCGTGCATTGTCTTATCCCGCCTGGCACGCCGCTATCAGAAAAAGAG
GTCTCAACCCGTTTTAACGTTTCGCGTCAACCGGTGCGCGAGGCTTTTATAAAGCTGGCG
GAAAATGGGTTGATTCAGATCCGCCCTCAGCGCGGTAGTTATGTGAATAAGATTTCGCTT
TCTCAGGTACAGAATGGCTGCTTTGTTCGGCAGGCGATCGAATGCGCGGTGGTCCGCCGT
GCGGCGGCGATGATCACCGACAACCAGTGCTATCAACTGGCGCAAAACCTGCACCTGCAA
CATATTGCTATCGAGCGCAAACAGATTGATGATTTTTTCCAGCTTGATGACGATTTCCAC
CAAAAGCTGGCGCAGATCGCCGATTGCCAGTTAGCGTGGGATACCATTGAAAATATCAAA
GCGACAATCGACCGGGTTCGCTATATGAGTCTGGATCATGTTTCTCCGCCGGAGATGTTG
TTGCGCCAGCACCATGATATTTTCTCCGCGCTGGAAAATCGTGACGGCAATGCGGTTGAG
AGCGCAATGACCCAGCATTTGCAGGAAATTAGCGAATCCGTCCAGCTTATCCGTCAGGAA
AACAGCGGCTGGTTTAGCGAAGACTAA
>EECEBICD_01850 NADP-dependent 3-hydroxy acid dehydrogenase YdfG
ATGATCGTTTTAGTAACTGGAGCAACCGCAGGGTTTGGCGAGTGCATTGCACGCCGTTTT
GTTGAGAATGGACATAAAGTCATTGCCACGGGACGTCGTCATGAACGCCTGCAAGCGTTA
AAAGATGAGCTTGGCGAGAACGTATTGACCGCCCAACTGGACGTGCGCAACCGCGCGGCC
ATCGAAGAGATGATGGCCTCTCTGCCTGCGCAGTGGCGCGATATTGATGTGCTGGTCAAT
AACGCCGGTCTGGCGCTGGGTCTTGAACCGGCGCATAAAGCCAGCGTAGAAGACTGGGAA
ACTATGATCGATACTAATAATAAAGGCTTAATTTATATGACCCGCGCCGTGCTGCCAGGA
ATGGTCGAACGTAATCGCGGTCATATCATTAACATCGGTTCAACCGCCGGCAGTTGGCCT
TATGCTGGTGGCAACGTCTATGGCGCGACGAAAGCCTTTGTGCGCCAGTTTAGCCTGAAT
TTGCGCACGGATTTACACGGCACGGCGGTTCGCGTGACCGACATTGAACCGGGCCTGGTT
GGCGGTACCGAGTTTTCCAGCGTTCGTTTCAAAGGCGACGATGAGAAAGCGGGTAAAACC
TACGAAAATACCACCGCGCTCACGCCAGAAGACATTACCGAAGCGGTATGGTGGGTGGCC
ACACTCCCCGCGCATGTGAATATTAATACCGTTGAAATGATGCCTGTTACCCAATCTTTC
GCAGGGCTCAGCGTCCACCGTAGCTAA
>EECEBICD_01851 Dipeptidyl carboxypeptidase
ATGTCGACGAATCCTTTGTTAGACCAGAGTATGTTGCCCTATCAGGCACCGCGTTTTGAT
CGGATCAAAGATTGCCATTATCGTCCTGCTTTTGATGAGGGCGTTCGGCAAAAACGCGTG
GAAATCGAGGCCATCGTCAATCATCCGGCGGCCCCTGACTTTACGAATACGCTTCTGGCG
CTGGAGCAAAGCGGGGCGCTTCTGTCACGCGTCACCAGCATTTTTTTCGCGATGACGGCC
GCGCACACTAACGATGAACTCCAGCGGTTGGATGAGGCCTTTTCTGCCGAGCTGGCGGCG
CTCTCCAACGATATTTATCTGAATAGCGCGTTATTCGCTCGCGTGGATGCCGTCTGGCAA
CAGCGTCACTCACTGGGGCTGGATGATGAGTCGCTACGGTTGGTCGATGTGATCCATCAG

CGTTTTGTGTTGGCAGGCGCGCAGCTTGCCGAAGAGGATAAAGCGCAACTGAAGGTATTG
AATACTGAATCCGCGACCTTAATGAGTCAGTTTAATCAGCGTCTGCTGGCGGCAAGTAAA
GCGGGCGGGCTGGCGGTCGATGACGCGCATTGCCTGGCAGGATTAAGCCCGGAAGAAATG
ACCGTCGCTGCTGAAGCGGCGCGTGAAAAAGGCCTGGAGGAGCGCTGGTTCATTCCGCTC
CTTAATACGACGCAACAGCCTGCGCTTGCTACGCTGCGCGATCGCCAGACTCGCGAAAAT
TTATTCGCAGCGTCATGGACTCGGGCGGAAAAAGGAGATGCCCACGATACCCGCGCTATC
GTTCAGCGTCTGGTAGAGATTCGTCGCTGTCAGGCAAAACTGCTGGGTTTCCCCAATTAT
GCCGCATGGAAAATGGCCGATCAGATGGCGAAAACGCCGCAAGCCGCACTGAGCTTTATG
CGTGGCATTGTGCCGCCAGCGCGTCAGCGTGTACTCAATGAACAGGCGGAAATTCAGAAC
GTCATTGATGGTGAGCAGGGCGGCTACACCGTTCAGGCCTGGGACTGGATGTTCTATGCC
GAACAGGTACGGCGGGAAAAATATGCGTTAGATGAAGCGCAACTGAAGCCCTATTTTGCC
TTAAATACGGTGTTGCAAGAGGGGGTTTTCTGGACCGCCAACCAGCTATTCGGCATCACC
TTCGTCGAGCGTTTTGACATTCCGGTGTATCACCCTGATGTTCGGGTGTGGGAGATTTTC
GATTCCGATGGTGTCGGCATGGCGTTATTTTATGGCGACTTCTTCGCGCGGGACTCGAAA
AGCGGCGGCGCGTGGATGGGGAATTTTGTCGAGCAATCCACACTCAATGAAACCCGGCCC
GTTATATACAATGTATGTAACTATCAGAAACCGGTTGATGGGCAGCCTGCATTACTGCTT
TGGGACGACGTTATTACGCTCTTCCATGAGTTTGGTCATACGTTACATGGTTTGTTTGCC
GTTCAGCGCTATGCCACGCTTTCAGGTACCAATACGCCCCGCGACTTTGTCGAGTTTCCC
TCCCAGATTAATGAGCATTGGGCGAGCCATCCACGCGTGTTCGAACGCTACGCGCGTCAT
GTCGACAGCGGTGAAAAAATGCCCGCTGATTTACAGGAAAAAATGCGCAAGGCGAGTTTA
TTTAATAAAGGTTACGATATGACTGAATTGCTCGGCGCCGCATTGCTGGATATGCGCTGG
CATATGCTGGAGGAGAGCGTAGCAGAGCAGTCTGTCGCTGAGTTCGAGCAGCAGGCGCTG
GCCGCCGAGCATCTTGATTTACCGGCAGTGCCGCCCCGCTATCGCAGCAGTTATTTTGCC
CATATCTTCGGCGGCGGCTACGCAGCGGGATACTACGCCTACCTGTGGACGCAAATGTTA
GCGGATGACGGTTATCAATGGTTTGTAGAGCAGGGCGGCCTGACGCGTGAAAACGGGCAG
CGTTTTCGTGATGCTATCCTTTCCCGGGGGAATAGTACTGATTTAGAAACGCTTTATTCA
GCCTGGCGTGGACATGAACCGCATATTGACCCCATGTTGCAATATCGTGGGCTCGATCGC
TAA
>EECEBICD_01852 hypothetical protein
ATGGCAGAACATCGCGGCGGTTCCGGTAATTTTGCAGAAGATAAAGAGAAAGCCTCAGAA
GCAGGTCGTAAAGGCGGCCAGCATAGCGGTGGGAACTTTAAAAACGACCCACAACGTGCA
TCTGAAGCGGGTAAAAAAGGTGGCCAGAATAACCACAGTGGCGGACGGAAATCCGGCAAT
TAA
>EECEBICD_01853 Nicotinamide-nucleotide amidohydrolase PncC
ATGAGATTAGAACAGCACGATATTGTCACCCTTGATGAAGCCGAATCCGTCGATAGCCTG
ACAAAAAAACTTGGTGATTTACTGGTTAACCAGAAACTCCGTCTGACCACTGCAGAATCC
TGCACTGGCGGGAAACTGGCTTCAGCATTGTGCGCAGCAGAAGATACGCCCTCATTTTAC
GGTGTGGGGTATGTCACCTTTACTGATGAAGCCAAAGCAAAAATCTTACGCGTACAACGC
CACAGCCTTGCCGAACATACCGCCGTCAGCGAAGCGGTCGTTACTGAAATGGCACAGGGC
GCTAAGGATCAGGCTGAGGTCAATATCAGCATAGCGATCAGCGGCTATGGCGGTCCGGAA
GGCGGCGAAGATGGCACGCCGGCGGGCACCGTTTGGTTTGCCTGGAATATTAACAATACA
ACTTTTACAAGCCGACAACATTTCAACGGAGATTGTCAGGAAGTATTAGAAAAATGCGTA
CACTTCGCCCTCGCTGAATTGCTTTTTCTGTTAACGAAGAAAGCATAA
>EECEBICD_01854 Protein YgiW
ATGACCATCGAACAGGCAAAAACCCTGCATGATGGCGCGACCGTTTCGCTGCGCGGCAAC
CTTATTGATCATAAAGGTGACGATCGCTATGTATTTCGTGATAAATCCGGCGAGATTAAC
GTGATAATTCCTTCCGCCGTATTTGATGGGCGGGAAGTTCAGCCGGATCAAATGATCAAC
ATTAACGGTAGTCTGGACAAGAAAGCGAAGGAACCGCTTGTCCGCGTGAACCGACTGCAA
AAATAA
>EECEBICD_01855 Small protein MgtS
ATGTTAGGCAGTATTAACCTTTTTATCGTTGTATTAGGAATTATTTTATTTTCCGGATTT
CTGGCCGCCTGGTTCAGTCATAAATGGGATGATTAA
>EECEBICD_01856 hypothetical protein
ATGGTCGTTGCCGATAGCGGCTTCGCGGAAAAAGTGGTAGCCGTCGTCCTGCCGGTTAAT

GCGGTTATTGTGGTCAGTTTGCAATACGCCGTGGGCCGTCGACTTACCGCCGTCAATATT
CGACCAATGATGACGACCGGCACCGTCTTTTTATCGCCGGGCTCATCGGATTTATTTTT
TCAGGCGACAATCTGTTTTTTTGGGGGCTTTCAGCGGCAGTATTCACGATTGGCGAAATT
ATTTATGCCCCCGGCGAATATATGTTAATTGACAACATTGCGCCTGCCGGGATGAAAGCC
AGCTATTTTTCAGCGCAATCGCTGGGCTGGCTGGGTGCGGCGGTAAACCCGCTGGTCAGC
GGCGTCATCCTGACGACGCTTCCCGCCTGGTCGCTTTTCGTTGTCCTGATAATCGCCATT
GTGTTCGCATGGGCACTGATGTTAAAAGGAATGCGGATTACCCCTACGCAACAGGCTATC
ACCTGTTAA
>EECEBICD_01857 hypothetical protein
ATGAATCCAGGATTGAGACGTTCGACGTTGGCTCTACTCGCCTCATCGCTGTTATTGACA
ATTGGACGCGGTGCCACGCTCCCCTTTATGACCATCTATCTTAACCGTCAGTACGGTTTA
GGCGTGGATCTGATTGGCTACGCGCTGACCAGCGCGTTAACGATCGGCGTCATCTTTAGT
CTTGGCTTTGGGATTCTGGCGGACAAGTTTGATAAAAAGCGCTACATGTTACTCGCGATT
ATCGCCTTCGCCTGCGGATTTATCGCCATCCCAATGGTGCATAATGTCGTACTGGTTGTC
CTGCTTTTTGCGCTTATTAACTGCGCCTATTCGGTTTTTTCTACCGTCCTGAAGGCCTGG
TTCGCCGATAACCTCACCGCGACAACAAAAACACGGATTTTTTCGCTCAATTATACCGTA
CTCAATATCGGCTGGACGGTTGGCCCGCCGCTCGGCACGCTGCTGGTGATGCAAAGCATC
AATTTACCCTTCTGGCTTGCCGCCATCTGTTCCGCATTCCCGCTGGTCTTTATTCAGGTA
TGGGTGACACGATCGGTTGCCGCCAGCGAGGGGAAAAATGCCGCTATCTGGTCTCCCTCC
GTTCTGCTGCGCGACAAAGCATTATTGTGGTTTACGCTTTCGGCATTTCTGGCCTCCTTT
GTGGGGGGCCTTTGCGTCCTGTATTTCACAGTACGTTATGGTCGTTGCCGATAG
>EECEBICD_01858 putative amino-acid metabolite efflux pump
ATGATGTCGCGTAAAGACGGTTTTTTGGCGTTACTGGTGGTTGTCGTATGGGGGCTCAAT
TTTGTTGTCATCAAAGTAGGCCTGCACCATATGCCGCCATTATTGCTGGCAGGATTACGA
TTTTTACTGGTGGCGTTTCCGGCAATTTTCTTTGTCGCCCGGCCTAAAGTCCCGCTTACG
CTGTTGCTGGGCTATGGGCTGACGATAAGCTTTGGGCAGTTCGCCTTTCTCTTTTGCGCG
ATAAAGTTTGGTATGCCCGCAGGACTGGCGTCGTTGGTATTACAGGCGCAGGCCTTTTTT
ACCATGGCTTTGGGGGCGTTTGTTTTTAGTGAGCGGTTACAACGTAAGCAGTTAGCGGGG
ATCGCCCTGGCGATTATAGGTGTACTGGTATTGATTGAAGCCAGTCTCAACGGACAGCAT
ATAGCGATGTCGGGATTTATGCTGACTCTGGCGGCGGCATTTAGCTGGGCCTGCGGCAAT
ATCTTTAATAAAAAAATTATGCAACATAGTCCGCGCCCGGCGGTGATGTCGCTGGTGGTC
TGGAGCGCGCTGATCCCGATCCTGCCGTTCCTGCTGTCATCGCTGCTACTGGAGGGGGCG
GATCACATTACGCAAAGCCTTATTACCATCGATATGACGACGATATTGTCGCTGCTTTAT
CTGGCTTTTGTCGCCACTATACTTGGCTACGGCATCTGGGGCGCGTTACTGGGGCGCTAT
GAGACCTGGCGCGTGGCGCCGTTATCATTGCTGGTGCCGGTAGTCGGGCTGGCGAGCGCC
GCGGTGTTACTCGGGGAAACGTTGACCGGGATGCAGCTTGCGGGCGCAGTACTGATTATG
GCTGGACTCTATATCAATGTGTTTGGATTTCGTGTGCGCCGTACTGCCCGCGTCAGGGGA
TGA
>EECEBICD_01859 hypothetical protein
ATGCTGTTTCCCGCCCTGCCGGGTCTGTTACTTATCGCCTCCGGATATGGCATTGCAGAA
CAAACTTTGTTACCTGTGGCGCAAAATAGCCGCGATGTGATGCTGCTGCCCTGTGTAGGC
GATCCGCCAAATGACCTTCACCCCGTGAGCGTGAACAGCGATAAGTCAGATGAATTAGGC
GTGCCCTATTATAACGACCAACACCTTTAA
>EECEBICD_01860 Multiple antibiotic resistance protein MarA
ATGTCCAGACGCAACACTGACGCTATTACTATTCATAGCATTTTGGACTGGATCGAGGAT
AACCTGGAGTCGCCGCTCTCACTGGAAAAAGTGTCTGAGCGTTCAGGATATTCCAAATGG
CACCTGCAACGGATGTTTAAAAAAGAGACCGGTCATTCATTAGGCCAATACATCCGCAGC
CGTAAAATGACGGAAATCGCGCAAAAATTAAAAGAGAGCAACGAGCCCATTCTCTATCTG
GCGGAACGCTATGGCTTTGAGTCACAGCAAACATTGACCCGGACGTTCAAAAACTACTTT
GATGTGCCGCCACACAAATACCGGATCACCAATATGCATGGCGAATCACGGTATATGCTG
CCGCTGAACCATGGCAACTACTAG
>EECEBICD_01861 Multiple antibiotic resistance protein MarR
GTGCTTTGCTCGATACGCTGCGCGGGATGTATTACCCCGGTTGAACTTAAAAAGTGCTG
TCTGTCGATCTCGGCGCATTGACGCGAATGCTCGACCGCCTGCTGTGCAAAGGCTGGATC

GAAAGACTGCCGAATCCTAATGACAAACGCGGCGTACTGGTGAAGCTAACGCCGGACGGC
GCGGCAATTTGTGAGCAATGTCATCAACGACCAGGGCAAGACCTGCATCAGGAATTAACA
AAAAACTTAACGGCGGACGAAGTGGCAACGCTTGAGTATTTGCTCAAGAAAATTCTGCCG
TAG
>EECEBICD_01862 hypothetical protein
ATGATGGATTTGTTTAAAGCGATTGGATTGGGGCTGGTCGTACTGCTCCCGTTAGCCAAT
CCGCTAACCACCGTGGCGCTGTTTCTTGGCCTTGCGGGCAATATGAATAGTGCGGAACGC
AACCGGCAGTCCTATATGGCTTCGGTTTATGTCTTCGCTATTATGATGGTGGCGTACTAC
GCCGGGCAGTTAGTTATGAACACCTTCGGTATTTCGATTCCAGGGCTACGGATCGCCGGG
GGGTTAATCGTGGCGTTTATCGGCTTCAGAATGCTTTTCCCGCAGCAGAAGGCGCATGAG
TCGCCGGAAGCGAAAAGCAAATCGGAGGAGCTGGCAGACGAACCGACGGCTAATATTGCA
TTTGTTCCACTGGCTATGCCAAGCACCGCAGGACCGGGGACCATCGCAATGATCATCAGT
TCCGCTTCCACGGTGCGTCATGGCGGCGAGTTTCCCGACTGGGTCATTATGGTCGCGCCG
CCGATTATTTTTCTTGCCGTGGCGGTGATCCTGTGGGGATGCTTACGCAGTTCTGGCGCT
ATTATGCGGCTGGTGGGTAAAGGGGGGATTGAAGCGATCTCCCGCTTAATGGGATTCCTG
CTGGTGTGTATGGGCGTGCAATTTATTATCAACGGCGTGTTGGAAATTATTAAAACGTAC
CACTAA
>EECEBICD_01863 Sugar efflux transporter
ATGACAATCAATCCAGTTTCCCGCAAGGTCGCGTGGCTACGGGTAGTTACGCTTGCTATT
GCCGCTTTTATTTTCAATACCACAGAATTTGTGCCGGTTGGTTTGCTATCAGACATTGCT
GAAAGCTTTCATATGCAAACCGCGCAGGTCGGCATCATGCTAACCATTTACGCCTGGGTA
GTTGCCGTCATGTCGCTGCCTTTTATGCTTCTTACCAGTCAAATGGAGCGCCGTAAGCTA
CTAATTTGTCTGTTTGTTTTGTTTATTGCCAGCCATGTGCTCTCCTTTCTGGCATGGAAC
TTTACCGTGCTGGTCATTAGCCGTATTGGTATCGCTTTCGCCCACGCTATTTTCTGGTCG
ATAACCGCCTCGCTGGCGATCCGCCTGGCCCCTGCCGGGAAACGCGCGCAGGCATTAAGT
CTGATAGCGACCGGCACCGCGTTGGCCATGGTGCTTGGGCTGCCGATAGGTCGCGTTGTT
GGTCAGTATTTTGGCTGGCGCACCACCTTTTTTGCCATCGGCATGGGCGCATTAATTACG
CTGCTATGTCTGATTAAGCTTCTACCGAAATTGCCGAGCGAGCATTCCGGCTCACTAAAA
AGTTTACCTTTGCTATTCCGGCGCCCGGCGCTGATGAGCCTCTATGTGCTGACCGTCGTT
GTGGTGACTGCGCATTACACAGCTTATAGCTATATTGAACCGTTCGTTCAGAATGTCGCC
GGGCTCAGTGCGAATTTTGCTACCGTCCTGTTACTGATCCTCGGCGGCGCCGGGATCATC
GGCAGCCTCGTGTTTGGTAAACTGGGTAATCGCCATGCCTCGTCGCTCGTCAGTATCGCT
ATTGCCCTGCTGGTTGTCTGTCTGCTTCTGCTGCTGCCTGCGGCGGATAGCGAAGCGCAT
CTGGCCATACTGAGCATTTTCTGGGGCATCGCGATTATGGTGATAGGGCTCGGTATGCAG
GTGAAAGTACTGGCGCTGGCCCCGGACGCGACCGATGTCGCAATGGCGCTATTTTCAGGC
ATCTTTAATATTGGTATTGGCGCTGGCGCGCTGGTAGGCAATCAGGTGAGTTTGCACTGG
TCAATGTCGGCCATCGGCTATATTGGCGCCATACCCGCCTGTGCCGCGCTGGTATGGGCT
GTGCTCATATTCCGCAAATGGCCAGTGACGCTGGAAGAACAACCGCATTAA
>EECEBICD_01864 HTH-type transcriptional regulator GltR
ATGGACCTGACGCAACTGGAAATGTTTAACGCTGTCGCTCTGACGGGCAGCATTACGCAG
GCGGCGGCAAAAGTACACCGCGTTCCGTCCAACCTGACCACGCGTATCCGCCAGCTTGAA
GCCGACCTGGGCGTCTCTCTGTTTATTCGCGAAAACCAGCGACTCAGGCTGTCACCAGCC
GGGCATAACTTTTTACGTTACAGCCAGCAGATCCTGGCGCTGGTCGATGAAGCCCGCATG
GTGGTTGCCGGAGAGGAACCGCAAGGACTGTTTTCACTCGGCGCGTTAGAAAGTACTGCC
GCCGTACGTATCCCCGCGCTGCTGGCGGGTTATAACCAGCGTTACCCTAAAATTCAGTTC
GCGTTAACGACCGGTCCTTCCGGCGCCATGCTGGATGGCGTGCTCGAGGGAAAGCTGAAC
GCCGCCTTTATTGATGGCCCGCTAATGCATCCGGGGCTGGAAGGTATACCGGCTTACCAG
GAAGAGATGATGATCGTCGCGCCACACGGCCATTCCGTCGTTTCGCGGGCCAGCGAGGTG
AATGGTTATAACATCTATGCTTTTCGCGCCAACTGTTCTTACCGACGGCATTTCGAAAGC
TGGTTTCATGCCGATGGCGCGACCCCCGGTACGATTCACGAAATGGAGTCATATCATGGA
ATGTTGGCGTGCGTAATTGCCGGCGCAGGTATTGCGCTGATGCCAGCAAGTATGCTCAAC
AGTATGCCAGGACATCATCAGGTGGAAGCCTGGCCGCTGGCGGAGAAGTGGCGCTGGCTT
AACACATGGCTAATGTGGCGGCGCGGGGCGATGACGCGTCAGCTTGAGGCCTTTATCGAG
TTACTTAACGCCCAACTGGCGAGCGTCGACTGA

>EECEBICD_01865 Succinate semialdehyde dehydrogenase [NAD(P)+] Sad
ATGACCATGATGACTGCAACACAGGCGTTATCCGTTAATCCTGCGACCGGCCAGACGCTG
GCGGCAATGCCCTGGGCTAACGCGCAAGAGATTGAACATGCGCTGAGTCTGGCGGCGAGC
GGTTTCAAAAAGTGGAAAATGACATCAGTAGCGCAACGGGCGCAAACATTACGCGATATT
GGTCAGGCGCTTCGCGCTCATGCAGAAGAGATGGCGCAATGTATTACGCGTGAAATGGGT
AAACCCATAAAGCAGGCGCGTGCGGAAGTGACGAAATCCGCCGCGTTATGCGACTGGTAT
GCGGAACATGGCCCGGCAATGCTGAATCCTGAGCCTACGCTGGTAGAAAATCAGCAGGCG
GTGATTGAATACCGTCCGCTGGGCGTCATTCTGGCGATAATGCCGTGGAATTTTCCTTTA
TGGCAGGTGTTACGCGGGGCAGTGCCGATACTGTTGGCGGGGAATAGCTACTTGTTAAAA
CATGCGCCCAATGTCACGGGTTGCGCGCAGATGATAGCCCGAATCTTGACGGAAGCCGGT
ACTCCCGCTGGCGTCTATGGATGGGTGAACGCCAATAATGAGGGCGTGAGTCAGATGATT
AACGATCCGCGAATCGCGGCGGTGACGGTGACCGGTAGCGTGCGCGCCGGCGCGGCGATT
GGCGCTCAGGCTGGGGCGGCGCTGAAAAAGTGCGTACTGGAACTGGGGGGCTCGGATCCG
TTTATTGTACTCAATGATGCCGATCTCGAGCTGGCGGTAAAAGCTGCGGTTGCAGGACGC
TATCAAAACACCGGACAAGTCTGCGCCGCCGCCAAACGTTTTATTGTCGAAGAGGGCATT
GCGCAGGCGTTTACCGATCGTTTTGTGGCGGCTGCCGCAGCATTAAAGATAGGTGATCCT
CTGGTTGAAGAGAATGACCTGGGGCCGATGGCGCGCTTTGATTTGCGTGATGAACTCCAT
CAACAGGTGCAGGCTTCCGTCGCCGAAGGCGCACGGCTACTGTTGGGCGGGGAGAAAATC
GCCGGGGAGGGCAACTATTATGCCGCGACCGTTCTGGCGGATGTTACACCTGACATGACT
GCATTTCGTCAGGAACTTTTTGGGCCTGTTGCCGCCATTACCGTCGCAAAAGATGCGGCG
CACGCCCTCGCGCTGGCGAATGACAGTGAGTTTGGCCTGTCGGCGACGATCTTTACCGCC
GACGATACGCTGGCCGCGGAGATGGCGGCGCGTCTTGAGTGCGGCGGGGTATTCATTAAT
GGGTACAGCGCCAGCGATGCGCGTGTGGCATTCGGCGGGGTGAAGAAAAGTGGCTTCGGT
CGCGAGCTTTCGCACTTTGGTTTACACGAATTTTGTAATGTGCAGACCGTCTGGAAGAAC
CGCGTCTAA
>EECEBICD_01866 Glutaminase 2
GTGGCCTGGGCCATGGATAATGCAATTTTAGAGACGATTTTACAGCGCGTTCGCCCCCTC
ATTGGCCAGGGCAAAGTCGCTGACTATATTCCGGCATTAGCGTCCGTCGAGGGTTCAAAG
CTGGGGATAGCGATTTGTACCGTGGACGGGCAGCATTATCAGGCGGGCGATGCGCACGAA
CGCTTCTCTATTCAGTCTATTTCGAAAGTGCTCAGTCTGGTGGTGGCGATGCGTCATTAT
CCGGAAGAGGAGATCTGGCAGCGCGTCGGAAAAGATCCTTCCGGCTCGCCGTTCAATTCG
TTGGTTCAGTTAGAGATGGAACAAGGCATCCCGCGTAATCCATTTATTAATGCGGGCGCG
CTGGTGGTATGCGACATGTTGCAAGGCCGGCTTAGCGCGCCGCGTCAGCGGATGCTGGAA
GTTGTGCGCGCGCTTTGCGGCGTATCGGATATTACGTATGACGCTACGGTCGCGCGCTCT
GAATTTGAGCATTCCGCCCGTAACGCCGCTATTGCTTGGCTGATGAAATCATTCGGTAAT
TTTCATCATGATGTGCCTACCGTGTTGCAAAATTACTTCCACTACTGTGCGTTGAAAATG
AGCTGCATGGAACTGGCGAGGACGTTTGTCTTTCTGGCGAATCAGGGCGAGGCGTTTCAT
CTCGACGAGCCGGTAGTGACCCCCATGCAGGCCCGGCAAATTAATGCGCTAATGGCGACC
AGCGGGATGTATCAGAATGCCGGAGAGTTTGCCTGGCGGGTAGGATTGCCCGCTAAATCC
GGCGTGGGCGGTGGTATTGTCGCCATTGTGCCGCATGAAATGGCGATTGCCGTCTGGAGT
CCGGAGCTGGATCCTGCGGGAAACTCTCTGGCAGGTATCGCCGCGCTTGAACAATTAACA
CAAACGTTAGGACGTTCGGTATACTGA
>EECEBICD_01867 hypothetical protein
ATGAATGCGCTTGAACCGCTCTTTGCCCGCCTGGCGCGATCGACATTTCGTTCGCGTTTT
CGTCTCGGGATAAAAGAAAGACAATATTGCTGGGATAAAGGCGCGGAGGTGATTGATAAA
CACGCCGCCGATTTTATCGCCCAGCGACTGGCGCCAGCTCACCCGGCGAATGATGGTAAG
CAAACGCCGATGCGGGGCCATCCGGTATTTATCGCGCAACATGCGACGGCAACCTGCTGT
CGCGGGTGCCTGGCGAAGTGGCACCAGATTCCTCAGGGAGAACCGCTGAGCGAAGCGCAG
CAACAGTATATTGTCAGCGTTATCCATTACTGGCTGGTCATACAAATGAATCAGCGGTAG
>EECEBICD_01868 hypothetical protein
ATGACAGTGCTTTTTTACCTGCTGAAGATAATCATGATCGTTAGACCACAACAGCACTGG
ATTCGCCTGATATTTGTCTGGCATGGTTCCGTGTTATCTAAAATCTTTTCACGACTGCTC
CTTAACTTTCTTCTTTCTATCGCCATTATTATCATGCTGCCCTGGTACACAATGCTGGGT
ATTAAGTTTACCCTTGCGCCGTTTAGCATACTGGGCGTCGCTATCGCCATTTTTCTGGGG

TTTCGCAATAATGCCTGTTATGCGCGTTACGTCGAAGCGCGACATTTGTGGGGGCAGTTG
ATGATCGCTTCACGCTCAATACTGCGTGAGGTGAAAACAACCTTGCCTGATGAACGCGGT
ATCGAGGATTTTGTTCGGTTACAAATCGCTTTTGCCCACTGTTTGCGTATGACATTGCGT
CGACAGCCTCAAACGCAAGTTCTGGGCAACTATCTTGACCAGGAGGCGTTACAGAAAGTT
GTCGCTTCCCATTCCCCTGCTAACCGTATCTTATTGCTGATGGGAGAGTGGCTGGCGATT
CGCCGCCGCAGCGGAAAGCTTTCCGATATTTTATTTCATAGCCTCAACAACCGTTTAAAT
GATATGTCGTCAGTGTTGGCCGGCTGTGAACGCATCGCCAACACGCCGGTGCCTTTTGCC
TATACGTTAATTTTGCATCGTACGGTTTATCTTTTCTGCATTATGTTGCCATTTGCACTG
GTGGTTGATCTGCACTATATGACGCCGTTTATTTCGGTGCTTATTTCTTATACGTTTATC
GCGTTGGATGCGTTAGCGGAAGAGTTGGAAGATCCGTTCGGTACTGAAAATAATGATTTA
CCGCTGGATGCGATATGCAATGCGATAGAAATCGATTTACTGCAAATGAATGATGAAAGG
GATATTCCGGAAAAACGGATACCGGATAAGCGCTATCAACTGACCTGA
>EECEBICD_01869 hypothetical protein
ATGCAAAAAACGTCACTATTGTTTTCGGCCGCCGCGATAACGTTAACTCTGATGGTGTCA
TCGGCATCAGCGGCAACGCCAACCGCCGCACAAAATGAAGCTACGTCGACCGTTAAACAA
GAAATTACAGAAGGCATTAACCGTTACCTTTATTCAATAGATAAGGCAGACCCAACGTTA
GGCAAGCAGCTCTTTTATGTTTCACCGGAAACCAGTTTTATTCATCCACGCGGTCACGAA
CGCGGATGGTCGCAGATCGCTGAAAATTTTTATGGCACCACTATGGGTAAAACTTTCAGT
AAACGCACATTAAAACTGGACGCTCCCCCTGCTATTCATGTGTATGGCAATGCCGCCGTT
GCAGAGTTCGACTGGCATTTTACCGCGGTACGCCGTGATAATGGGCAGACTCAACACACG
ACAGGCCGCGAAAGCCAGGTGTGGGCGAAGATACCAAACACAGGATGGCGAATCGTTCAT
GTCCACTACTCCGGTCCAGCCAAAACCGGCGTTGGAGAAGGCTATTAA
>EECEBICD_01870 Outer membrane porin C
ATGAAAAAAGTAGTGGTGTTATCTGCGGTAGCCGCAGCCGTAATGATGGCTGGAGCCGCC
AACGCAGCAGAAATCTATAACAAGGATGGCAATAAACTGGATTTGTATGGCAAGGTCGAC
GGCCTGCATTATTTTTCCAGTAATCATAGTACGGATGGCGATCAATCTTATATCCGTATG
GGTATTAAAGGCGAGACTCAAATTACCGATCAACTAACCGGTTTTGGACAGTGGGAGTAT
CAGGTCAACGCCAATCGTCCGGAAGATGGTGACTCCAGCGGTTCCCCGCAAAGCTGGACG
CGTCTCGGTTTTGCTGGTTTGGCATTTGCCGATATGGGGTCTGTTGATTATGGTCGTAAC
TATGGCGTGTTATACGACATTGGTTCATGGACTGACGTACTGCCTGAATTTGGTAATGAT
TCGTATGAAGCTTCCGATAATTTTATGACCGGACGCGCGAATGGCGTTCTGACTTATCGC
AATAATGATTTCTTTGGTCTGGTTGATGGTCTGAATATTGCGTTGCAGTATCAGGGTAAA
AATGACGGCCTGTCGAAAGAAGGCGATCCTTTAAGTAATAATGCCAGAAAAAGTATCGCG
TACCAGAATGGCGATGGTTTCGGGGCATCAGCGACATATGATTTGGGCATGGGGGTGAGC
CTGGGGGCAGCCTATACCAGTTCCAAACGCACGCTTGATCAGATGACGCAAGATAAATAC
GACAATGGCGACCGCGCTGAAGCCTGGACTGGCGGCGTGAAGTATGATGCCAATAATATT
TACCTGGCGGCAAACTATACCCGTACTTATGATATGACCTATATGGGCGATACATTGGGC
GGCTTTGCACATAAAACCGATAACTGGGAAATGGTAGGGCAATATCAATTTGATAATGGC
TTACGCCCGTCACTGGCATTCCTGCAATCCAGAGCCAATGATGTTGATGGACTGGGTTCG
TTCGACCTTGTGAAATATATTGATGTGGGTTCCTATTACTACTTCAATAAAAATATGTCC
GCGTATGTTGACTACAAAATCAATCTGCTAAAAGATGGCAACCCATCGAATCCTAATACA
GACAACACCGTGGCGCTCGGCTTAGTCTACGAGTTTTGA
>EECEBICD_01871 Hydrogenase maturation factor HybF
ATGCATGAATTAGCCCTGGCGCAAAATATTATCGAATTGCTGGAGGAACAGGCAGTAAAT
CACCAGTTCAGCAAGGTAAAGCAAGTCTGGCTGGAGATTGGCGTTATGGCCTGTGTTGAG
GTGCCGGCGCTGCATTTTGGCCTGGACGTCGCCGCTCGTCATACCCTTGCCGACAATGCG
CGTTTCCACATTACTATCGCACCGGCGCAAGGTTGGTGTCTGTCCTGTAATCAACCCTTC
ACCTCTCAAACATCCACACTCTGTTGTCCCTTTTGTCATTCAGGCAAAGTGCAGATAGAT
GACAGTAGCAGAATGCGCATTCGCGAGATCGAAGTCGAATAG
>EECEBICD_01872 hypothetical protein
GTGCAAAGATTGACCGTCTATTCTCACCCGCTACGGATTATCTGGCAGGAGGCGCCGATC
GGTCGTTTGTTACAGGGCGCAACGCCGGTATACGCAAAAACGCTTATTTCACGTTTGTTT
ACGTTGTGCGCTCAGGCGCACAGCGCGGCGGCGGCTTTACTGTTGTTTCCTGAAAAGAAG
CCAGATATGCAGGCCGCACAACAAGAGCTGGCGCGAGAAACGCTCCGTCGCGCGTTGACA

GACTGGCTCCCCCTGTTTAGCCACCGACAGGCAACCGCTGAAGAATGGGCGTTGTTACGT
CGCGGAGAGTTATCCCCGCTGGCAAGCACAATCTTTTTTGACGATGATCCTCAGACCTGG
CTTGCCGCTGGCGTAAAGGGCTGGGAAGCCTGGTTTTTGCAAGAGCGTTCTGAAACCGCC
CGCTGGCTGGCGGCAGTACAGAATATCATTACACCTACACTACCGATGGCATCGTCCCCG
GACCATACGTTAATAACCCATGGTCCCCTGGATGTCTCGCCTCTGGCTATCGAATATCCG
CTGCTGTCAGCCTGTTGTCTTAGCGGTAAAACGACTGCGCTACGTCTGCTGGCCCGCTGC
ATTACGCTCGCCCGAAGTTTGAGCGCCCTTCCCACCCTGCGCTGGAATCGTTTTGATGAT
GGCGAATGGAAAATAGCGGTAGTGGAAACCGCGCGAGGATGGTTAGTTCATCAGGCCAGA
CTGACGACGTCAGGCAATATTCTTGATTACCGTATTATCTCGCCTACCACACGTCACGCC
CAACCTGACGGCGTCATTGCTCGCGAACTGGCCACTATTCCACTCTCATTGTGGTCGCAA
CAGTTACAGGTAATTGATCCTTGTGTCGCCGTAAATATTGTTGAATGA
>EECEBICD_01873 Anaerobic nitric oxide reductase flavorubredoxin
GTGAATTATTCCAGAACTATTCCAGTCGTTAATATTGCCGGACCAGGCTCGCAGCCAGAA
GAGGAAGACTTTAACTTTCTTCCTATCCCCGCCGGCATTAATCTCCCGCTGACGCCGGTT
TTACCGGAACAGGCGCTGCCCGCTGAGCTCCGTGTTGCCAGACACATCCTGACTACGCTT
ATTCGCGATATGGATAACCCAGTGGCAACGCTCCCCTTTCCCCTGAGCTATAAGCTGAAT
GCCACTGAGCAACAGAATAGCGGTTTATTGGATCAACTGCTCGGCGAAGGTGAAATCTCC
GCCCGGGTACTATTATCCGATGGAAAAGAACAGCGTATTCAGGAGACGGTTTTTACGGGC
GTCTGGCGTGTGCGTGAATATAACGCTGACCAGCAACGGGTTGCCGATGAAATTATCATT
GGCCCGATCCCAGAGAGCATCTGGCAGACGCATCCGCAGCCGCCGATTACGCCAGAATTG
CCGCCACAACCGGCGGGATTGATGAATGGTGCCTTTATCGCGCATGAAATAGCCGAGCGC
GTAAAACAGCCGGTAAAAGAGCCGCATATCATTAACTTAACGCTGTTGCCAGTAAACGAT
GCCGATCGCGAGTATCTGGATCATTTTTTAGGCGAAGGTTGTAGCGCTATTTTTTCACGC
GGATATGGTAAATGCCGGATTGTAAGCACGCATTTTCCCGGCGTATGGCGGGTCAATTAT
TTCAATGATATGAACACATTACTGCAAGATATGATTGAGATAGCGGACATTCCTGATATC
GCCGTTGCAGGCATCGATGATATCGAAGATGCCTACGCGGGGCTAAAAAATACGTTGGAA
TGGTTGAAAGAATACCCGGTTACAGAAAATGAGCCAGTGGTGCGCATGGAGTGCAAAGTA
TGTTGGTGGGTTTACGACCCTGCGCTGGGCGATGACGTATGGCAAATTCCACCCGGTGTG
CCCTTCAGCCAGTTACCTGATTACTGGTGCTGTCCGGTTTGCGAAACCAGTAAGTCCGGG
TTTATGGTGATCGATGAAGGTAATAGTTCGTGCAAAGATTGA
>EECEBICD_01874 hypothetical protein
ATGACTATCTCACCCCAAAATCCTGTTTGTCCACTGGCGGATAAAGGCTTTCTGGTTACC
GATGCGCAAACGCTCCCGTCGTTGATTAATGCGCATCCGACTGTGCTGGTTTTGCTGCAA
AGCGACCCCAACAAACATCCGGAAGTCGCCGACAGTTGGGTCATCATCCCGGAGATCCTC
AAGCAATTTCCGGCGACGTCTTATACCGCGGCATTTGCGGATAGCGAACAGAGTGAACTG
ATCGCCCGGGAGTATCGCATTCTGAAATATCCGGCGCTGCTTTTCTTTCGCCAGCACCGT
TTTGTCGGCAGTCTGGCGGGATTATATAGCTGGCAAGAATACTCTCAGCGGGTGGCCGCA
TTGCTGACCACACCGGGCTATCGTCAGGATATTCCCGTTATCACGCAATAG
>EECEBICD_01875 Hydrogenase 1 maturation protease
ATGGCAGAAGTAACAATCTTAGGGCTTGGCAACCTGCTTTGGGCAGATGAAGGGTTCGGC
GTCAGAGCGGCGGAAAAGCTGTTTGAACAGTATGCCGATAACGAGAAAGTTGACGTTGTT
GATGGCGGTACGCAGGGGCTGGCGCTGCTGCCCTGGCTGCAACAGACCGAAAAACTGTTA
ATCATGGATGCTATCGATTTTGGTATGGCGCCAGGTTCGCTGGCGATGTTTCGTGATGAA
CAGGTTCCCGCCTATCTGACGGCGAAAAAACTCAGTTTACATCAGACCAGTTTTTCTGAA
GTGCTGGCATTGCTGCAATTAACCGGCGGCCAGTTATCGGAGATTGTGCTGATTGGCGTG
CAGCCGGAATGTCTGGATGACTATGGCGGGAGTCTTACGCCGCAGGTAAAGGCGCAACTG
ATGCCTGCGGTCTATCTCGCGCAGGAAGTGCTGGCGCAATGGGGTATCACCGCCTCTTCA
GCGGCCTTGCCCACTGAAAGACTCAATCATTACAGCCTGTGTATGGAACGCTACGAAGAT
GAACGCCCAGATGCGCAAAGCGCTTGCCGGGTCGGCGATATTCGGGTGCTACAGAGAGAG
AAATCATAA
>EECEBICD_01876 putative Ni/Fe-hydrogenase 1 B-type cytochrome subunit
ATGCATCTCAAAGAAATAACCAGCCAGGGGTACTATATCTATGAAGCTCCGGTCCGTTTA
TGGCACTGGATCACGGCGTTATCCATTGTCGTCCTGGCTGTTACAGGATATTTCATTGGC
CGCCCCCTGCCATCGATTCAAGGTGAAGCGACCTTTATGTTTTGGATGGGCTGGATACGA

CTGATCCATTTTACCACGGCGTATATTTTTACTGTCGCGCTGCTGTTTCGTATTTACTGG
GCATGTGTCGGCAATGAGTACGCCAGGGAGATGTTTCTGGTTCCGTTCTGGCGCCGCGCC
TGGCGCAAAGGCGTTATCAGCGAAATCCGCTGGTATTTTTTCCTCGAAAAAGAGGCCCAT
CGTTACTATGGACATAATCCGGTAGCGGGACTGGCGGTAATGTTCTATTTCTGGATGTCC
GTACTGATGGTGTGTAGCGGCTTTGCGCTCTATGGCGAAGGACTTGGAACAGACAGTTGG
GCGTATCAATGGTTTGGCTGGATGATTCGCCTGACTGGCAACGATAGCCTCGCGCTGCAT
TTCTGGCACCGGCTGGGCATGTGGTTCATTATCGCCTTCGTCATTGCGCATGTTTATACC
GCTATCCGCGAAGACATTATGAGCCGACAAAGCGTGATTTCCGTCATGATAAGCGGCTGG
CGCTGGTTCAGGTGA
>EECEBICD_01877 Hydrogenase-1 large chain
ATGGCATATCCTTATCAGACTCAGGGTTTTACGCTGGACAATAGCGGACGGCGAATTGTG
GTCGATCCGGTTACTCGCATCGAAGGGCATATGCGTTGCGAAGTGAATATCGACAGTAAT
AATGTCATTACTAACGCCGTGTCGACCGGCACCATGTGGCGCGGCCTGGAGGTTATCCTG
AAAGGTCGCGATCCGCGAGACGCCTGGGCATTTGTTGAACGCATTTGCGGCGTCTGCACC
GGTACACACGCTCTGACCTCAATCCGTGCCGTAGAAAATGCGCTGGGAATCGCGATTCCG
GACAACGCAAACTGTATCCGTAATATGATGCAGGCTACGCTACACGTTCACGATCACCTG
GTGCATTTTTATCATCTGCACGCGCTGGACTGGGTTGATGTGGTTGCCGCCCTGAAAGCC
GACCCGCATCAGACCTCCGCCATCGCGCAAAGCCTTTCCGCATGGCCGCTATCCTCGCCG
GGATATTTCCGCGATTTGCAAAATCGACTGAAGCGGTTTATCGAGTCCGGTCAACTTGGC
CCTTTCCGTAATGGCTACTGGGGACATCCGGCGATGAAACTTCCGCCGGAAGCCAATTTG
CTGGCGGTCGCCCACTATCTTGAAGCGCTCGATTTTCAAAAAGAGATCGTAAAAATTCAC
ACCGTTTTCGGTGGTAAAAACCCGCATCCTAACTGGCTGGTGGGCGGCGTTCCTTGCGCC
ATCAACCTTGACGAAACGGGCGCGGTCGGCGCAGTGAATATGGAACGCCTGAACCTTGTC
AGTTCCATCATCCAAAAAGCTCGCCAGTTCTGCGAACAGGTTTATCTTCCGGACGTCTTG
CTGATTGCCAGTTATTACAAAGACTGGGCAAAAATCGGCGGCGGGTTATCGAGCATGAAC
CTGCTGGCTTATGGCGAGTTTCCGGATAACCCTAACGATTATTCCGCCAGTAATCTTCTG
CTTCCGCGTGGGGCTATCATTAACGGTCGTTTCGACGAAATTCATCCTGTCGATTTAACC
GCCCCCGATGAGATTCAGGAGTTTGTCACTCACTCCTGGTATACCTATGGTAACGGCAAT
AACGATAAGGGCCTGCATCCCTGGGATGGTCTGACCGAACCGCAACTGGTCATGGGCGAA
CACTATAAAGGCACAAAAACTTTTATCGAACAGGTGGATGAGTCCGCTAAATATTCATGG
ATTAAATCGCCGCGCTGGAAGGGACATGCGATGGAGGTAGGTCCGCTGGCGCGTTATCTG
ATTGGATATCATCAAAATAAACCGGAATTTAAAGAGCCCGTGGATCAACTGCTGAGTGTA
TTAAAGCTGCCTAAAGAAGCGCTCTTTTCTACATTAGGACGTACTGCCGCGCGTGCGCTG
GAATCCGTATGGGCAGGCAATACGCTGCAATATTTCTTCGATCGTCTGATGCGTAACCTC
AAGTCCGGCGATACGGCGACGGCAAACGTCACGCTCTGGGAACCCGATACCTGGCCTACA
TCGGCAAAAGGCGTTGGTTTCAGCGAGGCGCCGCGTGGCGCGCTGGGGCACTGGATAAAA
ATTGAAAATCAGAAAATCGACAGCTATCAGTGTGTGGTACCGACAACCTGGAATGCCGGG
CCACGTGACGATAAAGGTCAAATTGGCGCGTACGAAGCGGCGTTAATGGGGACTAAACTC
GCCGTTCCCGATCAACCATTGGAGATTTTACGTACTCTGCACAGCTTCGATCCCTGCCTG
GCCTGCTCCACCCATGTTATTGATAATCATGGAGGGGAGCTGGTCAGGGTTCAGGTACGA
TGA
>EECEBICD_01878 Uptake hydrogenase small subunit
ATGCAAACACAAGATACATTTTATCAAGTTATGCGTAGACACGGCGTGACAAGGCGTAGC
TTCCTCAAGTTTTGCAGCCTTACCGCAACGTCTTTAGGCCTGAGCAGTTCGATGATTCCT
CAGATAGCTTATGCCCTGGAGAATAAACCGCGTACGCCAGTGATATGGCTGCATGGTCTT
GAATGTACCTGCTGTACCGAATCTTTCATTCGCTCTGCGCACCCGTTGGCAAAAGATGCC
ATTTTGTCGCTCATTTCCCTGGATTATGACGACACCATTATGGCCGCCGCAGGGCAACAA
GCTGAGCAGGCGTTGGCGGATGTGATGCGTGAGTACAAAGGAAATTATATCGTTGCCGTG
GAAGGTAACGCGCCGCTGAATGAAGATGGGATGTTTTGCATACTGGCTGGCGAGCCGTTC
CTGGAGAAGTTAAAACGCGTCAGCGCGGATGCTAAAGCGATTATCGCCTGGGGGGCCTGC
GCATCATGGGGCTGCGTGCAGGCAGCCCGTCCCAACCCAACTAAAGCCACGCCGGTTCAC
AAATTAATTACCGATAAACCCATTATTAAAGTGCCAGGCTGCCCGCCTATACCTGAAGTG
ATGTCCGCCGTTATCACCTATATGCTGGCATTTGATCGCATCCCTCCCCTTGATCGTCTT
GGACGACCAAAAATGTTTTATGGCCAGCGAATTCATGACAAATGTTACCGACGCGCGCAT

TTCGATGCCGGTCAGTTTGTGGAAGCGTGGGATGACGAAGGCGCCAGAAAAGGGTATTGC
CTGTACAAGATGGGCTGTAAAGGCCCGACCACCTATAACGCCTGTTCAACCGTTCGCTGG
AATGATGGCGTGTCTTTTCCTATCCAGTCGGGTCATGGATGCCTCGGATGTTCGGAAGAT
GGATTCTGGGATTACGGTTCTTTTTACAGCCGGGCAACGGGCATTCCGCAAACCGGCATT
GAAGCTACCGCCGATAAAATCGGACTGGGCGTTGCCGGCGTCGCGGGCGCTGCCGCCATC
GCTCACGCCACGGTGAGCGCCATAAAACACGCCCGCAATAAAAACAACACGTCATCAGAA
AACGCTCCGGAAGAGAAAAAATAA
>EECEBICD_01879 hypothetical protein
ATGAAAAAAAGTAAAGCAATTTTATTTATCGCTCCGGCGCTGTTTTCATACGCCATGGCT
GCACAGGCCTGCACAACCCTTGCTATTCAGGATAAACAAGGCGATATTTTTCATGGGCGT
ACGCTGGAATATATGCAGGATTTACCCTCATGGTTAACGTATTACCCGGCGGGAACACAG
TTTGATAAAAAAACGCCGGATGGCAGCCAGGGGATAAGCTATCAGGCGAAGTATCCGATA
CTGGCCATTACTTCCACCATTACTGATGGCGATAGCAGAGACATACTTGAAGGCATGAAT
AGCGCGGGATTGTCATTTAGTGAAAACATGATCATGAATGCGCAGTTGCCGCCATTGCCC
GCGAGTGAATATAAACAGGCAATTCCTGTCACCTCGTTGGGAGAATGGGCGCTGGCCCGT
TTTGCTACTGTCGGCGAGGTAAAACAGGCAATAAAAGAGGGCAAATTCTGGTCACCGGAA
CTGCATCGGTTTGGCGATCTGAAATCGCCATTTCATTACGCGTTTTACGACAAGAAAGGA
GGAAGCATTGTCGTTGAAGTGGAAAATGGCAAATTCCATGTCTATGACAATCCCACTCGC
GTGATGACAAATGGTCCCGCATTCCCCTGGCATCTGACAAATCTGAATAATTATACGCAA
TTAACCAATGTGGACCGCTCTTCAGGCACATTGGGCGGGATAAAAGTGATGCAGCCTGAT
AGCGGGATTGCTATTGCAGACTTGCCATCGTCCGATACGTCGGTTTCCCGTTTTATACGC
GGCGTTTATTACACCACATATGCGCCGCAAGCGACATCCGCCCACGACGCAATGAATACG
CTTGCACATATTATGAGCCGTTTCGATCGTCCGAAAAATATTACGGTTGATTATATGGGG
AGTGAAGGGGAAGGCAACGCTACCCGCAAACCGGTCTCAGAATATACGGTCTGGACAACG
CTGTCTGACTTAACGCACGGTGAGATGATGGTGCGCGGCTACAATGATATCAACTATAAA
ACCTGGTCTCTTTCTCAGTTTAAGAACGCGACAGCGCCCGTGTTCGAGAAAATCAACGTC
AAAGGATAA
>EECEBICD_01880 Pyruvate dehydrogenase complex repressor
ATGGTTATACCTGAGGTCAGGCAGGAGAGGCTCTATCAGAAGATAGCCAACCTCATCATT
AAGCTGATCAATGATAATATTTTTCCCCCGGGAACTTTTTTACCGCCAGAACGCGAACTG
GCAAAACAGCTTGGCGTGAGCCGCGCCTCCTTGCGAGAAGCCCTTATTGTACTGGAGATC
TCCGGATGGATCGTGATTCAGTCCGGTAATGGGGTTATCGTTTCAGATAAAAAACACCTT
GCCAGCGACTATACCATCGAAGAAATTCTCTCTACTCGCGAGCTGGTTGATAGCCATTGT
GCGCGCTTAGCCGCACAAAACGATAACGTTGACGTCATCAACCAAATAGAGGCGATTTAT
CATCGTATGGATCAAGCCATTAACGATAATAACGTCCACGGCTTTTACTCGCTGGATAAA
GAGTTTCACCTTGCGATCTCTGAGGCTAGCCGTAACCGGGTGTTGTTCGATATGTCCCGT
ATGTTATGGGAACAACGGATTAATATCCCTTATGCCGGGCTTGATGAGCAGTCTGGAGAC
AGAAATGTTTTACTTAATCTCAACAAACAACATAAAGCGATAGTGGATGCAATGCGCCAG
TCTGATGCTGACAGCGCTTATGAGGGATCGCTGGAACATTTACGCTATGTGAGAAAGATT
GTCGGCGGCTAA
>EECEBICD_01881 putative zinc-type alcohol dehydrogenase-like protein
YjmD
ATGAAAGTCAAAGCTGTGTTCATTAATAAGCCTGGTGAAGTTGAAACCCGCTGGGTGGAT
TATCCCCATAAGAAAGAAAATGAAGTATTAATTAAGGTTGATGCTGCAGGCATTTGCGGA
TCGGATATCGGGGCTTTTCGTGGAACGAACCCACTGGTTACTTACCCAAGAATTATTGGG
CATGAAGTGACCGGAATTGTTCTTCAGGAAGGCGCCGGAATGCCGGACAATATTAAAGAA
GGTGACCGCGTAATTGTTGACCCCTATATTTATTGTGGTCACTGCTACCCTTGCTCTGTC
GGCAGAACTAATTGCTGTGAAAATTTAAACGTCATTGGCGTGCATGTCGATGGCGCAATG
CAGGAGGTGGTTACTCATCCCGCACATTTAATTCATAAAATACCAGATAATGTGCCTTCT
GAAATGGCGCCGCTGGCAGAACCGCTGACCATCGCTCTGCACGCGCTGCATCGGGCAAAC
GTTAAGGCTGGGGAATATGTTGCCATCATCGGCGCGGGGGCCATTGGTCTTATGGCGGCA
TTAAGCGCGCGTCATTACAAGGCAACGCCTATTCTGATCGATATTGTGGAAGAAAGGCTA
CGCTATGCGCAAAAGCTTGGCGTTCCTTATGTGCTGAACGCCGCTGACGATCAGGTTATC
GACGCTATTAAAAATATCACCCACGGCACCATGGCGCAGGTCGTGATAGAAGCTTCCGGC

GCAAATAGCGCTATTCGAAATATGCTTGATTTAGCATCGTTTGCTGGTCGTATTTCGTTT
ACCGGATGGCCAAAACAGGAAACGTCGCTGCCGACGAACCTGATCACCTATAAAGAACTG
GATTTACGCGGTTCCCGCACCAGTGCCGGTGAATTTGATGAGGCATTACGCATGCTGTCA
ACGCTGGAGATTAATCCGCAAGATGTTGTCAGTAAGGTCGTCAATCTTGATGAGATCCCT
GATGCAGTTAAAGAACTTGATCGCTATCCGGAGCGCTACTTAAAGATTAATGCTGTTTTC
CATTGA
>EECEBICD_01882 Hexuronate transporter
ATGGGCGACAACTCTGGCTTTTTTCAGCTACAACTATATTCTTTTTTCTTCCTGACCTGG
TTCCCAAGTTACCTGAACCATTCGTTACATCTCGATATTAAAGAAATTAGTATCGCCACG
GTAATTCCCTGGGTCATCGGCGCCATTGGTATGGTACTCGGCGGCGTCTGTTCTGATGTA
ATTTATCGGATTACCGGTAATGCTCTGCTCTCACGTCGACTTATTCTGGGCGTATGTCTG
GCAGGCGCGGCGGTATGTGTTGCCGTTTCCGGTACCGTTAGCACTATTGGTAGCGCAATT
ACGCTCATGTCAGTTTCACTCTTCCTGCTCTATTTAACAGGCCCAATTTATTGGGCGGTT
ATTCAGGATGTTGTTCATAAAGATAAGGTAGGGAGCGTCGGAGGAGCCATGCATGGCCTG
GCTAATATATCCGGTATTATCGGTCCGCTTGTCACCGGTTTTATCGTTCAATTTAGCGGT
AAATATGACTATGCGTTCTATCTGGCTGGTGCTATCGCTATCGTTTCAAGCCTGTTGGTC
TTCGTGTTTGTAAAAAGTAAAGGATTCAAAGCCAATGAAAGTCAAAGCTGTGTTCATTAA
>EECEBICD_01883 Hexuronate transporter
ATGAAAAGAAAAACAATGGGATGGCTTATCGTTTTTCTTCTATTTATAGTTTACATGCTC
AACTATATGGATCGTTCAGCATTGTCGATAACTGCCCCCTTGATCGAAAAGGAGTTAGGA
TTTAACGCCGCGGAGATGGGGATGATCTTCAGCGCGTTTTTTATCGGATACGCCCTGTTT
AATTTTATTGGTGGCTGGGCCAGCGATAAAGTTGGGCCAAAAACGGTTTTTCTCATCGCA
GCGTTACTATGGTCTGTATTTTGTGGCTTAACTGGTTTGGTCACCGGATTGTGGACAATG
CTTATCGTGCGCGTTCTTTTCGGTATGGCTGAAGGACCGGTCAGCGCTGCCGGAAATAAA
ATCATCAATAACTGGATTTCCCGAAAGGAGTCTGCCACGGCGATCGGAATTTTTAGCGCC
GGTTCCCCGCTCGGCGGCGCGGTGTCCGGGCCGATAGTGGGCCTGCTGGCGTTATCCTTA
GGCTGGCGCCCCGCATTTGGCATTATTTTCTTATTTGGCCTGGTCTGGGTACTACTGTGG
TACTTTATCGTTAGCGATAAGCCAACAATGAGTAAACGCCTGGCGCCGGAAGAACGCATT
GATTTTGAAAATCATGAGGACGTTATTTTATCTGATGATGGCCGGGCTACGCCTTCACTC
GGCTATTACATGAAACAACCAATGGTATGGGCGACAACTCTGGCTTTTTTCAGCTACAAC
TATATTCTTTTTTCTTCCTGA
>EECEBICD_01884 hypothetical protein
ATGAAAAAAACATATCTCGTTGTTATCGTACTGTTTTTCATTTCTACAAAAGTTTATACA
CTACTTCATAACAACATCTTCTTTTGTCGAAACAGCCCGGAGTGTGATTTGTCTCATGTT
CTCCCCGACTACCGGGAGCAGATTTCAGGCACACCGCTAAAATATACCTTGATTAATACA
GCTCCATTAGCACAGGTGGTAGTCAGACACTATGAATTATTATCGCAACACTGGTCGCCA
GATGACATGGTAACCCCTGCGCAATGGCGACATAACGTTGATATTTATATCCCTGAAACG
GCAAAAGAGCACCATGCTTTGGTGGTTGTAAACAACGGAATTAATTATGATAAAGGCGTA
CAAATCACTGGTAAACCCGGTGATTTCCCCCAGGAAACACTGGCAAGCATCTCCCGTGAT
ACGAATACCATCGTAATATCAGTGAGTGATATACCTAACCAATATTTAACCTTTCAGGAT
GATAAAAAACCACTGAAAGAGGACGAGAGCGTTTCCCGAAGCTGGGCATTATTTATGGAA
GCGCCGGAGAAACGTGAGTTAATGCCTTTAAATATTCCTATGGTCACTGCCCTATCGCAA
GCGATGAGATTAGCAAAAAAGGAGTTAACGCAATGGAATATAAATTCCTTTATCATTACA
GGTATTTCAAAGCGTGGGTGGACCACCTGGTTGTCAGCTATCGCCGATCCTGATGTTGAA
GCAATTGTTCCCTTCGCCATTGATCTACTTGATATTGATGCTTCACTTGAGCATATTTAT
CAATCATACGGTGGAAATTGGCCCATTACATTCTACCCCTACTACCAGCAAGGCATAGAT
GAGAAAATAAAATCCCCCACATTTACCCAACTAAGGCAAATAATAGATCCGTTAAGATAT
CTCAATACTATTTATCAACCCCGACTTGCAATACCGAAATATATTATTAATGCAAGTGGC
GATGATTTTTTCGTACCAGACAACACTCGCTTTTATTATAGCAAACTTCCTGGCGTAAAA
TCATTACGTATAGTGCCTAATATGAACCACTACTCAATTAACCAGTTCGCTGAAGAAAGC
CTCGTTCCTTTTATTAATAGGTTTCAAAGTAAAAAAACATTACCACAGTTGATCGGCCTT
ATTCACCACCATCTTCTTACGGTCTATTTCTCAGAAGCGCCGGTAAAAGTCGTTCGCTGG
ACAGCTAACAATCCAAATGCGCGTGATTTTCGTTATGCCTGTGGCATCCGCTATAAGCCG
CTTACAATAGATATTCCCGCCAACAATAAGATCAGTATTACGTTAAATGAACCGAAGACA

GGTTGGGAAGCTACCTATATCGAAGCCACTTTTAATGATGGCTATGTCGCCACGAGTCAG
GTTTATATTACGCCCGATGAAAAATACCCACAGACAGCACCACCTTCCGTTAACGCCGCA
TGTCAAACGTTACCGGGGCGTGGATTAGGGGAAAACGATAGCCCCGATTGA
>EECEBICD_01885 Tetracycline resistance protein, class C
ATGAATACTAATGTCTATGAGAACACCGACAGCGAAACTATCACCCCGCTCAACAAGCGG
CGTATTTTGCCTGTTTTCCTGCTTGTCGGCCTTTACGCCGCCAGTACAGCGGCTGTAATG
TCGGTACTGCCTTTTTATATCCGCGAGATGGGCGGTTCGCCGCTTATCATTGGAATCATC
ATCGCCACTGAAGCTTTTAGCCAATTTTGTGCGGCGCCCCTGATTGGCCACCTTTCCGAT
CGCGTTGGCCGCAAGCGAATACTGATTGTCACGCTGGCTATTGCGGCGATAAGTTTACTA
TTACTCGCCAACGCGCAATGTATCCTGTTTATCCTGCTCGCCCGCACGCTTTTTGGCATT
AGCGCCGGGAATTTGTCAGCCGCCGCAGCCTATATTGCCGATTGTACGCACGTCAGAAAT
CGGCGTCAGGCAATCGGTATCCTCACAGGCTGCATTGGTTTAGGCGGTATTGTCGGGGCA
GGCGTTTCCGGGTGGTTATCGCGTATCAGTCTGAGCGCGCCGATCTACGCCGCCTTTATA
CTTGTCCTTGGGTCTGCCCTGGTCGCGATTTGGGGGTTAAAAGACCCTTCCACAACATCA
CGTACCACAGATAAAATAGCGGCGTTCTCTGCCCGCGCTATTTTAAAGATGCCTGTCCTT
CGCGTCTTAATCATCGTAATGCTTTGTCATTTCTTCGCCTATGGCATGTACTCTTCACAA
TTACCTGTTTTTCTTTCTGACACCTTCATCTGGAATGGGCTTCCCTTTGGGCCAAAAGCG
TTAAGCTATCTGTTAATGGCGGACGGGGTTATTAATATTTTCGTTCAGCTATTTCTGTTA
GGTTGGGTGAGCCAATATTTTTCGGAGCGAAAGCTAATTATCCTCATCTTCGCCCTTCTT
TGTACTGGATTTCTCACTGCGGGTATCGCCACGACCATACCTGTGCTTGTTTTTGCTATC
GTTTGTATTAGCATCGCTGATGCGCTAGCCAAACCCACTTATCTTGCCGCCTTGTCCGTC
CATGTATCGCCTGCCCGACAAGGTATCGTCATCGGAACGGCGCAGGCATTAATCGCAATC
GCTGATTTTATATCCCCCGTATTGGGCGGATTTGTCCTGGGTTATGCTCTGTATGGCGTC
TGGATCGGTATAGCTATCTCTGTCGCCATTATTGGTCTGGTGACGGCAATGATTTACCTT
TCAAAAAGTTCAGTGCTAATAGTGAAACCAGAAACAGAATAA
>EECEBICD_01886 Flavin-dependent monooxygenase
ATGACAAGATACACCACTACAGATGTACTGATCTGCGGCGCTGGCGTTACCGGGCTTACT
TTAGCCATCGAACTGGCGCGTCACGGGGTCTCATTTCGCCTGATTGAGAAAAGAACGACG
CCTTTTATCGGCTCCCGCGGTAAAGGTATTCAGCCGCGAACGCAAGAAATCTTCGAGGAT
CTTGGAATACTCAATAAAGTGGTCGCCGCCGGCGGGCTCTATCCCCGACTACGAACCTAC
CGTCACGACGGAAGCTATGTAGATTCAGATATTGCGCATCATACAAAACCGACCCACGCC
GAACCCTACCATCTGCCATTGATGGTTCCACAGAACGTGACAGAAACGATTATGCGAGAG
CAATTAAAAGCATGGGGACATCGGGTGGAGTTCGGCTGCGAGTTACGACATTTTGCGCAA
ACCCCACGCACAGTCACCGCGTATGTTGCCGGGCCGGCTGGAGAAGAGGTCATCATCGCG
CATTATTTGATAGGTGCGGATGGCGGCGGAAGCTTTGTCAGAAAAAAATTAGGCGTCAGC
TTCCCCGGTCGTACGTTGGGAATCCATGCGCTTGTGGCTGACGCTTCACTTTCCGGTCTA
AACCGGGATGTATGGCACCATTTTAACGATGGCGATATGACGCGGATGATAACGATTTGC
CCTCTCGCCGGCACGCAGCTCTTTCAAATCCAGGCGTTGTTGGCCCCGGACGACTCGCAG
AATTTCTCAGCCGACGTGCTCACGGCTTTTCTTACTGAACGAATCGGGCGGACGGATGTT
CGCATCCATTCGATTCCCTGGGTATCGAAGTACCAGATGAATGCGCGCATAGCGGAACAT
TACCGTGTGGGTAAGGTATTTTTAGCCGGTGATGCCGCTCATGTGCATCCGCCGACGGGT
GGGCAAGGATTAAATACCAGCATCCAGGATGCCTATAACCTGGGGTGGAAAATGGCAGCC
TCGCTGCGGGGCGCAGGAGAAGAGCTTCTCGATAGCTATGAACAGGAACGCCGCCCGGTA
GCCGAATCACTGCTCCACCTTTCGACCCGATTACTTGACTCTCAAAAACAGGGTGGCATC
AAACGAGAACGCGACGTTCAACAACTTGACATCCAGTATACCGACTCCCCCTTAGCCCAC
ACGTTGCCCGAACGTCAGCATGGGTTACAAGCGGGAGAAAGAGCTCCGGATGCTCCTCTT
TTGGGCGCCGGCGGCCAATCATTACGATTATTTCAGTTACTACAAGGTCCGGACTGGAAT
CTGTTGGCCTACGAAACCCACGGTAAGGTCATCGATGCGCGGCGAGGACTGCGTATTCAT
CACATTGGCGAGCAGGACGAGCTCATCGATACCCTGGGGCACTTCCGGGAATCCTATCAT
CTGGCGCCGGGTCAATGCGTGGTGATTCGACCCGATGGCTATGTCGGCGCATTCTTTCAC
GGCAAACAGAGCAACGATATTGAAAATTATCTCTCTCGTTTTGCCATAGGGATTAAAGAT
GAATACTAA
>EECEBICD_01887 hypothetical protein
ATGTCAGAAAATAAAAATGTGCAAGATACACATATTTCCGAGCAAATGAAGGCGCTTCAC

GGTGCGTTGATTCGCGTGGTGAGCGCGCTTAACCGGCCTCGTAATGATGAGAAATTAATT
GCAGAGGCGGGATACAACTCGATCGTGCGCTATTTTCGATCCTTATTAGTATCGAACGT
TTAGGGCCGATTGGCGTTGTTGAGTTAGCCGAACGTGCCGGTCGTGATTATACGACGGTA
AGCCGACAGGTCGCCAAACTGGAGAAGTTGGGATTGATAATACGGCAACATAATGCCATC
GATCGTCGTATACGCGAGGCCGTTATCTCCCGTACAGGAAAGGCGATGACGGAGCGTATA
GACGCCGCTCGAGAGCAAATGGGCAAAGTTGTTTTTAAAGACTGGCCTCAGGATGAACTT
GATGTCTTTGTTCGGCTAATGCAAAAGTTTGCCGATGCAATGGATAGCGATGCTTCAATG
CCGGAATAA
>EECEBICD_01888 S-adenosylmethionine:tRNA ribosyltransferase-isomerase
ATGCTTACCGAAAAATATGATTTTCGCATTACCGATCAGATGACTATCCCACTCAGACCT
CATTGGATAGCCAATGATTCATACAGGGAAAAGTGTAAAATGCTTGTCCTCAACCGGAGT
AAAGGGGAAATTCATAAGGTAGACTTTAGTAAATTAACAGATTATATCAAAGAAGGTGAT
GTAATCTGTTTTAACGACAGTACTATAATAAACCATATGTTTATCTGTAAAACAAGACAG
AATAGGCTAATTAAAATTGTTTTAGAGGGATTTTTACCAAATAATAGAGTAATTATTTCC
GGACTTTTAAAGGAAAGGCTTAACGCGAATGATGTTTTCTATTTGGTTGATAACCCTGAA
ATTTCCATTAAGATAGAGCAAAGGTTTAGCGAGGAGAGCCAGTACCGGGCCGTCGTGGAA
AATCATGAGGCCTTAATTTGCTATTTAGCCAGTCATGGTGAGCGTCTTGATGAGTATGTG
GACTCCAGCTTGTTTTATAAATATCCAGATGCTTACCGTTCCGTTTTTTCTAAAAAGTAT
GGTTCTTTGGAAATACCTTCTGCCGGCATTCATTTTACCTGGGATCTTATCCAAAAAATC
AAAGATAAAGGTGGGTTAATATCCTTCATCACTTTACATGTTGCATCAACAGAAATGTTG
TCCAACAGAAAAAATACAAACAAAATGTGTAGAGGAAGTGACTATCAACGAAGAATACTAC
GAGGTTCCGCAAGCAACAGCGGATATCATTAATACTGCGAAGCAAAATGGCGGACGTATT
TTTGCCGTAGGAACTACCGTTACACGATGCCTGGAGTCCGCGTATTCGAGGGAGCACAAT
TGCCTCAAAGCAAGTTCTGGCTGGACTGCGCTTTATATACATCCAGGCTATCAACTGAAA
GTGGTTGACTGCCTGCTAACGAACTTACATCAGCCAAAAACGACGCATATGGTGTTAACT
GGTCAGTTTGCTGGGGTTGATCTACTCATGAAGGCCTATGCCTCAGAAGATATCCAGTCA
TGTCAGTTCGATATGTTTGGTGATTGCATGTTAATCATACAGGATGAAGGGTAG
>EECEBICD_01889 hypothetical protein
ATGGTAAGTGATACTCTTGAACAGCGGATTTATGAACTGGTAAGAAGCCATGATGGAATT
TACCTCTTTAAGAAAAAAGAACTGACACCGTCAACAGATTTGGATTCAGATCTACGGCTG
GAAGATGATGAAGCACTGGCATTGATGGATGATTTTTTTACGACATTTAATGTAGATAGA
GGGAGTTTCTCGATCACAACATACTATCCGCCAGAACCACCGCTAAAGCATTTGCTTAAT
CCGTTTCGCAAAAACGATATTCCTCAGGTGGCGGATTTCACTATAGGTATGCTCATTGCG
TCTGCCAGGGCTGGTCGCTGGTTGTATGACTGA
>EECEBICD_01890 hypothetical protein
ATGGCCGCGTATGACATCGAAAAGGACATCGGGCCGCGGGTTCAGATTATCCAGGAGATA
CACGAATTAATTCTGGCGGGTGAAATGGAAGTCGCCAAAAGGATGCTGCGCACGCTGATT
AAAGTCACGTTCGCTTATCCTGCGATTTTTGACGAGGTTCGGCAGGAATGCCAAATCAAC
AATGCGCATGTTGTCGCCGGATGTGGATCCGGGTATCAAAGCGTTTATAGCCGTTGTCAG
AGCTGCAGAACATCAGTCAGAAAAAGCAGCGGACCAGGGTAA
>EECEBICD_01891 HTH-type transcriptional regulator GalS
ATGGCCGGTGTATCAACCGCGACTGTATCACGCGTCATTAACCAGACCGCGTGGGTTGAA
CCCGTCACGCGCGAACGTGTAGAGAAAGCCATGCGCGATCTCAATTATCGCCGGAATGCC
GCCGCTATCGCGCTGGCGAAACGTAGCGGCGATATGCTCGGATTGTTAACCGGCAATCTG
GCCGATCCTTTTTTTGCCCGCCTTGCTCGCGGCGTAGAAGATGTTTCTCGTAAGCAGCAG
TATCGCTTGATGGTATGTAGCGGCGGCCACGACGAAGAAATGGAAAAAGCGGGACTGGAT
TTTCTGGTTAATCAAGGCTGTGAGGCTATTGTTGTACACGCCAGCCGATTACCGGATAAA
GAGCTGCTGCGCTACGCAGCCCACTTTCCAGCGCTGGTTGTTGTTAATCGTTATATCGCC
GGTATGGCCAACCGGTGTATCTGGCTGGAAAATCGCAGCGCCGCCAGGGAAGCGACCCGA
TATCTCCTGGCGAACGGGCATCGGCGCATCGCCTGTGTGACATCTGACTTGCCAATTATC
GATCGTCAGGAACGCCTTGATGGTTACCGTCAGGCGCTGGAAGAATATGGTATTTCTCCA
GACCCTCGCTGGGTGATTAGCGTTCCGTTTAACGAAGAAGGCGGCGAGCGGGCCGCGCAT
CAACTTATTAATAGCGGGCTTCCCCTTACGGCAGCGGTCACGTTTAACGATGTGATGGCC
GCTGGCATTATGCGTATTCTTCATCAACGCGGCGTTCAGCTTCCGCAACAGCTCTCTATT

GTCGGCTTTGATGATGTGGTGCTGGCCCGGTATCTTTATCCGGCGTTAACAACAATGCAT
TATCCGGTCGAACAAATGGCGCGTTGTGCCGCGCAACTGGCGATACAGTTATACCAGGGC
ATTACGCCGCCGCCCAGCAGCAATCATTTCAATGCCGAACTGGTGATCCGCGATTCTGTC
GCGCCCTATTTTTCACGATGA
>EECEBICD_01892 hypothetical protein
ATGAGTGCATCACAAAACAAGAAGAAAACGCTGTCATTGGGTTTAGCGCTAATCCCAGTC
ATCTCCATGTTGTTATTGTTGATTATCGGTTACGGCATCATGGGGCTACGTATCGAACCG
CTCCTTCTGTGCTCTGCGGCGGTAGCGGCAGGCATTGCATGGTGGCAAGGATACTGTTGG
GAAGACATCATCAACTCAGTGGTGGATAAGCTGGCTAAAGCAATGCCGGTTATCATGATC
CTGATCTGCGTGGGCGGTTTGATTGGCACCTGGATGTTTAGTGGCACCATTCCCTACATG
GTGTACTGGGGCTAA
>EECEBICD_01893 Malate-2H(+)/Na(+)-lactate antiporter
TTGATTGCAGCCTTCTTTTTAACCAGCGTGGTCTCCGTCTGTACCGGTACATCATGGGGA
TCCGCCGGGACGGTTGGCGTTGCGTTAATGGGGGTTGCCGCCGGGCTGGACGTGTCTCTT
GCAGCAGCAGCTGGCGCCGTTGTTTCTGGCGCTTATTTCGGCGACAAAATCTCACCGTTG
TCCGACTCGACTAACTTTGCCGCGATTGTCGCAGACACTACTCTGTTTGAGCATATTCAG
CATTTACTTTGGACGACCCTGCCAAGTTTCCTGCTTGCCGCCGTGGTTTATCTTATCGCC
GGACACAGTAATATGCTGGGCGAGGTGGCTACGCCGCAGCGCGTTACGGACATCATCCAC
TCGCTGGAGTCGCTATACCATTTTAATATTATTCTTATTTTGCCGCCGGTGATCGTACTG
TGGGGCGCAATTCGCAAAAAAGCCTGTCATCCCATTAATGTTAAGCGCCTGCGTACTTGC
CTTGTTCCTCGGCGTCATCATGCAGGGACTGAGCATCAAACAGGGACTGGACGCTTTCAT
TGA
>EECEBICD_01894 hypothetical protein
ATGCAGGGACTGAGCATCAAACAGGGACTGGACGCTTTCATTGATGGATTTGATATCGCC
ATGTTCCCACAAGGCGCAGAAGGTGTCGTGGCCGATGTTCCGCGACTGCTAAACCGGGGC
GGAATGTTCTCGATGATGGGAACGATACTGTTAGTTTTCTGCGCTTTTTCATTTGCCGGA
GCGCTAACGTTAACCGGCGCGTTAACGATCATCATTAACCGACTGCTGACCATTATTCAT
AGCGTTGGTCAACTCATTGCCGCCACTATCGGCACCACAATTTTGGTCACAGGAGCGACC
AGCGACGGTAAACTGGCATTGTTAGTTCCCGCCGAACTGTTTAAAGATGCCTATCGTCGT
ATGGGGCTGGATACTAAAAACCTTTCACGGACGATAGAAGATGCCGGTACCGTTATCGAA
CCTCTCCTGCCGTGGACCTCGGCTGGCGTCTACATGGCAACCACGCTGGGCGTTAGCACG
CTTGATCTTCTGCCCTGGGCTATCCAGTGCTATGCGGCGATCTTCTTCGCATTAATTTAT
GGTTTTAGCGGAGTTGGTATTGCCAGAACTGCCTCCGCTTCAGAAAAGTCGCCGCAAACG
TCAGTGACGAAATAA
>EECEBICD_01895 Cystathionine beta-lyase PatB
ATGGATTTTAACCAGATTATCAATCGCAACAATACTGGTTCGGTAAAGTGGGATTTTATT
GAGCGTCACTTTGGCGACGGCGCTGGGAAACTGCTGCCGATGTGGGTTTCTGATTTCGAT
TTTGCCTGCCCGCCTGAGGTTCAGGCGGCATTGCATCAGCGAATCGAGCACGGCGTATTT
GGCTATAGCGAACGTGATGAGGCCTATTTTAACGCCCTGCTTCACTGGTTTTCGTCACGC
CACCAGCTTACGTTAAAACAGGAATGGGTATGCAGCGTGGAAGGCGTCGTACCAGGGTTG
GCGCTCTTAGTGCAAATGTTGACGCATCCAGGCGACGGCGTAGTGGTACAGGGACCATAC
TATGGCTCCTTCGCCAAAATAATCACGCTTAATGGTAGAAAACTGCTGGAGAATCCCCTT
AGCGAGTCGCCGGACGACGGCTACCAGATGGATTTTTGCCAACTGGAAAGGCTGTTTCGC
CATGAGCGCCCTCCGCTGATGATTTTGTGTAATCCACATAATCCAACCGGGCGTTGCTGG
TCGGCCAACGAGCTGGAGCAACTGCTTTTATTATGTGAAGCTTATGATGTCACTCTCATC
TCGGATGAAATATGGGCCGATCTATTGCTGCCCGGCGAAACCTTTACCTCGGTACTGCAT
TTAGGGGAGCGCTGGCATAAACGGGTCATCTCCGCCACTGCGGCCAGCAAAACGTTTGGC
TTATCGTCGTTGCGAATTTCTAATTTTCTCATTCCCGATCCTACGCTTCGCCAGCGATTC
CTGTCTCGTCTGGACGCTCATGGGCTGGACGTCTTTAACGCCCTCTCCGTTCAGGCGGCG
ACTACCGCATGGAATGAAAGCAGACAGTGGCTGGATTCGCTACTGGATTACCTGGCGGAA
AACCGCCGCTGGTTTGTTGAACAGGCGACAGAGCACCTTCCCTGGGCAAGAATTGTCCCG
GCGCAGGGAACCTATTTATTATGGATGGATTGTAAAAAACTGGGACTGGATGACGAACAG
CTAAAATGGGTAATGGCGGATGTCGCGGGTATTGCTCCTTCAATGGGCAGCGGCTTTGGC
CCGGCGGGCAGCGGATTTATCCGTTTAAATCTGGGATGTCCACGTACCTACCTTGAAATG

GCAATAGACGGCCTGAAGCGAATATCGGTGAAGACAATCAAAGGAATACCAACAGGCGGA
TGA
>EECEBICD_01896 Glycogen operon protein GlgX
ATGGTGCGCGCTTTCCATGATGCCGGCGTCGAAGTGATACTGGACGTGGTCTATAACCAT
ACGGCAGAAGGGAATGAGCTTGGCCCCACGCTGTCATTTAAAGGTATCGATAACTTCTCT
TACTATCGCACCATGCCCGATCAGCATCGCTATTACATTAACGATACAGGGACAGGTAAT
ACCGTCAATACCTCTCATCCACGGGTACTGCAAATGGTGATGGATTCCCTTCGCTATTGG
GCGGAGTCAATGCATGTCGACGGCTTTCGTTTTGATTTGGGCACCATTCTTGGTCGTGAG
CCGGAAGGCTTTGATCAGAGAGGCGGTTTTTTTGATGCGGTAACGCAAGACCCGGTGCTG
GCAAAACTGAAACTGATTGGCGAACCCTGGGATATCGGACCGGGCGGCTATCAGGTTGGC
GGCTTTCCACCAGGGTGGGGCGAATGGAATGATAAATACCGCGATACGGTACGCGAATAT
TGGAAAGGTGACAACGTCACAAATGATTTCGCCGCACGCCTGCTGGGTTCCGGCGATCTC
TATGATTTGCGCGGCCGTCGCCCCTGGTCAAGCGTTAACTTTATTACTGCCCATGACGGC
TTCACGCTGAACGATCTGGTGTCTTATAACGACAAACATAATGAAGCCAATGGCGAAGAT
AATAATGACGGGCATAATGATAACCGCTCCTGTAACTATGGCGCAGAAGGGCCAACCGAC
GACGAGGGCATCAATGCGATACGCGAGCGACAGAAACGTAATTTCCTGACCACCTTGCTT
TTCTCGCACGGCACGCCGATGTTATTAGCGGGCGATGAGTTTGGCCGCAGTCAGATGGGC
AATAATAATGGCTACTGTCAGGACAGTGAAATATCGTGGGTCCACTGGGATAATTTGCCG
GAAACCGCCAATGCGCTACGGGAGTTTACCCGTCATCTGATTCAACTGCGCGCCACGCAA
CCGCTGCTGCGCCGGGAAAGCTGGCGTGACGGTCTGGAGATCCGCTGGTTTAACGCCGGC
GGAGGGGCGCAGCAATCCGAACAATGGGATGAGGGATCGACCATAGGCGTATGCATTAGT
CGTCCCGATCTTCAGCCGGAAGCGGGGATCTGGCATGACGCCTTACTGCTTTTCAATCCG
TTTGAAGGCAGCGTACCGTTCCGTATTCCGATGTGGGGAGAAGGCGGCTGGGTACTTGAA
CTGACGACTGCCGATAACGCGCAGCAGGGAATACGGATTACCGAAGAAATGGATTTCGAT
CTGGCCGGGCGCAGTATCGTGTTATTCAGACGCCCCTGA
>EECEBICD_01897 Glycogen debranching enzyme
ATGACAACAAACAGGGCTTTTGAAATCAGATCAGGATACAGCCATACGCTTGGCGCAAAT
TATGACGGCGAAGGCGTGAATTTCGCTATTTTTTCCGCCCATGCGGAACGCGTCGAGCTT
TGTCTTTACGATCCGAGCGGGGAGCATGAAATCGCTCGTCTGGAGTTACCGGAATATACC
GACGAAATATGGCACGGCTATGTCCCGAAGTTGCAGCCCGGCGCGCTGTATGGGTACCGC
GTTTACGGTCCTTATGACCCGGAAAATGGTCATCGTTTTAACCCCAACAAGCTGCTTATT
GACCCTTATGCCCGTGAGCTGGTGGGCGATATTCAGTGGAACGATGCGCATTTCGCGTAT
CAATTACTTCATGACGATAAAGATTTGACCTTTGATGACCAGGATAGCGCGCCATTTACG
CCTAAATGCCGGGTGATTGATCCGGCTGAGGCGAATTGGGAGGATAGACAACGGCCTTCC
ATACCGTGGTCGAACGCCATTATTTATGAAACGCATGTAAAAGGCTTTACCCAGCTTAAT
TCCGCGATCCCTGAGCCTCTACGCGGTACGTTTGAGGGCATGGGACATAAAGCCAGCGTG
GATTACATTAAAAGTCTGGGTATTACCTCCGTCGAGCTTTTACCCGTTCACTGGTTCCCG
GACGATCAGCATCTGCTCGATAAAGGACTGAAAAATTTCTGGGGATACAACTCGCTGGGC
TTTTTGCGCCGGCGACGCGCTACTATGGTCCAAAAGGTATTCAGGGATTCCGCGATATGG
TGCGCGCTTTCCATGATGCCGGCGTCGAAGTGA
>EECEBICD_01898 Maltooligosyl trehalose synthase
ATGGCGAAATCGGTAGAGGATACGCTTTTCTTTCGTCAAAACATGGGACTGGCGTTAAAT
GAAGTCGGCGCCGAGCCCGTCACGCATCACTTCTCCATCGAACGCTTTCACCATGAGATG
AAAACCCGACAGGCGCGACAGCCTGATGCGTTATCCGGTACGTCGACCCACGATACCAAG
CGAGGCGAGGATGCGCGAGCACGCCTGTATACGCTTACCGAAGCGCCGGAACAGTGGAGC
GAATGCCTTGCCCGCTGGCGGCAGATGAACCAGACCCATGTCAAATTTTTGAATGATGGC
ACTGCGCCAAAATCGGCGGATACGTGGATGTTATATCAGGCGCTGACCGGCGTCTGGCCC
CCGATGCTACAACCACAGGACGAAACAGGTTTAAACGCGCTGAAAATACGCTTTGAAGCC
TTTGTGGAGAAAGCGCTACGCGAAGCGAAGCTACGTACCGATTGGGTGGACAGTAATGAG
GCTTACGAAACGGCAATGCTTGATTACGCCCGTTATTTACTGGCGCCGGATAATCAGACA
TTTTTGCAGGATTTTTATCGTTCCTTGCAACCCTTCATCCGCGCAGGGCTGGTTAACAGC
CTGACGCAGACCGTTATCAAGTTAACCGCGCCGGGAGTACCTGATATCTATCAGGGAAGC
GAGGCGTTGAATTTTAGTCTTGTCGATCCCGACAACCGCCGCGAACCGGATTTCGCCACG
CTGGCGCAGCAGTTAGACCAACTAACGCCGGGAGTTTTCTCGCGCGAAGAGAGTTGGCTG

AACGGGCAGGTGAATCAGTATGTCACCGCCGCCTTACTGCGTTTGCGTCAGCAGAATCAT
GAGCTCTTCCGTTTTGGCGACTATATACCGCTGCGGGCTGTTGGTCAGCGTGCCGACAAG
GTCATCGCTTACGCCAGAGTCAATCATGATGATGCGCTGATTGTTGTTGCGCCCCGACTG
GTGTTCGCCGAATGTGACGGCTTATTGTCACAATCGCATAGCGGTTTCTGGTCAGGAACC
GACATTATTATACCCGGACAGCTTAATCAGCATCGTTATCGCAATGTGCTTACCCAGGAG
AGGTTAATGCCTGGTGAACGCCTGTCGTTGGCCTCACACCAGGGCGGCGTACTGGTTTTA
ATGAGCGACTGA
>EECEBICD_01899 Maltooligosyl trehalose synthase
ATGATCCCAACTGCAACGTATCGTCTTCAGTTTCGTAATGGCATGACCTTCGATCGCGCT
GCGGCGCTGGTGCCTTACCTTAAAAATCTGGGTATCAGCCATCTTTATGCCTCACCCATC
TTTACCGCCACCAAAGCGTCAACGCATGGTTATGATGTCACCGATGCGAATGAGATTGAG
CCTTCTATTGGCGGGCGCGAAGGATTTGAAAGACTGGTGGCCGAACTTAAAGCGCAAGGT
CTGGGATTGATCATCGATATTGTGCCTAATCACATGGCCAGCTCGCTGGAAAACGCCTGG
TGGCGCGACGTGCTTGAATATGGCAAGGAGAGCCGTTACGCGCGCCATTTCGATATTGAC
TGGTCGCGCCGACTGACGCTGCCTTTTTTAGGCGACACGTTTGACGCCGTGCTGGAAAAC
GGAGAAATTGCGATCAAGCCCGATCCCGCTACGGGTAAACCCGCGTTCGCCTATTATGAT
AACTATTATCCGCTTGCGCCGGCGACGTGGCAGGGGCGCGAGGCCGAAATACTGGCGCTT
ACCGATAAAGCCGCTATTGCTGATTTGCATGAGCGCCAGCCGTGGAAATTAATGTCCTGG
CGCGATGCCGCTCGCTCGCTTTCATATCGGCGTTTCTTTGAGGTCACAGGGCTGGTAGGA
ATGCGGGTGGAAGATAAAACCGTCTTCGACGATACACATCGGCTGATTCTGGAGCTGGTC
AGAACGGGGGCGGTTGACGGTTTACGTATCGATCATATCGACGGACTTGCCGATCCGAAA
GCCTATCTTGCGCGTTTACGTCAGGAAGTGGGGCCGGCGTGCTATATCACCGTGGAAAAA
ATTCTCGCTAAAGGCGAGCAACTTCCTGATGACTGGCCGGTTTCCGGCACCACAGGCTAT
GAATTTATCGCCTCGCTGGCGGAGGTGCTGGTTGACGATGAGCAGATAGACAATCTGCGC
CAGGCTTACGAGACCGTTAAAGGCGCGCCAGTGGATATGCGGGCGGAATTACGGGCGGCT
AAATTGCTGATGGTCGATCGCAATTTCGAGGGGGAATTTACCCGGCTGCTCGCGCTTGCG
CTGTCGATTGCCAGCGAGCTACAGATCGCACAGGAAGAGAGCATCGTTCGGCAGGCGTTA
CGCGAGTTATTAATTGCCTTTCCCGTATATCGCACTTACGGCACGGCAGAAGGGCTACCG
CCGACCGATATTTGCCTGTTACACCGTATTGTGGAACGGGTAAAGACGCTTGAAAAATCC
TCCGCAGCCAGAAGCGCTTACATTTCTGAGCCGTCTTCTTACTGGCGATGTTCCCGCCTC
CAGCCAGGAGGAGGCGACACAGTTTCGCGTTCGGTTCCAGCAACTCACCGGTCCGCTGAT
GGCGAAATCGGTAGAGGATACGCTTTTCTTTCGTCAAAACATGGGACTGGCGTTAAATGA
>EECEBICD_01900 Malto-oligosyltrehalose trehalohydrolase
ATGAGTTCAAAAATTTTTTGCAAAAGCTGGGGGGCTGAATATATCGCCGCTGATGTTGTC
CGCTTTCGTCTTTGGGCCACCGGTCAGCAAAAGGTTATGCTCAGGCTTGCGGGTAAAGAC
CAGGAAATGCAGGCGAGCGGTGACGGCTGGTTTACGCTGGACGTCTCCGGGGTGACGCCA
GGTACGGAGTATAACTTTGTACTCAACGATGGCATGGTGGTCCCCGATCCGGCTTCCCGC
GCCCAAAAAACTGACGTCAACGGTCCGTCATATGTGGTTGATCCAGGCAGCTACACGTGG
CGCAACACCGGGTGGAAAGGTAGCCGTTGGGAGCAGGCCGTGGTGTATGAGATGCATACA
GGCACGTTCACTCCGGAAGGCACCTTCCGCGCCGCAATAGCGAAGCTGCCTTATCTCGCT
GAACTCGGCGTTACCGTTATTGAAGTGATGCCCGTTGCGCAATTTGGCGGCGAGCGTGGC
TGGGGCTATGACGGCGTACTGCTTTACGCGCCGCATTCTGCCTATGGGACGCCGGATGAT
TTCAAGGCGTTTATTGACGCCGCGCATGGGTATGGTCTTTCCGTCGTCCTGGATATTGTG
CTGAACCATTTCGGCCCGGAGGGAAATTATTTACCGCTATTGGCGCCGGCGTTTTTCCAC
AAAGAGCGCATGACGCCGTGGGGAAATGGTATCGCCTATGATGTCGACGCCGTGCGGCGC
TATATCATCGAGGCGCCGTTATACTGGCTGACAGAATACCATCTCGACGGCTTACGCTTT
GACGCTATCGATCAGATTGAGGACAGTAGCGCCAGGCATGTGCTGGTTGAAATCGCACAA
CGTATTCGGGAAGACATTACCGACAGACCCATTCATCTGACTACCGAAGATAGCCGCAAT
ATTATTTCTCTGCATCCCCGTGATCAGGATGGCAATGCGCCGCTGTTTACCGCCGAATGG
AATGACGATTTTCATAATGCCGTCCACGTTTTTGCGACCGGAGAGACCCAGGCCTACTAC
AACGATTTTGCTGATACCCCGGAAAAACACCTCGCGAGAGCGCTGGCCGAAGGATTCGCT
TATCAGGGAGAAATTTCCCCCCAAACCGGCGAACCTCGCGGCGTAAAAAGTACCGGACAA
CCTCCGGTCGCCTTTGTGGATTTTATTCAGAACCACGATCAGGTCGGTAACCGCGCCCAG
GGCGACAGACTGATAACCCTGGCGGGCGCTGAACGAACAAAAGTATTGCTCGCCACGTTG

CTGCTTTCACCGCATATTCCGCTGCTTTTTATGGGCGAAGAGTATGGCGAAAGCCGTCCT
TTTCTTTTTTTACCGATTTCCATGGGGATTTAG
>EECEBICD_01901 hypothetical protein
ATGAAAATTTTACCGCTGGCACTCTTTATCATTCCTTTTCTGGCCGGATGCGGCGCCAAT
AATACGCCGCCGCAAACGCCTATTCCCGGGGAAAAAACCTCTGCCAAATTACGTACCCTG
GAAACAGGCGCGGCGGCTATTCAATCCAGACCACCTGTCGATGCCATCAGTACCTACCTT
GACGGGTTCCATTTTTATAGCGGTGATAAAAACGGACAGATGGAAGCGCACCATTACGTT
ACCGTCCTGAACGAAGATGTCATGCAGGCGGTGATTTACGACGGCAATACGAAAAACGCG
CGCCTGATGGGGGTGGAGTACATTATCAGCGAACGTTTATTTAAAACGCTTCCTCCCGAG
GAGAAAAAACTGTGGCACAGCCACCAGTACGAGGTGAAATCCGGTAGCCTGGTGGCGCCT
GGCTTACCGCAGGTCGCTGATAAAGCGTTGATGAGTAAGATTGTTAATACTTATGGCAAG
ACCTGGCACACCTGGCATACCGACCGGGATAAAACCCTGCCAATGGGTATCCCTGCGCTG
ATGATGGGCTTTACTGGCGACGGGCAGCTTGACCCCGCGCTGCTGGCCGATCGGATCGC
CGTCTGGGAATTGATACCCAGGCCATTAAACGCGAGCGGCAGGATCTGCCTGAACATCCC
GTTGTTAAGGGGGCTAACGCCTGGGAGCAGGGGGAGGTTATCCAGCTACAGCGTGTTCAG
GGCTCTGGCGAACACGGGCGAGGCGATACCGCGCACTTCGGTACATCTGAGCAATCCCGA
CAATAA
>EECEBICD_01902 Acid stress chaperone HdeB
ATGAATAAATTCTCCCTTGCTACAGCAGGTATTATCGTGGCAGCGCTGGTAACCAGTGTT
AGCGTGAATGCGGCAACAGATACTACTAAAACAAACGTTACGCCTAAAGGTATGAGCTGC
CAGGAGTTTGTTGACCTCAATCCGCAGACGATGGCGCCAGTCGCTTTCTGGGTGCTGAAT
GAAGATGAAGATTTTAAAGGCGGGGACTACGTAGATTTCCAGGAAACTGAGACGACAGCA
GTGCCGCTAGCCGTTGAGCTTTGTAAGAAAAACCCGCAGAGTGAATTAAGCAAAATAAAA
GACGAAATCAAGAAAGAACTCTCAAAATAA
>EECEBICD_01903 Peroxiredoxin OsmC
ATGACAATCCATAAGAAAGGTCAGGCACACTGGGAAGGCGACATCAAACGGGGTAAAGGT
ACTGTATCAACAGAAAGCGGCGTGCTTAATCAACAACCGTATGGATTCAATACCCGGTTC
GAAGGCGCGCAGGGAACCAACCCGGAAGAGTTAATTGGCGCCGCACATGCGGCCTGTTTC
TCCATGGCGCTATCACTCATGCTGGGAGAGGCTGGATTTACGCCGACATCCATTGACACC
ACCGCTGACGTATCGCTTGATAAGGTGGAGGCGGGCTTCGCCATTACCAAAATTGCGCTA
CAAAGCAAAGTCGCGGTAGCAGATATTGATGCTTCAACTTTCGATCAAATCATCCAGAAA
GCAAAAGCCGGGTGCCCGGTATCGCAGGTTCTGAATGCGGAAATCACCCTCGACTACCAG
CTTAACGCCTGA
>EECEBICD_01904 Protein bdm
ATGTTTACTTATCACTCAGCAAATACCTCAGCAGCACAACCGGCCTTGGTCAACGCGATT
GAACAAGGATTACGCGCGGAGTTAGGCGTTGTCACTGAAGATGATATTTTAATGGAGCTG
ACCAAATGGGTTGAGGCTTCCGACAATGACATCCTCAGTGACATCTACCAGCAGACCATC
AACTATGTGGTAAGCGGGCAGCACCCGACTCTGTAA
>EECEBICD_01905 Stationary-phase-induced ribosome-associated protein
ATGAAATCGAACCGTCAGGCTCGTCATATACTTGGACTGGATTACAGAATTTCCAACCAG
CGTAAGGTTGTTACTGAAGGCGACACCAGCAGCGTGGTGAATAACCCAACCGGCAGAAAG
CGCCGCGCCGACAGCCAAAAGTAA
>EECEBICD_01906 NAD-dependent malic enzyme
ATGGAAACTATAACCAAGAAAGCACGTTCCCTTTATATCCCTTACGCTGGCCCTGTATTG
CTGGAATTCCCCCTGTTAAACAAAGGTAGCGCTTTTAGCGTAGAAGAGCGCCGCAACTTC
AATTTGTCAGGGCTGCTGCCAGAGGTCGTCGAGTCGATTGAAGAACAGGCCGAACGCGCC
TGGCTCCAGTATCAGGGGTTTAAAACAGAAATCGATAAGCATATTTACCTGCGTAACATC
CAGGATACCAACGAAACGCTCTTTTATCGACTGGTGCAAAACCATCTTGAAGAGATGATG
CCGGTTATCTATACCCCGACCGTGGGCGCGGCCTGTGAGCGCTTCTCTGAGATTTATCGC
CGGGCGCGCGGCGTATTTATCTCTTATCCGAACCGTCACAATATGGACGACATTTTGCAG
AATGTGCCTAACCACAACATTAAAGTTATTGTGGTGACGGATGGCGAGCGGATTCTGGGG
CTTGGCGATCAGGGCATCGGCGGGATGGGGATCCCTATCGGTAAGCTGTCATTATACACC
GCCTGCGGCGGCATTAGTCCGGCCTATACCCTTCCCGTTGTGCTGGATGTCGGCACCAAC
AATCAACAGTTACTCAACGATCCGCTTTACATGGGCTGGCGTCATCCGCGCATTACCGAC

GATGAGTATTATGCGTTTGTCGATGAGTTTATTCAGGCCGTTAAGCAGCGCTGGCCAGAT
ATCCTGCTACAGTTTGAAGATTTCGCGCAGAAAAACGCAATGCCGCTGTTGACGCGCTAT
CGTGATGAGATTTGCTCCTTTAACGATGATATTCAGGGCACCGCAGCCGTGACCGTCGGT
ACGTTGATCGCCGCCAGCCGCGCTGCCGGCAGTCAGCTTAGCGAGCAGAAAATCGTTTTC
CTCGGCGCGGGTTCCGCCGGATGTGGGATTGCTGAACAAATCATCGCGCAGACTCAGCGT
GAAGGTTTAAGCGAAGATGCCGCTCGACAGAACGTTTTTATGGTCGATCGCTTCGGCCTG
TTGACGGACCGGATGCCTAATTTATTGCCGTTCCAGGCAAAACTGGTGCAGAAATGCGAC
AATCTCCAGCACTGGGATACCGAAAATGACGTGCTGTCGCTGCTGGATGTGGTGCGTAAC
GTGAAGCCGGATATCCTGATCGGGGTTTCCGGCCAGACCGGGCTCTTTACTGAAGAGATC
ATCCGCGAGATGCATAAGCATTGCCCTCGCCCGATCGTCATGCCGCTCTCTAATCCCACC
TCTCGCGTGGAGGCCACGCCACAGGACATTATCGCCTGGACGGAAGGCAACGCGCTGGTG
GCAACCGGCAGTCCATTCTCGCCGGTCATCTGGAAAGATAAGATTTATCCGATCGCACAA
TGTAATAACGCCTATATTTTCCCCGGCATCGGTCTGGGGGTTATCGCTTCCGGCGCATCG
CGCATTACTGATGAAATGCTGATGTCAGCCAGTGAGACGCTGGCGAAACATTCTCCGCTG
GTCAATAACGGCGAAGGATTAGTGTTACCAGCGCTTAAAGACATCCAGGTCGTGTCGCGC
GCTATCGCTTTTGCCGTAGGTAAAATGGCGCAGCAACAAGGCGTAGCGGTGAAAACCTCT
GCGGAAGCGCTGCAACAGGCGATTGACGATAACTTCTGGAAACCGGAATACCGCGACTAC
CGTCGGACTTCCATTTAA
>EECEBICD_01907 Alcohol dehydrogenase, propanol-preferring
ATGAAAGCTGCTGTTGTCACACAAGATCATCAGGTTGATGTTACTGAGAAAACGCTGCGT
CCCCTTCGCCACGGTGAAGCTTTGCTAAAAATGGAGTGCTGCGGCGTATGCCATACCGAT
CTACACGTGAAAAACGGCGATTTCGGTGACAAAACCGGCGTCATTCTGGGACACGAAGGC
ATTGGCGTCGTGGCGGAAGTCGGTCCTGGTGTCACCTCGCTGAAACCCGGCGATCGCGCG
AGTGTCGCGTGGTTTTATGAAGGATGCGGACACTGTGAGTATTGCAATACGGGCAATGAA
ACCTTATGCCGCAACGTGAAAAATGCCGGTTACACGGTAGATGGCGGCATGGCCGAAGAG
TGTATTGTCGTCGCGGATTATGCGGTGAAAGTACCGGAAGGTCTGGACTCTGCGGCGGCA
AGTAGTATTACCTGCGCGGGCGTTACCACGTATAAAGCGGTGAAGATTTCTCATATTAAA
CCTGGCCAGTGGATTGCCATCTACGGTCTGGGCGGTCTGGGTAATCTGGCCTTGCAGTAC
GCGAAAAACGTTTTCAATGCGAAAGTGATCGCCATTGATGTCAATGATGGACAACTGAAA
CTGGCCGAGGAAATGGGCGCAGATCTGACCATTAATTCACGTACGGAAGATGCGGCTAAA
ATTGTGCAGGAAAAAACCGGTGGCGCGCACGCAGCGGTGGTTACCGCGGTCGCTAAGGCG
GCGTTTAATTCCGCAGTAGATGCCGTTCGCGCCGGAGGTCGCGTGGTGGCAGTCGGGCTG
CCGCCAGAAGCCATGAACCTTGATATCCCTCGCCTGGTGCTGGATGGCATTCAGGTTGTC
GGTTCGTTGGTGGGGACACGCCAGGATCTGACCGAAGCCTTCCAGTTCGCGGCGGAAGGA
AAAGTCGTGCCAAAAGTCGCTTTACGTCCGCTTGAAGATATCAACGTTATTTTTAAAGAG
ATGGAACAAGGGCAAATTCGTGGCCGTATGGTCATCGATTTCCGTCGTTAA
>EECEBICD_01908 Formate dehydrogenase, nitrate-inducible, cytochrome b556(Fdn) subunit
ATGAGTAAGTCGAAAATGATTGTGCGCACGAAATTTGTCGACCGCGCCTGTCACTGGACG
GTGGTGATCTGCTTCTTCCTGGTAGCGCTGTCGGGAATTTCGTTTTTCTTCCCGACGCTA
CAATGGTTGACGCAAACTTTCGGCACGCCGCAGATGGGACGTATTTTGCACCCGTTCTTC
GGTATTGCCATCTTCATCGCGCTGATGTTTATGTTCGTGCGTTTCGTCCACCACAACATC
CCGGATAAAAAAGATATTCCGTGGCTGAAAAATATTGTCGAAGTATTGAAAGGCAATGAG
CATAAGGTGGCGGATGTCGGCAAATACAATGCCGGACAGAAGATGATGTTCTGGTCCATT
ATGAGCATGATTTTTGTGCTGCTGGTGACTGGCGTGATTATCTGGCGTCCGTATTTCGCG
CAGTATTTCCCCATGCAGGTGGTGCGTTATAGCCTGTTAATTCATGCGGCGGCGGGCATT
ATTTTAATGCACGCGATCCTCATCCATATGTATATGGCATTTTGGGTGAAAGGCTCGATT
AAAGGCATGATCGAAGGGAAGGTCAGCCGTCGCTGGGCGAAGAAACATCATCCACGCTGG
TATCGTGAAATCGAGAAGGCGGAAGCAAAAAAAGAGAGCGAAGAAGGTATTCAGTAA
>EECEBICD_01909 Formate dehydrogenase, nitrate-inducible, iron-sulfur subunit
ATGTCTATGGAAACGCAGGACATTATTAAACGGTCCGCAACAAACGCCATCACGCCGCCT
CCGCAGGCGCGTGATTACAAAGCCGAAGTCGCGAAACTGATCGACGTCTCCTCCTGTGTC
GGCTGTAAAGCCTGCCAGGTAGCCTGTTCTGAATGGAACGATATTCGCGATAACGTTGGT

CATTGTGTCGGGGTGTACGATAACCCTGCCGATCTCAGCGCCAAGTCCTGGACGGTGATG
CGCTTTACCGAAACCGAGCAGAACGGCAAGCTGGAGTGGCTGATTCGTAAAGATGGTTGT
ATGCACTGTGAAGATCCAGGCTGTCTTAAAGCCTGTCCATCTGCCGGGGCTATCATCCAG
TACGCGAACGGGATTGTTGACTTTCAATCTGAACACTGTATCGGCTGTGGTTACTGTATC
GCCGGGTGTCCGTTTAATATTCCGCGCCTCAACAAAGAGGACAACCGCGTTTATAAATGT
ACGCTCTGCGTCGATCGCGTCAGCGTGGGCCAGGAACCGGCCTGCGTCAAAACTTGTCCG
ACCGGGGCTATTCACTTCGGCACCAAAAAAGAGATGCTGGAACTGGGCGAGCAACGCGTG
GAGAAGTTAAAAGCGCGTGGCTTTGAACATGCCGGTATCTACAACCCGCAGGGCGTCGGC
GGCACGCACGTCATGTATGTGCTGCACCATGCCAATCAGCCGGAGTTGTACCACGGCTTG
CCGAAAGATCCGCAGATCGATACCTCAATCAATCTGTGGAAAGGGGCGCTAAAACCGCTG
GCCGCAGCGGGCTTTATCGCCACTTTCGCCGGTCTGATTTACCACTACATCGGTATTGGC
CCGAATAAAGAAACGGACGACGATGAGGAGGATCATCATGAGTAA
>EECEBICD_01910 Formate dehydrogenase, nitrate-inducible, major subunit
ATGACCAACCACTGGGTGGATATTAAAAACGCCAACGTCGTGATGGTGATGGGCGGCAAT
GCCGCAGAAGCGCATCCGGTGGGTTTCCGCTGGGCGATGGAAGCGAAAAATAATAACGAT
GCGACGCTGATTGTCGTTGATCCACGCTTTACGCGTACGGCGTCGGTGGCGGATATCTAT
GCGCCAATACGTTCCGGTACAGACATTACCTTTCTGTCCGGCGTTTTGCTGTACCTGATC
GAAAATAACAAAATCAACGCTGAATACGTTAAGCATTACACCAACGCCAGCCTGCTGGTG
CGGGATGATTTTGCCTTTGATGACGGTCTTTTCAGCGGCTATGACGCCCAAAAGCGTCAG
TACGATAAATCCTCCTGGAACTACCAGTTCGATGAAAACGGCTATGCCAAACGTGATGAA
ACGTTGACGCACCCACGCTGTGTGTGGAATCTGCTGAAGCAGCACGTTTCGCGCTACACG
CCGGACGTCGTGGAAAACATCTGCGGTACGCCGAAAGCGGATTTCCTGAAGGTGTGTGAG
GTGTTGGCCTCTACCAGCGCGCCGGATCGCACTACCACCTTCCTGTATGCGCTGGGCTGG
ACGCAACATACCGTCGGGGCGCAGAACATCCGTACTATGGCGATGATCCAGTTGCTGCTC
GGCAATATGGGGATGGCTGGCGGCGGTGTGAACGCCTTGCGCGGCCACTCTAATATTCAG
GGTTTAACGGATTTAGGACTGCTCTCCACCAGCCTGCCGGGTTACCTGACGCTACCGTCA
GAAAAACAGGCCGATCTGCAAACTTATCTGGCAGCGAATACCCCCAAAGCGACGCTGGCC
GATCAGGTCAACTACTGGGGTAATTATCCTAAGTTCTTCGTCAGCCTGATGAAATCTTTC
TATGGCGATGCGGCGCAGAAAGAGAATGACTGGGGCTTTGCCTGGCTGCCTAAGTGGGAT
CAGTCCTATGACGTGATCAAATATTTCAACATGATGGATAGCGGGAAAGTCACCGGCTAT
TTCTGTCAGGGCTTTAACCCGGTGGCGTCCTTCCCGGACAAAAACAAAGTGGTGCAAAGC
TTAAGCAAACTGAAGTATCTGGTCGTTATCGATCCGCTGGTGACTGAAACGTCAACGTTC
TGGCAGAACCACGGCGAATCTAATGATGTCGATCCGACAACGATTCAGACCGAAGTCTTC
CGTCTGCCGTCTACCTGTTTTGCGGAAGAGGATGGATCTATCGCTAACTCCGGTCGCTGG
TTACAGTGGCACTGGAAAGGTCAGGACGCGCCGGGCGAAGCGCGTAACGACGGCGAGATT
CTGGCCGGTATTTACCATCGTCTGCGTGAAATGTATCGCGCAGAAGGCGGTAAAGGCGCC
GAACCACTATTGAAGATGAGCTGGAATTACAAGCAGCCGGATGAACCGCATTCGGAAGAA
GTGGCGAAAGAGAACAACGGCTATGCGCTGGAAGATCTCTATGACGCCAACGGTACACTG
CTTGCCAGGAAAGGCCAGTTGTTGAGTAGTTTTGCATTACTGCGCGATGACGGTACGACA
TCCTCCTCCTGCTGGATTTATACCGGTAGCTGGACGGAGCAGGGCAACCAGATGTCCAGA
CGTGATAACGCCGACCCGTCAGGGCTTGGCAATACACTGGGTTGGGCGTGGGCGTGGCCG
CTAAACCGTCGCGTATTGTATAACCGCGCTTCGGCGGATCCGCAGGGTAAACCGTGGGAT
CCAAAACGGATGCTGATCCAGTGGAACGGCGCGAAGTGGACAGGGAACGATATTCCGGAC
TTTAACAACGCGGCGCCGGGGAGCGGGACCAATCCGTTTATCATGCAGCCGGAAGGGCTG
GGACGTCTGTTTGCTATTGATAAAATGGCGGAAGGGCCGTTCCCTGAGCATTACGAGCCG
ATGGAAACGCCGCTGGGCACTAACCCGCTGCACCCGAATGTGGTCTCGAACCCGGCTGCG
CGTCTGTATGAAGAAGATGCGCTGCGCATGGGTAAGAAGGAGCAGTTTCCGTACGTGGGC
ACGACTTACCGCCTCACAGAGCATTTCCATACCTGGACCAAACATGCGCTACTTAACGCT
ATCGCTCAGCCGGAACAGTTTGTGGAGATCAGTGAGACGCTGGCGGCAGCGAAAGGTATT
GCTAACGGCGACTATGTGAAGGTGAGCAGTAAACGCGGGTTTATTCGCGCGGTCGCGGTG
GTAACTCGTCGTCTTCGCACCCTGCATGTCAACGGTCAGCAGGTTGAAACGGTGGGGATC
CCGATTCACTGGGGCTTTGAGGGTGTCGCGCGCAAAGGCTATATCGCCAATACTCTGACG
CCAAATGTCGGTGATGCCAACTCGCAAACGCCGGAATACAAAGCGTTTTTAGTTAACATC
GAGAAGGCGTAA

>EECEBICD_01911 Formate dehydrogenase, nitrate-inducible, major subunit
ATGGACGTCAGCCGCAGACAATTTTTTAAAATCTGCGCGGGCGGTATGGCGGGAACAACG
GTGGCCGCACTGGGTTTTACCCCTAAAATGGCCCTGGCTCAGGCGCGAAACTATAAGCTG
TTGCGCGCAAAAGAGATCCGTAACTCCTGCACATACTGTTCCGTAGGTTGCGGGCTATTA
ATGTATAGCCTGGGAGATGGGGCAAAAAACGCGAAAGAAGCGATTTACCATATTGAAGGC
GACCCGGATCATCCGGTAAGCCGTGGCGCGCTGTGCCCGAAAGGGGCCGGTTTGCTGGAC
TATGTTCACAGTGAAGATCGCCTACGCTATCCGGAATACCGCGCGCCGGGTTCCGACAAG
TGGCAGCGCATTAGCTGGGATGACGCTTTCACTCGTATTGCTAAATTAATGAAAGCCGAC
CGCGACGCCAACTTTATTGAAAAGAATGAGCAGGGCGTCACGGTTAACCGCTGGCTCTCT
ACCGGGATGCTGTGCGCCTCGGCGGCCAGCAATGAAACGGGTATGCTGACGCAGAAGTTT
GCGCGTTCTCTCGGTATGCTGGCGGTAGATAACCAGGCGCGCGTCTGA
>EECEBICD_01912 Methyl viologen resistance protein YddG
ATGACATCACAAAAAGCAACGCTAATAGGGCTTGTTGCCATCGTTCTGTGGAGCACGATG
GTTGGGTTAATTCGCGGCGTCAGCGAAGGTCTCGGACCGGTAGGCGGCGCAGCCATGATC
TATTCATTAAGCGGTCTGCTGCTGATTTTTACCGTCGGATTACCTGATATCCGCCGTTTC
CCGGGGCGATACCTCATCGCAGGCAGCGTACTGTTTGTCAGTTATGAAATATGCCTGGCG
CTTTCTCTCGGTTATGCCGCCACTCGCCATCAGGCTATCGAAGTCGGCATGGTTAACTAT
TTATGGCCCAGCCTGACCATTCTGTTTGCCATTCTTTTTAATGGGCAAAAAACTAACTGG
CTTATTGTTCCTGGATTGCTTATTGCACTTACTGGCGTTTGCTGGGTTCTGGGGGGCGAG
AATGGTCTTAATCCAGGAGAAATTATCAGCAATGTCGCCACCAGTCCGCTAAGTTATTTA
CTGGCATTTCTTGGGGCGTTTATATGGGCGACGTACTGCACCGTCACAAATAAATATGCG
CGTGGTTTTAACGGTATCACAGTCTTTGTGTTACTCACCGCCGTGGCGCTGTGGTTCCAC
TATTTTCTTACGCCGCAACCTGCCATGATATTTAGCCTACCGGTGATTGCTAAACTTTTT
ACCGCGGCATTAACGTTAGGGTTTGCCTACGCCGCATGGAATGTGGGCATACTTCATGGC
AATGTGACTATCATGGCCGTAGGGTCTTATTTTACCCCCGTCATGTCCTCTGCTCTGGCC
GCGCTGTTACTCAGCTCACCACTGTCATTCTCCTTCTGGCAAGGCGCAGTCATGGTTTGC
GTAGGTTCGCTACTTTGTTGGCTGGCAACGCGGCGGCGCTAA
>EECEBICD_01913 Outer membrane porin protein OmpD
ATGAAACTTAAGTTAGTGGCAGTGGCAGTGACTTCCCTGTTGGCAGCAGGCGTTGTAAAT
GCAGCCGAGGTATATAACAAAGACGGCAATAAACTGGATCTGTACGGTAAAGTTCACGCT
CAGCATTATTTCTCTGATGATAACGGAAGTGATGGTGACAAAACTTACGCGCGTCTGGGC
TTTAAAGGCGAAACTCAGATCAACGATCAGTTAACCGGTTTTGGCCAGTGGGAATATGAA
TTCAAAGGCAACCGTACTGAAAGCCAGGGCGCTGACAAAGACAAAACCCGTCTGGCGTTT
GCTGGTCTGAAATTCGCTGACTACGGTTCTTTCGACTACGGTCGTAACTACGGCGTTGCT
TACGACATCGGCGCATGGACCGACGTTCTGCCAGAGTTCGGTGGCGATACCTGGACTCAG
ACTGACGTCTTCATGACTGGCCGTACTACTGGTGTTGCCACCTACCGTAACACTGACTTC
TTTGGCCTGGTTGAAGGTCTGAACTTTGCCGCGCAGTACCAGGGCAAAAACGACCGTGAC
GGCGCGTACGAGTCTAACGGCGACGGTTTCGGTCTGTCCGCAACGTATGAGTACGAAGGT
TTTGGCGTAGGTGCGGCCTATGCGAAGTCTGACCGTACTAACAACCAGGTGAAAGCAGCG
AGCAACCTGAATGCTGCGGGTAAAAACGCTGAAGTCTGGGCTGCTGGTCTGAAATATGAT
GCGAACAACATCTACCTGGCGACCACCTACTCCGAAACGCTGAACATGACCACCTTTGGT
GAAGATTCAGCGGGTGATGCGTTTATCGCGAATAAAACCCAGAACTTTGAAGCAGTTGCT
CAGTATCAGTTCGACTTCGGTCTGCGTCCGTCCATCGCTTACCTGAAATCCAAAGGTAAA
AACCTGGGTACTTACGGCGACCAGGATCTGGTTGAGTACATCGATGTCGGTGCTACCTAC
TACTTCAACAAAAACATGTCCACCTTCGTTGATTACAAAATCAACCTGCTGGACGACAGC
GACTTCACCAAAGCGGCTAAAGTGTCTACCGACAACATCGTTGCTGTTGGTCTGAACTAC
CAGTTCTAA
>EECEBICD_01914 hypothetical protein
ATGGTCGGGTGGAATAGCCATGTATCTGGAAGCCTGCGGGCAACCACAGCTAGCCCCCTT
TCCCCGGAACCGCCATACTTTTCATCCTCTTCGCGCGGCGAACGCTACTTCTGTTCCGGC
TGCGGGTGTCCGCTGTGGTTTCGGCTTACCGATACCGATCGCTATTTTATTCCCTGGACC
TTACTGGAACTGAACGAAGTTGATCGCCGTCGGCTGATTCTGGCTGCGGAAATTTATACC
GAAACCCAGCCTGCCTTCTGGCGACTGACGGGGCAATACGCACGCCTCAGCGGTAAAGAG
GTTGAGGAAATGGACTCCCCTGCCAGCTAG

>EECEBICD_01915 Methyl viologen resistance protein SmvA
ATGTTTCGTCAGTGGTTAACGTTAGTCATTATTGTGCTGGTCTATATTCCTGTCGCCATT
GATGCCACGGTCTTGCATGTCGCCGCCCCGACACTGAGTATGACACTGGGGGCCAGCGGC
AACGAGCTGCTGTGGATCATTGATATTTATTCTTTGGTCATGGCTGGCATGGTGTTGCCG
ATGGGCGCGCTTGGCGATCGTATCGGTTTTAAACGCCTGCTGATGCTGGGCGGGACGCTC
TTTGGCCTGGCGTCATTGGCGGCAGCGTTTTCGCATACCGCCAGTTGGCTTATCGCCACC
AGGGTATTACTGGCTATTGGCGCGGCGATGATTGTACCGGCGACGCTGGCCGGAATACGC
GCTACCTTTTGTGAAGAGAAGCATCGCAACATGGCGCTGGGCGTCTGGGCAGCGGTAGGT
TCGGGCGGAGCGGCGTTTGGGCCGCTCATCGGCGGCATATTATTAGAGCATTTTTACTGG
GGATCGGTTTTCCTGATCAACGTGCCGATTGTGCTGGTCGTCATGGGCTTAACCGCCCGC
TATGTTCCTCGCCAGGCGGGCCGTCGCGATCAACCGCTCAATCTTGGCCATGCGGTGATG
CTGATTATTGCCATTTTGCTGTTGGTCTATAGCGCTAAAACCGCGCTGAAAGGGAATTTG
TCGCTGTGGGCCATCTCGCTTACCCTGCTTACCGGCACGCTGCTCCTGGGACTCTTTCTC
CGCACACAGCTTGCGACATCGCGTCCGATGATTGATATGCGACTATTTACCCATCGCATT
ATCCTGAGCGGCGTCGTGATGGCGATGACCGCGATGATCACTCTGGTGGGTTTTGAGCTG
CTGATGGCGCAAGAGCTACAGTTTGTTCACGGGCTATCGCCTTATGAGGCCGGGGTATTT
ATGCTGCCGGTGATGGTCGCCAGTGGATTCAGCGGGCCGATTGCGGGCGCGCTGGTCTCG
CGTCTGGGGTTACGGCTGGTCGCGACGGGCGGCATGGCGTTAAGCGCGCTGAGTTTTTAT
GGCCTGGCGATGACGGATTTCAGCACCCAACAATGGCAGGCCTGGGGGCTGATGGCGCTG
CTGGGATTTAGCGCCGCCAGCGCATTGCTGGCTTCCACGTCGGCAATTATGGCCGCTGCG
CCGGCAGAAAAAGCGGCGGCGGCCGGCGCGATAGAAACGATGGCTTATGAACTGGGCGCG
GGACTGGGCATTGCCATTTTCGGTCTGTTGTTAAGCCGTAGCTTCTCCGCGTCTATCCGT
CTGCCTGCCGGGCTTGAGGCGCAAGAGATTGCCAGAGCGTCATCTTCAATGGGAGAAGCC
GTGCAGTTGGCGAATAGCCTACCGCCCACGCAGGGGCAGGCAATACTGGACGCCGCCAGA
CATGCCTTTATCTGGTCGCATAGCGTGGCGTTAAGCAGCGCCGGGAGTATGCTTCTTTTG
CTGGCGGTAGGGATGTGGTTCAGCCTGGCAAAAGCCCAACGCCGATAA
>EECEBICD_01916 hypothetical protein
ATGAGTTACCTTAACCGGGATGAACGCCGCGAAGTGATCCTGCAAGCGGCGATGCGCGTG
GCGCTGGCAGACGGGTTTGCCGCCATGACGGTGCGACGAATTGCATCAGAAGCCGATGTC
GCCGCCGGCCAGGTGCATCACCACTTCAGCTCGGCGGGCGAGCTCAAAGCGCTGGCCTTT
GTCCATTTGATCCGCACCCTGCTTGATGCCGGGCAAGTTCCGCCGCCCGCCACCTGGCGT
GCACGGCTTCACGCTATGTTGGGTAGCGAAGACGGCGGCTTTGAGCCTTACATAAAATTG
TGGCGAGAGGCGCAAATTCTGGCCGACCGGGATCCGCACATCAGAGATGCTTATCTCCTC
ACGATGCAGATGTGGCATGAGGAAACGGTCACAATTATCGAGCAGGGCAAACAGGCAGGA
GAATTTACGTTTACTGCTAACGCCACGGATATCGCCTGGCGGCTTATCGCACTGGTGTGC
GGGCTGGACGGTATGTATGTACTGGGGATTCCGGAAATGGCCGATCCCGCGTTTAAATTC
CATCTGGATCGTATGATTACGCTTGAACTCTTTGCGTAA
>EECEBICD_01917 Nitrate/nitrite transporter NarU
ATGACACGACAAAACGAGAATTATAACCGTTATCTCTTGAGTGACTGGCGACCCGAAAAT
CCAGCCTTCTGGGAGAATAAAGGGAAAGGCATCGCCAGAAGAAACTTATTGATTTCAGTA
AGTTGTTTGCTCCTTGCATTTTGCGTCTGGATGTTGTTTAGCGCTGTTGCCGTCAACCTC
AATAAAATTGGATTTAATTTTACCACCGACCAACTCTTTTTATTAACCGCACTACCTTCT
CTTTCCGGCGCAATATTACGCGTTCCCTACTCCTTTATGGTACCACTGTTTGGTGGCCGT
AAATGGACCGTATTAAGTACCGTAATATTGATTATTCCCTGCACCTGGCTGGGATTCGCC
GTGCAAAATCCTGCCACCCCTTTCGGGGTATTTATGCTCATCGCTCTGTTGTGCGGTTTT
GCGGGGGCAAACTTCGCGTCCAGTATGGGCAACATCAGTTTTTTCTTTCCAAAAGCCAGA
CAAGGTAGCGCGTTGGGTATTAACGGCGGACTGGGAAATCTTGGCGTCAGTGTGATGCAG
TTGATTGCGCCGCTGGTTATTTTTCTGCCGATATTTACTTTTTTAGGCGTCCGGGGCGTG
CCGCAACCTGATGGCTCCCTGCTCGCCCTCACTAACGCAGCCTGGATCTGGGTGCCGTTA
CTGGCTGTCGCGACGCTTGCCGCCTGGTTTGGTATGAACGATATCGGCAGTTCAAAAGCG
TCTGTCGCCTCGCAATTGCCGGTGCTAAAACGTCTGCACCTGTGGCTGCTTAGCCTGTTG
TACCTGGCGACATTCGGGTCATTCATCGGCTTTTCCGCAGGTTTCGCCATGCTCGCCAAA
ACGCAATTTCCCGACGTAAACATTCTGCAACTGGCGTTCTTTGGCCCCTTTATCGGCGCG
CTGGCGCGATCGGCAGGCGGCGTGATCTCCGATAAATTCGGCGGCGTTCGCGTCACGCTC

ATTAATTTTATCTTTATGGCGCTGTTTACCGCCTTGCTATTCCTCACACTGCCAGGCTCC
GGCGCAGGCAGCTTTAGCGCGTTCTATCTTGTTTTTATGGGCCTGTTTTTGACAGCGGGT
CTCGGCAGCGGTTCTACCTTCCAGATGATTGCGGTGATCTTCCGTCAGATCACGCTCTAC
AACGTCAAACTGCGCGGCGGGAGCGATGAGCAGGCGCAGCGCGAAGCGGTGACGGATACC
GCCGCCGCGCTCGGTTTTATCTCCGCTATTGGCGCTGTGGGCGGCTTCTTTATTCCAAAA
GCGTTTGGCACCTCTTTGGCCTTGACCGGCTCGCCGGTGGGCGCCATGAAAATCTTTTTG
CTGTTTTACATCGCTTGCGTCCTGCTGACCTGGCTGGTCTACGGGCGTCGTAAGCCTAAA
CAACAATAA
>EECEBICD_01918 Respiratory nitrate reductase 2 alpha chain
ATGAGTAAACTGTTGGATCGCTTTCGTTACTTTAAACAAAAAGGCGATACCTTTGCCGAC
GGCCATGCTCAGGTGATGCATACCAACCGCGACTGGGAAGACAGTTATCGCCAGCGCTGG
CAGTTTGATAAGATCGTGCGCTCAACCCACGGCGTCAACTGCACGGGCTCCTGTAGTTGG
AAAATTTATGTCAAAAATGGTCTGGTGACGTGGGAAACCCAGCAAACCGACTACCCGCGC
ACGCGTCCCGATTTACCGAACCATGAACCGCGCGGCTGCCCGCGCGGCGCCAGCTACTCA
TGGTATCTCTATAGCGCCAATCGTTTGAAATACCCTCTGGTGCGTAAGCGGCTGATAGAA
CTGTGGCGCGAGGCCCTTTCCCGGCACAGCGATCCGGTACTGGCATGGGAATCTATAATG
AACGACCCGCAAAAATGCCAAAGTTACAAACAGGTGCGCGGACATGGCGGCTTTATTCGT
TCGAACTGGAAAGAGCTGAATCAGCTCATTGCCGCTGCTAACGTCTGGACAATCAAAACC
TATGGTCCGGATCGTGTGGCGGGGTTTTCACCTATTCCGGCAATGTCGATGGTTTCTTAC
GCCGCCGGAACACGTTATCTGTCATTGCTTGGCGGTACTTGTCTGAGCTTCTATGACTGG
TATTGCGATCTTCCACCCGCGTCGCCAATGACCTGGGGCGAGCAGACTGACGTGCCGGAG
TCCGCCGACTGGTATAACTCAGCCTACATTATTGCCTGGGGCTCTAACGTGCCGCAGACG
CGTACCCCGGACGCCCACTTCTTTACCGAAGTGCGCTACAAAGGCACCAAGACGATCGCC
ATCACCCCGGACTATTCCGAAGTCGCTAAACTGTGCGATCAGTGGCTGGCGCCAAAGCAA
GGTACCGACAGCGCGCTGGCGATGGCCATGGGTCACGTTATTTTAAAAGAGTTTCATCTC
GATAATCCCAGCGACTATTTTCTTAACTATTGCCGCCGTTATACCGACATGCCAATGCTG
GTTCTGTTGGATGAGCGCGCAGACGGCAGTTATGTGCCGGGGCGTATGATGCGCGCGTCG
GATCTGGTGGATGGACTGGGAGAAGCCAATAATCCGGAATGGAAAACCGTCGCGCTCAAC
AGTACGGGCGAACTGGTCGCGCCCAATGGTTCTATCGGTTTTCGCTGGGGCGAAAAGGGA
AAATGGAACCTGGAACCGGTGGCGGCGGGCGTCGAAACTGAACTGTCGCTCTCTTTACTC
GGTCAGCATGATGACGTGGCGGGTGTCGCCTTCCCCTATTTTGGCGGTAACGAAAACCCG
CATTTTCGCAGCGTGCGGCAGGAACCGGTGCTGGTACGTCAGTTACCGGTGAAACGCCTG
GCTCTGGCGGACGGTAGCGAGCGGATGGTCGTCAGCGTTTACGATCTGGTGCTGGCTAAT
TATGGGCTGGATCGTGGTCTGGATGACGGCCATAGCGCCAATAATTATAACGACGTAAAA
GCCTACACGCCGGCCTGGGGCGAACAGATTACCGGCGTGCCGCGTCGACATATCGAAACT
ATTGCACGTGAATTTGCCGAGACCGCCCATAAAACTCACGGTCGTTCGATGATCATCCTC
GGCGCGGGAGTGAATCACTGGTATCACATGGATATGAATTACCGTGGGATGATTAACATG
CTGGTGTTCTGCGGCTGTGTTGGACAAACCGGCGGCGGCTGGGCGCACTATGTCGGCCAG
GAGAAGCTGCGGCCGCAAACCGGCTGGCTGCCGCTGGCCTTCGCGCTGGACTGGAATCGC
CCGCCACGTCAGATGAACAGTACCTCGTTTTTCTACAACCATGCCAGCCAGTGGCGCTAT
GAAAAACTGACTGCGCAAGAGTTGCTGTCGCCGCTGGCCGATCCGGCTAAATTTTCCGGA
CACCTGATCGATTTCAACGTGCGCGCGGAACGTATGGGCTGGCTACCCTCGGCGCCGCAG
CTTAATCTTAACCCGCTCAGCGTGAAAGCGAGCGCCGATAAAGCCGGGCTGTCTGCGGCG
GATTATACCGTGCAGGCGTTGAAATCAGGCGCTATCCGTTTTGCCTGCGAACAGCCTGAC
AGTGGTCATAATCACCCGCGAAATTTATTTGTCTGGCGCTCTAACCTGCTCGGCTCCTCC
GGGAAAGGTCATGAGTACATGCTCAAATATCTGCTGGGCACCGACAGCGGTATTCAGGGC
GAAGCGCTGGGTTCCAGCGAAGGAATTAAGCCTGAAGAGGTGGAATGGCAGTCCGCCGCG
ATTGAGGGCAAGCTCGATCTGTTGGTCACGCTTGATTTCCGTATGTCCAGTACCTGTCTG
TTTTCCGATATCGTTTTGCCGACCGCCACCTGGTATGAAAAAGACGATATGAATACCTCG
GATATGCATCCGTTTATCCACCCCCTTTCCGCCGCGGTCGATCCTGCCTGGGAATCCAAA
AGCGACTGGGAGATCTACAAAGGTATCGCCAGCGTCTTTTCTGAAGTGTGTGTCGGACAT
CTCGGACAGGAAACCGATGTGGTGTTACACCCGCTCCAGCATGATTCCCCGGCGGAACTG
GCGCAGCCATTTGATATTCTCGACTGGCGTAAAGGCGAGTGCGAACTTATTCCGGGCAAA
ACCGCCCCCAATATCGTCGTGGTTGAACGCGATTATCCGGCCACCTATGAACGTTTTACC

TCGCTGGGTCCCCTGCTGGATAAATTAGGCAACGGCGGTAAGGGTATTGCCTGGAATACG
CAAGATGAAGTCGATTTCCTCGGTAAGCTCAATTACACCAAACACGATGGCCCGGCGAAA
GGACGTCCGCGGATCGACACCGCGCTGGATGCTTCGGAAGTGATTCTTGCCTTAGCGCCG
GAAACTAACGGCCAGGTGGCGGTGAAAGCCTGGCAGGCGTTGGGCGAGATGACCGGGCGC
GAGCACACCCATCTGGCCATCAATAAAGAAGATGAGAAAATTCGCTTTCGTGATATCCAG
GCGCAACCGCGCAAAATTATCTCCAGCCCGACCTGGTCAGGGCTGGAAAGCGAGCATGTC
TCTTATAACGCCGGCTATACCAACGTACATGAACTGATCCCGTGGCGCACGTTGTCGGGG
CGTCAACAACTCTATCAGGATCATGCGTGGATGCGCGCTTTTGGCGAAAGCCTGGTGGCA
TATCGTCCGCCGATTGATACCCGCAGCGTCAGCGAAATGCGAGAAATTCCGCCCAACGGT
TTCCCGGAAAAAGCGCTTAACTTCCTGACGCCGCACCAGAAATGGGGAATTCACTCCACC
TACAGCGAAAACCTGCTGATGCTGACCTTGTCGCGCGGCGGGCCGATTGTCTGGATCAGC
GAAGCCGATGCCCGTGAATTGGGTATTGAAGATAACGACTGGATTGAAGCCTTCAACGCT
AATGGCGCCCTTACCGCTCGTGCGGTGGTCAGCCAGCGCGTCCCGCCGGGTATGACCATG
ATGTACCACGCCCAGGAGCGGATTATGAACATTCCGGGTTCGGAAGTGACCGGGATGCGC
GGCGGGATCCATAACTCCGTGACCCGGGTCTGTCCAAAACCGACGCATATGATCGGCGGC
TATGCGCAACTGGCTTACGGCTTCAACTATTACGGAACCGTGGGTTCGAATCGCGATGAA
TTCATCATGATCAGGAAGATGAAAAACATTAACTGGCTGGATGATGAGGGCCGGGATCAG
GTACAGGAGGCGAAAAAATGA
>EECEBICD_01919 Respiratory nitrate reductase 2 beta chain
ATGAAAATACGCTCACAGGTTGGGATGGTACTGAATCTTGATAAATGTATTGGCTGTCAC
ACCTGCTCCGTCACCTGTAAAAACGTCTGGACCGGACGTGAAGGGATGGAATACGCATGG
TTCAATAACGTCGAAACTAAACCGGGTATCGGCTATCCCAAAAACTGGGAAGACCAGCAG
GAGTGGCAAGGCGGTTGGGTTCGTGATGTTAATGGCAAGATAAGGCCCCGTCTGGGCGGC
AAAATGGGGGTAATTAGCAAAATTTTCGCCAACCCGGTGATTCCGCAAATCGATGATTAC
TATGAACCCTTTACCTTCGATTATCAGCATTTACATAATGCGCCGGAGAGTAAGCATCAG
CCCACTGCCCGTCCTCGTTCACTGATCGACGGAAAACGGATGGACAAGGTGATCTGGGGG
CCAAACTGGGAAGAGCTGCTCGGCGGCGAGTTTGAAAAACGCGCCCGCGACCGCAACTTC
GACAACATACAAAAGGAGATGTACGGTCAGTTCGAAAACACCTTCATGATGTACCTGCCG
CGCCTGTGCGAACACTGCCTCAACCCCAGTTGTGTCGCCACCTGCCCAAGTGGCGCTATT
TATAAGCGTGAAGAAGACGGCATCGTGCTAATCGACCAGGATAAATGCCGGGGCTGGCGT
TTGTGTATCAGCGGCTGCCCGTACAAAAAAATCTACTTTAACTGGAAGAGCGGCAAGTCT
GAGAAATGCATATTTTGCTATCCGCGTATTGAATCCGGCCAGCCGACCGTTTGCTCGGAA
ACCTGCGTCGGGCGCATCCGTTATCTTGGCGTTCTGCTCTACGACGCCGACAGGATTGAA
GAAGCGGCCAGTACCGAACACGAGACGGATTTGTATGAACGTCAGTGTGATGTGTTTCTC
AATCCGCACGATCCGGCGGTCATCGAAGAGGCGCTTAAACAGGGCATCCCGCAAAACGTC
ATCGACGCTGCCCAACGGTCGCCGGTGTACAAAATGGCGATGGACTGGAAGCTGGCGCTT
CCGCTGCACCCGGAATACCGTACCCTGCCGATGGTCTGGTACGTACCGCCGCTGTCGCCC
ATCCAGTCATACGCCGACGCGGGCGGGTTACCGCACAATGGCAATATTCTGCCGGCGGTG
GAGACGCTGCGTATTCCGGTCCAGTACCTGGCCAATATGCTCAGCGCGGGCGATACCGGT
CCGGTCATTCGGGCTCTAAAGCGGATGATGGCGATGCGCCACTACATGCGCTCGCAGACG
GTTGAAGGCGTGACGGATACCCGCGCGATTGACGAAGTCGGTCTGAGCGTACAACAGGTT
GAAGAGATGTATCGCTACCTGGCGATTGCCAATTATGAAGATCGTTTTGTCATTCCCACC
AGCCACCGGGAAATGGCGCGCGATGCCTTTCCGGAGCGCAACGGCTGCGGCTTTACTTTT
GGCGACGGTTGCCACGGGTCGGATACCAAATTCAACCTGTTCAACAGTAGCCGTATTGAT
GCCATCAATATCACCGAAGTACGTGATAAAGCGGAGGGGGAATAA
>EECEBICD_01920 putative nitrate reductase molybdenum cofactor assembly chaperone NarW
ATGCAAATCCTCAAAGCGATTGGCCTGTTGATGGAGTATCCGGACGACGAGTTGTGGGAA
TGTCGGGATGAAGCGCTTGCCCTTATCCAACACGATGCGCCAATGCTGGCCGACTTCACT
CGCGAGCTGCTTTATGCGCCGTTACTGGATAAACAAGCAGAATGGTGTGAAGTGTTTGAC
CGCGGGCGCGCCACTTCATTGCTGTTATTCGAGCATGTTCATGCGGAGTCCCGCGATCGC
GGCCAGGCGATGGTCGATTTGTTAAGCCAGTATGAAACCGTGGGCCTGCAGCTCAACTGC
CGCGAACTGCCGGATCACCTGCCGCTCTATCTGGAATACTTAAGCGTGTTGCCGGAAGCG
GAAGCGCGCGAGGGATTACAGAATATTGCCCCTATTCTGGCGCTACTGGGCGGTCGTCTG

AAACAGCGAGGAGCGCCGTGGTATCAACTGTTTGACGCCCTGCTGACGCTGGCGGGCAGT
ACGCTTACAAGTGACAGTGTCACCAAACAGATTGTTCAGGAATCGCGGGATGATACCCGT
CAGGCACTGGATGCCGTCTGGGAAGAAGAACAGGTGAAGTTTATCGAAGATAACGCCACG
ACCTGTGACAGTTCACCGTTACACCATTATCAACGACGCTTTAGCCAGGACGCGGCGCCG
CAGTACGTCGACGTCAGCGCGGGAGGCCCGAAATGA
>EECEBICD_01921 Respiratory nitrate reductase 2 gamma chain
ATGATACAGTACCTGAACGTCTTTTTTTACGATATCTACCCCTATATTTGCGCCACGGTC
TTCTTCCTCGGCAGTTGGCTGCGCTATGACTACGGACAATATACCTGGCGCGCGTCGTCC
AGCCAGATGCTGGATAAGCGTGGGATGGTGATATGGTCGAATCTGTTCCATATTGGCATT
CTGGGGATCTTTTTTGGCCATCTGTTTGGAATGCTGACGCCACACTGGATGTATGCCTGG
TTCCTGCCGATTGCGGTAAAACAGCAGATGGCGATGATCCTCGGCGGCGTGTGCGGCGTA
TTAACGTTGATCGGCGGCGCGGGATTACTGTGGCGTCGTCTGACCAATCAGCGTGTGCGG
GCGACATCCACTACACCAGATATTATCATCATGAGCATTTTGCTGATTCAGTGCCTGCTT
GGCCTGAGCACTATTCCGTTTTCGGCGCAATATCCGGATGGCAGCGAAATGATGAAGTTG
GTGGGCTGGGCGCAAAGTATTGTCACCTTCCGCGGCGGGTCTTCGGAAATGCTGAGCGGT
GTGGCGTTTGTTTTCCGCGTTCATCTGGTGCTGGGAATGACTATTTTCCTGCTCTTCCCG
TTCACCCGTCTGGTTCATGTCTGGAGCGCGCCGTTTGAGTATTTCACCCGTCGCTACCAG
ATTGTTCGTACCCGCCGCTAA
>EECEBICD_01922 putative isomerase YddE
ATGAAACCGCAGATTTACCATGTTGACGCCTTCACCAGCGAACCCTTTCGCGGCAACTCT
GCTGGCGTTGTCTTACATGCGGATACGCTAAGCGACGCGCAGATGCAGCTGATTGCCCGT
GAACTTCGTCACTCGGAAACCGCCTTTTTGCTAAAAAGCGAAGAGAGTGATGTGCGCATA
CGCTACTTTACACCGACAGTAGAAGTCCCCATCTGTGGTCATGCCACCGTGGCCGCGCAT
TATGTTCGGGCAACGGTACTGGGGTTGGGAAACACCACCGTCTGGCAAACCTCGTTAGCG
GGCCGTCATCGCGTTGAAATTCATGCCGAACACAACGACTATCGCATCACGCTGGAACAA
GGTCAGCCCTCTTTTGAACCACCGTTAGTCGGAGAGATTCGGGCTGCCATTATCACCGCA
CTGAACCTGACTGAAGATGATATCGTGCCGGGAGCGCCAATTCAGGTGGCTTCAACAGGA
CATGCAAAAGTGATGATCCTGCTCAAACCAGACGTTGATATTGATGCCCTTTCTCCGAAT
CTGGCAGCCCTGACGGCAATCAGCCAGCAAATTGGCTGTAACGGCTTTTTTCCTTTCCAG
ATTCGTCCGGGAAAAAACGAAACCGATGGGCGAATGTTTTCCCCGGCGATAGGAATTGTG
GAAGATCCGGTAACGGGAAACGCCAATGGCCCGATGGGGGCATGGCTCGTCCATCATGGC
CTGATGGCGCATGACGGTAAAACGCTGCAGATTCAAGGCCATCAGGGGCGCGCTTTAGGC
AAAGAGGGTACTGTTGATGTTACCGTCACCATTCGCGATAACCAGCCGGAAAACGTCACC
ATCTCCGGCCAGGCGGTCATTCTGTTTCATGCCGAATGGGCCATTACTTTTTAA
>EECEBICD_01923 Arylamine N-acetyltransferase / N-hydroxyarylamine O-acetyltransferase
ATGACCTCTTTTTTACATGCTTATTTTACGCGATTACATTGTCAGCCGTTGGGGGTTCCT
ACGGTTGAAGCGCTCAGAACGCTTCACTTAGCGCACAACTGCGCGATTCCTTTTGAAAAT
CTGGATGTGCTACTGCCTCGTGAAATACAGCTCGATGAGACGGCATTAGAGGAGAAACTG
CTTTATGCCCGACGCGGTGGATACTGTTTTGAACTGAATGGTCTGTTTGAACGTGCGTTA
CGCGACATCGGTTTTAACGTCCGAAGCTTATTGGGGCGGGTCATTCTGTCTCATCCCGCC
AGTTTACCGCCGCGCACGCACCGTTTGCTGCTGGTTGATGTTGAGGATGAACAGTGGATC
GCTGACGTGGGGTTTGGCGGCCAAACGCTAACCGCGCCGCTTCGTCTGCAGGCTGAAATT
GCCCAGCAAACGCCGCATGGCGAATACCGCCTGATGCAGGAGGGAAGCACATGGATTTTA
CAATTCCGCCACCATGAGCACTGGCAGTCGATGTACTGTTTTGATCTGGGGGTGCAGCAG
CAGAGCGATCATGTGATGGGCAATTTCTGGTCGGCTCACTGGCCGCAATCCCATTTTCGT
CATCATTTGTTGATGTGCCGCCATTTGCCTGATGGCGGCAAGCTGACATTAACGAATTTC
CACTTCACGCGTTACCACCAGGGACATGCCGTTGAACAGGTCAATGTACCGGATGTACCG
TCGTTGTATCAGTTACTTCAGCAGCAGTTTGGTCTCGGCGTAAATGATGTAAAGCACGGT
TTTACCGAGGCGGAGCTGGCCGCGGTAATGGCTGCATTTGATACCCATCCGGAGGCGGGA
AAATAA
>EECEBICD_01924 hypothetical protein
ATGGCAGCATATAATGCCTTACCTCAGGCATTAGGCGCCATTCCGTGTTTAGAATATTAT
ATCGCTCGGGCCAGCAACATGCAGGAGGCGAAAGCGCAATGGGCGGCCGACATAAAAGCT

CGCTACCATAACTATTTGGACAATTATTAA
>EECEBICD_01925 hypothetical protein
ATGCCATATACATCAGTTTCTACCTATGCCAGAGCTTTATCAGGAAATAAGCTACCTCAT
GTTGCTGCAGGAGATTATGAAAATAAATTATCAACAAAAATCATGAAAGGCATCTTGTAT
GTGCTTACCGCAGGACTGGCCTACGGATTTACGCGAGTAATTGAACATTATTGCAATGTT
ACGCCTAAAGTTGATGAGTTCTGTGCCAATGCAGGGAATATCCATAATCACCTTGCTGAT
GCTGTGTGTGATGGGCTGTATACTATAGATGTCGACCTTTCTGATGGTCGGAGGTTAACA
TTCGAACAACTCTCGCTGATGGCTGGACGTGAGCCAATCGTCAGGATATCGGATGGCGAA
CATACCGTTGAGGTTAAGGGGTCCTTTGAAGAGATTTGTATGAGACTGGAAGAGGGCTTT
TTGAAGCGCCAGCGTACTATGATTATGATATTGATGAAAAATATAAAACCGTCAGAGAGC
GAATGGCAGCATATAATGCCTTACCTCAGGCATTAG
>EECEBICD_01926 L-asparagine permease 2
ATGAAAACACAGACTACGCACGCCGCAGAACAACATGCGGCGAAACGACGCTGGCTGAAC
GCCCATGAAGAGGGGTATCACAAAGCGATGGGGAATCGTCAGGTTCAGATGATTGCTATC
GGCGGCGCGATTGGCACCGGTTTATTTTTAGGTGCAGGCGCCCGATTGCAAATGGCCGGA
CCGGCACTGGCGCTGGTCTATCTGATATGCGGCATTTTTTCTTTTTTCATTTTACGCGCG
CTGGGCGAACTGGTATTGCATCGCCCTTCCAGCGGGAGCTTCGTCTCCTACGCCCGTGAA
TTTTTGGGTGAAAAAGCGGCCTACGTCGCAGGCTGGATGTACTTTATTAACTGGGCGATG
ACCGGGATCGTCGATATCACCGCAGTGGCGTTATACATGCACTACTGGGGCGCATTTGGC
GACGTTCCGCAATGGGTGTTCGCGCTCGGCGCGTTAACCATTGTCGGTACCATGAACATG
ATCGGCGTAAAATGGTTTGCCGAAATGGAGTTCTGGTTCGCGCTGATAAAAGTACTCGCC
ATTGTTATCTTCCTGGTGGTAGGCACGATTTTTCTCGGTACCGGTCAACCGCTTGAGGGT
AATGCCACCGGTTTTCACCTGATTACAGATAATGGCGGCTTTTTCCCGCACGGCCTGTTG
CCCGCGCTGGTACTGATTCAGGGCGTAGTGTTCGCTTTTGCGTCGATTGAACTGGTCGGT
ACCGCCGCTGGGGAGTGCAAAGATCCACAGACGATGGTGCCAAAAGCCATCAATAGCGTG
ATCTGGCGTATTGGTCTTTTCTATGTAGGATCGGTCGTATTGCTGGTTTTGCTGCTGCCG
TGGAACGCCTACCAGGCGGGGCAAAGTCCGTTTGTCACCTTCTTTTCTAAACTTGGCGTG
CCTTATATCGGCAGTATTATGAATATCGTGGTGTTAACCGCCGCGCTTTCCAGCCTGAAC
TCCGGGCTTTACTGTACGGGGAGAATTTTACGTTCAATGTCGATGGGGGGTTCCGCGCCG
AAATTCATGGCGAAAATGAGCCGTCAGCATGTGCCCTATGCGGGTATACTGGCGACTCTG
GTGGTTTACGTGGTCGGCGTATTCCTGAATTACCTGGTCCCCTCCCGCGTGTTTGAGATC
GTTCTTAATTTTGCCTCGCTCGGCATTATCGCGTCGTGGGCGTTTATCATGGTGTGCCAG
ATGCGTTTACGCCAGGCGATAAAAGAAGGCAAAGCCGCAGACGTCAGTTTTAAACTGCCC
GGCGCGCCTTTTACCTCCTGGCTAACGCTGCTGTTTTTACTAAGCGTGCTGGTACTCATG
GCGTTTGACTACCCGAATGGAACTTACACTATCGCCTCGTTGCCGCTGATCGCCATTCTG
CTGGTCGCCGGCTGGTTTGGCGTGCGACGTCGCGTGGCGGAGATCCATCGCACTGCGCCA
GTGACGGCGGATTCGACAGAGAGTGTCGTATTGAAAGAAGAAGCAGCGACATAA
>EECEBICD_01927 putative lipoprotein YgdR
ATGAAAAAGTTATTTATATGCTCAGGTTTGGGCATGATGTTTTTTATGCTTGCGGGTTGT
ACAACCAACTATGTGATGACGACGAAGAATGGGCAAACGATCGTAACTCAGGGTAAGCCG
CAGTTAGATAAAGAGACGGGGATGACCAGTTATACCGATCAGGAAGGCAACCAGCGGGAA
ATTAACAGCAATGATGTGGCGCAGTTGATTAAGGCTGACTAA
>EECEBICD_01928 putative protein YncE
ATGCACTTACGTCATCTTTTTTCGCCGCGCCTGCGTGGTTCTTTATTGTTAGGTTCGCTC
CTCGTGGCATCCTCATTTAGCACGCTGGCGGCGGAAGACATGCTGCGTAAAGCAGTAGGC
AAAGGCGCTTATGAGATGGCCTGGAGTCAGCAAGAAAACGCGCTCTGGCTGGCTACATCG
CAAAGCCGTAAACTGGATAAAGGCGGCGTAGTTTATCGTCTCGACCCGGTGACGCTGGAA
ATCACGCAAGCGATTCATAACGATCTCAAGCCGTTCGGCGCCACCATCAATGCCGCGACC
CAAACGCTGTGGTTTGGCAATACCATTAACAGCGCTGTTACCGCGATTGATGCCAAAACG
GGTGATGTAAAAGGTCGTCTGGTACTTGATGCGCGCAAACGTACTGAAGAGGTTCGTCCG
TTACAGCCCCGTGAGCTGGTTGCCGATGCGGCGACCAACACGATCTACATTAGCGGTGTT
GGTAAAGAGAGTGCTATTTGGGTAGTGGATGGCGAAACCATCAAACTGAAAACGACGATC
GAAAATACCGGCAAAATGAGTACGGGTCTGGCGCTCGACAGTAAAGCGCAACGCCTGTAC
ACCACCAATGCGGATGGCGAATTTATCACCATCGATACCGCCAGCAATAAAATTCTCAGT

CGTAAGAAGTTGCTGGATGACGGTAAAGAACACTTCTTTATTAACCTGAGTCTCGATACC
GCAGGTCATCGCGCGTTTATCACCGACTCGAAGGCGACTGAGGTTCTGGTTGTCGATACC
CGTAATGGCAATATTCTTGCCAAAATCGCGGCGCCTGCTTCTTTGGCCGTCCTGTTTAAC
CCGACACGTAACGAGGCGTATGTGACACATCGTCAGGCAGGTCAGGTCAGCGTGATCGAT
GCGAAGACCTATAACGTTGTTAAAACGTTCGATACGCCGACGTACCCGAATAGCCTGGCG
CTATCGGCAGACGGTAAAACGCTCTACGTCAGCGTGAAGCAGAAATCGACACGTGAACAG
GAAGCGACGCAGCCGGATGATGTTATTCGCATTGCTCTGTAA
>EECEBICD_01929 Fe(3+) dicitrate transport protein FecA
ATGTCACGTTTTCCGTTGATACGCTTACATTTATCCGGCATTTTCATGAAAATTATTTCC
GTTAGACAGCGACTTTATCCCGCCCTCTTATTGCCTCTGACCTTCTCCCCGGTCCTTCAG
GCCGCCAGCGCGTCCAATGAACAAACCATGATAGTCACCGCAACGCCGCAAACCGTATCC
GAACTGGATACACCTGCCGCCGTTAGCGTCATTGAGGGAGAAGACATGCGTCTGGCGACG
CCGCGCGTTAATTTATCTGAGACTTTAACCAGCGTCCCGGGATTGCAGGTGCAGAACCGG
CAGAACTACGCGCAGGATCTGCAAATCTCTATCCGTGGATTTGGCTCGCGTTCCGCCTTT
GGCGTGCGCGGCATTCGTCTGTATGTTGATGGCATTCCCGCCACCATGCCGGATGGACAA
GGCCAGATTTCGAATATTGATATTAACAGCATACAAGACGTTGAAGTGTTACGCGGCCCC
TTCTCAGCGCTATACGGCAATGCTTCCGGCGGCGTAATAAATGTCACCACCGAAACCGGG
ATTGAGGCCAGCAGCTACTACGGCAGTTATGGAAGCTGGCGCTATGGGCTAAAAGCCACG
GGCGCGATGGGTGACGGCACACAGCCTGGCGACGTGGATTACACGGTATCAACTACCCGT
TTCACCACCCACGGCTACCGCGATCACAGCGGCGCGCGAAAAAATCTGGCTAATGCCAAA
CTGGGCGTGCGCCTTGACGATGTCAGTAAACTTTCGCTGATTTTTAATAGTGTGGACATT
AAGGCGGACGATCCCGGCGGACTTACCGAATCTGAATGGAAAGCCGATCCGCAACAGGCG
CCGCGCGCTGAACAGTACAATACGCGTAAAACCATCAAACAAACTCAGGCAGGATTGCGT
TATGAACGTCAGCTCAGCGCGCAGGATGATATCAGCGTGATGGCCTACGCCGGAGAGCGG
GAAACCACGCAATACCAGTCTATTCCCCTGGTGGCACAGCTAAAACCAGCCCAGGCTGGC
GGCGTAATTACACTGCAACGCCACTATCAGGGAATTGATTCCCGCTGGACTCACCGGGGA
GAATTAGGTGTGCCGGTCACCTTCACTGGCGGGTTAAACTATGAAAACATGAGCGAAAAC
CGCAAGGGTTACAATAACTTCCGTCTCAACAACGGCCCCCCTGAGTTCGGGCATAAAGGC
GATTTACGGCGGGATGAGCGTAACCTGATGTGGAATGTCGATCCTTATCTGCAAACCCAG
TGGCAGCTTACGCAAAAACTCTCGCTGGATGCTGGCGTGCGCTACAGTTCCGTGTGGTTC
GATTCTAACGATCACTATATCGCGCCCGGCAATGGCGATGACAGCGGCGACGCCAGCTAT
CACCACTGGCTACCCGCCGGATCGCTAAAATATGCGTTAACTGACGCCTGGAATCTCTAT
CTTGCTGCCGGTCGCGGATTTGAAACTCCCACTATCAATGAACTTTCTTATCGCGCTGAC
GGCCAAAGCGGGTTCAACTTTGACCTCAAACCGTCGACCAATGATACGGTGGAAGTCGGC
AGTAAAACGCGGATTGGCAATGGCTTGTTGACCGCCGCGTTATTTCAAACCGATACCGAT
GATGAAATTGTCGTCGCCAGTAGTATGGGAGGACGCACTACCTACAAAAACGCGGGCAAA
ACGCGCCGCCAGGGCGCAGAGCTCGCGTTGGACCAGCGCTTCGCCGGCGACTGGCGGGTG
AAAGCATCATGGACCTGGCTGGATGCGACCTATCGCAGTAACGTTTGTCAGGGGCAAAAC
TGTGATGGAAACCGGATGCCCGGCATCGCCCGTAATATGGGATTCGCCTCATTAGGTTTT
ATCCCGGATGAGGGGTGGTACGCCGGAACAGACGTTCGGTATATGGGCGATATCATGGCC
AATGATGAAAATACCGCCAAAGCGCCTTCGTATACCGTTGTTGGACTAAATACAGGGTAT
AAATTTAATTACAGTCAGCTTACCGTCGATATTTTTGGGCGGGTAGATAATCTATTTGAT
AAAGAATATATCGGTTCGGTGATCGTTAATGAATCTAATGGCCGATATTATGAGCCAGCG
CCAGGGCGTAACTATGGCGTGGGGATTAATCTGGCATGGCAATTTGAATAA
>EECEBICD_01930 HTH-type transcriptional regulator McbR
ATGCCGGGTACGGAAAAAACACAACATATAAGCCTGACCACACAGGTTGAAAATCGGTTA
AAGCATCAATTGTCTATTGGCGCATTAAAACCAGGCGCAAGGCTTATCACTAAAAATATC
GCTCAAGAGTTAGGCGTCAGTATTACGCCAGTGCGTGAGGCCCTGTTAAGGCTGGTCTCT
TCCAGTGCTTTGGCGGTGGCGCCAGCACAGGCGTTTATGGTGCCGGAGATCTCGCTGGAA
AGTTTGCTGGAAATTAATACGATTCGTACTGCCCTGGAGGAGATGGCGGTGGTCGCCGCT
GCGGGTAAGATTACCCCGGATCGCGAGCAGACGCTGAATGCGCTATTAGATGAATTTCAA
CAGGCGCTGGAAAGTGGCGTTATGGAGAAAATTCTTTTGGCCAACCGCGCATTCCGCTTT
GAGATCTACCACTACGCGGATATGCCAACGCTGTATGCCATGATCGAGCAATTATGGGTA
CGCCTGGGGCCAAGTCTGCATTTTTTATATGATAATTTTAAACTTGATGATTATCAAAAC

GGCGTTAATCTGTACAGAAAATTGCTTAATGCGTTAGTGACTGGTGATAAAGAAGCCAGT
CGCCACTGCCTACAGAACGTTTTGCAACAAAACGTAGCGACGATTAAAAATCAGTACTTC
ATGTAA
>EECEBICD_01931 NADPH-dependent curcumin reductase
ATGAAGCAACAGACCAACCGAAATCGTCGTTGGGTTTTGGCCTCGCGTCCCCACGGCGCG
CCGCAGATGGATAACTTCCGCCTCGAAGAAGATGACGTCGCGACGCCCGGTGAAGGGCAG
GTGCTATTGCGTACCGTTTTTTTATCACTCGACCCTTATATGCGTGGCCGCATGAGCGAT
GAACCCTCATATGCGCCGCCTGTTGAACCTGGCTGCGTTATGGTTGGCGGTACGGTCAGT
CGCGTTGTCGCGTCAAACCATCCTGATTACCGACCAGACGAATGGGTGTTGAGTTACAAT
GGCTGGCAGGATTATGACATTTCAGATGGCGAAGGGTTAGTAAAGCTGGGTGATAATCCG
CAACATCCTTCCTGGTCATTGGGTATTTTAGGAATGCCGGGGTTTACCGCCTATATGGGG
TTACTGGATATCGGCCAGCCGAAAGAGGGCGAAACGCTGGTTGTCGCCGCCGCCACCGGC
CCCGTAGGCGCAACGGTTGGGCAAATTGGTAAGCTGAAGGGCTGCCGGGTCGTGGGTATT
GCTGGCGGCGCGGAAAAATGCCGTCATGCGACAGACGTTTTGGGATTTGATCTGTGCCTT
GATCATCACGCTGATAATTTCCCACAACAGCTTGCGCAGGCCTGCCCGCAGGGTATCGAT
ATCTATTATGAGAATGTCGGTGGTAAAGTCTTTGACGCCGTCTTACCGCTACTGAATACG
TCGGCGCGGATTCCGCTCTGCGGTCTGGTCAGCGGCTACAATGCGACAGCGTTGCCGGAC
GGTCCAGACAGACTGCCTCTGCTGATGGCGACATTACTTAAAAAGCGTATTCGTCTGCAA
GGTTTTATCATCAACCAGGACTATGGTCACCGGATACATGAGTTTCAGCAAGAAATGGGG
CGTTGGATCAAAGAAGGGAAAATACATTACCGGGAGCAGATTACTGATGGGCTGGAAAAT
GCGCCAGAAGCGTTTATGGGGCTGCTGGCGGGGAAGAACTTTGGCAAAGTCGTGATACGT
TTAGCGGACGATGCGTAA
>EECEBICD_01932 L-methionine sulfoximine/L-methionine sulfone acetyltransferase
ATGACGATTCGCTTTGCCGATAAAGCAGACTGCGCCGCGATTACCGAAATCTATAACCAT
GCGGTATTACATACTGCGGCCATCTGGAACGATCGGACGGTCGATACGGATAATCGCCTT
GCCTGGTATGAGGCCCGTCAATTACTGGGCTATCCGGTGCTGGTAAGCGAAGAAAATGGC
GTGGTGACTGGCTATGCATCATTTGGCGATTGGCGCAGTTTCGACGGTTTTCGCTATACC
GTCGAGCACTCGGTTTATGTTCATCCGGCGCACCAGGGGAAAGGACTGGGGCGAAAGCTG
CTCAGCAGACTGATCGACGAAGCCCGACGCTGCGGCAAGCATGTGATGGTGGCCGGTATT
GAGTCACAGAATGCCGCCTCGATACGCCTGCATCATTCGCTGGGATTTACCGTTACCGCG
CAAATGCCGCAGGTCGGCGTAAAATTTGGCCGTTGGTTAGATCTTACGTTTATGCAGCTC
CAGCTTGATGAACACGCCGCGCCGGACGCCTGCTGA
>EECEBICD_01933 Inner membrane protein YdcZ
ATGAACCAGTCTTTGACGCTTATCTTTTTGATCGCCGCCGGCGTCGGGCTGGTGGTACAA
AACAGTATTATGGTGCGTATTACGCAGACATCATCGACGATTCTGATCGCTATGCTGTTG
AACTCGCTGGTAGGGATCGTTTTATTTGTGACGATATTGTGGTTTAAGCAAGGCGCTGCG
GGTTTCGGCGAGCTGGTCGCCAGCGTTCGCTGGTGGACATTGATCCCCGGTCTGCTGGGG
TCATTTTTTGTATTTGCCAGCATTAGCGGGTATCAGAACGTTGGCGCCGCGACGACTATC
GCGGTACTGGTCGCCAGCCAGTTAATCGGCGGACTGGCGCTGGATATCGCGCGTAGCCAC
GGCGTCACGTTACGCGCAATGGTCGGGCCGGCGTTTGGCGCGCTGTTGCTGGTCATTGGC
GCATGGCTGATTGCGAAGCGCCAGTTTTAA
>EECEBICD_01934 putative protein YdcY
ATGTCGCACCTGGAGGAAGTGAGCGCCCGCGTGGATGCCGCCATCGCGGAGAGCGTAATT
GCCCATATGAATGAATTGCTGATTGCCTTGAGCGATGACGCTGAATTACGTCGCGAAGAT
CGTTATGTGCAGCAGCAGCGTTTACGCACGGCTATCGCGCATCACGGCAGGCAATATCAA
GAAGATCGGGACGCGCGCCCGCGAGCAGCTCACCAAAGGCGGCACGATCCTGTAA
>EECEBICD_01935 hypothetical protein
GTGGCAAAATCTCTCTTGCGCAGCGGCAATCTGGATGATTATCAGGCCGTGGGCGGCGGC
GGTCAGGCCGTATTTGAATCAGCATTGCAAATTCGCGAAACGCTTCGTCTGCGTAAACAG
CAGGCGATGGTTGACTGTCTGGCGATTCCGCAACTTAACGATAATGGCGATCGGGTTGAC
TGGTATTCGCCGATTGAAGGTCAGGCGATAGCGTGGAAAGCGGCTGACGAAGAGACACGT
TCTCGGGCTTTACGTTATTTAGCCAGCACTTTTGAAAGCGCGGCTGCACTCAGTCGCAAA
AGTCTGCAATCCGGTAAAACAGCGCTCCAGCTATTTGGGTCGCTACTTGAGAAAGCGACG

CAATTTCCCGGCGAAAATCATGTTTTCCTGGTTAACGGCAAACCGGTTATCACCTTCTGG
GGATTCGTTAATCTCAACGAAAATACACGCGATGATGTGCTGGACTGCCTGCGGGTAACC
GAGGCAATCCCCGACATCCCTCTGGTCGAGCCGGAGCCGGAGCCCGAAGAAAAGCCGCTG
GTAGAGGCGGCATTTAGTCAGGCGGATGAACCTTTGTTAACGTCTGTCATTGAGCCGCCG
AAAATGCCGGAGGAACCTGTTGCGCCGCCCGTTATCGTTAGCGAGCCGAAGCCGGCCACA
CCCATCCCGGTTGCTGAGGCGAAAAGAGCTCGCCGTCTACCGCTGTGGAGCTTACCTGTC
GCTGCCGTGGTTATCGCCGCCGTCGCCACGCCTTTTTTCTGGCCATCTTCATCCGTTGAC
GGGTCCTCTCCTGTTCGCGCCGCGCCTGTCGCTACCGCGAAAACTGACGTTACCCCATTG
CCTGAACTCACCGCGCATTTGCCGCTGCATCGCGCTGAGGTGACGCCCGCCCCCAAAGCG
GCGCCGCTGCCGGAAGCGCCTGTTATCATTGCCGCTATTCCCAAAGACGCGCTGGTGATG
GACAGTACGCAGATGAAGCTTGGAACCACGCGCTTTTTAAACGGTAGCTGGCGGGTCAGC
GTGGATGTCAAAGATCCGATTACCGGCAAGCCGCCCTCTCTGCGTTATCAGATCCAAAAT
AACAAAGGTATCGCGCGCGTAGTACATGGCGATAACGTGGTTTGTCGGGCGGAAATTTTC
TCTGGTCTGCATCAAACGGGCGAGCTGATGATTAAAAGTCGCGGCAATGCGCGCTGCACC
GACGGTTCACGTTATCCGATGCCGGAAATTACCTGTAAAGCAGGCGTCAATGACGTCGCA
ACCTGTACCGCCCGTTATGGCGACCACGCGGCAATTCCGTTGACGTTTAAAAAGATAGGT
GCCTGA
>EECEBICD_01936 hypothetical protein
ATGTTGGTCAATCTGTGTGATTACAAACAGAGCGTCACGCTCATTGCAAACAGTGGCGTC
CAGTTTCTTGATTTTGGCTTAACGCCGCAAGAGTCGGCCCACTACGGACGTTTTGTGCGC
AAAACGGCAAACGGCCCGCTACTGCGCCTCGATTTTGATCTGACCAGCGGGCGCTACACG
TTGCCTGGACGTGCTGGCGGCCAACCCGAAGTCGTGAAACCGGAAAGCACTCAGACGCTG
CACTATTCTCTGGATGTACTCGACGGAATCTGGCTTCCCCTGCCGTTCCTGCGTTTTAAT
CCGCCGCGTACCTTTATTGACGGGCCGGATAACTGGGCGCGCATTCAGGTACGTAAACTC
AGCGAGCCAGATAGCGCCGGAAACACACACCGGATCACCCTCGCCTTCGACAGTCAACTG
GCAAAAAATATGCCTGCGGCGTTGGCGCCTTGCGAAAACGATCTGCTCAATGGCACACGT
TTTGCGCTCGCCTGGCAGGATGAAGAAGTTGCCGATTTTCTTGACCAGACATGGATTGAC
GGCTGGCTGCGCGAGTCGTTTCTTCAGTATGCCTCACAGGTGGAAAACCGTTCGGAACAG
GCCATACAACAAGCGCTGCGTAGCTTTGAGTATCAGGCGCACTGGTTGAATTTACTGACG
CTGTTAGGCGAACAGCTTACGGTGCCGGAAGTAAAGTTTGTGACACATACGTTAAGCACG
CCCGCAATCCCGGTCGATCTGATTCTGGATGTCGGCAATACGCATACCTGCGGCGTCTTG
ATCGAGGATCATGGCGATGCGAATGACGGGTTACGCCAGACGGCAGAGCTTCAGGTGCGT
TCTTTAAGCGAGCCGCAGTATCTGAACGATCCTTTATTTACCAGCCGGGTGGAATTCTCG
GAAGCACGCTTTGGCAAGCAACACTTTTCTGTCGAAAGCGGACGAGACGACGCCTTTGTC
TGGCCTTCCATTGTGCGTGTGGGCGACGAGGCTCGCGCCCTTGCTATGCAGCGTGTGGGC
ACCGAAGGCAGCAGCGGCATCTCCAGCCCGCGTCGCTACCTGTGGGATGAAACCCCCGCC
TTGCAGGACTGGCGTTTTAGCCAGATCCACGGTAAAACGCAGCGCGAAGCGCTTGCCACC
GCGTTTCCGCTGATGAATCTGATGAACGATGACGGACAGCCGTTATTCCGCCTGCCGCAT
GAAGAAAGGCTACCGGTTTTTTCGCCGCAATACAGCCGTAGCACCTTGATGACGCATATG
TTGTGCGAAATTCTGGCTCAGGCGCTGGGGCAAATTAACAGCGTCGCCACCCGGCTGCGG
CTCGGCTTTCCCGCCTCGCCGCGCCAGCTACGCACCCTGATTTTAACGCTGCCTTCCGCC
ATGCCGAAACAGGAGCGAGAAATTTTCCGCCAGCGAATGTTTGAAGCGCTGGCGCTGGTC
TGGAAAGCCATGGGCTGGCATCCGCAGGATGAGGATTTCACCACGCCTAAGCAGCGTGAA
AAAAGCGTGGTGCCGGTACCAGAAATACAGATGGAATGGGACGAAGCAAGTTGCGGCCAA
CTGGTATGGCTTTATAACGAAGCCATTTCGCATTACGCCGGGCGTACCGAATCCTTTTTT
AATGCGCTCGCCAGACCGGATCGCCAGCCTGAACCCGGCGTCGTCCCCGGACGCGCGTTG
CGCGTGGCATCGATTGATATCGGCGGCGGCACGACGGATATGGCGATCGTTCACTATCAG
TTAGATGACGGCGTGGGGGCCAATGTAAAAATTACGCCTCATCTCCTGTTTCGCGAAGGT
TTTAAAGTCGCCGGGGACGATCTGCTATTGGATATCATTCAGCGCTGTGTACTCCCTTCC
CTGCAAACGGCGTTGCAACGGGCAGGCGTTACGGATGCCGCCGCCCTGCTGGCGACCTTA
TTTGGCGATTCGGGACGAATTGATACTCAGGCCATTTTGCGTCAGCAAACGGCGCTGCAA
TTATTTATGCCGCTGGGCCATGCGGTACTTTCCGCCTGGGAGCAAAGCGACATTAACGAT
CCGTTTGCCGGTTTACACGCGACATTTGGCGATCTGCTGATCCGCCGCCCAACGAGCAAC
GTGATGAACTATATCCAGCAGGCGATCGATCATGCCTTACCGTCTGGTTCGCCGACATTT

GATATATTCAATGTGCCGTTGCAAATCCAGTTTAGCCAGCTTCAGGAGGCTCTGCTGGCC
GGTCAGTTTACGCTAACAACGCCGCTTCATGCGGTCTGCGAAGCGATCTCCCACTATCAC
TGCGATATCTTGCTGGTGACGGGGAGACCGACCTGCTTACCCGGCGTACAGGCGCTCATC
CGGCACTTACAACCGGTGCCGGTTAATCGCATTGTGTGGATGGATAAGTATCAGGTACAT
GAATGGTATCCCTTCAGTCAGCAAGGGCGTATCGGTAACCCAAAATCCACTGCCGCGGTG
GGCGCGATGTTATGCAGCCTGGCGCTGGACTTACGTCTGCCGCGTTTCAATTTTAAAGCG
GCTGATATCGGCGCGTATTCGACTGTACGTTATCTCGGCGTACTGGATAATACCGTTAAT
ACCCTTCGCGATGAAAACATCTGGTATCACGAAATCGATTTGGATAAACCCGGCGCAACG
CTGGACGCTCGCTTGCATTTTCCGCTACGCGGTAACGTGACGTTGGGCTTTCGTCAACTG
GCGAACAGTCGCTGGCCGGCAACACCACTTTATTGCCTGAGCATTAATTCCGCCGAACTG
GCGAAAACCATCGCTGGCGACGGCGTACTGAATGTGCGACTGAAATTACGCGGCAGTAGC
AAAGATAGCGCCCCGGAATCGTTCATATTAAGTGATGCCTGGCTCCAGGACGGGACGCCG
GTCGCCGCCGATGCGCTGACATTAAAATTGAATACGCTGGCTGACCGCCGCCACAGCGGC
AGCCACTACTGGATAGATAGCGGGAGTGTATACCTGAAATGA
>EECEBICD_01937 hypothetical protein
ATGACAAAACCGTTAAACGCCACCCAGGCTGTTATCGAGTGGGTTAACAACACGCGCCGT
TACGCAACGCGACTGGATGACGAAGCCGACGCTCTGCTGGCGCAACTTACGCTTGCCGCG
GCGGATGAGTCAGCGCTCAACGCCGCCTGCGCGTCACATGGCTGCGTTGGTCTGTACGGG
TATGCGCAATCCGCAAAAGCGCATCTGTTAACGACGCTATGCGGTAACGAGAATGGCAAG
CTGGAAATTATCACGCCGGACCGTGACTATGATTATTTCAGCCACATCAATCCGGGTCAT
GCGCCAGCGAATATGGCTATTCGCTTTACGCGCGACATTTTTTCAAACGAGAGCGGCTGG
CCGTTGCGCCTGCGCTTGATCAGTGAAGCCGAACTGGTGCAGATCTTTATTGCCTGGACC
AGCGCGTCCCCCATTTGTCGCCAGGTCGAAAAATCCATTATCACATCACGCCTGGAGAAA
TGGCAATCGCTTCGCCAACCGCAGCCCGTCCCCGGCGTTACCGCTGAAGAGGTAGCGACC
ATCGCGAGTTTCTGGCGCTCTTGCCTGCCGTCCGCCAGACAACATATTGATGATGCGACA
TGGCAACACTTTGCCTCGCTCTTGCCTGCGCTGGATCTCACGACACGGGCGCACGCCTGG
GCATTACTGTGGGGAGAGCAACCGGAGATCACCCAGCAATGGCTGGCGCTGGCGCATATG
TTGCAACAAACCGGCCATGCCGGGGAACTGGCGGCGCCGCTCAGCCTGCTGGTCGATCAT
TTTGGCTTACCGGCGGAAAACTTCCTGACGCAAATGGCGCTAACCGCCAACGATACGCAA
ATCGATGTGGTGGTGCATCCGGTTAAGGAAGGCCGACTGCTCAATGCGGTCAGCCTTTCG
CTTGATTCTCTGGCGCTGCTTACCCGCGAACTGGTGCTAACCGTTGAAAACAACGTACTG
GATAACGTAGATTTACTGGATATTCCGGTCGCGCCAGACAGTCATCCTCATCCGTTATGG
CGGGCGAAATTAGGCTGGATGCTGGCCCATTACCGCCAACAGGTACAGCCCGATGTTCTG
GTGATTTGCAATGCTCTGGCATCTCGCTCGCAAACGTCCACTGCCGCCCGTCACTTACTG
GAGTGGGTTAACGCCACGCAGCCGCAGCATGAATCAGCGTTACCCGGCGTCGTCTGGGCT
ATTACTCCACAGGATGCCCGTTTTGCCACCCAGCAAAATCTGGATGAAGCCGTCCAGCAG
TTGATGGGTAAACCTGGCGTGCATTGGGGAACGTTGCAGGCGCTGGATAAACACAGTATG
CAGCGTTTGGTGGAATGGTTATCGCAGGCGACATCGGCACCACAGCGACAGGCGCGCTTA
CAGGCGTTACGCGAACAACTGCGAGGCCGCGTGCGCGATCTGTTACCGATGTTTGATGAC
GCGCGGCTACCGGTTGAAACCGTGATCCGGCGTCTTCAGGCCCAGGCCGCCAGACATGGC
GACCTGCTGGCCGGGCTGTTACCGCCAGTACAGAATTTCGAAGCGTTGCTGCGCACCCGA
CAGTCACGAGAAGAACAGGTCTGCGGTTTATTTAATGACGCGATCGATCTCTTTGCCGAC
GAACCAACGCGCGCTTCAGCGTCAGAAGGTCACGAAACAGGCTACCAGGCGCACAAAATG
TGGATCAATCATCTGCGCCAGTGGGCCCACTGCCGGGATAATGCGCAGCGTCTGGGACTG
GAACCGCAGATGCTCAACGCCGTGGCGGAGATTTTAATTACCGCCAGCTATCGCCTCGGT
TTACCTCAGCAGTTACAGAAAACCATGCAGCGCGAGGAGGTGAGCGGCGCGCAGCTTCAT
GCCATCATTGGTAACTTCATCGCCTGGCTCGGCTACGCCAATATCGAGGAGGCCCAACGC
CCGGCCAGCCGCGTTCAGAAAGGCGCAGCCATTTTCGCCGCAACACCGCGTTCGACAATG
TTGCGTCTAACAAAGCTTGACGAACAGCCCGTTCACGCCGCCAGCCGCTATGTCTATGAC
TGGCTTGTTGCGCTGTATACCCTGGCCAATGAGAACGCAGGATATCGGCATCCGCAGGAC
GTTACCGACGTAGACAGAGCGCAATTAATTGCCCTGATAGCCTGA
>EECEBICD_01938 Orphan toxin OrtT
ATGACCCTCTTTCAAAAGATATTAATTTTTTACGCCGTCATGGCGGTGATATCATCCCTG
ATCACCTGGTTTTTGACTCATGACAAAAAACGAATTCGTTTCCTGAGCGCCTTTTTGGTT

GGCGCGACATGGCCAATAAGTTTCCCCGTCGCATTGCTGTTTTCGCTTTTCTGA
>EECEBICD_01939 Gamma-aminobutyraldehyde dehydrogenase
ATGCAATATCAATTACTGATTAATGGCGTGCTGGTGGACGGTGAGGGAGAAAGGCAGTCT
GTTTATAACCCGGCGACAGGCGAGGTCATACTTGAGATTGCGGAAGCGTCGCCCGCGCAG
GTTGACGCCGCAGTGCTGGCCGCCGATAGCGCGTTTGCCGAATGGGGGCAGACCACACCC
AAAGCACGGGCGGAATGTCTGTTAAAACTGGCGGACAGCATCGAGCAAAACGCCCTTGAG
TTCGCCCGGCTTGAATCGCAAAACTGCGGTAAGCCTTTGCACTGCGTAATTAATGATGAA
ATTCCGGCAATCGTGGATGTCTTTCGCTTTTTTGCCGGCGCGGCCCGTTGTTTGAGTGGG
CTGGCGGCGGGCGAATATCTGGAAGGACATACGTCGATGATCCGCCGGGATCCGATTGGC
GTGGTTGCCTCTATCGCGCCGTGGAATTATCCGCTCATGATGGCGGCCTGGAAGCTGGCG
CCAGCGCTGGCGGCAGGTAACTGCGTGGTGATTAAGCCTTCTGAAATCACGCCGCTGACG
GCGCTTAAACTTGCCGCGCTGGCGAAGGATATCTTTCCGCCGGGGGGGCTCAATGTCCTG
TTCGGACGAGGGCAGACTGTTGGCGATGTATTGACCGGTCATGAAAAAGTACGCATGGTC
TCTTTAACCGGTTCTATCGCGACCGGGGAGCATATTCTGCGCCATACTGCGCCAGCGATA
AAACGTACGCATATGGAACTCGGTGGTAAAGCCCCGGTGATTGTTTTTGATGACGCCGAT
CTGGATGCGGTGGCGCAGGGCGTCCGGACTTTTGGTTTCTATAATGCCGGACAGGATTGT
ACTGCGGCGTGCCGTATATACGCGCAGCGCGGTATCTATGATGCGCTGGTCGAAAAGCTA
GGGAACGCGGTGAGCAGCCTGAAAATGGGCGCGCCGGAGGATGAGTCGACAGAGCTGGGG
CCGTTAAGTTCGCTGGCGCACCTGAAGCGCGTCACAGCGGCTGTAGAGGAGGCGAAGGCC
CTCAGCCATATCCGTGTGATTACTGGCGGAAGTCAAACGGAAGGTAAAGGTTACTATTTC
GCTCCGACGTTGCTGGCAGACGCAAAACAGGAAGATGCCATCGTGCAACGCGAGGTCTTC
GGCCCGGTGGTCAGTATAACCGTCTTTGATGATGAAGATCAGGTTCTGAGGTGGGCCAAC
GATTCTCGCTATGGTCTGGCATCCTCCGTCTGGACTCAGGACGTTGGGCGGGCGCACCGG
CTTAGCGCCAGATTGCAGTATGGCTGTACCTGGATTAATACCCATTTCATGTTGGTTAGC
GAGATGCCGCATGGAGGGCAAAAACAGTCGGGATACGGCAAAGATATGTCATTGTATGGT
CTGGAAGACTACACACTTGTCCGTCACATCATGGTTAAGCATTAA
>EECEBICD_01940 2-aminoadipate transaminase
ATGAAAAAATATCAACGTCTGGCGGAGCAAATTAGAGAACAAATCGCCTCTGGCGTTTGG
CAACCCGGCGATCGATTACCCTCGCTGAGGGAGCAGGTCGCCAGTAGCGGCATGAGTTTT
ATGACTGTCGGTCATGCGTACCAGTTGCTGGAAAGTCAGGGACGGATTATCGCCCGTCCG
CAATCTGGTTATTATGTCGCGCCGCATCCGGTTTGTCGGTCAGTCGCGACGGCAGCGCAC
GTTATTCGGGATGAAGCCGTAGATATCAATACCTATATTTTTGAGATGCTGCAGGCGAGC
CGTGATACCTCTGTGGTGCCCTTTGCCTCAGCGTTTCCCGATCCGCGTCTGTTTCCCTTG
CCGCAATTGAACCGCTCTCTGGCGCAGGTCAGTAAAACCGCGACGGCAATGAGCGTCATC
GAAAACTTGCCGCCAGGTAATGCAGAATTGCGCTATGCGATAGCACGCCGTTACGCGCAG
CAGGGGATTACCGTTTCTCCTGACGAGATTGTCATTACCGCCGGCGCGCTGGAAGCGTTG
AATCTTAGTTTACAGGCGGTGACGGCGCCGGGAGACTGGGTGGTGGTGGAGAACCCCTGT
TTCTACGGTGCGCTACAGGCCCTGGAGCGATTGCGCCTGAAAGCGTTGTCGATACCTACC
GATGTTAAAGAAGGTATCGATCTTATGGCGCTGGAACAGGCGTTACAGGAATATCCGGTG
AAAGCGTGCTGGTTAATGACCAACAGCCAGAATCCGTTAGGGTTCACGCTGAGTGCTGAG
AAAAAAGCGCGGCTGGTTGCGTTACTCACGCACCATAACGTGACGCTAATTGAAGACGAT
GTTTATAGCGAACTCTACTTTGGCCGCGAGAAACCTCTGCCTGCCAAAGCATGGGATCGC
GACGATACCGTGTTGCATTGTTCGTCTTTTTCCAAATGTCTGGTTCCCGGTTTTCGCATT
GGCTGGGTTGCGGGAGGAAGCCATGCCAGACAAATTCAGCGTTTGCAGTTAATGAGCACA
TTATCCACCAGTTCCCCCATGCAATTGGCGTTAGTGGATTATCTCTCGACACGTCGATAT
GACGCGCATCTGCGACGATTGCGAAGGCAGCTTGCCGAACGTAAACAGCAAGCCTGGCAA
ACCCTTTTGCGCCATCTCCCGGCGGAAGTAAAAATTCACCATAACGATAGCGGTTATTTT
CTTTGGCTGGAGCTACCTGAGCAGCTTGATGCCGGTGAACTTAGCGCCAAAGCGCTTGAA
CACCTTATCAGTATCGCGCCGGGGAAAATGTTTTCAACGTCCGGCGCCTGGACGCGTTTC
TTTCGTTTTAACACCGCATGGCATTGGGGAGAACGGGAAGAACAGGCCGTAAAACAGCTT
GGCAGCCTGATTCGCGAGATGCTGAGCGCTAAAAGCCTTGTGTGA
>EECEBICD_01941 D-alanyl-D-alanine dipeptidase
ATGGTAAAATGCGTATCTTCTTTCCTCCTGTTCAGCCTGTTGTCCGTTCAGGCAATGTCA
GCTGAAAACCATATTGATCTCCACCAGCCAAAAGACTTTGTCGATATTACTACGGTCGCC

CCCGACGTACAGGTAGACATGCGATACTTCAGTTCCCATAACTTTATTGGTCGCCCCATT
AAAGGCTATAACGCGCCTGTTTGCCTGTTAACACGACCAGCCGCGAACGCAGTGAAGCAG
GTCGCCGATCGTTTACGCCCCTTCGGACTTACCTTAAAAATATATGATTGCTACCGTCCG
CAAAGCGCAGTGAACGACTTTATCGCGTGGGCCAAAGATCCTTCTCAAAACCAAATGAAA
AACGAATTTTATCCGCAGGTAGAGAAAAATCGTCTGTTTGAGGAAGGTTATCTTGCCGCC
AGATCCGGCCACAGTCGGGGAAGTACGCTTGATCTAACGATTGTTCCACTTGACAGTAAA
ATACCAATATACGATCCCGGACGACCACTGGTGAATTGTACTGCGTCCGCGGCGCAACGC
TCGCCAGATAATAGTCTGGATTTTGGTACCGGCTTTGACTGTTTTAGTCCGTTATCCCAT
CCCGATAATGTCATGCTTACCGCTCAGCAACGCGCAAATAGGTTGTTATTACAAACATTG
ATGCGTGATGCGGGTTTTACGCCACTGGATACTGAATGGTGGCACTTCTCTCTTACTCAT
GAACCATACCCGAACACTTGGTTTGACTTTCCCGTTAAGCAGAGACCCTGA
>EECEBICD_01942 hypothetical protein
ATGAAGAAATCAGATGGTGAAATTCACGAAAAGACAGCATCCTGGGGCATTTTGCAGTCA
GAATGGCTAAGAAAATGTGGACGGCTATTATTGCTGTTACTTTACCGTTTCGTTATCGGA
TGGGCTTTTTTTCAATTACTTGCCATGATCGTGGCAGGGATATTTTTGTTAGGCGTCTTA
TTATTTCATCCCATAATATTTGTACAGACTATCGCAATCACTGAGAAGTTAAATCATGCT
TCGCTTGATTTATGGCATATCCTTAAGTTATGCCTATGGCATTACGGTATCATCGCAGGG
TTTATTTTTATGGCGGAGTGTACGTTAAGTAAAAGTATACGGCAGGTTCAGCGCTTGTCC
AAAAAATTTGGGGCACAGGATTTTTCTTCACGCCCGTGA
>EECEBICD_01943 hypothetical protein
ATGTTGCCTCTCCGCCTGACGCGCAACGTTTATTTGATTTATTTTGCGCGTTATATGAAT
TATCATCTCCGTCATGCAGAGGAAATTTCCATTTTCAGCACTATAAAGATGCTGAGTGTC
AATATACAAATCTCTTTATTAAAGATGGCGAAGATATTCCGTTATGCATTGTGA
>EECEBICD_01944 hypothetical protein
GTGATACGTCAGGATCACTACTATTATGATATAATGAATAGAACAGTGCTCTGTGTTGAT
ACTCAGCCTGCCCACCTGAAAAGATATTCTGATATTACTATAAAAGCTAGCACTTATGTT
TGTGAAGAGCTATGTTGCTTGTTCCCTGAGCGGTTACTACTCTCGCTATCCGGCGGTATA
ACATTTCCCGTTGACCTAAAAAATATAAAAGAGACGCTTATCGCCATGGCAGAAAAGGGT
AATTTATGTGACTGGAAGGAACAGGAAAGAAAAGCCGCCATCTCCTCCAGGATTAATCTG
GGCATTGCCCAGGCAGATGTACCACCTATTGACGACGCTATCAAAAACAAAATAGCCGCA
AAGGTTATTGAGAATACTAACTTAAAGAATGCAGCGTTTGAACCAAATTATGCACAAAGC
TCAGTTACTCAAATTGTTTATTCATGTTTGTTTAAGAATGAGATACTAATGAATATGCTG
GAAGAAAGTTCCTTTCATGGCCTGCTATGTTTAAACGAGCTTACTGAATATGTAGCGCTA
CAAGTTCATAACAGCCTTTTTTCTGAAGATTTATCTTCACTCGTTGAAACAACAAAAAAT
GAGGCTCATCACCAGAGTTGA
>EECEBICD_01945 hypothetical protein
ATGCTTACAAAAAACGTTATCAGTAGTTTTACTGACCTGTGCGCTGTTTTCAGGCCAGTTA
CTGGCGAAACAGCAAGACCACGCGTTCGTATGGTTTGCGACGGGAGGGCACCAGCTTAGA
CACGAAGCCGACAGCGATGAATTACGGGCGGCAGCGGAAGAATCCGCGGAAGGCTTGCGC
GAGCACCACAACTGGCAGAAATCGCGCAAACCAGAAAGCTATTTTCGTTAA
>EECEBICD_01946 23S rRNA 5-hydroxycytidine synthase
ATGCGCCAGCAACCTCACTATCTCGAATTATTAAGTCCGGCCCGTGACGCCGCAATTGCT
CGCGAAGCGATTTTGCATGGCGCAGATGCCGTCTACATCGGCGGACCCGGTTTTGGCGCA
CGTCATAACGCCAGTAACAGTCTGCGCGATATCGCCGATCTGGTTCCGTTTGCTCACCGT
TATGGCGCCAGGATTTTTGTCACGCTGAATACTATCCTGCATGATGATGAGCTGGAGCCC
GCGCAGCGGTTAATCACCGATTTGTACGACACCGGGGTGGATGCGCTGATTGTGCAGGAT
ATGGGCATTCTGGAACTGGATATCCCGCCGATTGAGCTTCACGCCAGTACACAGTGTGAT
ATTCGCAGCGTGGAAAAAGCGAAGTTTCTTGCCGATGTCGGTTTTTCACAGATTGTACTG
GCGCGCGAGCTTAATTTGAGTCAGATAGCGGCTATTCATCAGGCTACTGACGCCACGATT
GAGTTCTTCATTCATGGCGCGCTGTGTGTCGCTTATTCTGGGCAGTGTTATATCTCTCAT
GCGCAAACCGGGCGCAGCGCCAATCGGGGCGACTGTTCGCAAGCCTGTCGCTTACCGTAT
ACGTTAAAAGACGATCAGGGGCGGGTGGTCTCTTACGAAAAACATTTGCTATCGATGAAA
GATAACGACCAAACGGCTAACCTCGGCGCGTTGATCGATGCAGGCGTACGTTCCTTCAAG
ATTGAAGGGCGCTATAAAGACATGAGCTATGTCAAAAACATCACCGCGCATTATCGTCAG

ATGCTGGACGCGATTATCGAGCAACGTGGCGATCTGGCGCGTGCGTCGGTTGGTCGGACC
GAACACTTTTTTGTTCCCTCCACGGAGAAAACTTTCCATCGCGGCAGCACCGACTATTTT
GTTAACGCGCGTAAAGGTGATATTGGCGCATTTGATTCACCAAAATTTATTGGCTTGCCG
GTAGGTGAGGTGCTGAATGTGGCGAAGGATTATCTCGACGTAGAAGCTACGGAGCCGTTG
GCGAATGGCGATGGTCTGAACGTGTTGATTAAGCGTGAAGTGGTAGGTTTTCGCGCCAAT
ACGGTGGAGAAAACCGGTCATAACCGCTACCGCGTTTGGCCAAATGATATGCCTGCCGAC
CTGCATAAAGTCCGTCCGCATCATCCGTTGAATCGTAATCTGGATCATAACTGGCAGCAA
GCGCTGACAAAAACCTCCAGTGAGCGCCGTGTGGCGGTTGATATCATGCTGGGCGGCTGG
CAGGAACAGCTTATTCTGACGCTGACCAGTGAAGACGGTGTCTGCATCACGCATACGCTT
GACGGGGTATTTGAGGAAGCCAACAACTCTGAAAAAGCGTTGAATAACCTAAAAGCCGGA
CTGGCGAAGCTGGGACAGACGCCTTACTACGCGCGTGATATGCAGGTGACATTACCGGCG
GCGTTGTTCGTGCCAAATAGCCTGCTCAATCAGTTCCGTCGGGAGGCGATTGATATGCTT
GACGCGGCGCGGCTGGCCCATTATCAACGAGGTCGTCGTAAACCCGTGGCGCAGCCTGCG
CCGGTCTATCCGCAAACGCATCTCAGCTTTCTCGCTAATGTCTACAACCACAAAGCACGG
GAATTTTATCACCGTTACGGCGTACAATTGATTGATGCGGCCTATGAGGCGCATCAGGAG
AAGGGCGAGGTACCGGTCATGATCACCAAACACTGCCTGCGTTTTGCGTTCAACCTTTGT
CCAAAGCAGGCGAAAGGAAATATTAAGAGCTGGAAAGCCACGCCGATGCAGTTGGTGCAT
GGCGATGAGGTACTGACGCTAAAATTCGACTGCCGCCCTTGCGAAATGCATGTCATTGGC
AAAATTAAAAACCACATCTTAAAAATGCCCCAGCCCGGCAGCGTTGTCGCTTCAGTGAGC
CCTGAAGCGCTGATGAAAACGCTGCCGAAGCGCAGGGGCGTTTAA
>EECEBICD_01947 HTH-type transcriptional regulator SutR
ATGGCCTTACTGGGGGGGAAAATGGATAACCTGACACACTATCTGGCGACCACGTTAAGA
ATGTTACGCCATCAGCGAGGCTGGAGTTTATCGCGTCTGGCGGAGATCTCCGGCGTCTCA
AAAGCCATGTTGGGGCAGATAGAGCGTAATGAATCCAGCCCGACAGTCGCAACGCTGTGG
AAAATTGCGACGGGCCTGAACGTTCCATTCTCAGTGTTTATTTCGCCGCCGCAGGCCGAA
TTTCCTCCGACCTTCGATCCCCAGCAGCAAGCGATGGTAATCACACCGTTGTTTCCATGG
GATCCTGAACTCTGTTTTGACTATTTTTCCCTATTGCTGGCGCCTGGCGCGGTCAGCGAA
TCAACGCCGCATAAAGCCGGCGTTGTCGAACATGTGGTGGTGATTCAGGGTCGGCTGGAC
ATGTGTACTGGTGGGGTATGGCAAACCATAGACGCCGGTAAAGGATTGCGTTTTGCCGGT
GATATGGCGCATACCTATCGCAATAGCAGTGACCAGACGGTTCATTTTCACTCGCTGATC
CATTACCCGCGTGGTTAA
>EECEBICD_01948 Inner membrane protein YdcO
ATGCGTCTGTTATCACTGCCGTTACCTACTGTATTATCCGGTCTGGTCGCCGTTCTGGTC
GGCTATGCAAGTTCCGCCGCCATTATCTGGCAGGCGGCGCTTGCCGCTGGCGCAACACCC
GCAGAGATCGCCGGCTGGATGACCGCCCTCGGTATAGCCATGGGCATAAGCACACTAACG
TTAACCTTGTGGTATCGTGCGCCGGTTTTAACCGCCTGGTCAACGCCTGGCGCCGCTCTG
TTGGTAACAGGGCTTCAGGGATTATCGTTGCCAGACGCGGTGGGCATATTCATTGTCGCC
AATGCCCTGATTGTGCTGTGCGGCGTCACGGGATTGTTTGCGCGTTTGATGCGGATAATC
CCCCATTCTCTTGCCGCTGCAATGCTGGCGGGAATACTGCTACGCTTCGGCCTGCAGGCG
TTCGGGACGCTCAACGGAGAGTTTGTCATGTGCGGCGGTATGCTATTGGCGTGGCTACTC
TTTAAAGTATTCGCGCCCCGCTATGCCGTTATTGCTGCCATGGTGATGGGAATAACGGTG
GCGCTCATCCAGGGTAAGGTGGCGATGAGCGGTATTCATTTCGCGCCGGTGTGGCCCACC
TTCGTTCCCCCCCACTTTTCATTCGCTCAAAGCCTGAGCGTCGCGGTACCGCTCTTCCTG
GTGACGATGGCATCGCAAAACGCCCCCGGCGTCGCCACAATGAAGGCGTCTGGCTATCAG
CTTCCTGTCTCTCCGTTAATGATTTTTACCGGATTGCTGGCGCTGCTGCTTTCGCCATTT
GGCGTTTATTCTATCTGCATTGCCGCCATTACAGCCGCCATTTGTCAAAGCCCGGACGCG
CACCCCGATCCAACGCGTCGCTGGTTAGCGGCCGCCGCCGCAGGCGTTTTTTATTTACTT
GCCGGGTGGTTTGGCGGTTCGATAACCGGGCTGATGGTCGCCCTGCCGGTTAGCTGGGTG
CAAATGCTGGCTGGTCTGGCACTGTTAAGTACGATTAGCGGTAGCCTGTATCAGGCGCTT
ACTCACGAAAGTGAGCGTGACGCGGCAGTTATCGCATTTCTCGTCACCGCCAGCGGGCTA
ACGCTGATGGGCATCGGATCGGCATTCTGGGGGTTGATCGCTGGCGGGATAGGCTATGCA
GTATTAACGAGAACGCGCCGCCCATCGTTGTCTGGCTGA
>EECEBICD_01949 hypothetical protein
ATGCGTACTCATACTTTATTTAAAGTTGCAGTGCTTACTGGCTTGCTGGCGTTATCCGGC

TGTGCGTCTAAAGTCACACAGCCGGATAAGTATTCTGGATTCTTAAAAAATTATTCGGAC
CTGCAGGAAACTACCTCCGCCACGGGGAAACCGGTGTTGCGCTGGGTAGATCCACATTTC
AATGACTCTAACTATGACAGTATTGTTTACAATCCGATAACATACTATCCCGTCCCCAAA
CCAACGACCCAGGTCGGGCAGCAAGTTCTTGATAAACTGCTGGCTTATACGAACACTAAA
GTAAAATCAGCAATTGAACAGCGCAAACCGCTGGTCACGACGCCAGGGCCGCGCAGTTTG
ATTTTCCGCGGGGCGATTACGGGCGTGGATACCAGCAAAGAAGGGCTGCAATTCTATGAG
GTGATCCCTGTAGCGCTGATTGTGGCGGGCACGCAAATGGCGACGGGCCACCGGACCATG
GATACCCACCTCTATTTTGAAGGTGAGCTGATAGATGCCGCGACCAACAAGCCGGTCGTC
AAGGTAGTACGCCAGGGTGAAGGTAAAGACCTGAGTAACTCCAGTACGCCAATGGCGTTT
GAGACGTTGAAACAGGTCGTGGATGATATGGCGACGGATACCTCTATGTTTGACGTTAAC
AAAAAATAA
>EECEBICD_01950 Tellurite methyltransferase
ATGACCGTTCGTGACGAAAATTACTTTACTGAGAAATACGGTTTAACCCGCACGCATTCG
GATGTGCTGGCGGCGGCAAAGGTTGTCGCGCCAGGCCGAACGCTGGATTTGGGATGTGGC
AATGGCCGTAATAGTCTGTATCTGGCCGCGAACGGTTATGACGTTACCGCGTGGGATAAA
AATCCGGCCAGTATGGCTAATCTTGAGCGTATTAAAGCGGCAGAAGGGCTGGATAATCTG
CAAACCGACCTTGTCGATCTGAATACATTGACCTTTGACGGTGAGTACGACTTTATTCTC
TCGACGGTGGTTATGATGTTTCTTGAGGCGCAAACCATTCCCAGTTTAATTGCCAATATG
CAACGCTGCACAAAACCCGGCGGGTATAATTTGATTGTTGCGGCAATGGATACGCCGGAC
TTTCCCTGTACGGTGGGATTCCCGTTTGCTTTTAAAGAAGGCGAATTACGCCGCTATTAC
GAAGGATGGGACATGCTGAAATACAACGAAGATGTCGGCGAGCTTCACCGCACGGACGAA
AACGGCAACCGTATCAAACTGCGCTTTGCTACGATGCTGGCGAGAAAAACGGCCTGA
>EECEBICD_01951 Tellurite resistance protein TehA
ATGCGTAATCACAAGCAGAGCGATCGCGTGCTCAATCTGCCTGCGGGCTATTTTGGCATC
GTCCTTGGCACTATCGGTATGGGATTCGCCTGGCGTTATGCCAGCCAGATATGGGGAATA
AGCCACTGGCCCGGCGACATCATGGTGATCCTGGCGATGATTATTTGGGCACTCTTGACC
CTGGCGTTCCTTAGCCGTCTGGTGCGCTTTCCCCATAGCGTGATGGCTGAAGTGCGACAT
CCGGTAATGAGCAGTTTTGTCAGCCTGTTTCCCGCCACCACGATGCTGGTCGCGATCGGT
TTCGTTCCCTGGTATCGCCCGTTGGCCGTTGCGCTATTCAGCGTGGGGGTCGTCATACAA
CTGGCGTATGCCGCCTGGCAGACGGCGGGACTTTGGCGCGGAGCGCATCCGGAAGAAGCG
ACAACCCCGGGGCTTTATCTGCCCACGGTAGCGAATAATTTTATCAGCGCTATGGCCTGT
GGAGCGCTCGGTTACAATGATGCCGGACTGGTGTTTTTAGGCGCAGGCGTCTTTTCGTGG
TTAAGTCTGGAGCCGGTTATTTTGCAGCGACTGCGTAGCTGCGGCGAATTACCGGCGGTG
CTGCGTACGTCGCTGGGTATCCAACTGGCGCCCGCGCTGGTCGCCTGTAGCGCCTGGCTG
AGCGTCAATGGCGGGGAGGGCGACACGCTGGCGAAAATGCTATTCGGCTACGGTCTGTTA
CAGCTTTTATTTATGCTGCGATTAATGCCCTGGTATTTATCACAACCGTTTAATGCTTCC
TTCTGGAGTTTTTCGTTCGGCGTTTCCGCGCTGGCGACCACGGGGTTACATCTTGGTCAC
GGTAGTGAATCAGGGCTTTTCCATATTCTGGCCGTCCCGCTGTTTATTTTCACTAACGCT
ATTATCGCTTTATTGCTGGTTCGTACATTTCTATTGCTGGTGCAGGGGACGTTGCTTATT
CGCACTGAGCGCGCTGCACTGTTGAAAACGGAGGAAAAAAATGACCGTTCGTGA
>EECEBICD_01952 hypothetical protein
ATGATCAAATATCGACTGAGTGAAGAACCTCGTGCTTTTACTTATCAGGTAGACGGTGAG
AAGAAAAGCGTCTTATTACGACAAGTTATTGCCGTAACGGATTTTAACGATGTTAAAGCC
GGAACCTCCGGCGGCTGGGTTGATGCCGATAATGTGCTGTCACAACAGGGGGATTGCTGG
ATTTATGATGAAAACGCGATGGCCTTTGCGGGCACGGAAATTACGGGTAACGCGCGTATC
ACGCTGCCGTGCACGCTCTACAATAACGTGCGAATAGGCGATAACGTCTGGGTTGATCGC
GCTGATATCAGCGATGGCGCACGGATAAGTGACAACGTCACCATTCAGTCCTCTTCCGTG
CGCGGAGAGTGTGCGATTTATGGCGATGCCCGCGTGTTAAACCAGTCAGAAATTCTCGCC
GTTCAGGGACTAACGCATGAACATGCGCAGATTTTACAAATTTACGATCGCGCCACCGTG
AACCATTCGCGGATTGTCCATCAGGTACAGCTTTATGGCGACGCCACTATCACACACGCT
TTTATTGAGCACCGGGCGGAGGTCTTTGATTTCGCGCTCATCGAAGGCAACAAAGACAAT
AATGTCTGGATCTGTGACTGCGCCAAAGTCTATGGCCATGCGCGGGTTATCGCCGGTACA
GAGGAAGATGCGATCCCTACCCTTCGCTACAGCTCGCAGGTAGCGGAACATGCGTTAATC
GAAGGCAACTGTGTGTTAAAACATCATGTGCTGGTAGGCGGACATGCTGAGGTACGCGGA

GGCCCGATTTTACTGGACGACCGCGTGCTGATTGAAGGCCATGCCTGTATACAGGGCGAA
ATTCTGATTGAGCACCAGGTCGAGATTAGCGGACGGGCGGCGGTGATAGCCTTTGACGGC
AATACTATTCACCTGCGCGGCCCAAAAGTGATCAATGGCGAGGATCGTATTACGCGTACC
CCCTTAGTCGGTTCCCTTTAA
>EECEBICD_01953 Ribosomal-protein-serine acetyltransferase
ATGGTGGAAATTATTCCTGTTAGCACGACGCTGGAACTGCGAGCGGCAGATGAAAGTCAT
GTTCCGGCGCTGCACGAACTGGTACTCAAAAACAAAGCGTGGCTACAACAGTCGCTCGAC
TGGCCGCAATATGTCACGTCGCAGGAGGAAACGCGTAAGCATGTGCAGGGTAATATACTG
TTGCACCAGCGTGGTTACGCCAAAATGTACCTGATTTTCTGCCAGAATGAGATGGCGGGC
GTACTCTCATTTAATGCCATTGAACCGGTAAACAAAGCCGCCTATATCGGTTACTGGCTG
GATGAGTCCCTCCAGGGACAAGGGATAATGTCGCAATCATTACAGGCGCTGATGACACAT
TATGCCCGGCGCGGCGATATTCGGCGCTTCGTCATTAAATGCCGCGTTGATAATCAGGCC
AGTAACGCGGTCGCACGGCGCAACCATTTTACGCTGGAAGGCTGTATGAAACAGGCAGAG
TATCTTAACGGCGACTATCACGACGTTAATATGTATGCCCGCATTATTGATGCGGATTAA
>EECEBICD_01954 Putative aminopeptidase YsdC
ATGACCTTTTCTGTGCAGGAAACGCTTTTTTCTTTACTGCGGCTAAACGGGATTTCAGGA
CATGAAAGCAGTATTGCAAACGTTATGCAGCACGCGTTTGAACAGCAGGCCAAAGACGTC
TGGCGGGATCGCTTAGGCAATGTCGTCGCCCGTTATGGCAGCGACAAATCCGACGCGCTT
CGCCTGATGATTTTTGCGCATATGGATGAAGTCGGTTTTATGGTACGCAAGATCGAACCC
TCCGGCTTTTTACGTTTTGAACGCGTGGGCGGCCCGGCGCAAATTACTATGCCCGGTTCG
GTCGTGACGCTTGCCGGACGTTCAGGCGATATCATGGGCTGTATCGGTATTAAAGCATAT
CACTTCGCGAAGGGTGACGAGCGCACCCAGCCTCCCGCGCTCGATAAACTCTGGATTGAT
ATCGGCGCAAAAGATAAAGCGGATGCCGAACGAATGGGTATTCAGGTGGGGACGCCAGTA
ACCCTTTACAACCCGCCGCACTGTCTGGGCAACGACCTGGTGTGCAGTAAGGCGCTGGAT
GACAGGCTGGGGTGTACGGCGCTACTGGGCGTCGCCGAGGCCCTCGCCTCCACACCGCTC
GATATCGCGGTATTCCTGGTCGCGTCGGTACAGGAAGAGTTCAATATTCGCGGTATTGTT
CCCGTTTTACGACGCGTGCGCCCCGACCTGGCGATTGGTATTGATATCACCCCCTCCTGC
GACACGCCTGACCTGCAGGATTACTCTGATGTGCGGGTCAACCACGGCGTCGGCATCACC
TGTCTGAACTATCACGGACGCGGTACGTTGGCGGGACTGATTACGCCGCCGCGTTTGCTG
CGGATGCTGGAGACCACCGCGCACGAAAATAATATTCCCGTACAGCGAGAAGTCGCGCCA
GGCGTCATCACCGAAACCGGCTACATTCAGGTTGAACTGGACGGTATTCCCTGCGCCAGT
CTTTCTATTCCCTGCCGCTATACCCACTCGCCAGCCGAAGTCGCCAGCCTGCGCGACCTG
GCTGATTGTATCCGTTTACTGACTGCGCTGGCTAACATGTCGCCAGAACAGTTTCCCATT
GAGCCTGAAACAGGCGCTACACAAGAGGCACGACCATGA
>EECEBICD_01955 PTS system galactitol-specific EIIB component
ATGATGAAAAAGATCCTTGTGGCATGTGGTACCGGCATGTCGACATCAACCATGATTGCA
CAAAAACTGCAGGACTTTCTTGCGGAGCAAGGCATTGCGGCAACCACGGCGCAATGTTGT
CTGAATGAGATCCCGTTGAATTGTAACGGTATGGATCTCATCGTAACGTCCATGCGTACG
CACAGTGACTATGGCATCCCGACGCTCAACGGCGCCGCCCTGCTGACAGGCATCAACGAC
GACGCATTAAAACAAGAAATCAAGGCGCTGTTAACACAATAA
>EECEBICD_01956 PTS system galactitol-specific EIIC component
ATGTTTGATTACATCCTGTCTCTCGGCGGCACCGTTTTTGTGCCCATCATTATGATTATC
ATCGGCTTAATCTTTCGTATTCCCTGGCTGCAGGCGGTGAAAGCCGGGGTGACGGTCGGC
ATCGGTTTTGTCGGCATGGGGCTGGTGATCGTCATGGCGATCGACAGTCTCAGTCCGCCG
ATTAAAGTGATGATTGAGCGTTTTGGCCTGACGCTGCATGTGTTTGACGTCGGCGCCGGG
CCCGCATCCGGCGTCGGCTATGCGACGGCCATCGGCGCGATGATCATTCCCGTTATCTTT
CTGCTCAACGTCGGTATGCTGGTGACTCGTCTGACCAAAACCATGAACGTTGATATTTAT
AACTACTGGCACTACGCCATTACCGGAACGGTCGTCCAGTTGATGACCGGCAGTCTGATT
TATGGCGTACTGGGCGCGATTTGCCACGCCGCCTTGTCGTTGAAAATGGCCGACTGGACG
GCGAAACGAGTACAGAACATTGTGGGTCTGGAAGGGATTTCTATTCCGCAAGGGTATGGC
TCCAGTTCCGTGCCGTTGTTCGTATTGCTGGATGCGATATACGAAAAAAATACCGTTTATG
AAAGGACGCAATATTGATGCTCAGGAGATCCAAAAACGGTATGGGATGGTTGGCGACCCG
GTGATCATTGGCGTTGTTCTCGGGCTGATCTTCGGACTTGCCGCAGGCGAAGGCTTTAAA
GGGTGCGCAACCTTAATGATTACCGTGGCGGCGATTATGGTGCTGTTTCCGCGCATGATT

CGCCTTATCGTTGAGGGATTAATGCCCATTTCCGACGGCGCGCGTAAGTTCTTCCAGAAA
CACTTTAAAGGGCGCGAAGTCTTTATCGGTCTGGATACCGCAGTGACGTTAGGCCACCCG
ACCACCATCGCGGTTGGACTGCTGTTAATTCCCATCATGTTAATTCTGGCGAGCATACTG
CCGGGCAATAAAGTCCTGCCGCTGGCTGATTTACCGGTTGCGCCGTTTTTTATCTGCATG
GCCACGGTTATTCATCGCGGCGATCTGATTCGTACTCTGCTCAGCGGCATCATTGTCATG
ATTACCGTTTTGCTGATCGCCACCCAGTTCGCGCCGTACTTTACCGATATGGCGCTCAAA
GGCGGCTTTAGCTTCGCCGCTGAAAACGCGCAAATTACCGCGCTGTCGGTGGGAAATATG
TTTGGCTGGTCAATATCTGAATTGATGTCGCTGGGCATGATTGGTGTGGTTATTGTGGTC
GGCATTGTCGCCAGCATCATACTTGTCTTACGTAAACGTGAACTACCGGAATAA
>EECEBICD_01957 hypothetical protein
ATGAGTTGGCTAAAAGAGGTAATAGGAACGGAAAAAGCCGTTATCGCCATGTGCCATTTA
CGGGCGCTGCCGGGCGATCCCGGGTTCGATACCAGGAAAGGCATGAATTGGGTAATTGAC
CGCGCCCATGACGATTTAATGGCGCTACAAAATGGCGGCGTGGATGCGGTGATGTTCTCC
AACGAATTTAGTCTGCCTTATTTAACGAAAGTCAGACCGGAAACGACCGCCGCGATGGCA
AGAATCATCGGTCAGTTAATGAGTGAAATCCGCGTACCGTTTGGGGTAAACGTGTTGTGG
GACCCTGTGGCCTCGTTTGATTTGGCGATGGCGACCGACGCAAAATTTATTCGCGAAATT
TTCACCGGCGCTTACGCCAGCGATTTTGGCGTCTGGGATACGAATGTCGGCGAAACCATT
CGCCATCAGCATCGCATCGGCGCAGGCCACGTGAAAACGCTGTTTAACATCGTGCCGGAA
GCGGCGGTGTATCTGGGAAACCGGGATGTCTGTTCAATTGCGAAATCGACCGTATTTAAC
AATAACCCCGATGCCCTGTGCGTCTCGGGGCTGACGGCGGGCGCTCGCACGGATAGCGCC
ATACTGAAGCGGGTAAAAGAGACCGTCCCCGACACGGTCGTGCTGGCGAATACCGGGGTG
TGTCTGGAAAACGTCGAGGAGCAGCTCTGCATTGCCGACGGATGCGTAACGGCAACCACG
TTTAAAAAAGACGGCGTCTTCGCCAACTTTGTCGACCAGGCGCGCGTGGCGAAGTTTATG
GAGAAAGTACGCCATATCCGACAATAA
>EECEBICD_01958 Ascorbate-specific PTS system EIIA component
ATGATTAATGATGTTAAGTGGGTTCAGGCGCAACGCCATGCCAGCGACTGGCGACACGCG
CTGGATATCGCCGTTCGTCCGCTGATTACCTTCGGCGCGGCGGCCCCCTGCTACCTGGAC
GGTATTATTGAGAACACCCTTAAATGGGGGCCATATTATCTTATCGCGCCGGGGATTGCG
TTGCCCCATGCCAGACCAGAGCAAGGCGCCAACCATAATCAAATCAGCGTCACTACGTTA
GCAACGCCGGTCGCTTTCGGCAATGCGGATTGCGATCCCATCTGGCTGTTGCTTTGCGCC
AGCGCCACGGATGCCGAAGCACATATTCGCACCATTCAACGCATCAGCCAGTGGCTAGAC
TCGCCGGAAAGCGTCGCGATGCTGCAAGATGCGCCAAATGACGCCGCGCTTTTCGCGCAA
TTAACCGCATTGCGCTGA
>EECEBICD_01959 Ribulose-phosphate 3-epimerase
ATGATCCTGCACCCTTCACTGGCCTCCGCCGATCCGCTGCGCTATGCCGAGGCGTTGACC
GCTTTACACGATGCGCCCCTCGGCTCGTTGCATCTGGATATCGAAGATACCAGTTTCATC
AACAATATTACCTTCGGTATGAAAACCATTCAGGCAGTCGCACAACACACCCGCCATCCG
CTCTCATTTCACCTGATGGTCTCATCACCGCAACGCTGGCTACCCTGGCTTGCAGCTATC
CGCCCCGGCTGGATTTTCATTCATGCCGAAAGCGTGCAAAACCCCCCGGAGATTCTGGCG
GATATTCGCGCTATCGGGGCGAAAGCAGGACTGGCGCTTAACCCGGCAACGCCGCTATTG
CCTTATCGTTATCTGGCATTGCAACTGGATGCGCTGATGATCATGACCAGCGAACCCGAC
GGACGCGGACAGCAATTTATCGCCGCGATGTGTGAAAAAGTGAGCCAGGGCAGAGAGCAT
TTCCCTGCTGCGGAATGCTGGGCGGACGGCGGTATTACACTTCGCGCCGCACGGCTGCTT
GCCGCCGCAGGCGCACAGCATCTGGTGATTGGCCGGGCGTTATTCACGACGGCGAACTAT
GATGTGACGTTGTCACAATTCACCGCGCTATAA
>EECEBICD_01960 Glucitol operon repressor
ATGAGCCAACAGCGCCCCGACCGTATCAAACAGATGTTGCATTACCTTTGGCAACACAGG
CACCTCTCAACACAGCAAGCTATTGAGCTTTTTGGCTATGCAGAGGCAACCGTGCGCCGG
GATTTTCACTATATTGCCAGCCGTTATCCCGGTATGGTTCGCGGCCATGGTTGTATTGAC
TTCGACGACAGTACGGAAGATAAAGAATATGTTTTCGACGTTAAGCGGACGTTACAAAGC
GAGGCGAAACGCGAAATCGCTGCCCTCGCCCGCACGTTCATCAAGGATGGAGACTGCTTT
TTTCTCGACTCCGGCTCCACCTGTCTGGAACTGGCAAAATGTCTGGTCGACGCCAAAGTA
AAAGTCATTTGCAATGACATCAAAATCGCTAATGAACTGGGTGGCTTCCCACACGTAGAG
AGTTACATTATCGGCGGACTGATTCGCCCCGGTTACTTTTCGGTGGGCGAGAGTCTGGCG

CTGGAGATGATTAACGCCTTTGCCGTTGAACGCGCCTTTATCTCCTGCGATGCGCTGTCG
ATTGAAACGGGCATCACCAACGCTACCATGTTTGAAGTGGGCGTTAAAACGCGCATTATT
CAGCGCTCACGCGAGGTCATATTGATGGCCGATCACTCAAAGTTTGATACTGTAGAACCC
CATGCGGTGGCGACGCTGTCCTGTATTACTACCATTCTTAGCGACAGCGCGCTCCCGTCT
GCTATCGCCCGACGTTATCAGCAGGCGGGGTGCCGTCTCATCATGCCAGACCCCTCATCA
GGAGTGCGTTAA
>EECEBICD_01961 Aminoglycoside N(6')-acetyltransferase type 1
ATGGACATCAGGCAAATGAACAAAACCCATCTGGAGCACTGGCGCGGATTGCGAAAACAG
CTCTGGCCCGGTCACCCGGATGACGCCCATCTGGCAGACGGCGAAGAAATCCTGCAAGCC
GATCATTTGGCATCATTTGTTGCGATGGCAGACGGGGTGGCGATTGGCTTTGCGGATGCC
TCAATCCGCCACGATTATGTCAATGGCTGTGACAGTTCGCCCGTGGCTTTTCTTGAAGGT
ATTTTTGTTCTCCCCTCGTTCCGTCAACGCGGCGTAGCGAAACAATTGATTGCAGCGGTG
CAACGATGGGGAACGAATAAAGGGTGTCGGGAAATGGCCTCTGATACCACGCCGGAAAAT
ACAATTTCCCAGAAAGTTCACCTGGCATTAGGATTTGAGGAAACAGAGCGCGTCATTTTC
TACCGGAAGCGTTGTTGA
>EECEBICD_01962 D-galactonate dehydratase family member
GTGAGTAACCTGAAAATTACCAACGTGAAAACGATTCTGACGGCGCCGGGCGGCATTGAT
TTGGCAGTCGTTAAGATAGAAACCAACGAGCCAGGGCTGTATGGGCTGGGATGTGCGACA
TTTACCCAGCGTATCTTCGCGGTAAAAAGCGCGATCGATGAGTACATGGCGCCTTTTCTG
GTTGGCAAAGATCCGACCCGTATTGAGGACATCTGGCAGTCAGGCGTGGTGAGCGGCTAC
TGGCGCAATGGCCCGATCATGAACAATGCATTGTCCGGCGTGGACATGGCACTGTGGGAT
ATCAAAGGTAAGCTAGCGGGGATGCCAGTTTATGATCTGCTGGGCGGCAAATGTCGTGAT
GGGATCCCACTGTACTGCCACACCGATGGTGGTGATGAAGTTGAAGTGGAAGATAACATC
CGAGCCAGAATGGAAGAGGGTTACCAGTACGTTCGCTGCCAGATGGGAATGTATGGCGGT
GCGGGGACCGATGACCTTAAACTGATCGCCACGCAGCTGGCACGTGCGAAGAACATTCAG
CCGAAACGTTCACCGCGCAGCAAAACGCCCGGCATCTACTTTGACCCGGACGCCTACGCC
AAAAGCGTTCCGCGTCTGTTCGACCACCTGCGTAACAAGCTCGGTTTTGGCATCGAATTT
ATTCATGATGTACATGAGCGCGTGACCCCGGTGACTGCCATTAATCTGGCCAAAACGCTT
GAACAGTATCAACTATTTTATCTTGAGGATCCGGTGGCACCGGAAAACATCGACTGGCTG
AAAATGCTGCGCCAGCAGTCTTCCACTCCGATATCCATGGGTGAGCTGTTTGTGAATGTG
AATGAGTGGAAGCCGCTCATCGACAACAGGCTCATCGACTACATTCGCTGCCACGTGAGT
ACCATCGGCGGCATTACGCCGGCGAGGAAACTTGCTGTCTACAGCGAACTGAACGGTGTG
CGCACCGCATGGCACGGGCCGGGCGACATTTCTCCAGTTGGGGTTTGCGCCAATATGCAT
TTGGATTTGAGTTCACCGAACTTTGGCATTCAGGAATACACGCCAATGAACGACGCGCTG
CGTGATGTCTTCCCGGGCTGCCCTGAGATTGACCACGGTTATGCCTATCTGAACGATAAG
CCGGGTCTGGGTATTGACATTGATGAAGCCAAAGCGGCGAAATATCCGTGCGAGGGGGAA
TCCCATCCTGGACAATGGCGCGCACACCAGACGGTACGGCCTCAAGACCGTAAGCAATGA
>EECEBICD_01963 5-keto-D-gluconate 5-reductase
ATGACCGCTTTATTTGATTTAACTGGGAAAACGGCGCTGGTAACGGGTTCTGCACGAGGA
CTGGGCTTTGCCTACGCAGAGGGTCTTGCCGCTGCGGGTGCACGGGTTATCCTGAATGAT
ATTCGCGCCACGCTGTTGGCCGAATCAGTGGATACGCTGACCAGAAAAGGCTACGACGCG
CATGGCGTGGCTTTTGACGTCACCGATGAACTGGCGATTGAGGCAGCTTTTAGCAAACTT
GATGCAGAAGGGATCCATGTTGATATCCTGATCAATAACGCCGGTATTCAGTACCGCAAA
CCAATGGTCGAGCTGGAGCTGGAAAACTGGCAGAAGGTGATCGACACTAACCTGACCAGC
GCATTTTTAGTCTCCCGCTCGGCGGCAAAACGGATGATCGCCCGCAACAGCGGCGGCAAA
ATTATTAATATCGGCTCGCTTACCAGCCAGGCGGCCCGCCCGACTGTTGCCCCGTACACG
GCAGCAAAAGGCGGCATCAAAATGCTCACCTGCTCGATGGCTGCTGAATGGGCGCAGTTT
AATATCCAGACTAACGCCATTGGACCTGGCTACATTCTGACCGACATGAACACCGCGCTT
ATTGAAGACAAGCAGTTCGATAGCTGGGTGAAAAGCAGCACCCCTTCTCAACGTTGGGGT
CGTCCGGAAGAGCTCATCGGCACCGCCATCTTCTTATCATCCAAAGCATCAGATTATATC
AACGGGCAGATTATTTACGTCGATGGTGGCTGGCTCGCAGTTTTATAA
>EECEBICD_01964 D-galactonate transporter
ATGAAGGCTTCAAGACAACGACTCTTTATTCTCACATTGCTGTTTATTGTGACTGCAATT
AATTATATGGATCGCGCCAATCTTGCCGTCGCTGGTTCGAATATCCAGAATGATTTCAGT

CTGACGCCAACACAACTGGGTTTGCTTTTCTCCATGTTTACCTGGGCCTACGCTGCCAGT
CAAATTCCTGTCGGCTATGTTCTTGATCGCATTGGCTCACGTATTCTTTATGGTGGTGCG
ATTATTCTGTGGAGTATTTTTACCTTTATGATGGGGTTCGCCTCACACCATTTATTCGCG
ACGGCAACCGCTTCATTTGCAATGCTTCTGGCCTGCCGCGCATTAATTGGTGTTGCCGAA
GCGCCATCCTTCCCGTCTAACACAAAAATAATCGCCACCTGGTTCCCGGACCATGAACGT
GCCCGTGCGACCGCGATTTATTCCAGTGCACAATATATCGGTCTCGCGCTGCTGACGCCT
GCCCTCGCCTTTATTGTGGCTAACTACGGTTGGGAAATGTCGTTTTATCTGTCCGGTGGC
GCCGGTATTCTGTTTGGCATCTACTGGCTTATGTATTACCGCGATCCACAGCACAGCACT
GCTGTGAACCAGGCCGAACTGGATTATATCAAAGCAGGCGGCGGCTATGGCTCGGAGAAC
CAATCCTCCGTGAGTGCAAAAATCAGCTGGCAAAATATTAAATTCTTCCTCAGCAAAAAA
ACGATTTGGGGTTTGTTCATCACCCAGTTTGCCTGCTCGTCTACGCTCTATTTTTTCCTG
ACCTGGTTTATTGTTTACCTGGAAAAAGGACTGCATCTCTCTATTTCCAAAGCCGGGATA
GGCGCCATGCTGCCCTATATTATGGCGATGCTTGGGGTGCTCTGTGGCGGTACGCTAAGC
GACATGCTGCTGAAAAAAGGAAAATCCCGAACGCTGGCACGAAAATTACCCGTTATGGCT
GGCCTGTGCGTCACCATGATTATTGGCCTGGTCAATTTCTTTGAAAACCAGCCAGTTATT
GCGATTGTCATTCTGTCTGTTGCGTTCTTTGCCAACGCCTTTTCAAACCTGGGCTGGGTG
GTCTGGAGCGATGTAATTCCCCGTAATTTCTCGGGACTATGGGTGGCTTTTTAAATATT
TGCGGCAACCTTTCGGGGATCGTTAGCCCAATTGTTATTGGGGTTATTCTCCAGCGCACG
CAAAACTTCCAGTATGCCATGTGGTATATCGCAGGCGTCGCCGGACTGGGCTTATTAGCC
TACATATTCCTGGTGGGCAAAATTGAAGTGATCCTGCCTGGAAAAAAGAATGCCGACACT
GTGGATAAGAATGCCATTAACCCGGCAACTGCCAACAAATAA
>EECEBICD_01965 L-idonate 5-dehydrogenase (NAD(P)(+))
ATGCCGACACTGTGGATAAGAATGCCATTAACCCGGCAACTGCCAACAAATAATGAGGTC
ATTATCAAAATGGAAAAAATAACCTGTAACGCTTGTCTGGCCCATGCGGAAAAAGACGTA
CGCTTTGAATCACGTGAAATCGAGCACAGTGAACATGACGTCGTCGTGAAGGTCGCCTGC
GGCGGCATCTGTGGCTCTGATATTCACTACTATCAGCATGGCCGCGCGGGGATGTCTGTC
CTCAAGCATCCGATGGTGATTGGCCATGAGTTTGTTGGCGTGATCAGCAAAGTGCCAGCA
GGCAGCGACCTGAAAGTGGGCCAGACGGTTGCGGTGAACCCGTCCAGCCCATGCAATCAG
TGCGAGATGTGCCTTTCCGGCCATCAAAATCTGTGCGGCTCCATGCGCTTTATGGGCAGC
GCGCAGTTCAATCCGCATGTGAATGGCGGTTTTTCTGAATACGTGGTGGTAAAACCGGAG
CAATGCATTCCTTACGACAGGCGCGTGCCCGCAAACGTGATGGCCTTTTCGGAGCCGCTG
GCGGTCGCCATTCATGCAGTAAAAAAGGCAGGCCAGTTGACCGGCAAACGCGTACTGGTG
ATTGGCGCAGGCCCCATTGGTTGTCTGATCCTCGCGGCGGCGCGCAGTGCCGGAGCATCT
GAACTGGTAGCATCCGATCTCAGCCCACGCTGTCTGGAACTGGCACGCCAAATGGGGGCC
ACAGCGGTGATGGATCCGCGCGATGAAGAACAGGTTGCCCATTATCAGCAGCATAAAGGC
TACTTTGATGTCGTGTTTGAGGCCTCCGGCGCGCCGATTGCCGTGGCATCCACCGTCGAC
TTCACCCGCCCTGCCGGCACCATCGTTCAGGTGGGGATGGGCGCCAGCCCGGTAAGCTGG
CCGGTGTCAACGATGCTGGTTAAAGAACTCAACTGGGTCGGCTCATTCCGTTTTATCGGT
GAGTTCATCACCGCGGTACGCTGGCTGGAAGATGGGCGCGTCGATCCTCGCCCGCTTATC
AGCGCCGAGTTCCCGCCCCAGCAAATTGAAGACGCGCTGATTACCGCCACAGACAAAAAT
GTCTCTGCTAAGGTACTCATTCGTTTCGATTAA
>EECEBICD_01966 HTH-type transcriptional repressor NanR
TTGAGCATAAAATCCATTCAAAAACAGAATGTTGTAAATGAAATTTATGATCAGATAAGT
AGCAAACTGCTGGACGGCAGTTGGGCGCCGGGTAGCCGTTTGCCCTCAGAAGTGGAACTG
ACCGCCTCATTTAACGTCAGCCGGGTCAGCGTTCGCAGCGCAGTACAGCGTTTTCGTGAC
CTGGGGATTGTGGTGACGCGTCAGGGCAGCGGCAGCTACGTGAGCGAAAACTTCACCCCG
CAGATGTTGAGTAACGATCCCCGCCCAATCATGCACCTTAGCCGCGAAGAGTTTCACGAT
ATGATGATTTTTCGTCAGACCGTGGAGTTCAAATGCGTGGAGCTCGCCGTCACACACGCC
ACCGATGATGACATTCGCCAGCTCGAGGAAGCATTGAACAACATGCTGATCCACAAAGGT
GATTATAAAAAATACTCGGAAGCGGACTACGAGTTCCATCTGGCGATTGTCAGGGCATCG
CACAACAGCGTGTTCTACAACGTGATGAGCTCGATTAAAGACATCTATTACTACTATCTT
GAAGAGCTTAACCGTGCGCTGGGTATTACCCTTGAAAGTGTGGAAGCCCATATCAAGGTC
TACATGTCGATAAAGAATCGCGATGCCAGCACGGCCGTCGAAGTGCTCAATGAAGCGATG
TCAGGCAATATTATTGCGATCGAAAAAATCAAATCTACAGAGACATCAGGGACAAAATAA

>EECEBICD_01967 Protein YceI
ATGAAAAAGAGCTTTCTTGGTGTTGTCCTGTCTCTGGGTTTATTAAGTGCTGCTCATGCA
GATGTATATAAATTTGATAATACGCATACCAATGCGGTATTTAATATCGATCATTTCCAG
ACCTCAACCAATCATGGCGGGTTTTATGCTATTAGCGGCGAACTGAAATATCAGCCGGAA
AAGCAAGATGCAGAGATGCGCGTGACGATTCCCGTGAGCGCCTTAAATACCGGGGTGGAT
GCGTTTGATAACCATATTCGCAGTAGCGATATTCTGGATGCGGAAAAATATCCAGAAATG
GTATTTAAATCAACAAAATGGCATTTTGAAGATAATAAACCGGTTTCTATTGACGGTTTG
CTAACAATGAAGGGAGTAACGAAGCCCGTCACCTTAACCACCACCAAGTTTGGCTGTTAT
ATGAGCCCGATATTCAAGGCGCAGGTTTGCGGTGGTGATTTTGTCACGCAAATAGATCGC
ACCCAGTGGGGGGTAGATTATCTGGTCGATATGGGGATGACGAAAGTTGTCGATATAAAA
ATTCAGGCAGAAGCGGTTAAGCAATAA
>EECEBICD_01968 Glucans biosynthesis protein D
ATGGCCGCCGTGTGCGGTTCCAGCGGTATTGCTTCACTCTTTTCTCAGGCGGCGTTTGCC
GCGGAATCCGATATTGCGGATGGTAAAATTGTCCGTTTTGATTTTGCTGGTCTGCAATCA
ATGGCCCAGGCGTTAGCGAAAAAGCCCTGGGGCGGCGCGCCGGGACCCTTGCCGGATACG
CTCGCCAATCTGACGCCGCAGGCCTATAACAGCATTCAGTATGACGCGGCGCATTCACTC
TGGAACGGTGTTGCCAACCGGCAGCTCGATATTCAGTTTTTCCACGTAGGGATGGGCTTT
CGTCGTCGCGTACGCATGTTTTCCGTTGATACGACGACGCATCTTGCTCGCGAGATTCAT
TTTCGCCCGGAACTGTTTAAATACAACGATGCCGGCGTCGATACGACGCAACTGGAAGGG
CAGAGCGATCTCGGTTTCGCCGGTTTCCGTGTCTTTAAAGCGCCGGAACTGGCGCGGCGC
GATGTCGTCTCCTTCCTGGGCGCCAGTTATTTCCGGGCGGTAGATGATACTTATCAGTAT
GGCCTGTCGGCTCGCGGGCTGGCGATAGATACCTATACTGACGGTCAGGAAGAGTTCCCT
GACTTCACCGCATTCTGGTTTGACACCGCGAAGCCGGGCGATACTACGTTTACCGTTTAC
GCTCTGCTGGACAGCGCTAGCGTGACGGGCGCGTATAAATTTGTGATTCATTGCGAAAAA
TCGCAGGTGATCATGGATGTAGAAAACCATCTCTACGCCCGTAAAGATATAAAGCAACTT
GGCATTGCGCCGATGACCAGTATGTTTAGCTGTGGGAATAATGAACGTCGGGTATGCGAC
ACCATTCACCCGCAAATTCACGACTCCGATCGGCTGGCGATGTGGCGGGGTAACGGCGAG
TGGATTTGCCGCCCGCTGAATAATCCGCAGAAATTGCAGTTCAATGCATATATGGACGAT
AACCCAAAAGGGTTCGGCCTGCTGCAACTCGATCGCGATTTCTCGCATTATCAGGATGTG
ATGGGCTGGTACAACAAACGTCCGAGCCTGTGGGTGGAGCCGCGTAGTAAGTGGGGGAAA
GGCGCAGTTAGCCTGATGGAGATCCCAACCACTGGCGAAACTCTGGATAATGTGGTCTGT
TTCTGGCAGCCGGAAAAAGCGATCAAAGCCGGGGATACGCTGGCGTTTAATTATCGTTTG
TACTGGAGCGCGCAGCCGCCGGTACAATCTCCGCTTGCGCGGGTCATGGCGACCCGTACA
GGGATGGGCGGCTTTCCCGAAGGTTGGGCGCCGGGCGAACATTACCCGGATAAATGGGCG
CGCCGTTTTGCTATTGATTTTGTCGGCGGCGATCTGAAAGCGGCCGCGCCAAAAGGCATT
GAGCCGGTGATTACGCTTTCCAGTGGCGAGGCGAAGCAGATTGAGATCCTCTACGTCGAG
CCTTTCGACGGTTATCGTATCCAGTTTGACTGGTATCCGACCTCGGATTCTACGGCACCG
GTGGATATGCGTATGTTCCTGCGCTGCCAGGGGGAAGCTATCAGCGAAACCTGGCTGTAT
CAGTATTTCCCGCCCGCGCCGGATAAGCGCCGTTATGTCGACGATCGTATCATGCGTTAG
>EECEBICD_01969 Para-nitrobenzyl esterase
ATGTTGAATCCCTCCATCCCGCTGGTGGCGACGCGTCACGGTAAAATCGTGGGCGTCGTA
CAGGAAGAAACCCATATCTGGCGCGGTATACCTTATGCCGCGCCGCCAACAGGGGAGCTA
CGCTGGCGTGCGCCGCAGCCTGTTACGCCCTGGCAGGATGTGCGCCAGGCGGATTGTTTC
TCCTGCGCCAGTTGGCAGGATATTACCTGGTGTCGCGAACTCGGCGGCGGCGATCCGGGA
AATTTTTCCGAAGACTGTCTCTATCTGAACGTCTGGGCTCCCGCCGTGCGTCATGAACCG
TTGCCGGTCATGGTCTGGCTACACGGCGGCGGTTACACCATTGGCGCGGGGAGTTTGCCG
CCCTATGACGGTCAAGCGCTGGCGAAGCGCGGCGCAATCGTGGTGACGGTCAATTATCGG
CTTGGCCATCTGGGCTTTTTCGCGCATCCGGCGCTGGAAGGCGAAGGGGCGGAGTGTATT
CATAATTTCGCTTTACTCGATCAAATCGCTGCGCTGCGTTGGGTGCAGGACAATATTGCC
GCATTTGGCGGCGATACGCAGAATGTGACGTTATTTGGCGAATCCGCCGGCGCCCCGCAGC
GTGCTGTCGTTGATGGCTTCGCCGTTGGCGAAGGGGCTTTTTCATAAAGCGATTATTCAG
AGTGGTTATACGCTGCCGGATACGCCCCGCGAGGTGGCGCTAAAAAAAGGCGTTGCGCTG
GCGGAACATCTTGGTTTAGCCCATGCCACTGCCGAACAGCTACGCGCTTTACCGGCAGAG
ACGTTTTGGCCGCTGGACGCGCCTTTTAAAATTGCGCCGACGCCGATTTCCGGCGACGTC

GTGCTGCCGCATCCGATGCTGGAAACCTTCTTTGCGGCGAAACAGCATCCAATACCGGTG
ATGATCGGCTCGAATAGCGATGAAGCCAGCGTATTGGCCGTTTTTGGCGTCGATATTGCC
GGTCAAATTCAAAAAATGCGCCGTGAGCGGCGTGTTGGCCTGGGGTTAATCCGGCTGCTT
TATCCTGGAGTGAAAGGCGACGAAGCGCTAGGGCGGCAGGTCTGTCGGGATATGGTATTT
ACCACGTTGGGTTATGTCGTCATGCAAGCGCAGCAGCGGATCGGCGAACCGTGCTGGCGT
TACTGGTTTGATTATGTCGCCGAGGCGGAGCATAACACCTACGCGAATGGCGCCTGTCAT
GGCAATGAAATACCGTATGTATTCGATACGTTAACGCGGGCTGAGCCCACATGCCATTAT
GTGAATGAGAATGACCTGGCCTTTGCCTCGCAGGTGGCGGATTACTGGGTGAATTTCGCT
CGTCATGCCAGCCGCACGCGCGACGTGCTGCACGGCCCGGTTCGCTGGCCCGCCAGCATT
CGGGGGCGAGATCGTCTGTTGCGGATTGGGCTGAACAAACTTGCCGGTTTTAAAGTGGAA
AACCGCTTTATGCGTGCGCGTCTGGCGCTGTTTAAACGAGTCATGAAACATCACGTTAGT
CTGGAATAA
>EECEBICD_01970 putative protein
ATGGCGAACACCATCACGGCAGATGAGATTCGGGAACACTTTTCGCAGGCAATGTCGGCA
ATGTACCAGCAGGAAGTTCCGCAGTACGGTACGTTACTGGAACTTGTGGCTGACGTGAAT
CTGGCGGTGCTGGAAAATAATCCCAAACTGCATGAGCAGTTGGCTAATGCCGATGAACTG
GCGCGATTGAACGTGGAACGGCATGGCGCCATTCGTGTTGGCACCGCAGAAGAACTTTCT
ACCTTGCGTCGGATATTCGCCATCATGGGGATGTACCCAGTAAGTTATTACGATCTCTCT
CAGGCGGGCGTGCCTGTGCACTCCACGGCATTTCGTCCCATTGACGAGGCTTCGCTCTCG
CGTAATCCCTTTCGCATGTTCACTTCGCTATTACGCCTGGAGCTCATTGAAAACGCGGCA
TTACGCCAGCGGGCGGCGGAAATCTTATCGCAGCGGGATATTTTTACATCGCGCTGCCGA
CAATTGCTGGATGAGTACGACGAGCAGGGCGGTTTTAGTGCTGCTCAGGCCGAAGAATTT
GTCCGTGAAACGCTGGAGACTTTTCGCTGGCATCGTCAGGCTACCGTTGATGAAGAAACG
TACCGCAGCCTGCATCGTGAGCATCGTTTAATTGCCGACGTGGTCTGTTTTCCGGGATGT
CACATTAACCATCTGACGCCGCGTACTCTGGATATCGACCGGGTACAGGCCATGATGCCG
GAGTGCGGCATTACGCCTAAAACTCTTATCGAAGGTCCGCCTCGCCGCGAAGTGCCTATT
CTGTTGCGGCAAACCAGTTTCAAAGCCCTGGAAGAGCAGGTGCTCTTCGTTGATGAAAAG
CAGGGTACGCACACGGCGCGCTTCGGCGAAATTGAGCAACGTGGCGTCGCGCTGACGCCA
AAAGGTCGTCGATTATATGACGAGCTGTTGCACAAGGCGGGAACGGGTAAGGATAACTTC
ACGCATCAACTCCATTTGCGGGAGGTCTTTAACGCCTTTCCGGATAGCGAATTTCTGCTT
CGCCAGCAGGGGCTTGCCTGGTTCCGCTATCGCTTAACGCCTTCCGGTGAGGCGCATCGT
CAGGCGATCCATCCTGGCGACGATCCGCAGCCGCTTATTGAGCGCGGTTGGGTAATCGCG
CAGCCGATTACCTATGAAGATTTTCTTCCGGTCAGCGCAGCCGGCATTTTTCAGTCGAAT
TTGGGAAATGAAACGCTGGCCAGAAGCCACGGCAATGCCAGCCGCGACGCCTTTGAACAG
GCGCTGGGATGTGCTGTACGGGATGAATTTTCGTTATATCAGGAAGCGGAAGAACGCAGT
AAACGCCGTTGCGGTCTGCTTTAA
>EECEBICD_01971 HTH-type transcriptional regulator GbpR
ATGGAAAAAAATGGTCTGTTTAGTCAGCGCATTCGGTTGCGCCACCTTCATACATTTGTA
GCCGTTGCGCAACAGGGAACTCTGGGGCGCGCGGCTGAAACTCTTAACCTGAGCCAGCCA
GCGCTTTCTAAAACGCTGAACGAACTGGAACAACTCACCGGAACCCGTCTGTTTGAACGC
GGGCGACTGGGCGCGCAATTAACGGTTCCTGGCGAACAATTTTTGACTCATGCGGTAAAA
GTATTAGATGCATTGAATACCGCCGGGCAGGCGCTGAATCGCAAAGAGGACGCCAGCGCC
GATGTGGTGCGTGTTGGCGCGCTCCCCACCGCTGCGCTGGGTATACTCCCGGCGGCTATT
GGGCGGTTCCATCAACAGCAAAAAATCGACCTCGTTACAGGTCGCGACCATGAACAATACG
ATGTTGCTTGCGGGTCTGAAATCAGGCGAGATCGATCTGGGTATTGGCCGCATGTCAGAC
CCGGAATTAATGGGCGGTTTAAATTACGAACTGCTGTTTTTAGAATCGCTTAAATTAGTG
GTACGCCCCGGTCATCCCCTGTTACAGGAGACGATCACGCTCAGCCGGGTTATGGAATGG
CCGGTAGTCGTCTCGCCAAAAGGCACGGTACCGCGCCAGAATGCGGAAGCCCTGTTGCAA
AGCCAGGGGTGTAAAATGCCTGCCGGATGTATCGAAACGCTATCCGCATCGTTATCACGA
CAACTGACCGTAGATTACGATTACGTCTGGTTCGTCCCCTCTGGCGCGGTTAAAGAAGAT
CTTCGCCAGGCGACGCTGGTCTCATTACCCGTGCCGACCCAGAGCGCGGGCGAGCCGATA
GGTATTCTTACGCGAGTTGATATCCCGCTCTCAACCGGGGCGCAAATGCTGATTGCGGCG
ATCCGTAAATCGATGCCGCTGTGA
>EECEBICD_01972 Methyl-accepting chemotaxis protein III

ATGGGCAACACATTCTCGATGCAAGCCTCGCATAAACTGGGCTTCCTGCATCATATTCGG
CTGGTTCCGCTGTTTTCCTCCATTCTCGGTGGCATCCTTCTTCTTTTTGCTTTGAGCGCC
GGGCTGGCAGGCTATTTTCTGTTACAGGCCGATCGCGATCAGCGTGATGTCACGGACGAG
ATTCAGGTACGGATGGGATTATCCAACAGCGCTAACCACTTACGTACCGCACGTATCAAT
ATGATCCACGCTGGCGCGGCGAGCCGGATTGCTGAAATGGATGAAATGAAAGCCAATATT
GCCGCTGCGGAAACGCGGATCAAGCAGTCTCAGGACGGCTTTAACGCTTATATGTCCAGA
GCGGTGAAAACACCCGCTGATGACGCGTTAGATAACGAGTTAAACGCCCGCTACACCGCC
TATATCAACGGCCTGCAACCGATGCTGAAATTTGCCAAAAATGGCATGTTCGAAGCGATC
ATTAATCATGAAAACGAGCAGGCAAAACAACTGGATGCAGCATATAACCATGTCCTGTTG
AAAGCGATTGAACTGCGCACCGAGCGGGCAAGACTACTTAGCGAGCAGGCTTACCAACGT
ACCCGACTCGGTATGATGTTTATGATTGGCGCATTTACGTTGGCGCTGGTATTGACGTTA
ATGACCTTCATGGTGCTGCGTCGCACGGTGATACAACCCTTGCAGCAGTCTGCGTCACGT
ATCGAACGCATTGCGGCCGGCGATCTGACCATGGCTGACGAGCCCACAGGACGCAGTGAA
ATTGGTCGCCTGAGTCATCATCTTCAGCAGATGCAGCATGCGTTGCAACAAACGGTAGGG
GCGGTGCGTCAGGGGGCGGAGGAAATTTATCGAGGCACCAGCGAAATTACCGCTGGTAAT
ACGGATTTATCTTCTCGTACTGAACAGCAGGCTGCCGCCATTGAACAAACCGCGGCCAGT
ATGGAACAGTTGACGGCAACCGTTAAGCAGAATGCGGATAATGCGCATCACGCCAGCAAA
CTGGCGGAAGATGCTTCCGGTAAAGCCAGCCGCGGAGGCCAGATGGTTTCCGGGGTCGTA
CAAACGATGGGGAATATTTCCACCAGCTCGAAAAAAATCTCGGAAATTACCGCCGTCATT
AACAGTATCGCTTTCCAGACCAACATCCTGGCGCTGAACGCGGCGGTCGAGGCGGCACGC
GCCGGGGAACAAGGACGCGGTTTTGCGGTGGTCGCCAGTGAGGTTCGTACCCTCGCCAGT
CGCAGCGCGCAGGCGGCAAAAGAGATTGAAGGCTTGATCGGCGCATCGGTCAGCCTGATT
GAACAAGGATCTGAAGAGGTTATCGCTGCGGGGAGCACCATGAATGAGATTGTGGATGCC
GTCAAACGCGTCACCGATATTATGCTCGATATCGCGGCAGCCTCTGATGAGCAAAGTCGC
GGTATTGTTCAGGTCAGTCAGGCGATTTCTGAGATGGACAAGGTTACGCAACAAAACGCG
TCGCTGGTCGAAGAGGCGTCCGCGGCGGCGGCTTCGCTTGAAGAACAGGCCGCCCGCTTG
ACGCAAGCCGTTGATGCGTTTCATTTACAGGATACTGGCGCGACGATGCGATCATCCTTC
CTTTAG
>EECEBICD_01973 S-(hydroxymethyl)glutathione dehydrogenase
ATGAAATCACGTGCAGCAGTTGCTTTTGGTCCCGGTCAGCCGTTAAAAATTGTCGAGATT
GACGTTGCGCCGCCGAAAAAAGGCGAAGTTCTGGTCAAAATCACTCATACCGGCGTTTGC
CATACCGATGCTTTTACCCTTTCCGGCGATGATCCGGAAGGCGTATTCCCGGCAGTCCTG
GGTCACGAAGGCGGCGGCGTGGTAGTCGAGGTCGGCGAGGGGGTCACCAGCCTGAAACCC
GGCGATCATGTTATTCCACTGTATACGGCGGAATGCGGCGAGTGTAAGTTCTGTAAGTCC
GGTAAAACTAACCTTTGTCAGGCCGTACGCGCCACTCAGGGAAAAGGGCTGATGCCGGAT
GGCACTACCCGCTTTTCTTATAACGGCGAACCCGTTTATCACTATATGGGAACCAGCACG
TTCAGCGAATATACCGTTTGCGCGGAAATTTCTCTGGCGAAAGTGAACCCGCAGGCGCCG
CTGGATAAAGTGTGTCTGTTGGGCTGCGGCGTGACGACTGGTATTGGCGCCGTCCATAAC
ACCGCTAAAGTGAAAGCGGGCGACACCGTTGCCGTTTTTGGTTTAGGCGGTATTGGGCTG
GCGGTTATTCAGGGCGCGGTTCAGGCTAAAGCCGGGCGTATTTTAGCTGTCGACACGAAT
CCGGAAAAATTTACGCTGGCGGGCGAAATGGGGGCCACGGACTTTATTAATCCGAATGAT
TACGACAAACCGATCCAGGACGTTATTGTTGAGCTTACTGATGGCGGCGTAGATTTCAGT
TTTGAATGTATCGGCAACGTTAACGTGATGCGAGCAGCGCTGGAATGTTGTCATAAAGGC
TGGGGCGAAAGCATTATTATTGGCGTAGCGGGGGCAGGGCAGGAAATCAAAACCCGTCCC
TTCCAGCTAGTGACCGGTCGCGTCTGGCGTGGCTCCGCCTTTGGCGGCGTGAAAGGGCGC
ACGCAGCTGCCAGGGATGGTCGAAGATGCGATGAACGGTAAAATCCGTTTAGATCCTTTT
ATTACTCACCGCCTGCCGCTGGAGCAGATTAACGATGCCTTTGAACTGATGCATCAGGGT
AAATCCATCCGTACTGTTATCCACTTTGGCGATAACTGA
>EECEBICD_01974 Transcriptional repressor FrmR
ATGCCGCATTCACCTGAAGATAAAAAACGTATCCTTACCCGTGTTCGTCGCATACGCGGG
CAGGTCGAAGCGCTGGAGCGGGCGCTTGAGTCTGGCGAACCTTGTCTGGCGATTCTGCAA
CAGATTGCCGCCGTGCGCGGCGCGTCTAATGGCCTGATGAGTGAAATGGTTGAAATCCAT
CTGAAAGATGAGCTGGTCAGCGGGGAGACTACGCCAGATCAGCGCGCGGTTCGGATGGCG
GAAATCGGCCATCTTCTTCGCGCTTATCTAAAATAA

>EECEBICD_01975 hypothetical protein
ATGTTTCAGGCCAAACCCAAGTTGAACTGGAGCGCTCAGAAGCCGATTACCTACATAACA
AATATTGGCCGCAAGCTTATGCTGTGGGCGGAATATACGACCGCTATTATCCAGACTGGC
TGTTAA
>EECEBICD_01976 hypothetical protein
ATGAATGATGATTCAATTATTAAAAACTCTTCGATGCTTTTGGCCTATTTAGGTGGTTTG
GGATGGGGAAGCGCCTATTTTTATGGCTGGGGCGTCTCCGCGTACTATGGTTTCCCGTGG
TGGTATGTAGGCGTCGGCCCTGACAACATTGCCCGAAGTCTATTCCATGCGATTAGCCTG
ATGGCTATATTCATAATAGCGTGGGGAGTGGGTATTCTATTATTTTTTCTAGTAAAGCAA
AAAGCACGTATCCACGACCTTAGCTTTTTACGTCTTTTTTTGGCAGCGGTTTTGTTCTTT
ATTCCAATTGTTATCGAGTTTTCTCTCTTAACAGAATCGTTTTTGTGGGAGCTTTTTTTT
ATCATTTTACTGGTGGCTTTATGCTTATCGGTGGGGATGCGTTTTTATAGTAAATTGATG
CCGGTGATTTGTTTTACGCAACTCAGTTGGGTGCGAAGGCATTGTTTCACTATAGTTATG
CTTGGTTTTATTATATATTTTTTTATATTTTCTTTTTTTGTTGGGATCTATAAACCACAA
TTAAAGAAAGAATACGAAATGATATTGTATGATGGAGGCTGGTACTACGTCCTGGCCAGA
TATCATGATAGTTTTATTTTGTCGAAATCCTTTACTAAAAATAACAATAGATTTATTATT
TTTAGACCAGAAGATGGGCACTCTTATGAAATTACTCTGGTAAAAGTCAGGCTTTAA
>EECEBICD_01977 Secreted effector protein SseJ
ATGCCATTGAGTGTTGGACAGGGTTATTTCACATCATCTATCAGTTCTGAAAAATTTAAT
GCGATAAAAGAAAGCGCACGCCTTCCGGAATTAAGTTTATGGGAGAAAATCAAAGCATAT
TTCTTTACCACCCACCATGCAGAGGCGCTCGAATGTATCTTTAATCTTTACCACCATCAG
GAACTGAATCTAACACCGGTACAGGTTCGCGGAGCCTACATCAAACTTCGAGCCTTAGCG
TCTCAGGGATGTAAAGAACAGTTTATTATAGAATCACAGGAACACGCCGATAAGTTGATT
ATTAAAGATGATAATGGTGACAATATTTTATCTATTGAGGTTGAATGTCATCCGGAAGCT
TTTGGTCTTGCCAAAGAAATCAATAAATCACATCCCAAGCCCAAAAATATTTCTTTGGGT
GATATTACCAGACTGGTATTTTTTGGCGACAGCTTGTCTGACTCCTTAGGGCGTATGTTT
GAAAAAACACATCATATCTTACCCTCCTATGGTCAATACTTTGGCGGAAGGTTTACTAAT
GGATTTACCTGGACTGAGTTTTTATCATCTCCACACTTCTTAGGTAAAGAGATGCTTAAT
TTTGCTGAAGGGGGAAGTACATCGGCAAGCTATTCCTGCTTTAATTGCATCGGTGACTTT
GTATCAAATACGGATAGACAAGTCGCATCTTACACCCCTTCTCACCAGGACCTGGCGATA
TTTTTATTGGGGGCTAATGACTATATGACACTACACAAAGATAATGTAATAATGGTCGTT
GAGCAACAAATTGATGATATTGAAAAAATAATTTCCGGTGGAGTTAATAATGTTCTGGTC
ATGGGGATTCCCGATTTGTCTTTAACACCTTATGGCAAACATTCTGATGAAAAAAGAAAG
CTTAAGGATGAAAGCATCGCTCACAATGCCCTGTTAAAAACTAATGTTGAAGAATTAAAA
GAAAAATACCCCCAGCATAAAATATGCTATTACGAGACTGCCGATGCATTTAAGGTGATA
ATGGAGGCGGCCAGTAATATTGGTTATGATACGGAAAACCCTTATACTCACCACGGCTAT
GTACATGTTCCCGGGGCTAAAGACCCTCAGCTAGATATATGTCCGCAATACGTCTTCAAC
GACCTTGTCCATCCAACCCAGGAAGTCCATCATTGTTTTGCCATAATGTTAGAAAGTTTT
ATAGCTCATCATTATTCCACTGAATAA
>EECEBICD_01978 hypothetical protein
ATGTATCTTAACCGCTTTTGTTATTTACTCCTGATCTTCGCTGCGGTCGGAAGCATTTTT
ACATCGCATCCCGTCGCGATGTGGTTCCTGCTCGCCAATGTGCTAACGCTGGCGATCTAC
GGTATAGACAAAACGGCGGCACGTAAAACCTGGCGCAGAGTGCCGGAATCCACTTTGTTG
GTGTTTGGGGTTGTCGGTGGGTGGCCTGGGGCGATTGTTGGTCAACAACTCTTCCGTCAT
AAAACGCAAAAGCAGCCATTCAAAACATACTTTATTGTCAGTGTGATAGTGAGTATTTTA
GTTACGGTGGCGATTTATCGACTCTATCCATTTTTGTCTTACTGA
>EECEBICD_01979 L-cystine-binding protein FliY
ATGTTATCCAAAAAATTTGGCTTATCGATGATAGTGTTGGGAATAATGTCGTCTTCAGCG
TTTGCTGATAGCATCGTTGAAGGGAGAACACTCAATGTCGCAGTATCACCAGCCTCTCCC
CCTATGTTATTTAAAAGTGCTGATGGCAAATTGCAGGGGATAGATCTGGAGCTTTTTTCG
TCTTACTGTCAGTCACGGCATTGTAAGCTAAACATCACCGAATATGCCTGGGATGGAATG
CTTGGCGCAGTGGCGAGTGGTCAGGCAGATGTAGCATTTTCGGGAATTTCAATCACAGAT
AAACGTAAGAAAGTTATTGATTTCTCTGAACCTTATTACATCAACTCTTTTTATCTGGTC
AGTATGGCCAATCATAAAATAACGCTCAATAACCTCAATGAATTAAATCAATATTCTATT

GGATACCCTCGAGGAATGGCTTACTCCGACCTTATCAAAAACGATCTGGAGCCCAAAGGA
TACTACTCGTTAAGTAAAGTAAAATTGTATCCAACCTATAATGAGACGATGGCTGATCTA
AAAAATGGCAATCTTGATCTGGCTTTTATAGAAGAGCCGGTTTATTTTACCTTCAAAAAT
AAGAAAAAGATGCCGATAGAAAGCCGTTATGTTTTCAAGAATGTTGATCAGCTTGGCATT
GCCTTTAAGAAAGGCTCTCCCGTGCGCGATGATTTCAACTTATGGCTTAAAGAGCAAGGG
CCACAAAAAATATCTGGCATTGTCGATAGCTGGATGAAATAG
>EECEBICD_01980 putative amino-acid permease protein YxeN
ATGAACCTCAGCGTCGGCTTAATCAGTGGGAGCTATCTTGCCGAGGTGTTCAGAGGGGCG
CTTAAATTAGTTGAGCCATACGAAATTACGGTAGCGAAAGTAGCAGGAATGCGTCAATTA
CAGGTTATAATAAATATTGAACTGCCACAGATGTTGCGATTTTCTGTGCCCGGTATTATC
AATGAGTTTTCTTCTGTATTAAAAGCGACGCCCTTTGCTTATACTGTAGGTATCGCTGAA
ATAACGAAGCAGGCAATGTCACTGACAGCCATCACGCTGAACGGGTTACAGATCTATACT
CTTGACGGGGTATTATATTTCATTATTTATAAAGTTTTTACTCTTCTGGCCGGAGTGTTT
GAGAAAAAAATATCGCATCAGTTGA
>EECEBICD_01981 hypothetical protein
ATGGCTTTACTGAAACTAAAAGAGATAAGTAAGGAATATTCAGGAAAAAAGGTTCTTGAT
ACGGTTAGTCTGAAAATTCAATCGGGAGACATGAAAGTAGTCATGGGGCCATCAGGTTGT
GGAAAAAACGACGCTTTTACGCTGTCTGCTTCGATTAGAAGAACCTGATTATGGCAATATT
TATTTTCACGGGAAAAATATTTACGACAAAGAATTTACTATTCTTGAGTTCAGAAAAAAG
TTGGTTGTGTCTTCCAGAATTACGCTTTGTATCGCCATCTTAACGTCATGGATAATATTA
CCCTTGCCCTATGTAAAGTGTTTGGCATGCCGGGACAACTAG
>EECEBICD_01982 Histidine transport ATP-binding protein HisP
ATGGATAATATTACCCTTGCCCTATGTAAAGTGTTTGGCATGCCGGGACAACTAGCCCGG
GAGAAAGCCTTACATGAGCTACAAAAACTTGATATGGCCTCGCATAGCGCGAAATACCCT
TCGCAATTATCGGGCGGCCAGCAACAGCGCGTCGCCCTGGCCCGTACAATGGTCACCGAC
CCAGAACTCATTATTTTTGATGAGCCGACGTCTGCGCTTGATCCGCTCATGACTCGTGAA
GTCGGAATGTTGATCAAGCAACTTCACGACAATGGCGTCACTATATTATGCGTCACTCAT
GATATTCGCCTCGCCAGATTACTAAGCGATAACGTAACGTTTCTTAATCATGGCAAGATT
CGTGCAGAAGGCGCTTTTACCGATCTTGCCTTACGAGAAACGGATCCAGATATCCACTGT
TTTTTTAGCGAGGCACAACGATGA
>EECEBICD_01983 L-cystine transport system permease protein YecS
ATGATCGCTGGCTGGTCTCTCTTCTTTAATGACCTGACTGAACAACTGCCACTTGTTGTG
GACGGTATTAAAGAAACCTGTAAACTTGCGCTGATTGTTTCCATCACCGGTTTTTTGTGG
GGAATTATTATTTTCTTTCTGAGCCTGAGCCATCGCCCCGTGGTAAAAGCCATAACTCGA
CTGTACATGGATTTCTTTATCGGTACGCCACTCATTCTTATTCTATTTGTTATCTATTAT
GGTTTACCTCAATCTGGCATTCACCTGTCCTCTTTTACCGTGGCGGTTACAGGTTTTACC
TTGAATGTCGGCGCATATAATGCCGCGTACATGACAACGGCCTATAACGCATTGAATAAG
TATGAAACGGAGGCAGCAGTCGTTCAGGGGTTGAACAAACGGCAGGTCTTTTTGTGGATT
ATACTGCCTCAGGTATTGCTGTCTTCCATTCCAGCCTTAACGAATCAGGTGATTAATAAC
CTTAAAGACAGCACCATCGTTTTTCTTATCCAATATACTGAGTTTTTCGCGCGAATTCAG
GAGGTTGCTGCAACCAGCTTTAAATTCTTCCATGCTTACCTTTTTGCCGCCATAGTGTAT
CTTATTGGCGTTACCTTTCTCGTCGGTTTGACCCGGTTTTTAGAGCATAGACTGCTTCGC
CATTACGGTCAGGGTTACTGA
>EECEBICD_01984 hypothetical protein
ATGGCGCTACCACAACTATCAATATGGCAGCCCGGCGATGGGTTAAGAAAAAATAAAGTAT
AAATATCATGTTTGTCTCATTGTCATATTCTCCGTCCTTATACGGTTGCTGTTTTTAACT
GACCGCTACCTTTGGTGTGACGAAGCGTCCAGCGTGCTGATTAGTCGACATAGCGTAGAT
AATCTGCTATTCCATACCGCTTTTGACGTGCATCCCCCGCTTTATTATCTGTTACTGCAT
GACTGGATCATATTATTGGGTGATAGCATTGCAGCGGTACGCTCTCTCAGTCTACTCTTT
GGGATCCTGACAGTTGTGCTGGTTATCGCGCTTACGCGATGGCTGGCAAATGAACGTACA
GCCTTATTGGCGGGATGGTTCGCCGCCCTGATGCCGATGGCAGTTCATTACAGTCAAGAA
ACGCGCATGTACGCGTTAATGGGAATGCTAACGCTCGCTGCCGCGTTAGCATTGATGATG
TGGATAAAAACACCCACCCATAATCGCTATCTTATCGCGTATGCTTTACTTATGACGTTC
AGCTTTTACACCCATTATTTCACCCTATTTACACTTATTGCCCACTGGATCGCAGTAACA

ATATTATCCTGCCAAAGCCATAAAACGGCCTGCTACCTTAAACTTCCTGGCTGGTGGTTT
GCTAACCTGGCGATTGCTGTCGCTTATCTCCCTGGTTGCCCGTATTGTTTAATTTACTGA
>EECEBICD_01985 hypothetical protein
ATGCTGGTCTTCATCATTTCCTGGTGGATGCCGTTATTTATCGATCGTTATTTCTTCTTC
TCATCGCTGAGCATTCCTCCCCTGCTCGCGGTATTACTTACCCGCGCAAAAAAAGCGGTT
TGTGGCTTCTGGTTTATCTTATTCACCTTATTATTCGGCTATGGTTCATATCATAATAAC
CCTGAGCACATAGACGAGTTTAAACCACTGGTAAACTATATTAATACCCGTCACCAGCCT
AATGATGCCGTGGTAGTCAGTAAAATGTTCGATTATTTAAGTTATGTGTATTATAATAAG
CGAGATTATCGTACGTTTCTTTATACACCGCCTAACGCTCATGGAACATCAGGCAGACCG
AATGCCTATGGATTTGGTTCACTGTTTTATGCGCAAGCCGATCAGACATATATTGATACT
CTGACAACGCTTTCAAAAAGTTACCATCGTGTCTGGTTGGTAAGCGGCGGTAACTTTAGT
CAGGATTATCCCTTGCCGTCAGAATGGCAAAATATCGCAAACTTCAGAAGTGGTAGATTT
CAGGTACAGTTATTTGTAATACCGACTCAACAGGCGCGTCAAATGCAATGA
>EECEBICD_01986 hypothetical protein
ATGATTGAGGTTTTTGAAGCGTTTGGCTGGACCTTACTTTTAGGCGGTCTGGCAGTGGCA
GTACTGTTTATCACTGCACTGGATAAAAAACGGTAA
>EECEBICD_01987 hypothetical protein
ATGAAGCATACACAACAATCAGGCGCAAAAGTCTATAACCCATTCACGCTTAGCTTATAT
GACTGGTGGGTACTCAATATCTCAAACAAATATGCATGGAAGTGTCCTACTGATACGCGA
CTACTTCCTTTCTTCCTGCATCACATGGGCGAGACTCACCTGGATATTGGCGTAGGTACA
GGTTATTACCTGAAACATGCACCTGCGACTCATGCCATTTCGCTGATGGATTTAAATCCG
GATAGCCTGAAAATAGCCGCCAGCCGAGTGGGTAATACGAAAATCCGCGCCACACTACAA
CACGATGTTTTCGACACGTTCCCGGCGGACTGGCACGGGCGCTTCGATTCCGTTTCGATG
TACTATCTTCTGCACTGCTTACCCGGTGAGATGACAACAAAAGCGAAGGCAATTAAAAAT
GCCGGTATGGCGCTTAAACCCGGCGGCACACTTTTTGGCGCCACGATTCTGGGCAAAGAG
GTTCCTCACAATGCTTTTGGTAAGAAATTAATGGCTGTTTATAACAAGAAAGGAATATTC
AGTAATACCAATGATTCAGCCGATACGTTACGCAGCGTGTTGGACGAACATTTCGCTAAG
GTGACTTTGGAGCAACATGGCGCCGTTGCGCTATTTTCCGCCACTATGCCACGTTCATTG
TAA
>EECEBICD_01988 Cytochrome b561
ATGGGCAATAAGTATTCCGGCCTGCAAATTGGTATTCACTGGTTAGTCTTTTTTCTGGTT
ATTGTGGCTTACGCCGCGATGGAACTGCGCGGTTTTGCGCCGAGTAGTTATCGGCCCTGG
TTTAATATGACGCATGTTTCATGTGGTATCACTATTTTGCTTTTAATGGTGGCGCGCTTG
ATTATTCGGCTGAAATATCCCACGCCGCCCATTGTTCCTCGTCCAAAACCGATGATGACC
GGAATGGCGCATTTAGGACATCTGGTGATTTACCTGTTATTTATCGTGCTGCCTGTTATT
GGTCTGGTCATGATGTATAACCGCGGCAATCCCTGGGTCGCATTTGGCATTACTATGCCG
CATGCCGCCGAAGCTAACTTTGAACGCGTAGATATGCTGAAATCCTGGCACGTCACGCTG
GCGAATCTGGGCTATTTTGTTATTGGACTTCATGCCATCGCTGCTTTGATGCATCACTAT
TTCTGGAAAGACAATACGCTGTTACGGATGATGCCGCGTAAGCGTAACTGA
>EECEBICD_01989 Protein YdcF
ATGAATACAACGCCATTTCCGGCGCTCTCTGCGGAAACATTGCTGGCGGTGAATACCGTC
GGACAGTGGCTGGCGCAAAATGATTTTTCCGGCGAACAACCTTATTCGTCGGATTGCGTC
GTCCTGGCGGGCAATGCGGTGATCCCCACTATCGACGCGGCCTGTCGCATCGCCAAAGCG
CAAGGCGTTCCTTTACTGATTAGCGGCGGTATAGGTCATTCGACGCCGTTTTTGTACGCC
GTGATTGCCCGGCATCCGCGCTACCACACTATTCGCACAACCGGACGCGCTGAAGCCGCG
ATCCTCGCGGATATCGCGAACCAGTTCTGGCATATTCCGGCTGAGAAAATTTGGCTGGAA
GATCGGTCAACTAATTGCGGCGAAAATGCCCGTTTCTCTTGCGCACTGATCCGTCAGGCG
AAAGAAAACATTAACACGGCTATCGTTGTGCAAGACCCCACCATGCAGCGGCGCACTATC
GCGGCATTCCGGCGCGTGACAAACGACGACACCGATGCGCCGCGCTGGCTGAGTTTTCCT
GGTTTTGTTCCGGTACTGCGCCACCTTAACGGCGGTACACGTTTTGCCAACGTTGAAGAG
GGCATCTGGACGGTGGAGCGTTATCTGTCTCTGATTGCCGGCGAGCTGCCACGTTTGCGT
GACGATGAAACAGGGTATGGACCGCGCGGTAAAGATTTTATCATTCACGTTGATATCCCA
CGCGATATTGAAAACGCATGGCAGGTATTGCAGGCAGATACGACGCTGCGCAGTGCGTTA
GAACAGAGAGCGTTACGCTAA

>EECEBICD_01990 hypothetical protein
ATGCTGCCCAAGAGCTTTATGACTGAACAATCTACGTTGACGCTTCAGGTGTTACAGCAG
CGGCTGGATGCACTGATGCTGCGCGACAAACAGCGCTTTGCGCGCCGCCTGCATGGCGTA
AAGAAAGTAAAAAATCCTGATGCGCAACAGGCCATTTTCCAGACGATGGCGAAAGAAATT
GAACAGGCTGTGGCGCAAGTCGCCCTCCGTGAAGCGGCGCGCCCCTCAATCACTTATCCG
CAAAATCTGCCTGTTAGCCAGAAGAAACAGGACATTCTGGAAGCGGTCCGCGATCATCAG
GTAGTGATCGTAGCAGGGGAAACCGGTTCCGGTAAAACCACCCAGTTGCCGAAAATTTGT
ATGGAACTGGGACGTGGAATTAAAGGACTTATTGGTCATACTCAGCCGCGTCGCCTGGCG
GCGCGCACCGTCGCTAACCGCATTGCCGAAGAGTTGCAAACGGAGCCAGGCGGCTGCATC
GGCTATAAAGTTCGTTTCAGCGATCATGTCAGTAGCAACACCATGGTCAAACTGATGACG
GACGGTATCCTGCTGGCGGAAATCCAGCAGGACAGACTATTGATGCAGTATGACACCATC
ATTATTGATGAAGCGCATGAACGCAGCCTGAATATTGACTTCTTGCTCGGCTATCTGAAA
GAGCTTCTGCCGCGTCGCCCCGATCTGAAAGTGATTATTACCTCTGCGACCATCGACCCG
GAACGCTTCTCGCGCCACTTTAATAACGCGCCGATTATCGAAGTCTCTGGCCGAACGTAT
CCCGTAGAAGTTCGTTATCGCCCCATCGTGGAAGAGGCTGACGACACTGAACGCGATCAG
CTACAGGCAATTTTCGACGCTGTTGATGAACTGGGGCGTGAAAGTCCCGGCGATATCCTG
ATTTTTATGAGCGGAGAGCGGGAAATCCGCGATACCGCCGATGCCCTGAACAAGCTGAAC
TTACGGCATACCGAAGTGTTGCCGCTGTATGCACGCTTGTCGAACAGCGAACAGAACCGC
GTTTTCCAGTCGCACAGCGGACGCCGTATCGTGCTGGCGACTAACGTGGCGGAAACCTCG
CTGACGGTGCCGGGTATCAAGTATGTGATCGATCCTGGCACTGCGCGCATCAGCCGCTAC
AGCTACCGCACTAAAGTGCAGCGCCTGCCGATTGAACCGATCTCACAGGCTTCCGCCAAT
CAGCGTAAAGGGCGTTGCGGACGCGTATCGGAAGGGATTTGCATTCGTCTGTATTCGGAA
GATGACTTTTTGTCACGTCCGGAATTTACCGACCCGGAAATTCTACGTACCAACCTGGCG
TCGGTTATTTTGCAGATGACCGCACTGGGGCTGGGTGACATCGCCGCCTTCCCGTTTGTT
GAAGCGCCGGATAAGCGCAATATTCAGGATGGCGTGCGTTTGTTGGAAGAGCTGGGGGCG
ATTACCGCCGACGAACAGCAAACGGCATATAAGCTGACGCCGCTGGGCCGACAATTATCC
CAGTTGCCGGTCGACCCGCGCCTGGCGCGTATGGTTCTGGAAGCGCAAAAGCATGGCTGT
GTGCGCGAAGCGATGATCATTACTTCCGCACTCTCTATTCAGGATCCGCGCGAGCGCCCA
GTGGATAAGCAGCAGGCTTCCGATGAGAAACACCGGCGCTTTCATGATAAGGAATCCGAT
TTTCTGGCGTTTGTTAACCTGTGGAATTACCTCGGCGAGCAACAAAAGGCCCTGTCGTCG
AATCAATTCCGCCGTCTGTGTCGCACCGACTATCTGAACTATTTACGCGTGCGTGAGTGG
CAGGATATCTATACCCAACTGCGTCAGGTGGTAAAAGAGCTGGGGATTCCGGTAAACAGC
GAACCCGCAGAGTACCGGGAAATTCACGTTGCGTTGTTAACGGGGCTGTTGTCGCATATC
GGGATGAAAGATGCCGATAAACAAGAGTACACCGGCGCGCGTAACGCCCGATTCTCTATT
TTCCCCGGATCCGGTTTATTCAAAAAACCGCCGAAATGGACAATGGTCGCAGAACTGGTG
GAGACCAGCCGTTTGTGGGGGCGCATCGCTGCCCGCATTGAGCCAGAATGGGTGGAACCG
GTAGCCCAGCACCTGATTAAACGTTCATATAGCGAACCGCACTGGGAGCGGGCGCAAGGC
GCGGTGATGGCGACGGAAAAGGTTACTGTCTACGGTCTGCCGATTGTCGCCGCGCGCAAG
GTGAATTACAGCCAGATTGACCCGGCGTTGTGTCGTGAGTTGTTTATCCGCCATGCGCTG
GTGGAGGGGGACTGGCAAACCCGTCATGCCTTCTTCCGTGAGAATCTGAAATTGCGTGCG
GAAGTCGAGGAACTTGAGCACAAATCTCGCCGCCGCGATATCCTGGTGGATGACGATACC
CTGTTTGAGTTTTATGACCAGCGTATTAGCCATGACGTTATTTCCGCCCGCCACTTTGAT
AGTTGGTGGAAAAAAATCAGCCGCGAGACGCCGGATCTGCTCAACTTTGAAAAAAGTATG
CTGATTAAAGAAGGCGCGGAAAAAATCAGCAAACTGGATTACCCGAATTTCTGGCACCAG
GGCAATCTCAAGCTGCGTTTGAGCTATCAGTTTGAACCGGGCGCGGATGCTGACGGCGTG
ACCGTCCATATTCCGTTGCCGCTACTTAACCAGGTTGACGAGAGCGGTTTTGAATGGCAG
ATCCCGGGGCTGCGCCGTGAATTAGTGATTGCGCTGATTAAATCGTTGCCAAAACCGGTA
CGTCGCAACTTTGTTCCGGCGCCAAACTATGCTGAAGCGTTTTTGGGTCGCGTAACGCCG
CTGGAGCTGCCGCTGCTGGACGCTCTGGAGCGTGAACTGCGCCGGATGACCGGCGTCACG
GTCGACCGGGAAGACTGGCATTGGGATCAGGTGCCCGGGCATCTGAAAATCACGTTTCGC
GTGGTGAATGACAAAAACAAGAAACTGCAGGAAGGGCGTTCGCTCGCCGAGCTTAAAAAT
GCGCTGAAAGGCAAAGTGCAGGAGACGCTCTCGGCGGTGGCGGATGACGGCATCGAGCAG
AGCGGGCTGCATATCTGGAGTTTCGGCGAGCTGCCGGAAAGCTACGAACAGAAACGCGGT
AATTATAAAGTGAAGGCGTGGCCAGCGTTAGTGGATGAGCGTGACAGCGTCGCTATCAAA

TTGTTTGATAATCCGCTGGAACAACAACAGGCGATGTGGTGCGGTTTACGCCGTCTGCTG
TTGCTCAATATTCCTTCACCGATTAAGTATCTGCATGAGAAATTGCCGAATAAAGCCAAG
CTGGGGCTGTACTTTAACCCGTACGGCAAAGTACTGGAGTTGATTGATGACTGTATCGCC
TGCGGGGTAGATAAGCTGATAGATGCCAACGGCGGGCCGGTTTGGAGCGAAGCAGGTTTC
ACCGCGCTGCACGAAAAGGTTCGCGCCGAGCTGAATGATACTGTGGTGGATATCGCGAAG
CAGGTTGAGCGGATCCTGACGACCGTATTCAATATCAATAAGCGTCTGAAAGGGCGGGTG
GATATGAGCATGGCGTTGGGGCTTTCCGATATCAAGGCGCAGATGAGCGGTCTGGTATAC
CGTGGTTTTGTGACTGGCAACGGCTTTAAACGTCTCGGCGATACGCTGCGTTATCTGCAG
GCAATTGAAAAGCGGCTGGAGAAACTGGCTGTCGATCCGCACCGCGATCGGGCGCAAATG
CTGAAAGTCGAAAGCGTCCAGCAGGCGTGGCAACAATGGATAAATAAATTGCCTCCAGCG
CGTCGCGAAGATGATGATGTTAAAGAGATCCGTTGGATGATTGAAGAACTGCGTGTCAGT
TATTTCGCCCAACAGCTCGGTACGCCTTATCCTATTTCCGATAAGCGTATTTTACAGGCC
ATGGATCAGATTACGGCCTAA
>EECEBICD_01991 FMN-dependent NADH-azoreductase
ATGAGCAAGGTATTAGTTCTTAAATCCAGTATTCTGGCAGGGTACTCTCAGTCTGGTCAG
TTGACTGACTATTTTATTGAACAATGGCGCGAAAAACACGTCGCAGATGAAATTACTGTC
CGCGATCTGGCGGCCAACCCTGTTCCGGTGCTGGATGGCGAGCTGGTGGGCGCGATGCGT
CCGGGCGATGCGCCTTTAACGCCACGTCAGCAGGACGCTCTGGCGCTGTCTGATGAACTC
ATTGCTGAACTGAAAGCCCACGACGTCATCGTTATTGCGGCGCCGATGTACAATTTCAAT
ATCCCGACGCAGCTGAAAAACTACTTTGATCTGATTGCCCGCGCCGGTATCACTTTCCGC
TATACCGAAAAAGGCCCGGAAGGTCTGGTAACCGGTAAACGCGCGGTGGTTCTCTCCAGC
CGCGGCGGTATCCATAAAGACACCCCGACGGATTTGATTGCGCCTTACCTGAAAGTGTTC
CTTGGTTTTATCGGTATCACCGACGTGAATTTTGTCTTTGCCGAAGGCATCGCCTACGGG
CCGGAAGTGGCGGCTAAAGCTCAGGCCGATGCTAAAGCCGCGATTGACAGCGTCGTGGCC
GCTTAA
>EECEBICD_01992 hypothetical protein
ATGACGTCTGAAAAATTATCTGCTGCTTGCCATTGTGGCAGCGTCGTGTTTACCGTTCAA
CTTAGCGATGGCTTTCATACCGCCAGGCGCTGCAACTGTTCGTTTTGCCGGATGCGCGGA
GCGGTGGCAGTGTCTGCGCCGCTATCGGGTATTAAAGTGCTAAAAGGGCAGGATAAGCTT
ACCGAATATCGCTTCAATACGGGCAAGGCGGTGCATTTTTTCTGTTCGGTATGCGGGATA
TATACTTTTCATCAGCGCCGATCCAATCCCGATCAATATGGGGTCAATGTGGCGTGCATC
GAAAATGTGTCGCCTTTTGATTTCGCCTGCGTTGAAGTCAATGATGGGGTAACGCATCCC
AGCGATGGCGGCAGTAGCGGCGTGGTAGGATATTTGCGCTACGAACCGGAGACGTTGCCT
CCCGTCGAAACGGGAGGCAAAAATATTTAA
>EECEBICD_01993 hypothetical protein
ATGAAAAAGTATTTAATGCTATGGGTGCTGACGCTGAGCTTGCTCACACCTTCTGTGTGG
GCCTTAACCCTGGACGAAGCGCGCACGCAGGGCCGGGTCGGGGAAACGCTGAATGGCTAT
CTGGTGGCCTTAAAGAATGATGCCGAAACCCAGAAACTGGTGCTTGATATTAATCATGCC
CGCCGCGCCAGTTATCAGCAGTTAGCGGATAGCAATCATCTTCCCGTGGACGAGGTGGCG
AAGATGGCAGGGCAAAAACTGGTTGAGCGCGCCAGGCCGGGAGAATATGTCCAGGGAATT
AATGGCAAATGGATGCGTAAATAA
>EECEBICD_01994 hypothetical protein
ATGAAAATGAGTATTGCCATGCTGTCAGCGCTTGCGTCATTTATCGTGGTGGGCTGCACG
CCACGTATTGAAGTCGCCGCGCCGGAACAGCCGATTACCATCAACATGAATGTAAAGATC
GAGCATGAAATACATATCAAAGTCGATAAAGATGTTGAAGAACTCCTGAAATCACGCAGC
GATCTATTCTGA
>EECEBICD_01995 hypothetical protein
ATGAAGGGTAAATATAAAGCCGCCCTTGCGCTACTGTTACTGCTGATTCTTATCCCTCTG
ACGCTACTGATGACCCTCGGGCTGTGGGTTCCCACTCTGGCGGGAATTTGGCTACCCGTC
GGTACCCGTATCGCTCTGGAACAAAGTCCACGCCTGACACGTCATGGGTTAGTGATTCCC
GATCTTCGCTATCTGGTCAATGATTGTTCGTTAGCGCATATTACCCAGGCGGAACTGACG
CATCCCAGCCGTTGGTTGCTGAATATTAAAAGCCTTAAACTGGATGCCGCATGCCTGGCG
AAACTGCCCGCTACGGAAGCTTCTCCCGCCGCGCCGCGTACGCTGGCGCAGTGGCAGTCA
ATGCTGCCCAATACCTGGATTAACATCGATAATGTCATTCTCGCGCCCTGGCCGGAATGG

CAGGGCAAGCTGGCGATCTCCATGACGCCGGTCATTCAGCAGATACGTTATCAGGGCGAA
AAAGTAAAATTTCAAGGGCAGCTACGCGGTCAGGCATTGACGGTGAGCCAACTGGAGATC
GCCGCGCTGGCCAACCAGCCTCCCGTTAGCCTGGCGGGGAATTTGTGTTGCCGCTGGTA
TCGGATGGCCTGCCGGTCAGCGGTCATGCGGCAGCGACCTTGCGTCTGCCGCAGGAACCG
TCGCTGGTGGATGCGGAACTGGAGTGGCGTGATAACGCCGGACAGCTCATCGTGATGGCG
CGAGGTAATCCTGACCCGATTCTCGATCTCCCCTGGGCGGTAACGCGTCAGCGGCTTACC
ATCAGCGATGGCCGCTGGAACTGGCCATATCAGGGATTCCCCTTGAGCGGACGTCTGGCT
TTTAATATCGATAACTGGCAAGCCGGGCTGGATAATGCGCAAGTCAGCGGACGCCTGAAT
ATTCTGACCCAGGGCGATGCCGGTAAAGCGAACGCTGTACTGACTATCGGCCCGGGAAAA
CTCAGCATGGATAGCAGTGAGATGCCTCTACAGCTTACCGGCGAGGCTAAACAGAAGGAT
TTAATCTTTTATGCCGTATTGCCCGCTATGTTCCGCGGCAGTCTGGCCGATCCGCAGCTC
ACCTTTGCACCTGGCGCATTGCTACGCTCGCGCGGGCGGGTGATTGATGCGTTAGATATT
GATGAAATTCGTTGGCCGCTGGCGGGCGTTAAGGTGACGCCGCGCGGCGTTGATGGCCGA
TTACAGGCTATTTTACGGGCGCACGAAAATGAGATGGGCGATTTCGTTCTTCATCTTGAT
GGCCTGGCCAATGATTTTCTCCCTGATGCTGGTCGCTGGCGGTGGCGCTATTGGGGGCAA
GGAAGCTTTACGCCAATGCGCGCCCACTGGGATATTGCCGGACAGGGCGAATGGCATGAT
AACACCATCAGGTTGACCAGTTTGTCCACCGGTTTTGATCAGTTGCACTATGGCGCAATG
ACTGTCACATCGCCAAGACTGGCGCTGAATAAACCTATTGTCTGGGTTCGCGACGCCACA
ACGCCGTCGCTACAGGGCGCGCTGTCTCTGGTGGCGGGGAAAACCGTATTTACCAGTGGG
AGCGTGCTGCCGCCTTCTACCCTTAATTTTAGCGTTGAAGGGCGAGAGCCAACCCTGTTC
CAGTTTAAAGGCGACCTTCGGGCGGGGGCGATAGGGCCGGTACGGCTGAATGGGCGCTGG
GATGGCGAACGCCTTCGTGGACAGGCCTGGTGGCCGAAGCAGTCGCTTATCGTTTTCCAG
CCGTTGCTTCCACCGGACTGGAAAATGACGCTGCGCGAAGGCTCACTGTATGCGCAGGTG
GCTTTTTCCGCCGCACAGGGGCAGGGGTTTGAAGCAGGTGGACACGGCGTGTTAAAAGGC
GGCAGCGCCTGGATGCCGGATAACAAAATCAACGGCGTCGATTTCATTCTGCCATTTCGC
TTCCACAACGGCGCGTGGCAATTGGGGACGCGCGGGCCCATCTCGCTGCGTATCGCCGGG
ATAGTTAACCAGGTCACGGCGAAAAATATCACCGCTGATTTACAGGGCGGTTATCCCTGG
AGCGAGTCGAATCCATTACTGCTAAGCGATGTCAGCGTAGACGTGCTGGGCGGTCAAATC
ATTATGAAACAGTTGCGAATGCCGCAGCACGACCCGGCGCTTTTGCGGGTGCAAAATATT
TCATCCAGCGAATTGATTAGCGCCATAAATCCAAAGCAATTCGCCATGTCTGGTCCGGTC
AGCGGCGCGCTGCCGCTGTGGCTGAACAATGAAAAATGGATAATCAAAGATGGCTGGCTT
ACGAATCCTGGGCCAATGACGTTGCGTATCGATAAAGATACTGCGGATGCGGTGGTGAAG
GATAATGTCACGGCAGGCTCGGCGATCAACTGGCTACGTTATATGGAGATTACGCATTCG
TGGACGAAAATTAACGTTGATAACCTGGGCGTACTAACGATGCAGGCGGCTATTACCGGC
AAAAGCCGGGTAGATGGTAAAACCGCGATCGTTAACTTGAACTATACCCATGAAGAAAAT
GTGTTTACGCTGTGGCGCAGTTTGCGTTTTGGCGACAATTTACAGGCATGGCTTGAACAA
AATACAGTGTTGCCGCAACCTCCTTGCCGGAAAGACAAGGACTGTGAGGATAAATAA
>EECEBICD_01996 D-lactate dehydrogenase
ATGAAACTCGCCGTCTATAGTACCAAACAGTATGACAAGAAATATCTACAGCAAGTGAAC
GAAGCGTTCGGTTTTGAACTGGAGTTTTTTGATTTTCTGCTAACCGAGAAAACGGCGAAA
ACCGCCAATGGATGTGAAGCAGTTTGTATCTTCGTGAATGACGACGGGAGCCGACCGGTA
CTGGAAGAGCTGAAAAAACACGGCGTGAAATACATCGCGCTGCGTTGCGCCGGATTTAAT
AATGTCGACCTGGATGCCGCAAAAGAGCTCGGTTTGCAGGTGGTGCGCGTACCGGCTTAT
TCTCCGGAAGCCGTCGCTGAACATGCGATTGGCATGATGATGACATTGAACCGCCGTATT
CACCGCGCTTATCAACGCACCCGCGACGCTAACTTTTCCCTTGAGGGACTGACCGGCTTT
ACCATGCACGGAAAAACCGCTGGCGTCATTGGTACCGGTAAAATCGGCGTCGCCGCGCTG
CGTATCCTGAAAGGCTTTGGAATGCGTTTACTGGCGTTTGATCCCTATCCCAGCACCGCC
GCGCTTGATCTCGGCGTCGAGTATGTGGATCTGCAAACGTTGTTTGCCGAATCGGATGTC
ATTTCCCTGCACTGCCCGCTCACGCCGGAAAACTACCATTTACTGAACCATGCGGCATTC
GACCAAATGAAAAATGGCGTTATGGTCATTAATACCAGTCGTGGCGCGCTGATTGATTCC
CAGGCGGCTATCGAAGCTCTGAAGAATCAGAAAATCGGCTCGCTGGGGATGGATGTGTAT
GAAAACGAACGCGACCTGTTCTTTGAAGATAAGTCAAACGATGTTATCCAGGATGATGTT
TTCCGCCGTCTGTCGGCCTGCCATAACGTACTGTTTACGGGGCACCAGGCATTCCTGACC
GCAGAAGCGCTGATCAGCATCTCTGAAACCACGTTACAAAACTTAAGCCAGCTTGAGAAG

GGCGAAGCCTGCCCGAACGCGCTGTTCTAA
>EECEBICD_01997 Heat shock protein HslJ
ATGAAGAAAAGCGTCACGCTGGTCGCCCTGAGCATCATAATGACAGGGTGTGTGAGCAGT
GGAAAAGTGTCCGTCAAACGCGAGCAACTGGAGCACCACCGCTTTGTTCTGGAAAGCGTA
AACGGTAAAACGGTCACCGGCCCGGAGCTCAGCTTTGGCGAGGACATGACGGTATCCGGC
AAAATGTGCAATCAATTCACCGGCGAAGGAAAACTGTCGGACGGCGAATTAAAAGTGAAA
AATTTAGCGATGACCCGAATGATGTGCGCCGATCCACAGCTTAACGCGCTGGACGGCACC
CTCAGCAAGCTCTTCAGCAAGGGCGCACAGGTCGATTTAACCGCGAACCAATTAACGCTG
GCGACGGCAGAAACAACGCTAATGTATAAACTGGCGGATTTAGCGCACTAA
>EECEBICD_01998 hypothetical protein
ATGCGCGCAGCGTTTTGGGTGGGATGTGCCGCGTTATTATTGTCGGCTTGCAGTAGCGAA
CCTGTTCAGCAGGCAACCGCGGCACATGTTTCGCCAGGGATGAAAGCGGCGATGTCCAGC
GCCGGCGAAGCGAATTGCGCTATGATTGGCGGTTCGCTTTCAGCGGCCCGGCAGCTTGAT
GGTTCAGTAATTGGTATGTGCGCGCTACCCAACGGCAAACGTTGTAGCGAACAGTCGCTT
GCCGCCGGAAGCTGCGGGAGTTATTAA
>EECEBICD_01999 Pyruvate-flavodoxin oxidoreductase
ATGCAAACAATTGACGGTAATGGCGCAGTAGCGTCGGTGGCGTTCCGTACCAGCGAGGTT
ATCGCCATTTACCCGATTACGCCCAGTTCAACAATGGCTGAACAAGCGGATGCCTGGGCC
GGCAATGGGCTTAAAAATGTCTGGGGCGATACGCCGCGTGTGGTAGAGATGCAGTCGGAA
GGCGGCGCGATCGCCGCCGTTCACGGTGCGTTACAAACCGGTTCGCTTTCTACGTCATTT
ACATCATCGCAGGGGCTGCTGCTGATGATTCCAACGCTGTATAAACTGGCGGGGCAGCTC
ACGCCGTTTGTACTGCACGTTGCCGCGCGCACTGTCGCCACACACGCGCTTTCTATTTTT
GGCGACCATTCCGATGTGATGGCGGTCCGCCAGACAGGCTGCGCGATGTTATGCGCCGCC
AGCGTTCAGGAAGCGCAGGATTTTGCGCTGATCGCACACAGGGCTACGCTGAAAAGCCGC
GTTCCGTTTATTCATTTTTTTGATGGCTTCCGTACATCGCATGAAATCAACAAAATTATA
CCGCTAACGAATGAAACCATTCTGAACCTGATGCCGCAGGCGGAGATCGACGCTCACCGC
GCCCGCGCGCTGAATCCGGAACATCCGGTGATTCGTGGAACTTCAGCGAATCCGGACACC
TACTTCCAGTCTCGCGAAGCGACCAACCCGTGGTACAACGCCGTTTATGATCATGTTGAA
GAGGCGATGAAGGCATTTGGCGACGCTACGGGCCGTCAGTACCAGCCATTTGAATATTAT
GGCCATCCCCAGGCTGAACGCGTCATCATAATGATGGGCTCCGCCCTCGGCACCTGCGAA
GAGGTCGTGGACGAGCTACTGATACGCGGAGAAAAGGTCGGCGTTCTGAAAGTCCGTCTG
TTCCGCCCCTTCTCCGCCAAACATTTGTTACAGGCATTACCGGAAACCGTTCGCGCTATC
GCCGTGCTGGATCGCACCAAAGAGCCTGGCGCCCAGGCCGAGCCGCTTTATCTGGACGTG
ATGACCGCACTGGCGGAGGCCTTTAATAATGGCGAGCGCGAAACGTTGCCGCGCACCATT
GGCGGCCGTTATGGGCTATCATCCAAAGAGTTCGGCCCGGCGTGCGTACTGGCGGTCTTT
AATGAACTCAGCCGTGCGAAACCCAAGCCACGGTTTACGGTGGGCATTTATGATGACGTT
ACTAACCTCTCGCTGCCATTACCGGAAAATACCCTGCCCGGCAGCGCTAAACTTGAAGCG
TTATTTTACGGTCTGGGAAGCGACGGCAGCGTATCCGCCACGAAAAATAATATTAAAATT
ATCGGTAATTCTACGCCGTGGTATGCGCAAGGTTATTTCGTTTATGACTCTAAAAAAGCT
GGCGGGCTCACGGTATCGCACCTGCGCGTCAGCGAAAAACCCATTCGTTCCGCTTACTTA
ATTGCTCAGGCGGATTTTGTCGGCTGCCATCAATTACAGTTTATCGATAAATATCAGATG
GCGGAGCGTCTGAAGCCCGGCGGTATCTTTCTGCTTAATACGCCGTACAGCGCCGACGAG
GTCTGGTCGCGTCTGCCGCAAGAAGTTCAGGCGGTGTTGAATCAGAAAAAGGCGCGTTTT
TACGTGGTTAACGCGGCGAAAATCGCCCGCGAATGTGGACTTGGCGCCCGCATCAATACC
GTCATGCAAATGGCTTTCTTCCACCTGACGCATATTCTTCCGGGCGATAGCGCCCTGGTT
GAGCTGCAGGGCGCGATTGCCAAAAGCTATAGCAGCAAAGGACAGGATCTGGTTGAGCGT
AACTGGCAGGCATTGGCTCTGGCGCAGGAATCGCTGGCGGAAGTGCCGTTGCAGGCGGTT
AATCCGCATAGCGCGCATCGCCCGCCGGTCGTCTCCGACGCCGCGCCGGATTTCGTGAAA
ACCGTTACCGCCGCCATGTTGGCGGGCCTCGGCGACGCACTGCCTGTTTCCGCCCTGCCG
CCGGACGGAACATGGCCAATGGGCACCACCCGCTGGGAAAAACGCAATATCGCCGAAGAG
ATTCCTGTCTGGAAAGAGGAACTGTGTACACAATGTAATCACTGCGTCGCCGCCTGCCCT
CACTCGGCGATTCGTGCAAAAGTCGTTTCCCCGCAGGCGATGGAGAACGCGCCAGCCAGT
CTGCATTCGCTGGACGTGAAGTCCCGCGATATGCGCGGCCAGAAATATGTGCTGCAGGTC
GCGCCGGAAGACTGTACTGGCTGTAATTTATGCGTAGAGGTCTGCCCGGCTAAAGATCGG

CAAAATCCACAGATCAAGGCTATTAACATGATGTCGCGCCTGGAGCATGTGGAAGAAGAG
AAAGTGAATTATGACTTTTTCCTCGACTTACCGGAAATCGATCGCAGCAAACTGGAACGA
ATTGATATCCGCACATCGCAGCTTATTACGCCGCTGTTTGAATATTCCGGCGCCTGTTCC
GGCTGCGGTGAAACGCCGTATATTAAACTGCTGACTCAGTTATATGGCGACAGAATGTTA
ATCGCTAACGCCACGGGGTGTTCCTCTATTTACGGCGGTAACTTACCCTCTACGCCTTAT
ACCACCGATGCCAACGGACGCGGCCCGGCGTGGGCGAATTCGCTATTTGAAGATAACGCG
GAATTTGGCCTCGGTTTCCGTCTGAGTGTGGATCAGCACCGGGCGCGCGTCATGCGTTTA
CTGGCGCAATTCGCCGATCGCATTCCGGCGGAATTAAACGATGCGCTGCATGCCGAAGCA
ACGCCTGACGTTCGGCGTGAACAGGTCGCCGCATTACGCCAACATCTGAAATCGGTTGCG
GGCGCAGAGGAACTGTTAAAGGATGCTGATGCGCTGGTAGAAAAATCAATCTGGCTGATT
GGCGGCGATGGTTGGGCATACGACATCGGCTTTGGCGGTTTAGACCATGTGTTAAGCCTG
ACGGAGAATGTCAATATTCTGGTTCTGGATACGCAGTGTTATTCCAATACTGGCGGCCAG
GCGTCGAAGGCGACGCCGCTTGGCGCCGTCACTAAGTTTGGCGAACATGGCAAGCGTAAA
GCCCGTAAAGATCTTGGCGTCAGCATGATGATGTATGGTCATGTTTATGTCGCGCAAATT
TCGCTGGGCGCGCAGTTAAACCAGACGGTGAAAGCCATCCAGGAGGCCGAAGCCTGGCCA
GGACCGTCGCTGATCATTGCCTATAGCCCCTGTGAGGAACATGGTTACGATCTGGCGCTA
AGCCATGACCAGATGCGCCAGTTAACGGCGACCGGTTTCTGGCCGTTATACCGCTTTGAT
CCCCGCCGTGCGGATGAAGGCAAGCCGCCGCTGGCTCTGGACTCTCGGCCACCGTCTGAT
GCCCTGGCGGAAACGTTGCTTAATGAACAGCGTTTCCGTCGCCTGAATGCGCAACAGCCT
GAAGTAGCTGAACAATTATGGCGAGATGCCGCATTGGATTTGCAAAAACGCTATGACTTT
CTTGCCTTGCTGGCAGGAAAAGCGGAAAAACCCGGCGCAGATTAA
>EECEBICD_02000 Universal stress protein F
ATGAACAGAACGATTCTTGTACCCATCGATATTTCAGATTCAGAATTAACTCAACGCGTA
ATTTCGCATGTTGAAGCTGAAGCAAAGATTGACGACGCTAAAGTGCACTTTTTGACTGTA
ATCCCGTCTTTGCCCTATTACGCTTCACTGGGACTGGCTTATTCAGCAGAGCTTCCCGCA
ATGGACGATTTGAAAGCCGAAGCCAAATCTCAACTGGAAGCGATTATCAAGAAATTCAAC
CTTCCTGCGGACCGCGTGCAGGCTCACGTTGCAGAAGGCTCTCCTAAAGATAAGATTCTG
GAAATGGCAAAAAAATTACCGGCCGATATGGTGATTATCGCCTCGCATCGCCCGGATATT
ACTACCTATCTGTTGGGTTCCAACGCCGCAGCCGTTGTGCGTCATGCGGAATGCTCCGTA
CTGGTGGTACGCTAA
>EECEBICD_02001 Multidrug transporter EmrE
ATGACTAAAGAAGCTGTAATCTTTCTATTTATCGCCATCGTGGTAGAAGTTATCGCCACG
ATCTCATTAAAATTATCAGATAGTTTTACGCGTCTGGTACCGAGCCTCGTTACTATCATC
GGATATTGTATTGCGTTCTGGTGTCTTACCATCCCAATGCGAACCATCCCTGCGGGTATC
ATTTATGCTATTTGGTCTGGGGTAGGGATTGTTCTTATTGGATTGATAGGATGGTTATTT
CTCGGCCAAAAACTGGATATGCCGGCTATTATTGGCATGTTGCTTATCATCTGCGGCGTA
ATCGTAATCAATCTGTTTTCAAAAAGTGTCAGTCACTAG
>EECEBICD_02002 hypothetical protein
ATGCCGCGAATGGCGGCGAAATGGGCACTGGTAGCGATACTGGTGCCTTTTTTACTGGTG
GGGTGCGCCAGCCTGGATAAGGCGCGCCAGATATTCGAAGCTGCCGGGCAGAGTCGTAGT
GAACGTCTTTTGGGCCTGAAAAGTAAATCCCCAGTTGTTGCTGACAACTGGGGATTTTTA
TAA
>EECEBICD_02003 hypothetical protein
ATGAAACCGAAGGACGAAATTTTTGATGAAATTCTGGGTAAGGAAGGCGGCTACGTCAAC
CATCCGGACGATAAAGGCGGGCCGACAAAATGGGGTATTACGGAAAAAGTTGCCCGCGCC
CACGGATACCGTGGTGATATGCGCAATTTAACCCGTGGACAGGCGCTGGAAATTCTGGAG
ACCGACTACTGGTACGGTCCCCGCTTTGACCGGGTGGCGAAGGCCTCGCCGGATGTTGCT
GCCGAACTGTGTGACACGGGCGTGAACATGGGGCCGTCGGTGGCAGCGAAAATGTTGCAG
CGCTGGCTGAACGTGTTCAACCAGGGCGGGAGGCTGTATCCGGATATGGATACGGACGGG
CGCATCGGGCCGCGAACCCTTAACGCGTTACGTGTTTATCTGGAAAAGCGCGGTAAGGAT
GGCGAGCGTGTACTGCTGGTGGCGCTGAACTGCACGCAGGGGGAGCGCTATCTGGAGCTG
GCGGAAAAGCGGGAGGCTGACGAGTCGTTTGTCTATGGCTGGATGAAAGAGCGCGTATTG
ATATGA
>EECEBICD_02004 hypothetical protein

ATGGAAAGTAATCTGACCGGCACACTGAATGCGGGCCTGTGCCTGGTGACAGTGCTGGCC
CTTTTTCTCTACCGCCGGAACGGCGCCAGATACAAACCGGGAATAGCCTGGCTGTCGTAC
CTGCTGATGCTGGGCTATGCGCTGGTTCCGTTCCGTTTTCTGGCCGGACATTACCCGTCT
TCATCCTGGCCTGTGGTGCTGATGAACGCGCTGTTCTGCGGGCTGGTGCTGTGGGCGCGG
GGTAATGTGTCGAAAATACTTTCACTGCTGAGGCTGCGATGA
>EECEBICD_02005 hypothetical protein
ATGTTAGGTATTTTCAGAAAGAAAACCCGCAAGGCTATTGTTGAAGTGAAGAAGATGGAG
AACCGGGATGCGGTGGAGGCGACCGTCTGGGGCGCATATTCCATTGCATACGCCGATGGC
ACCTGTGACGCGAAAGAAATTGCAGTGCTGGAGAAAACCATCGCGGCACTTCCTGCCTTT
GCGCCGTTCTCCGGCGAGATTGCCCAGATGAGTGCCAATATCCGCGCCCGCTATGAGGCG
TCACCGCGTAGCGCGAATGCTCAGGCACTGCGTGAGCTGGCTGACGTGGCAGGAACAGCA
GAAGCGGTTGATGTGCTGTGCCTGTACTGGCCTCGTTCCTGTCCCGCCTTGCTGACTACA
ACGGTAAACCGCTGGATGCGCTGTGTGCAGTGGTGA
>EECEBICD_02006 hypothetical protein
ATGATTTACCCAACAAACACCGGAAAAAGCGGAGAACACCTTCGTCTCAGCACGCTGGAA
AGTGTGTGGATTCAGGGGAAATTGCGTATGTGGGGGCGCTGGTCATACATTGGTGGCGGC
AAAACAGGGAATATGTTTAACCAGTTGCTGGCGTCCAAAAAACTGACGAAGACGGCCATT
AACGATGCTTTGCGCCGTATGAAAAAAGCGGGGCTGGAGAAACCTGAACTGGAAGTGTTC
CTGAGAGAGATGATCAACGGAAAGCAAAAAAGCTGGCTGGCACACTGTACGGATACGGAA
GCGCTGATTATCGATCGGGTTGTAGGCGAGGTACTGACGGATCATCCGGGGCTGCTTGGT
ATCCTGAACCAGCGTTACGTGGGGCGGGGGATGAGTAAGAGAAGGATGGCCGAGTTACTA
AACGAACAGTACCCAGAGTGGGCGTTGATTACATGCCGACGCCGTGTTGAGCAGTGGTTG
AGTATCGCTGAGTTCATTTTGTATTCACCTATGAGAAAAGCGTTCGATTATGCTTAA
>EECEBICD_02007 Putative nuclease YbcO
ATGGCGGATTTACGTAAAGCGGCGCGGGGCCTGATGTGTACGGTAAGAATTCCCGGCCAT
TGCAACCATAATCCTGAAACGTCCGTACTGGCACATTACCGGCTGGCGGGTACGTGCGGA
ACGGCGACAAAACCAAACGATATGCAGGCAGCAATTGCCTGTAGCTCGTGCCACGATATT
GTCGATGGGCGGGTAAAAATCGACGACTTCACGAAAACAGAAATTCGCCTGATGCACGCA
GAGGGCGTTTTCCGCACGCAGGAAATCTGGAGAGAGAAAGGCATTTTATGA
>EECEBICD_02008 hypothetical protein
ATGGCGCATCTACAACTTGTTAAACAAACCTCATCAGGGCTTCTGCTCCCGGCAACGCCG
GAGAGTGAGGACTTCCTGCGCTCAGTAAAAATCGGTGCGTGGATACACGCCGATTTTAAG
CGAGTACGTAACTACGCGTTCCACAAACGTTTTTTCAAGCTCCTTCAGCTTGGTTTCGAT
TACTGGACTCCGATCGGCGGGGCGATCCTGCCTCAGGAACAGGAGCTGATTACCGGCTTT
GTCGATTTCCTGTGTGAGTCAGCAGCGCAGGGCCACAGTCCCGCACTCAGTGACGCGGCG
GAACAGTACCTGCATAAGGTTGCTGTCAACCGAACGCTCGATGTTGCGCTGCTCAAGTCC
TTCGACGCTTTCCGCGAGTGGGTAACCATTCAGGCCGGGTTTTATACTGAGCATTATTAT
CCGGATGGCAGCCGTGGGCGCCGGGCGAAATCCATCGCTTTTGCGAATATGGACGAAACC
GAGTTTCAGCAGGTTTATAAGGCCGTACTGAACGTCCTGTGGAACTGGATTCTGTTTCGT
AAATTCTCCTCTCCGGAAGAGGTCGAAAATGTCGCAGCGCAACTGCTGGAGTTTGCGTAA
>EECEBICD_02009 hypothetical protein
GTGAAAAAACTCAAGGATTTATTAGAGTTAGATGAAGACGGGCTTTATGCAGTACGTGTA
AAAAATGGTGAAATCTCATTCTGTACGCTAATTCCTGACGACCATCTGATTCTGTCTGTT
GAAGCGTTTATTGATTATCTGATAAGACTGGGTTTCACTGTCAGTTATTAA
>EECEBICD_02010 hypothetical protein
ATGCTTTGCACATGTAGGTTAGCCTCTTACGTGCCGAAAGGCAAGGAGAAGCAGGCTATG
AAGCAGCAAAAGGCGATGTTAATCGCCCTGATCGTCATCTGTTTAACCGTCATAGTGACG
GCACTGGTAACGAGGAAAGACCTCTGCGAGGTACGAATCCGAACCGGCCAGACGGAGGTC
GCTGTCTTCACAGCTTACGAACCTGAGGAGTAA
>EECEBICD_02011 hypothetical protein
ATGCACAGCGTTAATTTCTATTCATTCCGCGTATTGACCCATAAAGGCAGTCGAGCCAGC
AAAAAACTTAATGACTTAGGTTTAAGTAATAAAAAAACGGCATATGAACTTTTTGTTGAT
TATTTTACTCTTTATAAAAACACCCCCATCGAGTTCGGCGTTTCAAAAACTAAAATATCT
CTGGAACAACATACTAAACTTCACTTTGATAACACAAAAAAAATCATATATGGTTATATA

AAAGTTGGAAAATATGGAGAAAGCAGTGAAATAAAAGATGTAAAACTCAAAAAAGTCCAT
TACAGAACAACTGCTTATGATGTAACACTCAAAGAGCGTTATATTTTAATATATCTACCA
GATAATCTTGAAGAAGGAATTATTGCATTCCATTCATGCGATAATATTTCTGCTCGAGGT
GTTCTTTCTGATTCTATCACTGAATATCTAAAAAAACAATTTCAACTAGAAGCAAGAATC
AATCCATTACATCATAAGAAAATCCCTCAATACATTCTCAATTCTGAATTGAAACAAATT
AAAGCTCAAGGATATAAAGCACCAGAAGATATTGCTGATTCCTTTGGTAAAAACAAAACA
AACATCAAGACAGACTTAATAATAAAAGCAAACGATGGCATATTCGGAAGTTTCAGGGAT
TTAAGAAACAAGAATATAGGAAACATCATTGAGATTATTGAAGATAAATGTGATGCAATA
AAAGTAAGCTTACAGCTCGGCAGTCGGACTGTCGTTTTCAATTATGATACCATACTAAAA
AAAGGAATTTCAGCAGAGTTAGATGATAATGATCTAAAAATCAACCCATTAACAGGTATA
CCTGATCTAACAGCACTTCATGACACGATAAAAAACCTTTCCAATGATATATTGGAAGAA
CTGCACTGTGGAAATAAAGGGGTGATTATATGA
>EECEBICD_02012 hypothetical protein
ATGAATAAAATAAATGTGCTGGGTGTAATAATAAAACACTACAAAACAATGTCAGATCAG
CGTGGAACAATGTTGATGAGCGACATTACCGTACATTTTATAGTTCCTCTATCTCTTTCT
TTCGTTCTGTGCTGGACATACGGAATAATGAAACCGGCAATTGCTTCCGTCTTCGTTAAC
TTCGGGGCTATTACAACAGCACTATTAATGAGTGCAGTAATAATGATTTATGAACAAAAA
CAAAAAACCATTACTAAGATATCAGATATAATTGAAGGAAACAAATCAAGAGACAAATTG
ATATCATTAAACACTAACAAAACCATATATGAGCAGTTATGCCACAACGTCGCTTATGCA
ATATTAACTTCAATAGTATTGGTTATATTTTCAGTAATAATATATTTCCTGCCTGACAAT
GCAGTGGATTTAATGAAATGGTATTTTCGCGCACCCGCATATATCGTTAGCTTTTTAGCC
TATACATCCTTTTTTATCACTGTCATAACTTTCTTAATGGTAATAAAAAGATTTAGCACG
ATTTTAGACAATTAA
>EECEBICD_02013 hypothetical protein
ATGAACTCCGGCCTGATAACACTTACGGAGCTGAGGAGGATGACGGGGTTAACCATTTAT
TCGACCCGCCACTACCTGGACAAGGCAGAACGTTGTGGGGATGTGTACCAGGCGGGCAGA
AGAGGGGGGATTTTCCCGTCAGAAGAGGCTTATCGTGCCTGGAAGAAACAGGCGAAAGTG
GACGCTGACCTGATTTGGAAGCTGCCTGACGGTGAGGTACGTCGTTACGACAGGCACCAC
AACGTAATTTGTCGTGAGTGTCGTAAAAGCGAGTACATGCAGCGGGTACTGGCGTTTTAT
CGGGGAAACTTTCAGGAGGTGCTGTTGTGA
>EECEBICD_02014 hypothetical protein
ATGAGAAAAAAAACGTGAAGAAATAGCTCCACCAGAAGCTACCCAGCGCTTACGCGCCATC
TGGGACGCCAAAAAGCGAGACCTCAAACTTACTCAGGAGATCGCCGCTGATCTTATGGGC
TTTGAGACACAATCTACCGTCAGTCACTATTTGAACGGTAAGGCACCTCTCAACACTGAT
GCGGCCTTAAAATTTTCTGTTCTATTGAGAGTTAAACCCGAAGAGTTACGACCGGATTTG
GCCGATCTAATGAACTACGTTCGTTCTTCTGGTACTTACGATGACAACTTCGAAGGTGGT
GGCTGGCGAATGGTGAGCAGACAACAGGCTGATTTACTAAACCTTTTTGATATACTCCCT
GAATCTGAGAAAGAAAAACTCATTGACCGGCTTAAAGGTCAGAATGAGCTATATAAAGAG
GCTTTCCAGAACATGCTCGCTGCACAAAAGCGCCTAAAAAAATCAGTGA
>EECEBICD_02015 hypothetical protein
ATGGGAAGGAATGAAGTGATTCAGTATTTGATGGATAGTTGCAACGTCAGCTTTAGCGCA
GCTCTCCAAGCATTGCGCGACAATGGATGGGATATGTTTTTGGCTCAATGCGAGCTACAG
GAACAGTATTATCCGGGGTGA
>EECEBICD_02016 putative protein YdfB
ATGGATTTCGACACAATCATGGAAAAGGCTTACGAAGAATACTTTGAAAGCCTTGACGAA
GGAGAAGAAGCACTCAGTTTCAGTGAGTTTCTGCTGGCGCTTTCAGCTAACGGCTAA
>EECEBICD_02017 hypothetical protein
ATGTCAGAAACCGCTCTGGTTATCGTAAAATTCCTAATTGGTAAATCCGTCGGACAATTT
ATGCTCACAGTGGCTTTATTTTTCTTAATTATCATCTTCATTCCTAGAGATATTACGGAG
CTTATTGAGGCGCGTAGCGATTTACCATATGCCGTTCAGATTTTTAGTTTTGCTGTGGCT
TACCTTATAGTGCTGATCCTCAAAGTCACTGGTTATTTTTTCGTGTCGGCGCTGCCGTTG
TGCCAGCGTAGGGGCAGGGCAAAACGCATGTTAAAAACGCTTAATTCATTGAGTACTGAA
CAGCTGTTTTTACTTGAACCCTTTCTTAAAACTCATTCTCCCACTTTCCGGGCGTCCTGG
GATAACCCTGATGCAGATGCTCTGGTTAAGGCAGGTATCGTTCGTCCGGCTGGTTCGTGT

ATCGACGGTGTTTCTGTGATGTTCAAAATCGAACCCGAGTATGAGTCGTTAATGCTTTCC
ACCTGGAATCCCTGCACAAAACGGTTCGATATTAGCCGTTAG
>EECEBICD_02018 Killing protein KilR
ATGATTGCTCATCACTTCGGAACGGATGAAATACCGCGTCAGTGTATTACGCCGGGAGAT
TATGTTATCCATGATGGTCGTACTTATATCGCCTCAGCGAATAACATTAAAAAACGCCGT
TTATATATCCGTGATTTAACAACGCAAAGATGTATTACCGATTGCATGGTAAAAGTCTGG
CTGAACAGAAATGGTCTGCCTGCCAAAGCTGAATCATGGTAA
>EECEBICD_02019 hypothetical protein
ATGAAAAATGTACCTGAATCAGTAATTGCAGAGCTCCGCCAGCTTTCAGGAAAAATTCGT
ACGTTATGTATCGAAAACAATATGCCCTGTGTTGTTTCTTATGCCCGGGACTGTGACGAT
GAAAGTGTTTCCAGAACTCTTGTTGCATATACAGACAGTGAAACAGGCGCATATGACAGG
TCAATAACAGCCGCAATAATGCTGTTAAAAATGAACGAAGCTCCTCCCGAAAATTTTATT
TCATTGTTGAAACTGATGGAGTGTAAAGAGCTCGTCACGAACGCATTCTGCTCAATGAAA
AATGAAAGTCTTCATTAA
>EECEBICD_02020 hypothetical protein
ATGAGCGACAATAAAACAGAATACTCATATTATATTAAGGTTAAAAATGAAAGCGCCCGG
AAACGTCTCGGCTTCCCTTTTGCTTTCTGGTGGAAAACTGAAAGCAGCGAAGCCGCTGCC
ACAGCACGCCTTGCCGTATCAATGCTTGACGCCGGATTCGAACCGACAGATTTTGCAAAA
CCGGTTCGCGTTAATTCCCCCGCTGTTAACGAACTTCCGCCGGAGGGAAGTTTTGATACC
ACCTTCTGTCAGAAATATGAGCTGGGCGGCGAAGATGGCAAAACATTTATGCTCATCCCC
GGCACGCCCGCTACTGACGCCCACGACGAAAAAACGGAGGAATGCGCCGACGACGCTGGC
ACCGAAGAAAGCGGGACAGACACCAGTGACAACGACGAATGTCAGGACTGCGAAGTTTCC
GTCGCCACCCTGCCGTTCCCCCAGCGCGTGTTGCACATTTTTACTTACGCTGCCACAGAC
AAAAAATATTTGCATCACGCCACCCGCGCTCAACGCAGGCATATTACCGTTCTCGAAATG
GAACAGGAAAACAGCTATATCCAGAACCTGTTAATGGTATTGCGGAAGTCTGAACAGGTT
CATGCCCAGGATGAGTAA
>EECEBICD_02021 Putative defective protein IntQ
ATGAGTAAAGGAAAACAGAAACCTTATTACTCGGTAAGCAGTTTAGGGGCAAGGTGGAAT
GCGGCAGTAAAACGTGCTGGTATTCGCCGCCGTAATCCGTACCATACGCGACATACTTTT
GCCTGCTGGCTGTTGACGGCAGGAGCGAACCCGGCATTTATCGCCAGCCAAATGGGGCAT
GAAACTGCGCAGATGGTGTATGAAATTTACGGTATGTGGATTGATGACATGAACGACGAA
CAAGTAGCGATGTTGAATGCACGGTTATCGTAG
>EECEBICD_02022 tRNA-cytidine(32) 2-sulfurtransferase
ATGCAAGAAAATTCAAAAGAATACAAAGAAAGAACAATACAACCTCAACAAGTTGCAAAAG
CGCCTGCGCCGTAACGTTGGCGAAGCGATTGCCGATTTTAATATGATTGAAGAAGGCGAT
CGCATTATGGTTTGCCTTTCTGGCGGCAAAGATAGCTATACGATGCTGGAAATTTTACGT
AATTTGCAGCAAAGCGCCCCGATCAATTTTTCACTGGTCGCCGTCAACCTCGATCAAAAG
CAGCCAGGTTTTCCGGAACATATCCTGCCAGCCTACCTTGAGCAGCTGGGCGTAGAATAT
AAAATCGTCGAAGAAAACACCTACGGCATTGTGAAAGAAAAGATTCCGGAAGGAAAAACC
ACCTGCTCGCTGTGCTCGCGTTTGCGTCGGGGTATCCTGTATCGTACGGCGACTGAACTG
GGCGCGACCAAAATCGCCCTGGGCCACCATCGCGACGATATTCTGCAAACCCTGTTTCTG
AATATGTTCTATGGCGGAAAAATGAAAGGGATGCCGCCGAAACTGATGAGCGATGACGGC
AAACATATCGTGATCCGCCCGCTGGCTTACTGCCGCGAGAAAGATATTGTCCGTTTTGCT
GAGGCCAAAGCCTTCCCTATCATTCCTTGTAATCTGTGCGGTTCGCAACCAAACCTGCAA
CGCCAGGTGATTGCCGACATGCTACGCGACTGGGATAAGCGCTATCCTGGACGGATCGAG
ACGATGTTTAGCGCCATGCAGAATGTCGTGCCGTCTCACCTTTGTGACACTAACCTGTTC
GATTTCAAAGGAATCACTCACGGTTCCGAGGTCGTCGACGGCGGCGATTTAGCGTTCGAT
CGTGAAGAGATTCCCTTGCAGCCCGCTGGCTGGCAGCCGGAAGAAGATGACACCGCCTTA
GAGGCGTTGCGGCTTGATGTTATCGAAGTGAAATAA
>EECEBICD_02023 ATP-dependent RNA helicase DbpA
GTGACCGCTTTTTCAACCCTGAATGTTTTGCCCGCCGCCCAGCTCAATAACCTTACTGAG
CTGGGCTATCTTGAGATGACGCCTGTTCAGGCCGCAGCATTACCCGTCATTCTGGCGGGT
AATGATGTGCGTGTGCAGGCCAGGACCGGTAGCGGCAAAACGGCGGCGTTTGGTCTTGGG
CTCTTGCATCGAATTGACGTCACTCTGTTCCAGACACAGGCATTAGTGCTGTGCCCGACG

CGGGAGCTGGCGGATCAGGTTGCCGGAGAGTTACGTCGCCTGGCCCGTTTTCTGCCAAAT
ACCAAAATTCTGACCTTGTGTGGCGGGCAACCCTTTGGCGCACAGCGCGACTCGCTTCAG
CACGCTCCGCATATCATTGTCGCGACGCCGGGGCGCCTGCTGGATCATTTACAAAAAGAA
ACCGTATCGCTGGATGCGCTGCATATTCTGGTAATGGATGAAGCAGACCGAATGCTGGAC
ATGGGATTCAGTGACGCCATTGATGAGGTGATCCGCTTTGCGCCTGCGACGCGCCAGACG
TTATTGTTTTCAGCAACCTGGCCTGAGGCCATCGCGGCGATTAGCGGTCGTGTACAGCAG
CAGCCAATACGTATTGAAATCGATACGGTAGATGCGCTACCGGCTATCGAACAACAGTTC
TTCGAAACGTCTGCGCATGAAAAAATTTCGCTGCTACAAACGTTGCTTAGCCAGCATCAG
CCAGCGTCCTGCGTGGTATTTTGCAATACCAAAAAAGATTGTCAGGCCGTTTGTGATGCG
CTTAATGCGGTAGGACAAAGCGCGTTGGCGCTCCACGGCGATCTGGAACAACGCGACCGC
GACCAGACGTTGGTGCGTTTTGCAAACGGTAGCGCGCGCATTCTGGTTGCCACCGACGTT
GCCGCGCGAGGATTAGACATTAAATCGCTCGAACTGGTGGTTAACTATGAACTGGCCTGG
GACCCGGAGGTGCATGTCCATCGTATTGGCCGTACGGCGCGCGCGGGAAGCAGCGGCCTG
GCGATCAGTTTCTGCGCGCCGGAAGAGGCGCAGCGGGCGAATATTCTTTCAGAAATGCTG
CAACTCAAGCTGAACTGGCTGAATGCGCCCGCCCGGCAGCCGTCACTCCCTCTGGCCGCA
GAGATGGCTACCCTATGCATTGACGGCGGCAAAAAAGCGAAAATGCGTCCGGGAGATATT
TTGGGCGCGCTGACCGGCGATATTGGATTAGACGGGGCGGATATTGGCAAAATTAACGTG
CATCCAATGCACGTTTACGTCGCCGTACGTCAAGCAGTAGCGCAAAAAGCCTGGAAGCAG
TTGCAAAACGGGAAGATCAAAGGCAAGTCATGCCGGGTACGGCTATTAAAATGA
>EECEBICD_02024 Zinc transport protein ZntB
ATGGCGGATGAAATTGCCCAGGTGATGCAGGAGTCTCTGGCGCGCAGAACTTATACCATG
TCGCTTATGGCGATGGTTTTTCTGCCCAGCACCTTCTTAACAGGATTATTTGGCGTTAAC
CTGGGCGGTATTCCTGGCGGCGGATGGCGGTTCGGCTTTTCTCTGTTTTGTATTCTGTTA
GTTGTCCTGATCGGTGGTGTTACTTTATGGTTGCATCGCAGTAAATGGTTGTAA
>EECEBICD_02025 Zinc transport protein ZntB
GTGGAAGCCATTAAGGGATCGGATGTGAACGTGCCGGATGCCGTTTTCGCATGGCTACTG
GATGGTCGTGGTGGAGTAAAACCGCTCGAAGATAATGATGTGATCGATAGTCAGCATCCC
TGTTGGCTACATCTTAATTATACCCACCCGGACAGCGCCCGCTGGTTGGCTTCAACGCCT
TTACTCCCCAATAACGTGCGTGATGCGCTGGCTGGTGAAAGCTCGCGCCCGCGCGTAAGT
CGTATGGGGGAAGGGACGCTCATTACGCTGCGCTGTATTAATGGCAGTACTGACGAACGC
CCGGATCAATTAGTGGCAATGCGTTTATATATGGACGAGCGATTCATTGTGTCTACCCGT
CAGCGAAAGGTTCTGGCGCTGGACGATGTGGTCAGTGATTTGCAGGAGGGCACCGGGCCC
GTAGATTGCGGCAGCTGGTTAGTAGATGTGTGCGATGCGTTAACCGATCACGCCAGTGAA
TTTATTGAAGAGCTACACGATAAAATTATCGATCTGGAAGATAATCTTCTCGACCAGCAA
ATCCCACCACGCGGTTTCCTCGCATTGCTACGTAAACAGCTAATTGTTATGCGCCGCTAT
ATGGCGCCGCAGCGCGACGTCTATGCCCGGCTTGCGAGTGAACGGCTGCCGTGGATGAGC
GACGATCACCGGCGCAGAATGCAGGATATCGCCGACAGGCTGGGAAGGGGTTGGATGAAA
TTGACGCTTGTATCGCGCGTACTGGCATCATGGCGGATGAAATTGCCCAGGTGA
>EECEBICD_02026 Methyl-accepting chemotaxis protein IV
ATGTTGAGAAATATCAGCGTCAGAACATGCATTATTCTATTCATGGTATGTACGTTTCTT
TTGGTGGATACTTTACAAATAACCTTTCTGCACGATCTCCCTATTTTAATTACGTGTAAT
ATCATTTATCTGATCTCGACATTACTGCTTTGGTGGTATATGACCTGCTATCTGGTAGTG
CCGATTAATACCGTGAAGAAAAGTATTGAAGAGGTCGCAGCAGGAAACTTGTCTATTCAT
ATCTCCGAATTTGGCAATAATTGCGCCGGGCGACTCATCCCCGGAATTAATAGCCTGTCG
GAGAATATTTCCGCGCTGGTACGCGAAATCAGATCCTCTTCGCAAACGGCAATGACCCTT
TCCGAACAACTGGCGGCGCGCAGTATGTCGCTATCCGTAAAGACGGAACAGCAGTCGGCC
TCGTTAATTCAAACTGCCGCCAGTATGGATGAAATGGCGGCGAGTACCAAAAACAATGCG
GATAATACCCGAATGGCGAGCATACAGGCGGATTGTGCAACCCAATGCGCCCGTAAAGGC
GGTGAATTAATGGTGCGGGTTACAGAAAATATGCGTTCTATTACCGACTGTGCCTCGCAG
ATGACGGAGATTATTTCATTGATTGACGGTATCGCATTCCAGACTAATATTCTGGCGCTT
AACGCGGCGGTAGAGGCTGCGCGGGCGGGCGATCATGGAAAAGGTTTCTCCGTGGTGGCC
GGGGAAGTGCGTAACCTGGCGCATCGTAGCGCCGAGGCGGCAAAAAGTATCAAGGCGCTG
ATCGACGTTACGCATGAGAATGTGCGGCAGGGGGCCGCCATAGTGCAGGAGGCTGAAAAA
AATATGCAGGAGATTGTTGGCGGCTCCGGGCAATTAAACGTGCTGATGAGTGAAATTTCC

ACCACCACGCGGGAGCAGGAAAAAGGCATTAACCAGATAACACTGGCGTTGAGCGATCTG
GAAAGCGCAACCCATAGCAATGTTTTAATGGTTGAAGCGTTATCTGCTTCTTCGGATGTT
TTAAAGGCGCAGGTGATCGAGTTACAGACTAAAACCGACAAATTTCGTTTAAGCCAGCCG
GGTTACAGTGAACATGCGCTGTCGCGCTCTCATGTATCACCTCTTTCGACCATCACCAGG
CGCGGCCAGGCCTGA
>EECEBICD_02027 putative DNA endonuclease SmrA
ATGAACCTTGACGATAAAGCGCTGTTTCTTGACGCCATGGAGGATGTCCAGCCGCTGAAA
CGCCATACTGATGTACACTGGCAGCCAACGCGTAATCTTAAAACGCCCCAGCGTATCGAC
ACGCTTCAGCTTGATAATTTCCTGACGACGGGCTTTCTGGATATTCTGCCGCTGAATGAA
CCGCTGGAATTTCGTCGGGAAGGATTGCAGCAGGGTGTCATCGACAAGTTACGCAGTGGG
AAATACCCTCAGCAGGCCAGCCTCAATTTGTTACGCCAGCCGGTCGAAACGTGCCGAAAA
ATGCTATTTCGTTTTATCCTGGAGGCGCAGAAAGAGGGGTTGCGTAATGTCCTGATCATT
CATGGTAAAGGGCGAGAGGCGAAGTCGCATGCCAATATCGTCCGAAGCTATGTGGCCCGC
TGGTTGACAGAGTTTGAGGATGTACAGGCTTATTGTTCGGCGCTGCCGCATCATGGCGGC
GGCGGGGCATGTTATGTCGCGTTACGTAAAACGGTGCAGGCTAAACAGGATAACTGGGAG
CGCCATGCGAAGCGTAGTCGGTAA
>EECEBICD_02028 Methylated-DNA--protein-cysteine methyltransferase
ATGCTGAGATTACTTGAAGAGAAGATAGCCACGCCATTAGGTCCGTTATGGGTGGTTTGC
GATGAGCAGTTTCGACTGCGGGCCATTGAGTGGGAACAGTACCGCGATCGTATGGAACAA
CTGCTAAATATCCACTACCGTCACGAAGGCTATGAACGCGTTTCTGCAACTAACCCCGGT
GGACTCAGCGATAAGCTTACAGATTATTTTGCAGGCAATCTCGCCGTAATTGATACCCTG
GAAACCGCCACGGGGGGCACACCTTTTCAACGGGAAGTATGGCAGGCGTTGCGCGCTATC
CCCTGCGGGCAGGTGATGCACTATGGTCAGCTGGCGGCGCAATTGGGACGACCGGGCGCC
GCACGCGCAGTCGGTGCTGCGAATGGCGCTAACCCCATCAGTATTGTTGTTCCCTGCCAT
CGCGTCATTGGGCGTAACGGCACTCTGACCGGATACGCCGGTGGCGTGCAGCGAAAAGAG
TGGCTATTACGCCATGAAGGCTATCTTTTATTATGA
>EECEBICD_02029 Fumarate and nitrate reduction regulatory protein
ATGATCCCGGAAAAGCGAATTATACGGCGCATTCAGTCTGGCGGTTGTGCTATCCATTGC
CAGGATTGCAGTATCAGCCAGCTTTGCATCCCGTTCACACTTAACGAGCATGAGCTTGAT
CAGCTTGATAATATCATCGAGCGCAAAAAGCCCATTCAGAAAGGGCAAACTCTGTTTAAA
GCAGGTGATGAATTGAAATCGCTCTATGCTATCCGCTCCGGAACGATTAAGAGCTACACC
ATTACCGAGCAAGGCGATGAGCAGATTACTGGATTCCATTTAGCAGGCGACCTGGTGGGT
TTTGACGCCATTGGCAGCGGTCATCATCCGAGCTTTGCACAGGCGCTGGAAACCTCAATG
GTATGTGAAATACCCTTTGAGACACTGGATGACCTGTCCGGCAAAATGCCTAACCTGCGT
CAGCAAATGATGCGTCTGATGAGCGGTGAAATTAAAGGCGATCAGGATATGATTCTGTTA
TTGTCGAAGAAAAACGCCGAAGAACGTCTGGCTGCGTTTATTTACAACCTGTCCCGCCGC
TTTGCGCAGCGTGGTTTTTCACCGCGTGAATTCCGCCTGACGATGACCCGTGGCGATATC
GGCAACTATCTGGGCTTAACGGTCGAAACCATTAGCCGTCTGCTGGGGCGTTTCCAGAAA
AGCGGCATGCTGGCGGTGAAAGGTAAGTATATTACTATTGAAAACAGCGATGCGCTGGCG
GCCCTCGCCGGTCATACCCGCAACGTCGCTTAA
>EECEBICD_02030 Universal stress protein E
ATGGCTATGTATCAAAATATGCTGGTGGTCATCGATCCCAATCAGGACGACCAACCCGCA
TTACGGCGAGCAGTTTATTTGCATCAACGGATTGGCGGCAAAATTAAAGCTTTCCTGCCT
ATTTACGATTTCTCGTATGAGATGACCACCCTGCTTTCTCCTGACGAACGCACCGCAATG
CGTCAGGGCGTAATAAGCCAACGCACAGCCTGGATACGTGAGCAGGCGAAATATTATCTT
GAAGCCGGTGTCCCCATTGAAATTAAAGTGGTCTGGCACAATCGTCCCTTTGAGGCCATC
ATCCAGGAGGTTATCGCCGGCAGTCACGATCTGGTGTTGAAAATGGCGCATCAGCACGAT
CGACTGGAAGCGGTCATTTTTACCCCTACCGACTGGCATTTGCTGCGTAAATGCCCAAGC
CCCGTGTGGATGGTCAAGGATCAGCCGTGGCCAGAGGGCGGCAAAGCCCTGGTCGCCGTG
AACCTGGCAAGCGAAGAGCCTTATCACAATGCTCTCAACGAAAAGCTGGTAAAAGAGACC
TTACAATTGGCGGAGCAGGTTAATCATACCGAAGTGCACCTGGTCGGCGCCTACCCCGTT
ACGCCAATCAATATCGCCATTGAGCTGCCGGAATTTGATCCCAGCGTGTATAACGACGCC
ATCAGAGGCCAGCATTTACTGGCGATGAAAGCGCTGCGCCAGAAATTCAGCATCGATGAA
AAAGTTACGCATGTAGAAAAAGGCTTGCCGGAAGAAGTTATCCCGGATCTTGCTGAACAT

CTCCAGGCGGGGATCGTTGTGCTTGGCACTGTTGGACGTACCGGACTCTCGGCCGCGTTT
TTTGGGGAACACGGCCGAACAGGTCATTGA
>EECEBICD_02031 hypothetical protein
ATGATCATGATGAAGCTGAAATCGGCGAAGGGGAAAAAATTTCTGTTATGCCTGCTGGCA
GTGTTTATCGTTGCGGCATCGGTGGTAACGCGCGCGACTATCGGCGGTGTGATTGAGCAG
TATCATATTCCACTGTCCGAGTGGACAAGCTCTATGTATGCCATTCAGTCGGCAATGATT
TTCGTTTACAGTCTGGTGTTCACTATTCTGCTGGCGATTCCTTTGGGGATCTATTTCCTC
GGCGGAGATGAGTAA
>EECEBICD_02032 Low conductance mechanosensitive channel YnaI
ATGTTCACTGAATTTTTTCTCAAAAACGCATTTAATCTGGCGATACTTTTCAGTTGCGGT
ATGGCGTTGCTGGTCGTGAGATTTTGGCTCAGCCGTAACGTACAGTGGAAGAAAGGATTC
ACGTTCCATGCGGCACAGTTTTTATTTACGCGATTATTATTGGAACCATCGGGAGCATC
CTGAATAATGCTATTGAAGATTATAATTTGCGTTTTATCTCTTCCGGGGTCATTGATTTT
ATCTGCACCTCGCTCATTGCGCTTATTCTGACGATAAAACTTTTCCTGATTATTAACCAA
TTTGAAAAAGCACAGGTCAATAAAGGTAGAGATGTCACCAGTACCCGTATTCTGGCACGG
GTCATAAAAATAACCATTATTGTCGCGATTGTCTTACTGTATGGCGAACATTTTGGCATG
AGTCTGTCCGGTCTGCTGACCTTTGGCGGTATCGGCGGTATCGCTGTTGGTATGGCGGGC
AAAGATGTCCTGAGCAATTTTTTCTCCGGTATTATGCTCTATTTCGATCGCCCCTTCAGT
ATCGGAGACTGGATTCGTTCTCCTGACAGAAATATCGAAGGTACGGTCGCCGAAATCGGC
TGGCGAATTACGCGAATTAACACATTCGACCACCGCCCGTTGTACGTGCCTAATTCCGTC
TTTTCATCCATTAGCGTTGAGAATCCGGGACGGATGACCAACCGTCGAATAAAAACGGTT
ATTGGATTACGTTACGAAGATGCCGACAAAATTGGCCTCATTGTTGACGCTATCAGAAAC
ATGCTGCAGGCGCACAGAGATATCGATCAGAAACAGACGCTGTTGGTCTATTTTAATGAA
TTCGCCGACTCTTCGCTGAATATTATGGTGTATTGCTTTACCAAAACAACAGTATGGCAG
GAATGGCTTGCCGTACAGCAGGATGTTTACCTGAAAATTATTGCGATTGTCCAGGAAAAC
GGCGCTGACTTCGCCTTCCCAAGCCAGACGCTCTATATCGATGACCCGGAGGCCGCGCCT
GCGACAAAATAA
>EECEBICD_02033 HTH-type transcriptional regulator SutR
ATGACCAAAAAAGTCAACTTATCGACCACGACGGGCGCCGACGTCAGCACAATAAATGAA
GCTGTTTCCCAACGTATCAAACAATTTCGTAGCCAAAAAAAGATGTCGCTGGATGAGCTT
GCGCGGCGTTCCGGCGTCAGTAAAGGAATGCTGGTGGAAATTGAAGGCTGTAAAGCCAAT
CCAAGCATTGCGCTGTTATGTAAGATCGCGGCGGCAATGGGAGTGTCCGTGGCGGATTTT
GTCAACGTCGCCAGCGAGCCGATGGTTCATCTTATCGACCGGGATGCTATTCCGGTGTTG
TGGCGCGGCGAGAAAGGCGGCAGTGCAAAACTAATGGCGGGAACCAGCGGGCCGGATATG
CTGGAACTCTGGCAATGGATAATGCATCCTGGTGAACAGTTTGAATCGGCAGGGCATCCG
GCAGACACCTGTGAATTACTGTTTGTTAATCAGGGAACACTGACATTAACAGTCAATGGT
AGACGCTTTATTATTCGGGAGGGCTGCTCCGCTGTAGCCAGAACCGACATGCCTCATGCC
TACGTTAACGACACTAATGAGGTGCTTGAATTTACCATGACGGTGAATGAAAAGAGCCGT
TGA
>EECEBICD_02034 hypothetical protein
ATGTCTTCTATGTTACCGTCAATCAGCCCGGAACTGGCCCGTATCGCTCCCGGCTTTCGT
GCATTAAGTATTAATGTTATTGCCGCTCCGATTCGCGATGCGCAGGTCGGAGAAATCGCG
CTAAAAGAGGCCTGTCAGGCCGTAATTAACGGCCAACCGACGTGGGCACAGGCGCATATA
GACGCCTGGAATGCGGTGTTTAAAGCATTCGGCGCAAAACCGAAGCGCACCCCCTGCTCG
GCTGAAGCGCTACGAAAACGCGTGTTGAAGGATGGAACAATGGCGGCGCTGGACCCGGTC
GTTGATCTTTATAACGCCGTGAGCCTGCGCTATGCCGTACCGGTAGGCGGCGAAAATAGC
GCGGCTTATTGTGGATCGCCGCGGCTGGTTTTCGCAGACGGAAGTGAAACATTTGATACC
CTGAAAGAAGGACAACCCGTCACCGAGTCGCCAGAACCGGGCGAAGTTATCTGGCGCGAT
GACAGAGGCGTCACCTGTCGGCGCTGGAACTGGCGTCAGGGAGTACGTACCCGACTAAGC
GCTTCAGATAAAGCGATGTGGTTTATCCTTGAAAGCTTGCCGGAAATGCCGGTTGATGAA
TTGTACGCGGCAGGAAACATGCTGACTGACGGCCTTGAAAAAATGATGCCAGGTTTACGT
TTTGAATCCACGTTGATGGGTGTTTGA
>EECEBICD_02035 hypothetical protein
ATGTCGCTGTTTCTGACAAGTTTTATTTTCGAAAAAAAGAGTACGACGAAGAGTTTTATC

AACTGGATGGCTGGATTGAGCGCTATACACAGACTATTGATGGCTATATTGGGATGGAAA
GCTATACCGATGCCGCCAGCGGAAGAATGGTTAATAATTATTACTGGCAGACCCGCGAAT
CAATGGAGCTATTGA
>EECEBICD_02036 Thiol peroxidase
ATGACACCAGCAACAACACAAGATTATATTTCATTGGGTCAGGAACACGCTGTCACCTTT
GGCAAAACGCAACTGACCCTCAAACCCGGTATACTGGCTGAAGGCGAACCATTACCCTGC
ACAAAGGGGCTGGTGAGCCATAATTTATTACCGGGTTACTGTATCCCGGGGATAAAAAAG
CGGATTATTGTCGTCCCGTCACTGGATACGCCGGTTTGTGAATGGCAGGTAAAAGACTAT
AGTAACCGCCTTAAATCAGCGGGTAGCCACTCAAACCGGGCGGTATATGTGCTGAGTATG
GATACGCCATTTGCTCAGGCCCGTTTTATTCTGGAACATGATATTCATCCCGGCATAACC
TTTGTTTCTGACTACGCTTGCCGCCAGTTTTTGGATAATTCCGGGCTGAAAATTAATGAG
CTCAGTATTTTTGCCAGAGCGTTGATAGAGTGCGATGAAAATAATGTGGTTACGCGTGTT
ATTGTACCACGAGATATCACTCATCTTCCTGTTTACTAA
>EECEBICD_02037 hypothetical protein
ATGTCGAAACATAATCTTATTATCTATAGCCTGCTTCTGGCGGCTGCGCCGATAAGCGTC
TTGGCGGATTCGTCGACAACTTCCGCGGCAACCGTCGGGATGTTTTCTCCTCCTACGGCG
CCGAGCGTTGGGCACAGGCCTGGAACACCTCGCGATGGCTACGAATTGAAGTTTAATGGA
CTTGACTTTTCTATTGAAAAGTATACACCTTTTCCGGGAATGACTATTGAGAGGTATGCC
AGAAATGTAGGCATCAGAGATCCGGATAATAACTATCAGTCTGCGGAAACTTTTACTACA
GTATGTACGTATTATCAGATAAATAAAGATAACTCTCAGCATATACTTAAACGTGAAAAT
CCATGTGAGCATAAATTTAATATCCAAAAGGAACATCGGGGTTCAAAGATTAAACTAGAA
ATTTATAATGAAACGGATATGGCTTCAGCGTCAGGGTACACGCCTGCGCCATCGGTATCT
GAATTTCAGTATATTGAAACAGAAACTATATCTAATACGCCAAGTCGAGATTTAACAGTT
ATGAGTATAGATAAGTCTGTATTATCGCCGGGTGAAAGTGCCACCATCACAACTATTGTT
AAGGATATCGACGGGAATCCTATAAATGAAGTTTACATTAATAAGTCTGTAGCGTGTGAA
ATTTTAGAGTGCTTATGGGATTATGGTCCTCTTAAGAAAGAAAATGCTCCAGGTAAATAT
ACACAAGTTATAACCTATCGTGGGCATAGTAATGAGCGTATTGACATATCTTTTAAATAT
TCAGCGGCTTTTACGAAAACAATTTCCATCAGAGGAAGACCATAA
>EECEBICD_02038 Invasin
ATGTTTATTCCCGTCCAGGAAACAACATCGTCTTTACTCTTTGGTCAGCTTGGCTTTCGC
GACCACGACAACAGCAGTTTCGATGGCCGCACCTATGTTAACGTTGGTATGGGTTACCGC
CAGGAGGTCAATGGATGGCTGCTCGGCGTCAATACGTTTCTGGATGCCGACATCCGTTAT
TCCCACTTACGTGGCGGCATTGGCGGGGAAGTTTATAAAGACAGTCTGGCCTTTTCCGGC
AACTATTATTTCCCTCTCACCGGCTGGAAAACGTCAGCAGCGCATGAATTACATGATGAG
CGTCCGGCATATGGCTTTGATCTGCGAACAAAAGGCACGCTTCCCGATTTTCCCTGGTTC
AGTGGCGAGCTCACATATGAACAGTATTACGGGGATAAAGTTGATCTATTGGGTAACGGT
ACATTAAGCCGGAATCCCCGCGCCGCAGGTGCGGATCTGGTCTGGAATCCTGTCCCCTTG
CTTGAGGTCAGAGCAGGATATCGGGACGCCGGTAACGGCGGCTCGCAGGCAGAGGGCGGT
CTGAGAGTCAACTATTCCTTCGGTACGCCGTTACATGAACAGCTTGATTACCGCAACGTT
GGTGCGCCGTCAAATACGACTAATCGGCGTGCCTTTGTCGATCGTAATTACGATATCGTC
ATGGCGTATCGGGAGCAGGCCAGTAAAATCCGCATTACGGCAATGCCGGTTTCAGGCCTG
TCCGGGACGCTGGTGACATTAATGGCGACGGTAGATAGCCGTTACCCGATTGAGAAGGTT
GAGTGGTCGGGTGATGCGGAACTTCTCGCCGGACTCCAGCTTCAGGGGAGTTTGGGCAGC
GGCCTTATCCTTCCACAACTTCCGCTGACGGTGACCGACGGTCAGGAGTATAGCCTTTAT
TTAACGGTGACTGACAGCCGGGGTACTCGCGTGACCTCCGAGCGTATCCCGGTAAGGGTG
ACACAGGATGAGACCAGTTTCCGTTCATGGATAAATGTTATCAATGACGATGTTCAGGTT
GAGGACGGCAACTTTGTTATCAGTACGCCGCTTCCTGCCGGCGAAGAGGGGAAGGTTATT
GAGTGGCACTACGTGCGCGAGCGCTCTGAAGAGGAGTGGGCGTCGCTCAAACCACGTCAT
ATTAAGTATCAATCAGATATGCCGGGTCTGGCATTTAAAGCGCTCGGTGGCACAGAGCGT
GACGGCCATTGGGTTGAACGGGTTCTGGTGACCCATATCGGCGATGATACCCGCTCGCTC
AAATTACATATTGAAGCAAGCGGACCAGACGATAAACATCCGGTGAAGGGGACCGTACTG
CTGCAAGCGCAGCCGGACAGCATTGCGCAGAAGGTCACTTCTGTGGAGGTGCTTTTTACG
CCGGGTACTGAAGAAGCCAACGGAAGCGTTACTGCGCCAGTCGTCGGAACAGAAATGCGA
GCCAGAACGCTTTGTGTAAATAACACGGACTGTACTGATGCGTTTAATTATCAGTGGGAA

ATCTCGGATGAAATGAAATCCTGGCAATCCGTACCGGGGGCGACAAAGGCCACCTGGCTT
ATCCCTTATAGCCTTAATGGGGAATCGCTACAGAATAAATATATCCGCGTCAGAATTATT
TCTGATAAAGAAAATGCCAAGAGTTCTACCGCAGCATCTGATGCGAATTAA
>EECEBICD_02039 hypothetical protein
TTGTACTTCAAAAGATCTTTATTAGCGTTAGCAATTTGTGCATCCGTGGTCTCAGTAGAG
ACGGTGGCGAATACCAGAGCCAATGATATGGCTTCTTCTGCTACACCTGCTTCTGTTGGT
CACCGTCCTGTGGCAACTACTAATGCAAAAAAAATAGAAGTGTCTGGGGAACTGACGACC
GGTTCAACCCTCCAGATAGCGGATGTTTTTATTAGGGATGAGGATGGGGATCCCTTATCG
CTGGATAAAATGAATAATGCCGACGATATTAAATGGTATCTGGTGGATAATGAGGCCGCC
GATCCTTCTGGAACCCCGGCGGCAACCGGGACGGCTTTTACCATTCCTGCTGATGCAGGC
GGTAAAAAAATTAAAATCGTTTACCGGATTAAGACGGCAACGGGGACGCCGGATAGTGCA
TTCTTGCCTGCCACTGTCTTATTAACTTCTGCATCTTCTGGGGTGAGCGGCGACTCCGCC
GCAGACGGTACAATTTCTAATAAGCTACGTTCTGTCACTATCAATGTGGTGAATGAAAGT
GGTAAACCGACTGATGAACTCAACGGTACCAATGACGCCAACACTCCAGTGGTAGGCGGC
ACCCTTGAAGCGGTACTGGAATGTGCCGCCACAGCGGCGGCTGAGTGTGAGGTCAGTAAT
TATAACTTTACCTGGCAGATGGCGGATGCCGGGACACCAGATAAGTTCACCGATCTGAAT
AATACCAACGCTGAAAAGCATAAATATATTATTAAGGGAACTGAGCAGAATAAACTGTTC
CGTGTCCACGTTACGCCTAAAACGACGAAAGCCACGCCGGAGAATAAACGCGCTATCCGT
CGCTGA
>EECEBICD_02040 Right origin-binding protein
ATGAAAAAAGCCATAATAATCTCAGTATTAGAGCAAATAGAAAAAAATCTTGAAGAGAGG
ATTGATATTGAAAAGCTGGTTGTTATAACAGGTTATTCGCGAAGGAGTTTGCAGGATTTT
TTTAAAGAAAAGTGTGGCGTCTCAATCGGAAAATATATCAGACAACGCAAGTTGAGCAGA
AGCGCCACTCTGCTAAAGCTGACCTCTCAGTCGGTCACAGATATCGCGTTCAGAATGGGG
TTTGATTCAGTGCAGTCCTATAGTCGAGAGTTCAAAAAAACATTTGGCGTCAATCCCAAT
AACTATCGCAAGGCTGATTTCTGGGATCTCAGAAACCTACGGCCTCCCTACTGGCTTGAC
TGTGATGAGCATTATCAGTTCGAAATTTGTCAGCTTACGTCAAAAGAAATTTTCGGATTT
CAAACGTCTCATCAGATCGCAACAAATGACCTTCCTAAAAAAGCGTCGCCTATAAAATGG
AAAATCATTCATGAGACGCTCAGAACGTGGGGCGAAAATGTGTATTGTCTCTCGTCTTTT
AAGCCGGATAATACCAAAGACCAGGTCATCGCCGTAAGCAGTTTTTTTGGTATGGAACAT
AATTCCGTCGATGGAGGCAAAATACCCATGTCGAGGGTAATAAAATGTGGTAAATATGCA
AAATTCCATTTCGTTGGTCATAAAGAACAATATCAACAATTTTCCAATACAATATATATG
TGTATATTGCCAAAACTGAATCTTATCAGGAGAGAAGGAGAAGATATTGAATACTTCCAT
TTGGCATCCGTCCAAAAGCAACAGAGTAATGAATCCATTGTCGATCTTTATTATTACATC
CCTGTACTGTAA
>EECEBICD_02041 hypothetical protein
ATGTATACAACCATCAGAAACACAACGCTAGCAATGGTAGCGTGTTTTTCGTATATCGCA
CATGCCAGTACCCACCCTCCTCTTATTATCACCAGGGGAGCCGGAGGAGACGCCTCCGGA
GCCACAGTCATTCATGATAATTGGCGGCATGGCACTCCTGACCTGGTCAATCTTACCGAT
ATCCCCATAGATAAAATCAGACCAGAAAAATACCGTTGCGTACTGATTATTGGGCAAGGA
GCTATAAAAGAGATGCTTCTTGCGAACAATGCCTCAGCAATACTTTCCGGAAAAAACTGTT
GGTCTTTACACTCATCTGATCGATCAGAACACGCTCAGATTACTTCGGCAATTGCAGAAC
AAAGTGAGATTCAATCTCTTTTTTACCCGCTCACAGATAACTCTACTAAAACTAAGGAAC
ATTTCAGAATATAATTTTTTGAGTTCTAAAGTAAATAATGTCTGGGGACAAGACTCACTA
GCAATTGAAACAGTGGCACCTGACAGGGGTAATATACCGGAAAAAAACGCTTCCCCTCAAA
ACAACAGATTATGTGATCTGGCTGGGTGGAAACTATACTACCTCCTCTGGTACGCAGCGT
ATCTTCACAAACGATCAGATTGTTGTAGCCCTGAAACCACTACATAATGTTATTTCATCA
AATGCATCGATTGCTATTATGCTTTCACCACGTTTTTTTGATAATAGCATGAGTAAAGAA
GCAAAAGTTAAACGACTGAAGGCAGTGCTTAACACCTTTTCGCGAAACAGGGTCACATTT
TATATGAGTAAGGAGATGCTTGCTAATCTGAAAGAATTTGATTTGCCTGTTCAACTATCC
CCTCCTTACGCAGAGCTGATGCGAATGCCCTGGGCCAGCGCTACCCAGCATTTTGCCTCT
GTGGATCAATATAACCTCTTTGCTGATCTCATACCTAAAGTCACGCCGTTTCTTCTTGAA
CCGAATGATGCCGATCAAGCTCTCTATGCGACGGATTATCTTAATACCCGGCGTGTAAGT
CTGACCCAAAATATTCTTAACCACGGTTGTGACTAA

>EECEBICD_02042 putative lipoprotein YgdR
ATGCAAAAGTGTAGTCTTATTACGGTACTTAGCTTATCAGTGTTGATGCTAGCCGGATGT
ACAACGACCTATACCATGACGACCCGGACGGGAGAGATTATTGAAACGCAGGGTAAGCCG
GAGGTTGACTCCGCAACCGGAATGACGAAATACGCTGATGTTTATGGTTATCATCGCGTT
ATTAAAACCAGTGAAATAGTGCAGACAACAGAAGGCGCATCGAAGCTGGACTGGTAA
>EECEBICD_02043 HTH-type transcriptional repressor BdcR
ATGACTACAAAACATAGCCGAACGCCGGGTCGCCCTCGCCAGTTCGATCCGGAGCAAGCC
ATTGAAACCGCTCAACACTTATTTCATTCGCGCGGATACGACGCGGTGAGCGTAGCGGAT
CTGACAAAAGCGTTCGGGATCAATCCTCCCAGTTTCTATGCGGCGTTTGGCAGCAAGCTG
GGGCTGTATACTCGCGTATTAAAGCGTTACAGGATGACCGACGCGATTCCGCTCGGGGCG
CTTCTGCGTCACGATCGCCCGACCGCGAAGTGCCTTATCGATGTGCTGATGGAAGCGGCA
AGACGTTACGCTGCCGATCCTGATGCCACTGGATGTCTGGTTCTGGAGGGCGCTCATTGT
AACGATAAACCGGCACGAGAGGCCGCCTGCGAGTTTTACATCGCTGCTGAAAACCTGATT
CGCACTTATGTCGCCATGCGGTATCCGCAAGAGGCTGACCGGACGACGGACTTTATGGGG
ACGCTAATGGCGGGGCTTTCCGCCAAGGCGCGAGCGGGGTATAGCCTGGAGCGTCTTCAG
GAGAGCGTGCTTCTGGCTGGGGATGTGCTGGAGCGATTACTGCCGGATTAA
>EECEBICD_02044 Cyclic-di-GMP-binding biofilm dispersal mediator protein
ATGATGCTATTTCGCAACAAATCTGTACTGGTTTTAGGGGGAAGTCGTGGTATCGGCGCG
GCTATTGTACGACGCTTTAGCGCTGACGGCGCATCGGTCGTTTTCAGCTACGCCGGATCG
CGTGAAGCCGCCGAAAAACTGGCAGCGGAAACCGGAAGCACCGCCATCCAGACGGATAGC
GCCGATCGGGATGCCGTCATCAGCCTCGTGCGTGAGTACGGACCACTGGATATTCTGGTC
GTCAATGCGGGCGTGGCTCTCTTCGGCGATGCGCTGGAACAAGATAGCGATGCGATAGAT
CGTTTGTTCCGTATTAATATCCATGCGCCTTACCATGCGTCAGTCGAAGCAGCCCGCAAC
ATGCCGGAAGGTGGACGCATTATTATTATCGGTTCCGTGAATGGCGATCGGATGCCCGTA
CCGGGAATGGCCGCGTACGCCGCCAGTAAATCAGCCCTGCAAGGGCTGGCGCGCGGCCTG
GCCCGTGATTTTGGCCCCCGCGGGATTACGATAAATGTGGTGCAGCCAGGCCCCATTGAT
ACCGACATCAATCCGGAAGATGGCCCCATGAAAGAGCTGATGCACAGTTTCATGGCAATC
AAACGACACGGAAGACCTGAAGAAGTGGCTGGAATGGTGGCATGGCTTGCCGGCCCGGAA
GCCTCTTTCGTCACCGGCGCGATGCACACCATCGATGGTGCCTTCGGCGCCTGA
>EECEBICD_02045 putative oxidoreductase YtbE
ATGCCTATGGTGGGTTTTGGCGTTTTTAAGGTAACCGACAAAGAAGAATGTCAGCAGTCA
GTGTTAAGCGCTATTCGCAGCGGTTATCGTCTTATCGATACCGCTGCGGTATACGGCAAT
GAAGATGCCGTTGGTGACGCTGTTCGTAAAGCTATCGCGACAGGTCTGTGTACCCGTGAG
GAGTTATTTATTACTTCTAAACTGTGGGTGCAAGATATGGCTAACTACGATCTGGCCAAA
GCAGGTATTGAAGCGTCGCTGAAAAAATCCGGGTTAGATTATTTGATCTCTATTTATTG
CATCAGGCAATGGGCGATTATTTTAGTGCGTGGCGCGCGCTGGAAGACGCTTATGAAGCC
GGTAAACTAAAAGCCATTGGCGTGTCCAACTTCTATGCGCATGTGTTAGCCAACTTCTGT
GAAACCGTCAGAATTACGCCAATGGTAAACCAGGTCGAATTGCATCCATACTTTGCACAA
CCCGCCGCGCTGGAAACCATGAAGCATTATAACGTTCAGCCTGAAGCGTGGGCGCCGTTA
GGCGGCGGGAGACATAAACCGTATGAAAATGTCATGCTTCAAAGGATTGCCGATGCCCAT
CAAAAAACCATCGCGCAGGTTGTCCTGCGTTGGAACGTGCAGCGTGGTGTCACGGTTATT
CCTAAATCCACTCGACAGGAACGCATTGAAGAGAACTTTGCCATCTGGGATTTCTCACTA
ACGGATAATGAAATGGCTCAAATCAACGCCCTTGATTTAGGCTACGTTGGCGAAGCGGTG
AAGCATTTTAATCCTGAATTTGTTCGCGGTTGTCTTGGCGTTAAAATCTACGATTGA
>EECEBICD_02046 HTH-type transcriptional regulator PgrR
ATGGACAAGCTGGAAGCGATGCAGGTCTATGTTTGCGTAGTGGATACCCATAGTTTTATC
AGAGCCGCCGAAGTGCTTGGCGTGCCGCGCTCAACGGTATCGCGAGTGGTTAAAGAACTG
GAATCCTGGCTTAAGATTCAACTTTTGCAACGTACAACCCGCAAACTTAGCGTGACCGCA
GAGGGGCGGCGTTACTACGAAGAGTGCAAAAGAGTGCTGGCAGATATCGCGGCCATGGAG
TCGTCATTTCCCGGCCGGGCGGCACAACCCAAAGGGCGTTTTAAAGTGGGGATGCCGCAA
TCTCTCGCCCGGCATTGCATTATTCCTACGCTCCCGGCCTTTTTGTGCCAGTATCCGGAG
CTGGAGCTGATACTTTGTTCCAGCGATAGCGTGGAGGATATTATCCTTCAAGGGTATGAC
TGCGTGATTCGGGCCGGCAGAATCGATGATTCCACCACGCTGGTTGCCCGTCCACTTGCC
AGCTTTAACTGGGTGATAGCAGCATCGCCCACTTACATTGAGCGTTACGGTAGCCCTGAA

AATTTAGATGATCTGCAAAAACACCATGCGGTCGGCTACCTGAACCACCGCACCGGGCGT
ACCACCGACTGGTTTTTACGCGTGAGGGGGAGGATTACGCGATGCGGGTGCAGGAGACG
CTGGTTGTCGATGATACGGACGCTTATATTCAGGCCGGAATACAGGGGCTGGGGTTAATT
CGCGTCGCCAGCTATCTGGTTGCGCCGTATCTGCACAGCGGGGCGCTGGTGACCTGCATG
GACAACCATTTTTATGATTTGCCGCTTGCGCTGGTCTATCCGCAAAACAGGTTTCTGCCG
CCTGCGGTACGCGCTTTTTATGACTGGTGCAAAACGGCGCTGAACCAGCCGGAGCCTCTT
CGCTAA
>EECEBICD_02047 hypothetical protein
ATGAAACAATCCAATAATCAGGTGGGTGAAAAAGTATTGGTGCTGGGCGCGGGACAACTG
GGGGCGTCAGTGCTGGCGTCGTTGGTTCCGGCGGTCACACAGCGTAATGGTTCCGTTTGC
GTTATTGTGTCAGGGCGATCCAGGGATAAACAGAGCAAACGACGTTCCAGCATTCATCAA
CAACTGGCGGATGCCGGTGCGCGGTTTATCCCGGTCGACATCGCAGGCAGTTCGGTTGCA
GCGTTAAAAGATCAATTCCACGGTTTTGATACGATCATCAATTGTATGGGGTTTGTCGCC
GGTGCGGGTACCCAGATAAAAATCACCCGCGCGGTACTGGAGGCGGGCGTTAAGCGCTAT
TTCCCTGGCAGTTTGGCGTGA
>EECEBICD_02048 hypothetical protein
GTGAATTACGATGTGGTCGGCAAAGGTAGTGGCCAGCCGGTGTGGGACGAACAGTATGAC
GTCAGGACGCTTTTGCGCGAGCAAAACGTCACGGAGTGGGTGATTGTTTCAACCGGTATA
TTTACCTCTTTTCTCTTCGAACCGGCCTTTGGTGTGGTCAACTTAGCCCAAAAAACCATC
AATGCCCTCGGCGGATGGGAAATGCAGGTCACCGTCACGTCGCCTACGGATATTGGTCGC
CTGACCACTGAAATTTATCTGCATCAGCCGCGTATCACTAACGAAGTCGTCTTTGTCGCG
GGGGAAACGACCTCTTATGCAAAACTGGCGGAAACGGTCGAACGCGTGACGCAGCAAACT
TTTACCCGAGGCGTATTGACGCTACCTGACTTACAGGAGCAGCTCCGTTTACATCCGCAT
GACCCAATGCTGCGCTATCGCGTCGCCTTCGCGCGTGGTGACGGTATGTGGTGGCCGATG
AGTGACACCTGGAATGCACAACATCATCTGCCGACACAGGATATTGCCGCCTGGCTGAAA
ACACAGCAATGA
>EECEBICD_02049 Periplasmic murein peptide-binding protein
ATGAGACATTCTGTTTCAGTAACCTGTTGTGCGCTGTTGGTTAGCAGCTTTTCCCTGGCA
TATGCTGCGGATGTTCCCGGCGGAACGGTACTGGCAGAAAAACAAGAACTGGTGCGTCAT
ATCAAAGATGAGCCCGCTTCGTTAGATCCAGCGAAAGCCGTTGGGCTGCCTGAAATTCAG
GTGATCCGCGATCTGTTTGAAGGGCTGGTTAACCAGAACGAAAAGGGTGAAATTATCCCT
GGCGTCGCCAGCCAGTGGAAGAGCAATGATAATCGTATCTGGACGTTTACGTTGCGGGAT
AATGCGCAATGGGCCGATGGAACGCCGGTGACCGCCCAGGATTTTGTCTATAGCTGGCAA
CGTCTCGTTGATCCCAAAACGCTTTCCCCCTTCGCCTGGTTTGCCGCGCTGGCTGGCATC
ACTAATGCGCAAGCCATTATCGACGGTAAAGTCACGCCGGATCAGCTTGGCGTCAGTGCC
GTGGACGCGCACACTTTGCGTGTTCAGCTTGACAAGCCGTTGCCCTGGTTTGCCAGTCTG
ACCGCCAGTTTTGCCTTTTATCCGGTCCAAAAAGCGAATGTCGAAAGCGGCAAAGACTGG
ATGAAGCCGGGAAAACTGATTGGCAATGGCGCGTATGTGCTTAAAGAGCGCGTGGTAAAT
GAAAAACTGGTGGTCGTGCCTAATACGCATTACTGGGATAACGCGAAAACGGTACTGCAA
AAAGTAACATTTTTACCCATTAACCAAGAATCGGCTGCGACGAAACGTTACCTTGCCGGT
GATATTGATATCACCGAATCTTTCCCTAAAAATATGTACCAGAAATTATTGAAGGATATT
CCAGGGCAAGTTTATACGCCGCCGCAATTAGGGACTTATTATTATGCGTTTAATACGCAG
AAAGGGCCGACGGCGGATTCCCGCGTTCGTCTGGCGCTAAGTATGACCATTGATCGCCGT
TTGATGGCGGAAAAAGTCTTAGGTACCGGTGAAAAACCGGCCTGGCATTTTACACCGGAT
GTCACGGCAGGATTTAAGCCCGATCCTTCACCGTTTGAACAAATGAGCCAGGAAGAACTT
AACGCCCAGGCGAAAACATTGCTGCGTGCAGCAGGCTACGGATCGCAGAAGCCGCTTAAA
TTAACTCTGCTTTACAATACCTCAGAAAACCATCAGAAAATCGCGATTGCGGTGGCGTCA
ATGTGGAAGAAAAATCTGGGGGTGGATGTGAAATTGCAAAACCAGGAGTGGAAAACGTAT
ATCGACAGCCGGAATACAGGTAATTTTGATGTTATTCGCGCCTCCTGGGTGGGTGATTAC
AACGAACCGTCGACTTTCTTATCCTTATTAACGTCCACGCATACGGGGAATATTTCACGC
TTTACTAATCCGACTTATGACAAAATCCTGACGCAAGCGACGATGGAAAATACCGCCGAA
GCGCGTAACGCGGATTACAATGCAGCGGAGAAAATTTTAACGGAACAAGCGCCTATAGCG
CCTATTTATCAGTATACCAATGGCCGGTTAATTAAACCGTGGGTAAAGGGATACCCCATT
ACTAACCCGGAAGATGTGGCCTATAGCCGTACAATGTATATCGTGAAGCACTGA

>EECEBICD_02050 Murein peptide amidase A
ATGACTGTCACCCGCCCCCGTGCCGAGCGCGGCGCATTTCCGCCAGGCACTGAACATTAT
GGACGTTCGCTACTAGGCGCGCCGCTAATCTGGTTTCCGGCACCTGCAGCCAATCGCGAA
AGCGGTCTGATACTGGCTGGCACCCACGGGGATGAAACCTCATCCGTGGTGACGCTCTCC
TGTGCGTTACGCACCTTAAATCCAACCCTTCGCCGTCATCATGTCGTACTTGCCGTTAAT
CCTGATGGCTGTCAACTGGGGTTAAGGGCAAATGCGAATGGCGTTGATCTAAACCGTAAC
TTTCCCGCCGCCAACTGGAAAGCGGGTGAGACAGTTTATCGCTGGAATAGCCGCGCGCAA
GAGCGTGATGTCGTGCTGCTAACCGGCGACCATCCCGGCTCAGAACCGGAAACCCAGGCG
CTTTGCCAGTTAATTCATCGACTCCAACCCGCCTGGGTCGTCTCTTTTCACGATCCGCTA
GCCTGCATTGAAGATCCACGTCGCAGCGAGCTAGGCGAGTGGCTGGCCGATGCGTTTGCG
TTGCCGCTGGTGACCAGCGTAGGTTACGAAACGCCCGGTTCGTTTGGCAGCTGGTGCGCC
GACCTCAACCTGCACTGCATTACCGCCGAGTTTCCCCCTGTTTCTGCCGATGAAGCCAGC
GAAAAATACTTGCTGGCTATGTCGAATCTGCTCCGCTGGCATCCTAAAGATGGAGTTGAC
CCGTCGTAA
>EECEBICD_02051 L-Ala-D/L-Glu epimerase
ATGAGATCTGTTAAGGTATATGAGGAAACCTGGCCGTTACATACGCCTTTTGTTATTGCG
CGTGGTAGCCGAAGCGAAGCGCATGTTGTCGTTGTCGAACTGGAAGAAGAGGACGTAAAA
GGCATCGGGGAATGTACGCCATATCCACGTTATGGTGAAAGTGATGCCTCGGTCATGGCG
CAAATCATGAGTATTGTGCCTCAGTTGGAAAACGGGCTGACTCGCGAAGCATTGCAGAAA
CTGTTGCCGGCAGGCGCGGCGCGCAACGCGGTGGATAGCGCCTTATGGGATTTGCAGGCC
CGCCAGCGGCAGCAAAGCCTTGCCGGGCTGCTCGGCGTGACGCTGTCCCCGGTTTTTACT
ACCGCGCAAACGGTAGTGATAGGTACGCCGGAACAGATGGCAGCCAGTGCGGCGGCACTA
TGGGAAAAAGGGGCGACGTTGCTGAAAATTAAACTCGATGCTCGTCTGATTAGCGAACGT
ATGGTGGCGATCCGCTCGGCGGTGCCGGAGGCGACGCTCATTGTGGACGCCAATGAATCG
TGGCGTCCGGAGGGGCTGGCGGCGCGTTGCCAGTTACTGGCGGATCTGAATGTCGCGATG
CTGGAGCAACCGCTGCCCGCGCAGGATGACGCCGCCCTGGAAAATTTTATTCATCCGTTG
CCCATCTGTGCAGATGAGAGCTGCCATACTCGCAGTAGTCTGAAAGCCCTGACGGGGCGC
TACGATATGATCAACATTAAGCTGGATAAAACCGGCGGTCTGACTGAGGCGCTGGCGCTG
GCAACGGAGGCGCGGGAGCAAGGATTCGGCCTGATGCTGGGCTGTATGCTCTGTACATCA
AGAGCGATTAGCGCCGCGCTGCCGCTTATGCCGCAGGTGAGCTTCGCCGATCTTGATGGC
CCAACATGGCTGGCGGTTGATGTTGAGCCCGCGCTACGCTTTACGACGGGTCAACTCCAT
CTTTAG
>EECEBICD_02052 Thiol peroxidase
ATGTCACAGACTGTACATTTCCAGGGTAACCCGGTCACCGTTGCCAACGTTATTCCGCAG
GCTGGTAGCAAAGCACAGGCTTTTACTCTTGTCGCAAAAGATTTGTCTGACGTTTCCCTC
AGCCAATATGCAGGCAAACGCAAAGTGCTGAATATTTTCCCAAGCATTGATACTGGCGTA
TGCGCCGCATCAGTTCGTAAGTTTAATCAACTGGCCACAGAAGTGGAGAATACCGTCGTA
TTGTGCGTTTCTGCTGACCTGCCTTTTGCACAGTCGCGTTTTTGCGGCGCGGAAGGCCTG
AGCAACGTTATTACGCTTTCCACTCTGCGTAATAACGAGTTTCTGAAAAACTACGGCGTC
GAAATCGTTGACGGCCCGCTGAAAGGTTTGGCGGCGCGCGCAGTCATCGTGCTTGACGAA
AACGACAACGTTATTTTCAGCCAGTTGGTGGATGAAATCACCCACGAGCCGGATTACGAC
GCCGCGCTTAATGTCCTGAAGGCATAA
>EECEBICD_02053 Transcriptional regulatory protein TyrR
ATGCGTCTGGAAGTCTTTTGTGAAGACCGACTTGGTCTGACCCGCGAATTACTTGATTTA
CTGGTGTTACGTAGCATTGATTTACGCGGAATCGAGATTGATCCCATTGGGCGAATTTAT
CTTAATTTTGCTGAGCTGGAATTCACCGACTTCAGCAGCCTGATGGCAGAAATCCGCCGT
ATTTCCGGCGTAACGGATGTCCGTACCGTTCCCTGGATGCCGTCCGAACGTGAACATCTG
GCCCTGAGCGCGCTGCTTGAGGCGTTGCCGGAGCCGGTGCTCTCATTGGATATGAAGAGT
AAAGTGGAGATGGCGAACCCGGCGAGTTGTCAACTTTTTGCCCAGAGCCAGGAGCGAATG
CGGCACCATACCGCCGCACAATTAATCAACGGCTTCAATTTTCAGCGCTGGCTGGACGGT
AACCCGCAAAGCTCCCATAACGAACATGTCGTGATCAACGGGCAAAACTTCCTGATGGAG
ATTACGCCGGTACATTTACAAAACGAAAATGACGAATACGTGTTGACCGGGGCGGTCGTG
ATGTTGCGTTCCACGATTCGTATGGGACAGCAGCTACAGAATTTGTCCACGCAGGATCTG
AGCGCGTTTAGTCAGATTATTGCCGTGAGCGCAAAGATGAAGCACGTCGTTGAGCAGGCG

CGCAAACTGGCGATGCTCAGCGCGCCGCTGCTGATTACCGGCGATACGGGAACCGGCAAA
GATCTTTTCGCCTATGCCTGTCACCAGGCAAGCCCTCGTTCAGCGAAACCGTATCTGGCG
CTCAACTGCGCTTCAATCCCGGAAGATGCGGTAGAAAGCGAACTATTTGGCCATGCGCCG
GAAGGTAAAAAAGGTTTCTTTGAACAGGCGAATGGCGGTTCGGTGCTGCTGGATGAAATT
GGCGAAATGTCGCCGCGTATGCAGGCGAAGCTGCTGCGTTTTCTCAACGATGGTACGTTC
CGTCGCGTCGGCGAAGATCACGAAATTCATGTTGATGTCCGCGTTATCTGCGCCACGCAG
AAAAATCTGGTGGAGCTGGTGCAAAAAGGACTGTTCCGCGAAGATCTCTATTATCGACTT
AACGTTCTGACGCTTAATTTGCCGCCGTTGCGCGATTGTCCGCAGGATATTATGCCGTTG
ACCGAACTGTTCGTGGCGCGTTTTGCCGACGAACAGGGCGTTCCGCGACCGAAACTGTCT
GCCGATCTGAGTACGGTCCTCACTCGTTACGGCTGGCCGGGTAACGTTCGCCAGCTTAAA
AATGCGATTTACCGGGCGCTGACGCAACTGGAAGGGTATGAGCTGCGTCCGCAGGATATC
CTGCTGCCTGACTACGATGCCGCGACGGTGGCAGTCGGCGAGGATGCGATGGAAGGCTCG
CTGGATGACATTACCAGTCGTTTTGAACGTTCTGTCCTGACCCAGCTTTATCGTAGCTAT
CCGAGTACGCGTAAACTGGCGAAACGGTTGGGGGTATCGCATACCGCGATTGCCAATAAG
CTGCGTGAATATGGTCTGAGCCAGAAGAAGGGTGAAGAGTAG
>EECEBICD_02054 hypothetical protein
ATGAGCGAACCGTTAAAACCGCGTATTGATTTTGCAGAACCGCTAAAGGAGGAACCTACG
TCGGCCTTCAAAGCGCAGCAAACTTTTAGCGAAGCGGAGTCGCGTACATTTGCGCCTGCA
GCTATCGATGAGCGCCCGGAAGACGAAGGCGTGGCAGAAGCGGCGGTCGATGCCGCGCTG
CGCCCCAAACGCAGTCTGTGGCGTAAAATGGTGATGGGAGGGCTGGCGCTGTTTGGCGCG
AGCGTGGTCGGGCAAGGCGTACAGTGGACAATGAATGCCTGGCAAACTCAGGACTGGGTC
GCTTTAGGCGGCTGTGCCGCAGGCGCGCTGATCATTGGCGCTGGCGTGGGATCGGTGGTC
ACGGAGTGGCGGCGATTATGGCGCTTGCGCCAGCGGGCGCATGAGCGCGATGAGGCGCGT
GAACTGTTACATAGCCATAGCGTCGGGAAAGGTCGCGCATTTTGCGAAAAACTGGCGCAG
CAGGCGGGGATTGATCAATCACATCCGGCATTACAACGTTGGTATGCCGCTATTCACGAA
ACGCAAAACGACAGGGAAATCGTCGGCTTGTATGCGCATCTGGTACAGCCGGTACTTGAC
GCGCAGGCGCGACGTGAGATTAGCCGTTTCGCCGCGGAATCGACTCTGATGATCGCCGTC
AGCTCGTTAGCGTTGGTGGATATGGCGTTTATTGCCTGGCGTAATTTACGCCTGATTAAC
CGCATCGCAACGCTGTATGGCATTGAACTTGGTTATTACAGCCGCCTTCGTCTGTTCCGT
CTGGTGTTGCTGAATATCGCGTTCGCGGGGGCCAGTGAGCTGGTGCGTGAAGTCGGTATG
GACTGGATGTCTCAGGATCTGGCCGCACGCCTGTCTACGCGCGCGGCGCAGGGGATTGGC
GCAGGTCTCCTTACCGCTCGACTGGGAATAAAAGCGATGGAGCTATGTCGACCATTGCCG
TGGATCGATAACGATAAACCACGTCTCGGTGATTTTCGTCGTCAGCTTATCGGTCAGCTA
AAAGAGACGCTGCAAAAGAGTAAGTCGTCGCCTGAGAAATGA
>EECEBICD_02055 putative protein YcjX
ATGAAGCGACTTAAAACTGAACTCAACGCGCTGGTCAATCGTGGGGTAGACAGGCATTTG
CGTCTTGCCGTAACCGGGTTGAGCCGCAGCGGTAAAACAGCGTTTATCACGGCGATGGTT
AACCAGTTGCTTAATGTCCACGCGGGCGCGCGCTTACCGTTGCTCAGCGCGGTACGCGAA
GAACGCCTGCTTGGCGTAAAGCGCGTGCCTCAACGTGATTTTGGTATTCCCCGTTTTACC
TACGATGAGGGGATACTTCAGCTTTATGGCAATCCTCCCGCATGGCCCACACCGACGCGC
GGCGTCAGTGAGATTCGGCTGGCGTTGCGCTATCGCTCCAACGACTCGCTGCTGCGCCAT
TTTAAAGATACCTCGACATTGTATCTGGAGATTGTGGACTATCCAGGGGAATGGTTACTC
GATCTGCCCATGCTGGCTCAGGATTATTTAAGCTGGTCCCGTCAGATGAACGGCTTGTTA
CAAGGACAACGCGCTGAGTGGGCCGCAAAATGGCGGCAACTTTGCGACGGGCTCGATCCG
CTGGCACCCGCCGATGAAAACCGGCTGGCGGAGATTGCCGCCGCCTGGACGGATTATTTG
CATCAGTGTAAATCGCAGGGATTGCATTTTATTCAACCTGGACGGTTTGTTTTGCCGGGC
GATATGGCGGGTGCGCCAGCGTTGCAGTTTTTCCCGTGGCCGGATGTGGACGCCTTCGGC
GAGTCAAAACTGGCGCAGGCGGATAAACAGACCAACGCTGGAATGCTGCGCGAACGCTTT
AACTACTATTGCGAAAAAGTGGTCAAAGGGTTCTATAAAAATCATTTTCTACGCTTTGAT
CGCCAGATTGTACTGGTCGACTGTCTGCAACCGCTTAATAGCGGGCCGCAGGCGTTTAAT
GATATGCGTCTCGCCTTAACGCAGTTGATGCAAAGTTTTCACTATGGCCAGCGGACTCTG
TTTCGCCGCTTGTTTTCGCCGGTGATTGATAAATTGCTGTTCGCCGCGACGAAAGCCGAC
CATGTCACCCTCGACCAACATGCCAATATGGTGGCCTTGCTCCAGCAGCTTATTCAGGAT
GCCTGGCAAAATGCCGCGTTTGAAGGCATTAGTATGGATTGCCTAGGGCTGGCGTCGGTA

CAGGCGACCACCAGCGGCGTGATTGAGGTCAACGGTGAAAAAATCCCGGCTTTACGTGGA
AACCGTTTGAGTGACGGCGCGCCGTTAACCGTGTACCCCGGCGAAGTGCCTTCGCGTTTG
CCGGGACAAGCGTTTTGGGATAGTCAGGGTTTTCAGTTCGAAGCCTTTCGTCCGCAGGTG
ATGGATGTTGATAAACCGTTACCACATATTCGTCTGGACGCTGCGCTGGAGTTTTTAATA
GGAGATAAGCTGCGATGA
>EECEBICD_02056 Thiosulfate sulfurtransferase PspE
ATGTTGAAAAAAGGAATATTTGCGTTAGCGTTATTCGTAGCCATGCCGCTTTATGCCGCA
GAATACTGGATAGATGTCCGCATTCCGGAACAATATCAGCGTGAGCATATTCAGGGCGCG
ATTAATATCCCATTGAAGGAGATTAAATCTCATATCGAAACGGTAGTTCCAGACAGGAAC
GATACGGTAAAGTTATATTGCAACTCCGGACGACAGTCGGGCATGGCAAAACAAATGTTG
CTTGATATGGGGTATACGCACGCGATGAATATGGGCGGCATCAGTCGTCTTGATATGCCG
AAAGTAAAAAAATAG
>EECEBICD_02057 Phage shock protein D
ATGAATACTCGCTGGCAACGCGTCGGGCAGAAGGTGAAGCCCGGCTTCAAAATAGCAGGT
AAGCTGGTTCTGTTGACCGCGTTACGCTACGGGCCTGCCGGCGTGGCGGGATGGGCGGTA
AAATCCGTCGCCCGTCGTCCGCTAAAAATGTTGCTGGCGTTTGCGCTGGAGCCAGTACTT
CGTAAGGCGGCGAATAAAATTTCGCAGCGTTACAAATAG
>EECEBICD_02058 Phage shock protein C
ATGGGTGGAATCAATCTGAATAAAAAACTATGGCGTATGCCGCAAAAGGGCATGGTGCGT
GGCGTTTGCGCCGGGATCGCCGACTACCTTGATGTACCGGTAAAACTGGTGCGTATTCTG
GTCGTGCTGTCGATTTTCTTTGGGCTGGCGTTTTTTACCGTAGTGGCCTATATCGTTCTG
TCTTTTGTACTGGACCCTATGCCGGATAATCTGGCCGCAGGCAAACCGCAGCCGACCAGC
GGCGAACTGCTGGATACCGTAGACAGAGAGCTTGCCGCAGGCGAAAAGCGTTTGCGAGAA
ATGGAACGCTACGTCACGTCAGATACGTTCACACTGCGTAGTCGTTTCCGTCAACTTTAA
>EECEBICD_02059 Phage shock protein B
ATGAGCGCGCTATTTTTGGCCATCCCGTTAACCATTTTTGTGTTGTTTGTGTTACCGATT
TGGCTGTGGCTGCATTACAGCAACCGCGCCGGTCGGGGAGAACTGTCGCAAAGCGAGCAG
CAACGCTTACTGCAACTCACAGACGATGCGCAACGTATGCGCGAGCGCATTCAGGCGCTG
GAAGACATTCTTGATGCAGAGCATCCGAACTGGAGAGAGCGCTAA
>EECEBICD_02060 Phage shock protein A
ATGGGTATTTTTTCTCGTTTTGCCGACATCGTGAACGCCAATATCAATGCGTTGTTGGAA
AAAGCGGAAGATCCGCAGAAGCTGGTGCGCCTGATGATTCAGGAGATGGAAGATACGCTG
GTGGAGGTTCGCTCGAACTCCGCGCGTGCTTTAGCGGAAAAGAAACAGTTATCCCGTCGC
ATTGAACAGGCTACGGTTCAGCAGACCGAGTGGCAGGAAAAAGCGGAACTGGCTCTGCGC
AAAGATAAAGACGATCTGGCGCGCGCCGCATTGATTGAAAAACAAAAGCTGACCGATTTA
ATTGCTACGCTTGAACAGGAAGTCACGCTGGTGGATGACACGCTGGCGCGCATGAAGAAA
GAGATTGGCGAGCTGGAAAACAAACTCAGCGAAACACGCGCTCGCCAGCAGGCGCTGATG
CTGCGTCATCAGGCGGCGAGTTCTTCCCGCGATGTCCGTCGTCAGCTTGACAGCGGCAAA
CTGGATGAAGCCATGGCGCGGTTTGAATCCTTTGAACGTCGTATCGATCAAATGGAAGCG
GAAAGCCATCGTTTTGGTAAGCAGCAATCGCTGGACCAACAGTTTGCCAACCTGAAAGCC
GATGATGAAATCAGCGAGCAGCTGGCGCAGTTGAAAGCCAAAATGAAGCAAGATAATCAA
TAA
>EECEBICD_02061 Psp operon transcriptional activator
ATGGCTGAATTTAAAGATAACCTGCTCGGCGAGGCAAACCGTTTTCTTGAAGTACTGGAA
CAAGTCTCCCGGCTCGCGCCGCTGGATAAGCCAGTGCTGATTATTGGCGAACGCGGTACG
GGTAAAGAACTTATCGCCAATCGCCTGCACTATCTCTCTTCACGCTGGCAGGGGCCGCTT
ATCTCGCTTAACTGCGCAGCGCTTAACGAAAATCTGCTGGACTCCGAACTGTTTGGTCAT
GAAGCGGGTGCTTTTACCGGGGCGCAAAAACGCCATCCCGGTCGCTTTGAACGCGCCGAT
GGCGGCACGCTGTTTTTGGATGAACTGGCGACAGCGCCAATGCTGGTACAGGAAAAGCTA
CTGCGGGTGATTGAATATGGCGAGCTAGAGCGCGTCGGCGGCAGCCAGCCGTTACAGGTT
AATGTGCGATTAGTATGCGCCACCAATGCCGATTTACCCGCCATGGTAAAGGAAGGAACA
TTTCGTGCCGATCTGCTCGACAGGCTGGCTTTTGACGTTGTACAACTGCCGCCGCTGCGC
GAACGTCAAAGCGATATTATGCTGATAGCCGAGCATTTTGCTATTCAGATGTGTCGTGAG
CTACGTCTGCCCTTATTTCCCGGTTTTACCGATCGGGCAAAAGAAACGTTGTTGCATTAC

GCCTGGCCGGGTAATGTCCGCGAGCTGAAAAACGTAGTCGAACGTTCAGTGTATCGCCAC
GGCAGCAGTGAACATCCTTTGGATGAAATTGTTATCGATCCTTTTCAACGCCATTCCGCC
GAACCGCCCACGCCCGCCCTGCCAGCGGCATCCGCCACGCCTGACCTGCCGCTCAAGTTG
CGGGAGTTTCAACTGCAACAGGAAAAAGCGCTGCTGCAACGTAGCTTACAACAGGCGAAA
TTTAACCAGAAACGAGCCGCCGATTTGCTGGCACTCACCTACCACCAGTTTCGCGCCTTG
CTTAAAAAGCATCAGCTTTAG
>EECEBICD_02062 Peptide transport periplasmic protein SapA
ATGCGCCTGGTTTTATCGTCTCTGATCGTGATAGCGGGTCTACTGAGTAGTCAGGCTACG
GCTGCGGCTGCGCCCGAACAAACTGCGAGTGCAGATATTCGCGATAGCGGCTTTGTGTAT
TGTGTCAGCGGGCAGGTCAACACCTTTAATCCGCAAAAAGCGAGCAGCGGCCTCATCGTC
GATACCCTGGCAGCCCAGTTATATGACCGCCTGTTGGATGTCGATCCCTATACTTATCGT
TTAGTCCCAGAGCTGGCAGAAAGCTGGGAAGTGCTGGATAACGGGGCAACGTACCGTTTT
CACCTGCGCCGCGACGTTTCCTTTCAAAAAACCGCCTGGTTTACGCCGACCCGAAAACTC
AATGCTGATGATGTCGTCTTTACCTTTCAGCGGATTTTTGATCGTCGGCATCCGTGGCAT
AACATCAACGGCAGTAGCTTCCCCTACTTTGATAGCCTACAGTTCGCCGACAATGTAAAA
AGCGTGCGTAAGCTGGACAATAACACCGTTGAGTTTCGCCTGACGCAGCCAGACGCCTCC
TTTTTATGGCATCTGGCTACACACTACGCTTCCGTCATGTCCGCTGAGTACGCCGCGCAG
CTTAGCCGAAAAGATCGTCAGGAACTGCTAGACCGCCAACCGGTCGGCACCGGGCCTTTC
CAGCTTTCGGAGTACCGTGCCGGACAGTTTATTCGTCTCCAGCGCCACGATGGGTTTTGG
CGCGGCAAACCGCTGATGCCGCAAGTGGTAGTGGATTTAGGCTCCGGCGGTACCGGGCGT
TTATCGAAATTACTGACCGGTGAATGCGATGTTCTGGCCTGGCCCGCCGCCAGCCAGCTA
ACTATTTTACGCGACGATCCGCGTTTACGTCTGACGTTGCGCCCGGGGATGAATATCGCC
TATCTGGCCTTTAACACCGATAAGCCGCCGTTGAATAATCCCGCAGTGCGCCATGCGCTG
GCCTTATCGATCAACAACCAGCGTCTGATGCAGTCGATTTATTACGGCACGGCGGAAACC
GCAGCCTCCATTTTACCGAGAGCCTCATGGGCTTACGATAACGATGCCAAAATTACGGAG
TACAATCCGGAAAAAATCGCGCGAACAGCTAAAAGCGCTGGGCATTGAGAATCTTACGCTG
CATCTCTGGGTGCCGACCAGTTCTCAGGCCTGGAACCCAAGTCCGCTAAAAACGGCGGAG
CTTATTCAGGCGGATATGGCGCAGGTTGGCGTAAAAGTGGTCATTGTGCCGGTTGAAGGT
CGTTTTCAGGAGGCGCGCCTGATGGATATGAATCACGATCTGACCTTATCCGGCTGGGCC
ACGGACAGCAACGATCCGGATAGCTTTTTCAGACCACTGTTAAGCTGTGCGGCCATCAAT
TCGCAAACCAATTTCGCCCACTGGTGTAACCCTGAATTTGACAGCGTGCTGCGTAAAGCA
CTGTCGTCGCAGCAGTTGGCTTCGCGCATAGAAGCATATGAGGAAGCGCAGAATATCCTG
GAGAAAGAGCTGCCGATACTGCCGCTGGCATCATCACTACGCCTGCAGGCTTACCGCTAC
GATATTAAAGGGCTGGTGTTAAGCCCGTTCGGCAATGCGTCTTTTGCCGGCGTCTCCCGC
GAAAAACACGAAGAGGTGAAAAAACCATGA
>EECEBICD_02063 Peptide transport system permease protein SapB
ATGATTATCTTCACCCTGCGTCGGTTATTGCTGTTGCTGGTGACGCTATTCTTCCTGACC
TTTATCGGCTTTAGCCTGAGCTATTTTACGCCGCACGCGCCGCTACAAGGCGCATCATTA
TGGAATGCCTGGGTTTTCTGGTTCAACGGTCTGCTGCACTGGGACTTTGGCGTGTCCAGC
ATTAACGGCCAGCTGATCTCCGAACAGCTTAAAGAGGTGTTTCCCGCCACGATGGAGCTG
TGCATTCTCGCCTTTGGTTTTGCGCTGATGGTCGGCATCCCGGTCGGGATGCTGGCTGGC
GTGACGCGTAGCAAATGGCCGGATCGTTTTATCAGCGCGCTGGCGCTATTAGGTTTCTCA
ATTCCGGTTTTCTGGCTGGCGCTTCTGCTCACACTGTTCTTTTCGCTCACGCTGGGGTGG
CTGCCGGTATCCGGGCGATTTGACCTGCTTTATGAAGTTAAACCGGTTACCGGTTTCGCC
ATCATTGACGCCTGGATTTCAGATTCGCCATGGCGTGATGAAATGGTGATGAGCGCCATA
CGGCATATGGTTTTACCGGTGCTCACGCTCTCCGTCGCGCCGACCACAGAAGTGATCCGC
CTGATGCGAATCAGCACTATTGAGGTCTACGATCAGAACTACGTTAAAGCGGCGGCCACG
CGCGGCTTATCGCGCTTTACGATTTTACGCCGTCACGTGTTGCATAATGCGCTGCCGCCG
GTCATTCCCCGTCTTGGTCTGCAGTTTTCAACCATGTTAACGCTGGCGATGATTACCGAA
ATGGTTTTCAGCTGGCCTGGGCTGGGACGATGGTTAATTCACGCCATTCGTCAGCAGGAC
TATGCCGCTATTTCCGCTGGCGTGATGGTCATTGGGTCGCTGGTTATTGTGGTCAATGTG
ATTTCCGATATTTTGGGTGCTATGGCTAACCATCTGAAACATAAGGAATGGTATGCCTTA
CGATAG
>EECEBICD_02064 Peptide transport system permease protein SapC

ATGCCTTACGATAGCGTATACAGCGAAAAGCGCCCGCCAGGCACCCTGCGCACTGCCTGG
CGTAAATTTTATAGCGACGCGCCTGCGATGGTGGGTCTGTATGGATGCGCCGGTCTGGCG
TTGCTGTGTATTTTTGGCGGCTGGATCGCGCCCTATGGTATCGATCAGCAATTTCTGGGC
TATCAGCTACTGCCACCCTCCTGGTCACGCTATGGCGAAGTCTCCTTTTTTCTGGGGACT
GACGATCTGGGGCGCGATGTGCTGAGCCGCCTGTTAAGCGGCGCGGCGCCCACGGTCGGC
GGCGCGTTTATCGTAACGCTGGCGGCTACGCTATGTGGATTAGTCCTCGGCGTAGTGGCA
GGCGCGACGCACGGTTTACGCTCTGCCGTGCTGAACCATATTCTGGACACGCTGCTTTCC
ATCCCTTCGCTCTTGCTGGCGATTATCGTCGTGGCATTTGCCGGCCCACATCTGTCACAC
GCGATGTTTGCCGTCTGGCTGGCCCTGTTGCCGCGAATGGTACGTTCCGTCTACAGCATG
GTGCATGATGAACTGGAAAAAGAGTACGTCATTGCCGCCCGTCTGGATGGAGCGACAACG
CTGAATATTCTGTGGTTCGCCATTTTACCGAATATTACCGCCGGTCTGGTCACCGAAATT
ACCCGGGCGCTGTCGATGGCGATTCTGGACATCGCCGCGCTGGGATTTTTAGATCTCGGC
GCGCAGCTTCCCTCTCCTGAATGGGGCGCCATGTTGGGGGATGCGCTGGAGCTAATCTAT
GTCGCGCCGTGGACCGTTATGCTACCCGGCGCGGCGATAACGCTCAGTGTTTTACTGGTA
AACCTGCTCGGAGATGGGATTCGTCGTGCGATTATTGCGGGGGTGGAATAA
>EECEBICD_02065 Peptide transport system ATP-binding protein SapD
ATGCCGTTACTGGATATCCGCAATCTCACCATTGAATTTAAAACCAGCGAAGGTTGGGTA
AAAGCGGTCGATCGCGTCAGTATGACATTAAGCGAAGGTGAAATTCGCGGCCTGGTAGGT
GAATCGGGTTCCGGAAAGAGCTTGATTGCTAAAGCTATTTGCGGCGTCGCGAAAGACAAC
TGGCGAGTCACTGCCGACCGTATGCGTTTTGATGATATCGATCTGCTGCGCCTCTCCTCA
CGTGAACGGCGTAAGCTGGTCGGACATAATGTGTCGATGATCTTCCAGGAGCCGCAGTCG
TGCCTTGATCCTTCCGAACGCGTCGGCCGCCAGTTAATGCAAAATATTCCCGCCTGGACC
TATAAAGGCCGCTGGTGGCAGCGTTTAGGCTGGCGTAAACGGCGTGCAATTGAATTACTG
CACCGGGTGGGAATTAAAGATCATAAAGACGCGATGCGCAGTTTTCCCTATGAACTGACC
GATGGCGAGTGTCAGAAAGTTATGATCGCTATCGCGCTGGCGAATCAACCGCGACTCCTG
ATCGCTGATGAACCCACCAACTCAATGGAACCGACGACGCAGGCGCAAATTTTTCGCCTG
TTGACGCGACTGAATCAAAACAGTAATACCACCATTCTGTTGATTAGCCACGACCTGCAA
ATGCTGAGTCAGTGGGCTGACAAAATCAACGTTTTATACTGCGGCCAGACGGTAGAAACC
GCGCCAAGTAAAGATCTGGTGACGACGCCACATCATCCTTATACGCAGGCGCTTATCCGT
GCGATTCCGGATTTTGGCAGCGCGATGCCGCATAAAAGCCGTCTCAATACGCTGCCGGGC
GCGATCCCGCTACTGGAGCAGTTACCGATAGGTTGTCGTCTGGGGCCGCGCTGCCCTTAC
GCGCAACGAGAATGCATTATCACGCCACGCTTAACCGGCGCGAAAAATCATCTCTATGCC
TGTCATTTTCCGCTGAATATGGAGAGAGAGTGA
>EECEBICD_02066 Peptide transport system ATP-binding protein SapF
ATGGTGGAAACTCTGCTTGAAGTGCGCAACCTGAGTAAAACCTTTCGCTACCGGACAGGA
TGGTTTCGCCGCCAGACCGTCGACGCCGTGAAACCATTAAGCTTTACGCTTCGTGAGCGT
CAGACCCTGGCCATTATCGGCGAAAACGGTTCCGGCAAATCCACGCTGGCAAAGATGCTG
GCCGGCATGATTGAACCCACCAGCGGCGAGCTTCTTATTGACGATCATCCCCTGCATTAC
GGCGACTATTCGTTTCGAAGCCAACGTATTCGAATGATTTTTCAGGACCCCTCAACGTCA
CTGAATCCACGTCAACGTATTTCGCAGATCCTTGACTTCCCCCTGCGCCTTAATACCGAT
CTGGAGCCGGAACAGCGGCGCAAACAGATTGTCGAGACGATGCGTATGGTGGGGCTATTG
CCCGATCACGTGAGCTACTACCCGCATATGCTGGCGCCGGGGCAGAAACAGCGTCTGGGC
CTGGCCCGCGCGCTGATTTTACGCCCTAAAGTCATTATCGCGGACGAAGCGCTGGCTTCG
CTTGATATGTCCATGCGCTCGCAGCTCATCAATCTGATGCTTGAATTGCAGGAAAAGCAA
GGCATCTCCTATATCTATGTCACGCAGCATATCGGCATGATGAAGCATATTAGCGATCAG
GTATTAGTGATGCATCAGGGGGAAGTGGTTGAACGCGGCAGCACCGCCGATGTGCTCGCC
TCTCCGCTACATGAGCTTACCCGCCGTTTAATCGCAGGCCATTTTGGCGAGGCGCTGACC
GCTGACGCATGGCGAAAGGATCGCTAA
>EECEBICD_02067 Putative anti-FlhC(2)FlhD(4) factor YdiV
ATGCGCTATTTTTTTATGGCAGAGCCGATAAGGGCAATGGAGGGGGATTTACTGGGTGTC
GAAATCACTACCCATTTTGCATCGTCGCCCGCTCGTCCGCTACACCCTGAGTTTGTTATC
TCGTCATGGGACAATTCGCAGAAGCGCCGTTTTCTGCTTGATCTGTTGCGGACAATCGCG
GCCAAACATGGCTGGTTTCTACGCCACGGCCTATTCTGTATCGTAAATATTGATCGGGGT
ATGGCGCAGCTTGTCCTTCAGGATAAAGATATTCGCGCATTACTACACGCCATGCTATTT

GTGGAGCTGCAGGTCGCTGAACATTTTTCATGTCAAGATAACGCCTTAATCGATCCTTTA
ATACACGCGCTTCATAAGCAACCTAATCCATTATGGCTGGGCGACCTGGGCGTAGGGAAT
GCAACTGCTGTGCCATTAGTTTGCGGGTGTTTTAGCGGCGTAAAACTTGATCGCAGCTTT
TTTGTCTCACAAATTGAAAAAATGACGTTTCCGCTGCTTGTCAAACATATCCGGCGTTAT
TGTGACAAGATCGTGGTGGGGGGCCAGGAAAACACACGCTATTTGCCGGTGTTAAAAACG
GCAGGCATTTGGGCAACCCAGGGCACGTTATTTCCTTCCGTTGCGTTAGAAGAGGTTGAA
ACATTGCTGTTGGGCAGTCGCATGAACACCCTCAGAGAATCGAACTGA
>EECEBICD_02068 Secreted effector kinase SteC
GTGACCGAGACGGAGTGTTCTGAACGTTGCGTCGATTTTTTAAAAGAAACGATGTTTAAT
TATAATAACGGGGAAATTACGCTTGCCGCTTTGGGTAATGATAATCTACCGCCGAGCGAG
GCAGGTAGTAATGGAATCTATGAGGCTTTTGAGCAACGTTTGATAGGCTTCCTGACCACG
CCCGCCACGGCGTCTGGTGATGAGAGCGGCGCTATTGATCAGACGGATGCATCACAACCA
GCGGCAATAGAGGCGTTTATTAACTCGCCGGAGTTTCAGAAAAATATTCGCATGCGTGAT
ATTGAAAAAAATAAAATAGGTAGCGGAAGTTATGGGACAGTCTATCGGTTACATGATGAC
TTTGTTGTTAAGATTCCAATTAATGAGCGGGGAATAAAAGTGGATGTTAACTCTCAGGAA
CATCGTAATTGTCATCCTGAGCGTGTCAGTAAGTATTTGAATATGGCCAATGGCGACAAG
AATTTTTCTCGTTCGGCCATCATGAATATTAATGGTAAAGACGTTACGGTTTTAGTTTCA
AAATATATTCAAGGTCAGGAGTTCGATGTTGAAGATGAAGATAACTATCGAATGGCGGAG
GCGCTTCTTAAATCCCGGGGGGTTTATATGCATGATATAAATATCTTAGGTAATATTCTG
GTTAAAGAGGGGGTTCTTTTCTTTGTGGATGGCGATCAAATCGTGTTATCACAAGAATCA
CGGCAACAGCGAAGCGTTTCTCTTGCAACAAGGCAACTGGAAGAGCAAATTAAGGCCCAT
CACATGATAAAATTAAAGCGGGCCGAGACCGAAGGAAATACTGAAGATGTTGAATATTAT
AAGTCACTTATTACGGATCTTGATGCGTTAATCGGAGAGGAAGAACAGACACCTGCGCCA
GGAAGACGTTTTAAACTAGCCGCGCCGGAGGAAGGGACTCTTGTGGCTAAGGTATTAAAG
GATGAATTAAAAAAATAG
>EECEBICD_02069 hypothetical protein
ATGCCGTTTACATTTCATATCGGAAATCATAGTTGCCAGATATCAGAAAGGTATTTAAGA
GATATCATCGATAATAAAAGAGAACATGTTTTTTCAACGTGTGAGAAATTTATAGATTTT
TTCAGGAATATATTTACCAGGCGAAGTTTAATTTCTGATTATAGAGAAATCTATAATCTA
CTGTGCCAAAAAAAGAGCATCCCGATATTAAAGGGCCTTTCTCTCCAGGACCATTTTCGA
AGCGAGATGAAGACTGTACACGATGGCGCCCCCTTCTTGGATATATTAAACTTATTGATG
CCTCCAGACCTGAAACAATAG
>EECEBICD_02070 Enoyl-[acyl-carrier-protein] reductase [NADH] FabI
ATGGGTTTTCTTTCCGGTAAGCGCATTCTGGTCACTGGCGTGGCCAGTAAACTGTCCATC
GCCTACGGTATCGCACAGGCGATGCACCGCGAAGGAGCTGAACTGGCGTTCACCTACCAG
AACGACAAACTGAAAGGCCGCGTAGAAGAATTTGCTGCCCAGCTGGGTTCCAGCATCGTT
TTACCGTGTGATGTCGCTGAAGATGCCAGCATTGACGCTATGTTTGCGGAGCTGGGTAAC
GTTTGGCCGAAATTTGACGGTTTTGTACACTCGATCGGTTTCGCGCCTGGCGATCAGCTG
GACGGCGATTATGTTAATGCGGTTACCCGCGAAGGCTTTAAAATCGCTCACGATATTAGC
TCTTACAGCTTTGTTGCCATGGCGAAAGCCTGCCGCACCATGCTGAATCCGGGTTCTGCG
CTGTTGACCTTGTCTTATCTGGGAGCGGAGCGCGCCATCCCGAACTACAATGTCATGGGT
CTGGCGAAAGCCTCTCTGGAAGCCAACGTGCGTTATATGGCAAATGCAATGGGGCCGGAA
GGCGTTCGCGTCAACGCCATCTCTGCGGGTCCAATCCGCACGCTGGCGGCGTCCGGTATT
AAAGACTTCCGTAAAATGCTGGCGCATTGTGAAGCCGTTACGCCAATTCGTCGCACCGTG
ACCATTGAAGATGTGGGTAACTCAGCGGCGTTTCTGTGTTCCGATCTCTCGGCAGGCATT
TCTGGCGAAGTCGTCCATGTTGACGGCGGGTTCAGCATTGCGGCAATGAATGAACTGGAA
CTGAAGTAA
>EECEBICD_02071 hypothetical protein
ATGGAACAATGCCGAAAAGCGGGTAAAAGCCACTGGTATCATGAAACACAATCCACTATG
TCGTCACAACCCCCGCTTTCGCTGATGCCGGAAGCCGCGTATGTTAACGATCGTTTTTA
CTGGATTTAACGGTATCAGAAACCGCGTTGACGCCTTTTGAGTCATGGTTAAAGCCCGCT
CACCAGTTGGCGGATGTCTTATTTCCCCGCACGGTTCTCAACGACCGGCTTCATACGTTT
AGCACCTATGAACGTATGAGCACCGCGCTGACCGCCGCTCAGGTATTTGGGGTTCAGCGT
TTGTGCCGCTATTATGCCGCGCGTCTGGCGCCGCTACCTGGCCCTGATGCCTCAAGAGAG

AGCAATCAACGGCTGGCGCAAATTACGCAATACGCTCGTCAGCTTGCCGGCTCCCCCTCC
GTTATCAATACGCGAGCCCGCGAACAGCTTGCAGAGGTGGGATTAACCGCCCGGATACC
GTACTCATCAATCAGATCATTGGGTTTATCGGTTTTCAGGCGCGGGTAGCGGCGATATTT
CAGGCGTTTTGCCGTCTGCCCGTGCGCGAACTGCCCGGCCAGGAGATGCAGCGCTTTGCC
AGGGCCGCGCGGTTTCAAAACCCCCAGACGATATGGCGGCCCGCCGCCAGTCTGGTAGAA
TATCCTCCAGCCCATACCAAAGTTCGGCGGCAATATTCGTCTTCACAATGCCAAATGATG
GCGCCGGTACTCATGCGCGATCCGTCTTCATTCGCGTTACTCGAACGCATACTGACCAGC
ACATTACACACTGCATCGCCCCCTTCTTTACTTCCACTTATCACGCTCCTCACATCACGT
ATCAACGGCAGCGCCGCCTGCTTTAACGAACAGGCAACCCAACCTGGCGCGTGGCGTCGC
GCTGTCATCACGTTACGCCAGGAAGACGACGACATCGCTCGTTGGGAACTTGAGCCTGCG
CTGACACAGGCGATACAATGGCTCACCAGAGCGCCCGATCGCTTTAGCGCCGCGCATTTT
TTCCCGCTACTCACCAGGGGAGTTTCATCGGAGCAGGCCATTAATATGTTAGGCTGGTGC
GGCGTGTGCGGATGGTTAAATCGTTTAAAAATCGCGTTGGGCGAAACCTATTAA
>EECEBICD_02072 Exoribonuclease 2
ATGTTTCAGGACAACCCGCTGCTAGCGCAGCTTAAACAGCAACTGCATTCCCAGACGCCG
CGCGCTGAAGGGGTCGTAAAAGCCACGGAAAAAGGCTTTGGCTTCCTTGAGGTTGACGCG
CAAAAAAGCTATTTCATTCCTCCGCCGCAGATGAAAAAAGTGATGCATGGCGATCGTATC
GTCGCGGTCATCCACACGGAAAAAGAACGTGAGTCTGCCGAGCCGGAAGAACTGATCGAA
CCGTTCCTGACGCGTTTTGTGGGAAAAGTTCAGGGTAAAAATGACCGCCTGTCCATCGTG
CCGGATCATCCGTTGCTGAAAGACGCGATTCCCTGCCGCGCCGCGCGCGGCGTTCAGCAT
GAGTTCAAAGAAGGTGACTGGGCGGTTGCCGAAATGCGCCGCCATCCGCTTAAAGGCGAC
CGTTCGTTTTACGCCGATTTAACGCAGTACATCACCTTTGCCGACGATCACTTTGTTCCC
TGGTGGGTTACGCTTGCCCGCCATAACCTGGAAAAAGAAGCGCCGAACGGCGTAGCGACG
GAGATGCTGGACGAAGGGCTGGAGCGTCAGGATCTGACCGCGCTGAATTTCGTCACTATC
GATAGCGCCAGCACTGAAGATATGGATGACGCGCTATATGCTGAGGAATTAGCGGACGGC
AGACTCCAGCTTACGGTGGCTATCGCCGATCCTACCGCCTGGATTGCCGAAGGCAGTAAG
CTGGATAACGCCGCGAAAATTCGCGCCTTCACCAACTACCTGCCGGGCTTCAATATTCCT
ATGCTGCCACGTGAGCTGTCTGACGATCTGTGCTCACTGCGCGCCAACGAAGTGCGTCCG
GCGCTCGCCTGTCGCATGATAATCTCCGCGGACGGCACCATTGACGATGATATCGCCTTT
TTTGCGGCCACCATCGAATCGAAGGCCAAGCTGGCGTACGACAATGTCTCCGACTGGCTG
GAAAATAACGGCACCTGGCAGCCGGATAACGAAGGCATTGCCCACCAGATTCGTCTGTTG
CACCGTATTTGCCTAAGCCGCAGTGAGTGGCGTCATCATCATGCGTTAGTCTTTAAAGAC
CGCCCGGATTACCGCTTTGTGCTTGGCGAAAAAGGCGAAGTACTGGATATTGTGGCGGAA
CCGCGCCGTATCGCTAACCGTATCGTAGAAGAGTCGATGATCGCCGCTAACCTTTGCGCC
GCGCGCGTACTGCGCGATAAGCTCGGTTTCGGTATTTACAACGTACATACGGGCTTCGAT
CCCGCGAATGCCGACGCGCTGGCCGCGCTGCTGAAAACGCATGGTCTGCATGTCGATGCC
GAAGAGGTGCTGACGCTGGAAGGCTTCTGTAAACTGCGTCGCGAACTGGATGCGCAGCCC
TCCGGTTTCCTTGACAGCCGCATTCGTCGCTTCCAGTCTTTTGCGGAAATCAGCACCGAG
CCGGGGCCGCATTTCGGTCTTGGTCTTGAGGCTTACGCCACCTGGACCTCTCCTATCCGT
AAGTATGGCGATATGATTAACCATCGCCTGCTGAAAGCGGTCATCAAGGGGGAGGCTATT
GCGCGTCCGCAGGAAGATATCACCCAGCAAATGGCGGAACGCCGTCGCCTGAACCGCATG
GCGGAGCGCGATGTCGGCGACTGGTTATATGCACGCTTCCTTAACGATAAGGCGGGGACA
AATACCCGTTTCGCGGCAGAAATTATTGACGTCAGCCGCGGTGGTATGCGTGTGCGACTG
GTCGATAACGGCGCTATCGCCTTTATCCCTGCCCCGTTCCTGCACGCCGTTCGCGATGAA
CTGGTATGCAGCCAGGAAAACGGTACGGTACAGATTAAAGGTGAGACGGTTTATAAAGTG
ACGGATGTCATTGATGTCACTATTGCGGAAGTTCGCATGGAAACCCGCAGCATTATTGCC
CGACCGGCAGCGTAA
>EECEBICD_02073 Cyclic di-GMP phosphodiesterase PdeR
ATGATGAAACAGATTCAGGAACAAACGGCGCTAAATCCCCTTCACTCTCACTGGCGTCTG
GCTGATGACCGCAACGTGCTGTATCTCTCCCCGACAGGTGAAACGGATACAGAAAAAACG
GTCGAGCTATCGCCGGAGCAAGCCGGGCGTATCCGGGAAATAACGGCGATTACGTCCAGT
CTGCTGATAACGTTGTTGATAGAAAAACAGGTTACCGCTGTGCACCTGGTAGGCCGTAAG
ATTAATAACCGTGAATGGGCTGGCAGCCTGGCAACCTGGCCTGATACGCCTGCCGTCGCG
CCAACGCAGGCGCAGGAGCTCTCTTTTGCCGAGCAAATCGTATCAGAAGCCAACTCAGTC

ATTGCGATACTGGATAGTCGGGGAAAAATCTGTCGATTTAATCGGTTATGCGAAGAGTAC
ACGGGCTTAAAAGAGCGAGATGTAATTGGTCAAAGCGTCTTTAAATTATTTATGAGTCGC
CGGGAGGCGGTTGCCTCCCGGCACTACATTGAGAATTTTTTTCGCAATGGCAATGCCTAT
GAAATCGAACGCTGGATAAAGACCCGTAAAGGCCAACGCCTGTTCTTATTTCGTAATAAA
TTTGTCCATAACGGTAGCGGAAAAAACGAAATTTTCCTGATCTGCGCAGGCACGGATATC
ACCGAAGAAAGGCGGGCTCAGGAGCGTTTACGCATACTGGCGAATACAGACACCGTCACC
GGCCTGCCCAATCGCAATGCGATCCATGAATTTATTAATCATGCTATTGCCTCGGCTGGC
GAGTCGCAAGTGGGTATCGTTTATCTCGATCTGGATAACTTCAAGAAAATTAATGACGCC
TATGGGCATATGTTTGGCGATCAGCTCTTACAGGCCGTTTCGCTGGCGCTGTTGAGTTGT
CTGGAGGAAGATCAATTGCTGGCCAGACTGGGGGGAGATGAATTCATTGTCCTCGCTGCC
CATACGTCTCAGGCCGCGCTGGAAGCAGTAGCCTCACGAATCCTTACCCGTCTGCGGCAA
CCGTTTCGTATTGGACTCATTGAAGTTTACACGGGATGCGCCATTGGCATTTCGTTGGCC
CCCCGGCACGGACAGGACAGCGACAGTCTCATTCGCACTGCCGACACCGCAATGTATAAC
GCCAAAGAAGGCGGCCGCGGTCAGTTCTGCGTTTTTTCGCCGGAAATGAATCAGCGCGTA
TTTGACTATCACTGGCTGGATACAAACCTGCGAAAAGCGCTGGAAAACGACCAGTTACTT
ATTCACTATCAGCCCAAAATTACCTGGCGCGGCGAAGTACGTAGCCTGGAAGCGCTGGTA
CGCTGGCAATCGCCGGAACGCGGATTAATTCCGCCGCTGGAGTTCATCTCATACGCCGAA
GAGTCAGGACTCATCGTCCCGCTGGGCCGCTGGGTGATTCTGGATGTGGTTCGCCAGATT
GCGAGATGGCGCGATAAGGGGATTAACTTGCGCGTAGCCGTTAACTTTTCGGCGCGACAA
CTGGCCGACCAGACGCTTTTTACTGCGCTTAAACAGGCTTTGTACGATCTGAATTTTGAG
TATTGCCCGATCGATGTCGAGCTAACGGAAAGCTGCCTGATCGAAAACGACACGCTGGCG
CTATCGGTTATCCAGCAATTTAGTCAGCTCGGCGCCCAGATTCATCTGGATGATTTTGGC
ACCGGGTATTCTTCGCTTTCTCAACTCGCCCGGTTCCCTATTGATGCCGTTAAACTGGAT
CAGGCTTTCGTCAGAGATATCCATAAGCAACCGCTGTCGCAATCTCTGGTTCGGGCGATT
GTCGCGGTGGCTCAGGCGCTAAATTTACAAGTGATTGCCGAAGGCGTAGAAAATGCTAAA
GAAGATGCCTTCCTGACAAAAAACGGCGTCAATGAACGACAGGGCTTTTTATTCGCCAAA
CCTATGCCCGCCGTTTCTTTTGAACGCTGGTACAAACGTTATCAGACGAAAAAAATGCGT
TAA
>EECEBICD_02074 hypothetical protein
ATGTCTGATATCGAAGCACAACGAATTGCCGCACGCATTGATACCGTGCTGGATATCCTT
GTTGCTGGCGATTACCACTCGGCCATCAATAACCTTGAAATATTGAGAGCAGAGCTGTTA
GATCAGGTTAAAGACGGTATCTCGCCATCCCAGGCTCCCGGGTCGCCCTGGGAAATCTGA
>EECEBICD_02075 HTH-type transcriptional repressor CsqR
ATGGTGGTTGATAAAGGCCAGATGAGCGTTGCTGAACTGGCGAAAATTACCGGCGTATCT
GAAGTCACGATTCGTCAGGATCTGAATACCCTGGAAAAGCAGAGCTATTTACGCCGTGCG
CACGGTTTCGCCGTTTCGCTTGAGAGCGATGACGTAGAGACTCGCATGATGACGAACTAC
ACGCTAAAACGCCGACTGGCGGAGTTCGCCGCCTCGCTGGTCAGTCCAGGGGAATCCGTT
TTTATTGAAAATGGCAGCAGCAATGCGCTATTGGCCCGGACGCTGGCGGAACAAAAAGAT
GTCACGATCATCACCGTGAGCAGTTATATCGCGCACCTGCTCAAAGAGACCCCTTGCGAA
GTCATTCTCCTCGGTGGAATCTATCAGAAAAAAGTGAAAGCATGGTAG
>EECEBICD_02076 hypothetical protein
ATGGTAGGGCCGTTGACGCGACAGTTTATCCATCAAGTGCATTTCAGCAAAGCATTTATC
GGTATTGATGGCTGGCAGGCTGATACCGGTTTTACCGGACGCGACATGATGCGATCGGAT
GTGGTTAACGCTGTTCTGGAAAAAGGCTCAGAAGCGATTGTCCTCACCGACAGCTCCAAA
TTTGGCTGTGTGCATCCCTATCCCCTGGGCCCGCTCTCTCGCTTCCATCGTGTCATTACG
GATTCCAAAATTTCCGCCAGCGATCAAATGCAGTTAGAACATGCGGGGCTGCTAGTCAAT
GTTATTGGCTCATCCGTTTAA
>EECEBICD_02077 Osmotically-inducible lipoprotein B
ATGTTTATGACGAGCAAAAAAATGGCCGCTGCTGTGCTGGCAATCACCGTAGCAATGTCT
CTGAGCGCCTGCTCAAACTGGTCCAAGCGCGATCGCAACACGGCTATTGGCGCAGGTGCG
GGGGCGTTAGGCGGCGCAGTGTTAACGGACGGCAGTACATTAGGCACCCTGGGCGGCGCG
GCGGTCGGCGGCGTGATTGGTCACCAGGTTGGAAAATAA
>EECEBICD_02078 putative protein YciH
ATGAGCGATTCCAATAGTCGTCTGGTTTATTCAACACAGACGGGGCGCATTGAAGAACCT

AAAACGGCGCCGGTTCGTCCCAAAGGGGATGGTATCGTTCGCATTCAGCGTCAAACCAGT
GGCCGTAAGGGTAAAGGCGTATGTTTGATTACCGGTATCGAGATGAATGATGCGGAATTA
ACCAAACTGGCGGCTGAACTGAAGAAAAAATGCGGCTGTGGCGGGGCAGTAAAAGAAGGG
ATTATTGAAATTCAGGGTGATAAACGCGACTTGATAAAATCATTGCTGGAAGCTAAAGGA
ATGAAAGTAAAATTAGCTGGCGGCTAA
>EECEBICD_02079 Orotidine 5'-phosphate decarboxylase
ATGACGTTTACTGCTTCATCTTCTTCCTGCGCTATTACTGAATCGCCTGTCGTTGTGGCG
TTGGATTACCATGAACGCGATAAAGCCCTGGCGTTTGTCGACAAAATCGATCCTCGCGAT
TGTCGCTTAAAAGTGGGCAAAGAGATGTTCACGCTGTTTGGTCCACAACTCGTTCGGGAT
CTCCAGCAACGTGGGTTTGATGTCTTTCTTGATCTAAAATTTCATGATATTCCCAACACC
ACGGCCCGGGCAGTCGCCGCAGCGGCAGACCTGGGCGTCTGGATGGTAAACGTTCACGCG
TCTGGTGGGGCGAGAATGATGGCCGCAGCGCGCGACGCGCTGGCGCCATTCGGTAAAGAT
GCGCCGTTGCTCATTGCCGTGACCGTGTTGACCAGTATGGAAACCAGCGATCTACACGAT
CTGGGCGTGACGTTGTCACCGGCGGAACATGCGGAGCGGCTGGCGCGCCTGACGCAGCAA
TGCGGGCTGGATGGCGTGGTCTGTTCCGCTCAGGAAGCTGTCAGATTTAAACAGGCCTTT
GGCGCTGCATTTAAGCTGGTGACGCCGGGCATTCGGCCCGCAGGCAGCGAGGCAGGCGAC
CAGCGACGCATTATGACGCCCGAACAGGCATTATCCGCTGGCGTCGACTATATGGTGATT
GGCCGCCCGGTTACGCAATCGGTAGATCCGGCGCAAACGCTGAAGGATATTAACGCGTCA
CTGAAACGGGAGGCATAA
>EECEBICD_02080 Lipopolysaccharide assembly protein B
ATGTTGGAGTTGTTATTTCTGCTGTTGCCTGTAGCCGCTGCCTATGGGTGGTATATGGGT
CGCAGAAGTGCGCAACAAACAAAACAGGATGAAGCTAACCGCCTGTCGCGCGATTATGTC
GCAGGGGTTAACTTCCTGCTGAGTAACCAACAAGATAAAGCGGTGGATCTGTTCCTCGAT
ATGCTTAAAGAGGATACGGGCACCGTTGAGGCTCATCTCACTCTCGGTAATCTGTTTCGC
TCTCGCGGCGAAGTCGATCGCGCCATTCGTATTCATCAAACGCTCATGGAAAGCGCTTCA
TTGACCTATGAACAGCGTTTACTGGCTGTTCAGCAACTGGGGCGCGACTATATGGCCGCC
GGTTTATATGACCGCGCGGAAGATATGTTTAACCAACTTACCGACGAAACGGAATTTCGC
GTAGGCGCGTTACAGCAGCTCTTGCAAATCTATCAGCTAACCAGCGACTGGCAAAAGGCG
ATCGAAGTAGCAGAACGGCTGGTGAAGCTGGGCAAAGATAAACAACGTATCGAAATCGCC
CATTTTTACTGTGAGTTAGCGTTACAGCAGATGGGCAACGACGACATGGATCGCGCGATG
GCGTTGCTGAAAAAAGGTGCCGCCGCAGATAAAAATAGCGCCCGGGTGTCTATCATGATG
GGGCGCGTTTATATGGCGAGAGGGGATTACGCCAAAGCGGTCGAAAGCCTGCAACGTGTG
ATCGTTCAGGATAAAGAGCTGGTCAGCGAAACGCTGGAGATGCTGCAAACCTGTTATCAA
CAGCTCGGTAAAAATGCCGAGTGGGCGGAGTTTTTACGTCGCGCCGTTGAGGAGAATACC
GGTGCTGGCGCTGAGTTAATGCTTGCCGATATTCTGGAAGCGCGTGAAGGTAGTGACGCA
GCTCAAGTCTATATCACGCGTCAGCTACAGCGACATCCTACCATGCGGGTGTTCCATAAG
CTGATGGATTACCATCTCAACGAGGCGGAAGAAGGGCGAGCGAAAGAAAGCCTGATGGTA
CTGCGTGATATGGTTGGCGAGCAGGTGCGCAGTAAACCGCGGTATCGTTGTCAGAAATGC
GGTTTTACCGCCTATACCTTGTACTGGCACTGTCCGTCCTGCCGGGCATGGTCGACCATT
AAACCTATTCGCGGACTTGATGGGCAGTAG
>EECEBICD_02081 Lipopolysaccharide assembly protein A
GTGAAATATTTACTCATTTTCTTACTGGTATTGGCGATTTTTGTTATTTCGGTGACGCTG
GGCGCGCAGAATGATCAACAGGTGACGTTTAACTACCTGCTGGCGCAAGGGGAGTATCGT
ATCTCCACGTTGCTTGCTGTGTTGTTCGCCGTCGGTTTTGCGATTGGCTGGCTGATTTGC
GGCCTGTTCTGGCTGCGGGTCCGCGTTTCCCTTGCCCGTGCTGAACGTAAAATTAAACGA
CTGGAAAACAAGCTCTCGCCAGCAACCGACGTTGCGGTCCCTGCCGACTCGTCGGTTGTA
AAGGAATAA
>EECEBICD_02082 Phosphatidylglycerophosphatase B
ATGCTTTCAATTGCGAGGCGCACCGCGGCAGGCGCCGCCCTGTTGCTTATCATGCCGCTT
GCCGTATGGGTATCAGGCTGGCAGTGGCAGCCAGGACATCAGGTCTGGTGGCTAAAAACG
CTATTCTGGATAACGGAAACCGTGACCAAACCCTGGGGCGTTATCACGCATGTGATTCTT
TGTGGATGGTTCTTATGGTGCCTGCGATTTCGCCTGCGCGCCGCCATTATGCTGTTTGCG
ATCCTCGGCGGGGCGATTATCGTCGGGCAGGGTGTGAAGTCATGGGTAAAGGAGCGCGTA
CAGGAGCCGCGGCCATTTGTGGTCTGGCTTGAAAAAACGCACCATATTCCCGTCGATGAG

TTCTACACTTTAAAGCGTACAGAACGCGGACATTTAGTGAAAGAGCAGTTGGCCGGGCAA
CAGAACATCCCCGTTTTCCTGCGTCAGCACTGGCAAAAAGAGACGGGCTTTGCGTTTCCC
TCCGGACACACCATGTTTGCAGCGAGCTGGGCGCTTTTGGCCGTGGGTCTGTTATGGCCG
CGCAGACGGACTTTCACCATCGCGTTTTTGTTAGTCTGGGCGACCGGCGTAATGGGCAGC
CGTTTGCTTCTGGGAATGCACTGGCCGCGAGATTTAGTGGTCGCCACGCTCATCTCCTGG
CTATTAGTTACCCTGGCGACCTGGCTGGCGCAGCGAATTTGCGGGCCGCTTATGCCGCCT
CGCGAAGAAGCGCAAGAAATTGCTGAACGCGAGCAGGAAAGCTGA
>EECEBICD_02083 GTP cyclohydrolase-2
ATGCAGCTTAAACGTGTGGCAGAAGCCAAACTGCCAACCCCATTGGGTGACTTTCTGATG
GTGGGATTTGAAGAACTGGCAACCGGACACGACCATGCCGCGCTGGTTTTTGGCGATATT
TCAGGTAAAACGCCGGTCTTGGCTCGCGTTCATTCCGAATGTCTTACCGGGGACGCGCTT
TTTAGTCTGCGCTGTGATTGTGGTTTTCAACTGGAAGCCGCGCTGACGCATATTGCGGAA
GAAGGACGCGGTATTCTGATTTACCACCGTCAGGAAGGCCGCAATATCGGCCTGCTTAAT
AAAATTCGCGCTTATGCGTTACAGGATCAAGGCTATGACACGGTGGAGGCCAATCATCAG
CTCGGCTTTGCCGCCGATGAGCGTGATTTCACCCTGTGCGCGGATATGTTCAAATTACTG
GGCGTAGATGAAGTTCGCCTGCTGACCAATAATCCGAAGAAAGTCGAAATACTGACCGAG
GCGGGAATTAATATCGTCGAACGCGTTCCGCTCATTGTGGGTCGTAATCCAAATAATGAG
CATTATCTCGATACTAAAGCGGCCAAAATGGGCCACCTACTAAGCAAGTAA
>EECEBICD_02084 Aconitate hydratase A
ATGTCGTCAACCCTACGAGAAGCCAGTAAGGATACATTGCAGGCCAAAGATAAAACGTAT
CATTACTACAGTCTGCCGCTGGCTGCCAAATCCCTGGGCGATATCGCCCGTTTGCCCAAA
TCACTTAAAGTGTTACTGGAAAACCTGTTGCGCTGGCAGGACGGCGAATCTGTGACTGAT
GAAGATATTCAGGCGCTGGCCGGTTGGCTTAAAAATGCCCATGCCGATCGTGAAATTGCC
TGGCGGCCCGCCCGTGTCCTGATGCAGGACTTTACCGGCGTGCCTGCCGTTGTCGACCTG
GCGGCGATGCGTGAAGCCGTTAAACGCCTCGGCGGCGATACGTCGAAAGTGAACCCGTTA
TCGCCGGTTGATCTGGTTATTGACCACTCTGTGACGGTCGATCATTTCGGCGATGACGAT
GCGTTTGAAGAAAACGTGCGGCTGGAAATGGAACGTAACCATGAGCGTTATATGTTCCTG
AAATGGGGAAAGCAGGCATTCAGCCGTTTCAGCGTGGTGCCGCCCGGCACCGGCATTTGC
CATCAGGTTAACCTGGAATACCTGGGTAAAGCCGTCTGGAGCGAATTACAGGACGGGGAG
TGGATTGCTTACCCGGACTCGCTGGTGGGGACTGACTCCCATACGACCATGATTAATGGT
CTGGGCGTATTGGGGTGGGGCGTGGGCGGTATCGAAGCGGAAGCGGCGATGCTTGGTCAG
CCCGTGTCGATGCTTATCCCGGATGTTGTCGGCTTTAAGTTAACCGGTAAGCTTCGGGAG
GGGATCACCGCTACTGACCTGGTGCTAACCGTCACGCAAATGCTGCGTAAGCATGGCGTC
GTGGGTAAATTTGTTGAATTTTATGGTGATGGTCTGGATTCGCTGCCGTTGGCGGATCGC
GCGACCATCGCTAATATGTCGCCGGAATATGGCGCCACCTGTGGTTTCTTCCCTATTGAC
GCCATCACCCTGGAATATATGCGATTAAGCGGACGTAGCGACGATCTGGTCGAGCTGGTT
GAAGCCTACGCGAAGGCGCAGGGAATGTGGCGTAATCCCGGAGACGAACCGGTATTTACC
AGTACGCTGGAACTGGATATGGGCGATGTCGAGGCCAGCCTGGCCGGGCCGAAACGCCCG
CAGGATCGCGTGGCGTTAGGCGATGTGCCGAAAGCCTTTGCCGCCAGCGCCGAGCTGGAG
CTGAATACCGCGCAAAGAGATCGCCAGCCGGTTGACTATACGATGAACGGACAGCCATAT
CAGCTTCCGGATGGCGCTGTCGTCATTGCCGCCATCACCTCCTGCACGAATACCTCGAAT
CCCAGCGTGCTGATGGCGGCGGGATTACTGGCGAAAAAAGCAGTAACGCTGGGGTTGAAG
CGTCAACCGTGGGTCAAGGCTTCTCTGGCGCCGGGGTCAAAAGTGGTATCTGACTATCTG
GCGCAGGCCAAACTTACGCCTTATCTGGATGAGCTCGGTTTTAACCTGGTCGGTTATGGC
TGTACGACCTGTATCGGGAACTCTGGTCCGTTGCCGGAGCCTATTGAGACCGCGATTAAA
AAAGGCGATCTGACGGTAGGGGCCGTGCTTTCCGGTAACCGAAATTTTGAAGGGCGTATC
CATCCGCTGGTGAAAACGAACTGGCTGGCGTCGCCGCCGCTGGTGGTCGCGTATGCGTTG
GCCGGAAACATGAATATTAACCTCGCGACAGACCCGCTGGGGTACGATCGTAAAGGCGAT
CCGGTATACCTGAAGGATATCTGGCCTTCGGCGCAGGAAATTGCCCGCGCCGTTGAACTG
GTATCATCAGATATGTTCCGTAAAGAGTATGCGGAAGTGTTTGAGGGCACGGAAGAATGG
AAATCGATTCAGGTTGAATCGTCCGATACCTACGGCTGGCAGTCGGATTCAACCTATATT
CGCCTGTCGCCTTTCTTTGATGAAATGCAGGCCCAGCCTGCGCCAGTCAAAGATATCCAC
GGCGCGCGTATCCTGGCGATGCTGGGGGATTCGGTAACGACCGACCATATTTCTCCGGCC
GGCAGTATCAAGCCGGACAGTCCCGCCGGACGCTATCTGCAAAACCACGGCGTTGAGCGG

AAGGATTTTAACTCCTATGGATCACGGCGCGGCAACCATGAAGTGATGATGCGCGGTACG
TTCGCCAATATTCGTATTCGCAACGAAATGCTGCCCGGCGTCGAAGGTGGGATGACGCGG
CATTTGCCGGGTACGGAAGCGATGTCGATTTATGATGCTGCGATGCTCTACCAGCAGGAA
AAAACGCCGCTGGCGGTAATTGCCGGGAAAGAGTATGGGTCGGGATCGAGCCGTGACTGG
GCGGCAAAAGGTCCGCGGCTGTTAGGTATCCGCGTGGTGATCGCCGAGTCGTTCGAACGT
ATCCACCGCTCAAACCTGATTGGGATGGGGATCCTGCCGCTGGAGTTTCCACAGGGCGTA
ACGCGTAAAACGCTGGGACTGACCGGGGAAGAGGTGATTGATATCGCGGATCTGCAAAAC
CTGCGCCCTGGCGCGACCATTCCGGTGACGTTAACGAGATCGGACGGCAGCAAAGAAACG
GTGCCTTGCCGCTGTCGCATTGATACCGCCACCGAGCTGACTTACTACCAGAATGACGGC
ATATTGCACTATGTCATTAGAAATATGCTGAACTAA
>EECEBICD_02085 Protein YmiA
ATGCCTTCTGAAAACCAGGAACCGCGCCGCGACCCTGAGCTCAAACGTAAAGCATGGCTG
GCGGTTTTTGTGAGTTCTGCGTTGTTCTGGGTAGTGGTAGCGCTTGTTATCTGGCATTGG
TGGGGCTAA
>EECEBICD_02086 HTH-type transcriptional regulator CysB
ATGAAATTGCAGCAGCTTCGTTACATTGTTGAAGTGGTTAATCATAACCTTAACGTCTCC
TCTACCGCTGAGGGGTTGTATACTTCCCAGCCGGGGATCAGCAAGCAGGTTAGGATGCTG
GAAGATGAGCTTGGCATCCAGATTTTCGCCCGCAGTGGTAAGCATCTGACTCAGGTGACG
CCCGCAGGTCAGGAGATTATTCGTATTGCGCGTGAGGTGCTTTCCAAAGTCGATGCCATT
AAGTCGGTCGCCGGCGAGCACACCTGGCCGGATAAAGGCTCGCTCTATATTGCCACTACC
CATACTCAGGCGCGTTACGCATTGCCAGGCGTGATTAAAGGCTTCATCGAGCGTTACCCG
CGCGTTTCGCTGCATATGCACCAGGGGTCGCCAACGCAGATTGCGGAAGCGGTATCGAAA
GGGAACGCCGATTTCGCGATTGCCACGGAAGCGCTGCATTTATATGACGATCTGGTGATG
TTGCCATGCTATCACTGGAACCGTTCGATAGTGGTGACGCCGGATCATCCATTGGCGGCA
ACGTCTTCCGTAACGATCGAAGCGCTGGCGCAGTATCCGCTGGTGACGTATACCTTCGGC
TTTACCGGGCGTTCCGAACTGGATACCGCTTTTAATCGCGCAGGATTAACGCCGCGGATC
GTCTTTACTGCAACCGATGCGGATGTGATTAAAACGTATGTCAGGTTGGGACTGGGCGTT
GGCGTCATTGCCAGCATGGCGGTCGATCCGCTTGCCGATCCTGATTTAGTGCGGATTGAC
GCGCACGATATTTTTAGCCACAGCACCACAAAAATTGGTTTTCGCCGTAGTACTTTTTTA
CGCAGCTATATGTATGATTTTATCCAGCGTTTTGCGCCACATCTGACGCGTGATGTGGTG
GATACGGCGGTGGCGTTGCGTTCTAATGAAGAAATTGAAGCGATGTTTCAGGATATAAAG
CTGCCTGAAAAGTAA
>EECEBICD_02087 DNA topoisomerase 1
ATGGGTAAAGCTCTTGTCATCGTTGAGTCCCCGGCAAAAGCCAAAACGATCAACAAGTAT
CTGGGTAATGACTACGTGGTGAAATCCAGCGTGGGTCATATCCGTGATTTGCCGACCAGT
GGCTCAGCAGCTAAAAAGAGCGCCGACTCTACCTCCACCAAAACGGCTAAAAAGCCCAAA
AAGGATGAACGTGGCGCTCTCGTCAACCGTATGGGGGTTGACCCGTGGCACAACTGGGAT
GCGCACTATGAAGTGCTGCCCGGTAAAGAGAAGGTCGTCTCTGAACTGAAACAGTTGGCG
GAAAAAGCCGACCACATCTATCTCGCAACCGACCTTGACCGCGAAGGGGAAGCCATTGCA
TGGCACCTGCGGGAAGTGATCGGCGGCGATGACGCGCGCTACAGTCGCGTAGTCTTTAAC
GAAATAACCAAAAATGCCATTCGCCAGGCGTTTGAACAGCCGGGCGAGCTGAACATTAAC
AGGGTCAACGCCCAGCAAGCGCGCCGCTTTATGGATCGCGTGGTAGGCTATATGGTCTCG
CCGCTGCTGTGGAAAAAGATCGCCCGTGGTTTGTCGGCAGGTCGCGTTCAGTCTGTCGCG
GTCCGTCTGGTCGTTGAACGCGAGCGCGAGATTAAAGCGTTTGTTCCGGAAGAGTTCTGG
GAAATTGACGCTAATACGACGACGCCGTCTGGCGAGGCGCTACCGCTACAGGTCACGCAT
CAGAACGACAAGCCATTCCGGCCGGTAAATCGCGAGCAGACGCTTGCTGCGGTGAGCCTG
CTGGAAAAAGCCAGCTATAGCGTGCTGGAGCGAGAAGACAAGCCGACCAGCAGCAAGCCA
GGCGCGCCGTTTATCACCTCCACGCTACAACAGGCGGCCAGCACCCGGCTGGGCTTTGGC
GTGAAGAAAACCATGATGATGGCGCAGCGTCTGTATGAGGCGGGCTACATCACCTATATG
CGTACAGACTCCACCAACCTGAGTCAGGATGCGGTCAACATGGTGCGCGGCTATATTGGC
GATAATTTCGGCAAGAAATACCTGCCGGATAACCCGAACCAGTACGCCAGCAAAGAAAAC
TCTCAGGAAGCGCACGAAGCGATCCGTCCTTCCGACGTTGCGGTCATGGCGGAGTCGCTG
AAAGACATGGAGGCTGACGCGCAGAAGTTGTATCAGCTAATTTGGCGTCAGTTTGTCGCC
TGTCAGATGACGCCAGCACAATATGACTCCACTACGCTTACCGTTGGTGCAGGCGAGTTC

CGTCTGAAAGCGCGTGGGCGTATTTTGCGCTTTGACGGCTGGACAAAAGTGATGCCGGCG
CTGCGTAAAGGCGATGAAGATCGCACATTGCCTGCCGTGAATAAGGGCGATGCGCTGACG
CTACTTGAATTGACGCCTGCTCAGCACTTTACTAAACCGCCAGCCCGTTTCAGCGAGGCT
TCTCTGGTTAAAGAGCTTGAGAAGCGCGGTATTGGTCGTCCGTCTACCTATGCGTCGATT
ATCTCGACCATTCAGGATCGCGGCTATGTTCGCGTTGAAAACCGTCGCTTCTATGCGGAA
AAAATGGGTGAAATCGTCACCGATCGGCTGGAAGAAAACTTCCGCGAATTGATGAACTAC
GATTTTACTGCGCAGATGGAAGATAGTCTGGATCAGGTTGCGAATCACCAGGCCGAGTGG
AAAGCGGTACTGGATAACTTCTTCAGCGATTTTACTCAGCAGCTCGATAAAGCCGAGAAA
GATCCGGAAGAAGGGGGGATGCGTCCTAACCAGATGGTGCTGACCAGCATCGACTGTCCA
ACCTGTGGCCGTAAAATGGGAATTCGTACTGCCAGTACGGGCGTTTTCCTTGGATGTTCA
GGTTATGCGCTTTCGCCGAAAGAGCGTTGTAAAACGACCATTAACCTGGTGCCGGAAAAT
GAAGTTCTGAACGTGCTGGAAGGCGACGACGCCGAAACCAACGCCCTGCGCGCCAAGCGT
CGCTGCCAGAAGTGCGGTACGGCGATGGACAGCTATCTGATCGATCCGAAGCGTAAATTG
CATGTGTGTGGTAATAACCCGACCTGTGACGGCTACGAGATTGAAGAAGGCGAGTTCCGC
ATCAAAGGGTATGACGGCCCGATCGTTGAGTGTGAGAAATGTGGTTCCGAAATGCACCTT
AAAATGGGGCGTTTTGGTAAGTACATGGCCTGTACTAACGACGAATGTAAAAATACCCGT
AAGATCTTGCGTAACGGCGAAGTTGCGCCGCCGAAGGAAGATCCTGTTCCATTACCGGAA
CTGCCGTGTGAAAAATCGGACGCGTACTTTGTGCTGCGCGATGGCGCCGCAGGGATATTC
CTGGCGGCCAATACGTTCCCGAAATCACGCGAAACGCGCGCGCCATTAGTCGAGGAGCTT
TACCGGTTCCGCGATCGCCTGCCGGAAAAACTGCGGTATCTGGCGGATGCGCCGCAGCAG
GATCCGGAAGGTAATAAAACCGTTGTTCGCTTTAGCCGCAAAACAAAACAGCAGTACGTT
GCGGCGGAAAAAGACGGGAAGGCGACGGGATGGTCAGCGTTCTTCGTCGACGGCAAGTGG
GTTGAAGGTAAGAAATAA
>EECEBICD_02088 Protein YciN
ATGCATAAAGAGACGCAACCTATCGATCGCGAAACACTGTTGCTTGAAGCCAACAAAATT
ATTCGGGAACATGAAGACACGATGGCAGGTATTGTCGCCACCGGCGTAACGCAGAGAAAT
GGCGTGCTGGTTTTCAGCGGCGATTACTTTTTAGACGAGCAAGGGTTACCGACCCCTAAA
AGCACCGCGGTGTTTAACATGTTTAAACACCTGGCGCATGTGCTGTCTGAGAAGTATCAC
CTGGTTGATTAA
>EECEBICD_02089 putative protease SohB
GTGATAGATTTCAAAGGCAGTATGGACGCTCACGAAGTTAATGCGTTACGCGAAGAGGTC
ACGGCGGTGCTGGCAGTGGCGAAACCCGGCGATCGGGCGGTTGTGCGTCTGGAAAGCCCC
GGTGGCGTTGTGCACGGCTATGGCCTGGCGGCATCGCAATTGCAGCGCCTGCGCGATAAA
AATATTCCGCTGACCGTGACGGTGGATAAAGTCGCGGCAAGCGGAGGCTACATGATGGCC
TGCGTGGCGGAAAAAATTATCGCGGCGCCGTTCGCTATTGTGGGGTCAATTGGTGTTGTC
GCGCAAATCCCGAACTTTAACCGCTTTCTCAAAAGTAAAGACATTGATATTGAACTGCAT
ACCGCAGGGCAGTACAAACGTACCCTGACTTTGTTAGGCGAGAATACGGAAGAAGGGCGG
CAGAAGTTTCGTGAAGATCTCAACGAAACGCACCATCTGTTCAAAGAGTTTGTGCAGCGG
ATGCGTCCGGCTCTGGACATTGAACAGGTCGCCACGGGCGAACACTGGTACGGTCAGCAG
GCGCTGGAGAAGGGACTGGTTGATGAGATTAACACCAGCGATGAGGTTATCCTCGGCCTG
ATGGAAGGGCGCGAGGTGCTGAATGTGCGCTATATGCAGCGTAAAAAACTGATCGATCGT
GTTACCGGCAGCGCGGCGGAAAGCGCGGATCGGCTGCTGCTGCGCTGGTGGCAGCGTGGA
CAAAAGCCGTTGATGTAA
>EECEBICD_02090 hypothetical protein
TTGTTGTCTGAATATGGCTTATTTTTGGCAAAAATCCTCACCGTTGTGGTGGCCATTGCC
GTCATTGTGCTGCTGATCGTGAATGCTACGCAACGCAAACGTCAGCGCGGCGAGCTGCGC
GTGACCAATTTGAGCGAGCAGTATCAGGAGATGAAGGATGACCTTGCTGCGGCGTTGATG
GATGGCCATCAGCAAAAACTGTGGCATAAAGCGCAGAAAAAAAGCATAAGCAGGAGGCGA
AAGCCGCCAAAGCGAAAGCGAAGCTGGGGGACATTGCGACATCGGACAAACCGCGCGTAT
GGGTGA
>EECEBICD_02091 putative oxidoreductase YciK
ATGCATTACCAACCGAAACAAGATCTATTGCAAAACCGCATTATTCTGGTCACTGGCGCC
AGCGATGGCATTGGGCGGGAAGCCGCGCTTACGTATGCGCGTTACGGCGCAACGGTGATT
CTGCTGGGACGCAACGAAGAGAAACTGCGTCGGGTCGCGCAGCACATTGCTGACGAGCAA

CATGTTCAACCACAGTGGTTTACGCTCGATCTGCTGACCTGCACCGCTGAAGAGTGCCGG
CAAGTGGCCGACCGCATCGCCGCGCACTATCCCCGCCTGGATGGCGTCCTGCACAATGCC
GGGTTACTGGGGGAAATCGGTCCAATGAGCGAACAAGATCCGCAGATCTGGCAGGATGTC
ATGCAGGTAAACGTTAACGCCACCTTTATGCTCACCCAGGCGCTGTTACCGTTATTGCTG
AAGTCCGATGCCGGCTCGCTGGTCTTTACGTCATCAAGCGTTGGCCGTCAGGGACGCGCC
AACTGGGGCGCCTATGCCACCTCGAAGTTTGCTACTGAAGGAATGATGCAGGTGCTGGCG
GACGAATACCAGAATCGTTCTCTGCGTGTTAACTGCATTAACCCGGGCGGCACGCGCACC
AGTATGCGCGCCAGCGCCTTTCCGACGGAAGATCCACAGAAATTAAAAACGCCTGCCGAT
ATTATGCCGCTTTACCTGTGGCTTATGGGCGACGACAGTCGCCGTAAAACCGGTATGACC
TTTGACGCCCAACCGGGCCGTAAACCAGGAATCGCCCAATGA
>EECEBICD_02092 Corrinoid adenosyltransferase
ATGAGTGATGAACGTTATCAGCAGCGCCAGCAGAAGGTAAAAGATCGGGTTGACGCCCGT
GTCGCCCAGGCACAGGAAGAGCGCGGTATTATTATCGTGTTTACTGGCAATGGTAAGGGC
AAAACCACGGCGGCTTTCGGAACTGCCGCCCGCGCCGTAGGACACGGTAAAAACGTAGGC
GTGGTGCAATTTATTAAAGGCACCTGGCCCAACGGCGAGCGTAATCTGCTGGAACCGCAT
GGCGTCGAATTTCAGGTGATGGCAACGGGATTTACCTGGGAGACGCAAAATCGCGAGGCA
GACACCGCAGCATGTATGGCCGTTTGGCAGCATGGGAAACGGATGCTGGCCGATCCGCTG
CTTGATATGGTGGTCCTGGATGAGCTGACCTATATGGTGGCGTATGACTATTTGCCGCTG
GAAGAGGTCATAAGCGCGCTAAACGCGCGCCCTGGTCACCAGACGGTGATTATTACAGGT
CGCGGCTGTCACCGGGATATTCTTGATCTTGCGGATACCGTCAGCGAACTGCGTCCGGTT
AAACATGCTTTTGACGCGGGCGTAAAAGCCCAGATGGGAATTGATTATTAA
>EECEBICD_02093 Ribosomal large subunit pseudouridine synthase B
ATGAGCGAAAAGTTACAAAAAGTGCTGGCGCGCGCTGGCCACGGCTCTCGCCGTGAAATC
GAATCCATTATTGAAGCGGGACGTGTCAGCGTTGACGGTAAAATCGCCACGCTTGGCGAT
CGTGTTGAAGTCACTCCCGGTTTGAAAATCCGTATCGACGGTCACCTGATTTCGGTGAAA
GAGTCGGCGGAACAAATTTGCCGCGTTCTGGCCTATTACAAACCAGAAGGTGAACTGTGT
ACCCGCAATGACCCTGAAGGGCGGCCAACCGTTTTTGATCGCTTACCGAAATTGCGCGGC
GCGCGGTGGATTGCGGTAGGACGTCTGGATGTCAACACCTGCGGGCTATTGTTGTTTACC
ACCGACGGTGAACTGGCGAACCGCCTGATGCACCCTAGTCGTGAAGTTGAACGTGAATAT
GCCGTGCGCGTGTTCGGTCAGGTGGATGAATCGAAACTGCGTGATTTAAGCCGTGGCGTA
CAGTTGGAAGATGGTCCGGCGGCATTTAAGACCATTAAATTCAGCGGCGGCGAAGGCATT
AACCAGTGGTACAACGTGACGCTGACGGAAGGGCGCAACCGCGAAGTTCGCCGACTGTGG
GAAGCGGTAGGCGTTCAGGTCAGCCGTCTGATCCGCGTTCGTTACGGCGATATCCCGCTG
CCGAAAGGCCTGCCGCGCGGCGGCTGGACTGAGCTCGACCTCGCGCAAACGAACTATCTG
CGTGGTCTGGTAGAGCTCCCGCCGGAAACCTCGTCGAAGGTTGCCGTCGAAAAAGACCGC
CGTCGTATGAAAGCGAACCAGATCCGTCGGGCGGTAAAACGCCACAGCCAGATCGCTGGC
GGACGCCGCTCCGGTGGGCGTAATAACAACGGGTAA
>EECEBICD_02094 putative protein YciO
ATGAGCCAGTTTTTTTATATCCATCCGGATAACCCACAGCAACGGCTGATTAATCAGGCC
GTCGACATTGTGCGTAAAGGCGGTGTTATTGTTTATCCAACCGATTCCGGCTATGCCCTT
GGTTGTAAAATTGAAGACAAAGGCGCAATGGAGCGCATTTGCCGCATCCGTCATCTGCCG
GACGGCCACAACTTCACGCTGATGTGCCGCGATTTGTCCGAGTTGTCGACCTACTCCTAC
GTCGACAACGTGGCATTTCGTCTGATGAAAAACAATACGCCGGGTAATTACACCTTTATC
CTGAAGGGGACAAAAGAGGTGCCGCGCCGTCTGTTGCAGGAAAAGCGTAAAACTATCGGC
CTGCGCGTACCGTCAAACCCGATTGCGCTGGCGTTATTGGATACGCTGGGCGAACCGATG
CTCTCGACGTCGTTGATGCTGCCTGGCAGCGATTTTACCGAGTCGGACCCGGAGGAGATT
AAAGATCGGCTGGAGAAGCAGGTGGATCTGATCATCCACGGTGGTTACCTCGGTCAACAG
CCTACCACGGTAATCGATTTGACCAATGACTCCCCGGTGGTGCTGCGCGAAGGCGTCGGC
GACGTTACACCTTTCTTATAA
>EECEBICD_02095 5'-3' exoribonuclease
TTGAGCGACACGAATTACGCAGTGATTTACGATCTGCACAGTCATACCACGGCGTCCGAT
GGACTGTTGACGCCAGAAACATTAGTACACCGTGCGGTAGAGATGCGAGTCGGCACGCTG
GCGATTACCGATCATGACACCACAGCCGCGATTCCTGCGGCAAGAGAAGAAATTTCACGT
TCCGGGCTGGCCCTGAATCTTATTCCCGGCGTGGAGATTTCAACGGTCTGGGAGAATCAT

GAGATTCACATTGTGGGTCTGAATATTGATATTGCGCATCCGGCAATGCGCGATTTTCTG
GCGCAGCAGACACAGCGGCGTCAGGCGCGTGGTCGGCTCATTGCGGAGCGTCTGGAAAAA
GCCCATATTCCCGGCGCGTGGGAAGGGGCATTGAGACTGGCCAATGGCGGCGCGGTGACG
CGCGGCCACTTCGCCCGCTTTCTGGTGGAGTGCGGCAAAGCCGCGACCATGGCGGATGTT
TTTAAAAAATATCTCGCGCGTGGGAAAACCGGTTACGTTCCGCCGCAGTGGTGTACAATA
GAACAAGCTATTGATGTCATTCATCATTCTGGCGGTAAGGCGGTGTTGGCCCACCCGGGA
CGCTACGACCTTAGCGCTAAGTGGCTGAAAAGATTAGTGGCGCATTTTGCCGACCATCAC
GGCGACGCGATGGAAGTGGCGCAGTGCCAACAATCACCCAATGAACGCACCCAACTGGCG
ACGCTTGCGTGTCAGCATCATTTATGGGCATCGCTTGGATCTGATTTCCATCAGCCCTGC
CCGTGGATTGAGTTGGGGCGTAAACTCTGGCTGCCCGCTGGCGTTGAAGGCGTATGGCAG
GCATGGGAACAGCCGCAAATATCACAGTGA
>EECEBICD_02096 Anthranilate synthase component 1
ATGCAAACACCAAAACCCACGCTCGAACTGTTGACCTGCGATGCCGCCTATCGGGAAAAC
CCAACGGCGCTTTTTCACCAGGTCTGCGGCGATCGCCCGGCAACGCTGCTGCTGGAATCC
GCGGATATCGACAGTAAAGATGATTTAAAAAGCCTGCTGCTGGTAGATAGCGCGCTGCGC
ATTACCGCTTTAGGTGACACTGTCACCATTCAGGCGTTATCTGATAATGGCGCCTCGTTA
TTGCCGCTACTGGATACCGCCCTGCCCGCTGGCGTGGAGAACGAAGTCCTGCCTGCCGGT
CGCGTTCTACGCTTCCCGCCCGTCAGCCCATTATTAGATGAAGACGCCCGTTTATGCTCT
CTGTCGGTATTTGATGCGTTCCGTCTGTTACAGGGAGTGGTGAACATACCGACGCAAGAG
CGGGAGGCTATGTTTTTCGGCGGTCTGTTTGCCTACGACCTGGTCGCTGGCTTTGAAGCG
CTGCCACACCTTGAGGCTGGCAATAACTGCCCGGACTACTGCTTTTATTTAGCGGAAACG
CTGATGGTCATAGATCATCAGAAAAAAGCACCCGTATTCAGGCCAGTCTGTTCACCGCAA
GCGACCGGGAAAAACAGCGCCTGA
>EECEBICD_02097 Anthranilate synthase component 1
ATGCGCTGCGAATGCAATCAGAGCGATGACGCGTTCGGCGCGGTGGTACGCCAGTTGCAA
AAAGCCATCCGCGCGGGGGAGATATTTCAGGTGGTGCCGTCGCGCCGCTTTTCACTGCCC
TGCCCGTCGCCGTTGGCTGCCTACTACGTGCTGAAAAAGAGCAACCCCAGCCCGTATATG
TTCTTTATGCAGGATAATGATTTCACGCTTTTCGGCGCGTCGCCGGAAAGCTCGCTGAAA
TATGACGCCGCCAGTCGTCAGATTGAGATTTACCCCATCGCGGGTACCCGTCCACGCGGT
CGCCGCGCCGATGGTACGCTGGACAGAGATCTCGACAGCCGTATTGAGCTGGACATGCGT
ACCGACCATAAAGAGCTTTCCGAACATCTGATGCTGGTCGATCTGGCGCGCAATGACCTG
GCGCGCATCTGTACGCCGGGCAGTCGCTACGTTGCCGATCTGACCAAAGTTGACCGCTAT
TCGTACGTGATGCATCTGGTTTCCCGGGTAGTGGGCGAACTGCGTCACGATCTCGACGCG
CTGCACGCCTATCGCGCCTGCATGAACATGGGCACCCTGAGCGGCGCGCCGAAAGTACGC
GCCATGCAGTTGATTGCCGATGCGGAAGGACAGCGCCGCGGCAGCTATGGCGGCGCGGTC
GGTTACTTCACCGCCCACGGCGATCTGGACACCTGTATTGTTATCCGCTCCGCGCTGGTG
GAGAACGGTATCGCCACCGTACAGGCGGGCGCCGGAATCGTGCTGGACTCTGTTCCGCAG
TCTGAAGCCGATGAAACCCGTAATAAAGCGCGCGCCGTATTGCGTGCTATCGCCACCGCG
CATCATGCACAGGAGACCTTCTGA
>EECEBICD_02098 Bifunctional protein TrpGD
ATGGCTGATATTCTGCTGCTCGATAACATCGACTCGTTTACCTGGAACCTGGCAGATCAG
CTACGGACCAACGGTCATAACGTGGTGATTTACCGTAACCATATTCCGGCGCAGACGCTT
ATCGATCGCCTGGCAACAATGAAAAATCCGGTGCTAATGCTCTCCCCCGGTCCGGGCGTT
CCCAGCGAGGCAGGCTGTATGCCGGAGCTGCTGACCCGACTACGCGGCAAGTTACCGATC
ATCGGCATTTGTCTGGGGCATCAGGCGATTGTCGAAGCTTACGGCGGTTATGTCGGTCAG
GCGGGAGAAATCCTGCATGGCAAAGCCTCCAGCATTGAGCATGACGGTCAGGCGATGTTC
GCCGGGCTGGCGAATCCGCTACCGGTCGCGCGTTATCATTCGCTGGTCGGCAGTAATGTT
CCTGCCGGGCTAACCATTAACGCCCATTTCAACGGCATGGTGATGGCGGTACGTCATGAT
GCGGATCGCGTTTGCGGTTTTCAATTTCATCCTGAGTCCATCCTGACGACACAGGGTGCG
CGTCTACTGGAGCAAACATTAGCCTGGGCGCAGCAAAAGCTGGAACCGACCAACACCCTA
CAGCCAATTCTGGAGAAACTCTATCAGGCGCAAACTCTGACGCAACAAGAGAGCCATCAG
TTGTTTTCGGCGGTCGTCCGCGGCGAACTTAAACCTGAACAGCTCGCCGCCGCGCTGGTG
AGCATGAAAATTCGCGGCGAGCATCCCAATGAAATTGCCGGCGCCGCCACTGCGTTGCTG
GAAAATGCCGCCCCGTTCCCGCGCCCGGACTACCTGTTTGCGGATATCGTTGGTACCGGC

GGCGATGGCAGTAACAGTATCAATATCTCTACCGCCAGCGCCTTTGTCGCAGCGGCCTGT
GGACTGAAAGTGGCGAAACACGGCAACCGTAGCGTGTCCAGCAAATCGGGGTCATCCGAT
CTGCTGGCGGCGTTCGGTATTAATCTGGATATGAACGCCGATAAATCACGTCAGGCGTTA
GATGAACTGGGCGTCTGTTTCCTGTTCGCGCCGAAATATCACGCCGGATTCCGTCACGCG
ATGCCGGTTCGCCAACAGTTAAAAACGCGAACCCTGTTCAACGTACTCGGCCCGCTGATC
AACCCGGCGCATCCGCCGCTGGCATTGATTGGCGTCTATAGCCCGGAACTGGTGCTGCCG
ATTGCGGAAACCTTACGGGTACTGGGCTATCAGCGCGCGGCGGTGGTACATAGCGGCGGC
ATGGATGAGGTTTCGCTCCATGCGCCGACGATTGTCGCGGAGCTGCATGACGGCGAAATT
AAAAGCTATCAACTTACGGCGGAGGATTTTGGCCTGACGCCTTACCATCAGGATCAGTTG
GCTGGCGGCACGCCGGAAGAAAACCGTGACATTCTGACGCGGTTATTACAAGGTAAAGGC
GATGCCGCGCATGAGGCCGCCGTCGCCGCCAACGTGGCGATGCTGATGCGTCTGCATGGT
CAGGAAGATCTCAAAGCCAACGCGCAAACCGTGCTTGATGTTCTGCGCAACGGCACCGCA
TATGACAGAGTCACCGCACTGGCGGCAAGAGGGTAA
>EECEBICD_02099 Tryptophan biosynthesis protein TrpCF
ATGCAAACCGTTTTAGCGAAAATCGTCGCAGACAAGGCGATTTGGGTAGAAGCCCGCAAA
CAGCAACAGCCGCTGGCCAGCTTTCAAAATGAGATCCAGCCAAGTACACGCCATTTTTAT
GATGCGCTCCAGGGCGCGCGTACCGCCTTTATTCTGGAGTGTAAGAAAGCATCGCCATCA
AAAGGCGTGATTCGCGATGATTTCGATCCGGCGCGTATTGCCAATATTTATCAACATTAC
GCCTCGGCAATCTCGGTGCTCACCGACGAAAAATATTTTCAGGGTAGCTTCGATTTTCTG
CCGGTCGTTAGCCAAAGCGCACCGCAGCCGATTCTGTGTAAGGATTTTATTATCGATCCC
TATCAGATCTACCTTGCCCGTTACTATCAGGCCGATGCCTGTTTACTGATGCTCTCGGTT
CTGGATGACGAACAGTATCGCCAACTCTCCGCCGTCGCGCACAGTCTGAAAATGGGCGTG
CTCACGGAAGTCAGTAATGACGAAGAACGGGAGCGCGCGATAGCGTTAGGCGCAAAAGTG
GTAGGTATCAACAATCGCGATCTGCGCGATCTGTCGATTGATTTGAATCGCACCCGCCAG
CTGGCGCCAAAACTGGGCCACGGCGTGACTGTCATCAGCGAGTCCGGGATTAACACCTAT
GGTCAGGTACGCGAACTGAGCCACTTCGCCAACGGTTTTTTAATTGGCTCGGCGTTAATG
GCGCATGACGATCTTAACGCCGCCGTCCGTCGCGTGCTGCTTGGCGAAAATAAAGTCTGC
GGCCTGACCCGCGCCCAGGACGCTAAAGCGGCCTGTGACGCTGGCGCAATATATGGCGGG
TTGATTTTTGTGCCCTCATCTCCACGCGCGGTGAGCGTTGAGCAGGCGCGAGAAGTGATA
AGCGGCGCGCCATTGCAGTATGTCGGCGTTTTCCAGAACGCTGATATCGCCGATGTTTGC
CAGAAAGCCGCCGTCCTGTCGCTTTCTGCCGTACAGCTACATGGCAGCGAAGACCAGGCG
TATGTCAACGCGCTGCGCGAGGCGTTGCCGCGCAATGTGCAAATCTGGAAGGCGCTGAGC
GTTAGCAATGCCCTTCCCGCACGCGATTATCACCATGTCGATAAATACATTTTCGACAAT
GGGCAAGGCGGCAGCGGGCAGCGCTTCGACTGGTCACTGCTACAGGGGCAACCGCTGGAT
GATGTGTTACTGGCGGGCGGGCTGGCGGCCGATAACTGCGTCCAGGCGGCGCAAGTCGGC
TGTGCCGGTCTCGATTTTAATTCAGGTGTGGAGTCACAGCCGGGCATCAAAGATGCTCGT
CTTCTGGCCTCGGTTTTTCAGACACTGCGCGCATATTAA
>EECEBICD_02100 Tryptophan synthase beta chain
ATGACAACACTTCTCAACCCCTACTTTGGTGAATTCGGCGGCATGTATGTGCCGCAGATC
CTGATGCCTGCGCTGAACCAGCTTGAAGAGGCCTTCGTCAGCGCGCAAAAAGATCCTGAA
TTTCAGGCGCAATTCGCCGATCTGCTAAAAAACTACGCGGGACGCCCCACCGCGCTGACG
AAATGCCAGAACATTACCGCCGGTACGCGTACCACGTTGTATTTAAAGCGCGAAGATTTA
CTGCACGGCGGCGCACACAAAACCAATCAGGTACTGGGTCAGGCGCTGCTGGCCAAACGG
ATGGGTAAAAGCGAGATTATCGCTGAAACCGGCGCCGGTCAGCACGGCGTCGCCTCTGCG
CTCGCCAGCGCCCTGCTGGGTCTGAAATGCCGTATCTATATGGGTGCCAAAGACGTTGAG
CGCCAGTCGCCGAACGTCTTCCGTATGCGTCTGATGGGCGCTGAGGTTATCCCGGTTCAT
AGCGGCTCCGCTACGCTAAAAGATGCCTGTAACGAGGCGCTGCGCGACTGGTCCGGTAGT
TACGAAACCGCGCACTATATGCTCGGCACGGCGGCAGGACCGCATCCCTATCCCACCATC
GTTCGCGAGTTCCAACGCATGATTGGCGAAGAGACGAAAGCGCAAATCCTCGACAAAGAG
GGCCGTCTGCCAGATGCCGTTATCGCTTGCGTCGGCGGCGGCTCAAACGCTATCGGGATG
TTTGCGGATTTTATTAATGATACCAGCGTCGGGCTAATAGGCGTTGAACCTGGCGGTCAC
GGTATTGAAACCGGCGAGCATGGCGCGCCGCTTAAACATGGTCGCGTTGGCATCTATTTC
GGGATGAAAGCGCCGATGATGCAAACAGCGGACGGCAAATTGAAGAGTCCTATTCCATT
TCCGCCGGGCTCGATTTCCCATCCGTTGGGCCGCAACATGCGTACCTGAACAGCATCGGA

CGCGCGGATTATGTCTCCATTACCGATGATGAGGCGCTGGAAGCCTTCAAAACGTTGTGC
CGCCATGAGGGAATTATCCCGGCGCTGGAATCCTCCCACGCGTTGGCGCACGCTCTGAAA
ATGATGCGCGAGCAGCCGGAAAAAGAGCAACTGCTGGTGGTCAATCTCTCTGGCCGCGGA
GATAAAGACATCTTTACCGTACACGATATCCTGAAAGCGCGAGGGGAAATCTGA
>EECEBICD_02101 Tryptophan synthase alpha chain
ATGGAACGCTACGAAAATTTATTTGCACAGCTCAACGATCGCCGGGAAGGCGCTTTTGTC
CCCTTCGTGACCCTGGGCGACCCTGGCATTGAACAGTCACTGAAAATTATCGACACACTG
ATTGACGCTGGCGCCGACGCTCTGGAACTGGGGGTTCCCTTCTCCGATCCGCTGGCCGAT
GGCCCTACCATCCAGAATGCGAACTTACGCGCCTTCGCCGCTGGCGTCACGCCGGCTCAG
TGTTTTGAAATGCTGGCGCTGATTCGTGAAAAACACCCGACCATTCCGATTGGCCTGCTA
ATGTACGCGAATCTGGTGTTCAATAACGGCATAGATGCGTTCTATGCCCGTTGTGAACAG
GTTGGCGTAGATTCCGTGCTGGTCGCAGATGTCCCGGTTGAAGAGTCGGCCCCCTTCCGC
CAGGCGGCGTTACGGCATAATATCGCGCCGATCTTCATCTGCCCGCCAAATGCGGATGAC
GATCTTCTGCGCCAGGTCGCATCTTACGGCCGCGGTTACACCTACCTGCTTTCGCGTTCG
GGCGTCACCGGCGCGGAAAACCGTGGCGCATTGCCGTTGCATCATCTCATTGAGAAGCTT
AAAGAGTACCATGCCGCGCCTGCGTTACAGGGCTTCGGTATCTCCTCGCCGGAACAGGTG
TCTGCGGCCGTGCGTGCCGGGGCGGCTGGCGCTATCTCCGGCTCAGCCATTGTCAAGATT
ATCGAGAAAAACCTCGCGTCTCCCGAACAGATGTTGGCGGAGCTCAGGTCCTTTGTCTCA
GCCATGAAAGCCGCCAGCCGCGCATAA
>EECEBICD_02102 hypothetical protein
ATGGCCGAACATCGTGGTGGTTCAGGAAATTTCGCCGAGAATCGTGAAAAAGCATCTGAA
GCGGGCCGCAAAGGCGGTCAGCATAGCGGCGGGAATTTCAAAAACGATCCGCAACGAGCT
TCTGAAGCCGGTAAAAAAGGTGGTCAGAATAGTCACGGCGGACGTAAATCCGATAATTCC
TGA
>EECEBICD_02103 Protein YciF
ATGAATATCAAAACCGTTGAAGACCTTTTTATCCATCTACTTTCAGATACCTATAGTGCA
GAAAAACAATTAACCAAGGCTCTTTCTAAACTAGCCAGAGCCACGTCCAATGAAAAATTA
AGCCAGGCCTTTCAATCTCATCTTGAAGAAACCCAGGGTCAGATTGAACGTATTGATCAG
ATCGTCGAATCTGAATCTGGCATTAAACTGAAAAGAATGAAATGCGTCGCTATGGAAGGG
CTGATCGAAGAAGCCAATGAAGTCATCGAAAGTACGGAGAAAAACGAAGTACGCGATGCA
GCGCTTATCGCCGCGGCGCAAAAAGTCGAGCATTACGAAATCGCCAGCTACGGCACGCTA
GCCACCCTGGCCGAGCAGCTCGGCTATAGCAAGGCATTAAAACTGCTCAAAGAAACCCTC
GACGAGGAAAAACAAACTGATTTAAAACTTACCGATTTAGCAGTCAGCAATGTTAATAAA
AGTGCTGAACGCAAATCGAAATAA
>EECEBICD_02104 Protein YciE
ATGAATTATACTGAACATTATCATGACTGGCTTCGTGACGCCCATGCCATGGAAAAACAG
GCAGAATCGATGCTTGAATCTATGGCCAGCCGTATTGAAAATTATCCTGATATAAAAGCC
AGAATTGAACAACATATTAGTGAAACCAAACATCAAATTACCATGCTCGAAGAAGTATTG
GACCGTAATGGCATTTCTCGTTCGGTGTTGAAAGACTCCATGAGTAAAATGGCAGCAATG
GGGCAATCTATCGGTGGCATGTTCCCTTCCGATGAAATTGTCAAAGGCTCAATTAGCGGT
TATGTTTTCGAGCAGTTCGAAATTGCTTGTTATACCTCCCTGCTGGCGGCGGCAAAAAAA
GCTGGCGACACTGCCTCAATTCCGACGATTGAAGCCATTCTGAAAGAAGAAATGCAAATG
GCTGACTGGCTGATCAAACATATTCCGCAGACAACGGAACAATTTTTACTGCGATCTGAA
GCAGATGGCGTTGAAGCCAAAAAATAA
>EECEBICD_02105 Manganese catalase
ATGTTTCGACACGTAAAACAACTTCAATATACTGTGCGAGTGAGCGAACCTAATCCTGGA
TTAGCGAACCTGCTGCTGGAACAGTTTGGCGGCCCGCAGGGCGAACTGGCGGCCGCCTGC
CGCTACTTCACGCAAGGTTTAAGCGATGATGATGCCGGTCGTAGAGAAATGTTAATGGAC
ATCGCCACCGAGGAGCTGAGTCATCTGGAAATTATCGGCTCACTGGTAGGAATGCTTAAT
AAAGGCGCTAAAGGCGAGCTGGCGGAAGGTACCGAAAACGAAGCGGAACTTTATCGCTCG
CTGACGCAGAATGGTAATGACAGCCATATCACCTCTCTGCTGTATGGCGGCGGCCCGGCG
TTGACGAACTCCGGCGGCGTACCGTGGACCGCGGCTTATATTGATACTATCGGCGAAGTC
ACAGCCGATCTACGCTCAAACATCGCGGCAGAGGCGCGAGCGAAGATCATTTATGAACGC
CTGATTAATCTGACCGACGATCCCGGCGTAAAAGACACGCTCAGTTTCTTAATGACCCGT

GAAGTCGCGCACCAGCTCTCTTTTGAAAAGGCGCTGTATTCGATTCGTAACAACTTCCCG
CCGGGTAAATTACCGCCGGTAGAACAGTATACCGACGTGTACTACAACATGTCGCAAGGC
GACGATCCGCGAGGTAGCTGGAATAGCGATGAGAACTTCAACTATGTCGCAGAACCGATG
CCTGCTGTTGATGGCGGCGATGGCCTGGCGACAGTGAAATTGCCGCGCGAGCAGATGGCG
CTGCTCAAAGCGATGGCAGAAAGAACGAAATCGGACCCCACCGTTGATCCTCTCACTGGC
GCAGAACTAGGCTGCGGCGAACCGAAAGAAGACAAGTGA
>EECEBICD_02106 Outer membrane protein W
ATGAAAAAATTTACAGTGGCGGCACTGGCGTTAACAACTCTTCTCTCAGGCAGCGCTTTC
GCGCACGAAGCCGGAGAATTCTTTATGCGTGCAGGTCCGGCAACCGTCAGACCTACAGAA
GGGGCGGGTGGTACGCTGGGGCATTTAAACGGGTTTGATGTGAGTAATAACACGCAACTC
GGCCTGACATTCACTTACATGGCGACGGACAATATCGGGGTTGAATTGCTGGCGGCCACG
CCGTTCCGCCATAAGGTGGGTACGGGCGCGACGGGAGATATCGCCACGGTTCACCTGTTA
CCGCCAACGTTAATGGCGCAGTGGTACTTCGGCGATTCCAGCAGCAAAGTGCGTCCTTAT
GTCGGCGTGGGGGTGAACTACACCACCTTCTTCGATAACGACTTTAACGATAATGGTAAA
AATGCCGGGCTGTCCGATCTGAGCTTTAAGGATTCCTGGGGCGCGGCAGGGCAAGTTGGG
GTAGATTACCTGATTAACCGCGACTGGTTAATTGGCGCCTCTGTCTGGTACATGGATATT
GATACTACCGCCAATTATAAAATGGGCGGCGTTCAGCATCATGACAGCGTGCGTCTGGAC
CCATGGGTATTTATGTTCTCGGCAGGCTATCGTTTCTAA
>EECEBICD_02107 hypothetical protein
ATGAGCGTTCTTAACCCATGCATGACATGCGGTGCCTGTTGTGCCTATTTTCGTGTCTCC
TTTTATTGGGCTGAAGGCGACGATGCCAGCGGACGCGTTCCGGCGTCATTAACCGAGCCT
GTTACCCCTTTTTTACGTTGTATGGCCGGTACCAACCAAAAACAGCCGCACTGTAAAGCG
CTAATTGGCACGCCGGGTGAAAACGTCAGTTGCGCTATTTATGAAAACCGCCCTTCAACC
TGTCGCGAATTTTCGATATCGGGCGAAGGAGGCGAAGTGAATGAGGCGTGTAATCGCGCC
CGCGCGCGATACGGATTACCGCCGCTTTACAAAGATATGCTTTTCCATACAACTGCTGAT
GCTGCCACTGTAGAGTTATCCAGGGTACAATTGCCCGCAAATTAA
>EECEBICD_02108 hypothetical protein
ATGTCTATCACGGCGAAGTCCGTTTACCGTGACGCCGGAAATTTTTTCCGAAATCAATTT
ATTACTATCTTGCTGGTTTCGTTGCTGTGCGCATTTATCACGGTGGTCTTAGGTCATGCC
TTTTCACCCAGCGATGCGCAAATCGCGCAACTCAGTGAAGGAGAACATCTGGCAGGCAGC
GCTGGCCTGTTTGAGCTGGTACAAAATATGACGCCGGAGCAGCAGCAGATCCTGTTGCGC
GCCTCTGCCGCCTCGACGTTTTCCGGGCTTATCGGCAACGCTATTCTGGCCGGCGGTATC
ATCCTGATGATTCAGTTAGTTTCAGCGGGACATCGGGTTAGCGCGCTGCGCGCTATCGGC
GCCAGCGCCCCTGCATTGCCTAAACTGTTTATTCTCATTTTTCTGACTACCCTGTTAGTG
CAAATCGGTATAATGCTGATTGTCGTTCCGGGGATTATTATGGCCATTGTGCTGGCGCTT
GCGCCAGTGATGTTGGTTGAAGAGAAAATGGGCGTGTTTGCCGCAATGCGCAGCAGTATG
CGTTTAGCCTGGGCTAACATGAGACTGGTCGCGCCTGCGGTGATAGGCTGGTTACTGGCT
AAAACATTGCTATTGCTGTTCGCGCCCAGCTTCGCTGTACTGACCCCAAATGTAGGCGCT
GTACTGGCGAATACGTTGAGCAATCTGATTTCCGCAGTATTGCTTATCTACTTATTCCGC
CTGTATATGTTGATTCGCCAATAA
>EECEBICD_02109 putative intracellular septation protein A
ATGAAGCAGTTTCTGGATTTTTTACCGCTGGTTGTCTTTTTTGCGTTTTATAAACTTTAT
GACATCTATGCGGCGACCTCCGCACTGATTGTCGCGACGGCAATCGTGCTGATTTATAGC
TGGGTGCGCTATCGGAAAATAGAGAAGATGGCGCTGATTACCTTTGTTCTGGTTGCCGTC
TTCGGCGGTCTGACGCTCTTCTTCCATAACGATGAATTTATTAAATGGAAGGTGACGGTT
ATTTATGCGCTGTTCGCCGGCGCGCTGTTAATAAGCCAGTGGGTGATGAAAAAACCGTTA
ATCCAACGTATGTTAGGTAAAGAACTGGCGTTGCCGCAGCAGGTCTGGTCAAAGCTGAAC
CTGGCGTGGGCGCTGTTTTTTATTGCGTGCGGTCTGGCTAACATTTATATCGCCTTCTGG
TTGCCGCAAAATATCTGGGTCAACTTTAAGGTCTTTGGACTCACGGCGCTGACGCTCATT
TTTACCCTGCTGAGCGGCGTCTATATCTACCGGCATCTGCCACAGGAAGATAAATCATAA
>EECEBICD_02110 putative acyl-CoA thioester hydrolase
ATGACAACAATGGATAATACTCCTCAGGGCGAGCTGGTTTTACGTACATTGGCCATGCCT
GCCGATACCAATGCGAACGGCGATATTTTTGGCGGCTGGCTGATGTCGCAAATGGATATT
GGCGGCGCGATACTGGCCAAAGAGATCGCGCACGGTCGGGTTGTAACCGTACGCGTGGAA

GGAATGACATTTCTGCGCCCCGTCGCGGTTGGCGATGTCGTATGCTGCTACGCGCGCTGC
GTTAAACGCGGTACGACGTCTATTAGCATCAATATTGAAGTCTGGGTGAAAAAAGTCGCG
TCAGAACCGATTGGACAGCGCTACAAGGCCACCGAGGCGCTGTTTATTTATGTTGCCGTC
GATCCGGACGGTAAACCTCGCCCGCTCCCGGTTCAGGGTTAA
>EECEBICD_02111 Protein TonB
ATGACCCTTGATTTACCTCGTCGCTTTCCCTGGCCGACACTCCTTTCCGTAGGCATTCAT
GGTGCTGTAGTGGCAGGTTTGCTCTATACCTCGGTACATCAGGTTATTGAATTGCCTGCG
CCAGCGCAGCCTATCACGGTGACAATGGTTTCACCCGCTGACCTGGAGCCGCCCCAGGCG
GTTCAGCCGCCGCCGGAGCCAGTGGTTGAACCCGAACCGGAGCCAGAGCCGATCCCTGAA
CCGCCTAAAGAAGCGCCCGTGGTGATTGAGAAACCCAAACCTAAGCCAAAACCCAAGCCA
AAACCGAAACCGGAACCGGTGAAAAAGGTTGAAGAGCAGCCGAAGCGCGAAGTCAAACCA
GCCGCGCCTCGTCCTGCCTCACCGTTTGAAAATAGCGCCCCGGCGCGGCCAACAAGCAGC
ACCGCCTCGGCGACCAGCAAGCCGGCGGTGAGCGTACCCACTGGACCTCGCGCTCTGAGC
CGTAACCAGCCGCAGTATCCGGCGCGAGCACAGGCGTTACGTATTGAAGGCCGCGTAAAG
GTTAAGTTTGATGTGACCTCTGCTGGGCGAGTGGAAAATGTACAGATTCTGTCCGCGCAG
CCAGCCAATATGTTTGAGCGTGAAGTTAAGAATGCGATGCGTAAATGGCGCTATGAAGCA
GGCAAGCCGGGTTCCGGGCTGGTGGTCAATATTATCTTCCGTCTGAACGGTACGGCGCAG
ATTGAGTAA
>EECEBICD_02112 Protein YciI
GTGCTGTATGTTATTTACGCTCAGGATAATGCCAACTCGCTTGAAAAACGCCTCTCGGTA
CGGCCCGCGCATCTGGCGCGCCTGCAACTTCTGCATGATGAAGGGCGATTATTAACTGCC
GGGCCGATGCCCGCAGTGGACAGTAACGATCCTGGGGTAGCCGGATTTACCGGGTCAACG
GTGATTGCTGAATTTGAATCTTTAGAAGCAGCGCAATCCTGGGCTGATGCGGATCCCTAC
GTCGCAGCGGGCGTCTATGCGCAGGTTTCGGTCAAACCGTATAAGAAGATCTTTTGA
>EECEBICD_02113 hypothetical protein
ATGAAGCGTAGTAGAACGGAAGTGGGGCGCTGGCGAATGCTACGTCAGGCGAGCCGCCGT
AAAGCGCGTTGGCTTGAAGGGCAATCACGCCGTAATATGCGTATCCATACTATCAGGAAA
TGCATCTTTAATCGTCAGCGTAATTCATTACTCTTTGCGATTCACGGTGTGTGA
>EECEBICD_02114 Cardiolipin synthase A
ATGACAACCTTCTACACCGTGGTGAGTTGGCTGGTCATTTTGGGATACTGGGTTCTCATT
GCCGGCGTCACATTACGTATTCTAATGAAACGGCGCGCAGTTCCCTCCGCAATGGCCTGG
TTGTTAATCATCTATATCCTTCCGCTGGTCGGGATCATCGCCTATTTGTCCGTGGGCGAA
TTGCATTTAGGCAAACGTCGCGCCGAGCGTGCCCGCGCCATGTGGCCATCTACTGCCAAA
TGGTTGAATGACCTCAAAGCCTGCAAACACATTTTCGCGCAAGAAAACAGTAGCGTCGCA
TCGTCCTTGTTTAAGCTGTGCGAACGACGTCAGGGGATTGCGGGCGTCAAAGGCAACCAG
TTGCAATTACTTACCGACTCCGATGATGTCATGCAGGCGCTTATTCGCGATATCCAACTG
GCGCGGCATAATATCGAAATGGTGTTTTACATCTGGCAGCCAGGCGGCATGGCGGATCAG
GTCGCTGAATCGCTAATGGCCGCCGCCCGCCGCGGCATCCATTGCCGGCTTATGCTGGAT
TCCGCGGGTAGCGTCGCTTTCTTCCGCAGCCCCTGGGCGGCCATGATGCGTAACGCTGGC
ATTGAAGTCGTAGAGGCGCTGAAGGTGAATCTGATGCGCGTCTTTTTACGCCGTATGGAT
CTTCGCCAGCACCGTAAAATGGTGATGATTGACAATTACATCGCCTATACCGGCAGTATG
AATATGGTGGACCCGCGTTTTTTCAAACAGGATGCCGGCGTTGGACAATGGGTCGATTTG
ATGGCGCGGATGGAAGGCCCCGTGGCGACAGCAATGGGGATTGTTTACTCCTGCGATTGG
GAAATCGAGACGGGTAAACGCATTCTGCCTCCCCCGCCGGATGTGAATATTATGCCGTTT
GAGCAGGCCAGCGGGCATACCATTCATACCATTGCTTCCGGTCCTGGTTTTCCGGAAGAT
CTCATTCACCAGGCACTATTAACCGCCACCTATGCTGCGCGCGAATATTTAATTATGACC
ACGCCATATTTCGTCCCCAGCGACGATCTGCTGCACGCCATTTGTACCGCTGCGCAGCGC
GGCGTGGATGTCAGTATTATCCTGCCCCGTAAAAATGATTCACTGCTCGTCGGCTGGGCC
AGTCGCGCCTTTTTCAGCGAGTTACTGGCGGCGGGCGTGAAAATCTATCAGTTCGAAGGC
GGGCTGTTGCATACTAAAAGCGTGCTGGTCGATGGCGAACTGAGCCTGGTCGGTACCGTG
AATCTCGACATGCGTAGCCTGTGGCTTAATTTCGAGATTACGCTGGTCATTGATGATACG
GGTTTCGGCGCCGATCTGGCTGCGGTACAGGACGATTATATTTCGCGCTCCCGTCTCTTA
GATGCGCGTTTGTGGGTAAAACGTCCGTTATGGCAGCGTATTACCGAGCGATTGTTTTAC
TTCTTTAGTCCATTGCTGTAA

>EECEBICD_02115 hypothetical protein
ATGGAAATGGATCTGAACAATCGCCTGACTGAAGACGAAACGCTTGAGCAGGCTTACGAT
ATTTTTCTTGAGCTGGCGGCGGATAATCTTGATCCGGCTGATATCATTCTGTTCAATTTG
CAATTCGAGGAGCGCGGCGGCGCGGAGTTGTTTGATCCCGCTGAAGACTGGCAAGAGCAC
GTCGATTTTGACCTGAATCCAGACTTTTTTGCCGAGGTAGTGATTGGCCTAGCGGATACC
GAAGACGGCGAAATTAACGACATTTTCGCCCGCGTTCTGCTGTGCCGGGAAAAGGACCAC
AAGCTGTGCCATATTCTCTGGCGCGAATAA
>EECEBICD_02116 hypothetical protein
GTGTCACTTTCTTTGTCGTCGGTTCGCCGTCGTCTTTATCATAATCTGTTCGATTTGACT
ACGCGCTCAGGTCGCCGTTTTGAAGGGTTATGCGCGCTGTTTGCGCTGCTTAGCGTACTG
GTCATTTTTGTTGAATCAGGCGTGGGAACGGAATATCACCTGACATTTGATGAATGGCAT
ATTTTTGTCTGGCTGGAGCTCTGCGTTACCCTGATATTTACCGGTGAATACTTGCTCAGA
CTGTTTAGCTGGCCCGAACCGGCAAAATACGTTTTTAGTTTCTGGGGATTTATTGATCTG
GTGACGATTTTGCCGCTGTACGTCATGTGGCTGTGGCCGGAAATTAGCCTGAACTATATG
TTTGCCTGGCGTGCTATGCGAGCGATTCGCGTGCTGCGTATTCTTAAACTGCTACGCTTT
ATGCCATCCCTGCGCGTATTCTGGAGCGCCATTATTAGCGCGCGTCATCAGCTTATTCTG
TTCTATTCATTTATTGCGATCGTGATGATTATTTTCGGTGCCCTGATGTATCTTATTGAA
GGACCGAAATATGGCTTCACGACGCTTAATGCCTCGGTGTATTGGGCGATAGTGACAGTC
ACAACCGTGGGGTATGGTGATATTACGCCGCATACGCCGCTGGGACGTATTGTCGCGTCG
GTGCTTATTTTGATTGGTTATTCGGTGATTGCGATTCCTACAGGACTGATTACCACGCAC
ATGAGCAGCGCGTTTCAAAAACGTCACTGGCAGCGAAAATGTCCGCAGTGCCAGCAAAGC
CAGCATGAACATAGCGCGCAATATTGTAACCGATGCGGCAGTAAGCTACCGGATTAA
>EECEBICD_02117 Vitamin B12 import ATP-binding protein BtuD
ATGAATGCTGTGATTGAACAACGTAAAGTTCTCCTCGAAATCGCCGATCTGAAAGTACAT
TTTGATATCAAAGAAGGGAAGCAGTGGTTCTGGCAACCGCCGAAAACTCTGAAAGCGGTG
GACGGCGTTACGCTACGCCTGTATGAAGGCGAAACGCTTGGCGTGGTGGGTGAATCCGGC
TGCGGCAAGTCGACGTTTGCGCGCGCCATTATTGGTCTGGTGAAGGCGACAGACGGTAAA
GTCGCCTGGCTGGGAAAAGATCTGTTGGGCATGAAAGCGGATGAGTGGCGCGAAGTGCGC
AGCGATATCCAGATGATTTTCCAGGACCCGCTGGCGTCACTCAACCCGCGAATGACGATT
GGCGAAATTATCGCGGAACCGCTGCGCACTTATCATCCTAAACTTTCGCGCCAGGACGTG
CGTGACCGCGTGAAAGCCATGATGCTGAAAGTGGGGCTGCTGCCGAATCTCATCAACCGT
TATCCTCATGAATTTTCCGGGGGGCAGTGCCAGCGCATCGGTATCGCCCGGGCGTTGATC
CTGGAACCTAAGCTCATTATTTGCGATGAGCCGGTGTCGGCGCTGGACGTTTCTATTCAG
GCGCAGGTGGTGAATTTGCTCCAGCAGTTACAGCGAGAGATGGGGCTGTCGTTGATTTTT
ATTGCGCACGACCTGGCGGTCGTAAAGCACATATCCGATCGTGTCCTGGTGATGTATCTC
GGTCATGCAGTAGAATTGGGCACCTATGATGAGGTGTACCATAATCCACTGCATCCTTAT
ACCAAAGCGCTGATGTCGGCGGTACCGATTCCCGATCCCGATCTGGAGCGAAATAAGAAG
ATACAATTGCTGGAGGGCGAATTACCTTCGCCCATTAACCCGCCTTCCGGCTGCGTGTTC
CGTACACGCTGTCCGCTAGCCGGGCCAGAATGCGCGCAAACGCGACCGGTGCTGGAAGGA
AGTTTTCGCCATGCCGTTTCCTGCCTTAAAGTAGACCCGTTATAA
>EECEBICD_02118 Oligopeptide transport ATP-binding protein OppD
ATGAGCTTATCAGAAACCGCAACTCAGGCGCCGCAACCGGCGAATGTACTGCTGGAGGTA
AACGATCTTCGTGTTACGTTCGCTACGCCTGATGGCGATGTGACGGCGGTCAACGATCTG
AACTTTACGCTTCGCGCTGGAGAAACGTTAGGTATTGTTGGCGAGTCTGGATCGGGGAAA
TCGCAAACGGCGTTCGCATTAATGGGACTGCTGGCGACGAATGGTCGAATCGGCGGTTCT
GCGACCTTCAACGGACGTGAAATTCTTAATCTACCTGAGCGCGAACTGAATACGCTGCGC
GCAGAACAGATTTCGATGATTTTTCAGGACCCGATGACCTCGCTAAATCCTTATATGCGC
GTTGGGGAGCAGTTAATGGAAGTCCTGATGCTGCATAAAGGGATGAGCAAAGCGGAAGCT
TTTGAAGAGTCCGTCAGAATGCTGGATGCCGTAAAAATGCCGGAAGCGCGCAAACGGATG
AAAATGTACCCGCACGAATTTTCCGGCGGAATGCGTCAACGTGTGATGATTGCGATGGCG
CTGTTGTGCAGGCCGAAACTGCTTATTGCCGATGAGCCTACCACCGCGCTTGATGTTACC
GTGCAGGCGCAGATTATGACCTTGCTTAATGAGCTAAAGCGCGAATTTAATACCGCCATT
ATCATGATTACCCACGATCTGGGCGTGGTCGCGGGTATTTGCGATAAGGTCCTGGTAATG
TATGCCGGGCGTACCATGGAATACGGTAAGGCGCGCGATGTCTTTTATCAGCCCGTTCAT

CCGTATTCGATAGGCTTGCTTAATGCGGTGCCGCGTCTGGATAGCGAGGGGGCGGAAATG
TTGACGATTCCGGGCAATCCTCCCAATTTATTGCGTTTGCCTAAAGGTTGTCCGTTCCAG
CCGCGTTGTCCTCATGCGATGGAGATCTGCAATAATGCGCCGCCACTTGAAGCGTTTAGT
CCTGGTCGCTTGCGCGCCTGTTTTAAACCGGTGGAGGAACTGCTATGA
>EECEBICD_02119 Oligopeptide transport system permease protein OppC
ATGATGTTAAGTAAGAAAAACAGCGAGACGCTGGAAAATTTCAGTGAAAAGCTGGAGGTG
GAAGGTCGCAGCCTTTGGCAAGATGCCCGTCGGCGCTTTATGCATAACCGTGCGGCGGTG
GCCAGTCTTATTGTCCTGTTTCTGATAGCCCTGTTCGTGACAGTGGCGCCTATGCTGTCG
CAATTTACCTATTTCGATACCGACTGGGGCATGATGTCCAGTGCGCCGGATATGGCGTCC
GGTCACTATTTCGGCACTGACTCTTCCGGGCGCGATCTGTTGGTGCGCGTTGCCATCGGC
GGGCGTATCTCATTAATGGTCGGGATTGCCGCCGCGCTGGTGGCGGTCATTGTCGGAACG
CTGTATGGCTCGCTGTCCGGCTATCTGGGCGGCAAAATTGATTCTGTGATGATGCGTTTG
TTGGAGATCCTCAACTCGTTCCCGTTTATGTTCTTTGTGATTTTGCTGGTGACGTTTTTT
GGGCAGAACATTCTGTTGATTTTCGTCGCCATCGGCATGGTCTCCTGGCTTGATATGGCG
CGTATCGTGCGTGGCCAAACCCTAAGTCTTAAACGTAAAGAATTCATCGAAGCCGCGCAG
GTGGGTGGTGTGTCGACTGCCAGCATTGTGATCCGCCATATTGTGCCTAACGTCCTGGGC
GTAGTGGTAGTGTATGCATCGTTGCTGGTGCCCAGCATGATTCTGTTTGAGTCTTTCCTC
AGTTTCCTGGGGCTTGGGACGCAGGAACCGTTAAGCAGTTGGGGAGCGTTATTGAGCGAT
GGCGCAAATTCAATGGAAGTCTCACCGTGGCTGCTGCTGTTTCCGGCTGGCTTTCTCGTG
GTGACGTTATTCTGTTTTAACTTTATTGGCGATGGCCTGCGTGATGCCCTCGACCCGAAA
GATCGTTAA
>EECEBICD_02120 Oligopeptide transport system permease protein OppB
ATGTTGAAATTTATTTTACGCCGCTGTCTGGAAGCGATTCCGACGCTGTTTATTCTTATT
ACCATCTCTTTCTTTATGATGCGCCTCGCGCCGGGTAGCCCTTTTACTGGCGAGCGCGCA
TTGCCGCCGGAAGTGCTGGCCAATATTGAAGCAAAATATCATCTGAACGATCCCATCATG
ACGCAGTACTTCAGCTACCTGAAGCAGCTGGCGCATGGCGATTTTGGACCATCGTTTAAA
TACAAAGATTACACGGTCAACGATCTGGTTGCCGCCAGCTTCCCCGTTTCGGCGAAACTT
GGCGCTGCCGCTTTTTTGCTGGCGGTCATTATTGGTGTCAGTGCTGGCGTGATTGCCGCG
CTAAAGCAAAATACGCGATGGGATTATACGGTGATGGGATTTGCCATGACCGGCGTCGTT
ATCCCCAGTTTTGTGGTAGCGCCGCTACTGGTGATGGTCTTTGCAATAACCTTGCAATGG
CTGCCTGGCGGCGGCTGGAACGGCGGGGCGTTAAAATTTATGATCCTGCCGATGGTCGCG
CTTTCACTGGCCTATATTGCCAGCATCGCTCGTATTACGCGCGGCTCAATGATTGAAGTT
CTGCATTCGAACTTTATTCGTACCGCGCGGGCAAAAGGATTACCGATGCGCAGAATCATC
TTCCGCCATGCGTTAAAACCTGCGCTGCTGCCGGTTTTATCCTATATGGGGCCAGCCTTT
GTCGGCATTATTACCGGCTCAATGGTGATTGAAACTATTTATGGCTTACCGGGCATTGGG
CAGCTTTTCGTTAATGGCGCGCTCAACCGCGATTACTCGCTGGTGTTAAGTTTGACCATT
CTGGTCGGGGCGCTGACCATATTATTTAACGCCATTGTCGATGTGCTCTATGCCGTTATC
GACCCGAAAATTCGCTATTGA
>EECEBICD_02121 Periplasmic oligopeptide-binding protein
GTGGGAATACTCACTGCGCTCATCGCCGCCAGCGCCGCGACTGCGGCAGACGTGCCCGCC
GGTGTTCAGTTAGCCGACAAGCAAACGTTGGTACGTAATAATGGATCAGAAGTGCAGTCT
CTCGACCCGCATAAAATTGAAGGCGTGCCGGAATCGAACGTTAGCCGTGATCTCTTTGAA
GGCTTATTAATTAGTGATGTCGAGGGGCATCCTTCACCGGGCGTCGCGGAAAAATGGGAG
AATAAAGATTTTAAAGTCTGGACATTCCATCTACGTGAAAATGCGAAATGGTCAGATGGT
ACGCCCGTCACTGCCCATGATTTCGTTTATAGCTGGCAACGCCTGGCCGATCCTAATACG
GCATCGCCGTACGCCAGCTATCTGCAATACGGTCATATCGCTAATATCGATGACATTATC
GCCGGTAAAAAACCCGCCACCGATCTGGGGGTAAAAGCGCTTGATGATCACACCTTTGAA
GTGACCTTAAGTGAGCCGGTGCCTTATTTCTACAAATTGTTGGTCCACCCTTCAGTGTCT
CCTGTACCGAAATCAGCGGTCGAAAAATTTGGCGATAAGTGGACACAACCGGCCAATATT
GTCACTAACGGCGCTTATAAGTTAAAAAACTGGGTGGTGAACGAACGTATCGTGCTGGAG
CGTAACCCGCAATACTGGGATAACGACAAAACCGTCATCAATCAGGTAACTTATTTGCCT
ATCTCTTCGGAGGTGACGGATGTCAACCGCTACCGCAGCGGCGAGATCGATATGACTTAT
AACAATATGCCGATCGAATTATTCCAGAAACTGAAAAAAGAGATCCCGAATGAAGTCCGC
GTAGACCCTTATTTGTGTACCTATTATTACGAAATTAATAATCAAAAAGCGCCGTTTAAC

GATGTCCGCGTGCGTACCGCGCTGAAACTGGCGTTAGATCGCGACATTATTGTTAATAAG
GTTAAAAATCAGGGCGATTTACCCGCTTATAGCTACACGCCGCCGTATACCGATGGCGCA
AAACTGGTAGAGCCTGAGTGGTTCAAATGGTCCCAGCAAAAGCGTAATGAAGAAGCGAAG
AAATTGTTGGCTGAAGCCGGCTTTACGGCGGATAAGCCCTTAACATTCGACCTGCTGTAT
AACACCTCCGATCTGCATAAAAAACTGGCCATTGCCGTGGCGTCTATCTGGAAGAAAAAT
CTGGGCGTCAATGTGAATCTGGAGAATCAGGAATGGAAAACCTTCCTGGATACCCGTCAT
CAGGGCACCTTTGATGTTGCCAGAGCCGGGTGGTGCGCAGATTATAATGAACCGACCTCA
TTCCTGAATACTATGTTGTCCGACAGTTCAAATAACACCGCGCATTATAATAGCCCGGCG
TTTGATAAGCTGATTGCTGACACGCTGAAAGTGGCGGACGATACGCAGCGCAGCGAACTC
TACGCTAAAGCGGAGCAGCAGTTAGATAAAGACTCGGCTATTGTGCCGGTGTATTACTAC
GTTAACGCGCGCCTGGTGAAACCTTGGGTGGGCGGGTATACCGGTAAAGACCCGCTGGAT
AATATTTATGTGAAAAACTTATATATTATCAAACATTAA
>EECEBICD_02122 hypothetical protein
GTGATTCAACCGCTTTTTGATTTTCCTGTTTACTTTAAGTTCTTTATCGGGCTCTTTGCG
CTGGTTAACCCGGTGGGGATTATCCCCGTTTTCATCAGCATGACCAGCTACCAGACGGCC
ACGGCGCGTAATAAAACTAACCTCACCGCGAATCTGTCGGTTGCGATTATCTTGTGGAGT
TCCTTATTCCTCGGCGATGGCATCCTGCAGCTGTTTGGTATTTCTATTGACTCGTTCAGA
ATTGCGGGCGGTATCCTGGTTGTCACCATTGCAATGTCGATGATCAGCGGGAAGCTGGGG
GAAGATAAACAAAACAAGCAAGAAAAGTCAGAGACGGCCATACGCGAGAGCATTGGCGTT
GTGCCGTTAGCGCTGCCGTTAATGGCGGGGCCAGGGGCGATCAGCTCCACTATCGTCTGG
GGGACGCGTTATCACAGCATCGCGCATTTGCTTGGTTTTTCAGTTGCCATTGCGCTTTTT
GCGCTTTGCTGCTGGGGCGTGTTCCGTATGGCGCCGTGGCTGGTTCGTTTGCTGGGGCAA
ACCGGTATCAACGTTATTACCCGAATCATGGGGTTGTTGTTAATGGCGCTCGGCATCGAA
TTTATTGTCACAGGAATAAAAGGCATTTTCCCCGGCTTGCTCAACTAA
>EECEBICD_02123 hypothetical protein
ATGGCTGTTACTAATGTCGCTGAACTTAACGCACTCGTAGAGCGTGTAAAAAAGCCCAGC
GTGAATATGCCAGTTTCACTCAAGAACAGGTCGACAAAATCTTCCGCGCCGCCGCTCTGG
CAGCCGCTGATGCCCGCATTCCGCTGGCCAAAATGGCCGTCGCCGAATCCGGTATGGGCA
TCGTGGAAGACAAAGTGA
>EECEBICD_02124 Aldehyde-alcohol dehydrogenase
ATGGCCGTCGCCGAATCCGGTATGGGCATCGTGGAAGACAAAGTGATTAAAAACCACTTC
GCTTCTGAATATATTTACAATGCTTATAAAGATGAAAAAACCTGCGGTGTGCTGTCTGAA
GACGACACCTTCGGGACCATCACCATTGCCGAACCTATCGGCATTATTTGCGGTATCGTT
CCAACCACTAACCCGACCTCTACCGCGATCTTCAAATCGCTGATTAGCCTGAAGACCCGT
AACGCCATCATCTTTTCTCCGCATCCGCGTGCTAAAGAAGCAACCAACAAAGCGGCAGAC
ATCGTTCTGCAAGCGGCTATCGCTGCCGGCGCGCCGAAAGATCTGATTGGCTGGATCGAT
CAACCTTCCGTAGAACTGTCTAACGCGTTGATGCACCACCCGGATATTAACCTGATCCTC
GCCACTGGCGGTCCAGGCATGGTTAAAGCTGCATACAGCTCCGGTAAACCGGCTATCGGC
GTAGGCGCAGGCAACACTCCGGTTGTCATTGATGAAACTGCGGATATCAAACGCGCTGTG
GCGTCTGTTCTGATGTCTAAAACCTTTGATAACGGCGTAATCTGTGCTTCTGAACAGTCT
GTTGTCGTTGTTGATTCCGTCTATGATGCCGTTCGCGAACGTTTCGCCAGCCACGGCGGC
TACATGCTGCAGGGCCAGGAGCTGAAAGCGGTTCAAAACGTTATCCTGAAAAATGGCGCT
CTGAACGCCGCTATCGTCGGTCAGCCAGCCTACAAAATTGCTGAACTGGCAGGCTTCTCC
GTACCAGAAACCACCAAGATTCTGATCGGTGAAGTTACGGTCGTTGATGAAAGCGAACCG
TTCGCACACGAAAAACTGTCTCCGACTCTGGCGATGTACCGTGCGAAAGATTTCGAAGAA
GCGGTAGAAAAAGCAGAAAAACTGGTCGCTATGGGCGGTATCGGTCACACCTCCTGCCTG
TACACTGACCAGGATAACCAGCCAGAACGCGTTGCTTACTTCGGTCAGATGATGAAAACC
GCGCGTATCCTGATCAACACCCCGGCCTCTCAGGGTGGTATCGGTGACCTGTACAACTTC
AAACTCGCACCTTCCCTGACGTTGGGTTGTGGTTCCTGGGGTGGTAACTCCATCTCTGAA
AACGTTGGTCCGAAACACCTGATCAACAAGAAAACCGTTGCTAAGCGAGCTGAAAACATG
TTGTGGCACAAACTTCCGAAATCTATCTACTTCCGCCGTGGCTCTCTGCCCATCGCGCTG
GATGAAGTGATTACTGATGGCCACAAACGTGCGCTCATCGTGACTGACCGCTTCCTGTTC
AACAACGGCTATGCAGACCAGATCACCTCTGTGCTGAAAGCGGCTGGCGTTGAAACCGAA
GTCTTCTTCGAAGTTGAAGCCGACCCGACTCTGAGCGTTGTGCGCAAAGGCGCTGAGCTG

GCGAACTCCTTCAAACCAGACGTGATCATCGCGCTGGGCGGCGGTTCCCCGATGGACGCC
GCGAAAATCATGTGGGTCATGTACGAACATCCGGAAACTCACTTCGAAGAGCTGGCGCTG
CGCTTTATGGACATCCGTAAACGTATCTACAAGTTCCCGAAAATGGGCGTGAAAGCGAAA
ATGATCGCCGTCACCACCACTTCCGGTACCGGTTCTGAAGTCACACCGTTTGCGGTTGTG
ACCGACGATGCAACCGGTCAGAAATATCCGCTGGCTGACTACGCCCTGACCCCGGATATG
GCGATTGTCGACGCCAACCTGGTGATGGATATGCCGAAGTCCCTGTGTGCGTTCGGTGGT
CTGGATGCGGTAACTCACGCCCTGGAAGCTTACGTTTCCGTACTGGCTTCTGAGTTCTCT
GACGGTCAGGCTCTTCAGGCTCTGAAACTGCTGAAAGAAAACCTGCCGGCGTCTTACCAC
GAAGGGTCTAAAAACCCGGTTGCGCGTGAACGTGTTCACAGTGCAGCGACTATCGCCGGT
ATCGCGTTTGCCAACGCCTTCCTCGGTGTATGTCACTCCATGGCGCACAAACTGGGCTCT
CAGTTCCACATTCCGCACGGTCTGGCGAACGCCCTGCTGATTTGTAACGTTATCCGCTAC
AACGCGAATGACAACCCGACCAAGCAGACCGCTTTCAGCCAGTACGATCGTCCGCAGGCA
CGCCGTCGTTACGCTGAAATTGCTGACCACCTGGGCCTGAGCGCGCCGGGCGACCGTACC
GCCGCTAAGATTGAAAAACTGCTGGCATGGCTGGAAAGCATTAAAGCTGAGCTGGGTATT
CCTAAGTCTATCCGTGAAGCAGGCGTGCAGGAAGCTGACTTCCTGGCACACGTTGACAAA
CTGTCTGAAGATGCCTTCGATGACCAGTGCACCGGCGCTAACCCGCGTTATCCACTGATC
TCCGAACTGAAACAGATTCTGCTGGATACCTACTACGGTCGTGATTTCACCGAAGGTGAA
GTTGCAGCGAAGAAAGACGTCGTTGCCGCACCGAAAGCAGAGAAAAAAGCGAAAAAATCC
GCTTAA
>EECEBICD_02125 Thymidine kinase
ATGGCACAGCTATATTTCTACTATTCGGCAATGAATGCGGGTAAGTCGACTGCACTGCTG
CAATCTTCATACAATTATCAGGAACGCGGGATGCGTACCGTCGTATATACGGCGGAAATC
GACGATCGTTTCGGCGCAGGCAAAGTCAGCTCTCGTATCGGTTTGTCGTCGCCGGCAAAA
TTGTTTAACCAAAATACATCCTTGTTTGAAGAGATTCGTGCCGAGAGCGCGCGGCAGACA
ATACATTGCGTGTTAGTCGACGAAAGCCAGTTTTTGACTCGCCAGCAGGTTTATCAATTA
TCCGAGGTTGTCGACAAATTAGATATTCCAGTACTTTGCTATGGGCTACGAACAGACTTT
CGTGGGGAGCTCTTTGTCGGCAGCCAATACTTGCTGGCATGGTCAGATAAGCTGGTGGAA
TTAAAAACTATCTGTTTCTGCGGTCGTAAAGCCAGTATGGTTCTGCGCCTTGATCAGGAC
GGCAGGCCTTATAACGAAGGCGAACAGGTGGTTATTGGCGGGAATGAACGCTACGTTTCA
GTTTGCCGTAAGCATTATAAGGACGCGCTGGAGGAGGACTCACTGACGGCGATTCAGGAG
CGTCATCGGCACATATGA
>EECEBICD_02126 DNA-binding protein H-NS
ATGAGCGAAGCACTTAAAATTCTGAACAACATCCGTACTCTTCGTGCGCAGGCAAGAGAA
TGTACTCTGGAAACGCTTGAAGAAATGCTGGAAAAATTAGAAGTTGTCGTTAATGAGCGT
CGTGAAGAAGAAAGCGCTGCTGCTGCTGAAGTGGAAGAACGCACTCGTAAACTGCAACAG
TATCGTGAAATGTTAATTGCCGACGGCATTGACCCGAATGAACTGCTGAATAGCATGGCT
GCCGCTAAATCCGGTACCAAAGCTAAACGCGCAGCTCGTCCGGCTAAATATAGCTATGTT
GACGAAAACGGTGAAACTAAAACCTGGACTGGCCAGGGTCGTACACCGGCTGTAATCAAA
AAAGCAATGGAAGAACAAGGTAAGCAACTGGAAGATTTCCTGATCAAGGAATAA
>EECEBICD_02127 UTP--glucose-1-phosphate uridylyltransferase
ATGGCTGCCCTTAATTCGAAAGTCAAAAAAGCCGTTATCCCGGTCGCGGGATTGGGAACC
AGGATGCTGCCAGCGACCAAAGCTATCCCGAAAGAGATGCTGCCGCTGGTTGATAAGCCA
TTAATTCAGTACGTCGTGAACGAATGTATCGCTGCTGGCATTACTGAAATCGTGCTTGTT
ACGCACTCGTCTAAAAACTCTATTGAAAACCACTTTGATACCAGTTTTGAGCTGGAAGCG
ATGCTGGAAAAACGCGTTAAGCGTCAGCTTCTGGAGGAGGTTCAGTCTATTTGCCCTCCG
CATGTCACTATTATGCAGGTACGTCAAGGGCTGGCAAAAGGCCTGGGCCATGCCGTATTG
TGCGCGCATCCCGTTGTCGGAAACGAACCTGTCGCTGTTATTCTGCCAGACGTTATTCTT
GACGAATATGAGTCCGACCTGTCTCAGGATAACCTGGCTGAAATGATCCGCCGTTTCGAT
GAAACCGGCAATAGCCAGATTATGGTTGAGCCAGTAGAAGATGTGACTGCATACGGCGTG
GTCGATTGTAAAGGCGTTGAGCTGGCACCGGGCGAAAGTGTGCCGATGGTTGGCGTGGTT
GAAAAACCAAAAGCGGATGTCGCGCCGTCTAACCTTGCGATTGTCGGGCGTTATGTGTTG
AGCGCGGATATCTGGGCGTTGCTGGCGAAAACCCCTCCGGGCGCCGGGGATGAAATTCAG
TTGACCGATGCTATCGATATGCTGATCGAAAAAGAAACGGTTGAAGCCTACCACATGAAG
GGTAAAAGCCATGACTGTGGTAATAAGTTAGGATATATGCAGGCATTCGTTGAATATGGC

ATCCGTCATAATTCGCTGGGTGCTGAATTTAAAGCCTGGCTTGAAGAAGAAATGGGTATT
AAGAAGTAA
>EECEBICD_02128 Regulator of RpoS
ATGATATGTGATATCGCTATGCCGAGAATGAACGGTCTCAAACTGGTAGAAAACTTACGT
AATCGCGGAGACCAGACGCCTATTCTGGTTATTTCCGCAACAGAAAATATGGCGGATATC
GCCAAAGCGTTACGCCTTGGGGTTGAAGATGTCTTGCTGAAGCCGGTAAAAGATCTCAAC
CGCTTGCGGGAAACCGTATTCGCTTGCTTGTACCCTAATATGTTTAACTCGCGGGTTGAA
GAAGAAGAACGCCTGTTTCGTGACTGGGATGCAATGGTGAGTAATCCGACCGCCGCAGCG
CAATTGTTGCAGGAATTGCAGCCCCCGGTACAGCAGGTGATTTCTCATTGCCGCATTAAT
TACCGGCAGCTCGTCTCCGCCGATCAGCCTGGACTGGTGCTGGATATTGCGCCGCTATCT
GATAACGAACTGGCATTTTATTGCCTGGATGTCACCCGGGCGGGCGATAACGGCGTGTTA
GCCGCATTGTTGCTGCGGGCGCTATTTAACGGCTTATTGCAGGATCAGCTCGGCCAACAA
AAGCACCGTTTACCGGAGTTAGGCGCGCTATTAAAACAGGTAAATCATCTGCTCAGACAA
GCCAATTTACCTGGGCAATTCCCGCTATTTGTCGGCTACTATCACAGTGAGCTCAAAAAT
TTGATTCTCGTCTCCGCCGGATTGAACGCCACCTTAAATACGGGCGCTCATCAGGTTCAA
ATTAGCAGCGGCGTGCCGCTAGGAACATTGGGTAACGCCTACCTTAACCAATTAAGCCAG
CGTTGCGACTCGTGGCAGTGTCAAATATGGGGCGCAGGAGGGCGTCTGCGATTGATGTTG
TCTGCGGAATGA
>EECEBICD_02129 Regulator of RpoS
ATGACGCAGCCATTGGTCGGAAAACAGATTCTTATTGTTGAAGACGAGCCTGTATTTCGC
TCGCTTCTGGATTCGTGGTTTTCCTCTTTGGGAGCGACAACGGCATTGGCAGGCGACGGG
GTAGATGCCCTGGAGTTGATGGGGCGTTTTACCCTGATCTCATGA
>EECEBICD_02130 putative NTE family protein
ATGAAAATAGGGCTGGCGCTGGGATCTGGCGCAGCGAGAGGATGGTCTCATATTGGGGTC
ATCAAAGCCCTCAAGCAAACAGGCATTGATATTGATATCGTTGCAGGATGCTCAATAGGT
TCGCTGGTCGGCGCCGCGTATGCCTGTAATAAATTATCGGCGCTTGAACAGTGGGTATGT
TCATTCCGCTACTGGGATGTTTTACGCCTGATGGATGTATCCTGGGGCAGAGGAGGGTTG
CTTCGCGGCGAACGCGTATTTAATCACTATCGGGATATTATGCCCGTCACGGATTTTGAC
CACTGTTCAAGACGTTTTGGCGCGGTCGCGACGAATCTTAGCACGGGACGAGAGCTATGG
TTTACTGAAGGCGATCTACACCTTGCTGTACGCGCGTCGTGCAGTATGCCAGGGCTGATG
TCTCCGGTTGAGCATAACGGTTACTGGCTGGTAGATGGCGCAGTCGTTAATCCGGTACCT
GTTTCGTTAACCAGGGCGCTGGGGGCTGATATCGTTATTGCGGTAGACCTGCAACATGAT
GCCCACCTCATGCAGCAAGATTTGCTCTCTGTAAATGTCGGGAATATCAATGAAGAGAGC
GATGATGCGCTGCCCTGGCATAAGCGTTTAAAAGAACGTTTCAGTAGCCTGACTTCACGC
CGTGGCGTGAGTTCGCCTGGGGCAATGGAAATCATGACAACCTCCATCCAGGTGTTGGAA
AATCGTCTTAAACGTAACCGTATGGCTGGCGATCCGCCTGATATTCTCATTCAACCATTC
TGTCCGCAAATCTCAACGCTGGATTTTCATCGCGCCCATGCCGCTATTGCGGCGGGTCAG
TTAGCCGTAGAGAAAAAGATGGACGAACTTATACCCTTGGTGCGTACCGACGTTTAA
>EECEBICD_02131 hypothetical protein
GTGTCTCAGCCTTGTCCCTGTGGTAGCGCTGACGAGTATAGCCTATGTTGTGGTCGTATT
GTGTCCGGAGAACGAGTAGCACCCGATCCGTCACATCTCATGCGCTCTCGTTACTGCGCT
TTTGTGATGAAAGACGCAGATTACTTAATTAAGAGCTGGCATCCAACCTGCAATGCGGCC
GCGTTTCGTGATGATATCATCGCCGGATTTGCCAATACCAGGTGGCTCGGCCTGACTATT
TTTGAACATACCTGGTCAGAAGCAGAAAATACAGGGTATGTTAGTTTTATCGCGCGTTTT
TCCGAACAAGGGAAAACCGGGGCGATTATCGAACGTTCTCGTTTTATCAAAGAAAACGGT
CAGTGGTATTATATTGACGGTACCCGCCCGCAGTTGGGTCGAAATGATCCCTGCCCGTGC
GGTTCAGGCAAAAAATTTAAAAAGTGCTGCGGCCAGTGA
>EECEBICD_02132 Formyltetrahydrofolate deformylase
ATGCAATCACTACAACGTAAAGTTCTGCGTACTATTTGTCCCGATCAAAAAGGCCTTATC
GCGCGGATTACCAACATTTGCTATAAGCACGAGCTTAATATTGTGCAAAACAATGAATTT
GTGGACCATCGCACCGGACGCTTTTTTATGCGGACCGAGCTGGAAGGCATCTTCAATGAT
TCCACTCTCCTGGCCGATCTGGATAGCGCCTTACCGGAAGGCTCTGTTCGTGAACTCAAT
CCCGCCGGACGCCGTCGGGTAGTGATTCTGGTTACCAAAGAGGCGCATTGTCTGGGCGAC
CTGCTGATGAAAGCCAACTATGGCGGGCTGGATGTTGAAATCGCCGCGGTCATTGGCAAC

CATGAAACGTTACGCTCGCTGGTTGAACGTTTTGAGATCCCTTTCGAACTGGTCAGCCAT
GAGGGATTAACGCGTGAAGAGCATGACACGAAGATGGCGGACGCCATTGATGCCCATCAA
CCGGACTATGTAGTGCTGGCCAAGTATATGCGTGTTTTGACGCCAGGTTTTGTGGCGCGC
TTCCCGAACAAAATTATCAATATCCACCACTCATTCCTGCCGGCGTTTATCGGCGCGCGT
CCTTATCATCAGGCCTATGAGCGCGGCGTGAAAATCATCGGCGCTACCGCGCACTATGTG
AATGATAATCTGGATGAAGGCCCAATCATTATGCAGGACGTGATTCACGTCGATCACACC
TACACCGCAGAAGATATGATGCGTGCCGGGCGCGACGTTGAGAAAAACGTATTAAGCCGC
GCGCTGTATCAGGTTCTGGCACAGCGAGTCTTTGTTTACGGCAATCGGACGATTATTCTT
TAA
>EECEBICD_02133 tRNA-Tyr(gta)
GGTGGGGTTCCCGAGCGGCCAAAGGGAGCAGACTGTAAATCTGCCGTCATCGACTTCGAA
GGTTCGAATCCTTCCCCCACCACCA
>EECEBICD_02134 tRNA-Tyr(gta)
GGTGGGGTTCCCGAGCGGCCAAAGGGAGCAGACTGTAAATCTGCCGTCATCGACTTCGAA
GGTTCGAATCCTTCCCCCACCACCA
>EECEBICD_02135 hypothetical protein
ATGCGTTTTTCATCAATTTTCAAAATAATAATATTAAATATATTTATTTCAAAAATGGCT
CACTCTGCTGTCTGCGAGGAGCGCTATCCCCCCGACTCAGAAGAATGCCAGTACGTTCAG
GAATTAGAGCAAAAAGCGGAACAAGGAGATGAAAGCGCGCAGTTCGCGCTTGGAAGCTGG
TATGCGGAGGGGCGATATGTTAAGCCTGATTATAAACTAGCCATAAAATGGCTGGAGAAA
GCGGGTAAACAAGGTTCTGATTTTTCCTATTTCATCCTTGGCTATCATTACAACTATGGT
GAAAATTTTCCACTTAGTCGACAAAAAGCGCTGGAGTGGTATCGCAAGGCGGCAGAGCTA
GGGGATAGTAGTACGCAAGAAATCCTCGGTGACGCCTATATGTATGGCGATGGGTTTCCC
CAAAATACCCAGCTAGCGCTGGAGTGGTATCGAAAAGCCGCCTCTCCAACCAATGATGCG
GGCGTTGTCCGCGGCCAGGGTTCAGCTTCGTCAGCACAATTTAAGCTTGGAGTAATGTAC
GCCCACGGTCAGGGCGTCCCTCAGGATTATCAGCAAACGGCGATCTTGATGCGTAAAGCG
GCGGAAAATATGTACTACCCCGCGCAACTTTATCTCGGTGTTGCCTATTTTTATGGTGAA
GGCGTGCCTCAGGATTATCGTCAGGCAGTTTACTGGCTTAATGAAGGTATACCAAGCAGC
TATACGCCAGGCCACATTCCGCTGAATGCGCTCTATGATAAAGCGCATCCCGCTGACCAG
GTTCACTCTCAGACGTGGTATCGAAAAACAGCGCAACGCGTGATGGCAAAGGTACAGTAC
AATTTTGGCGTATGGTATTACAACGGTTATCACTTATTGAAAGATCACAACCTGGCGCTG
GAGTGGTATCGCAGAGCCGCAGCGCAAGGATTGGCCGAGGCGCAGGATGCGATCGGCGTG
ATGTTTATGCAGGGTGAGGGCGTATCTCAGGACTACCAACAGGCGCTGGCGTGGTATCGC
AAAGCCGCACGCCAGGGACTGCCTGCTGCGCAAACGCATCTGGGCATCATGTCTGCTTTT
GGTCGCGGCGTCGCGCAAAGCGACAGACAGGCTATCGCATGGTATCGAAAAGCAGCAAAA
CAGGATTTTGCGAAAGCGCAATATCAGCTTGGCGTAGCATATAGCACGGGAAGAGGCGTG
CCTGAAAATAGCCGGAATGCGCTGAAGTGGTACCTTAAAGCGGCAGAGCAGGGGTTTACT
CCGGCACAATTAGCGCTTGGGGAAATTTATGCTCATGGGCGCCAGGGGGTGCCTAAAGAT
AATAAGCAGGCCTATATTTGGTATTACATGGCAAGTATATATACGGAAAAATCTAAGGAT
GATTGTTCAGCGCTCATTGCCGAAAGAAACCGACTCAAGGGAACGCTTACCCCAGATCAA
CTTAGCGAGACATATGCCGCCTTTGATCTCATCAGGCGAAAAATCAACCAGTCAAAGGAG
GCGAAAAAGATTGCCAGGAAGAAGTATTGA
>EECEBICD_02136 Respiratory nitrate reductase 1 gamma chain
ATGCACTTCCTGAATATGTTCTTCTTTGATATCTACCCGTACATTGCCGGATCGGTATTT
CTGATCGGTAGCTGGTTACGTTACGACTACGGCCAGTACACCTGGCGGGCGGCTTCCAGT
CAGATGCTGGATCGCAAAGGAATGAACCTGGCGTCAAACCTGTTCCACATTGGGATTCTG
GGTATTTTCGCCGGTCACTTCCTGGGCATGTTAACGCCGCACTGGATGTATGAAGCTTTT
CTGCCGGTCGACGTGAAGCAGAAGATGGCGATGATTGCCGGCGGCGCCTGCGGCGTGCTG
ACACTGGTTGGCGGTATTTTACTGCTCAAGCGTCGTCTGTTTAGCCCACGTGTCCGCGCC
ACCACTACCGGCGCAGATATTTTGATTCTCTCGCTGCTGGTGATTCAGTGCGCGCTGGGC
CTGCTGACCATTCCGTTTTCCGCCCAGCATATGGACGGGAGTGAAATGATGAAGCTGGTC
GGGTGGGCGCAGTCAGTTGTGACCTTCCACGGTGGCGCATCCGCACATCTGGACGGTGTG
GCCTTTATCTTCCGTATGCACCTGGTACTGGGGATGACGCTGTTCCTGTTATTCCCATTC
TCGCGCCTGGTGCATATCTGGAGCGTGCCGGTCGAGTACCTGACCCGCAAATACCAGATT

GTTCGTGCACGTCACTGA
>EECEBICD_02137 Nitrate reductase molybdenum cofactor assembly chaperone NarJ
ATGATTGAACTCGTCATTGTTTCGCGTCTGCTCGAGTACCCGGATGCTGCCCTGTGGCAG
CATCAGCAGGAGTTGTTCGATGCGCTCGCGTCATCTGAGAACCTGGAGAAAGAGGATGCC
CAGACTCTGGGCGTTTTCCTGCGCGATTTAACCGCGCAGGACCTGCTGGATGTACAGGCC
GCTTATAGCGAACTGTTTGACCGTGGTCGCGCCACATCGCTGCTGCTGTTTGAACATGTA
CACGGGGAATCCCGCGATCGCGGTCAGGCGATGGTTGACCTGATGGCACAGTATGAGCAG
CACGGTTTGCAGCTCGACAGTCGCGAACTTCCCGATCATCTGCCGCTGTATCTGGAATAC
CTTGCGCAATTGCCGAAAAGCGAAGCGTTAGGGGGTTTACAGGATATCGCGCCGATTCTG
GCGCTGCTCAGCGCACGTTTGCAACAGCGTGACAGCCGTTATGCGGTGCTGTTCGATCTG
CTGTTAAAACTGGCGAATACCGTTATCGACAGCGATAAAGTGGCTGAAAAAATTGCGAAT
GAAGCACGTGATGATACCCCGCAGGCGTTGGATGCGGTCTGGGAAGAAGAGCAGGTGAAA
TTCTTCGCCGATCAAGGGTGTGGTGAGTCGGAAATCTCCGCTCACCAGCGTCGTTTTGCC
GGGGCCGTGGCACCGCAATATCTGAATATCACCACCGGAGGACAGCACTAA
>EECEBICD_02138 Respiratory nitrate reductase 1 beta chain
ATGAAAATTCGTTCACAAGTCGGCATGGTGCTGAATCTCGATAAGTGCATCGGCTGTCAT
ACCTGTTCAGTCACCTGTAAAAACGTCTGGACCAGTCGCGAAGGTATGGAATACGCGTGG
TTCAACAACGTAGAAAGTAAGCCTGGCGTTGGTTTTCCGAATGACTGGGAAAATCAGGAA
AAATGGAAAGGCGGCTGGATCCGCAAAATCAACGGCAAACTACAGCCGCGCATGGGCAAC
CGGGCGCTGCTGCTGGGTAAAATCTTCGCTAACCCGCATCTGCCGGGCATCGACGACTAC
TACGAGCCGTTTGATTACGACTACCAGAACCTGCATACCGCGCCGGAAGGCAGCAAACAT
CAGCCGATCGCGCGTCCGCGCTCGCTGATTACCGGCCAGCGGATGGACAAAATTACTAGC
GGGCCAAACTGGGAAGAGATTCTGGGCGGCGAGTTCGAAAAACGCGCCAAAGACCAGAAC
TTCGAAAACATGCAGAAGGCGATGTACGGCCAGTTCGAAAACACCTTCATGATGTACTTG
CCGCGCCTGTGCGAGCACTGCCTCAATCCGGCGTGCGTGGCGACCTGCCCAAGCGGTGCC
ATCTACAAGCGTGAAGAAGACGGCATTGTGCTGATCGACCAGGACAAGTGCCGCGGCTGG
CGGATGTGTATTACCGGCTGCCCGTACAAGAAGATCTACTTCAACTGGAAGAGCGGCAAA
TCTGAAAAATGCATCTTCTGCTATCCGCGTATTGAAGCGGGTCAGCCGACTGTTTGCTCG
GAAACCTGCGTGGGCCGTATCCGTTATCTTGGCGTACTGCTGTATGACGCCGATGCCATT
GAAAGCGCCGCCAGTACGGAAAACGAAAAAGATCTGTACCAGCGTCAACTGGACGTGTTC
CTCGATCCGAACGATCCGGCTGTCATTGAGCAGGCGTTGAAGGACGGCGTGCCGCAGAGC
GTGATCGATGCGGCGCAGCAATCGCCAGTATACAAAATGGCGATGGACTGGAAGCTGGCG
CTGCCGCTGCATCCGGAATACCGCACGCTGCCGATGGTCTGGTACGTGCCGCCTTTGTCA
CCGATTCAGTCTGCCGCCGACGCGGGTGAACTGGGCAGCAACGGTATTCTGCCGGATGTA
GACAGTCTGCGTATCCCGGTGCAGTACCTGGCGAATTTGTTGACCGCTGGCGACACTCAG
CCGGTACTGTTGGCGCTGAAACGGATGCTGGCGATGCGTCACTACAAACGTGCGGAAACC
GTAGACGGTAAAGTGGATACCCGCGCGCTGGAAGAGGTGGGTTTAAGCGAAGCCCAGGCG
CAGGAAATGTATCGTTACCTGGCAATTGCTAATTATGAAGATCGCTTTGTGGTGCCGAGC
AGCCATCGTGAACTGGCGCGTGACGCATTCCCGGAGAAAAGCGGTTGTGGCTTTACCTTC
GGCGATGGTTGCCACGGGTCTGATACCAAATTCAATCTGTTCAACAGCCGCCGCATCGAC
GCTGTTGATGTGACCAGCAAAACGGAGCCGCACGCATGA
>EECEBICD_02139 Respiratory nitrate reductase 1 alpha chain
GTGAACGAACTGATCGCCGCTTCCAACGTGTACACCGTGAAAACCTATGGCCCTGACCGC
GTCGCGGGCTTCTCACCCATCCCGGCGATGTCGATGGTTTCCTACGCTTCCGGCGCACGT
TATCTTTCACTGATTGGCGGTACCTGCCTGAGCTTCTACGACTGGTACTGCGATCTGCCG
CCAGCCTCCCCGCAAACCTGGGGCGAGCAGACCGACGTGCCGGAATCCGCTGACTGGTAC
AACTCCAGCTATATTATCGCCTGGGGCTCTAACGTGCCGCAAACCCGTACGCCGGATGCC
CACTTCTTTACCGAAGTACGTTACAAAGGCACTAAAACCGTCGCGGTGACGCCGGACTAT
GCTGAAATCGCCAAACTGTGCGATCTGTGGCTGGCGCCGAAGCAGGGGACCGATGCGGCG
ATGGCGCTGGCGATGGGCCACGTCATGCTGCGCGAGTTCCACCTCGACAACCCGCGCCAG
TACTTCACCGATTATGTCCGTCGCTATACCGACATGCCAATGCTGGTGATGCTTGAAGAG
CGTGATGGTTATTATGCCGCAGGTCGTATGCTGCGCGCTGCCGATCTCGTTGATTCGCTG
GGGCAGGAAAATAACCCGGAATGGAAAACCGTCGCTATCAACAGCAACGGTGAAATGGTG

GCGCCAAACGGTTCAATTGGTTTCCGCTGGGGCGAAAAAGGCAAATGGAACCTCGAACAG
CGCGACGGGACTACCGGTGCAGAAACTGAATTGCAGCTCAGCTTGCTGGGCAGCCAGGAT
GAGATTGCTGACGTGGGCTTCCCGTATTTCGGCGGCGAGGGTTCAGAGTACTTTAATCAT
GTGGCGCTGGATAACGTCCTGCTGCATAAACTGCCGGCAAAACGTCTGCAACTGGCCGAT
GGTTCTTCAGCGTTAGTCACCACCGTGTATGACCTGACCATGGCGAACTATGGTCTGGAA
CGCGGCCTGAACGATGAAAATTGTGCCGCCAGTTATGATGATACCAAAGCGTATACGCCA
GCATGGGCCGAGCAGATCACCGGCGTGCCGCGGGCGCAGATCATCCGTATCGCACGGGAG
TTCGCCGATAACGCGGACAAAACGCACGGGCGTTCAATGATTATCGTTGGCGCCGGTCTG
AACCACTGGTATCACCTCGATATGAACTATCGCGGGCTGATCAATATGTTGGTGTTCTGT
GGTTGCGTTGGTCAGAGCGGCGGCGGTTGGGCGCACTACGTCGGCCAGGAAAAACTGCGT
CCGCAGACCGGCTGGCAGCCGCTGGCGTTTGCCCTTGACTGGCAGCGTCCGGCGCGTCAC
ATGAATAGCACCTCTTACTTCTATAACCACTCCAGCCAGTGGCGCTACGAGACGGTGACC
GCCGAGGAATTACTGTCGCCGATGGCGGACAAATCCCGCTATAGCGGCCATCTGATTGAC
TTCAATGTGCGCGCCGAGCGTATGGGCTGGTTGCCGTCGGCACCGCAGTTAGGTACCAAC
CCGCTGTATATTGCGCGTGAAGCGGAAAAAGCCGGTATGACGCCGGTAGATTATACCGTC
AAATCCCTGAAAGAGGGTTCGATTCGCTTTGCCGCCGAGCAGCCGGAAAACGGTAAAAAC
CACCCGCGTAACCTGTTCATCTGGCGTTCAAACCTGCTTGGGTCTTCCGGTAAAGGTCAC
GAGTATATGCTGAAATATCTGCTGGGTACTGAGCATGGTATTCAGGGGCTGGATCTGGGT
AAACAGGGCGGTGTGAAGCCGGAAGAAGTGGAGTGGCGTGACAATGGCCTGGACGGCAAA
CTGGATCTGGTGGTGACGCTGGACTTCCGCCTGTCGAGCACCTGCCTGTACTCCGATATC
GTACTGCCGACCGCCACCTGGTATGAAAAAGACGATATGAATACCTCGGATATGCATCCG
TTTATTCATCCGCTGTCTGCCGCCGTAGATCCGGCCTGGGAATCGAAAAGCGACTGGGAA
ATCTACAAAGGCATTGCGAAGAAATTCTCCGAAGTCTGTGTAGGTCACCTGGGTAAAGAG
ACTGACGTCGTGACGCTGCCGATCCAGCACGATTCTGCCGCTGAACTGGCGCAGCCGCTG
GACGTGAAAGACTGGAAAAAAGGCGAATGCGATCTGATTCCAGGGAAAACCGCGCCGCAC
ATCATGACCGTTGAGCGCGATTACCCGGCGACTTACGAACGCTTTACCTCTATCGGCCCG
CTGATGGAGAAAATCGGCAACGGCGGGAAAGGGATTGCCTGGAACACGCAGAGCGAAATG
GACTTGCTGCGTAAGCTCAATTACACCAAAGCGGACGGTCCGGCGAAAGGCCAGCCGATG
CTCAACACGGCGATTGACGCGGCAGAGATGATCCTGACGTTAGCGCCGGAAACCAACGGT
CAGGTGGCGGTGAAAGCCTGGGCGGCGCTGAGCGAGTTTACCGGTCGCGACCATACGCAT
CTGGCAACCAATAAAGAAGAAGAGAAAATCCGCTTCCGCGATATTCAGGCCCAGCCGCGC
AAAATTATCTCCAGCCCGACCTGGTCTGGTCTGGAAGATGAGCATGTTTCTTATAACGCC
GGTTATACCAACGTTCACGAGCTGATCCCGTGGCGTACGCTGTCTGGTCGTCAACAGTTG
TATCAGGATCACCAGTGGATGCGCGACTTCGGCGAAAGTCTGCTGGTGTATCGCCCACCG
ATCGACACCCGCTCGGTGAAAGCGGTGATGGGGCGTAAATCCAACGGTAATCCGGAGAAG
GCGCTCAACTTCCTGACGCCGCACCAGAAATGGGGTATCCACTCGACCTACAGCGATAAC
CTGTTGATGCTGACCTTGTCGCGTGGCGGCCCGATTGTATGGATGAGCGAAACCGACGCC
AAAGATCTGGGCATTGAAGATAACGACTGGATCGAAGTCTTCAACAGCAACGGCGCATTG
ACCGCACGCGCAGTGGTCAGCCAGCGTGTACCGACGGGCATGACCATGATGTACCACGCG
CAGGAACGTATCGTAAACCTGCCGGGGTCGGAAATTACCCAGCAGCGCGGCGGTATTCAT
AACTCGGTGACGCGCATTACGCCGAAACCGACCCATATGATCGGCGGCTATGCGCAGTTA
GCCTACGGCTTTAACTACTACGGTACCGTCGGCTCCAACCGCGATGAGTTTGTGGTGGTA
CGTAAGATGAAGAACATTAACTGGTTGGATGGCGAAGGCAATGACCAGGTACAGGAGAGC
GTAAAATGA
>EECEBICD_02140 Respiratory nitrate reductase 1 alpha chain
ATGAGTAAATTCCTGGACCGGTTTCGCTACTTCAAGCAGAAGGGCGAAACCTTTGCCGAT
GGGCACGGCCAGCTTCTCGAAACAAACCGGGACTGGGAGGACGGATACCGCCAACGTTGG
CAGCATGACAAAATTGTGCGCTCAACCCATGGGGTGAACTGCACCGGTTCTTGTAGCTGG
AAAATTTATGTCAAAAATGGTCTGGTGACCTGGGAAACACAGCAAACCGATTACCCACGC
ACCCGTCCCGATATGCCAAACCATGAACCTCGCGGCTGCCCGCGCGGCGCAAGCTACTCC
TGGTATCTCTACAGCGCTAACCGACTGAAATATCCGCTGATGCGCAAACGTCTGATGAAG
ATGTGGCGCGAGGCGAAAAAAACTGCATCGCGATCCGGTAGAGGCATGGGCCTCCATCAT
TGA
>EECEBICD_02141 Nitrate/nitrite transporter NarK

ATGGCGATTTTCAGCGGTCTGCTGTTCCTGACATTACCTACTGGCGGCGTTGGAGGTAGC
TTTATCGCGTTCTACGGGGTATTTCTGGCGCTATTTCTGACGGCGGGGTTGGGGAGTGGT
TCTACCTTCCAGATGATCTCCGTTATCTTCCGTAAACTGACGATGGACCGGGTTAAAGCG
GAAGGCGGGTCGGACGAAAGAGCCATGCGTGAAGCGGCGACCGATACGGCGGCGGCGCTG
GGCTTTATTTCCGCGATTGGCGCGATTGGCGGCTTTTTTATTCCGAAAGCGTTTGGTTCA
TCTTTAGCATTAACTGGTTCTCCAGTCGGCGCGATGAAAGTCTTTCTTATTTTCTATATC
GCCTGCGTCGTTATTACCTGGGCGGTATATGGACGTCATTCTAAAAATAAAAAGTAA
>EECEBICD_02142 Nitrate/nitrite transporter NarK
ATGAGTCACTCATCCGCCCCGGAAAGGGCAACTGGCGCTGTTATCACGGAATGGCGTCCG
GAAGATCCGGCATTCTGGCAGCAGCACGGCCATCGTGTCGCCAGCCGTAATTTGTGGATT
TCTGTCCCGTGTTTACTGCTGGCGTTTTGCGTCTGGATGCTATTCAGCGCAGTGGCGGTG
AACCTGCCGAAAGTGGGATTTAATTTTACGACCGATCAGCTCTTTATGTTGACTGCGCTG
CCATCTGTTTCCGGCGCGCTGCTACGGGTGCCTTACTCCTTTATGGTGCCGCTATTTGGC
GGACGTCGCTGGACAGCGTTCAGTACCGGTATCCTCATCGTGCCCTGCGTATGGTTAGGC
TTTGCCGTTCAGGATACCAGTACGCCATTTAGCACTTTCATTATTATTTCCTTGCTGTGT
GGTTTTGCCGGCGCGAACTTTGCCTCCAGTATGGCGAACATCAGCTTTTTCTTTCCTAAA
CAAAAGCAGGGCGGCGCGCTGGGTCTGAACGGTGGTCTGGGCAATATGGGCGTGAGTGTG
ATGCAGCTGGTTGCGCCGCTGGTGGTTTCGCTCTCTATCTTTGCCGCCTTTGGCAGCCAC
GGTGTAGAGCAGCCGGACGGTTCTCAGCTCTATCTGGCGAACGCAGCCTGGATTTGGGTG
CCTTTCCTGGCCATTTTTACGCTGGCGGCGTGGTTCGGCATGAACGAACTGGCGACGTCT
AAGGCCTCGCTAAAAGAGCAACTGCCGGTGCTCAAACGTGGTCATCTGTGGATCATGAGT
CTGCTGTATCTGGCGACCTTCGGATCGTTTATCGGTTTCTCCGCGGGGTTCGCTATGCTG
TCTAAAACACAATTCCCGGATGTGCAGATCCTGCATTATGCGTTTTTCGGTCCGTTTATC
GGCGCGCTGGCGCGTTCGGCAGGCGGCGCAATTTCTGACCGTCTGGGGGGACTCGCGTCA
CGCTTATCAACTTTGTCCTGA
>EECEBICD_02143 Nitrate/nitrite sensor protein NarX
ATGTTTAAACGGTGTTTCTCTCCTCTTACGCTGGTTAACCAACTGGCGCTGATTGTCATG
CTCTCTACCGCTATCGGCGTGGCGGGGATGGCGGTTTCCGGCTGGCTGGTTCAGGGCGTG
CAGGGTAGCGCTCATGCGATTAATAAAGCGGGCTCATTGCGTATGCAAAGCTATCGACTA
CTGGCCGCTGTCCCGCTGGATGCTAAAGATCAAAAGCTGCTGGATGAGATGGAGCAAACG
GCATTCAGCCCGGAACTGACCCGCGCCCGCTGAGCGAGACGGGCAGCAAAAGCAGCTTAAA
GCGTTGCAGGATTACTGGCATAACGAACTCTCGCCAGGGCTACAGCACGCGCAAAACGCG
CACGCGGTCGCAGAAGATGTCACGCGCTTTGTGGCGGGCCTGGATCGCCTGGTGACGTCG
TTCGACCATACTACTGAATTGCGTATCGAACGCGTGGTATTAGTTCACCGGGTAATGGCG
ATCTTTATGGCGCTATTGCTGGTATTTACGATTATCTGGCTACGTGTTCGCCTTTTGCAG
CCCTGGAAACAGTTGTTATCAATGGCGCGCGCCGTCAGTCAGCGCGACTTTACCCAACGT
GCCAATATCAGTGGACGCAATGAGATGGCCGCGTTGGGTTCTGCGCTCAATAATATGTCT
GAAGAACTGGCCGAAAGTTATGCGGTACTGGAACAGCGCGTACAGGAAAAAACAGCCGGT
CTGGAACATAAAAACCAGATTCTCTCGTTTTTGTGGCAAGCCAACCGCCGACTGCATTCA
CAGGCTCCGCTATGTGAGCGTCTTTCTCCGGTACTGAATGGCCTGCAAAATTTAACCCAG
TTGCATGATATTGAGCTGCGAGTCTATGACCTGGAAGATGAAGATAATCACCAGGAATTT
ACCTGTCAGTCGGACATCAGTTGCGATGATAAAGGCTGCCACCTTTGCCCACGTAGCGCC
CTGCCGATGATTAACGGCGGCACCACCTTAAAATGGCGGCTCACCGACTCTCACACCCAA
TACGGTATTTTGCTGGCGACGCTGCCCTATGGACGCCATCTCAGCCACGATCAGCAACAG
TTGGTGGACACGCTGGTTGAACAGCTTACCGCGACTCTGGCGCTCGACAGGCATCAGGAG
CGCCAGCAGCAGTTGATTGTCATGGAAGAGCGTGCCACCATTGCCCGCGAACTGCATGAT
TCTATTGCCCAGTCGCTCTCTTGTATGAAGATGCAGGTGAGCTGCCTGCAAATGCAGGGC
GACGTGTTACCGGAAAGCAGCCGTGAGTTACTCAGCCAAATTCGCAATGAGCTGAACTGC
TCCTGGGCGCAACTGCGCGAACTGTTAACCACTTTCCGCTTACAGTTGACGGAACCCGGG
CTACGTCCGGCGCTGGAAGCAAGCTGCCAGGAGTTCAGCGCCCGGTTTGGCTTTACGGTA
AAACTGGATTATCAGCTTCCCCCCCGCCTGGTGCCTTCACACCAGGCGATACACCTGTTG
CAAATTGCCAGGGAAGCCCTCAGCAACGCCCTTAAACACTCTCATGCCGACGATGTGGTG
GTGACAGTGACGCAATGTGGCAAACAGGTCAAACTAAAAGTACAGGATAATGGCTGCGGC
GTCCCGGAAAACGCCGAACGCAGTAATCACTACGGCATGATTATCATGCGCGATCGGGCG

CAAAGTTTGCGCGGCGATTGCCAGGTACGCCGACGCGAGACTGGCGGAACAGAGGTCACT
GTTACTTTTATTCCAGAAACAAACTTCACAGAAACCCAGGGAGATACCCATGAATAA
>EECEBICD_02144 Nitrate/nitrite response regulator protein NarL
ATGAATAATCAGGAACCGGCAACCATCCTGTTAATCGACGACCATCCGATGCTACGCACG
GGTGTAAAGCAGCTTGTGAGTATGGCGCCCGATATCAGCGTGGTCGGAGAAGCCAGCAAC
GGTGAGCAGGGTATTGACCTGGCGGAATCGCTCGATCCCGATCTGATCCTCCTTGATTTG
AATATGCCGGGAATGAATGGCCTCGAAACGCTGGATAAATTACGCGAAAAAGCGCTGTCA
GGACGTATTGTCGTTTTCAGCGTATCCAACCATGAAGAAGATGTCGTCACGGCGCTAAAG
CGCGGCGCGGACGGTTACCTGTTGAAAGACATGGAGCCGGAGGACTTGCTCAAAGCGTTA
CAACAGGCCGCCGCCGGCGAAATGGTATTAAGTGAAGCGTTAACGCCGGTACTGGCGGCC
AGCCTGCGGGCCAACCGCGCCACCACCGATCGCGATGTAACGCAACTCACCCCGCGTGAG
CGCGATATTCTGAAGCTCATCGCGCAGGGATTGCCGAATAAGATGATTGCCCGGCGTCTG
GATATCACCGAAAGTACGGTCAAAGTGCACGTGAAACATATGCTCAAGAAAATGAAGCTG
AAATCGCGCGTGGAGGCCGCCGTCTGGGTACATCAGGAACGCATCTTTTAA
>EECEBICD_02145 Invasin
ATGCCATTCCTCTTATCTTTTGTTGTAGAAGTTGCCGACACGTTGAGCCGTATTGTTTTT
CGTTCATTCTCACTCTCGCTTCTCTTGCTGGCTGCCAGCGGTACGATCCGTGCGCAGGCG
CAAGACCCTTTCACGCAAAACCGTCTGCCGGATTTAGGCATGATGCCGGAATCCCACGAA
GGGGAAAAGCATTTTGCCGAGATGGCCAAAGCGTTTGGCGAAGCCAGCATGAAAAATAAC
GATCTGGATACTGGTGAACAGGCGCGGCAATTCGCTTTCGGACAGGTACGCGATGTGGTC
AGCGAGCAGGTTAACCAGCAGCTTGAAAGCTGGCTATCAGCCTGGGGCAGCGCCAGCGTG
GATATTAACGTCGATAACGAAGGTCATTTTAACGGCAGTCACGGAAGCTGGTTTATCCCT
TTACAGGATAAACAGCGCTATCTGACCTGGAGCCAGCTTGGTCTTACGCAACAGACTGAT
GGACTGGTAAGCAACGTTGGCATAGGACAGCGCTGGGCGCAGGATGGCTGGCTTCTTGGC
TATAATACCTTTTACGATAATCTGCTGGATGAAAATCTGCAGCGCGCCGGCTTTGGCGCG
GAGGCGTGGGGAGAATATTTGCGTTTATCCGCCAACTATTATCAGCCTTTTGCCGACTGG
CAGACGCATACGGCGACCTTAGAACAGCGAATGGCGCGCGGGTATGATATCAACGCGCAA
ATGCGGCTGCCGTTTTACCAGCATATCAATACCAGCGTTAGTCTGGAGCAGTACTTTGGC
GATAGCGTGGATCTGTTTGACTCCGGAACGGGGTATCACAATCCTGTCGCGTTAAAACTG
GGTCTCAACTATACGCCAGTACCTTTGCTTACCATGACCGCCCAGCACAAACAGGGCGAG
AGCGGGGTCAGTCAAAATAACCTGGGGCTGACGCTTAACTACCGCTTCGGCGTGCCGCTC
AAAAAGCAGCTTGCCGCCAGTGAAGTGGCGCAAAGCCAGTCATTACGCGGTAGCCGCTAT
GATACGCCGCAACGTAACTCGCTGCCGACAATGGAGTATCGGCAGCGTAAAACGTTAACG
GTTTTTCTGGCGACGCCGCCCTGGGATCTTACGCCTGGTGAGACGGTTGCGTTAAAATTG
CAGGTGCGCAGCGTGCACGGTATTCGTCATTTGAGCTGGCAGGGCGATACACAGGCATTA
AGTTTGACGGCAGGAACGGACACCCGCAGTACCGAGGGCTGGACAATCATTATGCCGGCC
TGGGATCACCGCGAAGGCGCGGCAAATCGCTGGCGTTTATCGGTGGTGGTTGAGGATGAA
AAAGATCAGCGTGTCTCTTCCAATGAGATCACGCTCGCGCTGACTGAACCGTTTATCACG
ATGCCGGACGATAATCCGCATTGGCAACCGTTCCAGGAGCAATAA
>EECEBICD_02146 Protein YchN
ATGCAAAATATCGTCATTATCGCTAACGGCGCCGCCTACGGCAGCGAATCATTATTTAAT
AGCCTGCGTCTGGCCATTGCGCTGCGCGAGCAGGAAAGCCATCTCGATTTACGCCTGTTT
TTGATGTCAGATGCCGTAACCGCAGGGTTACGCGGTCAAAAACCAGCCGAAGGCTACAAT
ATTCAGCAAATGCTGGAAATTCTGACCGCGCAAAACGTACCGGTAAAATTATGCAAAACC
TGTGCGGACGGACGTGGTATCACTCCCCTGCCGCTGATTGACGGTGTGGAAATCGGAACG
CTGGTCGAGCTGGCGCAATGGACGCTGGCGGCGGACAAAGTGCTGACATTTTGA
>EECEBICD_02147 Putative cation transport regulator ChaB
ATGCCGTATAAAGCCAAAAGTGATTTACCCGATAATGTGCGGAATGTTCTTCCAACCCAT
GCCCAGGACATTTACAAAGAAGCCTTTAATAGCGCCTGGGAGCAATATAAAGATAAAGCC
GATCGTCGTGATGACGCCAGCCGCGAGGAAACCGCACACAAAGTCGCCTGGGCGGCGGTT
AAAAACGATTATGAAAAAGGCGAGAATGATAAATGGCATAAGAAAAAATAA
>EECEBICD_02148 Sodium-potassium/proton antiporter ChaA
ATGACACATGCCTGTGAGGCGGTGAAAACCCGCCATAAGGAGACTTCGCTTATTTTCCCG
GTTCTTGCGCTGGTAGTGCTGTTCCTTTGGGGAAGCAGCCAGTCACTACCAGTGGTTATT

GGCATCAATATCCTTGCACTTATTGGTATTTTAAGCAGCGCATTTAGCGTGGTCCGTCAT
GCCGATGTATTAGCGCACCGACTCGGCGAACCCTATGGATCGCTTATTTTAAGCTTGTCC
GTCGTTATTCTTGAGGTGAGTTTAATTTCCGCGCTGATGGCTACCGGCGATGCTGCGCCA
ACGCTTATGCGCGATACGCTCTACTCTATTATTATGATTGTCACCGGGGGACTGGTCGGT
TTCTCGTTGTTGTTGGGCGGGCGTAAATTCGCAACGCAATATATGAATTTATTTGGGATT
AAGCAGTATCTTATTGCGCTATTTCCGCTGGCGATTATTGTGCTGGTTTTTCCCATGGCA
TTGCCGCAAGCCAATTTCTCTACCAGTCAGGCATTATTGGTCGCGTTAATTTCCGCCGCG
ATGTACGGCGTATTTTTGTTGATTCAAACCAAAACGCACCAGAGCTTATTTATCTATGAA
CATGAAGATGAAGGCGATGATGACAATCCACACCACGGTAAACCCTCCGCGCACAGTAGC
GGCTGGCATACGGTCTGGTTACTCATTCATTTAATTGCGGTCATTGCCGTTACCAAAATG
AATGCCAGCCCGCTGGAGGCGTTGCTGACCAGCATGAACGCCCCGGTCGCCTTTACCGGT
TTCCTGGTCGCGCTGTTAATTCTCTCGCCGGAAGGACTGGGCGCGCTGAAGGCGGTACTG
AATAATCAGGTTCAGCGGGCAATGAATCTGTTCTTCGGATCGGTGTTGGCGACGATTTCA
TTAACCGTGCCGGTCGTCACGCTCATCGCCTGGGCGACAGGTAATGATCTGGTGTTTGGT
TTAGGCGCGCCGGAAATGGTAGTGATGGTGGCCTCGTTAGTGCTGTGCCATATTTCGTTC
TCTACCGGACGCACCAACGTGCTAAACGGCGCGGCGCATCTGGCTTTATTTGCCGCCTAT
CTGATGACGATTTTTGCCTGA
>EECEBICD_02149 2-dehydro-3-deoxyphosphooctonate aldolase
ATGAAACAAAAAGTGGTTAACATTGGTGACATTAAGGTGGCAAATGACCTGCCGTTTGTG
CTGTTTGGCGGTATGAACGTGCTGGAGTCACGCGATCTGGCAATGCGCATTTGTGAGCAC
TACGTAACCGTTACCCAGAAGCTGGGTATTCCTTACGTGTTCAAGGCCTCTTTTGATAAA
GCCAACCGTTCCTCTATTCACTCTTACCGTGGGCCGGGCCTGGAAGAAGGGATGAAAATC
TTCCAGGAACTCAAACAGACATTTGGCGTAAAAGTGATCACTGACGTCCATGAAGCCAGC
CAGGCGCAGCCTGTGGCTGATGTGGTTGACGTGATTCAGCTCCCGGCGTTTCTGGCGCGC
CAGACCGACCTGGTGGAAGCGATGGCGAAAACCGGCGCTGTGATTAACGTGAAAAAGCCG
CAGTTCGTAAGCCCGGGTCAGATGGGCAATATCGTGGATAAGTTCCATGAGGGCGGTAAC
GATAAGGTGATTCTGTGCGACCGCGGCGCGAACTTCGGTTATGACAACCTGGTGGTGGAT
ATGCTGGGCTTTAGCGTGATGAAAAAGGTCTCCGGCAACTCGCCGGTGATTTTCGACGTT
ACCCATGCGCTGCAATGCCGCGACCCATTTGGCGCTGCCTCGGGCGGTCGTCGTGGTCAG
GTGACCGAGCTGGCGCGCGCCGGTATGGCTGTCGGTCTGGCGGGCCTGTTCCTGGAGTCG
CATCCGGACCCGGCTAACGCGAAGTGCGACGGTCCTTCCGCGCTGCCGCTGGCGAAACTG
GAGCAGTTCCTGACCCAGATAAAAGCGATTGATGATTTGGTGAAAAGCTTCGACGAGCTG
GATACCGAGAACTGA
>EECEBICD_02150 hypothetical protein
ATGAGGTCGTTAGCTGATTTCGAATTTAACAATGCGCCGCTGTGTGATGGCATGATCCTG
GCATCAGAGATGATTCGCCTGGATTTTCCCACGCAGTTTGTCTACGACGAACTGGAACGT
CTGGTCAGTCTGGCGCAGGAAGAAATTAGCCAGCTCCTGTCTCAGGATGAGCAACTGGAA
AAATTGCTGGCACTTTTTTACGGCGAGTGGGGGTTCACGGACTCCCGCGGCGTCTACCGT
[truncated: 767,977 more chars]
