# Supplementary material for: Genome sequencing and analysis of Salmonella enterica subsp. enterica serotype Enteritidis PT4 578: insights into pathogenicity and virulence
Source: Access Microbiol. 2024 Nov 4;6(11):000828.v3. doi: 10.1099/acmi.0.000828.v3 (PMC11649194; doi:10.1099/acmi.0.000828.v3)
Supplement: Uncited Supplementary Material 3. [file acmi-6-00828-s003.pdf]

>contig_1
ACCGCACGTTAACGTCGGCACCATCGGCCACGTTGACCACGGTAAAACTACCCTGACCGCTGCAATCACCACC
GTACTGGCTAAAACCTACGGCGGTGCTGCTCGTGCATTCGACCAGATCGATAACGCGCCGGAAGAAAAAGCGC
GTGGTATCACCATCAACACTTCTCACGTTGAATACGATACCCCGACCCGCCACTACGCACACGTAGACTGCCC
GGGGCACGCCGACTATGTTAAAAACATGATCACCGGTGCTGCTCAGATGGACGGCGCGATCCTGGTTGTTGCT
GCGACTGACGGCCCGATGCCGCAGACCCGTGAGCACATCCTGCTGGGTCGTCAGGTAGGCGTTCCGTACATCA
TCGTGTTCCTGAACAAATGCGACATGGTTGATGACGAAGAGCTGCTGGAACTGGTTGAGATGGAAGTTCGCGA
ACTGCTGTCTCAGTACGACTTCCCGGGCGACGACACTCCGATCGTTCGTGGTTCTGCTCTGAAAGCGCTGGAA
GGCGACGCAGAGTGGGAAGCGAAAATCATCGAACTGGCTGGCTTCCTGGATTCTTACATCCCGGAACCAGAGC
GTGCGATTGACAAGCCGTTCCTGCTGCCGATCGAAGACGTATTCTCCATCTCCGGTCGTGGTACCGTTGTTAC
CGGTCGTGTAGAACGCGGTATCATCAAAGTGGGCGAAGAAGTTGAAATCGTTGGTATCAAAGAGACTCAGAAG
TCTACCTGTACTGGCGTTGAAATGTTCCGCAAACTGCTGGACGAAGGCCGTGCCGGTGAGAACGTAGGTGTTC
TGCTGCGTGGTATCAAACGTGAAGAAATCGAACGTGGTCAGGTACTGGCTAAGCCGGGCACCATCAAGCCGCA
CACCAAGTTCGAATCTGAAGTGTACATTCTGTCCAAAGATGAAGGCGGCCGTCATACTCCGTTCTTCAAAGGC
TACCGTCCGCAGTTCTACTTCCGTACTACTGACGTGACTGGCACCATCGAACTGCCGGAAGGCGTAGAGATGG
TAATGCCGGGCGACAACATCAAAATGGTTGTTACCCTGATCCACCCGATCGCGATGGACGACGGTCTGCGTTT
CGCAATCCGTGAAGGCGGCCGTACCGTTGGCGCGGGCGTTGTTGCTAAAGTTCTGGGCTAATTACTCGTTAAT
TAGTTTTGAATTGAAGAGGGCGCTTCGGCGCCCTTTTTGTTGCCTGCTATTTAGCCGCTGTTATATTTATTCA
CCCTTTTTAACGCTTCTCCCGTGGCGTAAATCGCTTTGTGCTATTGTAATCATAACCATTCTCATTTACACTT
TGTGCGGAAATTGAACGGGAGCGATCATGTACGTTTGTTTGTGTAATGGTATAAGCGATAAAAAAATCCGCCA
GGCTGTACGACAATTTCATCCACAGTCATTTCAACAGTTGCGTAAATTTATTCCTGTGGGAAATCAATGTGGT
AAGTGTATTCGCGCCGCGCGAGAAGTGATGCAGGATGAGTTAACGCAAATGCCGGAATTTAAAGAGATCGCCT
GAGTCACACTCTTTTTTTGACATCCCTGTAGCCCCATCTACGCTCTAGAGAGTGGAAGCGGAGGGACTATAAA
ATGAAAGGTGATGTTAAAATCATAAATTATCTCAATAAACTATTGGGAAATGAGCTTGTCGCAATTAATCAGT
ATTTTCTTCATGCCCGTATGTTCAAAAACTGGGGGCTGACCCGGCTTAATGATGTTGAGTACCATGAATCCAT
TGATGAGATGAAACACGCCGATAAGTATATTGAGCGTATTTTATTTCTGGAAGGCATACCGAACCTACAGGAT
CTGGGCAAGCTGGGCATCGGTGAAGATGTCGAAGAGATGCTACGATCGGATCTCCGGCTCGAACTGGAAGGCG
CAAAAGATCTACGTGAGGCAATAGCCTATGCCGATAGCGTTCATGACTATGTTAGCCGGGATATGATGATTGA
AATTCTTGCCGACGAGGAAGGCCACATCGACTGGCTGGAAACTGAGCTGGATTTGATTGCCAAACTTGGTATG
CAAAATTATCTGCAATCACAAATTAAGGTTACCGATTAACCGTCTGGAAAGAAAACTTTCCAACCTGTTATGA
AACCCGCCACCGCCAGCCATGGCCCAAAAGGTAGCGGGTTTATTAATGCCGATTTACCTCTCACCAGTAGCGC
GACGCCAAACCCGCCACAGGCCAACATAGCGGCAAGGAAAACCAGTAACGGCAGTGTTTCCCAACAATGCCAT
GCCCCTAAGGCAGCGAGGTACTTGACGTCGCCATATCCAAGCCCCTCTTTCTGATAACGCAGGCGATAGCCCC
AGTAAATTAACGCGAATCCGCCATAGCCGGCGATAGCACCCCAAAGCGCATCCGGTAAACGCTCCGGCAGGCA
AATCTGGTGATAAAGCAGACCACCCCACAGTAATGGACAGGTAAACCGGTCAGGCAGCAGACCGGTACGCGCG
TCGTATATGCCAAGTAATAAAGAGAACGACGCATAAAAAATGAGAAAGGGGAGGGCAACAAACATAAGCAAAT
CCTCGTCAAGGCTTGTGCTCAGCTTTATAGATTTATGCGACAGCGACAACGACGTATTGCCAGGTTTGCCTGA
TGCCTTCCAAAGAAAAGGTGCGCGTTGCCATCCCATGCGTACCATCTGGAATCATGCTCGCATAAGAATAAAG
CGCTTAATTTCTATCCGACACTTGCGTCAGGCTGAGATTTACGTATAATGCGCGGGCTTGTCGTAATTGACGG
CAGGTTCGATTTGAACCTCGAATAGCCGATTTGGCTATTTAACAATGCTCCCAATCGGGGAGCTACGTAAGAA
CGGTTACACTCTCCCATCAATCGTAATGGGTGTGAGGAGTAATCATTTTCGTTTATAAAATAATTGGAGCTCT
GGTCTCATGCAGAACCAAAGAATCCGTATCCGCCTGAAAGCGTTTGATCATCGTCTGATCGATCAATCAACCG
CGGAAATCGTCGAGACTGCCAAGCGCACTGGTGCGCAGGTTCGCGGTCCGATCCCGCTGCCGACCCGCAAAGA
GCGCTTTACCGTTCTGATCTCTCCGCACGTTAACAAAGATGCGCGCGATCAGTACGAAATTCGCACTCACAAG
CGTCTGGTTGACATCGTTGAGCCAACCGAGAAAACCGTTGATGCTCTGATGCGTCTGGACCTGGCTGCCGGTG
TAGACGTGCAGATCAGCCTGGGTTAATCAGGTCATCGAGCGATTGAGAGGTTGATACAATGATTGGTTTAGTC
GGTAAAAAAGTGGGTATGACCCGCATCTTCACTGAAGATGGCGTCTCTATCCCAGTAACCGTAATCGAAGTTG
AAGCAAACCGTGTTACTCAGGTTAAAGACCTGGCTAACGATGGCTACCGCGCTGTTCAGGTTACTACGGGTGC
TAAAAAAGCTAACCGTGTCACCAAGCCGGAAGCGGGTCACTTTGCTAAAGCTGGCGTAGAAGCTGGCCGTGGT
CTGTGGGAATTCCGTCTGGCTGAAGGCGAAGAATACACCGTAGGTCAGAGCATCAGCGTTGAACTGTTTGCTG
ACGTTAAAAAAGTTGACGTAACCGGTACCTCTAAAGGTAAAGGTTTCGCTGGTACCGTTAAGCGCTGGAACTT
CCGTACCCAGGACGCTACTCACGGTAACTCCTTGTCTCACCGCGTTCCGGGTTCTATCGGTCAGAACCAGACT
CCGGGCAAAGTGTTCAAAGGCAAGAAAATGGCAGGTCAGATGGGTAATGAGCGCGTAACCGTTCAGAGCCTTG
ACGTAGTACGCGTTGACGCTGAGCGCAACCTGCTGCTGGTTAAGGGTGGTGTTCCGGGTGCAACCGGTTGCGA

CCTGATCGTTAAACCAGCTGTGAAGGCGTAAGGGGATAGCAATGGAATTAGTATTGAAAGACGCGCAGAGCGC
GCTGACTGTTTCCGAAACTACCTTCGGTCGTGATTTCAACGAAGCGCTGGTTCACCAGGTTGTTGTTGCTTAT
GCAGCTGGTGCTCGTCAGGGTACTCGTGCTCAGAAGACTCGTGCTGAAGTAACTGGTTCCGGTAAAAAACCGT
GGCGCCAGAAAGGCACTGGCCGTGCGCGTTCAGGTTCTATCAAGAGCCCGATCTGGCGTTCCGGTGGCGTGAC
CTTCGCTGCTCGTCCGCAGGACCACAGTCAAAAAGTTAACAAGAAGATGTACCGCGGCGCGCTGAAAAGCATC
CTGTCCGAACTGGTACGTCAGGATCGTCTGATCGTTGTCGAGAAGTTTTCTGTAGAAGCGCCGAAAACTAAGC
TGCTGGCACAGAAACTGAAAGACATGGCTCTGGAAGATGTGCTGATCATCACCGGTGAGCTGGACGAGAACCT
GTTCCTGGCCGCACGTAACCTGCACAAGGTTGACGTACGCGATGCGACTGGTATCGACCCGGTTAGCCTGATC
GCCTTCGACAAAGTCGTAATGACTGCTGATGCTGTTAAGCAAGTTGAGGAGATGCTGGCATGATTCGTGAAGA
ACGTCTGCTGAAGGTGCTGCGCGCACCGCACGTTTCTGAAAAAGCGTCTACTGCGATGGAAAAAACTAACACC
ATCGTTCTCAAAGTTGCTAAAGACGCGACCAAAGCAGAAATCAAAGCTGCTGTGCAGAAACTGTTTGAAGTCG
AAGTCGAAGTCGTTAACACCCTGGTCGTTAAAGGGAAAGTTAAACGTCACGGACAGCGTATCGGTCGTCGTAG
CGACTGGAAAAAAGCTTACGTCACCCTGAAAGAAGGCCAGAATCTGGACTTCGTCGGTGGCGCTGAGTAAGTC
GGAGGAGTAATACAATGGCAGTTGTTAAATGTAAACCGACATCTCCGGGTCGTCGCCACGTCGTTAAAGTGGT
CAACCCAGAGCTGCACAAGGGCAAACCTTTTGCTCCGCTGGTTGAAAAAAACAGCAAATCCGGTGGTCGTAAC
AACAATGGCCGTATCACCACTCGTCATATCGGTGGTGGTCACAAACAGGCTTATCGTATTGTTGACTTCAAAC
GCAACAAAGACGGTATTCCGGCAGTTGTTGAACGTCTTGAGTACGATCCGAACCGTTCCGCGAACATCGCGCT
GGTTCTGTACAAAGACGGCGAACGCCGTTACATCCTGGCCCCGAAAGGCCTGAAAGCTGGCGACCAGATTCAG
TCTGGCGTTGATGCTGCAATCAAAGCAGGCAACACCCTGCCGATGCGCAATATCCCGGTTGGTTCTACCGTTC
ATAACGTAGAAATGAAACCAGGTAAAGGCGGTCAGCTGGCGCGTTCCGCTGGTACTTACGTTCAGATCGTTGC
TCGCGATGGCGCTTATGTCACCCTGCGTCTGCGTTCTGGTGAAATGCGTAAAGTCGAAGCAGACTGCCGTGCA
ACCCTGGGCGAAGTTGGCAATGCTGAGCATATGCTGCGCGTTCTGGGTAAAGCAGGTGCTGCACGCTGGCGTG
GCGTTCGTCCTACCGTTCGCGGTACTGCGATGAACCCAGTAGACCACCCACATGGTGGTGGTGAAGGTCGTAA
CTTTGGTAAGCACCCGGTAACTCCGTGGGGCGTTCAGACCAAAGGTAAGAAGACCCGCAGCAACAAGCGTACT
GATAAATTCATCGTACGTCGCCGTAGCAAATAATTTTAGAGGATAAGCCATGCCACGTTCTCTCAAGAAAGGT
CCTTTTATTGACCTGCACTTGCTGAAGAAGGTAGAGAAAGCGGTGGAAAGCGGAGACAAGAAGCCCCTGCGCA
CCTGGTCCCGTCGTTCAACGATCTTTCCTAACATGATCGGTTTGACCATCGCTGTCCATAATGGTCGTCAGCA
CGTTCCGGTATTTGTTTCCGACGAAATGGTCGGTCACAAACTGGGTGAATTCGCACCGACTCGTACTTATCGC
GGCCACGCTGCTGATAAAAAAGCGAAGAAGAAATAAGGTAGGAGGAAGAGATGGAAACTATCGCTAAACATCG
CCATGCTCGTTCTTCTGCTCAGAAGGTTCGCCTTGTTGCTGACCTGATTCGCGGTAAGAAAGTGTCGCAGGCT
CTGGATATTCTGACCTACACCAACAAGAAAGCGGCTGTACTGGTCAAGAAAGTTCTGGAATCTGCCATTGCTA
ACGCTGAACACAACGATGGCGCTGACATTGACGATCTGAAAGTTACGAAAATTTTCGTAGACGAAGGCCCGAG
CATGAAGCGCATTATGCCGCGTGCAAAAGGTCGTGCAGATCGCATCCTGAAGCGCACCAGCCACATCACTGTG
GTTGTGTCCGATCGCTGAGACTCTGGAGACTAGCAATGGGTCAGAAAGTACATCCTAATGGTATTCGCCTGGG
TATTGTAAAACCATGGAACTCTACCTGGTTTGCGAACACCAAAGAATTCGCTGACAACCTGGACAGCGATTTT
AAAGTACGTCAGTACCTGACGAAGGAACTGGCTAAAGCGTCTGTATCTCGTATCGTTATCGAGCGTCCGGCTA
AGAGCATCCGTGTGACTATTCACACCGCTCGCCCGGGTATCGTTATCGGTAAAAAAGGTGAAGACGTAGAAAA
ACTGCGTAAGGTCGTAGCGGATATCGCTGGCGTTCCTGCACAGATCAATATCGCCGAAGTTCGTAAACCTGAA
CTGGACGCTAAACTGGTTGCTGACAGCATCACTTCTCAGCTGGAACGTCGCGTGATGTTCCGTCGTGCGATGA
AGCGTGCTGTACAGAACGCCATGCGTCTGGGCGCTAAAGGTATCAAAGTTGAAGTTAGCGGCCGTCTGGGCGG
CGCGGAAATCGCACGTACCGAATGGTACCGCGAAGGTCGCGTACCTCTGCACACTCTGCGTGCTGACATCGAC
TACAACACCTCTGAAGCGCACACCACTTATGGTGTAATCGGCGTTAAAGTGTGGATCTTCAAAGGCGAGATCC
TGGGTGGTATGGCTGCTGTTGAACAACCGGAAAAACCGGCTGCTCAACCGAAAAAGCAGCAGCGTAAAGGCCG
TAAATAAGGAGCGTCGCTGATGTTACAACCAAAGCGTACAAAATTCCGTAAAATGCACAAAGGCCGCAACCGT
GGTCTGGCTGCAGGCGCGGATGTTAGCTTCGGCAGCTTCGGCCTGAAAGCTGTTGGCCGTGGTCGTCTGACTG
CCCGTCAGATCGAAGCAGCACGTCGTGCTATGACCCGTGCAGTTAAGCGTCAAGGTAAGATCTGGATCCGTGT
ATTCCCGGACAAACCGATCACTGAAAAGCCGCTGGCAGTGCGTATGGGTAAAGGTAAAGGTAACGTGGAGTAT
TGGGTTGCCTTGATTCAGCCGGGTAAAGTCCTGTATGAAATGGACGGCGTACCGGAAGAGCTGGCCCGTGAAG
CATTCAAGCTGGCAGCAGCGAAACTGCCGATTAAAACCACCTTTGTAACTAAGACGGTGATGTAATGAAAGCA
AAAGAGCTGCGTGAGAAGAGCGTTGAAGAGCTGAACACCGAGCTACTGAACCTGCTGCGTGAGCAGTTCAACC
TGCGTATGCAGGCTGCAAGTGGCCAGCTGCAACAGTCTCACCTGTTGAAGCAAGTGCGTCGTGATGTCGCACG
CGTTAAGACTTTACTGACTGAGAAGGCGGGTGCGTAATGACCGATAAAATCCGTACTCTGCAAGGTCGCGTGG
TTAGCGACAAAATGGAGAAATCCATTGTTGTTGCTATCGAACGTTTTGTGAAACACCCGATCTACGGTAAATT
CATCAAGCGTACGACCAAAATGCACGTACATGACGAGAACAACGAATGCGGTATCGGCGACGTGGTTGAAATC

CGCGAATGCCGTCCGCTGTCCAAGACTAAATCCTGGACTCTGGTTCGCGTTGTAGAGAAAGCGGTTCTGTAAT
ACAGTACGCATTCTCAACGAATAAACGGCTCAGCGATGGGCCGTTTATTTTTTCTACCCATATCTGATTTGCG
GTGTTATAATGCCGCGCCCTCGATATGGGGTTTTTTTAACGACCTGATTTTCGGGTCTCAGTAGTAGTTGACA
TTAGCGGAGCACTAAAATGATCCAAGAACAGACTATGCTGAACGTCGCCGACAACTCCGGTGCACGTCGCGTA
ATGTGTATCAAGGTTCTGGGTGGCTCGCACCGTCGCTACGCAGGCGTAGGCGACATCATTAAGATCACCATCA
AAGAAGCGATTCCGCGTGGTAAGGTCAAAAAAGGTGATGTGCTGAAGGCGGTAGTGGTGCGCACCAAGAAGGG
TGTTCGTCGCCCGGACGGTTCTGTCATTCGCTTCGATGGTAATGCATGCGTTATTTTAAACAATAACAGCGAG
CAGCCTATCGGTACGCGTATTTTTGGGCCGGTAACTCGTGAACTTCGTAACGAGAAGTTCATGAAAATTATCT
CTCTGGCACCAGAAGTACTCTAAGGAGCGAATCATGGCAGCGAAAATCCGTCGTGATGACGAAGTTATCGTGT
TAACCGGTAAAGATAAAGGTAAACGCGGTAAAGTTAAGAATGTCCTGTCTTCCGGCAAGGTCATTGTTGAAGG
TATCAACCTGGTTAAGAAACACCAGAAGCCGGTTCCGGCTCTGAACCAACCGGGCGGCATCGTTGAAAAAGAA
GCTGCAATTCAGGTTTCTAACGTTGCAATCTTCAATGCGGCAACCGGCAAGGCTGACCGTGTAGGCTTTAGAT
TCGAAGACGGCAAAAAAGTCCGTTTCTTCAAGTCTAACAGCGAAACTATCAAGTAATTTGGAGTAGTACGATG
GCGAAACTGCATGATTACTACAAAGACGAAGTAGTTAACAAACTCATGACTGAGTTTAACTACAATTCTGTCA
TGCAAGTCCCTCGGGTCGAGAAGATCACCCTGAACATGGGTGTTGGTGAAGCGATCGCTGACAAGAAACTGCT
GGATAACGCAGCAGCAGACCTGACAGCAATCTCCGGTCAAAAACCGTTGATCACCAAAGCACGCAAATCTGTT
GCAGGCTTCAAAATCCGTCAGGGCTATCCGATCGGCTGTAAAGTAACTCTGCGTGGCGAACGCATGTGGGAGT
TCTTTGAGCGCCTGATCACTATTGCTGTTCCTCGTATCCGTGACTTCCGTGGCTTGTCCGCTAAGTCATTCGA
CGGTCGTGGTAACTACAGCATGGGTGTCCGTGAGCAGATCATCTTCCCAGAAATCGACTACGATAAAGTCGAC
CGCGTTCGTGGTTTGGATATTACCATTACCACTACTGCGAAATCTGACGAAGAAGGCCGTGCTCTGCTGGCTG
CCTTTGACTTCCCGTTCCGCAAGTAAGGTAGGGTTACTAAATGGCTAAGCAATCAATGAAAGCACGCGAAGTA
AAACGCGTAGCTTTAGCTGATAAATACTTCGCGAAACGCGCTGAACTGAAAGCGATCATCTCTGATGTGAACG
CAACCGACGAAGATCGTTGGAATGCGGTTCTTAAGCTGCAGACTCTGCCGCGTGATTCCAGCCCGTCACGTCA
GCGTAACCGCTGCCGTCAAACTGGTCGTCCGCACGCTTTCCTGCGGAAGTTCGGGTTGAGCCGTATTAAGGTC
CGTGAAGCCGCTATGCGCGGTGAAATTCCGGGTCTGAAAAAGGCTAGCTGGTAATTGTCACCAATTGAATCAC
GGGAGTAAAGACAGATGAGCATGCAAGATCCGATCGCGGATATGCTGACCCGTATCCGTAACGGTCAGGCCGC
GAACAAAGCTGCGGTCACCATGCCTTCCTCCAAGCTGAAAGTGGCAATTGCCAACGTGCTGAAGGAAGAAGGT
TTTATTGAAGATTTTAAAGTTGAAGGCGACACCAAGCCGGAACTGGAACTGACTCTTAAGTATTTCCAGGGTA
AAGCTGTTGTAGAAAGCATTCAGCGTGTCAGCCGCCCAGGTCTGCGCATCTACAAACGTAAAGATGAGCTGCC
GAAAGTTATGGCCGGTCTGGGTATCGCAGTTGTTTCTACCTCTAAAGGTGTTATGACTGATCGTGCAGCGCGC
CAGGCTGGTCTTGGTGGCGAAATTATCTGCTACGTAGCCTAATCGGAGGAAAAAATGTCTCGTGTTGCTAAAG
CACCGGTCGTTGTTCCTGCCGGCGTTGATGTCAAAATCAACGGTCAGGTTATTACGATCAAAGGTAAAAACGG
CGAGCTGACTCGTACTCTCAACGATGCTGTTGAAGTTAAACATGCAGATAATGCACTGACCTTCGGTCCGCGT
GATGGTTACGCAGACGGTTGGGCACAGGCTGGTACCGCGCGTGCCCTGCTGAACTCAATGGTTATCGGTGTTA
CCGAAGGCTTCACTAAGAAGCTGCAGCTGGTTGGTGTAGGTTATCGTGCAGCGGTTAAGGGGAATGTAGTAAA
CCTGTCTTTAGGTTTCTCTCACCCGGTTGACCATCAGCTGCCGGCAGGTATTACTGCTGAATGTCCGACTCAA
ACTGAAATCGTGCTGAAAGGCGCTGATAAGCAGGTGATCGGCCAGGTTGCAGCAGATCTGCGCGCCTACCGTC
GTCCTGAGCCTTACAAAGGCAAGGGTGTTCGTTACGCCGACGAAGTCGTGCGTACCAAAGAGGCTAAGAAGAA
GTAAGGTAACACTATGGATAAGAAATCTGCTCGTATCCGTCGTGCGACCCGCGCACGCCGCAAGCTCAAAGAG
CTGGGCGCAACTCGCCTGGTGGTACATCGTACCCCGCGTCATATTTACGCACAGGTAATTGCACCGAACGGTT
CTGAAGTTCTGGTAGCTGCTTCTACTGTAGAAAAAGCTATCGCTGAACAACTGAAGTACACCGGTAACAAAGA
CGCGGCTGCAGCTGTGGGTAAAGCTGTCGCTGAACGCGCTCTGGAAAAAGGCATCAAAGATGTGTCCTTTGAC
CGTTCCGGGTTCCAATATCATGGTCGTGTCCAGGCACTGGCAGATGCTGCCCGTGAAGCTGGCCTTCAGTTCT
AAGGTAGAGGTGTAAGATGGCTCACATCGAAAAACAAGCTGGCGAACTGCAGGAAAAGCTGATCGCGGTAAAC
CGCGTATCTAAAACCGTTAAAGGTGGTCGTATTTTCTCCTTCACAGCTCTGACTGTAGTAGGCGATGGTAACG
GTCGCGTAGGTTTTGGTTACGGTAAAGCGCGTGAAGTTCCAGCAGCGATCCAGAAAGCGATGGAAAAAGCCCG
TCGCAATATGATTAACGTCGCGCTGAACAACGGCACCCTGCAGCACCCGGTTAAAGGTGTTCACACGGGGTCT
CGTGTATTCATGCAGCCAGCTTCCGAAGGTACCGGTATCATCGCCGGTGGTGCAATGCGCGCCGTTCTGGAAG
TTGCTGGGGTTCATAACGTTCTGGCTAAAGCATACGGTTCCACCAACCCGATCAACGTGGTTCGTGCAACTAT
TGATGGCCTGGAAAATATGAATTCTCCAGAAATGGTCGCTGCCAAGCGTGGTAAATCCGTTGAAGAAATTCTG
GGGAAATAAACCATGGCAAAGACTATTAAAATTACTCAAACCCGCAGTGCAATCGGTCGTCTGCCGAAACACA
AGGCAACGCTGCTTGGCCTGGGTCTGCGTCGTATTGGTCACACCGTAGAGCGCGAGGATACTCCTGCTGTTCG
TGGTATGGTCAACGCGGTTTCCTTCATGGTTAAAGTTGAGGAGTAAGAGATGCGTTTAAATACTCTGTCTCCG
GCCGAAGGCTCCAAAAAGGCGGGTAAACGCCTGGGTCGTGGTATCGGTTCTGGCCTCGGTAAAACCGGTGGTC

GTGGTCACAAAGGTCAGAAGTCTCGTTCTGGCGGTGGCGTACGTCGCGGTTTCGAGGGTGGTCAGATGCCTCT
GTACCGTCGTCTGCCGAAATTCGGCTTCACTTCTCGCAAAGCAGCGATTACAGCCGAAGTTCGTCTGTCTGAC
TTGGCGAAAGTAGAAGGCGGCGTTGTAGACCTGAACACGCTGAAAGCGGCAAACATTATCGGTATCCAGATCG
AGTTCGCGAAAGTGATCTTGGCTGGCGAAGTCACTACTCCGGTAACTGTTCGTGGCCTGCGTGTTACTAAAGG
CGCTCGTGCTGCTATCGAAGCTGCTGGCGGTAAAATCGAGGAATAAGTAGCAGATGGCTAAACAACCGGGATT
AGATTTTCAAAGTGCCAAAGGTGGCTTAGGTGAGCTGAAACGCAGACTGCTGTTTGTTATCGGCGCGCTGATT
GTGTTCCGTATTGGCTCTTTTATTCCGATCCCTGGTATTGATGCCGCTGTACTTGCCAAACTGCTTGAGCAAC
AGCGAGGCACCATCATTGAAATGTTTAACATGTTCTCTGGTGGTGCTCTCAGCCGTGCTTCTATCTTTGCCCT
GGGGATCATGCCGTATATTTCGGCGTCGATCATTATCCAGCTGTTGACGGTGGTTCATCCAACGCTGGCAGAA
ATTAAGAAAGAAGGGGAGTCTGGTCGTCGTAAGATCAGCCAGTACACCCGTTACGGTACTCTGGTGCTGGCGA
TATTCCAGTCGATCGGTATTGCTACCGGTTTGCCGAATATGCCTGGTATGCAGGGCCTGGTGATGAATCCAGG
CTTTGCATTCTATTTCACCGCTGTTGTAAGTCTGGTTACAGGGACGATGTTCCTGATGTGGCTCGGCGAACAG
ATTACTGAGCGCGGTATCGGTAACGGTATCTCAATCATCATCTTCGCTGGTATCGTTGCGGGACTCCCGCCAG
CCATTGCCCATACTATCGAGCAAGCGCGTCAAGGCGACCTGCACTTCCTCGTGTTGCTGTTGGTTGCAGTATT
AGTATTTGCAGTGACGTTCTTTGTAGTATTCGTTGAACGTGGTCAACGCCGTATTGTGGTAAACTACGCCAAA
CGTCAGCAAGGTCGTCGTGTCTATGCTGCACAGAGCACACATTTACCGCTGAAAGTGAATATGGCGGGGGTAA
TCCCGGCAATCTTCGCTTCCAGTATTATTCTGTTCCCGGCGACCATCGCGTCATGGTTCGGGGGCGGTACTGG
TTGGAACTGGCTGACAACAATTTCGCTGTATTTGCAGCCTGGGCAACCGCTTTATGTGTTACTCTATGCGTCT
GCAATCATCTTCTTCTGTTTCTTCTACACGGCGTTGGTTTTCAACCCGCGTGAAACAGCAGATAACCTGAAGA
AGTCCGGTGCATTTGTACCAGGAATTCGTCCGGGAGAGCAAACGGCGAAGTATATCGATAAAGTAATGACCCG
CCTGACCTTGGTTGGTGCGCTGTATATTACCTTTATCTGCCTGATCCCGGAGTTCATGCGTGATGCAATGAAA
GTGCCGTTCTACTTCGGTGGGACCTCACTGCTTATCGTTGTTGTCGTGATTATGGACTTTATGGCTCAAGTGC
AAACTCTGATGATGTCCAGTCAGTATGAGTCTGCATTGAAGAAGGCGAACCTGAAAGGCTACGGCCGTTAATT
GGTCGCCTGAGAAGTTACGGAGAGTAAAAATGAAAGTTCGTGCTTCCGTCAAGAAATTATGCCGTAACTGCAA
AATCGTTAAGCGTGATGGTGTCATCCGTGTGATTTGCAGTGCCGAGCCGAAGCATAAACAGCGCCAAGGCTGA
TTTTTTCGCATATTTTTCTTGCAAAGTTGGGTTGAGCTGGCTAGATTAGCCAGCCAATCTTTTGTATGTCTGT
ACGTTTCCATTTGAGTATCCTGAAAACGGGCTTTTCAGCATGGTACGTACATATTAAATAGTAGGAGTGCATA
GTGGCCCGTATAGCAGGCATTAACATTCCTGATCAGAAACACGCCGTGATCGCGTTAACTTCGATCTACGGTG
TCGGCAAGACCCGTTCTAAAGCCATCCTGGCTGCAGCGGGTATCGCTGAAAATGTTAAGATCAGTGAGCTGTC
TGAAGAACAAATCGACACGCTGCGTGACGAAGTTGCCAAATTTGTCGTTGAAGGTGATCTGCGCCGTGAAATC
AGCATGAGCATCAAGCGCCTGATGGATCTTGGTTGCTATCGCGGTTTGCGTCATCGTCGTGGTCTCCCGGTTC
GCGGTCAGCGTACCAAGACCAACGCTCGTACCCGTAAGGGTCCGCGCAAACCGATCAAGAAATAATCGGGGTG
ATTGAATAATGGCAAAGGCACCAGTTCGCGCACGTAAACGTGTAAGAAAACAAGTCTCTGACGGCGTGGCTCA
TATCCATGCTTCTTTCAACAACACCATCGTGACTATTACCGATCGTCAGGGTAATGCGCTGGGTTGGGCAACA
GCCGGTGGTTCCGGTTTCCGTGGTTCTCGCAAATCCACTCCGTTTGCAGCTCAGGTTGCAGCAGAGCGTTGCG
CTGACGCCGTGAAAGAATACGGTATCAAGAATCTGGAAGTTATGGTTAAAGGTCCGGGTCCAGGCCGCGAATC
TACTATTCGTGCTCTGAACGCCGCTGGTTTCCGCATCACTAATATTACTGATGTGACTCCGATCCCTCATAAC
GGTTGTCGTCCGCCGAAAAAACGTCGCGTATAACGCTTCGTTTTCCAGGTTAGTTGGAGAAAGAAAATGGCAA
GATATTTGGGTCCTAAGCTCAAGCTGAGCCGTCGTGAGGGCACCGACTTATTCCTTAAGTCTGGCGTTCGCGC
GATCGATACCAAGTGTAAAATTGAACAAGCTCCTGGCCAGCACGGTGCGCGTAAGCCGCGTCTGTCTGACTAT
GGTGTGCAGTTGCGTGAAAAGCAAAAAGTTCGCCGCATTTACGGTGTGCTGGAGCGTCAGTTCCGTAACTACT
ACAAAGAAGCAGCACGTCTGAAAGGCAACACCGGTGAAAACCTGTTGGCTCTGCTGGAAGGTCGTCTGGACAA
CGTTGTATACCGTATGGGCTTCGGCGCCACTCGTGCAGAAGCACGTCAGCTGGTTAGCCATAAAGCAATTATG
GTAAACGGTCGTGTTGTTAACATCGCTTCTTATCAGGTTAGTCCGAATGACGTTGTTAGCATTCGTGAGAAAG
CGAAGAAGCAGTCTCGCGTGAAAGCCGCTCTGGAGCTGGCTGAGCAGCGTGAAAAGCCAACCTGGCTGGAAGT
TGATGCTGGCAAGATGGAAGGTACGTACAAGCGTAAGCCTGAGCGTTCTGATCTGTCTGCGGACATTAACGAA
CACCTGATCGTCGAGCTTTACTCCAAGTAAAGCTTAGTACCAAAGAGAGGACACAATGCAGGGTTCTGTGACA
GAGTTTCTAAAACCGCGCCTGGTAGATATCGAGCAAGTGAGTTCGACGCACGCCAAGGTGACCCTTGAGCCTT
TAGAGCGTGGCTTCGGCCATACTCTGGGTAACGCACTGCGCCGTATTCTGCTCTCATCGATGCCGGGTTGCGC
GGTGACCGAGGTTGAGATTGATGGTGTACTACATGAGTACAGCACCAAAGAAGGCGTTCAGGAAGACATCCTG
GAAATCCTGCTCAACCTGAAAGGGCTGGCGGTGAGAGTTCAGGGTAAAGATGAAGTTATTCTTACCTTGAATA
AATCTGGCATTGGCCCTGTGACTGCAGCCGATATCACCCATGATGGGGATGTCGAAATCGTCAAGCCGCAGCA
CGTGATCTGCCACCTGACCGATGAAAACGCGTCTATTAGTATGCGTATCAAAGTTCAGCGCGGTCGTGGTTAT
GTGCCGGCTTCTACCCGAATTCATTCGGAAGAAGATGAGCGCCCAATCGGCCGTCTGCTGGTCGACGCCTGCT

ACAGCCCTGTAGAGCGTATTGCCTACAATGTTGAAGCAGCGCGTGTAGAACAGCGTACCGACCTGGACAAGCT
GGTCATCGAAATGGAAACCAACGGCACAATCGATCCTGAAGAGGCGATTCGTCGTGCGGCAACCATCCTGGCT
GAACAACTGGAAGCTTTCGTTGATTTACGTGATGTACGTCAACCGGAAGTGAAAGAAGAGAAACCAGAATTCG
ATCCGATCCTGCTGCGCCCTGTTGACGATCTGGAATTGACTGTCCGCTCTGCTAACTGCCTCAAGGCAGAAGC
TATCCACTATATCGGTGATCTGGTACAGCGTACCGAGGTTGAGCTTCTTAAGACGCCTAACCTGGGTAAAAAA
TCTCTTACCGAGATTAAAGACGTGCTGGCTTCCCGTGGACTGTCTCTGGGTATGCGCCTAGAAAACTGGCCAC
CGGCAAGCATCGCTGACGAGTAACCGGATCACAGGTTAAGGTTTTACTGAGAAGGATAAGGTCATGCGCCATC
GTAAGAGTGGTCGTCAACTGAACCGCAACAGCAGCCATCGCCAGGCTATGTTCCGCAACATGGCAGGTTCACT
GGTTCGTCATGAAATCATCAAGACGACCCTGCCTAAAGCGAAAGAGCTGCGTCGCGTAGTTGAGCCGCTGATT
ACTCTTGCCAAGACTGATAGCGTTGCTAATCGTCGTCTGGCATTCGCCCGTACTCGTGATAACGAGATCGTGG
CAAAACTGTTTAACGAGCTGGGCCCGCGTTTCGCGAGCCGCGCCGGTGGTTACACTCGCATTCTGAAGTGTGG
CTTCCGTGCAGGCGACAACGCGCCGATGGCATACATCGAGCTGGTTGATCGTTCAGAGAAAACAGAAGCTGCT
GCAGAGTAATCTGTAGTAACGTAAAAAAACCCGCTTCGGCGGGTTTTTTTATATCCTCCTGAACCCCACATAT
CTACAATAATTGTATTCTTTTCGTTCAATCCCTGGAGCGACGTATGTGGTTACTTGACCAGTGGGCAGAGCGT
CATATTATCGAGGCACAGCGTAAAGGCGAGTTTGATAATTTGCCTGGCCGTGGCGAGCCGCTTATTCTGGATG
ATGATTCTCATGTGCCGGCGGAACTTCGTGCGGGTTATCGCTTACTCAAGAATGCGGGCTGTCTTCCCCCTGA
ACTGGAGCAGCGCAGAGACGCTATTCAGTTACTTGATATCCTCAACAGTATCCGGGAAGATGACCCTCGATAC
CATCAGGTTAGTCGCCAGCTCTCCCTGCTTGAACTAAAACTTCGGCAGGCTGGGTTGAGTACCGACTTTTTAC
ACGGTGAGTATGCAGAAAAACTGCTGCATAAAATCAACGATAATTAACGGAGCTTGTATGTATCGCATTGGTG
AGCTGGCAAAGCTGGCAGACGTAACGCCTGATACCATTCGCTACTATGAAAAACAGCAGATGATGGATCATGA
GGTACGTACTGAAGGCGGGTTTCGTCTGTATACCGAAACCGATCTGCAGCGTCTCAAGTTTATTCGTTATGCG
CGACAGTTGGGTTTCACGCTCGACTCGATCCGCGAGCTTTTATCGATCCGAATTGATCCTGAGCACCATACCT
GTCAGGAGTCAAAAAGTATTGTGCAAGAGCGATTGCAGGAGGTTGAAGCCAGGATTGCGGAACTGCAAACGAT
GCAGCGTTCGTTACAAAGGCTTAATGATGCTTGTTGCGGAACGGCCCACAGTAGCGTCTATTGTTCCATTCTG
GAAGCCCTGGAACAGGGGGCCAGTGGAGCGAAATCAGGATGTTGATTTATTGAACCGGCGGGGCTACACTCGA
GCCGTTAATTTATACAATCGGGAGACATTATGAGTCGTTATCAACATAAGAAAGGGCAGATAAAGGACAATGC
CATTGAGGCATTACTGCACGATCCCCTTTTCAGGCAACGCGTTGAAAAAAATAAGAAAGGCAAAGGAAGCTAT
CTGCGCAAAGGTAAACACGGTAATCGAGGGAACTGGGAGGCCAGTGGCAAGAAAGTAAGTAACTTTTTTACCA
CTGGCCTTCTGCTTATTAAGAACGGTTATTTTGTTCTTTCAGCAGATCGCGGATTTCGCCCAATAGCACTTCC
TCTTTAGAAGGCGCTGGCGGAGCTGCCGGCTCTTCTGCTTTTTTACGGTTGAGTCTGTTGATAAGCTTAATGG
CAACAAAGATAGCAAAGGCCACAATCACAAAATCAAATACGTTCTGGATAAACACGCCGTAATGCATGACGAC
CGCCGGAATATCTCCCTGCGCTTCGCGTAACGTAAAAGCAAATTGTTTAAAGTCGATCCCGCCGATTAACAAT
CCCAACGGCGGCATAATGATATCGGCAACCAGTGAAGAAACAATCTTACCGAATGCCGCGCCAATAATGACAC
CCACTGCCAAATCCACAACATTCCCGCGCATCGCGAATTCGCGAAATTCTTTAATAAAACTCATTTTTATTCT
CCCTATGCAGCTGACGTTATAAGTTTAACAAATGCTAAGGCATTTTCCATTTACGGCTTCTGTTTAGCGAAAT
AAATTAAACCTTGATAAATAAAGGGAATTAAAAAGGGCGCTGAATGAGCGCCCCTTAAATTAGAGGAAGAAAG
GACTTGGCTGGAACAGACGTTCGACGTCGGTAATAAACTTTTTATCGGTCAAAAACATAATGACGTGATCGCC
TTGTTCAATGCGCAAATTGTCATTAGCAATCATGACATCATTACCACGAACGACCGCGCCAATAATGGTGCCT
GGCGGCAGTTTTATTTCGTCAATGACGCGGCCAACCACGCGGGAAGTGCTTTCATCGCCGTGCGCGACGGCTT
CGATGGCTTCGGCAACTCCGCGACGTAGTGAGGAAACGCCGACGATATCCGCTTTACGCACATGGCTCAGTAA
TGCAGAGATGGTGGCCTGCTGCGGAGAAATCGCAATATCTATAACGCTACCCTGCACAAGATCGACATAAGCT
CGACGCTGAATCAACACCATCACTTTTTTGGCGCCCATCCGCTTGGCCAACATCGCGGACATGATATTGGCTT
CATCATCGTTAGTGACCGCGATAAAGAGATCGACCTGATCAATATGCTCCTCCGCCAGCAATTCCTGATCGGA
GGCGTCGCCAAAAAAGACGATCGTGTTTTGCAGTTTCTCAGCCAGTTCTGCGGCGCGTTGTTGATCGCGTTCG
ATCAATTTAACGCTATAGTCTTTTTCCAGACGCCTTGCCAGCCCGGCGCCGATATTGCCGCCGCCCACCAGCA
TGATGCGCTTGTAGGGTTTTTCCAGACGCTGTAATTCGCTCATTACTGCACGGATATGCTGTGAAGCCGCAAT
AAAGAAGACTTCATCCCCTGCTTCCACGATAGTCGAGCCCTGAGGACGGATTGGTCTGTCATGGCGGAAAATT
GCGGCAACGCGAGTATCGATATGCGGCATATGTTCTCGCATTGTCGAGAGCGCGTTACCTATTAGTGGACCGC
CATAGTAGGCTTTTACTACTGCCAGACTGACTTTCCCTTCAGCAAAGTTTACAACCTGTAATGCTCCCGGATA
CTCGATCAGACGATAAATATTATCGATAACCAACTGCTCCGGCGCGATCAGATGATCAATCGGAACCGCTTCA
GAATGAAAGAGTTTATCCGCATCGCGAACATAGTCCGGGGAGCGAATGCGGGCGATACGGTTTGGAGTGTTGA
AGAGTGAATAGGCTACCTGACAGGCAACCATATTAGTTTCATCTGAACTGGTGACAGCGACCAGCATATCCGC
ATCGTCAGCGCCCGCTTCGCGCAACACGCGGGGATGCGAACCATGCCCCTGAACTACGCGTAGATCGAACTTA
TCTTGCAAACTGCGTAACCGTTCGCCGTTGGTATCAACCACCGTGATGTCGTTGTTTTCGCCGACCAGGTTTT

CCGCCAGTGTTCCGCCAACTTGCCCTGCGCCCAGTATGATAATTTTCATCAGTCGTGGCCCGTTATCTCATCA
CTTCTTGATTAGCTTAGCGTAAAAGAACCCATCACCTTCTTCGCCGCCCGGCAGATTTTGCTGTCCTGGCTGA
TCAGGTGTTCCCGTTTCGCTGAGCGCGGCATCGGGAGTGCGTTGCAAAAAGGTTTTGATCTGATCGCGGTTTT
CTTCCGGCAGTACGGAACAGGTGGCGTAAACCAGCGTCCCGCCAGGCTTCAGGCGCGGCCAGACGGCATCAAG
AATTTCCGCCTGTAATTGCGCCAGTTCCGCGATATCGCGATCGCGACGCAGCCATTTGATATCTGGATGACGG
CGAATGACGCCGGTTGCGGAGCAGGGCGCATCCAGCAGAATACGGTCAAACTGCTGCTCGCCGCACCATTGGG
CGGGATAACGTCCATCCCCTTGTTTAACTGTCGCTTTCATCCCAAGACGCTTTAGGTTGTCGTAAACGCGGGA
GAGTCGTTGTTCATCAATATCAACCGCCAACACGTCTGCTTCGGGGGCCACTTCGAGAATATGCGTGGTTTTA
CCGCCGGGCGCGGCGCACAAGTCTAAAATGTGCTCACCGTTCTGAGGAGCGAGAAAAACCGCACATCCCTGCG
CAGAGGCATCCTGGACAGTTACCCAACCCTCAGCAAAACCGGGTAAAGCATGTACCGGAGCTGGCGTTTCCAG
ACGTACAGCGTCGGGATAATCGGGATGGGGATATCCCTTCATGCCTGCGTCTTCAAGTAAGCCAAGCCAACCG
TCGCGGGTGTGGTGTGTACGATTTACACGTAGCCACATCGGTGGACGTTGGTTATTCGCTTCAATAATGTGTT
GCCACTGTGTGGGGTAAGCATTTTGCAAACGCTTTACCAGCCAACCTGGATGTAAAAAGCGCGCATCGCTGGT
CGCAAACTCATTAAGCAACGTTTCCTGCTGGCGCTGAAACTGGCGCAGGACGCCGTTAATTAACCCTTTCAAC
TGCGGACGTTTGATGGCGACCGCGCCTTCAACGGTTTCAGCCAGCGCCGCATGGGGAGGAACGCGGGTGTACA
GCAACTGATAAAAGCCGACCATAATTAAGTAATGCACTGTACGCTGCTTGCCGGTCATTGGACGAGACATCAG
CTTATTGATCAGCCATTCCAGCTGTGAAAGGGTGCGCAGGACGCCAAAGCACAGCTCCTGAAGTAGCGCTTTG
TCTTTATCGGCGACTTTTTGTTGCAGCGGCGGGAGCACATTACTTAATGATTGTCCCTGTTCGACGACCAGTT
CAACGGCCTGTGCTGCCAGACTACGTAAATTGTTTTGTTTTTTCATATGTATAAAATAAAAATGCCCGGTAAC
ACCGGGCTTATAAAGACGTGACCGTCAGGCAAGACGGTTACCAGGAATAAACCATTCCCGGCGTGAATTTAAC
AGATCCTGGGCGCTCATTGCTTTTTTGCCGGCAGGCTGAAGCGAAAGCAAATTTAATATCCCCTTGCCTGTTG
CAACCTGAATGCCCTGTTTCGTCGCGGCCAGAATGGTTCCCGGCAGCGACTGCGTGGCGTCCTCAATGACGGA
CGCCTGCCAGACTTTTACCGGCTGGCCGTCGATTTCCAGCCAGCTCATTGGCCACGGATTAAAGGCGCGAATG
CAACGCTCCAGCTGTACTGCGGAGAGAGACCAGTCGATACGCGCTTCTTCTTTACTGAGCTTTTCCGCATGAG
TGACCAGCGCTTCATTCTGCGCTTCAGGCGCCGCTGTGCCGTCCGCCAGTTGTTTTAGTGTGGTAATTAACCC
CTGCGGGCCAAGCTCAGCCAACTTATTATACAGTGAGCCACTGGTATCTTCTGCGGTAATCGGGCACGCAAGT
TTATACAGCATGTCGCCGGTATCCAGCCCTACATCCATCTGCATAATTGTGACGCCGGTTTCCGCATCGCCTG
CCCAGAGTGAGCGCTGAATAGGCGCCGCGCCGCGCCAGCGTGGAAGCAGCGAACCGTGAACGTTGATACATCC
GAGACGGGGCATATCCAGCACTGCCTTTGGGAGAATCAGACCATATGCTACGACAACCATGACGTCGGCATGT
AAGTCAGCAACCAGTTGCTGGTTTTCCTGAGGACGAAGCGACACGGGCTGGAAAACGGGCAGGCCTTTTTCTT
CCGCCAGCACTTTTACCGGGCTGGGCATCAACTTTTTGCCGCGACCTGCTGGACGATCGGGTTGGGTAAATAC
GCCGACAACGTTATGTCCAGAAGTTAACAGCGCGTCGAGATGACGCGCTGCAAAGTCAGGTGTACCCGCAAAA
ATAATACGTAGTGAATCTGACACGTTGGTTCTTGTCCTTTAAGCCCGGGCGTTCAGGCGGTCGAGTTTTTCTA
CTTTCTGACGAATACGTTGTTGCTTCAGCGGCGACAAATAATCGATAAACAGTTTACCGACCAGATGATCCAT
CTCATGCTGAATGCAAATTGCCAGCAAACCATCCGCTTCAAGCTCAAACGGGTTGCCGTCGCGATCCAGCGCG
CGGATTTTCACTTTCTCGGCGCGCGGCACTAAAGCGCGCTGTTCCGGAATCGACAGACAGCCTTCTTCTATAC
CCGTTTCGCCACTTTTCTCCAGCAATTCCGGGTTAATGAGTACCAGGCGCTCGTCGCGGTTTTCCGAAACGTC
AATCACGATGATCCGCTGGTGGATATCGACCTGCGTTGCGGCCAGACCGATACCTTCTTCCGCGTACATCGTT
TCGAACATATCATCGACGATACGCTGAATTTCTGCATTCACTTCTTCAACCGGCTTTGCGACTTTGCGAAGGC
GCTCGTCCGGAATATGTAACACTTGCAAAACTGACATAATTATCCAGAGTTGTGTTCAGGAGTTGAAAAGATT
ATTACCTCTATTCTAGACAAATCCCCACCTGATTGACAGCATCAGTGACCAATCGCAAAGATTGTTCAGACTG
CTTGTGGCAGGGGAGGGAAAGGATGGCCCGTACCGAAATTTGGTTACGTTTAATGTACGTCGGCGACCTTTAT
GGCGAGGCGATGTTGAACATGGCGAACTCGCTTATTCGTCAGCCTCAGATAAATCGCACGCACCTTCAGGAGG
CTGGTCTTACCGCGCGGCAAGCTGAACGTTTTTTACAGCTTCCAGCAGGTGTGCTTGATGAGACGTTACGCTG
GCTTGAACTACCGCAGCATCATTTTCTGTGTGCGGATAGTGAGATTTATCCTCCCCAACTGCGTGCTATTGAT
GATTATCCCGGCGCTATTTTTATTGACGGCGATCCTGCCTGTCTGCATACCTGCCAACTTGCCGTCGTAGGGA
GCCGGAGCCACTCCTGGTATGGAGAACGCTGGGGACGTCTGCTTTGCGAAAGCCTTGCGAAAAGCGGTTTGAC
GATCACCAGCGGCCTTGCCCGGGGAATTGATGGCGTAGCACACAACGCCGCGGTGAGTATGGGGGGAAAAAGT
GTAGCGGTGTTAGGGAATGGCTTGGCAAAGATTTATCCTCGCCGACATGCCATGCTGGCTGAAAACTTGATTG
CTACCGGCGGCGCAGTGGTCTCAGAATTTCCGCTTTCAACGCCTCCGCTACCGCAAAATTTTCCTCGCAGAAA
CCGTATCATCAGCGGACTGAGCAAAGGCGTGCTGGTGATTGAGGCGGCGTTGCGCAGCGGTTCGTTGGTGACG
GCGCGCTGCGCGCTTGAACAGGGGCGGGACGTTTTTGCATTACCAGGTCCTATCGGTAGTCCGGGAAGCGAAG
GCACGCACTGGTTAATTAAACAAGGGGCCACGCTTGTGACGACGCCGGAGGATATTCTGGAAAATTTGCAATA
CGGCTTACACTGGTTGCCAACTACGGCGGAAAATTCACTTTATTCACTAAATCAGGATGAGGCGGCATTGCCA

TTTCCTGAGCTCCTGGCTAACGTAGGAGATGAGGTAACACCTGTTGACGTCGTCGCTGAACGTGCCGGCCAAC
CTGTGCCAGCGGTAGTGGCTCAGCTACTCGAACTGGAGTTAGCAGGATGGATCGCAGCTGTACCCGGCGGCTA
TGTCCGATTAAGGAGGGCAAGCCATGTTCGACGTACTAATGTATTTGTTTGAAACATACATCCATAACGAAGC
CGAGCTGCGCGTGGATCAGGACAGACTGGAGCGTGATCTCACCGACGCTGGGTTTGATCGCGAAGACATCTAC
AATGCGCTGCTGTGGCTGGAAAAACTCGCTGACTATCAGGATGGCCTCGCGGAACCCATGCAACTTGCTTCCG
ATCCTCTTTCAATGCGCATTTATACGGTTGAAGAGTGTGAAAGGCTTGATGCCAGTTGCCGGGGGTTCCTGTT
ATTCCTTGAGCAGATTCAGGTGCTAAACCTCGAAACACGAGAAATGGTGATTGAGCGTGTGCTGGCGCTGGAT
ACCGCAGAGTTTGACCTGGAAGACTTGAAATGGGTTATTTTGATGGTGTTATTCAACATTCCGGGCTGTGAAA
ACGCCTATCAGCAAATGGAAGAATTACTCTTTGAGGTAAATGAAGGTATGCTGCATTGATAGGTGTTAATGCT
GCATGAGTGAATATGGCTAAATCAGCACTGTTTTCGGTGCGTAAAAATGAGCCCTGCCCGCAGTGCGGGGCCG
AACTGGTAATTCGCTCCGGGAAACATGGTCCGTTTCTCGGCTGTTCTCGCTATCCGGAATGCGATTACGTTCG
TCCGCTGAAAAGTCAGGCGGACGGACATATCGTTAAAATTCTGGAGGGGCAACCGTGCCCTGAGTGTGGCGCC
GTGCTGGTGCTGCGTCAGGGGCGCTTTGGCATGTTCATCGGATGCAGTCAGTATCCGCAATGTGAACATACAG
TGGTTATTGATAAACCGGATGAAACGGCGATTGCCTGTCCAGCCTGCCAGCAAGGACATTTGGTCCAGCGCCG
TTCTCGTTACGGTAAAATCTTTCATTCCTGCGATCGCTACCCGGAATGTCAGTTCGTCATTAACTTCACACCG
GTTGCGGGGGAATGCCCCGAGTGCCATTATCCGCTACTCATCGAAAAGAAAACCGCGCAGGGCGTGAAGCGCT
TTTGCGCCAGTAAACAATGTGGAAAGCCGGTTCCGGTGGAATAAACAAGTGAATAATAGCCTGCCAACAGGAT
CTATCGCCGCAGCGGTAGATCTCCTGAATAAAGAAAATGTCATCGCTTATCCAACAGAAGCCGTTTTCGGCGT
CGGCTGCGATCCCGATAGTGAAACAGCCGTTACGCGCCTGCTGGCGTTAAAACAACGGCCCGTCGATAAGGGA
CTCATTTTGATTGCCGCCAGCTTTGAGCAGTTAAAACCTTATATTGATGACAGCATACTTACCGCCGCACAGC
GTAAAGCGGTATTTGATTGCTGGCCCGGGCCGGTAACGTTTGTTTTTCCTGCGCCCGCGACAACGCCGCGGTG
GCTCACGGGACGTTTTGATTCCCTGGCCGTTCGTGTTACCAACCACCCGCTGGTGGTCGCGCTATGCAATGCG
TATGGCAAACCGCTGGTTTCCACCAGCGCCAATCTGAGCGGATTACCGCCATGTCGTACCATTGAAGAGGTGC
GTGCGCAATTTGGCGATGATTTTCCGGTGGTTGAAGGCGCAACGGGTGGGCGTTTAAATCCTTCAGAAATTCG
CGATGCTCTGACGGGCGAACTGTTTCGACAGGGGTAATATGATGGAAACCTATGCTGTTTTTGGAAATCCGAT
TGCGCACAGCAAATCGCCATTTATTCATCAGCAATTTGCTCAGCAGCTAGATATTGTTCACCCCTATGGTCGC
GTGCTGGCCCCTATTAATAATTTCATTAATACGCTTGATGCCTTTTTCGCGGCAGGGGGAAAAGGCGCAAACA
TCACAGTACCTTTTAAAGAGGAGGCGTTTGCGCGTTCGGATGAGTTAACGGAACGAGCATCGCTGGCGGGAGC
CGTCAATACATTAAAACGGCTGGAAGATGGTCGTTTGCTTGGCGACAATACTGACGGTATCGGTTTATTAAGC
GATCTCGAACGTTTAAATTTTATCCGCCCAGGATTGCGTATTTTGCTGATTGGCGCGGGCGGCGCATCCCGGG
GCGTGCTGTTACCCCTGCTTTCTTTGGATTGCGCGGTCACTATCACTAACCGTACAGCTTCACGTGCCGAAGC
GTTGGCGAAAATCTTTGCTCATACCGGCAGCGTTCATGCCACGGATATGGACAAGCTGGATGGTTGTGAGTTT
GACCTGATTATTAATGCGACCTCCAGCGGCATACGGGGCGAAATCCCGGCGATTCCGGCGTCACTTATTCACC
CTTCCCTCTGTTGCTATGACATGTTCTATCAAAAAGGGAATACGCCATTTCTCTCCTGGTGTGTACAACAGGG
CGCAAAACGCTACGCAGATGGGCTGGGAATGCTGGTGGGGCAGGCTGCACATGCCGTTTTGCTTTGGCACGGT
GTATTACCGCAGGTCGAGCCAGTGATTGAGCTGCTACAGCAGGAATTATTAGCGTGAACCAGGCCATACAATT
TCCTGACCGCGAAGAATGGGATACTGCCGCCAGTGCTATCATCTTTCCCGCCCTGGTTAATGGGATGCAACTC
ACGTGTGCAATAAAAAAAGATGTACTGGCGTATCGCTTTGGTGGAGAAACAGCGGAGCAATGGCTGGCCATTT
TTCGTGAGTATCGCTGGGACCTGGAAGAAGAGGCGGAAGCATTAATCCTGGCCCAGCAGGAAGATGATCACGG
TTGGATATGGTTATCCTGAGAAAGATAGTCGTCTTTCCACTTTACATAGTTATTGGCGGAGTACTGCAGGCCC
GATCTCTCAGCATCGCTCAAGGGGCGGATCTGTTTTACCGGACTGCCAAGATATAAATAGCCGCTTTCCAGCC
GTTTGTGCTGCGGCACCAGGCTTCCCGCGCCAATCATTACGTCATCTTCTATTATCGCGCCATCCAACACAAT
GGAGCCCATACCGACCAGTACGCGGTTACCGATGGTACAGCCGTGCAGCATAACTTTATGGCCAACGGTAACA
TCTTCGCCAATGATTAATGGGTTGCCGTGTGGATTGGATGAAGACTTGTGGGTTACGTGAAGAACGCTGCCAT
CCTGAATGTTCGTACGAGCCCCTATCGCAACGTAATTCACATCGCCGCGAATCACCACAAGCGGCCAGATCCC
GATATCATCCGCCAGACGGACATCACCAATAACCACGCTGCTGGTATCGATCATCACGCGCTGACCGATTTCA
GGAAAAAGATCCTTATAAGGACGTAATGTGTCAGACATGTCTACCTCATCATAAATGAATAGTCATGAAGACT
TTGGTTGCTTTAACGGCGTTGTGCAAGGGGAGATAGCATCAAAAAATCGCCACTTTGCGCAGGAATGGAGCGA
AAGGGATGAAAAATCAACAAACAGAAAAAAAGATCCAAAAAACGCTTGTGCAAAAAAATGGGATCCCTATAAT
GCGCCTCCATCGACACGGCGGATGTGAATCACTTCACACAAACAGCCGGTCGGTTGAAGAGAAAAATCCTGAA
ATTCAGGGTTGACTCTGAAAGAGGAAAGCGTAATATACGCCACCTCGCAACGGTGAGCGAAAGCCGCGTTGCA
CTGCTCTTTAACAATTTATCAGACAATCTGTGTGGGCACTCGAAGATACGGATTCTTAACGTCCTCGGACGAA
AAATGAATACCAAGTCTCAAGAGTGAACACGTAATTCATTACGAAGTTTAATCCTTTGAGCATCAAACTTTTA
AATTGAAGAGTTTGATCATGGCTCAGATTGAACGCTGGCGGCAGGCCTAACACATGCAAGTCGAACGGTAACA

GGAAGCAGCTTGCTGCTTCGCTGACGAGTGGCGGACGGGTGAGTAATGTCTGGGAAACTGCCTGATGGAGGGG
GATAACTACTGGAAACGGTGGCTAATACCGCATAACGTCGCAAGACCAAAGAGGGGGACCTTCGGGCCTCTTG
CCATCAGATGTGCCCAGATGGGATTAGCTTGTTGGTGAGGTAACGGCTCACCAAGGCGACGATCCCTAGCTGG
TCTGAGAGGATGACCAGCCACACTGGAACTGAGACACGGTCCAGACTCCTACGGGAGGCAGCAGTGGGGAATA
TTGCACAATGGGCGCAAGCCTGATGCAGCCATGCCGCGTGTATGAAGAAGGCCTTCGGGTTGTAAAGTACTTT
CAGCGGGGAGGAAGGTGTTGTGGTTAATAACCGCAGCAATTGACGTTACCCGCAGAAGAAGCACCGGCTAACT
CCGTGCCAGCAGCCGCGGTAATACGGAGGGTGCAAGCGTTAATCGGAATTACTGGGCGTAAAGCGCACGCAGG
CGGTCTGTCAAGTCGGATGTGAAATCCCCGGGCTCAACCTGGGAACTGCATTCGAAACTGGCAGGCTTGAGTC
TTGTAGAGGGGGGTAGAATTCCAGGTGTAGCGGTGAAATGCGTAGAGATCTGGAGGAATACCGGTGGCGAAGG
CGGCCCCCTGGACAAAGACTGACGCTCAGGTGCGAAAGCGTGGGGAGCAAACAGGATTAGATACCCTGGTAGT
CCACGCCGTAAACGATGTCTACTTGGAGGTTGTGCCCTTGAGGCGTGGCTTCCGGAGCTAACGCGTTAAGTAG
ACCGCCTGGGGAGTACGGCCGCAAGGTTAAAACTCAAATGAATTGACGGGGGCCCGCACAAGCGGTGGAGCAT
GTGGTTTAATTCGATGCAACGCGAAGAACCTTACCTGGTCTTGACATCCACAGAAGAATCCAGAGATGGATTT
GTGCCTTCGGGAACTGTGAGACAGGTGCTGCATGGCTGTCGTCAGCTCGTGTTGTGAAATGTTGGGTTAAGTC
CCGCAACGAGCGCAACCCTTATCCTTTGTTGCCAGCGGTTAGGCCGGGAACTCAAAGGAGACTGCCAGTGATA
AACTGGAGGAAGGTGGGGATGACGTCAAGTCATCATGGCCCTTACGACCAGGGCTACACACGTGCTACAATGG
CGCATACAAAGAGAAGCGACCTCGCGAGAGCAAGCGGACCTCATAAAGTGCGTCGTAGTCCGGATTGGAGTCT
GCAACTCGACTCCATGAAGTCGGAATCGCTAGTAATCGTGGATCAGAATGCCACGGTGAATACGTTCCCGGGC
CTTGTACACACCGCCCGTCACACCATGGGAGTGGGTTGCAAAAGAAGTAGGTAGCTTAACCTTCGGGAGGGCG
CTTACCACTTTGTGATTCATGACTGGGGTGAAGTCGTAACAAGGTAACCGTAGGGGAACCTGCGGTTGGATCA
CCTCCTTACCTTAAAGAACTGTTCTTTGAAGTGCTCACACAGATTGTCTGATGAAAAGTAAAAAGCAAGGCGT
CTTGCGATTGAGACTTCAGTGTCCCCTTCGTCTAGAGGCCCAGGACACCGCCCTTTCACGGCGGTAACAGGGG
TTCGAATCCCCTAGGGGACGCCACTTGCGCGGTAATGTGTGAAAGGCGTTGCCATCAGTATCTCAAAACTGAC
TTACGAGTCACGTTTGAGATATTTGCTCTTTAAAAATCTGGATCAAGCTGAAAATTGAAACACAGAACAACGA
AAGTTGTTCGTGAGTCTCTCAAATTTTCGCAACACGATGATGAATCGTAAGAAACATCTTCGGGTTGTGAGGT
TAAGCGACTAAGCGTACACGGTGGATGCCCTGGCAGTCAGAGGCGATGAAGGGCGTGCTAATCTGCGATAAGC
GCCGGTAAGGTGATATGAACCGTTATAACCGGCGATACCCGAATGGGGAAACCCAGTGTGATTCGTCACACTA
TCATTAACTGAATCCATAGGTTAATGAGGCGAACCGGGGGAACTGAAACATCTAAGTACCCCGAGGAAAAGAA
ATCAACCGAGATTCCCCCAGTAGCGGCGAGCGAACGGGGAGGAGCCCAGAGCCTGAATCAGCATGTGTGTTAG
TGGAAGCGTCTGGAAAGGCGCGCGATACAGGGTGACAGCCCCGTACACAAAAGCGCATGTGCTGTGAGCTCGA
TGAGTAGGGCGGGACACGTGGTATCCTGTCTGAATATGGGGGGACCATCCTCCAAGGCTAAATACTCCTGACT
GACCGATAGTGAACCAGTACCGTGAGGGAAAGGCGAAAAGAACCCCGGCGAGGGGAGTGAAAAAGAACCTGAA
ACCGTGTACGTACAAGCAGTGGGAGCACAGGTTTACCTGTGTGACTGCGTACCTTTTGTATAATGGGTCAGCG
ACTTATATTCTGTAGCAAGGTTAACCGAATAGGGGAGCCGGAGGGAAACCGAGTCTTAATTGGGCGTTAAGTT
GCAGGGTATAGACCCGAAACCCGGTGATCTAGCCATGGGCAGGTTGAAGGTTGGGTAACACTAACTGGAGGAC
CGAACCGACTAATGTTGAAAAATTAGCGGATGACCTGTGGCTGGGGGTGAAAGGCCAATCAAACCGGGAGATA
GCTGGTTCTCCCCGAAAGCTATTTAGGTAGCGCCTCGTGAATTCATCTCCGGGGGTAGAGCACTGTTTCGGCT
AGGGGGCCATCCCGGCTTACCAACCCGATGCAAACTGCGAATACCGGAGAATGTTATCACGGGAGACACACGG
CGGGTGCTAACGTCCGTCGTGAAGAGGGAAACAACCCAGACCGCCAGCTAAGGTCCCAAAGTCATGGTTAAGT
GGGAAACGATGTGGGAAGGCCCAGACAGCCAGGATGTTGGCTTAGAAGCAGCCATCATTTAAAGAAAGCGTAA
TAGCTCACTGGTCGAGTCGGCCTGCGCGGAAGATGTAACGGGGCTAAACCATGCACCGAAGCTGCGGCAGCGA
CACTGTGTGTTGTTGGGTAGGGGAGCGTTCTGTAAGCCTGTGAAGGTGTGTCGTGAGGCATGCTGGAGGTATC
AGAAGTGCGAATGCTGACATAAGTAACGATAAAGCGGGTGAAAAGCCCGCTCGCCGGAAGACCAAGGGTTCCT
GTCCAACGTTAATCGGGGCAGGGTGAGTCGACCCCTAAGGCGAGGCCGAAAGGCGTAGTCGATGGGAAACGGG
TTAATATTCCCGTACTTGGTGTTACTGCGAAGGGGGGACGGAGAAGGCTATGTTGGCCGGGCGACGGTTGTCC
CGGTTTAAGCGTGTAGGTGTGTGTTCCAGGTAAATCCGGTTCACTTTAACACTGAGGCGTGACGACGAGGCAC
TACGGTGCTGAAGCAACAAATGCCCTGCTTCCAGGAAAAGCCTCTAAGCATCAGGTAACATCAAATCGTACCC
CAAACCGACACAGGTGGTCAGGTAGAGAATACCAAGGCGCTTGAGAGAACTCGGGTGAAGGAACTAGGCAAAA
TGGTGCCGTAACTTCGGGAGAAGGCACGCTGACACGTAGGTGAAGTGATTTACTCATGGAGCTGAAGTCAGTC
GAAGATACCAGCTGGCTGCAACTGTTTATTAAAAACACAGCACTGTGCAAACACGAAAGTGGACGTATACGGT
GTGACGCCTGCCCGGTGCCGGAAGGTTAATTGATGGGGTCAGCGCAAGCGAAGCTCCTGATCGAAGCCCCGGT
AAACGGCGGCCGTAACTATAACGGTCCTAAGGTAGCGAAATTCCTTGTCGGGTAAGTTCCGACCTGCACGAAT
GGCGTAATGATGGCCAGGCTGTCTCCACCCGAGACTCAGTGAAATTGAACTCGCTGTGAAGATGCAGTGTACC
CGCGGCAAGACGGAAAGACCCCGTGAACCTTTACTATAGCTTGACACTGAACATTGAGCCTTGATGTGTAGGA

TAGGTGGGAGGCTTTGAAGTGTGGACGCCAGTCTGCATGGAGCCGACCTTGAAATACCACCCTTTAATGTTTG
ATGTTCTAACGTGGACCCGTTACCCGGGTTGCGGACAGTGTCTGGTGGGTAGTTTGACTGGGGCGGTCTCCTC
CTAAAGAGTAACGGAGGAGCACGAAGGTTGGCTAATCCTGGTCGGACATCAGGAGGTTAGTGCAATGGCATAA
GCCAGCTTGACTGCGAGCGTGACGGCGCGAGCAGGTGCGAAAGCAGGTCATAGTGATCCGGTGGTTCTGAATG
GAAGGGCCATCGCTCAACGGATAAAAGGTACTCCGGGGATAACAGGCTGATACCGCCCAAGAGTTCATATCGA
CGGCGGTGTTTGGCACCTCGATGTCGGCTCATCACATCCTGGGGCTGAAGTAGGTCCCAAGGGTATGGCTGTT
CGCCATTTAAAGTGGTACGCGAGCTGGGTTTAGAACGTCGTGAGACAGTTCGGTCCCTATCTGCCGTGGGCGC
TGGAGAACTGAGGGGGGCTGCTCCTAGTACGAGAGGACCGGAGTGGACGCATCACTGGTGTTCGGGTTGTCAT
GCCAATGGCACTGCCCGGTAGCTAAATGCGGAAGAGATAAGTGCTGAAAGCATCTAAGCACGAAACTTGCCCC
GAGATGAGTTCTCCCTGAGACTTAGAGTCTCCTGAAGGAACGTTGAAGACGACGACGTTGATAGGCCGGGTGT
GTAAGCGCAGCGATGCGTTGAGCTAACCGGTACTAATGAACCGTGAGGCTTAACCTTACAACGCCGAAGATGT
TTTGGCGGATGAGAGAAGATTTTCAGCACTGATTCAGATTACATCAGAACGTAAAAACGGTCTGATAACAGAA
TTTGCCTGGCGGCAGTAGCGCGGTGGTCCCACCTGACCCCATGCCGAACTCAGAAGTGAAACGCCGTAGCGCC
GATGGTAGTGTGGGGTCTCCCCATGCGAGAGTAGGGAACTGCCAGGCATCAAATTTAGTGTGCTGATATGGCT
CAGTTGGTAGAGCGCACCCTTGGTAAGGGTGAGGTCCCCAGTTCGACTCTGGGTATCAGCACCACTTAATACG
GTTAAAGTTCGGTATTTGAAAAAGAATTTGTCTGGCGGCAGTAGCGCGGTGGTCCCACCTGACCCCATGCCGA
ACTCAGAAGTGAAACGCCGTAGCGCCGATGGTAGTGTGGGGTCTCCCCATGCGAGAGTAGGGAACTGCCAGAC
ATCAAATAAAACAAAAGGCTCAGTCGAAAGACTGGGCCTTTTGTTTTATCTGTTGTTTGTCGGTGAGTAGTCT
CCTGAGGAGGACAAATCCGCCGGGAGCGGATTTGAACGTTGCGCAGCAACGGCCCGTAGGGTGGCGGGCAGGA
CGCCCGCCATAAACTGCCAGACATCAAATAAAGCAAAGGGCCCAATCGAAAGACTGGGCCTTTTTGCATTTAT
CTCTCTATGGAAAAAGTTATCGGATAATGACGCCGACGCGTTTTAAACATCTTCGTAGAGTGGATAAAAACCG
TTTTAGTCAACGTGATTTCCCGCCATATATCCGCCTGAACCATCGAATGTGAACCGTGCTGAAGTAGATGTAC
AAACGGCTTTTACTTACGCCTCTAATAAGCAATAGAGATATTCGCTGAGGACTAAAAATCTGGCCCATTAGGC
TATTTTATTTGCCAGTTTGGCCTAGGGACAGTGCTCAAAATCCTCATGTATTACGTGTATGTTCCGGTTTATA
TCCAGACTGTCTGTAATAATTACGCCTAATGATCACGCTCTAAGTCTTACTGTATAAAGCCTGTTTGTAATGC
GGCTTACGATGCTATCAAGCTATAATTTTGTCTGCATCGGACAAGGAATAGAATAAATACCATCGGGATGGGC
GAGTAAGAAAAGGGAGCGTTTTATCGTGTTAACTTTCGGGGGATAGACTGAACAGATTACGTCGCTGAGTATC
CTTACGACCTTTATATTATTACTGAAAGCGCGGCATTGCCGCGCCTGCATGTATCATTTAGAAAGCGCAATGA
TACCCATCGTCAACCCTGCTACCGCCGCAGCGCCGCCAGCGATAGCGCCTGCTGTCTCCCAGTTATCCTGCGT
TGTACCTTTCATACATGTATTGGTATGAACCAGCCGCCCTTGATCGTCATAAACCGGTACGCAGGGAGAATCA
TGTGCACAACCTGCCAATAAGGTGGTAAGCAATGCAACTGGAATTAGTCTTTTCATGGTGTTACCTCATTAAT
CAGCGTTTCGCGAAGTACAGCAACGTAAATGCTGGCTCTAACCGCTGCGAAAACGAGAGTTATTTTACCACCA
CAGAAAAAATGGCCGACATGAGGAATCATCTCAATTCATGTGAATTGTAAAAACTATGGAGAATGCAGAGGTA
AGCGGAGAGGGGGGAATAGCGGAGATAAAGACGTACCGTTTAAAAAGGCGTCCGAAGACGCCTCTGTTTACCG
GTTAATCATGATGGCGATTAAATCGGCGTTTTACAACAACGAAGAATACTGGCACGAAGAAGATAGCTAATAA
GGTTGCGGTTAACATTCCCCCCATAACCCCTGTGCCTACTGCGTTCTGTGCCCCACTACCTGCGCCACGACTG
ATAACTAACGGCATTACCCCGAGAATAAAGGCCAGCGAGGTCATTAGAATAGGACGTAAACGCATACGGGATG
CTTCCAGCGTCGCTTCAATCAATCCACGTCCTTCTTTTTCCATGAGATCTTTGGCGAACTCGACAATCAGGAT
GGCGTTTTTAGCAGAAAGGCCAATAGTGGTTAACAAGCCAACCTGGAAGTAAACGTCATTGTTCAGACCGCGC
AATGACGCTGCAAGCAGAGCGCCAACCACGCCGAGCGGTACGACCAGCATTACCGAGAACGGAATTGACCAGC
TTTCATACAGAGCGGCAAGGCAGAGGAAAACGACAATGAGTGAGATTGCGTACAGCGCCGGCGCCTGGTTACC
TGACAACCGTTCCTGATACGACATACCTGTCCAGTCATAGCCAATACCGTTTGGCAGCTGTGAAGCCAGGTTT
TCCATTAACGACATGGCTTCACCGGTACTTCGTCCGGGCGCTGCTTCACCGAGCAGTTCCATTGACGGCATCC
CGTTATAGCGTTCCAGGCGTGGCGAACCATAAATCCACCGTGCTGAGCTAAAGGTAGAGAAGGGCACCATCTC
GCCATTGGCGCTGCGTACATAAAGATTGTTGATATCTCCCGGCAGCATACGGAACTGAGCGTCAGCCTGAACG
TATACTTTTTTCACTCGTCCGCGATCGATAAAGTCGTTAACGTAATAGCCGCCCAACGCCGCGGAGATGGTTT
CGTTGATATCAGACAGCGAAACACCGAGCGCCTGCGCTTTTTCTTGATCGACATCCAGCTTGAACTGTGGCGT
GTCTTCCAGCCCGTTCGGGCGTACGCGCACTAATAGGTCAGGATGCTTCGCGACCATACCCAGGAGTTGATTA
CGCGCTTTTGTTAACGCATCGTGACCGAGTCCTCCCTGATCAATCAGTTCAAAGTCGAAACCTGTCGCGGTAC
CTAACTCGACAATTGCTGGCATGTTGAAGGGGAACACCAACCCGTCGCGAATCTGGCTGAAGGCGCGTGTCGC
TCTGGCGATAACGGCTTCGACGCTATTTTCTTCACCGTTACGCTCTTCCCAGGGTTTAAGGCTGACAAATGCC
ATCCCTGAGTTTTGTCCCTGACCGCTAAAGCTAAAGCCGTTTACGGTAAACACGCTTTCGACGTTCGCTTTTT
CATTATTCAGGTAGTAATGAGTGACTTGATCCAGCACTTTCTGCGTACGTTCTTGCGTAGCGCCAGAGGGGAG
CTGAATCATGGTCAGGAATACCCCCTGATCTTCTTCCGGCAGGAAGGAGGTCGGGAGGCGTAAAAACAGAACC

GCCATTCCGACTACAATAAGTAGATAGATAATGAGATAGCGACCCGTATTACGCACGATGCCGCTTACGCTGT
TAGTATAGTGGTTAACGCTGTGGTCAAACCTGGTATTGAACCAGCCAAAGAAGCCGCTTTTTTTCTCGTGATG
TTCAGCAGATACGGGTTTAAGCAGCGTAGCGCACAGCGCTGGCGTCAGAATCAACGCAACCAGAACGGATAGC
GCCATTGCTGAAACAATAGTAATAGAGAACTGGCGATAAATTGCCCCGGTCGAGCCGCCAAAAAAGGCCATCG
GGATAAATACCGCAGACAGTACCATCGCGATACCAACCAGCGCTCCCTGAATCTGCGACATGGATTTTTCCGT
CGCCTCTCGGGGAGGAAGGTTATCCTCCATCATTACACGTTCAACGTTTTCTACGACCACTATCGCATCGTCA
ACCAACAGCCCTATCGCCAGTACCATGCCGAACATCGTCAGGGTATTGATGGAATAGCCAAAGGCGGCGAGTA
CCGCAAAAGTGCCTAGCAACACGACAGGAACAGCGATGGTAGGAATCAGGGTTGCCCGGATATTCTGTAAGAA
CAGATACATTACCAGGAACACCAGAATAATCGCTTCAAACAGCGTTTTTACCACTTCGTGGATAGATATTTTT
ACGAAGGGCGTTGTGTCATAAGGATAAACCACCTTCATTCCCTGGGGGAAGAAAGGCTGCAGCTCCGCCAGTT
TCGCTTTGATTGCGGTTGCGGTATCCAGCGCGTTAGCGCCGGTCGCCAGTTTAATACCGAGACCGGAGGCCGG
TTTACCGTTAATGCGCGCTACAACGTTATAGTTTTCACCGCCAAGCTCAATACGCGCGACATCTTTGAGATGG
ACGACAGAGCCGTCGGTATTGACGCGCAACGTAACTTTGCCGAACTCTTCCGGATCTTTCAGACGCGTTTGGG
CGATGATTGAGGCGTTAAGCTGCTGGCCCGGTAATGCTGGCGTGCCGCCCAGTTGGCCTGCCGCAATCTGGTC
GTTCTGTACTTTTAACTGGTTGATGACGTCAACTGGCGTGAGCTGGTATTTATTTAGCAGATTCGCATCCAGC
CAGATACGCATGGCGTACTGTGCGCCAAATAGCTGAACGTCGCCCACACCATTCAGACGGCTGATAGAATCCT
TAATGTTAGAGGCGACATAGTCAGAGATGTCGTCCTGGGTAGTGTTCGGATTATCTGAGACGAACCCGGCGAC
CATCAAAAAGCTGCTGCTGGATTTTTCAACGCTAATCCCCTGCTGCTGGACTTCTTGCGGCAGTAAAGGCGTG
GCGAGCTGCAATTTATTTTGCACCTGAACCTGCGCGATATCCGGATCGGTTCCGGACTGGAAGGTCAGGGTGA
TGGTCACGCTACCAGCAGAGTCGCTGGTAGAGGACATATACATCAGGTTATCGATACCGTTCATATTTTGTTC
GATAACCTGAGTAACCGTATCCTGTACCGTCTGCGCATCCGCGCCAGGATAGGTTGCAGAAATAGAAACCGCT
GGCGGCGCAATGGTTGGATACTGCGCAACGGGAAGTTGCATTATTGCCAGTGCGCCAGCCATCATCAGGATAA
TGGCCAGAACCCAGGCGAAAATAGGACGTCTAATAAAAAAGTTTGCCATGTACGGTTACCTTATTGCGCCGTT
TTCGCTGCAGGAGCATCGGTAGTGGCTTTCACCTGGACGCCCGGTCGCGCTTTTTGTAAGCCGCTGACGATGA
CCTTATCGCCCGGTTTTAACCCTTCGCTGATGAGCCATTTATCGCCAATAGCCTGCGCCGCCACGACATTGCG
GGCTTCGACCTGGCTTTTATCGTTAACCACCATCACCATTGCGTCGCCGCGCGGCGTGCGGGTTACGCCCTGC
TGGGGGACAAGGATGGCATTGGGCTGGACGCCTTCATCAATGCGGGCGCGAACAAACATACCGGGAAGCAGAC
TGTGTTGCGGGTTAGGGAACACCGCCCTGAGCGTGATAGAGCCGGTGCTTTCATCTACGGTAACGTCGGAAAA
TTGCAGCGTGCCTTTAATCGGGTAGACCTGACCATTTTCCATTACCAGTTGAACCGTGCTACTGGCGCTGTCT
TTATGCAGGTTACCTTGTTCGACGGATTGCTTGAGTCGCATAAAGTCGTTGCTTGATTGCGTCACGTCGACAT
AAATCGGATCGAGTTGTTGCACGGTAGCCAGTTCAGTTGACTGACCATTAGTCACCAGCGCGCCTTCAGTCAC
ATTAGATTTTCCTATACGCCCGCTGATGGGTGAGGTGACTTTGGTATACGCAAGGTTAATACGCGCGCTTTCA
ACAGCGGCTTTTGCCGCCACAACGGCGGCATCGGCCTGGCGGGCGTCGGCAATCGCCTGATCATATTCCTGTT
GGCTGATATATTTTGTGCCGACCAGTGGAACATAGCGTTTGACCGTCAGGTGCGCGATAGCCGCAGCCGCTTC
GCTTTTAGCAAGTTCGCCTTTAGCGCTGTCATAATCAGCCTGATAAGTGGCAGGATCGATCTGATAGAGCGAC
TGCCCGGCCTCTACATCGCTACCTTCGGTGAAGTTTCTTTTAAGCACGATCCCGCTCACCTGGGGGCGAACCT
CCGCAATGCGAAATGCGGACGTACGTCCGGGAAGTTCGGTCGTTACGGCTAGCGGCGCTGTTTCGACCACATG
GACGGTGACTTGCGGCTCGCCGGCATGAGCCTGGGTATCTCCCTGGTCATTACAACCGGCCAGCAGCGCAGCA
GAGAATATGATGAATGAGGGCAGGAGTGAAAACCTGGCATGTTTCGTCATTACTGTTCCTTAAAAAACCGAAA
GCTCGTTATTCACCGGATAACGGTAAGGCGTTAACGCGCGAAAAATAGAATAGCAGTGGGGTATCCTACAAAT
TATTAGCTTGTGATGTAAGCAATACTTATTTTGGGATGGGGTTCACGGCACATAAAAGCAAAAAAAAAGAACT
ATTCCCTGTCGTTTCATAATGTCCCGATTAGTAAAATCATGATGTTGAATAACATAAATAGAAATATATATTT
TATATGAATGTGTTTTATTAATTTATTTGTTTTTATTATTGATTTGGCGATATTGTGCGAAATAAAAATAGCT
GTCTCTCGTCTGACTAAAAAGGACATGATAAAGTTGTTTTTTGTGCTGAGTATTTTATCAGCGTATTTATCGT
TTTTATGGTCCAGGATATCTTAATTATGGCGAAGAAAACGAAGGCGGATGCGCTTAAAACGCGGCAACATTTG
ATTGAAACCGCTATTGCGCAGTTCGCTTTGCGCGGCGTTGCCAACACGACGTTGAATGATATCGCTGATGCCG
CTGATGTCACGCGGGGCGCCATCTACTGGCACTTTGAGAATAAAACGCAGTTGTTTAATGAGGTTTGGTTACA
GCAACCACCTTTACGCGAACTTATTCAGGATAGGCTTACGGGATGCTGGAATGATAATCCTTTACAGGATCTA
CGAGAGAAATTTATTGCAGCCTTACAATATATTGCGGCAGTGCCTCGTCAGCAGGCATTAATGCAGATTTTAT
ATCATAAATGTGAATTCCATAATGGTATGATATCGGAACAGGCCATTCGGGAGAAGATGGGTTTCCATCATCA
GTCGCTGCTTGAAGTATTACAGCGATGTATGGATAAAAAACTGATCTCAGGCAGTCTTGATCTGGATGTCATA
TTAATTATTCTGCATGGTAGTTTTAGCGGAATAGTAAAAAACTGGTTAATGAATCCCACAAGTTATGATCTTT
ATAAGCAGGCGCCTGCATTAGTCGATAATGTATTAAAAATGCTAAGCCCGGATGGCAGTGTGAGGCAATTAAT
GCCAAATGAACAACAGGCGGAAGAAGCCTGAGATTATTTTGAGTTGATATTATATATTGACCTTGTGAATATA

TTGGCAGGATCCCGAATATCGCGAGCAATTTCCTCTGCCGTACGCGGCTTGCCAAGCAGGAACCCCTGAAGCG
TATGGCATCCTAACTGGGTCAAAAATTGTTGCTGCGTTTCATTCTCTACCCCTTCTGCCACAATTTGCAGATT
TAACGCTTTCGCCAGCGCGACAATAGCCGCAACAATGGTCGCGCCGTCGCCTGCCTCGCTGAGTTCATGTACG
AAAGCGCGATCAATTTTTAGTTCGCACGCCGGAAGACGTTTCAGATAGAGAAGGCTGGAATAACCGGTGCCAA
AGTCGTCAATAGAGGCCTTAACCCCCATTTCCGTTAATCTGGTGAGTATAGCGACGCTTTGCTCTGGCTTGTT
CATCGCCGTGGTTTCGGTGATTTCCAGAATCAATAAATCGGGACGGATACTGTGTCGGGCCAGGCTCCGCGTA
ATGGTGTCCACAAGCCCGGGCTGCTCAAATTGTAAGGCGGACAGATTTACCGCCACTGACCATAAGTCGTATC
CCTGTAAGTGCCACTCGCGGAGCTGTCGACACGCCTCGTCTATCACCCAGCTACCGATAGTGACGATAAGACC
CGTTTTTTCCGCCAGCGGTAAAAACTGGTCAGGATTCAGTACGCCTTGTTTTGGATGATACCAGCGCAACAGG
GCCTCAAATCCTACAATAGGGCCGGCAGAAGCCTGAAATTTAGGCTGATACACTAACCTGAGCTCTTGTCTTT
CCAGCGCCAGCCACAGGTCATTCATTAGCTGAAGTTGGGTTTGCGCCAGCATATTCATTGATGGCTGAAAGAA
ATGGTATCCGTTACGCCCCGTATGTTTCGTGTGGTACATTGCGGCATCGGCGTTGAACATCAGTTCCCGTTCG
TTTTTTCCGTCCAGCGGATAGAGCGCTATTCCCACGCTAAGGGTGACGACCAGTTCGTACGGATCTATTGTAA
AGGGCGCATCTATCGAATGTACCAGCGCGCTGGCAAGGGAAGCCGCTTCGTCAGGCGCGCTGACTTCTGCAAG
CAGCACAAATTCGTCGCCGCCAATCCGCGCCAGGGTAAACTGCCCGCTCAGCGGTTGGTTCAGACGATGCGTG
ACCGCTACCAGTAGCTTATCGCCGATATCATGACCATAAGCGTCATTAACGGTTTTAAAGCCATCCAGATCCA
TAAACAGTAGCGCGAAAGACGTGTTTTCCCGATTCGCTTTGCTGATGGCCTGTTCCAGACGATCCTCAAGAAG
CACCCGGTTTGGTAAACGGGTGAGCGTATCATGCAGCGCCAACTGCGCCAGTTCCTGGTTCGCCTGCGCCAGC
GAAGAGGCAAGTAGTGCGGTACGGGCCTGAAGCCGGGCGTCAAATAAAGAAACCAGCAGAGTAATACCCAGGA
TAGTCAGCGCGACCACGCTGACAAGAACAGCCAGCCAGTTGCTATCGACGCCACGATGCTCCATCGGCCAGTT
TTGGGGAAAGTGTGCGGCCTTCATGCCGGCGTAATGCATGCCGGCGATAGCTATCCCCATTAATACCGCGGCG
CCTGCTCGCCTCAGGGCGACATCTGTTCCCTCATGACGCAGGCGGAACGTGAGCCACAGCGCGCCGCAGGACG
CCAGTAGCGCGATAATGATAGAGAGGGCAACCCACGCGCTATTCCAGACGATAATGGAAGCAAATTGCAGCGC
CGCCATCCCGGTATAATGCATTGCGCTGATGCCCAGTCCCATCACCAGCGCGCCCGGCAGTAAACGGCGCAGG
CGCAGTTTTTCAGCGCTGACCAGCCATAGCGCAAACAGGGAGGAGCCGATCGCGATCAGCATGGAGAGGCCGG
TGAGGAACGGATCGAAGCGCATGTTCATCGCGTGATCCATCGCCAGCATCCCGACAAAATGCATCGCCCAAAT
ACCGACGCCCAGCGCGAAGCCGCCGCCCATTAGCCATATTCTGGCGTTTGATCGGGCGCTTCCTGCTACGCGT
CCGGCCATATTTAATGCGGTATAGGACGCAAAAATAGCGACAGCAAATGAAACCGCCACAAGGATGTGGTTGT
ACTCACTAACCGGCATGTATCATTAGCTCATTGGTTATGCAGGAAAAGAACAGGACGAGACAGTATCACCCCT
TCTTTTATCCTCAACGTGTTTTAATTCCCTGTTTTTATAAGGGATAAAAGAAATCCGGGTGATAAATAATCAT
AGGTTAAATCGCCCGGTTCGCTACTCCAGATCGGTACAATTGATCAATTTGAGAGGGTTAACCGAATCCATGT
TTCGGCACTTATCCTGTTCAGTTGCCATCAATTCGATCCATTGCATCAAAAGCGCATCAAAAAGAAAAACGGA
GACGGCAAAAACAACCAGCCACCAATACTTACGTATCATCGTCAAATCCTGTGTATTCATGCCCATGCGTGCT
GGGAATATACATGAAATTTTGACGCTGAAAAGCGGACTTACAATGCTTTTACTTCTCTGAAGATAGCGCGTTT
ATCCCGACATTTCGCTGCTTTTTGGACAGGTTTTGTGTTTTTCTCTTTTTGACTTTTGCAAGTTCATTTTCTG
AATAATGCGAAGTGACGCTCAATCTTCTGAGCCCCATTTTTACGTACTCATTATTGAGTTCAATACCGATAAA
CTTACGACCTGACGCCGCTGCGACAGCGCCTGTGGTGAAACTGCCCGCGAAAGGGTCCAATACGGTATCGCTC
GGGTTAGAAGAGGCCAGTATGATCCGTTTTAACAGCGCGCTGGGCTTTTGCGTAGGATGGTTTTCATATTCAT
CCATCAGATAACGTACGCGAGGAAATGACCAGACGTTGCCCGGCACTTTTTTCTGATTGTATGGTTGGGGCGG
GTTTTTTCGATAATCGATTAGCGCGCGCTTAGCGCCCGTAGTGGTTTCGACCAGAATCGCATCACGGTTAAAG
GTATAGCTTTTCGGGTTTTTTACCATCATCAGGATCGGTTCATACATAGAACCAAAGTATTTTTTCGCCTGCA
CCCCGGAACTATCATAGGACCAGACGATACGGCTTTTGATGGTAAAAAGCGTTCGGCATTTGAGATCGATATA
CGGCATATTTTCTGTGCTATTCATGATGTACATGGTGCCGTGTTTCTTCAGTACGCGGTGGCACTCATCAATG
CATTCATACAGCCAGGCCAGAAAAGACGCTTCGTCCCAGGACTCCACCATACCGTCGAAATCTTTTCCGATAT
TGTAAGGCGGATCGGCAAAAATTAAGTCAATGCTTTCAGAAGGCAGTTTTTTAAGTTCTGTTAGCGCGTCACC
GTGAATAATCTTCTTCGATTCATCGCCAAAGTACTGAGGTTCACATTCCGCTTTCATGACCAAATTCCATGTG
ATGCGTATAAAAAAGGCGCTTCCCCATGCCGAGTAGCGCCTTTTTAAACAAGCAGTTAGCTAATCGAAATTAG
TTCATGCCGTATTTTTTTAATTTTTTACGCAGCGTACCACGGTTGATGCCCATCATCAGAGCAGCACGGGTCT
GGTTACCACGGGTGTATTGCATCACCATGTCCAACAGGGGCTGTTCTACTTCAGCCAGTACCAGCTCATACAG
GTCATTAACGTCCTGACCATTCAGTTGAGCAAAATAGTTCTTCAGTGCCTGTTTAACCGAGTCACGCAGGGGT
TTTTGGGTTACCTGATCCTGAGAGTTAACGGTAGAAACGGTCAGTACGTCAGAATTTACGCGTTGTTCGAACA
TAGTTCTGTCAGCTCTTTATTTCTGTTTACGCAAAATTTTCGAAGTATGCCTCCAACGCCTCCAGCTGTTCGC
TGGCGTCCTCAATAGCGTTGAATGTGCGCCGAAACTGGTCGTCTGGAGCGTGCTCCTGGAGATACCAGGAGAC
GTGTTTACGCGCGATTCGGTACCCTTTTGCCTGACCGTAAAAGTCATGCAGTTCCCGAACATGCGTGCAAAGC

AAGCGCTTTACCTCTGCCAGCGGCAGCGGCGGAAGCAGCTCCCCAGTGTCCAGATAATGCTGGATTTCCCGAA
AGATCCAGGGTCTTCCCTGAGCTGCGCGGCCTATCATCAGGGCATCCGCCCCTGTATAGTCGAGAACAGCTCT
GGCTTTATGCGGGTTAGTAATGTCGCCATTCGCGATAATCGGAATGGAAACTTTCTGCTTAACTGCCCGAATA
CTGTCGTATTCAGCTTCTCCGTTGAACAAACAGGCGCGGGTGCGGCCATGGATGGTCAGAGCCTGAATACCAC
AATCTTCAGCCAGTTGGGCAATCTCTACGCAGTTACGGTGTTCCGGCGCCCAGCCGGTGCGAATCTTGAGAGT
CACAGGAACGTCCACTGCGTTTACGACCCCGATGAGTATAGACTTCACTAAATCCGGGTACTGCAAGAGGGCT
GAACCTGCCAGCTTACGATTCACTTTCTTCGCCGGGCACCCCATATTGATATCAATAATCTGGGCGCCGCTTT
CCACGTTAATACGTGCGGCATCGGCCATCTCAACAGGGTCGCTACCGGCAATTTGCACCGTGCGAATTCCTGG
CTCATCAACGTGCACCATCCGTAAACGAGATTTATCGCTTTCCCACACTTGCGGGTTAGAAGACATCATCTCG
GATACTGTCAATCCTGCTCCCATCTCATAGCACAGCGTCCGGAAAGGTCTGTCAGTAATGCCAGCCATGGGCG
CTGCGATCAGGCGATTTCTGAGCTGATATTGTCCGATGCGCATGAGTTAAGAAATGACCATACTGTGACTGCA
AGGCGGCGTATATTACGCATTTTTTGCACGAGATGAAAGGCCAAACTTTGAACAATCCACTGTTGTAGATCAA
TGAATCGTGCTTAAAAACAGCAAAATAAATTTATAATTCAGTAAAATCATAGGGTTATTTATTGTCGGCGGTT
TTGTACGCGCTTACAAATTCCCGTAACTTCTCTCTATTTCTTCGTCCAGAAACAAGATAAGCGGAATAATCTG
CTTTTTTTATAAGCTAATTACCCCAAATGAATGGGATCGCCGTCTCGTTTCAGGACAAGATCACTTCCCGCTG
CGCGTGATTTTTCTCCATTGCCAGTCGGAAGGGGGCGCACAGATGCGCTTTTGTGTTCATGAATGGCACGGAT
AAATTTTTCCAGCTGCGCTGAAGAGAGGGTTAGCGGATGCTGCAGAACAACCCGGACAGCATTACTAAGTACG
AGGGCGATGGCTGCGACCCGTTAAAACGCCAGTATCTTTTGTCCGCTGGTAGCAGCTGTTTATCCAGACGGAT
AGTGACAAAGAAGTTATTTCAGGCATTCATTTGATGAATTTAATGATGTCTCGGTGCCTGAAAGGCGTTAAGG
CAGTTGTGTGGAGAAAAGCGTGGCCGGGCGACCACGCTGACAAGAATTATTTCTTACGGCCAGTAATGCGGCA
CCATTCTTCTTTTTCGACGACCGGGTCGAGAGTGAAAAGCTCGGCGTAAGCATCGCAGACGCTTTCCGCCTGG
CTGGCGAGAATACCTGAAAGCCCCAGCAGACCGCCTTCAACGGGCAGTACGCTGATTAACGGGGCCAGTTCGC
GTAATGGGCCTGCCAGGATGTTAGCGACGACCACGTCGGCTTTCATGGCTTCTGGCTGGTCTTTCGGCAGGTA
CAGCTCCAGCCGGTCAGACACGCCGTTGCGTTCAGCATTATCGCGACTGGCCTGAATAGCTTGCGGATCGATA
TCAATACCAATCGCCTTTGCCGCTCCCAGTTTCAGCGCGGCAATCGCCAGAATGCCTGAACCGCAGCCAAAGT
CGATCACGGTTTTGCCGTTGAGATCGAGCCCGTCCAGCCATTGCAGACATAAAGAGGTGGTGGGGTGCGTACC
CGTACCGAACGCCAGTCCCGGGTCGAGCATCACGTTGACGGCATTCTCGTCCGGAATGTCACGCCAGCTGGGA
CAAATCCACAGACGCTCGCCAAAACGCATCGGATGGAAATTATCCATCCATTCGCGTTCCCAGTCTTTATCTT
CCAGTTGTTCGATTTTATGGGCAAATCCGGCACCCAGCAGCGGATGTTGCTCCAGAATCGCGACAACGTCTTT
CATGTCGGTTTCGGCGTCGAACAGACCGATCACATCGGTATCGCCCCACAGACGGGTTTCGCCCGGCAACGGC
TCAAAAACCGGCGTATCGTGCGTATCCTGAAAGGTAATGGAAACGGCGCCCGCTTCCATCAGCGCATCACTCA
GCTCTTCAGCATTCGCGCCGGTGGTATTCAGTTTCAATTGGATCCATGGCATGGCAAAACTCTTTATTTATCA
GTAGACAATACGGTGGCTTGCGAAGCGGAAGAGCCGAATCGGTTTCCGATCAGAAAAGCCAATAAACTTAACA
GTAACGCGGGCACAATCGGATGAAAGCCCAGGTACTGAATATTAAATGTGGCAAGCAGGGCATATAGCACGCC
GCCCACGATCATGGCGCTTAATGCGCCTGCCGCGTTTGCCCGCTCCCAGTAAAGGCCCAATACCAGCGGCCAT
AAGAACACCGCTTCCAGGCCGCCAAACGCCAGCAGGTTGAGCCAAATGATCATCTCTGGCGGTTTCCATGCGG
CCAGCAGCAACAGCGCGCCTAGCAGTAATGTAATCGCTGCCGACATACGCTTCAGCCGGATCTCATTTTGCAT
CTGATCGGGGCGCAGGTTGAGATAGAGATCTTTAATGATCGTCGCGGAACTTTGCAGCAGTTGGGCGTTAATT
GTTGACATGATCGCCGCCATTGGCGCAGCGAGGAAGATCCCGGCGGCAAACGGCGGCAAAACTTTCACCATTA
GCGTTGGGATCACCAGATCCGGCACCGTCAGATCCGGAAGCACCGCGCGACCCAGCGCGCCGGCAAGGTGCAT
CCCGAACATTAAAATCGCCACGACAATCGTACCGATAATAATGCCCCGGTGTACCGCTTTACTGTCTTTATAG
GAGATGCAGCGCACCGCCGTATGCGGCAGGCCAATCACACCAAAACAGACCAGTACCCAAAAAGAGGTCATAA
AGGCGGGAGAAAGTATGTCATCGGCGCCCTGCGGCGTTACCAGTTTGGGGTCGAGAGCGTGCAGCGTGTCAAC
GGCCTGACTTAGACCGCCCGCCGCATGGATCACGCCCACCAGCAGCACTATCGTGCCGACCAGCATCACCAGG
CCCTGTAGCGTATCGTTAAGTACGCTGGCGCGAAAGCCGCCAAACGCCGTATAGAGTGCGATACTGACGCCAA
ATATTAGCAGGCCGGTTTCGTAGGGAATTCCCGCCGCGGTTTCCAGCAGTCGCGCGCCGCCGATAAATTGTAC
GGTCATTGCGCCAATAAACGCGACCAATAAACTCAGGCTTGCCAACCACACCAGCAGGCGGCTTTGGTAACGC
GCGAATAGCATATCGTTAAGCGTGACGGCATTATAGCGACGCGCCAGGATAGCGAATTTTTACCCAGAATACC
CAGTGAAAGCCAGACGGCGGGAAGCTGGATCATCGCCAGCAAGACCCAGCCCAGACCGTATTTATAAGCCGCG
CCGGGACCGCCGATAAATGAACTGGCGCTAATATAGGTGGCGGTAAGCGTCATCGCGAGCACGATCCCGCCCA
TCGAACGGCTGCCAAGAAAATATTCGTTTAAAAAAGTGCCGGCGGTACGCTTGCGCATAGCGTAAATCGAGAC
CCCGAAGACCACGACCAGATAGGCTACCAGCGGTAGAATGACCTCAAGCTGCATCGTCATCCTCCAGCGAAAT
ATCGCGATAAATGAATTTCACCATCGCCCAGCACAGCAAAATAAAGACCAGCGGCGTCAATAAGCAGGCCATC
TCGAACCAGTGCGGCAGACCGGTAATTCCCGGCGAGTCTCCAGGTAAGTAAGCAGCCACTAACCATGCCGCCA

GATAGCAAAGGGTCAGCCACAGCGCCCAGCGCGCCTCTTTATGGGCCTGAACAAAACGAGCGTCCATTTTTTG
TCCCTTATGGGTGAAGAAAGCGGGAATTGTACATGATGGGGCATCGCTATCCCCAGAAATAAAAAAGGCCGGA
TTCACCGGCCTTTTGAGATTAACGTGCGATTACTTTTCCTGAAGACCGAGTTTTTTCTCCAGATAGTGGATGT
TGGTTCCACCGTGCTGGAAGTGCTCGTCATTCATGATGCGGGTCTGCAGATCGATATTGGTTTTGATACCATC
GATAATCAGTTCCTGCAGGGCATTTTTCATACGGGCAATCGCCACGTCGCGGTTTTCACCGTAGCAGATGAGT
TTGCCGATCATGGAATCATAGTACGGCGGCACCGTGTAGCCCGCGTAGATATGAGATTCCCAGCGAACGCCAA
AGCCGCCAGGCGCATGGAAGCGCGTGATTTTGCCTGGGCTTGGCAGGAAGGTGTTCGGATCTTCGGCATTGAT
ACGGCATTCTACCGCATGGCCTCGAACGACAACTTCGTCCTGTGTGATCGACAGCGGCTGACCCGCCGCGATG
CGCAACTGCTCTTTGATCAAATCGACGCCGGTAATCATTTCAGTCACCGGGTGTTCAACCTGAATACGGGTGT
TCATTTCGATGAAATAGAACTCGCCGTTTTCGAACAGGAATTCGAACGTCCCTGCGCCGCGATAGCCGATGTC
TACGCACGCTTTCGCGCAGCGTTCGCCGATATAGCGACGCAGTTCCGGCGTAATGCCTGGCGCCGGGGCTTCT
TCAACCACTTTCTGGTGGCGACGCTGCATGGAACAGTCGCGTTCCGCCAGATAGATAGCGTTGCCCTGGCCGT
CCGCCAGCACCTGAATTTCGATGTGGCGTGGATTTTCCAGGTATTTTTCCATGTATACCATGTCGTTGCTGAA
AGCCGCTTTCGCTTCCGCTTTGGTCATGGAGATGGACTGCGCCAGTTCAGCATCGCTACGAACCACGCGCATA
CCGCGGCCGCCGCCGCCGCCGGACGCTTTGATGATCACCGGATAGCCAATACGTTTGGCATGAGCGCGGTTCG
CATTCATATCGTCGCCCAGCGGGCCGTCAGATCCTGGTACGGTCGGCACGCCCGCTTTTTTCATCGCGGTAAT
CGCGGACACTTTATCGCCCATCAGGCGGATGGTGTCCGCTTTCGGGCCGATAAAGATAAAGCCGGAGCGTTCA
ACCTGCTCGGCAAAATTGGCGTTCTCAGAAAGGAAGCCGTAACCCGGGTGGATTGCCACCGCGCCGGTGATTT
CAGCGGCGCTAATGATAGCCGGGATGTTCAGATAACTTTTTACGGACGGTGCCGGGCCAATACAGACCGTCTC
ATCCGCCAGCAATACGTGTTTTAAATCGCGATCCGCGCTTGAGTGCACAGCGACGGTCTTGATGCCGAGTTCT
TTACAGGCTCGAAGAATACGTAGTGCGATCTCGCCGCGGTTGGCGATGACAATTTTATCCAACATGTTCGCCT
CGTTACTCGATGACGACCAGTGGCTCGTCAAATTCTACCGGTTGACCACTTTCGACCAGAATCGCTTTCACAG
TACCTGCTTTGTCAGCTTCGATCTGGTTCATCATTTTCATGGCTTCAACGATGCACAGGGTATCGCCCACGTT
CACTTTCTGGCCCACTTCGATGAACGCTTTCGCGTCCGGGCTTGGGGTGCGGTAGAAAGTACCAACCATTGGG
GAGCGTACGATGTGACCACTGATTTCCGCTGCGGCAGGCGCTTCCATTGCTGGAGTAGCCGCTGGCGCGACAG
CGTTAGACAGAGCGGGTTGCTGCATCATCGGCGCCGCATAAGCCTGCTGCATCACCGGAAAACCGGCGTTTGC
CGTTGTGCGGCTGATGCGAACAGATTCTTCGCCTTCAGAAATTTCCAGTTCGGAGATACCTGATTCTTCAACC
AGCTCGATCAGTTTTTTAATCTTACGAATATCCATGAGTGGGTTCCGTACTCTTTGTTTAGTGTGATTGTGAC
AGGCGTTTTAGCGCCGTCTGTAAAGCATGTGAATGACTGCCGCGGGTAAAATACCGCGCATCTTTGATTTGCC
TGTTATGCCGTAGCACACGAAGGTGAAATTCATGATTTGCCAGTCATTGTGTGGCTATCTTCTGCCATTTTCG
GGCAAAAAACAAAATATACCTTCATTGCTCGATTGTCACCCTTTCTGCGAACGAAAATCGCCGTTGGGGAATG
TAAAGTCGCACATTATAACTATTTCGTCGCAATTGGCAGCTAAATACTGGTCTTATCAGGGAAGATAATCAAC
CCGAAACATAAAAAGGACATTACCTGTTTTGCGTTTTGCAATCGACCGCACAGTTCACCAAACTAGCGCCACC
ACTGGCGAAACCTCCGGTAACGCAACGCTAAAAGGGCTAACGCTAATGCCGCGTAGATGACCGGTTGCGGTGA
TAAAATCTTTACCGACCACAGGTAATGTATGGGAGCCAGGATCGCGACAAGATAGACGACGTTATGCAGTGTT
TGCCAGCGTTTGCCCAATTTACGCTGCGCAAATTGCGTTGAGGTCAACGTCAGCGCCAATAACACCAGCCAGC
TAATGATCCCAAGCGTCAAATAAGGGCGTGAAATCAACTCGCTTCCTAACAACGCCAGGTTATGTATCCCCAG
TTCCAGTAAGGCATAGCTGGTTAAATGCAGCGTAGCCCAAACGAAACACCATAACCCCAACAGGCGGCGTGTG
CGTATCAATAATGGCTGTTTAGCGTAGCGCGCCAGCGGTGAAACCAGCAAGGTGGCGAGCAAAAAATTTGAGCG
CGGTTCTGCCGGTAAAGTGTTGGATATCCTTTACCGGGTCTGCGCTGAGTCCACCGTGATTTATCGCCCAAAA
GAGCCACAGAAGCGGCAAAAAACCGGCAAGATGCAGACAAACTTTTAGCCAGGTTATCTGTTTTGCTGTCAGA
CGCACTTAAAAATTCTCCCGCAAATTGAGACCGCGATACAGCGAAGCGACTTCATTGGCATAGCCGTTAAACA
GCAGCGTCGGCTGCCTTTGCACATCGAGGATACCGCCTGAACCAATAAAGCGTTCGGTAGCCTGAGACCAGCG
TGGATGATCCACATGCGGGTTCACATTGGCGTAAAAACCATATTCGTTGGGAGCCGACAAATTCCAGGTGGTT
AGCGGACGTTCGCGGGTGAGTTTAATGCTGACAATAGATTTAATACCTTTAAAACCATACTTCCATGGAACGA
TGAGTCGAATGGGCGCGCCGTTTTGCGGGGGTAACGCCTTACCATAGACGCCAACGGTCATCAGAGTCAGCGG
ATGCATGGCTTCGTCCAGACGTAGCCCTTCGACATAAGGGTATTTCAGTCCGCCGCCAATAAAACGATCTTTC
TGTCCTGGCATATCATCCGGCGCGTATAGCGTTTCGAATGCCACATATTTAGCGTGGCTGGTGGGCTGTGCCT
GCGCGAGTAGCTTATATAAAGGGAAACCAATCCACGGCACGACCATGGACCACGCTTCGACGCAGCGCATTCG
ATAGATACGCTCTTCTAATGGGAAACGATGTGTTAAATCGTCATAATCCAGCGTAAATGGCTTCGCGACTTCC
CCGCTGATTTTCAACGTCCACGGTTCGGTTTTCAGACTTCCGGCATTGGCCGCCGGGTCGGCTTTATCAAGGC
CAAACTCATAGAAATTGTTGTAGCCCGTCACCTTATCTTCCGGCGTTAACGCTAAATCGCTTCGCCAGGCGGC
AGGCTGACTAAACTCAAGCGGTTTACCGGCAGGCGCTTTCGGACGATCGTTGCCTTTAAACCAACTGAAGAGA
TCGGCCTGCGCCGTTGAGGGTAAGGATAAGGCGGCCGCGCTGATGCCTAATGCTTTTAGCACCTGTCGGCGCT

GCATAAAAAAAAGCCGATTCCGCAGTCACATCGGCTTCTGTTAATGGACGTATCTTTTTCATCACAGACTCCCG
GTCTACCCACCACAACATGGCGTGTAGTGAATATGACAGGGAGTGTGTCAATGTGTAAAAATAAATAGTTAAT
TAAGTGTAAATTTTTTTATACGTGACGTTATTTTATCTTCACCAGCGTACGGCCCTGAACCTGATTATTGATG
ATGGCATCGGCGAACTTCGGCGCATCCGCCAGCGTAATCTCTGTGGCGGCCTGAGCATAGAACGATTCCGGCA
GATCCTTAACCAAACGCGCCCAGGCTTCTGCGCGACGTGCCGGTGGGGTCATCACCGAGTCCACCCCTTGCAA
ACGCACATTACGCAGAATGAATGGCATAACGGTGGTCGGCAGCGCGAAGCCGCCCGCCAGGCCGCAGGCGGCC
ACGCAACCGCCATAGTTCATCTGTGCCAGCACTTTCGCCAGCACTTTATCGCCAACGGTATCAATTGCACCGG
CCCATAGCTGTTTTTCCAACGGGCGAGATTCAGCAAACTCATCGCGGCTGAGAATACGGTTAGCTCCCAGGCT
CTTAAGATAGCCGTAGGTGCTTTCACGTCCTGATACTGCTGCAACCTGATAGCCCAGCTTATGCAGTAATGCA
ACGGCAGTGCTGCCCACGCCGCCGCTGGCACCGGTGACGACAACCTCACCGTCTTGCGGGCGAATACCTGCAT
CTTCAAGCGCCATGACGCACAGCATGGCGGTAAAACCGGCGGTGCCGATGATCATGGCGTTACGGCTGCTCAG
TCCCGCAGGCAGCGCGACCAGCCAGTCGCCTTTAACGCGTGCGCGTTCGGCCAGGCCGCCCCAGTGGTTTTCG
CCCACGCCCCAGCCAGTCAGTAGTACTTCCTGGCCTGCATGAAAGCGAGGATCTTCGCTTGCGTGAACGGTGC
CGGCGAAATCAATACCAGGAATCATCGGAAAATGACGGATAATTTTCCCTTTTCCGGTGATAGCCAGAGCATC
TTTATAATTCAGGCTGGACCAGTGGACATCCACCGTCACATCACCTGCCGGCAGTTGACTCTCTTCGAGATGT
TGCACGGATGCGAGGGTTTTACCGTCCTGCTGTTCTAAGATCAACGCCTGCATAACCTGTCCTCACTTTACAT
GGTAGATTGGAAAAATAGCTACGAAGACTATACTCGCTAATTAAAACGTGATGCCGATGCAACGCAATAAATT
GCCAGATAGATCCATTTTGGTAGTATGCCTGCTTCATTGCGCGCCGTGGCGAATTCCGCCTTCAGATTCGCTT
TTTCATACTGTTTATAACCGTCGGAGTTAACTCAAGGATGCGATTAACGACGAAATTCTCAGCTTTTATCACG
CTGCTCACGGGGTTAACGATCTTCGTGACTCTGATAGGTTGCTCGCTAAGCTTTTATAACGCCGTACAGTATA
AGTATGTCAGCCGTGTGCAGGCGACGGCGACAGCCATTGATACGCACCTGGTGACCCATGATATTGTCTCGCT
GACGCCGCAAATCGACGAACTCATGATCGCTTCCGATATTGTGCGTGTCGATCTGTTGCAGGGGGAGCGCAGC
GTCTACAGCCATTCTCGTGCCAGAGGTTATCGGCCAGCCGGCACCAGCGACATGTATCGCGAGCTGGTCGTAC
CGCTGATAAAACATCCAGGAATGTCGCTACGTCTGGCTTATCAGGACCCGATGGGTAACTATTTTCACTCACT
GATCACCACGGCGCCGCTAACGCTGGCGATCGGTTTTATCGTTCTGATTTTGTTTTTATCGGTGCGCTGGCTG
CAGCGGCAGCTTTCCGGGCAGGAGCTTCTGGAAATTCGCTCAACGCGTATTCTGAACGGCGAACGCGGGGCGA
ATGTCCGCGGTTCTGTTTACGAATGGCCAGCGCGCACCAGTAGCGCGCTTGATGTATTGCTTTCCGAAATTCA
GTTTGCTCACGAACAGCGTAGCCGCCTGGACACCCTCATTCGCTCCTACGCCGCGCAAGACACCAAAACCGGA
CTCGGTAACCGTCTGTTCTTTGATAACCAACTGGCGACGCTGCTGGAAGATCAGGAAAAAGTGGGAGTGCATG
GCGTGGTGATGATGATCCGCTTGCCAGACTTCAATCTTCTGCGCGACAGCCTGGGAGGCAATCAGGCGGAAGA
GCAGATGTTTATGCTTATCAATCTGCTATCGACATTTATTATGCGTTACCCAGGCTCGCTGCTGGCGCGTTAT
CACCGCAGCGACTTTGCCGTGCTGCTACCGCATCGAACGCTGAAAGAAGCGGAAAGTATTGCCGGACAACTTT
TAAAAGCTGTCGATGCGCTACCGGCTAACAAAATGCTTGATCGTGACGATATGGTACATATCGGCATTTGCGC
CTGGCGCAGCGGACAGTCCACCGAGCAGGTGATAGAACATGCTGAAGCCGCGGCGCGGAACGCCGCCCTGCAG
GGGGGAAATAGCTGGGCTATCTACGACGACACGCTGCCGGAAAAAGGGCGCGGCAATGTGCGCTGGCGCACAT
TGATTGAGCAGATGCTGAACCGGGGCGGACCGCGACTTTATCAAAAACCGGCGGTCACGCGAGAAGGTCGCGT
ACATCATCGGGAATTGATGTGTCGCATCTATGACGGTAAAGAAGAGGTGAGCTCGGCGGAGTATATGCCGATG
GTGTTGCAATTTGGTTTATCGGAAGAGTATGACAGATTACAAATTAGCCGCCTGATTACGTTGCTGGGCTACT
GGCCGGATGAAAATCTGGCAATGCAGTTGACGGTCGAGTCGCTGATTCGTCCGCGTTTTCAGCGCTGGTTGCG
CGATACGTTAATGCAGTGTGAAAAATCACAGCGAAATCGCATAATTATTGAACTTGCGGAGGCAGATGTTTGT
CAACACATCTGTCGGTTACAACCCATCCTTCGCTTAGTCAATGCGCTTGGCGTTCGGGTGGCGGTGACACAGG
CGGGTTTGACGCTGGTTAGCACGAGCTGGATTAAAGCGCTCAATGTGGAATTACTCAAGCTGCATCCGAGTCT
GGTCAGAAACATTGAGAAGCGAACGGAAAACCAGCTTCTGGTTCAAAGTCTGGTAGAGGCCTGCGCGGGTACG
CCTACGCAGGTTTACGCGACGGGCGTCCGTTCGCGCGGCGAGTGGCAGACGCTGACAAAACGCGGCGTGGCTG
GCGGGCAGGGTGATTTTTTTGCCTCCTCCCAACTACTTGACACAAACGTGAAAAAATATTCGCAAAGATACTC
GGTTTAACCTGCCGTTTAATTTGTTTTCACGTAGAATAACGCGCGCTGCGCCTCATGGGGGTGTGCTTGTCTG
CTCGCCAGATTGTTGCAGCAAATATGCAGGTGAATGACCTTTGACAGGTTGCAAACGAAGGCGAGGATTGCTG
CTGATTTTTTCAGCCCTGAGGGACTCAGGCCGAGATTTGTAACAAAGGAAACGAACTGCACTAATTTTCACCG
TAGCAGATGATTTTTGCGCCTTGTCGCTGCTGCGTGTGGTTGGTAAAGTAAGCGGATTTTGTTTTCCGCCCCA
GCTTTCAGGATTATCCCTTAGTATGTTGAAAAAATTTCGTGGCATGTTTTCCAATGACCTGTCCATTGACCTG
GGTACCGCGAATACCCTCATTTATGTAAAAGGACAAGGCATCGTATTGAATGAGCCTTCCGTTGTGGCCATTC
GTCAGGATCGTGCCGGTTCGCCGAAAAGCGTGGCTGCAGTCGGTCATGATGCGAAGCAGATGCTGGGCCGTAC
GCCGGGCAATATCGCTGCGATCCGTCCGATGAAAGACGGCGTTATCGCTGACTTCTTCGTGACTGAAAAAATG
CTCCAGCACTTTATTAAACAAGTGCACAGCAACAGCTTTATGCGCCCAAGCCCGCGCGTACTGGTATGCGTGC

CGGTCGGCGCGACGCAGGTAGAACGCCGCGCAATTCGTGAATCCGCTCAGGGCGCAGGCGCCCGTGAAGTCTT
TTTGATCGAAGAGCCGATGGCGGCCGCGATCGGCGCAGGTCTGCCAGTTTCTGAAGCAACCGGTTCAATGGTT
GTGGATATCGGTGGTGGTACCACTGAAGTTGCCGTTATCTCCCTGAACGGCGTGGTTTACTCTTCTTCTGTCC
GTATTGGTGGTGATCGCTTCGACGAAGCCATCATTAATTACGTTCGTCGTAACTACGGTTCTCTGATCGGTGA
AGCCACCGCAGAACGTATTAAACACGAAATCGGTTCCGCTTATCCGGGCGACGAAGTCCGCGAGATCGAAGTG
CGTGGCCGTAACCTGGCGGAAGGCGTTCCACGCGGCTTTACCCTGAACTCAAACGAGATTCTGGAAGCGTTGC
AGGAACCGTTGACCGGTATCGTCAGCGCGGTAATGGTGGCGCTGGAACAGTGTCCGCCGGAGCTGGCGTCAGA
CATCTCCGAGCGCGGTATGGTGCTCACCGGCGGCGGCGCGCTGTTGCGTAACCTTGACCGTCTGTTAATGGAA
GAGACGGGCATTCCTGTCGTGGTTGCTGAAGATCCGCTGACCTGTGTGGCGCGCGGCGGCGGCAAGGCGCTGG
AAATGATCGATATGCACGGCGGCGACCTGTTCAGCGAAGAGTAGTTGGATACGGGCAGGATTATCCCTGGATT
GTCCTGCCTCTCCGACGCGAGAATACGCATAGCCTATGAAGCCAATTTTTAGCCGTGGCCCGTCGCTACAGAT
TCGCCTTATTCTGGCGGTGCTGGTGGCGCTCGGCGTTATCATTGCCGACAGCCGCCTGGGGACGTTCAGTCAA
ATCCGAACGTACATGGACACTGCCGTCAGTCCTTTCTATTTTATTTCAAACGGCCCTCGTGAACTGCTCGACA
GCGTGTCGCAAACGCTGGCTTCTCGCGACCAGCTTGAGCTGGAAAATCGGGCGCTGCGCCAGGAATTACTGCT
AAAAAACAGCGACCTGCTGATGCTGGGGCAGTACAAACAGGAGAACGCGCGGTTGCGCGAGCTGCTGGGGTCG
CCGCTGCGTCAGGACGAGCAGAAAATGGTGACTCAGGTTATTTCAACGGTTAACGATCCTTACAGCGATCAGG
TCGTTATCGATAAAGGCAGCGTTAATGGCGTATATGAAGGTCAACCGGTCATTAGCGACAAAGGCGTCGTAGG
GCAGGTTGTCGCCGTGGCGAAACTGACCAGTCGCGTACTGCTGATTTGCGATGCGACTCATGCGCTGCCGATT
CAGGTACTGCGCAATGACATCCGCGTGATTGCCGCCGGTAACGGCTGCACTGACGATCTTCAGCTTGAACATC
TGCCCGCCAATACCGATATTCGCGTTGGCGATGTGCTGGTAACGTCCGGACTTGGTGGGCGTTTCCCGGAAGG
GTATCCGGTGGCGGTGGTCTCCTCCGTCAAGCTGGACACTCAGCGCGCCTATACCGTCATTCAGGCACGTCCA
ACCGCGGGTTTGCAGCGTTTACGCTATCTGCTGCTGTTGTGGGGAGCCGATCGTAACGGCGCCAATCCGATGA
CGCCGGAAGAGGTTCACCGCGTCGCCAATGAGCGCCTGATGCAGATGATGCCGCAGGTGTTGCCTTCTCCGGA
TGCGATGGGGCCGCCGGCGCCAGTGCCGGACCCGGCGACGGGGATTACTCAGCCATCTGCGGGCCAGACGGCG
CCGGTATCAACGCAACCATCGCCTTCGGGCGCGACCACGCCGCCTGCGCGTGCGCCGGGAGGGTAATGGTGGC
GAGCTATCGTAGCCAGGGGCGCTGGGTTATCTGGCTCTCGTTTCTTATTGCGCTGTTGCTGCAAATCATGCCC
TGGCCGGACGACATCATTGTTTTCCGGCCAAACTGGGTTTTGCTCATCTTACTGTACTGGATTCTGGCCCTGC
CGCACCGCGTAAATGTGGGGACGGGATTTGTGATGGGTGCCATACTTGATCTCATCAGCGGTTCCACACTTGG
CGTGCGCGCTCTGTCGATGAGTATTGTCGCCTATCTGGTCGCTCTGAAATTCCAGCTCTTCCGTAACCTGGCG
CTCTGGCAGCAGGCGCTGGTGGTAATGTTGCTTTCACTGGCCGTGGACATTATTGTTTTCTGGGCCGAGTTTT
TAGTGATCAACGTCTCTTTCAGACCGGAAGTGTTCTGGAGTAGTGTAGTCAATGGCGTGCTGTGGCCCTGGCT
CTTTTTGCTGATGCGTAAAGTACGCCAGCAGTTTGCTGTGCAATAAAGGTCGATATGACAACTCTGTATCTTG
CTTCCGGTTCCCCGCGTCGCCAGGAATTATTGACTCAACTTGGATTTTCATTCGAGCAGGTCGTGCCTGGTAT
TGAAGAGCAACGGCGTGCTCAGGAAAGCGCGCAGCAGTATGTTGTTCGGCTGGCACGCGAAAAGGCGCAGGCT
GGCGTGGCGCTGGTTCCCCGTGATCTGCCGGTGTTGGGCGCGGATACGATAGTCGTGTTAAACGGCGAGGTGC
TGGAAAAGCCGTGCGACGCCGCTCATGCCGCAGAAATGCTGCGTTTGCTTTCCGGAAATACGCATCAGGTGAT
GACTGCTGTTGCGCTTGCGGACTCGCAGCAGACGCTGGATTGTGTGGTGGTAACGGAGGTGACTTTCCGCACG
CTTTCTGCGCAGGATATTACTGGCTATGTCGCCAGCGGCGAGCCTTTAGATAAAGCAGGTGCATACGGTATTC
AGGGGCGGGGTGGCTGTTTTGTCAGGAAGATAAATGGCAGCTATCACGCCGTGGTCGGCTTACCGCTGGTTGA
AACGTATGAGTTGTTGAGTCATTTTAACGCACTGCGTGATAAAAGGGATAAACATGACGGCTGAATTGTTGGT
AAACGTAACGCCATCGGAAACGCGCGTGGCGTACATTGATGGCGGTATTCTTCAGGAAATCCATATTGAGCGC
GAAGCGCGACGCGGAATCGTAGGCAATATCTACAAAGGTCGTGTAAGTCGTGTACTTCCAGGTATGCAGGCGG
CTTTTGTAGATATTGGGCTGGATAAAGCCGCATTTCTTCACGCTTCCGACATTATGCCGCATACCGAATGCGT
GGCGGGCGACGAGCAAAAACAGTTTACCGTGCGCGATATCTCCGAACTGGTGCGTCAGGGACAGGATCTGATG
GTGCAGGTAGTGAAAGATCCGCTCGGCACGAAAGGCGCGCGTTTGACCACGGACATCACGTTGCCCTCCCGTT
ACCTTGTTTTTATGCCCGGCGCGTCACACGTTGGCGTTTCCCAGCGTATCGAAAGCGAAAGTGAACGCGAGCG
CCTGAAAAAGGTGGTGGCGGAATACTGCGACGAACAGGGCGGGTTTATTATCCGCACCGCGGCGGAAGGCGTG
TGTGAAGAGGATCTCGCGTCTGACGCCGCCTATCTCAAGCGCGTCTGGACGAAAGTCATGGAGCGCAAAAAGC
GTCCGCAAACGCGCTACCAGATGTATGGCGAACTGGCGCTGGCGCAGCGCGTGCTGCGCGATTTCGCCGATGC
GCAGTTGGACCGAATTCGCGTCGACTCACGTCTGACCTATGAGTCGCTGCTGGAGTTTACCGCAGAATACATC
CCGGAAATGACCAGTAAGCTGGAGCATTACAGTGGCCATCAGCCGATCTTCGATCTTTATGACGTGGAAAATG
AGATCCAGCGCGCGCTGGAGCGTAAGGTTGAACTCAAGTCTGGCGGTTATCTGATTATCGACCAAACCGAAGC
GATGACGACGGTAGATATCAATACCGGCGCGTTTGTCGGACACCGTAATCTCGACGACACCATTTTTAATACC
AATATTGAAGCGACTCAGGCCATTGCCCGCCAGCTACGCCTGCGTAATCTGGGCGGCATTATCATTATCGATT

TTATCGACATGAATAATGAAGATCATCGCCGCCGGGTGCTGCATTCGCTGGAACAGGCGTTAAGTAAGGATCG
CGTGAAAACCAGTATTAACGGTTTTTCGCCGCTGGGGCTGGTGGAAATGACCCGTAAACGGACCCGTGAAAGC
GTGGAACATGTGCTTTGTAACGAGTGTCCAACCTGTCATGGCCGCGGGACGGTAAAAACGGTGGAAACCGTCT
GTTATGAGATCATGCGTGAAATTGTACGTGTTCATCACGCTTATGACTCCGATCGCTTCCTGGTTTATGCTTC
TCCTGCGGTGGCGGAAGCGCTGAAAGGCGAAGAATCACACGCGCTGGCGGAAGTCGAAATCTTTGTCGGCAAA
CAGGTTAAAGTTCAGGTCGAACCGCTTTATAACCAGGAGCAGTTTGACGTCGTAATGATGTAATTGAGTAGTA
GCACCAGCGCATGTAGGCCGGATAAGGCGCAAGCGCCGCCATCCGGCAATATACCCTCAACGGGTGTGAGTCG
TATTTTTTGGCAGACAAGGAGAGACGCGTGAGGCGATTGCCGGGGATTTTATTGCTCACTGGAGCCGCGCTCA
TCGTCATTGCAGCGCTGCTGGTTAGCGGGCTGCGCCTGGCGTTGCCTCATCTTGACGCCTGGCGTCCAGCTAT
CCTGAATAAAATTGAGTCCGTGACCGGCGTGCCGGTGGCCGCCAGCCAACTCTCCGCAAGCTGGCAAAATTTT
GGCCCTACGCTGGAAGCGCATAATATTCATGCCGCCCTGAAAGATGGCGGCGAGCTGTCGATAAAACGCGTTA
CGCTGGCGCTGGACGTCTGGCAAAGCCTGTTGCATATGCGCTGGCAGTTTCGCGACCTGACCTTCTGGCAACT
CAATTTCCGCACCAACACGCCTCTTCAAAGCAGCGACGGCAAAGGCATCGAAACCAGCCGTTTAAGCGATCTT
TTCCTGCGTCAGTTCGATCATTTCGATCTGCGCGATAGCCAAATCAGTTTTCTGACGCTTTCCGGACAACGCG
CGGAGCTGGCGATCCCACAGCTTACCTGGCTAAACGGCAAAGAGCGGCACCGCGCCGAAGGCGAGGTCAGCCT
CTCCAGCCTGACCGGGCAGCACGGCGTGATGCAGGTGCGGATGGATTTACGCGATGACGACGGTTTGCTGAAT
AACGGCAGAGTCTGGTTACAGGCCGACGATATTGACGTCAAGCCGTGGCTGGGCAAATGGATGCAGGATAATG
TGGCGCTCCAGACGGCGCGTTTCAGTCTGGAAGGCTGGATGACGCTCAGTAAAGGCGAAATCGCCGGAGGAGA
CGTCTGGCTGAAACAGGGCGGCGCAAGCTGGCTGGGCGATAACACGACGCACACGCTGTCTGTCGATAACCTG
ACGGCGCAGATTAGCCGCGAGCAGCCGGGCTGGCAGTTTTATATTCCGGACACACGGATTACGCTTGATGGTA
AACCCTGGCCGAGCGGAGCCTTAACCGTGGCCTGGCTCCCGCAGCAGGACGTCGGCGGCGAGAATCACACACG
TAGCGATGAGCTACGCATTCGCGCCAGTAATCTTGAACTGGCGGGGCTGGAAGCATTACGTCCGCTGGCGGCT
AAACTGTCGCCTGTGCTGGGCGAAATATGGCAGGCTACGCAGCCGAGCGGAAAGATCGCCACACTGGCGCTGG
ATATTCCGCTACAGGCGACCGAAAAAACGCGTTTTCAGGCATCGTGGGAAAATCTTGCCTGGAAACAGTGGAA
GCTGTTGCCCGGCGCGGAACATTTTTCCGGTACGCTGGCGGGAAGCGTAGAAGACGGGCAGATGAAGGTTGCG
ATGCAGCAAGCTAAAATGCCCTATGAAACCGTTTTTCGCGCGCCGCTGGAAATTGAAAACGGCGTCGCGACGC
TTAGCTGGTTGAAGAATGAGAACGGCTTTCAGCTTGATGGCCGCGACATTGACGTCAAAGCAAAGGCGGTACA
TGCGCGCGGCGGGTTCCGCTATCTGCAACCGACGGGCGACGAACCGTGGCTGGGTATTCTGGCCGGAATCAGT
ACCGATGATGGATCTCAGGCGTGGCGCTATTTTCCGGAAAAACCTGATGGGCAAAGCGCTGGTCGATTACCTTA
GCGGCGCGATTCAGGGCGGCGAGGCGGATAACGCCACGTTGGTTTACGGTGGTAACCCGCACCTCTTTCCCTA
TAAGCATAACGAAGGTCAGTTTGAAGTGCTGGTGCCGCTACGCAATGCGACGTTTGCCTTTCAACCCGACTGG
CCGGCGCTAAAAAATCTCAACATTGAACTGGATTTCCTGAACGACGGTCTGTGGATGCGTTCGGATAGCGTCG
ATTTGGGCGGGGTGAAGGCCAGCAAACTCGCGGCGACGATTCCGGATTATTCCAAAGAGAAACTGCTCATTGA
TGCCGATATTAACGGGCCGGGAAAAGCCGTTGGGCCTTATTTTGACGAGACGCCGCTAAAAGACTCGCTTGGC
TCGACGCTGGCGGAGCTCCAGTTAGATGGCGATGTGAATGCTCGCTTACATCTTGATATTCCGCTGGACGGAG
AACAGGTTACCGCCGAGGGCGATGTCTCGTTGCGTAATAACAGTCTGTTTATTAAGCCGCTAAACAGCACGCT
CAAAAATCTGAACGGTAAATTCAGCTTTGTGAATGGCGCGCTAAAAAGCGGGCCGCTGACGGCAAACTGGTTT
AATCAGCCGCTTAACCTGGATTTCAGTACGACGGAAGGGGCAAAAGCCTATCAGGTCGCCGTCAACCTGAACG
GTAACTGGCAACCAACGCGTATGGGCGTCTTACCGCCGCAACTGAATGACGCCCTGAGCGGCAGCGTGACGTG
GAATGGTAAAGTCGGCATCGATCTTCCGTATCACGCTGACACCACTTATCACATCGAGCTGAACGGCGATCTG
AGAAATGTGAGCAGTCACTTACCTTCTCCGCTAAATAAACCCGCAGGCGAAGCCATTCCGGTGAACATTCAGG
CTGACGGCAACCTGAAAAGTTTTGCGCTAACGGGGAGCGCAGGGAGTAAAAATCACTTTAACAGCCGCTGGTT
ATTAAATCAGAAGCTGACGTTGGATCGGGCTATCTGGACGACGGACAGCCGGACGATTCCGCCATTACCTGCT
CAACAAGGCGTTGAACTCAATCTGCCGGCGCTGGATGGCGCACAATGGCTGGCGTTATTCCAGAAAGGCGCGG
CGGATAACGTGAGCAGTTCAGCCGAGTTTCCTCAACGCGTCACGTTGCGTACTCCCGCGCTATCGCTGGGTGG
CCAGCAGTGGAACAATTTGAGCGTCGTTTCAGCCCCCTCGCTGAACGGAACAAAAATTGAAGCGCAGGGCCGT
GAGGTGAACGCCACGCTGCTCATGCGCAACCATGCGCCGTGGCTGGCGAATATTAAGTACCTGTACTACAACC
CTGGCGTCGCAAAAACGCACGCCTCGTCACCAACGCCGACATCGCCGTTGGCTTCGGCGAACACGATTAGCTT
CCGCGGCTGGCCGGACTTACAGCTTCGCTGCGAAGAGTGCTGGCTGTGGGGGCAAAAATATGGGCGTATTGAT
GGCGATTTCGCCATCAAAGGCAATACGCTGACTCTGGCGAATGGCCTGATCGATACCGGATTCGCCCGTTTGA
AAGCGAACGGCGAGTGGGTGAATGCGCCGGGTAATGAACGAACCTCGCTGAAAGGTAGTCTGCATGGTAGTAA
CCTCGACACGGCTGCCGGGTTCTTCGGTATCTCGACGCCAATCCAGAACGCGTCTTTTAACGTAGATTACGAT
CTGCACTGGCGAAACCCGCCCTGGCAACCTGAGGAAGCCACGCTCAACGGGATTTTACGTACGCGTCTGGGTA
AAGGCGAGTTTACTGATCTCAGTAGCGGTCATGCCGGACAGTTACTGCGGCTGCTCAGTTTTGATGCGTTGCT

GCGTAAGCTGCGGTTTGACTTCAGAGATACCTTTAGCGAGGGCTTCTATTTCGACTCTATTCATAGTACCGCG
TGGATTAAAGATGGCGTCCTGCATACTGACGATACGCTGGTGGATGGGCTGGAAGCGGATATTGCAATGAAAG
GTTCTGTTGATCTGGTGCGTCGTCGTCTTGATATGGAGGCCGTTGTCGCGCCGGAAATTTCCGCCACAGTGGG
CGTCGCCGCCGCGTTTGCCGTTAACCCGATTGTCGGCGCGGCGGTATTTGCCGCCAGTAAAGTGTTGGGGCCG
CTATGGAGCAAGGTCTCCATTTTGCGATATCGCATTACCGGTCCGGTCGATGCGCCGCAGATCAACGAAGTTC
TGCGCCAACCGATAAAAGAAAGCCAGCAATGATTTGACGGGGGCGAGGAATTGCCCCACTCTCAGTAAATAGT
CATGCCGGACGGCGCTGCGTTTATCCGGTTTGTACAATCTAACAACGTAGTCCGGATAAGGCGTGAACCGCCA
TCCGGCAGAAATCAACGAGTAGCAAAAACGATGAGTCTGAACCTGGTAAGTGAACAATTGCTAGCGGCGAATG
GCCTGAACCATCAGGATCTGTTCGCTATTTTGGGCCAACTGGCCGAACGCCGTCTTGATTATGGCGACCTCTA
TTTTCAGTCGAGCTATCACGAATCCTGGGTTTTAGAAGACCGCATCATTAAAGATGGTTCATATAATATCGAC
CAGGGCGTTGGCGTTCGCGCCATTAGCGGCGAAAAAACCGGTTTTGCTTATGCTGACCAGATAAGCCTCCTGG
CGCTGGAGCAGAGTGCGCAGGCAGCGCGAACCATTGTACGCGAGAACGGCGAAGGCAAGGTAAAAACGCTCGC
CGCCGTAGCGCATCAGCCGCTCTACACCACCCTTGATCCGCTGCAAAGTATGAGCCGCGAAGAGAAGCTGGAT
ATCCTCAGACGCGTTGACAAAGTGGCGCGAGAAGCCGATAAACGCGTGCAGGAAGTTAACGCCAGCCTGACCG
GCGTATATGAATTAATCCTCGTGGCGGCGACCGACGGGACGCTGGCGGCGGATGTCCGTCCACTGGTGCGGTT
GTCCGTTAGCGTGCAGGTGGAAGAAGACGGTAAACGCGAGCGCGGCGCCAGCGGCGGCGGCGGTCGCTTTGGT
TATGAGTATTTTCTTGCCGATCTCGACGGCGAGGTTCGCGCCGACGCGTGGGCGAAAGAAGCGGTACGCATGG
CGCTGGTTAATCTCTCCGCGGTCGCTGCGCCAGCGGGGACGTTACCGGTGGTTCTGGGCGCCGGGTGGCCGGG
CGTATTGCTGCACGAAGCGGTCGGTCACGGGCTGGAAGGTGATTTTAACCGTCGTGGGACATCTGTGTTTAGC
GGTCAGATCGGCGAGCAGGTTGCCTCCGCGCTTTGCACCGTAGTGGACGACGGCACAATGATGAACCGTCGCG
GCTCCGTTGCTATCGATGATGAAGGTACGCCAGGCCAGTACAACGTATTGATTGAAAATGGCGTGCTGAAAGG
ATACATGCAGGACAAGCTGAACGCTCGCCTGATGGGCGCTGCGCCGACCGGTAACGGGCGTCGCGAATCTTAC
GCGCATCTGCCGATGCCGCGTATGACGAATACCTATATGTTGGCGGGGCAGTCAACGCCGCAGGAAATTATCG
AATCCGTTGAGTACGGCATCTATGCGCCTAACTTTGGCGGTGGTCAGGTGGATATCACCTCCGGCAAGTTTGT
GTTCTCTACCTCGGAAGCGTATCTGATTGAAAATGGCAAAGTCACGACGCCGGTGAAGGGCGCGACGTTGATT
GGATCAGGCATTGAAACGATGCAACAGATCTCCATGGTCGGCAACGACCTTAAGCTGGATAACGGGGTGGGGG
TCTGCGGTAAAGAGGGCCAAAGCCTGCCGGTAGGCGTAGGCCAGCCGACGCTGAAAGTCGATAACCTGACGGT
TGGCGGCACCGCATAACTGCCCGCCTTATCAGGCCTGTGGTGTGTGCCGCCCAGGCCTGATAAGCGCAGCGCC
ATCAGGAAATGTCAGCCTCGGGTGGCATCTGGCCTACAATCGTCTTCAGCAATTACCCGGTGTTTTTATTTTT
CTTTTCCCCGCCCGTGCATCCCCTGAAACAGGTGTGCGACATCGACAAAATAGTCAGTTAACGCGTTAATCAC
TACCTGCACTTTAAGCGGCAGCTTATCTTTTTCGGTGTACAGCGCGTACACCGGACGGGGATCGGACTGATAG
CGAGGCAGCAGGATCTCCAGATCGCCACGATTGATCTCATCGATCACCCACATCAGCGGCACATAGGCGATTC
CGGTTCCGGCGGTCAGCCAGCGTACCAGCGTCATCGGATCGTTAGTGACGAACCGCCCCTGTGGAATCAGTCG
GGTAGAAATTCCTTCCGGCGCGATAAGTTCAAACTCATTATCCGGGCGCACGCTATATTCCAGCCAGGAATGG
CTACTGAGATCGGCGGGTTTTTCCGGGACGCCGTACTGAGCGAGGTAGGGCTTGGCGGCGCACACAACCATCG
GCATCGCGCCGAGTCGACGGGAAAACAGGCTGGAGTCTTGCAGCGCGCCCACGCGGATCACCACGTCCAGTCC
GTCGGCAATTAAATCCGGAGCCGGAATTCCTGTCACAAGATTGACCGCCAGACCGGGATACTCTTTTAATAAT
TTTGCGGTAAGCCCGGCCAGCACATTTTGCGCCATGGTTGAAGAACAGCCGATACGCAATGTACCGATGGGCG
TGTTATTAAAAGCGTAAAGCTGCTCATGAACATCCTGCACTTCATGCAGCATACGACGACAGCCCTGATAATA
GATTTTTCCAGCCTCGGTGAGCCCAATGCTGCGTGTGCTGCGGTTGAGCAATTTAACCTGAAGCTCATCTTCC
AGTTTCGCCACAGTCTGGCTGATGGACGATACGCTCATTTGCAGCTGTCTGGCTGCGGCGGTAAAAGAGCCAA
ACTCGACAACTTTGGCGAATACCGACATTCGTTTTAATCGTTCCATTATTCACTCTGGCTTAAAAGTGATTTA
GATCACATAATATAGATAACAGCATAACAATTACGTTAATATACTATTATTCGAATAAGAGCTATGTCGCTCT
CGCCTGGCGTCTCTTGCCCTTCTCTGCGGCGATGTAGCTTAACAATCTATCGCCTGCTCTCTCTCAGAAATCA
AGGTCAACATGAGTCTGTTTCCCGTTATCGTGGTGTTTGGTCTTTCCTTCCCACCGATATTTTTCGAATTGCT
TTTGTCACTGGCTATTTTTTGGCTGGTGCGTCGGATGCTTGTGCCGACGGGTATCTATGATTTTGTCTGGCAT
CCGGCTTTGTTCAATACCGCGCTGTATTGTTGTCTGTTTTATTTAATATCGCGCCTGTTCGTTTGAGGTTGAA
GTGAAAACACTAACAAGAAAACTCTCCCGTACGGCCATTACCCTCGTACTGGTTATTCTGGCCTTTATCGCCA
TTTTCCGCGCCTGGGTGTATTACACCGAATCTCCCTGGACGCGCGACGCGCGTTTTAGCGCCGATGTCGTCGC
TATTGCCCCGGATGTCGCGGGCCTGATTACCCATGTAAACGTGCATGATAACCAACTGGTGAAAAAGGATCAG
GTCCTGTTTACCATCGATCAGCCACGCTATCAAAAAGCGCTGGCGGAAGCGGAAGCTGATGTAGCCTATTATC
AGGTACTGGCGCAGGAAAAACGCCAGGAAGCCGGTCGCCGTAATCGCCTCGGCGTACAGGCGATGTCCCGGGA
AGAGATCGATCAGGCGAACAACGTCTTGCAGACGGTACTGCATCAACTGGCTAAAGCCCAGGCGACGCGCGAT
CTGGCTAAACTCGATCTGGAACGCACCGTCATTCGCGCGCCTGCCGACGGCTGGGTGACTAACCTTAATGTTT

ATGCCGGTGAATTTATTACCCGCGGTTCCACAGCCGTCGCCCTGGTGAAAAAGAACTCTTTTTACGTGCAGGC
CTATATGGAAGAGACCAAGCTGGAAGGCGTGCGTCCGGGATATCGTGCCGAAATCACGCCGCTTGGCAGCAAC
CGGGTATTAAAAGGTACCGTCGATAGCGTGGCGGCAGGGGTAACGAATGCCAGCAGTACCAGCGATGCGAAAG
GGATGGCGACGATAGACTCTAACCTCGAATGGGTGCGTCTGGCCCAGCGTGTCCCGGTGCGTATCCGACTCGA
CGAGCAGCAGGGGAATTTGTGGCCTGCCGGTACGACGGCAACCGTCGTGATCACCGGTAAGCAGGATCGTGAC
GCAAGCCAGGACTCCTTCTTCCGTAAACTGGCGCACCGGCTGCGCGAATTCGGTTAATCGTCATGGGCATTTT
CTCCATTGCCAACCAGCACATTCGTTTTGCGGTAAAGCTGGCGTGCGCCATTGTGCTGGCATTATTTATCGGC
TTCCATTTTCAACTGGAGACGCCGCGTTGGGCGGTGCTGACCGCCGCGATCGTGGCGGCAGGCCCGGCATTTG
CCGCCGGCGGGGAACCCTATTCCGGCGCTATCCGTTATCGCGGAATGTTGCGTATTATCGGGACGTTTATTGG
CTGTATCGCCGCGCTTATCATTATTATCTCTATGATCCGCGCGCCGTTATTGATGATTCTGGTGTGCTGTGTC
TGGGCCGGTTTTTGTACCTGGATCTCCTCTTTAGTACGGATCGAAAACTCTTATGCGTGGGGACTGTCAGGTT
ATACTGCGCTGATCATTGTGATTACCATTCAGACGGAGCCGCTGCTTACGCCGCAATTTGCGCTTGAGCGCTG
TAGCGAAATCGTCATCGGCATTGGCTGCGCTATTTTAGCGGATCTGCTTTTCTCGCCGCGATCGATAAAGCAG
GAGGTTGATCGCGAACTGGACAGTTTACTGGTAGCGCAATATCAGCTAATGCAACTTTGTATTAAGCATGGCG
ACAGCGAAGAAGTCGACAACGCCTGGGGCGATCTGGTGCGACGCACGGCGGCGCTGGAGGGAATGCGAAGTAA
TCTGAATATGGAATCTTCGCGCTGGGTACGCGCTAACCGCCGCCTGAAAGCGTTGAATACGCTTTCGCTCACG
CTGATTACGCAATCCTGTGAAACTTATCTTATTCAAAACACGCGGCCGGAACTGATTACCGATACATTCCGGG
AACTGTTCGAGACGCCGGTTGAAACGGTACAGGACGTTCACAGGCAGTTAAAACGGATGCGGCGTGTCATTGT
CTGGACCGGCGAGCGCGAAACGCCCGTCACGCTTTATAGCTGGGTAGGCGCAGCGACGCGCTATCTGCTGCTT
AAACGCGGCGTGATCAGCAACACCAAAATCAGCGCGACGGAAGAAGAGATCCTGCAAGGCGAGCCCGTAGTTA
AAGTGGAATCCGCTGAACGTCACCATGCGATGGTGAACTTCTGGCGCACCACGCTTTCCTGCATACTGGGGAC
ATTATTCTGGCTATGGACCGGCTGGACATCGGGTAATGGCGCGATGGTGATGATTGCCGTGGTGACCTCGCTG
GCGATGCGTTTACCCAATCCGCGCATGGTCTGCATTGATTTTATTTATGGCACTCTGGCGGCGTTGCCGCTGG
GATTGCTCTATTTCCTGGTGATTATTCCCAATACGCAGCAAAGTATGCTGTTGTTATGTTTGAGCCTGGCGGT
ATTGGGGTTCTTTATCGGCATTGAGGTCCAGAAACGGCGTCTGGGGTCGATGGGGGCGCTGGCCAGTACGATT
AACATTATTGTGTTGGATAATCCGATGACGTTCCATTTCAGCCAGTTCCTCGACAGCGCGTTGGGGCAAATTG
TCGGCTGTATGCTGGCGTTTATCGTCATCCTGCTGGTGCGTGATAAATCCAAAGATCGAACCGGTCGCGTGCT
GCTTAATCAGTTTGTTTCCGCTGCGGTCTCGGCAATGACGACCAACGTGGTACGTCGTAAAGAAAACCGTCTG
CCTGCGTTATATCAGCAACTCTTTTTGCTGATGAATAAATTCCCTGGCGATTTGCCGAAGTTTCGCCTGGCGC
TAACGATGATCATTGCTCATCAACGTCTGCGCGACGCGCCGATACCGGTTAATGAGGATCTGTCAGTGTTCCA
CCGTCAGCTTCGTCGTACCGCAGACCATGTTATTTCCGCCGGAAGCGATGATAAACGGCGACGCTACTTTGGT
CAGTTGCTGGATGAGCTGGATATCTATCAGGAGAAATTACGTATCTGGGAGGCGCCGACGCAGGTGACGGAAC
CGGTTAAACGCCTGACGGGCATGTTACATAAATACCAGAACGCGCTTACCGATAGCTAACCCACAAGAACCGA
TGCCAAAAGCGTCGGTTTTTTTATGACTATACTTATTTGAGATACAAAAACAGCGCAAGAGTGCTCCGGGGCT
AAAAGCGTTTCTGGAAGTGAAACCTCTGCATAATGATGCTTAGTAGTAAAACAGACAATTGTCTGTCGGGGAT
GGTGGCTGGCTATGAATGTCTATACTTTTGATTTTAATGATATTAAAAACCAAAGCGATTTTTATCGCGAGTT
TACGCAGACGTTTGGTCTTGCCAGTGAGAAAGTAAGCGATCTTGATACATTATGGGATGCGGTAATGAGCGAT
ATCTTACCTCTACCGCTAGAGATTGAGTTCGTTCACCTGCCTGATAAACTGCGCAGGCGCTACGGGGCGCTGA
TTTTACTCTTTGATGAAGCGGAAGAAGAGCTGGAGGGGCGGCTACGGTTTAATGTCCGGCATTGAAAAAGCAT
AAAAAAGCCCCCGACAGGCGGGGGCAAGTCGTCGGGTTAGACGACGAGGGTTTACTTATACAACTCGGCGGTG
GCGTGCCAGTTATCACCTTCTTTTAATTCGATGATGCGATAGCTATTAGCGCCTGCTTTTTCGGCTTTAGCGA
CAATCTCCTGGCGCATATCCATTGGCGTACTGCCAATCTGCGAGACGGAAATGGTGCCCATCGGTTGCAGATT
CTGCGCCTGTTCCGCGTTAACCTGATGTACTGCGGCATTTGCGCCGAAAGAGAGCAGAGTGGCCAGGCCGAGT
GATGCGATAATGTATTTCGTTTTCATGGTCTTTTATTCCTTTATAGGGTCAACCTCGCGGATTAATGGGCTTT
TCTTATGCGATATCTCCGCGAAGCAATGACACGTTATATGCGATGGCAGGTGAATTTTATTTGTACAGTTCGG
CAGTGGCGTGCCAGTTGCTACCGCTACGCGCTTCGGTAATGTGGTAGGCCGTAGCGCCTTGTTCATCCGCTTT
TTTGCTCAGCATGGCGTTCATATCCATAGGCGATGAGCCGATAGCGCTGACTGAAACCGATCCGATTGCTTCA
CGATTTTGCGCTTGCTCTGCGCTGATAGGTTCTGCGGCGAAAGCGCCAAAAGAGAGAACGGACAGAACACTTA
ATGTTGCAACAGTGGTTTTGATTTTCATGATTTTTACCTCGTCGAATTCCTTTACTGGGGTCTTTGTTTCGTG
ACCCTCATCACAAAATTAAGTATACACTAATCACGCGATTAATTAATACCACGCTAATTGTTTCTGCTTTTTA
AAGACATTAAAAATTTTATTTTTTATAACGTTATGGAATTAAAATTGCTGTTTTTCGCCGCTGCTGTTTAAAA
AATAAAAGAAAATCAATAGGTTAAAAAATTTGACATTTTGTGCGTTTTTTTGATTTGTCATTCATGCGGTGAA
TGTTTAAGGGCATGATTTGCCAAATGATAGCCGTACAGGAAAGCGGCGAGGGGAGAGGACAAAAAGGTGCCTC
CGCCGTACGACGACGGAGGAAGTGGGACTAAAGCTCTTGTTCAAAAAGTTCCAGAATGGCTTCGTACAGATCT

CTTACGGAAAAGCCGCTGGCAGGCGTAGTGAAGATAGTATCGTCGCCGGCAATCGTGCCGAGAATACCTTCTG
CTTTCCCCAGTGAATCCAGCAGGCGCGCGATGAGTTGTGCCGCGCCGGGGCTGGTATGAATCACCACGACAGC
GTCGTTATAGTCGATATCCAGCACCAGATTTTTCAACGGACTGGATGTTGTTGGGACGCCCAGTTCAGCCGGA
AGACAGTACACCATTTCCATTTTTGCGTTACGTGTACGCACTGCGCCGAACTTAGTCAACATGCGCGAGACCT
TGGACTGATTGATATTTTCAAAGCCCTGATCCTGCAACGCGAGGACGATTTCGCCCTGTGAGCTGAATTTTTC
TTCTTTGAGGAGCGCTTTAAACGCTCTTACTAGTTCTTCTTGTTTAGCCGAGCTTCGCATAAGTCACCCGTAA
TATGGCGGTAGAAACAACATTATTGTGCATACAGATGAATTTTTATGCAAACAGTCTGCCTTGTTTAAGGCTG
AAATAATGTTATGAAAGAGCGGAATTTTATCAAATTTCGTTATCAAGAAACATGCCTGTGTCACGCCTCGCAA
ATAAGATTAAAAGTAAATTAATTGTTATCAAAATGATGTTGTTTTGAGAGGATAGTAGGGTATATTGTCACCA
CCTGTAGGAACGTTGCTCTCTGGTTGCAGTCGATCCGGATTACGCAAATTAAATGCATAAAAGCCAAAATTGC
GCGACTCCGCATTCTTGATGAGTGAGGATTGTAATCATTGAATTTGTGAATTAAGGTCGCCGCCGCGGAGCAA
TAGACACTTAGCTAATCATATAATAAGGAGTTTAGGATGAAAGTCGCAGTCCTCGGCGCTGCTGGTGGTATCG
GTCAGGCGCTGGCATTACTTTTAAAAAACCAACTGCCTTCAGGTTCAGAACTCTCCCTGTACGACATCGCTCC
AGTGACTCCCGGTGTGGCCGTTGATTTGAGCCACATCCCCACCGCTGTAAAAATCAAAGGTTTCTCCGGTGAA
GACGCAACCCCGGCGCTTGAAGGCGCTGACGTAGTACTGATTTCTGCGGGTGTGGCGCGTAAGCCGGGTATGG
ACCGTTCCGACCTGTTTAACGTTAACGCTGGCATCGTGAAAAACCTGGTGCAGCAGATCGCTAAAACCTGCCC
GAAAGCGTGCGTGGGCATTATCACCAACCCGGTGAACACCACCGTTGCGATTGCGGCGGAAGTGCTGAAAAAA
GCAGGCGTATACGACAAAAACAAACTGTTTGGCGTTACCACGCTGGATATCATCCGCTCTAATACCTTTGTCG
CAGAGCTGAAAGGTAAGCTGCCAACGGAAGTTGAAGTTCCGGTGATTGGCGGACACTCCGGCGTGACGATTCT
GCCGCTGCTGTCGCAGATTCCAGGCGTAAGTTTACCGAACAAGAAGCGGCCGAGCTGACTAAACGTATTCAG
AACGCCGGTACTGAAGTCGTCGAGGCGAAAGCCGGCGGCGGATCGGCAACCCTCTCTATGGGCCAGGCTGCCG
CGCGTTTCGGTCTTTCTCTGGTTCGCGCTCTGCAGGGCGAAAAAGGCGTGGTGGAATGCGCCTATGTGGAAGG
CGACGGTCAGTATGCCCGTTTCTTCTCTCAGCCGCTGCTGCTGGGTAAAAACGGTGTAGAAGAGCGTAAATCC
ATCGGCACACTGAGCGCTTTCGAGCAACATTCGCTGGACGCTATGCTGGATACGCTGAAAAAAGATATTCAGT
TGGGTGAAGATTTTATTAATAAATAAGCTGTTCCTGCCCGATGCCGGGGCTTTTGCTCCGGCTTCTTTGACTG
TAAACAAGCCACGAAATCAAGTACTAAATCTGCTAAACTCCTCACGTCTTATCGGTTATTAACGAGAACGTCG
TGAAAAAGATCCAAAGAACGCAAACCCGCGATCACATTACGCAAATGCTTCGCTATGAAATTCTTTCCGGCAA
TATAAAAGCGGGAGAAGAACTCGCGCAGGAAAGTATTGCGGAGCAGCTTGGTCTTTCACGAATGCCGGTTCGA
GAAGCGCTACAGTCACTCGAACAGGAAGGTTTTTAATCCGACTGCCCAACAGGCACATGCAGGTCGCGCATC
TTGAGGCCGATCGCGTGAGTCATATTTTTCGCGTAATTGCCGCGATGGCCGCCGAAATGTTTTCGCTGATACC
CAGCGAAGTAGGCGATGCCCTCCTGATACGTGCGCAAGCGCTGGCTGTCGCAGAAGACAAATCCTGCGAACTG
GAGTGTCATGCGATGCTGATTTCTTATGTGAATAACCGCTATCTGGAAAAGGTGTATCAGCAGTTCCTGGACG
GTTATGTCTCTTATGTTATTTTGCATCTGAAAAAAGATAATCAGGAGTCGGCGCAGCTTTTCGCTGAACTGGC
TGACGTCATACGCCAGGGGCGGCGTGATGAGATTGGGCAAGTTATGCAGCGTTATTTCCTCTCTTTAGCAGAA
ATAATGCGTCAACATATGAAGGATTGGGAAAGTGCAGAAGCCTAAGTTAGGGAAGATTAAGCTTCTTTCAGCG
AAAGAACAGGTCGCGGCAGTGCTGCGTAAAGCGATTCTTTCGAGGGAGTTAGTAGAAGGGCAGGAGATTACGT
TAGAAGGCATTGCCAGAATGGTCGGCGTGTCCAGTATGCCAGTGCGCGAGGCGTTTCAGATTCTGGCGGCGGA
TGGGCTGATAAAAGTGCGCCCTAATAAAGGGGCGGTGGTGTTGGGCATCAATGAACAAACGATCCGTGAACAC
TATGAAATTCGCGCGCTGTTAGAAAGTGAGGCGGTGGCTAAAGCTTCTCGTCCTGGCACCGATATCTCGCGGA
TAGCCCAGGTGCATTATGCCGCTGAAAAGGCTCTCGCGGAAAATAACTCGGCGGAATACTCCGACCTGAATCA
GGCATTTCATATGGAAATATGGAATGTTGCTGGCAACGAAAAAATGAAAATGTTGCTCTGTAATATGTGGAAC
GGCCTGTCGATGGGCCATAAGGTCACCGAAGAAGAGTATGCCGTGATTTCCATTCAGGAACATAAAAGTATTC
TGCAAGCTCTTGAACTTCATGATGAAACATTGGCACGCCAGCGCATGCGCGAACACATTATTCGCTCAATGGA
GAATATGCTGACCCGCTATGTTGGTGACCCTTCTGCCTGATCTTTTCGCCTGCGTGAGCATCGCAGGCGCTTC
AACCAATTCTTCAATTCTATTAATCACGTCCAGTAACGATCTAACCACGATCTTGATGTAGATCCCACTCCTG
CATCTTTCCTGAGAAACGATTTGACTTAGTTTTTGTATGGGAATAATTTCTCCCTGTCAACATTGGATACAAT
ATCATATATCATTGATTGGATAATTATAAATATGGAACCCATTACCCTTACCTTATGTTTATTGGTGTTCGCT
ATCGTCATGTTTGTATGGGAAAAAGTGCCACTTGCCGTCACTTCCATGATTGTCTGCGTGGCCTTAGTGATCA
CCGGCGTTCTGAATATCAAACAGGCCTTTGCTGGCTTTATCGACACTAACGTTATCCTCTTTGTCGCGATGTT
CATCGTGGGCGGTGCGTTGTTCGAGACCGGTATGGCAAACAAAGTGGGCGGCGTCATCACGCGCTTCGCGAAA
ACAGAAAAACAGCTGATCTTCACCATTATGGTGGTGGTGGGTTTGATGTCTGGCGTCCTCTCTAACACCGGTA
CCGCTGCGGTTTTGATTCCGGTGGTGATCGGCGTGGCGGCAAAATCGGGCTTCTCGCGTTCTCGTCTACTGAT
GCCGCTGGTTTTCGCCGCTGCGTTGGGCGGTAACCTGTCGTTAATTGGCGCGCCGGGAAATCTTATCGCGCAG
TCTGCGCTACAGAATATTGGCGGCGGCTTTGGCTTTTTTGAATACGCAAAAATCGGTCTCCCGATGCTCATCT

GCGGCATTCTCTACTTCCTGACTATTGGCTATCGCTTTCTGCCGAATAATGCGACCGGCGGTGAGGTGGGGAG
CGTCGGGGAGCAACGTGACTACAGCCATGTTCCACAGTGGAAACAACGGCTCTCACTGGTGGTGTTGATCGCA
ACGATCCTCGGCATGATCTTTGAGAAGAAGATCGGTGTCAGCCTGGCCGTGACCGGTTGTATTGGCGCTCTTG
TGCTGGTAGTTACCGGCGTACTCACGGAAAAACAGGCCTATAAAGCCATTGATTCACAGACTATTTTTATCTT
TGGCGGCACCCTGGCGCTGGCAAAAGCGCTGGAAATGACCGGGGCAGGCAAGCTGGTTGCGGACTATGTAATC
GGCATGCTGGGGCAAAACTCTTCACCGTTCATGTTGCTGATCGCCGTTTTCGCCTTATCCGTTGTGATGACCA
ACTTTATGTCCAATACCGCAACGACAGCGCTATTGGTGCCGGTGAGTTTGTCCATTGCCGCAGGTATGGGGGC
CGACCCGCGTGCGGTTCTGATGGCGACGGTCATTGGCGGTTCCTGCGCCTACGCAACCCCTATCGGTATGCCT
GCCAACATGATGGTGCTCTCCGCGGGTGGCTATAAATTTGTCGACTACGCTAAGGCCGGTATCCCCCTGATTA
TCGTTTCAACCATTGTCAGCTTGATCCTGCTGCCAATCCTTTTCCCTTTTCATCCGTAATTAACGGAAGCTGT
TCTGACTAAGGAGCATTTATGAGCAAAAGTGAACAGATATCCCATATGACCGACGTTATGGCCAAGTTTGTGG
GATATACCGGCAAAGTTTTGCCTGATGATGTCACTGCAAAACTGGAAGATTTGCATAAGAAAGAGACCAGTAA
ACTGGCCGACGTCATCTTTACTACCATGATTGAGAACCAGCGTCTGGCGAAAGAGCTGGATCGACCTTCCTGC
CAGGACACCGGTGTCATTCAGTTTCTGGTGGAGTGCGGGACGAACTTTCCGCTGATTGGCGAGCTGGAAGCGT
TGTTGCGTGAGGCGGTGATCAAAGCAACTGTCGATTCTCCGCTGCGCCACAACAGCGTAGAAACTTTTGATGA
ATACAACACCGGTAAAAACGTGGGTAAAGGTACGCCGACGGTCTTCTGGGAGATCGTTCCCAATTCCGATCAG
TGCAGCATTTATACCTATATGGCGGGCGGCGGTTGTTCCCTGCCGGGGAAAGCGATGGTGCTGATGCCGGGTG
CAGGCTATGAAGGTGTGACCCGCTTTGTACTGGATGTGATGACCAGCTACGGCCTCAACGCGTGTCCGCCGCT
GCTGGTTGGCGTGGGCGTAGCGACCTCTGTTGAAACGGCGGCACTGCTATCGAAAAAAGCGCTGATGCGTCCG
ATCGGTTCGCATAACGAGAATGAGCGTGCAGCCTCGCTGGAAAAAATGCTGGAAGAAGGCATTAACAAAATTG
GCCTGGGGCCGCAGGGGATGTCCGGTAATACCTCCGTTATGGGCGTGAACATTGAAAACACCGCGCGTCATCC
TTCTACCATCGGCGTGGCGGTAAACGTCGGCTGCTGGTCGCACCGTAAAGGGCATATCGTTTTCGATAAAGAT
TTGAATTACACCATTACGTCTCACAGCGGAGTGAATTTCTGATGACTAAAAAAATCCTGACTACGCCAATTAA
AGATGAAGATTTAGCGGATATTAAGGCTGGCGATATTATTTATCTCAATGGTCATATTGTTACCTGCCGTGAC
GTAGCCCATCGCCGTTTAATTGAAGGTGGTCGCGAGCTTCCGGTAGATGTTCGCGGCGGCGCTATTTTACACG
CTGGCCCCATTGTCCGCCCAATTAAAGGCGAAGACGATAAGTTTGAAATGGTCTCCGTTGGACCAACCACCAG
CATGCGTATGGAGAAATTCGAAAAAGAATTTATTGCGCAGACCGGCGTGAAATTAATTGTGGGTAAAGGCGGT
ATGGGTAAAGGTACCGAAGAGGGCTGTGCTGAACACAAAGCGCTGCACTGCGTATTCCCTGCCGGTTGTGCGG
TTGTAGCCGCCGTGTGCGTGGAAGAAATTGAAGATGCCCAATGGCGCGATCTGGGGATGCCGGAAACTCTGTG
GCTCTGTCGGGTTAAAGAGTTTGGCCCGCTGATTGTTTCTATTGATACCCATGGGAATAATCTGTTTGAACAG
AACAAAATTATTTTTAATCAGCGTAAAGAGATCGTCGCTGATGAAATATGCCAGAACGTAAGTTTCATCAAAT
AAACCTGATTACGTAAATTGCACGATAATGGTGGTAATACGTTATGACGATAATAATAACCAAGGGCTCTGGC
CGGGGGCGGCTCAGGGAAATCCACTCTTATTATTTCATCGTCATAAATAGCCCCATTATCGTGCCTCCTGCCT
GAAGGCTATTTTATGACTAATGCAGCTCTGCTATTAGGTGAAGGCTTCACCCTGATGTTCCTCGGCATGGGGT
TCGTGCTGGCGTTTCTGTTCCTGCTGATTTTCGCCATCCGCGGCATGTCTGCCGTGATTACTCGCTTCTTCCC
GGAACCAGTCGCCGCGCCCGCACCGCGCGCCGTTCCCGTCGTGGACGACTTCACCCGCTTAAAGCCGGTGATT
GCCGCCGCCATTCACCATCACCGCCTTAACGCTTAATTCCCGGAGGGATCCATGACCATTGCCATTACCGACG
TCGTCCTGCGTGACGCCCACCAGTCCCTGTTCGCCACCCGCCTGCGCCTTGATGACATGCTGCCGATTGCCGC
CGCGCTCGATGACGTGGGCTACGGCTCGCTGGAGTGCTGGGGCGGCGCCACCTTTGACGCCTGTATCCGCTTT
CTCGGCGAAGACCCGTGGCTGCGCCTGCGCGAGCTTAAAAAGGCCATGCCGAAGACTCCCCTGCAGATGCTGC
TGCGCGGCCAGAACCTGCTCGGCTACCGCCATTACGCCGATGATGTGGTGGAACGCTTCGTTGAGCGGGCGGT
GAAAAACGGCATGGACGTGTTCCGCGTCTTCGATGCCATGAACGACCCGCGCAATATGAAGGCCGCCCTGCAG
GCGGTGCGCAGCCACGGCGCGCACGCCCAGGGCACGCTGAGCTATACCACCAGCCCGGCGCACACCCTGCAGA
CCTGGCTGGATTTAACCGAACAGTTGTTAGAGACCGGCGTCGATTCCATCGCCATCAAGGATATGTCCGGCAT
TCTCACGCCGATGGCGGCGTATGAGCTGGTGAGCGAAATCAAAAAACGCTATGACGTGCGCCTGCACCTGCAC
TGCCATGCCACCACCGGGATGGCGGAGATGGCGCTGCTGAAGGCCATCGAGGCGGGCGTCGACGGCGTGGACA
CGGCGATTTCCTCCATGAGCGCCACCTACGGCCACCCGGCCACCGAAGCGCTGGTGGCGACGTTGGCAGGTAC
TGAGCACGACACCGGGCTGGATATCCTGAAGCTGGAAAATATCGCCGCGTACTTCCGCGAGGTGCGCAAAAAG
TATCACGCCTTTGAAGGCCAGCTGAAGGGCTACGACAGCCGTATTCTGGTGGCGCAGGTGCCGGGCGGAATGC
TCGCCAACCTCGAAAGCCAGCTGAAGCAGCAGAACGCGGCGGACAAACTCGACCAGGTGCTGGCGGAAATCCC
CCGCGTGCGCGAGGACCTCGGCTTTATCCCGCTGGTGACCCCGACCTCGCAGATTGTCGGCACCCAGGCGGTG
CTCAATGTCCTGACCGGCGAGCGCTACAAAACCATTGCCAAAGAAACGGCGGGCATTCTGAAAGGCGAATACG
GCCACACCCCGGTGCCGGTGAACGCCGCGTTACAGGCCCGCGTGCTGGAAGGGGGCGCTCCGGTGACCTGCCG
CCCGGCGGACCTGCTGAAGCCGGAACTGGCTGAACTGGAAGCGGACGTCAGGCGCCAGGCGCAGGAGAAGGGC

ATTCAGCTTGCGGGAAACGCCATCGACGACGTGCTCACCGTGGCGCTGTTCCCGCAGATTGGCCTCAAATTCC
TCGAAAACCGCCACAACCCGGCGGCGTTTGAGCCGGTACCGCAGGCGGAAGCCGCGCAGCCGGTGGCAAAAGC
AGAGAAGCCTGCCGCCTCCGGTATCTACACCGTGGAAGTGGAAGGCAAAGCCTTTGTGGTGAAGGTCAGCGAC
GGCGGCGATATCAGCCAGCTGACGGCGGCTTCGTCTGCGCCGGTTCAGGCGGCATCCCCGGTTGCCCCGGCAG
GCGCCGGCACCCCGGTCACCGCGCCGCTGGCGGGCAACATCTGGAAGGTGATTGCCACCGAGGGCCAGAGCGT
GGCCGAAGGCGACGTGCTGCTGATTCTGGAAGCCATGAAGATGGAAACCGAAATCCGCGCCGCGCAGGCCGGG
ACGGTACGCGGTATCGCGGTGAAGTCCGGGGACGCGGTCTCCGTGGGCGACACCCTGATGACGCTGGCGTAAC
GGAGAACTGAAATGGAAAGTCTGAACGCCCTGCTTCAGGGCATGGGGCTGATGCACCTTGGCGCAGGCCAGGC
CATCATGCTGCTGGTCAGCCTGCTGCTGCTGTGGCTGGCGATTGCGAAGAAGTTCGAACCGTTACTGCTGCTG
CCGATTGGCTTCGGCGGCCTGCTCTCCAATATCCCGGAAGCGGGTATGGCGCTGACCGCGCTGGAGAGCCTGC
TGGCGCACCACGACGCCGGGCAACTGGCGGTGATTGCCGCGAAGCTCAACTGCGCGCCGGACGTGCACGCCAT
TAAAGAGGCATTAGCGCTGGCGCTGCCGTCGGTGCAGAGCCAGATGGAGAACCTGGCGGTGGACATGGGCTAC
ACGCCGGGGGTGCTGGCGCTGTTCTATAAAGTGGCGATTGGCTCCGGTGTCGCGCCGCTGGTCATCTTTATGG
GCGTGGGCGCGATGACCGACTTCGGCCCGCTGCTGGCCAACCCGCGCACCCTGCTGCTGGGGGCGGCGGCGCA
GTTCGGCATCTTCGCCACCGTGCTGGGGGCGCTGACGCTGAACTACTTCGGCCTGATTTCCTTCACCCTGCCG
CAGGCGGCGGCAATCGGCATTATCGGCGGCGCGGACGGCCCGACGGCGATTTATCTGTCGGGCAAGCTGGCCC
CGGAGCTGCTGGGGGCCATCGCGGTGGCGGCGTACTCGTATATGGCGCTGGTGCCGTTAATCCAGCCGCCGAT
TATGCGCGCGCTGACCAGCGAGAAAGAGCGCAAAATCCGCATGGTGCAGCTGCGCACGGTGAGTAAGCGGGAA
AAAATCCTCTTCCCGGTGGTGCTGTTACTACTGGTGGCCCTGCTGCTGCCGGACGCCGCGCCGCTGCTGGGGA
TGTTCTGCTTTGGCAACCTGATGCGTGAAAGCGGCGTGGTGGAGCGTCTGAGCGACACGGTACAGAACGGGCT
GATTAATATCGTGACCATCTTCCTCGGGCTGTCGGTGGGCGCGAAGCTGGTGGCGGATAAGTTTTTGCAGCCG
CAGACGCTGGGCATTCTGCTGCTGGGTGTGATTGCGTTCGGGATTGGGACGGCGGCCGGGGTGCTGATGGCGA
AGCTGATGAACCTGTGCAGTAAGAACAAAATCAACCCGCTTATCGGTTCGGCGGGGGTGTCGGCGGTGCCGAT
GGCGGCACGCGTATCGAACAAGGTGGGCCTGGAGTCGGACCCGCAGAACTTCCTGCTGATGCACGCGATGGGC
CCGAACGTGGCGGGGGTTATCGGTTCGGCCATTGCCGCGGGCGTGATGCTGAAGTACGTGCTGGCAATGTAAT
CCAATCTAATCGGGAGCAGTGATTCTGCTCCCGCAAAATGAGGACACAGTAATGAGTGAAATGGTTGCGTTTC
GGCAAGGTACATCCATGCCATCCAGAGAAACCATCTTGCGTTATGTTGTTGAGACGGTGAATCAGATAACTGA
GCTTGAGCCAGCACTGCATTTGCTGCCGTGGTCTGGCGTCAACTCAGCAATATATGAGCAACGTTTTGCACAG
TGTTACGACGAGGGGCTTTGTGCCGCACAGACGTCGGCACCGAATGTTCCGCAGGGGATTCTGCCATCGACCG
ATTGGGCTCAGGGTATCGGGTTACTCTGTTTTGCCGCAGGGTATATGAGCGCAGGCGAGCGGCCATTAACACA
CAATCAGTTATGCGATTTCGTCAAACAGGCGGCTGTTGGTTTATCGCCGATTGAGGGGGAGGCGGCGAGCGGT
TTCTCGACGGTACGTAGTATTGCATTACCCGTATTCAGGCGATTACAACGCGATGGTCACGCGTCTCGGATCC
TGCTGTTACAAACGTTGCTGCATCTGGTTGCCTGGAAAAGCGCATCGCAGTATGCGCGTCAACAGGCGCAACG
ACTGCTCTGGATGGGCGGCATTTTGGGGGAAGGGGGGAGCATAGCCTGCTTGTACTGGATAAGGCGCTGCGTG
AAGAAGCCGTCGGGGAGAAAAGTCTCCCCGCTCTGCTGATCTTCACGAGTTTCCTGGCGCATTTTCCGGCAGG
GCCAGTCTTTATTGACTAGTCGTCGAACGACGCGGGCAAAAGCCGCGTCGTTTTAGTTCGACGCCGGGTATTC
CTGCACCGTCACCTGGAACGTGAGCTGCTTATCATCCCGCATTACCACGACCGGAATGACGGAGCCCGGGCGG
ATTTCCGCCACCTGATCCATCGTCTCCAGCGCGGACACAGCGGGTTTATTATTGACCGAAATAATCAAATCAT
TAACCTGAATACCGGCAAGCGCGGCGGGGCCGTTTGGCGTCACTTCATTAACGACAATGCCCTGAATCGGGTC
CATGCCGCTACCCTGCTGCGCGTGCAGCGGCGCGATTTCTCGTCCGCCAATACCGATATAGCCGCGAATCACG
CGACCGTCGCGGATAAGCTTATCCATAATTTTCGTGGCTAGCTGGAAGGGAATCGCAAAACCAAGGCCTTCCG
GCGTTTCGCCATCGTTACTCTTATCAAAAGAGAGGGTGTTGATCCCCATCAGTTCGCCTAACGAGTTGACCAG
CGCGCCGCCGGAATTACCGTGGTTAATCGAGGCGTCGGTCTGGAGAAAATTCTGTCGCCCCGTCGGGTTCAGG
CCGATACGACCCGTTGCGCTGATGATCCCCTGGGTGATGGTCTGTCCCAGATTATATGGGTTGCCGATAGCCA
GTACGACGTCGCCAATATGCGGTGTACGCTTTGTATTAATCGGGATGGTAGGCAGCCCGCCAGTGGCGTTGAT
CTTCAGCACCGCCAGATCGGTAAGCGAATCGGAGCCAACCAGTAGCGCTTCAAAGACGCGGCCATCCTGTAGC
GCGACGATAATCTGATCGGCATCGTTAATCACGTGCTTGTTGGTAATAATATAACCGCGTTGATCCATGATCA
CGCCGGAACCTAGCGTGCGGATCTCCAGTTGATTATGCGCGGTACTGTTCATACTGCGGTTATAGACGTTGAC
GACGGCAGGCGCGGCGCGGCGAACCGCAAAATTATAACTGGCTGGCGTCTCATCGGTACTGTCGAATTGCGGG
ACGGCGATAGGATTAATTTTGCGCAAAGAAGGCATGACGGCCAACAGAATAGCGCCGACAATTAAACCTATTG
CGACCGAACGTAAGAGCTTCACAAACATGATGGAGGCGTCGTTAAAAAAGGGAACGGCAGCAGCATACCATGA
GTTAACCGGACATCACATCGCAGGCTGATGCCCGGTTTAAGACAGGATTAGCGCAGCAATAGATAAATGTTCT
CGTTGCCGCGTACTACCTGAAGAGCAATGATGGACGGTTTTGCCGCCATCACTTTGCGCATTTCGGCGATAGA
ACTGATGCGATCGCGGTTAACGCCGATGATAACATCATCTTTTTGCAAACCGGCCTGCGCGGCAGGACTGCTT

TTTTCGACGCTATCAACCTTAACGCCTTTCGTCCCGTCTTTCAGCTGGCCGTCGCTCAACGTCGCGCCTTGCA
ACGCCGGGGCGATCATTTCGGCGCTGGCGGAAGAAGAGGTATTGGAATCCAGCGTGACTTCCACCTCCAGCGG
CTTACCATCGCGCAGCAGGCCCAGCTTCACTTTCGTGCCCGGTTCGGTGGTGGCGATACGCGAACGCAGTTCG
GCAAAGCTATTCAGCGGCTTACCGTTAAGACTGATAATCACGTCTCCGGATTTCACCCCGGCCTTCGCCGAAC
CTGAATTGGGTAAAACCTCGCTGACAAAAGCGCCACGCTGAACGTTCAGTTTGAATGCCTTAGCGATATCAGC
GGTCATTTCAGTGCCTTTAATTCCCAGCAATCCGCGTTTGATTTCGCCGAACTGAATCAACTGCTGCGCCAGC
GTCTGCGCCATATTGGAAGGAATAGCAAAGCCAATGCCGATGCTCCCGCCCCCTGGCGCGAGGATCGCGGTAT
TAATCCCGATCAGCTCGCCGTTCAGGTTAAGCAGCGCGCCGCCGGAGTTGCCGCGGTTAATAGAGGCATCGGT
TTGAATAAAGTTCTCAAGCCCTTCCAGATTAAGCCCGCTGCGTCCCAGCGCTGAAATAATCCCGGAGGTGGCG
GTTTGTCCTAGACCAAACGGATTACCGACCGCCACGGCGAAATCGCCGACGCGGAGTTTGTCGGAATCGGCGA
TGGCAATTTGCGTTAACTTGCTGGGATTCTGAATTTGTAACAGAGCGATATCGCTCTGGTCGTCGCCGCCGAT
CAGCTTCGCGTCGAATTCGCGTCCGTCATTCAGTTGAATACTGATCTTCTGTGCCTGATTAATCACATGATTA
TTGGTTAATACATAGCCTTTCGCGGCATCGATAATCACCCCCGAACCGAGTCCTTCAAACGGCTGGGACGGCT
GGTCTGGCAGATCCTCGCCAAAGAATTTTTTAAACTCCTCCGGCACTTTTTGGCTCTGGGCGGCGGTTCCCTC
GACTTTTACGCTGACGACAGCAGGCAGCACTTTCTCCAGCATAGGGGCAAGGCTTGGCAGCGTCGCCTGGCCT
GGCACCTGGCCTGGTATCGATGCAAGGGCTGGAAACGGCGCCGAAAGAGTTAACCCGACACTTAACGCTAATG
CACTTAACAGCTGGGTGTGTTTTTTCATGGATGCTGCTCTCGTACCTTGAATGAACGGATAGACAATTCAAAA
CAGATTGATGTTATTGAATTTTCAGCCTGGTAACAATGATAATAGCTGGGACAGGAAAGATAGAGAGAGGGCG
CAAGCGATGCGCCCGATAAATTTCTTAACGGCGTGCGATTAATCGCGCTTTGCGCCGCTACGTAACAGGCCAG
ACGCGCCTTCAGAATAATCACGTGGCATTTGTACTGGCGCCTGATCGTTGCTCGCCTCAGACTCCGCCAGACG
GTTACGGAACGGATTCGACTCCGCAGACATTTCCGGCAGCAGGCTGCTGGAGCTTTTCGCCATATGCTGATAC
AGCTGGCGGTAATCATGCGCCATGGTATCCAGCAGTTCCGCGCTGCGGGCAAAGTGGCTAACTAGCTCTTCAC
GGTACTCTTCCAGCTCAGCTTTGTTTTTTTCCAGTTCGTACTGCAATGCCTGCTGTTGACGTAACTTACGATT
ACCAAAACGCATGGCCACGGCACCAATGATGATACCGACGACTAATCCAATTAGCGCATATTCCCAGGTCATG
AACATCTCCCGTTGTCTTATGTTTCCGTAGGGTGTTGGCTCCAGGCTCCATGCCCGCGCCTGATTATGCCACT
ATAACCGCTAATTCCGTAGAAGTGGAATCCCGACAGCATATCGCGTAGTGTAGAACGGCCTTTTTTTCGTCAG
CCGTGAACGACGGCACATCGATTTTCAAGGAATAACAATAACATTATGCAAAGCCTTTCCCCTACATCGCGTT
ATCTCCAGGCACTCAACGAAGGCACCCATCAGCCTGACGACGTACAGAAAGAGGCGGTCGACCGTCTGGAAAC
CCTTTATCAGGCGCTCACCGCGAAAAAATCGTCGGCTACGCCACCCGGCGGACTGATAGCGCGACTGGGGAAA
TTATTAGGCAAAAACGAACCGGACGCGCAAATACCGGTTCGCGGGCTGTATATGTGGGGGGGGCGTGGGTCGC
GGCAAAACCTGGCTCATGGATCTCTTCTACCACAGCCTGCCGGGTGAGCGAAAATTACGCCTGCATTTTCATC
GGTTTATGCTGCGCGTGCATGAAGAACTTACCGCGCTACAGGGACAGATCGATCCGTTAGACATTATTGCCGA
TCGGTTTAAAACCGAGACGGACGTACTGTGTTTCGATGAGTTTTTCGTCACCGATATTACCGACGCCATGCTG
CTGGGCGGTTTAATGAAAGCGCTGTTTGCACGCGGCATTACGCTGGTCGCGACCTCCAATATTCCGCCGGATG
AACTCTACCGCAACGGTTTGCAACGCGCCCGCTTCCTGCCGGCTATCGATGCCATTAAACAGCACTGCGACAT
TATGAATGTGGACGCCGGCGTCGATTACCGGCTGCGGACGCTGACGCAGGCCCATTTATGGCTAACGCCGCTT
AATGATGAAACCCGGCGGCAAATGGATAAACTGTGGCTGGCGTTAGCGGGGGCCGCGCGCGAGCACGCTCCGA
CGCTGGAAATCAATCATCGCTCTTTATCCACCCTGGGCGTTGAAAACCAAACGCTAGCGGTCTCTTTCGCTAC
GCTGTGCGTCGAGGCCCGCAGCCAGCATGATTACATTGCGCTCTCAAGGTTATTTCACACGGTTCTGCTGTTT
GACGTGCCGGTCATGACGCCGCTGATGGAGAACGAAGCGCGCCGCTTTATTGCGCTGGTGGATGAGTTTTACG
AGCGCCACGTTAAGCTGGTGGTCAGCGCCGCCGCACCGTTATATGAAATTTATCAGGGGGAACAGCTCAAGTT
TGAATTTCAGCGCTGTCTGTCACGCCTGCAAGAGATGCAGAGTGCGGAGTACCTCAAGCGGGAGCATATGCCG
TAAGCCGGACAAGCGCAGCGCCCCGGCATTATGCGGTATTATATCTGTGCAACCTCCCCGACCGAGGGTGAAC
CAGGCCTGAACAAAACCCGCGGCAAATCGCAAAAAAGGGGTCGATCTTTGACCTGGACTTCTCTATAATCCTG
CGACCCCACGTTACAAGAGAGTTTTTTCCCAAACTTTCTCTGTGCCGGCCAAACATATCCGAGGGGGTAGGTT
TACCGGACTTTGTCGTGTGAACCTCAACTATTTAAAACGTTTGGGTGTTCACCAACGTGTAACTTATTTATTT
GGGTAAGCTTTTAATGAAAACTTTTACAGCTAAACCAGAAACCGTAAAACGCGACTGGTATGTTGTTGACGCG
ACCGGTAAAACTCTGGGCCGTCTGGCTACTGAACTGGCTCGTCGCCTGCGCGGTAAGCACAAAGCGGAATACA
CTCCGCACGTAGATACTGGTGATTACATCATCGTTCTGAACGCTGACAAAGTTGCTGTAACCGGCAACAAGCG
TACTGACAAAGTGTACTATCACCACACCGGCCACATCGGTGGTATCAAACAAGCGACCTTTGAAGAGATGATT
GCCCGCCGTCCTGAGCGTGTGATTGAAATCGCGGTTAAAGGCATGCTGCCAAAAGGCCCGCTGGGTCGTGCTA
TGTTCCGTAAACTGAAAGTTTACGCGGGCAACGAGCACAACCACGCGGCACAGCAACCGCAAGTTCTTGACAT
CTAATCGGGATTATAGGCAATGGCTGAAAATCAATACTACGGCACTGGTCGCCGCAAAAGTTCCGCAGCTCGC
GTGTTCATCAAACCGGGCAACGGTAAAATCGTTATCAACCAACGTTCTCTGGAACAGTACTTCGGTCGTGAAA

CTGCCCGCATGGTAGTTCGTCAGCCGCTGGAACTGGTCGACATGGTTGAGAAACTGGATCTGTACATCACTGT
TAAAGGTGGTGGTATCTCTGGTCAGGCTGGTGCGATCCGTCACGGTATCACCCGCGCTCTGATGGAGTACGAC
GAGTCCCTGCGTGGCGAACTGCGTAAAGCTGGCTTCGTTACTCGTGACGCTCGTCAGGTTGAACGTAAGAAAG
TCGGCCTGCGTAAAGCACGTCGTCGTCCGCAGTTCTCCAAACGTTAATGGGGTTCTGCTTCGGCAGACAACTG
TTAGCGAAAAAACCCGCTTCGGCGGGTTTTTTTATGGCTAAAGTCCGAATCCAGTCTCTTTTCCTCCCTATCT
TTAGCATTATTCTCATCTTATTCATCTTTCATGCACGCTCAGAGAAAAGGAAGTCGCAGTGGGATTTTTTAAC
TCATTGTTTACTAAGAAAGAGTCGTTAATGCTGTCGAATAAATGGGAATTCTTTATAACAACGGTGGATGACC
ATGTTACGGGTATACGTGTGGATATCGGCGCCATTCAGGATGAAAAATTTGATCGCCTTATCCATACCGGGTT
CTTACGTGTTCATTACACCAACTGTTATGAAAATGGTTTACCGCAGCCCGATGAAACACAGCGTTTAAATCGT
ATAGAGGACTGGCTTGATGAAAAGGGGAAGACGTTTCCGATCTGGCTGGTGGGTGTGGTTACCCAACAAGGCT
GGCGCGATTTTGTGTTCATGTCGGAAGAGGATCTTAACTGGGAAAAGACGCTGGATAAATTGCTCGCTGGTGG
CCCGGAAATCTCTTTTAGTTATAGAGAGAGTCACAATGATAAAGGAAACTTTTACCGCCAGTTCTTGTATCCA
ACCCGATACGACTGGAACTGGATACACGATTCCCGTGTGTGCCGGGGATTACAGGAACAAGGTGATGATCTGA
CGCTTCCGCGCGCTATTGATTATTATGCGACATTGCCTACAGAGGTCGCAGCGCGCGACTTGGCGCAAGATAT
TGCTGCATTACCGTATGGCATAACGCTGGTCAGTATTCGAATGAATGACCCGCAACAGGGGTTTATGGCGTCT
TTTATCAGCACCGATGCTCCGCAACAATGGCATATGACAGAAATCACTTGCCAGCTTACCGATCTGGCTGAAA
AACATGGCGGCAGTTTTGATGGCTGGGGCGCACCGGTAGTACAGGCATAACAGGCGAAAGACAATATCTCAGG
TGGGGCGAGCATTTCAGACCAGGAATATCTCTTTTTCCTCATTTCCAGAATCGACTCACCACAAAGGTCACAA
AATCTGGTAAACTATCATCCAATTTTCTGCCCAAATAGCGGGGAATGTTCATTTTTTGTCTCGTTTCTGAGCA
ATGATGGCTTTTTTCGCCCTGGGTGACATAACATCTGGCTGGTCACATTGTGGCTGGTAGCAGTAAAAATTCT
GAATATACCTGGAGGTTTTCATGGCTGTCGCTGCCAACAAACGTTCGGTAATGACGCTGTTTTCTGGTCCTAC
TGACATCTATAGCCATCAGGTCCGCATTGTGTTGGCTGAAAAGGGTGTTAGTTTTGAGATCGAACATGTGGAA
AAGGACAATCCACCTCAGGATCTGATTGACCTCAACCCGAATCAAAGCGTTCCGACACTGGTGGATCGTGAGC
TGACCCTGTGGGAATCTCGCATCATTATGGAATATCTGGATGAGCGTTTCCCTCATCCGCCGCTGATGCCGGT
TTATCCGGTCGCGCGCGGTGAAAGCCGTCTGTACATGCACCGTATTGAAAAAGACTGGTATACGCTGATGAAC
GTCATCGTAAACGGGTCTGCTTCCGAGGCCGACTCTGCGCGTAAACAACTGCGTGAAGAGCTGCTGGCGATTG
CGCCGGTATTTGGTCAGAAACCGTATTTCCTGAGCGACGAATTCAGCCTGGTTGATTGCTACCTGGCGCCTCT
CTTGTGGCGTTTACCGCAGTTGGGTATTGAATTCAGCGGCGCAGGGGCGAAAGAGCTGAAAGGCTATATGACC
CGCGTCTTTGAACGTGATTCTTTCCTGGCTTCTTTAACCGAAGCTGAACGTGAAATGCGCCTCGGTCGGGGTT
AACCTGTATGGATTTGTCACAGTTGACGCCGCGTCGTCCCTATCTGTTGCGTGCTTTCTACGAGTGGTTGCTG
GATAACCAGTTGACTCCGCATCTGGTGGTGGATGTGATGCTGCCTGGCGTGCATGTCCCTATGGAATATGCGC
GCGATGGGCAAATCGTCCTCAATATTGCGCCGCGGGCGGTAGGTAATCTGGAGCTGTCTAATGATGAAGTGCG
TTTTAATGCGCGCTTCGGCGGCGTTCCCCGTCAGGTTTCTGTGCCTTTGGCTGCGGTGCTGGCGATTTACGCC
CGTGAAAACGGCGCAGGCACCATGTTTGAGCCTGAAGCCGCCTATGATGAAGACGTTGTCAGCTTAAATGATG
ATGACAATACCGCAGGCGCGGAAAGCGAGACCGTGATGTCCGTCATTGATGGCGATAAGCCCGACCACGATGA
CGACAGTAGTCCTGATGACGAGCCGCCTCCGCCGCGTGGCGGACGTCCGGCACTGCGCGTAGTAAAATAATCA
ACAGGCCCTGAAGGGCCTGTTTTGTTTGGCCTCCTCGCGGGTTCACCCTGCTTTTGAAGCTGTGTTACGATAA
GCTTGCTATTATTTGCCACCGACCACTCATGAATGGACGTTATGAACGCATTTGATTCGCAAGCAGAAGATTC
ACCGACATCCCTCGGGCGTAGTTTGCGCCGCCGCCCGCTGGCGCGTAAAAAACTGTCTGAAATGGTTGAGGAA
GAGCTGGAGCAGATGATCCGCCGTCATGAGTTTGGCGAGGGAGAACAGCTACCGTCTGAACGTGAATTAATGG
CCTTCTTCAATGTGGGACGTCCCTCCGTGCGTGAAGCGTTAGCTGCGCTGAAGCGTAAAGGTCTGGTGCAAAT
CAATAACGGCGAACGCGCCCGCGTCTCGCGCCCCTCTGCTGATACCATCATCAGCGAACTGTCGGGTATGGCG
AAAGATTTTCTTACTCATCCTGGCGGCATCGCTCACTTTGAACAGCTACGGCTTTTCTTTGAATCCAGCCTGG
TACGCTATGCGGCGGAACATGCCACCGACGAGCAGATCGCGTTGTTAACGAAAGCGCTGGAAATTAATAGCCA
GTCGCTGGATGACAACGCGCTGTTTATTCGTTCGGATGTCGAGTTTCACCGGGTGTTGGCTGAAATTCCCGGC
AATCCGATCTTTATGGCTATTCATGTTGCTCTGCTGGACTGGCTAATCGCCGCTCGCCCAAGCGTTCCCGATC
GTGAGCTGCATGAACATAATAACGTGAGTTATCAACAGCATATTGTGATTGTTGATGCCATTCGCCAGCGAGA
TCCCGATAAAGCGGATCGCGCTCTGCAAACCCACCTCAATAGCGTTTCCGCTACCTGGCACGCACTCGGTAAA
AAAAGTCAAAAAATGCGGTAGCGATCGCACCTCCGATCCCGGTTTAGTGAAGCAGATCGCACTATAAGCGTTC
TGTATTATTTGCTGCTTATTTGATCTGGTATAACAGGTATAAAGGTAGGTAGTTAATATATTCATCATCCGTA
GAGGTAGGTATGGCAAAAGCATTGCAAGGCGTAATGGCGGCGTTGTTGACCCCATTTGATCATCAGCAGCAAC
TGGATAGCGAAAGCCTGCGCCGTCTGGTGCGTTTTAACATTGGGCAGGGTATCGACGGGCTGTACGTTGGCGG
TTCCACCGGGGAAGCATTCGTACAGAGCCTTGCTGAAAGAGAGCAGGTACTGGAGATTGTCGCCGAAGAGGCG
AAGGGAAAAATCACGTTGATCGCCCATGTCGGGACAGTAAGCACCGCAGAAAGCCAGCAGCTTGCCAGCGCCG

CAAAGCGTTACGGTTTTGATGCGGTCTCTGCGGTGACGCCTTTTTATTACCCTTTCAGTTTTGAAGAGCACTG
TGACCATTATCGGGCAATCATTGATTCCGCAGATGGATTGCCGATGGTGGTATACAACATTCCGGCGTTAAGC
GGCGTAAAACTTACCCTCGATCAGATCAATACGCTGGTGACATTACCGGGCGTGAGCGCGCTGAAGCAAACCT
CCGGCGATCTCTTCCAGATGGAGCAGATCCGCCGCGCGCATCCGGATCTGGTGCTGTACAACGGTTATGACGA
AATCTTCGCTTCCGGTCTGCTGGCGGGCGCGGACGGCGGTATCGGCAGCACATACAACATTATGGGCTGGCGT
TATCAGGGGATTGTTCAGGCATTACGTGAGGGCGATGTGGCGAAAGCGCAGCGCCTGCAAACCGAGTGCAATA
AGGTTATTGATTTACTGATTAAAACCGGCGTATTCCGCGGCCTGAAAACGGTCCTGCATTATATGGACGTGCT
ATCGGTGCCGCTGTGTCGTAAACCGTTTGCGCCTGTTGATGAAAAATACCTGCCGGCGCTCAAGGCGCTGGCT
CAACAACTGATGGAAGAAAAGGCGTAACAGCATAATGCCGGGGGCGCTACGCTTGCCCGGCCTACAACAGTGC
GAACAGTAGGCCGGATAAGGCGGAGCCGCCATTCGGCATAACGACAACAAAAATCGTCGGGAGAGTAAAATGA
GTACTTCTACCCAGAACATCCCGTGGTATCGCCATCTCAACCGGGCGCAGTGGCGGGCATTTTCCGCTGCCTG
GCTGGGATATCTGCTTGATGGTTTTGATTTTGTGTTGATTGCTCTTGTACTGACTGAGGTACAAAGCGAATTT
GGGCTGACGACGGTACAGGCGGCAAGCCTGATTTCTGCGGCTTTTATCTCTCGCTGGTTCGGCGGGTTATTAC
TGGGCGCGATGGGCGATCGCTATGGGCGTCGTCTGGCGATGGTCAGCAGCATCATTCTGTTTTCGGTGGGAAC
CCTGGCATGCGGGTTTGCGCCCGGTTACACCACCATGTTCATCGCCCGACTGGTGATTGGTATGGGCATGGCG
GGCGAATATGGTTCCAGCGCGACCTATGTGATTGAAAGCTGGCCAAAACATTTACGCAATAAAGCCAGCGGTT
TTCTGATTTCCGGCTTCTCCGTCGGCGCGGTCGTTGCCGCGCAGGTGTACAGCCTGGTGGTGCCTGTCTGGGG
CTGGCGCGCGCTGTTTTTCATTGGCATTTTGCCAATTATCTTCGCTCTCTGGCTGCGGAAAAACATTCCGGAA
GCGGAAGACTGGAAAGAGAAACACGCGGGTAAAGCGCCGGTACGCACGATGGTCGACATTCTTTATCGGGGCG
AGCATCGCATCATCAACATTTTAATGACTTTCGCCGCCGCCGCTGCGCTGTGGTTCTGTTTTGCCGGTAACCT
ACAAAATGCTGCGATTGTGGCGGGGCTGGGACTACTGTGCGCGGTTATCTTTATCAGCTTTATGGTGCAGAGC
AGCGGTAAACGCTGGCCCACTGGCGTCATGCTGATGCTGGTGGTACTGTTTGCTTTCCTCTATTCCTGGCCGA
TTCAGGCGCTATTACCCACTTATCTGAAAACCGAGCTGGCCTACGATCCGCATACGGTGGCGAATGTCCTGTT
CTTTAGCGGATTTGGCGCGGCGGTTGGTTGCTGCGTAGGCGGTTTTCTTGGCGACTGGCTGGGAACGCGTAAA
GCATATGTCTGTAGCCTGCTGGCCTCGCAAATCCTCATTATTCCGGTCTTTGCGATTGGCGGCACAAACGTCT
GGGTTCTGGGTCTGCTACTGTTTTTCCAACAGATGTTGGGGCAGGGGATTGCCGGGATTCTACCGAAACTGAT
CGGCGGTTACTTCGATACCGATCAGCGCGCGGCGGGGCTGGGCTTTACTTATAACGTCGGCGCGCTCGGCGGC
GCGCTGGCGCCGATCCTGGGAGCGCTGATCGCTCAACGTCTGGATTTGGGCACTGCGCTGGCATCGCTCTCTT
TCAGCCTGACGTTTGTCGTGATCCTGCTTATTGGGCTTGATATGCCGTCTCGCGTACAGCGTTGGCTACGTCC
GGAAGCGTTACGCACCCACGATGCTATTGACGACAAACCGTTCAGCGGAGCCGTACCGCTTGGCAGTGGTAAA
GGTGCCTTTGTAAAAACGAAAAGTTAATCCGCTTGCCCGGTTTTCAACCGGGCAATATTTGTAAGGGAGTAGT
ATGTCGTTACTTGAACAACTGGATAAAAATATTGCCGCTTCTGGCGGCCTGATTGTCTCCTGCCAGCCCGTTC
CGGGCAGTCCGCTGGATAAGCCGGAGATTGTAGCGGCAATGGCGTTGGCAGCGGAACAGGCGGGCGCGGTTGC
GGTGCGTATTGAAGGCATAGACAACCTGCGTATGACCCGCTCGCTCGTTTCGGTACCCATTATCGGCATTATC
AAACGCGATCTGGACGACTCTCCAGTGCGCATTACCCCTTTTCTCGACGATGTAGATGCGCTGGCACAGGCCG
GAGCAGCTATTATCGCCGTTGATGGAACCGCTCGGCAACGGCCGGTAGCCGTTGAGGCGTTGTTGGCGCGCAT
TCATCACCACCATCTACTGGCGATGGCCGACTGCTCATCCGTGGACGATGGTCTCGCCTGTCAACGGTTGGGC
GCGGACATTATCGGCACGACAATGTCCGGCTACACCACCCCCGATACGCCGGAGGAACCCGATCTGCCCTTAG
TCAAAGCGTTGCATGACGCCGGATGCCGGGTAATAGCCGAAGGGCGCTATAACTCGCCAGCACTGGCAGCGGA
GGCGATCCGCTATGGCGCATGGGCGGTGACGGTGGGGTCCGCCATTACCCGTCTGGAACATATCTGTGGATGG
TATAACGACGCATTAAAAAAGGCGGCGTCATGACTACGTTAGCAATCGATATTGGCGGTACTAAACTTGCCGC
CGCGCTCATCGACAAAAACTTGCGCATTAGCCAGCGTCGCGAGCTACCGACCCCCGCGAGCAAAACGCCGGAT
GCGTTACGTGAAGCATTAAAGGCGCTGGTTGAACCACTACGGGCAGAGGCGCGTCAGGTAGCGATAGCTTCTA
CCGGGATCATTCAGGAAGGGATGCTGCTGGCGCTAAACCCCCATAATCTGGGCGGATTGCTACACTTTCCGTT
AGTACAAACACTGGAAACGATCGCTGGCCTGCCGACGCTGGCGGTGAACGACGCGCAGGCGGCGGCCTGGGCG
GAGTATCACGCGCTCCCTGATGATATCCGCGATATGGTATTTATCACCGTTTCAACCGGCGTTGGCGGCGGCG
TTGTCTGCGACGGCAAGCTCCTTACCGGAAAAGGCGGTCTGGCCGGGCACCTGGGACACACCCTGGCGGACCC
ACACGGGCCCGTATGCGGCTGTGGACGCGTTGGCTGCGTAGAGGCCATCGCCTCTGGACGAGGGATGGCCGCG
GCGGCGCGGGACGATCTGGCGGGATGCGACGCCAAAACCCTATTTATTCGCGCTGGCGAAGGTCATCAGCAGG
CGCGACATCTGGTAAGCCAATCCGCGCAGGTAATAGCGCGAATGATCGCCGATGTGAAAGCGACTACCGACTG
CCAGTGTGTGGTCATTGGCGGCAGCGTAGGGTTAGCTGAGGGATACCTGGAGCAGGTTCGCGCGTTTTTGATG
CAAGAGCCTGCGCCTTATCACGTGGCGCTGAGCGCCGCTCGCTATCGGCATGACGCAGGATTATTAGGCGCTG
CGTTGTTAGCTCAAGGAGACACATTATGATGATGGGGGAAGTACAGTCGTTGCCTTCTTGCGGGTTGCATCCG
CGATTGTTGGATGCCTTGACTCTCGCGCTGGCGGCAAGGCCGCAGGAGAAAGCGCCGGGGCGCTATGAATTGC

AGGGCGACAATGTCTTTATGAATGTAATGCAGTTAACGACGCAAATGCCGGCCGAGAAGAAAGCGGAATTGCA
TGAACAATACATTGATATTCAGCTACTACTAACCGGCGTCGAGCGTATTGCGTTTGGGATGTCCGGTGCCGCT
CGCCAGTGCGAGGAGATGCACGTTGAAGAGGATTACCAGCTTTGTAGTCAAATTGTCGACGAGCAGACTATTA
CGCTGCAAGCGGGGATGTTTGCTGTGTTTATGCCAGGAGAACCGCATAAACCGGGATGTGCAGTGGGTGAGCC
GGATGACATCAAGAAAGTGGTAGTGAAAGTACGCGCCAGTCTGCTGGCGGCCTGAATCTCTTGCCGGAGCAGT
CTTCCGAACGCAGCCCCTTACGGGCTGCGTGGTGAATGTGCCGGAGAGCGCGGGTATGACGGGTTAACGCTTA
AACGTCACGGCTTCCGGCTGGGTCAGGTGCAGCGTCGTCTGGCTCGGCACCGTCTCGGCAATCACTCGCCCGT
GGCGAATCGAGTAACGCGCAGGCGTCTGGCGACGTACCGCATCGAATCCATTCTCAGCGGGTAAGATAACCAG
ATTCGCGGCATTGCCGACGCTCAGACCGTAGTCCTGTAAATGCAGGGTTTTTGCGCTGTGGGTAGTGATCAGA
TTTAACCCGTCGTTGATTTGCCCATACCCCATCAACTGGCAAACGTGTAACCCCATATGCAGCACCTGCAGCA
TATTTGCCGTGCCTAATGGATACCACGGATCGAAGACGTCATCATGGCCAAAGCAGACGTTGATCCCCGCTTC
CAGCATCTCTTTCACTCGCGTGACGCCACGACGTTTCGGGTAAGTGTCAAACCGTCCTTGCAGATGAATATTC
ACCAGCGGGTTGGCGACGAAGTTAATCCCCGACATTTTCAACAGACGGAACAGGCGTGACGCATACGCGCCGT
TGTACGAGTGCATAGCCGTGGTGTGGCTGGCGGTCACGCGCGCCCCCATGCCATCGCGATGCGCCAGCGCCGC
GACCGTTTCGACAAAGCGAGACTGCTCGTCATCAATTTCGTCGCAGTGCACATCGATCAAGCGATCGTATTTT
TGCGCCAGGGCGAAGGTTTTGTGCAGCGATTCAACGCCATATTCACGCGTAAATTCAAAGTGCGGAATGGCGC
CAATAACGTCAACGCCCAAACGCACGGCTTCCTCTAACAGCGCTTCACCGTTGGGATAAGACAGAATACCTTC
TTGCGGAAAGGCGACAATTTGCAGGTCTACCCACGGCGCAACCTCTTGCTTCACTTCCAGCATGGCTTTCAGC
GCGGTGAGCGTAGGATCTGAAACGTCAACGTGAGTACGAACGTACTGGATGCCGTTAGCCATCTGCCATTTCA
GGGTCTGCATCGCGCGCGCTTTCACATCTTCATGCGTGAGCATTGCTTTACGTTCTGCCCAGCGCTCAATCCC
TTCAAACAGTGTGCCGGACTGGTTCCAGCTCGGTTCACCCGCCGTTTGCGTGGTATCCAGGTGGATATGTGGC
TCAACAAACGGTGGAATAGCCAGGCCGCCTTCGGCATCAAGCACTTCGCCCTGCGGTAACGATGCGGCTTCCT
GAGGCTCAATCCGGCTAAAGCGCCCGTTTTCAATCGTAATCTGCCATAATCCTTCGTGTCCCTGCAGACGCGT
CTGACGAATAGTGATGTTATTGTTCTGCATGGCTGACCTCTGGTGATTTAGCAAGGCTGCGGTTGAAAAGTGG
ATTCAGCAGGATATAGCTGAAGACGCCGCCGAGTACGGCGTTGACGGGCACAATACCCGGAACATAATGTCCG
GCGGCGATGCCCAGCGCGACGGAAAGAATAGCAATCCAGTTAACGGGAATAAAGCGCACGGTGTTGAAGTCGG
CATAGCGGCGGCGGTTCAACAGATAGTCGGCAATAATCACTCCGCCAATCGGTGGGATGGCAGATGACAGGAA
CGTCAGCCAGCCGACAAAATTATTGTAAAGCCACAGCGCGCACACGGTACCGATAATCCCGTTCACCACCGAC
AGCGTACGGCTGGAAAGACCGGTAATATTGGCGAAGCCCAGACCCGATGCGTACAGCGCGTTATCGTTGGTGG
TCCAGATATTCAGGCCAAGCACCACAATCGCGGGCAGCAGCAGCCCCTGCGCTATCATCACGTCAGAGATATC
CGCCTGACCGACGGCGGCAGCGCCTGCCGCGCCAAAGATAAACATCAGCGAGTTGCCGAGGAAAAAAGCGACC
ATCGCAATCAGTACGGCGCTTTTGGCATGACGCCCGAAGCGGACGAAGTCGGCGGTCAATGTACCGGCGCTGA
CAAACGAGCCCACCACCAGCGCCAGCGCGCTGGAAAAATCCAGCGGCGTCTGCGGCACTATCGTTTTTAAATG
CTCCAGCCCACCCACGCCGCTGACCGCCAGCCAGACGGAGTAGCTGCCCAGTATAACGATCGCGGGTACGGCA
ATGATAGACAAAATGGTCAACGCCGAGATGCCGAAAAAAATGGTCAGGGTCATCAGTAGACCCGAAATGGCAA
TCAGAATATTGGCATCAATGCCCGTCGCTTTACTGACCGGAATAGCGAACATCGCTACGCCAACGCCAAACCA
GCCTACCTGTGTGCCGCCTAGCAGCAGCGAGGGAAGCCATGATCCTTTAACGCCAAAGGAGTAACGTGCAAGG
AGGTGGGTGGAGAGTCCGGTTTTTGCGCCGATGTAACCAAGAAATGCAGTGTAGATACCGAGGAGGAGATTAC
CGAAGAGAACTGCGAGGAAGAAATCGTGATAGGTAAGACCGGTGCCGAGTGTCCCTCCGGTCCACATACTGGC
GGAAAAAAACGTTAAACCCAACATGACAAACGTCAGTGGAATCACGCCCTTCCGCGCCGCCAGAGGGACGGGG
CCCTGGCTATAATTGTTGTCCTGCGACACAACGCCTCCATGAATTTTGCCCAAAAAATCCGCCGCATAATATA
CAGTTCTCAGAATAAAGCAAACGTTTTCGTATGATATTTTTTTGATAATTATTCATGATATTAATGGTTCGTT
AGTTTTTCTCCGTTGTCAGTGAACCACCGCGTGTGAGTTGAGTTTCCAGCATGGTTTGCGAACGAATAAGTTC
GTCTGTGAGTAATTTTTCGTCGGGCAACTGCACTTTGTACTCGCTCGCCATGATGGTGTTTGGCAGGCCAGTC
AGCGCGTAATGCGCTTCCCCCGCGCCTTTCCCTGCGCACAAAACCAGGCCGACTGGCGGGTTTTCTCCGGGCA
TTGTCCAGTGCTCTTTGGCATAGTTCAGGTACATATTCATCTGCCCGGCATCCGCATAACCGAATTTGCCTAC
TTTCAGGTCAACGAGCAACAGGCAGCGCAAGCGACGATGGAAGAACAGCAGATCGACGCGGAACCAGCTATCG
TCTATGCGTAGTCGGCGTTGGCGGCCAACAAAAGCAAAATCGTTACCGAGCTCCAGCATAAAGTCCATCAGGT
GGCTGAGCAGCGCGTCTTCGAGATCTGATTCGGAATACTCGTCCTTCAGGTTAAGAAATTCCAGAATGAACGG
GTCGCGTATGGCTTGCTCCGGCAAAACGTTTGGCTCGGCGGGTGCAGGCTGCTGCAACATGGCGGATTTGTCG
TGGGACAGTAGCGTTCGTTCGTAAAACTGAGTGGCGATTTGCCGATCGAGCTGCCTAACTGACCAGCCGTTAC
GTAGCGTCTCTTTCTCGTAAAAAGTGCGGGCATCGGGATTTTTTACCGACAAGAGGCGGACATACGCTGACCA
GGGAAGAGGGAATGTTTTGGCGAGCGGTATGAGATTTGAAAATTCCGCAGACAGTGTCTGCGGAATTTCAATG
TGTTGAAAACAAAGATAAAATGTACGAATTTGCCATAGGTTTCTGGTGGAAAAACCGCGCTTATAGCGCTGAC

TTAAATCTACCGATAATCGTTCGATAAGCTGAGTACCATAGGCCGCTCGCGCTTCTCCTCCCTGTTCAAATTC
GACAATGCGTCGGCCAATTTCCCAGTAGGTGGCAGTCATAATGGCATTGATGCTGCGCACGGTCTCGGTACGT
GCCGTATCGACCAGACGAATAATACCGTCATGAATTTGCTGATATTCATCAGACTGTGGCACTAAAGTTGGAT
TCGTCATGTCATCCCTTACGTGATAGTTTTATTGTGAAATTAGAACGCTATCAATACGCTTCAGGCGTAAACG
GATCAATCCATGAACGGATTTTACATGGCATTACATCTCGATTTACGAGCGGGTTTCCAGAATCGGCAGGAGA
GATATTGAGGAGAGGAATATCGGCTCCCCAGCCATCAGCCAAGGCAGTAAGCGGGGAGCCGGAAGGGTTATAC
TTCGAGGTAGTTCATAATCCCGTCAGCCGCTTTACGGCCTTCGGCAATCGCCGTCACCACCAGATCGGAACCG
CGCACGATGTCGCCACCGGCGAAGATTTTCGGGTTGCTGGTCTGGAAAGCATTATCGCTGCGTTCCGGCGCGA
TAATTCGTCCCTGCGAGTCCAGTTCCACGCTGTGTTTCGCCAGCCATTCCATGCTGTGTGGACGAAAACCAAA
CGCCATCACCACCGCATCAGCCGGAACCACATGCTCGGAACCGGCGACGATCTCTGCCCGCCGACGGCCTTTG
GCATCCGGTTCGCCCATTTCAGTGCGCACCATTTTTACGCCGCTCACTTTGCCGTTGGCATTGACTTCAATGC
CCAGCGGTTGAACGTTAAACTGAAATTCGACACCTTCCTCGCGCGCGTTCTTCACTTCGCGTCTGGAGCCCGG
CATGTTCTCTTCATCACGACGATAAGCACAGGTTACGTGCGTTGCGCCCTGACGAATGGAGGTACGTACGCAG
TCCATTGCGGTATCGCCGCCGCCCAGCACCACCACGCGTTTGCCTTCCATGCTGACATATGGTTCGTCGCTGG
TTTCGCCAAACCCCATGATCTGCTTCGTATTGGCGATCAGGAACGGCAGAGCGTCGAAAACCCCGTCCGCATC
TTCATTTTCCAGCCCGCCGCGCATCGACTGATAGGTGCCGACGCCAAGGAATACCGCATCGTAATCTTTCAGC
AGATCTTCAAGCTGTACATCACGACCCACTTCGGTATTAAGTTTGAACTCAATACCCATGCCGGTGAAGATCT
CACGGCGGCGGGTCATGACTTCTTTCTCCAGCTTGAAGGCCGGGATACCGAAGGTCAGCAGACCACCGATTTC
CGGATGGCGATCAAAGACCACCGCCTTCACGCCGTTGCGGGTCAGCACGTCGGCGCACGCCAGCCCTGCCGGA
CCTGCGCCGATTATCGCTACCCGTTTGTCCGTCTGGCGTACGCCGGTCATATCCGGGCGCCAGCCCATCTCAA
ACGCTTTATCGTTGATATAGCGCTCAATGTTACCGATGGTCACTGCGCCGAACTCATCGTGCAGCGTACAGGA
GCCTTCGCACAGGCGGTCCTGCGGGCAGACGCGGCCACACACTTCCGGCAGGGTGTTGGTCTGGTGCGATAGC
TCAGCCGCCTCAAAAATACGCCCTTCGTTAGCCAGTTTCAGCCAGTTCGGGATGTAGTTATGTACCGGACATT
TCCACTCGCAGTAGGGGTTCCCGCAGGACAAACAGCGATCCGCCTGGGCTTTCGCCTGACCTTCAGAAAACGG
TTCGTAGATTTCAACAAACTCGATCTTACGGAGCTTCAGCGGTTTCTTCGGCGGATCAACGCGCTGCAGGTCG
ATAAATTGATAAACATTCTGGCTCATTTCAATTCCTTACTGCGCCTGCACGCGCAATTCTGCCGCACTACGAC
TACGGTGGCCCAACAGGGCTTTGACATCGCTGGACTTGGGCTTAACTAACGCGAATTGGGTTGAGAAGCTTGA
CCACCTGGACAGGATCTCTTCGCCGCGTTGCGAACCAGTATGCTGCACGTGCTCGGTGATCAGGCCGCGCAGA
TGCTCTTCGTGGATAGCCAGCGAATCAACGTCCAGTACTTCTACCAGTTCCGGGTTCACGCGTTTACGGAATT
CGCCATCTTCGTCCAGCACATAGGCGAAGCCGCCGGTCATCCCCGCGCCGAAGTTAACGCCGGTTTTACCCAG
CACGCAGACGATGCCGCCGGTCATGTATTCACAGCCGTTGTCGCCGATGCCCTCTACTACGGTAATCGCCCCG
GAGTTACGCACGCCGAAACGTTCGCCCGCGCGGCCCGCCGCGTACAGACGGCCACCGGTGGCTCCATACAGGC
AGGTGTTGCCGATAATGCTGGCTTTATGGCTGAGGAAGGCGGAACCTACCGGTGGTCTGATGGCGATCAAACC
GCCTGCCATACCTTTACCGACATAGTCGTTGGCATCGCCAGTCAGATATAACTCTACGCCGCCCGCGTTCCAC
ACGCCAAAGCTCTGGCCTGCGGTACCGCTGAAGTGCGCTTTAATCGGATCGGAAGCCAGCCCCTGATCGCCGT
GGGTCTGTGCGATGTAGCCGGAAAGCGACGCGCCGACGGAACGATCGGTGTTGCGGATGTCGAACCAGAAGGT
TTTGCTCTGGCGCGCATCGACAAACGGTTTCGCCTGCTGCAACAGTTGCGCATTCAGCACGCCGTTATCAAAC
GGCGGGTTATTTTCGGTGCAGTACAGCGCTTTACCCGGATGAGGTTCAGCGGTTTCCAGTAGACGAGACAGCG
CCAGCTTCTGCTGTTTAGCGGTGAAGCCCTCCAGCTCTTTCAGCAGGTCGGTGCGGCCAATCAAATCCACCAG
GCGGGTCACGCCAAGCGAAGCCATCAGCTCGCGCACTTCGCGGGCGATAAATTCAAAGTAGTTGGTTACCTTG
AACGGCAGGCCGTGATAGTGGTTCTTACGCAGTTTTTCATCCTGCGTCGCCACGCCGGTTGCGCAGTTGTTCA
GATGGCAAATGCGCAGGTATTTACAGCCCAGCGCCACCATCGGGCCGGTGCCGAAGCCGAAACTTTCCGCACC
GAGAATTGCCGCTTTAATGATATCGACGCCGGTTTTCAGACCGCCGTCCACCTGCAGACGAATCTTGTGACGC
AGACCGTTGGCGACCAGCGCCTGCTGGGTTTCAACAAGTCCGAGTTCCCACGGACAGCCCGCATATTTCACCG
AAGAGAGCGGGCTGGCGCCGGTACCGCCGTCGTAGCCGGCGATAGTGATCAGATCCGCATAGGCTTTCGCCAC
GCCGGTCGCGATGGTGCCGACGCCCGGTTCGGACACCAGCTTCACGGAGATCATCGCCTTCGGGTTGACCTGT
TTGAGGTCGAAAATGAGCTGCGCTAAGTCCTCGATAGAGTAAATATCGTGGTGCGGCGGCGGGGAGATCAGCG
TCACGCCCGGCACTGAATAGCGTAGTTTAGCGATATACGGAGTGACTTTATCCCCCGGCAACTGACCGCCTTC
GCCCGGTTTCGCGCCCTGCGCGACTTTAATCTGAATCACATCGGCATTGACCAGGTACGCTGGCGTCACGCCA
AAACGGCCGGAAGCCACCTGCTTGATACGCGACACTTTATTGGTGCCGTAGCGCGCCGGGTCTTCGCCGCCTT
CGCCGGAGTTGGAGTTGCCGCCGAGGCTGTTCATCGCTTCCGCCAGCGCTTCGTGCGCTTCCGGACTTAATGC
GCCGATAGACATGGCTGCGGTATCAAAGCGTTTAAACAGCTCACTGGCGGGCTCAACCTCGTCGATAGTCACC
GCCTCGCCGTCTGGATGTATCGCCAGCAGATCGCGCAGCGTTGCCGCCGGACGCTCATTGACCAGTTTGGCGT
ATTCCTGATAATCGCTGTATTCGCCGCTCTGTACCGCCTGTTGCAGGGTACGCACGACATCCGGGTTATAGGC

GTGATATTCGCCGCCGTGGACGTACTTCAGCAGGCCGCCCGGGCTAATGGGTTTGCGCGCCAGCCAGGCGCGT
TTGGAGAGATTCAGCAGATCCTGCTGGAAGTCATCAAAGCTCGCGCCGCCGATACGGCTGACCACGCCCTGGA
AGCACAGGTTTACCACATCATCGTGCAGTCCGACCGCTTCAAACAGTTTGGAGCAGCGGTAAGAGGCAATGGT
CGAAATGCCCATTTTGGACATAATCTTGTACAGCCCCTTGTTGATGCCGTTACGGTAGTTCTGCATCACGGTA
CGGTAGTTTTTGGCGATGGCCTGGGTATCAATCAGACGCCCCAGCGTTTCGTAGGCCAGGTACGGATAGATAG
CCGTTGCGCCAAAGCCGAGCAGTACCGCAAAGTGATGCGGATCGCGGGCGCTTCCGGTTTCTACGATGATGTT
GGCGTCGCAGCGCAGGCTTTGTTCTACCAGACGCGTTTGCACCGCGCCCACCGCCATCGGCGCGGGGACCGGC
AGGCGATTCTTACCGATATTGCGATCCGAGAGGACCAGCAGCACGGTGCCGTTGCGCACCATCTGTTCCGCTT
TATCGCACAGCGCCTTAACCGTCGCGTTCAGTGTGGTTTCGGTCACGTCGAAGGTGATATCCAGACGGTCAGC
GCGATAATGCTCCTCTTTCATGGTGGTGAGCTGTTTGAAATCGGAGTACAGCAGAATTGGCGATTTAAAACTC
AGGCGATGGGCCTGACCTTCCGCTTCGCAGAAGACGTTCATTTCGCGACCGATGCTGGTGGCGAGCGACATGA
CGTGCGCTTCACGCAGCGGGTCGATAGGCGGGTTAGTGACCTGCGCGAACTGCTGGCGGAAGTAGTCGTAAAT
AATACGCGGCTGGCTGGAGAGCACGGCAAAGGGGGTATCGTCGCCCATTGAACCGACGGCTTCCTGGCCGTTT
TCGCCCAATACGCGGATAACCGAGTCCAGCTCTTCGGCGCTGTAGTTAAACTGTTTCTGATAGCTGGCGAGCA
GGTCATCGTCCAGTTCGCGGCTACCAACTTCTTCATCAGGCAGCTCTTCAAACGGCACCAGGCGGCGGACGTT
TTTCTCCATCCACTCTTTATATGGATGGCGGCTTTTCAGATCGTCATCGGTTTCCGCGGAATGCAGAATACGT
CCGCCGCGGGTGTCGATCACCATCAGCTCGCCTGGGCCGACGCGGCCTTTCTCGACCACTTCATCCGGCTGAT
AGTCCCAGATACCGACTTCGGAGGCGCAGGTGATCAGCTTATCTTTGGTAATGACGTAGCGTGCCGGGCGCAG
GCCGTTACGGTCAAGGTTACAGGCGGCGAAGCGCCCGTCGGACATCACGATGCCTGCCGGACCGTCCCACGGC
TCCATATGCATGGAGTTAAAGTCAAAGAAGGCGCGCAGATCCTGGTCCATATCGGGGTTGTTCTGCCAGGCGG
GCGGCACCAACAGGCGCATGGCGCGGATGATGTCCATCCCGCCTGCCAGCAGCAGTTCCAGCATGTTGTCCAG
CGAGCTGGAATCCGAACCGGTTTCGTTAACAAACGGCGCAGCGGACTGTAAATCCGGGATCAGCGGCGTCTGG
AATTTATAGGTACGGGCGCGCGCCCACTGGCGGTTGCCGGTGATAGTGTTAATCTCGCCGTTATGCGCCAGGT
AGCGGAACGGCTGCGCCAGCGGCCAACGCGGCACGGTGTTAGTGGAAAAGCGCTGGTGGAACAGACAGATGGC
CGATTCCAGACGCAGATCCGCAAGATCCAGGTAGAAACGCGGCAGATCCGCCGGCATACACAGACCTTTATAA
ATGTTGACCAGGTTCGACAGGCTGCATACGTAGAAATCCTTGTCGTTCTGCAGGCGTTTTTCAATGCGTCGGC
GGGCGATAAACAGACGGCGCTCCATATCGCGCGGACGCCAGCCCGCCGGAGCGTTGACAAAAATCTGCTCAAT
ACGCGGCAGGGAGGAGAGGGCGATTTCACCGAGCACGCCTTCATTGGTGGGCACATCGCGCCAGCCGACAATC
GACAGGGTTTCCTGCTGTAATTCTTCTTCGACAATATGGCGTGACGCGGCGGCCAGTTCAGGATCTTTATTCA
AAAAGATCATCCCGACGGCGTAGTTTTTGGCTAAGCGCCAGCCGCGCTCTTCGGCCACGATGCGAAAGAAACG
GTCAGGTTTTTGTAACAGCAAGCCGCAACCGTCGCCGGTTTTACCGTCGGCAAGGATCGCGCCACGGTGCTGC
ATCCGGGCCAGCGCGTGTATGGCGGTACGCACTACCTTGTGGCTAGGTTCGCCTTCTATGTGGGCGATCAGGC
CGAAACCACAGTTATCCTTCTCAAGGGATTTATCGTACAACATATCAGTGAACCTCCCCAGGCTCTACGGGAC
GCTCTCCCAACCGACGGCGCGCGGGCACAGCAAGAGCATGGCGACGGGGCTGGCGCATCATACGCTTACCTCG
CATTCGCCCTCTTTGTGTCCTTTGCGCATTGGTAAATACGGTTAAGAGGGAATCTCAATTACTGCATAAATAT
GATGAGCAGACGGCTCATCCAGAAAGCTTCCAGCGGATCCCCAACTTATCGGGAATCCACACCCAGGTCAAAT
GGCAAACTGATTTATGCAAAAGTGTGCTGTAGCACAGATAACTTTTTGAATATATTGAAATTAAAAGGTTTTT
TATAGAGGGGCTGCTAATACGGAGCGGCAGAAAGCCTGTGATCTGTCTCACTATAAGAGCCGCTCGCCAGACG
TTATCTGACCTTTCTTTTTCAAAAATCTTGGCATTTTATGCTGTTTTAAGCGTGAGCAGTATTGAATGCTACT
GTTTTTATCGATGACCCAGCAGACGCTGAAAAAAGAGGGGAATATCAGGAAAAATCATCAACGGGATTATGAA
TGAAATGTCGTTTATTTTGCTTGATTATGCAACGGATAAAAGCCTTCCGGGACGATTGATCCAGGTCATCGCC
AAAGGAGACTAAAAATGGCAGGCTTTGGCCTCTTTTTAAGAGGCGGTCTATCAGATTATGCAGTTACAGAAAT
TAGTCAATATGTTTGGTGGGGATCTTTTACGTCGGTATGGGCAAAAGGTTCATAAGCTGACGCTTCATGGCGG
TTTTAGCTGCCCTAACCGCGACGGCACCATCGGGCGTGGCGGTTGCACCTTCTGTAATGTCGCCTCTTTTGCC
GATGAGGCGCAGCAGCATCATTCGATTGCCGAGCAGCTTGCGCATCAGGCGCATCTGGTGAACCGCGCTAAAC
GTTATCTGGCCTATTTCCAGGCCTACACCAGCACCTTTGCGGAAGTGCAGGTGCTGCGTTCCATGTATCAACA
GGCGGTCAGCCAGGCCAGTATCGTCGGGTTATGCGTCGGTACGCGTCCGGATTGTGTCCCGCAGGCAGTAGTA
GATTTACTGTGTGAATATAAAGATCAAGGCTACGAGGTATGGCTGGAACTGGGATTGCAAACCGCGCACGATA
AAACCCTGCACCGAATTAACCGCGGGCATGACTTCGCCTGCTATCAGCGTACCACCCGGATTGCCCGGGAGCG
CGGGCTGAAAGTGTGTGCGCATCTGATTGTGGGGCTGCCGGGAGAAGGGCAGGCAGAATGCCTGCAAACCATG
GAGCGTGTGGTGGAGACGGGCGTTGACGGTATTAAACTGCATCCGCTGCATATTGTGAAAGGCAGTACGATGG
CCAAAGCCTGGGAAGCGGGGCGGCTGAACGGCATCGAACTGGACGACTACACGCTAACGGCAGGTGAGATGAT
TCGTCATACGCCGCCGGAGGTGATTTATCACCGAATTTCAGCCAGCGCTCGCCGTCCGACGCTGCTTGCCCCG
CTGTGGTGCGAGAATCGCTGGACGGGGATGGTTGAGCTGGACAAATACCTGAATGAGCATGGCGTACAGGGCT

CTGCGCTGGCCCGTCCCTGGATACCACCTGTAGCGTAGCGTGATGAGGGGCGCTACGCATATTTCGCACAATA
TTCAACGCGGTTCCCATAATTGGGTATTATGTGCGAAGTTGTGGTGAAGGAATCCTCTATGAAGCAAATTCGT
ATGCTGGCGCAATACTATGTCGACCTGATGATGAAATTGGGTCTGGTGCGCTTTTCCATGCTCCTGGCGTTGG
CATTGGTGGTGCTGGCGATTGTCGTGCAGATGGCGGTGACCATGGTGCTGCATGGTCAGGTTGAAAGTATCGA
CGTTATTCGTTCCATCTTTTTTGGCCTGTTAATTACGCCCTGGGCGGTCTATTTCTTATCCGTTGTTGTTGAA
CAACTGGAAGAGTCAAGGCAGCGACTGTCGCGTCTGGTGCAAAAGCTGGAGGAGATGCGCGAGCGCGATCTCA
AGCTTAACGTACAGTTGAAGGACAATATTGCCCAGCTTAATCAGGAAATTGCCGATCGCGAAAAAGCGGAAGC
GGAGCTGCAAGAGACATTTGAACAGCTAAAAGTAGAAATTAAAGAGCGTGAAGAAGCGCAAATCCAGCTAGAA
CAGCAATCCTCTTTTCTGCGCTCCTTTCTGGACGCCTCGCCGGATTTGGTTTTTTATCGTAATGAGGATAAAG
AATTTTCCGGCTGTAACCGCGCAATGGAATTGCTAACCGGCAAGAGCGAAAAACAGCTGGTGCATTTGAAGCC
GGAAGATGTGTACTCTCCCGAAGCGGCGGAGAAAGTCATTGAAACCGATGAAAAAGTATTCCGCCATAACGTC
TCCCTGACCTACGAACAGTGGCTGGATTACCCGGACGGACGAAAGGCCTGCTTTGAAATTCGTAAAGTGCCTT
ATTACGACCGTGTCGGTAAACGTCATGGCCTGATGGGATTTGGTCGTGATATTACCGAACGTAAGCGTTATCA
GGACGCGCTGGAACGCGCCAGCCGCGACAAGACCACATTTATCTCTACCATCAGTCATGAGCTGCGCACCCCG
CTTAACGGCATCGTCGGTTTAAGCCGCATCCTGCTGGATACCGATCTTACCGCCGAACAGGAAAAATACCTTA
AGACAATCCATGTCTCCGCCGTGACGTTAGGCAATATTTTCAATGATATCATTGATATGGATAAGATGGAGCG
GCGTAAGGTTCAGCTTGATAACCAACCGGTTGATTTCACCAGCTTTATGGCCGATCTGGAAAACCTTTCCGGT
TTGCAGGCGCAGCAAAAAGGGCTACGTTTTGTGCTTGAGCCGACGCTGCCGCTGCCGCACAAAGTCATTACCG
ACGGAACGCGTCTGCGCCAGATTTTATGGAATTTGATCAGCAATGCGGTGAAATTTACTCAGCAGGGCCAGGT
GACGGTGCGGGCGCGCTATGATGAAGGCGACATGCTGCACTTTGAAGTGGAAGATTCCGGGATCGGCATTCCG
CAGGATGAGCAGGATAAAATTTTCGCCATGTATTATCAGGTGAAAGACAGTCATGGCGGTAAACCGGCGACGG
GTACCGGTATCGGGCTGGCGGTCTCCCGTCGGCTGGCGAAAAATATGGGCGGCGATATTACCGTTTCCAGTCT
GCCGGGCAAAGGATCGACCTTTACGCTAACGGTTCATGCGCCAGCGGTGGCGGAAGAGGTGGAGGACGCCTTT
GATGAAGACGACATGCCGCTGCCGGCGCTGCATGTTCTGCTGGTCGAAGATATTGAACTGAACGTGATCGTAG
CGCGTTCGGTACTGGAAAAACTGGGTAACAGCGTGGATGTCGCGATGACCGGCAAGGCGGCGCTGGAGATGTT
CGCGCCAGGCGAATATGACCTGGTCTTGCTGGATATTCAGCTACCGGATATGACGGGTCTGGATATTGCCAGA
GAACTGACCCGTCGCCACACGCGGGAAGATTTACCGCCGCTGGTAGCGCTTACGGCTAACGTTCTGAAAGATA
AAAAAGAGTATCTGGACGCTGGTATGGACGATGTGCTGAGTAAGCCGCTGTCGGTGCCAGCCTTAACCGCGAT
GATTAAAAAATTCTGGGATGCCACCGATAAAGAGGAGAGTACCGTGACGCCTGAAGAGAGCGATAAAGCGCAA
GCGCTGCTGGATATCCCGATGCTGGAACAGTACATCGAGTTGGTTGGGCCTAAATTGATCACCGACGGTCTCG
CGGTGTTTGAAAAAATGATGCCCGGCTATTTAAGCGTGCTGGAGTCCAATCTGACGGCGCGTGACAAAAAAGG
CGTCGTTGAGGAAGGGCATAAGATTAAAGGCGCAGCCGGTTCGGTAGGGCTGCGTCATCTGCAACAGCTTGGC
CAGCAAATCCAGTCGCCGGATCTTCCTGCCTGGGAAGATAATGTCGCGGAATGGATTGAAGAGATGAAGCAGG
AGTGGCAGCACGATGTCGCGGTGCTGAAAGCCTGGGTGGCAAACGCGGAAAAAAAATGACCCCGGTTTGACCG
GGGTGCGCGAATACTGCGCCAACACCAGGGAAATCGTGGCTGCGCCGTGCTTATTATGATGATGTCAAAGGCG
CAGTCCTGTTTTCAGGCCGCACGCAGTTAAGATAGCAAATCCTAAATGATTTGTTACATGAATCATTTAAATG
TGTGAAGCGTAGCATTTTAATCAAAATTATTAATGGGCTTGAGTCGGTTGCAGGAGGATGTTCACCATGAAAA
AAATTGGCGTAGTGCTCAGCGGATGCGGCGTCTATGACGGCACCGAAATTCATGAGGCCGTTCTGACGCTGTT
GGCCATTGCCCGCAGCGGCGCGCAGGCTGTCTGCTTCGCGCCGGATAAACCACAAGCGGATGTGATTAATCAC
CTGACCGGCGAAGCGATGGCGGAAACGCGTAACGTCCTGATTGAGGCGGCGCGTATTACGCGGGGCGATATTC
GTCCCCTGTCCCAGGCGCAGCCAGAGGAACTGGATGCGCTCATTGTGCCCGGTGGTTTCGGCGCGGCGAAGAA
CCTGAGTAATTTCGCCAGCCAGGGGAGCGAATGCCGGGTGGATAGCGATGTCGTCGCGCTGGCGAAGGCCATG
CACCAGTCCGGCAAGCCGCTGGGATTCATCTGTATCGCGCCCGCAATGTTGCCGAAAATCTTTGACTTCCCGC
TACGTCTGACAATAGGGACGGATATTGATACCGCTGAAGTACTTGAAGAGATGGGCGCCGAACACGTACCTTG
CCCGGTTGATGATATCGTTGTGGACGAGGATAACAAGGTCGTGACCACCCCTGCCTATATGCTGGCGCAGGAT
ATCGCCCAGGCGGCAAGCGGCATTGATAAATTAGTGTCGCGCGTGCTGGTTCTGGCGGAATGAGCAAACGACG
CATCGCGCCGTTAACGTTTTTGCGCCGTCTACTTTTACGTATCCTTGCTGCGCTCGCCGTCTTTTGGGGCGGC
GGCATTGCGCTGTTTAGCGTGGTTCCGGTGCCTTTTTCGGCGGTGATGGCGGAACGGCAGATTAGCGCCTGGC
TCGGCGGAGAGTTCGGTTATGTGGCGCATTCTGACTGGGTCAGTATGGCGGATATCTCGCCGTGGATGGGGCT
GGCGGTAATTGCGGCGGAAGACCAGAAGTTCCCTGAGCACTGGGGCTTTGATGTTCCGGCGATTGAAAAAGCG
CTGGCGCATAATGAGCGGAATGAGAGCCGTATTCGCGGCGCCTCTACGCTTTCGCAGCAAACGGCGAAAAATC
TCTTTTTATGGGATGGACGCAGTTGGCTGCGCAAAGGTCTGGAGGCCGGGCTAACGCTGGGCATTGAAACTGT
CTGGAGCAAAAAGCGCATTCTCACGGTTTATCTGAATATCGCGGAGTTTGGCGATGGTATTTTTGGCGTGGAG
GCTGCGGCACAACGGTATTTTCATAAACCTGCCAGTCGCTTGAGTGTGTCTGAAGCCGCGTTGCTGGCGGCGG

TATTGCCAAATCCGTTACGCTATAAGGCGAATGCGCCGTCTGGCTATGTGCGTAGCCGGCAGGCGTGGATTAT
GCGCCAGATGCGACAACTGGGCGGGGAATCTTTTATGACGCGCAACCAGCTTAATTAATTTGCCGATTGGCG
GCGCGAACGCCTTATCCGGCCTGCGATCTGTGTCGTCCAGGCCTGATAAGCACAGGCCATTAAGCTTCGTGAG
AAGTAAGCGCCAGCCGTTTCTTGTCTTTTGGCTGCTCGTCCCTCTCCAGGGCAACGGCCAGCGCAGGCTGGGC
GATAAACCGCTGCCACACTCTCTCCAGTTTGCGTTCACAGCACCATGTTGACCAGGTCGCCAGCGGGATAAAG
GTACTTTCCCCCAGATTGTCGCAAACCACCGGAACCACTTCTGACTCGCTGATACGCTGGCACAGGATATTTT
GCGGCTTCAGCGACATCGTCACAATATGGTTATCCAGCAGGTAACGTTTCAGTTTTTTCAGTAGCCGCCGCAG
GACAGCGACATCCTCTTCATAACGACATTGCGCGGCGAACTCGGTCAGGGTAATAGAGGGCGCGCCGTTGAAA
TCGGTAATCATGTCGTAGACATAGCCGGTGCCGCAATCGGTTTCTACCGTGCCGTAATAACGGGGGATAGCGC
TCCAGTCCGTCAGGTAGCGGGACAGATGCGCATAGTAGCTCAGTTCACGCCGAATCTCTTTATCACCGCCGTG
GTCACGGTTGTAAATAACTTTTATGCAACGCCGGGCATTATCCGGATGCGTATAGCATTTACGATGTCGACCA
GCGCCCAGCGGAGTCTGTTCGGATAAAAGGATCATGGCAAGTCCTGGAAAAATTTATTCACATAAATTTAGTA
TGGACAAAGAACTTAAACAAAACCTAAACGAAATTTTTAAGGTTTTCTTAATGTTTTTATGCATAGGTAAAG
CGCACGAAATGTACAAATCTTACGAAAGGGTGGGGCTGCGGGACAAGTGGCAGGCAGGCGTAACACGCCCGGT
TTTGGGGTGACTGTCATCAAATACCGGGACGGCGTTGGGGGAAAATAGGGTATAGAGGGGGGAGGTTTAGTCTT
CGTCAAAGCCGGAATTGAACAGCGCGATAACCGCCGCCAGCGCTTCAACTTCTTGCGGGCCGGTGGCTTCAAT
TTCGATCTGACGCCCTTTGGCGGAGTCGAGCATTAACAGAGCGATGACGCTGTTTGCTTCCGCTTCGGTGCCT
TCGTCATTGCGTAATAGCACCTCGGCGTCAAACCCCTGCATCAGTTCGAAAAGCTTCATGGCAGGCCGGGCAT
GCATGCCCAGCTTATTGGTGACTTCAACAGTTTGCTTTACGGTCATGTTTTGCGTTTTTCCAGCGTGCGATGG
CGAGACTGGACATTTTTACCGCGCGAGCGGAAGTAGTCTGCCAGCTGTTCTGCAATATACACCGAACGGTGTT
TCCCGCCGGTACAACCGATAGCGACGGTAAGGTAGCTGCGGTTGTTGGTCTCCAGCATCGGTAACCATAGCTC
AAGATAGCTGCGAGTCTGATAGATAAAATTGTGAACTTCTGTGTGTCGGTCAAGAAACGCGGCTACCGGCTTA
TCAAGCCCGGTCATGGGGCGCAGTTTTGGATCCCAGTGCGGGTTGGGCAGAAAGCGGACATCAAACACATAGT
CCGCATCAATCGGGATACCATGTTTAAAGCCGAAGGACTCAAATACCATCGTCAGTTCGCGCTCGCGTTTGCC
CAACAGCCGGGTACGCAGCATCTCCGCCAGCTCATGGACGGACATTTCTGAAGTGTCGACGATCAGATCCGCA
CGTGAGCGTAACGGCTCCAGCAAGTCACTCTCTTTATCGATCGCACTCTCTAAAGAAAGATTTTTGCTGGAAA
GCGGATGCAAACGGCGCGTGTCGCTGTAGCGGCGAATCAAGGTATTGCGGTCGGCATCAAGAAACAGCAATTG
CGGCGAGAAAGCGCCAGGCAGATTATTCATCGCCTGTTCAAAAATCTCAGGTGATTCAGGCATATTGCGCACA
TCAATGCTGACGGCGGCGGAAATCTGGCGATCGGCCAGCGTTCGCGCCAGATCGGGCAACAGCACGACGGGGA
GGTTATCCACGCAGTAAAATCCCATATCTTCCAGCGCGCGCAGAGCGACAGATTTACCTGATCCGGAACGCCC
GCTGACGATCATCAGTACCATGTACCGTTTCTCCTCACAACGACAGAAAGAAATGCCATTGAGTTGTTATGCC
TCATTCTGCTCACCTTCGGTGTCAGTAATGATTTGATACAGCTCTTCATCATTCAGCGCCGCGCGCAGGCGGC
GGCAGATCGTTTTATCCGCCAGACGTTTCGCCACCAGCGACAGCGTGTGCAGATGCGTTTTAGTTTGATCGGC
GGGCACCAGCAGGGCGAAGAGGAGATCGACCGGCTGGTTATCGATGGCGTCGAACGCGATCGGCGTTTCGAGC
TGAACGAACACGCCGACGGCGCGTAAGGTATCTTCTTCCAGTTTCCCGTGCGGGATGGCGATACCATTACCAA
TACCGGTACTGCCCATTTTTTCGCGTGTCAGAATGGCTTCAAACACGACCTGAGGAGGTAAACTGAGCTGTTT
TGCCGCCAGTTCGCTGATGATTTCCAGCGCGCGTTTCTTACTCTGACAATGAACGCCGCTACGCGTACACTCC
TGGTTAAGTACACTGCTCAGTTGTAGAGTCGTATCGTTATTTATCATAATTTCACCTAAGAACCTGTCCTGTC
AGGTTGATGGCCAGGTTCGGAGCGCTAACTGTACAAATGGCCCGTTGTGTCACACAACAGGCCGTCCTGCACT
GGCTAACTGCCCGGACAATTAGTGTTGTTTCAGTTTATCTTTATGCCTGGTTAACTGCCTTGCCAGTTTATCA
ATCAAGCCGTCAATGGCGGCATACATATCCTGGCCTTCCGCGCTGGCGTGAATTTCGCCGCCGTTAACGTGTA
GCGTCGCATCCGAGATATGCGTGACCTTCTCCACTTTCAACACCACGTATACCTGATTAATCCGCTCAAAATA
CTGTTCAAGTTTGGCGAATTTTGTCGTCACAAATTCACGCAGGGCTTCAGTAATTTCGACATTATGTCCGGTG
ATATTGAGCTGCATAGTGTCTTCCTTATCGGTTGGGTCAAACCAGCTGTTTGCGTTGGTTTGACGGCGGAATG
GATAAAGACTCTCGGTACTTCGCAACAGTGCGGCGCGCCACCATGATACCTTGTTCTGACAGCAGAGAGGTTA
ACTTGCTGTCGCTCAGCGGTTTCGCGGGGTTTTCCGCCGCAATTAACTTCTTCACCAGCGCGCGAATCGCGGT
GGAAGAGGCTTCGCCTCCGCCTTCGGTATTGACGTGGCTGGAAAAGAAATATTTGAGTTCAAAAATACCGCGC
GGGCTGTGCAAATACTTCTGCGTGGTCACGCGGGATATAGTGGATTCGTGCATCTCGACGGCCTGGGCGATAT
CCGCCAGTACCATCGGTTTCATATATTCTTCGCCCTGTTCAAAAAAGGCTTGCTGTTGCTCGACGATGCAGCG
ACTGACGCGCAGCAGCGTGTCGTTGCGGCTTTCAAGGCTTTTTATCAGCCATTTCGCATCCTGTAAATTACTG
CGGATGAACTGGCTGTCGGCATCGTTGCGCGCGCTATTGCACATGGCGGCATAGTGCTGGTTAATCTGTAAAC
GGGGAATACTGTCGCTATTAAGCTCCACCGTCCAGCGACCGTTATGCTTGCGTACCAGCACATCCGGGATAAC
GTACTCCGGCTCGCCGGTCTGGATCGATTGACCGGGTCTGGGATCGAGCGACTGAATCAGGTTTACCGCCTCT
TTCAGCACCTCTTCTTTTAGTCGGGTAACGCGCATCAACGTGCGGAAATCGTGGTTGGCCAGCAGATCAAGAT

GATCGCAGATAATGAGCCGCGCCTCTTCCAGCCACGGCGTGGATTTGTCGAATTGTGAAAGCTGGATCAGCAG
ACAGTCGCGAAGATCTTTTGCCGCGACGCCTACCGGATCAAAACGCTGAATACGCTTCAGGACGGCTTCGACC
TCATCGAGATCCACCTCTACATCGCCCATGCTTTCGCGAATTTCGTCCAGGGATACGGTGAGATAGCCGGTAT
CATCTACCGCATCGACAATGGATGTCGCAATAGCGCGATCGGTGTCCGAGAAGGGCGTTAGCTCAACCTGCCA
CATCAGATAATCCTGCAACGACTGCGTCGTTTCACCCTGATAGACGGGCAGCTCATCGTCGATATAATCGCCG
CTGGGGCCGGACGGCGTCCCGGCGGTGTAAATTTCATCCCAACTGGCGTCAAGCGGCAGCTCTTCCGGCATCT
CTTTTTGTTCGAGCGCGTCGGCGGTATCGAGAGGATCGTTATCCTGAGGTTGCTGAGTGTCGATTTCGTCATG
AAGATCGGTTTGCTCAAGCAGCGGGTTATTTTCCAGCGCTTGCTGGAGTTCCTGCTGAAGTTCCAGCGTAGAC
AACTGCAACAGACGGATGGCCTGTTGTAGCTGAGGCGTCATTGCCAGTTGTTGGCTAAGCCTGAGTTGCAAAC
CTTGCTTCATATTCAGAGTCGTTTTCTCCCGCTAGAGCAGCATAGCCCTCTACCCTATCAGAGTCTGAAGTCT
TCCCCAAGATATACACGCTTAACGTGTTCGTCTTGCAGGATTTCTGTCGGCGTACCGTGGGCAATAAGGTGGC
CCTGGCTCACGATATAGGCGCGTTCACAGACTGCCAGCGTCTCACGGACGTTATGGTCGGTGATCAGCACGCC
GAGCCCGCTATCGCGCAGATGCTCGATAATGCGTTTGATGTCGATAACGGAAATCGGGTCAACGCCGGCAAAG
GGTTCATCCAGCAGGATAAATTTGGGATTAGCGGCCAACGCGCGGGCTATTTCTACACGACGGCGTTCACCAC
CGGACAGCGCCTGTCCCATGCTGTCGCGCAGATGCTCGATATGAAACTCTTCCATCAGCTCGTTGGCGCGGTC
TTCTCGCTGTTCTGCGGTCAAATCGTCACGAATTTGCAGTACCGCCATCAGGTTATCAAATACGCTGAGACGG
CGGAAAATAGAAGCTTCCTGCGGCAAATAGCCGATACCGCGGCGCGCGCGCGCGTGCAGCGGCAGCAGACTGA
TATCTTCATCATCGATGATGATATTGCCTGCGTCACGCGGCACGATACCGACGACCATATAAAGGTGGTGGT
TTTACCCGCGCCGTTTGGACCGAGCAGCCCGACAATCTCCCCGGAGTTAACGGTCAGACTGACATCTTCCACT
ACGCGACGGCCTTTATAGGCCTTCGCCAGATTCTTTGCAGTTAATGTTGCCATAACGAATTAGTTACTCTTCT
TTTGCGCCGGAGTCTGGCCTTTGTTTTTGTCTTGCAACTGCGACGGCACCAGAACGGTGGTGACGCGTTTGCC
TTTCTCGCTGAAGGCCTGCATTTTCTGCTCTTTCACCAGGTAGGTGATTTTGTCGCCGGTAATGTTGCTGTCA
AGCTGCTCCAGATAAGCGTTGCCGGTCAGCACCACAAAATCTTTCGCCAGCTCGTAATGCATATGGGAAGCGT
GGCCTTTCACCGGCTTACCGTTGTCCTGCATCTGGTAGAAGGTGGCCGGATTGCCGTAACCGTCGATCACCTC
TTTCCCCTGTTCGCCGCCAGGGCGGGTAACCACGACTTTATCGGCGTTGATCTTAATCGTGCCCTGGGTCATC
ACCACGTTGCCCGTAAAGGTCACGACGTTGCCTTGCATATCCAGCGACTGTTGATCCGAGTCAATATGAATAG
GCTGTTCGGTATCGCCCGTGACGGCGAAAGCCGGAATGCTGGCGGCCAGCAGCGAGCCGGCAAGCATAAGATT
AAGGCTGAGTTTGTTTGTTTTGAATTTCATAAGAGGTTCTAACCTTTTCAATCAGCTCGGCGTTCTTGCTGCG
TAAGTTACCGCGCATTTTCAGTCCGCTGGAGTTAAATGTTGTGCCGTATAACGTCACCAGATCGTTCGAGGTA
ACATCCTGCGTCACCAGATTAATCTGCGCGTTATCGGTGGTAATTCTGCGTAATTGAGCGTCAGGCACCAGGG
CGTTGACTTCAACATGACCATACAGATAGAGCATCCGATCGTTAGTCAATTTGGCTTTATCGGCCTTGATCGA
CCATGTCGGCACCTTATCCTTATCAAAGGTGGTTAATACCGGTTGCGTAAACCATGAGACCGCCTGATCGGAA
AAATATTCAACGTGCTGCGCGATCAGACGATAGCTCAACGCGCCTTCCGGACTGTACACGACGGTATCCGTAT
GCTCGCTTTTATACGTGGGATCGTTGCTGTTGACCATCACGGCAGCAGGATCGTCTTTATCCGCCAGATTGAT
ACCAATCAGTACCAGAATGGCCAGCGACAGTAGAATGATAACCCAACGTCTGGTTTTACTCATATCGATTGCC
CTTTGGCCTCATCCAGCTTGCCCTGCGCCAGCAGCAACAAGTCGCACACCTCACGTACCGCGCCGCGCCCGCC
GGCAATGTGCGTCACATAGTCAGCGCGGGGGATCAGCAGCGGATGCGCATCCGCAACGGCAACGCTTAACCCC
ACTTTTTCCATCACTGGCCAGTCAATCAGATCATCGCCGACATAGGCAACATTTTCCGGCGCAATGACCAGTT
TTTCCAGCAGATCGCTAAAGGCGATCAGCTTATTCGACTGTCCCTGATAAAGATGGACAATCCCCAGCGTGGC
GCAGCGATCTTCTACAAGTTTAGCCTTTCGTCCGGTAATAATGGCAACCTCGATATTGGAGGTCAAAGCACAG
CGAATACCATAGCCGTCACGAACGTTGAAAGCCTTCAGCTCTTCGCCGTTATTGCCCATATAAATCAGGCCGT
CAGACAATACGCCATCCACGTCCAGGATGAGCAGGCGGATATTCTCCGCTTTGGTCATGACATGGGTGCTGAC
GGGGCCATAACAGGTCGCAAGCGACGCACCTGCTTTACTCATCGTTAATTATCCTTGAGTTTCTACACGACGC
CTGCACGCAGGAGATCATGCATATGTAACACACCGAGTAACTGGTCGCCATCAGCAACCAGTACGGAGGTGAT
ATGGCGGGACTGCATTAAATTCAGCGCATCGACAGCGAGAATACCGGGACGCACGCGAATGCCCCCTGGCGTC
ATCACCTCGGCGATCCCCAACTGGCGCATATCGCCGCCCATATCGAATACGCGACGTAAATCGCCGTCGGTGA
AGATACCGTCGATCTTCATGGATTCATCGCAAATGACGGTCATACCGAGATTTTTACGCGTAATTTCTAACAG
CGCATCACGCAGGGTGGCATGTTTATTAACATGCGGGATCTCATCGCCAGTATGCATAATATCGCTTACTCGC
AGCAGTAGCTTACGTCCCAGCGCGCCGCCAGGATGCGACAGAGCGAAATCCTCGGCGGTAAAGCCGCGCGCTT
TTAACAACGCCACAGCGAGCGCGTCGCCCATCACCAGCGTTGCGGTGGTACTACTGGTAGGCGCCAGTCCTAA
CGGACACGCTTCTTTGGGCACTTTAACACACAGATGCACATCTGCCGCACGCGCCATGCTGCTTTCCGGTCGC
CCGGTAATGCAAATTAACGGCACGTGCAGACGTTTAAGTACCGGTATTAGCGCCGCGATCTCGCTGGATTCGC
CGGAGTTGGAGATAGCGATAACCACATCCTGCGGCGTAACCATCCCCAAATCGCCGTGCGCGGCTTCGCCTGG
ATGTACAAAAAAGGAAGAGGTGCCGGTACTGGCGAACGTGGCGGCCATTTTCCGTCCGATATGACCGGATTTT

CCCATCCCCATGACCACCACTTTTCCGGTGCAGTTGAACATTTTTTCACAGGCCAGCGTGAAATGCTGGTTGA
TATATTGATCAAGCTCCGCCAGGCCTTCACGTTCAATTTCCAGGACCTCTTTGCCTGCCTGCTGAAAGTCAAA
CCCCGGTTGCAACGCTAAATGCGACATAATCCGTTTCCAGTTATCCAACAAGAAGTGGCGATAGCCAATAGAG
CATCGCCAGCCATACGATAAACCCGCCGGTCAGCAGTATACCTGCGCCCCGACCAATCTGACGCGGATGCCGC
CAGCAAAGTAAAGCGAAAACCACGCTAACCAGCAACATAACGCTATAATCACGCCCAAATGCCAGCGGGTTGA
TCTCCCCCGGCGCGATCAGCGCAGGCAGTCCCAGCACGATGGCGAGATTAAAAATATTGGCGCCAATGAGATT
ACCAACGGCAATATCATTTTCGCCTTTTCGCACGCCGGCAATCGCCGTCGCCAGTTCTGGCAGACTGGTGCCG
ACGGCGATGACTGTCAGACCGAGCGTTAATTCACTCATGGCGAAATAGTTCGCCAGCACCGTAGCGTTATCGA
TTACCATGCGCGTCGCCATCGGCATGATGACCAGCGCAATGCCCAGCCATAAGAACGCGACGGGGAGCCCGCC
TTCGCGCGGCAGCTCCGCCAGTTGTTCGCGGGTGAGGCTGTCATTCCCCTGGCGTTCGGCCAGACGCGCGATT
TTAACAATGAATAACAGCCATAGCACAGCCAGAAGCAGGAGAAAGATACCATCACTGCGGCTTAACTGTCCGT
CATGCAGTACGGAACCGGCTACCACGCTAACAAATAACATTAATGGCAATTCGCGACGTAAAACATCGGAATG
CACGGTAAATGGACGAACCAGCGCCGCCAGCCCTAAGATCAGCAAAATATTGGTGATGTTAGAACCGAGCGCC
GCGCCGACGGCCAAATCGAGCTGACCGTGTAGCGATGCGGCGACCGACACAATAATTTCCGGCAGCGAGGTGC
CGATACTCACGACCGTCATCCCGATGATCAGCGGGGGAATACCGAATGTACGGCATAAAATAGAGGCCGCAAA
CACCAGACGATCGGCGCCGTAGGCCACCAGAAGCAAACCAATTATGAGTAGCGCCATCGCTAAAAGCATCTAA
AGTCCTTTCTTCAGGTATACTCGCCGGTCCCCTGGAAGGGATATCTGGAAGCCGTAACGAAGTCCTAATTTTG
ACTTTATGCGGCTAAAAAGTAAAACGAATGCCAGCTTTCGCTAACCCTGTCAGTTAATATTCTGTAATTATGT
CGAACCCATGGTTAAATTCAGCGCCATGGTTACCTCAACAAGGCAATACGAAGGATGAATCAATGGGTCAGTC
TGCGGCCAATTTAGTCGATATGCGCGACGTCAGCTTTTCCCGTGGCGAACGCTGCATTTTCGATAATATTTCC
CTGACGGTGCCGCGCGGGAAGATCACGGCGATCATGGGGCCGTCGGGCATCGGTAAAACCACGCTGTTGCGCC
TGATTGGCGGACAGATCCCGCCGGATAAGGGCGAGATCTTATTTGATGGCGAGAATGTGCCGGCCATGTCCCG
TTCGCGGCTCTACACCGTCCGCAAACGTATGAGTATGCTGTTTCAGTCGGGAGCGCTGTTTACCGACATGAAC
GTGTTTGACAATGTGGCCTATCCGCTACGGGAGCACACCAATTTACCCGCGCCGCTGCTAAAAAGCGTCGTGA
TGATGAAACTGGAAGCTGTCGGGCTGCGCGGCGCGGCAAAACTGATGCCTTCCGAGCTCTCCGGTGGGATGGC
GCGCCGCGCCGCGTTGGCGCGCGCCATCGCTCTGGAACCGGATCTCATCATGTTCGATGAGCCGTTTGTCGGC
CAGGACCCGATTACCATGGGCGTTCTGGTGAAGCTGATTTCAGAATTGAACAGCGCGCTGGGCGTGACCTGCG
TGGTGGTCTCGCATGATGTGCCAGAGGTGCTCAGTATTGCTGATCACGCCTGGATCATGGCGGACAAAAAAAT
CGTCGCTCACGGTAGCGCTCAGGCGTTGCAGGAGAATACGGACCCGCGCGTGCGTCAGTTCCTTGACGGCATT
GCCGACGGGCCGGTTCCGTTCCGCTATCCGGCGGGCGACTATCACCTTGATTTACTCGAAACAGGGAGTTAAG
CCACTCATGCTGTTAAATGCGCTGGCAGCACTCGGACACAGCGGGATCAAAACCGTCAGGACGTTCGGGCGCG
CCGGGTTAATGTTATTCAATGCGATTATCGGTAAGCCGGAGTTTCGCAAACATGCGCCGCTGCTGGTGCGCCA
GCTTTATAATGTGGGCGTCTTGTCGATGCTTATCATTATTGTCTCCGGCGTATTCATCGGTATGGTGCTGGGG
CTGCAAGGCTATCTGGTTCTGACCACTTACAGCGCGGAGACCAGTCTTGGCATGTTAGTGGCGCTTTCGCTGC
TGCGCGAGCTGGGGCCGGTGGTCGCGGCGTTGCTGTTTGCCGGGCGGGCCGGGTCGGCGTTGACGGCGGAAAT
CGGCCTGATGCGGGCGACGGAGCAGCTCTCCAGTATGGAGATGATGGCGGTCGATCCATTGCGCCGGGTGATT
TCGCCGCGCTTTTGGGCGGGCGTGATTTCGTTGCCGCTACTGACCATTATCTTTGTGGCGGTCGGTATCTGGG
GCGGCTCGCTGGTCGGCGTAAGCTGGAAAGGAATCGACGCCGGGTTCTTCTGGTCCGCGATGCAGAATGCCGT
CGACTGGCGTATGGACCTGGTGAACTGTCTGATAAAGAGCGTGGTGTTCGCCATTACGGTAACATGGATTGCG
TTATTCAACGGTTATGACGCAATACCAACTTCTGCCGGGATTAGCCGGGCAACGACGCGTACCGTGGTTCACG
CATCGTTGGCCGTTCTGGGGCTGGATTTTGTGCTGACCGCACTGATGTTTGGGAATTGAGTTCATGCAAACGA
AAAAAAATGAAATTTGGGTAGGCGTTTTTTTGCTGGTAGCGTTGCTGGCGGCGCTGTTTGTTTGCCTGAAGGC
GGCAAATGTGACCTCTATGCGCACGGAGCCGACCTATAAGGTTTATGCGACCTTCGATAACATCGGCGGTCTG
AAAGTGCGTTCCCCGGTACGCATCGGCGGGGTGGTTGTCGGGCGGGTTGAAGATATCTCGCTTGATCCGAAAA
CATACCTGCCGCGCGTAACGCTCGACATCGAAGAGCGTTATAACCATATTCCCGATACCAGCTCGTTGAGTAT
TCGTACTTCCGGGCTGCTGGGGGAGCAATATCTGGCATTAAACGTCGGTTTTGAAGATCCTGAGCTGGGAACG
TCTATCCTCAAAGATGGGAGTACGATTCAGGACACCAAGTCTGCAATGGTGCTGGAAGATATGATTGGCCAGT
TCCTTTATAACAGCAAAGGCGACGACAATAAGAATTCTGGCGATGCGCCAGCCGCAACGGAAGGCCATACTGA
AGCCACAACGCCCGCGGGCGAAACGAAATAATCTCAGGAGAATTGAGGCATGTTTAAACGACTGATGATGGTG
GCCCTGCTGGTCATTGCGCCATTAAGCGCAGCAACGGCAGCGGATCAGAGCAATCCTTATAAACTGATGAACG
AGGCGGCGCAGAAAACGTTTGATCGCCTGAAAAACGAACAGCCGAAAATTCGTGCCAATCCGGATTACCTGCG
AGACGTTGTTGATCAGGAACTTCTGCCGTACGTGCAGGTAAAATATGCCGGCGCGCTGGTGTTGGGCCGCTAC
TACAAAGAGGCGACCCCGGCGCAGCGTGAAGCCTATTTTGCCGCGTTTCGCGAATACCTGAAACAGGCATACG
GTCAGGCGCTGGCGATGTACCACGGTCAGACTTACCAGATAGCGCCTGAGCAACCGCTGGGCGATGCGACCAT

TGTGCCTATCCGTGTAACGATTATCGATCCCAACGGTCGTCCGCCGGTACGCCTGGATTTCCAGTGGCGCAAA
AACACTCAGACCGGCAACTGGCAGGCGTATGACATGATTGCGGAAGGCGTCAGCATGATCACCACCAAGCAAA
ATGAGTGGAGCGATCTGCTGCGTACTAAAGGCATCGACGGCCTGACCGCACAACTGAAATCTATCTCTCAACA
GAAAATTACTTTGGACGAGAAACAGTAATGACGCCGCAACTCACCTGGACGCGTGAGGCGGACACTCTGGTGC
TGGCGGGAGAGCTGGATCAGGACGTGTTGGCGCCGCTATGGGACGCGCGCGTAGAAGCGATGACTGGCGTTAC
CCGTATCGACCTGAGCCAGATCTCCAGGGTGGATACGGGGGGACTGGCGCTGCTGGCGCACCTTGTGAACCAG
GCAAAAAAGCAGGGCAATGCCGTGTCGCTGTCTGGCGTTAACGACAAGGTTTACGCGCTGGCGCAATTATATA
ACTTGCCCGAAGACGTACTTCCCCGTATGTAATGAATGCCGGATGGTTTATCCGGCCTACACTTCGCATGATC
CCCGTAGGCCGGATAAGTTGCGTTAGCAGCACCATCCGGCACTTTTACCGCGCTGATTTATCAGCGTAATCTT
TTGCTTATTTAAGACGGCGCGACTTTGCTCTAAGATGTTGGGCTGTTTTCACTATTTGACGAATAAAGAACCC
ATGGAAAATCATGAAATTCAGAGTGTGCTGATGAATGCCCTCTCCCTCCAGGAAGTCCACGTCTCCGGCGATG
GCAGTCACTTTCAGGTTATTGCCGTGGGCGAGATGTTTGACGGCATGAGCCGGGTCAAGAAACAGCAAACGGT
CTATGGGCCGCTAATGGAGTACATTGCGGACAACCGTATCCATGCCGTATCGATCAAAGCATATACCCCGGCG
GAATGGGCGCGCGATCGTAAACTTAACGGATTTTAAGCTACGGGTAACCTGCCGGTAGCGGAGATGAATGCTT
AACTGAGAATACAATAATGGATAAATTTCGTGTACAGGGGCCAACGACGCTCCAGGGCGAAGTCACAATTTCC
GGCGCCAAAAACGCCGCGCTGCCGATCCTGTTCGCCGCTCTGCTGGCAGAAGAACCGGTAGAGATCCAAAACG
TTCCAAAACTGAAAGACGTGGATACGTCGATGAAGCTCCTGAGCCAATTAGGCGCGAAAGTGGAACGCAATGG
ATCGGTACACATTGACGCCAGCCAGGTGAACGTGTTCTGCGCGCCTTATGATCTGGTTAAGACCATGCGCGCC
TCGATATGGGCGCTGGGGCCGCTAGTTGCCCGTTTTGGTCAGGGACAAGTCTCTCTGCCGGGGGGCTGTACTA
TCGGCGCGCGTCCGGTTGATTTGCATATTACCGGTCTGGAACAACTGGGTGCGACCATCAAGCTGGAAGAGGG
CTACGTAAAGGCCTCCGTCGAAGGACGTCTTAAAGGGGCGCATATCGTGATGGATAAGGTCAGCGTCGGCGCG
ACCGTCACCATTATGTGCGCCGCCACGCTGGCGGAAGGGACGACCATCATTGAAAACGCCGCGCGCGAGCCGG
AAATTGTCGACACCGCTAACTTCCTGGTTACGCTGGGGGCGAAGATCGCCGGGCAGGGCACCGATCGTATTAC
GATCGAGGGCGTAGAGCGTCTGGGCGGCGGCGTTTATCGTGTGCTGCCGGATCGTATCGAAACGGGGACTTTC
CTGGTGGCGGCAGCGATCTCTCGCGGCAAAATCCTCTGCCGTAACGCGCAACCGGACACTCTGGACGCAGTAC
TGGCGAAACTGCGCGATGCTGGCGCGGATATCGAAGTGGGCGAGGACTGGATTAGTCTGGACATGCACGGCAA
ACGACCGAAGGCGGTCAATGTCCGTACCGCGCCGCACCCGGCATTCCCGACCGATATGCAGGCGCAGTTCACG
TTGCTGAACCTGGTGGCGGAAGGGACGGGCTTTATCACTGAAACTGTCTTTGAAAACCGTTTTATGCACGTGC
CTGAACTCAGCCGCATGGGCGCGCGTGCGGAAATTGAAAGCAATACCGTCATATGCCATGGCATAGAAACGCT
CTCTGGCGCCCAGGTTATGGCGACAGATCTGCGGGCATCTGCGAGCCTGGTGCTGGCGGGCTGTATTGCGGAA
GGCACGACGATTGTGGATCGCATTTATCACATCGATCGCGGTTATGAGCGCATCGAAGACAAACTGCGCGCGC
TAGGCGCGAATATTGAACGCGTGAAAGGCGAATAACCTTCTGTACGCTGAATAACCCCCGCGAACGCGATTGC
GGGGGGTATTTTACAGGCGCTTACTCTACATTCTGTTTGTCTTTATTACGCGCTCTCATAAGCCGCGTTCTGT
CGATAAACTCATGCGTGCGCGGATCGTGATAACGCGATGGCCAAATAACCCAGGGTTCCGTTCCCAATGCTTT
CGCGATAATCAACTCTCCTTTCGGCCACGGGCGCGTTAATGCATTGGCCAGCGTGGATGAACTCAATCCATTC
CGACGTGACTCAGCGGCCATTGAAGTCCCTTTTTTACGTAGTCCGGCAATGATATCGGCCGGATGCCAGTCAA
TAAGCTTGCTTTCCATACATCCATCACTCCTTAGGTTAATCACTTACGGTTTTAACAACCCGACGACGTTCGA
TACTATAACGCTTCGTGAAGGAATGTTCTAAAAAGTTCATGTAAGTGGCGGCAATATTCAGAATATGCCAGGG
GAAGTCCTGGATGAGTATGTGAAAAACAGGCGATAGGGTATCTGGAGTTTATTAGAACTTAATGGGATATCCC
GCGCAGTACGCGGGATAAGAGAACGGCATTAACGATCGCGCTGCACCGCGATGTGTGCGAGACCGATTAGCGC
CTCACGCCATGGGGTATCCGGCAATATCTGCAACGCGGATATCGCTTTGTCGGCTTCTTCTTCCGCTCGCTGA
CGCGTCCATTCCAGCGAACCGCAGGTGGTCATCGCTTCCAGAACCGGTTCCAGAAGATGACGGCCATTGCCTT
GTTCAATAGCGGTACGGATCATCGCTGACTGTTCTGGCGTACCGTGGCGCATGGCGTGAAGCAACGGTAAGGT
AGGTTTGCCCTCATTGAGGTCATCACCCACATTTTTACCGAGATGCTCGCCGTCGGCACTGTAATCCAGCAGA
TCGTCAATGAGCTGGAAAGCCGTACCAAGGTAGCGGCCATAGTCCTGCAACCCTTTCTCTTGCTCAGGCGTAC
AACCGGCAAGAATACCGGAGCACTGGGCGGCGGCTTCGAACAGACGGGCCGTTTTACTGTAAATCACCCGCAT
GTAATTTTCTTCGGTAATGTCCGGGTCATTCACGTTCATCAATTGCAGCACTTCACCTTCTGCAATCACGTTG
ACCGCTTCCGACATCACTTCCAGCACTTTTAACGAGCCGAGGCTGGTCATCATCTGGAAGGCGCGGGTATAGA
TAAAGTCGCCGACCAACACGCTGGCGGCATTACCGAAAGCGGCATTTGCCGTCGCTTTGCCGCGACGCATATC
AGATTCATCCACCACATCATCATGTAGCAGCGTCGCCGTGTGGATAAACTCGATCAGAGCGGCGATAGTCACG
TGCGCGTTACCCTGATAACCCACCGCGCGTGCGGCAAGAACGGCAATCATCGGACGAATGCGTTTTCCGCCGC
CGCTAATAATGTAATATCCTAGCTGATTAATCAGTTGAACGTCGGAATTAAGCTGTTCAAGGATTGTCGCATT
GACACCCGCCATATCTTGCGCGGTTAACTCGTTGATTTTTTCTAAATTCATCGCAAAAGCCGGGCTTATCGCC
CCGATTATCCCATGTCTGAATGAGGTAACGTAGGGTTTATGGTTAGAAATATACCCTGATTGTACTGAAAAAA

CGCCGCAGATAAACGTTACCGTATGCGTTGTGTTTTTTTCTTCATGTATTGATGGTAGCACTTGTCAAAGCCG
TGCAATTTGCGTAATATTCGCGCCCTATTGTGAATATTTATAGCGCGCTCTGAATCATTGAAAGGTGTGCGCG
GAAGCGGAGTTTTTTATGTACGCGGTTTTCCAAAGTGGTGGTAAACAACACCGAGTAAGCGAAGGTCAGACCG
TTCGCCTGGAAAAGCTGGACATCGCAACTGGCGAAACTATCGAGTTCGCTGAAGTTCTGATGATCGCAAACGG
TGAAGAAGTCAAAATCGGCGTTCCTTTCGTTGATGGCGGCGTAATCAAAGCTGAAGTTGTTGCCCACGGTCGT
GGCGAGAAAGTTAAAATCGTTAAGTTTCGTCGCCGTAAACACTACCGTAAGCAGCAGGGCCATCGTCAGTGGT
TCACTGATGTGAAAATTACCGGCATCAGCGCTTAAGATTAGGGGAGCAGATTAAATGGCACATAAAAAGGCTG
GCGGCTCGACTCGTAACGGTCGCGATTCTGAAGCTAAACGTCTGGGCGTAAAACGCTTTGGCGGTGAAGCAGT
TCTGGCAGGCAGCATCATCGTTCGTCAACGTGGTACTAAATTCCACGCTGGCACCAACGTTGGTTGCGGTCGT
GACCACACTCTGTTTGCTAAAGCAGACGGTAAAGTGAAATTCGAAGTTAAAGGCCCGAAAAACCGTAAATACA
TCAGCATCGTTGCTGAGTAAGTTTTTTGTGGTCCGGTAACGGATGAAAGCCCCGCACCTGTTGCGGGGCTTTT
TACATTCGATGGCCGACTAAAATATACCCGTGTGGCTGTGCGAATGCCGTTAACGGGTCTTTGCAGGGAACAC
GGCATGAAGCAGCAGGCAGGCATTGGCATTCTTTTAGCGCTCACTACCGCGATGTGCTGGGGAGCGTTGCCAA
TAGCAATGAAGCAAGTGCTGGAGGTGATGGAGCCTTCAACCATTGTCTTCTACCGCTTCTTAATGGCCAGCAT
TGGCCTCGGGGCGATACTGGCAGTAAAAAGAAAGCTGCCGCCGCTGCGTATTTTTCGTAAGCCGCGTTGGTTG
GTGTTATTGGCGATAGCGACCTGCGGACTGTTCGGTAATTTCATTTTGTTCAGTTCTTCCCTGCAATATTTAA
GCCCAACCGCGTCGCAGGTTATCGGCCAGCTCTCGCCGGTTGGCATGATGGTCGCCAGCGTCTTTATTCTGAA
AGAAAAGATGCGCGGTACGCAGGTGATTGGCGCGCTGATGTTATTGAGCGGGCTGGTGATGTTTTTTAACACC
AGTCTGATCGAGATTTTTACCCGACTGACCGACTACACCTGGGGCGTTATTTTTGGCGTAGGCGCGGCGATGG
TGTGGGTGAGCTATGGCGTGGCGCAAAAAGTATTATTGCGACGTCTGGCCTCGCAGCAGATCCTGTTTTTACT
GTACACTTTATGTACGATAGCGCTCTTGCCGCTGGCAAAACCGATGGTGATAGCGCAACTGAGCGACTGGCAG
CTCGCCTGTTTGATTTTCTGCGGGCTAAATACGCTGGTAGGATATGGCGCCCTGGCGGAAGCGATGGCGCGTT
GGCAGGCGGCGCAGGTGAGCGCGATAATCACGCTTACACCGCTATTTACCCTGCTGTTTTCAGATCTTTTATC
TATGGCCTGGCCCGATTTCTTCGCCAGACCGATGTTAAACCTTTTAGGTTATCTCGGTGCGTTTGTCGTGGTT
GCGGGCGCGATGTATTCCGCCATTGGTCATCGTATTTGGGGCGGTTTACGCAAGCATGAAACGGTGGTTTCGC
AACCCCGCTCAGGCGAATGATTTACGGAGAATAAAATGAAGTTTGTTGATGAAGCATCGATTCTGGTCGTTGC
AGGCGATGGCGGTAATGGCTGTGTGAGCTTTCGCCGCGAAAAATATATCCCGAAAGGCGGCCCGGACGGCGGT
GATGGCGGCGACGGCGGCGACGTCTGGATGGAAGCCGATGAAAACCTGAATACGCTGATCGATTACCGCTTTG
AAAAATCGTTCCGCGCTGAACGTGGTCAGAATGGCGCGAGCCGCGACTGTACCGGTAAACGCGGTAAAGACGT
GACCATCAAAGTGCCGGTCGGTACGCGCGTTATCGACCAGGGAACCGGTGAAACGATGGGCGACATGACCAAA
CACGGTCAGCGTCTGTTGGTCGCGAAGGGCGGCTGGCATGGGCTAGGCAATACCCGTTTTAAATCCTCGGTTA
ACCGTACGCCGCGCCAGAAAACCAATGGTACGCCGGGCGATAAGCGCGACCTGCTGCTGGAACTGATGCTGCT
GGCGGATGTCGGGATGCTGGGGATGCCAAACGCCGGTAAATCAACGTTCATTCGCGCGGTATCGGCAGCAAAA
CCGAAAGTCGCGGACTATCCGTTTACCACGCTGGTGCCGAGCCTGGGCGTGGTACGTATGGACAGCGAAAAGA
GTTTTGTGGTGGCCGACATTCCGGGGTTGATCGAAGGCGCGGCGGAAGGCGCGGGCCTGGGGATTCGCTTCCT
GAAGCACCTCGAACGTTGCCGCGTACTGCTGCACCTCATTGATATCGATCCGATCGACGGTTCCGATCCGGTG
GAAAACGCCCGTATCATTATTGGCGAACTGGAAAAATACAGCCAGGATCTGGCGGCTAAACCGCGCTGGTTAG
TGTTCAACAAGATCGATCTGATGGATAAAACCGAAGCGGAAGAGAAAGCGAAAGCGATCGCCGAAGCGTTGGG
CTGGGAAGGGAAATACTATCTGATTTCCGCCGCCAGTCAGCTAGGGGTGAAAGATCTCTGCTGGGATGTGATG
ACCTTTATCATCGAGAATCCGATCGCGCAGGCTGAAGAAGCAAAACAGCCGGAAAAAGTCGAGTTCATGTGGG
ACGATTATCATCGCCAGCAGTTGGCTGAAGTGGAAGAAGACGCAGACGATGACTGGGATGACGACTGGGATGA
AGACGACGAAGAAGGCGTCGAATTCATCTACAAACGTTAATCTCGTAGCCGGATGGCGCTAAAGCTTATCCGG
CCTACAAAAGCCAACGGGCCGCATTTGCGGCCCATTTTATTATGTTCGTTGTAGGCCGGATAAGACGCGTTAG
CGCCGCCATCCGGCAATAATGCACTCAGTTAGTCTGATAAATATCCTTATACAACCGACTCTCGAAGCGTACC
AGCGGGATTCGGCGATTACGCTGATCGGCAGGTGGAACCGCGTAGCCGGACAAATACTGTACAAATGCCATCC
GTTGTCCGCTTGCGGTGGTAATAAAGCCCGCCAGATTATACACGCCCTGCAGTGATCCGGTTTTCGCCGAAAC
CTTACCGTCTACGCCCGCCTGATGCAGCCCGGCGCGGTATTGTAATGAACCATCATAGCCCGCCAGCGGCAGC
ATAGAGATAAAGTTCAGCTCGTTATCGTGTTGGGCGATATATTGCAGCACCTGCATCATCGTGGCGGGAGCGA
TCAGATTATGGCGAGAAAGACCGGAACCGTCGGCAATAATGGTATTGCCGATATCAATTCCCGCCTGTTGGCG
TAGGATCTGGCGAACGGCATCCGATCCCGCCCGCCAGGTGCCAGGCACGTTAAACCTGACATGACCAATCATC
CGAAAAACAGTATCGGCGATCATGTTGTCTGACTTTTTCAGCATAATCTTAAGCAGATCGTGCAGTGGCGCCG
ACTGTTTACTGGCGACGATCGTTCCGGGCTCGTTAACCTGCGTCTGGCGCAGCAGCGTGCCGCGGTAAGTTAT
CCCGGCCTCTTTCAGTTCCTGCTTGAGGATCGCGCCCGCGTAGCTGGCGCCGTCCTGAATAGCAAAGGCCAGC
GGCAGCGGGTCGGCGCGCTGTGGTAAACAGCCGGTAAGCGTATAACGGTTCAAATCGCCAGGCACAACATCCA

GCTCGCAATACTGCGCGTCTGCCGAACCGCGCGGTAGAGTCCGTACCTGGCTGAACATCGTTACCGGATAATA
AGAAGCCACGCGAATAAAGGCTAAATCGTTTGGTTTTTGCGCGCTATAAAGCGACACAGAAAAACAGTTGCGG
TCAACAATGGCGGCGGCGGGAGGCGCGCTGAAACACTGCGTCAGGTCGTTCCACGGCCAGCCTGGCGCTTTAT
CGTGGCTGGCGAAAATCGACGTGTCGATAAGCACATTGCCGTCGATTTGCGTGACGCCGGACTTTTTCAGCGT
CGCGACCATATTGCGAATATCCTGACGTTTTAGCGTTGGATCGCCACCAAAGCGCGCAATTACGTCACCTTTC
AGGATGCCGTTATCGACGTTGCCTTTAGTCTCCAGCGTGGTCGTAAAACGGAAATCCGGGCCGAGTTGAATCA
GCGCCGCCAGCGCGGTAATCACCTTTTGGGTACTGGCGGGAAGCGCCATCTGCTGACTATGATAATCAATAGC
CGGCGCGGGCGCGCCAATCTTTTGCACCATCAGCGCGAGGTTGGCGCCGGCCGGAAGCTGTTTGATGTATTCG
TCAATATTCGCGGCCTGAACGCTGAACGCTATACTGGTGGTCAATCCGATGATAAATCTGGAAAATCGCATAA
TCTCGCGCTAACACCCTGGAAACAAGCCGTCATACTACGGCGCAACGCCCTAAAAAGTAAACGATGACCCATA
GGGAACTCCAGGGTAAAATGGTTATCAAAGAGGAAATTGTAGCTGACCTGGGACTGCGCGCCCGGGTTGGTGT
TTTTTTGCTTCTGACCCTGGTATGGCATATGCCGGGGCTGGCTTAGCAGCCGGGGTAGAGTTCATTGCCCCCT
ACAGGAATGTTCAAGAGGTATAACAAATGCAAGCTATTCCGATGACCTTACGCGGAGCCGAAAAACTGCGCGA
AGAGCTGGATTTTCTGAAATCTGTGCGCCGTCCTGAAATCATCGCCGCTATCGCCGAAGCGCGCGAACATGGC
GATTTAAAAGAAAACGCGGAGTATCATGCTGCACGCGAACAGCAGGGTTTCTGCGAAGGGCGCATTAAAGATA
TCGAAGCAAAACTGTCGAATGCGCAGGTGATTGATGTCACTAAAATGCCGAACAATGGCCGCGTGATTTTCGG
CGCTACCGTCACGGTGCTGAACCTGGACACCGACGAAGAGCAGACCTACCGCATTGTTGGCGATGATGAGGCG
GATTTTAAGCAAAATCTCATTTCGGTAAACTCGCCAATCGCTCGCGGCCTGATCGGCAAAGAGCAGGATGACG
TCGTGGTGATCAAAACGCCTGGCGGCGATGTGGAATACGAAGTGCTTAAAGTGGAATACCTTTAAGTAGTACT
CAATACGCAAATTGTTGGTGTATTGTAAAGAAAGGAAAAAGGCCGCATAGCGGCCTTTTATCAACGGAGAGAG
CGTGGCATTTTGCTCTCCTGCTCGTGGAAAACCCCTCGTTTTACACAGATTTTGTGTTCGACTTTAGGATATC
CTTAACGCGGCAGCGAGATTTTACGCTCTTTAGTTGGGCGATACAGCACCAGCGTTTTACCGATGACCTGTAC
GTTACAGGCGCCGGTTTCGCGCACGATAGCGTCCACGATCAAGGTTTTTGTTTCGCGATCTTCCGAGGCGATT
TTCACCTTGATAAGTTCATGGTGCTCTAACGCTTGTTCAATCTCGGCCAGTACCCCTTCGGTCAAACCATTAT
TGCCAAGCATAACAACAGGCTTGAGCGGATGTGCCAGACCTTTTAGGTGCTGTTTTTGTTTAGTACTCAGATT
CATCGTATATTTTTGCTTACGTTGGGATTGAAAACGGGTCATTCTACCGCCATCTCCCTTATATCGCCAAATA
GGCGTGTAGAAATTTACGCCATTGGCTACGATGAACTGCCCCGACGGGAAAGTTAAATGACAGGTAAAAAGCG
TTCTGCCAGCTCAAGCCGCTGGCTTCAGGAACACTTTAGCGATAAATATGTTCAACAGGCGCAGAAAAAGGGG
CTACGCTCCCGTGCCTGGTTTAAACTTGATGAAATACAGCAAAGTGACAAACTTTTTAAGCCGGGAATGACG
GTAGTCGATCTCGGCGCTGCGCCAGGAGGCTGGTCACAGTATGTGGTCACGCAGATTGGCGGCAAAGGCCGCA
TCATCGCTTGTGATCTTTTACCTATGGATCCTATCGTTGGTGTGGACTTTCTTCAGGGCGATTTTCGTGATGA
ACTTGTCATGAAAGCGTTACTGGAACGCGTGGGTGACAGTAAAGTACAAGTTGTCATGTCGGATATGGCACCA
AACATGAGCGGAACACCGGCGGTGGATATCCCCCGCGCCATGTATCTGGTGGAGCTGGCGTTAGAGATGTGCC
GCGATGTGCTGGCGCCAGGCGGAAGTTTTGTAGTGAAGGTGTTCCAGGGCGAAGGTTTCGATGAGTATCTAAG
GGAAATTCGCTCCCTGTTTACGAAGGTTAAAGTTCGTAAGCCGGACTCTTCGCGTGCGCGTTCGCGTGAAGTG
TACATTGTAGCGACCGGGCGAAAGTGATAACCGGCAGATTTCAGACGTAAGGTTTGAAAGAAACTGGATATAG
AGTATCCTGACGCTGTTTTTAACACAGTTGTAATAAGAGGTTAATCCCTTGAGTGACATGGCGAAAAACCTAA
TACTCTGGCTGGTCATTGCCGTTGTGCTGATGTCAGTATTCCAGAGCTTTGGGCCCAGCGAGTCTAATGGCCG
TAAGGTGGATTACTCTACCTTCCTGCAAGAGGTCAATCAGGACCAGGTTCGCGAAGCGCGTATCAACGGACGT
GAGATCAACGTTACCAAGAAAGATAGTAACCGTTACACGACTTACATTCCGATTAATGATCCGAAGCTGCTTG
ATAACCTGCTGACTAAAAACGTCAAGGTTGTTGGCGAACCACCTGAAGAGCCAAGCCTGCTGGCTTCTATCTT
CATTTCCTGGTTCCCGATGCTGTTGCTGATCGGCGTCTGGATCTTCTTCATGCGTCAGATGCAGGGCGGCGGT
GGCAAAGGCGCCATGTCGTTCGGTAAGAGCAAGGCGCGTATGCTGACGGAAGATCAGATCAAAACCACGTTTG
CTGACGTCGCGGGTTGTGACGAAGCGAAAGAAGAAGTGGCGGAACTGGTTGAATACCTGCGTGAACCGAGCCG
TTTCCAGAAGCTGGGCGGTAAAATTCCGAAAGGCGTCCTGATGGTCGGCCCGCCGGGTACCGGTAAAACCCTG
CTGGCAAAAGCCATTGCGGGTGAAGCGAAGGTGCCATTCTTTACGATTTCCGGTTCTGACTTTGTGGAAATGT
TCGTCGGTGTCGGCGCGTCTCGAGTGCGTGACATGTTCGAACAGGCCAAGAAAGCGGCGCCGTGCATTATCTT
CATCGATGAAATCGACGCCGTAGGCCGCCAGCGTGGCGCAGGTCTGGGCGGTGGTCACGATGAACGTGAGCAG
ACGTTGAACCAGATGCTGGTTGAGATGGACGGCTTCGAAGGTAACGAAGGTATCATCGTTATCGCGGCAACCA
ACCGTCCGGACGTTCTGGACCCGGCGCTGCTGCGTCCAGGTCGTTTTGACCGTCAGGTGGTGGTAGGCCTGCC
GGATGTTCGCGGTCGTGAGCAGATTCTGAAGGTGCATATGCGTCGCGTACCGTTAGCGACGGATATTGATGCG
GCGATCATTGCGCGCGGTACGCCGGGCTTCTCCGGTGCTGACCTCGCGAACCTGGTGAACGAAGCGGCGCTGT
TTGCGGCTCGTGGTAACAAGCGCGTTGTGTCGATGGTTGAGTTCGAGAAAGCGAAAGACAAAATCATGATGGG
CGCCGAACGTCGCTCCATGGTGATGACGGAAGCGCAGAAAGAGTCGACCGCGTACCACGAAGCGGGCCACGCG

ATTATCGGTCGCCTGGTGCCGGAACACGATCCGGTGCACAAAGTGACGATTATCCCGCGCGGTCGTGCGCTGG
GCGTGACCTTCTTCCTGCCTGAAGGCGACGCGATCAGCGCCAGCCGTCAGAAGCTGGAAAGCCAAATCAGCAC
GCTGTACGGTGGCCGTCTGGCGGAAGAGATTATCTACGGCGTTGAGCATGTTTCTACCGGCGCGTCGAACGAC
ATTAAAGTCGCGACTAACCTGGCGCGTAACATGGTCACCCAGTGGGGCTTCTCGGAGAAACTCGGTCCGTTGC
TGTATGCGGAAGAAGAGGGCGAAGTATTCCTCGGCCGTAGCGTCGCAAAAGCGAAACATATGTCTGATGAAAC
TGCGCGTATCATCGACCAGGAAGTGAAAGCGCTGATTGAACGTAACTACAATCGCGCTCGTCAGATCCTGACT
GACAATATGGATATTCTGCACGCGATGAAAGATGCGCTGATGAAATATGAAACCATCGATGCGCCGCAGATTG
ATGACCTGATGGCGCGTCGTGAAGTGCGTCCGCCTGCGGGCTGGGAAGATCCAAACGGCACCAATAACTCTGA
CAGCAATGGTACGCCTCAGGCGCCGCGTCCGGTTGATGAACCACGCACGCCGAACCCGGGCAACACGATGTCA
GAGCAGCTGGGCGACAAATAAACGTTTGTTGTACAACATTATGTTTAGCGCTCACCGAAACCCTGGGGCTTGC
CCCGGGGTTTTTCTTTTTTAACTCACCACAAATAAAACAGGGATTTTGACCATGAAACTCTTCGCTCAGGGC
GCCACGCTCGATCTCACTCATCCGCATGTTATGGGTATCCTGAACGTAACGCCGGATTCGTTCTCCGACGGCG
GCGCGCATAACACGCTGATTGAGGCGGTGAAACATGCGAATTTAATGGTGAATGCCGGTGCGACAATTATTGT
GATGGTGGGGGAATCAACGCGACCAGGCGCGGCGGAAGTGAGCGTGGAAGAAGAGCTGGATCGCGTTATTCCG
GTACTGGAAGCAATCGCGCAACGTTTTGAAGTGTGGATTTCTGTGGATACCTCTAAACCCGAGGTGATCCGTG
AAGCGACAAGGGCGGGCGCGCATATTATCAATGATGTCCGTTCGCTCTCCGAGCCCGGCGCGTTGGAAGCGGC
AGCGGAAACCGGCTTGCCGGTCAGTCTTATGCACATGCAGGGCAACCCCAAAACCATGCAGGAGGCGCCGAAA
TATGACGATGTCTTTGCCGAGGTGAATCGCTACTTTATTGAGCAAATAGCACGTTGTGAGAAGGCCGGCATCG
CAAAAGAGAAATTGTTGCTCGACCCCGGATTCGGTTTCGGTAAAAATCTCTCTCACAATTATACCTTACTGGC
GCGACTGGGTGAGTTTCATCATTTAACCTGCCGCTGCTGGTGGGCATGTCACGTAAAACGATGGTTGGTCAG
TTACTTAACGTGGGACCATCAGACCGTCTGAACGGCAGCCTTGCGTGCGCGGTAATTGCCGCGATGCAGGGCG
CGCAGATCATTCGCGTCCATGACGTCAAAGAAACCGTAGAGGCGATGCGGGTGGTAGAAGCCACTCTGTCTGC
AAAGGGAAACAAACGCTATGAGTAATCGTAAATATTTCGGTACCGATGGGATTCGCGGACGTGTAGGCAATGC
CCCCATTACACCTGATTTCGTGCTTAAACTGGGTTGGGCTGCCGGAAAAGTGCTGGCGCGTCATGGCTCGCGT
AAAATCATTATCGGTAAAGATACGCGTATTTCCGGCTATATGCTGGAGTCCGCGCTGGAGGCGGGCCTTGCCG
CTGCGGGGCTGTCGGCCTCGTTTACCGGACCTATGCCAACGCCAGCCGTCGCCTATTTGACGCGGACTTTCCG
CGCGGAGGCGGGTATCGTTATCTCGGCTTCGCATAACCCGTTCTATGATAACGGCATTAAATTCTTCTCTATC
GACGGTACGAAACTGCCTGACGATGTGGAAGAAGCCATCGAAGCCGAAATGGAAAAAGAGATCACCTGCGTAG
ACTCCGCCGAGCTGGGGAAAGCGAGCCGCATCGTCGATGCCGCCGGACGCTATATTGAGTTCTGTAAAGGCAC
CTTCCCGAACGAACTGAGCCTCAATGGCCTGAAAGTGGTTGTGGATTGCGCCAACGGCGCTACCTACCATATC
GCACCGAATGTGCTGCGCGAGCTGGGCGCGACCGTTATCGCCATCGGCTGTGAGCCAAACGGCGTCAACATTA
ACGAAGAAGTGGGCGCGACCGACGTGCGCGCGTTGCAGGCGCGCGTGTTGGCGGAGAAGGCGGACCTCGGCAT
CGCGCTTGACGGCGACGGCGACCGGGTCATCATGGTTGACCATGAAGGCAATAAAGTCGATGGCGATCAGATT
ATGTATATCATCGCGCGCGAAGGGCTGCGCCAGGGGCAACTGCGCGGCGGCGCGGTGGGGACGCTGATGAGCA
ATATGGGGCTGGAGCTGGCGCTCAAGCAGCTTGGCATTCCGTTTGCCCGGGCGAAAGTGGGCGACCGCTACGT
GCTGGAAAAAATTGCAGGAAAAAGGCTGGCGCATCGGCGCGGAAAACTCTGGTCACGTTATTCTGTTGGATAAA
ACGACCACTGGCGACGGTATTGTAGCGGGGCTACAAGTTCTGGCGGCGATGGTGCGTAATCATATGAGTCTGC
ACGATCTGTGCAGCGGTATGAAAATGTTCCCGCAGATTCTGGTTAACGTGCGTTACACCGCCGGTAGCGGCGA
TCCGCTGGAAAATGAAGCGGTGAAAGCGGTAACGGCGGACGTAGAAGCCACGCTCGGCAATCGTGGGCGGGTA
CTGCTACGTAAATCCGGTACCGAACCGTTGATTCGCGTGATGGTGGAAGGCGAGGATGAAGCGCAGGTTACGG
CATTCGCCCATCGTATCGCGGATGCGGTAAAAGCCGTTTAAGCGTTTAAGTGACTAAAAAGGCGGCGGTTGCC
GCCTTACGGCCCGCAAAAAACAACGCTTTTGCTGTTTTTTTCCGCAGTTGATACAATGCGCTTAAATTGCCCT
TGCGAAGGTCATTCGCTTTGGTTAGTATTCACACCCGCTTCAGTGGGAAACAGTAAAAATCCCGCTTTATTGG
TAGAAGCATTGGTACGCGGCAACTCCGCAAGGAACAGGTTGATTATGTACGAAGCTCTTTTAGTTGTTTTCCT
TATTGTGGCAATTGGCCTTGTAGGTCTGATCATGCTGCAGCAAGGTAAAGGCGCTGATATGGGAGCCTCCTTC
GGAGCAGGCGCTTCCGCTACGCTATTTGGTTCAAGTGGTTCTGGTAACTTCATGACCCGAATGACGGCGGTAT
TGGCGACGCTGTTCTTCATCATCAGTCTGGTGTTGGGTAATATCAATAGCAACAAAACCAATAAAGGAAGCGA
GTGGGAAAATCTGAGCGCGCCGGCGAAGACCGAGCAGACTCAACCTGCTGCACCGGCCCAGCCGACCAGCGAT
ATCCCGCGCTAATAGCAGTATCCGTGCCGAGGTGGTGGAATTGGTAGACACGCTACCTTGAGGTGGTAGTGCC
CAATAGGGCTTACGGGTTCAAGTCCCGTCCTCGGTACCAATATTCCAGGAAAAAGACGCTGAAAAGCGTCTTT
TTTTCGTCTGTTGAAAACGGAGGCCCTTTACTAACTTAGGGCCGCCTCAGTTATCTTCCTATGTGTAAGGCGC
TTTCATCCAACTCGAAATCTGTAATTTCAAGTTTTTCCATAATTACAGGGAGAACTTGGTGAATGATTCGCAT
ACCAGTCAGTATTTCATTCATGTAGATATCTGGGTGGATTCGTGATTTATTTAAATCTAAGCTTTCAGCCTCA
GCTGCAACACTCAGATCATCTGCACTATCGTAAACCATCTGGAGGAGCTCTGCTCGTAATGGAATTCCTGCAC

GGAATGCCAGAAGTTTGAGTTGGCTTAACCAGTTTTCATAATCCTTCTCAACTTCATAACGGAATTGTCGAAA
ATTCGGTGCTGTTGCTGAATCAATCTCAATTTTGCAAGCTGCAAGCGACATCAAAGAATACATAATATTGATG
GCATCAGTTGAATAGATATCCAACCCTTCATCTTGTATTAATTCATCAACATAAGCAGCATAAAAGCCATAGA
CATCATTCAGCGAGTTATAGATTTTTTCAATAATTGAACTGTGAGGGTTATTGCTGATGGAGTCCCATAGATC
TTCAGTTTCTTCATCATCTGTATTCTCATAGTTGATATCTAATTCAACAGGGAACGATTTTGGAGCGGATAAC
CCCATTTTTTCCAATGTATCAATCGTTTCTTCACATAAGAACCCTTCTTCGTCGAGTAGAGGTGTTGTGACGT
AGCCGGTTTCTGCTCTGTCATGTACTCTATCAGCAATAAAATGCATCAGACGATCCCATTTTTCTGCATTACT
GACGGAGCCTGCCCACTCAACAAGCAGTGGAGAATACTCGCCTATGTTGGTGATTTTTCTGAATTTCTTTTCC
ATATCATCAGACATATGTTCACCTTTTTTCCACTTGCTAATTTGAGTGGAGGAAACCCCTAGCTTTCCAGCAA
GTTCTTTTTGGCTGCATTTAAGGATATCTAAGGCTAGGCGTACAAAGTGTTCACTGTTCATTTTTCTACCTTT
TTAGTAAATAAAACCGTTAATTAAGTGAATTTATTATTGATTTTATTTTAGTAAATTTCAATTTAGTTTTACT
TAAAAATTATGTGAGCCTAAGCTGGCAATAAAAAAGGCCGCTTTCGCGGCCTGTTGCATCAACGACGCGGGCT
TATTTGCCTTTGTTTTCCAGATTCTCAACCTGCGGCAGACCGGTCGCGGAAGAGGCGGTTAACAGACCCGCTT
TCGCGTAGCCGAACAGTTTCTCGCGGGTATCGGTAATGTCCAGATTACGCATGGTCAACTGACCGATACGATC
GTCCGGTGAGAATACGGACTCGCCTTTCTCCATGGTCAGACGCTCAGCCTTATAGGTCAGGTTGTCGGACACG
GTGTTCAGGATGGAGTAGTCGTTGCCGCGACGCAGCTCCAACGTCACTTCACCGGTAATCTGGCTGGCGACCC
AACGTTGCAGGCCGTCACGCAGCATCAGCGCCTGCGAGTCGAACCAGCGGCCCTGATACAGCAGTTTACCCAG
CTGACGACCATGAGAATGATACTGTTCAATGGTGTCTTCGTTGTGAATACCGGTCAACAGACGCTCGTAAGCG
ATGTGCAGCAGCGCCATTCCGGGGGCTTCATAAATACCGCGGCTTTTTGCTTCAATGATGCGGTTTTCAATTT
GGTCGCTCATGCCCAGGCCGTGACGGCCGCCGATGCGGTTTGCTTCCAGCATCATCTCAACATCATCGCTGAA
GGTTTTACCGTTCAGGGCAACAGGGTGGCCCTGTTCGAAGCGGACGGTCACCTCTTCTGCCGGGATCTTCACG
CTCTCGTCCCAGAACTTCACGCCCATAATCGGGTTGACGATCTTGACGCTGGAGTTAAGAAACTCCAGATCTT
TCGCTTCATGCGTGGCGCCGAGCATGTTGGAGTCGGTGGAGTAGGCTTTTTCAACGGACATTTTATAGTCGAA
ACCGCAGGCAATCATAAATTCAGACATCTCATGGCGACCACCCAGCTCATCGATAAAGTCGGTATCCAGCCAC
GGTTTGTAAATCTGTAATTCAGCATTCGTCAGCAGGCCGTAACGATAGAAACGTTCAATATCGTTGCCTTTAT
AGGTACTGCCGTCGCCCCAGATATTCACGCCGTCTTCTTTCATCGCGGCGACCAGCATCGTGCCGGTTACGGC
ACGGCCCAGAGGCGTGGTATTGAAATAGGTCAGTCCGCCAGTCGTATTATGGAAAGCGCCGCACTGAATCGCT
GCGATCCCTTCTGCAACCAACTGCTTGCGGCAATCAATCAGGCGAGCATTCTCTGCGCCATACTCCATTGCGC
GACGAGGAATGGCGTCATAGTCATCCTCATCCGGTTGTCCCAGATTCGCAGTATAAGCATATGGGACAGCTCC
TTTTTGTCGCATCCACAGCAGCGCGGCGCTGGTGTCCAGACCGCCGGAAAAAGCGATACCAATACGTTGACCT
GCTGGAAGATGCTTAAGAATCGTCGTCATAGAATAGAACCCTGCTTGATTGACTGATAATGAGATCGGTTTCT
GCTTTAGTGCATTTTTATGCAAAATAACTGAGTTTTCATTTAATCATCTTTTAGCAGAGACAGGAAGCGTTTA
GTGAAGGTTTACTGAAATAACGTTGTGAAATGCGTGTATAAGCCGGAGAGGTGGGATGAGTATAACGAAGAGT
GATATTCATTCATCTGCATATTGGGAATAGGGTTGACATAACTCATGCCTGGTCACTATACTGTGCGCAGATT
TTACGTCCCGTCCTCGGTACCAAATCCCAGCAGTATTTGCAAATTTTGCCCAAAACGAGTAGAATTTGCCACG
TTTCAGGCGCGGGGTGGAGCAGCCTGGTAGCTCGTCGGGCTCATAACCCGAAGGTCGTCGGTTCAAATCCGGC
CCCCGCAACCACTTTCCCTTAGAGTTCTTTTTCAAATATACTGTGAAGATTATGTCCTTCGTAGCTGGATTTG
AAAAAAATTCTTTCGGAAGGTACACCAGACCGCCGTTGCGGTTATAGGGTTCAGTTATCTAAAGCCCCGATTT
ATCGGGGTTTTTTGTTATCTGACTACAGAATAACTGGGCTTTAGGCCCTTTTTTTATGTCTTGGGGGTGGGCT
TGTCCACATTAGAGCAAAAATTAACAGAGATGATTACAGCACCAGTTGAAGCCTTAGGCTACGAGCTGGTCGG
CATCGAATTTATTCGCGGTCGCACATCCACACTGCGCATCTATATTGATAGTGAAGATGGCATCAATGTTGAT
GATTGTGCTGATGTGAGCCACCAGGTAAGCGCGGTGCTGGACGTTGAAGATCCTATCTCCGTGGCTTACAACC
TTGAAGTCTCGTCACCGGGTCTCGACCGTCCTATGTTCACTGCCGATCATTATGCGCGTTTCCAGGGTGAAGA
AGTCGCGCTGGTTCTTCGCATGGCGGTACAGAACCGTCGCAAGTGGCAGGGCATTATCAAAGCGGTGGACGGT
GAAATGATTACCGTCACAGTCGAAGGCAAAGATGAAGTGTTCGCGCTGAGTAATATCCAGAAGGCGAACCTGG
TTCCCCACTTTTAACAGTCTGGATGAGGTGAAAAGCCCGCGATGAACAAAGAAATTTTGGCTGTTGTTGAAGC
CGTCTCCAACGAAAAAGCGCTGCCACGCGAAAAAATTTTTGAAGCGCTGGAAAGTGCGCTGGCGACAGCAACA
AAGAAAAAATATGAGCAGGAGATCGATGTTCGTGTAGAAATCGATCGTAAAAGCGGTGATTTTGATACTTTCC
GCCGTTGGTTGATCGTTGAAGAAGTGACCATGCCGACGAAGGAAATTACGCTGGAAGCGGCGCGTTTTGAAGA
CGAAAGCCTGAATGTCGGCGACTATGTTGAAGATCAGATTGAATCTGTCACCTTTGACCGTATCACCACGCAG
ACTGCGAAACAGGTTATCGTACAGAAGGTCCGTGAAGCTGAACGCGCGATGGTTGTCGATCAGTTCCGCGACC
AGGAAGGCGAAATTGTCACTGGCGTGGTGAAGAAAGTGAACCGCGACAATATCTCTCTGGAAATTAAATCCGA
AGGGATGGCCGGTAACGCTGAAGCGGTGATTCTGCGTGAAGATATGCTGCCGCGTGAAAACTTCCGTCCGGGC
GACCGCATCCGCGGTGTTCTGTACGCTGTACGTCCAGAAGCGCGTGGCGCGCAGCTGTTCGTCACCCGTTCCA

AGCCGGAAATGCTGATCGAACTGTTCCGCATCGAAGTGCCGGAAATCGGCGAAGAAGTGATTGAAATTAAAGC
GGCGGCTCGCGATCCGGGTTCTCGTGCGAAAATCGCAGTGAAAACCAACGATAAACGTATCGATCCGGTCGGC
GCTTGCGTGGGGATGCGCGGCGCGCGCGTTCAGGCGGTCTCTACCGAACTGGGCGGTGAGCGTATCGATATCG
TGCTGTGGGATGATAACCCGGCGCAGTTCGTCATTAATGCGATGGCGCCGGCAGACGTCGCGTCTATCGTGGT
GGACGAAGATAAACATACCATGGATATCGCCGTTGAAGCCGGTAATCTGGCGCAGGCGATCGGACGTAATGGT
CAGAACGTCCGCCTGGCTTCGCAATTGAGCGGCTGGGAACTCAACGTAATGACCGTTGATGACTTGCAGGCTA
AACATCAGGCTGAAGCACATGCCGCTATCGAGATCTTTACTAAATATCTTGATATTGATGAAGAGTTCGCGAC
CGTTCTGGTAGAAGAAGGTTTCTCCACGCTCGAGGAACTGGCCTATGTGCCAATGAAAGAACTGCTGGAAATT
GACGGCCTTGATGAGCCGACCGTTGAAGCACTGCGCGAGCGTGCTAAAAACGCACTGGCCACTCTGGCGCAGG
ACCAGGAAGCAAGCCTCGGTGATAACAAACCGGCTGACGATCTGCTGAATCTGGAAGGATTAGATCGCGATAT
GGCTTTCAAACTGGCGGCTCGTGGTGTTTGTACGCTGGAAGATCTCGCCGACCAGGGCATTGATGATCTGGCT
GATATCGAAGGGTTGACCGACGAAAAAGCCGGTGAGCTGATTATGGCTGCCCGTAATATTTGCTGGTTCGGCG
ACGAAGCGTAATAAACTGTAGCAGGAAGGAACAGCATGACAGATGTAACCCTAAAAGCGCTGGCCGCAGAGAG
ACAGGTTTCCGTGGATCGCCTGGTACAGCAGTTTGCTGATGCAGGTATCCGGAAATCTGCTGACGACTCTGTG
TCCGCACAAGAAAAACAGACTTTACTGGCGCACCTGAACCGCGAAGCGGTTTCTGGTCCCGATAAACTGACGC
TGCAGCGTAAGACGCGCAGCACCCTGAACATTCCAGGTACCGGTGGAAAAAGTAAATCGGTACAAATCGAAGT
CCGCAAGAAGCGCACCTTTGTGAAACGCGATCCGCAAGAGGCTGAACGCCTGGCCGCGGAAGAGCAGGCGCAG
CGTGAAGCGGAAGAGCAAGCCCGTCGTGAGGCAGAAGAACAGGCCAAACGCGAGGCGCAACAAAAAGCTGAAC
GCGAGGCCGCAGAACAAGCTAAGCGTGAAGCCGCTGAAAAAGCGAAACGTGAAGCTGCGGAAAAAGACAAAGT
GAGCAATCAACAGACTGACGATATGACCAAAACCGCCCAGGCCGAAAAAGCCCGCCGTGAGAATGAAGCTGCA
GAGCTGAAGCGTAAAGCGGAAGAAGAAGCACGTCGCAAACTCGAAGAAGAAGCGCGTCGTGTAGCAGAAGAAG
CTCGCCGCATGGCGGAAGAAAACAAATGGACCGCAACGCCTGAGCCAGTAGAAGACACCAGTGACTATCATGT
CACCACTTCTCAGCACGCTCGCCAGGCCGAAGACGAAAACGATCGTGAAGTTGAAGGCGGTCGTGGTCGTGGC
CGTAATGCAAAAGCAGCGCGTCCGGCGAAAAAAGGCAAACATGCCGAATCCAAAGCCGATCGCGAAGAAGCGC
GTGCTGCGGTTCGCGGCGGTAAAGGCGGCAAGCGTAAAGGGTCTTCTTTACAGCAAGGCTTCCAGAAGCCCGC
TCAGGCCGTTAACCGTGACGTGGTGATCGGTGAAACCATCACCGTTGGCGAACTGGCGAACAAGATGGCGGTT
AAAGGCTCTCAGGTCATCAAAGCGATGATGAAGCTGGGTGCTATGGCCACCATCAACCAGGTTATCGACCAGG
AAACCGCACAACTGGTTGCCGAAGAGATGGGCCACAAAGTTATCCTGCGTCGTGAAAACGAACTGGAAGAAGC
GGTAATGAGCGACCGTGATACCGGCGCTGCGGCTGAACCGCGCGCACCGGTTGTGACCATCATGGGCCACGTT
GACCACGGTAAAACCTCTCTGCTGGACTACATTCGTTCCACGAAAGTAGCCTCTGGCGAAGCGGGCGGCATTA
CCCAGCACATCGGTGCTTACCACGTTGAAACTGACAACGGGATGATCACCTTCCTGGATACCCCGGGTCACGC
CGCGTTTACTTCTATGCGTGCTCGCGGCGCCCAGGCAACGGATATCGTGGTTCTGGTGGTAGCGGCAGACGAT
GGCGTGATGCCGCAGACTATCGAAGCTATCCAGCACGCGAAAGCGGCAGGTGTGCCGGTCGTGGTTGCGGTGA
ACAAAATCGATAAGCCGGAAGCCGATCCGGATCGCGTTAAGAACGAACTGTCCCAGTACGGCATTCTGCCGGA
AGAGTGGGGCGGCGAGAGCCAGTTCGTTCACGTGTCTGCGAAAGCGGGCACCGGTATCGACGAACTGCTGGAC
GCTATCCTGCTGCAGGCCGAAGTTCTGGAACTGAAAGCCGTTCGTAAAGGTATGGCGAGCGGTGCGGTGATCG
AATCCTTCCTGGATAAAGGTCGTGGTCCGGTGGCTACCGTTCTGGTACGCGAAGGTACGCTGCATAAGGGCGA
TATCGTGCTGTGTGGCTTCGAATACGGCCGCGTGCGTGCGATGCGTAACGAACTGGGTCAGGAAGTGCTGGAA
GCGGGGCCGTCCATTCCGGTGGAAATCCTCGGCCTGTCCGGCGTTCCGGCTGCGGGTGACGAAGTGACCGTCG
TTCGCGACGAGAAGAAAGCGCGTGAAGTTGCTCTGTATCGTCAGGGTAAATTCCGTGAAGTTAAACTGGCGCG
TCAGCAGAAATCTAAACTTGAGAACATGTTCGCCAACATGACCGAAGGCGAAGTTCACGAAGTGAACATCGTG
CTGAAGGCCGACGTACAGGGTTCTGTGGAAGCGATCTCCGACTCTTTGCTGAAACTGTCTACCGACGAAGTGA
AAGTGAAGATCATCGGTTCTGGCGTAGGTGGTATCACCGAAACCGACGCGACCCTGGCTGCGGCGTCCAACGC
CATTCTGGTTGGCTTCAACGTTCGTGCCGATGCCTCTGCGCGTAAAGTGATCGAATCTGAAAGCCTGGATCTG
CGTTACTACTCCGTCATCTATAACCTGATCGACGAAGTGAAAGCGGCGATGAGCGGTATGCTGTCTCCGGAAC
TGAAACAGCAGATTATCGGTCTGGCTGAAGTGCGCGATGTGTTCAAATCGCCGAAATTCGGCGCGATCGCGGG
CTGTATGGTTACCGAAGGTACGATTAAACGCCATAACCCAATCCGCGTACTGCGCGACAACGTGGTTATCTAT
GAAGGCGAGCTGGAATCCCTGCGCCGCTTCAAAGATGACGTTAACGAAGTCCGTAACGGCATGGAATGTGGTA
TCGGCGTGAAGAACTACAACGACGTTCGCGTTGGCGATATGATTGAAGTATTCGAAATTATCGAGATCCAACG
TACCATCGCTTAATCTTCGTCGTCTTTTGGGCCGCAACGGCGTTGGCTGCGCTCGCTCACCCCGGTCACATAG
TTATCTATGCTCCCGGGGATTTACGAGCTTGCCGCCTTGTTGCAATCCAAAATCCTTAGAAGATGATTAGGCG
TAGGCCGGGTAAGCGAAGCGCCACCCGGCAAGAAATTTAAAAAGGGGCTTATGGCCCCTTTTTTGTCTTTATT
CTGGAGAATTTATTATGGCGAAAGAATTTGGTCGCCCTCAGCGCGTAGCGCAAGAGATGCAAAAAGAGATCGC
AATCATCCTGCAGCGCGAAATTAAAGACCCGCGCCTGGGCATGATGACTACCGTCTCCGGTGTCGAAATGTCC

CGTGACCTGGCCTATGCCAAAGTGTTTGTCACCTTTTTGAACGACAAAGATGAAGACGCAGTAAAAGCGGGCA
TCAAAGCGTTGCAGGAAGCGTCTGGTTTCATCCGCAGCTTGCTGGGTAAAGCGATGCGTCTGCGCATCGTGCC
GGAACTGACCTTCTTCTACGACAACTCGCTGGTGGAAGGGATGCGTATGTCCAACCTGGTAACTAACGTGGTG
AAACATGACGAAGAACGTCGTGTGAACCCGGACGACAGCAAGGAGGACTGATGAGTCGTCCTCGTCGTCGTGG
CCGCGACATTCACGGCGTTCTGCTGCTGGATAAGCCGCAGGGTATGTCCAGCAATGACGTGCTGCAGAAAGTT
AAGCGCATCTACAATGCCAACCGCGCCGGGCATACCGGCGCGCTGGACCCGCTGGCGACCGGCATGTTGCCGA
TTTGCCTCGGCGAAGCGACAAAATTTTCGCAGTACCTGCTGGATTCTGACAAACGCTATCGTGTTATTGCCCG
TCTGGGGCAGCGCACGGATACCTCTGACGCCGATGGTCAAATCGTGCAGGAGCGTCCGGTGACTTTCAGCGCT
GAGCAGCTTGCGAGCGCGCTGGAGACCTTTCGTGGGGATATCGAACAGATTCCCTCGATGTATTCGGCGCTGA
AGTATCAGGGCAAGAAGCTGTACGAATATGCCCGCCAGGGTATCGAAGTGCCGCGCGAAGCCCGCCCGATTAC
CGTCTATGAGCTGCTGTTTATTCGCCATGAAGGTAACGAGCTGGAGCTGGAAGTTCATTGCTCCAAAGGCACC
TATATTCGTACCATTATTGATGATTTAGGTGAAAAACTGGGCTGTGGCGCGCACGTAACGTACCTGCGTCGTC
TGACGGTCAGCAAGTATCCGGTGGATCGTATGGTGACGCTGGAGCATTTGCAGACGCTGGTAGCACAGGCGGA
GCAGCAGGGCGTTCCGGCGGCGCAGTTGCTCGATCCATTATTGATGCCAATGGACAGTCCAGCTTCGGACTAC
CCTGTTGTTAATCTGCCGTTAACATCTTCGGTATACTTCAAAAATGGCAACCCGGTTCGTACCACGGGCGCGC
CGTTGAAGGGACTGGTTCGTGTGACGGAAGGTGAAGACGATAAATTTATCGGTATGGGCGAAATTGACGACGA
AGGCCGTGTCGCGCCTCGTCGGCTGGTAGTAGAGTACCCAGCGTAATGTCGCTTCGTCTTGCGATAAAAGAGC
GCTGCGAGTAGAATACCGCCGCTTAACGTCGCGTAAATTATTTAACAGTTTACGCGGCGTACACTGGGATCGC
TGAATTAGAGATCGGCGTCCTCTCATTCTTAAATACTTTGGAGTTTTAAAATGTCTCTAAGTACTGAAGCTAC
AGCTAAAATCGTTTCTGAGTTCGGTCGTGACGCAAACGACACCGGTTCTACCGATGTTCAGGTTGCTCTGTTG
ACTGCACAGATTAACCACCTGCAGGGTCACTTTGCAGAGCATAAAAAAGATCACCACAGCCGTCGTGGTCTGC
TGCGTATGGTTTCTCAGCGTCGTAAACTGCTCGACTACCTGAAACGTAAAGATGTTGCACGCTACACCGCGCT
GATTGAGCGTCTGGGTCTGCGTCGCTAATTCTTTCGAGTTTCAGAAAGAGGGGGCCTTCATGGCCCCTTTTTT
CCAGCAGATGGCAGCAATTCGCTGGAAACTAATGTATTGTTGCTATGAATGATCTTCCGTTGCAGAGGTTCGC
GCGGCTAATGAGAGGCTTTACCCATATCAGGGTTAGGGTTGTCATTAGTCGCGAGGATGCGCAGAAGATCGGG
TATTAACGACGGCGCGTATTATGCCGTCAAAAATTGGAAAGGATATTATTTTGCTTAATCCGATCGTTCGTAA
ATTCCAGTACGGTCAGCATACCGTTACGCTGGAAACTGGCATGATGGCGCGTCAGGCCACTGCCGCTGTTATG
GTAAGCATGGATGACACCGCCGTATTCGTTACCGTTGTCGGCCAGAAAAAAGCCAAGCCAGGCCAGGACTTCT
TCCCACTGACCGTTAACTATCAGGAGCGTACCTACGCTGCTGGTCGTATCCCGGGCAGCTTCTTCCGTCGTGA
AGGCCGTCCAAGCGAAGGCGAAACCCTGATTGCGCGTCTGATTGACCGCCCGGTTCGTCCGCTGTTCCCGGAA
GGCTTCGTTAACGAAGTGCAGGTTATTGCGACCGTCGTTTCCGTTAACCCGCAGGTTAACCCAGACATCGTTG
CCATGATCGGCGCATCCGCTGCGCTGTCGCTGTCCGGCATTCCGTTCAACGGCCCAATCGGCGCGGCGCGCGT
AGGTTACATCAATGACCAGTACGTGCTGAACCCGACCCAGGACGAGCTGAAAGAAAGCAAGCTGGACCTGGTG
GTTGCCGGTACCGAAGCGGCTGTGCTGATGGTGGAATCCGAAGCTGAACTGCTGAGCGAAGACACTATGCTGG
GCGCGGTGGTATTCGGTCACGAGCAGCAGCAGGTTGTTATCCAGGCGATCAACGATCTGGTGAAAGAAGCCGG
TAAACCGCGTTGGGACTGGCAGCCGGAAGCCGTCAACGACGCGTTGAACGCGCGTGTTGCTGCGCTTGCTGAA
TCTCGTCTGAGCGACGCGTACCGCATCACCGATAAACAAGAACGCTATGCTCAGGTTGACGTAATCAAATCCG
AAACCATCGAGCAGCTTATTGCAGAAGATGAAACTCTGGATGCTAACGAGCTGGGCGAAATCCTGCACGCCAT
CGAGAAAAACGTGGTGCGTAGCCGCGTACTGGCAGGCGAGCCGCGTATTGATGGCCGTGAAAAAGACATGATC
CGTGGTCTGGACGTGCGCACTGGCGTACTGCCGCGTACTCACGGTTCCGCTCTGTTTACCCGTGGTGAAACGC
AGGCGCTGGTTACCGCGACTCTGGGTACTGCCCGTGACGCGCAGGTGCTTGATGAACTGATGGGCGAGCGCAC
TGACAGCTTCCTGTTCCACTACAACTTCCCTCCGTACTCCGTAGGCGAAACCGGCATGGTCGGTTCTCCGAAG
CGTCGTGAAATTGGTCACGGTCGTCTGGCGAAGCGCGGCGTGCTGGCAGTGATGCCGGACATGGATAAATTCC
CGTACACCGTTCGCGTGGTGTCTGAAATCACCGAATCCAACGGTTCTTCCTCTATGGCTTCCGTGTGCGGCGC
TTCTCTGGCGCTGATGGACGCTGGCGTGCCAATTAAAGCCGCCGTAGCAGGTATCGCTATGGGTCTGGTGAAA
GAAGGCGACAACTACGTTGTACTGTCTGACATTCTGGGCGACGAAGATCACCTGGGCGATATGGACTTCAAAG
TGGCGGGTTCCCGCGACGGTATCTCTGCGCTGCAGATGGATATCAAAATTGAAGGTATCACCAAAGAGATCAT
GCAGGTTGCGTTGAACCAGGCTAAAGGTGCGCGTCTGCACATCCTGGGCGTGATGGAACAGGCGATCAACGCG
CCGCGCGGCGATATCTCTGAATTTGCGCCGCGTATCCATACCATCAAGATCAGCACGGACAAGATCAAAGACG
TGATCGGTAAAGGCGGTTCTGTGATCCGCGCGCTGACTGAAGAGACCGGCACCACCATCGAAATCGAAGATGA
CGGTACTGTGAAGATCGCGGCGACCGACGGTGAAAAAGCGAAATACGCTATCCGTCGTATCGAAGAGATTACC
GCAGAGATCGAAGTCGGCCGTATCTACAATGGTAAAGTGACCCGTATCGTTGACTTTGGCGCGTTTGTTGCCA
TCGGCGGCGGTAAAGAAGGTCTGGTTCACATCTCTCAAATCGCGGATAAGCGTGTAGAGAAAGTGACCGATTA
TCTGCAGATGGGTCAGGAAGTCCCGGTTAAGGTTCTGGAAGTTGACCGCCAGGGCCGTGTACGTCTGAGCATT

AAAGAAGCAACCGAGCAGTCTCAACCTGCGGCTGCGCCGGAAGCTCCGGCGAGCGAACAGGCCGAGTAAGGTA
GCCTTTTGCCCTCCGCAGTGCGGAGGGCTTTAGACCTGGCTGGACGCCTTGTTGAGCCACCGGGGAACAGGAC
GTTCATTCAATCGTTGTCTTCGGGAGTGGGAAATGAAGCCTTTTTTGCGCTGGTGTTTCGTTGCGACAGCACT
TACGCTTGCAGGATGCAGTAATTCCGCCTGGCGTAAAAGTGAAGTCCTCGCAGTACCATTGCAACCGACTTTA
CAGCAGGAAGTGATTCTGGCGCGTATGGAACAGATTCTTGCCAGTCGGGCTTTAACCGATGACGAACGCGCAC
AGCTTTTATATGAGCGCGGAGTGTTGTATGATAGTCTTGGTTTGAGGGCATTAGCGCGAAATGATTTTTCACA
AGCGCTGGCAATCAGACCGGATATGCCTGAAGTATTCAATTACTTAGGCATTTATTTAACGCAGGCAGGCAAT
TTTGATGCTGCCTATGAAGCGTTTGATTCTGTACTAGAGCTTGATCCAACTTACAACTACGCGCACTTAAACC
GCGGTATCGCATTGTATTACGGCGGCCGTGATAAGTTAGCGCAAGATGATCTGCTGGCGTTTTATCAAGACGA
TCCCAATGATCCTTACCGCAGCCTGTGGCTGTATCTTGTTGAGCAGAAGCTTAATGAGAAGCAGGCGAAAGAA
GCGTTAAAAGCACGCTTTGAAAAATCGGATAAAGAGCAATGGGGATGGAACATTGTCGAGTTCTACCTGGGCG
ACATTAGCGAAGCAACGCTGATGGAAAGGCTCAAGGCGGACGCAACGGATAACACCTCGCTCGCTGAGCATCT
CAGTGAAACCAACTTCTATTTAGGTAAGTATTACCTAAGTCTGGGGGATTTGGACAGCGCCACGGCTCTGTTC
AAACTGGCGGTGGCCAACAACGTTCATAACTTTGTTGAGCACCGATACGCATTGTTGGAATTATCGCTCCTGG
GCCAGGACCAAGATGACCTGGCAGAATCGGACCAGCAATAGCTGACGAACATATCAGCCCGTAATCTTTTTGA
TTGCCATCACCTTCGCGGGTGAGGGCGTTGTTGTTCGTTAATACACCTACTTTGAGCCGGTTCACACTTTTCA
ATGAAAATTGCAGATCAATTTCATGATGAGTTATGTAGACTGGCCGCCATTAATTTTGAGGCACACGTACTAC
ATGGCTGAATTCGAAACCACTTTTGCAGATCTGGGGCTGAAGGCTCCTATCCTTGAAGCCCTTACCGATCTGG
GTTACGAAAAACCATCTCCAATCCAGGCAGAGTGCATTCCGCATCTGCTGGGCGGTCGCGACGTGCTGGGCAT
GGCCCAGACCGGTAGCGGCAAAACCGCAGCGTTCTCTTTACCGCTGCTCAACAATCTTGATCCTGAGCTGAAG
GCACCTCAGATTCTGGTGCTGGCGCCAACCCGCGAACTGGCGGTACAGGTTGCCGAAGCCATGACGGATTTCT
CTAAACACATGCGCGGCGTAAACGTGGTTGCCCTGTACGGCGGTCAGCGTTATGACGTGCAGTTACGCGCCCT
GCGTCAGGGGCCGCAGATCGTTGTCGGTACGCCGGGACGTCTGCTTGATCACTTAAAACGTGGCACTCTCGAC
CTCTCTAAACTGAGTGGTCTGGTGCTGGATGAAGCCGACGAAATGCTGCGTATGGGCTTCATCGAAGACGTTG
AAACTATTATGGCGCAGATCCCGGAAGGTCATCAGACCGCTCTGTTCTCCGCCACCATGCCGGAAGCGATTCG
TCGCATTACCCGCCGCTTTATGAAAGAGCCGCAGGAAGTGCGCATTCAGTCCAGCGTGACTACCCGTCCTGAC
ATCAGCCAGAGCTACTGGACTGTCTGGGGCATGCGTAAAAACGAAGCGCTGGTGCGTTTCCTGGAAGCGGAAG
ACTTTGATGCGGCGATTATCTTCGTGCGTACCAAAAACGCGACCCTGGAAGTAGCAGAAGCGCTGGAACGTAG
CGGCTACAACAGCGCCGCGTTGAATGGCGATATGAACCAGGCGCTGCGTGAGCAGACTCTGGAACGCCTGAAA
GATGGTCGTCTGGACATCCTGATCGCTACCGACGTTGCGGCACGTGGTCTGGACGTTGAACGTATCAGCCTGG
TGGTGAACTACGATATTCCGATGGACTCCGAGTCCTACGTTCACCGTATCGGTCGTACCGGCCGTGCGGGTCG
CGCCGGTCGCGCGCTGCTGTTCGTTGAGAACCGCGAGCGTCGTCTGCTGCGCAATATCGAACGCACCATGAAG
CTGACCATTCCGGAAGTGGAACTGCCGAACGCAGAACTGCTGGGTAAACGCCGTCTGGAAAAATTCGCCGCGA
AAGTTCAACAGCAACTGGAAAGCAGCGATCTGGATCAGTACCGTGCGCTGCTGGCGAAAATCCAGCCGTCTGC
TGAAGGCGAAGAGCTGGATCTCGAAACGCTGGCCGCCGCACTGCTGAAAATGGCGCAAGGCGAGCGTCCGCTG
ATTCTGCCGCCGGATGCGCCGATGCGTCCTAAGCGTGAATTCCGTGACCGTGACGACCGTGGTCCGCGCGAGC
GTAATGACCGTGGCCCGCGTGGCGACCGTGAAGAACGTCCGCGTCGTGAACGTCGTGACGTTGGCGATATGCA
GTTATACCGCATTGAAGTGGGCCGTGATGACGGCGTTGAAGTTCGTCATATCGTGGGCGCTATCGCTAACGAA
GGCGACATCAGCAGCCGTTACATTGGTAACATTAAGCTGTTCGCGTCTCACTCCACCATCGAGCTGCCGAAAG
GTATGCCGGGCGAAGTGTTGCAGCACTTCACGCGTACCCGCATTCTGAACAAGCCGATGAATATGCAGCTGTT
GGGTGATGCGGTTCCGCACGCGGGCGGCGAACGTCGCGGCGGTGGTCGTAGCTTCAGTGGCGAACGTCGTGAA
GGCGGTCGTAATTTCAGCGGCGAACGTCGTGAAGGTGGTCGTGGTGATGGTCGTCGCTTCAGCGGCGAGCGTC
GTGAAAGCCGTGGTCCGCGTCGTGATGACTCTACCGGTCGCCGTCGTTTCGGCGGTGATGCGTAACTCACGCT
GACCTGCGCATTCACAGCGTAAAGTAAAATATACAGCCCCGATCGTCACAATCGGGGCTTTTTTTATTTCTTT
TGTACTCGTGTACTGGTACAGTGCAGCGTATTGAGAGATAGTCACACTATAAATGACTGGAGAGAGAGCTGAA
TGGCAACACTTACCACCACCCAAACGTCACCTTCGCTGCTTGGCGGCGTGGTGATTATCGGCGGCACCATCAT
TGGCGCGGGGATGTTCTCCCTGCCGGTGGTCATGTCCGGGGCGTGGTTCTTCTGGTCGATGGCGGCGCTGGTC
TTTACCTGGTTTTGTATGCTGCACTCCGGGCTGATGATCCTCGAAGCCAACCTTAACTACCGCATTGGTTCGA
GTTTCGACACCATCACCAAAGATCTGTTGGGGAAAGGCTGGAACATTGTCAACGGTATTTCCATTGCCTTTGT
GCTCTACATCCTGACTTACGCCTATATCTCGGCGAGCGGCTCGATTCTGCATCATACCTTTGCTGAGATGTCG
TTGAACGTTCCGGCGCGAGCGGCAGGTTTTGCCTTTGCGCTGCTGGTGGCGTTTGTAGTGTGGCTGAGTACGA
AGGCGGTCAGTCGGATGACGGCGATTGTGCTGGGCGCGAAAGTGATTACTTTCTTCCTGACCTTCGGCAGCCT
GTTGGGGCACGTTCAGCCTGCCACGTTGTTTAACGTGGCTGAAAGCCACGCGTCATATACGCCGTACCTGCTG
ATGACGCTGCCATTTTGTCTGGCCTCTTTTGGTTATCACGGTAACGTACCGAGCTTGATGAAATATTACGGCA

AAGATCCGCGTACCATCGTCAAATGCCTGATTTATGGGACGTTACTGGCGCTGGCGCTCTATTCCGTCTGGCT
GCTGGGGACGATGGGCAACATCCCGCGTCCGGAATTTATCGGCATTGCGCAGAAAGGCGGCAATATTGATGTG
CTGGTACAGGCGTTGAGCGGCGTGCTGAACAGCCGCAGCCTGGATCTGCTGCTGGTGGTGTTCTCTAACTTTG
CGGTCGCCAGTTCCTTCCTTGGCGTGACGCTGGGGCTGTTTGACTATCTGGCGGATCTGTTTGGTTTTGATGA
CTCGGCAATGGGGCGTTTTAAAACGGCGCTGCTGACTTTCCTGCCGCCGATGATTGGTGGCCTGCTGTACCCG
AACGGTTTTCTGTACGCCATTGGCTATGCAGGTTTAGCTGCAACTATCTGGGCGGCCATTGTGCCTGCGCTGT
TGGCACGTAAATCTCGCGAACGTTTCGGCAGCCCGAAATTTCGCGTCTGGGGCGGTAAACCGATGATTGCCCT
GATTTTGGTCTTCGGTGTTGGCAACGCGGTTATCCATATTCTGTCGAGCTTTAATTTGCTGCCGGTGTACCAG
TAATTGAACGAGCCGGATGAGCTGTTTTCATCTGGCAATATTTCACAAGTCCGCGAAATGGTATCAAATAAAA
CCCTGTTTATTATAAAGTTCTTCGGCGAGTGCTTCCCCGTGTTCATCGAAAAAAGCATCGGCGATAGTTAAAT
TTTCTGCCTTAGGATTTGCCGTTATTCTGCTACCATTGGCGCCAAATTCGAGCGCGATGGCGATCTCCGAACC
CCACCAGTAATAATAAATTAGCGCAAAATTATTTTCCCCTAGCGTAATAAATTTCTCGATTCCCCGCACACAG
GCCATGAGTGGCATGAAGATTTCATAGCTCTCTGAGAGAAATATGTGATCCATCGTGACTATCTGTGTTTCTT
TATTAAAGTTGATATGTTCAGCATAAATATTCTTTTCACTTTCACCAGAATTACATGTGGCAAGATTGTTAAG
CATTTTTGCAGGATCGCCGTACAACCTTTGCTCCTCAGTAAACCATGGCAACCAGTCGTCATAGTCTTTAATA
TTTGTTGCCGGTGAATGGAAAAATTTCTTGAGGTTACTCTCGGTAATATGTGCATGAAGAAAAAAGGAAGCAG
GTTCACTCATGATGATTTTCTCTGGTAATAAGTCGATTTATTCATATCCCTGTTCGGCCATGATAAAACGATT
ACTATTTTATAAAAGTACAGAACTGGACCTTTCCTCTATTTCATATCATAGGAAAATATATACTTGAACTGGT
TCAGAATAAGCGGAATACGAAAATGGGGCGTAAAGGACTACTCGCTATTGTATTATTGAGCCTGTTTATCGCT
TTTATTTTAAAATTTTTCTGGCTCACTCCGTACGATGAAGACGTTTACCTGCCAGTGGAAAAACCTGTCGCAT
CGTCTTTAAAAATCATTCACCCCGGCGACCAGTTATTTATCCGCATTCTGAAAGCGGAAGATAAGCTGGAACT
GTGGGCAAGCGCCAATAACAAGCCTTACAAACTCTACAAAACCTGGACTATATGCGCATGGTCTGGTGGCTTA
GGGCCAAAACATAAACAGGGAGACGGTAAAAGCCCGGAAGGCTTTTATGCCACCAACAAAGGGTTGCTTAACC
CAAACAGCCGCTATCATCTGGCATTTAATATTGGCTACCCGAATGCTTACGATCGGGCAAATGGCTACACCGG
TGATTTCATTATGGTTCACGGGAACTGCGTTTCGGCCGGTTGCTATGCCATGACCGATGCCGGTATTGAAGAG
ATTTATCAACTGGTGGCGCAGGCGCTAAACAGCGGGCAGAAGCGCGTGCCTGTCCATATCTTCCCATTTACGA
TGAATGACGAAAATATGCGCCAGGCGCAAGCATGGCCGGAATATAATTTCTGGCGTATGTTGAAGCCCGGCTA
TGATTACTTCGAAAAAAATCGCCGATTGCCAACCATCACCGTTGAAAATCGGCGTTATAAAATCAGCCCAACA
ACTCTTCCTTAACATCCATCGCTAAATCAAACGAATGCAGGCGTGCCTGGTGGTCGAAAATCTGCCCGTTTAC
CATAATTTCATCCGCCTGGGTCTCCCGCAGGATGGATTCCAGCCCGTGGCGGACTTTCGCTTTATCACCGACC
AGCGACATACTCAGCGCCTGCTGCACGCCATACTGCTCGGATGGCGACCAGAAGGTCTCCATATTCTCAATCG
GCGGCGGCAGTTGGCCGGTTTCCCCACGGCGTAGCTTCACGAAAGCCTGCTGCATGGAGGTAAACAGGAATTC
GGCGTCGCGGTTGCTGTCGGCGGCGATAATATTGATACACACCATCGCGTACGGTTTTTCCAGCCGCGCTGAA
GGTTTGAACTGGGTACGGTACAGATGCAGCGCCTGGAACAACATATCCGGCGCGAAATGCGAAGCAAAGGCGA
ACGGCAGGCCAAGCTGGGCCGCCAACTGTGCGCTATACAGGCTGGAGCCCAACAGCCAGACCGGGATCTGCTC
GCCGTAGCCGGGCACCGGGCGCACATGCGGATTAGGGGCGCGAGCGTCGAACCAGTCCACCAGCTCTGCGACA
TCGCGTGGAAAGTTATCGATATCGCCGCTCATATGGCGACGCAGCGCGCGCATCGTCGGCTGATCGCTACCCG
GCGCGCGTCCCAACCCTAAGTCGATACGTCCCGGGTAGAGCGTATTCAGCGTGCCGAACTGCTCGGCAATCAC
CAGCGGTGAATGGTTAGGCAGCATCACGCCGCCGGAGCCAAGATGTAATGTTGTGGTATTGGCCGCCAGATAA
CCAATCAATACCGAGGTCGCCGCGCTGGCGATACCGGTCATGTTGTGATGTTCCGCCAGCCAGTAGCGGTGAT
AACCGCGCTTTTCAGCCAGCCGTGCGAGATCAAGCGAGTGCGTGAAGGCTTCTTTTGCCGAAGATCCTTCAGG
GATCGGCGCCAGATCCAGCACCGAGAACGGAATAGTTTTGTCAGTCATAACGGCTCACTTTGTTACCGGTTAA
TCTTTTAATACTGCATTAAAGATTAGCGAATGTTGTTAACAAAGTGAGCTTTATGAGACTGACGGCAAAAACA
ATGCCCGATGGCGCTGCACTTATCGGGCCTACGGGATCGCTATTTGTAGGCCAGATAAGGTCTTTATGCCGCC
ATCCGGCTAGGCCTGTAGCTCCAGCCCGGCCAGCCGCCTCCAGTAGCCGTTGCAGTCGCTATGCGCGGCAAGC
GCTAATGGCGCGCCGCCGTTTTCGTTAGCGCGAAAGGCATCAAGCATGGCAAATGTCTCCGTACCCAACGGCG
ACAGCCGCACGATATCGACCAATCCCTGCATCGACGTCAGTTCGTTGCCAAGGTTATAGACATAGCCGCTCAT
GGTCTGAATACCGTTAAGCACAAACACCTGCTGATTCTCTTGCGAGAGCACGTCACGACCGTTTGGATACTTG
ATACAGCAGGTTTCGCACTCATCTTTCGGGCGGTCTTCCGAACGGGCGGTAAAGCAGCGAGCGGAGTAGGCCA
GCGGCAGATGCCCGTAGCTCAACACTTCCACTTCAAACTGATTGCGGATGCCCAGTTCATCGCACTGATTAAG
CAAATTCACCAGCCAGTCGCGGGACAGCTCCACCGGCATACACCAGCGTACCATGCCTTCTTTCAGCAACCGG
CGCAGCGTGACCGCGTTGTAGCAGTTCAGCGCATGGCCGGCGACAAACGGCAGTTTGCGCTCGGCGCACAGAT
TCACCACGCCAAGATCGCTGGCTTCCAGCAGAAAATCGCCGTTGTCCACATAGCGTTTCAGCTCGCTTAATTC
AGATGACGCCTGTACCAGCGCCAGGGTGGAGAGCACCACCTGCTTACCGCTGGCGGCGAGGGATTTCGCCATT

TCCAGCCAGTCGCCGACTTTGGTCGCGCGACGCTTGCTGCACACCGCTTCGCCCAGATAAATGACATCAGCGC
TGCTTTTTGCGGCCTGCTGATAAAAATCTTCCAGCGTCTCTTTCGGCCAGTAATAAAGCACCGGTCCTAAGGA
ATATTTCATAGCTCCTCTCACTGCCATTTACGGTGATATGCGCCAAGCGTGGTTTGGGTGCCTTCGGACATCG
CGCCGAGCGTCTCCATCCAGTCGCGCTGTGGAACGAAGTTTTGCGGGGCGGCTTTGCAGCGATCGATCGCCTG
GCGCCACACTTTCGCCACCTGGCTGACGTAGGCCGGGCTGCGCTGGCGGCCTTCGATCTTCACCGAAGCGATG
TTCGCCGCCATGAGCTCCGGCAGCAGTTCCAGCGTGTTCAGGCTGGTTGGCTCCTCCAGCGCGTGATAGCGCT
CGCCGTCCACTAAATAGCGGCCTTTACACAGCGTTGGGTAGCCTGCGTTTTCGCCATCCTGGTAACGGTCAAT
CAGGACATCATTCAGGCGCGATTCCAGCCCCTGCGGCGTTTGCTGCCAACGGACAAAGCGGGCGGGAGAGCAG
GCGCCGACGGTATTGGGCGACTCCCCCGTCAGGTAGGAAGAAAGATAGCAGCGACCTTCCGCCATAATGCACA
GGCTGCCAAACGCGAATACCTCCAGCGGCACCGGCGTGACGCGGGCCAGTTGTTTTACCTGGTGAATCGACAG
TACACGCGGCAGTACTACACGGTGGACATCAAAGTTGCGGTGATAAAAGCGAATCGCCTCTTCGTTGGTTGCC
GAGGCCTGAACGGAAACATGGCGCTCAATATGCGGGTAACGCTCTGCGGCATATTCCAGCATAGCGAGGTCGG
CGAGAATCAACGCGTCGGCGCCAAGCTGCGCCGCCATATCCACGGCGCGCTGCCAGCGGGCATAGCCGTCCGG
ATGCGCAAAAGTATTAATGGCGATGTGTAATTTGCGGCGATGCTGGTGAACAAAACTCACCGCTTCCTGCAAT
TTTTTTTCGGTAAAGTTAAGGCCGGCAAAATGACGGGCATTGGTATCGTCTTTTAACCCGATATAAACGGCGT
CAGCGCCGTTTTCGATGGCCGCCTTCAGCGCCGGGAGATTTCCGGCAGGGCAGAGCAGCTCCATAATTTATCC
TGAGGGTTACGTCTGAGACGCAAAATTGTTAACGAACAGGGATTTTAGTTAACCTTGCTGCGACAATTTTTGA
TTTAAGGCAGTTAAAGGCGTATTGGTAACACCAATAATGGGGTAGAAAATCAGACCCAATTGTTGATTTACGC
CGCCATGTGATTTATTGGATATGGCAAAATAACAGGACTATTGAAACAGGGAGTAAAGCTCGTGTTAGATAAA
CTGCGTTCACGCTTAGTCCATGCTGGCCCGTCTCTGATGAGTGTACCGGTTAAACTGACGCCCTTTGCGCTAA
AGCGCCAGGTTCTGGAACAGGTTCTGAGCTGGCAGTTTCGTCAGGCGCTGGCCGATGGAGAGCTGGAATTCCT
CGAAGGTCGTTGGTTAAGCATTCATGTTCGTGACATCGACTTAAAATGGTATACCACGGTTGAAAATGAAAAG
CTGATCGTTAGCCAGCAGGCGGATGCTGACGTCAGTTTTAGCGCCGACGCCAGCGATCTGCTAATGATCGCCG
CCCGTAAACAGGATCCGGATACGCTCTTTTTCCAGCGTCGACTGGTCATTGAAGGCGATACTGAGTTAGGGTT
GTATGTGAAGAATCTGATGGACGCCATCGAACTGGAGCAGATGCCGAAAGCGTTACGCGTTATGCTGCTGCAA
CTGGCGGATTTTGTTGAGGCGGGAATGAAAAACTCGCCGGAAACCAAACAGACCTCGGTAGGTGAACCATGCT
AATTCGAGTAGAAATTCCCATTGATGCGCCTGGTATTGATGCGCTGTTACGCCGTTCATTCGAAAGCGATGCG
GAAGCGAAGCTGGTTCACGATTTGCGTGAAGATGGTTTTCTGACGCTCGGACTGGTGGCCACGGATGATGAAG
GTCAGGTGGTGGGCTATGTCGCCTTTAGCCCGGTTGATGTGCAGGGCGAAGATTTACAGTGGGTCGGCATGGC
GCCGCTGGCGGTCGATGAAAAGTATCGCGGGCAAGGGCTGGCGCGCCAGTTAGTGTATGAAGGGCTGGATTCG
CTCAACGAGTTCGGCTACGCGGCGGTCGTGACGCTGGGCGATCCGGCGTTATATAGCCGCTTCGGTTTTGAAC
TGGCCGCACATTACGATCTTCACTGCCGTTGGCCCGGTACGGAAAGCGCTTTCCAGGTACACCGTTTGGCTGA
GGATGCGCTGGAGGGCGTGACGGGGCTGGTGGAGTATCACGACCACTTCAATCGTTTTTAAGCACCGGCGTTT
GCAGGCTGGACAGCAGCGCCTCAAACGCTTCGCGTTCCGTCACAAGGCGCTCCTTCTGGCGCTTTGTGAGCTG
TTTGATGCGATACTCTATGCGCAGGGCCAGCGAGCGATTGCCGACCTGCGCCGCAAACGCCAGCGTTAACTCG
CCTTTTCCCCGTAATGCTTTTGCGCCTTTTCCCGTTTGATGCTGCCTGTAACGACGCGCCACATCGGTGGTTA
TTCCGGTGTAAAGCGCATTGTCTGCAGTACGAATCAGATACAGATACCAGGGTGTCATTGTCGCCATCAGCAT
GATAATTTTCAGGTTCCCAGTATAAGCGAGAATAAAGATGGACACACTTACCGCTATTGGCCGTTGGCTGGCG
AAACAACACGTGGTGACCTGGTGCGTACACCATGAGGGAGAGCTGTGGTGCGCCAATGCGTTTTATTTGTTTG
ATGCGCAAAACGTCGCGCTGTATCTGTTAACGGATGATAAAACCCGCCATGCGCAAATGTCTGGCGCGTGCGC
GCCAGTTGCGGGAACGGTCAATGGTCAGCCGAAAACGGTGGCGCGCATTCGCGGCGTACAGTTTAAAGGGGAA
ATTCGCCGTCTGGAGGGGCAGGAGAGCGACGCCGCGCGTAAAGCTTACCTTCGTCGCTTCCCGGTCGCCAGGG
TGCTGCCAGCGCCGGTATGGGAAATCCGGCTGGATGAAATTAAATTCACCGACAACACGCTGGGGTTTGGTAA
AAAACTGCACTGGTTACGCGACTCACGCGCCCAGCAGGCGTAGCGCCTCGCGGTTAAATGCCGGCAGGTCGTC
AGGCGTGCGGCTGGTCACCAGCTGATCTTTATCGACGACGACTTCCTGATCGTAAAATTCCGCGCCCGCATTC
TTCACATCGATAATAATCGGTTTCACCGCCGTGAGTTTGCGGCCACGAATGACATCGGCGCTGATCAACAACT
GTGGGCCGTGGCAAATGGCAAAAACCGGTTTACCGCTGTTAACGAAATCGCGGGTAAAATCCACGAAACGGCT
GTCGCCGCGCAGGTAGTCCGGCGAATGACCTCCCGGCAATAGCAAGGCGTCGAACTCGTCGGGACGAACGTCA
TCAATAGCCTTATCAATGGTGACGCTGGCCTCGCCTTTTTTCCTTTCACCGTTTTACCCGCCTCTTTTTCGAT
GGTGATGACCTCATGGCCGGCCTGACGGAATTCTGCTGCCGGCGAGGTAAATTCTGAATCTTCAAATTCATCG
GTAATCAAAACGGCAATTTTCTTACTCATGCGTTTCCTCCGCAGTGGTGGCGTCGGGTAAGGCAAATTTTCCA
CAATGATGGATACTTACCTCGTAGACTATTAAGCCTGGTCGATAGGGCCGAGAGTGCAAGGTGGACAAATCTG
TAAAGGAGCGAGGATGAGTCAGGTACTGATTACCGGCGCGACCGGACTGGTCGGCGGACATCTGCTGAGAATG
TTAATCAACACGCCGCAGGTGAGCGCTATCGCCGCGCCGACGCGTCGCCCGCTGACGGATATTGTCGGCGTTT

ATAACCCTCACGATCCGCAGCTTACCGACGCGTTAGCTCAGGTGACCGACCCGGTGGATATTGTTTTTTGCTG
CCTGGGCACGACCCGGCGTGAAGCGGGAAGTAAAGCGGCGTTTATTCATGCGGATTATACGCTGGTGGTTGAT
ACCGCGCTGACCGGGCGTCGACTGGGCGCGCAGCACATGTTGGTGGTCAGCGCAATGGGAGCAAACGCGCACT
CTCCGTTTTTCTACAATCGGGTGAAAGGAGAAATGGAGGAGGCGTTAATCGCGCAAAATTGGCCTCGTTTGAC
GATTGCCCGGCCTTCTATGCTGCTCGGCGACAGAACAACGCGGCGGGTGAATGAAACGCTTTTCGCTCCGCTA
TTCCGGCTGTTGCCAGGAAACTGGAAATCGATTGATGCGCGGGATGTGGCGCGGGCCATGCTGGCGGAAGCGC
TGGCGCCAGCACAAGAAGGCGTGACGATTCTGACCTCATCGCAACTGCGGGAAAAAGCGGAATAGCGAAGCCG
GATGATTTACCGGATGGCGACGCGGTTTACACATTCTTATCGCCACCCGGCACACCGGTTACTTAATATACGT
AAAGGCGGTGGTCACGCGTTTCACGCCGCTCACCCGGCTGGCGATGTCCGCTGCCGCTTTACCTTCACGTTCA
GTCACCAGGCCCAGCAGGAACACTTCGCCATTTTCGGTGGTGACTTTCACGTTTGACGATTTTACCTGATCGC
TGGTCAGTAGCTGTGAGCGAACTTTCGTGGTGATCCAGGTATCGTTAGAGGCGGTCCCCAGGCCGATAGGCTG
ACCCTGGCGAATCTCGTTATAGACCTCGGTGGTGCCTTCAACGCCCATCGCTATCTGTTTAGCGCGTGCGGAG
AGTTCGCTGTTTGGCGACTGGCCCACTAACAAAACTTTGCCCTGATAAGCCGTGACATTGATGCGCGTCTCTT
TTTTGATTTGCTCATCTTTCGACAGCGCGCTGCTGACACGCAGTTCCAGCGTTCCGTCATCCACCTGGGTGCC
CACGCTACGCGGGTCGGTTGCGGCTTTGGTGCCGACGGCTGCCGTACCCACTACCGCGGCGGCGACGCAACCT
TGCAATAGCAACGCAGAAATAAGGACTGCGAGCGGCGAAAATGCCTTCATGTGTACTCCTTAATCATCCTGGT
GAGGAAAAAGCGTGTTATCGATCAAATCGCACAGGCAGTTTACCGTCAGCATATGCATTTCCTGAATGCGCGC
GCTGTGGTGTGAAGGGATACGAATTTCGACATCCTGCGGCCCTAATAGTCCGGCCAGCTCACCCCCGTCATAC
CCGGTTAGCGCCACAATGGTCATGTCACGTGTGACGGCTGCTTCAACGGCTTTTACGATATCGCGACTATTAC
CACGCGTAGAGATCGCCAATAAAACATCGCCTGCATGTCCCAGCGCCCGGACTTGTTTTGCATAAACTTCATC
GTGCAGGCGATCGTTAGCGATCGCAGTTAAGACCACATTATCGGTATTTAGTGCAATGGCGGGTAAACTGGGT
CGCTCCGTTTCAAAGCGGTTAATCATGCTGGCAGCAAAATGCTGTGCGTTGGCCGCGGACGTCCCGTTTCCAC
AACAGAGGATTTTGTTGCCGTTAAGCAGGGAATGAACCAGCGTCATGGCGGCGCGGGAAATGGCATCCGGAAG
CGCTTCCGCCGCAGCAATTTGAGTTTGAATGCTTTCTGTAAAGCAAACTTTGATTCTTTCGAGCACGCTAATC
CTTAAAATCTTCAATTATGAGTGGTCGTTAAACGCATCCCGAAACCACTCAATCTCATTTCCGGTGAAGGCTA
ACACATCGAACCGACAATCCACAGTATCAAAACTCCCATTCTGGCGGGCGAGCCACAAGCGGGCAGTATGTAA
TAATTTGTGTTGCTTGCTGCGGGTCACACTAGCCGCCGCGCCGCCATAAAGCCCGGAGCGCCGATAGCGGACC
TCAACAAAAACCGTCGTTTTTCCGTCGCGCATGATCAGATCGATTTCGCCGCCGCGCTCGCGCACGTTGGCGG
CGATAAACCGCAGTCCTTTGCTTTCCAGCCAGCGACGCGCGGCAGCTTCCCACGCGTCGCCAGCCTGTTTACG
CGTTAGCTGGCGGGAACAATCTCCCCTTGCTGGTATTTGAGCCATGATAACTTCCTGTTAATCACACAATCCG
GGCTGGCGGTTAATGCGCCGGTATTACCATTGATCTCAAATCCCTGCACCTGACGCATCTGCGAAAAGTGGTT
CGCCAGCGTCCAGGCATCCACGCCCATGGCGTACATCCGCGCCAGAGAATAGTCGTTATGTACAGCGCTCAGC
GCCTGCTGCATCAACGGCATATTGCCGCCTGCCAGCATAGGAATTTCACTGTATTGTAAACCCTCCATTTCCA
GACGGAAGTCGGGGCCGGAGGTGCCTTGCGCGCTGCGAGAGCTGGCATACAGCGTCGCGCCGCTCTGGGTGCC
GTTACGCATGGCGATCATCGGTTTGATAAAGCCAATCTCTTCCGGCGTAGCCAGAATATAGACCGCGTCTACG
CGTCCGCCGCCGGTGATTTGCGCGTCGGTCGGCGGCGCAGGGATGGTCAGACCGCCAATGGTGACGCCAGGCT
GCGCAGGCACGCTGGCGGCGACCGGACTGCCCGTCAACGCGATACCCGCGCCGCCGTTCACGCCCATTTTCAG
CTCGGCTACGGAGCCGAATTTTTGCTGTAACACGATGCCGCCGCCCAGTTTTTGCCACTCCTGGGTGAAGGCG
TTCGCCACGCGATCGCCAAGCGCGCTGCGTGGAATCAACAGCAGCGGCGACTGCTTGCCCTGGTCATAAATAT
GATGCGCCGCGTCACGGGCTTCATCTTCTGGAGAGAGCGCGAAATAGCAGATATTAGGGAAGCTACGTACCGT
TTCCGGCTGGTTGAGCGCCAGCACGTTGAGCGGCGTGTTGCTTTTCATCAGCGCTTCCACATTGTTTTTCAAC
AGCGGCCCGACCACGATACTGGCGCCGTCTTGCTGAACCTGAGCGAGCACCTGATCCAACGGCTGGGAAGAGG
TATCGTAGATTTTTAATTCAGCGGAGGGATCCGCCGGCGCGCTTGCTGTAGCAGGGGGAGTTTGTGGCGCGCT
GACTGGCGTAGCGGACTGGGCTGGAGCGTCATCAGTCAGATCGCTCACCGAGGCCTGCGACGGACTGGCGACG
CCGTTGGTCATTTGTGGTTGCGTCTCCTCCACGCCAGGTTCGACCGGCGCGTCAGGAGCGGCGGCAGGCTGCA
TCTCCACCGCCTGGGTGCCGAGGTTTTTCGCGGCTTCGAAACCTTGCTGGATGGTACGGCCAAACACGGCAGC
CTGACCGTTCAGCGGCAGCAGCAGAGCGATTTTGCTGGTGGAAGCCGGTTTGAAACGTTGCACATTGACGAGC
TGCGTCGGCAGCATTTTCGCCCCCGGATTTTGCGGGTAGCGTTTTTGCCAGTCGGCGATCCCGGCTTTCAGCA
TGTCCGGATCGTTGCGGTTGTCAAACCAGACGCGTTGCAGATCCAGCCAGCCCTGAAGCACGTTTTCATCGGC
GTTGATAACTAACGTCTTGGCCTGATCCGGCGTCATGGCGGAGAGCGCCTGCCAGGTGGCGTCGATATTTTTT
TGTTTATCTTTCGCCGCCAGTAGCGGTTCCTGGGCGATTAACGCCCGCAACAGGGTAAGCGACGGGCGTCCCT
GGCTGGCAACGATCTGCGCCTGCCAGTAACGCGCTTGCTGATTTGGCGCAAAGTCGGCAGGTTTTAGTTTATC
CAGCAAGGCCTGCGCGCCTGCGACATCTTTTTGCGCCAGTTTGATTTCTACCGCTAATAAAGACTGTTCGCGA
CGCTGGGCATCGTTCAGATTTTGCGGGAGTTGGTTGAACAGGTCGACGGCCTGCTGGCTTTTTCCTTCTTTCA

GCAGTGCATGAATGGCGAGTAATTGCCAGTTGGTCTTGCTATCATCTGCGCTTTGCTGCATCTGATGCAGGTA
AAAGGCGGAGTCAGCTTGCGCTGAACCCTGCATATAGGCTGCGCTTTGATCTGGCGCCTGGGTGCCGCACCCG
GCGAAAAGTAGCGCAGCCAGGACGACAGGCAGCGCGCGCGGCGTTCAAACGAGAAAATGTTGAGGGTACCA
TACTGTATCCAGTGATATTTTTTTACGCAATGCTCAATATTAAATCGGCAATACGGACGACACAATGAAACAA
AACGAATCGGCGGATAATTCTCAGGGCCAACTCTTTATTGTACCTACTCCTATCGGTAATCTGGCGGATATTA
CCCAGCGTGCGTTGGAGGTGTTACAGGCTGTAGATCTGATTGCCGCCGAGGATACTCGTCACACCGGTTTATT
GCTGCAACATTTTGGGATTAATGCCCGATTGTTCGCGCTGCACGACCATAACGAACAACAAAAAGCGGAAACG
CTGGTCGCGAAGCTAAAAGAAGGGCAGAACATTGCGCTGGTTTCCGACGCCGGTACGCCGCTGATTAACGATC
CGGGCTACCATCTGGTGCGTACTTGCCGTGAAGCGGGGATTCGCGTGGTGCCGCTACCAGGGCCGTGTGCTGC
TATCACTGCATTAAGCGCCGCGGGTTTACCATCAGATCGTTTTTGTTATGAAGGCTTCCTGCCTGCCAAATCA
AAAGGCCGCCGTGATGCGCTAAAAGCCATTGAAACGGAACCGCGAACGCTGATTTTTTATGAGTCAACCCACC
GCCTGTTAGATAGCCTGGAAGATATCGTCGCGGTATTGGGAGAATCCCGTTATGTGGTGCTGGCGCGTGAGCT
GACCAAAACCTGGGAAACCATTCACGGCGCGCCCGTTGGCGAGCTGCTGGCGTGGGTCAAAGAAGATGAAAAC
CGTCGCAAAGGCGAAATGGTGCTGATTGTCGAAGGCCATAAAGCGCAAGAAGACGACCTGCCCGCCGACGCGC
TGCGCACGCTGGCGCTGCTACAGGCAGAACTGCCGCTGAAAAAAGCGGCGGCGCTGGCAGCAGAAATTCACGG
CGTGAAGAAAAATGCGCTGTATAAGTATGCGCTGGAGCAGCAGGAAGAATAAAAGCGAGCGCCCGGGAACCGC
CTTTTATCGCCATCGCGTTAATACTCAATCAGGTGATTGATGTGGGTAAATTGATGGGGGCATTGGGCGGTGA
TAGCGGGCCGGACCTTTCCTTCGGGCTTACTCTCGCGATCAGCAACGCATAACCGACTCCTTTTTAGACGATC
ATTAATTCCACGCCTGTTTTCGCTAATGCCGCTTTATCCTGTTTGCTGATGGTGTCATCGGTTATCACACAGT
CGATCTTGTCCATCGGCAATACCTGGTTAAAGCCGCGACGGTTAAATTTGGTCGCGTCCAGTACCGCGATAAC
TTTGTGTGCCGCCGCCGCCATGACGCCGCTAATGGAATAACCTTCGTTAAACGTGGTAATACCGTTTGTCGCA
TCAATGCCATCCGCCCCGACAAACATTAAATCGGCGCTGATGCCGTGTAGCGATCGTTCCGCAATCGTGCCGT
GCATTGAGTGGGTTTTATGCCGTACCGTACCGCCGCAGACCACCAGCGTGAGATCTTTATTTTCTGATAGCGT
AAAAGCGGCAGGCAAGCTGTTAGTGATCACCGTAATATTGCTTTTTCGCGCGAGCGCTTCCGCAATCAGTAAT
GTTGTGCTCCCGCTATCCAGAATGATGGTCATTCCTTCTTCAACCATTGCCGCAGCCGCCTGGGCAATCCGCT
TTTTCGGATCGCTGGCGAGTTGATACCGATCTTCCAGAATGACTTCCTGATTGTCGCCTTCCGCCAGATGACT
TCCCGGTTTTGCCGCGCCGCCATGAAAACGGGTGACAAGGCCTTTCTCTTCCAGCAGACGCAGGTCAGCACGG
ATAGTCACTTCAGAAATACCAAATGTATTTGAAAGGTCCATCACCAGCACGCTGCCTTGCGTATTAATCAGGT
CGACAATTTTATTTCTTCGCTCAAATGAGTTCATATTGATTTGCCTGATAATTAACTGTAACTAGTTTAAACG
AAGGATTGCGAAAGGTGAACGCTAAATTTCGAAAGGAAATGTGAGCCAGCGCAGGTTGCTGGCTCGTACCATC
AGGAAAGTTGAAGTAGGATTTTGCCCTGCATGGGCGCGCCATTAAGCGCTTTGACTGCTTCTGCAAAGCTTTC
CGCATCGCCGCGATGGGCAATGAGAGGGGTCAGTTGAAGACGTTTTTCAGCCAACAGGCGCGCTGCGGTTTCC
CATTCTTCTCCTGGCCATGGCGCGGAGTAGTTCATCCAACTGCCGAGGAGCGTGAGCTCTTTACGCAAAATTA
GCCCGAATGTACGCGTTGTCAGGGTGAGATCGTGATGTAACGTCCCCACCAGCGCCAGTTGCGCGCGCGGCCC
GGCGATATCGATAGCCAGCGAGACGGTCTGGGGAGTTCCTGCCGTTTCGAGAACCAGTTGATCAAACTGAATG
TCGGACAACGCGGTCTGGATGTCGTCGGCGGTCATTTCCCGGCTATTACAGGTATGCGTTGCGCCGAGCGCTT
TCGCAAGTTCCAGTTTCTGCGGATTGATATCAATCGCCGTTACGCTTCGCGCGCCCAGTTCCCGCGCGCACTG
TAAGGCCAGCAGGCCAATTGTCCCGGCACCAACGATAATTACGTTTTTCCCCTCGCAGCCCTGCGCCAGATGA
AAGGCGTGTAAGCCCACGGTAATTGGTTCGATAAACGCGCCATCTTCAATCGGCATATCAGACGGCAAGCGGA
AAAGATTGGCCCGTTTGACCACCACATATTCCGCATTACCGCCTTCGCTGCGCGATCCTACAAACTGATACTG
TTTGCATAAAGAAAAGTAACCGCGTTCGCATTGCGGACAGCGAAAGCAGGGAAGAAGCGGGACACAAGCGACG
GCATCGCCAGGCTGCATATCCGTTACGCCTGTGCCGTAGGATTCAACGTAGCCGCTGAATTCATGGCCTAACG
TAATAGGATAATAATGGGCGCCCTGCGCGAAAATACGCGGGATATCCGACCCGCAAAGCCCTGAGCTAACGAC
TTTGACCAACACATCGTCCTCGGCTTGCAGTTGTGGTAAGGGACGTTCTTCAACGCGCACGTCTCCCTCAGCG
TGAATAACCACTGATTTCATAACATTCTCCAGGAAGGTGAGGGGAGCGATTGCTCCCCTAAAATTAAGAGGCG
ATTTGACTTTGCTGTAGCGCCGTGGCTTTCTCAGCCGCGACAAACTGTCGGGCGCGACGCCAGGTCAGCAGTA
CGCCGGCCAGATAGATAATGGCAATGACGGCGAAGCCGACGATATTCTGCCAGGTAAAAAGTTGAATCAGCAG
CCAGGTGATGGGAGAACCGCCCTGATCCAGCGAGGCGACTTGTCCGCCAGCTTTTAGCGCTCCGGCATTGGCA
GCCAGTTGGGTATGCAGGCCAATCGTCTGGGTGGCTATCCACAGGGTGATACCCATAATAATGACACCTGAAA
TCAGCGTGCGGAACAGGTTGCCCTGGTGTACCGCGACCGCCATCGCAATAAAGAAACCGATGGTGGCGAGGTC
GCCGAACGGCAGCACCTGGTTCCCCGGTACTAAGACGGCAATCAGGATGGTCAGCGGAATGAATATCAGGCTC
GCGGAGACAACAGAGGTATGACCGAGCAGTAGCGCTGGATCAAGGCCTATCAGGAACTCCTGCCCGCCAAATT
TAGCCTGTAGACGTTTACGCGCATGTTTCGCGATAGGCGTTAGGCCATCCATAATAGGTTTAATGACGCGTGG
CATAAGCAGCATCACCGCTGCGGTTTTGACCGCCAGTTGCAGAACGGCTTTGGCGTCGTAGCCTGCCAGTACA

CCAATCACCAGCCCCATCACGAAGCCGACAGTCACCGGCTCGCCAAACGGTCCGAAGCGTTTCTGGACATCGT
CTGCGCTAAAGTGAATACGATTGAGACCAGGAATTTTCTCGATAATGGTATCAACGAGTACCGCCACGGGGCC
CAGGTACGCGGATGAACCGTGTGGGATAGCAATCCCCTCGAGGCCAAAATAGTCGCGGGTATCTTTGGCAAAC
CAGTCCCCCAGTTTGTAGACAAAGGCGGCATGAACCACAACGCCCAGAATCCCCAGCCAGTATGAACCGGTCG
CCAGATGCAGCATGGCGCCCGTGAAGGTCATGTGCCAGATATTCCAGATATCAACATTCACCACGCGGGTCAT
GCGGGTCACCAGCATCAGGACGTTAACCCCGATGGCGACAGGGATCGCGACCAGCGCGATTTGTGACGCCCAG
GTCATGGGCGATGAACCTGGCCAGCCGACGTCAATAACGTGGAGATTGATTTGGAAATGTTCCGCCATGGCTT
TCGCCGCGGGGCCGATAGAATCCAGCATCAGGCCGATGACCAGACCAATACCCACGAAGCCGATACCAATATG
CAAACCCGATTTAAAACAATCCCCAAGCTTCATTCCCAGCAGTTTAGAGAACACGATGATCACCAGAGGCAAC
ATCACCGTTGGGCCTAAGTCGAGGATATAACGCATTATTTCGCTAAACATACGTTACCCCATGAGAATGGACA
GAATTTTTTGCTGTAATGCTTCAATACCCACGCCGGAAACAAAAGGCATACCGTGAACGACGGGAATATCGCC
AAAGGCGCGATCGACCCTGGCAGTGGTACAGATAAGATCGGCGCCGTCCATATAGGTTTCGATTTCGGTGACC
CGGCATTGCACTAAGTCGAGCTCAATATGGTTGGCATCACATAGCTCTTTAATTTCTTCCGCAGCCATCGTTG
AGGTTGCAACCGCACCGCCACAGGCCACAATTACTTTACGTTTCATAGGGTATTCCTCATTATTATCAATTTA
TTATCAAACCTGGACGCACTGCTCAGGCTCAAGGATGGTTTCCTGAAAGCGTATCGCCAGTTCGGCATCCGGC
GCGCTTAACAGGGCATCGAGGGTGCTCGGGTTCTGTAGCTCACTAAACAGGCGGCGCAGAAGTTTTAGCTGTG
CCGCCGGGTTTTCAACGATTAAGGCGATGATTAACGAAACGGCAATCTCTTCGTCATCATCCGCCTGTTGAAA
CATGACCGGCTTATCTGGACGAATCAGATAGATAGCGGGTGATTTCGCATGAACCGCCTCGCAATGAGGGATA
GCGACGGCGTGACGCTCCAGCGCAATGCCGGTAGGAAAAGACGCTTCACGTTCCACCAGCGCCTGCGGATAAC
TATCATGTACCACGCCTTTCGCCAGCATCTCTTTGCCAATGTGCACCAGCGCCTGTTGGCTCGAGTCGAATGT
GATTCCGGTTCGGACAAATAGTTGGCTCATAAATTCTCCATTATTCAGGCGACATCCGGCGTGCAACCGAAGC
GATAAGCGCGCAGAACATCCTGAATTTTGTCAATAATCAGGTTATGCGGCGTAGGGGTGAGCACGCCCTCAGA
CAGGCGTTCAAACTGTACGGGCATAAACTGGCTAATTAATCCCAGCGGCAATGTGACGTTGTTCAGATTGGCG
ATTAACTTCTCTACGCTTTGACGAATACGCGGGTGCGGCCAGTAATAGCGAATACGGTCAGACAAACTAAAGT
GGATGTCGACCATCGCCTGGCTCCAGGTTGGACGGTAGTATTTTTTCCAGTAGCCCGGTTCGTTTAGCATAAC
TTCATCAATGACCTCCAGTACCCGACTACGTTGTTCCGGGGAGATCAGCTCGTTTTCCATTTGCGCAAGTGCG
AAAATGGCTTCGCGCAGCGCGAAAGTGAGCGCCGGGCCGACCTTCAGAATGGCGTAATGATCGCGTACCAGCG
CACGGTAGGCCTGTCGCGTCTGATAATCAGTCGAATGCGCTTCATAGACCATCGGCGTTTCTTTTATCCATGC
GGAGAGCGCCTGTGCCGCCTGCGGCTGATAATGAATAATCTGGGTATGATCAAATTCAACGCCCGGCTGTACG
ACAATCGCGATAACCCGGTTCAATGCCTCATCCAGCCCCAGGGCGCGAAAAGCAGCCTGATGCGTTTGCAGCG
TGCGGGCCGCATCCTGTTCGCGGGTAACATGCACGGCGTTAATGGCGCTGGCTTCCCCGCCCGGAACCGGAAC
TTCAGTGCCGATAACATAGGTTAAATGGCGTTTTTGTTCGTCGGTCGCCGTCGTTTCCGCTGCCTGGCAAAGC
AAGGCGGCGCGTTTAGCGACAACCATTGGGTCCAGCGGCGTCGGGTCGTCAGCGCAGGACATAGAGGCATCAA
GATGGATTTTGCTAAACCCGGCAGCGACGTAAGCCTTAATGAGTTCGACGGATTTTTCCATCGCCGTATCCGC
AGGTTCGTTTTGCCAGCAGTTAGGTCCCAGGTGATCGCCGCCTAAAATAAGCCGTTCGCGTGGAAAACCCACT
TCCTGGGCGATGCCATAGACAAAATCACGAAAGTCAGCCGGTTTCATGCCGGTATAACCGCCAAACTGATTAA
CCTGGTTAGAGGTCGCCTCGATCAGAACTTTGTTCCCGCTATTCAGGTCAAAACGTAATGCGGATTCAATCAC
CAGCGGGTGTGCTGAACATACGGAACAGATCCCTATCTGTTCCCCTGCTTTGTGACGAGAAATTATTTCTTTC
ACTTCTGGCTCCGATATCTTTTGTTTATCTTCATAATATTTTTGGTTTGAAGGCAAAGGATAATAATGGCTTT
CGTGATCCAGCTATGAAAACGCCCTTTGCCGTGCTAAATCCGGGGGCTTTAGAGCTTCCCTTCACAACCGCAG
TCGGCAATCACTTTACGTACAACCTCTTTCATGGCCGCTTTCGCCGGGATCATGTAATGGCGGGGATCGCTGG
CCTCTGCGTGTTGCGTTAAATAGTTTTTAAGCGCTCCGGAAAAGGCGATTTTAAGCTCGGTCGCGACGTTAAC
TTTGCAGATGCCCAGCGAAATAGCGCGGGTAATATCGCGGGTCGGCAGGCCAGAGGCGCCGTGAAGGACTAAG
GGGACATCAACCCGCTGACGAATTTCCGTCAGTCGTTCAAAATCAAGTTTTGGTTCAGCGGTGTAGAGGCCGT
GAGCGGTGCCGATAGCAATGGCTAACGAGTCGATACCCGTTTTTTCTACAAATTCCCGGGCCTGTTCCGGATG
GGTATAAAGCGCATCTTTACCGTCAACGATAAGATCGTCTTCCTGCCCGCCGAGACGCCCAAGTTCAGCTTCA
ACGCTGACATCGTAGCGATGACAGTAATCAACCACACTTTTCACCAGCGCAATATTGTCGGCAAAGGGGAAAT
GCGACCCGTCGATCATGACCGAGCGTACCCCGGCGGCGACTTTCATTTTGATGTCGGCCAGTTTTTCATGATG
ATCGAGATGTACCGCGAGAGGATGGTTCCAGCTTTTCGCCAGTTCTGCGGCGATGGCCACGATATTACCGACG
CCTGCGTAGCTAAAGGTGCCTGGCGTACCGGCGACAATCAGCGGAGAGCGCAATTCTGCGGCGGTTTCCACCA
CCACTTGCAATGTCTCCAGGTTGTGGATGTTAAACGCCGGCACAGCATAGCCTTCCTGCTGCGCCTTCTTTAG
CATTGTTCTGCCTGAAATGATAAACATCCTTCCTCCGAAATCTTTCGTTTGTTTTCTTTGAAAGAATTTATAG
GCTTTCGGAATGTTTTTTTGTGATTTTGATCTGATAAATGAAAGTTTCGAAATAAAATGGAAGTTATCGAAAG
TCAGGAGTAAAAAAAGCATGTCGGGCAGACATACTTCAGGCTACAGACTAAGGGCGAAAAAATACCGGGTAGC

GCTGGCGCTTACCCGGTCTACAGATTATGCGGTTATACCCTGGTCGGAGAAGGGTACTGACTGGATCAGGTCA
CAGGTGCGGGGTTAAACACTGCCAGCTGGTTATGCAATCCCCATTGATCTGAGAAAGTCTTTTTCCGGCCGCT
GGCGACATCGAGAATAAAGTGAAATAGCTTCCAGCCCACATCTTCGATTGTCTCTTCACCCGTGGCGATGGTG
CCTGCATTGATATCCATTAAGTCATACCAGCGGTTCGCCAGCTCGGTACGCGTCGCCATTTTAATGACCGGCA
CCGCCATCAGCCCGTACGGCGTGCCGCGTCCGGTGGTAAAGACCTGTACGGTAATGCCGGAAGCGACCTGCTG
CGTGCCGCAGACGAAATCGCTGGCGGGCGTCGCGGCGTAAATCAGGCCGCGTTTGGTGGGGCGCTGGCCCGGC
GATAATACTTCGACAATGGCGCTCTTACCCGATTTAGCGATAGAGCCCAACGCTTTTTCCACGACGTTCGCCA
GGCCGCCTTTTTTGTTGCCAGGGGACGGGTTAGCGCTGCGATCGGTTTTCCCCATATCAAGATAGTTATCGTA
CCAGGCCATTTCATCCAGCAGGCGTTTACCCACGGCTTCGTTGATGGCGCGCGGCGTCAGAAGATGAATGGCA
TCGCGCACTTCGGTGACCTCCGAGAACATGACGGTCGCGCCGCAGCGTACCAGCAGATCGGAAGCATAACCCA
CCGCCGGGTTTGCCGTGACGCCAGAAAACGCATCGCTGCCGCCACACTGCATCCCGACGACCAGCTCGGAGGC
CGGGCAGGTTTCGCGCTGGCGCTGGTTCAGTTTCGTCAGATGGCGTTCGGCGACCCGCAAAATATCGTCGACC
ATTGATTTAAAGCCGACATGCTGCTCGTCCTGTAAACGCACAATACTGGCGCTTTCAACGGCGATGGCGGGAA
CATCCTCGGTGCCTTCCAGCAGCCGTTCCGGCTGCAGTTTTTCGCAACCGAGGCCAATCACCATCACTTCGCC
GCCAAAATTGGGGTTGAGCGCGATATTATGAATCGTGCGAATCGGCACGACCGCCGCCGGCGCGTTGATTGCC
ACGCCGCAGCCGTACAGATGGTTTAGCCCTACAACGCCATCGACATTCGGGTATTTGGGCAGGAGATCGCGCT
CAATCACTTTCACCACATAATCGACCACGCCCGCCACACAGTGCACGCTGGTGGTAATGCCAAGCAGATTTTT
GGTGCCGACGCTGCCATCGGCGTTACGATAGCCCTCAAACGTATAGCCTTCCAGCGGCGGCAGCGGTTCCGGT
ACTTTGGTCGCCAGCGGTAGCGTATTCAGCGGCGGCGCTTTTGGCAGTTCAACCAGCGACTCGTCGATCCAGC
TTCCGCGCGGGATGTCGCGTACCGCATAACCGATGACTTCGCCATAACGGATGATTTCGCCGTGAGCGGGGAT
ATCCGTCAGAGCAACTTTATGGCCTTGCGGAATATGTTCAGTCAGTTCCAGGCCATCCGGAAAACGTGTTCCG
GCTTTAAGACCGTGGTCATTAACAATAATTGCGACATTATCGGTTTCATGTACTTTTATATAAAACGCCGATG
GCGATTCCTGGCGAATTTCGATATTGGCCATTGTTCAACATTCTCCAGCCTGGATGATAGAAATAGGATTATC
TATTTTGGTTATGGTTAAATCGTTAAATCTCAATATAAGAAATAAAGTTCATGCATAATGAAATAAGAATGAC
AGGAGTATAAATATAAGAATGTGATCCAGATCACCGCAAATTATGAAATTTTTTATGTAGGCCGGGTAAAGCG
TAAATGTGTGCATTAGCGCCCAGTATCATGGGCGTATGCTCAAAAATAAGCGTTAAATAATCCCTTTTTAATG
AGCGTTTGCACAATGATTATCCGCCATAGGCTGTTTATACTGAATGGCGAATTCAGTGTATCTGACGAGTTAT
TTGAAAATACCATGATAATAATAAAAGACGATAAAGATATCAGAACGGTATATTGTGCTACAGGATGTGTACC
ACATTAGAAATAGAATGCGGAGACCCATTCTGATTTCAGATAAACATTTTATTGTCGTTGCTATAAGTTATGG
TGGCGGCATCACATGTAGAGAATTAAAGAAATAAGCGAAGGTATAAGCGATGAATAACGCTATTTTCCCGAAT
AAGTTTAAAGCGGCGCTGGCGGCGCAACAGGTACAGATTGGCTGCTGGTCTGCACTGGCAAGCCCAATCACCA
CCGAAGTTCTCGGTCTGGCGGGATTTGACTGGCTGGTGCTGGATGGCGAACATGCGCCGAACGATGTGACCAC
CTTAATTCCGCAGTTAATGGCATTGAAAGGCAGCGCCAGCGCGCCTGTGGTACGTGTGCCAACCAATGAACCG
GTCATTATCAAACGGATGCTGGATATTGGGTTTTATAATTTCCTGATCCCCTTTGTTGAAACGCAAGAGGAAG
CGGCTCGCGCCGTCGCCTCGACGCGTTACCCGCCGGAAGGCATTCGCGGCGTCTCCGTTTCTCACCGCGCGAA
TATGTTCGGTACGGTGCCTGATTACTTTGCGCAGTCGAACAAAAACATCACCATTATTGTTCAAATCGAAAGC
CAGTTGGGTGTCGACAATGTTGATGCCATCGCCGCAACGGAAGGCGTTGATGGCATTTTTGTCGGCCCAAGCG
ATTTGGCCGCCGCGCTGGGTCATCTCGGCAATGCTTCTCACCCTGACGTGCAGCAGACGATTCAACATATTTT
CGCCCGCGCTAAAGCGCACGGTAAACCGTGCGGCATTCTGGCGCCGGTAGAGGCGGATGCCCGCCGTTATCTG
GAATGGGGTGCGACCTTTGTCGCCGTTGGCAGCGATTTAGGCGCATTCCGCGCCAGCACGCAGAAGCTTGCCG
ACACCTTTAAAAAATAATCCTGGTATGGAAAGAGAGAACGCTATGACGATGAAAGTTGGCTTTATTGGCTTAG
GCATCATGGGTAAGCCCATGAGCAAAAACTTACTGAAAGCAGGGTATTCGCTGGTGGTGTCCGATCGTAATCC
TGAAGCGATTGCCGACGTCATTGCCGCAGGCGCGGAAACGGCCTCTACGGCGAAGGCGATCGCTGAACAGTGT
GACGTCATCATCACGATGCTGCCTAACTCTCCGCATGTGAAAGAGGTTGCGCTGGGCGAGAACGGTATCATCG
AAGGCGCGAAGCCGGGAACCGTATTGATTGATATGAGTTCTATCGCGCCGCTGGCAAGCCGTGAAATCAGCGA
TGCGCTGAAGGCGAAGGGCGTTGAGATGTTGGATGCGCCGGTCAGCGGCGGCGAGCCGAAGGCGATTGATGGC
ACGTTGTCGGTGATGGTGGGGGGCGATAAAGCCATTTTCGACAAGTACTACGACCTGATGAAAGCGATGGCGG
GTTCCGTGGTGCATACTGGCGATATCGGCGCAGGCAACGTCACAAAACTGGCTAACCAGGTGATCGTGGCGTT
GAATATCGCCGCCATGTCTGAAGCGCTGACGCTGGCGACTAAAGCGGGCGTCAACCCGGATCTGGTCTATCAG
GCGATTCGCGGCGGTCTGGCTGGCAGCACGGTACTGGATGCGAAAGCGCCGATGGTCATGGACAGAAACTTTA
AGCCAGGTTTCCGTATCGATCTGCACATTAAAGATCTGGCGAATGCGCTGGATACCTCCCACGGCGTTGGCGC
GCAGCTCCCGCTGACCGCCGCCGTTATGGAAATGATGCAGGCGTTACGCGCGGATGGACACGGTAATGACGAC
CATAGCGCGCTGGCGTGCTACTACGAAAAACTGGCGAAAGTGGAAGTCACTCGCTAACGACGTTGTGCCCGGA
TGGCGTGTAGCGATCCGGGCCTGAATTTCAGGCTGGATAAGTGAAACGCCATCCGGCGTTGTGCAGTAACAGG

CTACCACTATGAAAATCGTAATTGCCCCCGACTCCTACAAAGAAAGCCTTTCTGCCGCCGAGGTAGCGCAGGC
GATAGAAAAAGGATTTCGGGAAATTTTCCCCGATGCGCAATATGTCTCTGTTCCGGTTGCCGATGGCGGCGAA
GGAACGGTCGAAGCCATGATTGCCGCGACGCAGGGCGTTGAACGCACCGCATGGGTGACCGGGCCGTTGGGTG
AAAAAGTGAAAGCCTGCTGGGGAATGTCCGGGGATGGTAAAACCGCGTTTATTGAAATGGCGGCGGCCAGCGG
ACTGGCGCTGGTTCCACCAGAAAAACGCAACCCTTTGATTACTACCTCGCGGGGAACGGGGGAGCTGATATTG
CAGGCGCTGGAAAGCGGCGCAAGCAATATCATTATCGGCATTGGCGGCAGCGCGACAAATGACGGCGGCGCAG
GGATGATGCAGGCGTTAGGCGCAAAACTGCGCGATGCAAACGGCGCGGATATCGGTTACGGTGGCGGAAGCCT
TCATTGTCTAAGCGACATTGATATTTCAGAGCTCGATCCCCGTTTAAAACTCTGCGCGATTCGTGTCGCATGC
GATGTATCTAATCCTCTGATCGGCGACAACGGCGCATCACGAATTTTTGGGCCGCAGAAAGGCGCGACCGAAG
AGAACATTGTTGAGCTGGACAGAAATCTTGCTCACTATGCCGACATCATAAAAAAATCGCTGAATGTCGACGT
AAAGGCGGCGCCCGGCGCAGGCGCGGCAGGCGGTATGGGGGCGGCGTTAATGGCTTTTCTCGGTGCGGAATTA
CGCAGCGGGATCGAAATTGTCACGGCGGCACTGAACCTGGAGGAGCATATCCACGACTGTACGCTGGTGGTGA
CGGGAGAAGGGCGGATTGATAGCCAAAGTATTCGCGGTAAAGTGCCGATTGGCGTCGCGAATGTGGCGAAAAA
ATATCATAAGCCAGTGATTGGCATTGCCGGCAGCCTGACGCACGATGTAGGGATAGTGCATCATTATGGCATC
GACGCTGTCTTTAGCGTACTCACGCGCATCGTGACGCTGGAGGAGGCTTTTCGGGGCGCTTTTGACAATATTT
ATCGGGCATCCAGAAATGTTGCTGCCGCTCTGGCGATAGGAATGCGCAGCGCAGGGTGACAAAGTCGCGCGAA
CCCTCTATACTGCGCGCCGAAGCTGACCAGACAGTCGCCGCTTCGTCGTCGTCCTTTCGGGGAGACGGGCGGA
GGGGAGGAAAGTCCGGGCTCCATAGGGCAGGGTGCCAGGTAACGCCTGGGGGGAAACCTACGACCAGTGCAAC
AGAGAGCAAACCGCCGATGGCCCACGCAAGTGGGATCAGGTAAGGGTGAAAGGGTGCGGTAAGAGCGCACCGC
GCGGCTGGTAACAGTCCGTGGCACGGTAAACTCCACCCGGAGCAAGGCCAAATAGGGGTTCATAAGGTACGGC
CCGTACTGAACCCGGGTAGGCTGCTTGAGCCAGTGAGCGATTGCTGGCCTAGATGAATGACTGTCCACGACAG
AACCCGGCTTATCGGTCAGTTTCACCTCTTCATAAACCCGCTTCGGCGGGTTTTTGCTTTTACAGGGCGGCA
GGATGAATGACTGTCCACGACGCTATACCCAAAAGAAAGCGGCTTATCGGTCAGTTTCATCTCTTCATAAAAC
CCGCTTCGGCGGGTTTTTGCTTATCTGGCCGGTGAAGGGTATAGAGGTTGCAGAAGACGCCAGCGTCACTATC
CGGCAATTAATTGACAGCCGTGGTGACATAATATTATTCGTTTTTGTGATGAGAAACACAAAACGCTATGGAT
TGCGCTCTTTTTAGTTGAAGTGAAAAGCAATCGATTTCTTTCACAAAATTACATCCTGAAAAACAGCAAGAAA
GCGGTGTAAATTATTTGTTGTAAATGAAAATTAAATGTATGAAATATTTTGGTTAAAATTTTATTAAATGTTT
CTTTGCAAACAAATAAGGTAAAAAAATGTGACGCGTAACGGTTTTTAAGCGCATTTACCTTCGGTTTATACTT
AAATTTGAGAAATTACGCTTTAAATTTAACGCTGGAATTGCCGATAAAATGTCCCGTAATTGATTGAAATATC
GTCTTCATGGGATGTCGAAAATAGGAGATAAAATAGGCTGGTTATCGCCATAATATATCGTTATCCCGACCCT
ATTTTTTGATTGAAATCAGGCTAAGTTGCTGTTTTATTTTTGTAACTAATTGAAGTAATAGGTTTTCTTTATT
TAATAAAGACCATGTAAAAATTGCGGTAAGTTAATTTGTGAGTGGTCGCACATAACCTTTAAGATTTAATTTG
CTACACTTCCTGCCGTTCACAATGTTATTTCAGAGCGGTAGATATGAATACTCTTGTTCTCCCTAAAACACAG
CATTTGGTGGTCTTTCAGGAAGTCATTAGAAGTGGTTCTATCGGTTCTGCCGCAAAATCGTTAGGGTTAACCC
AACCCGCCGTCAGTAAAATCATTAGCGATGTTGAGGCCTATTTTGGCGTTGAGCTAATTGTACGCAAAAATAC
CGGCGTTACGTTGACCGAAGCGGGACAGGTACTGCTTTCCTGGTCTGAATCTATCACCCGCGAAATGAAAAAC
ATGATCAACGAGATGAACAGTATGACGTGCAATACCGTTGTCGACGTCTCGTTTGGTTTTCCTTCGCTGATTG
GCTTTACCTTCATGTCCGATATGATTCATAAGTTCAAAGAGGTATTTCCAAAAGCGCAGGTATCCATGTACGA
AGCCCAGCTCTCTTCATTCCTGCCGGCCCTGCGCGACGGGCGGCTGGACTTTGCTATCGGTACGCTCAGTAAT
GAAATGCAACTACAGGATCTGCATGTCGAGCCGCTGTTTGAATCAGAGTTTGTCCTGGTAGCCAGCAAGTCCC
GAACATGCACCGGCACCATCACACTGGAATCGTTAAAGGACGAACAGTGGGTGTTGCCGCAAACGAATATGGG
CTACTACAGCGAATTACTCACCACTCTACAACGAAATGGCATCAGCATCGAAAACATTGTTAAAACAGATTCC
GTTGTCACAATATATAATCTTGTTCTCAATGCTGATTTCTTAACTGTTATTCCCTGCGATATGACCACGCCTT
TTGGCTCTAATCAATTTATCACGATACCCATAAAAGACACGTTACCCGTCGCACGTTATGCGGCGGTATGGTC
TAAAAATTATCGTATCAAAAAAGCCGCTTCAGTATTAGTAGAATTGGCTAAACAATATTCATCATATAATGGA
TGCAGACGCAGGCAGTTAATAGAAATTGAATGATATGGAATAAACATATTTAGCGAAATTGACGTCACCTGTT
TCTTTTGAACAGTGGGCAACGGTAACTTATTTTTAATACCCAGGTTAAGAGGTTATAATGCACATTACATACG
ATCTCCCGGTTGCAATTGAAGATATCCTCGAAGCGAAAAAAAGACTGGCGGGGAAGATATATAAAACGGGAAT
GCCTCGCTCTAACTATTTTAGTGAACGTTGCAAAGGCGAAATATTTCTCAAGTTTGAAAATATGCAGCGTACG
GGTTCATTTAAAATTCGTGGCGCTTTTAACAAACTTAGCTCGTTAACGGAGGCGGAAAAACGTAAAGGCGTCG
TGGCCTGTTCCGCAGGTAATCATGCACAGGGCGTTTCTCTTTCTTGCGCAATGCTGGGTATTGACGGCAAAGT
CGTGATGCCGAAGGGCGCGCCAAAGTCAAAAGTGGCGGCCACCTGTGATTATTCGGCGGAAGTGGTGCTCCAC
GGTGACAACTTCAATGACACCATCGCTAAGGTGAGTGAAATTGTCGAAACCGAAGGCCGCATTTTTATTCCGC
CTTACGACGATCCAAAAGTCATTGCCGGACAGGGCACTATTGGTCTGGAAATTATGGAAGATCTGTACGATGT

CGATAATGTCATCGTGCCTATTGGCGGCGGCGGTTTAATTGCAGGTATTGCCATTGCGATTAAATCGATTAAC
CCGACCATTAAAGTTATCGGCGTACAGGCCGAAAATGTTCACGGTATGGCGGCGTCTTATTACGCTGGCGAAA
TAACCACGCACCGAACGACCGGCACCCTGGCGGATGGTTGTGATGTCTCCCGCCCGGGTAATTTAACCTATGA
AATCGTTCGTGAATTAGTCGATGACATTGTCCTGGTTAGCGAAGATGAAATTCGTAACAGCATGATCGCTTTA
ATTCAGCGCAATAAGGTTATTACTGAAGGCGCTGGCGCTCTGGCTTGCGCCGCACTATTAAGTGGGAAATTAG
ATAGCCATATTCAGAACAGAAAAACGGTCAGTATTATTTCTGGCGGCAATATCGATCTTTCTCGGGTATCACA
AATTACGGGTTTAGTTGACGCTTAATCTTTTCGTGAGGATAGGATATGAGTACTACTGATAGCATTGTATCCA
GCCAGGCAAAACAGTCGTCATGGCGTAAATCGGACACGACGTGGACGCTGGGTTTATTTGGTACCGCCATCGG
CGCCGGGGTGCTGTTTTTCCCTATCCGCGCCGGGTTTGGTGGGCTTATCCCCATTCTGCTGATGCTGGTTTTA
GCTTACCCCATTGCGTTCTATTGTCACCGGGCGCTGGCGCGTCTGTGTTTATCCGGATCTAATCCGTCCGGCA
ATATTACGGAAACGGTCGAAGAGCACTTTGGTAAAACGGGCGGGGTAGTTATCACGTTCCTTTACTTCTTTGC
GATTTGTCCGCTGCTGTGGATTTATGGCGTCACCATCACTAACACGTTCATGACGTTTTGGGAAAACCAGTTG
CAGATGCCAGCGTTGAATCGCGGTTTCGTGGCGCTGTTCTTGCTGCTGCTGATGGCCTTTGTGATCTGGTTTG
GTAAAGATCTGATGGTTAAGGTGATGAGTTACCTGGTATGGCCGTTTATCGCCAGTCTGGTGCTAATCTCATT
ATCGCTGATTCCTTACTGGAACTCGGCGGTTATCGATCAGGTTGATCTCAGTAATATCGCGCTGACTGGGCAC
GATGGCATACTGGTTACGGTGTGGCTGGGCATCTCTATCATGGTGTTCTCCTTTAACTTTTCGCCCATCGTCT
CTTCCTTCGTGGTGTCTAAGCGTGAAGAGTATGAGAAAGAGTTTGGCCGCGAATTTACCGAGCGTAAATGTTC
ACAGATCATCTCGCGCGCCAGTATGCTGATGGTCGCCGTGGTGATGTTCTTCGCCTTTAGCTGTCTGTTTACG
CTCTCCCCGCAGAACATGGCGGATGCGAAAGCGCAGAACATTCCGGTACTTTCCTACCTGGCTAACCATTTCG
CCTCTTTGTCGGGGACAAAATCTACTTTTGCAACGGTGCTGGAGTATGGCGCGTCTATCATCGCGCTGGTTGC
TATTTTCAAATCTTTCTTCGGTCACTACCTGGGGACGCTGGAAGGGCTGAACGGCCTGGTGCTGAAATTCGGT
TATAAAGGCGATAAAACCAAAGTCTCTATGGGCAAGCTTAACACCATCAGCATGATCTTCATTATGGGGTCTA
CCTGGGTCGTAGCTTACGCAAACCCGAACATTCTGGACCTGATTGAAGCGATGGGCGCGCCGATTATCGCCTC
GCTGTTATGCCTGCTGCCGATGTACGCCATTCGTAAAGCGCCGTCGCTGGCGAAGTACCGTGGTCGTCTGGAT
AACGTGTTCGTCACCCTGATTGGTCTGCTGACCATCCTGAATATTGTCTACAAATTGTTCTAATCCTCACGTT
TAGCCTGCGTTCCGCCCCAGGGCGTGGACGCAGGCTGACATCGACGGAGGCACTACATGAATGAATTTCCGGT
CGTTCTGGTCATTAATTGCGGATCATCTTCTATCAAATTCTCTGTACTGGATTTCGCTACCTGTGATGTTTTA
ATGGCGGGAATAGCAGATGGAATGAATACTGAAAACGCCTTTTTATCTATTAATGGCGATAAGCCGATAAACC
TCTCGCACTCTAATTATGAGGACGCGTTAAAAGCCATTGCCTTTGAGCTGGAAAAACGTGATTTAACGGATAG
CGTGGCATTAATTGGTCACCGTATCGCGCACGGTGGAGAGCTATTTACGCAGTCCGTCATTATTACCGATGAG
ATTATCGATAATATTCGTCGGGTATCACCTTTAGCGCCGCTGCATAATTACGCTAATTTAAGCGGCATTGACG
CGGCGCGGCACCTTTTCCCGGCCGTGCGTCAGGTGGCGGTATTTGATACCAGTTTCCACCAGACGCTGGCGCC
GGAAGCCTATTTGTACGGATTGCCGTGGGAATATTTTTCCAGCCTGGGTGTGCGTCGCTACGGCTTTCACGGT
ACGTCGCATCGCTATGTTTCGCGGCGCGCGTATGAACTGCTTGACCTCGATGAAAAAAACTCGGGTCTGATTG
TCGCGCATCTGGGCAATGGGGCGTCTATCTGCGCGGTACGCAATGGTCAGAGCGTGGATACCTCAATGGGGAT
GACGCCGCTTGAGGGGCTGATGATGGGCACTCGCAGTGGCGACGTTGATTTCGGCGCGATGGCGTGGATAGCC
AAAGAAACCGGGCAGACGTTGAGCGATCTGGAGCGAGTGGTGAATAAAGAGTCCGGTTTGCTCGGCATCTCCG
GGTTGTCATCCGACCTTAGAGTCCTGGAAAAAGCCTGGCACGAAGGACATGAGCGCGCCCGGTTAGCGATAAA
AACGTTTGTCCACCGTATCGCCCGTCATATTGCCGGACACGCGGCATCGTTACATCGGCTGGATGGCATTATT
TTTACCGGCGGTATTGGTGAAAATTCAGTATTAATTCGCCAGTTGGTCATTGAACATTTGGGCGTACTGGGGC
TTACGTTAGACGTTGAAATGAATAAGCAGCCGAATTCTCACGGCGAGCGCATTATTTCCGCTAACCCTTCTCA
GGTTATTTGCGCTGTTATTCCCACGAATGAAGAGAAAATGATTGCCCTTGATGCCATTCATTTAGGCAACGTG
GAAGCACCAGTGGAATTTGCATAACTTAACGGCTGTTCTGTAGAGAGATTATTTTTCATGAAGGTAAATATCG
ATACCAGCGATATGCTGTATGCCGAAGCATGGCGTGATTTTAAAGGTACGGACTGGAAAGAAGAAATTAATGT
CCGTGATTTTATTCAGCATAACTATACGCCCTATGAGGGGGATGAATCTTTTCTCGCCGACGCGACGCCAGCG
ACCACGGCGCTGTGGGAAAAGGTGATGGCGGGAATACGTATCGAGAACGCGACCCATGCGCCGGTGGATTTCG
ATACCAACATCGCGACGACGATTACCGCGCACGATGCGGGGTATATTGAAAAAGAGCTGGAAAAAATCGTCGG
TTTACAAACGGATAAGCCGCTTAAACGCGCTTTGCATCCGTTTGGCGGCGTTAACATGATCAAAAGTTCATTC
CACGCCTATGGCCGCGAAATGGACGCCGATTTTGAATATACCTTTACCGACCTGCGGAAAACCCATAATCAGG
GCGTATTCGATGTTTACTCGCCGGATATGCTGCGCTGCCGTAAATCCGGCGTATTAACCGGTTTGCCGGATGG
TTATGGTCGCGGGCGCATTATTGGCGATTATCGTCGCGTCGCGCTGTATGGCATCCGTTATCTGGTACGCGAA
CGCGAGCTGCAATTCGCCGACCTGCAGTCGAATCTGGAGCAGGGACAGAATCTTGAGGCGACGATTCGCCTGC
GTGAGGAGTTGGCGGAGCACCGTCGCGCGCTGCTGCAAATGCAGGAGATGGCGGCGAAATACGGCTATGATAT
CTCCCGCCCGGCGCGGAACGCGCAGGAAGCGGTACAGTGGCTCTACTTCGCCTACCTGGCAGCGGTAAAATCG

CAAAATGGCGGTGCAATGTCGCTGGGAAGAACCGCGTCGTTCCTTGATATCTACATTGAGCGCGATTTCAACG
CGGGCCTTCTGACAGAGCAGCAGGCGCAGGAGCTGATCGACCATTTCATCATGAAGATCCGCATGGTGCGCTT
CCTGCGCACGCCGGAATTTGATTCGCTCTTTTCCGGCGATCCGATCTGGGCCACCGAAGTTATTGGAGGGATG
GGGCTCGATGGCCGCACTCTGGTGACGAAAAACTCCTTCCGTTATCTGCATACCCTGCACACGATGGGACCAG
CGCCGGAGCCGAACCTGACCATACTGTGGTCAGAAGCGTTGCCGGTGGCATTCAAGAAATATGCTGCGCAGGT
CTCCATCGTGACCTCATCTTTGCAGTATGAAAACGACGATTTAATGCGTACCGACTTTAACAGCGACGATTAC
GCCATCGCTTGTTGCGTCAGCCCGATGGTCATCGGTAAGCAGATGCAGTTCTTCGGCGCGCGCGCCAACCTTG
CCAAAACGTTGCTGTACGCTATCAATGGCGGCGTTGATGAGAAACTGAAAATCCAGGTCGGGCCGAAAACCGC
GCCATTAATGGATGAGGTGCTGGATTACGACGCGGTAATGGAGAGCCTCGACCACTTTATGGACTGGCTGGCG
GTGCAGTACATTAGCGCGCTGAACATCATTCACTACATGCACGATAAGTACAGCTATGAAGCTTCGCTGATGG
CGCTGCACGACCGTGATGTGTATCGGACGATGGCGTGCGGCATCGCCGGACTGTCGGTGGCGGCGGATTCCCT
GTCCGCCATCAGGTACGCTCAGGTGAAGCCGATTCGCGACGAAAATGGCCTTGCCATCGACTTTGCTATTGAA
GGCGAGTATCCGCAGTACGGCAACAACGATGAGCGCGTGGACAGCATCGCCTGCGACCTGGTGAAACGCTTTA
TGCAAAAAATTAGCGTGTTGCCGACGTATCGCAATGCGGTGCCGACGCAGTCCATTTTGACCATCACCTCGAA
CGTCGTGTACGGGCAGAAGACCGGCAATACGCCGGATGGACGCCGCGCCGGAACGCCGTTCGCGCCGGGGGCG
AACCCGATGCATGGTCGTGACCGCAAAGGGGCAGTGGCCTCGCTGACGTCGGTGGCGAAACTGCCGTTTACCT
ACGCCAAAGACGGTATTTCGTACACCTTCTCGATTGTGCCTGCGGCGCTCGGGAAAGAAGATGCGGTGCGGAA
AACCAATCTTGTCGGCCTGCTGGACGGCTATTTCCATCATGAAGCACAGGTAGAAGGCGGGCAGCATCTGAAC
GTCAATGTCATGAACCGTGAAATGTTGCTGGATGCGATTGAACATCCGGAAAACTACCCGAACCTGACGATCC
GCGTTTCCGGCTATGCCGTGCGCTTCAATGCGCTGACGCGTGAGCAGCAACAGGACGTTATTTCGCGCACCTT
TACTCAGGCGATGTAACGTGAACGGGGCGATCGTGTGGATCGCCCCGCGACGGATCTTCACTGAACGGTAATC
TTAGGGGGAGATATGATTAGCGCATTTGATATTTTTAAGATTGGCATTGGACCTTCAAGCTCCCATACCGTAG
GGCCAATGAATGCAGGAAAATGTTTTATCGATCGGCTGATCGACAGCGGCGACCTGCCGCGAACGACGCGCAT
CACGGTTGATCTTTACGGATCGCTATCGCTGACGGGCAAAGGCCACGCGACCGATACCGCCATCATTATGGGC
CTGGCGGGCAATACGCCGCAGGATGTCAATATTGATAGCATTCCGGCGTTTATCCAGGAGGTTGCCCGCAGCA
GCCGTTTATCGGTGGCGGGTGGCGCGCATGTGGTGGATTTTCCCGTCGCCGACAGTATTCTCTTTCACGCTGA
AACCCTGGCGCGCCATGAAAACGGTATGCGTATTACCGCCTGGAACGGACAAGCGCCGCTTTTACACAAAACG
TATTACTCTATTGGCGGCGGCTTTATTGTCGAAGAGGAGCGTTTTGGTCAGTCGCACGACGTAGAAAAATCGG
TGCCGTATGATTTTCATTCCGCCAGCGAACTGTTGACGCTGTGTGAAAGACAGGGGCTCTCGGTTTCCGGCCT
GATGATGCAAAATGAACTGGCGTTGCGGAGCAAAGAGCAGATTGACGCTGGCTTTGCCCGCATCTGGCAGGTG
ATGGCGACCGGAATTGAACGTGGCATGAATACTGAGGGCGTGCTGCCGGGGCCGCTGAACGTTCCACGCCGCG
CGGTAGCGTTACGGCGTCTGCTGGTCTCCAGCGATAATCTCTCCCGCGATCCGATGAACGTCATTGACTGGAT
TAACATGTTCGCCCTGGCCGTGAGCGAAGAGAACGCGGCGGGCGGGCGAGTGGTCACCGCGCCGACCAACGGC
GCGTGCGGCATTATTCCCGCGGTGCTGGCCTATTACGACAAATTCCGTCGCCCGGTCAATGCCAACTCTATCG
CCCGCTATCTGCTGTCGTCGGGGGCGATTGGCATGCTGTACAAGATGAACGCCTCGATATCCGGCGCGGAAGT
GGGGTGTCAGGGTGAGGTTGGCGTCGCGTGTTCGATGGCGGCGGCGGGGCTGACAGAACTGCTGGGCGGCAGC
CCTGCGCAGGTCTGTATTGCGGCGGAAATTGCGATGGAGCATAACCTTGGCCTCACCTGTGACCCGGTGGCGG
GTCAGGTACAAATCCCTTGTATTGAACGTAATGCGATTAACGCCGTTAAAGCGGTGAATGCGGCGCGAATGGC
GCTACGCCGTACCTCTGAGCCGCGGGTTTCGCTCGATAAAGTGATCGAAACCATGTACGAAACGGGTAAAGAC
ATGAACGACAAATATCGCGAGACGTCGCGCGGCGGTCTGGCGATTAAAGTCGTTTGTGGATAGGCCCGACAGG
CGCAGCGTGAGCAGGCTATGGGATAACGCAGAAGACAGAAGCTGAGAAAAGGGCGCACCAACGTGCGCCCTTT
TTTGTTGTGATACGCGTCAAATAGAGAAAATAACAATATTAAATAAATAGTAAATAGAAAATAATTCTTAACC
CACAAGAAATGCAGGCGTTTTATTGGTAAGTTTTTCTCTTATCGTGCGCGTATGATTTTCTTATTACAAGAAA
GTACCTGCACCTCACGTTCTGCATATCGCCTTGCTGCGGCACGAAAAATAAAAAAACCTCTACCTACACATTG
AGCGAGAATTTTATGGAATCGGCTAGTAATACCAGCGTAATCCTCGACGCATCAGCCCCGGCGCGTCGGGCAG
GGATGACAGAAAGCGAATGGCGTGAAGCCATCAAATTTGATAGTACCGACACCGGTTGGGTCATTATGAGTAT
CGGGATGGCGATCGGCGCAGGGATCGTTTTTCTTCCTGTCCAGGTTGGCCTGATGGGACTTTGGGTCTTTTTA
CTCTCTTCTATTATTGGCTATCCGGCCATGTACCTGTTCCAGCGGCTGTTTATTAATACGCTGGCGGAATCCC
CGGAATGTAAAGACTACCCCAGCGTGATCAGCGGCTATCTGGGCAAAAACTGGGGTATTTTATTAGGCGCGCT
GTACTTTGTAATGCTCGTCATCTGGATGTTCGTCTATTCTACGGCAATTACTAACGACAGCGCCTCTTATCTG
CATACTTTCGGCGTAACGGAAGGATTACTTTCCGATAGTCCGTTTTATGGTCTGGTGCTGATCTGCATCCTGG
TGGCGATTTCCTCACGCGGTGAAAAATTACTGTTCAAAATCTCCACCGGCATGGTGCTGACCAAGCTACTGGT
AGTGGCGGCGCTGGGCGTCTCTATGGTGGGTATGTGGCATCTGTATAACATTGGCGCGCTGCCGCCGATGGCC
ATGCTGATTAAAAACGCCATTATTACCCTGCCATTTACGCTGACCTCGATTTTATTTATCCAGACGTTAAGCC

CAATGGTGATCTCTTACCGTTCACGCGAAAAATCTATTGAGGTCGCACGCCATAAAGCGCTGCGTGCCATGAA
TATTGCGTTTGGCATCCTGTTCGTTACCGTATTTTTCTATGCGGTTTCCTTCACCCTGGCGATGGGGCATGAC
GAAGCGGTGAAAGCCTACGAGCAGAATATTTCCGCCCTGGCGATCGCCGCGCAATTTATTAGCGGCGATGGCG
CAGGCTGGGTCAAAGTGGTTAGCGTAATTCTGAACATCTTTGCCGTCATGACCGCCTTCTTTGGCGTTTATTT
AGGATTCCGCGAAGCGACGCAAGGCATTGTGATGAATATCCTGCGCCGTAAAATGCCCGCCGAGAAGATTAAG
GAAAATCTGGTACAGCGCGGCATTATGATTTTCGCTATTTTGCTGGCCTGGAGCGCCATTGTGCTCAATGCGC
CGGTACTGAGCTTCACCTCTATTTGTAGCCCTATCTTCGGTATGGTGGGCTGTTTAATTCCGGCATGGTTGGT
CTACAAGGTTCCCGCATTGCACAAATATAAAGGCGCTTCGTTGTACCTGATTATTATTACCGGTCTGCTGCTA
TGCGTTTCTCCGTTCCTGGCATTCTCTTGATATACCTGAGATTAAGGTTGTTGTTATGTTTGAATCTAAAATA
AATCCATTGTGGCAAAGTTTTATCCTGGCCGTACAGGAAGAAGTAAAACCGGCGCTGGGGTGTACTGAGCCAA
TTTCACTGGCGCTGGCGGCAGCGGCGGCGGCGGCTGAACTTGACGGCACAGTTGAACGCATTGACGCGTGGGT
TTCGCCTAATCTGATGAAAAATGGTATGGGTGTTACCGTTCCAGGAACGGGAATGGTAGGGCTACCGATCGCC
GCGGCGTTGGGCGCGTTGGGCGGTGATGCGAAAGCCGGGTTGGAGGTGTTAAAAGACGCTTCCGCTAAAGCCG
TTGCCGATGCAAAAGCGATGCTGGCCGCTGGACATGTCGCGGTGATGTTGCAGGAGCCATGTAACGATATCCT
GTTTTCACGGGCGAAAGTGTATAGCGGCGATAGCTGGGCATGTGTCACGATCGTCGGCGATCATACAAATATT
GTGCGGATAGAAACCAACAAGGGTGTGGTATTTACCCAGGCCGATAATGCGCAGGAAGAAGAAAAAAACTCGC
CACTGGGAGTGCTGTCTCATACCTCGCTGGAAGAGATCCTGGCGTTTGTGAATGCGGTGCCCTTTGACGCTAT
TCGCTTTATTCTCGATGCGGCCAGGCTAAACGGCGCGTTGTCGCAGGAGGGACTGCGTGGTTCCTGGGGGCTG
CATATCGGTTCGACGCTGGCCAAACAGTGCGATCGCGGTCTGCTGGCGAAAGATCTCTCCACGGCGATTTTGA
TCCGTACCAGCGCGGCGTCAGACGCCAGAATGGGCGGCGCCACGTTGCCCGCGATGAGCAACTCCGGTTCTGG
CAACCAGGGGATTACCGCCACGGTGCCAGTCATGGTGGTTGCTGAACATGTCGGCGCCGATGACGAGCGCCTG
GCGCGCGCGCTAATGTTATCGCATTTGAGCGCTATCTATATTCACCATCAGCTTCCGCGTTTGTCGGCTCTGT
GCGCGGCAACCACGGCGGCGATGGGCGCGGCGGCGGGTATGGCGTGGCTGATAGATGGTCGTTACGACACTAT
CGCAATGGCTATCAGCAGTATGATTGGTGATGTCAGCGGGATGATTTGCGATGGCGCGTCGAATAGCTGCGCG
ATGAAAGTCTCTACCAGCGCGTCGGCGGCGTGGAAAGCCGTACTGATGGCGTTGGACGATACGGCGGTGACCG
GAAACGAAGGGATTGTGGCGCACAATGTCGAACAATCTATTGCGAATTTGTGTTCGCTGGCGTGTCGCTCAAT
GCAGCAGACAGACAAGCAGATCATCGAGATTATGGCCAGTAAAGCGCATTAATAATAACGCCCCGGCATGACG
TCGGGGCAGCGTGTTGCCCGATGGCGCTGCGCTTATCGGGTCTACCCGTTATCAGCCGGATCGGGCGCTTGCG
CCGCCATCCGGCATGATTTCAGGCTGCGGCCTCTTCTTCTGCCAGCCGGGCTTCGGCGTCCGCCACGATGGCT
TCCAGTTCGGTTAGCATCTCTTCATAGCCCAGCGGCACAGTGGCGCTCATCGTCTCCTGTACGGCAGGTTTTA
CCACGGGCTGACGTTTCTTCGCCGATTTTTTGCTCATCTTGCGACTCCTTTTCTGGCTGGCTACACCGGTAAA
TCTACCAGTAATGCGCGCAGCGGCGTATCGGCAACCAGCGTAATGTTAGGTTCATCACGAATAAATGCGCCAT
CTCCGCAGGTTAACGCCTCGCGTTCTTCGTTGTGCGTCATCGCGTGGAACGTACCGTGAATCGACTGCAAATA
GGCGCGCGGCCCGTGAAGCTGGAAGTTAAGCGATTCGCCTTTCTCCAGCGTAATATGATGTACCCAGACCTGT
TGGCGCAGTTGTAAGCTGTTTTGCTCACCGTCGGGCGAGGCAAGAAGCTGTTGTTGCGCCGTCGAGAGTGGGA
TTTTTTGTACCAGCGCGTTTTCCCGTTCAGGACAGGCGTCAAGCCACAGTTGCATTCGGGTTAACGGCTTAAC
TTTGCTGAGATTATGTTCGCTGTAGCTAATGCCCGGCTGCGCGGCGAGCAGCAACGCTTCGCCCGCTTTAGCC
TGTACATGGTTGCCGTCGCTGTCGCGGTATTCCGCTTCGCCGTCCAGAATCAGGTTTAAAATATCCACCTTTG
GATAAGTTCGAGGCTGAAAAGAGGCGCCCGGCGCCAGCACTTCCTGGTTCAGTACGCGCAACGACGCGTAGCC
CAGCAGTGTAGGGTCGAAATAGTGTCCAAAGGAGAAGGTATAGCGGGCCTGTAGCCATCCGTAGTCTGCCTGT
CCACATTGTTTGGCGGTTCGGGTCGTAATCATCGTTATTAACCTCTCTTTACTTCCTTTTATGTTAAGGGGCT
GGACGCTGCATTGTTAGCCAGATATTCTGCCCGGTATGTTCAAATTTCCTGAATGAGAACGTTATGGCTAAAG
AGAGAGCGCTGACGCTTGAAGCATTGCGTGTGATGGACGCCATTGACCGCCGGGGCAGTTTTGCCGCAGCGGC
TGATGAGTTGGGGCGGGTGCCCTCCGCGCTGAGTTACACCATGCAGAAGCTGGAAGAAGAGTTGGACGTTGTA
CTGTTCGATCGCTCCGGCCATCGTACAAAATTCACCAACGTCGGACGTATGCTGCTGGAACGCGGGCGGGTAT
TGCTGGAAGCGGCAGACAAGTTAACGACGGATGCTGAAGCGTTGGCCCGCGGTTGGGAAACGCATCTGACGCT
GGTCACTGAAGCTCTGGTACCGACGCCTGCTTTTTTTCCGCTGATCGACCGGCTGGCCGCTAAAGCCAATACG
CAGCTTTCATTGATTACCGAAGTACTGGCTGGCGCATGGGAGCGGCTTGAGCAGGGACGGGCGGATATCGTTA
TCGCCCCGGATATGCATTTTCGATCGTCGTCGGAAATTAACTCCCGTAAGTTGTATTCGTTAATGAATGTGTA
TGTCGCCGCGCCTGACCACCCTATTCATCAGGAGCCGGAGCCGCTTTCGGAAGTGACGCGGGTTAAATACCGC
GGCGTAGCGGTAGCGGATACCGCGCGCGAGCGGCCGGTATTAACGGTGCAACTGCTGGATAAGCAGCCACGTC
TGACGGTAAGCACGATTGAAGATAAACGTCAGGCGCTGCTGGCCGGGCTTGGCGTCGCGACTATGCCATATTC
GATGGTGGAACAGGATATTGCCGAAGGGCGGCTACGCGTGGTCAGCCCGGAATCGACCAGCGAAATCGATATT
ATTATGGCCTGGCGGCGCGACAGTATGGGGGAGGCAAAAGCCTGGTGCCTGCGGGAAATACCTAAGCTATTCG

CCGGAAAATAAATAGCGCGCGGCGCTTTAAAAGCGGCGCCGCGCCGCCATCAGGAAAAACGTTTCGGATCGGG
GCCGAAGCGGTTGTCGCCCGGCGTACCATTCTGGCAGTTGAACGCGATGATGATAAGCCAGCCGATAATGGGG
ATAAGTAGCAACAGTAGCCACCATGCGGAGCGGTCAGTGTCATGCAGACGACGGAACTGCACGGCCCACCACG
GTAAAAAGATCAGAACGCCGTAAATGGTGGTCAGAACGCCTTCACCCCCGGCGCGTTGCCAGCCAAGCATGGC
GTCCAGCAGGCCCAACACAAAGGTAAAGATGATATTGACCAGAATAAACATCCAGTACTCTTTGCGTCGCGCG
CGGCCGCCAAATCCCAGATAATTTTTTAAAACCTTTAAATACCAATCCATTTTCGCCTCAATAATGGTTCAAT
CACTTGTTTTTAAAGTAATCAAACTAAGAATAGCCGCGAATGAAATGTTGGTAAATATTTGTGGTATTTATTA
ATAAGGCGTCCCGGAGGACGCCTGAAATTAGCCAAAGCGTACGTCGCGCCCGTGAGGTTCGAGCAGATCCTGC
CACGGTCCGATGGAAATGATACCCGTGGGGTTAATCGTTTTATGGCTACGGAAATAGTGGTGACGAATATGGT
CAAGATTAACGGTTTCAGCAATGCCCGGTATTTGATAGATATCGCGCAGGAAACCGTACAGATTCAGGTAGTC
GCTGATTCGGTATTTGTCGCATTTGAAATGAGTCACGTAAACCGGATCAAAGCGTACCAGGGTCGTCCATAAG
CGGATATCCGCCTCGGTCAACTGGTCGCCCGTCAGATAGCGGTGCTGTCCGAGAATCTGCTCCAGGCGCGCCA
GCGCAGTAAACACCTTTTCTACCGCTTCATCATACGCTTGCTGGCTGGTGGCGAAGCCTGCTTTATAAACGCC
GTTATTAACGTTGTCATAAATCCAGCCGTTTAACTCGTCAATTTTGCTTTGCAGCGCGGGGGGATAGTAATCC
CCGGCTTTAGCGCCCAGCCCATCGAAGGCGGAGTTAAACATACGAATGATTTCGGCGGACTCGTTGCTGACGA
TAGTATGATTTTTTTATCCCATAAGACCGGTACGGTGACGCGGCCGCTGTAGTGTGGATCGGCGTGCAGATAC
AACTGGTAGAGAAATTCATGCTGATAGAGGGTATCGCCGGTGGCCGCCGGAAAAGTATCATCAAACGTCCAGC
CGTTTTCCAGCATCAATGGATTCACGACGGAAACCGGAATAAACGGCTCCAGACCTTTCAGTTTACGGAAAAT
GAGCGTGCGATGCGCCCAGGGGCAGGCAAGGGAAACGTACAGATGGTAACGATCTTTTTCTGCGGCGAATCCG
CCTTCGCCGGAAGGTCCGGGCGCGCCGTCAGCGGTAAGCCAGTTGCGGAAAGCCGACGCTGAACGCTGAAATT
TCCCCCCGGAAGATTTGGTGTCATACCAGGTGTCGTGCCAGACGCCATCGATCAGTTGTCCCATGTCTCTCTC
CTTCAGATGACAAAAGCGAGGCGCTATTCCCTCGCTTTTTTAATTGAGTATAGATTGCTTACCACTTTTTATT
CAGCAGACGGTCAAGGCTGAACGCGCCAGGCCCGGTTAACGCCAGTAATAAGAATCCGCCGGCAATGGTCAGG
TTTTTCATAAACATCAGCGAGTTTACACCTTCTGCAAAATTGCTATGGAAGATCAATGCCGTCAGCAACGTAA
AACCAGCGGTAAAAAGCGCAGTGGTGCGGGTGAGGAATCCCAGCAGAATCGCCAGACCGCCGCCGAACTCAAG
CAGTATGGTGAGCGGCAGCAGGAATCCTGGTACGCCCATCGCTTCCATATACTGCTGTGTGCCCGCATAGCCG
CTAATTTTTCCCCATCCTGCGGTAATAAACAGAACTGGCATTAAAATACGCGCTATCAGTACACCAACATCTT
CTAATTTTTTCATTTTATTCTCCAGGGGGCCGACTCGGCGGCTGCTGCTATTTTCATGAGCAATGACGCGGTT
TGGCGCGTCATTGTTATCGCTGGAGAGAATCATAAACGTGGCATACAGAAATTGTTAGCAAGGAAAATTGCCA
AATTTTTTCAAAAAATTTGAGTGATTGGTAAAGAGACTATATGCATAAAAGTATCGCGCCACCAGGTGTAATC
GGGTGATAACCTGATGGCGCGATGCTATAAAAGAAAAGATATTAACCGCGGAGCTGTTGTTGACGGAGGGTAG
TTTTTACCAGGCGCCAGGCGCTCCAGATACCCAGGCCGCGTTTCGCCCAGCGTACCAACATATTAGGATGACG
GATGGTCCAGATGGCCATTACGCTGCTACCGACCAGCGCCCAGGAGCGCAGGCTTAACACCGTATTCCAGCCG
CGATCGTACGCGCCGGTGGTCTCCAGCCAGTCGCGACGGCTGGCGGACAGATCCAGCCGTTGCTGCTGGATCT
GACTGAGAAGCAGCGCTTTACGTTTTTCGCGTTCGCCTTTGCTGCTCACGACTGATCGTCCTCCAGAATTTGC
CGATCGTTAGCCAGTTCGTGCCGCGTATGGCGCAATAGCGTGGACTGACGCGCTTTGCGCAAAGTCCAGATGC
CGCCGATTAGCGCAAGCACTAACAGCACAACAGTGGTAGCGATCATCGCGTTCAAACGGTACTGAGGATCGAT
AGCCCAGATGACCAGAACCATCAGGCTCATTAAGCCAAACGCCGCGAAGAGCATGGTAAGACCTACCATCAGC
AAAAGCTGAAAGAGGTTCGCCTTCTCTTCTTCCAGTTCTACTACCGCCAGTCGCAGCCGGGTTTCCACCATCT
CAACGATAATGGTGACAATCCGCTGCCCGATACCCAGTACGCTTTTGCCTGGTCCTTGTGCCTGTCGAGAGTC
CGCCATAATTAACGACGCGTCAGCAGTACGCCCAACACCAGACCGACAGCGGCGCCAATGCCTACGCCTGTCC
AGGGATTTTCGCGCACATAATCATCGGCGCGGGCGGCGGCGACGCGAGTCTGTTTGGCAATCACATCACCCGT
TTCGCCCAGGCGGTAACGGCTCTCTTTCAGCGCGCGCTCGGCTTTGCTACGAATCTTACTCAACTCTTCTTTC
GACTTTTCGCCCGAAGAGCTCAGCACTTCTTCAAGCGTATCGGTCAGGGATTTCAACTCAGCGCGCAGATGTT
CCGTAGTGTTATCTTTCGACATAGTTTTCTCCAGGTGAGTGAGTATTTACATTCGGCGTTAATTTAGTAGTCT
CTGGCCTCAAGTTTTTTTAGCTCATCCTGAGCCTCGGCCAGCTTACGTTCACGTTTGTCTATTTTATCCGCAT
CGCCTTTTGCTTTTGCCTCGGCTAAATCACGTTGGCGTTCGGCTACCTCTTCTTTTTGCTCAGCGATCTTTTT
CTGATGCTCGGCGCGCAGCTTGCTGTCAGTACAATTGGCGCGTACTTCGCTGAGCGCCTTGTTCAGACCCTCA
ATACGGCGCTGATTGTTGTGCTTTTCAGCGTAGCTAATTTCCTTTTGTATATCCTGCTCTTTTTCCTGACAAA
GCGAATTAGCGTAGCTACCGGCGCTAAGTGTAAAAAGCGTGATAGCCAAAGCGATGCGGTATTTCATTCCTGA
ACTTTCCCTTATGTTCGGACCCGTTGTCCGGGGCGGTGATCCCGATAATAACGCGGTGGGTTAAACACCGCTT
TATTAATCATAGTCATTAAACAATGAAATCACCAAAGTGAGTGATATGATTCGGATTCCTGGAGATTAGCCAA
AGCGGTGTGAGCTTTCCCGTAAGCGGGACAGCCACTGTACCGTTTCGTTGTCATCGTCCTGCTGAGCGACGAC
ATAACCGTGTGGTAACGTTTGATCAAGCACTGTTTTTGCTGCGGCGCTTTGCGCAGGGGAATCGAAGGTAATG

AGCAACATGTCATTTTTAGGCGTAATGCTTTTAAAATGAATGCCGTTCGCATCCAGATGATGCAGAACTGAGA
AGCCGTCTGGCATACTGGCGCCCTGGTTAACCGCGCGAATGGCCAGCGTCGACTCTTGCTGACGCAGCGTTGA
CCACGTCATCATGAGTATGGTCAGCATCAGTAGAAAAGCGCTGATCCAAATGAGTTGTCGGGCAGTTATGCGT
GGTTTAAGCATAAGTTATCCCCGGCTCCCATACTTCTTTTTCCACAGCATGACCAGTGAACCCGCCAGGCCAA
AAAAGAGCAGCACCACGGGCAGCAGCATCAGGCATGACATTAACTGATCTTCATATTTCATAAAGACGGGCGT
TTTGCCCAGCAGATAGCCCAGCGACGTCAGGATAAGCACCCACAACAAGCCGCTCATCCAGTTGAAGAACTGA
AAGCGCGCGTTATTCAGGCCGGATATGCCGGCAATGGTGGGCAGCAGTGTTCTGACAAACGCGATAAAACGGC
CTATCAATAATGCCGACAGGCCGTGTTTATGGAAAAGGTGATGGGCGCGTTGATGATAATGTGCGGGGAGATG
AGAGAGCCAGTTTTGCACGGTGCGGGTATTCCCCAGCCAGCGGCCCTGAATATAGCTCACCCAGCAACCCAGG
CTGGCCGCGACGGTAAGCAGCAAAATGGTTTGTGGAAAACCCATCGCGCCTTTGGCGATCAGCACGCCGACCA
GGATCAACAGGCTGTCGCCAGGCAAAAAGGCGGCAGGGAGCAGCCCATTTTCCAGAAACAAAATCATAAATAA
CACAAAATATAACATGCCAATCATGGAAGGATTGGCCAGTGTTTCAAAGTCCTGAGCCCACAGGGCATTCAGC
AATTGAGTCAGTAGTTCCATTCAGTATTCCTGGAGAATCGGTTAACGTCACGCCGGTGATCTGAGGTTTGCAC
TGCCGCAACAGAGTTGGAGTTTTTATCAGCGTTGCGGAAACGGCAAATTCGCGGCCTGAACTATTCCTTGTTA
AATTGTGAAGTAAGCAAGCACTAACACACAAAAATTCAAAATGCATCTAATTGTAACAAAGGTCAACGAAGTC
CGTTATAGAAATTACGCGTCGTTGAGCTTTGATTTTGCTTATCGCAAGGGAGGGTGGCGAGAGGATTTACACA
GGCTGACACTTTATACTTTACGCTGACCGAGCGCCGTGGCAGACGCCATCTACATATAACGTTACGCGAGGAT
GTTGCGCGTGAACATCAATTCGCTTTCCATCACAGCCTATGGATTGGGAGATTCAGATCTATCGTTACGCCGG
ATGGCATACGCCATCCGGCATCGATAATCACTTAGCTACGCAAGGCGTTATTCGCCAGACGTTCGTCCTCTGC
CTGGCAGGCTGCCGCAGTGAACAGAACATCGGTAGACGAGTTCAGCGCCGTTTCACACGAATCCTGCAATACG
CCGATAATAAAGCCGACCGCTACTACCTGCATCGCGATATCGTTCGGGATGCCAAACATGTTGCACGCCAGCG
GGATCAGCAGCAATGAACCGCCTGCCACGCCGGAGGCGCCGCAAGCACACAGCGAGGCCACCACGCTCAGCAG
CAGCGCTGTAGGCAGATCCACCGGGACGCCTAACGTATGGACTGCCGCCAACGTCAGAACCGTAATGGTAATT
GCCGCGCCGGCCATATTGATAGTGGCGCCCAGCGGAATGGAAACGGAGTAAGTATCCCTGTCCAGATTCAGCT
TTTCGCACAGCGCCATATTCACTGGAATATTGGCGGCAGAGCTGCGGGTAAAGAAGGCATAAACGCCGCTTTC
ACGCAGGCAGGCGAACACCAGCGGATAAGGGTTGCGACGAATTTTCCAGAATACCAACAGCGGGTTGACCACC
AGCGCGACCAGCAGCATACAGCCAATCAAGACGACCAGCAGGTGCGCATAACCCCACAGGGTAGAGAAGCCGG
TGGTTGCAAGCGTTGATGAAACCAGACCAAAAATACCGACTGGCGCGAAACGGATCACCAGTTTCACCATAAA
GGTCACGGCGTTAGACATATCGTTCACGAGGTTTTTGGTGGTTTCATTGCCATGACGCAGGGCAAATCCCAGA
CCGACGGCCCACACCAGAATCCCGATATAGTTAGCGTTCAGCAGCGCGTCGATGGGGTTCGACACCATGCTCA
TCAGCAGACCGCGCAAGACTTCTACAATGCCGGACGGCGGCACGATATCCTGGGCGCTACTGGACAGATGCAG
CGTTGAAGGGAAGGCGAAGCTGAACACAACGGCCGCCAGCGCGGCTGAAAAGGTGCCTAGCAGGTAAAGAAAG
AGAATAGGGCGAATATTGGTTTTTTGGCCGTGCTGGTGGTTGGCGATCGAGGCCATTACCAGCATCAGCACCA
GAACGGGGGCGACCGCTTTTAAGGCGCCGACGAACAGGGTGCCAAGCAAGCCAACCGCTTCTGCCGCAGGCTT
AGAAATCCATGCCAGTAAAATCCCCAGTACCAGACCCACTAAAATTTGTTTAACCAGACTGCCTTGCGCTAAA
CGCTGCAGTAGCCCTGATGCTCGTTGCGTAGCCATTTCATGTTCCTTGTATTGTGTTGCTGGTCATCCCCTGC
ACGCTCTTACGGCGCATCTTTCAGGATGTAACAAAGTGTTTGCCTGGTTGAGTATAAGGAAAGTTGAAGGTTC
GGGAAGCGAAATATCCTGGATTTTTTGCTGTGATCATGTTTTATAACTTTATATTAACAGTATTGTGACATGT
TAAACAAAAACGCCCGGCAGGTGTGCGCTGCCGGGCGTTTTTGATGGGGCAGGTTTACCCCGATAAAAGATTA
TTGCGCTCGTAGTTTCTTATCGCGCTGATGGTTAACCCAGGCGTTAATCACCAGCGTAATGGTCAGAATGCCA
AACACTACGCCCAGCGAAATCGCGATAGGAATATGGTAGAAGTCGACGATCAGCATCTTAATGCCGATAAAGA
CCAGAATGACCGCCAGACCATATTTCAGCATCGAGAAGCGTTCCGCCACGCCGGACAGCAGGAAGTACATCGC
GCGCAGTCCTAAGATGGCAAACAGGTTAGAGGTCAGTACGATGAACGGGTCGGTGGTGACCGCAAAGATCGCC
GGAATACTGTCAACGGCGAAGATCACATCGCTGAACTCAACCATAATCAGCACCAGCAGCAGCGGCGTGGCAT
ACAGCAGGCCGTTTTTACGTACGAAAAAGTGCTCGTTCTCGATCGTATCGGTCATGCGCAGATGCCCGCGCAG
CCAACGCACCATCGGTTTTTCGCCAATGCCGGATTCATCTTCCTTCGCCAGCGCCATTTTTACGCCGGTAAAC
AGCAGGAAGGCGCCGAACACATACAGCAGCCATTCGAACTGAGTGATAAGCCAGGTACCGGCGAAAATCATGA
TAGTACGCAACACGATCGCGCCAAGAACCCCATACACCAGCACCCGGCGTTGCAGAGCCGGAGGGACGGAGAA
ATAGCTAAACAGCATCAGCCAGACAAAGACGTTATCTACGGCCAGCGACTTCTCAATCAGATAGCCGGTAAGG
AACGCCAATGCCTGCGGGTCAGCAACTTCGCGCCCTTGCGTTTCAGCCAGATACCACCAGAAAGCGGCGTTAA
ACAGTAGCGAGAGCGTTACCCACAGGATTGACCATCCTGCCGCTTGTTTCATCGACATCGCATGTGCGCCACG
GCGTCCCTGCAACAACAGGTCGATGGATAGCATTATCACCACAACAACCGCGAATCCACCCCATAATAATGGC
GTACCAACAGTATTCATTATTTCTCCCTATGCAAAAAAAAACGGCTAACGCCGAATGGCATTAGCCGTTGCTT
TTATGCATAGACCTTGCCATTCGGCAAGGTCTCACTTACAACAATAAAGGAATACGCTGACGCATTCCTTTAT

GGCTGCCCGGTGACCGGATGTGGTTTTGCACACATCGTAATGACGATCGACCGGCAATGAAGTTACTCCCCTT
TGCGCGTAACAAAGTATGACAGGCCACGCGTAGAGTCAATGGCCTGTAACATATGATTTACATAATTTTACGC
AGTAAGGGGATGAGTCAGATCGTCCGCCGGGAAGATGACCCCGGTTTGACGGCGTATTTCTGTCAGCCATTTT
GCCGTGGCGCGACTTAGCGACAACCCCGGATGTTCCACGGCATGGTTTTCAATAAGCTGGGCAAACGCCCCGG
CTTCATACAGCATGGTATTAATATGCTGAGGCTGGGTCAGATCCTGCATCAGCGCGCCGCGCGGCGCAAAACA
CACTTTCTGGCATTCCGACAGCTTTTCAATGACCAGCGATCCCCTCTCGCCCTGAATTTCACTGGCCAGTACC
GAGTCGCTGACTTTGGAATGCTGTAGCGTGACGCTAAAATCGCCGTAATCCATAACCACGACGCCGTGTGCAT
CTACTCCGCTTTCCAATAAATTCGCCGACGCCTGAACGCTGCGCGGCTCGCCCCATAGCGCGATCGCCGACGC
CAGACAGTAATACCCGATATCCATAATCGAACCATTGGAGAAGGCCGGATTAAACGTATTCGGATTTTCACCG
TTCAGATAGCGCTGATAACGTGAAGAGTACTGGCAGTAATTTAGGAATGCTTTGTGCATTCTGCCGATTTTCG
GCAACGATTCCCGCAGCAGCAGGAAGTTGGGCAGGCAGGCGGTTTTAAAGGCTTCAAACAGTACCCGCTGATT
ATCGCGCGCGCAGGCGATAGCGGCATCAACCTCCGCCAGGTTAGAAGCCAGCGGCTTTTCGCACATGACATGT
TTTTTATGCTGCAAAAAGCGCTGCGTCTGGGAGAAATGCAGGGAATTCGGGCTGGCGATATAAACCGCCTCAA
TCGCGTCGCTTTGCGCCATCGCTTCCAGCGAGGTAAACAGATGCTCGACGGGATAATCATTTGCGAAAGACTG
CGCCTGTTCAAGACTACGGGAGTAAACGGCGGCGAGCCTGAATTTGCCGGTTTCATGGGCGGCGTCGACAAAC
TGGCGCGTGATCCAGTTAGTCCCAATTACAGCGAAACGTATCATAAACGTCAGCGGACTCCAGAAAGGGATTT
TTCGTCAATGTAGCACGCCTTTCCGCCCTTGCCAGTGCGCTGCGACGCACTCTGGCATTTTACTGGCGAGAAG
GGCACCCGTGTGCGCTATCAGCCCCAATAGTGACGCCAGCGCCCAGATATTTAGGCTGACGGTGCAAAATTTT
GGCATCCAGCACCGCGCTAACCCGCGCCAGCTTTTCCCGAAGTCGTGTGTAGGTTCCGTGCCTTTTATTCAGA
TCCGGCGCGTTAACGCCGATAGCGTCTATGCCATATTGTTGCGCCAGCCAGATCGCCCGTTGATTATGGAATG
CTTGCGAGATAATCGTAATGCGGCTTTCGCCAAACACTTTCCGGGCGCGAACTACGGAGTCCAGCGTGGAGAA
TCCGGCATAATCACAAAAAATGGCCGCTTCCGGCACACCTTTCGCTATCAGCGCCTGCTGCATCGCCGAAGGT
TCATCGTACTCTTTTTTGCCATTGTCGCCGCTGACTAACAGCCACTTCACTTTACCGGCGTGATACAACGCGG
CGGCAGTATTAATCCGCCGGGTAAAATACCGATTGCCGGGTTTAGCGCCTAACACCAGCCCGACGTTGCGGGC
GGGAACGGTCTGAATGCCATTCCATGTCAGATGCTGTGAGGCGTTGACGATAAGCCAGTTGGAATAGAAAAGC
GCGACGCCTGCTGCGACAAGCAGAAAACACGCCACTGTTAACGCCCGGCGTGTAAAACAGAGGCGGCGGATAA
AACGGGCACATTGTGAGAGCATGGTTGTTGCGCGGTTCCGTTGGCGGTGGATAAAAAATACCCTCTACAGATC
GTCATCCTTGACGTGGCGGCAGGCAATGAGCGAAATGTGAACGCGCCGATTTTATGCCCACGCCGTTAAAAAG
ATACGGGGGAAGGGGCATTTTCAGAATTTATTGTGCCGACAGCGCCTGATGCGTCAGCCACAGTCGCGTATCA
AACTCCAGTTGATGGTACTGCGGTTCCATATGGCAGCAGAGCTGATAAAACGCTTTGTTATGATCTTTCTCTT
TCAGGTGTGCCAGTTCGTGAACCACAATCATCCGTAAGAATGGCTCCGGGGCGTTGCGAAACACGGTGGCGAC
GCGGATCTCCGCCTTTGCCTTGAGCTTGCCGCCCTGAACGCGCGAGACAGCGGTATGCAGTCCCAGCGCATTT
TTCAGCACATGGATCTTGCTGTCATACATCACTTTATTGATGGGCGGCGCATTGCGCAGAAACTGACTTTTCA
GTTCCTGAGTATAGTGATATAGCGCTTTGTCAGTAGCGTAATCATGCGCGCCGGGATAACGTTTTTCCAGCAC
TGCTCCCAGGCGCTGCTCGGCGATTAAGGCGCGCACCTGGGCAAGGAGATGTTCCGGATACCCCTGAAGGTAA
GTTAGTGAGGTCATCAAAGCTCCATGTTGCTGATAAAAAGTGTATACTCACGCACCCTTTTCAGGGATAACGC
CGCAATTTTACCATTCAGGAGGCCTAATGAGCCACGTAGACGACGGTTTCCGTTCACTGACGCTGAAACGTTT
TCCGCAAACGGATGATGTTAACCCGCTTCTGGCGTGGGAGGCGGCCGATGAGTATCTGCTGCAACAGTTGGAT
GAGACGGAAATTCGCGGCCCGGTGCTGATCCTGAATGATACGTTTGGCGCGCTAAGCTGTGCGCTGGCGGAGC
ATTCGCCATACAGTATTGGCGATTCGTATTTAAGCGAACTGGGTACGCGAGAGAATTTACGCCATAACGGCAT
CGCGGAGTCCAGCGTAACGTTTCTCGACAGTACCGCTGACTACCCGCAGGCACCGGGCGTGGTATTGATTAAA
GTACCGAAAACACTGGCGTTGCTGGAACAGCAGTTACGCGCTTTGCGTAAGGTCGTTACGGCGCAAACGCGTA
TTATCGCCGGCGCGAAAGCGCGTGATATTCATACCTCTACGCTGGAACTGTTTGAAAAAGTGCTGGGCCCAAC
CACGACGACGCTGGCGTGGAAGAAAGCGCGCCTGATTAACTGTACCTTCAGTCATCCGCAACTGGCCGACGCG
CCGCAAACGCTAAGCTGGAAACTGGAAGATACAGGCTGGACGATTCACAACCACGCGAATGTGTTTTCTCGTA
CCGGATTGGATATCGGCGCGCGCTTCTTTATGCAGCATTTGCCGGAAAACCTCGACGGGGAGATTGTCGATCT
CGGCTGCGGCAATGGGGTGATTGGCCTGAGCTTACTGGCGAAAAATCCGCAGGCGAACGTAGTATTTGTCGAT
GAATCGCCGATGGCGGTAGATTCCAGCCGTTTGAATGTAGAAACGAACCTGCCGGAAGCATTCGAACGCTGCG
AATTTATGATCAACAACGCGCTCTCCGGCGTGGAGCCGTTCCGTTTTAACGCTGTGTTCTGCAACCCGCCGTT
CCACCAGAAACATGCGCTGACGGATAATATCGCCTGGGAGATGTTCCATCATGCCCGCCGCTGCCTGAAAATC
AACGGCGAACTGTATATTGTGGCGAACCGCCATCTGGACTATTTCCACAAGCTGAAGAAGATTTTCGGTAATT
GCGCGACGATAGCAACCAACAATAAGTTTGTTATTTTGAAAGCGGTGAAGCAGGGACACCGTCGATAAGGCGC
TCAAGCGTCATACCGGCCTATAAAACCGGTATGACGGCGTAAGCCCGATAAGCGCAGCGCCATCAGGTAAAGT
CGTTAAATCTCCAGCGCCAGTCGGATGCCCTGGGCAATCGCCCGTCGGGCATCCAGCTCCATCGCGACATCGC

ATCCGCCGATAAGATGTACCGTTTTACCTGCGGCGCGTAACGGGTCCGCCAGCTCGCGGCGAGGTTCCTGTCC
GGCGCAGATCACCACATGATCCACTTCCAGCAACTGCGGTTCGCCGCCGATCAAGAGATGTAATCCGTCGTCG
TCGATCTTCTGGTAACTGACCGCCGGGATCATCTTCACGCCGCGCGCCAGTAACGTCGCGCGATGGATCCAGC
CGGTGGTTTTACCTAACCCTTCGCCCGGCTTGCTGGCTTTGCGTTGCAGCATGACGATTTGCCGTGGACTACG
TGCCAGGCGCGGGCCTTCCGGGCGCAGACCGCCTGCCTGTTGCAGGCTGGTATCAATTCCCCATTCCGTACAA
AATTCAGCAATATTTTGGCTGGTGGATTCGCCATGCTGGCTCAGATACATGGCCGTGTCAAAACCGATCCCGC
CGCAGCCAATGATCGCCACTCGCTTACCAACCGGCGCTTTATCGCGCAATACCTCCAGGTAGGTCAATACTTT
TGGATGATCGATACCGTCTATCGGCGGGCGGCGCGGTACTATTCCGCAGGCGAGGATCGCTTCGTCAAACGGC
TGAAGATCCTCTGCGTTCACCCGTTGGCTCAGTTTCAGCGTAACGCCAGTAACATCGATCATTCGACGGTAGT
AGCGCAGCGTTTCGTAAAACTCCTCTTTACCCGGGATCTGCCTGGCGATAGTAAACTGGCCGCCAATTTCACT
CTGGGCGTCAAACAGCGTGACATGATGGCCGCGCGACGCGGCGTTAATCGCGAAAGCCAGCCCCGCCGGGCCT
GCGCCGACAACGGCCAGATTCTTTTTGCGTATGGCCGGCGCTATCGGCATGTGGGTTTCGTGACAGGCGCGTG
GGTTCACCAGACAGGAGGTCACTTTGCCGATGAAGATCCGATCCAGACAGGCCTGATTACAGCCGATACAGGT
GTTGATCTCATCTGCGCGTCCGGATTGCGCTTTAGTCAGGAATTCGGCATCGGCAAGGAAGGGACGCGCCATC
GACACCATATCGGCATCGCCGCGCGTCAGGATCGTCTCGGCGACCTGCGGATCGTTAATGCGATTGGTCGCGA
TCAGCGGAACCGACACATGACCTTTGAGTTTCCGCGTCACCCAGCTAAAGGCGCCACGCGGTACTGGCGTGGC
GATGGTCGGGATTCGCGCCTCATGCCAGCCAATGCCGGTATTAATTAGGCTTGCGCCTGCGGCTTCAATGGCT
TGCGCCAGTTGTACCGTTTCGTCGAACGTGCCGCCATTTTCCACCAGATCGAGCATCGACAGGCGATAGATAA
TAATAAAGTCGTTCCCTACCCGCTGGCGTACCGCGCGTACTACCTCAACCGCGAAGCGCATACGGCTGGTATA
GTCGCCGCCCCACTCGTCATCGCGATGGTTAGTTCGCCGGGTGAGAAATTCATTAATCAGATAGCCCTCTGAT
CCCATCACCTCTACGCCGTCGTAGCCTGCTTCCCGCGCCAGTTGCGCGCAATGCGCGAAGTCGTCGATTAACT
GCAAGATTTCATCATGCGTCAGTTCATGCGGCATAAAGCGGTTGATTGGCGCCTGAATTGCCGACGGCGCGAC
CAGGTGCGGCTGATAGCTGTAACGTCCGGTGTGCAGAATTTGCAGCGCGATTTTACCGCCTTGCGCATGTACG
GCATCGGTCACCACGCGGTGAGGCGTTAATTGGCTGGCGTCGTTCAGCATCGCGCCGCCGGTCATCACGACCC
CGGAGGGAACGGGCGCAATACCGCCGGTGACGATTAACGCTACGCCGTGTCGCGCGCGCTCGGCATAAAATGC
CGCCAGGCGCTCGGCGCCATCAGGATGTTCTTCCAGACCCGTGTGCATCGATCCCATCAGCACGCGGTTTCTG
AGAGTAGTAAAGCCCAGATCGAGCGGGGCGAACAGCGATGGGTAGCTCATAAGCGTATCCAGTATGTAAAATT
ATTGTTATGTGGTCGGATGAGTTACTACTGTAGCCGTCGCGAGATAAAAGGGAAAAGGGGAATGTGGTGATTG
TGATGGGATTCAAAAAAGAGGCCGCGACCCGGAGAAAACAGGCCGGATAAGCACAGCGCCATCCGGCATCGTT
TTTAAACCTCTTCAACGCTCACCTGCATTGCCGCCAGCGCATTACGCGCTGATTTCAATACCTGTTCGCACAG
TTCAATGGTTAGCGTGAGCGGTGGCTCGATACGAATTGTTTTCGCATTATTCAGCGTACCAGCGACCAGCACG
CGTTGACGGAACATCTCACTGGCAAAACGGTAGCCCGTTTCGTTATCGACAAATTCAATGGCTATCAACATAC
CTTTGCCGCGCGCATCGTGTACCAGATTGGGATATTCACGCGCAAGTTGTCGAAAACCATCCAGGAGCGTATC
GCCTTTTTGTTCCGCCTGGGCCGGGAGATTTTGCTCCAGCAGGACGTTAATGGTCGCCAGCGCGGCAGCACAA
GCCAGCGGATTACCGCCAAAAGTGGTGGTATGCAGGAAGGGATTGTCGAACAGAACCGAAAACACCTCTTCAG
TCGCGATGGTGGCGCCAATCGGCATCACGCCGCCGCCCAGGGCTTTCGCCAGACACAGAATATCAGGCTGCAC
ATTTTCGTGCTCACAGGCAAACATCTTGCCGGTACGCCCCATGCCGGTTTGTACTTCATCCAGAATCATCAGC
GCGCCGAATTCGTCACAGAGCTTACGTACCTCTGTCAGGTATCCCTGCGGCGGCAGGATCACGCCGCCTTCAC
CCTGAATAGGCTCCAGAATCACGGCTGCTATCTCGTCGCCCGTTTTTTTGCCTTCGCTAAATGCCATGCTCAT
CGCGTCGATATTGCCAAACGGCACATGGCGGAAGCCCGGCAGTAAAGGCATAAAGGGTCTGCGGAAGGTGGAT
TTGGCCGTGGCGGAGAGCGCGCCCAATGATTTGCCGTGAAATGCGCCGCTGGTGGCGATAAAGGTAAACTTGC
CGCGTGGCGACTGATACGCTTTTGCCAGTTTTAGCGCCGCTTCGACGGATTCGGTGCCGCTGTTACAGAAAAA
GCTGTACTTGAGTTTCCCCGGTGTCAGCGCCGCCAGCGTTTTTGCCAGCATCGCCCGCAAGGGGTCAAGTAAC
TCCTGGCTGTGGAGAGGTTGTTTTGCGAGTTGATTCTGGACGGCGGATACCACAACAGGATTACGGTGCCCCA
CGTTAAAGATGCCAAACCCCCCAAGACAGTCGATAAACTCCTGCCCCTGGGTGTCGACAAGCGTATTCAAACT
GCCCGCTTGCCACTCTACGGCTCCGTAATCCCCGCCGGCGGTAACAGATTTCCGGTATTCCAGAAATCCTGGA
TTTACATGCTCTTTAAAGTAGTCAATGACCTCTCGGTTTAGTGCTTTCATTTCCTCATGGTTTAGCGTTCGCT
TCTCGATGAGATTCAGTGCGTGCGCGCTACAGGCCAAAGCCGATGCGCTGGAAGGTAACCTGTTCAAAATGAG
CTCCAGAGGGCCGTGTATCACATGATACTGAATTAAGTATTGCAGGGATTAGCGGGGGTCTAAGCCCATAGTG
AAAATCGGGATAAACACCAGGGATAATCACATTTACACAACATTTAATGTCATGTGTTAACAATTGGATGCAT
TTTGAGCAGATCTTGTTTTATGGTTTACGGTGTGTTGCGTTAGCAGCGGCGTGAGAAAAAGGTGAGCCAGGGC
AGATTTTAAAGAAAAAGGTTGTCCACGCTAAACAATTTCATAATGAAAATAAGGCGCAGGATGAGGTGAAAAT
CGTACAAATATTAAGGATTAATCGCAAATTGCGATCCAGAGCAATTTTAACAACTAAAGATAAATAGATTAGC
GCCGAAATAACATCTGAGCGAAAAATTAACATCCAGATAACCTGCACAGGACGCTATCATGTCTTCTCATCCC

TACGTCAGCCAGCTAAATACCCCGCTGGATGATGATACCACTCTGATGTCTACGACCGACCTGGAAAGCTATA
TCACTCACGCCAATGACACTTTTGTCCAGGTGAGCGGCTATCAGTTAAACGAGTTACTGGCGCAGCCACATAA
TCTGGTGCGTCATCCGGATATGCCGAAAGCTGCCTTCGCAGATATGTGGTACACCCTAAAACAGGGCGAACCG
TGGAGCGGCATTGTGAAAAACCGGCGTAAAAACGGCGACCATTATTGGGTGAGGGCCAACGCGGTACCGATGA
TACGTGAAGGGCGTGTGACGGGATATATGTCGATCCGTACCCGCGCCACGGATGATGAGATTGCCGCCGTCGA
GCCTTTATATCAGGCGCTAAATGAAGGGCGGTGTAGTAAACGTATTCATAAAGGCCTGGTGGTTCGTCAGGGC
TTGCTGGGCAAACTGCCCGCTATGCCTGTTCGCTGGCGAGTGCGTAGTATTATGGGGCTAATGGCCGTAATGC
TGGCGTTGGCGCTGTTCGGTACGGATGCCTCATGGCAGGCGTTGTTGTTGGGCGCGTTGGCGATGCTGGCAGG
TACGGCGCTATTTGAATGGCAAATTGTGCGTCCCATTGAAAATGTGGCGACGCAGGCGCTGAAAGTGGCGACC
GGCGAACGCAACAGCGTACAACACCTTAATCGTAGCGATGAGTTGGGGCTGATGCTGAGGGCCGTGGGGCAGC
TTGGCTTGATGTGCCGCTGGCTGATCAATGACGTATCAAGTCAGGTTTCCAGCGTCAGAAACGGCAGTGAAAG
GCTGGCGAAGGGTAACAATGATCTGAACGAACACACCCGTCAGACCGTGGAGAATGTTCAGGAAACGGTAACG
ACCATGAACCAGATGGCGGAGTCCGTGAAGCTCAATTCCGAGACGGCTTCCGCTGCGGATAAGCTTTCCATGG
CGGCCAGTAGCGCGGCGACTCAGGGAGGTGAGGCGATGGATACGGTGATTAAAACGATGGATGATATCGCTCA
CAGTACGCAACGTATCGGGACGATCACCACGCTAATTAACGATATCGCTTTTCAGACGAATATCCTGGCGCTG
AATGCGGCGGTAGAAGCGGCGAGAGCGGGCGAGCAGGGGAAAGGGTTTGCCGTGGTTGCTGGCGAGGTACGTC
ATCTTGCCAGCCGTAGCGCTAATGCGGCGAACGATATTCGTAAATTAATTGACGCCAGCGCAACAAAGGTGCA
GTCAGGCTCCGAGCAGGTTCACGCCGCAGGCCGTACCATGGATGACATTGTAGCCCAGGTGCAAAATGTCACC
CTGCTTATCGCACGGATCAGCCAGTCGACGCAGGAACAGACAGATGGGCTTTCCAGCCTGACTCGCGCCGTGG
ACGAGTTGAACCGCATAACCCAGAAGAATGCGGCGCTGGTGGAAGAGAGCGCACAAGTCTCCGCAATGGTAAA
ACACCGTGCCAGCCGGCTGGAGGATGCGGTCACGGTACTGCATTAAGTTTATTTGTAGGTAGCAACGGTAATG
ACATCCGCCCGGTAGCAATAAGCTTACCGGGCGGATGCGCTCTGATAGAAGGCGGTGCCTGCCACCATAGAGA
GTACCAGTGGCCTGTGATGTTAAAAATAATGTGATATTTATTCCTTCTCCCTTTCCTACCTATTAATAGGTAT
AAACTCAGTTAATAAATAAGAAATGGTTAACCTTTTAGCGTATTTCTTTCATTATTAGTGCCAATTTATGTCA
CTTTTGGTTCATCTTTCTGTGAACAATAAAAATTACTTACTAATAATGTTCAGAAATAAAAAGCGATCAATAT
CATAAAGTTAGTAACATTATTGCCGATAAATCTTCCTGTATGAGGATATGCAATCCCAGGGAGAAAATATGTT
TTTGCATAACATTAAAATACGTTCAAAATTATTTATGGCCTTTGGCTTATTCATTGTTCTCATGGTGGTGAGT
TCCGCTCTGTCTTTGTTTAGCCTTGATCGGGCTAATACGGGTATGCAGGACATTATTACCAATGATTATCCCA
CCACGGTAAAAGCCAATCTGTTAATCGATAATTTTAATGATTTCATCATCGCGCAGCAGCTCATGTTACTGGA
TGAAGAGGGGCGCTGGAGCCAGAGCTCGCAGAAAGAACTCAGTGAGATAAGCCAGCGCATTTCGGCGCTACTG
GATGAGCTTTCCAGGGAAAATAGTCACGATGCGGATTCACAGAAAATCATTAATGAGATCCGTGAAGCGCGCC
AGCAATACCTGGAGTCCAGATTCCGTATTTTGAAAGATATTCAAAGCAATAATCGTCAGGCGGCCATTCAGGA
GATGATGACCAGAACGGTGCAGGTGCAAAAAGTCTATAAGGACAAAGTCCAGGAACTTATCGCTGTTCAGGAC
GCGCAGATGCATGAAGCGAGCGTGCAGGTCAAAGAGGATTTTAAAAATAATCGGACGCTGTTAATCACTTTGG
CGCTGATAAGCATCGCCGCCGGAGGCGTAATGGGATGGTATATTGTGCGTTCTATTACCCGGCCGCTTGATGA
CGCAGTACGCTTTGCCGAGGCGATTGCCGATGGCGATCTGACTCGCCATATCACCACCGACTATAAAGATGAA
ACAGGCGTACTACTGCAAGCGTTAATGGCGATGAAAACGCGTCTACTGGATATCGTACAGGAAGTGCAAAACG
GTTCGGAAAGTATCTCCACAGCGGCGGCGCAAATTGTCGCCGGTAACCAGGATTTGGCGGCGCGTACGGAAGA
GCAGGCCAGCTCGGTTGAAGAAACGGCGGCGTCGATGGAACAGATTACCGCCACGGTTAAAAATACGGCTGAC
CATACCAGTGAAGCGACCAAACTCTCTGCCGGCGCCGCCAGCGTAGTGAAAAACAATGGGGAGATGATGAATC
AAGTGACGCAGAAAATGCGCGTCATTAACGATACGGCAAATCGTATGTCGGATATCATCAATATCATTGATTC
CATTGCCTTTCAGACCAATATTCTGGCGCTGAACGCGGCGGTTGAAGCGGCGCGCGCGGGCGAACATGGACGT
GGTTTTGCCGTTGTCGCCGGAGAGGTTCGCCAGTTGGCGCAAAAGAGCGCCTCGTCAGCCAGTGAAATCCGTA
ATTTGATTGAAGATTCAACCAGTCAGACTCAGGAAGGGATGCACCTGGTGGAGAAAGCCAGCGCCCTGATTAA
TGGCATGGTGGATAACGTCGAAGAGATGGATGTGATATTACGTGAGATTGGGCAGGCCAGCCGCGAGCAAACT
GACGGTATTTCGCAGATTAACAGCGCGATTGGCCTGATTGACGCCGCCACGCAACAAAACTCCTGCCTTGTGG
AAGAGTCTGTTGCCGCCGCGGCGTCGCTGAACGAACAGGCGTTACATTTAAAAGAGCTGGTTAACGTGTTCCG
CGTCCGCGAAGAGGACACGCAGCCCGCTTAATCCAGTTCGGCGATTTCCAGCGCCGCGCGATCAATCACGCCG
ATAATGCGTTTGATTTGCGCGTCGTTGATATCGCTGTGGTTTATCCGCAGATCCAGCACGGCCTTGAAGTTAT
CCAGCGCCCGCTTCATCTGCGGATTTTTACGTAACTCCATGCCCACACAACGGGCTTTTAGCCGCGCCTGAAT
ATGCGTCAGGTGTTCGCGATTTTCATCAAGCCATTGCGCGCCGTTCGCGGTAATCGCGATTTTTTTCCGCCCG
CCTTCTTCGTCGCTAATCGTGATAAATTGCTGATCCTGTAAAAAATCCAGCGTAGGGTAGATCACGCCAGCGC
TTGGCGTATAGCCGCCCCCCGTCAGGTTTCTATCGCTTTAATCAGTTCATAGCCGTGGCTGGCGTCCCGGGT
CAGGATATCAAGAATGACCAGCCGAAGTTCCCCGTGGCCAAAAAATCGCTGACGTCGACCACCGCCCTGTCCA

TGACGACCACAGCCGTGTCCGTGACGGTGCTCGCCGCCGCAACTGGCGTTCTCATGCTGATGCGCGCTATGGC
AGCCTTCATGCTGATGTTCTCGATCTTTGCAGCAACCATCGTGCATATGATCCCGGTTTTTGCAACACCCTTC
ATGTTGATTTTGCATCATTGCCTCCTGCTTATTTAGATATATCTAATTTATATCTAAATGATGGTTTTTAGCA
AGGATAAAAGCGCTTGTTGATATAATCTATTAATTTATAACGTTTTATTTTGAAGTTGTGATTAGCGATGTGT
TGCCAGAACACTATCAAAAAACAGTTGCATTTTTACTGATTAACAATCATTATCATTTAAAACTTTAATTTAG
ATATATCGTATTACTTCACGAAGGCAAAAAATGACGACATCATCCGTACGCTACCCACAGCGTGTTCGTAATG
AGCTGCGTTTTCGCGAGCTGATCGTTCTGCGGGTAGAACGTATTAGCGCAGGATTTCAGCGGATCGTACTTGG
CGGCGAGGCGCTGGACGGTTTCATCTCCCTGGGCTTTGACGATCACACGAAAGTTTTTTTCCCTGAGCCAGGC
TGTCGCTTTACGCCTCCGACAGTCACTGAGGAAGGCATTATCTGGGGGGAGGGCGTTCGCCCGGTCTCCAGGG
ATTACACGCCGCTGTATGACGAGGCGCGTCGCGAGCTGGCGCTGGATTTCTTTATCCACGATGGCGGCGTAGC
CAGCCGTTGGGCAATGGAAGCCCGTGAAGGCGATACACTGACGATCGGCGGGCCGCGCGGTTCGCTGGTGGTG
CCGGAAGAGTATGCCTGTCAGGTTTATGTCTGCGATGAGTCCGGTATGCCTGCGCTACGTCGTCGTCTGGAAT
CGCTTAGCCGTCTGCCTGTGCGCCCTGCGGTAACCGCGCTGGTGAGCATTCAGGATGCGGCATACCGCGATTA
TCTCGCCCATTTGACGGATATTACTGTGGAATATGTTGTTGGCGGCGATGAACAGGCAATGCAAACGCGCCTG
TCGCAGCTGACAATACCAGAATCAGACTATTTCATCTGGATTACTGGCGAAGGCAAAACGGTAAAACGTTTAA
GTCAGTGCTTTGAAAAGGGGTTTGATCCACACCTGGTACGTGCGGCAGCCTATTGGCATCGTAAATAGCGGTA
TGGGGCGAGCCCTGTCAGAAACAAAAAAGCCACTCTTTAGAGTGGCTTAATTATATGATTTTAAAGCTAAAAT
TTGGTGGCCCCTGCTGGACTTGAACCAGCGACCAAGCGATTATGAGTCGCCTGCTCTAACCACTGAGCTAAGG
GGCCGTGGCGGTGGATTATAATGTAACTTTACGCTGTAATCCAGCCGTAACCGTGCGGGTGCTGTTTTTATAA
ACAATGTGTTTTCAACCCGTTATACTTAAAGAACGTTGTATTGCTCAGGAGTAATAATGGTTAAGGACATACT
GGCGCCAGGGCTACGGGTGGTTTTTTGCGGCATCAATCCGGGACTCTCCTCGGCGAATACCGGCTTTCCGTTT
GCGCATCCCGCGAATCGTTTTTGGAAAGTTATTCATTTGGCCGGTTTCACCGATCGACAGTTGAAACCGGAAG
AGGCGGAAAAATTACTGGATTTTCGCTGCGGGGTAACGAAACTCGTCGATCGACCGACGGTACAAGCGACTGA
GGTGAAGTTGCATGAATTACGTAGCGGCGGACGCAACCTGATTGAAAAAATAGAGGACTACCAGCCCGCAGCG
CTGGCGGTGCTTGGTAAACAGGCTTTTGAACAGGGATTCAGCCAGCGTGGTATCGCCTGGGGTAAACAAAAGA
TCGCCATTGGCGCGACGATGGTGTGGGTGTTGCCGAATCCCAGTGGCTTAAACCGAATTAAAACGGAAAAACT
GGTCGAGGCCTATCGCGAACTGGATCAAGCGCTCATTATGCGTGGGCTATAGCAATGGGGAATGAGGGCGCGA
ATACTCGCGCTGTGTAGAAATTCTGCAGGCCAAATAGGGCGCTTGCGCATGGCCTGATAAAAAAACGCCGCCT
TGCGGCGGCGTTTATACGTGTGCGGCGAATTAATCGTCGAGGAAGCTGCGCAGCACTTCAGAACGGCTCGGGT
GGCGCAGTTTACGCAGCGCCTTCGCTTCGATCTGACGGATACGTTCGCGGGTAACATCGAACTGTTTACCCAC
TTCTTCCAGCGTGTGGTCGGTGTTCATATCGATACCGAAACGCATACGCAGCACTTTCGCTTCACGAGCGGTC
AGGCCAGCCAAAACGTCGTGAGTGGCGGCACGCAGGCTCTCGGTAGTGGCAGAGTCCAGCGGCAGCTCGAGGG
TGGTATCCTCGATGAAATCACCCAGATGCGAATCTTCATCGTCGCCGATCGGCGTTTCCATGGAGATTGGCTC
TTTGGCAATCTTCAGCACCTTACGAATTTTATCTTCCGGCATCAGCATCCGTTCAGCCAGCTCTTCCGGCGTT
GGCTCGCGGCCCATCTCTTGCAGCATCTGGCGAGAAATACGGTTGAGCTTGTTGATGGTCTCAATCATATGCA
CCGGAATACGGATGGTGCGCGCCTGATCGGCGATAGAACGGGTGATCGCCTGACGGATCCACCAGGTTGCATA
GGTGGAGAACTTGTAGCCGCGACGGTATTCAAACTTATCTACCGCTTTCATCAGACCGATGTTGCCTTCCTGA
ATCAGATCAAGGAACTGCAAGCCACGGTTGGTGTATTTCTTAGCGATAGAAATAACCAGACGTAAGTTCGCTT
CAACCATCTCTTTCTTCGCACGGCGGGCTTTCGCTTCCCCGATGGACATTCGACGGTTGATGTCTTTCACCTG
TTCGATGGTCAGACCGGTCTCTTCTTCAATCTGCCGCAGTTTTTGCAGACAGCGTTGCACTTCTTCCGCGACA
TCGTGCAGTTTTTCCGACCACGGTTTGTTCATCGCGATAGCGGCGTTGAACCAGGTTTCGCTGGTTTCGTTAC
CGGTAAACAGCGTGATAAAGTTCTTCTTCGGCATTTTGCACTGCTCGACGCAGAGCTTCATGATCAGACGTTC
CTGGGTACGCACGCGATCCATCATCACGCGCATACTGTTGACCAGATAGTCGAATTGCTTCGGTACCAGACGG
AACTGTTTGAACACTTCAGACAGCTTCAGAATCTCTTCCTGCGCGGCAGCATGGCTGCGGCCTTTCGCTTTGA
TGGTGTCGCGCGTAACGACGTATTGCGCGCGCAGCTCAGCGAATTTTTCGCGTGCCAGTTCAGGGTCAATGCT
GTTGTCGTCATCGGCGGCGTCATCGTCGCCGTCTTCTTCATCTTCGTCTTCGTCATCATCCAGGTCTTCCTGG
GAGAGTTCAGAACCGACGTGAGTTGCGGTCGGCGCCATCTCTTCTTCCGCGTTCGGATCGACAAAGCCGGTGA
TAAGATCGGACAAACGAGCCTCTTCAGCCTCAACGCGATCGTACTGTTCCAGCAGATAGGTAATGGCTTCCGG
GTATTCGGCAACGGAGCATTGAACCTGGTTGATCCCGTCTTCGATACGTTTAGCGATGTCGATTTCGCCTTCC
CGGGTCAACAGTTCAACAGTGCCCATTTCACGCATATACATGCGTACCGGGTCAGTCGTACGACCGATTTCAG
ACTCAACACTGGACAGAACTTGTGCAGCAGCTTCTTCCGCATCTTCATCGGTGCTGGTGGTATTTTCAGCCAG
CAGCAGATCATCGGCATCAGGCGCTTCTTCCATTACCTGAATACCCATGTCGTTGATCATTTGGATGATATCT
TCAATTTGATCTGAATCGACGATATCTTCCGGCAGATGGTCATTGACCTCAGCATAGGTCAGATAGCCTTGCT
CCTTACCACGGGTGACAAGAAGTTTCAGCTGTGACTGCGGGTTTTGCTCCATAAGACGGTATCCACACTTATT

CGTTTGATTGGTGTCGACGGTATAGCTGTCGACATTTAAAGCGAGGGCATACTTATATTTTGGCCGCTGCCTC
TCACTGCGGCTGTCGGGGGCTTCCCGATCGATATGCGGCATTTAAGCCGTTAAATTCATTTTCTGGCCAGCTC
CTGGTTTAACGTCCAGAGCTCTCGGCGTTCTTCGCTGCTTAAACCGTGAGTGCGATCGCGAGCGATCAGCTCT
TCTTGTCGCAGTTGCAGCAGCGAATCAAACATATGGTTGAGTGAGTCGGTAAAGGTCTTTTCGGCAATCGCCT
TATCTGCTATATCGTCCCACATCGATAATTTTTCAAGGGTCGCAGCATCATTTGTGCCACGGTAGAGTTCCAG
AAGTTGTCCGGTAGTAAGACCTGGCTGAGCCAAACAAGTTTTGACCAATTCTTTAAATAAGCCAAGTCCAGGC
AGCTTATTCTGATCCAGCGCGTCTAAGGGCGGCACTAACGGCGCGAGATCCGGATTTTGCACCAGTAATCCTA
TCAGTATACGCATGGTCGTGCGTTTTAGCTGCGGAGCCGGGCGGGAAACGCCGCTTTCCGCCTGTTTTGGCAC
TAAACGATCAAGCTGACTATCATCGAAAATGCCTAACTTCAGCCCCAACGTCTGGCGCAACTGAATACGGTGT
GCGTCGCCAGGCACCTGATTAATGAGCGGCAGCGCCAGTGCGGCTAACTGCGTGCTGCCATCCGGCGAGCTTA
AATCCACCTGCGGCAGGAGGCTATTAAACAGAAACGTAGAGAGCGGCTGCGCCTGCTCCATCCGCGCTTCAAA
AGCCGCCTTGCCTTCTTTACGCACCAGCGTATCCGGGTCTTCGCCGTCAGGTAAAAACATAAAGCGTACCTGC
CGACCGTCGGTCATATAAGGCATTGCTGTTTCCAGCGCTCTCCAGGCGGCATCACGGCCAGCCCGGTCGCCGT
CGTAACAGCAAATTACGTTGTTCGTCGCCCGGAATAACATATGCATATGGTCTGCCGTGGTTGAGGTACCCAG
CGAAGCGACCGCATAGTTAATGTCGTATTGCGCTAGTGCGACGACATCCATATAACCTTCGACCACAAGCAAG
CGTTGCGGTTCAGCACTATATTGCTGAGCCTCATAAAGACCATATAGCTGGCGGCCTTTATGGAAAATGTCCG
TTTCCGGAGAGTTAAGGTATTTCGGCGTGTCGTTCCCCAGTACTCGACCGCCAAAACCAATCACCCTGCCGCG
TTTGTCCCGAATAGGGAACATGACCCGATTACGGAAACGATCGTAAGTGCTGCCCTGCTCGTTATTGACCAGC
ATCCCGGCATCCAGCAACAATGCCTTGTTATCGCTGTTATTACCGAAGCGCTTTAACGCGTTATCCCACCCTG
GCGGCGCGAAGCCAATGGCGAAACGCTGAATAATTTCAGCGCTCAGACCGCGCTTTTGCAGATAGTCACGCGC
AGGTTTTGCCGCAGGATGGGTAAGCGACTGCTGATAAAAGTCATTCAGACCATTCATCAACTGATAGAGGTTT
TGCCGTTGATGGCGCTCTATCTGGCTAAGCCCGGTGCCTGCTTCATAGGGAATTTCCAGGTTATGCATGGCCG
CTAATTCTTCGACGGTTTCGACAAATTCGAGTTTGTCGTAATTCATTAAAAAGTCGATCGCATTGCCATGCGC
GCCGCATCCAAAGCAGTGATAAAACTGTTTTTCACCGTTTACGGTGAAAGAAGGGGTTTTCTCATTATGGAAT
GGACAACACGCGTGATAGTTCTTGCCCTGTTTCTTGAGTTTTACGCGCACGTCGATCAGATCGACGATATCGG
TTCTCGCCAGCAGGTCATTAATGAATACGCGTGGGATTCGTCCAGCCATATGCCCCTTTTATACACGTCATTC
TTCAGGCTGCGTAGTGTTGGCTACGTTTGCTCACCCCTGTCGCTGACTTGCGTAAGCGCCGGGGACTTGCTCT
CTTGCCGCCTGGACGCAGCATGAATGATTTTGTGTATATGCTAATGCATAAACGAAAACAAGCCGCGCATTCC
TTTCGGAAGCACGGCCTTACAACTACAACTCGATCTGAATTGAGAGCAGCCCGCTCTCAAAAGATTAGTACAG
ACGAGTACGGCGTGCGTTTTCGCGAGCCAGTTTCTTCGCGTGACGTTTCACTGCAGAAGCTTTAGCGCGCTTA
CGTTCGGTCGTCGGTTTTTCATAGAACTCACGACGACGAACTTCCGCCAGAACACCTGCTTTTTCGCAGGAAC
GCTTGAAGCGACGCAGAGCTACGTCGAACGGCTCGTTTTCACGTACTTTAATTACCGGCATGTGCCTCTCACC
TTTGATTAATTCGGTTTGCCGCTGGCATCAACGCCAGCTTATTTCAAAATGGTGCGGAATTTTACTGCAATTG
CTGCTGCTTTGTAAAGCACCGCGGCCTTTTTTGAACGGGACTTTTATAAGGTGAGGGAGTATACACGAAGTCG
GGACTGAGGGTATACATCCACCCGCATTCGACCTACACTGCGCGGTATTAGAGCGAGGTAAAACAAGTCATGC
GTGTACTGGGTATTGAAACATCCTGCGATGAAACCGGCATCGCTATTTACGACGACAAAAAAGGTCTGTTAGC
CAACCAATTGTATAGTCAGGTGAAATTACATGCTGACTACGGCGGCGTAGTGCCTGAACTGGCTTCCCGCGAT
CATGTGCGTAAAACCGTGCCGCTGATTCAGGCGGCATTAAAAGAAGCCGGTCTGACGGCGAGCGATATCGACG
CGGTGGCCTATACCGCAGGCCCGGGCCTGGTCGGCGCGCTGCTGGTCGGCGCAACCGTCGGGCGTTCGCTGGC
ATTTGCCTGGAATGTGCCGGCCATTCCTGTACACCATATGGAAGGTCATCTGCTGGCGCCAATGCTGGAAGAT
AATCCCCCGGAATTCCCGTTTGTGGCGCTACTGGTCTCCGGCGGACATACGCAGCTCATTAGCGTGACCGGAA
TCGGTCAGTACGAACTGCTGGGAGAGTCGATTGACGATGCCGCCGGTGAAGCGTTTGATAAAACCGCCAAATT
GTTGGGGCTGGATTATCCTGGCGGCCCGATGCTGTCGAAAATGGCGTCGCAGGGGACGGCGGACGTTTTGTC
TTTCCGCGCCCGATGACCGATCGCCCGGGGCTGGATTTTAGTTTTTCCGGTCTGAAAACCTTTGCCGCTAACA
CCATTCGTAGTAATGGCGGCGACGAACAAACTCGCGCTGATATCGCGCGCGCTTTTGAAGATGCGGTCGTGGA
TACGCTGATGATCAAGTGCAAGCGCGCGCTGGAAAGCACCGGTTTTAAGCGTCTGGTCATGGCGGGCGGCGTC
AGCGCTAACCGCACGCTGCGCGCGAAGCTTGCCGAAATGATGCAAAAACGCCGCGGCGAAGTGTTCTATGCGC
GTCCGGAGTTTTGTACTGACAACGGGGCGATGATCGCCTATGCCGGAATGGTGCGGTTTAAGGCGGGCGTTAC
GGCGGATCTTGGCGTAACGGTACGTCCGCGCTGGCCGCTGGCCGAGCTGCCGGCGGCGTAAAAGCGGAGAAAG
GGGGCGACATCATGCCGCCCCAACGGGTTTACAGCAAAACGACGGGCATCCACAACAGCCTGGAGACGATCAG
CAGCACCAGGAAGATCAGGCCGAAAATCGCGCCTAACCACGCCAATGGTGGAAGGGTGACGCGCGGCGGATTC
GATATGTACGCCCATCACCAAGCTATTGCCGGTCAACCCTTGTGAGCCGATCCCCAGACGGTTGAGGCCCTCT
TCCAGACGCAGTTCCAGCTCAATCTTTCTGGCGTTTCTTTTTCAGCTTCGTCCAGATTTTCGTCTCCTGGCGA
CGCCACAGACGCTGGATGTTGTCATGATGGCGCAGCAAAATGAGACATGACAGCATCGATACCGGGAAGGTGA

ACTGCGGTTTAAACCACCAGACGTAAAACGGCGCAATCAAGGCGCTGACAATCGCCCCTAACGATGAATAACC
GCTTAACAGGACCGTCAGCAGCCAGGTGCCAGCCATGACGCCGGTTAAATCCCAGCCGATGGGCGCAATGGCG
CCAAACGCGGTTGCTACGCCTTTCCCGCCTTTAAAGCCAAAAAAGACCGGCCAGATATGCCCCAGACAGGCGG
CGATAGCGATAAGCCCCAGCCAGAAAGGGGTAACGCCTAACGCATACGCGCCCCAGACGGGCAACATGCCTTT
AAGGATGTCAAAAATAAGTACCGCGACAGCCGCTCCCTTGCCACCGATTCGTAATACGTTCGTCGCGCCCGGG
TTGCCGGAGCCGCTTTCTCGCGGATCGGGCAGCCCGGCGATGCGGCAAACCAGAATGGCACTGGAAATTGAGC
CGCAGAGGTACGCGAAGAGGATCATTCCAGGCGCGATTGCACTCATAAGCTGTTCCGTATTGAAAGTGTCGTT
TTATTTACTATTTCTGTGGATAATACGCACATTTCGCCGGAAGTGGTATCCGGGTTAGCCAAAAAGCAGGCAG
GTCGTGATGGATATTGTATTTATAGAGCAACTTTCGGTAATCACCACTATCGGTGTTTACGACTGGGAACAGA
CTATCGAGCAAAAACTGGTGTTCGATATCGAAATGGCGTGGGATAACCGTAAGTCGGCGAAAAGCGATGATGT
CGCAGATTGTCTAAGTTATGCGGATATCGCCGACACGGTGATAAATCATGTCGAAGGCGGGCGTTTTGCTCTG
GTCGAGCGCGTGGCGGAAGAGGTGGCGGATCTGCTGCTTAGCCGATTCAATTCTCCGTGGGTTCGCATCAAAC
TGAGCAAGCCTGGCGCCGTCGCGCGCGCGGCGAACGTAGGGGTAATTATTGAGCGTGGCAATAATCTGAAATA
AAAATAAATAATTTTATATTTTTTAAACCAAACGGTTATATATGGGGTCTTAACGCGAGATCCATTTTTGCAG
TGTTTTTCATTTTAGGGGTTTATTGATGAGCGATATGCACTCGCTGCTGATAGCGGCAATTTTGGGTGTGGTC
GAAGGATTGACGGAGTTTTTGCCGGTATCCAGCACGGGCCATATGATTATTGTGGGTCATCTGCTGGGGTTTG
AAGGCGATACGGCCAAGACATTCGAAGTGGTGATTCAACTTGGATCTATTCTGGCGGTCGTGGTGATGTTCTG
GCGGCGGTTGTTTGGCCTCATCGGTATTCACTTTGGCCGCCCGCTACAGCGTGAAGGTGAAAGTAAAGGTCGA
TTAACGTTGATTCACATCCTGCTGGGCATGATTCCGGCGGTGGTGCTGGGGTTAGTCTTTCATGACACGATCA
AGTCGCTATTTAACCCAATTAACGTGATGTACGCGCTGGTGGTAGGCGGTCTGTTGCTGATTGCCGCCGAGTG
CCTGAAGCCGAAAGAACCGCGCGCGCCGGGGCTGGATGATATGACGTATCGTCAGGCGTTTATGATTGGCTGT
TTCCAGTGTCTGGCGCTGTGGCCGGGCTTCTCGCGTTCCGGAGCGACCATTTCCGGTGGGATGCTGATGGGCG
TCAGCCGTTACGCCGCCTCTGAGTTCTCGTTCTTACTGGCTGTGCCGATGATGATGGGCGCAACGGTTCTGGA
TCTCTACAAAAGTTGGTCATTCCTGACCGCAGCGGATATTCCGATGTTCGCAGTGGGCTTTGTGACGGCGTTT
GTGGTGGCGCTGATCGCTATCAAGACCTTCCTGCAACTGATTAAGCGTATCTCGTTTATTCCGTTTGCGATTT
ACCGCTTTGTGGTGGCGGCTGCCGTTTACGTTGTTTTCTTCTGATGATTGACCTGCCGGATGGCGGCGTACCG
CCTTGTCCGGCCTAACTGTTTTGTCGGTCTGATAAGTGCAGCGCCATCAGGCAATTTATCGTGCTGGATGGCG
TTTAGCTGGCGGGGTTCGGGCAGCGCTTCTCTTTCCAGTTGGCGACCGCTGCAATTCGTCGCTTCGTCAGCTC
CTCGCGAATCTCAATGCCCTTAAATCCGGCCTCCACCACCTCTTTCGTCGGTACCGCTTGGGCCACCTGCCAG
GCTTCGCGCAGCCAGCGCCCCTGCGGGTAATCCGACGCTTCAAAGCCGGTACGTCCACGGACATCGGCTTCGC
TGGTCAGCGCAATCTGCTCGACGCGCTGCGGTTTACGCCATGCATCAATGGCGTCAAACAGCTTAACGATCGT
TTTCGGCTGGAGTATCGGAAAGGTGTGGATCAGATCATGGTACTCGGCGACCAGTTTAGCTAAATCACGTAGG
TCATTGGGGACGCGCAAACGCTGGCATAGCTGTTCGACCAGTTTCACGCCCGCCGGGCCGTGGCCATGATGGC
GCGGCCAGAGGTTTTTCGGCGTTAAGCCTTTGCCCAGGTCATGACAAAGCGTGGCGAAACGAACATCAAGCTG
CGGACTGAGCATGGCGGCCATCGACAGCGTCATCAACGTATGAACTCCGGTATCAATTTCCGGATGCCACTTT
GCCGGCGCGGGTACGCCAAACAGGGCGTCGATTTCCGGAAACAGGACGCGCAGGGCGCCGCAGTCGCGCAATA
CCTGGAAGTAAACCTGCGGATTACGGGTGGTAAGCGCATTTTCCGTCTCTTTCCATACCCGTTCCGGCGTCAG
ATGTTCCAGCTCGCCTGCCGCGGTCATTTCGCGCATTAGCGCCAGTGTTTCATCGGCGATACGGAAACTAAGA
TGAGCGTAACGGGCGGCAAAACGCGCCACGCGCAATACGCGCAAGGGATCTTCGCCAAACGCGGGAGAGACAT
GTCGCAGTAGACGCGCCTCCAGATCCCGGCGCCCGTGGTAAGGGTCGATAATCTGGCCGTCATCGTCGCGCGC
CAGAGCATTAATGGTCAGATCGCGACGCTGGAGGTCGGCTTCCAGGGTAACGTCCGGCGCGGCATAACAGGTG
AAGCCCGTGTAGCCGGAACCGGATTTACGCTCGGTACGCGCCAGCGCATATTCTTCGTGAGTTTGCGGGTGGA
GAAACACAGGAAAATCACGGCCTACCTGCTGGTAGCCCGCATCAAGCATCTCCTGCGGCGTGGCGCCGACAAC
GACCCAATCTTTATCTTTAACCGGTAGCCCTAACAACGCATCCCGAACCGCACCACCGACCAGATAAATCTTC
ACGCCACTTTCCTTTTAAAAACGATCATCCTCTAATGATAAGACAGTGCCGCAGAGAAGGCGATTTAGTTCAT
CCAGCGGTCTTTGCGTTTACGGCTTGGGATCATATGCGGCAGCACCAGCCCTAACAGCAAGCCAATACCCAAT
ACGCCGCCGCCATACATAAACCACTGCATAATAATGGTGCGCTGTTTGTCATCCAGTTGCAGATTCGCGGCGC
TAACTTTTTTCTGCGCGACAATCAGCTCGTTTTTCAGCTTCTGGTTCTCTTCTTTTAGCCCGTTGATGATGCT
GTCGCTTTGCGCCACTTTCTGCTGCATATCGGCGGTGCGCTGATTCCACGTGGTATCAATATTGTTGAGTTTA
TCGGTCAACGTTTTGACCTGATTTTCCAGATCCGGCACGCGGGTACGCAGGCTTGGTGTGGTATTCAGTTCTT
TTAACGGTATCCAGGCAGTACGACCAGAACTGTCTTTAATTTGACCATAGTTGGCATCGCTCTGTAAGAGCGT
GACTTCCTCGCCGGCGTTGACGGTACCCACAAGGCGATAATTATCTCCCGGACCGCTGCGGACCCAGGTATTC
AGTTCATCAGAAACGTAACGCGTCTCTTCAGCATGAGAGACGGCGGTGGCGCTAAGCGCAAGTAAAGTTAGTC
CAATCAGGCGTAATTTTGGCATCAGGCTGTCGTTATTGTCAGAGAAAGTGGAACGATAGTAACGGCATCAGTG

TCGCTACGCAAAGTAGTCGACATCAATCGGAATCATCTGTGTCGCCTTTGCCGCTACGTCAAACTTTTACGCC
CGTCAAATTGCTCGTTGCATGGCAATTTGGCGCAAAATACTATCTACTGACAAAAATAATGCGGGTAAGTTCG
CCAAGAATCTTCCCAGATGTGACATAACCCATAAGTACAAGGCCATGGCTCAGGAAATCGAATTAAAGTTTAT
CGTCAATCACGACGCCGTAGATGCGCTGCGAAATCATCTTCATACGCTCGGCGGCGAGCATCATGCGCCCAGC
CAGTTGCTGAATATCTACTTCGAAACGCCTGATAACTGGCTACGCCGCCATGATATGGGGCTGCGTATTCGTG
GTGAAAATGGGCGCTATGAAATGACCATGAAAATTGCCGGCCGGGTAACGGGCGGGCTGCACCAGCGACCCGA
ATACAATGTCGCGCTAAGCGAACCCGTGCTGGATCTGACGCAACTTCCGGCGGAGGTCTGGCCGGATGGTAAC
CTGCCCGCCGGCCTGGCATCAAGCGTACAGCCGCTGTTCAGTACCGATTTTTACCGGGAAAAATGGTGTCTGG
ACGTTGATGGCAGCAGGATTGAAATTGCGCTCGATCTCGGCGACGTAAAAGCGGGCGAGTTTGCCGAACCGAT
TTGTGAGCTGGAGCTTGAGTTGCTTCGCGGCGATACGCGCGCCGTGTTAAAACTGGCGAAGCAACTGCTGTCG
CAAACCGGTTTGCGTCAGGGCAGTCTGAGTAAGGCGGCGCGAGGTTATCACCTGGCGCAGGGGAATGCGCCGC
GAGAGAATACACCAACGGCAATTCTGCGGACTGCCGCGAAAGCCACGGTAGAGCAAGGGCTGGAGGTCTCGCT
GGATCTGGCGTTGTCACAGTGGCAGTACCATGAAGAGCTCTGGCTGCGCGGCGACGAGTCGGCGGAAGAACAT
GTGCTGGATGCTATGGGATTAGTGCGCCATGCCTTAATGCTTTTTGGTGGCATTGTTCCACGTAAAGCGAGCG
CTCACTTACGTGATCTACTTACCCAGGCGGAAGCGACGATGACATCCGCGGTGTCCGCCGTCACAGCCGTTTA
TAGTACGCAAACGGCGATGGCGAAACTGGCATTGACGGAATGGCTGGTGACGAAAGCCTGGCAGCCATTTCTG
GACGCGAAGGCGCAGGCCAAAATGGCCGATTCTTTTAAGCGTTTCGCCGACATTCATCTTTCTCGTCATGCTG
CTGAATTAAAAAAAGTTTTTGGACAACCGCTGGGTGATAAGTATCGCGACCAGTTGCCGCGTTTGACTCGCGA
TATCGACAGCGTTTTACTGCTGGCCGGGTATTATGATGCGATGGTCGCGCAGGCATGGCTGGAAAACTGGCAG
GGATTGCGTCACGCCATTCTTACCGGACAACGTATTGAAATTGAACATTTCCGTAATGAGGCCATTAACCAGC
AGCCGTTCTGGTTGCACAGCGGAAAACGATAATCAGAACCGTAGAAAGCGCCTGATGACGCTGCGCTTATCAG
GCTTACGGTATGCGGACTTGTAGGCCGGATCAGGCGAAACGCCGCCATCCGGCATTATTTAAAGGATATCCCC
TAAATGACGCCGCTCTCTTCACCCTTATCGCAGTACTGGCAGACCATTGTTGAGCGCCTACCAGAAGGATTTA
CTGAGACATCGCTTAGCGTGCAGGCGAAGTCAGTACTTACATTTAGCGATTTTGCGCTGGATAGCGTGATAGC
GCATCCGGAGTGGCTTGCCGAACTGGAATCCGCATCGCCGCAGGCTGATGAATGGCGACATTATGCTGGCTGG
TTACAGGAGGCGTTGGCAGGTGTCTGTGACGACGCCTCGCTGATGCGTGAACTGAGGTTTTTTCGTCGTCGCA
TCATGGTACGCATCGCCTGGGCGCAGACGCTGTCGCTGGTGGACGATGAGACTATTTTACAACAGTTGAGCCA
TCTGGCGGAGACGCTCATTGTGGGGGCGCGCGACTGGCTGTATGCCGCCTGTTGCCGCGAATGGGGAACCCCC
TGTAACCCGCAGGGCGTTCCGCAGCCGCTGTTGATTTTAGGCATGGGCAAGCTGGGCGGCGGCGAACTGAATT
TTTCGTCTGATATCGATCTGATTTTCGCCTGGCCGGAACATGGCGAGACTCGGGGCGGGCGTCGTGAACTCGA
TAACGCCCAGTTTTTTACGCGTCTCGGGCAGCGGCTGATCAAAGCGCTGGATCAGCCCACAATGGATGGTTTT
GTCTATCGGGTGGACATGCGCCTGCGACCGTTTGGCGACAGCGGCCCGCTGGTACTGAGTTTTGCCGCGCTGG
AGGATTACTATCAGGAGCAAGGACGCGACTGGGAACGCTACGCTATGGTGAAAGCGCGGCTAATGGGCGATAA
CGACGACGCCTGGTCTCGTGAATTACGCGCCATGCTGCGGCCTTTCGTTTTCCGCCGCTATATCGATTTCAGT
GTCATCCAGTCGCTACGTAATATGAAAGGGATGATCGCCCGCGAAGTGCGACGCCGCGGTTTGAAAGACAACA
TTAAACTTGGCGCGGGCGGTATTCGGGAAATTGAGTTTATCGTGCAGGTATTCCAGCTCATCCGCGGCGGGCG
CGAGCCGTCGCTACAGTCGCGTTCGTTACTGCCAACGCTGGATGCTATCGCTGCGCTGCATTTGCTGCCTGAG
AATGACGTGGCGCAACTGCGCGTGGCCTATCTTTTTCTACGTCGTCTGGAGAATCTGCTGCAAAGCATCAATG
ACGAACAGACGCAAACGTTGCCTGCCGACGATCTGAATCGGGCGCGGCTGGCCTGGGGAATGAAGGCGGAGAA
CTGGCCGCAGCTTGTTGGCGAACTGACGGATCATATGGCTAATGTACGCCGCGTATTCAACGAATTAATTGGC
GATGATGAAGCCGATACCCCGCAGGAGGAAGAACGTTCAGAGCCGTGGCGTGAAGTATGGCAGGATGCGCTAC
AGGAGGATGATTCGACGCCAGTGCTGGCGCATTTAGCCGATGAGGATCGGCGACAAGTTCTGACCCTCATCGC
TGACTTCCGTAAGGAACTGGATAAACGCCCTATCGGCCCGCGCGGGCGGCAAGTGCTGGACCAACTGATGCCG
CACCTGCTTGCTGATGTCTGTTCTCGCGAAGACGCCGCTGTTACGCTATCGCGGATAACGCCGTTGCTGGCGG
GGATTGTCACTCGCACCACCTATCTGGAGCTGCTGAGCGAATTTCCCGGCGCGCTGAAACACCTGATTATGCT
CTGCGCCGCTTCGCCAATGATAGCGAGCCAACTGGCGCGTTATCCGTTGTTGCTTGATGAACTGCTCGACCCC
GGCACGCTTTATCAGCCGACGGCTACCGACGCTTACCGCGACGAATTACGCCAATATTTGCTGCGCGTGCCGG
AAGAGGACGAAGAACAACAACTGGAGGCATTACGCCAGTTTAAACAGGCACAGCTTTTGCGGATTGCGGCGGC
GGATATTGCCGGTACGCTGCCGGTGATGAAAGTAAGCGATCACCTCACATGGCTGGCGGAGGCGATGATTGAT
GCTGTGGTGCAGCAGGCGTGGACGCAAATGGTTGCGCGTTATGGTCAGCCAGCGCATCTGGACGAGCGCCAGG
GGCGGGGGTTTGCGGTTGTCGGTTATGGCAAGTTGGGCGGATGGGAGCTGGGCTACAGTTCCGATTTGGATCT
CATCTTCCTGCATGACTGTCCGATGGACGTCATGACCAACGGCGAACGCGAAATCGACGGGCGGCAGTTTTAT
CTGCGCCTGGCGCAGCGCATCATGCATCTGTTCAGTACGCGCACTTCCTCCGGCATTTTGTATGAGGTGGATG
CGCGTCTACGCCCCTCTGGCGCGGCGGGAATGCTGGTGACTTCAGCGGACGCGTTTGCCGATTATCAGCAGCA

TGAAGCCTGGACCTGGGAACATCAGGCATTGGTACGCGCTCGCGTGGTATATGGCGACCCGCAGCTTACCTCA
CAGTTTGATACGGTGCGCCGCACTATCATGACGACGGCGCGCGACGGCAAGACATTGCAGACTGAAGTGCGTG
AAATGCGCGAGAAAATGCGGGCGCATCTCGGCAATAAGCATCGTGATCGTTTTGATATTAAAGCTGATGAAGG
CGGTATTACGGATATTGAGTTTATCGCGCAATATCTGGTACTGCGCTACGCTCATGAAAAACCTAAATTAACG
CGCTGGTCTGATAACGTGCGCATCCTGGAGCTTCTGGCGCAAAACGGCATTATGGATGAACACGAGGCGCAGG
CGCTGACGGTAGCCTACACCACACTGCGTGATGAACTGCACCACCTGGCGTTGCAGGAGCTTCCCGGTCATGT
GGCGCAGACGTGTTTTAGCAAAGAGCGCGCGCTGGTGCAGGCAAGCTGGCGGAAGTGGCTGGTAGCCGTTTGA
ATTATGCTATTATCGCGCGCAAATTTTGAATCTCTCAGGAGACAGGAATGAAAGTAAATCTGCCAGCGTTTGA
ACGTGCAGGCGTCATGGTTGTGGGTGATGTAATGCTGGATCGCTATTGGTATGGCCCCACTTGCCGTATTTCA
CCGGAAGCGCCGGTGCCCGTGGTTAAGGTAAATACCGTTGAGGAACGTCCGGGCGGCGCGGCGAACGTGGCGA
TGAACATTGCGTCTCTGGGAGCGAACGCCCGTCTGGTCGGCCTGACGGGTATTGATGACGCCGCGCGCGCGCT
GAGCAAAACGTTGGCGGAGGTCAATGTGAAGTGCGACTTCGTTTCTGTGCCGACGCATCCGACAATTACTAAA
CTGCGCGTGCTATCACGTAATCAGCAGCTCATTCGTCTTGATTTTGAAGAAGGCTTTGAGGGGGTGGACCCGC
AGCCGTTGCATGAGCGTATCAACCAGGCGCTGGGATCGATCGGCGCGCTGGTGTTGTCCGATTATGCCAAAGG
CGCTCTGACCAGCGTGCAGACTATGATTTCCCTGGCGCGCCAGGCGGGCGTGCCGGTGCTCATCGATCCGAAA
GGAACGGATTTTGAACGTTACCGCGGCGCCACGCTGCTGACGCCAAACCTTTCTGAATTCGAGGCGGTCGCGG
GGAAATGTAAAAGCGAAGACGAACTGGTTGAACGCGGCATGAAGCTCATTGCCGATTACGACCTTTCCGCGCT
GTTGGTCACGCGTTCCGAACAGGGAATGACGCTGCTGCAACCGAATAAAGCGCCGCTACATATGCCGACGCAG
GCGCAGGAAGTTTATGATGTTACCGGTGCGGGCGATACGGTGATCGGCGTGCTGGCGGCGACGCTGGCGGCGG
GAAATACCCTGGAAGAGGCGTGTTATTTCGCCAATGCGGCGGCGGGCGTAGTGGTAGGTAAACTCGGGACGTC
AACGGTTTCCCCTATTGAGCTGGAAAACGCAGTGCGCGGACGCGCGGATACCGGCTTCGGCGTTATGACCGAA
GAGGAGTTGAGACAGGCCGTCGCCAGCGCGCGTAAGCGTGGCGAGAAAGTGGTCATGACCAACGGCGTTTTCG
ATATTCTGCACGCGGGCCACGTCTCTTATCTGGCGAACGCGCGCAAACTGGGCGACCGCCTGATTGTTGCGGT
CAATAGTGACGCCTCGACTAAACGTCTGAAAGGCGACAGCCGTCCGGTTAATCCGCTCGAACAGCGTATGATC
GTGCTGGGCGCGCTGGAGTCGGTCGACTGGGTTGTCTCTTTTGAAGAGGATACGCCGCAACGACTGATTGCCG
GTATTCTGCCGGATCTGCTGGTAAAAGGCGGCGACTATAAGCCGGAAGAGATCGCGGGCAGCGAAGAGGTCTG
GGCCAACGGCGGCGAAGTCATGGTGCTGAACTTCGAAGATGGTTGTTCCACGACCAATATCATCAAAAAGATC
CAGACCGAGAGCGAGAAGTAAAACCGCGTCATCAGGCGTTACAGACGCTGCGGGCGCTGATTTTGAAGGTGCG
TCGGACATTTTGTTTCGACGCGTATCTCGCGGTTGACGCTGCTTTTTTTTCGACCTAAAGTAAAGGAATAAGG
GTGAGAGGGGATCTCTCCCCCCTCTGATTGGCTGTTAGTAAGCTGGGAAACTTATCAGTAACAGAACAATCAG
TATGATGACCTGGTGCATCATAACCCTTTCCTTATTACAGCCCCTTCCTCGGGAGGGGCTTTCCCGTTTCAGC
CCCTTGCTGACATCGCGATTGTTGGTGAGTGGCCTGTCTGGCGCAAGGGCTTTATCCCGCCGATTTTGCGTTA
CTGGATCGCCCACGCAAAATGGCCTGCTGGTCATAACGCTTTGTACAGACAAGGTGGAAAAAAGATAAAAAAA
TCCCCGGCCTTATGGCCAGGGACAGGAGATATAGCGGGGAGTTATTGCTCTTTTACCGTTGCTTCGCGATTAA
TCTCATCCGTTGCCGATGAAACCAACGGCGTGGTCAGCGCCTTTAGCGAGCCGCCAGCGATGCCAATCTCGTT
TAACAGCGAATCGATCAGCGGTGCCTGGGTACGATAGGTGAGCGCGGCAGATAACGCTTGTTCCGCCAGGTTT
CCCCCGTTAGCGCCGCCAGCGGCGACGTCGCCCGCCGTCGCGCCGCGATTGAGGCCATCGACCTGAATAATCT
TAATGCCGTCGATGGATTTCATCGGTTCAACAGACTTCTCAATAACAGCAGGCAACGATTGTAAGAGCGCTAG
TTTAAATTTGAGGCTGGTTTGTTCGTCAGATAGTACGTTGATAGCGTCATTCAGCGCCCGTTGCGCTTCGGCT
TCCGCCAGACCTTTTTTACGCGTTGCTTCCGCCAGTTCGATGATGGCGGCGGCTTGCAATTCTGCCGCTTCTT
TCTCTGCTTTTGCCCGAACGGTCAGTTCGACCGCTTTGGTTTCCGCATCCTGCGCCGCGGCGATCAGAGCGAC
CTGCTTGGCGCGATCCGCTTCGGCGGTCTGGCGGGTGGTTTCTACGTTTTGCTGGGCGCGAACCGCATCGGCC
AGCGCGTCGTTGGCGCGCGCTTCTGCTTGTGATTGCTGCTCGGATTTGGCGGCAATGGCGATAGATTTCGTCT
GGTTGGCGATTTCTGTCACCTGTTGCTGTTCGATCTCTTTAATACGTACTTCACGCTCGGCCTCAACTTTCCT
GGACCGTACTGCTTGCTCACGTTCGATCTCCGTCTCCTGGATCTGCCGCTCAGCAAGAATGCGCGTCTGTTCT
GCTTCACGATGGCGCTCGGCCTCAAAAGCGGCAATTTTAGCGTTTTGTTCGGCGGTGCGGGTTTTAACCTGTT
GCTCCTGCTCCAGAGTCATAAAGGCTTCTTGTTGCTCGATTTCCAGCTTGCGCTCAAGCGCGTCGCGGTTCTT
TTCGCGCACCGCCACTTCCACATCCTGTTCCACTTCATTACGTTCACGACGGCGGCGTTCGGTTTCCTGCGTC
AGTTTCGTCAGACCTTCGGCATCAAAGGCATTGTTTGGATTGAAATGCTCTTTGGACGTTTGGTTAAAGTTGG
TTAATGAGACACTTTCCAGCTCCAGGCCGTTTTTCGACAAATCTTCCGCAACGGTGTTTTGTACGCCCTGAAC
GAAGTTTTCACGCGTATCCTGTAGCTCATGCATCGTCATCTGGGCCGCGGTAGCGCGTAAGGCGTCAACGAAT
TTATCCTCAACCAGCATACGAAGATCTTCCGGCGACAGCGTTCGCTGGCCAAGCGTTTGTGCGGCGGTGGCAA
TACCTTCAACAGAGGGTTTTACACGTACGAAGAAAGCAACGACCACGTCAACGCGCATACGATCTTTGGTGAT
CAGGCTGTCGACGGTAGCACGGCTGACTTCAAGTTTTAGGGTATTCATATTGATCGGAATAATTTCGTGAAAG

ATTGGCATCACAATAGCGCCGCCGCTCATCACCACTTTTTGCCCGCCAAGCCCGGTACGGACAAACGCCTGTT
CCGCAGATGCGCGGCGATAGAGGCGAGCAAAAATGATCCCGATAATTAGTAAGACGATAACCGCAACGATGGC
GGTAAACATCCATGATGGCAATATGCCAAAAACATCATCCATTATGTAAGTTCCTCTCAATTAAACATAGAAG
GTACGCTCAGCCAAATAACGCGTTGCGGAAAGTCGGCAGACAATCAGCACCTTGTCTCCTTTTTTAAATTCTT
TTCCCTTTTCGGGTTCCAGCAGTAAATAGTGAATCTGGCCGAACTTATCGGTAAATTTTCCTTCGCAAGGTGT
ACCGGCGACGGCGGCGTGGCCGGTAATAATCGCCATGCCGCCGATAAATTCCTCTTCCCGGAGGGCGGAGCTT
TCATCTCTGGGTAGCCAGGGCGCGAGGATTTTTCCGCTGTAATGCACGGCAAAGACCGACAGGACAATGCTCA
ACGGGACCAGCAGCAGATTGCTGAGCGGCGCTTGCCAGAGCATGATCCCGCCATGCTGGATGAGGATACCGAA
GAGGCCGAAATAACCGGCCAATAGACAGAGGACGACCAGGGCGGGAACGCGTCCAATATTCAGATAATGAAGC
GCCTGGCCCGCAGGGCCGGATGACAGGGCGTCATAATGATCGAGATGGGCGTCGAGCGCGCCTGAAAGAAAGT
GGCCAAAAATCAGCGAAATCATCTCCAGCACGCCGATAAAGAAAACGAACGCGATGGCAAACAGATAGGGGCT
GTTATACTCCGCAAATAAGGTCATATTTTCTATCCTTGTCGCCGCCTTCAGAAGGATGTTCGCGTGATAATTT
TCATGCTTGTTATTTCATTGCGTTACAATATCATGTGACCCATATCTCCTTAAGTCCGAAAAGCGATTTATCC
GTCGTTATCTTTGGTGAATATTGTGATTTTGCGCATTAAATTTGCGCACGTTATGTATAAAACCGCCGTACAT
ATAGGGAAATACTGAAATTAGAAAAAGCATATAGCCTTAACAGAATTCAATAACTGGAGGGTATGCTGATGAA
CAACAATAACGTCTATTCACTAAATAATTTTGATTTTCTGGCGCGCAGTTTTGCCAGAATGCAGGCGGAGGGG
CGTCCTGTTGATATCCAGGCGGTAACCGGCAATATGGACGAGGAGCATCGCGACTGGTTTTGCAAGCGTTACG
CGCTTTACTGCCAGCAGGCGACTCAGGCGAAAAGGTTAGAATTAGAACACTAATGCAGGCTCCCTTATACAGG
CGCAGGACATTATTCAGATCTTATATGAGCGGGCCGTCAGGCCCGTTCACGTTTTTACTCTTGTGGATCGACT
GGCGGGATGGCGGGCGCAGGTTTTACTTCTTCAGGCTTGTCGCGAGCTTCCAGTTCGCTGAGGCGTTGCTCCA
GCAGCGCCAGTTTTTCCCGGGTGCGCAGCAAGACCTGAGTCTGGACATCAAACTCTTCGCGGCTCACCAGATC
GAGACGCGTCAGCTGAGATTGCAGCGTTTGACGGATTTTTTTCTCGATATCTTCCCCGAACTCGCGAATACCT
TTCGGCATTGACTCATGAACCTGGCGTGCGATTTGCTCAATTTTTTTCGGGTCAATCATTGTGGTTTCCCTGA
ACTGGTAAGTGTTAGCTCTTATTGTAGTGCGATATGTGGACGCCATAAACCAGAATTGTGTGACGTCATACGG
AGTAAAGGCGTTGATGAAGGTAATCAATAGCGTTATAGTTAACCCGCTTATTCTCAGGGCGGGGCGAAATTCC
CCACCGGCGGTAAATCAGCGATGTTTAACGCATTTCTCATCACGGCGCGCCGTGACGGAACGCGGAGAGCAGG
TTGAAAGCCCGCGAGCGCTTTTTGCCATAGCGAAAAGGTCAGCAGATCCGGTGTAATTCCGGGGCCGACGGTT
AAAGTCCGGATGGGAGAGGGTAACGATCCAGTCGGGCATGGACCCGCTCACGTTATTTTTTTGCCGCTTGTCG
GCACTCCTAAGACTGCCCTGATTCTGGTAACCATAATGTTAATGAGGTTTTTTTACCATGAATCAGACGCTAC
TTTCCTCTTTTGGTACGCCTTTTGAACGTGTTGAACTTGCACTGGATGCGCTGCGTGAAGGACGCGGTGTGAT
GGTGCTTGATGATGAAGATCGTGAAAACGAAGGCGATATGATTTTCCCCGCAGAGACGATGACCGTAGAACAG
ATGGCGTTAACCATTCGTCACGGTAGCGGTATTGTTTGCCTGTGTATTACTGAAGATCGTCGTAAGCAGCTCG
ACTTGCCAATGATGGTGGAAAACAACACCAGCGCCTACGGCACCGGTTTTACCGTGACCATTGAAGCCGCAGA
AGGCGTGACCACCGGCGTTTCCGCCGCTGACCGTGTCACTACGGTGCGTGCGGCAATTAAGGATGGCGCAAAA
CCGTCCGATCTTAACCGTCCGGGGCACGTTTTCCCACTGCGCGCTCAGGCGGGCGGCGTGCTGACGCGCGGGG
GTCATACGGAGGCGACCATCGATCTGATGACGCTGGCCGGTTTTAAACCTGCAGGTGTACTGTGCGAGTTGAC
TAACGACGATGGGACGATGGCGCGCGCGCCGGAGTGTATCGCGTTTGCAGGCCAGCATAATATGGCGGTAGTC
ACGATTGAAGATCTGGTGGCTTACCGCCAGGCGCATGAGCGCAAAGCCAGCTGATCGAACGGATCATGGTTGA
AAAAAGCCGATGAGATGTCATCGGCTTTTTTGTATTCCTTCGGTGCCATATTGTTCAGTTTCAAAGAACGACG
AATAAGCTGAATGGTAAAATTATTGTGAGAAAAGCAGAGGCCTGAATGCAGACCTCTGCTTTGGTTTATCTAT
TCAGGTTATTTGCCTCTGGCCAGTTTCAATGCCCAGGCGCCGCTCATCACCAGCAGCAGAATCAGGCACAACC
CAAAGGCCAACATACATGCCTGCGCCATGTTCATAAAGTGCCAGGGCGGAAGCAGATACCAGCCTTCGGACTG
CTGGTAGAGATCGACAAACCACTGCTGTACGCTACTGAGCGTTACGCCATCAGGGACGATAGGCGCGTCATAA
CCGCAGTCTCCGGTAGGTTTAAACCACTCCGGTGCCCATTCCGCCAACGGTAAGTTGAAAGGAAAGGTCGGGT
CAGTCGAACATCCTTGCACGCCAAAAAGTGAATCGGGGTCCGCATTATGTACCGCATGATGGATACCATTAAG
CTTAATAGAAAACTTAATGCCCATAATGCTGCCATAAAACGCCGCGATGCAGCCAATCAGCTTCAGCACGATA
TTTTTGGGATTTATCGCCGCAATCACCCCGCCAATTACCATCACAAACATCGCGTAACGAATGTAAACACACT
GTTCGCACGGCGCCATGTAGAGATAGATTTGGAAGAACGAGTGCGCCAGGATAATTAACCCTCCCATCGCGAT
GGCCATTAACAGCCACAGAAAGCGCTGTTCCTGCCATCTCACCAGCGTATCTACTGGTCTTGCGCGTAGGTCT
CGCCATAATCCCTTAATAAAATCCATAATCGTTTCCCTGCTTATTTTTAGTGGCCAGTTCCCGAACGAGTTC
AGCCATAGAATCGATGGATTTGATGTTTTTGGTATAGATAAGGTACTTGCCGTTGACGACGTAAGCCGGAACA
CCCTGAATTTTGGCAACGTCATAGGCGGCTTTCCATTTTTCCAGAGTCTCCTGAACGGCAGGATCTTTCAGCG
CCGCTTCAAAATCAGCCTGGCTCATGCCTGCGGCGTCCAGCCCGGTTTTAATAAATGCCGCCGGATCTTTCCC
ATCTGACCAGCGCTCTTTCTTATCATGGTAAGCCGCATACCAGGCGAACTTCGCTTTTTTGAATTGTGATTTA

GCATCAAATAACGAAATCCCGGCAGCTTTATCTTTGGCGATCAGGACTGCAAATACTTCGCTGGCCTGTTTGC
CATATTCGCCTTTTGTTTCCAGGTGGAACGGAGTGAACGTAACGAGATCGGCTACTTTATCAGAAACCGGACC
TGTTACGGCTTTGTCATATTTGTAGCAGAAAGGGCAGGCGTAGCTAAATACCTTAATCAGCGTTTTATCCGCA
TTAGGAATCGGTTTCTCCAGAACCATATAATCTGTGCCCTCGGTAAAAGCCGATGCCGCAAAAGGAGTGACTA
ATGCCGCGGTTAATACTACGCTTTTAAATAAAGAGGTAATCCATTTAGATGACATATTAATTCCTTCCCGTTA
TTTATTTAAACATTTGCGTTGGATGAACCAGTAATGCACGATAGTGAGTCTGGTTGGGTTTATCCGACAGCAC
ATCAATTTCAACTTTCACTTCTTTCGTTTTATAGTCAATCTCATTCAGTTTGCCGACTGTGGGTTTACCAACG
TCGAACAGGTTAATAGAACCGCCAAAGCCGAACATGGTGTCGCGGTCTTTTTGATATTCAACAACCGAGGTAA
TAGGACTATAGAAATCATATCCACGTTCTTTACCGTATTCCCAAACTTGTTGTACGGTGCCTTTCTTCTCATC
GATCTTATATTCGACAAAACGGGAATATTTCATGGTCGGTAGAGCCGGTTGCTCCAGGCCGCGACCATCGCCG
TTATCAAAGACCGTTAACGTGCCTTTGCTGGAAAGCCATGCCGTATGTTGGGTATAGGTGAAATCGAAATCGG
TGTCCTTACACTTGCCGTTTTCGTCACAGGTCAACGGCTTACCATGATCGTCTACCGGTTTCAGCAATTTACT
GGCTAGCTGCTTATTCCAGCCTTTAGACGGTGCCAGTATCCATTTCACCTGCTTATCGCGACCAATTTTTACA
ATACCCTGATGGCGGGAAGAAAGGATGATGGAGTCGTCTTTCGCGTCATAAGCGATAGAGTTGACGTGCGCCC
AGTTACGACCGGCACCAACGCCAAGCGCATCGCCATACGGCGTATCCGGTTCGAGTTTCGCCTGCTGTCCGGC
ATGGGCCAGATCGACGTTCACGCATACTGCGCCCGCATCCAGCGCGCCGAGCAGCGCATCACGCATAGGGTCG
AGGATTTTGGTTAAATCCCAAACGTCTACTACGCGGCCAGACTTATCAACCTCAATGATTTGGTCACGAATCG
TATGAACATGTATGCCGTCTTCTTTGCGGTAATCGCGTTTGCCGACGCGCAGCAGTACGGTGCCGTTCACGGT
TTCGATGGATTCATGAGACGCATCCAGGAACCCGCGCGGCAGTTTGTGATCGGCAAGAATTTGCCCCATCATG
TCAAACTCGTACCAGTGTTGGCCCTGCACCGCGGTAAAGGTGCCGCGAGGCGTTTCACGAATACCCATCAGGT
AGCCGCGTTTGTTGATATTCATATCGTGGCCGTCGTAGAAGGTATCTTGATCCAGCCACCAGCGGTATTCACC
CTGGGTGTCAACCACAAACGTATAAGGGGCGATATCGAAAGGCAAAGCACCGCCCGCCGGACCGGCATCAAGA
ATACCCGCATTTTTATCTTTTTCGCCGTGCCAGTGAAATTCAGCGCCCTGCGGCGTAAAGGTATGGGTATTTA
CCAGATAAAGGCGATCTTCAAATCCTGGCGCGACTTTAATAACTTTCGTTTGCTGAAGATCTGAAATAGAACG
GTTATCCATATAATGGTTGACGATGGCGGACGTCTGCACCACATAGTCATCCTTCATGGCTTTGCCGTTTTCT
TTATATTCTACCGTGACGTTGTTGGCAAATTTCTGATAAAGGCCAAAAATAGGAATACCGTCATAGGTTTCTA
AAGACTCTTTCCCAACGGTATAAGTAACAGGAACGCCTTTTTCCCCTTTGCCATGTACAGTAACTTTAACGTC
TGAAATAATATGGCTATCTAATTCCACCAGCGCGGTGAGAGGGGCATTTCCGTAAGGATCGACAACGACTGCG
CCTAATTTCCCTGCGGGCTGTGCCGGTTGAAAACCTGCGGCAAACGTACTGGCTGCGGTGAGTCCGCATGTCA
GTGCGACGGCGCCGGCAAGTATTGTTTTCCGGTATTGGTCAAACATGGATATATCTCCTTATATACAACTTCT
AAATATAATACTTCTGAATATACTATGTTTTTATGAATAATAAATGAGGTAAATGATTGGTCTTTAAATATAA
GTCATGATGCTGTTACTTATTTTAAAGTATATTCTGTATCAATATTTTTTAATTGCCTGAGTGAGTCGATAGT
AGGAGTGTGATATTTTAAGAACAATATAAATTAGTACGAAGTGTGATCTTATCGACATTGTTTTCTGCTTAAT
ACCTCTTTCATTATTGTGTTTCACCACTGATTTATATGATCAAAATCATTTATTTGTTCCACAAATATGCCAG
TATATGAATACGTCAAAATAAGGGTATGTAAAATTTTAACGTTAAATGTTTAGTGATGTTAAATTTTAAGGGT
AAAAAGGATGTTTATTGCCTGGTATTGGATAATATTAATCGTTCTGGTGGTAGTAGGGTATATTTGCCATATG
AAGCGTTATTGCAGAGCGTTTCGGCAGGACAGAGATGCATTACTTGAAGCGCGAACGAAACGTTTTCGCCAGA
CGAGCGAAGGCGACAGCGTGATGAATGAGCAGAAATAGCGCATATCCTTGACGGGCAGCCGGATGTTGCTTGG
CTAAAGGCCTGCATCCGGCGCGACGTTTCTCTTAACCGATACCTATCGTCTGCAAAATGACGAGACTGAGCCC
CATGATGGACATACCGCAAAGCACGCCATAGCTGGGGTTATTGTTAGGATCGATCTCTTTTGCCAGCGGCATC
AGTTCATCGACGGAGAGCGCCACCATAATGCCGGCGACTGCCGCCATGATAGCCGCCATAACGATCGGTGAAA
CCAGGCTGCCCAAAATCAGCCACGCCAGTACGCCGCCAAGAATTTCCGCCATGCCGGAGATACCGGCCCAAAA
AATCGCGGTACGTTTTGAGCCCGTCGCGGCATAAACCGGGCCGGCAACCGCCAGACCTTCAGGAATATTGTGC
AACGCCACCGCCAGTGCGATGCCGAAACCCAGTTCAAGATTGCTGCTGGCAGTGACAAAGGTGGCGATCCCTT
CCGGAAAGTTGTGCAGGCTAATGCCGAGCGTCAATAAAATCGCAGTGCGTTTTATCGAGCCGGGAAGCGGCTG
CTGTCTTTTTTGCACCAGATCCTGCGGATGAGCGTGAGGAAGCAGGCGGTCCAGCCCGAAGTAGCCCAACAGG
CCGATAATAAACATTCCGTAGCCCAGTACAGGCGACATCCCCTCGGTATCAAGCGCGGCGGGCAGCATCTCCA
TCAGCGAGATAAGCAGCATGATCCCTGCGGCGAAGCCCAGCGAAAAAGCCAGCACGCGGTTAGACGGTTTTTG
GCCGAGAACGCCAAGAAACGCGCCAATAAAGGTGGCGGCGCCCGCCAGTAAGGTCAGAATAAGTGGTACTGAC
ATCCATTACTCCTTATCAATTTGCCGTCCGAACCTGACCTTCAAAATAACTGATGATCTTCATCATAGTTATC
TGAGTTCTTCCTCTGTGAAGAAAGCGTATGGTGATGTTAACAAAATGTCTTTCCTGTAAGGATAACATCATGT
CTTTAACGTGTATGCCAGCATTGTTTTTGGGGCATGGCAGCCCGATGAACGTGCTGGACGATAATGATTATAC
CCGCGCCTGGCGGCGATTGGGGGAAGCCCTGCCGCGACCACAGGCGATAGTGGTGGTGTCGGCGCATTGGTAT
ACCCGCGGGACGGGCGTCACAGCGATGGAAAGGCCGCAAACTCTCCATGATTTTGGCGGTTTTCCGCAGGCGC

TGTATGACACGCATTATCCGGCCCCTGGATCGCCTGCGCTGGCGCAGCGTCTGGTTGAACTGTTGGCTCCCGT
TCCCGTCGCGCTCGATAAAGAAGCGTGGGGATTTGATCACGGCTCCTGGGGCGTATTGATCAAGATGTATCCG
AATGCGGATATCCCGATGGTGCAGCTTAGTGTTGATAGTACCAAACCGGCGGCATGGCATTTTGAGGTGGGCC
GTAAACTGGCGACGCTGCGTGACGAAGGCGTGATGCTGGTGGCCAGCGGGAATGTGGTTCATAACCTGCGGAC
TGTGCGTTGGCACGGTGACAATATACCGTATCCCTGGGCGGCGTCATTTAATGACTTTGTGAAAGCCAATCTG
ACGTGGCAGGGGCCGGTAGAACAGCACCCGCTGGTGAATTATCTGCAACATGAAGGGGGGGCCTTATCGAACC
CGACGCCGGAGCACTTTTTACCTTTGCTGTATGTACTTGGCGCCTGGGACGGCAAGGAACCTATCACCATCCC
GGTCGACGGTATTGAGATGGGCAGTATAAGTATGTTGTCGGTGCAGGTGGGGTAGGCCGGATAAGGCGCTGAA
TGATTGCCCGATGGCGCTACGCGCATCGGGCTTACAGGAGAATCTATCCTTCGACAAAAATATGCGGATAGAA
GCGGGAGAGATCCTGGGTGATCAGCGCCCGATCTTCACGGATACCAATTCCGGCGGGCTGATCGTTAATCAGC
CAGCTACCAATCAGGGTGTAACTGTCGCCGAATTTTGGCAGCGGATAGAACTGTTGCACGATCATACCCTCTT
CGCCATACGGGCCTTCCACGGATTCGATCGTTTTACCGTTCTCAATAATCGATACGTTCGCCCCTTCGCGGGA
GAAGATCGGTTTCACGACATATTTATCCATTGGCGGATGTTCGTCTTCGGCGAAATACGCAGGCAACAGATTC
GGATGGTCAGGGAACATCTCCCACAACAGTGGCAACAACGCTTTATTGGAGATAATGCTTTTCCAGGCCGGTT
CCAGCCAGCGCACGCCGGCATCTTCCAGCTTGGTGGAAAACATCTCGCGCAGCATAAATTCCCACGGATAGAG
CTTGAACAGGTTCGCAATGACCTGATCCTGCAGATCCGTAAATTGGCCTTTTTCCCCCAGACCAATATCGTCG
ATATAGAGAAATTCGGTGGCGATTTCCGCTTCGGCCGCGCAGTCCTGTAAATACTGAATGGTGCCGCGATCCT
CAACCGTATCGCGACAGCAGGTGAGATGCAGCAACTGAAAACCGTACTGCTCGCGAAGTTCAGCAAAGCGTTC
AATCAGTTTTTCCTGCAGGCTATTAAACTGATCGCTGCCTTCCGGCAGATTGCCCGCGTTGATCTGATCTTCC
AGCCAAATCCACTGAAAAAACGCCGCTTCATACAGCGATGTTGGCGTGTCGGCATTATTTTCGAGCAGCTTAG
GCTCGCCGATGCCGTCCCAGGCCAAATCGAGGCGGGAATAGAGCGACGGTTGTTGCGTTTGCCAGGACTGGCG
AACAAAACCCCAGGTATGTTTTGGAATACGAAACTTCGTCATCAGCTCATCGCTGGCGATGACGCGTTCTACT
ACCTTGAGGCACATCTGGTGCAGCTCGGCGGTAACGTCTTCCAGTTTTTCCACCTGGGCGAGCGTTAACTTGT
AATAAGCGTCTTCACACCAGTACGGTTCACCATACATAGTGTGAAAATTAAACCCGTATTCAGTCGCTTTATC
GCGCCAGTCCGGGCGCTCGGTAATACTGACTCTTTCCATGCGTATCAGCCGCCCATCGAACGTGTTGAAGTAC
CGGCAGCGCTACGCTGCATGGTGCTCTGTTTGGCGACGGATTCGCCAAAACCGCCGCGGGTAACGGTTGTTGT
GGTGGCAGGTTTCGGCGCCATCGCGGTTTTCGGTACGGTCATTGTCCGGCCCGGTTGCGCCGCGCCGTAGTTT
TTACCTGCCGCATCGGTATATTTGCCGTATGCAGGGCTGGCCGGGTTTTTCGAGCTAAACAGCGGCTGTTGCG
CAAAGCCTGCGCCGCCGCCCATCAGACGCCCCATCATGTAACCTGCCATAAGCGGCATCCAGAAACTGCCGCT
GGATTGTTGGGCCTGCGCCTGATTTTCCGGCGCCATGCCAGCCTGTGCGGGCGCTTGCTGGCACTGACCTTCG
CCAAACTCAGCGACGCAATCTTCGCGTGTAGCGTACTTAGGCGCAGTACGTTCGGCCTCTTTCAGCGCATTGT
TATACGCGGTTGTACACTCCGCGCTTTTGCCCGGATTCGCCGCTGAACAGTCATCAGCGTTTTGATACAGCGA
TACGGTGTCATCGCTTTTTTCACAGCCAGCCAGCATAAAAACAGCCGTAACCGCCAGGGCGACCGGCGTTAAA
TGCCGCGCGCTCCAGCTTTTGCGAAATGATGCGTGATGGATGGATTTTGTCCGTTTCATATTTGTCTTCCAGG
ACCCAGTGGTAAATACAACATGCGTTCAGAATAGAGGATGACTGGTCGAAATTGAAGCGAGAAAAGGCGAGAA
TGCGGCGGAATAGCAGGATCTTTACGTTGTCTTACGTTCAGGCGCGGCTTTATGACAGGTAGCGCCGCGCTTA
CCAGACCTACAAGGGCACAGGTCTGATAAGCGCAGCGCCAGCGAATAACTTATCAATGCCGGAATGGATTGCC
GTTATTGCTGTTGGCGCGAGCGGCGGTCGGCTGTACTGCCGGCGCGGCGCTATGGGCATTATAACCGTCTGCG
GCAGCATCCTGATCTGGCGTTTCCGGCGCTACGCTTTCCGGCGACGTCGGGATAGGTTTACCCAACGTACTGT
TAAGCGCGAGCAGATCCTGCTCGTTCAGCGTACCGAGCGCATATTTGATATTTAACTGATTAATCAAATAGGT
ATAACGCGCGTTGGCCAGTTGCTGCTTGGCATCATACAGAGTGGTGGTGGCATCCAGTACGTCAACAATGGTA
CGTGTACCGACCGAGTAACCGGCTTCCATTGCATCCAAAGAACTTTGCGCGGAAACGACTGCCTGTTTATACG
CGTTGATGCTGCTGATGGAGGCGTTAATATTGTTAAAGGAAGAACGTACGGTCTGCACCACGCTACGGTGCGC
GCTTTCCAGCTGTTCGCTTGCGCCGACGAAGTTATACTGCGCCTGTTTTACCTGCGAGTTAACCATCCCGCCT
TGATACAGCGGCAGGGAGAAGTTCAGGCCGATTTTATTCTGCCCCATGTTGCTATCGTCGTACTGGGCGGCGT
TGGTTTTAGAACCGCTATAAGAGGTATCAGAAATGCCGGTTGAGGCCGTTAAATTCAGCGTCGGCAGATGACC
ATCCTGCGCCTGACGGATTTGCTCGCGCGCCAGATCCTGACTTAAACGCGCCTGCAACAGCGACAGGTTACGG
TTTTCCGCTTCCTTCAGCAGCGCATTAACAGCTTTAGGCTTGTCGGTTTTAAAATGCTCGACGTTAAGCGACG
CCAGCTCCGGGTAATAATTGCCGGTTACCTGGCGCAGCTCTTCTACCGCGTTATCCAGGTTGTTGCGGGCGGT
CACTTCATTCGCCAGTACGGTATCATATTGCGCACGGGCGTTTTGCACGTCGGTAATGGCGACCAGACCCACG
TTAAAACGTTGCGTCGTTTGATCTAACTGACGGTAGATAGCCTCTTTTTGCGCCTGGGTATAGGAAAGCACAT
CAATAGCGTTCAATACCTTAAAATACGCGTTCGCGGTATTGAGGATCAGCGTCTGCTGATCGGTCTGATAGGT
GACATCCTGAATGCCTGCTGCTTTTTCTTGCAGGGTGAGCCCACGCCATTTCGACATATCAAACAGTGTCTGC
GTTAATTGCAGGGAAGCGCTGGTTTCATTGGAGTTGATACCGTTCGCATCGCGATAACCGTTGCTGTAGGTGT

AGTCGGCACCTAAACCCAGTTGCGGCAGTAAAGGACTACGCGCTTCGTTAATTTTTTCGAATGCAGCATCGCG
ATCGGCAGCGGATTTACGCAATTCCGGGTTGCTCAGGCGTGCTTGCTGATAAACTTGCATCAGGTTCTCTGCC
TGGCTTAGTGTGCTGAACCCCGACAGGCTCAGGCCGATAAGGATGGGGAGCAATTTCTTCATTTGCATTCCTT
GTTGTGAAGCAGTATTTAGCGCTGATCAATTTGTAAAAATAATTGCTGATTCTAGCAGAATCCGCCACTGCAA
AAAGTTGGCGTTACGTGCCATACGAGCCTTAATTGGCACCAATTTACCACAAGGGTGATAAAACAGCAGTCAA
CCACCCTTGAAATTCATCATTTAATCATTATTGCTAGCAGGGCAGCACAATGCGTAAATCAGACAACTTGCCA
GTTACTTTCACCAAAAGCGATGTAGAAATTATTGCACGGGAAACGCGCTATCGCGGTTTTTTTTCATTGGATG
TGTATCGATTCCGCCATCGTTTATTCAACGGAGAAATGAGTGGCGAGGTAAAGCGCGAAATTTTTGAGCGCGG
GCACGCCGCCGTCTTGCTACCCTTTGACCCTGAACGCGACGAGGTGGTGCTGGTCGAACAGATTCGTATTGCC
GCGTACGACACAAGCGTTACGCCGTGGCTGCTGGAGATGGTCGCCGGCATGATAGAAGAAGGCGAAACCATTG
AGGCGGTCGCCCGGCGGGAGGCGATGGAAGAAGCGGGGCTGACCGTAGGCCGCACCCGACCGGTCATCAGTTA
CCTGGCGAGTCCAGGAGGAACCAGCGAGCGCTCATCTATTCTGGTGGGTGAAGTGGACGCCACGACCGCGGTA
GGTATTCATGGTCTGGCGGATGAAAACGAAGATATTCGGGTTCATGTGGTAAGCCGGGAACAGGCATACCAGT
GGGTAGAAGAGGGGAAAATCGACAACGCAGCCGCAGTCATCGCTTTGCAATGGCTTCAGTTGCATCATCAAGA
GTTAAAAAACGAGTGGAAAAAATGAAGCGTTATACACCTGACTTCCCAGAAATGATGCGTTTGTGCGAAACCA
ATTTTTCGCAATTGCGCCGCCTGCTGCCGCGCAATGACGCCCCCGGTGAAACGGTGAGCTATCAGGTGGGCAA
CGTACAATATCGGTTAACGATTGTGGAATCGACCCGATACACTACGCTTGTAACGATCGAGCAAACGGCGCCA
GCCATCACCTACTGGAGCCTGCCGTCGCTGACGGTGCGCCTCTACCATGATGCGATGGTGGCTGAAGTGTGTT
CAAGCCAGCAGATCTTTCGCTTCAAAGCGCGGTATGATTATCCAAATAAAAAGTTGCATCAACGCGACGAAAA
GCATCAAATTAATCAGTTTCTGGCCGACTGGCTACGATTCTGTTTAGCACATGGAGCGATGGCGATTCCGGTT
TATTAGCGTCGTGAAACCTAAGGACACCATTTGGAAAGCCTGTTAACCCTACCTCTGGCTGGTGAGGCCCGAG
TCAGGATTTTACAAATTACAGATACTCACCTGTTTGCCGAAAAGCATGAAACGCTGTTAGGCGTCAATACCTG
GGAAAGTTACCAGGCCGTGCTGGAGGCTATTCGGGCGCAACAGTATGAATATGATCTGATCGTGGCCACCGGC
GATCTGGCGCAGGATCAATCCGCTGCGGCTTATCAGCATTTTGCTGAGGGTATCGCAAGCTTTCGCGCGCCTT
GCGTTTGGCTACCGGGCAATCATGATTTTCAGCCGGCAATGTACAGCGCGCTGCAAGAGGCGGGGATTTCCCC
GGCTAAACGCGTGTTAATCGGTGAACAATGGCAGATTTTGTTGCTGGATAGCCAGGTGTTTGGCGTCCCGCAT
GGCGAACTGAGCGAGTTCCAACTGGAATGGCTTGAGCGTAAGCTCGCCGATGCGCCAGAGAGACAGACATTGC
TCCTGTTGCACCACCATCCGTTGCCGGCAGGGTGCAGTTGGCTGGATCAGCACAGCTTGCGTAATGCTGGTGA
GCTGGATAGCGTGCTGGCGAATTTCCCGCGTGTAAAGTATCTGCTATGCGGACATATTCACCAGGAGCTGGAT
CTCGACTGGAACGGACGGCGTCTGCTGGCGTCGCCGTCAACCTGCGTACAGTTCAAACCGCACTGCGCGAACT
TTACGCTGGATACTATCGCGCCGGGCTGGCGTACGCTGGAGCTCCAGGCGAACGGCGTCCTGGAGACGGAAGT
GCATCGCCTGCAGGATACCCGGTTCCGACCCGATACCGCTTCGGAAGGATACTGATGTCTACGCTTCTCTATT
TGCACGGATTCAACAGTTCCCCTCGCTCGGCAAAAGCGTGCCAGCTAAAAAACTGGCTGGCGGAGCGTCATCC
GCATGTCGAGATGATCGTCCCTCAACTACCGCCGTATCCTGCCGATGCGGCGGAGTTGCTGGAATCTCTCGTG
CTTGAGCATGGCGGTGCGCCATTAGGGCTGGTAGGATCGTCGCTGGGTGGTTATTACGCCACCTGGCTGTCGC
AATGTTTTATGCTGCCGGCTGTGGTGGTGAATCCCGCCGTGCGGCCCTTTGAATTACTGACCGACTATCTCGG
TCAGAACGAGAACCCCTACACCGGGCAGCAATATGTGCTAGAGTCTCGCCATATTTATGATCTTAAAGTCATG
CAGATTGACCCGCTGGAAGCGCCGGACCTGATCTGGCTACTGCAACAGACGGGCGATGAAGTGCTGGATTACC
GCCAGGCGGTGGCATATTACGCCTCCTGCCGTCAGACAGTGACCGAGGGTGGTAATCACGCATTCACGGGCTT
CGAAGATTATTTCAACCAGATTGTCGATTTTCTTGGACTGCACAGTTGCTGACAATCAATTCGAATTGCTAAT
TAAACCATGACGCAAACTTATAACGCTGATGCCATTGAGGTACTCACTGGGCTTGAGCCGGTACGCCGCCGCC
CGGGGATGTACACCGATACGACCCGTCCCAACCATTTGGGTCAGGAAGTAATTGATAATAGTGTGGATGAAGC
ACTGGCAGGTCACGCCAAACGCGTGGATGTCATTTTACATGCCGATCAATCGCTGGAAGTGATTGACGACGGA
CGCGGGATGCCGGTGGATATCCATCCGGAAGAGGGGGTTCCGGCGGTAGAACTGATCCTCTGTCGGCTTCATG
CCGGCGGTAAATTCTCCAATAAGAACTATCAGTTCTCTGGTGGTCTGCATGGGGTGGGGATTTCGGTCGTGAA
TGCCCTGTCAAAGCGCGTGGAAGTGACCGTGCGCCGCGACGGTCAGGTCTATAACATCGCGTTTGAAAACGGC
GAGAAAGTGCAGGATTTGCAGGTTGTCGGCACCTGCGGTAAACGTAATACTGGCACCAGCGTCCATTTCTGGC
CGGACGAAAGTTTCTTCGACAGCCCGCGTTTTTCTGTCTCTCGCTTAATGCACGTTCTGAAAGCAAAAGCGGT
GCTGTGTCCCGGCGTGGAAATCACTTTTAAAGATGAAGTGAATAACAGCGAGCAGCGCTGGTGCTACCAGGAT
GGTCTGAACGACTATCTGGGCGAAGCGGTAAACGGCCTGCCGACGCTGCCGGAAAAGCCGTTTATCGGTAATT
TTAACGGTGAAACGGAAGCTGTTGACTGGGCGCTATTGTGGCTGCCGGAAGGCGGCGAATTACTGACGGAAAG
CTACGTCAACCTGATCCCGACCATGCAGGGGGGACGCACGTCAACGGTCTGCGCCAGGGCCTGCTCGACGCGA
TGCGCGAATTTTGCGAATACCGCAATATTCTGCCGCGCGGCGTCAAACTGTCGGCGGAAGATATCTGGGATCG
CTGCGCTTATGTGCTCTCCGTGAAAATGCAGGACCCGCAATTTGCCGGGCAGACCAAAGAGCGTCTGTCGTCG

CGTCAATGTGCGGCATTTGTTTCCGGCGTGGTGAAAGATGCCTTCAGCCTGTGGCTGAACCAGAACGTGCAGG
CGGCGGAACAACTGGCAGAGATGGCGATTGCCAGCGCGCAGCGGCGACTGCGCGCCGCAAAAAAAGTGGTACG
CAAAAAGCTCACCAGCGGTCCGGCGTTGCCGGGGAAACTGGCGGACTGTACCGCGCAGGATCTTAATCGGACC
GAGCTGTTCCTTGTGGAAGGGGATTCGGCGGGCGGTTCCGCCAAGCAGGCGCGCGATCGCGAATATCAGGCGA
TCATGCCGCTCAAAGGTAAGATCCTTAACACTTGGGAAGTCTCTTCCGATGAAGTGCTGGCCTCGCAAGAGGT
GCATGATATTTCCGTGGCGATCGGTATCGATCCGGACAGCGACGATCTGAGTCAGCTGCGCTACGGCAAGATC
TGTATCCTGGCGGATGCGGACTCCGATGGTTTGCATATCGCTACTCTGCTTTGCGCGCTGTTTGTCAGACATT
TCCGCGCGCTGGTGAAGAATGGTCATGTCTACGTCGCGCTGCCGCCGCTATACCGTATCGATTTGGGTAAAGA
GGTCTATTACGCGCTGACGGAAGAAGAGAAGGCGGGCGTACTGGAACAACTGAAGCGTAAGAAAGGCAAGCCG
AACGTACAGCGTTTCAAAGGCCTGGGTGAAATGAACCCGATGCAGTTACGCGAAACCACGCTTGATCCAAATA
CTCGCCGCCTGGTGCAGCTCACCATTAGCGATGAAGACGATCAGCGTACTAACGCGATGATGGATATGCTGCT
GGCGAAGAAACGTTCTGAAGATCGACGCAACTGGCTACAGGAAAAAGGCGATCTCGCGGATCTCGACGTGTAA
CGATTGTTGCACGCTTTATCCGGTCTGCGCGCCGCAGGCCGGATAAGCGAATTCGACATGACGCCGCAGCCTG
ATGGCGACACTTGCGCATCGCATCAGGGAGACGTCAGACGAGGGTAATAACTTAAACGCCGGATTCCAGAATA
CGAATATTCATTTCCAGCACATCGTCTTTTACCGCGTCGCTGTAGGCTTTCATATGCGGGGTTTGCAGATGCG
CCTCAAGGTGCGCAACGCTTTCCCACTGTTCAATCATCACGATCGAGTCAGGCGCCAGAGTCTGAAAACTCAC
GCCCGCGGCATGATCCACCATCGGTGCGTAACCGTGGCACCCCTCTTCTTTAAGAACGGTTGGAATAATTTTC
GCAAACTGGTCGAGGACGGCCTGACGGTGATGTTGGCCCGGACGCGTGCGAATTTCGGCAATGACGGTAAGCA
TGGTTAACTCCTTCTAATTCCAGGATGACCAGTTAAGCAAAAATTTCGCTCAGATGCTTGCGATATTCTGCGG
TATAGCGCGGAACATCCGGCATTTTAATCACGTCGTTGGCAATAAACGTTGGCAGCGCGTCCATGCCCAGGAA
CTGGTTGGCTTTATGGAATGGCAGATAGACGCCATCTACGCCTACGCCGTGGAAAAAATTGATCTTTTTCGGTA
AATGCTTCCATCGGCGCGTTCCACGTCAGTGACAGCATATATTTTTTCCCCTGCACCAGACCGCCGGAACCGT
ACTTTTTAGCGGCATCGGAACGCGTGCGGCCATCGCTGGCATAGAGCGTGCCGTGACCTTCGGTAAAGACATC
ATCCATATATTTTTTTACTGTCCATGGCGCGCCCATCCACCAGCCTGGCATCTGCCAAATCACCACGTCAGCC
CAGACGAAATTCTGCACCTCTTCTTTAATGTCATAGTCGCTGTCGGCGCGGACGATCTTAACCTGATGACCCA
GATCGCGCAGCAGCCCATCGGCAACTTCGGTCAGGGTGTCATTTAACTGGCCGTTGGAGTGCGCGAATTTTTT
CGCCCCGTTGATAATCAGGATGTTACTCATTTTGTGTCCTCAAGAAATGCGTTTGCTGCTGATTCTACGCTGA
CGGGCGGTGCGGTGAAATTAGCAAAATGTGCAAAGTCTTTTGCGTTTTTGGCAAAAGTCTGTGTTACCAACTT
ACTACGGCCTCAAATCCACCTTCCGCGGCGTTGCCAAACGACACCGTCATGCCGTGCAGCGTAGCGATGCGAC
GGACGATTGATAATCCCAGACCGCTGCCGGTCACACTTTGGCCAGGCGGGCGGTAAAAACGCTCGCCGATATG
CGTCAGAATCTCTGGCGCTACACCAGGGCCATTATCTCTGACGGTGAAGCTGCGGGCATGTAAGGTAACGTCG
ACGACGCTGCCTTGCGGACTGTAGCGAATCGCATTATCCAGCAGGTTACGCACTAACAGGCTTAAGAGTAGCG
GCTGGCCGGTACGTATGACGTCATGGGCGTTAAGCTGTAAACGCACGTCGATATTCGCCTGCTGCGCCGGATG
ATAAATATCCATTACCGCGGACTGCAGCAGCTCTTCCAGCGAAATCTCCGCTACATCCTGCAAATTATTGAGC
GAGTCGAGACGCGATAACGTCAGTAGCTGATCGACCAGCCGCGTGGCGCGATCGATCCCCGCATGGAGCTGGG
TCAGCGCTTTATCACGCGATAGCGGATCGTCGCCCGAGAGCTGCGCCACCTCGGTTTGTACTTTCAGCGCGGC
CAGCGGGCTGCGCAATTCATGGGCGGCGTCGGAGGTAAATCGCCGTTCGCGCACCATCATGGAGTGGATGCGG
CTAAAAAGCTGATTAAGCGCTTCGACCAGTGGGCGAACCTCGCTCGGTACGCCTTTGGCGTCGAGCGGCGTTT
CCGATTCCGGCGACCGGAAGCGTAGCGCCTGCGCCAGTTTTTTCAACGGTCTGAGCTCGCGGTGAAGCAGGAG
CAATAAAATCAGCAGCATGAAGGGAAGGGCGATCAGCCACGGCGTTAATTGCGCCGCTACGATCGCCAGCGCC
ATATCTTCGCGGTAATCCCACTCCTGACCGACGACGATACGATACTTGCCGTCCGCCGAGTTCAACCACAAAA
AACGCCAGAGGTCGTTGTCATCCTTCAGATAGCCATTGTCGAAACCTTCCCGGCGATAGCGATACGGAATATC
CTGGCCGTTGTCGCCATCATGCAGCAACATTTTGCCATCAGCGGAAAAGATGGCGAACGCCAGCGCATCGTCA
TCGATATGCCCATGTTTTAATTTCTTTGGCGTATGCGCCATGCGCTGCGGGGCGTTGAGCTCGTTAAGATCGA
GGGTGCTAAGCCGTCTGGCGAACAACATCAACTGTGTGTCAAACAGCTCGTCGACATTATCCGTCGTCTTTCT
CCAGGCCACAAAACTGGAGATTGCCCAGGTGATGGAGACCAGAATCAGGAAAATAAGCGTCAGCCTGACGCGC
AGGCTGAGACGTTGCGTCAATTTCATGCGTCACCCAGGGTGTAGCCGATGCCGTGCACGGTGCGAATAAATTC
GCTGCCGAGCTTGCGGCGTAAATGATGAACATGGACTTCCACTGCATTGCTGGACACGTCGTCATCCCAGTTA
TACAGCTTCTCTTCAATAAGCTTGCGCGGCAGCACGCGACCTTTGTTGCGCAGTAAGAGTTCCAGCAACGCGA
ACTCTTTTGGTTTTAGGGCCAGCGGTTCGCCGGCAAGCGTCGCGACGAGATTGCCTGGATTGAGCGTCACCTG
GCCGTGGCGCAATTCGCTACTGGCCTGACCGCTGGCGCGGCGTACCAGCGCCTCCAGACGGGCGGCGACCTCA
ATGAGCGCGAAAGGTTTACATAAATAGTCATCGGCGCCCAGACGTAGCCCCTCGACGCGCTCTGCTAACGCGT
CGCGCGCCGTCAGGATCAGTACCGGTTCCTGTTTACCCTTTTCGCGCCATTCGCGCAAAATATCCCGTCCGTC
CATGCCTGGCAGCGTCAAATCTAAAATAACCGCATCATAAGGCGCGCTGTAAAGCGCCTCTTTGCCCGGACGT

CCCTCGGTAAACCAGTCGACGCTGAAACCCATTTTACTCAGGCCCGCTTTTATGCCATCGCCAATAAGTGTGT
CATCTTCTACCAGTAAAATTCGCATCTGTTCTTCCTTGCGGTAACTCGCGTTACCGTCAGTTAACCTTGTCGC
CGCAGGCGATGTACAGAAAAAAGATTCTTTTTTTGCCTTAAGAAGTTGTTAAGGATTACCTTATTAAATGGAT
CTGAAACGACATGAAAGGGAAAAGTAATCATGAAAAAATTAGCTGCAATCGTTGCCGTTATGGCACTGTGTTC
TACCCCGGTTCTGGCGGCTCAGCAGGGCGGTTTCTCCGGCCCGTCGGCGACGCAAACGCAAGGCGGCGGATTT
GTCGGCCCGAACGGCAGCAGCACGACGGTAGAGAGCGCTAAATCGCTGCGTGACGATGCGTGGGTAACGTTGC
GCGGGAATATCGTCGAGCGCATTTCCGACGACCTGTATGTGTTTAAAGATGCGACCGGTACTATCAATGTGGA
TATTGACCACAAGCGCTGGAACGGCCTGACCGTAACCCCGCAGGATACCGTTGAAATTCAGGGTGAAGTGGAT
AAAGACTGGAACTCTGTCGAAATCGACGTAAAACAGATTCGTAAAGTGAATCCGTAATCCCACATTGCGCAAG
ATCGGTCAGGATTCACAGCCGCCCGGTGGGCAGAATAGGCCACAAGGAGGCAGTGATGAATGACCTGATCAGC
GCGGCTTATTCCGAACGTCTGCGGCGCGTTTGCGACCACATTGAGCGTCATCTGGATGAGCCTCTGTCGCTTG
AGGCGTTAAGCCGGATGGCGCACAGCTCGCCGTTTCATTTTCACCGGCAGTTTACCGTCTGGAGCGGGCTTCC
GCTCTATCGCTATATCCAGTGGCTGCGTTTGCGCCGGGCGTCATGGCGGCTGGCGTTTAATCCGCAAGATAAA
GTTATCGATATCGCGCTGGATGCCGGATTTCAGAATCCGGAGTCGTTCACACGTGCTTTCAAAACCGCGTTTG
GTCAAAGCCCGTGCCGGTTTCGGCAATCGCCGGACTGGCTGGCCTGGCACCAACGCGTTCCTAAACTTGCGTT
ACAGGAGCAGCATGTCATGGACGTAAAAATCGTTGAATTTCCACCTACCCGCGTCGCGATGCTGACGCATCTG
GGGCATCCGGATAAGGTCAACGCCAGCGCGGCGAAATTTATTGCATGGCGACGCGAAACGGGCCAGTCGCCGA
TTGCCAGCAGTCAAACTTTTGGTATTGCCTGGCACGACCCGCAGACGACGCCGCCGGCTCAATTCCGCTTTGA
TATCTGCGGCAGCGTCCGCCAGCCGATAGCCGAAAATGACGTTGGCGTGGTGAATAGCGAGATTCCTGGCGGG
CGCTGCGCGGTGGTGCGCCATCAGGGATCGCTCGACAGCCTCCCGGAGAGCGTGTGGTATTTATTCCGCGAAT
GGCTACCCGCCAGCGGTGAGACGCCGCGCGATTTTCCGGTCTTCTTCCAGTACCTGAATTTCGTCCATGAGGT
CGCGGAACATGAGCTGCTGACGGATATCTATCTGCCGCTTCGTTAACTTCCTCGCCACCTGTCTCACGGTTTG
TGACTCAGACCGTGAGATAGGGTATTATCTGCGGCAATATTGCCGTCCGAACGGTCAGCATGCAGATTGAGGA
TCCCCTGTTAATGAGCGATATGGCAGAGCGCCTTGCGCTACATGAATTTACGGAAAACGCCTACTTAAACTAC
TCCATGTACGTGATCATGGATCGTGCGTTGCCGTTTATTGGCGACGGCCTGAAGCCGGTACAGCGCCGCATCG
TCTATGCGATGTCAGAGCTGGGGCTGAACGCCACCGCTAAATTTAAAAAATCCGCCCGTACCGTTGGTGACGT
ACTGGGTAAGTATCACCCGCATGGCGACAGCGCCTGCTATGAAGCCATGGTGCTGATGGCGCAGCCGTTCTCT
TACCGTTACCCGCTGGTCGATGGCCAGGGGAACTGGGGCGCGCCGGATGATCCGAAGTCATTCGCGGCGATGC
GTTATACCGAATCCCGCCTGTCCAAATACGCCGAGCTGCTGTTAAGCGAACTCGGTCAGGGGACGGCGGACTG
GGTGCCAAACTTCGACGGCACCATGCAGGAACCGAAAATGTTACCGGCGCGTCTGCCGAACATCCTGCTGAAC
GGCACCACCGGTATTGCGGTGGGCATGGCAACAGATATTCCGCCGCATAACCTGCGCGAAGTGGCGAAAGCGG
CGATTACGCTGATTGAGCAGCCGAAAACGACGCTGGATCAGTTGCTGGATATCGTCCAGGGGCCGGATTACCC
GACCGAAGCGGAGATCATTACCCCTCGTGCGGAAATTCGTAAAATTTACGAAAATGGGCGTGGCTCCGTGCGT
ATGCGCGCGGTATGGACCAAAGAAGACGGCGCCGTGGTGATCACCGCGTTGCCGCATCAGGTATCTGGCGCAA
AAGTGCTGGAGCAGATTGCTGCGCAGATGCGCAATAAAAAACTGCCGATGGTGGACGACCTGCGCGATGAATC
GGATCACGAAAACCCGACGCGTTTAGTGATTGTCCCGCGCTCCAACCGTGTGGATATGGAGCAGGTGATGAAC
CATCTGTTCGCCACCACCGATCTGGAAAAAAGCTACCGTATTAACCTGAACATGATCGGTCTGGATGGTCGTC
CGGCGGTGAAAAACCTGCTGGAGATCCTCACCGAGTGGCTGGCGTTCCGCCGCGACACGGTGCGCCGTCGTCT
GAACTATCGTCTGGAGAAAGTGCTTAAGCGCCTGCATATCCTCGAAGGTTTGCTGGTGGCGTTTCTCAACATC
GATGAAGTGATCGAGATTATCCGTAACGAGGATGAGCCGAAACCCGCGCTGATGTCGCGTTTCGGCATCAGCG
AAACCCAGGCGGAAGCGATTCTCGAACTGAAACTGCGTCATCTCGCCAAACTGGAAGAGATGAAAATTCGCGG
CGAGCAGGACGAGCTGGAAAAAGAGCGTGACCAGTTGCAGGGCATTCTCGCGTCCGAACGCAAAATGAATACC
TTGCTGAAAAAAGAGCTACAGGCAGATTCCGATGCCTATGGCGACGATCGCCGTTCTCCGCTGCGTGAGCGCG
AAGAAGCTAAAGCGATGAGCGAACACGACATGCTGCCGTCCGAACCGGTGACTATCGTGCTGTCGCAGATGGG
CTGGGTGCGCAGCGCCAAAGGTCATGATATTGATGCGCCGGGGCTTAACTATAAAGCGGGCGACAGCTTTAAA
GCCGCGGTGAAAGGTAAGAGTAATCAACCGGTGGTGTTTATTGATACCACCGGGCGCAGCTATGCTATTGATC
CCATTACGCTTCCGTCGGCGCGTGGGCAGGGCGAGCCGCTGACCGGCAAACTCACGCTGCCGCCGGGGGCGAC
CGTAGAGCATATGCTGATGGAAGGCGATGACCAGAAACTGCTGATGGCGTCGGATGCGGGCTACGGCTTCGTT
TGTACGTTTAACGATCTGGTTGCCCGTAACCGTGCCGGTAAGGCGTTGATTACACTGCCGGAAAATGCGCACG
TCATGCCGCCGCTGGTGATTGAAGACGAGCACGATATGCTGCTGGCGATTACCCAGGCCGGACGGATGTTGAT
GTTCCCGGTAGACTCTCTGCCGCAGCTGTCGAAAGGCAAAGGGAATAAGATCATTAATATCCCCTCTGCAGAA
GCGGCGAAAGGCGATGATGGACTGGCGCACCTGTACGTGCTGCCGCCACAAAGCACTCTGACTATCCATGTCG
GGAAGCGCAAAATCAAACTGCGCCCTGAAGAGTTACAAAAGGTAGTCGGTGAACGCGGACGCCGTGGCACATT
AATGCGCGGCCTGCAGCGTATCGATCGCATTGAGATTGATTCGCCGCATCGCGTAAGTCATGGCGACAGCGAA

GAGTAAGACAGGGGCGCCCGTAGGCCGGACAAGGCATCTAAGCCGCCATCCGGCGCTCTGCCGTTGCCGGATG
ACGACGCAGTAGCGTCTTATCCGGCCTACGGTTTCAGATCGGATTAACATTAAAATTTCCCGTAAAACCGTTG
TTTATTCGTGCATTGCGATTAACAATACGTTTTTCCAGAGAGCCACACTTACCAAAATCCTAACCTGAATTTC
AGGTGACGTAGAATGCCGGGCTCTTAGGCCTTCAGAGGTTGTTATGCTATATATTTTTCGTCTTATTGTTACC
GTTATTTACTCCATTCTGGTCTGTGTTTTCGGTTCTATTTACTGCCTTTTCAGCCCACGTAACCCAAAACACG
TGGCGACGTTTGGACATATGTTCGGTCGTCTGGCCCCGCTGTTTGGCCTGAAAGTGGAGTGCCGCAAACCCGC
CGACGCGGAAAACTATGGCAATGCTATCTATATTGCTAACCACCAGAATAATTATGATATGGTCACGGCGGCG
AATATTGTGCAGCCTCCAACGGTGACGGTGGGAAAAAAGCCTGCTTTGGATCCCGTTTTTTGGGCAACTGTA
CTGGCTGACCGGTAATCTGTTGATTGACCGTAATAACCGCGCCAAAGCGCATAGCACCATTGCGGCAGTGGTT
AACCATTTTAAAAAGCGCCGTATTTCTATCTGGATGTTCCCTGAAGGTACGCGTAGCCGGGGACGCGGTCTGC
TGCCGTTTAAAACCGGCGCGTTTCATGCGGCTATCGCGGCTGGTGTGCCGATCATTCCTGTCTGCGTTTCCAA
CACCTCTAACAAAGTCAATCTAAACCGCCTGAACAACGGACTGGTTATTGTGGAAATGCTGCCGCCAGTCGAT
GTCAGCGAATATGGTAAAGATCAGGTTCGCGAGCTGGCGGCCCACTGCCGTGCGTTAATGGAGCAAAAAATCG
CGGAACTTGATAAAGAGGTTGCGGAACGTGAAGCGACCGGTAAGGTTTAAGCAGTCTTACTCTGTTATTGTAG
AAATCATTTTTTCAGGCACAACTCTTTAGTCTGTTTTACATGGAGCTTATATGTCATTCAGTCGGCGTCAGTT
TCTTCAGGCATCGGGAATCGCACTATGTGCAGGCGCGATTCCCCTCAGGGCGAATGCTGCCGGTCAGCAACAG
CCGTTGCCTGTTCCGCCGTTGCTGGAGTCCCGTCGCGGGCAGCCGTTGTTTATGACGCTACAGCGAGCGCACT
GGTCCTTTACGCAAGGCACGCGCGCGCCGGTCTGGGGAGTGAACGGTCGTTATCTGGGGCCGACAATCCGCGT
CTGGAAAGGCGATGACGTTAAACTCATCTATAGCAACCGCCTGGCGGAAAATGTCTCCATGACGGTTGCTGGT
TTACTGGTGCCGGGGCCGCTGATGGGTGGCCCGGCGCGTATGATGTCGCCGAACGCGGACTGGGCGCCGGTGT
TGCCGATTCGTCAGAGCGCCGCGACGCTGTGGTATCACGCTAACACGCCTAACCGCACTGCGCAGCAGGTGTA
TAACGGCCTGGCCGGAATGTGGCTGGTCGAAGACGACATTAGTAAAACGCTGCCCATTCCGAACCATTACGGC
GTGGATGATTTTCCCGTCATTATCCAGGATAAACGGCTGGATAACTTTGGGACGCCGGAGTACAGCGAACCGG
GAAGCGGTGGTTTTGTCGGCGATACGCTGCTGGTGAACGGCGCGCAAAGCCCCTATGTCGAAGTGTCCCGCGG
CTGGGTGCGGTTACGGTTGTTGAACGCCTCGAACTCGCGGCGCTATCAGCTTCAGATGAGCGATGGCCGGGCG
CTGCATGTGATTTCAGGCGATCAGGGCTTTTTACCTGCGCCGGTTTCCGTCAAACAGTTGTCACTGGCGCCCG
GCGAGCGACGTGAAATCCTGGTTGATATGACCAACGGCGACGAAGTCTCGATCACCTGCGGGGAAGCGGCCAG
CATAGTCGATCGGATTCGCGGTTTCTTTGAGCCTTCCAGTATTCTGGTTTCGACCCTGGTGCTGACGTTACGG
CCAACCGGATTGCTGCCGCTGGTGACCGATAATCTGCCGATGCGTCTGTTGCCGACGGAGATCATGAGCGGCG
CGCCTGTCCGCAGTCGGGACATCAGTCTTGGCGACGATCCGGGGATTAACGGCCAACTGTGGGATGTGAATCG
CATTGATATTACCGCCCAACAAGGCACCTGGGAGCGCTGGACGGTACGCGCCGATATGCCGCAGTCGTTCCAT
ATTGAGGGCGTCTCTTTCCTGATTCGTAACGTCAACGGCGCTATGCCGTTCCCGGAAGATAGAGGCTGGAAAG
ATACCGTCTGGGTGGATGGGCAAGTCGAATTGCTGGTCTATTACGGTCAGCCGTCCTGGCCGCACTTCCCGTT
CTATTTCAACAGCCAGACGCTGGAAATGGCTGACCGCGGCTCGATTGGGCAAATGCTGGTGAATCCGGCGTCG
TAAAGCGTCCGAGTACGGGTCTGAATAGCGCAGACCCGTCAGGGTTTTCAGGAAGGAGCAAACAGCGTGGGAA
GCAGCAGACTCAACTGCGGGAAGTAGCAAATCATCGCCATGACCACAAAGAGTGCCAGAAACATCGGTAATAG
CGGTTTGACGACTTTATCAATACTGATTTTGCCGATACTGCAACCGACGAACAAAATAGTGCCTACCGGGGGC
GTACACAGTCCGATGGTTAAATTGAAAACCATAATAATGCCAAAATGGACAGGGTCGATTCCCAGATGTTGAG
CAATGGGAAGGAAAATAGGCGTAAAGATCAGAATAGCCGGGGTAATATCCATAAACGTCCCGACGAGTAACAA
AATGAGATTGATAATCAGCAAGATGACCCATTTATTTTCGGACACGCTGGTAATCATTTCATTAATCAGTTCA
GGAACATCAGCATTGGTCATCGCCCATGACATTCCCATTGAGCAGCCGACCAGTAAGAGAACAATAGAGCTGG
TTACTACCGAATCCAGCAGAATATCGCGCAACTTACTCACATTAATTTCTCGATAAATGGCCGCCAGCGCCAG
GCTATAGATTACCGCGATCGCAGAGGCCTCTGTTGGCGTAAATACGCCGCCGATAATGCCGCCGATAATAATA
AATATCAGCAACAGGCTGGGCAATGAATCGCGGAATACCGACAGAAACTGCCGCAGGTTGATGCGTTCGGCAA
CCGGGTAATGGTGGCGATGGGCATACAGCGCCGCTACCGTCATTAAAGCCAGCGCGGTTAAGATGCCGGGCAG
GTAGCCCGCCAAAAACAGGGCGGCGACAGAGGTGCCGCCGCTAGCCAGCGAATAGACAATTAGCACATTGCTG
GGCGGGATCATCAGGCCAATCGGCGCCGAAGCGACGTTGATCGCCGTAGAGAACGCCGGAGAGTAGCCCTCTT
TTTCCTGTAATGGCGACATGATGCCGCCCACCGCGGCCGCAGAAGCGACAGCCGAGCCGGATATCGCGCCAAA
CAGGGTGTTCGCCAGAATATTGCAGTGCGCCAGCGAACCGGGAAGGCGGCCAACTAACGCCTGAGCCAGATTC
ACCAGGCGCCGGGCGATTCCCCCGGTATTCATGATATTGCCGGCTAACACAAAAAAGGGGATCGCCAGCAGCG
TAAAGCCATCGAGACCGACGGTCATTTTTTGCGAAATAACCGAGATTGCCGCATCCGGGGGAAGGGCTATCAG
GATTGAGAATAAGGACGACAGCCCGATAGCGAATGTAATAGGAACGCCGACCGCCAGCATAATGGCAAAGCTG
CCAAATAAAACAATTATTGGGTAAATATATTCAAAGTCCATATATCCCTCGTTAGCGAAGATGGCGCTGATGA
TAATTATCAATAACGTTCACAATGGTGTAGTACACCAGGATGACGCCGCTAATCGGCATGACCCAATAGACTT

TATCCATAGGCCATTTTAATACCGGGGACATTTGGCTGACGTGCGCCGCTTTTTCAACAATAATATCACCGCC
GTACACCATAAAAATCAGCGCGAACACGGTAATAATCACCTGAATGATACTGTCGAGCACGAGGGCGGGCGTT
CGGGAGAGTTTTTGCGCTAACAAATCGATAGCTAAATGTTTTTTTAATCCGGCGGTATAGACGCCGCCTAACA
GAATCATCCACATAAAAAGAATCTGGGTGAATTCGTCGAGCGTGCTGCTTGGCGCGTTGAATATAAAGCGCGC
CGCCACCTGCCAGGATACGCAGGCAACCATGATCGCCAGAATAATACAGCAGCACCCGGCCAGTATTTTGTTC
AGTGCATTGGTGAGTATTCGCATATTACATCCTGTAAAGGGCAGGAGAACCCTGCCCGGCAGATTTACATATT
CTCGATGTATTCCAGATCCTTCGCTGATTGAGCATCTTTCGCGCGGAACTCATCAAACAAGGGCTTAACACGC
TGTTTGAACGCGGATTTATCAATATCAACAACGGTCGCATTCATGGCCTTCATGCTCTCATAAGCCTCTTTTT
CCGCGGCGGGCCAGATGACGTCTTTCATTTGCTGCATGGATTCATCTGCGGCTTTATATAACGCTTTCCGCTC
TTTATCGCTCAATTTGTTAAGTACTTTGGTACTGATGACCAGGACATCCGGCACCATCGTGTGCTCGTCGCGG
GAATAGAATTTCGCCACCTCCGCATGACGCATATCGGCATAAACCACGGGGTTGTTTTCGCCGCCATCGACCA
CGCCTTGCTGGAGCGCGGTATAGAGTTCGCCTTGCGCCATCGGCGTCGGCACGCCGCCGAGCGCTTTCACCAT
CGCGATAGCGCTGGGGCTTTGCTGCACTCTGATTTCATACCGGCTAAATCGTCGGGTTGAGTGATGGGCTTA
CCAGCGTAAAAACTGCGGGCGCCGCCGTCGTACCAGGTTAAGCCGACGAAGCCTTTGCTTTCGGAGGACGCGA
GAATGCGTTTCCCTAAGTCGCTTTTCAGTACGTTGTAGTAGTGATCGCGGTCGCGGAATAAAAACGGCAGGCT
AAACACGCTATATTCCGGCGCAAAAGACTCCAGCGATGCCGCATTGACTTTCACCATTTGCAGAGCGCCGGAG
TTCATCAATTCCAGCGATTCACGTTCGTTGCCCAGCGTGGCGTTGGGGTAAATACGAATATTTAATTCGCCGT
CCGAATAGGCTTTCGCTTTATCGGCCAGATAGCTCATCGCTTTGTGAACGGCGTGGCTTTTATCCTGATTGTG
GCTCAGTTTCAGCGTGGTTTTGGCGAGCGCTGGAAATGCCAGTAGCAAACAGCCGGCCAGTAATACCGCTGAT
GTTGTGAATGAACGTGTGTTTTTCATATCTGCTTCCTTATAGGTTGGGATTCCTGTGGTTATCGTTGATTAAT
TAATAACCTGCTGATACGTAAGGTATTGCGTAGGGGAAAGGCGTGGTGATGGTGTTCTTTATCCCCTGAACTC
TGTCAGCCAATTTAATTTAATCAATAGACCAAAGAAAGAGATCTGGTTCACAGAAGAAAGTCATGGTAAAAAC
AGTGCCGTGAGCAATTCAGCGAAGTTTTTTTATATATTACTGATAATATGATATTTTTTTACTTTTTGTTGTT
ATGGCGATACTGCGTTTTTAGACGTGGGTATAATTAATTGCATACCAATTTGGAGATATTCATAAGACCAATT
TCAAACACATCAGGCCAGGCCGTTTAAAGTTGTTTCTATTTCGCGCCAGCCCTGCATATAAGGCGATAATCTC
CTTCATTTTCGCTATAGATACGGCTTATAATCCCCGACCTTTGATTATTTTTTTGCCAATCGTTTTGGAAGCA
ATATGAGCGCTATCTCCCTGATCCAACCGGATCGCGACCTTTTCTCCTGGCCGCAGTACTGGGCCGCCTGCTT
TGGGCCGGCGCCGTTTTTGCCGATGTCACGTGACGAGATGGATCAACTTGGCTGGGATAGCTGCGACATTATT
CTGGTAACCGGCGACGCCTACGTCGACCATCCGAGCTTCGGCATGGCGATTTGCGGTCGTATGCTGGAAGCGC
AGGGCTTCCGCGTGGGGATCATCGCCCAGCCTGACTGGAACAGCAAAGACGACTTTATGCGTCTGGGAAAACC
GAACCTGTTCTTCGGCGTCACCGCCGGCAACATGGACTCGATGATCAACCGTTACACCGCCGACCGTAAGCTG
CGCCATGACGACGCCTACACCCCGGATAACGTCGCGGGCAAACGCCCGGACCGCGCCACGCTGGTCTATACCC
AGCGTTGCAAAGAAGCGTGGAAAGATGTCCCGGTGATCCTTGGCGGCATTGAGGCCAGTCTGCGCCGTACCGC
GCATTACGACTACTGGTCTGATACTGTGCGTCGTTCGGTGTTAGTCGATTCCAAAGCCGACATGCTGATGTTT
GGCAACGGCGAGCGTCCGCTGGTGGAAGTGGCGCACCGTCTGGCAATGGGCGAAACCATCGATCAGATTCGCG
ACGTGCGTAACACTGCGATTATGGTTAAAGAAGCGCTGCCTGGCTGGAGCGGCGTCGACTCCACTCGTCTGGA
CACGCCGGGTAAAATCGATCCAATTCCCCATCCCTATGGTGAGGATCTGCCGTGCGCGGATAACAAGCCGGTC
GCGCCGAAAAAGCAGGAAGCTAAAGCGATTACCGTGCAGCCGCCGCGTCCGAAGCCGTGGGAAAAAAACCTATA
TCCTGCTGCCATCCTTTGAAAAGGTGAAAGGCGACAAGGTGCTGTACGCCCATGCGTCGCGTATTCTGCATCA
CGAAACTAACCCTGGCTGCGCGCGGGCGCTGATGCAAAAACACGGCGACCGCTATGTCTGGATCAACCCGCCC
GCCATACCGCTCTCCACTGAAGAGATGGATAGCGTGTTTGCGCTGCCGTATCAGCGTGTGCCGCACCCGGCTT
ACGGCAATGCCCGTATTCCGGCCTATGAGATGATCCGCTTTTCGATCAATATCATGCGCGGCTGCTTTGGCGG
CTGCTCTTTCTGCTCGATCACTGAACATGAAGGGCGCATCATTCAGAGCCGCTCGGAAGACTCTATTATCAAC
GAGATCGAAGCGATCCGCGACACGGTGCCGGGGTTTACCGGCGTGATCTCCGATCTGGGCGGTCCTACCGCGA
ACATGTATATGCTGCGCTGTAAGTCGCCGCGTGCGGAGCAGACCTGTCGTCGCCTGTCATGCGTCTATCCGGA
TATCTGTCCGCATATGGATACTGACCATACGCCGACGATCAATCTTTATCGCCGCGCCCGTGAGCTGAAAGGC
ATTAAGAAAATCTTAATTGCCTCCGGCGTGCGCTACGACATCGCCGTAGAAGATCCTCGCTACATTAAAGAGC
TGGCGAGCCATCATGTGGGCGGCTATCTGAAGATTGCGCCGGAGCATACTGAAGAAGGTCCGCTGTCGAAAAT
GATGAAGCCGGGTATGGGCAGCTACGATCGCTTTAAAGAGTTATTTGACCTTTACTCTAAACAGGCCGGAAAA
GAGCAGTATCTGATCCCGTATTTTATCTCCGCGCATCCGGGCACGCGGGACGAGGATATGGTGAATCTGGCGC
TGTGGCTCAAGCGCCACCGCTTCCGTTTGGATCAGGTGCAGAACTTCTACCCGTCGCCGTTGGCGAATTCGAC
CACCATGTATTACACCGGCAAAAACCCGCTGGGTAAAATTGGTTATAAGAGTGAAGATGTGGTGGTGCCGAAG
GGAGACAGGCAACGTCGCTTGCATAAGGCGCTGCTGCGTTATCACGATCCGGCGAACTGGCCGCTGATTCGCC
AGGCGCTGGAAGCGATGGGGAAAAAGCATTTGATTGGCGGTCGCCGTGAGTGTCTGGTGCCTGCGCCGACCAT

TGAAGAGATGCGCGAAGCGCGTCGCCAGAACCGCAATACCCGCCCGGCGTTGACCAAGCATACGCCGGTGGAA
CATCAGCGGCAGGGGCTTGCCGCTAATAAGAAGCGCGGGAAAGGCGCAGGGCGTTAATTACCTTTGAATCTGT
TGCTTCTGGAAGCTAATGTTTTTGGAAGTAACAGACATCTAAATATATTGTATCGGTCTTTAGTAATTGCTAA
ATTACGGGGAGCTTTATATTTTATTTAAATATTATTGATTGTATAAATTAATTATATATTACCTTAATGATTA
AGGCATGATGCTTTTATACAGCCCCAGAGGAGGGGATATCATGGGTATGATTATTATTCCCTGTTTTATCGTA
ATTGCTATAATAGCTGTTGTTATATTCATAATGGCTATTAAAGCATGTAGTATGGAAAAACGTGAAGATAGGC
AAATGACTCTTGCAGGGATAATTTTAATGGGTGTTATTGCCGCTATTCCTGGATGGTTTTTGTACGAAATATT
TAGCCACGCGCCGTAATATTTAACCTACCAGGGTTGATATATTAAAATAGATAAGAAGTATATAATCTAAGGT
ATAATGCCTGAAGGTGCTGCGCTTATCAGGTCTATGAATAGCACAGTTTTGTAGGCCGGATAAGGTGCTTACA
CCGCCATCCGGCAGAAGTGCTCCGTATTTATGCCTCACGAATTCTGGCCGCCAGCGCGGCCAGTTTTTCATCA
TCAGCCCGTTCATGATCGTGTGACGACAGATATCCTTCATACTGTTCGAAAAATTTATTCTGCCGCGATTCAA
TCGGTTTCCAGTGGGTTAAATCGCCAGTACCGTGCATGGGGCTTAATTTACTGTGCACATGGTCTACGCCGAA
ACCTTCGAAGAACATTCCCGTACGACTGACATCCGGGAAAGCCTTATCCAGTTTCTTAGCCACTTTTTGGGTC
GCCAGCATCAGGTCCGCTAACGCCTGCGGCGGTAGATCGAACGCGTAGCTGGGGTAATGTTTTTTCGGGATCA
CTACGGTAAACCCATCGGTATTCGGAAAAATCGACAAAAAAGCCAGGTGATGCTCATCTTCCCAAACTTTATG
ACAAGGTGCTTTACCTTCAACTATTTGACAGAAAATGCAGGTCATTCCATTTCTCCGTTAACCGAGGGGCTTA
TCGATGAGTTAAGCGTTTAATACAACCCTAACACTTTCCACCATGCGAGGCCGACTGTCAAAAAGACCGCCTG
ATTAAAGAGACTGATAATAAATCCCGTTCGCCACCAGACGCCCGTCGGTACATAGCCGGCGCCAAAAAGAATG
GGGCCGCGTGCGTGGGTATACTGCGTAAGAGAGCAGTACAGGCTGCTGGTAAACGCCAGCATGAGCGCCATCG
GCGCCGCCGGAATATTCAGATGCAGACCGACGCCCAGAAATACCGCATACAGCGCGGCGATTTGCGCGTTGCC
GCTGGCGAAAAAATAGTGGGTATAAAAATAGGCTGCGTTAAGCAGCAGCAGGATGATTATCCAACTGGTTCCA
TGCATCGTGCTGCCGATGCTGTCGCCAATCAGGTTGCCAAACCAGCTGGTGAATCCTAGTTTCTTCAACTGGT
TAGCCATCATCAGCAGCGCGGCAAACCAAATCAACGTATCCCAGGCGCCTTTTTCGCTTTTAACGTCTTCCCA
GGTGAGCACGCCGCTCAGTAATAAAATAGAGAGACCGACAAAAGAGGCCGTCGTGGCATCCACGCCGAGCGAG
CTGCCAAAAATCCATAGCACCAGCAACACGCCGACGGTCGCCAGCATGAGCCACTCGCCGCGCGTCATACTGC
CCATTTGCGCCAGCTCTTTGCGCGCCAAATCCGGCGCATCCGGCGTATGTTTAATTTCCGGACGCGTCAGCCA
GTAGACCAGCAGCGGGACGATAAGAAAAGAGACCAGGCAAGGCAGGAGCGCGGCGATAAACCAACTTCCCCAG
CTCAACGTAACGCCGGCGTTCGCCGCCAGTTTTACCGCCAGCAGGTTACCCGTATAACCGGTCATAAACAGTG
CGGCGGTGACGTCGTTAACATTGCCAATACAGGTGATCAGAAAGGTGCCGATTTTGCTGCGAGACTCATCTTC
CGGATGTGAATCAAAGCTGCGCGCCAGCGAGTCGGCGATCGGGTAAATCACGCCGCCGCAGCGCGCGGTGTTA
CTGGGCATCGCGGGGGAGAGGATCAGATCGGCAAACGCCAGACCATAAGCCAGACCCAGCGTTCGTTTACCTA
ACAGACGGATCATTTGTAGGGCGATACGCCGACCCAGCCCGGTTTTGATAAACCCACGGGCAATCATAAAGGC
GACGACGATAAGCCAGATCAGCGAACTGTTCAGCTCGCTCAAGGCCGTGGTAATCGCGCCGCTGGCCGTTTTG
TCGCCGGCAGCATAGGCGAGCGCAAAGACGGTGATGCCGATAATACCGACTGCGCCAATAGGCAGCACTTTCG
CCACGATAGAGGCGATCGTCGCGACAAAAATAATCGCGGAATGCCAGGCCGGCGCGCTTAACCCGGAAGGGGG
CGTTAGCTGCCACAGTCCGGCGGCGATAACCAGAATAAGAATAAATGGGAGCCATTTGACGCCCTGAGATTTT
TTTCCGCCACAATTACACATCCATAAATATTTTATATGTATTAAGTAAATTCATAATAAATATGAATAAATAT
TCACAAAATCAGAATAATTCTGCGCTTGTGTGTGGGGGAAATATACTGTTAAATGCGCGTAATTGTTTCTTAA
TGTTTCGGAATGCAAAAAAGCCCGACGACATCGTCGGGCATGGCGTGCGATAAATCCGGCTTAACCGCCGAAC
TGATCCGGATCGGGACCGAGGCGTTTTCCTTGATCCAGTTTTGCGATCTCGCCTAATTCATCTTTATCGAGAC
GGAAATCCCAGACAGCAAAGTTTTCCGCGATACGGGAAGGGGTGACGGATTTCGGAATGACGACCAGCCCGCA
GTCCAGATGCCAGCGAATAACAATCTGGGCGGGCGTTTTGCCGTATTTATCCGCCAGTTCGCGAATCACTTTC
TGATCGAATACGCCTTCGCCACCCTGCGCCAGCGGGCTCCAGGACTCGGTCTGGATTTTATGCGTCGCATTCC
ACGCATGTAGCTGGCGCTGTTGCATCAAGGGGTGAAGCTCGATCTGGTTGATCACAGGGGTAACGCCGGTTTC
ATCGATCAGACGCTGGAGGTGATGGATCTGAAAGTTACAGACCCCGATACTCTTTACCAGCCCCTCTTTTTGC
AGCGCGATCATGCCTTTCCAGGCGTCGACATAGTGATCGATAGCCGGTACGGGCCAGTGCATCAGATAGAGAT
CGAGATAATCAAGCTGCAGTTTTTTCAGACTCTCCTGCAGGGCTTCGCGGGGACGTTTTTGATCGTCATTCCA
CAACTTGGTAGTGATAAACAGCTCTTCCCGCGCCACGCTCGCTGCTTTTAGCGCTTTGCCGACGCCTTCTTCA
TTCTGATATGCTGTGGCGGTATCAATCGATCGATAGCCCACCTCCAGCGCTTTATGAATCGCCGCGATCACCT
CTTCATTGCTTGCTTTCCAGACTCCCAGCCCAAGTTGTGGCATTACGTTGCCATCCTGTAGCCTGATAATGGT
TGGATTAGCCATGATGTGTTCCTCTTTTAATTGGCTCGCCGGAGTCGGGTCCGGCGAGACTAAGTGACTTAAG
TCTGGACGAAATGGCCAAAAACGAAAGTCAGAGGCGAAAAAACCTTAGCGCGCCGCTTCATAAATCCGACGGC
TGACATCCAGCGTAATATCCTGATTTTCACCTAAATTTGTGCATCCATGCGCCTCAAGTTTAGCCAGTAGCGC
CGGAATAGTGCTGCCATCCAGACCGTAATCGGACAGACGGGTAGGCACGCCCATCTGTTCAAAGAAACGGCGG

GTGGCGGCAATGGCGGCATCAATACGCTCGTCGTCGGAACCGTCGGTGATATTCCATACACGTTCAGCATATT
GCAGGAGTTTTGCACGTTTAACGTCTCGTTTTTCGTTCCATAGCGCAGGCAGAATGATAGCCAGCGTCTGGGC
ATGATCGAGACCGTGCATCGCGGTAAGCTCATGGCCCAGCATATGTGTCGCCCAGTCCTGCGGAACACCAGCG
CCAATCAGGCCATTCAACGCCTGAGTAGCGGCCCACATCACGTTAGCGCGGACGTCATAATTTTCAGGCTCTT
GCAGCGCTTTGGGACCTTCTTCGATCAGTGTGAGTAAAATACCTTCGGCGAAACGATCCTGAATTTTGCCGTT
AACCGGGTAAGTGACGTACTGCTCAACGGTATGGACGAAAGCATCCACGACGCCGTTCGCAACCTGGCGCGGC
GGCAATGTATAGGTGTAGACCGGGTCCAGTACGGCAAACACCGGTTGCACGAACGAGGAGTGGAAGGCCTGTT
TGTCGCCAGTGGTTTTACGGGAAATGACTGCGCCGGCGTTCGATTCTGAGCCGGTTGCCGGTAGGGTCAGCAC
AGACCCCATCGGGATTGCGCTACGAATCTCAGTACCGCCGGTTTCCAGAATGTGCCACGGATCGACGCCGTCC
GTATACTGCGCCGCTGCGGCGATAAATTTGGTGCCGTCCAGTACGGAACCGCCGCCAACGGCCAGCAGGAAGG
TAATATTTTCGTCGCGCACAAGCTGCACGGCCTTCATCAGCGTTTCATAGGACGGGTTCGGTTCAATACCGCC
AAACTCCCGAACATCCAGTCCTTTTAACGCTTCCTGAACTTGCGCCAGTACGCCGGTCTTTTTCACGCTACCG
CCGCCATAGGTAATCAGCACGCGGGCGTCCTGCGGGATTTGATCGCGAAGATCGACGATAGCGCCTTTACCAA
ACAGAATGCGGGTAGGGGTATGTAATTTGAAATTGTTCATTGCTCATTCCCTTATGTGGGTAAAAAGAGGGCA
GGCGGCTGTCTGCCTGATGGTCATCATTGTGGTCTTGTGAGGGATGTCTCTCAATGCACATTTCTGCCAATGT
TTTGCCTATTTCTCCAGAATGCTGGAGAAAATCGCTAAAAATGCGCACAATCCAGCGGAGTGGATTTGATGAA
AAAATGGCGCGTTATGAATCGTGAAGCAATTTGCTTACAGTTGGCGGATAAAATTAATCATTTAAAAAATAAT
GATAAAATAATCAGCGAGCGGCTGGCGGGCATTCGTCTGTTGTATGGTGTTGAACCGGGGCCGCGCACGCCGG
TCATGTATCAGCCTGGCATCATATTTCTCTTTTCCGGCCATAAAATTGGCTATATTAACAAGCGCAAGTTTCG
TTATGACGCTAATGAATATCTCTTACTGACCGTGCCGCTCCCTTTTGAATGCGAGACCTGGGCAACGCCGGAG
GTACCACTGGCCGGTATTCGGCTCGACATTGATGTGCTGCAACTGCAAGAGTTGCTCATGGACATTGGCGAAG
ATGAACGTTTCCAGTTGCCGATGGCGGCCAGTGGGATTAACTCCGCCACGCTGTCGGACGAGATTTTATGCGC
GGTGGAAAGATTGCTGGATGTGATGGAGCGCCCGCTGGATGCGCGGATTCTGGGAAAACAAATTATTCGCGAA
ATTCTTTATCATGTGCTGATGGGACCGCGCGGCGGGGCGCTGCTTGCACTGGTTAGTCGCCAGACGCACTTTA
GCCTGATCAGTCGCGTACTTAAACAGATCGAAATGAAATACACCGAGAACCTGAATGTTGAGCAACTGGCGGC
AGAAGCGAACATGAGCGTGTCGGCGTTTCACCATAATTTTAAGGCGGTCACCAGCACCTCGCCGTTGCAGTAT
TTAAAAAGCTACCGACTGCATAAAGCGCGGATGATGATGATTCACGACGGTATGAAGGCCAGCGCCGCGGCGA
TGCGGGTCGGCTATGAGAGCGCATCGCAGTTTAGCCGGGAGTTTAAACGTTATTTTGGCGTTACGCCGGGCGA
AGATGCGGCAAGAATGCGGACAATGCAAGGGAGCTAAGTGGGGCATCCCGCCACGGGTCAACGAAGCGTAATC
GGGCCATCCTGGCCGATTATGGATACGTCAGGCGTTACAGTATTTCTTTTTAATCACCACGACCAGCGTACCT
AACAGTCCGGCGACCAGTAGCGCGACGGGAAGGATCATTAAAAATGTCATTACCTGGTCTTCATGGCGCTTAA
CAAACGGGATCATACTGAGCGCGTAGCCGAAGCTGGTCACGACAGTCACCCACAGCAGGCCGCTTAACCAGTT
AAAAAACTGAAACCGGCGGTTGGACAGGCCGGAAATACCCGCCATTGTTGGCAGCAGAGTGCGGACAAACGCC
AGAAAGCGCCCGGCAAGCAACGCTAACAAACCGTGGCGGTCAAACATGCAGGTGGCGCGCTGATGATATTTTG
CCGGAAGCTGCGCCAGCCAGCCTTTGACGGTACGCGTATTACCGAGCCAGCGTCCCTGGATATAACTTAGCCA
GCAGCCGAGACTGGCCGCGGTGGTGAGAATGCCAATCGTCGGCAAAAAATGCATCACATCCTGGGCGATCAAC
GCGCCTGCCAGTAGCAGCAGACTATCGCCAGGTAAGAATGATGCTGGCAGCAGACCGTTTTCTAAAAATAGCG
TGGCGAACATGACAAAGTAGACGACGCTAACAACGTGTGGATTCGCCAGCGCGGCAAAATCATGTTGCCAGAG
CGCAGCGATAATATCTTGAATGACAGCCATGAACTTTCCTGTGGAACAGCATTTATGCTTTTATTCGCCTATT
GTACTCCTGAATCGTCCGGGATGCCTTGATCCCGGACGCAACAAACGCAGACTTTTCCACGGAAATTGTCTGC
ATATATGTCCATCCCCGGCAACTTTACACAATTCTGGCGAAGCCGGCGGCTAAATCAGCGATCAAATCATCAA
CACTCTCTAAACCAATATGCACCCGGACCAGCGTACCGGTAAAATCTACGCCGCCTGCGGGACGAATCGCGGC
GATCTGTTCCGGCTGATTAGCAATAATCAGCGATTCGTAGCCTCCCCAGGAGTAGGCCATACTGAACAGCGAA
AAGTTATCCAGATAGGCTGATAATTCTGCCTCGGTCAGCTTTTTATTGAGCACAAAGGAGAACAGGCCGCTGC
TGCCTGTAAAGTCTCGTTTCCAGAACGCATGACCTTTGCTACCCGGCAGGGCCGGATGATTAACGCGCGCGAC
CTGAGGATGGTTCGCCAGCCATGCGGCGATTTTGAGGCTGCTTTCTTGATGCTGGCGCAGTCGCACGCCGAGT
GTGCGCAGACCGCGGCTGGTCATATAAGCCGTGTCGGCGTCGAGCATTTGCCCCATCAGATAGGCGTTTTCCC
GCAATTGTTCCCAGCAGCGCGCATTTGCAACGGCAGTACCGACCATCGCATCAGAATGACCAATCAGATACTT
AGTGCCCGCCTGGATAGAGATATCAATATCGAATTCCAGAGCTTTGAACAGAACGCCAGCCGCCCAGGTATTA
TCAATCATGATGACGGCTTCTGGCGCGACGCGTCTGACCGCACTAACAATGCTCGGAATATCATGTACTTCCA
TGGTAATAGAACCGGGGGATTCCAGAAATACGACTTTCGTGTTCGGTTGAATATGTTGCGTGATATCAGCGCC
GATGAGCGGGTCGAACCAGCTTGTCGTCACGCCCAGTTTGCCGAGAATTTTGCTGCAGAAATCCTGGCTGGGT
TCATAGGCTGTGTTGGTCATCAGGATGTGGTCGCCCTGCTCAACGAATGCCAGAATGGTGTTGGCGACCGCCG
CCGTGCCGCACGGGAAGAGCGCGCATCCGGCGCCGCCTTCCAGTTCACACATGGCTTCCTGGAGCGAAAAGTG

GGTCAGCGTTCCCCGGCGTCCGTAAAATAATTCGCCGTTGGCGCGATTGCGGGTAGCGTGTTTTTTGGCCTCG
ACGGTGTCAAACACCAGTGAAGAAGCGCGTTGAATCACACTATTCACTGAGCCAAGCGTGTATTTTTTGCTGC
GTCCTGCGTTTACCAGTTTGGTATCCAACTGTTTATCCGTCATGTTGCGTTTCCTGATTTTAACCGTCTGGAT
GTCTAAACTAGCATGAATATAACCATCTGCACCCTGGCGACGTGTGATACAGATGAAAATTTTGCCAAAATAA
AAACAGGTGGCTTTTTTAATGCGTAAGCGCAAAGAAAAAGCAGTAAAGGTGCGGCAGTATGTAAATAGTAATG
AGAACGACTATCAATTCGACGTTGTTTTGATATTATTATGCTCAGATTTTGTGATTTGCGTCCTGGAGATACA
GAGTGGGTAATAATTTGATGCAGACGGATCTTTCCGTCTGGGGCATGTATCAGCACGCCGATATTGTCGTGAA
GTGCGTGATGATTGGTTTGATTCTGGCGTCAGTCGTCACCTGGGCTATCTTCTTCAGTAAAAGCGTTGAATTT
TTCACGCAAAAGCGCCGCTTAAAGCGCGAACAACTGCAATTAGCCGATGCGCGCTCTTTAGATCAGGCTTCCG
ACATTGCGGCAGGTTTCAGCGCAAAAAGCCTCAGCGCACAGCTCATCAATGAAGCGCAGAATGAACTGGAACT
CTCTCAGGGCAGCGAAGATAACGAAGGGATTAAAGAACGTACCGGCTTCCGTCTGGAACGTCGCGTCGCAGCG
GTTGGCCGCTACATGGGACGAGGCAACGGTTATCTGGCGACGATCGGCGCCATTTCCCCCTTCGTCGGGTTGT
TTGGCACCGTATGGGGCATTATGAATAGCTTCATCGGTATTGCGCAAACGCAGACCACCAACCTGGCGGTGGT
CGCTCCTGGTATTGCGGAAGCGCTGCTGGCGACGGCGATTGGGCTGGTAGCCGCGATCCCGGCGGTCGTCATT
TACAACATCTTCGCACGTCAGATAGGCAGTTACAAAGCGACGCTGGGTGACGTGGCGGCGCAGGTATTGCTGC
TGCAAAGCCGCGATCTTGATCTGAATGCGAGCGCCAGTGCGCAGCCGGTACGCGCCGCGCAGAAATTACGGGT
AGGGTAATGCGTCATGGCAATGCGTCTTAACGAGAACCTGGATGACAACGGCGAAATGCATGAAATCAACGTG
ACGCCGTTTATCGATGTCATGTTGGTTCTGCTGATTATCTTTATGGTGGCTGCGCCGCTGGCGACGGTGGACG
TCAAGGTGAATCTGCCCGCTTCTACCAGTACGCCACAGCCGCGTCCGGAAAAACCGGTTTATCTGTCGGTGAA
AGCGGATAACACCATGTTTATCGGTAACGATCCGGTGACGGATGATACGATGATTGCCGAGCTAACGGCCCTG
ACGGAAGGGAAAAAGGATACTACGATTTTCTTCCGTGCGGATAAAACCGTAGAGTATGAAACCCTGATGAAAG
TGATGGATATGCTGCATCAGGCGGGCTATCTCAAGATTGGTCTGGTCGGCGAAGAGACCGCGAAAGCGAAATA
AGTATTCTGCTGACGCTGTAGGCCGGGTAAGGCGAAGTCGCCACCCGGCTTTTTTATTGCCTAAGATGGTGCC
TTGCTTAACAGAATTGCGCCACCTTAATCGACTTCATGCGTTAGCCTAAATGCTCCCCGCCGCACACGCCGTG
CACTTCTGCGGTAACGTAACTGGATTCCTGGCTTGCCAGATAGACATACACCGGCGCCAGCTCTGCCGGTTGG
CCGGCCCGCTTCATTGGCGTCTGCTGACCAAACTGCGGAATTTTATCCTGGGTTTGACCGCCGGAAATTTGCA
GCGCTGTCCAGATAGGGCCAGGCGCGACAATATTCACACGAATTCCTTTTTCCGCCACCTGTTTCGCCAGGCC
ACGGCTGTAATTGAGGATTGCCGCCTTGGTTGCCGCATAATCCAGCAGGTGCGGACTGGGCTGATACGCCTGG
ATAGACGACGTCGTGATAATACTGGCGCCTTTTGGCAGCAGCGGAATCGCTTCCTGGGTTATCCAGAACAGGG
CAAAAACGTTAACGGCGAAGGTTTGCTGAAATTGCTCGCTGGTCAGATCTTTTATCTCCGGGATTGCCGTCTG
TTTACCGGCTACCAGCGCCAGAATGTCCAGCCCGCCCAGCGCTTCACGCGCCTTATGGACCAGCGAACGGGCG
AAGGATTCGTCGCTAAGATCGCCTGGCAATAACACCGCTTTACGACCGCACTCCTCAATCAGCGCTTTAACCT
GCTGGGCGTCCTCTTCTTCCGCTGGCAGATAGTTGATCGCGACATCTGCGCCTTCACGCGCGTAAGCGATAGC
GGCAGCGCGCCCAATCCCGGAGTCGCCCCCTGTCACCAGCGCCTTACGATCTTTAAGGCGACCACTGCCGACA
TAACTTTTTTCGCCACAGTCAGGAACAGGCGTCATTTTCGCCTGGACGCCGGGCGCCGGCTGTTTTTGCTTAG
GATATTCGCCGGTGTAATACTGCGTGGTAGGGTCGTATAGATGAGACATTGAGCTTCTCCCTTCAGGTTGAAC
ACCCTTTAAGCGTAGACACTTTTACTACCGTAAGAGGGAGAATCAGAACGATCCTTATGCTTCGCCCGTATTC
ACAACAGAGTTAACTAGCCATGAAGGTTATTTTTCTTGCCGTGCGACGGTGGTGACGTTGCGCGTCCGGTAGT
CGCCATCGGAAAGCTTACGCGTCAGACGTAAGGTTGCCGTCTCGCGAGCCAGCAGATGCTGCCAGTCACTTTC
AAGACGAGACATAATTTTGTCTCGTAATTCAGGATGCTGCTCGATAATAGCATTGATGGTCGCGCCATAAATC
TCCTCTTTGACCTGTAACGTATACATCTCTCGTCGGTTTTGCCACTTCAGGCGCACTAACGCCGCCGGTATGG
ATACCACCTCCTGCGTTACCTTTTCCAGGGTGGCATTTTTACTGGCGATTAGCGCTGCAAGATTTTGCTTTAA
GATAAATTCATCACTTTTATGGTTGTCTAAAGCCAAATAAACGTTATTGTCTTCAACTTCTTTCATCTTTATC
TTCCAGTGAATAAAATAGCGGCATATTGGTTTCGCGAGTGATTAAATTAAGCCAAATGGCATTACCTTTTTCA
TTTATCATTGCGGTTTTATCCGCCAGTATTTTACTTAGTGTTTGGGCATCAATATTGCCTTCTGCCTGTAGTG
CGTTTATCAGGCGGGCAAACGTTTCAATTTTAGCCACACAATACAGACGTTCTTTAATATGACGGTCGCGTTC
TTCAAATAATACGTTTCTGTGTTGCCCGGTTGCGTTAGCGCAAATTAAAATATCGGGCTCGTAATCGCTGAGT
TTTGTCTTTCCCTGAGGCAAATTTTTACTGTTTTCTGTGGCTTCAGGGGGCGGCGATGACAGGCGAAAATGAG
AGGAAACCAGATGCTCAGGAAGCATGACTAAATATCCTTTACGATCAGTGGTATACTATTTTTAATAGTGTAG
GTTTCGTTGACAATAACATGGCGTAATTTTAAAATCAAAAAAAAATGGAGCCTGCTATGAACAGTATTTTTTAT
TCTGTTATTATATTGCTATTACTGACCGGCGCGATACTCTTCCTGATGTGGGAGGTTAATAAAAAACGTCCTG
GTGGAAAAAATCGTAAATTTAAATCAAACTGAACCCATGACAAAGGAAGAGGGCGAAGATCATTTCTCAGTATT
AATGAATTCGATAACGCCGGTATGGTACTGGCGCGTTAACCATGAATATATCGATTTCCTTCATGCAACGATA
AAACGAATGACAATGACTGAGTTGAATGAAACGCCTGGATTATTTGACGCACAGCGTCGTTGCAGCGACCTTA

ACTCTGCCGTTTATAAGTATTACGACAATATTAAGAAGCGTTGTCTGAACGGAGAAAAGGTGCCGTATTCGGA
TTTGGACGTCCTTAATTTACGCCAGTGTTTTCGCGAGTTCAGTCTTGAAGCCTACCCGGCATTGGTCGCGCTG
GTATGGCCGGAATATCAGCGCCCGCAGGTTAACCCGGATGAGATTTAGAGCCTATCCCATTAGGGCTATTTTA
CTTGCCATTTTGGACCTGGGCAGTGCTCGCCAAAACGCGTTAGCGTTTTGAACGCCGCTAGCGGCGGCCCGAA
GGGCGAGCGTAGCGAGTCAAACCTCACGTACTACGTGTACGCTCCGGTTTTTGCGCGCTGTCCGTGTCCAAAC
TGGCTGCGCCAATAACGCCTGGTGGGATAGGCTCTTAACTGGCATGTTGATCTGATGTTGCATGGTTGAGATA
TACTGTTGCTGGTTTTAGTTACCTGACAGGTCCCTATGGAACGCTTTCTTGAAAATGTAATGTATGCGTCTCG
CTGGCTGCTCGCGCCGGTGTATTTTGGCCTCTCTTTGGCGCTGATCGCTTTAGCGCTAAAATTTTTCCAGGAA
ATTCTACACGTTCTGCCGAACGTTTTTGCCCTGGCGGAGGCCGATCTGATTCTGGTGCTGTTATCGCTGGTTG
ATATGACGCTGGTCGGCGGATTGCTGGTGATGGTCATGTTCTCCGGGTATGAGAATTTTGTCTCTCAGCTGGA
TATCTCTGCGGGCAAGGAGAAGCTGAACTGGCTGGGGAAAATGGATGCGACGTCGCTAAAAAAATAAGGTGGCG
GCATCGATAGTCGCTATCTCTTCCATTCACCTGTTACGCGTGTTTATGGACGCGAAAAACGTCCCGGATAACA
AATTAATGTGGTATGTCATCATCCATCTGACCTTTGTCCTGTCGGCATTCGTTATGGGCTATCTGGACAGATT
GACGCGGCATAACCATTGAGCCGCCGTCAACGGCGGGGCAGTCGGGCAAAGGACGCTGAGCCCGGCTGTAAAA
CAGCGCTTCTTTAACGGCGGATAACCGTCTCCCTGCAGCCTTACGCAATATTTTTACTACATCTATTCATTTA
CTCAGTAATAAGACTAAAAGATTTTCTCTGTGTTCACAGATATATAACATCTGCGGGTACGTAAACGGCGATA
ACTGTAAAAAAATGGAGAATGCATTATATATTCTCCGCGCCTTGCCGATAACGAGAGTGCGTCATCATCAGAT
GACTAATCTCTTATATTCAGCAAGGAAATTCATATGCGTTTGCTGCAAAACTTTACTATTCGTATGGTCATGC
TGACGATACTGGGACTTTTTTGTCTACTCTGGTCGGGGGTGGGGTTATACAGCGTCCATGCTTTATCCGAAGT
TTCGGAAGGAAATGATATTGACCGGCATCTTGTGCGGCAAATGACGGTGCTAAGCCAGGGTAACGATCAATAT
TTCCGCTTTGTTACCCGCCTTAGCCGCGCGATGGATGTAAAAATCGGCGGCGGCACGCCTGATTTTGCGCCCG
CCCGGCAGTCGCTCGAGAATATGCGTCAGAAGCTGGAGGAGATGAAAGCGCTATCGCCAGGGCCGATGAACCC
TGATATCTCAAGGGAGGTCTTGTCCAACTGGCAGGCCTTGCTGGAGAAAGGCGTCGTTCCTCAAATGCAACTG
GTGCAGCAAGGATCGCTAACCGCCTGGAGCGAACATGCCAGTACGGTCACGCCCGCACTCAGTCGCGCGTTCG
GCGCCAGCGCGGAACGGTTTAGTCATGAAGCTGGCGCCATGCTGGATAACACCCGTGTGATGGTCGATGGTAA
AACCTATACCATCCGCATCCTGCTTATCACGGCGGTGATTCTGGGCATTGCTATCCTGATTTTTACCGATCGC
TATCTGGTTGCAATGATGGTGAAACCGCTGGAGCGTATCCGTCAGCAGTTTCAGCGTATCGCGCAAGGCGATC
TTAGCCAGCCGATTGAAGCGTTGGGGCGTAACTGCGTAGGCCGGTTGGTGCCGCTACTGCGCGCGATGCAGGA
CAGTTTACGCGAAGCCGTGAGTACGATCCGCGCCGGAAGCGACAATATCTGGCGCGGCGCGACGGAAATTTCT
ACCGGTAATAACGATCTCTCTTCCCGAACGGAAGAACAGGCCGCCGCGCTGGAAGAAACTGCCGCCAGCATGG
AACAGCTTACCGCAACGGTGAAAATGAACGCCGAACACGCTCGCCAGGCGAGTCAGCTTGCGGATGCCGCCTC
GCTAACGGCAGGAAAAGGCGGTGAACTGGTTTCCGATGTGGTAGAAACAATGAACGGTATTTCCGCCAGTTCG
CAGCAGATCGCGGAAATTACTACCGTAATTAATAGTATTGCTTTCCAGACCAATATTCTGGCCTTGAACGCAG
CGGTAGAAGCGGCCAGAGCGGGCGAGCAGGGACGCGGCTTCGCGGTGGTCGCCGGTGAGGTACGTAACCTGGC
CAGCCGCAGCGCGGGGGCGGCGAAAGAAATTGAAGCCTTAATTGGCGAATCGGTACGTCGCGTAGCGCAGGGC
GCGCAGTTGGTCCAGGAGACCGGCGCGACGATGGACGCCATCCTGCGCGGCGTCACGGAAGTGACGACGATTA
TGAAACAGATCGCGTCCGCCTCCGAAGAACAGAGTAAAGGTATTTCTCAGGTTGGCGTGGCGATCACGCAAAT
GGATAGCGTTACGCAACAAAACGCCGCGCTGGTTGAGCAGGTTTCCGCGGCGGCGGCGGCGCTGGAACGGCAG
ACGGAGGATTTGCAACGCTCCGTACAGCAGTTCCGGCTTTCCGCTAGTGAGCCGCAACAACGCGTAACGGCGA
AAGCGGCGCCTGGCGTTCAGAGAATGGCGTCGGCTCCCGCACAGTCTACTGATGAATGGGTTTCCTTCTGATG
TGAAGCAAAAAAGCGCGTAATGCGCTGGCCTTCATTTAATCAGTCGCTATATAATTTATTATATAGCGACTAA
GGAGGTCAGATGAATAATCATTTTGGGAAAGGGTTAATGGCTGGGTTGCACGCGCCATATGCATATAGCGCGC
ATCATGCGGTGAATTTCTGTTCTGAGTATAAACGTGGCTTTGTATTGGGTTTTACACACCGTATGTTCGAAAA
GACCGGCGATCGTCAACTTAGCGCGTGGGAGGCCGGAATTCTGACGCGTCGCTATGGTCTGGATAAAGAAATG
GTGATGGATTTCTTTAAAGAGAATCATTCCGGGATGGCGGTTCGCTTCTTTATGGTCGGTTATCGACTCGAAG
GTTGATCCAACAGGGGTATTATCTTGCTTTAATTAATTACACTATCGCCTCTTCCCTTCGTTTTACTGTTCCG
TCGAAGTATTATGGCGCACATAACCATAAAAAAAGTGTGGTAAATGGCGTACCGATCGCAATAACTTCTTCGC
GAACAGTGCAAAATAAAAGCCAGGTCTTCGTAACGGAATAACTATAAAATGACTGGAGATAATACTCTCATCA
CTTCTCACGGCATTAACCGTCGTGATTTCATGAAGCTTTGTGCAGCACTGGCCGCTACTATGGGGCTCAGTAG
CAAAGCCGCCGCAGAAATGGCAGAATCGGTATCCAATCCACAGCGTCCGCCCGTTATCTGGATTGGCGCTCAG
GAGTGTACCGGTTGTACCGAATCACTGCTTCGTGCTACACACCCAACCGTTGAAAACCTCGTTCTGGAGACTA
TCTCTCTGGAATACCACGAGGTACTTTCCGCCGCATTCGGTCACCAGGTCGAAGAAAACAAACATAACGCTCT
GGAGAAGTATAAAGGGCAATATGTTCTGGTGGTGGATGGTTCTATCCCACTAAAAGATAACGGTATCTACTGC
ATGGTTGCCGGCGAGCCGATCGTGGATCACATCCGCAAAGCCGCTGACGGCGCAGCCGCGATTATCGCTATCG

GTTCCTGCTCGGCATGGGGCGGCGTTGCTGCGGCTGGCGTAAACCCAACCGGCGCTGTCAGTCTGCAGGAAGT
CTTACCGGGCAAAACGGTTATCAATATTCCAGGTTGTCCGCCAAACCCGCATAACTTCCTGGCGACCGTCGCG
CATATCATCACTTACGGCACGCCGCCGAAGCTGGATGCGAAAAATCGTCCAACCTTTGCCTATGGCCGTCTGA
TTCATGAGCATTGCGAACGTCGTCCACACTTCGACGCAGGCCGTTTTGCCAAAGAATTTGGCGACGAAGGCCA
CCGTCAGGGCTGGTGTCTCTACCATCTTGGCTGTAAAGGGCCGGAAACCTGGGGCAACTGTTCTACGTTACAG
TTCTGTGACGTTGGCGGCGTCTGGCCAGTGGCGATCGGTCATCCTTGCTATGGCTGTAACGAAGAAGGTATCG
GCTTCCATAAGGGCATTCACCAGCTTGCTCATGTCGAAAACCAAACTCCGCGTTCAGAGAAACCTGACGTCAA
TATGAAAGAAGGCGGCAATATCTCTGCGGGCGCTGTCGGTCTGCTTGGCGGCGTAGTCGGTCTGGTTGCCGGC
GTCAGCGTGATGGCGGTACGTGAACTGGGGCGTCAGCAAAAGAAAGATAACGCTGACTCACGGGGAGAATAAC
CGTGAACAGACGTGACTTTATTAAAGCAGCCTCCGGTGGAGCATTGCTGTTGGGCGCCGCGCCGTCTGTCAGC
CATGCGGCTGCAGAAAACCGTCCGCCGATTCCAGGTTCGCTGGGTATGCTGTATGACTCCACTCTGTGCGTGG
GCTGTCAGGCCTGCGTCACAAAGTGTCAGGATATCAACTTCCCGGCGCGTAACCCGGAAGGGGAGCAGACCTG
GTCGAACAACGACAAGCTGTCGCCGTATACCAATAACATTATTCAGGTATGGCGTAGCGGCACGGGCGTGAAC
AAAGATCAGGAAGAGAACGGCTATGCCTACATCAAGAAACAGTGTATGCACTGCGTTGATCCTAACTGCGTTT
CCGTTTGCCCGGTGTCGGCGCTGAAAAAAGATCCGAAAACCGGCATCGTCCACTATGACAAAGATGTCTGCAC
CGGCTGCCGTTACTGTATGGTCGCCTGTCCGTACAACGTACCTAAGTACGACTACAACAACCCGTTTGGCGCG
CTTCACAAGTGTGAGTTGTGTAACCAGAAAGGCGTTGAGCGTCTGGATAAAGGCGGTTTGCCTGGGTGTGTGG
AAGTTTGTCCGGCAGGGGCCGTCATTTTCGGTACTCGTGAAGAGCTGATGGCGGAAGCGAAAAAACGTCTGGC
CCTCAAACCAGGCAGTGAATATCACTATCCGCGCCAGACGTTGAAAACGGACGACACCTATCTGCATACGGTG
CCTAAATACTACCCACACCTCTACGGTGAAAAAGAGGGCGGTGGTACTCAGGTGCTGGTGCTGACAGGCGTTC
CTTATGAAGATCTGGATCTGCCGAAGCTGGATGACCTTTCAACCGGAGCGCGCTCCGAGCATGTTCAACACAC
GCTTTATAAAGGCATGATACTACCCCTGGCTGCGCTGGCGGGCTTAACCGTGCTGGTTCGTCGTAATAGCAAA
AACGACCATCACGATGGAGGAGACGATCATGAGTCATGATCCAAAACCGCTGGGCGGAAAAATCATCAGTAAA
CCGGTCATTATTTTTGGACCGTTAATCGTCCTGTGTATGCTCCTTATCGTGAAGCGTCTGGTCTTCGGGTTGG
GCTCCGTTTCCGACCTGAACGGCGGTTTCCCATGGGGCGTCTGGATTGCCTTTGACCTGTTGATCGGCACTGG
CTTTGCCTGCGGCGGTTGGGCGTTGGCATGGGCGGTATATGTCTTTAACCGTGGGCAATACCATCCGTTGGTG
CGCCCGGCGTTGCTGGCAAGCTTGTTTGGTTACTCGCTGGGCGGCCTGTCGATCACTATCGACGTCGGTCGTT
ACTGGAACCTGCCGTACTTCTACATTCCAGGTCACTTCAACGTGAACTCGGTACTGTTTGAGACGGCGGTCTG
TATGACCATCTACATCGGCGTGATGGCGCTGGAGTTTGCGCCTGCCTTGTTTGAACGTCTGGGCTGGAAAGTG
TCGCTCAAGCGTCTGAATAAGGTGATGTTCTTTATTATCGCGCTGGGCGCGCTGTTGCCGACGATGCACCAGT
CCTCAATGGGGTCATTGATGATCTCGGCGGGCTATAAAGTGCATCCGCTATGGCAAAGCTATGAAATGCTGCC
GCTGTTCTCGGTTCTGACCGCGTTTATCATGGGCTTCTCCATTGTCATATTTGAGGGTTCGCTGGTTCAGGCA
GGCCTGAAAGGAAACGGTCCGGATGAGAAAAATTTGTTTGTTAAGCTGACGAATACCATCAGCGTGCTGCTGG
CGATTTTCGTCGTCCTGCGCTTTGGCGAACTGATTTATCGCGACAAGCTGTCCTATGCGTTTGCCGGCGATTT
TTACTCCGCTATGTTCTGGATTGAAGTCGTCCTGATGGTCTTCCCGTTAGTGGTGCTGCGTGTGGCGAAACTG
CGTAATGACTCTCGTATGCTGTACCTGTCGGCGCTGAGCGCGCTGTTGGGCTGCGCGACGTGGCGTCTGACCT
ATTCGCTGGTGGCATTCAACCCGGGTGGCGGCTACCACTACTTCCCAACTTGGGAAGAATTGTTGATTTCTAT
TGGTTTTGTGGCTATTGAGATTTGTGCATACATCGTACTCATTCGTCTACTGCCGATACTTCCTCCTTTAAAA
CAAAACGATCATAATCGTCATGAGGCGAGCAAAGCATGAGCCAGAGAATTACTATTGATCCGGTAACCCGTAT
TGAGGGTCATTTACGCATTGACTGCGAAATTGAGAACGGCGTCGTATCCAAAGCATGGGCGTCCGGCACCATG
TGGCGCGGTATGGAAGAGATCGTGAAGAACCGCGATCCGCGCGATGCGTGGATGATTGTGCAACGTATTTGCG
GCGTTTGTACGACAACTCACGCGATCTCCTCCGTTCGTGCGGCGGAAAGCGCGCTGAATATTGATGTTCCCGT
CAACGCGCAGTATATCCGTAATATTATTCTGGCGGCGCATACCACCCATGACCACATTGTCCATTTCTACCAG
CTCTCTGCGCTGGACTGGGTGGACATTACCTCTGCGCTGAAAGCCGATCCGGAAAAAGCCTCCGCGATGCTGG
ATGGCGTGTCGAGCTGGCACCTGAACAGCGCGCAAGAGTTCACGAAAGTTCAGAACAAGATCAAGGATCTTGT
CGCCAGCGGTCAGCTAGGTATTTTCGCCAATGGCTACTGGGGGCACCCGGCAATGCAGTTACCGCCGGAGGTC
AACCTGATTGCGGTCGCCCACTATCTGCAGGCGCTGGAATGTCAGCGTGACGCTAACCGCGTGGTGGCGTTGC
TGGGCGGTAAATCGCCGCATATCCAGAACCTGGCGGTCGGTGGGGTGGCGAACCCGATCAATCTCGACAGCAT
CGGTACGTTGAACCTTGAACGCCTGATGTACATCAAATCGTTTATCGATAAGCTGAGCGATTTTGTCGAGCAG
GTGTATAAAGTGGATACCGCAGTCATCGCGGCCTACTATCCGGAATGGCTGGAGCGCGGCAAAGGCGCGGTGA
ACTACCTGAGCGCGCCGGAATTCCCGACCGACGGTAAAAACGGCAGTTTCCTGTTCCCGGGCGGTTACATTAC
CAATGCCGATCTGTCGACATACCGTCCGATCACCTCTCACTCCGACGAATTCCTGATTAAAGGTATTCAGGAG
AGCGCGAAGCACGCCTGGTACAAAGATGAAGCGCCGCAGGCGCCGTGGGAAGGCACTACGATCCCGGCTTACG
ACGGTTGGTCGGATGACGGTAAATACTCATGGGTCAAGTCGCCGACATTCTACGGCAAAACCGTCGAAGTCGG

GCCGCTGGCGAACATGCTGTGTAAACTGGCTGCAGGTCGTGAATCCACGCAGACCAAGCTCAATGAAATCATT
GCGCTTTATCAGAAGCTGACCGGCAAAACGTTGGAAATGGCGCAACTTCACTCTACGCTGGGTCGTATCATCG
GGCGTACCGTTCACTGTTGTGAACTGCAAAACATATTGCAGGATCAATACAACGCGCTGATTGTAAATATCGG
CAAAGGCGATTACACCACGTTTGTGAAGCCGAACATTCCGGCGACGGGCGAGTTCAAAGGCGTGGGCTTCCTG
GAGGCGCCGCGCGGTATGCTGTCTCACTGGATGGTGATTAAAGACGGTATTATCAGTAACTATCAGGCGGTGG
TGCCATCGACCTGGAACTCAGGTCCGCGTAACTTCAACGATGACGTGGGGCCGTACGAACAGTCGCTGGTCGG
TACGCCGATCGCCGATCCGGCGAAGCCGCTGGAAGTGGTACGTACCATCCACTCCTTCGACCCTTGCATGGCG
TGCGCGGTACACGTGGTGGACGCCGATGGCAATGAAGTGGTCTCGGTGAAGGTTTTGTAATGCGGATATTAGT
CTTAGGGGTCGGCAATATTTTGCTGACCGATGAAGCCATCGGGGTGCGTATTGTTGAAGCGTTAGAGCAACGA
TACACCCTGCCGGATTTCGTTGAGATTCTGGATGGTGGTACGGCGGGAATGGAACTTCTTGGCGATATGGCGA
GTCGGGATCATTTGATCATTGCCGACGCCATCGTGTCGAAAAAGAACGCGCCGGGAACCATTATGGTACTGCG
CGACGATGAAGTTCCGGCGCTGTTCACCAACAAAATCTCCCCCCATCAGCTTGGACTGGCCGACGTATTGTCG
GCCCTCCGCTTTACCGGCGAGTTTCCGAAGAAACTGACCCTGGTCGGCGTTATTCCACAGTCGCTGGAACCGC
ACATCGGCTTAACGCCAACCGTCGAGGCGATGATTGAACCTGCGCTTGAGCAGGTTCTTGCTGCGCTGCGAGA
ATCCGGCGTCGAAGCAATTCCTAAGGAGACGGCGCATGTCTGAAACGTTTTCCGGATTCGACACGGCGCCCGT
CGCCCGGGTTCAGGCGGCGTTTGAAGAGATAGCCCATCGTTCGATGCACGATCTTTCGTTTCTGCATCCGACT
ATGCCGGTGCATGTCTCTGATTTTACGTTGTTTGAAGGACAATGGACGGGCACGGTAATTACGCCGTGGATGT
TAAGCGCATTAATCTTCCCTGGCCCGGACCAGATCTGGCCCGGGCGTACCATCGGTGAAAAACTCGGTCTGCA
ACTGCCTTATGGCACGATGACCTTTACCGTTGGCGAACTGGAAGGCGTTTCACAATATCTTGCCTGCTCGTTG
ATGTCGCCGCTTTCCCGTAGCCTGTCGCCGGAAGAGGGCGTCCGGCTGGCGGATGACTGCGCGCGGATGCTGT
TATCCCTGCCGGTAAGCAATCCTGATGCGCCGCAGACAAGCCGGCGTGCGTTGCTGTTTGGCCGTCGGAGCTG
TGAGAATGCATGAGCTGTCTCTTTGCCAAAGCGCCGTTGAAATTATTCAGCAGCAGGCAGAACAGCACGGCGT
GGCGCGCGTCACCGGCGTATGGCTGGAGATAGGCGCGCTTTCGTGCGTTGAAGAGCGCGCGGTTCGTTTCAGC
TTTGACATCGCCTGCCAGGGAACTTTAGCGCAAGGGTGCGAATTGCACATCGACTACAGACCGGCCCAGGCCT
GGTGCTGGGATTGCAGTCAGGTGGTGGAAATTCTCAGGCATGACGCGCAATGTCCGCGTTGTCATGGCGATCG
CCTGCGGGTGGATACCGGCGATTCGTTGAAAGTAAAGAGTATTGAGGTCGAATAACCCGCATTTTATTTCAGG
TGGTTAATTATGTGTATTGGCGTCCCCGGTCAGGTGCTGGCCGTCGGAGATGATATTCACCAGCTTGCGCAGG
TGGAAGTGTGTGGCATCAAACGTGATGTCAATATTGCTCTGATTTGTGAAGGTAAGCCAGCCGATCTGGTAGG
GCAATGGGTATTGGTACACGTTGGTTTTGCGATGAGCATCATTGACGAAGACGAAGCGAAAGCCACGTTAGAT
GCGCTGCGCCGCATGGAGTACGACATCACCAGCGCGTGATCCCTTAATCACCTCTATTATATCGCTCTATAAT
CGCTGTATAATTATTTATATATCGATTGTGGGCGAAACCTATGAATAAATTTTTGCGCCAGCTCTCTCTGCT
GGCGTTGCTTTTTTGCTGGCCGTTAATGTCACAGGCGGCGCGTACTTTTACCGATCAACTTGGCCGCCAGGTC
ACGGTTCCCGACACCGTCGACAGAGTGGTGGTGCTCCAGCATCAAACGCTCAATCTCCTTGTACAGATGAATG
CCACTGACAAAATCGTTGGGGTCATGGCGAACTGGAAACAGCAATTAGGCGACGGCTATGCGCGTCTGGCGCC
GGAACTGGCGCAAAAAGCCTCGCTGGGCGATCTAACGCACGTTGATCCTGAAAAACTGGTGGCGCTGCGCCCG
CAGGTGGTGTTTGTCACGAATTACGCTCCGCAGGAGATGATCGACAAAATCAGCCGTCTTGGCATTCCGGTGG
TGGCGATTTCGCTGCGCCATGATGTTGCTGGCGAGCGGGCTAAAATGAACCCGACCCTGGCCGATGAAGAGCA
GGCTTATAATCGCGGGCTGCGTGAAGGAATAACGCTGATTGGCGATATCGTCAATAAACCGCAGGAGGCGAAA
GCGCTGATTGAGGCGATGGATAAAGGTCGCAAGATGGTCAGCGACCGTTTGCAGTCGGTTCCTGAAAATGAGC
GGGTTCGCGCTTATATGGCGAATCCGGAGCTGACCACCTACGGTTCCGGTAAATACACCGGTCTGATGATGGC
CCATGCGGGGGCGCTGAATGTAGCGGCATCCTCGGTGAAAGGCTTTAAGCAGGTGACAATGGAGCAGGTGATC
GCCTGGGATCCACAGGTAATCTTTGTCCAGAATCGCTATCCAAAGGTCGTCAATGAGATCCTGCACAATCCCC
AGTGGCAGGTCATTGACGCGGTGAAGCATCATCGGGTTTACCTGATGCCGGATTATGCCAAAGCCTGGGGATA
TCCGATGCCGGAGGCGGTGGGAATCGGCGAACTGTGGATGGCGAAAAAACTCTATCCGGAAAAATTCCACGAT
ATTGACATGCAAAAGGCCGCTAACGACTGGTATCAACGTTTTTACCGTACTGACTATCAGGGCGTACAGTGAT
GCGAATACTGGCTGCGGGGAGCTTGCGCGTCGTCTGGCCGCAGCTAATGGCGGCATTTCAGGCGGATGCGGTA
TGTGATTTCGGTCCGGCGGGGCTGTTACGTGAACGCATCGAAGCGGGCGAAGCGTGCGACTTTTTTGCTTCGG
CGAACCTGGCGCATCCGCAGGCGCTGCTGGAAAGCGGGCGGGCGCTGCGGGTCGCGCCTTTTACGACGAACCG
TTTGTGCCTGAGCGTGCGGGCACAGGCGATGCGTGAAGGCGAGGACTGGCTGTCGCTATTAACCCGGCGGGAT
CTGCGGATCGGCACCTCAACAGCAGGTTGCGATCCTTCCGGCGATTATACGCAGCAGCTTTTTTCGCGGATGG
GCAAGGAGGGCGAAGCGGTCCGAAAACGCGCGGTAGCGCTGGTTGGCGGCAGGCAGACTTTGCCGTTACCGGC
GGGCCGGCTGGCTGCGGAATGGCTCATCAATCATGATTACACCGATATCTTTATCGGCTATGCCAGCTATGCT
CCCAGGCTACGGCAGGTTAATTCGCTGCGAGTGGTTGATATCCCGGAACCTTATAATCCCGTGGCTGAATATG
GCTTTGCCTGCCTGAGCGAGCAGGGAAAAACGCTTGCCGACTTTTTACTGTCAGCACGGGCGAGGTTGATTCT

CATGCAACATGGGTTTAGTGAAGCGCCGAATATGACACACTCGCAGAACTGATAAGCGAAGCGTCAGCAGGGA
ATACCATGCCGGATGACGACATTACTACCTCATCCGGCATCATGGTTTAACTTTGACGTTTATCTTCGGTATT
GTTTTCGAAGTCGCTGGCGTCATGGCGTTCGTGAAGCTGTTCGTTCAATGGGCCGTTGGTCCGGTTGACAATG
CGCCCCCGCTTTACCGCCGGACGTTCGGCTACCTGCTTCGCCCAGCGTTGTACGTTTTGATAACTTCCGGCAT
CCAGAAACTCCGCTGCATCATAGACATTCCCCAGCACCACGTTGCCGAACCACGGCCAGATGGCCATATCGGC
AATAGTGTACTCTTCTCCCGCGACATACGGGTGGTGAGCTAACTGCTTATCCAGCACGTCCAGCAGACGCTTG
GCTTCCATGGTGAAGCGATTAATGGCGTACTCAATTTTTACCGGCGCATAATGATAGAAATGACCGAAACCGC
CACCGAGGAACGGCGCCGCGCCCTGTAGCCAGAACAACCAGTTAAGGGTTTCTGTGCGCTTAGCCAGATCCTG
CGGCAGGAAATGGCCAAACTTATCCGCCAGATAAACAAGAATTGCGCCCGACTCAAAAACTCGAATCGGCGGG
TTGTGTGAGTTATCGCGCAGCGCCGGGATTTTTGAGTTCGGATTGATGTCAACAAAGCCGCTGGAGAACTGAT
CGCCCTCGCCGATGCGAATCAACCAGGCGTCATATTCGGCGCCGGTCACGCCCAGCGCCAGGAGCTCTTCCAG
CATAATAGTCACTTTCTGGCCGTTAGGCGTCCCCAGCGAATAAAGCTGTAACGGATGTTTGCCGACGGGCAGC
GTTTTGTCGTGCGTCGGGCCGGAAACCGGGCGATTAATGTTAGCGAACGCGCCGCCGCCGGATTTATCCCACG
TCCAGACTTTTGCGGGTTGATAAGTATTATCTGCCATATTGACCTGCCTTCACAGTGGTGGTGTTGAAGCAGT
GTAGCAGCTTGCTAGTCGGACATTTAACCGATTGGGACATTCCGGCGAGTGAAATTTTAACCTTCTGGCCTCA
GTGTGCCTTATTGAGGTGTATGATTATGTCGACCTCTGTCCGGTTAGTGTCACAGAGCAGACAATCAGAGCGA
AATCACGCCAGGCTGCTCAGGCGGTCTTGTTTTGAGGTAAGTGCATGAGCAAAGGAACCACCAGTCAGGATGC
CCCCTTCGGGACATTATTGGGTTACGCCCCCGGCGGCGTAGCGATCTACTCTTCAGATTACAATTCCCTCGAT
CCGTGGGACGACGACGACGCAGCTTTTCGTAGCTACATCGACGATGAATACATGGGGCATAAATGGCAGTGCG
TGGAATTTGCCCGCCGTTTTCTCTTTCTGAATTATGGCGTTGTTTTTACTGACGTCGGCATGGCCTGGGAAAT
TTTCTCGCTGCGTTTTCTGCGCGAGGTCGTGAATGACAATATTTTGCCTTTGCAGGCCTTCCCGAACGGTTCG
CCGCGCGCGCCGGAGGCCGGGGCGTTGCTCATCTGGCAAAAGGGCGGCGAGTTTAACGAAACCGGGCACGTTG
CGATTATCACCCAACTCCTGGATAACAAAATTCGTATTGCTGAGCAAAACGTTATCCACACGCCATTGCCGCC
AGGGCAGCAGTGGACTCGCGAACTGGAAATGGTCGTAGAAAACGGGTGTTACACCCTGCGCGACACCTTTGAT
GACACGACGATTTTAGGCTGGATGATCCAGACCGACGACACGCAATACAGTTTGTCACAGCCTGACATCGCGA
ATCAGTCGCTGGCGATTCGCGGCGCAAGACTGCCAGAGAAAGGTCAGTTTGACGGTCCGTGGCTGGATGAGCG
CGATCCGTTACAAAAAGCCTATGTACAGGCGAACGGACACGTTATCAATCAGGACCCGTACCAGTATTTTACC
ATCACCGAGAGCGCTGAACAGGAGCTTATCAAAGCGACCAATGAGTTGCATTTGATGTACCTGCACGCCACGG
ACAAAGTTTTAAAAGACGATAATCTGCTGGCGCTATTCGATATCCCCAAAATTCTTTGGCCGCGCCTGCGTCT
CTCCTGGCAGCGCCGTCGTCACCATATGATCACCGGTCGTATGGATTTCTGCATGGATGAACGCGGGCTGAAA
GTCTATGAATACAATGCCGATTCCGCATCATGCCATACCGAAGCGGGATTAATTCTCGAAAAATGGGCTGAGC
AGGGCTATACCGGTAAAGGGCATAACCCGGCGGAGGGCTTAATTAACGAGCTGGCAGGCGCCTGGAAACACAG
TAAAGCGCGTCCTTTCGTCCATATCATGCAGGATGATGATATCGAGGAGGACTATCACGCGCAGTTTATGCAG
CAGGCGCTGCATCAGGCCGGTTTCGCGAGCAAAATTCTCCGCGGTCTGGGCGAGTTGCGCTGGGATGACGCCG
GGCAGTTGATCGATGGCGACGGACGGCTGGTGAATTGTGTCTGGAAAACCTGGGCGTGGGAAACGGCGATGGA
GCAGATACGCGAAGTCAGCGAGACGGAGTATGCCGCAGTGCCTATTCGTACCGGGCACCCGGAAAATGAAGTC
CGGCTGATTGATGTATTACTGCGCCCGGAAGTGTTGGTGTTTGAACCGCTGTGGACGGTGATCCCGGGTAACA
AAGCGATCCTGCCCATCCTGTGGTCGCTCTTCCCGCATCATCGCTATTTGCTCGACACGGATTTTACCGTTAA
CGATGAGCTGGTGCAGACCGGCTATGCGGTAAAACCGATTGCCGGACGTTGCGGTAGTAATATTGATTTGGTG
AGCCATCAGGAAGAGCTCCTGGATAAAACCAGCGGCAAGTTCGCCACGCAGAAAAATATCTATCAGCAGTTGT
GGTGCCTGCCGAAGGTCGCCGGGAAGTATATTCAGGTCTGTACGTTTACCGTGGGCGGTAACTACGGCGGTAC
GTGCCTGCGCGGCGATGATTCATTAGTCATTAAAAAAGAGAGCGATATTGAACCGTTAATCGTCATCAAAGCG
TAACGGGGTCATTCTCGTCGGGATATCGGCGTGGGTGCCGATATCCCGCCTGTTGTGTTTACCAGCGAACGGC
TATCAGCCTGCCATTTACTGGTAACATATGAGAAATAATATCACAAATTATTCTTAACTTTTCTGACGCAGGC
GGTGACGCTCGCCGCGTTGGCGCATCCGTTTCCTGATTTTCGACTACTCGATACTTTTTGCGGCTGGTTACGC
TGCCCCCATTCCTTTGGATTTAGGTCATGCCTGTTTGGCTGGTAAACGGACAGCATAATATATTGATGAGTCC
AAATGATATTATTGGTCATATAAAAAGTTTACGTTAGGTTATTTTTCTATTGCAAAAGTTTGCGTTTAAACCC
TGTCTGCAATCTTCGTGGCGAGATATAGCTACACCTAAGTAAATGCAGTTAATTATTATTCAGCTATTGTTGT
CAGCCTTGTAAATAATATTTCCGTATAAAGCGAAGCGACTTCCATTATGACTATTAATGGTATTATTGAATAG
TTTACTCTGTAATTATTTAAGTTTAGTGGACGTTTTGTCTTATTCTGGTGAACGTATGGTTATGGCAGATGAC
AGCCGATTTTTCATTATGTTAGCATGAGTTGATAATACTTTATCCAGGCATAGATCAGGATGTCCACATATAA
TAAGGAGAATGGTATATGTTTAAAAAAGTATTAGGCTTGCGCCACTGGAATAATAATGTTGTAAAAATACCGC
CGCCAGCAGAATCTGGCGCTAATGCATCTGACGTGGTGGTGGATACTCCTGAGCCTTATGAGCGGATACTGTG
TCAAAAAATTCTGATGGGGATCTCGACGATCGATATCATTCGTAATGCCATCATTAAGTCCTGTGAGCAGCTC

AACATTGAAAAAGAACGCATTAATGAACTTAACGAACAGAATGATAAAGCCAGAAGTTCATTAAAATCGCTCG
TGGAATTTATCACCGAAATTGGCACGACTTCATCGGATATTGGCTGTCGTATGGGCGACTTAAATACCTCCTT
AACGCAAATTAATGCGTGTATTAAAGAGATACAGAAGATCGCTAACCAGACCAATTTGATAGCGATTAACTCC
GCGATCGAGGCCGCGCGCGTAGGGGATGCCGGGCGTGGTTTTAGCGTTATTTCGAAAGAGGTGAAAAACCTGT
CCGAAGATGTTAAACACAGTTCGAAAAGCGTCAGTACGTTAACCTCTGTAATCAAAGATAATACGGCCAGAGT
GAGTGAGGTACTGGATAACCAACAGCCGGTTATTGATAATATTACGACTAACATAAATCAAATTGTGGAGTCT
ATTGGCATCGTCATCGATAAATCGCTGAGCATGAAATCGGTCATGCAGTACATCTCTACGGTGCAGTTTTTGA
ATATTGTCAAAGTTGACCATGTTATCTGGAAAATGGAAGTCTACAAATTACTCTTAAATAAAGACATAAACAG
TAAAATAACGATGCACGATCAGTGTCGGCTGGGGAAATGGTACTACGGTTTTGAAGGACAGCAGTTTAGCAAT
TACTACAGTTTCCGTAGTCTGGAAGCGCCTCATAAAGAGGTGCATACCGCTGGCCACTCTGCGCTGAACTATT
TTGCCGCAGGCGATATGAATGCGATGTCGCAAGAACTCGACAGAATGGAGCGATCCAGTAATGAGGTGGTTAA
CCAGCTGGAAATGCTGGCTGTCGATTTGTTAAAAGAAACCACACTGTGAAGTCATGAGTTATTACCGTATAAC
CAACAGGAGCCATCATCAACAGGACTGATGTATCGCACTTTCTGCACTGGAGTTATTCCGGACAGAGAAAAGA
AGCATGACGCAGAAGATGGCTGTGTTTGTTCACCCGAATCACTTACCTTAGTAAGTGATTCGGGATTAATCAG
CGACATCTTTGCTTTTTATCGGCGGCCAGACTTTGGCTGGAAAAGTGCGTTACTGCTAAATTTCTCACTCTCT
TACCGCCTTCCTGCCCCTCGAAATACCTGAGACCTGTGCTCACAGATTAATAGCGATTATTTAATCGTGAAAT
AACGCTGGGCATTATTGAAGCAGATATCCTGAACCATACGGCTTAGCATCGCTTCATCATCAGGAATCTCTCC
GTCCTGCGCCCACTGTCCCAGCAGGTTACAGAGAATACGACGGAAATATTCATGTCGCGTATAAGAAAGGAAA
CTGCGGGAGTCGGTCAGCATCCCGACAAACTGACTTAACAGTCCCATTTGCGACAGTTGCTCCAGTTGGCGCA
GCATACCGTCTTTCTGATCGTTAAACCACCAGCCGGAACCAAACTGCACTTTTCCGGCAATTCCCGGCCCCTG
GAAGTTACCGATCATGGTCGCCAGGACTTCGTTATCACGTGGGTTCAGGCAATAGAGGATAGTCTTGGGCAGT
TCATTGGTCACATCCATACTGTCGAGCAAACGGGAGAGCGCCCAGCTAATGTTATTATCGCCAATGGAGTCAA
AGCCGGTATCCGGCCCCAGCAGGCGGAACATCCGGGTATTATTATTACGGATCGCGCCAATATGTAGCTGCAT
CACCCAGCCGCGCGCGGCGTACTGACGGCCCAGCCAGACCAGCACCGCCGTGGTAAACTGGGCGATCTCAAGT
TCGCTCAGCGTTTCGCCAGCCAGACGTTTGCCCAGAATGGCGTCAAGCTGCGCGTCGTCGGGCACCGGCGCAA
ATCGCAGCGTTTCAATGCCATGATCCGACGCGCGGCAGCCGCAGGCGGCGAAATGGTCGAGGCGGCGAGTGAG
CGCCTGACGTAAATCGTCGAAACGGGTAATGCTGACATCTGCCGCCGCTTCCAGTTTCCTCAGGTAATCGACA
AAGCCGTCCAGTTCGATTTTGAAAACTTTGTCGGGGCGCCAGCTTGGCGCGACTTCAATATCAATGCTGTCGT
CGGCGGCTATCTGGCGGTGATATTCCAGAGAATCTATCGGGTCGTCGGTGGTTCCGACCATCCGCACATTCAT
CTGCTGCATAATACCGCGCGCGGAAAAGGCCGGCGTCGCCAGTTTCTCATTACACTGCGTCCAGATACTTTCC
GCGGTATCCGGTCCGAACAGCGTACCTGTAATGCCAAATGGACGGCGTAGTTCAAGGTGCGTCCAGTGATACA
GCGGATTGCCCAGCGTTTTTGGTACGGTATTGGCCCAGGCCATATATTTTTCATAATCGCTGGTCTCTTTGCC
GGTGATCAGCGACTCATCCACGCCTGCGCTTCGTAGCGCTCGCCATTTATAGTGGTCGCCTTCCAGCCAGATC
TGACCGAGGTTATCGAAACGGCGATCGTCGGCGATTTCCTGCGGACTTAAATGGCAGTGGAAGTCATAAATCG
GCATGGGCGCGGCGTATTTATGGTACAGCGTGCGGGCAATGTCGTTTTTAAGTAGAAAATCTTCAGTCATAAA
CGTAGCCATGATTTATCTCCTTACTTAGCCAGCGCTTCTACCGTGGCTTTCGCGCCGTTCTGCAATAGCTGTT
GGTAAGCGTCGGTAACGGCTTGCACAAATTCGTGGTTCTTCGGCAGGTCGCTGCCAAAGATAGACTCGATGGC
CAGTAGCCCGCGAACGCGTTCTTCCGGCGTCTGATATTGCTGATGAATCGCCTGATACTGCGCGAGCAGCGGA
TCGACGACATCAATGGTTTTACCTTGTTCATCGATACCGCCGACGTAACGCATCCATCCGGCGACGCCCAGCG
TCAGGCGGCGATAGTCATCGCCCTGTTGCAAGTGCAGACGTACCGGGTCCAACAAACGCTGCGGCAGTTTCTG
ACTGCCATCCATCGCGATCTGCCAGGTGCGGTGTTTCAACGAGGGGTTAGTGAAACGCGCGATCAACAAATTC
GCATACCCTTCCAGATCGGTGCCTTCCGGCATTGATAACGTCGGCGCTTGTTCATCAAGCATCAGCGCCAGCG
CCGCGCGACGGTAAGCCGGGTTGGTCATGGTATCGGCGATAGTGTCATACCCGCCGAGATAACCCAGATAGGC
GAGGAAAGAGTGACTGCCGTTAAGCATACGCAGCTTCATCATTTCAAACGGTACGACATCTGCAACGAATTGC
GCGCCGACGGTGTCCCAGTCCGGGCGACCATTCACAAAGTTATCTTCGATGACCCACTGGCGGAAGGGTTCGC
AGGCGATAGCGCAAGGATCATAAACCCCTAACTGGTCGGCAACCAGTTGTAGCGTCTCTTCGGTCGCCGCCGG
AACAATGCGGTCAACCATGGTGCAGGGGAAGGTCACATTGTCGGCAATCCATGCGGCGAGGGCGGCATCGCGA
GCTTTTGCCAGGCCGAGGACGGCAGCCTTCGCGACGTGACCATTCTCGCGTACGTTATCGCATGACAACACGG
TAAAAGCTTTCAGTCCTTGCTCCCGGCGCATGTTTAGCGCTTCGACAATATAGCCAATGGCGGATTTAGGCTG
CTGTGGATGGGCAAGATCGTTTTGGATCAGCGGGTTATTGACATCAAGCTCGCCGCTGGCGGGGTCGGTGCAG
TAGCCTTTTTCGGTCACGGTTAAGGAGACGATGGCGGTTTCCGGGCGCGCCATTGCCGCCAGAATCCCTGCAT
GGCCGTCGAATTCAGGGTGCAGCGCTTCTTTCATTGAACCGATAATTTTCAGTTCGGTGCTTTCTGCGCCGCG
TTCTGCAACGGTGTACAGCAGATTTTGCGCTTTCAGGTTCGCGATCAGCCGCGCGTCGTTACCCGGCATCAGG
TTCACTTCACAAATGCCCCAGTCGCTGTCGCTCTTTTCCAGTAAATGATGGGTAAAGAGCGCCTGGTGCGCGC

GGTGAAACGCCCCGCAGCCCAGATGCACAATACGGGATACCAGACGTGATTTGTCCCAGTTTGGGCGGGCGAC
GGAAACCTGGGCGGTGGCGATATTCTGTTCCATTGCAAACTCCTGTCATTCGACGTCTTCGCCCGCGATCTCC
GGGGCGAGGCATGTTGCTATTGCATTGAGTAGGGGAAATCGCATGACGCGTCGCGCCATGCGAAAGAAGGTTA
TTTGCTAAAGAAAGCGCGCTGGATAGCCAGTTCGACGCCGCGGACTTCCGCAAGCCCTTTCAGACGGCCAATG
GCGGAATAACCCGGATTCGTTTTCTTCTTCAGATCGTCCAGCATCTGATGACCGTGGTCCGGGCGCATTGGGA
TCAGATCGTCGCTACCTTCGGCTTTACGACGGTGCTCTTCTTCCACAATCGCTTTAACGACTTCATACATATC
CACATCGCCGTGCAAATGGGCGGCCTCGTGGAAGGTCTTCGGATTCTCTTCGCGCAGCGTAGAGCGCAGATGC
GTAAAGTAGATGCGCGGACCAAACTGTTTGATCATATCAACCAGATCGTTGTCGGCGCGCACGCCGTAAGATC
CGGTACACATGGTGAAGCCGTTCGCCATGCTATTAACGGTTTCCACCATCCATTGCATGTCTTCAATGGTAGA
GACAATGCGCGGCAGGCCGAGAATAGGGCGCGGCGGATCGTCAGGGTGAACGGCCATACGCACGCCAACCTCG
TCGGCAACCGGAATAATGGCTTTCAGGAAATAGGCAAAATGTTCACGCAGTTTTGCTTTATCGATATCTTTAT
ACGTCGCCAGGTGTTGACGGAACTGATCCAGCGTATAGCCTTCTTCCGCGCCAGGTAAACCGGCAATAATGTT
GCGGGTCAGACGTGCTTTGTCTTCCTCGCTCATGGTGGCGAAACGTCGCTCTGCCTGAGCAATTTCTTCTGCC
GTATAGTCGGCTTCTGCTCCCGGACGCTTCAGGATATGCAGTTCGAACGCGGCGAATTCAATCTGGTCAAAAC
GCAACGCTTTTGAACCATCCGGCAATACGTATTCCAGATCCGTACGTGTCCAGTCCAGCACCGGCATAAAGTT
ATAGCAAACCGTATAGATACCGCATTGCGCCAGGTTACGCAGCGTTTGCTGGTAGTTTTTGATCCATAAATCG
TACTGACCGGTGTGGGTTTTGATATCTTCGTGGATAGGTACGCTCTCTACCACAGACCACTCCAGACCCGCCT
CTTCAACGATAGCTTTACGTTTCTGGATCTCGTCTATCGACCAAATTTCTCCATTCGGGATATGGTGTAACGC
CGTTACCACGCCGGTTGCGCCAGCCTGGCGTACATCTGACAGCGTTACCGGGTCGTTAGGTCCGTACCAGCGC
CAGGTTTGTTTCATATCTCGTTTCCTCTTCTTGCGATAACGTCTTCGTGGTTGACCCATTGCCAGCCAACATC
GAAACGTGCTTTGTAAACCCGTTCTGACCCCTAAATTCAACCAAAATTTTTCTCATGTCAACCTTATTGTCTA
AATTGGCTAACCAAATCACAAATATCATCATTCACGGTCTGCCAATTTTATTTATTTGATCTGTGTCAATTTT
TGCTGGGTGAAAAGCATTCACCATTCAACTTGAAATGAGTTGATGTATTTATTTCAAGAATATTAAGGGCGGG
AGTTGCCGCCAGATTTTGACCGGTCCGGATGAGAAAATATTGATTGGTCAACCAATTTTTGTGATTTCAGTTT
TCCCGCTACAGGTCAGACGGCGCGGAGCTAATGTTTTTTAACGAGGCTTTATCATGAAGATGACAAAATTAAG
ATGGTGGATTATCGGCCTGGTCTGCGTAGGGACCATCGTAAACTACCTGTCCCGTAGCTCGCTTAGCGTTGCG
GCGCCAGCGATGATGAAAGAACTCCATTTTGATGAGCAACAATATTCCTGGGTGGTGAGCGCCTTCCAGCTTT
GTTACACCATTGCGCAGCCGATCACCGGCTATTTGATGGATGTCATCGGACTGAAAATCGGCTTCTTTATCTT
TGCCTTGTTATGGTCGCTGATCAACATGGCGCACGCGCTGGCCGGCGGCTGGATAAGTCTGGCATTTCTGCGT
GGTCTGATGGGGCTAACCGAAGCGTCAGCGATTCCGGCCGGGATTAAAGCCAGCGCCGAATGGTTCCCGACGA
AAGAACGCGGTATTGCGGGGGGACTGTTTAATATCGGTACTTCGATTGGCGCGATGCTGGCGCCGCCGCTGGT
GGTCTGGGCGATGCTGACCTTTGCTGATAGCGGCATCGGTACTGAAATGGCGTTTGTGATTACCGGTGGGATC
GGCGTGCTGTTCGCCATCACCTGGTTCCTGATTTATAACTCGCCAAATAAACACCCGTGGATCACCCACAAAG
AGCTGCGTTATATCGAAGACGGCCAGGAAAGCTATCTCCAGGACGATAATAAAAAACCGGCCGTTAAAGAGAT
CGTTAAAAAGCGTAACTTCTGGGCGTTGGCGATTACCCGTTTCTGGCTGACCCGGCATGGGGAACGCTGAGT
TTCTGGATGCCGCTGTATCTGATTAACGTGATGCATCTGCCGTTAAAAGAGATCGCCATGTTTGCCTGGCTGC
CGTTCCTGGCGGCGGACTTTGGCTGCGTTGCAGGCGGTTTCCTGGCGAAGTTCTTCATGGAAAAGATGCACAT
GACCACGATCAATGCGCGTCGTTGTAGCTTTACCATCGGCGCTGTTTTGATGATTTCGATCGGTTTTGTCAGC
ATTACGACTAACCCGTATGTCGCCATTGCATTGATGAGTATTGGCGGTTTTGCGCACCAGACGCTGTCTACCG
TCGTGATTACCATGAGCGCTGACCTGTTCAAGAAAAATGAGGTCGCGACGGTTGCCGGTCTGGCAGGATCTGC
GGCATGGATGGGACAGTTAAGCTTCAACCTGTTTATGGGGGCATTGGTCGCTATTATCGGTTACGGCCCGTTC
TTCATTGCACTCAGTCTGTTCGATATTATCGGCGCCATCATCCTGTGGGTACTGATTAAAGATCCAGAAAAGC
ACCACCCGCCAATGACGGAACAGCCGCTGGCGTCTCACCGCTAAAGCTGTCTCGGTTTCACTGGCCTGATAAG
CGAAGCGCTACCGGGTTCTAACGGCCACCTTCGGGTGGCCGTCTTCATTACCCTTATCGGTAAAAATACGCGT
TGGTTTTATACCAGCGCGTTATCATTTAGTTTGCCTGATTATTTTCAGATGCTGAAAATAATATTGTGGTAAG
GGTTATTGTTTTTCGCCTGGATTTTTATCGACTCTCTGTCTCGCTGCGATCATCGATAAAGTCCCCTTTATGT
AAATAAAGGTTAAATGCTTCTACTCTTTTCCCCATATATTTTGCGCAGCTTCACAAAAAATAACCTGATGTCG
TAATGTCCGCTCCCTAATATTCTCCATGCGCTATACGTTAATTTAAGTTTAAATTTCATGCCGGGGATATCAC
GATGTCACGTAATTTTACTGTTTCAGCCTGTCAGTATATTGTTACCGAAATAAACACTTTTGAAGATTTCATA
ACAAAAGTAAGGATACTTCTTAATAAAAGTCAGGGTGCTGATGTCGTTATCTTCCCGGAGCTATTTACCATTG
AGTTATTTACATTATTGAAAAAATGGCAAGAAAGACCCATTTCACACCTTACTTTAATTGATCAGTTCACGGA
TGCCTATAAGCAGCTTTTTCAACAGGAAGCGAAAGAAAGAGGACAGTTCATTATTGCTGGCTCTCATCTGGAG
CAGACTGGAGCAGATCGCTATGAGAATGTTGCGCATATATGGGGACCCGATGGCGAACATTATGCTCACAGTA
AGACGCATATCTTCCCGGCTGAACGGGGTTGGTATACTCAGGAAGGCGATAAAATGGCTGTTTTTCAACTGCC

ATTTGCAAAGGTGGGTTTTAATATTTGTTATGAAGCAGAAATACCGGAGTGTGCCGCGACGCTGGCGGAACAG
GGTGCGGAGTTAATTTTAACGCCTTCCGCTACGTTTACTGAACAGGGATTTTGGCGAGTCAGGCATTGTTGCC
ACGCACGTTGTATTGAAAATCAAATTTATCTTGTTCACTGTTGCCTGGGCGGAAACCCGGGCGGGCCACTGCC
AGGCTGCTGGGCCCGAAGTTCAATTTTAAGCCCCTGTGATGTCGTGTGGAAAAATCCACAAGGTATTATCGCA
GAGGCGCACGTCAATCAAGAGGATGTCATTTCAGGAGAAATTAACCTCGATGTTCTATATGAAAATAGACTTG
GGGGGGCAGCAACAACGTTCAAGGACAGACGCAGAAAAGCAGGCATATATAATATCTGGCCATCACACATTAA
ATAACAGAATGTGTCCCTTCTATGTATTAGCTGGAGGTCGTTATGGGATATTTAAAATTACCTGAGGGTAAAA
GGATTGCGGTCAATCTTGGTGTTGATGTAGATGCTCAGTCTTTATGGCTGGGCGGATTTAATCGACCGTCGCC
ATCCTTTATGTCACGAGGAGAGTTTGGTGCTCAAGTTGGCGTACCAAGGCTTCTGAAATTGTTCAAAGAAAAT
AATATCAAAACCACCTTTTTTATTCCCGGACACACTGTAGACACCTTCCCGGAAATCATTAAAGCAATTTTTG
ATGCCGGGCATGAAATCGCTCACCATGGCTACTATCATGAAAATCCGACGCTGGTGAATCGTGATACAGAACG
CCGACTTATGGATCTGGCGTTCGCCTGTTATAAAAGACATTTCGGCATACGCCCTGTGGGATACCGTTCTCCC
TATTGGGATTATAGCGAGAATACGCTGGATCTTCTGGAGGAAGCCGGTTTTCTCTATGACAGTAGCCTGATGG
CGAGAGATCTTGTGCCTTACCATCCTCAACGTTGGCAGGTGAATTGGGAGACGGGAAATATCGCAGGCCCGGC
CAGCGCGATTCTGGAAATTCCTGTTAGCTGGTATCTGGATGATTTTCCCGCACTGGCGTATACCGGCAACCAG
GAGGGGATGAGCGATACTGACGGTGTTCTGCGACGCTGGAAGGATATTTTTACCTATGCTTATCACCATCAGG
AAAACGGTTTATATGCGATGGCCCTTCATCCGCAGATAATCGGTCATGGGCATCATATGATGATGTTGTCACG
TTTGATTGAACATATTAAAGGGCATGATGGCGTATGGTTTGCAACCTGTGAAGAGATAGCCAGTGTCTGGGTG
GATGACGAAGAGGATCGACAAAAAATGCTCCGTCCTGATGTTCGTGGCGTAGCGCCGGTTCCGGATGATTATG
GCTGGCCGGGCAAATGATATCACCTCATGACAATGTTAATATCAGTCGGGTAACGGATTACCCGGGCAGAAGT
TACCTGTTTGCCCGCTCGTTAATGCATATTGTCCATCAGAAAGGGAGACTATCCCGGGACGTATAAAATCTTT
TTTATTTCGGTCAAAACGTTAATCAACGAGAATAGTTTGTTTTTTGATTTAAATCATTTTTTAGTTTTCACAG
GATAATAAGCAAATAACATTTATAATGTGAGTATTAAGAAAAATAAAATTTCCATAGTGATTAATTTTCATTT
AACCCTTTGATTTTTAATGTTTTTGTGTGTGTTAATTGATGTAAATCAATTTGGATTTTTTTATTAAATAAGT
AAAAATTAAGTTGATATTTGCTTTTTGATGTGTTTTTTATATATAGCTGATTATTATTCTGAAATGTCAATTT
TTGAATAACGACGCTGTGATACCGGCACCAGGGATACTTTGCGATAAAAGGGATTGAATATGGCAGAGAGCGT
TCAGTATAACCTCAATTGTATTTGGCCAAAGGGGCCCCAACCCCCTCTGTTGCGTGGAAAAAGGGGAAAAAAC
CCCCCGAAGAGGGGGGAAAACGCCGGCAAACCCCCCGCACCCCGGGCCCTTTCCCCGTTGGGTTTTTGGGGGG
GCGGCTTTTAATAAGAGTCACAAGCGCTGGCCGCCGGTATTCGTCTGGCTGCTGAACTGCCACCTGATCAACA
AGGAGATAAATCATGGATGCGATTTTTTTAAAGCTCGACAATATCAAAGGCGAAAGCCAGGTTGAAGGCTTTG
AAGATCAGATAGAGATCATGTCTTATAGCCACAATGTGGCAATGCAGGTAAATAACGATGTTAGCCTCACTGA
GCGGACTTCCGGGCGCGCGCATGTTGGTGAAATGTCGCTCACCAAATTTGTCGACCTCGCCACGCCTGTGCTC
AATGAGTACTGCTGTAGCGGGAGGCTAATTAGAACCGCGGTGCTGACGTTGTGCCGCAATGACGACGGCAAAA
TGCGGTCGCTTATCGTTTATACCCTGACAAACGTGCTGATATCACAACTCAGTGTTAGCGGCGGCGCCGGCGG
TAAGCCGGTGGAAACGATGTCGCTGAACTTCACGAAAATCGAATGGCAAATCACTGCCGAGAAATCAGACGGC
GCTCAGCAGGAGTCCAGAACGTCAGTATGGGATCTGGCGATGGACCAGATAGGGAAATGATGATGACGCCAAC
CATTCCGGTCGCGCTATTCGACAGGCTCTTGGTTGAGGGGATAAGCCCACATGAATTGGTACGACGCAAGTTA
ATGTGTTTATTCAACTCTTGCGCCGTGCCAGGTGGCGAGACATTACCGCCGCTGCTCACTCGGGGTATGCCGG
AATGGCACGAGGTGAATGTGGGTGACAAACGGGTTCTCAACTGGTTTTGCCGTGAGCTTCGCGCTGCTATTTT
ACGTTATGAACCGAGCATCAACATGCTGAAAGTCAGTGTAAAAGATGCCCATCATCAGACGCTGGCGTTGTCT
TTGGAAGCGATGTTACAGGATGAGTCTGAACCGTTAAGGCTGGAGATAGCCTACTCTAACGGTAGATGGCGCT
AGTAATGCAGGAAGGAACGCCGGGCCGGGCGTTTAACATTATCCCAAAAGCAAAAAAACGCAGCGAGCGAACC
TGATAATCAGGCAGACAGTCCACCTTTCGGTATGGTTGATCTGCCTGCATCATTTTCAGGATGGTTAATAACG
GACCATGACAGATTTAAGTTCGGTATAGTCATCAATAAACGCGCTGCCAAATTCACGACCACTACCCGATCCT
TTCATCCCTCCAAAGGGTACCGCGGGATCGAGGAAGGTATGCATATTGACCCACACCATGCCAGCATCAATAT
CTTCAGCGTAGCGTAATGCCTTAGACATATTTTCCGACCACACACTTGCAGCAAGGCCGAATGGCGAAGCGTT
GATATGCGAAAGCGCCTCTTTTTCATTCTGATAACCGATAAAGCTACAGACAGGGCCAAAGGTCTCCTCGTGC
ATCAGCGTACTGTTTTTACTACGCACTTTTACCGCTGTCGGCTGTAAAAGAACCCTCCGCCGGGTAAAGTTTC
ACCGCCGTAAACAATAGTATCCCCTTCTTCCCGTGCGCGTTGAATCAGATGCAGCACTTTTTCAAGCTGAACC
TGATTCGCCAGCGGGCCCATCACCGTTTTTTCATCTAAAGGCGAGCCGGGCACGATCTCCGATAACCGTTGCC
TGAGGAGCGTCATGACCGTATCCAGTTTTTCCTGGGGCAGATAAAAACGCTCTGCGGCAGCACAAATCTGTCC
TTGATTCAGATAACCGGCTTCAAGAATACCGTTGACCATGGCTTGTGCCGAGAGATCATTGAGAAACAGCGCC
GCATTTTTCCCTCCCAGTTCGAGGGTAACGCGTTTTCCTGACGATGTTGCCGAACGCCGGACTTTCTCACCTG
TCTCAACTGACCCGGTGAAACTCACTTTGGCGCAATCTGGATGCGCGATCAGCTGTTGCGCTATCTCACCGCC

AGCACCGTTTACCACGTTAATTACGCCATCAGGGAAACCTGCCTCTTTAGCCAGTTCAGCGACTCGCAGCAGT
GTCAGCGGGGTATATTCACTTGGTTTAATGACGATGGTGCAGCCACATACCAGCGCTGCGGCCAGCTTCCAGA
TAGCAATCATAATTGAGAAATTCCACGGCACAATACCGACGACCACGCCAATGGGTTGGCGTTGGGTAAACGC
TGTGTATCTCTCTTCTCCCATTGATGGCAGGGAGACATTCAGCGTTTCACCGGTTATTTTTCCTGCCCAACCG
GCAAAGTAACGCAGGAAGGCCACTGACTGATCGAGTTCAAGACCGCGCGACAGCGTAATCGTTTTACCTGAAC
AGACGCTTTCCAGTTGAGCTAACTCTTCACGATGAGCGGCAAGAGTATCAGCCAGTTTCAGCAGTAATGCGCC
GCGTTGTAACGTTGGCATTTTACGCCAGACATCAAACGCCCGTCTGGCGCTCTGCATGGCCTCTTCTGCCTGC
GCAGGGGTTGCCGACACAACGTCGGCGATGATTTTGCCGGTGGCTGGGTTAGTGACCGAGAAGTTCGTGCCGT
TTCCGGCCTGACGTTCACCTGCAATAAATTGTCCGTGTCGTTGGCGTAAAAAAGTCGTTACTTCGGCAAGTAA
TGTCACGTCGCTCATCAGAAGTGCCTGTAGGTCAGGAATGTACGTTTTTCCATCTCAACATACTTGCCTGGAA
CTGAATATCGTTCCAAAGAACGTCATTCTTCCGGACGATATTTTGTAAAACGTTACCGTGCTGTGATAGCGGT
CTTAGTCAAGCGTGATGACAAGGTAACGCGATGAATACAAAAATCGATGCGTTGAATTACTACGGCGCGACGA
AAAAATACCCGTTGCATTTTCCTGCTCTGCAAGACGATATTGATGCCGATGTGGTGATCATTGGCGGTGGCTT
TTCAGGCATCAATACCGCGCTGGAACTGGCGGAGCAGGGGATCACTAATGTTGTCGTTCTGGAAGCCCGCCAT
CTTGGGTATGGCGGCACCGGGCGAAACGGCGGTCAGGTGATGGCCGGTATAGGTCACGACATCGAAGCCGTTA
AAAAATATGTAGGCGCAGCGGGAATGGAGACGCTGTTTAAAATCTCGAACCTCGGCGCAGGTATTATTCGGGA
GCGAATTCGTAAATACCATATTGAGGCTGATTTTGTCCCCGGATATGGTTATCTGGCCTACAACCAGCGGCAG
TTAAAAACGCTGCAACAATGGGAAAAAGAATTCAAAGCTGCTACGCCGGAAGATGATATTGAACTCTATACCG
GTAAAGACGTGCAGCAGGTTATTGGTTCCGACGTCTACTGCGGTGTTCTCAAGCATATGGGGGGAGGGCATGT
CCACTCGCTGAATTTATTGCTGGGCTCCGCTCAGGCTGCGAATTCGCTGGGGGTGAAGATATTCGAATATTCA
CCTGTGGTTGAGGTCAGTTACGGTAAAACTGTGCGGGTACGTACTGCGATGGGCAGCGTAAAAGCGGCCAAAT
TGCTGTGGGCCTGTGATAGTTTTCTCAATAATCTGGAACCAGAAATTTATAAGAAAACGCTGGTTACTTATTC
TTATCAGGTCTCTACAGAACCTCTCTCCAGCGAGCTTGTTGAGCGGATCAGTCCCTTGCGCGGGGCTTTTAGT
GATATCCGCCCGGTGATAAATTACTACCGTCTCACAAAAGAAAACCGTCTGCTGTTTGGCAGCGCCACAAGGT
TTCTTGAATATACCCCGCACGATTTTGCTGCCTGGAACCGCACTCTGTTGACGGAGGTCTTTCCTTATTTGAA
AGACGTTAAAATCGATTTTGCCTGGGGCGGACCGATGGCCTGTAGCGCGAATCTGTTTCCGCAGATTGGCACG
CTTCGTGAGCACAATAATGTTTTTTATGTTCAGGGTTATTCCGGATTTGGCGTCACGCCTTCCCATATTGTAT
GCAAAATCCTGGCCGAAGGAATAAATGGCGGTTCCGAGCGGTATCGTCTGATGAGTAGCATTCCCCACGCGAC
AATTTATGGTCGCGACAGTCTACGTCTATTGCTGGTCAGTGCGGGAAAACTGATACATCAAACAGCCGGTTTC
TGGAAAGGCCGGAATTAACCAGGAGAACATTAATGCACGCTTTTAAACTCAATCAACCTGTTCCTGAATTACA
GCCAATCGGGAGTGTCAGCCTGTTGGGGGCGTTGCCAACAGAAGGCGATCCCCAGGTCGCCGTTGCGATGATT
TACGGTAAACCTGAGGACGTATTTACCTGTGGACTGTTTTCATCGACCCGCGGCGGTTTCACTATGATTTATC
CCTTTACCGAGCACGCAACCGTGCTGGAAGGGGAAGTGGAACTCACTGTTGCGGGTCAGGAACCGATACGTTT
TTCACCAGGCGATAGTTGGTTTGTTGAACAGGGAACCGAAGTGGCATGGAAAGTTTTAACGCCCCGTTTTGTA
AAACATTATCTGGCAAAAGTTGAAAGCCATAAGCAGGGTTGAATCCTTTAATTGTTCGTCAGTAGCGTTGGCA
TCACTGGGGAGAATAACGCGCTCCCCACTTGCTATTAGCGCATGCGGGGACGGCGTCACAACGTTATTTACCG
TTATCCCTCTTCAGGACGATACCTGTTAGATACGGTGCCTGCCATAAATAAGCGTTATTCCGGAGCGGTAATT
TATTGTTGTAATAATAGTGAAGAGGTTGACCATGTTTAAAAACAGACTTGCCAGCCATCTTGAACGTGGCGTT
ATTGGTTTCCCCACGACGCTTGCCAGCTCCGTCGGGCTGATTATGGCAAGCCCGGTGATCCTGACCGTGACCA
GTGGTTTTGGCTCAGGGGGAGATACCTTTGCTCTTGCGGTATTACTGGCATTTATTATGATGCAAGCGCAGAT
TACCACCTTTGCCGAAGCGGCAACGCTTATTCCCACGACTGGCTCCGTCTACGACTATATTTCCTGCGGGATG
GGGCGTTTTTTCGCTATCACCGGTGCGCTTTGCGCTTACCTGATCGTACACATTTTTGCCGGCACGGCAGAAA
CGATTCTCGCTGGCGTCATGGCGCTGGTGAACTTTGAGTCTGTTAGCGAACACGTGGCCATGCAGCACAATAC
CTGGATGGTTGGCGTCGGCATGGTAGTGGTTTTCGGGTTACTGAATGCCATTGGTGTTGAAGTATTTGGCAAG
GTGGAAGTGTTTCTTACATTCGGTATGTGGACTACCCTGACCATCTTCGGTTTATGCGGGATCCTGTTATCCC
CGGTCACTCATCATTCCGGGTGGTTTGGCGCACCGCTTGATGTCAGTAATCTTTCTGGTCTGTTTGGTTATAT
TGGCATGGCGATGTTTATGTTCGTCGGTTGTGAACTGGTCACGCCAATGGCGCCGGAAATTAAAAAAGCGCAT
CGTACGATTCCCAGAGCGATGGCGCTTGGCCTTTTAGCGGTGGCAGGTTGTATGTTCCTCTATGGCGCTGCGA
TTAACCGGCAGGTAAATAATGTGGTGGTCGATGCGGCAAACCATATCTTTTTACTGGATACGCCGATGGCTAT
TCCCGCCTTTGCCAATCGTGTCATGGGACATGTCGGGCAATACTGGCTGGGCGTGGGGTTGTTGTTAGCGGGA
TGTGCGACGATTAATACGCTAATGGCGGCAGTACCGCGTATTATATACGGCATGGCGCTGGACGGCGCTTTGC
CGCGGTTTCTGACCTGGTTGCACCCCCGCTTTAAAACACCGGTCATCGCCATCGCATTCGGTGTTGCGATTCC
CTGTTTACATGCCTGGTATCTGAACGGTGATATCGACCACATTGTTCCGCTGATCCTCGCTGCGGTCTGCGCC
TGGGGAGTAGCTTATCTGCTGGTAACGTTATCGGTCGTTATCTTACGTATTCGGCGCCCCGATTTACCACGCG

CTTACCGGGCCCCCTGGTTTCCCTTACCTCAGATTATTTCGAGCGTGGGTATTATTATCGCCATTATTAATAT
CGCGCCTCCAGGAATGAACAGTCGGGATGTACTTATACCATTCGGTATTATGCTTGGATTGACTGCCGCCTAT
GCCTTATTCTGGACATTATGTGTGCAACGGGTTAATCCTTTCCTCCCGCTTCCGGTAGAAGAGGTCGTGGAAC
GGGCTATGGGCAATTATGAAAAGCGAAACAATGAAGAGATGACGCCAGATGTCCTCCGTCCCCTGGTTTAAGA
ACGCATTAATGAATATGGTGTTACGTGATTTGAGCGGCTGGCGTTGTGAAAAATTGACCGAACATAGCGCCGT
GCTGCATCTGAATGCATTTACCCAGGTTATCTGCCATGTTCAGCAAAAACGGTTATTCATGGCCAGTATTCAC
AGTTGTGAATTCAGGGTGAAAGGCACAATCAATTATCCTTTGCAGGGCAAAATTCGCGTTCATCAGCCCGGAT
GGCTGAAACGTTATCCCGTCATTTTTACTGGCAGCAAAAGTACCGCCGGGCTGATTAACTATCTTAATTGTTT
CCCCAATTTACAACAGGCACTGAGTGAACTGGATTATCGTCGGTTTACGCTGGTTTTACATCATAAGGAATGG
TATTGCGGCATTGAGCTATGGGCTGCTAGCGAAGTGGTTTGTAAAATGCCGCCGCTGCGCCGTTATCTGCGGC
TTGAGCGGCACCAGCGTGTTCTTCTGCTTAGTGTGATAAATATGATCAATCAGGCCATGAATCAATGGCTGCA
ACAGGATACCGACGCCAGATAATATGACATTTTATTGAGGCATCAGCTCAGAGAGTGGTATTAATTCAGGCAT
ATATGACAATGGGGAAAAGACCCGCAATTGACGCGGGTTAAAAAGCACGCTTTCACATATGCTGTGAATGTTG
TACCGAAGACATTCTGGTTCGGGAAACCAGTTCCGTGCGATTTATCACTTCTGTTTTAGCAAAAATATTACGT
AAATGCGTTTTCACTGTTGAGAGAGAAATATTCAACTGACGGGCAATCAATTTGTTGCTGGCCCCTTCGCAGA
CCATTCCCACAATTTCACGCTCTTTGGCGGTCAATATTGACGCCAGATCGTTGTCTTCCTGAAGAGAATCATG
AATTGCCAGTCCCAGTAATGGTTGTACTGCCTGGGCTCGCAAACGCTCTTCGCTGGAAAAAGGTATATCGCGC
ATTAATGAAATGCCCGCGATAATACGATGGCCCCGACGAATAAAGATCTCAATCATGTCGCACACATGATTAG
GTAGCATAAATTCATGATAATAATGGCGATGACGAGCACAGATGCCGGGCGTCATCGTCGCTACTGTAATAAC
CTGTTGGCCGAATCGGGAAGGCGATAGCGGGTCTAATGGTTGAAAATGCTCCAGATATTGTTGGTGTGTGCTA
TTAGAAATATTATGTAAAACATAGGCTTCCGGCTTTAAATAAGCATTAACACGATAAAAAACCGCCGACGACA
GAGGAATGAGTTGCGCAATGCTCTGTAAGCAATGGAGAGTGATCCCGTCATTATGGCCAGTAGTTGATAAGGC
TGACATGTTATCTCCTTCCATCCGGATGAGCGCGTCGGGGCGCAGTAAAATAGTGGTATGTTGAAACATACAA
TTTACATTCGGTTAACACAATCATTCACATGTTAATAATGTGACGTATGTTGCGGTATTTTTTATAACATATT
GAAAATAAATGATTTTAATTTAAATCCTGTCTGGTTATTCTAAAAAATAATTTAAAATTATTAATATAATTTT
TTCTGTCGCGGTTGACTCATCTCCTGAAAATGCCGTGAGTTCTCGTCCTTCAGGACGATATTCTTTTGAGACG
CCTGTTCTATGATTTTCTGTAATAGCGCAGGTTAACAGAGACTTAATTTCTGGCTCAGACAGGATTTGCTAAA
TAACATTTCAATAGCGTCATGCGCTATGCAGAAAACCGGCGTATCAATAGATACAGCCTGCTCTCGGCATTCT
GCCCGCAGCGCCAGACGAACGCTATTGCGAAATGTGAGTCATGTCTCCGCATCAGAAATGATAACTACAGGAC
AGAATAATGTTGAATCTCAACACATTACGGCAACAACAAATCCCCGTAATGACGGAATATCGCGCGCAGATAC
CCTTTCATATTCTGGCAAAACCCATAGGCCCTGCATGTAATCTGGCCTGCCGCTATTGTTATTACCCACAGGG
CGAAACGCCCGTAGAAAAAATGAATGAATCAACGCTGGAGGTTTTTATTTGTCGCTATATTGCGGCGCAACCT
GCCAGTGCGCGTGAAATTAATTTTGTCTGGCAAGGCGGTGAACCGCTTTTAGCCGGAATCGGTTTTTATAAAA
AGGTAATAGCGCTTCAACAACGATATGCGCCTGACGGCGTGACGATCAGTAATAGTCTGCAAACGAATGCGAC
GTTGTTAAACGATGCCTGGTGCCGTCTGTTTCGCGACAATAATTTTACTATTGGCATCAGTCTTGAGGGCAGT
GAAGACTTGCAAAATCATCATCGTCCGGGCAAACGCGGCGAGGCCAGCTATCCGGCGGTGTTGCGGGGAATCA
CATTGTTACAACACTATCGAGTCGATTTTAATGTACTGATTGTCGTGCATGATGACATGGCTCGCCATGCGGC
AGCCATCTACGATCATGTTGTTAGCCTTGGCGCTCGTTATCTGCAATTTCAGCCACTGATGAACGAAGGCAAC
GCCCTACAGCAACGTTACCAATTGAGTGCGGATAACTGGGGACGTTTCATGATTGATATCTGGCGTCAATGGC
GCAAACGCGGCGATATGGGACGGGTTTTTGTGATCAACATTGAACAGGCATGGGCACAATATTTTACGCATAT
CAGCGCCACCTGTGTCCATTCCGCCCGCTGCGGAACGAATCTGGTCATGGAGCCGGACGGCAAACTCTATGCC
TGCGATCATCTGATTAATAGCCAGCATTACCTGGGACAGCTTGCTAATAATACGTTAGCGCCAGCCGTAGATT
CCGCAACCCGGCTTCCCTTTGGTATTAAGAAAAGCCAGCGCCGGGAGTGTCAACGGTGTTCTGTGAAAATAGT
CTGCCAGGGAGGCTGCCCCGCACATATCAACAGTGCCGGCTACAACCGACTTTGTAGCGGCTATTACTCTTTT
TTCACGGAGATTCTGGCTCCGCTACGCGCCTGGCCCCGGGATCTGAATGGACTGAAAGCCTGGCGTGCTGACG
TTATGGGTAGATTTTCGGGCTGATGGTTCCTCTTGCCGCATTTGCCCGCCGCTCTTGTACAACATCCGTAGTC
TGAAATGGAGAATAAGCCGTGAAAAAAGAAGTAACACTTGCCACACTCAGCATTATCTTCTCCGGTACGGCGC
ACAGTACGCAAAACGAACGTCCTGATATTATCGTGATTATCGCTGATGATATGGGATATTCTGATATCACTCC
CTTCGGTGGGGAAATCCCAACGCCTAATTTGCAGGCGATGGCTGAGAACGGCGTGCGGATGAGTCAATATTAC
ACGTCTCCCATGTCTGCTCCCGCCCGTGCGATGCTATTAACCGGGAACACCAGTCAGCAAGCGGGTATAGGCG
GTATGTGGTGGTATGAAAATACCATAGGTAAGGAAGGCTATGAATTGCGCCTGACTGATCGGGTGACGACCAT
GGCTGAACGCTTTAAAGATGCTGGTTACAATACGCTGATGGCGGGTAAATGGCATCTTGGTTTTACGCCAGGC
TCGACGCCAAAAGATCGGGGCTTTCGTCATTCTTTCGCCTTGATGGGGGGAGGCGCCAGTCACTTTGATGATG
CCGTGCCGCTGGGAACCGTGGAGATATTTCATACCTATTATACCCGTGACAATCAGCGCATTTCACTGCCCTC

TAGTTTTTACTCCAGCGAAGCCTATGCCAGCCAGATTAATCGCTGGATCAGCGAGACGCCACGGGAACAACCT
ATCTTCGCGTGGTTGGCCTTTACTGCGCCACATGATCCTCTGCAGGCGCCGGATGAATGGATTAGTCGTTTA
AACGTCAGTATGAACAGGGCTATGCAAACGTCTATCGTCAGCGTATTGCTCGTTTGAAGAAACTGGGTTTCCT
GCGTGATGACATACCTCTGCCAGGACTGGAACTTGATAAAGAATGGCAGGCAATGACCCCGGAACAGCAGAAA
TATACGGCGAAGGTGATGCAGGTTTACGCTGCTATGATCGCCAATATGGATGCACAGATTGGCACCGTTATTG
AGACATTAAAAAAGACCGGGCGCGATAAAAACACGATTCTGGTCTTCTTAAGTGATAATGGGGTGAATCCGGC
AGAGGGCTTTCACTATGAATCTGAACCAGATTTTTGGAAGCAATTCGATAATCGTTACGAAAATATTGGTCGT
AAAAATTCATTTATCTCTTATGGTCCCCACTGGGCTGATGTCAGCAATGCGCCTTATGGTCGCTATCACAAAA
CGACCAGCGGTCAGGGGGGAATTAATACCAGTTTTATGATTTCCGGTCCTGGTATCATCCATCATGGCGCCAT
AGATAACGCCACGATGGCGGCGTATGATGTTGCGCCCACGCTCTATGAATTTGCAGGTATTGATGCCAGTAAA
TCATTATCTGAAAGACCGACACTGCCAATGATCGGCGTGAGTTTTAAACGCTATCTGACCGGTGAAAGTCTGC
ACGCGCCTCGCACACAATATGGTGTTGAACTCCATAATCAGGCGGCCTGGATAGATGGGGAATGGAAATTGCG
TCGTCTTGTCACAGTATTCCCACAGGCGGGTAATGCTCCATGGGAATTATTCAACCTGCAACGTGACCCCCTG
GAAACGCATAATCTCGCAGCAGATTATGTGGATAAAGTGAAAATACTGAGCAGTGCATATGAGGCATTTGCAA
AACAGACAATGGTGCTTTATGCCAAAGGCAAGCTTATCGATTATGTGGGTATCGACAGTAAAACCGGGCGTTA
TCTGGCTGTCGATCCACAGACATTGCAGCCAGTTCCTGCTCCGTTAGCGATTCCTTTAGACACAAAATCGGAC
CAATAATTATTGGTTACGTTTCAAATGATTATTCAACGCTCACATAACCGCATTCAGCCAGTGACTTTCAGGA
TGAAGTTTCTCAGCGTTGGCGATGTATCGCCGCGCCGGTATGCCAGAGCCAGCTTTGCGACGGGCATCTGGTC
TGCAACGGGGCGGTATACTACTCCCTTGACATTAACCTGTCTCATTGAATCTGGAACCATCGACACGCCCATC
TCTGCCGACACAAGGTTAATGACTGACGAAATTTGCGGCACGAGTTGACCAATTTTCGGTTCAAAGCCCGCTT
TACCGCAGGCCTTGATCACTGCGTCATAAAGCGACAGGCCGATTTCACGTGGGAAGAGTAAAAAAACTCATCT
TTGAGAATCGAGAGTGCAATCTGTTTACACGCCGCAGCAGGATGCGTTTCTGCCAGCACAATAACCATTGGCT
CTTCAGACAGTAACCTGAGCTGGAACCGTTCATTGCCTGTAAAACCGGGTCTCAGAAAGGCAACATCCAGCGA
TCCTTCATTCAATTCATCAGCCAGTTGAGTTGTATTTCCTTCCTCAAGCTGCAAACGAACGTCAGGATAAGCC
CGCTTGAAAGTACGGATTGCGCCGGGCACCACACTGTTAAATGCCGCAGAGGCAGTAAAACCCACCCTTAACA
CGCCCGATTCCCCCCTGGCAACACGCTGCGCCGCCAGCACGGCTCTTGTCGCGGTAGCAGGCATCCCTTTAAC
AACATCATAAAATGCTTTTCCAGCCTCGGTTAATTCCGCGCCATGAGGCACTCTCCGGAATAGCTGCGCTCCC
AGCTCTCTTTCCAGATCCTTAATCTGTTGGCTTAGCGGCGGCTGACCTATGCCTAATTTTGTCGCAGCCCGCG
TGAAATGCCGCTCTTCAGCGACGGCGAGAAAATAGCGAATATGGCGCAGTTCCATCCTCATCTCCAAAAGCAA
TTAAGAATGGCACCATCATATATTGGAAGAATGAAACCAGCGAGCGCATAGTGAGGTTAACAGGAGGTGCAAT
ATGCCCGATATTTCTCATACACCGACACGTAGCTGGCTTTTTACCCCAGCAATTCGACCAGAACGTTTCATAA
AAGCAGTTGAAAGTGCGGCGGATATTTCTATTATCGATCTTGAAGATTCCGTCACCCCGAATGACAAGGCGCA
GGCCCGGAAAATTGCGATGCAGTTCCTTTCATCCCGACCAAACTCATCGCTGAAAATAGCATTAAGAATTAAT
GGAATGAATACTCATGCTGGCATTGAAGACCTGCATATGTTACTGGAATGCCGTTTTTTTCCTGATTACATTA
TTCTTCCGAAAACAGAGTCAGCCGCGCACCTGCAAATCGTGGATAGTCTTATCATGATGGCTGGTTCTGATAC
TCGCCTTATTGGCATTATTGAGTCAGTTGTTGGGCTTAATGCCGTAGAGTCTATTGCGGACGCCACCCCCAGA
CTCTGCGGGTTGATGTTTGGCGCCGCAGATATGGCGGCTGACATTGGCGCGACACCCGCCTGGGAGCCTCTTG
CGCTGGCGCGGGCCAGAATAGTGGCTGCCTGCGCTATGAAAGGGCTTCTTGCAATAGATGCTCCCTTCTTTGA
TATAGGCGATTTTTCAGGTCTGAAAGAGGAAACGCTGCAGGCGCTCAGTTTTGGTTTTTCTGCAAAATCGGCT
ATTCACCCCGCTCAGATTAGTGTAATCAACGCCGCATTTACTCCTACAACGGCAGAAATTAATCATGCTCGTG
CAGTACTGACAGAGAATGCCAAAGGCGTCGGGATCGTTAGCGGCATGATGATAGATGCAGCCGTTGCCCGTCA
GGCCCGGAGATTACTCGCCCGGGCAGGGATCTTTTCCTGATTTTATTAACAGAGGAATTGATTATGAACACGT
TATTACCCGCTTATAAAACCATTAGCGAGGGGCGCTACCGAGAAATTTCGGGTCTTTACTGGGAGGATTTTCA
TCCCGGTGATGTTTTTGAACATCGTCCCGGCCGGACGGTTCTGGACGCCGATAATGTGTGGTTTACCCTGCTC
ACTCTCAATGTGCAGCCGGTCCATTTCGATGCGGCTTACGCCAGCAAAACGGAATGGAAAAAGTTACTGGTTG
ATTCAACGTTCACTCTGGCGCTTTTAACCGGTATGAGCGTGCGTACTGTCAGCGCCAAGGTCGTGGCCAATCT
TGGCTGGGATAAGGTACAAGCCGTCCATCCGGTTTTCGCAGGGGATACACTTTATGCTGAAAGCACTGTACTC
AGCAAACGCCTGTCAAATAGTCGCCCGGGACAAGGCATCGTTACCGTTCGGACATGTGGTATTAACCAGAATG
GTGTAGAGGTCATGCGCTTCGAACGAACAATGTTAGTTTATTGCTGTGGATGTTCGCCTGAAGAAGACGCCGC
TTATTGAGAAATCGCCCTGAGGTTTAGAGTCATGATGAATGTTAATGCAGTGTATGCCGAAAAATGCGTGACG
CCTGACGAGGCGGTTACTTTAATTACCTCCGGCAGTCACCTCTCTATGGGAATGTTTGCAGCAGAGCCTCCAG
CTTTGCTGAATGCGCTGGCAAAGCGAGCGAAAAGGGGAGAAATCAACGATCTGCGGGTTTACTGTTATGAAAC
AGCCAGCATTGCCGGGAACACTATTTTCCGTTATGAGCTAAGTGACTATATTCATCTCTACAGTATGTTTATT
ACCGGTATTGAACGGGCTTTAATACGTCAGGGGATTGAGAGTTCCGGGCGAAAAATAGTTAACTATGTACCTT

CAAATTTTCATCAGGCGACGCGATTACTAGCTGATGATATTGGTATTGATACCTTTATTCACACCGTGTCCCC
TATGGATAAGTATGGCTATTTTAATTTTGGAACTGGAAACGATTATTCCACCCGGATTGCGAGGACAGCTAAA
AAGCTTATTGTCGAAGTTAACAAATATATGCCGCGCGTTCATGGAGAAGGTGCTGCAATCCATATTTCAGAAA
TTGACGCCATTGTTGAAAACCATGTCCCTTTAATTGAACTACCAATACGTACTGCCGTAGCCGAAGATATTGC
TATCAGTCAGATTATCGCTTCTCTTGTGCCAGATGGCGCTTGTCTCCAGATGGGGGTCGGCGCATTACCGGAG
CTCATCTGTAATGCGCTCAAGGAACATAATGATCTGGGGGTCCATACAGAGGCGCTGAATCCGGGGTTGGTAA
GTTTGATTCAACAAGGCGTCGTTACCAATCAGCGTAAAAATATCGACCGGGGAATGTCTGTATTTACTTTTGC
TATGGGACAAAAGGATATGTATGACTATCTCAACGACAACCCCTCTTTTTTTAGTCGGCCTGTCGATTATGTT
AACGATCCGGGGATCATTGCGCAAAATGAGAATGTTGTCTCCATTAACGCTACCTTACAAATCGATCTCACTG
GTGCCTGTAACTCTGAATATTTGCTTGGACACCAGTACAGCGCTTCAGGAGGGCAATTGGATTTTGTCAGAGG
CGCATATGCATCTAAAGGAGGTCGTTCCATTATTACGACCCGCTCAACGGCCGCGAATGATACGATTTCTCGT
ATTGTTCCCCGCCTTGAGGGGCCTGTAACAACCCCACGAACGGATACGCACTGGATAGTCACTGAATTTGGCT
CCGTAAACCTGAAGGGGCTCTCTTCAACCGAGCGTGCGCTCAGCATCATTGAATTGGCACATCCCAATTTTCG
GCAGCAACTTAGAGTAGATGCAAAAAAAATGCATCTGATTTAAAAGTAGGTTAATGCTATTTTTTAATGTAGC
ATCCCTAAAATATAAACATCATGAGTCTATTCAAATGATAATTGACAGAATTGATCACCTTGTATTAACGGTA
TCTGATATATCAACCACAATAAGATTCTACGAAGAGGTACTTGGTTTTTCTGCGGTAACATTTAAACAAAACA
GAAAAGCGTTAATATTTGGAGCGCAAAAAATTAACCTCCATCAGCAAGAAATGGAGTTCGAACCAAAAGCGAG
TAGACCAACGCCGGGATCTGCCGATTTATGTTTTATTACATCGACACCGATAAATGATGTTGTGTCTGAGATC
TTACAGGCAGGCATTCCCATTGTTGAAGGCCCGGTTGAGAGAACTGGAGCCACAGGCGAGATAATGTCTATCT
ACATCAGGGACCCTGATGGAAACTTAATTGAAATTTCGCAGTATGTTTAATGTTAAAACTTACTATTACTGCC
TTTATTATGATCACGCATCCGTTTATATTGGTCTAATGGAGATCATATGGCAATAATTTCTGGAAAGAGTCTA
TGTTCAAACTTATTGACTTGAAGACATCGAGGTATTCATAAGAAAGCATAAAAAAGGAAAGGTGGTGCCCGGA
CTCGGAATCGAACCAAGGACACGGGGATTTTCAATCCCCTGCTCTACCGACTGAGCTATCCGGGCAACGAGGC
GCATTAAACCGTAATCGCCTCGCTTCGTCAATGAAATTTCTTCAAATCAACGTAGACTGCACAATCTATCACC
ACTTTGTTGTTATTGCACGCAGAATCAGGCAAAAAACATCGCCCATGCGGCGGAGATCGGCATCGCCAGCAAC
AGCGCCGGGAGCATATTCACTACCGGAAACATTTTGATTCCACATACACGCAAGCCGGTAGCCACCAGCAGCA
GTCCACCCACGGCGCTAAAGTCGCCCATCATTGCCGGCGTGGTCAGTGGAAGAATGAGGGTGGCGCAGGCGGC
AAGCGTTAACTGGATAATCAACATCGGCGCGCAAATCGCCGAAACGGCGATGCCAAGCGAACAGGCGAAAATA
ATGGCAGTGAAAAAATCCAGAAAAGATTTAGCGATCAAAATGTTTGGATCGCCGGTCATCCCTTCATGCATCG
CGCCGAAAATACCGGTGCCGCTGGCGCAAAAAGTACAATGATAGCGACGTAACTTTGAATAAATGAGTCGTGT
GTCGGCTTCTTACCCGATGCCATAAATAATTGTTGGATTTTCGCGACTGCGCCATTAATGCCTTTTTCCAGCA
AACAAAACTCGCCGATAAGCGCGCCGACCAGCGTCGCCAGCACCATCACCGGAAGATTAGCGCACTTCATCAC
CAGCAGAATGCCAATTCCCAGAGAGCACAGACCAAAAATAGACGTCATTGAGACGCGAATCCGTTCTGGCAGA
CGCTGACTTAACAGCGCGCCAATAACGCCGCCGAATAAAATTGCGCCAGCGTTAATAAAAGGTCCAATTACCA
CAATGACTCCTGTTATTTGTCTTTCAAATTGCCTTATTATCACGCGATTAAGCTGTTAATAGCTTGATTATCT
GTGACGTATAGAGCGCTGATTTTACCTTGCGCCGCCTGGTGAAAGGTGCCATGATTGCGCGAAATTTCTCCTG
CCAGCATGGGGCTCGCCGGGATGAACATAGCGCCTTTATATTGTCTTCACTGTCGCCTGACTGCGCGAGGTTT
GCCCCTTTCTTTCCTTTTTTTGTCATCCCCGCTATGCGGTGAGGGCAGCGCACGCTCACTGCAGGACAACAGT
AAAATCAGAAACAGTCTGTTTTTACTGATGTCTGGCGGTCGGAGCTGGTATCCAGTTTGACCCGGCATCTCGT
TGGGCAAGGCATCGGGCCTTGTCTGGGGTTACTCTACTCCCGAAACGGGTATTTGCGCTTATGAAATCAATGA
ATATTGCCGCCAGCGGTGAACTCATCCCCCGTCTATCCACCCACCGAAACGTAGTGGCGCTGGACAGTACCGA
CTTTACCGATGTGGCCGCAGTCGTCATTACCACTGCCGACAGCCGTAGCGGTATTCTGGCGTTGCTTAAACGC
ACCGGTTTTCACCTGCCGGTGTTTATGCTTGCGGATGAACCCGTCAGCGCGCCTGTCGGCGTTACGGCGGTAA
TCGGCGGGAACGCGCAGGAATGGCTGGAGTTGGAAAACGCCGCCAGTCGCTACGAAGCGGAACTTCTGCCGCC
CTTTTATGACACCCTGACCCAATATGTCGACATGGGGAATAGTACCTTTGCTTGTCCGGGGCACCAGCATGGC
GAGTTTTTTAGAAAACATCCCGCAGGCCGCCATTTTTATGACTTTTTTGGTGAAAACCTGTTTCGTGCGGATA
TGTGTAACGCCGATGTGAAACTTGGCGATCTGCTAATACATGAAGGTTCTGCCAAACACGCGCAGAAATTTGC
GGCAAAAGTGTTTAATGCGGATAAAACCTACTTTGTGCTGAATGGGACTTCGGCGGCGAATAAAGTGGTCACC
AACGCCCTGCTGACGCGCGGCGATCTGGTGCTGTTTGATCGTAATAATCACAAATCCAACCACCACGGCGCCC
TGATTCAGGCCGGGGCGACGCCGGTCTATCTTGAAGCGGCGCGTAACCCGTTTGGTTTTATCGGTGGTATTGA
TGCGCACTGTTTTGATGAAACGTACCTGCGCGACCAGATTCGTGACGTGATGCCGGAAAGCGCCGATGCGCTG
CGTCCGTTCCGCCTGGCGATCATTCAGCTGGGTACCTATGACGGCACTATTTACAACGCCCGTCAGGTGGTGG
ATAAAATCGGGCATCTCTGCGATTACATTCTGTTTGACTCCGCGTGGGTAGGCTATGAGCAGTTTATCAATAT
GATGGCGGATACCTCGCCGTTGCGGCTGGAGCTTAATGAAAACGACCCAGGGATCTTTGTGACCCAATCGGTA

CATAAGCAACAGGCGGGGTTCTCACAAACCTCGCAGATCCACAAAAAAGATAACCATATTCGCGGCCAGGCGC
GTTTTTGCCCGCACAAACGCCTGAACAACGCCTTCATGTTGCATGCTTCAACCAGTCCGTTCTACCCGCTGTT
TGCGGCGCTGGATGTCAATGCCAAAATCCACGAAGGGGAGAGCGGTCGCCGCCTGTGGGCGGAGTGCGTTGCG
CTGGGCATTGACGCGCGTAAAGCCATTCTCGCCCGTTGCAAACTGCTTCAGCCCTTTATCCCACTGGTGGTTG
ACGGCAAGCCGTGGCAGGCATATCCGACGGAGACCATTGCCAGCAATCGTCGTTTCTTCAGTTTTGAGCCCGG
GGCGAAATGGCATGGCTTTGAGGGTTATGCGGATGACCAGTATTTTGTCGACCCCTGTAAACTGTTACTGACC
ACGCCAGGTATTGATGCCGATAGCGGGCAGTACACCGAATTCGGCATCCCGGCGACCATTCTCGCCCACTATC
TGCGTGAGAACGGTATTGTGCCTGAGAAGTGCGATCTCAACTCGATTCTGTTCTTGCTGACACCCGCCGAGAG
CGAAGAGAAACTGGCGCGGCTGGTCGCGATGCTGGCGCAATTTGAACGGCACATTGAAGACGATACGCCGCTT
GCGGATGTATTGCCGACGGTTTTTCAGAAATACCCGGTGCGCTACCGCGATTACACCCTGCGTGAGCTATGTC
AGGAGATGCACAATCTGTACGTTAGCTTTGACGTTAAGGATCTGCAAAAAGCGATGTTCCGCAAAGAAAGCCT
GCCGCATGTGGCGATGAACCCACAGGACGCCAACAGCGCCTTTATTCGCGGCGATGTAGAACTGGTGCGTATC
AGCGAGGCGGGCGGGCGTATTGCTGCCGAGGGGGCATTGCCATACCCGCCGGGCGTACTGTGCGTGGTGCCCG
GGGAGATTTGGGGCGGCGCGGCGCAGCGTTATTTTTGGCGCTGGAAGAGGGCATCAATCTGTTGCCCGGTTT
TTCGCCGGAATTGCAGGGGGTATATAGCGAGACCGATGCCGATGGAATTCAACGCTTGTACGGATATGTTTTA
AAGTAACGTTGCCTGCAGGCCGGATAAGGCATTTACACCTTATCCGGCCTGCGAAACAGGGCAAGGATTAATG
TGCAACGGGTTGCGAACTTGCCGGAACGCGAACGTGTTTGTATTTGAACAAGGCTACGAACGCGAAGGCCAGC
ACCAGCGAGTAGCCTGCGAAGATGAGCCACACGGTCTGCCAGTCGGTAATGCCGTTTTGAGTGTAATACTCCA
CCACTTTACCGCTCACAATGCCGCCCAGGATACAGCCGAAGCCATTGGTCATCATCAGGAACATCCCCTGCGC
GCTGGCGCGGATTTCCGGGCGTACTTCTTTTTCGACAAACACCGAGCCAGAAATGTTGAAGAAGTCGAAGGCG
CAGCCGTACACAATCATCGACAGTACCAGCAGAACGGTACCGAACGGCGTCGGGTCGCCATAAGCGAACAGGC
CGAAACGCAGCATCCACGCCACAATACTGATAAGCATAACGTTCTTAATACCATAACGGCTCAGGAAGAACGG
AATGGTCAGGATGAATAACGTCTCAGAAATCTGCGAAATCGACATCATCACCGAGGCGTGCTGCACGATAAAG
CTACTGGCGAATAGCGGATCTTTATCAAAGCTATGCAGGAAGGTGTTGCCAAACATGTTGGTGATCTGCAGTT
CCGCGCCCAGCATCATGGAGAAGATGAAGAAAATCGCCATCCGCTTATTTTTAAACAGCGCAAAAGCGTCAAG
GCCCAGCATTGATGTCCAGCTCTGGTTTTTCTGTTGGTTCGCCACCGGAATGTGCGGCAGGGTAAAGGTAAAC
AGTACCAGCAGAACGGAAAGCGTCGCGCCGATATAAAGCTGCATATGGCTCAGCTCGAAACCGGAGAAACTCA
CGCCCCACATCGCCAGAATAAAGCCAATGGTGCCCCAGATACGGATAGGCGGGAAGTCAGTCACGATATCCAT
GCCGGCAGACTGCAGGCGGTAATACGATATGGTATTGATCAAGCCCAACGTCGGCATATAGGCCAACGAGTTA
AGCAGGATCACAAAGAACATCGCCCCAGGCGTAGTGACTTCCGCGGCCATGAATAGCGTGATGGCGCGACGAC
ATGACACAGGGCATAGACCCATTTCGCGCTCAGCCATTTGTCAGCCACAATGCCTAACAAGGTCGGCATAAAG
ACGGCGGCAATCCCCAGTGAACTATAAACTGCGCCAATAGATGCGCCGTCAAATTTTAAGGTGACGAACATAT
ACGAGCCCAGGGTAGTGAGCCAGCTTCCCCACAGACAGAACTGCAGAAAAGAGAGTATTTTAAGCTGCAGCTT
AAGATTCATGTTAATTTCCTCACACCTGAGAGCGGATGAATGTTTTAAAAGGCACATTTGCCGTTGTGATTTG
GTGTGATTCTATCAATGAGAGGCGGGCTTTTTTTGTTACCTGTGACAAATATTTGCAAACAATTTCTGATTGC
AAAGCAAAAGGGTGACGGAGTTAACACTTTTGCCTTTCCCGCCTCTGTCATTTGCTGGCCAGAGGCGGGATGC
GATGGTTTATCGGCGGCGATACGATCGCTGGGCGGAATTCACCTTATACAGATAGCGGCGCGACTCCGCAGAT
GGATGTCGGGTGGTTAATATCTGGTACACATCCCCCGGCGACATGCGGTTAATCATATTCGCGGCCTGAATCT
TATCGTTCGAGAATACGCGTAATACGCTGCCTGCGCCGCCATTATAGGCAGTAATGACCGCATAGCGCCGCGA
CGTCGGATTCTCGATACCGCTCAGATAGACATTATTCAACATCGCCAGATACGCTGTGCCGGTATCGATATTG
CTGGCGGGGTCGAACAGGAAGTTACGGCTTGGCGTGCCGGACTTACCCTGAGAACGGAAAACATCTTTCCCGG
CGCTGTGTTGCACCACCTGCATCAGGCCCAGCGCATCCGCGTGGCTGACCGCATACGGGTTAAACGAGGACTC
CGTTTGCATAATCGCCAGAATCAGCGACTCGTCAACGCCGTATTTACGGGAGGCCTGGCGTACCATGCCAATG
TATTTATGCGCGCGTTTATCAAGGTGGTTCGGCACCAGATTAATGGTGACGCTGTAAATAATACGCAGGCCGT
TACTGCGGCTTTTGAGCCGCGTTTTCAGCAAGTAATCCGCGAATGTCGTGGCGCGGCCTTCCCAGCGAATAGG
CTGGCCGGTATTGTCGAGGACCTGACCATACAGGAAGGGTTCTTTAGAAATTTTTATGTCATCGACATCGGAG
TAAAGATCGACGGAGGTTGGGTCGTCACCCATGAGCAACGTTTTAATAATAGCCCGGCGCAGATGTGCGGTAG
GTTCCGTCCCGGCGATGGTTTCAACGGTAATGGTCCCATCATCAAAGTTGATGTGGCTACGGGTCTGGAACTG
ATCGGTATATTTGACGTAGTCTTTCGGCCCGGCAATCAGGACTTCCTTATATCCCCACAGATTTTCAATATTG
TTGGCAAATTGCCCCATCAAAATATCAAAGCCGTTGGTATCCTTGACCCAGGCTTCATTATACGTCTCGCCTT
TTTTGGTCGAACTGGAGCAGGAGATGAGCAACGGCGCAATAACAGCAAGCGCGAGTAATTTTTTCATCATTCC
GGGAGTGCATGTTGTGTTGTCGTTATGCCATGTTGCCGGGCAGCGGCGCTAAGGGCTTATCCGGCATAACTCA
CAGGCCGAATAAGCGCAGCGTCATTCAGCAACGGGCCTATTTTTTATCTTCCGGCGTATAACCTTCGATATGA
ACGTCTTTGCCTTCGAACAGGAAGCTCACCATCTCCTGTTCCAGCAGCTTACGGTGCTCGGCGTTCATCATGT

TGAGCTTTTTCTCGTTAATCAACATGGTCTGCTTATGCTGCCACTGCGCCCACGCGTCCTTAGAGATCTCGTT
ATAGATACGTTTTCCCAGTTCGCCAGGGTAGAGCTGAAAATCTTGCCCTTCAGCGTCGCGTTGCAGGTAAGTA
CAAAAAATCGTTCTGCTCATAAGTCATCCTCTTCATCGACCGGGGCGTTAAACTGGCGCTCCGGTACGTAACT
GCTGTAACAAGCGCTCCACGGGGGCCGCCAGTCCGACTGACGGCGGTTGCGCTAAGTTATACCAGAGCGCGCT
GCCTTCATCCATGCAGGCGTCCAGTGACGACACGGGAAGCCACATAGGCACAATATCCAGGTGGAAATGGCTA
AATGTGTGGCGAAACGCGTTAAGCTGGGTCAAATTATCAGCGTTAACATGCCGTTGCGCCAGCCATTCACGTA
ATTCATCTTCGCTGGCGAACTGCGGAAAGCAGTAGAGTCCGCCCCATAAACCGCTGGGAGGGCGCTGCGCCAG
GAAAATCTCCTGATTATGCTGTAACAATAAAAAGTAACCCGTCCGCTCCGGCAACGTCTGTTTCGGTTTCTTA
CCCGGATAGCATGACCAGCTTTCATGCGCAGCGGCGATACAACCGTTTTGCAGCGGACACAGGGTGCACTTTG
GCTTTGAACGCGTACAAACCATCGCGCCCAGATCCATCATCGCCTGATTAAAACGCTCCACGCCGCGTGCGGG
CGTCACTTGCTCGCTCAACGTCCACAGCGTATTCTCCACCTCTTTTTTCCAGGCCAGCCGCTAACAGCATAAC
AGCGAGCCAGCACACGTTTAACGTTTCCATCAAGAATCGGATAATGTTTACCTAACGCGAGGGAGAGAATTGC
GCCGGCGGTTGAACGTCCGACGCCGGGGAGCGCGGCGATTTCGGCAAAAGTTTGCGGGAATTCTCCACCGTGA
AGCGTCGCCACCTGTTGCGCCGCTTTATGCAAATTACGTGCGCGGGCGTAATAGCCGAGCCCGGTCCATAAAT
GGAGTACTTCATCCAGCGGCGCATTCGCTAAATCCGTTACCGTCGGAAAGCGCGCCATAAATCGCTCAAAGTA
AGGAATCACCGTCGTCACCTGCGTTTGTTGCAACATGACTTCCGAGAGCCATACTTTGTAAGGCGTCTTGTTA
ATTTGCCAGGGCAGCGTTTTCCGCCCGTATTTGTCGTACCAGTCCAGAACCTGGGCTGAAAATTGAGACGCTT
GCATGGAAACTCATTCATTATTGCCGGGGGCAAGATTGCAGCACAGAGGCCGCAGGGTGTAAACCGGAACTTT
CCGCAGTGCGCTTCATGCTTGCATCCGGCAACTAACTTTGGATAATGCCCGTTTTCAGAACTCAAACACAGCA
GACTACACTTTTATGAAAAACGACGTCATTTCACCGGAATTTGATGAAAACGGTCGCCCGCTGCGCCGTATTC
GTAGTTTTGTTCGTCGCCAGGGGCGACTGACAAAGGGGCAAGAACACGCGCTGGAAAACTACTGGCCGGTGAT
GGGCGTCGAGTTCAGCGAAGCGCCGGTAGATTTCGCCACGTTGTTTGGTCGCGAAGCGCCGGTGACGCTGGAG
ATTGGTTTTGGCATGGGGGCGTCTCTCGTGGCGATGGCGAAAGCGCGCCCGGAGCAGAATTTCCTCGGCATTG
AAGTGCATTCACCCGGTGTCGGCGCGTGTCTGGCATCCGCTCATGAAGAGGGCGTTGAAAACCTGCGCGTCAT
GTGCCACGACGCGGTAGAAGTGTTGCATAAAATGATTCCTGACAATTCTTTATCGATGGTTCAGCTCTTTTTC
CCTGACCCGTGGCATAAAGCGCGTCATAATAAACGCCGTATCGTTCAGGTGCCGTTTGCTGAACTGGTATTGA
GCAAGCTGAAATTGGGCGGCGTTTTCCATATGGCGACCGATTGGGAAGCGTATGCGGAACACATGCTTGAAGT
CATGTCTTCTATCGACGGGTACAAAAATCTGTCAGAGAGCAATGATTATGTACCGCGCCCGGAATCGCGCCCG
GTAACGAAATTTGAACAACGTGGCCATCGTCTTGGTCATGGCGTATGGGACTTAATGTTCGAGAGGGTGAAAT
AATGGCAAAGAACCGTAGTCGTCGTCTGCGTAAAAAAATGCACATTGACGAATTCCAGGAATTAGGATTTTCG
GTGGCATGGCGTTTTCCGGAAGGTACATCTGAAGAGCAGGTCGATAAGACTGTCGATGACTTTATTAATGACG
TTATTGAGCCTAATAAACTGGCGTTTGACGGCAGCGGCTACCTGGCCTGGGAAGGGCTGATTTGTATGCAGGA
AATCGGTAAATGTACCGAAGAACATCAGGCGATTGTGCGTAAGTGGCTGGAAGCGCGCAACCTTGAAGAAGTC
CGAACCAGCGAACTTTTCGACGTTTGGTGGGACTAATAAGTACAGGGTCGGCAAATGCCGGCCCGCTTTGTCT
TGAGGGAAAGTTATGATGCGCAAAACGCTGCTGGCGGCAGTGCTAACGTTTACGGCGATGGCCGCTCATGCAG
ATTACAAATGCAGCGTCACGCCGCGTGACGATGTGATCCTGAGTCCACAAACGGTGCAGGTGAAAGGTGAAAA
CGGCAACCTGGTGATCACGCCGGACGGCAATGTGATGTACAACGGTAAGCAGTACACCCTTAGCGCCGCGCAA
CGCGAGCAAGCGAAAGATTACCAGGCGGAACTGCGAAGCGCGCTGCCGTGGATTGATGAGGGCGCCCGATCGC
GCGTCGAAAAAGGCCGCGTGGCGCTGGATAAAATCATCGCTAAAGAGGTGGGCGAAAGCAGCAACATGCGTAG
CCGTCTGACGAAGCTTGATGCGCAATTAAAAGCGCAAATGAACCGCATTATTGAACATCGTACGGACGGCCTG
ACGTTCCACTATAAGGCTATCGACCAGGTGCGTGCCGACGGTCAGCAGTTGGTGAATCAGGCGATGGGCGGTA
TTTTGCAGGACAGCATTAACGAAATGGGCGCCAAAGTAGTGCTTAAAGGCGGCGGCAATCCTTTACAGGGTGT
GATGGGCAGCCTCGGTGGTTTGCAAACCGCTATCCAGAACGAATGGAAAAACCAGGAGAAAGATTTCCAGCAG
TTTGGCAAAGATGTCTGTAGCCGCGTAGTGACCTTAGAAGATAGCCGCAAAGCGCTGGTTGGCAGCCTGAAGT
AATCTTTCCTTTTTAAACAATGGCGCAGATCGTATCTGCGCCTTTTTATTCTGTTTTTTCCTGCATTTTGTTA
TCCATCTCTAAAAAATACTCTCTGTCGGATATATATCGCTGGTTAATCGTATGGCGTCACATTATTCGTCTGC
AATATAGAGATAATGCGACCAGTTGACATAACTGGAGATATAACATGGAGTTTTTCAGGAAAACGGCATTAGC
TGCTCTGGTAATGGGTTTCAGCGGCGCAGCGTTCGCTTTACCGAATATCACCATCTTAGCGACCGGCGGAACG
ATCGCTGGCGGCGGTGATTCAGCAACAAAATCAAATTATACGGCAGGTAAAGTCGGTGTCGAAAATCTGGTGG
ATGCCGTTCCTCAATTGAAGGACATTGCGATAGTGAAAGGCGAGCAGGTCGTTAACATTGGCTCGCAGGATAT
GAACGATGAAGTCTGGCTCACGCTGGCGAAAAAAATCAATACCGAGTGCGACAGCACCGACGGTTTCGTGATC
ACCCACGGTACTGATACGATGGAAGAGACCGCTTATTTCCTCGATCTGACCGTGAAATGCAATAAACCGGTGG
TACTGGTCGGCGCGATGCGTCCCTCCACGTCAATGAGCGCAGATGGCCCGTTCAACCTCTATAATGCGGTAGT
AACAGCGGCTGATAAACAGTCTGCCAACCGCGGCGTGCTGGTGGTAATGAATGATACCGTCATGGACGGACGC

GACGTCACTAAGACCAATACGACTGACGTGGCGACGTTTAAAGCGGTTAACTACGGCCCGCTGGGTTATATCC
ACAACGGTAAAATTGACTACCAGCGTACGCCAGAGCGTAAACATACCACCTCCACCCCGTTCGACGTGTCGAA
GCTGACCGCGTTGCCGAAAGTCGGTATCGTTTATAACTATGCGAATGCGTCCGATCTGCCGGCAAAAGCGCTG
GTAGACGCGGGTTATGACGGGATCGTGAGCGCAGGCGTGGGTAACGGCAACCTGTATAAAACGGTCTTTGATA
CCCTGGCGACCGCCGCGCACAACGGTACGGTAGTGGTGCGTTCTTCACGTGTGCCGACTGGCGCAACCACGCA
GGATGCGGAAGTTGACGATGCAAAATACGGCTTTGTCGCTTCCGGCACGCTGAACCCGCAAAAAGCGCGTGTC
TTACTGCAACTGGCGTTAACCCAGACTAAAGATCCAAAACAGATCCAGACGATGTTCAATCAGTATTAATGAT
AATGCCCCGGTCGGAAGGCCGGGGCTATTTTTGGAAGACCTCTCGCACAACCAACACTATTATCTTGCTAATT
ATCATCATGGTTATGATAATTGTGGGCGTCATGTCTCTGGACGGGAGGTCCACAAGTGGTTCGACAATGGATT
GCTGGCGCAGCGCTGTTTGCCCTGATTTCAGGCTATAGTTGGGCAGAGGTTGCTCAACCCTCCGATAACATCT
TAAAAGAACAGTTTAGTAAGCAGTACCACGGCATACTTAAACTGGATTCCATTACCTTAAAAAATCTTGATTC
GACGGGCAATCAGGCCACCTGGTCTGCCGAAGGGGATATTTCTTCCCGTGAAGATATGTATACCGGCGTTGGT
ATGGCGGCGGATTATTATTTTGTTGAAAAAACCTGGACCAAAGATCGTCCCGTTAAATTTTCTGCCATGTTAA
CCAGTAAAGGGACGCCAGCCTCTGGCTGGACGGTGAACTATTACTCATTACAAATGGCAGCCAGCGATCAGGG
CCGGGCTATTGACGACATTAAAACGAATGATAAATACCTGATCGTCAACAGCGATGATTTTAACTATCGGTTT
GGCAACATTGAAGCCTCCTGGCGTGCGCAGAAAGCGTCGATCCCCGGTCTTGAAGAACAACTCTCTGCCCTGG
ATAAGAAAATAGCGGTAGCTAAAAAAGAAGCGGACGCGTATTGGGGAAAAGGCGCTGATGGGAAACCGTTGAC
CCGCGCTGAGGCGTTTAAAAAGACGTTAAAAGAGCGCGATGATTATGTAAAAGCCAACGATAGCAGCGTATAT
GCCGAGAAATACGAAAAAGAGGTCTATCAACCGGCATTAGACGCGTGCCGTAAGCAGAGTGAGCCGTGCAATG
AAGCGGCTATTCAGCAGAAAAGAGATTTAGACATACACGAGCAACGGCGGCAGGTATTTTTGAAATCAGAAGA
GCTGCGTCGAAAAGCGCAGAATGACTGGATAACGTTAGAGAAAGGACAATATCCGCTGAATATAGCGGTACAG
AAACTTCAGATGCAACAGAGTGATATCCGTGTGAAAATTATGGATATTAATGACGGTTACGAGCGCTGGAAAA
AAGATACGGACGATTTGCGTCGTAAAGGAGTTATTAAATAATGCGCTTGCCGGCGGCGCATTAACCGCCACAG
GTCGGTAAGCGTCGCGCCATCAGGTATTTTACCCGCTGGCGCGACAGGCTTATCCTTGTTATTCCGCGAGAAA
CAACTCAAGAAGAGAGTTTAAAAACAGTTTACCGTGCCGGGTAATCTGCCAGTATTGCTCGCATTCGGTCAGG
TAGCCCTGGGCAATAGCCTCGTCGATTGGCTGACGAATAACCGCTTCCGTAAGCCCGGTATAGTCGACAAATT
CGGCGCGAGGCGCGCGCTCCAGCAACCGAAAACGGTTCATAAAGAACTCAAAGGGTTTATCGTCATCACTCAC
GTCACGCTGGCTTTCCAGGTAACGTCCCTGCATATAACCGCGTGGGTGACGGGTTTTGGTGGTGCGCAGGATC
CTGCCGCCCGGGAAGGTGACTTTACCGTGGGCGCCGCAGCCAATCCCCAGATAGTCGCCAAAGCGCCAGTAGT
TCAGATTATGCTGGCACTGATAACCGGGTTTGGCATAGGCCGACGTTTCGTATTGCTGATAGCCCGCGGCGGT
TAATAACTGGTGGCCCTGCTCAAAGATATCCCACAGAGCGTCATCGTCCGGTAAAACCGGCGGACGCGAACCG
AACAGAGTGTTGGGTTCAATCGTCAATTGATACCATGAGAGATGCGGCGGATTAAGCGCAATTGCCTGTCGCA
AATCATTCAGCGCCTCTTCCAGCGTTTGGTCCGGCAATCCATGCATCAAGTCGAGGTTAAAGCTGCGTAGCCC
AAGCCCATTTGCCAGTCTTGCTGCCCGCATCGCCTCTTGTGGACCGTGAATACGGCCAAGACGTTTCAGCTTA
GGCTCACTAAAGCTCTGCACGCCAATGGAGATCCGGTTTACGCCGGCGCGCTGATAGTCGATGAAGCGGTCGG
CTTCGACCGTGCCGGGGTTCGCTTCCATAGTAATTTCCGCATCCGCCGCCAGATTCAGGCGCGCACGCACGCC
GTCCAGCAGCGTTTGCATCGCCGGCCCGGAAAGCAGGCTTGGCGTACCGCCGCCAATAAAAATGGTCTTTACT
TCACGCCCTTGCGCCCAAGCGACGTCGGCATCCAGATCGTTTAACAGATGCTGGACGTAGTCGTCATGTGGCA
CCTCGCCCTTCAACGCATGGGAATTGAAGTCGCAATATGGACATTTTTGTACACACCAGGGAATATGAATATA
AAGACTGAGCGGCGGTAGTTTAGCCATTGCGTAACGCATCCAGCAGCAGCTTGAGCGCTTGTCCACGGTGGGA
AATCGCGCTTTTTTCTTCACGGGTCAGTTCTGCGGCGGTTTTGCCTTCAGACGGTACGAAGAAAATGGGATCA
TAGCCAAATCCGCCATTTCCGGCCGCCTGGCGAGTAATGACGCCAGGCCAGCTACCGTGACAGACTATCGGTG
TGGGATCTTCCGCATGACGCAAATAAACCAGTACACAATGGAATCGCGCCTGGCGTTTGTCGTCCGGAACATC
CCGTAGAGTATGCAGCAGTTTTTCCAGATTTTGCTGATCTGTCGCGTTTTCGCCAGAATAGCGCGCGGAATAG
ATACCCGGCGCGCCGCCTAAAACGTCAACGGCCAGACCGGAGTCATCGGCAATGGCGGGCAGGCCGGTCATTT
TGGCGGCATGGCGCGCTTTGAGAATCGCGTTTTCAATAAACGTCAGGCCGGTTTCTTCCGCGGAATCGACGCC
CAGTTCCGTTTGCGCTACCACATCAAGCCCAAAATCGCTTAACAGCGACGCGAGCTCGCGCACTTTACCGGCG
TTGCCGGTTGCGAGAACAACTTTTTGCATGGAATACCTACTCAGTTAACGCCGCAATTTCCGGCGGGATCTGT
TGCGGGTGAATAATTTTTACCTGTTTGTGACGGCCCAGCTCGCCTTTTTCGATGACGATCTGACTTTTCGCCA
CGCGAAACTGTTTACCAAGAAACTTGGTCAGATGGCTGTTTGCCTGACCGTCAACCGGCGGGGCAGTAATGGC
GACTTTCACCTCGTCGCCATGTAAGCCCACAATACTGTCGCGGCTGGCTTTCGGCTGAATATAAAGCCGTAAC
ACCAGACCGTCTTCGCAGCGAGTCACAGCACTCATAGCGCCATCCACAGCCCCGGCAGCAACATGTTGCCTGT
CGCTTGTAAGACTTCAGCGATCCCCATATTAACGACATACAGCAGCAGCACCAGAACCATCGGCGAGAAGTCG
ATGCCGCCCATCGCAGGCAGTATACGGCGAATCGGGCTGAGCAGCGGCTCGGCAAGCTGAATCAATACATATT

CAATCGGGCTACGACCCTGGCTCACCCAGCTCATGATAGCCATGACCAGCAGCACCCAGAAGATCAGCAGGCC
GATTGTTTTAAGCAAGATAAGCACTGCGGCAATCCAGATAATCGCCTGAAACGTAATGACCTTAAACAGCACT
ATCGCCTTAATAACGCTAAGAATAAACGCCACTAGCAGGGAAGCGCTGTCAATCGGCCCCATTGGCGGGATAA
TACGACGCAGCGGTCCGATGATGGGCTGCGTAATTTTCACCACGAACTGGGAGAAAGGGTTGTAAAAGTCACA
GCGGGCCCACTGCATCCACACGCGCAATAATAGCACCATGGTATACAGCTCAATTACCGTTGAGAGCAGGAAG
GTCAACGTATTCATGGCGTTCCTCAGTTTTCCTTAATTTTTTGTGTAATCACGAGCACCAAAGATAGCGGTGC
CGATGCGCACCATTGTGCTACCTGCCGCAATCGCGGCTTCCATATCGTCGCTCATACCCAGAGATAGGGTATC
AACGTCAGGGTAGCGCGCTTTCAGCCCGGCAAATGCTACAGCCATTTGACGTGCGACTTCAAACTGCCTTACA
TAATCTGACTCTGGCGCCGGGATCGCCATCAGTCCGCGAAGCCGCAGACGCGGCAGTGTAGCCACCGCAGCGG
CCAGCTCATCCAGTTCCGCCAGCGGAATGCCTGATTTGCTGTTTTCATCGCTGATATTAATTTGGATCAGAAC
ATTTAGCGCCGGCAGATTATCCGGACGCTGTTCGCTCAGCCGCGAGGCGATACGCAGGCGATCGATGGTATGA
CACCAGTCAAAATGCTCGGCCACCAGCCGACTTTTGTTGGACTGTAAAGGGCCAATAAAGTGCCAGTGCAGGC
CTTCCACTTTCGCTTCCTGAAAGTGGCGGATTTTTTCCACCCCCTCTTGTACATAATTTTCACCAAATGCCCG
TTGTCCGGCGGCAATGGCTTCTGCGATGTCGCTCGCAGGTTTGGTTTTACTCACTGCAAGCAACGTAACTTCT
TCTGAAGAGCGGCCGCAACGCGTCGCCGCGGCGGAGATTTTGTCCCGGATGTATGCCAGGTTATGCGCGATAT
CTTTCATTTTCCGAGGATGTTTATATGAATATGGAAGAAATTGTGACGCTTAGTGTAAAGCATAACGTCTCGG
ATCTACACCTGTGTAACGCCTGGCCTGCGCGATGGCGCAAACAAGGACGCATGGAGAACGCGCCTTTTACCGC
GCCTGATGTCGACAGGCTTCTGCTTGATTGGCTTAATGATGCGCAACAATATCAGTGGCGAACGCACGGCCAG
CTTGATTTCGCCGTCTCGCTGTCTGGCACGCGGCGCCTGCGCGCCAGTGCGTTCACACATCAACAGGGAACGT
CGCTGGCGCTACGGCTATTGCCCGAGCGTTGTCCTGATCTGGCGGAAATCCAGACGCCGCCGATAGTACCCGC
ACTGCTTGCCAGCGAGAACGGACTGATTCTGGTCACTGGCGCTACTGGCTGCGGGAAATCCACTACGCTGGCG
GCGATGGTGGGACATCTTAATCAACATGCCGATAAACACATCCTGACGCTGGAAGATCCTATTGAGTACCGTT
ACACCAGCAAGCGTTGCCTGATACAGCAGCGGGAAATCGGCCAGCATTGCGCCACTTTTGCCGCCGGGCTGCG
CGCGGCGTTACGTGAAGATCCCGATGTCATTTTACTGGGAGAGCTGCGTGACAGCGAGACCATTCGCCTGGCG
CTGACGGCGGCGGAAACGGGGCATCTGGTACTGGCAACCCTGCATACTCGCGGTGCGGCGCAGGCGGTGGAAA
GATTAGTGGATAGTTTTCCGGCGCAGGAAAAAGAACCGGTACGCAGCCAACTGGCGGGTAGTCTGCGCGCCGT
ACTATCACAAAAACTGGAGGTCGACAGACAGGATGGGCGCGTGGCGTTGTTTGAATTACTGATTAATACGCCA
GCCACAGGGAATTTGATCCGTGAAGGCAAACTCCATCAGTTGGCCCACGTGATACAAACCGGGCAGCAGCAGG
GGATGATGACATTCGCCCAGAGCGCGCAATGGCGTCAGGCGCAGGGACGACTGTAGCGAAGATGGCGGCGTAG
GGGGGTTGTAAAGTGTTGACTGGCGCGGTGGATTGAACGGCATGCAGGCTTGTATCCTGGCGAACCGGCGTTA
AAATCGCAGCTTTGTTAAAACCACAGCGAAGCCATTTAACGATGTCATCACAACCGAATCAAAGCCTGATAGA
TGGCATTCGCTGTCTGCAATATCTGGTCTCCAGCGACCGTGCTATCGGATGTCGCGAGCTTGCCCGGCTGATG
GATATTAATACCACGCGCGTTAATCGCCTGTTAATGACGATGGCCTCTATTGGACTCACGATGCAGGATGAGC
ACCGACGCTATTTACCCGGGCCAGGCATTCATGCTTTGGCGGCGCAGGCGATTCGTGGTTCAGCGCTGTTCTC
CCATGCGTTGCCTATCCTTGAACGCCATGCGCCCAAAGACATCGTGGTGGCATTAGGCGTGCTATGGGAAGAC
CAGATTATCTATATTTATCACTCTACGCCGGGTTCGCAGGGAAGTCAGGCGCTGGCGGGGTTTCGCATGTGCC
CGGCCTGGCAGTCGGTAACAGGAGTGGCGTTACTGGCGGCAGAAAGCGATGAGGCGCTAATGCAGCGTTTTAC
TCCGGAGCAATGGCGTAATCTGGCGCCGCATGTGGCGCAGCAGCGCCAGCGGGGATACGTGCTGTGGCATCAC
GCTGATGGTGAAGTTTCTATGGCGCAACCGCTAGGCAAGCACGCCGCAGCGCTGGCCTTTGCGGGAATGTGGC
GGATTGATGAGGCGGAGGCGGCTGCGCGTTTACAGGCGTTGAAGGCATTAAACCAGCTTATTGCCCAGTAAGC
CTGGCAACCGTGACCGCGTTATCCGCCTGCTGACAAAAACGGCTTTAATACCCCTGTTCAAAATAGCTTTCGA
GGATAATCACGGCGGAGGCCGAGTCCACTTTGCCTTTGTTCAGTGCGCGATACCCACCTCGTTCAAACAGACC
GGAGCGTGCTTCAACGGTGCTGAGTCGTTCATCGTGCAGCGTGACAGTTACCCCGAAACGCCCATGAATACGG
TTGGCGAATTTACGTGCGCGCGCCGTCAGCGGCTGTTCAGTGCCATCCATATTGAGCGGTAGCCCGACAATAA
TTTCGTCCGGCTGCCACTCTTTCAGCAAACGTTCAATGAGCATCCAGTCGGGCGTGCCGTCCTGTGCTTTGAT
TGCCGGAAGCGGACGGGCGGTGCCGGTAATGCGTTGTCCTATTGCAACGCCGATGCTTTTTGTGCCGAAGTCA
AAGGCCAGTAAGGTGTCGCTCATCAGGCGTGCCCCGCAACGCCAGGCATGGTCAGAATATCAATGCCAATAAG
TTTTGCCGCTTCGCGCCAGCGCTCGGCAATGGGTGTTTTGAACAAAATATTGAGATCGGCGGGCGCGGTAAGC
CAGGCGTTATCAAGCAGCTCTTGTTCAAGCTGGCCTTTATCCCAGGAGGCGTAGCCCAGCGCGACCAGTACAT
CGGAAGGCTGTTGTTGGGTACCTAACGTTTCCAGCACATCGCGGGAGGTGGTAATCACGGTATTGTCTGAGAT
ACGAATGCTGGAGGCGAAACGTGACGGCGGAGTATGGAGAATAAATCCGCGATCTTCCGCCAGCGGGCCGCCG
AGCATCACGGCTTTATCAAGACGAATTGACGAATCGCGCGGTTCCGGCGTAATTTTTAGTTTTTCCAGAATCC
CTTCAATTTGCAGATTTTCCAAAGGCTTATTAACAATAATCCCCATAGCGCCGTCCTGGTTGTGCTCGCAAAT
ATACACCACGGAACGGCGGAAAATCGGGTCCTGGAGCGCAGGCATGGCAATAAGAAAGTGATGCTGTAAATTC

ATTGTCAGAGGTTCTGTTTCCTGGTTCAAAAAAACGACAGTGTTCAGTATGTGGGGAAAGCGGACGGTTGTCA
CTCATATAAGCCGTCTTTTGCCAAAGGACGGTGGCCTTATCGGGCAATAGACTGCCGGATGGCGATGCTGATG
CGCCGCCTTATCCGACCGGGGCGGCGTGATTGCTCAGGCCCGGTAAGCGCACGCCACCGGGCAATAAACCTTT
ACTTCGCCAGACGCGCTTCGATGGCGTCCATCAACATCCCGGTGATAGAGATCGGGTATTCCGCTTCAATTTC
ACGCACGCAGGTTGGGCTGGTCACGTTAATTTCCGTTAAGCGGTCGCCGATAATATCCAGACCGACGAAAATA
AGCCCTTTGGCTTTAAGCGTTGGCCCAACGCGGCGGGCGATTTCCCAGTCGCTTTCGCTCAACGGACGGGGTT
CGCCGCGACCACCAGCCGCCAGGTTGCCGCGGGTTTCGCCGCCCTGCGGAATACGCGCCAGGCAGTAAGGCAC
CGGCTCGCCGTCGACGACCAGGACGCGCTTATCGCCATCTTTGATCGCGGGCAAATAGTTCTGTGCCATGCAG
TAGCGGTTGCCCAGCTCGGTCAGCGTTTCGGCAATAACGCCTATGTTAGGATCGCCTTCCTTAACGCGGAAAA
TGGACGCGCCGCCCATACCGTCCAGCGGTTTCATAATGATATCGCCGTGCTTTTCCCAAAACGCTTTGAGCTG
CGCTTTATTGCGCGTTACCAGCGTTTCCGGCGTCAGATCGGCAAACCAGGCAGTATACAGTTTTTCATTACAG
TCGCGCAGGCTTTGCGGTTTGTTGACGATCAGCGTCCCTTCTTCTTCAGCGCGTTCCAGAATATAGGTCGCAT
AAATAAACTCGGTATCAAACGGCGGATCTTTGCGCATCAAAATGACGTCCAGATCGGCGAGTTTAATCTCCTG
CTCGCTGCCGAACTCGTACCATTTATCGTAATTTTGCTCCACGCTCAACGTTCGCGTGCGGGCGCGGGCTTCG
CCGTTAATCAGATAAAGGTCGGCCATCTCCATATAATGAAGTTCATAACCACGACGTTGGGCTTCCAGCAGCA
TAGCGAAACTGGTGTCTTTCTTGATGTTGATGTGTGCGATGGGGTCCATCACGATGCCGAGCTTGATCATTAT
TTTCTCCGTTAACGCTTCATCCTAAGTCGCCAAAACGCACCTGTAGCGCGGTAATGGCGGTGAGCGCGGTTGT
CTCAGTACGCAGAACGCGAGGTCCTAACAGAATATCAGTAAACTGATAGCGCGCGGTCATCGCGATTTCGTCC
GCCGACAGCCCGCCTTCCGGGCCGATCAGCAGTCGGACGCGTTCAACCGGCAGCGGCAGAGTGTTGATGCTGG
CATGGGCGCGCGGATGAAGATTGAGCTTCAGCCCGGAATCTTGCTCGGCGCACCAGGCTTCCAGCGCCATCGG
CGGGCGAATTTCCGGCACCCGGTTACGCCCGCATTGTTCGCAGGCGGCGATGGCGATCTTTTGCCACTGCTGG
CGCTTTTTGTTCAGACGTTCATTATCCAGTTTAACGCCGCAGCGCTCAGAGAACAGTGGCGTAATGAGGCTTA
CACCTAGTTCGATCGATTTCTGGATAGTAAATTCCATTTTTTCACCGCGCGACATCACCTGGCCCAGATGGAT
ATGTAGCGGCGATTCACGATCGTCGATTTCGCCTTTCATCACTTGCACTTCAACGCTTTTCTTACTGGCGCTA
ATGATTTCAGCATCGAATACCTGATTACTGCCGTCAAACAGCTGTAACGCTTGTCCCGCTCCCATGCGCAGTA
CACGACCAATATGGTTGGCCGCATCTTCGCATAACGAAATCTGCGTACCGGACGTCAACAATTCAGGGTGATA
AATGCGGGGAATACGCATAATAAAAAAATCCGCGTCATGGCCCCGGCGTTTGGCTATCCAGGGCATGGGTTAA
CAAGAATCCCGCTAGTGTAGGTTAGCTCTTTCGCGCCTGGCAAGCGCGTTGCACATAGGGATTATGATTACCC
TGGACCTTCGCGATACGCGCATCGCGCTCGCACTCCCAGGCGGTAACGGGGTACTGCTTATCCCAGACGTTAA
AAAGCTGCGTTTGTTGGCGGGAAAGTTTCAGTTGGTATTGGTCGCGCATATAAAAATAAGTGCGGGCGATTGC
GCCACGGGCGCGGGCGGGCGGCTCGGCGAGCTTCGCTTTGAAATCTACTTTCATGGCGCACTGCCCGTACTGA
CCTTCGCCGCCGTTCCACTGGCTATACATAAAGTTGCCGCGATCGCCATTCACTTCGCCAATCGCGGGTTGCA
GGTTATGCATATCGCTTTCCATTTTGCGGTAGACCGGGTCTTTAGCGCAGTTTTTTCGCCCGCCGTCCTGCCA
GCACTGGCGCTGATGACCGAATTGCCAGGCGGGGACAACGTGCTCCCACTCAATGCGTCTGGCGCGATTCTCG
TTTTTACGCACCTTATAGCCGCAGGACTCCAGGTCTACGACGCCTTTTTTACCCTGCCAGCGGATTTGGCACC
CGCAGTAAAAGCTGCCGGGCGCGTCGGCATTGACTTTGACGCTCGCCGCTTTGGCCTGAGAAAAATTGTTAAT
GCCATCGGCCAGGGCCTGGCCTGAAAACGCTGCGGCCAGCAACGCAGCGGCAAAAGAAAAATTACGGTACATT
GCACACTCCATAGCAAAACGAGCCCGCAACGTAACGAAAGTTGTGTCTGTATGCAATCAGTCCGCTCAGGAAA
GTTCCCGGACGTTTCGGGAAACTACCCGGCGACCAGTGGTTCGCCGCAGTGAACGCAACGATAAACCGCTTCG
CCGCGTACTACCCGATTATGACGGCGGACGGTGAGTTGATGTTGCTGGCATTGGCAATGGTAGGGAAAGGTGT
TGCGCCGTACGGATTGCAGCGCAAATTGATGAGTACGTCTGGCCGGAACGCCCAGCACGCTTTCCATCATCCA
CTTCCATTCCTTGCCATGCGGAGCCTTGCGTCCGAAGTGCTTCCACACCAACAGATGCGCCAGTTCATGCGGC
ACGACCTCTGCTATAAAGGTGTCGATGTTTTCCAGCAGTAACACCGGGTTGAGGCGGATTTCGTAGCTCTCCA
GCCAGGCGGTGCCCGCCGAGGTGCCGCGTTGCGTATACACCAGTTTCGGCTCCGGATAATGTCTATCGAGCTT
GAGGTTCGCCTGGGCGAGATTTTCCCGCAGACGGCGCATCACGGCCTGCTGGATAGCGATAGGGAGACGAGGG
GTTTTCATAGCGGCAGAGAATAGTGCGACGGAAGAGGGACTGCAAGAAAAACGCTTCCTCCTGTGGCAGGAGG
AAGCGTGGAGGGATTAGTCGTGGGCGCCGATTTCGCGCAGTTTACGACCTTTCATCAGGTTACGTTCAATGTG
TTCCAGCGAAACGTGTTTGGTTTCCGGCACCAGCCACAGGGTCAGCAGGATAAACAGCACGTTCAGGCCAGCG
TAGACCCAGAAGGTGTTGGCGTTACCCAGATTGTTCAGCATGGTCAGGAACGTTGCGCCGACGATCATATTGG
CGATCCAGTTGGTCGCGGTGGAGCAGGTGATGCCGAAATCGCGGCCTTTCAGCGGCTGGATTTCGGAGCACAG
TACCCAAATCAGCGGACCAGCGCTCATCGCAAATCCAATGATGAACATCAGCAGCATAGCGATAGCGAAGTAT
TGCGCAGACGGCGAATGGATGCCGATGTGCATCATCGTACCCAGAATCCCCATACCAATCGCCATGACCAGGA
AGCCCAGCGTCAGGGTCGGCTTGCGGCCCCAGCGGTCAACCAGGCCGATAGCGATGAAGGTTGCCAGCACGTT
AGTCAGACCAACAATAACGGTGCCCCACATCTGCTCCGTGGTATTCGTGTACCCCGCCAGTTCGAAGATTTTC

GGCGCGTAATACATGATGACGTTCATCCCGGTAAATTGCTGCATGACCTGCAACAAAATGCCAAGAAACACGG
CGCGGCGGAAGTTGCTGTTCTCTTTGAACAGCGCCCAACCGCTCTGTTTAACCTGCAAACTTTCGCGAATTTC
ATCGAGCTCACGTTTTGCTTCCGCACTGGTGTCACGCAGGCGCAACAGGACGCGTTCAGCATCATGGAAGCGG
CGTTTTGCCGCAAACCAGCGCGGGCTGTCCGGCAGGAAGAAGACGCCGATCAGCAGCAGAATGGCCGGGATGA
TGATCACGCCGAGCATCCAGCGCCATGCGCCGCTGTAGCTGAACGCGGTATCTGACAGATAAGCGCCCAGAAT
CCCGATGGTGATCATGAGTTGGTACATTGAGATCATACTGCCGCGAATTTTTTCCGGCGCGATCTCGGACAGG
TACAACGGTGCAGTGTAGGAAGCGACGCCGACGGCCAGACCCAGCAGCACGCGAGAAATAATCAGGACTTCAA
CGTTAGGCGCCGCAGCGGAGAACAGCGAACCGGCGACGAACAGGATAGCGCCGATCATCAGGCTTTTTTTACG
ACCCAGCTTAAAGGAGAGCCAGCCGCTGCCGACAGCGCCGACGGCCGCGCCAAACATCATGGAGCTGACCACC
CATTCCTGAGTGTGTGCGGTAATCTGAAACTCGTCGGTAATGAAGGGTAAAGCACCGGCGATAACGCCGATAT
CCAGGCCAAAAAGTAATCCTGCCAGGGCTGCAAGAAAGCAGACAAAAAAATGTCATGGCCTTGTTGGAACGCCC
TTGTTTTTTATTGTCAGGCATTATGCCCTCCGGTTGGGTTAGCAATTTTATCGATGTTAAGAGTAGGTGAGTA
CGCCGTAAAAAGATGTGAGGCTAATCACAATGGTGTAATCGTTTACACTACTATTTATCTCGGTGATTATTTA
ATTATCTGAAAAATAATAGTTTTAATAATCTTATAACCATATCAATCGCCTGGTATTCAGACAAAGTTGAAAA
ATTATGTAATATTTTCATCGTAGAATTAGCATACCGATAGTGAGGCGGTCAGTTCTTAATGAAAATGAATGTA
ACCGCTTTCATGATGACTGAAACTGAATGATAAAAAGTGTAGTCGAAGAAACGAAAGGGGAGAGTGGATTGTG
CTGCGCATCACAAAAAGGTCAGTATAAATTCACGCTGGAATATCTGATAACGTTGCGTGTATCAGCAATGATA
AAGGCCAGCAGGGCGCTGGCCTTGATAAGTACGGTATGGTTTACCTGGCTGAAAAACAGCGTCAGGCGCGGGC
GATTATTTCAGACCGGCAGCATCGCGCAGCAGTTGCGCTTTGTCGGTTTTTTCCCATGGGAAGTTTTCGCGAC
CGAAGTGGCCGTAAGCCGCAGTTTCTTTGTAGATCGGGTGCAGCAGATCCAGCATCTGAATCAAGCCGTACGG
GCGCAGGTCGAAGAATTCGCGCACCAGCAGAATCAACTGTTCAGCAGGCACTTTTTCAGTACCGAACGTTTCC
ACCATGATGGACGTCGGCTCCGCTACGCCGATAGCGTAGGAGACCTGGATTTCACAGCGATCCGCCAGACCTG
CCGCCACGATGTTTTTCGCGACATAACGCGCAGCGTAAGCTGCAGAACGGTCAACTTTAGACGGATCTTTACC
GGAGAAGGCGCCGCCGCCGTGACGCGCCATACCGCCGTAGGTATCAACAATGATCTTACGACCGGTCAGACCA
CAATCGCCCATCGGGCCGCCGATAACAAAACGACCGGTTGGGTTGATAAAAAACTTAGTGGACGTATTTAACC
ATTCAGACGGCAGAATGGGTTTGATGATCTCTTCCATCACCGCTTCTTGCAGCGATTTTTGGTCGATATCTTC
TGCGTGCTGCGTAGAGAGAACCACGGCGTCGATACCGACGATTTTGCCGTCGTCATACTGGAAAGTGACCTGG
CTTTTGCATCCGGACGCAACCATGGCAGGGTGCCATTTTTACGCACTTCAGCCTGACGCTGTACCAGACGGTG
CGCGTAGGTGATTGGCGCAGGCATCAGTACGTCGGTTTCATTTGTCGCGTAGCCAAACATCAGGCCCTGGTCG
CCCGCGCCTTGTTCCAGCGGGTCTGCGCGGTCAACGCCCTGGTTGATATCCGGAGACTGTTTGCCAATTGCGC
TCAGTACCGCGCAAGAGTTGGCGTCAAAGCCCATGTCGGAATGCACATAGCCAATTTCGCGCACCGTATTACG
GGTAATCTCTTCGATATCGACCCAGGCGCTGGTGGTGATCTCACCGCCAACTAAAACCATGCCGGTTTTAACG
TAGGTTTCACAGGCGACGCGTGCTTTCGGATCCTGTTGCAGGATAGCGTCCAACACGGCATCAGAGATTTGGT
CTGCAATTTTGTCAGGATGCCCTTCTGATACGGACTCAGACGTAAAAAGGTGTTTTGCCATATTTAATTTCAC
CTAAGGAGAATTTAGTTAGCTCAAACTGTTGTGCGGAATAGTCATGACAGAAGATTCTACCATGAGTCTGAAG
GTCGCTGACACTGGCAGTCTGAGTGTTAATCAGTATGGATGGATTAACATCTGGACGGCTATTTTAGGTCACT
TCTTCGCCCAATTTCCAGCTTTTTTTGAAGGCCGTCGCAATGTCGGTCAAAATCAGGCGATAAACGTTTCTGA
TAATGCTGACATTATGCGCATTTATGCTTTGCTTTTTTTCTGCGTTCTGGGTATAAAACGCGACGCGCGGCTC
ATACAAAAGCACACGACGTTTTCTTCGTGTCGCCACTTCCAGCCGGGTTAAAACGTTGCTTTGGCTTGTGGGT
TCGTCTTACGAGGCGGCCGTGGAGGTGATACGAGATAATGAACCGTTGTATTCTGCTGAATCTGTCGCATCGT
TCTGGTGCGCGCATGTCCCCCGCAATCTGCATCTCTGCTTTGCATACCTGCCGATGTTATACCCATCTCGGCG
CTTCTCAGGATTCCAGAGCCGGCAATGGCGCTGAAGACTGAACAAGGGCGCTCTTGTTAACGCAAGAGTTTTC
TCGTGGTTTCGCCGAACCGTTATCTGTGAGTTCGGTTACGTGTTTACAATGATATGAAAAAGAAACCGGTCGC
GCAGGCGGAGCATCAGCGCTATCTGCTGGAAAATCCGCTTGTCTATGGGTTGTTATCGCGTCTTCGGATTGCG
ATAGTAGTCAACTGTTTTACACTTGCTAATAAAAATTGAGGTTCGCTATGTCTGACGACATGTCTATGGGTTC
GCCTTCGTCAGCAGGCGAACAGGGTGTACTACGCTCCATGCAGGAGGTTGCAATGAGCTCCCAGGAAGCCAGC
AAGATGCTGCGCACTTACAATATTGCCTGGTGGGGCAATAACTACTACGACGTCAACGAGCTGGGCCATATTA
GCGTATGCCCCGATCCCGACGTACCGGAAGCGCGTGTCGATCTTGCCAAACTGGTGAAAGCGCGCGAAGCGCA
AGGTCAGCGTCTGCCGGCGCTGTTCTGCTTCCCGCAGATCCTGCAACACCGTTTGCGTTCAATTAACGCGGCG
TTTAAGCGTGCGCGTGAATCTTACGGTTATAACGGCGACTACTTCCTCGTTTATCCGATTAAGGTCAATCAGC
ATCGCCGCGTGATCGAGTCCCTTATCCATTCCGGTGAACCGTTGGGGCTGGAAGCGGGGTCAAAAGCGGAATT
GATGGCGGTGCTGGCGCATGCCGGCATGACCCGTAGCGTGATCGTCTGTAATGGTTATAAAGACCGTGAATAC
ATTCGGCTGGCGCTGATTGGCGAGAAGATGGGCCATAAGGTTTATCTGGTCATCGAAAAGATGTCTGAAATCG
CGATTGTGCTGGAAGAGGCCGAGCGCCTGAATGTGGTTCCTCGCCTGGGCGTGCGTGCGCGTCTGGCCTCTCA

GGGGTCCGGTAAGTGGCAATCCTCCGGCGGCGAAAAATCAAAATTCGGCCTGGCGGCGACCCAGGTGCTGCAA
CTGGTGGAGACCCTGCGTGACGCCGGGCGTCTGGACAGTCTGCAACTGTTGCACTTCCACCTGGGATCGCAGA
TGGCGAACATTCGCGATATCGCGACCGGCGTGCGCGAGTCCGCGCGTTTCTATGTTGAGCTGCATAAGCTGGG
CGTTAATATCCAGTGCTTCGACGTGGGCGGCGGTCTGGGCGTGGATTATGAAGGTACCCGCTCGCAGTCCGAC
TGTTCGGTGAACTATGGCCTGAACGAATATGCCAATAACATCATCTGGGCGATTGGCGATGCCTGCGAAGAGC
ATGGTTTACCGCATCCGACGGTGATTACCGAGTCTGGCCGCGCCGTCACTGCGCACCATACGGTACTGGTCTC
TAACATTATCGGTGTGGAGCGTAACGAATACACGGATCCGACTGCCCCTGCTGAAGATGCGCCGCGCGCGCTG
CAAAATCTGTGGGAAACCTGGCAGGAGATGCATAAACCTGGCACCCGCCGCTCGCTGCGTGAGTGGCTGCATG
ATAGCCAAATGGATCTGCACGATATTCATATCGGCTACTCTTCCGGCGCGTTTAGTTTGCAGGAGCGCGCCTG
GGCGGAGCAACTTTATCTCAGCATGTGCCATGAAGTGCAGAAGCAACTGGACCCGCAAAACCGTGCGCATCGC
CCGATTATCGACGAACTGCAAGAGCGGATGGCGGACAAAATGTACGTCAACTTCTCGCTGTTCCAGTCGATGC
CGGACGCGTGGGGAATCGATCAGCTCTTTCCGGTGCTGCCGCTGGAAGGGTTAGATCAGGTACCGGAACGTCG
TGCCGTGCTGCTGGATATAACCTGTGATTCCGATGGCGCTATTGACCACTATATCGATGGCGACGGTATCGCC
ACGACGATGCCGATGCCGGAATACGATCCAGAGAATCCACCGATGCTCGGCTTCTTTATGGTCGGCGCCTATC
AGGAGATCCTCGGTAACATGCACAACCTGTTTGGCGATACTGAAGCGGTTGACGTGTTTGTCTTCCCGGATGG
TAGCGTCGAGGTTGAGCTGTCGGACGAAGGCGATACCGTTGCGGATATGCTGCAATATGTGCAACTGGATCCA
AAAACGCTGTTAACGCATTTCCGCGACCAGGTAAAACAGACGGATCTGGACGATGCGTTGCAGCAGCAGTTCC
TTGAGGAATTTGAAGCGGGACTGTACGGTTATACTTATCTTGAAGATGAGTAATTAAGAGTATTCCGCTTGTG
GGTCGATTTAATTATTTAAATCGACCCTTTTATATTTTATAAATTCTTAATTATCAGCGGTGCCGCTTCTCTA
TGTAATTTTATGGGCCTACAATCCCCCTAATAATTAAAAAGGGGGTTAAGTATGAGCAAATTTAAATTTAATG
CCGTCATTGTTGGCTTGTTTTTGCTGTTAGGCGGTTGTTCCAGCGGAATGCAATCTAATAATGGCTCTTCATC
CGACGTCGGCACCGCCTGGGGCGGCGACGTGCATTCATCGGTACAAAGCGTTTCGGTAGAACGTGCCGATCGA
GAACCTGCCGAAATGGTGATAATAAATTACTCCACTCAATATCCGTCAGGTTACGATAAAGTTTATAGTATAC
GTATCAGCGATCTTGAATATGCTGTGCGCGACGCTAATTTTAATTCGATTCCAATTACTCGTCGCTACAACGC
ATCAATGGGACAATGGCAATATAGTATTCCTGCAAGATCAGGGATGAACTATCAGTTATATATTCGTAATTAC
AGTCATGATACCAATTACGAAATCGTCGCAACGGTTGATGGCCTGGACGTGTTGAACGGAAAAGCAGGCTCGC
TGAATCATCATGGCTATATTGTTAACGCCGGCGATTCACTGGCGATTAAAGGGTTTCGCAAAGATAAACACAC
TGAGGCGGCATTCCAGTTCGCCGACGTAGCTGACGCTTATGCGGCCCACTCCGCGCAGGGCGATGTACGCAAC
ATCGGCGTCATCGGCTTTGCCGCTTTTGCATTGCAGGGTAAAGCAACAAATACGCTACCGCCTTGTTCCTCGC
AGGCTTTCCCGGCGGACAACAACGGGTATGCGCCGCCGCCCTGCCGCAAATAAACCCGGCATCAAACGTCAAA
TATGCGTTATGGGAATTAGCGCCTTTCCTGCCGAGTCTGGTGGGGAAGGACTGTGTGACGCGCATAGCCGTAG
GTTGCGGGAAAGAAACGACCACTCTCTGGCAGGGAAATTGTTTACCATAAACATTAGGATTTGTTTACACTAC
CCTCAAGTGGAGCAATTGCTGTCTACATAGGTATGTGCATGGAACAAATTTTAACCAAGCGGCGCTATTTTGA
TATAGGGCTGCAAATAGAAGAACTACTCTATTCAGGGGTATTTAAAGCTGGCGAGCGACTTCCGGCGGAGAGA
GAACTCAGCGAGCGTTTTCAAACCAGCCGCACGACGATTCGTGAGGCGATTATTATGCTTGAGCTGAAGGGGG
TGGTAGAGGTTAAGCAAGGCGCAGGCACTTACTTTATTGATAGTCTGGAAAAAATCAATCAAAAAGCGCTCTT
GCCTTACTCAGACATTGGCCCCTTTGAATTGCTACAGGCCCGGCAGGTTATCGAAAGTAACATCACCGGTTTT
GCCGCTACGCAAATAAGATTTAATGAGCTTAAGCAGCTTAAACGTATTATTGAGCAGCAGGAAAAGCAGATTG
GCGGCGACAGCGACAAATTTGAAGAACTGGATCGTCAGTTCCACAATATCATCGCGGAATCCACGCAAAATCG
TGTATTAATGAAACAATCTGCCGAGCTGTGGCGGGCTGTCAGGACCGAAAATCCGCGCTGGAAGCAGTTAAAC
TATAAGTATTTACATAAGAAAGAGTTAAGAATGAAATGGGTGGAAGATCACCGTAGTATCTTCCTTGCATTGC
AAAAACGCGATGCTGAGCAGGCCAGACAGGCCTCGTGGACCCATCTTGAAAACAGTAAAAATGAGCTGGTAAA
AATCTTCCAACAGGATGATTCGCTGGAAGATTTTGATGATTTTTTCTTCGCCACCTGACGGCTATTTTCAGGC
GGATGACATTTTTGTCAGCGATTAACCGGGCCTGCCGCCAAAAACAAGCCCGGAGAGAGGGAATCAGGCTTCG
TAAACGCTCAGCTTATCCCAGTCAAACTCTACGCCAATGCCGTAGGTGTCCGGTGCCACGGCGCGATAATTGT
CGTCCACCAGCGGATGTTTGGTATATTGATCGATCGGGAAGCTGTGAACTTCCAGTCAGCCAGTCTCGAATGC
GGAAACCAGTCTGACGTGTAATTCCTGCATACCGAGCGAGCATACCAAACGACCGCCATTCGTCGACAGACGC
GCCGCCTTCAGCCAGCCATTTTCCACAGCGGCAGTTTTTCACGTTTGCCTTTCAAATCCCAAAGAGCGATATC
CATCGCAAACGATGCGATGCCTCCGCGACCCACATAGTGAATATGCTATTCCATAAAGTCATAGATATCGTCA
ATCGCAGCGCCTCTTTCCCTGACAGTACCGGGGCAAGATCGTGGTCGAACATCGCTTTAATTGCATACCCTCC
CTTTCCGCCGGTATAGGAATATCCGGTGCCAGTTGAACCGTCTTCCAGCGTAATCGTCGTGGTTACCAGTTCG
AAATGGCTATTATGCCATGTTTTGCGTCAACTAAGACCTTGGCCAGAGGGGCTTTGAAAGCTTTGTTTCAACG
TGCCTAATCTGCGTCTTCATCAATTTTTTCCTGTGTTCAATCAATGGATTAATTTGCCAATTACGTCTAAAGG
CGGCTCACTCCTCATCGCTTTAGCAGCGACCCGGATGATCAAAACCGTAACTGATGTTCTTAGGTTAACGTTG

ATGCTGATAATAAATTGAGTTCATTAATTCACTAGACAAATTATCAGATTTGGTTTGCTGTATTTCGGGTAAT
TATTGAACCAATCATGCGTGACATATACGCTCGCAGCGGGCGTGCTATTCAATAAGGTATTGTTTTTCATGGT
GTTTAATGATTGCAGTTGTGTGGGTGGTATGCCAATTCGTGGCCGATTTTTCTAATCAAAATCACGCTTTTGT
GAATCGTAAAATTTATCCTGACAAGCGTATTTTATAACCAATTGATAAATAAATTATTTTTATTCATTGGCTA
TTAGTCAGCATACCAGGGTGAAGAACCCGGTGCCGTGAGTGTGATCATATTCACGGATTTTGGTATGCTGTTT
AAGCAGAATTGGTAAACAAAACAGGAGTGGGAAAAATTCATGGACACCATAGCTCGCCAGTTAGACGCCGCAA
GGCACCAATTCAAAACGACTTATTCACGTCAGGGAATGGAAGCCAACATCGTACATATTGGATTTGGCGCCTT
TCACCGTGGTCATCAGGCGGTTTATAACGATCTCACGAATGAGATAACCGGCAACCGCTGGGGGATTTTTGAA
CTGAATCTGTTCGGCGATGCGGAGCTGGTGGATGCGCTAAATGCGCAACAGGGGCTGTTTTCAGTGGTGGAAA
CGTCAGCCAGCGCCATCAATAGTCGCCTGGTTCGTACCATCACCGGCGCGTTGCATACGCCGAAGTCAGGTAT
TCAGGCGGCGATCGAGAAGCTGACTGAGCCGCAGGTAAAAATTGTTTCGTTGACGATAACGGAAAAAGGTTAC
TGCACCGATCCACGGTCCAGAACGCTCGATCTCAGCCATCCGCTCATCAAACACGATCTTGCCGATCCTGAAC
ATCCCCGCTCAGCACTCGGACTGATCGTCGAAGCGCTACGGATACGCCGCCAGCAAGGGCTTCCCCCGTTTAG
TGTACTGTCGTGCGATAACATTCCGGAAAACGGTCATTTGACCAAAACGGCGATCGTTACGTTTGCCGATAAT
CTCGATCCGCAACTGGCGGCCTGGATAGCGAAGCATGTCACCTTTCCTGGCACCATGGTCGATCGCATCGTGC
CGGCAATGACGCCAGAGCAATTTGACATGATTAAAGACCAGATCGGTTTTGCCGATCCCTGCGGCATCGTATG
CGAGGATTTCCGTCAGTGGGTTATCGAAGATAATTTCGTTGCCGGTCGTCCTGACTGGGATAAAGCAGGGGCG
ATGTTCGTCTCGGACGTGCTTCCCTATGAAGAAATGAAACTTCGTATGCTGAATGGCAGCCATTCGTTTCTCG
CCTATAACGGCAGCCTGGCTGGATATGAGTTTATCTATCAGTGCATGGAAGACGACGCTTTTAAAACCGCCGT
ACATCATTTAATGACGGAAGAGCAGGCAAAATCGCTGCGCCCGAATTTGGCGGTAGATGTACATCAATATGCG
CAGTTGCTCATTGAGCGATTCAGCAACCCGAACATTAAACATAAGACCGGACAGATTGCGATGGACGGTTCTC
AGAAGCTGCCTCAGCGGGCCGTCGATCCTTATCTGACGCTTCAGGCGCGCGGCGTTAAGGGCAGGGCGTTAGC
GACGCTGATTGCGGGCTGGCTCCATTACGTCATTAATACACTCTCTCAGGGGCAGAGTGTGGCCGACCCGCTC
AATGATACCTTCAATGCGGCTATTAAAAATAAAAACACGCGATGGGAACAAGCGCTGTCTTTGCTGCAGATAA
ATAGCATCTTTGGAACGCTGGCCGGCGACAACGCAGACTTTTTAAACGACATTCAGCAGGCCTTTAACCATAT
TGAGTTACATGGCGTCACGGCCACCATTCACCGTTTATCTTCAAAGGGTTAAGTATGAAAACGCTTATCTGTA
ATAATCCGGGAAATATCGAATACATTGAAAGAGATATTCCTCATTTAAAAGACGATGAAGTACTGTTAAAAAT
TAAAGCGGTTGGTATTTGCGGCACCGATATCCATGCGTTTGCCGGTCGCCAGCCCTTTTTTGCCTATCCGCGC
GTGCTGGGTCATGAAATTTGCGGCGTGGCGGAAATATTGGGTAAATCGTGCAGCACGGCGAAGGTCGGCCAAC
GCTATAGCGTTATTCCCTGTATTCCTTGCGGCGCTTGCGCAGCCTGCCGGGAAGGAAAAACCAACTGCTGTGA
GAACGTCTCGCTGTATGGCGTTCACCAGGACGGCGGCTTTAGCGAATATCTGGCCGTTCGTGAACAAAATCTG
GTTGAACTGTCGGATAACCTCACCGACAGCGCGGGCGCGCTGGTTGAGTGTTTCGCCATCAGCGCCCACGCAG
TACGCCGCGCTGACGTGAAGCCGCAGCAAAACATTGTGGTGGTCGGCGCCGGGCCGATTGGGCTGGCCGCCGC
CGCGATAGCAAAAGCCAAAGGCGCTCGCGTCGCGGTAGCTGATATTGACGCCGAACGCCGTCGTCTGGTCGCC
GAAAAGGTAGGCGTTGCAACGCTCGATCCGTCGTCAGACGACTATATTGATGTGCTGAAAGCCTGTTTCTCCG
GTGAATTAGCCGGCATTGTGCTTGATGCCACCGGCAATAAATCCTCCATGAGCCGCGCTGTTGATCTGATTCT
TCACGGCGGAAAAATCGTCTTCATCGGGCTATATATCGGCGAGCTCGTCATTGACGATCCGACCTTCCATAAA
AAAGAGACCACGCTATTGAGCAGTCGTAACGCGACGCGGGAAGACTTCGAATGTGTCATTGAGCTAATGGCTC
AGGGCGCTATTAGCGAAACAATGATGAAAAACCAGGAATTCGATTTCTATACGTTCGGCAACCAGTACCAGAA
GAACGTGGTAGAAAACAAAAAATTGGTTAAAGGCGTTATCAAATTTTAATAGCGGGATAGTTATGTCAACAAT
ATTAGTCAAAGAAAATGAATTGAAAGCGCTGGCGTTTAATAAACTCACCCAGGCGGGCCTTGACGCGCAAACG
GCGCAGCAGGTAGCCGACGTTTTGGTTCATGCCGATATTACCGGCGTTCACTCCCACGGCGTTATTCGCGTAG
AGCACTATTGTACCCGCCTGAACGCAGGCGGGCTCAATCCAAAAGCAACATTCAGCATCGAACAGATTTCGCC
TTCTGTCGCTATTCTGGACTCTGATGATGGAATGGGGCACTGCGCGCTGATAAAGGCGACAGACCACGCTATT
AGTCTGGCGAGAGAGACGGGGCTGGGCTTCGTCAGCGTCAAAAATACGTCCCATTGCGGCGCGCTCTCGTGGT
TCATTGAGCAGGCGACAAGCCAGGGAATGGTGGCTATCGCCATGACGCAAACGGATACCTGCGTTGCGCCGTA
TGGCGGCGCGGAGCGCTTTCTGGGGACTAACCCAATCGCCTTCGGCTTCCCCGTTAAAGACAGCCATCCGATG
ATCGTCGATATGGCGACCAGCGCCATTGCGTTTGGTAAAATTCTGCACGCCAAAGAAACCGGGAAACCAATAG
GTCATGGTCTGGCGCTGGATAAAGAGGGACATATCACTACCGATCCGCATAAAATTGAAAATCTGCTGCCCTT
TGGCGGACACAAAGGTTCTGGTATTGCCCTGGCGATTGATGCGCTCACGGGCGTCCTGATGGGGGCAAACTTT
AGCAACCATATTGTTCGGATGTATGGCGACTATGACAAGATGCGCAAGTTAGCGAGTCTGGTTATCGTTATTG
ATCCGCAGATGTTGGGCAATCCGCTGTTTTCCTCAATAATGAGCACAATGGTCAATGAGCTAAGGGCTGTAAA
ACCGATGCCCGGTGTTGATAAAGTGCTGGCGCCAAACGATCCGCAAATCGCCTATAAAGAAAAATGTTTAAAA
GAAGGTATTCCTGTGGCTGAAGGTATTTACCAGTACCTGATTGGCTGATAATTATAATCACGACCATTATTTA

GGATAACTATAGTATGCTATATGGAAATATAGAGCAGCTTACTTTATTACCTTACGTTAACCACATTATTAAA
AAACTTATCATCGAAGCGGTGAAGATAGCTGAAGATCAGCCTGCTGGTAGATATGAACTTTCTTTCCCGGAGT
CATTTCTTATGATAAGCGAAGGAGAAACACACTCTTCGTTAAACCGCAAAGCTGAACTTCATAAAAAATATAT
CGATGTTCAGATTTTACTAAGCGGTTATGAGGAGATTGGCTATTCCAACAAAATTGATACCAGGATAAAGGAG
CTTGAGCATCTTCCTGATGACATCATCTTTCCTGAATGTGTCGCTAATGAGCAGTTCGTTACATTAAATCCTG
GCGATTTCGCGCTTTTTTATCCCAACCAGATCCACCGTCCGCTTTGTACGCGTGGTAAACCTGCGCCGGTTAA
AAAAGCGATTGTGAAAATTCCGGCCACGGCATTTAGCGAGTCAAGCTTGCCCTGTCAGACGAAGGAATAAAGA
AAGCGCCAAATGCAATTCAGGAAGAGACCTTCAAGTCATCCTGAATTACAGCCAGAATGAATAAGGTGGCACT
TTTCTGACTTCAGAGCCTTTTCGCGTTGAGGCTAATCGCGGAAAGGCTTTTTACGTTCTGTAAAAAGAGGATG
CGGGGTCTCCCCGGTAAATAAGTGAAAATGATACAAATCGATTTTGATGTTATTTTTGTTCAATCTGAATAAT
ATGGTCTTTTACACGGAAAGTGATTGGCGAGGTGTTATGAATCATATTTTTAAATTAGCATTTATTTTTATTA
TCAATATTATATTTTTAATAAATGCTTCAGCAACAACGATGGACAAAGATAAATTTGTGGAAGCGAATGGTAT
CAAAATTCATTACGTTGAAGAGGGCGAGGGGCCTCCGCTGCTGTTGATTCACGGTGGTGGTCTGACGGCGAAA
AGTTGGCAGGGATTAGCGAAAGAGGCATCTCGATATTTTCGCGTCATCATGCCGGACAGTCGTGGACATGGAT
TAACCAATAACCCGCAAGGAACGTTTAGCTATGATCTGATGACGGAAGATATGGCGGCTTTTGTTAAAGCGCT
GAAGCTGGAAAAACCGCTGGTGATGGGTTACAGCGATGGCGGCATGGTCGTTCTGAAGTTAACCAGCCGATAC
CCGGATCTAGCGCGCGCTGCTATCGTCGGCGGCGCAACGCATCGCTTTGCTACGACCCATTACATGCAGGGGA
TGGAAATTTTTTATGGTAAAGGAATGCCTTAGGGGCAATTGACGGATCGAGATCTGGATAAAATGGCTTCGGA
TGCGCCCGGGATGGTTAAATTTTATCAGAACATGCATCATCCAGAGCAAAAAGACTACTGGCGCACGTTTCTC
GAAGGTGTCTGGCCAATGTGGACAACGCCCACATCGCTGACGGAAGAAGAGGTGAAAAAAATCCACATTCCTGT
GCTATTACTGGACGGCGACCGGGATGAATTTTTCACCGTTGAAGAAGTTACCGAGCTGTACCGGCTGCTGCCT
CAGGCGGAGATGACCCTCATTCCCGGTAGCGGTCACGCCATTTTCCAGACGCCGGGGAAAACACCGCTGTTTT
ACGCATTAGTGCTTGAGTTTTTACAGCGGCAACTCCCTAAGGCCTCTTAACTGACCGCGATGGACGTATGAGC
CTACTGATTTGCGCTGGATATATATTTTTTTCGGGCGTTGTCTACTGTACCTGGCTAAACTGACTTCAATAAT
GCGGTCTGTCAGAGTAAACTTTGAGCCGTAAACAAATTCAAACCCTTCCTCGTCGGGCCTAACGACGCGGGAG
GGTTTTTTTATATTGACTAAGAAGAGGTTTTTGCCATGAGCACCTTAGGTCATCAGTACGATAACTCACTGGT
TTCTAATGCGTTTGGTTTTTTACGTCTGCCAATGAACTTCCAGCCGTATGACAGCGATGCCGACTGGGTGATC
ACTGGCGTACCGTTTGATATGGCAACGTCCGGTCGCGCTGGCGGCCGTCATGGCCCGGCGGCGATCCGTCAGG
TGTCGACCAACCTCGCCTGGGAACATCACCGTTTCCCGTGGAGTTTTGACATGCGCGAGCGCCTGAACGTCGT
GGACTGCGGCGATTTGGTGTATGCGTTTGGCGATGCCCGTGAGATGAGTGAAAAATTACAGGCGCACGCTGAA
AAACTGCTGTCTGCAGGCAAGCGTATGCTCTCTTTCGGCGGCGACCACTTCGTCACGCTGCCGCTGCTGCGCG
CCCACGCGAAACATTTTGGCAAAATGGCGCTGGTACATTTTGATGCGCATACCGATACCTACGCTAACGGCTG
CGAATTCGATCACGGCACGATGTTCTACACCGCGCCGAAAGAAGGCCTGATCGATCCGCATCATTCGGTACAG
ATCGGTATTCGTACTGAGTTTGACAAAGACAATGGCTTTACCGTGCTGGATGCCTGCCAGGTCAACGATCGCG
GCGTGGATGATATTCTCGCTCAGGTGAAACAGATCGTCGGCGATATGCCGGTCTATCTGACCTTTGATATCGA
CTGTCTGGACCCGGCGTTTGCGCCTGGCACCGGTACGCCGGTGATCGGCGGTTTGACCTCCGATCGCGCCATT
AAACTGGTACGCGGTCTGAAAGATCTGAACATTGTCGGTATGGATGTAGTGGAAGTCGCGCCGGCTTACGATC
AGTCGGAGATCACCGCTCTGGCGGCCGCGACGCTGGCATTAGAAATGCTCTATATCCAGGCGGCGAAGAAAGG
CGAGTAAGTTGTTTTGCCGGATGGCGCTGCGCTTATCCGGCCTACAGCTGATTGATGCTTGTAGGCCGGATAA
GGCGTCCACGCCGCCATCCGGCACTGTTCGGGGATTATTTGATCCCGTCCGCGCTCATACGATCGCGCACATG
CTGTGCGCGTGCGGCGGACGCCGGGTGATCGTCGAACATCGAGCTTTGACGACCCGCTTCCAGTTTTGCCAGT
TTCTCAAAGCTGGTGGCAAGTCCTGCCGGACTGATACCGCGTTTGCGCAACAGATCGTAAGAATAATCATCCG
CTTCGGACTCCTGACGCTGGGAGAACTGCGAGTTCACCAGTTTTTCGCCAAGATCGCCAAGCTGTGATTGCGA
CAGGCTGCCGACAACGCCTCCTGCCGATGCCGCCGCGACGCGCACGGCGTTAGTGCCCAGCGCGACCTGCATC
CCTTTCTTTACATGACCCAGCGCGACATGGCCCATTTCATGGCCGATCACGGCTTCAACTTCGTTGTCGGTCA
TCATATCCATCAGTCCGCTGTAGACGCGAATACAACCGTTCGCCATGGCAAACGCGTTGACGTCTTTCGTCTC
GTAAACCTTATAGTTCACCGGCTGGCCGTTAATGTTATCCCCCAGCGCAGCGGCAATTTTTGCCAGTCGTTTA
GCGTATTCGCTGCTCGCCGGCGCGATTTTCGCTTTGCTGTCGAGCTCTTGACAGGATTGATCGCTCAACGCTT
TTACCTGCGCATCGCTTAACGTATAGGCCTGAAAAGCTTCAGCGCCCGAAGAAAGCAGTCCATTAGAGTCCAT
ATTCTGGCAACCGGTCAGCACCGTCGCCATGCCCAAAGCAAGCAGTAAAGCGCGAATTTTCATTTTTCTCTTC
CATACGAATTAATATTATTTTGAAATGCGCTCGTTGCAGATGAATTAGCGCCTGGGTTAAGTATAAAGAAAAG
CATGGCAGGCAAGTTAGCTAATTGCGCAACATACGAGCACGATCCAGAGATTTCTGAAGCGGTAAAAGGATGT
TCCATGTACATGACGCGCGGCTTGGGGTAAATTGTTGGCAAATTTTCCGGCGTAGCCCAAAACGCGCTGTCGT
CAAGTCGTTACGGGCATGGCCTTTCAACATCCGATCTGGAGTCAAAATGTCCTCACGTAAAGAGCTTGCCAAT

GCTATTCGTGCGCTGAGCATGGACGCAGTACAGAAAGCCAAATCCGGTCACCCGGGTGCCCCGATGGGTATGG
CTGACATTGCCGAAGTCCTGTGGCGTGATTTCCTGAACCACAACCCGACCAACCCGTCCTGGGCCGACCGCGA
CCGCTTCGTGCTGTCCAATGGCCACGGCTCAATGCTGATTTACAGCCTGCTGCACCTCACCGGTTATGACCTG
CCGATGTCCGAGCTGCAGAATTTCCGTCAGCTACATTCAAAAACCCCTGGTCACCCGGAAGTGGGCTACACCG
CTGGCGTTGAAACCACTACTGGCCCGCTGGGTCAGGGTATTGCCAACGCCGTCGGGATGGCGATTGCCGAGAA
AACTCTGGCGGCGCAGTTTAATCGTCCTGGCCACGATATCGTTGACCACTTCACCTACGTCTTTATGGGCGAC
GGCTGCATGATGGAAGGCATTTCTCACGAAGTGTGCTCTCTGGCTGGTACTCTGAAACTGGGCAAACTGATCG
CGTTCTATGATGACAACGGTATCTCTATCGATGGTCATGTTGAAGGCTGGTTCACCGATGACACCGCGAAACG
TTTTGAAGCCTACGGCTGGCACGTTATCCGTGGCATTGACGGTCACGATGCCGACGCCATTAAACGCGCCACG
GAAGAAGCTCGCGCCGTGACTGACAAACCGTCCCTGCTGATGTGCAAAACCATCATCGGTTTCGGTTCGCCGA
ACAAACAGGGTACCCACGATTCCCACGGCGCGCCGCTGGGCGACGCGGAAATCGCGCTGACCCGCGAACAGTT
GGGCTGGAAATACGCGCCGTTCGAAATCCCGTCTGAAATCTACGCCCAGTGGGATGCGAAAGAAGCCGGCCAG
GCGAAAGAATCCGCATGGAATGAGAAATTCGCGGCTTATGAGAAAGCCTTCCCGCAGGAAGCTGCTGAATTCA
CTCGTCGTATGAAAGGCGACATGCCGGCTGACTTCGATGCGAAAGCGAATGAGTTCATCGCTAAACTGCAGGC
GAATCCATCCAAAATCGCCAGCCGTAAAGCGTCCCAGAATGCCATCGAAGCCTTCGGTCCGCTGTTGCCGGAA
TTCCTCGGCGGCTCCGCTGACCTGGCACCTTCTAACCTGACCCTGTGGTCCGGTTCGAAAGCGATTAACGAAG
ATGCCGCAGGTAACTACATTCATTACGGTGTGCGCGAATTCGGTATGACCGCTATCGCCAACGGTATCGCCCT
GCACGGCGGTTTCCTGCCGTACACCTCCACCTTCCTGATGTTCGTCGAATATGCCCGTAACGCAGTGCGTATG
GCAGCGTTGATGAAACAGCGTCAGGTGATGGTCTATACCCACGACTCCATCGGTCTGGGCGAAGATGGCCCGA
CGCACCAGCCGGTCGAGCAGGTGGCAAGCCTGCGCGTAACGCCGAACATGTCCACATGGCGTCCGTGCGACCA
GGTGGAATCCGCGGTGGCGTGGAAATATGGCGTAGAGCGTCAGGATGGCCCAACCGCGCTGATCCTCTCCCGT
CAGAACCTGGCGCAGCAGGAACGTACTGAAGAGCAACTGGCGAATATCGCCCGCGGTGGTTACGTACTGAAAG
ATTGTGCGGGCCAGCCGCAGATTATCTTCATTGCGACCGGTTCAGAGGTTGAGCTGGCGGTTGCCGCTTACGA
AAAACTAACTGCCGAAGGCGTGAAGGCGCGCGTGGTTTCCATGCCGTCTACCGACGCATTCGACAAGCAGGAT
GCGGCTTACCGTGAATCCGTGCTGCCGAAGGACGTCTCTGCGCGTGTAGCGATTGAAGCGGGTATCGCTGACT
ACTGGTTCAAATACGTCGGCCTGAACGGCGCTATTGTCGGGATGACCACCTTCGGTGAATCCGCCCCGGCAGA
GCTGTTGTTTGAAGAGTTTGGCTTCACGGTGGACAACGTGATCGCCAAAGCGAAAGCACTGCTGTAATCCTTA
ATACGGCATAAAACGGGTCGCTTCGGCGACCCGTTTTTTATTCTCCCTTTGACAACCTGCCGTTTTCCAGCCG
TACCACCCGTGAAAAATGACGCCGGGTAAACGCAGCGTCATGGCTAATCGCCACGACGCTGGTTCCCCGTGCC
CGGCATGTCGCCAGCCAGTGTTCAAATACGGTTAGCCAGTGGGCGTCAAAATCCCGGCTGGGTTCATCCAGCA
ATAACACAGGCGGCGCCATCGCCTCCAGACTGGCGACCGCCACCATCCGGCGCGGTGCGGCGTGCAGATCAAG
CGGGTGCGCCTCGGCGACGTCCGTTAGCTGGCATAACCGCAGCGCGGCCTGCGTGCGCCGTGAAATTTCCTCT
GCCGATAAGCGTTGTAGTTTCAGCCCAAAAGCGACCTCTTGCGCGACATTGCTGTGGAAAATCTGGTTTTCCG
CTTCCTGAAAGAGTACGCCGATAGCGGCGGCGCGTTGACGATTTTTAAGCTGCGCAATCGGCTCTCCATTAAG
CGTTACCGAGCCGGAAGTGGGGGAAAGCAGGCCGGCCATAATCCGTAACAGTGTTGATTTTCCCGCGCCGTTA
TCGCCGGTCAACGCCAGCCATTCGCCGTCACGAAGTTCAAGCGAGATAGCGTGCAAACAGTCGGCTGCGGCAT
CCGGCCAGCGATAAGCGACCTGATTTAACGTCAACATAATGGGGTTAGCGCTCCGTCGCGCAATTGCCATACC
CGCTGGCATCGCGTCAGAAAAGGAAAATGGTTACGCTCAAAAAGCACAATGAGAGAATGACGTTCCAGCGCCC
ACTGTTGTAGCCGTTCCAGCAGCATCCCGGTGGCCGCTGACGTCAGCCGACTGAAGGCTTCATCAAGAACTAA
TAGCCTGGGCTGCATGGCAAGCGCGCTGGCAATCACTACCCGCTGCGTTTCGCCGCCGGAGAGCGTGCCGGGA
TGGCGATGGCGTAACGACTGGCAGTTTGTCAGAGTCAGCGCCTCGTCAATGCGCCGTAGAATTTCCGCTTCAT
GGAGGCCGAGATTCTCCGGGCCAAACGCCACCTCTTCCTCTACGCTAAAGGTACAGCCGGAAAGCTGTAAATA
AGGCGATTGCTGCACCAACTGGATGGTGGGCGACTGTTCTACCAGCGGGAGCTGCCCGATAGGGACGCCAAGC
AGTAAGCCTGTGCCGTCAATATCACCGGGAAGATAATCCGGATACCAGCCAGCCATCAGTTGCGCCAGCGTAC
TTTTTCCGCTGCCGTTATCGCCGACTATCGCCACTATTCCGGGTGTTGAATAGTGGAAATCAAAACATGCCGG
CGGGCGTGTCGCATCGGACGGAAGATAACGAAACTGCTCTAACGTAACCATATCCACACTCCCGCTTCCGCCA
GTATTAGCAGTACCATGCCATACCGCGCAACCCGTTGTAACATGCTATCTTTCGGCGCCCACAGCGTGGTTCG
GGCTCGATGCAGCCGAAACCCCCGCATATCCAACGCCGCGCCGCGTATTGTCAGATCGTTAAGCGCATTTTGC
GTAAGCGGCACGATCAGCGCAGGCATTGCCCGTAGCCGCTGATACCAGCCTTCATCTAGCGGTACGCCACGCG
CGCGCTGGGCTTCATGAACGATCGTCAGTTGTCGTTTGAGCTGTTCGACGACCAGCAGCGGGCCAGCGAACAG
GTAAGCGATGCCGGGCGGCAAACGTGAAGCGAACAGGGCGCGAATAAACCGCTGAACCGGCACATACTGCATC
CATAGCTGCGACGTCGACACGATCGCCAGCAGGCGTAGCCATAGCGTAACGGCATATATCCAGCGCTGCGGAT
CGCAAGGCTGCCCGCTTATCCATTCCGTTAGCCAACCGCCATGTACCAGCCATAGCCCAAACCCTAAAGACAG
CATCAGCCAGGCGACATACTTCGCCCGCCGCCGCGTTGATTTCAGCGCCAGCAGACAGAGGAAGGCCGCCGCG

CTATAAACCGGGAGCACGGTCTGCGCAGGGAGTAGCAGCGTGGTGCAGGCCGCCAACGCCCATAAGGTTAACG
AGGTAAACGGATGCATTTAGCGTACGGCTGACATCGCAGGAAAATGCCGTGTCGTACGTAACGGTAGCTGACG
CAGCAGCACCCAAACGATGATCGCCGTCAGAATTTTATCGACCAGATTAGCGCCTATGACGGTAATCGCGACT
GACTCCACCAGGTTTTGGCCCATTGAATGCATCCAGGCGACGAACAAATCAGCGCCGCTACCGGTAACGCCGC
CAAAAAGCGCGGTACGAAGCGGTACGGCGACCAGCGTCACCGCCAACGTAATGACGACCCCGCTGACGATCAC
TTTGGGCAGGGTACGAAACCAGCCTGCGCGCGCCAGCCACCCGGCGACCAGACCGATAACCATCGCTACCGGG
GCAAACGCGGCAGCGATAGGGTCGGTAAGCAGCCCCCACAGCAAGTTGGTCAGAAGCCCAGTCAACATGCCGA
TGAACGGCCCTAACAGTACCGCGCTAATTAAGGTGCCGATCGAGTCGAGAAAAATAGGCAGCTTCAACATGCT
AATAAGCTGTCCGCCAATCATATTGATGGCAATAGCGATGACAATCAGCACCAGAGACTGGCTGGAAAACGGG
CGACGCGCCATAAAGTACCTCTTAAAGAGTCGTCAATTAACGGGAAGAAATATGCAGTTCGTCATAACCGGAA
ATGGCGCACGGTTGACGGCTGAACGTCAGCGCTGGCAGCTCGCCGATAACCAGTTCCAGCGTGGTACGCACGG
TACACCCTGGCATCATATGGCCGCCGAGCATGGCCCCGTAGGGATCCGATACCGCGAGATGCAGATGCTCGCC
GGTCAATTCAAGCGTGCCGTTTAGCGAGATAACTTCAAACGTGCCGGTGAGCGATGTGGTCGCTTCTTGCCCG
GCATAACGCAGCGCAACGTCGGTCAGACTGCCGGTACACCCTGCAATCCAGGCGGCATGTAATTGGTTCTGTT
GCACAAAGGCGTGTAATTGGGAAAACACTTCCTGGCCGGGAAGCAGACGAAGCGCGTAAAAGCGCGCTGTAGA
GGCATTGTGGTGAGATACAGTCATCGTTACAGGACTCCGAGACTCGGACGTGGCGCTGATAATATAGACGCTG
GCGACAGCGCTTTCTGATATAAATCAGTATTTGTGACGCGACGATGAAAAGATCTGACGGATGTACTGGTGAT
TCTTAAATATGGAATAAATTGCAGCGTAAGCGGCCATGAAATGTGATGCAGGTCAGATAACTGTCTTCTTCGA
CTGGACAAACATTCCTTTTATTCCCCGTTTCGCTTATTCTAGCTGAAGCGTTTCAGTTAATTAAATGTTCGAC
AATTAACCAATCAGTCGCAGTTTGCGACAGGTGAGGTTTTACTCGACGATGAGCTGCATTACTCTGTCAGCCT
CATCTGAATCGGGATAGCCTTGCAGGAGACCTATGACCGTACGCATAGCGATTAATGGCTTTGGTCGCATCGG
ACGTAACGTGGTTCGTGCTTTGTATGAATCCGGACGTCGCGCGGAAATTACCGTGGTGGCCATCAACGAGCTG
GCGGATGCCGCAGGCATGGCGCATTTGTTGAAATACGATACCAGCCACGGGCGTTTTGCATGGGAGGTTCGCC
ACGAGCGCGAGCAGCTTTTTGTCGGCGACGACGTCATTCGTATTCTGCATGAACGAACGCTGGCGGATCTGCC
GTGGCGCGAACTGGGCGTGGATGTCGTGTTGGATTGTACGGGCGTATATGGCAACCGGGAGCATGGCGAGGCG
CATATTGCCGCTGGCGCGAAGAAAGTGCTCTTTTCTCATCCGGGCAGCAACGATCTTGACGCCACCGTCGTTT
TTGGCGTGAACCAGAATGAACTGCGTGCGGAACATCGTATTGTCTCAAACGCATCCTGTACCACGAATTGCAT
AATTCCCGTCATTAAATTGTTGGATGATGCTTACGGCATCGAGTCTGGTACCGTCACGACGATCCACTCCGCG
ATGAACGATCAGCAGGTGATCGACGCGTATCACTCCGATCTGCGGCGCACGCGCGCGGCCAGCCAGTCGATTA
TCCCGGTAGATACAAAACTGGCGGCAGGCATTACGCGTATATTCCCGCAGTTTAACGACCGTTTTGAGGCGAT
TGCCGTGCGCGTTCCAACCATTAATGTGACGGCGATCGATTTAAGCGTAACGGTGAAAAAACCAGTAAAAGCC
AGTGAAGTCAACCAGTTGCTGCAAAAAGCAGCACAAGGTGCATTTCATGGTATAGTTGACTATACGGAATCAC
CGTTGGTCTCGATAGATTTTAACCACGACCCGCACAGCGCCATTGTTGATGGCACGCAAACCCGGGTTAGTGG
CGCCCACCTGATCAAGACGCTGGTCTGGTGCGATAATGAATGGGGCTTTGCTAACCGGATGCTCGACACCACG
TTAGCGATGGCCGCAGTTGGTTTCAGGCTCGACGCGTCAGCGTCGACAAAACTTTAAGCATCATTGAGAGGAT
TCACCATGTCTGTAATTAAGATGACCGATCTGGATCTGGCCGGGAAACGCGTTTTTATCCGTGCGGATCTGAA
CGTACCTGTTAAAGAAGGGAAAGTGACCAGCGACGCGCGTATCCGCGCCTCTCTGCCGACCATCGAACTGGCT
CTGAAACAGGGCGCGAAAGTGATGGTAACTTCCCACCTGGGTCGTCCTACCGAAGGCGAGTACAACGAAGAAT
TCTCTCTGCTGCCGGTTGTTAACTACCTGAAAGACAAACTGTCTAACCCGGTTCGCCTGGTCAAAGATTACCT
GGACGGCGTTGACGTGGCCGAAGGTGAGCTGGTGGTTCTGGAAAACGTTCGCTTTAACAAAGGCGAAAAGAAA
GACGACGAAGCGCTGTCTAAAAAATACGCCGCCCTGTGTGACGTATTCGTGATGGACGCCTTTGGTACTGCGC
ACCGCGCGCAGGCTTCTACCCACGGTATCGGCAAATTTGCTGATGTTGCGTGCGCAGGCCCGCTGCTGGCCGC
TGAACTGGATGCGCTGGGTAAAGCGCTGAAAGAACCGGCTCGTCCGATGGTGGCTATCGTTGGTGGTTCTAAA
GTATCTACCAAACTGACCGTTCTGGATTCCCTGTCTAAAATCGCTGACCAGCTGATTGTCGGCGGCGGTATCG
CCAACACCTTCGTTGCCGCTCAGGGCCACAGCGTCGGTAAATCCCTGTACGAAGCTGACCTGGTTGACGAAGC
AAAACGTCTGCTGACCACTTGCGATATCCCGGTTCCGACCGATGTTCGCGTTGCCACCGAGTTCTCCGAAACC
GCACCGGCGACTCTGAAATCCGTTAACGACGTGAAAGAAGACGAGCAGATCCTGGATATCGGCGATGCTTCCG
CTCAGCAGCTGGCTGAAATCCTCAAGAATGCCAAAACCATTCTGTGGAACGGCCCGGTTGGCGTGTTTGAGTT
CCCGAACTTCCGTAAAGGGACTGAAATCGTGGCGAATGCCATCGCTGATAGCGAAGCGTTTTCCATCGCTGGC
GGCGGCGACACCCTGGCGGCAATCGACCTGTTCGGCATTGCTGACAAAATTTCCTACATTTCTACTGGCGGCG
GCGCATTCCTCGAATTCGTGGAAGGTAAAGTTCTGCCAGCCGTAGCGATGCTCGAAGAGCGCGCTAAGAAGTA
ATCGTTACGGGCAGGGATTCCTGCCCGTTGATTTATCGCCGATCTGACATTCAGGGCGGCCCGTGTTTTTTCC
CAAAGCCCGACGATACAGGACAACGAAACATGTCTAAAATTTTTGATTTCGTAAAACCAGGCGTGATCACTGG
TGATGACGTACAGAAAGTGTTCCAGGTAGCAAAAGAAAACAATTTTGCTCTGCCGGCAGTTAACTGCGTGGGC

ACTGATTCCATCAACGCTGTTCTGGAAACCGCTGCAAAAGTTAAAGCACCGGTCATCGTTCAGTTCTCCAACG
GCGGCGCGTCCTTCATCGCGGGTAAAGGCGTGAAAACTGACGTACCTCAGGGCGCGGCAATCCTGGGCGCTAT
CTCTGGCGCGCATCACGTTCACCAGATGGCTGAACACTACGGCGTGCCGGTTATCCTGCACACTGACCACTGC
GCGAAGAAACTGCTGCCGTGGATCGACGGTCTGCTGGACGCGGGTGAAAAACACTTCGCGGCTACCGGTAAAC
CGCTGTTCTCTTCTCACATGATTGACCTGTCTGAAGAGTCTCTGCACGAAAACATCGAAATCTGCTCCAAATA
CCTGGCGCGCATGTCCAAAATTGGCATGACTCTGGAAATCGAACTGGGTTGCACCGGTGGTGAAGAAGACGGC
GTGGACAACAGCCACATGGACGCTTCTGCTCTGTACACCCAGCCGGAAGACGTTGATTACGCGTACACTGAAC
TGAGCAAAATCAGCCCGCGTTTCACCATTGCCGCTTCCTTCGGTAACGTACACGGCGTTTACAAGCCGGGTAA
CGTGGTTCTGACCCCGACCATTCTGCGTGATTCTCAGGACTATGTTTCTAAGAAACACAACCTGCCGCACAAC
AGCCTGAACTTCGTTTTCCACGGCGGTTCCGGTTCTACTGCTCAGGAAATCAAAGACTCCGTAAGCTACGGCG
TAGTGAAAATGAACATCGATACCGATACCCAATGGGCAACCTGGGAAGGTGTTCTGAACTACTACAAAGAAAA
CGAAGCGTATCTGCAAGGTCAGCTGGGCAACCCGAAAGGCGAAGATCAGCCGAACAAGAAATACTATGACCCG
CGCGTATGGCTGCGTGCTGGCCAGACTTCGATGATCGCTCGTCTGGAAAAAGCATTTAAAGAACTGAACGCGA
TTGACGTTCTGTAATATAGTCTGCTGATTGAAATGGCTTCCCTTGGGAAGCCATTTTTTTATCGTTATTTCCG
GAGAGCGGCGTTAGTTAGCGCTTTCATCCGGCCAACTCACAATGTACCTGCCTTTTTTGGTTTTGGGATGATG
CCGAAAAATGGCATATTCCTCTGTTTTTGTTTACCTTTAAAAAGTTCTTTTCGTGAGAATGATCTCATTTGCT
TGTTTTGCTATGACCAAGGAATATTGAATGGAAGATTTGAATGTTGTCGACAGCATAAATGGTGCGGGCACCT
GGCTGGTACGCAACCAGGCGTTATTGCTCAGCTATGCTGTGAATATTGTTGCGGCTATCGCTATTATCATCAT
CGGCTTGATTGTAGCGCGCGTGATTTCCAATACGGTAAATCGTCTGATGCGTGCGCGTCATATTGATGCGACA
GTGGCCGACTTTCTTTCTGCGCTGGTGCGCTATGGGGTTATCGCTTTTACGCTGATTGCGGCTCTGGGTCGCG
TGGGGGTTCAAACCGCCTCGGTTATTGCCGTACTGGGCGCCGCTGGTTTAGCCGTTGGCCTGGCATTACAGGG
ATCGCTTTCTAACCTTGCGGCTGGGGTATTGCTGGTTATGTTCCGTCCGTTCCGCGCCGGTGAGTATGTCGAT
TTAGGCGGCGTTGCCGGTACGGTACTTAACGTGCAAATCTTTTCCACGACAATGCGCGCGGTAGACGGCAAAA
TTATTGTGATTCCGAATGGCAAAATTATCGCGGGAAATATCATCAACTATTCTCGTGAGCCGGTTCGCCGCAA
CGAATTCATTATTGGGGTGGCCTACGATTCGGATATCGATCAGGTGAAACAGCTGCTCACGACGATTATCGAG
TCCGACGATCGTATTTTAAAAGATCGCGAAATGACGGTGCGTTTGAATGAGCTGGGCGCCTCATCAATTAATT
TTGTGGTGCGTGTCTGGAGTAAAAGCAGCGACCTGCAAAATGTCTACTGGGACGTGCTGGAACGTATTAAACG
TGAATTCGACGCCGCTGGTATTAGCTTCCCGTACCCGCAGATGGACGTCAATTTTAAACGCGTGAAAGATAAC
GCCGCGGAGTAATTTTCCTCCCGTGCTGCCTGCCAGTGCTGCGTTTATCCGCCTCCAGTATTCTCTGGTTATT
GAGCGGATAAGCGTAGCCGCCATCCGTTATTCAGTCTCTTCATTAGCTTTTCTTATTAGTGATTAAAATTATC
AATTTCCGCTAATAATATTCCCGCGCTATAGTCTGCCTTTAAGAAAAACAGCTCAGGGTAGTTAACGTGATAT
CTTATTATTTTCAAGGGTTTGCCCTTGGCGCCGCCATGATCCTTCCGCTTGGCCCGCAAAATGCGTTTGTGAT
GAATCAGGGAATCCGTCGCCAGTACCACCTCATGATAGCGCTGCTTTGCGCGTTAAGTGATTTAGTCCTGATT
AGCGCCGGTATTTTTGGCGGTAGCGCGTTGCTGATGCAGTCGCCGTGGCTGCTGGCGCTGGTCACATGGGGCG
GGGTTGCGTTCTTGTTATGGTACGGTTTAGGCGCGCTGAAAACGGCAATGAGCAGTAATCTGGAGCTGGCCAG
CGCCGAGGTTATGAAGCAGGGGCGCTGGAAAATTATTGCTACGATGCTGGCGGTAACGTGGCTGAATCCGCAC
GTTTATCTGGATACGTTTGTCGTGTTGGGCAGTCTCGGCGGACAACTGGCTATGGAGCCAAAGCGCTGGTTTG
CTCTCGGCACGATTAGCGCGTCGTTCTTGTGGTTTTTTGGGTTAGCGCTACTGGCGGCCTGGCTTGCGCCTCG
ACTGCGTACGGCGAAAGCTCAACGCATTATCAACATTCTGGTAGGGGTGGTGATGTGGCTTATCGCCTTCCAA
CTGGCGCGGGAAGGCGTCGCGCATATGCACGCGCTTTTCAACTAAGCGTTGTCTGATGGACAACAGGCCGATA
TCCGCTAAGCTTGCCTGCATGCGCCCTGTATATCAGGGCAATGACTGCAATATGGAGGAGAGACTGTGAAGTT
CAAAGTGATGGCCCTGGCGGCATTAGTGGGTTTAAGTGCAATGTCGGCGCAGGCAAGCGAATTGCCGGAAGGC
CCGCACATCGTGACTTCCGGTACGGCAAGCGTCGATGCTGTGCCTGATATCGCCACCTTAGCGATTGAAGTGA
ACGTCGCGGCGAAGGACGCGGCGACGGCGAAGAAGCAGGCCGATGAGCGCGTGGCGCAATATCTTTCCTTCCT
TGAGCAAAATCAGATTGCGAAAAAAGACATTAGCGCGGCGAATCTGCGCACGCAGCCGGATTATGATTACCAA
AACGGTAAAAGCATTCTGAAAGGGTATCGCGCGGTGCGAACCGTTGAGGTGACGCTGCGTCAGTTAGATAAAC
TCAATTCGCTGCTGGACGGCGCGCTGAAGGCGGGTCTGAACGAGATTCGTTCGGTATCGCTGGGCGTGGCGCA
ACCGGACGCCTATAAAGACAAAGCCCGCAAAGCCGCCATTGATGACGCGATTCATCAGGCGCAAGCGCTGGCA
GCCGGTTTTAACAGCAAATTGGGGCCGGTTTACAGCGTTCGTTACCATGTCTCTAATTATCAGCCGAGTCCGG
TGGTGCGAATGATGAAAGCCGCAGATGCTGCGCCGGTATCCGCGCAGGAAACTTACGAGCAGCCGACCATTCA
GTTCGACGATCAGGTCGATGTGGTATTCCAGCTTGAACCTGGAACAGGGCAGACGTCGACCACGGCCGCCAGC
ACGCAGTAATCTCGCCCGGCCTGGGCGCTTCCAGGCCGCATTTTCTCCCGGTGTTCGCGTTTTTTGCTCCGTA
TTACGCATTGAAATGAAGATTTGCTAATACGGTGAGCGTTTTGAATCGCCTTGTCGCCAGCAAAAAATAGCCA
ATGGCGCGTCGTTTTTTGCCGCCTGAACGCAATGATTTTGTGATGAACTTCAAAGAAGTGACGAGGGAATAAT

GGGTCATGCAGGCCGGATAAGACGTTCACGCCGCCATCCGGCATTCATAGAGAGGCTAATCCTGACGCAGCAC
CTTGTGCCCGTACTCCAGCAGCGCGTCGGTCACTTTGCGCATCATCCGGCTTTCCGGGGCAAAGCGGTGCCAG
TAAAGCATCCGGCGCTGCAGCAAACCAGGCGTTAAGTTGATCAACTCGCCGCTTTCCAGCTCTTTCTCAATCT
GCAGGTGAGGGATCATGCAGCAGGTCGTGCCCTGACGCGCAAGCTGTACAAATGCTTCGGATGAATTAACGAT
ATGGCACGGCACGCTGCCGGGCGGTAGATCGAAATTCTGTTGCAAAAAGGCCTGATGCATATCATCCAGATGG
TCGAACGCGACGGCGGGCGCTTTCAGCAGCGACGACCGGGTAACGCCATTGGGGAAATAGCGCTCGGCAAACG
GCCTTGACGCGACAAACAGATAGTCGAGCGCGCCCAGTTTGTCTACCAGGCAACTGGGCAGCGCCTGATGCTG
AATACTCACCGCGCCCACCACCTCGCCGCGCCGCAGACGTTCTTGCGTGCGCGTTTCATCTTCCACCTGTAAA
TTGAGACGGATAGGGGAGTCCGCCAGCACCGGCGCCAGCGCCGGAAGCAGCCAGGTCGCCAGACTATCGGCGT
TAACCGCCAGCGACAGCAGCAGCGGTGTAGAGCCGGTTTGTTCATCGCCCAGCCACTCTTCTTCCAGCAGTTC
AACCTGACGCAGCAGAGCCAGTAATTTTTGCCCTTGCTCCGTAGGGCGAGGAGGAACGGTACGCACCAATAAA
GGTTGTCCGAACATATTCTCAAGCTGTTTTATGCGCTGTGAGACGGCGGATTGCGTGATGCACAGCTTCTGGG
CGGCGCGCTCGAACCCTCGTTCACGTATAACGGCATCCAGCGCCTGTAGTGTTCTGTAGTCCGGACGTTTCAT
TGTTGTGTCATGCTCCTGTTATTTTTTTCCGCACTATGACATAAATTTGCCGTCGGTACAGATGAAATCCGTA
GCCTGTGTACGGACACGGAGATTTTTCCCGACAATGTTCTATAATGCGGCTGTATTTTCACACCACAGGCGAA
ACGATCTATGACGCAGGATGAACTGAAAAAAGCGGTAGGATGGGCGGCACTTCAGTATGTACAGCCCGGCACC
ATTGTGGGCGTTGGTACAGGCTCAACGGCCGCACACTTTATTGATGCGCTGGGCACAATGAAAGGCCAGATTG
AAGGCGCGGTCTCCAGCTCGGACGCCTCGACTGAAAAACTCAAAGGCCTCGGTATTCACGTGTTTGATCTCAA
CGAAGTGGATAGCCTGGGTATCTATGTCGATGGCGCGGACGAAATCAACGGTCATATGCAGATGATCAAAGGC
GGCGGCGCGGCATTAACCCGTGAGAAAATTATTGCTTCCGTGGCGGAGAAATTTATCTGTATTGCGGATGCCT
CTAAGCAGGTGGATATTCTTGGCAAATTCCCGTTGCCGGTCGAAGTGATTCCGATGGCGCGCAGCGCGGTCGC
TCGTCAACTGGTGAAGCTGGGCGGGCGTCCGGAGTACCGTCAGAACGTGGTCACGGATAACGGCAACGTGATT
CTGGATGTTTATGGTATGGAAATCCTTGATCCCATCGCGCTGGAAAACGCCATTAACGCGATTCCGGGCGTCG
TGACCGTGGGGCTGTTCGCCAACCGTGGCGCCGATGTCGCACTGATCGGCACGCCGGACGGGGTGAAAACCAT
CGTAAAATGATCGCGCCAGGCGTATTTCCTGCCGCGTAAAAAATTCGGTCAGGGCAAATTTGGTGACATGTAT
CACGTTTAATCGTTTGCCCTGCCGATCCCTCCAAAGAAAAAGTTATTCCCGGTATTTTCATCTGCTATTTCCC
GCTGTATGCGATTCCTTCACGACGCTGGCGCAAACGTTCATATTGCCGTGATAATTTTTTTGATATGTTGAA
GGACGGATGCAAATCCACACACAACATCAGATGGCAAAAAAAGACAGGATCGGGGAAATGGCAAAGGTATCGC
TGGAGAAAGATAAAATTAAATTTCTGCTGGTTGAAGGGGTGCATCAAAAGGCGCTGGAAAGCCTTCGTGCGGC
AGGTTACACCAACATTGAATATCACAAGGGCGCGCTGGACGCTGAACAGTTGAAAGCGTCCATCCGCGATGCC
CACTTCATCGGTCTGCGATCCCGTACTCATCTGACAGAAGAAGTGATTAACGCCGCAGAGAAGCTGGTGGCGA
TTGGCTGTTTTTGCATCGGCACCAACCAGGTGGATTTAAATGCGGCGGCAAAACGCGGTATTCCGGTCTTTAA
CGCGCCATTCTCGAATACCCGTTCCGTGGCGGAGCTGGTGATTGGCGAGCTGTTGCTGCTGCTGCGCGGCGTG
CCGGAAGCGAACGCCAAAGCGCACCGCGGCGTATGGAATAAACTGGCGGCGGGATCGTTCGAAGCGCGCGGTA
AAAAGCTTGGCATTATCGGTTACGGCCATATAGGAACGCAGTTGGGCATTCTGGCGGAATCGCTGGGGATGCA
TGTCTATTTCTATGATATTGAAAATAAACTGCCGCTCGGTAACGCAACTCAGGTGCAGCACCTGTCTGAATTG
CTGAATATGAGCGATGTGGTCAGTCTGCACGTACCTGAAAACGCCTCCACCAAAAACATGATGGGCGCAAAAG
AGATTGCCCTGATGAAGCCGGGTTCGCTGCTGATTAACGCCGCGCGCGGTACGGTGGTAGATATCCCGGCTTT
GTGCGACGCGCTGGCGACGAAACATCTGGCGGGGGCGGCTATCGACGTCTTCCCGACGGAACCGGCCACTAAT
AGCGATCCGTTTACCTCTCCGCTGTGCGAATTCGATAACGTGATCCTGACGCCGCATATCGGCGGCTCGACGC
AGGAGGCGCAAGAAAACATCGGTCTGGAAGTCGCAGGAAAACTGATTAAGTACTCGGATAACGGCTCGACGCT
GTCCGCCGTGAACTTCCCGGAAGTGTCGCTGCCGCTGCACGGCGGTCGTCGCTTGATGCACATCCACGAAAAC
CGCCCAGGCGTATTGACCGCGCTTAACCAGATTTTTGCCGAGCAGGGCGTTAACATTGCCGCGCAGTATCTGC
AAACCTCCGCCCGAATGGGGTATGTGGTAATTGATATAGAAGCCGATGGCGATGTGGCGGAGAAAGCGCTGCT
GGCGATGAAGGCGATTCCAGGTACCATTCGCGCCCGTCTGCTGTACTAATTTCCTCTAAATGGGCAGGAGATG
ACCTGCCCATCCTTTCTCTGGTTTCCCTGCCTCAACATCTCTTCGTGTAACGCATACATTCTGTTTTCGAGCC
TTGCCGCAATTGGTAATTAAAGGCAGCGTTGCGTGTTGTACAGCAATAAAAGGAATTTACGATAAGGGCTCAG
CATAGCGCTGAAACGGGAAAGCCCCTGGCGAAGCAGGGGCTCTAAAAAGGAAAGGGACGATGATGAGATACGT
CATCATGCTGCTGATCCTGTTAATCATCGCTACGCCAGCATATTAACAGAGAGTACGCAGGAGGGAGGTTCGC
CTCCCTCACCCTTCACCGAGAAATTTAGGTCAAAAAAGCGACGCTGTCAATCGACAAAACACGGCTACCACTC
CCAAATTTTGGACGGCGTCACCACCGCAGGCAGCGGGATATCCCACTCTTCAACTGGCAGTTTTTCTACTAAC
TGGCAATCATGCGCGTAGCCCACGGGCTGGATCTTATGCTGCTGCCAGTTTTGCAACGTACGGTCATAAAATC
CGCCGCCCATCCCCAGGCGCTGCCCGCCTTCGTCAAACGCCACTAATGGCGTTACCAGCACATCCAGCTTTGC
CAGCGGCAATACGTCGCGGACATCAAGCCGTGGCTCCCGGATCTTTAAGCGGTTGGTCACCAGCGCACTCTGC

GGATGATAGTGCAGGAACAGGAGGTTGCCAGGGCTGAAGGGATGAAGAACCGGAAGGTAGACGCGCTTGCCTG
CTCGCCATAGCTGTTCAATCAGCGGCTGGGTGTCGAGTTCGCCATCAAAGGAGAGAAACACGGCGACAGTGTG
CGCCATCACGACGGGAGGAAAGCTGAGCATTCGGGCGGCGGCCTGTTGACCAAAGCGGCGTTGCTGCTCTGGC
GTCAGCGCACGACGACGTTGCCGAATCATTCGGCGGATTTCTTGTCGTGATAAAGAGAGTTCGGGAAGTTGCG
TCATGGCTGTCGCCAGTCAGAAGGGGAATCTCCGAGATGCCGCCGCAGGCTGTAACCCTTGAACCCTTGGTTC
AAGGTGAATGTGTCGTCACAGTTTTAAGGCTTCTCGGACGAACCGAGCATGCTCACCAACCGCGGAGCGCCAC
ATTCTTGTGGTATGAAATATCGGCTCAGGGGACTGGCCCGCTTGCAAACATCTCAGAGAAATTCTGTTTTCAC
AATCACTTTACCATAGTAAAGCGAAAAGTGTTATTCAAAGTTTTGGCCCGTTTTTTCGGTTATGCGACCTTGA
TCAAGCAACGCCTGTTCGATGGTCTGTTGTAGCATCCGAATACGTTGTTCCATGCTCGCCGCGTAATCGCGGG
TCTTCGCTTTTTCCTGAGTTAACTCATAGCTGATATTCAATGCGGCAATGAAAACCAACTGCTCAGTATTTGT
GACTCTAGTGCGAACTTTCAGATCTTGCAACCGCTGATTCAGATCGTCCGCAGCCTGATTCAACGCATCCCTT
TGGTCAGGCGGGCAATTCACTCGCAGTGAACGGCCAAATATTTGGATATCGACGGGTTGTGCAGACATGCCAC
CTTCCTGCTGATTGACTGCGCTGCCTTCGTCTCCAGACCCAGGGTCCGCGAAGGGGCGACACTATAGCTACCC
TGGTGTGAAGATACAAGCCCTTTTCTGGTTCACCAGGGTCCAAAGTGGTAGCATATCATGAATATCCTCCCTT
TGATGACGAATGCTTATGTCTATACAGAACGAAATGCCTGGTTACAACGAAATGAACCGGTTTTTGAATCAAC
AAGGGGCCGGTCTGACCCCGGCTGAAATGCATGGTTTAATCAGCGGGATGATTTGCGGCGGCAACAACGACAG
CTCATGGCAGCCGCTGCTGCACGACCTGACGAACGAAGGTCTGGCCTTCGGGCATGAGCTTGCTCAGGCGCTG
CGCAAAATGCACGCGGCGACCAGCGACGCGCTGGAAGACGACGGCTTTCTTTTTCAGCTTTATCTGCCTGAAG
GCGACGATGTCAGCGTATTCGATCGCGCAGATGCCTTAGCCGGGTGGGTTAACCACTTTTTGCTTGGCCTCGG
CGTAACGCAGCCTAAACTTGATAAAGTCACCGGCGAAACGGGTGAAGCGATTGACGACCTGCGTAATATTGCG
CAGCTTGGTTATGATGAGAGCGAAGATCAGGAAGAGCTGGAGATGTCGCTCGAAGAGATCATCGAATATGTCC
GTGTTGCGGCGCTGTTATGTCATGACACTTTCACGCGCCAACAGCCGACCGCGCCGGAAGTGCGTAAACCGAC
GCTACACTAAGAAAAAACGAAATGGAGGCCATGTCATGACTCAGCAGGAATACCAACGCCGTCGCCAGGCATT
ACTGGCGCAAATGCAGCCCGGCAGCGCCGCGCTGATCTTTGCCGCGCCGGAGGCGACGCGCAGCGCAGACAGT
GAATATCCGTATCGCCAGAGTAGCGACTTCTGGTATTTCACCGGTTTTAACGAACCGGAAGCCGTGCTGGTAC
TGATTAAGAGTGATGACACCCACAACCACAGCGTTTTGTTCAACCGCGTTCGCGACCTGACTGCGGAAATCTG
GTTTGGTCGCCGTTTAGGACAGGATGCCGCACCGGAAAAACTGGGCGTTGACCGGGCGCTGGCGTTTAGCGAA
ATCAACCAGCAACTCTTTCAGTTGCTTAATGGTCTGGATGTGGTGTACCACGCGCAGGGCGAATATGCGTATG
CCGACGAGATTGTTCTGGCTGCGCTGGGGAAGCTGCGTAAAGGCTCCCGCCAGAATCTGACCGCGCCGGCCAC
CATGACTGACTGGCGACCGATCGTCCATGAGATGCGCCTGTTCAAATCGCCGGAAGAGATTGCGGTCATGCGC
CGCGCCGGGGAAATTAGCGCTCTGGCGCATATCCGCGCGATGGAAAAATGCCGTCCGGGGATGTTTGAGTATC
AGCTGGAGGGTGAAATTCACCACGAATTTAATCGCCATGGCGCGCGCTATCCCTCCTATAACACCATTGTCGG
CAGCGGCGAAAATGGCTGTATCCTGCATTACACTGAAAACGAAAGTGAAATGCGCGACGGCGATTTAGTGCTT
ATCGACGCGGGTTGTGAATATAAAGGTTACGCGGGCGACATCACGCGTACTTTCCCGGTGAACGGGAAATTTA
CGCCAGCTCAGCGTGAAATTTATGACATCGTTCTGGAATCGCTGGAGACCAGCCTGCGACTGTTCCGTCCTGG
TACCTCTATTCAGCAGGTGACCGGCGAAGTCGTGCGCATCATGATAACCGGGCTGGTGAAGTTGGGGATTTTG
CAAGGAGAGGTTGATCAACTGATTGCCGAGAATGCGCATCGTCCTTTCTTTATGCATGGCTTGAGCCACTGGC
TGGGGCTGGATGTTCATGATGTCGGCGTTTATGGGCCGGATCGCTCCCGCATCCTGGAGCCGGGCATGGTGCT
GACCGTAGAGCCAGGCCTCTATATCGCGCCGGATGCCGACGTGCCGGAAGCGTATCGCGGCATTGGCGTTCGA
ATTGAAGATGACATTGTCATTACCGAAACCGGTAATGAAAACCTGACCGCTGGCGTTGTGAAGAAGGCGGATG
ACATTGAGGCATTAATGGCGGCGGCGCGGCAGCAATGAGCGTCATTATCGTTGGCGGCGGCATGGCAGGCGCA
ACGCTGGCGCTGGCCATCTCTCAGTTCAGTCATGGGACGTTGCCGGTACACCTGATTGAAGCGAAAGCGCCTG
AAGCCGACGGTCATCCCGGCTTTGACGCCAGAGCGATTGCGCTGGCGGCGGGCACTTGCCAGCAACTGGCGCG
AATTGGCGTCTGGCAGGCGATTTCCGATTGCGCAACGGCAATCAACACCGTACACGTCAGCGATCGCGGTCAT
GTCGGATTTGTGACTCTCGACGCGCAGGATTACAGCCTGGCAGCGTTGGGTCACGTTGCGGAGCTGCATGATA
TCGGGCTGCGTCTGTTTGCGCTCTTGCGTAAAGCGCCAGGCGTTACGCTGCACTGCCCGGAGCGCGTAGCCAG
CGTGTCGCGTACCCAACAGCAAGTTAACGTCACTCTGGAAAACGGCAATGTGATAGCTGGCAGCGTGCTGGTG
GCGGCTAACGGTACCCACTCTGCGCTGGCGTCAGCCTGCGGCGTTGACTGGCATCAGGAGCCTTACGAACAGT
TAGCGGTGATAGCGAATGTCGCGACCGCTATCCCGCATCAGGGACGCGCCTTTGAACGTTTTACTCCCAATGG
CCCGCTGGCGATGTTGCCGATGTCGCATGGCCGATGTTCGCTGGTTTGGTGTCACCCACTCGACCAGCGCGAT
GAGGTGCTGAGCTGGTCCGACGCGCAGTTCTGCCATGCATTGCAGGCCGCGTTCGGCTGGCGGCTGGGGCGAA
TTACTCAGGCCGGTAAGCGCAGCGTGTATCCGCTTGCCCTGACTACGGCGGCAAAAGTGTTTACTCACCGGAC
TGTCATGGTCGGCAATGCGGCGCAGACGTTGCATCCTATCGCCGGACAAGGGTTTAATCTCGGTCTGCGCGAT
GTCATGAGCCTGGCGGAAACGCTGATCGCGGCACAGGACGCGGGCGAGGATATTGGCGCCTGGCACGTGTTAT

CGCGCTATCAGCAGCGTCGTCAGGTTGACCGGGATACGACGATTGGCGTAACGAACGGTCTGGTCCATCTGTT
TGCAAATCGTTGGGCGCCGCTGGTTATTGGGCGCAATATCGGTTTAATGGCGATGGAATTATTACCGCCTGCG
CGCGATGCGCTGGCACAGCGGACCTTAGGTTGGGTTGCGCGTTAAGCCGATCTGTAGGCCGGATAAGGCATTT
ACGCCGCCATCCGGCATCGTGTTCGTAAAAATACCGGATGGCAGCTTCGCCTTATCCGGCCTACGGTTTTGAT
TGTTCCGGTTTCCAGGAGTAAATAGTGCAAAGTGTTGATGTAGCAATAGTTGGCGGAGGCATGGTGGGACTGG
CTGTCGCCTGCGGCTTGCAGGGGAGCGGTTTGCGCGTTGCGGTACTGGATCACGTTGTCCCCCAGCCGTTGGC
GAACGATGCGTCGCCTCAGCTTCGGGTGTCGGCGATTAATGCCGCCAGCGAAAAACTGCTTACCCGTCTTGGC
GTATGGTCAAACATTGTGGCGCGACGGGCAAGTTGCTACCACGGTATGGAAGTCTGGGATAAAGACAGCTTTG
GCCGTATTACGTTTGATGACGCAAGCATGGGATATAGCCATCTGGGGCATATTGTTGAAAACAGCGTCATTCA
CTACGCATTGTGGCAAAAAGCGCAGCAAGCGGCGGATATCACCTTAATGGCGCCAGCGGAGTTACAGCAGGTC
GCCTGGGGGAGAACGACGCCTTCCTGACTTTAAAAGACGGCGCGATGTTAACCGCGCGTCTGGTGATTGCCGC
GGATGGCGCCAACTCCTGGCTGCGTAACAAAGCGGATATTCCCTTAACTTTCTGGGACTACCGTCACCATGCG
CTGGTAGCGACTATCCGCACCGAAGAGCCGCACGGCGCGGTTGCTCGCCAGGTGTTTCATGGTGAAGGTATTC
TGGCGTTTTTGCCGTTAAGCGATCCGCATCTGTGTTCCATTGTCTGGTCGCTTTCTCCGCAGGATGCGGAGCG
TATGCAACAGAGCGGCGACGAGGCGTTTAACCAGGCGTTGACTATCGCCTTCGATAATCGGCTGGGCTTATGC
CGCCTGGAAAGCGAACGGCAGGTTTTTCCGCTGACGGGCCGCTATGCTCGCCAGTTTGCCGCGCATCGTCTGG
CGTTGGTCGGCGATGCCGCGCACACCATCCATCCGCTGGCAGGGCAGGGCGTCAACCTCGGCTTTATGGACGC
GGCCGAACTGGTGGACGATCTGCGCCGGCTTCAGCGCCAGGGCAAAGACATTGGCCAACATCTTTATCTCCGT
CGTTATGAGCGTAGCCGTAAACATAGCGCGGCGATGATGCTTGCCGGGATGCAAGGTTTTCGTGACCTGTTTG
CCGGCGAAAACCCGGCAAAAAAATTGCTGCGCGACATCGGGCTGAAACTGGCGGATACGCTCCCCGGCGTCAA
ACCCCAACTGCTTCGCCAGGCAATGGGGCTAAACGACCTTCCGCAATGGCTTCGTTAAGCGTCTGACTGCACG
CCAGCAAGAGTTTGAAACCCGTCACACTTTGCTTTCCCGGCTATCGCGCCGGGGGGCTTTCCTCGTTTGAAAT
AATCTAATTTCACCGCTTATTTCGCATTATATTTTCTAATGACACTATTTTTGGCGAACGCGATTTTCGACAT
TTTTTCCGCATTTAATATTAATTTATGTTGTGATAAATCGTATCAGCTTCATTTTTATGGAGGTTATTTAGCG
TAATAGTGGTTTTTTGTGCTGATGGGGTTTTGGCGTTTTTTAGTCATAAGCTAATGTGATGGTAAATTCAGCC
TTATGGTTTAGCGCCTGGTTCGGTGGTAAGTTCAGGAGCAAGAGAACGTTTGCGTCGGCGCAGGAAAAAGGGC
GTCGATGCCAGGTTTCGTGGCGAAGATTTCCCATGAAAATGGTTGTTAGCCCCCGCTAATTCAACGAGGAAAA
GATGGCCCAACAGACGCCTTTGTACGAACAACACACGCTATGCGGCGCCCGCATGGTCGATTTTCATGGCTGG
ATGATGCCCCTGCATTACGGCTCTCAGCTTGATGAACACCACGCTGTGCGCACCGATGCCGGGATGTTTGATG
TATCGCATATGACCATTGTCGATCTTCATGGCAGCCGCACACGAGAGTTTCTACGTTATCTGCTGGCCAACGA
CGTCGCGAAGCTAACGAAGACGGGTAAAGCCCTCTACTCCGGTATGCTTAACGCATCGGGCGGCGTGATTGAT
GACCTTATCATCTACTACTTCACTGAAGATTTCTTCCGTCTCGTTGTTAACTCCGCCACCCGCGAAAAAGACC
TCTCCTGGATTACCCAACACGCTGAACCTTATGCCATCGACATTACCGTTCGTGACGACCTGTCGCTGATTGC
GGTACAGGGGCCGAATGCGCAGGAAAAAGCGGCGACTCTGTTTACTGACGAACAGCGCCATGCAGTAGAAGGT
ATGAAGCCGTTCTTCGGCGTTCAGGTGGGCGATCTGTTTATCGCCACCACCGGTTATACCGGCGAAGCGGGCT
ATGAAATCGCCATGCCGAATGAAAAAGCGGCGGATTTCTGGCGTGCGCTGGTGGAGGCGGGCGTGAAGCCGTG
CGGTCTTGGCGCGCGCGACACGCTGCGTCTGGAAGCTGGCATGAACCTGTACGGTCAGGAGATGGATGAAGGT
ATCTCTCCGCTGGCGGCTAATATGGGATGGACTATCGCCTGGGAACCTGCTGACCGCGACTTTATTGGCCGTG
AGGCGCTGGAGATGCAGCGTGAAAAAGGCCATGAGCAGCTTGTCGGTCTGGTGATGACCGAGAAAGGCGTATT
GCGTAATGAACTGCCGGTGCGTTTTACCGATGCGCAGGGCAATCAGCAAGAAGGCATTATCACCAGCGGGACG
TTTTCCCCAACGTTGGGCTACAGCATTGCGCTGGCGCGTGTTCCGGCGGGCATCGGCGAGACGGCGATTGTAC
AAATTCGTAACCGTGAAATGCCGGTAAAAGTGACTAAACCTGTTTTTGTGCGTAACGGCAAAGCCGTCGCGTG
ATTCATCCTTTTTTGGAGATTAATTGATGAGCAACGTACCCGCAGAACTGAAATACAGCAAAGAACACGAATG
GCTGCGTAAAGAAGCGGATGGCACTTACACTGTTGGCATTACTGAACACGCTCAGGAGCTGTTGGGCGATATG
GTTTTTGTTGATCTGCCGGAAGTCGGCGCGACCGTAAGCGCGGGCGACGACTGCGCCGTGGCGGAGTCCGTAA
AAGCGGCGTCCGACATTTATGCGCCGGTAAGCGGCGAGATCGTGGCGGTAAACGATGCGCTGAGCGACTCCCC
GGAGCTGGTGAACAGCGAACCGTATGCCGGTGGCTGGATCTTTAAAATCAAAGCCAGCGATGAAAGCGAACTT
GAATCGCTGCTGGATGCCACCGCGTATGAGGCATTGTTAGAAGACGAATAATGTTGTGCCGGATGGCGCTAAT
GCTTATCCGGCCTACTCACTGCAACGCGAATGATTTTGCGTATTCGTTTTCGCACGCGTAGGCCCGGTAAGCG
TCAGCGCCACCGGGCAATTGAGTTAGAGACAATCACGATTCACTGCACGTTTCAGGAACCATCGCCCATGACA
CAGACGTTAAGCCAGCTTGAAAACCGCGGCGCTTTCATTGAACGCCACATCGGACCGGACGCCGCACAGCAAC
AAGAGATGCTGAATGCGGTTGGCGCCGAGTCGTTAAACGCGCTGACAGGTCAGATTGTGCCGAAAGACATTCA
GCTCGCGACTCCGCCGCAGGTTGGCGAGGCGGCAACCGAATATGCCGCACTGGCGGAATTAAAAGCCATCGCC
GGGCGTAACAAGCGCTTCACGTCGTACATTGGCATGGGCTACACCGCCGTGCAGCTACCGCCGGTTATCCTGC

GCAACATGCTGGAAAATCCAGGCTGGTACACCGCTTATACCCCGTATCAACCGGAAGTCTCTCAGGGGCGTCT
GGAAGCGCTGCTCAACTTCCAACAGGTGACGCTGGACCTGACGGGGCTGGACATGGCTTCCGCCTCGCTGCTG
GATGAAGCGACCGCCGCCGCTGAAGCGATGGCGATGGCAAAGCGCGTCAGCAAACTGAAAAATGCCAACCGGT
TCTTTGTCGCCTCTGATGTCCATCCGCAAACTCTGGACGTCGTGCGTACTCGCGCCGAAACCTTTGGCTTTGA
CGTCATTGTGGATGACGCGGCGAAAGCACTGGATCACCAGGATGTGTTTGGCGTGCTGTTGCAGCAGGTTGGC
TCCACGGGCGAAATTCACGATTACAGCGCGCTGATTAGCGAACTCAAAGCCCGTAAAGTGATTGTCAGCGTCG
CCGCCGACTTTATGGCGCTGGTGCTGCTTACCGCGCCGGGTAAACAGGGCGCGGACATTGTTTTCGGCTCTGC
GCAACGCTTCGGCGTACCGATGGGGTACGGTGGCCCGCATGCGGCGTTCTTTGCCGCCAAAGACGAATTTAAA
CGCTCCATGCCAGGCCGTATTATCGGCGTCTCAAAAGATGCGGCGGGCAATACCGCACTGCGCATGGCGATGC
AGACTCGCGAGCAGCATATCCGTCGTGAGAAAGCGAACTCCAACATTTGTACCTCGCAGGTACTGCTGGCTAA
CATCGCCAGTCTGTATGCCGTTTATCATGGTCCGGTTGGTCTGAAGCGCATCGCTAATCGTATCCACCGCCTG
ACCGATATTCTGGCGGCAGGTCTGCAACAGAAAGGCTTAAAGCTGCGTCATGCGCACTACTTCGACACCCTGT
GTGTGGAAGTGGCTGATAAAGCTGCGGTACTGGCGCGTGCGGAAGCGGCAGAGATCAATCTGCGCAGCGATAT
TCATAACGCGGTTGGTATTACGCTCGATGAAACCACCACCCGTGAGAATGTGGCGCAGTTGTTCAATGTTTTG
CTGGGCGGCAGCCACGGTCTGAACATTGAAACACTGGATAAAGACGTGGCGCTTGATAGCCGTTCCATTCAGC
AAAGTATGCTGCGTGACGATGCGATTCTGACGCATCCGGTATTTAACCGTTACCACAGCGAAACCGAGATGAT
GCGCTATATGCACTCGCTGGAGCGTAAAGATCTGGCGCTGAACCAGGCGATGATCCCGCTGGGTTCCTGCACC
ATGAAGCTGAACGCCGCCGCCGAGATGATTCCTATCACCTGGCCGGAATTCGCTGAACTGCACCCGTTCTGCC
CGCCGGAACAGGCCGAAGGATATCATCAGATGATTAGCCAGCTTTCTGACTGGCTGGTGAAACTCACCGGGTA
CGACGCCGTCTGTATGCAGCCTAACTCCGGCGCACAGGGGGAATACGCGGGGCTGCTGGCGATTCGTCACTAT
CACGAAAGCCGCAACGAAGGGCATCGCGATATCTGCCTGATCCCGGCCTCTGCTCACGGCACTAACCCTGCTT
CTGCACATATGGCAGGAATGCAGGTGGTGGTGGTCGCCTGTGATAAGAACGGCAACATCGACCTGGACGATCT
GCGGGCAAAAGCAGAACAACATGCTGCGAACCTCTCTTGCATCATGGTGACTTATCCTTCCACCCACGGCGTC
TATGAAGAAACGATCCGTGAAGTGTGTGAAGTTGTGCACCAGTTCGGCGGTCAGGTTTACCTTGACGGCGCGA
ACATGAACGCCCAGGTCGGCATTACCTCACCGGGCTTTATTGGCGCGGACGTTTCGCATCTTAACCTGCATAA
AACTTTCTGCATTCCGCACGGCGGCGGTGGCCCAGGCATGGGACCGATCGGCGTGAAAGCGCATCTGGCACCG
TTCGTACCGGGCCATAGCGTGGTGCAAATCGAAGGCATGTTAACCCGTCAGGGCGCGGTTTCTGCGGCGCCGT
TCGGTAGTGCCTCTATCCTGCCAATCAGCTGGATGTACATCCGCATGATGGGCGCAGAAGGCCTGAAGCAGGC
AAGTCAGGTCGCGATTCTTAACGCTAACTACATTGCCAGCCGTCTGAAAGATGCCTATCCGGTACTGTATACT
GGTCGCGATGGCCGTGTGGCGCACGAATGTATTCTTGATATTCGCCCATTGAAAGAAGAAACCGGCATCAGCG
AGCTGGATATTGCCAAGCGTCTGATCGACTACGGTTTCCATGCGCCAACCATGTCGTTCCCGGTGGCGGGCAC
GCTGATGGTAGAGCCGACGGAATCTGAAGGTAAGGCTGAGCTGGATCGCTTTATCGATGCGATGCTGGCTATT
CGCGCAGAGATTGACCAGGTGAAAGCGGGTGTCTGGCCGCTGGAAGATAACCCGCTGGTGAACGCGCCGCACA
TTCAGAGCGAACTGGTCGCCGAGTGGGCGCATCCGTACAGCCGTGAAGTTGCCGTATTCCCGGCAGGCGTGGC
AGACAAATACTGGCCGACGGTGAAACGTCTGGATGATGTTTATGGCGACCGTAACCTGTTCTGCTCCTGCGTA
CCGATTAGCGACTACCAGTAATTTACTGATTCGACTGTTTTCTAAAGGCGCTTCGGCGCCTTTTCAGCTTGTT
AATAAAGTGCGCTTTGTTTATGTCGGATGCAGTGTGAGTGCCTTATTCAGGCTACAAAGTGTTAATAATTCAA
TATATTACACTAAATCGTTGGCTCCTGTCAGGCAACCTGGCGTTTGTATCAGTGTATTAGCCAGAATTAAATT
ACCGCTTTTTTAGAGCCTAATGATTCATTGTGCATCATTTTTACCTTGCTGTTATTTAATCAAAACAACACAG
TATATTTACATGTAGTTACAAATAGTTTCTCTCCGGAATCGTTATTTACCTCTCAATATAGTTACAAAAATTT
CCGTATATTAAATTCATCAAGTTGTTAATTTAAATTATAAATTAAAAATAAACATAAATAACGTAAATATTTA
TTAACTTAGCGCTTTGTTTTTTATACCTGGAGGGAATAATGAAACGATTTATATTTCTGCCATTAATGACATA
TTTTCTATTAATGAATGCAATGGCAGCTAACGGAGAAAATAATCCCAGTAATCTATTAGGTGCACCAGTTAAT
ACTGCTATTTCTGGTGGTTCAGTATTGCCGGAAGGAAAGTTATTAACCGCCGTGAATAGCTCATTTCGCGATA
AAGATCATCAAATCGAGGGGCACGGAAGTCCGGATGTTTACTCCCAGATATGGTTATTGAAGATTCGTTACGG
TTTGACCGACAGACTGGAATTATCAACTGTGGGTTCCTATATCAATAACAAACGAGACAATCTGTCTCCTGAG
CACATAGAGGGAATGGGCGACCAATCAGTGGGGGTCAATTATGCTTTAATGAGCCAGCGAAGAGGTGATCCGT
TTTGGGTTACGGTAGGTGGGGCAGTATTACTACCCACCGGGCAAGATGGAGATAATCATTTGCCTGGGAATAG
CGCCTGGGGAGGAAGAGTAACACTCACTTTAACAAAATTATTTACTCCTAACATTAAAGGGGATATGGGTTTT
GTCTATCAGGGACCATTTGAGCGCGGTAACCAGGACGTGAAACGAGGTAACGAATTCCAGTGGAATACCCAGG
TGCGGTATATGTTTAGCGATTTACCGCTGGATCTTGGTCTGGAAAGTGCCTACAGCAATAATGCCTCTGGAAC
GAAAAAACTGTCTAATGGCAGCGTAATTAATAACCATTCTGGAACTACCGAATGGGTTGTTGGTCCGTCATTT
AATATTGCCGTCGATTCACTGAAATTATGGTTTGGTGCCGGAGCCTTCTTCCCGGTAATGCAGGAAGCGAAGT
CGCCAACCAAAATGGAAGATGTACGCTGGGAATTTAAAATAGGTAAGACCTGGTAAGACTCGTTCAAAGTGTT

ACCCGCCGACTGGCGGGTAACGTGCAGGAGTTAAAGCTTCTCGCCGTTGCTGGCAATCACCTCTTTGTACCAG
TTGAAGCTCTTCTTACGCGAACGCGACATATCGCCCGTACCGTCATCGTGCTTGTTCACGTAGATGAAGCCGT
AGCGCTTGCTGTACTGACCGGTGGTGAACGACACGCAGTCGATGCAGCCCCACGGCGTGTAGCCCATCAGGTC
GACACCATCGTAAGTCACCGCTTTTTTCATCTCTTCAATATGGGCGCGCAGGTAGTCAATTCGGTAGTCGTCG
TTGATGCTGCCATCTTCTTCTACTTTGTCGTAAGCACCAAAACCGTTTTCGACAATAAACAGCGGCTTTTGAT
AGCGTTCGTACAGTTCACACAATGAATAACGCAGGCCCACCGGATCAATCTGCCAGCCCCAGTCAGATGCTTT
AACATAGGGGTTCGGTACGCTGCCTTCAAAACCGGAAATCGCATCGCCGCTACCGCCTTCGGCTTTGACCGCG
TTGGTCATGTAATAACTGAAACCAAGATAATCGCAGGTGCCTTCGCGCAGCACTTCAAGATCGCCATCTTCCA
TTTTGATGTTAAATCCGCGGCGCTCCCACTCGTTCAACACATAGGACGGGTAATAGCCGCGCAGCTGCACATC
GGTAAAGACGTAGCGTTCACGCATCGACTCCTGAGCAAACATCACATCTTCTGGTTTACAGGAGAAAGGATAC
AGCGCGACCATCGCCAGCATACAACCCACTTTCATCTGCGGGTTAATGCGACGTGCCGCTTTTACCGCCAGCG
CGCTGGCGACAAACTGATGATGTAAGACCTGATACATGGTTTCTTCTGGATTCTCATGCTCGGTATACACCAC
GCCGGAACAGCAGTAGCCAAACAGCGGCGCGCGCCAGTTTCGCTGGTTGTTAATTTCGTTGAAGGTCATCCAG
TATTTGACCTTATGTTTGTAGCGTTCAAACACGACTTCAGCAAAACGGACAAAGAAATCAACGACCTTACGAT
TGGTCCAGCCGCCGTAGTGCTGTACCAGATGTAATGGCATTTCAAAGTGGGAAAGGGTGATGACCGGTTCGAT
GTTGTATTTGAGTAACTCATCAAACATGTCGTCGTAGAATTTCAGACCGGCCTCGTTTGGCTGGGATTCGTCG
CCATTCGGGAAGATACGCGTCCAGGCAATAGAGGTACGGAAGCATTTGAACCCCATTTCGGCGAAAAGACGGA
TGTCTTCTTTGTAATGTCCGTAAAAATCCACCGCCTCGTGGTTCGGATAGTATTTGCCAGCAACGACATTCTG
GGTGATTTCGCGTGGCACGCCGTGTGCGCCGCCGGTTAGCACGTCGCAGATGCTGGGGCCTTTACCGTCTTTA
TTCCAGCCGCCTTCAACCTGGTGTGCAGCAACCGCGCCGCCCCATAAAAAATCTTTTGGCAAGGTGAGTTTTC
TCATTGTTGCATAATCTCAATTTATTTAGTCTGTTATCATTCATTCTGCACCAAATGACATGTAAAGCGTACG
CAAAATTCCAGGGTGCTCGCGTGTCGGGACAAGAACATATTCAGATCGTAACATTCACTTCAACGGGGCATAT
TCAAGGGATAACCACATGCAGCCAAACGACATTACTTTCTTTCAACGTTTCCAGAATGACATTCTGGCAGGGC
GTAAAACCATCACTATTCGTGATGCCTCCGAGTCCCACTTTAAAGCAGGCGATGTGCTACGCGTGGGGCGGTT
CGAAGATGATGGCTATTTTTGCACAATTGAAGTGACAGGAACGTCAACTGTGACGCTGGATACGCTGAACGAA
AAACATGCGCAACAGGAAAATATGTCGCTTGATGAGCTGAAACGGGTCATTGCTGAAATCTATCCCAACCAGA
CGCAATTTTATGTGATTGATTTTAAATGCCTTTGATAAATATGAACGCGTGTTAGTTGAGTTTCTGTGATTAA
ATTATTGAATAAGAAAGAAAATTTAATTTTGCGAATTTATTTTAGCTAACATGTGTTCACTGGAACCATTCTC
AGTTACGCTAGAGGCGAAGTTCATCGTTATGAACGTTTCTCCGGAGTAAGTTATGGTGCAAAAACCATTAATG
ACGCAGGGATATTCGCTGGCTGAGGAAATCGCCAACAGTATTAGCCACGGCATTGGATTGGTATTTGGTATTG
TCGGCCTGGTGCTGTTGTTGGTTCAGGCGGTAGAGGCGAATGCGGGTATGACGGCTATCGCCAGCTACAGTTT
ATATGGCGGGAGCATGATCCTGCTTTTTCTTGCCTCAACGCTTTATCATGCCATTCCTCATCAGCGGGCAAAA
ATCTGGTTAAAGAAATTTGATCACTGCGCTATCTATCTGCTCATTGCCGGAACCTATACGCCATTTCTGCTGG
TAGGTCTGGATTCGCCGCTGGCGCGCGGACTAATGATTGTGATCTGGAGCCTGGCGCTATTCGGTATCCTCTT
CAAGTTGACGATTGCGCACCGATTTAAAGTGCTTTCACTGGTTACGTATCTGGCAATGGGCTGGCTGTCGCTG
ATAGTGGTTTATCAGTTGGCGATTAAGCTGGCGATAGGCGGCGTGACGCTGCTGGCGGTAGGTGGCGTCGTTT
ACTCGCTAGGCGTAATTTTCTATGTTTGCAAACGCATACCTTATAACCACGCCATCTGGCATGGCTTTGTGCT
GGGCGGCAGCGTGTGCCACTTCCTGGCCATCTATTTGTATGTGGGACAGGCATAATTCCCGGATGCCCGTGCC
GGATGGCGGCGCTGTCGTACCTTATCCGGCCTACGAGAACAGCACGTGCGAGCCTGATGGCGTTGCGCTATCA
GGCTATCCGGCTTTTAACGTGATATCACTCTTCCAGCGAGTAGGGCAGCGGGACGATATGCAGGGTGTTGGCA
TCGTCGCGAACGCGGAAGACGCTTTCGGCTTCTAAATCGTTATTCATGACGGCCTGAACCAGTAACTGACCAT
CGTCCAACTGCGTCGCCGCCAGAATAGCGCCGGTACGACGCCAGTTCTCGCCCATCTGTAGCTCCAGATCTTC
TCCTGCTTCCGGTACCCGGCTGGCTTTGCCCGCCAGCAACCAAAGCGCGCGTTTATTGGCGCCGCGGAATTTT
GCCCGTGCGACCATCTCCTGTCCGGTGTAGCAGCCTTTCTTGAAGCTGATGCCGCCCAGCGCCTGTAGGTTAG
TCGCCTGGGGGATAAACTGACCGCTGTTCGCCGCGTCAATCACCGGAATGCCTGCTTCAATATCCAACGCCAG
CCACTGTTGACTGTTATTCAGTTCGGCTTCGCCATGCAACTTCTCCGTTAACATGTTAGCCGTCGCGACATCG
GTGACAAGCAGGAATCGTTCCGCAGGATGTTCAAACCATAGCAGCGTGGAGGCGCCGTCTCTGACCACCTGAT
TTTCGCTATTGGGCAGTTCGCTAAAGACATTAGCCAGCGCCGCGCGGGCCTGAAAACCTGCCACGCCAAGCAA
TACGCGTTCGTCATCCGGCGCGATGACTACTTTGGAAAATACGGCGTACTTTTTCAGCTCCGTTAATTGCGCT
TCACGCACGCTACGGCGTTCAATCCAGGCGAAACCGTCGCGCTCACGGAACAGGCGCAGAGTGCTCCACATCT
TACCTTTAGCATCGCAGTGGGCGGCGAGAAGGTGTTGCTGTTCAGTCATCTGGCTTACGTCAGCGGTGACCTG
GCCCTGAATATACTTTTCACTGTCTACGCCGGTAATGGTCGACAGCGCCCAGTCGTCAAGCGCGATCAGCGTC
AGCGGCAGGCGGGCTGACGATGAAGGATGACGTGGTGGAAAGGAGATAAAAGCCATAAATAATGTCCTGGTTA
GCTGCAACAATAATGGTTAATGGTAAAAGAGCCATCGGTCATTGCAAGTGCTTTAAATAGCCCATTGAGAGCG

GATTGATCGTTTTGCGAGGCGGTATCCAAAACGGATGAACATATTGATTCCCAGCCTTTTATTTCGCGATCGC
GTTCCAGATTACTGAGATTCCCGTAAAACAACGCCAAAAACACGTTACAATAGCCCGGTACTTCATTTTCGCG
AGGCAGGAAGAACATGGATATTCACAATAAAGCACGTATTCACTGGGCATGTCGTCGCGGTATGCGCGAACTT
GATATTTCCATCATGCCGTTTTTTGAACATGAGTACGATAGTTTAAGTGATGAAGAAAAGCGTATTTTTGTCC
GTTTGTTGCAGAGTGACGACCCGGATTTATTTAACTGGTTAATGAATCACGGTAAACCGGCTGACGCTGAACT
GGAACAGATGGTCCGACTCATTCAGACACGGAATCGGGAACGTGGTCCTGTGGCAATCTGATTTACGCGTCTC
ATGGCGCGCTCAGTGGATCTCATTGCTCATTCATGGGCTGGTTGCCGCGGTGATTTTATTGATGCCCTGGCCG
CTGAGTTATACCCCGTTATGGATGATACTACTGTCGCTGGTCGTGTTTGACTGTGTACGTAGCCAGCGCAGGA
TTAATACCTGCCAGGGAGAGATCAAGCTACTCATGGACGGGCGCTTACGCTGGCAGGGGCAGGACTGGACGCT
CTTGCGTCCGCCCTGGTTACTGAAGAGCGGGATGGTGTTGCGTTTACGCGCAGAGTCTGGACGTCATCAGCAT
TTATGGCTGGCGGCGGATAGCATGGAAGAAGCGGAATGGCGTGAGTTGCGTCGGATACTGTTACAGCAGCCGA
TATCAGGCCAACACTAACGGAACGGCGTAAACGCGGTTCCGTCGAGAGGGAAGTACAGCGAGTAAGGTTTCAG
GCGTAGTGTTCGGCCATTTCGCCGAGAATTTGTTCACACCAGGTCTGTATACGCTCATCGCTGAGATCGTACT
GGTTCGTTTCATCCAGCGCCAGGCCGACAAACAGTTGCCCATCCGCAATGACCGGTTTATTGCTGGTAAATTC
GTAGCCTTCTGTGGGCCAGTAGCCGACAAATTTAACGCCTTTGAGGGCAAGTTTGTCATGCAACATACCCAGC
GCATCCAGGAACCACTCGCCGTAGCCGAGCTGGTCGCCCATACCGTAAAGCGCAACGATTTTGCCTTCCAGAT
TGAGATCGTCCAGTTGCTCCCAGACGGCTTCCCAGTCCTCCTGAATTTCGCCAAAATCCCAGGTGGGGATGCC
TAAAATGAGCACGTCGTACTGCTCCATTAAGGCGGGGGCGTCATCCTTGAGGTTATGTAATGTCACCAGCTCC
GGGCCGAGGATATCGCGAATTTTCTCTGCGGCCATCTCGGTGTAGCACGTGCTGGAACCGTAAAAAAGACCCA
TGTTCATTACGTTACATCTCGATTCTGCTATAGCGTTGCGGGTAGTGTAACAGAATCACTGCATTCACGGCAC
AATGAGGCATAATGCACCAAAAGTAAGCGTGAAGTAGGGAGCGCGAATGGAACAGGATTTAGCGCGTATTGAG
CAGTTTCTGGATGCCCTGTGGCTGGAACGAAATCTGGCGGAAAATACATTAAGCGCGTATCGTCGCGATTTAT
CGATGGTCGTTGCGTGGTTGCATCATCGCGGAAAAACGCTTGCGACGGCGCAGGCTGATGATTTACAAACGCT
ATTGGCTGAACGGGTGGAGGGCGGATACAAAGCGACCAGCTCCGCACGCCTGTTAAGCGCTATGCGGCGCTTT
TTCCAGCATCTGTACCGTGAAAAGTATCGTGAGGATGATCCGAGCGCGCAGTTGGCCTCGCCTAAGCTTCCGC
AGCGTTTACCGAAAGATCTTAGCGAGGCGCAGGTTGAACGGCTCTTGCAGGCGCCGCTTATTGATCAACCTTT
GGAGCTGCGCGATAAAGCTATGTTGGAAGTGCTGTATGCCACCGGTTTGCGCGTGTCGGAACTGGTGGGGCTG
ACAATGAGCGATATCAGTCTGCGGCAGGGCGTGGTGCGGGTTATCGGTAAAGGCAATAAAGAGCGGTTGGTGC
CGTTAGGTGAAGAGGCGGTATATTGGCTGGAAACGTATCTGGAGCATGGGCGTCCCTGGTTACTCAATGGCGT
ATCGATTGACGTATTATTCCCCAGCCAGCGTGCGCAGCAGATGACGCGGCAGACCTTCTGGCACCGTATTAAA
CATTATGCGGTACTGGCTGGAATCGATAGCGAAAAACTATCGCCGCATGTTTTACGTCACGCTTTCGCCACGC
ATTTGCTGAATCACGGTGCCGACTTGCGCGTTGTACAAATGCTACTTGGGCATAGCGATCTTTCGACGACACA
AATTTATACGCATGTCGCAACGGAGCGTTTGCGTCAACTTCATCAACAGCACCATCCGCGGGCATGAGTGCTG
ACAGGAAAGGATAGTTTATGAAAAAGCGTTTTATGATGTTCACTTTACTGGCGGCGGCGTTTTCAGGCGTGGC
CCATGCGGATGATGCGGCTATCCGTCAGTCGCTGGCTAAGCTGGGCGTTCAGAGTACGGAAATTCAGGCCTCG
CCAGTGGCAGGGATGAAAACCGTCCTCACTCACAGCGGCGTACTGTACGTCACTGATGACGGCAAACATATTA
TTCAAGGGCCGATGTATGACGTGAGCGGCGCGCATCCGGTAAACGTGACGAACAAACTGCTGATGAGCCAGCT
AAATGCGCTGGAAAAAGAGATGATCGTCTATAAAGCGCCGGATGAGAAACATGTCATTACTGTTTTTACCGAT
ATCACCTGCGGCTATTGTCATAAGCTACATGAAGAGATGAAAGATTATAACGCGCTGGGGATCACGGTACGTT
ATCTGGCCTTCCCGCGCCAGGGGCTGGAAAGCCAGGCGGAGCAGGATATGAAATCTATCTGGTGCGCCAAAGA
CAAAAACAAAGCGTTTGATGACGCCATGGCAGGTAAGGGCGTGAAACCGGCAAGCTGTGACGTGAACATTGCT
GATCATTATGCGTTGGGCGTGCAGTTGGGCGTCAGCGGTACGCCAGCCATTGTATTGAGTAACGGCTATGTGG
TGCCTGGCTATCAGGGACCGAAAGAGATGAAAGCGTTTCTTGATGAGCATCAAAAACAGACCAGTGGTAAATA
ATACGCGTGAAACAACAGAGACAACTTCGTCGGCGCGAGGCTGATGAGACGGCGGAACTCCCCGCCGATCTTC
CTCCATTACTACGACGTTTATATGCCAGCCGGGGCGTTCGTAGCGCCCGCGAACTGGAGCGCAGCGTGAAAGG
AATGCTGCCCTGGCAACAGCTTAGCGGCATAGATAACGCGGTGGAGATCCTCTACAACGCCTTTCGCGAAGGT
ATCCGCATTATTGTTGTTGGCGATTTTGACGCCGACGGCGCGACCAGTACTGCGCTAAGCGTATTGGGAATGC
GTGCGTTGGGATGTGACAACATCAGTTATCTGGTGCCTAATCGCTTTGAGGACGGCTACGGTTTAAGCCCGGA
AGTGGTCGATCAGGCGAAGGCGCGCGGCGCGCAGCTTATCGTCACCGTAGATAACGGCATCTCATCCCACGCC
GGCGTAGCGCATGCAAAGACGTTGGGGATTCCGGTGATCGTGACCGATCACCACCTGCCTGGCGATACATTGC
CGGAGGCCGAAGCGATTATTAATCCCAATCTGCGCGACTGCGAATTCCCGTCTAAGTCGCTGGCGGGCGTCGG
CGTGGCGTTTTACCTGATGCTGGCGTTGCGAACATTTTTGCGCGACAAAGGATGGTTCGACGAGCGCAACATT
GCGCCGCCGAATTTGGCGGAGCTGCTGGATTTAGTGGCGTTGGGAACGGTAGCAGACGTTGTGCCGCTGGACG
CTAACAACCGTATTCTGACCTGGCAAGGGCTAAGTCGTATTCGTGCCGGGAAATGCCGTCCGGGAATTAAAGC

GTTGCTGGAGATATCGAATCGCGATCCGCAGCAGCTTGCCGCCAGTGATTTAGGCTTCGCGTTGGGGCCTCGC
CTGAATGCCGCCGGCAGGCTGGATGATATGTCCGTTGGCGTGGCGTTACTGTTGTGCGACAACCTCGGCGAAG
CGCGTGTTCTGGCCAGCGAGCTGGATGCGCTTAACCAGACGCGCAAAGAAATAGAGCAGGGGATGCAGGCGGA
AGCGCTTATCCTGTGCGAAAAGCTTGAGCGCAGTAGTGAAACGCTTCCGGGCGGTCTGGCGATGTATCATCCT
GAATGGCACCAGGGCGTGGTCGGAATTCTGGCGTCGCGCATTAAAGAGCGTTTTCACCGCCCGGTGATCGCCT
TTGCGCCTGCGGGCGACGGCACGCTAAAAGGCTCAGGCCGATCGATTCAGGGGCTGCATATGCGCGATGCGCT
GGAACGGCTGGATACCCTTTACCCTGATCTGATGATCAAGTTCGGCGGCCATGCGATGGCGGCGGGATTGTCG
CTGGAAGAGCATAAATTCGAGCAGTTTCAGCAACGGTTTGGCGAGCTGGTGACGGAATGGCTCGATCCTGCCC
TGTTGCAAGGCGAGGTGATCTCCGATGGTCCATTAAGCGCGGCGGAGATGTCTATGGAAGTGGCGCAACTGTT
GCGGGATGCCGGACCGTGGGGGCAAATGTTCCCGGAACCGTTATTCGATGGTCGTTTCCGTCTGCTACAGCAG
CGTCTGGTGGGCGAGCGTCACCTCAAGGTGATGGTGGAGCCTGTCGGCGGCGGCCCGCTGTTGGATGGCATCG
CATTTAATATTGATACGACCTGTTGGCCGGATAACGGCGTGCGGGAGGTAGAACTGGCTTATAAACTGGACAT
TAACGAGTTTCGCGGCAACCGTAGTTTACAGATTATTATCGATGATATTTGGCCGCTATGATCGGGGCGATTT
AACAACACTTTCGCTGTAATTAAGGGGCGAAATCCGCTAATATTTCGCCCTTATAACCGCATTTTGACAAGTC
AAATAAAAGAAATCAGACCATGTTTGAAATTAACCCGGTGAATAACCGCATTCAGGACCTCACGGAACGCACC
AACGTTCTTAGGGGGTATCTTTGACTACGATGCTAAGAAAGAGCGTCTGGAAGAAGTAAACGCCGAGCTGGAA
CAGCCGGATGTCTGGAACGAACCTGAGCGCGCGCAGGCGCTGGGAAAAGAGCGTTCATCGCTCGAAGCGATCG
TCGATACGCTTGATCAAATGACTCAGGGGCTGGACGACGTTTCCGGGCTGCTGGAACTGGCGGTAGAAGCTGA
CGACGAAGAGACGTTTAACGAAGCCGTCGCGGAACTGAATACGCTGGAAGAGAAACTGGCGCAGTTGGAATTC
CGTCGGATGTTCTCCGGCGAGTACGATAGCGCCGATTGCTATCTCGATATTCAGGCCGGTTCCGGTGGTACTG
AAGCGCAGGACTGGGCCAGCATGTTGCTGCGTATGTATCTGCGTTGGGCGGAAGCGCGCGGCTTCAAGACGGA
AATCATTGAAGAATCGGAAGGGGAAGTCGCCGGTATTAAGTCCGCGACTATCAAAATCTCCGGCGAATATGCC
TATGGCTGGCTGCGCACTGAAACTGGCGTACATCGTCTGGTGCGTAAAAGCCCGTTCGACTCCGGCGGTCGCC
GTCATACCTCGTTTAGCTCCGCGTTTGTGTACCCGGAAGTGGACGACGATATCGATATCGACATTAACCCGGC
GGATCTGCGTATCGACGTCTATCGCGCGTCTGGCGCAGGCGGTCAGCACGTTAACCGTACGGAATCCGCCGTG
CGTATCACCCATATTCCCACCGGGATTGTGACGCAGTGCCAGAACGACCGTTCGCAGCACAAAAACAAAGACC
AGGCCATGAAGCAGATGAAAGCGAAGCTTTATGAACTGGAGATGCAGAAAAAGAACGCTGAAAAACAGGCGAT
GGAAGACACTAAGTCCGATATCGGCTGGGGAAGCCAGATTCGTTCCTATGTCCTGGACGATTCCCGCATTAAA
GACCTGCGTACTGGGGTGGAAACCCGCAATACACAGGCGGTACTGGACGGTAGTCTGGATCAATTTATCGAAG
CAAGTTTGAAAGCAGGGCTATGAGGAATCAACATGTCTGAACAAAACGCACAGGGCGCTGACGAGGTAGTCGA
TCTTAACAATGAAATGAAGGCACGTCGCGAGAAGCTGGCTGCACTGCGTGAGCAGGGTATCCCGTTCCCGAAC
GATTTCCGTCGTGACCGTACCTCAGACCAACTGCACGCAGAGTTTGACGCGAAAGAAGCTGAAGAACTGGAAG
CGCTGAATATTGAAGTGTCCGTCGCGGGCCGTATGATGACTCGCCGTATTATGGGTAAGGCCTCTTTTGTTAC
CCTGCAAGACGTAGGCGGCCGCATTCAGCTGTATGTGGCGCGCGATGATCTGCCGGAAGGCGTTTACAACGAG
CAGTTCAAGAAGTGGGATCTTGGCGATATCCTCGGCGCGAAAGGTAAGCTGTTCAAAACCAAAACCGGCGAGC
TGTCTATCCACTGCACCGAACTGCGCCTGCTGACGAAAGCGCTGCGTCCGTTGCCGGATAAATTCCACGGCTT
GCAGGACCAGGAAGCGCGCTATCGCCAGCGTTATCTGGATCTCATCTCTAACGATGAATCCCGTAACACCTTC
AAAACCCGTTCGAAGATCCTGGCCGGTATTCGCCAGTTTATGGTTGCTCGCGGCTTTATGGAAGTCGAAACCC
CGATGATGCAGGTGATCCCAGGCGGCGCGTCTGCCCGTCCGTTTATCACCCATCACAACGCGCTGGATCTGGA
CATGTACCTGCGTATCGCGCCGGAACTGTACCTCAAGCGTCTGGTGGTTGGCGGCTTCGAACGCGTATTCGAA
ATTAACCGTAACTTCCGTAACGAAGGCATCTCCGTGCGCCATAATCCTGAGTTCACCATGATGGAACTCTATA
TGGCGTATGCGGACTACAAAGACCTGATCGAGCTGACGGAGTCTCTGTTCCGTACGCTGGCGCAGGATGTTCT
GGGCACCACCCAGGTTCCGTACGGTGATGAAGTGTTTGACTTCGGTAAGCCGTTCGAAAAACTGACCATGCGT
GAAGCGATCAAGAAATATCGCCCGGAAACGGATATGGCTGATCTGGACAACTTCGATTCCGCGAAGGCGATTG
CTGAAAGCATCGGCATTCACGTCGAGAAAAGCTGGGGTCTGGGCCGTATCGTCACCGAGATCTTTGACGAAGT
CGCTGAAGCGCATCTGATTCAGCCGACCTTCATCACCGAATATCCGGCTGAAGTGTCTCCGCTGGCGCGTCGT
AATGATGTGAACCCGGAAATCACTGACCGCTTCGAGTTCTTTATCGGCGGTCGTGAAATCGGTAATGGCTTTA
GCGAGTTGAACGATGCGGAAGACCAGGCGCAGCGTTTCCTGGATCAGGTTAATGCAAAAGCGGCGGGCGACGA
CGAAGCCATGTTCTACGACGAAGACTACGTGACGGCGCTGGAACACGGGTTACCGCCGACAGCGGGTCTGGGA
ATTGGTATCGACCGTATGGTGATGCTGTTTACTAACAGCCACACGATTCGCGACGTTATTCTGTTCCCGGCGA
TGCGTCCGGTGAAATAAGATGTAAATTGCCGGATGGGGCATTAGCCGCCATCCGGCATCTCTTCGTTCAGACG
TAGCGCCATCAGGCAATTTTTAACGCTGGCAGTAATTGAGCAGTCGTTCACGCGCTTGTTCGTCTGACGCTTG
CATGACCATCCACGGGCTAAATGCCCACGGCGTAGCGAGAAGGCTTTTCCATACGCTTTTAAATTCGCTCCAC
TGATAGTCCATCACCTCTTCGCTGTTAACCTGTAACACACTGGTTGCCCGCGCGGCAAATACCGGACAGACTT

CATTTTCCACAATACCGTTTGGATCTGTCGCTCGATAGCTGAAGTGCGGATAGACGGGGGTGAGATCGGTAAT
TTCCACGCCGAGTTCGAACCGACAACGGCGGATGATCGCCTCTTCGGTGGTTTCGCCTTGTTGCGGATGACCG
CAAACCGAATTCGTCCACACGCCTGGCCAGGCTTTCTTGCTCAGCGACCGTCGTGTTACCAGCAACTGGCCAT
CTTCGTTAAATAGCCAGCAGGAGAAAGCCAGATGCAAAGGCGTATTAAGCGTATGAGCCGCATACTTTTCCAG
TGTGCCGGAAGGCTTATCCTGCTCATCCAATAAAACGACATGTTCCTCAGTCATGAAAGCTCCTGAAAATTAA
AATGACCCGGCAGCATGGCCAGGCCATATACTCAGACGATCAGCGTAGCGCATAACAGAAAGAAAGGCATTAC
CAAAACAGGCCCTGACAGAACGCAGACGAGCGCCATTGTGCCGTGAGTCGATATTATGCTGGCATATCGGATA
TTCGCGGCATCATCATCCACGCGGACCCTTTCCGGCGTCAATGGATTGGGCATACGGCATTTGGCTGCTATCA
TAAGGCGTCTGTCAATAGAGTGAGGAATGGTTTTGAGCGCAGGACGCCTGAACAAAAATAGCCTGGGTATTGC
GGTGCTGTTATGCACAGGGCTGCTGCTGGCGGGGTGTTCCAGTAATTCCGGCTCAGGAACGTATTCCGGCTCC
GTCTATACCGTTAAGCGCGGCGATACGTTGTATCGTATATCGCGCGCTACGGGAACCAGCGTGAAAGAACTGG
CCCGACTGAATGGCATTTCTCCGCCTTATACTATTGAGGTGGGGCAGCGAATTAAAGTGCGCGGCAGCGCAAA
GAGTAGCAGTTCAACACGCAAAACCAGCAATAAAACAGCAACAAAAACTGCTGCGGTCAGACCGTCATCTTCG
GTGCCAAAATCCTCCTGGCCGCCGGTGGGGCAGCGTTGTTGGGTATGGCCTGCTAACGGTAAAGTGATTTTAC
CTTATTCCACCGCGGAGGGCGGAAATAAGGGGATCGATATTGCGGGCGCGCGCGGAACGCCTGTTTATGCGGC
GGGAGCGGGTAAAGTTGTCTATGTCGGGAACCAGCTGCGCGGTTACGGCAATCTCATCATGATTAAGCACAAT
GAAGATTACATTACGGCTTACGCTCATAATGACACCATGCTGGTTAATAATGGACAAAGCGTTAAAGCGGGGC
AAAAAATCGCTACGATGGGCAGCACGGATGCTGCATCCGTAAGACTACACTTCCAGATTCGCTACCGGGCGAC
GGCCATCGATCCGCTGCGTTATCTGCCGCCGCAGGGGAGTAAACCGAAGTGCTGACGGCGAATTAATCGCCAG
TCAGCCAGTAAGTCTCTTGCCAACCAGAGCGTAAGGTTTATAATGCCTTACGCATCTCAAAGCGGGCGTAGTT
CAATGGTAGAACGAGAGCTTCCCAAGCTCTATACGAGGGTTCGATTCCCTTCGCCCGCTCCAGACAAATATCT
TCTAGTATTTACTAAAACCACGAATTTACAGCAATTTCCCGTGTCGGCCCCACAGTACACAGGGTTTCAATAC
CTATGTCGGACGGCTATTATCAGCTGGCGACTCCGGTAATGACTTTATCATTCAGCCCGCAACACAACGATGC
CCGGTCTGGAATGCATGTATTCCATAAACGGTGTATCCACCACTTTTCTGTTTGCGGCCAATGAGGAGGCGTT
CCTGAACCACACCTGTTCATCATGGCGAACTACGATACCCACATGTGAAACGTCCAGCCCGTCAAGCGGGGAG
TAGACACCGATATAATCCCCGTTTTTTAGGTGGTTCATGACCTGCTGGTTGATGGCTCTTCCGGGAATGTAAT
TTATTTTACGAGGATGGATGCCCAGACCAGGAATGTACTCGCCGCCATCAGGTTTATGGTTAAGCTGCTTGTC
AACAACGACATAATCAGGGCTGATATCCGGCGTTACATCCCGTGCATTACGCGGTGTTGCGGCGAACCAGTCA
GAGAAGAAGTGCCGGCGGCTGAGATAAGCAACTTTGCCCGCGGCGTAGCGAGTCCTGGCAAGATTATGCAGAA
ACGATTTCTGATTATCGCTGCGGGCCAGGGCTTCAACGTAATCAGCCAGTGTAAAGCAGTCCACGCCGTTGAA
ATTCGCTACAAGGACTTCGGGGATGCCGGGCCCACCGATGAGCGTATCCGCCTGATAAGGTGTTCCGAGAAAA
GCAGACGATACGCGGCTGATAACTTCTCCATGGTTTTCGCCTTTATAAGGAATGACATCCGACTGGATAATCC
GGCTTATTTTTTCTGCCGTAACGCTGTCCATGCTGGTCTGATATTTTTCTGTGCCAGGGACGGCACAGGCCGT
CAGATGCGCCGACATAAGCAAAATTGTCCATGTATTTACCTTCATTATCATCTCCGGAAAGGTTCAAATCCCT
TTTTTACCTTTACCGGAAATGGTTGTGATGAATACAGAAAAACGCCAGAGGTTGATTACAGGTTGAGTAATAT
GAACGCTGATGATGATAAATGTGAAGATCATCATACGACTTTATTTTATTCGCCGTTGTTTTTTGCCTGATCG
TCAAAGCCAACATGCCCCTTCAGGCTGGCTTATAAGTCAGAGTGTAGATTGCGACGTGTCCGATCAGCGATTC
TGACGCGTCCACCTCGGTGTGGCGGTCGTCGCTGACGATCACTTTGACATGGAGAATTTTGTACTCACTGAAT
GTTCGAACCGGACCACCTCCCAAGAGGTGAAGGAATAGTCCATCAGACGGATTTCCTGTCGCGCGGTCTCAAT
TATCTTAAGAACAAGCTGGAGATCTGTACCTTCCGGATAAAATCCTGCCTCAATGATGGCTGCGTAAGTATGT
GGTACTGGCGCACACAGAGCCAGACCGAGCAAACCGGGCAGAAGCCATCGGCACATATGTTTTTTATGCATTG
TTTGCATGGTATTTCCTTCATTGGATGTATTCAGAAATGCCGGAGTCGGGGAAACTCGTTTTCCCCGATTGCC
GGGGTTGGACAAGGATGCCCTGCGGAGTGCGCGTTAAGCGACTGGATTATTTTTTCCTGTATATCTCTTTGAC
ATATACATCGCCTCTACTCATAATGGATATACGCGTTGTATATCCAGAAAGGAGGCAGTTATGCACACAACAC
TTTTTTTTAGTAACCGGACCCAGGCAGTCAGATTGCCTAAATCGATATCCTTTCCGGAGGATGTGAAACATGT
CGAAATCATTGCGGTTGGTAGAAGCAGAATCATTACTCCAGTCGGAGAATCCTGGGACAGCTGGTTCGATGGC
GAAGGCGCCAGTACTGATTTTATGAGTACCAGAGAACAACCCGCAGTACAGGAACGGGAAGGATTTTGATGCT
GAAATTCATGCTTGATACCAATACCTGTATTTTCACCATCAAAAATAAGCCCGAACACATCAGAGAACGCTTC
AACCTCAATACATCCCGAATGTGTATCAGCTCCATCACCTTAATGGAGCTGATTTACGGTGCTGAAAAAAGCC
TGGCGCCGGAGCGTAATCTTGCCGTCGTGGAGGGATTTATCTCCCGCCTTGAGGTTCTGGATTACGATACACA
GGCAGCGATACATACCGGTCAAATCCGTGCCGAACTGGCCCGCAAGGGAACACCTGTCGGGCCTTATGACCAG
ATGATTGCTGGCCATGCCGGTAGCCGCGGACTGGTCGTCGTCACAAACAATCTCCGCGAATTTGAACGCATTC
CGGGTATCCGAATCGAAGACTGGTGCTAATTTCTGCATCAGATGTTATCCCCGGATTCAAACTCTTTTCCGGA
TTTAGTGGCACAGTCAATTTGGTTAGCTGCTTAATGGTGGTTTGCATCACATTTTTAGCTGGTGGTATTTTTT

TATGTTGACTGGAGATATCTGCCAACAGCATAACTTTTACGGCCAGATAATAAATAAAAGAGTACCAAAATGT
CGTATTCTGACATGTTTACGAAGGGAGTACCGCTCCCTTCTTTTTTTGTTTCCTGGATACTCACTGAACAATG
CCGTTACTGCCCGATAACTGCCGCCCCCCCCCTCTCCATAGCCGTTTCCTGCAACTTAAATCACAGACGTTGA
TTTACCAGGCCGATATGCCTTCTTTCCCGTTATATATCCTTCATACTTCAAGTTGCTTATGTGTTGGCTACGG
ATTACTCGGTCCATCCATGGGCCTCGCCCTGACGGGCCGTCGCACGCGACGTTCAAATCTGCTCCCGGCAGAA
TTGTCGCTCACCCCAGTCACAGAGTTATCTATGCTCCCGGGGACTCACTCCCTTGCCACCTTTAAGCAACTCG
AATTATTTTGGGTATATATCAGTGAACTTCTATTTTTGTCAGTTTTTATTTTTCAGTTTATTTATTATTAAAT
TCTGTTTCTGGCCTGGATATATTTTCGTGAATAGCCTCCCGACAGCCGGAGATAAAGGTATTTGCTGGCGTAA
CAGCGCTCATAATTTAAAAGATTTATTTATAGTTTGTTATCACGGAAAGATTTTAACTTAATACATGCCAGTA
AGGCATCAGGTAAAAATAACCGGAGAGAAGAAGATGAACAAAATGCTATTAGCCGGAAGTGCCGGTATTGTTC
TGCTGTCTGCTGCAGCCAGTCCGGTATGGGCAGACGATAATGCCAGTACATTTTCACTTGGATACGCCCAGTC
CCATACTAATCACGCCGGGACGTTGCGTGGGGTACGGCTGGCAAACAACTATGAAATGTCACCGGACTGGGGG
CTGACAACCTCATTTGCATGGCTAAATGGAAGCCAGCGCTATTCCGATGAAAGCAGTAATGGTCGTGTAACAA
CCCGTTATTACTCCCTGCTTGCAGGCCCCCCTGGGAAAAAAAAAAACCATTTAAGTCTGTTAAGCAAAGGGGG
CCCCGTATTATTGCATCAGCGTGATCACGGGATAAATGAGTCTGACAGTAAGGTCGGATATGGTTATTCTGCT
GGCGTGGCTTATACCCCGGTCAGTAGTGTCGCTATAACTCTGGGTTATGAGGGAGCGGATTTTGATGCTACCC
ATAACAGCGGAAGCCTTAATAGCAATGGTTTTAATCTTGGCGTGGGTTACCGGTTCTGAATAGGATAATCCGA
TAAAAAATGTTACTTTGTGGAGATGATGGATTATGAAAAGCCATATTATGCCAGGCGTTGCGCTGATGGTTAT
CCTGTTTTCAGGAAATATTATGGCCGCAACAAACATGACTGATAACGTTACTCTGAATAATGACAAGATATCC
GGACAGGCCTGGCAGGCCATGCGTGATATCGGAATGTCGCGATTCGAGTTGTTTAATGGTCGCACCCAAAAGG
CGGAACAACTGGCTGCGCAAGCAGAAAAACTCCTGAACGATGACAGCACTGACTGGAATCTGTATGTCAAATC
AGATAAAAAAGCCCCAGTTGAAGGAGACCACTATATTCGGATAAATTCATCGATTACGGTAGCAGAAGATTAT
CTCCCTGCGGGCCAGAAAAATGATGCTATTAATAAAGCAAACCAGAAAATGAAAGAAGGAGATAAAAAAGGGA
CTATAGAGGCCCTGAAACTAGCAGGCGTCAGTGTTATTGAAAATCAGGAACTCATTCCTCTTCAGCAAACCCG
TAAGGATGTGACCACAGCGCTTTCCTTGATGAACGAAGGAAAATACTATCAGGCAGGTCTATTACTGAAATCA
GCTCAGGATGGCATAGTTGTAGACTCTCAGTCAGTTCAGGAATCACCCACGCACTCCGTGCAGCACGACGCCG
CGCATTAATATCCCCCAGCCTGCTGTATACATTACAGCAGGCAGCATTATGTCTGACAGCTATTACCAGTTTG
TTCTTTGCTGTCGGCATTGTTTTTAAATTTCATCCGCGAAGTTTAGAAATGTCAGGGTACCTGCATTTATATC
TATTGTGATGCTCATCACAAATACAGAAGAATATCATTTCATTTATTATTTTTTATTCCTTCCACTTATGATG
GTTCATAATGATTTATTAAGTTAATGTTTTGGAATTAAATGCCTTTTACTTGCGTTATATCCAGTTTTTTATG
CTGCCAGTAATTTCATTGTTATATATGATTAGTATTATGTTTTGTAAATATCCAGTGTAATGGAAAAGCAAAA
CATACATTCCCGATAATATATTACAAAATATTTCATGTCCGCTGCGAATATCTGTCTGTTAGGTATTCACCCG
CTTATAATTACATTAAAAAGTATTTCTTTGATGATTATTCTTAAATTACGATCATAATCATCGATCGTATCGA
TCTATTTTTATTGTTTTAATTTGAATAAGAATTTTCTTACAACTGATTAGGCATTAGCTGAAATCAATATTTT
TCGATGGAAAGTTCAGGTGCTTCGTTTAACACCAGGCGTTTATTATTCATACGAATCTTTTCTGAACGCTTTC
ATTAATACCCTCAGAGTTGTTTTCAGCCTTTGCAAAATAATTCTCATTTACCCAAAGGACATATTATCTATGC
GTAATAAAATAATACTTGCCATGGCGGCTGCCGGTATGATGTATGGCGCTTCTGTATATGCAGTCGATCCACC
CACAGCAGGCCCGTTCGGCTCAGGTAAAATTACCTTCACTGGCACCATCACCAACTCCCCCTGTGATATCGCA
CCGGGCGACGATGCGATCACCGTTCCGTTTGGACAGATTTCTTACCGTAAACTGAATACCGCCGACGCAACTA
CGGAATCAAAACCGTTCACCATTCATCTACAGAACTGTGCTTTCGATCCGAATACCCCGGACACAGCTGGCTC
CGCAGGGAAAATGTCCAAAGTAACCGTATCTTTCTCTGGCGCAGCGAATACCAGCAAAAAGGCTTACACCACC
AGCGGTATCGCACAGCATGTTGGTGTCCAGCTACTGAAGAGTGACAACGCCACAATTATCGATCCCAACACCC
CGATGCCGGATGGCGACGCTCAGCAACTGCAGGCCGGAAACAACGAACTCAACTTCTTTGCCCGACTAATAGC
ACTGGATAATGGCGTCACTCCCGGTGACTTCAACGCATCCGTCACCTACACCCTGAAATACCTGTGATTTCAC
GCGGTAAATAACGACAGGGAGAAGCCGTCCACGGAGGGGATCGGACGGCTTTTATATCTGACTGTGCCTGCGG
GCTGTCTGTTACACGGACGACAACACGAAAGCGCCATGCCATGAAAATTACCCGGCTTGCCATTCTGATTACC
CTGACATTTTCCGTCCTGAAGTCGCAGGCCACGGAGTTCAATGCCAGCCTGCTGGACAGCGGGAACCTGTCTA
ACGTGGACCTCACGGCCTTCAGCCGCGAGGGGTATGTGGCGCCCGGTAACTATATTCTCGATATCTGGCTCAA
TGACCAGCCGGTACGGGAACAGTATCCGGTCAGGGTTGTACCGGTTGCCGGACGCGACGCGGCCGTCATCTGT
GTGACAACAGACATGGTGGCCATGCTGGGTCTTAAGGATAAGATTATTCATGGCCTGAAACCCGTTACCGGTA
TCCCGGACGGGCAATGCCTTGAGCTGCGCTCCGCTGACAGTCAGGTACGGTACAGCGCTGAAAATCAGCGTCT
GACCTTCATTATTCCCCAGGCCTGGATGCGTTATCAGGACCCGGACTGGGTGCCCCCTTCACGCTGGAGCGAC
GGCGTCACCGCCGGCCTGCTTGATTACAGCCTGATGGTCAACCGCTATATGCCGCAGCAGGGGGAAACCTCTA
CCAGCTACAGCCTCTACGGCACCGCCGGGTTTAATCTCGGCGCCTGGCGATTGCGGAGCGATTACCAGTACAG

CCGTTTTGACAGTGGGCAGGGCGCATCACAGTCCGATTTTTATCTGCCGCAGACGTATCTGTTCCGTGCCTTG
CCAGCACTGCGCTCAAAACTGACGCTCGGACAGACGTACCTCAGCTCCGCTATTTTTGACTCCTTCCGCTTTG
CCGGGCTCACTTTGGCCAGCGATGAGCGCATGCTGCCGCCCTCTCTTCAGGGGTATGCCCCGAAAATTAGCGG
CATTGCGAACAGTAATGCACAGGTCACCGTCAGCCAGAATGGTCGCATTCTGTACCAGACCCGGGTGTCGCCG
GGGCCTTTTGAGCTTCCCGACCTGAGCCAGAATATCAGCGGCAACCTGGATGTCAGCGTCCGGGAAAGCGACG
GTAGCGTCCGGACCTGGCAGGTCAACACGGCCAGCGTCCCCTTTATGGCACGCCAGGGACAGGTTCGCTATAA
GGTGGCCGCCGGACGTCCGCTGTACGGCGGGACGCATAACAACAGCACGGTCAGCCCTGACTTTCTGCTCGGG
GAGGCCACCTGGGGAGCATTTAACAACACGTCGCTGTATGGCGGCCTCATTGCCTCCACCGGCGACTACCAGT
CCGCGGCGCTCGGGATCGGCCAGAATATGGGGCTGTTGGGCGCTCTGTCTGCGGACGTCACCCGCTCAGATGC
GCGGTTGCCGCATGGGCAGAAGCAGTCCGGTTACAGCTACCGTATCAACTATGCCAAAACGTTTGATAAAACC
GGCAGTACCCTGGCGTTCGTCGGATACCGCTTCTCGGACCGCCATTTTCTGTCCATGCCGGAGTACCTGCAGC
GTCGGACCACTGACGGCGGCGATGCATGGCATGAAAAACAGAGCTACACCGTGACTTATAGCCAGTCCGTTCC
GGTACTCAACATGAGCGCGGCCCTCTCGGTAAGCCGGCTCAATTACTGGAACGCACAGTCCAACAATAACTAC
ATGCTCAGCCTCAACAAGGTGTTCAGTCTCGGCGACCTTCAGGGACTCTCGGCCTCGGTGTCGTTTGCCCGTA
ACCAGTACACCGGCGGGGGTAGCCAGAACCAGGTCTACGCCACCATCAGTATCCCGTGGGGAGACAGTCGCCA
GGTGAGTTACAGCGTACAGAAAGATAACCGGGGCGGCCTGCAGCAGACCGTGAATTACAGCGATTTCCATAAC
CCGGACACTACATGGAACATCAGCGCCGGTCATAACCGTTATGACACCGGCAGCAGCTTCAGCGGCAGCGTGC
AGAGCCGTCTGCCGTGGGGCCAGGCGGCAGCGGACGCCACCCTGCAGCCCGGACAGTACCGCAGCCTGGGCCT
GAGCTGGTACGGCTCGGTGACTGCCACCGCCCACGGTGCCGCTTTCAGCCAGTCGATGGCCGGGAATGAGCCC
CGCATGATGATAGATACCGGAGACGTGGCCGGTGTGCCGGTGAACGGCAACAGCGGGGTGACCAACCGCTTCG
GGGTGGGCGTGGTGAGCGCCGGCAGCAGCTATCGCCGCAGCGATATTTCGGTTGATGTGGCCGCCCTGCCGGA
GGACGTGGACGTCAGCAGCTCCGTCATCAGTCAGGTACTGACGGAGGGCGCGGTGGGCTACCGGAAGATTGAC
GCCAGCCAGGGGGAGCAGGTGCTCGGTCATATCCGGCTGGCTGATGGCGCAAGTCCGCCGTTTGGCGCCCTGG
TGGTGTCCGGGAAAACCGGCAGAACCGCGGGGATGGTCGGAGATGGCGGTCTGGCCTACCTGACAGGTCTCAG
CGGGGAAGACCGCCGGACCCTGAATGTGTCCTGGGATGGCCGGGTGCAGTGCAGACTCACCCTGCCGGAAACG
GTGACACTCAGCCGGGGGCCGCTGTTACTGCCCTGCAGATAAAAATATCCCTGTCACAGAGCAGGTCTAAAGG
AGAAAACAGGTGAAAAACAGCAGAAGAAGCCGTGTGATTTTACTCGCACTGGCGGCAGCATGGAGCCAGTACA
GCCCGGCGGCCGTCAATGTTGACCGGACCCGTATTATCATGGATGCGCCGCAGAAAACGGTGGCCATCACGCT
GAATAACGATGACAAAACCACGCCGTTCCTGGCGCAGTCCTGGGTGACGGACGCTGACGGGGTGAGAACCGAT
GCGCTGATGGCGCTGCCGCCGCTGCAGCGTATTGATGCCGGACAGAAATCCCAGGTCCGCATCACCCAGGTTC
GCGGGCTGACAGATAAGCTCCCTCAGGACAGGGAAACACTGTTCTGGTTTAACGTGCGGGGCGTTCCCCCGAA
GCCAGAAGACGACAACGTTCTGCAACTGGCCATGCAGAGCCAGCTGAAACTGTTTTACCGTCCGAAAGCCATT
ATCAGAAGCAGCAGTGACCAGCCGGAAAGAAAGCTGACTGCTGAGCGGAATGCGGGACATCTGACCCTCAGAA
ACCCGACGCCGTACTACATCACTGTGGCGTGGCTGGGAGCAGACCGTAGCCACAGGCTCAGCGGTTTCCGCGA
GGGCGTGATGGTACCGCCCCTGGGGAACCTGCCTTTAAAGGCTGTTCTGCCGGCAGAAACCCGGACGCTGTGG
GTGGGGTATATTGATGATTACGGGGGACTGCAGATGAATCGCTACACCTGCGATGCCCTGAACTGCGCGTTTA
AGGACGCAGGGGCGACATCATGACAGAATGGATTTTTAATCTGAAAACAAAACTGACCGTGCTGGTGATGATG
TTGTGCTCTTTATGTGTGACTAAAGTGTATGCGGTTGAGCTAGGTATAAATGAATGTGCAGTCACTTCCGGTC
AGAATATTAATTTAAGAAGTATAAATTTGACAACCGATGATTTTAAACCTGGCACGGATTCGGTTATCTATAC
AATCAATCAGGATGCAGTTTTTAAATGTTACATGGGATATGACACGCAATTTCCACAGCTGGTATTTAATCAG
GGCTACTTCAGTAAATTTACCAAGACACTAGATGCGATGGGACTTGGATTCAGAATGTCCATTAAAGAAACAG
GAAATGCTTCAAGTGCTGTTTCGTTCTCCTGGGATGAAATAAAGAGTACTCAGTCAGGAAATGAGTTACGAAA
AGAGTTTGGCACAAAACTACCTGTTGGGACTACAGAACGAAAAGTAAGGATAACACTGGATTTCTTATATACA
AAAGCATATTCAGAGAGTAGTGCAGTTACAGCTTTTACGGGGATAAGTAATGTTTTAAATATTGTACCTTTTT
CTTATTCTTTGAGACAAAACGGATTTATTTTATCTGGTTTTAATGTGAGAATTCTCAGAAACGGATTGGGTAA
AGTTGACATTGTTCCATTACAGGTCAACTTTGGGCATATTTATACGACTTATGAACCTTCACAGACCAGACAG
GCAAATTTTACAGTGATCGCCACGCAGGTTTTACGACCGGCTATGGGACAGGAATTCACGATCCCTCTGGCCA
TCACCTTCGGGAAAGGTGCACTGACACAGGATACCGGGCAAACTCTGAATCTTGTCAGCCTGGATGGCCCCAA
CAAAGGTCAGCCTAATGGACTCCGGCTTTCAATAAAAGATGATAAAGGAAAAGAGATTACTTTTGATAAACAA
GAAGTACTGGGGGATATTACTATTACAGGAGCTGTCACCGGTAATGTGTCGAAGGTATATACTGCAGTAATAA
CCCCGACCCCGGGGGGAAGTGTAAAGACAGGAACGTTTTCTGCTGCAATACCCGTTACTGTTACCTATAACTA
ATCGGGGGGGCAGATAATAAAAATAAAAGAACTGACTGAGTTATCGGGGAACTAATCCCGAATAAACCCACAT
TAATAATAAATACTATTTAAGAGTTGTTCTTCACCTCCGGGAAATCATTTACCCAGTTATGGAGAGGTTTTAT
GTGCCCTGATAATACACACGCAAAAAAGCAGTACCTTACCCCTGGTAACGATATTCATTATCCCGGACAGACC

AACCATGATGCCTGTTTTATTCCGGAAAGCGTCCGCCAGTATGCCGACGAGCCCCTGTATATCATTGTGGCAC
ACTGGTGCCTGTTGCAGCAGAACTGGGTTCAGCGTAACCAGATAGCAGAGGCGTTTCACATCACGGCCAGACG
GGCCAGCTACCTGATTGCCTATCTGCGAAGCAAAACATCGCGGGTGGTCAGCATCTGCCGTCACCAGACCCTG
CCTAATAAAGCACGACGCTATGAGATTTATGTTATCCGGGTTCTTGACAGTCCCACCCCGTCCACGCGCCGTG
AAAAAGCCGGACCGCCGCTGGTATCAAAACGCCGGGTCGGTAATGGTGACAGGTCGATGGCAAATGAACTGTG
GAACCGGCTGTGCAGTAACAGAAATGCGGGGAAAATCCTGAAAAAGAAGGAGGATGAGGATGACGGAACATAA
CCGGATGCCGGCCCGGCAGATTATCGTTTACGGTGACTGCTGGCCGGTGACGATCGCCGTGGCACACCTGGTC
CGGAGATTTCTGCCGGGCTGCAACTGTGAAACCGCATACAGTCTGCCTGTTCTGTTGCAGCAATTACGTCGGA
AACCGGAGGCCATACTGATACTGTGTCTGCGTCCCCGTGAACATCTCTTTTTGTTCTATTCCCTGAGACAGAT
ACTGCCGGACTACCCGGTTATGATTATCAGCGATGAGCTGTTTTTCAGCGACCGGGTGGTACTGAAGGTATAC
GGCGGCATCCCGGCGCTGCTGGAGCAGGAACTGGCGGAGATACTTATTCGGTGGCGGCGGGATGAACAGTGGG
CCGGGGGCGCACGCCTGCGCAGGACAGGAGGGCTGGACGCCTTTTTGTTATCTCCGGATCCGGTGACGGGCTT
TCTGGAAGTGCCACCGATATTTAACAATCCGAAGCGGTTGATGAACTATATGGATCAGTTGATGCACCGGGAG
ATACTGGCATGCGGAGTGAGCCTGGCCCAGTTACGCTTGCTTCAGGAAGTTTACCGTGGACGAGGAAGGTTGT
CAGCGTTGTGTGGAAGGCTGAATACACAGGAGAAGCAAATCTGGCAGGACAAATACCGGTTACTGGTGAAGCT
GGGAATGCGGAACCGATTACGGGAACTGCTGTTTGGTACCCGGTTTTGTAAATCACTGCAGCGAACTCCGTTT
ATCGCTCCGCAATAACCCGCCTCCGGCGACCCTATCCAGAAACACTGACACGCCGGCTAAACCCGGTGGCGGT
GGGGTATTTTTTGCCTGTGGTGTAGTGGAGTAAAATACAGAATTCAGGCGGGACGCTGAATGTATGGCCTTTC
ATACCGTTTGATAAGTAAAAACGCTTCTGGAATGAAACCCGATGTGAATGATACGGCCTGAGCAGAAGCGTTA
CATATTTACTGCATGATCCCGGTATAGCCATGAATTCCCATATACACGCCGACCAGACCAATCAGAATGCTCG
AAAAATAGGGCGCCCGCCGGGCAAGCGTCGAAAAACCACTCCAGCGCTTTGCTGCCTGTTGAACGCTTATCGC
CGCGCCAACGCCTACCGTCACCAGCGTTAATGCCAGGCCAAGACTAAAGCTCAGTACCATCGTGGCGCCCAGC
GTAAACGCTTTAAGCTGGATACAAATCAGTAAAACGGTGATCGCAGCCGGACAGGGGATAAGCCCTCCGGTCA
GGCCGAACAGCAGGATCTGTCCATTAGTCACTGTTTGACCATCAAAACGGCGTTGAATATCGGCAGCATGGGC
GCGTTCATGGGCATCCTGATACGCTTTTGACGTTGCACCTTCCGGATGTATGTGACCATGATGGTCATGGTCA
TGGTCATGGTCATGGTCATGGTCATGGTCATGGTCATGGTCATGGTCGTGATGGTGGTTTCCCGCCAGCCACT
GCTGCTCGCCTCGCCATGTCCGCCAGAACATCCAGCACGCGGTGCTCAGAATAATAATCGCAGAAATTAACTG
CAGCCATGGTTCCACTGATTGTGCGGTGAATGCCCGGCTAAGATACATCCCACCCAGCGCGATTAACCAGACG
ATCGCGGTATGAGAAAGCGTTGCTGCCAGACCGAGCATGACGGCCTGTTTAACCGTACCTTTAATGGCAATAA
TAAAAGCCGCCATCATAGTTTTGGAGTGCCCTGGTTCAAGCCCGTGCAAAATACCTAATAAAATGGCGCTGGG
AATGAAGAACCAGCCGTTTCCTTGCTGAAGAAGTGTCGAAAATTCACCCATGAGAATGATTCTTAGTTGTTTT
ATGTGGGTGTCATTCTACTCCCCCCAGTATGGAATACTACCCCCCAGTAGCATCGTAATGCTATAATTTGTAT
TCGTTAATGTTAGTGAGGTGTTGCAATGTCACATACCATCCGGGACAAACAAAAGCTTAAAGCCAGAACCAGC
AAGATTCAGGGGCAGGTGATAGCGCTAAAAAAAATGCTGGATGAACCGCATGAATGCGCGGCCGTGTTACAAC
AGATCGCGGCGATTCGCGGCGCGGTAAATGGCTTAATGCGCGAGGTGATTAAAGGGCATCTGACCGAACATAT
CGTTCATCAGAGTGATGAAGCCAGACGTGAAGAAGATCTGGACGTAATATTAAAGGTGCTGGATTCTTATATA
AAATAGCCGGGAGTTATATCCCGGCTATCTTTTACTTTAGCCGAAAAACATCACCGATACGCAAAGTAAACCG
ACGACCAACGTAATGAAATTACCTACTGAACGGTAGGGGTTCAATGCCGGTATCAGGTACGTTGACAACGTGG
GCATAATAAACAGAATCATCGCAATGAGCGGGCCGCTAATGGCATAAATCATAGAGATGGCATTCGGGTTAAT
ACAGCAAATAATAAACGTAATCCCGGAAACCAGCATGATAGACAGCGCGCGATTGAACGCCCGGCTTTTTTTC
ACGCCAACCTGCTGTAGGGTGGTTCTTACCATTTCGGTTGCGCCCTCGATGACGCCGAAATAGGTGCCGAGGA
AAGATTTCGACATGGCCACGACGGCAACGATAATACCGGAAATAGAGAGCCAGGCCGGCGCATTGGGCATCAT
CGAGAGCGCGGAAAGGATGGTAACGCCTTCGTTGCGCGCGTCTTCAATGTATGACGGCGGAATGGAGAGCAGG
CAGCTAAAGACAAAGAATAGCACGCTCAGGCAGATAATCAGGTAGGCGACCTTCATGATCTTTTTGCATTTAT
CCATGGCCTGATCGCCAAAATTTTCCCGTCTGTCGATGGCAAACGTCGAAATAATGGGCGTATGGCTAAAAGC
AAAAACCATCACCGGAATAGAAATCCATACCTGGTGCAACGTGTGACTATCCAAAGACATCTGCCCGGTCAGT
AACGAGGGTTGCCAGCTTCCGGTTAAGTAGAGCGAAAGGAACAAGAAGTAAGCGATAAGTGGAAAGACTAAAA
AGCCCATTACGCGAAGAGTGGCATGTCTTCCCATTAAGAAAATCATATTCAGGATAAGTACTACGCCGAAGCT
AACTGCCATCCGGATGCGAATATCTATCTGAATATGCTTCGCTAATTGCTCTGTCAGGGAATTGGTAATCGCC
ACCGCATAAATTAATACCACGACAAAAAACGCAATAAAATAAAGCGTGGTAATAATACTGCCGATTTTCTTGC
CGTAATAATGAGTAACGGCGCCAGTAATTCCCTCACCGGCAGAAGTCTTTGAGGAAAGAATAAACTGACACAG
CGCCTTATGCGGCCAGTATGTCAGAGGCCAGGCGACCAGCGCGGTAATAAATAGCACGATTGCGCCTGCCGAT
CCCAACTGGATGGGAAGGAAAAGAGTGCCTGCGCCTACAGCCGTTCCGTATAATGCAAAACTCCAGAGAGTCT
CATCTTTTGACCAAATTTTAGACATTATTTGAATTTATCAACTATAAAACACACAAAAAGAAGCGCAATTTAC

CATAAATAGCGTCAGTTGGGGGGCGTTTTAAGCAGATGGATGTCGTGGAGGCGGAGGGCCGGGAGCGTTGCGA
TCCCGGCAAAAAAGAGCAATATCATGCACTAACGCGCTGTTGGCGGCGAGTCACTATTCGTTGGCGTAAAACA
TCGTATGCCCAGTTGTACAGCATCGTGTACGGCAAAAAGAACAGGAAGAAACCAATTTCCAGGGTGAATGCTT
GTAGCAGGCTGACATTCAGTACCCAGGCGACAATACTCACGCCAATGACAATAAACCCACTTTCAAATCCTAA
CGCATGGAGTGCGCGCACTTTGGCGGTTCGTCTCACCTGATGCGCAGGCCACAGTCGATCAAACAGAGCGTTA
TAAATGATATTCCAGATCATGGCCGTCGTTGCCAGCAATATCGTCAGGCCGCCCATCTCTAGCACCGAGCGCT
GCATTAGCCAGGCGGTCGTTGGCGCCAGTATCGCAGTCGCAATACCTTCAAAACAGACAGCATGGAAAATACG
CTCCGGCAGCGAGCGACGTTGTATGGCGTCATGTTGCATAAACGAAAAAACCTTACTCTTAATCAAAATAAAG
AAATTTCCGCTATTTTATCGTTTAATGTGATACCTAAAAGATAATATCCATCGATAAAGTAGATAGGTTATGC
GTTACTCACCTGAAGCGTTAATGGCATTTGTTGAGACCGTCTCTTGCGGTTCGTTTTCTGCGGCGGCGCGCCG
CCTGCGTAAAAGCCAGTCCACCATTAGTACCGCCATTGCGCATCTGGAGGCGGATCTGGGATTTTCATTATTT
GATCGATCTTCGCGCCAGCCCGTTCTGACCGAAGAAGGAAAACGGGTGCTTAGCTATGTTCAGGCTATTTTAT
CGGCGAGCGATCGTTTGGATGAGGTGGCGCTGACGTTATCCGGCGAGACGGAGGCAAGACTCACGTTCGTCCT
CTCTGATACCCTGCACCCTGACAGTCTGGAAGAGTTAATGGCGCAGTTTGATCGTCAATTTCCGCATACCGAG
TTTGAATGCTTGATCGGAGAAAATGATGATGTCATCGACCTGTTGCAAAAAGGGCGCGCCCAGATAGGCCTGA
TTGAAGCGCGTGATGACTATCCCACAGACATTGGCGCGACGCGTCTGCCTGTTCAAAGCTGGATGGGGGTTTA
TGTGGCGACCACGCATCCGTTGGCGTCGCAGACGAACCTGCAATGGGAGCAACTGCATACCTGGCGCGAGCTG
CGTCTGAATACCTACCTTGAATCTAATGCCAACATTACGCGCGGCCCGGCCTGGTCAGCGCCAAACTATTTAT
TACTGTTGAGCATGGCGGTTCAGGGATTTGGCTGGTGCGTATTGCCCTGCGCGCTGGTGGAAGAGTTTGCGGC
GGAAAAACCGCTGGCCCAGCTGGAAGTTCCTGGCTGGCCGAGGGCGATTGCTATTGATTTGCTGTGGAACAAG
AAGACGCCGCCCGGAACGGCAGGGAACTGGCTTCGCTACCATTTGCAGCAGAAATAAGCTGCGTCACCGTCAA
ATCAGCGTCATTTCCTTTGTGATAAAACTCACTTTTTTAAACAATCAAGATAACTAATTTTAACAATGCTCTA
CTATGGCGGTGTCATTTTGCGCTGAGGTGGTAATGAAAGAGGTTGTGATAGTGGGGGCTTTACGGACACCCAT
CGGCTGTTTTCAGGGAACGCTGGCCCGTCATTCCGCCGTCGAGCTGGGGAGCATGGTCGTAAAAGCGTTAATT
GAGCGCACCGGCGTGGACGCTAACGCTATCGATGAGGTCATCCTCGGTCAGGTGCTGACGGCTGGCGCCGGGC
AGAATCCGGCGCGCCAGTCAGCCATCAAAGGAGGGCTGCCGACTACCGTTTCCGCCATTACTATTAATGACGT
TTGTGGTTCCGGTTTAAAAGCGTTGCACCTGGCGACGCAGGCTATTCAGTGCGGCGAAGCGGACATTGTTATT
GCGGGCGGGCAGGAGAATATGAGCCGTGCGCCGCATGTCCTCAATGACAGCCGTACCGGCGCGTTGCCTGATG
CCGACAATCTGGTGGATAGTCTGGTACATGATGGCTTATGGGATGCCTTCAACGATTATCACATTGGCGTGAC
GGCAGAAAACCTGGCGCGGGAATATGGCATCAGCCGCGAGTTACAGGATGCTTATGCGCTCAGTTCGCAGCAA
AAAGCCAGGGCCGCGATTGATACCGGGCGTTTTAAAGATGAGATTGTCCCCATCGTCACGCAACGTAATGGGC
AGACCGCAATTGTCGATACGGATGAACAACCGCGGGCCGACGCCAGTGCGGAGGGGTTAGCCCTGCTGCATCC
GGCGTTTGATAGTTTAGGTTCGGTGACGGCGGGTAATGCCTCCTCAATCAACGATGGCGCCGCCGCGGTGATG
ATGATGAGCGAGGCGAAAGCCCAGGCGCTGGGGCTGCCGGTGCTGGCGCGCATCCGCGCGTTTGCCAGCGTCG
GCGTCGATCCTGCGCTAATGGGGATTGCGCCGGTATATGCTACCCGGCGCTGTCTGGAGCGTGCCGGCTGGCA
ACTGACGGAGGTCGACCTTATCGAAGCCAACGAAGCGTTTGCCGCCCAGGCGTTGTCGGTAGGAAAAATGCTG
GAATGGGATGAGCGACGGGTAAACGTTAATGGCGGCGCGATTGCGCTGGGGCATCCCATTGGCGCTTCCGGTT
GTCGAATTCTGGTTTCACTGGTGCACGAAATGGTTAAACGCGACGCGCGAAAAGGTCTGGCGACGCTGTGTAT
CGGTGGCGGCCAGGGCGTTGCTTTAACCATTGAACGCGACTGATCCTGCGCCAGCCATCCTTTTCCTGCTTCA
GTATTCCGTTGTAAAAAGCCCTCTATCACGAGGGCAAAGCCAGTGACAGAACCCCCTTTTTCGTTATAGCGCA
GAACGTCTTGCCGCGTCGGGCATTGCAGACGCATACCGCTTTTAATTCAATTCGCACATCAGACCCCGTTCCA
AAACAAGCTCCAATTTTTATCTGGGTAACCGCATACAGCATCTGGCGCACATGTCTGAAAATCAGACATGTGC
GTAAGTTTTAGCGCCGTTCGTGATCGCTACAGCAGTTTTAAAAATAACCAACCATATAAATGAAACGTTGTTT
TATTTATTGTTGAAAACCTTTATCCAGGCGACTATGATGCCGTTAAATAAATCAAAACAGTGTTTTGAATTTT
ATGCCATTGCGATCGATTTATCATTGGAGGGAAAAGTGGACGTAAGGCAAAGCATCCATAGCGAGCACGCTAA
AACGCTGGACACCCAGGCGCTGCGACGCGAGTTTTTAATAGAAAATATTTTTGTCGCTGATGAATATACGATG
GTCTACAGCCATATCGACCGTATTATTGTCGGCGGCATTATGCCCGTCAGCCATTCTGTTGAAATCGGCGGAG
AGGTGGGTAAACAGCTTGGCGTTTCCCGGTTACTGGACCGCCGCGAGCTGGGCGTCATTAATATTGGCGGCGC
GGGCGCCATTATCGTTGACGGGCAGCGCCATGATATTGGTCATCGTGATGCTCTGTATATTGGTAAAGGGGCG
AAAGAGCTGGTCTTCGTCAGCAATGAGGCGAGCAGACCGGCGAAGTTTTATTACAACTGCGCGCCAGCGCATA
CCGCTTATCCGACGAAAAAAGTGTCGCCTGCCGACGTTGCGCCCGTCACCTTAGGCGATAACCTCACCAGTAA
CCGTCGCACCATCAATAAATACTTCGTACCGGATGTACTGGAAACCTGTCAGCTCAGTATGGGACTCACCGAA
CTGGCGCCCGGTAATCTATGGAACACCATGCCATGCCATACCCATGAGCGCCGTATGGAAGTTTACCTCTATT
TCAATATGGAAGAGGACAGTTGCGTTTTCCATATGATGGGGCAGCCTCAGGAAACCCGTCATATTGTGATGCG

TAATGAACAGGCGGTTATTTCCCCAAGCTGGTCAATACATTCTGGCGTTGGCACCAAAGCCTATACGTTTATT
TGGGGCATGGTGGGAGAAAATCAGGTCTTCGATGACATGGATCACGTTGCGGTACAAGATCTGCGCCAGTCGC
GACCGGTGATAACGCCTGTGCGTGACAGGCATGACAAAAATAAGGATTAACAATGATTTTGAATGCATTTTCT
CTTGAAGGTAAAGTCGCGGTTGTGACGGGGTGCGATACCGGATTGGGGCAGGGAATGGCGTTAGGGCTGGCGG
AAGCGGGCTGTGACATTGTCGGTATTAACATTGTTGAACCGACGGAAACTATTGCGCGCGTCACCGCTCTGGG
ACGTCGTTTTTTAAGCCTGACAGCCGATCTGCGTCAAATCGATGGTATTCCGGCATTACTGGAACGGGCGGTG
GCAGAATACGGTCATATTGATATTCTGGTGAATAACGCGGGTTTAATCCGTCGTGAAGATGCGATTGATTTCA
CGGAAAAAGACTGGGATGACGTCATGAACCTGAACATCAAGAGCGTGTTCTTTATGTCTCAGGCGGCGGCAAA
ACATTTTATTGCCCAGGGCAACGGCGGCAAAATTATTAATATCGCGTCTATGCTTTCCTTCCAGGGCGGCATT
CGCGTTCCGTCTTATACCGCGTCGAAAAGCGGCGTAATGGGAATAACGCGTCTGATGGCGAACGAGTGGGCGA
AACACAATATCAATGTAAACGCCATCGCGCCGGGCTATATGTCCACGAATAATACCCAGCAATTGCGCGCTGA
TGCACAACGTAGCGCCGAAATTCTCGACCGTATTCCGGCTGGACGTTGGGGGCTACCGGGCGATTTAATGGGT
CCGGTAGTCTTCCTGGCATCCAGCGCCGCTGATTATGTTAACGGCTATACCATAGCGGTAGATGGCGGTTGGC
TGGCGCGTTAATCCGCTACTGACAAATGCGTTTCTGGGAAAGTCGACTCTTTGGCCGAAAAAACTGATAAAGC
CCTGTCCTCGTACAGGGTTTTTTTATGATCTCATTAGCATAGTCCATATTGTGGGTTTAACTTAATCCATATA
TTGTTAAATAATAGCTATGATCAATGCTTTAACTTATTGAAATATTGGTGGTTTGAAAAAATACCCGGCAACG
GCGTTAAATTTAAAAAGTGTAATATCCATCACATATCGCTATAGCGTAGCCATTTAATCCATATTTATGCCGT
TTCCAGCCTGACACTTGAGGAAGAGTATCCCGTACTTTCAGGCTATGTCTTACTCTGTTGTGGCAGGAAAATA
TGGTCTCTATTAATCATGACTCTGCTTTAACGCCGCGTTCGCTTCGCGACACACGACGTATGAATATGTTTGT
TTCGGTTTCTGCAGCGGTAGCGGGACTGTTATTTGGTCTGGATATCGGCGTTATCGCCGGGGCGCTGCCTTTT
ATTACCGACCATTTCGTACTGACCAGCCGGCTGCAGGAATGGGTCGTCAGCAGCATGATGCTTGGCGCGGCAA
TTGGCGCATTATTTAACGGCTGGCTTTCATTCCGGCTGGGGCGTAAGTATAGCCTGATGGCTGGCGCGATTTT
GTTCGTGCTCGGCTCGCTGGGGTCGGCGTTTGCTTCCAGCGTGGAAGTATTGATTGGCGCCCGCGTGATACTG
GGCGTAGCAGTAGGGATTGCCTCCTATACCGCGCCGCTTTATCTCTCTGAAATGGCTAGCGAAAATGTTCGCG
GCAAAATGATCAGTATGTATCAACTGATGGTGACGTTAGGCATTGTGCTGGCTTTTTTATCCGATACGGCATT
CAGCTACAGCGGCAACTGGCGCGCGATGTTGGGCGTGCTGGCGCTGCCTGCGGTGTTGCTCATTATTCTCGTG
GTATTCCTGCCGAATAGTCCGCGTTGGCTGGCGCAAAAAGGTCGCCATATTGAAGCGGAAGAGGTGCTGCGTA
TGCTGCGCGATACCTCGGAAAAAGCCCGTGATGAACTGAATGAAATTCGGGAAAGCCTCAAACTCAAGCAGGG
AGGGTGGGCATTATTTAAGCTAACCGCAATGTTCGCCGCGCCGTGTTCCTCGGTATGCTGCTACAGGCAATG
CAGCAGTTCACCGGCATGAACATCATTATGTACTATGCGCCGCGCATTTTTAAAATGGCCGGCTTTACCACCA
CGGAACAGCAAATGATCGCCACGCTGGTGGTCGGACTGACTTTTATGTTCGCGACGTTTATCGCCGTCTTTAC
GGTCGATAAGGCCGGGCGTAAACCGGCGTTAAAAATCGGTTTCAGCGTAATGGCGTTAGGGACATTGGTGTTG
GGCTACTGCCTGATGCAGTTTGATAACGGTACGGCATCAAGCGGTCTCTCCTGGCTTTCCGTTGGGATGACGA
TGATGTGTATCGCCGGTTACGCGATGAGCGCCGCTCCGGTGGTGTGGATACTGTGTTCGGAAATCCAGCCGCT
GAAATGCCGTGATTTTGGCATTACCTGTTCAACCACGACAAACTGGGTATCGAACATGATCATCGGCGCGACA
TTCCTGACACTGTTGGACAGCATTGGCGCGGCAGGTACATTCTGGCTCTACACCGCGCTGAATATCGCTTTTA
TCGGCATCACTTTCTGGCTGATTCCGGAAACCAAAAATGTCACCCTGGAGCACATCGAACGCAAGCTGATGGC
GGGCGAGAAGCTAAGAAATATTGGCGTGTAATCACCCCTCCCATGCCGGATGACGCCTGTTATCCGGCATGAT
GAAAAATAGACTGGAAACGGATGTGTAAGTTTGCTTCACTGCCATAATGCTTTACAAAAAGGAGAGCGCAGTG
AAAACGATCGGACTGTTGGGGGGGATGAGCTGGGAATCGACTATCCCTTATTACCGTTTAATCAATGAAGGCA
TTAAACAGCAGCTGGGAGGCCTGCACTCGGCGAGCTTACTGCTGCATAGCGTAGATTTCCACGATATTGAAGT
ATGTCAACGCCGCGGCGAGTGGGATAAAGCGGGCGATATCCTGGCGCAGGCCGCGCAGGGGTTACAGCAGGCG
GGCGCAGAAGGCATTGTGCTGTGTACCAACACCATGCATAAAATCGCGCACGTTATTGAATCTCGTTGTTCTC
TGCCTTTCCTACACATCGCGGATGCTACCGGGCGAGCTATCGCCCGTCAGGGGTTGCATCGCGTGGCGCTATT
AGGGACCCGCTATACGATGGAGCAGGATTTTTATCGCGGGCGGCTGGAACAGCAATTTGCGATTGAGACCATC
GTGCCGGAGGCGGACGATCGGGCGCGTATCAATCAGGTCATTTTTGACGAGCTCTGTCAGGGGACATTCACCG
ACGCGTCGCGCCACTATTATTTACAGGTTATCGAACAACTGGCTGCGCAAGGCGCGCAGGGCGTTATTTTTGG
CTGCACTGAAATAGGTTTGCTTGTTCCCCAGACGCAAAGCGCGCTACCGGTTTTCGATACCACGGCAATTCAT
GCTGCAGATGCCGTGAGCTTTATGCTTTCGTCATCGGCCCCAGAATAACCTCCAGCGGTTCGACCAGGCGCGA
AAGATGCGTTTGTAAATGCTTACTGAAGGCGTCAACCAGCGCGGAGGCGGGGCGGTGTAGCGGGCGAATCAGG
CTGACGGTAAAGGGCACGTCAATGCTGAAGCGGCGGACGGTGACGCCGCTGGCGGCATAGCCCAGCGCGGTCA
GCGGGTTAACGATCGAAACGCCCGCCCCGGCGCGCACCATCGCGCAGACGGAGGCCGCGCTATGCGTTTCCAC
CACCATTCTGCGCTTCACCTGATGTTCGGCAAATAACGTATCCAACAACTGTCGGTAGCTGTCCAGGCGGGAG
AGGCTGATAAAGTTTTCCCCCTGAAAATCGTCTGGCGTCAGTACCGTTTTGGCGGCAAGCGGATGGTCGCAGG

GCAGTACGCACACTTCATTTAGCGTTAGTAACTCGGTGCGGGTTGTTCCTGCCGGGGCATGAAGCGTCTCCGT
CAGCCCTAAGTCGTGGCGCTGGGCGGAGAGCCACTCTTCCAGCAACGGCGACTCCTGCGGCACAATATTCAGG
CTGACATCCGGATAACGCGCCAGAAAAGGCGGTAGTAACGGCGGCAAAAAGGATTGTGAAAAGACCGGCAGGC
AGGCGATAGAGAGCTCGCCCTGACGGAACTCGCGCAGACTTTCCGCCGCGCTGACGATGCGATCCAACCCATA
CCAGGAGCGTTGTACCTCTTCGAACAGGCGTAGCCCTTGCACCGTCGGATGTAAGCGCCCACGTGTGCGCTCA
AACAGCGTTAATCCCAGCACTTTCTCAAAACGCGCCAGCTCGCGACTGACGGTAGGCTGCGAGGTATGCAGCA
GCCGCGCCGCTTCGGTGAGATTTCCGGCGGTCATTACGGCATGAAAAATTTCAATGTGACGTAAATTAACGGC
AGCCATACGTTTCCCTGAATCATTTATCCCATATCATTTTTGCATAGACTGACAATAAAACGATATTTTTTAT
TCTTTCTAAGGTATGGCGTAATCGTTAAAAAGCCTGTTCCGGAGTTTGTTATGTCATTGCCGCATTATTACGC
TGAAACTGACCTTAACGCTGAAAATTTGCTCAGATTGCCTGCTGAATTCGGCTGTCCGGTCTGGGTCTATGAC
GCGCACATTATTCGCCGTCAGATAGCCGCCCTGCAGCAGTTTGACGTCGTGCGTTTTGCGCAAAAAGCCTGTT
CGAACATTCATATCCTGCGCTTAATGCGCGCGCAGGGCGTCAAGGTGGATTCTGTTTCATTGGGGGAGATCGA
ACGTGCGCTGGCGGCGGGCTACGATCCGCAAACGTGCCCTGATGATATCGTTTTTACCGCCGATGTGATTGAC
GCCGCCACGCTGGCGCGCGTCAGCGAACTGCATATTCCGGTCAATGCCGGCTCGGTAGATATGCTGACGCAGC
TTGGTCAGGTTTCGCCTGGGCATCGCGTCTGGTTGCGCGTGAATCCTGGCTTTGGTCACGGCCACAGCCAGAA
AACCAACACCGGGGGCGAAAACAGCAAACACGGCATCTGGCATAGCGATCTTCCGGCGGCGCTGGCGGTAATG
CAGAAATACCGACTGAAACTTGTCGGTATCCATATGCACATTGGTTCCGGCGTCGATTATGGCCATCTGGAGC
AGGTTTGCGGCGCGATGGTACGCCAGGTGCTGGAATGTGGTCAGGATGTGGAGGCGATTTCGGCAGGCGGCGG
ACTCTCGATACCTTATCGTGAGGGAGAAGAGCCCGTTGATACGCGCCATTACTATGGTCTGTGGAACGCGGCG
CGCGAGCAAATCGCCCGCCATCTGGGACATGCGGTCAAACTTGAGATCGAACCCGGACGTTTTCTGGTGGCGC
AATCGGGCGTGCTGGTAACGCAGGTGCGTAGCGTAAAACAGATGGGAAGCCGCCATTTCGTGCTGGTAGATGC
GGGTTTTAACGATCTGATGCGCCCGGCGATGTACGGTAGCTATCACCGTATTTCGGCGCTGGCGGCGGATGGT
CGCGCGCTTGAAAATGGCCCGTGGGTCGAGACGGTGGTCGCCGGGCCGCTATGCGAATCAGGCGATGTTTTTA
CGCAGCAGGAAGGGGGAATGGTTGAAACACGCGCCTTACCTGCGGTCATCCCCGGCGATTACCTCGTATTGCA
CGATACCGGCGCGTACGGCGCGTCTATGTCATCGAATTACAACAGCCGCCCGCTCCTGCCGGAAGTACTGTTT
GACAATGGACAGGCGCGGCTGATCCGCCGTCGTCAGACCATTGAAGAGCTGTTAGCGCTGGAGATGCTCTGAA
GCGCCTCATTAGGCCGGACGTTGAAAAAACGGGCCCGCGGCGACGGACTCGCGCAGAATCAGCGTGCCGGTAA
AGGGCGGGATCGGGGCGACCGGCTCGCTGTTCGCCAGGCGAACGGCCTGATCGATCGCGGTGGCTATCATATC
CTCGATGGGTAAATAGACTGTTGAAAGCCCCGGTTCAAGCCAGGTTGCGCCCGGCGCATCGTCAAAACCAAAC
AGCGACACATCCTGCGGGATGCGTAATCCGGCCTGGCGCAGCGCTTTTGCCGCGCCCAGCGCCGTGTCATCAT
TACAGGCGAATAGCGCGCTAAACGTGACGCCTTCCTCCAGTAATTCCCGGCAGGCGTCATAGCCGCGCGTCAG
CGTGTAATCGCCGTATTTCACTTTTGCCGGTTGCCAGGGAATACCATACTTTTCCAGCGCCTTGCGATAACCC
GCTACGCGTGATGTGCCAGTGGGAGTATGAACAGGCAGCGTAATACAGGCGATATCGCGATGGCCCTGCGTAA
TCAGGTATTCCACCGCCTGGAATGCCGCCGTCTCCTGCTCGAAGAAAATAGCGCGATCGCGGGCCTGAGTGAC
GTTGCGATTAATAATCACCAGCGGCATTTCCGTGGCGTCAATCAACGACAAAATCGCCGGCTCATCCATATAG
CGAGTGTAAAGAATAATAGCGTCGCACTGTCTGTCGGCCAACATTTGTACGGCTTTCTGCTCTCGTTCCGGCG
TATCGTGACCATCGGTGACGATCAACTGCTTGTTATGAAACTCCGCCTGGCGCGACGCCCGGCGCAACAAACT
GCCGAAATAGAAGCCGTCAAACGTAGAGACGACCAGACCGATGCTGTTGCTGGTGCGATTCGCCAGCGAGCGT
GCCAGCAGGTTGGGGCGATAGCCCAGAGCCTGCATCGCCGTAAACACTTTCTGGCGCGTGCTTTCTTTTACCT
GCCCCGTCCCATTCAGCACTCGTGAGACGGTGGCCTTTGATACGCCCGCATGGCGGGAAACATCCAGCATTGT
CGCCATCATGCTACCCTGATTAATCGGTTGTCGACAGGTGCCCGGTATCCGTCGGCGTGGATACCGAATGTCG
GCGTACCAGTGTCGGACTAAATACATGAGTTACTTCTGGCGTAGGGCATTTCCCCGCCAACGCTAACGCCAGC
TCCGCCGCCTGTGTCGCCATGGTGACGATCGGATACCGAATGGTGGTCAGTCGGGGACGCACATAGCGTGAGA
CCAGTACATCATCAAAACCGATGAGCGATACTTCGCCCGGCACCCCCACGCCATTATCATTTAATACTCCCAT
CGCGCCGGCCGCCATCGAGTCGTTATAGCAGGCCACCGCGGTAAAATGTCTGCCGCGGCCTAATAACTCAGTC
ATCGCCTGCTCGCCGCCGCTTTCATCCGGTTCGCCGAACGTCACCAGCCGATCGTTAGCCGGGATATGGCTTT
CCGCCAGCGCATCGTAATAGCCCCTCAGGCGATCTTCGGCATCAGAGATAGTGTGATTGGAACAGATATACCC
AATACGCGTATGACCTTGCTGGATCAGATGCCGGGTCGCCAGCCATGCCCCGTAACGGTCATCCAGCGCGACA
CAGCGGTGTTCTAACCCCGGTAAAATGCGGTTAATCAGCACCATGCCGGGGATTTGCTTCATTAATGAGGCCA
GGTCCGCATCCGGAATCATTTTGGCGTGTACCACTAACGCTGCGCAACGATGACGAATCAACTGTTCAATAGC
CTGACGCTCTTTTTGTTCGTTATGATACCCGTTGCCAATCAGTAAAAAATTGCCGGTGTGATACGCCACCTGT
TCAACGGCTTTCACCATCGCGCCGAAAAAAGGATCGGAAACGTCGCCGACCACCAGACCGAGGGTTTCCGTTG
CCTGCTGTGCCAGCGCGCGCGCGTTGGCGTTAGGGTGATAGCTCAGGGACTCCATTGCGCTGGTCACCGCCAG
CCGGGACGCTTCGCTGGCTTTTGGCGAATCGTTAATAACGCGAGAAACGGTGGCGACTGAAACACCGGCCAGT

CGGGCTACATCTTTTATGGTCGCCATGATAATACCTATAGTGGGTAAACGCTTACATTACGCCAGTTTTACGG
AAAATGAACGTCTGTTCAAGCCTCTTGCCCCGCCGTGTCGGGCAAATGTGACCTGCACGGGAGAAATGCGTCT
GTAAAGTGACAATACAAATCTTTCGTTACAGTTTGCGAAGCGCTGTTGCGATTGTCTGCTGGCAGGCCAGCGA
GTCAGGTTGATAGAGTGGTGATCCCAGAGGTATTGATAGGTGGAATCAACGTCATTGTTGATCACACGAATTA
CACCAACCTGCGTAGAGATGCGCAGGTTTTTTTTTGTTTCGCCTGAACCTGTAACAGTAACGGATAATTTACA
CAACTCGTTGCATTTCCGTTTATTCCTTTGCGTTTTCGGACTGGCGATGCAGACGCGCGAAGCCCACAATCAA
GATCCCAGAGGTATTGATTGGTGAGATTATTCGGTACGCTCTTCGTACCTGTCTCTTGCACCAACCTGCGCGG
ATGCGCAGGTTTTTTTTCGAACCTATTTTGCTATCGCTCCTGTTACGCAACTCGTGTAATCTGCCACAATTCA
TGTTTTAGCGAATTGTCGGCCAAAGGGAGTTGAAATGCTGTTTGGATTTTTCCGTAACCTGTTTCGCGTGCTT
TATCGTGTGCGCGTCACTGGCGATGTCCGGGCGCTACAGGGGAACCGGGTCTTAATTACCCCCAATCACGTTT
CATTTATCGATGGTATGCTACTGGCGCTGTTTTTGCCGGTACGGCCTGTCTTTGCGGTATATACCTCCATCAG
CCAGCAATGGTATATGCGCTGGTTAACGCCGCTGATCGATTTTGTGCTGCTCGATCCCACTAAACCCATGTCC
ATTAAACACCTGGTGCGTCTGGTGGAACAGGGGCGTCCAGTGGTGATTTTCCCGGAAGGGCGGATTTCCGTGA
CCGGTTCGCTGATGAAAATTTATGACGGCGCAGGCTTTGTGGCGGCAAAATCGGGCGCAACCGTCATTCCGCT
CCGCATTGACGGCGCGGAGCTTACCCCTTTCAGTCGCCTGAAAGGTCTGGTTAAACGGCGCTTATTCCCCCGC
ATTCAGCTTCATATTTTACCGCCGACGCAAATACCGATGCCGGAAGCGCCGCGCGCGCGCGACAGACGTAAGA
TAGCGGGTGAGATGCTGCACCAGATCATGATGGAAGCGCGTATGGCGGTGCGCCCGCGCGAGACGTTATACGA
ATCGTTGCTGGCGGCGCAATATCGTTACGGCGCGGGTAAAAACTGTATTGAGGACATTAACTTTACGCCGGAT
ACGTATCGTAAGTTACTGACCAAGACGCTCTTTGTTGGCCGGATTCTGGAAAAGTACAGCGTTGAGGGCGAGA
AAATTGGTTTAATGCTGCCGAATGCCGCCATCAGCGCGGCGGTGATTTTCGGCGCGGTTTCTCGTCGCCGCAT
TCCGGCCATGATGAACTACACAGCGGGGGTAAAAGGGCTGACCAGCGCGATTACCGCGGCGGAAATTAAAACG
ATTTTTACCTCCCGCCAGTTCCTCGATAAAGGTAAATTGTGGCACCTGCCGGAGCAGCTAACGCAGGTTCGCT
GGGTTTACCTGGAAGATTTAAAAGCGGACGTCACACCCGCCGACAAGCTGTGGATTTTTGCCCACCTCCTGGC
GCCGCGACTGGCGCAGGTAAAACAGCAGCCGGAAGACGCGGCGATCATTCTTTTTACCTCCGGCTCGGAAGGG
CACCCGAAAGGGGTGGTGCACAGCCATAAAAGCATTCTGGCGAACGTCGAGCAGATAAAAACGATTGCGGATT
TCACCGCCAATGACCGTTTTATGTCGGCGCTGCCGCTCTTTCACTCGTTCGGTTTGACGGTGGGCCTGTTTAC
GCCGTTGTTGACCGGCGCGGAAGTGTTTCTTTATCCAAGTCCGCTGCACTACCGCATTGTGCCGGAACTGGTC
TATGACCGTAACTGTACCGTGTTGTTTGGTACGTCAACCTTCCTCGGCAACTATGCGCGCTTTGCCAATCCGT
ATGATTTTTATCGCCTGCGCTATGTGGTGGCCGGTGCGGAAAAACTTCAGGAGAGCACCAAACAACTGTGGCA
GGATAAATTCGGTCTGCGCATTCTTGAGGGCTACGGGGTAACGGAATGCGCGCCAGTAGTCTCGATTAACGTG
CCGATGGCGGCTAAGCCTGGCACTGTTGGCCGCATTCTGCCTGGCATGGACGCGCGTCTGCTGGCGGTGCCCG
GTATTGAAAACGGCGGTCGGCTACAGCTGAAAGGGCCGAATATCATGAATGGCTATCTGCGCGTGGAAAAACC
GGGCGTGCTGGAGGTGCCGTCGGCGGAAAACGCCAGAGGCGAAACCGAGCGCGGGTGGTACGACACGGGCGAT
ATTGTACGCTTCGATGAAAATGGTTTTGTGCAGATTCAGGGGCGCGCGAAACGCTTTGCGAAAATTGCTGGCG
AAATGGTCTCGCTGGAGATGGTCGAGCAACTGGCGCTTGGCGTTTCGGCGGACAAAATGCACGCTACCGCCAT
TAAAAGTGATGCCAGCAAAGGTGAAGCGCTGGTACTGTTTACGACTGACAGCGAACTCACCCGAGAAAAGTTA
CAGCACTATGCTCGTGAACACGGTATTCCGGAACTTGCCGTCCCGCGTGATATCCGTTATCTGAAGCAGCTTC
CCTTGCTTGGCAGCGGGAAACCCGATTTTGTGACGCTGAAAAGTTGGGTGGATGCGCCGGAACAACATCATGA
GTGAGTCAGTACGTACTAACACCTCCATCTGGTCGAAAGGGATGCTGTCGGTGATTGTCGCGCAGTTTCTCTC
GGCTTTTGGCGATAATGCGCTGCTGTTTGCGACGCTGGCACTGCTGAAAGCTCAGTTTTACCCGGACTGGAGC
CAGCCTGTTTTGCAAATGGTGTTTGTGGGCGCTTACATTCTTTTTGCGCCGTTTGTCGGCCAGATGGCGGACA
GCTTTGCCAAAGGCCGGGTGATGATGGTTGCCAATGGTCTTAAACTGGCGGGAGCGGCAGATATCTGCCTCGG
CGTAAATCCTTTTGTCGGTTATACGTTGGTCGGATTGGCGCGGCGGCTTATTCTCCGGCGAAATACGGTATT
CTGGGCGAACTGACGACGGGTGATAAGCTGGTGAAAGCCAATGGCCTGATGGAAGCCTCTACGATTGCGGCGA
TTTTGCTCGGTTCCGTCGCGGGCGGCGTATTGGCCGACTGGCATGTCATCGCCGCGCTGGTTGCCTGCGCGTT
GGCCTACGCTGGCGCAGTGGCGGCTAACCTGTTTATCCCGAAGCTTGTGGCGGCGCGTCCAGGACAATCCTGG
CGGTTGTCGGCGATGACGCGTAGTTTCTTCAGCGCTTGCGTTGTCCTTTGGCGTAACGGTGAAACGCGTTTTT
CGCTGGTGGGGACCGGGCTATTCTGGGGCGCAGGCGTGACGCTGCGTTTTCTGCTGGTGCTGTGGGTGCCAGT
GGCGCTGGGCATTACGGATAACGCCACGCCGACTTACCTGAACGCCATGGTGGCGGTAGGCATTGTGGTCGGC
GCGGGCGCGGCGGCAAAACTGGTGACGCTGGAGACCGTATCGCGCTGTATGCCCGCCGGGATTTTGATTGGCG
TTGTGGTTGCGATCTTCTCATTACAGCACGCATTGCTGCCTGCTTATGCCTTGTTGCTGTTGATAGGTATGCT
GGGCGGCTTTTTTGTGGTGCCGCTCAACGCCTTGCTCCAGGAGCGCGGTAAGAAGAGCGTTGGTGCCGGGAAT
GCCATTGCCGTACAAAATCTGGGCGAGAATAGCGCCATGCTATTAATGCTGGGACTGTACTCGCTGGCGGTGC
TGGTTGGCGTTCCGGCAGTGGCGATAGGTATCGGCTTTGGCGTGCTGTTCGCGCTGGCGATCGCGGCGCTGTG

GATCTGGCAGCGCCGGCAGGCATCATACTGAGTGAATATGCCGGGCGGCGAGTGCGCGTTATCCGGCTTACAT
TGTCTCTGTAAGCCCGGTAGCGTAGCGCCACCGGGCACTGACTATCACGGCGCCGGGTAGGTATAAACCTGAT
GCGCCGCCTCTATCTCCGCCAGGACCTCTTCGCTTAACGTCAGATGCAGACTTTCCACATTCGTCTTGAGCTG
CGCCATCGTGGTTGCGCCCAGTAACGTACTGGCGACAAACGGCTGGCGGCGAACAAAGGCCAGCGCCATCTGC
GCCGGATCAAGATTGTGACGCTTCGCGATATCGACGTAGGCGGCAACGGCTTTTTGCGCCTGTTCGCCGCTGT
AACGTGTGAAGCGGCTGAACAAGGTATTCCGCGCGCCTGCGGGTTTGGCCCCGTTCAGGTATTTACCGGTTAA
TGTGCCAAACGCCAGACAGGAGTAGGCGAGCAATTCAACACCTTCATACTGGCTGACTTCCGCCAGCCCGACC
TCGTAGCTGCGGTTTAACAGGCTATAGGGGTTCTGGATCGTGACAATACGCGGCAGGTCATGTTTTTCCGCCA
GATGCAGATAGCGCATAACGCCAAACGCTGTCTCATTTGAAACGCCAATATAACGAATCTTACCCGCTCGCTG
AAACTCACTTAGCGCGTCTAACGTCTCCAGTAGACTTACTACTGGCGTGGAGTCCGTCCAGTTATAGCCGAGT
TTACCGAAGCAGTTAGTGGGGCGCTGCGGCCAGTGCACCTGATACAAATCCAGATAGTCGGTCTGCAATCGGG
TCAAACTGTCGTGTAGCGCTTCGCGAATATTTTTACGATCCAGCGCCTGATGAGGCCGGATGCCTTGATCATT
ATTACGTGCCGGTCCGCTAACTTTGCTGGCGATGATCAGTTTTTCACGGTTGCCGCGTTTGGCGAGCCAGTTA
CCGATATAGGACTCCGTCAGCCCTTGTGTTTCCGGGCGCGGCGGTACCGGATACATCTCGGCGGCATCGATAA
GGTTAATGCCATTTGCGATGGCATAGTCGAGCTGTGCGTGAGCGTCGGCTTCGCTGTTTTGTTCACCAAATGT
CATTGTGCCCAGCCCTAGCGTACTTACTTCGAGCGAGCTGTGGGGTATACGGTGATAGTGCATAGCCGTTTCC
TTTTTATTATGAAGTCAGAAATTCCCGACAAAGGACAATAAAAATGGCAGAGGGAAGGCAAAAGGGAAAGTAA
AATTGCAGGCTGCGCGGCGTAATTGTTACCGGGCGTGGTCGCCACGCACCGTTACGGGACGCAGCCTGAAGGC
TTGCAGAACGCTGACCTCGATCACAGGCCACCGCTTAACGTTCGATGATTTGAGACACTTCGTCACGGTTGAT
TTGCATCTCGTTTCCCTGTGCATCGGTATAGCTGACCAGACCGGTATCATCGTCAACTTGCGGTTTTCCATCG
GTCAGGATCATACGACCATCTTTCGTCGCCATTACATAATCGCTGCTGCATCCGGATACCGCAAAAGCCAACC
CTACTGCGGAAATTACAACTGCCCACTTTTTCATCCGTTTGTCCTCACGTAGCCTGCATCTGATAATAGTCTG
TTAATCATATGTCTGATAATAGTCTAGTAATAACGCGCGGCTCTGCATGTCGGAAGCAGCAAAATCTTAATCT
GTGAGAAGAAAGGAAATAAAGTGAGTATTCGCTCCCCGGCGGGGGAGCGAGACAGGATTACAGCGGATTCTTT
TTATTACGCAACAGGTTCAGGCTCTCTACTGCGATAGAGAAGAACATGGCGAAATAGATATACCCTTTCGGAA
CGTGAACGTCGAAACTTTCCAGAATCAGGGTAAAGCCAACCAGGATCAGAAAGGAGAGCGCCAGCATTTTTAC
CGACGGATGACGATCGACAAACTCGCCGATGGGCCGTGCCGCAAACATCATGACGCCCACGGCAATAACGACT
GCCGCCATCATAATAAACAGATGATCGGAGAGGCCAACCGCTGTGATAACTGAATCAAGGCTGAAAATAATAT
CCAGCAGCATAATCTGCACTATTGCGCCCAGGAAAGAAGAAACCCGGGTTTTTAACCCTTCTTCTTCGCCTTC
GATGGACTCATGAATCTCCTTGCTGGCTTTCCAGATTAAAAATAACCCGCCCAGTAACAGAATGAGATCGCGG
GCGGAAATCGCTTCGCCGAAAAGCTCAAACAGCGGATTAGTCAGCCGAGTGACCCAGGCGATTGACGCCAGCA
GCGCCAGGCGCATAACCATCGCCGCCGCCAACCCAAGCCGCCGTGCGTGGTTCCGTTGCGCCGTAGGAAGTTT
TGCTACCACCAGAGAAAGAAAAATAATGTTGTCGATCCCAAGTACAATCTCCAGCAGCGTCAGCGTACCGAGC
GCCAGCCAGGCATTAGGATCGGTTATCCATGCAAATAACATCGAACAAAGTCCTGCCAAAAAAAGAAGCGGTA
ATTATAGGCCCGTCGGGTGACGGGCGAAAGCCCAGGCTGAATTTGCGCAACCAGGAACGGTGTAAAATAGCGG
CGACAATACGCTTACGTTTATCGGGCGTCGGTCCGACGCGTAGCCATAGCGGAGGCGCATTTGCGTGTAATGG
ACGTTACGGATTCTGCAATAAAAAATGGCGAGCCAGCAGCGCCTGCGTAAAATTTTTCAGATAAAATCCACG
CGGTAGCGTCAAAATAGGTTCCCCGCGCGCACCGATAGCCTCGGTGAGCGCCCTGGCATTGGCGGCTTTGGGC
CGCAGTTGCAAGACTTCGCCATGGCGGGCGGTAATGCGTTCCACCTGGCCCAGCACTATCATGTCCATCAGCT
CTTCCCAGTCGAGGCGTAGCTGCCTGTCCTCTTCTTCACTCGGACTCCAGAGTAGCGGCGAACCTACGCGGCG
TTCGGCTAACGGTATGCTGCGATCGCCTTCGACCGGCACCCATAGCACGCGCTTCAATTTATGCCGTACGTGA
CTTGTCTCCCAGGTCACGCCGCTGTTACCGGTTAACGGCGCCACGCAAACAAAGGTTGTTTCCAGAGGACGAC
CCAGACTATCCACCGGGATGGTTTTCAGTTCTACGCCTAATGCGGCGAAATCCTGCTCCGGCTTGCTCCCGGC
GCTGGCGCCGAGCCAGATCTCCAGCAAGACGCCGATCCAGCCTTTGTCTCGCTTCAAATCTTTAGGCGTGGTA
ATGCCGGCCATCGCCGCCAGCTCGCCTAACGTATAGCCGGAAAGCTGCCGCGCCTGGGCTAACAGCAATGCTT
CCGAAGCAGGCGGAGTCAGTAGCGGACAAAGAGCGGACATGACAATTACCTTAGGTCAAAAAATGAGCAGTGA
TTTTATACCGTATGGGCAGAGTTTATCTTTTTCTGTGTCTTATATTCAATAGACTGATTTTTATTGCCTTTTT
TGTGTTGTTTCTTCGTGTTGCGCAACGCTGAAGCGGGTTTTCCAATTCTGGTCACTGACAATAAACAGGATCT
TACACTATGTTATCCACAGAAAGTTGGGATAACTGTGTAAAACCACCACTACTGTTTCCATTTACAGCCTTGA
CGTGCGACGAAAATCGAAAATTCAGACAAATAGTGCGTAAGTTATTGGCACAATCTGTGGATAAAACCGACGC
TGGTTGATCTTTCATCAGCATAAGGATCTTAGATGTGATGCTGGTCACGCTTTATGAAGGTAGCATGTAAGAT
TTGTCTATAATGCAATGAATATAAGGCACTATTTTTACTGTCTCCAGCATGAGGAATTCAGACTGTTCTGGGT
TTTCCCGTGGTAATTCCATACTTCTTCACAACTATATCCACAGAAAAGGTGAATAAAATGGCGCAAGGGCGTC
ATCATCTGTTTATAACTCGGCTATTTACTGTGAGTTATTCAATTGTTATTAGGCTGTGTCACTATTACAGAGT

GGTTTACCGCCTCACTGGAGTATGAAACAATCGTTCATATATAAGGTTTATGTTGAGGTAGTCCGGTGATTGA
TGACGATGGCTACCGCCCAAATGTAGGTATCGTAATTTGTAATCGTCAGGGGCAGGTGATGTGGGCCCGGCGA
TTTGGTCAGCACTCCTGGCAGTTCCCGCAGGGCGGGATAAATCCAGGGGAGTCGGCAGAACAGGCGATGTACC
GGGAACTGTTTGAAGAAGTTGGGTTAAGCCGTAAAGATGTGCGGATCCTTGCTTCTACTCGCAACTGGTTGCG
TTACAAGTTACCAAAACGTTTGGTGCGTTGGGACACAAAGCCGGTATGTATCGGCCAGAAACAAAAGTGGTTT
CTTTTGCAGTTAATGAGCGCCGACGCCGAAATCAATATGCAAACCAGCAGTACGCCGGAGTTTGACGGCTGGC
GCTGGGTAAGTTACTGGTATCCGGTTCGACAAGTGGTGTCATTTAAACGCGATGTCTACCGCAGGGTAATGAA
AGAGTTTGCAAGTGTTGTCATGGCGCTTCAGGATAATCCCCCTAAGCTACAAAGCGCGCCTGCTTATCGACGT
AAAAGAGGTTAAGCCACGCAAATTATGCTCACTCGCCTGCGCGAAATAGTCGAAAAAGTGGCCAGTGCGCCGC
GTCTGAACGAGGCGCTGAATATTCTGGTCACGGATATCTGTCTTGCGATGGATACTGAGGTTTGCTCGGTTTA
CCTGGCCGATCATGACCGACGCTGTTATTACCTGATGGCGACGCGGGGGTTGAAAAAACCGCGTGGTCGAACC
GTTGCGCTTGCGTTTGATGAGGGGATCGTCGGTCTGGTGGGCAGACTGGCGGAACCCATTAACCTTGCCGATG
CGCAAAAACATCCCAGCTTTAAATACATTCCTTCGGTAAAAGAGGAGCGTTTTCGCGCGTTTCTCGGCGTGCC
GATCATTCAGCGTCGCCAACTTCTCGGCGTGCTGGTCGTACAGCAGCGCGAATTGCGCCAGTACGATGAAAGC
GAAGAGTCTTTCCTTGTCACGCTCGCTACGCAGATGGCGGCTATCCTGTCACAGTCGCAGGTCACAGCGCTGT
TCGGGCAATACCGGCAGACGCGAATCCGCGCGCTTCCTGCGGCGCCGGGCGTGGCTATCGCAACGGGCTGGCA
AGACGCGACCATGCCGCTGATGGAACAGGTTTACGAAGCGTCTACGCTGGATACCTCTCTTGAACGTGAACGA
CTGACCGGCGCGCTGGAAGAGGCGGCGAACGAATTCCGACGATACAGCAAACGCTTTGCCGCCGGCGCGCAAA
AAGAGACGGCGGCCATTTTTGATCTGTACTCACACCTGCTTTCAGACGCCAGACTGCGTCGCGAACTCTTCGC
CGAGGTCGACAAAGGCGCTGTTGCGGAGTGGGCCGTTAAAAAAATCATCGAAAAATTTGCCGAGCAATTTGCG
GCGCTAACCGATAACTACCTCAAAGAACGTGCGGGAGACTTACGCACGCTGGGGCAGCGGCTGTTATTCCACC
TTGATGATTCAGTTCAGGGGCCAAACGCCTGGCCGGAACGTTTTATTCTGGTCGCGGATGAGCTGTCGGCGAC
CACCCTGGCGGAGCTGCCGCAGGACAGGCTGGCGGGCGTTGTGGTGCGCGACGGCGCGGCAAACTCGCATGCG
GCGATTATGGTGCGCGCGCTCGGTATCCCGACCGTCATGGGTGCGGATATCCAGCCTTCGGTGCTACATCGCC
GTACGTTGGTGGTGGATGGCTATCGCGGTGAATTACTGGTCGATCCTGAACCCGTACTTATTCAGGAATATCA
GCGGCTCATTAGTGAAGAGATAGAACTCAGTCGTCTGGCGGAGGACGACGTCAATCTTCCCGCCCAACTGAAA
AGCGGCGAGCGTATCAAAGTGATGCTGAATGCCGGGCTCAGTCCCGAGCATGAGGAAAAGTTAGGCAGTCGGA
TCGACGGTATCGGCCTGTACCGTACTGAAATTCCCTTTATGCTGCAAAGCGGGTTTCCCTCGGAAGAAGAGCA
GGTGGCGCAGTATCAAGGGATGCTGCAAATGTTTAACGATAAACCGGTGACGCTGCGGACCCTGGATGTGGGA
GCGGATAAGCAACTGCCCTATATGCCGATCAGCGAGGAGAACCCGTGTCTGGGCTGGCGCGGGATCCGCATTA
CGCTGGATCAGCCGGAGATCTTTTTGATCCAGGTCCGCGCGATGCTGCACGCCAATGCGGCGACTGGCAACCT
CAGCATTTTGTTGCCGATGGTCACCAGTATTGATGAAGTCGATGAAGCGCGGCGTCTGATCGAACGCGCCGGG
CGCGAGGTCGAGGAGATGATCGGCTATGCGATCCCGAAACCGCGTATCGGCATTATGCTGGAAGTACCGTCAA
TGGTATTTATGCTGCCGCATCTGGCTAACCGGATCGACTTTATCTCGGTCGGCACCAACGATTTAACGCAATA
TATCCTGGCGGTAGATCGCAACAATACCCGCGTGGCCAGTATTTACGACAGTCTGCATCCGGCTATGCTGCGC
GCACTGAGCATGATTGCGCAGGAAGCCGAAAAAAATGGCCTCGATCTTCGCTTGTGCGGCGAGATGGCGGGCG
ATCCGATGTGCGTGGCGATTCTTATCGGTCTGGGATATCGCCATCTTTCGATGAATGGCCGTTCGGTAGCGCG
TGTGAAATATCTGCTGCGGCATATCGATTTTGAAGACGCGCAAACCCTTGCCAGACGCAGCCTTGAGGCGCAG
ATGGCGACCGAGGTGCGTCATCAGGTGGCGGCGTTTATGGAGCGCCGCGGGATGGGGGGATTGATTCGCGGAG
GGTTGTAACGACTGGAGCGGGAAAAGAGAGTGACTGTGGATTCACGGCGCAGACAACACGCCTGCAAGATGAA
AGACAATGCGTATACATATCTTTTAACGGTAATCGGCATCTCGCTTTTAACCCTTGTGCTATTATTCGCACCT
TTGGAGCGTCTGCAACGCGCAGGCGCGCTTATCAATCGCTATCTCTTCAGCGAAATAACAAGAAATTGTGGTG
ACAGATGACCAGTAGCTATCTGCATTTTCCGGACTTTGATCCGGTCATTTTCTCAATTGGGCCCGTCGCGCTT
CACTGGTATGGCTTGATGTATCTGGTGGGGTTCGTTTTCGCGATGTGGTTGGCGGTGCGTCGCGCTAACCGTC
CGGGAAGCGGTTGGACCAAAAACGAAGTTGAAAATTTACTCTATGCAGGTTTCCTGGGGGTCTTCCTGGGGGG
ACGTATTGGCTATGTCCTGTTTTATAACTTCCCTCTGTTTCTGGATAACCCGCTCTATTTATTCCGCGTCTGG
GACGGCGGCATGTCCTTTCACGGCGGGCTTATCGGCGTGATACTGGTGATGATTATCTTCGCCAGGCGCACGA
AGCGCTCGTTCTTTCAGGTGTCTGATTTTATTGCGCCGTTAATTCCGTTCGGCCTGGGCGCCGGGCGTCTGGG
CAACTTTATCAACGGTGAATTGTGGGGGCGCGTCGATCCTGACTTCCGGTTTGCCATGCTCTTTCCTGGCTCA
CGCGCGGAAGATATTGCGCTGCTGCCGTCACATCCGCAATGGCAGCCTATTTTTGATACCTACGGCGTATTGC
CGCGCCACCCTTCCCAGTTGTATGAGCTGGCATTAGAAGGCGTGGTGCTGTTTATCATCCTTAATCTCTTTAT
TCGCAAACCGCGTCCGATGGGCGCAGTCTCCGGATTATTCCTGATTGGCTATGGCGCGTTTCGTATCATTGTT
GAATTCTTCCGCCAGCCGGACGCGCAATTTACCGGCGCGTGGGTACAGTACATCAGCATGGGGCAGATTCTCT
CTATCCCGATGATTATCGCTGGCGCGATCATGATGGTTTGGGCATATCGCCGCCGCCCGCAGCAACACGTTTC

CTGAGGAACCATGAAACAGTATTTAGAACTGATGCAAAAAGTGCTGGATGAAGGCACACAGAAAAACGACCGT
ACCGGCACCGGCACGCTTTCCATTTTTGGCCATCAGATGCGTTTTAACCTGCAGGAAGGCTTTCCTCTGGTGA
CGACAAAACGCTGCCATTTACGTTCAATTATTCACGAACTGCTATGGTTTTTGCAGGGCGATACCAACATCGC
TTACCTGCACGAAAATAATGTCACCATCTGGGACGAATGGGCCGATGAGAACGGCGATTTAGGCCCGGTTTAT
GGTAAGCAGTGGCGTGCATGGCCAACACCGGATGGCCGCCATATCGATCAGATCGCCACGGTCTTAAGCCAGC
TTAAAAACGACCCGGACTCTCGTCGTATTATCGTTTCTGCGTGGAACGTCGGCGAGCTGGATAAGATGGCGCT
GGCGCCGTGTCACGCCTTTTTCCAGTTTTATGTCGCAGATGGAAAACTCTCCTGTCAGCTTTATCAGCGCTCC
TGCGATGTGTTCCTCGGTCTGCCGTTCAACATTGCCAGCTACGCGTTACTGGTGCATATGATGGCGCAACAGT
GCGACCTGGACGTCGGCGATTTTGTCTGGACCGGCGGCGACACGCACCTGTACAGCAACCATATGGAGCAGAC
GCACCTGCAATTAAGCCGTGAACCTCGCGCGCTGCCGAAATTGGTGATTAAGCGTAAACCCGATTCGCTTTTT
GACTACCGGTTCGATGATTTCGAAATTGAAGGCTATGATCCGCACCCCGGTATTAAAGCGCCGGTCGCTATCT
AAGCGCTGACGATCCTGCCATAACCGACGTCATTGCGCGTCGGTTTTTTTTACCCTTCGTCTCAATCTTCGGC
GCGGATTCCTGAAATTTTGCAACGCGCTGCAAGACATGAACTTTTTTCCGTAACGCGCCGAACAATCTGTATT
TGGCGCTCGTTATTTTTCTTCCGCCCGCCATACTGCCGACATGAAAAAACAACAGGGCTATACGCTCATTGAA
ACGGTGGTCGCGATGTCGCTCATTATCATTCTCAGCGCCACAGGACTTTATGGCTGGCAACGCTGGCAGCAGC
AACAGCGCCTGTGGCAAACCGCATGTCAGGTGCGGGATTACCTGCTGCAACTTCGTGAGGATGCTCACTGGCA
TAACCGCGATCACATTCTTCGCGTGGTCAGCGAAGGCGGTTCATGGTGCTTTGTCAGCTCTGTTGCTACGCAA
ACATCGTGTACGCCATCTTCCCCCTTTGTGTTTACGCCCGACTGGCGTGATGTCGTCCTTGCCGATATCACGC
CGTCGTTAGCCTTCTTTGGCTTGCGCAATACGGCATGGGCAGGGCACATCGTACTGAAAAACGCTGCCGGAGA
GTGGCGTCTGGTCGTCTCCTCCTGGGGGCGAATCAGGCTATGCGAGCGTAACGAGGCCGCCACGTGTCAATAA
CCACGCGGGGGTTCTCGCTACTGGAGGTCGTGCTGGCGATGGCGATAGGTTCCATTTTACTGCTTGGGTCCGC
ACGATTTTTACCGGCGCTACAGCGTGAGATCTGGCACAACACGCGACAGTTATCGCTGGAAGATGAAATCTGG
CAGCGAACCTATACCGTAGCGAAGCATCTTCAACGTGCGGGTTACTGTCATGGCCACTGTATTGGCGAAGGGT
TACATCTTGCCGAACAGGGGCAATGCGTCATCGTTCAGTGGGACGGGAATAATAACGGTGTCTGGGATACCAT
ACCGGCGAAAGAGGCCGACCAGACCGGGTTTCGTGTGAAGGATAACGTTCTGGAGACATTACGCGGAGCAACA
TCTTGCCAGGGTAAGGGATGGGAAAAAATGACCGATCCCAATACGGTACTCATCACGGCGTTTACCGTTGAGC
GCCGGGATATAACGGGATTCTCGCCAGTGCTCATGCTGCATCTGCGCGGCGCCAGCAAAGCCGAGCCTCAGAC
GGTTATTGACGCGCAATACAGCGTAACGGGATTTAATCTGTGAATCGGGAACGTGGCGCTTCTTCGCTAATAC
TGGCGCTTTTAATACTGATTCTGGGCAGTCTGCTACTGCAAGGCGTAAATCAGCAGCAGGCCAGCTATGCCAC
CCGCGTGACGACGCAGAGCCTGGCGATACAACGCCAGGCGCTCGTACAGTCGGCGCTGGAATGGGGGCGGGGG
CAGCTATGGTCAGGCGTAACCGAAATGGAATGTCGGCGCTATTCCTCTTCAGGAGCGAGAGTCTGTTTGCGGA
GGTTACCGGGGGATGAGGTGGTGATGGCGGCGCAAGATGACAGCATGACGTTATGGCGTTTGGGCAACGTCAT
TCAGGGAAGCATTGTCTTTTCTCCCCACGGCTGGAGCGATTTTTGTCCGTTAAAAGAGGTGGCGTTATGTCGC
ATCCCCTGAATTTACAACGCGGGTTTAGCCTCCCGGAAGTTTTAGTGGCGATGGTTCTAATGGTAATGATCGT
CACCGCGCTGTCAGGCTATCAGCGGGTATTAATGCACAGTTTTGCATTACGACACCAGTATCTTCAGATCTGG
CGGCAGGCGTGGCAGCAAACGGCGCTGTATCCATTTTCGCCTGCTGAAGGCTGGAAGGCCAACCGGATGCAGA
CAACGCAATCGGGATGTGTCAGCATCAGCGTCACGATGGTTTCACCCTCTGGCAGGCAGGGGCAGATGACGCG
CTTGCATTGTCCAAATCGTTAATCGTCAGGAGTAATTATGTTAAGGGTCTACCACTCCAATCGTCTGGATGTG
CTGGAAGCGTTGATGGAATTCATCGTCGAACGCGAGCGGCTGGATGATCCCTTTGAACCAGAAATGATTCTGG
TACAAAGCACGGGTATGGCGCAGTGGCTGCAGATGACCCTTTCACAAAAGTTTGGTATTGCGGCGAATATCGC
TTTTCCGTTACCGGCGAGTTTTATCTGGGAGATGTTTGTCCGCGTGTTGCCGGATATCCCCAAAGAAAGCGCT
TTCAGCAAACAAAGCATGAGCTGGAAACTGATGACCTTGCTGCCGCAGTTGCTGGATAAGGATGAGTTTGTAC
TGCTGCGCCATTATCTGACCGACGACACCGATAAACGCAAACTCTTTCAGCTTTCCGCGCGCGCCGCCGACCT
GTTCGATCAGTATCTGGTTTATCGGCCTGACTGGCTTACGCAGTGGGAAGCGGGAAAAACCGTGGAAGGTCTG
GGAGAGGCGCAAAACTGGCAGGCGCCGTTGTGGAAAGCATTGGTGGAATATACCGCCGCGCTGGGGCAGCCGC
GCTGGCATCGCGCCAATCTCTACCAACGCTTTATTCAAACGCTGGAAAGCGCGACCGCCTGCCCGCCAGGGTT
GCCGTCCCGCGTTTTTATTTGCGGCATTTCCGCGTTACCGCCAGTGTATCTCAGAGCATTGCAGGCGCTGGGT
AAACATATTGAAATCCATCTCCTTTTTACTAATCCGTGTCGGTATTACTGGGGGGATATTAAAGACCCAGCCT
GGCTGGCAAAACTCATGGCTCGCCAGCGTCGTCACAGTTTTGAAGACCGTCATTTACCGTTGTTCCGTGAAAA
CCAGAATCCGGAAGCCTTATTTAACAGCGACGGTGAACAGGATATCGGTAATCCGCTACTGGCCTCATGGGGA
AAACTGGGACGGGATTATATCTACCTGCTATCTGAACTGGAAAACAGCCAGGAGCTGGATGCGTTTGTTGATA
TTACGCCAGATAATCTTTTGCATCGGATTCAGGCGGACATCCTTGAACTGGAAAGCCATGCGGTGGCGGGCGT
AAATCTTGAGGAGTATTCGCGCAGCGACAATAAGCGTCTGCTCGATCCCGAGGATAACAGCCTCTCTTTTCAC
GTTTGTCACAGCCCGCAGCGCGAGGTGGAGATACTGCACGATCGCCTGCTGGCGATGCTGGAAGCCGATCCGA

CACTAACGCCGCGCGACATTATCGTTATGGTGGCTGATATTGACAGCTATAGCCCGTTTATCCAGGCGGTGTT
TGGTAGCGCGCCAACGGAGCGCTATCTGCCTTATGCCATTTCCGATCGGCGGGCGCGTCAGTCACATCCGGTG
TTACAGGCATTTATCAGCCTACTTTCGTTACCGGACAGCCGCTTTGTCTCCGAGGATGTTCTGGCGTTGTTAG
ACGTACCGGTATTGGCTGCCCGCTTTACGATCAACGAAGAAGGGTTGCGTTATTTGCGCTTGTGGGTCAATGA
GTCAGGCATTCGCTGGGGAATTGACGATGACAACGTACGCGAGCTGGAACTTCCTGCCACGGGGCAGCATACG
TGGCAATTTGGCCTGACGCGTATGCTGCTGGGGTATGCGATGGAAAGCGCCCAGGGCGAGTGGCAGTCGGTTC
TGCCTTATGACGAATCCAGCGGTCTGATTGCTGAGCTGGTCGGGCATCTGGCTTCGCTGCTGATGCAGCTTAA
TATCTGGCGGCGCGGTCTGGCGCAGGAGCGACCGCTGGAAGAGTGGCTGCCCGTTTGTCGCGATATGCTCAAT
GATTTCTTCCTGCCCGATGCGGACACGGAAGCGGCAATGACATTGATTGAACAGCAGTGGCAGGCCATTATCG
CCGAAGGCGTCGCGGCGGAATATGGCGACGCGGTACCGATTTCGCTACTGCGGGATGAACTGGCGCAGCGTCT
GGATCAGGAACGTATTAGCCAGCGCTTCCTGGCGGGCCCCATCAATATTTGTACGCTGATGCCGATGCGTTCC
ATTCCGTTTAGAGTCGTTTGCCTGCTGGGAATGAACGACGGCGTATACCCGCGCCAGCTTGCGCCGTTGGGCT
TTGACCTGATGAGCCAAAAACCCATGCGCGGGGATCGTAGCCGTCGCGACGATGACCGCTACCTGTTTCTGGA
AGCGTTAATCTCGGCGCAGCAGACGCTGTATATCAGCTACATCGGTCGCTCCATCCAGGATAATAGCGAACGC
TTTCCCTCTGTACTGGTGCAAGAATTGGTGGATTACATCGGACAAAGCCACTATTTGCCGGGCGACGAAACGC
TGACCTGTGATGAGAGCGAAGCACGAGTCAAAGCGCATATTACGCGTCTGCATACCCGTATGCCTTTTGATGC
GCAAAATTACCAACCCGGCGAACAGCAAAGTTATGCCAGAGAGTGGTTGCCCGCGGCGAGTCAGTCGGGAAAA
GCGCATTCTGACTTTGTGCAGCCGCTTCCTTTTACGATGCCGGAAACATTGACTCTGGAAAGTCTGCAGCGTT
TTTGGGCCCATCCGGTACGCGCATTTTTTCAGATGCGGCTTCAGGTTAATTTCCGCTCGGAAGAGAGTGAGAT
TCCTGATGCGGAACCGTTTGAATTAGAGGGATTGACGCGATATCAACTTAACCAACAGTTGCTCAATACGCTG
GTTGAAGAGGACGACGCCGAACGCTTGTTCCGCCGTTTCCGGGCTGCGGGAGAGTTGCCTTACGGCGCGTTTG
GCGAAATATTCTGGGATGCGCAATGTCAGGAGATGCAGCAACTGGCAAGCCGGGTCATCGCCTGCCGTAAGCC
GAGCCAAAGCCTGGAAGTGGATCTGCTTTGCAATGGCGTTCAGCTAACCGGCTGGCTGCCGCAGGTACAGGAG
GATGGATTATTGCGCTGGCGTCCCGCTTTAATCAGCGTGGCGCAAGGTGTGCAACTTTGGCTGGAACACCTTG
TCTATTGTGCCAGCGGCGGGAGTGGCGAAAGTCGCCTGTTTTTGCGCAAAGAGGGAGAGTGGCGATTTCCGCC
TCTTGATAAAACGCAGGCAATGGCTTACCTGGCGCAACTGATTGAAGGCTACCGGGAAGGCATGTCTTCTCCG
CTATTGGTACTGCCGGAAAGCGGCGGCGCATGGATAAAAGCCTGTTATGACGCTGAAAATGATGTCATGTTAG
ACGACGATGACACCTTACAGAAGGCCAGAACAAAGTTTCTACAGGCTTATGAAGGCAACATGATGGTGAGCGG
CGAAGGCGAAGATATCTGGTATCAACGTCTTTGGCGTCAGCTGGAATCTGAAACGTTGCAGGCTATCATTGCG
CAGTCACGGCATTACCTGTTACCGCTGTTTCGTTTTAATCAGTCTCGTTGATTGTATGAAAATCGCGCCATTA
AAAGGCTTCGACTATGATGCGCTCTTCGTCACGGACTGATTACTGATAAAAAAATGCTGCCTGGTTCAGCACA
ATAATATCCCGTTCCGCTCAGGTCGATGGTTTACGTGACCATACAGATGAGTGGGAATGAAAGTTAATGATGA
GGTCCGTGAATGCCCCGCAGCACCTGGTTCAAAGCGTTATTGTTATTGGTCGCCCTCTGGGGGCCTGCAGTTC
AGGCGGATATCGGTTGGCAACCGCTGCAAGAAACTATCCGTAAAAGCGATAAAGATACCCGGCAGTATCAGGC
GATACGTCTTGATAATGACATGGTGGTGTTGCTCGTATCCGATCCGCAGGCTGTAAAGTCGCTTTCAGCGTTA
GTGGTGCCCGTTGGATCGCTTGAAGATCCTGAGGCTCATCAGGGGCTTGCTCATTATCTTGAGCATATGTGCC
TGATGGGGTCAAAAAAATATCCGCAGGCGGATAGCCTTGCCGAATACCTTAAAAGACATGGCGGTAGCCACAA
CGCCAGCACCGCGCCTTATCGAACAGCCTTCTACCTGGAGGTTGAAAACGACGCGCTTCCAGGTGCCGTAGAT
CGACTGGCCGACGCCATTGCCGCGCCATTGCTCAATAAAAAGTATGCCGAACGCGAACGAAACGCGGTGAATG
CCGAGCTGACGATGGCGCGCACCCGCGACGGTATGCGTATGGCGCAGGTAAGCGCCGAAACCATTAACCCGGC
GCATCCAGGCTCGCACTTTTCTGGCGGCAATCTGGAAACGCTAAGCGATAAGCCGGGAAATCCGGTGCAACAG
GCGTTGATCGCTTTTCATGAAAAATACTATTCATCTAATCTGATGAAGGCGGTGATTTACAGTAATAAACCCT
TGCCGGAACTGGCGAGTATTGCCGCCGCAACCTATGGTCGCGTGCCGAATAAACAGATTAAAAAACCGGAAAT
TACCGTACCTGTCATCACCGAGGCGCAGAAGGGCATCATTATTCATTACGTGCCGGCGCTACCGCGTAAAGTG
CTGCGCGTGGAGTTTCGTATTGATAACAATAGCGCGCAGTTCCGTAGCAAGACCGATGAACTGGTCTCTTATC
TGATTGGCAATCGTAGCCCGGGGACGCTCTCTGACTGGCTGCAAAAACAAGGACTGGTCGAAGGTATTAGCGC
GGATTCCGATCCGATTGTTAATGGTAACAGCGGCGTATTTGCCATTTCGGCAACGCTGACTGATAAAGGTTTG
GCCAATCGTGATGAAGTCGTCGCAGCTATCTTTAGCTATCTCAATACGTTACGTGAAAAAGGGATAGATAAAC
GTTACTTTGACGAGCTGGCGCATGTGCTGGATCTCGACTTCCGCTATCCGTCGATTACCCGCGATATGGACTA
TGTCGAGTGGCTGGCGGACACTATGATCCGCGTCCCGGTAGCGCATACGCTGGATGCGGCGAATATCGCCGAT
CGTTACGATCCTGCCGCTATCAAAAATCGCCTGGCGATGATGACGCCGCAGAATGCGCGCATCTGGTACATCA
GTCCCCAGGAACCCCATAATAAGATTGCGTATTTTGTGGATGCGCCTTATCAGGTCGATAAGATTAGCGAACA
GACGTTTAAAAACTGGCAGCAAAAAGCACAGGGCATTGCGTTGTCGCTGCCGGAGTTAAACCCCTATATTCCT
GATGATTTTTCGCTTGTTAAGAATGACAAAAACTACGTGCGGCCAGAACTGATTGTCGATAAAGCGGATTTGC

GCGTGGTTTATGCGCCGAGTCGTTATTTCGCCAGCGAGCCAAAAGCTGACGTGAGCGTGGTATTGCGTAACCC
GCAGGCGATGGACAGCGCCCGCAATCAGGTACTGTTCGCCCTTAATGATTATCTGGCGGGAATGGCGCTCGAT
CAGCTCAGCAACCAGGCGGCAGTGGGCGGCATTAGCTTTTCCACTAACGCCAACAATGGTCTGATGGTCACTG
CGAACGGTTATACCCAGCGTTTGCCGCAGCTTTTCCTGGCGCTGCTGGAAGGATATTTTAGCTACGACGCCAC
GGAGGAACAGCTGGCGCAGGCGAAATCCTGGTATACGCAGATGATGGATTCTGCGGAGAAGGGAAAAGCCTAC
GAACAGGCGATTATGCCGGTGCAGATGATTTCGCAGGTGCCTTATTTTTCCCGTGATGAACGTCGCGCTTTGC
TGCCGTCCATTACGTTAAAAGAGGTGATGGCCTATCGTAATGCGTTAAAAACGGGCGCTCGTCCGGAATTTCT
GGTTATAGGGAATATGAGCGAAGCCCAGGCGACCTCTCTGGCGCAAGATGTCCAAAAACAGCTCGCGGCGAAC
GGATCGGCGTGGTGTCGTAACAAAGACGTGGTGGTTGAGAAAAAGCAGTCCGTAATATTTGAAAAAGCGGGCA
GTAGCACCGACTCCGCGTTAGCAGCGGTCTTTGTCCCGGTCGGCTACGACGAGTACGTCAGCGCCGCCTACAG
CGCGATGTTAGGTCAGATTGTTCAACCGTGGTTTTACAATCAGCTACGAACCGAAGAGCAACTGGGATACGCT
GTTTTCGCCTTTCCGATGAGCGTTGGCCGTCAGTGGGGAATGGGATTCCTGCTACAGAGCAACGATAAACAGC
CCTCTTACCTGTGGCAACGCTATCAGGCATTTTTCCCTGACGCCGAGGCGAAGCTGAGGGCGATGAAGCCGGA
AGAGTTCGCCCAAATTCAGCAGGCGATCATTACGCAAATGCGCCAGGCGCCGCAAACGTTGGGCGAAGAAGCA
TCCCGTTTAAGCAAGGATTTCGATCGGGGTAATATGCGCTTTGACTCGCGTGATAAAATCATCGCTCAGATAA
AATTGCTGACGCCACAAAAGCTTGCCGATTTCTTCCACCAGGCGGTGGTGGAACCACAAGGTATGGCAATATT
GTCACAGATTGCCGGTAGCCAGAATGGAAAAGCAGAATACGTGCATCCGACAGGCTGGAAAGTGTGGGATAAC
GTCAGCGCTTTGCAGCAAACGTTACCTCTAATGAGCGAAAAGAATGAATGATGTCGCCGAGACTCTTGATCCT
CTGCGCTTGCCCCTTACAGGCGAGCGTTTGATTGAAGCATCAGCGGGCACAGGCAAAACGTTTACTATCGCGG
CGCTGTATCTGCGGCTACTGCTCGGTTTAGGCGGCAGCACCGCATTTCCCCGTCCGTTGACCGTTGAAGAACT
GCTGGTGGTAACCTTTACGGAGGCCGCCACTGAAGAGCTGCGTGGGCGCATCCGCAGTAATATCCATGAGTTG
CGCATCGCCTGTCTGCGGGAAAGCACGGATAATCCGCTGTATGCCCGCTTGCTGGAAGAAATTAGCGACAAAA
AGCAGGCCGCGCAGTGGTTATTACTGGCAGAACGACAAATGGACGAGGCCGCGGTCTTTACCATCCACGGTTT
TTGCCAGCGTATGCTCAGCCTGAACGCTTTCGAATCCGGTATGTTGTTTGAGCAACAGCTTATTGAAGACGAA
TCTTTGTTGCGTTATCAGGCCTGCGCCGATTTCTGGCGGCGTCATTGCTATCCGCTGCCGCGCGACATCGCGC
AGGTGGTGTTTGACGTGTGGAAGGGACCGAAGGCGCTCTTAAAGGATATCGATCGTTATCTGCAGGGCGAAGC
GCCGGTCATTAAAGCGCCGCCATCGCAGGAGGAGACGCTGGCATCGCGTCATGAACAGATCCTGGCGCGCATC
AACCAGGTGAAGCAGCAGTGGTGTGAGGCGGTTAGCGAGCTGGACGCGCTGATCGAATCATCCGGCATCGATC
GCAGAAAGTTCAACCGCGGCAATCAGGCGAAATGGATCGAAAAGATCACCGCGTGGGCGCAAGAAGAGACGAA
AAACTATCAATTACCCGAGGCGTTGGGAAAATTTTCACAGCGTTTTTTAGCTGAGCGCACCAAAGCTGGCGGC
GTGACGCCCCAGCACCCATTGTTTGTCGCCATTGATAACCTGTTGGGCGAACCGCTATCGATTAAAGATCTGG
TTCTTACCCGAGCGCTATCAGAGATCCGTGAAACGGTGGCTCAGGAAAAACGCCGGCGCGGCGAGCTGGGGTT
TGACGATATGCTCAGCCGTTTAGATACCGCGCTGCGTAGTGAAAGCGGCGAGGCGCTGGCTGCGGCGATTCGT
ACCCGTTTTCCGGTGGCGATGATCGATGAATTTCAGGATACCGACCCACAACAGTACCGTATTTTTCGCCGTA
TCTGGCGACATCAACCCGATACGGCGCTATTGCTGATTGGCGATCCTAAACAGGCGATTTATGCGTTTCGTGG
CGCGGATATCTTCACCTACATGAAGGCTCGCAGCGAAGTGAGCGCGCACTATACGCTTGACACTAACTGGCGC
TCCGCGCCGGGGATGGTGAATAGCGTTAACAAGCTGTTCAGTCAGATGAACGACGCCTTTATGTTCCGCGATA
TTCCCTTCAGTCCGGTAAAGTTTGCGCCCAGAAACCAGTCGCTGCAATTTAAAGTGAATGACGCGCCTCAACC
GGCGATGACCCTGTGGCTAATGGAGGGGGAAAGCTGCGGTTCGGGCGATTATCAAAGTTATATGGCGCAGGTG
TGCGCCACGCAAATTCGCGACTGGCTGCGTGCCGGGCAAACGGGCGACGCGCTGCTGACGAACGGCGATTCGT
CGCGGCCCGTTCGTGCCTCGGATATCAGCGTGTTGGTGCGCAGCAGGCGGGAAGCCGCGCTGATACGGGATGC
GCTGACCTTACTGGCGATCCCCTCCGTCTATCTTTCTAACCGCGACAGCGTATTCGAAACGCTCGAAGCGCAG
GAGATGCTGTGGGTGTTGCAGGCGGTGATGGCGCCCGAACGTGAGAATACGCTGCGTAGCGCTTTGGCGACAT
CAATGATGGGCCTTACCGCGCTGGATATCGAAACGCTTAACAATGATGAAAATGCGTGGGATGCGGTTGTCGA
AGAGTTTGATGGCTACCGACAGATTTGGCATAAACGCGGCGTGATGCCTATGCTGCGCGCGCTAATGTCGGCG
CGCAATATTGCTGAAAACCTGCTGGCGACGGCGGGAGGCGAACGTCGGCTGACGGATATTTTGCATATTAGCG
AGCTGTTACAGGAAGCCGGTTCGCAGCTGGAAAGCGAACATGCGCTGGTGCGTTGGCTGGCGCAGCATATCCT
TGAACCCGATAGCAATGCTTCCAGCCAGCAACTACGTCTGGAAAGCGATAAGCATCTGGTGCAAATCGTCACC
ATTCATAAATCGAAAGGTCTGGAATACCCGCTGGTCTGGTTACCTTTTATCACCCATTTCCGCGTGCAGGACC
AGGCTTTTTATCACGACAGGCACTCCTACGAGGCGGTGCTGGACCTTAGTCACGCCGAAGAGAGCATAGCACT
GGCGGAAGCCGAACGCCTGGCGGAAGACCTGCGCCTACTTTATGTCGCGCTGACCCGTGCAGTGTGGCATTGC
AGCCTCGGCGTCGCGCCGCTGGTGCGGCGTCGTAGCGATAAGAAAGGAGAGACGGATGTGCACCAAAGCGCGC
TTGGGCGACTCCTGCAAAAAGGCGAACCGATGGATGCCGCAGGCCTTCGCGCCTGTATTGAGGCGCTATGCGA
CGAGGATATCGTGTGTCGAACGCCAGGCAATACGGATAACGACCGCTGGCAAATTGCCGCTGCATCGCACGCG

GAACTCAGTGCCAGAACGCTGCAACGCCTGCTTTATGATAGCTGGCGCGTCACCAGTTATTCCGGTTTGCAGC
AGCGGGGACATAGCGTGGCGCAAGATCTTATTCCTCGTCTCGACATTGACGCCGCAGGCGTTGGCGAAGCGGC
GGAAGCGCCGGCAATGACGCCGCATCACTTTCCTCGCGGGGCATCGCCGGGAACATTTTTGCACAACCTGTTT
GAAGAGCTTGATTTTACTCAACCGATAAACCCCCAATGGGTACAGGAAAAACTGGAGCTGAGTGGTTTTGAGA
CGCGATGGGAGCCCGTACTTACCCGCTGGCTGGACACCGTGTTGCACGTTCCCCTTAACGAGACGGGCGTTAG
TCTGAGCGTGCTGACCGAGCGTGAAAAACAGGTCGAAATGGAGTTTTATCTGCCGATTGCCCAGCCGCTTACC
GCCGGAGAGCTGGATGCGTTGATTCGTCGATACGACCCGCTTTCAGCAGGTTGCCCTGCGCTGGATTTCATGC
AGGTTCGCGGCATGTTAAAGGGCTTTATCGACCTGGTTTTTCGTTATGAGGGGCGCTATTACCTGCTCGACTA
TAAATCTAACTGGCTGGGCGAGGATAGCGCCGCTTATACCCAGGCGGCGATGGCTACGGCGATGCAGGCGCAT
CGCTATGATTTGCAGTACCAGCTTTATACGCTGGCATTGCATCGTTACCTTCGTCATCGCATGGCGAATTACG
ACTATGAACGCCATTTCGGCGGCGTCATCTATCTCTTTTTACGCGGGGTGGATAGCGAACGTCCGCAGCAGGG
TATTTTTACCACTCGCCCTGCGGCGGCGTTAATTAACCAACTGGATGATATGTTTGCAGGTGAAATGAGTGAG
GAGGCGCAATGACAATCCAGGAGCGGTTGCTGGAGGCCGTTGAACAAAAACTCTTACGGCCCATTGATGCCCA
GTTTGCTTTAACCGTCGCCGGTAACGACGATCCCGCCGTGACGCTGGCGGCAGCGCTGCTTAGTCATGACGCA
GGTGAAGGTCACGTGTGTCTGCCGTTGTCGCGTTTAACGTTAACGGAGGAGGCGCATCCCTTACTGGTCGCCT
GTATAAGCGAAACGGCTACGCCAATCGACTGGAAAAAGCGGTTACTGGCGTCTGCGGCGGTTAGCTGCGGCGA
TAGCCCCGCGCCGTTAATTCTGTGCGGCGAGCGTCTCTACCTGAATCGCATGTGGTGCAACGAACGTACGGTT
GCGCGCTTTTTTAACGAGGTTAACCAGGCCATCGCCGTAGATGAAGATCAGTTATCCCGTATTCTGGACGCGC
TATTCCCTCCGACAGACGAGGTGAACTGGCAAAAGGTGGCCGCCGCCGTAGCGCTAACTCGCCGTATCTCCGT
GATTTCAGGCGGTCCCGGCACCGGTAAAACCACCACCGTCGCGAAGCTGCTGGCGGCATTAATTCAAATGGCG
GATGGCGAACGTTGCCGTATCCGGCTGGCAGCGCCGACCGGGAAAGCGGCCGCACGCCTGACGGAGTCGCTCG
GCGCGGCGTTGCGTCAGCTTCCACTTACCGATGCGCAGAAAAAGCGTATACCGGAGGACGCCAGTACGCTGCA
CCGACTGCTAGGCGCACAGCCCGGCAGCCAGCGATTACGCCATCATGCGGGCAACCCGCTACATCTGGACGTG
CTGGTGGTCGATGAAGCATCAATGATTGATTTGCCGATGATGTCACGTTTGATTGACGCTCTGCCGCCGCACG
GGCGGGTCATTTTTCTTGGCGATCGCGATCAGTTGGCATCCGTTGAGGCGGGCGCTGTGTTGGGCGATATTTG
CGCCTATGTCAACGCAGGGTTTACGGCGGAACGCGCCCGACAGCTAAGTCGACTCACTGGTAGCGCCATTCCG
GCGGGGGCCGGAACGCAAGCCGCATCTTTACGCGATAGCCTCTGTTTGTTGCAAAAGAGCTATCGTTTCGGCA
GCGATTCTGGCATCGGCAAGCTGGCGGCGGCAATTAACTGCGGCGACAGGTCGGCAATCCAGGCTGTTTTTCA
GCAGGGGTTTAGCGATATAGAGAAGCGTACATTGCAAAGCAGCGACGATTATGCCGGGATGCTGGATGAAGCG
CTGGCGGGCTATGGTCGCTACCTGCGGTTGCTGCATGAAAAAGCGACGCCGGAGGCAATCCTTCAGGCTTTCA
ATGAGTATCAACTGCTTTGTGCGCTGCGCGAAGGCCCATTTGGCGTGAGGGGATTGAATGACCGCATTGAGCA
GGCGATGGTGCAGCAACGAAAAATTCAGCGGCACCCGCATTCTCGCTGGTACGAAGGGCGTCCGGTAATGATT
GCGCGCAACGATAGCGCGTTGGGGCTATTTAACGGCGATATTGGTATTGCGCTCGATCGCGGGCAGGGGTTAC
GCGTCTGGTTTGTGATGCCGGACGGCACGATCAAGTCCGTTCAGCCCAGCCGTCTGCCGGAACATGATACAAC
CTGGGCGATGACGGTACATAAATCACAGGGGTCTGAATTTGATCACGCCGCGCTGATTTTACCCAGCCAGCGT
TCGCCGGTCGTGACGCGGGAGCTGGTGTATACCGCTGTTACGCGAGCGCGGCGGCGGTTATCGCTGTATGCCG
ATGAACGGATCCTGGCAGGCGCGATTGTGACGCGTACGGAGCGGCGCAGCGGGCTAGCCACCTTATTTGATGA
AGTCAGTCGTACCGGATAAGACGAGTTCTCGCCGGATGGCGACGCGTAGCGTCTTATCCGGCCTCCAAATGAT
GCTTTCGAGCGTAGGCCTGATAAGCGCAGCGCCATCAGGCCTCCCTCGTTACCCTAAATCCGCCATCAGCACC
TTTGAACGTCGCTGATAGTTATACATTTTTTTCTTGCTCTCAGGCAGTAACTCAATATCGACAGGCGTGAAAC
CGCGCTCCTGGAACCAGTGAATACTGCGCGTCGTCAGCACAAACAATTTACGCAGCCCCATTTGCCGCGCCTG
GGCGGCAACCCGCTCCAGAAGCACTTCTCCACGCGATGAGCTGCGGTAATCCGGATGTACCGCTACGCAGGCC
ATTTCGCCTATTTTCTCTTCCACAAAAGGATACAGCGCCGCGCAGGCGATAGTCATATTATCGCGCTGAATAA
TGGTAAATTTATCGATCTCCATCTCCAGTTGTTCACGGGAACGGCGTACCAGAATGCCCTGCTGTTCCAGTGG
GCGGATAAGCTCCAGAATACCGCCAATATCGTTGATGGTAGCGCGGCGGATCTGCTCGGCGCTCTCCATGACG
ATCTGCGTACCGATACCGTCACGCGAGAACAATTCCTGTAATAGCGAGCCATCTTCCTGATAGCTGATCAGAT
GGCAACGACGAACGCCGCTACGACAGGCTTTTACCGCGCCGCGCAAAAACGTACGGTTCCGGAGTTGTAATCG
CCCTGCGCTTCCAACTCTTCCACCCGCGCCTGTGCTTCATTCGGGAAGAGTTCAGAAATGATGCCGCCCTCGC
TATTGGTAACGCCCTGAGAAGAACAGAAACCGATCATTTTTTCCGCTTTCAGTTTAACGGCGAGTTGAGTGGC
AATCTCTTCTGAGGTCAGATTAAAACTTTCGCCGGTCACTGAGACGGCGACCGGCCCCATCAGAACGATCGCG
CCGTTATCCAACTGGCGGTTGATCGCATCTTCATCAATGCGCCGGATACGGCCGCTATGACAGTAGTCCACGC
CATCATCAACGCCCAGCGGCTGCGCAATGGTGAAATTGCCGCTGACGACATTAATGTGCGCGCCCTGCAACGG
CGTATTGTTCAGACTCATCGACAGACGGGCGGTAATATCAAGCTGGAGTAAACCGGCGGCCTGTTTCACCAGC
TCCAGTGCTTTGGCGTCCGTCACCCGCGTGTTTTTGTGGTAAATCGGTTCATGATGATGCGCGGCCAGATTGG

CGTCGATTTGCGGTCGCGCGCCATACACCACGACAAGGCGAATACCGAGGCTATGCAGTAGCCCGATATCGCT
AACGATGCTGGAAAAATTGTCATGCTCAATGGCTTCGCCGCCGAGCATAATGACAAAGGTTTTTCCGCGATGG
GTGTTGATATAGGGAACTGAATGGCGAAACCCTTCTACCAGTTCGGTTTTACGTTCCTTTATCATTGCGCGCC
CCACTAGTGAATTATTATTCGAAATTAGTGTATTTTTATTCTGTTTTTGCCCTCTGTGCAAGCGTAAATTTTA
TGCGGTCCGATCGCTTTTCTTAGTAATGCCGTGAATATAAAAAGAATGTTTACCCTTTTATAGATGACAGATT
ATGCGTCATTCGCTAAAGTTTCCGGTCAATTTGGATGAACTTTGTATGATCTCTATTTAGTTTTTGCTCGGGA
GAAGCATGTCGGGAGCCAATTCAGCAATCAGCCGTCGCCGTTTACTACAAGGCGCGGGCGCCATGTGGCTATT
AAGCGTGAGCCAGGTCGGGCTGGCTGCCGTAAGCCAGGTCGTTGCGGTGCGTATCTGGCCTGCGTCCAGCTAT
ACCCGCGTCACGGTAGAGTCGAATCGTCTGCTGAAATATAAACAATTTGCCTTAAGTAACCCGGACCGCGTGG
TAGTAGATATTGAGGATGTGAATCTGAACTCGGTGCTTAAAGGCATTGGCGCGCAGATTCGCAGCGACGATCC
GTATATCAAATCCGCAAGGGTAGGGCAGTTTGATCCTAAGACTGTGCGTATGGTCTTTGAGCTAAAACAAAAC
GTGAAGCCGCAGCTATTTGCGCTGGCGCCTGTGGCAGGCTTTAAAGAACGTCTGGTGATGGATCTTTATCCTG
CCAATGCGCAGGATATGCAGGATCCGCTGCTGGCGCTGCTGGAAGACTACAATAAAGGCGATCTGGATAAACA
GGTGCCGCCTTCGCAAAGCGGGCCGCAGCCCGGTAAAGCGGGGCGCGATCGTCCCATTGTCATTATGCTTGAT
CCGGGACACGGCGGCGAAGACCCCGGCGCGATAGGCAAATACAAAACGCGTGAAAAAGACGTGGTGCTCCAGA
TAGCCCGCCGCCTGCGCGCGTTGATCGAAAAAGAAGGCAACATGAAGGTCTATATGACGCGCAATGAAGACAT
CTTCATTCCGTTAAAAGTCCGGGTGGCGAAAGCGCAGAAACAGCGCGCCGACCTGTTTGTCTCTATTCATGCG
GATGCCTTCACCAGCCGGCAGCCTAGCGGTTCATCCGTATTCGCGCTATCGACCAAAGGCGCCACCAGTACCG
CGGCGAAATATCTGGCGCAAACGCAGAACGCCTCGGACTTAATTGGCGGCGTGAGCAAAAGCGGTGACCGTTA
TGTCGACCATACGATGTTCGATATGGTGCAATCGCTGACCATTGCCGATAGTTTGAAATTCGGTAAAGCGGTG
TTGAAGCAGCTTGGTAAAATTAATGACCTGCATAAAAACAAGGTCGAGCAGGCGGGGTTTGCGGTGTTAAAGG
CGCCCGATATTCCGTCTATTCTGGTCGAGACGGCGTTTATCAGTAACATTGAAGAAGAACGCAAGCTCAAGAC
CGCGACGTTCCAGCAACAGGTCGCAGAGTCGATTCTGGCGGGCATTAAAGCGTATTTTGCCGATGGGGCGACG
CTGGCGAGAAGAAGTTAACCTGAGATGACGGCGAGTTTATTGCGCGGGTAAGTAGGATGCTGCCCGCACTGTC
GTTGCGGACCAGACGACAGATACAAAAAAACACCCGTAAGGGTGTTCATTAAGTTGGTTGCGGGGGCCGGATT
TGAACCGACGACCTTCGGGTTATGAGCCCGACGAGCTACCAGGCTGCTCCACCCCGCGTCCGTCTAAACTTGC
ATTCTCATTGTAATTGGTTGCGGGGGCCGGATTTGAACCGACGACCTTCGGGTTATGAGCCCGACGAGCTACC
AGGCTGCTCCACCCCGCGTCCGTGGATGCGCACTATACTCCGCTGAGCTTTTGTTGCAACCCTTTTTTCATAT
AAATCGCTAATTTCTCATGAATTGCGCAAATACACCGCAGATAGCACGATTTTTAAACGAATTTACATAATGA
GATCGCGATCGCATTTCATTACTTCGGACGGTTTGTTATCTTCACCGGGCACTTTCGATTACGCAGAAGACGA
GACACCATGAAAGGACGTTGGGCAAAATATGTGGCGACAGGCGTAATGCTGGCAATGCTGGCTGCCTGTTCAT
CAAAACCGACCGATCGCGGTCAGCAATATAAGGACGGGAAATTTACCCAGCCCTTCTCCCTGGTAAACCAGCC
GGACGCAGTGGGCGCGCCGATTAATGCTGGAGACTTTGCCGAGCAGGTCAACCAGATCCGTTCCGCGTCGCCG
CGTTTATACACCAATCAAAGCAACGTCTATAACGCTGTCCAGAACTGGTTACGTTCCGGAGGCGATACGCGCA
CTATGCGTCAGTTTGGTATCGATGCCTGGCAGATGGAAGGCACCGACAACTACGGTAACGTCCAGTTCACGGG
CTACTATACGCCCGTCGTTCAGGCTCGCCATACGCGCCAGGGCGCGTTCCAGTATCCTATCTATAGTATGCCG
CCAAAACGCGGACGCTTACCGTCCCGCGCTCAGATCTACGCAGGCGCGCTGAGCGATAAGTACATCCTGGCCT
GGAGTAATTCGCTGATGGATAACTTTATTATGGATGTCCAGGGCAGCGGTTATATTGATTTCGGTGACGGTAG
TCCGCTGAACTTCTTTAGTTATGCCGGTAAAAACGGCTGGCCTTATCGCAGTATTGGTAAAGTGCTTATCGAT
CGCGGTGAAGTGAAAAAAGAAGATATGTCGATGCAGGCTATACGCGAATGGGGAGAGAAGCACAGCGAAGCGG
AAGTGCGTGAATTACTGGAGCAGAACCCGTCGTTCGTCTTCTTTAAGCCGCAATCTTTTGCGCCGGTCAAAGG
CGCCAGCGCTGTACCGCTGATTGGGCGAGCATCCGTCGCCTCCGACCGCAGCATCATTCCGCCTGGCACGACC
TTGTTGGCGGAAGTACCGTTGCTGGATAACAACGGTAAATTTAGCGGTCAGTATGAGCTACGTTTGATGGTCG
CACTGGATGTCGGCGGAGCGATTAAAGGCCAGCACTTTGATATTTACCAGGGAATTGGCCCGGATGCCGGGCA
TCGTGCCGGATGGTATAATCACTATGGTCGCGTCTGGGTGTTAAAAAGCGCGCCGGGCGCCGGTAACGTGTTT
AGCGGCTGATTGTGGTATTCTGCGACAACCGGATTGCCCGATAACGCGATCGCTATCGGGCCTGAAAATAGTG
AGCAGGGGTGAGGAATAACCTCACCTTTTTTTATTCTGAGGTCATATGTCAGTGGTGATAAGTGATGCCTGGC
GCCAACGCTTTGGCGGCACCGCGCGTCTGTACGGTGAAAAAGCGCTGCAGCGGTTTGCTGAGGCGCATATCTG
CGTCGTGGGAATCGGCGGGGTGGGATCATGGGCGGCAGAGGCGCTGGCGCGTACGGGCATCGGCGCGATTACG
CTGATTGATATGGACGATGTCTGCGTGACCAATACGAACCGTCAGATCCATGCGTTGCGTGATAACGTCGGGT
TGGCGAAAGCGGAAGTGATGGCCGAACGGATTCGGCAAATTAACCCGGAGTGCCGGGTAACGGTTATCGATGA
TTTCATCACGCCGGATAATGTGGCGGGTTATATGAACGCGGGTTTTACCTATGTCATTGACGCCATTGACAGC
GTTCGTCCAAAAGCCGCGCTGATTGCGTACTGTCGCCGATATAAGGTTCCTTTAGTGACCACAGGCGGCGCGG
GCGGGCAGATCGATCCGACGCAAATCCAGGTCGCTGATTTGGCGAAAACTATCCAGGATCCGCTGGCGGCAAA

ACTGCGTGAGAGATTGAAACATAATTTTGGCGTAGTGAAGAACAGTAAAGGTAAACTGGGCGTGGACTGTGTT
TTTTCCACCGAAGCGCTGGTTTACCCACAGGCGGACGGTTCCGTATGCGCGATGAAAGCTACCGCAGAAGGGC
CAAAACGGATGGATTGCGCCTCTGGCTTCGGCGCGGCGACGATGGTGACGGCAACGTTCGGTTTTGTCGCCGT
TTCCCATGCGCTGAAGAAGATCATGGCTAAAGCGGCGCGACAAGAATAAAAGGACGGGGCGGAGTGTCACGCA
GACGCGTATCCGGCCCTGCGCCCTTCGTTAGCGAAAGAAGCGCTGTACCCTGCCGAACACCGGTTTCAGACGG
GCGCGGAGATAATGGGAATATAAATTTTCAGATATGCATAGAAACAACCCGAACCATACTGCACAAAAGCAGA
GCAGTTCCGCGTAATTAATGTGCTCGCCAAATATCATAATGGCAAGTAAGAACTGTAAAGACGGCTCTATATA
TTGCGCCAGTCCGGTGACAATCAACGAGGTGTGATTTAACGCGATGGCAAATAATACCAGTGGAATGATCGTC
ACTGGCGCCGTCATGACATACAAAAAGAAAGGAGTATTAGCGGATATAATTGGTCCAGTATCGCTAAAGAGAA
AAATTAATAGTGAGACAGGCAATGCCCATAATGTTTCAATCGTTATCGATGTCATCACATCATAATGTATAAA
CTTTCGCGCCAGGCCATATAGCGCAAATGATAATCCCATGGTTAGCGCCAGCGCCGGGAAATGCCGTAGCATG
ATAATTTGAAAAGTCAGGCCGCAGACACCGGAAAAAACGGCGATGGCCTGAAACAACGACAGTTTTTCTTTAA
GAAAAATGCATCCCAGCGCAATAACAAATAGCGGGTTGATAAAATAGCCGAGGCTGGCATCAAGCACCAGATG
ATGGGTTAGCGCATAAATAAAAGATGACCATGATACAATCATCAAAAGTCCGGCGATCATGCAGAAGAAAAAG
GATTTTTTATCTTTCCAGACATCATGAAATCGCGTTCGCTGCCGAAATAAGAACCTTACTGCCAGTAATAATG
GAATTGACCAGAATACCCGGTATATCAATATTTGTGCGAGGTTCCCCCCTGATAAATATTGATAGTATAAAGG
TGTTAACCCCCATAATATAAATGCCAGCACCACAAACATGGTACCGTTACGCAGATATTTTTCCACTTATCCA
TATCCTGTTATAACTTCACCCGATGCGTATGAATTTTCCAGTTCTCCTGCTACGCTACATCGAGTGGCTTTAT
TTACTCTAAAATCTTCACGCCGTTTTAACATGGATTCTAACATTGTAATTTCATTGTGATAGCTGATAAAAAT
CAAAAGAATGTTTATCAGGGAAATCTGACGCCGAAACTAATAAAGTGACTGGATTATTTTATCTGCATCGCCT
GACCGCCGCGGACGGCGCTGTCAGGCGACAGAATATTTACGCTATTTCAACCCTTATGCGTTGCGGCAACAAT
CGCTTCGCTCAGCGCGTTCAGCCCCTGACTGCGTGAGGCGCTAAGCTGGGCGCGTAAACCCAGCTCATCAAAC
AGCGCCAGCGGTGAACGAGCCTGTAATTCGGCGGCGTTTTTTCCTTCCACTGCGGTGAGCAATACCGCCAGCA
GGCCGCGGGCAATCCGCCCCTCACTGTCGCCGAAAAAGTGCATCGTTCCGTTATCTGACTGGGTAAAGCCCAG
CCAGACGCGGTTCTCGCAGCCGGCTATCTCTTTCGCCTGCGCTTTGCATTCATCCGGCAGAGCGGGGAGCTGT
TTTCCTAACAGAATCAACTGACGATATTTATCCTCCCACTGGGTCAGCGGGAGAAAGATAGCGCGTAACGTCT
CTTCCGTTACCGTTGTGCCGAACGGGTGTCCGGTATAAAGAGGGTTTGTCATTAATCCACCAGCAGTTCCAGC
GCGCGGTCAACGGCGTTAACCAACGCATCCACATCATGTTGGGTATTATACGGCGCAAAAGAGGCGCGCAGAG
TCCCTGTGACGCCAAGTTCCGCCAGCAATGGCTGGGCGCAATGTTGCCCGGCCCGGAGCGCAATACCGTATTC
CGCCAGCAGCGTCACCATATCGCCGTGGTGCACGCCGACAAAATCAAAGGCCAGCAGGCTGGAGTCCTGGCAG
CGGAACGAACGAAAGCCCGGACGTTTCGCCAGTGCGTCTTCCGCCAGCGTCGCCAACCCCCGGCTCCAGTTTT
CGGCCTGTTCAATATCGATATCGGACAGCCATTCCAGCGCAGCGCTCAGGCCGATGACCCCGGCGACGTTCGG
CGTCCCCGCTTCCAGCTTCCAGGGAGCGCTTTGAGTGGTGAAGCCTTCAAAGCTAACGTCACGGATCATCTTG
CCGCCGCCGAGCCAGGGCGACATCGCCTCAAGAAGCTCCGGCTTACCGTACAGCACGCCGATACCGGTCGGGC
CATACAGTTTGTGAGCGGAAAAAGCATAAAAATCGATATCAAGCTGCTGGACATCCGCCGGGAAGTGTACCGC
GCCTTGCGCGCCATCTACCATCACGACCATTCCCGCTGCGTGAGCGGTGCTGATAGCGCTTGCGAGATCCGGG
CAGCCGCCCGTTACGTTCGACATTTGCCCCAGCGCCAGAATCCGGCTGCGCGACGTGATCAGTTCCGGCAGAC
GCTCAACATCAGGAAGACGTCGGTCATTAAGCGGCAGTTTTATGACCTGCGCGCCGGTTTGTTGCGCCACCAT
CAGCCAGGGCACAAGGTTGGCGTGATGCTCGGCGACGCTAACGATAATTTCATCGCCGGGGCGCAGACGAGGA
CGGGCGTAACACTGCGCCACCATGTTGATCGCTTCGGTGGTGCCGCGTGTCCAGACGATACTTTTTTCATCGG
GCGCGTTTAACAGCCGCGCTGCTTTTGCTCTGGCCGCTTCATATTGCGCCGTCAGGCGCTGCGCCTGCGCAAA
CTGGCTACGATGAACGTTACCGGCGCTCAAACAATAAAACTGGTGCGTGGCGTCAATGACTGCCTGTGGCTTT
AATGCCGTGGCGGCGCTATCGAGATAAACACCCGCATCGGCTAGCGCGGGAAACTGCGCGCGAAACTGCGTGG
GATTAAAAGCGTTCATGATATTCCTCGGTTCAGTTTACCGATCGTGGCGTAAAAGCGCGGCTGATACAAGGTT
TGTCCTAACCATCGGAATCGTCTGTATATCCTGGCGAATTATGTAAAGGAGGTTATGCTGAATAATGTTAGGC
GAATGTTTAAGCCTGTTACGGAGTCCGCTTTAAATAGCACAAACAGCTTTAAGGAGAAGAAGATGAAAAAGAC
TGCCGCAATTATCTCTGCATGTATGCTGACTTTTGCCCTGAGCGCCTGTTCCGGTCCTAATTACGTTATGCAC
ACCAATGATGGACGTAGCATTGTGACCGACGGTAAACCCCAGACCGATAACGATACCGGTATGATTTCGTATA
AAGACGCTAATGGCAACAAACAGCAGATCAATCGTACTGACGTGAAAGAGATGGTGGCTCTGGAAAACTAACC
GTTTGTTATACAAAAAAAAGCACCGCAATATGGCGGTGCTACATTAATCACTATGGACAGACAGGGTAAATGT
ACAGGAAGTGAAAAAGGGTAGTCTCGCTACCGTGGTCTGAATCGCAGACCAATTGCAAACACAACAACACAAC
ATCACAACCGTAAGCCAAAAGCCCATCAGAACACGCATTCCGATAAAACTTTTCGTTCCGGCTCAGGAAGTGC
CGCCACTATAGGTATTTGCTGGTAGAACCTCAACGGACAATTTATAATGTCTCAGATTAAAAAAACTAATAGG
TTACATATTGTTACCTATTTGTTAAATTCATTCGACATTAAGTCCAGAGGCCATGTCAAAACGATTACCTCCT

TTAAATGCGTTACGCGTTTTTGATGCTGCCGCGCGCCATTTGAGCTTTACGCGAGCAGCGGAAGAACTTTTTG
TGACGCAGGCCGCAGTAAGCCACCAAATTAAGTCACTTGAGGATTTTTTGGGGCTAAAACTGTTCCGCCGCCG
CAATCGTTCGCTGCTACTGACGGAAGAAGGCCAAAGCTATTTTCTCGACATTAAAGAGATATTTTCCCAGCTC
ACGGAAGCAACGCGTAAGCTCCAGGCGCGCAGTGCCAAAGGGGCGCTGACGGTGAGTTTACCGCCCAGTTTTG
CCATCCAATGGCTGGTGCCCAGGCTCTCCAGCTTTAACTCAGCTTATCCGGGAATTGACGTTCGGATACAAGC
CGTTGACCGTCAGGAAGATAAACTGGCAGACGATGTCGATGTCGCGATTTTTTATGGCCGGGGAAACTGGCCG
GGCTTGCGCGTCGAAAAATTATACGCAGAATATTTACTGCCCGTCTGTTCTCCTTTATTACTCACAGGCGAAA
AACCGTTAAAAACGCCGGAAGATCTGGCGAAACACACGTTATTGCACGATGCCTCCCGGCGTGACTGGCAGGC
CTATACTCGTCAGTTAGGGCTCAACCATATTAACGTTCAGCAAGGGCCGATATTTAGCCACAGCGCGATGGTG
CTACAGGCCGCTATTCACGGACAGGGGATCGCGCTGGCGAATAATGTTATGGCTCAGTCGGAAATTGAAGCCG
GGCGACTGGTGTGTCCATTTAACGATGTTCTGGTGAGTAAGAATGCTTTTTATCTGGTTTGTCATGACAGCCA
GGCAGAACTGGGTAAAATAGCCGCCTTCCGTCAGTGGATTTTGGCGAAGGCGGCGACGGAGCAAGAAAAATTC
CGTTTTCGTTACGAACAATAATTAACTTAGGGTATGACCATGACCAGCCGTTTTATGCTGATTGTCGCCGCTA
TCAGCGGTTTTATTTACGTTGCTCTGGGCGCGTTCGGGGCGCATGTTTTGAGTAAAACCCTGGGCGTGGTTGA
AATGGGCTGGATTCAGACCGGCCTGCAATATCAGGCGTTCCATACGCTGGCGATCTTTGGCCTGGCGGTGGCG
ATGCAGCGCCGTATCAGCATCTGGTTTTACTGGAGCAGCGTTTTTCTGGCGCTGGGCACGGTGCTGTTTAGCG
GCAGTCTGTACTGCCTTGCGCTTTCGCATCTGCGCCTGTGGGCGTATATCACGCCTGTCGGCGGCGTCAGTTT
CCTGGTGGGCTGGGCGCTATTGCTGATTGGCGCCATTCGTATGAAACGTAAGGGCGCGAGCCATGAATAAAGT
TGTATTGCTCTGCCGTCCGGGGTTTGAAAAAGAGTGCGCTGCAGAAATTACCGATAAAGCGGGAAAACGGGAA
ATATTTGGTTTTGCACGCGTAAAAGAGAACGCAGGCTATGTCATTTATGAATGCTACCAGCCGGAAGATGGCG
AAAAGCTGATTTCAGAGCTGCCGTTCAGTTCGCTGATTTTCGCCCGCCAGTGGTTTGTGGTGGGTGAACTGCT
GCAACATTTACCGCCGGAAGACAGAATTACGCCGATTGTCGGCATGTTGCAGGGCGTAGTGGAAAAGGGCGGC
GAGCTGCGAGTTGAAGTCGCCGACACCAATGAAAGTAAAGAACTGATGAAGTTCTGCCGCAAATTTACGGTGC
CGCTGCGCGCGGCTTTGCGTGATGCGGGCGTATTGACGAATTATGAAACGCCGAAGCGTCCGGTCGTTCATGT
CTTTTTTATCGCGCCAGGATGCTGCTATACCGGTTATTCGTTCGCGCATAACAATTCACCGTTTTATATGGGC
ATCCCGCGTTTGAAGTTTCCGTCTGATGCGCCGAGCCGCTCAACGCTGAAACTGGAAGAGGCGCTCCACGTTT
TCATCCCGGAAGATGAGTGGGATGAACGTCTGGCGAACGGCATGTATGCGGTGGATTTGGGCGCCTGTCCCGG
CGGATGGACGTACCAGTTAGTGAAGCGCAATATGTGGGTTTACTCCGTAGATAACGGCCCGATGGCGCAAAGC
CTGATGGACACCGGACAGGTGACCTGGTTGCGTGAGGATGGCTTCAGGTACCGTCCGAACCGGAATAATATTT
CATGGATGGTCTGCGATATGGTGGAGAAGCCGGCCAAAGTCACCGCGTTAATGGCGCAGTGGCTGGTGAACGG
CTGGTGCCGGGAGACGATTTTTAACCTTAAACTGCCGATGAAAAAACGCTATGAAGAGGTATCGCATAACCTG
GCGTATCTTCAGGCGCAGTTGGATGAGCATGGCGTTAATGCGCAGATCCAGGCGCGTCAGCTTTATCACGATC
GCGAAGAAGTGACGGTACACGTGCGTCGGTTGTGGGCGGCTGTTGGCGGGCGTCGCGACGAGCGATAAAAACG
CGCTGCCGTCATCCGGCGCGTCATCAGCGCCGGATGACGCACGCTTATCCGGCCTATGACTGCGGCGCATCTG
AAACAATATGCGTCACGTCATCCAGATGGCCGATGCGCGCCTCTCCTGAACGATTAAATTTACTTTTATCAAT
CAATAATAAAGCCTGCGAGGCACGCCTGAGCAGGATAGATTTAAAATCGGCGTTGATCGCATTGGAGTCCCAC
AGATCGCCGCCGCCGTCGATCCCCTCACAGGAAAAGATGAACAGATCGATATCAAGCGATTTTAGCTGTGAGA
TAAGCGACGGATTAACATAACAGCCATATTTACGTTCAAGCTGGCCGCCGGAACTTATCAGCGTAATGCGTTC
GCGTTTTCCCAGCTCCTGACAGATAGGATGGCTATTGGTAAAAACCTGAATGGGAATATCCGGCAACTGGCGG
GCCAGATACCAGCAGGTTGAACTGGCATCCAGCGCAATCGTCATTCCTTCTTCTATCCACGCTAACGCTTCAC
GGGCAATATCGGCTTTGTGCGCATAATGGCTTTTCAGGCGGATATGGAATGGATCGCCGCTGTCCTGATTTTC
ACGATGAATATATTTAGCTCGTCCGTGATTACGCAGCACTTTTCCCTGCGTCTGTAGTTCACTGAGATCGCGG
CGGATGGTCTCTTTACTGACGTTAAGCCGGGTGGCCAGCGCTTCGGTGGTCAGACTTTTATGATTGATCAGCA
GATCGACTATCGCTTGTTGGCGTGCCGCTTTCATTTTTCTCACCCCATCATTCGATGAACGCCCGCGTCATGC
GGGCGCACCGGCAAGAGATTACGGCGTTACCCCTTTTTTTAAAAGAATATTTCCGTACTTCGCGCACTCACCT
GTGATAACGATCGCAAACGCTTTTTGCGCCCGCTCATAAAACGCATAACGGTCGATACGAACGATATCCGGGC
ACGGCGCTTCTAATGACAAAGCATCGCGATAGCGCGCTTCAACGCTGGGGTCGAGCGTGTCGCCTTCCACGGC
GGCCATCATTACCAGCGGCGGGGCGTAGCTATCCAGCTCAAAGAGCGGAATAATTGCCCGCAGTAGATCGCTG
ACGCTAAGACCATCGGCGCGAATCACCTGTGGGCCCAGGCTATGCGCGGGAAAGTGAGCGTCGGAAAAAAATGA
TTTCGTCCCCGTGGCCCATCTCGGCCAGCACTTTGAGCAGTGTCGGGGAAATTAACGGTGAAATGGTTTTTAG
CATTTCATACTCCCTCAATAATTTCAGGTTCGGTTTGCGGCCAGAAATAGCGATACTGATAATTGACCTGCGC
GCGCGCCTGTTCCGGGCTACTAAATTCGCCTACGCCGTACCAGCCAAACATGGCAGCGCCGGCCACGGTTGTT
TCCGCGTCGTCCAGCACTTTGATAGGGATATCAAGCTGATTCGCTTTAATTTGATTCCACAAAGCGTTGCGAC
TGCCGCCGCCAACCAGTAACAGTTCTGTGGCGTTAAAATGACCGATTTTCTCCAGCGTGCGCAGATTACGCTG

CAGTTGCGCCGTCAGGCCTTCCAGCGCGGCGCGATAGAAATGGCCGCGGGTGGTATTGAGCGTCACGCCCTGC
CAGCCTGCATTTTGGCAGGCGAGCAGATCGCACTGCATCCGTACGCCTTCCGCGCCAGCGGAAATAGCGCGCG
CTTCGTCGATCAGCGTTTGCCATGGTGTTTCCGGCGTCCACAGCAGTTTTCGTACCCATTCGAGTACACCGGA
GGCCAGCCACTGCATTCCGGGGTTATAAAGCCCCGACTGACTGTCCAGTTCACAGGTCGAGCCCGGATACTGA
CTGAGCAGCGAGGTATCCACCTGACCGCTGCGTACCATCAGAATCTCCCATGTTCCGGAGGAGAGCACCGGTT
CACCTTGCTGCGCGCCAGCGCCGAATAACGCGAACTGAGTATCGTGTCCGGCAGAGATCACCGGCACACCCGC
CGGGAGACCGAGTAGCCTGGCCGCGTCTGTCTGTAGCGTGCCTATCTGGGCGCCTGCTTCCACGATACGCGGG
AACAGGCGGCGCGCCAGGCCCGTCGCCTGCAGGATTTCCGGACTAAAGTCGCGCTGATGGATATCCAGTAGCT
GGCTGGTGCCTGCCATAGTGATATCGGTTGTAAACTCGCCGGTCAGACGGTGGTTAATCAGCGACGAGATAAA
CAGCCAGCAGTGAGCTTGTTCCAGCAGGCGCGGATGATTCTCTTTAAGCCAGACCAGTTTATACAGCGTGTTA
AAGCTGAACGCGCCGACGCCGGAGAGCGTCTGCAATTGCCGGGGCGAGATAAAGCGCTCAATCGTTTCCATCA
CGGCGGCGGTTCGCGGGCATTTCCAACTGATGACCGGGTAGAGCAGCTTACCCTGCGCATCGACCAGCGCGCC
ATCGACGCCGAACGTAGTCACCGTAATACCGCGAACAACACACTCCGACAGGGCGCTGGAGAGTGACCTGCAG
CAGTCGGCGAAACGTTGCAGGATGGCGTCCAGCGACCACTGATGCCAGGCGCTATTTTCTGCGGCAATGTCGC
TGGCGTTAGCGGTTGAGGCGCGGGCGACAATTTTCCCCTGGCGATCAACGGCGATCGCCCGAACGTTAGTCGC
ACCGCAGTCGAGAACCAGGATAACGTCTTGTTTCATAGATAACTCCATAAACGCCGCCCGATGGCGCTACGCT
TATCCGGCCTACGGTTCACGCGCCCGTAGGCCCGGTAAGCCTGCGCCACCGGGCTAAGCCGCATTAACGTTTA
TACAGCGGGCCATAATTTTGGCAGGCGCGATAATCCTGGCCTTCGATATCCATACCGTGAGCCGCCCAGGCGG
AAGGACGGTAGATCTTCGCCTCCTCCACGTTGTGCATACATACCGGGATACGCAGCATCGCGGCGAGAGTAAT
AAAGTCTGCCCCTACATGGCCGATGGTGAGTACGCCGTGGTTAGCGCCCCAGTTCGCCATCACCGAATAGACA
TCGGTAAACGGGCCTTTGCCGGTAAGACGCGGCGCAAACCAGGTGGTAGGCCAGGTAGAGTTGGTGCGGGCAT
CAAGCTGATCGTGCATCGCTTTCGGCAGTTCGACGCTCCAGCCCTCGGCGATTTGCAGTACTGGTCCCAGGCC
TTTGATGATATTCACGCGAGTCATGGTGAATGGCACGCCGCCTTCGGTCAGAAAACGGGATGAGTAGCCGCCG
CCGCGGAAGTATTCGTGAATCGCCGGGCACCATTCGGTGGCCGCCAGACACGCATCCGCCTCTTGTTGGCTGA
TTTCCCAGTGCGGTTTCATGGTCGGCTTGCCTTCGCTGTCGCGCTGTTTACAGGCACCGTCCAGAGCGGCAGA
ACCGGAATTAATCAGGTGAATAATGCCGTGTTCCGCCAGGCCGCTGAGCGCCTGGCCGGTGACGCGTTCTACG
GCTTCCGGCGACCAGTAGGTTCGCACGTCGGCGAATATCTGCGCGGTACCAGTGAGTTGATGACCGAACAGCA
TCGCTACGCCGTTCAGACTGTCATTTTCGGTGGCGACGACAAACGGTTCGCGTACGCCGTTCCAGTCAAAAGA
ACTATTGAGCAGCGCTTCGGCGGTATCGCCGTTCGGGTATTGATCGGTCCAGTGGCGCTGGCCCTGGAAGCCG
GCGGCAATGGCGTTGTAGCCCAGCGATTCTTCAACTAACCCTTTGTCCGCCAGCGTCTTGTTGCCCTGCATCA
TATCGCGAATACACATCGCCATCAGCAGGCTCTCTTTTAACACCGCGCGGTTCTGGGCTTCATTGCGCTTATA
CTGCGAGGCGTTCTGATCTTCGCCGTAGCGGAAGTTTTTATCCGCCCACGCCAGCGCCATCTCCAGTTCGGCC
TCATCATAAATTTTTTGATCGATACGGCGGCGCAGTTCGGTCATATCTACCGCTTGCACCTTCATGCCCAGCC
AGGATTCAAAGAAGTTATGATCGACAATGGAACCCGCGATCCCCATCGACACGCCGCCGACGGAGAGGTAGCT
TTTGCCTTTCATGCTGGCTACCGCTAACCCTGCCCGCGCAAAACGCAGCAGTTTTTCTTCGACGTCGGCAGGA
ATGGAGGTATCGTCGGCATCCTGCACGTCATGGCCATAAATGGAGAACGCTGGAATGCCTTTCTGGCTGTGCG
CCGCCAGCGCCGCGGCAAGGTAAACCGCCCCAGGACGCTCTGTGCCGTTAAAGCCCCAGATGGCTTTTGGACG
CATCGGGTCCATATCAATGGTTTCGCTGCCATAGCACCAGCACGGCGTAACGGTAATGGTCACGCCCACGTTC
TGGCTACTGAATTTTTCTTCACAGGCGGCGGATTCCGCCATTCCGGCAATACAGGTATCCGCAATGACGCACT
CCACTTGTGCCCCACAGGCGTGGCGCATTTTCTCGGTGATGAGCGCCGCCGTCGCTTTCGCCATGTTCATGGT
TTGCTCTTCGAGCGACTCGCGTACGCCCATACGACGTCCATCAATCACCGGGCGGATACCGATTTTCGGTAAG
CTGATTTTTTTCATGACAGATTCCTCACGTTATTCGGATAAATGTTCAGTTTGTCGCCGCTTGTGAACGGAAA
CGGGCAAAAATGAAGATGACGGCAAAGCACAATGCCGGAACCAGTTCGGCGGTCGGGATTTTGCCTGCGGCGT
CGCTAACGAAGCCCATTACTGGCGTGACAATACCGCCGCCAATAATGGTCATGACGATAAAAGACGAGCCGTA
CTTAGTGTCCTGTCCCAGATTTTTGATACCCAACGAGAAGATGGTCGGGTACTGGATTGACATAAATGCGCTA
CACAACGTCAGCGCCAGCAGGCCGATATGTCCGCCGCTAAAGGCGGAAATCAGACACAGGAGCATGGCAAACA
GGGCGTAGGCGGCCAGCACTTTATGCGGCGCGAAGCGGCTGATAAGCCAGGTCCCGGTGAAACGGCCGATAAA
GAAGCACACCATCGTGCCGGTCAGGTAGTTGGCGGCGAAACCGGGCGTCATTCCAGGAATCTCCTCAATGGCG
TAGCGGATCAGATAGCTCCAGCAGGCGGTTTGCGCCCCCACGTAGCAGAACTGCGCCAGCACCGCCCAGCGCC
AGTGGCGAATACGGATGAGTCGGGAGAGAGAAGATAAGAAAGAGCTTTGTTTAGCATCGCTATGATCGTCACT
TTGCAGGGCCGGAAATTTGGTCAGCATAATCAGCAGCGCAACTACTAATACGATGGCGACGATAATCATGTAT
GGCGTTTGTACCGATAACACCAGGCTGTGTTTATAGGCGCTAAGCTGATCCGGCGTCATTTTATCAAGCGCTT
CTTGTGATTGATGCGGCACGTTAGACAAAATAAGGCTTTGCCCAAACACAACGGCGATAATAGCGCCAAAGGA
GTTAAAAGTTTGCGCCAGATTAAGCCGGAAATGTCCGCCGCTTTCTGGACCTAATACCGTAACAAAAGGATTG

GCCGCAGTTTCAAGGCAGCCTAAACCGGCGGCGATGATAAACAGGCCAATTAAAAATAATGTGTAATTCATTA
TCTCGGCGGCAGGCCAGAATAATGCGGCGCCAACAGCATACAAAAAAAGCCCGGTGATAATCCCTGCTTTATA
ACTGAGCTTTTTCATCAAAATCCCAGCGGGAATTGGAATGACGAAATAACCGAAATAAAAGGCTGACTGAATA
AGTCCGGCCTGGAAGTTAGTTAGCGTAAAAGCTTGTTGAAACTGCGGTAATAAAATGTCATTCAGATTATTGG
CGACGGCCCACAGAAAAAAATAGCGAGCAAAGTAAGGCGAATGGAATAATGTAGCGCTTGCTTTTGCTTTGCTC
TGCATCCACAGCACGAAAACTCTGTGTTTGTATTGTTGTGTTTCCCATAGCATCCTCATCGGTTCAGAAGCTT
TTGCGTGCGGTCGGTTCTGGTCACCAGACCGTAAAATTAGGTGAGTTCGCTTTTCTTACCGGTAATAAATTGG
CGTCCGGAATGAACTCATGGAGTCATTTTCAAATAAAAAAGGCCTTTTAATATGATGGCGGTCACATTTATAC
CTGAGAGCAGAGCGTTCTGTGATTATCATCACTTTAATTTCATAAAAAAATGACCGTTTGAGATGTTTAATT
TAAAAAACCACAGACTGAATGCCCGAATTAAATTATTGCCACGAAAACGGGCAGTAGTAACTTATTCTTCATC
TTTGCCCGTTTTAAAAAATATTATTAAGAGCGGGCATTTATCGGCGAGAGAAACGGTAGTCGTCTGGTTTATC
GTGGCGAATTAATTGATGTGAATCACAAAATATCGTTATTTAAATTCCCTTCTCTTTATTCAGTATGATGATA
GTTGCTAACTATTCGATTATTTCAACAGGACAGCAGCGCGATGCGAAAGGCGGTTTTGTTGTCTCATTTTGAA
TCACAGAGAGAGGTAAATAATGGAACGAAATAGACTGGCGCGGCAGATTATTGATACCTGTCTGGAAATGACG
CGGTTAGGGTTAAATCAGGGGACGGCAGGTAACGTGAGCGTACGTTATCAGGGCGGATTGCTGATTACGCCGA
CGGGGATCCCCTACGAGAAGCTGACAGAGTCGCACATTGTCTTTATTGATGCTGACGGTCAGCATGAGCAGGG
CAAGCTGCCTTCCAGCGAGTGGCGTTTTCATATGGCGGCATACCAGACGCGCCCTGACGCCAATGCCGTGGTA
CATAACCACGCTGTCCATTGTACGGCAGTCTCGATCCTGAATCGCCCGATTCCGGCGATTCACTACATGATTG
CCGCCGCAGGAGGAAACTCTATTCCCTGCGCGCCCTACGCCACTTTTGGCACCCGCGAACTTTCTGACCATGT
TGCCGTGGCGCTTAAAAATCGTAAAGCGACGCTGTTACAGCATCACGGGCTTATCGCTTGTGAAGAAAATTTA
GACAAAGCGTTGTGGCTGGCGCATGAAGTGGAAGTGCTGGCGCAGCTCTATTTAAGTACGCTGGCCATTGTCG
ATCCGGTACCGGTACTGGATGACGAGGCGATCGCTATCGTACTGGAGAAATTTAAAACTTACGGATTACGTAT
TGAAGAGTGATGTCAGGAGACAGGCGATGGCGAACAGAATGATTCTGAATGAAACGGCATGGTTTGGCCGGGG
AGCGGTGGACGCATTAACCGATGAGGTGACGCGCCCGCGGTTATCATAAAGCGCTGATTGTTACCGATAAAACG
CTGGTGCAATGCGGTGTGGTCGATAAAGTGACAAGCAGAATGGATGCCGCAGGGCTGGCGTGGGAGATCTATG
CTGGCGTGATCCCTAATCCTACGATTTCCGTTGTCCAGGAAGGGCTTAAGGTCTTTACCCAAAGCGGCGCGGA
CTATCTCATCGCCATTGGCGGCGGTTCTCCGCAGGATACCTGCAAAGCGATCGGTATTATCAGTAACAATCCG
GAATTTGCCGATGTGCGCAGTCTGGAAGGGCTATCGCCGACCCGTAAACCCAGCGTGCCGATTATGGCGATCC
CGACAACGGCGGGAACCGCGGCGGAAGTCACTATCAATTATGTGATTACCGATGAAGAGAAACGGCGCAAATT
CGTTTGTGTCGATCCGCATGATATTCCGCAGGCGGCATTTATCGATGCCGATATGATGGACGGTATGCCGCCC
GCGCTGAAAGCGGCAACGGGCGTGGATGCGCTAACCCATGCTATTGAAGGCTATATTACCCGGGCCGCATGGG
CGCTGACGGATGCATTACATATCAAAGCCATTGAGATTATTGCTGGCGCGCTGCGCGGCGCCGTGGCCGGAGA
AAAAGAGGCCGGTGAGGCGATGGCGCTGGGGCAATATGTTGCCGGGATGGGGTTTTCCAATGTGGGGCTGGGG
CTGGTGCATGGGATGGCGCATCCGCTGGGCGCGTTTTACAACACGCCGCATGGCGTCGCGAATGCTATTTTGC
TTCCGCATGTGATGCGTTTTAATGCCGGGTCTACCAACGAGAAATTCCGTGATATCGCCCGGGCGATGGGGGT
GAAGGTAGAAGGATTGAGTCTTGAAGAGGCACGTAATGCCGCAGTGGAAGCCGTATTTACGCTAAATCGCGAT
GTGGGAATTCCGCTGCATTTGCGTGATGTCGGCGTTCGAAAAGAGGATATCCCGGCGCTGGCGCAGGCGGCGT
TCGATGATGTCTGTACGGGCGGCAATCCGCGTGAAGCGAGTCTGGCGGACATTGTCGAACTGTATCATATCGC
CTGGTAATTTTGCCGGATGGCGCTGCGCTTATCCGCTCTACATTTCGTGCAGTCCTGTAGGCAGGATAAGGCG
TTCACGCCGCCATCCGGCAACAAAACTACCGCGCCAGCCGTAGCTGCTGTAAGTTTCCGTCGATATGTAAATC
GGTTTGCAGACGTGCAATATCGCGGCAAAGAAATGCCATTTCTTTATGCGTCTCAAGCTTTTTGCGCCACTTT
TCCGGCACCTCGTCCAGATGGGCGTAAATTCCCTCCAGATTTTGAAACTGAATCAACAGCTGGGTCGCGCTTT
TGGGGCCGATACCGGCAACGCCGGGGACTTTCGAACTGCTGATTCCTGCCAGACCCCAGTAATCCGGCAACTG
CCGGGGAAGTACGCCAAACTCTTTTTCAATAAACGGCGCGTCCAGCCAGCGTTTCTGGAAGTAGTCGCGAATG
CGCAATCCCGGAGAGAGCAACTGGCAATAGCCTTTATCGGTTGACACAATGGTTGCCTGGTGTCCGGCTTCGG
TAACTTTTAGCGCCAGCGTCGCCGCCAGATCGTCCGCTTCGTTACCGTCTGATGCCCAACAGCGTATGCCGCG
CTGTTCAAATGCGGCGCGTAGGGCGGGCATTTCATTATGTAAATCGTCAGGCATCGGCGGACGTCCTGCTTTG
TAGTCCGGTAAACGCTGATGACGCCAGCCGCTGCTGCGCGCGTCGTCATCAAATACCGCTACGGCATGGGTTG
GCTGGCTGTGGATAATCAATTGATCGAGCGCGTGTTGGCAGGTTTCCACACAGGGCGATCCCTGAACGGCGTG
AATACGGCGAATCAAATTGAGCGCGTCGACGATAAGCAGATGAGCAGCCACGGTGTTCTCCCTTACAAACCTG
CGCGTAGGGTAACATTCACGGCGGGGGGAGGCGAGCATTGCGAAATAGCGCTATCCGTAGGCCTGATAAGCGA
AGCGACATCAGGCAACAAAGCCGGATGGCGGCGAGCGCCTTATCCGGCCTACAGATCCTAAAATGTGCTGAGA
ATTAATCGCAGGTAACGATCTTCATCGCCAGGCCGCCGCGCGAAGTTTCGCGGTACTTAGCGTTCATGTCTTT
ACCGGTTTCGTACATGGTCTCAATCACTTTATCGAGGCATACACGCGGTTCGCTGGTACGGCGCAGCGCCATA

CGCGCGGCGTTGACCGCTTTGACTGAAGCAATCGCGTTACGCTCAATGCAGGGAACCTGTACCTGACCGGCAA
CCGGATCGCAAGTTAACCCCAGATTATGCTCCATGGCGATTTCTGCGGCGATACACACCTGCGCCGGACTACC
GCCCAGCAGCTCGGCCAGACCCGCTGCCGCCATGGAACAGGCCACGCCGACCTCTCCCTGACAACCCACTTCG
GCGCCGGAAATAGAGGCGTTCATCTTATACAGCGAGCCAATCGCGCTGGCGACCAGCATATAGCGAGCAAGTG
AGTTAGCATTTACTTCGCGGATAAACTTGTCATAGTACGCCAGCACCGCCGGCACAATACCACATGCGCCGTT
AGTCGGCGCAGTGACTACTCGTCCGCCTGCGGCATTCTCTTCGTTCACCGCCAGCGCGAACATATTGATCCAG
TCGACGACCGCCATCGGATCGGTGGTGGTCTTATCCTGACTGACCAGCACCCGACGCAGCGCCGCCGCGCGAC
GCGGAACGCGCAGTTTGCCCGGCAATACGCCCTCGGTGGTGATACCGCGCTCAATACCGCTGCGCATTACCTC
CCAGACCCTGGCGAAGTGCTGCTCCAGCGCTTCTTTACTGTGCAGAGCCAGTTCGTTTTGCATCATCAGACCG
GAGAGCGACAAACCCGTCTCCTGGCAGTGGCGCTGTAAATCCGCGGCGGTTTTGTAAGGGTACGGTACGTTAA
CGGGTTCGCTATTCGTCAAACCAAAGTGCTCTTCATCGACGATAAAGCCGCCGCCGATGGAGTAGTAGGTTTG
GCTGTAGAGCACCTTATCGCCCGCCAGCGCGGTAATACGCATCCCGTTCTCATGCAGAGACAGGTTGTCGGCG
TGGAAATTCATGCACTTATCGACCGGAAATTCGACTTCATGCTGCCCATTAGCCAGTAGTAAACGCCCATGGG
TGTTCACATCCTGGATAAAGGACGGAATGGCGTCGATATCGACGGTGTCCGGCAGATTCCCCGCCAGGCCCAT
AATAATGGCGATATCAGTGTGGTGGCCTTTACCCGTCAATGACAGAGAGCCGTAGACATCCACCACGACGCGG
GTGACATCTGTAAGGATATGGCGGGCAATCAGATCGTCCGTGAACTGCTTGCCTGCTTTCATTGGTCCGACGG
TGTGAGAACTGGAAGGGCCAATGCCGATTTTAAAAATATCGAATACGCTAATCATAGGGCATCCATTGCGGTT
GTATCATAGAGAAGCGCCGCGGTAAGCGGCGCTAAAGGACTTAGCTGAACAGTGAGTAGAAAATAGCGGAGAT
GGCAATCAGGCCCATAATCACAACGAATACGTTGCTGATATGACCGCTGTACTTACGCATTGCCGGTACTTTC
TGGATGGCATACATCGGCATCAGGAACAGAATCATCGCGATGATCGGGCCGCCCAGGGTTTCAATCATGCCCA
GGATGCTGGGGTTCAGCGTCGCCACAATCCAGGTGGTGACCAGCATGAACAGCGCGGTAATTTTATTCAGTTT
GTTGATTTCGATGGATTTACCTTTACCACGCAGCGATTTAATCACCATCCCGTTAAAGCCTTCACGCGCGCCC
AGGTAGTGGCCGAGGAAGGATTTGGTGATAGCAATCATCGCAATGATCGGCGCCATCCAGGCGATGATCGGCG
CGTTAAAATGGTTCGCCAGGTAAGACAGAATAGAGATGTTCTGCTCTTTTGCCGCTGCCAGATCTGCCGGCGT
CAGGCTCAGCACGCAGCTGAAGACGAAGAACATCACGGTCAGCACCATCATGATGTGGGCGAAGGCCAGAATT
TTAGAACATTTCTTCTCTGCGCCTTCACCGTACTCTTCACGCTTCGCAACCGCGAAGGAGGAGATGATCGGGG
AGTGGTTGAAAGAGAAAACCATCACCGGAATCGCCAGCCACAGCGTCATCCACAGACCGTTACCGGTAGACGC
GGCGGAATCAAAGGACAGAGTTTCCAGCGCCGCGCCGTTCCACTGCGGGATCAGGTACAAGGCCAGCAGCATC
AGCGCAGCGACGAACGGGAATACCAGGATACTCATCGCCTTAACGATCATTTGCTCGCCGAAGCGCACGATGG
TCATCATGCCAACAATCAGGATCAGGGAAAGAATCGCCCGCGGCGGCGGATTAATCGCTAACTGGTGGGTCAG
GAAGCTTTCCACGGTATTAGTAATCGCCACGCTGTAAACCAGCAGGATGGGGTAAATCGCGAAGAAGTAGAGC
AGGGTAATCAGTTTACCTGCGCCGATACCGAAGTGTTCCTCAACGACTTCAGTGATGTCTTCACCCGGGTTTT
TACCGGACAGCACGAAGCGTGTTAAGCCACGGTGTGCGAAAAAGGTCATCGGGAAAGCGAGAATCGCCATGAT
GATGAGCGGAATCATGCCGCCGACGCCTGCGTTGATCGGCAGGAACAGAACGCCCGCGCCGATAGCCGTGCCG
TAAAGGCCCAGCATCCACATGGTATCCGTTTTGCGCCATGCGCTTCGAGAGTCAATCGAAGCAATAGTGCTGG
TCTGAGTGGTTTCCATCTATTTCTCCTGGAGGATGCTAAAAATCAGGTTGCTGGTCTAATCCAATGAAAAAAG
ACCTGATCGTAAAATGAAATCTGCTTAGGGACGCTTTTTATATAATTTTTCGCCGTAATAATTTTCGGGCGGA
AAGATACATTTAAGTGATGAATCTATCAGTGATCCCGATCTCAAATGCAATAATCCGCTTCTTACATGGATAA
ATTTGGACCATTAATCTATTAAAAAAACGTCATAACGAATTTCCGGGCGCGAAGGCTACCATTAACGATATAA
GGCCGGAAAGATTGGGGCGCAAGATACGGAGGTTTGACTATAAAAATCAAGGTTTTTAGTGGATTCTTGCGAA
GAATGAGAGTTTGAACCGATAAATCACGGTAAGAGGAGTCAGGTAAACGTTTGCCTGGCAATCTATTTATGGT
GAATTATAAATGGTGTTTATAAAACAATGCGGGCTATGCCTCTGATGATCATCATAAAAAATGAAGGCATACG
TGAATCGCCATACCGCAATGAAAGGAATGGGAGAAGCGCCCGGCTGCTGGCGGCAACCGGGCATAAGCGTTAA
GCGCAGATCTCGTAACAGGGGATGTAGGCGGAACCCGGCAATTTCATACGATGCTGCGCGACGAATCCCTGCA
GCAGATCGTCCATGCGACGCATCATCTCTCGATCGCCGTGAATTTTATACGGACCATTAGCTTCAATGGCGCG
TATCCCGACCTCCTTCACGTTCCCCGCGACAATACCGGAGAAGGCGCGCCGCAGATCCGCCGCCAGAATCTCA
ACCGGCTGATCCGGGTACAGTTTCAGGTTGGCCATATTCTCATGCGACGGCTCAAACGGCACCTGCAGATCCG
GTGCAATACGCATCGACCAGTTAAAACTATAGGCGTCGCCAGTATCGCGGCGATTCTCTTTCACTTGCGGCAT
CGCTTTCTTCATCAGCCGCGCTACCTCCGCCGCATCGTCAATGATGATGCGGTAATGACGGCGGGCCGCTTCG
CCCAGCGTATGGGTAATAAACTCATCCAGCACGCGGAAGTAATCGGCGCTCTCTTTTGGTCCGGTCAGGATCA
GCGGCAGCACCTGATTTTTATTGGCTGGATTCATCAGAATCCCCAGCAAATAAAGCAGCTCTTCCGCCGTACC
CACGCCGCCCGGGAAGATGATGATACCGTGTGCGATACGGACGAACGCTTCCAGGCGTTTTTCGATATCCGGC
ATGATGATCAGTTCGTTCACCAGCGGGTTAGGCGGTTCAGCCGCAATAATGGAAGGTTCAGTCATGCCGATAA
AGCGGCTGTCTTTATAGCGCTGCTGTGCGTGACCGACCGCCGCGCCCTTCATGGGGGCTTCCATCGCCCCAGG

GCCGCAGCCGGTGCAGATGTTCAGCTCGCGTAATCCCAACTGCGTGCCGACGCGACGGGCATACAGGTACTCG
TTTTCGTTAATTGAGTGGCCGCCCCAGCATACCACCATGTTTGGCGCTTCGCCGACGTGCAGCGCCCGGGCGT
TGCGCAAAATGGAGAACACCAGATTGGTGATATGTGTTGAGCTTTCAAGATCCAGATGCTGGAAACGGCCGGC
ATTATGGATCTGCCCATTCACAAAGAGAATGTCGCGCAGCACCGCGAACAGGTTAGCCTGTAGCGCACGAATA
ATCCGCCCGTCAACAAAGGCGTCTTCAGGCGGATTAATCAATTCCAGTTTCACGCCGCGCTCGCGGCGCAGTA
CGTTAATATCGAAATTTTCAAAGCGGGACAGCAGCTCTTTGCTGTTGTCGGTCAGGCTGCCGGAGTTAAGAAC
GGCAAGTGAACAGTTACGAAACAGTTGATACAGGTCGCTACTGGCCGTGCGTTTCAGCATGTCCACTTCCAGT
TGCGACAACATATCCATTGAGCCAAGCGGGCTAATATGTGTAATCAAGTAAACTCCTTATGGGACGCAACACG
CCATTCCCTGTTGATTACAATAGCCCTGGCTGATGACCTTTCACAACCTTACATGCGCAATCCGGCACGCAAA
ATTGACAAAAAAATTACTGTCGCGCCAGCCTGCCCGTGGCGGGCACGAAATCGGTATTGCTGCGCCAGGGATT
AATGTCCAGCCCGCCGCGGCGGGTATAGCGTGCATAAACGCTTAACGTTTCCGGCTGGCAGAAGCGCAGAATG
TCATTGAAAATACGCTCTACGCACTGCTCGTGAAATTCATTATGGTGACGGAAGGAGACCAGATAACGCAGCA
GTTTTTCCCTGTCGATTTTGCGTCCGCGATACTGAATTTGTATCGAGCCCCAGTCCGGCTGGTGGGTGATCAG
GCAGTTGGATTTCAACAGATGACTGACCAGCGTTTCTTCTACTTGCTTCTCGCCGCTGACCGCATGTTGCAGA
TAGTCGGTAGTAAACTGGTAGTTGTCGATACTGATATCCTGATCGTCAATACAGGTGCCGTGGAAATGGGCGA
CAGGCTGTCCTTCCAGCTCATCAAGACGATGAAGCCTCACGCTAACGTTGCCCTGCGCGCAGGCGCGCAGATC
GCGCTCCAGCGTCTGGCGCACCGTCTCCCAGGTGTCAAAGCGCGTCTGGTTAAAGCTGTTCAGATACAGTTTA
AAGCTTTTGGATTCGATTAAATTAACGCTGGTGTAATCCAGTTCGACATGCCCGACGGCGACCTGCGGAAGAC
CCTGGCTGTTAAGCCAGGAAAGTTCATACAACGTCCAGATATCCGCGCCGTGGAACGGCAAATTATCGGCTGT
TAATCCGAGCGGGTCGCGATTCAAACTGCGCGGTACGCCCTGCAGCAGGCTGGCGTCATAATTGTCCCGGTAA
TCCGTTGATTTTCCGAGAGTCAGGCCATCGAGAGCCTGATGGTTTTCATAAGAAGACATGTTTCACCGTCATA
AACCAGATACACTATGCGTCAAGTGTATCCTGGCTTTTATGAAGAGAGAAACTGGTGGACGAACTGACAGCGC
AGGCATTGAAAGCCTTTACGACGCGTTACTGCGACGCGTGGCAAGAAAAACACGGTAGCTGGCCGTTGAGCGA
AGAACTGTATGGCGTACCTTCTCCGTGCATTATTTCTTCAACCCGCGATGCCGTTTACTGGCAGCCGCAGCCT
TTTGAAGGCGAAGAAAATGTAAACGCGGTTGAACGCGCCTTTGATATTATGGTACAACCTGCGCTCCACGCGT
TTTACACCACGCAGTTTGCTGGCGACATGCCCGCGCAGTTTGCGGATGAAAAGCTTACGCTACTGCAAACCTG
GAGTCAGGATGACTTTCGGCGCGTGCAGGAAAATCTGATAGGCCACCTGGTCACGCAAAAGCGGCTCAAGCTT
CCTCCCACGCTTTTTATTGCGACGCAGGAAAATGAGCTTGAGGTCATTTCGGTCTGTAATCTGTCCGGCGAGG
TGATTAAAGAAACGCTGGGCACCCGTAATCGTACCGTTCTTGCCGCAACGCTTGCGGAATTCCTTACGCAACT
TAATCCTCTTCTGTAATCGTTTGCCCTAACGGGTGTGAGAGATCTCTTACAAAGTCTGTAGGAGATCGCTAGG
ATAGCAGCGGATACTGAGCCCCGTTTTCTTATATAAATATATATAAAACAACATGTTAAAATGTTTTTGCGCA
CTTTCATATGAAAATTTACTTAAGGTATTGTCTGTAAGCGTCTTGTAAGACAAGGTGAAACAGGCGATTCTAT
CTTCGTCGACAGGGAGTCGTACAACGAAGCGAACGTCAGGATGATGACGCTTCGGCAGGACACGCCAGGATGG
TGTTACAAGGAAAGGCTTCAGGATGAAGCAAAGTGGAAAGCGCAGGATGCGTTAAAGGACACCTCCAGGACGG
AGAACGAGAGCCGATCAGGATGTTCGGCGGGTCTGGATGACCAGGGACGCTTCAGGAAGAAGCTATCACATCG
GGCGATGTGCGCAGGATGCAAACGTTCAGGATGAACAGGCCGTAAGGTCACAGGAAAAGTTGTCACGGATGAG
CAGGGAGCACGAAAAGTAGCTGGAATGCTGCGAAACGAACCGGGAGCACTGTTTATACAGTGCTCCCTTTTTT
TGTTATTCTTCGCGCCAGATTTTCATCATTGAGGTTTTTAACATGACGACTCATGACCGTGTGCGTCAGCAGT
TACATGCGCTTGAAACGCTGCTGCGCGAGCATCGCCACTGGCGGCAGGATGCGCCGCAGGCGCACCTGTTTAC
CAGCACCCAACCATTTTTTATGGATACCATGGAACCGCTGGAATGGCTGCAATGGGTATTGATTCCACGTATG
CATACCCTACTTGATAATGCGCAGCCGCTGCCCGAGGCATTTGCCGTCGCCCCTTATTATGAAATGGCGCTGG
CGGCGGATCATCCGCAGCGGGAGGCGATCCTGGCGGTTTTGCAGGATCTGGATGCGCTATTCGTCCGCGATAA
ATCCTGATGCTGGAGATCCTCTATCAAGACCCGTGGCTGGTTGCCGTGAATAAACCCGCAGGCTGGCTTGTTC
ACCGGAGCTGGCTGGATCGCGACGAAAAAGTCGTGGTCATGCAAACGGTGCGCGACCAAATCGGCCAGCATGT
TTTTACCGCCCACCGTCTCGACAGACCCACGTCGGGCGTACTGTTAATGGGGCTGTCCAGCGAAGCGGGACGC
CGTCTGGCGCAGCAGTTCGAGCAGCACCACATCCGTAAACGCTACCATGCCATTGTGCGCGGCTGGCTGATGG
ATGACGCGGTACTGGATTATCCGCTGGTGGAAGAGCGCGATAAAATTGCCGATAAATTCGCCCGCGAAGATAA
AGCGCCCCAGCCGGCTGTAACGCAGTATCGCGGACTGGCGACGGTCGAAATGGCGGTACCGACCGGGCGTTAT
CCCACCACGCGTTATGGTCTGGTTGAGCTAGAACCAAAAACGGGACGTAAGCACCAGCTCCGTCGTCATCTGG
CGCATCTGCGTCATCCTATCATCGGCGACAGTAAACATGGCGATTTGCGACAAAACCGCAGCGCCGCAGAGCA
TTTTGCCTGTCGTCGCCTGATGCTTCATGCCAGTCGGCTTGAGCTGACGCACCCCTTCACCGGACAGCCGTTG
ATGATTCAGGCCGGGTTGGATGAAACCTGGATGCAGGCGCTGACACAGTTTGGCTGGCGTGGACTTCTCCCTG
ACAATGAAAGGGTTGAGTTTACGGCGGCGTCCCGGCAGGATGAGACTCATCAGGCATAATTCAGGGAGATACG
CATAATGGCAGAAATTGGTATTTTTGTCGGGACCATGTATGGCAACTCACTGCTGGTGGCGGAAGAAGCGGAA

GCGATCCTCGCCAGACAGGGGCACAGCGCGATCGTGTTTGAAGATCCTGAACTGTCCGACTGGCAGCAATATC
AGGATAAGGTTGCGCTGGTCGTTACCTCGACGACCGGGCAGGGCGATCTACCGGATAGCATTGCGCCGCTCTT
TCACGGCATTAAAGATACGTTGGGTTTTCAGCCAAACCTGCGTTACGGGGTGATTGCGCTAGGTGATAGCAGC
TACCCCAATTTCTGTAATGGCGGCAAACAGTTTGATGCCCTGTTGCAAGAGCAAAGCGCGCAGCGGGTGGGGG
AGATGTTATTCATTGACGCCAGCGAACATCCGGAGCCGGAGAGCCAGTCCAATCCCTGGGTTGAAAACTGGGG
AACTTTACTTTCCTGAGGTAAATCCCTCCCCCCCTACGGGAGGGTACCTTTTCGTTTGCTTGCGTTGCCAGTA
AGCAAAATCACGACCTGTACACAGTGTAAAGTAACTGAATCGTGTTAGCTTTGGGCATATGCCCGCAAAAGCG
GCAGTTTTTTACGGGCGTTTTCATGTAATCAAGCGACCTGTTTCACATTCTTCTCTTTTTATTCCTCCTGCGT
CGACGCCGACGCCTTCTGATTTCATTTCCGTGAAGTGGCTTCCACTGTCCTGGGCTTTTGCCATAAACAGGCG
TAATTCATCGCCAAAATACTGTGTTGTTGCACGGTGAGTGTGTGTGGCGCGCTTTTTATACTTCTCCTGCCAG
TGAATAAAAGCATGCAGCGTGCAAAGCAAACGACCTAATAAAAGCTGCAACAAGGAAACGTTATCTCTGATTC
CCTACCGGTTGTGCAGTTCAGAGTGAGCGTAGCTAACGCGAAATTTCAGGAGTGCAACAATGAGTTCATTAAG
TCACGCGGCGAGCAGTGCGGAGAAACGCACGAACGCCCGCTACTGGATAGTGGTGATGCTGTTTATCGTCACA
TCCTTCAACTACGGCGACCGCGCCACATTGTCCATTGCCGGCTCAGAAATGGCCAAAGATATCGGCCTTGATC
CGGTAGGCATGGGCTATGTTTTTTCTGCTTTTTCATGGGCCTATGTTATCGGGCAGATCCCGGGCGGCTGGCT
GCTGGACCGCTTTGGTTCCAAACGCGTCTATTTCTGGTCTATTTTCATTTGGTCAGTCTTCACCCTGTTGCAG
GGTTTTGTCGATATCTTTAGTGGTTTCGGCATTATTGTCGCCCTCTTTACGCTTCGTTTCCTGGTCGGTCTGG
CGGAATCGCCTTCTTTCCCGGGCAACAGTCGCATCGTTGCGGCCTGGTTCCCGGCGCAGGAGAGGGGAACGGC
GGTGGCCATCTTTAACTCCGCGCAATACTTTGCCACGGTTATTTTCGCGCCGATTATGGGCTGGCTGACGCAC
GAAGTGGGCTGGTCGCATGTCTTCTTCTTTATGGGCGGGTTAGGGATCATCATCAGCTTTATCTGGTTGAAAG
TTATCCATGAACCAAATAACCATCCGGGCGTAAATAAGAAAGAGTTGGAGTACATCGCAGAAGGCGGCGCGCT
GATCAATATGGATCAGAAAGACACCAAAGCGAAAGTACCGTTTAGCGTCAAATGGGGCCAGATCAAACAGCTG
CTGGGCTCTCGTATGATGATTGGCGTTTACATCGGTCAGTACTGTATTAACGCATTGACCTATTTCTTTATTA
CCTGGTTCCCGGTCTATTTGGTCCAGGCGCGCGGCATGTCGATTCTCAAAGCGGGCTTTGTCGCCTCGGTTCC
TGCAGTCTGCGGCTTTATCGGCGGCGTGTTGGGCGGCATCATTTCCGACTGGCTGATGCGTCGTACTGGCTCC
CTGAATATCGCGCGCAAGACGCCGATTGTGCTGGGAATGTTGCTGTCAATGGTTATGGTGTTCTGTAACTATG
TGAACGTGGAATGGATGATCATCGGCTTTATGGCGTTGGCCTTCTTCGGTAAAGGTATCGGCGCGTTGGGCTG
GGCAGTAATGGCGGATACCGCGCCGAAAGAGATCAGCGGTCTTTCCGGCGGCTTGTTTAATATGTTCGGCAAT
ATTTCCGGTATTGTGACGCCAATCGCGATTGGTTACATCGTTGGCACCACCGGTTCCTTCAACGGCGCGTTGA
TCTATGTCGGCGTACATGCCCTGGTGGCCATCATCAGCTATCTGGTGCTGGTAGGCGATATTAAGCGTATTGA
ACTGAAACCTGTCGCAGGACAATTATCATGACAACGCAATCCAGTCCCGTTATTACGGACATGAAGGTCATTC
CGGTTGCCGGACATGACAGTATGCTACTCAACATCGGCGGCGCGCATAACGCGTACTTCACCCGTAACATCGT
CGTCCTCACCGATAACGCCGGGCATACTGGCGTCGGCGAGGCGCCGGGCGGAGAAGTGATTTATCAGACGCTG
GTCGATGCTATTCCGATGGTATTAGGCCAGGAAGTCGCCCGCCTGAACAAAGTGGTGCAGCAGGTGCATAAAG
GCAACCAGGCTGCTGATTTTGACACCTTTGGCAAAGGCGCATGGACGTTTGAACTGCGGGTTAACGCGGTGGC
GGCTTTGGAAGCGGCGCTGCTTGATCTACTGGGACAGGCGCTTAATGTGCCGGTCTGTGAACTGTTAGGGCCA
GGTAAACAGCGCGATGCCGTCACCGTGTTGGGCTATCTCTTCTATATTGGCGATCGCACTAAAACCGATCTGC
CGTATCTGGAAAGCACGCCGGGCAGTCATGAATGGTATCGTCTGCGCCATCAGGAAGCGCTGAACAGCGACGC
GGTCGTGCGGCTGGCGGAAGCGTCTCAGGATCGCTACGGCTTTAAAGATTTTAAACTCAAGGGCGGCGTGCTG
CCGGGCGAACAAGAGATTGATACTGTTCGCGCGCTAAAAAAACGCTTCCCGGACGCGCGTATTACCGTGGATC
CGAACGGCGCCTGGCTACTGGATGAAGCCATTGCGTTGTGCAAAGGGCTGAAAGATGTACTGACCTACGCGGA
ATATCCGTGCGGCGCGGAGCAAGGATTCTCTGGTCGTGAAGTCATGGCGGAGTTCCGCCGCGCGACAGGACTG
CCGGTGGCGACCAATATGATCGCCACCAACTGGCGCGAAATGGGGCATGCGGTGATGCTCAACGCGGTAGACA
TTCCTCTTGCCGACCCGCATTTCTGGACTCTCACCGGCGCCGTTTGCGTGGCGCAACTGTGCGATGACTGGGG
GCTAACCTGGGGCTGCCATTCGAATAACCATTTTGATATTTCACTGGCGATGTTTACCCATGTCGGCGCGGCG
GCGCCAGGCAAACCCACCGCTATTGATACGCACTGGATCTGGCAGGAGGGCGATTGCCGCCTGACTAAAAATC
CGCTGGAAATTAAAAACGGCAAAATTGCCGTTCCGGATGCGCCAGGACTGGGCGTGGAGCTTGACTGGGAACA
GGTGCGAAAAGCGCATGACGCGTATAAAAAGCTGCCAGGCGGCGCGCGTAATGATGCAGGCCCGATGCAATAC
CTGATCCCCGGCTGGACTTTTGACCGCAAACGCCCTGTTTTTGGTCGTCACTAATTCTGAATAAGGACACAGT
TATGAATACTCAATTTACGACGCCTGTAGTGACTGAAATGCAGGTTATCCCGGTTGCGGGTCATGACAGTATG
CTGATGAACCTGAGCGGCGCGCACGCCCCGTTCTTCACGCGCAACATCGTCATTATTAAAGATAACTCCGGCC
ATACCGGGGTCGGCGAGATTCCGGGCGGCGAAAAAATTCGCAAAACGCTGGAAGATGCGATCCCACTGGTGGT
GGGAAAAACGCTGGGTGAATATAAAAATGTCCTGACCGCCGTTCGCAACCAGTTTGCCGATCGCGATGCGGGC
GGACGCGGTTTACAAACATTCGATCTCCGTACCACTATCCATGTGGTGACGGGTATTGAAGCGGCAATGCTTG

ACCTTTTGGGCCAACACTTGGGCGTCAACGTCGCTTCGCTGTTAGGCGACGGTCAGCAGCGCAGCGAAGTCGA
AATGCTGGGTTATCTGTTCTTTGTCGGTAATCGCAAGGCTACGCCGCTGCCGTATCAGAGCCAGCCGGATGAG
CAATGCGACTGGTATCGTCTGCGCCATGAAGAGGCGATGACGCCGGAAACGGTAGTGCGTCTGGCGGAAGCCG
CCTATGAAAAATACGGCTTCAACGACTTCAAACTGAAAGGCGGCGTGCTGGCGGGCGAAGAAGAGGCCGAGTC
AATCGTGGCGCTGGCGAAACGTTTCCCACAAGCGCGCGTCACGCTCGATCCAAACGGTGCCTGGTCGCTGAAC
GAAGCGATCAGCATTGGTAAATACCTGAAAGGTTCTCTGGCCTATGCAGAAGATCCGTGCGGCGCGGAGCAGG
GTTTTTCCGGACGTGAAGTGATGGCGGAATTCCGTCGCGCGACCGGATTACCGACGGCCACCAATATGATAGC
GACCGACTGGCGTCAAATGGGGCATACGCTGTCGCTGCAATCCGTCGATATCCCGCTGGCGGACCCGCACTTC
TGGACTATGCAAGGCTCTGTACGCGTGGCGCAAATGTGTCATGAGTTCGGTCTGACCTGGGGCTCGCACTCTA
ACAACCACTTTGATATTTCGTTGGCGATGTTTACCCATGTTGCCGCGGCGGCGCCGGGCAAGATCACCGCGAT
CGATACCCACTGGATCTGGCAGGAAGGCAACCAACGTCTGACTAAAGAACCGTTTGAAATTAAAGGCGGCATG
GTGCAAGTACCGACCAAACCGGGTCTGGGCGTTGAGCTCGATATGGATCAGGTGATGAAAGCGCATGAGCTCT
ATCAAAAACATGGCTTAGGCGCGCGTGACGACGCGATGGGAATGCAGTACTTAATTCCTGGCTGGACGTTTGA
TAATAAGCGTCCTTGCATGGTGCGTTAAGTCAGAACCGGTTCCGTAAGGAACCGGTTCTTCTCTGGGTGTATG
CTTAACAGGATCAACATGCGTAAGGACGACTTATGAAAATAGTGATCGCACCAGACTCGTATAAGGAAAGTTT
GAGTGCTCTTGAGGTAGCGACCGCCATTGAACAAGGATTTCGCGAGATCTGGCCTGACGCCGATTACCTGAAA
TTGCCGCTGGCTGATGGCGGAGAGGGTACTGTCGAAGCGATGGTTGAAGCGACGGCGGGTCGAATCGTGCATG
TTGAGGTCACCGGGCCGTTGGGGCATCGCGTCAATGCGTTTTATGGGCTTTCCGGCGATGCGCGTTCCGCATT
CATTGAAATGGCGGCCGCCAGCGGGCTGGAGCAGGTGCCGCCCGCGCAACGCGATCCGTTGAAGACCACGTCA
TGGGGAACCGGCGAGCTGATCCGTCATGCGCTGGATGCGGGCGTTGAGCATATCATTATTGGTATCGGCGGTA
GCGCCACCAACGATGGCGGCGCGGGGATGGTACAGGCGCTGGGGGCCAGGCTACGGGATGCGCAGGGCAATGA
TATTGCGCAAGGCGGCATTGGGCTGGAAACACTCGCCAGCATTGATATCAGCGGCCTGGATAAGCGTTTGTCG
GCGTGCCATATAGAGGTCGCCTGCGATGTCACGAATCCCCTTACCGGCAAAGAGGGCGCGTCTGCGGTTTTTG
GTCCGCAAAAAGGGGCGACGCCGGAAATGATTGAACGCCTGGAGACGGCATTGACTCGCTATGCGCATTTGAT
TGCTCGCGATCTGCATGTCGATGTGTTGGATCTGGCAGGCGGCGGCGCGGCAGGCGGCATGGGGGCGGCGTTA
TACGCGTTTTGCGGCGCGCAGCTACGGCGTGGCATTGAGATTGTCACCGATGCGCTTCATCTGGAAGCGTGTC
TTGCGGATGCTGATTTAGTCATCACCGGCGAGGGACGCATCGACAGCCAGACCATTCACGGTAAAGTCCCTAT
TGGCGTGGCAAACATCGCCAAACGGTATAACAAGCCGGTAATTGGTATTGCGGGTAGCCTGACGGAGGACGTT
GGCGTGGTTCATGAACATGGGCTTGATGCCGTTTTCAGCGTGATTTATACCATCTGCACGCTGGAGGACGCGC
TGAAGAATGCGAGTGAAAACGTACGAATGACCGCGCGCAACGTGGCTGCAACGCTGAAAGCGGGGCAACAATT
ACGCTGAGTTGTAGGCCGGATAAGGCGGTCACGCCGCCATCCGGCGAATATCAGCCTAATATCTTCTTCGCTT
CACGCGCGACATTATCCATCTCATCCAGCAGCTCCAGAAACTCAGGCTCCAGCTCCTCTTCGTGGACGCCGCT
GCGAAGCTGTTGCTCAATAAGCTGGCAAAGGTTCTTCATCCGCGGTACGCCGCTATAGCCGCAGCTCCCGTGT
AGCTTATGGACCAGATCGACCAAACCGTTGGGATTTTCTCCCACCAGTTGTTCTTCAATTTTGTTGCGCACTT
CCGGCAGAAAATCAATCAGCATTTGCAGCATATCCCGTGCCAGATCGGGCTTACCGGCAGCCTGGCGGAGCGC
AAGCTGCCAGTCGAGCGTTGCATTCGGATTGAAGATAAACTCAGCCGGTTCCGGCGCCATCAGGCGCGCTGCT
ACGTTGGCGCCAGGTTTATAGCGCAGCAACAGATTATGCAACTTCTCTTCTTCTATCGGTTTAGCCAGATAGT
CGTTCATGCCCGCGCTGAGCAATTTCTCTTTTTGCCCGGCCATCGCATGTGCCGTAACGGCAATAACCGGTGT
TTGCTGCTGATGAGGAAGCTGGTGAATCAATTCGCAGGCGCGTATGCCGTCCATATCCGGCATCTGAATATCC
ATCAAAATCAGATCAAACTGCATTTGTTTCGCCCGATCCACCGCCTGATGTCCGCTATCACAAAGTTCTACGT
GCTGGACTTTATCTTCCAGTAACGCGCCGATAAGCTTCAGATTAGCGGGATTATCATCAACCGCCATAACCGT
CATGGTGATTTTACTGGTATCCATTAGCAGCGGTTCAGGATGGTGATTCAACTGGCAATATTCCGTCAGCGCT
GGCAACAGGCGCGTTGACGTCAATGGTTTTAACAGACAGGCCGCCGCGCCTCCTTGTTTCAGCTTTTCGGCGT
TAATTTGCGCATGGCAAGGTAGCGCCAGCAGTAGAAAGTCCGTCATTGACGCTGCTTTCGCCAGACGTTCATG
CTGCATGGTGAGCGGCTCGCGGAAGGTCACCGGAACGCTCAAAATCATAATATCGTAGTGCGCTAACGGCAGC
GCGGAGAAGGTCGGGCTGTAAACCACTTCCACCGGCGTGTCGCTCAGCAGATCCAGGGTACATTGCGCGGCGG
TAGCATTCGGTTCGATATAAGCCAGTCGTTTCCCGGCCAGACACGCGGTCGACGGCCCGTCAATAATGACATT
TGGGTTAAGATCAAGATGAATATGAAACCAGAAGGTCGAGCCGCGATTAGGCTGGCTGTGGAAAGAGATATCC
CCGCCCATTTCGTTGACCAGCTTTTGCGTAATCACCAGCCCAAGCCCGGTGCCGCCGTGACGGCGAGAAATAC
TGGCATCGGCCTGGCGAAACGCCTGAAACAGTCGAGACTGGTCGCGTTCCGGAATGCCGATCCCCGTATCGCG
GATCTGCACTTCAATCTGTACTTTGGTGTTACTGAGCGCCCGCTTTTCTACCAGAATATCGATATTGCCACTC
TCGGTGAACTTAATGGCATTGCCCACCAGATTAGTAATGACCTGTTGCAGGCGCAGCGGGTCGCCAATCACAT
TATCCGGGACGTCGTTTTTAATATTTAACGTCAACTCCAGCCCTTTATCATGCGACGAGTGAGCCAGCAGCGT
AACCACTTCATCCAGCGTGTTGCGCAGTGGAAAAGGGATACTTTCCAGAATGAGCTTACCGGCTTCCAGCTTG

GAGAAATCAAGCACGTCATTAATGATCGCCAGCAGATTATTCGCGGAACGCTCAATGGTGTTCAGATGGTCGC
GCTGGGTGGGGTTCAGCTCCGTTTTTAATGTCAGGCGGGTAAAGCCAATGACGCCGTTCAGCGGCGTTCGCAG
TTCGTGCGACATGTTTGCCAGGAACTCCGACTTAATACGCGCCGCTTCCTGGGCGCGCTTTTTCGCCAGATCC
AGCTCAACGTTTTGGATTTCCATCTGCTCAAGGGTTTCACGCAGGTCCGAGGTGGCCTGATCGATATTATGCT
GCATCTCTTCGTGATAGGCGGCAAGCGACATCGCCATGGAATTAATGCCGTTTTTCAGCATGTCCAGCTCGCC
CAGCATAAATCCTTCCACCCGGCTATCCAGTTGTCCGCGGCGAATGCGGTCAACGGTATTCACCATATTACGG
ATAGGCCCGGTGACATCGCGCATAAGCCGCCAGCCAAAGATCAGCGCAATGCCAATACAAAAAAGCATCATCA
CGCTGGAGATAAAAATCTCTTTGTATTGCTGTAGCCTGACCGACTTGAGATCCAGTTCAAGCGCCACATACCC
CAGCATATTTTTGGTATTTTTTGCGTCGGCAATCGCTGACTCGTCCGGCGAATAGCTCTCCGAGATAATCGGG
GTGCGCAGAATCATAATATCGCCGTGGCGATCGACGCTCAGACGACGTGGAAACGGCGCTCCGGCGGGAAGCT
GCATCTGTGAGGGGGCCAGATGGAAATTAGACGTTACAAACAGGCGGTTATGATCGTCATAAACGGAAATTGC
CCGCACAATATCAGAGTGTCTGCGGTGCAGGACGCTGATAAGTTGGCCGATAGACTCTCGGTTTTGTAAGTTC
ATACCATATTCGCTGGAGACGGCGAGCGGTTCAATAATACTGGCGCCGGCATCTTCCAGTTGACGCTGCAGGT
CGTTATAGCGGTGCACTACAAAAAAGATACTGAGCAGCAAACCTATCAGGACGGTCGGGGCCAGGATCAGAAT
CATCATGCGTGCGCGCAGGCTGTAGTTGGTCATGGAGTTCCGTTATGGGACAATTAGGGTCACACTGTTAAAT
TGAGAAAAAGCTGGTCGATGGCGCAATTCTACTCTGCAAAACGGCGCGTGACGACGCGTCAGATCATAACCGT
TAAAGTCAATGACCTCGATTCATTTGGTCAGGGCGTAGCCCGTCATAACGGAAAAGCGCTGTTTATCCCCGGT
TTATTGCCTGAGGAAAGCGCAGAAGTCATTATTACAGAAGATAAAAAGCAATTTGCCCGCGCACGCGTTTCGC
GCCGTTTGAATGATAGCCCGGAACGAGAAACGCCGCGCTGCCCGCATTTTGGCGTCTGCGGCGGTTGTCAGCA
ACAGCATGTCAGCATCGCCCTGCAACAACGCAGCAAAAGCGCGGCGCTGGCGCGTTTGATGAAACACGAGGTG
AATGACATCATCGCCGGCGCGCCCTGGGGTTATCGGCGTCGTGCGCGGTTAAGTCTGAATTGCCCGCCGGATA
AACCGCTGCAAATGGGATTTCGCAAAGCGGGTTCCAGCGATATCGTCAATGTCGAACAGTGCCCTGTTTTGGC
GCCCCAGCTTGCAGCATTGCTGCCCCGAATCAGGGCGTGTCTGGCGAGTTTACACGGCACCCGACATCTTGGG
CATGTTGAGCTGGTACAGGCGGGAAGCGGTACGCTGATGATTCTGCGCCATACCGCGCCATTAAGTGCGGCGG
ATAAAGAAAAACTGGAACGCTTTTCGCATTCTGAGGGATTGTCTCTGTTTCTGGCGCCGTTTAGCGAGATACT
GGAAACGGTATCTGGCGAAGCGCCCTGGTATGATTCGCACGGCTTACGTTTAGCCTTCAGTCCGCGAGACTTT
ATTCAGGTTAATGAGGCGGTAAACCAGCAAATGGTCGCCCGCGCGCTGGAGTGGCTTGATGTGCGCGCCGAGG
ATCGTGTCCTGGATCTGTTCTGCGGAATGGGGAATTTTACGCTGCCGCTGGCAACGCGCGCGGCAAGCGTGAT
TGGGGTTGAGGGCGTTCCGGCGCTGGTAGAAAAAGGCCGTGAAAACGCCATCCGCAATGGTTTACATAATGTG
ACATTCTTCCATGAGAACCTGGAGGAAGATGTCACGAAGCAGCCGTGGGCGAAAAACGGCTTTGACAAAGTCT
TACTCGATCCTGCGCGTGCGGGGGCTACAGGAGTGATGCGACATATTATAAAATTAAAACCTATTCGCATTGT
TTATGTATCCTGTAACCCGGCGACGCTGGCGCGCGATAGTGAAGCGCTGGTCAATGCGGGATATGAGGTTACG
CGTTTAGCGATGCTCGACATGTTCCCGCACACAGGACATCTGGAATCAATGGTTCTGTTCGAGCGCATGTAAT
GATTACCGGCTTACCGACTTCGGTAGGCCTGGTCCCTTAAGGAGAGGACGATGGTCGCGGTAAGAAGTGCACA
TATTAATAAAGCTGGTGAATTTGATCCGAAGAAGTGGATCGCAAGCCTGGGAATTTCCAGCCAGCAGTCGTGT
GAGCGCTTAGCCGAAACCTGGGCGTATTGCCTGCAACAGACACAAGGACATCCGGATGCGGATCTGTTGCTGT
GGCGTGGCGTGGAGATGGTAGAAATTCTTTCCACGCTGAGTATGGATATCGACACGCTGCGGGCGGCGCTACT
GTTCCCTCTGGCCGACGCCAACGTAGTCAGCGAAGATGTACTGCGCGAAAGCGTCGGCAAATCTATCGTTACC
CTGATTCATGGCGTGCGCGATATGGCGGCGATCCGTCAGCTAAACGCCACTCATAACGACTCTGTTTCTTCGG
AGCAGGTTGATAACGTCCGTCGAATGTTATTGGCGATGGTGGATGATTTCCGCTGCGTGGCGATCAAACTGGC
CGAGCGAATCGCTCATTTGCGCGAAGTGAAAGAGGCGCCGGAAGATGAGCGCGTGCTGGCGGCGAAAGAATGT
ACCAACATCTATGCGCCGCTCGCTAATCGTCTGGGCATCGGGCAACTGAAGTGGGAACTGGAAGACTACTGTT
TCCGCTACCTGCATCCGGCGGAATACAAACGCATCGCCAAACTGCTGCATGAGCGCCGTCTCGATCGCGAACA
TTACATCGAAGAGTTTGTTGGACATCTGCGCGCCGAAATGAAAAACGAAGGCGTGCAGGCGGAGGTCTACGGA
CGACCAAAACATATTTATAGCATCTGGCGCAAAATGCAGAAAAAGCATCTGGCGTTTGATGAACTCTTTGACG
TGCGCGCCGTGCGTATTGTCGCTGAACGTCTGCAGGACTGCTACGCCGCGTTGGGGATAGTGCATACGCACTA
TCGTCACCTGCCGGATGAATTCGATGATTATGTCGCTAACCCGAAACCGAACGGTTACCAGTCTATCCACACC
GTGGTCCTGGGACCGGGCGGTAAAACCGTTGAGATCCAGATCCGTACCAAACAGATGCATGAAGACGCCGAAC
TGGGCGTGGCGGCACACTGGAAGTATAAAGAAGGCGCCGCGTCCGGCGGCGTGCGCTCCGGTCATGAAGACAG
AATTGCGTGGCTGCGTAAGCTGATCGCCTGGCAGGAAGAGATGGCCGATTCCGGCGAAATGCTGGATGAAGTG
CGCAGCCAGGTGTTTGACGATCGGGTCTACGTTTTTACGCCAAAAGGCGACGTGGTTGACTTGCCTGCCGGAT
CTACGCCGCTCGATTTTGCTTACCACATCCACAGCGATGTTGGGCACCGCTGCATTGGCGCTAAAATCGGCGG
CCGTATTGTGCCATTCACCTATCAGTTGCAGATGGGTGATCAAATTGAAATTATCACTCAGAAGCAGCCGAAT
CCCAGCCGCGACTGGCTGAATCCAAACCTGGGCTATGTGACGACCAGCCGCGGACGCTCGAAAATTCACGCCT

GGTTCCGCAAGCAGGATCGTGACAAAAATATCCAGGCTGGACGGCAGATCCTCGACGATGAGCTGGCGCATTT
GGGGATTAGCCTGAAAGAGGCAGAAAAACATCTGCTGCCGCGCTACAACTTTAATGAGCTGGAAGAGTTGCTG
GCGGCGATAGGCGGCGGCGATATCCGTCTTAATCAGATGGTGAATTTCCTGCAATCGCAGTTCAATAAGCCGA
GTGCGGAGGAGCAGGATGCAGCGGCGCTGAAACAGCTTCAGCAAAAAACATACGCGCCGCAAAATCGTCGTAA
AGACGACGGGCGCGTGGTGGTGGAAGGGGTTGGTAACCTGATGCACCACATTGCCCGCTGCTGCCAGCCGATT
CCTGGCGATGAAATTGTCGGCTTCATTACTCAGGGGCGGGGAATTTCCGTGCACCGGGCCGACTGCGAACAGC
TGGCGGAACTGCGCTCCCATGCGCCGGAGCGGATCGTAGAGGCGGTATGGGGCGAAAGCTACTCGGCGGGATA
TTCGCTGGTGGTGCGCGTCCAGGCCAACGATCGCAGCGGCTTGCTGCGCGATATCACCACTATTCTGGCTAAC
GAAAAAGTCAACGTGCTGGGCGTCGCCAGCCGCAGCGACATTAAACAGCAGATCGCCACCATTGATATGACCA
TCGAGATCTACAACCTGCAGGTGCTGGGCCGCGTGCTCGGTAAGCTGAACCAGGTGCCGGATGTGATTGATGC
ACGGCGACTGCACGGGGGGTAAAGTGAAACGTTATTATCTACAAAGTAGTCTGATATATCTACCCGTTATACT
TTACGTTGCCTGTGTGTTGGCATGGCCCGGCTCTCCGGGCCTCGCCCTAAAGGGCCGCCGTAAGTGGCGTTCA
CATCGGCCTTGCCAATTTGTCCTCGCTCCCCCCAGTCACTGACCAGCGTAAGCCCCGGGGATTGATGGGGGAC
ATCCTTGTCCCCCACCGCAGGCAGCCTTCGGCTGTTCAAATTCGTATCAGACGAATTTGTCACTGCGTCGCCG
CCTGCCTGCAACTCGAATTATTTAGGGTATATGAGTATAAAAGTATGCAATCGTAAACTCAGATCTATTAAGA
AAAATACTATTGCCAGGGCAAAATAACTGTCACGCTGCAATTAGTATCAACGTTTATTCGTTCCCAGTTGCCG
ATTATTCTTCCCAGTTCGCCATTGTTGCATGCCCCCATATCACCATTCCTACATTGATAATCTACATTTGTTT
TAATCCTGCTATCAGCACAAACTACATTGATCGTGCTATTTTCCAATGTATAATCGAATCCTTCACCCCACGT
GACATTTTTCTCCTTATACATCAAATAAATTTTCCCTAGTTCTATATTTTCTGCCTTATCGTTATAATGAAGT
TCATTATAATTTAACACGGAGTTCTTATCCAAAGACGCAGTTATGAAACCAATCGGCGATGCAACCGTATAAG
TTGAATTGTTATTCGTAAAAACAGAACCTGTCCCAGAATGATATTTGTATTGACCGCATCCAGGTACCGTAAA
AATAAACCCGGGATCATATAGTGGTGGCATTGTTATGTTAGAAATAACACCCGTATTAGGGGTTGGTGTTATT
GCGCCTGTATTAATTACTACTGCACCACCAATCGCCATACCCTGAAAGCCACTGCCATCCATCGGGATCTCTG
TTATATATCTCATTGTTCCTGTAGCATATAATTTACTTTCGATAATTGACATGCCATTATCTGTCCTTACAGG
TACAGCTTTGTCTGTGTCATAACCAGTAACAAAGCATGTTATGCCGTAATAACCAGGAAAATTACCATTGAGT
AATGTACACTGGGCATCTGTATTAACTTTGACATCTGCTGTTGTATATAAATTAACTGTTATGCCACCAATAA
ACATAGATATGAGACTTCCATTAAGCAGGGTTGCCTCATATTTTGCTGGAATAACAGTGCTATCATGCGGCCA
GCTTTTGCATTGTGAGAATTCAGCCTGTTGAGCCTGAGGTAGTACTAAATAATTTGTTGAGTCACGTATTACT
AATGGTTCAATCATTACATTTTGGTTAATGGCCTGAGTTATCCCAGGATATGCAATTATACTTAGAAAAAATA
ATACTTTTTGATTCATTTAAATTTCCATTATTTAAATAAAATATTAGAAATGAGTATATAACTTGAGAAGTTA
CTGATATTCAAAACTCACTGTTGCGCTACCCGAAAATTCCCCCGGCATAACCTCTTCACCAGCGTATTTTTGC
AGCCTGGCCTGCAAGTTGATGGTGGTAATCCCTTCTTTCAGCACAAAAGCTGTACCCACTGTTGCATTGATAC
TCACTGGTTGCTGTGCAGCCGTTTCCACGCCAATCGCAAAACCACTGGCTTCCGACGAGGGCTCCAGCGCCAG
CAGTCCGGGCAGGGTACTGTCTTCAACGCCCTGAAAGGTCACCAGCACGCCGTTGGCTAGCGCTGTTTTGCAA
TCCTGGAGGCTAAGTGTGAATGGAACAGGAGTCGTTTGCCCGCTGGCGTCGAAATTGTCACTGCCAATAGTAT
GAAAATCCACCGTCGCCATCGTGTCATTATCCACTACCAGGTTACAGGCGCCGGCAACCAGCTTACCGCTGTA
GTGCACATTAGGCGTGGCGGCAGGTAGCGTATTGCAAACCGCCCATAACATTACGCCTCCGATCAAATGCCCG
GCAGTTTGAAAAATACGGTTCATTGGTAATCCACCTGTAACGTGGCAAAAGCCTCAAAATCGCCTTCGGCAGG
CTCTTTACCGGGCTGTTTTACCGGCACGGCTTTTAGTGTGGGAAGCGATGACTCATTGATGGTTAGCGACGTG
CCGGGAGGAAACACCGTGCCGTTCTGTTGAAGTTCAATGCCCAGCTCAGGGACGCTGGTGGTTAAGGCTTTTG
CGGAGTAAGGCGTCGCCGTGCCCAGATAGCTCAACGTAAAAGTTAATGCGTCATCGCGAAAGCTGCTGTCGCA
GGTAAGGGTCCACGGGATCTCGGTCAGGTAATTGACGCCGTCAATATTATCAATAATGACGCTACCAAAACTG
ACCTCCAGCATTTCACCGTTATTGATAGTACAGGCGGGCGGGTTTATCAGTTTGCCGTGAAAGGTCAAATTGT
CGGCGCAGGCAAACTGGGTGATTAAAAGCGTCAGGATAAGCACCCTTTTCACTATGAATCTCTCCATCACTGG
TAATCCACGACTAACGTCGCACTGGCGTTAAAATCGCCACCCGCTAACTGCGTGGTGGGCTCTTTAACCGGTA
CGGCTTCCAGCTCTGGCCCATTGCTGCCGCTCAGGGTGAAGTTCAGCCAATCCGTTATGCCAACCGGAACCAG
TTGCTTATTGCCCGCCTGCTGAATACGGATGCCTAACCCCTGCACCGAAGTTTGCAGAACCTGCTCCCCGCTG
ATGGTGGTGGTGGTTCCCTGAATTTGTAGCTTCAGATAGTCACTCGCTCTGCCATCGCAGTTCAGGCTGTAGC
CCACAGGTTTCGTTTGAGAAGCATCGCCAACTTTGGTGGTCAGGACATCGCCAAACTCTACGGAAGCGCCGCC
TACCGTACAGGGCGGTGGTTGAGTGACGACCAGATGCAGCTCCAGTTGCACGGTTTTTGTGCCGGCAGCCTCG
GTGCCGGTAACGAATCCACCGCACAGCAGCACCAGCATTTTCCAGCGAGTCAACATCGTCATAGATCTCTTTC
GCACAGGATTACTTATTCGCTTTCTTCTCAGCGTCACGGGTTTTTCCTCATTGACCTTGCAGGTATTCCCTG
AGCAGGTAAAAACCAGCAGGCGATGGCCGCCGTAATCATCGACGTAGCCCACCACCGGCGAGCTGCCGAGCGA
GGCACCTTTGATGGGAGAGGTCACTTTTTCACGCGGGGCAATCAGCAGGTTTTTAAAGCCGGAGGCGGTTTCG

TTCTCTTTGCTACCGGCATTGCTGATGACGAGGTAAAACGGTGTGGGGTTGTCGAAGATCACACCATCGCCCT
GGTAGGTCAGCGTGAGCTTCGTCTGCCACGGGTGCGTCTTATCCACCTGCCGGGCGACGGCTACCGGGCGGTA
GAAAAACTTAATACGCGTTTGCAGCGCCAGCTGGAGCGTATTAGGCTTATCGCTTTTCGGCGGAATTTCCCGC
ACGTTGAAATAAAACAGACTTTCCCGGTCCTGCGGTAGGCTAGCCGCGGCTGGTAACGGCTGGACTTTAACCT
GCCCGTCGGATTTTGGCTCAATACGCTGCAGCGGCGGCAGGGCGGTAAAATAGCGGGTATCTTTGGCGAACTT
ATCGTCTTCCAGCCACGCCTGTGCGAGGTAGGGCAGAGTGGCGTTGCCGTTTTTCAGCGTGACGGTGATTGAA
TTTTCATTGCCGTTAAAAATAACGCGGGTACGATCCGGCACAATCGCGCTGTGCGATACCAGCGGCGCAGCCA
GTAATACCGAAGCGATAAGCGCCTGGCGCTTAAAATGGTTGGTCTTGTTCATTACAGGTTCCCTTTATTCCAT
ATCCTTGCGATTCACCGCATGGTTGTCAGCCAGCGGTATGGGCGGGGTTTGAGTGGTTGTCGCCACCGGCGCG
GCCGGTTGGCCATCGGTTTGCGTATGTTCCTGGATTAGCGGTTGCGCAGGCAGCGGAGAACTTTTCTGCGGTG
CGGTGGGGGGCTGACCTTCCAGCATTTTGCACGGCAGCAATAGCGCGTTGGCCAATAGCTCCGGTGTAAACGT
GGACGGGAGTGATGCTTCACACTGCGCCGCGCCATCCCAGAAGACTTTCAATGTTTCCCCGGCTTTTACGCCC
GCCAGCCAGGCGTTGCCGTCATCGGCCACCAGGCCCAACTGCTGCTGGCGCTCGTTTTTCACTTCGGCGCCGA
ACGGTGGGAAGTCGCCGTCTGCCAGCCGGAAAACCGCCATCATTTTCTCGCCGCTGACCACGTCAAAGTGACG
GTAGCCGATGGCGCCTTCGGTGAGCGTGATCTGCACAACCGACTTAGTAGCTTCCGCCTTCTCCGGCAATTTG
TTCAGATCGATACGCGCCAGGCTGCGCGAATAGCTTCCGACATCCGCAATCACGGCCTTGCCGAAAATGTTGG
TTGAGGTTGGCGAGCCGTTACCGCTAACGGGGACGTCCGCAATGCCGTCGGTATCAACCAGCAAACGTGTGCT
GCCCGCTAAACTTCCCCGGTGTAGCGCGCCGCCCTGGGTGGTCAGCGTCATGCCGCCGCGCAGCGACATGCCC
AGCGAACGGTACGATCCTTCTTCATAATCCGCGCTCAGATCGATGTCCGTCAGCGCGCCCTGATGGCTGTAAT
AACCGTCTGCCTGTGCGCCTTGTTCATCCTGCCCGACGTGAAGCTGCCAGTTATCGCCGTTCTGGCTGTGGCC
GGAATAGCCGAGCTGATTACGGTGTTGACTACCGTTAAACGTACCGTTGTAGCTGATGGAATCATTGCCCCAG
GGAACAGACAGCGAGATATAAACGCCATCATCTTTATCACCGTTATATTCGCTGCGATAGCCGTTCAGCGACG
CACTGAGATTTTTTATCGACCCTAAGTCGAAGTACCAGGAAAGAGACAGGTTATAGTTACTCTGATCCGGGCT
GTCCCAGAAGGTACTGCGTGAGTAGCTGAAATAGGCGCTCATGCCCTGTTCACGGAAGTTCTGGTTATAGGTG
ACCGTATAGCGTTCTTTTTCGTGACCGGCATTGAGATGATGATAGGTTTTCGCATCCAGATAGTCGCTCATGC
TCATAAAGTTCTTTTCTGAGAAGCGGTATCCGGCAAAGGTTAGTCGGCTGTCGATTTCATCAAAGTCACGGGA
GTAGCTGATGCGATATGAGTTACCCTGCAGCGTTTCGCCGTCAAACCCGTCGTCTTGCGGCATGTGGGCGATG
GAGTGCGTAATGTCGACCGCCACCGCGCCCACCACGCCAAGATCCTTACCGCTTCCCAGCGCCACGGCCTGAT
AGTTGCTTTCTCCAATTGCGCCGCCATAGAGCGACCAGCCGTTGGTGACCCCCCACGAAGCTTCCGCCGAGGC
GAATGTGCCGGTAATGGGGTGATGATCCCAGTCCTGCGGGCGGCCCAGCGCCATCTTGTAGCGCACCATGCCG
GGGCGCGTCAGGAAGGGAACCGATGCGGTGTTAACGTCAAACTCCTGGGTCTGACCGTTCTGCTCTTCCACGG
TGACGTGCAACGTACCGGAAACGGACTGGTTGAGATCCTGAATACGGAACGGTCCTGCGGGCACCTGCGTTTC
ATACAGTACGCGCCCCTGCCAGGAGACTTTGACTTTGGCATTAGAGCGCGCAATGCCCACGATCTCCGGCGCA
TAGCCGCGCAGTTTCGGCGGCAGCATCTGATCGTCAGAAACGACGCTGGCACCGATATAGTTAAAGCTGTCGA
AAACATCGGACTGGAGATAGCTTTCGCCCAGCGTAAGTTTGGCCCCGAGCGTCGGTAGTGCGCGATAGGCGTA
GTAGCGGCTCCAGCTCTGATCGTGGAGGGTGGAAGTGTTCTCGTCATCGTCACGATGGTCGTAGCTGGCCTGC
CAGTCGGCCCGCAGTCGCCATGCGCCCAGGTTCGCGCCTAACGTCCCGTTGCCGCTGATGCTTTGCTCTTCGC
TGCCGCTTTCTTGATCGTGGCGGAGCTGATTATTAATGTTGTAATCCAGAATGATGCCGGGAATACCATTATC
CCAGCGGGCGGGCGGGTCCCAGTCGACATCGCTGTATTCCATATACGCCTGGGGTAAATTCACCAGCAGCGTG
GAGTGTCCCAGGTCAGCCTGAAAATCCATGCCTTTCAGTGAATCTGGCGCCAGACATTGTCGTTCACCGTGCG
TTATCCAGACGAGTTTTTCCGCGAGTTCCGGCTTTATACCCAATTGCGTAACTAATTCTGGTTCTGCGCAAAC
CTCACTTCCTTGTGGATCATGTTCTGGAATAACCCAGTTAACAGGAAATTCCTGCGGCAGAGTCTGTCCGTTA
ATTTTAATTTGTAAAAGGTAGACGCCTGGCAGAATAAAACCCTTACGAGAAAACTGTGCAATGTTAACGTTAT
CGCGATTTTTTACATCGAGGAAATCGGTATTAAATTCGATGTCTTTGCCTGACGCGCTATAAGGGGAGAGGCT
TAGAAGCATTCCCACTCCGCACGCTATTGGGCAAAGAACACTTTTCCATGTGTTATTCATCAAATATATTCCG
CAATAAAACTCAACAGGAATACCCTACCTAAAGCGGCAGGGTATTATCGTCGTGACTCTGTTAACTGAATTAC
AGGTAAGAGATAGTGACGTTGGCGGCGCTGCTGAATTCACCTGTGCCCACGGTACCGGTTGCTGCGCCAACCA
GCCAGGCGGTGAAATTCAGGCTTTGCTTCTCATCAAGAATGGTATCTCCGTCCAGGTCCGCAATGGCCTGTGT
ATAGGTATCGCCCAGTTTGATATTGTTTTTGCCAGCATCGCCGATTGCCAGGCTTACGTTGCTAATAGCGCCA
GTGCCGTCAGTCTTCATCAGCGCGAGATTATCCGTGGCGCCTGTAGCCGCTGTGGTTCCACTACCTGTAGTGA
ATACCACATTCATATTTTTCTCGGTAGCCCAGGTACAACCCTCAAGATCGATATGAAAGGTACGGGCATTGTT
GATGGTCGTTTTACCATTACTCAGCGTGCCATTACCGATACTTGAACCCATATTGACTGTTTGATCTTCCGTA
TCCAATGTGACGGAGCAGGGGGAGTCAACAATATTACCAAGGAAAGTGACCTTCGCTGTGTTGCTACCCGCGG
CTTGAGCGCCGGAGACAATTCCCATAACTAAAGATGCAGTTACTGCCAGTTTACAAAAATGAGATGACTTCAT

CGTGTTATCCTTAATTAACGTGTTTTATGGCAACATTTTGTACGCGTATACCAAGGGATATGCGGCATAGAGC
CATGCTGCGGGCTTTGGGTAAAAAATAGGTCATAATAAAAATATTATATTTCTACATTTTTATGAGTATATTC
TTATGAGCTGAATGATCGTACCTGCTCATAGAATATTATATGTAATACTTTCAATATATTTATTTCATGTATG
TTCCAGAAAAAACAAAAAAGCGGTCTTTTTTGAATATTAAACTGGCGTGCATGCTACTCGTTTTTCCGTAAAA
GTGAAATAATTTATTTTTTTTGAAAGGGAAGGCTCTTTGGGGGGGGGGAGGCTTATAGTGTTTGCATAATTTT
TTTACTTAATTGATGTTATTTATAATGCCTGGTGCAAATATCTGTTTTTTAATAAATTAGTTATATGATATAT
TATATTGTTGTGTATCGATTAATAATTTTAAAGTTATACGGGAAAGGTAGCATTCAACGCCTGTTAACAATAT
ATTTTATTATATAGGCGTTGTCGTAGTATCTCTGTGCGTGAATGGTTAAGAATAATACGTTACAATTGTCGCT
AGTATTTACAACCGATTAAAAACCATGACAACTAACCATCAAATTGACCGTCTTCTCACCCTCATGCAGCGCC
TGCGCGACCCGGAAAACGGCTGCCCGTGGGATAAAGAGCAGACCTTCGCCAGCATCGCGCCTTATACGCTGGA
AGAAACCTATGAAGTACTGGATGCGATTGCGCGTGAGGATTTTGACGATTTGCGCGGCGAACTGGGCGACCTG
TTGTTCCAGGTGGTGTTTTACGCGCAGATGGCGCAGGAAGAAGGCCGCTTCGATTTTAATGATATCTGCGCTG
CCATCAGCGACAAGCTGGAGCGCCGCCATCCGCATGTTTTCGGCGAGCTTTCCGCGGATAATAGCGAAGAAGC
ATTAGTTCGCTGGGAGCAAATCAAAACGGAAGAGCGCGCGCAAAAAGCGCAGCATTCCGCGCTGGACGATATT
CCGCGCAGCTTACCGGCCTTGATGCGCGCGCAAAAAATCCAGAAACGTTGCTCAAACGTGGGCTTTGACTGGA
CGACGCTGGGACCGGTCGTCGATAAGGTCTATGAAGAGATCGATGAGGTGATGTTCGAGGCGCGGCAGGCGGT
TGTTGATCAGGCTAAACTGGAAGAGGAAATGGGCGATTTGCTCTTTGCCACGGTCAACATGGCGCGTCATTTA
GGTACGAAAGCGGAGCTCGCTTTGCAAAAAGCGAACGATAAATTTGAACGTCGTTTTCGCGAAGTTGAACGTA
TTGTCGCAGCCAGAGGCCTGGAAATGACCGGGGTAGACCTGGAAACAATGGAAGAAGTTTGGCAGGAAGTAAA
ACGCCAGGAAATTGATCTCTAAAGGTTTTAGGCGGTCAAGAGCGATTTGTGTGATTTTTTAAATAACAAGCGC
TTGATTTACGTCAAAAACATTTACCTCAAAGGGGCTATTTTCTCCCTCCCTGTGTGAATGTCAGCCTGACGAA
GAGGAGGAATAATGAAAGTTTGTGGCGTAGGTCATGTTCGGGTATACTGCTTTCCCGTCCTGGTTATTCCATC
GTCTTTTAAACCTAACTTCTCAGGTTCCGCATGACAACGAACTATATTTTTGTGACCGGCGGGGTCGTATCCT
CTCTGGGTAAAGGCATTGCCGCAGCCTCCCTCGCAGCCATTCTGGAAGCCCGTGGTCTCAACGTGACCATCAT
GAAACTGGATCCGTACATCAACGTCGATCCGGGTACTATGAGCCCAATCCAACACGGGGAAGTGTTCGTTACT
GAAGACGGCGCTGAAACCGACCTGGACTTGGGGCACTACGAGCGTTTCATCCGCACCAAGATGTCTCGCCGCA
ACAACTTCACGACTGGCCGCATCTACTCCGACGTTCTGCGTAAAGAACGCCGTGGCGACTATCTGGGCGCAAC
CGTACAGGTAATCCCTCACATCACTAACGCGATTAAAGAGCGCGTGCTGGAAGGTGGCGAAGGCCACGATGTG
GTACTGGTGGAAATCGGCGGTACCGTCGGTGATATCGAATCGCTGCCGTTTCTTGAGGCGATTCGTCAATTGG
CGGTAGATATCGGTCGTGAACACGCGCTGTTTATGCACCTGACGCTGGTACCTTACCTGGCGGCTGCGGGCGA
AGTGAAAACTAAACCGACTCAGCACTCCGTGAAAGAGCTGCTGTCTATCGGTATTCAGCCCGATATTCTGATT
TGTCGTTCCGACCGTGCGGTTCCTGCCAACGAGCGTGCAAAAATTGCATTGTTCTGTAATGTGCCGGAAAAAG
CCGTTATTTCAATGAAAGATGTCGATTCCATTTATAAAATTCCAGGCCTGTTGAAATCTCAGGGGCTTGATGA
TTATATTTGTAAACGATTCAGCTTGAACTGTCCGGAAGCTAACCTGTCTGAATGGGAACAGGTCATTTACGAA
GAAGCGAACCCGGCAGGCGAAGTGACTATCGGCATGGTCGGCAAATATATTGAACTGCCGGATGCCTATAAGT
CGGTGATCGAAGCGCTGAAACACGGTGGTCTGAAAAACCGCGTTACCGTCAACATCAAATTGATCGATTCGCA
AGATGTTGAAACGCGCGGCGTCGAAATTCTGAAAGATTTGGACGCTATCCTGATCCCCGGCGGCTTCGGCTAT
CGTGGCGTTGAAGGTAAAATCGCCACTGCGCGCTATGCGCGTGAAAACAATATTCCTTATCTGGGCATTTGCC
TGGGGATGCAGGTTGCGTTGATTGAGTTCGCTCGTAACGTGGCCGGCATGGACAACGCCAACTCAACGGAATT
TGTGCCAGACTGTAAGTACCCGGTTGTGGCGTTAATTACCGAGTGGCGCGATGAAGACGGTAACGTTGAAGTC
CGTAGCGAGAAAAGCGATCTGGGCGGCACCATGCGCCTGGGCGCGCAGCAGTGCCAGCTTAGCGATGACAGTC
TGGTACGTCAGTTGTACGGCGCGTCAACGATTGTTGAACGTCATCGCCATCGTTACGAAGTCAACAATATGTT
GTTGAAACAAATTGAAGCAGCGGGTCTGCGTGTCGCAGGCCGTTCCGGTGATGATCAGTTAGTCGAGATCATT
GAGGTACCGAATCATCCGTGGTTCGTGGCCTGTCAGTTCCATCCGGAATTTACTTCCACGCCGCGTGATGGAC
ATCCGTTGTTCGCTGGCTTTGTTAAAGCCGCCAACGAGCATCAGAAACGTCAGGCGAAGTAAAAAAGTTAGAA
CGGCGGTGCACCCTCAGCGGTGCACCGTTTGTCTGAAGTTTTAGTTTAACTAGTGACTTGAGGAAAACCTAAT
GTCCAAAATCGTTAAAGTCATCGGTCGTGAAATCATCGACTCCCGTGGTAACCCGACTGTTGAAGCTGAAGTA
CACCTGGAAGGTGGTTTCGTAGGTATGGCGGCGGCTCCGTCAGGTGCTTCTACTGGTTCCCGCGAAGCGCTGG
AACTGCGCGATGGCGACAAATCCCGTTTCCTGGGTAAAGGCGTAACCAAAGCTGTTGGCGCGGTTAACGGCCC
GATCGCTCAGGCTATTCTTGGCAAAGACGCTAAAGACCAGGCTGGCATCGACAAAATCATGATCGACCTGGAC
GGTACTGAAAATAAATCTAACTTCGGTGCAAACGCCATTCTGGCTGTCTCTCTGGCTAACGCCAAAGCTGCTG
CTGCCGCTAAAGGTATGCCGCTGTACGAGCACATTGCTGAACTGAATGGCACGCCGGGCAAATACTCCATGCC
GGTTCCGATGATGAACATCATCAACGGCGGCGAGCACGCTGACAACAACGTCGACATCCAGGAATTCATGATC
CAGCCGGTTGGCGCGAAAACGGTTAAAGAAGCCATCCGTATGGGTTCTGAAGTTTTCCATCACCTGGCAAAAG

TGCTGAAAGGCAAAGGCATGAACACCGCTGTGGGTGACGAAGGCGGCTATGCGCCGAACCTGGGTTCCAACGC
TGAAGCGCTGGCTGTTATCGCTGAAGCGGTTAAAGCGGCTGGTTACGAGCTGGGTAAAGACATCACCCTGGCG
ATGGACTGCGCAGCATCTGAATTCTACAAAGACGGTAAATACGTTCTGGCTGGCGAAGGCAACAAAGCGTTCA
CCTCCGAAGAATTCACCCACTTCCTGGAAGAACTGACCAAACAGTACCCGATCGTTTCCATCGAAGATGGTCT
GGACGAGTCTGACTGGGACGGTTTTGCATACCAGACCAAAGTACTGGGCGACAAAATCCAGCTGGTTGGTGAC
GACCTGTTTGTAACCAACACCAAAATCCTGAAAGAAGGCATCGAGAAAGGCATCGCTAACTCCATCCTGATCA
AATTCAACCAGATCGGTTCTCTGACCGAAACGCTGGCTGCAATCAAGATGGCGAAAGATGCTGGCTACACTGC
TGTCATCTCTCACCGTTCTGGTGAAACTGAAGACGCTACCATTGCTGACCTGGCTGTTGGTACCGCTGCAGGC
CAGATCAAAACCGGTTCTATGAGCCGTTCTGACCGCGTTGCTAAATACAACCAACTGATTCGTATCGAAGAAG
CTCTGGGCGAAAAAGCACCGTACAACGGTCGTAAAGAGATCAAAGGCCAGGCGTAATCCTGCTTTTACCTCTT
AATGTAAAAACCCGCTTCGGCGGGTTTTTTTGTATCTGTTCAATAGCGCAGCCGCTGTGCATGACCGGGCAGA
CGAAATCTGCAATAATTAACGATTATCCCCTTAACAGAGAACGCTATGCAGTACCCGATTAACGAGATGTTCC
AGACTCTACAAGGCGAGGGTTATTTTACCGGCGTCCCCGCTATTTTTATTCGTTTACAGGGATGTCCGGTCGG
CTGTGCCTGGTGTGATACTAAGCATACCTGGGATAAACTGAGCGATCGGGAAGTGTCTCTGTTCAGCATTCTG
GCAAAAACCAAAGAGAGCGACAAATGGGGTGCTGCGAGCAGTGAAGACTTGCTGGCAGTAATTAACCGTCAGG
GCTATACCGCGCGTCACGTTGTCATCACCGGTGGCGAACCCTGTATTCACGACCTCATGCCGCTCACCGATCT
GCTTGAAAAGAGTGGTTTTAGTTGCCAGATTGAAACCAGCGGGACGCATGAAGTGCGCTGTACGCCTAACACC
TGGGTCACGGTTTCACCAAAAGTGAATATGCGCGGGGGATATGACGTGCTTTCCCAGGCGCTGGAGCGCGCCA
ATGAAATCAAGCATCCCGTTGGGCGAGTTCGTGATATTGAGGCGCTGGATGAACTGTTAGCCACGTTGAGCGA
TGATAAACCGCGCGTGATTGCTTTGCAGCCCATTAGCCAGAAAGAAGACGCGACGCGTCTGTGTATTGAAACG
TGTATTGCACGTAACTGGCGGCTGTCTATGCAAACGCATAAATATTTAAATATCGCCTGATGCATTACTAAGC
TTACAGACGGCCTGCCGATGCCGTCTGTGACTCATCCATTACCTTGCATTGTTTATTTTCTCTATGTGAATTT
CGATGAGTGTATAAAAGCGCTGATAAATTTTTCCATAGCGATGCACGGATCGCGCTATTTTGGTAAATTTAAA
GAAAAAATCATTCTATGAACTTTTTTGCATCAAAATCAGCAAATTAGCTGTTCTTTAATAATTTAAATTGTTG
CGATTATGTTGGTGGAATGTGGTGCTGATAAAAAGTAGTTTATAAACAATGACATACGTTTAGTGTGTTCCCC
GCGCCAGCGGGGATAAACTGAGGATTTCGCCGCGCTGTTGGCCTCCAGATTCGTGTTCCCCGCGCCAGCGGGG
ATAAACTGCCGTGCCTGTCCAGGACAAATTGCCGATTATTGTGTTCCCCGCGCCAGCGGGGATAAACTGCTGC
CGGTCTGTGCTGTTGTCGTCAATAATCAGTGTTCCCCGCGCCAGCGGGGATAAACCGGCATGTGACAGTCTGA
TTTTTATAGCGCATGAGTGTTCCCCGCGCCAGCGGGGATAAACCCAATTTAGGGGCCGGAACTCCGGGAAAGG
CAGCGTGTTCCCCGCGCCAGCGGGGATAAACCGCAAACACCAAGCTCTTCCGCCGCGCGTTCCTGAGTGTTCC
CCGCGCCAGCGGGGATAAACCGTTGCTCTCATTAAAGGGGTTTCCATGTTTGATGTGTTCCCCGCGCCAGCGG
GGATAAACCGCGGTCGCAGCCTGGCCTGTTGCCGTAGAATCGGTGTTCCCCTCGCCAGCGGGGATAAACCGAG
TTACTGATGCAGACTGCGGATCTTAATCGGGTGTTCCCCGCGCCAGCGGGGATAAACCGTGCGCCAACGACTG
GAATTTTTGCGTGTAGCCGTGTTCCCCGCGCCAACAAGGATAGCCGTTGCATAATCATAATCTGTGTACCAGT
AATGGCTGGTACAAACCTGTAAAGTAAAAAGGCCGCGTTTTCCCGGGGAGGCTTTTCTACAGGAGAAGGCCAT
GGCGTTAAGGATCAGGGTATTGCTCGAAAATCATAAGGGAGCTGGAGCGGATAAATCGTTGAAGGCCCGGCCA
GGATTAAGCCTGTTAGTCGAGGATGAGTCTACGTCGATCTTGTTCGATACCGGCCCTGATGGCAGTTTTATGC
AAAACGCGTTGGCGATGGGGATCGACCTGTCCGATGTGTCTGCTGTGGTGCTTTCGCATGGTCATTACGATCA
TTGCGGCGGCGTGCCGTGGCTTCCTGATAACAGTCGAATCATCTGCCATCCCGATATTGCGCGCGAACGTTAT
GCGGCAATGACTTTTCTCGGTATTACCCGAAAAATAAAAAAATTGTCGTGTGAGGTGGACTATTCACGCTACC
GAATGATGTACACGCGTGACCCCCTGCCGATTGGCAAAAACTTTATCTGGTCAGGGGAAATACCGGTGGTTGC
GCCTGAAGCCTACGGCATTTTTGGCGGCCACGATGCGGAGCCGGACTCCATTTTGGATGAGGGCGTACTGATT
TACCAATCGACAAAAGGATTAGTCATCATTACGGGATGCGGGCATCGGGGGATCGCAAATATTGTCCGGCACT
GTCAGAACATTACCGGTATAAAGCGGATTTACGCGCTTGTCGGCGGATTTCATCTCCGCTGCGCATCACCGTT
CACGCTGTGGCGAGTCAGGCGCTTTCTGCAAGAACAAAAACCTGAAAAACTATGCGGTTGTCACTGTACAGGG
GCCTGGGGGCGGTTGTGGCTACCGGAAATAACCGCACCTGCAACCGGTGATGTGCTGCGCTTTTAACACCGCG
CTGGACAATGTTTTCACTGATAAAACGTCGTGTGGATAGGCTGGAATCAATTTGACATCCAGCCCACGCGTTA
ATAAACCTTATTCGCCGCGATAGACGCAGCCGGCTGTACAGGTCTCTTTCACCATCACGGCGCTCAGTAGCGG
CACGACGGGCTTAACCTGATCCCAAATCCATTTTGCCAGCACTTCGCTGGTGGGATTGGACAGCCCTGGAATA
TCATTCAGATAGTAGTGATCAAGACGATCGTAAGTCGGTTTAAACGCCGCTTTGAGATCCGCGAAATCCATAA
TCCAACCGGTATGCGGATCGACTTCACCGGTAATTTCGAGACGCACCATAAACGAGTGTCCGTGCAGGCGGCC
GCACTTATGCCCTTCAGGCACGTGAGGCAGGCGGTGAGCGGCTTCAAAGGTGAAATCTTTATACAACGTGGTG
GACATGATGAACTCTCAGGCAATAAAAAAACCGCCGTAGGGTACCGGAAAGCGCATTTTTTATCATTAACTAA
AACCATTCTCCGCCTTGTGACGGGCAATTAATTTAAGAATTTCCAGAAAATTTAATAATTATCAATCAATTAT

AAAATCGATTTTGGCGTTAATAACGATAACTAAAACAGGTTAGTTCATTTGGTTATTTGTTATTTCCAACCCT
TCTTTAATTGTTATTCCTCTCACCGTTAACCTTATCCTCAGTTTGGGATTTATCGCTTATACATTTCATCTTT
CAAGCCGCATCTGTGTTGACTGCGTTTACTCACCCCAGTCACGTATTTATGTACGCTCCTGGGGCGCGCTCAG
CTTACGGCGAACTTGAATGATTTTGTGTATAGATCCGCTTTGCTTACTGGAACATAACGACGCATGACGACAC
CGGCTCCACTGACTGGGTTGCTTCCGCTTAATCCGGAGCAACTGGCGCGCCTTCAGGCCGCGACAACCGACCT
TACTCCCGAACAGCTTGCCTGGGTCTCCGGCTATTTTTGGGGCGTACTGAACCCGCGCTCAGGCGTTGTTGCC
GTCACGCCTGTTCCTGAGCGGAAGATGCCGGGGGTTACGCTTATCTCCGCTTCGCAAACCGGCAATGCGCGTC
GTGTGGCGGAAGCGCTGCGTGATGATCTGCTGGCGGCCAACCTTAATGTGACACTGGTGAACGCCGGCGACTA
CAAATTCAAACAGATTGCCAGTGAAAAACTGTTGGTGATAGTGACGTCCACCCAGGGAGAAGGCGAGCCGCCG
GAAGAGGCCGTCGCGCTGCATAAATTCCTGTTCTCCAAAAAAGCGCCGAAACTGGAAAACACCGCTTTTGCCG
TGTTTAGCCTCGGCGACACCTCTTATGAATTTTTCTGCCAGTCCGGAAAAGATTTCGACAGCAAACTGGCGGA
ACTGGGCGGTGAACGTCTGCTCGACCGTGTCGATGCTGACGTTGAATATCAGGCAGCTGCCAGCGAATGGCGC
GCCCGCGTGGTTGATGTTCTGAAGTCTCGCGCACCTGTCGCGGCACCTTCGCAATCCGTCGCTACTGGCGCGG
TAAACGACATCCACACCAGCCCCTATACTAAAGACGCGCCGCTGATCGCGACCCTCTCCGTGAATCAGAAAAT
CACCGGTCGCAACTCGGAAAAAGATGTGCGTCATATTGAGATTGATTTAGGCGACTCCGGCCTGCGCTATCAG
CCGGGCGATGCGCTGGGCGTCTGGTATCAGAACGATCCGGCTCTGGTGAAGGAGCTGGTTGAGTTGCTGTGGC
TGAAAGGCGATGAGCCGGTGACGGTTGATGGTAAAACCCTGCCGCTCGCCGAAGCGCTGGAGTGGCACTTCGA
GCTGACGGTGAATACCGCCAACATTGTTGAGAACTACGCGACCCTGACGCGCAGTGAGTCGCTGTTGCCATTA
GTGGGCGACAAAGCGCAGCTCCAGCACTACGCCGCGACCACGCCCATTGTTGATATGGTGCGTTTCTCTCCGG
CGCAGCTCGACGCTGAAGCATTAATCGGCCTGCTGCGTCCGCTGACGCCGCGTCTGTACTCCATCGCCTCCGC
GCAGGCGGAAGTCGAAAGCGAAGTACACGTAACCGTCGGCGTCGTGCGCTACGATATTGAAGGCCGCGCCCGC
GCGGGTGGTGCTTCCAGCTTCCTCGCCGACCGCGTGGAAGAAGAGGGCGAAGTGCGCGTCTTTATTGAACACA
ACGATAATTTCCGCCTGCCAGCGAATCCGCAGACTCCGGTGATTATGATTGGCCCAGGTACGGGTATCGCGCC
GTTCCGCGCCTTTATGCAGCAACGCGCCGCCGACGGTGCGGAAGGCAAGAACTGGCTGTTCTTCGGCAACCCG
CACTTTACCGAAGATTTCCTCTATCAGGTGGAGTGGCAGCGCTACGTGAAAGAAGGCGTGCTGAGCCGGATCG
ATCTGGCCTGGTCCCGCGATCAAAAAGAAAAAATTTACGTACAAGACAAGCTGCGTGAACAGGGTGCCGAGCT
GTGGTGCTGGATCAATGATGGCGCGCATATTTATGTCTGCGGCGATGCCAGGCGCATGGCCGCAGATGTCGAA
AAAGCATTGCTGGAAGTGATCGCTGAATTCGGCGGTATGGACCTCGAATCGGCAGATGAATATTTAAGTGAGC
TGCGCGTCGAGCGCCGTTATCAGCGAGATGTCTACTAATGAGCGAAAAACATCCAGGCCCACTGGTGGTCGAA
GGTAAACTGTCCGATGCCGAGCGCATGAAGCTGGAAAGCAACTATTTGCGCGGTACCATTGCGGAAGATCTGA
ACGACGGTCTCACCGGTGGTTTTAAAGGCGATAACTTCCTGCTGATCCGCTTCCACGGCATGTACCAGCAGGA
TGACCGTGACATTCGTGCCGAGCGCGCCGAGCAGAAGCTGGAGCCGCGTCACGCGATGCTGCTGCGCTGCCGT
CTGCCGGGCGGGGTGATTACTACGACGCAATGGCAAGCGATCGACAAATTCGCGGCGGATAACACGATTTATG
GCAGTATCCGTCTGACCAACCGCCAGACCTTCCAGTTTCACGGCATTCTGAAGAAGAACGTCAAGCCGGTGCA
CCAGATGCTGCACTCGGTGGGGCTGGACGCGCTGGCGACCGCTAACGATATGAACCGTAACGTGCTGTGCACC
TCCAATCCCTACGAGTCTCAGCTGCACGCTGAAGCCTACGAGTGGGCGAAGAAGATCTCCGAACATCTGCTGC
CGCGCACCCGTGCGTATGCGGAGATCTGGCTCGATCAGGAGAAGGTCGCGATCACCGATGAAGAACCGATCCT
CGGGCAGACCTATCTGCCGCGTAAGTTCAAAACCACGGTAGTGATCCCGCCGCAAAACGATATCGATCTGCAC
GCCAACGACATGAACTTCGTGGCGATTGCCGAAAACGGCAAGTTGGTCGGCTTTAACCTGCTGGTGGGCGGCG
GTCTGTCTATTGAGCATGGTAATAAGAAAACCTACGCTCGTACGGCCAGCGAGTTTGGCTATCTGCCGCTGGA
GCACACCCTGGCGGTGGCCGAAGCGGTAGTGACGACGCAGCGCGACTGGGGTAACCGTACCGACAGGAAAAAT
GCCAAAACCAAATACACCCTTGAGCGCGTGGGACTGGAGACATTTAAAGCGGAAGTCGAGCGTCGCGCGGGGA
TCAAATTTGAGCCGATCCGCCCGTATGAGTTTACCGGTCGCGGCGATCGTATCGGTTGGGTGAAAGGGATCGA
CAACAACTGGCACCTGACGCTGTTTATCGAAAACGGTCGTATCCTGGATTATCCGGGGCGCCCGCTGAAAACC
GGCCTGCTGGAGATCGCGAAGATTCATCAGGGCGAGTTCCGTATTACCGCTAACCAGAACCTGATTATTGCCA
GCGTGCCGGAAAGCCAGAAAGCGAAGATCGAGACGCTGGCGCGCGACCACGGGTTGATGAATGCGGTGAGCGC
GCAGCGTGAAAACTCAATGGCCTGTGTATCGTTCCCGACCTGCCCGCTGGCGATGGCGGAAGCCGAGCGTTTC
CTGCCGTCGTTCACGGACAAAGTGGAAGCGATTCTGGAAAAACACGGCATTCCTGATGAGCATATCGTGATGC
GCGTGACCGGCTGCCCGAACGGCTGTGGTCGCGCGATGCTGGCGGAAATTGGCTTAGTAGGGAAAGCGCCTGG
TCGTTATAACCTGCATCTTGGTGGAAACCGTATTGGGACACGTATCCCACGGATGTATCAAGAAAATATCACC
GAGCCGGATATTCTGGCCTCGCTGGATGAACTGATTGGGCGCTGGGCGAAAGAGCGTGAAGCGGGTGAAGGCT
TCGGCGACTTTACGGTGCGTGCGGGCATCATTCGCCCGGTGCTCGATCCCGCGCGTGATTTCTGGGAATAACC
TGAATTGCCGGATGGCGGCATAAATGCCTTATCCGGCCTACCGCCCGCGTAGGCCTGATAAGCGAAGCGCCAT
CAGGCAAGGTATACCGCGAGGAACTTATGTCCAAACTCGATCTAAATGCCCTGAATGAACTGCCAAAAGTCGA

TCGCGTACTGGCGTTGGCGGAAACCAACGCTCAACTGGAAACGCTGACTGCCGAAGAACGCGTGGCTTGGGCG
CTGGAAAATCTGCCCGGCGAATACGTGCTTTCTTCAAGCTTTGGCATCCAGGCGGCGGTGAGCCTGCACCTGG
TGAATCAGATTCGCCCGGATATCCCGGTGATCCTCACCGATACCGGCTATTTGTTCCCGGAAACCTATCAGTT
TATTGATGAGCTAACGGACAAACTCAAACTCAACCTGAAAGTTTACCGCGCGGGCGAAAGTCCGGCCTGGCAG
GAAGCGCGCTACGGCAAGCTGTGGGAGCAGGGCGTTGAGGGCATTGAGAAATACAATGAGATCAACAAAGTCG
AGCCGATGAACCGGGCGCTAAAAGAGCTGAAAGCGCAGACGTGGTTTGCGGGTCTGCGTCGCGAGCAGTCCGG
CAGCCGCGCGCATTTACCTGTTCTCGCTATCCAACGCGGCGTGTTTAAAGTGCTACCGATTATCGACTGGGAT
AACCGCACGGTTTACCAGTATCTGCAAAAGCACGGTCTGAAATACCATCCGCTGTGGGATCAGGGCTATCTGT
CGGTGGGCGATACGCACACCACCCGCAAATGGGAGCCGGGTATGGCGGAAGAAGAGACACGCTTCTTCGGACT
GAAGCGCGAGTGCGGGCTGCATGAAGGGTGATTGTCCTTACGCGGTATGATTATACTTGCTGTTATGAAAAAT
CCGGCAGGCAGCCGGATTTTAAATTGGTTATATTACTGACTATCTTTATGTCAGTAATATATTACGACTGCAC
CCATCTTTACCAATGTGCAAAGAACGTTCATGGGTTAGATATAGCTTTTCAAGAATTACATCAATAAACATTT
TTTTTGCATGATAGTCGTTATAATCCTGATTAAGTTTTTTTAAAAAACCAGAATTTGTTGGTTTAAACTCTGC
AAACGGCGAATAGGTATGTAGTAAATAGGAGATACACGGATGCATCATGCTAAAAGCGACTTCACCTTGTACC
TCCTGATAACCTATTTTGCAAAACTGAGTTGTAGGCTCAAAGTTACCTGCATTAATACAGGCCAGCATCGATT
TTTCATCTTCAGTAAGCGAGTCCTGCCATTCGACAAGCGGGTGGTCTGGAGCCTCTGCGGGACGCATCATCTC
ATAAACCGCCGCTTTGATTTGTGCATTGCCCGTCACTTCTTTTCGTAGTGTAGAAATCGACTCCTGAAGTAAT
TCCGGGTGCCAGGTCATCTTCGCACCAATCACCTCGCATATTTGTAAAAATAACTGTCTCTCCTCTCTACTCA
TAGATTCTATCACTGCATCATCTGATATATTGAGGATGTCCTTAATATTGCTTTGGCTGATCACCGTGTCGCC
GACCATAAGTAAAAATTGTGTCTGAGAAGCGTCCATCTTGATAGTAAACAGATCTTGATGAGCCGGGCAGGCC
AGATGTTGTAGCCGTTTAAAGGCGTAAATTTTATTTATATCCTCTACGTTAACTTCCATCTCTCCAGGTTCAC
CACGGCCCTGCCCGTGAATGATGGAGTGTAACACTTCCAGCGCATGGTCCTTTTTTTCCGCTCTAAAATGATC
CTGGACTTTATCCCAACCCCCCATATGGATTGCTTTTTCTTTATCTGCGCTTAACAGATGAGCAAGCCGACTT
TCATTAAGCGTATAATTTTGATGATTACCGAAGCTTAAAGTGACTGGCATAATATTTTCCTTCAAATTTATAT
TATTCAATATCCGGAGTAAAATATATCGTTCAATTCTCTCTTTTTTATTAAACAGATAAAGTTTACTACAGGA
GAGAATTTGAACGACATTATTAATACAGCACATTTATGGTTGGTAATCCTTTGTGGTGACGATAATTCAGACA
AAAGGTAGATGATTCGCCGCAAATGGTCGTTTTTTTTAAATTTTGGTTTATGCAGAAGGCATTGGAGGAAGCC
TCTTTGAGCGCTTCTTCCAGAAGAAAAAGGCGGAAAAAATTTATAATAATTATTCGTGGGGTAAATATTTCTGA
AGAATATATAAAATTGAGCGGCATATTTAAATATAATCTCAGGAAGTGCAATTTTTGTGAGGTATATCAACAA
AAAAGACCATTAAAAAGGCGTAGAGTGCTTTTATTATCCACATGCTGGAGTTTACGTGTCGATATATCACTAT
TGGGGAAAGTCTCGAAGAGGAGAAACTGACGGCGGTGATGATTACCATTTGCTTTGCTGGCATTCTTTAGATG
TTGCGGCTGTGGGTTACTGGATGGTGATAAATAATATTTATTTTATTGACCACTATCTAAAAAAAATTAGGCAT
CCAGGATAAGGAGCAGGCGGCGCAATTTTTTGCCTGGATTTTATGTTGGCATGATATTGGAAAGTTTGCTCAT
TCCTTCCAGCAACTATACCGTCATGAGGCTTTAAATATCTTTAATGAGCCTACACGGCATTATGAAAAAATCG
CGCATACCACGCTGGGATACATGTTGTGGAACTCCTGGCTAAGTGAATGCCCTGAATTGTTTCCTCCTTCTTC
GCTTTCAGTTCGTAAAAGTAAGCGCGTTATGGCGCTTTGGATGCCAGTCACTACAGGTCATCATGGACGCCCT
CCAGAGGCAATCCAGGAGCTGGACCATTTTCGCCAGCAGGATAAAGACGCGGCAAGAGATTTTCTTCTGAGAA
TAAAAGCGCTCTTTCCTTTAATTACTTTGCCTGAAGCCTGGGATGAAGATGAGGGTATCGACCAATTTCAGCA
ACTTTCCTGGTTTATTTCCGCTGCGGTTGTACTGGCTGACTGGACTGGTTCTGCCAGCCGTTATTTTCCGCGT
ACTGCGGAAAAAAATGCCTGTTGATACCTACTGGCAGCAAGCTCTCGCTAAAGCACAAACTGCCATCACGCTAT
TTCCCTCAGCGGCGAATGTGTCTGCCTTTACGGGCATAGAAACGCTTTTCCCTTTTATTCAGCATCCCACACC
GTTACAACAAAAGGCGCTTGAGCTGGATATCAACGTGGATGGCGCCCAACTCTTTATTCTTGAAGATGTCACC
GGGGCCGGAAAAACAGAGGCGGCGCTCATATTAGCTCATCGACTGATGGCGGCAGGTAAAGCGCAGGGACTCT
ATTTTGGACTGCCGACAATGGCGACAGCCAACGCGATGTTTGAACGTATGGCGAACACCTGGCTGGCGCTGTA
TCAGCCGGACTCCCGTCCCAGCCTGATTCTGGCGCATAGCGCGCGTCGCTTAATGGATCGTTTCAATCAGTCA
ATATGGTCGGTCACTCTTTCTGGTACGGAAGAACCCGATGAAGCGCAGCCTTATAGTCAGGGATGCGCCGCCT
GGTTTGCCGACAGCAATAAAAAAGCGTTGTTGGCGGAGGTTGGCGTAGGCACGTTGGATCAGGCGATGATGGC
GGTAATGCCATTTAAACATAACAACCTGCGGTTACTGGGTCTTAGCAACAAGATCTTACTGGCTGATGAGATC
CATGCCTGTGATGCCTGGATGTCCCGAATACTTGAAGGTTTGATCGAACGGCAGGCCAGTAATGGCAACGCCA
CTATTCTGTTATCTGCGACGCTATCGCAGCAGCAGCGAGATAAGCTGGTGGCGGCATTTTCCCGTGGGGTGAG
GCGTAGTGTGCAGGCGCCGTTGCTAGGCCATGACGATTATCCCTGGCTGACTCAGGTCACACAAACAGAGCTG
ATTTCTCAGCGGGTTGATACACGCAAAGAGGTTGAGCGTTGCGTAGATATTGGCTGGCTACATAGTGAAGAGG
CGTGTCTTGAACGTATAGGTGAAGCAGTGGAAAAAGGAAACTGTATCGCCTGGATACGTAACTCCGTTGATGA
TGCGATTCGTATCTATCGCCAGCTTCAACTGAGTAAGGTCGTCGCCACGGAAAACCTTTTACTCTTCCATAGT

CGCTTTGCTTTTCACGATCGTCAGCGGATTGAGTCGCAGACGCTGAATCTCTTTGGCAAACAGAGCGGCGCGC
AACGTGCCGGTAAGGTCATTATCGCCACGCAGGTAATCGAACAAAGTCTGGATATTGACTGCGATGAGATGAT
CTCTGATTTAGCGCCGGTGGATTTATTAATTCAGCGGGCCGGTCGACTACAGCGTCATATTCGCGATCGTAAC
GGTCTGGTGAAAAAGAGTGGGCAGGATGAGCGAGAGACGCCAGTGCTGCGCATTCTTGCTCCGGAGTGGGATG
ACGCGCCGCGAGAGAACTGGTTATCCAGCGCCATGCGTAACAGCGCCTATGTCTATCCCGATCATGGGCGCAT
GTGGCTGACGCAGCGCATATTACGTGAGCAGGGGACGATTCGGATGCCGCAATCTGCCCGCTTGTTGATTGAG
TCGGTCTACGGTGAGGATGTCAACATGCCGGTTGGATTTGCAAAAACCGAGCAATTGCAGGAAGGCAAATTTT
ATTGCGACCGGGCATTTGCCGGCCAGATGCTGCTTAACTTTGCGCCGGGCTACTGTGCTGAAATTAGCGATTC
TTTACCGGAGAAAATGTCAACGCGGCTGGCGGAAGAGTCTGTCACGCTGTGGCTGGCGAAAATCGTGGATAGC
GTCGTAACCCCTTATGCCAGCGGTGAACACGCCTGGGAGATGAGCGTGCTGCGAGTACGTCAGAGCTGGTGGA
ATAAACATAAAGACGAGTTTGAAAAATTAGACGGCGAACCCTTGCGTAAGTGGTGTGCGCAACAGCATCAGGA
TAAGGATTTTGCCACGGTGATTGTGGTGACGGACTTTGCCGCTTGTGGTTATTCGGCGAATGAGGGATTGATT
GGCATGATGGGGGAATAACGAATACAGATATGGACAATTTTTCACTTTTAACAACGCCCTGGCTCCCCGTCCG
TTTCAAAGACGGTTCCACGGGCAAGCTGGCCCCCGTCGATCTGGCGGATGAAAACGTGGTGGACATCGCCGCA
ACGCGAGCAGATTTACAGGGAGCGGCCTGGCAGTTTCTGTTGGGATTGCTGCAATGCAGTATCGCGCCGAAAA
GATACAAAAATTGGGAGGATATCTGGTTTGATGGATTGCATGCCGATGTGCTCCATAAGGCATTAGCACCGTT
AGAACACGCTTTTCAGTTTGGCGCGGAATCCCCCTCGTTTATGCAGGATTTTGAACCGTTAAGCGGCGAAAAA
GTCTCTATTGCCTCATTGTTGCCGGAAATACCTGGCGCGCAAACCACGAAGTTCAATAAAGATCATTTTGTCA
AACGCGGCGTAACGGAACGTTTTTGTCCGCACTGCGCGGCGCTGGCGCTGTTCTCGTTGCAGCTTAACGCGCC
TGCGGGCGGCAAAGGCTATCGTACCGGGCTGCGCGGCGGCGGGCCACTGACCACGCTGGTTGAATTGCAGGAA
TATCAGGGCGAGCGGCAAACGCCGATCTGGCGCAAGCTGTGGCTCAACGTGATGCCGCAGGATACTGCGGATC
TGCCTTTACCAGACCAGTGTGATGCGACCGTTTTCCCGTGGCTTGCCGCGACGCGGACCAGCGAGCAGGCGAA
TGCCGTTACCACGCCGGAGCAGGTCAATAAACTCCAGGCGTACTGGGGGATGCCGCGTCGTATCCGCCTGGAT
TTTGCCACCTTACAGTCAGGTTGCTGCGATATTTGCGGCGCTGAAAGCGATGAGCTTCTTGGCTTTATGACCG
TCAAGAACTACGGCGTTAACTACGATGGCTGGCGGCACCCGCTGACGCCTTATCGCGCCCCGGTAAAAGATCA
AAACGCCTTCTTTTCCGTTAAACCGCAGCCCGGCGGCCTTATCTGGCGCGACTGGCTGGGATTAAGTCAGAAC
AACCAGACGGAAGCGAATTACGAATCTCCCGCGCAGGTAGTCAAGGTGTTTAACGCCCGCTCGCTGACTGACG
TTAAAGCGGGGATCCGGGGCTTTGGCGCGGATTTCGACAATATGAAAATCCGCTGCTGGTATGAGCATCACTT
CCCGTTGCTGATGACGGAAGGTCTGATCCCTGATTTACGTAAGGCCGTGCAAACTGCGGCCCGCCTGTTGAGC
CTGCTTCGCAGTGCGCTAAAAGAAGCGTGGTTCACCAATGCGAAGGATGCGCGGGGTGATTTCAGTTTTATCG
ACATTGATTTCTGGAACCTGACGCAGGGGCGCTTTCTCAATCTGATCCACGATCTGGAAAACGGACACAAGCC
GGACGAAAGGCTGAATAAATGGCAAAGAGAACTTTGGCTGTTTACCCGTTGTTACTTCGATGATCACGTCTTT
ACCAACCCCTACGAGAGCAGCGATCTGGAGCGCATCATGAAGGCGCGCAAAAAATATTTTACTTCATCGGCGG
AAAAGCAAAGCGCAAAAGCCGCCAAAGCAAAGAAACAGGAGGCTGCTGAATGAGTGTTGTGACGAAAGATGAT
AAAGCCACTTTGCGTCAGTGGCATGATGAACTACAAGAAAAACGTGGATTACGTGCCAGCTTACGACGTAGTA
AAACGGTGAATGATGCCTGTTTGGCTGAGGGGCTTCATTCATTGTTAATGCAAACACATTCTTTATGGAAAAA
CAAAGCACCCTGGAATGTTACTGCATTGGCAATCACAGCGGCACTGGCTGCACATATCAAATTTATTGATGAG
CAAAAGAGTTTTGCTGCGCAGCTTGGACAGAAGAAAGGTGGCGACACTCCGGTGATGTCAAAATTACGCTTCT
CCCACCTATTAGCGGTAAAAACGCCGGATGAACTGCTGCGCCAGTTACGGCGTGCGGTGAAGCTGTTAGATGG
TTCGGTCAACCTGTTTTCTCTTGCAGACGATATTTTCTGCTGGTGCCAGGAGCAGAATGACTTGCTGAACCAC
CATCGCCGCCAGCAGCGGCCAACCGAATTTCTTCGTATCCGCTGGGCGCTGGAATACTACCAGGCTGGCGATA
CCGATAACGAACAAGATTAACAGGGATATCTTTATGACAACGTTTATTCAGCTCCACTTACTGACCGCTTACC
CCGCCGCTAACCTGAACCGTGATGATACCGGTGCGCCAAAAACCGTGGTGCTTGGCGGAGCAACACGTCTGCG
TATCTCCTCTCAGAGCCTGAAACGCGCCTGGCGTACATCTGAGTTATTTGAACAGGCATTAGCTGGCCATATT
GGTATTCGTACTGGTCGCATTGCTCGTGAGGCGGCGCAAATCCTCGTTGATAGCGGCATTGACGCCAAAAAAG
CGGTTGAGTACGTCAAGAACATCGCCAACTGCTTTGGCAAGGTAAAAGAGGATAAGAAACCCAAAGATGAGTT
GACGAATGCTGAAACCGAGCAACTGGTGCATATCAGCCCTGCTGAGTTTGAGGCCGTGAAAGCGCTGGCGCGC
CGTCTGGCAGAAGAAAAACGTCCGGCAACAGAAGAGGAAGCGGAACTGTTACGTCACGATCGCATGGCCGTCG
ATATTGCCATGTTTGGCCGGATGTTAGCGAAGAAAACTGATTTTAACGTGGAAGCCGCCTGCCAGGTCGCCCA
CGCCTTCGGCGTCAGCGAAACGATCATCGAAGACGATTTCTTTACCGCTGTGGATGACCTACGCCAGGCATCG
GCAGAAGATGCAGGCGCAGGCCATCTCGGCGAAACCGGCTTTGGCTCCGCGCTGTTTTACACCTATATCTGCA
TCGACAAAGATCTGCTGGTGAAAAACCTGAACGGCAATGAAGAACTGGCAAACAAAACGCTGCGCGCCTTTAC
TGAAGCGGCGCTGAAAGTGTCGCCGACCGGCAAACAGAACAGCTTTGCCAGCCGTGCCTATGCCTCGTGGGCG
CTGGCCGAAAAAGGCACCGACCAACCACGTTCACTGGCGGCCGCGTTTTATGAACCGATCAACGGTACAGACC

AATTGAACGTTGCGGTTAAGCGTATTACCGCGCTGCATGAAAATATGAATGAGGTCTATGCACAGGAGACGGC
GTTCAAAAACTTTAACGTGATGAATCAGCAGGGAAGCATGAAAGACGTGCTTGACTTCATCTGCGCGTAAGGG
GCGGCTTATGAGCCAATATCTGGTTTTTCAGCTTCATGGGCCGATGGCCTCCTGGGGCGTGGATGCGCCAGGC
GAAGTGCGCCACAGCCATGAACTTCCTTCGCGTTCTGCGCTGTTGGGGTTATTAGCTGCGGCGCTGGGTATTC
GCCGCGATGAAGAGGAACGGCTAAACACCTTTAATCGTCATTACCAATTTTTGCTGTGCGCCAGCGGCAACCC
GCGCTGGGCGCGGGATTATCACACGGTGCAGATGCCGAAAGAGGTACGTAAGGCGCGTTACTTCAGCCGCCGT
GAAGAGTTGCAGGATCCTGAGTTACTGAGCGCGCTGATTTCCCGACGCGACTACTACACCGACGCCTGGTGGA
TGATCGCTGTCAGCGCGACGCCGGACGCGCCTTACACGTTGGCGCAGCTTCAGGCGGCGTTACAACATCCTGT
GTTCCCGCTGTATCTGGGGCGCAAAAGTCACCCTCTGGCGTTGCCGCTTGCGCCGCAACTGCTGGAAGGGAAT
GCGGCGGATGTCCTGCGTGAAGCATATCGTTGGTATCAGGATCAATTCAATGCGCTGAAACTGACTCTCCCCG
GATTACAGAATGAATGCTGGTGGGAAGGTGAACACGATGGCTTAACGGCGAACAAAATTTTACGTCGTCGCGA
CATGCCGTTGAGCCGCCAGCAATGGCTGTTCGGTGAACGTAGCGTGAATCAGGGGCCGTGGCTCCGCAAGGAG
GACGCGTGTATCTCTCAAGAATAACGCTGCACACCAGCGAGCTTTCGCCGGCACAGCTCTTGCATCTGGTGGA
GCGCGGCGAGTACGTGATGCACCAGTGGTTATGGGATCTCTTTCCCGGCGGCAAGGAACGGCAGTTTCTTTAT
CGCCGCGAAGAGTTACAGGGCGCGTTTCGCTTTTTTGTTCTCTCACAGGAACAGCCTGCCGCCAGCACGATTT
TTGACGTACAGACTCGCCCGTTTGCGCCAATGCTGAGCGCGGGACAGACGCTGCGGTTTAACTTGCGAGCCAA
TCCGACAATTTGCAAAAACGGTAAGCGTCACGATTTGCTGATGGAGGCGAAACGCCAGCGTAAAACGCAGGGC
GATAGCCAGGATATTTGGTCTTATCAGCAGCAGGCGGCATTGGAATGGCTGGCGCGCCAGGGGGAGCAAAATG
GCTTTACTCTGCGCGAGGCGAGCGTTGATGCTTACCGGCAGCAGCAGATACGTCGAGAGAAATCCCGGCAGAT
GATTCAGTTTAGCAGCGTGGACTATACCGGCGTGCTGGTGATTAACGAGCCCGCGCTTTTCCTGCAACGTCTG
GCGCAGGGGTACGGTAAAAGCCGGGCGTTTGGCTGCGGAATGATGATGATCAAACCCGGAGACGACGCGTGAC
GTTCGTACCGCTCAACCCGATCCCGTTAAAAGATCGAACCTCGATGATCTTCCTCCAGTACGGTCAAATTGAC
GTGCTGGATGGGGCATTCGTGCTGATCGATAAAACGGGAATCCGTACGCATATTCCCGTCGGTTCGGTCGCTT
GTATCATGCTGGAACCGGGAACGCGGGTTTCCCATGCGGCAGTGCATTTGGCATCAACGGTCGGCACCCTGTT
GGTATGGGTGGGCGAGGCGGGAGTGCGGGTCTATTCCTCCGGACAACCCGGCGGCGCACGAGCCGATAAGTTG
CTTTACCAGGCAAAGCTGGCGTTAGATGATGACCTGCGGCTTAAAGTAGTCCGCAAAATGTATGAACTGCGTT
TTCGTGAGCCGCCTCCCGCCCGTCGTTCCGTTGAGCAACTGCGCGGTATTGAAGGATCCCGTGTGCGGGCGAC
CTATGCATTACTGGCGAAGCAGTATGGCGTGAAATGGCATGGTCGTAACTATGATCCGAAAGACTGGGAGAAG
GGGGATGTCGTCAACCGATGTATTAGCGCGGCGACATCGTGCCTGTACGGGATTTCAGAAGCGGCTATCCTGG
CGGCGGGATATGCGCCGGCTATCGGTTTTATCCATAGCGGTAAGCCGCTTTCTTTTGTTTATGACATTGCCGA
TATCATCAAATTTGAATCGGTGGTGCCCAAAGCATTTGAGATCGCCGCTCGTCACCCGGCGGAACCTGATAAA
GAAGTGCGCCTGGCCTGCCGGGATATTTTTCGCAGTTCGAAGCTGACCGGAAAATTGATCCCGCTGATCGAAG
AGGTGCTCGCTGCCGGTGAAATTGAACCACCTCAGCCTGCGCCGGATATGCTGCCGCCAGCAATACCGGAACC
TGAATCACTGGGTGATAGCGGCCATCGGGGGCATGGTTGATGAGTATGGTGGTTGTGGTCACGGAAAACGTAC
CGCCGCGCCTGCGGGGACGTCTTGCCGTCTGGTTACTTGAGGTTCGTGCAGGTGTTTATGTTGGCGATACCTC
GAAGCGTATTCGGGAAATGATTTGGCAACAGATCACACAGCTTGGCGGAGTCGGAAACGTAGTAATGGCCTGG
GCAACAAATACTGAGTCTGGTTTTGAGTTCCAGACCTGGGGTGAAAACAGACGTATTCCGGTAGATTTGGATG
GACTGCGTTTGGTTTCTTTTCTTCCTGTTGAAAATCAATAAGTTGAATGTTCTTTAATGATAAGGAATTGTTA
TCTTACCGTTGGTAGTTTGTTATGTAGTAAAAAAGGGCTTTTAGAACAAATATATAGTTTTAGTGTGTTCCCC
GCGCCAGCGGGGATAAACCGACGTTGGCTGAAAACGGTTTTTCGGTCCGCCTGTGTTCCCCGCGCCAGCGGGG
ATAAACCGAATATATGGCGCTCACGCGCATGAGCATTCTCGTGTTCCCCGCGCCAGCGGGGATAAACCGCAGG
GCAAATTCATCCGCCGCTGACCACTGGTGTGTTCCCCGCGCCAGCGGGGATAAACCGGACGCTTACATCTCAC
CGAGAGATTTTGAGGCGTGTTCCCCGCGCCAGCGGGGATAAACCGGGAACTGGTTTAGCTATCGCTGCCGGGG
CTATGTGTTCCCCGCGCCAGCGGGGATAAACCGGATTGCTCAGATTGGGAATTTGACCAGCGGCCGTGTTCCC
CGCGCCAGCGGGGATAAACCGTCACGAGGGCCCCCTTATTGGGTCGGGCAGGTGTGTTCCCCGCGCCAGCGGG
GATAAACCGGTTGGGTTGCATAGATGACACGCTTATAAATAGTGTTCCCCGCGTCAGCGGGGATAAACACGTT
TCACCAGCATATCAGGACGTTTTTTCCGCCTTCGCCAGCTCTTTTACCAACGGCAGCATTATCCGTACTACAT
CGCGGCTACGGCGCTCAATTCGCCCTGGCAGCGCCTTGTCAATATGCTGTTGATTATCAAGCCGTACGTCGTG
CCAGCTATTGCCGTTCGGGAAGGAGGCATTTTTCACGCGTTGCTGGTATCCGTCTTTTTTACCCAGATTCCAG
TTCGTCGCCTCAACAGAAAGCACCGATATTCCCGCTTTATCGAAAACCTCCGCATCATTACAGCAACCCGTTC
CTTTGGGGTAGGATGGATTGCGTCCCGGATTGGTGTTGGCGGCGATACCATAACGGCGCGCAATCGCTAATGC
CCGATCGCGGGTCAGTGTACGCACCGCTTCCGGCGTATTTTTCCCGCTATTAAAATAGAGCTTGTCGCCAACA
ATCAGGTTATCGAGATTAATCACCAGCAGCGTATTTTTCTTCTCAGCGTCACTCATTCGTTTGAGTAAATTTT
CCGCGCCTAGCTTTCCCTCTTCTTCCCCGCTGGTGGCGATAAAACGAATCCCGTAATGGGTCGGTATATCTTT

CAGACGCGCCGCCAGTTCCAGCATAACGCCTAATCCCGCGGCATTATCATCCATTCCCTGTAACGTTAAACCG
CCCAGATTGGCATCGACATCAGCGTCGCTCTGCGGAGCGTACGTATCCAGATGCGCCATAATGATGATCTGTT
GCGGCACGCGCCCTTCATGGGCGGCGATGACCGTACTGCCCGTCACGTTATGCCAGTTTTTGCGATTATCCTT
CGTGGTATAAATAAATCGGCTATTAAACGTTCGAATATCGCTTTGATATCCCATCTGGGTAAATTGTTGGCGT
AAATAATCGGCAGACAACATCTCGGCGGGGGAGCCAGTCATTCTCCCGGGAAAAAAGGTGGCGATATGTCGTG
CCTGAGTATTCGCTATTTCACCAGGCCCTGGTGATGCTGCTTGTGCAGGAAGGATAAAGCCTACGCCGAGCGC
CAGAATGACGGCAAAGCGGCGCGTTGCGGAAAACATAATGAGTCCTTAAATACAGAACAAAATTTTACGTGGC
TAGTATGAAACCGCGATCGTGTTAATACAATTTCATTTGCTCCTAAATGCTGGCAATCCGGTACGTTAAGCAT
TTTTTGATATTGGTTTTTGGGCCGCGTTATTCCTTTGAGGAACTACTCATTCCAATTCGTCATTTCATTCACT
CTCCTGCGCTCCCTATAGTCGTGTAATCGAAATGTTTAGCAAAAAACGGTATTGCAAAGGAACGGTTATGGAT
CAAAAACGACTCACCCACCTGCGGCAACTGGAGGCGGAAAGTATCCACATTATCCGTGAAGTGGCAGCGGAAT
TCGCTAATCCGGTCATGCTGTACTCCATTGGTAAAGACTCCAGCGTCATGCTGCATCTGGCGCGTAAGGCGTT
TTATCCGGGTACGTTGCCGTTCCCGCTATTGCACGTTGATACCGGTTGGAAATTCCGTGAGATGTACGCCTTT
CGCGATCGTACCGCCAACGCATATGGCTGCGAGTTGCTGGTACATAAAAATCCGGAAGGCGTGGCGATGGGCA
TCAATCCGTTCGTTCACGGTAGCGCTAAACACACCGACATTATGAAAACCGAAGGGTTAAAGCAGGCGCTGAA
TAAGTACGGTTTTGATGCCGCGTTCGGCGGCGCACGGCGCGATGAGGAAAAATCCCGTGCGAAAGAACGTATT
TACTCCTTTCGTGACCGTTTCCATCGCTGGGACCCGAAAAATCAGCGTCCAGAGTTGTGGCGTAACTATAACG
GTCAGATTAATAAAGGTGAAAGTATACGCGTCTTCCCGCTCTCCAACTGGACAGAGCAGGATATCTGGCAATA
CATCTGGCTGGAAAACATCGACATCGTGCCGCTGTATTTGGCTGCTGAACGCCCGGTTCTGGAACGTGACGGC
ATGTTGATGATGGTGGATGACGATCGTATCGATCTCCAGCCTGGCGAAGTCATCAAAAAAAGGATGGTGCGCT
TCCGTACACTTGGCTGTTGGCCGCTCACCGGGGCGGTGGAATCCCACGCGCAAACGCTGCCGGAAATTATCGA
AGAGATGCTGGTTTCTACCACCAGCGAGCGCCAGGGGAGGATGATTGACCGCGATCAGGCGGGGTCGATGGAG
CTTAAAAAACGTCAGGGGTATTTCTAAGGAGCCGCCATGAACACCATACTTGCACAACAAATCGCCAATGAAG
GCGGCGTCGAAGCCTGGATGATTGCCCAACAGCACAAAAGCCTGCTGCGTTTTTTAACCTGTGGCAGCGTTGA
TGACGGTAAAAGTACGCTGATAGGTCGTTTGCTGCACGATACTCTGCAAATTTATGAAGATCAGCTCTCTTCT
CTGCATAATGACAGTAAACGTCATGGCACGCAGGGAGAAAAACTCGATCTGGCGCTGCTGGTAGATGGGCTGC
AGGCCGAGCGTGAGCAGGGCATTACTATTGACGTCGCGTACCGCTATTTCTCCACGGAAAAACGAAAATTTAT
TATCGCCGACACGCCGGGCCATGAACAATATACCCGTAATATGGCGACCGGGGCGTCCACCTGCGATCTGGCG
ATCCTACTGATCGACGCGCGTAAAGGCGTGCTGGATCAGACGCGTCGCCATAGCTTTATCTCCACTCTGTTGG
GGATCAAACACCTGGTGGTGGCAATCAACAAAATGGATCTCGTTGACTACTGCGAAGAGACCTTCGCCCGTAT
TCGCGAAGATTACCTGACCTTTGCTGAACAGTTGCCGGGCGATTTGGACATTCGCTTTGTCCCGCTTTCGGCG
CTGGAAGGGGATAATGTCGCCGCGCAGAGCGCGAATATGCGCTGGTACAGCGGTCCGACGCTGCTGGAAGTCC
TGGAAACGGTAGACATACAGCGTGCGGTAGACCGTCAGCCGATGCGCTTTCCGGTACAATACGTTAATCGCCC
GAATCTTGATTTTCGGGGGTATGCCGGCACTCTGGCGTCTGGTAGCGTCAAAGTCGGAGAGCGCATTAAAGTG
CTGCCGTCCGGCGTGGAATCCAGCGTGGCGCGTATCGTCACTTTTGATGGCGATAAAGAAGAAGCCTGTGCGG
GCGAAGCCATTACCCTGGTACTCAACGACGACATTGATATTAGCCGCGGCGATCTGCTGCTGGCGGCAAACGA
GACGCTGGCACCTGCGCGGCACGCCGCGATCGACGTGGTATGGATGGCGGAACAGCCGCTGGCGCCGGGCCAA
AGCTACGACGTTAAACTTGCGGGTAAGAAAACCCGCGCGCGTATCGAGGCTATTCGCTATCAGATTGATATCA
ACAATCTGACCCAACGAGACGTCGAGAGCCTGCCGTTAAACGGTATTGGTCTGGTGGAGATGACATTCGATGA
GCCGCTCGCGCTGGATATTTATCAGCAAAATCCGGTGACGGGCGGGCTGATTTTTATCGACCGACTTTCTAAC
GTTACCGTGGGCGCTGGCATGGTGCGGGAACTGGACGAACGCGGAGCGACGCCGCCCGTGGAATACAGTGCGT
TTGAGCTGGAGCTGAATGCGCTGGTACGCCGTCATTTTCCGCACTGGGACGCCCGAGATTTGCTGGGAGATAA
ACATGGCGCTGCATGATGAGAACGTGGTCTGGCACTCTCATCCCGTTACTGTCGCCGCCCGTGAACAGCTCCA
CGGCCACCGTGGCGTGGTGCTGTGGTTTACCGGACTCTCCGGTTCGGGAAAATCAACGGTGGCCGGCGCGCTG
GAAGAGGCGTTACATCAGCGTGGCGTCAGTACCTACCTGTTGGATGGCGATAATGTGCGCCACGGTTTGTGCC
GTGATTTAGGCTTTAGCGATGCCGATCGCCAGGAGAATATCCGGCGGGTAGGCGAAGTGGCCAGCCTGATGGC
GGACGCCGGCCTGATTGTTTTGACGGCATTTATTTCTCCGCACCGCGCCGAGCGGCAACTGGTAAAAGAGCGC
GTCGGCCACGATCGTTTTATCGAGATTTATGTCAATACGCCGCTGGCTATCTGTGAACAGCGCGATCCTAAAG
GGTTGTATAAAAAAAGCGCGTGCGGGCGAGTTGCGTAACTTTACCGGCATCGACGCCATTTACGAAGCGCCTG
ACTCGCCACAGGTTCATCTTAATGGTGAACAATTAGTAACAAATTTGGTTTCCCAATTATTAGACCTGCTGAG
ACGGCGCGATATTATCAGATCCTGAGACACCACCGGGCTTGTATGCCCGGTATGGTCAAGGTTACAGGATTTA
ATATGCGTAATAGCCATAACATTACTTTCACAAGGTCAGACGCCTTCATGGTCGACGATGACGCCACCAGTGC
ATTTCCGGGGGCGGTGGTCGGCTTTGTGTCATGGCTGCTGGCGTTGGGGATCCCTTTTCTGCTTTATGGCCCT
AACACGCTGTTTTTTTTCCTCTATACCTGGCCCTTTTTCCTCGCGTTAATGCCGGTATCGGTGATTATCGGCA

TTGCGTTGCATCTGCTGGTAAAGGGCAAAATACTTTTTAGCATTATGTTTACTCTACTGGCCGTAGGCGCGCT
GTTCGGCGCGTTATTTATATGGCTATTAGGGTAGACGGGTTATCACTGCGCCTCCGGTCATAATTCAAACCGT
AACCAACAATGAGATTATGTTCTCCTTACTCGGGTATACGTAACATCCGGCAAATGTGGTACATTTGCCGGCG
TTGTCGCGGCAATCCCGTCCTGAATGTGGAGTCACAGAGGACGTTATGGGATGATGATGCCGTTTTTCAGGGG
GCAGGATGGGTAAACTAACGCTGCTGTTGCTGGCTTTACTGGTCTGGCTACAGTACTCGCTGTGGTTCGGTAA
GAACGGCATACATGACTATAGCCGCGTCAACGATGATGTGGTCGCGCAGCAGGCGACAAACGCCAAACTTAAA
GCGCGTAACGATCAGCTTTTTGCCGAAATTGACGATCTCAATGGCGGCCAGGAAGCGATTGAGGAGCGCGCGC
GCAACGAACTTAGCATGACCAAACCGGGCGAAACCTTCTATCGTCTGGTGCCTGACGCCTCAAAACGCGCGGC
AACGGCGGGGCAAACTCATCGATAAACCATCCAGGGATTTAACATGGCAGCCACTTTATTGGACGTTTGCGCC
GTGGTGCCGGCGGCCGGATTTGGCCGCCGAATGCAAACAGAATGTCCTAAGCAGTATCTCTCAATCGGTAATA
AAACCATTCTGGAGCACTCAGTTCATGCGCTGCTGGCGCATCCTCGGGTGACACGCGTTGTTATTGCTATCAG
CCCTGGCGATCATCGCTTTGCCCAACTTCCGCTGGCGAATCATCCGCAAATCACCGTTGTGGATGGCGGCAAC
GAACGCGCTGATTCTGTTTTGGCCGGGTTGCAGGCCGTTGCAAAGGCGCAGTGGGTGCTGGTGCACGACGCGG
CAAGACCCTGTTTGCATCAGGATGACCTTGCGCGGTTGCTGACCATCAGCGAAAACAGCCGGGTCGGCGGTAT
CCTCGCCAGTCCGGTGCGCGACACCATGAAACGCGGCGAACCGGGGAAGAACGCTATTGCCCATACCGTAGAG
CGCGCCGATTTATGGCACGCGCTCACCCCGCAGTTTTTCCCCCGCGAACTCTTGCACGATTGTCTGACGCGTG
CGCTGAATGAAGGCGCGACCATCACCGACGAAGCGTCAGCGCTGGAATATTGTGGTTTTCATCCGGCGCTGGT
AGAAGGTCGCGCGGATAACATCAAGGTCACCCGTCCGGAAGATCTGGCGCTGGCGGAATTTTATCTGACCCGA
ACCATCCACCAGGAGAAAGCATAATGCGAATTGGACACGGTTTTGACGTACACGCGTTTGGCGGAGAAGGGCC
GATTATTATTGGCGGCGTGCGTATCTCTTATGAAAAAGGGCTATTGGCGCATTCCGATGGCGATGTGGCGTTA
CATGCGCTGACCGATGCGCTGCTGGGTGCGGCGGCGCTTGGCGATATCGGCAAGCTCTTCCCGGATACCGATC
CGGCATTTAAGGGAGCGGATAGCCGCGAACTACTGCGTGAAGCCTGGCGTCGTATTCAGGCGAAGGGGTACAC
CCTCGGCAACGTAGATGTCACCATTATTGCGCAGGCGCCGAAAATGCTGCCGCATATTCCGCAAATGCGCGTC
TTTATCGCCGAAGATCTCGGCTGCCATATGGATGATGTTAACGTGAAGGCGACGACGACGGAAAAGCTCGGAT
TTACCGGACGCGGAGAAGGCATTGCCTGCGAAGCGGTCGCGTTGCTGATGAAGGCGGCGAAATGACAGAGTTT
GACAATCTCACCTGGCTGCACGGAAAACCACAAGGTAGCGGATTGCTGAAAGCGAATCCGGAAGATTTTGTGG
TGGTTGAAGATTTAGGCTTTACGCCAGATGGTGAAGGCGAGCACATTCTGCTGCGTATTTTGAAAAACGGTTG
CAATACCCGTTTTGTCGCTGACGCGCTGGCGAAATTCCTGAAAATTCACGCTCGCGAAGTGAGCTTTGCCGGG
CAAAAAGATAAACACGCCGTTACCGAGCAGTGGCTGTGCGCGCGCGTGCCGGGGAAAGAGATGCCCGATTTCA
GCGCCTTTCAGCTGGAAGGTTGTAAAGTGCTGGAATACGCGCGTCACAAGCGTAAGCTGCGTTTAGGCGCGCT
GAAAGGCAATGCCTTTACGCTGGTGTTGCGTGAAATAAGCGATCGCCGCGATGTCGAAACACGATTGCAGGCG
ATTCGCGATGGCGGCGTGCCGAACTATTTCGGCGCACAGCGATTCGGCATTGGCGGCAGCAACTTGCAGGGCG
CGCTGCGCTGGGCGCAAAGTAACGCGCCGGTGCGCGATCGCAATAAACGCAGTTTTTGGTTGTCGGCGGCGCG
TAGCGCGTTGTTTAATCAAATTGTTCACCAGCGGCTGAAGAAACCAGACTTTAATCAAGTTGTTGACGGCGAT
GCGCTACAATTAGCGGGGCGTGGAAGCTGGTTCGTCGCGACATCTGAAGAACTGCCTGAATTGCAGCGCCGGG
TAGACGAAAAAGAGCTGATGATTACAGCGTCGTTGCCGGGCAGCGGAGAGTGGGGAACACAGCGTGCGGCGCT
GGCGTTTGAGCAAGATGCCATTGCCCAGGAAACGGTGTTGCAATCGCTGTTGCTGCGCGAAAAAGTAGAGGCG
TCGCGCAGAGCGATGCTGCTTTATCCGCAACAATTAAGCTGGAACTGGTGGGATGACGTTACCGTCGAGTTAC
GTTTCTGGCTGCCCGCAGGCAGTTTTGCCACCAGCGTAGTAAGGGAACTGATCAACACAATGGGTGATTATGC
GCATATTGCTGAGTAACGATGACGGGGTTCACGCGCCCGGTATACAAACGCTGGCGAAAGCGCTGCGTGAGTT
TGCTGATGTACAGGTCGTAGCCCCGGATCGTAACCGCAGCGGCGCGTCTAATTCCCTCACGCTGGAATCTTCG
CTTCGTACTTTTACCTTTGATAATGGCGATATCGCCGTACAGATGGGGACGCCGACCGATTGCGTCTATCTGG
GCGTTAATGCCTTAATGCGTCCGCGTCCGGATATTGTCGTTTCCGGTATTAACGCGGGTCCGAATCTGGGCGA
TGATGTGATCTATTCCGGTACTGTCGCCGCGGCGATGGAAGGTCGTCATCTCGGCTTTCCGGCATTAGCGGTC
TCCCTTAACGGCTATCAACATTATGATACGGCTGCGGCCGTGACTTGCGCGCTTTTGCGAGGGTTAAGCCGGG
AGCCGTTGCGTACCGGGCGTATTCTCAACGTGAATGTCCCGGATCTACCGTTAGCGCAGGTTAAAGGCATCCG
CGTGACTCGCTGTGGTAGCCGCCATCCAGCGGATAAAGTAATCCCGCAGGAAGATCCGCGCGGTAATACATTG
TACTGGATTGGCCCGCCGGGCGATAAATACGATGCCGGGCCGGATACCGATTTCGCGGCGGTGGATGAAGGCT
ACGTCTCCGTCACGCCGTTGCATGTGGATTTAACCGCGCACAGCGCGCATGATGTGGTTTCAGACTGGTTAGA
CAGCGTGGGAGTTGGCACGCAATGGTAAGTGGACGTGTACAGGCTCTTCTTGAACAATTGCGCGCGCAGGGCA
TCAGAGATGAGCAGGTGCTTAATGCGCTTGCCGCAGTGCCGCGCGAGAAATTTATTGATGAAGCGTTTGAACA
TAAGGCCTGGGAAAATATCGCTTTGCCGATAGGCCAGGGTCAGACGATTTCGCAGCCCTACATGGTGGCGCGA
ATGACGGAACTGCTCGAACTGACGCCGCAATCCAGGGTGTTGGAAATTGGCACCGGTTCCGGCTATCAGACGG
CGATTCTGGCGCATCTGGTACATCACGTTTGCTCCGTTGAGCGGATTAAGGGGCTGCAATGGCAGGCGCGCCG

CCGCCTGAAGCAGCTCGATTTACATAATGTTTCTACCCGTCATGGCGATGGCTGGCAAGGCTGGCAGGCGCGT
GCGCCGTTTGACGCTATCATTGTGACGGCCGCGCCACCGGAAATTCCTACCGCGCTCATGGCACAGTTGGATG
AAGGCGGCATTCTTGTTCTGCCCGTGGGCGATGAGCAGCAGTTTTTGAAACGCGTGCGTCGCCGGGGCGGCGA
ATTTATTATCGATACCGTGGAGGCCGTTCGCTTCGTCCCGTTAGTCAAGGGAGAGCTGGCCTAGTTTGCCCTG
CAAACACCCACTCTTACGCCTGATGAAGCCGAAACGAATACCTGGAGTTTTCCTGGTTATTCCCGGTGGGTTA
AGCGTATCGTGAACACATTTTCCCGTGTTCACTTAATGCGTTGCACGGTTCTTACGTCACTGGTTGTTAACCA
AATTTTTTACTGGGGGATAAATGAGCGCGGGAAGCCCAAAATTCACCGTTAGTCGCATTGCGGCACTATCACT
GGTTTCACTATGGCTGGCAGGTTGTACCAGTTCGTCAAACCCTCCGGCGCCGGTCACGTCTGTGGACAGTGGG
TCGTCATCAAATACCAATTCCGGGATGTTGATCACACCGCCGCCAAAAATGGGCGCGACGACGCAACAAACGC
CGCAACAAGCGCCGCAAATTCAACCCGTTCAGCGTCCTGTAACGCAGCCCATGCAAACTCAACCGGTCACGGA
ACAACCCGTGCAGATGGAAAATGGGCGAATTGTATACAATCGCCAGTATGGGAATATTCCGAAAGGTAGCTAT
ACGGGTGGCAGTACTTACACCGTGAAAAAGGGTGATACGCTTTTTTACATCGCCTGGATTACCGGGAACGATT
TCCGCGATCTGGCCCAGAGAAACAGTATTTCTGCCCCGTATAGCCTGAATGTAGGGCAAACTTTGCAAGTTGG
TAACGCATCCGGTACGCCAATTACTGGCGGAAATGCGATCACCCAGGCGGATGCAGCACAGCAAGGAGTTGTG
ACCAGGTCTGCACAAAATTCCACCGTTGCAGTTGCGTCTCAACCAACAATTACGTATTCTGAGGGCTCAGGTG
AACAAAGTGCTAACAAAATGTTGCCAAACAACAAGCCTGCTGGGACGGTTGTCACAGCGCCTGTAACGGCACC
AACGGTAAGCACGACCGAACCAAATGCAAGCAGTACGTCAACCAGCGCGCCGATTTCCGCATGGCGCTGGCCG
ACGGATGGCAAAGTGATCGAAAACTTTGGCGCTTCCGAAGGGGGCAATAAAGGGATCGACATTGCAGGCAGTA
AGGGACAGGCTATCGTCGCAACCGCTGATGGGCGCGTCGTATATGCCGGTAACGCACTGCGTGGTTACGGTAA
TCTTATTATCATCAAACATAACGATGATTACCTGAGTGCCTACGCCCATAATGATACGATGCTGGTCCGGGAA
CAACAGGAAGTTAAGGCGGGGCAAAAAATCGCTACTATGGGTAGCACCGGCACCAGCTCTACACGCTTGCATT
TTGAAATTCGTTACAAGGGGAAATCCGTAAACCCGCTGCGTTATTTACCGCAGCGATAAAGCGGCGTAACCAG
GCTTTGACTTGCTAGTTCCGTCAAGGGATCACGGGTAGGAGCCACCTTATGAGTCAGAATACGCTGAAAGTTC
ATGATTTAAATGAAGACGCGGAATTTGATGAGAACGGAGTAGAGGCTTTTGACGAAAAAGCCTTGAGTGAAGA
GGAACCCAGTGATAACGACCTGGCTGAAGAAGAGCTGTTATCGCAAAGGGGCCACACAGCGTGTGTTGGACGC
GACTCAGCTTTACCTTGGTGAGATTGGGTATTCACCACTGTTAACAGCCGAAGAAGAAGTCTATTTTGCGCGT
CGCGCACTGCGTGGAGATGTCGCTTCTCGCCGTCGCATGATTGAGAGTAACCTGCGTCTGGTGGTAAAAATTG
CCCGCCGTTATGGCAATCGTGGACTGGCGTTGCTGGACCTGATTGAAGAGGGCAATCTGGGGCTTATCCGTGC
AGTCGAGAAGTTTGACCCGGAACGCGGGTTCCGCTTCTCAACATACGCAACCTGGTGGATTCGCCAGACAATC
GAACGGGCGATTATGAACCAAACCCGTACGATTCGCTTGCCGATTCACATTGTTAAAGAGCTGAACGTATACC
TGCGCACCGCACGTGAGTTGTCGCATAAACTGGACCACGAACCGAGTGCGGAAGAAATTGCAGAGCAACTGGA
TAAACCGGTTGATGACGTCAGCCGTATGCTTCGTCTCAACGAGCGCATTACCTCGGTAGACACCCCGCTGGGC
GGTGATTCCGAAAAAGCGTTGCTGGACATCCTGGCCGATGAAAAAGAGAACGGTCCGGAAGACACCACGCAAG
ATGACGATATGAAACAGAGCATCGTCAAATGGTTGTTCGAACTGAACGCCAAACAGCGTGAAGTGCTGGCGCG
CCGTTTCGGTCTGCTGGGATATGAAGCTGCGACACTGGAAGATGTAGGCCGTGAAATCGGTCTTACGCGTGAG
CGTGTTCGTCAGATTCAGGTTGAAGGCCTGCGCCGTCTGCGCGAAATTCTGCAGACGCAGGGGCTGAATATCG
AAGCGCTGTTCCGCGAGTAAGTACCCTTGTCAAAAAAAGGCCAGTCGACAGACTGGCCTTTTTTTACCGTTTG
TCTTCTGCCAGCAGCGGCGGGATACTCGGCACCATTGGCGCGTCATCAATATCTTTTTGCGTCATGCGAAACG
CTTGTGGATAGTGTTCGCGGCTGGTACGGCGTAACGGTTCGGTATCGCGCCAGGTATACAGACAATGCTGACA
CTGATATACCGTCCAGACTCCTTTTACCGGAGAAGTCGCCATGAGTTCAATATGTGCATCGGCACAGCGTGGA
CAAATCATCGGTTACTCCTTATTTACGGTTGGCCAGCATGGCGGTCAGCTTTTCAGCCCAGGCTTTAGTTTCC
GGTAAATCAACAACCGGCTGGCTATAGTGACCACGGTTATCTGGCGCAACCGGCGTGGTGGCGTCAATGATCA
GCTTGTCGGTAATCCCCGCCGGGCTTGAGCCCGGGTCCAGTTCCAGGACGGACATATTCGGTAGCTGTACCAG
ATCGCCTGCCGGATTCACTTTCGACGACAACGCCCACATCACCTGTGGCAGATTGAATGGATCGACATCTTCA
TCGACCATGATCACCATCTTCACATATCCCAGACCGTGCGGCGTCGTCATCGCTCGCAGGCCCACCGCACGGG
CAAAGCCGCCGTAGCGTTTTTTGGTCGAGATGATGGCCAGCAAGCCGTGGGTGTACATGGCGTTTACCGCCTG
CACTTCCGGAAATTCGGCTTTCAGCTGTTGATACAGTGGCACGCAGGTCGCCGGCCCCATCAGGTAGTCGATT
TCCGTCCACGGCATCCCCAAATAGAGCGATTCAAAAATGGGTTTGCTGCGGTAAGAGACCTTATCGATACGCA
CCACGGTCATGTTGCGACCGCCGGAATAGTGGCCGGTAAATTCGCCGAACGGTCCTTCAATTTCACGTTTACG
GCTTTCGATGACCCCTTCAAGAATGACTTCAGATCCCCACGGCACATCAAAACCGGTCAGCGGCGCGGTGGCG
ATAGGATAGGGGCTTTCACGCAGCGCGCCTGCCATTTCATACTCTGACTGATCGTATTTCAGCGGCGTTGCGC
CCATCAGGGTAATGATCGGATCGTTACCCAACGTGATCGCAATCGGCAGATCTTCCCCGCGCTCTTCAGCTTT
GTGCAGGTGCAATGCGATATCATGCATCGGCACTGGTTGCAGACCCAGCTTACGCTTGCCTTTAACTTCCATG
CGGTAGATGCCGACATTCTGCTTGCCGAAGTTATCGGGATCGAGCGGATCGCGTGATACCACACAGGCTTTAT

CCAGATAGAATCCGCCGTCGCCGTCGTTCAGACGAAACAGCGGCAGGATATCAAACAGGTTGATTGCGTCGCC
GTCGACGGTATTTTCCGCCCAGCCCGGATTCGCCCGTCGTTCCGGCGCTACCGGAAAATTGTCCCAGCGGCGA
ATAAATTCATCGATCTGTTTTTAACCGGCGTGTTAGGCGGCAAACCCAGTGAGATGGCATGGTTCTGCCAGG
AACCGATGGTATTCATCGCCACGCGGGCGTCGGTAAAACCACGAATATTGTCAAACCACAGCGCGGGCGCGCC
ATCGCCGATGCGTCCCGTTGCGTTGGCAGCGGCGGCGAGATCCGGCTCCGCATTCACTTCCTCGCTGATTTTC
AGCAGTTGTCCCTGCTGATCCAGCGCATGTAAAAAACTGCGCAAATCATCAAATGCCATTATCCATTCTCCTG
TGAAAAATTTGCTGCCTGTCGCAGTCCTTGCCAGCGGCGCGTGCGCGTATGCTCCAGACCAAACTGGTCGAGA
ACGCGCGCTACGATGTGCTGGATAATATCGTCAACGGTTTGCGGCAGGTTGTAGAAGGCGGGCATGGGCGGCA
CTATCGCCACCCCCATACGAGAAAGCGCTAGCATGTTTTCCAGATGAATCGTGCTGAGCGGCATTTCCCGCGG
CACAAGCACCAGCTTGCGACCCTCTTTCAACACGACATCGGCAGCGCGTCCCACCAGCCCCTCGGCATAGCCG
GCGCGAACCCCCGCCAGTGTTTTCATACTGCAGGGGATAATAATCATGCCGTCGGTACGAAATGAGCCGGAGG
AAATGGTCGCCGCCTGATCGGCCGGGCTATGACAATAATCTGCCAGGGCGGCTACCTCAGCGGGCGTGTAGGG
CGTCTCCAGTTCAATAGTGGTTTTCGCCCACTTTGACATTACCAGATGCGTTTCAACATCAGGTATCGCCCGC
AATGCCTGGAGTAGCTCGACGCCAAGCGGCGCGCCGGTCGCACCGGTCATCCCCACAATCAATCTCATTTCTT
ACCTCAAATATCGTTCGTATACGAACATTTTGATCAAAATACGCCCACACGGCCTTTGCGACAAGATCGCTCC
CGACGCCTGCGCACGTTTAGCTAAAGCATTCGTCATTAAAAAACCGTGCATAAGTCGTTATGATAGAAAAAGT
TAATTTATCCGGAGCGCTCATGGAGTTACGAAACACCGCGTTTCATCTGTTACGCCAACTTTTTCAGCAACAT
ACCGCACGCTGGCAGCATGAACTACCGGAGCTCACCAAACCTCAATATGCGGTGATGCGCGTTATTGCTGAAC
ATCCGGGTATTGAACAGGTCGATCTGACCGAAGCGGCGGTCAGCACTAAAGCGACGCTGGCGGAGATGTTAAG
CCGGATGGAAAATCGTGGGCTGGTGAAGCGAGAAAACGATCCGCTGGATAAACGTCGGCGTTTTGTCTATCTC
ACCGTTCAGGGCCAAACGTTGCTCGCCGCCGCGATACCACTGGGGGATCGCGTTGACGATGAGTTTTTGGGGC
GACTGAGCGCTGAAGAACGCGAACAGTTTACGCAACTGGTACGCAAGATGATGACGTAAGCGGCTTGCGCCGT
TTTGCTATTGTTTGGCCATCAGCAGTGAGATATCCACTTTGGCCAGCGCCCGAATTGCGCTTTCCGGCAGGCC
GTCATCGGTGACGATTTGGTTGAATTCCGCCAGCGGTAACGCCAGCCATGTAGCGACCTGGCCGTATTTGGTC
GCGTCGCACACCAGAATTTTTTGGCGGCTGGCGCTGGCGACGGCCCGTTTCACCGTGACTTTATCTTCTGCCG
GCGTTGAAATACCGCGCACGCTCCAGGAAGAGGCGGAAATAAAGGCCTGGTCAATCATCAACCCACGCAAAAG
CGTCGCCGCGGCTTCACCGACGCACGACCGGTTTTCCCGGCATACCGCCCCGCCAGTGTGAATAATTGTGCAA
TCGCTATTGTCCATCAGGTAATCGGCGATAACGAAATCATTGGTCACCACCGTAAGTTTATTCATCGTAACCA
GCTGCCTGGCGATCGCCAGCGTGGTAGTACCTGCATCAAGGTAGATACAGCTTTCTGGCTGTACCAGGCTGGC
GGCCAGTTTGCCAATCGCCGCTTTCTGCGGCGTCGCCAGTTCGGTTTTGATCTGATGCGAGGGTTCGTGCGCC
ACGCGAGTCGATGATTGCACGCCGCCAGAGACCTGGATTACCGCGCCTTGCTGCTCCAGTTTTTGTAAATCAC
GGCGGATAGTCATGTGGGATACATGCATGCGCTCGGTCAGTTCAGCGATACTGACGATTCCTTTCTCCGCCAC
CATATCAAGAATGATTTGTCGACGCTCAGTAGGTATCAACGTCTGCTCCTTTCTTTAGTCCTGTCGCGATTTT
ACGCGATTTCTCGTCACAGCGTGTGAAGTTCTATGAGGATTGCCTTCACAAAAAATAACATATTTTGTTATTT
TTTGTGAAACAACTCACCGCAATTGTTTACAAAAACGCCTATATTCACATAACAAAACATAATATCACGTAAA
TTAACATGGAGTGATAATGACAACAGGAACTGATTTTCATGTCGGCATTGTGGGGCTTGGCTCAATGGGAATG
GGCGCTGCGCGTTCATGCCTGCGCGCCGGGCTTTCCACATGGGGGGCCGATCTTAACCCGCAAGCGTGCGCGA
ATCTGCTCGCTGAAGGGGCTTGTGGGGCTGCCGCCAGCGCCAGGGAGTTCGCCGGCGTCGTGGATGCGTTAGT
CATTCTGGTCGTCAATGCCGCCCAGGTCAGACAGGTGTTATTCGGTGAGGATGGCGTCGCCCATCTGATGAAA
CCCGGAAGCGCGGTGATGGTTTCGTCTACGATCTCAAGCGCCGACGCGCAAGAGATCGCCGCGGCGTTGACGG
CATTGAATCTTAACATGCTGGATGCGCCGGTTTCCGGCGGCGCGGTAAAAGCGGCGCAGGGCGAAATGACGGT
GATGGCCTCCGGGAGCGAGGCGGCGTTTACCCGCCTGAAGCCTGTGCTGGACGCCGTTGCCAGCAACGTTTAT
CGCATCAGCGATACGCCGGGCGCAGGCTCAACCGTCAAAATCATTCATCAGCTGCTGGCTGGCGTGCATATTG
CGGCGGCGGCCGAAGCGATGGCGCTGGCCGCGCGGGCTGGTATTCCTCTGGACGTGATGTATGACGTCGTGAC
CCATGCCGCCGGCAACTCCTGGATGTTCGAAAACCGTATGCAGCATGTCGTTGACGGCGACTATACCCCGCGT
TCGGCCGTCGACATTTTTGTCAAAGATTTGGGGCTGGTTGCCGACACGGCGAAAGCGCTGCGCTTCCCCTTAC
CGCTGGCCTCTACGGCGTTAAATATGTTCACCAGCGCCAGTAACGCCGGTTATGGCAAAGAGGATGACAGCGC
TGTGATCAAAATTTTCTCAGGGATTACTCTGCCTGGCGTCACGCCTGAGGAGCCGTCATGTTAAAAATTGGCG
TCATTGCCGACGATTTTACCGGCGCGACTGACATCGCCAGTTTTCTGGTCGAAAACGGGATGCCGACAGTGCA
GATCAATGATGTCCCAACCGGGACGCAACCGGAAGGGTGCGACGCGGTGGTTATCAGCCTGAAAACCCGCTCA
TGCCCGGCGCAAGAGGCGATAAAACAATCGCTGGCGGCGCTGGTATGGCTGAAAAAACAGGGCTGCCAGCAAG
TCTATTTCAAATATTGCTCGACTTTCGATAGTACCGCCGAAGGCAATATCGGCCCGGTCACCGATGCGCTGAT
GGTGGCGCTGGATACCTCATTTACCGTGATTTCTCCCGCGCTGCCGGTTAACGGACGCACGGTTTATCAGGGC
TATCTGTTTGTCATGAACCACTTGCTGGCGGAGTCCGGTATGCGCCACCACCCAATCAATCCGATGACCGACA

GCTACCTGCCGCGTCTGATGGAAGCGCAGGCGCAAGGGCGCTGCGGCGTTATTCCGGCTCAGACGCTTGATGA
AGGCGTTGCCGCGACCCGTGCGGCGCTGTCGCGTTTACAGCAGGAAGGATATCGCTACGCAGTACTTGACGCG
CTCAATGAGCGGCACCTGGAAATCCAGGGCGAGGTTTTGCGTGATGCTCCGCTGGTGACCGGCGGTTCTGGGC
TGGCAATGGGGCTGGCGCGTCAGTGGGCGAAGCACGGCGTTTCTCAGGCCCGTTCCGCAGGCTATCCGCTGAG
CGGCCGCGCGGTGGTGCTTTCCGGTTCCTGTTCGCAAATGACGAATCAGCAGGTGGCTTTCTATCGACAACAT
GCTCCCACACGCGACGTTGATGTGGCGCGCTGCCTGTCATCCGAGACGCGCGAGGCCTACGCTGAAGCGCTGG
CGCAGTGGGTGCTCAGTCAGGACAGCGAACTGGCGCCAATGATTAGCGCCACCGCCTCCACGCAGGCGCTGGC
CGCCATCCAGCAGCAATATGGCGCTACCGAAGCCAGCCATGCGGTAGAGGCGCTCTTTTCCCTGCTGGCTGCT
CGCTTAGCGGAAGGCGGTATCACCCGGTTTATTGTGGCGGGCGGCGAAACCTCGGGCGTGGTGACGCAAAGCC
TCGGTATTACCGGTTTTCACATCGGACCGTGCATTTCACCCGGCGTGCCGTGGGTCAACGCGCTCCATGCGCC
AGTCTCGCTGGCGCTAAAGTCAGGTAATTTTGGCGATGAATCCTTTTTCATCCGCGCTCAAAGGGAGTTTCAG
GTATGACGACACTGGCAAAAGAAGAGCACGCTTTGCGAGAAGAGATGGTCAGGATAGCCGCCTCGTTTTTCCA
GCGCGGCTATGCCACCGGCTCGGCAGGCAACCTCTCGCTGTTACTGCCGGACGGCAATATTCTGGCGACGCCC
ACCGGTTCGTGTCTGGGTAATCTCGACCCGCAGCGGCTATCCAAAGTGGACCCGCAGGGCGAATGGCTCAGCG
GCGATAAACCGTCTAAAGAGGTGCGTTTTCATCTGGCGTTGTATCGCAATAACCCTTGCTGCAAGGCCGTGGT
ACATCTGCACAGCACCTGGTCAACGGCGCTTTCCTGTCTCGAAGGGCTGGACCCGCAAAATGTGATTCGCCCG
TTTACCCCCTATGTGGTGATGCGGATGGGCGACATTCCGCTGGTTCCCTACTATCGCCCGGGGGATGACCGAA
TTGCCCGCGATTTGGCTGCGCTGGCGGCCCGGCATCAGGCCTTTTTACTGGCTAATCATGGGCCGGTCGTGTG
CGGGGAGAATTTGCAGGAAGCCGCTAATAATACCGAGGAGCTGGAAGAGACGGCAAAGCTGATTTTTATCCTT
GGCGAACGCCCGATTCGTTATTTAACCACCGAAGAAATTACGCAGTTAAGGAGATAACCCGATGCCGCGTTTT
GCCGCCAATTTGTCGATGATGTTTACCGAAGTGCCGTTTATCGAGCGTTTTGCCGCCGCTGCCGAAGCCGGTT
TTCAGGCCGTTGAGTTTCTGTTCCCCTACGGCTTTGCCGCCAGCGAGATTAAAGCCCAGCTATCGCGTCATGA
TCTGACGCTGGCGCTGTTTAATACTTCGGCGGGCGATACCGCGGCGGGCGAGTGGGGACGTGCCGCGCTACCG
GGGCGCGAACACGACGCGCGCGCCGATATTGAACTGGCTCTGGAATACGCTCTGGCGCTGGAATGCGAGCAGG
TTCACATCATGGCGGGCGTAGTGCAGGATGGCGCTGATGGCGCACGTTATCGCGCCACGTTTATCGATAATTT
GCGTTATGCCGCCGACCGGTTTGCCGCCCACGATAAACGTATTTTGATCGAAGCGCTGAGTCCCGGCGTTAAA
CCCGGCTACCTCTTTTCCAGCCAATATCAGGCGCTGGGCATCGCAGAAGAGGTTGACCGCCCGAATGTGTTCA
TCCAGCTCGACACTTTCCACGCGCAAAAAGTGGATGGCAATTTGAGTCACCTGATTCGCGAGTACGCGGGCCG
CTATGCCCATGTACAAATCGCCTCGCTACCGGACAGACATGAACCGGATGATGGCGAGATCAACTACCCGTGG
CTGTTCCGGCTGTTCGATGACGTAGGTTATCGCGGCTGGATAGGCTGCGAATACCAACCACGGAATACCACTC
AGGACGGGCTTGGCTGGTTTAACGCCTGGCGCTAACCCTGAACTACCCTACACAACAATAAAGAGGACATCCG
GATGCAGATTATCATTACCGGCGGGGGCGGCTTTTTAGGCCAGAAACTCGCAAGCGCCTTATTAAACTCATCG
CTGGCGTTTAACGAACTGCTTCTTGTTGATTTAAAAATGCCTGCACGGTTATCAGATTCCCCTCGTTTACGCT
GCCTGGAAGCTGACTTAACCCAGCCGGGCGTGCTGGAGAATGTGATTACCGCTAATACCTCTGTTGTTTATCA
TCTCGCTGCGATTGTCAGCAGCCATGCGGAAGACGATTTCGATCTGGGATGGAAAGTTAACCTGGATCTTACA
CGCCAGTTACTTGAGACGTGTCGTCGACAACCGCAGAAAATTCGTTTTGTCTTCTCCAGCTCGCTTGCCGTTT
ATGGCGGTACGCTGCCGGAATGCGTCACCGATACCACCGCGCTCACGCCGCGCTCGTCTTATGGCGCGCAGAA
AGCCGCCTGTGAACTGTTGGTCAACGATTATACCCGCAAAGGCTATGTGGATGGGCTGGCGCTGCGTTTGCCG
ACGATCTGTGTTCGCCCGGGTAAACCAAACCGCGCCGCTTCTTCTTTTGTCAGCGCGATTATTCGTGAACCGT
TGCAGGGCGAGACGACCGTCTGCCCGGTGTCGGAAAGTTTGCGGCTGTGGATTTCCAGCCCGGCGACGGTGAT
CCATAACCTGTCGCTGGCCGCAACGTTACCCGCGCCTGGCGAGGCGAGCAGCATCAACTTACCCGGGATCAGC
GTAACCGTGGGCGAGATGCTGGAAACGTTGCGTCAGGCGGGCGGCCAGGCGGCGCGCGATCGGGTTACGCATC
AGCGCGATGAAGGCGTCGAGAAAATTGTCGCCTCCTGGCCGGGACGTATCGATAACCAGCGTGCGCTGGCGTT
AGGTTTTGTCGCCGATAAACGCTTCGATGACATTATTGAACGCTTTCGACAAGATGATATGGAGGGGAGGTCA
TGACAAGTTTACCGACCCCAATTATTGGCCTGATCGTTGCCGTTTTCGTGCTGGTATGGCTGGTGTTACGCAC
CCGTGTTCATGCGCTGATCGCGATGCTGGCGGCGGCCTGCATCGCCGGTCTGTTGGGGGGAATGGGCATTGAT
AAAACGCTCTCTGTCATCACCAGCGGATTCGGTACGACGCTGGGCAGCATTGGCCTGGTGATTGGTCTCGGCG
TAATGATGGGGCGCTTGCTGGAGGTTTCCGGCGCGGCGGAGCGTATCGCCTGGAGCTTTATCAAATGGCTGGG
GAAACGCCGTGAAGAGTGGGCGCTGGCGATCACGGGTTATATTGTCAGCATCCCCATTTTCGTCGATTCCGCT
TTTGTCATTCTTTATCCGGTGGCGAAAGCGCTGGCGAAAAGCGGTAAACGTTCGCTACTGACGCTGGGCGTCG
CGCTGGCGGGCGGGCTGGTGGTCACTCACCATACGGTACCTCCGACGCCCGGTCCGCTGGGGGTAGCCGGGAT
TTTTAACGTCGACATCGGCGCAATGTTATTGACCGGGATGGCGCTGGCGGTGCCCTGTGTGATCGGCATTGTG
TTCTATGCGCAGTGGCTGGATAAACGCTACCCTGATTTCGTGCCGCGCACGCTGAACGCCGATGAAGTCAATG
CCGCGCTGGAGCAGTACAATAAAGAGAAAGAGCAAAAGGATCTGCCGAGCCTGACGCTCTCTCTGCTGCCTAT

TGTGGTGCCGATCGTGCTGATCTTCCTGAAGGCTATCTGTTCCACCCTCGCCACTGTAGAAGGATGGTCCGGC
CTGGCGACGCATCCGGTGGTACAGGCAATCAACTTTGTGGGAAGTCCAGTGATCGCGTTGGCCATCAGCGTTC
TGCTGGCGGTGTATACGCTGGTGCCGCGCATGGATAAACATACCACCGCAGAGCGTCTGGAAGAGGGCTTGCA
AAGCGCCGGGATTATCTTACTGGTCACGGGCGCTGGCGGCGCGTTGGGCGCGATCCTGCGCGACAGCGGCGCG
GGGCAACAGCTGGCGGAGCAGGTTGCCAACCTGCCGATCTCGCCGATCCTGATCCCGTTTATCGTGGCGACGT
TGGTACGCCTGATTCAGGGATCGGGGACGGTGGCGATGATCACTGCCGCCTCGATTTCCGCGCCGATCCTGGC
GCAGATCCCCGGCATCAATATGTTGCTTGCCGCTCAGGCCGCAACGATGGGGTCGCTGTTCTTCGGCTACTTC
AACGATAGCCTGTTCTGGGTGGTTAACCGCATGATGGGCGTCTCTGATGTCAAACAGCAGATGGTGGTTTGGT
CTGTACCGACGACTATTGCCTGGGCGATTGGCGGCACCGGCGTGGCGCTGATTAACCTGCTGTTCGGTTCCGG
CGGCAGTTGGCTCGATCCGTTACTGCCGATTGTCGTTCTGGCGGCAATTATGCTGTGGGTGCGTTGGCAGGCG
CAGGGGATCAAAGACAAGCTGGTAGTTAAGGATTAAGACGGTATTATCCGTCCGGGAGCGCAATGCTCCCGGG
CTGGCTACAATGATGGGGCGTGCTCCCCGCAATCCCATTGCGAACGTAAAAAATCAATAAATGCGCGTACTTT
TGTCGTTACATGGCGGGCGTCCGGGTAAACGGCATAAATGCCCTGACGGGGAAAATGATAGTCGGGTAACACG
TGAATTAACGTTCCATCCTCCAGTGCGGTGTTGACTAACCATTGCGGCAGTAATGCGACGCCGCTTCCCGCGA
GAGCGAAGGCCATCAGCGAGCGCGCGCTGTCGACAGAAATTTTGCCGGCTTTGCTTATCTCCAGGCGTGAGTG
TTGGCCATGATTATTCGTTACTGACCACCGTAGCGGCGTAGGTAATCGCTCATGAATAATCCATTCCGCCTGA
GCCAACGACTCCAGCGACGACACGGGGTGGCGGGCGAGCCACTGCGGCGCGGCGACCGGCAGAATGGTAAAGC
GGGATATCAGCGCGGCGCGATAGCGGGAGTCGGCCAGCGAGCCAAGCCGAATCGCCACATCAAAGCGCTCGGC
AATTAAATCGGCATGATGTGATGAGGACATATGCCGAATACGGAGATCGGGATGGCGCTGACTAAACTGCGCC
AGTAACGGAATGATCACCTGTTCGCCAAACTCGGGCGTAGTGGTAATACGTAACTCGCCGCCCAGCCCACTAT
GGCTGGCACGCACTTCATCCTGCAAATTTTTCGCTGCGTTGAGTAAATTCACGCCTTTTTGGTAAAAGAGGAC
GCCGGCGTCTGTGAGCGTCAGTCGTCGGGTGGAACGTAGCAGTAACGTTACGCCAAGCTCTTTTTCAAGCTGC
CGAATATTAAAACTCACTACCGCTTTTGTTTGGCCCGATGCGGCCGCCGCCGCCGTGAAGCTGCCGCTATCTA
CCACGGCGATAAACAGCGACATACGCTGCAAATTGAGCATGGCGAGCTCCTTTACTGTCAAAATTATTTTGAC
AGTATATCGTTGAAGCGCGGACTTATCTGGCTTTCTCCGGACGGCTACATTAGCGCCTCTGACGGAGGATGCA
TAATGACGTATCGCAGTAAAGTGGCAGTGGTTTACCTGCTCGGCTTCTTTCTTGATCTGATTAATCTGTTTAT
TGCCAGCGTGGCTTTTCCTGCGATGTCCGTGGAGCTGCATACGTCCATATCGGCGCTGGCATGGGTGAGTAAC
GGTTATATCGCAGGATTAACGCTGATCGTTCCGTTTAGCGCGTTTCTTTCGCGTTATCTCGGCGCACGACGGC
TGATTATATTTTCGCTCATTCTGTTTAGCGTTGCCGCCGCTGCCGCAGGTTTTGCCGATTCCTTACACAGCCT
TGTTTTCTGGCGTATTGTGCAGGGGGCGGGCGGTGGCTTATTGATCCCGGTTGGTCAGGCGTTAACCTGGCAA
CAGTTTAAACCGCATGAACGCGCTGGCGTGTCATCGGTTGTCATGATGGTGGCGCTACTGGCGCCGGCATGTT
CGCCTGCGATTGGCGGGCTGCTGGTGGAAACGTGCGGCTGGCGCTGGATATTCTTTGCCACGCTGCCGGTGGC
CGTTCTCACTTTATTGCTCGCTTATCGTTGGCTGAACGTCGCTTCGACGACAATGGTGTCAGCAAGATTGCTC
CATCTGCCGTTGTTGACGGACAAGCTGCTGCGTTTTGCCATGATCGTCTATCTGTGTGTGCCGGGTATGTTCA
TTGGTATCAGCGTGGTCGGGATGTTTTATCTACAGAACGTCGCGCAGCTATCGCCTGCGGCGGCGGGGTCGCT
GATGCTCCCCTGGTCGATCGCTTCATTTGTCGCCATTATGCTTACCGGACGTTATTTCAATCGGCTTGGGCCG
CGTCCGCTGATTATCGTCGGCTGTCTTCTTCAGGCCGCAGGGATATTGCTTTTAACTAACGTTACGCCTGCGA
CATCGCACCGTGTACTGATGATGATATTCGCCTTAATGGGCGCTGGCGGCAGTTTGTGCAGCAGTACCGCGCA
GAGTGGCGCTTTCCTGACTATTGCTCGCCGGGACATGCCTGACGCCAGCGCGCTATGGAATCTTAATCGTCAG
ATCAGCTTTTTCCTTGGCGCGACGCTGTTGACGCTGCTGTTAAATGCGTTCCAGCGCGTTATGTCGCTTGAGG
TTGCTTATCGCTGGACCTTTATTGCCGCCGCCGGTATCACCCTGCTGCCCCTTATTTACGCTGTCTGTCTGAA
CAATAGACAGGCGCTTCTGTGCCTGAAAAAGGAAAGACCATGAATCCATATAAAGAAGAAATTATTCACGCCC
ATGCGGCGATTGAGAACTGGCTTAGTAAGGGGATGGGTAGCCTGGAGGCGTTGATAGCGCGTTTCGCTGTTGA
TTTTACGATGATAACGCCAGGAGGTATTTGTCTGGATTATCCGGCGCTGGGCGCTTTTTTCCAGGCGCAGCGC
GCGTGTCGCCCGGGGATGGTTATCGTGGTTGAGCACATTGACCTTGTGGCGGAGTGGCCGGAAGGCGCCGCGC
TGCGTTATCGCGAGCGACAGCAACTGCCGGGCCAGGCTGAGACGGTACGCTGGTCGACGGTCATTTTAAAAAG
GGAAAGAGGACGGATAGTCTGGCGTCATTTACATGAAACGACAGCCACAGCCTGAAAAGCCTGTGCCGGCGCG
TGGGCCCGATAACGCGGCGCTATCGGGCCCGACACGTTTACACCAGACTTTTCAGCCGATAGATCCACTCCAG
CGCCTGGCGTGGCGTCAGGGAGTCCGGATCGAGATTCTCCAGCGCTTCAACGGCAGGCGATGTCTCCTCCGGG
GCGGCAAGCAACGACATTTGCGTGCCGTCCACCTGCGTTGCCGCCGCATTGGGCGAGATGCTCTCCAGCTCGC
GGAGTTTTTGGCGCGCGCGTTTGATGACTTCTTTAGGGACGCCCGCAAGCGCCGCGACCGCCAGGCCATAGCT
CTTACTTGCCGCGCCGTCCTGCACGCTGTGCATAAACGCGATAGTATCGCCGTGTTCCAGCGCATCCAGGTGG
ACGTTCGCCACGCCTTCCATTTTCTCCGGTAATTGGGTCAGCTCGAAGTAGTGGGTAGCGAACAGCGTTAACG
CTTTAATCTTATTCGCGAGATTCTCCGCGCAGGCCCAGGCCAACGACAGCCCGTCATACGTAGAGGTACCGCG

CCCGATTTCATCCATCAATACCAGACTGTTTTCCGTGGCATTATGCAGAATGTTCGCGGTTTCGGTCATCTCC
ACCATAAAGGTCGAACGCCCGGAAGCCAGGTCGTCCGCTGCGCCGACGCGGGTAAAAATACGGTCGATGGGGC
CGATTTCCACGTTTTGCGCCGGAACGTAACTGCCAATATAGGCCAGCAGGGCGATCAATGCCGTTTGTCGCAT
ATAGGTACTTTTACCGCCCATATTGGGGCCGGTAATGATCAACATCCGGCGCTGCGGCGACAGATTGAGCGGG
TTAGCGATAAACGGCTCATTCAGTACCTGTTCAACCACCGGGTGGCGACCTTCGGTAATACGGATACCGGGTT
TATCGGTAAATGTCGGGCAGGTGTAATTCAGCGTCCAGGCGCGTTCGGCCAGGTTAACCAGCACATCCAGTTC
CGCCAGCGCGTTGGCGCTCTGCTGTAAATCGGCCAGATGCGGCAACAGCAGATCAAACAATTCGTCATATAGC
TGTTTTTCCAGCGCCAGCGCTTTGCCTTTCGAGGTCAGAACTTTATCTTCATACTCTTTTAGCTCAGGAATAA
TGTAGCGTTCGGCATTTTTCAGCGTCTGGCGACGCACATAATTGATGGGCGCCAGATGGCTTTGACCACGGCT
AATCTGAATGTAATAACCATGTACCGCGTTATAGCCGACTTTCAGCGTATCCAGCCCGGTACGCTCGCGTTCG
CGAATTTCCAGACGATCGAGATAATCGGTGGCGCCATCCGCCAGCGCGCGCCATTCGTCCAGCTCTTCATGGT
AGCCGGGCGCAATAACGCCGCCGTCGCGGACCAGTACCGGCGGCGCGTCAATAATGGCGCGTTCCAGGAGGTC
GCGCAGCTCGGCGAAATCGCCCATTTTTTTACGCAACGCCTGTACCGGCGCGCTGTCAACGGTTTCAAGCTGA
GCGTGCAATTCCGGCAGCTGCTGGAAGGCGTGACGCATTCGGGCGAGATCGCGCGGACGCGCGGTGCGCAGCG
CCAGACGCGCAAGGATACGCTCCAGATCGCCGACCTGACGCAGCACCGGTTGCAGTTCGCTGACGGTGTCCTG
CAAGGCGCCGATGGTCTGCTGGCGTTCACGTAATATGTCGGTATTTCGTACCGGCATATGCAGCCAGCGTTTA
AGCATTCGGCTACCCATAGGCGTCACGGTACAGTCAAGCACCGCGGCGAGGGTATTTTCGACACCGCCGGCCA
GGTTCTGGGTAATTTCCAGATTGCGGCGGGTCGCGGCATCCATAATGATGCTGTCCTGCTGGCGTTCCATCGT
AATGGAACGAATATGCGGCAGGGAGGTGCGCTGGGTATCTTTTACGTACTGTAACAGGCAGCCTGCCGCACAT
AATCCACGCGAGGCATTTTCGACGCCGAAACCGACCAGATCCCGCGTACCGAATTGCAGATTTAACTGCTGGC
GGGCGGTATCAATCTCAAACTCCCACAACGGGCGACGGCGCAGACCCCGGCGTCCCTCTATTAACGCCATTTC
AGCAAAATCTTCGGCATACAACAGTTCGGCGGGATTGGTGCGCTGCAGCTCTGCGGCCATCGTTTCACGGTCG
GCGGGTTCGCTCAGACGAAAGCGCCCGGAGCTGATATCCAGCGTGGCGTAACCGTAGCCTTTACCATCCTGCC
AGATAGCCGCCAGCAGGTTATCCTGACGCTCCTGTAACAGGGCTTCATCGCTGATAGTGCCAGGCGTAACGAT
ACGCACGACCTTACGTTCAACGGGACCTTTGCTGGTGGCCGGATCGCCAATCTGTTCGCAAATAGCGACGGAT
TCGCCCTGATTGACCAGTTTCGCGAGGTAGTTTTCTACGGCGTGGTGCGGGATACCCGCCATAGGGATAGGTT
CGCCAGCCGATGCGCCGCGTTTGGTCAGCGAAATATCGAGCAACTGCGACGCACGCTTTGCGTCGTCATAAAA
CAGCTCGTAAAAGTCTCCCATACGATAAAAGAGCAGGATCTCCGGATGCTGGGCTTTCAGCTTGAGATACTGC
TGCATCATGGGGGTGTGGTTGGAGAAGTCCTTATCAAATGACTCATTCATATTGATTTTACTCGTTTTTTTAT
TCTTTCAAGTGCTCATGTGGCCCCAGGTAAAACCCTTATAAACTCACATAAACCCATAAATTTGTAGCTCACC
TGTAGCCTAAAATGTGACAGGTTACATTCTCATCATTCCTGGACTACAACAGGGGTGATACTTTGAAAACAAC
TCTCAGCCAGCCTTTCATTATCAACAAGTTAAGTATCAATGTTAAGTCTGCACTCAGTCGCTCTGGAAAAATC
GTTTTCGAAGCTAACCCAGCTCAAAAACTCTACATTGTTTTCGATGACCACAGAGAAGCTCCTGCTGGTTTCG
GGGTAAAAGCCAGTTTGACGAAAAAAACCTACGTAATCCAGCGAAGAGTAGCCTCGTCAGATCGTAATGTCAG
TGAGGGTAGGAAGCCAAGTTCTGTCTTGAAAGTAAAAGTTGGAAATGTGTTCGATTTTCCAAATATTGACGAA
ACAAGGCAAGTAGCCAGGCAATTAGTTCAGACCATGCTCGCAACAAAGAGAAATCCCAACAAAATTAAGAGGG
GAGCGGATGCTTCCGAACTCAAAATGAGACTGTAATTAAAATTGTGTAATTGCCTGATTTTGATATGTTCGAT
TCAACATTAAAAGCTGGTTGCTTTATGGACGAAAAACAGTTGCAGGCTCTGGCTAATGCACTGGCCAAAATCT
CAAAATACCTGAAGATCTCAGCCCGTTCGATCGTCTGCTGAAGAAAAACCAGACCCGCATTTCCGGCATAGAT
AACCAACATGCTATCAAAAGCGTACGGTGTTTCCGACGGACGAGTCAGTGAAAAAGGTCGTGTGACTGGCAAT
TCAGGCCGACATCCGAAAATGGACAATGTCGCTAAGGGACTGGCTCATGACAACGAGTCGCTTTATTATCACG
CTCGGGTTCGGTAGCCGCCTGGACGTTCACTTCTGAGAAAGGCATTTACACCGAATGGTGTATAGGCTCGTGG
TTGGTCTTCGGTGAGAGGCAGGATGTCTCTCATTAATCTCCGGGAGCGTACAATAGTTGCTCCGGCAGCCTGA
AGGATGAAGTGTATATAACCTAAAACATAACGGATATAGCTTCCTTACTCAACCTGACGTAATACGCCGACAG
TAAAATCAGGGATACCATACCATCATGGAAAGTCGTCAATACACCCGGCATCTTTCGCTTTCGGAGCTGAAAT
GGTTTGCTATCGGGATCGGTTTTTTTATTCTCAGCATAGCAACAGCCACCGTGAATTATCGGCTTTCCGGTAT
ATCGCTACTGGTGGGCCTGTTGTTTATCATCTGGAAATTCAGCGTCACAGTTCTGTTTCTGTTTACGCCGCGC
AGGATGACGTTAACCGAAACCGCGTTGCAGGCCGGGCATCGTGTTATCCATTACGATGCGCTTGAATCAATGC
GTCTGCTCCACCAATCCGACAAACTCATCTTGCGCCACAGTGGCGGCAAGAAGTACGTCATCTACCTTGATTT
CTGGAACGATGGCAACGGAATTTACGACCGACTGGCTGCTGAACTTGTCCGGCGACACGGTTCTGCGCTGGGG
GCGCGCCTCGCCGCCGATGGACGTCTTAAATTCGGCAAAGTGACCGCGCTGGCGGATCGGCTTGAACACAAAA
ACCGGGCGGTGCCGTATGCGCAGATCGCCAGTATTCGCACCCAGCGCGAGGAGGGGGCTGGCAGTTCAATGAG
CTATCTGATGATTAGCACCGCTACGGGCCGGATCTGCAAAATCGACCGCTCCACTATCGTCAATGAGCCGCTG
CTGCTCAACTTTCTTTCCCAGCGTTTACCCGCCTGACGACATTTTTATACAAGGATGAATCAATGGAACTGTA

CGTATCAAAAACCAGCCAGCAAAACATGCCTGTGGAGTTCAATATCTATTATTCCAGATCTAAAACGTTGGTT
GGTATTGTGTTCTGGATTTGTATGATGGGAGTTTTATTGCGCTGGCGGTGAGTATGGCCACCGATCCCGAGG
AGCCCAAAACTGGCGGTGCCGTCTTTATGGGGCTTATTTGTTGCGGCAGTCTGTTATTTATTGTGCGCCTGGC
TCGCTCATTTTTAAGCGCGGCAAAGCCGGCAGTCACCATCAGTCGTGAAGGTATTCGCTTCTGTAATGGCAAA
TTCGCGGCCTGGACAAATATCGCCGAAAACACCTGGCATTCACAGAGCATTAACTTCATCCCGGCCGCCGCCG
GTATTAAGATTGTGTTGCATGAAGGCAAGCCGATCCATTTCGCCACCACGGTTATTGCGCTTTCTTCCGATAG
GTATCTTGAGATGTGCGATCTTTACTCGTCTCAGGCGCTCGGGTAACTACCTGCGTCGGATTACGGCTTCGTT
TTTTTGGCCTGCGATCTTAATGCTGAAATGGGCAGAAAAACGCGAAATTTAGACGCTATTACGAATGGGCAAT
GGTACTTATTGAAGGCTATATCATTGCCCATAATGCAGGGAGGGTTAATATGCTATGATAGAATTCTATGTTT
GAATATATATCCTCAGAATACAAAATAAAGTAACGCTATCGTTGCGTATATTACATAATTCTATTTCCAGATT
ACTTTATTTTAAAAAAAGACAGATTGCCTGAACAGAAAGAACCTGTGTCAATGTAGATTTGATTGGCGAATGT
TGTTGTGTAGTCAACAATCATATGGCCGAAGATGAAAGTATCCGCACCGCGTATTGGATGGATATTCCCCTGT
AACGAACCAAGCAGACGATCGCGTGACCATAGAACGCTATCAATATCAACTTGCTTCCCATAATCGTAGCTGT
CATCTGGATAATCGGCATGGGCAATCACGTACTTCTTTGAGGACGTTTCAACCTCTATGATGTGAGGACGCTG
CTTAAAAGTTAGTAATAGCTCAGTGGCTTCTTGCCTGTCTTTTTCGGTGACCGAGTCGTACCAGTAACCACCG
TTTACATACCAGAAATTGCCATCCTGTGTTTCAAAAGCGTCAAGCGCCATAGCTTCATGATTACCCACTACAG
AAATAAACCATGGGGTATTTAACAGTCTCAGTGTTTCAAGGTTTTCTTTTCCTCGATCAATGTTATCTCCAGT
GGAGATCAAGAGATCGGTATCAGGAGAGAAATTTAATTGAGCCAGTCTGGTTAACAATAAGGAGTAACATCCA
TGAATATCACCAACGACCCAAATGTGGCGATAGAGATCGCTGTTGATGTCAGCATAACGGATTAATTCCATGA
TGTTCTTATAGCACAATTTTGGAGGCTGGTATTATAACAAAGAAGTAGGGTAATATTAATTTTTATTGTTTAA
AATACACAGTTATTAAAATAGCCTACAAGCTAGAAATGAACAGAGAATATATATTTTATGAATTGGTTAATTA
AAGTAGACGATGTGCTTGAAAGATGCACGATTTCTTGCGCAACACTTTATTGATTAATTGCAAAGGCATACTT
TCCCATTCAGCGGAAACTCTATCCCGAAGGGGCTTTGTTGAATAAATAGAACTTTCAGGAGATTGGCGGATCT
AATCGTTACTGTCGTTTCAGCATCGGGTTCCGATGGCAAAGCAAAAGTTTAAAATCAACATCGGAGCGGCAGC
AATGAGGTCTGGAAAAGAAAGTGGGCTATCACCGTCGTTCAGTAGTGGAAACAGCGATGTTCCGTATCAAAAC
CTTGCTGGGCGGCCATCTGAGCCTGCGGAACTATGACGCGCAGGTGGGTGAAGCGATGGCGATGGTCAAAGCG
CTGAACCGGATGACGTTGTTGGCAATGCCGTCCAGCGTCAGGCTCGTATAACGAGTGCTCCGGTGGGAGTCCT
GTATCCTTATCGTTGGCCGATTCTGAATTATTCAACAAAGCCTCATGTTGTCATAACCTACCAGATGCAGATA
AAGCAGCATAAAGCGTTTTAAACGACAGCAACAAGGAAAGGGGTTGATCGTTTAATGGTACTGCGCCAAATCC
TTTGTACCAGTCGCAGACCTGTTTATTTTTGGCATCAATAAGTAGGGCTACGCCACCGACCTGCAAAGCCGCC
TGTATGCAACGCTTTCCGGCAGATAACAAAAGTTGTGCTCCCAGCCCTTGCCCCTGCATAGATTTGTCTACGG
CTAAACGACCCAGACGAAACACTGGCACATCGTATCTCCCTAATCCTTTGCTGATTGCGCCGGGAACCTGTGC
ATATATCAGTGATGCAGGACTGACTGAGTAAAAGCCGTGGATACGTGTAACATCCGAATTATCAAGCGCAACA
TAGGTTTTCGTTGTCCCTTTCTCATGATTTTGTCGTGCATGGCGTTGCAGGAATTGGTTCAGATCGGCATCCC
CACAATCAAAATTCATTCGATTGTGGGTTTTCCCTATCGCGGCCTCATGCCAGTCTGTAAACATCATTTCTTC
ATGTCCGGCAGGGCGAAAGCAGCGGCAAGAAGCTTTTCATTCGGCGCTGGCGGGTTATCTAAAATTTCCATGA
TCATTTGAGTATCGCGCTCCGTCAGATATACCCGTTCGGCGGCATCAACAATCTTCTGTGCGACAGGTAGTAC
GTTACTGACCACAAAATCCGTCAGGTTGGTTTGCTGGATTGCCGCAGCGCGGACTATCAACTTTTTGGCATCC
GTCGATACACGCAACGACAGCCTTTCGTTAGATTCTATAGCTATCTGAGGCATGGTTTTCATCCTCCTGTTTA
TGAACAATATGTACAATTATAATACATATATGTACGCTTTCAAGGCGTACATATGAGGCTGATATAGTCCAGG
CATGGCAAGAAGGTCGTTCACTGTCTTTAAACAGGGGATTAACTTCGGTTTATCGCGATTGCATTACTTTAAT
CTCACTGATTACGTATCATCAGGGAGTTTGCTAAATATATTCATGATTCTCTTTTTTTTGCCGTAACTGCTTGA
TTGATCCTACCCGCGTAATAGGGACACAACCCTAAGCGGCGGACATCTTCTGCTCACGTTCAGAAGAAGAGAG
CTGAGTTTGCTGTTTGCTTCGCCAGCTGTAGATCTGTGATTTATACAGGTTAAGCTCGCGGGTGGCAGCGGCC
ACCCCAATGCGTTCCGCCAGTTTCAGGGCTTTCAGGCGTGTGCTGCTTGCGTGGCTTATTGGTGGTTGATGCT
GGTTTTGTCATGTGAGTCACCTCTTGTTTGAGCGTTTACTCACTTAGTTGTGTGTCCACTATTGCTGGGTAAG
TTCAAAAGAACGTTATCCGCGAAAAGTGGATGGGGTATCGTAAGAATGCCAACATGTGGGGCAAGAGGGGGAT
GTCAGTTTATCGCGATATACGAGACGATAGTTTGTCAGTATAAGTTTCGGCTATTTTCCCGAGTTTGGGAGGC
AGTTCTTTATCACAAATAAAAAAAGCAGCAGCGAGGTGCTGCTGTATCAAAAAGCAGATTGATAAATGCAACG
TAAGAGACAAATGGCCAACCGTAAAAGTGACCATAGCTATAATAATTATATTGATATTAAGTGGCAATGCAAA
ATATGGTCTTAATTATATCATGATGAGTTCAGCCAACGGTGATATGGCCTTATAAGGATTGCAGTCTTTCATG
GGCAGCAAGTAACGTCTGATATATGCTTAAATTCTTACTTCCGGGTTCAAGAAGAACTTTTTTAAATTCGGTC
ATGAGTTGCTCTTCATCTTCTTTCGAACGCATGTATTGTGGATGTTCCTGGAAGAAGGTGAGCGCCTGCTCTT
TGGTTTGTTTATATTTTTCGCAAAAAATGCTTGAGCTGATTGCGCTATTTTTTGATGCGGAATTATCAGCGTT

CTGGTTTGATAATGATTTATTCTTCGCAAGGTCTGACGGCACATACGGAAGAGACTGACACTCATCAATACTA
TTTGCGTTGGCCAGTTGCTCTTTCTGAGCGCCAGGTTGCTGTACCGGTTTGCTCACGGAGGAAGGGAGGGGCA
CCTGGGCACAGCCGATCAGTAAAAAGACAGGAAGACAGCTATAAAATTTTTTCATATTAACTCCAACTACAGA
AGAATGAGGCGCAATGTTTTTACACAACCGTCAGAATGTTATCTGACAATAGTGATAAGAGGCGAATGCGTTT
CAGGAATGCTAGCAAAAAAAACTCCTGATATAAATAATTAATTCTCCATCCAGATGACAATATCTGAATTTTT
GCTGCTGCAGAACAATAAGCCAGAATTTATACACTATGAGCTAATTTTCTATTTTAAATCAGCAAGCACTGTG
CAAGTTGGCCAATTTAATTAGCGGGCGGCATCAGTTTCATAATGATTGCATCAGGATTTTGCCACCCGCTCCC
GGTATTGTTTACATATTAAAATGATTTTTAACTGGTGCTGACAACTATGCTAAATACGCAGGAAGTACTTAAA
GAAGGAGAGAAGCGGAAAATCCGCAGCCCGGAAGCATGGTTTATACAGACGTGTTCCGCGCAAAAGCTGCATA
TGTCATTTTCTGAAAGCCGACACAATGAAAATTGCCTGATTCAGGAAGGCGCGCTGCTTTTTTGCGAGCAGGC
CGTTGTCGCACCAGTATCAGGAGACCTGGTTTTTCGACCGTTAAAAATTGAAGTACTCAGCAAATTACTGGCA
TTTATCGATGGCGCAGGATTAGTGGACACGACATATGCTGAATCCGATAAATGGGTTTTGCTGAGTCCTGAGT
TTCGCGCTATTTGGCAAGATCGTAAACGCTGCGAGTACTGGTTTTTGCAGCAAATTATTACGCCTTCTCCGGC
CTTCAATAAGGTACTGGCGCTGTTACGAAAAAGCGAGAGTTACTGGTTGGTTGGCTATTTACTCGCTCAGTCA
ACCAGCGGCAACACGATGAGAATGCTGGGAGAAGACTATGGCGTTTCTTATACCCATTTTCGTCGTTTGTGCA
GCAGAGCGTTGGGCGGAAAAGCGAAGAGTGAATTACGAAACTGGCGTATGGCGCAATCGCTGCTGAATAGTGT
AGAAGGCCACGAGAACATCACCCAATTAGCCGTTAATCATGGTTACTCATCGCCTTCACATTTTTCTAGTGAG
ATCAAAGAGCTGATCGGCGTTTCGCCGCGGAAATTATCAAATATTATTCAATTGGCAGACAAATGAAGACACA
TATTCTTTTGGCCAGAGTGCTGGCATGTGCCGCGCTTGTTCTGGTTACACCTGGTTATTCTAGTGAAAAAATA
CCTGTAACGGGAAGTGGGTTTGTTGCGAAAGACGATAGCCTGCGGACATTTTTCGATGCCATGGCGCTACAGC
TAAAGGAGCCTGTCATTGTTAGCAAAATGGCGGCACGAAAAAAAAATTACGGGCAACTTTGAGTTTCACGATCC
TAACGCATTACTGGAGAAGCTTTCCCTACAACTGGGGCTGATTTGGTATTTCGATGGGCAGGCTATCTATATT
TATGACGCCAGTGAAATGCGCAATGCCGTGGTTTCTTTACGCAACGTCTCACTCAATGAGTTCAACAATTTTC
TAAAACGCTCAGGTTTATATAACAAAAATTACCCGCTACGTGGCGATAACCGTAAAGGAACATTCTATGTTTC
AGGGCCACCCGTCTATGTTGATATGGTGGTCAACGCCGCCACCATGATGGACAAGCAAAACGATGGTATTGAG
CTGGGACGTCAGAAAATAGGGGTGATGCGTCTGAACAATACCTTCGTGGGCGATCGTACCTATAATCTGCGCG
ATCAGAAAATGGTTATCCCCGGTATTGCCACGGCCATTGAAAGGTTGTTGCAGGGAGAAGAGCAACCTTTAGG
TAATATTGTCAGTAGCGAACCCCCGGCGATGCCAGCGTTTTCAGCGAATGGAGAAAAAGGTAAAGCAGCAAAT
TATGCCGGTGGCATGAGTCTGCAGGAAGCTTTAAAGCAAAATGCCGCGGCGGGCAATATTAAAATCGTGGCCT
ATCCGGATACCAACAGTTTGTTAGTAAAGGGAACGGCTGAGCAGGTGCATTTTATCGAAATGCTGGTTAAAGC
GCTGGATGTCGCCAAACGTCACGTAGAATTATCCCTGTGGATTGTCGATCTTAATAAAAGCGATCTGGAGCGT
TTGGGCACTTCATGGAGCGGCAGCATTACTATTGGGGACAAACTTGGCGTGTCATTAAACCAGTCTTCAATAA
GTACCCTCGATGGCAGTCGATTCATCGCCGCGGTCAATGCGTTAGAAGAGAAGAAACAGGCGACGGTGGTTTC
ACGCCCGGTATTACTGACCCAGGAAAATGTTCCCGCTATTTTTGATAACAACAGAACGTTTTACACCAAGCTG
ATTGGGGAACGTAATGTGGCGCTTGAGCATGTAACATACGGAACAATGATCCGAGTGCTGCCCCGTTTTTCCG
CAGATGGTCAGATAGAAATGTCGCTGGACATTGAAGATGGCAACGATAAGACGCCGCAATCCGATACTACCAC
CTCCGTAGATGCGTTACCCGAAGTCGGGCGAACGTTAATTAGCACTATTGCGAGAGTGCCGCACGGAAAAAGT
TTGCTGGTCGGTGGTTATACACGGGATGCAAATACCGATACTGTCCAAAGTATTCCGTTTTTAGGCAAATTAC
CGCTTATTGGTAGCCTGTTCCGCTATTCCAGTAAGAATAAAAGTAATGTTGTTCGTGTGTTCATGATTGAACC
AAAAGAAATTGTCGACCCGTTAACGCCGGATGCCAGCGAATCGGTAAACAATATTCTGAAGCAAAGCGGTGCC
TGGAGTGGGGACGATAAGTTACAGAAATGGGTTCGTGTTTATCTGGATAGAGGTCAGGAGGCAATTAAATGAT
TCCTGGCTCAACCTCCGGTATTTCATTTTCCAGAATATTGTCCCGGCAGACATCTCATCAGGATGCGACCCAG
CATACTGATGCGCAACAGGCGGAAATACAACAGGCCGCAGAGGATTCGTCTCCAGGGGCGGAAGTACAAAAAT
TTGTCCAGTCGACGGACGAAATGTCAGCGGCGCTGGCGCAATTTCGTAACCGTCGCGATTATGAAAAAAAATC
CAGTAATTTATCTAACAGTTTTGAACGCGTGCTGGAAGATGAGGCTTTACCGAAGGCGAAGCAAATCTTAAAG
CTAATTAGCGTACATGGCGGCGCGTTAGAAGATTTTTTACGTCAGGCGCGTAGCTTATTTCCTGACCCCAGTG
ATTTAGTCCTTGTGTTACGCGAATTGCTTCGTCGTAAAGACCTGGAAGAGATCGTGCGGAAAAAGCTGGAGTC
GTTACTTAAGCACGTTGAAGAGCAAACCGATCCGAAGACCCTCAAGGCAGGGATTAATTGTGCGTTGAAGGCC
CGGCTTTTTGGGAAAACATTATCGTTAAAACCAGGCTTATTGCGCGCCAGCTATCGGCAATTTATCCAGAGTG
AATCACATGAAGTGGAGATTTACTCTGACTGGATAGCCAGTTATGGCTATCAACGTCGACTGGTGGTACTGGA
TTTTATTGAGGGTTCGCTATTAACCGATATTGACGCGAATGACGCCAGTTGTTCGCGCCTGGAGTTTGGCCAG
CTTTTACGACGCCTGACGCAACTTAAAATGTTGCGCTCCGCTGACCTACTGTTTGTGAGTACATTGTTGTCGT
ATTCGTTTACCAAAGCGTTTAATGCGGAGGAGTCGTCGTGGCTACTACTGATGCTTTCGCTATTGCAACAGCC
ACATGAAGTGGATTCGCTGTTAGCCGATATTATAGGTTTGAATGCGTTATTGCTTAGTCATAAAGAACATGCA

TCCTTTTTGCAGATATTTTATCAAGTATGTAAAGCCATACCCTCTTCACTCTTTTATGAAGAATATTGGCAGG
AAGAATTGTTAATGGCGTTACGTAGTATGACCGATATTGCCTACAAGCATGAAATGGCAGAACAGCGTCGTAC
TATTGAAAAGCTGTCTTAATTTAATATTAACAGGATACCTATAGTGCTGCTTTCTCTACTTAACAGTGCTCGT
TTACGACCTGAATTACTGATTCTGGTACTAATGGTGATGATCATTTCTATGTTCGTCATTCCATTACCTACCT
ATCTGGTTGATTTCCTGATCGCACTGAATATCGTACTGGCGATATTGGTGTTTATGGGGTCGTTCTACATTGA
CAGAATCCTCAGTTTTTCAACGTTTCCTGCGGTACTGTTAATTACCACGCTCTTTCGTCTGGCATTATCGATC
AGTACCAGCCGTCTTATCTTGATTGAAGCCGATGCCGGTGAAATTATCGCCACGTTCGGGCAATTCGTTATTG
GCGATAGCCTGGCGGTGGGTTTTGTTGTCTTCTCTATTGTCACCGTGGTCCAGTTTATCGTTATTACCAAAGG
TTCAGAACGCGTCGCGGAAGTCGCGGCCCGATTTTCTCTGGATGGTATGCCCGGTAAACAGATGAGTATTGAT
GCCGATTTGAAGGCCGGTATTATTGATGCGGATGCTGCGCGCGAACGGCGAAGCGTACTGGAAAGGGAAAGCC
AGCTTTACGGTTCCTTTGACGGTGCGATGAAGTTTATCAAAGGTGACGCTATTGCCGGCATCATTATTATCTT
TGTGAACTTTATTGGCGGTATTTCGGTGGGGATGACCCGCCATGGTATGGATTTGTCCTCCGCTCTGTCTACT
TATACCATGCTGACCATTGGTGATGGTCTTGTCGCCCAGATCCCCGCATTGTTGATTGCGATTAGTGCCGGTT
TTATCGTGACTCGCGTAAATGGCGATAGCGATAATATGGGGCGGAATATCATGACGCAGCTGTTGAACAACCC
ATTTGTATTGGTTGTTACGGCTATTTTGACCATTTCAATGGGAACTCTGCCGGGATTCCCGCTGCCGGTATTT
GTTATTTTATCGGTGGTTTTAAGCGTACTCTTCTATTTTAAATTCCGTGAAGCAAAACGTAGCGCCGCCAAAC
CTAAAACCAGCAAAGGCGAGCAGCCGCTTAGTATTGAGGAAAAAGAAGGGTCGTCGTTGGGACTGATTGGCGA
TCTCGATAAAGTCTCTACAGAGACCGTACCGTTGATATTACTTGTGCCGAAGAGCCGGCGTGAAGATCTGGAA
AAAGCTCAACTTGCGGAGCGTCTACGTAGTCAGTTCTTTATTGATTATGGCGTGCGCCTGCCGGAAGTATTGT
TACGCGATGGCGAGGGCCTGGACGATAACAGCATCGTATTGTTGATTAATGAGATCCGTGTTGAACAATTTAC
GGTCTATTTTGATTTGATGCGAGTGGTAAATTATTCCGATGAAGTCGTGTCCTTTGGTATTAATCCAACAATC
CATCAGCAAGGTAGCAGTCAGTATTTCTGGGTAACGCATGAAGAGGGGGAGAAACTCCGGGAGCTTGGCTATG
TGTTGCGGAACGCGCTTGATGAGCTTTACCACTGTCTGGCGGTGACGCTGGCGCGCAACGTCAATGAATATTT
CGGTATTCAGGAAACAAAACATATGCTGGACCAACTGGAAGCGAAATTTCCTGATTTACTTAAAGAAGTGCTC
AGACATGCCACGGTACAACGTATATCTGAAGTTTTGCAGCGTTTATTAAGCGAACGTGTTTCCGTGCGTAATA
TGAAATTAATTATGGAAGCGCTCGCATTGTGGGCGCCAAGAGAAAAAGATGTCATTAACCTTGTAGAGCATAT
TCGTGGAGCAATGGCGCGTTATATTTGTCATAAATTCGCCAATGGCGGCGAATTACGAGCAGTAATGGTATCT
GCTGAAGTTGAGGATGTTATTCGCAAAGGGATCCGTCAGACCTCTGGCAGTACCTTCCTCAGCCTTGACCCGG
AAGCCTCCGCTAATTTGATGGATCTCATTACACTTAAGTTGGATGATTTATTGATTGCACATAAAGATCTTGT
CCTCCTTACGTCTGTCGATGTCCGTCGATTTATTAAGAAAATGATTGAAGGTCGTTTTCCGGATCTGGAGGTT
TTATCTTTCGGTGAGATAGCAGATAGCAAGTCAGTGAATGTTATAAAAACAATATAAGGGCTTAATTAAGGAA
AAGATCTATGCAACATTTGGATATCGCTGAATTAGTTCGTTCCGCACTGGAAGTAAGTGGTTGCGATCCTTCA
CTCATCGGAGGAATAGATAGCCATTCAACAATTGTTCTGGATTTATTTGCATTGCCAAGTATCTGTATCAGCG
TCAAGGACGATGATGTATGGATCTGGGCGCAATTGGGTGCTGACAGCATGGTGGTATTACAACAGCGGGCTTA
TGAAATCTTAATGACCATCATGGAAGGATGCCATTTTGCCCGCGGCGGGCAATTACTACTGGGGGAGCAGAAT
GGGGAGCTAACGCTTAAAGCCTTAGTGCATCCGGATTTTTTATCTGACGGTGAAAAGTTCTCTACTGCCTTGA
ATGGGTTTTACAACTATCTGGAAGTTTTTAGTCGGTCGCTAATGAGATGAAAACACCTCGTTTACTGCAATAT
CTGGCCTACCCACAAAAAATAACCGGCCCAATTATTGAGGCGGAATTGCGCGATGTGGCCATTGGCGAACTGT
GTGAAATACGCCGTGGCTGGCACCAAAAACAGGTTGTTGCACGTGCGCAGGTGGTTGGCTTACAGCGGGAACG
CACCGTGTTGAGCCTTATCGGCAATGCCCAGGGGCTGAGCCGCGATGTCGTGCTTTATCCCACTGGACGTGCG
TTATCGGCGTGGGTGGGATACTCGGTATTAGGCGCGGTGTTGGATCCGACAGGGAAAATCGTTGAGCGTTTTA
CCCCTGAAGTGGCGCCGATTAGCGAAGAACGCGTTATTGATGTCGCACCGCCGTCTTACGCTTCACGCGTTGG
CGTCCGTGAACCGCTGATTACCGGTGTGCGCGCGATTGACGGGTTATTGACCTGTGGCGTAGGCCAGCGAATG
GGCATTTTTGCCTCCGCAGGATGCGGTAAGACCATGCTGATGCATATGCTGATCGAGCAAACGGAGGCGGATG
TCTTTGTTATCGGTCTTATCGGTGAACGAGGCCGTGAGGTCACTGAATTCGTGGATATGTTGCGCGCTTCGCA
TAAGAAAGAAAAAATGCGTGCTGGTTTTTGCCACTTCCGATTTCCCCTCGGTCGATCGCTGCAATGCGGCGCAA
CTGGCGACAACCGTAGCGGAATATTTTCGCGACCAGGGAAAACGGGTCGTGCTTTTTATCGATTCCATGACCC
GTTATGCGCGTGCTTTGCGAGACGTGGCACTGGCGTCGGGAGAGCGTCCGGCCCGTCGAGGTTATCCCGCCTC
CGTATTCGATAATTTGCCCCGCTTGCTGGAACGCCCAGGGGCGACCAGCGAGGGAAGCATTACTGCCTTTTAT
ACGGTACTGCTGGAAAGCGAGGAAGAGGCGGACCCGATGGCGGATGAAATTCGCTCTATCCTTGACGGTCACC
TGTATCTGAGCAGAAAGCTGGCCGGGCAGGGACATTACCCGGCAATCGATGTACTGAAAAGCGTAAGCCGCGT
TTTTGGACAAGTCACGACGCCGACACATGCTGAACAGGCATCTGCCGTGCGTAAATTAATGACGCGTTTGGAA
GAGCTCCAGCTTTTCATTGACTTGGGAGAATATCGTCCTGGCGAAAATATCGATAACGATCGGGCGATGCAGA
TGCGGGATAGCCTGAAAGCCTGGTTATGCCAGCCGGTAGCGCAGTATTCATCCTTTGATGACACGTTGAGCGG

TATGAATGCATTCGCTGACCAGAATTAAAGTATTGCAGCGGCGCTGTACGGTATTTCATTCACAGTGTGAGTC
GATATTACTTCGCTATCAGGATGAGGACCGCGGGCTGCAGGCCGAGGAGGAGGCGATCCTTGAACAAATAGCG
GGTCTGAAATTGTTATTAGATACGCTGCGTGCAGAAAACAGACAGCTCAGTCGTGAGGAAATTTATACGTTAT
TACGTAAGCAGTCTATTGTTCGCCGGCAGATAAAAGATTTAGAACTCCAGATTATACAAATTCAGGAAAAACG
GAGCGAGCTGGAAAAGAAAAGGGAAGAGTTTCAGAAAAAAAGTAAATATTGGTTGCGCAAAGAAGGGAACTAT
CAACGCTGGATAATCCGTCAGAAAAGATTCTATATCCAGCGAGAGATACAGCAGGAAGAGGCCGAGTCAGAGG
AGATAATTTAATGGGCGATGTGTCAGCTGTCAGTTCATCCGGGAACATTTTACTGCCGCAGCAGGATGAGGTT
GGCGGTTTATCAGAAGCATTAAAAAAAGCGGTGGAAAAACATAAGACAGAATATTCCGGTGATAAAAAAGATC
GCGACTATGGCGATGCTTTCGTAATGCATAAAGAAACGGCTTTACCGGTATTACTGGCGGCATGGCGACATGG
CGCGCCAGCGAAATCAGAACATCACAATGGCAACGTTTCTGGTCTGCATCATAACGGAAAAGGCGAACTCAGG
ATTGCTGAAAAACTGTTGAAAGTCACTGCTGAAAAATCTGTCGGTTTGATTTCTGCGGAGGCCAAAGTAGATA
AATCCGCAGCGTTGCTATCGTCTAAAAATAGGCCGTTAGAAAGCGTAAGCGGTAAAAAATTATCTGCTGATTT
AAAAGCCGTGGAGTCCGTTAGTGAAGTAGCCGATAACGCCACGGGAATCTCTGACGATAATATCAAGGCATTG
CCTGGGGATAATAAAGCCATCGCGGGCGAAGGCGTTCGTAAAGAGGGCGCGCCGCTGGCGCGGGATGTCGCAC
CTGCCCGAATGGCCGCAGCCAATACCGGTAAGCCTGACGATAAAGATCATAAAAAGGTTAAAGATGTTTCTCA
GCTTCCGCTGCAACCAACCACTATCGCCGATCTTAGCCAATTAACCGGCGGCGATGAAAAAATGCCTTTAGCG
GCGCAATCAAAGCCGATGATGACTATTTTTCCGACTGCCGATGGCGTGAAAGGAGAGGATAGCTCGCTGACTT
ACCGTTTTCAGCGCTGGGGAAATGACTATTCCGTCAATATTCAGGCGCGGCAAGCAGGGGAGTTTTCGTTAAT
ACCGTCAAATACGCAGGTTGAACATCGTTTGCATGATCAATGGCAAAACGGTAATCCCCAGCGCTGGCACCTG
ACGCGAGACGATCAACAAAATCCGCAGCAGCAACAGCACAGACAGCAATCTGGCGAGGAGGATGACGCCTGAT
GTCATTGCGTGTGAGACAGATTGATCGTCGCGAATGGCTATTGGCGCAAACCGCGACAGAATGCCAGCGCCAT
GGCCAGGAAGCTACGCTGGAATATCCGACGCGACAAGGAATGTGGGTTCGGTTGAGCGATGCAGAAAAACGGT
GGTCGGCGTGGATTCAACCTGGGGACTGGCTTGAGCATGTCTCTCCCGCTCTGGCGGGGGCGGCGGTTTCTGC
TGGCGCTGAGCACCTGGTCGTTCCCTGGCTTGCTGCGACAGAGCGACCGTTTGAGTTGCCCGTGCCGCATTTG
TCCTGTCGGCGTTTATGCGTAGAGAACCCCGTGCCGGGAAGCGCGCTGCCGGAAGGGAAATTGTTGCACATTA
TGAGCGATCGGGGCGGCCTGTGGTTTGAATATCTTCCTGAACTGCCTGCAGTCGGGGGCGGCAGGCCGAAAAT
GCTGCGTTGGCCGTTGCGCTTTGTAATCGGTAGCAGTGATACGCAGCGTTCGTTGCTGGGCCGAATCGGGATC
GGAGATGTACTCCTGATTCGTACTTCCCGTGCGGAAGTTTATTGCTACGCGAAAAAGTTAGGTCATTTCAACC
GTGTTGAAGGGGGAATTATTGTGGAAACGTTAGATATTCAACATATCGAAGAAGAAAATAATACAACTGAAAC
TGCAGAAACTCTGCCTGGCTTGAATCAATTGCCCGTCAAACTGGAATTTGTTTTGTATCGTAAGAACGTTACC
CTCGCCGAACTCGAAGCCATGGGGCAGCAACAGCTATTATCACTGCCGACCAATGCTGAACTTAACGTTGAAA
TTATGGCGAATGGTGTTTTGCTGGGTAATGGCGAACTGGTACAGATGAATGACACCTTAGGCGTTGAGATCCA
TGAATGGCTGAGCGAGTCTGGTAATGGGGAATGATATCTCATTAATTGCCTTACTGGCATTTTCCACCCTGTT
GCCATTTATTATTGCGTCAGGAACCTGTTTCGTTAAATTTTCTATTGTATTTGTCATGGTGCGTAACGCCCTG
GGATTGCAGCAGATACCTTCAAATATGACGCTTAACGGCGTCGCATTGCTGCTTTCTATGTTTGTTATGTGGC
CAATAATGCATGATGCCTACGTCTATTTTGAGGACGAAGATGTCACCTTTAATGATATTTCGTCATTAAGTAA
ACACGTTGATGAAGGTCTGGATGGTTATCGCGATTATCTGATCAAATATTCAGATCGCGAGTTAGTTCAGTTT
TTTGAAAACGCGCAACTGAAGCGTCAGTATGGAGAAGAGACCGAGACGGTAAAGCGTGACAAAGATGAAATTG
AAAAACCATCAATATTTGCGTTATTACCTGCTTATGCGCTGAGCGAAATAAAAAGCGCGTTTAAAATTGGTTT
TTATCTCTATTTGCCCTTTGTTGTCGTCGACCTGGTGGTATCCAGCGTGCTACTGGCGCTGGGGATGATGATG
ATGAGTCCGGTGACGATATCTACACCTATTAAGTTGGTGCTTTTTGTCGCGCTTGATGGCTGGACCTTACTGT
CTAAGGGATTGATATTACAGTATATGGACATTGCAACATGACATCATTACGAGACGGGATAGTTAAATGGATG
ATTTAGTGTTTGCAGGTAATAAGGCGCTCTATCTTGTTTTGATCCTGTCAGGGTGGCCGACGATTGTCGCAAC
GATTATCGGCCTCCTGGTAGGGTTATTCCAGACGGTAACGCAATTACAGGAACAGACGCTGCCTTTTGGCATT
AAATTACTTGGCGTGTGTTTATGCTTGTTTTTACTGTCTGGCTGGTATGGCGAAGTTTTACTCTCTTACGGGC
GTCAGGTGATATTCCTGGCGTTGGCTAAGGGGTAAAAAATGCTTTACGCGTTGTACTTTGAAATTCATCACCT
GGTTGCGTCTGCGGCACTAGGGTTTGCTCGCGTAGCGCCGATTTTTTTCTTCCTGCCGTTTTTGAATAGCGGG
GTATTAAGCGGCGCGCCGAGAAACGCCATTATCATCCTGGTGGCATTGGGAGTATGGCCGCATGCATTGAACG
AGGCGCCGCCGTTTTTATCGGTGGCGATGATCCCGTTAGTTCTGCAAGAAGCGGCGGTAGGCGTCATGCTGGG
CTGTCTGCTGTCATGGCCTTTTTGGGTTATGCATGCGCTGGGTTGTATTATCGATAACCAGCGAGGGGCAACG
CTAAGTAGCAGTATCGATCCGGCAAACGGTATTGATACCTCGGAAATGGCTAATTTCCTGAATATGTTTGCCG
CTGTCGTTTATTTACAAAACGGCGGTCTGGTCACGATGGTTGACGTGTTAAATAAAAGCTATCAGCTATGCGA
TCCGATGAACGAGTGCACGCCTTCATTACCGCCGCTATTAACGTTTATTAATCAGGTGGCTCAAAACGCCTTG
GTTCTGGCCAGTCCGGTGGTATTAGTGCTGTTGCTGTCAGAAGTATTCCTGGGTTTATTGTCGCGCTTTGCTC

CGCAAATGAACGCTTTTGCGATTTCACTGACGGTAAAAAGCGGTATTGCCGTTTTAATTATGCTGCTTTATTT
CTCTCCGGTACTACCGGACAATGTACTGCGACTCTCTTTCCAGGCCACAGGGTTAAGCAGTTGGTTTTACGAG
CGAGGGGCGACGCATGTCCTCGAATAAAACAGAAAAACCGACTAAAAAACGGCTGGAAGACTCCGCTAAAAAA
GGCCAGTCATTTAAAAGTAAAGATCTCATTATCGCCTGCCTGACGCTGGGAGGAATTGCCTATCTGGTGTCGT
ATGGCTCATTTAATGAGTTTATGGGGATAATTAAGATCATTATTGCGGATAATTTTGATCAGAGCATGGCTGA
CTACAGTTTGGCCGTTTTTGGGATAGGGTTAAAATATCTGATTCCATTTATGCTGCTCTGCTTAGTGTGTTCC
GCATTACCGGCGTTATTACAGGCCGGTTTTGTGCTGGCGACAGAAGCATTAAAGCCTAATTTATCGGCGTTAA
ACCCGGTAGAAGGGGCAAAAAAACTTTTTAGTATGCGCACGGTTAAAGATACGGTCAAAACCCTACTGTATCT
CTCATCCTTTGTGGTGGCCGCCATCATTTGTTGGAAGAAATATAAGGTTGAAATCTTTTCTCAGCTAAATGGC
AATATTGTAGGTATTGCCGTCATTTGGCGTGAACTTCTCCTCGCATTGGTATTAACTTGCCTTGCTTGCGCAT
TGATTGTCTTATTATTGGATGCTATTGCGGAATATTTCCTGACCATGAAAGATATGAAAATGGATAAGGAAGA
AGTGAAGCGTGAAATGAAGGAGCAGGAAGGGAACCCAGAGGTTAAATCTAAAAGACGTGAAGTTCATATGGAA
ATTCTGTCTGAACAGGTGAAATCTGATATTGAAAACTCACGCCTGATTGTTGCCAACCCCACGCATATTACGA
TCGGGATTTATTTTAAACCCGAATTGATGCCGATTCCGATGATCTCGGTGTATGAAACGAATCAGCGCGCACT
GGCCGTCCGCGCCTATGCGGAGAAGGTTGGCGTACCTGTGATCGTCGATATCAAACTGGCGCGCAGTCTTTTC
AAAACCCATCGCCGTTATGATCTGGTGAGTCTGGAAGAAATTGATGAAGTTTTACGTCTTCTGGTTTGGCTGG
AAGAGGTAGAAAACGCGGGCAAAGACGTTATTCAGCCACAAGAAAACGAGGTACGGCATTGAGCCGCGTAAAG
CAGTAGCGATGTATTCATTGGGCGTTTTTTGAATGTTCACTAACCACCGTCGGGGTTTAATAACTGCATCAGA
TAAACGCAGTCGTTAAGTTCTACAAAGTCGGTGACAGATAACAGGAGTAAGTAATGGATTATCAAAATAATGT
CAGCGAAGAACGTGTTGCGGAAATGATTTGGGATGCCGTTAGTGAAGGCGCCACGCTAAAAGACGTTCATGGG
ATCCCTCAAGATATGATGGACGGTTTATATGCTCATGCTTATGAGTTTTATAACCAGGGACGACTGGATGAAG
CTGAGACATTCTTTCGTTTCTTATGCATTTATGATTTTTACAATCCCGATTACACCATGGGACTGGCGGCGGT
ATGCCAACTGAAAAAACAATTTCAGAAAGCATGTGACCTTTATGCAGTAGCGTTTACGTTACTTAAAAATGAT
TATCGCCCCGTTTTTTTTACCGGGCAGTGTCAATTATTAATGCGTAAGGCAGCAAAAGCCAGACAGTGTTTTG
AACTTGTCAATGAACGTACTGAAGATGAGTCTCTGCGGGCAAAAGCGTTGGTCTATCTGGAGGCGCTAAAAAC
GGCGGAGACAGAGCAGCACAGTGAACAAGAAAAGGAATAATTATGGTAAATGACGCAAGTAGCATTAGCCGTA
GCGGATATACCCAAAATCCGCGCCTCGCTGAGGCGGCTTTTGAAGGCGTTCGTAAGAACACGGACTTTTTAAA
AGCGGCGGATAAAGCTTTTAAAGATGTGGTGGCAACGAAAGCGGGCGACCTTAAAGCCGGAACAAAGTCCGGC
GAGAGCGCTATTAATACGGTGGGTCTGAAGCCGCCTACGGACGCCGCCCGGGAAAAACTCTCCAGCGAAGGGC
AATTGACATTACTGCTTGGCAAGTTAATGACGCTACTGGGTGATGTTTCGCTGTCTCAACTGGAGTCTCGTCT
GGCGGTATGGCAGGCGATGATTGAGTCACAAAAAGAGATGGGGATTCAGGTATCGAAAGAATTCCAGACGGCT
CTGGGAGAGGCTCAGGAGGCGACGGATCTCTATGAAGCCAGTATCAAAAAGACGGATACCGCCAAGAGTGTTT
ATGACGCTGCGACCAAAAAACTGACGCAGGCGCAAAATAAATTGCAATCGCTGGACCCGGCTGACCCCGGCTA
TGCACAAGCTGAAGCCGCGGTAGAACAGGCCGGAAAAGAAGCGACAGAGGCGAAAGAGGCCTTAGATAAGGCC
ACGGATGCGACGGTTAAAGCAGGCACAGACGCCAAAGCGAAAGCCGAGAAAGCGGATAACATTCTGACCAAAT
TCCAGGGAACGGCTAATGCCGCCTCTCAGAATCAGGTTTCCCAGGGTGAGCAGGATAATCTGTCAAATGTCGC
CCGCCTCACTATGCTCATGGCCATGTTTATTGAGATTGTGGGCAAAAATACGGAAGAAAGCCTGCAAAACGAT
CTTGCGCTTTTCAACGCCTTGCAGGAAGGGCGTCAGGCGGAGATGGAAAAGAAATCGGCTGAATTCCAGGAAG
AGACGCGCAAAGCCGAGGAAACGAACCGCATTATGGGATGTATCGGGAAAGTCCTCGGCGCGCTGCTAACCAT
TGTCAGCGTTGTGGCCGCTGTTTTTACCGGTGGGGCGAGTCTGGCGCTGGCTGCGGTGGGACTTGCGGTAATG
GTGGCCGATGAAATTGTGAAGGCGGCGACGGGGGTGTCGTTTATTCAGCAGGCGCTAAACCCGATTATGGAGC
ATGTGCTGAAGCCGTTAATGGAGCTGATTGGCAAGGCGATTACCAAAGCGCTGGAAGGATTAGGCGTCGATAA
GAAAACGGCAGAGATGGCCGGCAGCATTGTTGGTGCGATTGTCGCCGCTATTGCCATGGTGGCGGTCATTGTG
GTGGTCGCAGTTGTCGGGAAAGGCGCGGCGGCGAAACTGGGTAACGCGCTGAGCAAAATGATGGGCGAAACGA
TTAAGAAGTTGGTGCCTAACGTGCTGAAACAGTTGGCGCAAAACGGCAGCAAACTCTTTACCCAGGGGATGCA
ACGTATTACTAGCGGTCTGGGTAATGTAGGTAGCAAGATGGGCCTGCAAACGAATGCCTTAAGTAAAGAGCTG
GTAGGTAATACCCTAAATAAAGTGGCGTTGGGCATGGAAGTCACGAATACCGCAGCCCAGTCAGCCGGTGGTG
TTGCCGAGGGCGTATTTATTAAAAATGCCAGCGAGGCGCTTGCTGATTTTATGCTCGCCCGTTTTGCCATGGA
TCAGATTCAGCAGTGGCTTAAACAATCCGTAGAAATATTTGGTGAAAACCAGAAGGTAACGGCGGAACTGCAA
AAAGCCATGTCTTCTGCGGTACAGCAAAATGCGGATGCTTCGCGTTTTATTCTGCGCCAGAGTCGCGCATAAA
AACTGCCAAAATAAAGGGAGAAAAATATGTTAATTAGTAATGTGGGAATAAATCCCGCCGCTTATTTAAATAA
TCATTCTGTTGAGAATAGTTCACAGACAGCTTCGCAATCCGTTAGCGCTAAAGATATTCTGAATAGTATTGGT
ATTAGCAGCAGTAAAGTCAGTGACCTGGGGTTGAGTCCTACACTGAGCGCGCCTGCGCCAGGGGTATTAACGC
AAACCCCCGGAACGATCACGTCCTTTTTAAAAGCCAGTATTCAAAATACCGACATGAATCAGGATTTGAATGC

TCTGGCAAATAATGTCACGACTAAAGCGAATGAGGTTGTGCAAACCCAGTTACGCGAGCAGCAGGCAGAAGTC
GGAAAGTTTTTTGATATTAGCGGAATGTCTTCCAGTGCCGTTGCGCTGTTGGCTGCCGCGAATACGTTAATGC
TGACGTTGAACCAGGCTGATAGCAAACTGTCTGGTAAGTTGTCATTAGTCAGCTTTGATGCAGCTAAAACGAC
AGCAAGCTCCATGATGCGCGAAGGGATGAATGCGTTGTCCGGTAGTATTTCCCAGAGCGCGCTTCAGTTGGGG
ATCACTGGCGTGGGCGCCAAACTGGAATATAAGGGGCTGCAGAATGAAAGAGGCGCGCTTAAACATAATGCCG
CGAAGATCGATAAACTGACCACTGAAAGCCACAGTATTAAAAACGTGCTGAACGGGCAGAATAGCGTCAAACT
CGGTGCTGAAGGCGTCGATTCTCTGAAATCGTTAAATATGAAGAAAACCGGTACCGATGCGACAAAAAATCTT
AATGATGCGACGCTTAAATCTAATGCCGGAACCAGCGCCACGGAAAGTCTGGGTATTAAAGACAGTAATAAAC
AAATCTCCCCTGAACATCAGGCTATTCTGTCGAAACGTCTTGAGTCTGTCGAATCCGATATTCGTCTTGAGCA
GAATACCATGGATATGACCCGAATTGATGCGCGCAAGATGCAGATGACGGGCGATCTGATTATGAAGAACTCG
GTCACGGTCGGTGGTATTGCAGGGGCGTCCGGGCAGTACGCCGCTACTCAGGAACGTTCCGAGCAGCAAATTA
GCCAGGTGAATAACCGTGTTGCCAGCACCGCATCGGACGAAGCCCGTGAAAGTTCACGTAAATCGACCAGCCT
GATTCAGGAAATGCTGAAAACAATGGAGAGCATTAACCAGTCGAAAGCATCCGCACTTGCTGCTATCGCAGGC
AATATTCGCGCTTAATCTGAAAGGTCATCTATACGCCATCATGGGTGTGATTTAATCGCGCTCCTGATGGCGA
ACTGGGGATATTATGCTTAATATTCAAAATTATTCCGCTTCTCCTCATCCGGGGATCGTTGCCGAACGGCCGC
AGACTCCCTCGGCGAGCGAGCACGTCGAGACTGCCGTGGTACCGTCTACCACAGAACATCGCGGTACAGATAT
CATTTCATTATCGCAGGCGGCTACTAAAATCCAGCAGGCACAGCAGACGCTGCAGTCAACGCCACCGATCTCT
GAAGAGAATAATGACGAGCGCACGCTGGCGCGCCAGCAGTTGACCAGCAGCCTGAATGCGCTGGCGAAGTCTG
GCGTGTCATTATCCGCAGAACAAAATGAGAACCTGCGGAGCGCGTTTTCTGCGCCGACGTCGGCCTTATTTAG
CGCTTCGCCTATGGCGCAGCCGAGAACAACCATTTCTGATGCTGAGATTTGGGATATGGTTTCCCAAAATATA
TCGGCGATAGGTGACAGCTATCTGGGCGTTTATGAAAACGTTGTCGCAGTCTATACCGATTTTTATCAGGCCT
TCAGTGATATTCTTTCCAAAATGGGAGGCTGGTTATTACCAGGTAAGGACGGTAATACCGTTAAGCTAGATGT
TACCTCACTCAAAAATGATTTAAACAGTTTAGTCAATAAATATAATCAAATAAACAGTAATACCGTTTTATTT
CCAGCGCAGTCAGGCAGCGGCGTTAAAGTAGCCACTGAAGCGGAAGCGAGACAGTGGCTCAGTGAATTGAATT
TACCGAATAGCTGCCTGAAATCTTATGGATCCGGTTATGTCGTCACCGTTGATCTGACGCCATTACAAAAAAT
GGTTCAGGATATTGATGGTTTAGGCGCGCCGGGAAAAGACTCAAAACTCGAAATGGATAACGCCAAATATCAA
GCCTGGCAGTCGGGTTTTAAAGCGCAGGAAGAAAATATGAAAACCACATTACAGACGCTGACGCAAAAATATA
GCAATGCCAATTCATTGTACGACAACCTGGTAAAAGTGCTGAGCAGTACGATAAGTAGCAGCCTGGAAACCGC
CAAAAGCTTCCTGCAAGGATAACAGAAGAGGATATTAATAATGGTTACAAGTGTAAGGACTCAGCCCCCCGTC
ATAATGCCAGGTATGCAGACCGAGATCAAAACGCAGGCCACGAATCTTGCGGCGAATCTTTCCGCAGTCAGAG
AAAGTGCCACAACGACGCTGTCAGGGGAAATTAAAGGCCCGCAACTGGAAGATTTTCCCGCGCTGATCAAACA
GGCGAGTCTGGATGCATTGTTTAAATGCGGGAAAGACGCTGAGGCGTTAAAAGAAGTTTTTACCAATTCAAAT
AATGTCGCCGGTAAGAAAGCGATAATGGAGTTTGCCGGGCTCTTTCGTTCAGCGCTCAACGCCACCAGTGATT
CTCCTGAGGCGAAGACGCTACTGATGAAGGTGGGGGCAGAGTATACCGCGCAAATCATAAAAGATGGCCTGAA
AGAAAAGTCAGCTTTTGGGCCATGGCTGCCAGAAACAAAGAAAGCGGAAGCGAAGCTGGAAAACCTGGAAAAG
CAGCTGTTAGATATTATCAAAAATAACACTGGTGGTGAATTAAGTAAATTATCGACGAATCTTGTTATGCAGG
AGGTGATGCCCTATATTGCCAGCTGCATTGAACATAACTTTGGCTGTACGTTAGATCCGTTAACCCGCAGCAA
TCTTACGCACCTTGTTGACAAAGCGGCGGCGAAGGCGGTTGAGGCGCTTGATATGTGCCACCAAAAATTAACG
CAAGAGCAGGGTACCAGCGTAGGACGGGAAGCCCGGCACCTTGAAATGCAAACGTTGATACCCCTGCTGCTAC
GTAATGTTTTTGCACAAATTCCTGCAGATAAACTGCCTGACCCTAAAATTCCGGAGCCTGCGGCTGGACCAGT
ACCTGATGGTGGGAAAAAAGCAGAACCTACGGGTATTAATATCAATATTAATATTGATAGCAGTAACCATAGC
GTGGATAACAGTAAGCATATTAACAATAGCCGAAGCCATGTCGATAATAGCCAGCGCCATATCGATAATAGCA
ACCATGATAATAGCCGGAAGACGATTGATAATAGCCGAACATTTATTGATAACAGCCAACGCAATGGCGAGTC
ACACCATTCGACTAACAGCAGCAACGTAAGTCATAGTCATTCGCGTGTGGATTCGACTACGCATCAAACGGAG
ACGGCACACAGCGCCAGCACAGGGGCAATTGACCATGGCATCGCGGGTAAAATTGACGTCACGGCTCATGCGA
CGGCAGAGGCTGTGACCAACGCTTCATCCGAAAGTAAAGATGGAAAGGTGGTCACGTCAGAAAAGGGCACTAC
GGGTGAAACAACCTCTTTTGATGAAGTCGATGGCGTAACCAGCAAGAGCATTATCGGCAAGCCGGTACAGGCC
ACGGTGCACGGCGTTGATGATAATAAACAGCAAAGCCAGACGGCAGAGATTGTGAATGTGAAGCCGCTTGCCA
GTCAACTGGCTGGCGTCGAGAATGTTAAAACCGATACCTTACAATCAGACACGACGGTAATTACAGGTAATAA
AGCCGGTACGACCGATAATGATAATAGTCAGACAGATAAGACTGGCCCATTTTCTGGTTTGAAGTTTAAGCAA
AATAGTTTCCTCTCAACAGTACCGAGCGTTACGAATATGCATTCAATGCATTTCGACGCCCGTGAGACGTTTT
TGGGTGTGATACGTAAAGCCCTGGAGCCAGATACCTCCACACCGTTCCCTGTACGCAGAGCGTTTGACGGCTT
GCGTGCGGAAATATTACCCAATGACACGATAAAGAGTGCAGCGTTGAAAGCGCAATGTAGCGATATTGACAAG
CACCCAGAATTGAAAGCGAAAATGGAGACTCTCAAAGAGGTCATTACTCATCATCCACAAAAAGAAAAACTGG

CTGAGATTGCACTGCAGTTTGCCAGAGAGGCGGGGCTGACCAGGCTAAAAGGGGAAACTGACTATGTGTTAAG
TAATGTGCTGGACGGCCTTATCGGAGACGGTAGCTGGCGAGCTGGCCCGGCTTACGAGTCATACCTGAATAAA
CCTGGCGTGGATCGGGTTATTACTACCGTTGATGGCTTGCACATGCAGCGTTAATTAACCGGGAAAGATGCGA
TGAATATGGATATTGAAGCAAGAGTCAAAAAAGTGATCACCTCCTGTATTGCCGTTGATGTTGATAGTATCAA
TGGTCAGACCCATCTGGTTGAGGATCTTTACGCTGACTCATTGGATTTAATTGATATTGTATTTGGTCTTAGT
GAGGAGTTTGACATTAGTTGCAATGAAAACGATCTTCCTGATATGACGACCTTTGCGGATATATGCCGTGTTG
TTAAAAAAAGTCTTGAGTCCAGGGTGTAGTATTTTTCTCTCGCTTGACAACAATTGAATAAGATTAATTGTTA
TGAAAATCATCACACATGTAGTACCCGGTTCAGGTATGGCTGCAATATATGATGATATTGCTGACAGTTCGCG
TTTTGTTATTAAAGGCAAGTTACGTCATGTTGAAAATGATCCGAAGGAGCTGCTGATATGTGTTCCGATGCGT
AGTGAATGGCTATTCTACTGGATTAAAGGAGAGAAATATTGCGCCCGGCGATGGGCGCGAAAAAGTATCAAGA
CATTGTGTAACCAGATTAAATTTGAAGAAGCAATCTTATAAAAGATATAATTCAAGAGGAAAGTAAATTGCAA
GCACACCAGGATATTATCGCTAATATTGGTGAGAAATTGGGTTTACCGCTCACTTTTGACGACAACAATCAGT
GCTTATTATTACTCGATAGCGATATTTTTACGTCTATTGAAGCTAAAGATGATATCTGGTTATTGAACGGTAT
GATTATACCGTTATCGCCTGTTTGTGGCGATTCTATCTGGCGGCAGATTATGGTGATTAATGGTGAACTGGCT
GCGAATAATGAAGGTACGTTAGCGTATATTGATGCCGCAGAGACGTTGTTGCTTATACATGCAATTACCGATC
TGACAAATACTTACCATATTATATCGCAGCTTGAGTCATTTGTGAATCAGCAGGAAGTGCTCAAAAACATACT
GCAGGAATATGCTAAAGTATGAGGAGAGAAAATTGAATAATTTAACGTTGTCTTCGTTTTCAAAAGTTGGTGT
GTCGAATGATGCCCGACTTTATATTGCTAAGGAAAATACTGATAAGGCATATGTTGCGCCTGAAAAATTTTCG
TCAAAAGTATTAACCTGGCTTGGAAAAATGCCGTTATTTAAAAACACTGAAGTGGTGCAAAAACATACGGAAA
ATATCAGAGTACAGGACCAAAAGATTTTACAGACATTTCTCCATGCACTAACGGAAAAATATGGGGAAACAGC
GGTTAATGACGCACTGTTAATGTCCCGTATAAATATGAACAAACCCCTTACCCAACGTTTAGCAGTGCAGATC
ACGGAGTGTGTAAAAGCTGCTGACGAAGGGTTTATAAACCTTATTAAGAGCAAGGATAATGTTGGTGTCAGGA
ATGCCGCTTTAGTCATAAAAGGCGGCGATACAAAAGTGGCAGAAAAAAATAACGATGTTGGAGCAGAAAGTAA
GCAACCTTTACTCGATATCGCGCTAAAGGGACTTAAAAGAACATTACCGCAACTGGAACAAATGGATGGAAAT
AGCCTGCGAGAAAACTTCCAGGAGATGGCTTCAGGTAACGGCCCGCTGCGTTCATTAATGACGAATTTACAGA
ATTTAAATAAAATTCCAGAGGCTAAACAACTTAATGATTATGTTACAACGTTAACAAATATTCAGGTCGGTGT
AGCCCGGTTCTCACAATGGGGAACCTGTGGAGGAGAGGTTGAGAGGTGGGTTGATAAAGCCTCTACTCATGAA
TTAACCCAGGCTGTAAAAAAATTCACGTTATTGCTAAAGAACTGAAAAATGTTACAGCTGAACTTGAGAAAAT
AGAAGCCGGCGCGCCAATGCCACAGACGATGAGCGGACCGACACTGGGCCTGGCACGGTTTGCGGTCAGCAGC
ATTCCAATTAATCAGCAAACCCAGGTGAAACTGAGCGATGGAATGCCTGTGCCAGTGAATACGTTAACTTTTG
ACGGTAAGCCTGTGGCATTAGCCGGTTCGTACCCAAAAAATACGCCGGATGCGCTGGCAGCGCATATGAAAAT
GCTTCTTGAAAAAGAATGCTCATGCCTGGTGGTGTTAACGTCGGAAGATCAGATGCAGGCAAAACAATTACCA
CCCTATTTCAGAGGGAGCTACACCTTTGGCGAGGTGCATACCAACAGCCAAAAAGTGAGCTCAGCCAGTCAGG
GAGAAGCGATAGACCAATACAATATGCAACTGTCCTGCGGGGAAAAGCGGTATACAATCCCGGTATTGCATGT
GAAAAATTGGCCAGATCACCAGCCGTTACCGTCTACGGATCAGTTAGAATACCTGGCGGATAGGGTGAAAAAT
AGTAACCAAAATGGCGCGCCGGGGCGTAGTTCATCAGATAAGCATTTACCGATGATTCATTGTCTGGGCGGAG
TGGGGAGAACCGGAACGATGGCGGCGGCCCTTGTACTTAAGGATAATCCTCATAGTAATCTGGAGCAGGTACG
TGCAGATTTCCGGAATTCACGGAACAATAGAATGCTGGAAGACGCCTCACAGTTTGTACAACTAAAAGCAATG
CAAGCCCAGTTGCTTATGACGACGGCACGCTGATAAAAACATAGCTTACTTTTAGAACTATCTGAAAGTAAGC
TATTTCTGTATAATTATTTGTTTGACGCGATAGAAAGTCTTTTGTTTTTCTCTTCTGCTGACATTCCTTTTAA
TTTTCTGTAATTCTCCCAAATTTTTTTAGCATAACGTTTCCTTATATCCGATCGTTTCGGCGACGTCCCGGCA
TTATAAGCGCCAACGGCCTCCCAGCTATAACCGTAGATTTTCATCATATCTGATAAAATGGAAGCGCCCACAA
TGACAGAAATGCAGGGGTCCTGTAACAACTGTTTTTCACTAATCCCCATTTTTTTCAGCCTTTTCATATGGAA
GCTGTTAATTTGCATCAGGCCAAGATCGGTTGAACCATCTCGGTTATGACCAATGGCGCCAGGTTTCATCGCC
GATTCCTGCTGGGCGATAGCGTAAAGTAGTTCGGATTCAATATTGAACATTTTTTCAGCCTGAAGCCAGCAAT
CAGCCCATGCCGTATTTATGCTAAGCAACCAGATTACGATGATAAAAAAATAATGCATATCTCCTCTCTCAGA
TTTTACCGTAATTTAATCAAGCGGGGATCCTGTTTCCATCTTTTGAACCAAATATTGTCTTCGTTTTTAAATT
TATTCCACATTTTCTCGGCAATAGCTTCGCCGTGGGCAACCAGCACTAACGGTAATAATCCCGGATTATTATC
TATACGCTGTTCACTTTCCAGAAATTCTCTTATCGTCGGTAAGGCACGCTCACTATTCTGACAATATTCAGCG
TAAAGAAGATTAACAGCAATAAGTCCTGTTATTTCCTGCGTGGATATTTCTTTAGTTAATTTTCGTGCCAGTT
CATGTTTACCTTTAAGCGAAAGAAACATAACCTGCATACTTAATAATATTGGATTATCCTGCAGGTGTTGTGA
GCGTAATTCATCGCCTAAACGTATAGCATCATCAATACCGGTATGATAATAGGTAATCCACAGCTTAGTGATC
CCTGCGGCTGCGCGCGTTGGGTCCAATTTTAAACACTCGTTAATCGTTTGTAAGGCCTCCTCCAACTGACCAG
CCATGAAAAGATTCCAGCCATAATAATATTTAATATCTGCAGAAATGGGCGAAAGTAAGTTAGCTTGTTTGAA

TAGCAAACTCCCGACGATGTATTCTGAGTGAATCGTATTAATTAGCCCCAGTAATCCTAAAGCTTGTGGATTA
TTGTGGTCCAGCTCTGTCGCCTTAATCGCATGTTCTTTAGCTTTGATCATAGCGTTTTGTTTATCAAAAATCC
CCATTTGCGCCATGCTGAGGTAGCATTCTGCCAGCGCACAGTAAGGCGCAATGCTGTTTGGCGACATGTTAAC
GCATTGAGTCAGCAATTTAAGCGCTTGCTGTAAGCTATAGGGGGTGTATTGATTTAACTCATGCTTACCGCGT
AAGTAAACCATAGTACTGTCAATCGAATTTAGCTCGCTAATCTGCTTTGTGTCCCAGCGAAGTCCGGGAATAC
ATCTGAGCAAAAGATTCGCAATTTTGTTTTGCAAGAGAGAAGCGGGTTGGTGTTCTATCAACTTAATGCTTTC
CTGGTGCAGCAGGTGATAACCTTTAACCCGAACTATCTCAATCTGTACAATATTATCATTACCATCGGGTATC
ATCTGCCCGGAGATATAATAATCGGGGCGTAATTGATCCATGAGCTCAAGAATATCCTTAACACTGCGGCAGT
TCTTCGTAATGGTCACCGGCAGCACGCTCAGGCCAAAGGGCGCATACTGCGATAATCCCTTCACGATAGAGTA
ATGCAGACTCTCGGATTGAACCTGATCCTGCATCTGAAAAGGAAGTATCGCCAATGTATGAGTCGTAGGTTGC
GGCGCTGGCGGAGACACCACTACGACCGGACGATTAAACCGATAGCCCTGTCCGTACAGTGTTTCAATGTAAC
GATGCTCTTTATCTTCCGACAGAATACGTCGTAAGGCATAAATACAGCGGGTAAGAGATTCTTCGTTAACTTC
CGCGTCGCCCCATACCTGGTCCAGTAAGGTGTTTTTACTCACAATCTCGCCGGCGGCTTCGAGCAGGATGAC
CAGAACGGCATATTCTTTTGGCGGAATATTGACTTTCTTTTCTGAGCGTAGCAGGGAGCCGTCCATGTTGAGT
ATGAAATCATCAAAGACGAATTTTTTATTCGATACAGGAACAGGATTAAAATGTGGCATGATAATAGTGTATT
CTCTTACAGGGTGAAAAGTTATAATAACTAATACAAGTCGTTAGGATAGAATCTACTGTTTCAGTAATTCTGC
TGTTAGTACTAACGGTCAGGTTGAGCTTTTATTATGAGCTTAATGTCATATTCTACGGGCTCGGGTTTATATG
TTTTTAACATTTGATTATTTAGCTTATGCCTGTGCTTTAAATAACTGACATGTTGGTAATAATATTGTTATAA
CTAACCGTGATTATCGAATGGACGCTGCCGTATATCCTGGTGCAGAGAGAGTCAGCGCCTGGCATCTAAATAT
TTACATATTTAACTTGGCCTCATTGCAATAGAGATTAGTTTTGTAGCTATCTTACTGCATTTTTTTGGGGTGT
AAATGCTGCTTATTATATCTTCATGGTTAATGGTCTGATAATTACACCATCAGAACCATTTTTCTCGAAGATT
TCCACTAAATGACCATTCGTTGAAAGTTTAAGAGTGTAATTTGTCTTAGGTTCCAAAATATAAATGAAAGACT
GTTTTTAATGGTGCGCTTATTTTTATATTTTTTTGCAAAATAATGATATCGTCTTTTCTTTACTCCCTTCAGT
TATAGCGTCAAAAAGGCAGGGGGTTTATGAGCAGTATCATCCTCAGGCTGGCCCCAGGCATTATATCGATATC
AGGAGGAAGAGAGGAGGTATGCCTGGCAGAAAGCTAACAAGCGTGACTGTTTCGGTATAAACCGTTCAGGCAA
ATAGTTCTCAGAGGGAACGGATGATGTATAAATATGAATAAAATGCCGGCCTTAATCCACAGGGTTAAAGCCG
GAAGGAGATAGTGTCATCTGGACGAATTCTTACGCTGTGAATAAGGCAATATCGTTTTACCAGTCATCATAGC
GATGACAAGTTCACAAAAATCGTGTTATATAATCAGCCTTACTTTAAATTACCAGAGATATCAGTATAAGCTG
TCTTCCGGACAGAACAATGATATTGAATAGCCTCCCATCCTGATAGAGCGTGTTAATGCGCAGTCTGAATTTT
AAATTATTACAATTTTAATAAAAATCTTTACTTAAGTGACAGATACAAAAAATGTTAATGGTTCGCCATTTTT
ATGAATGTCGATGGCGTAGTTTTAAAATATTTTTTGAAACATTGAATGAAGTAGGACGTGCTATCATAACCAC
ATTTTAATGCTACAGCATTAACATTATGGTTGCCTATGCGTAAAAGTTTTGCTGCCTGATTCATTCTTGCCGA
TAAGTAGATGTCGCTAAAGCTGGTACCTTCTTCTGCAAGTTTCCGTTTGAGCGTTGACGTACTCATAAATATA
TGATCGGCAACATCCGTAAGCTTCCACTGTCTACTGGGTGACGAAGATATAATGTTGTAAACGCGCTCCTTTA
ACGTTATCTGAGCCGAGCTAAGGATGATCTCATAACTCTCCGGGAGTCGGAGAAAAGCAGTAAGCAGGAACAG
CAGAAAATAAGGAATTAACTCTTCATTTTCAATACAACTTTTGTTGTTACAGTTACTGCAACCTTTGCTTGTA
TCCTTCGTACAGGAGAACGCCGTTTTCAGATGTTCAAATACCTCTCTTCTGGCAGGAAAGTCAGGCGTATAGA
AGATTTTCTGCGCTTTCTCTGTGGGTACCGCCATTTTGGTTTGCTGCTCGTTTGGGATAAGTGCATAGAGAGC
GCCAAGTCGTTGCGTCGGTATCTCGAGTATATCGAAATCCATGTGGCCATTGACCTCTTCAAGCGTCACGTTA
ACTACCTGCTCTTTTTTGAGCACCAACATCCCAGGTTCGTCACAGTAAATCGTATCGTGATTATTGCTAATCG
TCAGTTTACCGCTCCGAAAGCAAACTAAAGTGAAACTGCTTACATAAAGATTTTTGATGGTAACCTGCTGAGT
CTGACTTTTAATTTGCTGCCGGGTATTTGTCAAAAGTGATTTTAATTTCTGTAAGTTATCTGCGGCAGGACGC
TGATGACTATTACTTACAAAGGTTACATTTTCCATATTATCCCTTTGTTGATGTTATTTTAATGTTCCTTACT
GGTATCCTACTGAAAAAATCTGAGTTGTAAATGCTCTTTATTAGCGTGTGTTGGCAATGGTCTGATTGTTACA
CCAAAAGAACCCAAATTTGGGTAATTTATCTACAGTAGTTTAAGCCCCAATGGGGATGATGGTTCTTTTAATA
TGTGTTGAGACGCATTATACAGAATAAATTGATTTTATTTCTCACTTTTCATTCTATTTTCATCAGGAATCCC
TGTGTCCTGTGCGGTAATCTGCTGCTATCGAGAACGACAGACATCGCTAACAGTATATATGGAAACATCAAAA
GAGAAGACGATAACAAGCCCTGGGCCATACATAGTTCGATTACTTAACAGCTCACTGAACGGCTGTGAGTTTC
CATTGCTGACAGGCCGAACACTCTTTGTGGTAGGTCAGAGTGATGCGCTCACTGCTTCAGGCCAACTCCCTGA
TATACCTGCCGATAGCTTTTTTATCCCGCTGGACCATGGCGGAGTAAATTTTGAAATCCAGGTGGATACGGAT
ACGACCGAAATTATACTCCATGAGCTGAAAGAAGGAAATTCTGAATCTCGTTCGGTGCAATTAAATACGCCAA
TACAGGTCGGTGAATTGCTTATCCTGATTCGCCCGGAAAGCGAGCCGTGGGTGCCCGAGCAGCCTGAGAAGTT
AGAAACGTCTGCAAAAAAGAACGAGCCGCGTTTTAAAAACGGAATTGTAGCAGCACTGGCCGGGTTTTTTATA
TTGGGAATTGGGACTGTGGGGACGTTATGGATACTTAACTCGCCGCAGCGGCAGGCCGCAGAGCTCGATTCGT

TATTGGGGCAGGAGAAGGAGCGTTTTCAGGTGTTGCCAGGCCGGGACAAAATGCTCTATGTCGCTGCGCAAAA
TGAAAGAGATACGCTGTGGGCTCGTCAGGTTTTAGCGAGGGGCGATTATGATAAAAATGCGCGAGTGATTAAC
GAAAACGAAGAAAATAAGCGTATCTCTATCTGGCTGGATACCTATTATCCGCAGCTGGCTTATTATCGGATTC
ATTTCGATGAGCCGCGTAAACCCGTTTTCTGGCTAAGCCGCCAGCGAAACACGATGAGCAAGAAAGAGCTCGA
GATGTTAAGTCAAAAGCTGAGAGCGCTAATGCCTTACGCGGATTCGGTTAACATCACGTTGATGGACGATGTT
ACCGCAGCAGGCCAGGCGGAAGCGGGGCTAAAACAGCAGGCGTTACCTTATTCCCGCAGGAATCATAAGGGGG
GCGTAACGTTTGTTATTCAGGGGGCGCTCGATGATGTAGAAATACTCAGAGCCCGTCAATTTGTCGATAGCTA
TTACCGCACATGGGGAGGACGCTATGTGCAGTTTGCGATCGAATTAAAAGATGACTGGCTCAAGGGGCGCTCA
TTTCAGTACGGGGCGGAAGGTTATATCAAAATGAGCCCAGGCCATTGGTATTTCCCAAGCCCACTTTAATTTA
ACGTAAATAAGGAAGTCATTATGGCAACACCTTGGTCAGGCTATCTGGATGACGTCTCAGCAAAATTTGATAC
GGGCGTTGATAATCTACAAACGCAGGTAACAGAGGCGCTGGATAAATTAGCAGCAAAACCCTCCGATCCGGCG
CTACTGGCGGCGTATCAGAGTAAGCTCTCGGAATATAACTTGTACCGTAACGCGCAATCGAACACGGTAAAAG
TCTTTAAGGATATTGATGCTGCCATTATTCAGAACTTCCGTTAATCAGTTATAAGGTGGATTATGTCGATTGC
AACTATTGTCCCAGAGAATGCCGTTATAGGGCAGGCGGTCAATATCAGGTCTATGGAGACGGACATTGTCTCG
CTGGATGACCGGCTACTCCAGGCTTTTTCTGGTTCGGCGATTGCCACGGCTGTGGATAAACAGACGATTACCA
ACAGGATTGAGGACCCTAATCTGGTGACGGATCCTAAAGAGCTGGCTATTTCGCAAGAGATGATTTCAGATTA
TAACCTGTATGTTTCTATGGTAAGTACCCTTACTCGTAAAGGAGTCGGGGCTGTTGAAACGCTATTACGCTCA
TGATTCGTCGATATCTATATACCTTTCTGCTGGTAATGACCCTTGCCGGCTGTAAGGATAAGGATCTTTTAAA
AGGACTGGACCAGGAACAGGCTAATGAGGTCATTGCCGTTCTGCAAATGCACAATATAGAGGCGAATAAAATT
GATAGCGGAAAATTGGGCTATAGCATTACCGTTGCTGAGCCTGATTTTACCGCTGCGGTGTACTGGATTAAAA
CTTATCAGCTTCCTCCCCGGCCACGGGTGGAAATAGCGCAGATGTTCCCGGCGGATTCGCTGGTATCGTCTCC
GCGAGCTGAAAAGGCCAGGTTATATTCGGCTATTGAACAGCGACTGGAACAGTCATTACAGACGATGGAGGGC
GTGCTCTCCGCCAGGGTCCATATTAGTTATGATATTGATGCTGGTGAAAATGGCCGTCCGCCAAAACCTGTTC
ATCTGTCGGCATTAGCCGTATATGAACGAGGTTCGCCGCTTGCGCATCAGATCAGCGATATCAAGCGTTTCTT
AAAGAATAGTTTTGCCGATGTGGATTATGACAACATTTCTGTTGTGTTGTCAGAACGTTCTGATGCCCAATTA
CAGGCTCCCGGCACACCAGTAAAACGTAATTCTTTTGCAACCAGTTGGATTGTGTTGATTATTTTGTTATCCG
TGATGTCAGCAGGCTTTGGCGTCTGGTATTACAAAAACCATTATGCCCGCAATAAGAAAGGCATAACGGCTGA
TGATAAGGCGAAATCGTCAAATGAATAGGCAGCCATTACCCATTATCTGGCAAAGAATCATTTTTGATCCGTT
ATCGTATATCCATCCTCAGCGGTTGCAGATAGCGCCGGAAATGATTGTCAGACCCGCCGCCAGGGCGGCGGCA
AATGAGTTAATACTGGCGGCATGGCGGCTTAAGAACGGAGAAAAGGAGTGTATTCAAAACTCACTGACGCAGC
TGTGGCTGCGTCAGTGGCGCCGACTGCCGCAGGTAGCGTATTTACTCGGTTGCCATAAACTGAGAGCCGATCT
GGCAAGGCAGGGAGCCTTGCTTGGCCTGCCGGATTGGGCGCAAGCATTTTTGGCAATGCATCAGGGAACAAGT
TTATCTGTCTGCAATAAGGCGCCGAATCACCGGTTTTTACTTAGCGTCGGGTATGCACAGTTAAATGCCCTAA
ATGAATTTTTACCTGAATCTTTAGCACAGCGTTTTCCTTTGCTTTTTCCTCCATTTATTGAGGAGGCATTGAA
GCAGGATGCTGTAGAAATGTCAATTTTGCTACTGGCCTTACAATATGCTCAAAAATATCCCAATACCGTCCCC
GCTTTCGCCTGTTGAGGGGATACTGATAAAGCGTAAAACGCTGGAACGTTATTTCTCTATTGAAAGATTAGAA
CAGCAGGCGCATCAACGGGCTAAGCGTATTTTGCGAGAGGCGGAAGAAGAGGCAAAGACCTTGCGGATGTACG
CCTATCAGGAAGGTTACGAGCAGGGAATGATAGACGCGTTACAGCAGGTCGCCGCTTATCTTACTGATAATCA
GACAATGGCCTGGAAGTGGATGGAAAAAAATACAGATTTATGCCCGCGAGTTATTTTCAGCTGCGGTCGACCAT
CCCGAAACGCTTTTAACCGTCCTGGATGAGTGGCTAAGGGATTTCGATAAGCCTGAGGGGCAACTTTTTTTAA
CACTGCCAGTTAATGCGAAAAAAGATCACCAAAAACTGATGGTGTTGCTTATGGAGAACTGGCCAGGCACTTT
TAATCTTAAATATCATCAGGAACAGCGCTTTATCATGAGTTGCGGCGATCAGATCGCAGAATTTTCACCTGAA
CAATTTGTTGAAACGGCAGTAGGCGTCATTAAGCATCATCTTGATGAACTTCCACAAGACTGCCGGACAATTT
CTGATAACGCCATTAACGCACTTATTGATGAATGGAAGACAAAAACGCAAGCGGAGGTTATAAGGTGATACCG
GGTACGATTCCGACTTCTTATTTGGTCCCGACAGCAGATACTGAAGCGACGGGTGTGGTTTCGTTGAGTGCCA
GGGCCGCTATGCTGAATAATGTGGATAGCGCGCCTTTAAGCAATGGCGGCGACGTTGATCTTTATGATGCATT
CTACCAACGACTTCTGGCGCTTCCTGAGTCAGCCTCTTCTGAAACGCTTAAAGATAGCATTTATCAAGAGATG
AATGCCTTCAAAGATCCTAACAGTGGTGATTCGGCTTTTGTTTCTTTTGAGCAGCAAACGGCTATGCTACAAA
ACATGTTAGCAAAAGTTGAACCGGGCACCCATTTATATGAGGCTTTAAACGGGGTATTAGTTGGTACAATGAA
CGCACAGTCACAAATGACCTCCTGGATGCAGGAGATTATTTTATCGGGCGGGGAAAACAAAGAGGCAATTGAC
TGGTGAACCCCCCATTGCTGGCGATTTGTGATAAGGTATGGTGTATATATCAGCAAATAATGCAGAAAATGCAC
CATGCCAGTGTGTCTATCTTATTGATGTATATGGATTATTAAGATGTCTGCCAGCCGTTTTATTCTGAAATGA
TTATATTAATATTGTAAGCTTTCATTACAAAATTGTGCATAAAGTTTGTAAGGGTATCTTTTTGTGACGAAAT
GAACGCGCGTTGGAATGTAATTATTGGCTATAATAATAAAAAAATCGGATTTAAATCATCTCTGTGAGTTTTA

ATTATTTGTTTATAAGAATATAACGATTTTGAGTTCCTTATAGCACACAGGATAAAATATGGTATTGCCTTCA
ATGAATAAATCAGTTGAGGCCATTAGCAATAATCACCTTCAACAGCCGAACAAATTTCCATTAATAAATGGAT
TAGCTGACGTAAGAGACTATTATGTCGCAAACTGCTTATTGTTTAAACTTAATAAAGGCAGTTTGCGAATTGA
AAACGAATTTGGGGAGTTCATCGAACAATCTGCGCCGTGTTTATTTTTATTGGAAAAAGATCAAACAATCACG
CTTAGTATGAGCGAAATAGAAGGGCATATTGATTTTTCTTCACTGGAAGTTTCCTATGACTTAATGCAAAAAT
TCTACAAAGTTTTTTACAGTACAAGAAACTATAATGATCGAGAGCTATCATTAAAAACGAAACCAAAGTATTT
TTTTCATGCGGACTTGTTGCCAGGGATGAGTGATACTTTTGACTCTATTTTGCATGGTGTGGCATGTCCACGG
GTTTGTAGTAATGTGAGTATTGATGATCATGATTATTCATATTTCTCATTGATGTATCTTATATCGGCATTTG
TACGTAAGCCCGGTGGGTTTGATTTCCTTGAGCGAGCAATAAAAATTACGACAAAAGAGAAAGTTTATAACAT
TATTATCAGCGATCTCACCCGCAAATGGTCACAGGCTGAGGTGGCGGGAAAGCTGTTTATGAGCGTATCAAGT
CTGAAGCGAAAACTGGCCGCTGAAGAGGTGAGTTTTAGCAAAATATACCTAGATGCTCGTATGAATCAGGCTA
TAAAATTATTACGTATGGGGGCTGGAAATATTTCACAAGTCGCGACGATGTGTGGCTATGATACGCCTTCTTA
TTTTATCGCTATTTTTAAACGGCATTTTAAGATTACACCGCTTAGCTTTATGCGTACGATGAACCATTGATTT
GTGAAATTATTTTAAACGTTACTATCTGTTTGCGTTACAGATACCAAATATTTTTTTAATCGTAGTGTATATT
TCAGTATCGCTACACCAGTGATATGTAGTTAATGAAAGGGAATCGAAAGCGTTCATTGTCAGACATACATGGC
CACCAGGCGCCGGGCAATCGTTGTACTCTAAGAGATTACAGTAAAGTAAAACAGCAAATTTAATCATTATCAG
ATTTGTAAAAGGAATTCATTTCGGTAACGACATTGCGCATGTAATAACGTATCTGGTCTTATTACCTTTGTAG
TAAGAAGGCGTTATCTACTTGCGCAGTAGTCGCCCAGGAAGAAAAATCATTACCGAAGCTGTGGTCAGTTTTC
AAAAAAGGATAAATAAATGAGAAATGTAATTATATACGGTATTAACTGGACTAATTGTTATGCCCTTCAGTCT
ATTTTTAAGCAAAAATATCCGGAAAAGTGCGTTAAAACGTGTAACTCATTAACCGCACTTTTGCATTCTCTAT
CCGATATGCCGGACGCCGGGCTGATTTTAGCTTTGAACCCGCATGAGCATGTTTATCTTTTTCATGCGTTGCT
GACGCGGTTGCAGAACCGTAAAGTTTTGGTAGTGGCAGATCGCTTGTATTATATCGATCGCTGTGTGCTGCAA
TATTTTGGCGTTATGGATTATGTTTTAAAAGACGAACTATCCTGCGCTATCCGCTCGGAGCGAGAGAAGCTCC
GTCTTCCGGAAGCCTGGCTGCGTTTCTGCCACAGGCCACAAAAGAAAACCGTGGCTGCTACGTATGCTTTTAA
TGCCGGCGAGACGCCGGAAGAGGTATTGTTTAATATCAATCAATACGCCTGGTGGAATCTACCTCCCGGTGTC
ACGCAGGCGAAATATGCGCTGCTGATATTATTATCCTCCGGACATCCGGCAATTGAACTGGCGAAAAAATTTG
GTCTGGGAACAAAAACCGTCAGTATTTATCGTAAGAAGGTTATGTATCGTTTGGGGATGGACTCTTCACCGCT
ATCGCTGTTCCGGGGACTGAAACTAGACGCGCATTTACAGCGCACTGCTTTTGCACATAACCCTGCTGTACCT
GACGATAATTGCGCGTTACCGATTGCGGTAGGAATGAATTAAAATATTTTTTATCATTTATGACCATGTGTTG
AGCTTTTATTATAAAAAAGATTTTTTGAGTAGTAATTCTTATATATAATCATCCGGAGGTGGTTGGTAGCCTG
GCTCAATCATTGAGGCATATTTTTGCAGGCAATATATTGAATCTGAAAAGTTAAAGATGATATTTTCGGTGCA
GGAGCTATCATGTGGAGGGAAAAGTATGCTAAGTCCTACGACTCGTAATATGGGGGCGAGTTTATCGCCTCAG
CCTGACGTCAGCGGGGAGCTAAACACCGAAGCATTGACCTGTATTGTTGAGCGTCTGGAAAGTGAAATTATAG
ATGGCAGCTGGATTCATATCAGTTACGAGGAAACCGATCTCGAAATGATGCCTTTTCTTGTTGCACAGGCCAA
TAAGAAGTATCCAGAGTTAAATCTTAAATTTGTTATGTCAGTCCATGAGCTTGTTTCCTCTATAAAGGAGACC
AGAATGGAAGGCGTTGAATCTGCCCGATTTCTCGTAAATATGGGAAGTTCAGGTATCCATATTTCAGTCGTCG
ATTTTAGAGTTATGGACGGAAAGACATCGGTGATTTTGTTCGAACCAGCAGCGTGTAGCGCTTTTGGACCTGC
TTTACTGGCGTTGAGGACCAAAGCAGCTCTTGAACGTGAACAACTGCCTGATTGTTATTTTGCTATGGTCGAG
CTGGACATTCAACGAAGCTCTTCTGAATGCGGTATTTTTAGCCTGGCGCTCGCCAAAAAACTTCAGCTTGAAT
TTATGAACTTAGTAAAAATTCATGAAGATAATATTTGTGAACGTCTGTGTGGTGAAGAACCTTTTCTCCCGTC
CGATAAAGCAGACCGTTATCTGCCGGTGAGTTTTTACAAACATACTCAAGGCGTACAACGATTAAATGAATAT
GTGGAGGCCAATCCGGCGGCGGGAAGCAGTATAGTAAACAAAAAGAATGAAACGCTTTATGAGCGATTCGATA
ACAATGCCGTTATGCTAAACGATAAAAAACTCTCTATATCCGCTCATAAAAAAAGGATAGCTGAATATAAGTC
TTTACTTAAATCGTAATGAAATTATAAGGTTGCCTGCCAGAGGAAATCCTTCCAGCGCCATAAGACTACTCTA
TATTATGACCGTATGTTTCCTGAATAGCAGGCAAAAAAAGTGTTAACCTTGCGCCTCTGGTACGATTTCATTA
CGCCTGTCGCGCCATGTTGCGTAAATAAATACCGCAATAAACACGATGGCAAATAATACCACGATGGTCGGCG
CTGGCGCGCTATCAAGATAAAAAGACAGATAGACGCCCGCAAAAGCGGTAATAACTGACAGACTCACCGCTAA
TCCCAGCGCGCGCGCAAAGCGTCGCGTCAGCAAAATGGCAATTGCGCCGGGGGCGATAAGCAATGAAATCGAC
AGAATAATACCCACCGATTTCAGCGTGGCGACGATAGTCAGCGCAATCATACATAGCAGGCCATAATGCAGTA
GCGTGATATTCAGCCCGCTGGCTTTCGCCTGGTGCGGGTCAAAAGCGTGTAGCAGGAGATCTTTCCATTTCAG
CCCAATGATTAACGCAATACCCAGTGCAATAACTGACGTTTGTACGATATCCCCCAGCGATACGCCCAGCATA
TCCCCAAACAGGATGTGATCCAGATGCACTTCCGATTGAATAGAAACATAGAGCACCAGACCTGCGCCAAACA
TCCCGGAAAAGACGATGCCCATCACCGTATCACGTTTGATGCGGCTGTTATCGTCCAGATAGCCGGTCGCGAT
AGCGCAAAATAATCCGGCAATGAAAGCGCCAATCGCCAGGGGATCCCCACAATATATGCCAGCACGACCCCGG

GGAATACCGCATGACTCATTGCATCGCCCATTAACGCCCAGCCTTTTAACACTAAAAACACGGACAGTAAGGC
GCAGGGAATCGCCACAATGACCGAAACCATAAGGGCGTTCACCATAAAATCAAACTGAAAGGGTTCCAGTAAC
GTTGTTAAGAACATGTCGGCTCCTTCTGCAGGCGGGCGCGTCGACGGTTGGCTAACAGACCGTGTTTCGGGGC
GAAAATAAAGGCTGTGATGAACAGTAAGGTTTGCATGACGACAATAATTCCACCCGTGGCGCCATCCAGCCAG
TAGCTAAGCCATGCGCCCAGAAAACTGGTCAGGCTGCCAATCACGACGGCAATCATGAGCAGACGTGGAAAAC
GGTCGGTGAGTAACCAGGCCGTCGCGCCCGGCGTGACGACGAGACAGATCACCAGGAACGCCCCAACGGTTTG
CAGGGCCGCCACGGTAGAGACGGACAATAGAGTGAAAAACAACAGCTTTAGTCGACCCGGATTAAGCCCGATA
GAACGCGCATGGGTTTCATCGAAAAAGACGACCATCAGATCTTTCCACTTTAACAGCAGAATCGTCAGCGAGA
CTGCGCCGATAATCGCCAGTTGCGCAATATCCGCTGGCGCGATCGCCAGCACGTTGCCGAGAATAATCGTCTG
GATATTCACCGACATCGGATTGAGCGACACCATAAAAAGGCCGACGCCAAAAAAAGAGGAGAAGATAAGCCCG
ATAATCGCGTCTTCTTTCAGGCGTGAGCGTTGGTTAAGAAAGAGCATACTGCCTGCCGCCAGTCCGCCGGAAA
GAAACGCGCCGAGCGAGAAGGGCAGCCCTAACATCCAGGCGCCAGCGACGCCAGGCACAATAGAGTGAGATAG
CGCATCGCCAATGAGCGACCAGCCTTTGAGCATCAAATAGCAGGAGAGAAACGCGCACAGACCGCCCACCATC
GCGGAGACCCACATCGCGTTGAGCATATACTGGTAGCCAAACGGCTCAACAAGCCAGTTCATGACGATTTTCC
TCCGCTGGCCACGCGCCGGGAAATAAAGGGGCGCTCGTCATCGGTGATGATGTGCTCTTCACCTCCGCTTAGC
GCGATATGACGTAGAACGCCGCTAAACGCCTGTTCCAGATTCGCGGCGGTAAACGTGGTTTCGGTAGGGCCGC
TGGCCAGTACGGTTCCTTTAATCATTACCGTATAGTCGCAAAACTCGGTGACGGAGCCGAGATTATGCGTTGA
GACCAGCATCGTGCGGCCTTCATCGCGCAGTTCCCGTAGTAGATCGATGATTCGCGCTTCGGTTTTTACATCC
ACGCCGGTAAAAGGTTCATCCAGCAGGATTACCTGTCCATCCTGAGCGATGGCCCTGGCAAGAAACACACGTT
TTTTCTGCCCGCCGGACAGCTCGCCAATCTGCCTGTGACGATATTCCTGCATATCCACCCGCGCCAGCGCGGC
GTCAACACAGGCGTGATCATGCGCAGTGGGACGGCGTAGCCAGCCCATATGGCCGTAGCGTCCCATCATCACC
ACATCTTCTACCAGCACCGGAAATGACCAGTCCACTTCTTCCGATTGCGGAACATAAGCAATGAGGTTTTTTT
TCAGCGCCTTGTTGACTGACTGTTGCAGGATAGTGATATCTCCCTGCGCAAGATGCACAAAGCCCATTAACGC
TTTAAATAACGTAGACTTACCGGAACCATTTACGCCTACCAGCGCGGCGATTGAACCGCCAGGCACCTGAAAA
GTGGCGTCCCGTAGGGCGGTATGACCGTTGCGATACGTCACCGTGACTTGATCAACGGTAATCGCAGATTGAC
TCATTGTTGACTCCTCAGTCCGTCGTTAATGCCGTTGACGATGGTTTCGGTCGTGACGCGCAGCAGATCCAGA
TAGGTTGGCACAGGGCCGTCAGCGGCGCTCAGAGAATCGACATACAGTACGCCGCCATAATGCGCGCCGGATT
CACGCGCGACCTGGCGGGCGGGTTTATCGGAAACCGTACTCTCGCTAAAGATGGCGGGAATATGGTGCTTTTT
AATGGTATCAATCACTTTACGCACCTGTTTTGGCGTACCCTGTTGATCGGCGTTAATTGGCCAGAGATAAAGC
TCTTTCATGTCGTTATCGCGCGCCAGGTAAGAGAACGCGCCTTCACTGGTGACCAGCCAGCGCTGATCGGCGG
GAATTTTTTCCAGTTCGGCACGCAACGGATCGGCCATCTGGCGAATTTTCGCTTTATAGCGTTCGGCATTTTG
CTTATAGATCTGCGCATTATCCGGATCGTACTTCACCAGGGCGTCGCGAATGTTATCGACATAAATCAGCGCG
TTTTCTGCCGACATCCAGGCGTGCGGGTTCGGTTTACCGTTATACGGGCCTTCGGTAATGCCCATCGGTTTGA
CGCCGGTGGAGACGATGACTTCCGGTACGCCGGAAAGGTGCTGATAAAAGCGGGCGAACCATCGCTCCAGGTT
CAGACCATTCGCGAGGATAAGCTGCGCCCCCTGCGCTCGTTTAATATCGCCGGGCGTTGGCTGATACTCATGG
ATTTCAGCGCCGGGCTTGGTAATTGAGCTGACTTCCGCTGCGTCGCCCGCCACGTTTTTCGCCATGTCGGCAA
TAACGGTAAACGTGGTGATGACTTTAAATTTCTCTTTTGCATAGGCTGGCGATAGCGCGAGTATCGCGACAAT
ACCGGCAATCAGGAGTGTTTTCAGACGATGTAGATTCGTCATAGTATCCCTCGCAACAATGTGGTTAATTAAT
CATCGAATATAGCCTTTGCTATGTTATATAGCACAAGGTATGAATAAATGAAAATAATTCTTATTTGCGAAAT
GGCGACGATATGCAGTGATAGTGCTAAAGAGGGATAAGGTGTAAAATAAATGCACCTTAATGTTCGGAGAGAG
CACGATGCCTGGTAAACGTATCGCTCGTGAAAAACTGACGATCAAAAAAATGATCGCGCTGTATGAAAGCCAG
TGTCCACAGGCGTCAGCGGTGCAGGGGCATTACGACGCGCTGTACGCCTACGCGCAAAAACGTCTTGATAAAT
GCGTATTTGGCGAGGAAAAACCCGCCTGTAAGCAGTGCCCGGTGCACTGCTATCAGCCGGCTAAACGCGAGGA
GATGAAGCAGATTATGCGTTGGGCAGGTCCGCGAATGCTTTGGCGACACCCTGTCCTGACCGTGCGGCACCTT
ATCGACGATAAGCGCCCCGTTCCGGAATTGCCGGAAAAATATCAGCGCAAGAAGTGAAGGGGAAGCGCCAGGC
CTACGGCGCGCATCATCTCCAGGCCGGATCAGATCCGGCAACCCGTTACGCTAACGCGTCTTTATCAATACCC
AATCGCTTCATACGCGACAGCAGGGTGGTTCGTTTAAGCCCCAGACGCTGCGCCGCGCCTTTTGGTCCAGCCA
CTACGCCATTGGTCTCTTTCAGCACGCGTATAATAAGCTGATATTCATCTTCGCCTTCTTTGGCGCTTTCAGT
CGCAACAGGCGAGGTATCGGGCGTTACGGCGGTAATATCCGGCAGCGACAGTTGTAGCACATTGCCGCGCGTC
AGCAGCACGGCGCGCTCGACGACATTCTCCAGCTCGCGAACGTTCCCCGGCCATTCCATGCTGCTGAGCGTGC
GCAGCGTTTCGGCGGGAATGCTGTCGATATTACGCCCCATTCGGCGGGCGATTTTGAAAGTGAACGCTTTGAC
CAGTAGTGGAATATCTTCCGGACGTTCGCGCAACGGAGGAAGCTGGATCGGAAAAACATTCAGCCGATAGTAG
AGATCGTTACGGAATTCGCGATCGGCGACCATTTTTTTCAGATCGCGGTTAGTCGCGGCGATCAGCCGCACGT
CGGTCTGGATCAGTTTATTGCTGCCGAGACGTTCAAACTCTTGCTCCTGAAGGACGCGCAGCAGCTTAGGCTG

TAGCTCCAGCGGCATGTCGCCCACTTCATCCAGAAACAGCGAGCTTTTATCCGCCAGCTCGAAACGGCCAATC
CGCTGCGCGCTGGCGCCGGTAAACGCGCCGCGCTCATGACCAAACAAATCGCTTTCCAGCAGCCCCGCCGGCA
TCGCGGCGCAGTTCATCTTTACCATCCTGCGCCCGCTACGTCCGCTCAGGTTGTGAATGGCGCGGGCAATCAA
CTCTTTACCGGTTCCCGTTTCGCCCAGAATCAGCACGGTACTGTCGCTCTGCGCCACCATCTCAACCTGCTTT
AGCACGTTGTACATCGCTTCGCTGCGCCCGATGATCTCGCCAAACTCGCTGTCGACGTTATTAAGCTGCTCCG
TGAGCGCCAGGTTTTCGTCGACCAGGCGTTCCTTCAGTCGGTGGATTTCCTGATAGGCCAGCGCATTATCCAC
GGCAATGGCTACTCGTTCGGCAATCTGACGCAGCAGCTTGAGGTTGGCGGTGGTAAACACTTTTTCTTCGCAC
TGGGCCAGTTTGAGCACGCCAAGCATCGTCTTGCCGGACATCAACGGCAGCAGGCAAAGCGTCTGGATCTGAT
TGCCCCAGGTATCGAACAGCATGCGTTCGTAGGGAGCAAGCGGATCGCGTTCGTTGAGGTTGATAAGCAGCAT
CTCTTTACTTTTAAAGACGCGCTCCGTGAGCGTGCCGGCTTCGTCGACTTCGCTCTGTTCGTGCGCCGGATGA
TGCTCATCAAGGTAGTGGGTGGAGTAGATATTCAGCTTATTTTTGCGATGGCTGCGCAACACGATGCTGATTG
CGTCGATATTGAAATAGTGGTGGATCTCTTTTGCGACTTCGCTTACCAGCTCATCAATATCCAGACGCGACAG
GACGGCGTTGGTGATCGCAACCAGAATACGGAAGTTATCGCGTTCGCGGCACAACAGATCATAATCGACATTA
TTGTTTACCCGGTTTTGAATTTGCTCGGCGACCACGCCGACAATTTGGGTAAACGTATGCAGACGGTCATATT
CCTTTTCACTCCAGGGGCGATCTTCCTGACGGATGAATTCGCAGCCGCCGAAAATACGGCCCTCCGCGGCTAA
AGGAAGCAGACAGTAATGACCAAATTCCGGGTAGAGGCCGCTCGCGGCAAGCTGCGGCCAGGTTTCGGTAAAC
TCATGGAAATTACAGTGCAATGCATCGGGGCGAGAAAGGATGCGACGTACCGGGCCGTGCGCCAGTACGGTTT
CATCTTCATATTCAACAGGACGGCCATTTTCTCGCGTCGCATAATATTGTGCGCGTTGCGACAGCGCCTGCCA
TAGCACAATGCCGGCGCTGTCGGCGAGCGCTGAACGCTTTACCAGTTGCGAAAGCGCTTCACTGAGCGACGCC
AAATCGGGCTGCTGCAATAAGGTACGAGTGATATCGAACAGGCCTTGCTGTCCAAGATCGCTCATGGGTGTAT
ACGACATGTTGCTTTTCCAGGAAAATACCGCAAAACGGTGCGAAAACGGTAAATGAATTATAGGTATAAGTCG
TAAGTTATTGCCGGATGGCTGACCGCAAACTCGCTGGTCACACACTCCGTAGGCCTGATAAGCAAAGCGCCAT
CAGGCGTTCATGCAGCGTTAATGCCGGATGTCGACGCAAATGCGTCTTATCCGGCCTACAGTATGCTGCCAAC
AGGCTGCTAGCATATACGAGGTAATGGTTCGGCGTGTGGCAAATCAAGGGTTCGCTTCACGCCATAGAGTCCG
GCTAAGCGAACGCCTTTGCGTTCCACGACTTCGCCAATCAGCGCTGCATCACGTCCCAGCGGATGCGCGCGTA
ATGCCGCCAACGCCCGATCTGCGGCCTGCCGTTCAACGGCAATCACCAGTTTTCCTTCGTTGGCAAAGTTCAG
GGCATCCAGCCCCAACAGCTCGCAGACGCCGCGCACGGCAGGTTTGAGCGGCAGTGCGGATTCGGACAATTCA
ATACCGTAACCGCAGGACGTCGCAAACTCATGGGCGACCGCATTCACGCCGCCGCGCGTGGCGTCACGCAATG
CCTTCACTCCGTCAATATGACGCAGCGTCTGAATAAGCGGCGTTAATACTGCGCAGTCGCTCGCCAGCTCGCC
ATCCAGTCCCAGTTGCTCACGCAGGTTGAGGATAGTGGCACCGTGATCGCCAAGCGTACCGCTGACTAACAGC
ACATCGCCAACGCTCAGCGTTTGCGCGCCCCAGCGAATATCGGCGGGAATCGCCCCCATGCCGGCAGTGTTGA
TAAATAATTTATCCGCCGCGCCGCGCTGCACAACTTTGGTGTCGCCGGTTACAATGGCGATATCCGCTTCCCG
CGCGGTCGCCGCCATGCTATTGACCACGCTTTTTAACGTCTCCATCGGCAGCCCTTCTTCAAGGATAAAGCCG
CAGGAGAGATAGCGGGGGATCGCGCCGCTCACCGCAACGTCGTTCGCCGTACCGCAAATCGCCAGTTTGCCGA
TATTGCCGCCGGGGAAAAAGAGCGGATCGATCACATAGCTATCGGTAGAAAACGCCAGACGGTCGCCCTCCGC
CGTCAGTTGCGCCAGCTCCAGACGCGCCTGATCTTCTTGTTCCGCCAGCCACGGGTTGGCAAAGGCCTCCATA
AACAGGCTATTAATCAACTGTTGCATAGCCTGGCCGCCGCTGCCGTGGGCGAGTTGTATATTGTTCAAACTTC
ACACTCCTGCTGACGATACTGATACCGGGCGGCGCATGCGCCTTCGGAAGAGACCATCAGCGCGCCGAACGCG
GTCTCTGGATTACATGTTTTACCAAATAGCGGGCACTGATGCGGCTTGCATCTGCCGGTCAGCACTTCGCCGC
AGCGGGCGCGCGGATCGTCATAGACCTGTTGTGGCGCTGGGCGGAAATGCGCTTCGGCGTCAAAGCGCTGATA
CTCTGGCGTCAGGTGGACGCCGGAGGATTCAATCACGCCCAGACCGCGCCACTCGCTGTCGCCATTAACGCAG
AACACATCGGCGATGGCCTGCTGCGCCAGCATGTTTCCGGCATCCGGCACCACGCGACGGTATTGGTTTTCAA
CCTGGCTTAGGGCCGCTATTTTCTGCTCAACCAGCATGACCACGCCTTGCAGTAGATCAAGCGGTTCGAATCC
AGCCACCACCAGCGGGCGATTAAAATCGGCGGCGATAAACTGGTACGCCTCGGTGCCGATGACCATGCTGACG
TGGCCTGGCGCAAGAAAGGCGTCAATGCCGTTGTCCGGCTGCTCCAGTAGGCTACGCAGCGTCGGGATCAGCG
TAATGTGCTGGCAGAAAAAGTAGAAGTTTCGCACGTCACGCTGTTTTGCCTGCTGCAGCGTGATGGCGGTGGT
CGGCATGGTGGTTTCAAAGCCCAGCCCGAAAAATACCACTTTGCGGGTCGGGTTATCCTGCGCCAGCTTCAGC
GCGTCCATCGGAGAGTAAACAATACGGACATCCGCGCCGCGCGCTTTAGCCTGCAACAGAGAACCCTGTTTCC
CCGGCACGCGCATGGCATCGCCAAAGGTGCAGAAAATCACCTCCGGATGGCTGGCGATTTCCACGCAACTGTC
GATGCGTCCCATCGGCAGCACGCAGACCGGACAACCGGGGCCGTGAATAAATTCCACGTTTTCGGGCAGTAAC
TGATCGAGACCAAATTTAAAAATTGCGTGGGTATGACCCCCGCAGACTTCCATAATGCGCAACGGACGTTCGG
CAGTGTAAGGAAGAAGGGCGGCGCGTTCACGCAGGTGTTCAATGAGCTGCATTACCTGTTCCGGCGCACGGTA
CTCGTCAACAAAACGCATTATCTCTCCTCACCATACAGCAGTGCGCCGACGTCCGGCTCAACATCAAACATAT
TTTGTAGCGCGTCGAGCGTGTCGCGCGCTTCCGCTTCGTTAATGACGCTCATGGCGAATCCGACATGAACCAG

CACCCACTGACCCAGACGCGGCTGACCGTTTTCATCACAACTGCCGACTAATGTGAGATCGACGTCGCGCTGA
ATGCCGCAGACGTCAACTTTTGCCTGATTGCCGTCAATGGCGCGGATTTGACCCGGCACGCCTATGCACATCG
CTGCGCCTCCAGCCAGGCCAGCCACTGATCCATGCCTTCGCCGCTGGTGGCGGAAATCAGGATAATTTCAATC
TCCGGGTTCACTTCACGCGCGCTGGCGATGCACTTCTCAACGTCAAAATTGAGGTAGGGCAGCAGATCGACTT
TATTGAGCAGCATGAGCGACGCAGCGGCAAACATATGCGGATATTTCAGCGGCTTATCTTCACCTTCGGTCAC
GGAAAGCACTGCGACTTTATGCTTTTCGCCCAGATCGAAGCTGGCCGGGCAGACGAGATTACCGACGTTTTCG
ATAAACAGAATACCGTGGTCATCCAGCGGCAGGCGCGGCGCGGCGTCGGCGATCATTTGCGCGTCAAGATGGC
AGCCTTTACCGGTGTTGACCTGAATGGCGGGCGTGCCGGTAGCGCGGATGCGGGCGGCGTCGTTCACTGTCTG
CTGGTCGCCTTCAATCACCGCGCACGGTACACGGTCTTTCAATTTCATCAGCGTTTCGGTCAGCAGGGTGGTT
TTACCGGACCCGGGGCTGGACACCAGGTTGAGCACCAGTTGCTGGCGGGCGGCGAAACGGGCGCGGTTGCGCT
CAGCCAGACGATTATTTTTGTCCAGCACGTCGATTTCAACCTCAAGCATCCGCCGCTGACTCATACCAGGCGC
GTGGGTTCCGGCTTCGCCGTGGCCGTAATGCAGATCGCCTTCTGCGGTCTGACTTGGGGCAAAGTCAGGCGTT
TTAATGCCGGTAATATTCAGCGCCGGGCGGGCCGCTGGGGCAAAGGGCGCGCTACGAAACGCTGAGTGCGGAT
TATGTTCATCGCCTTCTATATAAAGGTTGCCTTCAGCGCAACCGCAAGTCGTACACATCGCTTACTCCTGATC
TTTTTCTTGTTACAGGCTTTCTGCCGGATGGCGACGCGAGCGTCTATCCGGCATTAATCTTCAGTTTCATCAA
TCTCTATGCGCCGAATTTGTAAGCCGTCATCGGCCACAATTCGCAGCGTATCGCTATGACATTGCGGACAACG
ACGTACACGGTGGGTTAACAACGTGACATATTGTTGACAATGCTCACACCAGCATTCGGCTTCCTGTTCTTCG
AGGTGCAGTTTACAACCTTCTGCTATCGTGCCGCGGCATACCAGATCAAAACAAAAGGAAAGAGCGCTGGTTT
CCACGCAAGAAAATGCGCCAATTTTGATCCAGACGGCTGTCACACGTTTTGCACCGTATGCTGAAGCCTGTTG
TTCAATCAATTCCAGTGCCCGTTGGCAGAGGGTGATTTCGTGCATATCGCCTCCCGTAGTGGTTGCCCCTGTG
AAGCAATATTTATGCCAATATTTTTATCCCCTGGCAGGGTAATGTCGACGATGACGAAATGACATGTCGTCAC
CGCGTCGAAACGGCAGTAACTGCGCTCGTCTGCTATTAAAAACAATAAGTTAAAAATTGGCATGGAAAATGCT
TAACGCAGGCATCTCTGTTAAACGGGTAACCTGACATGACTATTTGGGAAATAAGCGAAAAGGCCGATTACAT
CGCGCAACGGCATCGTCGCCTACAGGACCAGTGGCATATTTACTGCAACTCGCTGGTTCAGGGGATCACCCTC
TCGAAAGCGCGCCTGCATCACGCAATGAGCTGCGCGCCGGAAAGGGATCTTTGCTTTGTCCTGTTTGAGCACT
TTCGTATTTACGTCGCGTTGGCGGATGGTTTTAACAGCCACACCATTGAGTACTACGTTGAAACCAAAGATGG
CGAAGATAAGCAATTGATTGCACAGGCGCAGCTCGATATCGACGGCAAGGTTGATGAACGGGTTAACAACCGC
GATCGCGAGCAGGTGCTGGAGCACTATCTGGAAAAAATCGCCAGCGTTTACGACAGCCTCTATACCGCTGTAG
AAACCAACTCGCCGGTTAATTTGCGTCAATTGGTAAAGGGAAACAGCCCGGCGGTATAAGCCTGAGGTGTTGC
CCGCTTTGATTCGCGATGTGGCGTTGCGCCACGTCATGAATGTTTTGGGGATGGGCGTGTCGATAAGTGTCGA
AAATGACATTTCAACGGCATGTTTTCGTCAAAAATGACAATCACCTGAGGAATGCCTGGTGAATCGTTTTGTA
ATTGCTGACTCCACTCTCTGTATCGGCTGCCACACCTGTGAGGCCGCCTGTTCAGAGACGCATCGCCAGCATG
GCCTGCAGTCAATGCCGCGCCTGAAAGTGATGCTGAATGAAAAAGAATCTGCGCCGCAGCTTTGTCACCACTG
TGAAGATGCGCCATGCGCCACGGTATGCCCGGTTAACGCGATTAATCGCGTTGACGGCGCTGTCCAGCTTAAT
GAAAGCCTGTGCGTGAGCTGCAAACTGTGTGGTATTGCCTGTCCGTTTGGCGCTATTGAGTTTTCCGGCAGCC
GTCCGCTGCATATTCCGGCGAACGCCAATACGCCGAAAGCGCCGCCCGCGCCGCCAGCGCCTGCGCGCGTCAG
TACGTTGCTCGACTGGGTGCCGGGCGTACGCGCTATCGCGGTGAAATGCGACCTGTGCAGCTTTGACGAGCAA
GGCCCGGCCTGTGTGCGGATGTGCCCGACTAAAGCGCTACACCTGGTGGAGAACACGGATATCGCCCGCGCCA
GCAAACGTAAACGTGAGCTGACGTTTAATACCGATTTTGGCGATCTCACCTTGTTTCAGCAGGCTCAGAGTGG
GGATGCATAATGAGTTCACTTTCACTGATTACTAGCGGCGTCGCGTGGTTTGCCGCCGCCGCCGTTCTGGCGT
TTCTGTTTTCGTTTCATAAAGCGCTGAGCGGCTGGATTGCCGGTATCGGCGGCGCGGTCGGCAGCCTGTGTAC
GGCTGGCGCGGGGTTCACCGCACTGACCAGCGCCGTTACGGTGAGCGGCGTGATGCCGTTTACCGGTCACATG
CTGCAAATAACGCCGCTGAATGCTATCTGGCTGATTACGCTGGGGCTGTGCGGCCTGTTCGTCAGCCTGTTCA
ATATCGACTGGCATCGCCATCCACAGGTAAAAGCCAACGGACTGTTGATCAACCTGCTGATGGCGGCAGCCGT
CTGCGCCGTGGTCGCCAGTAACCTGGGGACGATGGTGGTGATGGCGGAAATCATGGCGCTCTGCGCCGTGTTC
CTCACCGGCGGTAGCAAAGAGGGCAAACTGTGGTTTGCGCTGGGCCGTCTCGGTACGCTGCTGCTGGCAATCG
CCTGCTGGCTGGTGTGGCAGCGCTACGGCACGCTGGATCTGGGGCTGCTGGATCAACGCGCTCAGCAACTGCC
GCTGGGCTCCGATATCTGGCTGCTCGGCGTTATCGGCTTTGGTCTGCTGGCCGGGATTATCCCGCTACACGGT
TGGGTGCCGCAGGCGCACGCCAACGCCAGCGCTCCCGCCGCCGCGCTGTTCTCGACCGTGGTGATGAAAATCG
GGCTGCTGGGCATTCTCACGCTATCTCTGCTTGGCGGCAATGCGCCGCTGTGGTGGGGCGTGGCGCTGTTGGT
GCTTGGGATGATCACCGCCTTTGTCGGCGGTCTGTACGCGCTGATGGAGCATAACATTCAGCGCCTGCTGGCC
TATCACACGCTGGAAAATATCGGCATCATTTTGCTCGGTCTGGGCGCAGGCGTTACCGGGATTGCTCTCAATC
AGCCGGTACTGATCGCGCTGGGTCTGACCGGCGGGCTCTATCACCTGCTGAACCATAGCCTGTTTAAAAGCGT
GCTGTTTCTGGGCGCGGGCAGCATCTGGTTCCGTACCGGTCATCGCGATATCGAAAAACTGGGCGGTATTGGT

AAACGGATGCCGGTTATTTCTATCGCCATGCTGGTCGGTCTGATGGCGATGGCTGCGTTGCCGCCGCTGAACG
GCTTTGCCGGCGAGTGGGTGATTTACCAGTCCTTCTTCAAACTGGGCAACAGCGGCGCGTTTGTTGGTCGTCT
GTTAGGACCGCTGCTGGCGGTTGGCCTGGCGATTACCGGCGCGCTGGCGGTGATGTGTATGGCGAAAGTTTAC
GGCGTGACGTTCCTTGGCGCGCCGCGCACGAAGGAAGCTGAAAACGCCAGCTGCGCGCCTATCCTGATGGGCG
TCAGCGTGGTTGCGCTGGCTATCTGCTGTGTGATTGGTGGGGTTGCCGCGCCGTGGCTGCTGCCGATGATTTC
CACTGCGGTACCGTTGCCGCTGGAGACCGCGCACACCACCGTATCGCAGCCGATGATCACGCTGCTGTTGGTC
GCCTGTCCGCTACTGCCGTTCATCATTATGGCGATGTTCAAAGGCAACCGTCTGCCGTCGCGTTCTCGCGGCG
CGGCCTGGGTGTGCGGCTACGACCATGAGCAGTCGATGGTGATTACCGCGTACGGTTTCGCCATGCCGGTGAA
AGAGGCCTTTGCCCCGGTACTCAAATTGCGTAAATGGCTGAACCCGGTATCGCTGGTGCCGGGCTGGCAGAAC
GCGGCGGCCGCGGTGCTGTTCCGTCGTCTGGCGCTGATTGAGCTGGCGGTGCTGGTGGTGATTGTGGTTTCAC
GAGGAGCCTGAAAATGAGTGTGTTTTATCCGTTAATTCAGGCGCTGGTGCTATTTGCCGTTGCGCCATTGCTG
TCCGGTATTACTCGCGTGGCGCGCGCCCGTCTACACAATCGCCGTGGGCCGGGCGTGTTGCAGGAGTACCGCG
ACATTATCAAACTGCTCGGTCGTCAGAGTATTGCGCCCTCTGATTCCGGCTGGGTTTTTCGCCTGACGCCGTT
TGTGATGGTCGGCGTGATGCTGACTATCGCCACCGCGCTGCCGGTCGTCACCGTTGGATCTCCGTTGCCGCAA
CTGGGTGATTTAATCACCCTGATTTACCTGTTCGCCATCGCCCGTTTCTTCTTCTCTATTGCAGGCCTGGATA
CCGGCAGTCCGTTCACCGCGATCGGCGCCAGCCGTGAAGCGATGCTCGGCGTGCTGGTGGAGCCGATTCTGCT
GCTGGGTCTGTGGGTCGCCGCGCAGGTGGCGGGTTCAACGCATATCAGCAATATCGCCGATACCATTTATCAC
TGGCCTGTCGCGCGCAGTATTCCGCTGATTCTGGCGCTGTGCGCCTGCGCCTTCGCCACCTTTATCGAAATGG
GCAAGCTGCCGTTCGACCTGGCGGAAGCGGAGCAGGAGTTGCAGGAAGGGCCGTTGACCGAATACAGCGGTAG
CGGCTTTGCGGTGCTGAAATGGGGCATCAGCCTTAAGCAACTGGTGGTGCTGCAAATGTTCGTCGGCGTGTTT
CTGCCGTGGGGACAGATGGAAACCTTCAGCGCAGGCGGACTGTTGCTGGCGCTGGTGATTGCCATCGTCAAGC
TGATTGTCGGCGTACTGGTGATCGCCCTGTTTGAAAACAGCATGGCGCGACTGCGTTTTTGCGCCACTTCACG
CGTGACCTGGGCCGGTTTTGGGTTTGCGTTTTTAGCCTTCGTCTCCTTGCTGGCGGCGTGATTTAAGAGAGTT
TGAGCATGTCTGAAGAAAAATTAGGTCAACAATACCTTGCGGCGCTGCACCAGGCGTTTCCGGGCGTCGTGCT
GGACGAAGCCTGGCAGACCAAAGATCAGCTGACTATTACGGTGAAAGTAAACTATCTGCCGGAAGTGGTGGAG
TTTCTTTACTACCAGCAGGGTGGGTGGCTGTCGGTACTGTTCGGTAATGACGAACGCCAGTTGTGCGGCCACT
ATGCCGTTTATTACGTGCTGTCGATGGAGCAGGGCACGAAGTGCTGGATTACCGTCCGCGTTGAAGTGGATGC
CAATAAGCTGGAATTCCCATCCGTTACGCCGCGCGTGCCGGCTGCCGTGTGGGGTGAGCGCGAAGTACGCGAC
ATGTACGGTTTAATCCCGGTCGGTCTGCCGGACGAGCGCCGTCTGGTGCTGCCGGACGACTGGCCGGATGAAC
TCTATCCGCTGCGTAAAGACAGTATGGATTATCGTCAGCGCCCGGCGCCGACCACCGATGCGGAAACCTACGA
GTTCATTAACGAGCTGGGTGACAAGAAAAATAACGTGGTGCCGATTGGCCCGCTGCATGTTACTTCCGATGAA
CCGGGCCACTTCCGTCTGTTCGTCGATGGCGAAAACATTATCGACGCCGACTACCGCCTGTTCTACGTCCACC
GTGGTATGGAAAAACTGGCGGAAACCCGCATGGGTTATAACGAAGTCACCTTCCTGTCGGATCGCGTGTGTGG
TATCTGCGGCTTCGCCCACAGCACCGCCTACACCACTTCCGTGGAAAACGCGATGGGCATTCAGGTGCCGGAG
CGTGCGCAAATGATCCGCGCTATTCTGCTGGAAGTGGAACGCCTGCACTCGCATCTGCTCAACCTTGGCCTGG
CCTGCCACTTTACCGGCTTTGACTCCGGCTTTATGCAGTTCTTCCGCGTGCGTGAAACCTCCATGAAGATGGC
GGAAATACTGACCGGGGCGCGCAAAACTTACGGTCTGAACCTGATCGGCGGGATTCGCCGCGATCTGCTCAAA
GAGGACATGATCCAGACCCGTCAACTGGCGCAGCAGATGCGTCGTGACGTGCAGGAGCTGGTGGACATGCTGC
TGAGCACGCCGAATATGGAACAGCGTACCGTGGGTATCGGTCGTCTGGACCCGGAAATTGCCCGCGACTTCAG
TAATGTCGGCCCGATGGTGCGTGCCAGCGGTCACGCCCGCGACACCCGCGCCGACCACCCGTTTGTCGGCTAC
GGTCTGCTGCCGATGGAAGTACATAGCGAGCAGGGTTGCGACGTGATTTCTCGTCTGAAAGTCCGTATCAACG
AAGTCTACACCTCGCTGAATATGATCGATTTCGGTCTGGATAATCTGCCGGGCGGCCCGCTGATGGTGGAAGG
CTTTACCTATATTCCGCACCGTTTTGCGCTCGGCTTCGCTGAAGCGCCGCGTGGTGACGATATCCACTGGAGC
ATGACTGGCGACAACCAGAAGCTGTACCGCTGGCGCTGCCGTGCGGCGACCTACGCCAACTGGCCAACGCTGC
GCTATATGCTGCGCGGCAACACCGTTTCTGATGCGCCGCTGATCATCGGCAGCCTCGACCCGTGCTACTCCTG
TACCGACCGGATGACCGTGGTCGATGTGCGTAAGAAGAAGAGCAAAGTCGTGCCGTACAAAGAACTTGAGCGC
TACAGCATTGAGCGTAAAAACTCGCCGCTGAAATAAGGAATCGCCATGTTTACCTTTATCAAAAAAGTTATCA
AAACCGGCACGGCGACCTCGTCTTATCCGCTGGAGCCGATTGCGGTTGATAAAAACTTCCGCGGCAAGCCGGA
ACATAATCCGCAGCAGTGCATCGGTTGCGCCGCCTGCGTTAATGCCTGCCCGTCGAACGCGCTGACGGTGGAA
ACCGATCTTGCGACCAATGAGCTGGCGTGGCAGTTCAATCTTGGCCGCTGCATCTTCTGCGGTCGCTGCGAAG
AAGTTTGCCCGACGGCGGCGATCAAACTGTCGCAAGAATACGAACTGGCAGTGTGGAAGAAAGAAGACTTCCT
GCAACAGTCCCGCTTCGCGCTGTGCAACTGCCGCGTATGTAATCGTCCTTTCGCGGTACAGAAAGAGATCGAC
TACGCCATTGCGCTGCTTGCGCACAACGGCGACAGCCGCGCGGAAAACCACCGCGAAAGCTTTGAGACCTGCC
CGGACTGTAAGCGTCAGAAATGCCTGGTGCCGTCCGACCGTATTGAACTGACTCGCCATATGAAAGAGGTCAG

CTAATGAGCAATTTATTAGGCCCGCGTGACGCCAACGGCATTCCGGCACCGATGACGGTGGATGAATCCATCG
CCAGCATGAAGGCGTCTCTACTGAAAAATATTAAGCGTTCGGCATACGTTTACCGCGTGGACTGCGGCGGCTG
TAACGGCTGTGAAATCGAAATCTTTGCGACGCTTTCGCCGCTGTTTGATGCCGAGCGTTTCGGCATCAAAGTG
GTACCGTCGCCGCGTCATGCCGATATTCTGCTGTTTACCGGCGCGGTGACCCGCGCCATGCGCTCTCCGGCGC
TGCGCGCCTGGCAGTCGGCGCCGGACCCGAAAATCTGTATCTCTTATGGGGCCTGCGGCAACAGCGGCGGCAT
CTTCCATGATCTGTACTGCGTCTGGGGCGGTACGGATAAAATCGTACCGGTGGATGTGTATATTCCAGGCTGC
CCGCCAACGCCTGCGGCGACGCTGTATGGCTTTGCGATGGCGCTTGGCCTGCTGGAGCAGAAAATCCACGCCC
GCGCGCCGGGTGAGCTGGACGATCAGCCCGCAGAGATCCTGCATCCGGATATGGTGCAGCCGCTGCGCGTGAA
GGTGGATCGCGCGGCGCGCCGGCTGGCGGGTTATCGCTATGGTCGTCAGATCGCCGATGATTACCTGACACAG
TTGGGGCAGGGCGAGCAGCAAGTGGCGCGTTGGCTGGAAGCGGAAAACGATCCGCGTTTAACCGAAATTGTCA
CCCATCTCAATCATGTTGTTGAAGAGGCGCGTATCCGATGAGCGAACAGGTGGTGTTCAGTCAACTGAGCCGT
AAGTTTATTGATGAGAACGATGCGACGCCTGCCGAGGCGCAGCAGGTTGTCTATTACAGCCTGGCGATTGGTC
ACCACTTGGGGGTTATCGATTGTCTGGAGGCCGCGCTCACCTGCCCATGGGATGAGTATCTGGCGTGGATCGC
CACCCTGGAAGCCGGTAGCGATGCCCGGCGTAAAATGGAAGGCGTGCCGAAGTATGGCGAGATCGTTATCGAT
TTCAACCATGTGCAAATGCTGGCGCGCGCGTTTGATGAGGCGCGCGCCGCGCAGACCCCGCAGCAGCAAGAAT
GGAGCAAACTGATGCTCAGTATGCTGCATGACATTCATCAGGAAAGCGCCATCTACCTGATGGTGAGGAGGCT
TCGTGACTGACGTTTTACTCTGTGTCGGCAACAGCATGATGGGCGACGACGGCGCAGGCCCATTACTGGCGGA
AATGTGCGCGGCGCAGCCGAAGGGGAATTGGGTGGTGATTGATGGCGGCAGCGCGCCGGAAAACGACATTGTG
GCGATTCGCGAACTGCGCCCGCAGCGTCTGTTGATTGTCGACGCCACCGACATGGGACTTAATCCGGGCGAGA
TCCGCATCATCGATCCGGATGATATCGCCGAAATGTTTATGATGACCACCCACAATATGCCGCTCAACTACCT
TATCGATCAGCTGAAAGAGGATGTCGGCGAGGTGATTTTTGTTGGTATCCAGCCGGATATCGTCGGGTTTTAT
TATCCGATGACTCAACCGATTAAGGACGCGGTAAATATCGTCTATCAGCGCCTGGAAGGCTGGCAGGGAAACG
GCGGATTTGCCGCGCTGGAAGCGCCGGAGGCGTAAAATGCGCACATATTTAGGGAGAATTTGTGTTACACGGC
AAAGGTTGTAAGAAGGACGTGAAGATTATCGTATGGAGCGGACTGCTATTTCTCTGCCTCGCCGCACGCGCAC
TGGCCACCGGTGTTGTCGGTTACCTGCCGATGAGCGACGGTGAATATGCGCAAAAGCGTGCGCTGAAGCCGCT
GTTGACCCTGCCTTATTCTGTATCGCCCGACCAGACCTGGCATTTTCGCCAGGTCGGAGTCAGTGGCGTGACG
CTACTGCCGGAGCCGAAAAAAGATAACGAATGGCGGATTAGCGGAAAAGACAGAGCCGGTAATTCGTGGGTTG
TCCCGGTGGGAAGGCTGATTAATTTGGCGGGTAATGCGCAATTTTATCGCGCTGACCTCGATCGTAACGGCAT
TCAGGATCTGGTTATCTGGTTGGGCAATCCTGGGTTGGGCCTGGCGCCGAGCGCGCAATACATCATTTTCACC
ATTTCTCAAGAATGGCCGCCCCTGCGTCTTTGAACCGTGGGGGTTTTATACCGCGACCGATACTGGCGTTGAT
GATTTGCTTGATTTACAGGGCAATGGGCGCACGCAACTGTTGGATATGCAATTTGACTCAGGCTACTGGATAA
CCAATTTATATCAGGTGAAGGACGCCCGCTGGCAGCGCGTACACGGCTGGTTTGGCCGGTTGGGTTATCCGGC
GCTGACCCGTTTTAATCACTATCCTGGCCGTAAATTAATTATCAAACCTATTGCCGGAAGGAATCCGCAAACG
GACGATCTTTCCCTTACGCAGCGCTGTTTAATACGCGGTAACGTATTGCCAGGCGTTAATCAGGATTGATGCC
AGTTGCGGTGCAGAGTTGGCTGGCTATCACTGCAAATCTTGACCACCACTGGCGACATTACGTGTCATCGACA
CTTGTGACGATGACACGTAAATGCTCACGAAAATAGAAATAATATTCTTAAATAACAATGAAATAAAAATTGG
CACGATTCGTGTATATATCGGCACATCATCTGTTATTGCTGGAGAATTTGATGAACCGTTTCATCATTGCAGA
CGCGAGTAAGTGCATTGGTTGCCGTACCTGTGAAGTGGCTTGCGTCGTATCCCATCAGGAAAATCAGGATTGC
GCATCGCTGACCCCTGAAACCTTTTTACCTCGTATCCATGTCATCAAAGGCGTGAACGTTTCCACGGCAACGC
TGTGTCGCCAGTGTGAAGACGCGCCGTGCGCCAACGTTTGCCCGAATGGCGCCATCAGCCGTGACAAAGGCTT
TGTTCATGTGATGCAGGAACGCTGCATTGGCTGTAAAACTTGCGTAGTGGCATGTCCTTACGGCGCCATGGAA
GTGGTGGTACGTCCGGTGGTTCGCAATAGCGGCGCGGGGCTGAACGTGCGTGCCGAAAAAGCCGAAGCCAACA
AGTGCGATCTGTGTCACCACCGTGAAGCCGGTCCGGCCTGTATGGCCGCTTGCCCAACGCACGCCCTAATCTG
CGTGGACCGCAATAAGCTTGAACAGCTGAGCGCGGAAAAACGCCGTCGCGCGGCGCTGGATTCAACGGCATCG
CTGCTTTTCTGATTTCTGCGATTGCGCGTAGAATACGCCACAAAGCATTGCCGGATGGCGGCATCAATGCCTT
ATCCGGTCTACGGTCCGTGCGGTTGTAGGCCTGATAAGCGTCGCGCCATCAGGCATCCGGGTTCGATGATGGC
GGGAAAATAAGATGGCGATAGACACACCTTCCGGCGTACAGCTACGCATTCGGGGCAAAGTACAGGGCGTCGG
TTTTCGTCCTTTTGTCTGGCAGCTGGCGCAGCAGTTGCGATTACACGGCGACGTGTGTAATGACGGCGACGGC
GTCGTCGTTCGGCTGCTGGAAGAGCCGTCGCAATTTATTGCCGCGCTCTATCAGGATTGCCCGCCGCTGGCGC
GCATTGACAGCGTTGAACACGCGTCGTTGGTATGGGAGCGCGCGCCGACGGATTTCACCATTCGTCAGAGCGC
AGGCGGTTCGATGAACACGCAAATCGTGCCGGATGCGGCGACCTGCCCGGCATGTCTTGCCGAGATGAATACC
CCAGGCGAGCGGCGCTACCGTTATCCTTTCATCAATTGCACCCACTGCGGACCACGCTTCACCATTATTCGCG
CTATGCCCTATGACCGGCCATTTACGGTGATGGCGGCGTTTCCCTTGTGTCCGGAATGCGACAGCGAATACCG
CGATCCGTACGATCGCCGTTTTCATGCCCAGCCCGTTGCCTGTCCGTCATGCGGGCCGCATCTTGAGTGGCGG

AGCCAACATGAGCGAGCGGAAAAAGAGGCGGCTTTGCAGGCGGCGGTCGCCCAACTGAACGCCGGAGGCATTA
TTGCCGTTAAAGGGCTGGGGGGTTTCATCTGGCCTGCGATGCGCGCAACGATAACGCAGTGGCGATGCTGCGG
GCGCGTAAACATCGCCCGGCGAAACCATTGGCGGTGATGTTGCCCACGGCGCAAACGCTGCCGAGCGCGGCGC
GTTCGCTGCTGACCACGCCAGCGGCCCCGATTGTACTGGTGGATAAGCAGTATGTACCTTCGCTGAGTGAGGG
CATCGCGCCAGGACTTACGGAAGTGGGCGTGATGCTGCCAGCCAACCCATTGCAACACCTCTTGTTGCAGGCG
CTCAATTACCCGCTGGTGATGACATCCGGCAACCTGAGCGGCAAACCGCCCGCCATCACCAACGAACAGGCGC
TGGACGATTTACACGATATTGCCGATGGTTTTCTGTTGCATAATCGCGACATTGTACAGCGCATGGACGACTC
TGTCGTGCGCGACAGCGGCGAAATGCTGCGTCGTTCGCGGGGATACGTGCCGGACGCGATTGCGTTGCCGCCC
GGATTTCGCGATGTGCCGCCGATACTTTGTCTGGGCGCGGATCTGAAAAACACGTTCTGTCTGGTACGCGGCG
AACAGGCGGTTGTCAGCCAGCATTTAGGCGATCTCAGCGATGACGGTATCCAGGCGCAGTGGCGCGAGGCATT
GCGTCTGATCCAGTCAATCTACGATTTTACGCCAGAGCGTATCGTCTGTGATGCGCATCCGGGCTATGTTTCC
AGTCAGTGGGCCAGTGAGATGCGTCTGCCGACAGAGACGGTGTTACACCATCATGCCCATGCGGCGGCTTGCC
TGGCCGAGCATGGTTGGCCGCTGGACGGTGGAGAGGTGATTGCCCTGACGGTAGACGGTATCGGCATGGGTGA
GAATGGCGCGCTATGGGGCGGAGAATGTTTGCGGGTCAATTATCGCGAGTGCGAACATTTAGGCGGTTTACCC
GCCGTGGCGCTGCCGGGAGGCGATCTGGCTGCCAAACATCCGTGGCGTAATCTGTTGGCGCAGTGCCTGCGCT
TTGTGCCGGACTGGCAGGATTACCCGGAGACTGCGGGGCTGCAACAGCAAAACTGGAATGTCCTGGCGCGTGC
CATTGAGCGCGGCGTCAATGCCCCGTTGGCCTCTTCCTGCGGGAGGTTGTTCGACGCGGTGGCTGCCGCGCTT
CGCTGCACGCCAGCATCGCTTAGCTATGAGGGCGAGGCCGCCTGCGCGCTGGAGGCGCTGGCCTCTCAATGCG
CTAACGTTGAGCATCCGGTAACGATGCCGCTTAACGGCGCTCAACTGGACGTCGCCGTTTTCTGGCGGCAATG
GTTGAACTGGCAGGCCACGCCCGCGCAACGCGCCTGGGCTTTTCATGATGCGCTGGCGTGCGGGTTTGCCACG
CTAATGCGCCAGCAGGCTACGGCGCGGGGGATAACCACTCTGGTCTTCAGCGGCGGGGTGATACACAACCGCT
TACTTCGCGCGCGTCTTGCCTTTTATCTTTCTGATTTTAAATTGTTATTTCCGCAGCGGTTACCGGCGGGCGA
CGGCGGACTGTCGTTTGGACAGGGCGTGATTGCCGCAGCGCGAGCGTTAAGGGAAGTGTAGGGCACCGGATGG
CGGCTGCGCCTTATCTGGCCTGCAAACAGATGCTCACTCGTAGGCCGGATAAGACGCGAAGCGGCGCCATCCG
GCATCACAGGCTTATACGGACAACGTTTTCAGCAGTGCAAAGGCCTCTTTCATGCGGTCTTCACTCACCACAA
AGGCGCGTAGTTGGCCTTCTCCGCTCATGCCTTTGGCAATCATACCGTCAGATTCTGCGGTAATCTGCCAGCT
CAAATCGCTGCGCTGAGTTTCTCCCGCCAGATGCAGCGGCAGTTCCGGCGTTTTGACTTTGACCAGCATCGCC
GGTAGCTTCAGCGGCGCGTTGCCGCCAAGCAGATTTTTCGCCAGGTACATCGCGCTTAACTGGATCGGTTGCA
GGAAGGGCAGCACCTGACCGTTGATCTCCGCGCAGTCGCCGATCGCATAAATATCCGGGTGACTGGTCTGTAG
ATAGCTATCCACGCAGACGCCGCGGTTGACGGCTACCCCGGCGCGGCGCGCCAGAGCGGTTTCCGGGCGTAAG
CCGGTGGCGGCAATCACCGCATCGACCTCAATACTATGCTGGCTTACCAGCGTAGCGCGGATACCGGCTTCGG
TTTTCTCCAGTTTTTGCAACTGCGATTTCAGCAGCAGATGCACCCCCATATCGGTCAGGTGATGCTGTAAGCG
ACTGCTCACTTCTGGTGGCATCAGTGAGGCGAGAAGGCTGGCGGCGTTATCCATCAGCGTTACCGTTTTCCCG
GCGCGGCAGAAATCCATCGCTAATTCACTGCCAATGAGACCGCCGCCGACAATCAGCACTCGCTGCGCGTCGC
GCAGTTGTGTCTCACAGGCGCGGTACTCCTGCTGGCTATTCAAAGTAAGCATTAACTCACGTCCTGCAATCGG
CGGCACAAAGGCTGCCGCCCCGGTCGCCAGTACCAGCTTGTCGTACTGCCACTGCTTATCCTGACTTTTCACC
ACATGGGCATCGGCATCAATGTCAGCAACCCAGGTATGCGGGAACAGTCGCAGATTAAACTGTTCAGCAAATT
CCCCCGCTAACTGGCGGGTGAGATCATCGGCGCGCTGCGACTGGCTGATAACGTGGCTGAGATCGGGCTTGTT
GTACTCATCCATGCTGTCCGCCGCAATCAGGGTTAACGGCACATGCGCATCTTGTTTACGGATATTTTTGACG
AGCTGGCGGGCGGCGAAGCCCGACCCAATAATGATGATTCCCCGACTCATTTTGCCTCCGTCGCCAGTACGTC
GAACACGTCTTTACCTAATGAACATTCCGGGCACAGGAAGTTATCCGGCACGTCGTTCCACGGCGTACCCGGC
GCGACATCCTGCAACGGCTCGCCCAGCGCAGGGTCGTAAATCCACTGGCATACGCTGCATTGCATTTTGGGGC
CGAAGTCTGCGGCGGCGCAGGTTGTGGTTTCTTCGACTGGCGCGGTTTTCTGTGTGGTTTCCGGCAGCGGAGC
AAGCGCCCACTGACGCGCGATGTCGCGACCGTGCTGACGGCACAACTCCAGCGCATCCAGATCCGGACGCCAT
TTCGCTTTCAGACTCAGGGACATTTCAAAACCGGCATCCTGTAAGCGCGTGGACAGTCGATCCACCGCGCCGC
CGCTCCAGCCATGAGAACCGAAGGCGCTGGCGCGTTTGTTGCGAAAGCGCAGGCCGGTCATCTCTTCCACCAG
ACCAGCGATTTTCGGCATCATCACGTTGTTCATGGTAGAAGTGCCGACCAGCACGCCTTTCGAACGGAAGACA
TTGGTCAGTATTTCATTTTTATCGCTGCGGGCGACGTTAAAGATTTTGACCGCCACGTTCGGGTCCACTTCAT
TGATACCCTGCGCGATGGCATCCGCCATCATGCGAGTATTGTTCGACATGGTATCGTAGAAAATAGTGATACG
ATCTTCCTGATAATCTGCCGCCCATTTCAGATACAGTTCCACGATTTGCGTTGGGTTGTCGCGCCATACCACG
CCGTGTGAGGTGGCAATCATATCCACCGGCAGGTTGAAGCCGAGGATTTCGGTAATTTTTGGCGTTACCAGAC
GGCTGAACGGCGTCAGGATATTGGCGTAGTAGCGCTGGCATTGTTCAAACAGTTCGGTTTGGTCCACTTCATC
ATTGAACAGGCGCTCGTCGCAGTAGTGCTGACCAAAGGCGTCGTTGCTGAATAACACCGCGTCGCCGGTCATA
TAGGTCATCATGCTGTCCGGCCAGTGCAGCATCGGCGTTTCCACGAAGATCAACTGCTTGCCGTTGCCGATAT

CCAGCGTATCGCCGGTTTTCACCACCTTAAAATTCCATTCCGGGTGATGGTGATGGCCGTTGATAGAGTCAAT
GGCGTTGGCGGTACAGTAGATAGGCGTGTCCGGGATCTGCGCCATCAGTTCGGTCAGCGCGCCTGCATGGTCT
TCTTCGGCATGGTTAATGATGATGTAGTCGATATCCGCGAGGTCGATTTCACTGCGCAGATTTTGCACAAACT
CACGGCTAAACTTATGATCGACGGTATCGATCAGCACATTCTTTTCTTCACGAATGAGATAGCTGTTATAGCT
GCTGCCGCGCAGCGTTTTGTATTCAGTGCCGTGAAAATCACGTACTTCCCAGTCGCGCTGGCCAACCCAATGA
ATATTATTTTTAACCAGAATAGACATAACCACCTCAATTCATTCAACGTTTACAAAAAGTTGTTTTGCTTATT
GCAGGTAGTGTGCCAATTTTACATCTTATTGATTTATATGAATTTATAATAAGTTTGTATTTTTGATATAGTC
AAAAAGACTATAAATGATGCTGTCAATATGACACTATAGGTTGTCAAAATGACAATGAGGTAACCATGAGTTT
TTCCGTGGAGGTATTGGCGGGCATCGCCATTGAGTTACAACGCGGCATTGGGCATCAGGATCGTTTTCAGCGG
CTGATTACTACGCTGCGTCAGGTTCTGGCGTGCGACGCCTCGGCGCTGTTGCGTTATGAATCACGACAATTTA
TTCCACTGGCGATTGATGGCCTGGCGCAGGACGTTCTCGGCAGACGTTTTACCCTGGAAGGGCATCCGCGCCT
GGAAGCCATCGCTCGCGCGGGGGACGTGGTGCGTTTTCCGGCTGATAGCGATTTACCCGATCCGTATGACGGG
TTAATTCCCGGACAGGAAAGCCTGAAAGTCCACGCCTGTGTTGGTCTGCCGCTGTTTGCCGGACAGAACCTCA
TCGGTGCGCTGACCCTTGATGCTATGACGCCGGAGCAGTTCGAGGTATTTAGCGATGAAGAGCTGCGGCTTGT
CGCCGCGCTGGCCGCCGGGGCATTGAGCAATGCGCTGTTAATTGAACAACTGGAAAGCCAGAACATGCTGCCG
GGGTCGTCAGGCGTGTTTGAACCGATAAAAGAGACGCACATGATCGGCCTGTCGCCGGCGATGACGCAGTTGA
AAAAAGAGATCGAGATTGTGGCGGGCTCCGATCTTAACGTGCTGATCGGCGGTGAAACGGGGACTGGTAAAGA
GCTGGTGGCGAAAGCCATTCACCAGGGCTCCCCCAGAGCGGTCAATCCGCTGGTCTATCTCAACTGTGCCGCG
CTGCCGGAAAGCGTGGCGGAGAGCGAGTTGTTTGGTCATGTAAAAGGGGCTTTTACCGGGGCTATCAGTAATC
GTAGCGGTAAGTTTGAAATGGCGGATAACGGCACGCTGTTTCTGGATGAGATTGGCGAACTGTCGCTGGCGCT
ACAGGCCAAGCTACTTCGCGTGTTGCAGTATGGCGATATTCAGCGCGTCGGCGACGATCGTAGCCTGCGCGTG
GATGTGCGGGTGTTGGCGGCGACCAACCGTGACCTGCGTGAAGAGGTGCTGGCGGGGCGTTTTCGCGCCGACC
TGTTCCATCGCCTGAGCGTTTTCCCGCTCTTTGTGCCGCCGCTGCGCGAGCGCGGCGATGATGTGGTGTTGCT
GGCGGGCTATTTTTGCGAGCAGTGCCGACTGCGGCTGGGGCTGTCCCGCGTGGTACTGAGTCCCGGCGCGCGC
CGCCATTTGCTCAACTATGGCTGGCCGGGCAACGTCCGGGAGCTGGAACATGCCATTCACCGTGCGGTTGTAC
TGGCGAGAGCGACCCGGGCAGGGGATGAGGTGGTACTGGAGGAGCAGCATTTTGCCTTGTCAGAGGACGTTTT
ACCCGCGCCGTCTGCGGAAAGCTTTCTGGCGCTGCCTGCCTGCCGCAATTTGCGCGAATCGACGGAGAACTTC
CAGCGAGAGATGATTCGCCAGGCGCTGGCGCAAAATAATCACAACTGGGCGGCCAGTGCGCGGGCGCTGGAAA
CCGACGTCGCCAACCTGCATCGGCTGGCGAAGCGTCTGGGACTGAAGGATTAGATAATCCCCGCCTGGTAGAA
ATCCTGCAGGTTAATGGCGCCGGTGAGTTTGCCGTTTTCATCGACCACCGGCGCGGCGGTAATTTTGCGTTTC
ATCAGGAGCTCTTTGGCGTCAATGGCGCGGCTTTGCGCCTGGAGCGTAATACCGTTGGGCGTCATGGCTTCGC
TTACCGGCGTGGTGAGCGCGCCGCCGCCCACCAGCCAGCGACGCAGGTCGCCGTCGGTAAAGACGCCTTTCAC
AACATGTTGCTCATCGCAAACCGCCACCAGCCCCAGCCCGGTACGGCTAAGTTCCAGCATGGCATCCATCACG
CTGGTGGCGAGCATTACCTGCGGTATTGCATCGCCCTGGCGCATCAGGTGATGCACATTATTGAGCAAACGCG
CGCCCAGTGCGCCAGCCGGATGCGAACGGGCGAAATCTTCTTCGTTAAAACCGCGCGCCTGCATGACCGCCAT
CGCCAGCGCATCGCCCATCATCAGCGTATTGACGGTACTGGATGTCGGCGCCAGATGCATCGGACAGGCTTCA
CGCTCGACGGAAATATCCAGAACGGCTTTTGCCGCTCGCCCCAGCGGAGAGAGAGGTTTACCGGTCATCGCCA
GCAGCGCGACGGATTTATCTTCCAGACGCGGGATGATGAGGTCGAGTTCTTTTGCGCCGCCGGAATAGGAGAT
AAATAACATCACATCGCGGCTTTCAATCATCCCCAGATCGCCATGCAGTGCTTCCGCCGGATGAACAAAAAAA
GCGGGAGTACCGGTACTGGCAAGCGTCGCGGCGATTTTTTTACCAATATGACCTGATTTACCAATACCGGAAA
CGATCACTTTGCCTTCACAGTGAATAATGATATTGGCGGCGCGGACAAAATCATCGCCCAGACGCTCCGGCAG
ACGGCTGGCTTCTTGTAGCTCCAGCATTAAGGTCTGACGGCCCGCGTTTAGTAGTGCATCACTCATTGCTTTC
TCCGGTTATGATTACTTCAATCCCTTTTGCTTCCAGCGCCTGACGAAATGCCGGATCGATACCCGCGTCCGTG
ATCAGTTTGTCGACGGTTTCCAGACTGCACACCACATTGGGGCTTTTACGACCAAATTTTGAGGAGTCCGCCA
TCAGAATCACCTCGCGTGCTGCATTGCACATTGCGTTACTGACGGTGTAAACCTCATTGAACGTGGTCACACC
GACGTTCAGGTCAATACCGTCAGTTCCCATGAAAAGTTTATCAAAGCTGAATTGCTCAAAGGCGTTTTCCGCC
AGTTGACCGTGAAACGACGCCGATTTTTTACGGAAGGTGCCGCCGGGCATCAGGATAGTTTGTTCGTTGTCCA
GTTCCGATAGCGCGTTGACAATATGGAGGCTGTTGGTCATCACCGTGATGTTGCTAAAACGCGAAAGCAACGG
CACCATCTGTAACACGGTACTTCCGGCGTCCAGAATAATGGAATCGCCATCATGGATATATTTCACAGCGGCT
GCGGCTATCAGCGCTTTTTTGTGGGTGTTGATAAGCGTTTTGTGATCAATAGGCGGATCGGACTCTTCTTTAT
TCAACACCACGCCGCCATAGGTGCGGATGACGGTTCCGGCATTTTCCAGAATGACCAGATCTTTACGGATAGT
GGTGCCTGTCGTGTCAAAGTACTGGGCCAGTTCTTCAACCGAGCATTTTCCCTGTTTTTGCAGGTGCTCCAGA
ATCGCGGCCTGACGCTGACGAGGTTTCATAGGCGTGATTACCCTGACTTATGTATTTACCCGTCATACTTCAC
GCTGCATGTGCGTTGGCTTTCCTCGCTCACCCCGGTCACGTACTGATGTACGCTCCTGGGATTCACTGCGTTG

CCGCCTTCCTGCAACTCGAATTATTTAGGGTATTGGTTGCAAAATGATAATTTCGCAAGCTATACGCATTCAC
AACGAAATTACCCATGTTTCAGTTTAAGCGCCAATGATAGTGCATTCTGCGCGAGCGAATCATGGGGAAAGAT
CACATCAGGCTGTAATTCATGCAAATGTTTTCCTTGCATTGCGGCAATTTTAACCGGTCTGGCGAATACCGTA
AGGCCTTTCATCAATAAAGTGTCCGTCACCCGCTGCTGTTCGTCCAGCGCCACGGCAACCACCACCCGGGGCT
TAAAACGCCCGCCGGAGCGCCCGACGCCGACCCGCCCCTGTTGGCTAAGTTTATCGAAGGCCCGATTAAAGCG
GGAAATCTGCCAGCCGCCCAGCGCCAGTTGCGCACACCAGGCGATAACGGCGACGGTAATCAGAGTGGAAACC
ATAATGTGCTCCTGTTGTTTTGCCGGATGGCGGCGTAACCGTCTTATCCGGCTTACGGCGCCATCCGGCAATT
CACGGTCAGAACATCACTTGACCGCCGGTTACGTTGATCGACTGCCCGGTGCAATAAGACGCTTTCGGGCTGG
CATAAAATAGCAGCATGTTCAGCACATCCTGATAATCACACCCGCGTTTGAGCGGTACTTTATCAATGTAATA
CTGTTCCACCTCGTCGGGCTTAATGCCCAGTTTAGTGGCGTATTGCGGCAGCAGTGACTGGAACATCGGCGAT
TTCAACAGGTTGCCGAGCATCAACGAGTGTACGGTAATGCCGTATTCCGCCAGATCCAGCGCCAGAGACTGGG
TTAACCCCACGCCGCCGAATTTCGCCGCGCTGTAGCCGGAGTTGTGTTTACTGCCCACTTTGCCGGATTTAGA
GTTAATCTGAATAATGCGCCCCTGAATGCCGTCGCGGATCATCAGGCGGGAAAACTCACGGGCGCAGAGGAAA
TAGCCCACCAGATTCACCTGTAGCGAGCGGTCAAAATCGCCCAACTGGAAATCGCTGATAAACGCCGCTTTGG
CGATACCCGCGCTGTAAACCAGCAGATCGACGCGCCCGAAAATCTCGTCTACGCCGCGGGAAAGCGCCAGCAC
GCTCTGCTCGCTGGTGGCGTCGGCGCCAAAACCGTACGCCATACCTTCGCCAAAATCGGCGTTAATTTCCTGG
GCGACATTCGCGGCTTTATCGCTTTGAATATCAACGACCGCCACGCGGTATCCTTCTTCTGCAAGCCCACGGC
AAAGGAATGCCCCCAGGGTCTGGCCTCCGCCAATGACAACGGCAACTTGATTCATGTTTTTTCTCCTTAAGCG
ACAAACGTCAAAATACAGCCCGGCGCGATATCGTCCGGTACAGGCCCCGCAACGTGTACGGTGCCGGGAAATT
CTGCTTCGCGCAGACCATCGAAGCGCAAAGTGATATGCCCCAGTTCACGCAGGTTTTGCTCGGCTACGCTGCC
GACCGCCGTGACCGGATAGCAATGTTGACCCAATTCACACCGCGCGCCAGGCTGCAGCGCGCCGGTCAGTTCG
CCATGACAATGGATGAAGCAAAATTCTTCGATATCCGCCGGCGCGCCTTCCCGAAACGTAATCAGCATCTGTT
CGCCGAGCGCCTCCTTCGCGCTCTGGCCGATACGGGTGATGGTGGTTTGATAAATCACGCTCATGTCTGAACC
TCTTATTGATAGATAAAGCCGGAAACAAACCAGGCGATAAGTACCGTGGGCGCGCCAGTCAGGAAGCGTCCGA
CCAGCACAGAAGGTACGCCGACGCGAACGGTGTCTTGTTTCGCTTCCGCCAGTGACAGGCCGACCGGGATAAA
GTCGCAGGCCGCCTGCGCGTTAATCGCGAACAGGGCGGGAAGCGCCAGATGCGGGGGGATATTGCCCAGGCCT
ATCTGAACGCCGATCAGGACGCCAATGACCTGAGCGATAACCGCGCCAGGGCCGAGAAAGGGCGACAGCAGCG
GGAAAGAGCAGATCAATGCCAGCGTCACCAGCCCCAGTGGATGGCTGGCTAATGGCGCCAGGCCGTGGGCGAT
CCAGTCGCCAAGACCTGAGGCCATAATGATGCCGATCAGCGCCGAAACGAACGCCATAAACGGCAGGATTGTT
TTCAGGACCGTATCAATGGTGTCGCGACCGGACTGGAACAGTACCGCCACGGCGGAGCCCATTCCCATACCGA
CTTTTGCCAGCAGCCCGTCGCTCTGCTCGGTGATTTTTTTGCTGGTGTCGTAGTCACGTCCTAATGTCGTTTT
GGCAGGCTGAGGACTGGCAGGCGTTTCGCCGACAAGAGTAATGTTTTCTTCTTTTACGCCGGAGACATAAATA
TCCTCAACAATATACTGCGCCAGTGGGCCGGACTTACCCGTCGAGTGAATATTAATGGTTGGAATCCGGCGTT
TCGGATAGATACCGCAGCGCAGCGTACCGCCACAGTCGATAATGGCCGCGCCGATCTCCGCTTCCGGCGGTTC
GCCTTCTTTAAAGCCGTCCACCGCTTGCCAGCCTGTTAGTTGCGCCAGTTTGTCGACGATCGCCGGGCGCGTA
CCGGCTGTGATATAGACGATCTTTTTTGCCTGGCGTAACGTCCAGTTCAAGCGGGCCGCCCCAGCCGCCGGCG
CCTTTCTCAATACGAACCCGTGTCATGATACCGCTCCTGTCAGATGAACTTTCTGCTCAAGTTGAATCCCCAT
TTTTTCTCAAAAATAGCGGTGGTGAGATCGGTGACCCAACCGCGGAAGAAGTTCGTCACCAGACCTACCAGCA
GATAGCTCACCGCGAGCGGGCCGAGAGGAAGCCCTAACGTGGTGAGTCCGCTGGCGATCCCCAGATAAACAAA
CAGTTCGCCGGGATTGATATGCGGAAACAGACCGTTCATGGAGTGACAGCTATAGGAGGCCGCCGCGTAATAG
CTCGGCTTGTACTTCTCCGGCATAAAGCGTCCCAGACTCAGGGTCATCGGATTACAAAAAACAAACGTTCCGA
TAAACGGTAAAAGCAGGTAGCGGATAGCGGATTTCCGGCGCAGCGTTGGGCAAAACGCTCAATACGCTGCTG
GCCGATAAAATTGATGAGCGCATTCATGATAACCAGCAGGCTTATCAGTAGCGGAAGAATACCTGTCACCATA
CCGGTAAACACCTCTCCGCCTTTCTGGAATAACCCGATGAACCACTCCGCACCATGTGTAATGGTTTCGATCA
TTGTTCTCTCCTGTAGGGTTTTCAGTTTTCTTGCTAATTTCCTGTTCATTGTAATCATGTTGAAAGATTCAAA
AGTGTTATTGTGATCGCAAAATAAAAGAAAAATATTTTAAGTTGAAAGATTGCGGGAGTGGGGGAGCGGATAC
AAACTGAAAAGAGGAGAAAGGTGATTTATTCTCTTGTGCCGCTTCCTTGTGTGGCGGGGGATGCTTTACCATA
CTTCTCCCTGGCTGAATTTGTTTAATGGATGTCTCATGTTCAAGCGTCGTTATGTAGCGTTACTTCCACTTTG
CGTATTGCTTGCTGCCTGTAGCAGCACGCCAAAATCTTCCGAGACTCAAGCGACGACGGGCACGCCTTCCGGC
GGCTTTCTGCTTGAGCCGCAGCATAACGTCATGCAAATGGGCGGCGACTTCGCCAACAACCCTAATGCGCAGC
AGTTCATTGATAAAATGGTCAATAAACACGGTTTCGACCGTCAGCAGCTGCAGGAGATCCTCTCGCAGTCTAA
AAGACTGGATTACGTCCTGCGTCTGATGGACAGACAAGCGCCGACCACTCAGCCGCCCGCCGGACCAAATGGC
GCCTGGTTGCGCTACCGCAAACAATTTATTACGCCGGATAACGTGCAAAACGGCGTCGCGTTCTGGAATCAGT
ATGAAGACGCGCTGAACCGCGCCTGGCAGGTATACGGCGTACCGCCGGAAATCATCGTGGGGATTATCGGCGT

GGAAACCCGCTGGGGACGTATCATGGGGAAAACGCGGATTCTGGACGCGCTGGCGACGCTGTCATTTAACTAT
CCGCGCCGGGCGGAATATTTCTCCGGCGAGCTGGAAACCTTCCTGCTCATGGCGCGCGATGAGCAAGACGATC
CGCTCAATCTGAAAGGTTCCTTTGCGGGCGCGATGGGCTACGGCCAGTTTATGCCATCTTCCTATAAAGAATA
TGCGGTCGATTTCAATGGCGATGGGCACATTAACTTGTGGGATCCGGTAGATGCCATCGGCAGTGTGGCGAAC
TACTTCAAGGCGCATGGTTGGGTGAAAGGCGATACGATTGCGGTGCCAGCGAATGGTCAGGCGCCGGGACTGG
CGAATGGTTTCAAAACAAAATACAGCCTCTCTCAGCTTGCCGCCGCAGGCTTAACGCCGCAGCAATCGTTGGG
CAATCATCAGGAAGCCAGCCTGCTTCGTTTGGATATCGGTACGGGCTACCAGTACTGGTACGGCCTGCCGAAC
TTCTACACTATTACCCGATATAACCACAGCACGCATTATGCGATGGCGGTTTGGCAACTGGGCCAGGCGGTAG
CGCTGGCGCGGGTGCGCTAATTTACGCTTAGGGAGGGGAGCACCCCCTCCGCTATTATTATGGCTTTTTGGGC
TGATTCTCTGCCTGCAAGTGGGATATCTTCTTTGATAGTGCCAGACTATATCCCAGACATCCCAGGCCTAAGA
TAATTTGACCGCCATCATACGGTTCAAAATAGCCTGACGACAATATATTCAGTACTCCCATTCCTAATGAACA
TACTCCACCCACAATTAACCACAGCCGTAGTTGACAGGTTTGTTTCAGTTTGTGCTGTGTGCTTTCCGTTTTT
TTGAGTGACATCCTTGCTTTCTCTCCGTGCGTGTAAATTTAGCGACATGGTACTGCTATTTAGTCATGTGCTA
CCTCATTTCATTCTGCATAAATAACCTGCTGGCATTTTTTCGGAATCCGACTCGTAAAATCGACTGCTCGTCC
CCATCTCTGGTGAGAAAGTGCTATCTTGTGCGGCAGGATTTCTAACTGACAGAGGTCATGATGACTGACAGTG
AACTGATGCGGCTTAGCGAACAGGTCGGGCTGGCGTTAAAAGCGCGCGGGGCGACCGTAACGACAGCAGAGTC
CTGTACCGGCGGCTGGCTGGCGAAAGCCATTACCGACATCGCCGGAAGCTCCGCCTGGTTTGAACGCGGCTTT
GTCACCTATAGTAATGAAGCCAAAGCGCAGATGATTGGCGTGCGTGAAGAGACTCTGGCGCAGCATGGCGCGG
TCAGCGAACCCGTAGTGGTCGAAATGGCGATCGGGGCGCTGAAGGCCGCTCGCGCCGATTTTGCTGTCGCTAT
TAGCGGAATTGCCGGGCCGGATGGCGGCAGTGAAGAAAAGCCGGTTGGTACGGTGTGGTTTGCTTTCGCCAGC
GTCAGCGGAGAAGGGATTACGCGTCGGGAATGCTTCAGCGGCGACCGTGAATCGGTGCGTCGACAGGCGACGA
CGTACGCGCTACAAACCCTGTGGCAACAATTTCTACAAAACACTTGATACTGTATGAGCATACAGTATAATTG
CTTCAACAGTACGAATTCACTATCCGGTTCAATACCAAGTTGCATGACAGGAGTAATAATGGCTATCGACGAA
AACAAACAGAAAGCGTTGGCGGCAGCACTGGGCCAAATTGAAAAGCAATTTGGTAAAGGCTCCATCATGCGTC
TGGGTGAAGACCGTTCTATGGATGTGGAAACTATCTCCACCGGTTCGCTTTCACTGGACATCGCACTCGGTGC
GGGCGGTCTGCCGATGGGGCGTATCGTCGAAATTTACGGGCCGGAATCTTCCGGTAAAACGACCCTGACGCTG
CAGGTGATTGCCGCTGCGCAGCGTGAAGGTAAAACCTGTGCGTTTATCGATGCGGAACACGCGCTTGACCCTG
TTTACGCACGCAAGCTGGGCGTCGATATCGATAACCTGCTCTGCTCTCAGCCGGATACCGGCGAGCAGGCGCT
GGAAATCTGTGACGCGCTGGCGCGTTCAGGCGCGGTGGACGTCATTGTGGTCGACTCCGTAGCGGCGCTAACG
CCGAAAGCGGAAATCGAAGGTGAAATTGGCGACTCTCACATGGGCCTCGCGGCGCGTATGATGAGCCAGGCGA
TGCGTAAGCTGGCGGGGAACCTGAAACAGTCCAACACGCTGTTGATTTTCATCAACCAGATCCGTATGAAGAT
TGGCGTGATGTTCGGTAACCCGGAAACCACCACCGGCGGTAACGCGCTGAAATTCTACGCCTCTGTTCGTCTT
GATATCCGTCGTATTGGCGCGGTGAAAGAGGGCGATAATGTCGTGGGTAGCGAAACGCGTGTGAAAGTGGTGA
AAAACAAAATCGCCGCGCCGTTTAAGCAGGCCGAGTTCCAGATCCTCTACGGTGAAGGCATCAACTTCTATGG
CGAACTGGTTGACCTGGGCGTGAAAGAGAAGCTGATCGAGAAAGCGGGCGCATGGTACAGCTACAACGGCGAG
AAGATTGGCCAGGGTAAAGCGAACGCGACTACCTGGCTGAAAGAGAACCCGGCGACAGCGAAAGAGATCGAGA
AAAGGGTTCGTGAATTACTGTTGAGTAATCAGAATGCCACGCCCGATTTCGCCGTTGACGATAGCGAAGGCGT
TGCAGAAACCAACGAAGATTTTTAATCATCAGGTGATGGTGGGTCGGGCAAAACGAAACCGCCATTCAGCAAA
ACGTTAAATTACCATCAAGGGCTGCATTTATGCGGCCCTTTTGGCATTTCTCCCTGTTAAGGTTTTTTATGAG
TGAACCCACATCGCGCCGTCCCGCTTATGCTCGTCTCCTTGATCGTGCGGTACGTATTCTTGCTGTCCGCGAT
CACAGCGAGCAGGAATTACGGCGTAAACTCTCCGCGCCGGTGATGGGCAAAAATGGGCCGGAAGAGATTGATG
CGACGGCAGACGATTATGAACGCGTGATCGCCTGGTGCCACGAACATCACTATCTTGATGACGAGCGCTTCGT
GATGCGCTTTATCGCCAGTCGTAGCCGCAAAGGCTATGGCCCGGCGCGTATTCGCCAGGAGTTGAATCAGAAA
GGCATTTCCCGTGAATCGACTGAAAAAGCGATGCGAGAATGTGAAATTGACTGGAGTGAAATGGCACGTGAAC
AGGCCGTTCGCAAATATGGCGAGCCGCTCCCTTCAAACTTTTCAGAAAAGGTTAAGGTACAGCGCTTTTTGCT
CTATCGCGGGTATCTGATGGACGATATCCAGCAAATATGGCGAAATTTTGCAGATTGAGCGCATACGGGATTT
TACTTCCCGGTAAAGAAAACTTATCTTATTCCCACTTTTTCCGTCTGGACGCGGGTTGCTTAACGGCGAACAG
CGTCTGCGACGAATATTCCTTAAATACACAAAATCATTCAAGCCGCATCAAAGCGGCAAGTGAGCCTCGGGAA
CGCGTGACCGGGAAACTCGCAGCTAACGCAGAGGCGGCCTGAAGGATGAAGTGTAGCTTGATTTCAGGATAAT
TATGAGCAAGAGCACCGCTGAGATCCGTCAGGCGTTTCTCGACTTTTTCCATAGTAAAGGGCATCAGGTAGTT
GCCAGCAGTTCCCTGGTGCCTAACAATGACCCCACTCTGTTGTTTACCAATGCCGGGATGAACCAGTTCAAGG
ATGTTTTCCTTGGGCTCGATAAACGTAATTATTCCCGTGCAACCACTTCCCAGCGTTGCGTACGCGCGGGGGG
GAAACACAACGATCTGGAAAACGTCGGATACACTGCACGTCACCATACTTTCTTCGAAATGCTGGGCAACTTC
AGCTTCGGCGATTACTTCAAACACGACGCCATTCAGTTCGCATGGGAATTACTGACCGGTGAAAACTGGTTTG

CTCTGCCAAAAGAGCGTTTATGGGTCACCGTCTATGAAACCGACGACGAAGCCTATGAGATCTGGGAAAAAGA
AGTCGGCATCCCGCGCGAGCGTATTATCCGTATTGGCGACAATAAAGGCGCGCCATACGCATCGGATAACTTC
TGGCAGATGGGCGATACCGGTCCCTGCGGTCCGTGCACCGAAATCTTCTACGATCATGGCGACCACATCTGGG
GCGGCCCTCCGGGAAGCCCGGAAGAAGACGGCGATCGCTATATTGAGATCTGGAACATCGTCTTTATGCAGTT
CAACCGTCAGGCTGAAGGGACGATGGAACCATTGCCGAAACCGTCTGTAGATACCGGTATGGGGCTGGAGCGT
ATTGCCGCGGTACTGCAGCACGTTAACTCTAACTATGACATCGACCTGTTCCGCACGTTGATTGAAGCGGTAG
CGAAAGTCACCGGCGCTACCGATCTGGGTAATAAATCGCTGCGCGTTATCGCCGACCACATCCGTTCTTGCGC
ATTCCTGGTTGCCGATGGCGTGCTGCCGTCGAATGAAAACCGTGGTTATGTGCTGCGTCGCATTATTCGTCGC
GCGGTTCGTCACGGCAACATGCTGGGCGCGAAAGAGACCTTCTTCTACAAGCTGGTTGGGCCGCTGATTGAGG
TAATGGGCTCCGCAGGCGAAGAGTTGAAACGTCAGCAGGCGCAGGTCGAGCAGGTTCTGAAAACGGAAGAAGA
GCAGTTCGCTCGTACGCTGGAGCGCGGTCTGGCGCTGCTGGATGAAGAACTGGCGAAATTGCAGGGCGACACC
CTGGACGGCGAAACCGCTTTCCGTCTGTATGATACTTATGGTTTCCCGGTCGATCTAACGGCGGACGTCTGCC
GTGAGCGCAACATCAAAGTCGACGAAGCCGGTTTTGAAGCCGCCATGGAAGAGCAACGCCGTCGCGCGCGCGA
AGCTAGCGGTTTTGGCGCGGACTACAACGCGATGATCCGCGTTGATAGCGCTTCTGAATTTAAAGGTTATGAC
CATCTGGAACTGAACGGCAAAGTGACTGCGCTGTTTGTCGATGGTAAAGCGGTTGAAGCGATTAACGCGGGAC
AGGAAGCTGTTGTCGTGCTGGATCAGACCCCATTCTATGCGGAATCAGGCGGTCAGGTTGGCGATAAAGGCGA
GCTGAAAGGCGCTGGATTTACTTTCGCTGTGGACGACACGCAAAAATATGGTCAGGCGATTGGTCACCTCGGC
AAATTGTCTGCGGGCGCTCTGAAAGTGGGCGATGCGGTACAGGCTGACGTAGATGAAGCGCGCCGCGCACGTA
TCCGTCTGAACCACTCCGCCACGCACCTGATGCACGCGGCGTTGCGCCAGGTGCTTGGGACACACGTTGCGCA
GAAAGGTTCGCTGGTTAGCGATAAAGTGCTGCGCTTCGACTTCTCGCACAATGAAGCGATGAAGCCGTCGGAG
ATTCGTGAGGTTGAAGATTTGGTGAATGCGCAAATTCGCCGCAACCTGCCGATTGAAACGAACATCATGGATC
TCGACGCGGCGAAGGCGAAAGGGGCCATGGCGCTGTTTGGCGAGAAATACGACGAACGTGTACGCGTTCTGAG
CATGGGCGATTTTTCTACCGAACTGTGCGGCGGTACTCACGCCAGTCGTACCGGTGATATCGGTCTATTCCGT
ATTATCTCTGAGTCCGGCACCGCAGCGGGCATTCGTCGTATTGAGGCGGTAACCGGTGAAGGCGCTATGGCCA
CCGTTCATGCGCAAAGCGATCGCTTAAACGATATTGCGCATCTGCTGAAGGGCGACAGCCAGAATCTGGGCGA
CAAAGTGCGTGCCGTGCTGGAACGTACACGTCAGCTGGAAAAAGAGTTGCAGCAGTTGAAGGACCAGGCCGCA
GCGCAGGAGAGTGCAAATCTTTCCAGTAAAGCGGTCGATCTCAACGGCGTGAAGCTCCTGGTCAGCGAGCTTG
CTGGTATTGAGCCGAAAATGCTGCGAACCATGGTTGATGATCTGAAAAATCAACTGGGGTCTACCGTTATCGT
ACTGGCAACGGTTGTTGAAGGTAAGGTTTCTCTGATTGCGGGCGTGTCGAAGGATGTGACCGACCGGGTTAAA
GCAGGGGAATTGATTGGTATGGTCGCTCAGCAGGTGGGCGGTAAGGGTGGCGGTCGTCCGGACATGGCGCAAG
CCGGTGGTACGGATGCGGCAGCTCTGCCTGCAGCGTTAGCCAGTGTACAAGGCTGGGTCAGCGCAAAATTGCA
ATAATTATAAGCGTTCGTTGGCGCCGCAGCTCGTGCTGCGGCGTTTACGTCAACGCTATCGACAACGATAAAG
TCAGGTTGAAGTTTTGTATATCGGCTAAACTTAGGTTTAACAGAATGTAATGCCATGACTGCTTACACCTAAG
GTGTTTGTCATCGCTTACTTTTTGGCGTTATATGATGGATAATGCCGGGATACAGAGAGACCCGACTCTTTTA
ATCTTTCAAGGAGCAAAGAATGCTGATTCTGACTCGTCGAGTTGGTGAGACCCTCATGATTGGCGATGAGGTC
ACCGTGACAGTTTTAGGGGTGAAGGGCAACCAGGTGCGTATTGGCGTGAACGCCCCGAAAGAAGTTTCTGTCC
ATCGTGAAGAGATCTACCAGCGTATCCAGGCTGAAAAATCCCAGCAGTCCAGTTACTAAGGTTTTCGCGTCTC
ACTTTTCGGGTGAGACGCAGCCTCTCTTCTCGCTCGCCCGCGTTGTCCTTTCATGACTTTTTTCAACCTCACT
GTTGATATGGTGCGCTTTTGGCGGCCAGCGTGTCATCAAATTATCTGTTGATAAAACACTCTTTTTGGCTTTT
TTACAGACTAATTGAGCGTGAAGTGTGCAAACGATAAAAGCTCTGGAAAAATTGTTTGACTTATAAGTCCCAG
AAAGTAATATGTGCGCCACGCAGCGACGATGAGCAGAACAAGTTCTTCGAAGCACTCGTAAGAGGCGTGTGGT
GAGGTGGCCGAGAGGCTGAAGGCGCTCCCCTGCTAAGGGAGTATGCGGTCAAAAGCTGCATCCGGGGTTCGAA
TCCCCGCCTCACCGCCATTTGCATCCGTAGCTCAGCTGGATAGAGTACTCGGCTACGAACCGAGCGGTCGGAG
GTTCGAATCCTCCCGGATGCACCATCTCTTACTTGATACGGCTTTCCCGCGGTATCAAAATCTGCAGTAAAGT
AAGTTTCCCGATGCATCCGTAGCTCAGCTGGATAGAGTACTCGGCTACGAACCGAGCGGTCGGAGGTTCGAAT
CCTCCCGGATGCACCATCTCTTACTTGATACGGCTTTCACCGCGGTATCAATATCGGCAGTAAAGCAAGTTTT
CCGATGCATCCGTAGCTCAGCTGGATAGAGTACTCGGCTACGAACCGAGCGGTCGGAGGTTCGAATCCTCCCG
GATGCACCATATTCTCCGTACTTTCAGTGATGAAGATACGGATAAGATAGCGGTAATCACCGCTGGCACCAGG
AAGGATAACGTTGCGTTAGCAACGGCCCGAAGGGCGAGCCGTAAGGCGAGTAATCCTCCCGGATGCACCATCT
TATTTAGTGTGGCAGTAAATGCGACATCAAATCTTGTAGTAAACATCATTTCCAATGCATCCGTAGCTCAGCT
GGATAGAGTACTCGGCTACGAACCGAGCGGTCGGAGGTTCGAATCCTCCCGGATGCACCATATTCTCCGTACT
TTCAGCGATGAAGATGCAGATAAGATAGTTATAATCACCGCTGGCACCAGGAAGGATAACGTTGCGTCAGCAA
CGGCCCATAAGGCGAATAATCCTCCCGGATACGTTACTGCCCTGCAATCGCTTTTCCCCTTATCCTGAATACA
CCTCTTCATCTCTATTAGCGACAAAATCAACCAATTGCGATAAAGTACTGTCTGGTTATTCGGTTTGCGAGAA

CACGATGTACGCACGTTATGCTGGTTTGATTTTTGATATGGACGGTACTCTCCTGGATACCGAACCCACGCAT
CGCAAAGCGTGGCGTGAGGTTTTGGGGCGCTATGGTCTTCGTTTTGATGAACAGGCAATGGTCGCGCTTAACG
GTTCGCCAACCTGGCTTATCGCCCAGTCAATCATTGAGCTTAACCATGCCGATCTTGATCCCCTGTCACTGGC
GCGTGAGAAAACCGACGCGGTAAAAAGCATCCTGCTGGACTGCGTAGAACCGCTGCCGCTGGTTGAGGTGGTC
AAAGCGTGGCATGGGCGGCGTCCCATGTCGGTCGGTACCGGTAGCGAAAGCGCTATCGCGGAAGCCTTGTTAG
CGCATCTGGGGCTGCGTCGTTATTTTGATGCGGTTGTGGCGGCAGATCACGTACAGCATCACAAACCCGCGCC
TGACACCTTCCTACTGTGCGCCCAGCGGATGGGCATCATGCCGACGCAGTGCGTGGTATTTGAAGATGCCGAT
TTTGGTTTACAGGCGGCGCGGGCGGCGGGAATGGATGCCGTGGATGTCCGACTATTGTGAGTGACGGACTGTC
GCTCCTGTCTCTGTTTGCCAGTAGTTTTCTCAGCGCGACGCTGTTACCCGGTAATTCTGAGGTGGTTTTGGTT
GCTATGCTGGTCTCCGGCGTCAGTCATCCCTGGGTCTTAGTCTTAACAGCAACAATGGGTAATAGCCTTGGAG
GGGTAACTAACGTTATTCTTGGGCGTTTCTTTCCGCTGCGCAAAACGTCGCGCTGGCAGGAAAAAGCGACTGG
CTGGCTAAAGCGTTATGGCGCAGCCACACTATTATTAAGCTGGATGCCGGTCATCGGTGATTTGTTGTGCCTG
TTAGCGGGATGGATGCGCATCCCGTGGGGGCGGACGATCTTTTTTTGTGTCTTGGTAAGGCGCTGCGCTATGT
TGCGCTCGCAGCGGCGACGGTTCAGGGCATGATGTGGTGGTAATAATTGTAGGTTTGGCATGTATGGCCGCCA
TTACAGTTATGCTTATTAAAACGATTTGGACAGGCGGGAGGTCAATTTGATCCCGGACGTATCACAGGCTCTG
GCCTGGCTGGAAAAACATCCTCAGGCGTTAAAGGGGATACAGCGCGGGTTAGAGCGCGAAACGCTGCGTGTCA
ATGCTGATGGCACGCTGGCGACAACAGGTCATCCTGAAGCGTTAGGTTCAGCGCTGACCCATAAATGGATTAC
CACGGATTTTGCGGAAGCCCTACTGGAGTTTATTACGCCAGTCGATGGCGATATCCAGCATATGCTCACCTTT
ATGCGCGATCTCCATCGGTATACGGCCAGGAAACTGGGCGATGAGCGGATGTGGCCATTAAGTATGCCCTGCT
ATATTGCGGAAGGGCAGGACATAGAGCTGGCGCAGTACGGCACATCCAATACCGGGCGCTTTAAGACGCTCTA
TCGCGAAGGGCTGAAAAATCGTTATGGCGCCTTGATGCAAACCATCTCCGGCGTACATTACAATTTTTCATTA
CCGATGGCGTTCTGGCAGGCGAAATGCGGCGTTACGGAAGGCGAAGCGGCAAAAGAAAAAATTTCTGCCGGCT
ATTTTCGTCTGATTCGCAACTATTACCGTTTTGGCTGGGTCATCCCCTATCTGTTTGGCGCGTCTCCGGCCAT
TTGTTCCTCTTTCCTGCAAGGGAAGCCAACCACATTACCGTTTGAAAAAACGGACTGCGGCATGTACTACCTG
CCGTATGCGACGTCGCTGCGTTTGAGTGATTTGGGCTATACCAATAAGTCGCAAAGCAATCTCGGAATTACGT
TTAACGATTTGCATGAATACGTTGCAGGTCTGAAGCGGGCGATTAAAACCCCTTCCGAGGAGTACGCGCGGAT
TGGTGTGGAAAAAGACGGTAAGCGGCTACAGATTAACAGCAACGTTCTGCAAATTGAAAATGAGCTGTACGCG
CCCATTCGTCCCAAGCGCGTGACGCGCAGCGGCGAATCGCCTTCTGACGCGCTTCTGCGTGGTGGTATTGAGT
ATATTGAAGTTCGTTCTCTCGATATTAACCCGTTCTCACCGATCGGCGTAGACGAGCAACAGGTGCGCTTCCT
CGATCTGTTTATGGTCTGGTGCGTATTGGCCGATGCGCCGGAAATGAGTAGCGATGAGCTGTTATGTACGCGT
ACTAACTGGAATCGCGTTATTCTGGAAGGGCGTAAGCCGGGCCTGACGTTAGGCATTGGCTGTGAAACTGCGC
AGTTCCCGCTGCCCAAAGTAGGGAAAGATCTCTTCCGCGATCTGAAACGCGTGGCGCAAACGCTGGACAGTAT
CCACGGGGGAGAGGAGTATCAAAAGGTGTGTGACGAGTTGGTCGCCTGCTTTGATAATCCTGAACTGACATTC
TCCGCCCGAATCTTACGGTCTATGATTGATGAAGGCATTGGCGGCACCGGGAAAGCGTTCGGTGAGGCTTACC
GTAATCTGTTACGCGAAGAGCCGCTGGAGATTTTACAGGAAGAAGAGTTTATTGCTGAACGTGACGCTTCAGT
ACGTCGTCAGCAGGAGATTGAGGCGGCGGATACCGAGCCGTTTGCCGCGTGGCTTGCAAAACACGCCTGACCC
AAAAGAAAAAGGCCACTCACGGAGTGGCCAAAATTTCATCGCTGAAAACAGGGATGATGATAACAAATGCGCG
TCTTTCATATACTCAGACTCGCCCCGGAACAAAGAGTTCAGTTTATTTTTAAAAAATTATCGGAGGTGACTAA
ATGCCATTATTAGATAGCTTCGCAGTCGATCATACCCGGATGCAAGCGCCGGCGGTCCGGGTTGCAAAAACGA
TGAACACCCCGCATGGCGACGCAATCACCGTGTTTGATCTGCGTTTTTGCATTCCGAACAAAGAAGTGATGCC
GGAAAAAGGGATTCATACGCTTGAGCATCTGTTTGCTGGCTTTATGCGCGACCACCTCAACGGTAACGGCGTT
GAGATTATCGATATCTCGCCGATGGGCTGCCGTACCGGTTTCTACATGAGCCTGATTGGCACGCCGGACGAGC
AGCGTGTTGCCGACGCGTGGAAAGCGGCGATGGCGGATGTGCTGAAAGTGCAGGATCAAAACCAGATTCCGGA
GCTGAACGTTTACCAGTGCGGTACGTATCAGATGCACTCGCTCAGTGAAGCGCAGGACATTGCCCGTCATATT
CTGGAGCGTGATGTGCGCGTGAACAGCAATAAAGAGCTGGCGCTGCCGAAAGAAAAACTGCAGGAATTGCATA
TTTAGTCTTATTGGTGAGCGGTTCCAGTATTTAAATTAACCCCGGTTTATGACCGGGGTTTTTTATTGCTTAA
TATAATTTGTATTTATTATATTTGCGGCAAGAATACATTTTAAATTAAGTAATGCCTGGCCTGTTAAATAATA
AAATGTGATCTGGTAAACAGAAAAGGCGGATAACTCACGATGCCAGAGTGTATTGATCACATAAACTAAAAAT
CATAAATAATTAATAGAAAAACATAATGGAATGCGCCAGATATCCAGGTAATAACAAGACTCTACTAATAGCC
GGTCGATGGGGTTTTTAGCAAATCTTATCTGGTATACATCAAACTTAAGGGATAAAAAATGAAGGCTAAATAT
TTGGCGCTGATGATCGGTGCTTGCTTTTCTCATAACCTTTGGGCAGCGAATAATATCACTATTGAGCAGCGTC
TGGCTGAACTGGAGCAACGTGTTGTTAATGCTGAAAAACGGGCATCCGATGCCGAGGCGCAAATTCGCTCGTT
GAAACAGCAGCAGGTCGCCGCTACGCCGACGGTGAATGTCCAGTCCGCCGAGCCCATTGCAGCAGATAAAACG
CCGCCGAAGCTGACCTTATCCGGATTCAGCGATATTAAGTTCTATGGCGATGTCGAATTTAATATGGATGCGG

CAAGCCGTTCCGGTAGCCTGACCTCGATGAGAACGTCAGCGAATAAAGATTGGGCATCAGGAACCAATGAACG
CTGGGATATTAACGGGCGCTTGTTGCTGGGCTTTGATGGCTACCAGCGGCTGGACAACGGTAATTTTGCCGGA
TTCTCTGTACAGCCTCTGGCGGACCTGACCGGAAAAATGAACCTTGATGATGCCGTTTTCTTCTTTGGTCGTG
AGAATGACTGGAAAATTAAGGTTGGTCGTTTTGAAGCCTACGATATGTTCCCGCTGAATCAGGATACGTTTAT
TGAATATTCGGGGAATACAGCGAACGATCTTTACAGTGACGGTTACGGCTATATCTATATGATGAAAGAAGGA
CGGGGACGTAGCGACAGTGGGGGTAACTTCCTGCTGAGCAAAACCATCGACAACTGGTATTTCGAAGTTAACA
CATTGCTGGAAAATGGCAGTACGTTATATACAGAGAAGCAGTACCACGGAATGGATTTAACCAACGATAAAAA
CGTGGCTTACGTCCGTCCGGTTATCGCCTGGCAAAACGGGCGTTTTTCAACGGCGATAGCGATGGAAAGTAAC
GTCGTTAACAACGCCTATGGCTATTATGAGAATGGGAAGTGGATCGATCAGTCAGATCGTACGGGCTATGGTT
TTACCATGACCTGGAATGGTCAAAAAACTGACCCGGAAGATGGCGCAGTGATTAACCTGAATACCGCCTATAT
GGATGCGACCGATGAGACAGATTTTACCGCTGGGGTGAATGCGCTGTGGCATCGATTTGAACTGGGTTATATC
TATGCGCATAACAAAATCGAAGCCTTCAATGCTACCAATATCGATGCCGTTTGTGAGGACGATTGTTGGATCA
CCGATCCCGGCAATTATGATATTCACACTATTCATGCCTCATATTTATTCCCCAACGTGATGGATATGAAAAA
CTTTAACATCTACCTCGGTGCCTATGCTTCATGGGTAGAGGCCAATCCGAATAATGGCGATAACAGTGAAGAT
GCGCGTTACGGCGGGCGTCTGAGATTCAAATATTTCTTTTGATTGTTGGTTTACTAAAAAAGGGATCCGCAGA
TCCCTTTTATCAGATAGTTAGTGCGCGCCGCCACCGCCGCCGCCCGCACCGAAGGGCGGTTTGGCGAACCACA
CCAGGCCGAGCAGCACCAGGAAGATCCCCGCCGACATCCAGAAGATTTCGTTGGCGGAAATAATCAGCCCCTG
ATTCGTGATTTGCTGGGCTATCCAGCCTGACGCCTGTTGGTGCGTCATCCCTAAGCCTTCCAGTTTATCGTAC
ATAGCCTGAGCGTTTGGATTATACGCCGTAACCGACTCCGTGAGCTGAGCATGGTGTAGCGACTCACGATCGG
TCCACATAGTGGTGGTAATCGAGGTGCCGATGGAGCCCGCCAGCGTTCGCGTGAAGTTCGACAGGCTTGATGC
CGCCGCCAGCCGTTCTGGCGGTAACCCGGAGAGCGTAATGGTGGTCAGCGGCATAAAGAAGCAGGCGACGGCA
AATCCCTGAATAAACTGCGGCCATGCGGAAGCGCCGAAATCCATACCCGGTTCGAACGTCCAGGCGCGCCAGT
AAAAACAGACGGCGTACATAATGAAGCTGAACGTGACCAGCCTGCGCATATCCAGTTTATGCGCGAAGCGTCC
GATAATCGGCGACAAAATTACCGGAATAATGCCGACTGGCGCCGACGCCAGACCTGCCCACGTTGCGGTATAG
CCGTACACCTCTTGCAAAAGCTGCGGCAGTAGAACAATGGCGCCGAAATAGAGCATATAGGCAAGGCTGATGC
ACAAGCAGCCAATCGTGAAGTTACGCGATTTAAAAAGAGAAAGATCGACGATAGGGTGATCGTCGGTCAGTTC
CCAGACGATCAGGAAACTGATGGCGATGACGGCGACCACGGTCAGGATGATAATTTCCTGTGAGGAGAACCAG
TCCAGCTCTTTACCGCGATCGAGCATAATTTGCAGGCTGCCGATGCCAATCACCAGTAACGCCAGCCCGACGG
CGTCAATACGTCGCCGTTCGGTATGCGTTTCACGTCCGCGCAGCGTGTGTAACGTCATCAGGACGACGGCGAT
GCCAATCGGCACGTTGATGAAGAAAATCCATCCCCAGTGGTAATTATCACTGATGTAGCCCCCCAGAATCGGG
CCGCAAATCGGCGCGACAATGACGGTCATTGACCAGAGCGCCAGCGCAATGGAGCGCTTTGCAGGCGGATAGT
TATTCAGCAACAGGCTTTGCGAGAGCGGAATCAGCGGCCCGGCGACCACGCCCTGCACCACGCGGAAGAAGAT
AAGCATATTCAGGCTGCTGGATACGCCACAGGCCCAGGAGGCCGCGGCAAAGGCGACCGTCGACCACATGAAC
AGTTTGACTTCGCCAAAACGTTTCGCCAGCCAGCCGGTTAAGGGAATCGAGATAGCGTTCGCCACCCCGAAAG
AGGTGATAACCCATGTCCCCTGGCTTAGGGAAGAACCCAGGTTCCCGGCGATGGTCGGAATCGCCACGTTGGC
AATGGTGGAGTCCAGCACCTGCATGAATGTCGCCAGCGACAGCGCGATCGTCATGATGACGAGCTGCGCGCCC
TCCAGCGGTTTTTGCTGTTGCATCACACGCACCTCAGGATTAACCCGCGTTAGCCTGTACGATGTCGTCGATC
AGTTTATTGACCGGCGCGAGATTAATTTCGCGTGCGTTACTTTCGGCAACCGGCGTCGTTCGTACCTGGCTGG
CCAGTACCTGACCGTCGCGATTAGCGGTATCCACGGTGACCAGCGTCGATAAACCAATGCGCAGCGGATGTTG
TTCTAACTGGCGGGCGTCCAGTTCGACGCGTACCGGCAGACGTTGAACCACTTTAATCCAGTTACCCGTCGCA
TTTTGCGCGGGCAGCAGGGAGAAGGCGCTGCCTGTCCCCATATCCAGACCGACGACTTTACCGGTGTATTTTA
CGTCGTCGCCATAAATATCAGTAATCACCGTCACTGGCTGCCCAATGCGCATATTCGCTAATTGGGTTTCTTT
AAAGTTAGCGTCCACCCACAGATCGGTTGCAGGCACCACGGCCATCAGCGGCGTGGTGGGGCTGATTTGCGCG
CCAGGCTGGACGGCGCGGCGGGAGACATAACCAGTCATTGGGCTGACGATGCGAGTACGCTCCAGCGCCAGCC
AGGCGTTACGCACTTCGGTCGCCGCTTGTTGAACCGCAGGCTGATCTTCCAGATTACTGTTGAGTATCATTGC
CTGGTTGGCATTGTACTGTTGAATGGCGACATCCAGCTGTGCCTGCGCGCTGGCGACGGCATCGCGGGCGTGT
TGCAGCTCTTCCCGGCCAATAAGATTGGCATTACCCAGCGGCACACGACGGTTAAGGTCGCTCTGCGCTTGCG
CCAGGGCGGTTTTTTGCACGTCGATATTCGCCTGCAACTGCTTACTGTTAATCATCAACTGGTGCGTTTGGCG
CACGCTGGAGGCCAGCGCCGTTTTGGCTCTTTCAAACGCTTGTTTGGCGTCAGTCTGATCGAGCGTGACCAGA
ACATCGCCCTCTTTTACAAAGTCGGTGTTATCAGCCCAGACTTTCGTCACGCTGCCTGACACCTGCGCCATGA
TTTGAACCTGGTTCCCTGCCACGTAAGCATCATCTGTCTCTTCAATATGACGCAATACTAAAAACCAATAAAT
TCCATATGCCACGGCAATAATAACAAAGAGCAAGGTAAGAAGTAGCAGCGCTGTTTTGCGTTTGCCTTTCTTC
TTAGCCGGTTGCTGCGGGGTTTGGATCTCCGCATTTGCGCTCATGTTGTTCTCCACGATCTTATTATTCACAT
CGGCTGAGCCGACCTGTTGTCAGAAAGGCCAGCAGTAATGCGTGCCGGCCTGTCCGCTTTCCTGTTATTAATC

TGGATTTTTGAGCGGGTCGGCGCTTTAGCGCAGCGCCTCAAGAACAGTGCCTTCCTGCTCCATTTGATCAAGA
CGCGTCAGGAGTTTACGAGTGATGTGCTCAAGTTGATCTTTTTCGGCTGTGCTAAGTGATGACCAGAGTTGAT
GCAGACAATGATGCTGAGGCGGAAGCACTTCTTGCAAAAATGCCTGGCCTTTCTCCGTTAATTGCAGATGCAG
GCAACGGCGGTCGTTATCGCTCTCACGACGCTCAATCCAGCCGCGTTTTTCCAGCTCATCTGCAATGCGTGTC
GCATTGGTGCGAGATGAACCCAGCGCGCAGCTTAATTCAGACGGCTGAATACTGTGGTTTTCCTGAGACTCCA
GCGTAATCAACGCCATAAACAACGTCTCGTTAATCCCTTGAGCCTTCAGCATTTTATTGCGGTTTTCCAGGAG
CTTGCCTTGCATGTGCATACAAAGACGAGTCAAAAGAATTTCCTGATAAGGAAAGTCTTCGTGACGGCTGGCG
CGAAATTTTAGCATTTGTTCAATGGGCGTAAACGAACTATCCATTTGGGCATGACCTCATTAATTTCAGCCGA
TATAGTAACGACAGTGACAAATAAAGTAAATGTATTATTAATACAAAATCATTCTTTCAGGCTATAAAGCAAC
CAATGATACGCAGACTGAGACGCCAGCGGGAACATTGGGCCCGGTAAAGCGCCGCCTGCGGCAAATCCAGGAA
TGACGAGAAAAGCGTGAGTGCCTGCATGAAAAAATCAGGAGAGAACGATAAGCGGGAGGGGCGAAAGCCGGGT
AAACGGCCTTACAACCCGATGCAAAAGCCACCGGAATATCACGGATAGCTATCGGTGAACGACCTTCGAATCC
TTGAACGGGCGCTATTGGCCTGGGAGCAGATGGGGATGCAGGGCTTTCCATAGAATTATATTATTCTTCACCG
GTATAAAAATAACCATGATAAATGAATAGCATAACCTTAACAGACTGGGGAGCTCCTTAACACCCTAAGGAGC
TTAAACAAAGGTAAACGCTCACTATCCGCTTAATTTACGGCTTCCTGTCGGTGAAAACCTCGCCACCAGACCA
GTAAATTAAGTAAGGCTACCGTGACACCCGCCAGACAAACGCCGGCCCAGCCGGCATGTTGCCAGGCGGAGGC
GGAAATCAGCGACCCCGCGGCGCCACCGATAAAGTAGCTGGTCATATAGCCGGCGGTGAGCCGGTTACGCGCA
TCCGGATGCAAACGATAGATGACCGTCTGATTGGTGATATGTACCCCCTGAACGGTGAGGTCCAGTACCAGAA
TGCCAATAATCAGCGCCAGTACCGAGGTATGCCCAAGCCAGATAGCCAGCCAGGAGAGTAACAGCAGCAGTAA
GCCGAACGTGGTGGTGAGGTGAGATTTACCTTTATCGGCAAATCCGCCAGCCGGACGCGCGCCTAAAGCGCCG
GCGGCCCCCGCCAGGCCAAACAGGCCAATCATCCCCTCGGAATAGCTAAACGGCGGCGCGGCGAGCAAAAAGG
CCATTGACGTCCAGAGGATGCTGAAATTAGCAAAGGTCAGACAGCCCAGCAGCGCGCGGGTACGCAGCAGCTT
ATCGTGAATAAACAGGCTGAATACAGAACCCAACAGCTGCGGGTAGTTAAGATGGGTGTCGGATTTGAGCTTT
GGCAGTCCGCGCCACAACGCGACGGCCATCAGCGCCATCAGCGCCGACGCTACCCAAAATACGGTGCGCCAAC
CGCCGAGGTTTGCCAGCAGTCCGGCGACCGTTCGCGCCAGCAGGATCCCCAGCAACAGGCCGCTCATAATGGT
GCCGACCACTTTACCGCGGGTGGCGGGCGTCGCAAGCGTGGCGGCCAGCGGAACCAGAATCTGCGCCACCACG
GAGAACAGTCCGGTTAAGGCCGTTCCGAGTATCATCATGCTAAGCGACTGACTGCTGGCGGTGATCAGCATTC
CGCCAGCCGCCAGCAACGTCATGGAGACAATCAGCGTTCGGCGTTCAAACATGTCGCCGAGCGGCACCAGAAA
CAACAGGCCAGCGGCATAGCCCAACTGCGCGGCGGTAACGATAAACCCTGCGGAGCTGGCGGAAAGCGAAAAG
TGATGCGCGATGGTATCAAGCAGCGGCTGGGCATAGTAGTTGCTGGCGACCGCCAGACCCGTGGCCACAGACA
TTAAAACGATCAGCGCCGGGCTAAGCTCATGAGTGGGTTTCGTCATTATGGTAATCGTGATTCAGATAATTAT
TTCCTGGGAAAACAATCATAGCGGATGTTGATGAATAAAGGGGAACGGTGACGTGATGAATCGTGCGCTTTGA
GGAGAGATTGAATAGGCCGGATAGCGGCGCATGAACTGCGCTTTATCCGGCATAAAAAACGATCTTATTTCTG
CGCGGCCAGCGCTTCTTTCACCCAGCCGTCAAACTGCTGCTGGTGGGCGTTGATCCAGCCGTCCACGTGGCCC
TGAACATCGGCTTCAGACGATTTACCGGCATGCATCATGGCGTTCTGCGCGTTGATATCCGCCAGCGGCAGCT
TCATGATGGCGAACAGTTTCGCCGCCGCCGGGTTTTTCTCCGCCCATGCCTTATTGGCGACAATATGCATGGT
ATTAACCGGGAACCCATAGTTCGCGCCGTTCGGCAGTTTAGTATCAATATTTTTCTGTTCGCCCGGCAGAGAG
GAGAACGGCACCTGTAGCCAGACGACATCTTTACCCGGCTTCATTACGTCGCTCACCCAGTACGGGGTCCAGG
TGTAATACAGCACCGGCTTGCCTTCTTTAAAACGGGTAATGGTATCAGCCATCATCGCCGCATAGTTACCGTG
GCTGACCTCAACGGTTTTTTGCAGATCAAACGCTTTATTCTGATGATTGATCACGGCTTCGCAGCCCCAGCCC
GGCGAGCAGCCCATCATGTCCGCTTTTCCGTCGCCATTGGTATCGAAGATTTTGGCGATTTTCGGATCTTTTA
GCTGAGCGATATTGGTGATGTTGTACTGCTCGGCGGTTTTTTTATCGATCAGATAGCCCTGCGCAGCGCCGGA
GACGAAGACGCCCTCGCGGTAAAACTTGTTGTCTCCGCCTGCTGCGGCATACATATCATCATGCAGCGGCTGC
CAGTTCACGGCGGTAAACGTCGCATCGCCAGAGGCGATGGAGGTATAGCCTACGTTGTAATCGACTTCACTCG
GCTTATTTACGGTATAACCCAACTTTTCCAGCGCCCGACTGACCAGTAACGTCTGGAAGGTCTCTTCAGAAAT
CGTGCTTTGGATAGGTTGGACGGTAATGCCTTTGCCAGGCAGGTCGGCAGCGAAAGCGCTGGCGGTGACAAGG
GTGGCAAACGCTGAGGCAAATATCACGGTATGTCGCATCGTTGTTCCTTTAATTATGGTAGGGAGTTGTAAGC
CCGGTAAGCGCAATGTCACCGGGCGTCAGGAGAGTTACTTAACGAAAGGGCGGGTGATTAGCCCAACAGGACC
GGTGGTATACCAGCGACGGTTACCGCGGCTACGCGAGTCGCGCCCGACGGCCTGCGTCAGACGGTCCAGAATG
ATGGCGAGAATCACAATGCCGACGCCGCCGACGGTTGCCAGCCCCATATCAAGACGACCAATGCCGCGTAGTA
CCATCTGGCCAAGTCCGCCGACCGCAATCATCGAGGCGATGACGACCATCGAGAGAGCCAGCATCAGCGTCTG
ATTAACGCCTGCCATAATGGTGGGCATCGCCAGCGGTAGTTGCACTTTGAACAACATTTGGCGCGGGCTGGCG
CCGAACGAGCGCGACGCTTCAATTAAGTCGGCAGGCACCTGGTTAATGCCCAGGATCGTCAGGCGTACAATCG
GCGGTAGAGCAAAAATAATCGTCACCACCACGCCCGGCACGTTACCGATGCCGAATAACATGACAATCGGCAC

CAGATAGACAAACGCGGGCGTGGTCTGCATCGCATCCAGCAGCGGACGAACTATTTTGGCCGCACGCGGGCTG
CGCGCCAGCCAGATTCCCATCGGTAATCCGATCACGACGCAGAACAACAGGGCGGTCAACACCAGCGCCAGGG
TAATCATCGCCTGCGACCAGGCACCGATCGCGCCGATCGCGATCAGCGATATCAGCGTCGCGATCCCCATGCC
CACGCCGGAAACCTGCCAGGCAATCAGCGCAAAGAGAATAATCGCCACAGGGGCGGGCATTCCCAGCAGCAGT
TGCTGAAAGCCGTTGAGGATGTAATCCACCGGCACACGAATCCCCTGAAAAAGGGGACGGAAATGGGTGACGA
CCCAGTCGATTCCCTCTGTGACCCAGCTATCCAGCGGGATCAGCGTCTTATGGAACGGGTCCAGAAGAGAAAA
GTGTTCAGGGGCTGGCGCGGGCGCGCTGTTCAACCAGTCGGTACTGCCGCCGTCCGTGGCTACGCCTGCCGGT
GTTCCCCAGGCATCAGCCGTTTGCGTCGTAGTATCGGCCACCTGTGCGGTATCCCACGGATTCGTTTGATCAG
CCATTGTTACCCCCTCGCGATCTAAAGCCTGTAGCAACATACGTTTTGAAATAATGCCAACATACTGGTGTTC
TTCATCGACAACCGGCACCGCGCAGGGCGCCTGGCCGACGTGAGAGAGCAACTCGCTGAGTGGGGTTTGCGCA
TCAACAACTAAAGGGTCGTCGATAAGCGCCGCTTCAATCCCTTGCGCCTGGCTTAATGCCGCTTTTAATGAGT
CGATGGACACGACGCCCACGAATTTATTGCCACGCTCAATGACGTAACCATATTCACGGTCTTCGTCCTGTAA
TAATTTCAGTGCCGAACGGGGACCGAAACCTGGCGTTTTACGAATTAAGCCGACCGGACTGCGACGGGCAATA
TCTTTGGCGCTAAAGACCTGACTAATATCCACGCCACGGAAGAACGTGCGGACATAATCATTTGCCGGATTAT
TCAGGATCTCATCCGGCGTACCAACCTGTACGACCTCGCCATTTTGCATAATGGCAATCCTGTCGCCAATACG
CATAGCCTCATCAAGATCGTGGGAAATAAAGACAATGGTGCGCTGATGTTTCGCCTGTAATTTCACCAGTTCA
TCCTGCATTTCGGTACGAATTAATGGATCGAGGGCGGAAAACGCTTCGTCCATTAATAAGATATCAGGGTTGA
TTGCCAGCGCGCGGGCAAGTCCAACACGCTGACGCATCCCACCGGAAAGTTCATCCGGATAGGCATGAGCGTA
ATTTTCAAGCCCCACCTGACGCAAGGCGTCCAGCGCTTTTTCGCGACGCTCTTGCGCCGCGATGCCCGCTAAT
TCCATACCGAATGCCGTATTATCCAGCACGGTCATATGCGGCATGAGCGCAAATGACTGGAAGACCATCGCAA
TCTTTTTCCTGCGCACCTCGCGAAGCTCAGCGTCTGATATTTTGGCAATATCAACGCCGTCAATCAGTACCTG
TCCGCGGGTGGGTTCAATCAGGCGATTGAGAAGGCGTACCATTGTGGATTTACCCGAGCCGGATAATCCCATG
ATGACAAATATCTCGCCTTCTTCAATGGCCAGACTGGCGTCTTTAACGCCAAGCGATAGCCCCGTTTTTTCCA
GTATTTGCTCTTTCGATAGTCCCTTTTCAATATATTTGAAGGCACGCTGCGGATGCTCTCCAAATATTTTATA
CAGATTCTTCACTTCTAATTTAATTGCCATGCAATAGAATGATTCCTGTTATTTACTTATGTCGATATGTTAC
CTGATAGAAATAGTTACCTTTTTCTACCCTAACATACTCAGAATCTGAGACAACCCTGAATTTGCTTCTGGCA
TGAATATTGCGAGGTGCTATCGGCTGTGATTTCCCATGATATAAGGGCTGAACGCGAATGGGGAGGAAGAGAT
ATTTTTTGGAACAACCAGCAAAAATCCACCTGAATTGGAGGAATCCAGGTGAATAATGTCACTCGTATTGAGT
GTAGGACATGTTGTAATATTTGCGCGATTTAACCCGGTATTGCTTGAAATAAAGGCATGGAAAATGTAACGTT
ATTTCCCATGCCCGCAAGGTTAAAAATTCCAGTCTTCGTCTTCGGTTTCGACTGTTTTCCCCATCACATAAGA
TGAACCTGATCCGGAAAAGAAATCATGATTTTCGTCGGCATTCGGCGAGAGCGCGGCAAGGATTGCGGGATTC
ACGTCAGCCATCTCTGGCGGAAATAACGCCTCATAACCCAGGTTCATTAAGGCTTTATTGGCGTTGTAGCACA
AGAAGGCTTTGACGTCGTTAACCCAGCCGGTTTCCGCATATAACGCTTCTGTGTAGCAGATTTCGTTGTCGTA
CAGTTCCATCAACAAATCCAGCGCGAAAAGCTTTAACTCTTCACGCTCGATTGCCGATAGTTTTTGTAGCGCT
ATCTGATACTTATAACCAATATAATAACCGTGAACCGCTTCATCGCGAATGATTAAACGAATCAGGTCGGCAG
TGTTCGTGAGCTTACCGCGGCTGGAGAAATACATCGGCAGCCAGAAGCCGGAATAGAACAGAAAAGACTCTAA
AAAGACGCTGGCAATCTTTTTCTTTAGCGGTTCATCGCTGACGTAATGAGCCAAAATAATCTGCGCCTTACGC
TGAAGCGGCGGGTTTTCTTCGCTCCAGGCGTAGGCGGCATCAACCTCTTTCGTCTGGCACAGCGTGGAAAAAA
TAGAACTGTAAGAGCGGGCGTGTACCGCTTCCATAAAGCTGATGTTCGACAGCACTGCCTCTTCATGCGGCGT
GATGGCATCTGCCATTAACGACGGCGCGCCTGCGATGTTCTGGATAGTGTCGAGCAGCGTAAGTCCCGTAAAC
ACGCGAATGGTGAGCTGCTGTTCGGCGGCGCTCAGCGTCTGCCAGGCCGGAATATCATTCGATAACGGCACTT
TTTCCGGCAGCCAGAAGTTACTGGTCAGCCGGTTCCACACCTCCAGATCTTTGTCGTCCTGGATCTTGTTCCA
GTTGATGGCGCTAATACGAGATAATTTCATATGGCTTTCCTTATAGCGCGCAGGATACGCAGCCTTCAATTTC
AGTACCTTCCAGCGCCAACTGGCGAAGCCGGATGTAATACAGTGACTTAATACCTTTTCGCCAGGCATAGATC
TGCGCTTTGTTGATATCGCGGGTCGTGGCGGTATCGGGGAAAAACAGGGTGAGCGACAGTCCTTGATCGACGT
GGCGCGTGGCCTCGGCATAGGTATCAATAATTTTTTCCGGACCGATATCGTAAGCATCCTGATACATGTCCAG
ATTTTCATTGGTCATAAACGGCGCGGGGTAATACACACGCCCGGTTTTGCCCTCTTTGCGAATCTCAATTTTG
GCCACAATCGGATGAATGCTGGAGGTCGCATGATTAATGTAAGAAATCGAACCGGTCGGCGGCACCGCCTGCA
AATTTTGGTTATAGATGCCATAGCGCATCACATCGTCGCGCAGCTTTAGCCACATTTCTCGTGTGGGCAGCGT
AATGCCGCTGCGGGCAAATAGCGTTCTGACTTTCGCCGTTTTCGGTTGCCAGTCGTCCTGTAAATACTGCGTA
AAATAGTCGCCGCTGGCATAGCGCGACTGCGCAAATCCGGCGAAGGTTTTGCCGCGTTCGCGGGCCAGCCGCA
TTGAAGTATGCACGGCATGCCAGGTAATGGTGTAAAAATAGAGATTGGTGAAATCCAACGCCTCCGGCGAACC
GTAGGCAATGCCTTCTCTCGCCAGATAGCCATGCAGATTCATCTGGCCCAGACCGATGGCATGAGAGGCGGCA
TTACCGGCGGCTATTGAGGGTACGCTGCGTATATGGCTCATGTCCGACACCGCCGTCAGGCCGCGAATAGCGG

TTTCTACGGTACGACCAATGTCCGGTGAATCCATGACGTGAGCGATATTCAGCGAGCCGAGATTGCAGGAAAT
GTCATGCCCGATGTGGGTATAGTCAAGGTTATCGTCGTAACGGGAGGCGCTATTGACCTGTAAAATTTCCGAG
CACAGGTTGCTCATATTAATGCGCCCAGCAATGGGATTCGCGCGGTTTACCGTATCTTCAAACATGATGTAGG
GATACCCGGATTCGAACTGAATCTCCGCCAGTGTTTGAAAAAAGTCACGGGCGTTAATATAGGTTTTACGCAC
GTGCGGATCGGCAATTAATTCATCGTACCGTTCGCTAATGGCGATATCGCCAAACGGTTTGCCGTAGCGTCGT
TGTACGTCATAGGGCGAAAAGAGCGCCATTTGCGCGTTTTCTTTCGCCAGCCGGAAGGTGATATCCGGGATCA
CCACGCCGAGAGAGAGCGTTTTGATCCGGATTTTTTCGTCGGCATTTTCCCGTTTGGTATCCAGAAAACGCAG
AATATCCGGGTGGTGCGCATGGAGATAAACCGCGCCGGCCCCCTGGCGCGCGCCAAGCTGGTTGGCATACGAA
AACGCGTCTTCCAGCATTTTCATCACCGGGATCACGCCGGAAGACTGATTCTCAATGCGTTTGATCGGCGCGC
CCGCCTCGCGCAGATTGGAGAGTAAAAACGCGACGCCGCCGCCGCGTTTGGAGAGTTGCAGCGCCGAATTCAC
CGCCCGCCCGATCGACTCCATGTTGTCTTCGATACGGAGCAGGAAGCAGGAGACCAGTTCCCCACGCTGCTGT
TTACCGCAATTTAAAAAAGTCGGGGTAGCGGGCTGAAAGCGACCAGAAAGCATTTCATCGGTCAGTTGGGTGG
CCAGCGTTTCGTCACCCTGCGCCAGCGTCAACGCCACCATTGTCACCCGATCTTCAAAGTGTTCCAGATAACG
TTTGCCGTCGAAGGTTTTCAGCGTGTAACTGGTATAGAACTTCCAGGCGCCAAGAAACGTCTGGAAGCGAAAG
CCGCTGGCGTGGGCGTGCTCGAACAGGCGGAGGACGAAGGCGCGGTTGTAACGCGCGAGGACAGCGTCATCGT
AATACCCTTCCCGAACCAGCGTCTCCAGACGTTCATGCTGGCTGGCAAACGTCACGGAATGCGGGCGGACGTG
GGTGGCAAAGAAGGCGTCGATCGCCTGCTGGTCCTTGTCGAACTGAATATGGCCTGCTTTATCGTAAAGATTC
AGCATCGCGTTCAGGGCGTGGTAATCCATGGTTTCCTGCATTACGCGCTCCGGGATAGTTGTTGCCAAAATTC
ATTTACTCCTTTTCGGACGTTATCGATGTCGCGTTGTGTGCCCATGAGCTCAAAGCGGTACAGCCAGGGGACG
CCGCATTTTTGTGCTATCACATCGCCAGCGCATCCCCAGGCATCGCCGAAATTGCGATTACCGGAGGCGATAA
CGCCGCGAATGCGCGCCCGGTTGTGTTCATCATTTAAAAAGCGGATCACCTGTCGCGGCACCGCACCGGCCAT
CCCGCCGCCGCCGTATGACGGCACAACCAGAATGTACGGTTCGTCTACCTGAATTCGCTCCCGCTCATTGAGC
GGAATACGCGTGGCGGGCAGCCCCAGACGCTGCATAAAACGGTGCGTATTTTCAGAGCTGCTGGAGAAGTAGA
CGAGCGCGCTCATGCGTTTGCCGCGTGGGGTGTCGGGTGCAGACGGTTAATCATGTCCGGACGGAAGCCAGAC
CAGCTCAAATCGCCCGCTATCACCACCGGTAATTGACGAAACCCTTGCGCCCGGAGCGTATCCGCTGCATCCG
GCACCAGATCGACGTTCACCATCTCAAATTCAAATCCACGGCTTTCCATCGCCCGCTTTGTGGCGTGGCACTG
AACACAGTTATTGCGAGTGTAAATAGTAATGCTCATGATTCGTATTTCCATTTAAAATGAGAAAACGGCGCGA
TGAAACGCGTCGGATTGTCGTGTGTTGATACACGAAATACTAGATGTAGTTGAATTAAGATTCAACCATACAA
GATATGGGAATTTTCGATTGATTTTAACGTCGCTGATGAACGCAGCGGGGTAGGGGTGGCATGGCTAATTTTT
GCCGGATGGTGGCATGAACGCCCGCTCCGGCCTGCAGGTTGACGCATAACAGAGTTGGCTGACATCAGCGCCA
TCCGGCAACAATGCGTCACATTCGCATACCGATCGCCAGACGGTTAAAGCAGTTCATTAAACCAATAGCGAAG
GTCAGATCGCTGATTTCACGCGCGCTGAAATGATCGAGCAGCGGCAGGTAAACATTATCTTCGGCGTGGGTTT
CAGCAATGTGCGTCACCGATTCCGCCCAGGCTAACGCCGCGCACTCTTGCTCGTTGAAATGGTGGCTTACCCG
CCATCCTGCCAGCGCATCTAATTTAGCTTGATTCACGCCAGCCTTACGCAGCGCTTTACTGTGCATCTCCAGG
CAAAATGCACAACCATTTATTTGCGAAACACGCAGGTAAATCAGCTCCATCAGAGTGGGGTCAATCGTGCTCT
TTTCCAGCGCGTTTTTCGCCTGCACCAGAGCGCTGTAAACCTCCGGGCTCAGTTCATAATAGGGTTGGCGTAA
CGTCGTCATCATGTTTCTCCTCGTGTTGATGACGAGCACTGTAGCACTATAATGGTCTGTAAAAGAGAGCCAT
ATTTTTACAGAAAAAGCAGACCATATGCCGCGCTATCAGCACATCGCTCGTCAGTTAAAAACGGCCATTGAGC
AAGGAGAACTCGCGCCCGGAACGCGCTTGCCTTCCAGCCGGACGTGGGCGCAGGAACTGGGCGTTTCTCGCGC
CACGGTGGAAAATGCCTATGGCGAGCTGGTGGCGCAGGGCTGGCTGGAGCGACGTGGTCAGGCAGGCACGTTT
GTGAGCAACGCTCTACGGTTTGAGACGGCGCCGCCGATACCCGCTGTTTTTGCCGGAGAAAGTCCGGAACCGA
AACCCTTTCAGATGGGGTTACCGGCGCTGGATCTCTTTCCACGCGAGAAGTGGGCGCGAGTGATGGGGCGTCG
GTTGCGCACGCAGACGCGCTTCGATCTGGCATTAGGCGACGTCTGCGGCGAGGCGATTTTGCGCCAGGCGATA
GTCGATTACCTGCGGGTTTCGCGTAGCATTGAATGCCTGCCGGAACAGGTATTTATTACCTCCGGATATGCGG
ATTCTATGCGGCTAATCCTGCGTACATTGTCTGTGCCGGGAGACAGCATGTGGGTGGAAGATCCCGGTTTTCC
GTTAATTCGCCCGGTGATAACGCAGGAGGGGATTACGCTGGCGCCGATTCCGGTCGATGCGGATGGGCTGAAT
GTCGCGGCGGGGATGCGGGATTGCCCGCAGGGGCGCTTTGCATTGGTGACGCCCGCCCATCAAAGTCCGTTGG
GGGTGGCGCTGTCGTTAACTCGCCGACGGCAACTTCTGGCATGGGCGGCGAATGTGCAGGCCTGGATTATTGA
AGATGACTACGACAGCGAATTTCGTTATCACGGTAAACCGCTTCCGCCGCTCAAGAGTCTGGATGCCCCGCAG
CGAGTGATTTACGCCGGAACGTTCAGTAAGTCGCTCTTTCCGGCATTACGTACCGCCTGGCTGGTGGTGCCGA
TAAAGCAGATTGAGCATTTCCGCCAGCAGGCGTCGCTGATGCCCTGTAGCGTACCGTTGTTATGGCAGCACAC
GCTGGCTGATTTTATCCGTGATGGCCATTTCTGGCGGCATCTGAAAAAGATGCGTCAACATTATGCTCAGCGA
CGGTTATGGATTGAAGAGGCGCTGGCAGAACAGGGATTTGTCGTGACATTACAGAAAGGCGGTATTCAACTGG
TTATTGAGGTTGAAGGCGATGATAAAGCGCAGGTAGCGAAAGCGAATCAGGCCGGACTGGCGGTACAGGCGCT

AAGCCGTTGGCGAGTGGTTTCGTCAGGAAAGGGGGGCATTTTACTGTCGTTTACCAATATTACTTCCGCCGCC
ATGGCGAAACAGGTCGCATGGCAGCTTCGACAGGCGATACAGTAACCGTTTCGCCGAATGGCGCGACCTGGGC
CTGCGGCAATGATGCAGGCCCAATAAAGTATGTCGCCATCCGGCAGCGCACGTCGTTAGCGACGCAGGCTAAG
CAACGCCCCGATAAAAATTCCTACCGCTGCGGCGGTTCCTACGCTACACCAGGGTTTTTCACGCACAAACGTA
TCCGCGCACCCCATCGCATCACAGGCGGCTTGTTTTACGCGAGTACGTCCATGCATCCGCGCCCGGGTCTCTT
TCAGCAACGCCTGTGCTTTACGTCGCGCAGCCTCGGCTTCGTCTTTCGCATCGCTTCCCCAGGATTTAAGGAC
ATCTTCCAGACTGTCGGCTAATCGATTGACATCATTCTGAATATCCTGAACACCATCATCGACGTCGTTTCGG
TTCGGTTTGTTAAGCATATGATCCTCCATTGATTTCGATGTAGCTTTCAGTGTAGTTCATATTTTTACCTGCT
GAGGCTTGTTACGGCGATATCATGCGTTTCTGGACTTTTCTGAAAGCGTTGATAACGCGCTATCGGGTAAGGT
AAGCCCGCAGTAAAAGAGAATGAGGATAAAATATGTATTTACGACCTGACGAGGTGGCGCGGGTACTGGAAAA
GGCAGGGTTTACCGTCGATGTGGTGACGAATAAGACGTATGGTTATCGACGCGGCGAGAATTATGTTTATGTT
AACCGCGAGGCGCGTATGGGGCGTACCGCGCTGATCATTCACCCCAGATTAAAGACCGTAGCTCATCACTGG
CTGACCCGGCTTCGGATATTAAAACCTGCGATCACTATCAAAATTTTCCGCTCTATTTAGGCGGCGAGACCCA
CGAACATTACGGTATTCCCCACGGCTTTAGCTCCCGAATAGCGTTAGAACGTTATCTGAATGGGCTTTTTGGC
GACGAAAAAACCGACTAAAAGCGCGACTGGCAATGCCAGTCGCGACGCTAACTTACGCCTTAGCCTGATGATA
GCTGCTCACTTTAAACAGGCGGCGACAATAATCCAGGAAGTAGCCATAAACCGCGCCCATTAACATTGAAACA
ACAATGTTTGAACTCACGGCGGCCACTATCTGATGCCAGCCGGCACCGACGGTCAGCAAGATGATAATATAAA
CGGGTGACTGAAAGGTGACATAAGCCAATACATCCGCCAGGTTTTTGGCCCATCCCGCCGGGCTTGCCTTACG
CGCGACGCGCATAATCAGATCGCGGTAAACGCCGTATGGCCATGCAATCAAAATGTTGACAGGTATCGCCACC
AGCCTGGAAGAAAGCGACTGCTCAAAGCTCATTCCGGAGAGGAATATCTCGATCAACATGTTCACAACAGAAC
AGTAAACAACCATCGCAAACGTGTCTGCTACGGCATGACGCAAGCGTGACTGTGGAGAGAACATGTTAATGCT
CCTTGTAGCGTATCGATCAGAATGAGGTGATGGTTGGTAGTGAAATCATAAAGCACTTTTGTCTTTGCATAGA
GTATATCCCTATATAATAACTATCAACTAGTGTAATATTTTTATTTATAGGCTTTTTGTCGATAAAATACTCT
GTGATTGTGTGTTTTTTGCTCGCGGCGAGATTTTCGTTGTTGTTAGTCATTAATTACTTTTAAATCAATGCAT
TATAATGCATTGTTTTTCTGGCGATTTTCAGCCACATTCCAAATGTTGTCAGATGCGATGTATTCTCGGCTAA
CGGCACGCGCTCTACTTTTCTCAGCATAATTATAGCTATTGCAGGCTGATTTTTAGCATAAAGCTCTGATGAT
GATTTTTGAATGGTTAATCTCTTATTGTGATGAAATATTCACTTCTGTATAAGGTCAGCCACCGTGAGGGTCG
CCGTGGTTACTGAGATACGCAATACGCTATAAACAGCCGAACGGGAAACCTACAGATATACCGTAGGCAGAAT
AACCTGAAACACACACAGGGGGTAATGAATATAACCTTCTGGTTAACAGATTAATATAATTAATCGAGTTATACT
GAGTATATCTGTATGAACTTTAGGATCTGGAAATATAATCAATAATCGCATACAATACCGCCTGTAATAGTTT
TTGTTTTCTGCGTTAAAAGGTTTTTATTGATATGAATTTGATGTTACAGAACTTAAATAATATCCGCACGCTG
CGCGCTATGGCTCGCGAATTCTCCATTGACGTTCTTGAAGAAATGCTCGAAAAATTCAGGGTTGTCACTAAAG
AAAGACGCGAAGAAGAAGAATTGCAGCAACGCCAGCTTGCCGAGAAGCAGGAGAAAATTAATGCCTTTCTGGA
GCTGATGAAAGCAGACGGTATTAACCCGGAAGAGTTATTTGCCATGGATTCAGCAATGCCGCGTTCTGCTAAA
AAGCGTCAGCCGCGTCCGGCAAAATATCGTTTTACTGATTTCAATGGCGAAGAAAAAACCTGGACCGGACAAG
GCCGTACGCCTAAACCGATTGCCCAGGCGCTGGCGGCGGGGAAATCTCTGGATGATTTCTTAATCTAATCACG
GATAACCTTTAACAGATGGCGCTGCGTTTCCTGTCTCTATTAACCGGGGCTCAGGGAACGTTGCGCCATGCTC
ATTCTGATAAATCAGCACACGCCAAAATCGCCCTCTTCGCAATAGGGGGTGACATCTGCCGCTTTGAGGATCA
ATTTTTCGCCCTGCTCATTACCGACCTGTACCGCGACAATATGATCCTCGTTGACTTCAATCACTTTCAGTTT
CGGCCCACCCATTCGGGGCTGTACGATATCGCCTGGTGTGAACATCACGCTCTCCTGTTGAACAATGGTATAA
ACCCGCCTTCAGTCTACTACACACCTACCGACGCGCGGTGAAGGTACCGCTTAATGAGTACGGGTATTCCAGG
GCATTTTTTCCAGTAGTCGCGCCATTCCGCAGAAGCCGGTCATGCCTGCCAGCATTAACCCGGCCCCGACAAA
ACCGCTAATGAGGAAAAAGCCGCCGTGGACCGTATAGCCCAGAATCACCCCAAGCAACGTCAACCCGCCGGCG
GCGATTTGCACCTGACGCATCAGTGGCAACGGTTGCGATTTGTCTTCTGTGACGGGCAATCCTGCCGCTTTCC
ATCCGTCGATGCCGCCTTCCAGTAGACTGACCTGAGCAGGGGCGGCAATGGCCTGTAGTTTTGCTGCATTGCT
GCTGGTTCGTTTACCTGACTGACAGTGAAAAATAATCTGTTCCGCTCGCAGGTTGGCGGGCAAATCGCCCTGT
TCAAGCCGGGAAAGCGGCGCCAGTTGCGCATGTGGAATATGCTCGCGCAGATATTCGTCGGCGTCGCGTACAT
CAATCAGTTTCGCGCCTTGCGCTATCAGCGCTTGCGCCTCACGGGGGGAAACTATCCCAATGGACATCGTGCC
TCCTTATGGACAATAGAGATTCTTTAACGTGGCGATAATCGTATTAACCGCCTCATTTTTAATGAAATAGTGA
ATGCGCTGGGCATCGCGTTGACTATCAATCAGCCCTTCTTCCCGCATCCGCGCCAGATGTTGCGACGTCGCGG
AGGGGCTTAGCCCGGTAATGCGCGCCAGTTCGCCTGCGCTGGTTTTTGGCGAACCGCAAAGCATACATAAAAT
CAGTAATCTTTTTGGGTGGCTCATCGCTTTGAGCAGGGCCGCCGCCTGTTCTGCGCTGGCCTGAAGTTGTTCA
AGCTCTGTCATAGTTATTTAAGTATTTTCTAAATTTAGTTAATTCTAAAATAAACATCAACCTGATGCCAGCG
TAAAAAGCGTAAAGGAGGGTAAAAGCGCGGCGCGCGACTGGCAGGCTCAGGTAAACTGACTATGCTTAGAGAT

AAAATCTCTTTTTAAACAATGACTAATTTTGCTTGTATGGAGTGTATATGGGTTTCTGGAGAATTGTATTTAC
GATTATTTTGCCTCCGCTCGGCGTACTTTTAGGAAAAGGGTTCGGTTGGGCATTTATCCTTAATATCCTGCTG
ACATTACTCGGATATATTCCGGGGCTTATCCACGCATTTTGGGTTCAGATGCGTCATTAATTACCGGCCACCG
GCGGCGCATAAGCGCCGCTTATCAGGATAACCTCATTCCCTCTGCTACACTTTTCTTTGTGTGTATTTTTGAC
AAGCGAGGTGAATATGGGACTTTTCAATTTTGTAAAAGATGCAGGTGAAAAACTCTGGGACGCGGTAACCGCT
AATCATGATAAGGACGATCAGGCGAAAAAAGTCCAGGAACATTTAAATAAAACGGGAATACCCGACGCTGACA
AAGTGAATGTGCAAATAGCTGATGGAAAAGCGACAGTGACGGGGGATGGGCTGAGTCAGGAGGCAAAAGAAAA
AATTCTGGTTGCCGTGGGGAATATCGCCGGGATTAGCAGCGTTGACGATCAGGTTAAAACCACGACGCCAGCG
GCTGAAAGTCAGTTTTATACCGTAAAATCAGGCGACACCCTGAGCGCTATTTCAAAGCAAGTGTATGGTAACG
CCAATCTGTATAATAAAATCTTCGAGGCAAATAAACCGATGCTGAAAAGCCCGGAAAAAATATATCCGGGACA
AGTGTTGCGTATTCCTGAAGAATAATGGCTATCAGGTCGCGCCAGGCTACATTTTCCCTGCCATCGCCTGGCG
AATAATGGGAATCGGCGTCAGCAAATGCTGACGCATTAATTCGCTGGCGCGTGCCGCCTCGCGCGCCAGTATC
GCTTCGGTTAGCGTGTGATGCTGGATATGTTTATCTTCCAGCATCTCGACGGACAGAACGGTTTCACGTAACC
AAATAAAGCGGTAGCGGGCCGCCAGGTCAAATAAGCGTTCGCGCATCTGTAGCAGATAGTGGGAGCCGCACCC
GGCGACAATGGCGGAATGGAACGCCTGGTGGCGCTGATCCCATTCATCGAGCATATGTTCACTGGCATCGCTG
GCTTCCAGTTTACTGAGCATATGCGCTCGCGCCAGGATCTCCGCCTCCCACGCATCATCGCCGCGTTCAATCG
CCAGGCGTACCAGCATCGCTTCCATATTGGCGCGCGCGTCAAAAATATCGAGGAGTTCCGCCTCTGACATGGA
TGCCACGCGATAGCCTTTTTGATTCACCACGGTGACCAGCCGTTCCGCCACCAGTTGCGACAGCGCCTCACGC
AGCGGCCCTACGCCGAGCGCATAGCGGGCGGTTAATAAACTCATCCGCAGTTTCTCATCCGGCTGATAAGTGC
CGCGAATAATGTCATTTTTAAGCCAGCGATAGCCATCTATGGCCGTCGGTTGGGAAAGGGCGGTCATCTTTTA
CTCCTGAGAAGCTTCCCGGCTGACGCCGGGAATAACTAACGTAGGTTTTGCAGGGGGGCTTTTTGCCACCTTA
TCAGTTTTTTCCAGCGAGACATAATGGGGACAGTACCTATTATGCCCAGCCCTAACAGCCCCGTTGAAATGAC
TTCTAGTTGCTGTGCCGGTCGAAATAGCATCACCACCAGAACAAAGCAGATAAAGCCGATAACCAGCCAGGTG
AGCCACGGATAAAGCCACATTTTCAACTTAATTTCTCCGCCCTGCGCCAGCAGAATTTTACGCATCCGCAATT
GCGAAATCGCAATCACCAGATAGACCAGCAGCGCGATGGCGCCGGAACTGTCGATAAGAAATTTAAACACTTT
TGCCGGGGCGTAATAATTGACGATAACGGTTAAAAAGGCCGCGCCGGTAGAGAGGAGAACCGCCACCCAGGGC
GTTTTGCTACGGTTAGTTTTTCCCATTATCGCAGGCGCATCGCCGCGGCGGCTAAGGGAATAGAGCATCCGGG
AGGCGGTATATAATGCGGAGTTCAGACAACTGGTCACCGACAACAAAATCACGCAGTCCATAATGAATTTTGC
GTGCGGAATATGAAGCAGCTCCAGTACCGAACGGTACGAACCTACGCTTTTCAGACCCGGCATATTCCACGGG
ATCAGCGCCACCACCACAAAAATGGAACACAGGTAAAAGATGGAAATTCGCCAGATAACAGAATTGGTGGCGC
GTACGATATGTTTATCCGGCGTATCGGATTCCGCGGCAGCAATAGTGACGATTTCCGCGCCCATAAAAGAGAA
CATGGTGATCAACATGGCGCTTAACACCGCGCCGAAGCCGTTAGGCATAAAACCACCGTGATCCCATAGCCGG
GAGATACCGCTTACCTCAGCATACGGATAAAAACCGCTAATCGCTGTCGCGCCCAGCGCGATAAACGCCAGAA
TGGCAATTACTTTACATAGCGCCAGCCAGAATTCGAACTCGCCGTAATTTTTGACGCTCAATAAATTGCTTCC
GGTTAAGGCAAGCGTAATCACTAATGAAAATAGCCATACCGGAATACCCGGTATCCATGAATTCAGAATAATG
GCGGCAATATTGGCCTCCAGCGGAATAACCAGCACCCAAAACCACCAGTATAACCAGCCGATGGTATACCCGG
CCCAGGGGCCGATCGCTTTATCCGCATAGGTGGAAAAAGAACCGGTATCCGGTGTGGCGACGGCCATTTCAGC
CAGCATTCGCATAATCATGACGACTAAAAGTCCTGCGAACAGATAGGCCAGCAGTACCGCAGGGCCGGCCTCC
GCAATCGCCACGCTGGAACCGACAAACAGGCTGGCGCCGATGACGCCGGCGATCGATAACATGGTGACGTGGC
GTGATTTCAGCCCGCCCCCTAATGCATGTGATTCTGACAATTGCCCCATCCTAAAACCTCTTTATTGTTATTG
CGCTCGTTTACCGGATGGCGCCTGGCTTATTCGGCCTACAATCGAAACGGTTTTTGTAGGCCGGATAAGACAG
TTACGTCGCCACCCGACGCTGCTTATTGGACATTACGCTTGTTTCGCCTCATCAAAGCACTGGGCGATAATCT
CCAGGCCTTGCCGAATCTGCGAGGCCTCAATAGTGAGTGGAACGAGGATGCGCAAGATGTTGTAGTAAGGACC
GCAGGAGAGCAGAATTAACCCTTTCTCGCGGGCGCGGGCCACAATGTCGGCGGTCAGAGCCGCGTTCGGTTTG
CCGGGGTCGCCATTTTCGAACAACTCAATGGCGATCATGGCGCCCAGTCCGCGCACGTCACCAATCTCACGGT
GCGTTTCCGCTATCTCCATCAGGCCATCGCGCAGCGTTTTGCCAAGCGTATTCGCCTTTTGCAGTAGATTTTC
CTGCTCGAAAATGTCCAGTACCGCCAGCGCGGCGGCGCAGGCAATCGGGTTTCCGGCATAGGTGCCGCCCAGC
CCGCCCGGCGCGATAGCGTCCATCACGTCTGCCCGGCCGGTAACGCCTGCCAGCGGAAAGCCGCCGGCGATCG
ATTTCGCAAACGTGGTAATATCTGCCGCAACGCCCATCTGTTCCATGGCGAACAGCGTACCGGTCCGGCCCGC
GCCGCTTTGCACTTCGTCGGCAATCAACATAATGCCGTGTTGGTCGCATAGCGCGCGCAGTCGCTGCATAAAC
GCGGGAGAGGCGGCGTAAAATCCGCCTTCGCCCTGTACCGGCTCAATAATAATGGCGGCGATATCTTCCGGCG
CGGCATCATTTTTAAAGATGCGTTCAATGCTGGCGATCGCGTCGTCGTCGCTGACGTTGTGTAGCGGGCAAGG
ATAAAGCGCGCGGTAGACATGGCCTGGCATCAATCCCATACCAGCAGAGTACGGGTGTACTTTCCCGGTCAGA
GAGAGCGTGTAGTGGGTACGACCGTGGTAAGCGCCGCTAAACGCGATAGCGCCGCTGCGTTTTGTTGCCGCGC

GGGCGATTTTCACGGCGTTTTCAACCGCTTCTGAACCGGTCGTGACCAGCAACGTTTTTTTGGCGAAATCGCC
CGGTACCTTCTGGTTCATGCGTTCGCACAACGCCAGATACGGTTCATAGGCCAGCACCTGAAAACAGGTATGG
GACAATTTCTTCAACTGCGCTTCGACCGCTGAAACAATGCCCGGATGTAAATGACCGGTGTTCAGGACGGCAA
TGCCGCCCGCGAAATCGAGATACTCACGGCCTTCGACATCCCAGACCCGACAATTTTCCGCCCGCTCGGCGAA
AATTGGGTGAATCTGGCCCACGCCGCGAGGAACGGCGTTATGACGGCGCTGCATTAAAGCGTTATTGGTATTC
ATTAGCGACTCTCCTGTTAAAGGCCGATGCACATATATTTGATTTCTAAATAATCTTCGATGCCGTATTTGGA
ACCTTCACGGCCTAATCCGGAGGCCTTAATACCGCCGAAAGGCGCGACTTCGTTGGAGATAATGCCGGTATTA
ATACCGACGATGCCGTATTCCAGCGCTTCGCCGACGCGGAAAACGCGGCTTAAATCGCGTGCATAAAAATAGG
CTGCCAGGCCGAACTCGGTATCGTTAGCCTGTCTGATGACATCCGCCTCGTCGCTGAAGCGGAACAGAGGCGC
GAGCGGCCCGAATGTCTCTTCTTTGGCGACTTTCGCGTTATCAGGAACATCCGCCAGAATGGTCGGTTGAAAG
AAGTTGCCGCCCAATTTGTGCGCTTCACCGCCCGTAATGACGCGGGCGCCTTTTTCCAGCGCGTCGGCGATAT
GCTCCTGGACTTTCGCCACGGCTTTTTCATCAATCAGCGGCCCGATAGCGACGTCGGCTTGCAGGCCGTCGCC
GACCGCGAGCTTATTTACCGCCTGGTTGAGTTTTTCCGCAAAACGATCGTAAACGCCGTCCTGAACATACAAT
CTATTGGCGCAGACACAGGTCTGCCCGGCGTTGCGAAACTTCGAAGCCAGCGCGCCTTCGACGGCTTTATCCA
GATCGGCATCGTCAAAGACGATAAACGGCGCATTGCCGCCCAATTCCAGCGACACTTTTTTAATGTCTTTGGC
GCATTGTTCCATTAGCTGGCGGCCAATCTCCGTCGAGCCGGTAAACGACAGTTTGCGTACCAGCGGGTTGCTG
GTCAGCTCGCCGCCGATATCGCCTGCCGAACCGGTAACGACGTTAAATACACCTGCGGGAATTCCGGCGCGCC
GGGCCAGTTCCGCCAGCGCCAGCGCGGAAAACGGCGTCTGGCTGGCGGGTTTGAGTACCATCGTGCAGCCTGC
GGCCAGCGCGGGGCCGGCTTTACGGGTGATCATTGCTGAGGGAAAGTTCCACGGGGTAATCGCGGCGGTGACG
CCGATGGGCTGTTTAATCACCAGCAGCCGTTTATCGGCCTGATGACCTGGAATCGTGTCGCCATAGATACGCT
TCCCCTCTTCGGCGAACCATTCAATAAACGAGGCGGCGTAGCTAATTTCGCCTTTCGCTTCCGCCAGCGGTTT
ACCCTGTTCCAGCGTCATCAGATGCGCCAAATCGTCCTGATGTTCCATCATCAGATTGAACCAGCGACGCAGG
ATATTGGCTCGCTCTTTGGCGGTGAGCGCGCGCCAGGCGGGCAGGGCGCGATTGGCGGCGTTGATGGCGTCGC
GCGTCTCTTCCGCCCCCATTTTGGGCACATTGCCGAGCGGTTTCCCGTTGGCGGGGTTGCTCACGGGGATGAC
ATCGCCGCCGCGCGCGTCGCGCCAGTCGCCATCGATAAAGGCCTGCTGGCGGAACAGGGTAGAGTCGTTAAGT
TGCATAATTGCTTCCTGTCTGAAGGGTTAACGGGTAAATGCGGCGTGTAATGCGTCCACGCTGCGTGCCGCAC
GCAGCGTACGTCCGGGGTTGCTCTGGCTTGCGCGTAGCGCCTGAACTTTACTGACGATATGCGCGCCGATAGG
TATCGCGGATGTCGCCGCCGGGGAGGGGGCGTTACAGGTGTGAATGGAACGCGGCGTCGTGACAAACAGAAAA
TCATCAATCAGTTTGCCGTCCGGTGAAACCGCCTGCGCCCGCACGCCTGCCGGCCAGGGCTGGAGATCGTTGA
CGGTCAGGCTGGGGCAATACTTTTGTACTCGCCGCAAATAGCCGCTTTTGCACAGCGAGTTTTTCATCTCGCC
CAGCCCGGAAAGCAGATGGTTTTGCAGTACGCGGCGAATGCCGGCGGAGCGGAAAATCTCCAGGGTGTCGGTG
AAGGAAACGTCGCGCTTACGGTAGCCTTCGCGTTTAAGCGCCAGCACCGCATTCGGCCCGACCGTGACGCTGC
CATCGATCATGCGGGTAAGATGGACGCCGAGAAACGGCATTGCCGGATCGGGGATCGGGTAGATCAAATGGTT
AACGATCCGGTTGTGTCGCGGGGCCAGACGAAAGTACTCGCCGCGGAAAGGGCAGATAATAAAACCGGGCTCC
ACGCCCAGCATTTTGACCAGCCGATCCGCCATCAGCCCTGCACAGCCGATAAGCGTAGCGGTTTCTATTTCCC
GACCTTGCGAGGTGCGAATAACAATGCCAGCGGCATGTTCTGTTAGCGCGCTGACCTCGGCATGGTAGATGAT
CTCGCCGCCTTTGGCCTGAAAGCGGTTCGCCATGGCCGTCGCGACATCGCGATAGCTGACAATCCCGCTGGAA
GGGACAAAAATCCCGCCCAGGCCAATGATATTGGGTTCGCGTTCACGCAGCTCCGCCGCGCTTAACCACTCGC
GCTCTAAGCCGTTGGCCGCCGTCCGCTCCCATAGCGCGCGCATCCGCGCCATTTCCAGCTCCGAGGTGGCGAC
CAACATTTTGCCGCAGGTGTCGTAGCGGATGTTGTTTTGATCGCAAAAGGTTTTGGTCGCCTGATTGCCCGCC
AGACAAAACCGCGCTTTGAGGCTGCCGGGCGTGTAGTACACCCCGGCATGGCTCACGCCGCTGTTATGCCCGG
TCTGGTGGCAGGCTGGCGCGGACTCTTTTTCCAGCAATGCGATGCGCGCATCCGGGTAGACATCAATAAGTTG
CATGGCGGTCGACATGCCGATAATGCCGCCGCCGATAATCACAAAATCATACATCCGCGTAATTCCTTCGCCG
TGGCGCTTATTGGTGAGTTTGATAATGGCTGGCGGCGTAGGCGAAGTAACCGCGCTGGCGCATCAGTTCGCGA
CGCAAGTCAGGATGCGGCGTAAAACGATCGCGTCCATGTAGCCAGAACAGATTGTTAATCAGCAGGAATTTGC
CGACCGGTACCGGCACGGAGAGGATGTTTTGACTGGTTTCCAGCGCGTCTGAAAGCTCGCTGAGCCAGACGCC
TTCTTCAAAGTCTTTTGGCTGGACGAACTGGTCGATATAACGCATGACCGGGCGGCCTTGTTGGTCGACATCA
AACACCGGGTGCCAGACGTCATGGCTAACATTTTTGCTTGGCGGTGCCGCCCAGCGCATGACGCGACGCGCCA
GCGGATGGGTAAAAAACGACTCCAGATGTTCCCAGTCATCAAGGTGCAGCAGCAGCGAATTACCGCCTTCCAT
ATTTTGTTCGTCGATTTTCATCATCAGCACGTAGTCGGTCACCTCCTCAACATAGGTGCCGTCATTGTGCAGT
TCCATTACGCGGTGGGGCTGGCGCAGATAACTGTCGGAGTTGTCGACGTTTTTGACCACGAAACGCGCGTAAT
ACTGGCCGCTCATCGCATCGTAGTTGGAGCGACCAATAAGATGCGCCACCGCCGTCGCCAGTTTGACCATCTC
TTCTGCCTGTGCGACATCATCGAGCCCTTCGGCGCTGATCAACAGCGCGCCTTGTGCACGATTGAGTAGCGTT
TTGATCAACAGCGGTTGTAGCTGATTGCCGCACAGATCGTCGAGGATCTTGCCGATTTTGAAACGTAAGAAAG

ATTTGTATTCCAGCGCCTGCACCGGCCACTGTGCAACCTGATGGAGAAATTGTCTTGTGGTATCCGCCGTAAA
CGTTAACGCCAGCAGACGCGGCGACTGCGCCGAAGGGGCAAGGGTGAAACCAGTATGGCGTTGGGCTAAATCA
TCTGTGTTCGCTTTTACGGCAGTCAGTGCATTCATTAGAATCATTCCTCTCGGTAAGATGGTCAGGGAGACAT
GGCGATGCTCATTTCGTAGCCATAAATTAAAAATATCGACATTTTCCAGGAACGCGTATGAAATAACCGCTTT
TGTTTAATTATTGTGATCAAAAACAACAAATATTTAACAATACAAAGGGTGAGGTTAACGGCGCCACGAGCCT
GACAACGGCAAGAAGGGGTGTAAAATGAGAGCGTTATGGGGAAAGTGATGCTGGCGGGGTTGCCCGGCCTGAC
ACATGAGCGCGTAGGCCGGACAAGTACAACGTATTTCAGCAGTCAATTAACCGGCGTCAACCTGCGGTTTACG
GCTGTGTTTACGGAGCAGACGTAACACTGGCGGTACGACAATGACCATGATCGCCATGATCAGCAGGGCTTTG
GCAACGCCGCTTTGCCACAAAATCGCCATATTGCCGTTACTGATGGAGAGTGCGCGACGCAGGTTCTGCTCCA
GCATTTCCCCCAGTACGAATCCCAGGATCAGCGGCGACATGGGGAAGTGCATTTTACGTAAAATGTACCCTAA
CACGCCGAGCGCGACCATCAGCACCAGATCGAAGGTGGTGCTGTGTACCGCATACACCCCTACCGCCGATACG
GCAGCGATGGCGGGTACCAGGAACCACAGCGGAATGGTGAGCATACGGGTGAACAGACCGATCAACGGGATAT
TCATGATCAGCAGCATCACGTTCGCAATCAGCAGCGCAGCGATGAGTCCCCAGACGATATCCGGCTGTTCGGT
GAACATCGCCGGGCCTGGCGTGATGTTGTACAGCGTCAGCGCCCCCATCATCACTGCCGTAGTACCGGAACCG
GGAACGCCCAGCGTCAGCATCGGGATGAAGGAGCCGCAGGCTGAGGCGTTGTTTGCCGCCTCCGGCGCCGCGA
CGCCGCGAATATCCCCTTTGCCGAAGCTATCGCTGTTGCCGCTGAGTTTTTTCTCGGTCATATAGGTAATGGC
GCTGGCAATGGTCGCCCCGGCGCCGGGCAATACGCCGACAAAAAAGCCGATTACCGAAGAACGCAGGGTGGTG
CCGATACACTGCGCGCCTTCTTTCAGGTTGAACAACATTCGACCCGTTTTGCGGACCATTGTTTGCCCGCTGC
TGGTATGTTCCAGCATTAAAAGTATTTCTGATACCGAGAACAGGCCGATCACCACGACGATAAACTGTACGCC
GTCGGAAAGATGAACGCTGTCAAAGGTAAAGCGATAAACCCCGGTGTTGGCGTCCACGCCGACGGTCGCAAGG
CCAAGACCGATCAGCGCTGCCAGAAATGATTTAAGCGGGTTTTGCGCCATCATGCTGCCAAGACAGGCGATGG
CAAAAACCATTAAGGCGAAATATTCCGCAGGACCAAAGGCCAGCGACCATTGCGCCAGTAAAGGGGCGAACAG
AATGATGCCGCCGATAGCGATTAATGAACCGAAAAATGAGCTGACTGCGGAAATCGACAGCGCTACGCCGCCT
TTCCCTTGCTGCGCCATTGGGTAACCGTCCAGCGCCGTCATGATCGCCGCCGCATCGCCGGGGACGTTGAGCA
ATATGGAGGAGATCCTGCCGCCATACTCACAGCCAATGTACACCGTCGCCAGCAGAATTAGCGCCGACTCCGC
AGGCAGATGCAACGCAAAGGCCAGCGGCAGTAAGATTGCCACGCCGTTGATCGGTCCCAGGCCCGGCAGCAGA
CCGACGATCGTGCCCACGAAGCAGCCAATCAACGCGATAACCAGGTTTTCCGGCGTCATCGCCACCGCAAAGC
CCTGAGAAAGATATATCCAGGTATCCATCGTTGCTCCGTTAACTCAGCCAGGCGCCGAGAGGTAAGGTGACAT
CAAGCAGCCGGTCAAAGGCATACCACAGCAGAATACCCAATACCGCGCCGGAGATACCCGCCGCCGGAATCGT
GGCGCCGAACAGCATCCCGATAACCATGGTGAGAAGCGCGGTGGCGATGGGAAAACCCAGCCATTCGAATCCC
CAGGCATACATCAGCAGGATGATGATCATGGTAATTAGCTTTTGCAGAACATGGCGGCGCGGCCAGCTCACCG
TGTCGGGATGGCGCAATAACAGCGCCAGCGCGCAGAGCGCCATCAGGCCGATAATCCCCAGCGGAAAAGGGCG
GGGGCCGACGGGTTCATAGCTGTATTCGCTTTGGATCTGCCAGGCGATGAATAGCCCGGCGATACAGAGCAAC
AGCCAGATACCCGCAAAGATACGATCGCTCATCATTGCCTCCGCGATTATTTCGCGAGTCCGAAGGCTTTCGC
CTGTTCACGGTAATCAGTAACCTGTTTTTTCACGTAGTCATCGAGCTGCTGGCCGGTCATGTCGAACTCAAAC
AGGCCGCGCAGATCGCGCTGCTTTTTAAACTCGTCGGTTTGCTGGAGCTTCTTGAAGGTATCCACCCACCACT
GGTAATCGGCATCGCTGACTTTGGGCCCGACGTAGAAGCCGCGAATAATCGGCCACACCAGGTCGTAACCCTG
TTCTTTAGCGGTAGGGACATTGGCAAGCTGGCCCGGCAGACGATTTTCTGAAAAGACGGCAAGCACGCGGATT
TTGTCGCCGCCCAGATAAGGCACCATTTCACTGAGATCGCCGGAGACAACCTGAACATGGTTGCCCATTAATG
CCGTCACCGGCTCGCCGCCGCCCTCAAAGGCAACGTAGCGCATCTTGTGCGGGTCGACGTTCGCCTTTTGCGC
CAGCAACGCCGACTTCATCCAGTCCTGGCTGCCAATAGAGGCGCCAGCGCCAATGACCACGCTGTTGGGATCT
TTTTCCATCGCCGTCATCAGATCTTTCAGCGTTTTCCACGGGGAATCCGCACGCACGGCGATCATGCCGTAAT
CAGTGCCCACGCTTGCCAGCCAGCGCACATCATCCACGCCGTAGCGACCAAATTTCCCCTGCGACAGGTTGAG
CAGCGAACCGCCGGAAAAGGCGACCACAGTCCCGGGTTCGCCAGGGCGTTGAGCGACTATCGCGTTATAGGCC
ACAGCGCCGACGCCGCCGGGCATATACGTTACCCGCATGGGTTTCTCGATAGCGCCAGTCTCCAGCAAACTCA
CCTGAATCAGCTTACAGGTGAGGTCGAAACCGCCGCCAGGTTTGGCTGGCGCGATACATTCCGTTCGCGACGG
CGCCTCCTGCGCCAGAACAGAGGTACTCATTAATAAAATGCTTGCAGTAAGGGTACGAAGTAATTGTTTTTTC
ATTTTTTATCCTCACGCCGGACGTCCGGATTTTTCAGGTTCGGAAGAAGATTTTTGTGATTATGTGAGCCGAC
ATGTAGCAGTTCGGTTGCGTGATTGTTAAAACAATAACCTTTCAATTCCCTTTCAATGCGGCAGAAACTTTAC
AGGATGTGATATGCGTCTCTTATTGGCGGAAGATAACCGTGAGCTGGCTCACTGGCTGGAGAAAGCGCTGGTG
CAAAACGGCTTTGCCGTGGACTGCGTATTTGACGGCCTGGCGGCTGACCATCTTCTGCACAGTGAAATGTACG
CGCTGGCGGTGCTGGATATCAATATGCCGGGAATGGATGGACTGGAGGTGGTGCAACGGCTGCGTAAACGCGG
TCAGACCTTGCCCGTTTTGCTGCTCACGGCGCGAAGCGCGGTGGCGGATCGTGTAAAAGGTCTCAACGTCGGC
GCCGATGACTATCTGCCGAAACCCTTTGAACTGGAAGAGCTGGATGCCCGTTTACGGGCCTTGCTGCGACGCA

GCGCTGGGCAGGTTCATGAAGTTCAGCAACTGGGGGAATTGATCTTTCATGATGAAGGGTATTTTCTGTTACA
GGGACAGCCGTTAGCGCTGACGCCGCGTGAGCAGGCGCTCTTAACCGTATTGATGTACCGACGAACGCGGCCG
GTTTCGCGTCAGCAGCTTTTTGAACAGGTGTTCAGCCTGAACGATGAGGTAAGCCCTGAAAGCATCGAACTTT
ATATTCATCGACTGCGTAAAAAACTCCAGGGAAGCGATGTACGAATTACGACGCTGCGCGGCCTGGGTTATGT
GCTGGAGCGCGGCGATGAAGTGGGTTAAGCCGCAATCGTTGTACCTGCAACTCTTACTGTTCCTGGGGTTGCC
GCTGATTCTGCTATGGGGGCTGTCGGCGTTTAACAGCTATGTCAATGCGCTACAGGCCGCCACTCAGGCTTAT
GATCGTACGTTGCTCTCCTCGGCGCGAACGGTATCGGAACGGCTGGTGGTGCGCAATAACCATCTGGAAGTTA
ACGTGCCCTGGGTGGTGCTGGATAGCTTCGAGCTTAATATGAATGATCGACTCTATTACAAAGTCGTCGACCC
TTCCGGAAAGGTCATTTCCGGTTATGACGATCTGCCCGTCATGCCGCCCGCTACGCCCCGTACCCGGCTTTAT
CCGGCGCTGGCGTGGTTCTATCATACCGAGTATCGCGGCGAGGCGATCCGCGTGGCGCGTTTGCTACAGCCGG
TCAACGAAGGCGGCATTATCGGCATGGCGGAGATTTATGTCGCAGAGACGCTGCAGTCCCGTCGTTATCTGGC
GGGCCAACTGCTGTTCTCTTCCTGGATCTCACAGGGGCTGCTGGTGTTGCTGACCCTGGTGCTGGTTGGCTGG
TTATTACGCCGGATCCTGCGCCCTATGCGTCAACTTTCGTCATTAATGGTACGCCGCGAACCGGGTCTGTTAA
CGCCGCTGCCGGAACTGCTGCCGTGGTCGGAAACACGCTTGTTAATCGTGGCGTTTAATCGCTACATTGACAG
GCTCCGCGGCATTCTCTCCCGGCAGGAACGCTTTAGTGCCGATGCTTCTCACCAGTTAAAAACGCCGCTGGCG
GTGTTAAAAACGCAGGCGGCGGTGGCGTTAGCCAGCCAGCACCCTCATCATTGGTACGAAAGTCTGCAGGCGA
TGAGCGTAACGCTGGATAGTACCATTCAGCTCACGGAAAGATTGTTGCAACTTTCTGCGGTAAAGCATAAAGA
GCAGGGCGAGCGGCGCTTTTCCCCGGTGAATCTGTACGATATCGTACAGAGCGGCTGCTTTACCCGCCTGGCG
CAGGCGCGGAGTAAACATATCGACCTGGGCTACGAAGGCGAACAGGAAGCGATGTGGATCGAGGGGGATGAGG
TACTGCTTAGCGAGCTATGCGGAAACTTGCTGGATAATGCGCTGAAATACACGCCTGAGCAAGGTATCGTCAC
CGCGCGGCTTGAACGGGATGGCGATGCCGTGACGCTGGTTGTCGAAGATAGCGGCCCCGGCATTGATGATGAA
CACATCCATCTGGCGCTACAGCCGTTCCACCGGCTCGATAACGTAGGAAATGTGGCCGGGGCGGGCATTGGTC
TGGCTTTGGTCAATGATATTGCACGCCTGCACCGTACTCATCCCCATTTTTCCCGTAGTGAAGCGTTGGGAGG
GTTATACGTTCGGATACGCTTTTTAAGCCTTGTTCCGCAGTAATGCGCACAAATGTGTCAGCGGTAAAGCACG
TTTACAAAAAGTCGCATCTTGAGGTGTGACGTTTTAATCAAATAATCATACATTCCCCCTACAAACCGTAGCG
TTAATTAAAAATATAGTGGGTGCAGGGGCATGAATAATCATACTAAACGACGGGGAATAGCATTAACCGTCTT
TCTTGTCGGGGTTAACATCCTGGCCTGGATTTGGGCGTTCTGTGTTTTTCATCACCATGCAGTCATGCTAAGT
GCAGCCATACTGGCGTATAGCTTTGGGCTGCGTCATGCGGTTGATGCCGATCATATCGCAGCCATCGATACCG
TCACCCGTAAACTGATGCAGCAGGGTAAAACGCCTTTAGGCGTGGGGGCGTTTTTTTCATTGGGTCATTCGAC
CATTGTCGTTCTGGCATGTCTGGCGATTGTTGTGACGTCGATGGCATTTCGCGATCGTATTGATGTTTTGCAT
CAATATGGTTCGTTGATTGGTACGGCTGTTTCCGCTTTTTTTCTGCTGGCGATGGCCCTGCTTAACCTGTTTA
TTTTGTTCAACGTCTGGCGGCAATTTCGCTCGGTTACCCGCGGCGAGTCAGTGAGGGCGCATGACGAGGCCAT
ACCTGGCGGGCTGATGACGCGTATATTTCAGCGTACGTTTCGTTTGGTCACTTCCAGTTGGCATATGTATTTT
GTCGGTTTTCTGTTTGGTCTGGGATTTGATACCGCAACCGAGGTTGGGTTGCTGGGCATTTCCGCTTCAGCAG
CCAACCAGGGGCTATCTCTCTGGTCAATGATGATTTTTCCTGTGCTGTTTACCGCCGGGATGGCGCTGGTAGA
TTCCCTGGATAACTTTGTCATGGTCGGCGCTTACGGTTGGGCTTTCTCGCATCCATTACGCAAGCTTTATTAT
AATATGACCATTACCGCCGCATCCGTCATTGTCGCGCTGGCGATTGGCGGGCTTGAGGCGCTGGGGTTGATTG
ATGATGCGTTACAGTTAAGCGGAACATTTTGGCAGACCGTCAGCACGCTTAACGATCATATGGGGAATGTGGG
CTTCTGGGTAGTGGGTGCTTTTGTGCTATTCTGGCTGCTCTCGTTGTTGAATTATCGCTGGCGCGGCTATGAT
AAAATCACCCTCAATACCTGATTGATTTCTTTAAAAATAGCATTTCGGCAGTGGTGTCATATCATTGCCGAAG
CATTTTATCTCCTGAGAGTATATTCTTCCCGGTAAGTGCGTTATTTTTCATGCTTGCCTTTCGTTATCAATTT
AAGCAATCCGATATAACATACAATACCAAAAATAATAATAACAGTTGTATACCCAAGAATCAGCCAGTCTGCA
TGTGTCATAATATAATCCAGTTATCAGGTATCCCGTGTTAAGGCGATCTTTAAGCCTGTTGTCAGCAAGAACC
ACTGTACCCGTTCAGTGCTGTTGGCAATAACGTATTGTTTATGCACAAGAGCGTTGTTACAAATAACGTATAC
ATTGAGCATAGACCTCACCCACATTTTTGTAATGCCATAAAGCAAGGCTACACCTTTAATCTACTGACGGTAT
GCCAGGCCAGAAGAGATAAAAGGGTAGTTGTCATCATCATCTGTTCCTGACGCCAGGTTAATGACACACACAT
ATGAAAATGTATAACCGCTGCACGCTTTTTAGCACGTTATCTGTAACATACTGTGTTAATCAATTCCGTAAAA
CCGAGTGTAGTGTAAGTGAATTTCGAGTAATTGTCATTATGATTATATGGTGGTTAACTTATTTTTCAAATTA
TGATTAAACTTTCGTTTTTTATCAGTTCATCAGGACTTATGAAAAATCTGGACGTAAAAAACATATTTACGTC
CAGGCTTTCTTTTAGCAAATCGATTTTCCGGTAAAATAGGGGGTCGACCACATGCGCTTATATTCCCATTCAG
GCTTTAGTATGCCGATGGAAAAAAGCAATTTTTTTTTGTCTTTCCTGGCAGTAATGTCTGGCACGGTTAATATT
TAGCCACATTAGTATGCTGCCGGGACTGAGTGGTCTGAACTCATTTTTAATTGCACCATTAGATACGTCAAAG
TAGACGTTCATCTGGCTTTCAGATTTCAATACGATATCAAATGCACAGGGTATCCCTTCAATATAAAGAATAT
GCCCAAACAGAAGGTGATGCAGTTGACTAAAAAAGTTGGCAAGGTTATCAGCGGGATAGCAGGATGAAGTATT

GCCGAAACGAGACCGAAATAACTCGATAAAAATATGAGTCAGTTCATCAGAAGAACAATCAGCAACGCTTTTT
ACACTTCCGCCTTTTTTAAGAAATCGCTGATATTCATTACGGCGTGTTTTTTCAAACTTAGAAGAGAACGTTT
CTTTGACCAGACAATTTTGTTTCTTTCTGGCGAGTTTCCATATGGCATTGCGTATTTGTGGCTGATGCAACGC
CGAAAGTCGGTTAGTACGATCGGGTAAGAAGCAGCGTAAATCTGGTGCCATTGGAATAAGTATCTCATCTGAC
GATAGCGGGAAGGTTCTGCGCGTCAGGATGCCAATATTCTGATCATTACAGATAAAGTAGGCGCCTTTAATCT
TATCATCTTCTTTATAGTGGAAGAATTTAAAATGCCAGTTATGGCGCTTCATAAAATAATCAACAATATCTGG
GTGCATATTGATACTACCGCCGTATTGCGTAAACACCTCCCGGTATAGGGTAAAGGAAGACGGCTGCCAGCGG
GTTAATATACGCTTAACTTCTTGTATTTTCACCAAAACTCCTGACTTGGTTTACTGAAAAATCTATTTTTTTT
TTTTTTTTTTATTAACAAAAAAAAAAATTGGCAAATGTTTAGCTTGTATTTAATGGTTAACATGCTAATCGATG
GCGTTATGGTGAAGGGAATAAGATTAAGATGTTGATCATAGCATGAGTGAAAGCGACGGTTAAATGCCGCGCT
ATTCACTCGCAAGCCATTCTGTATTTAACCATCCAGTAGCGATACAGATTTCAAAGAGATCCGCTGAACGGTA
GCAGGATCTGCACATCCGCGTATGAAATAGTTCTACCGGGAGAGGCTGTTAACCTCTTGTATTATTGTATCCA
GAATATGATAGCGTTTCCGATACTCTGCGCGTTTTTTGCTGGGGATACTTTCAGGCGCTTTACGCATCACCTG
TGATGGCAGATGATATAAGGCACCTTTCTTAACGCCGTTTAGCGACTCCCAGAATTCACTATAGCTGGCCACA
AAAGTTTTTTTCTTCTGGAATAAGTACCTGAGTTGACGATAAACATGGTTGTTTTCACTTACCGCATAAATCT
CGTCGACATTACAGGCCTGCATCAATGTTGCAAAAGCTTCATACAGCATCCGTTTAGGAAAGAGACCGTAACA
ATCTTTGGTGGCGTTACGTATGACTTGTGGGCCGACATGTTTACTGGGCCCTTGTAACCCGGCAACCAGCGCT
ATTTGCTTCCCTTCATGGCGGATAAAGGAAAATGATAAACGTGCCAACGGCGTATCATTGAAACACAGCGTTA
ATGTCAGCTCACCTTCTCTGTCATAGCCACAGGGACCGCAGTAGATATCTACCAGAGCGTCGTTTTTTCCTTC
CAGGTGAGCGAGCAAGATTTGTTCCTCGGACAGGAGGATCTTTTTAATTTTACTTTCCGGAAAGGACTGAACG
AACTGGTAGTGTTCCAGAATAGCTTTTGCCCGGCAACGTATGCTAAGCCCGGTATACAAATAGGGCCGCTGGA
TTTTTGCCGGCAACAGTGGACGATGAATAATCAGCGTTCTGGGCTGAGACAGATGGCATAATTCATTGAAGTA
TTTAATTGTCGTTACGGGGTTAATAAGGCAACGTAATACAAATTTATAGCGAAACTTACGCTCTCCCCAGCGT
TTATGGCGATAAGGCGTCTGCGCCATGACTTCTTTTAACATTAATAATGGATTATATCTTTCCATATCACTTT
GCTGCATCGTCATACTACGTCCCTACTACTCGATAGACTTATAACCGAGACGGTAGCCTGATTGAGTTAAACG
TAAAGGCAACGAAGAAGGTCTGTACAGACGATGTTGTTAAACAGTTTATCAATGGCGGTAATAAAACGCCTGA
ACACGCTTTTTAATAGGTTTTCTGCTAATGAGTGGGATGAGTCCTGAACGTAGTATTAAAACGAATGCCGTGT
TTATTATTTTAAATGATGTTATATACTCTAAATAATTCGAGTTGCAGGAAGGCGGCAAGCGAGCGAATCCCCA
GGAGCTTACTAAAGTAAGTGACTGGGGTGAGGGAACGCGGCCGCAGCACATGCAACTTGAAGTATGACGAGTA
TAAGCCAATATATTTATTTGGCTGCTATTGTGTAAGCCAGACAGCAACGCGTCGTGATACGTTATTATGTAAC
CAGACGTAAAGGGGGTATTCACCTTATCTCTAAATGCAAATCTATATGATAAATTTTATCATGCACTGTGTTG
CTGTCTCTGGGAGAAAATATATGGAGCGTTCACTCGATAGTCTGGCTGGTATGGCTAAATCTGCTTTTGGCGC
GGGGACTTCTGCTGCTATGCGGCAAGCTACCTCGCCCAAAACCATTCTGGAATATATCATTAACTTTTTTACC
TGTGGTGGGATACGTCGGAGAAATGAAACACAATATCAGGAATTGATAGAGACTATGGCTGAGACATTGAAAA
GTACAATGCCTGACAGAGGTGCTCCGTTGCCAGAAAACATCATCCTGGATGATATGGATGGGTGTCGTGTCGA
ATTTAATCTTCCTGGTGAGAATAACGAAGCTGGACAAGTTATTGTACGAGTCAGTAAAGGCGACCATTCTGAG
ACAAGAGAAATTCCGCTTGCCTCTTTTGAAAAAATATGCCGAGCTTTACTATTCAGATGCGAATTTTCTCTCC
CTCAGGATTCTGTAATATTAACTGCCCAGGGAGGCATGAATCTTAAAGGCGCTGTCCTTACCGGAGCAAATCT
GACGTCAGAAAATTTATGTGACGCAGACTTAAGCGGCGCAAATTTAGAGGGGGCAGTGCTGTTTATGGCGGAT
TGTGAAGGTGCAAATTTTAAGGGCGCAAATCTATCGGGAACATCACTAGGCGACAGTAATTTCAAGAACGCCT
GTCTGGAAGATAGCATTATGTGTGGCGCTACCCTCGATCACGCTAATCTTACTGGCGCCAATTTACAACACGC
GAGTCTGTTAGGCTGTAGCATGATAGAATGTAATTGCTCCGGTGCAAATATGGATCACACTAATCTTTCAGGC
GCAACCCTTATACGTGCTGATATGAGCGGTGCGACATTACAGGGTGCTACTATAATGGCTGCAATTATGGAAG
ACGCTGTCTTAACCCGGGCAAATCTGCGAAAGGCGAGTTTCATTTCTACGAACCTGGACGGGGCTGATTTGGC
TGAGGCTAATTTAAACAATACCTGTTTTAAAGATTGTACACTAACCCATTTGCGTACCGAAGACGCCACAATG
TCTACAAGTACACAAACACTCTTTAACGAATTTTATAGTGAAAATATTTAGCCCTTTTTGACGTAAACCTGAA
ATAAAAGCAATGTAAGAATAACAATCTGCTACTGAATAGTAGCTGAGAGTAAATTATTTACAGTATCTTTTAT
TATTACAGGAGTATGCGACGGTAAAAATATCTGAATAACCATTCCTATTTTCTCAAGTAGAACTTCCAACTAA
AGCGAATTGTTTGAAAAGGAAAACGCTATGCCTGGATGTATAAGTGCCTGTTCGTGCGTTGTCAGCTCGACGC
CTACCGATCCTAAGGCCCCCCTAAGCCTAAGTGGATAGAGTTAAAAGGTTATTGTGTTACATGTACTAGTCAG
GAGGATATTCGTCCACCTAAAGAGGGTGAACCAAAGCAAAAAATGACACCCAAGGATTTAGTGCTGTTACGCG
GTCTTTTAAAAGACCTGGGGGTAACACAAAATCCCTAGCAAGACCCGACAGTAAAATCCACTAACTGTGGGTT
GTCATTTTTCCCGGTAGATGCTGGAGAACGAGAAACCGATGTTTGATGGTTTTCTGTCTGCAAAGCGCCTGAT
AAATATTACCAGGCGCTTTGTATTTTGGCGCCAACATCACTTTCATCGTCATATATCGAATGACTACAATCGT

ATCCGATCCCGCCATTACCCTGACGGAAAGCCTCTGGCAAACTGTAAGAACGGTTCGCCGTCGGCAGGGAAGC
GCCGATGGACTCTGAACCGCGCTATACCATTTTTCCTGGTTGTTTCTTGTCATTAACGCTTTGAAAAATAGTT
TTATTTTATCTATTCCTGTTTTGTAAAACCTCCGCTCAGTAGGCGCATTCTGCCACCCTTCCCGGATTTATTG
GCAAAGCGGAGCCCGGACAGAGAGTCATATTGCAAAATCCCGTTTCTGTTTTCTTATGACCAGATTTTTGCGG
TCGAAAGATTGCCTTTTTCTTAATTGAATGATAATTATTATCATTAGCATATGATAATAATTACTATATAGCC
GTAACCTGGCAAGGATGTGAGCTTGAGGGCAACAGCGCTACTTTAGACATTATTTAGGGAATGGGTATGAAAG
TTAATAAGTTCCTCTGGTTAATAACCGTGGTTTCTACAGGGGTTAATAGTCCATTATCAGCAGCAGAATCTAC
AGACGACAACGGCGAAACGATGGTGGTTGAATCCACTGCCGAGCAAGTATTAAAGCAACAGCCGGGCGTTTCG
ATTATTACTCGTGACGATATTCAGAAGAATCCTCCCGTTAACGATCTTGCCGATATTATTCGTAAGATGCCTG
GGGTTAATCTTACCGGCAATAGCGCCTCGGGGACGCGCGGAAATAACCGACAAATTGATATTCGTGGTATGGG
GCCGGAAAATACCTTAGTGCTGATTGACGGCGTGCCGGTGACGTCACGAAATTCGGTTCGCTATAGCTGGCGC
GGAGAACGCGACACCCGTGGCGATACGAACTGGGTACCGCCGGAGATGGTAGAACGTATTGAAGTGATCCGTG
GGCCCGCCGCCGCGCGCTATGGCTCCGGTGCCGCTGGCGGGGTGGTGAATATCATCACCAAACGTCCGACTAA
CGACTGGCATGGTTCGCTGTCGCTCTATACCAACCAGCCGGAAAGCAGCAAAGAAGGTGACACCCGGCGGGGT
AACTTTAGCCTTAGCGGCCCGTTGGCTGGAGATACGTTGACGATGCGCCTGTACGGTAATCTCAACAGAACCG
ATGCCGACAGCTGGGATATTAACTCCTCCGCCGGTACCAAGAATGCCGCCGGACGGGAGGGGGTCACCAATAA
AGATATTAATAGCGTCTTCTCATGGAAAATGACCCCGCAGCAAATCCTTGATTTTGAAGCGGGATACAGTCGG
CAGGGGAATATCTATGCCGGCGATACGCAAAACAGTACTTCGAACGCGGTTACTAAAAGTCTGGCGCAGTCCG
GGCGGGAGACCAATCGCCTGTACCGGCAGAATTACGGTCTAACTCATAACGGCATCTGGGACTGGGGGCAAAG
CCGCCTGGGGTTCTACTATGAAAAAACCGATAATACCCGCATGAATGAGGGACTCTCCGGCGGCGGCGAGGGG
CGCATTACCAACGACCAGACGTTCACCACTAACCGCCTGATCTCGTATCGCACCAGCGGCGAGGTGAATGTCC
CGGTGATATGGCTATTTGAGCAAACGCTCACCGTCGGCGCGGAGTGGAACCGCGATGAGCTGAACGATCCTTC
CTCTACCAGTCTGACGGTTAAGGATAGCAATATCGCCGGGATTCCCGGTTCGGCGGCTAACCGTAGCAGCAAA
AATAAATCGGAAATCAGTGCGCTGTACGTTGAAGATAATATCGAACCCATGGCCGGTACCAATATTATTCCGG
GTCTGCGTTTTGACTATTTGAGCGAATCCGGGAGTAATTTTAGTCCCAGTCTTAACCTTTCCCAGGAGTTAGG
TGAATACGTCAAGGTGAAAGCCGGTATTGCCCGGGCCTTTAAGGCACCGAACTTGTATCAAACCAGTGAGGGG
TATTTACTCTATTCGAAAGGCAACGGTTGTCCAAAAGATATTACGTCAGGCGGCTGTTACCTGGTCGGTAATA
AGAACCTCGATCCTGAAATTAGTATCAATAAAGAGATTGGGCTGGAATTTACCGTAGACGATTATCACGCAAG
CGTAACGTATTTCCGTAACGATTATCAGAATAAGATTGTCGCAGGTGATCAGATTATTGGCAGAAGCGCATCT
GGCGCTTATGTATTGCAGTGGCAGAACGGCGGTAAAGCGCTGATTGAAGGTATTGAAGCCAGCATGGCTGTTC
CGCTGATGCCGGACCGTCTGAACTGGAATACCAATGCGACTTATATGATCACTTCTGAGCAAAAAGATACCGG
TAATCCCTTATCGATAATCCCGAAATATACGGTGAATACCTTCCTCGACTGGACTATCACCAGCGCGCTCTCC
GCTAACGTCAACTGGACGTTATACGGCAAGCAAAAACCGCGAACACATGCGGAGTCGCGTAGTGAAGAGACCA
AAGGATTGTCCGGGAAAGCGCTGGGGGCTTACTCACTGGTCGGCGCTAACGTTAACTACGATATCAATAAGAA
CCTGCGTCTTAATGTCGGTATCAGCAATATCTTCGATAAACAGATCTATCGCTCCGCCGAAGGGGCAAATACC
TATAACGAACCGGGTCGGGCTTATTATGCCGGTGTGACGGCCTCGTTTTGATGGATTATGCCCGTCGCTGGCG
GGCATATTCTGGCGGCGGGTTAATGACAGCGTGGTTTTTGGCTTGCCGGTTCGCGACTGATATCCAGCAATGC
ACTGCGAAAAGAGGCGTTAAACATGGGCCCGTGGCCCAGGCCCGGGTAATGCTGCAGGGCCGCGTTAACGCCG
TTGTTCTCAAGCGTTCTCACGGTCTCCTGAACTTTTTGCAGGAGCTCGGCCATCTGGCGTTGCCTGCTATCCC
CATTCGACGCTGAACCTTCCATTATGATGAGTTTTTTATGGCAGAACGGCGACGGCTTCACTGCCGTAAGGCG
ATTCAACAGGGCAAAGTTATCCCTGCTGAGTGACGGGCTGGCGCTGTAATAGATATGAAAGAAGGATGATGAC
AGCCAGGAGTCTAAAACGAACAGGCCGCCATAAGAATGTCCCCATACCCCGCGCCGTTCGGGGTTGATGGTTA
TTCCTTGTTCCACCTGCGGCGCGATATGTCTCTCCAGTAGCTGGCGGAACGCTGGCCCGCCGCCGGTCTTACG
ATGAAAACGCGGGTTATTTTCACTGCCTTCGATTCCCGGCGCCGGCGTATAGTCATAAGCTCTGCCGTTAAGA
TCAAACGGCAGGTTAGTCTGATAGCCAATCGCCACAATCACCGGCGGCGAGTGGTCTGCCAGCTGTTTGAGCA
GCGTCTCCGGCAGTCTGTCCATCACTGCGTTGCCGTCCAGCATATATAAGACAGGATAACCCGACGGCGGGGC
GGCTTTATTGGGAATAGCGGTCCATACCCGGTAATGTCTTGCGCCATCGGCAGATTGAAAGTCATTGACCCTA
AAATGGTAATATCCCGAACCTTTATCAGCAATATTCGGTCCTAATGGCTGCATATCCGGCTGCGCATAACTCA
GCGCGCTAAAACAAAAACTCACTAAAAATAGCGCCATGCGATAGCACTTGTTGTGGTACTGGCGACCATACAT
CGGATGTGATTCCTTTTTGGCAGGTGAAGTTTTATCGGGGCAGTAGACGTAATCCGTCAATCAGCGCGCCGCG
CCACCAGGCATAATCGTGACCGCCGGTGTACACGCTGCAATGGCTTTCAACGCCGGCGGCGCGCAATTTTTCA
TGCAACTGTTTGACCTGCGGGACCGTCGAACCTTCCAGCGCTCCCACGCACAGATGCGTACGTACGGCTGGCG
AGGGCGCGGATAACACATGTTCGCTGACCCATGAGCGCTCTTCTGCGCTGAAATGGGGCGGGCGATTGCGGTT
GTCCGGCGTCCACCACATTGAGGGGGAGTGGCTCAGCACCAGGCCGAAGCTCTCTGGCGTGTGGCGGGCCGCC

ATCAGCGCGGTCACGCCGCCGAGGCTTTGCCCTGCCAGCACCGTTTGTGTACGGTCCGCCCACCGCCGTTCCG
GGTAGTTGGCGCGAAGAGTTGGCAGCAGGCGTTCGGCAATGTCGAGCACCAGCTCGCGGCGACCGCCGAGGAT
TGCGACGCGTTCACGCGCGTTGATGTTATCAATGCCGAGTACGGCCACCGGCGCGATGCGTCCGCTGCGGATG
GCGGCGTCAATGGCGGCGCTTACCCCAAGATGGTCGAACCAGATCTCGCCATCCGTGAGTACCAGCAAGCCCA
GCGGTTGTACGACAGGAACGTCAGGAAGATACAGCCGCACACGGCGACATTGTCCGGCGAGCCGATGCTCTGA
GGTGAAAAGCTGCCCGGCGTAAGCGCGACAGCCTGACCACTCCTCCTGTGCCGGGGCATGATCCAGCGCCAGA
ACGGACTCCTGCGCATTGCCCCGAACATTTATTCCCGGCGTGCTGTTCAGCGGGTCCGCCTTCCCGACAAGGG
AGGCGAAACGGCTTCCAAGCTGCAACACCGTCTCGTCCGGGGTTTCCGGTGGTATTTCGACCATCGTATAGGA
ACCGCAGTAGGACGCCGGTAAGCGAAGCGTCAGATGCCAGATATCCGTTGTGGGCAGTTGCGTCATCATACCT
TTCGCCACGTTATCTTTATCGGTGACACGGTTCAGGCGAACATAAACCCCCCCGCAGAGGAGAGGCGGCACGC
CACAGGAAGGTGACTTCCCGTTCATCGTCTGCGCCGTTAATGGCTGTCACCAGCGGCAGTGCGAGCGAGTTTG
CTTCTCGCCAGAAGTGAGAAGCGTCAACCTGGCTTCCGGCGGCGAGTTGGCGGCGAAGGCGCGTAGCGGTCTG
TATAGCGACAGGAGCGACGGGCGGGCTCAGTCTCATTCTTTGTATTCCATCTCATTTATATTTCGGTTAGTAC
TAATAGCTAAAGCGGCTGTTACAGGAACAGCCGCTTGCGGGCTTTCTTTTGCGTAAATCGTGGCGATAAAAAC
TATCCTATGACCTCTCCTTGCGTATCGCGTGTCTGGCCGATGCTGTCCCGCCACAGACGAGCATACAGGCCAT
GAGTCGCTATCAACTGCTCATGGCTGCCATATTCAGCGATACATCCTTTATCGATTACGACAATAACATCGCA
ACGGCGAGCGGTGGTCAGCCGGTGCGCGACGATAAGCGCGATGCGTTTCTCGGTATGCGTCACCCTGGTAAGC
GAGGTCATTAAGCGCTCCTCGGCGCTACGGTCGATACGCGCTGTCGCCTCGTCGAGCAGCAGAATATGCGCCT
GCGCCAGGTGGGCGCGGGCGAGGGCAATCAACTGACGCTGGCCCGCGGACAGATCCGTGCCGCCGTTATTGAC
CGGAGTACGGAACCCCAGCGGCAGATGTTGCACAGTTTCAAAGAGTCCCGCCTGTCGCGCCGCGATCTCCACC
TCCGTGTCGCTGCTGTCTGGCAGCGAATAGCGGATATTTTCGGCAATATCGCCGCTAAATAATGCGACATCCT
GAGTGACCAGCCCCGTCTGGCGACGATAATCACTAAGCGACGCGGCATCAATTAGGCGCTCACCGACACGGAT
TTGCCCGCTGCCGGGAGAGTAGAGCCCGGCCAGCAGCTTGATCAGCGTCGATTTGCCGGCGCCGCTACGTCCG
ACCACCGCGACTACCGTTCCTGGCGGAATGGTGAGCGAGAGATTATCAAGCACAGGTTGCGTTTTTTCGGGAT
AGCGGAATGTCAATGCCTCCAGACGTAGCGCGCCGCCAGTGCCAGGCGTTATGGAGGACGGTTCAATATTCTC
CGTTTCCTCAGTCGCCAATAGCGCATTGATATGTTTGCCGCTGGCGGTCGCCTGCTGCCAGGAATCAACAATG
CCTGACAACTGCTGTACCGGGCCATAAAATTGTCCCAGTAGCAGGAAGAACGCCGCCAGTATCCCGGGCGTCA
TTTCGCCTCCGGCGACCCGGGTAGCCCCGATTAAAAGCACGGCGGCATAGGCGGCCTCGGTGCAGAGGGTCAG
GAACGGAAAATAGACAGCAAGGTATTTTTGCGCCCGCACGCGGGTGGCGCGGAAGTTGTCTGATAACGCCCGT
AGCCTGGCGGCTTCCTGCTTCTGTTGACCGTGTGACTGCACTACCCGCATACCGGAGACCTTTTCCTGAAGCG
TACTGTTCACCTTACCAATTTCCAGCCGTGCCTGGGCGTAGGCAGGCGAGCTCAAACGGCGGTAAATCCACGT
CGCCAGTATCACTACAGGTACGGCGCTTAATGCCGTTAACGCAAGAATGGGGTCAAGCCAGAACATTGCCGCG
GCGATAGCGACCATCGTCACGATGCTGGTGGCCGCGCTGGCAAGACCGTTTTGCAGAAATCGGGCGAGTGAAT
CAACGTCGACGGTCATGCGAGTAAGACGCGAGTCGATGTGCTTCTCGTGCCAGGGAAGACTCAGGCGTAGCAG
GTGGCTGAAGCTGCGTAAGCGTACCGTATGCTGTACCGACTCTGCCGCGCGGGCGGCGAAGATCGTTTGCAGC
GCATAGCAGCACCAACCGATCGCTACCAGACACAGCGCGAGCAGAGCGCATATGCCAATAGTCGACATATCCC
CTGCGGCAACGCCTGAGTCAATACCGCGCTGCAAAAGCACCAGCACGCCAACGCCTGCGGCGGAATCAAGCGC
GATAAGCAGGGCGGCGATGACGAACATCCAGGCTACTGGCGTCAGCAGCGATGAGACTTGACGCCCGTGGCCT
GCCAGCGCCATCTGTACGGCGCGTTCCGGAACGCGCGTCATCCGGTCGACAAAACCTTTTCCGGCGGGTGTCT
GGCGCTCATGCGCGTCGTCGGATTTTGCCGCCTGCGTTGTTGGCCATAGCGTCCGTTGTTCTGCCGGGGCAAG
GGCGAGAAAATCACCCTCGCCGCTCATCAGCGAGCGAAACGTCGGACACCTTGCATCCAGCTCCGCCTGGGTA
CCGATATCCACGACGCGGCCTTTATCCAGCACGACGATCCGATCGGCTAACTGCAGCGTTGAACGGCGGCGTG
CAATCACCAGCAGCATATGCTCATTATCGGCATAACGACCAAGAGCCGTGTTAATTTCCGCTTCAGTACCGGC
ATCGACCGCCGAGGTTGTGTCATCGAGAATCAGCAGTTCCGGCGCGGTAATCAGTGCCCGGGCTAGCGCAATA
CGCTGGCGTTGGCCGCCGGAGAGGTTGCTTCCGCGTTCGGCCAGTCGGGTGTTGAACCCCTGCGGTAGCGCAT
TGATGAACCCGCTGGCGCCTGCGGCATCGGCGGCGCGTCGAATGTCGTCCTGAGTCGCTTGCGGGTGCCCATA
GGCGATGTTTTCCGCCACCGTACCGGCAAACAGAAACGCGTCTTCAAACACTACGCCAACGCGACGACGAAGC
GCCGTCAGTTTCAGGTCGCGAATATTCTGTTGACCCGTAGTGGTGTTGAGCCACACCCCGCCGGAGGTGGGAT
CGTAAAAGCGCGCCAGCAACATCAGCAACGTTGATTTCCCGGAGCCGGAGGCGCCAACCACTGCCACGGTTTC
ACCGGCGTGAATGGAAAACGAGATCTCATTGAGGATACGGTTACCGTTGTCGTAGTGGAAACTCATCTTCTCC
ACGTCCAGCCCGATAATCTGACCTTCAACCGACTCAGTACCGTCTTCCATGCGAGAACGCGTGTCGATAAGCG
CGAAGACGCGCTCCACCGACGCCTGTGTGCGCTGTGCGATAACCAAAAACGATGCCAGTACGCGGGTAGGGCC
GGTCAGCATAGCGAGAAAACTCGCGAACGCAACGAAGGTTCCCAGATCGATGCGCCCATTCATTACCGACCAG
CCGCCATAGCACAGCAGGACGATTTGCCCTATCACCGGTAGCGCCAGCATGGTTGCGCCCGGCATCGCCTGCG

AGATAGCGGCGCCGATGCGTACCCGCACCATCTGCCGCGACTGACCATCGAGCCAACGCGTTTCGCGTAACTC
CGCCACGCAGGACTTCACCACTGAGATCTGTTCCAATACTTCGCGCATATGTTCCGTCATGTGCGCCAGCCGG
TCTGAGGCAAGCCCCGTTTGTGCGAAAACACGGCGACGGGCGCGCAGGGCGGTAATAGCAAGGGCGGCCAGTA
CGCAAATCACGATAAGCGTCATGGATGGCGACATCCATAGCATCACCGCGATACCGGCCACATAGTAAGTGAG
CACCGCCAGCGGCACCGGACACATCTGTAGCAAGGTGTGTACCTGTTGAAGATCGCTGTTGGTACGCGAAATG
ACCTGCCCGGTACGCAAGGCGTCCTGGCCTGCGCCGTCCAGTTTTTGAATACTGTCGAACGCACGGCTACGTA
ACGTATGCTGAACCCACAGCGAGAGTTCCCCGGCATAGCCGCGGCGCACGTAATTCCCGATAAAATCAAAAAG
GGCAATTAACAGCAGGCCGCAGGCAAGTAACGGCAGGTGGGTCGTATCGCCTGCTATCGCGTCATTGACGGCC
TGACGCGTTATTAGCGGCGTCAGCGCAGCCAGTAAAATGGTCGATACTGATGCTACGATGACAATGATGCTCA
GTGTTTTACGTTCCCAACACACGCGGGCAAGGCGAACTATCCAGGCACGAGCGGGCATGGGGGAATGAGTCGC
GGGCATTAAGCCTCCTTATCGGTGAAAGATGAGATATGGCGACACACGCTGGGTTCAGCCGCCATATTGTCAT
TGCGCTGCCGCGGTTAGCCGTGTTGCAGCATGGCGATCAGTTTTTTTGCCACCTCGGTCGGGCAAGGCTGGGC
CGCCATTTCCGCCGCGACCTCCTGCGACGCCTCGCGAAGCGCGCGATTACCGAGGAAGGTATTGATCATGCTG
CTCGTAAGCCCGCTCTTACCGGGAATAATGCCGCATCCGCGCTCGACCACGGCGCGGGCATTGACGGGTCTGT
CGGCACCCTGGCCAAAGACTATCTGCGGAATGCCAGCATGTAGCGCCGTCAGGGTGTTGCCTGCGCCGCCATG
GTGGATAAAACCGTCGGCGCCGTTAAGGAAAACGCCCATCGGAAGCCAGTCGACCAGACGGACATTCGACGGC
AGCGAACGTAAATCCGAGCGGGCGTTTGCCGGAAGATGCAGGATGATTTCGGCATCTACTTCGCCGGCAGAAT
CCATTACCCAGGAAATCAGATCCAGGCCATCCACCATCGGTTTGACGGTGCCGAGGCTAACCAGAAGACGCTT
GCGATCAGGTGTACGTTCCCACCATTCTTCCCAGACGGCGCCGCCGTTATACGGGACGTATTGCATGGAGATA
ACCGGCTCTCCGTCATTTTGCAGTATGCTCATGCTGGGCGGTGTGACGTCTATCCACGCCAGATCTCTTGGTG
GCGCGCTGACCCCATGGCGGCGGTAGGCGTTAGAAAGTGATTTCGTCACGCCTTTGATGTGCCAGGGCGTATG
ACCGAAGCCGACGGTTTGCATCACTACCGGAATGTCATACTTAGCGGCAATCAGTGGTCCAACGACCCCAAGG
GGAGGGTAGACGATGAGATCTGGTCGCCACTGCCCGACGAACGCGACCAGCGGGTCAGTCATCTCTTCGCTGA
AGAATGAGAAGTTCCCCATTTTTGTTCCAATGTTATTTTCTTTTCGTAATGCCTCCTGACGGCGATAACCCGC
TTCCGAATCGAAACCAGGCGCAGCGTCAAATACCACCAACCCAGCTTCGGCTGCTTTCTGTGCAAATTTTCCA
CCGCTTGCAATCAGCACTTCATGGCCATTAACACGAAACGCTTGCGCCAGGGACAGCACAGGGTATAGCAGTC
CATACAGTGGTGGACCGACAAACAGAATACGCATGAGAAATCCTCTCTCTCCACGTCATAAATCGAACAAGTC
ATTAATGAGAATGATAATAATTACCAATATCATTTGAAAAATGCAATAACAAATACGACCCTTGATCATGTGA
TGATTTTTTTCATAAAAATCGGGGAAGGGGAGAGCGTTAAGCGGTGAAGTTTGGTTTCAGGCTTATTTACGCG
CGTATGGTCGCACAGGCAAAGCGCCAGGATTGGGGAACTGGATTCGCATACCAGACGCCCGTGGATTATTAAT
GATAACTTCGCAAATAACCTTTCAGACAATGAGGTAAACGTACCGACAGCAAGGTAACGGGAAACGTAGGGAG
TAGTATTGATAACACGTTGCGGGGCAATTTAATGGTGTCCGGCGGCAGATCGTGTTCTGTGCAGACTTTCCGT
GCAGTGATGTGACGGACGCCTCTTCCTAAAGTAGTCAGTGCCATACTTTTAATGACTGCATTAATCCTGGCGT
TAAATCCTTTAATTTGGTGGACGTAAAGTGGACATAGACAATAAATATCCTCCGGCATAGCCGGAGGTTTTTC
TGATGCGCCTGTAAGGCTCTCTTACCAGCCGCGCCCTAACAGGCGCATACGATCTGACATTTGCATCAAACTT
CGTTACTTACGGCCCGTAAACGGGCTGCCCGGATAAGGGATCGATAATTGCTCACCCATTTTATCCTCTTCAA
GCTGGTGCTTTATGTAGTCCTGTATCTTCGCCGTGTTCTTACCCACCGTATCGACATAGTACCCTCTGCACCA
GAACTCCCTGTTCCTGTATTTGAATTTTAGATCCCCAAACTGCTCGTAAAGCATCAGACTACTTTTACCCTTC
AGATATCCCATGAAACTCGACACACTCATCTTCGGCGGGATCTCCAGAAGCATGTGAATATGATCTGCACAAC
ATTCTGCTTCCAGAATTCGTACGTTTTTCCATTCACACAATTTTCTTAATATGCTGCCTACTGCCCTACGCTT
CTCTCCATAGAACGCTTTGTAAGTATCCCGCATAATCGTGCCATTCACATTTAGAGATCATCCGGCATAATCA
ATCTGCCAACGAAGGAGATCGCTATGCGTAAAGCCCGTTTTACTGAGCATCAGATCATCACTGTGATTAAGTC
GGTTGAAGCTGGACGAACCGTTAAAGATGTCTGCCGGGAGGCCGGTATCTCTGAGGCCACCTACTATAACTGG
AAGTCCAGATACGGCGGCATGGAACCTTCTGATATTAAAAAGATAAAAGATCTTGAGGACGAGAACCGACGCC
TCAAACAGATGTTTGCCGACCTGAGCCTTGAGAACCGGGCGCTGAAAGACGTTATCGAAAAAAAGCTTTAAAA
CCAGCCTTTAAGCGTGAGCTGGTCACTCATCTGATAACGGCATTCGGACTCAGTATCCGTCAGGCCTGCCGGA
GCCTGAACCTGAGCAGAACGGTTTATCATTACCGTCCGGATAACACACGTGACGAACCCGTTATTACCGCACT
GCAGGCGGCAGCTGAACGGTATCCACGATACGGTTTTCCGAAGCTTTTTCAGGTTCTGCGGCGGCAGGGATAC
ATGTGGAATCACAAAAGGATCCACCGTATTTATTGTCTGCTGAAGCTGAATTTTCGCCGTAAGGGCAAACAAC
GGCTGCCGGTGCGTAACCCTTCGCCACTGGCCACGCCGGAAGCGCTGAACCAGAGCTGGTCTGTTGATTTTAT
GCATGATGCCCTGGTCTGTGGGCGTCGTTTTCGCACGTTCAATGTCGTTGATGACTTTAACCGTGAGGCGTTG
TCGATTGAAATAGATCGGAATCTGCCAGCCCCACGAGTGGTCCGGGTGCTTGACAGGATCGCGGCAAACCGTG
GCTATCCGGCCATGCTTCGCATGGATAACGGCCCAGAATTTATCTCTCTGGCACTGACTGAATGGGCAGAGAA
ACATGCCGTAAAACTGGTGTTTATCCAGCCGGGTAAGCCGACACAAAATGCTTTCATTGAGCGTTTTAACAGG

ACATATCGCACAGAAATACTCGATTTTTATCTGTTCAGAACGCTGAATGAAGTACGTGAAATCACGGAAAAAT
GGTTATCAGAATATAACTGTGAACGTCCGTATGAATCGCTGAACAATATGACACCGGAGGAATACCGACAGCA
CCATTATTTGGCCGGGAACTCAAAAAATGTATGGAACTAAAACGGGTCTATTTACAGCTTGTCTTCGGTATTT
GGGCGCGAAAACTATGTGATATTTACAGTTCCATCGGGTGTGCGCTAAGCTCTTTTCGTCCCCCATTGGGACC
CCCTTTTGATTTCTTGTTGAACTTTTGCAGTTGCCAGACCGCAAGATGTTTTAACAAATCAAAAGGGGTTTTA
ATAACTGGCTTAAAGCTGAAAGCTTTCCGGAACCCCCAGCCTAGCTGGGGGTTTTCCATAGACAACAAAAAGG
GGCTACGTTTTCACGTTGCCCCTTGTTTTATTTGCTGGCGTCCTTGTAATTGGTGGGGGAGGTGGTTGAGTTT
GTTGGGGATTTTTATTTTTTGCGCGAAGGCGCAAAAGAGTGGCGATGGTGTTGATATGGAAGGGGTTTTGTAT
ATTTTTTGATTTTTAAGTTGATTATTGAGAGGCGGGGTTTTGCGTGTTGACTAACTGTCCTGAATGGCAGTTA
AAGTGTTGTCGCTTCCTTTTAGTCCGTCTTGTGGCAAGGCCAAGAGAGGACGTTTCACAACTCTACTTAGATA
GCTAAAATTAGGGCACATTTATAGGTCAATAGTTTTTAGGTTGAGATAGATAAGATAGATAAGTTGTATCTTG
TTAAATTATGATTTTCTTAAGGATTTGTTGAAGTCATATTGGTTACGGGGTATGCTACTTATAACGAGTTTAA
GATTTTGGGGATCTAGAGGCGGCCCCCCGAAAGTTGATACCGCCTAGTTGGTAACTTCGTCGAATGACTGGGA
CTCCAACCGTGCCGGCTGAGAGGTCGGATCTACTATTTTCGTGTGTTGGGAAGGGCGTAAGTGAGCTTAACTT
CTTGGGTGACAATATCCCTTGGTCCTTTTGACCTGTCGAAGAAAAACCAAATATTGAATTTACGTTCTGTTGG
CCAAGTATCATGCATCAGTGAGTCCCATCCCAAGTAGGATGCAACATTGTTTGCGACGGCTAGCTTTGCCTCA
GAAGTTGCTTTACCTTGATAAGCGCACATGACCGCACCTAGCAAAAAATCTGCTATCTGAATATGTTCTGACG
CCTTTGAGTCTTTACTTACTACGCTTTTGATAATATCTTTGCGCCCAAACCTACGTGCCAAAGTGTGGTTGGT
GATTACATGGAATTCCTCATCAGCTTTTTTGTAGCGGGATGGTAATGGATCTACTTCAACACGAAACTCACAC
TCTCTGTCGGGGTGGGCTTTGATGACATTGCCAATTTTAGTCTCTATTAGCTTGCCAAAATGCTTACGCATTG
CAAGATCATAGTCGCCACCGTGGAAAGATTTTTCTACCTTCGACTTTTCCACGACTATGCAATGAAAAGCTAG
CCAAGGGTGTTTGAAGAAGGTCTCGATTAAATCCTGATAGAATGCAGCGTTACGCTTGGAATGAGCTTTCTGC
CATTTAATTTCATCGCTACAGTTATGTTTCTCACGAAGTTCGCGGACAATACGCGCAAAATCGCCACGCCTCT
GGTATTTCATCCATAAGCTACCAAATCCATAAAATCGCTGACCATCTATCCCTGATTCATCGCAGGCGACATG
CCAAATCAGCTTTCCAGGATCGTTCGATTCCATGTTTACCCTTCATGAATGGATATTAGACGTAAGGTTATGA
TTTAACCACATTCTTAACGGGTTGATAAGGATAAGATTTGATTTCCATTGAGGATGGAGCCGTGTTGAAAGTG
CTGGTGGGGTTTTGGCTAGCCAGAAACGCAAATCCGCGCACGGGTTTGCATGAGCTCCCGTGGAGTGTTTCAT
CGTCCTAGCAAGTATTGATCCGGATCGCTACGATTGCTACAGGTGCATGAAATCCACCACATTAAGAGGGCTG
ACGTGGAAGGAATATGAAAAACAATCTTATCAACGGCTCGGAAATGTCTAGATTATCCGTGGCGATTCAGCCA
GCAGGCTTTGCCTGAAGTCTAAATTTATTTGGCTCACTAGTTTGCATAGCGGGCAGCTATGCAAAGCTAGGAG
TCTGTTGCTGTTGCGGCTGACTCTATCCTATTTAGGGCTTCAAAAATGAGTTCGTCTAACCGATAAAGGCAAT
GGCTATAAGGTAGTTCAGTTATTGGGTGAATCCAACCTTGTTGTCTAAGGTAATTGATGGCCGAATTCGTATC
TGAGTAGCCCTTGTGGCCCCTTCGTAGCTCATAGACAAAATATTTATAGCGGGGGGCTATGAGGGGCCATGCC
AGTATGAAATTTTCAATAAACTTTTCTTGGATTCTCTTGATTTCCTTGCTCTCAATCTCTTGTTTGCGGGCGA
GTTCTTTTCTTAATTTCCTCCTTTCTATGAATTTTTCGAGCTTGAGATACATCCAAGCTACTAGTTCAAAGGT
TAGAGTTTCCGCAATAAGTGAAAACAAAATCGTTAGAGCAAAATGCGCTGCATAGGAATACCCCGGCAAGTCT
TGTTCAGTTAGAGGCGCAATGTCCTTAAGTATTGGGAGTATAAAGCGGATAGAAAGGACAATTGCTACAGAGA
GGAATCCATACCTAACAGCCGTCTTTGTCGAAATGGGTGAAGCCAGAATTTTCCACAAACTTTCAGGTGTTGG
CATTTTTTATCCCTATCTGGTCACCTGATCTTAGGTGGACGCTAAGTGGACACTGGACATTAAAAAGGGGCTA
CGTTTTCACGTAACCCCTTGTTATATTTGGTGGAGCTGGCGGGAGTTGAACCCGCGTCCGAAATTCCTACATC
CTCGGTACTACATGCTTAGTCAGTCTTTACATTCGCCTGGCACCTGCGGACGGACACGCCACTACCAGACTAG
CCTGATTCGTTTTAACGCTTCAACCCCAGGCAGGGCATCCACGCGATCTCTTTTGGGTTTGACCTCTCTTGAT
CCCCGTCCTAAGAGCGGAGGCTAGGGAGAGAGGGCTCTAAGCAGGTTATTAAGCTGCTAAAGCGTAGGTTTCG
TCGTTTGCGACTATTTTTTTGCGGCTTTTTACGAGGCCAACCGCCCCTCGGCATGCACCTTGGGTTTCGCAAA
TCCCGTCGAATCCAGAATCAGCCCCAATGTGTAAGGTAAGTATAACAGACTTGTGACACGCGTGACCAGCCCC
CGTATCTTCTTATCTTCAACGATTTACCGCTCGCGCAGCGCTTCTTTCGCGCGATTAAACGGTTTGATTAAAT
AGTCGACAATGGTTTTTTCACCGGTTTTGATATCCACCGTGGCGATCATGCCTGGAACAATCGAAAAACGGCG
TCCTGATTTATTTTGTAGATAGTCCTGGTGGGTGCGGATAAACACGCGATAGTAGAAAATTTCCGGTTTCACT
TTATCCTGAATGGTATCCGGTGAAATGGTCTCCACCACGCCGTCAAGCCCGCCGTAAATGGCGTAATCGTAAG
CAGTAATTTTAACCAATGCGCGTTGGCCGGGATGAATAAAGGCGATATCACGCGGCGAAAGGCGGGTTTCAAT
CAACAGACGATCGTCTACCGGCACTATCTCCATCATCTCACCGTTAGGCGGGATCACGCCGCCAATCGTCGTG
ACCTGGATATTTTTAACAATGCCGCGTACCGGCGAACGTATGGTAAGGCGGGTGACGGAATCCTCGCGTCCTT
TTAAAATGGCGGAGAGCATATCGACCTCAGCGTTCGCTTTTGATAACGCTTCGCGTGCCTGCACATAATATTG
TGAGCGCAGATCGGTAATTTTTAAGCCTAAATCGCTTTTTTGTCGTTGCAGGCGCAGCACTTCAACATGACTG

GCCGCGCCGCTTTTCTCCAGACGCTGGGTAATGGCCAGCTCTTTATTAACCGACGCCAGCGCATCCCGTAGCT
CGGCTTCGGTATCGGCCAGCTGCGCGCGGCGGCTTTTATAGAGACGCGTCTCTGCGGCAATCAGATCCGGCCA
GCCGTTCAGCTCAGCGGGGAAGGCGAGAGGTAAGTCGTTGACTTCCGCGGTTAACCGTGCGCTGGAGGCGAGT
GAAGCGCGATATTTTGCCGCACTTTCACCCACATTGGACGCCAGACGCGTCGGATCAAGCCGGGCGACAATCT
GGTTAGCCTGAACTCTGTCGCCTTCCCGCACCGTCAACTGCGCCAGAATGCCGCCATCCAGCGACTGCAGAAC
CTGTTCGCGTGAACTGGGGATCACTTTCCCGTTGCCGGTGGAAACCTCATCCAGTCGGCCAAACCACGCCCAG
ATGCCGAGAATGAGAAATAACAGTGAGCAGATCAGAACAATACGACCCGCGCCGGAAAACGCCCGTTCACGCT
GAATATCGGGATCGTCCATCGCGGCATCATGCTGATTGATTTTCATTTTTCCACTCCCGACGGTGACTTTGCA
TCCGATCCGCGTTAAGCGCCTGCGCTTTTGGCGCATCCATCACCAGTTGTCCTTCTTTCAGGACAACGACACG
CTCAACCAGCTCCAGTATTGGTACCCGGTGCGTCGCGACGACCAGGGTACGGTTGCCAAGCCACTGATGTAAC
CGCTGAATAAACTCTCGTTCCGTATGCTCGTCCAGCGAGGCGCTGGGTTCATCAAGTAGCACAATATTGGGGG
AGCGCAGCAGCATTCTCGCCAGCAACAGGGACTGGCGCTGCCCACCGGACAGGCCGTTACCGCCCTCCATAAT
GGGATGATCCAACCCTTTTGCCAGCCGCCTGACGAAGACGGCGCCGCCGCTGACTTCGAGCGCGTCAAAAATC
TGTTCATCGTTGGCGTGCGGCGCGCCGAGCGTCAGGTTCTCTCGTAGGGTGCCGAAGAAAAGCCGCGCGTTCT
GGCTAAGAAAGCCGATGTTGCGGCGTAAATCCGCCATATCGATGTGCGACAAGCTGAGATTATCAAGCCGGGC
ATCTCCCTGAATCATCTCCAGGCCGCCCGCCATCGCCTGTAAAAGTGTGGATTTACCCGCGCCGTTACGCCCC
AGTATCGCAATCCGCTCGCCTGGCATGATCTCCAGACGCACGAGGCGCAGCGGTATGCGTTGATCGTCGTTAT
GGTAACGAAACTGAGCATTTTCAAAGAGGTAATGCCCGTGGAGGATGTCGCGGTGTATCAGGCTATCGTCATG
CTGTGTCTCGGTGGGCAGTTGCATAATGTTGTCGAGACCCATCTTCGCCGCTTTCACCTGCTGCCAGCGCGCC
AGCACGCCGCACAGGTTAGCCATTGGCGCGATCATCCGTGAGCCGAGCATCGACGCGGCGACCACGGCGCCGG
TAGTCATACTGCCTTCAATGACCATCGGCGCGCCGAACATAATCACTGCGGCATACACCAGACTTTGTACCGA
CATCCCCCAACTGATAAGCCCTTGCGTGAGTTTGCGGGTGCGTAGACCCGACTCGCCGGTAATACGGATATAG
CTATTCCACTGCTGAAGAAAGCGGTTCTCCGCCTGCATCAGTTTAATATCCTCCAGCCCCTGAACGCTTTCGA
CCAAGACTGCATTGCGTAGCGTCGCTTCGTGGGCGGCCTGGTTGGCGAGGACAGCCAGTTTTTTTGCAGTGCG
ACGCCCGGCAGGATCATCAGCAGAGCCGCCACGGGCGCTATCCATGCCAGCGGTGGGGCAATAATCGCCAGGA
CTATCATAAACAGGAAAAAGAAAGGCAGATCGACAATGGTCGCCAGCGTTGAAGAGGTAATCATCTCGCGGAT
CTGCTCTAGTTCGCGTAACTGCGAGATAAAACTGCCGGTGGAACGGGGGATAGCGCTGTTGCGCAGCCTTAAC
GCATGACCGAATACCCGATCGGAAATGCGCATATCGGCGCGTTTACCGAGCACGTCCATAATGTGCGTACGCG
CTTCACGCAGCAGAAAACCGAACAGCACCGCTACCAGCACGCCGAAGGAGAGCACGTACAGGGTAGGATATGA
CTGAGCGGGGATCACCCGATCGTAAACCTGCATAGAGAACACAATCCCGGCCAGCGACAGCACGTTAATCAGA
AAGGCGGCGACCATTACCGGTAAATAAGGGCGAATATCCTGCAGAACCAGCTCGCGCATCCAGTCGGGTTTAA
AGCGGGAGATATAGCGATCGACGCGGCTATCCTTGAGCGCCGATAGCGGGCGTAGCGCGGCAACATAGAGGAT
CTCCGGCAATAACTCGCTGAGCGTCAGGCGGTTACGCTGACCCTCTTCTTCAATCACAAAAACGTCTACCGCA
TCTTCGCCATTAACATGCTCGATGACCAATAACTGCCCGTCCCGGAGCTCGACAACCAACGGCAATCGCCACT
GACTAAATGCCGTTTTGTCTATGTCCGGCGCATGAAAGGATAAACCCGCCTGCCGCGCGAGCTGCGTTAAGGC
GGTCGTTCTGCTTTTACCCCTAAACCACGGGGCGTTGGCCTGAATTGAACCGGGAGAACAGGCAACGCGATAA
TGACCGGCGACATAGCTGATGGCCTGCGCCCATTGGCTTAGCGCGCGCTCACTGAGTACCTCTTCTACATCGG
GGGCGGCGCGGGTCATGGCTGGATCTCCACGGATTGAATGCTGCGATGATTTAACGCGAACGCCTGACGAAGC
GCGCCGGTGTTATACAGACAGTTCAGTTGCAACTGATGCAACTGGCTTTCCGTTTGCAGTTCGGCAAAACGCG
CCTGGTAAACTTCCTGCTCCGCATTAAGCACGTCGAGCAGCGGGCGGGAACCGAGGTTAAGGTATTGCTGCTG
ATACAGTTCGCGCGTGCGTTCGCTAAGCTGCTGTTGCCGACGAAGGATTTGTAACGCGCTGGCAAGACTCATT
GCCTGGCTGCGCGCCTCCATCAGTTTCTGGCGGACATCAAGCCGGGTGCGCTGAATGGTCGACTGAGCCGCGT
CTACCGCATGGCTGGCGGCGTTGCGTCGGGCAGTCAGCCCTCCGCCCTGGTAAAGCGGCATCTCAACTTTCAC
CCAGGTGGAATACTGGGTTTTATCCAGCACTTCATGACTGGGATATTTATCATTAAGATAATGTTGTACAGAA
GGTTCGAGCGAAATCGTCGGCGTCATTTGCGCGCTGGCATAGTCCAGATTCGCCCGCGCAACGTTAGCCTGCG
CCCAGGCGGCCAGTACCGCGGGCACCAGTCGATCGTCGGGAGTCGCCGTCTCACAGCTGCGAGCAAGTTTCGC
CGGGAAGTCATTATTGATGCCGTTAAGCGAATTCCAGCCCAGCCAGCTCATCAGTGAGGCTTTCGCACTGTCG
AGATTGGCCTGATACTGCGCCAGTTGCGAGCGGGCGGATTCGATTCTGGCTTCGGTTTGCACCACGTCAGATA
GCGACGTAGCGCCTTCATCGCTGCGCTGCCGGATAAGTTTACCGATGCTGTCCAGCGCGACGAGCTGTTCTTC
CGCCGCGTCTACCATTTGCTGCCAGCTCTGCGTTTGTACAATGGCGTTGGCGGTTTCGTGCGCGACGGTATCA
ATACTGAGCAACACATTGGCCTGTTGCTGCGCTGCGCCTGCGGTTTCGGCGCGAACCTGGCTTGCCACTTTGC
CGAAGTCATAAAGCATTTGCGATACCGACAGCACCAGCGAGGGGCTATACCCGTGATCGGTGTAGGTATTGCT
GTACCCATTGTTCACGCCAGCGCTGACTTGCGGATAATATTTCGATTTGGCGACCTCTATTTGTTCATTCTGT
GCGAGCAGTTTGCCGACGGCTTCGCGAATAGAAGGATGCCAGTTAACGGCGCGATTGACCGCGTCATTGAGCG

TCAGGTTGCCCGGCGCGGCGGCGCTGAGCGGCAACTCAGCGGCGGAGCCATCCAGCGAAGGAAGCATCTGGTC
CGTTGCTAATCCTTCGGATGTAATCATTGGCGGCGCTTCTGCGCCCCGGGGCTGGTGATGAAACAAAGCAAAT
GCCAGTACGATGGCGACAGGCGCGACTCTTCCCATAGTTTATCCCTTTTAAATATCACCGCTTGTTGCCCCGA
ACGTCGTGGCGTTCGGGGCTTTTTTATCAACCGCCGGTTATCAGGTGGTTGTTCTGCAACAGATCATTTAGCG
TGAGCTGCACATTCTCCAGAGTGACAAGGTCAGTGGATTTAAACGCACTGCCGGCGCCGTCGCGATCGACGGA
TATCACCGTATTGGCGCCGCTGGTATGAACCTGCACAAAGTTACCCAGCGTCGCCGCCTGGTGATCCCAGCCG
GTCAGCAGTTCGTGGATATCGATCTTGTCGCCCTGCGCGGTAGAGAAGTTTTGCCAGCGATCGACGCCGTTGC
CGCCGGTCGCGTCCGCGCCGTTTAACAGATGGTAAATCAGCGTATCGCCATAGGCGGAGCCGATGAGAACATC
GTTTTGGTTGGTACTGACGATTTGTGGCGCCATATCAATGGTCAATGTGGCGCTATCCGTATGACCCATCTTG
TCATCCAGTTGATAGGTAAAGACCTCTTTACTGGTGATGGACGACATCGCGACGCCATTATTCAGGGTGTAGG
TGTAAGCGCCATCGAGGTTAATTTGCAATGTGCCGTAATGTCCCTGAATCGTGGCGCTGGTGGTATTCGCCGC
GGCGTCCAGCGTGGCGGCGCTGCCGTTATACCCGCTAATGCTCAGGCGGGTATTCACCGTATTAAGCTGATCC
ATCGCCCCCGCCGCGTCGCTGCCGTCAAAAATATTGCCGAGAACGGTATGCGTTCCGGACGTTTCAAAATTAT
CCAGATCGACGGTGGTGCCGATGACGCGTGGCGTGATCGTCGCCGCCCCGGCCAGGGTATTGGTGCCGGTAAA
GTTAAGCGTGTACGTTCCGGCATCCAGCTCCAGACCGCTCAGGTTGACGGTCACCGTCGCGCTGCCCAGCGTG
ATATTCGCCACCGGAACGGTTCCGTTGCGTATGACGGTCCCGTTCTCCTGAATGGCCCAGCTAATATTCAGAT
TGCCCAGCGTAATGAGCGTGGAGACATCAAAGACCAGTGACGCGCCTTTAAGTACGGTGCCCGTTGCAACGTC
AAAGGTGCCGCTCCCGCTGCCCGATTTGCCGAGTAGCCCCCAACTGGCGCTGCCGACGCTGGAGTCCAGCCAG
GCAGCGGTATCCTGAAGCGTGGCGACGCTGAGGGTATCGCTGACATCATTAATCGCATCCAGCGCCCTGGCGG
TTGGCGTGATATTGAGCGAGGCCGTATCGGTATCGCCGTTTGGCGCTTTGACCGTATAGATAAAGCTGTCCGG
CGTTTTAATGCTGTCGGCGCCGACGCCGTTCTTCAGCGTGTAGGTATAGTTGCCTCTTGCGTCAATGATGAGC
GAGCCATACAGCCCGTTTACCTCCGTTGTGCCGGTCGCCGCAATCGCCACGCCGTTTACTTCAGTGAGGAGGG
CGTCCGTAGGGACGACATCATTGGTCAGTACGTTGCCGGTGGTGTTGGCGGTGATACTGTCAACGGCATAGGT
GTGGTCCTGGGAAATCGCCAACGTATAGCCAGTGAGGACGCTAATGCCGCTGGCGGTATTCAGCACGAACAGG
TATTCGCCGCCAGGCAACGTCAGGGTCAGCGGCTGGGACTGTCCGGCTAACAGCAGGGTGTTAATCCAGCCCT
TTTCCACCCGGAACTGCTCATATTGCTGAATGGCATCGTTGAAGCGATAAACGTACAGATCGAACGTGGAAAC
CAGTGAGACGCCGCCGACGGTTCCCTGTAACGTCATGGTTCGCGTAGCGCCTTCTTCAACGTTAAAGATGATC
GGATTAGTCATATCATCAAGCAGATTCGCGTTCAGTACATTACCAAGTCCGACGCTTAATACCGTGACGCCGC
TTTGTGTCGAGGGACCGTTGTTGACGTAGCTAACGTGAGTATCAAAGACCAGCGAGGCGTTATTGTCTGTCGC
TATCACGCTGCCCGGTACAGGCGCGGGACCCAGATTGATAACCAGTTGCGCGGCGCTGCTACCGACGCCATTC
TGGGTAATGGTGTAGGTGAAGCTCTCTTTATGTCCGTATCCTGCCGTGGGCTTAGCCAGCGTGTAGGTGTAAC
TGCCGTCCTGATTAATGTGCAGCGTGCCGTATTGTCCCTGGATATCCACGCCGCCCGCGCCGACCGGCGTACT
GACGCCGTTGGCGTTGGTGATGGCGGTGACCGTGGTGCCCGTCGGCGCGGTATCATCAGCCATGACGTTGCCG
GTACTGATGGTTGGCCCGCTAATAATACCTGCGCTGGTCTGGTGTACATCGACATCCAGGCTGGTATATGACC
CGGTGGCGAGCAGACTGGTGTTATAAGTGAGTACCCGGTACTGGCCTTCGCCCAGGCCGCTCAGGTTCAGGGT
AACGCCGCTCCCGGTCAGGGTAAGCAAATTCGCGAAGTCGCCAACGGCGGTGTTGACGATCGTCGTCCAGGCG
CCGAGGCTGGTGTCGTAGCGCTGTACCACAATCTCCTGAGTACTGAGCAGCGAAAGCACGATTCCCGTTGCGG
CGGCATCGATGGTGACGTCGCCCGTACCACCTTCAGCAATGGTGAACTCAACCTGAGCGGTATCGTTACCCAA
AACCGAGGCGACATTGCCAAGCGCGCCAACCAGCAGCAGGCCGTAATCCGAATAGCTTTGCGTGCTGGTGACG
GCGGTAGAGGTCAGATCAAGGCTGGTGATGTTATCCCTTGCCGCCAGCGGCAGAACCGGCGCGGTAATGCCTA
ACGTGCCGGAGGCGTTACCCGCAGCGTCAGTGGCCGTCACGTTAATGAGCTGACCTTCAATTTGTTTTGTCGG
CAAGGTGAGAGACCATGCGCCGTTGCTGCCAGCGGTGGTGGTGAGCGTCACGCCCCCCGTCAGCGTGACGGTT
ACGGTGCTGTTTGCCTCTGCCAGCCCGGAAAGTGAACCGCCATCGGCGCTGAGCGTACCTGACGGCGTACCGG
GCGCCAGCGTATCGACAATCACCGTCGCGGCGGTGGAGACGCTGCCTGTACCGTTGGCGTTAGTCGCGGTGGC
GGTGAAGGCGTGGCTGCCTTCGGTCAAATTGCCTGTCGGGGTAAAGCTCCAGTTTCCGCTGGCGTTCGCCGTG
GTGGTGCCGAGCAGCGCCCCGTTGTTATAAATGCTCACCGTGGCGCCCGCCTGAGCGGTACCATTCAATGTGG
GTTGTGCGTCATTAGTGACCTGGCCGTTGGCGATAGCGCCGGTATAAATACCCACATCATCTACTACGTTGGT
GATGATCGGTGCTTCCGGCACACGCGTATCGGGCGCGGTAAATCCGGCGGCAATGCCTGTGTTGCCTGCTTTA
TCCTGGGCAAATGCCAGTAGCGGCTGACCATTGGTCTGCGCGGGCGTGAGTGTGGCGGTGAAGCTGCCTGTAC
CGTCGGCGGTGGCGGTTCCCAGTACCACACCAGTATCAGAGGTAATCGTCACTGTACTGCCTGCTTCCGCGGT
GCCCGTCACCCGATTACCGGTAGCATTGATGACCAGGTTGCCGGGCGCGCCGGGCGCAATGGTATCCACGATA
ATCGTTATCGATGGCGAAGCGGCGCTAACGTTGCCAGCCGCATCGGCGGCGCTAACGCTATAGGCGTGACTTC
CCTGGCCGAGCGCGGCGGTTGGCGTCCAGCTCCAGTTGCCGCTGGCATTGGCGTTCACGGTCGCGACCAGCGC
GCTATTATCGAAGATGCTTACCGCGCTGTTGGCTTCCGCAGTACCGTTGAGCGTCGGCTGTGTGTCGTTGGTC

GATTGTCCATTGCCAATGGCGCCGGTATTGTTCGGGACATCATCAACGATGCTGGTTATAAGCGGAACCGTTG
GCGCAAGGGTATCGACCACGATAGTAAAACCGCTGGTGGCCGGGCTAACGTTGCCTGCCGGGTCGGTGGCGGT
CACTGTTAATGTGCGCGTGCCGTCGTTTAACCCTGTCGTCGGCGTGAAGCTCCACGCGCCGCTCGCGTTAGCC
GAGGTGACGCCAAGCAGAGTGTCGCCATCATAGATACTGACCACGCTGCCCGCTTCGGCAGTGCCGTTCAGCG
TGGGGCGGTTATCATTGGTTATCTGACCATTAGCGAGATTTCCTGTGACCCCACCCGCGACGTCATCCACCAC
GGACGTCAGGATTGGCGTTTGCGGCGCGGTGGTATCGACCGTCAGGGTGAAAGCCGTACTGTTCGGGCTGCTG
TTTCCTGCGGCATCGGTGGCGACGGCGGTGATGACGTGCGTTCCGCTTGCCAGCGTTGAGGTGGAGACGCTCC
ACGCGCCGTCAGGCTGAGCCGTTGTCGTGCCAATGACCGTACCATTTTCATAGAGCGTGATCGTGGCGCCGGC
TTCTGCGGTGCCGCTGAAGGTTGGCGTAGGATCGTTGGTATTTTGGCCGTTCGCGATCGTGCCGAGGCCTGGC
GCCTGATCGTCACTGACTGATGTGATTACCGGCGTCAGCGGCGGCGTAGTATCGACGACGAAATTAACGCTGT
CCGAAACGGGGCTGGCGTTGCCTGCGGCATCAACGGCAATGACGGTAATTACATGGTTGCCCGTCGCCAGCGA
GGCGTCGGGCGTGTAGCGCCAGCTACTGCTATTGTCGGCGGGAATTTCAGCCAGCAGCGTGCCGTTATCGTAG
AGACGAATAGTTGTGTTGGCTTCACGGGTGCCGCTAATGACAGGGCGCGCCTCGTCTGTGATCTGTCCTGAAC
TCAGATTGCCGGTAATGGGGCCCGTACCGTCGTAGACCGTATCAATAACAGGCGCGGCGGGAGCCGTGGTATC
AATAGTAAACGAGCGTATTTCGGACGCGGCGCTGGTATTGCCTGCCGCATCGGTTGCTGTCGCCGTCAGGTTC
CAGACGCCCGCGCCCAGCGAGGTAGACGGTGTAAAGCTCCAGCTACCGTTGGCCTGAACTTGCGCCGTACCGA
CCAGCGCGCTGCCATTATAAATCGAGATGGTTGTACCCGCCTCACCGGTACCCGAGAGTGTTGGTCGGGTTTC
GTTCGTCGAGCCGCCGTTGGGGACGATGCCCGTTACCGGCGCCGTATTATCTGCCACGCTGGCGATAACCGGC
GCGGCGGGCGGTGTGGCGTCAATCGTCAGCACAAATCCGCTGGACACCGAGCTGCTATTACCCGCCGGATCGG
TGGCGCTAAGGGTGAACGTGTGGCTACCGTTTGCAAGCGGCGTCTGCGGCGTGAAACTCCACGTGCCGTCGCT
ATTCACCGTCGTGGTACCGATGGACGCGGGATTACCGTCAAGATAGACATTAATCGTCGCGCCTGCCTCTCCG
CGCCCGTTCAGGGTGGGCGTCGCATCGTTCGTTAGCTGCCCGCTAACCAGGTTGCCGGTAATTCCGGGCTGGT
CATCCACGACGGAGGTGAGAACCGGGATCGCCGGAGGCGTAATATCGACCACGATCGTCCACGGCGCGGAGAC
CGCGCTGGTATTGCCCGCCGGATCGGTAGCGCTAACCGTCAGATTATGGCTTCCCTCTGACAAATTGCGGGGC
ACCGGGAAGGTCCAGCTACCGTTTTCGCCTACCTGAGCCGTGCCAAGCGGCGTACCGTTATCAAAAATGGTGA
TTGTCGCGCCAGGTTCGCCAGTTCCGTTCAGTATTGGCTGCCGATCGTCGGTGGATTGCCCCGGTAACACCGG
AACGGTCGGTTGATTGTTATCGTCAATCACGGACGTGATAACCGGCACATCTGGCGCCTGCGCGTCAATGTTC
AGCGTAAAGCCGTCCGAAGGCTGGCCGCTATTGCCAGCGGGATCGGTCGCCACGGCGGTAAAGATGTGCGCGT
TGCTCTCTAGCGGCGCGTTCGGGGTGAAGCGCCAGTTGCCGCTTTGATCTACCGTGGCGGAACCGATTTCTAC
GCCGTTATCCAGAATGCGGATCGTGGCGCCAGCTTCTCCCGTTCCGTTGAGCGTCGGGCGTGAATCATTGGTG
AGCGCGCCGTTATCCAGCGTACCGGTCATGCCGGGGACATCATCCGTTGCGCTGGTAATAACCGGTTGCGCCG
GCGCGCTAAGATCGACAGTGATCCCGACCGGTGCGGACAGGCCGCCCGGGCCATTCGCATTGGTTGCCTGGAC
GGTGAAGTAATGTGTGCTTTCGCTTAATGGCGTCGCGATCTCAAAGATCCAGTTCCCGCTGGCGTCAACGGTG
GCAAAGCCTGCCGAATCGCCATCAATGAAAACTTCAACGGTAGAGCCGATATCCGTGGTGCCGCGCAGCGTCG
GCATGGTGTCGTCCGTGGCGCCGCCCGCCGCAATATTCCCCTGTACGCTCCCGACATCATCTTCAATCGCCGT
GATGGCCGGGACGGCGGGCAGACCGCTGTCGGAAGCGGGAAAATTCGTAGCCGGGCCGTTATTCCCCGCGCGA
TCGATAGCAATTGCCGTCAGCGTACCGCCATCGGTTTGCGCCGGACTGAGCGTAACGACGAATTCACCGGTGC
CATTAGCGACAGCGCTGCCGACCAGGGTGCCATCGGCATGGTGAATTTCAACGGTAGCGTAAGGATCGGCAAG
TCCGGCGACGCGCGTGCCATCGTCGTCCACCGTCTGGATCGTTGGCGCGGGCGGGGCAGTGATATCCACGGTA
ATAGTGATCGGCTGGGTTTGCGCCGTGGTATTTCCAGCATCGTCAGTCGCGCTGAAGGTTAGCGCATGGCTGG
CTTCTGATAATTCGGGCGTATTGAATGTCCAGACCCCGTCGGTGACGAGCGCCGTCCCGATTGCCACGCCATT
GTCGTAAATCGTGACGGTGCTGCCGTTTTCGCCGCTGCCGTTAATAGTCAGCGTCCGATCGTTAGTAAAGGCG
CCATCGGTTAATGGCCCGGTCACTGGCGCAACATCGTCAAGGATTTCATTGACGACGGGCGGCTGCAACGGAA
CGGTGTCGACGGTGAAGGTAAAGCTGTTCGATACGCCGCTGCTGTTTCCGGCGGCATCGGCGGCAATGGCAGT
CAGGGTGTAGACGCCATCCGGCAGCGCGCTGGTCACCGCTAGCGTCCAGGTGCCGGTCTCATCGACGGTGACT
ACGCCTAAAACGCTGCTGCCATTATAGAGCGTAATAATGCTGCCGATGTCGCCAGTGCCGTGAATGACCGGTG
TATTGTCGTCAGTGGTTGCGCCGCTGACAATAGTATCCTGGACTTCGCCGACGTTATCTTCGGCAAAAGTGAT
AACCGGATTGGCGGGCGCAGTGGTGTCTAGCGTAAAAGTGACTGAGGCGGAAGGTGGACTGATATTGCCCGCT
CTATCGGTTTCCGTGACGGTAATGGTATAGGTTTCTGGCGTCAGTGCCGCAGGCGTAAAGTTCCAGCTACCGT
TATCGCCTACCGTTGTGCGACCAATTTCACTGCCATTATTGTAGAGAATTATCGTGCTGCCGATCTCTCCCGT
GCCGCTGAAGGTCGGCGTGGTATCGTTCGTTGCGCCATTATTGCCGATGTTGCCAATAACCGGCGCGACGTTA
TCGCTGATTAGCGGCGCGTCAGGCTGCGCTGGAGGCTGGGTATCAAGGGTAACGGAGAAAACGCCGGAGGTCG
GGCTACTATTGCCTGCGGCATCCGTGGCGGTGGCCGTCAGGATGTTTAACCCTTCGCTCAGATTACGGGTTAG
CGGATAGGTCCAGCTACCCTGTGGATTGACCTGGACGGTAGCCAGTTCTACGCCGTTATTGTACAGGGTGATG

ATGGTGCCGGGTTCGCCGGTGCCGCTGAGGGTCGGCATACGCTGATTGGTAATACTGCCGTTTGCCAGTAGCA
CCTCGCCTTCTCCGCCGGTGATGGCGGCTGACACGATCTGCGGCGCATCCGGCGCCTGGGTATCGATGCGTAG
GGTAAAGCTGATGGATTCGGGACTTTCGTTCCCCGCCGGATCGTTGGCGCGAGCGGTCAGATGGTGGTCGCCG
TCGCCCAGCGCGGCAGGCGGCGTAAACTGCCAGCGCCCGTCCGTGCCGACCAGCGCCTGACCTATGGCATTGC
CATTATCGTAAAGGATGATTGTGTCGCCTGCGGTGCCGCTACCGCTCAACCGGGGCTGGGATTCCTGGGTAAT
CTGATTGGTATCAATCGTATTTCCGTCGCCGTCAGCTACGCCGATAACGCCGGGGGCGGCAGGGGCCTGGGTA
TCAATGGTAATCGTAAAATCGGTCGAAAAAGGCGTGGTGTTGCCGTTACTGTCGCTGGCCGTCACGGTAAAGG
TGTAGGTGTCGTCCGCCAGTGGCGAGGCCGTGGTGTAAGCGTAGCTCCAGACGCCGGAATTACCGACGATAAT
ATCTGCTATCCGGGCGCCATTCTGATACAGGTGTACTGTGGAACCCGGTTCACCTGTACCATTAATCGTTGGC
GTGCTGTCGTTAGTGAGCCCCAGATGCGCGACGCTACCGAGAATACCCGGCGCATCATCTGTAATGAAGGTGA
TTACCGGTTGCTGCGGCGATTGCGTATCGACCACCAGGTCGAATGACGCGGCCTGACTGGCATTGCCTTTAGC
GTCGGTTGCGATTACGGTCAGTCGATGCGCGCCTTCACCTAAATTTTGCGACGGCGTAAACGTCCAGGCACCG
TTGGGCTGTAGCGTGGCGAGTCCCAGTGAAACGCCGTTATCAAAGACTTCGACACGCGCGCCGGCCTCCGCCG
TACCGCTAAGGGTGGGACGGTTGTCGTTGGTGGTTTGTCCGTTAACCAGCGGCCCGGTGAAGCTTTCAACATC
GTCCAGTACCTGAGTGATAATGGGCTTATCCGGGGCGGTGATATCGGGCGCTTGCGCTGTGGTTGGCGGGCTG
ACGTTGTTGGCGCTGTCGGTGGCGGTGACGGTAACCTGTTCGCCGTTGGTCTGTGCGGGGACGAGGGGAAGGG
TAAAATGACCTTCGCCATCTGCTTTGCCGCTGCCAAGCGGCTTGCCGTCCGGCGTGGCGATCGTGATGGCGCT
ACCCGGTTCGGCGGTGCCGCTGATACTGGTGCCATCCTCACTAATTACCACGTTTTTCGGCATTTCCGGCGCG
GTACTGTCCGGCGCGATAACCGTCGCGGCGGCGCTTTCGTTTTTCGCGACATCGGTGGCGGTAGCGATAAGCG
CCTGGCCGTTAGTCTGCGCCGTATTCAGCGGAACGATAAATTGTCCGTTGCCATTAGCGACGGCGCTGCCAAG
TACGGTGTTGCTACTGTCGCGGATAGTGACCGTTGCCCCGGCTTCCGCCTGACCGCTTACGGACAGACCGTCG
GTCGCCACGGTAAGACCGTCCGGTGCCGCAGGCGCAGTTTTATCCGGCGCGTTCAGCGTTTCTACCGGACCGC
TATTCGCGGCTTTATCGGTAACGCTAATGCTTAATGTTTCGCCGTTAATTTTCGCCGGGCTAATACCTATTGA
GAAGGTACCGTCGTCGCCTGCCGTTCCACTCCCGAGCGTATTTCCCAGGCTGTCTTTGATAACCACAGTACTG
CCAGCTTCCGTAGTGCCGGTGAGTCTGTCGCCGACGTCGCTAACCGCCAGGCCTTCCAGCACCGGCGGCTGCG
TGTCGATATCAATGGTAAAGGAGACGGACTGCCCGCTGGTGCCGCGATCGTTGGTCGCGGTAGCGGTAAAGAC
ATGGTCGCCATCGGCAAGCGCCAGCGAGGGGGTAAAGCTCCATTTGCCGTCAGCGTCAGCGACAATGGGCGGA
AAGGTCGCTACAGGAAAGCCATTATCGTTAATCGATATCGTACTGCCTGGTTCAGCAGTACCGCTGAGGGTTG
GGCGGTTATCATCTGTGGCATCGCCATTTTTCAGCAGCCCGGTAACGGCGCCAGCGTCATCCGTCACGGTAAG
GATAGTCGGCTGGGCAGGATACTGTGAGTCAGAAGCGGTAAACTCCGTGGCGGGGCCGATGTTACCGACGGCG
TCCTGAATACGCGCTTCCAGACGCTCGCCGTGGGTCTGAGCCGGATTCAGCGTGATGCTGAAGTGGTTATTTT
CTCCGGTAATCGTGGAGCCCAGGTAATTTCCCGCCGGGTCATAGATCGACACAGTACTGCCAGCTTCAGCGGT
GCCGCTAACGGTTGTGCCGTCTTTCGCAACATTATCAATGATAGCGGCGTCAGGCGGCGTGGTGTCCACAATG
ATAGTAGGCGTTGTCGTCACGCGGCTCGTGTTTCCGGCTTTGTCGCTTTGTTCGACGGTAAAGGTATGTTCGC
CATCGCTTAACGGCGTAGTCGGCGTAAAGGTCCAGCGTCCATTTTCGTCTACGGTAGCGCTGCCGATAACGGT
TGAACCCTCTTTAATCGTGATGGTATTGCCTGCCTCTCCCTGCCCGGAAAATAGCGGTTTTGCCTCGTCAGTT
CGGTCATTATTCTGTAGCGGCCCGGTGAGCAAACCGGTGTTATCGGCAATCAGTTCAACACGTGGGGCATCGG
GGATGAGTGTGTCGATAGTCAGCGTAAAGCGGGCTGATTCGCTACTTTCGCCTTTCACGTTAGTCGCAATGGC
GGTGAAATGATTTTCCCCATCCGCTAGCGCGCTGGTAATGGCAAAATGCCAGTTTCCGCTGGTATCAACCGTT
GTTGAGCCTATTTCCTGACCATTGGCGTAAATATGGATGACTGTGCCTGCGCTGCCGGTGCCCTCCAGAGTGG
GGGTATTGTCGTTCGTAAACTGGTTATTGCTCAGCGTGCTGGTGAGGGGAGCGGCATCATCGATAGCGGCGGT
GATAGCTGGTGTATCGGGGAGAGGAATGTTGGGCGCATCGATGGTTGCGGATGGGCCGGTATTGCCGGCATCG
TCAGTCGCGGTCACGGTAAGTTGTTCGCCGCTAATCTGTGGTGCTGTCAGATCAATACTAAATTTACCGTCGC
TATCCGCTTTGCCCTCGCCAACGACGTTGCCTGAGGGATCTTTGATGGTAATGGTACTGCCCGCTTCGGCTTG
CCCGCTTACCGTTTTTCCGTCCGCTGAAATCAATAATTCGCCGGGAGCGAGAGGCGGGGTAGTATCTACAGGA
TTACTGCTACCTGGAGTATTGCCGTTGGGGGTACTGCCGCCGGGATTGCTGTTATCAGGGGGATTACCGTCAG
GATTGGTGTTATCGGGGGGCGGATTATCGCCATCCGAATGATGGTGGGAATCGCCGCCGCCGGAAGACGCGAT
AGCGGCAATGCCGCCAGCCGCCACCGCGCCAGCCAGTACCCACGGCCAAACGGCGCCGCCTGCTTCATGGGAA
GCGCCGGAAGTGACCAGCAGTTCATTAATATCAGCGATTTGTTCAAAATGTAGCCCGGCTTGCGGATTTTCTA
CCCACCATAACGTGCCATCGTTTTCCGCCAGTACCAGCTGGCTTGCGCCCAGATCGTTGGTAACGTAAAAGTT
TTTAATCGTGATCGTTTCGCCGGAATGGAGCGTCACCACCAGATCCTGATTAATGCGCGTAAGTTGACTAATT
TCCTCCCGGGCCACTGATAATTTAACAATTGACGGGGCGTTAAGAGTGACCGCTGAGGATTCCACAGTGGTGG
AGACGCCAGTCAATTTCGAAACCACGGCGAGTAGACGCATAAATTAGCTCCTGATGGATTTGCTCTGTGTATT
AATTAACGGGCACGACGTTCAGGGCAAAATGCGTAGACGAGCCTTCCCGTTCATCATGAGAGATGAATTTCCG

TAGCCATATTCTTTTTTATGAGCGTAGGTGAAGGTCTGGTTTTCTCGCCGAATAATCCTTTGTTTTTTGTGCC
TGACTAGTGCTTTCGTATTAGATGATATAGTCAGAAAAAAATCTTTCTGCAAATTAAGATCTATTGATAAAGA
TGTTAGTGCGTCTGAAATATAACGGAGTTTGTGTTAAGGAGAGGTGGGGCAGGCTAACAACGCATGATAGTTT
TTTATTTATCTTTATGTTTTATAATGAATTTTAATTTCTTCTTCTTTTTCTTTTTTAACATAAAAACAATACC
TTTTGTGAAGGTATTCAGCAATATTAAATTTTATAAAAATCATTTGAAGTATTGTGTTATTAGGCTATAAAAA
TTTTTTAAACGAAGAATATATTCTTGTTGTGGTATTTATTGCTATTGATAAGCGTCAGACACAGAGAATATAT
TTTCGAGGCATGCATGAGATATGAACATTGACCAGCTTCATACTGAGAAGCTGGTCACAAAAAATGTCTATTA
TCGGCCTGCATTTTTCATAATGCGTGCTTTATCCAGTTGCCACTCACGTTCTTTAAGATCGGAACGTTTATCA
TGTTGTTTTTTACCTTTCGCCACGCCGATCTTCACTTTGCACCAGGCATTTTTCCAGTACAGAGAAAGCGCGA
CGACAGTATAACCTTCACGATTAATACGGCCATACAGTGAATCAAGCTCACGCTGATTTAACAACAGTTTGCG
GGTTCGGGTTGGGTCGCATACGACATGTGTCGAGGCGACCGCCATCGGCGTAAAGTTTGCGCCAAACAGCCAG
GCCTCGCCATCTTTGAGGATAACGTAACTGTCGCCGATATTGGCTTTTCCTGCGCGCAGAGATTTTACTTCCC
AGCCTTGCAGGGCGAGACCAGCCTCGAACTCTTCTTCGATAAAGTATTCGTGCCGGGCGCGCTTATTCAGCGC
GATGGTGGCTGAGCCCGGTTTATGTGCTTTTTTCTTCGTCATAAGTGTCGTGAATTCATAGGTAATCTGATAT
CGAAAACACCCCATTACGTCCTGATGAGGTGCAAAAATCTATCTTAGCATGAGTTGGGCTATGCACGCACAAT
TAAGGCATGACGGTTGGGGACAGCATTTTTTTTACTCGCTGATAAGTGGTATTATTTACACGATTTATGTTGA
TGGGAATTGCTATGCCTCAGATTAGTCGGACTGCTTTAGTCCCCTACAGTGCGGAACAGATGTATCAGTTAGT
GAATGACGTGCAATCGTATCCCCAGTTTTTGCCTGGGTGTGTTGGTAGTCGCGTCCTGGAGTCATCGCCAGCA
CAGATGACGGCGGCTGTTGACGTGTCGAAGGCGGGTATCAGCAAGACGTTTACGACGCGTAATCAGCTAACGC
GTAACCAGAGTATTCTGATGCATCTGGTGGATGGCCCCTTTAAAAAGCTGATCGGCGGCTGGAAATTTACCCC
GCTCAGCCCCGAGGCGTGCCGCATTGAGTTTCAGCTTGATTTTGAGTTTACCAATAAACTCATTGAGCTGGCG
TTTGGTCGTATATTCAAAGAGTTAGCGTCCAACATGGTGCAGGCGTTTACGGTTCGCGCCAAAGAGGTTTACC
GTGCCGGATAAACTTGTGGTTGAAGTGGCTTATGCGCTGCCGGAAAAGCAGTATTTGCAGCGCGTAACGCTGG
AGGAGGGCGCGACGGTCGAAGAGGCGATTCGCGCTTCCGGCTTGCTGGAATTGCGCACGGATATCGATCTGGC
GAAAAATAAAGTAGGTATCTACAGTCGTCCGGTAAAACTCACCGATACGGTGCAGGATGGCGATCGGGTTGAA
ATTTACCGTCCCTTAATCGCCGATCCAAAAGCGTTACGCCGCCAGCGCGCCGAAAAATCGGCTGGCAGATAAT
TAGCCTCTTTTGCCGGATAACGGTGTGGAAGCCGTACCACACCTGAAGCACAGGCGTGCCTGTAAGCCAGATA
AACGTAGCGCCATCAGGCAAATTCGTACCAAATGGCGGGGGAGAGGTTTGCAGGCCTGATGAACGTAACCTCA
TCAGGCGGTATCACAAAATTACTTCGTCAACGCCGGTTTGTTATCAATATTGGTTAACACGCCGCTGCTGTTA
AAGGTGAGCGTCAGAGTCTGCTGCGTCACGTTCTCATGTCCTGGCTGCTGGCGGAAGACATAAAACCAGGTGT
TCGTACCAAAAGGATCGGTCATCATCGGCGTGCCTAAAGCATACGCAACCTGTTGTTGCGTCATACCAACACG
CACTTTGGCGACATCCGTAGGTGTAAGATAGTTCCCCTGATTGATGTCAGGTCGGTAAACCACTCGCTCCAGA
GTGGAACAGCCTGCGGTCAACATCAGGAGTACTGCTGCGGCAGCAGTCAGCGTTTTACAGCGCATAGTGATTG
GGTTCCTTTTCGGGCCTGAGCAGTACGTGGCTCATATTAATATGCCGATGATAATAGACCTTTCATCACTTTA
AAACCTTTTGCTGTCGGGTCTTACGGCGTTTTTGTTGTGAACTCAGACCTTCGGGAGATAAAAAAGTTTACGC
CGCCAGCAGTTCTTTTGCGTTTGCCAGCGTATTACGCGTAACTTCACTTCCTCCCAACAGGCGGGCCAGTTCC
TGTAAACGCGCACGTTTATCCAACGGTTGCATATGGGTTTCGGTCATTGCCCCGTCTGTTTCTTTACTGACAA
AGAAGTGCTGGTGACCGCACCCGGCTACCTGTGGCAGGTGAGTGACGCACATCACCTGAGTGGATTCGCCAAG
CTGACGCAGGAGTTTACCGACCACGGCGGCGGTTGGGCCGCTGATGCCAACATCGACCTCGTCAAAAATTAGC
GCCGGCGTTTCCATTTTGCGCGCGGTAATCACCTGAATAGCCAGCGCAATGCGCGACAGTTCACCGCCAGACG
CGACTTTAGCGATAGGCTGCAGCGGCTGACCCGGGTTTGTCGTCACTTTAAACTCCACGCGATCGGCGCCGTC
GTTGCTCAGATGATGCTCATCAAATTTCACGTCGATAGTAAACAGACCGTGCGGCATCGAAAGTAAATGCATA
CTTTCAGTGATCAACTGCCCCAGCTCCTGGGCGTAAAACTGACGCTGTTGATGCAGCGCTTGCGCCGTTTCCA
GCGCTTGTTGGTGATGCTTATTAACCGCCAGCGTTAGCGTTTCCAGCGAATCGGCCTGATCGTCGAGCTGCTG
CTGTTCTTCGAGCAGTGACTGATAAAGCTGCGGCAGCGCTTCCGGGCTGACGTGATGTTTTCGCGCCAGCGAA
ATTTGTTTAGCGATGCGCTGTTCAAGCTCAAACAGGCGATTGGGGTCTAAATCCAGACGCTCGCAATAGTGGC
GTAATTCATCGCTGGCTTCGGTGAGTTGGATAGTGGCTTCTTCCAGCATATCCAGGATGCCGGAAAGCTTGCT
GTCCATACCTACCAGCTCGCTAACCAGTTGTTTTGCGCTATATAACTGGCTTTGCAGGTTAACGTCTTCGCCG
TCCGCGAGTAGCGCCAGAGCGTTCTGGCTGGTCGTAAGCAACTGCCCGCTGTTGGCCAGCCGTTTGTACTCTT
CGTCAATTTGCTCGAATTCACCGGCCTGCGGATTAAAGTCGTTCAACTCTTTTAACTGATATTGCAGCAGTTC
TGCGCGCGCGGCGCGTTCCTGGCTTTGTTGCTGGTGATGGGCAAGATCGCGGCAGCTTTGATGCCATAATTGG
TAGCGGGCAGCCATCTGCTGCGCTAAAGCGGCTTCGTTGGCATAGCTATCCAGCAGCGATTTTTGCTGTTCGG
GTTTGGTAAGCTGCTGGTGCGCATGTTGACCATGAATTTGAATCAGCAACTGACCGAGCTCGCGCAATTGAGA
CAGGGGCACAGCGGTGCCGTTAATAAAGCCCCGTGAGCGGCCATCGCTGCTGATAACGCGGCGAAGTAAGCAT

TCGCGACCTTCTTCAAGCTGGTTTTCTTCCAGCCAGCGCAATGCCGCAGGCGTGTCTTTCAGCGCAAAGCGGG
CACACAAATCGGCGCGGGTCGCTCCGGTACGGACCATATCGGCCTCAGCGCGACCGCCCAGACACAGACCCAG
GGCATCAATCGCAATAGATTTACCCGCGCCGGTTTCACCAGTAATGACGGTCATTCCGCTCTGAAAATCGATC
TCAAGCTCGCGAACGATAGCAAAATTGCTGATGGTCAGTTGCGCCAACATAGCTGTTTTCCTGTATGAAAAAC
CATAACTGTAATTAAATACAGTATAAACTGGTTTTTTATACAGTAAAGAGGCTAACGTAAAATTAGAATAATT
TTTTTGACCAGCCGAGTTTTGTGCTTAATGTATTGAAATAGCTGTAGTCTTTGGGATGTATCAGGTTGAGATG
GTAGTCGCAGCGGCGAATCAGCACATCTTCTCCTTCCTGAATAGGCAGGGCTATCTGGCTGTCGCAGCTAATT
TCCAGATCGCTACGGCGATGCGAAAAACGCAAACGAATCGTGCTACTGCTGTTAATGACTAACGGGCGCGCCG
ACAAGGTATGCGGGAACATCGGCACCAGGGTGATGGCATCCAGTGAAGGGGTCAGAATAGGGCCGCCTGCGGA
GAGCGAATAGGCGGTGGAGCCGGTCGGCGTAGAAATGATCAGACCATCAGATCGCTGTGAAAAAGCGAAGGTT
TCATCAATATAAACCTCGAATTCGATCATATGCGCCACCTTTCCCGGATGCAGCACCACTTCATTAATGGCTG
TGCTGATACGCTTCTGGCGGTCCTGTTGGCAGACCTGCGCTTCCAGCAGAAAACGTTTTTCGGAGATATAGCG
TCCTTCCAGCACATCAGATAATTGTTGCAGGGCGTTGTCCGGATCGAGATCGGTCAGGAAGCCAAGATTACCG
CGGTTAATGCCAATCACATTGATGTTATAACGCGCCAGCGTACGCGCTGCGCCAAGCATATTGCCGTCGCCGC
CCACGACTACCGCCAGATCCGCCTGTTGACCAATTTCCGCCAGCGTGCCGGTTGGCACATTTTTTAGCTGTAA
TTCGTGGGCGATTTGTTGCTCCACAATGACCTCATAACCCTGAGCGCATAGCCAACGGTAGAGCATTTCATGT
GTCGTGAGTGCGGTGGGATGACGCGGATGCCCCACAATGCCGATACACTTGAAATGATTATTCATTTTTCTGA
GGTCCTTGTAGCGAAGATTGATGACAATGTGAGTACTTCCCTTGAAACCCTGAATCTGATCCCCATAATAAGC
GAAGTTAGCGAGATGAATGCGAAAAAAAACGCGGAGAAATTCATGAGTAGTAAAGAACAGAAAACGCCTGAGG
GGCAAGCCCCGGAAGAAATTATCATGGATCAGCACGAAGAGGTTGAGGCAGTTGAACCAAACGATTCTGCTGA
GCAGGTGGATCCGCGCGATGAAAAAATTGCGAATCTGGAAGTTCAGCTTGCAGAAGCCCAGACTCGCGAACGC
GACACCGTGTTGCGCATCAAAGCGGAAATGGAAAACCTGCGCCGTCGTACTGAACAGGATATCGAAAAAGCGC
ATAAATTCGCTCTCGAGAAGTTCGTCAACGAATTGCTGCCGGTCATCGATAGCCTGGATCGCGCGCTGGAAGT
CGCAGACAAAGCCAATCCGGATATGGCGGCAATGGTCGAAGGGATTGAGCTGACGCTGAAGTCTATGCTGGAT
GTGGTGCGTAAGTTCGGCGTGGAAGTGATTGCTGAGACCAACGTGCCGCTGGACCCGAACGTGCATCAGGCGA
TTGCGATGGTGGAGTCGGAAGAGGTTCCGGCAGGCAATGTGCTGGGTATTATGCAGAAAGGCTATACGCTGAA
CGGTCGTACCATTCGCGCGGCGATGGTGACTGTAGCGAAGGCGAAGTAAAAGTCTGATGGCCGATGTAAAGTC
GGCCATTTTTTTAGCTTAAAAAATCCTGCACTTCCAGCCACGGCGTGTTGAAGTCCTCTATTTTTTTATATTC
GCTCATTGGCAGAACGACCACATTATAGGTGTCAGATCCTTCATTCAGATCAAAAATCTGGAAGCCCCTCTTA
TTGAGTTCGGTCTGGATGCGTTCAAAGATCTCGTCAAGGTAACGGTCTCCGGCTTCACAGACATCGGAGATTT
CATCCTCTTGGGTTATTTCGACAAGAAGATTTCTTAATTCCTCAGAATTCGTCAGGTTTTTTGTGAGTTCATA
AAAGCGATCTGCGGCAAAATTAGCCAGTTCGCCTTCTTCATCTTCACCTTTCCAGTCAACGAGGTGAATAAAT
CTTTCGCCGCGTAGAACTTGTAAAAATGCCTGTGCCAGCTCGTTATTTCCGAGATCGCCACCGCTACAATCCA
GGACGTCCTGCAACCCATATTCGTCTTCATCTTCGTCTTCAAAGATTTCAGGTTGCGTCGAAAGTTGAGAGAA
AATAGTGGGCCATAAGGTTGGGTTGTGTGATAAAAAAACGTTGGCGACGACGTTGAGGTCGTCTGCAAAATTC
TTATCCCATTCAATTACATAATCTTCTGACATAACATCAATATCCTTTCTGAATGCCTGAAAAGACATTATTG
CATTGTTAAGTAAATGGCAGGAATTGATGGAATCTTAAAAGACGGCGATAAAAAAGGCGAACCGGAGTTCGCC
ATTGTGTTACTCCGCCACACTCTCGCGCAGCGGCTTTACCGGTACAACCTTTACCTGCTTAATCATATTTTCC
TGTACATCGAGAATATCGATATCGTACTGCTCAATGCGCACGCGCGTGCCAGCAACCGGAATTTCCTCCAGAG
CCTCCAGAATCACCCCGTTGACGGTGCGCGCGTCGTCTTCCGGCAGGTGCCAGTTAAACGCTTTATTGATTTC
CCGGACGTTGGCGGTACCGTCAATAATGACCGAACCGTCGTTTTGCGGCGTGACCTCTTCCGCCAGGGTCGGC
GACATCGATGTGGTGAAGTCGCCAACGATCTCCTCGAGAATATCTTCCACGGTGACCAGCCCCTGGATATCGC
CATATTCGTTGACGACCAGTCCGACTTTCTTTTTATTGCGCTGAAATTTAATCAGTTGTGTACTGAGCGGCGT
ACCTTCCGGGACGTAATAGATTTCATCGGCGGCGCGCAGCATCATCTCTTTGGTGAACTCTTTTTTCTCGGCC
ATTAACCGCCAGGCTTCACGCACGCGCAGCATACTGATGGCGTCATCCAGCGAATCGCGATAGAGCACAATGC
GTCCGTGTGGCGAGTGGGTGAGCTGCCGCTCGATAGACTTCCAGTCGTCATTGATATCGATCCCGATAATTTC
ATTACGCGGCACCATGATGTCATCCACGCTGACCTTTTCCAGGTCGAGAACCGACAGCAGCATATCCTGATTG
CGGCGAGAGATTTGCGAACGGGATTCATGCACGATAGTGCGTAATTCCTCTTTGCTTAACGACCCGCTAACCA
CGATGTCCGTTTTAATTCCCATCAGACGCATCAGCAGGCGGGTGATGGTGTTAAGTAACCACACCAGCGGCAT
CATCAGTATCTGTAACGGCGCCAGCAGGAAGCTGCTGGGATAGGCCACTTTTTCCGGATAGAGCGCGGCGATA
GTTTTGGGCAGGACTTCGGCAAAAACTAACACGACAAACGTCAGTACCCCGGTGGCGATAGCCACGCCAGCGT
CGCCGTAAAGCCGCATACCCACAATCGTGCCGAGCGCCGAGGCAAGAATGTTGACCAGGTTATTGCCGATCAG
CACCAGGCTTATCAGTCGGTCGGGTTTACGCAGTAATTTTTCTACCCGTTTTGCCGAGCGATTACCTTGCTTC
GCCATATGGCGTAAACGGTAACGGTTTAGCGTCATCATTCCAGTTTCGGAACCGGAAAAATAGGCCGAGATGA

CCACCATGATGATTAATATGATAATCAGCGTGGTGGTGGAGATGTGTTCCAGGGGAAACTCCTTTAAAAGCTT
AGCTTACAAATTGCTGTAATATGCGGCTACCAAAATAGGCCAGCGTCAGAATACCCGCGCCTGCGACGTTAAA
CCACACGACCCGCCGACCCCGCCAGCCCTCATGGTAATGCCCCCACAGCAGAACGATATAGACAAACCATGCC
ACAATAGAGAGCACGGCTTTATCAATATTTTCAGTACTGAACAGGTTATGCATGTAAAACAGGCCGGTACACA
GAGTAAGGGTCAGCAGTACGACGCCAATCTGCGTGATATGAAACATTTTGCGCTCAATGCTCATCAATGGCGG
CATTTCATTACTAAACGCCAGCTTTTTGTTCTTCAACTGATAGTCGATCCACGCCAGTTGTAGCGCATACAGC
GCGGCGATTATCAGCGTCGCGTAAGAGAAAAGCGACAGACCGATGTGCACCATCATGCCTGGGGTCGCTTCCA
GATGCGTAATATATTCATTAGGCATGAACGTGGCGAAGGCCAGGTTAATCAGCGCAAAAGCATAGACAATGGG
CAGAAGTAGCCAGCCGCGATTACGCGACGCGACAATGGTCATCACCGTGCAGATCATCAGACTGACCAGCGAA
CCGACATTCAGCAGGCTCAGGTTTTGTCCACTGTCGCCGCCGGGCAGAATACGCGATTCCAGCGCGACAGCAT
GGCACACCAGCGCGATGACCGCAGAAAGAATAGCCATACGCCGCCAGCCGCTGTTTTTTTGCAACAGTCCGGG
AACGATCAGCGCGAGGCTGAAGGAGTAGGCGACAAGGGCAAGTAAAGCAAAAACGGGCATAGTTGTGTCGACA
GTTGACCAGTGAGAAAGAGAAGCAGTATAGCGTTACGTGACCGCAGCTCCAACCGTTGCATAACAACAAAGAC
GGCTTCATGGTATACTGTGGCGAAATTCTGTAACTGTGTCGCTGCGGCGACCAACCGTTTCCACCCCAGGCGA
GAGACAATGTTTGATAATTTAACCGATCGTTTGTCGCGCACGCTGCGCAATATCAGTGGCCGTGGGCGCCTTA
CTGAAGACAACGTTAAAGAGACGCTGCGCGAAGTGCGCATGGCGCTGCTGGAGGCTGACGTTGCGCTGCCGGT
AGTGCGTGAGTTCATCAATCGCGTAAAAGAGAAAGCGGTTGGTCATGAAGTTAACAAGAGCCTGACCCCAGGG
CAGGAGTTCGTCAAGATTGTCCGTAGCGAACTGGTTGCGGCGATGGGCGAAGAGAACCAGACGCTGAATTTAG
CTGCGCAGCCGCCAGCCGTAGTATTGATGGCGGGTCTGCAGGGGGCGGGTAAAACCACCAGCGTCGGTAAGTT
GGGTAAATTCTTGCGCGAGAAGCACAAGAAGAAAGTGCTGGTCGTCTCTGCCGACGTCTATCGCCCGGCGGCG
ATCAAACAGCTCGAAACGCTGGCTGAACAGGTTGGCGTGGATTTCTTCCCGTCTGATGTCGGCCAGAAACCGG
TTGATATCGTCAACGCCGCGCTGAAAGAAGCGAAGCTCAAATTCTACGACGTGCTGCTGGTGGATACCGCCGG
TCGTCTGCACGTTGACGAAGCGATGATGGACGAAATCAAACAGGTCCACGCTTCTATCAAGCCAGTAGAAACG
CTGTTTGTCGTCGATGCGATGACCGGTCAGGATGCGGCGAATACCGCAAAAGCCTTTAACGAAGCGCTGCCGT
TAACCGGCGTGGTGCTGACCAAAGTTGATGGTGATGCTCGTGGCGGTGCGGCGCTCTCTATTCGTCATATCAC
CGGCAAGCCGATTAAATTCCTCGGTGTCGGCGAGAAAACCGACGCGCTGGAACCGTTCCACCCGGATCGTATC
GCCTCGCGTATTCTCGGTATGGGCGACGTACTGTCTCTTATCGAAGATATCGAAAGCAAAGTTGACCGCGCAC
AGGCTGAAAAGCTGGCGACTAAACTGAAGAAAGGCGACGGTTTCGACCTGAACGACTTCCTGGAACAGCTCAA
ACAGATGAAAAACATGGGCGGTATGGCCAGTCTGATGGGCAAATTACCGGGCATGGGGCAGATTCCGGACAAC
GTTAAATCGCAGATGGATGACAAGGTGCTGGTGCGTATGGAAGCGATCATCAATTCGATGACGCTGAAAGAGC
GCGCGAAGCCGGAAATCATCAAAGGTTCCCGCAAGCGTCGTATCGCGCAGGGCTGTGGGATGCAGGTGCAGGA
CGTTAACCGCCTTCTGAAACAGTTCGACGACATGCAGCGCATGATGAAGAAGATGAAGAAGGGCGGGATGGCG
AAGATGATGCGCAGTATGAAGGGGATGATGCCGCCAGGCTTCCCGGGTCGCTAATTTCCCGGCCTGGCTCACT
TGCTATTTCGGCACCGGGGTGTGCTCGCCATCCTCACGTACTGCGTGTACGCTGCGGTGGCTGCGCGCACGCC
GGCACCGAACTCGCTGCGCCGCCGACGGCCTTCTACAGCAGAGTTATTAACTGTTGATTGCAATTCTCCCAGA
AATCAGTAAAATTTTCGGGCTTTTAATATGACGCCGGGCTCCGTTCCTCGATGGGGCCCGGTTGTTTTATTCA
CACAAGAGGATGTTATGGTAACTATTCGTTTAGCTCGTCACGGCGCTAAAAAGCGTCCGTTCTACCAGGTTGT
TGTCACCGACAGCCGTAATGCACGCAACGGTCGCTTCATTGAGCGCGTTGGTTTCTTCAACCCAATCGCTAGC
GAAAAAGAAGAAGGCACCCGCCTGGATCTGGATCGCATCGCTCACTGGGTTGGCCAGGGCGCAACCATTTCCG
ATCGCGTTGCGGCGCTGATCAAAGAAGTAAAAAAAGCAGCTTAATCTGTCACGGTGGTCATGATGAGCAAGCA
ACTCGCCGCACAAGTACCCGCTGAACCGGTTGTACTGGGGAAAATGGGTTCTTCATACGGTATCCGTGGTTGG
CTCAGAGTGTTTTCCTCCACTGAAGACGCCGAAAGCATTTTTGACTATCAGCCCTGGTTTATCCAGAAGGCGG
GTCAGTGGCAGCAGGTACAGCTGGAAAGCTGGAAGCACCACAATCAGGATCTGATCATCAAGCTGAAAGGCGT
TGACGATCGAGATGCCGCGAATCTTCTGACCAATTGCGAAATTGTCGTGGATTCCTCGCAACTTCCTGCGCTT
GAGGAGGGAGACTATTACTGGAAAGACCTGATGGGTTGCCAGGTAGTCACCGCTGAAGGCTACGATCTCGGTA
AAGTTATCGACATGATGGAAACCGGGTCGAATGACGTTCTCGTCATTAAGGCGAACCTGAAAGATGCATTTGG
TATCAAGGAACGACTAGTACCGTTCCTCGATGGGCAGGTTATCAAGAAAGTCGATCTCGCTACTCGTACTATC
GAAGTAGACTGGGATCCTGGTTTTTAAACCACCGAATAAACGGTAATAAATGGCGTTATGGGGATTGGCTTGT
GTTTATTGGCATCGTTAGCCTGTTTCCTGAAATGTTCCGCGCAATTACCGATTACGGGGTAACTGGCCGGGCA
GTAAAAAAAGGCCTGCTGAACATCCAAAGCTGGAGTCCTCGCGACTTCGCGCATGACCGGCACCGTACCGTGG
ACGACCGTCCTTACGGCGGCGGACCGGGGATGTTAATGATGGTGCAACCCTTGCGGGACGCCATTCACGCAGC
AAAAGCCGCGGCAGGTGAAGGCGCTAAAGTGATTTATCTGTCGCCTCAGGGACGCAAGCTTGATCAAGCGGGC
GTTAGCGAGCTGGCCACGAATCAGAAGCTTATTCTGGTGTGTGGTCGCTACGAAGGCGTAGATGAGCGCGTAA
TTCAGACCGAAATTGACGAAGAATGGTCAATTGGCGATTACGTTCTCAGCGGTGGCGAACTACCGGCAATGAC

GCTGATTGACTCCGTCGCCCGGTTTATACCGGGGGTTCTGGGGCATGAGGCATCAGCAATCGAAGATTCGTTT
GCTGATGGGTTGCTGGATTGTCCGCACTATACGCGCCCTGAAGTGTTAGAGGGGATGGAAGTACCGCCAGTAT
TGCTGTCGGGAAACCATGCCGAGATACGTCGCTGGCGCTTGAAACAGTCGCTGGGCCGAACCTGGCTTAGAAG
ACCTGAACTTCTGGAAAACCTGGCTCTGACTGAAGAGCAAGCAAGGTTGCTGGCGGAGTTCAAAACAGAACAC
GCACAACAGCAGCATAAACATGATGGGATGGCATAGGCCCCCGAATATCAGTTTACCCAGGATAAGAGATTAA
ATTATGAGCAACATTATTAAGCAACTTGAACAAGAGCAGATGAAGCAGAACGTACCTTCCTTCCGTCCGGGCG
ATACCGTGGAAGTGAAAGTATGGGTTGTTGAAGGTACCAAGAAACGTCTGCAGGCATTCGAGGGCGTGGTTAT
CGCTATTCGTAACCGCGGTCTGCACTCTGCATTCACTGTTCGTAAAATTTCCAACGGCGAAGGCGTTGAGCGT
GTCTTCCAGACTCACTCTCCGGTAGTTGACAGCATTGCTGTTAAACGTCGTGGTGCTGTTCGTAAAGCTAAAC
TGTACTACCTGCGTGAGCGTACTGGTAAAGCGGCTCGTATCAAAGAGCGTCTTAACTAAGAACGCGCTTTCGC
AACATCCGAAAGCTTGTAGAAAAGAAGGGGCTGGCATTACGCTGGCCCCTTTTTTATGGGGTTGCTCCACGCC
ATGGACGGTTGCATGATTAGGCTGGGTCTGTGGTCTCAACGCTGAGTCAGAAACGGCCAGGCCCGTTCCTTAA
TTTAGCGAGCGTTCCGCACGCCGGTGTTTAGCCTGATACATATTTCGATCGGCCAGTTCTTGTAGTTTTTCGG
CAGTGGCATGTTCCCATGTCAGCGCAAAGCCAATACTCAGGGTCATCGTTATCCGCTGGCCGTTATGCAGTTC
AAAAGGTCGATTAAACGCCTGGGATAGCGCTGCGCAAATACGTTGTACTTCATACTCCGAATGTACATCGTAA
AGCACCATCGCAAATTCATCGCCGCCGAGTCGGTAAGTTTGATAACGGCTACCACCGAATTCCGCTAATCTTT
TGGCAACCTCTATAAGTACGCGGTCGCCTGCCGCATGTCCCCAGGTATCATTAATATATTTAAAGTTATCGCC
ATCCAGAAATAACAATGCCGAACTGCTACGAGCGGAATTGTCCTTCATCAGCGCGTTAATACAGCTGCGAAAT
GCCGCGCGATTGGCAAGCCCCGTCAGCGGATCGTGCAGCGCGGTACGTAGTAACTGGGCATTTTTAGCCTGAA
GCCGTAGCTGCCATTCTTCCATCTCATCCAGAAGGCTATTGAAATCCTGCGCAAACAGGTGAAATTCCGCAAT
ACGCTCATCAGGTACCCGGCGTGAAAAATTTCGGTTAGTGCGAACGTCGTGTACAACTTCAGTAATATTTTGC
AGCGCATCCACAACGCCATTGTGCAAATAACGCGTGAGCAGCAGGGCAATGCCGGATGCCAGCAGAATACATC
CTGTCAGCACTGCCAGCGATAGCCAGATAAAATGACCAATAAGGCTGTCGCGGGCGACAAGCCGTACTTCGCC
GATGGCCCTGCCGTTGTGCCAGACGGGTTGTGATACCGGCAATGGAAAAAGCCAGTGGCTAATTAAGCCGATG
AGCTTATCGTCTGCGGCTCGCGCATCATAGCGCCATGAGGCGATAGTACGGCCATTTTTATCGCGGACCTCCG
CCGCTGAAAATTGTCCCTGGCGTCCCAGTGTGGCGAGCGTTTCCGCTGCGGCCGCGTTATCGGAAAATACCAG
TGCCGCTTCAAGGCTATGGGCCATTGTGGCGGCGGTCAAATCGAGATTTTTTTGCGCATACTGTTTGAGCGTA
AGGACAGACGCAACGCAAATTAATAGCCAGATCAATGTCATTGTAAGCAGCACGCTGATTATACTAATCCGCC
GTAGTGTGCGTTTAAATGTTGGTCTGGACAGAGAAAATTCCTTATTCATGCTTTTGATTCCGTGCAAGCATTA
ATACTTCTGGATTTACTCTCACGCCGCTATGCGAGAGGGAGTCCAGGTTGACGGAAAATTTGACTTCATCATT
GTTAATGATCAGACAAAATGCGCTGCCAATAATACATTCGGTATTTTGCTCGGCAATTAATAACAAGGCATTA
ACTGGATAATGACGCGTTAACTCCACCTGATAAGCTGGCGATTCATTGCCAAAGTAAAAACCATCACAGCGGG
CGCTCAGGGCTTCTTGTGTGGTACGGATGACAAGCGGTTGATAAGGGAAAGCCCAATCAGCCTCTTCGCTAAG
TACCCTGACAAAACGCGCAGATGAAAATATGCATAGCCTTGGCGGCCCGGATAACGCCGGCCAGTGTGTATAA
CTGACGATACCGGAGACGATGGCGCGTACGGATTTCTCTTCTTCCGTGACGCGTTGAGCATACAGAGGCAAGC
TTGCTAATAATAGCGATAACAGAAGGATAAATCGGTGAGAAAAACGCATTAACAAGTCCCCACCATCTTCAGT
CGTAAAGCATTAAAATACATCCGTGATTGTTTGTAGCAAAATAACATTATTTTGCCATATCGTCATTATGTCT
TATTGATAATATGAATAATTTAAGTGGCGTCCCTGATATTAAATTCTTGTGATAATGGTATTAATCAACCTAT
ATCAGAAAGTAAAAATATGTGTGCGGAGAAAAAATAATCAAAAAATAGCGAGCATCGCTCATTTTACCGTTTT
TCTGAGGCTGGGAATAAAATAATTCACTTGTTGTGTAAAGTTTTTGATACGAAATGGAGGGGGTTAATCGACG
ATTGCGCATGGTGTAAAGTTTATTTTACGTAATATGGATTGAAATCTTTACTTTTTGTGGTATGGTTTAAACA
TCCTCGTGGAGGATCAACTATCGCAAACAAGAAAAGACAGGATCGCCATCATGCAAAAAGACGCGCTGAATAA
CGTACGTATCACCGATGAACAGGTATTAATGACGCCGGAGCAGCTTAAAGCGGCCTTTCCGTTGAGCCTGGCG
CAGGAAGCGCAGATAGCGCAGTCCCGGGGAATCATTTCTGACATTATTGCCGGGCGCGATCCGCGTCTGTTGG
TGGTATGCGGTCCTTGTTCTATTCACGATCCTGAAACCGCTCTGGAATATGCCCGTCGATTTAAAGCCCTTGC
CGCAGAGGTCAGCGATAGCCTCTATCTGGTAATGCGCGTCTATTTTGAAAAGCCGCGAACTACCGTCGGCTGG
AAAGGGCTGATTAACGATCCTCACATGGATGGCTCATTTGATGTGGAAGCCGGGTTGAAAATAGCGCGTCAGC
TACTGGTGGAACTGGTGAATATGGGGTTGCCATTGGCGACCGAAGCGTTGGATCCGAACAGCCCGCAATACCT
GGGCGATCTGTTTAGCTGGTCGGCGATAGGCGCGCGCACAACCGAATCGCAAACCCACCGCGAAATGGCGTCT
GGTCTTTCTATGCCGGTCGGCTTTAAAAACGGCACGGATGGCAGCCTGGCGACAGCGATTAACGCCATGCGCG
CCGCTGCGCAACCTCATCGTTTTGTTGGCATTAACCAGGCCGGTCAGGTTGCGTTATTGCAAACCCAGGGAAA
TCCGCATGGCCATGTGATTTTGCGTGGCGGCAAAGCGCCAAACTATAGCCCGGCAGATGTCGCTCAGTGTGAA
AAAGAGATGGAACAGGCGGGACTACGTCCTTCGCTGATGGTAGATTGCAGTCATGGTAACTCCAATAAAGATT
ATCGCCGCCAGCCAGCCGTTGCCGAATCTGTGGTTGCGCAGATTAAAGATGGCAATCGTTCAATCATTGGCTT

AATGATTGAAAGTAATATTCATGAGGGTAATCAGTCTTCCGAACAGCCGCGCAGCGAAATGAAGTATGGCGTT
TCCGTCACCGATGCTTGTATTAGCTGGGAGATGACCGATGCCCTGTTACGTGAAATTCATAAAGATTTGAGCG
GCCAGCTGGCGGTGCGCGTCGCATAAGAGGTTGTTATGGTTGCTGAATTGACCGCGTTACGCGATCAAATAGA
TGATGTCGATAAAGCGTTGTTGAATTTACTGGCTAAGCGCCTGGAACTGGTTGCCAAAGTCGGCGAGGTGAAA
AGCCGTTTTGGCCTGCCTATTTACGTGCCGGAGCGTGAGGCCTCTATGCTGGCTTCACGACGGGCGGAAGCAG
AAGCGATCGGTGTCCCGCCCGATCTCATTGAAGATGTCCTGCGCCGGGTAATGCGTGAATCTTACTCCAGCGA
AAATGATAAGGGGTTCAAAACGCTTTGTCCTTCTCTGCGTCCGGTCGTCATTGTGGGCGGCGGCGGACAGATG
GGGCGTCTGTTTGAAAAAATGCTCACGCTGTCGGGCTATCAGGTCCGTATTCTGGAACAGCAGGACTGGCCGC
GCGCCAGGGACATTGTCGCCGATGCCGGAATGGTGATCGTCAGCGTGCCGATTCATGTTACTGAACAGGTCAT
AGCGCAACTGCCGCCCCTGCCGTCCGACTGTATTCTGGTCGATCTGGCATCGGTGAAAAGCGATCCGTTACAG
GCAATGTTGGCGGCCCATGATGGCCCCGTGTTGGGCTTGCATCCGATGTTTGGCCCGGACAGCGGGAGCCTGG
CGAAGCAGGTGGTGGTCTGGTGTGACGGGCGTCAACCGGAAGCGTATCAGTGGTTCCTTGAGCAAATCCAGGT
GTGGGGCGCTCGGTTGCACCGAATTAGCGCTGTCGAGCACGATCAGAACATGGCTTTTATCCAGGCGTTGCGC
CATTTTGCTACCTTCGCTTATGGGCTGCATCTGGCGGAAGAGAACGTCCAGCTTGAGCAGCTTCTGGCGCTAT
CATCGCCGATTTATCGACTGGAGCTGGCGATGGTCGGGCGTCTGTTCGCCCAGGACCCGCAGCTGTATGCGGA
CATTATTATGTCGTCGGAGCGCAATCTGGCGCTTATCAAGCGTTACTATAAACGTTTTGGCGATGCGATCGGG
TTACTGGAACAAGGTGATAAGCAGGCTTTTATCGACAGTTTTCGCAAAGTTGAACACTGGTTTGGCGATTATG
CCAGACGCTTCCAGAATGAAAGCCGTGTGTTATTGCGTCAGGCGAATGACAGCCGACCATAATGATGACATCT
ATACTAAAAGCCAGCTTTGCTGGCTTTTTTCGTTTAAGGAACCGTTATGACTACGCCGCACGTTTTATTTGAT
TATGCCGGACATTTACCAGAATGCCCCACATGGAGCGAGGATGAAAGCGCGCTGTATTGGACCGATATCCTGG
AACAGGAGATCCATCGTTACCATCCGGCGAGCGGGACGCATAGCGTGCTGGCGTTTCCGGAAGAGGTGGGCTG
TTTTGCCTTGCGTGAGCAGGGCGGATTTATTGTGGCCATGCGTCATGCTATCTGGCTGGCCGATAAAAATGGA
TTATTGCAGCGTAAGGTTTGCGACAATCCCTCTAATACGAAACTGGCGCGTTTCAACGATGGCGGCACTGATG
GCGATGGACGTTTTTATGCCGGAACGTTCTGGGCGCCAGGTGACTATAATGGCGCGTTGCTGATGCGGATCGA
TCATGACCTGACGGCAAAAGTGATCCAGTGCGATATCCAGGGACACAACGGCCTGGCGTTCAGCCCGGATAAC
CAGTGGATGTATACCTCCGATACGCCGAATGGCGTGATATACCGCACGTTACTTGATAAGCATGGCGATCCGG
GCAAGCGTGAACTTTTTCGCCATTTTGGCGAAGGAGAAGGACTTCCCGACGGCGCGGCGATGGATAGCGAAGG
GTGCTACTGGAGCGCGATGTTTGACGGCTGGTGCGTGGCGCGTTTCTCACCGCAAGGAGAGCAGCTGGAAGAG
TACCGGTTGCCGGTACGTTGTCCGACGATGGTTTGCTTTGGCGGCGCAGATATGAAAACGCTGTTTATTACAA
CTACCAGAGAAAATATGTCTGCTCAGGAAGTGGCGGATTATCCGCTCTCCGGCGCCATCTTCACCCTGCAGGT
AGCCGTCGCAGGGATGAAGAAAAGCCGTTTTATTGAACGTCAGGCAGGTTCTACCGGCACGACGTTTTCGCTG
GGATAGCAGCCAAGCACTTTCATTGAGCGCGTGATCTCGCCCAGCTCTTTTAGCGCGCTTTGCATTACCTGCG
ACTCCAGGTTCGCCTGAATATCGAGATAAAACATCTCTTCCCACGGATTGCCGTGAATGGGGCGCGACTCCAG
TTTCGTCATGATGAGATTGTGGTTACGCAGCACCAGCAGCGCTTCGACCAGCGCGCCAGCTTGCTGCCCGGTG
GCGATTAACAGAGTGGTTTTTGCCGGAACCTGATCGGAAACGTTGATGGCTTTGCGCGCCAGTACCAGAAAGC
GGGTGATATTCTGCGTCTGGTTTGCGGCAATGCGTTCCAGCACCTGTAAACCGTGCAACATGCCGCCTGCCTC
GCTGCCGAGCGCCGCGACGCGCGGAGAGTTTGCCTGCGCGACTTTTTCCATCGCTGCCGACGTACTCTCGGTA
TAGTCGATTTTCCAGTGCGGATAGCGGCTCAAAAATTTACTGCACTGCTGAAACGGCTGCGGATGGCTGTACA
CCGTTTCGATGGTATTCAGATCTGTAGCGCCGGAAACCAGCACGCAGTGATCGATAGTGACAGTCATCTCACC
GACAATCGACAGACTGGTGTGTTGCAATAAGTCGTACACATCGTTGATAGCGCCGGAGCTGGTGTTCTCTATC
GGAACCACGGCGTAATCGGCCTGGCCGGTTTCGACCTGATGAAAAATATCGGCGAATTTTGCGCAGCCGCTCT
CAATAAATTGCTCAAAATGGCGTGCAGCATACTGGCGCGCCGCGAGATGAGAATAGGAGCCTTTCGGCCCAAG
AAACGCAATACGTGCCGAATGAGGGTGAGTATTATTCAGATGTTGTTGCAGCAGCGCCTGCTGAGTAAGCACG
GAGTCTTCAATGATAAGCTGGAACAGACGGGTAATGTAGTGTGCGTCGAGATGGTGGGCTTTACCGAGATGGA
TGAGTCTGTCCAGCAGCGCGCGTTCACGATCGATATCCCGAACCGGACGATGCGACAGTAGTTTTGCTTGTCC
CACTTCAATCGCCAGCGCGCGTCGTTTTGCCAGTAAGGCCAGTAACTCTTCGTCTAAAGCGCTGATTTTATCT
CGCAGCGCCAGTAATGGGTTTTCCGATGTCATAGTGTTGCCTTTTTTGTTATCAATAAAAAAGGCCTCCCGGT
GGGGGAGGCCTTCTTGTTCGTCTTCGCATTCTTTATCACATGACGAAACGCCTCCCAGTCAGGGGAAGATAAA
AAAGAATGCGAAGAAAAACCGGGTTAGCTTCATAACTGTTTCCTTTGTGTACGTAAGTACAGTACCCGTACTG
TTTTAATCCTGTCAACAAAAAACGCGCCCGAAGGCGCGTTGGCGATACACTCAATGTAAGGGACTACTCTTCT
TCTGCTTCGACGAAGCTGGCATCTTTCACCGATGCGGTGGCACGACGGGCTTCGCCTTTATGCTGCACTTTAT
TGAGCTGCCGTTCCAGCTTGTTGATCAATTCATTAATAGCGGTGTACATATCTTCGTGTTTTGCGCTGGCGAC
CAGATGTCCGTTCGGTGTATTAATGGTGGCGTCAGCAATAAAGCCCTGTGGCTCTTTAGACAGAATGATATGA
GGATTAATCAGGTGAGTTTGCCATTTTTCCAGTTTGGCGAGACGGTCTGCGACATGTTGGCGGATTGCCGGAG

TAATTTCCATTTGTTTACTGGTAATGTTCATTGTCATAAATTTTACCTCTTGTCTTTCCCGTCTTGGTGATTC
CAGCATACCGTTCTTAATGTCAAAATGTGTGATGTAAATCACATTATTTTGTCACTTTTTGTCAAGGCGAACT
TTTTGTGAGGCATCGCAGGAAGAAGCGATCTTTTACTTGAGGAATGGCTGAAAGCAGGCCATATTTGACTGGA
TGCGTTGCAGTGAAATCATTTCTGTTGCATCAGATATCGAATAAAAAAACGGCAGCCGGAAGGCTGCCGTTTT
GCTTGTTGGTATTGCTTCAGGTGTTTTTGCTGTTAGCGGCAATAATTTTCGCGACTTTGTCAGCCTGCGCATT
CAGCTGCATCTGACGATAGGCGTTTTCCATCAGCGGCAACGCATCGCGCGTGGCCTGCGTATCAGGATAATTG
CGTAGCATACCTTCTACCCGGTTTACGACCGCAACCCATGCGCCGCGCGCGGTATAGTACTCTGCGACGGAAT
ATTCATATTTCGCCAGACGGTCTTTCAGGAATACCAGACGCTTAGTGGCGTCGGTGGTGTACTGGCTATTAGG
ATAGCTGCGTACCAGTTTCGAAAAGTCATTGAACGCCGCCCGCGCATGTTGCGGATCGCGATCGCTGCGATCG
ACGCCAAAGAAACCTTGCAGCGCGCTATCATCCAGCGCCATGTTCGTCAGGCCGCGCATGTACATGACATAGT
CAATATTAGGGTGCGTTGGATTAAGACGCATAAAACGATCGATGGCGGCCTGCGCTAGCGGCAAATCGGCGTT
TTTGTAATAGGCGTAGATAAGATCCAACTGCACCTGCTGAGAATACGGTCCGAATGGATAACGATTATCTAAC
GCTTCCAATTGCGTTATTGCCTGTTTCCAGTTACCGTCCTGCAGCTTTTGCTGAGCAGTCGCGTAGATTTCAT
TCGGCGGATTATCGGGCACCTCTTCCTTTGAACCCGAGCAACCCGCCAGAAACAGGCTCAACGTGGCTGCTGC
CACCAGATATTTCATGCGCGTCATGACGTTTTGACTTTCCTCAAAATGTTTTACGGGAGATTCTCTGTTCCTG
CTCCCGGTTAAGACCAGCTACAATAGCACACTATATTAAACGGCAAAGCCGTAAAACCCAACGTTAAACGAAG
AAGCTGTATATGGCACAACGAGTACAACTCACCGCAACGGTCTCCGAAAACCAACTCGGTCAACGCTTAGATC
AGGCTTTGGCCGAAATGTTCCCGGATTATTCACGTTCGCGTATAAAAGAATGGATTTTGAATCAGCGCGTGTT
GGTCAATGGTCAACTTTGCGATAAGCCAAAAGAAAAAGTGTTAGGCGGCGAGCGGGTGGCTATTGATGCGGAG
ATCGACGAAGAGATTCGTTTTGAAGCGCAGGATATCCCGTTAGATATTGTTTATGAAGATGACGATATTCTGG
TTATCAACAAACCACGCGATCTGGTGGTACATCCTGGCGCGGGTAACCCGGACGGTACGGTACTGAACGCGTT
GCTACATTATTATCCGCCGATTGCGGATGTCCCGCGCGCGGGCATTGTGCATCGTCTGGATAAGGACACGACG
GGGCTGATGGTGGTGGCCAAAACCGTTCCGGCGCAAACGCGCCTGGTGGAGTCTTTGCAGCTGCGCGAGATCA
CCCGCGAATATGAAGCGGTCGCGATCGGACATATGACGGCGGGAGGAACGGTGAATGAACCCATCAGTCGTCA
CCCGACCAAACGTACCCATATGTCGGTGCATCCCATGGGCAAACCGGCGGTGACCCACTACCGCATTATGGAA
CATTTCCGTGTGCACACGCGTCTTAGGTTGCGTCTGGAGACCGGGCGTACGCACCAGATCCGCGTGCATATGG
CGCATATTACGCACCCGTTAGTGGGCGATCAGGTCTACGGTGGTCGTCCACGTCCACCGAAAGGCGCATCGGA
AGAATTTATTTCCACGCTGCGTAAGTTTGACCGCCAGGCGCTGCATGCAACAATGCTGCGTCTCTACCATCCT
GTATCGGGTATCGAAATGGAGTGGCACGCGCCGATTCCACAAGATATGGTGGACCTTATCGATGCGATGCGCG
CCGATTTTGAAGATCATAAAGATGATGTGGACTGGTTATGAATGCACTGATTGTCCCGCAGTGGCCGCTGCCG
AAAGGCGTCGCAGCCTGTAGTTCTACCCGTATTGGCGGGGTGAGTTTACCGCCTTACGACTCGCTGAATCTGG
GCGCGCATTGCGGCGATAACCCGGAGCATGTTGAAGAGAACCGCAAGCGTCTGTTTGCGGCGGGCAATCTGCC
TTCGAAACCTGTCTGGCTTGAACAGGTTCACGGTAAAAATGTGTTGAGGCTTACCGGAGAACCTTATGCCTCC
AAACGTGCGGATGCGTCTTACAGCAATACGCCAGGAACCGTCTGCGCGGTAATGACGGCAGACTGCCTGCCTG
TGCTATTCTGTAACCGTGAGGGAACAGAAGTGGCGGCGGCTCATGCGGGCTGGCGCGGATTATGTGAAGGCGT
ACTGGAGGAAACTGTCACCTGCTTTGCTGACAAACCAGAAAATATCATCGCCTGGTTAGGTCCGGCGATCGGC
CCGACCGCGTTTGAAGTGGGGCCGGAAGTGCGTGACGCATTTTTAGCAAAAGATGCGCAAGCCGATAGCGCAT
TTTTGCCGCACGGGGAAAAATTTCTGGCGGATATTTATCAGCTTGCATGCCAACGTCTGGCGAATACCGGCGT
TGAACATGTCTATGGCGGGGATCGCTGCACCTTTAGCGAAAGTGAGACTTTCTTCTCTTATCGTCGCGACAAG
ACGACAGGTCGTATGGCAAGTTTTATTTGGCTGATATAACCTAAAGAATCAAGACGATCCGGTACGGGTAACT
TTCTTTTCACATAATTCAGGTCATTCACCTTGAATAATTGAGGGATGACCTCATTTAATCTCCAGTAGCAATT
TTGACCTGTTATGGGAGGAGTTATGCGTCTGGATCGTCTTACTAACAAATTCCAGCTTGCTCTTGCCGATGCC
CAGTCGCTCGCGCTGGGGCACGACAACCAATTCATCGAACCTCTTCATTTAATGAGCGCCTTGCTGAACCAGG
AAGGGGGATCGATACGTCCTTTATTAACCTCCGCCGGCATTAATGCTGGCCAGTTGCGCACCGCTATCGATCA
GGCGCTGAGCCGTTTACCGCAGGTGGAAGGCACCGGCGGCGACGTACAGCCTTCTTCGGAACTGGTACGCGTA
CTGAACCTTTGCGACAAGCTGGCGCAAAAACGGGGAGACAATTTTATTTCGTCAGAGCTGTTTGTTCTGGCGG
CGCTTGAGTCTCGCGGCACGTTAACCGATCTGCTGAAATCAGCCGGTGCGACCACGGCCAATATCACTCAGGC
AATTGAACAGATGCGCGGAGGTGAAAGCGTGAACGATCAAGGGGCTGAAGACCAACGTCAGGCCTTGAAAAAA
TATACCGTCGATCTGACCGAGCGGGCTGAACAAGGCAAGCTTGACCCGGTAATTGGTCGTGATGAAGAAATTC
GCCGTACCATTCAGGTACTGCAACGTCGTACCAAAAACAACCCGGTGTTAATCGGTGAGCCAGGGGTCGGTAA
AACGGCAATTGTTGAAGGACTGGCGCAGCGCATTATTAACGGTGAAGTCCCTGAAGGCTTAAAAGGTCGCCGC
GTACTGGCGCTGGATATGGGCGCGCTGGTGGCGGGGGCGAAATATCGCGGCGAATTTGAAGAGCGTCTGAAAG
GCGTGCTGAACGATCTGGCGAAACAGGAAGGCAACGTCATCCTGTTTATTGACGAGCTGCACACGATGGTTGG
CGCAGGTAAGGCTGATGGCGCCATGGACGCCGGGAATATGCTGAAACCGGCGCTGGCGCGTGGTGAACTGCAC

TGTGTGGGCGCCACCACGCTGGATGAATACCGTCAGTACATTGAAAAAGACGCGGCGCTGGAACGTCGTTTTC
AAAAAGTGTTTGTCGCAGAACCTTCAGTGGAAGACACCATCGCTATTCTGCGTGGCCTGAAAGAACGTTACGA
GCTGCACCACCATGTGCAGATCACTGACCCGGCGATTGTGGCGGCGGCAACATTATCTCACCGCTATATCGCC
GACCGTCAGTTGCCGGATAAAGCTATCGACCTCATCGATGAAGCGGCATCCAGCATTCGTATGCAGATTGACT
CTAAGCCGGAGGAGCTGGACAGACTCGACCGCCGGATTATTCAGCTCAAACTGGAACAGCAGGCGTTGATGAA
AGAGTCTGACGAGGCGAGTAAAAAACGTCTCGATATGCTCAACGAAGAACTGGACGATAAAGAGCGTCAGTAC
TCTGAGCTGGAAGAAGAGTGGAAAGCGGAAAAAGCATCGCTTTCTGGGACGCAAACTATTAAAGCGGAGCTGG
AGCAGGCGAAGATCGCCATCGAGCAGGCGCGTCGCGTTGGCGACCTGGCGCGAATGTCTGAACTGCAGTACGG
CAAAATTCCGGAGCTGGAAAAACAGCTGGAAGCCGCGACCCAGTCGGAAGGTAAAACCATGCGTCTGTTACGT
AACAAAGTAACGGATGCGGAAATTGCCGAAGTGCTGGCGCGCTGGACCGGTATTCCGGTTGCCAGAATGCTGG
AAGGCGAACGTGAAAAACTGTTGCGTATGGAACAAGAGTTACACAGCCGTGTGATCGGGCAGAATGAAGCCGT
TGAAGCAGTATCGAACGCCATACGTCGTAGCCGTGCTGGGCTGTCCGATCCGAACCGTCCGATTGGTTCCTTC
CTGTTCCTGGGGCCGACGGGGGTCGGTAAAACCGAACTGTGTAAAGCGCTGGCTAACTTTATGTTCGACAGCG
ACGACGCGATGGTGCGTATCGATATGTCCGAGTTTATGGAGAAACACTCCGTGTCTCGTTTGGTCGGGGCGCC
TCCGGGATATGTCGGCTATGAAGAAGGCGGTTATTTGACGGAAGCGGTACGCCGTCGTCCTTATTCCGTCATC
TTGTTGGATGAAGTAGAAAAAGCGCATCCGGATGTATTCAACATTCTGTTGCAGGTGCTGGACGACGGTCGGC
TGACTGACGGGCAGGGGAGAACGGTCGACTTCCGTAATACGGTGGTCATTATGACTTCTAACCTCGGTTCCGA
TCTCATTCAGGAGCGCTTTGGCGAACTGGATTACGGTCGAATGAAAGAGATGGTGCTGGGTGTGGTTAGCCAA
AACTTCCGTCCGGAATTTATCAACCGTATTGATGAAGTTGTGGTATTCCATCCGTTAGGTGAGCAACACATCG
CTTCTATTGCTCAGATCCAGCTGCAGCGTCTGTACAAACGTCTGGAAGAACGTGGTTATGAAATCCATATCTC
CGACGAGGCGCTGAAACTGTTGAGCGCCAACGGTTACGATCCGGTCTACGGGGCGCGTCCGTTAAAACGTGCT
ATTCAACAGCAGATCGAAAACCCACTGGCGCAGCAAATCCTGTCTGGTGAACTGGTTCCTGGCAAAGTGATTC
GCCTTGAAGCCAGTGATGATCGTATTGTGGCAGTGCAGTAAGTCACAAATTTGAATAAAACGGGCCCTGACGG
GCTCGTTTTTGTTTAAAAACCAGGCGAAAATAGGCTTTTGGTTCAAATGTTGTTTGAAAAGTGAACGGTTGAG
CTTTTTTTTGCATTTAGGTCTTGTCAGGCTCTAATAACTCCCTATAATGCGCCACCACTGACACGGAACAACG
GCATACAGGCCGCCGGGTCAGAAAGGCTTCGTTAAGAAGCCTGTGAGGAAAGCGAAAATAAAGGCTTGACTCA
AAAAGAGGAAAGCGTAATATACGCCACCTCGCGACGGTGAGCTGTAAGCCGCGTCGCACCTGCTCTTTAACAA
TTTATCAGACAATCTGTGTGGGCACTCGAAGATACGGATTCTTAACGTCCTCGGACGAAAAATGAATACCAAG
TCTCAAGAGTGAACACGTAATTCATTACGAAGTTTAATTCTTTGAGCATCAAACTTTTAAATTGAAGAGTTTG
ATCATGGCTCAGATTGAACGCTGGCGGCAGGCCTAACACATGCAAGTCGAACGGTAACAGGAAGCAGCTTGCT
GCTTCGCTGACGAGTGGCGGACGGGTGAGTAATGTCTGGGAAACTGCCTGATGGAGGGGGATAACTACTGGAA
ACGGTGGCTAATACCGCATAACGTCGCAAGACCAAAGAGGGGGACCTTCGGGCCTCTTGCCATCAGATGTGCC
CAGATGGGATTAGCTTGTTGGTGAGGTAACGGCTCACCAAGGCGACGATCCCTAGCTGGTCTGAGAGGATGAC
CAGCCACACTGGAACTGAGACACGGTCCAGACTCCTACGGGAGGCAGCAGTGGGGAATATTGCACAATGGGCG
CAAGCCTGATGCAGCCATGCCGCGTGTATGAAGAAGGCCTTCGGGTTGTAAAGTACTTTCAGCGGGGAGGAAG
GTGTTGTGGTTAATAACCGCAGCAATTGACGTTACCCGCAGAAGAAGCACCGGCTAACTCCGTGCCAGCAGCC
GCGGTAATACGGAGGGTGCAAGCGTTAATCGGAATTACTGGGCGTAAAGCGCACGCAGGCGGTCTGTCAAGTC
GGATGTGAAATCCCCGGGCTCAACCTGGGAACTGCATTCGAAACTGGCAGGCTTGAGTCTTGTAGAGGGGGGT
AGAATTCCAGGTGTAGCGGTGAAATGCGTAGAGATCTGGAGGAATACCGGTGGCGAAGGCGGCCCCCTGGACA
AAGACTGACGCTCAGGTGCGAAAGCGTGGGGAGCAAACAGGATTAGATACCCTGGTAGTCCACGCCGTAAACG
ATGTCTACTTGGAGGTTGTGCCCTTGAGGCGTGGCTTCCGGAGCTAACGCGTTAAGTAGACCGCCTGGGGAGT
ACGGCCGCAAGGTTAAAACTCAAATGAATTGACGGGGGCCCGCACAAGCGGTGGAGCATGTGGTTTAATTCGA
TGCAACGCGAAGAACCTTACCTGGTCTTGACATCCACAGAAGAATCCAGAGATGGATTTGTGCCTTCGGGAAC
TGTGAGACAGGTGCTGCATGGCTGTCGTCAGCTCGTGTTGTGAAATGTTGGGTTAAGTCCCGCAACGAGCGCA
ACCCTTATCCTTTGTTGCCAGCGGTTAGGCCGGGAACTCAAAGGAGACTGCCAGTGATAAACTGGAGGAAGGT
GGGGATGACGTCAAGTCATCATGGCCCTTACGACCAGGGCTACACACGTGCTACAATGGCGCATACAAAGAGA
AGCGACCTCGCGAGAGCAAGCGGACCTCATAAAGTGCGTCGTAGTCCGGATTGAGTCTGCAACTCGACTCCAT
GAAGTCGGAATCGCTAGTAATCGTGGATCAGAATGCCACGGTGAATACGTTCCCGGGCCTTGTACACACCGCC
CGTCACACCATGGGAGTGGGTTGCAAAAGAAGTAGGTAGCTTAACCTTCGGGAGGGCGCTTACCACTTTGTGA
TTCATGACTGGGGTGAAGTCGTAACAAGGTAACCGTAGGGGAACCTGCGGTTGGATCACCTCCTTACCTTAAA
GAAGCGTACTTTGCAGTGCTCACACAGATTGTCTGATGAAAAGTAAATAGCAAGGCGTCTTGCGAAGCAGACT
GATACGTCCCCTTCGTCTAGAGGCCCAGGACACCGCCCTTTCACGGCGGTAACAGGGGTTCGAATCCCCTAGG
GGACGCCACTTGCTGGTTTGTGAGTGAAAGTCACCTGCCTTAATATCTCAAAACTGACTTACGAGTCACGTTT
GAGATATTTGCTCTTTAAAAATCTGGATCAAGCTGAAAATTGAAACACAGAACAACGAAAATTGTTCGTGAGT

CTCTCAAATTTTCGCAACACGATGATGAATCGTAAGAAACATCTTCGGGTTGTGAGGTTAAGCGACTAAGCGT
ACACGGTGGATGCCCTGGCAGTCAGAGGCGATGAAGGGCGTACTAATCTGCGATAAGCGCCGGTAAGGTGATA
TGAACCGTTATAACCGGCGATACCCGAATGGGGAAACCCAGTGTGATTCGTCACACTATCATTAACTGAATCC
ATAGGTTAATGAGGCGAACCGGGGGAACTGAAACATCTAAGTACCCCGAGGAAAAGAAATCAACCGAGATTCC
CCCAGTAGCGGCGAGCGAACGGGGAGGAGCCCAGAGCCTGAATCAGCATGTGTGTTAGTGGAAGCGTCTGGAA
AGGCGCGCGATACAGGGTGACAGCCCCGTACACAAAAGCGCATGTGCTGTGAGCTCGATGAGTAGGGCGGGAC
ACGTGGTATCCTGTCTGAATATGGGGGGACCATCCTCCAAGGCTAAATACTCCTGACTGACCGATAGTGAACC
AGTACCGTGAGGGAAAGGCGAAAAGAACCCCGGCGAGGGGAGTGAAAAAGAACCTGAAACCGTGTACGTACAA
GCAGTGGGAGCACAGGTTTACCTGTGTGACTGCGTACCTTTTGTATAATGGGTCAGCGACTTATATTCTGTAG
CAAGGTTAACCGAATAGGGGAGCCGGAGGGAAACCGAGTCTTAATTGGGCGTTAAGTTGCAGGGTATAGACCC
GAAACCCGGTGATCTAGCCATGGGCAGGTTGAAGGTTGGGTAACACTAACTGGAGGACCGAACCGACTAATGT
TGAAAAATTAGCGGATGACCTGTGGCTGGGGGTGAAAGGCCAATCAAACCGGGAGATAGCTGGTTCTCCCCGA
AAGCTATTTAGGTAGCGCCTCGTGAATTCATCTCCGGGGGTAGAGCACTGTTTCGGCTAGGGGGCCATCCCGG
CTTACCAACCCGATGCAAACTGCGAATACCGGAGAATGTTATCACGGGAGACACACGGCGGGTGCTAACGTCC
GTCGTGAAGAGGGAAACAACCCAGACCGCCAGCTAAGGTCCCAAAGTCATGGTTAAGTGGGAAACGATGTGGG
AAGGCCCAGACAGCCAGGATGTTGGCTTAGAAGCAGCCATCATTTAAAGAAAGCGTAATAGCTCACTGGTCGA
GTCGGCCTGCGCGGAAGATGTAACGGGGCTAAACCATGCACCGAAGCTGCGGCAGCGACGCTTATGCGTTGTT
GGGTAGGGGAGCGTTCTGTAAGCCTGCGAAGGTGGCCTGTGAGGGTTGCTGGAGGTATCAGAAGTGCGAATGC
TGACATAAGTAACGATAAAGCGGGTGAAAAGCCCGCTCGCCGGAAGACCAAGGGTTCCTGTCCAACGTTAATC
GGGGCAGGGTGAGTCGACCCCTAAGGCGAGGCCGAAAGGCGTAGTCGATGGGAAACGGGTTAATATTCCCGTA
CTTGGTGTTACTGCGAAGGGGGGACGGAGAAGGCTATGTTGGCCGGGCGACGGTTGTCCCGGTTTAAGCGTGT
AGGTGTGTGTTCCAGGTAAATCCGGTTCACTTTAACACTGAGGCGTGACGACGAGGCACTACGGTGCTGAAGC
AACAAATGCCCTGCTTCCAGGAAAAGCCTCTAAGCATCAGGTAACATCAAATCGTACCCCAAACCGACACAGG
TGGTCAGGTAGAGAATACCAAGGCGCTTGAGAGAACTCGGGTGAAGGAACTAGGCAAAATGGTGCCGTAACTT
CGGGAGAAGGCACGCTGACACGTAGGTGAAGTGATTTACTCATGGAGCTGAAGTCAGTCGAAGATACCAGCTG
GCTGCAACTGTTTATTAAAAACACAGCACTGTGCAAACACGAAAGTGGACGTATACGGTGTGACGCCTGCCCG
GTGCCGGAAGGTTAATTGATGGGGTCAGCGCAAGCGAAGCTCCTGATCGAAGCCCCGGTAAACGGCGGCCGTA
ACTATAACGGTCCTAAGGTAGCGAAATTCCTTGTCGGGTAAGTTCCGACCTGCACGAATGGCGTAATGATGGC
CAGGCTGTCTCCACCCGAGACTCAGTGAAATTGAACTCGCTGTGAAGATGCAGTGTACCCGCGGCAAGACGGA
AAGACCCCGTGAACCTTTACTATAGCTTGACACTGAACATTGAGCCTTGATGTGTAGGATAGGTGGGAGGCTT
TGAAGTGTGGACGCCAGTCTGCATGGAGCCGACCTTGAAATACCACCCTTTAATGTTTGATGTTCTAACGTGG
ACCCGTTACCCGGGTTGCGGACAGTGTCTGGTGGGTAGTTTGACTGGGGCGGTCTCCTCCTAAAGAGTAACGG
AGGAGCACGAAGGTTGGCTAATCCTGGTCGGACATCAGGAGGTTAGTGCAATGGCATAAGCCAGCTTGACTGC
GAGCGTGACGGCGCGAGCAGGTGCGAAAGCAGGTCATAGTGATCCGGTGGTTCTGAATGGAAGGGCCATCGCT
CAACGGATAAAAGGTACTCCGGGGATAACAGGCTGATACCGCCCAAGAGTTCATATCGACGGCGGTGTTTGGC
ACCTCGATGTCGGCTCATCACATCCTGGGGCTGAAGTAGGTCCCAAGGGTATGGCTGTTCGCCATTTAAAGTG
GTACGCGAGCTGGGTTTAGAACGTCGTGAGACAGTTCGGTCCCTATCTGCCGTGGGCGCTGGAGAACTGAGGG
GGGCTGCTCCTAGTACGAGAGGACCGGAGTGGACGCATCACTGGTGTTCGGGTTGTCATGCCAATGGCACTGC
CCGGTAGCTAAATGCGGAAGAGATAAGTGCTGAAAGCATCTAAGCACGAAACTTGCCCCGAGATGAGTTCTCC
CTGAGACTTAAAGTCTCCTGAAGGAACGTTGAAGACGACGACGTTGATAGGCCGGGTGTGTAAGCGCAGCGAT
GCGTTGAGCTAACCGGTACTAATGAACCGTGAGGCTTAACCTTACAACGCCGAAGATGTTTTGGCGGATGAGA
AGACGATTTTCAGCAGTGATTCAGAGTTGAGTACGCAATACTTTGCGCAGCAGCAAGGCGGCAAGCGAAGGAA
AGGAAGGAGCATACAGAAGTATGTGACTGAGTTTACGAGCGCAGGCAACGCCGCTGATGCGATAAAGAATTGC
GTACAGAGCACAAAGAATTTGCCTGGCGGCACTAGCGCGGTGGTCCCACCTGACCCCATGCCGAACTCAGAAG
TGAAACGCCGTAGCGCCGATGGTAGTGTGGGGTCTCCCCATGCGAGAGTAGGGAACTGCCAGGCATCAAATAA
AACGAAGGGCCCTGTCGGAAGACAGGGCCTTTTGTTTTTGTCTGTTTCTGCCGGATGGCGGCTCCGCCTTATC
CGGCCTACAGGAATTATCCCCCCTACGAAGCTGGTGTTCTTGTAGAACCGACAAGAGTACATCCCACCCAATC
GTTAACGATGAACTATCCTTTACACGTGCTTATATAAGCAGTGAGGATTTTCATTGGCTATCAAACCTTTTAA
CTACCAGCAGGACTTTTCCAGCATTGACTTCCGCCAGCAGCCTGAATTGTATCAGGTTGGACGAGGCGAGCAG
GGGGTGCTACTGGTTGAACCCTACAAAAGCGAAATTCTTCCTTTCTGGCGCTATAAAGATGAAGCATCGGCGA
TGAAATCCGCAGAACAGGTCTACCAGTTGTTCGAAGCTTACCGGCAGCAGGATGATTTCGTTGGTATGGACAT
GGCGCGTAAATTTATTCAGATGGGATACACCCGCGCCAGGCGATACGCTAACTATAAAGGCGGTAAAAAGTAT
GCAGAAGACGGCAGCCTGAATACACGGGGTAATGATCCTATTAAGGCCGCGGCGGCAACGGTTTTCAAAGGTT
GGTGGGATAAAATCCGTCAGGACGAAGACTATCTGAAACGGAAACGCCAGCATCAGGCACGGTGGGGATAAAA

ATTAGCAAACAAATAATTAACACTTTATTTACTTTTGGGTCTCGCGTAATCTGAATCTCCAAAATAAAAGGCC
GATTAAGCCTGGCAACAAGGAGAACAGTATGGCAGAAATTATTCAGCGTATAGATAAAAATAACGCTGAGGCT
CCGGAGATGCGGCGACGCATATGGGCTATCGTGGGGGCCTCTTCCGGAAACTTAGTGGAATGGTTTGATTTCT
ACGTCTACTCTTTCTGCTCGCTCTATTTTGCACATATCTTCTTTCCTTCTGGTAATACCACTACTCAGCTTTT
GCAAACGGCAGGCGTCTTTGCCGCCGGTTTTTTAATGCGGCCTATCGGTGGTTGGCTGTTTGGACGCATTGCC
GATCGACGCGGGCGCAAAGCCTCAATGTTGATTTCGGTGTGCATGATGTGCATGGGGTCGCTGGTGATTGCCT
GCTTACCTGGTTACGATACCATCGGCACCTGGGCACCGGCGCTGCTACTTATTGCAAGGTTATTTCAGGGGCT
CTCGGTAGGGGGAGAGTATGGGACCAGCGCGACATATATGAGTGAAGTTGCGCTTGAAGGTCGTAAGGGATTT
TTCGCGTCGTTTCAGTACGTTACGCTTATCGGTGGCCAACTACTGGCATTGCTGGTCGTTGTGATTTTGCAGC
AGGTTCTGGAGGATGCTGAGCTTCGTGCCTGGGGATGGCGTATTCCCTTTGCGCTGGGCGCCGTGCTGGCGGT
TGTGGCGTTATGGCTACGTCGCCAGCTGGATGAAACCTCGAAGCATGAGACTCGCGCATTAAAAGAAGCCGGT
TCGTTGAAAGGTTTGTGGCGTAATCGCAAAGCCTTTTTGATGGTGCTTGGTTTCACCGCGGCAGGCTCGCTGT
GCTTTTATACCTTCACCACCTATATACAGAAATATCTGGTAAATACAGCAGGTATGCATGCCAATGTCGCCAG
CGGCATTATGACGGTCGCGCTGTGTGTTTTCATGTTTGTGCAGCCGTTATTCGGCGCGTTATCCGATAAGATT
GGCAGGCGCACCTCTATGCTATGTTTTGGCGCACTCGCGACGCTTTTCACCGTTCCAATTCTCTCCGCGTTGC
AGAATGTGACGTCGCCTTATGCCGCATTTGCTTTAGTGATGTGCGCGCTATTGATTGTGAGTTTCTATACCTC
GATTAGCGGGATCCTGAAAGCAGAGATGTTTCCGGCGCAGGTTCGCGCCCTGGGCGTCGGTTTATCTTACGCG
GTAGCAAACGCGTTATTTGGCGGCTCTGCGGAATATGTCGCGCTGTCGCTAAAATCTGTCGGTATGGAGAGTT
CGTTCTTCTGGTATGTTACCGCGATGGCCGTTCTGGCTTTCCTGGTTTCGTTGATGTTGCACCGTAAAGGAAA
GGGCTTACGCCTTTAGTATCGCGCCATTTGCCAGATAGCGTAACCGGTTGTCGCGCCAGCGACATCCCAGACA
AAATCTTTCCAGCTCCAGCCGCTGCCTTCCGGGCGGCTGTCCCAGAGTTCTTTTGAGGCTCCGAGACTCAAAG
AAAACATCAGCCCTATCGCTGCGCTGCGGTCAGGGCTTACTCCCTGGTGACGCGCATACTCATTACCGGCTGC
CGATAGCATCGCTGATGCCATAAAATGCTGGGCTTTATCCTGACCGCTCCAGTGGTCGTTAGCCAGATGGCTG
CACCCGGAGAGAAACAACACCGTAAAAGGAATCAACACGCGCACCAGTTCACTCCTAAGAGAAAACCCCGTAC
AGGACGGGGCTTCGATTACAGGATACGACTAATTAATCTGTCGATGCGGATCCGGCGCAGACGGCGAATCAAT
TTACGCACTTTTATCGGATAGTCGGCAATGCTCTGCAGGTCACGATAATGTTTTACGATGGTTGTGTGCGTTC
GGATAAGTTCCAGCTCTTTTTCCCGCTGAGGGGCAAGCTCCTGCTTAGGATCGTGGATCAGAATGGCGTTTTC
CAGATCCAGACGCCAGGCGCGGGGATTCAGATTGTTGCCGGTGAGTAGCATCCATTTATCATCTACCCACATC
CCTTTCAAATGATAGGTATTGTCGTCATCTTTCCACAGACGCACCACAAGCTGATCGGTATTGACGTAATACT
GTAAACGGCTCAGGAAGCGGCGCAGGTTAATCTCATAGAGATAGGGCAACGCGCCAATGATCTTGAACGGTTC
ATCTTCCGGAATGTAAAAATCATTCGCGGTTTTATCGCCAACAATGATTTCGACTTTCTTTCCGTCGCGCAGT
AGCTGAATAATATTCCGCACCAGCACCGCTGGTAGATTGAAATAAGGGGTACAAATGGTGAGCTTATGCTCCG
CACAGGGCATGAGATGGAAAATGGTTTTGTTCAGCAGACTGGATTTGCCAAGCCCGACCAGCGGCGTAACGGA
GAGTTGCTCGTCATTAGCGTCACCCTGAAAATGATATGACGCATCACGCAGCTCCTGACGGTATAAGCGGATA
TCGTTTTTAATCTCCGGGCTTTTCGGCCGCTGGGTATTATCCAGACGATTCACACCACGGCCATTCATCAGAT
TTTGCGTCACCCAGTCAAACATGATGTCTGCCATTTGCCGATTGCGAATCAGCTGGTAGCGATCGTAGCGATA
TTTGTCATGCTGATGGAGATAGACATCGTTCAGGCTGGCGCCGCTGTACAGGACGCTATCATCAATGATAAAG
CCTTTAAAATGCAGTACGCCAAGCGCTTCTCGCGTATTAATCGGTACGCCGTAAACCGGGACATCAATGCCGG
GGTTTTCCTGCGCCAGTCGGCAATACCAGTCTGCATTGGTATTCGAGGCCGCTGCGCCAATACGCCCGCGTTG
CGCGCGATGCCAGTCGACCAGCACCCTCACGTCCAGTTCAGGACGCTGCCGTTTAGCCGCATAGAGCGCGTCG
AGTATACCTTTACCCCCATCGTCCTGTTCCAGATACAGGGCAACGATACAAATACGCTGCGTCGCGCTGGCGA
TTTTCTCCAGCAGTGTCTCCCGAAAAGTAGCAGGAGTATAAAAGAAATCTACATCATCAACTGACTGAGAAAT
CTTAGGGAGTTGGGCAAGGTGTTGTTGATGTTTATTACGCTTAAATTTTGACAACATCACAGTGCATTTCTTC
TCTGTTCATTGAAGGGTCCTCTGTGCTATGCAGACGACATAAGCGAGCAATAATAACATCAGTACCCGTTAAA
GTGGTCAACATTTCCAGTACATTACTCACGATTCATCACATTTGGCCAGATTCAGCGTCAATCCCACGATGCC
CTCTTCGAGCTGAATATCGACCTGAAATCCCAGTTTTCTGGCCAGCGCGACCATGCCGCGATTGTTTGGCATC
GTAATACCGTTCAGCCGCTTCAATCCGTGATCGCGAGTATAGGCAATCAATTTCTCCATTAAACGGCGTCCTA
AACCCAACCCTTTGAGATCTGAACGTACCAATACGGCAAATTCGGCATCTACGTTGTCAGGATCGGAGATCGC
GCGCGTTACGCCGAGGATCTCTTCAGCATTGTCCATCCGCCTCACGGCCACAAAGGCCATTTCTCGATCGTAG
TCGATCTGCGTCATGTTGGCTAAATCTTCATGGGTGAATTCGTTGATCTCGCTGAAATAACGGTAGTAAAGAT
CCTCTTTGGTGACCTGTGCGATGAATTGTCGCAGTTGGGGCTCATCTTCCGGCAGGATAGGACGGAACAGGCA
GCGATCGCCATTTTTCATCTCCACCCACTCTTCAAGCTGGTGGGGATAGGAGCGTACCGCAAGTCGACTTTCG
TTATCGCCATCAAACGGGGCAATATCCAGCGTCACATCCAGCGCGGTAAACTCACTGGCGGAAGCCAGCAGCG
GATGGATATCCAGACGCTGAATTTCCGGGCAGTCGACAATCAGGTTTGAAACCTGGACCAGCAATTGGCTTAA

ACCGACAATATCCAGCGGACGCAGCGCGCTACGGGCGCGAATTTTCCGCTGTTTAATGCCCTGAATCACCAGA
TAGCGCGCCAGGTTCATGTTGAGCGGCGGCAGCGCGACGACAGCCTGCTCTTCCGGACGCCACTCTACGCCGC
CTTCACCCAACATAATCAAAGGACCAAACACCGGATCGTGCTCGACCACCACACGAAGCTCCTGCGCGCCGGC
GCGGTTAGCCATGCTTTGTACCAGCAAACCGTGAATCCGCGCTTGCGGCCAGGCCATCTTTACACGATCAAAA
ATGGCGTTCGCGGCCTGTTGTACCTCGCTTGCGGTCCGCAGGTAAAGCATGACCCCCTGAACTTCAGATTTAT
GCGGAATGTCGGGCGAGCGCAGCTTGAGAGCTACCGGATAGCCTATCTGTTCGGCGATATGCACCGCTTCAGC
GCTGTCGCTGGCAATCCAGGTTGGGAGCGTGTGCAGCCCATAGGCGTGTAAAATCGGCTGTACTTCATGGGTA
TCCAGTGAGGCGGCGCCTTCCGCAATCGCCCGCTGTAACAGATTATGCGCCTCAGCGGTATTGGACGTCAGGT
TACTCGGCAACGCTGGCGTTTCTCGCAGTTGCTTCTGGTTACGCCGGTATTCCACCATATGCATAAACGCGGT
AATCGTGCCTTCCGGCGTACGGTAGGTTGGTAATCCGGCTTCGCTGAATAGCCGTCTTGCCTCCTGAGACGAG
AACTCGCCGCACCAGTTTGTCAGCAGCGTAACAAGCTTGCCTCTGGGGTGGCGCTTAATCGTCTCGATCAGGG
CATGGGCGCTTTCCGTACCCGGCGCGGCAGCGCTGGGAGAGTGGATAACCATAAGCGCGTCAAAATCCTGACT
GGCGAGCAGGATATCCAGCGTTTTGACGTAATGTTCGCTGCTGGCGTCATCACACAGATCCAGCGGATTGGCA
ATATCAATGTGCGCGGGAAGCGCCTGCCGTAGTTGCAGGCAGGTCTCTTCGCTCAACGTCGCCAGCTTGCCGT
TACGCGACCACAACTCATCTAACGCCAGCGCGGCAGGCGCGGCGCCATTGCTGATGATCATCAGTCTGTCGCC
GCGTAGCGGACGCATATGGCTCAGTGTTTCGACGGCGGAAAAAAGCTCGTGCGTATCCTGGACTCGCAGCAGG
CCTGCGCGCTGGATGGCCGCATCCCACGCAGGGTCCATTCCCGCGCTGGTATTAAGTAAACGCTGGGCTGCCG
GGCTTCGGCCGCTTTTAATCACCAGAATCGGTTTGTTACGTGAAGCGCTACGGGCGGCGGAAACAAAACGGCG
GGCGTCGCTTAACTGTTCCAGATAGAGCAAAATCGCGCTGGTCTTGCTGTCGCGCGCCAGATAGTCCAGTAGT
TCATCGACATCAATATCCAGGCTATCGCCCAGCGCGATAAAGTAGGAAAAGCCCATTTCACGCTGTTGCGCCC
AGTCAAGAATAGTATTGGAAACGGCGGCAGACTGGGAAATAAAAGCGAGCTTGCCCTGTTTAATCGGGACGGG
AGAAAAGCTGGCATTCAGCCCTTGCCACGGCGCGAGGAGCCCAAGACTGTTTGGACCCAGCAGACGCATTTTA
TAATGCCGGGCACAGGCAAGAAGTTCTTCATGTTGCGACGTGGGAGCAGAAAGGATAATGCACGTTTTACACC
CTTTCGCGCCAAGCGCGTCCAGTAATGCCAGGTTACGGCTGGCGTTAGTGCATAAAATAGCCAGATCGGGGGT
GAAAGGGAGACTGGCGATATCCGGCCAGGCCATGACGCCTAAAACGGCTTTCCAGGCGGGCGTCACGGGAAGG
ACGGGGCCATTGAATCCGCCCGCCAACAAGTTACGCATCATCAGGTAACCCGCGCGGTGGGGCTTCATTGATG
CGCCAATCACCGCGATCGATTTTGGCCGCAGTAGCGCTTCCAGTCCTTGCTGGCTCATACCGGTTTCCCTTAC
ACAGAAGTGACCGGATAATTTTAAACGCTTTCGCTATTTTATGCTGTGACGTTTCCCGGATGTTGCGTTTTTC
CTGCCAGATAGCGCGTTTTAAAACGGGTGAAATGTTCACCTAAACTGGTTGCCGCCTCGGTATCTCCCGCCAG
ATCTAACAACGCTATCGCGACTTCGGCGGTACAATATTGCCCTTCGGCATGGATTTCACGCAGACGATAAGCT
GAAAGACGCGATAAATCGACGGAAATGACCGGCAGATGATCCAGATAAGGGCTTTTACGAAACATTTTACGTG
CTTCGGGCCAGGTGCCGTCCAGCATAATAAACAGCGGCGGTTTCCCGGCGGGCGGGGTGGAAATGACCTCGCG
CGCTTCGCCGGCGTAGGACGCAGGAAAGACCACTATCGGTTGATAATCCGGATGCTGTACCAACTCCAGCAAC
GCCTGTGGCGGCTCGGTACGCGACCACTGAAACGCCGCGGTATCCGGCAAAATATCGGCTATCAGCCGGCCGG
TATTGCTGGGTTTCATCGGTTCAGTGTCAAACATGACCAGACAAAAACGGCTTTTCGCCTGAGAAGGCGTTAA
CGTATCACACAGGCAAGATTTTAGCGGCAGCAGGCAGCGTTGACAGCGGCGAACCCGATTGCCTCTGGCAAGA
AAAGGGCGAGTAGCGCGCGCAAGCCGTTCGGCGCGTAACTGAAGGACAGCGTTATTGGTCATAGAAAGCGTTT
GCGGAGAAAAACGCTATTGTGGCAGAGGTGAAAACGGGGCACAAGATGCGCCCCGTGGCGTTTACAGCGCTTC
GTTCAGCCAGCTGTCGAAAGGCGCTTTAGGCACTGCGCCATTAAGCATATCGACCACCTGTCCATGTTTAAAA
ATCATAATCGTCGGGATGCTGCGGATACCGAAACGGGCGCTGAGTTCCCGTTCCGCCTCGGTATTCACTTTGA
CGAAACGGACTTTACCGCTGCGCTCTTCTGCGACATCCTCGAAGATTGGCGCAAAGTTACGGCAAGGGCTGCA
CCAGGGGGCCCAAAAGTCGATCACCACTGGAAGATCGTCCTTCAACAGTTTGTCCAGCGTTTCGCCGGTCGCA
TTAATGACTTCTCCGTCAAACAACTCATGACCGCAGCGTCCACATTTTGCCGCATCCTGAAGCCGATCGTCGG
GGATGCGGTTAATGGCCTGACAATGGGTACAAACGGTGTTCATAGCTAACCTCTGAATACGTCGGAAGACGTC
CCGCTACGCGCGGGATGTTTCTATAATGTTACATATTATCGAGGAGTCTGTGCTAAACAACAATGTGTTTTAT
TCAATGAGTACAATAGTCGCAAGCTTTTAAACGAAATCATGGCTAACCGGATGCACATCGGGTAATCTTCGCG
CTTCGCGCAGCGCTGGTGGAGAAAAGCATGAACGATGAATTAAAAAACAAAAGCGGCAAAGTCAAAGTGATGT
ATGTCCGTAGTGATGATGACTCTGATAAACGCACCCATAACCCGCGTACCGGGAAAGGGGGAGGCCGCCCAGC
AAAGTCTCGCACTGACGGTGGACGTCGCCCCGCCCGTGATGAGCGAAATAATCAGAGCCGTGACCGTAAACAT
GAAACTTCGCCGTGGCGCACCGTGTCCCGCGCGCCAGGCGATGAAACGCCTGAGAAAGTCGATCATGGTGGTA
TTAGCGGTAAAAGTTTTATTGACCCTGAAGTTTTGCGTCGCCAGCGTGCGGAAGAGACGCGCGTCTACGGTGA
AAACGCCTGCCAGGCGCTATTCCAGAGCCGTCCTGACGCCATCGTGCGCGCATGGTTTATCCAAAGCGTAACG
CCGCGTTTTAAAGAGGCGCTGCGCTGGATGGCGGCGAATCGTAAAGCCTACCATGTGGTGGATGAAGCCGAAC
TGGCAAAAGCTTCCGGGACTGAGCACCACGGTGGCGTGTGTTTCCTGATTAAAAAGCGTAACGGTACGACCGT

TAAGCAATGGGTAAAGCAGGCGGCGGATCAAGACTGCGTGCTGGCGTTGGAAGATGTCGCTAACCCGCATAAT
CTGGGCGGCATGATGCGTAGCTGCGCGCACTTCGGCGTGAAAGGCGTGGTGGTACAGGATGCGGCGTTGCTGG
AGTCCGGTGCGGCGATTCGTACTGCGGAAGGCGGGGCGGAGCATGTCCAGCCGATTACTGGTGAAAGCATTGT
TGACGTACTGGATGATTTTCGTCAGGCGGGGTACACCGTGGTGACGACGTCCAGCGATCGCGGTCAGGCGCTC
TTTAGTACGACCCTGCCGGAAAAGATGGTGCTGGTCTTAGGCCGTGAATATGACTACCTGCCGGAAGCGGCGC
GCGAACCCGACGATCTGTGCGTGAAAATTAACGGCACCGGGAACGTGGAAAGCCTGAATGTGTCGGTTGCGAC
AGGCGTGCTGCTCGCCGAGTGGTGGCGACAGAATAAAGCGTAAGGGCTGCAGCCCGGTGACGCAACGCGCGCC
GGGCCGATTATGTACTTCCTACCGCCCGGTCAGACAAGGCGCTTTCGCGCTGTTATCCGGGCGATAAAACGGC
GAGCCGCCAACGTTATTCACTCTCAGCCGGTAATACTGGCGTCCAGTCGATAGTTTTCTCGCCGTGTTGTTCC
AGCCATTGGTTCGTTAGCGCAAAATGGTTACAGCCAAAAAATCCGCGGTGCGCAGAGAGTGGCGAGGGATGCG
GAGCTTTCAGAATATGGTGGCGCTGTGGATCAATAATCGCGCCTTTTTTCTGGGCGTGCGAGCCCCACAGCAA
GAAGACGACCCCTTCGCGGTGTTGGTTAATCAGGCTTATCACTTTATCCGTAAAGGTTTCCCACCCCAGGCTG
GCATGAGAGTGCGCCTGGCCTGCCCGTACCGTGAGCACCGTATTCAGTAACAGTACGCCCTGGCGCGCCCAGC
TTTCCAGATAACCGTGCGCTGGGCGAACAAAGCCGGGAATGGAGGCTTCCAGCTCTTTGTACATATTCACTAA
TGACGGCGGGGGAGCGATCCCAGGGCGCACGGAAAACGCCAGACCATGTGCCTGGCCGGGGCCGTGGTAAGGA
TCTTGTCCCAGGATCACCACCTTAACGTCGCCCAGTTCCGTGAAGCGAAAGGCGTTAAACACATCTTTTTGCG
GCGGGTAGACCGTGATACCCGACTGACGCTCGCCAGCAACAGTGTGAAGCGTATTGATAAAGTAAGGTTGCTG
TTTCTCATCAGCCAGCACATCGTGCCAGGTTAACTCAGTCGCCATCTCGCTCTCCTGCGAATTTCCAGTTCCC
TTAGCTTAACTGCTTCTTTCTGCAGTGCAAAATCCTGCGTGCTACAAAAACGGTTACCTACAAAAGATAGTTG
AAAATTTATGTAAAAAGTTTATAGGTCTGAATGGTGTAAGTTGATGTAAAACAATAAATTCCCTTCATCACCC
TTTTTATGGTGTGGTTTTTATTGATTTAAATCAAAGAATGAAGGGTGATCGAAGGGTATATATACACTCAAGC
AACAATGGTTTTACCAATTGGCCGGGATTAGGCCGACCGAAATCAAATAATTTTGCCTGGGGAGGCATCACAT
GATTACAGGTATCCAGATTACTAAAGCCGCTAACGACGACCTGCTGAATTCCTTCTGGCTGCTGGACAGCGAA
AAAGGCGAAGCGCGCTGCATCGTTGCAAAATCCGGCTTTGCTGAAGATGAAGTTGTGGCTGTCAGCAAACTGG
GCGAGATTGAATACCGGGAAATTCCGATGGAAGTTAAACCGGAAGTACGCGTTGAAGGCGGTCAGCATCTGAA
CGTTAACGTTCTGCGTCGTGAAACTCTGGAAGACGCGGTTAAGCATCCGGAAAAATACCCGCAGCTGACTATT
CGTGTTTCTGGTTATGCGGTGCGTTTTAACTCCCTGACGCCGGAACAGCAGCGCGACGTTATCGCTCGTACCT
TTACCGAAAGCCTGTAAGGCTTATCCGGTAATAACAAGCGGAAGAGGCCGGGCCTTTTCCGCTTTTGTTTTAT
TTGGGTCAGTAAAAAATCCGCACCGCGCAGTACACCAGCAGTAACGCCAGAATGATATTTAACTGGCGACCAT
AATGGCGAAACGCCCGCTGAAACAGATGTCCCGCCAGCGCCCAGCACACATTACCGAACGTGCCGATCAATGC
CAGCAATATACTGACGCCGATGACCCAGTTCAGCGCCTGCGTTTGCGGTAAAACAAATGTAGACAGAGCGGTA
ATGCCGTACAGAATAATTTTGACGTTCACAAACTGCAGGCCAAAGCTTACCCAAAAGCCAACCGGTTTTGGTC
TGACCTTCTCATCCGCCGCTGGACTGGTGGCAATTTTCCACGCCAGCCACAGGATATACGCAGCCCCTACCCA
ACTGAGCAAATGAATGATTGCCGGATCGATAACCGCAAGGGAAAACGCGATCCCGGCGCACAGCAGCATGACG
ACCAAAAATCCCAGACTCATACCTGCCAGTACACGAATACTCTGCCGAAAACCATGTGCAGTTGCTGCGCTAA
GCGCGAGAATATTGTTTGGGCCGGGGGTAAGGGCGGTGATTAATGTATAAGTCCAGAAAGCGCTTAAAAGCAT
CGGTGTCACTTGTCATCTCCTCTGTTTTAGCAGACAGTACCCGATATGAAATATTTAAAAAAATGAATGTTTT
TTAATTCTGAATTCGAGATTTTCGATAGGTTGGTGGTTATGGATCTGCGTCGGTTTATTACGCTGAAAACGGT
AGTGGAAGAGGGCTCGTTTCTGCGGGCTTCGCAAAAGCTGTGCTGCACTCAGTCGACGGTAACTTTTCATATT
CAACAGCTTGAACGTGAGTTTTCCCTGCAACTGTTCGAGAAAATAGGGCGCCGAATGTGCCTGACCACTGAGG
GGAAAAAGCTGATGCCGCATATCCATGAACTGACGCGTGTGATGGAGTTGATTCGTGAAGCCGCAAGGCAGGA
TGCAGAACCCGGCGGAGAGCTGCGCGTGGCGACAGGTGAAACCTTACTGGCGTACAAAATGCCGCAGGTTCTA
CAACGTTTTAAGCTGCGAGCGCCGAATGTCAAATTGTCGCTACAGTCGCTGAATTGCTATGTGATACGTGATG
CGTTGCTGAATGACGAGGTCGATCTGGGCGTTTTTTATCGCGTAGGTAATGATGACGCGCTCACGATGCAGCA
ACTGGGGGAGCAGTCGCTGGCGCTGGTGGCCTCTCCACTGCTACAGGATGCTGATTTTACGCAGCCAGATCAG
CATATCCCCTGCAGCTTCATCATCAATGAACCGCAATGTGTCTTCCGCCAGCTATTTGAAAGTACGCTACGGC
AGAGGCGGATTACGCTGGAAAACACCATTGAACTGTGGAGTATTGAAAGTATTAAACAGTGTGTGGCTGCTAA
CCTGGGCATCAGCTTTTTACCACGTTTTACCGTTGAACGAGAACTTAGTACCGGTCAGTTGAAAGAGCTGCCG
TTTGGCGCGCCGTCGCTATCGATCATGGCGCTGTGCGCCCATCATGCCGGGAAAGCGGTCAGCCCGGCAATGC
AGATATTTATACAGTGTATGGAGGCGTGTTTTACCGTGGAAGATAAAAAAATGCCGGGTTAACGAACCCGGCA
TTATCATCACTCTTCGCTGGATTGCTCCTGCATTTTTGTACCGCTTGGCTTGCGGCGCTTACCGATATTTTTT
GTATCGCGGTGACGTTTCTTCACGCGCGGCTTCTCTTTTTCTTTCTCTTTTTTCTTCTCGGCGCGTTTCGCCA
GCACTTTTTTCGACGGTTTGCCGGTCAGCTTTTCGCTGGGCGCACGCGTGGTCGGGCGAAGCTCATCAATGAC
GCGCGCTTTTAGCGGCTCTTCGATGTAGCGGCCGATTTTCAGCAGCAACAGATGGTCGTGGGCTTCTACCAGC

GAGATTGCAGTGCCTTTGCGGCCAGCACGGCCTGTACGACCAATGCGATGCAGATAGGTATCGCCACTGCGCG
GCATGTCGAAGTTAATGACGTGGCTGACGTCAGGAATATCAATCCCACGTGCCGCGACGTCGGTCGCGACCAG
CACGTTAACGCGACCGTCGGTGAGACGCTTAATACCTTCGTTACGTTTGATCTGCGCCATCTCACCTTCAAGA
TAGCAATTGTTGATCCCTGCCAGACGCAGCGTTTCGGCCAGTTCATGGACGCGTTCACGTTTACGTACAAACA
CGATAGAACGGGTTGCATCGTCTTGTTTTAGCAGGTGCTTCAACAGCGCAACTTTATGTTCGAAATTGTCGGC
ACGGTAGTACCACTGGTGAATTTTCTTACGCTCGCGGGTTGACGGATTGGCGGAAACCTCAACCGGGTCTTCC
AGCAGGCGCTCGGCAAAATCTTTAATCGCATCGCCTTCCAGCGTGGCGGAGAACAGCATCGTCTGTTTACGCC
AGCGGGTCTCACCAGCGATATGTTCAATGTCCTGAGCGAAGCCCATGTCGAGCATCCGGTCAGCTTCGTCAAG
GATCAGTGTTTCAACCGCCCGGCAGTCAAAGTTCTCTTCTTTTATATATTGCAGCAGACGGCCCGTCGTGGCG
ACCACGATATCCTGGTTTTCGCTAAACACTTCAGCATGGTTCATATAGGCCACGCCGCCGGTAATCGTGGCGA
TATCCAGATGAGTATGCTTAGCCAGTTCACGAGCGTGATCGGCAACCTGCATTGCCAGTTCGCGGGTTGGCGT
CAGGATCAGAATGCGCGGCGGCCCCGATTTTTTACGCGGAAAGTCGAGCAGGTGCTGCAACGCCGGCAGCAGA
TATGCCGCCGTTTTACCGGTGCCTGTCGGCGCAGAACCGAGTACATCACGGCCATCGAGCGCAGGCGGAATGG
CGGCAGCCTGAATGGCGGTCGGGCGAGTGAAACCTTTATCCTGGAGGGCATCCAGCAGGCTTTCGTCGAGTTC
AAGTTCGGAAAAAGTCGTTACAGTCATGTTCTACCTCTGTGTGGGGCGCTGATTATAGACGTTACGGCCGCAA
TCTTCATCTGTTTGTATGGATATCCCTTCTCAGGTATTGTTTTCACTCGGCGCTGTCGCCGTTTTTTGCAAGG
TTGTTTTTACATGTCTCAGTCTGGCTCCGTTCTTCGTCGTAACGGATTTACGTTTAAACAGTTTTTTGTCGCA
CACGATCGGTGTGCGATGAAAGTGGGAACTGACGGTATTTTACTCGGGGCGTGGGCGCCGGTCGCGGATGTAA
AGCGAATTCTGGATATCGGTACTGGAAGCGGTCTGCTGGCGTTAATGCTGGCGCAGCGGACGGATGACAATGT
GCCTATTGACGCCGTGGAACTGGATGCCGGGGCTGCCATGCAAGCGCAGGAAAACGTGGCTCACTCTCCCTGG
CCGCATCGAATAACGGTGCATACTGACGATATTCAGAGTTGGGCGCCTCGCCAGACGGTACGCTTTGATCTTA
TTATCAGCAACCCGCCTTATTACGAACCCGGTGTGGAATGCGCCACGCCGCAGCGGGAGCAGGCGCGTTACAC
CGCTACGCTGGATCACCAGACGTTATTGGCTATTGCGGCGGATTGTATTACCGAAGATGGCTTTTTTTGCGTA
GTCTTGCCGGAACAAATAGGTAACGCTTTTACGCAGCAAGCGCTCAACATGGGATGGCATTTGCGGCTACGTA
CTGACGTTGCGGAAAACGAAGCGCGCTTGCCGCACCGGGTTTTGCTCGCATTCTCCCCGCAAGCCGGGGAGTG
CTTTAGCGACAGATTAGTAATTCGCGGGTCAGACCAGCATTATTCTGAAAGTTATACGGCGCTGACCCAGGCA
TTTTATCTGTTTATGTGAGGCGTTAATGGCGAAAGGATCGATGGGCCTGATTCAGCCAGTTGTTGCGGATAAT
CCAGCGTAAAGTGCAGCCCCCGGCTCTCTTTACGCATCATCGCGCAACGCACAATCAGTTCGGCCACTTGCAC
CAGATTGCGCAGCTCCAGTAAATTATTCGAGACGCGAAAGTTGGCGTAATATTCGTCAATCTCTTGTTGCAAC
ATAGTAATACGCCGTAGGGCGCGCTCCAGCCGTTTCGTCGTGCGCACAATCCCCACATAGTCCCACATCAGCA
GACGCAGTTCGTGCCAGTTATGCTGAATCACCACCCGCTCATCGGCGTTCTCCACGCGGCTTTCATCCCAGGC
GGGAAGGACGTCAACGCTGTGGACAGAAGGCATTCGCCTGTCGATATCCATTGCTGCGGACCAGCCGTACACT
AAGCATTCCAGCAACGAGTTGGACGCCATACGGTTAGCGCCGTGCAGGCCGGTGTAACTGACTTCGCCAATGG
CATATAAGCCGTCTACATCGGTACGACCATAATCATCGACGACCACGCCGCCGCAGGTATAGTGCGCGGCAGG
AACAATGGGAATCGGTTCTTTGGTTAAATCCATCCCCAAATCGAGCAGTTTCGCATAGATCATCGGGAAGTGC
TGGCGGACAAAATCGTCAGGCTTGTGGCTGATATCGAGAAACATACAGTCTGCGCCGAGTCGTTTCATCTCAT
GGTCAATAGCGCGGGCGACAATATCGCGCGGCGCCAGTTCCCCGCGTTCGTCAACGTCCGGCATAAACCGTGA
ACCGTCCGGACGTTTAAGGTATGCGCCTTCGCCGCGCAGCGCTTCGGTAAGGAGAAAATTACGCGCCTGCGGG
TGATAAAGGGCGGTGGGATGAAACTGGTTAAATTCCAGATTAGCGACCCGACAGCCTGCGCGCCAGGCCATCG
CGATACCGTCGCCGGAGGAAATATCCGGGTTAGTGGTATATTGATAAACCTTTGAGGCGCCGCCGGTCGCCAG
CACCACCGACTTCGCATGACAGGTTTCAACCCACTCTTTATTGCGATTCCAGATCCAGGCGCCGACGACCCGG
CGAGGCCCCGGCAGACCCATTTTATCGGAGATAATTAAATCAACGGCGTTGCTGCGTTCCAGCACCTGAATAT
TAGGATGATTTTGCGCCCGACTGACCAACGTGGTTTCGACTTCCTTTCCGGTAGCGTCGGCGGCATGAAGGAT
ACGGCGATGGCTATGGCCGCCTTCACGCGTGAGATGATAGCTCTCCTTGCCGTTTGGCTGGACATGCGTATCA
AACAGGACGCCCTGATCGATAAGCCACTGCACACAGGTACGGGCGTTGCTGGCGACAAACTCCACCGCGTGGC
GATCGCAAATTCCGGCGCCAGCAATTAGCGTATCTTCAACATGCGACGCAATACTGTCGGTTTCATCAAACAC
GGCGGCAATACCGCCCTGCGCATAAAACGTGGAGCCCTCGCTCACCGGCCCTTTACTCAGGACGATAACCTTA
TGCTTTTCAGCCAGACGCAGCGCCAGCGAGAGCCCGGCCGCGCCGCTGCCGATAATTAACACATCACAGGACA
GTTCAGGAGTTGTCATCATGGTTTTTTGTTTAATTTACTAAACACGGTTGCGGCAGATTAGCACTATATGGCG
CTAAAAAGCACGTGATGATTTCGCTGGTGTTGTGCTTATTAAACATAAAATAAAAATACGGCGAGAGATAAAC
GGCAAAAAATATTTTATAGGGCAGGGTGTTAGCGTCGGATTTTGTAGTGAAATAAAGATGCTGTTAGGTTACT
CTGCAAACGACGAAATGGGCATTTCTGTACAGATAGTGCGTTGTTCAGCATCTGTAGACTTATAATGGAAGAT
AAGACCTGTCTACAACATGACAAACAAAAACAAATGCGTAACGGAACTTTACGAAACATAGACACTCCAACCT
GTTGCTTGCTCATAGTGCGGCTTATGGAGTGGCGTTTCGAAAGCGCGTGGAAATTTGGTTTGGGGAGACATTA

CCTCGGATGAGCGAGCAGTTAACGGACCAGGTCCTGGTTGAACGGGTCCAGAAGGGAGATCAGAAAGCCTTTA
ACTTACTGGTAGTGCGCTACCAGCATAAAGTGGCGAGTCTGGTTTCCCGCTATGTGCCATCGGGCGACGTTCC
CGATGTCGTACAGGAATCATTTATTAAGGCCTATCGCGCGCTGGATTCTTTCCGGGGGGATAGTGCTTTTTAT
ACCTGGTTGTATCGTATTGCGGTCAATACTGCGAAGAACTACCTGGTTGCGCAGGGGCGTCGTCCGCCTTCCA
GTGATGTAGACGCGATTGAAGCAGAAAACTTTGAAAGCGGCGGCGCGCTGAAAGAAATTTCGAACCCTGAGAA
CTTAATGTTGTCAGAAGAACTGAGACAGATAGTTTTCCGAACTATTGAGTCCCTCCCGGAAGATTTACGTATG
GCAATCACCTTACGGGAGCTGGATGGCCTGAGCTATGAAGAGATAGCGGCTATCATGGATTGTCCGGTGGGGA
CGGTGCGTTCACGTATCTTCCGGGCGCGGGAAGCTATTGATAATAAAGTTCAACCGCTTATCAGGCGTTGACG
ATAGCGGGATACTGGAAAAGGTATTAGGCATGCAGAAAGAAAAACTTTCCGCTTTGATGGATGGCGAAACGTT
GGATAGTGAGCTGCTCAAAGCGCTTACGCACGACCCGGAAATGCAGAAAACCTGGGAGAGTTATCACCTGATC
CGCGATTCAATGCGGGGGGATACGCCTGACGTTCTCCATTTCGATATATCCGCTCGCGTGATGGCCGCTATTG
AAAACGAGCCAGTACGTCAGGTGTCGCCATTAATCCCTGAGGCCCAACCCGCTCCGCAGCAATGGCAGAAAAT
GCCGTTCTGGAAAAAAGTGCGTCCGTGGGCGGCGCAGCTTACCCAAATGGGCGTGGCGGCGTGCGTTTCACTT
GCAGTTATCGTCGGTGTCCAGCACTATAATGGGCAATCTGAAACATCCCAACAACCTGAAACGCCGGTTTTCA
ATACCCTGCCTATGATGGGGAAAGCCAGCCCGGTAAGTCTGGGAGTGCCTTCTGAAGCAGCGCCTGTCGGCAG
TCAGCAACAGCAGGTACAGGAGCAGCGTCGTCGCATTAATGCCATGTTGCAGGACTATGAACTGCAGCGCCGA
CTGCACTCCGAACAGCTTCAGTTTGAGCAGGCGCAAACCCAGCAAGCCGCGGTACAGGTGCCAGGAATCCAAA
CTTTAGGAACGCAATCGCAGTAATGAAGCAACTTTGGTTTGCCATGTCCCTTGTGGCGGCTAGCCTGTTCTTC
TCAGCAAACGCCTCGGCCGACCCTGCGTCCGGGGCGTTATTGCAGCAGATGAACATCGCCAGTCAGTCACTGA
ATTACGAGCTGTCATTCGTCAGCATCACGAAACAAGGCGTTGAATCTCTGCGTTATCGACACGCACGCCTGGA
TGGTCGCCCGCTTGCGCAATTGCTACAGTTGGACGGTCCCCGCCGGGAGGTCGTACAGCGTGGTAATGAAATC
AGTTATTTTGAACCGGGCCTTGAGCCGTTCACCCTGAACGGTGATTATATTGTCGATTCTCTGCCTTCCCTGA
TTTACACCGACTTCAAGCGCCTGGCGCCATACTACGACTTTATTTCTGTCGGACGCACCCGTATCGCAGACAG
ATTGTGCGAAGTTATCCGCGTTGTGGCGCGCGATGGTACGCGTTACAGCTATATTGTCTGGATGGACATGGAC
ACCAAACTACCGATGCGCGTTGATCTGCTGGATCGCGATGGCGAGACGCTGGAGCAGTTCCGCGTTATTGCGT
TCACGGTGAGTCAGGATATCGGCAGCAACATGCAGGCGCTGGCGAAGGCGAATCTGCCGCCGCTGCTTTCTGT
TCCCGGTGGCGAAAAAACGAAATTTAACTGGAGTCCATCCTGGGTGCCGCAAGGCTTTAGCGAAGTTTCCAGC
AGCCGTCGTCCGTTGCCGACGATGGATAACCTGCCTATCGAATCGCGGCTTTATTCTGACGGTCTGTTTAGCT
TTTCCGTCAATGTGAACCGGGCGACGCAAAACAGCAGCGATCAGATGCTGCGCACCGGGCGCCGAACGGTTTA
TAGCAGCGTTCGCGACAACGCGGAAATCACCATTGTCGGCGAGCTTCCGCCGCAAACGGCGAAGCGCATTGCC
GACAGTATCAAATTCAGGGCAGTACAATGATTAAAGAGTGGGCAACCGTTATCTCCTGGCAGAATGGTCAGGC
GGTGGTGAGCTGCGACGTTAAAGCGTCATGCAGCAGCTGCGCCTCCCGTGCGGGATGCGGCAGTCGCGTGCTG
AATAAATTAGGGCCGCAAACCACCCATACCATTGTTGTGCCAAGCGCGGAGCCGCTGGCGCCGGGGCAGAAAG
TCGAGTTGGGCATCGCCGAAAAAAGCCTTTTAGGTTCGGCGTTGCTGGTCTATATGTCGCCTTTAGCGGGGCT
ATTTCTTTGTGCGGCGCTTTTTCAGATGCTGTTTGGATCTGACCTTGCCGCATTAAGCGGGGCCGTGCTGGGC
GGCGTGGGCGGTTTTCTGGTTGCCCGCGGCTATTCCCGAAAACTGGCGGAGCGCGACGCCTGGCAGCCGGTGA
TTTTAAACGTTGCCCTCCCGCCGGACCTTGTTCGCGTCGAAACTACCTCAATCGAAACGCGCCAGTAAACGTT
TTGCCCAACGGTACACCTGCCGCGTTGGGTTGTTGTCCCGATAAACATGCGCTTTTCCTGCGTTGCGGTGTTT
TACAGGAAAAAGCGAGGCGTTTTCCAGTTCCCGCTCTCCTTACACTATGAACATTTCCTCGCCTTAGCGTTGT
AGTGTAGAATGCGGCGTTTCAGTTAAAACAGACGTTAAGCTCAGAACAGCGACCTCTAAAGTCTGGTCAACCA
GGCGTAAGGCATAATAATTTATCTCTATGAAGAACATACGTAACTTTTCGATCATTGCTCACATTGACCACGG
TAAATCGACGCTGTCTGACCGTATTATCCAAATCTGCGGTGGCCTGTCTGACCGTGAAATGGAAGCTCAGGTA
CTTGATTCGATGGATCTTGAGCGTGAGCGCGGTATTACTATTAAAGCCCAGAGTGTGACGCTGGATTTTAAAG
CGTCTGATGGTGAAACTTATCAACTGAACTTTATCGACACGCCGGGACACGTTGACTTTTCCTATGAAGTTTC
CCGTTCGTTAGCCGCCTGCGAGGGCGCGCTGCTGGTGGTGGATGCCGGCCAGGGCGTAGAAGCGCAAACGTTG
GCGAACTGCTACACCGCGATGGAAATGGATCTTGAAGTGGTGCCGGTGCTTAACAAGATTGACCTGCCGGCCG
CCGATCCGGAGCGTGTGGCGGAAGAAATCGAAGACATTGTCGGTATCGATGCGACGGACGCGGTACGCTGCTC
CGCCAAAACGGGTGTCGGCGTGACGGATGTTCTGGAACGCCTGGTGCGCGATATCCCGCCGCCGCAAGGCGAT
CCGGACGGCCCGCTGCAGGCGCTGATTATTGACTCCTGGTTCGATAACTACCTGGGCGTGGTATCGCTGGTGC
GTATTAAAAACGGCACCATGCGTAAAGGCGACAAAATTAAAGTGATGAGCACCGGGCAGACCTACAACGCTGA
CCGCCTGGGGATCTTCACGCCAAAACAGGTTGATCGTACCGAGCTGAAGTGCGGCGAAGTAGGCTGGCTGGTC
TGCGCCATTAAAGATATCCTCGGCGCGCCGGTTGGCGATACCTTAACCTCAGCGCGTAACCCAGCGGAAAAAG
CGTTGCCGGGCTTTAAAAAGGTGAAACCGCAGGTCTATGCAGGTCTGTTCCCGGTCAGCTCCGACGATTATGA
AAGTTTCCGCGACGCGCTCGGCAAGCTGAGCCTGAACGATGCCTCACTGTTTTATGAACCGGAAAGCTCCTCG

GCGCTGGGCTTTGGTTTCCGCTGCGGCTTCCTCGGCCTGTTGCACATGGAGATCATTCAGGAGCGTCTGGAAC
GCGAATACGATCTGGATCTGATCACCACTGCGCCGACCGTGGTTTATGAAGTAGAAACAACGGCGAAAGAGAC
TATCTATGTTGATAGCCCCTCCAAGCTGCCGCCGTTGAATAACATTTATGAACTGCGCGAGCCTATCGCCGAG
TGTCATATGCTGTTACCACAAGCCTATTTAGGTAACGTTATTACGCTGTGTATTGAGAAACGCGGCGTACAAA
CTAACATGGTGTATCACGGTAACCAGGTCGCGTTGACCTATGAAATCCCGATGGCGGAAGTGGTGCTCGACTT
CTTTGACCGTCTGAAATCAACGTCGCGCGGCTATGCGTCTCTGGATTATAACTTCAAGCGCTTCCAGGCTTCC
GATATGGTGCGTGTTGATGTGTTAATCAACAACGAGCGTGTCGATGCGCTGGCGCTGATCACGCACCGTGATA
ACTCGCAAAGCCGTGGTCGCGAGCTGGTGGAGAAGATGAAAGATTTGATTCCACGCCAGCAGTTTGATATCGC
GATTCAGGCGGCGATTGGTACGCATATTATTGCCCGTTCGACGGTAAAACAGTTACGTAAAAACGTGCTGGCG
AAGTGCTACGGCGGTGATATCAGTCGTAAGAAAAAACTGCTGCAGAAACAGAAAGAAGGTAAGAAACGCATGA
AGCAGATCGGTAACGTCGAGCTGCCTCAGGAGGCGTTCCTCGCCATTCTGCATGTCGGTAAAGACAATAAATA
ATCTCTAAGGAGTTGGCATGGCGAGCATGTTTGCCCTGATTCTGGTGATAGCCACACTGGTGACGGGCATTTT
ATGGTGCGTTGATAAGTTTGTTTTCGCGCCAAAACGTCGGGCGCGCCAGGCTGCCGCGCAAACGGCGTCGGGA
GATGCGCTGGATAACGCTACGCTCAATAAAGTGGCGCCTAAGCCGGGCTGGCTGGAGACCGGGGCGTCGGTTT
TCCCGGTTCTGGCGATCGTTCTGATCGTTCGTTCATTTCTTTATGAACCCTTTCAGATCCCGTCAGGCTCAAT
GATGCCGACACTGCTTATCGGCGATTTTATTCTGGTGGAAAAATTTGCCTACGGCATTAAAGATCCGATCTAC
CAGAAAACCCTGATTGAAACCGGTCATCCAAAGCGCGGGGATATTGTGGTATTTAAATATCCGGAAGATCCTA
AGTTAGATTACATCAAACGCGCAGTCGGTTTGCCGGGCGATAAAATCACTTATGATCCGGTTGCGAAAGAGGT
GACGATTCAGCCTGGCTGTAGCTCCGGTCAGGCGTGCGAAAATGCGCTGCCGGTTACCTACTCTAACGTTGAG
CCGAGCGATTTTGTACAGACCTTTGCCCGCCGTAACGGCGGAGAAGCGACCAGCGGTTTCTTTGAGGTTCCGC
TAAACGAGACGAAAGAAAACGGCATTCGCCTGACCGAACGTAAAGAGACGTTAGGCGATGTGACGCACCGCAT
TCTGATGGTGCCGATAGCCCAGGATCAGTTGGGCATGTATTACCAACAGCCAGGACAACCGCTGGCGACCTGG
GTTGTACCACCGGGACAATATTTCATGATGGGCGACAACCGCGATAACAGCGCGGATAGCCGTTACTGGGGAT
TTGTTCCGGAAGCGAATCTGGTCGGTAAAGCGGTCGCTATCTGGATGAGCTTTGACAAGCAGGAAGGGGAGTG
GCCGACAGGCGTACGCCTGAGTCGTATCGGCGGTATTCACTAACTGTGATGAAATGATCGTTCACGCTGCCGT
CTTTTTAGCGGCAGCGTGAATTATTTCCTGGATAAATTCCCTAAGACTAACGACATCCCCTGTCGTTGTGTAT
AGAATATTCCCCCGAAGTTTTAGGTTGGCGCCGTTTGGTCGCCACGGCACACGAAACAGCGTTGGTTATAGAC
AACCTTCTTTCCGCTGCAGCGATGCGGCAGGACAGATGACGTGTATCAGGTCTGTTTCGTGTGCTGGATTGTT
GACGCATTCATTTATTGGTATCGCATGAACCCCATCGTAATTAATCGGCTTCAACGGAAGCTGGGCTACACTT
TTAATCATCAGGAGCTGTTGCAGCAGGCATTAACTCACCGCAGCGCCAGCAGTAAACATAACGAACGCCTGGA
ATTTTTAGGCGATTCAATCCTGAGCTTCGTTATCGCTAACGCGCTTTATCACCGTTTTCCACGGGTGGATGAG
GGAGATATGAGCCGTATGCGCGCCACGTTGGTACGTGGTAATACGCTGGCGGAATTAGCCCGTGAATTTGATT
TAGGCGAGTGCCTGCGTTTAGGGCCGGGCGAATTAAAAAGCGGCGGATTCCGTCGAGAGTCCATTCTGGCGGA
CACCGTGGAAGCGTTAATCGGCGGAGTATTTCTCGATAGCAATATCCAGACCGTCGAGCAGTTAATCCTTAAC
TGGTATAAAACCCGTCTGGATGAGATAAGCCCGGGCGATAAACAAAAAGATCCTAAAACGCGCTTGCAGGAGT
ATTTGCAGGGTCGCCATCTGCCGCTGCCATCTTACCTGGTTGTGCAGGTACGCGGCGAGGCGCACGATCAGGA
ATTTACTATCCACTGCCAGGTCAGCGGCCTGAGCGAACCGGTGGTTGGCACGGGTTCCAGCCGTCGTAAGGCG
GAGCAGGCTGCCGCCGAACAGGCGCTGAAAAAACTGGAGTTGGAATGAGCACTGACAAAACTTACTGCGGATT
TATTGCCATCGTCGGACGTCCGAACGTTGGCAAATCCACCTTGCTGAACAAACTGCTTGGGCAGAAGATTTCG
ATCACTTCCCGTAAAGCGCAGACCACGCGCCACCGCATTGTCGGTATTCATACCGAAGGCCCGTATCAGGCAA
TTTATGTCGATACCCCGGGACTGCATATGGAAGAAAAGCGCGCCATTAACCGTTTGATGAATAAGGCGGCGAG
CAGTTCGATTGGCGACGTTGAGCTGGTGATTTTCGTTGTGGAAGGCACCCGTTGGACGCCGGACGACGAAATG
GTCCTGAACAAACTGCGCGATGGCAAAGCGCCGGTTATTCTCGCCGTTAACAAGGTGGATAACGTGCAGGAAA
AAGCCGATTTGCTGCCGCACCTCCAGTTCCTGGCAAGCCAGATGAACTTTCTTGATATCGTACCCATCTCTGC
TGAAACGGGTATGAACGTCGATACCATTGCCGGTATCGTGCGTAAACATTTACCGGAAGCGATTCATCACTTC
CCGGAAGATTACATCACCGATCGCTCTCAGCGCTTTATGGCGTCTGAAATCATTCGTGAAAAGCTGATGCGTT
TTCTCGGCGCCGAGCTGCCGTATTCCGTTACCGTTGAGATTGAACGTTTTGTTACCAACGAACGCGGCGGCTA
TGATATCAACGGGCTGATCCTCGTCGAGCGCGAAGGGCAGAAGAAGATGGTGATTGGCAACAAAGGCGCCAAA
ATCAAAACCATTGGTATTGAAGCGCGTAAAGACATGCAGGAGATGTTTGAGGCGCCGGTACACCTGGAACTGT
GGGTGAAAGTGAAATCCGGCTGGGCCGATGACGAACGCGCTCTGCGCAGTCTCGGTTACGTAGACGATCTGTG
AGTTAACACAGCGTGGAAGGGTGGCAGCGCGCATTTGTCCTGCACAGTCGCCCCTGGAGCGAAACCAGCCTGA
TGCTGGACGTCTTCACGGAAGAATCGGGGCGCGTGCGCCTTGTCGCCAAAGGCGCGCGATCTAAACGTTCCAA
TCTGAAAGGCGCGTTGCAGCCTTTTACGCCGTTATTGCTACGCTACAGCGGACGCGGCGAGGTGAAAACCCTG
CGCAGTGCCGAGGCAGTTTCTTTGGCGCTGCCGTTAAGCGGTATTACGCTCTATAGCGGCCTGTACATCAACG

AACTCCTCTCTCGCGTACTGGAATATGAAACGCGCTTCTCCGAACTCTTTTTTGATTATCTGAACTGTATTCA
GGCGCTGGCGGGAACCACCGGCTCGCCTGAACCGGCGTTGCGACGTTTCGAACTGGCGTTGCTGGGTCATCTG
GGGTATGGCGTCAATTTCACCCACTGTGCAGGCAGCGGCGAACGGGTGGATGACACCATGACCTACCGTTACC
GCGAAGAAAAAGGCTTTTTCGCCAGCGTCGTCATCGATAACAACACCTTTACCGGACGGCACCTGAAAGCGTT
GGAGGAGCGGGAATTTCCGGATGTAGATACCCTGCGTGCCGCTAAACGCTTTACCCGTATGGCATTAAAGCCG
TATCTTGGGGGAAAACCGTTAAAAAGCCGGGAGCTGTTCCGGCAATTTATGCCCAAACGCACAGTAAAAATGA
AGAAAGATTAACGAGGATTGTCATGGCTGAATTACTGTTAGGCGTCAATATTGACCACATTGCCACGTTACGT
AATGCGCGCGGCACCGACTATCCGGATCCGGTACAGGCGGCGTTTATTGCTGAACAGGCAGGCGCGGACGGCA
TTACCGTACACCTGCGCGAAGATCGTCGCCACATTACCGATCGCGATGTGCGCATTCTGCGTCAGACGCTGCA
TACGCGTATGAATCTGGAGATGGCGGTGACCGAAGAGATGCTGGCGATCGCCGTAGAAACCAGGCCGCATTTC
TGTTGTCTGGTGCCGGAAAAACGCCAGGAAGTCACCACCGAAGGCGGTCTGGATGTGGCCGGACAGCGCGATA
AAATGCGTGATGCCTGTGCGCGCCTGGCGGCGGCTGGCATCCAGGTTTCGCTCTTTATCGATGCCGATGAAAC
GCAAATCAACGCGGCGGCGGAAGTCGGCGCGCCGTTTATCGAAATCCATACCGGCTGCTACGCCAACGCAGAA
ACCGATGCGGAACAGGCAAAAGAGCTGGCGCGTATCGCCAGCGCCGCGACCCTGGCGGCCCGTCTGGGGCTAA
AAGTAAATGCGGGTCATGGTCTGACCTACCATAACGTCAAAGCTATTGCCGCACTGCCGGAAATGCACGAGCT
CAATATCGGTCATGCCATTATTGGTCGGGCGGTGATGACGGGGCTGAAAGAGGCGGTGGCTGAAATGAAACGT
TTGATGCTGGAAGCGCGCGGCTAATGGCGATTCTCGGCCTGGGAACGGATATTGTAGAGATTGCCCGCATTGA
GGCAGTGATCTCCCGTTCCGGCGAACGTCTGGCAAGGCGTGTGCTCAGTGACAACGAGTGGGCTATCTGGGAG
ACGCATCAGCAGCCGGTGCGTTTTCTCGCCAAGCGTTTTGCGGTCAAAGAGGCGGCAGCGAAAGCTTTTGGCA
CCGGCATCCGCAACGGTCTGGCGTTCAATCAGTTCGAAGTGTTTAACGATGAGCTGGGAAAACCACGTTTGCG
GCTGTGGGGCGAGGCGTTAACGCTGGCGGAAAAACTCGGTGTGGCGCATATGCATGTCACGTTGGCCGATGAA
CGCCATTACGCCTGCGCTACGGTCATTCTGGAAAGTTAGATTTGCCGGATGGCGGCGTAAACACGTTATCTGA
CCTACAGGCCGATATATTGTCGGCCTGATAAGCATAGCGCCATCAGACATTGTTGCGTTGGAAATGCGTTAAC
CCGCTTACAGTTTATCTGCGTGATGCATCAGAACAAACTTATCCCACAACTGTTCTTCGGTTTCGACATGGGC
GGGATCCTGCAAAATCGTATTGGGGATCGGGCAGACTTTCTGACAGGTCGGCGTTTCGTAATGGCCTACACAC
TCGGTGCATTTGTCGCTGTTAATCTCATAAATGCTGTCGCCCATTGAAATCGCCTCATTCGGGCACTCCGGCT
CGCACATATCGCAATTGATACATTTTTTGGTGATTAACAGCGCCATCAGGAAATTCTCAGAAACAACACAAAT
CGGGCGGGCATTATACGCCTATTTTCTGCTCGGGCCAGCTCTTTTCTGGCGCTGATTTGTTATCTACCGTGTA
AAAATTTTTGATCTGCTTGAGCAGGCAGATGGTTGCGTTATCCAGTGAAAGGTCATTACTGTGCACCAGCATG
ACAGGAATAAAGAATGCGGGGATGCCCAGTGAAACCAGGCGAACATTTTTGGGTGTCGATAAATGGGTTAGCG
AGCGGGTAATAAATGCGATGCCAATCTGCGCTTCGGCATACTCAAGGCATGAGTAGTGGTTATCCAGTTCGCA
GATGACGTTAGGAACGACTTTGCGGTTATTAAATTCTGTGGTGATGATCCCTCTTAATAGCAGAGAGTCATTA
AGCACGATAATGGGCTGGTCGTTTAAATCTTTTAAATGCAGACGTTCGCGTGTCGCCAGAGGATGAGAAAGTG
GGATTGCAGCGATAATTTCATCTTCAAACAGCATGGTCGAGCTTAGCTGGGCAGGGAGCGCTGTTCGATAAAC
AATGCCAATATCAACGGCGCTATCCGTCAGCTTTTTAATGGTTTGCTCAGTTGAGAACTCTTTAACATTTAAA
GATATTTCCGGATGGTGCGCCATAAAGTTGCGAAAAGCGGGCAGGACCAGGTAACTGCCTGACACCCCGATTG
AAATAGTATCGCGCTGTTGGTCCTGATAGGGAAGAAGTTCATTTTCGGCATTTTTTAAATCCAGCATTATTTT
GTTAGCGTGTCGTTGCAAAATAATGCCTGCTTTTGTTAATTTAATTCCTCTACCTTTTCGCTTAAATAATATT
GTTTTAAGTGATGACTCAAGTTGTTTGATTTGCTGACTTAATGATGGCTGAGTGATAAACAGGCGACTGGCTG
CCCGACCATAATGTAATTCATCACACAATATCAGGAAGGAGTGTAGTTGTTTGATATTCATTAAAATTTAATG
CCAATAGATTGGGTTTTGTTATAACGATTATAAAAAATACCAAATTGTTTGCCTATTTTGCTTTTTTTAAACT
GTGATCAGTGCATTTTAAAAAGGGAAAATTATGTCGGCATCAATCAACCGCTGGGGAATGCTTGCCGCTCACG
TGTGCATCAATTTTGTGCTCGGGGGCGTCTACGCATTTAGCTATTTCAAAACACCACTCATGGCGCAATATCA
CTGGGATCCGGCTACGCTGGCGTTAGCATTCTCTATCAATATGGGGATCATTCCTTTACCGATGATTTGGGGC
GGGAGAATGATCGACAATGGTAAAGGAAAGCAGGCGATAGTTATCGGTGGTATCCTGTTTTCTTTAGGTTTTA
TCTTGTCCGGGTTTGTGGATAATTTGCCCATGCTGTTTTTAACCTACGGCGTCATTGCCGGGTTGGGATCGGG
CCTGGCTTTTACGGGTAATCTTAATAATATTCTGAAATTTTTCCCTGACCGTCGCGGTCTTGCCAGCGGTATC
GTACTGGCGGGTGTTGGCGTCGGGACGCTACTTTGCACCCGCCTGGCCGAATATTTTATGGCGCAAACTCACG
ATGTTAGTCGGGCGTTGTTATATCTGGGTATTGTTTATCTGGTGGTTATTTTTATCGTCCAGTTCTTTATTCG
TAGCGCGCCAGCAAAAGATAGCGGAGGAATTAAAGCGTCGCCACTGGATAAAGACTATCGGCACATGCTGAAA
GATCTGCGCTTCTGGCTGCTGTTTATGATTCTGGCGCTGGGCGTGTTCTCTGGGATGGTAATTAGCTCAAGCT
CTGCGCAAATTGGTATGACGCAGTACGGTTTACTGTCCGGTGCATTAGTCGTTAGCCTGGTCTCGATATTTAA
CTCGATCGGTCGCCTGTTCTGGGGAGGGTTAACCGATAAATTAGGCGGCTATAATACGCTGGTTATTGTTTAC
CTCTTTACCTGCTTGTGTATGCTGCTGTTATTTTTCTTCAATGGCAATACTTCGGTATTTTATTTCAGCGCTC

TGGGCGTGGGCTTTGCTTATGCCGGTATATTAGTTATCTTCCCTGGTTTGACCAGCCAGAATTTTGGTATGCG
TAACCAGGGGCTAAACTACGGCTTTATGTATTTTGGTTTTGCCGTCGGTGCGGTTATTGCTCCTTATGTAACG
TCCGCTATTGCAAAATATACCGGAAGCTACAATACAGTATTTATTTTGACAACGGTGCTATTGCTTATTGGAG
TCGTGTTGACCCTGATAACGAAAAAATATGTCGCAACGGTTTTAGCCAAAATTCATTAATCAGATAATAAGGA
AATAAAAATGAAAATTGCAATCGCAGGTGCAGGCGCTATGGGGTGTCGTTTTGGCTATATGCTGCTGGAGGCC
GGGCACGACGTGACGCTTATCGATAGCTGGCAGGAGCATGTCGACGCTATTCGTAGCAAGGGGTTGTTTGTCG
AAACGGAAACGACTCAGAAGTATTACCCCATCCCTGCTATGTTGGCTGATGAATCCCAGGGGGAGTTTGAACT
GGTTATTCTGTTTACCAAAGCCATGCAGCTGGATAGCATGTTACTGCGTATTAAGCCGCTGCTGCCAGCCGCG
AAGGTCGTGATGATTTTATCTAACGGCCTGGGAAATATTGAAACGCTGGAGAAATATGTCGATCGGCAGAAAA
TCTATGCGGGTGTGACGTTATGGTCCAGCGAACTGGAGGGGGCTGGGCATATTATGGCCACCGGTACCGGAAC
GATTGAACTGCAGCCGATTGCCAGCCAGGATTCGGCTCAAGAGGCTAAGGTCATTGCCACTCTTAATAGCGCG
GGATTGAATGCTGAAATAAGCCCTGACGTATTATTATCGATCTGGAAGAAAGCAGCCTTTAATAGCGTAATGA
ACACCTATTGCGCGCTACTGGATTGTAATGTCGGCGGATTTGGTCAGCGGCCTGGTGCTTTAGATTTAGCGCA
AGCGGTAGTTGATGAGTTTGTATTGGTTGCTGCCAGCCAGAATATTCCGTTGACTGAGCAAATGGTGATGAAT
ACGGTGAAGAAAGTGTTCGATCCGCGTGAGAGCGGCCACCACTATCCTTCTATGCATCAGGATTTACATAAAG
GCCGACTGACTGAAATCGACTATTTAAATGGTGCGATTGCGCGAATCGGCGCTCAGAACAATATTGCCGTACC
GGTTAACACACTCCTGACGCAATTGATTCACGCGAAAGAAGCGCAATAAACTTGATGGCTCTTCTGCATGAGG
CGGAAGAGCCATTTTCAAACCAGTTTTTTTACCAGCGCCTCGCTATGGCGAATACGCTCCGGCGCCCGCTCTA
AATCCTGTTGTACCAGCGCCATAAACAGTAAGTCGGTCAACATCATTTGCGCATGTGTCGAGGAGATAGCGGC
GCTGCGCGTCGCCTGCTCTTCGGCAATCGTATACAGACAGCGGGTGGCGCGCTGCTGCAAGGCGTTGGGAGAA
AAACCGGTAATGGCCAGGATCTTCGCGCCTGCGCGTAATGTTTCGTCCGCCGCCAGGTTCAGCTCGCGCCGTT
CCCCGGAATAAGAGATCGCCAGCAGCAGATCCTCCGGCGCCAGCGCCTGCACTGTTGCCAGCAGGGCGTGCAT
ATCGCGCTCAACCACCGCGTTAATGCCAATCTTCAGTAGCTTCCAGGCAAAATTTTGCGCCACCAGCCCAGAG
GCGCCAATCCCGGTGATCACAATACGGCGGGCGTGGCGCAGCATCGTGACGCTTTCTAACAGCTTTTCTTCAC
TGTTAACGTCCAGCGTGGCGTGCATCGCCGCCACATTCTCTTTAATTAATTTTTCCCCGACCAGGCGCATCGG
ATCGTCGCCGCGAATCTGGTTATGAACGGGAACGGAATGCGGATTAGGATTGCTGGCCAACGCTTCGCTGATA
GCCAGTTTCAATGCAGGAAACCCTTTAAACCCCAGTTTTTGCGCGAATTTGACTACGCTGGATTGGCTGACGC
CCGCTTCGGCCGCCAGTTGCTGCGAGCTCAGATGTCGCGCTGTATCCGGTTGCGCGAGCAGATAATCCGCCAG
TTTCCTGTCGCTTTGCGCGAGATCCGGATAACGCTGGCGAATACGAATCAAACAGTTCATGGGCAGCTCCTGC
GAAACGTGATGGCGACGACAAAACGAAATTTCACTCGCCTGGGGGAATATTCTATTCCATCATGGACGCTTAT
TATGAATTAAAAATTCCAGAGGGACAAAAATGAATCTTGGTACCTTAGTGTCTGAAACCCGTAACCCGCAAAC
GATGGATCTGGATGCGTTGCCTACTCCTGAATTAGTTAAGCGTTTTAATGAACAGGATACGCGGGTAGCGGAA
GCGGTAAAAGCCACTCTGCCTGACGTTGCGCGTGCGGTGGATGCCGCTGTGGCGGCGCTGAAGTCTGGCGGAC
GTATCATCTATATGGGCGCGGGCACCAGTGGTCGGCTTGGCGTACTGGATGCCTCAGAATGCCCCCCGACGTT
TGGCGTTCCGCACGGTCTGGTCGTCGGGCTGATTGCCGGTGGGCCGGGCGCGTTGCTGAAGGCGGTCGAAGGC
GCGGAAGACAGCCAACAGGCAGGCGAAGACGATCTTGTCGCGCTAAATCTGCAAGAACAGGATCTGGTCGTGG
GGCTCGCGGCGTCGGGTCGTACACCCTATGTGATTGGCGGCCTGCGCTATGCGCGACAGTCCGGTTGTACCAC
TGTTGCCGTCTCCTGTAATCCAGATTCGCCTATTGCGCGGGAGGCTAATATCGCCATTTCGCCCGTTGTCGGG
CCGGAAGCGCTTACCGGCTCCACGCGGCTGAAATCCGGCACCGCGCAGAAAATGGTACTCAATATGATTTCTA
CCGGCGCGATGGTGAAGTTCGGCAAGGTCTATCAAAACCTGATGGTGGATATGAAAGCCACCAACGTCAAACT
GGTCGATCGCGCGTGTCGGATGGTCGTTGAAGCGACCGGGATAGGTCGTGAAGAGGCGGAAACGTTGCTGAAA
CAGACCGATTTTGAGGTTAAGCCCGCCATTTTGATGGCGCTTACGGGGCTGGATGCTGCAGCCGCCAGGGAAA
AACTCGCCGCTCATCAGGGCTTTTTAAGGGCGGCGTTAGAACACTAAAGAGGTGTGTATGGATAAGACGGCAG
CGCTCGCCAGCGACATTTTACGTGGTATCGGCGGAGAGCAAAATATTCTGCGCCTGGAAAACTGTATGACGCG
AGTCAGGGTGGAGGTACAGGACGACAGCCAACTGGATATTCCGCGCTTAAAAGCGTTGCCTGGCATCAGTGGA
TACGTAAAGCAAGGGGAGCAACATCAGCTGATCGTTGGACCGGGGAAGGCTGCACAGGTGGTTGATGCGATGC
GCGTGCAGATTGCCGTAGGTGGTGTAAAGCCGGATGACGCTATGGCGCGTACCAAATCCGAGGCGAAAGCGAA
GTATAAAGCGCCGATGAGCGATGCGTTGCGTAAGCTGGCGAATGTTTTTATACCGCTTATCCCCGCCTTTATC
GCATCCGGCTTGATTACCGGCATTATCAACATTCTTAAACGTCCGGATATCGTCGGCGATGTTGCCGTCCACT
ATCCTAACCTGCTGGGCCTGATGGGGATATTTGGTAGCGCGGTGTTCGCTATCATGAACATTCTGGTTGGCGT
CAATACCGCGAAAGTCTTTGGCGGATCGCAGGCGTTAGGCGGCGTGATGGCGGGGATCCTTTCCAGCCCGCAA
CTGGCGCAGATTACGCTCTTTGGCGAGGCGCTTCAGCCGGGACGGGGCGGCGTTATCGCCGTCCTGCTGGTGG
TGGCGTTAATGTGCTGGATTGAGCGCCAGTTCCGTAAGCTGCTGCCGGGGTCGCTGGAGTTAATTCTTAACCC
TTTATTGACGACTGTTATCACCGGCGCGGTAGCGATTGTCGCTCTGCAACCGCTTGGCGGCTGGATTTCAGAC

GCTATCGCTCACGGCGCGTCCTGGGCTATCGATCGCGGCGGTTTTCTGGTGGGCGCGGTGCTAGCCGGAACTT
TCCTGCCGTTGGTACTGACCGGGCTGCATCAGGGGCTGGTGCCTATTCATGTGGAGCTGGTGCAAGCGCACGG
CTATAACGCGCTGTTTCCCATCCTGGCGATGGCGGGCGTTGGTCAGATAGGCGCGGCCATTGCCGTACTGATG
AAAACCCGCAATGCGCGGCTCAAAAAGGTGATTAAAGGCGCGCTTCCGGTCGGACTGCTGGGGATTGGCGAGC
CGCTGATTTTTGGCGTCACGCTGCCATTGGGTAAGCCATTTATTGGCGCTTGCCTCGGCGGCGCGGTAGGCGG
GGCGTTGATCAGCTACTGGAAAGTGGCGACGGTAATTACCTTTGGTATTTCCGGTTTGCCGCTGGCATTAACA
ATTGTTGCCGGAAAAGTCCTGTTCTATCTGTTAGGCTATTTAATAGCGGTAATTGCCGGGTTTATTTTTACCT
GGCTGCTGGGGTTCAATGATCCAGAGGAGTAAGGTTTGGTCAGCCACGAGCGTCGTGTCGTCTTTTTTGATTT
GGATGGAACGTTACATCAACAGGATATGTTCGGCAGTTTTCTGCGTTATTTGCTGCGCCGTCAGCCGCTAAAT
GCGCTGCTCGTACTGCCGCTGTTGCCCATTATCGGTATCGGTTTACTGGTAAAAGGGCGTGCGGCCCGCTGGC
CGATGAGTCTGTTATTGTGGGGCTGTACTTTTGGCCATAGCGAAACGCGTTTGCAGGCGCATCAGGCGGATTT
TGTCCGTTGGTTTCGCGCTAACGTCACGGCGTTTCCGGTAGTACAGGAGCGTCTGACCACCTATCTATTGAGC
TCTGACGCGGATATCTGGCTTATTACCGGTTCTCCGCAATCATTGGTAGAGCAAGTCTATTTTGATACGCCGT
GGCTGCCGCGCGTTAACCTCATCGCCAGCCAGATGGCGCGACGGTATGGCGGCTGGGTATTAACCGTGCGCTG
TCTGGGCCATGAAAAAGTCGCGCAACTGGAACGTAAAATCGGTACGCCGCTGCGTCTTTATAGCGGCTACAGC
GACAGCAAACAGGATAACCCGCTGCTTTATTTTTGCCAGCATCGCTGGCGGGTGACGCCGCACGGCGAGTTGC
AGCAGCTCGAATAGTGAAACGCATAGCGTCTGTGTATAATGCCGCCCGCTTTCATTACCGGAGTTACCTCTTT
GTCTGATGTCGAATTAGATCACGAATACTGGATGCGCCACGCGCTGACGCTGGCGAAACGCGCCTGGGACGAA
CGCGAGGTTCCGGTTGGCGCGGTATTGGTGCATAACCATCGCGTCATTGGCGAAGGCTGGAACCGACCCATTG
GCCGCCACGATCCTACTGCGCACGCTGAAATAATGGCGCTGCGTCAGGGCGGTCTGGTATTGCAGAATTATCG
GTTACTGGATACCACGCTGTATGTCACACTGGAACCCTGTGTGATGTGCGCAGGCGCTATGGTGCATAGCCGC
ATTGGACGCGTTGTCTTTGGCGCGCGCGATGCAAAAACCGGCGCGGCCGGATCGCTAATTGATGTGCTGCACC
ATCCGGGAATGAATCACCGGGTCGACATTATTGAAGGGGTGCTGCGCGATGAATGTGCGACGCTGCTCAGTGA
TTTTTTCCGTATGCGTCGTCAGGAAATTAAAGCCCTGAAGAAAGCCGACCGTGCGGAAGGCGCGGGGCCGGCA
GTATAGCGCAATGCCGGATAAGAGGTTGCGCGTCGCCATCCAATAAGACCATCGCGCTATTTTTCTTCTTTCT
TCTGCGGCGTAAACAGTAAAGACGGCGAATGCGTCAGGTAGCCTGATGACTGCGATAAAAACGACAGGAAGGG
GAAGGGTGCTTTATTCAGCTCCTCCGGCGACACCGCCGGGTAATCCTGCGCCAGTTTCATAGCTTCCGCTTCC
TGCTTTTCTTTTTCCTGCAAATACCCCACCAAACTTATCTGGTATTTACGGATATTTTCCACATAAGCATAAG
CCTCATGTCCACGCGCGTAACCGTAGGTTAACTTACTGTAATACGGTTTTTGGCTTAATAGCGGCAAACGCTG
CTTCACATCGGTCCAGCTATCCGGATTGCCTTTGGTTTTTACCGTCAGCGATCTGGCATCGAGCATGTGCGCA
TAACCCATATTGTAGGCGGCTAAGGCAAACCAGATACGCTCGTCTTCCGGAACGGTTTCGGGAACTTTAGCCA
TCATATCTTCAAGATAGCGAGCGCCGCCGCTGATGCTTTGCTCCGCATCGGTACGGTCGGTCAGCCCCAGACT
TTGGGCGGTATTTTTAGTCAGCATCATCAGGCCGCGCACGCCGGTTGGCGAGGTCGCCAGCGGGTCCCAGTGT
GATTCCTGATAAGAAATCGCCGCCAGCAGGCGCCAGTCTATCTCTTTGGCATATTTTTTAAATAACGGTTCCA
GCTCCGGCAGGACGTTATCTACCGCTCGCAGGAAAGAGCGGGTATCAACGTAGTCGAAATCATCGCCATGTCC
CAGATATTTCTCTTCGATCCGCGCCAACGACCCGTCTTCATTGATCGAATTGAAAAAATCGAGCAGCGCGGCG
GAGAGCGTATTATCGTCGTCCAGTCGGCTAAACCAGGTCACGGGCTGTTCATCCGTGACATCCAGCGCCACGG
CCAGCTCGGGATGAACGCGCTGAAACAGACTGATCGCCACTGAATCGGCAATGGTGTAATCCAGTTTGCCGCT
GATGACCTCTTCCAGCAGCGTCGTCGAGCCTTTTTTATCATCCACTTTCCAGCTCAGGTCGGGAAACTTTGTC
TCTTTTAAACGCTGTAAGTCATTGACCACGACGTGCCCTGGCGCAATGGTAAGCTGATTCTCGTTAACGGTCG
CCAGCGAACGTGGGCGATATTGTCCGACCCGGTAGACGAGCTGTTGCGAAACCGAGTAATACATGGGACCGGG
CTGATAGTTTTTAACGCGTGCGCTGTCGTAGACCAGCCCTGCCGCGAGCAGGTCGGCATTGCCGTTATCCAGA
TCGTCAAACAACTGGCTGATATTCTGGCGCACCGTCACTTTCAGCTTCACGCCGAGATAGTTGGCGAACTGCT
GCGCCAGTTCATAGTCCAGACCATATTTTTTACCGTTGATAACGGAATAGGTTAATGGCGAGTCGATCGTACT
GACGCGCAGTATCCCCCGGGCTTGAATCGCGGCGATATGGTTTTCGGTTTTACCGAACCAGGGGATTGAGGGC
CAAAGGGCTGCTGCCAGCAGCAGCGTCAATATGCCGATGAACAGATAATTAATCTTTAATTTTTTCAATTAGT
TAATTCTCTGGATCGTGCGTTGCCTCGGGCTGAAAAAGATGTTTTGCTGCAATAGATAAGTCATTAATGAGCG
GCATTTTGCGTAACAATGCGCCAGTTGGCAACTTATTCGCAACGATAGCCGCACCGTATGACAAGAAAAAGCG
GTGATTTTATTTCTACGCAAACGGTTTCGTCGGCGCGTCAGATTCTTTATAATGACGGCCGTTTCCCCCCTTG
CGCACACCAAAGCTTAGAAGACGAGAGACTTATGATGGAAATTCTGCGTGGTTCGCCTGCACTGTCTGCATTC
CGTATCAATAAACTGCTGGCGCGCTTTCAGGCTGCCAACCTCCAGGTCCACAATATATACGCTGAGTATGTCC
ATTTTGCTGACCTGAACGCCCCGTTAAATGATAGCGAGCAGGCGCAGCTTACCCGTCTGTTGCAATACGGCCC
GGCGCTTAACAGCCATACGCCGGCGGGTAAACTCCTGTTGGTCACACCGCGTCCTGGTACCATCTCCCCTGGT
CTTCAAAAGCAACGGATATCGCCCACAACTGCGGCCTGCAACAGGTTGATCGGCTGGAGCGCGGCGTAGCGTA

CTATATCGAGGCTTCGACGTTGACGGCGGAACAGTGGCGACAAGTGGCCGCAGAGCTGCACGACCGTATGATG
GAGACGGTGTTCTCTTCTTTAACCGATGCGGAAAAACTGTTTATCCACCATCAGCCTGCGCCGGTTTCCAGTG
TCGATCTGCTGGGAGAAGGCCGTCAGGCGTTAATTGACGCCAACCTGCGTCTTGGTCTGGCGCTGGCGGAAGA
TGAAATCGACTATTTGCAGGAGGCGTTTACCAAACTGGGGCGCAACCCGAACGATATCGAATTGTACATGTTC
GCCCAGGCGAACTCCGAGCACTGCCGCCACAAGATTTTTAACGCCGACTGGATTATCGACGGTAAACCGCAGC
CGAAGTCGCTGTTTAAAATGATTAAAAACACCTTCGAAACCACGCCGGACTATGTGCTCTCTGCCTATAAAGA
CAATGCCGCGGTGATGGAAGGATCTGCGGTGGGGCGCTACTTCGCCGACCACAATACTGGCCGTTACGATTTT
CATCAGGAGCCCGCGCACATTCTGATGAAGGTCGAAACGCATAACCACCCAACCGCCATCTCTCCGTGGCCGG
GGGCGGCGACCGGTTCCGGCGGCGAAATTCGTGATGAAGGCGCCACCGGGCGTGGCGCGAAACCAAAAGCCGG
GCTGGTAGGCTTCTCCGTTTCCAACCTGCGTATTCCGGGCTTTGAACAGCCGTGGGAAGAGGATTTTGGCAAG
CCGGAACGTATTGTCACCGCGCTGGATATCATGACCGAAGGTCCGCTGGGCGGCGCGGCGTTTAACAACGAAT
TTGGTCGCCCGGCGCTGACCGGTTACTTCCGTACTTATGAAGAGAAAGTGAACAGCCACAACGGCGAAGAGCT
GCGCGGCTACCATAAGCCGATCATGCTGGCGGGCGGGATCGGTAATATTCGCGCCGATCACGTGCAGAAAGGC
GAGATCGTCGTGGGCGCGAAGCTGATCGTCCTTGGCGGCCCGGCGATGAATATCGGCCTCGGCGGCGGCGCGG
CATCGTCAATGGCTTCCGGCCAGTCTGATGCGGATCTCGATTTTGCCTCTGTTCAGCGCGACAACCCGGAAAT
GGAACGCCGCTGCCAGGAAGTGATCGACCGCTGCTGGCAGTTGGGCGACGCTAACCCAATCTTGTTTATCCAT
GATGTGGGCGCGGGCGGTCTCTCCAATGCGATGCCGGAGCTGGTGAGCGACGGCGGGCGCGGCGGGAAATTCG
AACTGCGCGACATTCTTAGCGACGAGCCGGGCATGAGCCCGCTGGAAATCTGGTGTAACGAATCCCAGGAGCG
CTACGTACTGGCGGTCGCGGCTGACCAGTTGCCGCTGTTCGACGAACTGTGTAAGCGCGAACGCGCGCCGTAT
GCGGTCATTGGCGATGCTACCGAAGAACAGCATCTCTCGTTGCATGATAACCATTTCGACAATCAGCCGATCG
ACCTGCCGCTGGATGTCCTGCTGGGCAAAACGCCGAAAATGACCCGCGATGTGCAAACATTGAAGGCGAAAGG
CGATGCGCTGAACCGCGCGGATATCACGATTGCCGACGCGGTAAATCGTGTTCTGCATCTGCCGACCGTGGCG
GAAAAAACCTTCCTTGTCACTATTGGCGACCGTACCGTCACCGGTATGGTGGCGCGTGACCAGATGGTTGGCC
CGTGGCAGGTCCCGGTGGCTGACTGCGCGGTCACTACCGCCAGCCTCGACAGCTACTACGGCGAAGCGATGTC
GATTGGCGAGCGCGCGCCGGTGGCGCTGCTGGACTTTGCCGCCTCCGCCCGTCTGGCCGTCGGTGAAGCGTTG
ACTAACATTGCCGCGACGCAGATTGGCGATATCAAGCGCATTAAGCTTTCCGCTAACTGGATGGCGGCAGCCG
GTCATCCTGGCGAAGATGCCGGTTTATATGACGCGGTTAAAGCGGTGGGAGAGGAACTCTGTCCGCAGCTTGG
CCTGACGATTCCGGTGGGCAAAGATTCGATGTCGATGAAAACCCGCTGGCAGGAAGGCAACGAACAGCGCGAA
ATGACCTCGCCGCTGTCGCTGGTGATTTCCGCGTTTGCCCGCGTGGAAGACGTGCGTCACACCCTTACGCCGC
AGCTGTCAACGGAAGATAACGCCTTGTTGCTGATCGATCTTGGCAAGGGCCACAACGCGCTGGGCGCAACGGC
GCTGGCGCAGGTCTATCGTCAGCTTGGCGACAAACCCGCGGACGTGCGTGACGTCGCGCAACTGAAAGGCTTC
TATGACGCCATGCAGGCGCTGGTCGCTGCGCGTAAATTACTGGCCTGGCACGACCGTTCCGACGGCGGTCTGC
TGGTGACGCTGGCCGAGATGGCCTTTGCCGGCCACTGCGGCGTGCAGGTCGATATCGCCCGCGCTGGGCGATGA
CCATCTGGCCGCACTGTTCAACGAGGAGCTGGGCGGCGTGATTCAGGTACGCGCTGAAGACAGAGACGCGGTC
GAAGCGCTGTTGGCGCAGTACGGTCTTGCTGACTGCGTCCACTACCTTGGTCAGGCCCTGGCTGGCGATCGCT
TTGTGATTACCGCTAATGACCAGACGGTATTCAGCGAAAGCCGCACTACGCTGCGTGTCTGGTGGGCGGAAAC
CACCTGGCAGATGCAGCGTCTGCGCGACAATCCGCAATGTGCTGATCAGGAACATGAGGCGAAAGCGAATGAT
GCCGATCCGGGCCTCAACGTGAAGCTGTCGTTTGATATTAATGAAGATATCGCCGCGCCGTACATTGCGACAG
GCGCACGACCGAAAGTCGCTGTACTGCGCGAGCAGGGGGTGAACTCTCACGTGGAGATGGCGGCGGCGTTCCA
TCGTGCGGGCTTTGACGCGATTGACGTCCATATGAGCGACCTGCTGGGCGGTCGTATCGGTCTGGGCAACTTC
CATGCGCTGGTGGCTTGCGGCGGCTTTTCCTACGGCGACGTGCTGGGGGCGGGCGAAGGCTGGGCGAAATCCA
TTCTGTTTAACCATCGGGTGCGCGACGAATTCGAAACCTTCTTCCATCGTCCGCAGACACTGGCGCTGGGCGT
GTGCAACGGTTGCCAGATGATGTCGAATCTGCGCGAGCTGATCCCGGGCAGCGAACTGTGGCCGCGGTTTGTA
CGTAACCATTCTGACCGCTTCGAAGCCCGCTTCAGCCTGGTGGAAGTGACGCAAAGCCCATCGCTGCTGCTGC
AAGGAATGGTAGGCTCACAGATGCCGATTGCGGTTTCCCATGGGGAAGGGCGCGTTGAGGTACGTGACGATGC
GCATCTTGCCGCGCTTGAGAGCAAAGGCCTGGTCGCTCTGCGCTACGTTGATAACTTTGGTAAGGTGACTGAA
ACCTATCCGGCTAACCCGAACGGTTCGCCGAACGGCATCACGGCGGTGACGACCGAAAACGGTCGGGTGACGA
TTATGATGCCGCATCCGGAACGCGTATTCCGCACCGTCGCCAACTCCTGGCACCCGGAAAACTGGGGCGAGGA
TAGCCCGTGGATGCGTATCTTCCGTAATGCGCGTAAGCAGCTTGGTTAATTAGCCCGATAGCCATTAAGCCCC
TATTCACGAGGGGATTTTTTGTAGACGGCGCTTATCAGGCCCACGGGGAAGCGCGGTTGAAGCTACCGTCTTG
AACGTCAAGCGGTAGGCCGGATAAGGCGTTTACGCCGTCATCCGGCAATAAACTAGTGCTTATGCCGCAAGAT
TAAAGTGTCGGGAAATCCCGACATGAGTGGGGGATAGGTTGTCGCCAAAAAGAGACATTTAACTATTTGATTT
TAAATTGTTTTATTTTTAGGTGTTGCAGGTGTTGCTGATTGGCGACAGAAAGGGCAAAAAACAGACAGAGCCA
TTTTTAGCATGAATAACGATATTTTTCATATCTATCATTGTGTTAACAAAGTCTTTTGAATATTGGCACAATT

ACTGCATAATGATAACCAGTGGCTCATTCACCTTCTTATGTCAGCCCCTTAGGGACGCGCTACATAAACTTCG
AATGACGCACAACAAGGTGCCTGCCGTCCAACTTCTGATAATAGCGTAGCTTTATCAACCTCCGGGCGAAACG
TCGAGTTAGGCACCGCCTTATTCCATAACAAAGCCGGGTTTAGCCCGGCTTTGTTGTATCTGGCGCTTCCCTC
GGTTAGCATCTTTTTTATTCTTCTTTTATGTGAGCGACACGTTGAAGCGCTGGTCTGTTTTCCCCCGTTCTTT
ACGACAATTGGTCATGCTGGCCTTTTTGCTGATCCTGCTGCCGTTGCTGGTTCTGGCCTGGCAGGCGTGGCAA
AGCCTTAACGCGTTAAGCGATCAGGCTGCGGTGACTAACCGCAGCACGCTTATTGACGCCCGGCGCAGCGAGG
CGATGACCAACGTCGCGCTGGAGATGGAGCGAAGCTACCGACAGTATTGTGTGCTTGACGATCCGACGCTGGC
AAAAGTGTATCAAAGCCAGCGTAAGCGCTATAGCGATATGCTGGACGCCCATGCCGGCGTCTTGCCGGATGAT
AAGTTGTATCAGGCGTTACGCCAGGATTTAAACGCGCTGGCGCAATTGCAGTGTAAAGATAGCGGCCCGGAAG
CGGCAGCGGCCGCGCGTCTGGAGGCCTTTGCCAACGCGAATACGGAAATGGTGCAGGCGACGCGTACTGTGGT
TTATTCGCGTGGGCAACAGTTACAACAAGAAATCGCCGAGCGCGGACAATTTTTCGGCTGGCAGGCGCTGGTG
CTATTTCTGGTGAGCCTGGCGATGGTGCTACTTTTTACCCGCATGATTATCGGGCCGGTGAAAGGAATTGAAC
GGATGATTAATCGCCTGGGCGAAGGCCGTTCTTTAGGGAATACCGTCACCTTTACCGGTCCGCGTGAATTACG
ATCGGTGGGGCAGCGCATTATCTGGCTGAGTGAGCGTCTGGCGTGGCTGGAATCCCAGCGCCATCAGTTTCTA
CGTCACTTGTCCCATGAGCTGAAAACCCCGCTGGCCAGTATGCGCGAGGGGACAGAGCTGCTGGCCGATCAGG
TGGTTGGGCCGCTGACGCCGGAGCAAAAAGAGGTGGTCGATATTCTGGATGACAGCAGTCGTAATTTACAAAA
ATTGATTGAGCAATTGCTTGATTACAACCGGAAACTGGTAGACAGTGCCACAGAGCTGGAAGCGGTCGATATC
GCGCCGCTTGTGGATATGGTGGTTTCCGCCCACAGTTTGCCTGCCCGCGCTAAAATGATGCATACCGATGTCG
ACCTGGAGGCCGAACGCTGCATAGCAGAGCCCATGTTGCTGATGAGCGTGCTGGATAATCTCTATTCCAACGC
GGTGCACTATGGCGCTGAATCCGGTAACATTTGTATCCGTAGTCGCTCTCAGGGCTCCACGGTTTATATCGAT
GTGGTTAATAGCGGCGAGCCGATTCCGCAAACCGAAAGAGAAATGATTTTTGAGCCTTTTTTCCAGGGAAGCC
ACCAGCGAAAAGGGGCGGTAAAAGGCAGTGGGCTAGGGCTGAGTATCGCCAGAGATTGTATTCGTCGAATGCA
GGGAGAGATCCAACTGGTGGATGACAAAGCGCAGGAAGTCTGTTTTCGCATCTCGCTGCCGCTGCCTGCATCT
GACAAACACTAATATGAATTTAAGCCTGGTGCGTATGTCACACGTTTTCGTCCAAACTATTAAACGATGCCTT
TTGCGGTGGGGAATACCGGTGGGCATATCGTGTCTGGCGCTGACCGCATGTGTTCCCCATGCTTCACAGCAAC
TGCCCGGTAGCGCAGCGCAGGACACGCTCCCCCATTATCAACTGGCGGATTATCTCCCGACGGCGTGCGCGGA
TATCTGGTCGCTACGCGGGCAGGCGGTGGAGACGAATCCGCTGTACTGGCTACGCACGATTGACTGCGCCGAT
CGTCTGATGCCGGTTCAGTCGCGCGCAGAGGCGCGCGCGTTGACGGATGATAACTGGCAAAATGCCTTCAGGC
GCGGGATTTTACTGGCGGATGCAAAAATTACGCCGCCGGAGCGGCGCGCCATAGTGACCCGTCTGGAGGCGTT
AAGCGCGCAAATACCGGCTCAGGTACGGCCCGTCTACCAAATCTGGCATGACGGTCAGGCGCTGCAATTAGCA
CTTTCTGCCGAACGTCAGCGTTATAGCAAGCTCCAGCAAACGTCAGATAGTGAGCTTGATGCGCTGCGCCAGC
AGCAACAGGCCTTGCAAACGCAGCTCGATCTTACCACCCGTAAGCTGGAGAGTTTAACCGATATTGAGCGCCA
GCTTTCGACACGAAAACCGGCAGGGAATTACAACGCCGATACGCCGCATACGAATGACAAACCCGCGACGTCT
GAAGATGGCGCGGCGCCGTCGCCGTCACAAGATGAGGTAACGCCATGATAAGCCGTAAACCGGCGCACCTGCT
GCTGGTCGATGACGATCCCGGATTACTGAAATTGCTCGGTATGCGCCTGACCAGTGAAGGATACAGTGTAGTG
ACCGCAGAGAGCGGCCAGGAAGGACTGCGGGTACTGCATCGGGAAAAAGTGGATCTGGTAATAAGCGACCTGC
GCATGGATGAAATGGATGGTATGCAACTGTTCACAGAAATTCAGAAAGTACAGCCGGGAATGCCGGTTATCAT
TCTGACGGCGCATGGTTCTATTCCGGATGCGGTGGCCGCTACCCAGAAAGGCGTATTCAGCTTTTTGACGAAG
CCGATCGACAGAGACGCGTTATATAAAGCGATAGATGAAGCGCTGGAGCAGTCCGCTCCCGCGACGGACGATA
GCTGGCGCAACGCTATCGTTACCCGTAGCCCGCTGATGCTGCGACTGCTTGAACAGGCGCGCATGGTCGCGCA
GTCGGATGTCAGCGTATTGATTAACGGACAAAGCGGCACCGGGAAAGAGATTTTCGCACAGGCGATTCATAAT
GCCAGCCCGCGCAGTAACAAGCCGTTTGTCGCCATCAACTGCGGCGCGTTGCCGGAACAGTTGCTGGAGTCCG
AATTATTCGGCCATGCGCGCGGCGCCTTTACCGGCGCAGTGAGCAATCGTGAAGGGTTGTTCCAGGCGGCGGA
AGGCGGCACGCTGTTTCTGGATGAAATTGGCGATATGCCCGCGCCGTTACAGGTGAAGCTGCTGCGGGTGTTA
CAGGAGCGTAAAGTCAGGCCGCTGGGCAGCAATCGCGATATTGATATCGACGTGCGAATTATTTCCGCGACGC
ACCGCGATCTGCCGAAGGCGATGGCGCGGGGCGAATTTCGTGAAGATTTGTACTACCGGCTGAATGTCGTGAG
CCTGAAAATTCCGGCGCTGGCGGAGCGTACCGAGGATATTCCGCTGCTGGCTAATCATCTGCTCAGACAGTCG
GCACAGCGGCATAAACCGTTTGTTCGCGCCTTTTCTACTGATGCAATGAAACGGCTGATGACGGCAAGTTGGC
CGGGCAACGTGCGCCAACTGGTCAACGTCATTGAACAATGCGTGGCGTTAACCTCTTCGCCGGTGATCAGCGA
TGCGTTGGTCGAGCAGGCGCTGGAAGGCGAAAACACCGCGTTGCCAACTTTTGCCGAAGCGCGAAACCAATTT
GAACTGAACTATCTGCGCAAGTTACTGCAAATTACCAAAGGTAACGTGACGCACGCGGCAAGGATGGCGGGGC
GCAACCGCACGGAATTCTATAAGCTACTCTCCCGCCACGAACTGGATGCGAATGATTTCAAAGAGTAGTTTTC
ATGCCGTAAAGTAGCGCGAAATATGTTACGTTTAACCGATCAAAACACAGGCGACCTTTTCAAGGAATAGCAT
GAAAAAGATTGATGCGATTATTAAACCCTTCAAGCTGGACGATGTCCGTGAAGCGCTGGCCGAGGTAGGTATT

ACCGGGATGACGGTCACTGAAGTCAAAGGCTTTGGCCGTCAAAAAGGCCACACCGAGCTGTATCGCGGCGCGG
AATATATGGTGGATTTTCTGCCGAAGGTAAAAATTGAGATCGTTGTGCCTGACGACATCGTGGATACCTGTGT
TGACACCATCATTCGTACCGCGCAGACGGGGAAAATCGGCGACGGTAAGATCTTTGTCTTTGATGTCGCCCGC
GTTATTCGTATCCGTACTGGCGAAGAAGACGACGCGGCTATCTGATGTGCCGATTCGCTGCCCGTCCCCTCCT
TGTGGGCAGCGAACCACCGTTTAATCGCCCTGCGGTAGCGCCGATGTAGTCCGGGTGCAAACCCATCTCAGGC
CGACAATGGCGACGATCAGCGCCACGCAACTTAGCGTCCACTCGCCCATTTGCGAGAAAAAGTGTCCGTAGGC
GGCTACCGCCGTATCACTAATTTGCGATTCCGTGGTTTGTTGCGCGACGACGCCAGCCAGCCAGTTAGCGACC
GCGCCGGTCGCCAGCATATAAATACCGGTCAGCACGCCCGTGACGCCAGGCAGGTTGAGGCGGGTAATTTGCG
CCATCGCGACCGGATCAATAAACAGCTCTGCAAAGCCCATCAGTGCCAGCCCTGCGATCATCATTCCCATTGA
TGCCTGCCCGTCTAACCGGGCCTGATGCGCGTTTAGCGCCAGCAGCATAAAACCGCCGCCCATTAATACCAGC
CCCACGGCAAATTTAAGCCAGACGCGCAGCACCGAGCGGGCGCTCTCTTTCGGGCTGCTCAGCCACGCCAGCA
CCACGCCCGCCGCCATCACCGCAATCGCATTGACCGACTGGAACAATGCGGTGGGGACGGTCATATTCAGCCA
GTGGCGGTTGACAAAACGGTCGATGAATAAACTGATCGAACTTCCGCCCTGCTGCGCCAGAACCCAAAAGAGA
GTGCCGGTGATCATTAACAGAACGATTTGCCACAAGGCGCGACGGTGCTCGGGAAATTTAACCATAATACGGG
CGATAAGTTGCGCCGCGAAGACGCAGACAATGGCCAGCACATAGCCAGACCAGTTATTTTCCAGCAGGAGGGT
GAAGAATACCGGCGCGATACACAGCATTAATACCAGCCAACCCCAGGTTGGAAGGGCGAATTTAACCGCCCGC
AGCGCGGGGCGATTAACCCCACGCGTCTGCTGAAAGTGGCGATGACCGCTTAAAAAGATGAGCAGGCCGATGA
ACATTCCCACGCCCGCCAGCGCAAAGCCGACATGCCAGCCATACCACTGCGCCGCCAGACCACAGGCGATCGG
CGCGGCTATCGATCCAATATTCCCGGCGGCGTAGAGCAGCGAGAATCCCCCGTCGCGGCGGTTGTCATCAGGG
GCGTAAAGTTCGCCCAGCAGACAGCTAATATTGGATTTAAACAAGCCGTAGCCGCAGATAATAATGGCCAGCG
CCGCATATAAACTCAGCGTTGAATCAGATTCTAAGCCCAATACCACATGACCCAGCGTCATCAATAAGGCGCC
GGTGATAACCGCCACGCGGTTGCCAAGCAGGCGGTCGGCAAGCCAGCCGCCAAGAATAGGGGTAACGTAAACT
AAAGAGGCGTAGGCGCTGAAGAGGTTGATCGCGTGGCTGTCATTAAAACCAAGCTGGTGGGTAAGATAAAGGA
TGAGTAAGGCACGCATGCCGTAAAAGCTGAAGTATTCCCAAATTTGAATCGCCACGATGTAAAAAATCGCGCG
CGGTTGTGAGGGTGTTTTCATAAAGTCTCCTTGCTGCGTGAAAAAAGGGAAGTGGCAAGCCACTTCCCTTTGG
TACTTATTTCGTATTTTCTTTCAGCACTTTAACGGTGTAACGTCCGTCTGCCTGACGATAGGCGCCGTGGATA
TCGGTTTCGAAGCCCGGATAGTGGGCGCCGATTTCGCACAGCATTTGCAGGAATTCCAGTACCGGACGGCTTT
CCTCGGTGATCATTTCACCAGGCATCACCAGCGGCACTCCCGGAGGGTAAGGAAGGATCATGTTGGCGTTGAC
GCGTCCAACCATCTCTTCAAGATACACCTCTTCCGTTTCGCCATGCAGCTCTTTCTGGAACGCAGTATACGGC
GTCATCACCATTTTCGGCAGCACTTCAAACGCACGGTACATTAAGTCCGGCAGATTATGATGTTCGACCAGTT
TATGAATATTCTGCGCCAGTTCCTGGATGCGCATATTTTCATAAAACTCAGGCGCTTCGCGGTACAGTGCCGG
CAGAATGTTTTTGACGCGCAGGTTCAGGTCAAAGGCGCGTTTAAATTCAGTGAGCGCGCGCAGCAGGCTCAGA
GCTTTGGTTTTGTCGATACCAATGCTGAACAGGAACAGCAGGTTGTATGGACCGGTTTTTTCTACGATGATGC
CGCGTTCATCGAGATATTTGGCGACCAGACTTGCCGGAATACCGAATTCATCCATCGTACCGTCTTTCTTCAT
CCCCGGCGTCAGGATAGTGACTTTGATCGGGTCGAGGTACATGTGTTCGTTATCGATATTTTTGAAGCCATGC
CATGCGCTATCTGAACGCAGCGGCCAGCACTCAGCTCCATCGATATGTTCCGGCTGCCATACGTCAAAGAACC
AGCCGTCGGATTCGCTTTTCAGGCGTTTAATTTCTTTACGGAATTTAATCGCACGTTCAATAGAGCCGTTAAT
CAGGCGTTTACCCGCATTACCTTTCATCATCGCCGCTGCGGTTTCAGTAGACGCTACGATACCGTAGTGCGGA
GAAGTGGTGGTATGCATCATGTAGGCTTCGTTAAAGGTTTCTTCGTTAATGTCGCCTTTAACATGAATCATGG
ATGCCTGTGAAAACGCCGCCAGCAGTTTATGCGTGGACTGGGTTTCATAAATGATTTTGCCTTCTACACGGTC
GCCGCTCATACCGCATTTACCCTGATAAATAGGGGAGAAATTGGTATAAGGCACCCAGGCGGAGTCAAAATGA
ATGGACTTCACATCCAGAGTTTTCTTGATGTAGTCAGTGTTATACAACAGACCGTCATAGGTTGAGTTAGTAA
TAACAGCATGGACCGGCCAGGTCGCGTTAGGCGTCTCTTTTACCCGTTTAGCGATAGTGGCGTGCTGGAACTC
GCTTTGCGGAATACCGCCAAGAATACCGTAGGCGTTACGGGTTGGGCGGAAATAAATCGGCGTAATGTCGCTC
ATCATCATCAGATGGGTCAGCGATTTATGGCAGTTACGGTCAATCAGTACCGTGCTGCCTGCCGGCGCGGAGT
ACATACCGACGATTTTGTTCGCCGTAGAGGTGCCGTTCGTCACCATGTAGCTACGTTCGGCGTTAAAGACGCG
GGCGATATACTCTTCCGCTTCTTTATGCGGGCCGGAGTGGTCCAGCAGCGAACCCAGTTCAGAAACGGAAATC
GAAATATCGGATTTCATCGTGTTCGGGCCGAAGAAATCATAAAAGATGCTGCCGACGGGGCTTTTCTGGAAAG
CGGTCCCGCCCATGTGGCCAGGCGTACAGAAAGTATATTTCCCTTCGCGCACATATTTGAACAGCGCTTTTGT
CAGCGGCGGTAGAATATTGTCGATGTATTCATCGGTATTCTGGCGAATTTTCGCCGCGATATCGGCTGCGGCG
CCTAAAGCATATTCAAAGAAGCGAACCTGCATTCTCAGATCGTTGAGGCTGACGTCCAGCGTAGAGTAGCTGT
TGGCGAAGGCGTACAGCGGCATATACTCGTTCAGCTTGCTAATCTCTTCGCAAAGCTCAAGGTTATATTTATC
CCAGTCGAAAATGACGCCGCAAAGGCGGGAGTTATTTTCAATCAGCTTCAGCAGGTCTTCTCGGTCGTTTGGA
TAGACGATACGGAAATTTAAACCTTCCAGTGCACGATGCAGTTCACGAATAGGCTCTTCTTTAAAGTAGACGC

CCATGTGGTTCATGATAGCAATAACGTTCATAGTCTTGTTCCTGGTAAAGTGGGCCCCTCCCGCTACGCTATA
GCAGGTGACGAAAGGGGCTTTGAGAAAAAGGAGTTAGCGGTTAAAGCGCTTCAGCCGTATTGTTGTCGCTGTC
GTTGTTCTGGCGCTGGTGCATTTTGCGACCGTAGAACATCAGGATGATCAGACTGACGATAAAGGTGCCGGAG
AGTTCGAAGGAGCTGGCGCCCATCAGTGCGATGAAGCAAAATGCGCAGCCCAGTACAGAACAAATCAGGCTTA
CCGAGTTACGGATGTTGAACCCTTCGAAGCGAATCAGGTCAACACAGGAGTAGAAGTACGGCAGCATGGTAAG
CAGTACGGCAATCCCGGTCAGTTCGCCGAACAGGTCGGACGCTTTACCGCCGCTGGAGTTCATGATGGTGATC
AGAATCATCAGCGCCGTCATTTTCACCGCAGCCAACAGCAGACCTTTTTTCGGAATACCGTTTTTATCCATTT
CACCGTAAATTTTCGGGAAGTTGCCGTCGTTGGCGGCGCGAACCCCAGCCTGGCCGACCAGCATCATCCAGGA
GCCCAGAGACGTCAGGCAGGCGAAAGCGGTAAAGGCGGAAACCAGCGGCGCAGCCCAGCCACCCAGAATCGTG
GAGGTACTGATAGCGAACGGCGCGCCGGAAGAGGCCATAACGGAAGCTGGGAACATCCCGGCGATAACCTGAG
TCGCGGCGATATAGATGATGCCTGCCAGCGCGGTACCCAGCATGGTTGCCAGCGGTACGGTGCGTTTCGGGTT
TTTCACCATGCCGGTGCTGACGGCGGCAGACTCAACGCCAACGAACGCCCACAGGCAGAGCAGAATACTTTTA
ACAATGGCGTGAGAGTCGGTCGTCGTGGACGTGTTCCAGTTCGCGTGATAGGTCGCGATATCGAACCAATGCC
AGCCGGCGACTGCGGTCAAGACTACCGGGATCAGCACCAGGAATAGGCCAATCGTTGTCAAGCGGCTGACCCA
GGCCCCACCCAGCATATTAATAAAGGTGAACAGCCAGACGATAGCAATACAGGCAATACCTGCCGGTATTGGA
TTATTTAATGCCGGGAAGAACGTAGAAAGATAAGAAACAGCGGTAATGCCGATGGCTAAGTTACCAATCCAGT
TTGCGTGATAATAAAGAACGCCGGTCTGGAAACCAAACGCCGGAGATATTTCCCCAGCATAAGCGATAGGGCC
ACCCTGTTGAGGGTTTTTGGTTGCCAGCCTCGCATAAACATAAGCCAGCGACATTGCCCCAATAATAGAAATT
ACCCAGCCCCAGATGGCAATGCCACCAATACTGGCAAGGTTGGCGGGTAATAATGCAATCCCGCTCCCCATCA
TATTACCGGCAACGACGCCAGTACAGGCAAATAGCCCGATCTTTTTGACAGAACTCATGTTCATTTCTCCTGA
GCTGTATTTTTAAGAACCGGGCTTATGCTTCATATATGCTGCGTGCCCGTTCAACTGTCGACAAGATTAACGA
ATGGGTTGAGATAAGTCCTAAAGAAAAAGTGAAATAAACACAAACATGAAAAAACTGATAGATTGATTTTTTT
GAATTAACATATAAATATCATGGAAAATATCATGAAAGGATAAACATCGTTTAATTACAAAGAGTTATATTTA
CATTTCTTTGCGATGAACATGTTGCCAAAATTAACATAAGCTATTTTTGGCAACATGTTCTTCACAAAAATAA
AAGCGAAAAATTTAATATCTTTTCGCTTTTATTTGTCATGGTTCAGCGTTAAATTTAATCTTCTGCCAGAAAA
CTATTCAGATAAGGGACAATTTTCTGAACGGATGTCTGAAATACGCCGTTCTCGATCCAGTACAGCGTGTTTT
CACCTGGACGTAAATTAAAGGCGGTCAGATAGGCATCAGCGGCCAGACGATTCTCACCTTTCATTTCGTAAAC
TTTCCCCAACAGGACATAGTTAAACCACGACATTTCAAGCTCGATGCTTTTATTGATTTCCTCATACGCTGCA
TCGATATCACCTTTGCCCAAAAGATCAACGGTCTTGATTTTATAATAAACAGACGTTTTCTCAACGCCCGGAA
TTTGATCTATTTTTTTAAACTCTTCGTTCAGCGCAGCCACTTGTTCTTTATCAAACGGCTGTTGTGATTGCCG
TAATACATCTACCAGCACTTTATATTCATAGGCATAGTTGAAATCAGGCGTTTCCTTAATAATGCCATTCAGA
ATATCGCTGGCTTTACCTAGCGCCTGCGCATCGCCGCTCAGAATAAGCTGGCGCGCCTCATAAAAACGCATCA
GCGCGGTATTTTGCGGCGTTCTGAACAGGGCCAGTTGTTCTCGCATACGATTAGGCCAGGGCTGTGTTAAAAC
GAAAGACAGGCTATTAAACAGATCATCCTGGATGGACAACTGGTTAGTATTGGTGACGAAATAACGCTTATCC
AGCATGGTTGAACCATCGGCGTTATCAATGAGCACCACAGACATAAAGCACTGCTGCGCCCGATAGTGGCGCT
GATTGACAAACTGAATGGTTAACGTCTTACCGGAGCTGCTTGGCTCATTGACCCGGTAATTGGTTTTATCATG
TACCATAAAGGTCGAGTAGGTGTTCAGCAAGTCGGTGATTAAGCCGCCCAGACCGATAGCGTAGGACTCCTGC
GAGACCCAGTTATTACAGGAATTGCCGCCTTCAAAACGAACATCTATATCTCGTGGGTTAAGTAATAACCGCG
TTTTTGTTACCGCGGCATTATGATCTACCACCGACATCACGATAAACACCACCAGCGTCGCCAGCGAGAGCAG
GAACATCACCCACGTCCAGAAAGCGGCGATACGCGGCTTTTTCGCCTTTTTAGTCGGCGTTTGCAAAGAGGCG
GGCGGCACAGGCGTAGCGGCAGTAACCCCCTCTGTCGGTTCTGCATTCGTCGCTGCGATCGGCGGCGGCGGCG
ACGTTGATGAATTATCGATCTCATCGCTATTTTCTTCGCACCAGATAACCGGCGCCGTCAGCTTATAGCCGCG
TTTAGGTACGGTAACGATGTATTCAGCATTGCTGTCTCCGCCATCTCTTAATGATTTGCGTAGTTCAGAAATG
CTTTGGGTGACAACATGGTTGGTCACGATGGTGCGCATCCAGACATGATCAATAATGTTATCCCGGCTTAACA
CTTCATCCGGGTGATGCGCAAAATACATCAGAAGATCGATCAGCCGTGGTTCAAGAGTAATCTGGCGTCCCTG
ACGACTGATCTGATTAACAGAAGGTGTAACCAGCCACTCTCCAATGCGTACAACAGGTTGCTGCATAAAAAGA
TGCCCTAACGAGCTAAGTCAAACGTTAATAATAACACGATTTGCACAGACTTTTTTTATTCCCCTTTTTTGTA
AAAGAAAGGCATAAAAAGCAGCGCAATAAGCAGGGGAACGGGGAGGAAAACGGGCGTTCGCCTTAACGATAAC
GCCCTTTTTTTCAGAGGATTTCTTGCAATTACAGCACTTTATGCGGGCCGAAGCATTCGTAATGAATGTTTTC
GTGATTCACGCCAAGTGAAACCAGTTGTTTTGCGGCAAACTGCATAAAACCTACCGGGCCGCAAAGATAGAAC
TGCATTGCCGGATCGCTGATCGCAGCTTCCAGTTTGCTTAAATCCATCAGCCCTTCGCTGTCGAAGAGGCGTT
GGGCGCGATCGGCCTCAGTCGGCTCGCGGTACCAGGTATGGGCAGTGAAACGCGGCAGTGTACGGCCCAGCTC
GCTCACTTCGTCGGCAAACGCATGGACGTCGCCGTTCTCCGCCGCGTGGAACCAGTTCACCTGCGCGGTATGC
TGTTCTTTCGCCAGCGTATCGAGCATCGCTAACATCGGCGTCTGGCCGACGCCAGCGGAAATCAGCGAAACGG

GGGTATCAGCGGCGACATTCATAAAGAAGTCACCTGCCGGCGCAGCCAGATGCACCACATCGCCCACGCTGGC
GTGATGGTGTAACCAGTTTGATACCTGGCCGCCGTCTTCACGCTTCACGGCAATACGGTACCCTTTGCCATCG
GGTTTACGGGTCAGTGAATATTGGCGGATCTCCTGATGCGCAAAACCTTCCGGCTTCAGCCAGACCCCCAGAT
ACTGCCCGGGACGGTATTCCGCCACCGTACCGCCGTCGACCGGTTCAAACTCAAAGCTGGTGATCAGTGCGCT
ACGCGGGGTTTTCGCAACGATGCGGAAGGGGCGCGTGCCTTCCCAGCCGCCGTCTTTGCTGGCGTTCTCGTGA
TAAATCTCGGCTTCCCGATGAATAAAGACGTTAGCCAGTACGCCATAGGCTTTGCCCCACGCGTCCAGCACTT
CCTGGCCCGGGTTGAACATTTCGTCCAGCGTCGCCAGCAGATGCGTCCCGACGATGTTGTACTGCTCCGGCTT
AATCTGGAAGCTGGTGTGCTTCTGCGCGATTTTTTCTACCGCCGGCAGCAACGCCGGTAAATTTTCGATATTG
CTGGCGTAGGCCGCGATAGCATTAAACAGGGCTTCACGCTGATCGCCGTTACGCTGGTTGCTCATATTGAAGA
TTTCTTTGAGCTCCGGGTTATGCGTAAACATACGGTCGTAGAAGTGGGCGGTCAGTTTCGGTCCTGTTTCAAC
CAGCAGGGGAATGGTGGCCTTTACTGTAGCGATGGTTTGTGCGTCAAGCATACGTGCTTCCTTTATGTGAAAA
TCTAATGATGTATATCAAATGCATCTTATAAAAATACCCTTGCATTGTAAATGGATCTTCTCTGCTTTACGTT
ATGGAGGTAACAACGTGAAAAATCTGCATCACAAAGCTGAAAAGAAATCCGTTGAAATTCGTCAGGCTCTCGT
TCAGGAAACCCTTATCTGACGCATAGGTAATCGTTTGCGTAAAAACCTTTGTCAAGACCTGTTATCGAAGAAT
GATTCGGTTATACTGTTGGCCGAAGTCCCACGGACTGCCTTTTCAGGCACAAATTTTATTGTTAGCTGAGTCA
GGAGATGCGGATGTTAAAGCGTGAAATGAACATTGCCGATTATGATGCCGAATTGTGGCAGGCTATGGAGCAG
GAAAAAGTACGTCAGGAAGAGCACATCGAACTGATCGCCTCCGAAAACTACACCAGCCCGCGCGTGATGCAGG
CGCAGGGGTCTCAGCTGACCAACAAATACGCTGAAGGATATCCGGGCAAGCGCTACTACGGCGGTTGCGAATA
CGTTGATGTCGTAGAGCAGCTGGCTATCGACCGCGCGAAAGAACTGTTCGGCGCCGACTACGCTAACGTGCAG
CCGCACTCCGGTTCTCAGGCTAACTTCGCTGTTTACACCGCGCTGCTGCAGCCGGGCGATACCGTTCTGGGTA
TGAACCTGGCGCAGGGCGGCCACCTGACTCACGGCTCCCCGGTTAACTTCTCCGGTAAACTGTACAACATCGT
ACCTTACGGTATCGATGAGTCCGGTAAAATTGACTATGACGAGATGGCGAAGCTGGCCAAAGAGCACAAGCCG
AAGATGATTATCGGTGGCTTCTCTGCCTACTCCGGCGTGGTTGACTGGGCAAAAATGCGTGAAATCGCTGACA
GCATCGGCGCATACCTGTTTGTCGACATGGCGCACGTGGCGGGCCTGATTGCCGCAGGCGTTTACCCGAACCC
GGTTCCGCACGCTCACGTTGTCACCACCACCACCCACAAAACCCTGGCGGGTCCGCGCGGCGGCCTGATCCTG
GCGAAAGGCGGCGATGAAGAGCTGTACAAAAAACTGAACTCCGCCGTCTTCCCAAGCGCGCAGGGCGGCCCGC
TGATGCACGTGATCGCGGGTAAAGCGGTGGCACTGAAAGAAGCGATGGAGCCGGAGTTCAAAGTTTACCAGCA
GCAGGTGGCGAAAAACGCCAAAGCGATGGTGGAAGTGTTCCTGAACCGCGGCTACAAAGTGGTGTCTGGCGGC
ACTGAGAACCACCTGTTCCTGCTGGACCTGGTGGATAAAAACCTGACCGGTAAAGAAGCTGACGCCGCGCTGG
GCCGCGCTAACATCACCGTGAACAAAAACAGCGTGCCGAACGATCCTAAGAGCCCGTTCGTGACTTCCGGTAT
CCGTATCGGTTCTCCGGCGGTCACTCGTCGCGGCTTCAAAGAAGCGGAAGTGAAAGAGCTGGCTGGCTGGATG
TGTGACGTGCTGGACAACATCAATGATGAAGCCACCATTGAACGCGTGAAAGCGAAAGTGCTGGATATCTGCG
CCCGCTTCCCGGTTTACGCGTAAGCGCTTGTGTTGTGAGAATGGCCCGATGGCGCTGCGTCTGTCGGGCATTA
CTTATGCGCCATAACGTGACGCCGCCGGAAGTCATTATCCGGCGGCGTTGCCGTTTGAACTTCTCCCTCCGCC
TGTTAACCTTATGTTACCCTTGCCAGCGCAACCGTTTACCGCCAGACTATCGCCTCCAAACGGGAGGGAATCA
TGGCATTGCATTCCACGCGCTGGCTGGCGCTCAGTTATTTCACCTACTTCTTTAGTTACGGTATTTTTCTGCC
CTTCTGGAGCGTCTGGCTCAAAGGTCTTGGGCTAACGCCGGAAACCATCGGTCTTCTGCTGGGCGTGGGTCTG
GTCGCGCGTTTTCTCGGTAGTCTGCTCATTGCGCCTCGCGTAAGCGATCCTTCGCGGTTGATCTCCGCGCTGC
GCGTACTGGCATTGCTGACGCTGGTATTTGCGCTGGCATTTTGGGCAGGAACGCATGTCGCGTGGCTAATGGT
GGTGATGGTTGGGTTTAACCTCTTCTTTTCACCACTGGTGCCGCTGACCGATGCGCTGGCCAATACCTGGCAA
AAGCAAATTACCCTGGACTATGGTCGGGTGCGGCTGTGGGGGTCCATCGCCTTCGTGATAGGGTCGGCGCTGA
CCGGTAAGCTGGTGAGTTTATACGATTACCAGGCGATTCTGGCGCTACTGACGCTCGGCGTCGCCTCGATGTT
GCTGGGTATGTTGTTACGTCCCAGCGTGCCGCCGCAAGGGGAAAGCCGTCAGCAGGAGAGCGCAGGCTGGCCC
GCCTGGCGTACGCTGGTGGCACAAAGTTGGCGTTTTCTCGCCTGCGTCTGTTTGCTGCAAGGGGCGCACGCCG
CCTACTACGGTTTTAGCGCCATCTACTGGCAGGGGGCTGGCTATTCAGCATCGGCGGTCGGCTATTTATGGTC
GTTGGGCGTGGTGGCGGAAGTGATCATTTTCGCCCTGAGTAAAAAGCTATTTCGCCGGTTTAGCGCCCGCGAC
TTGCTGTTGCTTTCCGCTGTGTGCGGCGTGGTACGCTGGGGACTGATGGGCTGGAGTACGGCGTTGCCGTGGC
TGATTGTGATACAAATCCTGCACTGTGGCACGTTTACCGTTTGCCATCTGGCCGCCATGCGCTATATCGCTGC
GCGTCAGGGAAGCGAGGTGATTCGTTTGCAGGCCGTCTATTCCGCTGTGGCGATGGGCGGCAGCATCGCGATT
ATGACGGTATTTGCCGGTTTTCTGTATCAACATCTGGGCGGCGGCGTGTTCTGGATTATGGCGCTGGTGGCAT
TGCCCGCTATCTTCCTTCGGCCCAAAGTGGTTGCCGCGTCATGATTCCAGGATCTTGCGGATCTGCTGCTGTT
GGTGCTCTGTGAGGGTGCGATCCGCATGAATTAATGGCGGTGAGAAGAGCGGTAGCGGCGTGGCGTAAGGGGT
AACAATCAGCGCTACGCCGCGCGGGCATCCCTCTTTTTGAAATGCCTGGAGCGATAGCCGTCTGATATTCAGC
GGCAACAGGGTCAGTTCACGCAGTTGCTGCTCAATATGCGTCTCCAGCGCATCATTTTTATCCGCTAACAATA

CGATCTGTCTCTCATGCAAATCGTTATCCTGCATTAGCCAGGCGCCGAAAATCACTGCCACCAGCCCCGTCTC
TTCCTCGGAAAAGTGGATACCATATTCGGCTTCAAACCCGGCGAGCGCCTCACGTGTGGTGCGAACCAGACGC
GGATAAAGACGGTTGAACTCTTCCGGAAGCGTATTATCGATGCCAATCGTGAATAAACTTCGGTTCAGCGCCT
GGGCGAGATGGACATACAGCTGATCGTTAAGCCCTTGTTCGTCGCTGAAGCGTACGTTCCCTTTTTCTCGAAA
GCGCAGCACCAGCCGCGCCACCTCCAGTCGCAGTTGCTGCGCCCGTTGATGCGTGTCGCGAATCGGGTCGGGG
AGGCGAATCATTGAAAACAGTAACGCCATAAACAGCGCTTCATTGAGCGGTGCGGCCTGCATCACGTGGCGCT
GCCAGTGACGACCAATTTCCTGGGCGAGTGGATATTCCGCGCACGACTGCGCCCAAATCTGCTGCACAGGGTT
GAATTCCGGGGTGATGCCGGCATGGTGTTGTAGCAGACAATACTGTAAAAAGAGACGCAGGAACTGCACGTCG
CGATGTTCAAATGGTTTTTGCAACCGGCGGGCGCACAGATTGATGAGCGCGTGCAGATTAATATCGTCATACA
GCGGACGCGCGATACCGCGCTGCTTTAGCGCGTTTTTTAAGGCCGGGGTAAACTGTTGCGTGACGAAGGTCGG
GCATAAACGCAGGCCGCGCCTGAGCCAGTGTAAAAGGCAAAGGCGCTGATCGAGTGCTGTACCTTCGATCCGA
TAGCAACCGTTCTGGCATGTTGTGATGGCAAGGCGATGATAGCGCTGGATCTCCAGGCTTGTCTCAGTGATAT
CCTCACGGGCAGTATCATCATCAACGCCATTAAGCGCGCTGAAGATTTCCACCGTGGCAATTGGCTCCGGCTG
GAAAAGCGTCAGCAATACCTGACAGCGGCGCTGGGGAGCGGAAAGTACAGATGGTGGGGCAATTGTCGGCATC
ATCTGTCAGGTCTCCCGTTAGTTTGTCTGGTAAGGATAGCTAAAGTTTGCGTGTGAAAAATGTGATAAGCGTG
CTTTTAGGCCAGGAATGCTGAGGAGTGTCACAGAATTTTTATCGCGAGGAACAGATGAAGCCAGTGAAACGCA
GCGCCTTAACACTATTTCTTGCCGTTTTATCTTTCGTGGCGGCGGCGCATCCGCACAGCTTCATCCGTCTGCA
AACGCAGGTGGTCAGTGAAAATGAGCAATTTGTGGCGTTAAAAATGCGCTGGACAATGGACGCGCTGACGTCC
GCAGATCTGCTGTATGACGCCGGAAACGCGGCACCGGGGTCAGAAATATGGAAAAAGTTGGCGGCGGAAGTGA
TGGCTAACGTACTTGGGCAGCACTACTTTACAGAAGTGTGGCGCAATGGCGCGAAAGTGAAGTTTAAAAACAG
GCCGACAGAATACGGTATGACGCGTGACGCGCATCAGGCGGTATTGACCTTTACGCTACCGCTGGCTGAACCG
CAGCCGTTAAGCGGACAGACTTATACCTTTTCAACGTTTGATCCCTCTTATTATGTCGATATGCATTATGACC
AGGACAGCGATATCACGATGCCGGAACCGCTGCGCGAAAAGTGTCGGATTCAGGTTTATACTCCTGCGCCCGG
CGAAGAGACGCTACGTTTCGCCCAGTCGCTGGATAAAGAGGACGCGCCGCCGGAGGATATGGATTTAGGTAAA
CAGTTTGCCCAAACGGTGACCTTGCAATGTCAGTAATTTTTTCTCAACGCACTCCGGGGCGACGCTGGCTCTC
GCTGTGGCCATTGGCGTTATTCCTGCTGCTGATGCTGGTTGGCGGTCTGTGGATATGGCAGGCCTGGCCGCAG
GTGATGTTGAAAAGCGCTCTTTGGCAGCGGGACGTAAATCAGCAGTTGAGCGCGCTGCTTAATGCGGTGGCGA
CGCATCCTGAGCGGGCGGGCGGATCGTTGCTTCTGCTGAGTTTTATGTATGGCGTGCTTCATGCGCTGGGGCC
GGGACACGGCAAAGTGGTGATCGCGACATGGCTGGCGACGCATCCTTCGAAGTTGAAATCGAGTATCGTGTTA
ACCCTCGCCGCCGCGTTATTACAGGGGCTCGTGGCTATCGGGTTGGTGGTGGGCGTGCTGACCGTATTGCAAC
TTCCGGCCAGGCAGCTCCATCTGAGCGGCTTCTGGCTGGAAAAGGGGAGCTATGCGTTAGTTGGCGGGCTGGG
CATTTTGCTGTGCTGGCGGGCCATAAAAAGGTTACGCGCGCTGCTACGTAAACCTGTCTTTATCGCTTTTACG
CCGCGCCATGTTCACCATGAAAAATGCGGCTGCGGACATCAGCATTTACCGACGCAGGAACAATTGCATAGCG
GCGATGACTGGCGCGCGCGACTCATGATCGTACTGTCTATGGGGATGCGGCCCTGTTCCGGCGCGATAATGGT
GCTGTTATTCAGTAAAGTGATTGGCGTTTTTAGCTGGGGAATGGCGTCGGTACTGGCGATGGCGGCGGGAACC
TCGCTAACGATTACCTCGCTGGCGCTGCTGGTACACACTTTTCGTGATCTGGCGGTGAAACTCAGTGGAAACA
AAGCGCCGGCGTTATGGCGGCAGGTAGGCTGGTCAACGCTGGCGCTGGCCGGAGGGGGCATACTGCTTGTTGC
GGCGCTGGTCATGTGGTTTAGCGTGCCGCAGCCTGTGGGCGGGCTGCGGCCATGGCGTGGTTGATTAGTGTCC
GGCGGGTTTGAGATGCATCCGCGCGACGGATTCGTCTTCGGCCCAGTAAATGCGTTCGGCGACCATCGCTTCC
GGATTGAGCTGAACGCCGCGCAGTACCCGTTCTTTAAAACGCAGATAGCCGCCTTTGTCGATCAGATGGCCCA
TATGTAGATAGATCGGTTTTCCGCCGAGCATCTCTTTTTCAAATTCATACAGGTTAACGATCACCTGCTTTAT
CACATCTTCAGTCACCCAGTTGAGGAAGAGCTTGCCGACGCGCGGCGTCTTTTTACTGGTGCGTCCGCCCAGA
CGGACTTGCCAGAGCTGATCCGGCTTACGTTGCCAGGCCAGCGTCGGACAGGCCAGCACGCACTCGCCGCAGC
CGATACAGGCGGACTCTTCTTTGACCGCTTTGCCGTTCTTCAGCGCCAGGCAGCCTACCGCGTGGTGACTACA
GGCCTTCACGCAGGCGCCGCAGCCGATACAGCGATCGGCAGTGAAGCGCATTTTCGCCACGCCAATAATTCCC
AGATCGGCCATTGACGCCTTCGCGCAATCATTCGGGCAGCCAACGATGACCGTTTTGAGATGATAAGGGCTGG
GATAGACCAGTTTTTCAAGACGACGGGACAGACCGGTCGTGTCAGTGTTGGCTTTCTGGCAAATGCGGTTCCC
CTGACAGGCGACAATATTTCGCCCGCCAATAGCGAGATAGCCCGCTTTGGTATCCTCAACCTGGACGTCGCAC
AGTTCTATTTCAATCTCGCGAAGAAACGGCTCCAGCGCAGCGTTCACGTTGTCGATATCTTCGTAACGGATAC
CCGGCATCGCCAGCTTCTGGCGGGTAGTGAGGTGAATTTGTCCGTTGCCCCAGGTTTCGGCGATGTCACGCGC
CACCGTTAGCAAATGCGCGGGCAAAATACCGCCGGGAATGCGGACGCTGATCATGGCTTCGCCGCGCACTTTT
GACAGGCGATATTCGTTTTTCGCACGGGCTTTAATGATATCAATATCAATGCTCATGATGTTCTCCTTAATCG
GCAAAACGTTGCGCGACGGCGTAGTTAAATATCGGACCGTCGGTGCAGACATACACTTCGCCCATACGGCAGT
GGCCGCACTTCCCGACGGAGCAGGCCATCCGGCGTTCGTAGTCCACCCAGATTTGCTCCGGCTTGAGCCCTTT

TTGCAGCAGCATTTTTACGGTAAAGGTAATCATTATTGGCGGCCCAACGACGATGGCCTGCATAGTGTCGATA
TCGCTAAGCGTCATGTCGGCAAGCCGATCGGTCACCCGGCCAATCTGGTAACGGTCATCAGCCTCGCCTTCAT
CCAGCGTGAGTACCAGATTATGTTTGCCGCGCCACGTCGCCATTTCTTCTTTGTACAGCACGCAATCGCGATT
TTTATAGCCGAGAATCATATCCAGTTGACCAATTTCCTGGGGATTCTCAACGAAATAGCGCATTAAGCCTTTC
ACCGGCGCGACGCCCGTACCGCCTGCGACAACCAGTAAGGGCTTATGGCGCAGCGTATCGACCGGATAGCCAT
TGCCATAGCAGCCGCGTAGCCACACGTTGTCGCCTTCTTTCAGCGTAAAGAGGGCGCTGGTCACTTTTCCGAC
ATTACGAATCAGCAGATCTATCCAGCCGTCGCCGTAGTCGGAAACGGAGATGGGCGCTTCGCCGACGCGCGGC
AGCGACACCTCAACAAATTGCCCCCAGTGCGCGGGAAAATCGACGGCCACGCGGAAATTCCACTCCAGCGGCG
TGTGGCGAGTAATACTGAGGATGCGGTACGCCGCAGGCAGCAAACTGTGCTGCGGTTTATCATGACAGGAACA
ATGTGACATGCGTTATGCCTCCTCTGCCAGCGCCTGCCGAACGGCAGCGGTCATTTTATTGATAATCAAAGAA
AATTTGATGTATTGCGGGCAGCGATCGTCGCAGCGGCCGCAGCCGACGCACATATGTTCAATGCCGTTGCGCG
CTTTGTAGTCATTGACCTTATGCAGGGCGCGGTAGCGCAGACGCTCGCCGGGTTTTTCGCGAAAACCATGACC
GCCGGCCATGTCGCTAAAGCCCGGCACCATGCAGCTTGCCCACTGGCGACGGCGTTCGCCGCGCTGCGGATTT
TCATCATAGGCGACGTCAAAGACGCTATAGCAGGTACAGGTTGGGCACCCGGTAGTGCAGCGGCCACAACTGA
TGCAGCGGCTGTCGTAGGCGTCCCATAGCGGGTGGCGGGTGAGAATGTCGCGAATTTTTTGCGGATCGCGGCA
AACGCTGTCCGGCGTGACGACGGTTTCCCGGTTTTCGCTGACGAAAGAGGGGGTATAGTCAGCCTCCTGCCCC
AGCCCTTGTATCGCCGCCTCGATAAAGGGATCGCGGATGCTGACAAGCGCGCCCTCATCGCTAAAACGCATGG
CGGCGCTATAACAGTCAGTTTTATTGGTACCCATTGAGACGCAGAAACAATTTTCGAAGCTTTCTTCACACTC
AATAAGGACGAAACGAATATGCTCCCGTAGCAGTTGATAACTGTAATCGGAATTATTCCCATTTGATAAATAC
ATATAATCCAGCCGCGACATGGCGTTAATGTCGCAGGCGCGGGCAAAGATGATTATCGGGGAAATGTCTGTCT
CGGCAATTTGAATGGTGTCTTTATCGAAATAAAAGAGCGTTTCGGTGATAGGGGCGATAATGGTATTCGGCGA
CATGTGCGATTTCTCGTGCCAGATGAGATCGCGCCAGCCGCTAATCCGTTGATAAATAATGTTGTCAGTGTCG
GAAAAACGTCCGCCGCGAAACTCTGCGGACGGGGCAAACACGCGCCATTTTTTGTTTAATCGCTGGATAAGCA
AACTAAACTCGTCAGGCGTAATTTTGATAGCCATCACATACTCCACAGTAATATTCAGGAAAAACAAATGAAA
ATAAATTGTTATAAAAATCAGTTGAATATTACTGTTACGCTAGCGCTAATCCATCGCCTTCGCTAACGGGAAA
AAATGGTTTTTACTATCGGTATAATAAATATATTCTTAATCATAAAATATGCATATATACAGCGTCGCGCTAT
TTAGCGGATAGCGCTGTCTGAATAAATTTAATCATTGCATCACGGTAAAGGTTTGCATAACGTCCGGAAAAAG
CATCAATGTGATCGCCGTCAGGAATGAAGATTTTTTGTTTGGGCTCCCGGGCGAGGGCATACAATTTCTCACT
GTCCTGCCAGGGAATAACATGGTCTGCCGTGCCATGAAGAATCAATACCGGAATGGGGCTGACGCTGGCAATA
TTGCGATCCGCGCTATAGCGGTCATCAAGCAAATAGCCGCTGCCCGGGATCATCTGGTTGGCGATGGAGGAAT
AGGATAAAAACGTTGAGTCAAGGATAATCGCACGTATGCCCGCCTGGTCGGCGTAGCGCATATTTGCGCAGCC
TACGCAGTGGCCTACGGCGGCCAGTACATTATTGCCGCCCAGGCTCTGCCCCAGCAGTACCAGGCGCTCGGGG
TTAACGTCGGCGCGATGCCGCACATAATCTATCGCGCTTTTCGTATCGTCCAGTAGCCCCTCTTGTGATGGGG
TACCCTCCGATTCGCCAAAACCACGGTAATCAAACATAAACAGGTTTACGTTGCGTTCTGGCAGCCAACTGAC
TAACGGCCAGTGCGCCGACATATTTCCGGCGTTGCCATGGACATGAATCACCGTCGCGACGGCGTTTTCCGGC
CTGCCGAAAGCAGTGGGAATAAACCAGCCGTGCAGATGCGTGCCATCTTTGGCGGTAAAGGTAATGGATTCAG
CTTCGGCAGGGGTTGGCCCATAGACTTTATTGTCCGGGTAATAAAAGGCATTGATCGCTACGCGGGGTAGCAG
ATAAATACAGACCACAATGATAAACAGAATAAAAAGGGATTTGACGATGCGTCGGGTATGTTGAAGGGTCATG
TTCGCTTCCTTGCCGCTTTATGCCAATAGCGGAACAGGGAGCGATCGTACAAAAACATGCAGGCAAGTAAAGC
GATGAGGCTGTGTTTAGGAAAAACGCCTGAAAAGGCACGGCGGATGAGTGGCCCCATCCGCCGAAGAGACTTA
GCGCTTCAGCGCATCGCTCAGTTCGTCGCGCATATTCGCCAGCATGGCTTTAACGACGCGCGGATTCCCCGCC
ACGATATTACCGGTCATCATGTAGTTATGACCTCCGGTGAAATCGCTGACGATACCGCCCGCTTCACGAACCA
GCAGTTCGCCCGCGGCGAAATCCCACGGACGAAGGCCAATTTCAAAGAAACCATCGACACGACCTGCAGCCAC
ATAGGCCAGATCCAGCGCGGCGGAACCGGTGCGGCGGAAATCGGCGCATTCGCTGAACAGCTTGCCGATAATA
TTAATGTAGGTGGTCGCGTATTGTTTGGCTTTGAACGGGAAGCCAGTAGCGAGGATAGTGCCGTCCAGATCGC
GCGCGGTACTGCCGCGCAGACGGTAGCCGTTCAGCTGTGCGCCCTGACCGCGGGTGGCGGTGAACAGTTCGTT
ACGCATCGGATCGTAAACGACAGCCACTTCAGTACGGCCTTTAATGCGTACAGCAATGGAAACAGAGAAGTGT
GGCAGACGTTTGATGAAGTTGGTGGTGCCATCCAGCGGATCGATAACCCATTGAACATCCTGATCTGTGCCAA
CGTGCTCACCGCTTTCTTCGGTGATAATAGTGTGTTGCGGGTAAGATTTGCGAATGGTGTCGATAATCACCGC
TTCAGCGGCCTTATCTACATTGGTCACAAAATCGTTACTGCCTTTTTGGCTCGCTTCTACAGCGTCCGGAGTT
TCATAGTTTTTGGCAATTACATTACCCGCCTTGCGCGCTGCGCGCACGGCGATGGTCAGCATCGGATGCATCG
GTCTCTCTCACTGGATGTTAAAGAACGGGAAAACGGCGCAGAGTATAGCAGCGAGATCGGAATCTGTCTTGGT
TTTATGGTAAGATGTCAGAATAATCTTTAGCTGAAGAAAGACAATGCTGCAAAACATTCGAATTGTGCTGGTG
GAAACCTCCCACACCGGCAATATGGGCTCCGTGGCTCGCGCTATGAAAACCATGGGCTTAACTAACCTGTGGC

TGGTAAACCCGTTGGTGAAACCGGACTCCCAGGCCATCGCCCTGGCGGCAGGCGCCAGCGATGTGATCGGTAA
TGCGCAGATCGTCGATACGCTGGATGAAGCGTTGGCTGGCTGTAGCCTGGTGGTCGGCACCAGCGCCCGTTCC
CGCACTTTGCCGTGGCCGATGCTTGATCCGCGCGAGTGCGGCTTAAAAAGCGTGGCGGAAGCGGCAAATACGC
CGGTTGCGCTGGTTTTTGGTCGTGAGCGTGTCGGGCTGACTAACGATGAGTTGCAAAAATGCCATTATCACGT
CGCTATTGCCGCTAACCCGGAGTACAGCTCGTTGAATCTGGCGATGGCGGTACAGGTCATCGCGTATGAAGTC
CGCATGGCCTGGCTGGCGACGCAGGAAAACGGCGACGCAGCAGATCATGAAGAGACGCCGTATCCGCTGGTGG
ACGATCTTGAACGTTTCTACGGCCATCTGGAGCAGACGTTGCTCTCCACTGGCTTTATTCGCGAAAACCATCC
GGGGCAGGTGATGAATAAATTGCGCCGTCTGTTTACCCGCGCCCGACCGGAAAGCCAGGAGTTGAATATTCTG
CGTGGGATTCTGGCGTCTATTGAGCAGCAGAATAAAGGCAAGTAACCGCATCAATATCGCACGGAACGCTAAA
TACCTGACTAATTTACTCAAGTAAATAGTTGACCAAATTACTCGGGAATGTCAGACTTGTCCCTGCTATGCAA
TACCCCCATTTTACAATAAAAAACCCCGGGCAGGGGCGAGTTTGAGGTTAAGTAAGACATGAGACTGACATCT
AAAGGGCGTTATGCCGTGACCGCAATGTTAGACGTTGCGCTCAACTCCGAAGCGGGCCCGGTACCGTTGGCTG
ATATTTCTGAACGTCAGGGGATTTCCCTTTCTTATCTGGAACAGCTATTTTCCCGTTTACGTAAAAACGGTCT
GGTTTCCAGCGTACGCGGGCCGGGCGGCGGTTATCTGCTGGGTAAAGATGCTGGCAGCATTGCGGTCGGCGAA
GTGATCAGCGCGGTTGATGAATCCGTTGACGCGACCCGTTGCCAGGGCAAAGGCGGCTGCCAGGGCGGCGATA
AATGCCTGACCCATGCGCTGTGGCGTGATTTAAGCGATCGCCTGACCGGTTTTCTCAACAATATTACCTTAGG
CGAGCTGGTGAATAACCAGGAAGTTCTGGATGTGTCTGGTCGCCAGCACACGCATGACGCGCCGCGCGCCAGT
GGTCGTGCGCAGGACGCTATCGACGTTAAATTACGCGCTTAATTAAAATATTACGCCATCTCTTCTGGCGGCC
TCATGCGGTGAATGCCGCGGTCGTCTGAAGGATGAAGCGTAAAAAAGAATTCAGAATCAGGCCGGGACGCGGA
CGTCCCGTCAACACGGTCGTACATCCAGCCGGTTGCCTGATTCCTTGCATTGAGTGATATACGGAGTTTAAGA
GCAATGAAATTACCGATTTATCTCGACTACTCCGCAACCACGCCGGTGGACCCGCGTGTTGCCGAGAAAATGA
TGCAGTTTCTGACCCTGGACGGAACCTTTGGGAACCCGGCGTCTCGTTCACACCGTTTCGGCTGGCAGGCTGA
AGAAGCCGTCGATATCGCCCGCAACCAGATTGCTGAACTGGTCGGCGCCGACCCGCGTGAAATCGTCTTTACC
TCAGGGGCGACGGAGTCTGATAACCTGGCGATTAAAGGCGCTGCCAACTTTTATCAGAAAAAAGGCAAGCACA
TCATCACCAGCAAGACCGAGCACAAAGCGGTGCTGGACACCTGCCGTCAGCTTGAGCGCGAGGGGTTTGAAGT
AACCTACCTGGCGCCGCAGCGCAACGGCATCATCGATCTCAACGAGCTCGAAGCAGCGATGCGTGACGACACC
ATTCTGGTTTCCATCATGCACGTGAACAACGAAATCGGCGTGGTGCAGGATATCGCGACCATCGGCGAAATGT
GCCGCGCGCGCGGTATCATCTACCACGTTGACGCCACCCAGAGCGTGGGCAAACTGCCTATCGACCTGAGCCA
ACTGAAAGTGGATCTGATGTCCTTCTCCGGTCATAAAATTTATGGTCCGAAAGGCATTGGCGCGCTGTATGTG
CGTCGTAAGCCGCGTATTCGCATTGAAGCGCAGATGCATGGCGGCGGGCACGAACGCGGTATGCGCTCTGGTA
CTCTGCCTGTCCACCAGATCGTCGGCATGGGCGAAGCTTACCGTATCGCGAAAGAAGAGATGGAGACCGAAAT
GGCCCGTCTGCGCGGTCTGCGTAACCGTCTGTGGAACGGCATCAAAGATATTGAAGAAGTTTACCTGAACGGC
GACCTTGAGCAGGGCGCGCCAAACATTCTCAACGTGAGCTTTAACTACGTTGAAGGCGAGTCGCTGATCATGG
CGCTGAAAGACCTGGCGGTCTCTTCCGGTTCCGCCTGCACCTCCGCCAGTCTGGAACCGTCTTACGTGCTGCG
CGCGTTGGGCATGAATGACGAGCTGGCGCATAGCTCTATCCGTTTCTCTTTAGGTCGTTTTACCACTGAAGAA
GAGATCGACTACACCATTGATCTGGTTCGTAAATCCATTGGCCGTCTGCGTGACCTTTCTCCACTGTGGGAAA
TGTACAAGCAGGGTGTGGATCTGAACAGCATCGAATGGGCACATCATTAATCGGTATCGGAATCAGGAGAATT
TATAATGGCTTACAGCGAAAAAGTAATCGATCACTATGAGAACCCGCGTAACGTAGGGTCGTTTGACAACAAC
GACGATAACGTGGGAAGCGGCATGGTTGGCGCGCCGGCCTGCGGCGACGTCATGAAGTTGCAGATTAAAGTTA
ACGATGAAGGTATCATTGAAGACGCGCGCTTCAAGACTTACGGCTGCGGTTCCGCTATCGCCTCCAGTTCTCT
GGTAACGGAGTGGGTGAAAGGGAAATCCCTGGACGAAGCGCAGGCGATTAAAAACACCGACATTGCAGACGAA
CTGGAACTGCCGCCGGTGAAAATTCACTGCTCTATTCTGGCGGAAGACGCGATTAAAGCCGCTATTGCGGATT
ACAAAAGCAAACGCGAAGCGAAATAAAATAGAACGTTAACATTGAGGTTTTGTTATGTCGATTACACTTAGCG
ACAGTGCAGCAGCGCGAGTTAATACCTTCCTGGCCAACCGTGGTAAAGGGTTTGGTCTGCGTCTGGGCGTGAG
AACCTCCGGCTGCTCAGGTATGGCTTATGTACTGGAATTTGTTGACGAGCCAACGGCGGAAGACACCGTGTTT
GAAGACAAAGGCGTTAAGGTAGTGGTTGACGGCAAGAGCCTGCAATTTCTGGACGGTACACAGCTGGACTTCG
TAAAAGAAGGTCTGAACGAAGGGTTTAAATTCTCCAACCCGAATGTGAAAGATGAGTGTGGTTGCGGCGAAAG
CTTCCACGTGTAACCGTGTTCCGCTGTTATACCGAGCATTCGTGCTGTAGAAAAGCGGCAAGCGAGTGAATCT
CCAGGCGCTTACTTGAGTAAATGACTGGGGGAACCCGCGCAGACAACGCATCTGCGGCGCGAAGTAAGAAGGT
ATAACCCCACCGTGGTCGCCTGCGTGTGGGGTTTGTTCTGACAGGCTATCCCTGAGAATGTTATGGATTACTT
CACCCTCTTTGGCTTACCAGCCCGTTATCAGATTGACACCCAGGCGCTGAGCCTTCGTTTTCAGGATCTGCAA
CGCCAGTATCACCCTGACAAATTTGCCAACGGCACTCAGGCGCAGCAGCTTGCCGCCGTCCAGCAATCCGCCA
CGATCAACCAGGCCTGGCAAACGCTGCGTCACCCTTTGACGCGCGCGGAGTATCTGCTCTCCTTACACGGGTT
CGATCTGGCCAGCGAGCAACATACGGTACGCGACACCGCGTTTCTGATGGAACAACTCACGCTTCGCGAAGAG

CTGGACGATATCGAACAATCAAAAGACGACGTGCGGCTGGAAAGCTTCATCAAACGCGTACAAAAGATGTTTG
ATGCTCGCCTTCAGCAAATGGTGGAACAGTTGGACAACGCCGCGTGGGACGCGGCGGCGGATACGGTGCGCAA
ACTGCGGTTTCTCGATAAACTGCGAAGCAGCGCAGAACAACTCGAAGAAAAGCTGCTCGATTTTTAACTGGAA
TCTAACATGGCCTTATTACAAATTAGTGAGCCTGGTCTGAGCGCCGCGCCGCATCAGCGTCGTCTGGCGGCGG
GCATCGACTTAGGCACTACCAACTCTCTGGTTGCGACGGTTCGCAGCGGCCAGGCGGAAACTCTGCCTGACCA
CGAAGGGCGCCACCTGTTGCCTTCAGTGGTTCACTATCAGCAGCAGGGCCATACGGTCGGTTACGCTGCGCGC
GACAATGCCGCGCAGGATACGGCCAACACTATCAGCTCTGTGAAACGCATGATGGGACGCTCGCTGGCGGATA
TCCAGGCGCGCTATCCGCATCTGCCTTACCGGTTTAAGGCAAGCGTTAATGGACTGCCGATGATTGACACGGC
GGCTGGATTGCTCAATCCGGTACGCGTATCGGCTGATATTCTCAAAGCTCTGGCCGCGCGGGCTAGCGAATCG
CTCTCCGGCGAGCTGGATGGTGTGGTGATTACCGTTCCCGCCTATTTCGATGATGCCCAACGTCAGGGAACGA
AAGACGCCGCGCGGCTGGCAGGCCTGCATGTACTGCGCCTGCTCAACGAACCGACGGCGGCGGCGATTGCCTA
CGGTCTGGACTCAGGCAAAGAAGGCGTTATTGCTGTTTACGATCTCGGCGGCGGTACCTTCGATATCTCTATC
TTACGCCTGAGCCGCGGGGTGTTTGAAGTGCTGGCGACGGGCGGTGATTCTGCGCTCGGCGGCGACGACTTTG
ACCATCTGCTGGCGGACTATATTCGCGAGCAGGCGGGAATTGCCGATCGTAGCGACAATCGCGTTCAGCGCGA
ATTGCTGGATGCCGCGATTGCCGCGAAAATCGCCTTAAGCGATGCCGATACTGTTCGAGTTAACGTTGCGGGC
TGGCAGGGGGAGATCACTCGCGAGCAGTTTAACGATCTCATTTCCGCGCTGGTTAAGCGCACGCTATTGGCCT
GCCGTCGCGCATTGAAAGATGCCGGCGTTGATCCCCAGGACGTTCTGGAAGTGGTCATGGTCGGCGGTTCGAC
CCGTGTACCGCTGGTGCGTGAGCGGGTGGGCGAATTTTTTGGCCGCACGCCGTTAACCGCTATTGACCCGGAT
AAAGTGGTCGCTATCGGCGCAGCGATTCAGGCCGATATTCTGGTCGGCAACAAGCCGGACAGCGAAATGTTGC
TGTTGGACGTGATTCCACTCTCTCTTGGGCTGGAAACGATGGGCGGCCTGGTGGAAAAAGTCATTCCGCGCAA
CACCACTATCCCGGTGGCGCGCGCGCAGGATTTCACCACCTTCAAAGACGGCCAGACCGCGATGTCTATCCAT
GTGATGCAGGGCGAACGCGAACTGGTGCAGGACTGCCGCTCGCTGGCGCGCTTTGCGCTGCGCGGGATTCCGC
CGCTGCCGGCGGGCGGGGCGCATATTCGCGTGACCTTCCAGGTAGACGCCGATGGCCTGTTGAGCGTCACCGC
AATGGAAAAATCCACCGGCGTCGAGGCCTCCATTCAGGTCAAACCTTCCTATGGCCTGACCGATGGCGAAATC
GCCTCGATGATCAAGGATTCCATGAGTTTTGCCGAGCAGGACGTGAAAGCGCGTATGCTGGCGGAGCAAAAAG
TCGAAGCCGCGCGCGTGCTGGAAAGTTTGACCGGCGCGCTAACTGCCGACGCCGCGCTGTTAAGCGCCGCAGA
ACGTCAATGCATTGATGATGCCGCCGCACATTTAAGCGCGGTCGCACAAGGCGATGATGTTGACGCCATAGAA
CAAGCCATTAAAAACGTAGATAAACAAACCCAGGAATTTGCCGCTCGCCGCATGGACCAGTCGGTTCGTCGCG
CGTTGAAAGGCCATTCCGTAGACGAGGTTTAACATGCCGAAGATTGTTATTCTGCCTCATCAGGATCTTTGTC
CGGATGGCGCAGTTCTGGAAGCTGAGACCGGTGAAACCATTCTTGACGTTGCGCTGCGCAACGGCATCGAGAT
TGAACACGCCTGTGAGAAATCGTGCGCCTGCACCACCTGTCACTGTATTGTACGTGAAGGCTTCGACTCCTTG
CCGGAAAGTTCGGAAGAAGAAGACGATATGCTGGATAAAGCGTGGGGGCTGGAGCCGGAAAGTCGACTGAGCT
GCCAGGCCCGCGTTACCGACGACGATCTGGTTATCGAAATTCCACGTTACACAATCAACCATGCGCGTGAGCA
TTAACAGAGGGTAGTATGGGACTCAAGTGGACCGATAGCCGCGAGATCGGCGAAGCGTTATACGACGCATATC
CTGATGTCGATCCTAAAACCGTTCGTTTCACCGATCTGCATCAGTGGATCTGCGAACTGGACGATTTTGACGA
CGACCCTAACGCATCCAATGAAAAAATTCTGGAGGCGATTCTGCTAGTCTGGTTGGATGAAGCAGAGTAATTT
TTGCGTCATGATCCTCGCCGCAGGTGCGTTGGCTACGTTCGCTCACCCCAGTCACATAGTTTGCTATGCTCCC
GGGGATTCGCTCACTTGCCGCCTTCCTGCAACGCGAATCATTTAGCAAAAGATCTAACGGGCTGCCCTACGGC
GGCCCGTTTGCTAATAAGGAAAATAACAATGACAGAAGCCATGAAAATTACGCTTTCCACGCAGCCTGCCGAT
GCGCGCTGGGGCGATAAAGCCACTTACAGTATTAATAACGACGGTATTACCTTGCATCTTAACGGTAAAGACG
ATCTGGGGTTGATCCAGCGTGCGGCGCGTAAAATCGACGGCCTGGGAATAAAACAGGTCGCGCTGACAGGCGA
AGGATGGGATATCGAGCGTTGTTGGGCATTCTGGGCGGGTTATAAAGGACCAAAAGGCGTCCGTACCGTAATG
TGGCCGGATTTAGACGATGCCCAACGTCAGGAGCTGGATAACCGCCTGACCATTATTGACTGGGTGCGCGACA
CCATTAATGCTCCGGCGGAAGAGCTTGGACCGGAGCAACTGGCGCAGCGCGCGGTGGATCTGCTGTGCAGCGT
CGCGTGCGATAGCGTCACTTACCGTATCACCAAAGGCGAAGATCTGCGCGAGCAGAACTATATGGGGTTGCAT
ACCGTAGGCCGTGGTTCTGAGCGTCCGCCGGTGCTGCTGGCGCTGGATTACAACCCGACGGGAGACAAAGATG
CGCCGGTTTATGCCTGCCTGGTCGGTAAAGGTATCACCTTTGACTCCGGCGGCTACAGTATCAAACAAAGCGC
TTTTATGGACTCAATGAAATCCGATATGGGCGGCGCGGCGACAGTAACGGGCGCGCTGGCGTTCGCCATTACC
CGTGGGTTGAATAAACGCGTAAAACTGTTCCTGTGCTGCGCCGATAATCTGATCAGCGGCAATGCGTTTAAAC
TGGGCGATATTATTCGCTACCGTAATGGTAAGAATGTCGAAGTCATGAATACCGATGCGGAAGGGCGACTGGT
ACTGGCGGATGGCCTGATTGACGCCAGCGCGCAACACCCGGAGCTAATCATTGATATGGCGACGTTAACCGGC
GCGGCGAAAACGGCGCTGGGTAACGACTATCATGCGCTATTCAGTTTTGACGATACGCTGGCGGGGCGCTTGC
TGACCAGCGCGGCGCAAGAGAATGAACCGTTCTGGCGTCTGCCGCTGGCGGAGTTCCACCGTAACCAGCTGCC
GTCTAACTTTGCGGAACTGAACAACACCGGCAGCGCGGCGTATCCCGCTGGGGCAAGCACCGCCGCGGGTTTT

CTGTCGCACTTTGTTGAAAATTACCGCGAAGGCTGGCTGCACATTGACTGTTCCGCCACTTATCGCAAAGCGC
CGGTTGAGCAGTGGGCTGCCGGGGCAACCGGTCTGGGTGTGCGCACCATCGCGAATTTGCTGACCGCCTGAGC
GGTGTTTTTGTAGGCCGGGTAAGCGAAGCGCCACCCGGCATTTTGCCTGATGGCGACGCCGTGCGTCTTATCA
GGCCTACAACCCATATACATAATTTGTGATTCCTATGTCCGAAACTAAAAATGAATTAGAAATCTTGCTGGAG
AAAGCGGCGACTGAGCCCGCGCATCGTCCGGCATTTTTCCGTACCTTACTGGAGTCCACCGTCTGGGTGCCGG
GAAGCGCTGCCGAAGGCGAGGCGATAGTGGAAGACAGCGCTCTGGATTTGCAGCACTGGGAAAAAGAAGACGG
CACGACCGTCATTCCCTTCTTTACTTCTCTGGAGGCGCTGCAGCAGGCCGTGGAAGACGAACAGGCATTTGTG
GTGATGCCGGCGCGCACGCTGTTTGAAATGACGCTTGGCGAAACGCTATTCCTCAACGCTAAATTGCCGACCG
GCAAAGAGTTTATGCCGCGTGAAATCAGCCTGCTTCTCGCTGAGGAAGGCAGTCCGTTAAGTACGCAGGAAGT
GCTGGAAGGCGGCGAGTCGCTTATTTTATCCGAAGTGGCCGAGCCGCCGTCGCAGATGATTGATTCCCTCACT
ACGCTGTTTAAAACCATCAAACCGGTGAAACGCGCATTTCTCTGCGCGATTAAAGAACATGCGGACGCCCAAC
CCAATCTGCTGATTGGCATTGAGGCTGACGGCGAAATTGAAGAGATCATTCACGCAGCGGGCAATGTGGCGAC
CGACACGTTACCGGGTGACGAACCGATCGATATTTGTCAGGTGAGGAAGGGCGAGCAGGGTATCAGCCACTTT
ATCACTGAGCATATCGCGCCGTTCTACGAGCGCCGCTGGGGCGGGTTCCTGCGCGATTTTAAACAGAACCGCA
TCATCTGAAGAAAAGAAAGCCGCCCAACAGGGCGGCCTCCTGGCTTACCTGCTCTGGACGTTGAGCGTTACGG
GAACAGAGCTACCTGGGTAGCTTCTGTCTGCGTGAAGACACAACGTGCCAGAAACAAGAAGCACGACACAAAA
GCATCGTACCTTCATGTTGCGGTCCTTGCGAAGACCGCATCACGGCAGCACCGCTGGCGATAATGCCGTGATA
GCAGGCACTACGGTGTGCCTGCGCTTACGGCCATGGGTTATCCTCTCCTGAAGCCAGCGCCCGTTTGCGAGAC
GGCGGAAAGGGCCGGGAAAGATAATACCTCAAATGTTGAATAGTTTCCTGATAAATTATATTGACATTTGCTC
CCTTAAAAGGGAGTATTAATCCCCTACGGTGTGCCTGCGTTGCTATGGCAACAAAGCCTGCTGGAAAGGCCGC
GAACCGGACCAGCACAAAAAAACCGCTCAATTGAGCGGTTTTTTTGTGACCTGCTTAAACCGAATTTATGTAG
ATTCGTTTTAAATCAGCCTGAACGGTAGGCCGGATAAGCGTGAGCGCCACCCGACAGCATGTCGATTAATCGA
CGAGCGCGTCCAGTTTTGCCCAAAACGCCTCATGATAATCCTCCAGGTCAAGTAAAGCGGCATCGCGTTTTTC
ATTCATTTTTTGCTGTGCCTTCCATTGCGAAAGATGTAAATCCTCTTCGGTAAAGCCAATGTCTTTAGCGCCC
GAAATAATATTATAGAGATATTCCACCAGAGAAGACTCCGCCAGCCAGAATTCATTTCGTTCCATGCGGGCAA
TAGGGTATTCGCCGTTGGCATCCCGTTCTGTGATATAGATAAAAATGGCCTGATCGCCGACATGATCGATACG
AATTAATGCCGGAAGACGGGGATCGTTACTCCAGACAGGCATCAGATCTTCAATATCAATACCTGAGCTATCC
ATGCCAGCGCCGTAATACGCCTCTTCATCAGGGGAGTAAGAATGAATTACTCCCTCTTTATCAATAGATCTTT
GTAATGTTTCAATCACAGGCATATTATAATTGCTAAGTAGATCGCGGTCGTAGGCCAGTAGCGCCTTCAGGCT
TTCAGGTAACTCAGTGGTTTCCGTATTTTGCGGATCGCGATACTTACGCACTCGCAGACGACTTATTTCCTCG
GCAGGCATAGGATTAGGCACCTTATTGCGATTAACCTTTTTAACTAACTCAATTAAATTAGCGCGTGAAATAT
CCATTTTCCATCCTTAAAAATTATATTTACAGGAGTTATTTCCCGGTGAAAACCAAAACAGCGGTGATGATAC
TCCTTATACCCGGCGTAAAGTATTTACCGATAAAAAATAGGATTTTATTAAGAGAAAATAATGCCCGTTTGCA
GGACAGTGGAAAAGAGCTGAATAAAAACAATGTCTGTAGATCGTTTTACATCAGCCTGAACGGTAGGCCGGAT
AAGCGTGAGCGCCATCCGGCATGGCAGATATTAAGCGGGTTCAACCGGTAAATCGGTACGCGCGCCCCATTCG
CTCCACGCGCCATCGTAAAGCGTGACGTCAGGCACATCCAGCGTGGCGAGCGCCAGCACCACGACCGCGGCCG
TCACACCGGAACCGCAGCTGGCGATAATCGGTCTGTCGAAACTCACGCCGTGGCTGAAAAAAACCTCATTCAG
TTCGTCAGTGGTCTTTAATTCGCCCTCATACACCAGTTCCGTCCACGGGACGTTTAGCGCGCCAGGAATGTGT
CCGCGCCTCAGTCCCGGACGCGGTTCATCAGCCTGAGCGTTAAAACGCGCCGCCGGACGCGCGTCAACGATTT
GCGCGGTTTTTTCATGGCTTGCCAGCAATACGTCGGTGAGACGCACCACGGCTTGCGGCGCGAATTTCGCCTC
AAACTCGCCTCCTTCATGCGCTTCTTCGCCTTCGCGCAATAGCCATTCGTCGCGCTGCCAGCCCGCCAGTCCG
CCCGCCAGAATGGACACTTTCTCGGCACCGAACGTGCGCAGCATCCACCATGCCCGCGGAGCGGAGAACAGAT
TGCCTTCATCGTATATGACCAGGTGTTTATCCTGGCGAACACCCAGTTCACGCATGGCCACCGCAAACGCTTC
CGGGCGCGGCATCATATGCGGCAGCGGCGATGCGCGATCGGAAAGCGCTTCAATATCAAAAAACAGCGCGCCG
GGAATATGTCCGGCGCGATATTCGCCCGCCATATCACGATGTTCCTGTCCTGGCGGCGCCATACGGGCGTCAA
GAATCTGTATTTCCGGATCATCAATATGTTCGGCAAGCCAGTCGGCGGCGACAAAAAAGGCGGTGGTCATGGG
CATCTCCATTGTTACCTGATTATGGTCAAATTGTCGGACGTTTCCCGAAAAGGGACAAGTAGAAGAGGCTGTT
TTGGCTGGCGAAACCACATTTCTGCGTAAAATTTTCGCATCATCACAGTAATACGGACTTACACAATAAAATT
TCTCGCGCATAATATCTCATCAACCGTTGCTGACAGGCCGTTAAGGCCAGGGATGAAAGATGAAACATTTACG
CGTGGTGGCCTGCATGATCATGCTGGCGCTGGCGGGATGCGATAACAACGATAAAACCGCCCCGACGACTAAA
AGCGAAGCGCCAGCCGTAGCGCAGCCCTCGCCCGCGCAAGACCCGGCACAGCTACAAAAGCTGGCGCAGCAAA
GCCAGGGCAAAGCGCTCACGCTATTAGACGCCTCCGAAGCGCAGCTTGACGGCGCGGCGACGCTGGTGCTGAC
GTTTTCAATTCCTTTAGATCCTGAACAGGATTTCTCCCGTGTGGTTCACGTGGTTGATAAGAAAAGCGGCAGC
GTTGACGGCGCATGGGAGCTGGCGCCAAATTTAAAAGAGCTAAGGTTACGTCATCTGGAACCTGAACGCGTGC

TGGTGGTCACGGTTGATCCCGCCGTTAAGGCGCTGAATAACGCCACCTTCGGCAAGTCTTATGAAAAAACGAT
TACCACGCGTGATGTCCAGCCCAGCGTCGGCTTTGCCAGCCGGGGATCGCTGCTACCGGGGAAAATAGCGGAA
GGACTGCCGGTCATGGCGCTTAACGTCAACCACGTTGATGTGAACTTTTTCCGCGTTAAGCCCGGATCGCTGG
CGTCATTTGTCAGCCAGTGGGAGTACCGTAGTTCTCTCTCTAACTGGGAGTCCGACCATCTGCTGAAAATGGC
GGATCTGGTTTATACCGGTCGTTTTGATCTTAATCCGGCGCGCAATACGCGTGAGAAACTGCTGCTGCCATTA
AGCGATATTAAGCCGCTGCAACAGGCGGGCGTATATGTGGCGGTAATGAATCAGGCTGGACACTACAACTATA
GTAATGCCGCTACGTTGTTTACCCTTAGCGATATCGGCGTGTCCGCGCACCGTTACCATAATCGGCTGGATAT
CTTTACGCAAAGCCTGGAAAACGGCGCGGCGCAGTCGGGAATTGAGATCGTTCTTCTGAATGATAAAGGTCAG
ACGCTGGCGCAGGCGACGAGCGACGCGCAGGGACATGTGCAACTGGAGGCTGATAAAGCGGCGGCGCTATTAC
TGGCGCGTAAAGAGGAGCAGACCACGCTGCTCGATCTCACGCTTCCGGCTCTGGATCTGTCGGAATTTAATGT
CGCTGGCGCGCCCGGCTACAGCAAGCAGTTCTTTATGTTCGGCCCGCGCGATCTCTACCGCCCGGGCGAAACG
GTTATCCTCAACGGATTACTGCGCGATAGCGACGGTAAAACGCTGCCCGATCAACCCGTTAAGCTGGAAGTGG
TAAAACCAGACGGACAGGTGATGCGTACCGTCGTCAGCCAGGCGGAAAACGGGCTATACCGTTTGAATTATCC
GCTGGATATCAACGCGCCGACCGGCTTGTGGCATGTCCGCGCCAACACCGGCGATAATTTGCTGCGGAGTTGG
GATTTCCACGTGGAAGACTTTATGCCGGAGCGGATGGCGCTCAACCTGACGGCGCAAAAAACGCCGCTGGCGC
CTGCGGATGAGGTGAAATTCTCCGTTGTCGGCTATTACTTGTATGGCGCGCCTGCTAACGGCAATACCCTGCA
AGGGCAACTTTTCCTGCGTCCGCTGCGCGACGCTGTCGCGGCGTTGCCTGGCTTCCAGTTCGGCAATATTGCT
GAAGAGAATCTTTCGCGCAGCCTGGACGAAGTTCAACTGACGTTGGATAAAGGCGGACGTGGTGAAGTGAGTG
CTGCCAGCCAGTGGCAAGAAGCGCATTCGCCGCTGCAGGTAATTCTACAGGCCAGCCTGCTGGAGTCTGGCGG
TCGTCCGGTCACTCGTCGCGTAGAGCAGGCGATTTGGCCTGCCGATACGTTACCGGGAATTCGTCCACAGTTC
GCCGCCAAAGCGGTATACGACTACCGTACGGATACCACCGTTAATCAACCGATTGTCGACGAAGACAGCAACG
CCGCATTCGATATTGTTTACGCCAACGCGCAGGGCGAGAAAAAAGCGGTGTCCGGTTTACAGGTGCGGCTCAT
CCGCGAGCGTCGCGACTATTACTGGAACTGGTCGGAAAGCGAAGGCTGGCAGTCGCAGTTTGATCAAAAAGAT
CTGGTGGAGGGCGAGCAGACGCTGGATCTGAACGCGGATGAAACCGGAAAAGTCAGCTTCCCGGTGGAATGGG
GCGCGTACCGTCTGGAGGTCAAAGCGCCGAATGAGACGGTCAGCAGCGTTCGTTTCTGGGCCGGCTATAGCTG
GCAGGATAACAGCGACGGTAGCGGCGCGGCGCGTCCGGATCGCGTCACCCTCAAACTGGATAAAGCGAGTTAT
CGTCCAGGCGATACCATGAAATTGCATATCGCCGCGCCGGTCGCTGGTAAAGGTTATGCGATGGTGGAGTCCA
GCGACGGTCCGCTGTGGTGGCAGGCGATCGATGTGCCGGCACAAGGTCTGGAGCTCACGATTCCGGTGGATAA
AACCTGGAATCGCCACGATCTCTATCTCAGTACGCTGGTGGTGCGTCCCGGCGATAAATCTCGTTCCGCGACG
CCAAAACGCGCCGTGGGGTTACTACACCTACCGCTGGGGGATGAAAACCGCCGCCTCGATCTGGCGCTGGAAA
GCCCGGCTAAAATGCGCCCGAATCAGCCGCTCACCGTCAGGGTGAAAGCCAGCGTTAAACACGGCGAAATGCC
AAAACAGATCAACGTGCTGGTCTCCGCGGTCGATAGCGGCGTATTGAATATCACCGATTACGCGACGCCGGAC
CCGTGGCAGGCGTTCTTCGGTCAAAAACGCTACGGTGCGGATATCTACGATATTTACGGCCAGGTCATTGAAG
GGCAGGGGCGGCTGGCGGCGTTGCGTTTTGGCGGCGATGGCGACGACCTTAAGCGCGGCGGAAAACCGCCGGT
AAACCATGTCAATATCATCGCGCAGCAGGCGCAGCCGATCACGCTCAATGAGCAGGGCGAAGGGGTCGTAACG
CTGCCGATTGGCGACTTTAACGGCGAACTGCGGGTTATGGCGCAGGCATGGACAGCGGACGATTTTGGTCGCG
GCGAAAGCAAAGTCGTTGTCGCCGCGCCAGTGATTGCCGAGCTGAATATGCCGCGTTTTCTGGCGGGAGGGGA
TGTTTCGCGACTGGTGCTGGACGTCACCAATCTGACCGACCGCCCGCAGACGCTGAATATTGCGCTCGCCGCC
AGTGGGTTACTGGAACTGCTTAGTCAACAGCCGCAACCGGTCAACCTGGCGCCGGGCGTGCGCACCACCTTAT
TCGTTCCGGTACGTGCGCTGGAAGGTTTTGGCGAAGGCGAAATCCAGGCGACCATTAGCGGTCTGAATCTGCC
GGGAGAAACCCTCGGCGCGCAGCATAAGCAGTGGCAAATCGGCGTGCGTCCGGCCTGGCCTGCCCAAACGGTA
AATAGCGGCATTGCGCTGGCGCCGGGAGAGAGCTGGCATGTACCAGAGCAGCATCTGGCAAACGTCTCGCCAG
CCACGTTACAGGGACAACTGCTGTTAAGCGGAAAACCGCCGCTCAATCTGGCGCGCTACATTCGCGAGCTGAA
AGCATATCCGTACGGGTGCCTGGAACAAACCACCAGCGGGTTATTCCCGGCGCTGTATACCAATGCCGCTCAA
TTGCAGTCGCTCGGTATTACCGGCGATAGCGATGAAAAACGGCGCGCCGCGGTGGATATCGGCATCTCCCGCA
TACTACAGATGCAGCGTGATAACGGCGGTTTTGCGCTATGGGATGAAAATGGGGCGGAAGAGCCCTGGCTAAC
GGCCTACGCGATGGATTTCCTCATTCGCGCGGGCGAGCAGGGATATAGCGTCCCGCCGGAGGCCATTAACCGG
GGCAATGAGCGACTGCTGCGCTATCTGCAGGATCCCGGTACGATGCTGATTCGTTATAGCGATAATACCCAGG
CCAGTACTTTTGCCGCTCAGGCTTACGCCGCGCTGGTACTGGCGCGTCAGCAGAAAGCGCCGCTCGGCGCGCT
GCGCGAAATCTGGGAGCGCCGTAGTCAGGCGGCTTCAGGACTGCCGCTGATGCAATTGGGCATCGCGTTAAAC
ACGATGGGTGATGCCAGACGCGGCGAAGAGGCCATTACGCTGGCTCTGAATACGCCGCGTCAGGACGAACGGC
AATGGATAGCGGATTACGGCAGTTCTCTGCGCGATAACGCTCTGATGTTGTCGTTACTGGAAGAGAACAACCT
CAGACCGGACGCGCAAAACGCGCTATTAAGCTCGCTTTCTGAGCAGGCCTTCGGTCAGCGCTGGCTCTCTACC
CAGGAGAACAATGCCTTGTTCCTCGCCGCGCATTCGCGACAGGCCAGCGCGGGCGCCTGGCAGGCGCAGACCT

CGTTAGAGGCGCAGCCGCTGTCGGGCGACAAGGCGCTGACCCGTAATCTGGATGCTGACCAGCTGGCCGCCCT
TGAGGTGACGAACACCGGTAGCCAGCCGCTATGGCTGCGTCTGGATAGCAGCGGCTATCCCTCATCTGCGCCT
GAGCCTGCCAGCAACGTTTTGCAGATTGAACGACAAATACTGGGGACCGATGGTCAGCGTAAATCGCTGTCCT
CGTTGCGTAGCGGCGAACTGGTGCTGGTCTGGTTAACGGTAGTGGCCGATCGCAATGTGCCGGATGCGCTGGT
GGTGGACCTGCTCCCGGCCGGGCTGGAGCTGGAAAACCAGAATCTGGCTGACAGCAGCGCCAGCCTGCCGGAG
AGCGGTAGCGAAGTGCAAAATCTGCTTAATCAGATGCAGCAGGCGGATATTCAGTATATGGAATTCCGCGACG
ATCGGTTTGTGGCTGCCGTCGTTGTCAATGAGGGCCAGCCCGTGACGCTGGTCTACCTGGCGCGCGCGGTAAC
GCCGGGGACGTACCAGCTTGCGCAACCGCAGGTGGAATCGATGTACGCGCCTCAGTGGCGGGCAACCGGCGCG
AGCGAGGGGCTGCTGATTGTGACGCCTTAAATGAACGGTTGGCGTGGTAAACGTGGCCGCTGGCTGTGGCTGG
CGGGCGCGCCGCTTTTTATTTTCCTGGCGTTATGGGCAGCGGATAAACTCTGGCCGCTGCCGCTCAATGAGGT
CCATCCTGCGCGGGTCGTGGTAGCCCATGACGGTACGCCGCTGTGGCGCTTTGCCGACGCCGGGGGGATCTGG
CGCTATCCGGTGACGATTGAAGAGGTCTCTCCCCGCTACCTGGAGGCCTTAATCAATTACGAAGATCGCTGGT
TCTGGCGGCACCCTGGCGTTAACCCTTTCTCCGTCGCGCGCGCGGCATGGCAGGATCTTACCGCCGGGCGCGT
TATTTCCGGCGGCAGTACGCTGACTATGCAGGTGGCGAGACTGCTGGACCCGCATTCGCGCACGTTCGGCGGT
AAAATCCGCCAGCTTTGGCGCGCCCTCCAGCTTGAATGGCATTTGTCCAAGCGCGATATCCTGACGCTGTACC
TGAACCGCGCGCCGTTCGGCGGCACGCTACAGGGTATTGGCGCTGCAAGCTGGGCCTACTTCGGCAAGCCGCC
CGCGCGCCTGAGCTACGCTGACGCGGCGCTGCTGGCCGTATTGCCGCAGGCGCCGAGCCGGTTACGCCCCGAC
CGCTGGCCTGCGAGAGCCGAAGCGGCACGAAATAAAGTGCTGGATCGGATGGCGGCGCAGGGCGTCTGGCCTG
CCGAAACGGTACGCGAGTCGCGTGAAGAGCCGGTCTGGCTGGCGCCGCGTCAAATGCCGCAACTGGCGCCGCT
TTTTGCCCGAATGATGTTGAGTAAAAGCCGGAGCGACAAAATTGTGACCACGCTGGACGCCGGGTTGCAGCGG
CAACTGGAAGATCTGGCGCGGGCATGGAAAGGGCGGCTGCCTGCACGCAGTTCATTGGCGATGATCGTCGTCG
ATCATACCGATATGAGCGTGCGCGGCTGGGTAGGATCGGTAGACCTTAATGACGACAGCCGTTTTGGTCATGT
CGACATGGTAACGGCGATCCGATCGCCGGGATCGGTACTCAAACCCTTTGTGTACGGTCTGGCGCTGGATGAC
GGCTTGATCCATCCCGCATCATTATTACAGGACGTTCCGCGCCGCACCGGAGATTACCGCCCGGGAAACTTCG
ACAGCGGATTTCACGGCCCGGTCAGCATGAGCGACGCGCTGGTACGCTCGCTGAATCTGCCTGCCGTTCAGGT
GCTTGAAGCCTATGGGCCGAAACGATTTGCGGCAAAACTGCGTAACGTCGGCCTGCCGCTATATTTGCCTGCT
GGCGCGGCGCCCAATCTTTCGCTGATTCTGGGCGGCGCGGGCGCGCGGCTTGACGAGATGGCGGCGGCATACA
GCGCGTTCGCTCGTCATGGGAAAGCGGCGAAACTACGGTTACAGCCGGACGATCCGCTGTCGGAAAGACCATT
AATGTCGCCGGGAGCGGCGTGGATCATTCGGCGGATTATGGCGGATGAAGCGCAACCCTTGCCGGATAACGCG
CTGCCGCGCATTGTGCCGTTGGCGTGGAAAACCGGCACCAGCTACGGCTATCGCGATGCGTGGGCTATTGGCG
TTAACGCGCGCTATATCATCGGCATCTGGACAGGCAGACCCGACGGCACACCTGTGGTGGGGCAATTTGGTTT
TGCCAGCGCGGTGCCATTGCTTAACCAGGTCAATAATCTGTTACTGGCGCATACGGGACGCCTGCCGGAAGAT
CCGCGTCCGCAGACGGTAAGCCGCGGCGTCATTTGCTGGCCGGGCGGACAGACTCTGCCTGCCGGAGACAGCA
ACTGTCGCCGCCGACTGGCGACATGGCTGCTCGATGACAGCCAGCCGCCCACGCTGCTGTTACCTGAGCAGGA
AGACATCAACGGTATTCGTTTCCCGGTCTGGCTGGATGATACGGGGCGGCGTGTCGCTGCCGACTGTCCGCAG
GCGTGTGCGCATACGTTTATCGTCTGGCCGCGGCCGCTGGAGCCGTGGCTGCCGCCTGCGGAAAGACGTAGCG
CCCGCTTGCCCGCGGCGTCCGACCACTGTCCGCCGCTACAGGGCAGTGATGCCGCGCCGTTAATGCTGTCAGG
AGTGAGGGATGGCGCGGTGATCAGGCAATTGCCAGGCCAGGAAAACGTTACGCTGCCCGTCTCGACTACCGGC
GGTAAAGGGCGTCGCTGGTGGTTTTTAAATGGCGAACCTGTTAATGGCGAAAATAATCGCCTTTCTTTATTAC
TCAATATCGCTGGACGTTATCAACTTGTCGTAATGGATGAGTCCGGTCAGGTTGCTGCGGTTAATTTTGAATT
AATACGTTAACAACTCTTCAGAATAAATTATGAATTTCATTTAATTTAAAGTTAATGAGAATTATTAATTTTT
CCGGAATTACCCTTAATGAGGGAGCTTGATAAATATCAAGAGATCTAACACTTAAAAACGTGAGAATGGGTTT
TTATTTGACGAGTCGGATCATGAAAAATATAAAAAGCCAGGGAAATGGCGAACAGCCCGCTATTTCTCGCCGC
CACTTTATCCAGGCCAGCTCCGCGCTTATTGCGTTACCGTTTGTCTCCTCGCCAGCTACCGCTCAGGCGCGCG
CTGTGACGGCCACTGAAAACAGGCCTGCGGAAAAAGTCGTTCAGACATGCAGCACTTTTGACTGCGGGGGCAA
ATGCGATATTCGGGCGCATGTTAGTGACGGTATCGTGACGCGAATTTCAACGCGACCGGATAATGCGCTGGAT
GCGCAAATGCCGGTGATGCGTGCCTGTGTTCGTGGACGCGCATACCGTAAATTTGTTTATCACCCCGATAGAC
TTAAATATCCCATGAAGCGTGTAGGAAAACGCGGTGAAGGAAAGTTCGAGCGAATTTCCTGGGATGAAGCGAC
AACCTTAATCGCCGATAATTTAAAATCGATTACTGAAAAATATGGCCCGGCTTCGCGCTATGTTCATGTTGGC
ACCGCCGTTTCCGGCGGCACCTTTTCCGGCGATAAAATGGCGCGTCGCCTGCTGAATCTGACCGGCGGCTATC
TCGAAAGCTACCATTCTGTGAGTATGGGCAATACGGCGGCCGCGACGCCTTATACTTACGGCATTGCCGCCAG
CGGCAGTTCGCTGGATACGCTTTTGGACACCAAACTGGTGATTCTGTGGGGCCATAACCCGACGGAGACCATT
TTTGGTCATACCAACTATTTCTTGCAGAAGATGAAGCAGAACGGTACGCGGTTTATCGTGGTCGATCCGCGCT
ATTCCGACACCGTTTCCTCGCTGGCGGATCAGTGGATCCCGCTGCTGCCCACCACCGACAATGCGCTGATGGA

CGCGATGATGTACGTCATCATCAGCGAGAATCTGCACGATCGCGCATTTATTGCGCGCTACGCTATCGGTTTT
GACGAAGACTCAATGCCGGAAGGCGTACCGGCTAACGAATCGCTGGTGGCGTATCTGACCGGCGCCAAAGACG
GTGTCGTTAAGTCCCCCGAGTGGGCGGAAAAGATCACCCATGTGCCGGCGCAAACCATACGCCAGTTAGCGTG
TGATTACGCGAATACCAAACCCGCCGCGTTGATTCAGGGGTGGGGACCACAGCGCCACAACTGCGGCGAGCGC
ACCGCGCGCGGCTCGACCCTACTGGCGACCATTACCGGTAATGTCGGCATCAAAGGCGGTTGGGCTGCGGGTT
ATGGCGGCTGCGCTAACCGTAAATTTGCCGCGGGTCCGGAGATGCCGGATAACCCGGTAAAAGCCAAAATTTC
GGTGATGAACTGGGTGCAGGCCGCAGACGACGCCAGCAAAGTGACGCCGGATATGGGGCTGAAAGATGCCGAT
AAGCTGGACAGTAACATCCGTATTCTGTTCTCGCTGGCCGGGAACTATCTGGCGAATCAGAACCCCGATCTGC
ACCAGGCGGTACGCGTACTGGAGGATGAATCAAAGATCCAGTTTATTGTCGCCAGCGATCTGTTTATGACGCC
GAGCGCGAAATACGCTGATCTACTGCTGCCGGAAACCAGCTTTATGGAACGCTGGAACATCGGTGAAACCTGG
GGCACGGCAAGCTACCTGATACTCTCTGAAAAACTGATCGAGCCTGAATTTGAGCGGCGCTCCGACTACGACT
GGCTACGTGAGGTGGCGGCGAAATTAGGCATTGAAAACGAATTTAGCCAGGGGCGCGATGAGAAAGCGTGGAT
TGAACACATCTGGGAACAGACGCGCCTGGCGATGCCGGACGAAAATCTACCGGATTTCGCCACGCTGCAAAAG
ACCCGTCAGCATCTGTTCAAAAGCGCGCCATTTATCGCCTTTGAAGACAATATTCGCGATCCGGACAATCATC
CGTTCCCGACGCCGTCAGGGAAAATAGAGATCTTCTCGAAGCGCCTGTACGACATGCAGCATCCGGAAATCCC
GGCGTTGTCGCACTACGTTCCCGCGCATGAAGGCCCGGAGGATGCGCTGGTGAAAGATTTTCCGCTCCAGTTA
ATTACGTGGAAAGGAAAAAACCGCGCCAACTCAACGCAATACGCTAACCCGTGGCTGATTGAAGTGCAGCAGC
AGACATTGTGGATCAACCCGCAGGATGCGCAAAAGCGCGGCATTACGCATGGCGACATGGTGCGTATTCATAA
CTCACGCGGGATTTGCGAAATCCCGGCGGAAGTGACGCCGCGCATTATTCCCGGCGTTGTCGCAATGCAGGCT
GGCGCCTGGTGGCAGCCGGATGAAAACGGCGTTGATAAAGGCGGCTGTGCGAACGTCCTCAGCTCGGCCCGTA
TTACCGCGCTGGCGAAGGGAAATTCACATCAAACCATGCTGGTGGAGGTGGCTAAAGCATGAGTCAGTTTACA
CATTACCCGCCGGTGAGCGATAAACAACTGGGGTTTTTCATCGACTCCTCACGCTGCTCCGGTTGTAAAGCCT
GCCAGGTGGCCTGCAAAGACAAAAACAATCTGGAAGTAGGCCGTCGTTTCCGCCGCGTCTATGAAGTCAAGGG
CGGAAGTTTCATCCCGACCGGGCAGGGCGGGGTCAGCAATAACGTATTTGCTTATACGCTTTCTATTTCCTGC
AATCACTGTGCGGACCCTGTCTGCACCAAGAATTGCCCGACTACCGCTATGCATAAACGCCCGGGCGACGGCA
TCGTGCGCGTTGATACCGACAAATGCGTCGGCTGTGGCTATTGCGCTTGGTCCTGCCCTTACGGCGCGCCGCA
GCTTAACGAGCAGACCGGCCAAATGTCCAAGTGCGACATGTGCGTTGATTTACTGGCGAAAGGTGAACCGCCC
GTCTGTGTTGCGACCTGTCCGCTGGAGGCCATCAAGTTTGCTCCGATTGATGAACTGCGCGCGAAGTACGGTA
GCGTATGCGACGTTAACGGGCTGCCAGATTCATCCATAACCAAACCTAATCTGGTTGTCAAAGCGCATCAGGG
CGCAGAAAAAGAGGGTAAACGCCATGCATGAGTTACCGCTGCTGATTTTTACGCTCTGTTTGCAAGGGTCTGT
GGGTGTGACAGTGTGGCTGGCGTTGGGCCGCCAGTACGCCGTAGAGGGACGCGTGCCTGCACGCGGCGCGCTT
CCCGCGATGGCGGGGGCGTTTGTACTGGCCTGCGTAGGGCTGCTTGCTTCAGCGCTGCACATGGGCTATCCCC
TGAATGCGCTAAATGCGTTACGTCATGTCGCCAGTTCGTGGCTGAGTCGCGAAATCGTCTTTGCCAGCCTTTA
TCTGGCGGCGCTGGGACTGGGGGTTGTGCTGCTGTTTTTCCGAAAGCCAGGCTGGCAGCCGCTGCTGGCGTTG
GCGGCAGCGTTCGGGCTGGTGGATGTATTCTGCATGGCGCAGGTTTATATCCATGCCTCGGTGGCGACCTGGC
AGCATAGCAACACGCTGGCGCTGTTCTTTGGCACATCAGGCATTATCGGCTCGGTAGTCATTGCGCTGGCTTA
TCTGCGCAACGCTGGCGCCGCAATGCGCTGCGCTGTGGTGGTGGTCGCACTGATGGTGCTGATTCGCCTGATC
ATGCAGCCGCTATGGCTGGCGGATATTAATGCAGTGGATACGACGGTCGTCACTTTCCCGCATCATCCGCTAC
AGGCGCTGGCGCAGTTGCGCGACGTCTATCTTCTCGGCTGGTGTGTCTCAGCGGCGGGAATGCTCTGCTTTGC
CGCAGGCGGTCTGCGAAACGCCAGAGGAACTCTGGTGGCGGGCAGCGTATTGCTGCTTATCGGCGAGATTATG
CTGCGCTACGTCTTCTTTAGTATTGGCTGATGGTTAACCTGACGATCGATCCGGCGGTAGACGTGACGCAGGC
TTGCGTCCGTCGTCGCTTTCGTTTTTCTTCCTGCCGCGCTTGCGCCGATGTTTGTCCGGCGCAGGCGTTTTCG
CTGGCGCAGGGGCAGGTCAGCATAGATACGACACGCTGCATAGCCTGTGGCGACTGTCTGTTTGTCTGTCCTG
TTGACGCCATTACCGGTATCAAACCGGTTAAACGCTTTGTGCAGGGGGATACGTTGGTGGGGCCGTTTTCGCT
ACAGGCGCCAACGGTCGATGAACTGCTTCTCTGGCACAGCCAGTACGGTATTCGCTTTATTGATATTGCGGTG
GAGCGATCGGCGCAGTGGCTGATGGCGCTGGCGGGGCTAAATTTAGCGCTGCGCCGCTACGGCGAGCCGGGCT
GGTCTTTCAAACACGTTGTCGGCGCGGAGATTAACGCCTCGCGGCGCACTCTGTTTCATGTTCCGCGCGACGC
CATTACCCCGTGTGCCGTCGAGCCGGGGAAACGGCGTCTCAGACAGGCGTTTTCCGCGTTCAGCGAATGCGTA
CCCGAAATCAGTCCGCAAGAGTGCAGGATGTGCGGCGCCTGCTGGCGAAGCTGCCCGGAGAACGTTATCCAGT
TTGACGACAATACCCTGACGATAGCGGCGGCGCGTTGTACGGGATGCGGCGGCTGTGCGGCGGTATGTCCGCA
TCAGGCGCTGCGGCTGCGGTTTGACGTGGAACCCGCCTCAACGCGGCATAGTGCGGCGCATACGCTCACCTGC
GACATCTGCAAACGTACTTTCCATGCTCTCACGCCTGAACACACGCACTGCGTGCTGTGTCAGTCCCCTGAGT
TTGCTGTGCGTCTTTAACTTTTGTTGCTAATGCTCATTTTATTTTAAAAAAATGTTACCTATATCCATAGGCA
ATTGATGGGATGCTCTTTATAATCCGCGCCTGACATAACAACAGAACATATTTCAGAGGTAAACATGGCTATT

GAACGGACTTTTTCCATCATTAAACCCAACGCGGTGGCAAAAAACGTTATTGGCAGCATCTTTGCGCGCTTTG
AAGCTGCAGGGTTCAAAATTGTTGGCACTAAAATGCTGCACCTGACCGTTGAGCAGGCGCGCGGCTTCTATGC
TGAGCACGACGGCAAACCGTTCTTTGACGGTCTGGTGGAGTTCATGACCTCTGGCCCTATCGTGGTATCCGTG
CTGGAAAGTGAAAATGCGGTACAGCGTCACCGCGATCTGCTTGGCGCCACCAATCCGGCGAACGCGTTGGCGG
GTACGCTGCGCGCCGACTACGCTGATAGCTTAACTGAAAACGGCACCCACGGTTCGGATTCGCTGGAATCCGC
GCAGCGCGAAATCGCCTTTTTCTTCGGTGAAGGCGAAGTGTGCCCGCGCACCCGTTAATCTCCTTCGTCTTTC
ACGCTGTAAATGCGTTGGCGGTAAATTATCGGTTCAAATATTATTTTTGCTAATGCCGCGTGCAGACGTGGCA
TCCTTACGCCAGAATTTGTACAATGCAGCGCCCCCGGACGAGCAGTATGCTTACCGGGGCGTTTCTTTTTTCA
ACCCTCCAGGGGCCATAACGTGTAATAACGAGGCCGGAATAGCATATGTCTGAACAAATTGTCACACCTGAAA
GCAGTACCCCGGTAGTTCTTAACAACGAAACAAAAATCAACCTGCTGGATCTCAACCGTCAGCAGATGCGCGA
GTTTTTTAAAAACTTAGGTGAAAAGCCTTTCCGCGCCGATCAGGTGATGAAATGGATGTATCACTATTGCTGC
GATAATTTTGATGAGATGACCGACATCAACAAAGTGTTGCGCGGCAAATTAAAAGAGGTCGCGGAAATCCGCG
CGCCGGAGGTCGTTGAAGAGCAGCGTTCCTCAGACGGCACCATTAAGTGGGCGATTGCTGTCGGCGATCAGCG
CGTTGAAACGGTCTATATCCCGGAAGACGACCGCGCTACGCTGTGTGTCTCTTCACAGGTAGGATGCGCGCTG
GAGTGCAAGTTTTGTTCAACTGCGCAGCAGGGCTTTAACCGCAACCTGCGCGTATCGGAAATTATCGGTCAGG
TCTGGCGTGCGGCGAAAATCGTCGGCGCGGCCAAAGTCACCGGCCAGCGTCCGATCACCAACGTGGTCATGAT
GGGGATGGGCGAGCCATTGCTTAACCTGACCAATGTCGTACCGGCGATGGAAATTATGCTCGATGACTTTGGT
TTCGGCCTGTCCAAACGTCGTGTCACGCTCTCAACGTCAGGCGTGGTGCCTGCGCTGGATAAACTGGGCGATA
TGATAGATGTCGCGCTGGCTATCTCCTTACATGCGCCGAATGACACTATTCGCGACGAAATCGTCCCGATCAA
CAAAAAATACAATATCGAAACGTTCCTGGGCGCGGTTCGCCGCTATCTGGAGAAATCCAACGCCAATCAGGGG
CGCGTGACGATTGAATACGTGATGCTTGACCACGTCAACGACGGCACTGAACACGCGCATCAACTGGCGGAGC
TACTAAAAGAGACGCCTTGTAAGATTAATCTGATTCCGTGGAACCCGTTCCCTGGCGCGCCGTATGGCCGCAG
CTCTAACAGCCGTATCGATCGCTTTTCTAAAGTTTTGATGAGTTATGGTTTCACGACCATCGTACGCAAGACG
CGTGGCGATGACATTGATGCGGCATGCGGACAGTTGGCAGGCGATGTGATTGACCGTACTAAACGTACCCTGC
GTAAGCGTATGCAGGGAGAGGTGATTGACATCAAGGCTATTTGATAGCAGTTGTGCCACGGGGCATTCATTAT
ACCGTGGCGCAATGTCAGTTGAATAATGTCTGGCGTTGTACTTGTCTGGTCAATAATAACGGTGCGTTTTTGT
CGACTTTAAGGCAGTATGTAATGGTGCAACAAGAGTTTAGCCTTGTGTCTTAGCGGTTTTGTCATCTGCGGCC
TGACAGGCTTATACTGTCTGACTAAGGTTAACTAACCTGATGTTTCGCGGTGTGGGCGGGCATTACGGCTCGT
CGGCGCCTGAATCCTAATTTACACGTACCTGTAGCTGTAGCGAATGAATACTGAAGCCACGCACGACCAAAAT
GAAGCACAAACCACCGGCGTTCGTCTGCGCAATGCCCGTGAACAACTCGGACTCAGCCAGCAGGCTGTCGCTG
AGCGACTTTGCCTGAAGGTTTCTACGGTACGCGATATTGAAGAAGATAAGGCGCCGTCCGATCTTGCTTCAAC
CTTCCTGCGCGGATATATCCGCTCATATGCGCGTCTGGTGCATGTTCCAGAAGAGGAACTGCTGCCGGGGCTG
GAAAAACAAGCTCCGCTGCGCGCCGCTAAAGTCGCCCCGATGCAGAGTTTCTCATTAGGAAAGCGTCGTAAAA
AACGCGACGGCTGGTTAATGTCTTTTACCTGGCTGGTACTGTTTGTGGTCGTAGGGCTGACCGGCGCCTGGTG
GTGGCAGAACCACAAGGCGCAGCAGGAAGAGATCACGACGATGGCCGATCAAAGCACGGCTGAACTGAACGCA
GATAAAGACAGCGGCCAAAGCGTACCGCTGGACACCGGTGCCGTGACAAGCCAGGATACGACGCCAGCGCAAA
CGGCTCCGGCGCCTGCGACGCCGGTTGATTCGACGGCGGCGACGCAAACGCCAGCGCCAACGGCGGCGGCAAC
GCAAAATACGGTAGTCGCGCCTTCGCAGGCGAATGTCGATACGGCTGCGACATCTGCCGCGCCAGCGGCAACG
GAGACGCCGTCCGCGCTGCCGACCAGTCAGGCTGGCGTAGCGGCGCCTGCTGCCGATCCGAACGCGCTGGTGA
TGAATTTTACCGCTGACTGCTGGCTCGAAGTCACTGACGCCACGGGTAAGAAGCTGTTTAGTGGTATGCAGCG
TAAAGATGGTAATTTAAACCTAACCGGTCAGGCGCCTTATAAACTTAAAATTGGCGCGCCGGCCGCGGTGCAG
ATCCAGTATCAAGGAAAACCTGTCGATCTGAGCCGCTTTATCAGAACTAATCAGGTTGCGCGTCTGACCCTTA
ATGCCGAACCGACACCGGCGCAGTAACAGACGGGTAACGCGGGAGATTTTTCATGCATAACCAGGCTCCGATT
CAACGTAGAAAATCGACACGTATTTACGTTGGGAATGTGCCGATTGGCGATGGCGCCCCCATCGCCGTCCAGT
CGATGACTAACACGCGTACCACTGACGTGGAAGCGACGGTTAATCAGATCAAAGCGCTGGAACGCGTTGGCGC
AGATATTGTTCGTGTTTCTGTACCGACCATGGACGCCGCCGAAGCGTTCAAGCTTATCAAACAGCAGGTTAAC
GTCCCGCTGGTTGCCGATATTCACTTCGACTACCGCATTGCGCTGAAAGTAGCGGAATACGGCGTTGACTGCT
TGCGTATCAACCCCGGCAATATCGGTAACGAAGAACGCATTCGCATGGTGGTGGACTGCGCGCGCGACAAAAA
CATCCCTATTCGTATAGGCGTAAATGCCGGATCGCTGGAAAAAGATCTGCAGGAAAAATACGGTGAGCCGACG
CCGCAGGCGCTGCTGGAATCAGCCATGCGCCATGTCGATCATCTCGATCGTCTCAACTTTGATCAGTTCAAAG
TGAGCGTAAAAGCGTCCGATGTATTCCTTGCCGTTGAGTCTTATCGTTTGTTGGCTAAACAGATCGATCAGCC
GCTGCATCTGGGGATCACCGAAGCGGGCGGCGCGCGTAGCGGGGCGGTCAAGTCGGCGATCGGTTTAGGTCTG
CTGTTATCGGAAGGTATCGGCGACACGCTGCGCGTATCGTTGGCGGCCGACCCGGTGGAAGAGATCAAAGTCG
GTTTCGATATTCTGAAGTCGCTGCGCATTCGCGCGCGCGGCATCAATTTCATCGCCTGCCCGACCTGTTCTCG

CCAGGAGTTCGACGTTATCGGCACGGTGAACGCGCTGGAGCAGCGTCTGGAAGATATCATCACCCCGATGGAC
GTCTCGATCATCGGTTGCGTAGTGAACGGGCCGGGCGAAGCGCTGGTATCGACGCTGGGCGTGACGGGCGGCA
ATAAGAAAAGCGGCCTGTATGAAGACGGCGTGCGTAAGGACAGACTCGATAACGACGATATGATCGCGCAGCT
TGAATCCCGTATTCGCGCGAAAGCAAGTCAACTTGATGAAGCGCGTCGGATTGACGTGCTGCAGGTTGAAAAA
TAAGAACGTGATGGGAAGCGGCGCGCCTCCCGTGTATGATTGAACCCGCATGGCTCCCGGTTCGTTCGGGGAA
GCGCTGAGGGTTCATTTTTATATTCAGAAAGAGAATAAACGTGGCAAAAAACATTCAAGCCATTCGCGGCATG
AACGATTATCTGCCTGGCGAAACCGCCATCTGGCAGCGCATTGAAGGCACACTCAAAAACGTGCTCGGCAGCT
ACGGTTACAGTGAAATCCGCTTGCCGATTGTAGAGCAGACCCCGTTATTCAAACGTGCGATCGGTGAAGTTAC
TGACGTGGTTGAAAAAGAGATGTACACCTTTGAGGATCGCAACGGCGATAGCCTGACATTGCGCCCTGAAGGT
ACGGCGGGCTGTGTACGCGCCGGCATCGAACATGGTCTCCTGTACAATCAGGAACAGCGTTTGTGGTATATCG
GGCCGATGTTCCGTCATGAGCGTCCGCAAAAAGGCCGCTACCGTCAGTTCCACCAGCTAGGTGCCGAAGTCTT
TGGCTTACAAGGCCCGGATATCGACGCGGAACTGATTATGCTGACCGCACGCTGGTGGCGCGCGCTGGGCATC
TCTGAACACGTTAGCCTGGAGCTGAACTCTATTGGTTCCTTAGAGGCGCGTGCGAACTATCGCGATGCGCTGG
TCGCGTTCCTCGAACAGCATCAAGAGACGCTGGACGAAGACTGCAAACGCCGTATGTATACCAATCCGCTGCG
CGTGCTGGATTCAAAAAATCCGGACGTGCAGGCGCTGCTCAACGACGCGCCCGCTCTCGGCGACTATCTCGAT
GACGATTCACGCGAGCACTTTGCCGGCCTGTGTAAATTGCTGGACGCGGCGGGGATTGCCTACACCGTCAACC
AGCGTCTGGTACGCGGTCTGGATTACTACAACCGCACCGTATTTGAATGGGTAACAAACAGTCTGGGGTCACA
AGGCACCGTCTGTGCGGGTGGTCGTTATGACGGTCTGGTGGAACAACTGGGCGGTCGCGCTACCCCGGCAGTG
GGCTTTGCGATGGGCCTGGAACGACTTGTTTTGTTAGTTCAGGCAGTTAATCCGGAATTTATTGCCTCTCCTG
TTGTCGATATATACCTGGTAGCTGCCGGCGCACAAACGCAGTCTGCGGCAATGACGCTGGCGGAGCGGCTGCG
CGATGAAATGCCAGGCGTGAAGCTAATGACAAACCACGGCGGCGGCAACTTTAAGAAACAGTTTGCCCGCGCC
GATAAGTGGGGCGCCCGTATTGCACTGGTTCTTGGCGAATCTGAAGTCGCCGATGGGACTGTTGTAGTGAAGG
ATTTGCGCTCCGGTGAGCAAACGGCAGTGGCGCAGGACAGCGTCGCCGCGCATTTGCGCACTTTATTGGGCTA
AGGAGAAGGACAGCGTGGAAATTTACGAGAACGAACACGACCAGGTGGATGCGATTAAACGCTTCTTTGCCGA
AAACGGCAAAGCGCTGGCTGTCGGAGTGATTTTAGGGGTTGGCGCGCTGGTTGGCTGGCGTTACTGGAGCAGC
CATCAGACGGAGTCCGCGCGTGCTGCTTCTCTGGCGTATCAAAATGCGGTGACGGCGGTAAGCGCCGATAAGC
CGGATAGCATTCCCGCCGCTGAAAAATTCGCGGCTGAGAATAAAAACACCTATGGTGCGCTGGCGTCTATGGA
ACTGGCGCAGCAGTTTGTGGATAAGAATGAACTTAAAAAAGCCGAAGCCCAGTTGCAGCAGGGGTTGGCGGCC
ACCAGCGATGAAAATCTCAAAGCGGTGATTAATCTGCGTCTCGCTCGCGTTCAGTTGCAGCTTAAGCAAGCCG
ACGCCGCGCTGAAAACTCTGGATGCCGTTAAAGGCGAAGGATGGACAGCCATTGTCGCTGATTTGCGCGGCGA
AGCGTTGCTGAGTAAGGGTGATAAAAAAGGCGCGCGCAGCGCATGGGAAGCGGGCGTCAATAGCGATGCTTCT
CCGGCGTTGAGCGAAATGATGCAGATGAAAATCAATAATTTGTCCATCTGAGAGGGACCCGATGCAATTGCGT
AAATTACTTCTGCCAGGGTTGCTTTCCGTTACCCTGCTCAGCGGCTGTTCACTGTTTAGCGGCGAAGAAGATG
TCGTCAAGATGTCTCCATTACCGCAGGTTGAAAACCAGTTTACCCCGACCACTGTCTGGAGCACCTCTGTCGG
TAACGGAATTGGCGAATTTTATTCTAACCTTCATCCGGTAATGGCAGATAACGTGGTCTATGCAGCCGATCGC
GCGGGCGTGGTAAAAGCGCTCAATGCGGATGATGGCAAAGAGATCTGGTCTGTGAATCTGGGCGAGAAAGACG
GCTGGTTCTCTCGTTCGTCCGCGTTGTTGTCCGGCGGCGTCACTGTCGCGGGCGGTCACGTCTATATCGGCAG
TGAAAAGGCCGAGGTTTATGCGCTGAATACCAGCGACGGTACCACAGCATGGCAGACGAAGGTCGCAGGCGAA
GCGTTGTCTCGTCCGGTTGTCAGTGATGGAATCGTTCTTATCCATACCAGCAACGGTCAGTTGCAGGCGCTAA
ATCAGGCGGATGGCGCAATTAAATGGACGGTGAACCTGGATATGCCTTCGCTGTCGCTGCGCGGCGAATCTGC
GCCGGCAACCGCGTTCGGCGCGGCTATCGTTGGTGGTGATAATGGCCGCGTCAGCGCCGTGCTGATGCAACAA
GGGCAGATGATTTGGCAGCAGCGTATCTCCCAGGCCACTGGCCCAACGGAAATTGACCGTCTGAGCGACGTCG
ATACCACGCCGGTTGTGGTAAACGGCGTCGTTTACGCGTTGGCGTATAATGGTAACTTAACGGCGCTGGATCT
GCGCAGTGGTCAGATTATGTGGAAACGCGAGCTGGGTTCGGTCAATGACTTTATCGTCGACGGAGACCGTATC
TACCTGGTCGATCAGAACGATCGCGTGCTGGCGCTGACCACTGACGGCGGCGTAACGCTGTGGACGCAAAGCG
ATTTGCTGCACCGTTTGCTGACCTCCCCGGTGCTGTATAATGGTGATTTAGTCGTCGGCGATAGCGAAGGTTA
TCTGCACTGGATTAATGTTGATGACGGGCGTTTTGTGGCCCAACAAAAAGTAGACAGTTCCGGTTTTCTGACC
GAACCGACGGTGGCGGATGGTAAACTGCTCATCCAGGCCAAAGACGGTACGGTCTACGCGATTACGCGTTAAT
CGTCACGGTTGTGTGTTTTAAAAAACGGCTCCTGGTGTAGGGGCCGTTTTCCTGTTTTTAACAACGGCGTAAA
AATGACGCGCGTTGTCTGATGATTTTTTAAATGAGGCTTTAAACATGGTACCTGTGGTCGCGCTTGTCGGGCG
CCCTAACGTCGGAAAATCCACGCTATTTAACCGTCTGACCCGCACCCGAGATGCGCTGGTTGCGGATTTCCCG
GGTCTGACTCGTGACCGTAAGTACGGTCGTGCGGAGGTTGAAGGCCGCGAGTTTATCTGTATCGACACTGGCG
GTATTGACGGCACCGAAGACGGCGTCGAAACGCGGATGGCGGAACAGTCGCTGCTGGCGATTGAAGAAGCGGA
TGTGGTGTTATTCATGGTAGATGCGCGCGCTGGCCTGATGCCGGCGGATGAAGCGATTGCCAAACATCTGCGT

TCCCGTGAAAAACCGACCTTCCTGGTGGCGAATAAAACGGATGGTCTCGATCCCGATCAGGCGGTTGTCGATT
TTTACTCGCTGGGATTAGGCGAAATTTACCCTATCGCCGCCTCACATGGTCGCGGCGTATTGAGCCTGCTGGA
ACATGTGCTTCTGCCGTGGATGGATGATGTCGCGCCGCAGGAAGAGGTGGATGAAGACGCGGAATACTGGGCG
CAGTTCGAGGTGGAACAAAATGGTGAAGAAGCGCCGGAAGATGATTTTGACCCGCAAAGCCTGCCGATAAAGC
TGGCGATTGTCGGTCGCCCGAACGTAGGTAAGTCCACACTCACTAACCGTATTCTCGGCGAAGAGCGCGTGGT
GGTATACGACATGCCGGGCACCACTCGCGACAGCATTTATATCCCGATGGAACGCGACGAACGGGAATATGTG
CTGATTGACACGGCAGGGGTGCGTAAGCGCGGAAAAATCACCGATGCGGTAGAAAAGTTCTCGGTGATTAAAA
CGTTGCAGGCGATTGAAGACGCTAACGTCGTACTGTTGGTTATCGATGCGCGCGAGGGGATCTCCGACCAGGA
TTTGTCTCTGCTGGGCTTTATCCTCAATAGTGGGCGCTCACTCGTTATCGTAGTCAACAAATGGGATGGCCTG
AGCCAGGAAGTCAAAGAGCAGGTGAAAGAGACGCTGGATTTCCGTCTGGGCTTTATTGATTTTGCCCGCGTTC
ATTTTATCTCTGCGCTACACGGCAGCGGTGTCGGCAACCTGTTTGAGTCCGTACGCGAGGCTTACGACAGTTC
CACTCGCCGGGTGAGCACTGCGATGCTGACCCGTATCATGACGATGGCGGTGGAAGATCATCAGCCGCCGCTG
GTGCGCGGTCGCCGTGTGAAACTGAAATATGCCCACGCTGGCGGCTATAACCCGCCGATTGTGGTTATTCACG
GCAACCAGGTAAAAGATCTGCCAGACTCCTATAAGCGCTATCTGATGAACTACTTCCGCAAGTCGTTGGAAGT
GATGGGGACGCCGATTCGCATTCAGTTTAAAGAAGGGGAGAACCCGTATGCGAACAAGCGTAACACACTGACG
CCAACGCAAATGCGCAAACGTAAGCGTCTGATGAAACATATCAAGAAAAGCAAGTAATGCTTTTTCAGTAATA
TTCCAGGCCTGACAAGCGCTGCGCTATCAGGCCTTTCAAGTAAAAGTGAGTCATGCGCCTGAGGAGAATGTTA
TGGAAATAACCTGCCCCGTTTGCCATCACGCGCTGGAGCGCAATGGCGATACTGCGCATTGCGAGACATGCGC
TAAAGATTTCTCGTTACAGGCACTTTGTCCTGACTGCCGCCAGCCGTTACAGGTTTTGAAAGCCTGCGGCGCA
GTGGACTATTTTTGCCAGAACGGTCATGGTTTAATTTCAAAAAAACGGGTTAATTTCGTCATTTCCGATCAAT
AAAACCGCTGTCGACGTTGCGCCGCAGAGCAGAGGCGCCGACGTCTTATCCGGCTAAGGGCAAAAGCGTTATT
CGATACTCCCGTAATATCAGCATAGTATCCTGCCCGGCGGTTATTTTATTCTTTTTATTGATTCAACTATTTA
TAGGGAATATTATTCCCGAAAATTAATATTAGAAATAAATAACAGCTTTCTTTTTATTTATTATATCAATATT
ATAAATCTGTTTCATATTAAACTTTTCTTTTTAGGAATATATTTAGGCCTGTTAGTTATGATTCCATCTGAAT
CATCCATATAATATCTTTTCATAGAGGCGTGTTTATTTAACGACAGGGATATCATATGGTGCGAAGGATTCGC
ATTCTGGTTTTGCTATTAGTGTCTGGAGTCTCAGTTGCGGCTTCTGGCGCAGATAACTCTGCATCTGCAAATG
ATGAAAGCCACTCCTCCCTGCCTATTTGCAAAACCAAATCACTTCCTCAGCACAGTCATATCTTGAAGGTGTA
ATGTCACCTTATGGCAAGATCCGTACCAGCCTGTCCGTGGGCGAAGGCGGCGATCTGGACGGCAGTTCGCTGG
ATTATTTTATTCCCTGGTATGACAACCAGAGCACGCTTTTCTTTAGCCAGATTTCGGCTCAGCGTAAGGAAGA
CCGCACCATTGGTAACTTTGGGCTTGGCGTTCGCCAGAACGTCGGCAACTGGCTGCTTGGCGGCAACGCATTC
TATGACTACGATTTTACGCGCGGTCACCGTCGATTAGGTCTGGGAACCGAAGCCTGGACGGATTACCTGAAAT
TCTCCGGCAACTATTACCATCCTCTTTCTGACTGGAAAGACTCCGAAGATTTCGACTTTTATGAAGAACGACC
CGCGCGCGGCTGGGATATCCGCATGGAAAGCTGGCTGCCGTTTTATCCGCAGCTTGGCGCTAAATTAGTTTAC
GAACAGTACTACGGCGATGAAGTAGCGCTGTTTGGCACCGATAATCTGCAAAAAGATCCTCATGCCGTCACTT
TGGGGCTGGAATATACGCCGGTACCGCTGGTCACGGTTGGTACGGATTACAAAGCCGGTACTGGCGATAGTAA
CGACTTTAGCGTTAACGCGACCGTGAACTACCAGATAGGCACGCCGCTGGCGGCGCAGCTCGATCCGGAAAAC
GTTAAAATTCAGCACTCCTTAATGGGCAGCCGCACCGATTTTGTCGATCGTAACAATTTCATCATTCTCGAAT
ATCGTGAGAAAGATCCGCTGGACGTCACGTTGTGGCTGAAGGCTGACGCCACCAACGAACACCCGGAATGCGT
GATTGAAGATACGCCTGAGGCCGCGGTGGGGCTGGAGAAATGTAAGTGGACGGTCAACGCGCTGATTAACCAC
CATTACAAAATTATTTCTGCCTCCTGGCAGGCGAAAAACAACGCCGCCCGTACGCTGGTGATGCCGGTGGTAA
AAGCTAATGCGTTAACCGAAGGCAACAATAACAGCTGGAATCTGGTGCTTCCTGCCTGGGTTAATGCCGATAC
CGAAGAGCAGCGCACCGCCCTGAACACCTGGAAAGTACGCATGACGCTGGAAGATGAAAAGGGCAATAAGCAG
AACTCCGGCGTGGTGGAAATCACCGTCCAGCAGGATCGCAAAATTGAGCTGATCGTCGACAACATTGCCGATA
CCGATCGCAGCGATCACAGCCATGAAGCCAGCGCGCTGGCCGATGGCGAAGACGGCGTGGTGATGGATCTGCT
GATCACCGATTCCTTTGGCGACTCCACGGATCGCAATGGTAACGAACTGGTGGACGATGCCATGACGCCCGTG
TTGTACGACAGCAATGATAAAAAAGTCACCCTGGCCCAGACGCCCTGCACCACGGAAACCCCCTGCGTGTTTA
TTGCCAGTCGTGACAAAGAAGCGGGGACGGTGACGCTTTCCAGCACCCTGCCGGGGACTTTCCGCTGGAAGGC
GAAAGCAGACGCCTATGGCGACAGTAACTACGTTGATGTCACTTTTATCGGCGACAACCTGAGCGCGCTGAAT
GCGGTGATTTATCAGGTCAAAGCCGCCAACCCTGTCAACCTTATCGGCAAAGAAGATAAGCACCCTACGGTCA
ATAATGCTTACCGTTTCCTGCTGTGGCGCGACAAAAATAAAGACGGCGTATTCCAGATGTCCGAGCAGCTCAC
GGAAGAAGAGATGGCGCTGTACGACTACCAGTGGGAGTTTACCGGGCAGAGTACAAACGGACATACCGGCGCG
CTGGCCAACACCATGAATGAAGATCTTGTCCTGCCAGTAACCAATAAAGAGGCGGCGCAGAAGTTTGCCGCCA
ATGTGGAAGATGGCGTTCAGGGTTACGGCATACGCGTTACGTATAGTCAGAAATAGCGTCAGCGTTGTGGCTG
ACCAGCCAGCCGCTGAATTTTGAGAAAAAGGAGAGTCTGAATGCAAGCAACAGTAAAACGGCGGCTGACGAAG

GTCGCTTTAGCATTAGTAGTTGCCGGGTATTGCGCCGCGCCAGCGGTGGCGGCAAACGGTAATCTGAAATCGG
GGCAATGGCAGATTGTCTCTGAACAGACGGGCACCATTCAGGGTACGGTGCCGTGGATTACCCGTGCGGCGGA
TAAAACTGCGGATACCGACAAAGATCACGTAACGGTAACTATCGATCGCGGCGATCGTAAAATTGTGACCGAA
GGCGATAAGCAGTTCCACGTTGGCGATAAGGTCACGGTGAACTGGGCGATTGGCGATACGGAAGGCGACCTGG
ACACCGACAACACGGCAACTAAAGCCACCGTGAAATGGGTGAGCTTTAGCGATCAGAATGGCAGCGACCCCAA
AGATCTTGGTACTGGTGACAGTTATGAAATCCAGGCCGCTGATGCGGATCGTTATATCGGTATCAAAATCACG
CCAACCACTACCACCGGTGACCCTGCCGTGGCAACCGAACTGCTGTTGAAAGACTTGTCGACCGATGCGGGCG
GTGGTTCGGATGATGACGAAATTCCGGAAGGTCCGGTGGTGGATGAAAACGTCCATGTGGTTATTCATGAGAA
AGATTCGAATACCAACCTGTTGAAGAATTCAGGGACGACACTCAAAACGAATACTACCTATCAGGTATTGCTG
TGGAGCGATAAAAACGGCAACGGCACCTATGATGCCGGTGAAAACGTTACCGACCAGTATGACTACCGCTGGA
AATTCGTCGGCACCAGTAAAATTGCAGGTACCGGTACTGGTGGTATCGTCAACGAAAACTGGAACGATAAAGA
CCTTGTCATCCCGCTTACCAACGCCGAAGCGAAAGAGGCGTTCGAGGGTGCGGAAGGCGGCGTTACCGTGGGT
AGCGACGGTGTCCAGGGCTTTGGTCTGTCCATCGACTACAAACGCAAATAAGTTGTCCTCAACATACCACTCC
CCGCGGGGAGTGGTATTTTCCGCGCACTTTGCTTAGCCGCGCGCTAAGCAAAGTGGGTTGAATTACGACAGAG
AATGAAAACGGGTGGCTATCGCTCATGGACAGGAATCGGCAAGTGAATAAAGTTGTACATTTCTTACTGACAT
TACTGATCATGTTTGCAGTGTCGATCGCACCTGCGCAGGCGCTATTGAAGGGCGGAACCTGGCAAGAGCTGAA
CTCAGTCACCGGTGCGGTTAACGGCACCGCGCCGCTGGCCGACGGCGCTATTATCCCCCTTTACCAGGGAAGT
ACGCTGCTTGACCCGAGCAAAAACCACGATATTGAATTCAGCGCCATGCCGCGCGATTTTAGCGCGGATGCTA
CCTCAACCTCGATGCGGGCGGTCAATTCCACCGATACCGAAGGCGATCTCTTTAGCGATCCGCCCACCATCGC
CTGGGAAAACCGCCAGCCGCCCGCCATGGGCCTGGTATGGGCCGATGCCGCCACGCCCGACACGCCGCTCTCG
CCGCAGCCGGTTCCGAACCTGACCTTTTGCGCCCAAAACCTCGCCGGGCGACAGCTGGTGGCCTGGGCGCAGG
TGGAAGATGAAACCAACGTTCCCGCGCTGTGGCTCTTTACGCGTACCGGGGTGCCGAATTATGCGACCATTCC
GCTGTTAAGCCCGAAAGTGGCGCTGAATATCAAACCAGCGGTGAGCGACCCTGTTTCTGTGAGCGGCGATCAT
GTCGATGCCAGTTTTGAAGCATCGAAAGTTAAGGTCGGAGAAAGTATTACTCTTACTGTCACCACCAAAGCGT
GTGATGGCGAACCTGCGATCAATGCGCCGTTTGTGATCCGTCGCGAGGATGCAATAAATCGCCAGGGTGTGGT
CAATAACGCTAACCCGGTGCGCGTTGGCAATACCGAGTTAACGACTGCGCAAACAGAATATCATGGTGTAACG
GACGGGCAGGGCAACGCCACGGTGGTGGTAACGCAAGAGAACGGTCCGGGGGTAAAAACGCGACTTATCGTCT
CTTCGCAAAACTATCCCACGCTGACCGACAATGTCGACGTCATTTTCACCACCATCACCAGTCCAGACAGCGA
TAAGGCCAGTATGTACGGCCACATGATAGAGAGCGCTACCGCGACGCTGAACGGTATAACCTACACCTTCACC
CGCCCGAAGCTGGCGGCGGAAGCGAGCGGCGCTGACAAAAGCGTCGTCGATACCAACGAAACCTGGGCGCTGT
TCGACTGGAACGGCGCCGATAACCATTGCAATATTCTGCCGGATGCCGAACAACTGGTGCAGATGCGACATGA
GCACAGCACGCTGGCAACGTACACCGGCTGGCCGGCGACGGACGATGCCGAATACTGGTCGTCGACCAAAGAT
CAGATGAGCGGCTACCACGCTGCCGTACATATGAACAGCGCATCGGTCGTCAGGGCCGCCGACAGCGACACGC
TGCTGGTCAGTTGTGTGGATAAAGCCCAGCCTGCGGCGCACCCGCAGATTACTCTCTCCCCACAAGGACCGTA
TAAAGCGCAGGTGGGGGAGTCGATTGATCTGACGATGACCGTAGTTGACCGGGACACGCAAAAACCGCTGCCT
TACCGCTACATGGAGCTGTTTATCGACCCGGCGAAAAACCGCAAAGGGGAACATCAGGACGCATGGGATAACC
TGCGGGTCACCGTTGATTCCGAAGGTATGAGCGCCTCGTCGCCGGAGCACTATACCGGCGTGACTGACGTCAA
CGGCCAGGCGCACCTGACGTTAAAACATAACAGCGGCCTGGGCGTCGAAACGCCCATCCGCATCGTGATGCCG
GACGACGAAGGCGGCAACGTCGAGCTGTCCTTTAGCGTAATTTTCACCGTGGTCACCAGCCCGGACGTGGACG
GCGCCAACATGTGGGGCCATATGCGCGGCGTGGTGGACGCGGGCAACCTGTACAAACGTCCGCTGCTGGCGGT
GGAGGCCAGCCATAAAGACGGCCAATTCAGCGAAAATAACGAAGAGTGGGCGACGTTTAACTCCGTGGCGTCG
GCCACCGCGCAGTGCGGTGTCGGCCAGGTGCCGGATCAGTCGTCGCTGGCGCATTTGTACAGCGAACATGCGG
GCGGACAGATGGAAAGCGAGCACGGCTGGCCGACTGAAGATTATTTCATCGCCGCCGACAGCGACGCCTCCGG
GACGGTACACGTCAACCTCGAAAACGGCGATAGCGGCAAATTTACCGATACGCCCAACTACCTGACCTGCTCG
GCCAATGAAATGGTGGCGGTGCTGGACGTCTACTTCAACGACGACCCGGTGATCAAAAACGCCGACGCCAAAG
TGGGTGAACAGGTTAAGATGAACATCCATTCGCGCAACGCCCTTAACGGCATAGCGATCGGCAATACCGACTT
CACCATCACCATGGCGAACGGCAGGCGGCGTGACGGGCTGACGACCGGCTTTACCGATACCAGCAACGGCGAG
ATGCAGTTTGACGACGTGGGCTACGTTGCCGGGCAGGTTTATCACGGGATCACCGACGCTAACGGCGATGCGA
CCATTATCCTCACCCAGAAAAAGGGCGTGGGGCTGCTGACGCCGTTAAGCATCGTGCCCGTCAATTCATACAT
CAATACGCCGGTTTCCCGCAGCGTGAAGTTTACCGTCGCGACCAGCCCGGATACGGCAAAAGCCAAAATGTGG
GGCCATATGGCCGACACGATTACCGTGGGCGACTGGACTTTTGAGCGGCCCAAACTGGCGGGTGAAGTGAGCA
ACCCGCTGCGTACCCAGGACGAGAGCAATGAGACCTGGGCGCGCGTGGCCCATAGCGACGCGGTGGGCAACCC
GGATGCGGGCGGCTGCGCGGCGAACCGCCTGCCGCGCATCGACCAGCTTGAGGCGCTGTATAACGCCAACAGC
GGCGGCAAGATTCACAGCATTCAGGGCTGGCCGACCTATCTTAACTACTGGTCGTCCACCTACCAGAGCGCCA

CCACCTGGAAACTGATCGCGCTGACGAACGGCAGTGAATTTGCCAACAGCAACGTCTCGATTTACGCCAGCTG
CCTGGCCAGCGATAACCCGGTGGCGGCGTCCATCACCATTGAACCGGTCAATCCGTCGCAGTGGTACGACGGC
AGCGACGTCCACGCGGTCAAAGTGAAAAAGGGCGAGACGATGCAGCTCAAGGTGACGGTGAAGGACGCCAGCG
GCAACCCGATCCCGGAAGCGCCGTTTGTGCTCACCCGCGGCGACGGTTACGATCGCCGGGGCGAAAAGTATAC
CGCTCAGGACGGTGACGATCTGCAAGGCATCGTGACGCCCGTGGTGATCGACGGCGAATCGCTGGCATGGACC
ACCACCAAAATGGGGTCGCAGACCGGCACTGACGGCACCCGTATCATCAGCGTCACCCGCCCGGACACCCACG
GCACCCGCACCGCGATTAACGCGACGCTGTATGAAAATGCCGCCGTGAGTGCCAGCATCGACACTATCTTTAC
CGTCGTCACCAGCCCGGATGTCAGCGTCGCCCGCATGTGGGGGCATATGGCGCCATCGCTGACGGCGGCGGAT
GGCGCGGTGTACCAGCGCCCGCTGCTGTACGCTGAGCTGTCCAGTACGGACAATACTGCGTCTAAGCAAGAAA
CCAACGAAACCTGGGCGGTTTTTCATGGGCCAGCCAGTGAAGGGGCCAATCCGGCCCGTTGCGCGGCGGGCTA
TTACCCTGCCGTTGAGGCGCTGGATTCGCTCTACAGCAAATACCCGAGCCGAACGATAAATACCGCCCAGGGC
TGGCCGGTCTACTATTCTTACTGGAGCGGATCGAACTCGACGTCATTTTCCAGCGGGGCAAAACTTGACTTCT
ATTACGCAGTCGATCTGGCTGATGGTTCGCGTCGGTCAGAAAACAGCGCGACCAGCACTGCCTGGCAGTATCA
AATTTGCGCCACGACGCCGCTGCCGCAGGCCACGCAGATTACCCTCACCTCCCCGCAGGCGATGGATGACGCT
ATTCAGGCGGTGAAGGCGAAAAACAGCGAGTCGATCCCGCTACTCATCACCACTACCGATGCGATGGGCAACC
CGGTGCCTTATGCGACTTTTTCGCTTAAACGCGACGCCGGGAAGGCGCGCAACACCGACTATAATAAATTTGT
AGCCACTAACGGCACCAACATGACGGTGACGCCGCTCACCGGTGCGCAGCAACAGTTTTACTATGCCACATCG
GTTCTGACCGGCGCCACCGGCGCGGATGGTACGCTGGCCCTGACGCTTGCCGAACCCGGCGGCATAGGGTTGA
AAAACCAGCTGACCGCGAATCTGAACGATACGCCGACGGCGACCTCATCGCTGCCGGTGGTGTTCACCGTCCT
CACCAGCCCGGACAGCGACAAGGCCAACATGTACGGCCATATGCCGGAGACCTTCACCGCCAGCAACGGTGCG
GAGTTTAAGCGCCCGCTGGTGGCAGGCGAGCCCTCATCGGAGGCGCACACGGATACATATTTCGAAACCAATG
AAAACTGGATCATGGTGAATAGTTTTAATACCGGCAATTACGGCGGGTGTCCGATGAACCAGATGGCTGCGAT
AGATGATTTCACTGCGCTGTACAACGATCACCCAAGCGGCAAGGTGGCGACCGATATTGGATTGCCCGTCGGG
AAGCGGTGGTGGGCGGGGGATAGCCTGCTGAAAGGCTCAACCCTTTACTGGCAGTATAAAGATCTCAAGACGG
GGAAAAACTACAGCATGTCGGAAAACCCGGGTAACTACTACTTACAACTCTGCCTGACAACATCGCGCAGCGG
GCTGAATATCGCGCTCTCTTCCGACGCATGGAACGCCGATAAATCGGCGGCCGTGGCGAAAAAAGGCGAAACG
ATACCGATGACGGTAACGGTGACTAACGACGCCGGGCAGCCGCAGGCCGGGGTGGCCGTACTGTTGACGCGTG
ATTACGCTTATTCGCGCGGTGCGGTGGATAAGCAATACATTGAACCAGGCGTGATTGGCGAGCCGGTTCCGTT
TACAACTTCGCCTGCGAATATGATGCTGACGCCGGTTGCACCTGCGGGTACGGCAGTGGCGTTTAATAACCAG
AATGGCCTCAGCACAAAATGGTCTGGCTTCACCGGCGATGACGGTAAGTTGCGGTTTACGTTAACTCAGGATA
AATCGCTGGGGTTAAAAACGTCTGTCACGGCGGCGCTTGCGAATCAATTTGATGAGGCGGCGAGCGTAGATGC
AATCTTTACGGTACAAACCAGCCCGGACACGCCGTATGCGTCATACTGGGGTCATATGCCGGATACCGTGCAG
GTGAACGGCGTTACGCTGCGCCGCCCGTACCTGAAAGCCGAGCTCTCTGCCGCGCCCCGGGATACCTGGCCGT
TTAATAATGAAATCTGGGGGACGAACTATTATTATCAGTCCGAACACGTGGAAACGTCGTTGACGCATCTTTG
CGGATCGCAGGAAAATATAGCCTCGCTGGACGATCTGAAAGCGTTGCAGAGCGTTATCGGCACGTTGCAATGG
CCGACGACATCATCCTGGGACTATGTGTCGCAAGATGAAGGTCAAAGCAATAAGTACTACTGTTCATTTAACG
AAACGACCGGCCAGACAACCTGTACTCGTGAAAAAGCGACGACCTCAGGCTTAGGCTCGTGCCGGGTGCCGTA
GTGAATGGGGAATGGGGCCGGGAGCGCGTTTGAATGTTGGGTGACGCTTTGCGGCGCCCAACCTGGGTATCCC
TTGTAGCTGGCGCTGTTCCGGTAGCGATGTATTTTTACGTTTTTGTATTCATGGAATGAACTGCAACGAAATG
GACTTTTCTCAGGTACGTCAGTGAAATCGAAAAAGTGTGTCGGTTTTTTACCCTGGCTGCTGCTATGCGCCCT
CAGTTTAACGCCTTTCTTCGCGCAGGCCGTGCGAAGCAGCGGCGCATGGCAGGAACTCACTGAGCAAACTGCG
GCTATTAATGGCACTCCGCCTCAGGCCGACAGTGTAACGATCCCGGTATATCAGGGCAGCGTGCAGTTGCGCT
CTGATGCGGCGAATCCTGTTGACTACTCCGCGAAGCCCAGCCAGTTCAGCACCAGTGATGCTGGCAGCGCGCT
GACCTTAACCAATCCGCACGACACCGAAGGCGATATCTTCGCCGTGCCGCCGCTGGTCTGGCAGGCTGAACGC
GCGCCCACCGTCACGCTGGTGTGGGCCGACGCCGCCACGCCGGACACGCCGCTTGATCCACAACCCGTCAGCA
ATATAAGTTTCTGCGCGCAGAATCTGGCGGGCCGCCATCTGGTGGTCTGGTCGCAACTGGATACCTCAACCGC
CATACCGCCGCTGTGGTTGCTTACCCGCACCGGCGTGCCTTATAACACGGCGGTCGAAGTGCTTGATCAAAAG
TTTGCCGTGGATATCGCGCCCGCCGTGGGCGACCCGGTAACGCTCGCCGCCGATCATCTGGACGAGTCGCTCA
ACGCCGCGAAGGTCAAAGCGGGCGAAAGCATCACGCTGACGGTAACGACCAAAGACTGCGGCGGCGAACCGGA
GGGCAACGTTGATTTCGTGATTACCCGCGGCGACGCGCAAAACCGCCAGGGCGTGGTAAACAATGCCGCGCCG
GTGCGCGTCGGCAACACCGAACTGACCACCACCGCCACGGAATATCACGGTACGACCAACGCCGAGGGCGTGG
CGACCGTGGTCGTCACCCAGGCGAACGGCCCGGGCGTTAAAACGCCGCTGATAGCGCACCCGTCGAGCGCGCC
AGCGCTGAAAGCCAGCGTGGACGTGATTTTTACCACCCTCACCAGCCCGGACAGCAGCAGCGCCAACATGTAC
GGTCATATGGCAGAGACGTCCACCGCCACGGTGGACGGCGCAAGCTACACCTTTTACCGCCCCAAACTGGCGG

CGGAAACCGACGGTGAAGACCGCACCGCCAGCATCAACAATGAAACCTGGGCGCAGTTCAACTGGGGCCACGC
CGACGCGCACTGCGATATTCTGCCGGATGCACGCCAACTGGAAGGGTTAAAAATTGCGCGCGGCGATCTGGCC
ACCACCCTCGGCTGGCCGGTAGGGCTGACATCCGGTGATGAAGAGTACTGGTCCTCCTCCCAGGGATCGACTG
CCACTGACCATATCAGCATTGATATGCGCAGCCGCAGCCTCACGCAGATGCCGGACGCCACGCAATCGCTGGT
TAGCTGCGTGGATAAAGCCCCGCCTGCGGTCACGCCGAAATTAGTGATTAGCGCCGACAATTTTGATTCAACG
GTCAACGCGGCGAAAGTAAAGGTGGGCGAAGAGATCAACATGAAGATCACCGTCACCGACAGCGCCACCAATA
AACCGCTGCCTTATCGCTACCTTAACGTCTACCTGGGCGACGAGCAAAATCGCCAGAATCAAAAGAATGCCGA
TATTGATAGCGGGCATCAGTGGACGGATGATCCGGTAATTATCACCAATCTGGAAGGCAGCGACGGTCACTAT
CACGGCGTCACCGACGCCAACGGTCAGTTCTCGCTGGTGTTGACTCAGGACAAGGGGGCGGGCGTATTAACCC
CGGTGCGCGTGGTGCTGTTCGACGGCACGGAAGCGACGCAGAACGTGATTTTTACCGTCGTCACCAGCCCGGA
CGTGGCGCAGGCGCGCATGTGGGGCCATATGCAGGGCGTGGTGGAAGCGGGCAATATCTACAAACGCCCGCTG
CTGGCGGACGAAGCCGCGCAGGATACCGGTTCCGAATTTGAGAATAACGAATACTGGGCGACGTTTAACTCGG
TAACGGCGGCGACGAACCAGTGCGGTACCGGCCAGGTGCCGGGGCAACTGTTGCTGGATACGCTGTATCAGGC
CCATTCCGGCAACGCGATGGAAACGACCTACGGCTGGCCGACGCAGAAACACAGCTATATCGCGGCGGATACT
GATGGTTCCACCACCGCGCACGTCAACCTGGCGACCGGTGCGGACAGCACGTTCAGCGGCACCGAACCGAACT
ATCTCTCCTGCTCCGGCAATGAGTTGGTGACCAGTCTGGACGTTTACTTTAATGGCGATGAGTCGTTACGCAA
TGCGGTAGCCAAAGTCGGCGAACAAATCACGATGAACGTCCATTCCGTCAATGCGCTGAATGGGTTAAGCGTG
CCCAACGCCAGCTTTATCGTCACCATGGCGCACGGCAAAAATCGCGACAACGCCACGACGGGCTTTACCGACT
CCAGCGACGGAACGCTGGTGATGGGCGGAACGCCTTTCGGCTCTTCCCAGGCGTCGATGACGTATCAGGGCAT
GACCGACAATGAGGGCAACGCCACGCTGGTGATTGAACAGCCGCAGGGCGTGGGCTTGCTGACGCCGCTGAGC
GTACTGCCGGTCAACTCACTGATTACCACGCCCGTCAACCGCAGCGTGAAATTTACCGTTCCCACCAGTCCCG
ATACGCCGGAGGCGCAGATGTGGGGTCATATGGCGGACACGCTCACCGTGGGTGATATGGAATTCCAGCGGCC
AAAGCTGGCGGCGGAAGCGACTGCCGCCACCCGTACCCAGGAGCAGGATAACGAAACCTGGGCGCGTGTCAGC
CACGCCGACGCGCTGAATAACCCCAACGCCGGGGGCTGCGAGGCCGGCCATCTGCCGCGCGCCGATCAGCTTG
CCGCACTGTATGCCTCCAGCGACGGCAATAAAATCCACACAGTATCCGGCTGGCCGACGACGTATGACTACTG
GTCTTCCACCTTCGCCAGCGCCGCTACATGGCAGGCGGTATCACTGGCTGCGGGCGGCTATACCGCTTCCGGC
GATGCGTCGGACTACGTGAGCTGTCTGGTCAGCAAAAATCCGACGGCGGCGTCTATCACCATTGAGCCGGTGG
ATGCGGCATTGTGGTATAACGCAAACAGCGAACACGCGGTGAAGGTGAAAAAGGGCGATACGCTCCAGCTTAA
AGTGACGGTAAAAGACGCCAGCGGCAATCCGCTGCCGCAGGCGCCGTTCGTGTTAAGCCGCGGCGACGGCTAT
ACCCGCCAGGGGGAGAAACATATCGCCGGTAGCGGCGACGGCATCGTCTCGGCGGTGGTGATTGACGGCGATT
CGCTCAACGACAGCGCCACCCAAATCGGCGGTATGACCGGTGAAAACGGCAGCAAAATCATCAACGTCACCCG
CCCGGATGCCCACGGGACGAAAGTCGCGATTACGGCGGCGCTGTACAATAACGCCAGCGCGACGGCCAGCATT
GATACTATTTTTACCGTGGTCACCAGCCCGGACAGCGACAAAGCCAAAATGTGGGGCCATATGCCGGAAACGA
CGACGGCGGCCAACGGCGAGGTGTTTAAGCGCCCGCTGTTATCGGCTGAAATCGCCAGCGGCGTTACCCACGG
CGACAATACGGAAAACAACGAAGCGTGGGGAATCGTCGATTTTGAAATGGCGAACGACGCCTGCGGCGCGGGA
TACGTGCCGACCCTTGCCGATATGCAATCGCTGTACGACGCCAGGCCCGGCGGCGCCATGAATACCCAGCAGG
GCTGGCCGCTGGATGGTAAAAACTACCAGGACAGCACCGCCGATTTGAGCCGGAGTACGCAAAACCGCTACGT
TAAGTCGATTAATCTGCGCGACGGCGGCGTCGGTTCGCTATTGTGGAATGAACAACTCTATTTTGTCTGCCTG
CAAAACGCCCATCCGGCGGCGACGCAGATAACGCTCACTTCGCCTGTGTATAACGACAGCGACGGATTCGCCA
AAGCGAAAGTCGGCGAGACCATCCCGGTCATCATCACCACGCGGGATGCCCAGGGCAACCCGGCCGCCGACAC
GCCGGTTATCTTTACGCGCGGCGACAGCGTCGGGCGCGCGAATCAGGAGGTGAATAGCTCGGCGGCGGCGGAT
ATACAGATAAACCACAGCGACGGGCGTAGCAGCGGCGCTAACTATTACACCGCCACGGGCGCCGACGGCACGC
TGACGCTGAATATCAGCCAGGACAGCGGCGCGGGCTTTAAAACGCCGCTGACGGCGGCAATAGAGCACAACGG
CGTAACGAGCGCGCCGCTGCCGGTCATCTTTACCGTGGTCACCAGTCCGGACACGCCGAAGGCCAACTACTGG
GGCCATATGGCGGAAACGCTGACCGACAGCAGCGGCGTGGCGTACAGGCGTCCGCTGCTGGCCAGCGAGTTTA
GCGCTACGCCCGGCAAAACGCTGACCATCGCCAACGGCTACTACGACAAAGGCGAAACCTGGGGCATGATAAC
CGTCGATAAAGCCTGGAACGGCACCGGTGGCGGCTGTGGGCGCGACACGCTACCGACCGTAGCCAACCTCCAG
ACGCTGTACGGGACATACCCGGATAACGCCATGCGCAGCCGTAACGGCTGGCCGATGACCAGTTCAGGCAACA
ACAACGTCAGCCGCTACTGGTGGGCGGGGGATTACGTGATATCGTCAGACGGCACGAAGTCGCTCTACGCGGC
GGTCAACCTGTTTAACGACGGCAATGATGTCAAAACGACGACCAGCACCTCGATGTACTACATGCAGACCTGT
CTTGCCTCGCCGCGCAGCGCCGCCGCCAGCCTGACGCTGACGCTGGCAGGCCAGGATGAAACCACCGGAAGCG
CAAAGGCGAAGAAGGGCGAGCAGATGGCCGCCACCATCGACGTGAAAGACGCCGCAGGTCAGCCGATGAAAAA
CGTGATGGTGAAAATCAGCCGCGGATCTTCGTACAATCGCGCCAACAGCGCCACATCGTCCTCAAGCGTCACA
GACGATATTACGCTGCGTAATGTGATGCCGTCCGGCCTGGCGACATACCTGCTGGATACCTCGGCGAAATATC

TGTATGCGCAAACTGATGCCCAGGGACAGGTGACATTTACCCTTGCGCAGGACAGTACCGCCGGTTTAAAAAC
CACTATCAGCGCCGCCACGATGGACGGCAGCAACCTGACAGACAGCAAAGACGCAATCTTTACCGTAGTGACC
AGCCCGGACAGCGATAAGGCCAAATACTGGGGCCATATGCCGGAAACCTTTACCAACAGTAAGGGGGTTTAAT
TTAAGCGACCGCTGCTGCGCGCTGAACTTTCTTCAACGGCGGACACGTCGGGTTATACGGAAAATAACGAGAC
CTGGTATACCTGGAGCCGTTATCCGAATATGTATCAGGACACCGCCAGCCCGTGCGATCGGCTGGGGTTGCCG
ACGGTAAATGATTTACAGACGCTATATACCGATTATCCCAATGGCGCACTCACGACAACGCTTGGCCTACCGG
TGGCATCAGGAAAATATTGGGGGGCGGGTAACTCAGTACCGGATGCCACGCACTCAGACAGTCAGTTCCAGTA
TGTCAGACTCAGCGATAATAATACGCTGACAACCAAAGCCAATACCGCCACGGCGCAGCTTTGTCTGGCAAAG
CGCCGGGACTTGTCGATAGAACTCACCTCCAGCGCCATGGATGCGGATAAAGGCGCTCCAGTGGCGAAGAAGG
GCGAGAGCCTGCCGTTGACCGTCACTGTGAGGGATGGTTCCGGCACGCCGCAGCCCAATACCACTATCCGTTT
GGGACGAACGCTTTCGATAGACCGGGCGGGTGTCGTTGATGGCAGTTCCGGCGGAGGCATGGTACTCACATCG
GTTGCGCCGTCTACGGGCTCAATGACGTTTAACTGCACGGTATCTTCGTGCACAAGTTATTGGTATGGCATTA
CGGATGAGGATGGTAAAGCGCAACTCGAAGTGACGCAGGATGATTCCCGTGGCCTGAGAACACCGCTGCAGGC
GATGCTTGTCGACGATCCGCTGACAGTTAGCGATATGGACGTCATTTTCACCGTCATTACCAGCCCGGACAGC
GATAAAGCCAAATACTGGGGACACATGCCGGAAACCGTGACCAACAGCGCGGGCGTGAAGTTCCGGCGTCCGC
TGCTGGCGGCGGAGATGACGTCGAATAGCGGTACTTACCTGGTTAACAATGAAACATGGCCCTTAGTCACCGC
AGCGAACACTGAAAAAGCTGGCGCAACCGGCTGTGATGCGGAATACCAGCCGTTGTCGGGCGATCTGCAAACG
TTGTATAGCGACAATCCTAACGGCGCAATCGGTACAAATTACGGCTGGCCGGTGGCGGGGAACAAATCCTGGT
GGGCGGCGGATCGTGCGCCGAATACCGGATATTACCAATTTATAAACCTGAATAGCGGCGGTAAGGGCACCGC
CAGTTCATCGACTGCCACCGGTGCTCAGGTGTGTCTGGTCGAGCCGCGTACTTCTACTCCTGCCAGTATTACG
CTGACCTCGACGGCGATGGACAGCGCGAAAAATGCGGCGGTAGTGCCGAAGGGAAGCGCGATGCCGTTAACGG
TGACGGTAAAAGATAGCAGCGGTAACCCGGTCGCCAACGTTGGTTTTACGTTGTCACGTGGCGATTCGAAAAA
TCGGGCAGGTATGGTGATAACGGATGGCGACGTGGCAGCGGATGCCGGAGCAGACGATCTCATGCTTAAAGAG
CTGACTCCCGCAAGCGCCAGCCAAAGTATGACCACCACCGGGATTGTCTTTACCGGCACTACCGGCAGCGACG
GTACGGCAACGTTTACGCTTAATCAGGATAAAAGCCTGGGGTTGAAAACGCCACTCACGGTGAAAGTGACGGA
CAATACGACTCTTCACGCATCGCTGGATGTGATTTTTATGGTACTTACCAGCCCGGATACGGATAAAGCGTTG
TTCTGGGGAAATATGTCGGATACGACATCCGTCAACGGAAAAACGCTGCATCGTCCGTGGCTTCAGGCTGAGA
TGCTGTCAGGCGTGACGCCAGTATTTACCAACGGAGTGCATGCAAATAACGAGTACTGGGCGATGGCGCATAC
GGTGGACAACACGAAATGGGATATTGCCAAACAGTGCGGTAGCCTTAGCAAAGCACCGGATAATAACGACCTG
TTGACGCTGTATCATTCAATTAGCTCGTTAGGCTGGCCGACGCTGGGCTATCCCTATTTATCTAAATCAACCT
CGAGCAGCGGCATGTATTGCGGCGTAGATGAGAATACCAAGAGTCAAAACTGCGCCATTAAGCCGGCAGGCAC
AGCGGGTTATGCGACGTGCGTGGAGTAACGTATTATGCGTCTGGCACGAAAATAATCTGCTTTTCACGTTCAA
TTGAGCTTCATGAAAAAGTGAAAGTAGCATTCATTGAAAAGTATATGTTCTGCTAATTGGCGCCACCACCGTT
TGCCGCGCGTGTGTAAAAGCGCCGTCTCCCTTCTGCAACCCGGCATCTGGCGCTACGTATATACTCTAAATAA
TTCGAGTTGCAGGAAGGCGGCAAGCGAGCGAATCCCCAGGAGCTTACTAAAGTAAGTGACTGGAGTGAGGGAA
CGCAGCCGCAGCACATGCAACTTGAAGTATGACGAGTGTATTTAAAATAACGCCAGGTGTCGCATGATAATCA
CACCTTTTTGTTTCACGCATTTATTTATTTTAATATATGAAGTTATTAAATGTTATTCACGACATAAATATTT
ATTAAATGGATGAATGTATTTATATCAATTATAAATCAACCAGAAATTGACCTGGCATTGAAATAAGCCTTGA
AATTAGTTAATAATTTGGCTTGGATAATATATTGTTTCAGGTGATTGCAACCATTCATAGAGCTGTATTTTTG
TTATTTATTATTTAACTCATTGATAATGTAGCATTTAAAGGCTATTTGCAGCCCAATCAAAATCCAGTTTGCT
TCGTGAACGTTATTAAAGGGAAATTTAAAAATTGAACAGGACTTACAGTATTGTCTGGAGCGCCGTGCGAAAT
ATGTATGTCGTGGCTTCAGAACTGGCGCGAGGCCACAGCAAAGTTAAAGCGCAAGTATGCGCGAGTGAAATCC
ATTCTCCAAACCAAAAGTCCGAATATGGACAAATAATAAAAGCAACGCGCGCGGCGCTGGCTTGCGCCGTGGC
TGCAGCGCTCGGATTTTTCAGCCCGCTGGCCATGGCCGACAATCAGGTATCATATGCTGATGCGCAGACCCAC
GTTCTGGATGCGTCAACGCCGCCGATGAGCTATTCGGGCACCGATGAAGGCGCGGCGCTATATGTCTCGGGTG
TCGCCACCGTCGGCTGGCAGCCCACCACGGTCACAGGGACGGGGCTCGTCATTGAAACAAGCGGAGGCGGAGC
TGATGATCCGGATGGCGGTAAGTATGTTTCCAACGCAATTTCGCTGGATCATTACGCTATCCTTGAGCTTACC
GACGCGAAGATAACCACTACGGGCATTTATACCCAGGGTATTTCCGCAGCGGATGGCAGCACACTGACGTTAA
CAGACAGTACGCTAACGATTGACGGCAATTTCGGCGTAATGACGTTGTATACGGGTTCAGAAGCAACGCTGGA
CGGTACCATCGTTGAGGCAGCTAACAGCAGCAGCGCGCAGGTGCAACAAGGCAGTACGCTGAACGTGCTGGAC
GGTAGCACAATCACCCTGGCGCAAGGCCAGATAAACGTTGTTGCCGGAAACACCGCGACAGACGAGGGTTCCA
CCCTGAACCTGTCTGATAGCTCGGTCAGCTCTGCAGGTACGATGAGCACCATCCAGGGTACCAATAAGGCCGC
TCTTAATCTCACCAACGCGACCATAACACATACCAATGCCAGCGGCGCGGCGGTACAGGCCAATAACGCCACT
ACCCTGGATATTACAGGCGGCAACATTACCTCTGCCGGTACGGGCGTGTATATTCTGGCGTCGGACGCCAGGA

TCGACGGCGCCACCATCAACGCCGACGGCGACGGTATCTTTATAACCAGCAAAAGAAAACTCGACGGGTATGA
AGATCTCAACGCGCTGACGGTCAACAAAGCACAGGTTAATTCAGACACGATTGCGCTGCATGTGGACACTGGC
ACGACGATTAACGCTCCTATCGTGCTGACCGACAGCACATTTGAAGCGCCGGAAGTGATCAAGCTTGGCAGCA
AGGCGGTCATTCAGGCCAACAATACGACATTGATCGGTGACGTTGCTCAGAGTGACATGTCATCGTCATCGCT
CTCTTTAAGCCAGGGGCCACGTTAACCGGTAGTGTAGATGCGATGTTTACTACGCTGTCGCTCGACGACACCA
GCCAGTGGAACATGACCGACCCGTCAACCGTCGGCAATCTGACCAACGACGGCGATATTACGCTGGGCAACGC
GAGCGGTTCGACAGGTACGCTGCTGACGGTGGACAATACCCTGACCTTACAGGATAACAGCCAGATAAACGCG
ACGCTGGATACCGCCAACAGCGCGCCAATTATCAAAGCCGCCAACGTCACGCTGGACGGGACGCTGAACCTTA
GCTCAACGGCCACCTTTGTCGCGCCCGAAACCGACGAACACTTTGGATCGATCACGCTGATTGATTCACAAAG
CGCAATCACTACCGATTTTGACAGCGTAACACTGGATGCTGACACCTCCGCTATGCCGGATTATCTGACGATC
AACGCGGGCGTTGATGCGAATGATAATACCAACTACGAGTTGTCTACGGGCTTGAGTTGGTACGCGGGCGCAA
ATTCTGCGCGCGCTGCGCATGGCACTTTTACCGTTGATGCAGGCAGTACCTTTACCGTCACCAGCGAACTGGA
CGAAACCACGGCGACCTCCAACTGGAACGGCAGCAAGCTAACCAAACAAGGCGACGGCACGCTGATCCTCAGC
AACACCGGCAACGACTACGGCGATACTGAAATTGACGGCGGCATTCTGGCCGCCAAAGACGCCGCCGCGCTCG
GCACTGGCGATGTGACGATTGCAGAAAGCGCAACGCTTGCGCTGAGCCAGGGCACGCTGGATAACAACGTGAC
CGGCGAGGGCCAAATTGTCAAAAGCGGTAGCGATGAGCTGATCGTGACCGGCGATAACACTTACAGCGGCGGC
ACCACTATTTCCGGCGGTACGCTGACGGCTGACCATGCGGATTCGCTGGGTACCGGGGCTGTTGCTAACAGCG
GCGTGCTTCAGGTCGGCGAAGGCGAGCTGGAAAATACGCTTTCCGGCAGCGGCTCGCTGGTGAAAACCGGCAC
AGGCGAGCTGACGCTAAGCGGCGACAACACTTACTCCGGCGGCACCACCATTACCGGCGGTACGCTGACGGCT
GACCATGCGGATTCGCTGGGCAGCGGCGATATCGATAACAGCGGCGTGCTTAAGGTGGGCGAAGGCGATCTGG
AAAATACGCTTTCCGGCAGCGGTTCGCTGGTGAAAACCGGTACCGGCGAGCTGACGTTAAGCGGCGGCAACGA
TTACTCCGGCGGCACCACCATTATCGGCGGTACGCTGACGGCTGACCATGCGGATTCGCTGGGTACCGGAGCT
GTTGCCAACAGCGGCGTGCTTCAGGTCGGCGAGGGCGAGCTGGAAAATACGCTTTCCGGCAGCGGTTCGCTGG
TCAAAACCGGTACGGGCGAACTGACGTTAAGCGGCGATAACAGTTACTCCGGCGGCACCACCATTATCGGCGG
TACGCTGACGGCTGACCATGCGGATTTGCTGGGTACCGGAGCTGTTGCCAACAGCGGTGTGCTTCAGGTCGGC
GAGGGCGAGCTGGAAAATACGCTTTCCGGCAGCGGTTCGCTGGTCAAAACCGGTACGGGCGAACTGACGTTAA
GCGGCGATAACAGTTACTCCGGCGGCACCACCATTATCGGCGGTACGCTGACGGCTGACCATGCGGATTCGCT
GGGTACCGGAGCTGTTGCCAACAGCGGTGTGCTTCAGGTCGGCGAGGGCGAGCTGGAAAATACGCTTTCCGGC
AGCGGTTCGCTGGTGAAAACCGGTACGGGCGAGCTGACGTTAAGCGGCGATAACAGTTACTCCGGCGATACCA
CCATCGCCGACGGCACGCTGATTGCCGCCAACGTCAACGCGCTGGGCAGCGGCAATATCGACAACAGCGGTAC
GCTGATGCTGGATGCCAACGGCGCATTTGAGCTTGCGAACATCACCACCCATACCGGCGCCACGACCGCGCTG
GCGGCGGGCAGCACCCTTGACGCGGGCCAGCTCACCCAGGAAGACGGCAGTACGCTAAGTATTGACCTCGGCG
CGGCCACCGACGACGCCGTGATTACCGCCGACAGCGTCACGCTGGGCGGCACTCTGAATGTTACCGGCATCGG
CAGCGTTACAGACAGCTGGACGCCTGAGGCATACACCTATACGCTGATTGACAGCGACAGCGCCATTACGAGC
GATTTCGACGATCTGACCATTGCAGGGATGAACCGCGAAGATGTTGACTTCCTGACTATCGACGGAAAAGTCG
ATGAAGCAGACAACACCCACTATGATCTGACGGCCTCCCTCTCCTGGTATGCGGACAGAGACAACGCCACTAC
CGACGCCCACGGCACCTTTACCCTGAGCGACCCTGACGGCAGCTTTAACGTTGCGGCTACACTGACAGACGTT
GATGACACGCTGGATCCGGGTAGCAGATGGGACGGTAAATCGCTGACCAAAGAAGGCGCAGGCACTCTGATCC
TCTCCGGCGACAACGACTACAGCGGCGGCACCACCATCAATGAAGGTACGCTGGTCGCCGCCAGTACGACGGC
GCTGGGCACCGGTCTGGTAGACAATAACGCCACGCTGGTGCTGGACGTAGACGGTGAAGTCAGCGCGGTGGGC
GGCATCACCACTCATAGCGGCGCGACGACCCAACTGGCGCTCGGCACCTCGTTGGATCTCGGCGACAGCGCGT
TAATTCAGCAAGATGGCAGTACGCTGAACGTTGAGCTGAATAGTGACTCAGTGCAACCGCTGATCACCGGCGG
CAGCGCCACGCTTGGCGGCGATTTGGTCGTCAGCGACGCCAGCCTGCAAGCGCGCGCCTCCGACGCGGAGTTC
CAGTCCTTTAAACTGATGGATATGGACAGCGATATCAGCGGCGACTTTACCAGCCTGACGATGAACCTCACCG
ACCAGCCGGACTATCTGACCGTAACCGGGACAATCAATCCTGCAGATGCGTCAGAATATTTGCTTACCGAAGG
GCTGAGCTGGAATGCGACCGCCACGTCAGCGACGCCAGCACACGGCACGTTTACCCTGGGCGCAGGCGACAGT
TTTGAAGTCACCAGCGTACTGGGCGATAAAACCGGCAACGGCGACTGGGACGGTAAATCGCTGACCAAACTGG
GCGCGGGTAAACTGACGCTGAGCGGCGCGAACACCTACACCGGCGATACTAACGTACAGGAAGGCACGCTATG
GCTGTCCGGAGACGGCTCGATTGGCGAGATGGGCAGCCAGCAGGCGGTGAACGTCGCCTCCGGGGCGACCTTC
GGCGGCAGCAATGGTACGACCGTTAACGGTAAGGTGACCAACGAAGGGACGCTGGTCTTCGGCGACAGCGAGG
AGACAGGCGCTATCTTCACCCTCAATGGCGATCTGATCAACATGGGGACGATCGCCAGCGGCAGCACCTCCAG
CACGCCGGGCAATACGCTTTATGTTGATGGCAATTACACCGGCAACGGCGGCTCGCTTTATCTGAATACCGTC
CTGGGCGACGATGATTCGGCGACCGATAAGCTGGTGATTACCGGCGATGCCTCCGGCACCACCGATCTGTACA
TCAACGGCATCGGCGATGGAGCACAGACCACTAACGGTATTGAAGTGGTGGATGTTGGCGGCGTATCGACCAG

CGATGCGTTTGAGCTAAAAAATGAGGTTAACGCCAGTCTGTATACCTACCGTCTGTACTGGAATGAAAGTGAC
AACGACTGGTATCTGGCGTCAAAAGCACAGTCTGATGATGACGACAGCGGTGGTGATGACAGTGATGTCACGC
CTTCGGATGGCGGTGACGATGGCGGTAATGTCACGCCTCCGGACGACGGCGGCGATGGCGGCGATGTCGCACC
ACAGTACCGTGCGGATATCGGCGCGTACATGGGCAACCAGTGGATGGCGCGCAACCTGCAAATGCAGACCCTC
TATGACCGCGAGGGCAGCCAGTATCGTAATGCCGATGGCAGCGTATGGGCGCGCTTCAAAGCGGGTAAAGCAG
AATCAGAGGCTGTCAGCGGCAATATCGATATGGACAGCAACTACTCCCAGTTCCAGTTAGGCGGCGACATTCT
GGCCTGGGGCAACGGCCAGCAGAGCGTTACCGTTGGCGTCATGGCGAGCTACATCAACGCCGATACCGACAGC
ACCGGTAACCGTGGCGCGGACGGTAGCCAGTTCACCAGTAGCGGCAACGTAGACGGCTATAACCTTGGCGTCT
ATGCCACCTGGTTTGCCGATGCCCAAACGCATAGCGGCGCGTATGTCGACAGCTGGTACCAATATGGTTTCTA
CAACAACAGCGTAGAGAGCGGTGATGCGGGATCTGAATCTTATGATTCAACCGCTAACGCCGTCTCGCTGGAA
ACTGGTTATCGCTACGATATTGCGCTTAGCAACGGTAATACTGTCAGTCTGATGCCGCAGGCGCAGGTTGTCT
GGCAGAATTACTCAGCGGATAGCGTGAAGGATAACTACGGCACCCGGATCGATGGTCAGGATGGCGACAGTTG
GACAACGCGTCTGGGTCTGCGTGTTGACGGCAAGCTGTACAAAGGCAGCCGTACGGTTATCCAGCCGTTTGCT
GAAGCTAACTGGCTGCACACCAGCGATGATGTGTCGGTATCGTTTGATGATGCTACGGTGAAACAGGATCTTC
CGGCTAACCGTGCGGAGCTGAAAGTGGGTCTGCAGGCAGATATCGATAAGCAGTGGAGCGTTCGCGCTCAGGT
TGCCGGGCAGACTGGCAGCAATGACTTTGGCGATCTGAACGGTAGCCTCAATCTGCGCTATAACTGGTAAGTT
AGACAAAACCGGGCGGGTGTTCCCTGCCCGGTTTTGAGGCGAATGACAAACCGGTTGATCGTCAGATTATGGC
TACACCTTATCCGACGTGGCGCGTAGGTTGGCTGAGGCGAAGTCGAAACCCAACATCGCGCTGCAAAGGGTGG
GGGAGTGTTGGATTACGTGCCCGGCGTGACGGATTTGACTTCACTTTCAAGCCAGCCGTCCTCCAGTCTCGTG
GTCATGATATCGCCTGCTTTGACCTGTTTGATCTTTTTCAGCACCTTGCCGTCCGTTGTCGTCGAAACCGTAT
AGCCGCGCGCCAGCGTGGCTAACGGGCTGACCGCTTCCAGATGCGTGACCGCGTTGCCAAAACGCTCGCGCTG
TTCGCTTAACCGTGAGCGGATATTCTCCGTCAGACGATATTCCAGCTGTTGGATGCGCGACTGCGCGCGGTGA
ATGCGCGGCTGCGGATTTTGCTGGCTCAGGCGTTGACTTACGCGCTGTTGGCGTTGATTGGCCTGCTTAATCC
TGGCCTCAAGCGCAAATCCCATCCGTTGGCGTAGACGTTCCAGCGCGGTTTGCTGACGGGCCAGACGCAGTTG
CGGATGTTGCTGTTGCAGACGGTGAAAAATCTGCGTGAAACGGCGGCTGCGATTGGCGAGGTAGTAGTCCATC
GCCATGCCCAGACGCTGCTGGGCGGACTGGATCTGACGCAACAACTCCTGCTGGTTACGGCTGACAATTTCTG
CCGCTGCTGATGGCGTAGGCGCGCGCAGGTCGGCGACAAAATCAGCTATCGTGACGTCCGTTTCGTGACCGAC
GGCGCTGACCACCGGAATGCGGCTGGCAAAAATCGCCCGCGCCACGCGTTCGTCGTTAAAACTCCACAAATCT
TCCAGCGAACCGCCGCCGCGTCCGACGATCAGTACATCACATTCGCCGCGCGCGTTCGCCAGTTCGATAGCAC
GAACGATCTGCCCCGGCGCGTCGTCGCCCTGGACTGCGGTTGGATAGATAATAACGGGCAGGGATGGGTCACG
ACGCTTTAGCACGTGGAGAATATCGTGCAGCGCCGCGCCGGTTTTCGAAGTGATCACCCCAACGCAGTGGGCC
GGGGAGGGCAACGGCTGTTTATGCTGCTGATCGAACAAGCCTTCGGCCTGCAGTTTGGCTTTTAGCTGCTCAT
ATTTCTGCTGCAACAAGCCTTCGCCCGCGGGCTGCATACTTTCGGCGATGATTTGATAATCGCCGCGCGGCTC
GTACAGCGTAATGTTGGCGCGTACCAGCACCTGCTGCCCGTGCTGCGGGCGGAACGTCACCCGGCGATTGCTG
TTACGGAACATCGCACAGCGCACCTGAGCGGTATCGTCTTTGAGCGTAAAGTACCAGTGGCCCGACGCAGGCT
GCGTGAAATTAGAAATCTCGCCGCTGATCCATACCTGTCCCATCTCCTGTTCTAACAGCAGACGAACCGTCTG
GTTAAGGCGGCTTACGGTAAAAATTGAGGAAGTTTGAGAGGATAACATGTGAGCGGGATCAAATTCTAAATCA
GCAGGTTATTCAATCGATAGTAACCTGCTCACGGGGGATCGCAAGCACTATTTGCAAAAAAATGTAGATGCAA
CCGATTACGTTCTGTATAATGCCGCGGCAATATTTATTAACCCCCCAGGTGAGATATTGCCCATGCTACGTAT
CGCTAAAGAAGCTCTGACGTTTGACGACGTCCTCCTTGTTCCCGCTCACTCCACCGTTTTGCCGAATACTGCC
GATCTCAGCACGCAGTTGACGAAAACTATTCGTCTGAATATTCCTATGCTTTCTGCGGCGATGGACACCGTGA
CGGAAGCGCGCCTGGCAATTGCCCTGGCCCAGGAAGGCGGCATTGGTTTTATCCACAAAAACATGTCTATTGA
GCGCCAGGCGGAAGAAGTTCGCCGCGTGAAGAAACACGAGTCCGGCGTAGTGACCGACCCGCAGACCGTCCTG
CCAACCACCACGTTGCATGAAGTGAAAGCCCTGACCGAGCGTAACGGTTTTGCGGGCTATCCGGTGGTGACTG
AAGATAACGAGCTGGTGGGTATCATCACCGGTCGTGACGTGCGTTTTGTGACTGACCTGAACCAGCCGGTGAG
TGTCTACATGACACCGAAAGAGCGTCTGGTGACCGTTCGTGAAGGCGAAGCCCGTGAAGTCGTGCTGGCAAAA
ATGCACGAAAAACGCGTAGAAAAAGCGCTGGTCGTTGATGATAACTTCCATCTGCTTGGCATGATTACCGTAA
AAGATTTCCAGAAAGCGGAACGTAAACCAAACTCCTGTAAAGATGAGCAGGGCCGTTTACGTGTCGGCGCGGC
GGTCGGCGCAGGCGCGGGCAACGAAGAGCGCGTTGACGCGCTGGTGGCGGCAGGCGTTGACGTACTGCTGATC
GACTCCTCTCACGGTCACTCTGAAGGCGTGTTGCAACGTATCCGTGAGACGCGTGCTAAATATCCTGACCTGC
AAATCATCGGCGGCAACGTTGCGACGGGCGCAGGCGCTCGCGCACTGGCGGAAGCCGGTTGCAGCGCGGTGAA
AGTGGGTATCGGCCCGGGTTCCATCTGTACGACTCGTATCGTGACTGGTGTGGGCGTTCCGCAGATCACCGCT
GTTTCTGACGCGGTGGAAGCGCTGGAAGGCACCGGGATTCCGGTTATCGCTGACGGCGGTATCCGTTTCTCCG
GCGACATCGCCAAAGCCATCGCCGCAGGCGCGAGCGCGGTAATGGTGGGTTCTATGCTGGCCGGTACCGAAGA

ATCCCCGGGCGAAATCGAACTCTACCAGGGCCGTTCTTACAAATCTTATCGCGGTATGGGTTCTCTGGGCGCG
ATGTCCAAAGGTTCCTCCGACCGTTACTTCCAGAGCGACAACGCCGCCGACAAACTGGTGCCGGAAGGTATCG
AAGGCCGCGTAGCCTATAAAGGTCGCCTGAAAGAGATCATTCACCAGCAGATGGGCGGCCTGCGCTCCTGTAT
GGGGCTGACCGGTTGTGCTACCATCGACGAACTGCGTACTAAAGCGGAGTTTGTGCGTATCAGCGGTGCGGGT
ATCCAGGAAAGCCACGTTCACGACGTGACCATCACCAAAGAGTCCCCGAACTACCGTCTGGGCTCCTGATTTT
CTTCGCCCGACCTTCGCGTCGGGCGATTTATTTAATCTGTTTCACTTGCCTCGGAATAAGCGTCAATGACGGA
AAACATTCATAAGCATCGCATCCTCATTCTGGACTTCGGTTCTCAGTACACTCAACTGGTTGCGCGCCGCGTG
CGTGAGCTGGGTGTTTACTGTGAACTGTGGGCGTGGGATGTGACAGAAGCACAAATTCGTGACTTCAACCCAA
GCGGCATTATTCTTTCCGGCGGCCCGGAAAGCACCACCGAAGAAAACAGCCCGCGCGCGCCGCAGTATGTCTT
TGAAGCAGGCGTGCCGGTATTTGGCGTTTGCTATGGTATGCAGACCATGGCGATGCAGCTTGGCGGTCATGTA
GAAGGTTCTAATGAGCGTGAATTTGGTTATGCGCAGGTCGAAGTGTTGACCGACAGCGCGCTGGTTCGCGGTA
TTGAAGATTCCCTGACCGCAGACGGCAAACCGCTGCTGGACGTGTGGATGAGCCACGGCGATAAAGTGACGGC
GATTCCGTCCGACTTCGTGACCGTCGCCAGCACCGAGAGCTGCCCGTTCGCCATTATGGCCAACGAAGAAAAA
CGCTTCTACGGCGTACAGTTCCACCCGGAAGTGACTCACACCCGCCAGGGTATGCGCATGCTGGAGCGTTTTG
TACGCGATATCTGCCAGTGTGAAGCGTTGTGGACGCCGGCGAAGATCATCGATGACGCCGTGGCGCGCATTCG
CGAGCAGGTAGGCGACGATAAAGTGATCCTCGGTCTCTCCGGCGGCGTGGATTCTTCCGTCACCGCCATGCTG
CTGCACCGCGCGATCGGTAAAAATCTGACCTGTGTTTTCGTCGACAACGGCCTGTTGCGCCTGAACGAAGCCG
AGCAGGTGATGGACATGTTTGGCGACCATTTTGGTCTGAACATTGTTCACGTACCGGCAGAAGATCGCTTCCT
GTCCGCGCTGGCTGGTGAGAACGATCCGGAAGCCAAGCGTAAGATCATCGGCCGCGTTTTCGTTGAAGTGTTC
GACGAAGAAGCGCTGAAACTGGAAGACGTGAAATGGCTGGCGCAGGGCACCATCTACCCTGACGTTATCGAAT
CTGCGGCGTCTGCAACCGGTAAAGCGCACGTCATCAAATCTCACCACAATGTCGGCGGCCTGCCGAAAGAGAT
GAAGATGGGGCTGGTTGAACCGCTGAAAGAGCTGTTCAAAGACGAAGTGCGTAAGATTGGTCTGGAGCTGGGC
CTGCCGTACGACATGCTGTATCGCCATCCGTTCCCGGGGCCGGGCCTCGGCGTGCGCGTACTGGGCGAAGTGA
AGAAAGAGTACTGCGACCTGCTGCGTCGCGCGGACGCTATCTTCATTGAAGAGCTGCGCAAAGCGGATCTGTA
CGACAAAGTCAGTCAGGCGTTTACCGTCTTCCTGCCGGTCCGTTCCGTAGGCGTGATGGGCGATGGTCGTAAG
TACGATTGGGTTGTCTCTCTGCGTGCTGTCGAAACCATCGACTTTATGACCGCACACTGGGCACATCTGCCGT
ATGATTTCCTGGGTCGTGTTTCCAACCGCATCATCAATGAAGTCAACGGGATTTCCCGTGTGGTGTATGACAT
CAGCGGTAAACCACCAGCTACCATTGAGTGGGAATAAGCCGATAATCCTTCCTGTGTTTTCATGAACAGGTAA
AAGTGAATTTAACCCTCTGTTTTTGCAGAGGGTTTTTATTTATGTGCATAGATGAGTTTTCTGTGTAGTGCAC
AGCTTCTGGCGGCAAATTTGACGTAATCATGAACCCACGAAGGTTGGCTGTATCTTGCCGTGGTCGTTGATTT
GTTCTCGCGCAAAGTTATCGGTTGGTCAATGCAACCACGAATGACAAAAGACATTGTCCTGAATGCGCTTCTG
ATGGCCGTGTGGCGACGTCATCCCCAAAAACAGGTGCTGGTTCAGTCTGATCAGGGTAGTCAGTACACCCGCT
ATGAATGGCTGAAATCGCACGGACTGGAGGGCAGTATGAGCCGTCGTGGCAACTGTCATAATGCGGTTGCAGA
AAGCTTTTTCCAGCTACTGAAGCGTGAGCGGATAAAGAAAAAGATCTACCAACGACTCAGAAGTGTCCAGGTT
ATCCGTGGCGATTCAGCCCAAATAAGGGGTATTTAAAATATAATAACTTTATTCTTCAACTATATAACCAACG
TCCAGTTTTAGCACTTTCAACGAGCATACCAACTGTTAGTGGCTTACGCGGATCAGGCTCTTTACTTCCAAAG
TACTCTGGATTCAGATAGTTGAGTATGTCAAATCCTTCTGAATCTACATTCCAACGCTTGAAGTACTCTTGTA
GAAGTTCTTCACTATCTTCAAAAACGAGTTTTAGATCATCCCTAATACTGATATCAAGCGTGATGGTTCGCTT
TGGCCCACAGAGAAAGTACTTTTTAGTGTTATATTCCTGATCGATAAAATCGATAATTTCTTTCTCAATATCT
CTCATTAGAAGTACGTCCACTGAATGCGATCTTTAGGAACCGCCACACGATTAAATGTATTTTTAATTCACGC
TGGATATCATAAATCGTAGTGAATGTCATAACCCAACCGAGCCAGGGTACATTACGACCAACGAAACGACCTA
AATTATTCGTTGAGCTTAGCTGTAATGAGTTGATGGTTTTGCCTGTCAACGTTGGTAATCTGATCCCAGGTGG
CTCTTTACAAAACAAATTCTGACGTATAGGACTGGTCACCTTATTCTCCTCCCACGATGGTTATCTTCCTGAT
TTCGCATTGACTCTGGCGGATAGGTCGGAGCCGCAGGTGACCTTTGAGTATACGTTTACGGTAAAGCACTGCA
TCGACACAATAGCGGGTTTCCTGAAGTGCGTTGAGGTTGAGCCGCATCCGGTTATCGCAGAGATACTCACACA
GTTTGAGAGTACCGTACGTCAGTGCCCGCTGGGATTCCAGATTTGCCCTGAACTTGTGCCAGCTACCGGGAGT
TTAACCACGCTGAAGGCTATCGGGTTTTGTATTCGGTCGAGGGAATCTTAGTAACCGCGCATGTGATCTTATC
ACCCAGGCAGGATATCCCGCGGTTGCTCTTTAAGCGTCTGATCATGGCCTGAACCCATTGAGTGGGTTGTCTT
TTTGCGTTAACGCTAAAGCTTCTTTTTCTTGCGCGGGCTTTTTAACGGATAACAGCTTAACGTTGTCCCGGCG
TGGTGACAGAAAGCGATGACTGTTGCGCTTCTTTTTTATCCTGATGGTGCTGCCAGGCGCCGATAGAAGAGTA
GACGAAACGTCCAAAGAAGAAGAGAAAACTGATAAGCAGTACGATACGGGTCATGCGAGTGTTAAATCGATGT
CGTTTTCGCATACTGTTGGCCTGACTCACAAAGGGTTCCTTAACGTTGGCCCAGCGATATGGGCGGGATCAGG
CTGGGTTCTTCAAGGGTATTATCAAGGCGAAGAACGTAACAAGCCTGCGTTCACTCTACATTAAGGCATACTG
CGAACCGATGGTCAATTCCTCTTAATGTAAAATTGCGTCAGTTGATTTATCTTATATTGTTATGGAAGATAAT

TTTAGTATTTACATTAATAATAAATTAATGATAACAAAGAATAAAAAGGCGAATAAAGGCTGCAGGAGAAAGG
TAATAATTGACTTAAATCAAATGTTACTTTTGTTTAATAATTACAATCGATGACTATCGTGTGATGTATATCG
CATCATCAATTTAAAGTCAGGTGGGATGCCTGTCTGTTAACGCCCCTTTGGGATTGGGGGGTTCTGCTGAGCA
TCTATGAAACTAAATAAAACTTATATAAATATCAGAGATAAATGGTGGGGACTACCGCTCATTCTCCCTTCGA
TTTTACTGCCTGTTTTAAGTAGCGCAAATACTTATGCGCTTACCAGCACTGGAAATGTGGTTCTTTTTTACCT
GCCGTTAGCTTTTATGCTTAGCCTGATGCTCTTTTTCGGCTGGGCAGCACTGCCGGGCATTGTGCTTGCTATC
TTCTGGCGCAGATACCCGCAGACAGGGCTGTATGAAACCCTTTCTGTGACGATGCACTTTATCATTACTATCG
TGCTAAGCTGGGGCGGCTATCGTGTCTTTTCCCCCCGGCGAAACAATGTTTCCCACGGCGACGCCCACCTGCT
GTTTCAACGAATATTCTGGCAGGTATTTTGTTCAGCAACATTATTTCTGGTCATCTACCAGTTTGCCGCTTTT
GTTGGCATGTACGAAAGTAAAGCCAGCCTGATGGGGGTAATGCCCTTTAATATCAACACGCTGATTAATTACC
AGGCGTTACTGGTCGGTAACCTTGTCGGCGTGCCATTGTGCTATTTTATTATTCGTACTCTCCGTAATCCGCT
GCACCTGCGGGGGTATTATCAGCAATTAAAGCTACAGATTGATAGCAAGGCCACTAAAAAAGAGATCGTTATT
TGGCTGGCCGTATTGACTACACTAATGTTTATACTGTGTATGCCATTGACTGATAATAGTTCAATATTTAGTA
CCAACTATACGCTTTCGTTATTATTGCCGGTCATGCTGTGGGGTGCGATGCGCTATGGCTATAAGTTTATTTC
AATTATCTGGGCCGTCGTGCTAATTACGTCGATTCATTATTATCAGCGTTATATGCCCTGGTATTCAGGTTAT
GATACGCAATTAGCGATTACCTCTTCGAGCTATCTGGTTTTTTCTTTTATTGTTAATTATATGTCTGTACTGG
CCACGCGTCAGCGCGTAGTGAGTGGTCGCGCACGGCGGCTGGCCTATCTTGACCCGGTTGTACATCTTCCCAA
TTTACGTGCGTTAAACCGCGCGCTCCAAAATGCGCCCTGGTCTACGATCTGTTTTTTACACGTGCCGGGCCTG
GAGCTATTGGGTAAAAATTATGGCGTCATGCTACGCATCCAGTATAAGCAAAAGCTCTCTCACTGGATTACGC
CGATGCTGGCCTCAAATGAGTGCGTCTACCAGATGTCAGGGCACGACCTGGTGCTGCGTCTTAATACAGAGGC
GCATCAGCAGCGTATTGAAGCGCTGGATAAACATATTAAGCAGTTTCGGTTTATCTGGGATGGACTGCCGCTA
CAGCCGCCTGTCGGCGTGAGCTACTGCTGCGTGCGTTCTCCGGTTAGCCACCTTTATCTTCTGCTGGGCGAAT
TAAGCACCAGTTCCGATCTTTCGTTGACGACTAACGCGCCGGAGGATTTACAGCGGCGCGGCGCAATGCACCT
GCAGCGCGATTTGAAGGGCAGAATTGCGATGATGAATCGCCTGCAACAGGCGTTGGAGCACGATCATTTTTTC
CTGATGGCGCAGCCTATTTTCGGTGTTCGTGGGGACGTTTACCATGAAATTTTACTGCGACTCAGGGATGACA
ACGGCGACGTTATCAACCCGGACAATTTTTTACCCGTGGCGCATGAGTTTGGTTTGTCTTCCGCTATCGATCT
CTGGGTGATTGAAAACACGCTGCGGTTTATGGCGGCCAGCCGGGAATATATGCCCGCGCATCGTTTTGCCATT
AATCTGTCGCCAACATCGGTATGCCGCGCTCGTTTCCCTGCAGAAGTTAAGCAGTTGCTTACGCAATATCAGG
TTGAACCCTGGCAACTGGTTTTTGAGGTAACGGAAAGCAACTCTCTCACCAATGCTGAGCAGGCGCAGTTGAC
GCTGGGACAGCTACAGCAGTTTGGCTGTCAGATAGCGATTGATGATTTCGGTACTGGCTACGCCAGCTATGCG
CGGCTGAAAAACGTTGACGCCGATATTCTTAAAATTGATGGCAGTTTTATTCGCAATATAGTGTCAAACAGTC
TCGATTATCAGATTGTGGCTTCTATTTGTCATTTAGCGCGAATGAAAAATATGCAGGTCGTCGCGGAGTATGT
CGAGAGCGAAGAAATACGAAGCGTCGTGCTCTCGTTGGGGATCGACTATCTTCAGGGATATTTAATTGGCGAG
CCGCAGCTATTATCTGAGATTCAGTAAAGGCAGACGCCGCGGCGCGGCGTCTGGCTCACAATTTACGCGGCGA
TCTCCGGCGAGCTTTCTTCCTCAATATTGAGACGCCAGCCAGTTACAGCTTCCCAGTACTGCTGTTCTTTTTC
CAGATCAAGCAGTACCAGCGCATTCTGGCTGAACCAGTCATGCGGAAAACATAACGTCCAGTGGCTGTCATCG
GTCGTTAGTCGCAGCGTCGGCGGCGTAGTGGTCGCCTGCCGCTGGTTGTTCAGTAATACGCCCAGCCGAAGTA
GCTGAATTAACGGCAGATACTGTTTTTTCTTAAACAGCGTAAAGCGGGGCATATCATCCAGTTTTATGGCTTT
ACGATGGTAACGCACCAGCGTCGCCATCATCATTTGCTGCTCCTGATTAAAGCCGGGCAAATCGCTGTGTTGC
AGAATATAAGCCGAATGGCGATGTAAACCGCTGTGATTAATATTCAGTCCAACCTCATGCAGCATTGCCGCCC
AGCGGAGCAACGCTTCAAGCTGCGGATGCGCCAGTTTTGGCTGCTGGGCCTGCCACTGCTCGTACATCTGCAT
GGTGGTTTCCAGCACGCGTCTGGCCTGTTCTCTGTCAATGTTGTATTGATTGGCCAGACTTTTTGCGGTACGG
CTGCGAACATCCTGATGGCGGAAGCGGCCTTCCATTTCATACAGCACGCCTTCGCGCAACGCGCCGTCGGAAA
GGCGAAGCTCGCGGATAGCCAGAGCATCAAAAACGCCGCACAGAATCGCCAGGCCCGGCACAAAGACCGCTTT
TCGTTCTTCAGACAGACCCGGCAGGCTGAGCGCATTAAAGGAGCGGTGCTTCAACACTTCTGACTTCAGTTTA
TCGAGGCGATCCGGCGTAATGAAGCCATCTTTCTCACCCAGCGCCAGGAGAACTTCATGAGCGGCCTTAATCG
TACCGGAAGCGCCCATCGCTACGTTCCAACCCTGAATACGATACTGCCAGGTTAAGGTTTCCAGTTTTTGCGC
CGCCGCCATTCGGGCGCGCTGGAAGTTTTCTTTATTGATAACGCCGCCGGGAAAGTAGAGCTGCGCGAAGCTC
ACGCAGCCCATACGACGGCTTTCAACCAGCCTGGGTTCGAAGTTTTCGCCAATGACCAGCTCTGTTGACCCGC
CGCCGATATCGATCACCAGCTTGCGGCCTTTTTCCGGCTGCGTATGTTCTACGCCCATAAAAATCAGGCGCGC
TTCTTCGTTACCGGAAATAATCTCTATCGGGTAGGGAATAACCTTTTCCGCGCGTTTGAGAAAATCAGCGGCA
TTTTGCGCCTGACGTAACGTATGGGTGCCTACGATACAGACGCTGGAAGGGGAGAAACCTTGTAAGCGTTCAG
CAAACAGCGACAGACAGCTAAGCCCCCGTTCCATGGCTTCTTCGCTGAGTTTATTATCTGCGCCCAGCCCGTC
CGCCAGATGGACGCGCTGTTTTAAACGCCCGATAATCTGCATTGCGCCGTCAACCACGCGGGCAATGACCATA

TGAAAGCTGTTTGAGCCGAGATCGACCGCAGCGAACTCCTGCGGACGAGGGGATTTATCGTAAATTGGCATAG
CGTTAGTCTGGTTGCTCGAGTGATTTGATGTAGTCATAAATTGCCAGCTGCGCCTGGACTTTACGGCGATTGC
CGCGCGGTACGTAGCGGTTGCTGAGTTCTTTATCGATAAAACGCGCTTTTACCGTGTCGCTAAACAGAATGTC
AATGATATCCAGGACGCGCTGTTTAAGCCGCGGGTCGAGGATAGGCGTAGCGACTTCGATACGGTAGTCAATA
TTACGCGTCATCCAGTCAGCTGAAGAGAGCCAAACTTGCTTATCACCGCCGTTTTCGAAAATATAAACTCGAT
CGTGTTCCAGATAGCGATCCACGATGCTAATCGCGCGAATATTATCGCTAATGCCTTCCAGTTGCGGAATAAG
AGAACACATACCGCGTACCAGCAGATTGACCTGGACGCCCGAACCCGACGCGGCGTATAGCCTGTCGACCAAC
CCTTTATCAACCAGGTTGTTAAGCTTTAAGGTAATCCCTGACGGTAAGCCTTGCTGGGCATTGGCGATTTCAC
GATCGATCATCTCATACAGCAGACGCCGTGAGTTTTGTGGAGAAACCATCAAATAATCGAAAGTGACCGGGCG
ATAGGGGTTTTCAATAAAATTAAAGACCCGACGCACTTCATTGGTAATGCGCGCATCGGCGGTCAGCAGCGAA
TAATCGGTATACAGCCGCGCTGTTTTTTCGTTGAAGTTACCGGTGCCGATATGCGCATAACGCACCACGTCAT
CACCCTCTTTTCGTGAGATGAGAAAGAGTTTGGCGTGAATTTTCAGCCCGGGCGCAGAGAAAATAACATGGAC
GCCCGCTTCGGTTAACCGCTTGGCCCAGTGAATATTCGCCTCTTCATCGAAGCGCGCCTGCAACTCCACCACC
ACGGTAACTTTTTTGCCGTTATGGGCGGCATGGATCATCGAGTCAATAATGCGCGAGTCTTTCGCTACGCGAT
AAATATTGATTTTGATCGCCAGCACGCTTGGGTCAAACGACGCCTGACGCAGCAGTTCCAGCACATGTTCAAA
GGTGTGATAAGGATAATAGAGCAGCACATCACGTTCACGAATCGCGTCGAAACCGTTACGAAACTTCTCTTTA
TCAAACCAAAGATGGCGTAACCGCGGTAACGGTTTATTGACCAGGTTGGCTTTACCGACATTCGGAAAATTAA
TAAAGTCTTTAAAATTGTGGTAGCGCCCGCCGGGTACGATTGAGTCATACCGGGAGATCGTCAGTTTTTCCCG
CAGTACGTCGACCAGCGCGGCGGGCATATCGCGTTGATAGACAAAGCGCACAGGCTCAGCGGTCAGGCGCTGC
TTCAGGCTGGAAGACATCAGCTCCATCAGACTGGATTCCATCTCATGCACCAGGTCATATTCGGCATCGCGGG
TCATTTTCATCGAATAGGCATTAAGCGCGTCATAGTCGAAGAACCCTTTAAAAATGTCATCCAGGCAGTAGCG
CAGAATATTATCCAGCAGGATCATCGGCTTGCGTCTGCGCGGTGTCTCCGGCGGCAGATTAACGAAGCGTGGG
ACTTTGTCGGACGGTATCTCCAGCAGCGCATAGTTGATGGTGTCGCCGCGGATAATCTCCACCGCCAGATAGG
TGTAATCATCTTTTAAAAACTGAACAAGATCCGTCTCCCGGTTAATAAGAATCGGGGTAATATGCTGGCGCAA
ATAGTGCTTAAAATAGTGGCGCAACCAGCTTTGTTGGTTCACCGAGAGCTGACGCTCGTTGATCAGAAAGATT
TGATTACGCGCCATTTCCAGCAGTAATTCGTTGTACAGGCCGTCGAACTCCTGGTCTGCTTTCAACACGCGGG
ACTGAATTTTGCCTAACAAATGGCGTGAATGAGAATTTGAACCCTGTTCTTCGCTGATAATAATGCGTCGTTT
CAGTTCAGCGAAACGCACTTTGTAGAACTCGTCGAGGTTATTAGAATAGATTCCCAGGAACCGCATCCGCTCA
ATCAACGGGTTGGATTTGTCCGCCGCTTCCTGTAACACGCGTTCGTTAAATGCTAACCAGCTTAGTTCTTTCT
CGATATATAGCTTTTCCTGACCCATTACTACCTATACTCCGTTTTATTCACGGGACACTGCCTGATGATTATG
GCGAGCATTACGCGTTAATGTCCAACTGCGCCAGAAAAATATGACAATAAACTGATAGCATAAAGGAAGGGAA
AAGAGTAGGCCGGATAGGCATTTATGCCGCCATCCGGCAAGCAGACGCAGTCCTGTTCGATGACGTATTATTC
GTCAGACGCATAACCTTGCGGCGGCAGACGACGCCCGTCCAGCCAGGCGGCGTTATCGCGCATCTTTAGACGC
CCCTGCGCAAACCAGCTAATCACCAGCGGATAAATCGCATGTTCCTGAGTCTGTACGCGTGCGGTGATATCAT
CTTCGCTGTCGCCGGCAAAAACCGGCACCTTCGCCTGGAGAATGACCGGGCCGCCGTCGAGTTCGTCTGTCAC
GAAATGTACCGAGGTACCGTGCTCCTCATCGCCGTTTTCCAGCGCCTGGCGATGGGTATGCAACCCCGGATAT
TTTGGCAGCAGGGAAGGGTGAATGTTCAGCAGACGCCCGTAGTAATGCGCGACAAACATCGGACTCAGAATAC
GCATAAAACCGGCCAGCACGACCACATCGGGCGCATACGCGTCTATCTTGCGAATCAACTCGCGATCGAAGGC
GTCGCGGCTGTCAAACCGATCGGCAGTCAGCGCTTGCGCCGGAATGCCGGCTTCTCTGGCGCGTTCAAGGCCG
AACGCGTCGGCCTTATTGCTGAATACTGCCCTGAGGGTGCCTTTAATTTTCTTCGCTTCGCAGGCATCGATAA
TCGCCTGTAAATTGCTTCCATTGCCGGAAATCAGCACCACAATATTCATTATTCAATAACCACACGCTGTTCG
GAATCAAAGGCTTTGATGATACCGATTTTCCATGCGTTTTCACCTTTCTCGTTTAGCAGAGCAAGCGCTTTGT
CCGCTTCCGGAGCGGAGAGCGCAATCACCATGCCGACGCCGCAGTTAAAGGTACGGTACATTTCATGTCGGCT
GACATTACCGGCGGTTTGCAGCCAGGTAAAGATGGCGGGCCACTGCCATGACGACTCATTAATTACCGCCTGG
GTATTCTCCGGCAGAACGCGCGGAATATTTTCCCAAAAGCCGCCGCCGGTGAGGTGGGCGATAGCGTGCACAT
CGACGTTTTCAATCAGTTCCAGAACCGATTTTACGTAGATACGGGTCGGTTCAAGCAGATGATCGGCCAGCGG
CTTCCCTTCCAGCAGAGTGGTTTGTGGGTCGCAGCCGCTAACGTCAATAATTTTCCGCACCAGCGAATATCCA
TTCGAGTGCGGGCCGCTGGAGCCGAGCGCAATCAGCACGTCGCCTTCGGCAACCCGGGAGCCGTCGATGATTT
CTGATTTTTCGACTACGCCGACGCAGAAACCCGCCACATCGTAATCTTCGCCGTGATACATGCCCGGCATTTC
CGCCGTCTCGCCGCCGACCAGCGCGCAGCCGGATTGCAGGCAGCCTTCGGCAATACCGTTGATCACGCTGGCG
GCGGTATCGACATCCAGTTTACCCGTGGCATAGTAATCGAGGAAAAACAGCGGTTCCGCGCCCTGAACGACCA
GATCGTTTACGCACATTGCCACCAGATCAATACCGATAGCGTCGTGGCGCTTTAAGTCCATCGCCAGGCGAAG
TTTGGTGCCTACGCCGTCAGTGCCGGAAACCAGTACCGGTTCACGATATTTTTGCGGCAACGCGCACAGCGCA
CCGAAACCGCCCAGACCGCCCATAACCTCCGGGCGGCGAGTTTTCTTCACTACGCCTTTGATTCGATCAACCA

GAGCGTTACCCGCATCAATATCGACGCCGGCATCTTTATAGCTAAGAGAGGTCTTATCGGTCACTGCCTGGTT
CCCCACGTTTTTTTTGCGGTAAAAGTTAAATTCGGCGCAATTCTAACAGGGAAAGCAAACGTTTGCGAGCGTG
GTCTGCGCCCTGATTATCCGGGCGATTTTTTTTGCATGGTTTCGGTCGCTGCTTGAAAATTTCCCTGTTGGTA
AAAAAGAAAAGGATATCCAGCACTTGGTTTACGACAGGCGGTCGGGACAGGTCATTCACCCTTAAAATTGCTA
ATATTCAAACGGTTGTTAGCCTTTATCGCCTGTTTCAACGTGAGTGATTTATACTCACTTTTCCGCTATCAGC
GCTTTTGGTTGATCCAGGTCAAGCATACATTGTGTTGCGTCAGAGAGGAAAAGCGGTATAATCCGGCGATTTT
TTTTGTGGTTGCCAGTCATCTGAGGATAGGAGAAGAGTATGAAGATCGTGGAAGTCAAACACCCACTCGTCAA
ACACAAGCTGGGTCTGATGCGTGAAAACGACATTAGCACTAAACGCTTTCGTGAACTCGCCTCAGAAGTAGGC
AGCCTGCTGACGTATGAAGCGACAGCCGACCTGGAAACGGAAAAAGTCACCATCGAAGGCTGGAATGGCCCGG
TGGAAATCGACCAGATCAAAGGTAAAAAAATTACCGTTGTGCCGATTCTGCGCGCGGGTCTGGGCATGATGGA
AGGCGTTCTGGAAAATGTACCGAGCGCGCGTATCAGCGTAGTCGGGATGTACCGTAACGAAGAGACGCTTGAG
CCAGTACCTTATTTCCAGAAACTGGTATCGAACATTGATGAGCGCATGGCGCTGATCGTCGACCCGATGCTGG
CGACCGGCGGTTCTGTCATCGCGACCATCGACCTGCTGAAAAAAGCAGGCTGTAGCAGCATTAAGGTGCTGGT
GCTGGTCGCCGCGCCGGAAGGCATTGCGGCGCTGGAAAAAGCGCACCCGGACGTTGAACTGTACACCGCCTCT
ATCGATCAGGGGCTTAACGAGCACGGATACATTATTCCGGGGCTTGGCGATGCCGGCGATAAGATTTTTGGTA
CCAAATAAGTGAATAAATAATTAAAAGCCGACTTTAAGAGTCGGCTTTTTTTTGAATAAAACCACTCATAACA
AACACACTTAGAGGAAAACACTATGACGCGCCGTGCTATCGGGGTGAGTGAAAGACCGCCGCTTTTACAGACA
ATCCCGCTTAGTTTACAGCACCTTTTCGCCATGTTTGGCGCGACCGTGCTGGTGCCAGTTCTGTTTCATATCA
ATCCCGCGACGGTGCTGCTGTTTAACGGTATCGGAACGTTGCTGTATCTCTTTATCTGCAAAGGTAAAATTCC
TGCCTACCTCGGATCGAGCTTTGCCTTTATTTCCCCGGTACTACTGTTGTTGCCGCTGGGTTATGAAGTGGCG
CTGGGCGGTTTTATTATGTGCGGCGTGTTGTTCTGTCTGGTCTCTTTCATCGTTAAAAAAGCGGGCACCGGCT
GGCTGGATGTGATGTTCCCGCCTGCGGCAATGGGCGCAATCGTTGCCGTCATCGGTCTGGAGCTGGCTGGCGT
CGCGGCGGGGATGGCCGGATTGCTCCCTGCGCAAGGGCAGTCGCCGGACACGAAAACAATTATTATCTCCATG
GTCACGCTGGCGGTGACGGTGTTCGGCTCCGTACTGTTTCGCGGTTTCCTGGCGATCATTCCGATTTTGATCG
GCGTGCTGGCGGGCTATGCGCTGTCATTCGCGCTGGGGGTGGTCGATACCACGCCGATTGCCCAGGCGCACTG
GTTTGCGCTGCCGACCTTCTATACGCCGCGTTTTGAATGGTTCGCTATCCTGACGATTCTGCCCGCGGCGTTG
GTCGTGATTGCCGAGCATGTCGGTCATCTGGTGGTGACGGCGAATATCGTCAAAAAAGATTTAGTGCGCGATC
CCGGTTTGCACCGCTCGATGTTCGCTAACGGATTGTCGACGATCATTTCCGGTTTCTTCGGCTCCACGCCGAA
TACCACCTATGGGGAAAATATTGGCGTCATGGCGATCACCCGCGTTTACAGTACCTGGGTTATCGGCGGCGCG
GCGATTTTCGCCATTCTGCTTTCCTGCGTTGGCAAACTGGCGGCGGCGATTCAGATTATCCCGTTACCCGTGA
TGGGCGGCGTCTCGCTGCTGTTGTACGGCGTTATCGGCGCGTCGGGGATTCGCGTCTTGATCGAATCGAAAGT
CGACTACAACAAAGCGCAAAACCTGATCCTCACCTCGGTGATTTTGATCATCGGCGTGAGCGGCGCGAAAGTG
CATATCGGCGCGGCAGAATTGAAAGGGATGGCGCTGGCGACCATCGTCGGGATTGGCCTGAGCCTGATTTTTA
AACTGATTAGCCTGTTGCGTCCGGAAGAAGTGGTACTGGAGGCAAATGATGCGGAGCCTCCGCATCAGTAACG
GGTTGCCGGGCAGCGATGCTGCCCGGTTCTATCTCACGGGAATTATGTGGTAAACTCAGCGCGATTTTATGTC
ATCCTGGGTTGAGGTATCTCTGAACACACCGGCACAGCTCTCTTTGCCACTTTATCTTCCTGACGACGAAACT
TTCGCAAGTTTCTGGCCGGGGGATAACGCCTCTCTACTGGCCGCGTTACAAAACGTGTTGCGCCAGGAACATA
GTGGATATATCTACCTTTGGGCGCGTGAAGGCGCGGGCCGCAGCCATTTGCTGCACGCCGCCTGCGCTGAATT
GTCGCAGCGTGGAGATGCGGTAGGCTACGTCCCGCTCGATAAACGTACCTGGTTCGTGCCGGAAGTGCTCGAC
GGTATGGAACACCTCTCGCTGGTGTGTATTGATAACATTGAGTGTGTCGCTGGCGATGAACTGTGGGAGATGG
CGATCTTTGATCTCTATAACCGCATTCTGGAGTCCGGTAAGACGCGGTTATTGATCACCGGCGATCGTCCGCC
AAGACAGTTAAATCTGGGCTTGCCCGATCTCGCTTCCCGTCTGGATTGGGGGCAAATCTATAAACTCCAGCCT
CTTTCCGATGAAGACAAACTTCAGGCGCTACAGTTGCGTGCCCGGTTACGGGGGTTTGAACTGCCGGAAGATG
TTGGGCGGTTTTTACTCAAACGTCTTGATCGCGAAATGCGCACGCTGTTTATGACGCTGGATCAGCTCGATCA
TGCGTCGATCACTGCCCAGCGAAAACTGACGATCCCGTTTGTCAAAGAGATTCTGAAACTGTAATACGCCGCG
ACGGCGGCGTTCAACGGTTAACCGAGGATGTCCAGCACCTGCTCAGGCGGTCGGCCAATGCGCGCCTGACCAT
TTGCCACCACAATGGGGCGCTCCATCAGTTTAGGGTGTTCAACCAGCGCCTGGATTAACGCCTCTTCACTCAG
CTGGCTATCCGCCAGGTGAAGCGTTTTATAGAGATCTTCTTTCTGGCGCATCAGTTCGCGCGCGCTGGACATG
CCCAACATACGCAGCAGTTCCCGCACGGTGGCGGCGTCGGCGGGCGTATCCAGATAAAGCACCACTTCCGGTT
CCACGCCGTTAGATTTCAGTAGGTTCAGCGTGTCGCGGCTCTTCGAGCAGCGCGGGTTATGGTAGATTTTAAT
CGTGTCGGTCATGAGTTCTCCTTGTGACGCCATCCGGCTAATAAACGGCGATTACATTTTCGTGTATGGCTTG
AAGCGTTCCTGTAACTGGCGCAGCTGGTCGATACGCGCATCGTAACGCGCCTGTTGCTGGCTACCCAGTTTTG
CCTGGGCGCTGGCGCTACTGAGCAGTGAAATTGCCTGATCCAGTCGTCCCGCCAGCGCATAACTTTCAGCGCG
CGCCGCCAGCTCCTGATCGCGGTTGTTCAGCGCGGCTTCGGCCTGAGCAAGCAGATCCCAGCCGTTACCGTCA

TCTTTATGGCTAAAGGTGTAGCGATTCAGAATGGTTTCCGCCGCTTTTGGCTGGCCTCCCTGGAGGTACGCAT
TGGCGAGGTTTAACTGCAGCACAGGATTAACGCGCAGATCGCGGGCATTTTTCAGGCGATTAATCGCGTCGTT
GGCTCTTTTCTGCCCGAGGTCAATATCGGTGGCGAGGTCAAGATACCAGGCATTGTTCGGTTCTGCGCTTAAC
AGCGGCTGCAACGTTTTGCGCGCTTCATCGTACTTGCTCGCTTCCATCGCCTGCAACGCCCGGCCATATTGCG
CCGCATGTTGCTGACGCACGTTGCCTTTAGACCACTGATCCAGCAGGTCGCTGGTGAGCTGGTTACGTCCGGA
ATTGTACATTCCCAGGGCGCGCGCTTTGGCGAAATAAAAGTCGGCGGAAGATTGCACCACGACCGGGCGCATC
TGGTTGGCACGGTTGCGGGCATCCGCAAGGCGGCTTTCCGGTAAGGGGTGAGTGAGCAAAATTTCAGGCGGAC
GCGTGGAGTAACGCGCCTGGTCGAGCAGTTTTTCGAGGAAAGAGGGCATCGCCTGTGGGTCAAATCCGGCGCG
TTGCAGTACCTGAATACCAATACGGTCGGCTTCTTGCTCATTTTGCTGGGTGAAACTTATCATTCCCTGGCGC
GTTCCCGCCAGAGTACCGGTTAGCGCCGCCATACCGGCCTGTGGGCTGGCCATGGCCAGCAAAATGGAACCCA
GCGCGCCCACCCAGGTAAGCGGCGCGCTGCGCTTTTGATCTTCCATCGCGCGCGCCAGGTGGCGCTGCGTCAC
GTGGGAGATTTCATGCGCCATGACTGAAGCTAGCTGGCTTTCGTTATCCGCGTAGCGAAAAAGCGCCGAGTGC
AGCACCACATTGCCGCCAAAGAACGCGAAGGCGTTGATTTCGTCATTATTGATCAAGAAAAAATGGAAGGGCG
TTTTGACGGAGTCGGCGTGCGAGACCAGACGCATACCCAGCGCGTTAATGTATTGCACCAGCAGCGGATCGTT
GATTAACGGCGCGCTGCCGCGTAGCTGGCGCACATAAAAGTCGCCCATTTGCATCTCTTGTCCGATAGAAAGC
GTGCTTCCTGCCGAGGTTCCCATATCGGGCAGCGTGTCGGCAGGGTCGGCAAATGCGGGCGCGACCTGACCGA
GAGCCAATGCTGCAATGAGGGTTGCCACCAGGTTTTTTTTCAACTGCCTGAACATAACCTCTATCCTGTTGTT
TTGAGATAGCCCTTTGACCAACGTCGTGAAATATCGTTCCACGCTCCGACACTTCATAAGTGTAACTGTGATG
AGCACAGACTCACTATTTTAGTTTCCTGAATCAACGATAAAAAGCGAAGTCTTTTTTGTGACATTTCGATACA
ATTCGGCATCACAATCCCGCAGTTGAGTTCTGGGAAGGGTTTTATGCTCGAAATGTTAATGCAATGGTATCGC
CGTCGCTTCAGCGACCCGGAGGCGATTGCCTTGCTGGTCATTCTGGTGGCTGGTTTTAGCATCCTCTTTTTCT
TTAGCGGCCTGTTAGCGCCGCTGCTGGTCGCAATCGTACTGGCCTACCTGCTGGAGTGGCCGACGGCGCGCCT
GCAGGCGATTGGCTGTTCCCGGCGCTGGGCAGCCTCCATTGTCCTTATCCTCTTTGTCGGCATTCTGTTATTA
ATGGCGTTTGTGGTGATGCCGATTGCCTGGCAGCAGGGTATTTACCTGATTCGTGATATGCCCGGTATGCTGA
ATAAACTGTCCGATTTCGCCGCGACCTTGCCGCGACGCTATCCGGCGCTGATGGATGCGGGCATTATTGATGC
TATGGCGGAAAACATGCGTACCCGGATGCTTAACATGGGCGATTCGGTGGTGAAGTATTCGCTGGCGTCGTTA
GTCGGGCTACTGACGCTGGCGGTATATCTGGTGCTGGTGCCGCTGATGGTCTTTTTCCTGGTAAAAGATAAAG
AGCAGATGCTGAATGCCGTGCGCCGCGTGCTGCCGCGTAATCGCGGGCTGGCGGGGCAGGTGTGGAATGAGAT
GAATCAGCAGATCACCAACTATATCCGCGGAAAAGTGCTGGAGATGGTAGTGGTGGGCGTGGCGACCTGGCTG
GGCTTTTTGCTGTTTGGCCTCAACTATTCGCTACTGTTGGCGGTACTGGTGGGCTTTTCGGTCCTGATCCCCT
ATATCGGCGCCTTTGTGGTGACAATTCCGGTGGTGGGCGTCGCGCTGTTTCAGTTTGGCCTGGGGACGGAATT
CTGGAGCTGTTTTGCCGTTTATCTGATTATTCAGGCGCTGGATGGCAATTTGCTGGTACCGGTACTTTTCTCC
GAAGCGGTGAATTTGCATCCGCTGGTGATTATTCTGTCAGTGGTGATTTTCGGCGGTCTGTGGGGATTCTGGG
GGGTGTTTTTCGCCATTCCATTGGCGACGCTGATTAAAGCCGTTGTCCATGCCTGGCCGGATGGTCAGGTGAC
GGATGCCTCATCCTGAGCGGTCAGTACAAAAGTTGTAAAGCGTGAACGACATCATATGCCCTGCCGCCATTTT
GCGCACGCCTGATGAGGGACAAGCGCTTAGAAAGCCGCTCTTTTTTTTAATGCCGCACCGTGTTGCGGCATTT
TTATTGTCGGAACCTACAAAATTCTCGCTGTAAAGCCGCCAAAATTTGGTGACTTAGCCTAAGCATTCTTCTG
AGAAATTCGTTAATAAGTGACATCATTGATGCGAGATTGTTTTTTTAATGTGAAGCGATAACGAAACAGGGAA
TGAGTGTGTTGAGAAAACATTACTTGAAAGGGTATACCGCGCGGCAAATTGTACAGCGAGCCATGAAAATTAT
TCCTTACTCGGTAAACGTCATGGATGAGCATGGCGTCATTATCGCGTCTGGCGAACCTTCGCGGCTTCGCCAG
CGTCACGAAGGGGCCATTCTGGCGCTGAAGGAAAATCGTTTTGTAGAAATTGATTCCGCTACCGCCAATCAGC
TTAAAGGCGTGCGATCCGGCATTAATCTTCCCATCTCTTTTCATGAACAGCTTATCGGCGTGGTCGGCATTAC
CGGCGAGCCGGAGGAGGTTCGCGCGTATGCCGAGCTGGTTAAAATGGCGGCGGAGCTGGTGATCGAGCATATG
GTGCTGATCGAACAGCGACAATGGGATAAACGCTATCGCGAAGAGCTGATCAACCAATTGATTTTGCGAGAAA
ATTCAACAGAGTCGTTGCGCTCCATGGCGGCCTATCTGGGTATTGATCTGGCGGTTCCCAGAGTGGTGCTGAT
TATTGAACTTTCCCAGCCGGATCGCGAAGCGCTGCGCAATGTAATGGATTATTTCGAGAATCACGCGCGTAAC
CATTTGGTGACGTTTACCGAATTTAATGAATTAATCATTATTAAGCCTATCACGTTAAAAGAGGGAAAGTGGA
ATACCCGCCAGGAAATGGGCGAATTGCAGATTTTTAAATCATGGGCTGCATCATCGGGCTTTAGCCGTATTTT
GGTTGGGGGCTATTTTGCCGGCGAGACGGGATTGCACCGATCTTTGCTCACAGCGAGAGCCACGCAGGCGATG
GCGAAAAGACAAAAGCTGCGCAGCCAGTACATTTTTTATCATGACCACGCGCTTCCCGCGCTGCTAAGCGGGC
TGTCTGAAAGTTGGCAAGTGCAGGAGTTATCGCGTCTGTGGCTGCAACTGGCGCAACATGACGCGAAAGGCGT
ATTGCAACAGACGCTGCGGACTTGGTTTGAACATAATTGTGACCTGACGCAAACGGCCAAAGCATTGCATATT
CATGTGAATACATTGCGCTATCGCTTACAGCGCTGTGAGGATATTACGCACATAAAAATCAACGAGTTAAAAA
GTACGCTTTGGCTTTATATCGGTATGGAGCTTCAGGCCGAATCTGTACCGTCCGACAAGTTACCGCTGCCTGG

TCGAATCGAAATTTGTTGAAAGCTCCAGCGGATTTTTAGGCGGTTTTTTCCATACTGCGCGCTCCATGTTAAA
GGAGGCGCAGTATATGACGTCGATATCCACCCTGGGAGCCATTGCCGCATTGGTTGTGGCCATCGTATTAATT
TTAAGAAAAGTGTCGCCAGCCTACGGCATGATGGCGGGCGCGCTGGTCGGCGGTTTGATTGGCGGCGCCGATC
TTCTGCAAACCGTGTCGCTGATGGTGTCAGGCGCGCAGGGCATCGTTAATGCCGTATTGCGTATTCTGGCCGC
AGGCGTACTGGCGGGCGTATTAATTGAGTCCGGGGCGGCGAATACGATTGCTGAGACGATTGTCCGCAAAGTG
GGGGAAACCCGGGCGTTATTGGCATTAGCCATTGCCACACTATGTCTGACCGCCGTCGGCGTATTTATTGATG
TGGCGGTGATTACCGTCGCGCCGATTGCGCTGTCGATTGCGCGTAATGCAGGCCTGTCAAAAAGCGCTATTCT
GCTGGCGATGGTCGGCGGCGGCAAAGCGGGTAACGTGATGTCACCCAACCCGAACGCTATTGCCGCCTCCGAT
GCGTTTCATGTTCCTCTTACCTCTATTATGCTGGCGGGCGTGGTTCCGGACATCGTTGGGCTTATTATCGCCT
ATCTGTTAGCGAAACGGCTGAATAATAAAGGCGCGGGCGTCGCCGATCATGAAGTGACTCACCATGATGACTC
CGTCGCCAGACCCGGTTTTCTGGTGGCGATTAGCGCGCCGCTGGTCGCTATTTTCCTGCTTTCTCTCCGTCCG
TTTGCCGGCATTTCCATTGACCCGTTAATTGCCCTGCCCGTAGGGGGATTGGTCGGTCTGCTGTTGATGGGAC
GCGCGCGCCACTGTAACCAGTACATGGTCGCTGGTCTGATGCGTATGGCGCCTGTCGCTATTATGTTGCTGGG
AACCGGTACGCTGGCCGGCATTATCGCTAATTCCGAACTTAAAGATGTCCTGATTCATGGCCTCACCGCTTCG
GGATTGCCATCCTGGCTGCTGGCGCCCGTGTCTGGCGCCATGATGTCAATGGCGACGGCCTCAACCACGGCGG
GAACGGCGGTGGCGTCTGGCGTTTTCTCTCCCACGTTACTTGAACTTGGCGTTTCCGCCCTGGCGGGAGCCGC
TATGATCCATGCGGGCGCCACGGTGCTGGACCATCTGCCGCACGGCAGCTTTTTCCACGCGACGGGCGGCAGC
GTCAATATGCAAATTCATGAACGTTTAAAGCTAATGCCGTATGAAACGCTGGTGGGGTTAGCCATTACCTTTA
TTTCTACTCTGATGTTCGGTTTCTTTGGTTTTGCAGGATAATCCCTATGAAAATTGTTATTGCTCCTGATTCA
TTTAAAGAAAGTTTGTCCGCAATGGCGGTCGCTGAGGCGATAGAAAAGGGGTTCCGCGAAATTTATCCTGACG
CCGACTACGTTAAAGTGCCCATGGCGGATGGCGGGGAAGGAACCGTACAATCAATGGTGGAGGCCAGCGGCGG
GCGTTATATTGACCAGTGGGTACAAGGCCCGCTAGGGCAACCGGTGGCCGCGCGCTGGGGAATGCTGGGCGAT
AGCGATACCGCCGTGATTGAAATGGCGGCGGCATCCGGGCTGCATCACGTTTCGCCTGAATTGCGCAACCCGC
TTAATACCACCAGCTACGGCACCGGCGAGTTAATCGTCGCGGCGCTGGAGCGTGGCGTAAAGCGTATTATTCT
GGGAATTGGCGGCAGCGCGACGAATGACGGCGGCGCGGGGATGATGCAGGCGCTTGGCGTTATATTGCGCGAT
AAACAGGGGCGCTCGCTTTCTCCGGGCGGCGAGGCGCTGGCGGCGCTGGCCTCTATCGATCTGTCCGGTTGTC
ACCCATTGCTGCGTAAGGTATCGATAACCGTGGCGTGCGATGTCAACAATCCTCTGTGTGGTCCCCAGGGCGC
ATCGGCCATTTTTGGCCCACAAAAAGGGGCGACAGCGGAAATGGTGAACACCCTGGACGCGGCGCTGGAAAAC
TGGGGCCGACATATTTATCAGGCTACGGGGCGTGAGGTGATTAATGCTCCCGGCGCGGGCGCGGCAGGTGGAA
TGGGCGCCGCGCTTTTAGGATTGCTCAATGCGGAATTACGCGCCGGCGTAGAGATTGTCGTGGAAACGCTACA
GCTTGAGCAGGCGGTTAAAGATGCCGATCTGGTGATTACCGGAGAAGGGCGGCTGGACAGCCAAAGTATTTGC
GGCAAGACGCCGATTGGCGTCGCCAGAGTGGCGAAGCGGTATCATAAACCGGTTATCGCGCTCGCCGGTGGGC
TGCAACACGATCATCACGTTGTGTATCAACAGGGTATCGATGCAGCGCTGTCGATTTTGTCGCATATCGTTAC
GCTGCCGGAAGCCTTACACGAGGCGGAATATAACCTGAGCCTGTCGGCGCGCAATGTGGCGGCGATATGGCGT
CTGGCGCGGCAAGCCTGACGGCGCATTCCCTTTCCAGGCCGCACATCAGCGCGGCCTGAGGCTTTTACGCGTT
TTCTTTCAGCCAGTTCAGCACCACATCATGGTGATTGCTGGTTTTAAAATCATTAAAGACGTGTTCAATCTTA
CCATCAGCGTCAATCAAAAAGCTGATGCGGTGAATGCCGTCATACGTTTTGCCCATAAACGACTTTTCGCCCC
AGACGCCGAACTGTTCACACACCTGATGGTTTTCATCGGACAAAAGGGTAAAGTTCAGCAGCTCTTTTTCAGC
GAAACGGGAGAGTTTTTCAGGTTTATCGGTGCTGATGCCCAGCACGTCCACGCCTGCTTTTTTGAGCTCGTCC
ATGTTATCGCGTAAGCCGCAGGCCTGCACGGTACAGCCGGGTGTCATGGCTTTTGGGTAGAAATAAACCAGAA
CGCGCTGTCCCTGGAAGTCGGTCAAATTTACTTGTTCCCCGTCTTGATCCGGCAAGCTAAATTTCGGTGCGAT
ATCACCGGCTTTCAGTGGATTCATTACTTAACTCCATCCTGTTCATCATGTTGTGAGTAATTGACGACGTTTA
TACTGCCTTGCGCATTGAGTTCTGTACATAGTGCTTTAAAGGCTTGCTCAATATTTGCGGAATTTTGCGACGC
CGGGCTATGTGCGGTGATTTGAATAAATAATTGCGCAGCTTTATCACCTTCAGCCGGCTGCGTGCGGGAGACC
AGCTCCGCAATATTCATTTCATGACTATTAAACAGCGCGGTGAATCGCTCAATTAAATGCGGGGAGTCGGCGA
CCTCAACCTGAACCCATACCGTCGCGGGCATTGCCGGGCGGGGGCGGTCGGACGTCCGTTTCATCACAATCAG
CAGATCCAGCTCCGCGCCTTTTAACGGCAATGTCGATTCAATCAAGGTAATCGCGTTCCACGTGCCGGACAAC
AGCATAATAAACGTGAATTCATCGCCCAGCATCGCCAGTCGGCTGTCTTCGATATTACAGCCGCAACTGCTGA
CATGGCGAGTAATGGTATTCACAATACCGGGCCTGTCGGCGCCCAGCGCAGTGATAACCAAATAGTGTTGTGA
TGACGGTGTCAAACCAGTTATTCCTTTGAGCGGTGAGGTAATCTTAAGGAAACCATAAAAAAAACCTGCATAC
AACAATCAGAACGGCTTTGGTCGCTTGCTTTTATTGCCATACCAAACGTACCATTGAGGGACTTGTTTGCACA
GAGGATGGCCCATGTTCACGGGAAGTATTGTCGCGCTTGTTACGCCGATGGATGAGAAAGGTAACGTCAGTAG
GTCTTGCCTGAAAAAACTCATTGATTATCATGTCGCCAACGGTACCTCGGCGATTGTTTCGGTTGGCACTACC
GGCGAGTCTGCCACGCTAAGCCATGATGAACATGGCGATGTCGTGATGATGACGCTGGAACTGGCTGACGGAC

GTATTCCGGTTATCGCCGGCACGGGCGCAAACGCGACCGCGGAAGCGATTAGCCTGACGCAGCGTTTTAACGA
TAGCGGCATTGTTGGCTGCCTGACGGTAACGCCGTACTACAATCGCCCCACGCAGGAAGGTTTGTTCCAGCAT
TTCAAAGCCATCGCGGAACACACTGACTTGCCGCAAATTCTGTATAATGTGCCGTCCCGTACCGGTTGCGACA
TGTTGCCGGAAACCGTGGGTCGTCTGGCGGAAATAAAAAATATTATCGCTATCAAAGAGGCGACAGGGAACTT
AACCCGCGTTCACCAGATCAAAGAGCTGGTTTCAGACGATTTTATTCTGCTTAGCGGTGATGACGCGTCTGCG
CTGGACTTTATGCAACTGGGTGGTCATGGCGTGATTTCCGTTACGGCTAACGTAGCGGCGCGCGATATGGCTG
ACATGTGCAAACTGGCGGCGGAAGGGCAATTTGCCGAGGCGCGCGCTATCAACCAGCGTCTGATGCCGTTACA
CAACAAACTATTTGTCGAACCCAATCCTATCCCGGTGAAATGGGCATGTAAGGCATTGGGTCTTGTGGCGACC
GACACGCTGCGCCTGCCAATGACGCCTATCACAGACCATGGTCGTGACATCGTCAAAGCAGCGCTTCAGCATG
CTGGCCTGCTGTAAAGTTTAGGGAGATTTGATGGCTTACTCAGTACAAAAGTCGCGCCTGGCGAAGGTTGCGG
GTGTTTCGCTTGTTTTGCTGCTCGCTGCCTGTAGTTCCGATTCGCGCTACAAGCGCCAGGTAAGCGGCGATGA
ATCCTATCTGGATGCCGCGCCGCTTGCTGAACTTCACGCCCCGGCAGGAATGATCCTGCCGATAATGACCGGC
GACTACGTTATTCCGGTCACGAAGGGGAGCGGCGCGGTTGGTAAGGCGTTGGATATCCGTCCGCCAGCGCAGC
CGTTAGCGTTAGTGAGCGGCGCGCGTACCCAGTTCTCTGGCGATACCGCTACGCTATTGGTGGAAAATGGTCG
TAGCAGCACGCTGTGGCCGCAAGTCGTCAGCGTGATTCAGGCGAAAAATTATCCGATTGAAAAACGTGACGAT
GCCAGCCAGACCCTGACGACCGATTGGGTGAACTGGAACCGCCTGGATGAAGACGAACAGTATCGTGGACGTT
ATCAAATCTCGGTGAAGCCGCAGGGCTATCAGCAGGCGGTAACGGTAAAACTGGTTAATCTGGAACAGGCTGG
CAAGCCGGTCGCTGACGCGGCGTCGCTGCAGCGCTATAGCACGGAAATGATGAACGTCATTTCCGCCGGTCTG
GATAAGACCGCTACGGATGCGGCAAATGCCGCGCAGAACCGTTCCGCCGCAACGATGGATGTGCAGAGCGCCG
CTGATGATACCGGTTTACCTATGCTGGTCGTTCGCGGTCCGTTCAACCTCGTGTGGCAACGCCTCCCGGCCGC
GCTGGAAAAAGTGGGCATGAAAGTCACCGACAGCACTCGCTCTCAGGGCAGCATGGCGGTAACGTACAAACCG
TTGTCTGACAGCGACTGGCGGGATCTGGGCGCTAGCGACCCGGGCCTGGCATCCGGAGACTATAAATTGCAGG
TCGGCGATTTAGATAACCGCAGCAGCTTGCAGTTTATCGATCCTAAGGGGCATACTCTGACGCAAAGCCAGAA
CGACGCGCTGGTTGCCGTCTTCCAGGCAGCGTTCAACAAGTAATTTAACAGGGCTGGAGTAATCCGGCCCTTT
TTTCTGATAAGATACGCAAACGTGTGCGTCGGTACAAAAACGCGATTTAACCGCTATTTCGCACCACAATTAC
AGGCCGACGTCTCCGGCAGGCGTAGCAGGAAATGAATACTTATCGTTAATTCACACCCAGGAGTGTTAAAGAT
GCAAAAGCAAGCTGAGTTGTATCGTGGTAAAGCGAAAACCGTATACAGCACGGAAAACCCGGACCTGTTGGTG
CTCGAATTCCGCAATGATACGTCAGCAGGGGATGGCGCGCGCATTGAACAGTTTGATCGCAAGGGCATGGTAA
ATAACAAATTCAACCATTTCATTATGACCAAACTGGCCGAGGCGGGCATCCCGACCCAGATGGAACGTTTGCT
GTCTGATACCGAGTGTCTGGTGAAGAAGCTGGAGATGGTGCCGGTTGAATGTGTGGTGCGCAACCGTGCGGCA
GGGTCGCTGGTCAAGCGTCTGGGCGTGGAAGAAGGCATGGAGCTTAACCCGCCGATCTTCGATCTGTTCCTGA
AAAACGACGCGCTGCACGACCCGATGGTGAATAGTTCCTACTGTGAAACGTTCGGTTGGGTAAGTCAGGAAAA
TCTGGCGCGTATGAAAGAGCTAACCTACAAAGCCAACGACGTACTGAAAAAACTGTTTGATGACGCGGGTCTT
ATCCTGGTGGATTTCAAACTGGAGTTTGGCCTGTATAAAGGCGAAGTGGTGCTGGGCGATGAGTTTTCGCCGG
ACGGCAGCCGCCTGTGGGATAAAGAGACGCTGGACAAAATGGACAAAGACCGCTTCCGCCAGAGTCTGGGCGG
TCTGATTGAAGCCTATGAAGCCGTCGCGCATCGCCTGGGCGTGAAATTAGATTAATTCCGCTTCTGACCGGGG
GGCAACCGCTCTCCGGTATCATCCTTTCCTCATTCTGATAGCGTTTACCTGTGGTAATCCTGCGCCTGACCGC
CTACGATCATCTGTATAACAAAGAGAGAATGAGGTGATTATGCGCTGGCAAGGGCGTCGTGAAAGTAACAATG
TAGAGGACAGACGTAATCGGCCAGGCGGCCCCTCGCTGGGTGGTCCTGGCTTTCGTCTTCCGCGCGGAAAAGG
CGGCATCATTCTGTTGGTGGTGGTGTTGGTCGCAGGGTACTATGGGGTGGATTTAACCGGTCTGTTGACCGGT
CAGCCGGTTTCGCAGCAGCAGTCGACGCGTTCGATAAGCCCCAATGATGATGAAGCGGCAAAATTTACCTCGG
TGATTCTGGCGACGACCGAAGATACCTGGGGGCAACTCTTTCAAAAAATGGGGCGCGGCTATCAGCAGCCAAA
ACTGGTGATGTACCGTGGTATGACGCGCACCGGCTGTGGTGCGGGTCAGTCCGTGATGGGGCCGTTTTACTGC
CCGGCGGACGGCACCGTCTATATCGATCTCTCATTTTACGATGATATGAAAAATAAGCTGGGCGCGGACGGTG
ATTTTGCTCAGGGTTATGTCATCGCCCACGAAGTCGGCCACCACGTACAAAAACTGTTAGGTATTGAGCCTAA
AGTACGCCAGTTACAGCAAAACGCGTCGCAAACGGAGGTGAATCGCCTTTCGGTGCGCATGGAGCTACAGGCG
GATTGCTTTGCCGGCGTGTGGGGACACAGTATGCAGCAGCAAGGTGTACTGGAGGCGGGCGATCTGGAAGAGG
CGCTCAATGCCGCACAGGCTATCGGCGACGATCGCTTACAGCAACAGGGGCAGGGGCGCGTCGTGCCGGATAG
TTTTACCCACGGCACCTCAGAACAGCGCTACAGCTGGTTTAAGCGTGGATTTGACAGCGGTGACCCGGCGCAA
TGTAATACGTTTGGCAAAAACTTTTGATAAACCGGGGCAGGGATGTCTGATATCGATGCGCTACAGGCGTTAA
CGTCGCAAATGACACAGGAAGGCATTCGCCGTTTACTGGTCATCAGCGGCGATGCGGCGTGGTGCCGGGAGCG
GGCCGAGGCGATACGCGCTGCATTGCCCGGTGACTGGCTGTGGGTCGCGCCTGACGCGCCCGCCCAGCCGTGC
TGTACGCCACAGGCGTTACAAACGCTGCTGGGCCGTGAATTCCGTCATGCGATATTTGATGCCTGGCAGGGCT
TTGACGCCGCAGCGTTTGCCGCCCTGAGCGGGACCTTGCAGGCCGGAAGCTGGCTGCTTCTGCTGATGCCGCC

GTACGAGACGTGGGAAAGCCGGCCTGATACCGATTCACTGCGCTGGAGCGACTGCGCGCAGCCTATCCCCACA
CCGCAGTTTGCACAGCATCTTAAACGGACTCTCTCCCGCGATCCGCAAACGCTGCTTTGGCGACAGCACCAGC
CGTTTTGCTGGCCATCTTATCCTTTCCGTGGACGCTGGCGACCCGCCACCGGCGAGCCGCAGCCGGAGCAGGC
GGCTATCTTATCGCGTTTACGTGAAATGCCGCCCGGCGTGGCGACGGTGATTGCCCCCCGTGGGCGCGGCAAA
TCGGCGCTGGCCGGGCAATTCATCTCCCGGATGGCGGGCACGGCTATCGTGACCGCACCGGCGAAAACGGCAA
CGGATATCCTGGCAGCGTTTGCCGGTGAGCGGTTCTGCTTTATGGCGCCGGATGCGTTGCTGGCGAGCGGGGC
AAGAGCCGACTGGCTGGTAGTTGACGAGGCGGCGGCAATCCCCGCGCCGCTGCTGCTGCAACTGGTTTCGCGC
TTTCCCCGCATACTACTCACCACCACCGTTCAGGGCTACGAAGGCACCGGACGCGGTTTTTTACTTAAGTTTT
GCGCCCGTTTTCCGCAGCTTCACCGCTTCACGCTTCGTCAGCCTGTCCGCTGGGCGCCAGAATGCCCGCTGGA
AAATATCGTCAGCGAGGCGCTGATATTCGACGATGAAGCGTTTGCGCAGGCGCCACATGGCGCGATAGCAATC
TCGGCGTTTTACCAACAGGCCTGGGTGAATACGCCCGCGTTGCCGAGGGCTGTGTATCAGTTACTGTCGGGGG
CGCACTATCGTACTTCGCCGCTGGATCTGCGCCGAATGATGGATGCGCCAGGACAGCATTTCTTGCAGGCTAC
GGCGAATAACCGTGTGGCGGGCGCCCTCTGGCTGGTCGAAGAGGGCGGGTTATCCGCAGAACTCAGTCAGGCT
GTATGGTGCGGTTTCCGGCGGCCGCGTGGGAATCTGGTGGCGCAGTCGCTGGCGGCGCACGGTAGCGACCCTT
TAGCCGCGACGCTGGTTGGGCGTCGCGTAAGCCGTATCGCAGTGCACCCCGCTCGCCAGCGTGAAGGCATCGG
TCAGCAGTTGATTGCCTGCGCGTGCATGCAGGCGGCGCAGTGTGATTATCTTTCCGTCAGCTTCGGCTATACG
CCGGAGCTGTGGCGTTTCTGGCAACGCTGCGGCTTTGTACTGGTACGGATGGGCAACCATCGGGAGGCGAGCA
GCGGTTGCTATACGGCGATGGCGTTATTGCCGCTGAGCGACGCGGGAAAACGGCTGGCGCAGCAGGAACACCA
ACGTTTGCGCCGCGATGCGGATATCCTGACGCAGTGGAACGGCGAGGCGATACCGCTGGCGGCGTTAGATGAA
CAGGCGCTTAATGATGAAGACTGGCGTGAGCTGGTCGGTTTTGCGTTCGCGCACCGTCCGCTACTGACATCGT
TAGGTTGCCTGCATCGTTTACTGCAATACAGCGCATTGCCATTACCGGCGCTGAGAGGGCGGCTGGAGGAAAA
AGCCAGCGATGCCGAACTGTGCGCCCGGCTACGCATTTCAGGCCGTAAAGCTCTGCTGGCGTTGCAACGTGCG
CAGGCGGCGCAGGCGCTGATTGCGTTGGATGCCGGACGAACGCAGCGTTTACGCGACGTAATGCCCGGCGGTG
GGGAGCATGCCGGATAGCGATACCTGTCCGGCATTATATCCGGTTACTTTTTCGGCTGATCTTTCTTCGGCCA
GTCTTCTTCATCGTCCCACTTATCGTTAAAATCGCGATGCGGGGGCAGCTCAGGTTTATTGGCAAGGAATTTC
TTATGGTCGATGCGTTTGAGATCTTTGATCACATTCAGCAGTACGCCTACCAGAAACACCAATACCAGAATCC
ACCAATATTTTGCCAGCCAGTCCATGCTTATTTCCTCTTACCGGAACGCTCATCAGGCGACGAGCTGTTCCAT
AATGCGTTGATACATACGGGCCAGTAGCTGGAGATCGGCGGCATTCACACATTCATTAATTTTATGAATGGTG
GCGTTCACCGGCCCCAGTTCTACCACCTGCGCTCCCATACGGGCGATAAAACGTCCGTCGGACGTACCGCCCG
TCGTCAGTAATTGCGGTTTAATTTCATTATAGTGCTCAATGGCGTTTACCACCGCGTCCACCAGCTTGCCGCG
TGCGGTCAGGAACGGCTGGCCGGAAAGCCACCAGTCTACGGTATAGCGTAGCTGATGCTTCTCAAGCAGCGCA
TGTACCCGCTCTTTGATCATCTCATCGGTCAGTTCCGTGCTGAAACGGAAGTTAAACTGCACGAACAGTTCAC
CGGGAATGACATTATTGCTGCCGGTGCCTGCCTGGATATTAGCTACCTGCATGCTGGTGGCCGGGAAAAAGTC
ATTGCCCCGATCCCACTCGATAGCGACCAGTTCATTAAGAAAAGGCGCGGCGCGATGAACAGGGTTATCCGCC
AGATGCGGATAGGCAACGTGCCCCTGAACGCCATGGATAGTGAGGTTACAGGTGAGAGAACCGCGACGCCCGT
TTTTGACGACATCGCCCACAATTTCAGTACTGGAAGGTTCGCCGACCAGACAGTAATCCAGCCGCTCGTTACG
GGCCATCAGCGCTTCAACCACCTTTACGGTGCCATTTTTTGCGCTGGCTTCTTCATCAGAGGTAATCAAAAAT
GCGAGGCGGCCCCGGTGATGAGGATGCTGCGCAACAAAACGTTCAGCAGCCACCACCATGGCCGCCAGTGAAC
CTTTCATATCCGCCGCGCCGCGACCGAACAACATTCCGTCGCGAATCGTAGGTTCAAACGGCGGATTGATCCA
GCGATCGACATCGCCAGCCGGTACGACGTCAGTATGCCCGGCGAACGCCAGCGTTTCGCCACGCCCACGCCAT
GCCCAAAAATTTTGCGTGTCGCCAAAATCCATATGCTCAATGGTAAAACCGATTTTACGCAGGCGTTCAATCA
TTAATGCCTGACAGCCTGCGTCATCCGGACTCAGGGAGGGGCGACGAATAAGTTGCTGTGTCAGCTCAATAAC
CGGGCACGACATAGACTACACCTCATCAAAAAACTGCTGATAACGGGATTCACTGAAACCAAGCAGCATAGGC
TTCCCGGGCGCGCAGAGCAATGGGCGTTTGATGATTGCCGGCTGTTCAACCATGAGAGCAGCGGCGGAGTCGG
CATCGGTAATCCCGCTGCGGTCGCCTTCATCCAGTTTGCGCCATGTTGTGCCGCGCGTATTCAGTAACGGCTG
CCAGCCGAGTTCGGCAATAAAGGTATTAAGAAGAGGGAGGTCTATGCCATCCACACGGTAATCGTGAAAACGA
TAATCAATGCCATGCTCCTCCAGCCAACGGCGCGCTTTTTTGATCGTGTCGCAATTCTTAATGCCATAGAGGG
TAATCATATTGAATCCTTTAACGCGAAATTCGAATAAATAATCAATAGTATCGTCTGCGGGATAATAAGTGTG
GCTGTTAATGGTTATTTATCCAGCGCTGATCGGCAATCAATATAACATTGTTGAGTGAATGTGAATAATGATT
CCAAAACGTTCCAGATGTGGCTTGTTTATAATTCGCGAGGAAAATCCTATTTTGCGCACATGCAATTGATTGT
TTTTTAACCCGATTTGATAAAGTAGTAGAAAAGGTATTAATGTTGCTAAATATTGCAGTAAGTCACATAAAAT
TTACGGCTACAGCGCCATAGTAATATCATAGACAGCATAACCAGAGGATATCGCAACAATCGGTTAAATGAAC
TTCTCTCTATACCGCTAACTACGTAGAATACCCATAATAATAAGAGAGGCTATTATGATTGAACGTGAACTGG
GGAACTGGAAAGATTTTATCGAAGTCATGCTTCGTAAATAAATGCCTGGAAGGCACCGCGCTCCCATAGGGGA

TAAGAAAAGGCGACTCAACGAAAGTCGCCTTTTTTGTGCCCGACACCTCGTATCAGGCTGGCCGGGAGCTTAT
TCCGGGCGCGGCTTCAGCGGGAAGCGGCGGCGTATCAGCACGAAAAACAGGGGTACAAAGAAGATAGCCAGAA
TGGTTGCTGAGATCATTCCCCCCATCACGCCGGTTCCGACAGCATGTTGGCTACCCGAGCCTGCCCCGGTGCT
GGTGGCCATCGGCAATACGCCAAAGATAAACGCCAGCGAAGTCATCAGTATCGGTCGCAGGCGCTGGCGGCTG
GCGTACAGCGTGGCGTCTAACAGCGCGTGTCCCTTCTGATTCATTTCGTTGGCGAATTCCACAATCAGAATCG
CGTTTTTCGCCGAGAGGCCGATAACGGTCAACAGCCCCACCTGGAAGTAAACATCGTTTTCCAGCCCGCGCAT
CCAGGTAGCGAGTAGCGCGCCAATGACGCCGAGCGGCACAACCAGCATCACCGAGAAGGGCACCGACCAGCTT
TCATACAATGCCGCCAGACACAGGAAGACGACTAATAGCGAAATAGCGTACAGCGCGGGCGCCTGCGCGCCGG
AGAGCCGTTCCTGGTAAGACATGGCTGTCCATTCCAGGCCAAAACCGCCCGGTAGCTGATGCACCAACGACTC
CATGACATCCATCGCAGTCCCGGTACTGACTCCCGGCGCGGCCTCTCCGACAATTTCTACCGCCGAATAGCCG
TTATAGCGTTCCAGACGCGGCGAGCCGGTTTCCCAGCGCGAGGTGGCGAAGGCGGAGAAGGGGACCATGCCGC
CATCTTTGTTACGGACATACCAAAGATTAATATCATCCGGCAACATACGATATTTCGCTGCGGCCTGAACATA
GACCTTCTTCACGCGGCCCCGGTCCATAAAGTCGTTGACGTAGCTCGATCCCCAGGCTGTTTGCAGGGTGTCG
TTGATATCGTCAATCGATACGCCCAGCGCCTGCGCTTTTCGTTGGTCAATATCAATTTGCAGTTGCGGGCTGT
CGTCCAGGCCGTTGTGGCGCACGCGGGTCAAGGAACTGTTTTTCCCGGCCAGCTCAATGAGTTGATCTCGTGC
GGCCATCAGCGCGTCATGGCCTGCTCCGGCGTGATCCTGTAATTCCATATCAAAACCGGCGGAGCTGCCCAGA
CCGCTAATTGCCGGCGGGCTGCTGGCGAAGACGCGAGCTTCTTTAATCTGGTTAAATGCTTTTGTCGCCCGCT
CAATAATGGCGAACGAGGAGCCGGTGGTGGGATCGCGCGCGTCCCAGTCTTTCAAGCGAACAAACATGCGCGC
GACGTTTTGCCCGTTCCCGCCAGGGCCGGAACCGACCGTCGAGAAGACCGACATAATGTTGTTTTTCTCATGG
GTGAAGTAATAGTTTTCAACCTTTTCAACAACTTTCAGGGTCTGCTGTTGCGTAGAGCCGCTCGGTAGCTGGA
TAGACGTAGTGAACATGCCCCGATCTTCTTGCGGCAGAAAGGAGGTGGGGAGGCGCAAAAACAGGAACACCAT
TCCGCCAAGCAACAGAACATAAATCAGAATCCAGCGCAGGCTGCGATGCAAAATTTTCGCTACGCCTTTCTCA
TAACGTTCGGCATTACGATTGAAGGTACGGTTAAACCAGCCGAAAAATCCGCGTTGCCCGTGCTGCTCGCCTT
TGTGCAGCGGTTTTAATAACGTTGCGCACAGCGCCGGTGTCAGGATCATGGCGACCAGCACCGACAGCACCAT
TGCCGAGACAATGGTAATAGAAAACTGACGATAAATTGCCCCGGTGGTACCGCCAAAGAACGCCATCGGCACG
AATACCGCAGACAGCACCATCGCGATACCGACCAGCGCCCCCTGGATTTGTCCCATGGATTTGCGCGTCGCTT
CACGCGGCGTGAGCCCTTCTTCGCTCATGATACGTTCGACGTTTTCCACCACCACGATGGCATCGTCGACCAG
GAGCCCGATTGCCAGCACCATCGCGAACATGGTTAATGTGTTAATACTGTAGCCGAACGCGTAAAGCACGGAG
AAGGTGCCCATTAATACTACCGGCACGGCGATCGTCGGAATCAGCGTGGCGCGAAAGTTTTGCAGGAACAGGT
ACATCACCAGGAAAACCAGCGCGATAGCCTCCAGCAACGTTTTGACCACATCGATAATCGAGGCTTTGACAAA
GGAGGTGGTTTCATACGCGATCTTGTATTCCAGGCCGTGAGGGAAGTACTGCGCCAGCTCGTTGAGGCGATCC
AGCACCAGCTTCGCGGTCGCCATTTCGTTCGCGCCGGAAGCCAGCTTAACGCCAAGACCGGAAGCCGGATTGC
CGTTAAAACGGCTGAGGTAGTCATACTTTTCCGCCCCCAGCTCCACGGTGGCGACATCGCCCAGTTTGACCTC
GGAACCGTCCTGATTAACGCGCAGGGTGATATCGCGAAATTGTTGCGGCGTTTGCAGCAGTGACTGCGCATTA
ATGGTGGCGTTCAGCGCCTGCTTGTCGACCGAAGGCGTACCGCCAAGCTGCCCGACGGCGATTTGCGCATTCT
GCGACTCAATCGCATCGGTCACGTCTTTCGTGGTCATCTGAAAACTATTCAATTTGGCCGGATCGAGCCAGAT
ACGCATAGAGTACTGTGAACCATAAGCGTCAATATCGCCGACGCCGTTGACGCGGCTGAGCGGGTCCTGAATA
TTACTGGCGACGTAGTCGGCGATATCCTGCTTGTCCATAGAACCGTCGGTAGAGACGAAAGCGATGGTCAAAA
TATTGGTATCGCCCGTTTTGCGTACCGTGACGCCTTGATCCTGTACCGCCTGCGGCAGTTTACGCATCGCGGA
CTGTAACTGGTTTTGCACCTGCTGAACCGCCTCATCAGGATCGGTTCCCGCAATAAAGCTCAGGGTGATGGTC
GCCTGTCCGGTGCCGCTACTTTGTGATGACATGTACATCAGATTATCGAGGCCCGTCATATTCTGCTCAATAA
CCTGGGTTACGGTATTTTCCAGCGTTTGCGCCGACGCTCCCGGATAATTCGCGGTAATACGTACGTTGGGCGG
TGCCAGATCGGGATATTGTTCAACAGGTAAAGAAAAAATGGCTAACGCCCCTGTCAGACACAACAGGATAGCC
AGCACCCAGGCAAAAATGGGGCGATCGATAAAAAAATTCGCCATTAAAGGGGAACCTCGTGTTTCTACATATC
GTTATAGATTTGAACTGCTTCACTGTAGCGGCAAGCTACGAGCCAAACGTGGAGAAATAAAGGAGATAATGTA
AATTATCATGCGCTTTTACAGCATACTTTTTGTGCCAACGCCGGAAAGCCTGACATCTGGCCTTCCGGCGATT
TGCCGTTCTTTACGTTAACTGACTCTCTTCCTTGTTGGAGGAGCGAAAACTGATACTGACCAGTGTGCCGCCG
CCGGAGGGTTGCGAAAAATTCAGCGTGCCGCCAAGGCGCTCCGCGCGCTCACGCATTATATTCAGACCATAGT
GTCCGGCAGGCTCATGCGGTTCGCCGATACCAATGCCATTATCGCGAATATAAACGGTATGATCGCCGTCAGG
CGCGGTGACGCAGCTCACGGCGATTTCGCTCGCGTTGGCGTGTTTAATGGCGTTCAGGACCGCTTCACGCACA
ATTTGCAATAAATGCACCTGCATTTGCGCATCCAGCGCCAGCGTGGGTAAACGGCAATCCAACGTGAGTTTGG
CCGGAGTCTGACTCTGTAAATCTTCCAGCATCTCATGCAATGCGGAGGGTAAATCGGCCTGCTGTAACGTCAG
ACGGAACGTGGTGAGTAGCTCGCGAAGCTGACGGTAGGCGTCGTTCAGCGCTCTGGAAAAATCCGCCATGATG
CTTTGCGCGCCGGCGTTATCCTCAGGAATAGCGCGCTTCAAAAGCGTTAACTGAATACGTAAATAAGAAAGTA

CCTGCGCCAGCGAATCGTGTAGCTCACGGGCAATCGTCGCGCGCTCTTCCATTAACAAAAGTTGCTGGAAGTG
TTTTTGCGCCTGATTAAAGTACAAACCACGCCCCAGCATCGTGGAGACGCTGTTCAGCAGCGGCGTAGAGGCA
TTAACGTTGGGGCTTTGCCAGTGGAGTTCGCCATAGACCGTGTCCTGCATCGTCACTGGCAGCATTTGCATCG
GCAGGTCGGGGCTTTGCATGCCTTCGCTAATCCGCCAGTTATCGCCCACGGTTAACTCCAGATACCAGGCGGC
GTCATGTTCACGGACAATTTGCAAAATATGGCGAAAACAGTGGACGTCGATTTGGCTGGTATTCAGCGCCTGG
GAGCACTGGTATAACACCTCCAGGCGGCGGTGCGCTTCGTGCAGATCGTGCGTTTTTTCTTCCACCGAGGCTT
CCAGCGAACGATAAAGTTTATGCAGCTCGCTCGACATCTGACTAAACGTTTTCGCCAACAATCCCAGCTCATT
GGGTAAGCTGGTATCCAGCGGCAGCGGGGCGAACTGACCATGTTCAATTCGCTGACTGGCGGTGACCAGTTGA
TTCAGCGGGCGAACGACCTGCTGGCGAATACGTCGTAGGGTAAAAAAGACCAGCGTAAATATGCCGATACCGC
CCGCCAGCGATATGGCGACGACCAGCATGACTTTACGCTCGGCGTAATGCTGTAATGCCAGAACGAAGAGATC
AATTTGATCGACGTAATTGTTGATATTGGTCTGATACCAGGCAATGTCGCCGTCCTGTAGGCGGCTATTCATC
TCCAGCCAGTTGGCGTGCAGGCGCGCGTAGCGAGTTTTCACCGCCTGCGGCACATACCAGGCGTTGAGATTTT
GCAGTACCGGCGAGTTGAGCGCGTGCTGGAAAAGTTGACGATGCGCATTAATCTGCGGGCTACGGCTTTGCAG
ATCGTAACCCAGCCGATAGCTCTGCATTCGTAGCGAACCGGCGATATTGATCGCTTCGGCATCCCGTAAACTG
CTGGTCAGCGTCAGTAATGCGCTGCCGGTGGACAGAATAGACAACAGCACAATATAGAAAAAAGCCTTAGCCA
GACTGGCTGAGACCGGGCGTTTAACCGTCACGCTGGCACTCCCCGCATTTAGCTGAATCGCAATTGCATTTGC
TTTGAAATAAATTGATCTGCCACAGGTTCTGGACAGATAGTTGTGCTTCCTGGGCAGATGAACATTGCCGACC
CCGCCTTCGTCAGTTAATAACAATGCTCATATAAAGAATAAGGTTTTTACAACCAAAACAGAAGGATGCCATG
AATCGTTTTATTATGGCCAATAGCCAGCAGTGTCTGGGCTGCCATGCCTGCGAGGTCGCCTGCGTAATGGCTC
ACAATGATGAGCGACATGTCCTGACGCCACAGCGCTATCAGCCCCGAATTACTGTCATCAAACATCAGCGCCA
GCGCAGCGCGGTTACATGTCACCATTGCGAAGATGCGCCCTGCGCCCGTAGCTGTCCGAACGGCGCAATCGCG
CATATTAACGACAGCGTGCAGGTCAATGCGCAGAAGTGTATCGGCTGTAAATCCTGCGTCGTCGCCTGCCCAT
TCGGCACCATGCAGATGGTATTGACGCCCGTCGCGCCAAACCAGTTCAAAGCCAGCGCGCATAAATGCGATCT
GTGTCAGGGACGTGAACAGGGTCCGGCCTGTGTGGAAAATTGCCCGGCGGATGCGCTGCAACTGGTCACCGAA
GACTCGTTGACCCGTCTGGCTAAAACGCGACGGCTGCGTACCGCCCGGCAGGAGATTCGCCCCTGGCATACCG
TGGATACACAACACAGTGGGACAGCAAGCAGCAAAGTCGAGCGTATGCAGGCGACGCCGCCGCGCGGCGAACC
GGATAAACTGGCGATTGAAGCGCGTAAAACCACCTTTGAGGAAATTTATCTGCCGTTTCGCGCCGCGCAGGCG
GAGCGTGAAGCGGCCCGCTGCCTCACATGCGGGGAACACAGTATCTGCGAATGGACCTGTCCGCTGCATAACC
ATATTCCCCAGTGGATCGAACTGGTGAAAGCCGGCGATATTGATGCGGCGGTAGAGCTTTCCCACCAGACCAA
CTGTCTGCCGGAAATTACCGGGCGCGTCTGTCCGCAGGACCGGCTATGCGAAGGCGCGTGTACACTCCGCGAT
GAGTATGGCGCGGTTACCATCGGCAACATTGAGCGCTATATCTCCGATCGCGCTCTGAGCAAAGGCTGGCGTC
CCGACCTGTCGGACGTACAGAAGAGCGATAAGCGCGTGGCGATTATCGGCGCGGGGCCGGCAGGTCTTGCCTG
CGCTGACGTGCTGGCGCGCCATGGCGTAAGCGCCACCGTCTACGATCGCCATCCGGAAATCGGCGGTTTACTC
ACCTTTGGTATTCCGGCTTTCAAACTGGATAAATCTCTGCTGGCGCGCCGTCGGGAAATCTTCAGCGCGATGG
GGATTCGTTTCGAGCTTAACTGCGAAGTAGGGAAAGATATTTCTCTGGAGACGCTGCTGGAGAGTTATGACGC
CGTCTTTGTTGGGGTTGGAACCTATCGTTCCATGAAGGCTGATTTACCGAATGAAGATGCGCCGGGGGTATAT
GATGCGTTGCCATTCCTCATCGCCAATACCAAACAGGTGATGGGGCTTCCGGCGTTGCCTGACGAGCCGTTTA
TTGATACTGCGGGCCTTAACGTGGTTGTGCTGGGCGGCGGTGACACCGCAATGGACTGCGTACGTACGGCGCT
GCGTCATGGCGCTGCTAACGTCACCTGCGCTTATCGCCGCGATGAGGCCAATATGCCGGGGTCGAAAAAAGAG
GTGAAAAACGCGCGGGAAGAGGGCGCGAACTTTGAATTTAACGTTCAGCCGGTTGAGTTGGTGCTGGATACGC
ACGGGCGCGCCAGCGGTATTCGCTTTTTGCGTACGCGGCTTGGCGAACCGGATGGTCAGGGGCGACGCCGCCC
GGTACCTGTACCGGACAGCGAGTTCGTGATGCCGGCGGATGCGGTCATTATGGCGTTTGGTTTTCATCCGCAT
GGTATGTCGTGGCTCGAATCGCATGGCGTGAAGGTGGATAACTGGGGACGTATTGCCGCCAGCGTCGAGAGCG
AGTTTCGTTACCAGACGTCCAACCCGAAAATTTTCGCCGGCGGCGATGCGGTGCGCGGCGCCGATCTGGTGGT
GACGGCGATGGCAGAAGGCCGACATGCTGCGCAGGGGATACTCGACTGGTTAGCTAAATAAGCGTATTTGTAG
GCCGGTTAAGGCCTGGAACGGTTAAATGATTGAGTGATAAAAAATCTTAAACTTATTGGCTAACCTTACTCCG
TTTGCAGAAATGTTGACGCTCGCAGGGCTGAACGCCCTGCGATAAACATTTTCCCGTCGGGTCGACGTTTAAC
AGGCCTGATGAGGCCAATCTTAGTATTGAAAATAGAGTGTAAATTGAAGGAAGGGACGCTATCAGAATCCCTT
CTTAGAGCCTATCCCATTAGGGCTATTTTACTTGCCATTTTGGACCTGGGCAGTGCTCGCCAAAACGCATTAG
CGTTTTGAACGCCGCTTGCGGCGGCCCGAAGGGCGAGCGTAGCGAGTCAAACCTCACGTACTACGTGTACGCT
CCGGTTTTTGCGCGCTGTCCGTGTCCAAACTGGCTGCGCCAATAACGCCTACAGAGATAGGCTCTTAATTTTT
ATTTTAAGGAACCATATATCTGGTCAACAATCCAGCCATACGTCGCCGCAGGTAATAATCCACTGACAAAAAC
ATTCGGGTTCACTTTAGGATTTGCTACCGGCGAGTGCGGCCTGTTACCTAAAATAACAATCGCCATATGATTA
ACCGGATCGATAGACGTCAGCGTTCCGGTCCATCCGGTATGTCCATACGTTTGCGGACTGGCTAATACCCCGA

ACGTTGGGGTCATTGAGGCGTTGCCGTTAACGCGCCACCCCAGACCAAAGGTAGCATCTTCTGGCGAGTGGCG
AGTAAACTGCGCAACCGTTTTGTCGTCGAATAGCTTCAGGTTACCGTAGCCGCCCCCATTGAGCATGACTTGC
ATTAACACAGCCATATCATGGGTGTCAGAAAACAGTCCGGCGTGTCCTGAAACGCCGCCCATTGAGTACCAGG
CTTTTTCGTCATGCACCTGGCCCCATAGTGTATTGGTGCGAATATTGGGGAAATGAATCACGCCGTCACGCGT
ATTGCCGTGTAGTTCCGTTGCGGCGATTTGCGGCGGCGTGAAGCCTTTCATCAGCGGGTTAAAGACGGTATGT
TTTAATCCGAGCGGTTTATAAATGGTGGTTTCGACGTAGCGATCTAATGGCATTGCCGTTATCGATTCAATGA
TAAAACCAAGAATCATATAATCCACGTCGCTGTAGATATGCTTCGACCCAGGTTGATATTCCAGCGGTGTCTT
TTTAATCATCTCCAGAGTAGTGCTTTTACTCTGTGAAAATAATTTACCGGCGACGTTTTTATTGGGATATTGT
GGGTCGGCAGGAAAGCCCGCGACGTGATGAAGAATATCAATAATGCGGAGTTTATCTTTGCCTTTTATTTTAT
CCCCAGGCATATCCTTAAAGCCGGGGATATATTTTGATACCAAATCATTGACATCTATTTTTCCTTCATACAC
TAGTTTTTGCAATGCGAAATTAGTGGCATACATTTTTGTGTTTGAAGCCAGATCATACATCGTGTTAGTGGTC
GCCCGAATGGGATGGGCTAACAGCGTGGAACCGTCATATTTTTTGGCATAGCCCCAGGCTTTTTGTAGAACAA
TATGATTATCTTTTATCACCAGAAGATTGATGCTGGGATATCCGGCATCAATTTGATTTTGAATCCAGCCATC
AAGTCGGTGAAGTTTTTGGCTATCAAACCCCACCTGATCAGGGCTCGCGATGGTCAATACCGGATACTCAACG
GCAAAAGCTGAAAGGGAAAACGTCAGTAGAACCGTCGCAACGAGAGTAAATTTCATGATGATTCCCTCAGTCT
CACTCCGGCCGTCAAATCATATCGCGAGCATGGACTCTGCGGCCCGGAGAAGCAGAATATGGTCATGGCAGTG
ACAGAAAAATATGGCTGTACATCCGTTCAGTCGGCATTAAGGATTAACCCTTCAGCAGACAAAACCGACCTCA
CGGCGTAATATTAATGCTCTTTTTACACCCTGGAGCCAGCATGTCGCAAACCATTACGCTTATTAAGGATAAA
ATTCTTTCTGATAATTATTTTACTCTGCGCAACATTACCTATGACCTGACCCGTCGTAATGGCGAGGTTATCC
GTCACAAGCGTGAGGTTTACGATCGCGGGAATGGCGCAACCATTCTGCTTTACAACTCAACTAAAAAAACCGT
GGTTCTGGTTCGCCAGTTTCGCGTGGCGACATGGGTCAATGGTAATCAAGACGGTATGTTAATTGAAACCTGC
GCTGGCCTGCTTGATAACGACGAGCCGGAGGTCTGTATCCGTAAAGAGGCCATTGAAGAGACAGGGTATGACG
TTGGTGAGGTACGCAAGATATTTGAGCTGTATATGTCGCCAGGCGGCGTCACGGAGCTGATTCATTTCTTCAT
TGCCGAATATCATGATAGCGAGCGTGCCAGCATCGGCGGCGGCGTGGAGGACGAAGAGATTGAGGTGCTTGAA
CTGCCGTTTTCCCGGGCGCTGGAAATGGTGCGCTCAGGCGAGATTCGGGACGGTAAGACCGTGTTATTGCTTA
ACTATTTACAGACGTCTCATCTAATGGACTGAAAAAAAGAAATATATTTCTTTACACAAAAAAGACCAATCCG
ATAAATCTGATTGAGAACCCTGAGGCGCGCAAGGAAGATACCGCCTTAGCTGTGTTGTTCTGAATCTGGGGTT
GTTCCGTTCATGCGCTACCGCGTTATTCTTTTTGTCTGTTTGGCCTGTTGCCGGTCCAGCTACTGTGGGCTGC
GCCAGCGCAACGGACTTTTTCCGACTGGCAGGTCACCTGCAATAACCAAAATTTTTGCGTAGCGCGCAATACT
GGCGAACATCATGGGCTGGTGATGACATTAAGCCGCAGCGCCGGGGCGCGTACCGATGCGGTACTGCGTATCG
ATCGTGGCGGTTTGGCGCCGCCCGACGCTAAAGAAGCCGCAATTGCGCCTCGCTTATTGCTGGATGGCAAACC
GCTTTCGTTCAACAGTCCACACTGGCGCGTGTCGCCCTGGCATCTCATGACCGGCGATCCTGCCACCATCACC
GCATTTTTACAGACGATTCAGGATGCGCAGGCCATCACCTTAAAAAACGGCGTTCAGACGCTTTCGCTGGCGG
GATTAAAAGCGGCATTACTGTTTATCGACGCGCAACAAAAACGTGTGGGCAGCGAAACCGCCTGGATTGAAAA
AGGAAACGAACCGCCTCTCAGCGTGCCCCCGGCACCGGCATTAAAGGGGATCGCCGTGATTAATCCAACGCCC
GTACCGCTGAGTGAGGAAGAACGCGACGATCTGCTGGACTACGCAGCATGGCGGGTAAACGGCATTCGTTGTT
CGCTTGACCCGCTGCGTCGCGAGACGCAGGTCAGCGCGTTGACGGATGATAAAGCGTTATTGATTGTTAACTG
CGAAGCCGGCGCCTATAACACTATTGATCTCGCCTGGGTAGTTTCACGCAAAAAAACGCTCGTCTCGCGCGCC
GTACGTTTGCGGCTGCCGTTTAATCGCGGCGTCGAAAGTAATGACATGGAGTTGATGAACGCCTTCTTTGACG
AGAAAACGCGTGAACTGGTGACGTTGGCGAAAGGGCGGGGGTTAACCGACTGCGGTATCCAGACGCGCTGGCG
TTATGATGGCGATCGTTTTCGTCTGGTGCGTTATGCCGAAGAACCGAGCTGCGATAGCTGGCACGGGCCGGAT
GCCTGGCCTACGCTGTGGATCACGCGTTGAATCCTGGTTGTTATCCCCTTTTCAGAATAGGGATACACCTATT
GAGGTTTCATTTATGGTATAGAGATTGCTACGTTACGGTAGGATTGTTACTTTACCCGATAGCCTCACCACAG
CAGGAAATAGTATGAAAATAAATAATGGCCCCGTATTATGCCCCCATTGTGGCTGCCTGAGCGCTTATTATGA
AATAGATCGGCTGGCGGCGATAAGAGAAAAAGTGAATAAAGAAGGTGGATCAGCGGCGTGGGATTCAACATTG
CAGGCGCATAAAAAAAAGGCCTTTTGCCTGATGTGCCATAAATCCATTGATGAGGTCGTCATCGGGCAGTCAG
ATGCGCCTGAGTCTACGAAGTAATTTTGCACAGCCTACAGAACAGGTCTGCAGACTGTGCAAAGTCTGTGTTG
CTAACGCTCGCCTTAGCCTTTGATGTTCAGCACCCGGCGGGCTTTTTCTACAATATTTTCCACGGTAAAGCCA
AAATACGGGAACAGTTTGTCAGCCGGGGCGGATTCACCGTATCCCGTCATGCCGATAATTGCGCCTTTTAGGC
CAACATATTTATACCAGTAGTCGGCTATACCGGCTTCCACCGCGACGCGGGCGGTCACGTGCGCCGGTAGCAC
AGATTCGCGATATGCCTCGTCCTGCGCATCGAAAATATCCGTTGAAGGCAACGACACCACGCGAACGTTATGC
CCCTCGCCAGTCAATTTTTCCGCCGCCTGGAGGGTAATTTCCATTTCCGATCCGGTAGCGATCAGAATGATAT
CCGGCTTGCCGCCGCTATCTTTCAGAATATAACCGCCGCGGGCAATCGCTTTCACCTGTTCCGGCGTGCGTTC
CACCTGCGCCAGATTTTGCCGCGAGAGAATCAACGCCGTCGGCCCGTGCTGACGTTCAATGGCCAGCTTCCAG

CCGACCGCCGCCTCAACCTGATCGCAGGGACGCCAGGTGCTGAAGTTTGGCGTTAAGCGCAGGCTCGCCAGTT
GTTCCACGGCCTGGTGCGTCGGGCCATCTTCGCCTAAACCGATGGAGTCGTGGGTATAGACCATAATCTGCCG
GGCTTTCATTAAGGCCGCCATACGCGCCGCGTTACGCGCATATTCGACAAACATCAGAAACGTGGCGGTATAC
GGTACAAAGCCGCCGTGATGGGCGATGCCGTTGGCAATGGCGGTCATGCCGAATTCGCGCACGCCATAGTGAA
TATAATTACCTGCCGGATCCTCTTTCAACGAGGTCGAACCTTTCCAGATAGTCAGGTTGCTCGGCGCCAGATC
CGCCGATCCGCCCAACAGTTCCGGCAACAGGGGACCGTAGGCATTGAGCGTATTTTGCGATGCTTTACGGGTG
GCGATTTTGGCCGGATTAGCCTGCAAGTCGTTAATAAATTTTTGCGTGGCGGATTCCCATGCCTCCGGCAGTC
CGCCGCTCATCCGGCGGGTAAACTCCGCGGCCAGCTCAGGATAAGCCTTCTCATAAGCAGCAAACTTTTCCTG
CCACTGCTGCTGCGCTTTCTCGCCTTTTTCACGGCCATCCCACGCGCGGTAAATTTCTTTTGGGATCTCAAAC
GCCGGATGATGCCATCCCAGCTTCTGGCGAGTCAGCGCCACCTCTTCTTCGCCCAGCGCCGCGCCGTGCGACT
CCTCCTTCCCGGCTTTATTCGGCGAGCCAAAACCTATCACCGTCCGACAGATAATTAGCGAAGGTTTATCTTT
TACGCTCTGGGCTTCGAGAATGGCTTTTTTCACCGCCTCCGGATCGTGCCCGTCAATGTCATATACGACATGC
CAGTGGTAGGCTTCAAAACGTTTGGCGGTATCGTCGGTAAACCAGCCTTCGGTTTCGCCATCAATGGAGATGC
CGTTGTGATCGTAAAAGCCGATCAGTTTGCCCAGTCCCAGCGTACCAGCCAGCGAACACACCTCATGCGAGAT
CCCCTCCATCAGACAGCCGTCGCCCATAAAGACATAGGTGTAGTGGTCGACAATCTCATGGTCCGGCCGGTTG
AACTGTGCGCCTAACGTACGCTCGGCAATCGCCAGCCCGACGGCATTCGCCAGCCCCTGGCCCAGCGGTCCGG
TGGTGGTTTCAACGCCGGGCGTATAGCCAATCTCCGGGTGACCTGGCGTTTTAGAGTGCAGTTGACGGAAGTT
TTTTAACTCCTCCAGCGGCAGGTCATAACCGGTTAAATGCAGCAGACTATAGAGCAGCATGGAGGCGTGACCG
TTGGAAAGGATAAAACGGTCACGGTCATACCAGGTTGGATCGGTGGGGTTATGTTTAAGAAAATCGTTCCATA
GCACTTCAGCGATATCCGCCATCCCCATCGGCGCGCCGGGGTGCCCGGAATTGGCTTTTTGAACGGCATCCAT
GCTCAGCGCACGAATCGCATTGGCAAGGTCTTTACGGGACATAAATCACTCCATGCAAGGTTTATAGTTTGGC
GGCAAGCAGGTCTTCCAGTTTGCGCTGGTCAATGGCAAAAAGACGGATACCTTCCGACAGCTTCTCTACGGCC
ATCGCGTCCTGATTATGTTCCCAGCGGAACTCCGCTTCCGTCATTGGCGTAGGACGGTGGAACATCTGCGAGG
AAGGCACCAGTTTACGGATCACCGGTTCTTCTTTTTCTTTCAGCTCTTTAAGCAGGTTAGGGGAGATAGTCAG
TCGATCGCAGCCGGTTAGCGCGAGGATTTGCTCAGTACGGCGGAAACTTGCCCCCATCACGATGGTTTCATAG
CGATGCTGTTTAAAGTAATCGTAGATATTGCGTACCGATTTCACGCCGGGATCTTCCTCCACGACATACGGCT
CCAGCGGACTACGCGCCTGATACCAGTCGTAAATTCGGCCCACGAACGGAGAGATCAAATAGACGCCCGCTTC
GGCGCAGGCGCGCGCCTGGGCAAAAGAGAACAACAACGTCAGGTTGCAATTAATACCCTCTTTTTCGAGCTGC
CCGGCGGCGCGGATACCTTCCCAGGTCGCGGCGAGCTTGATCAGAATGCGTGATTTATCAACACCCTGCTGCT
GGTAGAGATCCACCAGATGGCGCGCTTTCTCGATACTTTTTTCTTTATCGAACGATAACCGCGCGTCGACTTC
AGTGGAGACGCGACCGGGAATACTTTTCAGTATTTCCGCGCCAAAATTAACCGCCAGTTTATCGCTGGCTGCC
GCAACCTGCTGTTCCTGCGTACCGCCGTGTTTTTTCCCCCAGGCAATCGCATCCTCAATCAAATGACCATACT
GCTCAAGCCCGGCGGCTTTTAACAGTAAAGAGGGATTTGTCGTGGCATCCTGCGGCTGGTAGTGGCGGATGGA
CTCGATGTCGCCGCTATCGGCGACGACGGTAGTGAATTGTTTGATGCCGTCTAGCTGGTTCATAGGAAATACT
CCTTGAAAAGTAAAGTGTTAGATGAGTGTGTTGATTCACACTTCTGAAAGATTGATACACTACATAACCAAAA
AGCATAGCAGACAGGCATGGTATTGCTGGTATAAGCAGGTAACATGGCCGTTATAAGTTGATAACAAACTCTT
GCGCCGCGTCGTGATAGTTTGCAGACCTTCAAAGTTTGTGTTGCGCAGTACGGTGATAATGTGCGGCGCACCA
GGCCGCGTCACTTTTTGAACGATACGTGAAAGGAACAACAAAATGGATGAGCAGTTAAAACAAAGCGCCCTTG
ATTTCCACGAATTTCCGGTACCCGGTAAAATTCAGGTTTCTCCGACTAAGCCCCTTGCCACCCAACGCGATCT
GGCGTTGGCTTACTCGCCAGGCGTGGCGGCGCCCTGCCTGGAAATCGAAAAAGATCCGCTGGCAGCCTACAAA
TACACCGCGCGCGGCAACCTTGTCGCGGTCATTTCCAACGGTACAGCGGTGCTGGGGTTAGGCAACATTGGCG
CGCTGGCCGGCAAGCCGGTCATGGAAGGCAAAGGCGTTCTGTTTAAAAAATTCGCCGGCATTGATGTGTTTGA
CATTGAAGTCGATGAGCTTGACCCGGACAAATTTATCAATGTGGTCGCAGCGCTGGAGCCGACTTTTGGCGGC
ATTAACCTCGAAGACATTAAAGCGCCGGAATGTTTCTATATTGAACAGAAACTGCGCGAGCGCATGAACATCC
CGGTTTTCCATGACGATCAGCACGGAACGGCGATCATTAGTACGGCCGCCATTCTTAATGGTCTGCGAGTCGT
AGAGAAAAATATTTCCGACGTACGGATGGTCGTCTCCGGCGCAGGGGCGGCGGCAATCGCTTGTATGAACCTG
CTGGTCGCGTTGGGGATGCAAAAGCACAACATCGTGGTGTGCGACTCTAAGGGCGTTATTTACAAAGGCCGTG
AGCCGAATATGGCGGAGACCAAAGCGGCCTATGCGGTAGACGACAGCGGCAAGCGCACGCTGGATGAGGTCAT
TGACGGCGCGGATATTTTCCTCGGCTGTTCAGGCCCGAAAGTGCTGACTCAGGAGATGGTGAAGAAAATGGCC
CGCGCGCCAATGATTCTGGCGCTGGCTAACCCGGAACCGGAAATTCTGCCGCCGCTGGCGAAAGAAGTGCGTC
CGGACGCCATCATCTGTACCGGTCGTTCCGACTATCCGAACCAGGTGAACAACGTATTGTGCTTCCCGTTTAT
CTTCCGCGGCGCACTGGACGTCGGCGCGACGGCGATCAACGAAGAGATGAAACTGGCGGCGGTCCGTGCGATT
GCCGAGCTGGCGCACGCCGAACAGAGCGAAGTGGTGGCCTCGGCCTATGGCGATCAGGACCTGAGCTTTGGGC
CGGAATACATTATTCCGAAACCGTTTGACCCGCGTCTGATTGTCAAAATCGCTCCGGCGGTCGCGAAAGCGGC

AATGGATTCCGGCGTGGCGACGCGCCCGATTGCTGACTTTGATGCCTATATCGATAAACTGACCGAGTTTGTC
TACAAAACCAACCTGTTCATGAAGCCGATCTTCTCGCAGGCACGCAAAGATCCGAAGCGCGTAGTGCTGCCGG
AAGGGGAAGAGGCGCGCGTGCTGCACGCAACCCAGGAGTTGATAACCCTGGGACTGGCGAAGCCCATCCTGAT
CGGACGTCCGAGCGTGATCGAAATGCGTATCCAGAAACTGGGGCTACAGATCAAAGCGGGCGTTGATTTCGAG
ATCGTGAATAATGAATCCGATCCGCGCTTCAAAGAGTACTGGAGCGAGTATTACCAGATCATGAAGCGTCGCG
GGGTAACTCAGGAACAGGCGCAGCGTGCGATGATCGGCAACCATACGGCCATTGGCGCGATCATGGTGCAGCG
TGGGGAAGCGGATGCGATGATCTGCGGCACCATCGGCGACTACCATGAGCATTTCAGCGTCGTGAAAGCCGTC
TTTGGCTACCGTGACGGCGTGCATACGGCAGGGGCGATGAACGCCTTACTGCTGCCAAGCGGCAACACTTTTA
TCGCCGATACTTATGTTAACGAAGATCCCACGCCGGAACAGTTGGCGGAGATCGCCGTAATGGCGGCGGAAAC
CGTTCGCCGCTTTGGTATTGAGCCGAAAGTCGCGCTGTTGTCGCACTCTAACTTTGGTTCCTCAAATTCGCTG
TCCGCCAGCAAAATGCGCGAAACCCTTGAGCGGGTACGCGAACGAGCGCCCGATCTGATGATTGACGGTGAGA
TGCACGGTGATGCGGCGTTGGTGGAAAGTATTCGTAATGACCGGATGCCGGATAGCCCGCTGAAGGGAGCCGC
CAATATTCTGGTGATGCCGAATATGGAAGCCGCCCGCATTAGTTACAACTTACTACGCGTCTCCAGTTCCGAA
GGCGTCACGGTCGGCCCGGTCCTGATGGGCGTGTCTAAACCGGTACATGTATTAACGCCGATCGCCTCTGTAC
GCCGTATCGTGAATATGGTGGCGTTAGCGGTAGTTGAAGCGCAAACCACGCCGCTTTAATAACGTATTACTCT
CTCTTCTGGCGCAGGTTCTCCTGCGCTTTTTTTATGCGCCGCTGGGCTTCGCCATGGCATGAAAGGCTCGAGT
GGGGATATTCTGGAACCTATTGCGTTACCCCTACTTATTTCACTGAATCTGTACTATGCACAAATCGAGTGAT
ACGCATTATTTTTATCAATAATAGTAATATAAAGAATAGTCGGTGGGTATTTATTGCGGAACAGGCTTAAATT
CAGAATGACGCCTGAAAATGTGATGCACCACGATATTTATCGTTAACACCTTCGACAGAAGTCCCTTTTATTT
TCTTTCTCTTAGTGATCTACCTCACCTTTTACATCACCTTGCCGAATTTTGTTATTTACTCCGACAAAAAATT
GCCACGATGACGGCAGTTTCAGTGGAGACGGTGAGCAATGAATAAAGAACGCATTATTCAGGAATTTGTGCCG
GGCAAACAGGTCACTCTGGCGCATCTCATTGCGCACCCTGGTGAAGAACTGGCGAAAAAGATCGGCGTTCCCG
ATGCTGGCGCGATTGGCATTATGACCTTAACGCCGGGGGAAACGGCGATGATCGCTGGCGATTTAGCGATGAA
GGCTGCGGATGTACACATCGGTTTTCTCGACAGATTTAGCGGCGCGCTGGTGATTTATGGCACCGTCGGCGCA
GTAGAAGAAGCGCTATTACAGACGGTCAGCGGGCTGGGGCGATTATTAAACTTTACCCTGTGTGAGTTAACAA
AAAGTTAATTGGTGGCGGTAATGAAACGTATTGCTTTTGTCGGCGCGGTCGGTGCCGGAAAAACAACGCTTTT
TAACGCGTTACGGGGAAATTATTCCCTCGCCAGAAAAACGCAGGCCGTGGAATTTAATGATCATGGCGATATC
GATACGCCGGGTGAATATTTTAGCCATCCCCGTTGGTATCACGCCTTAATTACCACGTTGCAGGATGTGGACA
CGCTGATTTATGTCCACGCGGCAAACGATAAAGAAAGTCGCTTACCTGCCGGGTTATTGGATGTTGGAACCCG
TAAACGACATATCGCCGTTATCAGCAAAACGGACATGCCGGATGCCGACGTCGCCGCTACGCGGCAGTTGCTT
TGCGAGATTGGATTCCGGGAGCCGATTTTCGAGCTCAACGGCCACGACCCGCAAAGCGTGCGGCAGTTGGTGG
ATTATCTGGCGGCTCTCAGTGAACAGGAGGAAGAGGCAGGTGAAAAAACTTATCACAGCTAACGATATTCGTG
CGGCCCACGCCCGCGGCGAACAGGCGATGTCGGTAGTGCTGCGCGCCAGCATTATTACCCCGGAGGCTCGCGA
AGTGGCGGAACTACTGGGCTTCACGATTACCGAATGCGACGAGTCCGTTCCGGCATCCACATCGGCGCAGGCG
TGTAAAAACGAAAGCCAGCGCATTCGGGAAGCCATCATCGCGCAGCTCCCGGAAGGGCAGTTTACTGAAAGCC
TGGTGGCGCAGTTGATGGAAAAAGTGCTGAAGGAAAAGCAGTCGTTGGAACTGGGGACGATGCAGCCGAGCTT
TACCTCCGTCACGGGTAAAGGCGGCGTGAAAGTCATTGACGGCAGCAGCGTGAAATTTGGCCGCTTCGACGGC
GCCGAACCGCACTGCGTCGGCTTAACCGATCTGGTTACAGAGCAGGACGGCAGCAGCATGGCCGCCGGGTTTA
TGCAGTGGGACAACGCCTTTTTTCCGTGGACGCTGAACTACGACGAAATCGACATGGTGCTGGAGGGCGAACT
GCACGTACGCCATGAAGGCGAAACCATGATTGCCAAAGCCGGAGATGTCATGTTCATCCCTAAAGGCTCCAGC
ATTGAATTTGGCACGCCAACGTCCGTGCGCTTTCTGTACGTCGCCTGGCCTGCGAACTGGCAATCCGTATGAA
CGATTTCATCACCGAAACGTGGCTCAGAGCGAATCATACGCTCAGCGAAGGATCGGAGATCCATCTGCCCGCT
GATGCACGACTGACGCCCTCGGCCCGGGAACTGCTGGAAAGTCGCCGCCTGCGCATTAAGTTTCTGGATCCGC
AAGGCAGGCTGTTTGTCGATGATGACGAACAGCAGCCGCAGCCGGTACATGGCTTAACCAGTAGCGATACGCA
CCCACAGGCCTGCTGTGAACTGTGCCGCCAGCCGGTGGTGAAAAAGCCCGATACGCTGACCCATCTGACGGCG
GACAAAATGGTCGCCAAAAGCGACCCGCGCCTCGGCTTTCGCGCCGCGCTCGACAGCGCCATCGCGCTGACCG
TGTGGCTACAAATCGAACTGGCGGAACCCTGGCAGCCGTGGCTTTTCGATATCCGCTCGCGGCTGGGCAACAT
TATGCGCGCCGACGCTATAGATGAACCGCTGGCGGCGCAGGCGATCGTCGGGCTCAACGAGGATGAGCTGCAT
CGGCTTTCCCACCAGCCGCTGCGCTACCTCGACCACGATCACCTGGTGCCGGAGGCCAGCCACGGTCGCGATG
CGGCGCTGCTTAATCTGCTGCGAACCAAAGTCCGCGAAACGGAAACCCTCGCCGCACAAGTGTTTATTACCCG
TAGTTTTGAGGTCTTGCGCCCGGACATTTTACAGGCGCTGAACCGTCTCTCCAGCACGGTTTACGTGATGATG
ATTTTGAGCGTGGCGAAACATCCGCTGACGGTCGCCCAAATTCAACAACGGTTGGGAGAGAAGCCATGATCAT
TGAACGCGCCCGCGAACTGGCGGTACGCGCCCCCGCCAGAGTCGTCTTTCCTGATGCGCTGGACGAACGCGTG
CTGAAAGCGGCGCATTACCTGCAACAGTACGGTCTGGCTCGCCCCGTTCTGGTCGCCAGCCCGTTTGCGCTGC

GTCAGTTTGCCCTGAGTCATCGTATGCCGATGGATGGCATTCAGGTCATCGACCCACACAGCAACCTGAGTAT
GCGCCAGCGTTTTGCGCAACGCTGGCTTGCCCGCGCCGGAGAAAAAATGCCGCCGGACGCAGTGGAAAAGCTT
AGCGACCCGCTGATGTTCGCTGCCGCCATGGTGAGCGCCGGTGAGGCTGATGTGTGTATCGCAGGCAACCTGT
CGTCAACGGCGAATGTGCTGCGCGCAGGGCTGCGGGTGATTGGCCTGCAACCTGGGTGCAAAACATTGTCGTC
TATTTTCCTGATGCTGCCGCAGTATGCCGGTCCGGCATTAGGGTTCGCCGATTGCAGCGTAGTACCGCAGCCG
ACGGCGGCGCAGCTGGCGGATATCGCTATCGCCAGCGCCGACACCTGGCGGGCCATTACCGGCGAAGAGCCGC
GCGTGGCGATGCTGTCATTTTCAAGCAACGGCAGCGCCCGTCACCCCAATGTCGCCAACGTGCAGCAGGCGAC
AGAGCTCGTTCGCGAACGTGCGCCGCAACTGCTTGTGGACGGTGAATTGCAGTTTGACGCCGCTTTCGTCCCG
GAGGTCGCCGCGCAAAAAGCGCCTGACAGCCCGCTGCAAGGCCGCGCCAACGTGATGATTTTCCCGTCGCTGG
AGGCGGGCAATATTGGCTACAAAATCGCTCAGCGTCTGGGAGGCTATCGCGCTGTTGGGCCGCTAATTCAGGG
GCTTGCCGCGCCGCTTCACGACCTCTCCCGAGGCTGTAGCGTGCAGGAAATTATCGAACTGGCGTTGGTGGCA
GCCGTGCCGCGCCAGGCTGACGTGAGCCGCGAACGCAGCTTACACACACTGGTTGAATGAACGGTCCCGTTCT
GGACCCCTTTAATGAGAGGAAAACACGATGGAAGCATTAGGAATGATTGAAACCCGGGGCCTGGTTGCGCTGA
TTGAGGCCTCCGATGCGATGGTAAAAGCCGCGCGCGTGAAGCTGGTCGGCGTGAAGCAGATTGGCGGTGGCCT
GTGTACGGCCATGGTGCGTGGCGACGTGGCGGCGTGCAAAGCCGCAACCGATGCTGGCGCCGCTGCGGCGCAG
CGCATTGGCGAGCTGGTCTCCGTACACGTGATTCCACGCCCGCACGGCGATCTGGAAGAAGTGTTCCCGATCA
GCTTCAAAGGCGACAGCAACATTTGATGGTAGGCCGGATAAGACGCGCAGCGTCGCCATCCGGCATGAATGCG
CGCCATTGCCTGATGGTGCGCAGATTCAAGGCCTACGACCCAATGGGCAATCCATGGAGGCGGATATGAAACT
GGCAGTCGTCACAGGACAAATCGTATGTACCGTCCGCCATCAGGGACTGGCGCATGACAAATTGCTGATGGTG
GAAATGATCGATGCCCAGGGCAACCCCGACGGGCAGTGTGCCGTCGCTATCGACAGTATCGGGGCGGGAACCG
GAGAGTGGGTACTGCTGGTCAGCGGCAGTTCCGCGCGCCAGGCGCATCGCAGCGAATTATCACCGGTCGATCT
GTGCGTCATTGGCATCGTCGATGAAGTGGTGGCTGGCGGGAAAGTGGTTTTCCATAAATAGGACTGAACATCA
TGAATCAACAGGATATTGAACAGGTGGTGAAAGCGGTGCTGCTGAAAATGAAAGACAGCAGCCAGCCGGCCAG
TACTGTTCATGAGATGGGCGTTTTTGCCTCCCTGGATGACGCAGTGGCGGCAGCAAAACGGGCCCAGCAGGGG
CTGAAGAGCGTGGCGATGCGCCAGCTTGCCATTCATGCCATTCGCGAAGCGGGCGAAAAACACGCCCGAGAAT
TAGCGGAACTTGCCGTCAGCGAAACCGGCATGGGACGCGTTGACGATAAATTTGCCAAAAACGTGGCGCAGGC
GCGCGGCACGCCGGGCGTCGAGTGTTTATCTCCGCAGGTGCTGACCGGCGATAACGGCCTGACGCTGATTGAA
AATGCGCCGTGGGGCGTGGTGGCCTCGGTCACGCCGTCCACCAATCCGGCGGCGACGGTGATTAATAACGCCA
TCAGCCTGATTGCCGCAGGCAACAGCGTCGTGTTTGCCCCGCATCCGGCGGCGAAAAAGGTCTCTCAGCGGGC
AATCACTCTCCTCAATCAGGCGGTGGTCGCCGCCGGTGGCCCGGAAAATTTGCTGGTCACCGTGGCGAACCCG
GATATCGAAACCGCCCAGCGGTTGTTCAAATATCCCGGTATTGGCCTGCTGGTGGTGACTGGCGGCGAAGCGG
TGGTGGACGCGGCGCGCAAACATACTAATAAACGCCTGATTGCCGCCGGTGCCGGCAATCCCCCGGTGGTGGT
GGATGAGACCGCCGACCTGCCGCGCGCCGCACAATCCATCGTCAAAGGCGCATCGTTTGATAACAACATCATC
TGCGCCGACGAAAAAGTGCTGATTGTGGTGGATAGTGTCGCCGACGAGCTGATGCGTCTGATGGAAGGTCAGC
ACGCGGTGAAACTGACCGCCGCTCAGGCCGAACAGCTCCAGCCGGTGCTGCTGAAGAATATCGATGAACGCGG
CAAAGGTACCGTCAGCCGTGACTGGGTCGGTCGTGACGCGGGCAAGATTGCCGCAGCCATCGGCCTGAACGTC
CCGGATCAAACGCGCCTGCTGTTTGTGGAAACGCCAGCTAACCATCCGTTTGCCGTCACTGAAATGATGATGC
CGGTACTGCCGGTGGTGCGGGTCGCCAACGTCGAAGAAGCCATCGCCCTGGCGGTTCAGCTTGAAGGCGGTTG
CCACCATACGGCGGCGATGCACTCGCGCAATATCGACAACATGAACCAGATGGCGAACGCCATCGACACCAGC
ATTTTCGTCAAAAACGGGCCGTGCATTGCCGGGCTTGGATTGGGCGGAGAAGGCTGGACCACCATGACTATCA
CTACGCCAACCGGCGAAGGCGTGACCAGCGCACGTACCTTTGTGCGGCTGCGTCGATGCGTGCTGGTGGATGC
GTTTCGCATTGTATAAGGGATGACTCATGGCGCACGACGAACAACTCTGGCTCACCCCAAGACTGCAAAAAGC
GGCGGCCCTGTGTAACCAGACGCCCGCCGCCAGCGACGCGCCGCTGTGGCTGGGCGTCGATTTGGGCACCTGC
GACGTGGTGTCGATGGTGGTCGACGGCAACGCCCAGCCGGTGGCGGTGTGCCTTGACTGGGCCGACGTTGTGC
GTGACGGCATCGTCTGGGATTTCTTCGGTGCCGTCACCCTCGTGCGCCGCCATCTCGACACCCTTGAACAGCA
GCTCGGCTGCCGCTTCACCCACGCGGCGACCTCGTTCCCACCGGGTACTGACCCGCGTATTTCGATCAACGTG
CTGGAGTCTGCTGGGTTAGAAGTCAGCCACGTACTGGATGAACCGACGGCGGTGGCGGATCTGCTGGCGCTTG
ATAATGCAGGCGTGGTGGATATTGGCGGCGGCACTACCGGCATTGCCATCGTCAAACAGGGCAAGGTGACTTA
CTCCGCCGATGAAGCCACTGGCGGGCACCATATCTCCCTGACGCTGGCGGGCAACCGACGTATTCCCCTGGAA
GAGGCGGAACAGTACAAGCGCAGCAACGCGCAGGAGATTTGGCCGGTAGTGAAGCCGGTGTACGAAAAGATGG
CGGAAATCGTCGCTTGCCATATTGCCGGGCAGGGAGTGACAGATTTATGGCTGGCGGGCGGTTCCTGTATGCA
GCCGGGCGTGGAGGCGTTATTTCGCCAGCGTTTCCCGGAGTTACAGGTGCATTTACCGCAGCACAGCCTGTTT
ATGACCCCGCTGGCGATAGCCAACAGCGGGAGAGCGAAAGCGGAGGGACTCTATGCAAGCTGAACTACAGACG
GCGCTGTTTCAGGCATTCGACACCCTGAATCTGCAACGGGTGAAAACGTTCAGCGTACCGCCGGTCACGCTGT

GCGGACTTGGGGCGCTCGGCGCCTGCGGCCAGGAAGCGCAAGCGCGAGGCGTAAGCCATCTGTTTGTGATGGT
CGACAGCTTCCTGCATCAGGCGGGAATGACCGCGCCGCTGGCACGCAGCCTGGCGATGAAAGGCGTGGCGATG
ACAGTCTGGCCGTGTCCGCCAGGCGAGCCGTGCATCACCGACGTTTGCGCGGCGGTGGCGCAACTGCGTGAGG
CGGCGTGCGACGGCGTAGTGGCCTTTGGCGGCGGTTCGGTGCTGGACGCGGCGAAAGCGGTCGCCCTGCTGGT
GACTAACCCTGACCAGACGCTGAGCGCCATGACCGAGCGCAGTACATTACGCCCGCGTCTGCCGCTGATTGCG
GTGCCGACCACCGCCGGAACCGGTTCTGAAACCACCAACGTGACGGTGATTATCGACGCGGTCAGCGGGCGCA
AGCAGGTGCTGGCACACGCGTCACTAATGCCGGACGTGGCGATTCTTGATGCTGCCGTGACCGAAGGCGTTCC
GCCAAACGTGACGGCGATGACCGGTATCGATGCGTTGACGCATGCGATTGAGGCCTACAGCGCGCTCAGCGCC
ACGCCGTTTACCGACAGCCTGGCGATTGGCGCGATAGCGATGATTGGCAAATCGCTGCCGAAAGCCGTGGGTT
ACGGCCACGATCTGGCGGCGCGTGAAAATATGTTGTTGGCCTCCTGTATGGCGGGAATGGCCTTTTCCAGCGC
CGGTCTGGGGCTGTGTCACGCGATGGCGCACCAGCCAGGGGCGGCGCTGCATATTCCGCACGGCCAGGCCAAC
GCCATGCTGCTACCGACGGTCATGGGCTTTAACCGGATGGTTTGCCGCGAACGCTTCAGTCAAATCGGTCGGG
CGTTAACCAATAAGAAATCGGACGATCGCGATGCGATTGCGGCGGTGAGCGAGCTGATTGCCGAAGTGGGCCA
GAGCAAACGGCTGGCTGACGCTGGCGCCAAACCCGAACACTACAGCGCGTGGGCGCAAGCCGCGCTGGAGGAT
ATTTGTCTGCGCAGTAACCCACGCACCGCCACACAGGCACAGATTATCGACCTGTACGCGGCTGCCGGGTAAT
GCCTGTAGGCCGGATAAGGTGCTCGCACCGCCATCCGGCAATAACACGGCGTTAATGCCGGAGGCGACGTAAA
CGTCTTATCCGGCTTACAAATCAAAAATGACAGGGAGTAGGGGCTATGGGAATTAACGAAATCATCATGTACA
TCATGATGTTCTTTATGCTGATTGCCGCCGTGGACAGGATCCTGTCGCAGTTCGGCGGGTCGGCGCGCTTCCT
CGGTAAATTTGGCAAGAGTATCGAGGGGGCTGGCGGCCAGTTTGAAGAGGGCTTTATGGCGATGGGCGCGCTG
GGACTGGCGATGGTCGGTATGACCGCGCTGGCGCCGGTGCTGGCGCATGTACTGGGGCCGGTTATTATCCCGG
TATACGAAATGCTGGGCGCGAACCCATCCATGTTCGCGGGTACGCTGCTGGCCTGTGATATGGGCGGATTCTT
CCTCGCCAAAGAGCTGGCCGGCGGTGATGTCGCGGCGTGGCTATACTCAGGGTTAATACTTGGGTCGATGATG
GGGCCGACCATTGTGTTCTCCATTCCGGTGGCGCTCGGCATTATCGAACCGTCTGACCGCCGCTACCTGGCGC
TTGGCGTACTGGCGGGTATCGTCACCATTCCCATTGGCTGCATTGCGGGGGGATTGATCGCCATGTACTCAGG
CGTGCAGATCAATGGTCAGCCAGTGGAGTTTACCTTCGCGCTGATCCTGATGAACATGATCCCGGTATTGATT
GTCGCGGTGCTGGTAGCGCTGGGGCTGAAGTTCATCCCGGAAAAAATGATCAACGGTTTCCAGATTTTCGCCA
AATTTCTGGTGGCGCTGATCACCATCGGTCTGGCGGCTGCGGTGATTAAATTCCTGTTGGGCTGGGAGTTGAT
TCCGGGGCTCGACCCGATCTTTATGGCGCCAGGCGACAAACCTGGCGAAGTGATGCGCGCCATTGAAGTGATC
GGCTCTATCTCCTGCGTGCTGCTCGGCGCGTATCCGATGGTGCTGTTACTGACCCGCTGGTTTGAAAAACCGT
TGATGAATGTCGGTAAGCTGCTGAACGTAAATAATATTGCGGCGGCAGGCATGGTGGCGACCCTGGCGAACAA
TATCCCGATGTTCGGCATGATGAAGCAGATGGATACCCGCGGCAAAGTGATTAACTGCGCATTTGCCGTCTCT
GCGGCATTCGCGCTGGGCGACCATTTAGGCTTCGCCGCTGCCAACATGAACGCCATGATCTTCCCGATGATTG
TCGGCAAGCTGATCGGCGGCGTGACGGCGATTGGCGTGGCGATGATGCTGGTGCCGAAAGATGACGCCGCCCA
GGTGAAAACCGAAGCGGAGGCGCAATCGTGAACACTCGCCAGCTACTGAGCGTCGGTATCGATATCGGCACCA
CCACCACGCAGGTGATCTTCTCGCGCCTGGAGTTGGTTAACCGTGCGGCGGTGTCGCAGGTGCCGCGCTACGA
ATTCATCAAACGCGACATTAGCTGGCAAAGCCCGGTCTTCTTTACGCCCGTGGATAAGCAGGGCGGTCTGAAA
GAGGCCGAACTTAAAGCGCTGATTCTGGCCCAGTATCAGGCCGCAGGCATCGCGCCTGAATCGGTGGACTCAG
GGGCTATCATTATCACCGGCGAAAGCGCCAAAACCCGCAACGCCCGTCCGGCGGTCATGACGCTCTCGCAGTC
GCTGGGCGACTTTGTGGTCGCCAGCGCCGGGCCGCATCTGGAATCGGTCATTGCCGGTTACGGCGCCGGAGCG
CAAAGCTTGTCTGAGCAGCGGATGTGCCGGGTGCTGAATATTGATATCGGCGGCGGCACGTCTAACTACGCGC
TGTTTGACGCGGGAAAAGTCAGCGGCACCGCCTGCCTTAACGTCGGTGGTCGCCTGCTGGAAACCGATGCTCA
GGGGCGCGTGGTTTATGCCCATCAGCCGGGGCAGATGATTATCGATGAGGTGTTCGGTTCGGGTACTGATGCT
CGCGCCTTAGCTGCCGCACAGTTGGGACAGGTGGCGCGGCGGATGGCGGATCTCATCGTCGAGGTTATCACTG
GCGCGCTCTCGCCGCTGGCGCAATCGCTGATGCAAACCGGGCTGCTGCCCGCCGATATTACGCCAGAGGTGAT
CACCCTTTCCGGTGGGGTAGGTGAATGCTACCGCAACCAACCTGCCGATCCGTTCTGCTTTTCTGATATCGGG
CCGCTGCTGGCGACCGCGCTGCATGAGCACCCGCGTCTGCGCGAGATGAACGTGCAGTTTCCGGCGCAAACCG
TGCGCGCCACGGTGATTGGCGCGGGGGCCCATACGCTGTCGCTTTCCGGCAGTACTATCTGGCTGGAAGACGT
CCAACTGCCGCTGCGCAACCTGCCGGTGGCGATCCCGCAGGATGACGCCGATCTGGTGAACGCCTGGCGGCAG
GCGCTTCTCCAGCTTGATCTCGACCCGCAAACCGACGCTTACGTGCTGGCGCTTCCCGCCACGCTTCCGGTGC
GTTACGCCGCATTACTCACGGTCATCAACGCGCTGACGGCTTTTGTCGCGCGTTATCCCAATCCACATCCCCT
GCTGGTGGTGGCCGAGCAGGATTTTGGTAAAGCGCTGGGCATGTTATTGCGCCCACAGTTACCGCAACTTCCG
CTGGCGGTCATCGATGAGGTGGTCGTGCGGGCGGGAGACTATATCGACATTGGTACGCCTCTTTTTGGCGGAT
CGGTTGTGCCGGTGACGGTGAAATCACTCGCATTTCCTTCCTGAGGGAACGACTTATGAAACTAAAGACCACA
TTGTTCGGCAATGTTTATCAGTTTAAGGATGTAAAAGAGGTACTGGCTAAAGCCAACGAACTGCGTTCGGGGG

ATGTGCTGGCCGGGGTTGCCGCGGCAAGTTCGCAGGAGCGCGTAGCGGCAAAACAGGTACTGTCGGAAATGAC
GGTGGCGGATATCCGCAACAATCCGGTGATTGCCTATGAAGAGGACTGCGTGACGCGCCTGATTCAGGATGAC
GTCAACGAAACGGCCTATAACCGGATTAAAAACTGGAGCATCAGCGAACTGCGCGAATACGTACTGAGCGATG
AAACCTCCGTGGACGACATCGCGTTTACCCGCAAAGGGCTGACCTCCGAAGTGGTGGCGGCAGTAGCGAAAAT
CTGCTCCAACGCTGACCTGATCTACGGCGGCAAGAAAATGCCGGTGATCAAAAAAGCCAATACCACCATCGGT
ATTCCGGGCACCTTTAGCTGCCGTTTGCAGCCGAACGATACCCGTGACGATGTACAGAGTATCGCCGCGCAAA
TCTACGAAGGGCTTTCTTTCGGCGCAGGCGATGCGGTGATCGGCGTTAACCCGGTGACCGATGACGTGGAGAA
CCTGACCCGCGTGCTCGACACCGTTTACGGCGTTATCGATAAATTCAATATTCCGACCCAGGGCTGCGTGCTG
GCGCATGTCACCACCCAGATTGAAGCGATTCGCCGCGGCGCGCCGGGCGGACTGATTTTCCAGAGCATTTGCG
GCAGCGAGAAGGGCTTAAAAGAGTTCGGCGTCGAGCTGGCTATGCTCGATGAAGCGCGGGCCGTGGGGGCGGA
GTTCAACCGCATCGCCGGGGAAAACTGCCTGTACTTTGAAACCGGACAGGGCTCGGCGCTGTCGGCTGGCGCG
AACTTTGGTGCCGACCAGGTGACAATGGAAGCGCGTAACTACGGGCTGGCGCGCCACTACGATCCGTTCCTGG
TGAACACCGTGGTGGGCTTTATCGGGCCGGAATATCTCTACAACGACAGGCAGATTATCCGCGCCGGTCTCGA
AGATCACTTTATGGGCAAGCTGAGCGGCATCTCGATGGGCTGCGACTGCTGCTATACCAACCATGCCGACGCC
GACCAGAACCTTAACGAAAACCTGATGATTCTGCTCGCCACTGCCGGCTGTAACTACATCATGGGGATGCCGC
TCGGCGACGACATCATGCTCAACTACCAGACCACCGCTTTCCACGATACCGCCACCGTCCGTCAGTTGCTGAA
TTTACGGCCGTCGCCGGAGTTTGAACGCTGGCTGGAAACGATGGGCATTATGGCAAACGGTCGTCTGACCAAA
CGGGCGGGCGATCCGTCACTGTTCTTCTGATGACGCGGGGATAACACCATGGATCAAAAACAGATTGAAGAAA
TTGTACGTAGCGTGATGGCGTCAATGGGACAGGACGTACCGCAGCCCGTCGCGCCGTCAACGCAGGAAGGCGC
AAAGCCGCAGTGCGCCGCGCCGACGGTGACCGAAAGCTGCGCGCTGGATTTAGGTTCCGCAGAGGCAAAAGCC
TGGATTGGCGTCGAGAATCCGCATCGTGCGGACGTGCTGACCGAACTGCGTCGCAGTACTGCGGCACGCGTCT
GTACGGGGCGTGCCGGGCCGCGTCCGCGCACCCAGGCGCTGTTGCGCTTCCTGGCGGATCACTCCCGTTCGAA
AGATACCGTGCTCAAAGAAGTGCCGGAAGAGTGGGTAAAAGCGCAAGGGCTGCTGGAAGTGCGTTCGGAGATC
AGCGACAAAAACCTGTACCTGACGCGCCCGGATATGGGGCGTCGTCTGAGCCCGGAAGCCATTGACGCGCTGA
AGTCGCAGTGCGTGATGAACCCGGATGTGCAGGTAGTGGTCTCCGATGGCCTCTCTACGGATGCGATCACCGC
CAACTATGAAGAGATCCTGCCGCCGCTGCTTGCTGGTCTGAAGCAGGCCGGGCTGAACGTCGGCACGCCGTTC
TTTGTGCGCTATGGCCGTGTGAAGATTGAAGATCAGATTGGCGAAATTCTCGGCGCGAAGGTCGTCATCCTGC
TGGTGGGCGAACGTCCGGGGCTGGGGCAGTCGGAAAGCCTCTCCTGCTACGCGGTCTATTCCCCGCGCGTGGC
GACCACCGTCGAGGCCGACAGAACCTGTATTTCAAACATTCATCAGGGGGGACGCCGCCAGTAGAAGCCGCCG
CCGTGATTGTGGATTTGGCCAAACGGATGCTGGAGCAGAAAGCGTCCGGCATCAACATGACCCGTTAAGGAGA
CATCATGCCTGCATTAGATTTGATTCGACCTTCAGTGACTGCCATGCGCGTGATTGCCTCCGTGAATGACGGC
TTTGCGCGGGAACTTAAATTACCGCCACATATACGTAGCCTCGGACTCATCACGGCAGATTCTGATGATGTGA
CCTATATTGCCGCTGACGAAGCGACCAAACAGGCGATGGTGGAAGTAGTCTATGGCCGCTCCCTCTATGCCGG
GGCGGCTCACGGGCCGTCGCCTACCGCTGGTGAGGTGTTGATTATGCTGGGCGGACCGAACCCGGCGGAAGTG
CGCGCCGGTCTGGATGCAATGGTCGCCAGCATTGAAAACGGCGCGGCGTTCCAGTGGGCGAACGATGCGGAAA
ATACCGCATTCCTGGCGCATGTGGTTTCGCGTACCGGCTCGTACCTCTCGTCTACCGCAGGTATCGCGCTGGG
CGATCCGATGGCCTATCTGGTGGCGCCGCCGCTGGAAGCGACATTCGGCATTGACGCGGCGATGAAATCCGCT
GACGTTCAACTGGTGACTTACGTGCCGCCGCCGTCGGAAACCAACTATTCGGCCGCGTTCTTAACGGGAAGCC
AGGCCGCGTGTAAAGCCGCCTGCAACGCCTTTACCGACGCCGTGCTGGATATTGCCCGTAACCCTGTCCAGCG
TGCGTAACGGAGGCTGCCAATGATCAATGCCCTGGGATTACTGGAAGTGGACGGAATGGTCGCCGCCGTCGAT
GCGGCGGATGCCATGCTGAAAGCGGCCAACGTGCGTTTGCTCAGTCATCAAGTGCTCGACCCTGGCAGGCTCA
CGCTGGTGGTGGAGGGCGATCTGGCGGCGTGTCGTGCGGCGCTGGATGCCGGAAGCGCCGCCGCGCAGCGCAC
AGGCCGTGTGATTAGTCGCAAAGAAATTGGTCGTCCGGAAGAGGACACCCAGTGGCTGATTGGCGGGTTTGCG
CGTGCGACAACGCCGACTGAGAAAGCGCCCCAGGCACCCGCAACGCCTGAGTTCGCTGAGGCGTTACTGGCGC
TGCTGGCATCAGTACGCCAGGGAATGACGGCGGGAGAGGTTGCCGCGCATTTTGGCTGGCCGCTGGAGCAGGC
CAGAAACGTGCTGGAACAACTCTTTTCTGACGGAGCGTTGCGTAAACGCAGTAGTCGCTATCGCATCAAAAAT
TAATCTGTCGGAGGGCCGGGCGTCCTGGCGGGCGTCCGGCATAGAAGATCATGAAAAAGACCCGTACAGCGAA
TTTGCACCATCTTTATCATGAAGCGTTACCCGAAGACGTTAAACTTACGCCCAGAGTCGAGGTGGACAATGTT
CATCAGCGACGGACGACGGATGTCTATGAACATGCTCTGACTATCACCGCCTGGCAGCAGATCTACGATCAGT
TACACCCGGGTAAATTTCATGGCGAGTTCACGGAAATCCTGCTTGATGAGATTCAGGTGTTCCGTGAATACAC
CGGCCTGGCGCTGCGTCAGTCGTGTCTGGTTTGGCCAAACTCTTTCTGGTTTGGTATTCCGGCGACGCGCGGC
GAACAGGGATTTATCGGCGCGCAGGGGCTCGGCAGCGCCGAGATTGCCACCCGACCGGGAGGCACCGAGTTTG
AACTGAGTACGCCGGATGATTACACCATTCTGGGCGTCGTTATCTCGGAAGATGTTATTTCCCGTCAGGCCAC
GTTTTTGCATAACCCGGAAAGGGTGCTGCATATGCTACGTAACCAGTTAGCGCTGGAGGTAAAAGAGCAGCAT

AAAGCGGCGCTGTGGGGCTTTGTGCAGCAGGCGCTGGCCACCTTTAGCGAGAGTCCTGAAACCCTGCATCAAC
CTGCGGTGCGTAAGGTCTTGAGTGATAATTTGTTGCTGGCGATGGGCACGATGCTGGAAGAGGCGAAGCCGAT
TCATAGCGCCGAAAGCATCAGCCATCAGGGATATCGTAGGCTACTGTCGCGGGCGCGGGAATATGTGCTGGAA
AATATGTCGGAGCCGCTGACGGTGCTCGACTTATGTAACCAGCTACACGTAAGCCGTCGCACGCTGCAAAATG
CGTTTCACGCCATTTTGGGCATTGGCCCCAATGCGTGGCTGAAACGTATTCGCTTAAACGCGGTACGCCGGGA
ACTGATTAGCCCGTGGTCGCAGAGCGCCACGGTGAAAGATGCCGCGATGCAGTGGGGATTCTGGCATTTGGGG
CAATTTGCCACCGATTATCAGCAATTATTTGCCGAAAAACCGTCGTTGACGCTGCATCAACGGATGCGGCAAT
GGGCTTGATATTGATGGGTAGGCTCTCCGGTTGCTGATAACGTGCGCGTGTCGGGAATTGCCCGGTGGCGGCT
GGCGCCTTACCGGGCCTACGGATACGCTGCTGTCTGTGGTTGGTAGCCCGGACAAGGCGCGCCAGCGCCGCCT
CCGGGGAAGCGCGCGCAATTTTGTCTCCGCAGATAAACCCGTAAAGCATCCCATCGGCATATTTCTCAATATC
TTCATAACTTATTTGTGCGAGGAACATCATCGTTTGCGCGCACGTTGGGCACGTTGGATATTCCGCATCCTGA
ACCCAGGCCGGGTGGCCGCCGAGCTGTGAGAAGGTCGTGGGTGAATTAGGAACGGCCGTGGCGAAAACGGTGT
TATATGCCGTACAACACTACCAGGTCAGAATGCGAATTACCGAATTCTTTATCTACAATGAACTCGTTTATTT
CTACGAGCCTGCTTTTATTCAACGACCAACCGGATAGGCGATAAACAAACCGGCTATCGTCAAAATACATGCC
AGTAGGCATAGCAAGATACAGTTAACGCTTTTACGCGGAATATGAATAATAAAGCAGAGTAATGCTATCAGGG
CCATGACCACAGGGATGGTCACCATATTGATAACCAGAAGCACGATCAGACCGAAGCTCACCCCTCCGCTATG
AGGGATGAATATTCTTTCTTCAAAATATATTTCTAGCGTAATGGCAAGCAGGCAGCCAATAAAACTTATACCC
AGATGGGGATGCCATTTGGCGTCACTGGTTACTACTTTCCGTGTATTTGTTTGCATTGTTTTACTTTATAAAT
AAGTTAAATCCAGTCGCGTACCTGAATAAACTCGCTCAGCGCCGCTTCCGGGCTACCCGCTTCCGGCTGCCAG
TCGTACTCCCAGCGCACCAGCGGCGGCATCGACATCAGGATAGATTCAGTGCGCCCGCCGGTTTGCAGACCAA
ACAGGGTGCCTCTGTCCCATACCAGATTGAACTCCACGTAGCGACCGCGGCGATAAAGTTGAAAATTACGCTC
CCGCTCTCCCCAGACCATCGCTTTGCGTCGTTCGACAATCGGTAAATACGCCTCGGTGTAGCCGTTACCGACC
GCCTGCATAAAAGCAAAACAGTGGTCGAAATCCGGCGTATTCAAATCGTCAAAAAACAGGCCGCCGATACCGC
GCTGCTCGTTACGATGCTTGAGGAAAAAGTAGTCATCGCACCACTTTTTATAACGCGGATACACATTGTCACC
AAACGGCTGGCACAGGTCGCGGGCGGTGCGGTGCCAGTGAACGGCATCCTCTTCAAAGCCGTAGTAGGGCGTT
AAATCGAGCCCGCCGCCAAACCACCATACCGGATCGGCGCCCGGTTTTTCGGCAATAAAAAAGCGCACGTTAG
CATGGCTGGTGGGAATATACGGATTGTGCGGATGCACCACCAGCGATACGCCCATCGCTTCAAAGCTACGCCC
GGCGAGTTCTGGCCGATGCGCGGTGGCGGACGCGGGCATTGCATCGCCGTGAACGTGAGAGAAATTAACGCCC
GCCTGTTCGAAAATCCCGCCGTTGCGTAATACCCGGCTGCGTCCGCCGCCGCCCGCTTCGCGTCGCCAGCTAT
CTTCGACAAAGTTTGCGCCATCCACAGCGGATAACTTTTGGCAAATATCATCCTGCAGGCGTAGCAGAAACTG
TTTTACGTGATGTGCATCGGGTTTCATGATTACCGTTTCTTCGTGTGTGCTTTCTGATTATCAAACCAGTGAA
AATAACTGATGATGCCGTTGGCGATCGCCGTAGCGATTTTCTGGCGAAACGCCGTCGTGCCCAGCAGGCGTTC
TTCTTCCGGATTGGTAATAAACGAGGTTTCGACCAGCACCGACGGAATTGACGGTGATTTTAGCACCACGAAA
GCCGCTTGTTCGGTAGTGCGGCTGTGTAATTTATGTATAGGTTTGATTTTCTTGAGAATATGCGAACCCAGCG
TCAGACTGTTTTTAATCGTATCGGTTTGCACCAGATCAAACAAAACTTGTTGTAATAGATGATCCCGGTCAGT
GGCTTTTTTACCCGCCACTTCGTCCGCGCGGTTTTCACGCTCGGAGAGATACTTCGCCATGGCGCTACTGGCC
CCGCGGTTGGAAAGCGCAAAAACGGAAGCGCCCGCTGCTTTCGGGTTAGTAAAACCATCGGCATGAATAGACA
TAAACAGATCCGCACCGTGCTTATGGGCGATTTCCACCCGATCGTACAGCGGAATAAAGGTATCCCCTGTCCG
GGTTAAACGCGCATCGATTCCGTGATTTCGCAAAATAGCGCGCACGTTTTTGGCAATTGCCAGTACGACGTGT
TTTTCCTGAGAACCGTTGCGGCCAATCGCGCCGGTATCGATGCCGCCGTGTCCGGGGTCGAGCATCACCAGCC
GTTTACTGCCGGTTTTTTTGGTTTTGGGTTTGCTGTGACCATTACTGGTTTTTAGCGTTTCTTCTTTGGCTAT
TGCGTGCGACATACCTGACAGCGTCAGGGCCGCCAGACCTGTTTTGAGTACCTGGCGGCGCGATGTGAGAGTT
TTTAGGAGTTTAAAAGTGCTCATACGGCCTGAGTTGTGAAAATTAAGTTCAAGATGTTATATCGTATCGCGTA
ATCCGTTACGACGGTTGGTGATGAGAATTGTTTTACTTTTCATTTCAATACGTGACACATCGCCATTATGCCA
TTTTTTCCGCTACATCGCGTCAGATATTGGCTGAATCAGATGTTTGCGCCATAATCTTGGTTTTTCCACCCGA
AAAGGCAACGAATACCATGGAAATACGCGTTTTTCGCCAGGAAGATTTCGAAGAGGTGATTACCCTCTGGGAG
CGTTGCGACCTGCTGCGTCCATGGAACGATCCTGAAATGGATATTGAACGCAAGGTGAATCACGATGTCAGCT
TGTTCCTCGTTGCGGAAGTTAGCGGCGAAGTCGTCGGTACGGTAATGGGCGGTTATGACGGGCATCGCGGCTC
GGCGTATTACCTGGGCGTTCACCCGGAGTTCCGCGGGCGCGGCATTGCGAATGCGTTGCTTAACCGGCTGGAA
AAAAAACTCATCGCCCGCGGGTGTCCGAAAATCCAAATTATGGTGCGCGATGATAACGACGTTGTGCTGGGCA
TGTATGAACGTCTGGGGTATGAGCATTCTGACGCCCTAAGTTTGGGTAAACGCCTGATTGAAGATGAAGAGTA
CTGAGTTTCATCCAGCCGATTATGATGTACACGGACGTTTACGCCTGCCGTTTCTGTTCTGGTGCGTACTGTT
ACTTCAGGCGCGCGCCTGGGTACTCTTTGTCATTGCCGGTTCATCCCGTGGACAGGGTAATACTTTGTTAAAC
TTTTTTTATCCCGACCATGACAATTTTTGGCTGGGGCTGTTGCCCGGCGTTCCGGCGGTAGTGGCATTTTTAC

TCAGCGGGCGGCGTGAAGCGGTTCCTGGTGTGTGGCGCTGGCTGCGCGGACTCCTGATCCTTGCTCAACTGGT
GTCGTTGTGCTGGCTGCCGGTGATGTGGCTGGGTGGCGACCCGGTCAACGGCGTCGGGCTGGCGCTGCTGCTG
GCGGACATCGTCGCGCTGATCTGGCTGTTAACCAATCAACGTTTGCGTGCCTGTTTTTCCCTTGAGAAAGAAT
AACGGCACTTTTTGGCGAACCCGCACTCCAACCAACGTTAATTAATAAAAGAAAGGAAGTGAGATGAAATCGC
TGCGTTTGACTTTACTCGCACTGCCGCTGGCGCTGACCGGCTGCTCAACGCTCTCCTCGGTCAACTGGTCTGC
CGCAAATCCATGGAACTGGTTTGGTTCCTCGACAGAAGTCACTGAGCAAGGCGTGGGAGCGCTGACCGCCGCC
ACGCCGCTGGAAGAGCCGGCTATTGCAGAGGCATTAGATGGCGATTATCGCCTGCGTAGCGGTATGAAAACCG
ATAACGGCAACGTGGTGCGCTTTTTCGAAGCGATGAAGGGCGACAGCGTCGCGATGGTGATCAACGGCGAGCA
GGGAACCGTGAGCCGTATTGATGTGCTGGACAGCGATATCCCAACCGCCGCGGGGGGCAAAATCGGTACGCCG
TTTAGCGACCTTTACAGCAAAGCGTTTGGTCACTGCGAGCCGGTTTCCAGCGATAGCCATACCAGCGTCGAGT
GTAAAGCCGAGGGAAGTCAGCATATTAGCTATGTTTTCTCCGGCGAGTGGAGCGGACCGGAAGGCTTAATGCC
TCCTGATGACGTCTTAAAAAACTGGGACGTGCGCAAAATTATCTGGCGTCGTTAATTTGCGCCTGAACAAACC
GCTTCGCTTAAAAACAAGGTAAAATAGCCTCCATTAATGCCACGACTGCGTGGCATTCTCATTTTCAGGAGGA
ATCATGTCTCAGGTTCAAAGTGGCATTTTGCCGGAACATTGCCGTGCGGCAATTTGGATTGAAGCCAATGTAA
AAGGCGACCTGGACGCCCTGCGCGCGGCCAGCAGAACATTTGCCGATAAACTGGCGACCTTCGAAGTCAAATT
TCCGGACGCCCATCTTGGGGCCGTTGTGGCGTTTGGCAACAATACCTGGCGTGCCCTGAGTGGCGGCGTCGGG
GCGGAAGAACTGAAAGATTTTATTCCTTATGGTAAAGGGCTGGCGCCGGCCACGCAGTATGACGTGCTGATCC
ATATCCTTTCCCTGCGCCACGATGTGAATTTTTCTGTTGCTCAGGCGGCGATGGAAGCCTTCGGCGACTGTAT
CGACGTGAAAGAAGAAGTTCACGGCTTCCGTTGGGTGGAAGAACGCGATCTGAGCGGCTTTGTCGACGGGACA
GAGAACCCGGCAGGCGAAGAGACGCGTCGTGAAGTGGCGGTGATCAAAGATGGCGTGGATGCCGGCGGCAGTT
ATGTGTTCGTGCAGCGTTGGGAACACAATCTCAAGCAACTTAACCGGATGAGTATTCACGATCAGGAGATGAT
GATCGGGCGTACCAAAGAAGCCAATGAAGAGATTGACGGCGATGCGCGTCCGGCAACATCTCACCTGAGCCGC
GTCGATCTGAAAGAAGATGGCAAAGGGCTGAAGATTGTGCGTCAGAGCCTGCCATACGGCACCGCCAGCGGTA
CTCATGGTCTCTATTTCTGCGCTTACTGCGCACGCCTGCACAATATCGAACAGCAGCTTCTGAGTATGTTCGG
CGATACCGACGGTAAACGCGACGCAATGCTGCGCTTCACCAAACCGGTGACTGGCGGCTACTATTTTGCGCCG
TCGCTGGATCGCCTGTTAGCGCTATAAGGTAAAACCCTGTCGGATGGCAGCGCAAACGCTTTATTCGACCTAC
GATTGGTAGGCCTGATAAGCGCAGCGTCATCAGGCAGAACAGCATAAAAATGCCCATCCCCGCCTCCGCGGGG
ATTTTTTTACCTAATATTTACCTGAATTAGTGAGCTATTCCTCAGCACTTATTTGTTATTACTGTATTTTCAG
TGTTAATGGAGTGTAAGACTCCATATTCAAAAGGAGACATTGAAAATGGGTAAACTCACGGGCAAGACAGCAT
TGATTACGGGCGCATCGCAGGGCATTGGCGAAGGGATCGCTCGCGTATTCGCACGCCACGGCGCGAACTTAAT
CTTGCTGGATATCTCCGATGAGATTGAAAAACTGGCGGATGAGCTGGGCGGGCGCGGGCATCGCTGTACTGCC
GTTAAGGCTGACGTCAGAGATTTTGCTTCGGTGCAGGCGGCGGTTGCGCGCGCCAAAGAGACTGAAGGTAGAA
TTGATATTTTGGTGAATAACGCTGGCGTGTGCCGTCTGGGCAACTTCCTCGATATGAGTGAAGAAGATCGCGA
TTTCCACATTGATATTAATATTAAAGGTGTCTGGAACGTCACCAAAGCCGTCCTGCCGGAGATGATTAAACGT
AAAGATGGCCGCATTGTGATGATGTCTTCCGTCACGGGAGATATGGTGGCGGACCCGGGTGAAACGGCCTATG
CGCTGTCAAAAGCCGCCATTGTCGGTTTAACCAAATCGCTGGCGGTAGAGTATGCGCAGTCCGGTATTCGCGT
GAATGCCATTTGCCCCGGTTATGTAAGAACGCCGATGGCGGAAAGCATTGCCCGTCAGTCTAATCCTGACGAT
CCGGAGTCGGTATTAACGGAAATGGCAAAAGCCATTCCGCTACGCCGCCTTGCCGATCCACTGGAAGTAGGGG
AACTGGCGGCATTTCTGGCTTCCGATGAGTCCAGCTATCTTACCGGAACGCAAAACGTCATTGATGGCGGCAG
TACCCTGCCTGAAAGCGTAAGCGTAGGCGTCTGATTGATGCCGGTCTCCTCCTCACGCCGCGTGGGGAGGGAT
TCTCTTATTCCCTTTTCAACTTTCAAATCATCAAACGGTATATAAAACCGTTACTCCTTTAGCGCTGGTTATA
AATATGATGACCATAAGAAAGTCATTAAATTTATAAGGGTGCGCAATGGCCGTTAATTTACTGAAAAAGAGAC
CCCTGACGCTGGCAGCAATGCTGTTACTGGCAGGGCAGGCGCAGGCAACGGAGCTGCTGAACAGCTCATACGA
TGTCTCCCGCGAGCTGTTTGCCGCCCTTAACCCGCCGTTTGAGCAACAATGGGCGAAGGATAACGGCGGCGAT
AAGCTGACGATTAAGCAGTCTCATGCCGGGTCATCAAAACAGGCGCTGGCGATTTTGCAGGGACTGAAGGCAG
ACGTCGTCACCTACAATCAGGTGACCGACGTACAGATTCTCCATGATAAAGGCAAACTGATCCCTGCCGACTG
GCAAAGCCGTCTGCCGAACAACAGTTCGCCATTCTATTCCACGATGGGCTTCCTGGTGCGCAAGGGGAACCCG
AAAAATATTCACGACTGGAGCGATCTTGTACGTTCCGACGTGAAGCTGATTTTCCCTAACCCGAAAACCTCCG
GCAACGCCCGTTACACGTATCTGGCGGCATGGGGCGCGGCGGATAACGCGGACGGCGGCGATAAAGCCAAAAC
CGAACAGTTTATGACCCAGTTCCTGAAAAACGTCGAAGTGTTTGATACCGGCGGCCGCGGCGCTACGACTACC
TTTGCCGAGCGTGGTCTGGGCGATGTGCTGATTAGTTTTGAATCGGAAGTGAACAACATCCGCAAACAATATG
AAGCCCAGGGATTTGAAGTGGTGATCCCGAAAACGAACATTCTTGCTGAATTCCCGGTCGCCTGGGTGGATAA
AAACGTGCAGGCCAACGGCACAGAAAAAGCCGCCAAAGCTTACCTGAACTGGCTGTATAGCCCGCAGGCGCAG
ACCATCATTACCGATTTCTACTACCGCGTGAATAACCCGGAAATCATGGGCAAGCAAGCAGATAAATTCCCGC

AGACCGAACTGTTCCGCGTGGAAGACAAGTTTGGTTCCTGGCCGGAAGTGATGAAAACGCACTTTGCCAGCGG
CGGCGAGCTGGACAAACTATTGGCGGCGGGGCGTAAGTAATGCTTGCCGTTTCTTCCCGACGCGTGCTGCCCG
GCTTTACGTTAAGCCTCGGCACCAGTTTGCTGTTTGTCTGCCTGATCCTACTGTTGCCGTTGAGCGCGCTGGT
GATGCAGCTCTCACAGATGAGCTGGGCGCAATACTGGGATGTCGTGACCAACCCACAGGTGGTGGCTGCCTAT
AAAGTGACGCTACTGGCGGCATTTGTAGCGTCGATTTTTAACGGCGTGTTTGGCCTGCTGATGGCGTGGATTT
TAACCCGCTACCGTTTTCCAGGGCGCACGTTGCTGGATGCGCTGATGGATCTGCCGTTTGCGCTGCCGACAGC
GGTGGCGGGGTTAACGCTGGCCTCGCTGTTCTCAGTGAACGGTTTCTACGGTCAGTTTCTTGCGCAGTTTGAT
ATCAAAATGACCTATACCTGGCTCGGTATTGCGGTGGCGATGGCCTTTACCAGCATTCCATTTGTGGTGCGCA
CCGTCCAGCCGGTACTGGAAGAGTTGGGGCCGGAATATGAAGAAGCGGCACAAACGCTGGGGGCCACCCGGCT
GCAAAGTTTTCGCAAAGTCGTGCTGCCTGAGCTGTCGCCTGCGCTGATTGCCGGCGTGGCGCTCTCTTTTACC
CGTAGCCTGGGCGAGTTTGGCGCGGTGATTTTTATCGCCGGAAATATCGCCTGGAAAACAGAAGTGACCTCGC
TGATGATTTTTGTGCGTTTGCAGGAGTTTGATTACCCCGCCGCCAGCGCCATTGCCTCGGTGATCCTTGCGGC
ATCCTTGCTGCTGCTGTTCTCAATTAACACTCTGCAAAGTCGCTTTGGTCGACGTGTGGTAGGTCATTAATGG
CGGAAGTGACTCAATTGAAGCGATACGACGTCCCCCGCATTAACTGGGAAAATGGTTTTTGATTGGCGTCGG
GATGCTGGTTTCGGCGTTTATCCTGCTGGTGCCGATGATTTACATCTTCGTGCAGGCGTTCAGCAAAGGGTTA
ATGCCGGTACTGCAAAATCTGGCCGATCCGGACATGCTGCACGCCATCTGGCTGACGGTGTTGATTGCCTTGA
TCGCAGTGCCGGTGAATCTGGTGTTCGGCATTCTGCTGGCGTGGCTGGTGACGCGCTTTAACTTCCCCGGACG
TCAGCTACTGCTGACCCTGCTGGATATTCCCTTTGCCGTCTCGCCGGTGGTGGCCGGTCTGGTTTATCTGCTG
TTTTATGGTTCCAACGGCCCGTTGGGCGGCTGGCTGGATGAGCATAATTTACAAATGATGTTCTCCTGGCCGG
GGATGGTACTGGTCACTATTTTTGTCACTTGTCCGTTTGTGGTGCGCGAACTGGTGCCAGTAATGCTCAGCCA
GGGCAGTCAGGAAGATGAGGCGGCTATTTTGCTGGGCGCATCCGGCTGGCAAATGTTTCGCCGCGTGACGTTG
CCGAATATTCGCTGGGCGCTACTCTACGGCGTGGTATTAACCAACGCCCGCGCGATTGGCGAGTTTGGCGCGG
TGTCGGTGGTGTCCGGCTCGATTCGCGGCGAAACGTTATCGCTACCGTTACAGATTGAATTACTGGAGCAGGA
CTACAACACCGTCGGCTCCTTTACCGCCGCCGCATTGTTGACGCTAATGGCGATTATTACCCTGTTTTTGAAG
AGCATGTTGCAATGGCGTCTGGAGAATCAGGAAAAACGCGCGCAACAGGAGGAAAATCATGAGCATTGAGATC
GCCAGAATTAAGAAATCGTTTGGTCGTACTCAGGTACTAAATGATATCTCGCTGGATATTCCTTCCGGCCAGA
TGGTTGCCTTGTTGGGGCCGTCAGGTTCCGGCAAAACGACGCTGCTGCGCATTATTGCCGGGCTGGAGCATCA
GTCCAGCGGCCATATTCGTTTCCACGGTACGGACGTTAGCCGCCTGCACGCGCGTGAGCGTAAAGTCGGTTTT
GTGTTTCAGCACTATGCGCTGTTTCGCCATATGACGGTGTTTGACAACATTGCTTTTGGCCTGACGGTGCTGC
CGCGACGCGACCGTCCAACTGCGGCGGCGATTAAAACGAAAGTGATGCAATTACTGGAGATGGTGCAACTGGC
GCACCTCGCGGATCGCTTCCCCGCCCAGCTTTCCGGCGGACAAAAACAGCGTGTGGCGCTGGCGCGCGCGCTT
GCCGTAGAACCGCAAATTCTACTGCTGGATGAACCCTTTGGCGCGCTGGATGCCCAGGTACGTAAAGAGTTAC
GTCGCTGGTTGCGCCAGCTACATGAAGAACTGAAATTCACCAGCGTCTTTGTCACCCACGATCAGGAAGAAGC
GACGGAAGTGGCGGATCGGGTGGTGGTGATGAGTCAGGGCAATATCGAACAGGCTGATGCGCCGGATCGGGTG
TGGCGTGAACCGGCAACCCGCTTCGTACTGGAGTTTATGGGCGAGGTGAACCGCCTGACAGGCACCGTCCGCG
GCGGGCAGTTTCACGTTGGCGCGCATCGCTGGCCGCTGGGTTATACGCCCGCGTACCAGGGGCCGGTCGATCT
GTTCCTGCGTCCGTGGGAGGTGGATATTAGCCGCCGCACCAGCCTGGATTCTCCGCTACCAGTACAGGTCATT
GAGGCCAGCCCGAAAGGTCACTATACACAGCTAGTGGTGCAGCCGCTGGGGTGGTATCACGATCCGCTGACGG
TGGTGATGGCGGGTGAGGATGTTCCGGTTCGCGGCGAGCGTTTGTTTGTTGGACTGCAAAAAGCGCGTCTGTA
TAACGGCGACCAGCGTATTGAAACGCGTGAAGAGGAACTTGCTCTGGCGCAATCGGCCTGATAGGTTAATTTG
TATAGTTATCGCCCGGTGGCGCTACGCATACCGGGCTTTTTTACGAGCTGAAAACGTGAATACATTAGAACAA
ACCATCGGCAATACGCCGCTGGTCAAATTACAACGTCTGGGGCCGGATAACGGCAGTGAAATCTGGGTCAAGC
TGGAAGGCAATAATCCGGCAGGATCGGTGAAAGATCGCGCGGCGCTATCGATGATTGTCGAGGCGGAAAAGCG
CGGGGAAATTAAACCGGGCGATGTCCTGATTGAAGCGACCAGCGGCAACACTGGCATTGCGCTGGCGATGATC
GCCGCGCTGAAAGGTTATCGCATGAAGCTGTTGATGCCAGACAACATGAGCCAGGAGCGTCGGGCGGCAATGC
GGGCCTATGGCGCGGAACTGATTCTGGTCACTAAAGAGCAGGGAATGGAAGGGGCGCGCGATTTAGCGCTCGC
TATGTCTGAGCGAGGCGAGGGCAAACTGCTCGATCAGTTTAATAATCCTGATAATCCCTACGCCCACTACACG
ACCACCGGCCCGGAAATCTGGCGGCAAACGTCAGGTCGCATCACCCATTTTGTCTCCAGCATGGGCACGACCG
GCACCATTACCGGCGTGTCGCGTTTTCTGCGCGAGCAGGAAAAACCGGTCACCATCGTTGGCCTACAGCCGGA
AGAGGGCAGCAGTATTCCGGGCATTCGCCGCTGGCCTGCGGAATATATGCCGGGCATTTTTAACGCCTCGCTG
GTAGATGAGGTGCTGGATATTCATCAGAATGATGCCGAAAACACCATGCGTGAACTGGCCGTTCGTGAAGGCA
TTTTCTGCGGCGTGAGTTCCGGCGGCGCGGTAGCGGGCGCGTTACGCGTCGCACGAGCCACGCCCGGCGCGAT
AGTGGTGGCGATCATCTGCGATCGCGGCGACCGTTACCTTTCGACCGGCGTGTTTGGTGAAGAGCATTTCAGC
CAGGGGGCAGGGATTTAGATATGATCGCCAATGCTATGAAATTGACCAGGAATAGCGCAGTTTTTCCTGCTAG

TTGCACCATGTCTCCTCTGTTCAAACTGAAACTCATTGGATAAGTGACTGCAAAAGGGATATGACCGCACAAT
TTCAACTGGGCCGGAAATATCACATTGTCGATGGTGCGGAATCAGACACACAGAAAGCTATCTTTAGGTATAA
AAACACGTCAAAAAGGATGATGACGCTACGTAATTAAAGGAATTAATTTCATGAAATTTGCTTTCCGGACACT
GATTTTTGTCGGTTTGTTTTACGCCATTGCCCCTCTTGCCTATGCAGAAACCTCGTTGGATGAGTTACAGCAA
CAGGCAGATCGAGGGGATATGAATGCGCAGTTTCAACTGGGCCGTAAATATCATATTGGCGATGGTGTGGAGC
GTGATGTGGAGAAAGCCGTTTTTTGGTATCAAAAAGCTGCAGCTCAAGGGAGCGCAAAAGCGACAAATAATCT
GGGCGTGTTATATGAGCATGGACATGTTGCCCCCGAGGATGAGCACCGTTCAGTAGAGTGGATAATGGAAAAA
TCATTTGCCTATTTTACGGATGCCGCTAAAAAGGGTCAGTGTACCGCCCAGCAAAATATTGGTGCGACTTATG
CTTATCGACACGAATATGTTCAGGCGTGGGCATGGCTCACGGTAGCAGCGACGCGAGGTTCTAAATGGGGATT
GATAGACAGGGGACGTTTTGAGAACAGTATGAGTTCCGATGAACGAAAACAGGCGGAGAAACTGGCGAAGGAA
TACCTGCAAAAATACGACACGAAAGAAGGGCGGTGTAACGAGCAGACAATTGATTTGAGTACTTATAAGCGTT
AATCCTGCTCTACTTTTTAAGCAACAGATCTGCCTGGCAGGTCTGTTGCATGAGAATAACCCATTGCGCACCG
CCAGCTTCATTGCTGCGGTTTACTTCCCTTACGGCGCAACCGTTTTATCCGTTTCCGCAATAAGCGCATGTAA
ATAAGGCTGCGCGTTTTCATCGCTTTTACCGGGAATTTTAACGATATAGGGCTTTCCGGTGATGGACGACGAC
GACGCGACTTTATCGATAAACTGTTCGGCGGTGTCGATACGATTGCGCGTGTTACCCAGTTTTAAGCGCAGGT
GCGATACGGCTTCCTCGCAGTTATGCGCATCACCGTTACGAACGAAGGTCAGATCCTTTTTCTGCGCCAGCGC
ATTCAGCATGGCGTTAATGCGCGCTTCTTCGTGGGCGTTTAGCTTCGCCCAGGCGGGTAGGGTCAGCAGGAGC
GCAACGACGGCGCAGACAATTTTCTTCATGGTAAAATCCTTTTTCATGCGATAATCACTGCACCTCAGACCGT
TAGCCGGGTAATAAGTTGCACTCCTGGTGGGGTTATTTTTGTGAGTGCGCCAGCGTGAGTTCATCCAGAAAAC
GCCACAGCTTTTCATTCATTTGCTGGTAATTCCATGCGCGCATTTCGGCAACCGAGCGGATAAAACGCTTCCC
TTGCGCGTCGGCCAGCTCCTGCTGCGAATGTTGGATTAATCCTTCAACCGCCTCGACGGTAAATTCCATATGG
CACTGGAAACCGTAGACAAAATTACCGTACTGCACTATCTGACGTGGACAGCCTTCGCTTTCGGCCAGCACGG
TCGCCTGATCGGTGAGTCCTGGCATATCGTTGTGCCAGTGGCCGACGGTTAACGGTGAGCCAAAGTGGGCGAT
AAGCGGATGCCGCAGACCGGCTTCAGTGAGCGTAATAGGGTAGTGGCCAATCTCTTTTTCCGGACTTTGGCAC
ACCGCGGCGCCCAGCGCCTCGCCAATGAGCTGCGAACCAAGACAAATGCCGATAACCATTCGCCGGGCCGTAA
TCGCCTGATTAATTAAGTGCTGTTCGGCGCGAGAGTCAAAGTACGGGCACTCTTCGCGGGTGGTGCGCGGCGA
CTGCGGACCGCCAAATACCACGAGCATATCGAACTCATCGGCATTGGGCGGTAGCGCTTCTCCGGCATAGACG
CGCGACCATGAAATGGTATAACCGCGATCTTCAGCCCATTTCAGATAAGCGCCAGCGGATTCAAACGACTCAT
GGACAACAAAATGAACTCGCACGTTATTTTCTCCTGTTAGCGATTTAGCGCCTGATGGATATCAGCCGCCAGC
GTGTTGATCTCGCTGTCATTTAATGTCGACAGCGTGATACGTAGACCGTGAGACGGCGCGCTTACGCCAAAGG
CTTCTCCCTCACGTACCAGCCAGCCGGATTTTGCCAGCGTAAACGCGGTAGCCTGGCTATGGGTGTCCAGCGG
GAGCCAGGCATTCACGCCGTCGCCGGGAGAAATGGCGATGCCATATTGCTGTAGGGCGCGGGCCAGTTTTTGT
TGCCGGGTGGCGTAAAACAGCCGCGTTTGCGCCAGCCTGTGCTGATACTCAGGGTCGGTCAGGCAGGCGTAGA
CCAGATCCTGTAACAGATGGCTGACCCACTGACTACCGGCATTTAGCCGCAGCCGAAGTTTTGCCGAGGTCGC
GCTGTCGCTGGCGACAATAGCAAGACGTAAATCGGGGCCGAGGGTTTTGGAAACGGAGCGTATCACCGCCCAG
TGTTGCGTTGTCTGCGCAATAACCGGCTGCCAGGGCGACGAAGAAAGCAGGGCGAAGTGATCGTCGATAATGA
CCACTACCTGAGGGTAACGCGCCAGTATATTTTGCAGCGCAGCGGCACGGCGAGCGCTCAGGCTACAGCCGGT
AGGGTTGTGCGCGCGCGGCGTTAAGATCACTGCCCGCGCGCCCTGGTTTAGCGCCTGTTCCAGCTTTTCAGGC
TGCATTCCTTCGCTATCCACACTGACCGGGCTGGCGCTAAAACCGGCATAGCGCAGCATATTGATACTGCTTA
AAAAACAGGGATCTTCCACCGCGACGCTGTCGCCCGGCAGCAGATGCGCGCAAAGCAGTCTTTCAATGGCGTC
GATAGCTCCGCTGGTGATATCAATTTCGCCTGCGACGGGGGTGGCATCCCGTAGCCAGCGCGCCGCCCAGGCG
TGTAGTTCAGGGGAAACGGGCGCATCGCCGTAGAGATGCGGCGTGCGGCTGAGACGGGCAAAATAACGGCTAA
GGTCAGGCAGACGCTGCGGATCGGGATTGCCGCCGGAAAGGTCGTGCAGAGGCGTATGCGGATCGCCGCCTTC
CAGCGCTACGGGAGACGGACTGCCTTTAATCACCGTCCCATTGCGGCCTAAACTTTGCGCCAGTCCCGCCGTT
ATCAGCCGTTTATACGCCGCTGCGACGGTATTGCGGTTCACCTTTAATTCGCTTGCCAGCTCCCGTACCGGCG
GGAGCGAATCCTCCGCCCGTAACGTGCCGGCGATAATGTGTTGACGGATACTGTCAAAAATTTCGTTAGCGGT
TTTTCCGTCGATCATTATTGACCTATGACAAAATGAATTTTAGCATATGACAATGATAATGAGGCGGTAGCCT
GCTGTCCAGCGAGAGGAAAATTCTATGGGACAAGAGAGTGATATTCAGTCAGTGCTCTTCGACGACAACCATC
GGGCATTACAGACCGATATCGTCGCCGTCCAGTCACAGGTCGTGTACGGCAGCGTAGGCAACAGTATCGCTGT
TCCGGCCATTAAAGCGCAGGGACTACGCGTGACGGCGGTCCCGACGGTGCTATTCAGCAATACGCCGCACTAT
AAAACCTTTTATGGCGGCATTATTCCCGCCGAGTGGTTTGCAGGCTACCTTACGGCGTTGAATGAGCGCGATG
CGCTACGTGAATTAAAAGCGATTACCACCGGTTACATGGGCAGCGCAGACCAAATTGTCTTGCTTTCGAAGTG
GCTAATGGCGATCCGGGCTTCGCATCCGGAGGTGTGTATTCTTGTTGATCCGGTCATTGGCGATACCGACAGT
GGAATGTATGTGCAGGCGGAAATTCCGCAGGCTTATCGCACGCATTTGTTGCCGCAGGCGCAGGGGCTAACGC

CGAACGTATTTGAGCTGGAGATGCTGAGTGGTAAACCGTGCCGCACGCTGGAAGAGGCGGTGGCGGCGGCGCA
ATCGCTGCTTTCCGATACGTTGAAATGGGTGGTTATCACCAGCGCGCCGGGCGAGTCTCTGGAGACCATAACG
GTTGCGGTCGTTACTGCGCAGGTCGTGGAGGTCTTTGCCCATCCGCGGGTGGCGACGGAGTTAAAAGGGACGG
GAGATCTCTTCTGCGCCGAGCTGGTCAGCGGTATTGTGCAGGGGAAAAAACTGACGACCGCGGCAAAAGATGC
GGCGCAGCGCGTGCTGGAGGTGATGACCTGGACACAGCAGTGTGGCTGTGATGAGTTAATTCTGCCGCCGGCA
GGGGAGGCGCGATGAAGAGTTATCGGCTGGTGGTTCGCCAGCAGGGACGTATCGTGGGGCATTTTGAAACGAG
CGGGCTGGACGCGCTGGAAGATATCTGCGTGGCGCGGGCGATGTTTGGCATCACTGGCGGCTATCAGTGCGAG
TTGCAAGTGTCCGATTCCGAACGCAGGATGCTGGAGAGTGGCCCGGAGGGGATGAAGATCCTGATGCGCGAGA
AGTGCTTTCGCCCGGTGACCTCGCAGTTGTAACCCGTGGATTGGCGCTGAAATGTTCGCAACCTTGTAGGCCT
GATAAGCGTAGCGCCATCAGGCAGTGCGCCACACCATCGCCGGATGGCGGCGCAAGCGCCTTATTCGGCCTAC
AGGCAAAAAAAATGGCGCCCAAAGGCGCCATTCTTCACTGCGGCAAGAATTACTTCTTAATGCGGATAACCGG
AGTTTCACCCACGGTCACGCTACCGGACAGTTTGATCAGTTCTTTGATTTCATCCATGTTGGAGATAACAACC
GGAGTCAGGGTAGACTTGGCTTTTTCTTCCAGCAACGGCAGATCGAACTCGATGACCGGATCGCCAACTTTCA
CGCGCTGACCTTCTTCAGCAATACGCTTGAAGCCTTCGCCTTTCAGCTCAACGGTGTCGATACCGAAGTGAAC
GAACAGCTCAATGCCGCTATCGGATTCGATAGAGAACGCATGGTTGGTTTCAAAGATTTTGCCGATGGTACCG
TCAACAGGGGCGACCATTTTGTTACCGGTTGGTTTGATAGCGATGCCATCACCAACGATTTTTTCAGCAAAAA
CTACATCCGGCACGTCTTCGATGTTGACGATCTCGCCAGAGAGCGGGGCAACAATCTCAATAGTTCCGGTGTC
TTTCTTATCATCAGAAACCAGAGATTTTAGTTTATCGAACAAACCCATGATCTTCTCCTAAGCAGTAAATTGG
GCCGCATCTCGTGGATTAGCAGATTGTTTTTTCTTCAATGAACTTGTTAACCAGCGTCATTAACTCGTCCGTT
GTCGGTTGAGCAAGAGCCTGCTCTGCTAACACCTTCGCATCTTCGAAGTTCGTGTTACGGATAATCTTCTTAA
TGCGCGGGATAGAAATGGCGCTCATAGAGAATTCGTCCAGACCCATCCCCAGCAACAGAAGTGTAGCACGTTC
GTCGCCTGCAAGCTCACCACACATGCCAGTCCATTTACCTTCTGCATGAGAAGCATCAATAACTTGCTTGATC
AAGTTCAGTACGGACGGTGACATTGGCTGGTAAAGGTGTGAAATCATATCATTACCACGGTCAACTGCCAGGG
TGTACTGCGTTAAATCATTGGTGCCGATACTAAAGAAATCAACTTCTTTGGCTAAATGACGCGCAATTGTCGC
CGCAGCCGGTGTTTCCACCATCACGCCAATCTCAATGCTTTCGTCAAATGCTTTACCTTCGTCACGCAGTTCC
TGTTTGTAGATTTCAATCTCTTTGCGCAGCGCGCGAACTTCTTCAACAGAGATGATCATCGGGAACATAATGC
GCAATTTACCGAAAGCGGAGGCACGCAGAATCGCGCGAACCTGGTCACGCAGGATCTCTTTGCGATCCATGGC
GATACGCACGGCGCGCCAGCCCAGGAACGGGTTCTCTTCTTTCGGGAAGTTCATGTACGGCAGCTCTTTGTCG
CCGCCAATGTCCATGGTACGGACGATAACCGCCTGCGAGCCGCACGCTTCAGCGACCGCTTTATAGGCGGCAA
ACTGCTCTTCTTCCGTCGGCAGCGCGTCGCGATCCATGAACAGGAATTCAGTACGATACAGACCAACGCCTTC
CGCGCCGTTACGCTCAGCGCCTTCAACGTCACGAACGGTACCGATGTTGGCGCAAACTTCAACCTGATGTCCA
TCCAGCGTGATTGCCGGCAGATCTTTCAGTTTTGCGAGTTCCGCTTTCTCGGTCGCAACCTGCTCCTGGACGG
CGCGCAGTTGCTCAATAACATCGTTGGTCGGGTTGACGTAAACCTGGTTGTTTACGGCATCCAGAATCAGATA
GTCGCCGTTTTTCACCTGAGCGGTGACGCTACCGGTACCTACGATCGCTGGCAGTTCCAGAGAACGCGCCATG
ATGGAGGTATGAGACGTGCGTCCGCCCGCGTCGGTGATGAAGCCCAGCACTTTCTGCAGGTTCAGCTGCGCGG
TTTCTGACGGCGTCAAGTCAGAGGCAACCAGGATAACTTCTTCCTGAATCGCGCTCAGATCGATAATGGCCAG
ACCCAGGATGTTGCGCAGCAGGCGCTTACCGATATCACGTACGTCAGCCGCGCGCTCTTTCAGGTATTCATCA
TCCAGTTCTTCCAGGGCAGTGGCCTGACCTTCGATAACTTCATGTGCGGCTGCGTCAGCCGTCATGTGCTTAT
CTTTAATCAGGGCTATGATTTCCTGCTCCAGCTCCTCATCTTCGAGCAGCATAATATGCCCTTCAAAGATGGC
TTCTTTTTCTTCACCGAACGTTTCACCAGCTTTTGTCTTGATCGCTTCCAGTTGCGCAGATGCCTTGGCACGA
CCGCTCAGAAAACGTTCAACTTCCTGATCAACCTTGTCGGCAGAAATTTTTTTCCGGTCAATGACGATTTCGT
CTTCTTTCAGCAGCAGTGCTTTGCCGAAAGCGATACCCGGGGATGCTAAAATGCCTGAAATCATAACCCTACC
TTACTTGTGACTGATTTTTAAAAGAACCCGGAAAAAAACTTACTCGAGTTCCGCCATCAGTTTGACCAGATGT
TCAACCGCTTTCTGCTCATCTTCACCTTCTGCGGAGATGGTGACGACGGTGCCTTGAGTCAGGCCCAGAGTCT
GCAGTTTAAACAGGCTTTTCGCGCTGGCGCTTTTGCCGTTGGAAGTCACAGTAATCTCAGAAGTGAAGCCTTT
TGCTTCTTTAACAAACTGAGCAGCAGGGCGGGTGTGCAGACCGTTCGGAGCGGTAATGGTAACTTCTTGCTGG
AACATTGTATTTCCCCAACTTATAGGTTTAGTGTTGTGGAACTAAAGTCTAGCCTGGCGGCTCAACTTTAGCC
TGTATTGTTAGCGCCGGCGTAAACGGGACGCGACACAAAAGGTGTTCTACGCAATCCGTTGCTGGCAACTTAT
GGCCGCTGACGTTTCGCTCGTCATTAAACATTATGCCGCGAAAGGAAGATTTGAACCAAATCATAAAATCGAT
TCAGCTAACGCTTTTCCTGTAACGATTAATTTCGCGCATCAAAATAATTAGTCTGGTTAAATACCAGTTCCGG
CAGGGTGAATCAATGCCAGGTGAGGTGAAAGTTTGAAGCAGGCCACAAAAAAGCACCCAAAAAGGTGCTTTTT
TACGCGTTTTTAACATGCTGGCATCACTGTTGCAGTTCTTTCTCAGTAAAGAGATCGGCGAACAACGCGGTGC
TCAGATAACGCTCACCTGATGAAGGTAGGATAACCACAATATTCTTATTGGTAAAGCTTTCATCTTCCTGCAG
CTTAAGCGCTGCAGCAACCGCGGCACCGGAAGAAATTCCTGCCAGAATACCTTCCTCTTCCATCAGACGGCGC

GCGGTAGAAATGGCTTCTTCATTGGTGATGCCAACCACTTTATCAATCAGTTTCAGATCCAGGTTGCCCGGGA
TGAAGCCTGCGCCGATGCCCTGAATTTTATGCGGGCCTGGTTTGATCTCTTCACCAGCCAGCGCCTGGGCAAT
AACCGGGGAGTCAGTGGGTTCTACCGCAACGGTGATAAGATCGGTTTTACCCTTCGTCCCTTTGATATAACGC
GTGACGCCGGTTAACGTACCGCCAGTGCCGACGCCGGAGATAAACACATCCACCTGACCATCGGTGTCTTCCC
AGATTTCCGGGCCGGTGGTTTTTTCATGGATTTCCGGGTTGGCAGGGTTGCTGAACTGCTGGAGCAGGAGATA
TTTTTGCGGATCGCTGGCGACAATTTCTTCGGCTTTCTGAATAGCGCCTTTCATCCCTTTCGCCCCTTCGGTC
AGCACCAGATTCGCGCCTAATGCTTTGAGTAGCTTGCGGCGTTCAATGCTCATGGTTTCCGGCATGGCCAGGG
TGAGTTTGTAACCGCGCGCGGCGGCGACATACGCCAGCGCAATACCGGTGTTGCCGCTGGTCGGCTCCACCAG
TTCCACGCCAGGTTTCAGTACGCCACGCTTTTCGGCATCCCAAATCATATTGGCGCCGATACGGCACTTGACG
CTGAAGCTCGGGTTGCGCGACTCCACCTTCGCCAGAATGCGTCCATTACCGATACGGTTCAGTCGAACCAGCG
GCGTATGACCGATAGTCAGCGAGTTATCTTCATAAATCTTACTCATGGCCTGTCCTTAACTGTATGAAATTGG
GATACCTTCCCAGCATACCTTCCAGCCAGATATGGGGAAGTAAGGATTTCGCATATCTATATGCTGATGGGAA
ATAATGGTTATCAGTATGGAATAAGAGACAGCATAAGCCATCTCTTATTTATACATTTTTACGTTATTTCCAT
AACGCGTGCTTAGCACGCCAGCAATCAACCCACATTGCCGTCGCGCCACAGACGGCGACAGGCATAATGAAAA
GGTTAAGCACCGGAATCATCGTAAAGAGGCTGGTGAGCGCGCCAAATTGCATATTCGCCACCTTCTGCGTGCG
CAACGCGGCGCGCATCGTTTTAAACGGCACTTTGTGGTTATCAAACGGGTAGTCGCAATACTGGATCGCCAGC
ATCCAGGCGCTGAACAAAAACCACAGCACCGGCGCGATGGTTTGGCCAATGCCAGGAATAAAATAGAGAACCA
GCAGCACAATAGCGCGCGGCAGATACCACGCCAGCTTTTGCCATTCACGTTTCATAATGCGCGGTACATCTTT
CATGATGCCGAGTATGCCGGTATCTGGCGGCGTGGCGCCGGTTAACCGCGCCTCAAGCTGTTCGGCTAAAAGG
CCGTTAAACGGCGCGGCGATCCAGTTGGCGAGGGTAGAGAAAAAAATAGCCAAAGACCAGCAGAACAGAGATAA
CCGCAATCGGCCACAGCAGATAGCTCAGCCATTGCAGCCAGTCCGGCACATGACTCATCAACGAAGGTATCCA
GGCGTCAAGCTGGGTAAACAGCCACCAAAATGCGCCGCCCATCAGCACAATGTTGACCAGCAAAGGTAAAATA
ACAAAGCGGCGGATTCCCGGTAGGGTGACTAACTTCCATCCTTGCGAGAAATAATAGACGCCGCTACGTGGTA
CGGTTGTGGATGATGAAACCATATTCAGGACATACTCCTTTTTCTTAAAGCCCTGGGTAAACGTGGGATATCT
TACCCGGTTATCAGACGGTGGATAGTTCGGAAATGTTCGAAAAAACAGCAAAAAGCACATTTTCCTTCATCTT
TATGCTGCGAAAGCCAACAGTACACTTGCACTTGACGTAATCGGCAAATACTCTTAGTGAGTAAATGTTTGCC
GTGGTGGCAAGGTGTTAGAACAACAGAGAATATAATGATGCAGGATTTGCGTCTGATATTAATCATTGTTGGC
GCGATCGCCATAATCGCTTTACTGGTACATGGTTTCTGGACCAGCCGTAAAGAACGATCTTCTATGTTCCGCG
ATCGTCCACTAAAACGCATGAAATCGAAACGCGACGACGACTCGTATGACGACGATGTCGAAGAAGACGAAGG
CGTGGGTGAAGTTCGTGTTCACCGCGTAAATCATGCCCCCGGCCAGTCGCAGGAGCATGATGCCCCTCGTCAG
TCGCCGCAACACCAGTACCAGCCGCCTTATGCGTCTGCGCAACCGCGTCCAGCGGCGCCGCCGCAGCCGCAAG
CGCCGATGCAACAGCCGGTTCAGCAACCGGTCCAACCTGCGCCGCAGCCTCAGCAGGTTCAGCCATCTGCGCC
GCCGGTACAGCCGCCGCAACAGCAGCCTGCACCGCCGTCGCAAGCGCCGCAGCCTGTTGCGCAGCCCGCGCCG
CCGCCATCAGCGCAAACCTTCCAGCCTGCGGAACCTGTGGTTGAGGCGGAACCGGTAGTGGAAGAAGCGCCGG
TTGTGGAAAAACCGCAGCGTAAAGAGGCGGTCATCATTATGAACGTCGCGGCCCATCATGGCAGCGAGCTTAA
TGGTGAAGTGTTACTGAACAGTATTCAGCAGTCAGGCTTTAAATTTGGCGATATGAATATTTTCCATCGTCAT
TTGAGCCCGGACGGCAGCGGCCCGGCCTTGTTCAGTCTCGCGAATATGGTCAATCCGGGAACGTTCGATCCTG
AAATGACGGACTTCACTACGCCTGGCGTCACTATCTTTATGCAGGTACCGTCTTACGGCGATGCGCTGCAGAA
TTTCAAGCTGATGCTGCAATCCGCGCAGCATATCGCTGATGAGGTCGGCGGCGTCGTGCTGGATGACCAGCGT
CGGATGATGACGCCGCAGAAACTGCGCGAATATCAGGACCGCATCCGCGAGGTGATGGACGCTAACGCCTGAG
GTTAACGCAGGTGTGACATTTTTAACGTAACCCCCGCCTGTCGGGGGTTTTTTATCATTGATGGTGTGATATG
GAACCAATCGAACAACAACTGACAGAACTGCGAACCACGCTTCGCCATCATGAATATCTGTATCATGTGATGG
ATGCGCCCGAGATTCCCGACGCCGAATATGACCGCCTGATGCGGGAACTGCGAGAGCTGGAGGCGCAACGTCC
GGACCTTATCACGCCGGATTCGCCCACGCAGCGCGTTGGCGCGGCGCCGCTGACGGCATTCAACCAGATTCGC
CATGAAGTACCGATGCTGTCGCTCGATAACGTATTTGATGAAGAAAGCTTCCTGGCGTTTAACAAGCGAGTGC
AGGATCGTCTAAAAAGCACGGAAAATGTCATCTGGTGCTGCGAGCTAAAGCTGGATGGTCTGGCGGTCAGCAT
TCTGTATGAAAACGGCGTGCTGGTGAGCGCTGCGACGCGTGGCGACGGCACCACCGGTGAAGATATTACTTCA
AACGTGCGCACGATTCGCGCCATTCCGTTAAAGCTGCACGGCGATAACATTCCGGCGCGTCTTGAAGTTCGCG
GAGAGGTATTTTTACCGCAGGCCGGGTTTGAAAAGATCAATGAAGACGCGCGCCGCACTGGCGGCAAAGTGTT
TGCTAATCCGCGTAACGCGGCGGCGGGCTCGTTGCGTCAGCTCGATCCGCGTATTACGGCGAAGCGACCGCTA
ACATTTTTCTGCTACGGCGTCGGTATTCTGGAAGGCGGCGAGTTGCCGGATACGCATCTGGGACGTTTGCTGC
AATTTAAAGCGTGGGGGTTACCGGTCAGCGATCGCGTGACGCTGTGCGATTCTCCCCAGGCGGTACTGGATTT
TTATCGCAACGTCGAAAAAGATCGCCCCACGCTCGGTTTTGATATCGACGGCGTGGTGATTAAGGTCAACTCG
CTGGCTTTGCAGGAACAGCTGGGCTTTGTGGCCCGCGCGCCGCGCTGGGCGGTGGCGTTTAAATTTCCTGCTC

AGGAGCAGATGACTTTTGTGCGCGATGTTGAGTTTCAGGTCGGGCGCACCGGGGCGATTACGCCGGTGGCGCG
TCTGGAACCGGTTCAGGTGGCTGGTGTGCTGGTCAGCAATGCCACGTTGCATAATGCCGATGAAATTGAGCGA
CTTGGCTTGCGCATTGGCGATAAGGTGGTTATCCGTCGGGCAGGCGATGTGATCCCTCAGGTGGTCAACGTGG
TTCTGTCCGAACGCCCTGAAGAGACGCGTCCGATTGTGTTTCCAACGCACTGTCCGGTGTGCGGATCGGACGT
AGAGCGCGTTGAAGGCGAAGCCGTTACCCGTTGTACCGGCGGGCTGATTTGCGGCGCGCAGCGTAAAGAGTCG
CTGAAACATTTTGTGTCGCGCCGGGCGATGGATGTAGACGGGATGGGCGATAAGATCATCGACCAACTGGTGG
AAAGGGAGTATGTCCATACGCCAGCGGATCTGTTCCGTCTGACGGCGGGTAAACTGACCGGCCTGGACCGAAT
GGGGCCAAAGTCCGCGCAAAATGTTGTAAACGCGCTGGAAAAAGCCAAAGCGACGACTTTTGCGCGTTTTCTC
TATGCGCTGGGCATCCGTGAAGTGGGTGAAGCGACGGCGGCGGGGCTGGCGGCTTATTTCGGCACGCTGGAGG
CGCTGCAGACCGCTACCATTGACGAGTTGCAGAAAGTACCCGACGTGGGGATTGTGGTCGCTACGCACGTCTT
TAACTTTTTTGCCGAAGAGAGCAATCGTGACGTGATTGGGCAGCTACTGGCGGAAGGGGTTCACTGGCCCGCG
CCGGTAGTCATTAATGTGCAAGAGGTCGATAGCCCGTTTGCCGGTAAAACCGTGGTACTAACCGGTAGCCTGA
GCCAGATGTCCCGCGATGACGCTAAAGCGCGTTTAGCGGCGCTGGGCGCGAAAGTGGCAGGCAGCGTATCGAA
GAAAACCGATCTGGTGATTGCCGGTGAAGCGGCGGGCTCCAAACTGGCCAAAGCGCAGGAACTGGGTATTACC
GTGATTGATGAAGCGGAAATGATCCGCTTACTGGGTGCCTGAGATGGAAAAAGAGCAGCTGGTTGAAATCGCC
AACACCGTGATGCCCTTTGGCAAATATCAGGGACGTCGATTGATCGACCTTCCGGAAGAGTATTTGCTGTGGT
TTGCCCGTAAAGATCAATTTCCGGCGGGTAAACTGGGGGAGTTGATGCAAATCACGCTGCTGATTAAAACCGA
GGGGCTGACGCAACTGGTACAGCCCCTGAAACGCCCGCTTTAAGCTTTCGCGGCGCGGCTTTCCTGCTGCGCC
TGTAACTTCTCGGTCTGGCGTTTGTAGCGCCGCGCCAGCCCTGCGCAGACCATCAGTTGGATCTGGTGGAAGA
TCATCAGCGGTAATACCATCATACCCAGCACTGACGTCGGAAACAGAATATTGGCCATCGGGATCCCGTTGGC
CAGGCTTTTTTCGAACCGCAGAAAACAATAGTGATTTCGTCGGCTTTATTGAAGCCGCATTTACGGGCGACAA
AGACGTTAATGGTGATGACGATCGCCAACAGTATGAGACTGACCACGACGATAAACAGCAGCGATCCCCAGCC
GACTTTGTGCCAGATGCCGTTGACCACCGCTTCGCTGAACGCCGAGTAGACCACCAGCAAAATAGAGGTCTGA
TCTGTTTTCGCGATCCACTTTTTGTTGCGCGCGACCCAGTTGCCGATCCACGGGCGAGACAGGTGTCCCAGCA
CAAAAGGCAGCAGCAGTTGCAGCATAATTTTGCCCACCTCTTCCAGACTGCCCTGCGCGCCATGAATATTCAT
TACCAGACCGACCAGCAGCGGCGACAGGAAAATGCCCAGCAGACTGGACGCAGACGCTGAGCACACCGCGGCC
GCGACGTTACCGCCCGCGAGTGAAGTAAATGCAATCGCCGACTGCACGGTGGCGGGCAAAATGCACAGATACA
GAAAGCCACTGTAGAGCATAGGGTCGACATTGACAGGCGCCCACCAGGCAAACAGTACGCCGAGGACAGGAAA
GAGCACAAAAGTGCTGCACATGACCCACAGATGCAACCGCCAGTGGCTGCCGCCAGCGATGATCGCTTCACGT
GAAAGCTTCGCGCCATGCATAAAAAACAGCAGAGCGATGGCCGCTGTCGTAATGCCTTCAACGACAGGCACAA
AGCCGCCTTCCGCCGGGAAAAAAGAGGCCAGTAGTACCACGGTGATAAGTGTCAGCGTGAAAGGGTCAAGGAT
ACGAAAAAGTTTCATAAAAACTCCTGAAAGTCGGTGCAGCTATTTTGCGTTTTTCGCTTTGAGAAATAAAATT
AATTTATTGCATCTATATATGAATCTAGTCGATGAACTATTCACTGCGTCAACTGCGTATTTTTATTACCGTC
GCCCAGGCCAAAAGTTTCAGTCGGGCAGGGGACAGGATTGGACTGAGCCAGTCTGCGGTCAGTCATAGCGTAA
AAGAGCTGGAGCGGCAAACCGGCGTCAGACTGCTGGATCGCACCACGCGTGAGGTGGTGCTGACCGAGGCGGG
GCAACAACTGGCGACGCGTCTGGAGCGGGTGCTTGATGAACTGAACAGCATACTGCGCGATGCAGGCAGAGTA
GGGACACAATTAACCGGCACCGTGCGCGTTGCCGCGAGCCAAACCATTTCTGCGCATCTTATTCCGCAATGCA
TTGCCCAAAGTAATTCACTTTATCCCGCTATTGATTTTGTATTGCACGATCGTCCGCAGCAGTGGGTGCTGGA
GAGCATCCGCCAGGGTGAAGTGGATTTTGGCATCGTTATCGATCCCGGCGCAGCGGTGGATTTACAGTGTGAA
GCCATCTTATCAGAGCCTTTTTTACTGTTGTGTCGCCAGGATCATCCGCTGGCGCATCAGGAATGGGTGAGCT
GGCAGGATCTTAAACAGGCCTCGCTGGTCTTACAGGATTATGCTTCCGGCAGTCGACCGCTGATTGATGCGGC
GCTGGCCCATTTTGCTATTGAGGCGGATATCGTGCAGGAGATTGGTCACCCGGCCACGCTGTTTCCAATGGTG
GAAGCGGGAATCGGCATCAGCGTGCTGCCCGCGCTGGCGTTACCGCTCCCGCAGGGCAGCCACTTGCAGGTTA
AACGGCTGACGCCCGTAGTTGAAAGGCAGCTCATGCTGGCGCGACGAAAAAACCGATCGCTCTCCACGGCGGC
GCAGGCGTTGTGGGACGTGGTGCGTACCCAGGCCAGCGAATTAACCGCCGCGAGGGCGAAAGATCCGCTGTAT
CAGCTATAAATATCTATCTGTCCGTGTGTGAGGTGCGGTCTTGCCCTGCCATTTTTGACAGGGCAGTGTGTTA
AAACAGGTATTTGATGGAATAAATAATGCCATCCTGCAGGAAGGATTCTCCTAACTTATTATCAGCGTAACGA
TATTGTACTCCTGCGGTCAGGTGAGGAGTCGCATTCCACCACAGCGCTACTGCGCCATTAATGCCATCTTTCC
CGCCATTGCCCGCAGCGTAGGACGCATCGCGGTCAAACTCATACTCATTCCAGTTCGTTACGCTGAATTTCTC
ACTACCCAACGAGAAGCTATAGCCGGCCACCCAACCTAATACATAACCATTATCGCCTGTGTAGTAGGTCTGA
TCGGTATAACGTTTGGCAAAGAAGGGTTTAAACCAGTAACCGTTACCTGTGAAGTTATAGCCTAAACCATAGA
GAAACATGTCATCATGGAAATTAGCGCGATGAGGTGAACCGTACGTCCCGTAGGCATGGAGATAGAGGTTTAA
TCCTGTATCGCCCAGATAAATACGGTTGGTATTTTTAAAGGTGTAACGCTGTTCGCTGCCTGGTTTAGCGTGG
CGGCCATTGTAAAAATTTTCCCAGTCGAAGAACCCGTACATTTCTCCCCAGCTAAAATTCGCTCCGCCTTCCA

GTTCCAGGTACCCAAAATCATCTTTATGGGATTTTTTACTGGTTTTTTCCGTGGTGTCTGATGTCCAGTCGAG
GTAATGGAGGCCGATATCGGCGAACCCGCCTTTAAATTCCGCATGGGTGAGTGCGGGAATAATGAGTAAAGAA
GAAAGCGTCAGAGTAAGAATTTTTTTCATAGCATATTTCCCTATGCCGATAATTATTAATGGGTTAATTAAAA
AGAGGCAATTTATAAATATGAAAGCGGCATATTGTTAATATGAAGTGCAAGCAAAAACCAATACTGTTTTCCC
TCTGTATTAATACAGAAGCGATCTAAATCTATCGATATTAAATGGCTATTGATGACACGTGAAAAGTGTATTG
GTCAGCAAGGGGCGATGGTCGTTATAAAAAGGAGTCAAATTAAATCCGATAGAACAAGGAAACTTATGCCTCA
CGCTCTTTTTTCTCATAATCCTGAGTATTGTATTGATATTATTCATGCACATAAACCGGCATTTACCCCGCGA
GTCGCGTTTATTTTAGGCTCCGGGTTGGGTGCTCTGGCGGATCAAATCGAAGATGCGGTAGCCATTTCTTACG
AGAAATTGCCGGGATTTCCCGTCAGCACCGTACACGGCCATGCTGGTGAACTGGTGCTCGGCCATCTGGCGGG
TGTTCCGGTGGCCTGTATGAAAGGTCGCGGACATTTTTATGAAGGGCGTGGAATGGCGGTGATGACCGATGCC
ATTCGCACGTTGAAATTACTGGGCTGTGAAGTGTTGTTCTGCACCAATGCGGCAGGATCTCTGCGTCCGGAAG
TGGGGCCGGGTAGCCTGGTTGCGCTTAGCGATCACATTAATACGATGCCCGGTACGCCGATGGTGGGGCCAAA
CGATGATCGTTATGGCGATCGCTTCTTCTCGCTGGCTAATGCTTATGACGCAGATTATCGCGCCATGCTTCAG
TCCGTTGCGGCAGAGGAGGGATTCCCCCTCACTGAAGGCGTATTTGTTTCTTATCCGGGTCCGAACTTCGAAA
CGGCGGCAGAAATCCGCATGATGCAGATTATTGGCGGCGATGTGGTCGGAATGTCCGTTGTCCCTGAGGTGAT
TAGCGCACGTCATTGTGGCCTCAAAGTTGTTGCCGTCTCAGCGATCACCAATCTGGCGGAAGGGCTGGGCGAT
GTAAAACTCTCCCATGCGCAAACGCTGGCGGCGGCTGAACTTTCCCGGCAGAACTTTATAAATCTGATTTGCG
GCTTTTTACGTAAACTGGCCTAAACACAATGACTACGGCCCCGTATTGGGGCTTTATCTCACGGCAAGGAATA
AAAAATGGGTATTACGTCCCGCTTAAAAGTCATGTCGTTCTTGCAATATTTTATCTGGGGGAGCTGGCTGGTT
ACCCTGGGCTCTTACATGATCAACACTCTGGATTTTACCGGCGCGAATGTCGGTATGGTCTACAGCTCAAAAG
GACTGGCAGCGATTATCATGCCGGGCATTATGGGGATCATTGCTGATAAATGGCTGCGCGCTGAGCGAGCCTA
TATGCTTTGCCATCTGGTTTGCGCGGGGGCGTTATTGTACGCCACCACCGTTACCGATCCCCAGACGATGTTC
TGGGTGATGTTGGTTAATGCGATGGCGTATATGCCAACGATTGCATTATCCAATAGCGTTTCGTACTCCTGTC
TGGCGAAAGCAGGTCAGGATCCGGTAACGTCATTTCCGCCTGTGCGCGTTTTCGGCACAATAGGTTTTATTGT
TGCGATGTGGACGGTGAGCCTGATGGGGCTGGAACTGAGCAGTGCGCAATTATACATCGCTTCTGGCGCATCG
TTATTGCTGGCCCTGTATGCGCTGACGTTACCGAAAATTCCGGTAGCCGAGAAGAAGGCGAACACCACGCTTG
CCAGTAAGCTCGGACTGGATGCTTTTGTTCTGTTTAAAAATCCACGCATGGCAATCTTCTTTTTGTTTGCGAT
GATGTTGGGGGCGGTGCTGCAAATTACCAATGTCTTCGGTAATCCGTTCCTGCATGATTTTGCCCGTAATCCT
GAGTTTGCCGACAGCTTTGTGGTGAAGTATCCCTCTATCTTGCTTTCAGTTTCGCAGATGGCGGAAGTGGGCT
TTATCCTCACCATTCCGTTCTTCCTTAAACGCTTTGGTATTAAAACGGTAATGCTGATGAGTATGCTGGCGTG
GACGCTGCGTTTCGGCTTCTTTGCCTTTGGCGATCCATCCCCGTTTGGCTTTGTGCTACTGCTGCTGTCGATG
ATTGTTTATGGCTGCGCATTTGATTTCTTCAACATCTCAGGGTCAGTATTTGTAGAGCAGGAGGTGGACTCAA
GTATTCGCGCCAGCGCGCAGGGGTTGTTTATGACCATGGTTAACGGCGTGGGGGCGTGGATTGGGTCTCTTTT
AAGCGGTATGGCCGTGGATTATTTTTCTATTGATGGCGTAAAAGATTGGCAAACCATCTGGCTGGTCTTTGCC
GCCTACGCTCTGGCATTGGCCGTTATTTTTGCATTGTTCTTTAAATATCAGCACCATCCAGAAAAACTGTCGA
CCAAATCATTAGCACATTAATTTTATTTGACTGGCGAGAGGATGTTCGCCAGTCCATTTTTATGAACAAGGAC
ACAGCATGAAAGAAATTATTCTCTCCTCCGGTATTGGATTTGGTATTGGCGCATTTTTTACTTTATGCCGTAT
ACCTATTCCCGCACCAAATGTTTTACCCGGGGTATTATCTATTGTATTTATGTATATTGGGTATCTTGTCGTT
AAATCTATTTTTTACTAGGTAATATGGTAAATTCCAGACGTTATCCCTTATTCCTGGAGTGCGATAGTGATGG
AACGAGCCCATCGTATTGATCTAAAGCTATTACGTTATTTCCTCGCTGTTGCAGAAGAATTGCATTTTGGCCG
GGCTGCTGCCCGGTTAAATATGTCACAACCTCCCTTGAGTATCCATATCAAAGAACTTGAACAGCAACTGGGT
ACATTGCTGTTTATTCGTCATTCCCGCAGCGTTGCATTAACCCATGCCGGGAAAGTTTTAATGGAGGAGTCAC
GTCGCTTGTTAGCCAGTGCAAATCAGGCGCTGGCGCGAGTGGAGCAGATTGGTCGCGGTGAAGCGGGGCGCAT
TGAACTGGGTGTGGTTGGTACGGCGCTGTGGGGAAGAATGCGTCCTGCGATGCGTCACTTTTTGAAAGCAAAT
CCCAATGTTGAAGTACTTTTTCGCGAAAAGTCTCCCGGTATGCAAATGGCGTTGTTAGAGCGTCGGGAACTTG
ATGCAGGGATCTGGCGTATGGCTATCGAACCGGCAGTCGGTTTTACCAGCATACGTTTGCATGAATCTGCATT
CATGGTTGCAGTTCCGGAAGATCACGATCTGGCATCCAGAGATTCTGTGCCGCTGTCGGCGTTGCGCAATGAA
TATTTTGTTACGTTGCCTTCCGTACATTCTGATTGGGGATTTTTACAACGTGTATGCCAACAGGCGGGTTTCT
CGCCAATGATTATTCGGGAAGTGGTTGAGCCGCAAACGGTGTTGGCGATGATCAGTATGGGGATCGGGATTAC
GTTGATGGCTGATGGCTATGCGCAAATGAGCTGGCCGGGTGTGGTGTTCCGTCCGTTAGAGGAACGTATTCCG
GCGGATTTGTATATTGTGTATGACCAGCAGCAGGCAACGCCAGCACTGGAGAAACTGGTGGCGGCGTTGACGG
TATGATGTCTGTGCCGGATGGCGACTAACGTCTTATCCGGCCTACGTGTGCGGTGTCGTCTGTAGGCCTGATA
AGCGCAGCGCATCAGGCAAGACCCGAACCTGCTGCAGGTTCGAATCTTCCATATCGCAGATAGCAAAAAAGCG
CCTTTAGGGCGCTTTTTTACATTGGTGGGTCGTGCAGGATTCGAACCTGCGACCAATTGATTAAAAGTCAACT

GCTCTACCAACTGAGCTAACGACCCGAAGTGGTGGGTGATGACGGGATCGAACCGCCGACCCCCTCCTTGTAA
GGGAGGTGCTCTCCCAGCTGAGCTAATCACCCACTTCGGTACTTCACAATAAGAAATTTAGCTGGCGAGAAAG
TGGTGGGTGATGACGGGATCGAACCGCCGACCCCCTCCTTGTAAGGGAGGTGCTCTCCCAGCTGAGCTAATCA
CCCACTTCTCAATTTCTTATCTACACGGCGGAGACTACATAAAGTAGTTGGTGGGTGATGACGGGATCGAACC
GCCGACCCCCTCCTTGTAAGGGAGGTGCTCTCCCAGCTGAGCTAATCACCCCCGCTGTGTGGAGTCGCATTAT
AGGGAGAGTTGAAAATGAGTCAACGCATTTTCTAAAGTTTTTGTTTGTTCGTCGTAAAATTAAACAAAACGAT
CGTGAATTACCCCGCGCTGCATGATTTCTATACGGAAATAGCAAAGCGCAGTGTTCTGACCGGATCAACATTG
AGGAATCAAGCCACAGTGATAGAATACAAGGCTATTCATCTTTCCAGGATTTGCCGGTTGTCGGCATCTTTAT
AAACGTAAGGCCATTTCATGAAAATCAAAACTCGCTTCGCGCCTAGCCCGACAGGTTATCTGCACGTCGGCGG
CGCGCGTACTGCTCTTTATTCCTGGCTTTTCGCACGTCACCACGGCGGTGAATTCGTGCTGCGTATTGAAGAC
ACCGATCTTGAGCGTTCCACGCCGGAAGCCATTGAAGCCATCATGGATGGTATGAACTGGCTGAACCTGGAAT
GGGACGAAGGCCCATATTTCCAGACCAAACGTTTTGATCGCTATAACGCGGTGATTGACGAGATGCTGGAGGC
GGGAACAGCCTATAAATGCTACTGCTCTAAAGAGCGTCTGGAGCAACTGCGTGAAGAGCAGATGGCGAAAGGC
GAGAAGCCGCGTTATGACGGGCGTTGCCGTCACAGTTACGAACATCATGCCGACGATGAGCCATGCGTGGTGC
GCTTTGCTAACCCGCAGGACGGTTCCGTTATTTTTGATGATCAGATCCGTGGCCCGATCGAATTCAGCAATCA
GGAACTGGACGATCTGATCATTCGTCGTACCGACGGTTCGCCGACCTACAACTTCTGTGTGGTGGTGGACGAC
TGGGATATGGAAATTACCCATGTGATTCGTGGCGAAGACCATATCAACAATACTCCGCGCCAGATTAACATTT
TGAAAGCGTTAAACGCGCCGGTGCCGATGTACGCGCATGTGTCGATGATTAACGGCGACGACGGTAAAAAGCT
GTCTAAACGTCACGGTGCGGTGAGCGTGATGCAGTATCGCGATGACGGTTATCTGCCGGAAGCGTTGCTGAAC
TATCTGGTGCGTCTGGGCTGGTCCAGCGGCGATCAGGAAATCTTCACTCGCGAAGAGATGATCAAGCTGTTCT
CCCTCGGTGCGGTGAGCAAGTCTGCGAGCGCGTTCAACACCGATAAACTGCTGTGGCTGAACCACCACTATAT
CAATACGCTGGCGCCGGAATATGTGGCGACGCATTTGCAGTGGCATATTGAGCAGGAAAATATCGACACCCGT
AACGGCCCGCAGCTTGCTGAACTGGTGAAGTTGCTGGGCGAGCGCTGCAAGACTCTGAAAGAGATGGCGCAGA
GCTGCCGCTACTTCTATGAAGACTTCAGCGAGTTTGACGCCGATGCGGCGAAAAAACACCTCCGCCCGGTAGC
GCGTCAGCCGCTGGAAGTGGTGCGCGACAAGCTGTCGGCGATTACGGACTGGTCAGCAGAAAACGTCCACCAC
GCGATTCAGGCCACGGCGGATGAGCTGGAAGTGGGGATGGGTAAAGTGGGGATGCCGCTGCGTGTGGCTGTGA
CCGGTGCCGGGCAGTCGCCTGCGCTGGATGTCACCGTACATGCGATTGGTAAAACCCGCAGCATTGAGCGCAT
CAACAAAGCGCTGGGCTTTATCGCCGAGCGTGAAAGCCAGCAGTAACGCCCGCTGAAACAGAATAAACGGCAG
GGCAGACGCCTGCCGTTTATTCTTTGTTAGTCCGCCTCATTGATACCTAAACGCGTCAGAAATCCGCGAATAC
CTTCCCGCGATAAAAACAGCGCCAGTTTTTCCTGTTCCTGAGCAGACATATTTTCAACGGCATCAATAATTTG
TCGATAAGCGTGACTGTGCGCCGCGTTCGCATATTCTGCGAAAGCCTCTTCCGCCTGATAAAACGCAGGCAGT
TTGCGAAAAGCCGGAATATTGAGAATAAACTCCCGCACGCTGGCATCAATATGGATGAGTCTTGCGCGTCCGC
CTTTGACGCCGGGAAATTTTTCGGTTTTCCAGTGTTGTTCTCTGATCCAGCGATTGACGGTTTGTCTGGCCAC
GCCGAGGCATTCAGCCAGCTCTTCAGTGGTCATTTTACTGCGTAATCTTTTCATATTATTTGCTATCGCGTAT
CCCTAAGCGTTGTAACAAACCGGTAATCCCTTCACGAACGAGGAGAGAGGTAAACTGTTTCTGCTCGGATGAG
GTCATCTCTTTGGCGAGCGTCATGAGCAGCGCTTCAAGCGATGCGTTGCTGGCAGGCGTGAATGTGTCTGCGT
GATGGTCAACTGAACGTTCAGCGCTGCGGATATATTCACGAACCTGTTCGTTTACATGAACCAGACGGGCTTT
ACCGCCCTGGACGCCAGGTTTGGGCGATGTTGCCCAGCCTTCTTTCCGTACCCATTTATTGATGGTTTGTCGG
CTGTAACCGGTGAGATTAGCGAGTTCTTCTGGCGTCATCCGTTCCTTGAACATAACGATTCCCTGAATAGTCG
TGGGGTTAATTAGTGGTTCATTTTATAGCACCATTTTAGTCGAAACCGTGACGTGTTTAACTACTGAATGCGA
GTTGTGCCGCAATGTCCTTTATTTGAATGCTTTTTCAGCGATTGAATTCGGCAAGTTCATTTTGCCGTTGACA
CATGCCGAGGGAATTCATATGATGCCGCCCGTCAACTCGACAAGCTTTACGTAGTGGGGCTATAGCTCAGCTG
GGAGAGCGCTTGCATGGCATGCAAGAGGTCAGCGGTTCGATCCCGCTTAGCTCCACCAAATTTACTCCCGATA
AATTTGGCGTATACGTAAAAGCACTATGGGGCTATAGCTCAGCTGGGAGAGCGCTTGCATGGCATGCAAGAGG
TCAGCGGTTCGATCCCGCTTAGCTCCACCAACATTCAAACCCTCGCTGAAAAGCGGGGGTTTTTGCTTTTTGT
CACAAAATTTATGGCCTCTGAGCTGCTCTTCAGGTGCGTTTGTTTGCCGCTGCTTTTGCGGTCAATATGAGCT
TTTGTTAGATTGCTTCATAATGCGAATATACTTATTATTCATGCGCCTTATATAATGATGTGAGCATTAAAGC
AATGCCGGATAAGTGTAACGTATTAAAAAATATAAAAATATTTTTACTGGCCTTCTGCCTGACCGTTCCGGCT
ATTCTGCTATCTCGTCTCATTTCTCCCCGCGCCACAATTGATTCCAGCTATATCTTTCTGGCCTGGCTACCGC
TCTGCGTCATGTTTTCCGTGCTGTTTTTATTTGGCCGCCGTGGCGTCGCGCCGATGGTAGGCGGAATGATGTT
GACTAATGAATGGAATTTTCATTTACCTCTGCCACAGGCGATGGTGCTGCTATTTTGCCAGACGTTTCCCGTG
TTGCTGGTTTGCGCTATCGTACGCTGGCAGCTGGGCGCGCGCTGGCGCTACGGAATACCCAACCAAGGCATCT
GGCTACGCGTTTTCTGGTTAGGGTTAATGACGCCGTTTGGCATAAAAATCAGTATGCATCTGGCGGGGCACTA
CCTGGCTTTCCCGGTGACAATCTCTACCTTCTTTGGCACCGGCACCGCTATTTTCTCGATTGTTGATTTATTG

AGTCTGATTTCTGCCGCGCTTATTTTTACCCTCTTTTTCTATTATCCGCTGCGGATGATAGTCAGCCCGCACT
ATATTCGTATTTTCTGGCGGAGGGATGTCGCTCCTTATCTCGCCAAAGAAAAACGCCTGTTTACGCTTCTCTG
GTTTGCCACTCTGGCGACGCTATTAGCGGTGCTGTGTACGCCTTTCGAGACAAAGTATATTGCTGGCTATCTG
GTGCCAGTTCTGTTCATTGGATTTACCATCGGCGTGGGGAAAATCAGCTATCCGCTGTTGAATTTTAGTTGGG
CTCTCACTGCGCTATTCCTGCTTAGCTATAACCGCAATTTTCTGCAGGGCGTGGGCTCAGAATATTCGCTGGC
ATTTATCCTTTCAGTTCTCATTTCTTTTAGTATTTGTTTGTTGTACATGGCGCGGATTAATCAGCGTAGCGAA
TGGTTGAATCGACAGTGGCACTCGCAGGCATTAACCGATCCGTTGACGCAACTGCCTAACCTGCGTGCGCTGG
AGCAGTTTCTTTTACAGGATGCCGGGCAAAGCGTGTGTTATCTACGCATGGAAAACCTGGAATTTCTGAGCCG
TCATTACGGTATGCAGATGCGCGTGCATTGCGAACGCGAGGTGTTTCGCGTGTTGCAGCCGCTGCTGCTGGAA
AAGGAGAAAATTTTCCATCTGCCGGGCAGTGAGTTATTGCTGGTATTGACGGGACCGGAGACGGAAGCCCGCC
TGCAATATATGCTCAATGTGCTTAATAACCGCAAAATTTACTGGAACAATACCGGTCTGGACATGGAATACGG
CGCATCGTGGGGAACATTCGATGGTCGCCAGGAAACCTTGCAACCGCTGTTGGGGCAATTAAGCTGGCTGGCG
GAGCAATCCTGTTCGCACCGTCGGGTGCTTGCACTGACCCAGAGTATTGAAGCCGCTTCCGGGCAGACGACGG
AACGGGTGCTGCGGTTGCAGAAAATCCGTCAGGCGCTGGAGCGCGGCGCGCTGGTTTTGTATGCGCAACCCAT
TCGCGACTCGCAGGGAAAAGGGTATGACGAGATTCTGACGCGGCTTCGGTGCGACGACGGTATCATGATGCCC
AATCAGTTCATTCCGCTTATCGCCCAGTTTAACCTGAGCGTACGGTTTGATATGCAGGTGATGGAAGCGCTCC
TGCAATGGTTGTCCGCGCATCCTTCGGCGGAGCAGGGGGCGCGTTTCTCGGTTAATTTGATGCCGCTGACGCT
GCTGCAAAAAGAGACGGCGTCGCGCATTATGCAGCTTTTTAAACGCTATGGCGTCCCGCCTGCTTCGGTCATC
ATCGAAATTACTGAAGAACAGGCCTTCTCTCATTCCGAAATCAGTATGCACAATATCAACCAGCTGCGTAAGT
TTGGCCTAAAAATCGCCATTGATGATTTCGGCACCGGCTATGCAAACTATGAACGGCTAAAACGCCTCAAGGC
GGACATCATTAAAATCGACGGTTGTTTCGTAAAAGATATTCTGACGGACTCCCTGGATGCGATGATCGTGAAA
TCCATCACTGATTTAGCGAAAGCGAAATCGTTGAGCGTCGTGGCTGAGTTTGTTGAAACGCCTGCGCAGCGCG
ATCTGCTATTGCAGCTTGGCGTACACAGTTTGCAGGGATATCTGATTGGCCGCCCGCGCCCGTTAGGTAAATA
AGTGGCATAAAAAAACCGGGGATAGCTCCCCGGTTTCTACATTAAACGCAGACTTACAGTACCAGCGCCGCGA
TAGAGGCGGACAGTACGCTCACCAGCGTAGAACCGTAAACCAGTTTCAGACCAAAACGGGAAACCACGTTGCC
TTGCTCTTCGTTCAGGCCTTTAATCGCGCCCGCAATAATACCGATGGAAGAGAAGTTCGCGAAGGACACCAGG
AAGACGGAGATAATGCCTTCAGCGCGTGGAGAGAGGGTAGCGGCGATTTTCTGCAGATCCATCATCGCCACGA
ATTCGTTGGAAACCAGTTTGGTCGCCATGATACTTCCTACCTGCAGCGCTTCACTGGACGGTACGCCCATTAC
CCATGCGACCGGGTAGAAGATGTAACCCAGAATGCCCTGGAAAGAGATGCTGTAGCCGAACCAACCGGTAACT
GTTGCGAACAGCGCGTTCAGCGCCGCGATCAGCGCGATGAAGCCGATCAGCATCGCCGCGACGATAATCGCCA
CTTTAAAACCTGCCAGAATGTACTCGCCCAGCATTTCGAAGAAACTTTGGCCTTCATGCAGGTTCGACATCTG
AATATTTTCTTCGCTGGCATCAACGACATAGGGGTTGATCAGCGACAGAACGATAAAGGTGCTGAACATGTTC
AGAACCAGCGCTGCGACCACATATTTAGGGTCCAGCATGGTCATATACGCGCCCACGATAGACATGGAAACGG
TAGACATTGCGGTCGCCGCCATGGTGTACATGCGGTTACGGGACATTTTGCCGAGGATATCTTTATAAGCGAT
AAAGTTCTCAGACTGGCCGAGGATCAGCGAGCTGACGGCGTTAAAAGACTCCAGTTTGCCCATACCGTTTACT
TTGGACAACAGGAAACCAATAGCGCGGATGACCACTGGCAGAACGCGGATATGCTGGAGAATACCGATCAACG
CGGAAATAAAGACGATCGGGCACAATACTTTCAGGAAGAAGAACGCCAAACCTTGATCGTTCATACTGCCGAA
GACGAAGTTTGTCCCTTCGTTGGCAAATCCGAGCAGTTTTTCAAACATCTCGGAGAAACCTTTTACGAAGCCA
AGGCCAACATCCGAGTTCAGGAAGAACCAGGCCAGTAATACTTCGATAACAAGTAACTGAATGACGTAACGAA
TGCGAATTTTTTTACGGTCGCTGCTAACCAGCAGTGCGAGTACCGCAACAACGGCAAGCGCCAGGACAAAATG
AAGGACGCGGTCCATATTTGCTCCAAATATGAGGCAGGTTTAATTTTCGTGCACATTCTATGCAACAGCCGTA
AAGAAAACGAGATCCAACACACACTATAATAAGGACCTGTGACGAGATTCAAAATTAGTGATCTGTAATACAC
TTTTACTGTACTGAATATGAAAATGAAAAGTTATATCAGTGTGCTAATCTTGTAATGTTAAGCCAAACTGTTC
TGATACAGGTCGTCATCGTATCGGTCTATCGTTTCACACTATCAAAGTAATCACCCGTACCCATTGAAATGCA
CTTGATAATCATTATCAATAAACATAGCATGAAACATAGCAAAGGCTATGTTTTTGAGGCAAAAGATGACTGA
CAATCGCGTAGAGAATAGCAGCGGACGGGCGGCGCGCAAGCTAAGGCTCGCATTAATGGGACCTGCGTTCATT
GCCGCGATTGGTTATATCGATCCTGGTAACTTCGCGACCAATATTCAGGCCGGCGCCAGCTTTGGCTACCAGC
TACTCTGGGTGGTGGTCTGGGCGAACCTGATGGCGATGCTGATTCAAATCCTCTCGGCAAAACTCGGAATCGC
CACGGGCAAGAACCTGGCGGAGCAAATTCGCGACCATTATCCGCGCCCGGTAGTCTGGTTTTACTGGGTACAG
GCGGAAATCATCGCGATGGCCACCGATTTAGCTGAATTTATCGGCGCGGCAATAGGTTTTAAGCTGATCCTGG
GCGTCTCTTTATTACAGGGCGCGGTATTGACCGGTATCGCGACGTTTCTGATTTTAATGTTACAACGCCGCGG
TCAAAAACCGCTTGAAAAAGTCATCGGCGGTTTACTGCTTTTTGTCGCCGCTGCTTACATTGTGGAGCTGTTT
TTCTCTCAGCCTGATATGGCGCAGCTTGGCAAAGGAATGGTTATCCCGGCGCTACCGAACCCGGAAGCCGTAT
TCTTAGCCGCCGGCGTGCTTGGCGCGACGATTATGCCGCACGTGATTTATCTGCACTCGTCATTAACACAGCA

TCTGCACGGCGGAACGCGGCAACAACGGTACTCAGCAACGAAATGGGATGTCGCTATTGCCATGACGATTGCC
GGTTTTGTCAATCTGGCAATGATGGCGACCGCCGCGGCGGCGTTTCATTTTAGCGGCCACACTGGCATCGCCG
ACCTCGATCAGGCATACCTGACGCTGGAACCGTTACTAAGCCATGCGGCGGCAACGGTGTTTGGTCTTAGTCT
GGTAGCGGCAGGGCTTTCCTCCACCGTGGTAGGGACTCTGGCGGGGCAGGTGGTGATGCAAGGGTTTGTCCGT
TTTCACATTCCGCTGTGGGTACGGCGCAGCATTACCATGCTGCCCTCATTTATTGTGATTTTGATGGGGCTGG
ACCCTACCCGAATTCTGGTGATGAGCCAGGTGCTGCTGAGTTTTGGTATTGCGCTGGCGCTGGTGCCGCTCCT
GATTTTCACCAGTAACGCTACGCTAATGGGCGAGCTGGTGAACACGCGGCGAGTAAAACAGATTGGCTGGATA
ATTGTCGTGCTGGTCGTCGCCCTGAATATCTGGTTGCTGGTTGGAACGGTGATGGGATTGTCATAAAGACAGA
AAGATAGCAGGCCCGATGCGTGGGCGTCATCGGGCTGTTATTCAGCAGGAGGCTTAGTGATGATGTTTGTGAC
CCCGGCCTCGCCCGCGATGATCGTCGCGATCGCGCCAGCCTTCCCGATAACCGCGTTCATACGCTTTACGCTT
ATCCCAGCCGCGATGATAGCCGTTGTCATGGCGCCACCAGCGATTTTTACGCCATTCATAATGGTTGTGCCAG
TAATCCCGGTCGCGCCAGCGACCACCGTCCCAGTAGTTACCGTAATCATCGCGATCGCCAATCTGTAATTTTA
TTGAGGGCAACAGGGTGATTTCGCCCGCATTCGCGGCAAGCGGTGTAAAAGCCAGCAAGGCTGCCGCCAGAAT
CAGTGACCTGAACATACTTTCTCTCCTTCACGATCGAGCCGTCGCCGGCCTGTTAATGCAATATTACAAAGCA
GTAAAGATTTGTTTCATCGCCTTAACTCTTAAATCATGAAGGAGAGCATTTTTTGCTAAAACCGGCCCTCCAG
AATAGCGTCAATTTCTGCACATTCCGCCGCCGAAAAACGGCGATTGGCCAGCATTCCTACGGCGTCTTCGATT
TGCGACGGTTTGCTGGCGCCAATCAGTACCGAGGTAACGTTATCGTTACGCAGCACCCAGGCGAGCGCCATCT
GAGACAATTTTTGGCCCCGTCGCGCGGCCAGTTCATTCAACCGGCGAACTTTTTCCAGTTTGTCGGCGGTAAT
CTGTTCTGGTTTAAGGAAACGGCTTCCGCTCGCCGCGCGGGAATCTTCCGGAATGCCATTCAGATAACGGTCA
GTGAGCTGCCCGCCCGCCAGCGGCGAGAAGGCAATACTGCCGACGCCTTTTTCCTGCAACAGCGCCAGCAGCC
CGTCCTCTACCCAACGCTCAAAAAGCGAATATTTAGGCTGATGAATCAGGCAAGGCGTGCCGAGATCCTCCAG
AATATCGATAGCTTGTCTGGCCAGATCGGCAGGATAGTTAGAGATCCCCACGTACAACGCTTTGCCGTGGCGC
ACAAGGTGATCTAACGCTTTCATCGTCTCTTTAAGCGGCGTTTCGGGATCGGGACGATGGTGATAAAAGATAT
CGACATACTCCAGCCCCATACGTTTCAGGCTTTGATCGAGGCTGGCGATAAGGTATTTCCGTGAACCCCAGTC
GCCATAAGGGCCATCCCACATGGTGTAGCCCGCTTTGGTGGAGATGATCAATTCATCACGCCACGGTAAAAAA
TCTTCCTGCAGAATGCGGCCAAAATTGCATTCTGCGGAGCCCGGCGGCGGCCCATAATTATTGGCGAGATCAA
AGTGGGTAATGCCCAGATCGAACGCGCGTTGCAGCAGCGCCCGGCTATTTTCTACCCGTGTCGTATCGCCGAA
GTTGTGCCATAACCCCAGCGAAATAGCGGGAAGTTTGACGCCGCTGCGTCCGCAGCGACGATACTCCATTGTG
TGATAACGATTCTCATCGGGCTGATAAATCATGACCTTTCCCCCTGTGGCGTGAATAAATAGTGTATACGGTT
ACACTCGCCAGGCGGTATCCGGCTTTTACTGTTTGCCGCTGTAAACATTGTTTTCCAGGCTTTCATCCCCACC
GTGCTGGACAGCCATCGGCATTCCTTAATACTCAACATAATATCAACGTCAGAAGGAAAGCTGTCATGCAAAA
CCCCTATACCGTGGCCGACTATTTGCTGGACAGACTGGCAGGATGCGGCATTGGCCATCTTTTTGGCGTACCG
GGCGATTATAACTTGCAGTTTCTTGACCATGTGATTGACCACCCGACCCTGCGTTGGGTGGGATGCGCCAATG
AGCTGAACGCCGCTTATGCCGCGGACGGCTATGCGCGCATGTCGGGCGCTGGAGCGCTACTCACCACCTTTGG
CGTGGGAGAACTTAGCGCTATTAACGGTATCGCGGGCAGTTACGCGGAATATGTCCCGGTCTTGCATATCGTC
GGCGCGCCCTGTAGCGCTGCGCAGCAGCGTGGCGAATTGATGCACCATACCCTCGGTGACGGCGATTTTCGTC
ATTTTTATCGCATGAGTCAGGCGATATCCGCTGCCAGCGCAATATTAGATGAACAGAACGCCTGTTTCGAGAT
TGACCGCGTGTTGGGTGAAATGCTTGCCGCACGCAGGCCAGGATACATCATGTTGCCCGCCGATGTGGCGAAA
AAAACGGCCATCCCACCTACGCAGGCGCTGGCGTTGCCCGTGCATGAAGCGCAAAGCGGCGTGGAGACGGCTT
TTCGTTACCACGCCCGTCAGTGCCTGATGAACAGTCGGCGTATTGCGCTATTGGCCGACTTTCTTGCCGGGCG
TTTTGGTTTACGACCACTGTTGCAGCGCTGGATGGCGGAAACGCCCATCGCTCATGCGACACTACTGATGGGG
AAGGGGCTTTTTGATGAACAGCACCCGAACTTCGTTGGCACCTATAGCGCTGGCGCCAGCAGCAAAGAAGTGC
GTCAGGCCATAGAGGACGCCGATAGGGTTATCTGCGTCGGCACCCGTTTTGTCGATACCCTTACGGCCGGATT
TACCCAACAATTACCGACGGAACGCACGCTGGAGATTCAGCCTTACGCGTCGCGCATCGGCGAAACCTGGTTC
AACCTCCCGATGGCGCAGGCGGTGTCTACGCTGCGCGAACTGTGCCTGGAATGCGCTTTTGCGCCGCCGCCGA
CGCGTTCCGCCGGACAGCCAGTGCGGATTGATAAGGGAGAACTGACCCAGGAAAGCTTCTGGCAAACCTTACA
GCAGTATCTCAAACCCGGCGATATTATCCTTGTCGACCAGGGGACCGCCGCCTTTGGCGCTGCCGCGCTGTCG
CTTCCTGACGGCGCGGAAGTTGTGGTACAGCCGCTGTGGGGGTCTATCGGCTATTCCTTGCCCGCCGCGTTTG
GCGCGCAAACCGCCTGTCCCGATCGGCGGGTGATTCTGATTATCGGCGATGGCGCGGCGCAGCTCACGATTCA
GGAGATGGGCTCGATGTTACGCGACGGGCAGGCGCCGGTCATCCTGCTGCTCAACAATGACGGCTATACCGTA
GAGCGCGCCATTCACGGCGCGGCCCAGCGGTATAACGACATCGCGAGCTGGAACTGGACGCAGATACCACCGG
CGCTAAACGCGGCGCAACAGGCGGAGTGCTGGCGGGTGACGCAGGCTATCCAACTGGCAGAGGTCCTCGAACG
TCTGGCGCGCCCACAACGTCTGTCATTTATTGAAGTGATGTTGCCAAAAGCCGATCTGCCGGAATTACTGCGT
ACCGTGACCCGGGCGCTGGAAGCCCGCAACGGGGGATAATGGCCCCCGCTGCGCCGGATTAGGGTTCGTGACG

GTTGGCGGCCAGCAACGGTTTTCCCGCCAGCAATAGCCAGGCGGGGAGCATCACAATGCAGAGCAGCGGCAGC
AGATTGGTATCCGGTACGACGACTGCCGCCATAAACAGACTGAGCCAGCCGTCGCGCGTCACTACCAGCACTA
AGCCAAGAATCGCGCAGGAGACGGTAATCGCCGCGGGTACGGCCTCAACATGGGCATGGAGCATGAGCCCCAG
CGCCGCGCCGATAAACACAGCCGGAAAAATGCGCCCGCCGCGAAAGCCGCTCGCCGCCGCGATGACCAGCGCG
GCGAGTTTGACGACGGCTAAGGTAAAATAATCTCCCGCGCCCAACGTCTGGCTAAACGCCATTTGTTGCATTT
CGTCCAGGCCTTTAAACAGGGTAAGCGGCCCGCCAATGACGCCCAGGATGCCCAGTATAAAACCGCCAATGCC
GAGAATCAGGACCGGATTTTTCAGGCGGTGCAGCAACTCATGCAGACGCGGAAGGCACCAGACGGCGACCATC
CCGGCGGCAATGGCGATCGCCGCGACGATCGCTCCGCTGGCAATGTCCACCAGCCGCATCTGCGTGTAATGGG
CGATGGGTAACGAAAAATGCGGATGAAAGAACAGACTGGTGGTGAGTGACCCCGCCGCTGCCGCCATTAACGG
CGCAAACAGGCGATCCCACATGGGAATATCGTTAGAGCCGCTAAGTGTTTGCGAAAAAATCAGCGCTGCGGCG
ACGGGCGTGCCGAACAGCGCGCCGATGGTGCCCGCTGAGGCCAGAATCGTCCAGTCTAACGCCGTGATACGCG
GAAACAGGCGGGAGCCGAAAGCCGCCGCCAGCGCGATATTTATTGTCATTATCGGATGCTCTGGCCCCAAACT
TACCCCACCAGCAAGGCCGATGATTAAGGCGAGAAGCAGCCCGGGTAGCGCAGATGGCGAGACCGGCATACTG
ATTAACGGTTCGATGGCCGGATCGGGGCCAGCGTGTCCGGGACTGTAACGGATAATCAAACCCACCACGATCC
CGGTTAGCGTGAGCATACCCACTATCCAGAACGGCGAATCATAAGCGATGCCGATGCTGGTGGGCAGTCGTTG
CCAAAGAAATTGCTGAAAAACCGACGCGACTTTCATCGCAGCGATTAAAACCAGGCTTGACGCTACGCCAATA
ATCAGCGCGGGGAGCGACAGCAGCAGCATCGTTCGGGCACGCGGGTGGAACATAATCTCTTCCTTACACGGGG
TCGTATACCTATTTTGCACAGCCTGCCCGACGGCATTGCTGCAATTGGTGCTGAAACAAGAAAGTAATTCTGT
GATGAAGATCTATATTTATAGGCAGTCAGCCTGATGGCCGTTGTTGTTATGCCCCCATGAATTTACAGTGTGA
CAAAGACTTATTTTGACTTTAGCGGAGCAGTAGAAGAATGACAAAGTATGCTTTAGTAGGAGATGTAGGCGGC
ACGAATGCGCGTCTTGCCCTGTGTGATATCGCCAGTGGAGAAATCTCGCAGGCCAAAACGTATTCCGGCCTGG
ATTATCCCAGTCTTGAGGCCGTGGTGCGCGTTTATCTCGATGAGCATAGCGTCAGCGTGGAAGATGGTTGTAT
CGCCATAGCCTGCCCGATTACCGGTGACTGGGTAGCGATGACTAACCATACCTGGGCTTTTTCTATTGCGGAA
ATGAAAAAAATCTCGGCTTTAGCCATCTGGAAATCATCAACGATTTCACCGCCGTGTCGATGGCGATCCCGAT
GCTGAAAAAAGAGCATTTAATTCAGTTCGGCGGCGGCGAACCGGTAGACGGCAAACCTATTGCGGTGTATGGC
GCGGGAACCGGCTTGGGCGTGGCACATCTGGTGCATGTGGATAAGCGTTGGATTAGCCTTCCGGGCGAAGGCG
GGCATGTCGATTTTGCGCCTAATAGCGAAGAAGAAGCTATGATTCTGGAAATATTGCGCGCCGAAATTGGCCA
CGTTTCCGCCGAGCGCGTGCTGTCTGGCCCGGGGCTGGTGAATCTTTACCGGGCGATTGTTAAGTCCGACAAC
CGTCTGCCGGAAAATCTGCGCCCGAAAGATATTACCGAACGCGCCCTGGCGGATAGCTGTATCGATTGTCGCC
GCGCGTTGTCGCTCTTTTGCGTCATCATGGGGCGATTTGGCGGCGATCTGGCGTTAACCATGGGGACATTTGG
CGGCGTTTATATCGCTGGCGGTATTGTCCCGCGCTTCCTGGAATTTTTTAAAGCGTCCGGCTTCCGTGGTGGC
TTTGAAGATAAAGGCCGCTTTAAAGATTATGTACACGGTATTCCGGTCTATCTGATTGTCCATGATAACCCTG
GCTTGTTAGGCTCCGGCGCGCATCTGCGCCAGACATTAGGCCATATTCTATAAGTCAGATGGCGGCATAAACG
CCTTATCCGGCCTACAAAAGTGTGTCATCTGTAGGCCTGATAAGCGCAGCGTCATCAGGCAATTAATCATTCT
CTTGTCAGTCTCAAACCGCGTGCCTCACGCGGTTTTTTTCATTGCCTTTCCTGTGCATGAATGCTTCAACTGA
CTGAATTATTTCACTTTACTCTTGCTGAGCGAAAGGTCAAACACCAGCGCGTTGAGTCATCACGTCATGGCGG
TTAGTCAGGCGAAAACAGGGATTTATTTTACTATACGTATCCGGTTCGCTGCCTTATTTACGGTTTCATCGCG
GCATCTTGCTGGAAAGGATCCTCATTTTCGGGGAAATGTCCCTGAAAAAATACCGCAATATGACATGATTGAA
TACCTTTAGCGTTAGTAAGGGTTTTGATTAAAGAGGAAAAACCACAAAATCAAAAGGGGATTATTTGAGATAA
GGTTTATTGACAGGAAATTATCGTCGGCTGTAACACATTAGGTAAATTCAATGATATCAAGTTGTTAATAATA
TGATTTGTTTGATGCTATAACTGACATGCTTAAAAGGTAAATTTTTACGCTTGTTCCTCTTTTTTCGTCGCTA
AAAAATATTTTCATTAGCCTCTCGACAGGAAGAAAATTTTCAGGCTATTTTCTAAAGGAGCCACCATCGTGGC
AGCGTCGCTCGGACGGTCCGGGCGCTAACGTTAATCTGAGGATATTATGGCTGACTTCCGCCCTGAACGTCGC
TTTACGCGTATCGATCGCCTTCCGCCCTATGTTTTTAACATCACCGCTGAACTCAAAATGGCTGCGCGTCGGC
GCGGTGAAGATATTATTGATTTCAGTATGGGTAATCCTGATGGCGCAACCCCTCCCCACATTGTCGAAAAACT
TTGCACCGTCGCGCAGCGTCCGGATACGCACGGTTATTCCACGTCCCGCGGCATACCGCGTTTACGCCGCGCG
ATTTCTCACTGGTATCAGGAACGTTATGATGTCGATATCGATCCGGAAACCGAAGCGATTGTGACCATCGGTT
CCAAAGAGGGACTGGCGCATTTAATGCTGGCGACGCTGGATCATGGCGATACGGTACTGGTGCCGAACCCCAG
TTACCCGATTCATATCTATGGCGCCGTTATCGCCGGGGCGCAGGTGCGTTCGGTTCCATTGGTAGAGGGAGTG
GATTTCTTCAACGAGCTGGAGCGCGCTATTCGCGAAAGTTATCCGAAACCGAAAATGATGATTTTGGGTTTTC
CGTCTAACCCTACGGCCCAGTGCGTCGAACTGGAATTTTTTGAAAAAGTGGTCGCTCTGGCTAAACGTTATGA
TGTGCTGGTGGTGCACGATCTGGCGTATGCCGATATCGTGTACGACGGCTGGAAAGCGCCATCCATTATGCAG
GTGCCCGGCGCGCGCGACGTGGCGGTTGAATTTTTCACGCTGTCGAAAAGCTATAATATGGCGGGCTGGCGTA
TTGGGTTTATGGTGGGCAATAAAACCCTGGTCAGCGCGCTGGCGCGCATTAAAAGTTATCACGATTATGGCAC

CTTTACCCCGCTTCAGGTGGCGGCTATCGCCGCTCTGGAAGGCGATCAGCAGTGCGTGCGCGATATCGCCGAG
CAGTATAAACGTCGTCGCGATGTGCTGGTGAAGGGGCTGCATGAGGCGGGCTGGATGGTTGAAATGCCGAAAG
CCTCAATGTACGTCTGGGCGAAAATCCCGGAGCAATATGCGGCGATGGGATCGCTTGAGTTTGCCAAGAAGCT
GCTGAATGAGGCGAAGGTTTGCGTTTCTCCAGGGATAGGCTTTGGTGATTATGGCGACACCCATGTACGCTTT
GCATTAATAGAGAATCGCGACCGCATTCGCCAGGCCGTCAGAGGGATTAAAGCGATGTTCCGCGCCGATGGCG
TGCTGCCGTCACATCCAAAACCCGTTGAAGCGAGCACGGAGTAGAAAAGCAAACAGGAGCCCCATGGCTCCTG
TTTTTCTTTGCATTGTACTTTTAAATCATCAGGGCGAAAGTACCGGCCCAAACGATGACCATAAATGAAATGC
CCATAAAGAAATATTTCACGTTTCATCTCTCCGCGTACCTGTCGTACGCCTAAATGAACTTACACTTACCTGA
TTATAGAGAGTTGATGCCGGACAATACGAAATTGAGAATTATTCGTGTTTCTACCGCCTGCATCACCTCAGAA
TTCTGCGCGTCATCGCTCCAGACGGCGCTAATGTACGCCTAAAAATGAGGTGAAACAAGAGGCATTTTCTTAT
TTACTTTTAGTAACTTACAAGTGTCGGGAATCGGCGACAGTTTAATTTCTGACAGTGATGAATCGCTATTGAG
CGACGCCGCCGACACAGTAAAAAATCGCCTGTAAACAGGAGATTTATCGCTAAAAGCAGGAATGAGTAATTTA
CGATATGAATATTATGACAATGCGAAATGCCAAAATAAAAGGCCTTGCGGCCTTTTTCAGATGTAGAGTGACG
CTTCCCCCAACGGGCGAGTCTTGAAACGGCGATGGATCCAGAGATATTGCTCCGGGGCGCGCATGATCTCTTT
CTCAATAATTTTATTCATATAGGCAGCGGCTTGATTTTCATCTGCCGGGTAACCTTCCATTTCAGGGGTAATA
TACAGACGATAACCCGAGTTATCGGATTTCCTTACCATTGTAACGGTCAACATGGCGGCGCCAGACAGCCTGG
ACAGTACATAGGTGCCGTTGGTGGTGGCGACGTTTTCAACCGCGAAAAATGGCGCAAAGGAGCTGCCTTTAGG
GCCGTAGTCCTGGTCGGGAGCAAACCAAACGGCTTCCCCTTTTTTTAGCGCGCCCACAATGCCGCGCAGATTG
TTTCTGCCGATCATCGCTTTATTTGAGCGCATACGACCGCGCGTCTGCACCCACTCCATGAGCGGATTGTTAT
GCGGACGATAGGTCGCCATCATCGGCTGGCAGAGACCCATTACCCGGCCTCCCAATTCCAGCGACATAAAATG
CACGCCAACGACCATTACGCCGCGATTTTGCTCCTGCGCACGGGTAAGGTTGTCCAGCCCGTCCACATCAAAC
CATTTGCGAACCCGGTTATCAGGCCAGAACCAGGCCATGCCCGTTTCAAGCAGCGCCATGCCAAGAGAGTGGA
AATTCTCGGCGATCAGTTTTTCCCTTTCTTCCCGGGAGAGGGTCGGAAAACAGAGTTCAATATTTTTCTGGGC
GATTGACTCACGTCGCTTCAGGAAGGGGCGCGCCAGTTTACCGGTGCGGGTGCCCAGAAAACGCAAAACCGGG
TAGGGAAGCTGCACCAGTAACCAGAGGACGCCAACGCCAAACCACGTCAGCCAGTAGCGCGGGTGCAAAAACG
CGCGTGAAAATTTGCTTTGAGGAAACATAAAATACCTTCTGTAGATACGAAAATAATGTATCGCAGAAGAGTC
ATTATCCGCAGCGGTACACTCTGAAATACGCATCATCCTTCAAGTTGCGTTAGCTTATCCGAAGCACTTACTT
GAGTAAGCTCATCAGGGTAACTTGAATGATTTACGTATAGTTAGGCCATCATGTACGACAATGGTTCCCGTAA
GGAGTGTAAAACTAAGTCAGTACTAAACCACGAGGGCATGAACGCCAGGCAGGCTAATGTACCACCAGTTATT
TACTCGCGCCCAGAAATTCTTTATCAAGAAGCGTAAATATTTCTCCCGTAGCAAAAATAAGCTAATCATTATT
TTTAATGCATTGTTTTTACAGTGATAATTTTATTTCAGACGAAGGTCATTGAAAAATGATTAAAAAAATAAAA
TGTGAGATAAATGTTATGGAAATGACGGGCTGATAAGGTTTATCTTAAAGGAGAAGTGAGAGGGACCGTAAGG
CTATATGGAAGCCTTGCCATAGACAGGAGGTATTATAGATGATCTATTTATGGACATTTCTTGCAATAAGTAT
ACTTGCCGTGAGTGGCTATATCGGTCAGGTGATGGGGGCTTTTTCTGCGGTTTCTTCTTTTACGGGAATGGTT
ATCCTTGCCGCGCTGATTTATTTACTCAATGTATGGTTGCAGGATGGCGATGACATCGTTAGCGGATTATTGC
TTTTTCTCGCACCGGCATGTGGGTTAATTATTCGCTTTATGGTGGGATACGGTAAGCGTTAGTCAGTACATAC
CCTTCATACTTCAAGTTGCTGATGTGTTGGCTACGGATTACTCGGCCCATCGATGGGCCTCGCCCTGACGGCC
GTCGCACGCGACGTTCAAATCTGTTCCCGGCAGATTTGTCGTTCACCCCAGTCACATCGTTATCTATGCTCCT
GGGGACTCACTCCCTTGCCGCTTTTAAGCAACTCGAATTATTTTGGGTATACACGATGTCAGCGAATAGATAG
CGCCGACAATGTCGGCGCTTCTGTTCCCCGGGAAGGCTAATCGTTTATTGCGCGTCCGCAAGCTGCAGTGAGT
CCTGATTATGTAACGCATTCTGGCGCTGGCGTTCCAGTTCCAGCGCGCCGCGATGGGAGAGGTAACAGAATAG
AATGCAGCACACGATGCCGCCCATCAGAAGATAAAATCCGCCGTACCAGCCAAGTTTATCCACCATTACACCA
AACAGGCTGGTGCCCAACGAGGCGCCGAAAATATAGCTCATGAACCCACGTAAACCGACGGCGGAACCTACGG
CAAAGCTGGGGACTATCTCCATTGTCTGTACGGACGCGAGGAACTGCGGGACGTAAATCAGACAACCTACAAT
GGCGGCGAAAATCGTTACCATCAGCAAAGATTCACTTTTCCAGTATCCGATCAGACAGACAAAAATCAGCGCC
ATGCAAATCATGGCTAACGGCATTCTGCGGCCCTTAAACAGTTTATCTGACAGCCAGCCTGCCAGTAGCGTGG
AGGGAATCGCCGCCCACTCGAAAAAGAGAAACGCGACGCTCATCTGTTCTTTTGAAAAGTGTTTTACTGTCAA
CAGATAGATAGGCAACCAGCTAATCATGCCAAACCGCACCATATAGACGAAGACATCCACCAGCGAAATATAC
CAGGCATTTTTATTGCGCAGCACATAAGTACAGAAGATTTGCCATGCGCTCATATTTTCCGGCGCTTTTGCCG
TATTCTTCGTTTTCAGTACGACTTTTTCTTCCGGCATCATCTGTTCCAGTGAGGGAAGACCTTCTTCGCGTGG
CGAACCTTTTCCCAAGACCAGAACAATTAAGGCAAAAATGACGGCGACGCAGGCCGGAACGATATAACTGGCG
CTTTGCCAGTGTTCGTTGCCCAATATCGCAAAAGCCGCACCGACGATAGGCGCGACGATCCCGCCGCCAACGT
TATGCGAGATATTCCAGAAGGCGCCTACGCGTCCGCGTTCCCGACGAGGGAACCAGTTTGCAATAGTAATAAA
CGAGGGGCCGACGCCCATGCCCTGAAAAAGGCCATTGAAGACCACCAGAGCGGCAAATATCCAGAATGCGCTA

CTGAATCCCAGCCCAACGTTAACAATCGCGCAGAGTACCAGACCGCACGCCATGAAGACTTTTGGGCTGGCTT
TATCCGCCAGACTGCTCATTACGCCTTTACTGATTCCGTAAGCAATAAGCATACAGCTACTCAGCAGGCCGAT
TTGCGTAGCGCTGAGATCCAATTGCTCTTTTAAATAAGGCGTCGATAATGTGAAATTGTTTCTGACTATATAG
TAAGCGAGATATCCCAGAAATACGCTTAACAATGCCTGTATACGATATCGACCATATGTGGCCTGGACTTTTT
CCGGTGGGACTTTATGCGCCGATTGCCCTGTTTTTAATATTGTTAACATGGAGTCACCTGTATTTTATAGTGA
GATGAACATCAGGAGGTTTTGTCGGTAGAGTGAAAGGAGTGACAATAAAATTCAAGTCGAAGAAATGTGTCAA
AATTGACTCAATTGGGTGAATTTAAAGGTTATTTTTGACCCATCCTGAAGGGGGTGGTGAAACTTTAATTATT
GGCATGGCCGTTTTATCGTTAGCATGTGCGGATATTAACAAATTTGCTGCCAGTGATCTGATACGCTTTATTG
ATGTTTTCGGGCGACGGACCCGTTATTATTCAAATAACGTTGTTCTGGATGCCAACAGAAATAAAAGGGTAAG
TTTTGTCTCAATATTTTCTTTATGTGATATCGATATTTATGAACGGGATCACAAATCGACTTTTCTCATGCTG
TAGTCGTTTCGCCAGAGTGGGCGCTTTTACGCTATGGCTGGCGGCAATGTTCGGTTCTTGCCAGGCGTACAGC
CGCGAGCTGGTGATGGCAACGACCTTTTCACCCAGCGCGACCGCCTGGATTATCCAGCGCTGGCAGACGGAGC
CGGGTTCCGTCATGATTCGTACGCTCAACCGTACCAGCGGTTCTTTAGAGCAATTACTTGATACGGCGAATGC
GGAAAATGTCGATCTGATCCTGACGTCATCCCCCATGCTGTTGCAACACCTTCAGGAGCACCAAAAGCTGGCG
CTATTAGATAGCGCGCCTGCCGCCAGCCAGAAGCTGGTGCCGCGTTCTATCCGCTCAACTTCAGTGGCGGTAG
CGGTATCCGGATTTGGGCTATTGATCAACCGTTCAGCGCTCGCTGCGCGCCATCTTCCGCCGCCTGCTGACTG
GCAGGATATGGGCCTGCCCAGTTACCAGGGCGCTCTATTAATGAGCAGTCCGTCACGTTCAGACACGAATCAT
TTGATGGTGGAGTCGCTGTTGCAGCAAAAAGGCTGGACGGCAGGATGGGCGACGCTGCTGGCAATCTCGGGTA
ATTTGGTCACGATTTCCTCTCGCAGTTTCGGCGTAGCGGATAAAATCAAAAGCGGGCTTGGCGTTGCCGGGCC
GGTCATTGATAACTATGCTAATTTGCTACTTAACGATCCTAATCTTGCTTTTACCTATTTCCCGTATTCTGCG
GTGTCGCCCACTTACGTCGCGGTGTTGAAAAACAGCCGTCATGCAGACGAAGCGCGTGCTTTTATTCATTACT
TATTAAGTCCGAAAGGGCAAAGGATACTGGCTGACGCGAATACCGGTAAGTATCCCGTCGCACCGCTTAGCGC
CGATAATCCTCGCGCCGCGCAACAACAGCGTTTGATGGCCCAGCCGCCGTTGAACTATCGTCTGATCCTGAAG
CGTCAGCAACTGGTACAACGCATGTTTGATACCGCGATCAGTTTTCGTCTCGCGCAGCTTAAAGATGCATGGC
GAGCGTTGCATAGCGCAGAAACGCGGCTTAAACGCCCCTTACCTGAAATCAGAGCCTTATTGACCAGCGTCCC
GGTGGATGCGGCAAGCAGCGAGGATGAAACCTGGCTGGCGCAGTTTGATAATAAAAGCTTTGCCGAACAAAAA
ATGATGGAGTGGCAGATCTGGTTTCTGAACAATCAGCGGCTAGCGATTCACAAACTGGAAGAGCTGAAATGAA
GGGGAGATTATTGCAACGGCTTCGTCAGCTCAGTATCAGCAACAGTTTACGGGGTGCATTTTTAACGGGCGCA
TTACTGACGCTGATTGTCAGTATGGTCAGCCTTTATTCCTGGCATGAGCAAAGTTCGCAGGTTCGCTATTCGC
TTGATGAATATTTTCCTCGCATCCACTCCGCTTTTTTAATTGAGGGGAACCTGAATCTGGCTGTGGATCAACT
CAATGAATTTTTGCTGGCGCCGAACACGACGGTACGTTTGCAGTTACGTACGCAGATTATTCAGCATCTGGAT
AAGATTGAGCGTCTCAGCCAGGGGCTTCAGCTTGCTGAGCGCCGGCAACTGGCCGTCATTTTACAGGATAGCC
GGACCTTGCTGGCGGAGCTGGACAACGCCCTCTACAACATGTTTTTGGTGCGGGAGAAAGTCAGTGAGCTTTC
CGCTCGCATCGACTGGCTCCATGATGATTTCACCACGGAGCTAAATTCGCTGGTACAGGATTTTACCTGGCAG
CAAGGGACGCTACTCGATCAAATTGAGGCGAATCAGGGCGATGCAGCGCAGTATTTGCAACGTTCGCGCGAGG
TGCAGAACGAGCAGCAGCAGGTATATACCCTGGCGCGAATTGAAAATCAGATTGTTGATGACCTGCGCGACAG
ATTGAATGAGCTCAAGTCCGGCAATAATGACGGGATGCTGGTGGAGACCCATATTCGCTATCTGGAAAACCTA
AAAAAAACGGCGGATGAGAATATTCGTGCGCTGGATGACTGGCCCAGTACGATCACGCTGCGGCAAACCATCG
ACGAACTATTGGAAATCGGGATGGTAAAAAATAAGATGCCCGATACCATGCGCGATTATGTTGCGGCGCAAAA
AGCCTTACTGGACGCCAGCCGCGCCAGAGAGGCTACGCTAGGGCGCTTTCGCACGCTGCTTGAGGCGCAGTTA
GGTAGTAGTCACCAGCAAATGCAGATGTTCAATCAACGCCTGGAACAGATTGTTCGCGTTAGCGGCGGGCTTA
TTTTAGTGGCGACGCTCCTGGCATTGCTGCTGGCCTGGGGACTCAACCACTATTTTATCCGTTCCCGGCTGGT
AAAGCGTTTTACCGCGCTTAATCAGGCTGTGGTACAGATAGGGCTGGGGAGAACCGACTCGACTATCCCGGTT
TACGGGCGCGATGAGCTAGGACGGATAGCCCGTTTGCTACGTCATACGCTGGGCCAGCTCAATATGCAACGCA
GGCAGCTTGAACAAGAGGTCGCCGAGCGCAAAGAGATAGAAGCCGACCTGCGCGCCATGCAGGATGAGTTGAT
TCAGACGGCGAAACTGGCGGTAGTCGGCCAGACCATGACCACGCTGGCGCATGAAATTAACCAGCCGCTGAAT
GCGCTTTCAATGTATTTATTTACGGCGGGACGCGCGATTGAACAAGGTCAGAGCGGACAGGCGAGAAATACGC
TCACCAAAGCGGAAGGGCTCATTAACCGGATTGACGCCATTATCCGTTCGCTGCGGCAGTTTACCCGCCGGGC
GGAGCTGGAAACGCCATTGTATCCCGTTGATCTGCGCCAAACCTTCGTGGCGGCATGGGAGTTATTAGCCATG
CGTCACCAATCCCGGCAGGGAGCGCTTTCACTGCCAACCGACACCGTATGGGTTAGCGGCGATGAAGTGCGTA
TTCAGCAGGTTCTGGTGAATGTGCTGGCGAATGCGCTTGACGCCTGTTCGCATGATGCGGCGATCGCGGTGAC
ATGGCAGACGCAGGGAGAGGCGCTGGAGGTTTATATTGCAGATAACGGCCCTGGCTGGCCTGTGGCGCTGTTG
CCCTCTTTACTCAAGCCGTTTACTACCAGTAAAGCTGTCGGGTTGGGTATTGGGCTGTCGATTAGCGTGTCGC
TGATGGCGCAAATGAAGGGCGACCTGCGTCTGGCGTCGACGCTGACGCGTAACGCTTGCGTGGTGCTGCAATT

CTCTGTAACGGATGTCGATGATGTTGAATGATGAATGTTCTATTTTGCTGATTGATGATGACGTTGATGTACT
TGACGCCTACACCCAAATGCTTGAGCAGGCAGGCTACCGCGTGCGTGGCTTTACCCATCCTTTCGAGGCCAAA
GAGTGGGTAGAAGCGGATTGGGAGGGGATCGTTTTGAGCGATGTGTGTATGCCGGGGTGTTCCGGTATCGATC
TTATGACGCTGTTTCATCAGGATGACGATCAACTGCCAATCTTATTGATTACCGGACATGGCGATGTACCGAT
GGCGGTTGATGCGGTGAAAAAAGGGCGCCTGGGATTTTCTGCAAAAACCCGTCGATCCGGGCAAGCTGTTGAT
ATTAATTGAAGACGCGCTACGCCAGCGCCGGTCGGTCATTGCACGGCGGCAATATTGCCAGCAGACGTTACAG
GTTGAACTGATTGGGCGCAGCGAGTGGATGAATCAGTTTCGACAACGGTTACAACAACTGGCGGAAACGGACA
TTGCCGTATGGTTTTACGGTGAGCATGGCACTGGACGTATGACTGGCGCCCGTTATCTCCATCAACTGGGGCG
TAACGCGAAAGGGCCGTTTGTACGCTATGAACTCACGCCGGAGAATGCCGGGCAGTTGGAGACATTCATCGAC
CAGGCGCAAGGAGGTACGCTGGTGTTGAGTCATCCCGAATATCTGACACGCGAACAGCAGCACCATCTGGCGC
GTTTACAAAGCCTGGAGCATCGGCCTTTTCGTTTGGTGGGCGTTGGCAGCGCTTCGCTGGTGGAGCAGGCGGC
AGCTAACCAGATTGCGGCCGAGCTTTACTACTGTTTCGCCATGACCCAAATCGCCTGCCAGTCTCTTTCTCAG
CGACCGGATGATATCGAACCGTTATTTCGCCATTATCTTCGAAAAGCCTGCCTGCGGCTCAATCATCCAGTGC
CGGAAATAGCGGGGGAATTACTGAAAGGAATAATGCGACGCGCCTGGCCAAGCAATGTGCGCGAACTGGCTAA
TGCGGCAGAGCTTTTTGCTGTTGGCGTGCTGCCGCTGGCGGAAACGGTCAACCCGCAGTTGCTTCTTCAGGAG
CCGACCCCGCTTGACCGGCGCGTTGAAGAGTATGAGCGACAAATTATTACCGAAGCATTAAATATTCATCAGG
GACGAATTAATGAAGTGGCGGAGTATCTGCAAATTCCCCGTAAAAAACTTTATCTGCGCATGAAAAAATATGG
TCTAAGTAAAGAGCATTATAAATTCTGATATTACAGTTACTTTCAATCTGGCTGACAACAACAGCAACGATGT
CGTTAGCCAGATAACGTCGCATCACCGTAATGGTAAATCATTCCATCATGATTATAGATTGCTTATTATTCAC
CTGAGCATCAAATTCTATTTCTTAACTTCAATATAAGGTAAAAATGCGTCAAGTTCTCTGGCGTAATAAATGT
ACTCTTGTCCGACGATTTGACAAGATGAAAACTTCATCACCTCTCCAGATTACATCTGAATATGAGGACAAGA
GAAATGAAAAAACATGCTATTGCAGTAATGATGATCGCCGTATTTTCTGAGTCGGTTTATGCGGAGTCTACCT
TATTTATTCCGGACGTCTCTCCTGATAGCGTCACGACATCCCTTTCTGTGGGGGTGTTAAATGGTAAATCCAG
GGAGCTGGTTTATGATACCGACACCGGGCGGAAGCTGAGTCAACTGGACTGGAAAATAAAAAATGTCGCCACG
TTGCAGGGGGATTTATCATGGGAACCCTATTCGTTCATGACGCTGGACGCCCGCGGCTGGACGTCTTTGGCGT
CGGGATCGGGTCATATGGTTGACCATGACTGGATGAGCAGTGAGCAGCCTGGCTGGACCGATCGTTCAATTCA
TCCGGACACCAGCGCCAACTATGCTAATGAATACGATTTGAATGTGAAAGGTTGGTTATTGCAGGGCGATAAC
TACAAGGCGGGCGTGACAGCGGGCTATCAGGAAACCCGTTTTAGCTGGACGGCAAGAGGCGGGTCTTATATTT
ATGATAATGGTCGGTATATTGGTAACTTTCCTCATGGCGTGCGCGGTATAGGTTATAGCCAGCGTTTCGAAAT
GCCCTATATCGGGCTGGCGGGTGATTATCGTATTAATGACTTTGAGTGTAATGTACTGTTTAAATACAGCGAC
TGGGTAAATGCGCATGATAATGACGAACACTACATGCGCAAACTTACCTTCCGTGAAAAAACGGAAAATTCAC
GATATTATGGCGCGTCTATTGACGCCGGATATTATATTACCAGTAATGCAAAAATCTTTGCTGAGTTCGCTTA
CAGTAAATATGAAGAAGGTAAGGGCGGTACGCAAATCATAGATAAAACCAGCGGTGATACGGCGTATTTTGGT
GGCGATGCCGCAGGTATAGCTAATAATAACTATACGGTTACCGCGGGGTTGCAGTACCGCTTCTAGACCACAT
CAGGATGTCATCGGTCATAACCGGCCGATGACGACTTTTTTGCTGAACGTATGGCATGTCCGGTGATATTGTG
TTTACCTCATATAGATCTGGCGGGTTTGGCGTTAAGACGTGGCAAGGAACAGTAGAGGGGGACCTCATCCAGA
TGTTATAAACTTCAATTCAGAAAGTTACTTTCCATATACTTTTTGTGGGACTGCTTCAACCTTTGGGCAGATA
TCGGAAATGAAAAAGATAGACCAGGCAACTATTCGCTATCAGAATATCCGGTACACCAACTACCAACTACCAA
CTACCAACTACCAACAAATCATTTAGTCGATGGTCTCGTTGCCATTGGTTCATAGAGTGTTGGTCTTGGTATG
GATGGCTGGGGAAGTTATGTATCGAACATTCTTATGCAAGATTGCGCAGGGTCTGGTGATCTATGGTACACAT
ATGGGAAGGCATTCACATATATTTCTGTAATCGATACTAAAACTTTAACACTAACTAATTGTTTGTAGAAAGT
GGTTGCATTATTAATGGTTTGAGACTCATTGACATAAAACAAACACCATCTGGTAATCTGTCAGACCCCGCAT
CCTTAATAGTTAACTATAAAGATTGTATGGTAGTTGAGATGTCGTTAGTTCTAATATCGTGTCAGATAAATTT
ATAAAAGATTACTCATGTTTTGTGTCACATGCAAAAATAATTCGGCTTCGTTAAGGTCTTTAGGGGAAATACC
TAATGGATAATTAGTTAGATTAACGTTAACAACACTTTGAACGTGTAATGAATATGGGGGTAAAATATAAGTA
TTGGGAGATTGTAATTAAAAATTATGTAATTGTCTGATTATTATATATTCACTCCAGCAAAGGAGAAAGGCAA
TTATGGACGAAAAGAAACTCACAGCTCTTGCGGCTGAACTGGCTAAAGGTCTTAAAACTGAAGCCGACCTCAG
TCAGTTTTCCCATATGCTGACGACATTAACCGTCGAAACGGCGCTCAATGCTGAGCTGGCTGACCATCTCGGG
CATGAGAAAAATGCTCCTAAAACGAGCTCAAACTCCCACAATGGCTACTCATTAAAAACGGTGCTGTATGATG
GCGGCGAGATAGTGCTGAACATGCTGCGTGACCGTGAAAATACCTTTGAACTGCAGCTGATTAAGGAGCATCA
GACGCGTATTACGAGATGGACAGTCAGATTTATCCCTTTATGCCAAAGGCATGACATGACTACCCGCGAAATC
ATCGCCACCTTCAAAGAGATGTACAACGCTGATGTGTCGCCCATGCTGATACTTAAGATCACCGCCGCAGTAA
AAGAGTTGGTCAAAGGGGCGTCAAAAAATGGAGTATGCCGATCCAGGACTGGTCGCTGGCAATGAGTCGCTTT
ATTATCGAGTTCGGTGACCGCCTGAGCGATCACCGTTAATATAGTGGCAGTTACACAGTATGACTGGCAGGTT

CTATATTGGATTTATATGTAATAGTAACAGCCTATCTATTTGATTATTTTATTTCCGATTTTATTAAAAAGGA
AATCAGAACCAGGCTTTGTTAAATGTCCCCAATCAACGGCAGTGATAAAATCAGGACCATTACCAACTCTTGT
AAGACATCCACTTTCGTTACATAATGCTTTGTATGCTGATATATATTCAATTCCCATTTTTGGAACATTGTTA
CTAAAGTAAGAGTCCCACTCGCTTATTTCACTATTTAATCCATATGTCATATACAATGGTGGGGTTTTTTTAA
ACTCACTCAGGTAGTTAGATATTATTTTAACTAAATTTGCATTCCATTCCGGGACTGGTCCAATGAAAATAAT
CCTTGAGTCAGGGGATGCCTCTTTAATTTTTTTTATGGTTAATGATAACGTATCAATTGCTAACTTTTTATCA
TGTACTCCATTTGTTCCTCGAACTGACCATGTCAGCAGAACCACCTCAGGCTGAACACGTTTAATTTCATTAA
TTCTATTATTGTTTAGAGTGATGACACTTCTCTGTAAATCATCTTTACCGTCAACAAATAGAGGAGGAGCGTT
ACCATCTGTCATTTGGCTTATTATATAATCAGAACCTTTATTATCTATATAATGAGAAAGTCCATTGAAAAGA
GCCGCCGCATAAGAATCACCAATGATAAATATATTATGCTTGCCATTTTTTATACATCCATTGGATATGGCAG
CAGTAAGTTGTACTGAGTGGCATATCCCTCCACGGAGTAGTTCTCCATATTTATAATAATTGTACACGTCAGT
AACAGAAGCATATTCACCTGCTGATTTATTGATTTCCCTGTCTTTAACTCCATTTATATGAAAAATAAATGCG
CCAATTAAACCTGTCCCAAATACTGATAATGCTAATAATATTGCTGTGATATATTTATTTCTGGCATTTCTCA
GTGGTTTTTCAATTAAATAATAAGTTAATATCGCCAAAAAGAACGATGATAATAAAAGAAGAATTAATTCATG
GTAGTCTGGTGAACCAGCAAATATTGAACGATAGAATGAATAAATAGGCCAATGCCACAAATAAAGAGGATAG
CTAATAAGACCAAAGAAAACAACAGGCCTAACACTAAGCAATTTCGACACAACTAAATCATTACCATTAGATG
CTATTATAAGAGAGGCGCCAAGTACTGGGATTATTGCTATATATCCAGGAAATGACATCTTTTCATCTATCAT
GGTTATTGATAATGCGATTAGTATAATTCCTAACAGGGACATTAATTTTGATAACGAAGTGTTTATTCCTATA
AATCTCAATGTGGATATAATCGCTCCAGCCATTAACTCCCAAAATCTTGATGCGGGAGAGTAGTAATTAGCTC
CGCCATCAGATGCCATTGTAAAAATGCTAATCGCATAGCTAATTATAAATATAGTTGCGCATGATAATACTAT
GTTTCTGTTATGGTTTTTGCTTCTAAAGCATAGCAATATAACTACTGGCCATATTATATAAAATTGCTCTTCA
ATTCCCAGTGACCACAAATGTAGTAAAGGTTTAAGGTATGACTTTGAATCAAAATAGCCAGACTCACTCCAAA
GAGTAAAGTTTGATATAAAGAATGAGCCGCTAAAAACATGCTTACCAAGTAATTTGTAATCATCCTGGAATAA
ATAAACCCAGCCAACAATAAGACATGATACAAGAACTATGGATAATGCTGGAAATATTCTAAGCACTCTCCTT
TTATAGAAATCAAGGTATGAAAATGATTTGTTTGATGCAGATTTTAATATTATTGATGTTATAAGGTATCCAG
ATATCACAAAGAATATATCTACTCCAACAAACCCACCCGGCAATAATGATGGGAAATAATGGAATATTACCAC
AGATAAAACCGCTATTGCGCGTAATCCATCTATATCAGGTCTGTATTTTAAGTGTTCCAACTTAAATTACCTC
AATTTTAAAAAAAGATTAATAAAATGGTTGTGCATCTTGCATCATTCCCGAAGTTTCGTGTAAACGAAAACGG
AATGACGAGTGGATCAGATACGGCTATTATTTTAATTATTGACTCTGTCACATCTTTACTTCCGTCATTAATA
AAAATAATCTCAACTTTATATTTTTCAAGTTCATTAAACTCATGTACCGTTCTATAGAAAATCGGTATCGTGT
CTTCTTCGTTAAAAACTAGGACGACAAGAGAGATTTTCATTTTTATCCCTGAAGATAAAGAATCTGGAATAGA
TAAAGCCGCATACCAGGCTAATTGCCGAGAAAGTGATGAGGGTAACCAATGGTGGCAAAGAACATTGGTCAGC
CATCCATCCAACAACAGCGCTCAGTGCTCCCATGAATCCCATATACATCATGTAGCGAATTGCAGTAGTGCTG
GCATTAAAGGTGAAGCGCGCATTAGCATAGAAGCTGAACGATACAGCGATAACAAAATCGGAAAAGTTCGTCA
GCGCCTGATGCGTATGCATCCCATACATACAGAAAGCAAATACTCTCCAATGAATGAGCATGTTAAGAACACC
GATCGATGTGTACTTAGCGAATAACTTCAACATTATGAAAATTATCAGATTCAGAAAGGTCTGTAGTGTAGCA
CTACAAATTGGTTTGATCGATATAAGCGATCAATAATTGTATTTTTAATAGTTTTAAACTATTGAGTTTTAAT
ATATTGATCGATGTTATCGATCAATTGGTATTGCTGATTGCCAAGCGTCTTGGAATAAAAACGGGACATGTAA
AGCTTTGCATCGTCTTACAAGGCTTTGCATTTTTTTCAGGGAGAGGTACTTGAAAGGGTGGAAGTGCTGGGGG
GAGGGGGAGCGTTAAAAATTCTGTATAATTTTAGTAACATAAAATAAAAAAGAATGGCACATGTCCCATCCCT
TCGATTTCGACAAAGCACTTAAAGCCCTTCAGGTCCGGCCAGGCATTAACGGGCAAAGATGGCATCTTAACGC
CATTAATCAAGTATTTAACCGAGTCTACCCTGTCTGCTGAACTTGATTCCCATCTGGCTCAGGATGTTGAGGC
AAACCGTAAAAATGGTTCCGGCAAAAAGCCATTAAAGCCCCAACAGGCAGTTTTGAACTGGCAACTCCGCGCA
ATCGTAACGGCACTTTTGAGCCATAACTGGTGAAGAAGCTTCAGACCCCCTGTACGACGAGATCGAGCGCAAT
ATCATTCGACTGTTTGCGCTGGAGATGAGTTATCAGGACATCGGCCGGGAGAGTGAAGATCTTTATGCCTTCA
GCGTTTCAACCGCCACCGTCAGTGCAGTACCGATAAAGTTATCCCTGAACTAAAACAGTGGCAACAGCGCCCG
CTGGAGAAGGTTTATCCCGTCGTCTGGCTGGACGCTATTCATTATAAAAACCGTGAGGATGGCCGTTATCAGA
GCAAGGCGGTTTATACCGTTCTGGCACTGAATCTGGAAGGCAAAAAAGAAGTTCTGGGCCTATATCTGTCGGA
AAGTGAAGGTGCTAACTTTTGGTTAAGTTCTAACAGCGAGAGGGTACTTTAAAGGGATGCTTTTCGTTATGTT
TATAGGCACTATTCGCTGGAAATCATAAGACATCAAAAACGCTGCAACGCCTTGTGTGGTGTGGGGTTGCTGA
GATTTGTGAGAGGTGGGTAAAAGAGGTCATGGTGTCCCCTGCAGGAATCGAACCTGCAACTAGCCCTTAGGAG
GGGCTCGTTATATCCATTTAACTAAGGGGACAACGCGGCGCCAGTATAGCGTTTTTTATTCGCCGGAGTAAGT
GTAGCGCCGCCTGACTGGTTAAACCGTCGCCACTCAGCGCTGTTTTTCCGCTTTTTTCCGCTCCCGTTCCAGG
CGCTCGCCGCGTAGCCTCGCTTCTTCCTTACGCTTATTGCTCATATCGTTGCGGATCTGCGCGTGGCTCATCA

ATGCGAAAATAAAAGTGCCGCCGCAGATATTTCCGGCAAGTGTGGGAAGGGCGAAGGGCCAGAGAAAGTCGCT
CCAGGGCAGCGTGCCGTTGAAAACCAAATACAAAATTTCAACGGAACCGACGACAATATGGGTGGTATCGCCC
AGCGCGATAAGCCAGGTCATCAAAATAATGACCACAATCTTTGCCCCGCCTGCTGCAGGAAACATCCATACCA
TTGTGGCGATGATCCAGCCAGAGATAATCGCGTTGGCAAACATCTCCGTTGGGCTATTTTTCATGACCTCCAT
ACCAATTTTGACAAAGGCGTCGCGGGTCTCTTCATCAAATATAGGCATATATTCAAATGCCCACGCCGCAACC
CCGGTGCCAATAAGGTTGCCCAATAAGACTACGCCCCACAAGCGCATCAGCAGGCCAACGTTACTCAGAGTGG
GATTTTGCATTACCGGCAACACGGCGGTAACGGTATTTTCAGTAAATAATTGCTGGCGGGCCATGATGACAAT
GATAAAACCAAAGGTATAGCCGAGATTTTCCAGTAAAAAGCCGCCGGGAACGCCTTCAAGCTGCACGTGGAAA
ATCCCTTTCGCCAGGAGTGATGCCCCCATAGAAAGTCCTGCGGCAATGGCTGACCAGAGCAAAGCCATCGCAT
CGCGTTCCATCTCTTTTTCACCATCCTGGCGAATATGTTCATGAATCGCCATGGCGCGGGAAGGAAGACGATC
TTCATCCACTTCAATCTCTTTACCGCTTTGTTTTTCTTCGCTTTCAACTTCCAGGTCACTGCTTTGCCGGTTA
ATTTTATCGTCGTTAAGGCTATCCATGATGATCTCTGCTGATTAACACATATTAATAAAGCGTAGCGGTTTTT
CATTTACCCAGGCCGGGGCATTCTCCGAAAAAATAAAAATGATCACGCTCGTTTTTATCTGTTTCGTTAGAAT
AGGGCCGTCACGTTGTTGCGTTACCAGGCTATGCTCAAAGCAATGAGATCGCGCATATAGACATCGTTTGACG
TGCTGTTAAAATCGCTCACACCTAAACAGGCGGATACGGTAGCGTTCCGTCATGGATGGCAATCAGCGATAGC
CATAATTCACAGGGAGACATCTATGAAGCTTCGCCTGTCGGCGCTGGCGCTGGGAACCACACTGCTGGTCGGG
TGTGCGAGTTCCGGTACAGAACAGCAGGGGCGTTCAGACCCATTTGAAGGGTTTAACCGCACCATGTACAACT
TCAACTTTAATGTGCTGGACCCGTATGTTGTTCGGCCGGTCGCGGTCGCCTGGCGTGATTATGTTCCACAGCC
TGCGCGTAACGGTTTAAGTAATTTCACCGGCAACCTCGAAGAACCGGCGATCATGGTGAACTATTTCCTGCAG
GGCGATCCTTATCAGGGTATGGTGCATTTCACCCGTTTCTTCCTGAACACCTTATTAGGGATGGGCGGATTTA
TTGACGTCGCGGGAATGGCGAATCCTAAGCTGCAGCGCGTTGAACCGCATCGCTTTGGCAGTACGCTGGGCCA
TTATGGCGTGGGGTATGGGCCTTATATGCAACTTCCGTTCTACGGCAGCTTCACGCTGCGTGAAGATGGCGGG
GATATGGCGGATACCTTGTATCCGGTGCTCTCGTGGTTGACCTGGCCAATGTCCATAGGAAAATGGACTATCG
AAGGGATAGAAACCCGCGCGCAGTTGCTGGACTCCGATGGTTTGCTGCGCCAGTCTTCCGATCCTTATATTAT
GGTGCGTGAGGCGTACTTCCAGCGTCATGACTTTATCGCCAATGGCGGAAAACTCAAGCCGCAGGAAAACCCG
AACGCGCAGGCGATTCAGGACGAACTCAAAGAGATCGACTCGGAATAAACGGCAGTAAATAAAAAGGTGAGCG
CAATGCTCACCTTTTTGATCCGTTTCAGAAAGAGATTAGAACGCGTAGTTAAAGTTAGTACCGAACAGCCAGG
CTTTCCCTTCAGATTCAAACTGATAAGGGCCTTCATTAATTTTCACGCTCTGACCATGCATATAAGAGACGCC
AACGTCGACAGACGCATCTTTATTAAAGGCGTAAGTCGTACCGGCGCTCAGCCAGAAACGGTCCTGGTCCGGA
ATAGAGATAGAACGGTTTTGCGCTGGTACCGGGCTGTCATCGAAGGCGATACCGGTACGGAACGTCCAGTTGT
CATCGTAGTAATAGGTGGTACCCAACGCAATGCGATAAGCGTCTTTAAAGCCTTCATGCTTCTCAAAGAGCGT
ATCGCCGGCGGTCGATTTTGCCTTCAATTCCTGGAACTGGCTCCAGCTGGTATAGGCCAGGCTATAGTGGATG
GCCCACTGTGGCGCCACACGGTTATAACCGGACACTTCCCACATTTCCGGCAGATTCAGCGTCAGGTAGCCTG
ACTGCGTCGCGCCGCCGGTTGCCGTTGGGATAGGCAGGTTAAATCGGTTTATAGCGATGGGAAGATCGCTGCT
GTAGTTGCCTTTAAAGTCAATTTTGACTTCCGAGCGGTAGGTCAGCGCGTAGCGGTTATTTTTATCCAGTTCA
TAAAGGATACCGGCATTCCAGCCAAAGCCCCACTGATTACCGTTCAGGTGGGCAATCTTGGTGTCGCTGGGAA
TCTGACCCGCGACCGGCGCCAGCGCCGGATTCTGTGCCGCGACCAACTGGCCTAAATCGCCCGCAAAACGTTC
GATTTTCGCACGCGCGTATACCGCATCAAAGCCAAGGCCGAAGCTCCAGGCCTCATTTAAGCGATAAGCGCCG
CTTAAATTCAGGTTCATGGTTTCAAGATCGGTCGTACCGCCAACCGAGCCGCCGGCGTATGTGTCGTTAAACT
CTGTCGCCAGACCATAGTTAGAGGTAATAGAAGCCCCCCAGCCAAACTGATCGTTAATCGGGGCGACAAAATG
GACGTTCGGCACCCAGGCCGTCGGCGCGATGTTATCCGCATCTAACGTCCGACGAGATGGCGATGTCCCGCTA
ATATTAACATCAGGATCAATATAAACCGCGCCCGCTGAAAACGTCGGGCGGTCAAACATGGTAATTAACGCCG
GGTTGCGGCTAACGTTACCCGCATCATCTGCGATTGCGCCTTCACCCGAATAGGCCCGGCCAAGGCCAGAGGA
AGAAAATTCGTTTAACTGAAAGCCTGCAGACCAGGCCTGGGTGGAGATGATTGCCACTGCGACTGCGAGAGCA
GACTTTGTAAACAGGGTTTTCTGGCTCATGACCATAACCTCATTGATTATTTTTATACAAACTATGTTACATG
CTGTAACAGGAGCGCGAAGTGTAGGGTCTGCGGTACGACTTAGAAATCAGACCAGTGGCGAGAGTATAGGTCT
GACCAGCTAGAATGTTGCAAGTATGTTTATAAGTTTTTTCAATTGTGATCTTAGAAACGAGATCTGGCTGGCA
AAATCGGCGGGCTTAACGGTCACCCGATCGCGCGATTTTTGTTTTAGATCATTTTTAAGTGTGATTTCGGTCA
CTTAACGCGTTTCCCACCATTAACGACTGGAGAGGCGAATGCCGGGAGCGTAAAATATCCCCATCGTTTATCT
GGCACCCTGGGTGCAAATACAGAGGAAATCTACAATGAGTAAATGCAGTGCTGATGAAACCCCGGTTTGCTGC
TGTATGGATGTTGGAACCATCATGGACAACTCCGATTGCACCGCGTCATACAGCCGCGTATTCGCTACCCGCG
CAGAGGCTGAAGGGACGCTGGCGGCGTTAACCGAAAAAGCGCGTAGCGTGGAGTCTGAGCCTTGCCAAATTAC
GCCAACCTTTACCGAGGAATCCGAAGGCGTTCGTCTGGATATTGATTTTGTTTTCGCCTGCGAAGCAGAAACG
CTGATCTTCCAGCTCGGCCTGCGTTAATCCGTTCATTCACGACGCCTCCGTCATCATCTGGCGGAGGCGTTTT

CTTTTTTTGTCGTTTCCCTTCACTGTGCCTTAGCTCCCACTTTCTTTTCTCTCTGCTTGGCTAAATGTAAAAA
ATTGGTTAAGACTGTGATCAGGTCAGACCACTAATTTATTTTTTACAGGGGAGTACTATGCGTCAGGCTTTA
CCGTTGGTCACCCGTCAGGGCGATCGCATTGCTATTGTCAGTGGGTTACGAACCCCCTTCGCTCGTCAGGCCA
CTGCATTTCATGGTATTCCCGCTGTTGATCTCGGCAAGATGGTGGTAGGCGAGCTGCTGGCGCGCAGTGAAAT
TCCAGCCGATGCCATTGAGCAACTGGTTTTTGGTCAGGTAGTACAAATGCCGGAAGCCCCCAATATTGCGCGC
GAAATTGTGCTGGGTACGGGAATGAATGTTCATACCGATGCCTATAGCGTAAGCCGCGCCTGCGCCACCAGTT
TTCAGGCGGTAGCGAATGTGGCGGAAAGCCTGATGGCCGGCACGATACGCGCGGGTATCGCTGGCGGCGCAGA
TTCGTCGTCGGTACTGCCAATAGGCGTGAGTAAAGCCCTGGCGCGGGTACTGGTTGATGTCAATAAAGCCCGG
ACGACCCGCCAACGGCTGACGCTTTTTTCTCGTCTGCGCTTACGCGACCTGCTGCCCGTGCCTCCCGCCGTTG
CTGAGTATTCCACCGGTCTGCGCATGGGCGATACCGCCGAACAGATGGCGAAAACGTATGGTATTACGCGTGA
ACAGCAGGACGCACTGGCGCATCGTTCACATCAGCGCGCCGCGCAGGCATGGGCGGAGGGTAAACTGGCGGAA
GAAGTGATGACGACCTATGTGCCGCCGTATAAAAACCCCTTTGCAGAAGACAACAACATTCGCGGCGCCTCCA
CGCTGGCGGACTACGCTAAACTGCGTCCCGCTTTTGACCGAAAACATGGCTCCGTAACCGCCGCCAACAGTAC
ACCGCTGACCGACGGCGCTGCCGCCGTTATTATGATGACCGAATCCCGCGCTAAAGAGCTGGGGCTGCGCCCG
CTTGGTTATCTGCGCAGCTACGCCTTTACCGCTATTGATGTCTGGCAGGACATGCTATTGGGGCCGGCCTGGT
CAACGCCGCTGGCGCTGGAGCGGGCCGGGTTGACGATGGCCGATCTGACGCTTTTTGACATGCATGAAGCGTT
TGCCGCCCAGACTCTGGCGAACTTACAACTGCTGGGCAGCGAACGATTTGCCCGTGAGGTGCTTGGTCGTGCG
CAGGCGACAGGGGAGGTGGATGACGCTAAATTCAACGTGCTCGGCGGCTCAATCGCCTACGGGCATCCTTTTG
CCGCAACGGGCGCGCGGATGATAACCCAAACGTTGCATGAATTACGGCGTCGGGGCGGAGGTTTTGGCCTGGT
CACGGCCTGCGCGGCTGGCGGTCTTGGCGCGGCGATGGTTCTGGAGGCGGAATAATGACTACCACATCAGCTT
TTATGCTTAACGTGCGTCTGGATAATGTCGCGGTTGTCGCTATTGATGTCCCTGGAGAAAAGGTGAACACCCT
GAAAGCGGAGTTTGCCGCGCAGGTACGCGCCATCCTGAAGCAGATCCGGGAAAATAAAGCGTTGCAGGGCGTT
GTTTTTATCTCAGCCAAAGCAGATAACTTTATTGCTGGCGCTGATATCAACATGATAGGTCACTGCCAAAACG
CGCAGGAAGCGGAAACGCTGGCGCGTCAGGGGCAACAACTGATGGCGGAGATCCAGGCGTTGCCTGTTCCGGT
TATCGCCGCCATTCATGGCGCCTGTCTGGGGGGCGGCCTTGAAATGGCGCTGGCCTGCCATCGACGTATCTGT
ACCGACGACGTTAAAACCGTTCTCGGACTGCCGGAGGTACAGTTAGGGCTTTTACCGGGATCGGGCGGAACGC
AGCGACTTCCACGGCTGGTGGGGGTCAGTACCGCGCTGGATATGATCCTGACAGGTAAACAGCTTCGCGCCAG
GCAGGCGCTTAAAGCCGGGCTGGTTGATGATGTGGTGCCGCAAACTATTTTACTGGAAGCGGCTGTCGAGCTG
GCTAAAAAAGAACGCCTGGCGCAGCGGACATTACCGGTCCGTGAACGTATCCTGGCGGGGCCGCTGGGGCGCG
CGCTGTTATTTCGCCTGGTGCGTAAAAAGACAGCGCAAAAAACGCAGGGTAATTATCCGGCGACGGAGCGGAT
CATTGATGTGATTGAAACCGGACTGGCGCAGGGCAGCAGTAGCGGCTATGACGCGGAAGCGCGCGCTTTCGGC
GAGCTGGCGATGACGCCTCAATCGCAGGCGCTGCGCGCTGTTTTCTTTGCCAGTACCGAGGTGAAAAAAGATC
CCGGTAGCGATGCGCCGCCCGGCCCGCTGAACAGCGTGGGCATCTTAGGCGGCGGGTTAATGGGCGGCGGTAT
CGCCTGGGTTACGGCCTGTAAAGGCGGTTTGCCAGTACGAATTAAGGATATAAATACTCAGGGGATCAATCAT
GCTCTGAAATACAGCTGGGATCTGCTTGAAACAAAAGTACGCCGCCGCCATATCAAAGCCAACGAACGCGATA
AACAGTTGGCATTGATATCAGGTTCGACCGATTACCGTGGTTTCAGTCATCGTGATCTGGTCATTGAAGCGGT
GTTTGAAGATCTGCCGTTAAAACAACAGATGGTGGCGGAAGTGGAACAAAATTGCGCTGCTCACACCATTTTT
GCCTCCAATACTTCTTCCCTGCCGATTGGCGATATTGCGGCGAATGCGGCCAGACCGGAACAGGTGATTGGAT
TGCACTTTTTTAGCCCGGTCGAAAAAATGCCGCTGGTTGAGGTTATTCCTCATGCGTCTACTTCCGCGCAGAC
CATCGCCACGACCGTTAAGCTGGCGAAAAAACAGGGTAAAACCCCGATAGTCGTCAGTGATAAAGCCGGGTTT
TATGTTAACCGAATTCTTGCGCCATATATCAATGAGGCGATTCGGATGCTGACTGAAGGCGAACGCGTCGAAC
ATATTGACGCCGCCCTGGTAAAATTTGGTTTTCCGGTGGGGCCAATCCAACTTTTGGATGAGGTAGGAATCGA
CACGGGCACTAAAATTATTCCTGTGCTTGAGGCGGCGTATGGAGAACGTTTTAGCGCGCCTGCAAATGTTGTC
GCTTCAATTTTGAATGACGATCGCAAAGGTAGAAAAAATGGTCGCGGTTTCTATCTTTATGGCGAAAAAGGGC
GTAAAAGCAAAAAACAGGTTGACCCTGCTATTTATAAGCTTATTGGCGTGCAAGGGCAGAGTCGATTGTCTGC
GCAGCAGGTGGCAGAGCGCTGTGTCATGTTAATGCTTAACGAAGCGGCGCGCTGTTTCGACGAAAAAGTGATT
CGCAGCGCGCGCGATGGCGATATCGGCGCCGTGTTTGGTATCGGTTTTCCGCCGTTTCTCGGTGGCCCGTTCC
GCTATATGGATGCTCTCGGGCCGGGTGAAATGGTGGCGACATTACAGCGCCTGGCCGCACTTTACGGTCCTCG
TTACGCGCCTTGTGAACAATTAGTGCGCATGGCGGAGCGCAGGGAGCATTTCTGGACGAATGGGGAAACTGAC
CAGGGAAATTGAGGTCAAAGAGAATATGAATCCGCACTGAATGCCTCCTTAGTGGCGAAATAATAATCAAGGA
TTGACTATACTTACGCCATTGAGGTAAAAAACAGCGTTTCATTCAGTGAATGGATAAGGCACAATGCCGGCCA
CCGCATTTTTGCCGCAGCCAATAGGGGCATGTGGTAAGCGTGTTGGTGAAACTGGTTAACAAAAGCGGTGCAA
TATGCAAGTTTTTATCATGCGTCACGGCGACGCAGCCCTCGATGCCGCCAGTGATTCGGTTCGTCCCTTGACC
CCTTGTGGGTGTGACGAGTCTCGCCTGATGGCGAACTGGCTGAAAGGTCAAAAAGTGGATATCGAACGTGTTC

TGGTGAGTCCGTTTCTTCGGGCCGAACAGACGTTAGATGTGGTCGGCGATTGTATGAATCTGCCGGCTCAGGT
GGATGTCCTGCCGGAGCTAACGCCCTGTGGCGATGTCGGTCTGGTCAGCGCCTACCTCCAGGCGCTGGCCAAT
GAAGAGATCGGATCGGTGCTGGTGATTTCTCACTTACCTCTGGTCGGATACCTGGTATCTGAGCTTTGTCCGG
GCGAAACGCCGCCGATGTTCACGACGTCCGCAATTGCCAACGTTACGCTTGATGAAAGCGGCAAGGGGACATT
CAACTGGCAAATGAGTCCCTGCAATCTGAAAATGGCGAAAGCGATTTAATCTTTGCTGCCTGATGGCGCTTCG
CTTATCAGGCCTGCGACAGCGTTGGCTGTTCGTAGGCTGGATAAGCCGCTTGCGCCGCCATCCGGCAATTCCG
CATCAGGGTAATTCGGGCGGTTGCCACTCTTCCACTTCAATCAACACCAGCAGCGCGGCGTCGCCGCCGTACT
CTTTCGGCGCCTGATGAAAGGCCATCACATGCGGATGCTGCGCCAGCCATAACGGCGTTTGCTGTTTCAGAAT
ATGTTTACCGTGTCCATGCATGACGCAGGCGCAAAAAATATGTTCCCGACGGCAGGCGGCAATCAGCGCCCCC
AGTTCCTGCTTGGCCTGAAGCTGCGTTAAGCCGTGTAAATCCAGAAACAGTTCTGGCGAATAGTCGCCGCGAC
GCATTTTTTTAGCTCGAAATGGCTGACATCTTCACGGACATACTTTACCGGCCCTTCCGTATTGAGCAGCGGC
TGGAACTCATCGGAAAAAATAGTGGCTGGCGTCCGCCTGTTCCTGAATTAACCTTCGGGTAGGGACTTCCGTGA
TTTTCTTGCGCAGCGGCCGATGGACGATAGTATCCTGTTTAATCTTTCGGGTGCCGACCATCAGCTGACGGAA
CAGCGCCTGGTCCTCCTCGCTGAGCGATGTTTTCTTTTTCATTCGTGGGTCTCGTCTGATCTTTTGCCTTAGT
TTACCCGACCCGGAGGATATCCGAACGGTCAAAACGGATTTTTCGTATTCTCCCGCCGCGTCAATGCTGATTT
ATCCCTGTCTTCGTGGCAAACTAGCCGCCGAATTTAATGCGAGCATGCCCTGGAGGAATACGTGGATAAAATT
TTCGTCGATGAAGCAGTAAGTGAACTGCATACCATTCAGGACATGTTGCGCTGGGCGGTAAGCCGCTTTAGCG
CGGCGAATATCTGGTATGGACACGGTACCGATAACCCGTGGGATGAAGCGGTACAACTGGTGTTGCCGTCTCT
TTATCTGCCGCTGGATATTCCGGAAGATATGCGGACCGCGCGGCTGACGTCCAGCGAAAAACACCGCATTGTC
GAGCGAGTGATTCGTCGCATTAACGAGCGTATCCCGGTCGCCTACCTGACCAATAAAGCCTGGTTCTGCGGCC
ACGAATTTTATGTCGATGAGCGCGTGCTGGTGCCGCGTTCGCCCATTGGCGAGCTGATTAATAACCACTTCGC
CGGCCTTATTAGCCAACAGCCGAAATATATTCTGGATATGTGCACCGGCAGCGGCTGCATCGCCATCGCCTGT
GCTTATGCTTTCCCGGACGCAGAGGTTGATGCGGTCGATATTTCGCCGGATGCGCTGGCTGTCGCCGAGCATA
ACATTGAAGAACACGGTCTTATCCATCACGTGACGCCAATCCGTTCCGATCTGTTCCGCGATCTGCCGAAAGT
TCAGTACGATCTGATTGTCACTAACCCGCCTTATGTCGATGCGGAGGATATGTCCGATCTGCCGAACGAATAT
CGCCACGAACCTGAGCTGGGGCTGGCGTCCGGCACTGACGGCCTCAAATTGACCCGTCGTATCCTGGGGAATG
CGCCGGATTATCTGTCCGATGACGGCGTTCTGATTTGTGAAGTCGGAAACAGCATGGTACATCTGATGGAGCA
GTATCCGGATGTGCCGTTCACCTGGCTGGAGTTTGACAACGGCGGCGATGGCGTCTTTATGTTGACCAAAGCG
CAGTTGCTCGCGGCCCGTGAACATTTCAACATTTATAAAGATTAAAACACGCAAACGACAACAACGATAACGG
AGCCGTGATGGCAGGAAACACAATTGGACAACTCTTTCGCGTAACCACTTTCGGCGAATCACACGGGCTGGCG
CTTGGGTGTATCGTCGATGGCGTGCCGCCCGGCATCCCGTTGACGGAGGCCGATCTGCAGCACGATCTCGACA
GACGCCGCCCCGGCACCTCGCGCTATACTACCCAGCGCCGCGAACCGGACCAGGTAAAAATTCTCTCCGGCGT
GTTTGATGGCGTGACGACCGGCACCAGCATTGGCCTGCTGATTGAAAACACCGATCAGCGCTCGCAGGACTAC
AGCGCGATTAAAGATGTTTTTCGTCCGGGACACGCGGATTACACCTATGAGCAGAAATACGGCCTGCGCGATT
ACCGTGGCGGTGGACGTTCTTCCGCGCGTGAAACCGCGATGCGCGTAGCGGCAGGGGCGATCGCCAAGAAATA
CCTGGCGGAAAAGTTCGGCATCGAAATCCGCGGCTGCCTGACCCAGATGGGCGATATTCCGCTGGAGATTAAA
GACTGGCGTCAGGTTGAGCTTAATCCGTTCTTTTGTCCCGATGCGGACAAACTTGACGCGCTGGACGAACTGA
TGCGCGCGCTGAAAAAAGAGGGCGACTCCATCGGCGCGAAAGTGACGGTGATGGCGAGCGGCGTGCCGGCAGG
GCTTGGCGAACCGGTTTTTGACCGACTGGATGCGGACATCGCCCATGCGCTGATGAGCATCAATGCGGTGAAA
GGCGTGGAGATCGGCGAAGGATTTAACGTGGTGGCGCTGCGCGGCAGCCAGAATCGCGATGAAATCACGGCGC
AGGGTTTTCAGAGCAACCACGCTGGCGGCATCCTCGGTGGTATCAGTAGCGGGCAACACATTGTGGCGCATAT
GGCGCTGAAACCTACCTCCAGCATTACCGTGCCGGGACGTACGATCAACCGGATGGGTGAAGAAGTCGAAATG
ATCACCAAAGGGCGCCACGATCCGTGTGTGGGGATTCGCGCAGTGCCGATCGCAGAAGCCATGCTGGCGATCG
TGCTGATGGATCACCTGCTGCGCCATCGGGCACAGAATGCGGATGTAAAGACAGAGATTCCACGCTGGTAAGA
AATGAAAAAAACCGCGATTGCGCTGCTGGCATGGTTTGTCAGTAGCGCCAGCCTGGCGGCGACGCCGTGGCAG
AAAATAACCCATCCTGTCCCCGGCGCCGCCCAGTCTATCGGTAGCTTTGCCAACGGATGCATCATCGGCGCCG
ACACGTTGCCGGTACAGTCCGATAATTATCAGGTGATGCGCACCGATCAGCGCCGTTATTTCGGCCACCCGGA
CTTGGTCATGTTTATCCAGCGGTTGAGTCATCAGGCGCAGCAACGGGGGCTCGGAACCGTCCTGATAGGCGAC
ATGGGGATGCCTGCCGGAGGCCGCTTTAATGGCGGACACGCCAGTCATCAGACCGGGCTTGATGTGGATATTT
TCTTGCAGTTGCCGAAAACGCGCTGGAGCCAGGCGCAGCTATTGCGCCCGCAGGCGTTAGATCTGGTGTCCCG
CGACGGTAAACACGTCGTGCCGTCGCGCTGGTCGTCGGATATCGCCAGTCTGATCAAACTGGCGGCACAAGAC
AATGACGTCACCCGTATTTTCGTCAATCCGGCTATTAAACAACAGCTTTGCCTCGATGCCGGAAACGATCGTG
ACTGGCTACGTAAAGTACGCCCCTGGTTCCAGCATCGCGCGCATATGCACGTGCGTTTACGCTGCCCTGCCGA
CAGCCTGGAGTGCGAAGATCAACCTTTACCCCCGCCGGGCGATGGATGCGGCGCTGAACTGCAAAGCTGGTTC

GAACCGCCAAAACCTGGCACCACAAAGCCTGAGAAGAAGACACCGCCGCCGCTGCCGCCTTCCTGCCAGGCGC
TACTGGATGAGCATGTACTCTGATGGACAATTTTTATGATCTGTTTATGGTCTCCCCGCTGCTGCTGGTGGTG
CTGTTTTTTGTCGCCGTACTGGCAGGATTTATCGATTCTATCGCCGGGGGCGGAGGGCTGCTCACTATCCCTG
CGCTGATGGCCGCCGGGATGTCGCCGGCAAACGCGCTGGCGACCAATAAATTACAGGCGTGCGGCGGCTCCCT
CTCGTCTTCGCTCTATTTTATTCGCCGTAAAGTGGTAAACCTGGCCGAGCAAAAGCTCAATATTCTGATGACG
TTCATTGGCTCGATGAGCGGCGCGCTGCTGGTGCAGCACGTGCAGGCGGATATTTTGCGCCAGATCTTGCCTA
TCCTGGTGATTTTCATCGGCCTCTATTTTTATTGATGCCGAAGCTGGGCGAGGAAGATCGCCAGCGCCGCCTG
TATGGATTACCGTTCGCGCTGATAGCCGGGGGATGCGTCGGGTTTTACGACGGCTTTTTCGGGCCTGCCGCCG
GGTCGTTTTACGCTCTGGCGTTTGTCACCTTATGTGGCTATAACCTGGCGAAATCCACGGCACATGCCAAAGT
GCTTAACGCTACCTCCAACGTTGGCGGCCTGCTGTTATTTATCATCGGCGGCAAAGTGATCTGGGCGACGGGC
TTCGTGATGCTGGTTGGTCAGTTTTTAGGGGCGCGAATGGGGTCGCGTCTGGTATTGAGCAAAGGCCAAAAGC
TTATCAGACCGATGATCGTTATTGTCTCGGCGGTAATGAGCGCCAGGTTACTTTATGATAGCCACGGACAGGA
GATCCTCCACTGGTTGGGGATGAATTAATGAACAATACGCACCATTACGAACAGTTAATTGAGATTTTTAACG
GCTGCTTTGCCGAAGAATTTAATACCCGTCTGATTAAAGGCGACGACGAACCGATCTATCTTCCTGCTGACGC
GCAGGTACCGTACCACCGCATCGTCTTTGCGCATGGGTTTTACGCCAGCGCGCTGCATGAGATTTCTCACTGG
TGTATCGCCGGTAAGGCGCGCCGCGAACTGGTTGACTTTGGTTACTGGTACTGCCCGGATGGACGCGACGCGC
AGACCCAAAGTCAATTTGAAGACGTCGAGGTGAAGCCACAGGCTTTCGACTGGTTGTTTTGCGTGGCGGCGGG
GTATCCCTTTAACGTCAGTTGCGACAATCTGGAAGGCGATATTGAGCCGGATCGCGTGGCGTTTCAGCGCCGG
GTTCATGCGCAGGTAATGGCGTATCTGGAGCAGGGCATTCCTGAACGTCCGGCCCGCTTCATCAAAGCGCTAC
AGAATTACTATCACACGCCCGAACTTAAGGCGGAACAGTTCCCGTGGCCGGAAGCGCTTAATTAAAGCCAGCG
CCACGTTTACAATGAGGAAAGAAGATGATCGCGGAGTTTGAATCACGCATTCTGGCATTAATTGACGATATGG
TAGAGCACGCCAGCGACGATGAGCTTTTTGCCAGTGGATACCTGCGTGGCCACCTGACGTTAGCCATTGCCGA
GCTGGAGAGCGGTGATGACCACTCCGTTGAGGCGGTCTATGCCAACGTTAGCCAAAGTCTGGAGAAAGCGATC
GGCGCCGGCGAACTGTCGCCGCGCGATCAGGCGTTAGTCAAAGCGATGTGGGACAATTTGTTCGACAAGGCAA
AACAATAACGCCGTGGGCCGGAAGGCGACGCTTGCCGCCTTCCGCTATTGACTGACCCATTACTTCACCGCTT
TTCCCTTCAATAACTTTCTCACCCATAACCGATTTGGATTTAACGCCGCCAGCGTACCGGCATCCAGTGGAAT
CGGCTCGTTACTCATCTGCGCCGCCAGAATTTCTGCGCATAATGGCGCTGAGCACAAACCGCGCGATCCCAGT
GCGCCAAGCACAAATAAACCCGGATACACAGGCGCAGGCGCCGCCGACGTTTTGTTATCGGCTAAATCGGCGT
AATGCGTCAGCGTGGCGTGGTAATCCGGGACGTTGCCCACCATCGGCAGATGGTCGCGGGTGGCGCAACGCAC
GCCGCAACGGGCGCTGTTGCCGCTAACGTCAACCTCTGTGGCCCATTTCGCATCCGGAAAACAGTCAAGCAGA
CGCTGTCGGTTCTGACGCTGATCTTCTTCGCGCCAGACGGTGCTTTCGTCGCCCCGGTGATAGCTGGCGCCAA
TACAGTGCTGCTGATTATGCGGATTTTGCGGCGTCAGATAGCCGTCGTAGCATAACACTTGACGCAACGCCGA
GAGGGCGGGGGTGGTGGGAATATGACTGACCTGCCCCCCGACGGCATACACCGGCAGCGGCCGGGTCTGATCA
AAACGGTTAATCTGATGGCCGTTCGCCAGGACGACGGTCTCATGGCTGGCGGTTTCACCTGACATAAAGCGCA
GTTGCCAGCGCGACTCCTGCGCAACGAGCGACGTGAGCGTATGGCGAAAGCGCGTTTGTAAACCCTGTTCGGT
CGCCAGTGCGATGACTGCGCGAGTTAATTGTTCCGGGCATAGCCAGCCGCCTGCCGGATAGGTAATTCCTCCG
CAGCGTGTCGTCACGCCAACCGCCTGTTCCGCCTCTTCGGCATTGAGCGCCGAGGCCAGTTCGGCGGCAGCG
CCAGGGACAGCATCTGGGCAATTTTCTGCTGGCTTTTCTCATCCCATCCGAGCTGTGTCACGCCGCACCAGTC
ATGATCAAAAGAGACGGGCAGGGCATCATACAGGCGACGGGCAAAGGTGAATGCGGTCGGGAAAAAGCGATTA
ATGGCGGCGTCATGTTTGCTAAGGAGTGGATAAAGCGCACCCTGTCGATTACCGGAGGCGCCCTGGGCGGGCT
GGTCGTCCGCGCAATAGAGCGTCACCTGCCAGCCGCGACGCAGTAACGCCAGTGATAACAGCGCGCTGGCGAT
GCCGCCGCCAATGATCGCCGTTTCACGTTTTTCGCTTCCGCTGCGGTAAAACCAGGGCGCGGAAAGGGTCGGC
ATGAGGTGTTGTTCCATCACTCCACAAAGCATCTCGCGTTTACGTCCGAAGCCTTTGCGTTTTTGCATGGTAA
AACCGGCTTCCTGTAATCCCCGACGAACGAAGCCCGCCGAGGTAAACGTCGCCAGCGTTGCGCCGGGCCGCGC
CAGCCGCGCCATCGCGTTAAACAGGTTTGGCGTCCACATATCCGGATTTTTCGCCGGGGCAAAACCGTCGAGG
AACCAGGCGTCGACTGTTTGATTCAGCGTGGCGTCCAGTTGGTCGGTCAGCTCATTAATATCGCCAAACCACA
AATCCAGCGTCACCCGACCCCGATCCAGCAATAGGCGGTGGCACCCCGGGAGCGGCAGCGGCCATTGCGCCTG
CAGCTGCTCTGCCCAGGGCGCGAGCTCCGGCCAGTGTTGGTGCGCCAGCGCCAGATCGTCGCGCGTTAACGGG
AATTTCTCAAAACTGATGAAATGTAACCTTTGCAGCGTCGCCTGTGGGTGCGCAGAACGAAAGCTGTCAAACG
CCTGCCACAGCGTCAGGAAGTTGAGTCCGGTGCCAAAACCGCTTTCCGCGACTATAAATAATGGATGTGAATG
TACGGGAAATCGCTCCGCAAGACGATTGCCGCCAAGAAAAACGTAGCGTGTCTCTTCGAGTCCATTGTCATTA
GAAAAATAGACATCATCAAAATCTCGGGAAACAGGTGTACCCTCAGCGTTAAATTCGAGTGTGGCGGGTTGTA
TGGCGTATTGTTTCACGTAAGTTACTCGTCTGACGGGCGGTATCGCGATCTTAACGATGTGTGCCTGATGGCG
CAAATTTCCGTACTAAATGGCTGATCGGACTTGTTCGGCGTACAAGTGTACGCTATTGTGCCAATCGAAACTT

AAAAATAGTGCGAATTACAGAGGTATTGAATGAAACGTGCAGTGATTACTGGCCTGGGCATCGTTTCCAGCAT
CGGTAATAACCAGCAGGAAGTCCTGGCATCTCTGCGTGAAGGACGTTCAGGGATCACTTTCTCTCAGGAGCTG
AAGGACGCCGGCATGCGTAGCCAGGTATGGGGCAACGTAAAACTGGATACCACTGGCCTCATTGACCGCAAAG
TCGTGCGCTTTATGAGCGACGCCTCCATCTATGCTTATCTGTCTATGGAGCAGGCAGTGGCCGACGCCGGTCT
GGCGCCGGAAGTCTATCAGAATAACCCGCGTGTCGGCCTGATTGCAGGTTCCGGCGGCGGCTCCCCGAAATTC
CAGGTCTTTGGCGCAGATGCCATGCGTAGCCCGCGTGGTCTGAAAGCCGTGGGCCCGTATGTGGTGACGAAAG
CAATGGCATCCGGCGTTTCCGCCTGCCTCGCTACGCCGTTTAAAATTTATGGCGTGAACTACTCCATTAGTTC
CGCTTGCGCGACCTCCGCACACTGTATCGGCAATGCGGTTGAACAGATCCAACTGGGCAAACAGGACATCGTT
TTTGCTGGCGGCGGCGAAGAGCTGTGCTGGGAAATGGCCTGTGAGTTCGATGCGATGGGCGCACTGTCCACCA
AATATAACGATACGCCTGAAAAAGCGTCCCGTACCTATGATGCGCACCGTGATGGGTTTGTCATCGCGGGCGG
TGGCGGTATGGTTGTTGTCGAAGAGCTGGAACACGCTCTGGCGCGCGGCGCGCATATCTATGCGGAAATCGTC
GGTTACGGCGCAACATCCGACGGCGCAGACATGGTTGCTCCGTCTGGTGAAGGCGCAGTACGTTGCATGCAGA
TGGCGATGCACGGCGTAGACACGCCGATCGACTACCTGAACTCTCACGGTACGTCCACGCCGGTAGGCGACGT
GAAAGAACTCGGTGCAATCCGTGAAGTGTTTGGCGATAACAGCCCGGCTATCTCCGCGACCAAAGCGATGACC
GGCCACTCGCTGGGTGCGGCTGGCGTGCAGGAAGCCATCTACTCTCTGCTGATGCTGGAGCACGGTTTTATCG
CGCCGAGTATCAACATCGAAGAGCTGGATGAGCAGGCTGCGGGTCTGAACATCGTGACCGAAACGACCGAGCG
TGAACTGACTACCGTAATGTCTAACAGCTTCGGTTTTGGCGGCACCAACGCCACGCTGGTGATGCGTAAGTTG
TAAGCCTGACTATTACGTTGTAGTATCAATAATGAAACAGGTCGCTAATGCGACCTGTTTTTTCATTCAGGGA
ACAACAAATTATATGGATATTATATAAATGCCAACTAATACATTAGATAAAATTAGGCATAGTTTGAGTTGTG
TGGCTGTTTTATTTGGTTTGTTCGGCATTTTTGTTTTTGCCTCATTTTCGCCTTCCTATGCCTGGCTTTATTT
GGGAGGTCTGGCTGCGCCTTTTATATATAGTATTGTTTTTGTCTATGCGATTGCCGCCTGGAGTATTTATTCA
AAGTATTACCCGTTTTTATCATTGGGTCGCTTATCATTCGTGGAGTGTTTTGTTCCGGCGCTGGCCCTGGTGT
GCCTTACCGTTCTCTACAATGTTTTTAGTGGCCCGGAACCCTGGATGGCCGAGTTATCAAGGCAATTTTTTCT
TCATAAATTTCTTAACACGCTGGCGATGTGCTTTCTGGCTCCTGTGGCGGAGGAAATTATATTCAGAGGGTTT
TTATTAAACAGCAGTATCGGTTGGGGTCGATACTCAAGAGCTTCCGGTATTATTATTACTTCACTCGCTTTTG
CATTTATGCATACACAATATCTCTTTGCGGTGACTTTTGTTTATCTGTTTGTTTTTTCATCGATATTATGCGT
TGTTCGAATGAGAAGTCGAGGGTTAATGATACCGATAATACTTCATATTTTAAATAATGCCTGGGTTATTTTC
GGCTTACTGTTTTCAGCGACGGAATAAGCGCCTGATGCAAGAGGCGCTTTAACCTCTCCGGTACGAACAAGAG
ACCGATGGCGGTTCCCTTTTTTAATGCTTCTTATATTCCGGCGCGACTTTCGCTGACCAGGCCTCAGCCTGGG
TGATGGCGCCTGCGTCATAAATCGGCTGTTCATAACCCCTGTCAGCATCCCGAACGCGGTAAATTTTCCAGCG
TCCCTCTTCCCAACGGGTATAAACTTCCAGGTGGATGGGCGTTGACTCTGTAGCCAACAGAACATCCACTTTT
TCTCCGCCGAGGAAAGGGTGTGCCTTCCCAACCCGTAACTGTGGAATCCACTCTGGCGCATAGTCCTGCGCAT
ACATAAAATAATCTGCCCCCACAATCTCCTGTTCATAAAGGGACTGTATTAGCGCCAGGCGATGAATAACCTC
TTTGGCAACGTAACGTTGCATTAAGGCGGTGGTGTCGTCAGGGGGATTCACATCGTTCGTGAACGTCGTCATC
CATGAGGTATAGAACTGCTTCACGGCCTGCGTACTGTCGTGATGAGGCGTGGAGCAACTACAAAGCAACAGGA
TAAGCGCGACAAGGGAGAACGTTTTCATTAATTCTTCCTGTAGATGGTATATGAGGGCCTGGCTGCCCGGTAG
CCCGGGCCGGCCCACATATCGCGTTGTTTAAAATCTGAATACCACTCTGTGCCATCGTATATCGCCATGTGAC
CGCTCGGGTTTCCGCCAGCGTAAGGTTGAATAATGATGACGTCACCCTCGCGCGGCATTTCATAGGTACCAAT
AGCGGTGAATCCGGCGCTTCTTAACATTGGGCCATAATCTTTCGCATGATACGTATGCCCCAGCGTGATACCG
CCCGCCTGAATTGCCTTTCGGGTAAATTCCGCACACCGGCCTTGAGAATGAGAACCTGCATGCGTTTTGGCAT
AATTAACCGCCACTCTTTTATCCCAACTCATAATGATTCCTGTTAATAAAATACGTAATAGCAATGACGTAAT
GCTTTAACAGTATTTGATTCGGGAAGCATGACAGATAGTGCTATTACTATTTTCAATAGAATAAGTTTAAATA
TTTATTAATTGTTGAGATTGATAAGTTAGGGCTAATTTATTCAGCATTAACTATTATTCTGCGATATCGGCAT
CATTTTTTATTTGCATATCATCATAGCGCGACGGCCAGATCTCCTGCGGCGTGATGCCCAGCGCGTCCGCAAT
AATCCGTTCAGCTCTCGGCCAGGAACGGGTAAAGACATTGCGCAGTGAATCAGGTGATAAACCGGCCTGACGA
GAAAGATCTCTGAGCGTAATACCTCTTTTTTCAAGTGCACTTTTAATATCTGCACGATGCCAGTCTTGGCTTT
TTGGTTCCATTCTGTAATCCTAAGAAGATGTGTTATCGGCACCGATATTTTTAAGGTAAAAATTAGCGCGAGA
AAAAGAAAGTTACTTTCTTTTTCACTCTGAGAATAGTGCCAGAAAGAAAGCAAAATTTCGCGAAAAAGAAGAA
ATATTTTTATTTCATCAGGGGAAAGGAAAGAAACCGATGGCAAAGGAATGGTTCACGGTTAAGGAATGTCTTG
GCTTACCGGGGTTTCCGGGAAGCGAGCCTGCGGTTCGCGAACGTTTATATAAGTACAGTGAAGGGAAAGCGGG
GGTCAGGCGTAAGCGTGTTAAATCAAAGGCCGAGGAGTTTCATATATCTGTTTTCCCTCTCTATGTTCATCGT
TATCTGGACGACAGCGGAGAGGAACCGACGCCAGAACCCATTTCCTTACAGGAAGCGGAGCCTGAAGATATCT
GGGAGATGATGTTCAGGTTACTGACGCCAGAACAGCGCAAGCAGGTGACCGGACGGTTTAAGGTGAGAGGGAT
GAAAGCGGTCTTTCCCTTTCTGTTTGATGACACGCCGCCACGTTGATACCCTGGCTGCAATTTCAAGGTTGAC

AAGTTTCGCGTCTCACAGGCAGACTTCACTCTCTACATTATCCTTCCGATAATGCGTAAGCGTTTAACGTTAT
CCAACGCACCTTAATCCTGACGTTCAGGTAGAGGGGGGCGTGGCCTTTCCCCGCCTTACTCTGCATTCTGGAG
TAATGCATGACAGCAGTTAGCCAAAAAACAACGACCCCGTCTGCGAATTTTTCGCTGTTCCGCATCGCCTTTG
CGGTTTTCCTGACCTATATGACGGTCGGCCTGCCGTTACCCGTCATCCCGCTTTTTGTTCATCACGAACTGGG
CTATAGCAATACGATGGTTGGGATCGCCGTGGGCATTCAGTTTTTTGCGACGGTACTGACGCGCGGCTATGCC
GGACGACTGGCGGATCAATATGGCGCTAAACGATCGGCGTTACAGGGGATGTTTGCCTGCGGGCTGGCGGGCG
CCGCCTGGCTCCTGGCCGCGCTGTTGCCGGTTTCCGCGCCGATAAAATTCGCGCTGCTTATTGTCGGGCGTTT
GATCCTCGGATTTGGCGAAAGCCAGCTGCTGACCGGAACCCTGACCTGGGGACTGGGGCTGGTTGGCCCAACG
CGTTCCGGTAAGGTGATGTCGTGGAACGGGATGGCGATTTACGGCGCGCTGGCGGCCGGTGCGCCGTTGGGCT
TGCTGATCCATAGCCACGGCTTCGCGGCGCTGGCGGGGACGACGATGGTTTTACCGCTGCTGGCCTGGGCATT
CAACGGCACCGTGCGTAAAGTGCCTGCGTATACGGGGGAGCGACCTTCACTGTGGAGCGTGGTCGGGCTTATC
TGGAAACCGGGCCTGGGCCTGGCATTGCAGGGCGTGGGCTTTGCCGTCATCGGTACGTTTATTTCACTTTATT
TTGTCAGCAATGGCTGGACGATGGCGGGATTCACCCTGACCGCGTTTGGCGGTGCGTTTGTACTGATGCGCAT
ATTGTTCGGTTGGATGCCGGACCGCTTTGGCGGCGTGAAGGTCGCCGTTGTCTCATTGCTGGTTGAGACGGCA
GGGTTGCTGCTCTTGTGGCTGGCGCCGACGGCCTGGATAGCGCTGGTAGGCGCGGCGCTCACCGGGGCAGGGT
GTTCACTGATTTTCCCGGCGCTGGGCGTGGAGGTTGTGAAACGCGTACCTGCACAGGTACGCGGTACGGCGCT
CGGCGGCTACGCCGCGTTTCAGGATATCTCCTACGGCGTTACCGGCCCGCTGGCGGGCATGCTGGCCACGTCA
TGCGGCTATCCTTCCGTATTCCTCGCGGGAGCGATCTCCGCTGTGGTTGGCATCCTGGTGACGATATTATCGT
TCCGGCGTGGTTAAGAAACCAGCCAGAAAAGCGCCAGTACAATTATTAGCGCGATAAACAACAGGCCTGGCCG
CGCAGACAGATTTTTCACGGTTTCAATCATCGGCGCAAACGGGCGGTAGATGGGTTGAATAGAGAGCGGTTCC
AGCTCATCAGTCTCCCGCTCTACGCGACGCAGGAAAATATGGTTAAGTAAATCCTGCACCTGGGCGGTGGTCA
GCACCGTTTGCGGCTGAATTTGATAAGTGTGCTGGGCATACTCTTTGATCGCTGTGAGTTCGTCCGGCTCAAG
CGGTTGTTTTAACGCAGCCTGAAGCGTATGTAGCGTAGGCGCGTGTTGTAGTGACAGCGTCTGCCGCGCCTGT
AGCCATGTCGCCAGATGGGTAAACTGCTTTGCCGGAATGAGCTCGCCGCTTTTCACGCCGGAGAGTTCCAGCA
TTGATTGCCAGATAAGTTTGTTTGACTCGCCGGTCGCGGCTGCCAGCTTCGTCACCAACTGATTCAACGTATT
ATGTTCAGCAGGCAATAATGGCCGATCCGTTGCCGGACGTTGCTGCGGCTGCGGAATGGAAAGCTGCCCCTGT
TGCAGGAGCGTCAGCACGTTTTTGAGTTGATCGGGCGTAAGCTGGCTAAGCGCGGTTTGACCGTATTGTTGAC
GGATAAAATCGCTGACGGCCTGACGATTATTCCCGACGCCAAGCAGCTCTGTTAGCTGTGAAAGCACCTGACG
ATTGGCATGATTTTGCTGCGCTACGCCCAGACGCTGGGTAAGATTCTGTTCAGCGGCGGGGAAGTGGCGCGAC
AGTAGCGGCGCGTCATTTTTTATCCCCAGATCGTGTTTCATTCCGGCCCATACTTCCGCGCTTTGCTGCTGGG
TCAGTGAAATCAGGCGCGTGATCAGCCTTTCCAGTACCGTACGCTGTTGCATGGAAAGCGGCTGCTCGCTCGC
AGCGGAAAGAGGATTACGCCCTTCGCCTGGAGGCTGTGCAGGGGCGCCGGAGATGGGATGCATAATTAATTAT
CCTTTAACCATAAATCTGAGCAATATATGCCTGGCGGCCAGATTATGGCACACTTGTCCGGTTAACTCTCGTT
CTCAAACAGGTACGACAGTCGTGAAAATTCTCGTTGATGAAAATATGCCTTACGCCCGCGAATTATTTAGCCG
CCTGGGTGAGGTAAAGGCAGTGCCGGGTCGCCCGATCCCCGTTGAAGAATTGAACCATGCCGATGCGTTAATG
GTGCGTTCGGTCACGAAAGTGAATGAATCGCTGCTGAGCGGCACGCCGATTAACTTCGTTGGTACTGCTACGG
CAGGCACCGATCATGTGGATGAGGCATGGCTGAAGCAGGCGGGCATCGGTTTTTCAGCCGCGCCCGGCTGTAA
TGCTATCGCCGTGGTGGAGTATGTGTTTTCCGCGCTGTTGATGCTGGCCGAACGTGACGGCTTTTCGTTACGC
GATCGCACTATAGGGATTGTCGGCGTGGGTAACGTCGGAAGCCGGCTACAGACCAGGCTTGAAGCGTTGGGTA
TCCGCACGTTGCTGTGCGATCCGCCACGCGCCGCACGCGGGGACGAGGGTGATTTTCGTACGCTGGATGAGCT
GGTGCAGGAAGCGGATGTCCTGACTTTCCATACGCCGCTCTATAAAGACGGACCGTACAAAACGCTGCATCTG
GCCGATGAGACGCTGATCCGTCGACTGAAACCCGGCGCCATTCTCATTAACGCCTGCCGCGGTCCGGTGGTGG
ATAACGCCGCGTTGCTGGCGCGACTTAATGCCGGGCAGCCGCTGAGCGTGGTGCTTGATGTGTGGGAGGGCGA
ACCGGATCTTAACGTCGCGCTACTGGAAGCCGTGGATATCGGCACGTCGCATATTGCCGGTTATACGCTGGAA
GGCAAAGCGCGCGGCACCACTCAGGTCTTTGAGGCGTATAGCGCGTTTATTGGCCGCGAGCAGCATGTCGCGC
TGGAGACATTACTGCCTGCGCCTGAGTTTGGTCGTATTACGCTTCATGGCCCGCTGGATCAACCGACGCTGAA
AAAGACTGGCGCATTTGGTGTATGATGTGCGCCGCGACGATGCGCCGCTGCGTAAAGTCGCTGGAATTCCGGG
TGAATTTGATAAATTGCGTAAAAATTATCTTGAGCGCCGTGAATGGTCGTCTTTATACGTGATGTGTGACGAC
GAAACGGCAGCCGCCTTGCTGTGTAAGCTAGGTTTTAACGCTGTTCATCACCCGGCACATTAATGTCTTCTTA
ATGCTCCCTGTCGAATAATGCGACAGGGACGCTTATGTATTTCTGGAGTAAATCACCATGTCTGAAGGCTGGA
ACATTGCCATTCTGGGCGCAACGGGCGCCGTAGGCGAAGCCCTGCTTGAAACACTGGCTGAACGCCAGTTCCC
GGTCGGTGAAATTTATGCGTTGGCGCGTCATGAAAGCGCGGGCGAGCACCTCCGTTTTGGTGGCAAGTCGGTC
ATTGTCCAGGATGCGGCGGACTTTGACTGGACGCAGGCGCAGTTGGCGTTTTTCGTTGCCGGCGCAGAGGCAT
CGGCTGCCTGGATAGACGACGCGACTAATGCAGGTTGCCTGGTGATAGATTCCAGCGGTCTGTTCGCGCTGGA

GCCTGATGTGCCGCTGGTCGTGCCGGAAGTTAACCCGTATGTACTGGCGGATTACCGTAACCGTAACGTGATT
GCGGTCGCCGATAGTCTGACCAGTCAACTGCTGGCGGCGCTCAAGCCGTTAATCGATCAGGGGGGACTGTCGC
GCATCGCCGTCACCAGTATGTTATCAGCGTCCGCGCAGGGGAAAAAAGCGGTCGATGCGCTGGCTGGACAGAG
TGCGAAGCTGCTTAACGGCATACCGATTGATGAAGATGATTTCTTTGGCCGCCAGTTGGCGTTCAATATGCTG
CCGCTGCTGCCGGATCGGGAAGGCAGCGTGCGCCAGGAACGTCGTATCGTCGATGAAGTGCGTAAAATTTTGC
AGGACGATGGCGTGATGATCTCCGCCAGTGTCGTGCAGTCACCGGTGTTCTATGGCCATGCGCAGATGGTGAG
CTTTGAAGCGCTCCGTCCGCTGGCGGCGGAAGAGGCGCGCGAGGCATTTTCCCGCGGTGAAGATATCGTACTG
TCAGAAGAGACGGATTACCCGACGCAGGTGGGCGACGCTTCCGGCAATCCACAGCTCTCTATTGGCTGTGTGC
ATAATGATTACGGTATGCCGGAACAAATTCAGTTCTGGTCAGTGGCGGACAACGTGCGTTTCGGCGGCGCGCT
GATGGCCGTTAAAATTGCGGAGAAGCTGGTGCAGGAGTATCTGTACTGATGTCCGGACAGCAATCATCGCCGG
TCTATAAAATTGCGCTGGGTATTGAGTACGACGGCAGCAAGTATTATGGCTGGCAGCGTCAGAATGAAGTGCG
CAGCGTGCAGGAAAAGCTGGAAAAAGCGCTCTCGCAGGTGGCAAATGAACCTATTAACGTGTTCTGCGCCGGG
CGCACCGATGCCGGCGTTCACGGCACCGGGCAGGTTGTCCATTTCGAAACGACCGCATTGCGCAAAGATGCGG
CATGGACGCTGGGGGTAAATGCGAATTTGCCTGGTGACATTGCCGTGCGCTGGGTGAAAACTGTACCGGATGA
TTTTCACGCGCGATTTAGCGCTACGGCTCGCCGTTATCGCTATATCATCTACAATCATCGACTGCGTCCTGCG
GTATTAGCAAAAGGCGTGACGCATTACTATGAGCCGCTGGATGCAGAGCGGATGCATCGCGCGGCGCAGTGTC
TGCTTGGCGAAAATGATTTTACCTCGTTTCGCGCGGTGCAGTGCCAGTCGCGCACGCCGTGGCGTAATGTGAT
GCACATCAACGTTACCCGCCACGGCCCTTATGTGGTGGTCGATATCAAAGCGAATGCCTTTGTTCATCATATG
GTCAGGAATATTGTCGGCAGCCTGCTGGAAGTAGGCGCTCACAACCAGCCGGAGAGCTGGATCGCAGAGCTGT
TAGCGGCCAGGGACAGAACGCTGGCGGCGGCAACGGCGAAGGCGGAGGGGCTATATTTGGTTGCGGTGGATTA
CCCTGACCGGTTTGACCTTCCAAAACCGCCAATGGGCCCGCTATTTCTGGCGGACTAATCCGAATCGTACACC
CGAAACGTGTCGGGTAGAAAGGCAAAGACAAATATGGACCTGATCTACTTTATTATTGATTTTATTCTGCACA
TCGATGTGCATCTGGCGGAGCTGGTCGCGGAGTACGGCGTCTGGGTGTACGCTATTTTGTTTTTGATTCTGTT
TTGTGAAACCGGCCTGGTCGTCACGCCGTTCCTGCCGGGTGACTCGCTGCTGTTTGTCGCCGGGGCGCTGGCT
TCGCTGGAAACCAACGATCTCAATGTGCATGTCATGGTGGCGTTAATGCTGATCGCCGCAATTGTCGGCGATG
CGGTAAATTACACGATTGGTCGACTGTTCGGCGAAAAACTGTTCAGTAATCCGAATTCGAAAATTTTCCGCCG
CAGTTATCTGGATAAAACCCACCAGTTTTATGAAAAACACGGCGGTAAAACTATCATTCTCGCTCGCTTCGTG
CCGATCGTCAGAACCTTTGCGCCGTTTGTGGCAGGGATGGGGCACATGTCCTATCGGCATTTTGCCGCCTATA
ACGTCATCGGCGCGCTGCTGTGGGTGCTGCTCTTTACCTACGCCGGTTATTTCTTCGGTACGATACCGTTGAT
TCAGGATAACCTTAAGCTGCTGATTGTGGGGATTATCGTGGTTTCGATATTACCTGGCGTCATTGAGATTGTC
CGGCATAAGCGCGCTGCGTCGCGTGCCGCAAAATAGAAAGTAACTCAGCGGTTCGACCACTTTTTTATCCAAA
GTTTCGGGCTGTTATGTTTTAATGTGCAACATTCATGGTCTGTTGGGGGCAAAAATGGCATTATGCGCCCCTT
ATAATAAAGCTGACTAAGGTTCAGGCAGAAAGGTCACCAATGAGCTGGATTGAACGAATTAAAAGCAACATTA
CTCCCACCCGCAAGGCTAGCATTCCTGAAGGGGTGTGGACCAAGTGTGATAGCTGCGGTCAGGTTTTATACCG
CGCTGAGCTGGAACGTAATCTTGAGGTCTGTCCTAAGTGTGACCATCATATGCGCATGTCGGCGCGCAATCGC
CTGCATAGCCTGTTAGATGAAGGTTCCCTTGTGGAGTTGGGTAGCGAGCTGGAGCCAAAAGACGTGCTCAAGT
TCCGCGACTCCAAAAAATATAAAGACAGACTGGCGTCCGCGCAGAAAGAAACCGGCGAGAAAGACGCGCTGGT
GGTCATGAAAGGGACGCTTCACGGTATGCCGGTTGTCGCCGCCGCGTTTGAATTCGCGTTCATGGGCGGCTCA
ATGGGGTCTGTCGTTGGCGCACGCTTCGTTCGCGCCGTTGAACAGGCGCTGGAAGACAACTGTCCGTTAGTGT
GTTTCTCTGCTTCCGGCGGCGCGCGTATGCAAGAAGCGCTGATGTCGCTGATGCAGATGGCGAAAACCTCGGC
GGCGCTGGCTAAAATGCAGGAACGTGGTCTGCCCTACATCTCGGTATTGACCGATCCGACAATGGGCGGCGTT
TCCGCCAGTTTTGCGATGTTGGGCGATCTCAACATCGCCGAGCCAAAAGCCCTCATTGGCTTCGCTGGCCCGC
GCGTTATCGAACAAACCGTTCGTGAGAAGCTCCCGCCGGGATTCCAGCGCAGTGAGTTCCTGATCGAAAAAGG
CGCTATTGATATGATAGTTCGCCGCCCGGAAATGCGCCTGAAGCTGGCGAGCATTCTGGCGAAGCTGATGAAT
CTGCCCGCGCCGAACCCGGACGCCCCGCGTGAAGGCGTGGTGGTGCCTCCGGCGCCGGATCAGGAGTCTGAGG
CCTGATAACTGAAAAGGGCAGGGCCGCAGGCTCTGCCCTTTTGCTTTTCACCGTAAAGAAGCAGCGGAATCAA
TGAAAAACAAATCTATACCTCAAGCTGCGTCGCCCCTGGCCTCGTGGCTTTCTTATCTGGAAAACCTGCACAG
CAAGAGTATCGATTTAGGCCTTGAGCGCGTAAGCCAGGTGGCAGCGCGTCTGGACATCCTCAAGCCTGCGCCG
TTCGTTTTTACCGTGGCGGGAACCAACGGTAAAGGTACGACCTGTCGAACGCTGGAGTCGGTGTTAATCGCGG
CGGGATACCGTGTGGGCGTCTATAGTTCGCCTCATCTGGTACGTTACACCGAGCGCGTGCGCGTGCAGGGGAA
AGAATTAGCGGAATCCGCGCATACCGCGTCCTTTGCGGAAATAGAAGCGGCGCGCGGCGATATTTCATTGACC
TATTTTGAATATGGTACGTTGTCGGCATTGTGGCTGTTTAAACAGGCTAACCTTGACGTGGTGATTCTGGAGG
TCGGGTTGGGCGGCCGCCTGGACGCCACGAACATTGTGGATGCCGATGTAGCGGTAATTACCAGCATCGCGCT
TGACCATACTGACTGGCTGGGGCCGGATCGCGAAAGTATCGGCCGCGAGAAGGCCGGGATTTTTCGCGCGGAA

AAACCCGCTATCGTCGGCGAACCGGAAATGCCGACAACCATCGCGGATGTCGCGCAGGAGACCGGCGCGCTGT
TGCGGCGTCGCGGCGTTGACTGGCGTTATGAGGTGACAGCGACCCACTGGGCGTTTACCGATGGTGACGGCAC
GCTTGCCGGACTGCCGTTGCCGCAGGTTCCTCAGCCGAATGCCGCCACCGCTCTGGCCGCGCTGCGCGCCAGC
AGGTTGAACATTGACGAGCAGGCGATTCGCGACGGTATTGCGCAGGCTACCTTGCCGGGACGTTTTCAAATCG
TGAGTGAGTCGCCGCGCGTTATCTTTGATGTCGCCCATAATCCCCACGCGGCGGAATACCTGACGGGTCGTCT
GAAAATGTTGCCGAAACGCGGGCGCGTGCTTGCGGTGATTGGTATGCTGCATGATAAAGATATCGCCGGCACG
TTAGCCTGGTTGAAAAGCGTGGTTGACGACTGGTATTGTGCTCCGTTAGAGGGGCCGCGCGGCGCGACGGCGG
AACAACTTCTGGAACATCTGGGGAAAGGGAATGTCTATGATAGCGTCGCGCAGGCATGGCAGGCCGCTATCGA
CGCGGCGCAGCCAGAAGATACCGTGCTGGTGTGCGGATCATTCCACACCGTCGCACATGTTATGGAAGTGATA
GACGCGGGGAGAATCGGTGGCGAGTAAGTTTCAGAATCGTTTAGTCGGCACGATTGTGTTAGTCGCGCTGGGG
GTTATTGTGCTTCCCGGTCTGCTGGACGGACAGAAAAAGCATTATCAGGATGAATTTGCTGCGATTCCACTGG
TACCGAAGCCTGGCGACCGTGATGAGCCGGATATGATGCCTGCCGCAACGCAGGCGCTGCCGACACAGCCGCC
GGAAGGGGCGGCTGAAGAGGTGCGGGCGGGAGATGCGGCAGCGCCATCGCTGGACCCGTCGCGCATGGCGTCT
AACAATGTTGAGCTTGATCCGATACCGGCGGAGACGCCTAAACCGAAGCCGGTTGAAAAGCCGAAACCGCAGC
CTAAACCGCAACAGCCGGTTGTCGCCGCTTCCACGCCGACGCCCGCGCCGCAACCAGTCGCCGACGATAAACC
TGCGCCGACCGGCAAAGCCTATGTCGTACAGTTGGGCGCGCTGAAAAACGCGGATAAAGTCAACGAGATAGTC
GGCAAGCTTCGTTCCGCCGGGTTCCGGGTTTATACTTCCCCCTCAACGCCGGTACAGGGTAAAATAACCCGTA
TTCTCGTCGGGCCGGATGCGTCGAAAGATAAGATGAAAGGATCGCTTGGCGAGCTCAAGCAGATTTCCGGGTT
AAGCGGCGTGGTGATGGGATATAGCCCGAACTAAGGCGCGTTTTGCCTGAGCGCGCAGCGTTAAGGCCTACGG
TGTTCATCTGGTAATAGCCGAAGTTCTGTAGGCTGGATAAGGCGTTTACGCCGTCATCCGGCAGAAGGAATAA
ACCTGGTATCCTGCGCTTATCAGGCCGATGGCAGCTCTTCTGGTCGTGCCCGAAGAGATAAAAAGTGTGATGG
CGTTGAATATTTTTTCAGCGCCATTTTTATTTACGCGTGGGAAGGAAATCCCTACGCAAACGTTTTCTTTTTC
TGTTAGAATGCGCCCCGAACAGGACGACAGGGCGTAAAATCGTGGGACATCTATGGTCTGGATTGATTACGCC
ATTATCGCGGTGATTGCTTTTTCCTGTCTGGTTAGCCTGATCCGCGGCTTTGTTCGTGAAGCGTTATCGCTGG
TGACGTGGGGTTGTGCTTTCTTTGTCGCCAGTCACTACTACACTTACCTGTCTGTCTGGTTTACGGGCTTTGA
AGACGAACTGGTTCGTAATGGGATTGCTATCGCGGTACTGTTTATCGCGACGCTTATCGTCGGCGCTATCGTG
AATTTTGTGATAGGTCAGCTAGTAGAGAAGACCGGCCTGTCGGGAACTGACCGGGTGTTGGGGATCTGTTTCG
GCGCACTGCGAGGCGCGCTGATTGTCGCCGCCATTCTGTTCTTCCTTGATACCTTTACCGGCTTGTCGAAAAG
CGAAGACTGGAGCAAATCGCAGCTCATCCCGCAGTTCAGCTTCATCATCAGATGGTTCTTTGACTATCTGCAA
AGCTCGTCGAGTTTCTTGCCCAGGACGTAACAGGTTCTGAGATGTGGCTTAACGAGGAAAATGACGTATGTGC
GGTATTGTCGGTATCGCCGGTGTTATGCCGGTCAACCAGTCGATTTATGACGCGTTAACAGTGCTCCAGCATC
GTGGTCAGGATGCTGCCGGTATCATCACCATTGATGCGAATAACTGCTTCCGCTTGCGTAAGGCAAACGGGCT
GGTGAACGATATTTTTGAAGCTCGCCACATGCAGCGTTTGCAGGGCAACATGGGGATCGGCCATGTGCGCTAC
CCTACCGCAGGCAGCTCCAGCGCTTCCGAAGCGCAACCTTTTTATGTTAACTCGCCGTATGGCATCACGTTGG
CCCATAATGGCAATCTGACTAACGCGCATGAGCTACGTAAAAAGCTGTTTGAAGAAAAGCGCCGCCATATCAA
CACCACCTCCGATTCCGAAATCCTGCTCAATATTTTCGCCAGCGAGCTCGACAACTTCCGTCACTATCCGCTG
GAAGCCGACAATATTTTTGCCGCGATTGCCGCGACTAACCGTCAAATCCGCGGCGCCTATGCCTGCGTGGCGA
TGATTATCGGCCACGGCATGGTGGCTTTTCGCGATCCGCACGGCATTCGCCCGTTAGTGCTGGGCAAGCGTGA
CGTTGGCGATGGTCGTACGGAGTATATGGTCGCTTCTGAAAGCGTGGCGCTGGATACGCTGGGCTTTGAGTTT
CTGCGCGATGTCGCGCCGGGCGAAGCTATCTATATTACCGAAAAGGGGCAACTGTTCACGCGCCAGTGCGCGG
ATAACCCGGTCAGTAATCCCTGCCTGTTCGAGTACGTTTATTTTGCTCGCCCGGACTCCTTTATCGACAAAAT
CTCCGTCTACAGCGCTCGTGTGAATATGGGCACCAAACTTGGCGAAAAAATCGCTCGTGAATGGGAAGATCTG
GACATCGACGTCGTGATCCCTATCCCGGAAACCTCCTGCGATATCGCACTGGAAATTGCCCGTATTCTCGGCA
AGCCGTACCGCCAGGGATTTGTCAAAAATCGTTACGTGGGACGTACCTTTATCATGCCGGGCCAACAGCTACG
CCGTAAATCCGTGCGCCGTAAGCTCAATGCTAATCGCGCGGAATTCCGCGATAAGAACGTCCTGCTGGTGGAT
GATTCCATCGTACGTGGCACCACATCAGAGCAGATCATCGAGATGGCCCGGGAAGCCGGCGCGAAGAAGGTTT
ATCTGGCGTCCGCCGCGCCGGAAATTCGTTTCCCCAACGTCTATGGCATTGATATGCCGACCGCCAATGAGCT
GATCGCGCATGGTCGCGAGGTGGATGAGATTCGGCAGATCATCGGCGCAGACGGGCTTATCTTCCAGGATCTG
AACGATCTGATCGAAGCCGTACGCGCGGAAAACCCGGATATCCAGCAGTTTGAATGTTCGGTATTTAACGGGG
TATATGTCACCAAAGACGTAGACCAGCAATACCTCGACTTCCTGGATTCATTACGTAATGACGACGCCAAAGC
GGTGCTGTTCCAGAACGAAATGGAAAACCTGGAAATGCATAACGAAGGCTAATGGCTTTGCCAGCGGCGCAAT
GCCGCTGGCCGATGTCAATTACGCACCACGCCGAAGCGAATAGCGTATTTTTGTACTTTGTACTGTAACGTGG
TGCGCGATACGTTAAGGCTGCGGGCCGCGGCGGCAATATTCCCCTGATGCGTATCCAGCGCGGTTTCTATTAA
ATGCCTTTCGTAGTTAGCAACCCGTACCTCCAGCGACCCTTCATACTGCGGATCGGGTGACGAGGGAAGGGGA

TTTTCCGGCGCGGTTTCCGGTACGCCTAAATTTAACTCGTCCTGTTCAAAAATGATGTGTTTCAGCAGCCCGT
CTTTTTCCTGCATGATCATGCTGCGTACAATCGCGTTTTCCAGCATCCTGACATTACCCGGCCAGGCGTGATT
GAGCAGATCAGCACGCGCCGTCTCGCTTAGTCCGTGAATGTCCTGCGGCACATCATTACGGTATTTATCAATA
AAATAATTTGCCAGTAAGGGAATATCTTCCGGGCGGGCGCGCAGCGGCGGTAACGTCAACATTCCGACGCTCA
ACCGATAAAAAAGATCCGCCCGCAGGCGTTCTTGTTGAATTAATTTGACGGGCGCTTCGTTCATGGCGGCAAC
GATCCTGACATCAGAGTGGAGTTGCTGTTGTCCGCCGAGCCGCCAGAAGGTTTTATCCTGCAAGAATCGCAGC
AGCTTACTTTGCATTTCTATCGGCATGGCGTTCAGCTCATCAAGAAAGAGCGTTCCCCCGTTTGCCAGTTCCA
GATAGCCCTGGCTATTTTCCGCGCCGGTATAGGCGCCGCGCACGGTGCCGAAAAGCGTACTTTCGATAAGCGT
AGGCGGTAGAGCGCCGCAGTTCAGGGCGATAAATGGCTTATTCGCCCGCTTGCTGCATTGATGTATCAGGCGG
GAAAATAGCTCCTTACCCGTACCCGTCTCCCCGACAATCGTCACCGGAATATTACTGGCGGCCAGCCGTTTGG
CTTTGGCGATATTACTGAGCATTTCCGGGTTTTCGGTAATAATGGCATGGTGCTTCTCATGGCGATCGGCATA
CAGCAGTTGGTTCAGTTCTACCACCTGCTCCTGGAGCCGCCTGACGCCGGACATATTCCTGCCGATTTCGATA
ACGCCGACCATGCCGCCATCGCTTGCATAGAGCGGTGCGGTGGTGTGCTGATAGTCAACGGCCTGGCCTCGCG
CATTGTAATAAATTTGATAATGGCCAATGTATTCCACGCCTTTTTTAACGATGAAAGCATCGTACTTTGCGT
CTCTTTCATGCCCGGATAGACATCCAGCATATGTTTTCCCATTGCCCGTTCAATGCTGTAGCCGTCCAGATCC
GCGCTTTCCTGATTGTAGTAAACGTATTCACCACGCTCATTAATGACGGCCAACGGCTGATGAATCAAATCGA
AAAAGCGGGAAAACATCCTGAGCGCCGAGGCCAGTTCCTGATTGGTGGATGCCATACATGCATTCCTCATATC
TATTGTTCATGTTGCCATTATAAAAGTGACGAATTTTCGTCGTTGTGATGAATAGCAAACTCAATGACGAAAA
TGAGAAATAGCTTATTTTTATAAGTAATTGATTATTTTGATAAAAAATAGTGGCATGCCTTTTGCTTTATCCC
TGTAAAGAATGCGTATTTTTACCATAAACATTAAGCAGAGGGCGTTATGACTGACTCGATTATGCAGAATTAC
AACCAGCTACGTGAGCAGGTAATCAACGGCGATCGCCGTTTCCAGCACAAAGACGGTCATTTGTGTTTTGAAG
GGGTCGATCTGGACGCACTGGCGCGCCAGTACCCCACGCCGTTCTATGTGTTTTCTGAGCCGGAGATTATTCG
TAACATTCATGAAATCCAGCAGGCATTTGCGGCGCATAAAAATACCAAAACCTTCTTTGCGTCAAAAACCTGC
TCGGTCATGGGGGTATTGAAAGCCATTCGTGACGCCGGAATTTGCGCGGAGGCAAACTCACAATATGAGGTTC
GCAAGTGTCTGGAGATAGGCTTTCGCGGAGATCAGATCGTCTTCAACGGCGTGGTGAAAAAGCCTGCCGATCT
GGAATATGCCATCGCTAACGATCTGTATCTTATTAATGTTGATAGCCTGTATGAGCTGGAACATATTGATGCC
ATCTCTCGCAAGCTGAAGAAAGTCGCCAATGTTTGCGTCCGCGTAGAACCGAATGTTCCCTCGGCGACGCATG
CGGAACTGGTTACCGCTTTCCATGCGAAGTCGGGTCTCGACCTGGAACAGGCGGAAGAAACCTGCCGCCGCAT
TCTGGCGATGCCTTATGTGCATTTACGCGGCTTGCATATGCATGTAGGCGATCAGGTGCCGGAGTCAGAGCCT
TTCGCAAAAGCGACTAAAGTGCTGGTGGATGAATCCCGTCGTCTGGAAGAGGTGCTCGGTATTAAATTTGATT
TGATTAACGTGGGCGGCGGAATTCCGGTTCCCTATAAATATGACGACGAGAATGGCGACCCGTTAAAAGATAA
TATGTATGCCGGTATTACCGCCCAGGATTTTGCCGATGCGGTCATTCGCGAAGTGCATAAATGGCGTACCGAT
GTGGAAATATGTATTGAACCGGGACGTAAAGTGACCGGGTCGGCGGCGGTTTTACTGACGGAAGTCTCCTGCG
AAAAACGTAAAACAAATTACGATCTTAACGGCAATGTGGAATGCCACGTTGAATGGAAATTTGTCGACGCTGG
TTACAGCGTACTCTCTGACAGCCAGCATTTTGACTGGTTCTTTTATGTCTATAACGCCTCTCGCATGACGGCA
GCGCACGACGCCTGGGTAAAATTAGCCGGGCCGCTGTGTGATGGCGGAGATTATTTCCACATGGGGGTGAAAG
GCGAAGAGTTTTTACTGCCGAAGGAGACTCATGTTGGCGATATTGTCGCGTTTCTTGATGCCGGGGCATACAC
CATTGAAAGTCAAACCGTTTATAACAACCGTCCGCGCACGGGGGTGGTGATGATCGATAAAAATGGTGAGACG
CGTCTGATTCGTCGGGAAGACAGTTACGAAGATATGGTCAAGTACGACATTTATTAATACTGTTTTGGGCTTC
AGTTTCATGCTGAAGCCCGTCGTCAGATGATAAGAAGAGGGCAGTGATGCCTGAATGCATAACAGGAGATGGC
CATGAGTAACACGAATTCTGGCTTACTTGGCATCAAAGACATTGTCTTTATGAATGTCATCGCTATTCTGAGT
TTGCGGCAAATCCCTAACGTGGCGCCTTACGGGGCATCGGCCATGTTGTTATGGGTGATCGCCGCATTCTGCT
TGTTTTTTCCTTTAGCGATGGTATGCGGCGAGCTGTCAACCGGCTGGCCGAAAGATGGCGGGATATTTGTCTG
GATTAAAGAGGCATTTGGTAAACGTATTGCATGGATTGTCGTGGTTTGTTTTCTCTTTTCCTGCGTACTGTTT
TTCCCATTAATGCTGCAATTCGGCTTTACCGCGTTGGGCTATATGATCGGCGGCGGCCTGGCGGAAAATAAAG
CGTTTATCGGTATTGGTTCCGCGGTAATTTTCTGGCTATTAACGCTGATGAATATCCGCGGGATGGAATGGAC
AAAAATTATTAATAGCATCAGCGCCTGGTGCGGCGTATTTATTCCCTCCGCCATCCTCATTTTGCTGGCGGTA
GTATGGCTATGTACCGGACACCAGATGCAGACGGACTACACTACAGCGAAAAACTGGATTCCGGATCTCGGAC
ACTGGGATACGATCGTTTTCTTATCCAGCATGATGTTTGCGTTTGCCGGACTGGAGGTCGCCCCGATGATTGC
CGGGCGTACCCGTAATCCACAGCGTGATTTTCCGCGAGCGATGGCCGTCTCCGCCGCCGTTATCGTCGGGATC
TATATGGTCGGCACCTGGGCGCTAAATACGCTTTTACCCGCCGGGAAAACCGACATTGTGGCAGGCGTCATGC
AGGCTATGCATGCTGCTGCGGATACGCTGCATATGCCGTGGTTAATTCCTGTTATGGCCATCTGTATGTTTTT
TGGCGCTCTCGGGCAGATTAACTCCTGGCTGGTGGGGCCGATTTATATGCTCCAGGAAGCCTCGCGTGAAGAT
AATCTGCTGGGGGACCGAATCGGCAAATTACATCCGGTGTGGAAAACGCCCGCCTTTGCCTTGACTGTACAGG

CGATTATTGTCACGGTCCTGTGTTTTTCGACTTTTATTTCGCCAAGCGTTGCGGCGGCTTACTGGATGTTAAC
GGCGCTAACGACCATCACCTATTTTATTCCTTACCTGGTGATGTTCCCGGCGTTCTGGCGTTTACGTAAGACT
CAGCCGGATACGCCAAGAAGTTTTAAAATTCCGGGCAAGGTTTTACCGGCTATTTTACCCGCGCTGGGCTTTC
TTTCTATCGCCTTTGCCGTTGCGCTGTTATTTATTCCGCCTTCTCAAATTGATATGGGCGGATATTTCCAGTA
CGCCGGGAAAATTATTGGCGGAGCCGTGCTGGCGGTTGTCGTCGCGGAGTACATTTACCATCGGGCGCAGAAA
CGTAATGCCCGTTTAAGCATGGCGGGAGGAAAATAAAATGTATATGCCAGTTCTGGAAATAAATCTACGCAAG
CTGGAAGAAAACGCCCGCACGGAAAAAGCTCTGCTGGCAAGCAGCGGTATTGACGTGATGGCTGTCAATAAAG
TTTTTGACGGCTGCGTGGAAACCGCGCAGGCGGTGTTCAACGGCGGAATTACCGTTATCGCTGAATCACGAAC
CTATAATTTGAAAAAAATACGCGAGACAGGATGTACGACCTGCCTGTTACGCAGCCCGTGTCTGAGTGAAATC
GAAGATGTGGTGCGCTACGCGGATATTAGTTTAAATAGCGAGCCGGTGGTATTACGGGCATTATCGCAAGAAG
CCCAACGCCAGGGGAAAACGCATCAGGTATTACTGATGGTGGATATGGGCGATTTACGCGAGGGTATCTGGTT
TGGTGAATATCAACGCATTCTTGAGACCGTGACTCTGATTACTGGTCTGCCCGGTCTGGAATTATATGGACTG
GGCACCAACTTTAATTGTTACGGCACCGTGTTGCCAACGGTAAAAAATGGCGAGGATTTTCTGGCGCTGGCCG
CCCGGCTGGAAGCTGACAGCGGTATTCCTGTCCGGCGGCTTTCTGCGGGAAACTGCACCAGCTATCACCTGCT
GGATAAAGGTATCTGGCCGCAGGGACTGAATCATCTGCGCATCGGCGGACTGCATGAATTCGGCATTGAATAT
GTGGACATGAAATACCTGAATGAATTTCACCACTCCGCAAAACCGGTCGATAAAGCCTGTTCCGATATGTATA
TCCTGGAAGCGGAAATCATCGAACTGAACAGTAAACCTACCGTCCCGGTCGGGGAGCTGGGCGTCGATGCCTT
TTTGCAAAGTAAAACCTTTGTCGATCGTGGCATACGCCGACGCGCATTGCTGGCGTTTGGTCGCCAGGATGTG
CCGTCGGATAACTGTGTGCCATGCGACGACGCTATCACTATCTTAGGACAAACCAGCGATCATACGCTGGTGG
ATATTGAGGATTGTCGTCAGTCGCTAAAAGTAGGCGATGTGGTTCGCTTTGAGCTGGATTATACGGGCCTGTT
AATGGCCTGCCAGACCAAAGGCGTCGCCTGGAGGTTTACGCGTTAACGCATAACCGCCGCGGCTATGTCGCGG
CGGCATCTTTGACCTCACGAAAGACAGGGAGTAACGATGGATACCCTGCAACAATCTCCGCCAGCAGAGCCTG
AAGGCGGTCTGCGGCGTAACCTGAAGAAGCGGCACCTGCTGATGATGTCGCTGGGCGGCACGATTGGCACCGG
CTTATTTATCGGCATTGCCGAACCGTTAAGCAGCGTCGGGCCGGCGGGAACCTTACTGGCCTATCTGTTTGCC
GGAACCATTATGCTGGCGACGATGATGTGTCTTGGCGAATTATCATGCGCTTTTCCTCACTCCGGTTCATTTC
AACATTACGCGCTGATGTTTATGCCGTCACCTTGCTGGAGCTATACCATTGGCTGGCTTTACTGGTTTAGCTG
GGTCTTTTCCTTGGCAGCCGACCTCACTGCCGCAGGGTTTATTGCTCACCAGTTTTTCCCCGCGGTTCCGGTA
TATATGTTCTGCCTGGCGATTCTGCTCATCCTGACGGCTATCAATCTCACCTCCGCAAAAAGTTTTGGCGAAT
GCGAATACTGGCTTTCGGCCATTAAGGTCTTCGCGATCGTGCTGTTTATCTGCGCGGGCGGGGTCATGATCTA
CTCGCTAATGGGCCATAGTGACTGGCACCCGACGTTAAAAATAGACGGAATGTGGTTTCCCCACGGTTGGGAG
CAAATTGTGGTCTGTATGACGATCGTGATTTACTCCTTCCAGGGGTGGAACTGGTCGGAAATGCGGCAGGGGA
GACCGAATCGCCGCACATTATTTTACCGAAAGTGATCCTGGGGATCGGTCTCAGAATCATTTTGTTTTACGGC
CTGGCGATTGCGGTGCTGGCGCTGGTCTATCCGCATGAACTGACGCCAAATGGACAAAGTCCCTTTGTATGGG
TATTCTCCCACGCGGGCATTCCGGGGGCGGATACGCTGATGACGCTGGTGATCTTTTCCGCCGCGGTTTCTGC
CGCTAACTCGGCCATTTACGCCAGTTCGCGTATGCTGTGGTCTATGGCGGGCGATCGCTTTGCGCCAGCCTGC
TTTGGTAAAACGAACGGCGGCGGCGTGCCGGTGTATGCTATTTTAATCACGGCCTTGTTGGCGTTAGTGTCGT
TGCTGACCCGCTATATTCCCGCACAGCAATTTTATTTATATCTGATCGCCAGTACCGGGCAGGTGGGTTGCCT
GGCATGGATAACCATCGGATGGTGCCAGTATCGTTTCCGACAATCGGTGCGTAATGGAACGTACGCCAGCGAT
TTGCTACGCTATCGATCGCCGCTTTTCCCCTGGACGGCCCGATTTGTCATTATCACCAATTTTGCCATTATGG
TCGGCACCTGGTTTAGTGAGCAAGGGGTAGTGATAATGTTGGTTGAGCTGGCTTTTATGATAGGGATTCTTTT
GTCATGGTATCTCTTCAGACCCACTTTGAGCCGATTACGTAACACAGTGGGATGAACGGCGCTCGTAAACGGT
AAGAGAACATGAAACGACTCATTGTAGGAATTAGCGGCGCCAGCGGAGCGATTTATGGCGTGCGCTTATTACA
AATTTTGCGCGATGTCGATAGCGTGGAAACGCATCTGGTGATGAGCCAGGCGGCCCGGCAAACGCTGGCGCTG
GAGACCCATTTTTCGCTGCGCGAGGTGCAGGCGCTGGCCGATGTGACGCACGATGCGCGCGACATTGCCGCCA
GCATTTCCTCGGGCTCTTACCCGACGGCGGGAATGGTTATCTTGCCCTGTTCGATTAAAACGCTGTCGGGGAT
TGTCCACAGTTATACTGACGGACTGCTTACCCGCGCGGCGGATGTGATATTAAAAGAGCGGCGACCTCTGGTG
CTGTGTGTGCGCGAAACGCCGCTGCATATCGGGCATTTGCGTTTAATGACCCAGGCCGCTGAGATAGGCGCGG
TGATTATGCCGCCGGTTCCGGCTTTTTATCATCTTCCTCAGACGTTAGATGATGTTATAAATCAAACGGTTAA
CCGAGTACTGGATCAGTTTGACATCCCCCTTCCGCACGATCTTTTTGTTCGCTGGCAGGGCGCATAGCGTTGT
CTGTTTAGCGTGCATTATTGTCCCTGTTGCCCTGTTTCAGGGCAATTTTGTAACCGCGATCATATCCTCAACA
TTTAATTCGTTAAAAGCCGCCGAAACGCTGCGGTTCAACCGTCATACCTGCTATCTTCAACATCAGGACAATA
TTGCAACGTTTTATTAACAAATTTAACGTCGAATCGTTTTGCTGACGTGAAAATGGCATAAGACCTGCATGAA
AAAGTCTGCAAACACACAACACCACGTAAAACATAAGAAAATGACGCCACTTGAGGGGTATGTATGAAGAAGA
CCGTTCTCGCTTTGTCTTTGCTGATAGGTCTGGGCGCGACGGCGGCCAGTTACGCCGCGCTCCCGCAAACGGT

TCGTATTGGAACAGATACCACCTACGCGCCTTTCTCATCAAAAGATGCCAAAGGCGAGTTTATTGGCTTTGAT
ATCGATCTCGGTAATGAAATGTGTAAGCGTATGCAGGTGAAATGTACCTGGGTCGCCAGCGACTTTGATGCGC
TTATTCCCTCACTAAAAGCGAAAAAAATTGATGCCATTATTTCATCGCTCTCTATTACCGATAAACGCCAGCA
AGAAATTGCGTTTTCCGACAAACTTTACGCGGCGGATTCACGCCTGATTGCGGCAAAAGGGTCTCCCATTCAG
CCGACGCTGGAATCGCTGAAAGGAAAGCATGTCGGCGTGCTGCAAGGGTCCACGCAAGAGGCTTACGCCAATG
ATAACTGGCGCACAAAAGGTGTGGATGTGGTGGCTTATGCCAACCAGGATCTTATCTATTCCGATTTAACCGC
CGGTCGTCTGGATGCCGCATTACAGGATGAAGTCGCCGCCAGCGAAGGTTTCCTGAAGCAGCCAGCGGGCAAA
GAGTATGCGTTTGCCGGTCCTTCCGTGAAGGATAAAAAATATTTTGGCGACGGGACGGGGGTTGGGCTGCGCA
AAGACGATACCGAGCTAAAAGCCGCGTTTGATAAAGCGCTGATCGAACTGCGCCAGGACGGGACTTACGACAA
AATGGCCAAAAAGTACTTCGATTTTAATGTCTACGGCGATTGAGGGAAGGGGATAACGCGCCGCGCTGGGGAA
CTATCACGGTGCAAAATAGATGTTTTGCACCTTTTTGGTGCATAAGCCGTTGCGGAACAGGCGCATAAATGCA
TACTTAATCGATTTTTATGCAAAATAAACCGGCTGACAAGGTAGAATGCTTTGCCTTGTCGGCCTGATTAATG
GCACGATAGTCGCATCGGATCTGTAAAGATCTCCTGGAAAGACAGTCTGTTGAGGATAGTTATGAAAAAACTG
GCGTTATCCCTTTCTCTGGTGCTGGCATTTTCCAGCGCTACCGCAGCATTTGCCGCTATTCCACAAAAGATTC
GTATCGGTACCGATCCTACATACGCACCGTTTGAATCCAAAAATGCACAAGGTGAATTGGTCGGCTTTGATAT
CGATCTGGCCAAAGAGCTGTGCAAACGTATCAACACACAGTGTACGTTCGTGGAAAACCCGCTGGATGCGCTG
ATTCCGTCTTTAAAAGCGAAGAAAATTGATGCCATCATGTCCTCGCTGTCCATCACTGAAAAGCGCCAGCAGG
AAATCGCGTTTACCGACAAGCTTTACGCCGCTGATTCCCGTCTGGTGGTGGCGAAGAACTCTGATATTCAGCC
GACCGTCGCGTCGCTGAAAGGTAAGCGCGTCGGCGTGCTACAGGGGACGACGCAGGAGACCTTCGGCAATGAG
CACTGGGCGCCGAAAGGGATTGAGATCGTCTCTTACCAGGGGCAGGACAACATTTATTCCGATCTGACGGCTG
GCCGTATTGATGCGGCGTTCCAGGACGAGGTCGCGGCCAGCGAAGGTTTCCTTAAACAGCCCGTCGGAAAAGA
CTATAAATTCGGCGGCCCGGCGGTGAAAGATGAGAAGTTATTCGGCGTAGGAACCGGTATGGGGCTGCGCAAA
GAGGATAACGAGCTGCGCGAAGCGCTGAACAAAGCCTTTGCTGAAATGCGTGCTGACGGTACTTACGAAAAGC
TGGCGAAAAAGTACTTTGATTTTGATGTTTATGGCGGTTAATCACCGTCGGTGCAGATAAAGCCCAAAGAATG
TTGACTGTCTGCATTGCCTGATGGCGCTGTGCGTGTCAGGCCTACGGGGAGCATCGTAGGCCGGGCAAGGCAC
AGCCGCCATCCGGCGTGATGTGAGGCAAAAATGCAAAAGAGGCTTCTTACTTCACCACACACAACAGGACAGG
CTGCATGTTGTACGGGTTTTCAGGCGTAATTTTACAGGGCGCGATCGTCACGCTGGAACTGGCGCTCAGTTCC
GTGGTGCTGGCCGTGCTTATCGGTCTCGTCGGCGCGGGGGCGAAGCTTTCGCAAAACCGGGTAACGGGGCTTA
TTTTTGAAGGGTACACCACCCTGATTCGCGGGGTGCCGGATCTGGTATTAATGCTGCTGATATTTTACGGATT
GCAAATCGCGCTTAACGTGGTGACCGATTCGCTGGGTATCGACCAGATAGATATTGATCCGATGGTGGCCGGT
ATTATCACCCTTGGTTTCATCTACGGCGCCTATTTTACCGAAACCTTTCGCGGCGCGTTTATGGCGGTGCCGA
AAGGCCATATCGAGGCGGCGACGGCTTTTGGTTTTACCCACGGGCAAACCTTCCGGCGGATTATGTTCCCGGC
GATGATGCGTTACGCCCTGCCGGGGATCGGCAATAACTGGCAGGTGATACTCAAAGCGACGGCGCTGGTGTCG
CTGCTGGGCCTGGAAGATGTCGTCAAAGCCACGCAGCTTGCCGGTAAAAGCACCTGGGAGCCTTTCTATTTTG
CCGTGGTCTGTGGACTTATCTACCTGGTATTTACCACCGTTTCCAATGGTGTGCTGCTCTTGCTTGAGCGTCG
CTATTCTGTGGGCGTGAAGAGGGCCGATTTGTGATTGAGATCATTCAGGAGTACTGGAAATCCCTGCTGTGGA
CGGATGGCTATCGTTTTACCGGCGTGGCGATTACGCTGTGGTTGTTAATTTCATCGGTGGTGATGGGCGGTCT
TCTGGCGGTGATTCTGGCGGTAGGTCGCGTTTCCAGTAATAAATTCATCCGTTTTCCCATCTGGCTGTTTACG
TATATTTTTCGCGGCACGCCGCTGTACGTACAGCTACTGGTGTTCTATTCGGGGATGTATACGCTGGAGATAG
TCAAAGGCACCGATCTTCTTAACGCGTTTTTCCGCAGCGGACTGAACTGTACCGTACTGGCGCTGACGCTGAA
TACCTGCGCGTACACCACGGAAATTTTCGCGGGGGCGATTCGTTCGGTACCGCACGGTGAAATTGAAGCGGCA
CGGGCCTATGGCTTCTCGTCATTTAAAATGTATCGCTGCATTATTTTGCCGTCGGCATTACGTATCGCCTTGC
CTGCCTACAGCAACGAAGTGATTTTAATGCTGCACTCTACGGCGCTGGCGTTTACCGCGACGGTGCCGGATTT
GTTGAAAATCGCCCGCGATATTAACTCAGCGACTTACCAGCCGTTTACCGCGTTTGGCATTGCCGCCGTGTTG
TATTTACTCATTTCGTATGTCCTGATAAGCCTGTTCCGTCGGGCGGAAAGACGCTGGTTGCAGCACGTGTCCT
CTAAATAATTCGAGTAACATGATGTCAGAAAATAAATTACACGTTATCGATTTGCACAAACGCTACGGCGGTC
ATGAAGTGCTGAAAGGGGTATCGTTGCAGGCCCGCGCCGGAGATGTGATTAGCATCATCGGCTCGTCCGGCTC
CGGTAAAAGCACTTTTTTGCGCTGTATTAACTTCCTCGAAAAACCGAGCGAAGGCGCGATTATCGTGAACGGT
CAGAACATTAATCTGGTGCGCGACAAAGACGGGCAGCTCAAAGTGGCGGATAAAAATCAGCTACGCTTGTTGC
GTACCCGCCTGACGATGGTGTTTCAGCACTTTAACCTCTGGAGCCACATGACGGTGCTGGAAAATGTGATGGA
AGCGCCGATTCAGGTACTGGGATTAAGCAAGCACGACGCGCGCGAGCGGGCGTTGAAATATCTGGCGAAGGTG
GGGATTGATGAGCGCGCTCAGGGCAAATATCCCGTCCATCTCTCCGGCGGCCAACAGCAGCGCGTTTCTATTG
CGCGCGCGCTGGCGATGGAACCTGACGTTTTACTGTTCGATGAACCCACATCGGCGCTCGATCCTGAACTGGT
CGGCGAAGTGTTGCGCATCATGCAACAACTGGCGGAAGAAGGCAAAACGATGGTGGTGGTCACGCATGAAATG

GGCTTCGCCCGCCATGTCTCTTCGCACGTTATTTTTCTGCATCAGGGGAAAATTGAAGAAGAGGGCGATCCGG
AGCAGGTGTTCGGCAATCCGCAAAGCCCGCGTTTACAGCAATTCCTGAAAGGCTCGCTGAAATAAAAGAAGCC
CGGCGGCGCATTGCGTACCGGGCAGGATACGGCGCTTTAGGAACTAGCGAATCACATCCGCCAGCGCCTCTTC
TAAATCATACCAGCGAAATGCGAATCCGGCGGCTTCAAGCCGTTTTGGCAGCGCCCGCTGTCCGCCCAGCACC
AGCACTGACGACTCTCCCATCAATAAGCGAATTGCCGTCGCCGGAATGCGAATAATCGCCGGGCGGCGCAGCG
CGCGACCCAGCGCATGAGCAAACTGCTCATTATGGACGGGATACGGCGAGACCATATTGAAGGGACCGCGAAG
ATCGTTATCCAGCAGCCACAGAATGCCGTTGACCATATCGTCGATGTGAATCCAGGCCAGATATTGTCGACCA
TTACCCACCGGACCGCCGAGTCCGAGGCGAAACGGCGGAACCATTTTCCCCAGAATCCCGCCTTGCGGCGCCA
GGACAACGCCGGTACGCAGCAGGCAGACGCGGGTCTGGTCGCTCTGCGCACGACAGGCAATCTGTTCCCAGCG
GGCGCAAAGTTTATGCGTAAATTCGTTGTGCGGCGGCTCATCTTCAGTGACGACAACGTCGCCCAGATCGCCG
TAATAGCCCGTGGCGGAGCCGGATATCAACACCGAGGGCGGCGTAGCGCTGGCATGAATTAAATCCACCAACT
TTTGCGTGATAGCCCAGCGGCTTTGGCACAACCGTTCTTTTTGTTGCGAGGTCCAGCGCTTGTCGGCAATCGG
TTCGCCCGCCAGATTGATGATGGCATCAATCTCATTGAGGTGCTCACGTTCCGCCAGCCCTTTCCATAATGTC
ACTCTGGAATCGAGAATCTGACGCGCGTTATCGGGATTGCGCGTAACGACCGTCACCTGATGCCCCAGTGTTA
ACAGCCGGGGAATGAGATGACGCCCTATCAGGCCTGTACCGCCGGTAATCAGAATCTGCATATGACCTCCAGA
GTAGGGAGGATTTCCTCTTCCCTAATGTCAGGTTTTTAATACGAATAAGGGTGCCGGACGCAGGTTTTTTCCG
TGTATACACCTCTACATCATACATGATGTATGCTACCGCGTTACGCTTTCGTACTGTTACAGTGCAGTTGCGC
TTGTAACAGTGCGCGCGCTGTCGCAGGACGCGTGCGAATGCGTTCAAACCAGTTATACACCGCCGGGTAGGTA
TCAAGATCGATTCGCTGACGCTGATGTGCGTTAACCCACGGCCAACTGGCGATATCCGCAATACTGTAGTGGT
CGCCGCCCAGCCACGGGGAGGTTTCCAGGCGTTTATTCAGGACGTTGTATAAGCGTTGCGTTTCAACCTGATA
GCGTTCAATTGCATAGGGGATGGCCTGCGGCGCAAAGTGGTTAAAGTGATGATTCTGGCCCAGCATCGGCCCT
AACCCGCCGACCTGCCAGAACAGCCATTGCAGCGTGGTATGGCGTTCACGCAGCTCCCCGCTAAGCAGTTTGC
CGCTTTTTTCCGCCAGGTAGAGAAGAATTTCGCCGGATTCAAAAAGACTAAGCGGTTGACCGCCGTCTGCCGG
GGCGTGATCGACGATGGCAGGAATTTTATTATTGGGAGAGATAGCCAAAAAATCAGGGCGAAACTGATTGCCC
TTGCTGATATCTACTTTTAGCAAACGATACGCCAGCTCCGCCTCTTCCAGAAACAGCGTTATTTTATGCCCGT
TGGGCGTGGGGGCGTAGTAAAGGTCAATCATTTTGGCTCCTGCATGTGCGTAAAGAGTCTGGGCTGAATTAAG
TATAGAAAGCCTCCTGATGTGTGTGAGTTTTCATCAGGTGACAGCAGGCGAAAGACGCTATATTTTGGGAAGA
AACCTAATACGCCCTCAAGAGGACATTATGAGTAAGCCTGTTATTGTGCTGTGGTCGGATGCGAACTTTTTCT
CTCCCTACGTCTTGTCCGCCTGGGTGGCATTACAAGAAAAAGGGCTTTCCTTTACTCTGAAAACTCGCGATCT
GGACCAGGGAGAACATCTGCAACCTGGCTGGCGAGGATATGCGCTGACGCAGCGCGTACCGGTGCTTGAGGCC
GACAATTTTGAGCTGAGCGAGTCGTCCGCTATTGCGGAATACCTGGAGGAGCGTTTCGCGCCGCCGCAGTGGG
AGCGTATCTACCCGCACGATTTACAAAAACGCGCCCGCGCGCGACAGATTCAGGCGTGGCTACGCAGCGATTT
ACTGCCGCTTCGCGAAGAACGGCCTACCGACGTGGTGTTTGCCGGGGCGAAAAAAGCGCCGTTAAGCGAGGCT
GGAAAAGCCAGCGCGGCAAAACTGTTTGCGACGGCTGAAGCGCTATTGGGGCAGGGGACGCAAAATTTATTTG
GTGAATGGTGCATTGCCGATACCGATCTGGCATTGATGATCAATCGTCTGGCGCTGCATGGCGATGACGTGCC
GACATCGCTGGCGGCATATGCGACATTCCAGTGGCAGCGTGCTTCCGTACAGCGTTTTATTGCGCTTTCCTCG
AAGCGTTCAGGCTGAAAATCTATTCATATTCCAGTATCATAACGCCGTTTTTCACAACGAGGAGTGAGCGATG
AAACTGATGTTCGCATCGGACATTCATGGTTCTTTACCCGCCACGGAACGCGTGCTTGAGCGGTTCGCGCAAA
GCGGCGCGCGTTGGCTGGTGATATTAGGCGATGTCCTTAACCACGGGCCGCGAAATGCGCTGCCTGAAGGCTA
TGCGCCCGCCCAGGTCGCCGAACGCCTGAACGCGGTGGCGACGCAAATCATTGCCGTGCGCGGCAACTGCGAT
AGCGAAGTGGATCAGATGCTGCTGCATTTCCCGATCACTGCGCCGTGGCAGCAAATATTGACGCAAGAGAGGC
GGCTATTTTTAACGCATGGGCACCTCTTCGGGCCGACGAATCTGCCCGCTCTGCATACTGGCGATGTGTTGGT
TTATGGTCATACGCATCTGCCGGTGGCGCAACAGCAGGAAGGACTGTATCACTTTAATCCTGGGTCAGTGAGT
ATCCCGAAAGGCGGGTATGCGGCAAGCTACGGTATTCTGGATGATAATGTGTTGAGCGTAATCGCACTCAATG
ATCAAAGTATCATTGCGCAGGTTGCAATTAACTCGTAATTTACCTCACAAACGTAAATGCGCCGTAAGAGCGC
CAGAAATAAGAAGGTTTCCTGATGGTGGAACAGCGTCGTTTGGCAAGTACGGAATGGGTGGATATTGTGAATG
AAGACAATGAAGTCATTGCACAGTCCAGCCGGGAACAGATGCGTGCGCAACGCTTACGGCATCGTGCGACGTA
TATCGTCGTTCATGATGGTATGGGTAAAATTCTGGTGCAGCGTCGTACCGAAACAAAAGATTTTTTGCCCGGT
ATGTTAGATGCTACCGCAGGCGGCGTGGTTCAGGCTGATGAGCAGCTACTGGAATCTGCCCGCCGCGAAGCGG
AAGAAGAGTTGGGTATTGCAGGCGTGCCGTTTGCCGAACACGGTCTGTTTTACTTTGAAGACCAACATTGCCG
GGTTTGGGGCGCGCTGTTTAGCTGCGTTTCCCACGGGCCTTTCGCGCTTCAGGAAGATGAAGTCAGCGAAGTG
TGTTGGCTGACCCCGGAGGAGATCACCGCGCGCTGTGATGAGTTTACGCCAGATTCGCTCAAAGCGCTGGCGC
TGTGGATGACCCGCAACGCCAAAAACGAAGCCGCGCTCCAGGAGAAACCGGAAGAGACGGAATAAGCGTTTTA
GCAGCTTTCCCGCATACATAAACTGGAGGCGATGGATTTTCGATCGCTCCAGTTTCCCTGATTTAACCTTTCC

AACAGCGCCCGTCCGGCTTCACTGCCAATCTTACGGTGCGGTACGGCCATGGTGGTTAACGGCGGCTGGCAGA
CCCGGCTGACATCGCTATCGCCAAACCCGACCACCGCTAAATCGTCCGGAACTTTGATCCGCCGTCGTTGGCA
CTCGTATAACGCGCCGCAGGCCAGCTCATCCGATACGCATACCAGCGCGTCCAGCTCCGGCCACGCCAGTAAA
AACTCCGGCAGTTGCGACGCGCCGGTAGAAAAATTGGGCGGCAAGGCGGCATTAATCACCCGGTTTGGCGACA
TATGGTGGCGAAGCATCGCTTTGTACCAGCCGTGCAGATGTTGCTGAAAAATCCACTGCTCCTGATTGGCGCA
TAGCAGCCCGATATTCTGATAACCGCGCTGAATCAGCATTTCCGTCAGCTCATACATTGCCGCAACGTTATCA
ATACCGATATTCATATCAATAGGATCGCTGCGAATCGCGCCCATCTCCATCACCGGGATAGAGGCATTTTTTA
GCCACTGGCGCACGGTAGTGCTATGTTCGACGCTAAGCAGAATCGCTGCGGCAATGTTGGAGGCCAGCAGCGT
TTCGAGCAGCTTCTCCTCTTGTTCCACGCGATGCTGCGATTCCGCCAGCATAATCTGGTAGCCTGCGGGCTGT
AAAATTTGTTGCAGCCCGGCGAACATTTCAGAACACCCCGCTTCCGCAAGGTTCGGTACGACCATCGCGATGG
TGTGCGAGGAGGCAGACGCCAGCGCGCTGGCGGCGAGATTAGGCATATAGCCCAACTCATGCACTGCCGCTTC
GATTTTTTCCCGGAGTTTATCAGAGACCTGTTCCGGCGTGCGTAAGGCTCGTGATACGGTCATCGTGCCTACA
CCGGCAAGTTGGGCTACATCGGCGATAGTCACTTTACCGGTACTGCGCCGTTTACGGGGTATCGACATACAAA
TTCCTGCGTCATTACAACATGTTGAGATGCTTTTTTATTGTCGTTCTTTGCTTCGCATGACTGGCAGTATACG
CCTTAAATCCGCTTTTTTGCTGTGATTTCACACCATGATTTTAATTTGATAGCGCTATCACAATTCTGAGTAA
TAGTCGCTGGTAGCGCTATCTTTGTGATAACGATCGCAGCTAATATCGAATGGCTTGAATAAAGTTTGTTCAT
AATTTCATACACCGTGGGGGCGAATAACATGCTGGGAACCTGGTTATCTGACGCAACCATCACGCTACGGGAG
AGCGTGGAAACGTGGTCGCAGGCGCTGGAAATTTGCGGCAAACCCCTGCTGGACGCAGGTGTTATCGCACCGG
AATACATTACGGCGATTGTTCAGCAGCACCAAAAGCTTGGGCCGTATTATGTATTAGCGCCAGGGTTAGCGAT
GCCGCATGCGAGGCCGGAAGAGGGGGCGAAAGGGTTAGGTTTATCGTTACTGAAACTACAGCACGGCGTTTCA
TTTGGCGCAGACGAATTCGATCCGGTTGACATCATTATTATGCTGGCGGCGCCTGATAAGCACAGTCATATCG
AAATGATATCGGCGCTGGCGGAATTATTTTCCAGTGATGTAGATATGGAAAAATTACATCAGGCAAAAAACCT
GGAGGACATTAAAACGATTATTGACCGTTTCTGATTTTATTCCATTTTCTTGCGGTAAATAACATTACGCCCG
TGCGTGATGCAGACCAACTCTACTTTTAAAAGGTGACAATAATGAAAATCATGGCAATTTGCGGTTCCGGCCT
GGGTAGTAGTTTTATGGTAGAAATGAACATTAAAAAAGTATTAAAGAAACTCAATATTGACGCCGAGGTGGAA
CACTCCGATCTTTCATCGGCCACGCCCGGGGCTGCCGATCTTTTTGTAATGGCCAAAGATATCGCGGCCAGCG
CCAGCGTACCGGAAAGCCAACTGGTGGTGATCAATAACATCATCGACATCAACGAACTGGAAACGCAGTTGCG
CGCATGGTTCGCTAAACAATAATTATTGCTGGCGAGGTGGATATGTTTATCCTTGAAACGCTGAATTTTGTTG
TTGATATTTTAAAAGTACCCTCGGTGCTTGTAGGGTTAATTGCGCTTATTGGTCTGGTGGCGCAGAAAAAAGC
GTTTTCCGATGTGGTAAAAGGCACGATTAAAACCATTTTAGGTTTTATTGTGCTTGGTGGCGGCGCAACCGTA
CTGGTGGGTTCATTAAACCCGCTAGGGGGGATGTTTGAACACGCTTTTAATATTCAGGGCATTATTCCTAATA
ATGAAGCGATAGTCTCAATCGCACTGGAAAAATATGGCGCGTCCACGGCGTTAATTATGGCATTTGGTATGGT
GGCCAATATTGTCGTGGCGCGCTTTACGCGTCTTAAATATATCTTTTTGACCGGGCATCACACCTTTTACATG
GCCTGTATGATTGGCGTAATCCTGACCGTGGCGGGGTTTGAGGGCGTAGGGCTGGTCTTCACCGGATCGCTAA
TCCTGGGGCTGGTGATGGCCTTCTTCCCGGCGTTGGCCCAACGTTATATGAGGCGGATTACCGGTACCGATGA
CATTGCGTTTGGTCACTTCGGTACCCTGGGATATGTGCTTTCCGGCTGGATTGGCAGCCTGTGTGGTAAAGGC
TCCCGCTCCACGGAAGAGATGAACCTGCCGAAAAACCTCAGTTTTTTACGCGACAGTTCTATCTCGATTTCGC
TGACCATGATGATTATCTATCTGATCATGGCAGTCAGCGCTGGGCGTGAGTATGTCGAAGCGACGTTTAGCGG
CGGGCAAAACTATCTGGTGTACGCCATCATCATGGCAATCACCTTCGCCGCCGGCGTGTTCATCATTCTGCAA
GGCGTCCGTCTGATTCTGGCTGAAATTGTTCCGGCCTTTACCGGCTTTTCAGAAAAACTGGTGCCAAACGCGC
GTCCGGCGCTGGACTGCCCGGTGGTGTATCCCTATGCCCCTAACGCGGTACTGATTGGCTTCCTGTTCAGTTT
TCTCGGCGGGCTGGTGGGGCTGTTCCTGCTGGGACAGATGAAGCTTGTGTTAATCCTGCCTGGCGTTGTGCCG
CACTTCTTTACCGGCGCGACCGCAGGCGTCTTTGGCAATGCCACCGGCGGTCGCCGCGGGGCGATGATCGGCG
CATTTGCTAATGGTCTATTAATCACTTTCCTGCCAGTGCTGTTACTGCCGGTTCTCGGCGCGATCGGCTTCGC
CAATACCACGTTCTCGGATGCTGACTTTGGCGTAATCGGTATTTTGTTGGGCAACCTGGCGCGTTATCTGTCG
CCGATGGCTATCACGGGGCTGGTGGTGGCGCTATTCGCGCTACTGGTGGCGTACAACGTGTTGGCCAAAAACA
AAAAAGCGACTGCCGAAGTGCAGGAAAATAGCGGAGCGAAAGAATGAACGTAACCGAAATCACCCAACTGGCG
CGCGATATTCGCGTCGCCACGCTCAAATCCTTAAACCATCTTGGCTTTGGTCACTATGGCGGCAGCATGTCGG
TAGTGGAGACTCTGGCGGTTCTGTACGGCGCGGTGATGAAAATCGACCCTGCCGACCCGGACTGGCCGGAACG
CGATTACTTCGTACTGTCGAAAGGCCATGCGGGCCCCGCGTTGTACAGTACCCTGGCGATTAAAGGCTATTTC
CCGCGCGAAGAGCTGAATACGCTTAACCAGAACGGCACCCGTCTTCCCAGCCATCCGGATCGGCTAAAAACGC
GCGGCGTGGACGCCACGACCGGCTCACTGGGACAGGGCATCTCTATTGCGGGCGGAATGGCGTTGTCGCATAA
ACTGGCGCGGCGACCCAACCGGGTATTTTGCATTGTCGGCGATGGCGAGCTCAATGAAGGGCAGTGCTGGGAG
GCGTTTCAGTTTATTGCCCACCACCGCCTGAATAATCTGACCGTTTTCATTGACTGGAATAAACAGCAGCTTG

ACGGCGAGCTGGAGGAGATTATCAATCCATTCGATCTGGAAGGGAAATTCCGTGCGTTTGGTTTCGATGTCGT
CACCGTCAAAGGCGATGATATCGCCGGACTACTGGCGGTGGTTCAGCCTGTGCTGCCCGCTGATGCCCGGCCA
CGGGTTGTGATCCTTGACAGCATTAAAGGGCAGGGGGTGCCATACCTGGAGCAGTTAACGAATTCTCACCACT
TACGCCTGACGGATGGCATGAAGCAGACGCTCAATGAGGCCATTCATCAACTGGAGGTCATGCATGATTAAGC
TTGCGCCCGCAGGGCTTAAAGACGATATCGAAATGCGCAAAGTTTACGCCGGTTTTGTCGCCGGGCAGATTGA
GGCCGGAAGCCCAATTATTGCGCTTGAGGCTGATTTAATGAGCTCAATGGCGATGGACAGCGTGGCGCGTGAC
TACCCGCAGCATGTCATTAACTGCGGTATTATGGAGGCGAATGTGATTGGCACTGCGGCGGGGCTGGCGCTGA
CCGGTCGTAAACCGTTTGTGCATACCTTTACCGCTTTTGCCAGCCGTCGCTGTTTTGATCAGCTCTTCATGGC
GCTGGATTATCAGCGTAACAACGTCAAAGTGATCGCCTCCGATGCCGGAGTGACGGCGTGCCATAACGGCGGC
ACCCATATGTCTTTTGAAGATATGGGGATTGTTCGCGGGCTGGCGCACTCCGTAGTACTGGAGGTGACGGATG
CGGTGATGTTCGCGGATATTTTACGCCAGCTAATGGACCTGGACGGTTTTTACTGGCTGCGTACCATCCGTAA
GCAGGCGACGAGTATCTATGCGCCAGGCTCTACCTTTACCATTGGCAAGGGCAACGTACTGCGCGAGGGCGAT
GATATTACGCTTATCGCCAACGGCATTATGGTGGCGGAGGCGCTGGAGGCGGCGCGCCAACTGGAGCAGGAAG
GCGTGAGCGCCGCGGTCATTGATATGTTCACCTTAAAGCCTATCGATCGCATGTTGGTGAAAAACTACGCGGA
AAAAACGCGGCGTATCGTCACGTGTGAAAACCACAGCATCCATAATGGACTGGGGTCAGCGGTGGCGGAAGTG
CTGGTGGAAAACTGCCCGGTGCCGATGCGCCGTGTTGGCGTCAAAGAGCGTTATGGGCAGGTAGGAACGCAGG
ATTTCTTACAACAAGAGTACGGACTCACCGCCGAAGCGATTGTAGAAGCCGCGAAATCACTGCTTTAATCATG
GAGATATTGCCGGATGGCGCTGATGCTCATCCGGCATTTTGGATTAATGATATCCCATCATCTGCGCGCCAAT
GACGACCACGCTGGACATAATAAACAGTAGTCCCAGCAGCGACGCTCCCACTTTCAGCCAGTTGCGAAAATCG
ACCCGGCACACGCCCAGCGTGGCCATCAGCGAGGCTGATGTCGGATAGATAATATGGCTGAAGCCATCGCCAA
ACTGGAAGGCCAGAACAGTCACCTGACGGTTAACACCGACCAGATCGCCGAGCGGCGCCAGTAATGGCATGGT
TAACGCCGCCTGACCCGAACCGGACGTGACAAAGAAATTAAATACCGCCTGGAAGAGCAGCATAAACCAGGCG
GCGATAGCATTATTAAGCCCGCTGATGCCATGCGCGATACTGTTAAGCAGCGTATTGAGCACGCTGGGTTCCC
CGGCTTCACCGTTGCCGACCAGCAGCAGAATGCCTTTAGCAAAACCCACCAGCAAGGCGGGCGCGATCATCAT
CCTCGCCCCTTCGGTGAATGAGGACGCCATCACGTTGACCGTCATACCGTTGAGGCGGAAGATAACGCCGATG
AGGCCGATCACCACGCCCATGGTGAAAAACTGGCTGGCGATCTCCGGAATAAACCAGGCGTGGACAATCACGC
CCCAGACGACCCAAATCATTACGCCGGTCAGGACCAGCAGTACCAGCCAGTCACCGAAGGTGAAAGGGCGCTG
GACGACTTCATCCTGCTGTTCGCGGAAATAGCGATCGGATTCGTGGACGCGGGACAGCAGCGGATTCTTTTTC
ACGCGTGAGGCGTAGACCAGTGTAAAAACCAGCCCAATCAGCGTGGCGACAATCCAGACCACAATACGCAGCC
CGGAGCCGGAAAGTACCGGCACGCCGGCAATCCCCTGGGCGACGACTACGCAGAAAGGGTTCATCCACGAACT
GGCAAAGCCAATTTGGGTGGCGATATAGGTCACCAGGACGGTGGTAATGCTGTCATAGCCCAGCCTGACCATC
AGCGGGGCTATGATAATTGCAAAGGCGACCGCCTCTTCGCCCATGCCAAATACGGCGCCCCCTAACGAAAAGA
GGACAAATAGCACCGGAATAAACAGGACTTCGTTGCCCCGCGTGTGGCGAATAAGCGCCAGAATGCCGTTATC
CACGGTGCCGGTACGCATCACGATGCCGAATGCGCCGCCAATCACCAACATAAACATAATGATGCCGACGGCA
GTGCCGAATTTCGATCCGGATGTTAACCCTTCAAACGGAAAATTCATCAGCCCTGGTCTTTCATCGCCGGTAG
TGAAAAATTGTACGCGATGATACTGCGCTTCCCCGGCCTCATTGGTCACAATGCGAAAAGAGTGCGGGTCGAC
GACTTTGCGGGTTTTTGTCTGACCATCGACCTGATACTGCACTTCCTGGCTATCAAACATCCCAACGGGTACG
ACCCAGGTGGCGATACTGGTTAAAATGGCGACAAAAAAGATGATGACCAGCGTATCGGGCATTGCCCATTTTC
TGGCTGTTTTTGATTCTGTAACGACAGACATGACAGACTATCCCTGTTGCAAAGCAAAGAGGCTAAGTATGAG
AAGAAGGGAATAGTTATTCATTGATGCAGCGCAGTTAAGCAAAAAATTCCGCTTTGCAGGGTAAATAGCAGAA
TGCCGGATGGCGATGCTAACGCGTCTTATCCGGCCTACAACGGTATCTCACCTGTAGGCCGGACAAGGCGTTC
ACGCCGCCATCCGGCATTAGCTTTTACTGTTACTGCTGCTGTTGGGAAGCCTGGATCGCCGTCAGGGCGATGG
TATAGACGATATCGTCAACCAGTGCGCCACGGGACAGGTCGTTCACCGGCTTGCGCATACCCTGCAGCATCGG
CCCGATGGAGATCAGGTCGGCAGAACGCTGTACCGCTTTGTAGGTGGTGTTACCGGTGTTCAGATCCGGGAAA
ATGAACACGGTAGCGCGACCCGCAACCGGCGAGTTCGGCGCTTTGGATTTCGCTACGTCAGCCATGACCGCGG
CGTCGTACTGCAACGGACCGTCGATCATCAGGTCAGGACGTTTTTCCTGCGCCAGACGCGTCGCTTCACGTAC
TTTCTCTACGTCGCTGCCCGCGCCAGAGGTGCCGGTGGAGTAGGAGAGCATTGCCACACGCGGTTCGATGCCG
AAGGCAATGGCGGAATCCGCAGACTGAATCGCGATTTCTGCCAGCTGCTCTGCAGTCGGGTCTGGGTTGATTG
CGCAGTCGCCGTAAACGTAAACCTGTTCCGGCAGTAGCATAAAGAACACAGAAGAGACCAGGGAGCTACCCGG
CGCCGTTTTAATAAGCTGCAGCGGCGGACGGATGGTGTTGGCGGTGGTATGAACCGCGCCGGAAACCAGGCCG
TCGACTTCGTCTTGCTCCAGCATCAGCGTGCCGAGCACCACGTTGTCTTCCAGCTGCTCGCGGGCAACCGGTT
CGGTCATGCCTTTGCTCTTACGCAGCTCAACCAGGCGAGCGACATAGCTTTCACGCACCACTTCCGGATCGAC
GATTTCAATACCTGCGCCCAGCTCAACGCCCTGAGATGCCGCGACGCGGTTGATTTCATCCGGGTTGCCCAGC
AGAACGCAAGTGGCGATGCCGCGTTCAGCGCAGATTGCCGCCGCTTTGACGGTACGCGGTTCGTCGCCTTCCG

GCAGCACTACGCGTTTACCGGCTTTACGCGCCAGCTCGGTCAGTTGGTAGCGGAACGCCGGCGGAGAGAGACG
ACGGCTACGCTCGGAAGTGGCGGTCAGCGACTCGATCCACTCAGCGTTAACGTAGTTCGCGACGTATTCCTGA
ACTTTCTCGATGCGCTCATGGTCATCAACCGGGACTTCCAGATTGAAGCTTTGCAGGCTCAGCGAAGTCTGCC
AGGTGTTGGTGTTCACCATAAAGACCGGCAGACCGGTGGCGAATGCGCGTTCGCACAGTTTAGAAATGCGTGC
GTCCATTTCATAGCCGCCGGTCAGCAACAGGGCGCCGATTTCTACGCCGTTCATCGCGGCCAGGCAAGCTGCG
ACCAGTACGTCCGGACGGTCAGCGGAGGTCACTAACAACGAGCCTGCGCGGAAATGTTCCAGCATATGCGGAA
TACTACGCGCGCAGAAAGTGACGGATTTAACTCGGCGGGTTTTGATATCGCCTTCGTTAATGATGGTGGCGTT
CAGGTGACGCGCCATATCGATAGCGCGGGTCGCAATCAGGTCGAAGCTCCACGGCACCGCGCCCAGAACCGGC
AGCGGGCTGGATTCCTGCAGTTTAGCAGGATCGATTTTGATCACCTGCGCTTTGGAAGAGTCGTCAAAAATCT
CCGACAGATCCGGGCGAGTACGACCTTGTTCATCAACCGGTGCATTCAGTTTGTTGATGATAACGCCGGTGAT
GTTGGTGTTTTTCGCGCCGCCGAAGCTGCTGCGCGTCAGTTCGATACGCTCGTTCAGCTGTTCTGGCGTGTCG
GTACCCTGAGACATGACAAAAACGATTTCCGCATTCAGCGTTTTCGCGATTTCATAGTTCAGAGACTGAGCGA
ACTGATGTTTACGGGTCGGAACCAGACCTTCAACCAGCACCACCTCCGCGTCTTTGGTATTCGCATGGTAGTT
CGCGATGATCTCTTCCATCAGCACGTCTTTCTGGTTGCTGGAGAGCAGAGATTCAACGTGGCTCATCTTCAGC
GGTTCAGCCGCCGGCAGGGTAGAGTTCGCGCGAACGATAGTGGTGGTCTGGTCAGGCGCATCGCCGCCAGCGC
GAGGCTGGGCGATAGGCTTAAAGACGCTCAGACGAACGCCTTTGCGTTCCATAGCACGGATGACGCCGAGGCT
GACGCTGGTCAGGCCGACGCTGGTTCCGGTAGGGATCAGCATAATAATACGGGACACGGTTTATCCTCTTTCG
TTACCGTCTTTTGGGGACGGGTTACAAAACAGCACCGCCAGCGAGGCTGGCGGTGTGAAATCAGGCAGTCAGA
CGGCTCGCGTCCTGCGCGATAACCAGTTCTTCGTTGGTTGGGATCACCACCGCCGGACGGGTACCTTCTTTGT
TGATGAAACCAGATTTGCCGAAGCGGGCCGCCAGGTTACGTTCGTGGTCGACTTCAAAGCCCAGCACGCCCAG
TTTACCCAGAGACAGTTCGCGAACCATCGCGGCGTTTTCACCGATACCGCCGGTGAACACGACTGCATCCAGA
CGACCGTCCATCAGAGCGGTGTAAGAACCGATGTACTTCGCCAGGCGGTGGCAGTAAACGTCCATCGCACGTT
TTGCGTCTTCTTTGGTGGCGTAGTTGTCTTCAACATAACGGCAGTCGCTGGTCACTTCAGTCAGACCCAGCAG
ACCGGACTCTTTGGTCAGCATTTTGTTGATCTGATCCACGCTCATACCCAGAGTGTCGTGCAGGTGGAAGATA
ATCGCAGGGTCGATATCGCCGGAACGGGTACCCATGACCAGGCCTTCCAGCGGGGTCAGACCCATAGAGGTAT
CCACGCATTTACCGTTACGGATAGCGGAAACAGAACCGCCGTTGCCCAAGTGGCAGGTGATAATGTTCAGTTC
TTCTACCGGCTTGTTCAGCATTTTTGCCGCTTCCTGAGTTACATAGAAGTGGCTGGTGCCGTGTGCGCCATAG
CGACGAACGCCGTGTTCTTTATACAGGCTGTACGGCAGGGCATACAGGTAAGATTCTTCCGGCATGGTCTGAT
GGAAGGCGGTGTCGAATACTGCAACGTTTTTATCTTTCAGCTGCGGGAAAGATTTCAGCGCTTCGGCGATACC
AATCAGGTGAGCCGGGTTATGCAGCGGTGCGAAAGAGGCGGAATCTTTGATGCCCTGAATGACGGACTCATCA
ATGACTACGGAACTGGTATACTTCTCGCCGCCGTGTACGATACGGTGACCAATTGCAGTTAACTGCGCAGACA
GTTCTGGTTTTTGTGCCAGAATAGTGTTAACGATAAAGTTCAGCGCTTCACTGTGAGCGGCGCCTGCACCTAA
AGCCGCTTCTTGTTTACTGCCGTCCATTTTCCATTTGATGCGTGCTTCTGGCAGATGGAAACATTCGGCTAAA
CCAGAAAGGTATTCATCACCATTAACTGCATCAATAATTGCAAATTTCAGTGAAGAACTACCGCAGTTCAGAA
CCAGTACTAACTTACTCGACATGGAAGTACCTATTTATGATACGTGGCTAAAAAAACGTCAGTGAGCCATAGA
GCGTAGCGCATGATAGCCCTGACATTTATGATTAACATCATGCGCAGGAGTTTTTTTGGGCATGATGGAAGAA
ATACCTTTGAGTCTATGCCATTTTGCGTATTTTTCAGCCAATGGCATACTATGTGTAATATTGACGACTCAAT
GATTTTAAACTACCGGCCTTTGACAGGCCGACACAGGATACCTGATAGTGGGTATCGAAGACAAAATGTTTTG
AACGTTGTCCCTCGCAGTTGTTGTTTTGCACAAATTTTTTAAATTTTATATGTGAAGTTGAGGTAGGCCATGT
CGACACCGGATAATCGCTCCGTAAATTTTTTTAGCTTATTTCGCCGGGGGCAGCACTACGCGAAGACGTGGCC
GATGGAAAAGCGGCTGGCGCCCGTTTTCGTCGAAAACCGCGTCATTCGTATGACGCGCTATGCCATACGATTT
ATGCCGCCTGTCGCCGTCTTTACATTATGCTGGCAGATAGCGCTGGGCGGTCAGTTAGGTCCGGCCGTGGCGA
CCGCCCTGTTCGCCCTGAGCTTGCCTATGCAGGGATTATGGTGGTTAGGGAAACGTTCCGTGACGCCGTTGCC
GCCTTCAATCCTTAACTGGTTTTACGAAGTTCGCGGTAAATTGCAGGAGGCTGGACAAGCGCTGGCGCCGGTA
GAAGGAAAGCCGGATTACCAGGCATTAGCTGACACGCTTAAGCGCGCCTTCAAACAACTGGATAAAACTTTCC
TGGATGATTTGTAATTGACCCCGGATTACGCATATAAAAGAAGGCATACCAATGCCTTCTTTTTTTCATCGCC
ATAGCGTGATACAGGAGTTCAAAATGGAAATGACCAACGCGCAACGTCTGATTTTGTCTAACCAGTACAAAAT
GATGACGATGCTCGATCCCACCAACGCCGAGCGTTACCGTCGCTTACAAACAATTATTGAACGCGGCTACGGC
CTGCAGATGCGCGAGCTGGATCGCGAGTTTGGCGAGCTGACGGAAGAGACCTGCCGTACCATCATCGATATTA
TGGAGATGTACCATGCGCTGCACGTCTCCTGGACCAACCTCAAAGATACGCAAGCCATCGACGAACGTCGCGT
CACCTTCCTGGGGTTTGACGCCGCGACGGAAGCGCGCTACCTCGGCTATGTCCGTTTTATGGTGAATATAGAA
GGACGCTACACTCACTTTGACGCCGGGACACACGGCTTTAATGCCCAGACGCCGATGTGGGAAAAATACCAGC
GGATGTTGAATGTCTGGCATGCCTGCCCGCGTCAGTACCACCTGAGCGCGAATGAAATCAATCAAATCATTAA
TGCCTGAGGAGGCTCGCGTGCAGTGTAAAGGTTTTCTGTTTGATCTGGATGGAACGTTGGTTGACTCATTACC

TGCCGTCGAACGCGCGTGGTGTAGCTGGGCCGATCGTTTTAACCTTGCTCATGATGAGGTGCTGGGCTTTATT
CATGGCAAACAGGCGATCACCTCGTTGCGGCATTTTATGGCGGGTAAATCGGAGGCGGAAATTGCCGCTGAGT
TTACGCGCCTGGAGCAAATAGAAGCCACTGAAACCGCAGGGATCACCGCGTTGCCGGGGGCAGTCGATTTACT
CAATCACCTGAATAAAGCGGGCATTCCGTGGGCTATCGTCACCTCCGGTTCAATGCCGGTCGCGCGTGCGCGC
CATCAGGTCGCGGGCCTTCCAGCGCCAGAGGTGTTTGTCACCGCCGAACGAGTCAAACGCGGCAAACCGGAGC
CGGATGCGTATTTGCTGGGCGCACAACTGCTTGGCCTGGCCCCTCAGGAGTGCGTCGTAGTAGAAGATGCCCC
GGCTGGCGTGCTGTCCGGGCTGGCTGCGGGCTGCCACGTCATTGCGGTCAATGCGCCGGCCGACACGCCGCGT
CTGGCGGATGTTGATTTTGCGTTGGATAGCCTGACGCAATTATCGGTCGCTAAACAACCAAACGGCGATGTGG
TGGTGTTAAGAAAGACGTGATCATGATATCGCCCCGCTTTGGCGGGGCATATTTATGGCAGAATCGCGTTATC
TTCTCATCTGACAAGGATATGTTGTGAACGGTGAATTGATTTGGGTTCTCTCTTTACTGGCGATTGCCGTCGT
GTTGTTTGCGACGGGCAAAGTACGTATGGATGCGGTGGCTTTATTCGTCATTGTTGCTTTTGTCCTTAGCGGT
ACCTTGACCCTTCCGGAAGCCTTCTCCGGCTTTAGCGATCCGAATGTCATTCTTATCGCCGCCTTATTTATTA
TTGGCGACGGCCTCGTGCGCACCGGCGTTGCGACGGTGGTCGGTACCTGGCTGGTTAAAATGGCGGGCAGCAG
TGAAATCAAAATGCTGGTTTTACTGATGATCACCGTCGCCGGACTTGGCGCTTTCATGAGTTCAACCGGCGTG
GTGGCGATCTTTATCCCCGTTGTTCTTAGCGTTTCTATGCATATGCAGACCTCGCCTTCGCGCCTGATGATGC
CGCTGAGTTTTGCCGGGCTTATCAGCGGAATGATGACGCTGGTGGCGACGCCGCCGAACCTGGTGGTTAACAG
TGAACTGCTGCGTGAAGGCCTGCACGGGTTCAACTTTTTTAGCGTGACGCCGCTGGGGATAGTGGTGTTGGCG
CTCGGCATTGTTTATATGCTGGTGATGCGCTTCATGCTAAAAGGCGATGCGCCCGGACAACAGGCCGGTAAGC
GGCGCACTTTCCGCGATCTTATCCGCGAGTACCGTCTGACGGGGCGCGCTCGCCGTCTGGCGATTCGCCCCGG
TTCGCCAATGGTGGGACAGCGGCTGGACGACCTTAAACTGCGCGAACGTTACGGCGCCAATGTCATCGGCGTT
GAACGCTGGCGGCGCTTTCGGCGGGTTATCGTCAATGTGAACGGCGTATCGGAGTTTCGCGCTCGCGACGTTC
TGCTGATTGATATGTCGGCGGCGGAGGTTGACCTCAGAGAGTTCTGCGCCGAGCAACTGCTGGAACCGATGGT
ATTGCGTGGAGAATATTTTTCCGATCAGGCGTTGGATGTTGGCATGGCGGAAATTTCGCTTATTCCGGAGTCT
GAACTGATAGGCAAGTCGGTGCGTGAAATCGCCTTCCGTACACGTTACGGGCTGAATATGGTCGGCCTGAAAC
GCGACGGCGTCGCGCTGGAAGGGTCGCTGGCCGATGAACCGTTATTGATGGGCGATATCATTCTGGTCGTGGG
GAACTGGAAGTTAATCAGCCTGCTGGGGCAAAAAGGCCGCGACTTTGTGGTGCTGAATATGCCGGTCGAGGTC
AGCGAAGCCTCTCCTGCGCACAGCCAGGCGCCGCACGCCATTTTCTGCCTGGTACTGATGGTGGCGCTGATGT
TGACCGATGAAATTCCCAACCCGATCGCGGCGATTATCGCCTGCCTGCTGATGGGGAAATTTCGCTGTATTGA
TGCTGAAAGCGCGTATAAAGCGATCCACTGGCCGAGTATTATCTTGATTGTGGGTATGATGCCGTTTGCGCTG
GCGTTGCAAAAAACCGGCGGCGTCTCTCTGGTCGTGCAAGGATTAATGGATATCGGCGGCGGCTATGGGCCGT
ATATGATGCTGGGATGCCTGTTCGTGCTGTGCGCCGTCATAGGACTGTTCATTTCTAACACGGCAACGGCGGT
GCTGATGGCGCCGATTGCGCTGGCGGCGGCGAAATCGATGGGGGTGTCGCCCTATCCGTTTGCGATGGCGGTG
GCGATGGCGGCATCCGCCGCCTTTATGACGCCGGTTTCATCGCCGGTCAATACTCTGGTTCTGGGGCCGGGAA
ACTATAGCTTTAGCGATTTTGTGAAGCTTGGCGTGCCGTTCACGCTTATTGTGATGGCGGTTTGTATTGTGAT
GATTCCGATGTTGTTTCCGTTTTAACCCCAGACAGGAAAAGGCCTGATGGCGCTACGCTTATCAGGCCTACGG
ATATCACGCTACGTCCAGGCCGGATAAGACGTTGACGTCGCCATCCGGCAAAACCGTTTAAAGCGGTGAATCC
TGGCTTATCTCGTCAAGAGAGAGATGGAAACTTGGTACGAACACCGCCATAAAATAGTCCATCTCCTGGCTGC
GACGTAATTCCAGCGTTTTTTCCAGCCGCGTTTTTGCCAGTCCAAACTCATTGTTTCCCGCTGAGAGCTCCTC
CAGGCATTTCAGGTAGGCGCAAAGCGCATCGGCCTGTTTTACGATTGCCTGTTCTTCTTCGCTCCAGGCATTC
TCATCAATAAGCGGTGCGAAAATATCGCGTAATTCATCCGGCGCCATATCAACCAGCTTTTGCTGGGCGATTT
TTTCGATCGCTTTATATTCCTGGGCTATCTGGGAATTAAAATATTTTACCGGCGTCGGCAGATCGCCGGTCAG
TACTTCCGAGGCATCATGATACATCGCCAGAAGCGCGATGCGTTCAGCATTGAGCTGACCGCCAAATTTACGG
TTTTTTATCGCCGCCAACGCATGGGCGACCATCGCCACCTGCAGACTATGTTCGGAGACGTTTTCCGTACGCA
CGTTACGCATCAGCGGCCAGCGGTTAATCAACTTCATGCGGGAGAGATGGGCGAAAAAATGGCTCTGCTTCAT
AGATTACCTTTTGTTTTCACTGCGGCAGACTTCATTGTGCGGGGAGGGTGATGAAGATGCAATTCAGGCTACC
GAAGTAGCCTGAATTTAAGATTATTGATGGTAACCTGAGAGGAAACGCCCAAAACGGTTGATGGCCATCTCCA
GGTCATCTTCACGCGGTAGCGTCACAATGCGGAAGTGATCCGGCCACGGCCAGTTAAAGGCGGTACCTTGCAC
CAGCAGCACCTTTTCCTGTAGCAGGAAGTCCAGCACCATCTTCTGGTCGTCGTGAATATTAAAACGTTTAGCG
TCGATCCTGGGGAACATGTACAACGCGCCGCGCGGTTTTACGCAGGAAACGCCGGGGATATCGTTAATCAACT
CCCAGGCGCGGTTACGCTGTTCATACAGTCGACCGCCCGGCAGAATAAATTCGCTAATGCTTTGATAGCCGCC
GAGCGCGGTCTGAATAGCATGTTGCGCCGGGACGTTGGCGCACAGGCGCATTGAGGCTAACATCTCCAGCCCT
TCAATATAGCCCTTCGCATGTTTTTTCGGCCCGTTCAGCACCATCCAGCCCTGGCGGAAGCCGGCGACGCGAT
AGGTTTTAGACAACCCATTAAAGGTGATGGTCAGCAGATCAGGTGCCAGTGCGGCGATAGAGTGATGCTCGGC
ATCGTCATAGAGAATCTTGTCATAAATTTCATCGGCGAAGATGATGAGATTATGCTCGCGCGCAATGTTGACA

ATTTCCATCAACAATTCTTTAGAATAGACGGCGCCAGTAGGGTTGTTTGGATTAATAATGACAATACCACGCG
TACGTGGGGTGATTTTGGCGCGGATATCGTCAAGGTCCGGGAACCAGTCGGAAGATTCATCGCAAAGATAATG
TACCGCCTTGCCGCTGGAGAGCGATACCGCCGCTGTCCATAACGGATAATCGGGCGCGGGAACCAGCATTTCA
TCGCCACTGTTAAGCAACGCCTGCATCGCCTGAACGATCAGCTCGGAAACGCCGTTACCGATATAGATATCTT
CCACGGTTACATCGCGCATTCCACGGGCCTGATAATGTTGCATAATCGCTTTACGGGCAGAATAAAGCCCTTT
CGAGTCGCAATATCCTTGCGCCGTCGGCAAATTACGAATGACATCGACCAGAATTTCGTCCGGCGCTTCGAAT
CCGAAGGGGGCGGGGTTGCCGATATTCAGTTTAAGAACTTTATTACCTTCTTCCTCAAGGCGTTTCGCTTCTT
TCAGAACGGGGCCACGAATGTCATAACAGACATTTTCGAGTTTGCTGGATTTTTCAATGGGGGACATGAATCT
TGACCTTTTAGCTGTTATTGCCACTCCCTGCCGTGGAAGTCAGCACGAAACAATGTACTCCCACGACGGTGAG
TTTTGAAGAGTGAACAGAAGAAGATTTTGTTTATTTTTAACGCGCTTTCGTGCTGTTTAAAACTTCATAAAAT
CATTTGGTTAGATGTATTTTCTATTTTTGTTTGTATGTATAGTGTTTTATTCTATGTGATTGCGCGTAATGTG
GTTTTTTATGTTGTCAGAAATTATCTGTTTTATGAGAGATAAGCATTCATCATTAGTGTCGCGTCGGCTATTT
CGGAAATACTGAGTATTAAAGGTAAAATGAGAAGGTTAGACCTTTCCGGCGGCGGGGGGTGTGAAACTATTGT
TAAGTATAATTAATGAATGGTAGAAATCTTAATTAATTATTATTTAATATTTAAAAAGCATTTACTAAAGTCG
TAATAATATCTTTCTGGTCCGATTAGTATAACATATCCCAGGTTAAGATTATTCGTAATAATACTTATAAATA
TAGCGGTTTTAGGTTAAGATAATGATCCGAAACGGGGGCGTAGTCTGCGTTGTCTGCTAATTATCATGACGCG
ATTTTATACATAACCTATTGTTCAATATATTGTTTTTGTATCTGTGCGTCTCGGTAAACTATTTGACGCTGTT
ACAAGATTTACACGTCCATCATTAGCAGAAATCTGTAGACGCCTAAAGGTATGACACATTCTCTTTGATATAT
CCAGATCGGTTGCATTTGGTATTATATCAGTAAGTAAGAGAAGTGTTTTGGTCGTATTTTCAATTACATTCTA
ACATTTACCTGGGTAACCAGGCGGTACTGCGAAGGATGCACATTACAAATGGAATTGCTTGTTTCTGAATGCA
CAGCATTAACCAGGTCAGCCCGATATGACCCGCCAGTAAGTGAAAAATTATGATAAATGCAAATCGTCCGATA
ATTAACCTCGATCTCGATCTGCTGAGAACGTTCGTGGCCGTTGCCGATCTGAACACCTTCGCCGCGGCGGCGG
CGGCGGTATGTCGTACTCAGTCTGCGGTCAGTCAGCAAATGCAGCGTCTTGAGCAACTCGTGGGAAAGGAATT
GTTCGCCCGCCATGGTCGTAACAAACTTTTGACCGAACACGGTATCCAGCTTCTGGGGTACGCCAGAAAAATC
CTGCGTTTTAATGATGAGGCGTGTTCATCATTAATGTTCAGTAATCTTCAGGGAGTGTTAACAATCGGCGCTT
CTGATGAATCAGCTGATACTATCCTGCCTTTTTTGCTTAGCCGAATCAGCTCGGTTTATCCGAAACTCGCGCT
TGATGTACGGGTAAAACGCAACGCCTATATGGTGGACATGGTTAAATCGCAGGAAGTAGATTTGGTGGTGACG
ACTAACCAACCTCATTCGCTGGACTGTCTGAATTTGCGGACCTCGCCCACCCATTGGTATTGTGCGGCTGAGT
ATGTTTTGCAAAGGGGAGAGCCCGTACCACTAGTCCTGCTGGACGATCCCAGTCCATTCCGCGATATGGTGCT
GGAGACGCTAAATGCAGCGGGTATTCCCTGGCGGCTGGCCTATGTGGCGTCTACATTACCGGCGGTACGCGCG
GCCGTTAAAGCCGGTCTTGGCGTAACGGCTCGACCTGTCGAAATGATGAGTCCCGACCTTCGCGTGCTGGGCG
CCGCAGACGGATTACCGCCTTTGCCGGATACGGAATATCTGTTGTGCCGCGATCCCAATAGCCAGAACGAGCT
GGCGATGGTGATTTTCCATGCGATGGAAAGCTATCAAAACCCATGGCATTACAACCAGTTTTCTGCAGAGGGC
GGAGATGATCCGCTGATGGTGGAAGGGGGTTTTGAGTAGCTCGCAGGCAAAAGGCGCTAAGTCGTAAATGTAA
AAAAGAGCCGCTGGCGATGAAAAAACGCTCAGTGGCTCTTTTTTTGTGCCCATCTGTACGCGCTTAACCGCAC
AAATTGTAAAAGTTGCGGTAAATCAGCCAGTAGCGATTCGCAGTTCAATGTGGGTAAAAAATAACCAGAACAT
CCATTTTATGAATTTGCATGTTAAAAAACTAGAGAAATTGTTGCTGTTTTAGGGGGTGAATTAACAAAAGCGT
GTCACAGATCAAGAAAATACTCCCATTTAGGGGGAGGCATCTTCACAAATTCCTGTCAAAATAGGTGTGTGTT
ATTTCTTTTGCTTCACAACGGACACGATTCAACAACATAAAATCCCCCCTGGTCGAAGAGTCATTTTCTCCAG
GATGGCACTTAATGTTAATGATGATGAATTGATGTAAATTAATGTGAAGGAACTTTTGTTAAAGTTGACAAAA
GGTTATAGAAAGGAGTAAAAAACCACATCAATTAGCTGTTTTTATCATTTTCTACAGTAATTGTAGGGTTTTT
TTATTCCTCCCCATGAATCGATGTGGCGTCCATCTGCCGTAAAGAGCAGAGGAACTGGCGCTACTTTTGATGA
GTAAGCAATGAGTATGTCAACATCCACTGAAGTCATCGCTCATCACTGGGCATTCGCTATCTTTCTTATCGTT
GCCATTGGCCTGTGCTGCCTGATGCTGGTAGGCGGTTGGTTTTTGGGCGGTCGCGCACGCGCGAGGCACAAGA
ACGTTCCGTTTGAATCAGGTATCGACTCGGTCGGCACCGCCCGCTTACGCCTGTCTGCCAAGTTTTACCTGGT
AGCCATGTTCTTCGTTATCTTCGACGTTGAAGCGCTGTATCTGTTCGCATGGTCGACTTCTATCCGCGAAAGC
GGCTGGGTTGGCTTTGTGGAAGCTGCAATTTTTATTTTTGTGTTACTGGCTGGTCTGGTTTATCTGGCGCGTA
TTGGCGCGTTGGACTGGACGCCCGCGCGTTCACGCCGCGAGCGTATGAACCCGGAAACGAACAGTATCGCTAA
TCGTCAACGCTAACCGCGAGGCATTAAGATGGATTATACGCTCACCCGCATAGATCCTAACGGTGAGAATGAC
CGTTACCCCCTGCAAAAACAGGAGATCGTAACCGACCCCCTGGAGCAGGAAGTTAACAAAAACGTGTTCATGG
GCAAACTGCATGACATGGTTAACTGGGGCCGTAAAAACTCAATTTGGCCCTACAACTTCGGCCTTTCTTGCTG
CTATGTAGAGATGGTGACCTCTTTTACCGCAGTGCATGACGTTGCCCGTTTCGGCGCGGAAGTGCTGCGTGCG
TCGCCACGTCAGGCTGACCTGATGGTGGTGGCCGGGACCTGCTTTACCAAAATGGCCCCGGTTATTCAGCGTC
TGTATGACCAAATGCTGGAGCCAAAATGGGTCATTTCTATGGGCGCCTGCGCCAACTCCGGCGGCATGTACGA

TATTTATTCGGTAGTGCAGGGCGTTGATAAGTTCATCCCGGTGGATGTGTATATCCCTGGCTGTCCGCCGCGT
CCGGAAGCGTATATGCAGGCGTTGATGCTGCTGCAAGAATCGATTGGCAAAGAGCGCCGTCCGCTCTCATGGG
TGGTGGGCGATCAGGGCGTGTATCGCGCCAATATGCAGCCAGAACGCGAACGTAAACGCGGCGAGCGTATTGC
CGTCACGAACCTGCGTACCCCGGACGAGATTTAATTTGCGCCTGTCGGCAGCAGCACTTCACTTCGCTAACCA
CTATTTTAATAGCGCGAAGCACTGCCGACCACCACGGACCATTTGCAATGGTGAACAATATGACCGACTTAAC
CGCGCAAGACGCTGCATGGAGTACCCGCGATCATCTTGATGATCCGGTTATCGGCGAACTGCGCAACCGTTTT
GGGCCGGATGCCTTTACCGTTCAGGCGACCCGCACCGGTATCCCCGTTGTTTGGGTCAAGCGTGAACAACTAC
TGGAGGTTGGCGATTTCTTAAAGAAACTGCCGAAACCTTACGTCATGCTGTTTGACTTACACGGCATGGATGA
ACGCCTGCGTACGCACCGTGACGGGTTACCCGCCGCGGATTTTTCCGTTTTCTACCACCTGATTTCCATTGAG
CGTAATCGCGATATCATGCTCAAGGTGGCGTTGTCTGAAAACGACCTGCGCGTGCCGACCTTCACTAAACTTT
TCCCTAACGCCAACTGGTACGAGCGTGAGACCTGGGAGATGTTTGGTATTGATATCGAAGGCCACCCGCACCT
GACGCGCATCATGATGCCGCAAACCTGGGAAGGCCATCCGCTGCGTAAAGATTATCCGGCGCGCGCGACCGAA
TTCGACCCGTTTGAGCTGACCAAAGCCAAGCAGGATCTGGAAATGGAAGCGCTGACCTTTAAGCCGGAAGACT
GGGGGATGAAGCGCGGGACTGACAACGAGGACTTTATGTTCCTCAACCTCGGCCCGAACCACCCGTCTGCGCA
CGGTGCGTTTCGTATTATCCTGCAACTTGACGGTGAAGAAATCGTCGACTGCGTGCCGGATATCGGCTATCAC
CACCGCGGCGCCGAAAAAATGGGCGAGCGACAGTCCTGGCATAGCTACATTCCCTATACCGACCGTATCGAAT
ACCTCGGTGGTTGCGTCAATGAAATGCCGTACGTGCTGGCGGTAGAAAAGTTGGCAGGGATCACCGTCCCGGA
TCGTGTGAACGTCATCCGCGTGATGCTCTCCGAACTGTTCCGCATTAACAGTCATCTGCTGTACATCTCGACC
TTTATTCAGGACGTCGGCGCGATGACGCCGGTCTTCTTTGCCTTTACCGACCGGCAGAAAATTTATGACCTGG
TGGAAGCCATTACCGGTTTCCGTATGCACCCGGCCTGGTTCCGTATCGGCGGCGTCGCGCACGACTTGCCGCG
CGGTTGGGATCGCCTGCTGCGAGAGTTCCTTGAGTGGATGCCGAAGCGTCTGGATTCTTACGAGAAAGCCGCG
CTGCGTAACACTATTCTGAAAGGGCGTTCGCAGGGCGTTGCCGCTTACGGCGCGAAAGAGGCGCTGGAGTGGG
GCACCACCGGCGCAGGGCTGCGCGCGACCGGGATCGACTTCGACGTACGTAAATGGCGTCCATACTCTGGCTA
CGAAAACTTTGACTTTGAAGTGCCGGTGGGCGGCGGCGTTTCCGACTGCTATACCCGCGTGATGCTGAAAGTG
GAAGAGCTGCGCCAGAGTCTGCGCATTCTTCAGCAGTGCCTCGATAACATGCCGGAAGGGCCGTTCAAAGCGG
ATCACCCGCTGACCACGCCGCCGCCGAAAGAGCGCACGCTGCAACATATCGAAACCCTGATCACCCACTTCCT
GCAGGTCTCCTGGGGCCCGGTGATGCCGGCGCAGGAGTCCTTCCAGATGGTGGAAGCGACGAAGGGGATTAAC
AGTTACTACCTGACCAGCGACGGCAGCACTATGAGCTACCGTACCCGCGTGCGCACGCCGAGCTTTGCGCACC
TGCAGCAGATTCCCTCGGCGATCCGCGGTAGCCTGGTCTCCGACCTGATTGTCTATCTGGGTAGTATCGATTT
TGTTATGTCAGATGTGGACCGCTAATTATGCACGAGAATCAACAACCACAAACCGAGGCTTTTGAGCTGAGTG
CGGCAGAGCGTGAAGCGATAGAGCACGAGAAGCACCACTACGAAGACCCGCGTGCGGCGTCCATTGAAGCGCT
GAAAATCGTTCAGAAGCAGCGTGGCTGGGTGCCGGACGGCGCGATCTACGCTATTGCCGATGTGCTGGGTATT
CCGGCGAGCGACGTCGAAGGCGTCGCCACATTCTACAGCCAGATTTTCCGTCAGCCGGTCGGTCGCCACGTGA
TTCGCTACTGCGACAGCGTGGTGTGCCATATCACCGGCTATCAGGGGATTCAGGCCGCGCTGGAAAAAAATCT
CAACATCAAACCGGGGCAGACGACCTTTGATGGTCGCTTTACGCTGCTGCCGACCTGCTGCCTGGGGAATTGC
GATAAAGGGCCGAACATGATGATTGATGAGGATACTCACAGCCATCTGACCCCGGAAGCCATTCCTGAACTAC
TGGAGCGGTATAAATGAAAAACGTTATCCGTACTCCCGAAACCCATCCGCTGACCTGGCGTCTGCGCGATGAC
AAACAGCCGGTCTGGCTGGATGAATATCGTAGCAAAAACGGCTACGAAGGCGCGCGTAAGGCGCTGACCGGCC
TGTCTCCGGATGAAATCGTCAGCCAGGTAAAAGACGCCGGTCTGAAAGGGCGCGGCGGCGCGGGCTTTTCCAC
CGGTCTGAAGTGGAGCCTGATGCCGAAAGATGAATCCATGAACATCCGTTACCTGCTGTGTAACGCCGATGAA
ATGGAGCCGGGCACCTATAAAGACCGTCTGCTGATGGAACAACTGCCGCACCTGCTGGTGGAAGGCATGCTGA
TCTCCGCGTTCGCGCTGAAAGCGTATCGCGGCTACATCTTCCTGCGCGGCGAATATATCGAAGCGGCTGTACA
TTTGCGCCGTGCGATTGCTGAAGCCACCGAGGCCGGGCTGCTTGGCAAAAATATCATGGGCACCGGGTTCGAC
TTTGAACTGTTTGTCCACACCGGGGCAGGGCGTTACATCTGTGGTGAAGAAACGGCGCTGATTAACTCCCTCG
AAGGCCGCCGCGCGAACCCACGCTCCAAGCCGCCCTTCCCGGCGACCTCCGGCGTATGGGGCAAACCGACCTG
TGTGAATAACGTCGAAACGTTGTGCAACGTCCCGGCAATCCTCGCCAACGGCGTGGAGTGGTACCAGAACATC
TCGAAAAGCAAAGATGCGGGTACCAAGCTGATGGGCTTCTCAGGCCGCGTGAAGAATCCGGGCCTGTGGGAAT
TACCGTTCGGCACCACCGCGCGCGAGATCCTCGAAGACTACGCGGGCGGTATGCGCGATGGCCTGAAATTCAA
AGCCTGGCAGCCTGGCGGGGCGGGAACCGACTTCCTGACCGAGGCGCATCTTGACCTGCCGATGGAGTTCGAA
AGCATCGGTAAAGCAGGCAGCCGTCTGGGCACCGCGCTGGCGATGGCGGTAGACCACGAAATCGGCATGGTGT
CGCTGGTGCGTAACCTGGAAGAGTTCTTTGCCCGCGAATCCTGCGGCTGGTGTACGCCATGCCGCGACGGGCT
GCCGTGGAGCGTGAAAATTCTGCGCGCGCTGGAGCGTGGCGAAGGCCAGCCGGGGGATATCGAGACACTTGAG
CAACTGTGTCGTTTCCTCGGACCGGGTAAAACCTTCTGCGCGCATGCGCCGGGCGCGGTTGAGCCACTGCAAA
GCGCAATTAAGTATTTCCGCGAAGAATTCGAAGCTGGCATCAAGCAGCCGTTCAGCAATACCCATTTAATCAA

TGGTATTCAGCCGAACCTGCTCAAAGAGCGCTGGTGAGTTAACGATTTTTAATTTGGAAGCATGCTAATGGCT
ACGATTCATGTAGACGGCAAAGAATACGAGGTCAACGGCGCGGACAACCTGCTACAGGCATGTCTGTCCCTCG
GCCTTGATATTCCGTATTTTTGCTGGCACCCGGCGCTGGGAAGCGTCGGTGCTTGCCGCCAGTGTGCGGTGAA
GCAATACCAAAACGCGGAAGATACGCGTGGTCGCCTGGTGATGTCCTGTATGACGCCAGCCACCGACGGTACC
TTTATCTCTATTGACGACGAAGAAGCGAAACAGTTCCGTGAAAGCGTGGTGGAGTGGCTGATGACTAACCACC
CGCACGACTGTCCGGTCTGTGAAGAGGGCGGTAACTGCCATCTTCAGGATATGACCGTGATGACGGGTCACAG
TTTCCGTCGCTACCGTTTCACCAAACGTACCCACCGCAACCAGGATCTGGGGCCGTTCATCTCTCACGAAATG
AACCGCTGCATCGCCTGCTACCGTTGTGTGCGTTACTACAAAGATTATGCCGACGGCACCGATCTGGGCGTCT
ATGGCGCGCATGACAACGTCTACTTCGGGCGTCCGGAAGACGGTACGCTGGAAAGCGAATTCTCCGGTAACCT
GGTCGAGATTTGCCCGACCGGCGTCTTCACCGATAAAACGCACTCCGAGCGTTACAACCGTAAATGGGACATG
CAGTTTGCGCCGAGCATCTGCCAGCAGTGTTCCATCGGCTGTAACATCAGCCCGGGCGAGCGCTATGGCGAAT
TACGCCGTATCGAAAACCGTTACAACGGTACCGTTAACCACTACTTCCTCTGCGACCGCGGTCGTTTCGGCTA
TGGCTATGTGAACCTGAAAGACCGTCCGCGTCAGCCGGTTCAGCGCCGTGGCGACGACTTCATCACCCTCAAC
GCCGAGCAGGCGATGCAGGGCGCGGCGGATATTCTGCGCCAGTCGAAGAAAGTGATCGGTATCGGTTCGCCAC
GCGCCAGCATTGAGAGCAACTTTGCGCTGCGTGAACTGGTTGGTGCGGAAAACTTCTACACCGGTATCGCGCG
CGGCGAGCAGGAACGTCTGCAACTGGCGCTGAAAGTGCTGCGTGAAGGCGGCATTTACACCCCGGCGCTGCGG
GAAATCGAATCTTATGACGCGGTACTGGTCTTGGGCGAAGACGTTACCCAGACCGGCGCGCGCGTTGCGCTGG
CGGTACGTCAGGCGGTGAAAGGTAAAGCGCGTGAAATGGCGGCGGCGCAGAAAGTGGCCGACTGGCAGATTGC
GGCCATCCTTAACATCGGCCAGCGTGCCAAACATCCGCTGTTCGTGACCAACGTGGACGACACCCGTCTGGAT
GATATCGCCGCCTGGACCTATCGCGCGCCGGTGGAAGATCAGGCGCGCCTGGGCTTTGCGATTGCCCATGCGC
TGGACAATACAGCGCCTGCGGTTGACGGTATCGACAGCGACTTGCAAAACAAAATTGATGTCATTGTGCAGGC
GTTGGCCGGCGCGAAAAAACCGCTGATTATCTCCGGCACTAACGCCGGTAGCAGTGAGGTGATTCAGGCGGCC
GCCAACGTGGCGAAAGCGCTGAAAGGCCGTGGCGCCGACGTCGGTATTACTATGATTGCCCGCTCCGTGAACA
GCATGGGGCTGGGTATGATGGGAGGCGGTTCGCTTGACGATGCGTTGGGCGAGCTGGAAACCGGCAGCGCGGA
CGCGGTGGTGGTGCTGGAAAACGATCTCCACCGTCATGCAAGCGCTACGCGCGTGAACGCCGCGCTCGCCAAA
GCGCCGCTGGTGATGGTGGTTGACCATCAGCGCACGGCGATTATGGAAAACGCGCATCTGGTGCTTTCCGCGG
CAAGCTTCGCCGAAAGCGATGGTACGGTCATCAACAACGAAGGCCGCGCGCAGCGCTTCTTCCAGGTTTATGA
TCCGGCCTACTACGATAACAAGACGATTATGCTGGAAAGCTGGCGCTGGCTGCATTCGCTGCACAGCACCGTC
GAAAACCGCGAAGTGGACTGGACACAGCTTGACCATGTGATCGACGCGGTCATTGCCGCCATGCCGCAATTTG
CCGGCATTAAAGACGCCGCGCCGGATGCGACCTTCCGCATTCGTGGGCAGAAGCTGGCGCGCGAACCGCATCG
TTACAGCGGTCGTACGGCGATGCGCGCCAATATCAGCGTGCATGAACCACGTCAGCCGCAGGATAAAGACACC
ATGTTCGCCTTCTCGATGGAAGGGAATAACCAACCGACTGCCCCGCGATCTGAAATTCCGTTCGCCTGGGCGC
CGGGCTGGAACTCCCCGCAGGCGTGGAACAAATTCCAGGATGAAGTGGGCGGCAAACTGCGTCACGGCGATCC
GGGCGTGCGTTTGATTGAAGCGACTGAGGGCGGTCTGGATTATTTCACTACCGTGCCGGCAAGCTTCCAGGCG
CAGGACGGTCAGTGGCGTATTGCGCCGTATTACCACCTGTTTGGCAGCGACGAATTGTCTCAGCGTTCTCCGG
TCTTCCAGAGCCGTATGCCGCAGCCGTATATCAAACTTAACCCGGCGGATGCCGCGAAGTTGGGCGTCAATGC
CGGGACGCGCGTCTCCTTTAGCTACGATGGCAATACGGTGACGCTGCCGGTTGAAATCTCTGAAGGGTTAGCG
GCAGGGCAGGTAGGGCTGCCGATGGGTATGCCTGGCATCGCGCCGGTTCTGGCTGGCGCGCGTCTTGAGGATC
TGCGGGAGGCGCAACAATGAGTTGGATTACACCGGATCTGATTGAGATCCTGCTGAGCATTCTCAAAGCGGTG
GTGATTCTGCTGGTGGTCGTCACCTGCGGGGCCTTCATGAGCTTTGGCGAACGTCGTCTGCTGGGTCTGTTCC
AGAACCGTTATGGACCAAACCGCGTTGGCTGGGGCGGCTCGCTCCAGCTGGTCGCGGATATGATCAAGATGTT
CTTTAAAGAAGACTGGATCCCGAAATTCTCGGATCGCGTCATCTTTACCCTGGCGCCGATGATCGCATTTACT
TCGCTGCTGCTCTCCTTCGCCATTGTGCCGGTTAGCCCTAATTGGGTGGTGGCCGATTTAAACATCGGGATTT
TGTTCTTCCTGATGATGGCCGGTCTGGCGGTTTACGCGGTGCTGTTCGCCGGTTGGTCGAGCAACAACAAATA
CTCGCTGCTGGGCGCGATGCGCGCGTCTGCCCAAACGGTGAGCTATGAAGTGTTTCTTGGTCTTTCCCTGATG
GGCGTGGTGGCGCAGGCCGGTTCATTTAATATGACCGATATCGTCAATAACCAGGCGCATCTGTGGAACGTGA
TTCCGCAATTCTTTGGGTTTGTTACTTTCGCCATCGCGGGCGTAGCGGTCTGTCACCGTCACCCGTTTGACCA
GCCGGAAGCCGAGCAGGAACTGGCGGACGGTTACCACATCGAATATTCCGGTATGAAATTCGGTCTGTTCTTC
GTGGGGGAGTACATCGGTATCGTCACCGTTTCCGCGCTGATGGTAACGCTGTTCTTCGGTGGCTGGCATGGCC
CGTTCTTACCGCCATTCGTCTGGTTCGCGCTGAAAACCGCGTTCTTCATGATGATGTTCATTTTGATTCGTGC
GTCGTTACCGCGTCCGCGTTATGACCAGGTAATGTCCTTCGGCTGGAAAGTTTGCCTGCCGCTGACGCTCATC
AACTTGCTGGTAACGGCGGCTGTCATTCTGTGGCAGGCGCAATAAGGGACACAAAGACCATGACCTTAAAAGA
ATTATTAGTAGGTTTCGGCACTCAGGTACGCAGTATCTGGATGATCGGCCTGCATGCGTTCGCCAAACGCGAA
ACGCGGATGTATCCGGAAGAGCCGGTCTATTTACCGCCCCGTTACCGTGGCCGTATCGTGCTCACGCGCGACC

CGGACGGCGAAGAGCGCTGCGTTGCCTGCAACCTGTGCGCGGTAGCCTGTCCGGTAGGCTGTATCTCTCTGCA
AAAAGCGGAGACCAAAGATGGCCGCTGGTATCCGGAATTTTCCGCATTAACTTCTCACGCTGCATCTTCTGCG
GCCTGTGCGAAGAAGCGTGCCCGACCACGGCGATTCAGTTGACCCCGGACTTCGAACTGGGCGAATACAAACG
TCAGGATCTGGTGTACGAAAAAGAGGATCTGCTGATTTCCGGTCCGGGCAAATACCCGGAATATAACTTCTAC
CGGATGGCGGGTATGGCAATCGACGGCAAAGATAAAGGCGAAGCAGAGAACGAAGCCAAGCCTATCGACGTCA
AGAGCCTGTTACCGTAAGGAGAGGGCAATGGAATTCGCTTTTTATATCTGTGGCCTGATAGCCATCCTCGCGA
CCCTGCGAGTGATCACCCATACCAATCCGGTACACGCGCTGTTGTATCTGGTGATATCGCTACTGGCGATTTC
CGGGGTGTTCTTTTCGCTGGGCGCTTACTTTGCCGGTGCGCTGGAAATTATCGTCTACGCAGGCGCCATCATG
GTGCTGTTTGTGTTCGTGGTGATGATGCTCAACCTCGGCGGCAGCGAAATCGAACAGGAACGCCAGTGGCTGA
AGCCGCAGGTGTGGATTGGTCCGGCGGTTTTGTCGGCCATTATGCTGGCGGTCATTGTGTACGCCATTCTGGG
CGTTAACGACCAGGGCATCGACGGCACGCCGATTAGCGCGAAAGCGGTGGGCATTACGCTGTTCGGGCCTTAT
GTTCTGGCGGTCGAACTGGCGTCTATGCTGCTGCTGGCAGGCCTGGTGGTGGCGTTCCACGTCGGTCGTGAAG
AGCGTGCGGGCGAAGTGCTAAGCAACCGCGCCGATGACCGCGCGAAAAGAAAAACGGAGGAGCGCGCATGATC
CCCTTAACACACGGACTGATCCTCGCTGCGATTTTATTCGTCCTGGGTTTAACCGGTCTGGTTATCCGCCGCA
ATCTGCTGTTTATGCTGATCGGGCTGGAAATCATGATTAACGCCTCCGCGCTGGCCTTTGTGGTCGCCGGGAG
CTACTGGGGCCAGACCGATGGTCAGGTGATGTACATTCTCGCCATCAGCCTTGCGGCTGCGGAAGCGAGTATT
GGACTTGCGCTGTTGCTGCAACTCCATCGCCGTCGCCAGAACCTGAACATCGATTCAGTAAGTGAGATGCGTG
GATGAACATGCTTGCCTTAACCATTATTCTGCCATTGATTGGCTTTGTACTGCTGGCGTTCTCTCGCGGACGC
TGGTCGGAAAATCTCTCCGCGACTATTGGCGTGGGCTCCGTTGGTCTGGCGGCGTTGGTGACAGCGTTTGTCG
GGATGGATTTCTTCGCCAACGGCAAACAGGCGTTCAGCCAGCCGCTGTGGACGTGGATGTCGGTCGGTAACTT
CAACATCGGTTTTAACCTGGTGCTGGACGGCCTGTCGCTGACTATGCTCTCTGTGGTCACCGGCGTCGGCTTC
CTGATCCACATGTTCGCGTCCTGGTACATGCGTGGGGAAGAGGGCTACTCCCGCTTCTTCGCTTATACCAACC
TGTTTATCGCCAGCATGGTTGTTCTGGTGCTGTCCGATAACCTGCTGTTGATGTACCTCGGCTGGGAAGGCGT
GGGCCTGTGCTCTTATCTGTTGATCGGCTTCTATTACAGCGATCCGAAGAATGGCGCAGCGGCAATGAAAGCC
TTCGTGGTGACCCGTGTCGGTGACGTGTTCCTCGCCTTCGCGCTGTTCATTCTCTACAACGAACTGGGCACGC
TGAACTTCCGCGAAATGGTCGAGCTGGCGCCAGCGCACTTTGCTGACGGCAACAACATGCTGATGTGGGCGAC
GCTGATGCTGCTGGGCGGCGCGGTCGGTAAATCTGCGCAGCTACCGTTGCAGACCTGGCTTGCCGACGCGATG
GCCGGCCCGACGCCGGTCTCCGCGTTGATCCACGCCGCGACGATGGTTACCGCCGGTGTCTACCTGATTGCAC
GCACGCATGGCCTGTTCCTGATGACGCCGGAAATTCTGCATCTGGTGGGTATTATCGGGGCGATTACGCTGGC
CATGGCGGGTTTCGCCGCGCTGGTGCAGACCGACATCAAACGCGTGCTGGCCTATTCCACCATGAGCCAGATT
GGCTACATGTTCCTGGCGCTGGGCGTGCAGGCGTGGGATGCGGCGATTTTCCATCTGATGACCCATGCGTTCT
TCAAAGCGCTGCTGTTCCTGGCGTCCGGTTCCGTCATTCTGGCCTGCCATCACGAACAGAACATCTTTAAGAT
GGGCGGTCTGCGTAAGTCTATTCCGCTGGTGTATGCCTGCTTCCTGGTGGGCGGCGCGGCGCTGTCGGCGCTG
CCGCTGGTCACTGCGGGCTTCTTCAGTAAGGATGAGATCCTGGCTGGCGCGATGGCGAACGGTCATATCAATC
TGATGGTGGCAGGTCTGGTCGGCGCGTTCATGACCTCCTTGTACACCTTCCGGATGATTTTCATCGTATTCCA
CGGTAAAGAACAAATTCATGCTCATGCAGGGAAGGGGATTACCCACCATCTGCCGCTGATTGTCCTGATGATT
CTGTCTACCTTTGTTGGCGCGCTGATTGTGCCGCCTCTGCAGGGGGCTGCCGCAGACAACTGAACTGGCGCAT
GGTCGTGTGTTGACCCTGGAAATTACCTCTGGCGTAGTGGCGATTGCCGGCATCCTGATTGCCGCATGGCTGT
GGCTGGGTAAACGTACGCTGGTGACGTCGATCGCCAACAGCGCGCCGGGCCGTCTGCTGGGCACCTGGTGGTA
TAACGCCTGGGGCTTTGACTGGCTGTACGACAAAGCATTCGTCAAGCCATTCCTCGGTATCGCCTGGCTGCTG
AAGCGCGACCCGCTTAATGCGTTGATGAACATTCCGGCTATCCTTTCCCGTTTTGCAGGCAAAGGCCTGGTAC
TGAGCGAGAACGGTTATTTGCGCTGGTATGTGGCATCCATGAGCATCGGCGCGGTTGTAGTGCTGGCGCTGTT
GATGGTACTGCGTTGAGTTTGTTGGCTGGATGAACGACTGCCGGATGGCGCAGTGCTTATCCGGCCTACAAAC
CCAAAAATAATTTTTAGAATTCGTTGAAAATCTGTTCCTGAAGTACAGGCGTCATCGGCGAAGTCAGACCGGA
TACCGCCAGCGCACAGCAACCGGAGCGTACTGAAGTACGTGAGGATGATTCGCGAAGCTCACCCCTACGGGGC
CGTCCTTACGGACGTTCAAAACGCGCTGCGTTTTGTGTGAGCACTGGCAGGATTTGGGCTGACGAGTGAGATA
GCCTGAGCAGGAGTAAATATAAGGGACATACTTAGCTATGTTATTACCCTGGTTGATATTAATCCCCTTTATC
GGCGGCTTTCTGTGCTGGCAGACCGAACGCTTTGGCGTGAAGGTGCCGCGCTGGATAGCGCTGATTACCATGG
GGCTGACGCTGGCGCTCGGTCTGCAATTGTGGTTGCAGGGCGGCTACTCACTGACGCAATCCGCCGGTATTCC
ACAGTGGCAATCTGAATTTGTTCTGCCGTGGATCCCGCGTTTTGGCATCTCTATCCATCTGGCGCTCGACGGA
CTGTCGCTGCTAATGGTGGTGCTGACCGGCCTGCTCGGCGTTCTGGCGGTGCTTTGCTCCTGGCGAGAAATCG
AAAAATATCAGGGCTTCTTCCACCTGAACCTGATGTGGATCTTAGGCGGCGTTATCGGCGTGTTCCTGGCCAT
CGACATGTTCCTGTTCTTCTTCTTCTGGGAAATGATGCTGGTGCCGATGTACTTCCTGATAGCGCTGTGGGGC
CACAAAGCTTCTGACGGGAAAACGCGTATCACGGCGGCGACCAAGTTCTTCATCTACACCCAGGCGAGTGGTC

TGGTGATGCTGATTGCCATCCTGGCGCTGGTCTTTGTGCACTACAACGCGACCGGCGTGTGGACCTTCAACTA
TGAAGAGCTGCTGAATACGCCGATGTCCCACGGTGTGGAATATCTGCTGATGCTGGGCTTCTTCATCGCCTTC
GCGGTGAAAATGCCGGTGGTTCCGCTGCATGGCTGGCTGCCGGATGCGCACTCCCAGGCGCCGACCGCCGGTT
CCGTCGACCTGGCCGGTATCTTGCTGAAAACCGCCGCTTACGGTCTGCTGCGTTTCTCCCTGCCATTGTTCCC
GAACGCGTCGGCGGAGTTTGCGCCTATCGCCATGTGGCTGGGCGTTATCGGCATTTTCTACGGCGCGTGGATG
GCCTTTACCCAATACGACATCAAGCGCCTGATTGCCTATACCTCCGTTTCCCACATGGGCTTCGTGCTGATCG
CTATCTACACCGGCAGTCAGCTGGCGTACCAGGGCGCGGTGATTCAGATGATTGCACACGGTTTGTCCGCAGC
CGGTCTGTTCATTCTGTGCGGCCAGTTGTACGAACGTCTGCATACGCGTGACATGCGTATGATGGGCGGCCTG
TGGGGCAAGATGAAGTGGTTGCCTGCGCTGTCCATGTTCTTTGCGGTGGCGACGCTCGGTATGCCGGGCACCG
GTAACTTTGTCGGCGAATTTATGATTCTGTTCGGCAGCTACCAGGTGGTGCCGATGATTACCGTGATCTCCAC
CTTTGGGTTGGTATTCGCCTCTGTATACTCGCTGGCGATGCTGCATCGCGCTTACTTTGGTAAAGCGAAGAGC
CAGATTGCCAGCCAGGAACTGCCAGGGATGTCGCTGCGCGAGCTGTTTATCATTCTGTTGCTGGTGGTGCTTC
TGGTACTGCTTGGCTTCTATCCGCAGCCGATTCTGGATACCTCGCATTCTGCGATGAGCAACATCCAGCAGTG
GTTTGTTAATTCTGTTACTACTACAAGGCCGTAAATCGCCATGACAATAACTCCACAACACCTGATTGCGCTG
CTACCGCTGCTGATCGTCGGCTTGACGGTGGTGGTTGTGATGCTCTCCATTGCGTGGCGACGCAATCATTTCC
TCAATGCCACGCTGTCGGTCATTGGGCTTAACGCTGCTCTGGTTTCGCTTTGGTTTGTCGGCCTGGCTGGGGC
AATGGACGTCACGCCGCTAATGCGCGTTGACGGCTTTGCGATGCTCTACACCGGGCTGGTGCTGCTGGCGAGC
CTCGCCACCTGCACCTTTGCCTACCCGTGGCTTGAAGGCTATAACGATAACCAGGAAGAGTTCTACCTGCTGG
TGTTAATCGCCAGCCTGGGCGGAATTCTGCTGGCGAACGCCAACCATCTGGCGGCGCTGTTCCTCGGTATTGA
ACTGATCTCACTGCCGCTGTTCGGCCTGATTGGTTATGCTTTCCGCCAGAAACGCTCGCTGGAAGCCAGTATC
AAATACACCATCCTCTCCGCCGCCGCCTCGTCTTTCCTGCTGTTCGGGATGGCGTTGGTGTACGCGCAGTCCG
GCAACCTGTCTTTCGAAGCGCTCGGTAAGAGCCTCGGCGATGGTATGCTGCACGAGCCGTTGCTGCTGGCGGG
CTTTGGCCTGATGATTGTCGGTCTGGGCTTTAAACTCTCTCTGGTGCCGTTCCACCTGTGGACGCCAGACGTT
TACCAGGGCGCGCCTGCGCCGGTTTCCACCTTCCTGGCGACGGCGAGCAAAATCGCTATCTTCGGCGTGGTGA
TGCGTCTGTTCCTGTATGCGCCGGTAGGCGACAGCGAAGCGGTACGCGTGGTGCTGGGCATTATCGCCTTTGC
CTCTATCATCTTCGGTAACCTGATGGCGCTGAGCCAGACCAACATCAAACGTCTGCTCGGTTACTCTTCTATC
TCTCACCTCGGCTATCTGCTGGTGGCGCTGATTGCCCTGCAGAGCGGCGAGATGTCGATGGAAGCGGTAGGCG
TTTATCTGGCCGGTTATCTGTTCAGCAGCCTCGGCGCGTTTGGCGTGGTCAGCCTGATGTCCAGCCCGTTCCG
CGGCCCGGATGCCGACTCCCTGTACTCTTACCGTGGTCTGTTCTGGCACCGTCCGGTTCTTGCCGCGGTGATG
ACGGTGATGATGCTGTCGCTGGCAGGTATCCCAATGACGCTGGGCTTTATCGGCAAATTCTACGTGCTGGCGG
TCGGCGTTCAGGCCAGCCTGTGGTGGCTGGTCGCCGCCGTGGTGGTGGGTTCCGCGATTGGTTTGTACTACTA
CCTGCGCGTTGCGGTGAGTCTCTACCTGCACGCGCCGCAGCAACCGGGACGCGACGCGCCGACAAACTGGCAG
TACAGCGCAGGCGGTATCGTGGTGCTGATCTCCGCGTTGCTGGTGTTGGTACTGGGCGTCTGGCCGCAGCCGC
TGATTAGCCTTGTGCAACTGGCGATGCCGCTGATGTAATGCCGCGTTGATGATGTCAATAACCGCCGATTTAT
CGGCGGTTGAGGCTGATGAGCAATACCTGAGCCTCTAAATGTTGGATTGCATCCAACTTATGAAAACCTGTGA
CGCATAGAGACCATAGACTCGTTGGGTGAGGCGTAGCCGAAACCCAACACTTTTCCGATGAACGCAGAGCCGG
ATGTTGGGCGGTTCCGTAAGCGCCAGCGGTTTCATATGGGTAATTCACAATAATTGTCGAAGTGATAAACATA
TGGCCCATTACTCCGCTATAAAAATTATAACGTGACTGTCATTTCTCAGGCATCGCTGATAAAAACTCAGCAG
TTTGTCCATTTCTTGCATCAGGCAGTGGAACAACTCTCCCAACTGTTTTCGTGACCCGTTTCCCGGATAAATG
TCTATCTCATCGACTTCAATGGGAAAAATTTGATCTGAAGCGTATTTCTGTCAGCGTTTTTCAAGGCGTCCGA
TCGCGCTCACTTCATTATTTCATGTGACGGCGGCATATAATATCTTTCCATCAATAACGTGAGTGTCGGCACG
GCTTAATAGATTGCTGTTTGGTGGGTTGTAAAAGGAAGTATCGGCGAGGGCACAGTGCAGATAGTCCCCCATC
TTATATATTCACTTTTGGTAAATCGGTCACCTGTAAATAGTGTTCTTCACGCAGAATCTGTGGGTCGAACGCC
AGCAACTGATTAAGCCGTTCATCTTAAACGTAAATATTATTTACCTCAAAAATATAATTATCTTTTTTATTAT
TGGTGGCTATTAACGCAGGTAAAATTATTTTCTACTTTCGTCATGCTGATAATAAAAGTAAAGCGGAATATAA
TGCAGGACATCTGCAGAAAATTACCCGAATATAATTGAAAAGGAATTTGTATGCTTAATGGAAAAACATTAAT
GCTGTTGTTGGGTGGGGTTGTTCTCTCGGGCTGTGGGCCGGAGCCCTCCGACCCACAGGGAAATAATCCGGCT
GAGTTAAAACAGGAGCAGGCTATCCAGAAAGAAAATAGCGCTCAGGCCGGGGATGACACTGTCCAGAAAAGAC
AAGCCGAGGCCGCCCAACAGGCTGCGAAAAAAGCAGCCGAATATAAAGCGAATACTGAAGCCAAAGCCCTGGC
AGATGCCAAAGCGGCATCTCTGGCAACAGCCGAAGCACCGCAATATGAAATGCGGACACGTGCCGCGGCATCA
AAAGCGTTCGCTGCACAAGGCGGTAATGTAATGGGGACCGCGCGTTACGAACACTACGATGAAAATCCGATTA
AACAGGTAAGTCAGGCGCCGCTCGCCACGTTTAGTCTGGATGTAGACACTGGCAGCTACGCTAACGTGCGGCG
CTTTCTGAATCAGGGACAACTGCCGCCGCCGGAAGCCGTGCGGGTAGAAGAGATGCTCAACTATTTTCCCGCG
CCTCAGCCCGTTGCGGATAAGCAGGATAACACTAAACCCATTGCGGCCTGTATACCGATGCCATTTGCGGTTA

AATACGAACTGGCTCCCTCGCCGTGGAACGCGCAGCGTACGCTATTAAAAGTTGATGTTCAGGCCCGGGACAT
GCAGGTCAGGGATCTGCCACCTGCCAACCTGGTTTTTCTCATTGATACTTCTGGTTCTATGCAGCCAGCGGAA
CGCCTGCCGCTGATCCAGTCGGCGCTAAAACTGCTGGTGAACGATCTGCGTGCGCAGGATAACATCACGATTG
TGACCTACGCGGGCGGCACTCACGTCGCGTTGGCGTCTACAGCGGGAAATAACACAACCGCGATTAAAGCGGC
AATTGATAATCTGGATGCTTACGGGAGTACCGGTGGTGAAGCGGGATTACGGCTAGCCTACGAGCAGGCCGAA
AAAGGGTTTATCAAAGGCGGCGTTAACCGCATTTTGTTGACCACCGACGGTGATTTTAACCTCGGTATTACCG
ATCCCAAAGACATCGAAGCGCTGGTAAAAAAAGAGCGTGAGAAAGGTATTACCTTATCTACGCTGGGCGTCGG
CGATGACAATTTCAACGAAGCCATGATGGTGAGGATTGCTGATGTGGGTAACGGCAATTACAGCTACATCGAC
TCCCTCTCCGAGGCGCAAAAAGTCCTCAAGGATGAGATGCATCAAACGCTGGTCACCGTTGCCAAAGATGTAA
AATCGCAAATCGAATTTAATCCGCAGTGGGTGACTGAGTACCGGCAGATTGGTTATGAAAAACGCCAACTGCG
CGACGAGGATTTCAATAACGATAAGGTTGATGCCGGTGATATCGGCGCGGGTAAACACGTCACGCTATTCTTT
GAACTGACGCTTAACGGGCAGAAAGCCTCGGTGGATAAACTGCGCTACGCTCAGGACAAAGCCGCCTCAAAGC
CAACAAAATCAAGCGAGCTGGCGTGGATCAAATTGCGCTGGAAAGCGCCGCAGGGCAGCGAAAGTACATTAGC
CGAGTTCCCGGTCGTTATGGGAAAAATGCCGATCTTTGCTGACGCCTCTGAAGATTTTCGTTTCCGCGCGGCG
GTAGCGGCTTTCGGGCAAAAACTGCGTGGCTCAGAAACGCTGGCAGATACGACCTGGCCGCAAATTATTAAAT
GGGGTGAACAGGCGCGCGGGGAAGATAGACAAGGCTATCGCGCGGAGTTTATTAAACTGGTAAAACTGGCGGA
AGGCTTGTCTCACTAAGCGTCAGGGGATGCCTGTACTGTAGGCATCCCCTGTTACGTCAGGCGCTTTTGCGGC
TGATCAGTGGTCCCTGGCTGTTTGTTGCTGCGCGCTCCAGCACTTCCTGAATGACCGAAGAGAGTTCGTTAAT
CTCAAACTTGGCCACGTAGCCGTCGGCCTTCACCTTACGTATATGATCTTCGTTGGCGCTGCCGGAAAGGGAA
GAGTGAATAACGACCGGGATCTTTTTCAGCCGTTCATCGGTTTTAATTTTACGCGTTAACGTAAAGCCATCCA
TCTCCGGCATCTCAAGATCTGTCAGGACCAGCGCGATTTTCTCGCTAATTGGCTTACCTTCGGCCTCCGCTTC
CTGCGCCAGTTGCTGGATTCTCTCCCATGCATCCTTGCCGGTCACATGCATCTGGTGCGGAATTCCCATCGCG
TTCAGCCCTTTTTCCAGCATCGCGCGGGCGACTTTGGAGTCTTCCGCCACAATGGCTACCGCGCCAGGGGTAA
TATAAAATTTATTGGTCTTCAGATTGGTGGCGCGCAGGTCGTGGCTGGAGGGGACAATATCATAAAGGATCTG
CTCAACGTCGAGCACCAGCGCCAGGTTGTTGGTTTCTTTGTTATCATCCAGGCAGGCAATACTGGTGATGTAG
CGACCGTTGACGGCTTTCTCGGCGGTATGCACCTGTTGCCAGTCAAGACGCATAATATTTTCCACCGACTCGA
CGGCAAATGCCTGTACGCTGCGCGCATATTCGGTGATCAGCAAAATATTCAGCCCGGTTTCCGGCTTGCAGCC
CGCTACCGCTGGCAAATCAATCACTGGAATGACCTGATCCCGAATATTGACCATGCCCAATAGCGGCGCTTTC
ATGCCTGCCGGGCGCGTAAAGGCGGGCATTGGTACGATTTCGCGCAGCTTAAATACATTGATGCCGAACAGCT
CTGACTTCTGTTCATGCAGCGATGTACCAAGACGGAATAACAGCAGTTCAAAACGGTTGGACAGGGTTAAATT
GGCCCTGTCATCAATATCTTTCTGAAAATTGTCCATCCTAACCTCATTATGAAAGCCTGCCTTTTTTAGTGTT
ATCGGCAATGAGGACAAAAAATGGAGCGCTAAACCGGGAAAACAGAAAAATCGTAAGCCAGCTCCGTCGCCGG
GAAAATAGCGCGGCATTCCGCCAGTAAGCGCTGACAGCCTTTATCGTCATAGCGTGAGCTAATATGGGTCATG
ATCAGCCGACCGACGGCAGCTTCCCGCGCCAGCGTCGCGGTTTGACGGGTGGAGCTGTGTCCGCGAGCGTTGG
CTTTCTCCTCCATTGACGCATCCAGCGTGGTTTCATGGACCATCACATCCACGCCTTGCGCCAGCGCGAGGGC
GGCCTCGCAGGGCGCGGTATCGCCGAAAATCGCCACTGATTTCCCGGCCACGGCGGGAGCAAGGTAGTCGGCG
CCATTGATCTGCCTTCCATCGGCAAGTGTGACGGTTTTTCCTGCCTTCAACGCCTGAAACAGCGGGCCGGGCG
TAACGCCTGCGGCTTTTAACGCCCTGGCATTCAGCGCGCCAGGCTTGTCATGTTCAACAACGCGATATCCGTA
GCACTCCAGTGGATGCTCCAGCCTGAATGCGGTCACTTTACGCAGACCGTCGTCAAGAATATCGCCTGCGCTA
ACTTCTTCAATTTGCAACGGAAAATCGGTCCATGAACCGCTAAGACGCAGTGTGGTTGCAATAAACTCACGCA
CGCCTTGAGGCCCGTATACGGTTAGCGGATGGGGGTTCCCCGCCATCGACCGGCTGCATAGCAGACCCGGCAG
GCCAAACAGATGATCGCCGTGAAGATGGCTGATAAAAATGCGCTCAAGCTTACCGGGATGAAACGCGGTATTA
AGCATTTGATGCTGTGTCCCTTCGCCGCAATCAAAAAGCCATACTCCCGGCTGCGTTGGATGTTGCAGATGTA
ATAAAATTGCCGTTACATTGCGTGAGCGCGTGGGAACGCCTGCGGACGTACCTAAAAAAATTAATTCCATCTT
GTCCATACACGTTTGGTTATCGTTTAACCCAAGATTACCACAACACAGGAGCGCATCATGATTGACTGGCAGG
ATCTCCACCATAGTGAACTTACCGTTCCTCAACTTTACGCGTTACTCAAATTGCGCTGCGCGGTCTTTGTGGT
AGAACAGCGCTGTCCTTACCTGGATGTGGATGGCGATGATTTAGTCGGCGACAATCGGCATATTCTGGGCTGG
CATCAGGATGAGCTGGTGGCGTATGCGAGGATTCTGAAAAGCGATAACGAATCAGACCCGGTTGTCATTGGCC
GGGTGATCGTCAGCGATGCCTGGCGAGGGGCGAAACTGGGCCAGCAATTAATGGCGAAAACGCTGGAATCTTG
TGGGCGGCACTGGCCGGATAAGCCGCTATATCTGGGAGCGCAGGCGCATCTGCAACCTTTTTATGCGCGGTTT
GGTTTTATTCCCGTCACCGATGTTTATGACGAAGACGGCATTCCGCACCGGGGAATGGCGCGGGAGGTTCATC
AGGCATAAACCGAAACCGTTGACTATAGTTAGGAGCAGGTGTTAACACAACGGAGAATGAGAATGTCATATCA
ATTTGGTGAATCACGTGTTGATGATGATCTGACTTTGCTCAGCGAAACGCTGGAAGAGGTTTTACGTTCCTCA
GGCGATCCCGCCGATCAAAAATACATTGAGCTGAAAGCCCGCGCGGAACAAGCGCTGGAAGAGGTGAAAAACC

GCGTAAGCCATGCGTCGGATAGCTATTATTACCGGGCAAAGCAGGCCGTTTATAAAGCGGATGACTACGTTCA
TGAAAAACCCTGGCAGGGTATTGGCGTAGGCGCGGCGGTAGGACTGGTGCTTGGACTGCTGCTGGCGCGGCGC
TAAATCCCCCCACTGAACAGAATTGCAACCCTCCCTAAATGAGGTACTGCTTTTTTAGGGCAGTACCCCGTAT
AATGTGGGGTTTTTAACAGGGAGGGTCCGCGTGCATTCTATTACTACTGCGCTGGAAAATCTGACGCGTCAGC
TATCGCAAGAGATACCGGCGACGCCAGGGCTTTGCGTTTTCGATGCGCCTTTCCCTTTAAACGATGCCTTCGA
TGCATTGAGCTGGCTGGCCAGCCAGTCATCCTTTCCGCAATTCTACTGGCAACAGCGCAATGGCGACGAAGAA
GCTGCCGTTCTCGGGGCCATCACCGTATTTTCATCGCTCGATCTGGCGCAACGGTTTTTACGTCAGCACGCGC
AGCCTGACCTGCGTATTTGGGGGCTGAATGCGTTCGAGCCGAAAGAGGGCTACTTATTGCTGCCGCGGCTGGA
GTGGCGACGTAGCGGTGGTACGGCGACCTTACGCTTACATCTGCACAGTGACGTTTCGCTACGCGATGATGCG
CGCCAGGCGAAAGCGTTTCTCGCTTCTCTGGTCGGCATTAAGCCTGTTCCGGCGCTACGCCTTTCGCTCACGA
GCGAACAACACTGGCCGGACAAAGACGGTTGGGTGAAGCTGATTCAGCAGGCCACCCACACCATTGCGCAAGA
GGCGTTTGATAAAGTTGTGCTGGCGCGGGCCACCGACTTACAATTCTCCCGTCCGGTCAATGCCGCCGCGATG
ATGGCGTCCAGTCGTCGTTTAAATCTCAACTGCTACCACTTTTTTATGGCCTTTTCCGCCGATACCGCCTTTC
TGGGATCATCGCCGGAGCGGCTCTGGCGGCGGCGTGAAACGGCGCTGCGTACCGAAGCGCTGGCTGGGACGGT
GGCGAACCATCCGGATAACCATAAAGCCTGGCAGCTTGGCGAATGGTTAATGAAAGACGATAAAAACCAGCGA
GAAAATATGCTGGTTGTCGAAGACATCTGCCAGCGACTGCAAAACTGCACCCACTCGCTGGATGTGCTGCCGC
CGCAGGTGCTCAGACTGCGCAAGGTTCAGCATTTGCGCCGCTGTATCTGGACGGCGCTTAATCAGGCGGACGA
CACGCTCTGCCTGCACCAGTTACAGCCTACCGCAGCGGTCGCCGGGATCCCTCGTGAACCCGCGCGTTGTTTT
ATAGAACAACATGAGCCGTTCGAACGTGAATGGTATGCCGGGTCGGCAGGCTATCTCTCCACGCGGCAAAGTG
AGTTTTGCGTGACGTTACGTTCAGCCAAAGTGACGGCCAATGTGGTGCGATTGTATGCCGGCGCGGGAATCGT
CCGCGGCTCCGATCCGGAACAGGAATGGCAGGAAATAGATAATAAAGCGGCCGGGTTACGTACCTTATTGCAG
ATGGATGCGTAATGAGTCACATATTCCCGACCCATATCAATAATCCATTTTTGCAGCTCTTTATACTTAGTCC
CAATGATTGATACCGGACAAACTCATGTCAGTAAGTGCATTTAACCGGCGCTGGGCGGCGGTCATCCTGGAAG
CCTTAACCCGTCATGGCGTCAGGCATGTTTGTATCGCCCCTGGTTCCCGTTCCACGCCGCTCACGTTGGCGGC
GGCAGAGAATCCTGCTTTTATTCACCATACGCACTTTGATGAGCGTGGGCTTGGTCATCTGGCGTTAGGGCTG
GCGAAAGTCAGCCAACAGCCGGTTGCCGTTATTGTCACTTCAGGTACCGCCGTGGCGAACCTCTATCCGGCGC
TGATTGAGGCGGGGCTGACGGGCGAGAAGCTCATTTTACTTACCGCCGATCGCCCGCCGGAGCTGATTGACTG
CGGCGCCAACCAGGCGATCCGTCAGGCGGGGATGTTTGCCTCGCACCCCTCCCAAACTCTGTCGTTGCCGCGT
CCCACGCAGGATATTCCGGCGCGCTGGCTGGTGTCGACCATCGACAATGCGCTCGCCATGCTTCATGCGGGCG
CGCTGCACATTAACTGTCCTTTTGCCGAACCGCTGTATGGCGATATGAATGACACCGGTCTGGTCTGGCAGCA
GCGTCTCGGCGACTGGTGGCAGGATGAAAAACCCTGGCTGCGCGAAGCGCGTCGACTGGAAAGCGACAAACAG
CGCGACTGGTTTTTCTGGCGACAAAAACGCGGCGTCGTTGTCGCCGGGCGTATGAGCGCGGAAGAAGGCAAAA
AAGTGGCGCAGTGGGCGCAAACTCTGGGATGGCCATTAATTGGCGACGTATTGTCGCAAACCGGGCAGCCGCT
GCCGTGCGCCGACCTGTGGCTCGGCAACGCCAAAGCGGTGACCGAACTGCAGCAGGCGCAGATTGTCGTCCAG
CTTGGCAGTAGCCTTACCGGAAAACGCTTACTGCAATGGCAGGCAACCTGCGAGCCGGAAGAGTATTGGGTAA
TCGACAATATTGAAGGTCGTCTGGACCCTGCGCACCATCGTGGACGCAGACTGGTAGCGAAAATCGCCGACTG
GCTGGAACTACATCCGGCGGAAAAACGTAAACCGTGGTGTGTAGAGATCCCGCGTCTGGCGGAACTGGCGTGG
CAACGGGTGGTCGCGCAGCGGACACCTTTGGCGAAGCGCAGCTGGCGCACCGTATCCGCGACTATCTGCCGGA
GCAGGGGCAATTGTTCGTGGGCAATAGTCTGGTGGTGCGTCTGATCGACGCGCTGTCACAGTTGCCGGCTGGC
TATCCGGTATACAGCAATCGCGGGGCGAGCGGCATTGACGGCCTGCTTTCCACGGCGGCAGGCGTGCAGCGCG
CCAGCGCCAAATCGACGCTGGCTATCGTCGGCGATTTATCCGCGCTGTACGATCTTAACGCGCTGGCGTTACT
ACGTCAGGTATCCGCGCCGTTTGTGTTGATTGTGGTCAACAATAACGGCGGGCAGATCTTTTCCCTGCTGCCG
ACGCCGCAAAGCAAACGCGAGCGCTTTTATTTGATGCCGCAAAACGTCCATTTTGACCATGCGGCCGCCATGT
TTAACCTGCGCTATCATCGCCCGGAAAACTGGGAAGAGCTGGAGTCTGCGCTGGCTGGCGCGTGGCGAACCCC
GGCGACAACGGTGATTGAGCTGGTGGTAAACGATACGGACGGCGCGCAGACGTTGCAGCAACTTTTGGCTCAG
GTAAGCCATCTATGATGTTGCACGCGCAGCACATGCCCGGACAGCCTGGGGCGCCCTCACTGGTTTTCCTGCA
CGGTTTTTCTGGCGATTGCCGTGAATGGCAGCCGGTTGGCGAACAGTTTCACGGCTGTTCGCGGCTTTATATT
GACTTACCGGGACATGGCGGATCTGCGGCTATTCCGGTCGGCGGCTTTGCCGATGTCATCAGATTACTGCGCG
CTACGTTAATTAGTTACAACATACTAAAGTTTTGGTTGGTGGGATACTCCCTTGGCGGCCGGGTGGCGATGAT
GGCAGCCTGCCAGGGAATACCAGGCCTGTGCGGCCTGGTGGTTGAAGGCGGTCATCCGGGGCTGCAAAATGAA
CAGGCGCGCGCTGAAAGACGATTGTCCGACGGGCGCTGGGCAGAGCGTTTTCGTCATGAACCATTGACTGAGG
TTTTTCACGACTGGTATCAGCAGCCGGTGTTCGCCTCGCTGACGGCGCAGCAAAGACAGGCGCTAACGGCGCT
GCGTAGCCAGAATAACGGCGAAACGCTGGCTGCGATGCTGGAGGCGACATCGCTTGCCGTCCAGCCGGATTTA
CGCGAGGCGCTCAACGCGCTGGCATTTCCGTTTTATTATTTATGTGGCGAACGCGACAGCAAATTCCGCGCCC

TGGCGCAGGAAGTGGCGGCGACATGCCATGTCATTCGTAACGCCGGACACAACGCGCATCGGGAAAACCCCGC
TGGCGTGGTAGACAGTCTGGCTCAGATTCTGCGTCTCTGACTAAAGGACACACTATGATCTATCCTGATGAAA
CAATGCTTTACGCACCGGTAGAATGGCACGACTGCTCGGAGGGTTACACCGACATTCGTTATGAAAAATCCAC
CGACGGTATTGCAAAAATCACTATTAATCGTCCGCAGGTGCGCAATGCATTTCGTCCGTTGACCGTCAAAGAG
ATGATTCAGGCGCTGGCGGATGCCCGTTACGACGACAACGTGGGCGTGATTATTCTGACCGGCGAAGGCGATA
AAGCGTTTTGCGCGGGCGGCGACCAGAAGGTGCGCGGCGATTACGGCGGTTATCAGGATGACTCCGGCGTGCA
TCACCTGAACGTGCTGGACTTCCAGCGCCAGATTCGCACCTGTCCAAAACCGGTTGTCGCGATGGTCGCGGGT
TACTCCATCGGCGGTGGTCATGTGCTGCACATGATGTGCGACCTGACCATCGCGGCGGAAAACGCCATCTTCG
GCCAGACCGGTCCGAAAGTCGGCTCCTTTGACGGCGGTTGGGGCGCTTCGTATATGGCGCGTATCGTTGGTCA
GAAGAAAGCGCGTGAAATCTGGTTCCTGTGCCGCCAGTACGATGCGCAACAGGCGCTGGACATGGGGCTGGTT
AACACCGTCGTTCCGCTGGCGGATCTGGAAAAAAGAGACCGTGCGCTGGTGCCGCGAAATGTTGCAAAACAGT
CCGATGGCGCTGCGTTGCCTGAAAGCGGCGCTGAATGCCGATTGTGACGGTCAGGCCGGGCTGCAGGAGCTGG
CGGGCAACGCTACCATGCTGTTCTACATGACGGAAGAAGGGCAGGAAGGCCGTAACGCCTTTAACCAGAAGCG
TCAGCCTGACTTCAGCAAATTCAAACGGAACCCATAATGCGTAGCGCGCAGGTATACCGCTGGCAGATCCCCA
TGGACGCGGGGGTGGTTCTGCGCGACAGGCGGTTAAAAACTCGCGACGGGCTGTATGTTTGTCTGCGTGACGG
CGAGCGTGAGGGATGGGGAGAGATCTCTCCGCTGCCGGGCTTCAGTCAGGAAACGTGGGAAGAGGCGCAGACG
GCGCTCCTGACGTGGGTGAACGACTGGCTTCAGGGGAGCGAGGGATTACCGGAGATGCCTTCGGTCGCGTTTG
GCGCAAGCTGCGCGCTGGCGGAACTGACTGGCGTCTTGCCGGAGGCGGCGGACTATCGCGCTGCGCCGTTATG
CACTGGCGATCCTGACGATTTGGTGCTGCGGCTTGCCGATATGCCCGGCGAGAAAATCGCTAAGGTCAAAGTG
GGTCTTTATGAAGCGGTACGCGACGGCATGGTGGTTAATTTGCTGCTGGAGGCGATCCCGGATCTGCATCTGC
GTCTGGATGCGAATCGCGCCTGGACGCCGCTAAAAGCCCAACAGTTCGCAAAGTATGTTAATCCGGATTACCG
CGCTCGTATCGCCTTTCTCGAAGAACCGTGTAAGACGCGGGATGATTCCCGCGCCTTTGCCCGTGAAACCGGC
ATCGCGATTGCCTGGGACGAAAGTCTGCGCGAAGCGGATTTCACCTTTGAAGCCGAAGAGGGCGTCAGGGCTG
TGGTTATCAAACCTACGCTGACCGGATCGCTTGATAAAGTGCGTGAGCAAGTCGCTGCCGCCCATGCGTTGGG
ACTGACGGCGGTCATCAGCTCTTCGATCGAGTCCAGCCTCGGCCTGACGCAACTGGCGCGGATTGCCGCCTGG
TTGACGCCGGGAACGCTGCCCGGACTGGATACCTTGCATCTGATGCAGGCGCAACAGGTTCGCCCCTGGCCCG
GTAGCGCGTTGCCTTGTCTGAAGCGTGATGAGCTGGAACGACTGTTATGACATTCACGGATTGGCCGTGGCGC
CACTGGCGTCAGGTTCGCAGCCAGGCGCCCGCTCTGCGCCTGAACGATGAAGTGCTAAGCTGGCGCGCCCTCT
GTGAGCGTATTGATGCGCTGGCGGGCGGGTTTGCCGCGCAGGGCGTACGCGAGGGCGACGGTGTCCTGCTGCG
CGCCGGGAATCAGCCGCGCACTCTGTTGGCATGGCTTGCGCTGATGCAGTGCGGCGCGCGGGTTCTGCCCGTC
AATCCGCAGTTGCCCCAGACGTTGCTGGAGGCGCTGGTGCCGAAGTTAACGCTGCGTTTTGCGCTGACACTGG
AAGGCGAAAACGCGTTTTCCGGATTAACCGCGTTGCAGATACAAAAGAGCACAGCGGCGTATGCTGTGGACTG
GCAACCGCAGCGTCTGGTTTCCATGACGCTGACATCGGGTTCCACAGGATTGCCAAAAGCGGCTGTACATACG
TGCCAGGCGCATCTTGCCAGCGCGCAGGGGGTGCTTTCGCTAATGCCGTTTGGCCCTCAGGATGACTGGCTGC
TATCGCTGCCGCTGTTTCATGTATCCGGGCAGGGTATTATGTGGCGTTGGCTCTTCGCCGGGGCGCGCATGAC
GGTACGCGATAAGCAACCGCTGGAGCAGATGCTGGCAGGGTGTACTCATGCTTCGCTGGTGCCGACGCAGCTG
TGGCGGCTGCTGGCGAATCGGGCGGCGGTGACGCTGAAAGCCGTTTTGCTCGGCGGCGCGGTGATTCCGGTTG
AACTCACCGACCAGGCCAGCAAACAAGGGATTCGCTGCTGGTGCGGGTATGGGCTCACGGAGTTCGCGTCTAC
AGTATGCGCCAAGGAGGCCGATGGTTCTGATGATGTGGGCGCGCCGCTCCCCGGCAGAGAGATCAGAATCGTC
GATAATGAAGTCTGGCTGCGCGCCGCCAGTATGGCGGAAGGTTACTGGCGTGATGGCAAACTGATCCCGCTGG
TTAATGACGAGGGCTGGTTTGCCACGCGCGATCGCGGCGATCTGAACCACGGCAGGCTAACCATCGCCGGGCG
GCTGGACAACCTGTTCTTTAGCGGCGGCGAAGGCATTCAGCCGGAGGAAGTCGAACGCGTTATTAATGCGCAT
CCGCTAGTGCAACAAGCCTTCGTCGTGCCGGTAGAAGATAAAGAGTTTGGCCATCGTCCCGTTGCGGTTGTGG
AGTATGCATCGCAGGCCGGAGACGTGAACCTCGCTGAATGGGTCCGCGATAAGTTGGCGCGTTTTCAGCAGCC
GGTTCGCTGGTTGACGCTGCCGTCAGAACTGAAAAACGGAGGGATTAAAATATCTCGCCGTGCGCTCCAGCAG
TGGGTGTGTGAGAACTGTAAAAATTAATGTTAGGTTAATATACAACCATTCCATCGCTATTGCCGTTTTGTTT
ATCCGTTGATGTGTAACGTGTTATGAACAATCAGCGTGAAACGGGGGCGCTATGGAATGGTTGGTTAAGAAAT
CGCATTATGTCAAAAAGAGGGCGTGCCATGTTCTGGTGCTGTGCGATAGCGGCGGTTCGCTAAAAATGATCGC
CGAGGCGAATTCCATGATATTACTGAGTCCCGGCGATATCCTGTCGCCTTTACAGGATGCGCAGTATTGTATT
AATCGGGAAAAACACCAGACCTTAAAAATCGTTGATGCACGCTGTTATTCCTGCGACGAATGGCAGCGGTTGA
CGCGCAAGCCATCATGATCTGGCGGGCAGAAAAATCAGCATTACCCCCGCCATAATGCACAATACGCCGAGCA
TCGCTTTTAGGCTGAAAGCGCCTTGCAAACCGGGTAACAACATTGAGGCTACCCAGACAAGCACATAGCTGAG
ACTCAGCAGCGCATAGGCTTTGCTGAGCGCGAGCGTGTGTAATGTTTTATGCCAGCAGAAGACGGAAACCAGA
TACCCCGCCAAACCGGCGAATAGCGCCAGGGTCGCGGCATTAAGAGCGCCCAGCCCGGAAATAAACGCCAGTG

GATGCGCTATCGATGGCAGTCGCATCATGGCGAATCCTAAACTCAACTGGGCGAGGGAGGCAATCGCAACGCT
TATCAGTCCCCACATTACGCCCATTATGCCGCACTCCCCAGAATGATAATGCCGCTGATAATTAATGCGACGC
CAAACCAGTGGCGGGGCGGAACCTGTTCATGCCAGATTTTCCAGGCCGCCAGCGTGACCCAGACAAAATTCAG
ACTCAACATCGGGTAAGCAATTCCCACCGGCAGGGTTTGTAGCACCAGCAGCCACAGCACCATCGCTGCACCC
ATGCATATCAGCGCCAGGCCCAGCCACAGCATCAAGTGACGGCGGCGTCCGCCCACCGTCAATGGCCGTGTCG
CTTGCTTCTGGCAGAGCTGACCGCCAACGCTGAGCAGACTGGCAAGCACCAGAATGACGCCGATCATTTAGGC
CGATACTGAATTAACACCAGACGCCCCTGATAATCAACATTGTCCGCAGGGGGAAGGGAAAGAGCGCTGATGT
CTTCGTCTTTGGCTATTGAAAGTACTAGCGTAATAATGCCCTCCTGACGATGCTGGTTGAGCCAGGCGTTAAA
ATCGTCGGCTTTAACGAATTTATCCTGCACATCAGGATAGCTAAGACCATACCTGAGTTCGCCCGCGTGTCCG
TAGAGCATAATATCGTCTCGTTTCAGACTCCAGGCCAGACCGGCGGCGACGCCGACGTTGTCGGCAAGAATAT
AGCGGCTTGATGCCAAAGGCGCCTGGGTCATTTCGACAAAAAACTGTGGCTGTTTGGACTCCATCACCCTGTC
GGGGATGGAAAAGCCGAACAGCAGCGCCAGTCCCAGCGGACAAAACGCGGGTAATAGGTATTGTTGGCTATGA
CAGAGGCTATACCAGCCCACAAACGCCCAAACAGTAAAGACGCCCCAGACGCAAAACACTTTATAGGTTTCGA
TATGCGTCCACACCGGCGATTTCAGCGGGCCCCATGAGGAGACGACAAACGCCGCGACGATCCCGATTATCCC
GAACACCAGGTTGATCCCGCCGTTGACACGTAGCGCGGCGACGCCTTCTTTTACGTTATGCAGGACGAAGCGC
GCCATGAGTATCGCTATCGGTGCGAAACAGGAAAGAACATAGGTGGGCAGTTTCCCTTTCGCAATGCTGAAAA
AGAGAAGCGGCATGATCGTCCATCCGAGCAAATAGAATGCGCCGTTTCGCTCGCGCCAGCCCAGTTTAAGCGC
GCCAGGCAGTAACCCCAGCCACGGTAAACTTCCCGCGAGCAGTACCGGCAGATAGTACCAGAAAGGGGCTTTA
TGCTGGGCATCGCTCATGGCGAATCGCTGGATATGCTCCACCCAGAAAAAGTAATGCCAGAAATCGGCTTCGC
GTCGCGCAATGGCGATCGCCCAGGGAAGGACAACCACAAAGCAGCTCAAGACCGCCAGCCAGCCGTATAGCAA
AAAGTCCTTCCAGCGTTTCTGAACAATAACCCACGGCAGCACGCTCACCACCGGTACGGCCAGGGCGAGGAAC
CCTTTAGTTAATACACCCAGACCGCAGGTTGCGCCCAGTAGCAAAAACATCCCAATCTTGCCCGTTCGGGTCG
TGGCCTGCATCCCCTGCCAGAAACAACACATCCCGGCCGTAAGCCAAAGCGCGATCATCGGGTCGAGTACCGC
ATACGTCCCGATACTGTAAACGGCAAAGAGGGAGAGAAAGATAACGGAGGCGAGCAACGCGGTACGTTTATCC
CGCCATAAGCGGAACGTCAGCCACGCCACCAGCGCTGCGGCCAGCAATGTCGTTAAAATAGCGCCAGCCCGCA
CGCCAAAATTGGTAGCGCCAAAAAGCCACTGACCCAGACTGTTTATCCAGTAACCGGCTATCGGTTTTTCAAA
ATAACGTAATCCCAGAAAATGCGGCACGATCCAGTCGCCAGACGCCAGCATCTCCCGACTTATTTCGGCATAG
CGCGTTTCATCCGGCTGCCAGAGCAGGCGGCTATTGACAGGAATAACATAGTAGAGCGCGATAAATGCGGCGA
AAGCCAGATAATAGCGTATCGATTTCATCATGAGGCGCTCGCTGCCTGTTGGCACCCCAGCCAACCTTCACGG
CCAGGGATGTGCCCACGTACAATTTGCCCCAACGGCAGAGACTCAGGCGATGTCGGCAGCAGTTCGCCCAGAG
GACAAAAGGTGATCCCCGCGTCACGTGCACGAACGAGCAAATCTTCAAACAGCGGCTGATGGACAATACCTTC
GACTTCCGCATGGATGGTATATACCGGCGTGCCTTTGTCCTGCAACATACGGGAGATAATCCAGGTATTGAAC
GACTGCGCCTGAACGGCGGGGCCGATAACCTCATCCCACGTGGGTAAGGTGACCGGGATTTGTGGCGTCCCGG
TCTGGCCCGGCATCAGTAGCGGACGGAATAAGGTGGTTCCACGGCAATCGCTGTTATACCGCAGGTTGAACGG
CTCTTTCGCACGAACAACGCGCCCATCCGCTCGCCAGCCGGCCGCGGCTGAGCAGGTGACGGGTTTTCCGATA
ATGGCCTCAAGCGCCGTTATGCCGCGCGCGATATCTTCTTCAAGCTGGCGGATACTCCAGTGGCCGCTGTGAG
TCTGCCACGCATGGTGATCCCAGGCATGTAGTCCCGTCTCATGGTATGTTGCCGTTTCACGAATAATCCCGGC
ATTGGCGTTGCCGATATTTTTTCCCGGCCAGGCGGTACCGGCCAGCAGGATATCCCAGCCGTAGAGCGAAGCG
GCGTTTGAACGTAGCATTTTCCATAAAAATCGCGGTCTTATCAGACGCCAAAGATGACGTCCCATATTGTCCG
GCCCGACGCTGAAGAAAAAGCTGGCCTGAACGCCGTGGCGATGCAATGTCGCCAGCAGCCGGGGAACACCTTC
ACGCGTTCCTCGCAAGGTATCAACATCAATGCGTAAACCGACTTTCGTCATGATGCGCGTTCCGCGACATCTA
CGCTGCGCAGGAAGAAGTCCAGCGTTTCTTCAACCGTATCACGCATCGCAATAGACGGTTCCCAGCCCAGACA
GCGCCTGGCATTGTCGATACTGGGTTTCCGGTGTGCGACATCCTGATAACCCTTGCCGTAATAACTACGGCTT
TCGACGACCTGGAAACCGGCAAACGGTGGGAAATGGCAGCGCAGCGGATGCTTATCAAAACTGTCGAGCAACA
GCGTCGCCAGTTCCTGAATACTGGCTTCGTTGTCAGGGTTGCCGATATTAATGATTTTGCCATCGCACCGATC
GCCATCATTCACAATAATGCGGAATAATGCTTCGATCCCATCGCGAATATCGGTGAAACAGCGTTTCTGCTGA
CCGCCGTCGATAAGCTTTATCGGCGTCCCTTCTACCAGGTTCAGAATCAACTGCGTTATCGCGCGTGAACTGC
CGATACGCGCCGCGTTCAGGCTATCCAGCCGCGGCCCCATCCAGTTAAAGGGACGGAAAAGCGTGAAGCGCAA
TCCCTCTTTTTCGCCATAAGCCCAAATCACCCGGTCGAGAAGCTGTTTGGAAACGGAGTAAATCCAGCGTGGC
TTATTCACCGGCCCGACGATCAGATTCGATTTGTCCTCGTCAAAAGAGGCGTCCGTACACATGCCGTACACTT
CAGAGGTAGACGGGAAAACCACGCGTTTACGGTATTTCACGCAATAACGGATAATCCGCAGGTTTTCTTCAAA
ATCAAGTTCAAAGACGCGCAGCGGGTTGCGGGTGTATTCAATCGGGGTTGCAATTGCTACCAGCGGCAGTACG
ACATCGCATTTTTTGACATGATATTCGATCCATTCTGAGTGGATGCTGATATCTCCCTCCACGAAATGAAATC
GAGGGTGGAGCAGGAATCGGCTAATGGCGTTGCTGCCGATATCCATACCGTAGACCTCGTAATTCTCTTCATT

CAGTAACCGTTCGGTCAAATGATTGCCGATAAAACCGTTGACGCCGAGGATGAGAACGCGAATGCGACGTTTA
CCGCTGGTCGCTGGTGGACGGTTCAAACGCGCGCCCGCCACCAGACCAAGCGTCTGTGCAAGCTGCGAGCCCT
GTACAGTAATGCCGTCTCCGGCCTGGCCGGTAATAATCTCCAGCGCGCCATCAGCGCAGGCGACCCGCAGCGG
AGAGACCGAAATTACCGAGCCGGGAAGCGCGCCCTGCGCATCCGGACAAATTCGGGAAGACCAGATAGTGAAT
TTTTGCGAGCCGTTATAGCTGAACGCGCCGGGCCAGGGGGCGGCTACCGCCCGCACCAGGTTATGCACCGTGG
AAACCGGTTTGTGCCAGTCGATAAGGCCATCTTCCGGCCTGCGGCGACCATAATATGTAGCGTCAGACTCACG
CTGGGGAACAGAAGGTATATCGCCGCATTTCATGGTGGGCAGGATGCTATTAAGCAACTGGCGCGCGGCCTGA
CATAGCTTATGATGCAGGGTAAGCGCAACATCGTCCTGCGCAATAGCAACGCGTTGACTGGCGACAATCTCTC
CGGCGTCGGCACGTTTAACCATACGATGCAGCGTTACGCCCGTTTCGCTTTCGCCGTTAACCAGAACCCAGTT
TAGCGGCGCACGGCCACGATAGGCAGGCAATAGAGAACCGTGCAGATTGAACGCGCCAGCCGGGGCGAGGTGC
AGAATTTCTTCGCTTAACAGGTTGCGGTAATAGAACGAAAAAATAATATCCGGCGCAAGCTCAGCGATGCGGT
CGACCCAGATAGGATGGTTTACGTTATCCGGTGCATAAACCGGAATCCCTAATTCTGCCGCCAGCCGGGAGAC
GGAGCCAAAAAAGGTGTTTTCCGCAGGATTGTCGGCATGAGTGAAAATCGCGGCAATCTCATACCCGGCATCC
AGTACGGCCTGCACCCCCTGACATCCCATATCGTGATAGGCAAAAATAACGGCTTTCATTGGTGGCTTTCCTC
AGTAAACGGCGTGCTTTCCGGGTAGATAACTTGTTGAACAAAATAGCGGGGGCGGGCGCGGACATCGTTATAA
ATGCGACCGATGTATTCGCCCAGCAGTCCCATACCGATGAATTGCGCGCCGATGAAGGTAAACAGAACGGCAA
AGAGCATAAATACGCCTTCCGCCGCCCACTGCGGCCCCAGCGCCAGGCGAAGTACAATAAGCAATACCGACAG
GCTAAACCCGCCGATGGCGATAACGCTGCCCAGCAGACTCAACAGGCGCAGCGGCGTCGTCGTCAGGCAGGTC
ACCAGGTCATACATCAGGTTAATGAGACGCATAAAACTGTATTTAGAGTCGCCAAATTCACGTTCAGCGTGAT
GCACCGGGATCTCGGTAGCCCGACGAGCGAAAATGTTGGCCAGAATAGGGATAAAGGTGCTGCGTTCATGGCA
GCGCAACATAGTATCGATAATAGGGCGGCGATAGGCACGTAACATGCAGCCGTAATCGCCCATCGCTTTGCCC
GTTGTGCGCTGGATCAGTAGATTAATGATTTTTGACGCCGATTTGCGAAACAGGCTGTCCTGTCGGTTCTGGC
GCACGGTGCCTACCACATCGAACCCTTCATCGGCTTTCGCCACCAGGCGCGGGATCTCTTCCGGCGGGTTTTG
CAGATCGGCATCCAGCGTAATGATGAGATCGCCGCTTACATGGCTAAACCCCGCCATGATCGCTGCATGTTGA
CCGTAGTTACGGTTGAGTAAAATAGAGATGATATGGCTATCGGCTTCCTGGGACGCCTTAACCATCAGCTCCG
CCGAGCTATCGCTGCTACCATCATCTATCAATAAAATCTCCCACGCTTTGCCCAGACTCTCGCAGGCGGCGGT
GGTGCGTCTTATCAATTCCGGCAGGCTTTCCTGTTCGTTATAGACAGGAATAACCACCGAGACTTTTTTTATG
GGGGCGGCGTCAAACATGCTTATTGTCCTGCTATCTGATGAAGGGCGGTAATGACCCGATCAAAGTCACTCTC
GGTCATGTCCGGGAAGAGCGGCAGTGAGCAGATCCGCTCGCTGTTCCACTCGGTGTCAGGCAGCGTGAGCGTT
GGGAAACGTTCGCGATAATATTTTTGCGTATGCGCGGCGCGGAAATGCAGCCCTGTGCCGATGCCTTTCGTTT
TCAGCGATGCCATCAGAGCATCCCGGGTAATACCGCAACGGGCTTCATCGACCCGGATAATGAATAAATGCCA
GGCGTGAATATGTTCCCACGACGGAAGGCTCAGAGGCTGAAAAGGCAGGTCTGCCATTGCCTGGTGGTACTGC
GCTGCGATGGCGGCGCGGCGAGCGTTAAGCGCATCCAGTTTCTGTAACTGAGCAAGCGCGATAGCGGCGTTGA
GGTCGGGCAAATTATATTTGTAGCCCGGCGCCAGGACTTCCGCTTGTGGCGCGCGTCCTCCGCTCTGTCGATC
CCAGGCATCAACGCCAAGCCCGTGAAACTTGAGACTGCGCAGTTTATCGGCGAATTGCGGGTTATCCGTGACG
ACGATACCGCCTTCAGCGCAGGTGATGTTCTTAATGGCGTGGAAGGAGAAAATCGCGGTGCCCCGGGCGCCAA
TGTGGCGACCTTTGTAGCTGGTTCCTGTCGCGTGCGCCGCGTCTTCAATCACCGGGATGCCATAACGCTCGCC
AAGAGCGTAAATAGCGTCCAGATCGGCGGGCGCGCCAGCGTAGTGGACGGGGATAATTGCTTTGGTTTGTGGC
GTAATCGCGGCTTCGATATGTTCAGGCGTGACCATCAGCGTATCGCGATCGACATCGACCATGACGGGGGTCG
CGCCGAGTAAAACGATCATATTTAGCGTAGAAACCCACGTCATTGACGGTGTAATGACCTCGTCGCCTTCACC
AATGCCCAGCGCCATCAACGCGATGTGCATACCGGCAGTGGCTGAACTGACGGCAACGGCATATTGATTGCCA
GTTAGCCGACAAAACGCCGCTTCCAGTTCCTGATTTTTCGGGCCGGTTGTTATCCAACCTGAGTCCAGTACGG
TCTTAACGGCAGCGAGCTCTTCCGCGCCCATCGCCGGACGGGAGAAAGGCAAAAAGTCCGACATCATTTTTCC
TTCAGCCATTGAAAGCCGCTTTTCAAATATTTCGGCAATAGAAAAGGGCTTATTTTTTAACGCATTTAGCTGA
ACCCTACTGGATGAAGATTAAATTAACATTAAGAAATTAAGGTGAAGTAAAAAGATAATTTTTAACGGAATTA
TGCTTTGCTTTCGCCGAAAGTTTAAGGCATTTTATGCATGCCCAAAAGCTGTTTTTATGTTTCTGGCCTGGTC
TTCCGTCGCGGAATTTCATTTTAATTGTATTGCTATTAAGTTAGCTGCACTGTCTGCCTGAGAGGTTAATGTG
CTGGCATTTACCCTACGCTTTATTAAAAACAAACGCTATTTCGCGATTCTGGCGGGGGCGTTGGTTATTATCG
CCGGGTTGACCTCACAACATGCCTGGAGTGGTAACGGTCTACCGCAGATTAACGGTAAAGCGCTGGCGGCGCT
GGCGAAGCAGCATCCGGTAGTCGTGTTATTCCGCCATGCCGAGCGGTGCGATCGTTCCGATAATACCTGCCTG
TCAGACAGCACGGGGATTACGGTCAAGGGTGCGCAAGATGCCAGAGCGCTGGGTAAAGCCTTCAGTGCCGATA
TACAAAATTACAATCTCTATTCCAGTAATACGGTGCGCACCATCCAGTCGGCAACCTGGTTTTCTGCGGGCAG
GTCGCTTACGGTGGATAAAAAAATGATGGATTGCGGCAGCGGTATTTATGCTTCAATCAATACGTTACTGAAA
AAGTCGCAGAATAAAAATATAGTCATTTTTACCCATAATCATTGTTTGACGTATATTGCAAAAAAATAAGCGCG

GCGTAAAGTTTGATCCGGACTATCTTAATGCGCTCGTTATGCATGCGGAAAACGGAAAACTCTTTTTAGATGG
TGAATTTGTTCCCGGCTAGCCGCATGTTAAAAGCGTAAAGACAGCCTGAAGCGGCTGTCGTCATTATAATAAC
CCTTTCAATGCTAATGTGTGGCGTGTCGCTACATTCAAATCATACAGCGCCAGTTCTTCTGGCTTGACCCAGG
CGTAGTCCTGAAATTCGTCGTTAATACATATATCGCGATTAGCGGAAACACAGTCGAAGATGAGATAAATCAT
ATAAATCTCTTCTTGTCTGCCATCAGCGTAGGTTTTAACGCGGATATCGTCGCGGAAGGTCCACGGCGTAATA
TCGCTCAGAATCAGTTGTTCGCCGAGTTCTTCCCGAATCTCCCGACGCAGCGCCTCTTCAATGCGTTCGCCTG
GCTCGACCCCGCCGCCCGACAGCGCCCACTGACCGGGAAAGACGCCGCGGTTGTCGGCCATTTTGCACAGCAG
ATAACAGCCATCATTTTGAATTAATGGGCAAACTATAGTTCGTTGACGCATAAGTCGCTTCCTTAAACCACCA
GGTCATCAGCTTAACAAATCATTCTGCGAAATTCTTTGTGAAAAATGGAAGCGATAACGCAGACTGCGTTTTT
TGTTAATCCTGTTGATGCAGGCGTCACCATGTGATGGCGCAATGATGCGGCATGGCGAAAGTAGTATTTCGTT
ACAATATGTTTTTGGAATGGTTTCTTAGTGCATTGATTCCTCAGCGTCTGGCAGTACAATTCTGCCGTTTTGG
GGAGTTTTTCTTTATGTGCTTGCTGGAAAGGGTAAAAATGAAAAAAAGCATATTATTAGGTTTTGCCGGAATG
CTGTTCGTTTCTGCATCGGCGCAGGCCATCAGTATTAGCGGCCAGGCGGGCGAAGATTACACCAACATTGGCG
TGGGTTTTGGTACGGAATCGACGGGACTGGCGCTAAGCGGCAACTGGATGCACAACGATGATGATGGCGATGC
TGCGGGCGTGGGTCTGGGGCTGAACATTCCGGTGGGACCGCTGCTGGCGACGGTAGGCGGTAAAGGTATTTAT
ACCAATCCGAAAGACAGCGATGAAGGCTATGCGGCGGCTGTCGGCGGTGGGCTACAATGGAAAATCGGCAACA
GCTTCCGTCTGTTCGGCGAGTATTATTATTCACCAGATTCGCTGTCCAGCGGTATCGACAGTTATGAAGAAGC
GAATGCAGGCGCGCGTTTCACCATTATGCGCCCGCTGAGCATTGAAGCCGGTTATCGTTACCTGAACCTGGCG
GGTAAAGACGGCAACCGCGATAATGCGATTGCTGACGGCCCTTATGTTGGCGTAAACGCCAGCTTTTGATGCT
AACGGCGCGGCAATCGTCGCGCCATATTCCTCCTTTTCCCGTCATTCTTCTTTCCACGTCCTATTCCTCTTTG
CGTTATAGTGTTTTCATCATAAGAGCAGGAGAACACAATGCTAAATGTGGAAATGCTATCCACCGGGGATGAG
GTGTTGCACGGACAAATCGTTGACACCAATGCCGCCTGGCTGGCTGATTTTTTCTTTCATCAGGGCGTTCCGT
TATCACGGCGAAATACGGTAGGCGATAATCTGGACAGTCTGGTCGCTATCTTGCGTGAACGCAGTCAACATGC
TGATGTTTTGATTGTTAATGGCGGGTTAGGGCCGACCAGCGATGATTTAAGCGCGCTCGCCGCGGCAACGGCA
AAAGGCGAAGGATTAGTACTGCACCAGGCGTGGCTGACCGAAATGGAACGCTACTTTCAGCAGCGTGGCCGCG
TGATGGCGCCGAGCAACCGTAAACAGGCGGAACTGCCTGCCAGCGCGGAGTTTATTAATAATCCGGTCGGCAC
TGCCTGTGGATTTGCGCTCCAGCTCAACCGCTGCCTGATGTTTTTCACCCCCGGCGTACCGTCGGAATTTAAG
GTTATGGTGGAGAAGGAAATCTTACCGCGGTTACGCGCGCGTTTTTCGCTGCCGGAGCCGCCGCTTTGCCTGC
GGTTAACGACTTTTGGACGTTCAGAAAGCGACCTCGCGCAGCGTCTCGATTCACTGCCGTTGCCGCCCGGGGT
GACGATGGGGTATCGCTCATCAATGCCGATCATTGAACTGAAATTGACCGGCCCGGCTACGCAACGGGAGGCG
ATGCTGGCGTTATGGCCAGAGGTCAAACGGGTTGCCGGGCAGAACCTGATTTTTGAAGGTACGGAAAACCTGC
CGGCGCAAATAGCCCGCCGCCTGCAGGAAAGGCAGTTGAGTCTGACGTTGAGCGAGCAATACACCAGCGGCTT
ACTGGCGCTACAGCTTTCCCGCGCCGGGGCGCCTGTTCTTGCCAGCGAAGTCGTTCCTTCTCAGGAAGAGACG
CTGGCGCAAACGGCGCACTGGACGACAGAACGGCGCAGCAATCATTATGCCGGTCTGGCGCTGGCCGTGTCGG
GGCTGGAAAACGAACATCTTAATTTCGCGCTGGCGACGCCGGATGGCACGTATGCCTTACGCGTGCGTTTTAG
CGCCAATCGGTATAGCCTGGCAATACGTCAGGAGGTCTGCGCGATGATGGCGCTAAATATGCTGCGCCGCTGG
CTTAATGGCGAGGACATTACCAGCGAGCATGGCTGGATAGACGTGGTTGAATCATTGACATCATAATCCCTTT
CTCTTATCTGCGATGGGAACGACATCGTGAGCCGGATAAGGCCGCTATGCCATTATCCGGCAATATCATCTGC
TTTTTTGTCCAGCCAATTGTACTGAATACAAAGTAATACGTGCGTTAATTCACATTTATATGGTTAACCCTTC
CACTCCGTTGGAAGGCGGATAAGCTTTGACGCGCTTCACAATTTTTTCCCTACTGACCTTTCACACTGATTCC
AGTGTCTCAGACTGGAGTCCAATATGTTGGAATCAAGTAAAGTCCCGGCTCTTACTCGCGCAATCGACATCCT
CAATTTGATCGCGCGTATTGGCCCATGCAGCGCGGCAATCATTATTGAAACGCTGCGTATTCCAAAAAGCACC
GCGTACCTGCTGTTGAATGAATTAAAACGTCAGCGATTTATTAGCCTCGATCATCAGGATAACTACTGCCTGT
GGACTAAGCTCGTTGAATTGTCGGGTCATGCGCTCAGCAAGATGGATCTGCGGGAGCTGGCGCGTCCTCGTCT
AACACAACTGATGGATGAAACCGGGCTGCTGTGTCACTTGGGGATCATCGACCACGAAAATGCGTATTACATA
CTGAAAGTGGAATCCTCATCCACTATCAGCGTGCGCTCGCATGAGGGGAAAAGCCTCTCTCTTTATCGTTCCG
GCATCGGCAAATGCCTGCTGGCATGGCAGCCGGCCGCCGTACGCAAAGCCATTATTGAACAACTGGTATGGGA
GCGAGCCACGCTGACCACCATCACCGAGCCGCAACAATTGAATGATGAGCTGGAACGCATTCGCCAGCGTGGA
TGGAGCTTTGATAACGGGGAGGATTATCCGGATGTCCGCTGCGTTGCCGCACCGGTATTTAATGCCAGAAACG
AACTGACCGCGGCAATTTCAGTGGTCGGCACGCGCCTGCAAATCAACGAAGAGAATCTTGATTATTTGGCCGG
TAAAGCTATTGCCTGTGCCAAAGATATTTCACGCCTGTTGGGATGGAAAAGTCCCTTCGACTCACTCGCATCT
TAAAATGGAGAATATTATGACTCTTCCTAAAATTAAACACGTTCGAGCCTGGTTTATTGGGGGCGCTACCGCA
GAGAAAGGGGCTGGCGGCGGGGATTATCACGACCAGGGAGGAAATCACTGGATTGATGACCATATCGCCACTC
CCATGAGCAAATATCGCGACTATGAGCAGTCTCGTCAGTCTTTTGGAATCAACGTGTTAGGGACGCTGATTGT

CGAAGTAGAGGCGGAAAATGGGCAGACGGGGTTCGCCGTTTCGACAGCAGGGGAGATGGGCTGTTTTATCGTA
GAAAAACATCTTAACCGGTTTATTGAAGGCAAATGCGTAAGCGATATCAAACTTATTCACGATCAGATGCTCG
GCGCGACCATGTACTACTCCGGTTCCGGCGGTCTGGTGATGAACACGATTTCCTGTGTGGATCTGGCGCTGTG
GGATCTGTTTGGCAAGGTGGTTGGGCTGCCGGTTTATAAATTGCTGGGCGGAGCGGTGCGCGATGAAATTCAG
TTTTACGCCACCGGCGCACGCCCGGATCTGGCTAAAGAAATGGGCTTTATCGGCGGTAAAATGCCGACTCACT
GGGGGCCGCACGATGGTGATGCGGGGATCCGTAAAGATGCCGCGATGGTGGCGGATATGCGTGAAAAGTGCGG
GCCGGATTTCTGGCTGATGCTCGACTGCTGGATGAGTCAGGATGTGAACTATGCGACTAAACTGGCTCACGCC
TGTGCGCCATTTAATCTCAAATGGATTGAAGAGTGTCTGCCGCCGCAGCAGTATGAAGGCTATCGCGAATTAA
AACGCAACGCGCCTGCAGGGATGATGGTTACCAGCGGCGAACACCATGGTACGCTGCAATCCTTCCGCACTCT
GGCAGAAACCGGCATTGATATTATGCAGCCGGATGTTGGTTGGTGCGGCGGATTAACCACGCTGGTTGAGATC
GCCGCGCTTGCCAAATCGCGCGGGCAACTGGTGGTGCCGCACGGTTCGTCTGTCTATTCGCACCATGCAGTTA
TTACTTTCACTAATACCCCTTTCAGCGAGTTCCTGATGACCAGCCCGGACTGTTCCACCCTGCGTCCGCAATT
TGACCCGATTCTCCTGGATGAGCCGGTGCCGGTGAATGGACGCATCCATAAATCAGTGCTGGATAAGCCGGGC
TTCGGCGTCGAGCTTAACCGTGACTGTCACTTGAAACGCCCTTATAGCCACTAATAACTCGTGCGTTGCCTTT
GTACGGCAACGCCCTCATCCTGTTGAGGATATTGCTATGAGCATCACTTTACTTGACGGCGTAGTGAAGAAAA
ACCGCGCACGTTTAATTCCTTTTATGTTGGCACTGTATGTACTGGCGTTTCTGGATCGTTCAAATATTGGTTT
TGCCAAAGAGACCTATCAGATAGATACCGGGCTAAGTAATGAAGCCTACGCGCTAGGGGCCGGTATTTTCTTT
GTGGTTTACGCTTTCCTTGGCGTACCGGCTAACCTGCTGATGCGCAAATTTGGCGCAAGAACCTGGATCGGCA
CGACTACCCTGCTGTGGGGCTTTCTCTCTGCTGCGATGGCCTGGGCGGATAGTGAAGCAAAATTCCTGATAAT
CCGCACGCTATTGGGCGCGGCGGAGGCTGGATTTTTCCCCGGCATGATCTATCTAACTTCACAATGGTTCCCG
CAGCGTAACCGTGCCAGCATTATGGGGCTATTTTATATGGGCGCGCCGCTGGCGTTAACGTTGGGTTCGCCGC
TCTCCGGCGCTCTGCTGGAGATGCATGGTTTTATGGGGCACCCGGGATGGTTCTGGATGTTCGTTATCGAAGG
CCTACTGGCGATTGGCGCAGGGATATTCACCTTTTTCTGGCTTGATGACACTCCGCAGCAGGCGCGTTTTCTT
AGCCTGGAAGAAAAGAACGCGCTTATCAGGCAACTGGCGAGCGAAGAAGAGAAAAAGGTCACGTCAAGGCTTG
CGGATGCGCTGCGTAATGGACGCGTCTGGCAACTGGCTATTATTTATCTGACGATTCAGGTGGCGGTATACGG
ACTCATCTTTTTCCTGCCGACCTAGGTTGCCGCGCTATTGGGCACTAAAGTGGGCTTTACCGCTTCGGTGGTA
ACAGCGGTTCCCTGGGTGGCGGCTTTGCTTGGCACATGGCTTATTCCTCGCTACTCCGACCGTACCGGCGATC
GCCGTAACGTTGCGGCAGTGACGTTACTGGCTGCGGGTATTGGCATTGGCCTGTCAGGTCTGGTTTCTCCGGT
GCTCGCTATTCTGGCGCTCTGTGTTGCAGCAGTAGGATTTATTGCCGTTCAGCCCGTATTCTGGACCATGCCG
ACGCAGCTACTTTCCGGTACGGCTCTGGCGGCAGGAATTGGTTTCGTTAATCTGTTTGGCGCTGTGGGCGGTT
TTATTGCCCCAATTCTGCGCGTAAAAGCGGAAACGTTGTTTGCCAGCGATGCGGCGGGCCTGCTGACGCTGGC
CGGAGTCGCCATCATTGGCTCATTAATTATCTTCACGCTGAGCGTTAATCGTCCGGTGGCGCAATCCGGCGCT
GCGCATCATTAATCTTATCTACGTTGTAAGGATCGCTATGAACGCATTGTTATCAAATCCTTTTAAAGAAGGA
TTACGTAAAGGGGATACGCAAATTGGTTTGTGGCTCAGTTCAACCACCTCTTATATGGCGGAGATTGCGGCGA
CGTCGGGTTATGACTGGTTGTTAATAGATGGCGAGCACGCGCCTAATACGGTGCAGGACTTGTATCATCAGTT
ACAGGCCATCGCCCCATACGCCAGCCAGCCGGTGATTCGCCCGATCGAGGGAAGTAAAGCGCTCATTAAACAG
GTACTGGACATCGGGGCGCAAACGCTACTGATCCCGATGGTTGACACCGCAGAACAGGCGCGCCAGGTCGTTT
CCGCTACCCGATACCCGCCGTTGGGGCAACGAGGCGTTGGCGCGAGCGTGGCGCGTGCGGCGCGCTGGGGGAG
AATCGACAACTATATGGCACAGGCCAATGAGTCGCTCTGCCTGTTGGTGCAGGTTGAAAGCAAAGTGGCGCTG
GAAAACCTTGACGCGATTCTGGAGGTTGAAGGCATTGATGGCGTCTTTATCGGTCCGGCAGATCTCTCTGCTT
CATTGGGTTACCCCGATAATGCCGGACACCCGGAAGTGCAGCGAATCATTGAATCGTGTATTTATCGTATTCG
CGCTGCCGGAAAAGCGGCGGGTTTTTTGGCGGTCGATCCGGCAATGGCGCAGAAATGTCTGGCGTGGGGCGCG
AATTTTGTCGCGGTCGGGGTAGATACGATGCTCTACACCGAGGCGCTGGACAGCAGGTTGGCGATGTTTAAAT
CTGTTCAGTCAGTAAGCACGGCAAAACGTAGTTATTGAGAAAAATGCCGGATGGCACGTATGCCTTACGCATG
CGTTTTAGCGCCTATCGGTATAGTCTGGCAATACGTCAGGAGGTTTGTGCGGTGATGGCGCTAAATATGCTGC
GCCGTTGGCTTAATGGCGAGGACATTACCAGCGAGCATGGCTGGATAGACGTGGTTGAATCATTGACGGCATA
GAACTTTTAGTGGGAAGGCCTGGCCTTCCCATCCTTATATACTTATAAATTCTTGGCAGAGCATCATTTCAGG
ATAAGTACTCGCCATTACTGGAGACTGTATTCATATATTTGTCGCCGGGTTTGGGTGTTAAATAGTGTTTGTT
CCTCTACTGAAAGCGTTAAGAAATTTTCCGCAAACTCTCGTAGTGTGGTAGCAGGATCGTTTTGTCCTGCATT
CGATAAGAGTTGAATTGCATGGTAACAAAATAGACCACAGCCGTTGGGTACATTATTTTGTAAATTAGTTTCA
ATAAAATCAATATTTTCGTTTTCTGATATACCTGCAATTTTTGCTGCTTCTATAATCTCTTGCTTAGTATTTT
CATTTAAATCATAATAAGTATTAAATATAAGGCATTTTATTTTTTCTGCAAGTTTATAAAATAAACAGAGAAG
CCAGTGATCTCCTGTATTTACCAGGAAGACTTCATTGTGAGATAATCCTGATTGAGCAATCTTTTCAAGTAGC
TGAGTGCTTAATATATTTTCTTTGGTGGTAGGATGAATCAGCCCAATAGGATAGTTTACCGAGAGAAGAGGTG

ATTCTTGAACCAGACCTTGCGTTGCAGCTGCCAGCTCATCTGACGACAACATTCGCGCACCACACCAAAGATT
TTCAACTCCTACCTGTTCTTGATCTGTCTGAGCGAACTGGTCGCCCATAATTTTATGTGCTATTCCTATTTTT
TTAGAAAGGTCGGTTGTCGCTGAACCGGCCATATAAAGTAATCTGGAAGGGGCATGTAGCTTTTTCCACTCAG
TATTATTTTTTGTTATATCGTTATTGGCGATTTGATGTAATACAAGGCAAGCCTGATTGATTTTTTTGTCTAT
ATCTGGTGATCCCGATCTTTTTCCGCTAAAGAGATCAAAAAGTAATTTCTCAACTTTATGTCCCAGGTCATCG
TTGCGTAACGCAAGATTGCATAACAGATCAATACAATTCTGATTACCGTTCTCTACTTCGCCAATCAACAACG
CAAGCGCCTCATCGCTCACATCTTCTGAATATAAACTGTGAAATTGCGGTGTGCTCAATCGGTAAAGTGAATT
TACACATATATTCATTTTGCTCTGTTTCCTGGTAGGTGAAAGTAACGAATATGATGTCATGTATAATTATTTG
TAAAAATATTATTGTTAGTGGGAGATATATTAAATCAGGCATAGACCTGATTTTATTATTATTCACATAACAC
TCAATTATTATTGGAGATATTCAGTTATCCCCCTCTTTAAAGGAGGCTGGTAACGCGGCGATACAAGAGATAT
TGCTCTATAAACAGTAACGTCGTTATCTGTCATGCCACCGTGGGTTAGCGGTCATGGTTAACCAAATAGGGCA
GGTTATTGCAAAGAGACATCCGCGTTTATACCCAAAATAATCCGTAGCCAACACATAAGCAACTTGAAGTATG
AAGGGTATATGTCTGATGGAAAATCGTCCCTGGTGAGTAGGGTAATAGCGCCGGAGGGGCTCCGGCGCTTTTA
CTTAGCCGAGCGCCTGGGCCAGTAGCGTAATGGGGTGTTCGCAGCGTTTGCTTGTAGACATCTCAATTTGCCA
CTTACAGGTTTCACAATCGGTGACGACAATGTCGGCGCCGCTCTCTTCAATCTGGCGGAACAGTGGCGCGCCG
ATGGACTGTGATGTCGGATAGTTTTCCTTTTTAAATCCATACGTACCGGCAATGCCGCAGCACTGTGAATCAA
GTACCGTCAATTCAAGCCCCGGAATTTGCCGCAACAGTTCAAGCGTGTAAAGCGTCCAGCCCATTTTTTCCAT
ATGACACGGCGTATGGTAGACCACTTTCAGCGGTAAAGGATTAAGCGGCAGCGTTTTCCCGGCATCCAGTTTG
CGCCATAGCCAGCGGGTGGCCAGCTCGATATGTTCGCGCAGCCCGGCGTTGTCCACGTCCAGCACTTCAGGAT
ATTCATCGCGCAACGCAAACGTACAGGTGGATGACGTGGCGATGACCGGAATACCTTTGACGGCAATCGCCTC
GCGTAGCGACTCCACATTGCTGATAGCCTGTTTGCGAGCTTTATCCGTAAAACCGTTAGCGATCAGCGGTACG
CCGCAACACTTCTCTTTACTCAGTAACTGTACGCCAGTACCCATTGCGTTCAGCACCTTAATCAGATCTTTTC
CGAGCTGCGGATGGTTGTAATTCACAAAACAGCCATGGAAAAAGGCGACCTGATCTTTGTATTTAGCCTGCTG
CGCCGCCACGCTACGATACCAGCGGCGGAAGGTGCCGAAAGAGTATTTTGGTAAGGTGCGGCGATGATCGATT
TTGAGCGCGTAATCAAGCAACTGGCGCACTGGCTTTAATGCGGTGGCGGTATTGACCACCGGCGCGAACGGCG
TTGAAACGCTGCCCATCAGATCGGTATGGCTGAGAATAAAATTACGCAATGACGGGCGCGTCGTATCGTATTT
TGCGCGGGCGCGCTGGATGATATCGCCAATCTTCACGTCCGACGGACAAGCGACCTCACACCGTTTGCAGTTA
ATGCAATATTTCAGCGCCTCATCATACAGTGCGCCGTCTTTCAGACGCAGGCGCTCGCCATCCGGTCCGGCCT
GTTTAGGGCCAGGGTAGCCAGGATTAACGCGGCTGACCGGGCATGCGGTGGTGCACACCGTACATTTAATACA
ACTTTCAAAACGTGTGTCGCTCATTGCTGTTCTCCTGCACGCTCAGCGATATGGTGCGCGGCCTGGAGGGCGC
TGACTGCGCAGACACCGCCGCCGCATCCCTCAGCTATAGGATCGAATCCCGCCAGCACAGAACCTATTGCATA
CAGATTTTCTACCGTGTTGCCCGCAAGGGAGGGGCGGAAGGCGTCATCCGTCACGACGCCAAACTGCTGCCAG
GGCTGTGGGTCAAAGAAATGTTGTTGATACCATTCGGCGCGGGTGGCCGTTTGCTGTACGTCGAGCCCTAAAA
TAGGTTCGCGGATCCCTTCGCGCTCGGCAACCAGACCGCTACTAAAGAAGCTACCGCTGGCAAGTACGGCAAA
ACGCGGGCGCAGTGGGATATCGGCATGGTTGCGGGTCCATATTTCGCTCACGGTACCGCGACGGCAGGTGACT
TTCTTCACTTCATCGCCCGGCATCCAGATGCCGCCCTGACGGACAAACTGGCGCTGGAGCTGATTGTGCAGAC
GAATACCGAGCACCGATGGCGGCAGCGTTGGCAGCAGCGTGAGCGCGCAGGGCAGACGTTCGTTAAGCCAGCG
CCACAGCGTATCGTTCGCCAGCCCGAAACAGGCGGGCATGATGATCATGTCGCAGTTTGTCGCTATTGGCGAC
AGCGCGTCGTACAGCAGCGGCCATTTTTCTTCGTTATCAAGCAGACGCGCAATATTGACCGCGCGAAATTCCG
TCGGGTTATCACGCAGTACGTCCAGTTCCGGAAGGTCGATTTCTGCGGTTTCCACGTTGAGGTCGCGTTGGCG
CAGCGACGCTGCCGCAAGGTGCGCCTGGAAATCCAGTAATCCGCTAACGCCGACGACGCAAATTCGCTGTGCG
GATAACGGCCAGACCGGTACTTCCGGCGAGCTAAGCCAGGTTGAACGCAATGTGCCAAGCGGCGTGACACGCT
GGTGCGCCTGCTGTACGTCACCGTATAACTGCGCGCCGCTGGCGTTAAGTAGCGTTTGCGCCTGTTGCGCCAG
CGTAAGGACTTTTTGCGCGCCTAAACGCGAATAAGGATGTTCAGGCGCCTGGCGGCACAGCGCATCAAGCCCG
GCGGTAATGTCGGTCACCGGTTGGCCGTCGGGCAGGGCGCTGAGTAGATCCAGCGAGCCGGAGGAAAAATGCA
GCGCGCTTTGTCCGCGGGTGACGATGGCGCAGCGTAATCCATGCTGCTGGAGTTGCAGTCCGCATAGCAAACC
GGCGAGACCGCCGCCCATAATAACCGTATCAAATTTCATCCTGGTGCTCCTTCTCCAGACCGCATAATCCCTG
ATAAACCCAGCGCGTGAATTCGCTTTCGCGCAGCGCGTCTCCCCAGGCGACAGGTTGTACGCCTTTCCAGCGC
TCATTCAGAAATTCAGAGAGTTGCGTAATAGATTGCGCCGCGGTCGTCACATTAAAACGCTGCAATAGCCCGG
CCGCGCGGCAGGCGCACAGTTCGCCCTGGCAAGTGCCCATCCCTACGCGGGTGCGACGGCGCAGATCCAGCAG
GCTATTCACGGTTAAGTTTTCGACGGCATACTGCACTTCCCCCGCCGTGACGGCCTCGCATTCACAGACCAGG
CTACGGTGTTGGCGGCCTTCACTCAGCCACCCCGGCGTGCGATCGCCATGCCGATAAACCGCCGATCCGCGTA
GCGGCGCGGGAAGCGAGATAATGCGTTTCAGCGTATGTTCCGTGGACTCTTTTGAGCCAGGCAACGGCGTATC
CGCCGTGATGCAGGGGCGAGTATTGCCCAACTTGCGGCACACGGCGTCGGTCGCCCATTCCGCCATCAGGCGG

TAAGTCATTAGCTTCCCGCCGGTAATGGTAATGAAGCCATCAAGCCCGTCGCGTTCGGCGTGGTCGAACAACA
CAATACCGCGGCTGACGTTCCGACCGCTGGGATCGTCATCGCTGGCCACCAGCGGGCGGACGCCGGAATACGC
GCGCAGAATACGCGTTTTCGCCATGACAGGGGCCAGTTTTTCCCCTTCACGCAGCAGGATATCGACTTCATCT
GCGGTGACGCGGTTGCTGTCTATCTCATTGTAATCAATATGGGTAGAGGTAGTGCCGATCAATGAAATCGTAT
CGCCAGGTACCAGAATGTCGGCATCCGATGGTTTCCGGCAGCGGTTAATGACATGCTGGTTGATCCGATGGTC
CATGATCAACAGCGAGCCTTTCGCCGGGAACATGCGAATACTCAGGTCGGCGTATTCGGCGATCCGTTGCCCC
CAGATACCAGCCGCATTGACCACCACCGGCGCGTGAAGCGTCTGGGTTTCGCCAGTGAGATGGTTGCGCACAT
GGACGCCGCAGACCGTTGCGCCTTCACGAATGAGCCCGGTCACTTCATGCGCCGTTAACACGATTGCGCCATG
TTCTCTGGCATCCAGCATATTGGCGGCGGTAAGGCGGAAAGGGTCCACGGTGCCATCCGGCACTTTTACCGCA
CCGATGAGCGCAGGGTTAACGGAAGGTTCGATGATGCGAGCCTGCTGCGGATCGATGGCTTCAGCGCGAATGC
CCGCCGCTTCACAGGCGCGAATAAAGGTGGCCTGAAACGCAAGATCATCTTCCGGCAACGTAATAAACAGACC
ATCCGTCGGTTCGACGCAATGGCGGGCGATCCGTTTTAAAATCTGGTTTTCACTAATGCACTCGCGCGCGGAT
TCCGCGTCGGTAACCGCATAACGAGCGCCGCTGTGCAGCAGTCCGTGGTTACGCCCGGTCGCGCCGGTCGCTA
TATCATGCCGTTCCACCAGAATGACACGTAAACCGCGCAGCGCGCAGTCGCGGGCGATCCCTGCGCCTGTTGC
TCCACCGCCAATGATAATCACGTCACTTGCTTGCGAGTCGCGAGTTTTCATTGTTTTTCCTCACAGTTCGTTT
TTTATCATTTAGCCATACAAATCATATGGAATGTTTGATTTCGCGCATAATCGCTCACTATTCGAAAATGAAA
CGTGATTTCGTGCGCCTTTCTGAACATTAGTCATAAATCTGTAACAATATGTGCTGTAATTCACATTAACGTG
ACGTATCTTTACTTAACATCGCGGCCACACTGGGAGCAGCGGGGTTGTTTGAACGAACTGCGGTGTTTACCTC
TAAATAAAATATGGGCCACGGAGGCTACAATATGTTGAGTATTTTTAAACCAGCGCCGCATAAAGCGCGCTTG
CCAGCGGCGGAGATTGATCCGACCTATCGCCGATTACGCTGGCAGATTTTCCTGGGGATATTCTTTGGCTATG
CCGCGTATTATCTGGTGCGCAAGAACTTTGCCCTCGCGATGCCCTACCTGGTAGAACAGGGTTTTTCACGCGG
CGATCTGGGCTTTGCGTTGTCCGGGATTTCCATCGCTTATGGTTTTTCGAAATTTATAATGGGTTCGGTGTCC
GATCGCTCGAATCCGCGCGTTTTTCTGCCGGCAGGGTTGATTCTGGCCGCAGCAGTCATGTTGTTTATGGGCT
TTGTGCCGTGGGCGACATCCAGCATCGCCGTGATGTTTGTGCTGTTGTTCCTTTGCGGCTGGTTCCAGGGGAT
GGGGTGGCCGCCGTGCGGACGTACGATGGTTCACTGGTGGTCGCAGAAAGAGCGCGGCGGCATTGTGTCGGTC
TGGAACTGTGCGCATAACGTCGGCGGCGGGATCCCGCCACTGCTGTTCCTGCTGGGGATGGCGTGGTTTAACG
ACTGGAAAGCGGCGCTCTATATGCCAGCCTTTGGCGCGATTGTGGTGGCGCTGTTCGCTTTCGCAATGATGCG
CGATACGCCGCAATCCTGCGGTCTGCCGCCTATCGAAGAGTATAAAAACGACTATCCGGACGACTACAACGAA
AAAGCGGAAGAAGAGCTGACTGCGAAGCAGATCTTCATGCAGTACGTACTGCCGAACAAATTACTATGGTATA
TCGCTATCGCCAACGTGTTTGTTTATCTGCTGCGCTACGGCATCCTCGACTGGTCGCCGACCTACCTGAAAGA
AGTGAAGCATTTCGCACTGGATAAATCCTCCTGGGCCTACTTCCTGTATGAATACGCGGGTATTCCGGGCACG
CTGCTGTGTGGCTGGATGTCGGATAAAGTCTTCCGCGGCAACCGCGGTGCGACCGGCGTGTTCTTTATGACCC
TGGTGACTATCGCGACCATCGTTTACTGGATGAACCCGGCAGGGAACCCGAACGTTGATATGGCTTGTATGAT
TATCATCGGCTTCCTGATCTACGGCCCGGTAATGTTGATCGGTCTGCACGCGCTGGAACTGGCGCCGAAAAAA
GCGGCGGGTACGGCGGCGGGCTTTACCGGTCTGTTTGGTTACCTGGGCGGTTCTGTCGCTGCAAGCGCCATTG
TGGGTTATACCGTTGACTTCTTCGGTTGGGATGGCGGCTTTATGGTGATGATTGGCGGCAGCATTCTGGCGGT
AATCCTGCTGGTGGTCGTGATGATTGGTGAAAAACGCCATCATGATGAGCTGCAGTTAAAACGCAACGGAGGC
TAATAACATGAAAACCACACTGAAAAACCTTAGCGTGGCGTTAATGTTGGCAGGAATGACCATCGGAAGCGGC
GCCGTGGCGGCTGAAAAAGTCGTTATCGCCCACCGTGGTGCCAGCGGTTATCTGCCGGAGCATACGCTACCGG
CGAAAGCGATGGCGTATGCGCAAGGGGCAGATTATCTGGAACAGGATTTAGTGATGACAAAGGACGACCATCT
GGTCGTCCTCCATGACCACTACCTTGACCGGGTGACTGACGTCGCTGACCGTTTTCCGGACCGGGCGCGTAAA
GATGGGCGCTATTACGCTATCGATTTTACGCTGGATGAAATTAAATCCCTGAAGTTTACCGAAGGGTTTGATA
TTGAAAACGGCAAAAAAGTACAAACTTATCCGGGCCGTTTCCCGATGGGAAAATCTGATTTCCGCATTCATAC
CTTTGAAGAGGAAATTGAATTCGTTCAGGGATTAAATCACTCTACCGGTAAAAATATCGGTATTTATCCGGAA
ATTAAAGCGCCGTGGTTCCATCATCAGGAAGGGAAAGATATTGCCGCGAAAACGCTGGAAGTGCTGAAGAAGT
ATGGTTACACCGGCAAGCAGGACAATGTCTATTTGCAGTGTTTTGATGTCGCTGAGCTGAAACGTATTAAGAA
TGAACTGGAACCCAAAATGGGGATGGATCTCAATCTGGTTCAACTTATTGCGTATACCGACTGGAATGAAACA
CAGCAGAAACAGCCGGACGGTCGTTGGGTAAATTACAACTACGACTGGATGTTTAAGCCGGGCGCTATGAAGC
AGGTGGCGGAATATGCGGACGGTATTGGTCCGGATTACCATATGCTGGTTGCGGAAGGCTCAACGAAAGGGAA
TATCAAGCTGACTGGAATGGTGCAAGACGCGCATCAGAATAAGATGGTAGTGCATCCTTACACTGTGCGTGCC
GATCAATTGCCGGACTATGCCACAGACGTCAATCAGTTGTACGACATCCTGTATAACAAGGCGGGCGTCGACG
GGCTGTTCACTGACTTCCCGGATAAGGCGGTTATGTTCCTGCAAAAAAAATGACTAAAGGTTTGCCATAGTCGA
AGTGACGCGCCGGATGAGGGCATAAACGCCTCATCCGGCCTGGCGCAACGTTTGCCAGCGTAAGTCTGGTAAA
CGCAATGCCATCAGGCGGGGTTGTTTTATAGCAACCCCTCATCGGTCAGTGTTTCCATACATTTTTGTAATAA

AAATAACGGACTTCCTTCTACCGGCGGTTCTTTTGCTAACAGGGAAATTTGTCGATAAAAGGTGGGTTCCAGC
GCCACGGAGCATAATTTTCCTGTCGTGGCTTTTAAGGTTAATTCTGGCAATAAGGCGATACCTAATCCCTGGC
GAATAAAATTCAGCGCAGTATCGGGATGGTTAAATTCATATTTAAATATCGGCTCAATACCCTTTTCTTTAAA
CAACGCCATAATGCTTAATTCATAACGACCTTTACTGACAATAAGTGGTTCGTCCATCAGTTCTTCGACCGTT
ACCGTACTATTGGCCGCCAGGGGATGATTTTCAGGCACCACTACGGTGAATTTATCCCGGTAAATGGGGACGC
AATACATACCGTTGACCGGAAAAGGAACGAAACCGGCATCAATACTTCCGTCCTGCAGCGAATCAATAATAGC
CGTACTGTTCTCTTCATAGGGGATAATTTTTACATTTGGATGATGGCTTTCAAAATAGCGAATGACGCCGGGT
AAAATGCAGGCGCAGGCGCTGGGAAAACAGCCAATCCGTAGCGTACGGGCCGGGTTCTTTTTTCTTGTTCGGC
AATCTCTTTTACCGCGTTGACGTCGCGCTGGATCGCGCGTAAGTGCCTGACGATGCGGCTACCGGCGGCGGTA
AGCTCAATCTCTTTACGACGCTCACGAATCAATATGTCAACGCCGAGCTCCTCCTCAAGCGCGGAGATGGCCT
GGCTGACGGCGGATTGCGTCATGTATAGCCGTTTGCTGGCCTCGGTAAATCCGCCGTACTCCAGTACGGCAAG
CAGGGCGTGGATTTGAGTTAGTGTCATTAGTAAAACCTAATTAATATAATTAAAACGATTAATTTTTCTAATA
GAGTGCACTTGTTTATACTGCGATTCTATCGAATACATCACATTTTGACGCACCTGATAGGTGTTCAGTTGAG
CTGAGCGTTGCCTGCGTGCGCCATCTGAGCGGGTCGTTAAGAATGAAAGAAAAATTATGGACGAAGGATTTTT
GGGCAATCACCATAGTCAGTTTTATTATCTTTTTTGTTTTTTATGTACTGCTAACTTTATTACCCATTTATAT
CGCTGACCGCCTGCATGCTTCTGCAGATAAGGCCGGGCTGTTAGTGACATGTTTCCTGGCCGCGGCAATTGTT
ATTCGGCCATTTGCCGGACAATGGGTGGGAAAATATTCGAATAAAAAGATTCTGGTGCTCTCTTCTCTGGCTT
TTTTGGTGATTACCGCGCTGTATCCGGTTTGCCATTCTATCGAGTCGCTGCTCTTTATCCGCGTACTTCATGG
GATTACGTTTGGTGTTATTACCACGGTAAAAGGAACTATTTCAGCGCGGTTAATTCCGGCCTCCCGACGTGGA
GAGGGCATCAGCTTTTTCTCTTTAGCGATGGGCCTTGCGATGGTCGTAGGACCGTGGATCGGTTTGAATATGG
CCCGCTGGAACGCGTTCACTGCCGCATTCTGGCTTTGTTCCGCTGTAGCGGCGTTAGGGATTGTACTTTCCCT
GATTGTGACGGTCCCTCCGGTAATTCGTCATGCCGATGGTTCGAAACCGAATCTGGGTTTTGCCGCGATGTTT
GACCGCGCCGCCTTGCCTTTTGCGCTGGTCACCTTTTTTATGACTTTCTCCTATGCCGGCGTATCGGCTTTCC
TGGCGCTGTATGCGCGTGAGCTGGATTTAATGGCGGCGGCCAGTAATTTCCTGCTGTGCTACGCGATTTTCCT
GATGATATGCCGTACCTTCACCGGGAATATTTGTGATAAAAAAGGGCCGAAATATGTTGTTTTCCCATGCCTG
TTGGCTTTTACCATCGGTCTGGTGGCGCTGGGCTACACCAATGGCAGCATCATGATGATTATTTCCGGCGGTC
TCATCGGTATCGGCTACGGTTCGGTCACGCCGGTTTTCCAGACGCAAATTATCAGCTCGGTTGAACCGCATAA
AATCGGCGTGGCGAATTCACTGTTCTTTAACGCGATGGATGCCGGAATGGCGATTGGCGCCTTTATCATGGGA
ATGATGGTTGAGTCGGTTGGCTATCGTATGATTTATGTCGCTGGCGCGGTGCTGGTGGTACTCGCTGGCGCAC
TGTATGCCGTGCAGATGAAAAAACGTGGCGTGATGCCTTTGGTTTCCACCAGCGAGTTGCACTGAGTTACATC
TCGATTTCGATATCGCCTTTTGCCCTGCAGCAGCAGGGCAAAATCTCTCCTGGCTGGATAAACGCTAAAGGTT
CCGTAATCCAGTCAACCTGACCGGCAACCAGTCGGGTACGGCAGGAGCCGCAATAACCTTCGCGGCACTGATA
CTCCACCTCAACGTTATGCGATTCCAGCGCGGCCAGCAGAGAAGGGTGTTCATCCTGGCACAGCAGCTGTGTG
CCAGTGATACGCAGCGTGACGCGTCCCATCAGAGCTGGAAATTACTCAGATCGTCGGTATCGACTTCCGAATC
GATTTGTCCGACCAGATAAGAACTAACTTCCACTTCCTGTGGCGCAACCTGAACGTTATCGGACACCAGCCAG
GTATTGATCCACGGGATTGGGTTGGAGCGGGTCTGGAACGGCAGATCCAGCCCTACCGCCTGCATACGAATAT
TAGTAATATATTCAACGTATTGACACAGGATATCTTTATTCAGGCCGATCATCGAACCGTCGCGGAACAGATA
GTCCGCCCACTCTTTTTCCTGCTGCGCCGCCTGCACGAACAGGTCATAACATTCCTGTTTACACTCTTCGGCG
ATTTCCGCCATCTCCGGGTCGTCCACGCCGCTACGCAACAGGTTCAGCATATGTTGCGTGCCGGTCAGATGCA
GAGCTTCGTCACGGGCGATCAGACGGATAATTTTGGCGTTGCCTTCCATCAGTTCACGCTCGGCAAATGCGAA
AGAGCAGGCGAAGCTGACGTAGAAGCGGATGGCTTCCAGCGCGTTCACGCTCATCAGGCACAGATACAGTTTT
TTCTTCAGCTCACGCAGATTCACGACAACCGTTTTGCCATTAACGGTATGGGTGCCTTCACCGAGCAGGTGCC
AGTAACTGGTCATCTCAATAAGTTCGTCGTAGTAGGCGGAGATACCTTCGGCGCGCTTCTGAATTTGCTCATT
GGTCACGATATCGTCAAACACGACCGCCGGATCGTTGACGATGTTACGAATAATGTGGGTGTAGGATCGTGAG
TGAATCGTCTCAGAAAACGCCCAGGTCTCTACCCACGTCTCCAGCTCCGGGATGGAAATGAGCGGCAGCAGGG
CGACGTTCGGGCTACGTCCCTGAATGGAATCCAGCAGCGTCTGGTATTTGAGGTTGCTGATGAAGATATGTTT
TTCGTGCTCCGGCAACGCCTGATAATCGATACGATCGCGGGAGACGTCGACCTCTTCCGGACGCCAGAAGAAA
GAGAGCTGTTTTTCGATCAGTTTTTCGAAGATGTCATATTTCTGCTGGTCATAGCGTGCAACGTTGACCGGCT
GGCCAAAGAACATGGGTTCCTTGAGCTGATCGTTTTTCGTCTGTGAAAAAGTGGTGTATGCCATTGAAGTGAA
TCCTTTATGGGATGAGCGCCGGATGGCGGCGCAAGCGCCTTATCCGGCCTACAAACCGTACAATCGTAGGCCG
GGTAAGCATAGCGCTACCCGGCACTATTCATCAGATCTTACAAGCGCCGCTTTCGCAGCCATCGTCCTGAATA
GAAGGCGCCAAATCGTCCTGCGCATCTTCCGCGCCGTCGCGGGTATTTTGATAGTACAGAGTTTTCACGCCAA
ACTTATACGCGGTGAGCAAATCTTTCAGCAGTTGCTGCATCGGCACTTTTCCTGACGGGAAACGTGACGGATC
GTAGTTGGTATTGGCGGAAATCGACTGATCGATAAACTTCTGCATGATACCCACCAGTTGCAGATAACCGTCG

TTGTTCGGCATCTCCCACAACAGTTCGTAAGCGTCTTTCAGATGCTCATAATCCGGCACGACCTGACGCAAAA
TACCGTCTTTCGAGGCCTTGATGCTGACGTAGCCGCGCGGCGGCTCAATGCCGTTGGTGGCGTTGGAGATCTG
GGACGACGTTTCCGACGGCATTAGCGCCGATAGCGTGGAGTTACGCAGACCGTGGGTTTTGATCGACTCACGC
AGCTGTTGCCAGTCGTAGTGCAGCGGTTCGTTGACGATCGCATCCAGATCTTTTTTGTAGGTGTCGATCGGCA
AAATGCCTTTTGCATAGGTGGTTTCGTTGAACCATGGGCAGGCGCCTTGCTCTTTCGCCAGTTCGTTGGACGC
TTTAAGCAGATAGTACTGAATGGCTTCGAAGGTTTTATGCGTCAGATTATTGGCGCTGCCGTCGGAATAACGC
TTACCGTTTTTCGCCAGCCAGTAGGCGAAGTTAATCACGCCAATGCCCAGCGTACGACGGCCCATTGCGCCGC
GTTTGGCAGCCGGAATCGGGTAATCCTGATAATCGAGCAGAGCATCCAAAGCGCGTACTGCCAGAATAGCCAG
TTCTTCCAGCTCGTCCAGAGTTTTAATGGCGCCCAGGTTGAATGCAGACAGCGTACACAACGCAATTTCGCCG
TTTTCATCGTTGACATCGTTCAGCGGTTTGGTTGGCAGGGCAATCTCCAGACACAGGTTAGACTGGCGTACCG
GGGCGACAACCGGATCGAACGGACTGTGAGTATTGCAGTGGTCAACGTTCTGGATATAGATACGACCGGTAGA
GGCGCGTTCCTGCATCATCAGCGAGAACAGTTCGACCGCTTTCACACGCTGTTTGCGGATGCTGTCGTCGTGT
TCATATTTCACGTACAGGCGTTCGAATTCGTCCTGGTCGGCGAAGAACGCATCGTATAGCCCGGGTACATCCG
ACGGACTGAACAGCGTAATGTCGCCGCCCTTCAGCAGGCGGGTATACATCAGTTTGTTGATCTGTACGCCGTA
GTCCATGTGACGTACGCGGTTGCCTTCCACGCCGCGGTTGTTTTTCAGCACCAGCAGGCTTTCCACTTCCAGA
TGCCACATCGGGTAGAAGAGGGTTGCCGCGCCGCCGCGCACGCCGCCCTGCGAACAGGATTTCACCGCCGTCT
GGAAGTGCTTGTAGAACGGAATACAGCCGGTGTGGAACGCTTCGCCGCCGCGAATCGGGCTGCCCAGCGCACG
AATGCGTCCCGCGTTGATGCCGATACCGGCGCGCTGAGAAACGTATTTCACGATAGCGCTGGAGGTGGCGTTG
ATGGAATCCAGACTGTCGCCACACTCGATCAGTACGCAGGAGCTGAACTGACGGGTTGGGGTACGCACACCGG
ACATGATGGGCGTCGGCAGCGAGATTTTGAACGTAGAGACCGCATCGTAAAAACGTTTTACATAGTCAAGACG
CGTTTCACGCGGGTAGTTCGAGAACAGGCAGGCCGCTACCAGAATGTAGAGGAACTGTGCGCTTTCATAGATT
TCGCCGGTCACGCGGTTTTGCACCAGATATTTCCCTTCCAGCTGCTTAACGGCAGCGTAGGAGAAGGTCATAT
CGCGATCGTGAACGATAAACGAATCCATCTGCTTGAACTCTTCTTCCGTGTAGTCTTCCAGCAGATGATTGTC
GTATTTGCCAAGCTCAACCATTTTTACCACATGGTGGTAAAGTGCGGGCGGCTCAAACTGGCCGAAGGCTTTC
TTACGCAGGTGGAAAATCGCCAGACGCGCAGCCAGGTATTGATAATCCGGCGCATCGCGAGAGATCAGGTCTG
CGGCGGCCTTAATGATAGTTTCATGGATATCGGAGGTCTTAATCCCATCATAAAACTGAATATGCGAGCGTAG
CTCGACCTGAGAAACCGATACGTTATTCAGTCCTTCTGCCGCCCAATCAAGCACGCGATGAATTTTGTCGAGA
TTGATGCGCTCAGTACGGCCGTCACGCTTTGTCACCAGCAGACTCTGATTCATGTGGTTTTCTACCTGTTTAC
CTGTCCGTGAAAGAAAAATATCCCCCCGCTTATCCACAACGCCACGTTGTGACTAACTCTGTGGATAAATACT
ATATATAGGGGGTAATCGATAAGTGAAACGCTATATGGTGAGTATTCTAGTAGGGATTCTTTTGAGTACAAGA
GTTGATTTTGGCGTTAAATTGCGGTTGTAAAAGAGTAAGAATCGCTAAATGCGCGTCTGATAAGGCCTGGAGA
GGATGTCAACAATTCATAAAAAAAATCAAAATTTGATCGAATGCTGATTTCTTCATCGAAGAGAAAGGTTGCG
CCCGATGATCTAACGCAACCCTTATAGGAAAATTCTTTGATGTCAGGCTTTTTTAGCGCGGGTATGCAACATG
TAGTTAACGTCTACTCCGGGACCGAGTTTGAAAGTATTGGTTATCGGGTTGTAGTGCAGCCCGGTGATATGTT
GCTCTTTCAGGACGGTTTCATCTACCCAGCTTAACAGTTCAGCAGGCTTAATGAATTTCTTCACGTCGTGCGT
GCCTTTTGGCACCATGCGCAGAATATACTCAGCGCCGACGACCGCCATCAGCCAGGATTTCCCATTGCGGTTC
AACGTTGAGAAGAACACCTCGCCGCCGGGTTTAACCAGTTGGGCGCAGGCGTGGACGACCGATTGCGGATCGG
GAACGTGCTCCAGCATTTCCATACAGGTGACGACGTCGTATTGCTGGGCGTGTTTTGCCGCATGTTCCTCAAC
CGTCTCCTGCATGTACTCTACCTCGATGCCGCTTTCCAGCGCATGCAGTTTGGCGACCTGCAAAGGCTCAAAG
CCCATATCCAGCCCGGTGACGGTTGCGCCTTCGCGCGCCATGCTCTCCGCCAGAATACCGCCGCCACAGCCGA
CGTCGAGCACCTTTTTACCAAACAGGCCGCCTGAGCGCTCAGTAATGTAGCCAAGGCGTAGGGGGTTGATACG
ATGCAATGGCTTGAATTCGCCCTCCAGATCCCACCAGCGCGACGCGACGGCTTCGAATTTGGCGATTTCATTA
TGGTCAACGTTGTGAGCTACCGACGGTTTTTCAGTGTTCATGAGTACATGAGATTCCGCTATTGATCTATCGG
GGCAGTATAGCAGGTGCAAAGTCGCTGCGTTATATCTACTGCCGTCTTTATTACGCTTTCAGCGCAAGAAGTT
TTCTTGCGCGTTCTGTAAAACCGCTACCGATGTGGTGCGTGATAAAATTACAGACGCCCTCGACGTTGTTGTT
TTTTAACATATCCAAAATAGTGGCATGTCCACGGTTAGAGCTGGTGATATATTCAGTGGTAAAATCCAGTCTG
TAGCGAATTGCCAATAAACGTGCTGAGATCAACAGGTGTGCTTGTGAAATGTATTTATTATCGGCATATTTCA
CAAAAACATAATGAAAATCATGATCCAGTTTGAGGTAAGCTTGCGTATCGCGCTCTGCATTAATTTTTGCTGA
CTTCGATAAAATATTGCTTAATTCGACTATGACATTGTTAGCATTTTTCTCCATCGCTTCACGAATTGCGCCC
TGTTCAAGGATAACCCTCATTTTGATGAGATCATTGATATCCTCGTCGGTGACAGAAAAAACAAATGTCCCAC
TGCGGGGAATGATGTTGACTAAGTTTTCATTATAGCTCAGCCAAATAAAAGCTTCACGAATTGGTGTTTTACT
TACCATCAGAATATTTGATAGTTCATTTTCACTGATCGCTTGCCCAAGATCGAAATCGCCATGGATAATCATC
TCCCTTATTTTCTCTTTAATCACCTGGGTGATGGATTTGGGTATTTCAATTGATATGTGACTAAAGTCACGCT
TTAATCTATTTTTCATTTTCTTCTCCTCCATGGTTAAACCCGGCTCCATTGTAATTAATTTATTCAAAATTAA

CGGATCTAATATATCAGATATAAGAAAATTTACATACTTTATTGTTGTTATTGTATATTTCATTAAGTTTGCT
TGCTGATATATCAGATATGACACATTAGCTTTTGAATTGTATTGTTTTTTACGTAACATCAATTCCGGTCAAT
ACCCTTATTCATTGAATCATAAAAGGTGATGCAAAATGAGTAGTCCACAAGAAAATTTATACGATGCAATTCG
TATTGTAAAAAGGAAAATCATTCCTTTGGCCTTTATTCTTTACTTTTTTAACTATATGGATCGTGTCAATATT
GGGTTTGCTGCATTACGCATGAATGAATCTCTCGGTATTACGCCTGAGGATTTCGCTAATATTTCTTCTGTAT
TCTTCATATCATATTTAATCTTTCAAATTCCGAGTAGCATTGGCTTGCAAAAGCTTGGCGCAAGAAAATGGAT
TAGTTCCATTATTATCGGCTGGGGAGCGGTAACAGGGCTGATTTTTTTGCTAAAGATACTCAACATATTTTGC
TGGCTCGCATTTTTTTGGGGGTATTTGAAGCTGGCTTTTTCCCGGGCATGGTTTATTATCTTGCATGTTGGTT
CCCGGCTCGTGAACGCGGAAAAGTCAATAGTTTCTTCATGTTATCTATTGCTGTTGCATCGGTATTGGCTGCA
CCGATGTCTGGTTGGATCATCGAGCATTTGAATACCCCGGATTATGAAGGCTGGCGCTGGCTTTTTGCTATTG
AAGGTATCCCTACCGTATTCCTTGGCATCCTGACATTTTATTTACTGCCTGACAGTCCAGAAAAGGCTAAATG
GCTTACGCCACAGCAGATATCGGCCTTAGTTAATAAATTACGTACTGACAATGAAACCGCAGCTGCATTAAAT
AAAAATACCAACTCATCCTTTCTTTCCGTAATCAAAAATCCAGTTTTGCTTCAGCTTTCTTTCGCCTACATGT
TGATTCAGGCGGCTGCTCTGGCGGCAAACTACTGGTTGCCGGGGTTGGTGAAGGGATTTTCAGCTGATTTTAC
GGATACTGACGTTGGTTTAATCATGAGTATTCCGTTTATTTTCGCTATGTTCAGCATGCCCTGGTGGGGCTGG
CATTCCGATAAAAAGAATGAACGAAAATGGCATGCTGCATTACCCATGTTTCTGGCAGGCTGTGGTTTTCTGA
TGATTGCTCTTGTCCCCTCAATGTCGCTGAGAATGCTGGGGCTAACGTTCTATGGTGTTGGGATCCTCAGCTA
TTACGGACCTTACTGGGCGCTGCCTTCTGCATTATTGTCGCCGTCAGGGTTGGCTATCAGCATTGCGTTTATT
AACTCATGTTCAAGTCTTGGCGGATTCCTGATTAATAAGTCACTGGGATTTGTTTCTACTCATTATGGTGCGA
CAGGAATATTTATTGTAGAGGCAATACTTTGCTTCGCTGCGGTAGCCGTTTTGGCGTTAATGAAAATTGATGT
GAAAAAAGAAAAAGCCAACAAACAAATGTTGTATCACGAACCTGATAAATGGAGAAATTATGAAAATTACATC
TATTGAAGTTTTTGACTGTGAATTAAAAAAAGAGATCAAACGATGTCATCCTATAATCCAGTACTTATCCGGG
TGAATACCGACTCAGGATTAAGTGGTATTGGCGAAGTAGGACTGGCCTATGGCGCAGGCGCTAAAGCTGGCGT
AGGGATCATCAGAGATCTGGCGCCATTGGTTGTGGGTGAAGATCCATTAAATATCGAAAAAATTTGGGAGTTC
TTTTTCAGAAAGACTTTTTGGGGGATGGGGGGCGGAAACGTTTTTTATGCCGGAATGAGCGCTATTGATATTG
CTCTTTGGGATATCAAAGGTAAATATTTAGGTGTACCTGTTTATCAGCTCCTTGGCGGGAAAACTAATGAAAA
ATTAAGAACCTATGCTAGTCAGTTACAATTTGGTTGGGGGGATAAACGCCAGATATTAGTGACACCTGAGGAA
TATGCTGAGGCGGCGCGTGCGGCTCTTGACGATGGATATGATGCGATTAAAGTGGATCCACTCGAAATCGATC
GTAATGGCGATGACTGTGTTTTTCAGAATAAAAATCGTAACTATTCAGGATTGTTACTGGCCGATCAATTAAA
AATGGGGGAAGCGCGAATTGCCGCTATGCGTGAAGCAATGGGGGATGATGCTGATATTATCGTGGAAATCCAT
TCTCTTCTCGGTACAAACTCAGCTATTCAATTTGCGAAGGCGATAGAAAAGTATCGTATCTTTCTTTATGAAG
AACCGATTCATCCATTAAATTCTGATAATATGCAGAAAGTTTCCCGTTCAACAACGATTCCAATCGCCACTGG
CGAGCGTTCCTACACAAGATGGGGATACAGGGAATTACTGGAGAAGCAGTCTATTGCCGTAGCGCAACCTGAT
TTGTGTCTCTGCGGCGGAATTACCGAAGGAAAGAAAATCTGTGACTATGCTAATATTTATGACACCACTGTAC
AGGTGCATGTTTGCGGCGGTCCTGTTTCCACGGTAGCCGCGCTGCATATGGAAACAGCAATTCCCAACTTTAT
TATTCATGAGCATCATACCAATGCGATGAAAGCATCTATTCGGGAACTTTGTACCCACGATTATCAACCGGAA
AATGGCTATTATGTCGCGCCGGAACAGCCCGGATTGGGTCAGGAATTAAACGATGAGGTAGTGAAAGAATACC
TGGCCTATGTGATTAAATAGCATCTGGTGATACTGATTATGGTTTATGCCTCCCGGCATATCCCCTAAATAAT
TCGAGTTGCAGGATAAAATGGTTAACCGTCTTGAACAGCGCTTGCGCTAACCCCGACGGGGCGAGGCCCAGGG
ATGGGCCGAGTAACTTGCCGCCAACGCACATGAAACTTGAAGTATGACGGGTATACGCGGGAGGTATTGATTT
TCCAGCCTCTTCCTGGCCAGACCCCGATCTCCCCTGTGTCGCCTCTGAAACCGATCGCCGATAAACGCCGAGA
CCACGCTTATAAATGAAGAGAGCATGCGGCACCCGAATAAAGCATTGTCTGGCTGCATTCCGTTTACCAGTAC
GTGGGCGAATGTGTTATAATTTGCGACCTTTGAATCCGGGATACAGTAGAGGGATAGCGGTTAGATGAGCGAC
CTTGCGAGAGAAATTACACCGGTCAACATTGAGGAAGAGCTGAAAAGCTCCTATCTGGATTATGCGATGTCGG
TCATTGTTGGCCGTGCGCTGCCGGATGTCCGAGATGGCCTGAAGCCGGTACACCGTCGCGTACTTTACGCCAT
GAACGTATTGGGCAATGACTGGAACAAAGCCTATAAAAAATCTGCCCGTGTCGTTGGTGACGTAATCGGTAAA
TACCATCCCCACGGCGATTCCGCAGTGTATGACACCATCGTTCGTATGGCGCAGCCATTCTCGCTGCGTTACA
TGCTGGTGGATGGTCAGGGTAACTTCGGTTCTATTGACGGCGACTCCGCGGCGGCAATGCGTTATACGGAGAT
CCGTCTGGCGAAAATCGCCCACGAACTGATGGCCGATCTCGAAAAAGAGACGGTGGATTTCGTGGATAACTAT
GACGGTACGGAAAAAATTCCGGACGTCATGCCGACCAAAATTCCGAATCTGCTGGTGAACGGTTCTTCCGGTA
TCGCAGTAGGTATGGCGACGAATATCCCGCCGCACAACCTGACGGAAGTGATTAACGGCTGCCTGGCGTATAT
CGACAACGAAGACATCAGCATTGAAGGGCTGATGGAACATATTCCGGGGCCGGACTTCCCGACCGCCGCGATC
ATCAACGGTCGTCGTGGTATCGAAGAAGCCTACCGTACCGGTCGTGGCAAAGTGTACATTCGCGCCCGCGCGG
AAGTTGAAGCTGACGCGAAAACGGGCCGTGAAACCATCATCGTCCATGAAATTCCCTATCAGGTGAACAAAGC

GCGCCTGATCGAGAAAATCGCCGAGCTGGTGAAAGATAAACGCGTGGAAGGCATCAGCGCGCTGCGTGACGAA
TCCGACAAAGACGGGATGCGCATCGTGATTGAAGTGAAACGCGATGCGGTGGGCGAGGTGGTGCTTAATAATC
TCTACTCCCAGACCCAGCTACAGGTTTCCTTCGGTATTAACATGGTGGCGCTGCATCACGGCCAGCCGAAGAT
CATGAACCTGAAAGACATCATTTCAGCGTTCGTGCGCCACCGCCGTGAAGTGGTGACGCGTCGTACTATTTTT
GAACTGCGTAAAGCCCGTGACCGCGCGCATATCCTTGAAGCTCTGGCGATTGCGCTGGCTAACATCGACCCGA
TTATCGAACTGATTCGCCGCGCGCCAACGCCGGCAGAAGCAAAAGCGGCGCTGATTTCGCGTCCGTGGGATCT
GGGCAACGTTGCTGCGATGCTGGAGCGCGCTGGTGATGACGCTGCGCGTCCGGAATGGCTGGAGCCAGAATTT
GGCGTGCGTGACGGTCAGTATTACCTGACTGAGCAGCAGGCGCAGGCGATTCTGGATCTGCGTTTGCAGAAAC
TGACCGGCCTGGAGCATGAAAAACTGCTCGACGAATACAAAGAGTTGCTGGAGCAGATTGCTGAACTGCTGCA
CATTCTGGGCAGCGCCGATCGCCTGATGGAAGTGATCCGCGAAGAGATGGAGTTAATTCGCGATCAGTTCGGC
GATGAGCGTCGTACCGAAATCACCGCCAACAGCGCCGATATTAATATCGAAGATCTGATTAGCCAGGAAGATG
TTGTCGTGACGCTGTCTCACCAGGGTTACGTCAAATATCAACCGCTGACGGATTACGAAGCGCAACGTCGTGG
TGGGAAAGGTAAATCTGCCGCGCGTATTAAAGAAGAAGACTTTATCGACCGCCTGCTGGTGGCCAACACCCAT
GACACCATCCTCTGCTTCTCCAGCCGGGGCCGTCTGTACTGGATGAAGGTCTATCAGTTGCCGGAAGCCAGCC
GCGGCGCGCGCGGTCGTCCGATCGTTAACCTGCTGCCGCTGGAAGCCAACGAACGTATCACCGCGATTCTGCC
GGTTCGTGAATACGAAGAAGGCGTCAACGTCTTTATGGCGACCGCCAGCGGTACCGTGAAGAAAACGGCGCTG
ACCGAATTCAGCCGTCCGCGTTCCGCCGGTATTATCGCGGTGAACCTCAACGACGGCGACGAGCTGATTGGCG
TTGACCTGACTTCTGGTTCTGACGAAGTCATGCTGTTCTCGGCCGCGGGTAAAGTGGTGCGCTTCAAAGAAGA
CGCCGTCCGTGCGATGGGGCGTACCGCGACCGGTGTGCGCGGTATTAAGCTGGCGGGAGACGATAAAGTCGTC
TCTCTGATCATCCCACGCGGCGAAGGCGCTATTCTGACCGTAACGCAAAACGGCTACGGGAAGCGTACCGCAG
CGGACGAGTACCCGACCAAGTCTCGTGCGACGCAGGGCGTTATCTCTATCAAAGTGACCGAGCGCAACGGTTC
CGTTGTCGGTGCGGTACAGGTAGACGATTGCGACCAGATCATGATGATCACGGATGCCGGTACTCTGGTGCGT
ACCCGAGTGTCCGAGATCAGCGTCGTGGGACGTAATACCCAGGGCGTTATCCTTATCCGCACGGCGGAAGATG
AAAACGTGGTGGGTCTGCAACGCGTTGCTGAACCGGTAGATGACGAAGAACTCGACGCTATCGACGGCAGCGT
GGCGGAAGGGGATGAGGATATCGCCCCGGAAGCGGAAAGCGATGACGACGTTGCGGATGACGCTGACGAGTAA
TGTTATTGCTGTGCGAGGGCCGGATATCCGGCCCTTTTTCTTTGCCGTTGCGGGCTACGTCATTTACCGCTAC
CTTAGTCACACTCTATTTACATCCTGAGGCGGAGCTTCGCCCCTTTGAAATACCTTGCTTCCTTTCGAACTAC
GCTGAAAGTCTCGCGCTACTTATTCAGAGCGTTAGCGTTACTCATTTGGCTCTTAATCGCCTTTGTTTCGGTG
TTTTACATCGTCAATGCCCTGCACCAGCGGGAATCTGAAATTCGTCAGGAGTTCAACCTTAGCTCCGATCAGG
CGCAGCGTTTTATCCAGCGCACGTCGGATGTGATGAAAGAGCTCAAATATATCGCCGAGAACCGTTTAACGGC
GGAAAACGGCGTCATGTCGTCGCGTGCGCGTGATGACAAAATGGTGGTGCCGGACTTTGAACCGCTATTTGCC
GACTCAGACTGTGCGGCGATGGGCTCCGCCTGGCGCGGATCGCTGGAGTCGCTGGCCTGGTTTATGCGTTACT
GGCGCGACAACTTTTCAGCGGCGTATGATCTTAACCGCGTTTTCCTGATTGGCAGCGATAACCTCTGTATGGC
GAATTTCGGCCTGCGTGAAATGCCCGTGGAGCGCGATGATGCGTTAAAAGCGTTACATGAGCGGATTATGAAA
TACCGCAACGCGCCGCAGGAAGAAAGTGGGAATAACCTTTTCTGGATAAGCCAGGGCGCGCGTCAGGGCGTGG
GCTACTTTTATGCGCTGACGCCGGTGTATCTGGCCAACCGTCTGCAGGCGCTGCTGGGCGTCGAGCAGTCCAT
CCGTATGGAAAATTTTTTCACGCCCGGCAGTTTGCCGATGGGCGTCACTATTATTGATGAGAACGGCCATTCG
CTGATTTCGTTGACCGGTCCTGACGGCAATATTAAAGCGGAGCCGCGCTGGATGCAGGAGCGCTCCTGGTTTG
GTTATACGCCAGGTTTTCGCGAACTGGTACTCAAAAAAAGCCTGCCGCCATCCTCGCTCAGTATTGTCTATTC
TGTGCCTGTAGATTTAGTCCTGGAGCGTATTCGTATTCTTATCCTGAACGCTATCCTGCTGAACGTGCTGGTG
GGCGCAGGGCTGTTTACGCTTGCGCGGATGTATGAGCGGCGAATTTTCATTCCGGCGGAGAGCGATGCCCAGC
GTCTGGAAGAGCATGAACAGTTCAACCGTAAAATCGTCGCCTCCGCGCCGGTGGGGATCTGTATTTTGCGCAC
TATTGATGGTGTCAATATTCTGAGTAACGAACTGGCGCACACCTACCTGAACATGCTCACGCATGAAGATAGA
CAGCGCCTGACGCAAATCATTTGCGGCCAGCAGGTTAACTTCGTTGATGTATTGACCAGCAATAATACCAATT
TGCAAATTAGCTTCGTCCATTCCCGCTATCGCAATGAAAACGTCGCGATTTGCGTGTTGGTAGATGTGAGTAC
GCGCGTCAAAATGGAAGAGTCGCTGCAAGAGATGGCGCAGGCGGCGGAGCAGGCAAGCCAGTCTAAATCCATG
TTCCTTGCGACGGTCAGCCATGAATTGCGCACACCGCTGTACGGTATCATTGGCAATCTCGATCTGCTGCAAA
CCAAAGAGCTGCCAAAAGGCGTGGATCGGCTGGTCACGGCGATGAATAACTCCTCCAGTCTGTTGCTGAAAAT
TATCAGCGACATTCTCGACTTCTCGAAAATTGAGTCGGAACAGCTAAAGATTGAGCCGCGCGAATTTTCACCG
CGAGAGGTGATGAACCATATCACCGCCAATTATCTGCCGCTGGTGGTGCGTAAGCAGCTCGGCCTGTATTGTT
TTATTGAGCCTGACGTGCCGGTATCGCTGAACGGTGACCCGATGCGTTTACAGCAGGTTATTTCAAACCTGTT
GAGCAACGCCATTAAGTTTACCGATATTGGCTGCATCGTGCTGCATGTGCGCTGTGATGGGGATTATCTCAGC
ATTCGCGTGCGGGATACCGGCGTCGGTATTCCGGCAAAAGAGGTGGTTCGCCTGTTCGATCCCTTCTTCCAGG
TGGGAACCGGCGTACAGCGTAACTTTCAGGGGACCGGTCTGGGGCTGGCGATTTGCGAAAAATTGATCAGCAT

GATGGACGGCGATATTTCCGTCGATTCGGAGCCGGGCATGGGGAGCCAGTTTACGCTGCGCATCCCGCTTTAT
GGCGCGCAATACCCTGTGAAAAAGAGCGTGGAAGGACTCGCCGGAACCTGCTGTTGGCTGGCGGTGCGCAATA
CTTCTTTGTGCCAGTTTATTGAGACCAGTCTGGCGCGTAGCGGCGTTCATACGCAGCGTTATGAAGGGCAAGA
ACCCGCCGCGGACGATATTTTGATTGTCGATGACGCGCTGGAGCATACCTGGCAGGGGAGGGCTGCGGTCGTC
TTTTGCCGCCGTCATATCGGGATTCCGTTGGAAAGAGCGCCGGGCGAGTGGGTGCATAGTGTGGCGTCGGTGC
ATGAGTTGCCCGCGCTGTTGGCGCGTATCTATAGTATTGAGCTGGATAGTGAAGCGCTTTCCAGCGCATTGCC
GACGACGGATAAAACCGCAGATTCAAATGACGACATGATGATTCTGGTCGTTGACGATCATCCGATCAATCGC
CGCTTGCTGGCCGATCAACTGGGATCGCTGGGGTATCAGTGTAAGACAGCCAATGATGGCGTGGACGCTCTGA
ATGTGCTGAGTAAAAATGCTATCGATATTGTTCTGAGCGATGTGAACATGCCGAACATGGACGGCTATCGCCT
GACGCAGCGTATCCGTCAGCTTGGGCTGACGCTGCCGGTCGTCGGCGTGACGGCGAACGCGCTGGCGGAAGAG
AAACAACGTTGCCTGGAGTCCGGTATGGACAGTTGTTTGTCGAAGCCTGTGACGCTGGATGTGTTGAAACAGA
CGCTGGCGGTGTATGCCGAGCGGGTACGTAAAACGCGGGCATAAAAAATGCCGGATGGCGGCGTCGCCTGTCC
GGCCTGCAAAAGATGAGTCGACTGGTAGGCCTGATAAGCGTAGCGCCATCAGGCTGGGTAACGTAAAAGTGAT
TTATTCTTTGTCTGTCGGACTCAGGGTGACGGAAGAGAGATAGTTGAGCAGCGCGATATCATTCTCTACGCCC
AGTTTCATCATCGCCGATTTCTTCTGGCTGCTGATGGTCTTAATGCTGCGGTTGAGCTTCTTGGCGATTTCGG
TGACCAGGAAACCCTCGGCGAACAGGCGTAATACTTCGCTCTCTTTTGGCGACAGGCGCTTATCGCCGTAACC
GCCTGCGCTGATTTTCTCCAGCAGACGAGAAACGCTTTCCGGGGTGAATTTTTTGCCTTTTTGCAGCGCGGCC
AGCGCCTTAGGCAGATCGGTTGGCGCACCCTGCTTGAGTACGATCCCTTCAATATCAAGATCCAACACGGCGC
TCAGGATCGCCGGATTGTTGTTCATGGTCAGAACGATAATAGACAGGCTCGGAAAATGACGCTTGATGTACTT
GATCAAGGTGATCCCATCGCCGTATTTATCTCCCGGCATGGAGAGGTCAGTGATCAACACATGCGCATCTAAT
TTCGGCAGGTTGTTGATCAATGCTGTGGAATCTTCAAATTCGCCGACAACATTCACCCACTCGATTTGTTCAA
GTGATTTGCGAATACCGAACAGTACAATCGGGTGGTCATCGGCAATAATTACGTTCATATTGTTCATGTATTG
GGCTACCTTGCTACAGCAAGCTTTTGACGTAGGCGTCAATGTCGCTGATATATTTTTCTATACCTGGAGCATC
TTTCTCACGAATCAGATGTTCCAGCGTTTCACATAACTGCTTGCCGGGTACCAGATTAAGCATGGCAAACACC
CCTTTCAGGCGGTGGGCCGTTTGGGCCAGCGCAGCGAAATCGCTGGTTGCCGCCTCAGTATACAACCTCTTAA
CATCATCCGGTACTGTGTCTACAAATAGCGCATAATAGCCGCTGGCATGAAGCTCGGCATTTTCATCTCCGCC
GAGCGGGGAGGCGGGAATCTCTTCCTGCGCCAGCTGCTCTTCGATAAGTTGTAGTACAGCTTCCTGCATAGCA
TTGCTCATATTAAAGTTGACGCGCAACTGACCCGGGCCAATTTTCCGCACGCCTGACTCATCATCGCTTAAAA
GCAAGCCCGAGGCAGTAAGATTAGACGGATTATCCGTTAAAAAGAGATCATATTCTTGACTTATCTGCCTTTC
ATCCGGCGTGATGCAGGTGGCGCCCCAGTTTTCTAACTGACGCAGCACAATCGCGCGAACCTCGTTGGACGTC
ACATCGACCATCACGCTCACGTCGTCCAGCAGGCGCTCTTCGACCTGAGTATGCTGATCGTGAGGAAGCATTT
TGACATGAACGGTATAGCGCGTGCCCAGCGTTTCTCGCGCTTTAATGTTGAGATGACCGCCCAGTTTGCGCGC
CAGTTGATTGCACAGCCAGAAGGTGAGCGGATTCGCTTTGCCGTAACGATCGCCCTGCGTTTCATTCATATAC
GGGAAATGCAGATTATCAATCTCGTTTAACGTTACGCCTTCGCCCGTATCCAGAATACGGAAAGTCAGACGCT
CCGCGATAGATTCATCCTGCTCGACTTCAAGCGTAATTTTACCCATAGCGGTCGTTGTGACGGCATACTGAAT
CAACAGCAGCAAAATCCGACGCAGGGCGTCGCGATCGCCCCAGCGTTCATCGTTGCCGCTAAGATGGTTATTA
ATCAGCAGTTGCAGCCCTTTACGCTTAATGGCGGGCAGCACTTCCGGCACGACTTCGTCAATAAGATCCTGCA
GGGAGAACAGCGTGGCTTCGCTCTTCCATGCGTCATTTTCCAGCATATTCGCCAGTTGGATTTCATCGATCAT
ACGTACCAATACATCCGCCTGATCCGCCAGTTTCAGGCTTTCCGGCGTGGTGACTGCCGCGGCGTTAACCGCC
AGTTGGCGTACGGGCTCTTTAAGCGTGTTGCTGATATTTTGCATAAAGGCGGCGCGACCCTGCTGATTCTTCT
CATACAAACGCTGCGCCTGTTTAAGCTTCTTATTCACCAGCACTTCCCGGTCCTGATCGCGAATAATAAAGAT
CTGCGTGCGGGGAGCGATCTGGCTGCGAAACAGACGGATCTCATACAGTTCGTTATTAATTGTCGCCTGAATC
ACGCCCTGATGCTGTTCCGCCATGCTGGTAATATTTTGCAGGTTAAGATGCGGCAACAGATGATCGGCGATTT
TATTGCTGATCACGGTGCGGTTGCCTTCCTGATCGTAGACCAGCAGACCCAGCGGCAGCAGAGAAACGATCTC
TTCATTAATGGCGCGTAAGACGCGAAGCTCGTTATTCGCCGCGTTGCCGGATGTCGATTCCGTCGACCGTCCC
GGCTGATGACGGAACGTGGCGTAGCCAAATAACGCCAACGCCAGCAGCCCGATGTTCAGCAGTAAAGGCAGCA
GAATATTTTGCAACGTATCAAGCAAAAGCGTACCGAACGGTACCTGCCAGATCAGGCGCATTCCGGTAGAGTT
AAGCGCTGAGGAGATCTCAATTTTTGAACCGTTAAAGCTAATCGTGACGCTATCTGGCGCTTCCTTTTCACTG
CTTCGCCCCGTCGCCTGGGTTGCGTCAGGCTCTATCCGAAAACTGTCCAGCGGCATTCCCGGCGGGATTAAAT
CATTAATGGGCAGATCAAACGCCACCACGGTCGCCAGATGTCCTGGCTGATTGAAGGTCGTGCGCAGCGTAAA
ATAGTGACCGTTTTGCCAGGCCAGTTTTCGCAATGAGGAGAAACTTTCGCGCTCATCCAGCGCATTCGCCTGC
TGCAACATCTCTGCCCGGCGGGAATCGACGATATTGCCAATAGTGGACTCTTTAAAGCCGGAAGAGAGATCTT
TCAACGGTAGGGTAGAGATAAGAATCAGGCTGTTATCCTGTCCGTTGAGATAATACATTGACCACGGCACGTT
TTCCGCGCCCCACAGCGTATCCAGATAGGTTGACATACGCTGCGTCATTTCCAGCGTGGCGCTGTCATGGGAA

CCGAAAATCAGCGCTTCAGTTTTACGTCGCGGCTTTTCAAGATAATACACATCCTGTTTCAGACGCGTTTCCT
GAAGTCCTTCACCTGTCGATGGCGTAGTGGTGGCGGCGATGTTGTCATAAATTTGCCAGGTAACATACCGCCA
GGTGTCCACGCGTTTATGAATAGCGTGAGTGATATCGACAATTTGATAGCTTTTGTCTTTCAACCAGGCGTTG
ACGGCGCTCTGTACCATAACGCCCATCGTTACCAGCAGCACAATGATCAGCAATAAGAAGAAACGGGTAATGC
TTCCCGGTAGAAGGGAGAATCGGGTAGAGACCGTTGTGTCAGACTGACTCATTTATGTTTACGACCTGTAAAA
GCAACGCTGAAGGTGAAGGGCAGTATAAAGGGTAATGAATGAATTTCCAGACTCTTACGTCATGCCGGGCAAC
TTAACACGCGCTCCGTCGGCGGGACGGGAAAAGCGTACAACCTTCGCAGAACGGTACAAATATAAAGACTACT
TATAAATGTTTAGCACTAAATATCAAGAAATTATTTGCTAATGTCTTACGAACGGTCGAGCAGGGGAGGGTAG
TGAATAACCGTGATAGTTTTAGGCAGTAGCATAAATCTGCCGGGTGTGTCTGAAAAAACGTAAAAAAACCGAA
TGCGAAGCATCCGGTTGAAATAGGGGTAAACAGACATTCAGAAGTGAATGACGGTAATAAATAAAGTTAATGA
TGATAGCGGTCACTATTTTAGTTGCGAATGAAGATTCTGTTTTATCATTCAGTGCTATGAATTTCATCAATTT
AACCCATTGATTTTAAAAGTCTCGTGAATATATTTTGTCTATTTGTGCTTATTTTTACTTGATTTTTGCTTTA
AAAAAGTTCCGTAAAATTCATATTTTGAAACATCTATGTAGATAACTGTAACATCTTAAAAGTTTTAGTATCA
TATTCGTGTTGGATTATTCTGTATTTTTGCGGAGAATGGACTTGCCGACTGGTTAATGAGGGTTAACCAGTAA
GCAGTGGCATAAAAAAGCAATAAAGGCATATAACAGAGGGTTAATAACATGAAAGTTAAAGTACTGTCCCTCC
TGGTACCAGCTCTGCTGGTGGCGGGCGCAGCGAATGCGGCTGAAATTTATAATAAAGACGGCAACAAATTAGA
CCTGTTTGGTAAAGTTGATGGCCTGCACTACTTCTCTGACGACAAAGGCAGCGACGGCGACCAGACCTACATG
CGTATCGGCTTCAAAGGCGAAACGCAGGTTAACGATCAGCTGACCGGTTATGGCCAGTGGGAATATCAGATTC
AGGGCAACCAGACTGAAGGCAGCAACGACTCCTGGACGCGTGTGGCGTTTGCGGGTCTGAAATTCGCTGACGC
AGGTTCCTTCGATTATGGTCGTAACTACGGCGTAACCTATGATGTGACCTCCTGGACCGATGTTCTGCCGGAG
TTCGGCGGCGACACCTACGGCGCTGACAACTTTATGCAGCAGCGTGGTAACGGCTATGCCACTTACCGTAACA
CCGACTTCTTCGGCCTGGTGGATGGCCTGGACTTCGCGTTGCAGTATCAGGGCAAAAATGGCAGCGTGAGCGG
TGAAAACACCAACGGTCGCAGCCTGCTGAACCAGAACGGTGACGGTTACGGCGGATCGCTGACTTATGCAATC
GGCGAAGGCTTCTCTGTCGGTGGCGCTATCACCACGTCTAAACGTACTGCCGATCAGAACAACACCGCTGACG
AACACCTGTATGGTAACGGCGATCGCGCTACGGTCTACACCGGCGGCCTGAAATACGATGCGAACAACATCTA
TCTGGCAGCGCAGTATTCTCAGACCTATAACGCAACCCGTTTTGGTACCTCTAACGGCAACAATAAGTCCACC
TCTTACGGTTTTGCCAACAAAGCGCAGAACTTTGAAGTGGTTGCTCAGTACCAGTTCGACTTTGGTCTGCGTC
CGTCTGTGGCTTACCTGCAGTCTAAAGGTAAGGACATCAGCAACGGCTACGGCGCCAGCTATGGCGACCAGGA
CATCGTAAAATATGTTGATGTCGGCGCGACTTACTACTTCAACAAAAACATGTCCACCTATGTTGATTACAAA
ATCAACCTGCTGGATAAAAACGACTTTACCCGCGATGCGGGCATCAACACCGACGACATCGTAGCGCTGGGTC
TGGTTTACCAGTTCTAATCAGCAAAAGATGTTGCTAAAGGGCCTGCGGGCCCTTTTTTCATGCCTTATTCCGG
CGTACAAATACGACGTTTTGATATACGCTTGCGTCCATTCGAATGAGGATAAACAGCGATGAAAATGACTTTT
TGCCGGGCCGTGTGTCTGGCGGCGGCTTTTTTACTTATGGGCTGCGATGAGGCTCCCGAAACGACAACAGCGT
CACCTGCCGCTCAGGTGCTGGAAGGTAAAACGATGGGGACCCTCTGGCGGGTGAGCGTGGTTGGCATCGATGC
GAAACGCGCCGCAGAGTTACAGACTAAAATCCAGACTCAGCTTGATGCTGATGATTGGTTGCTTTCTACCTAT
AAAAATGACTCCGCGCTAATGCGTTTTAACCATTCACGCAGCCTTGCGCCCTGGCCGGTCAGCGAAGCCATGG
CGGATATCGTGACCTCGGCGCTACGTATTGGCGCGAAGACGGATGGCGCGATGGATATCACCGTGGGCCCGCT
GGTCAATCTGTGGGGGTTTGGGCCGGATCGGCAGCCGCTGCATATCCCAACACCAGCACAAATCGATGCGGCA
AAAGCGAAAACAGGCCTGCAACATTTGCAGGTTATCGACAGGGCTGGACATCAGTTTTTGCAAAAAGATCTGC
CGGATCTTTATGTTGATCTCTCCACGGTCGGGGAGGGCTATGCGGCGGATCATCTGGCGCGACTGATGGAGCA
GGAGGGCATTGCGCGTTATCTGGTCTCGGTGGGCGGCGCATTAAGCAGTCGCGGGATGAATGCGCAGGGGCAG
CCGTGGCGCGTCGCGATTCAGAAGCCGACCGACCGGGAAAACGCGGTGCAGGCGATTGTGGATATCAACGGGC
ATGGCATCAGCACCTCCGGCAGCTACCGTAACTATTACGAGCTGGATGGCAAGCGCGTATCGCACGTTATCGA
TCCACAAACGGGGCGCCCCATTGAACACAATCTGGTATCGGTTACGGTCATCGCGCCAACGGCGCTGGAAGCG
GACGGCTGGGACACCGGCCTGATGGTGCTCGGTACGCAAAAGGCGCAAGAGGTCGTGCGGCGGGAAGGGCTGG
CGGTCTTTATGATCATGAAAGAAGGTGAAGGCTTTAAAACCTGGATGTCGCCGCAGTTCAAAACGTTCCTGGT
GAGCGATAAGAATTAAAACGCAAGATTACGGGTTTTTGTGGGCGGATTTACGGACACACTGCATGTAAGCAAG
CACTATGCTTAATGAAGGAGTCTATGATGAAAAAAGCGTTACTTACCGATGATGAATGCTGGCTGCGGGTGCA
GGCGCGCGATGCCAGCGCGGATGGGCGTTTCGTTTTTGCAGTGCGAACCACTGGCGTTTTTTGCCGCCCTTCT
TGTCGCTCGAAGCGGGCGTTACGTAAAAATGTTCGCTTTTTTGCCAACGCGGAGCAGGCGCTGGACGCCGGTT
TTCGCCCCTGCAAGCGCTGTCAGCCGGATAATGCGCGCGCGCAGCAACGGCGGTTGGATAAGATTGCCTGCGC
CTGCCGTTTACTTGAGCAGGAGACGCCGGTAACGCTGGCGTCTCTGGCGCAGGCGGTGGCGATGAGCCCGTTT
CATCTGCACCGTCTGTTTAAAGTGAGCACCGGAATGACGCCGAAAGGGTGGCAGCAGGCGTGGCGCGCCCGGC
GGCTGCGTGAGGCGTTGGCGAAAGGAGAGCCGATCACGGCGGCTATTTATCGCGCTGGCTTCCCGGATAGCAG

TAGCTACTACCGTCATGCCGACCAGACGCTGGGCATGACGGCAAAACAGTTTCGCAAAGGCGGCGATAATGTC
TCCGTTCGCTATGCGCTGACGGACTGGGTTTACGGACGGTGCCTGGTGGCGGAGAGCGAGCGGGGGATTTGCG
CGATTCTCCCCGGTGATAGCGACGACGCGCTACTGGCTGAATTACACACCCTTTTCCCGACGGCCCGCCACGA
ACCTGCCGACGCGTCTTTTCAGCAACGGGTGCGGCAGGTTGTCGCGGCTATCAACACACGCGATGTGCTGCTC
TCGTTGCCGCTGGATATCCAGGGAACCGCGTTTCAACAGCAGGTCTGGCAGGCGTTATGCGCAATTCCCTGCG
GCGAAACCGTAAGCTATCAACAGCTTGCCGCGACTATCGGCAAACCCACGGCGGTACGCGCGGTCGCCAGCGC
GTGCGGCGCGAATAAACTGGCGATGGTGATCCCGTGTCATCGGGTCGTGCGTCGCGATGGCGCGCTCTCCGGT
TATCGTTGGGGCGTGCGTCGAAAAGCGCAGCTATTAAAGCGAGAAGCGCAAAAAGAGGAGTAGCAATGCTGGA
TCTATTTGCTGATGAAGCGCCCTGGCAAGAGCCCCTGGCGCCTGGCGCGGTGGTGTTGCGCCGCTTTGCGTTT
CGCGCGGCGCAGTCGCTATTAGACGATATCGGGTTTGTCGCCAGTCAGTCTCCGTTTCGTCAGATGGTGACGC
CGGGCGGCTACACCATGTCGGTGGCGATGACCAACTGCAGCGCGTTAGGGTGGACCACCGATCGTCACGGTTA
TTGTTATGCCGTGCGCGATCCGTTGACCGATAAACCCTGGCCTGCGCTGCCGTTATCGTTTGCCAGCGTATGT
CGTCAGGCGGCAATCGCGGCAGGGTATGCGAGCTTCCAACCGGACGCCTGCCTGATCAATCGCTACGCGCCCG
GCGCAAAACTGTCGCTGCATCAGGATAAAGACGAGCCGGATCTGCGCGCGCCTATTGTCTCAGTTTCGTTGGG
GGTGCCGGCGGTTTTTCAGTTTGGCGGCCTGCGTCGCAGCGATCCGCTCCAGCGGATCTTACTGGAGCATGGC
GATATCGTGGTCTGGGGCGGCGAGTCCCGTCTGTTTTATCATGGTATCCAGCCGCTTAAAGCGGGCTTTCATC
CCATGACCGGTGAATTTCGTTACAATCTCACCTTCCGTCAGGCGGCTGAAAAAGAATAAAAATAAGAATTATT
TGCTGTGCAGGTGGAGTTAGTTAAACTGCGGGCTGTTATTATCGGTCCGGGTTGTAAGCATGGAACTTCTTAT
TCTTGTCTGGCGGCAGTACCGCTGGCCATTTATTAGCGTGATGGCGCTGAGCCTCGCCAGCGCGGCGCTGGGG
ATTGGCCTGATCGCGTTTATCAATCAGCGCCTGATTGAAACGGTGGACACCAACGTCATGGTGCTGCCGGAAT
TTCTCGGCCTGCTCCTGTTGCTAATGGTCGTCACATTAGGTTCCCAACTGGCGCTCACCACTCTGGGACACCA
TTTCGTTTACCGCCTGCGCAGCGAATTTATCAAGCGCATTCTGGATACGCACGTTGAACGCATTGAGCAACTG
GGCAGCGCCTCTTTACTGGCCGGTCTGACCAGCGATATTCGCAATATTACTATCGCCTTTGTGCGTCTGCCTG
AGCTGGTGCAGGGCATTATCCTCACCGTCGGCTCGGCGGCGTATCTGGCGATGCTATCGACAAAAATGCTGCT
GGTTACGGCTATCTGGATGGCGGTCACTATCTGGGGCGGATTCGTGCTGGTGGCGCGGGTTTACCAGCATATG
GCGACCCTGCGCGAGACCGAAGATAAGCTGTACAACGACTATCAGACGGTGCTGGAAGGACGCAAAGAGCTGA
CGCTAAACCGCGAGCGTGCGGAGCAGATTTTTCAGCAATGCTATACCCCGGACGCCAAAGAGTACCGTCATCA
CATTATCCGTGCCGATACTTTTCATCTGAGCGCCGTCAACTGGTCGAATATCATGATGCTGGGGGCGATAGGC
CTGGTCTTCTGGATGGCGAATAGTCTCGGCTGGGCGGATACCAACGTCGCGGCGACCTATTCACTGACGTTGC
TGTTCCTGCGCACGCCGCTGCTGTCTGCGGTGGGGGCGTTGCCAACCCTACTGACCGCCCAGGTGGCGTTTAA
TAAACTGAATAAATTCGCGCTCGCCCCGTATAAGCCTGATTTTCCACAGCCGAAAGCCTTTCCCGACTGGAAG
ACGCTGGAGCTGCGCAACGTGGTTTTCCACTATCAGGATAACGCGTTTTCCGTGGGGCCCATCAACCTGACGA
TTCACCGCGGCGAACTGCTGTTTTTAATTGGCGGCAACGGTAGCGGGAAATCGACGCTGGCCATGTTATTGAC
CGGTTTATACCAGCCGCAATCGGGAACGATTTTACTTGACGGTCAACCTATTGCCGCCGGGCAGCCGGAAGAT
TATCGCAAACTGTTTTCTGCGGTGTTCACCGATGTGTGGTTATTCGACCGCCTGCTGGGGCCGCAGGGGAAAC
CCGCCAATCCCGCGCTGGTCGAAAAGTGGCTTGAGCACCTGAAAATGACGCACAAGCTGGAGCTAAACGACGG
GCGCATTTTGAATCTTAAACTGTCGAAAGGGCAAAAAAAGCGCATTGCGCTGCTGCTGGCGCTGGCGGAAGAG
CGCGATATTATTTTGCTGGATGAGTGGGCCGCCGATCAGGACCCGCATTTCCGCCGGGAGTTTTATCAGGTGC
TGTTGCCGTTAATGCAGGAAATGGGCAAAACCATTTTCGCCATCAGCCATGATGATCACTATTTTATTCACGC
CGATCGTCTGCTTGAAATGCGCAACGGACAGCTAACCGAACTCACCGGCGACGAACGTGATGCCGCCTCGCGT
GATGCGGTTGCGCGTACTGCGTAATGGCCGGATGAACCGCCTGATGGCAGCCCTACGTCCGCACGCGGGTAGG
CCTGATAAGATGCCACGCGTCGCCACCAGGCATCGGGCGAGCGGATTTCCAGACGTGTTTTGAAAAGAACCCC
GGAGCGCTGCGTGAGCCGGCGTGTTCAGATGCCCGGTAAGTGTGACCGGGCAGCGTAACGTTATCGCGCGACG
GCGTTCTGTACGTTAGCGTCCGCTTTCCAGATGCGGTATTTCACGTCCACATTCGCCGGGGTATACACCACGA
TCGGCAGCTTGCTGTTGTAGCGCAGCATCCCGTCTTCACCCAGCCAGGCGGTGACGAATTTTTGCTCTTTCTT
ACCTTCAGGGCAGGCCATCATGGTTGATACCGGAGAGGTGACGTTATCAAAGACATAATAGTCATAGCCCCAG
CCTTCCAGCGTTTTTGTTTCCAGCGTGCCGCCGAGGCGATGCTGGTTACAATCCACATTCAGCGTTTGACCAA
TCAACAGTTCCACTTTGAGGGTAGATTCATCCTGCTGAGGGGTAAGGGTTATCACTTGCCGCTTCATTCCTTT
TTCCGCCTGCGGATAGGGGGCGATTTTTTCCAGCGGCTGGGCGGTATCACCATTGTTAGCCCAGGCAGAGGCG
CTGGCGAGGGCGGCGAAGACAACGGCAGGGACAAACATCTTCATTTGATTGTTCACGGTATTTCCTTTCTGCT
GTTTTCGGTATGCACGGGCAACAGATTAACACATTCATATTATCTATTAATCTGTATTTACTCATATTTTCAA
GATTATTAATAAAATAGCGTCTTTTTATATAGTGGAATATTCCGATATTGATAATGTAAATGAAATTATCAAC
TATTAGTGATTTTCGTTATATAAGCATCTATATAACTCATTGAAATGTGAGAAAAAGCACGTTTACGGGTCTT
TACCTGGCACTCTTTTGATCCGGCAACAGCATTTACCTCTATCGGGGTATGCATCTCTTACACATCCTGAAAT

ATCTTATTTACAAAAACAACTACTCATAAATTGTACGGTTTTTATTGGTATTCACTCCCTATGGAAGCGTGTC
GTTATGGAAGGGCGATATCATGGTTGATTTATCCCGTCGAAGCATGTTGACCGGCAGTTGGCGCAACGCCAGC
AATGGGATTCTTCCGCCGTGGGCCAGAGAAACAACCTATTTTCTCGCACATTGTCTGCGTTGCGACGCCTGCA
TCCAGGCGTGTGAGGCCGACATCCTGCAGCGAGGGGCGGGGGGATATCCGAGCGTAGATTTCAAACGGGGCGA
GTGCAGCTTTTGTTATGCCTGTGCGCAGGCCTGTACTGAATCGCTTTTTCTTCCGCGCCACACCAGGGCCTGG
GATCTGATTTTTACGCTCACGGAAAACTGCCTCGCCAGGCAATCGGTTGAATGCCATCGCTGCCAGGACAGTT
GTGAACCTATGGCCATTACCTTTCGTCCCACGCTATCCGGCATTTACCAGCCGCAGCTTGACTCGCAGGCCTG
TAACGGATGCGGGGCGTGTGTGGCTATCTGTCCCGTGTCAGCCATCAAAGCGGAGAACCACCATGCGCACTAA
CTGGCAGGTCTGTAGCCTGGTCGTGCAGGCCAAAAGTCAGAACATCGCCGCCATTGGCACACAGTTGAACACG
TTGCCGGGTTGCGAAGTCGCGTTAAGCGACGTTGAAAGCGGGCAACTGATCGTGGTGGCGGAAGCAGATCAGA
GTGAAACGCTGATGCAAACAATTGAGTCAGTACGCAATGTCGAGGGCGTACTGGCGGTGTCGCTGGTTTATCA
CCAGCAGGATGAGCAAGGTGAGGAAACACCATGAAACTCAGTCGTCGTAGCTTTATGAAAGCTAACGCCGTTG
CGGCCGCTGCGGCGGCTGCCGGTCTGAGCGTGCCGGGCGTCGCCCGCGCCGTTGTCGGGCAGCAGGAAGCCAT
CAAATGGGATAAAGCGCCGTGCCGTTTTTGCGGAACGGGTTGCGGTGTGTTGGTGGGAACGCAGCAGGGCCGT
GTGGTCGCCTGCCAGGGCGACCCCGACGCGCCGGTCAACCGTGGTCTGAACTGCATTAAAGGCTACTTCCTGC
CCAAAATCATGTACGGTAAAGATCGTCTGACGCAGCCGATGCTGCGCATGAAAGACGGCAGCTATCACAAAGA
CGGCGAGTTTACGCCGGTGAGCTGGGAACAGGCCTTCGATGTGATGGAGGAGAAGTTTAAAACCTCCCTGAAA
GAGAAAGGACCAGAAGCGATCGGCATGTTTGGATCGGGTCAATGGACCATCTGGGAAGGCTATGCCGCCGCGA
AACTGTTCAAGGCTGGTTTCCGCTCTAACAACATCGACCCGAACGCGCGTCACTGCATGGCCTCGGCGGTGGT
GGGCTTTATGCGCACCTTTGGCATGGATGAACCGATGGGCTGCTACGACGATATTGAGCAGGCCGATGCGTTT
GTGCTGTGGGGTTCCAACATGGCGGAGATGCACCCGATTTTATGGTCGCGCATCACTAACCGCCGTTTGTCCG
ACCCGAACGTGAAGGTTGCCGTCCTCTCCACCTTCCAGCACCGCAGCTTTGAACTGGCGGACAACGGTATTGT
CTTTACCCCGCAAAGCGATCTGGTGATCCTTAACTATATCGCGAATTACATTATTCAGAACAACGCGGTAAAT
CAGGATTTCTTCACAAAGCATGTGAACCTGCGTAAAGGGGCGACGGATATCGGCTACGGCTTACGCCCGACGC
ACCCGCTGGAAAAAGCGGCGAAGAATCCGGGCTCCGACGCTTCCGAGCCGATGAGCTTCGACGAGTATAAAGC
CTTCGTGGCAGAATATACGCTGGACAAGACCGCTGAAATGACCGGCGTGCCAAAAGATCAACTGGAACAGTTG
GCACAGCTCTACGCCGATCCGAATAAGCGCGTGATCTCTTACTGGACGATGGGTTTTAACCAGCATACCCGCG
GCGTGTGGGCGAATAACCTGGTTTACAACCTCCATCTGCTGACCGGAAAAATCTCCCAGCCTGGCTGCGGTCC
TTTCTCGCTGACAGGCCAGCCTTCCGCCTGCGGTACGGCGCGTGAAGTGGGAACCTTCTCACACCGTCTACCC
GCAGACATGGTCGTGACGAACGAAAAACACCGTGATATCTGCGAAAAACACTGGCAGATCCCCGCTGGCACTA
TTCCGGCAAAAGTGGGTTTACACGCGGTGGCGCAGGATCGCGCGTTGAAAGACGGCAAGCTGAACGTCTACTG
GGTAATGTGTAATAACAATATGCAGGCCGGGCCGAACATCAATGAAGATCGGATGCCGGGCTGGCGCGATCCG
CGTAACTTTATCATTGTTTCCGATCCTTACCCGACGGTGAGCGCGCTGTCTGCGGATCTGATCCTGCCGACCG
CCATGTGGGTTGAGAAAGAAGGCGCTTACGGCAACGCCGAACGCCGCACGCAGTTCTGGCGTCAGCAAATTAA
AGCGCCGGGCGAAGCTAAATCCGACCTCTGGCAACTGGTGCAGTTCTCCCGTCGTTTTAAAACCGAGGAGGTC
TGGCCGGAGGCGTTGTTGGCTCAGAAGCCGGAGCTGCGCGGCAAAACGCTGTATGACGTTCTGTTCGCCACGC
CGGCGGTCAGCAAATTCCCGCTGAGCGAGCTGAAAGAGGATCAGTTAAACGATGAGTCTCGTGAGCTGGGCTT
CTATCTGCAAAAAGGGCTGTTTGAAGAATACGCCTGGTTTGGACGTGGCCACGGGCACGATCTGGCGCCGTTC
GACGATTACCACAACGCGCGCGGTCTGCGCTGGCCGGTCGTCGAAGGCAAAGAGACGCAATGGCGTTACAGCG
AAGGCAACGACCCGTATGTAAAAGCGGGGGAAGGGTACAAATTCTACGGTAAACCGGATGGCAAAGCGGTGAT
CTTCGCGCTGCCATTTGAACCCGCGGCGGAGTCGCCGGACAACGAGTACGATCTGTGGCTTTCCACCGGTCGC
GTGCTGGAACACTGGCATACCGGCAGTATGACGCGCCGCGTGCCTGAATTGCATCGCGCCTTCCCGGAAGCGG
TGGTCTTTATTCACCCACTGGATGCGAAAGCGCGCGATCTGCGTCGCGGGGATAAGGTTAAAGTGTCGTCCCG
CCGCGGTGAAGTGATTTCGATTGTCGAAACGCGTGGACGTAACCGTCCGCCGCAGGGACTGGTGTATATGCCG
TTCTTCGATGCCGCTCAGCTAGTGAACAACCTGACGCTGGATGCGACGGACCCGCTCTCTAAAGAGACGGACT
TCAAGAAGTGCGCCGTTAAGCTGGCGAAGGTGTAACGCGTTATGTCCCGTACAGCGAAACCCCAAAATGGCCG
CCGCCGCTTCCTGCGTGATGTTGTGCGCACTGCGGGCGGGCTGGCGGCGGTTGGCGTGGCGCTGGGCTTGCAA
CAGCAAACCGCGCGCGCATCGGGCGTGCGGCTGCGTCCGCCTGGCGCGCTGAATGAAAATGTCTTCGCCAGCG
CCTGTGTGCGCTGCGGGCAGTGCGTTCAGGCGTGTCCATACGACACGCTAAAACTGGCGACGCTGGCCTCCGG
GCTATCGGCAGGCACGCCCTATTTCGTCGCTCGCGACATCCCCTGCGAAATGTGTGAAGACATTCCGTGCGCC
AAAGTCTGCCCCAGCGGGGCGCTGAATAAAGATATCGCCTCCATTGATGACTCGCGCATGGGGCTGGCGGTGC
TGCTGGATCAGGAAAACTGCCTCAACTTTCAGGGGCTGCGCTGCGACGTTTGTTACCGCGAATGCCCGAAAAT
CGATGAGGCCATCACGCTGGAGCTGGATCGCAACATGCGCACCGGCAAACATGCGCGCTTTCTGCCCACCGTA
CACAGCGACGCCTGTACCGGCTGCGGCAAATGCGAAAAAGTCTGCGTACTGGAGCAACCAGCGATAAAAGTGT

TGCCGTTGTCATTGGCGAAAGGGGAGTTAGGACACCATTACCGCTTCGGCTGGCTGGAGGGGAAAGATGGCAA
ATCGTAAACGCGACGCCGGGCGAGAAGCGCGGGCGAAAAAAGGCTGGTGGCGTAGCCACCGCTGGCTGGTGTT
ACGCCGTATCAGCCAGTTTATGGTGCTGGGGATGTTTCTCAGCGGCCCGTGGCTGGGCTTTTGGGTTTTGCAT
GGCAACTACAGCAGTAGTTTGCTGTTCGATACCCTCCCGTTGACCGATCCGCTGATTACCCTGGAAAGTCTCG
CCAGTGGACATCTTCCCGCTACCGTGGCGCTGACGGGAGCAGTGATTATCGCTGTGCTGTATGCGCTGGCGGG
AAAGCGACTATTTTGCAGTTGGGTTTGCCCGCTAAACCCCGTTACCGATTTGGCTAACTGGATGCGCAGGAAA
TTTGACCTGAACCAGTCTGCGACGATTCCACGCCATATACGGTACGTCCTTCTGGTGGTGGTGTTAATCGGTT
CGGCGTTGACCGGCACGCTGCTGTGGGAATGGATTAATCCCGTGTCGCTGTTAGGCCGTAGTCTGGTTATGGG
ATTTAGTAGCGGCGCGTTGCTGATTATTGCTTTGTTTTTATTTGATTTATTAGTCGTTGAGCACGGTTGGTGC
GGCCATATTTGCCCGATGGGCGCGCTGTATGGCGTGCTGGGCAGTAAAGGCGTAGTGACCGTGACGGCCAAAA
AACGTGAAAAATGTAATCGCTGTATGGATTGTTTTCATGTTTGCCCGGAACCGCATGTGCTACGTGCGCCGGT
GCTGGATGAGCAAAGCCCGGCGCAGGTCACCCACCGCGACTGCATGACCTGCGGCCGCTGCGTGGATGTCTGT
TCTGAGGATGTTTTTACAATAACGATACATAACATCATTCGGGATGCATCGCGGCGGTAAGCGAGGAAATCCC
CAGGGGCATAGATAACGATGCGACTGGGATTTTGGAGCGCAGCCAACAAAGAGGCAGCTCGAAGGATGAAGTG
TAAATGCGATGGAGTTCGGGAGCGAAACAATGAAAAGCCATGACCTGATGAAAGCGCTGTGTCAATGGATGGC
CACGCTTGCCCTGGTGGTAAGTGGTGCGGTTTGGGCGGCAAACGGAGTGGATCTGAGCCAGTCGCCGGAAGTA
TCCGGGACGCAGGAAGGGGCGATTCGTATGCCGAAAGAGCAGGAGCGTATGCCGCTAAATTACGTCAACCAGC
CACCGATGATCCCGCACAGCGTCGAGGGCTACCAGGTGACGACCAATACCAACCGCTGTCTGCAATGCCACGG
CGTGGAAAGTTATCGTACGACGGGCGCGCCGCGTATCAGCCCGACGCACTTTATGGACAGTGACGGTAAAGTG
TCGGGCAACGTCGCGCCGCGTCGTTATTTCTGCCTGCAGTGCCACGTACCGCAGTCTGATACCGCGCCGATTA
TCGACAATACCTTTACCCCCTCGCAAGGTTACGGGAAATAAGAGGTCATTATGGAAAATTCTAACCGTAAACC
CGGCTGGATAAAGCGCGTCTGGCGATGGTGGCGCAGCCCCAGCCGCCTGGCGCTGGGGACGCTGCTGTTAATC
GGTTTTATCGGCGGCATCATCTTCTGGGGCGGCTTCAATACCGGAATGGAAAAGGCTAACACCGAAGAGTTTT
GTATTAGCTGCCATGAAATGCGCAACACGGTGTATGAGGAGTATATGGAAACCGTCCACTACAACAACCGTAG
TGGCGTTCGTGCCACCTGTCCTGACTGCCATGTGCCGCATGAGTGGGGGCCGAAGATGATCCGTAAGATCAAA
GCCAGTAAAGAGCTGTATGCGAAGGTATTTGGGCTGATAGATACACCGCAGAAATTTGAAGCTCATCGCCTGA
CAATGGCACAAAATGAGTGGCGGCGCATGAAAGACAACAACTCGCAGGAATGCCGTAACTGCCACAATTTCGA
CTTTATGGATCTGACCGCGCAAAAAGGCGTCGCGGCGAAAATGCACGATCAGGCGGTAAAAGACGGACAGACC
TGTATCGATTGCCACAAAGGGATCGCGCATAAACTGCCGGATATGCGTGATGTTAAACCAGGTTTCTGACAGG
ACGTTGATGAGTGGGAATGCTTGAAGCCAGAGATCTGCACTGCGAACGGGACGAGAGGACGCTGTTTCGCGGG
CTGTCGTTCACCGTGGACGCCGGGGAGTGGGTGCAGGTCACCGGCGGCAACGGCGCCGGAAAAACCACCCTGC
TGCGCCTGCTGACCGGGCTGGCGCGCCCGGACGGCGGGGAGGTGTACTGGCAGGGCGAGCCCCTGCGCCGCGT
GCGCGACAGCTTCCATCGCAGCCTGCTGTGGATAGGGCACCAGCCGGGGATTAAATCCCGCCTGACGGCGCGG
GAGAACCTGCACTTTTTCCACCCCGGCGACGGCGCGCGTCTCCCGGAGGCGCTGGCGCAGGCCGGGCTGGCGG
GATTTGAGGACGTGCCGGTCGCTCAGCTCTCGGCCGGGCAGCAGCGCCGGGTGGCGCTGGCCCGCCTGTGGCT
GACCCGCGCCGCGCTGTGGGTGCTCGACGAGCCGTTCACCGCCATTGACGTTAACGGCGTGGCGCGCCTCACC
CGGCGGATGGCGGCGCACACGGCGCAGGGCGGGATGGTCATTCTCACCACCCACCAGCCGCTGCCGGGGGCCG
CGGACACCGTCCGCCGCCTGGCGCTGACCGGCGGGGGGGCCGGGCTGTGATGTGGCGTGTCTTCTGTCTCGAG
CTGCGCGTGGCGTTCCGCCACGGCGCGGACATCGCCGGCCCGCTGTGGTTCTTCCTGATGGTCATCACCCTGT
TTCCGTTAAGCGTCGGGCCGCAGCCGCAGCTGCTGGCGCGTATCGCGCCGGGCATCATCCAGGTGGCGGCGCT
GCTGGCCTCGCTGCTGGCGCTGGAGCGGCTGTTTCGCGACGACCTGCAGGACGGCAGCCTGGAGCAGCTGATG
CTGCTGCCGGTGCCGCTGCCGGCGGTGGTGCTGGCGAAGGTGCTGGCCCACTGGGCGGTGACCGGCCTGCCGC
TGATGATGCTCTCCCCGCTGGTGGCGCTGCTGCTGGGGATGGACGTGTACGGCTGGAAAATCATGGCGCTGAC
GCTGCTGCTCGGCACGCCGGCGCTGGGGTTTCTCGCCGCGCCGGGCGTCGCGCTGACGGCCGGCCTGCGGCGC
GGCGGCGTCCTGCTGGGCATTCTGGTGCTGCCGCTGAGTGTCCCGGTGCTGATTTTCGCCACCGCGGCGATGG
ACGCGGCATCGATGCATTTACCCGTTGACGGCTATCTGGCGGTGCTGGGGGCGCTGCTGGCGGGCAGCGCGAC
GTTAAGCCCGTTCGCCACCGCGGCGGCGCTGCGCCTCAGCGTGCAGTAGCGCCCCGGCGCGACGGACATTTTA
CTGGTTTCAGGAGGTCAGGTGACAACGATGTGGAAAACCCTTCATCAGCTGGCGGCGCCGCCCCGGCTGTATC
AGATTTGCGGCAGGCTCGTGCCGTGGCTGGCGGCAGCCGGCATCATCGCGCTGGCCACCGGCTGGGTCCGGGG
CTTTGGTTTCGCCCCGGCGGACTACCAGCAGGGGAGAGCTACCGCATTATGTACCTGCATGTCCCGGCGGCCA
TCTGGTCGATGGGTATCTATGCGGCGATGGCGGTGGCGGCGTTCACCGGGCTGGTCTGGCAGATGAAAATGGC
CAGCCTTGCCGTCGCGGCGATGGCGCCGGTGGGGGCGGTGTACACCTTCATCGCGCTGGTCACCGGCGCGGCG
TGGGGCAAACCGATGTGGGGCACCTGGTGGGTGTGGGACGCGCGCCTGACCTCGGAGCTGGTGCTGCTGTTTC
TCTACGCCGGGGTCATCGCCCTGTGGCACGCCTTTGACGACCGTAAAATGGCCGGGCGCGCGGCGGGCATTCT

GGTGCTGGTCGGCGTGGTGAACCTGCCGGTTATCCACTATTCCGTCGAGTGGTGGAACACCCTGCACCAGGGC
TCGACGCGGATGCAGCAGAGTATCGACCCGGCGATGCGCTCGCCGCTGCGCTGGGCCATCGCCGGCTTCCTGC
TGCTCTTTATGACGCTGGCGCTGATGCGGATGCGCAACCTGATTTTACTGATGGAAAAACGCCGCCCGTGGGT
GAGCGAACTGATACTGAAAAGGGGGCACCGGTGAGTCCGGCATTTTCATCGTGGAGCGATTTTTTCGCCATGG
GCGGGTACGCCTTTTTTTGTCTGGCTGGCGGTGGCGATGACCGTGGCGCCGCTGGTGCTGCTGGCGCTGCACA
CGGTGCTGCAGCGCCGGGCCATTCTGCGCGGCGTGGCGCAGCAGCGGGCGCGCGAGGCGCGGATGCGTGCCGC
ACAGGCGCAACAGGAGGCCGCGTGAACCTGCGACGTAAAAACCGGCTATGGGTGGTCTGCGCGGTGCTGGCGG
GCCTGGCGCTGACCACCGCCCTGGTCCTGTACGCGCTGCGCGCGAATATCGACCTGTTCTATACCCCCGGCGA
AATCCTCTACGGCAAGCGCGAGACGCAGCAGCTGCCGGCGGTGGGCCAGCGCCTGCGCGTCGGCGGGATGGTG
ATGCCCGGCAGCGTCAGGCGCGACCCGGACTCGCTGAAGGTGAACTTCAGCCTCTACGACGCCGAAGGGTCGG
TGACGGTGAGCTATGAGGGGATACTGCCGGACCTGTTCCGCGAGGGACAGGGGGTGGTGGTGCAGGGCACCCT
GGAGAAGGGCAGCCACGTCCTGGCGCAGGAGGTGCTGGCCAAGCATGACGAGAACTACACCCCGCCGGAAGTG
GAAAAGGCGATGCAGGAAAACCACCGCCGCCCGCAACGCGCTGATAAGGACACCTCATCATGATGCCTGAATA
CGGCCACGCGCTGCTGTGCCTGGCGCTCGGCGTGGCGCTGCTGCTGTCCGTTTACCCGCTGTGGGGCGTGGCG
CGCGGCGACGCGCGGATGATGGCGTCGGCCGGGGTGTTCGCCTGGCTGCTGTTCATCTGCGTGGCGGGCGCGT
TTTTCGTGCTGGTGCACGCCTTTGTGGTTAACGACTTCACCGTGGCCTATGTCGCCGGCAACTCGAACACGCA
GCTGCCGGTGTGGTACCGGGTGGCCGCCACCTGGGGGGCGCACGAGGGCTCGCTGCTGCTGTGGGTGCTGCTG
ATGAGCGGCTGGACCCTGGCGGTGGCGGTGTTCAGCCGGCCGGTGCCGGCGGATATCGTCGCCCGGGTGCTGG
CGGTGATGGGGATGGTCTGCGCCGGTTTTCTGGCGTTCATCCTGTTCACCTCCGGCCCGTTCGCCCGCACGCT
GCCGGCCTTTCCGGTGGAGGGGCGCGACCTGAACCCGCTGCTGCAGGACCCGGGGCTGATTTTCCACCCGCCG
CTGCTGTACATGGGCTATGTCGGCTTCTCGGTGGCCTTCGCCTTCGCCATCGCCGCGCTGCTGAGCGGGCGTC
TGGACAGCGCGTTCACCCGTTTTGCCCGCCCGTGGACGCTGGCGGCGTGGGTGTTCCTGACGCTGGGCATCGT
GCTCGGCTCGGCGTGGGCCTACTACGAGCTGGGCTGGGGCGGCTGGTGGTTCTGGGACCCGGTGGAGAACGCC
TCCTTTATGCCGTGGCTGGCGGGCACCGCCCTGCTGCACTCGCTGGCGGTCACCGAACAGCGCGCCGGCTTTA
AGGCGTGGACGCTGCTGCTGTCCATCTGCGCCTTCTCGCTGTGCCTGCTGGGCACCTTCCTGGTGCGCTCCGG
GGTGCTGGTGTCGGTGCACGCCTTCGCCTCCGACCCGGCGCGCGGAATGTTTATCCTCGCCTTTATGGTGCTG
GTCACCGGCGGCTCGCTGCTGCTGTTCGCCGTGCGCGGGCACAGGGTGCGTTCGCGGGTGAACAACGCGCTGT
GGTCGCGCGAGTCGCTGCTGCTCGGCAACAACGTCCTGCTGATGGCCGCCATGCTGGTGGTGCTGCTGGGTAC
CCTGCTGCCGCTGGTGCACAAACAGCTGGGGCTGGGCAGCATTTCGGTGGGGGAGCCGTTCTTTAACACCATG
TTCACCTGGCTGATGGTCCCCTTTGCCCTGCTGCTGGGGGTGGGGCCGCTGGTGCGCTGGGGCCGGGACCGGC
CACGTAACATCAGGAAACTGCTGTGGGCTGCCGTTGTCACTACCCTGGTGCTGTCGGTACTTTTGCCGTGGCT
GCTGGAAGATAAAATCATCGCCATGACGGCGGTGGGGATGGCGATGGCCTGCTGGATTGCGGTGCTGGCGGTG
GCCGAAGCCGTACAGCGCGTGTCCCGCGGCACGAAAACCTCTCTCAGCTACTGGGGAATGGTGGCGGCGCACC
TCGGGCTGGCGGTGACGATTACCGGCATCGCCTTCAGCCAGAATTACAGCGTGGAGCGTGACGTGCGGATGCG
GGCGGGCGACAGCGTGACCATTCACGACTACCGCTTCACCTTCCGGGAGGTGCGGGACATCACCGGGCCCAAC
TACCGCGGCGGGGTGGCCCTCATCGGGGTGACGCGCCACGGCGAGCCGGAGGCGGTGCTGCACGCGGAGAAAC
GGCTCTACAACACCAGCCGGATGGTGATGACCGAGGCGGCGATTGACGGCGGGCTGACCCGCGACCTGTACGC
CGCGCTCGGGGAGGAGCTGGACAACGGCGCGTGGGCCGTGCGCCTGTACTACAAACCGTTTGTCCGCTGGATA
TGGGCCGGGGGGCTGCTGATGGCGCTGGGCGGGCTGCTGTGCCTGGTGGACCCGCGCTACCGCCGCCGTAAAC
CATTGCCGGAGGCCGGATGAAACGCAACGTACTGTTATTACCGTTACTGATTTTTCTGCTGATTGCCGCGGCG
CTGCTGTGGCAGCTGACGCGCAACGCGCAGGGGGATGACCCGACGAATCTCGAATCGGCGCTGACCGGGAAGC
CGGTGCCGGCGTTCCGCCTGGAATCGCTGGAGACGCCGGGTCAGTACTATGAGGCGGAGGTGCTGACGCAGGG
GAAACCGGTGCTGCTTAACGTCTGGGCCACCTGGTGCCCGACCTGCCGCGCCGAGCATCAGTACCTGAACCAG
CTTTCTGCGCAGGGTATCCGGGTGGTCGGGCTGAACTATAAGGACGACCGGGCGAAGGCGGTGGCCTGGTTAA
AGGAACTGGGCAACCCGTATGCGCTGAGTTTATCGGACAGCGACGGGATGCTGGGGCTGGACCTGGGCGTGTA
CGGCGCGCCGGAAACCTTCCTCATCGACGGCAGGGGGATTATCCGCTACCGCCATGCGGGCGATTTGAATGCC
CGGGTGTGGGAGAGTGAACTGAAACCGCTGTGGGACAGATACAGCCGGGAGGCGGCGCAATGAGACTGTTACC
GGGCATGGTGATGCTGATGCTGGTGCTGGTTATCTCCGGGTCAGCGCGGGCGACCACCGACGTGATGCCGTTT
AAAGATGAAGCGCAGGAGCAGCAGTTCCGCCAGCTCACGGAGCAGCTGCGCTGCCCGAAATGCCAGAACAACA
GCATTGCGGACTCGAACGCGATGATAGCCACCGACATGCGCCGCAGGGTGTATGACCTGATGCAGGAGGGGAA
GAGCCGCCAGGAAATCATCGATTACATGGTGGCGCGCTACGGCAACTTCGTCACCTACGACCCGCCGCTGACC
CCGTTGACGGTGCTGCTGTGGGTGCTGCCGCTGGCCGCCATCGTAGCGGGCGGGTGGATAATCGTTGCCCGCA
CGCGCCGGCGGGTGCGCCTGCGCCGGGAGCCGCTGCCGGCGGACACCCCGGTTTGCGGCGCGCGCGCCGGGTG
GGGCGTTTACGTGCCGGGGGCCGTCATTGCGCTGGCGGTCGGCGCCGGCAGCTACGCCCTGACCGGCAGCTAT

CAGCAGGTCAGGGCCTGGCAGCAGGCAACGGCGCAGACGCCCGGGCTGCTGGCGCGGGCGCTGGACCCGGCGG
CGCAGCCGCTGAATGAAGAGGAGATGGCGCGGCTGGCGCTGGGGCTGCGCACCCGCCTGCAGAATGATGCCGG
CAATGTTGAGGGCTGGCTCATGCTGGGGCGCACCGGTATGGTACTGGGTAATGCCGGTACCGCCACCGGGGCG
TATGCGAACGCCTACCGTCTGGCCCCGAAAAACAGCGATGCGGCGCTGGGCTACGCGGAGGCGCTGACGCGCT
CGTCCGACCCGGAGGATAACCGCCGCGGCGGGGAGCTGCTGCGTCAGCTGGTGAGCCGCGACCACACGGATAT
CCGGGTGTTAAGCCTGTATGCGTTTAACGCCTTTGAGCAGCGGCGCTTTGGCGAGGCGGTGGCGGCCTGGGAG
ATGATGCTGAAACTGCTGCCGGCGGGTGACGCCCGGCGGGCGGTGATAGAGCGCAGTATCCGGCTGGCGCAGG
AGAAATAAGACCCTGACGGCCCGGTGACGTGGCGCCATCGGGCCTGGCTTGTTACTGTATTCCGCGTGTTTGT
AAAAATAGAATGGTCGCCGCGACGCGTGAGCGCACGTTTAGCTTGCGAAGTAGATTACGAATGTGCACCTTAA
CCGTCTGCTCAGAAATATTCAGTACTGACGCAATCTGTTTGTTAGAAAGCCCCTGCGCCAGCTCATGCAAGAC
ATCTAACTCTCGTTCGGTCAGGATACTAAAGGGATCTTCCTGCGCGCCAAACCGTTCGCGCTCACGCAGATAT
TCATTGACCCGATCGCTAAAGACTTTACCGCCGTTAGCGCCTTTGCGAATGGCCTCCAGCAGCACTTCCGGAT
CGCTATCTTTGAGCAGATAACCGTCGGCGCCGGCGTCGATCAGCGCGTAGATGTCGCTGGCGGAATCCGAGAC
GGTAAGAATAATAATTTGCGCCGTCACACCATCCCGGCGTAGCGCGTTCAGGGTATCTAACCCGCTCAGACCT
TTCATATTCAGATCCAGCAGGATCAGATCGGGCTCAATGCGATTCGCCAGATCGATCGCACTCGCCCCGTCAC
CCGCTTCGGCAACGACATGAAAAGCCGGGTCCAGTTCCAGTAATTGACGAATACCTCGCCGCATAAGTGGATG
GTCGTCCACAATAAGCACCTGAAAAGGTGTTACTTCAGGCATGCTGTATCTCCTGAGTTTTATTAGAATGATT
ATTGTTTTCAGCGCGGAAATCGTCCAGATTTGCCACATTGTCAAGCGCGTAAATTTTACCCTAATTAGTGCAT
GGGTAAGTAGTAAAACCTGTGACAGGCGAGTAATTAGCTGAACGATAGAAAGGATAAAACAACCCGTTGTGAG
AGAAAACAGAATACTCCCTGAAGTTGAATACTGGTCTGGACGCTTTTTTCGGACAATGTGACATAACAGAAGT
GTGGCATATGTTGCTGCGCCATTGCACCCGTAAGTCCAGACTATCTTTACTGGCAGGAAATTCCTTATTAAAA
GTCATAGCGTCTAAAGCTTGTCATTATTAACAAATTATTTTAACTGAATTTATTTATGGTTTTATTAATGTCA
ATGAAATACATCTGAGCAGAACGAATAAACCGCGACATCTGTTAAATTAAATATTAAGTCAATAGCAGAGGTA
TTTTTATGCGATAAAGAGATTCTGACGATATTCTTATCATAAATGAAAATGTGGTCAGGAATGAGCGTCATTC
ACTTATAACTTATCCTGGATGTGCCTGAACAACACTCGCGGCGTTTTGAGCACCCGTTGGGGTGACTCAAGGG
GAGAACGTAGTGTGGATGCTATATCAGCCGTTTCTGTGAGCGTAAGCGTGGCGTAGAAAATTTTAAATATGTT
AGCCGGTTAAAAATAACTATTGACATTGAAATGGTGGTGGAGTGTATATGAAGAGAATATTTATATATCTATT
ATTACCTTGTGCATTCGCATGTTCTGCTAATGATAATGTTTTTTTTGGCAAGGGCAACAAGCATCAGATCTCT
TTTGCTGCGGGAGAAAGTATAAGAAGAGGAGGGGTTGAGCACTTATATACGGCTTTTCTGACATACAGTGAAC
CCAGCGATTTTTTCTTTTTACAGGCAAGAAATAATCTGGAGTTAGGAGGATTTAAGGCTAAGGGTAGCGATGA
TTGCAGTAAACATTCTGGCAGCGTTCCCTGCAATAAATATAACCAGGGCGTATTGGGTATCTCGAAGGATGTG
GCGCTGGTTCATTTCGCTGGTATCTATACCGGTATTGGTCTGGGGGCTTATATAAAATCTAAGTCGCGAGATG
ATATGCGTGTCAATTCTGCATTTACCTTTGGAGAAAAAGCGTTTCTTGGCTGGAACTTTGGGGCTTTTTCTAC
AGAAGCTTATATCCGGCATTTCTCGAATGGATCACTTACGGATAAAAATTCAGGGCATAATTTTGTAGGTGCT
TCAATTAGTTATAATTTCTGAAGTTGAATAACAATTAGCGAGTTGCTGGCTGAAGGAGTAATTAATCACCGTA
CGTTGTCTACAAAACGTGTTGTGAATAGCCGATCGTTATCTACCCTATGGCGACAGGGAGGCTAATTGTTAGT
GACAAAGAAGTGGTAGGCCGTTTACCTTACCAGAACGTTTTATTATTGCTGCGACAGATTGCTTACTCATTTA
CCTGCCACTACAAACATTTCTTCCAGCATAGGGTGTAGATGCGCTTTTTCTGTTTTAGTACAAACATGGTTTA
CGTTATTTGCCTGCATTGACTTTACGTTGACCACTGCACAGGAAAGATGTCCAATCAGTTCATGCAAAATCAG
TTCATGCAAAATAACGTCTCTGGCATCAGCTAACTTTTTAATTGTAGGCATAAAAAAAAACCAGCCCTGACAGG
CTGGTTTTCAAGGGGAATTTTGGTCGGCAGCTTCATCCGCAGCATCTGCGGAGAGCAGCGCAGGTACGGCGAC
CACAAAAGCCGGGGAGGCATCAGCCAGCGCGGCGTCGGCTGACACAGCCAGAACGGCGGCAGCCGCCTCGTCA
TCAGCATCGGAGGCATCCACCCACGCCGCCGCATCTGATACCAGCGCATCACTGGCAGCGCAAAGCAGAGCTG
CAGCCGGAGAATTGGCAACCAGAGCCGAAGAGGCCGCGAAACGGGCCGAGGATATCGCAGACGTGATTTCCCT
GGAAGATGCAAGCCTGACGAAAAAAGGTATCGTTAAATTAAGCAGTGCCACGGACAGTGACAGCGAAGCGCTG
GCGGCCACACCAAAGGCGGTAAAAACCGTTATGGGTGAGGTACGGACCAAAGCGCCGCTGGACAGCCCGGCAT
TCACTGGAACGCCGACCACACCGACGCCGCCAGGCGATGCTAAAGGGCTTCAGACAACAAACGCGGAGTTTGT
CCGCAAACTGATTGCCGCGCTGGTTGGTTCCGTACTGGAGCCACTGGACACCCTGCAGGAACTGGCTGACGCG
TTGGGAAATGATCCGAACTTTGCCACCACGGTACTGAATAAACTGGCGGGCAAGCAGCCGCTGGACGAAACCC
TGACGGCGCTGTCAGGAAAAAGCGTTGACGGTCTTATCGAATACGTTGGTTTGCGAGAAACCATAAGTCGTGC
CGCCGATGCATTATAAAAATCACAGAATGGCGGCGATATTCCGGACAAGGATTTGTTTGTGCGTCGTATCGGT
GCCGCGCGAGCGTTTGATGGCGCAGTTACTATCGGCTGTGATGATAATCCGTGGACGACGGCGGAGTTTATCG
TCTGGCTGGAGTCTCAGGGCGCATTCAATCACCCTTACTGGATGTGTCGTGGCTCCTGGTCTTACGCTTATAA
CAAAATCATCACGGATACTGGCTGCGGTACTATCTGTCTCGCTGGCGCAGTGATTGAGGTAATGGGAGTGCGT

GGCGCGATGACTATTCGGGTGACAACGTCCCATTCAGTATCTGGTTGGTGATACGTGGGTGACAGCCCCAAGT
GTATAAGAAGGAATAATTATGACAGCGGAAAAAAAATAAAAAGAACAAACAGTTTTTAAATATTAAAAATTTC
ATTCCGTATGCACCGGAACCAGATGACACATTATTCGCCGATGCGGCGTATCTTAAATCAGAGGATGGTCAGG
ACTGGTATGGGTGCCAGCAATTATTTTCAGCAGACACGCTGAAAATTACCTACGACGATAACGATGTTATTAC
GTGTATTACGCGTGATGTTTCCGGGCTGTGGCCTGCTGGCCAGAGCGTTGCAGAGTTGCCTGATACGGATGAA
AACCGTCGCGCTGATATTCATGCTGCTGGCAGTTTAAAGACGGTAAAGTCGTTCAAAGGGTTTATTCGCCGGA
AGAGCTGTGCAGGCAGGCAGAATCGAAAATTGAACGCCCGGGCGTTGATACCGGATGATCTGGTCATCGTGGA
AAGCGACCCTGAAAAAATCGACACTTTAGCTGTAAAATGACAGTCCCGCCATCCGGTCATCATAACGGATTTT
TCTTCTGCACCTTCTGAAGCCCGCCATGTCAGGACGGCCATGAATCCGCCGATAACCTTATTGTGAAATTAAG
ACCAGGAAGAGATGATGTCTGCCGGACAGATACTATATGTAAATTTATAAAGGTTTTTTGTTATGCCCTTTCA
TATTGGAAGCGGATGTCTTCCCGCCACCATCAGTAATCGCCGCATTTATCGTATTGCCTGGTCTGATACCCCC
CCTGAAATGAGTTCCTGGGAAAAAAATGAAGGAATTTTTTTGCTCAACGCACCAGACTGAAGCGCTGGAGTGCA
TCTGGACGATTTGTCACCCGCCGGCCGGAACGACGCGGGAGGATGTGGTCAGCAGATTTGAACTGCTCAGGAC
GCTCGCGTATGCCGGATGGGAGGAAAGCATTCATTCCGGCCAGCACGGGGAAAATTACTTCTGTATTCTGGAT
GAAGACAGTCAGGAGATATTGTCAGTCACCCTTGATGATGCCGGGAACTATACCGTAAATTGCCAGGGGTACA
GTGAAACACATCGCCTCACCCTGGACACAGCACAGGGTGAGGAGGGCACAGGACACGCGGAAGGGGCATCCGG
GACATTCAGGACATCCTTCCTCCCTGCCACAACGGCTCCACAGACGCCAGCAGAGTATGATGCTGTCTGGTCA
GCGTGGAGAAGGGCTGCACCCGCAGAAGAGTCACGCGGCCGTGCAGCAGTGGTACAGAAAATGCGTGCCTGCC
TGAATAATGGCAATGCAGTGCTTAACGTGGGAGAATCAGGTCTTACCACCTTGCCAGACTGTTTACCCGCGCA
TATTACCACACTGGTTATTCCTGATAATAATCTGACCAGCCTGCCGGCGCTGCCGCCAGAACTGCGGACGCTG
GAGGTCTCTGGTAACCAGCTGACTAGCCTGCCGGTGCTGCCGCCAGGACTACTGGAACTGTCGATCTTTAGTA
ACCCGCTGACCCACCTGCCGGCGCTGCCGTCAGGACTATGTAAGCTGTGGATCTTTGGTAATCAACTGACCAG
CCTGCCGGTGTTGCCGCCAGGGCTACAGGAGCTGTCGGTATCTGATAACCAACTGGCCAGCCTGCCGGCGCTG
CCGTCAGAATTATGTAAGCTGTGGGCCTATAATAACCAGCTGACCAGCCTGCCGACGTTGCCGTCAGGGCTAC
AGGAGCTGTCGGTATCTGATAACCAACTGGCCAGCCTGCCGACGCTGCCGTCAGAATTATATAAGCTGTGGGC
CTATAATAATCGGCTGACCAGCCTGCCGGCGTTGCCGTCAGGACTGAAGGAGCTGATTGTATCTGGTAACCGG
CTGACCAGTCTGCCGGTGCTGCCGTCAGAACTGAAGGAGCTGATGGTATCTGGTAACCGGCTGACCAGCCTGC
CGATGCTGCCGTCAGGACTACTGTCGCTGTCGGTCTATCGTAACCAGCTGACCCGCCTGCCGGAAAGTCTCAT
TCATCTGTCTTCAGAGACAACCGTAAATCTGGAAGGGAACCCACTGTCTGAACGTACTTTGCAGGCGCTGCGG
GAGATCACCAGCGCGCCTGGCTATTCAGGCCCCATAATACAATTCGATATGGCGGGAGCCTCCGCCCCCCGGG
AAACTCGGGCACTGCACCTGGCGGCCGCTGACTGGCTGGTGCCTGCCCGGGAGGGGGAACCGGCTCCTGCAGA
CAGATGGCATATGTTCGGACAGGAAGATAACGCCGACGCCTTCAGCCTCTTCCTGGACAGACTGAGTGAGACG
GAAAACTTCATAAAGGACGCGGGGTTTAAGGCACAGATATCGTCCTGGCTGGCACAACTGGCTGAAGATGAGG
CGTTGAGAGCAAACACCTTTGCTATGGCAACAGAGGCAACCTCAAGCTGCGAGGACCGGGTCACATTTTTTTT
GCACCAGATGAAGAACGTACAGCTGGTACATAATGCAGAAAAAGGGCAATACGATAACGATCTCGCGGCGCTG
GTTGCCACGGGGCGTGAGATGTTCCGTCTGGGAAAACTGGAACAGATTGCCCGGGAAAAGGTCAGAACGCTGG
CTCTCGTTGATGAAATTGAGGTCTGGCTGGCGTATCAGAATAAGCTGAAGAAATCACTCGGGCTGACCAGCGT
GACGGCAGAAATGCGTTTCTTTGACGTATCCGGCGTGACGGTTACAGACCTTCAGGACGCGGAGCTTCAGGTG
AAAGCCGCTGAAAAAAGCGAGTTCAGGGAGTGGATACTGCAGTGGGGGCCGTTACACAGAGTGCTGGAGCGCA
AAGCGCCGGAACGCGTTAACGCGCTTCGTGAAAAGCAAATATCGGATTATGAGGAAACGTACCGGATGCTGTC
TGACACAGAGCTGAGACCGTCTGGGCTGGTCGGTAATACCGATGCAGAGCGCACTATCGGAGCAAGAGCGATG
GAGAGCGCGAAAAAGACATTTTTGGATGGCCTGCGACCTCTTGTAGAGGAGATGCTGGGGAGCTATCTGAACG
TTCAGTGGCGTCGTAACTGATGCACCAGGTGAATGAGGTGCGGTGCGACAAAGATATTCCCGGACGAACAACA
TCAGACAGTACGGATGATGTACAGGTGAAATAGGGGAGACTTCTTCAGTCAGGGCGCGGCGAAACTTTTTCGA
TGATAACGCGCCGCGCGCCGGTAGCGAGAAGCCGATGGAAGTACTGGATCACCTGAATGCAAAGAAGGGCTGG
GGAAAGCTGTACTTTGCCGGGGAGGGTATCCAGCAACAGTGGGCTATGAAGAGAAACATACTGTCATCCCGAT
ATACCACCCGCTATGAAGACCTGCTTCAGGTTAAGTGACAGGTTTACCCTGATTTTCAAATTTCTGATGTGAT
GGTGTGCCGCAGCCACATTGTATGCAAGAACGGGCTGCGGCAAACTGGCGATCGTTCGATAGTGCGTGTATTG
AATAGTTACCAGTCGTGGCGGATTCTACTGGTTAAGGATGACTAATCAATGTATTTAAGTCATCTGTTCAACT
TTTCGCGTTGGAGGGAGCTTGAAGTCAATTTGCAGTGACCTTCGATAGCTATTTCACCATCATTTCACCATTT
GCAAATTGGATATATAAAAAAACCAGCCCTGGCAGGCTGGCTTTTAAGGGGAATTTTGGTCGGCACGAGAGGA
TTTGAACCTCCGACCCCCGACACCCCATGACGGTGCGCTACCAGGCTGCGCTACGTGCCGACTTGTGGGTGCT
AATACTACCTCTTTCCAACGCGAATGCAAGGGGAAACGGTGCTAACTGATTTATAATTAATCAGTTAGCGATA
AAACGTTTCTCTTCTGTTAGCACTTGCAGCAGCAGACTGAGCTGCGGCTTCTGATCTTTGATCTTCTCGCCGT

GTAAGTCATAGGTCTGATAATGACCGTTATTATTGAGCACCAGGGTCATTTGCGGCGTCGTGATTGCCAGCGT
ACTGCCGTCTGCCGCCGTTACCCAGTTATGACGACGTGGAACGGTAAAGATGTCCTGGCCCTGTGAATATTCG
TTCGCTGGCGTGCTGACATGTAACAGGCGTTGCATCAACGTGGTCATCACATCGGTATGATCGGTAAGCACAT
TAATACGCTGCGCAGGCGTCCCCGGCCAGTGGATCACCAGCGGTACTTGCAGATGACCTTGCGACCAGTCGAA
GCGATTTTCTTCCGGCGTCAACGGTATGCCGCGTCCTGCGGTAATGATCACGACAGTATTCTCGAATTTACCG
GCCTCTCGCAGCGCGTTCAGAACCCGATTGATTTGCGCATCCACGTCGCTGGCGGCACTGGCGTAACGCTTAA
CAAAATTTTTCTGGTTACTGTCATCAATATTAGTACCGTTAAACGATATCCATGAGAACCAGCGGTTATCTTC
CTGTGCGTAGCGCCCAAGCCAGTCTATCCACTGGCTGGCCGTTTGCGCATCAGACTGCGTTTGCGCTGCCGGC
ATCGAGAAATCGGACAGTAATGCCTGACGATAAAGCGGGCTGGCGAAGCCATCCGAAGAAAACAGTCCCAGTT
GGTAGCCTTGCTGATTCAACGCGGTAATGAGCGCCGCAGGCGTTCTGGTGGATAACACGCCATCCATATAGCC
TGGCGATATGCCATAGAACAGGCCGAAAATACCGTTATCAGTGGTATTCCCTGAACTCATATGACGGGTGAAG
TCGATGTTTTGCTCGGCAAATGTAGCCAGCTCCGGCATCTGCTTCTCAAAGCGAGAATAGTTCAGGCCGTCTA
CGGTAATGAGCAGCACATTCTGACCGGTGCCCATATCGCGATAGTGCAAATTGCTGAGCGGGTACTGAACGGA
GACCGCTTCCGGATTGCCTTGCTCTACCAGACGGCGCTGATATTCCTGCGCATCCAGCAGACCGTGTTTTTCA
AGAAAACGTCGCGCCGTCATCGGATAAGAGAGCGGCAGGTTTGCCCGCTGCATGGTAATCGGCCGGTAAAAAT
TAGCATCTGCCCAGATGTAGATAAGATGCGATGCGATAAAGGAGACGAAAAAGAATGCCGCAAGCGGCCTTGC
GAAATGGCGACGGCGCGTGAGACTGCGCAGCTTTTGCCAACTCCATGTCGCAAATAGCATCTCAATCAATAAG
ATAACCGGCACGCTAATAAACATAAGCTGCCAGTCGCGCGCCATTTCGTTCTGGTCAGGGTTGATGACCAGCT
CCCAGACAATGGGATTAAGATGCAGGTGAAAACGGGTAAAGACTTCGCTGTCGATAAGCAACAACGTCATACC
CGCGGTCGCCAGAATGGCTGATATAAACCGCATCAGCCGCTGGGACATCACGATAAACGTGAGCGGAAAAAGA
ATAAGCAAATAGGTGGCGAACACCAGAAAGCTAAAGTGCCCGACAATGCTCAGGTAGGAGTATATGCGACCCG
CAAGCGTTGTCGGCCAGTCGGCGACAAACAGGTAACGGCTGCCGAGCAACGTAGCCAACAGTATATTGAACAG
AGCGAACCAGTGCCCCCAGCTAACCATCTGGGAGACTTTTTCACGGTAGCGCTGACGATGAGTTACCATACCT
GTCGCTTGTTTCTCCGGAGTCGGCTTTTTAGTGCGCGTTGTCTTCGCTAATCGAAGACTGCAAGGCGCGGGCA
AAAGAGTTCGCAATCGCCTGGCGTTGAGCTGGCGCAACGCTGGTGTTGATAAGGTTGGTGACCATATTTCCCA
GCACCATCAGGGAAAGATCGGTCGGCGCCTTATGTTTTTCCAGTACACTGAGCAGTTCGCTCAGCAATTGTTC
AACATGTTCATCACTATAGCGGGAGAGTTGTGGCATAAATCAAAATCTGTTTGTTCATGAAAGGGCAACATAT
TACCGTAGCAACAGTTTTTTTTCTGCATTTTTATCCCCTAAATCATTCGCGTTACGGATAACGCCTGAAATGA
CAGGGCGATTGCTTGCGTCTTCCGGCAGGCGGTGGTTGAATACCGCCCGGTCTTAAAGGAGAGTTTATCATGA
GTCTGGATATCAACCAGATTGCCCTGCACCAGCTTATCAAGCGCGATGAGCAAAATCTTGAGCTGGTCTTGCG
CGATTCATTGCTGGAGCCGACAACCACCGTTGTCGAGATGGTGGCTGAACTGCATCGGGTCTATAGCGCCAAG
AATAAGGCGTATGGCCTGTTTAATGAAGAGAGTGAACTGGCGCAAGCGCTGCGGTTGCAACGTCAGGGGGAAG
AAGATTTTCTTGCCTTTAGCCGGGCGGCGACCGGACGCCTGCGTGACGAACTGGCGAAATATCCCTTTGCGGA
CGGCGGCATTGTATTGTTCTGCCATTATCGTTACCTGGCGGTGGAGTATCTGCTGGTTACGGTACTGAACAAC
CTGAGCAGTATGCGGGTCAATGAAAATCTGGACATTAACCCGACGCATTATCTTGATATCAATCATGCGGATA
TCGTGGCGCGTATCGATCTTACCGAGTGGGAAACTAATCCGCAATCGACCCGCTACCTGACGTTTCTCAAAGG
TCGGGTAGGGCGCAAGGTCGCTGACTTCTTTATGGATTTCCTCGGCGCCAGCGAAGGGTTGAACGCCAAAGCG
CAGAATCGCGGCCTGTTGCAGGCAGTGGATGATTTCACCGCAGAAGCGCAGTTGGATAAAGCAGAACGTCAGA
ACGTGCGTCAGCAGGTGTACAGCTACTGCAACGAGCAGTTACAAGCCGGGGAAGAGATTGAGCTGGAATCGCT
GTCTAAAGAGCTTTCCGGCGTCAGTGAAGTCAGCTTCAGCGAATTTACTGCCGAAAAAGGCTATGAGCTGGAA
GAGAGCTTCCCGGCAGATCGCAGTACGCTACGCCAGTTAACCAAATATGCCGGCAGCGGCGGCGGGTTAACGA
TTAACTTTGATGCGATGCTACTGGGCGAGCGGATTTTCTGGGACCCGGCGACCGATACCCTGACTATCAAAGG
GACGCCGCCGAATCTGCGCGAGCAGTTGCAGCGACGCACGTCGGGCGAGAAATAAAGGCATACGACATAAAAG
GACGCCGTAGTGAGTAAAGGAATACGACAGAAGCCGCCTGTTAATTTCAGTTTATTTCGGCAGGTTTTGTATG
CGCACATTGTCGCATTTTTGATGATGTTGGTGCTCGGCATGGTGTTCACCGTTCTCTCGTTGGTACTGTTTTA
TACTTATGGTGCCAACTGGTTACTGAGCTTGTTCATATGCCCGCTGTTCTTGTTAAGTGGTTTATTCATTACT
GGATTTGCTTTTAAATCAACTTGGTCCAGCATACGTTACTATTACGACAAGGGGCAGTTGAAACGGTATGGAC
TCAATCTTGATGCAACTTTGACGCATAAAGAGAAGGTGGAAATACGCATCGATAATGCAAAGCGTCAAGTGCG
TGTAGATGAACTGGAGCTCCATGTCTTATTTGATTTTCAGTTTGATAGCAAGACATGGAGCTGCGGCGACTTA
CTGACCAATGAAAAGGTATTTGATGCGTTGAATGACGGGCAAACTATACCAATACGAATTTTGCCATGGAAAC
CGGAAAGTGCCAGCGTTCGTCAACGGGCATTATTCAATCGGCTTAAAGGTATGAATACCGCGGCGGAAACTAC
AGATTCTCGATTAGGGGAGGCGCTAATTGAATGTGGCGAGGTGTAAGAAAGCAGAAAAGCAAAGTGGTTTCTC
GTTGCTCTGCATGTCGTCAAATTCAATTAAACGCATAAAAAACCCCGCCGGGGCGGGGTTTTTCTTCAACTTC
CAGGCGATTACGCGCGAACGAAGTCGATGTGAGTCAGCTTCGGTTTGTAAGCGTGACGCTGTACAGCCTGAGC

TTTAACTTTTACTTCTTTACCGTCAACAACGAGGGTCAGAACTTCGCTGTAGAATTCAGCTTTAGCTTGCATG
TTCATCACCTGGTCGTGGTCCAGTTCGATAGCAATCGGGGCTTCAGAACCGCCGTAGATGATTGCCGGGAACT
TGTTAGCGGCGCGCAGGCGGCGGCTCGCACCCTTACCCTGCTCTTTACGTACTTCTGCGTTGATAGTAAACAT
TTAAATCTCTCTTTAATAATTCCTGCTACAGGCGACCCAGCAACAGGTAAGTGATCTGCTTTGCGTATGCAAA
GGCGGGCGAGATTCTATACTCAAATCGCCGTTACATCAATGAAAAGTACGATTAACCACGTAATTCGTGCGCC
CGGCGGAAGCGGCCTTCATAGTCGAACACTTTTTCACGCACCTGCCAGTACTGGCCTTTCATCCGCGCGACCA
CAAAATCGGGATGCCGCAACAGCGCCTGCTGCGCGACAATATCCGCCGCCGTGATCCAGCGTAAAGGAACGCC
GGGCGTGCGGGTATGCGGGCGAATAAATAGCTGTTCGAAAGCGGTACGCTGGGCGGGCGTGTGCAAGCGGAAG
CGCTCACTGACATCCGCGCCGTCCTCGTCATAGTAAGTGATTTTCAGCCATTCGCCTTTCTCATCCTGCCCAT
GCTGCATCGTCATTCCGCTACAGCGCAGGACTAACGCATCCTTGAGTCTGAGCGCCGCTTTTAACATATCGTC
CGGGTCGACCAGAATAGCGTCACATTCCCGGCAGCGTCGGGCGGCAATATCGTTTTCGGCATTACACTGCGGG
CAGTTTTTGAAGCGAAAGCGAAAATCGCACTGCTCGCGATGGCCGTCGTCATCGTCAAACCAGCCCTGGCAGC
GACGGCCAAAGTGTTCAATCAGCGTGCCGTCGGCAGTGGTTTTCCCCCAGAAGGTGTTGGCAAAGCCGCAGGC
CGGGCAAAATACCTGGACAGGGACGTTATCGCTTTTTCCCTTCGGGCTACCGACCTCCGGGGCATACAGGTCG
TGCGGGTTGCCTGCGTAATCAAGAATCAGGCAATCGGTCTTTCCCGGCGCAAGGCGCAGACCACGCCCGACAA
TTTGTTGGTAAAGACTAACTGACTCGGTGGGACGTAGAATCGCGATGAGATCAACGTGTGGGGCGTCAAAGCC
GGTGGTCAGCACCGAGACGTTAACCAGATAGCGAAAACGCTGCGCCTTGAAATTATCAATCAGCGCGTCGCGC
TCGGGCCCTGGCGTATCGCCGGTAATCAGCGCCGCGTCGTCCGCCGGAAGCAGACCGACAATCTCTTTCGCAT
GTTCGACCGTGGCGGCGAAAATCATCACGCCTTTGCGCGTTTGCGCAAATTCCATAATCTGGCTGATGATGTG
CGGCGTAATCCGCTGCTGCTTTTTCAGCTCACGGTTCAGGTCGGCTTCGCTGAACAGCCCATTGCTTTGGGCC
TGCAGGCGGCTGAAATCGTATTGGACCACTGGCATATCAAGCCGCTCAGGCGGCGTCAGATAGCCGTGTTTAA
TCATATAGCGCAGCGGCAGTTCATAAATACAGTCGCGAAACAGCGCGTTGTCGTTGCCGCGCACCATACCGTG
GTAATGAAATTGATAAATCCAGCCTTTTCCGAGGCGGAAGGGCGTGGCGGTTAGCCCAAGCAGACGTAAGTGA
GGATTAACTTTACTCAGGTGAGTGAGGATTTGCTGATACTGACTGTCTTCATCGTCACCGATGCGGTGGCATT
CATCGACAATCAACAGCGAAAACTCCTCCTGGAAGGCGTCAAGATTACGCGCCACCGACTGTACGCTGCCGAA
CACGACTTTGCCCTGACTCTCTTTACGTTTGAGTCCGGCGGCGAAAATATCTGCTTCCAGCCCCAGCGCGCAA
TATTTGGCGTGGTTCTGCGCGACCAGCTCTTTCACATGCGCCAGCACCAGTACCCGTCCGCGGGCGACGCGCG
CCAGTTCGGCGATCACCAGGCTTTTACCTGCGCCGGTCGGCAGAACAATCACGGCGGGCGTACGGTGGCGGCG
AAAGTGGCTGAGCGTGGCGTCTACGGCTTCTTGCTGGTAGGGGCGGAGTGTAAAAATCATGGTCTCACTACGT
TAAACGGTTCCGGGAATAGTATGCCATGAATCATTTCCCTTGAGGGATATAGTTAGCCCGCTATACTGAGCGG
ATAGCAATTCCCTTTTTTCGGGTAGAATGCCCGATTTCCGTATTATTACAGGCTAAATCACACACATGCGACT
TGATAAATTTATCGCTCAGCAGCTTGGCGTCAGCCGCGCTATTGCCGGGCGTGAAATTCGTGGTAACCGCGTT
ACCGTCGATGGCGACATCATTAAAAATGCGGCCTTCAAACTGCTCCCGGAACATGCGGTTGCGTATGACGGCA
ATCCCTTAGCGCAGCAACACGGGCCACGCTATTTTATGCTTAACAAGCCGCAGGGATACGTTTGTTCAACCGA
TGATCCCGATCATCCAACGGTACTGTATTTCCTGGATGAGCCGGTGGCGTATAAGCTGCATGCCGCAGGACGT
CTGGATATCGATACTACCGGTCTGGTGTTAATGACAGATGACGGTCAGTGGTCGCACCGCATTACGTCGCCGC
GCCATCACTGTGAAAAAACCTATCTGGTGACCCTGGAGTCGCCGGTGGCCGACGATACGGTAGCGCAATTTGC
GAAAGGCGTGCAGTTGCATAATGAAAAAGATCTCACTAAACCCGCTACGCTGGAGGTGATAACGCCTGTGCAG
GTCCGTCTGACCATCAGCGAAGGCCGTTATCATCAGGTGAAGCGGATGTTTGCCGCAGTAGGCAATCGCGTTG
TGGAACTGCACCGCGAACGGATTGGCGCCATTACGCTGGATGAGAATCTGGCTCCCGGCGAGTACCGCCCGTT
GACTGAAGAAGAAATCGCCAGCGTCGGCTAACTATCTCGTTAAATTCAGGAGTTCGATGTGACCACCCGGCAG
CACTCTTCCTTTGCCATTGTCTTTATCCTTGGCCTGTTGGCCATGTTAATGCCGCTGTCGATTGATATGTATC
TTCCTGCGCTGCCGGTGATTTCTGCGCAATTCGGCGTGCCTGCCGGTAGCACGCAGATGACGCTCAGTACCTA
TATTCTGGGGTTTGCGCTGGGTCAGCTTATCTATGGACCGATGGCGGATAGCCTCGGGCGTAAGCCGGTCATC
CTGGGCGGGACGCTGGTATTTGCTGCTGCGGCGGTCGCCTGTGCGTTGGCGCAGACTATCGATCAACTGATCG
TGATGCGTTTCTTTCACGGTTTGGCGGCCGCGGCGGCAAGCGTCGTCATCAATGCGCTGATGCGGGATATTTA
TCCAAAGGAAGAGTTTTCGCGCATGATGTCATTTGTCATGCTGGTCACTACGATAGCGCCGTTAATGGCGCCC
ATTGTCGGCGGCTGGGTGTTGGTATGGTTAAGCTGGCACTATATCTTCTGGATACTGGCCATTGCGGCGATTC
TGGCGTCAGTCATGATCTTTGCTCTGATTAAAGAGACGCTGCCCGTTGAGCGGCGTCAGCCTTTTCATATTCG
TACCACAATAGGTAACTTTGCCGCGTTGTTTCGCCACAAACGCGTACTGAGCTATATGCTGGCGAGCGGGTTT
AGTTTTGCCGGTATGTTCTCTTTTTTGAGCGCGGGGCCGTTTGTTTATATCGAAATTAATCATGTTCCGCCGC
AGGATTTCGGCTACTACTTCGCGTTGAACATCGTATTTCTGTTTGTGATGACGATTATCAACAGCCGTTTTGT
CAGACGGGTAGGGGCGCTAAACATGTTTCGGGCCGGGCTATGGATTCAGTTTGCGATGGCGGTGTGGATGGTT
TTCAGCGCGCTGATGGGCATTGGATTCTGGGCGCTGGTGGTCGGCGTTGCGGCGTTTGTCGGCTGTGTGTCGA

TGGTTTCGTCCAATGCGATGGCGGTCATTTTGGATGAGTTTCCGCATATGGCCGGAACGGCGTCTTCGTTGGC
GGGCACTTTCCGCTTTGGTATTGGCGCTATCGTCGGCGCGTTGCTGTCGCTGGCTACCTTTACCAGTGCGTGG
CCGATGATCTGGTCGATTGCGCTTTGCGCCGCCTGTTCCATTCTGTTTTATCTCTACGCCAGCCGTCCCAAAA
AACGGTGACGGGCGCGCTGTCCCTCAATGGCGCCGAAGAATGCGCAGGCGGCGTTGGGGGATGCTATCCGCTA
CACGATGGCAAAGCCAAACGGCTTTCATAGTTGATGTATATCAATTACCAATTCATCATTTTCCTCCTTTATG
TTTATTTTATGTAAAATCCATTTATGTAAAAAGTCACATCATTGTAGTTAAAAAGGTTGAGTTAGATCGCAGA
AACGGGTACATATAGCCCGCCGCTATCTCCGCGTCGGTAAAACTATTTTAACTCCCGCTTGCTGTAGAGCCAC
TGACGGAGAGAAGGGCGTTAGCAATCTGTTTAAGGACGGGTTGTCAATGATGTGTTAATATAAATGTAAGCAA
ATTGTGAAGTAATTGTGCTTCCGGGGAAAGAAAGTGAATACATTACAGCTCTCAATTGTTCACCGTCTGCCGC
AAAACTATCGCTGGTCGGCGGGTTTTGCGGGGTCAAAGGTTGAACCGATTCCGCAAAACGGGCAGAGCACCGA
GAATAGTCTGGTGGCGCTTAAACTGTTGAGTCCGGCAGGCGATAGCGCGTGGTCAGTGATGCATAAGCTCAGC
CAGGCGCTAAGCGATATTGAAGTTCCTTGCTCCGTGCTGGAGTGCGAAGGGGAACCGTGCCTGTTCGTTAATC
GTCAGGATGAGTTTGCCGCCACCTGTCGGCTGAAAAATTTCGGCGTGGCGATAGCGGAACCTTTTTCTAACAA
TAATCCTTTTTAGGCGTCAGCTTAACGCGAGTAGCTGACGCGTATAGGCCTGTTGCGGTGCGGTAAACACGCG
CTCGCATTGCCCCTGTTCAACCACCTCCCCCTGCCGCAGCACGATAACCTGATGGCACAGCGCGTGTACTACA
TGCAGATCGTGGCTAATGAAGATATAGGCCAGACGGTGCTTTTGCTGGAGCGATTTCAGGAGGGCAAGAATCT
GCGCCTGAACGGTTTTATCCAGCGATGAGGTCGGTTCATCCAGAATAATAAGCGACGGTTTTAAAATCAGCGC
CCTGGCGACGGCGATACGTTGACGCTGGCCGCCGGAAAACTCAGCGGGGTAACGATGCCGCGTTTCCGGGTCC
AGGCCGACTTCCATCATGACCGCTTTCACCTGCTGTTCTCGCTGCGCGCCTGAAAGCGTAGGCTGGTGGACGC
GCAGGCCTTCTTCGATAATTTGCAACACGTTTAAACGCGGGTTTAGCGATGAGTTTGGGTCCTGGAATACGAC
CTGGATACGGTGGCGAACAGGTAAAAGCTGGCGGCGGTTTAACGTATCCAGCGATTGACCGTCAAACACGATG
CGGCCTTCGGAGCGGATAAGCCGTAACAGCGCCAGACCGGTGGTGCTTTTTCCCGATCCTGACTCGCCGACCA
GACCCAGCGTTTCGCCTGGATGCAGGGTGAAACTGATATTGTTAACCACCACATTATGATCCACGACGCGCTT
CAGAATGCCTTTGCGGATTGGGAAGGCGACGCGCAGCTTGTCCACTTCCAGCAACGGCGCCTGCCCGGCGGGG
AGCGGAACCGGATCGCCTGTGGGTTCGCTGTTGAGTAGTTTTTGCGTGTACGGATGGGTCGGCGCGGAGAGCA
GCGTGTCGGCGCGTTGGTTCTCTACGCACTTGCCGTGTTGCATTACCGCCACTGAATCCGCCAGTTTTTTTAC
AATGCTGAGGTTATGGGTGATAAACAGCAATCCCATATTAAGCTCGCGCTGGAGTTCCCGCAGTAGAGATAAG
ATCTGCGCCTGTACGGAGACGTCGAGGGCGGTGGTCGGCTCATCGGCGATCAGTAATTCCGGCCGTGTTAACA
GCGCCATGGCTATCATGACGCGCTGGCGTTCGCCGCCGGAAAGCTGATGCGGATAATCGCGCAGACGCTGGCT
CGCCTGGCGGATACCGACCCGATCCAGACAACCGATCATCTCCGCCCCTTGCCGCCTCCCGACGCATTCCCCGA
TGGAGCGACAGCACTTCATAGAGCTGTTTTTCCAGCGTATGCAGCGGGTTAAGTGAAACCATCGGTTCCTGAA
AAATCATGGCGATTTTATTGCCGCGCACGCCGCGTAGCGTCTGCTCGCTGGCATGAAGCAATGATTCGCCGTG
AAAGCGAATATCGCCGGAGAGGTAGACGGCGGGAGGGGTAGGCAAAAGACGCAGAATGGAAAGCGCCGTCACG
CTTTTCCCTGACCCGGACTCGCCGACCAGCGCCAGCGTTTCCCCGGCGTTCACCTGTAACGAAATGGCGTTAA
CGACAGGACGCACGTGCTGTTGCTGACGGAAACCTACCGATAAATTTTCAATCGCAAGCAATGGAGATGTCAT
CTTATACCGCCTTAGCAGGATCAAAGGCGTCGCGTACCGCTTCGCCGATAAAAATCAGCAGCGATAGCAGAAT
GGCGACAGACAGAAAGGCGGCGATCCCCAGCCAGGGAGCCTGTAAGTTGTTTTTCCCCTGTAAAAGAAGTTCG
CCGAGAGAAGGGGAGCCAAGCGGCAGACCGAATCCCAGAAAATCCAGCGACGTCAGGGTGGTGATGGAGCTAC
ATAAAATGAACGGTAAAAATGTCAGGGTAGCGACCATCGCATTAGGCAACATATGGCGCAGGATAATGTCACG
ATCGCTGACGCCAAGCGCCTGCGCGGCGCGGATATAGTCAAAATTGCGGGTCCGTAAAAACTCGGCGCGCACT
ACGCCCACCAGACTCATCCAGCCAAACAACACGGTTATGGCCAGCAGCCACCAGAAGTTAGGCTGCACTACGC
TGGAAAGTAAAATAATCAGAAACAGGGTCGGCATCCCCGACCAGACTTCGATGAGACGTTGCCCCCATAAATC
GACTTTGCCGCCATAATAGCCCTGTAGCGCGCCCGCCAGTACGCCCATGACGCTGGAGCAAATCGTTAACATC
AGGCCGAATAAAATAGAAATGCGGGTGCCGTACAGGATGCGGGCGAAAACGTCGCCGCCATTCGCATCGGTGC
CCAGCCAGTTTTTTCGCCGAAGGCGGGGAGGGGAAAGGCTGCGTGGTGGCAAAATTAATGGTATTGGCGCCAA
AGCGTACGGGGGGCCCACAACACCCAGCCCCGATTCTCAAGTTGCCGTTGTAGCCAGGGTCCTGATAATCTGC
CGTTGTCGCCAGCGGGCCGCCGAAATCGCGCTCGCTGTAATTTTTTACCAGCGGAAAATACCACTGGCCTTCA
TAGCGCACCAGCAATGGCTTATCGTTAGCAATCAGTTCCGCGCATAAGCTCAGGCTGAACACGACCAGGAAAA
TCCATAGTGACCAGTAGCCCCGGCGATTATGGCGGAAACGCGCCCAGCGGGCCTGATTGACCGGGCTTAATCG
CGGCATTATCGTCCCTCAAAATCAATGCGTGGATCGACCAGCGTATAGCTGATATCGCTCAGGATATTCAGTA
ATAAGCCAATCAGGGTAAAGATATAAAGCGTACCGAACATCACCGGATAGTCGCGCGAAACGGTTGCTTCATA
ACCCAGCAGCCCCAGGCCGTTGAGCGAGAACATGACTTCAATTAGCAACGAGCCGGTGAAAAACATACTGATA
AAAGTGGCGGGAAACCCGGCGATCACCAGCAGCATGGCGTTACGAAAAACATGTTTCCAGAGAATGTTTTTTT
CACTCACGCCTTTCGCGCGAGCGGTAACGACATACTGTTTACGCACCTCGTCGAGAAACGAATTTTTCGTCAG

CATCGTCAGGGCGGCAAAACCGCCGATGACGGTCGCCAACACCGGCAGCGTAATATGCCACAAATAATCGGTG
ATTTTCTGGTACCACGGTAATGTGTCAAAGTTGGCGGAGACCAGGCCACGTAGCGGAAAAATATCGTAGTAGC
TGCCGCCGGCAAAAAAGACGATTAATAAAATAGCAAACAGAAAGGCGGGGATCGCATAACCAATAATGATAAA
CGCGCTGCTCCACACATCAAAGCGACTGCCGTTATGGACGGCTTTGCGAATCCCCAGCGGGATAGACACCAGA
TAAATAATCAGCGTGCTCCATAATCCCAGGGTAATGGAGACGGGAAGGCTGTCTTTGATTAGCGTCAGTACGG
ATGCGCTGCGAAACAGACTGTCGCCGAAGTCAAAAGAGAGATAGTCCCGTAGCATCTTGAAATAACGCTCATG
CAGCGGTTTGTCGAAGCCGTAGCGATGGGTGATCTCCGCGATCACCTCCGGATCGAGTCCCCGGCCGCCGCGA
TAATGGCTGTCGCTGATATTGCCGACGCCGGTTTGCGCATGACTGGCGCGAACGCCTTCGCCGCTGGCGCCGG
GCAACGCGCCGCCCTGGCCAAATTCAATCGCGGCAATCGCCTGATCGACAGGGCCGCCGGGAGCGATCTGCAC
GATAAAAAAGTTAATGGTAATAATGGCCCATAGCGTGGGGATCACCAGTAACAGACGGCGGATAAGATAGGCG
CCCATTGATTCTTACTACCTCCTGGCTGCCGGTAGTTTGGCGGCTTTGTTGACATCATACCACCAGGTATCTA
ACCCGATGGTATATACCGGGCGAATCGCCGGATGGGAGAATTTATCCCACCAGGCGAGCCTGTCTTGCGCCAT
ATACCACATCGGCAGCATGTAATAGTTCCAGGTCAGCACGCGATCCAGCGCCCGTCCCAGCGGCACCAGTTTC
GCTTTATCACCCTGCGCTGCGATAATTTGCGCGATCAGTTTATCCACCACCGGGCTTTGTACGCCGGGAGCGT
TATAACTGGAGTCAATGTATTCCGACGCCCATGAGATTTGTAGATCGGAGCTGGGCCAGGGCATCGCCCGCCA
TAGCCTCGGCATCATGTCATAGTCGCGGCTGCGCATCCGGTTGGTGAGCTGAGAATTATCAACCTGACGGATA
GTCATCGTAATGCCCAGACGCTGAAGATTATGCTGGAAGGGCAGAACCCACTGGCTATTACTGCTGGCAGGAA
GGAGAAGTTCAAACGTCAGAGGCTTACCGGTGACGCTATTGACCCGTTGCTGTCCGTTGATCACCCATCCGGC
CTGCGTCAACAAGGCGTCAGCTTTAAGAAGATTTTCGCGATCGTAGCCGTCGCCGTTAGAGACCGGCGGCTGA
TAGATCTGAGTGAAGACTTCAGGAGGAAGATCTTTTTTCATCGGCGCGAGTAATACCAGCTCATCGGCGTCAG
GATAATTTCTGGCGGCGTACTCGGTATTCTGGAAGTAACTGTTGGTTCGGCTGCAGGCATTATAGAACAGCGC
TTTATTCATCCACTCAAAATCGAAGGCCAGGGTGACAGCTTCACGTACCCGCCGGTCTTTAAATACCGGGCGC
TGAATGTTAAAGGCCAGCCAGCGTGTGTCCTGCGCCGACTCGTTTTTCTGCTCTTCTTTAATGATGTAATGAT
TATCGAAATTTTTACCGATGTAGCGCGTTGCCCAGTTTTTAGCGTCGTTTTCCAGCCGTAGATCAAATGCGCC
CGCTTTAAACGCCTCGAAAGCGACATTGTCATCAAGGTAGTAATCGTAGCGGATAGTGTCGAGGTTAAAACGT
CCACGATTGACCGGCAGATTAGCCGCCCAGTAGTTTTTGACGCGTGAATAGACAATGTACTGGCCCATTTTCC
ACTGAGTAATCCGGTATGGGCCGCTGGCTAAGGGCGGCGTTGAAAGCGGATCGCTGAGCTTGTGATTTTTCCA
GAATTTTTCGGGCATGATCGGTAATGAAAACAGACTAAGCATGTCTTCTTTACCAGGCTTCGCCAGCTCAATT
CGCACGGTTAAAGGCGCAATCGCCTTCACGGTAGTACCTTTATAGACCAGACGGAACTGCGGTACGCCTTCGG
TCATAAACTTATGAAAGGTAAAGGCCACATCGCGGGCGGTAATGGGCGTGCCATCGTGAAAACGAGCGCGGGG
ATTAATCGAGATTTCCACCCAGGAATAGTCGGCGGCATAGCGGGCATGGTCGGCAATCAGCGGGTAATAGCTT
CCGGGTTCATCATCCGAGGTGGTAAAAAGCGTATCGTAAAGGGCTTCGGTACGTACGCCGGGATTGCCGCGCA
GCGAATAGCGATTGAAATTATCGAACGTACCAATGGCGGAAAGCGTCATCTGACCGCCTTTCGGCGCAGCAGG
ATTCACATAATCAAAGTGATCAAAGTTAAAAGCATACTTAGGTTCGCCGAGTACGGCGAAAGCGTAGCTTTCT
TTGATTTCCTGGGCCTGGGCGCCAGCGCTAACCAGTGCGACAAGCAGAAGCATTACGCGCGCAATCATCTGAA
GGCGGTTTCCTTATCGTATTGCCCGCTGCAAACCGGCAGGCGTTTCGCTGAATAATAAGTGACGTTGCGTCTG
ATGTGAATCCGTTAAAACCGTAAGGGGGCTACCATTCAGGCACAGAAGGCGCCGACATCCACAGCACGAAATC
TTGTAGGGGTAAAGGACGACTAATCCAGTACCCCTGCAAGTAATTAACACCGTGATCGCGTAGCCAACGGGCC
TGCTCCGGCGTTTCAACGCCCTCGGCCACCGTCAGCATATTCAGGCGTCTGGAGAGCGTCAGCACGGTATCCA
GAACCGGAGACGTGACGGTTTCCGTGCCGATCGCCTGGATAAAACCGCGATCGATTTTCAGATAATCTAAAGT
AAATCGCTCCAGATAAATCAGGGCGCTATGGCCTGTACCGAAATCATCTATAGCGATTTCAAACCCGGCGGAA
TGCAGCCATTCGAACAGTTTGGCCGCTTCATACTGCTGGAGCATATCGCGTTCAGTGATTTCCAGCACGATAT
GAAAATGGTGCGCGGGCAGGGCGTCCCTGAGCCGGTGCATATCCTCTTTAAAACTGTCGCTGTGCAGATGTTC
CGGCGCGATGTTAATGCCAAATTTGGCACCGACGGGCATCACCTTCTGTAACGTGGGCGCGTCGCGGGCGATC
AGCTCGAACAGATGCTGCGTTAACGGTACGATAAGTTGCTGCGCTTCGGCATAGTGGATAAAGGCATCCGGCG
GAATCTCTCCTGCCGTAGGGTGACGCCAGCGCAGTAAGACTTCAAGCCCGGTGACGCTCAGCGTACGAGTATC
GACGACGGGCTGATAAACCACATAAAACTGCTCGTGTTTGATCGCCGTTAAAATCTCTTTGCCCGGTCGCGTC
CGGAGGGCATAGATAAAGTAACAGATAAACCCCGCTACGATGCCGCTAATGCAGCCCAGCATCAACGCATACC
ACAAGTCATTGTAGGTCCAGCTATCGGCATAAAGCTGTATTTTAAGCGGCAGGCCGTTGATAGTTGCCTGTCG
TGAAGGCGTGCCCGGCAGCTCCGCGACGGTCAGCAGACGCGAAGAAAAGGTCGACAGCGCGGTATTGCCGACA
ATCAGCGCGATGCCGTTAAAATCATCCTGGCGCGTGGTATAGAGTAAGTAGGGCGTCAGGTTAATATTCAGCG
ATGTGAATACGCCGCTGTCATTTAATAATGGGTTTCGATACCAAATCACCATTGTCGGTTTGTTGGGCATCAT
TGGCGTGCCGGGCAGAATCGCCATAGCAACGTCTTTGCGGATATCTATATCCGGTACCAGTTGCTGAAGCGGC
ATATTCATGGCGCCAGTGGCGGAAGAGCAGAACACTTTTTTATCTTTAATCAATAAGAAGGCGCGGACATTAA

GGCTAAAAGCGGCTCTTGAGGTGAGTTCTGCGCCAACCTGCTGACAGGTGTTGATCGTCAACGGCTGAAGAAT
ATCGGTGGTGGCTTTCAGGTCGGCGAAATAGCTGGCCAGGTAATGTTGTACATCAGAAAGGAGCGTATTGTAT
TTAACATCCCGTTTATGCCACGACAGGAAAAATTGCAGGCTACCAATAAGTAATGCTGCCGCTATCCCCGTAA
TGATACAGGTAAGCAGGATTTTCCGACCGGAAGAGGAGTCGCGTGTAAGCATAATTCCTGGTGTGACCTTAAA
AGGCGGATGCCGGTTAAACTGGTTGATTTATAAGACTATAGCCTGGATTACCCCGGATAGTCATGCTTTTCTG
TGATGCGCAGGAAAGCGTATTGTCGCAGTAGTCGTTGGCGGAAACGACCTGAATGTCTGATGGCGCTATGTTT
ATCAGGCCTATGTTCGTGTGTTGTAGGCTGGATAAGAGGCGACACGTCGCTATTCGGTAAAACGGGTTCAGAA
GGGATAATAAGCAAAAGCACTGCCGAAGCAGTGCTTTTTATGTCGTCTCATCAGGTAAGCCAAGGGAGGTGCT
GCCTGATGAAGATTAACTGCGGCTCAGAACTCGACGCGCTTCATTGTAGCGTTTTTTCCAGTACGGTTCGTTC
ATACTGGAGATAATGACGCCGCTACTGGTAGACGCATGGACAAACTGGTTATTGCCGATATAAATACCGACAT
GACGACCAGTGGAACCGGCGCGGAACAGAACCAGATCGCCCGTACGCAGGTTATTGCGTGAAACTGCTTTGCC
CATTTCCTGCTGTTCATAGGTTGAACGCGGAAGCTCTAAACCAAACTGTTCACGGAAGGTGCGCTGTACAAAG
CTGGAACAGTCGACGCCTTTCTTGGTACTGCCGCCCAGGCGGTAACGCACGCCTTTCCAGTCAGCATACTGAT
CCATAATCCGCGACTTAACGTCGAGGTTACGCACCATATTTTCAAATTCATCCTGAGAGGCTTGCAGTGAAGA
GCTATCGCTATTGCCCACAGCATGCGTCTCAGAATGCATATTCTTTGCGGTATTGGTGGTTGTGCTACAAGCA
GAAAGCAGAACCGCAACTGCAATCGCAGGAATCCCGCGCAAAATATATCTCAAAATCGGTTGAGATTTGACCA
TGTTGTTTGTTTTCCCTTGAAGTCCTTAACGATAAATATCGTTATAAAAATGCCAAACGTGATGAGACTAATT
ACCTGCACAGCTTGAGACAATTCTTTGTGCACAAATCTGTGGCGGAACGCACAAATTTATTCGCAATGAGAAT
AATTATCTGGCGAGGCAAAACGAGTTTGAGATTACCCGATTGTTGCTGTCATTGCGAGCGATTTCGCGTGATT
TTTTATAAGAATTTATGATACGAAAAGTGAGCTATCAGCACGGGTCAAATTAAGCGCAAATCGTTTAAAAATC
GTGCAACCTGTTCATTTTGCAGAGGAATAATATGACCGAAAAATGATGGGCTGGGGGAATATCATCATAAATT
ATCTTCAACGATGTAAAACTATCGCTGAAGGTAATGTTAATTACATGGGTTATTTGTTTAAAATTTGTTTATT
TCCGCCCGGGAGATAATTCTCAAAAAGCGCTATCGCCCGGTCGCTGAGCGGGGTCATCAGCCACCAGGGGAGG
CCAATCAAAATAACCGTGAGCGACCCGACCACAATATCGGTGAACCAGTGAGCGCCAATCATAACGCGCGGAA
ACGCGAAAACCACAAATATAATAAGGGCGATAATCCCCGCCGTTTTGCCGAAATAACGCAGCATAAAGGCGGA
AAATATCAGCAGCATCATACCGTGATCGCCTGGGAAACTGTCTTTTGACGCGTCTTTTGTCGGGATGTGCAGC
AACTCGCTGACGCGATAAATATGCTCAAAAGAGAGCGTTGGGCTGGCGCGCTTGACGGGGATCAGCGCCTGAC
CGAGTTGGTTAAGCACCACCGCTGTTAGCAGCATGACCAATCCGATTATCACAATGCGCCGCCGGCCCGACGC
GTCTTCTTTTAGCCAGAAACTGAGCATCAGTCCGCCCATCGCCAGCAGCGAACAGCCGTCAAACGCCCGATTA
TTGGTAATAGCGACCCACCAGAGGAACGCGTGGCTTTCCACCAGTTTCTGGTTAAAGAAGTGGAATATGCCGG
AATCAATGGTGAACCAGAAACCGTGATTGACGGGAAGATACCATGACAAAAACAGCGCCAGACCGGCGGCATT
AAGCAGAATTATCAAGGAATAGCGGGTTTTCATCGTCATTTCGCCATAGTTTTTAAACGGTGGCAACCTTACG
CGTGCGTCGCTAAACGAAGCTTCAACAAGGCCGTCTGTAACGTATTCCAGTCGGTTTCTGTGTTTGAGATAAG
TTCAACCCGGCTATCCGGCGGCGCGACACTCTGTGTTTCGATGTGCAGGTCATCGCCCTGGCGATTGATGCGT
ACCAGCCCCTCTTGTATGCGCATAACGCCTTTCACCCGGCCCACCGGCGCCAGACGAGCCCATTCGAGGAGGC
CAATGGTGTCAAACACGGTATCGGCATCGAAAATCCAGCCGCAGGCCTGATGACCCTGTCCGCTATTAAGGCT
GCGTCGCCAGCGCTGCTGGGCGGGCAGATTTAGCGCGGCGAGTCCTTTTTTACTGGCATGAGTGTGAGAATGC
GCGGCGCTGGCCGGCAGTTCCGCCAGATTTTGTCGCGGTAAATCCAGAAGCTTACCGTCTATCTGTCCATGTT
CGGCATGAATCAGTTGACGATCGCCGCCGTACTGTCGCCACCACTGTTGCAGGGCGGCATCGCTCTGCGCCGT
GGCGCGATCGGTCTTATTGGCGATGATAATATCGGCTGAGGCGAGCTGATCGCGGAAATTTTCATTGGCGACG
TTCTGTTGGTCCAGTAGCAGGCGAGGGTCAAGGATGCAGAGCGTAGCGCGTAAATCAATCCACGGCTCATAAA
CCGGCGCAGTTAATAAATCCAGAATCTGTTTTGGGTGCCCCAGTCCGGTTGGTTCAATCAGCAACCGGTCAGG
TTTGCCCTGGCGCAGCAGCGTGTTGAGCCCCACCTGCATAGGCAATCCATTGACGCAGCACATGCAGCCGCCG
GGGATCTCTTTTAGCAGTGCGCCGCTGTCGGCAAGCAGCGCGCCGTCAATACCCACTTCACCAAATTCATTAA
CCAGGACGGCCCACTTTTCAGCCGGATCTTTATGAGCTAATAAATGAAGGATAGAGGTGGTTTTTCCGCTACC
GAGAAATCCAGTAATAAGATTGGTTTTGGTCACGTCAGCTCCAGAATATTGATACAACATAACAACTTTTAAT
AATCTTGGCGAATAAACGTGAAAAAGAGAAGTGCTGAGTTGACGAAGTCGTGCTTCTGCCATCTTAAGAGCAG
AAAATGTTAAATTTTTAACGTTTCTCTTGCCTGGTGCAGAACAGTCAATAAATAAAGGGGCAGACTGCGCCCT
GGGGCGCAGCCGAAAAATGTGAAACTTAATCCGCGCGCCCCATATAACGGCGCTCTTCGATATGAATGCGGAT
TTTCTCTCCTGCGCTCAGGTATTCGGGAACCTGAATCACCAGACCGGTGCTTAATGTCGCGGGTTTGTTACGT
GCGCTGGCGGACGCTCCTTTAATACCCGGCGCGGTTTCGACGATTTCCAGATCCACGGTCTGCGGAAGCTCTA
GCGCCAGCAGTTGGCCGTCCCAGGTCAGAACCTGCATATCCGGCATTCCGCCTTCAGGCATAAACAGCAACTC
TTCTTCAATCTGATCTTTGGTGAAGGTATACGGCGTATAGTCTTCTTTATCCATGAACACATATTCGTTGCCA
TCGACATAAGAAAAGTCAACGCCGCGACGGCTCAGGGTGACGGTATCAACGATATCGTCGCCTTTGAAACGTT

CTTCCACTTTCAGACCGGTGCGGACATCGGAAAAACGCATTTTATACAGTGTGGCGGCGCCACGGGCGGTAGG
CGACTGAATGTCGATATCTTTCACAATCAGCAATTTGCCGTTGTAGTTCAGTACCATACCTTTTTTGATTTCG
TTCGCTCTTGGCATCGAGGAGATCCTGTTGTAGAGATTATCTAAAATATCGCGTAAAACTACTCGCGCCGGGC
CATCCAGGCAAGTGGAATTGTCACTTTTGCTGGTGCAAAAAAGGTGATACTGTTCGCCCGCTTTCCGCTCCGC
GTTCAACGAGAATAGGCGATGGCATGTCGCCCGGAGGGGGGAAGTGTGCTGTGTTGTCCCTTCTGTTTCCAGC
CTTGTCTTCGGAATGTCTGAGGGCAGGTCTGATCATGATGACGTCCACATTTTAAATCTGGATTTTTCTTTGG
ACAAATTCTGTTATCTTTCATTTGGTTATAAATAGAGGGGCTGAGCATGGACTGTCGTAGCGGTTGTGGTGCT
TGCTGTATTGCGCCTTCAATTTCGTCGCCGATACCCGGAATGCCGAATGGCAAGCCGATGAATACCAGATGCG
TGCAGCTTTCGGAAGATAACTTGTGCCTTATCTTTGGCTCGCCTTTACGCCCAAAAGTGTGCTCTGGTTTGCA
ACCGACAGGGGACATGTGTTTAACGACCCGGGAAGAGGCCATTATTTATTTGCTGGAACTCGAACAGAAAACC
TTACCATGATCTAAGCCGCTTTTGATAAATGTGCAGGGGCTTACGTTATATCCCGAAATAGTCTGCAAAAGCT
GCGCATCAAATTACCTCCTTTTACCATTAAATAGCGTAATACATGCTTCCCTTATTTCGTGGCTTCTGAAGCG
GTAGATGCCGCCGCTCAACCCCGTTGGTGCCTGAACGTGTACCGAACAAACCGTCACATAAAACAAAAACGAT
CAATTTTATTTATATGATAGTTTTTATCTATTAAAAGAGGAATCTTGATCGATTTTAATTATTTATAACGAT
CGATATCATGAATTTGATAGTTTATATCTATCATTGATGTATTTACCGATCGATATAACCAGTGTGAATGTGT
TGTTATTTTCCTTTAAAATACTCGAATTCTTATGTGTGGGTTTTATCTTATGAAGAAATATACGTTCGCAGCC
AGGGCGTTTATTTTTACGCTGGTGTTGCTTTTTGTTATTTCAGTATTCGGGTTGACTATTAGCGTTTTAAAAG
GGATGTGAAATGCGGGGAGAAAATAGTGAAATTGATTTAGTCGACGTTTCCTTATTCCTTATAAGAAAATGGC
TAAGTATCGTGATCGTTACCTTTATTTTTGTCGCCATTGGCTATGTTTTTGTGAGCGTGCAGCATGATACAAA
GGTTGTGAGTATTACAATCAAACCTAATCTGGATACGCCTGCTACCTATGCGTTATGTGGCTATACTAATGAT
ATTCAATGTAAAACAACGGTTATCCTTAACCAGTTTAGCCGTTTCTTGCCTGCGGAATATAAAAATAAAATAA
GTTTTGACGCGAAAAATCAGCTTGTTCTTTTTAAGGCGCAGGGGAGGGAAGAAAGCGTCAATAATATTATTTC
CACCCTGAAAAATAGTGTCGATAAAATGGCGGCATGGTATATCGATGATGGTGACATTAAACGTTATAGCGTG
CATAAAAATATGTTACAAACGGAAACATATGCAAAGCTATCCTTGTTGTCGGAGGCGGTTAAAAATAATGCTA
ACATTGAAGTGATAGATGTTGCTGTCACACCAAAATATAAGATGCGGCTGGTGTTGGTTCTTTGCGGACTGAT
GGGCGTAATTTTCTCAATAACCGGTCTTTTATGTAGGAGGGCGCTTACAGAATATCGTTTTGAGAAAAAGACA
ATGAATGACCGCGCATGAACATTGCGCAGAGATAAACTCTGCGCTGAATGTGTCGAAGAGTAAAAGTATCAGG
CTTGCAAGTTAAACATCTTTAATACGCGCCAGGCAGAACATGGTGGCGACAATCATGACTAACGCAAACCAGA
AGACCGCATGATAATTCCAGATTTCTGCGGCGATTCCCGCCAGCGAACCGGCAATGATCCAGCCGACGCGAAT
GGTATTGGTATACAGCGTGGTGGCTGAACCCGCCTGGCCTGGCATGAGATCCTGAAAATAGAGCATGCCAATC
CCGCCAAGAATGCCGATGTAAATCGCGTTCAGCAACTGCAAGCCGAGTAACGTTGCCGGCGCGTGCGCCAGCA
ACATCCCCGCATAAAAGCACAGTCCGGCGACAACGGCGATGCACATTAACAGGCGTTTTCCCAACCGTTTGGC
GAAGTAACCTGCAATCAGCATTGTGGGAATCTCCAGCCCGGCGGCGGTGCCCATCATCACGCCTGCAAGTTTT
TCCGGAAGATGCAGCTCATTGATGATAAACAGCGGCATATTAATGATATACAGGCTGTTAGTTCCCCACATTA
ACGTACAGATGACAAACAGCAACAGCGTATCGCGGCGATTACGACGCGGTGCTTCCAGCGTGCCGGTTGCCAG
CGGCGCATCTTTTGCGCATTGACGGTAAAAAGAACCACACCATGACGCCGCAGACGATAAAAGCTACCGCCGC
GCTCAAATACATCACCGTAAAGCTAAAACCCATCGCCAGCGCATAGGCCAGCGGCGGCCCAATGACCCAGGCC
AATGAGACCTGGGCGCGGAGGATTGAGCTGAACATCACCGCTTCACGACCTGTACGATCGGCATGTTCACGGG
CGAGCGCAAACATCTGCGGGTTGGCGGTAGAGCCGAAGCTACTGAGAAAGACGCCGATAAACAGCAAAATAAA
GTAGTTGCGGTTCCAGGCAAACAGCACGCAGGCCAACATGCCCAGCACGCAACAGAAAACGATCAGTTTTTTG
CGATCGCCTTTTTTATCTGAGCGACCGGCCAGAAACTGGCTCACGATAATGCCGATAACCGCGCTGCCGGTAA
AAAAGAATCCCACCATGCCGGGGCGGGCGTGAACCTCATCCGTCAGAAAAATACTGAGCGTCGGCGTCTGTAG
CGCACCGGCAATACCGGTGAGAAAAGCGACGATCAAAAAGGCGGTAGACGTCAGGTCAAACGATTTAGGCGCA
GCGGCGGCGGGACTGTTGTGCATGTTTCTATATCAGTGGTGTGAAGAGAGGCGGAGTTTACGCTGCCCGTCAG
AAAATAAACAGGCGTCTGAATCAAAATCGACACGAAAAATCAAAATAGCATTCCAGTTTGTTACCAGGGCGTG
CTGACGCAAGCTGAAAGAAAAAAGCTGAAGCCTTTTATGCGTCAGCTATTGAGGGTCTGTTGGAGAAAAAATG
TGCTTCACCTCAAAAAATTGCTACAAAACCGGTAACTTTTCTTGCGTGCTGAAGCAGAAAGATCCAGACTTCT
TGAAACGTTTCAGCGCGATCTGGTTTTTTTGATAACTCAGATTGGCGCTTTTTTCTCATAGTGGCTGAATCGT
TTCAATTCAGCGGGAGAGGAGACACATGTTCCAGTTATCGGTTCAGGACATTCATCCGGGCGAGCAGGCCGGA
AATAAAGAAGAGGCCATTCGGCAAATCGCCGCCGCGTTAGCGCAGGCGGGGAATGTGGCGGGCGGCTATGTTG
ACGGTATGCTGGCGCGCGAACAGCAAACCTCGACCTTTCTTGGCAATGGTATTGCGATTCCCCATGGCACAAC
AGACACGCGCGATCAGGTGCTGAAAACCGGCGTTCAGGTCTTTCAATTTCCGCAGGGCGTTACCTGGGGAGAA
GGCCAGGTCGCCTACGTGGCGATTGGTATTGCCGCCAGTTCGGACGAACACCTCGGCCTGCTGCGTCAGCTGA
CGCATGTGCTGAGCGATGACTCCGTGGCTGAACAGCTGAAATCCGCGATAACGGCGGAAGAGCTGCGCGCATT

GCTGATGGGCGAGAAGCAAAGTGAACAATTAAAACTTGATAACGAGACGATGACGCTGGATGTCATCGCCAGC
TCGCTGGTCACATTGCAGGCGCTAAATGCGGCGCGTCTGAAAGAGGCGGGAGCGGTTGATGCGGCGTTTGTCG
CAAAAACGATTAACGATAGTCCGATGAATCTGGGACAAGGCATCTGGCTGAACGACAGCGCGGAAGGCAATTT
GCGCAGCGCTGTTGCGGTAAGCCGTGCGACCCAGGCGTTTGACGTAGAGGGTGAAAAAGCAGCCTTATTAGTC
ACTGTCGCGATGAACGACGAGCAGCCGATTGCCGTGTTAAAACGCCTCGGCGACCTGTTGCTGAACAATAAAG
CCGATCGTCTGCTGAGCGCCGACGCCGCGACGCTGCTGGCGTTGCTGACCAGTGATGATGCGCTGACGGATGA
CGTACTGAGCGCGGAATTTGTAGTGCGTAATGAGCATGGTCTCCATGCCCGTCCGGGCACCATGCTGGTGAAT
ACGATTAAACAATTTAACAGTGAAATTACCGTGACAAACCTTGATGGAACCGGCAAACCGGCAAACGGACGTA
GTCTGATGAAAGTGGTGGCGTTAGGCGTGAAGAAAGGCCATCGTCTGCGCTTTACCGCGCAGGGTGAAGATGC
TGAACAGGCGCTGAAAGCGATTGGCGATGCGATCGCCGCCGGTCTCGGGGAGGGCGCATAATGAGCAGGCGTG
TCGCCACCATTACCCTTAATCCGGCTTACGATCTGGTTGGGTTTTGCCCTGAAATCGAACGCGGCGAAGTGAA
CCTGGTGAAAACCACGGGTCTGCACGCGGCGGGCAAAGGCATTAACGTTGCCAAGGTGCTAAAAGACTTAGGC
ATCGACGTCACCGTCGGCGGTTTTCTCGGTAAAGATAACCAGGACGGTTTTCAGCAATTGTTCAGCGAACTGG
GCATCGCTAACCGCTTTCAGGTGGTTCAGGGCCGGACCCGCATCAACGTGAAGCTGACGGAAAAAGATGGCGA
AGTGACCGACTTCAACTTTTCCGGCTTTGACGTGACCCCCGCAGACTGGGAACGCTTTGTTAACGACTCCCTG
AGCTGGCTGGGCCAGTTCGATATGGTCTGCGTCAGCGGTAGCTTACCGGCTGGCGTGAGTCCGGAAGCGTTCA
CCGACTGGATGACGCGTCTGCGCAGCCAGTGTCCATGCATTATCTTTGATAGTAGCCGTGAAGCGTTAGTCGC
CGGTCTTAAAGCTGCGCCGTGGCTGGTGAAACCGAATCGCCGCGAACTGGAAATTTGGGCGGGTCGTAAGCTC
CCGGAAATGAAAGATGTAATTGATGCGGCGCACGCGTTACGCGAACAGGGTATCGCTCATGTGGTGATTTCGC
TGGGGGCGGAAGGCGCGCTGTGGGTTAACGCCTCAGGAGAATGGATCGCTAAACCACCGGCAGTTGACGTGGT
AAGTACCGTCGGCGCAGGCGATTCCATGGTTGGCGGATTGATTTACGGCCTGCTGATGCGCGAGTCCAGCGAA
CATACGCTGCGTCTGGCGACGGCGGTTGCCGCGCTGGCGGTCAGCCAGAGCAATGTGGGTATTACCGATCGTC
CTCAGTTGGCCGCAATGATGGCGCGTGTCGACTTACAACCGTTTAACTGACAGCAGGAGAGGCATAATGAAAA
CGCTGCTGATAATTGACGCTAATCTCGGCCAGGCTCGCGCTTACATGGCGAAAACCCTGCTTGGAGCGGCGGC
GCATAAAGCAAATCTGGAAATTATCGACAATCCGAATGATGCTGAGCTGGCGATCGTGCTGGGCGAATCTCTG
CCGAACGACAATGCTCTGAACGGCAAAAAGGTTTGGCTGGGGGATATTGGTCGCGCCGTCGCGCATCCGGAAC
TGTTTCTGAGTGAAGCCAAAAGCCATGCGACCCTTTACAGCGCCCCTGCTGCGGCTGCTCCTGCGGCGAGCGG
CGGTCCGAAACGCGTGGTCGCGGTAACGGCCTGCCCGACCGGCGTCGCCCATACTTTTATGGCGGCTGAAGCC
ATTGAAACAGAAGCGAAAAAACGCGGCTGGTGGGTAAAAGTCGAAACGCGCGGCTCCGTGGGGGCCGGCAATG
CCATCACCCCGGAAGAGGTGGCGGAAGCGGATTTAGTGATTGTGGCGGCGGATATCGAAGTGGATCTGGCGAA
GTTTGCCGGTCTCCCGATGTACCGCACGTCGACCGGCCTGGCGCTGAAAAAGACGGCGCAGGAGCTGGATAAA
GCCGTAGCGGAAGCGACGCCGTATCAACCGGCGGGTAAGGCATCGCAAGCGGCGACCGAAGGGAAGAAAGAGA
GCGCTGGCGCATACCGGCATCTGTTGACGGGCGTTTCTTACATGCTGCCTATGGTGGTTGCCGGGGGACTGTG
TATTGCGCTTTCCTTCGCCTTTGGTATTGAAGCCTTTAAGGTGCCGGGCACGCTGGCGGCGGCGCTGATGCAG
ATTGGCGGCGGTTCGGCGTTTGCGCTGATGGTGCCGGTACTGGCGGGTTATATCGCTTTCTCCATCGCGGATC
GCCCAGGCCTTACGCCAGGTCTGATTGGCGGTATGCTGGCGGTTAGCACCGGTTCTGGTTTTATCGGCGGGAT
CATTGCCGGCTTCCTTGCCGGGTATATGGCGAAGCTTATCAGTACCAAACTGAAACTTCCGCAAAGTATGGAA
GCGCTGAAGCCAATCCTGATCATCCCGTTAATTTCCAGTCTGGTGGTGGGGCTGGCGATGATTTACCTGATCG
GTAAACCGGTTGCCGGGATTCTGGAAGGGCTCACCCACTGGCTGCAAACCATGGGGACCGCAAACGCGGTGCT
GCTGGGCGCGATTCTCGGCGGGATGATGTGTACCGACATGGGTGGCCCGGTGAACAAAGCGGCGTATGCGTTT
GGCGTTGGTCTGCTGAGTACGCAAACTTACGCGCCGATGGCGGCGATCATGGCCGCAGGTATGGTACCGCCGT
TAGCGCTGGGTCTGGCAACGATGGTGGCGCGTCGTAAGTTCGACAAAGCGCAGCAGGAAGGCGGTAAAGCGGC
GTTGGTACTGGGGCTGTGCTTTATCACTGAAGGCGCTATTCCGTTTGCGGCGCGCGACCCGATGCGTGTACTG
CCGTGCTGTATCGTTGGCGGCGCGTTGACCGGGGCTATTTCTATGGCGGTAGGCGCAAAATTGATGGCGCCGC
ATGGCGGTCTGTTTGTTCTGCTTATCCCAGGCGCAATTACGCCGGTATTGGGATACCTGCTGGCAATTGTGGC
CGGTACGCTGGTGGCAGGACTGGCTTATGCCGTCCTGAAACGTCCGGAGACGGAAGTCGCGGCAAAAGCGGCA
TAAGAGAAACACGCTAAAAAAGGCAGAGCGCGCTCTGCCTTTTTTATTGCCTGAAGAACATTGAGCGTCAGGC
CATCGCTTCGGCAATTTGCTGGGCTTTTAACCACGCAATCTCTTCCGCCCAGATATCAGGATTGATGGTCTCC
AGTATCAGCGGAATACCGTCAAAACGCCCATCCTGCATAATCCAACGAAACGCATCGTGGCCGATATTGCCTT
CACCCAGACTGTGATGGCGGTCAACGCGGCTACCGAAGGCGCTTTTGGCGTCGTTCAGGTGCATTCCGCGCAA
ATACTGAAATCCGACAATTTTCCCGAATTCGGAGAATGTTTTTTCGCACGCCTCTGGCGTACGCAGATCGTAT
CCGGCGGCAAAGGCATGGCAGGTATCGATACAGACGCCAACGCGCGACTTATCTTCCACGCCGTCGATGATGG
CGGCTAACTGTTCAAACTCAAACCCCAGATTACTGCCCTGACCGGCTGTATTTTCGATAACCGCCGTAACGCC
CTCGGTCTGCGCGAGGGCAATATTGATGGATTCCGCGATCCGCGCCAGGCAATCCTCCTGTGCAATCTGCATC

AGATGGCTACCGGGATGAAAATTAAGCAAGGTTAAGCCGAGTTGTTCACAGCGCTGCATTTCATCGAGAAAGG
CATCGCGTGATTTTTCCAGCGCTTCACTGACCGGATGGCCCAGATTAATCAGGTAACTATCGTGGGGAAGAAT
TTGCGCCGCCGAGAAATGATACTTTTCACAGGCGATTTTAAAGTCATCAATGACCTGGGGAGTAAGGGGGGCG
GCACGCCACTGACGCTGGTTTTTTGTGAAAAGCGCAAAGGCCGTTGCGCCAATTTCAGCCGCGCGGGCAGGGG
CGTTAGCCAGACCGCCAGCAGCGCTGACGTGCGCTCCGATGTATTTCATAAAAGACTCCTGTTAAACCCGCTA
ACGGTTCATTACGAACGCTATGATAACGGGTTAACAGGCGTGAAACGTAATAATTTATGCGATAAGGCTATGG
ATTAGGACGTTAATGGCGCCACCGCCAATAATCAGCCAGGCGAATAGTGCCAGCGCCATCAGCAGCGGTTTCG
CCCCCGCTTTTTTCAATGCGCTAACGTGAGTGGTCAGACCCAACGCCGCCATCGCCATCGCCAGCAGCACTGT
GTCCAGCGTCACCAGCATATCCACCACGGCTTTCGGCAGCAGGTGGAAAGAGTTGAAGATCGCCACTACGATG
AAGAAAATAGCAAACCACGGAATGGTAATTTTGCTTTTTTCCGCGCCGGTCGCCGGTGAAAGCTGTTTAACGC
GTGCCGCCAGAATGATCAGGAACGGCGCCAGCATCATGACACGCAGCATCTTAGCGATAACTGCCGCATTTTC
CGCATCCGGGCTAACGGCATGACCCGCTGCGACCACTTGCGCGACTTCATGCATGGTTGAGCCGATATAGATG
CCGTAAGTTTCCGGGCTGAACCAGTGCGCCAGCAGCGGATACATTGCCGGGTAAAGGAAAATAGCGATGGTGC
CGAAAATAACGACAGTCGCGACGGCTACAGTAACCTTACTGGCTTCCGCTTTTACCACCGGTTCTGTCGCCAG
TACCGCCGCCGCGCCGCAGATACTGCTACCGGCGCCAATCAGCCAGCTGGTATGCCTGTCCAGACCAAAGACT
TTTTGGCCTAAGAAGCAGGCCAGCATGAACGTGCTGGAAAGCGTTAATACGTCAATCACAATACCGCTAATAC
CCACGTCGGCGATTTGTGAAAAGGTAAGGCGGAAGCCGTAAAGGATAATCCCTAAACGCAGCAGATGTTGTTT
TGCAAACAGCACGCCGCCGTCGCATTGTTTCCATATTTGTGGATAGACGGTATTCCCGATAACCATACCCAGC
AGGATAGCCAGGGTCAGGGCGCTGAACCCCGCGCCTGCAACGGCAGGGATAGCGCCGCCCCACAGGGCGACTC
CTGTAATGACGGCGCTCAGAGCGAGCCCCGGTATAAAATGCCACATTGTACGACGATGATTCTGCAAGGTGAG
TTCTGTCATAACCTTCTCCTGTATATGGACAATAAGGTTACGGTGATCTGGTTTAAAAATAAAATTGATTATA
TATTTATAATTAATCTTTATAAGTGGTAAGTGAAACGCTATCGGTGGGATACGACTATGCATATTACGCTACG
ACAACTTGAAGTGTTTGCTGAAGTACTGAAAAGCGGTTCAACAACCCAGGCGTCGGTCATGCTGTCATTATCG
CAGTCCGCCGTTAGCGCCGCGCTCACCGATCTGGAAGGTCAGCTCGGCGTACAGTTGTTTGATCGGGTGGGGA
AACGGCTGGTGGTCAACGAACATGGGCGCTTGTTATACCCGCGCGCGCTGGCATTGCTGGAGCAAGCGGTAGA
AATTGAGCAGCTATTTCGTGAAGATAACGGCGCGATACGGGTTTATGCCAGTAGCACCATCGGCAATTACATT
TTACCCGCCATGATTGCCCGCTATCGCCAGCATTATCCGGCCTTGCCGCTGGAACTCAGCGTGGGAAACAGCC
AGGACGTGATTAACGCGGTGTTGGATTTTCGGGTTGATATCGGCCTGATTGAAGGGCCGTGTCACAGTACAGA
GATTATCTCTGAGCCGTGGCTGGAAGATGAGCTGGTGGTCTTTGCCGCGCCGTCCTCGCCGCTGACGCAGGGG
CCGGTGACGCTGGAACAGCTTGCGGCATCCCCATGGATTTTACGCGAGCGCGGTTCCGGTACGCGGGAAATTG
TCGATTATTTGCTGTTGTCGCATTTGCCGCGCTTTCATATGGCGATGGAGTTGGGGAATTCAGAAGCGATCAA
ACATGCGGTGCGTCATGGACTGGGGATAAGCTGTTTATCGCGCCGGGTGATTGCTGAACAACTCCAGGCGGGA
ACCCTGGGCGAAGTCGCGGTGCCGTTACCGCGCCTGGTGCGCACGTTGTGGCGTGTTCATCATCGGCAGAAAC
ATCTTTCTAACGCGTTGCAGCGTTTTTTGAGTTACTGTGAATAAAGGTGAAGAGGAGGGCTGCCCGGGCGGCA
TCGCCATTTTTAGTATCCGCTTACGTATACGTTATTCTCTGCTTTACTTATAATCCGTGGCCGGTCATGAAGG
TCTCTTATAACCGCGCATTTGTGTCGGAAGGATAGTATTTCGTCTGTTACAATCGCGCCTCATTTTTTGGATG
GATAGCATTTTCACATGGGTTCCAAAACTAAAACCACAGAAGCGCCCGCGCTACGTCGCGAATTAAAGGCGCG
TCACCTGACGATGATTGCCATTGGCGGCTCCATCGGGACAGGTCTTTTCGTTGCATCTGGTGCGACAATTTCT
CAGGCGGGGCCAGGCGGCGCGCTGTTTTCTTATATTCTGATTGGTTTGATGGTCTACTTCCTGATGACCAGTC
TTGGCGAGCTGGCGGCCTATATGCCAGTATCTGGCTCGTTTGCGACATATGGTCAGAACTATGTGGAAGAAGG
CTTTGGTTTCGCGCTGGGCTGGAACTACTGGTACAACTGGGCGGTGACTATCGCTGTTGACCTGGTTGCCGCG
CAATTGGTGATGGGCTGGTGGTTCCCGGACACGCCGGGCTGGATCTGGAGCGCGCTATTCCTCTGCGTCATTT
TCTTGCTGAACTACATTTCTGTACGCGGTTTTGGCGAGGCGGAGTACTGGTTCTCGTTAATTAAAGTCGCGAC
CGTTATTATCTTCATCATCGTTGGCGTAGCGATGATTATCGGTATCTTTAAAGGTGTCGAGCCGGTCGGCTGG
AGCAACTGGACGACGGGCGATGCGCCTTTCGCTGGCGGTTTTGCGGCAATGATTGGCGTCGCGATGATCGTGG
GCTTCTCTTTCCAGGGAACTGAGCTGATTGGTATTGCCGCCGGGGAATCTGAAAACCCGGAGAAGAATATTCC
ACGCGCGGTGCGTCAGGTGTTCTGGCGTATTCTGCTGTTTTATGTTTTCGCGATTTTGATTATCAGCCTGATC
ATTCCTTATACCGATCCAAACCTGCTGCGTAACGACGTCAAAGACATTAGCGTCAGTCCGTTTACCTTAGTGT
TCCAGCATGCGGGCTTGTTGTCTGCGGCGGCGATAATGAATGCGGTTATCCTGACGGCGGTACTGTCTGCGGG
TAACTCCGGGATGTATGCCTCGACCCGTATGCTCTACACCCTGGCCTGTGACGGTAAGGCCCCGCGTATTTTT
GCGAAATTGTCCCGCGGCGGCGTACCGCGCAATGCGCTTTATGCCACCACCGTGATTGCCGGTCTTTGCTTTT
TGACCTCAATGTTTGGCAACCAGACGGTCTATCTGTGGCTGTTGAACACCTCCGGTATGACGGGCTTCATCGC
CTGGCTGGGGATTGCCATTAGCCATTATCGCTTCCGTCGCGGTTACGTGTTACAAGGGTATGATGTGAATGAT
TTGCCGTACCGTTCCGGGTTCTTCCCGCTGGGGCCGATTTTCGCCTTTGTACTGTGCCTGATTATTACGTTGG

GGCAGAACTATGAAGCGTTCCTGAAAGATACCATCGACTGGGGCGGCGTCGCGGCGACCTATATCGGTATTCC
GCTGTTCCTGCTTATCTGGTTCGGCTACAAGCTGATAAAAGGTACGCATTTCGTGCGCTATAGCGAAATGCAC
TTCCCGGAACGCGTTAAAAAGTAACGCGGTATTTTCGTCGTAGGCCGGATAGGCTTTTATGCCGCTATCCGGC
ACTGACTGCCTGAATCTGACTCTTCTAACCCTCTCATTCGAGAGGGTTTTTTGTTTTTTCTTCATGATTGGTT
CAAATGACATAATTATTAACAATTAAATTGATAATTATTATCGTTTGCTTTATCGTTACGATCGAAATCAGAA
AGGATGAAGATTCGGGCGCGACCGTCACTCATGAGCCCGACTCGCCTGAATAAAAAGAGTTCGCCGTGATAAC
CGTTATCGCAGGCGAAACGTAATGCGTTGTGAGCGATAACCGATTTTTTTGTCGTATTTACCTCATGGAGAAA
TGGAATGTTTAGGTTTAACCCTTTCGTTCGGGTAGGGCTTTGTATGTCCGCAGTTACGTTGGCCTGGCCGGTT
GCCGCGGCAACGGATGATGGTGAAACCATGGTCGTTACCGCGTCCGCCATCGAACAAAATCTGAAAGATGCGC
CAGCAAGTATCAGCGTCATTACCCAGCAAGATTTACAGCGCAGGCCCGTGCAAAACCTGAAAGATGTCCTGAA
AGAGGTGCCGGGCGTACAGCTCACTAACGAAGGGGATAACCGTAAAGGCGTGAGTATTCGTGGTCTGGACAGC
AGTTATACGCTGATCCTGATCGACGGTAAGCGCGTTAACTCCCGTAACGCGGTCTTCCGCCATAATGATTTTG
ATCTGAACTGGATACCGGTTGACGCTATCGAACGCATTGAAGTGGTGCGTGGCCCCATGTCCTCGCTGTATGG
TTCCGATGCGCTGGGCGGCGTGGTCAACATTATCACCAAAAAAATTGGTCAGAAATGGCACGGCAGCGTCACG
GTTGACTCCACTATTCAGGAACATCGCGACCGTGGCGATACTTATAACGGTCAGTTCTTTACCAGCGGTCCGC
TGATCGACGGCGTGCTGGGAATGAAAGCCTACGGTAGCCTGGCGAAACGCGAAAAAGACGAGCAGCAGAGTTC
CGCCACCACCGCTACGGGTGAAACGCCGCGCATTGAAGGATTTACCAGCCGGGATGGAAACGTCGAGTTTGCC
TGGACACCAAACGAAAATCATGACGTTACCGCAGGCTACGGTTTTGATCGCCAGGACCGGGACTCCGACTCGC
TGGATAAAAACCGTCTGGAACGGCAAAACTATGCGTTGAGCCATAACGGACGTTGGGATCTCGGCAATAGTGA
GCTCAAATTCTACGGCGAAAAAGTGGAAAACAAAAACCCCGGCAACAGTAGCCCTATCACGTCAGAAAGCAAT
TCGATTGACGGGAAATACGTTCTGCCGTTAGCGGCTGTCAACCAGTTCCTTACCTTCGGCGGCGAATGGCGTC
ACGATAAACTGAGCGATGCGGTCAACCTGACCGGCGGTTCCAGTACTAAAACCTCCGCCAGCCAGTATGCTCT
GTTCCTTGAAGATGAGTGGCGCATCTTCGAACCGCTGGCGCTGACCACCGGTATTCGTATGGATGATCATGAA
ACCTATGGCGATCACTGGAGCCCGCGTGCTTATCTGGTCTATAACGCAACGGATACCCTGACGGTGAAAGGTG
GGTGGGCCACGGCCTTTAAAGCGCCGTCGCTATTGCAATTAAGTCCGGACTGGGCGACCAACTCCTGCCGTGG
CGGATGCCGCATTGTCGGTAGCCCGGATCTGAAACCTGAAACCAGTGAAAGCTGGGAGCTCGGCCTTTATTAC
CGGGGCGAAGAAGGCATACTGGAGGGCGTGGAAGCGAGCGTTACCACGTTCCGTAACGATGTGGACAATCGCA
TCAGCATTAGTCGTACCCCTGACGTTAACGCCGCGCCCGGCTATTCCAACTTTGTCGGTTTCGAAACCAACAG
TCGGGGACAGCGCGTTCCGGTCTTCCGTTATTACAACGTGAATAAAGCGCGTATTCAGGGCGTGGAAACCGAG
TTGAAAGTTCCGTTTAACGAGGCGTGGAAACTGTCGTTGAATTACACCTATAACGACGGACGCGACGTCAGTA
ATGGCGGGAACAAGCCGCTTTCCGATCTGCCGTTCCACACCGCGAACGGTACGCTGGACTGGAAACCGGCGCA
GCTCGAAGACTGGTCATTCTACGTATCAGGGAAGTATACCGGGCGTAAGCGGGCCGACAGCGCAACAGCGAAA
ACGCCGGGCGGGTACGTGGTCTGGGATACCGGCGCGGCATGGCAGGCGACGAAAAACGTTAAACTGCGTGCGG
GCGTTCTGAACGTGGGCGATAAAGATCTCAAACGCGACGACTACGGTTATACCGAAGACGGACGTCGTTACTT
TATGGCGGTGGATTACCGTTTCTGACAGAGACTGGCGACAGCCGCGTTTTGCCGCTGTCGCCAGAATGTTATA
ACCGATGCAGCCTGGAGAGCAGCGCCAGCGACAGAGTCGTTAGCAGAATGGCGATCACCGCATAGATATAAAC
GCCCGACCACGCCCAGCTATTTTTCACCATAATTACCGGCACACCGGCCATTGCCGCGCCGACATAGCTGAAC
AATCCTCGAAAGCCGGTAATCGATCCCGCCGCATCCTTATGGGTCACATCCAGACAGCCGACGGCAAAAAGCA
TATGCGGTCCGTATAAGGCGAAGCCCATGATGGTGAACAGAATCGCGGTGAGGGTGTAAGAGGTATCCTGAAC
CAGCGGCACCAGCACGATGCATATGCACAGCGCTAACGCGAAGATTAATCCGGTCATCCAGCGGTTACTTTTG
AACATAAAGTCAGACAAGTAGCCTGCCAGCAACCCACCTGCCAGTCCGCCCGCCTCAAACCAGGAAATAATCG
AGTTCGCTTTTATCAGGCTCCAGCCGTGAACCTGCGAATAGTAAATCTGCGGCCAGTCGTTAAGGATGGTGCG
CGCAATATAAACTGACATATCGCCGATAATAATGATCCATACCAGTGGATTGGTCAGGATATATTTTACAAAA
ATCTGCCAGAAGCTCAGGTTGACCGGGCTGGCCTTGACCTGGGCCAGTTCTGTCGGGTCGTTACGCCACTGCC
CCACGTTGGGCAAACCTAACGTACCGGGACGATCTTTAATGGTAAATAGCGCCACTATCCCCATCAGCAGCGA
AATGGCGCCGGGAACATAAAACCCCATTTTCCAGCTACCGCTAAAGGCGATAGCAAAGCTGGTCAGCAGCGGC
GCGAGGCTGCCGCCGATATTGTGCGCCGCTTCAACGATAGCCCACCAGCGTCCCCGTTCGTTTTTCGAAAACC
ACGAGGCCATAATTTTCGCCATCGGCGGAAAGCTGGTGCCCTGTAAGATCGCGTTGAGGCTGTACAGTACGTA
GAATGCCGCCACACTATCGGAAAAGCCGAACAGAATATTTACAATCCCCACGCCAATCAGCACCGGGCCGGTC
ATGGCTTTCGGGTTAATTTTATCGACCAGCATCCCGCCGACAAACTTCATCGCGCCATAAAGAATGTAAAAAA
TAGAAGACATAATGGCGAAGTCTTCTGCGGTCAGCGACGTTTCCGTCAGCATGGCCGGCATCGCGGCGGAGAA
GTTTTTACGGGTAAGATAAAAGACGGCATAAGAGATAAAGACCGAGGCGATAATCTCAAAACGAAATTTTTTA
TAGGTCTGGTCTATCTCTTTTTTCTCTTTTACAATCGGCTTATCGGGCTGGCTGAAAATAAATGTTTTTATAC
TCATTTTAAAATTCCCTTCCGGCGAGGTGAGTCGCGTTATAATTCATCTTCAATAAGAAAAAGAAGCAATTCG

AAACCGTGATAATTAATCTGATGGTGGATTTTTTCCCGTACAACCGGCTTCGCTTTCCAGGCAATACCGGTGC
CAGCATGTTCTAACATCGGCAGATCGTTGGCGCCATCACCGCAGGCAATAATATTCTCCGTGGCAATATTTAA
CCGGGCAGCCAGGTCAACCAGAGTCTGTTTTTTGTTTGCGGCATTCATAATCGGCAAGGTAATGTTATCCGTC
AGAACGTTATCGCGTATTTCAACCGTATTCGAAAAGGCATAATCCAGTTGATACCGCGCTTTTAGACGCTGGG
TGAATATATCCAGTCCCCCGGAGATAATCGCGGTTTTAAATCCTTTGGCCTTGATGACTGGCAGAATAGTTAA
CAGGCCTGGCGAAAGGGTCATGCGATCGCATACGGCGTTCAGCACTGCTTTCGGTGTGCCTTTCAACATTCCG
ATACGTCGGGTAAAACTGGCGTTAAAATCAAGCTTGCCCTCCATCGCCTGTTGGGTAATGGCGGTGATTTGCG
TCGACATGCCAAGCTCGCGGGCAATTTCATCCACGCCTTCTTCCGCAATAAATGTCGAGTCCATATCAAACGC
GATAATACCGTTAGCAGGCAGTGGCTGCGGCTTAATAAAAAAATCAAACTGCCATTGCAATGAGAGTTCGCGT
AAATAGCGATAAAATTCAGGCGTCAGACATTCCGCTGGCAGCGTAAGCTTTAAATAAGCGGTATTGATAAGCG
CGGCATAACGTTTATCAAATAATAGCGGAAACGGCATGGTGACGGCATTAAATAACGTTTCCTGTAATTTTTT
CTGAAACGTATCGATATCCTGTGTAGTAATAAAATAAATAATGCTCATATGATAACTCCCTTAATTTACGGGT
ATTATAATGGCTCTTTTGCGGCGTGGAATCTCGTGCTATCACAGATATCACAGCCAGAAAAGGGCTAAAAAAG
CGTTGTTTTTGATACCAGGTAAAGTTCAGACACAAAAGGTATCACTTTTGATATGCCAACTGTGCGAGGATGT
TATTTATTACTGAACGCGAATGACACTGAAAGGATTGATATAACGATGGAGAGTTGCCTTGATATATTCAAAA
TAGTAATAGGTCCATCCAGCTCACGTACCGTAGGGCCGATGCGCGCCGCCTGTCACTTTATTTCATTGCTCAG
AGAACAAGAGACGTTGCCGCTAATTCGGGAAATTGAAATCGAGCTGTATGGCGCGTTATCGTTAAGCCGCAAG
TGCCACAATGTGGATACCGCCCTTTATCTTGGGCTGCTGGGTTGCCAGCCGGAGAATGTCGATCTCCGTTCGC
ATATGGCGGTGATTAAGCGTGCGGAAAATGAAAACAAAATCGAACTCCCGCTCTCCGACGCGGGCGGAATCAC
GATAAAAGTCAAGATTATCGCCAATCACCAGGCGCATCCAGGCCATCCTTATGCCATGACGTTTCGCGCCAGA
GATGATTATTTCACGGTGTATGAAGAGACCTGGTTTTCGACCGGCGCCGGACAGGTTCGCAAACATGGCGAGC
CGCTGACGCCATCGCTTCCTTTGCGTACAGTGTCGCCCTTTGAATTTAGTCATGCCGCGCAGTTGCTGGCGCT
GTGCCGACGTAATGGCCTGTCCGTGGCGGCGCTGATGATGAAAAATGAGCTGTGCCGCCACTCGCCACAGACG
TTGCAGAATTATCTGGCGCAAATATGGGACGTGATGCAGCAGGCGGTTTATCGCGGCCTGCATACTGAGGGGG
TATTACCCGGTCCCTATCAGGTACCGCGGCGCGCCTGTGCGTTACATAAGACGTTACAGGCTAACCGCTCCGC
CAGCGATTTTTTAACCGCGCTGAACTGGGTCAATGCCTTCGCGATTGCGGTAAGCGAAGAAAATGCCAGCGGC
GGACAGATTGTTACCGCGCCAACTAATGGCGCGTGCGGCATCATTCCGGCAGCGCTCTGCTGGTATGATAAAT
TTGTCACGCCGCTGGAGCCCGGTGCTCTGACCCGTTTCTTCCTGACCGCCGCCGCTATCGCGATATTGTTTAA
ACAAAACGCCTCTATTCTGGGATCTGAAGTTGGCTGTCAGGGGGAAATCGGCGTGGCGTGCTCAATGGCGGCG
GCGGGCCTGGCTGAACTGATGGGCGCATCCGTGGAGCAAACGCTGAGCGCCGCTGAAATTGCCATGGAACATC
ATCTGGGATTGACCTGCGATCCGCTGGGCGGGCAGGTACAAATTCCCTGCATAGAGAGAAATGCCATCTCTGC
CGTGAAAGCGATAAACGCGGCGACGATGGCGATGAGTCGCGTAAGCGAACCCTGTATTTCGCTCGATGAGATC
ATTGCTGCGATGTACGAGACCGGTAAAGACATGAGCGCTAAATACCGTGAAACGTATCATGGTTCGCTGGGGA
AAATTCAGCCGCGCAAGAGAGGGTAACAGCGTGAAATACAATACGATGAATAACGACGAGATTATCCTGTCGT
TGTGCGCCCGCTTAAAGGAGACGCGTCTGTCTCTTTCGATGACCCAGCAGCAGCTTGCCGATCGCGCGCACGT
TGGCATCGCCACCATCAAGCGTATCGAGAAGGGGGAGGGCTCAATCTTGATACGCTAATTTCTCTCCTGCGGG
CGCTGGATAAGTTGCATAATCTTGATGCGGTGCTGTTTGAATCCGAGCTGCGTAATTTTCACGAAAGTTATGA
AGGCGGAGAGGGCAGCGGCCGCCTTCAGGTACGCCAGCAGGCCGCGGATTTAAATAATAAATCTTCCGCGCCG
CAGTCGGAGGAGGTGAACTACAGCGCGGCGCTGGAAAATTCATTATGTTGGTGACGTCTCACGCTCGTCCCGT
AAATACCTGGCATGAAAGCGCAGATGGTCCTCGATGAAGGACGCAATAAAATAATAGCTATGATCGTAACCAG
GCTGGATTCGTAGCGTCATCGGCCAGGCGGTTTGCCGCGCGGCTTCCGCCAGTACGGCGGGCTGAAGCTGATC
GGCGAGGAATTGATCGCTGTCGCCCTGATCGATGAGCACAGGAATGGCGTCCTGCGGCTCGCTGGCCAGCATT
AATTCGCAGCTATCCCACTCAGTCCAGGCTGATTCATCTTCGCCGAGATAAGCGGTAAGCGCTTTGATTCCCC
ACGGCACGCGGCTGGGGTTGACGATAGGCGCAAAAGCAGAGACGCTGGTGTATTTGCCCGGATTTTTCAGCGC
CATGATTAATGCGCCGTGGCCGCCCATCGAGTGCCCGCTGATGGCGCAACGGTCGCTGACGTTAAACTGTGTC
TGGATAAGCGCCGGAAGCTCATCGCGCAGGTAATCGTACATGCGATAATGGCTGGCCCAGGGCGGCTGTGTGG
CATTGAGATAAAACCCGGCGCCGTGGCCTAAATCGTATCCGCTATCGTCGGCGACCTGTTCACCGCGTGGACT
GGTATCCGGCATCACCAATACAATGCCCAGTTCTGCTGCGATACGCTGCGCGCCTGCTTTGGTGGTAAAGTTC
TCATCATTGCAGGTCAACCCGGACAACCAGTACAGTACCGGAGGCGGTTCATTATCCTGAGTCGGCGGAAGAA
AAATGCTGAATGTCATGGCGCAGTTTAGCGTGGCGGCATGATGACGCCAGCGCTGCTGCCAGCCGCCAAAACA
TCGGTGCTCTTCGAGCATTTCCATGCGGGGGCTCCTTTTATAGTGAACCTGATAAGGTATGCAGATTCTTCAT
AATACAGATTATTCACCGCGTTGTGAGTAACTTTCACTTCCGTAATACATAAACATGCTTCATCATTTAAATA
ACGTTGCGTTAACAAATATGCCCTTTGTCCTGCCATCATTTTCGCACCGGAACAGATGTCATGAAAGGCAGCG
ACAATTTCAACAATTATGGGCGTGAATACTGGATTATGTGCGCTACCTCACGCACAATAATCACACTGTCACA

ATATTGACAAACTTTGCCCCATGCAGGGCAGAGGCGCCACACCTGCAGGAGAACACGATTAATGCCATCACTC
AGTAAAGAAGCGGCCCTGGTTCATGACGCGCTGGTTGCCCGGGGGCTGGAAACGCCGCTGCGCCCGCCTATGG
ATGAACTGGATAATGAAACGCGAAAAAGCCTTATCGCAGGGCATATGACTGAGATTATGCAGTTGCTGAATCT
CGATTTGAGCGATGACAGTCTGATGGAAACGCCGCATCGTATCGCTAAGATGTACGTTGACGAGATTTTCGCC
GGCCTCGACTATGCCAATTTCCCGAAAATTACCCTCATTGAAAATAAAATGAAGGTGGATGAAATGGTCACCG
TCCGCGATATCACGCTGACCAGCACCTGTGAACATCACTTCGTGACCATTGATGGCAAAGCGACCGTCGCGTA
TATCCCCAAAGATTCCGTGATCGGCCTGTCGAAAATTAACCGCATCGTGCAATTCTTCGCCCAGCGTCCACAG
GTTCAGGAACGTTTGACCCAACAAATTCTGACGGCGCTGCAAACCTTGCTCGGCACCAATAATGTAGCGGTAT
CGATTGATGCGGTGCATTACTGCGTGAAAGCGCGCGGTATTCGTGACGCAACCAGCGCCACGACGACGACGTC
GCTGGGTGGATTGTTTAAGTCCAGCCAGAATACGCGCCAGGAGTTTCTGCGTGCCGTTCGCCATCATCCATGA
TTAAGGCGGAGACGATGGAGCGTAATGTCACGCTGGATTTTGTACGTGGCGTCGCTATCCTCGGTATCCTGCT
TCTTAACATTAGCGCCTTTGGATTGCCGAAGGCGGCTTATCTCAATCCCGCCTGGTACGGCGCGATCGTTCCC
GAAGACGCCTGGTCCTGGGCGATTCTCGGTATTGTGGCGCAGGCTAAGTTTCTTACGCTGTTCGCGTTGTTGT
TTGGCGCGGGGCTACAAATGCTGCTTCCGCGTGGTAAACAGTGGATTCAGTCGCGCTTAACGCTATTGGTACT
GTTAGGTTTTATCCATGCGTTATTCTTTTGGGACGGCGACATTCTGCTGGCCTACGGTCTGGTCGGGTTAATT
TGCTGGCGACTGATACGCGATGCGCCTTCGGTAAAAAGCCTGTTCAATACCGGCATACTCCTTTATCTGGTAG
GGATTGGCGTGCTACTGCTGCTGGGCGTGGTATCGAGTAGCGAAACCAGCCGCGCCTGGACGCCGGATGCGTC
GGCAATATTGTATGAAAAATACTGGAAGCTGAACGGCGGCATGGAGGCTATCAGCAACCGGGCAGAGATGCTG
TCGAATAGTTTACTGGCGCTCGGCGCGCAGTATGGCTGGCAACTGGCGGGTATGATGCTGCTGGGCGCCGCGC
TAATGCGTAGCGGCTGGCTGAAAGGTCAGTACAGTCTGCGGCACTATCGCCGTACCGGGGCTCTGCTGGTGGC
GTTAGGGCTACTGATCAACCTGCCTGCGGTCATTTTACAATGGCGGCTGGACTGGGCGTACCGCTGGTGCGCC
TTTTTATTGCAGGCGCCGCGTGAACTGAGCGCGCCGTTGCAAACGCTGGGCTATGCCGCGCTGATGTTTGGCT
TCTGGCCGCAGCTTAGCCGATGCAGACTGACGCTTGCCATTGCCTGCGTAGGGCGAATGGCGCTGACCAATTA
TCTGCTGCAGACGATCATCTGTACCACGCTGTTTTATCAGTTTGGCTTATTTATGAAATTCAATCGACTGGAA
CTCTTGTTCTTTGTCGTTCCGGTATGGGCAATAAACCTTCTCTTTTCCGTCATCTGGCTACGTTTCTGGCGAC
AGGGACCGGTCGAGTGGTTATGGCGTCAACTCACGTTGCGCGCGTCAGGATCGCTCAGATAACGATCTGGATC
ACATTCATTAACAAAATGGCTGTAACCGTTTCCATCCCTGTGATGTTTCTCACGCAGCCCTGGGCAGGGTGCT
GACAAAATAGCCGCCTGCTAACGGGGCATTTGTGAGCATTTTTGAACTCAAACAGGGCAGTGAATATGATCAC
CATTCGTGATGTAGCGCGCCAGGCTGGCGTGTCTGTAGCGACCGTTTCCCGCGTACTGAATAACAGCGCGTTG
GTCAGTCCCGACACCCGTGACGCCGTTATGCAGGCCGTCACCCTGCTGGGATATCGGCCAAATGCGAATGCGC
AAGCGCTGGCCACCCAGGTGAGCGATACCATCGGCGTCGTGGTCATGGATGTTTCCGATGCCTTTTTCGGCGC
TCTGGTGAAAGCCGTAGATCTGGTCGCGCAGCAGCACCAGAAATATGTTCTCATTGGCAACAGTTATCATGAG
GCGGAAAAAGAGCGCCATGCGATTGAAGTCTTGATCCGTCAGCGTTGTAACGCATTGATTGTTCACTCAAAAG
CCTTAACCGATCGCGAGCTGAGCGACTTTATGGATCAGATCCCCGGTATGGTGCTGATTAACCGTATCGTGCC
GGGTTATGCGCATCGTTGTGTTTGTCTCGACAATGTGAGCGGCGCCAGAATGGCGACCCGAATGTTGCTGAAT
AATGGACATCAACGCATCGGCTACCTGGCCTCCAGCCACCGTATTGAAGATGACGCGATGCGCAGAGAAGGGT
GGTTACACGCGCTGCAAGAGCAGGGGATTGCTGCGTCGGAGAGCTGGATAGGCACCGGCACGCCGGACATGCA
GGGCGGCGAGTCGGCAATGGTTGAGTTGCTGGGACGCAATCTGCAACTGACGGCGGTATTTGCCTATAACGAT
AACATGGCGGCGGGTGCGCTGACGGCGTTAAAAGATAACGGCATCGCCATTCCCTTGCATCTGTCTGTCATCG
GTTTCGACGATATCCCTATTGCTCGTTATACCGATCCTCAGTTGACTACCGTGCGCTATCCTATTGCTTCTAT
GGCGAAAATCGCGACCGAGCTGGCGTTACAGGGGGCTGCAGGCACGCTGGATATCACGGCGACGCACTGCTTC
ATGCCGACCCTGGTACGGCGCCATTCGGTGGCATGGCGACAGAATGCGGTACTGATCACTAACTGATTACGCA
CCGCATGTAACCGTTTTCAATCTGTGAGTAAATTCACAGTTTATTAACATTGTGATAGCTATGATGACAACGT
TTGTCGCACTGTAACTAACGTGTAACAGTTAGTTGTCAGTTTTGCTGGGGTATTTCGCTTATAAAAACCGTTA
TCACAATATCCCGCGACTACCGGACAAAAATAAAGAGTTGAATAAGAGCTTATCCCATTAGGGCTATTTTACT
TGCCATTTTGGACCTGGGCAGTGCTCGCCAAAACGCGTTAGCGTTTTGAACGCCGCTTGCGGCGGCCCGAAGG
GCGAGCGTAGCGAGTCAAACCTCACGTACTACGTGTACGCTCCGGTTTTTGCGCGCTGTCCGTGTCCAAACTG
GCTGCGCCAATAACGCCTGGTGGGATAGGCTCTAAATACGCTTCGGCGTTCAGTAACACGCGTTAACGTGCTG
AACAGCCGGGCATTTTTTTACGCTATACCCTACATAATAAAACCGGAGCTACCATGAATAAGAAGGTACTGAC
CCTTTCTGCCGTGATGGCAAGTCTGTTATTCGGCGCGCACGCGCAGGCGGCTGATACTCGTATTGGCGTGACG
ATTTATAAATATGACGATAACTTTATGTCCGTGGTGCGTAAGGCTATTGAAAAAGATGGCAAATCCGCGCCGG
ATGTTCAGCTACTGATGAATGACTCGCAAAACGATCAATCTAAACAGAATGATCAAATTGACGTTTTATTGGC
GAAAGGGGTTAAAGCTCTGGCGATTAACCTGGTCGACCCGGCAGCCGCCGGTACGGTGATTGAGAAAGCGCGC
GGGCAAAATGTGCCGGTGGTGTTCTTTAACAAAGAGCCTTCCCGCAAAGCGCTGGACAGCTATGACAAGGCGT

ATTATGTCGGGACTGACTCTAAAGAATCCGGTGTGATTCAGGGCGACTTGATTGCCAAACACTGGCAGGCGAA
TCAGGGTTGGGATCTGAATAAAGACGGTAAAATTCAGTATGTTCTGCTGAAAGGCGAGCCGGGGCACCCGGAT
GCTGAAGCTCGTACGACGTATGTGGTGAAAGAGTTAAATGCTAAAGGTATTCAGACCGAGCAACTGGCGTTAG
ACACCGCCATGTGGGATACCGCGCAGGCAAAAGATAAGATGGATGCCTGGCTATCTGGCCCGAACGCTAACAA
GATTGAAGTGGTTATCGCGAATAACGATGCGATGGCGATGGGCGCGGTAGAGGCGCTGAAAGCGCATAATAAA
TCATCGATTCCGGTCTTTGGCGTCGATGCGTTACCGGAAGCTCTGGCGCTGGTGAAATCGGGCGCGATGGCCG
GTACGGTACTGAATGACGCCAACAATCAGGCGAAAGCGACATTCGATCTGGCGAAAAACCTCGCCGAAGGCAA
GGGCGCGGCTGACGGCACCAACTGGAAGATTGAGAACAAAATCGTGCGCGTGCCTTATGTCGGCGTGGACAAA
GACAATCTGAGCGAATTTACCCAAAAATAACTTTTCCGTCTGTGGGCGCAATTTTGTTGCGCCCTGAAACCGT
GGAAGGGTGACGTGCCGCAGACCGGATAACGTATTTCTTTTGCCATCCGGCGCGGATAGTCATAATTAAAAAG
GCCGACAGGGTATCATTATGGGCAGCACGATCTCTCCGTCGTCGGGTGAATATTTGTTGGAAATGCGTGGCAT
TAACAAATCATTTCCCGGCGTGAAAGCGCTTGATAATGTCAACTTAAACGTTCGTCCGCACTCTATTCATGCA
TTAATGGGCGAAAACGGCGCAGGCAAATCAACATTATTAAAATGTCTTTTCGGTATTTATCAAAAAGACTCCG
GCAGCATTGTATTTCAGGGAAAAGAAGTGGACTTCCATTCGGCGAAAGAAGCGCTGGAGAATGGGATTTCGAT
GGTACACCAGGAGTTAAACCTGGTATTACAACGTTCGGTCATGGATAACATGTGGCTGGGGCGTTATCCTACC
AAAGGTATGTTTGTCGACCAGGATAAAATGTACCAGGATACCAAAGCGATATTTGATGAGCTGGATATCGATA
TTGACCCGCGCGCGCGCGTAGGAACGTTATCCGTTTCGCAAATGCAGATGATCGAAATTGCGAAGGCGTTTTC
CTATAACGCTAAGATCGTTATTATGGATGAACCGACCTCATCGTTAACTGAGAAAGAGGTTAATCATCTGTTC
ACCATTATTCGCAAGCTGAAAGAACGCGGCTGCGGCATTGTTTATATCTCGCATAAAATGGAAGAAATTTTTC
AATTGTGCGATGAAATTACTATTTTGCGCGACGGTCAGTGGATTGCCACCCAGCCGCTGGAAGGGCTGGATAT
GGATAAGATCATTGCGATGATGGTCGGGCGTTCCCTGAACCAGCGCTTTCCGGATAAAGAAAATAAGCCGGGC
GACGTGATTCTGGAGGTTCGTCACCTGACCTCGCTGCGCCAGCCTTCCATTCGCGATGTCTCCTTTGATTTGC
ACAAGGGCGAAATTCTGGGCATTGCCGGTCTGGTTGGGGCAAAGCGTACCGATATCGTCGAAACGCTGTTTGG
CATTCGTGAGAAGTCGTCCGGGACAATCACTTTACATGGCAAGAAAATTAATAATCACACTGCGAACGAAGCG
ATTAACCACGGTTTTGCGCTGGTGACGGAAGAACGTCGTTCCACCGGAATTTACGCCTATCTGGATATCGGAT
TTAACTCATTAATTTCGAATATCCGTAATTATAAAAATAAAGTGGGGTTACTGGATAATTCCCGGATGAAAAG
CGACACCCAATGGGTCATTGATTCCATGCGGGTAAAAACGCCGGGGCATCGCACGCAAATTGGTTCGCTTTCC
GGCGGCAATCAGCAAAAGGTGATCATTGGTCGCTGGCTATTAACGCAGCCGGAAATATTAATGCTGGACGAAC
CGACCCGCGGCATTGATGTCGGCGCAAAGTTTGAAATTTATCAGCTTATTGCGGAACTGGCGAAAAAAGGCAA
GGGGATCATCATTATCTCTTCTGAAATGCCGGAGCTGTTAGGGATTACCGATCGTATCCTGGTGATGAGTAAC
GGTCTCGTTTCCGGCATTGTCGATACTAAAACAACAACGCAAAACGAAATTTTGCGTCTTGCTTCTTTGCACC
TTTAAGATCAGGGGCTCCTTATGAGTGCGTTAAACAAGAAAAGCTTTTTGACCTGGCTGAAAGAGGGCGGAAT
TTACGTCGTTCTTTTAGTGTTGCTGGCGATTATTATTTTCCAGGACCCGACTTTTTTAAGTTTGCTGAATTTA
AGTAATATTCTGACGCAATCTTCGGTACGTATTATTATCGCGCTGGGCGTGGCGGGACTCATCGTCACCCAGG
GGACAGACCTGTCGGCGGGGCGTCAGGTAGGGCTGGCGGCGGTCGTGGCGGCAACATTACTGCAATCAATGGA
AAACGCCAACAAAGTGTTTCCGGAAATGGCGACCATGCCGATTGCGCTGGTCATCCTGATTGTCTGCGCGATT
GGCGCGGTGATTGGCCTGGTGAACGGCATCATCATTGCTTACCTGAACGTGACGCCGTTTATTACCACGCTTG
GCACGATGATTATCGTTTATGGGATCAACTCCCTTTACTATGACTTTGTCGGCGCTTCGCCCATTTCCGGCTT
TGACAGCGGCTTTTCCACCTTTGCGCAGGGCTTTGTGGCGATGGGCAGTTTCCGGCTCTCATACATCACCTTT
TACGCTCTGATTGCGGTAGCGTTTGTCTGGGTGCTGTGGAATAAGACCCGTTTTGGTAAAAACATTTTTGCTA
TTGGCGGCAACCCGGAGGCGGCGAAAGTTTCTGGCGTAAACGTGGCGCTGAACCTGCTGATGATTTATGCGCT
CTCCGGCGTGTTTTATGCCTTCGGCGGCTTACTGGAAGCAGGGCGTATTGGTTCTGCCACCAACAACCTCGGC
TTTATGTATGAACTGGATGCGATTGCCGCGTGCGTGGTCGGGGGCGTATCGTTTAGCGGCGGAGTGGGGACGG
TCTTCGGCGTGGTGACCGGCGTCATTATCTTTACCGTCATCAACTATGGCCTGACCTATATAGGGGTAAACCC
GTACTGGCAGTACATTATCAAAGGCGGCATTATCATTTTCGCCGTGGCGCTGGACTCGCTGAAATACGCGCGT
AAGAAGTAGTTATCCAGACCTATTACTTTGCTTATCAGATAACCCGGCGCAGCGAAACTGCGCGCCGGGCTTC
ATACCGTGAGCTACAGCGTTAACGCGTGTTCTTTTTCGCCTTTTTTAAATTTGACCTCGCCTAAATCAATACA
GGCGACCGGGCAGACATGACCGCACAGCAGACAACCGACGCATTTTTCCGTATTGCAATGCGGCGTGCGGCTG
TGTTCATCCCACTCCATCGCCTGATGACCGCCGTCATAGCAGGAGATATAGCAACGTCCGCAGCCGACGCATT
TCTCCTGATTAATCCGCGGATAGACGATATAGCTGCGATCCAGGTCTTCCGCCGGGATAATATTGCCATTCGC
CAGCCCGATCATCTCCTGTAATGAGGCAAATCCCTGATCGGCCAGATAGTGGCTGAGGCCGCTCGCCATATCT
TCTACAATGCGGTAACCGTATTGCATAATGCCTGTCGTCACCTGTAGGGTCGCCGCGCCCAGCAGCAGAAATT
CGGCAGCGTCTTCCCAGGTTTCAATGCCGCCGATACCGCTAATCGGGAAATCGCGCAGTTCAGGGTGCATTCG
CAACTGCTGGATAAAACGCAGCGCAATGGGTTTCACCGCTTTTCCTGAATAACCGGAGATACTGGATTTACCG

TTAACCACCGGCATCCCGATTTTGCGGTTCAGGTCGATGTTAGTAATGGATTTCACGGTGTTGATGGTGGCGA
TACCGTCGGCTCCGCCGCGTTTGGCGGCCAGCGCGACTTCGCACATATCGCCAATATTCGGCGTCATTTTCGC
CAGCATTGGCAAAGATGAACCGCGTTTTACGGCGCGGCAGTATTTTTCAACCAGCTCCGGGCTTTGCCCTACA
TCGCTTCCCATGGCGTGCGAGGTCATCTGCGGGCAAGAGAAGTTACACTCAATCATATCCGCGCCGGCTTCTT
CAACCAGACGCGCCAGTTCCTGCCACTGCTGCTCATTTTCCCCCATGATGGAAGCAATCAGCACCTTATCCGG
ATAATCCTGTTTTAGCCGTCGAATGGCGGCCAGATTCTCTTCCAGCGGATGCTCAGCAATTTGCTCCATGTTT
TTGAAACCAATAAAACCGGTATCTTCTTTCGTCAGGTGATCAAAACGTGGAGAGACTTCGTTGGCAATAAAAA
AACCAATGGTTTTAAAAACGATCCCGCCCCAGCCGGTATCATAGGCTTTGGCGCACATCTCATAACAATTGCC
TACCGGAGAAGAAGAAAGACAAAACGGATTGGGAAACTTAACGCCGCAAAAGGTAACAGACAAATCTTTTGTT
AACATGAGCAGGCCTCCTCTAAATAATGATGAATAGCCTGAGCGGCTTCTTTTCCGGTTTTTACCGCATAAAC
GACGGTCTTATCACCTTTAACAATATCGCCAGCGGCAAAGATCGCCGGGTCGTCGGTTTGATAATTATGCGTG
TCGATAATATTATGCTGCGCTTTTATCTCCGCAAAGGTATCCAGTCGCGCGTGCTGCCCCACGGCCAAAATAA
TATTTTCCGCTTCCAGCGTCAGTTCTCCTGAGTGGCGTACATGGTGGAAGGTCACTTTATTTCCGCTGACGGC
GACAGGCGTAAAGCCATCAATAATCGATACGCCTAATGCCTGCGTGCTGGTAAATTCTTTTTCGCTGGCGGGA
AATTGGGCTAATTCTTCCCGCGCCACGCAGGTGACGGAAGGGCAGCCGAGAATTTTTAACGTACTGGCGACAT
CCATCGCTACGTCGCCGCCGCCAATAATTAATGCACTACGCGGAACGCTAATATCGCCGTCGGCCTGACGGGC
GCGCTGTAAAAAATCAACGGCAATCTCTACGTGACTGGCTTGCTCAAATAACGGTAAATCAGAGCCACAGGAC
ATCCCGACGGTCATCAGCACGGCGCGATATTCGGCTTTGAGTTGCGCAAGTGATAGCGACCCGCCCACGTCAC
AATTACATTTAATATTGACGCCCATTTCTACAATGCGGGCAATTTCCTGATCGAGTACGCTTTGCGGCAGACG
AAATGCCGGGATGCCGTGACGCAGCCATCCGCCGGGCTGCGGCTGTTTTTCGTAAATGGTGACGTCATAGCCT
AAATGTGTGAGCGTCACGCTTGCCTGCAAACCTGCCGGACCCGCGCCAATAATCGCGACTTTCCCGCGTGTTT
TACTCCCGGGCTGATAAATCTGCATGGCAGTCTGCTGTTCAAAATCGGTAATAAAACGTTGCAGGCGTGCAAT
ATCAATAGGTTTATCTATTCCGGAACGTGTACATCCGCGCTGGCATAATTTTTCCGTCGGACAGACTCTGGCG
CAGACAGCACCAAGCGCATTATTTTCCCGGATAGTTTCCGCTGCGCCTTTAAAATTGCGAAAGTAAATAGAAC
GAATAAATTTCCCCGGATCGGTTTGCGCCGGGCAATCCTGACTACAGGGCGCATCGTGACATAATAAACAACG
TGAAGCCTCTTTTATTGCCAGTAATGGCGTAAAACCAGGCGTGAGTTCATCCAGATAATTTTGTTGCGGCATA
TAATCTTCCTTAATGCTTGAATAAATTAAGGCGAAACGCAAATAACGCGTTTTAATGATCTAAAATCTACCGC
CAGCCTAAGCATTGAAAGAAGGGTATTCTGTGAAAGAATTCACAAAAAAATGAAACCAGAATGCAAAACAGCA
TTTGTTCCTTATATCATAATAATAACTATTCTCAATTTTAAAGGGATAATAAATAGATTAACGATAAAAAAGA
AGATAAATAATCATTCCAGTAAATAAAAAAACCGCAACCGGTATAAAGAAAAAGATAAGACCAATATTGTACGG
GATCGGCATGGCGATAGACATAAACCGGTTTTCGCGCATTTTTGACAACTTTATCTACCGCGCCGGTTCGCCA
GCCTTTCCAGGCCTCACGGAAGCAAAAAAGCGGGAGTAGCAGAAAGGCTAATATGACAAATATCGTCTGTACA
TCCATGATCATTTCCCCTTTTTTCTTTTCCAGTTCAAGCAATTGTTCCGGCGTAACTGCCGGATACCCCTGGG
CGTCATTCGGCACCTGATGCTGCGTGGGAATAGGTACCAGCGGGCCTAAAAAACGCGGTTCGCGTTTAAAAAT
ATAGAGATCCGCCAGCGCGCTAAAGCGGGCGCCAAATTCGCGCAGGCGCACTGTCAGCATATTTTTGGGCGAA
GGTACCGCGTAACACTGCGCCTGAATGCCCATATGCAGCGCAATAAACAAGGCGCGTTCGCAGTGGAAGCGCT
GAGTGATGATAATAAAGTCGTTGGTATCAAACACTTTGCGGGTACGCACAATGGAGTCAAGCGTACGGAAGCC
AGCGTAATCAAGCACAATGTCCGCAGGATCGACGCCCGCCGCGATAAGGTCTTTGCGCATGGTCATCGGTTCG
TTATAGCTTTGCAGGGCATTGTCGCCGCTGAGCAGCAGGTAATTGACTTTACCGCTATTGTAAGCGTTTAACG
CGCCCTGGATACGATAACGATAGTACTGATTAATGACACCCTTGCGATAGTATTTGGCTGTCCCTAACACGAC
GCCAACCTGGCGGTAGGGGAGATCCTGTAGCTCGTCATAGATATAGGGCGCTGTTTTCCAGCTCATCCACCGA
TCGAGGCCAAGCACCGTCAACAGCAGCAAGCCTACCAGGACCAACAGGCTGTAAAACACGCGCTTTAACATGC
AGGAAACTCGATACCAGGATGAAAGTACGATCAGGCTACTGTACCCGAAGACGTAGCGCAAGAAACCGCCGTC
TGATTGCGGAGATTTTCAGCATGATTTCCCTTGTATCGAGTCGCGGGAGAGGAGAGTCAGGCCGGATAAGACG
CCAGTGCGTCGCTATCCGGCACATTTGCTTAGCCGAGAAGGACGCGGTCAATATTATGGCAACCTAACGCTTT
CAGCGTTGCGGCGGTGGCATCCCATTGAATTAATGCTGCATCGCCTTTTTCCGCCAGAATCGCGCGCTGAATA
TCCGCATAGTCATAGCCATTGAGACTCAGCAGGTTTAGCGCGCCTTGCAGCGGGGGAAGCGTAGGGTTAAAGG
CGGCGTTTTCCGCATAGCTGCCGGTGAAAATACGGCCATCTTTACATTCCAGCGCCACGCCGCTCGGTGAGTG
GCTATAAGGCATATGGCTCTTATTGGCTGCCGTTATCGCCGCTTGCGTTAGCGTATCGCCCGTTAACGTAAAG
CCATGATCCTGCTCGTCCATCAGCAGCGTTTTGATCTCCAGATCTTTTGGCCCGAAAGCGTCAGGCAGATAAT
CGCGTAACGTATGCGGCGCGCGGCCCGGCAGATGAATGCGCAGATCCAGACCGCTGTTCAGCTCGTTCATAAA
CTGGCGACAGTGGCCGCAAGGCGTGTAGTTAACGGTGACGGCGGCGAGCCCTTTTTTCGCCGCGTAGCCAGGC
GTGGCTAATCGCGCTTTGTTCAGCATGCACAGTTTGTTGCATGGTGGCGCCCAGGAACTCCATATTCGCGCCG
AAATACCAGTTGCCGCTGACGCCGCGCGCAATCGCGCCGACGTTAAAATGGGACAAATCGGTACGGGCGCAGG

CCGCCGCCAGAGGTAACAGTGCGAAAGCCAGCGCGTCTTCGTCCAGTCCCGCCGTATTTTTAAGCGTCGAGAC
CTGCTCTGCGGTCAGCATGGCGGGAAAATGGTGGTCCGCCAGGATAGGGGCCAGCGCTGATTGCAAATTATCC
GCAAGCTGGGCAAAAGCAGTTTGAAAACGTGGATGCATGGCGTTGCCTCATAACAATGTAATGGAACCGTAGT
TTACGGGCCTGCTATGTCTTAATATGTGATCTAAATCTCATTGCTTATGCAACTTGTGAAACTTAAATGAAAA
ATGCGCGACGTATCGCAAATTTTAACCCGGTCTTTTGTCCCGTCGTTAGCGCATTACCGCCAGAATGAGCGGA
AACAAAAAGGGCGCTACCAGCGAGGTAATAATGCCGCAGATCACCAGCGCCAGGGAGCTAAACGCGCCTTCCT
GGTAATCCAGCTCGGCGCAGCGCGCAGTACCGAGCGCATGTGAGGCGGTCCCCATTGCCAGCCCGCGTGCGGC
CTTAGTACGGATGTGCATCGCGTTTAACAGCGTGTGGCCAAACACCGCGCCCAGAATACCGACAAAAATTACG
CATACCGCGCTGATCGCGGGGATGCCGCCGATGCTGCCGCCCACCGCCATCGCGATAGGCGTAGTCACGGATT
TAGGCAGTACCGATGCCGCGATTTCAGGCGTTGCGCCCATCAGCAAGGCAACGGATGTCCCGGTAATCATCGC
TACCAGACTGCCGACAAAACAGATGCTAATAATGGATTTCCAACGCGCGCGAATCTGGTGCAATTGTTCATAA
AGGGGATAGGCCAGTGCCACTACCGCCGGTTGCAGCAGATCGTTAAGTACTTCGCTGCCTTTGAAGTAGTGCT
CGTAGGGAATACCGGTCAGCAACAGAAAAGGAATAATGACTACCATTGCCACCAGCAGCGGATTCAGCAACGG
CATTTTAAAATGCGCCGCCAGTCGGCGGGCGGCGAAAAATACCGCCAGCGTCAGCGGTAACGACCACCAGATA
TATGTCATCATTTTTTCGTTCCCTTCTGCCCAACCACTTTACGCTCACCGTGTATCAGATGTGAACTCCAGCT
TACGACCACAAAAACGACCAGCGTACTGATAGCGCAAGAGACCACCACCGGGCCGAATTGCGCGCGCAGTAAG
TCAAAATATTGCATGACCCCAACGCCAATCGGCACAAACAGCAACGCCATATAACGAATCAGCACATAACAGC
CGGGGTTAACCCACTTTGCCGGCAGAATTTGCAGCGCCAGCAATACAAACAGAATTAACATCCCGATAATGCT
GCCGGGAATAGTGATAGGAAGCAGTGATGCAAGAAAAATGCCTGCGTACAAACAGGCATAAATCAATACAAAA
GCGCGTATATATTGCCAGATAATATTCAGTGACTTACTCATGTTTAAAGCCCTTGATGAGATGGATTCATCAT
ACAATTAAACGTTAAAACGTGCTACCGATCACATCATGAATTCTTAGCATAGTGGGTATAGACAGCAGGAACA
TCGGGCAATGCCCCTGGTCGGCGGCGTTAAACCTGGGCTAAAAAATACAAAAAGCGACTCAGGGCCAAAGACG
GCGTCTCATTCTTACGCCAGAACATCCCTACGCTGCCTTTTTGCTCAATTTTTGGCAAGTTTAAAATCGCCAG
TTGCCCCTGGCTGGCATAGCGTTGCGCCAGCCGTAAAGAAAGAATAGAGATCATATCGCTGCTTTGCAGCAAA
TTGGTGCTGACGTTCATTGACGCCGATTCAATGGTGTTTTCCGGGAGCATGACGCCATTATCGACGAGGGCGT
TATCAATACTGATGCGAATAGGGGTGCCGGTTGGCCAGACTATCCACCGCCAGTGCGCCAGATCGCTCCAGGC
GATCTGCGCTCGCGCGGCCAGCGGATGGTGCGGACGCGCCACAAAGCAGACCGGCTCGGTATAAAGTACCTGA
TAATTAAGCGGCAGTTGCAGGGCGCGGCCGCCGACACGCCCTACCACCACATCCACCTGACCGGCAAGCAAAT
CGTGCAGCAGCGGCGTCATTACCTTCTCTTCAATATTCAGATGCAGCGTCGGCATTTCGTTAAGCAGGCTCAG
AATCGCCTGTGATACGCAGTCAGTCGCCACGGGCGAACAGCCGATCTTCAGGCTGCCAACCAGGCCGCCCTGC
TTGAAACGGGCGATTTCATACTGTGAACGCTCCAGATCGTTAATTAGGCGCTGGGCGTGCTGGAGCAGTAATT
TACCGCCTTCCGAGGGGCGTAGTCCTTTACTGTGGCGCTCAAAAAGCGTGATGCCTATCTCATCTTCGAGTTG
AGATAGCCACTTCGATAGCGCCGGCTGGGAGATATTCATCATCCGGGCGACGTGCGTCAGGTTGCCCTGTTCG
CCCAGCGCAACCAGCGTTTGCAGGTGATGTAATTTCAATTTCTGCGCCCAGTTCGCCATTCGCTATAACCTCC
AGGTTATGTCTCTGCCTGAAAAGCCATTGTACATTTTATCGCTCACTCTACAAACTCGTAACATAGCAAACAT
AAAAACAACATCTATCTCCCTGCACTGAGGCATTAGCTATGACTCAACGACGTGATCTACAAGCCCTTATTGA
TGCCGCGCCCGTCGGCAAAATGCAGTGGCGCGTTATCATCTGCTGTTTTCTGGTGGTTATGCTCGACGGTTTT
GACACCGCCGCGATTGGCTTCATCGCCCCGGATATTCGTACTCACTGGCAGCTAAGCGCCAGCGAACTTGCGC
CGCTGTTTGGCGCAGGGCTGCTGGGGCTTACGGCCGGCGCGCTGCTATGCGGGCCGCTGGCGGATCGTTTTGG
CCGCAAGCGGGTCATTGAGCTTTGCGTGGCGCTATTCGGCGCATTGAGCCTGCTTTCCGCTTTCTCGCCGGAT
ATAGAAACCCTGGTGTTGCTGCGCTTCTTAACCGGTCTGGGACTGGGCGGAGCGATGCCGAATACCATCACCA
TGACGTCGGAATACCTTCCCGCTCGTCGACGCGGAGCGCTGGTCACGCTGATGTTCTGCGGTTTTACCCTGGG
GTCGGCGATGGGCGGGATTGTGAGCGCGCAACTGGTGCCGCTGATTGGCTGGCACGGAATTCTGGCGCTAGGC
GGCATCTTGCCTTTGATGCTGTTTTTCGGCCTGCTGTTCGCGCTGCCGGAATCTCCCCGCTGGCAGGTACGCC
GCCAACTACCGCAAGCCGTTGTCGCCCGGACGGTCAGCGCCATTACCGGCGAGCGCTATCACGATACGCAATT
CTTTCTGCATGAGACGGCAGCCGTCGCCAAAGGCAGTATTCGCCAGCTTTTTGCCGGGCGACAGCTTGTCATT
ACCCTGATGTTATGGGTGGTGTTCTTTATGAGCCTGCTCATTATCTATCTGCTTTCCAGCTGGATGCCGACGT
TACTTAACCATCGCGGTATTGATCTGCAACAGGCGTCGTGGGTGACTGCCGCATTCCAGGTTGGCGGCACGCT
TGGCGCGCTGTTACTCGGCGTGTTGATGGATCGGCTTAACCCGTTCCGGGTACTGGCGGTGAGCTATGCGCTG
GGCGCGGTTTGCATTGTCATGATAGGCCTGAGCGAAAACGGCCTTTGGCTGATGGCGCTGGCGATTTTTGGTA
CCGGCATCGGTATTAGCGGTTCACAGGTCGGGCTTAATGCTCTGACGGCGACGCTGTACCCCACCCAAAGCCG
GGCGACGGGCGTGAGCTGGTCGAACGCCATTGGACGCTGCGGGGCGATTGTCGGTTCGCTCTCCGGCGGCATG
ATGATGGCCCTCAATTTCTCTTTCGATACGTTGTTTTTTGTCATTGCTATTCCGGCGGCTATCAGCGCGGTAA
TGCTTACCCTGCTGACGGTGGTTGTCCGCCTTTCGATTTCTGTACCTGACGACCTGCCGCGTGCCAGCGTCGT

AAACGAATAAGGAGAATCTCCATGTATGAAATAAATCAGAACGTAAAAGATAGCCGTCAGCAGTATTACCAGC
ATATTTCCGGGCAGAATCTGACGCCGCTGTGGGAATCGTTACATCACCTGGTACCGCAGACGCCAAACGCCAA
CTGCGCGCCGGCCTACTGGAATTATCAGGAAATTCGTCCGCTACTGATGGAAAGCGGCAATGTCATTGGCGCG
AAAGAGGCGATCCGCCGGGTGCTGGTGCTGGAAAATCCGGCATTGCGCGGTCAGTCGTCGATCACGGCGACCT
TATACGCTGGTTTACAGCTGATCCTGCCCGGCGAAGTCGCGCCGAGTCATCGCCATAACCAGTCGGCGCTGCG
TTTTATCGTCGAAGGTAAAGGCGCATTTACCGCGGTGGACGGCGAGCGCACGCCAATGCATACCGGCGATTTT
ATCCTGACGCCGCAGTGGCGCTGGCACGATCATGGTAATCCGGGGTCAGAGCCGGTGGTATGGCTGGATGGTC
TGGATCTGCCGTTAGTCAACCTCCTGGGCTGTGGGTTTGCGGAAGACTATCCCGAAGATCAGCAGCCGGTGAC
GCGAAAAGAGGGCGATTATCTGCCGCGCTATGCAGCGAATATGCTGCCGCTGCGCCACCAGCGCGGGAACTCG
TCGCCGATTTTCAACTACCGTTACGACCGCAGTCGCGAGGCGTTGCACGATCTGACCCGTATGGGCGATCCGG
ATGAGTGGGAAGGCTACAAGCTGCGTTACGTTAATCCCGTCACCGGCGGTTATCCGATGCCGTCGATGGGCGC
GTTCCTGCAACTGCTGCCAAAAGGCTTTGCCTCGCGTGTGGCGCGGAGCACCGACAGCACTATCTACCACGTC
GTTGAAGGGGCAGGGCAGGTCACTATCGGCAACGAAACTTTTCATTTTTCCGCAAAAGACATTTTTGTGGCGC
CGACCTGGCATGAGGTGTCGTTTCGCAGCAGCGAAGACACGGTGTTATTCAGTTTTTCGGACAAGCCGGTTCA
GGAAGCCCTGGGGCTGTTCCGCGAAGCACGTTATTAATTAGGGAGAGAGAACATGACCAAATATGTATTCCAG
CCGCAGGCTCCGGTAACAGTACCCGTGGCGGGCAGTGATGAGCAGTTTCCGGTACGTCGCGTTTACTGCGTGG
GTCGCAACTATGCCGCCCATGCCCGTGAAATGGGCTTTGATCCCGACCGCGAACCGCCGTTCTTCTTTTGTAA
ACCAGCGGACGCTGTCGTCCCGGTAGCGGCGGGGGATACCCTAGAACTGCCGTATCCGGCGCAGACTGATAAC
TACCATTATGAAATTGAGCTGGTGGTGGCGATTGGTAAAAAAGGGAGCGATATTCCGCTGGAAAAAGCCCATG
AATATGTCTGGGGATACGCCACCGGTCTGGACATGACGCGTCGCGATCGTCAGATGGAAATGCGTCAGATGGG
ACGCCCGTGGGAAATCGGCAAAGCGTTCGATCTCTCCGCGCCCATTGCGCCATTACACAAAGCCGCTGAAACG
CATAATGTGGATAACGCCCCTATTTGGCTACAGGTTAACGGCGAAGACCATCAGCGCAGCGATATTCGTCACC
TGATTTGGTCGGTGAATGAAACCATCAGCTATTTGTCCGGCTTTTTTGAATTGCAGCCAGGCGATTTGATTTT
CACCGGTACGCCAGAGGGGGTGGGCGCCGTGGTGAAAGGGGATGTCATCACCGGGAACGTAGAAGGTCTGACG
CCGATTGCTGTGAAAATTGTCTGAGGTGAACGATGAAGCTGTACAGTTTCTTTAATAGTTCGGCGTCTTATCG
TGTACGTATTGCGCTGGCGTTAAAGGGGATTGATTACCAGACGGTGGGCGTCAATATTCGTATCGGCCAGCAG
AATGCGCTGGCCTACCGACGGATGAATCCGGTAGGCCTGGTGCCGACGCTGGTCACTGATGACGGCGAATCGT
TGGGGCAATCGCTGGCCATCGTCGACTGGCTGGACCGACATTTTCCGCAAACGCCGCTGCTGCCGACGAGCGA
CCCGGCGCGCAGCCAGGCGCTGGAAATTGTCTACGCTATCGCCTGCGATATACACCCAATCAATAACATGCGC
GTGTTGCGCTACCTGACCGATGAGCTGAAGGTTAGCGAAGAGGATAAAAAACGCTGGTACGCCCACTGGATAC
AGCAAGGGTTAAGCGCGGTAGAGCAGCTATTGCGTAAAAGTCAGTCCGGACGATTCTGCGTGGGCGACGCGCC
AGGACTTGCGGACTGCTGCCTGATTCCTCAGTGGGCGAATGCGCTCAGGATGGGCTGCGATTTATCAGGCTAT
CCGCGCTGTAAAGCCGTTTATGACGCCTGCGTACAGCTACCGGCGTTTATTGCCGCCGCGCCGGAAAATCAAC
AAGATAAAATCCCGGCCTGAAAAGGAGAATGACCATGACTCACGTGACAAGCGCAATTATTGTAGGTGGCGGA
ATTGGCGGCGCGGCGACCGCGCTGTCCCTGGCGCGCCAGGGAATAAAAGTCATGCTGTTGGAAAAAGCGCACG
AAATTGGCGAGATTGGCGCGGGTATTCAGCTTGGGCCGAACGCTTTTTCGGCGCTGGATAGTCTCGGTGTGGG
CGATGTTGCCCGCCAGCGTGCGGTGTTTACCGATCACATTACCATGATGGATGCCGTGAATGCCGAAGAAGTG
GTATGCATTGAAACCGGACAGGCGTTCCGCGACCATTTTGGCGGGCCGTATGCGGTAATTCACCGGGTAGATA
TTCATGCAACGGTATGGGAAGCGGTGCTGACGCACCCTGGCGTGGAGTATCGTACCTCGACCCATATTGTCGA
TATCCGCCAGACGCCGGATGACGTTACGGTGTTTGATGAGCAGGGAAATAGCTGGACGGCGGATATTCTCGTG
GGCTGCGACGGCGTTAAATCGGTCGTGCGGCAAAGTTTACTCGGCGATGCGCCGCGCGTAACGGGGCATGTAG
TTTATCGGGCGGTGATTGATTGCGACGATATGCCGGACGATTTACGTATTAACGCACCGGTGCTGTGGGCAGG
CCCGCATTGTCACCTGGTCCATTATCCGCTCCGTGGAGGTCAACAGTACAATCTGGTGGTGACATTCCACAGC
CGCCAGCAAGAAGAGTGGGGCGTGAAAGACGGCAGTAAAGAAGAGGTGCTCTCTTATTTTGCCGGCATTCATC
CCCGCCCGCGCCAGATGCTGGATAAGCCGACCTCCTGGCGCCGCTGGTCGACCGCCGATCGCGAGCCGGTCGC
GAAATGGGGAACCGAGCGTATCACTCTGGTGGGCGACGCCGCGCATCCGGTGGCGCAATATATGGCCCAGGGC
GCCTGTATGGCGCTGGAAGACGCCGTCACGCTGGGCAAAGCGCTGGAACGCTGCGACGGCGACGCGCAGCAGG
CATTTGCGCTGTATGAGTCGGTGCGTATTCCCCGCACGGCGCGTATCGTCTGGTCTACCCGCGAGATGGGACG
TCTCTATCACGCCGCCGGGGTGGAGCGTCAGGTGCGTAATCTGCTGTGGAAAGGGAAATCGCAGGAGGCGTTT
TATCGCGGTATCGAATGGCTGTACGGCTGGAAAGAAGATAACTGTCTTGAGCCGCGTTGAGCGCCTTACGTGA
CGTAGCGTAGCGTTTGCGCCGCCACCCGGCAAAAAGTTTTGCTCATCGCAGAGAGGCGTTATGATAGCGCCTC
TTTTTTTGCTGTGGATACCGATATGCGTGTTTTACTGGCGCCGATGGAAGGCGTGCTCGACGCGTTAGTGCGC
GAGCTGCTGACCGAAGTGAATGATTACGATCTCTGCATCACCGAATTTGTGCGCGTGGTGGATCAGCTGCTGC
CGGTAAAAGTGTTTCATCGCATCTGCCCGGAGTTGCATCACGCCAGCCGCACGCCGTCCGGCACGCCGGTGCG

TATTCAGCTTCTGGGCCAGCATCCGCAGTGGCTGGCGGAAAACGCCGCGCGGGCGACGGCGTTGGGATCGTAT
GGCGTGGACCTGAACTGCGGCTGTCCGTCAAAAGTGGTGAACGGCAGCGGCGGCGGCGCGACATTGCTCAAAG
ATCCCGAACTCATCTATCAGGGCGCGAAAGCGATGCGGGCCGCGGTACCGTCGCATCTGCCGGTGACGGTAAA
AGTGCGTCTCGGCTGGGATAGCGGCGATAGAAAATTTGAAATCGCCGATGCGGTGCAGCAGGCCGGCGCCAGT
GAACTGGTGGTGCATGGTCGTACCAAAGCGCAGGGCTACCGCGCCGAGCATATCGACTGGCAGGCGATCGGCG
AAATACGCCAGCGTTTGACTATTCCGGTTATCGCGAATGGCGAAATCTGGGACTGGCAGAGCGCGCAGGCATG
TATGGCGACCAGCGGCTGCGATGCGGTGATGATTGGCCGTGGGGCGTTAAATATTCCTAACCTGAGTCGGGTG
GTGAAGTATAACGAACCGCGTATGCCGTGGCCGGAAGTGGTGACGTTATTACAAAAATATACCCGACTGGAAA
AGCAGGGCGATACCGGTTTATACCATGTCGCGCGTATTAAACAGTGGTTGGGATATTTACGTAAGGAATATAT
TGAGGCGATAGAACTTTTTCAGTCGATTCGGGCGTTAAACCGTTCGTCCGAGATTGCGCGGGCGATTCAGGCT
ATTAAAATCTAATCTCTACAGCGTCCAGAACGTGTGCCGGGCAGATACTATCGCTGCCCGGCGCAGGAATCAG
AAAATCATCTTAAACACGCCCGTTACCACCAACAGCCCAATCAGAAAAATAATTAAGATTGCCCATAGAAGAA
TTTTCATTGTTTGTCCCTTTATTGGTAAATACAAACAAAGTATAGGAAAGTATTAAGCCATCTGCTGCCTTGT
TGGGCGCGGCGACGTTTAACTCATGAAGGCTGCTGGCGCGTATTGCAGCAAAATCGTTCATTTATGAACGGGT
TAGTGGGACTTTTCATTGAAGTATTACATCAAAAAATGTATCAGATGAAATTATTTGCTAATCATATAAATTT
CAAAATATGTCTTTTGTTGTCTGATGATGTATTGCCACGCGTAACTAAAAAATAGATCGCCGTAAGACGTACT
CTCTGCGCACCTTTTTTTATCAGAACACCTCCATGAATCGTAATTCTTTCCTTGCCGCGACAGCGAGCTTACC
GCTTTTTATTTTGCTGGCGGGTTGCGCCCCCATGCACGATACCCGCCAGTCGCTGACCCAGCAGACGCCCTCT
TCTCATGTTGATTCCAGCCTGCCGGCGGCATTGAAAAACGGTTGGCCGGATAGCCAGTGGTGGAAGGCGTATC
ACGATGCGCAGCTTGACGCGCTGATCGATAATGCCATACAGCATTCGCCGGATATGCAGGTGGCTGAACAGCG
TATTCAACTGGCGGAAGCGCAGGCGAAAGCGGTTGAGGCGCAGGACGGCCCGCAGCTCGACTTTTCTGCCGAC
ATTGAGCGCCAGCGAATGTCCGCGGAAGGGCTGATGGGGCCATTCGCGATTACCGACCCCGCGGCGGGAACGA
CCGGGCCGTGGTATACCAACGGTACATTCGGCCTGACGGCGGGCTGGGATCTGGATTTATGGGGGAAAAACCG
CGCTGAAGTTACCGCGCGGATCGGGGCGGTCAAAGCGCGCGAAGCGGAGCAGGAGCAGACCCGACAATTGCTG
GCAAGCGGCGTGGCGCGACTCTACTGGGAGTGGCAAACACAGGCAGCGCTGAAAAATGTGCTGATGCAAATCG
AGCATGAGCAGCAAAACGTGGTGGCGGTAAACCGGGAGTTGTATCAACACGGTATTACTTCTTCTGTTGAAGG
GGTCGAGACCGATATCGACGCCAGCAAAACCCAGCAGCAGTTGAATGACGTTAACGGTAAAATGAAGGTTATT
GAAGCGCGGCTCAGCGCGTTAACCAATACGCAATCGGCGGCGCTCAAGCTGCGTCAGGTGAGTCTCCCCGCCG
TTGAAAGCCAGTTACCGTCGCAACTGGGGTATTCGTTGCTGGCGCGCCGGGCGGATCTACAGGCCGCGCACTG
GTACATTGAATCCTCGTTGAGCAGCATTGACGCCGCGAAGGCCGCGTTTTATCCCGATATCAATCTGATGGCT
TTTTTACAGCAGGATGCGTTACATCTGAGCGATCTGTTCCGCCATTCCGCACAGCAATATGGCATTACCGGCG
GGCTGACGCTGCCGATTTTCGACAGCGGCAGGCTGAATGCCAACCTCGATATCGCCAAAGCGCAAAGCAACCT
TTCTATCGCCAACTACAACAAAGCGGTGGTAGATGCGGTTAATGATGTGGCGCGCGCCGCCAGTCAGGTAGAG
ACGCTGGCGCAGAAAAATCAGCATCAGCAGCAGATTGAGCATGACGCGCAGCGTGTGGTCGGTCTGGCGCAAG
CGCGCTTTAATGCCGGCATTATTGCGGGTTCCCGCGTGAGCGAGGCCAAAATCCCGGCACTGCGCGAGCAGTG
TAACGGCTTGCTGTTGCAGGGCCAGTGGCTGGATGCCTCTATTCAGCTTACCAGCGCGCTGGGCGGCGGTTAT
CATTCCTGATTTTTCCGGATGATTCAGACAGGGTTCCTATACTTAGGCTGTGTATGAATCATCCGGAGAGGAA
TATGACCAAAGTCGCAATTGTAACGGCATCAGATTCGGGAATTGGCAAAGCGTGTGCGTTGTTATTAGCACAA
AACGGTTTTGATATTGGTATCACCTGGCATTCCGATGAACGCGGCGCGCAGGAGACGGCGAAAAAAGCGGCGC
AGTTTGGTGTGCGGGCAGAGACCATTCATCTTGACCTGAGCCAGTTACCCGAGGGCGCGCAGGTCATTGAACA
TCTTATCCAGCGCCTGGGGCGCGTTGACGTGCTGGTCAATAATGCCGGGGCGATGACCAAATCCGCGTTTATT
GATATGCCGTTTACGCAGTGGCGGCAAATTTTTACCGTGGATGTCGATGGCGCGTTTCTCTGCGCGCAGATCG
CCGCCCGGCATATGATTAAACAAGGAGAGGGAGGGCGCATTATCAACATCACCTCCGTCCATGAGCATACGCC
GCTTCCGCAAGCCAGCGCCTATACCGCGGCGAAACATGCGTTGGGGGGATTGACTAAATCCATGGCGCTGGAA
CTGATTGAGCATCATATTTTGGTCAACGCCGTCGCGCCAGGCGCTATCGCCACGCCGATGAATGATATGGATG
ACAGCGACATTGAGCCGGGATCGGAACCCTCGATCCCTATCGCCCGACCGGGTTCTACGCATGAAATAGCCAG
CCTCGTCGCCTGGCTGTGTTCGGAAGGAGCAAGCTATACCACCGGTCAGTCGCTGATTGTCGACGGCGGGTTT
ATGCTGGCGAATCCGCAATTCAACGCCAAATAAACGCCGGGTGCCGGATGGCGGCGTTATGCCATCCGGATAC
CATCGGGTTTAACGGGCCTCCGCTTTGCCGCGTCTTTTCAGCCACCAGCGCACGCCCACCACCAGGACGATGG
CCAGAATCAGCCAGACCCCGTGCCTGAGATGCTGATCGAGGTCGTGCAGCCATGGCGCGATCACCTCGCCGCC
AAGATATCCCAGCGTGGTAAACAGCAGCGCCCAGATCAGCGCGCCTACAATATTCAGCGGCAGAAAAATTTTA
GGCGGCAGGCGGCTGGCGCCAATCAGCAGCGGGCCAACCACCCGGAAGCCGTACATAAAACGCGTGCCGATCA
CAAACAGATACGGATGGCGTTGGATCATTTTCTGCGCGCGACGAATCTTCGTATGATAACGCGGGAAACGACG
CAGGATCTTCCCGCCATAGCATCGTCCTAACAGGTAAAGTAGCTGATCGCCCATCATCCCTCCCAGCGCGACG

GCCGCCGCCACCAGCGGAAACTTGAGTAATCCCTGATGCGCCGCGACGCCCCCCAACAGGGTAACGGTTTCAC
CTTCGGCCATACTGCCGATAACCAGCGCCGCATAGCCATAATGCGTAATAAGCGTGTTGATATCCATGCGTAA
ACCACCTCCGGCATTAAGCATACACCCTGGCAAGAGGCGTGCGCGGCAAAGCCGACGTTTTGCCGGAGACTAT
TTTGCCATTGTTCATAAAATAACCAGAAACTGAATTATACTTGAACATACTGGCATCTGAGGCAGCAGAAGGG
GGCAGTATGAACCATGTATGGGGGCTTTTTCCCATCCCGATCGGGAAATGCAGGTGATCAAAAGCGAAAACGA
AACCGTTTCGCATCACTATACGCACCATGTTTTGCTGATGGCGGCGATTCCCGTCGTCTGCGCCTTTATCGGC
ACGACGCAAATCGGCTGGAATTTTGGCGACGGCAACGTGTTACAACTCTCGCTGTTTACCGCTTTTGCGCTGG
CGGTCTTGTTCTACGGCGTGATGCTGGCGGGCGTCGCGGTGATGGGACGCGTCATCTGGTGGATGGCGCGAAA
CTATCCGCAACGGCCATCGCTTGCGCGCTGTATGGTTTTTGCCGGCTATGTCGCGACGCCGCTGTTTTTAAGT
GGTCTGGTGGCGCTCTATCCCCTGGTATGGCTATGCGCACTGGTCGGCGCTGTCGCGCTCTTTTACACCGGCT
ATTTGCTGTATTTGGGTATTCCGACTTTCCTTAACATTAATAGAGAGGAAGGATTAAGCTTCTCCAGTTCGAC
GCTCGCTATCGGCGTACTGGTCCTGGAGGTGCTACTGGCGATCACCGTGATCCTGTGGGGATACGGCTACCGC
CTGTTCTGATGCGAAAAAAGGGCTGGCGCAAAAGCCAGCAATGCAGCATTTGCTGACGATTCTTCTGTAGCGG
CAACGCTGGCGTGCCGCTATGATGCCACTGCTAGCTTGAGTATAATCTTAGCTCAGGCGGTGTGCGTTACGAC
GCGCGTGAATCATTATCAGAAATCTCATCATGCTGAAATTCCGAGCTTCATTCCTGAGCCTTGCTCTGATGTT
GGCCGTTCCTTTTGCCCCGCAGGCTGTGGCGAAAACGGCAGCGACAACCGCGGCGTCTCAACCTGAGATTGCC
TCCGGTAGCGCAATGATTGTTGATCTCAACACCAATAAAGTGATCTATTCGAACCACCCGGATCTGGTGCGTC
CGATTGCCTCGATAACCAAATTAATGACAGCGATGGTCGTGCTCGACGCGCGATTGCCGCTGGATGAAATACT
GAAGGTCGATATCAGCCAGACGCCGGAAATGAAAGGGGTTTACTCCCGCGTGCGCCTGAACAGCGAAATTAGC
CGCAAAAATATGTTGCTGCTGGCGTTGATGTCATCCGAAAACCGTGCGGCGGCAAGCCTGGCGCACTATTATC
CTGGCGGTTATAACGCGTTTATTAAAGCGATGAATGCGAAGGCGAAAGCGCTGGGCATGACGCATACCCGCTT
CGTTGAGCCGACGGGGCTGTCGATTCATAACGTCTCGACCGCGCGGGATTTAACAAAATTGCTGATTGCCACG
AAGCAATACCCGCTGATTGGCCAGCTAAGCACCACGCGCGAAGACATGGCGACATTCTCGCATCCGGCGTATA
CGCTGCCGTTTCGCAATACCAACCATTTGGTCTACCGTGATAACTGGAATATCCAGTTAACGAAAACCGGCTT
TACTAATGCGGCGGGGCATTGCCTGGTGATGCGTACCGTGATCAATAACAAACCGGTCGCGCTGGTGGTCATG
GACGCGTTCGGCAAATATACTCACTTTGCGGACGCCAGTCGCCTGCGTACATGGATTGAAACCGGTAAAGTCA
TGCCCGTTCCGGCTGCCGCGCTGAGCTATAAAAAGCAAAAAGCCGCGCAAATGGCGGCAGCCAGCGCCAGCGC
CGGAGCGCAAACCGCACAGAACGATTAAGTCGCCGCAGGCCCGGCAGGCATCGCGCCGCCGGGCATTCACGGT
AAACCACTCGGTGTTAATGCGTTTCGTCGGGCGTATTTTCCTGCCAGAATTTCCGCTTACTCGTTTTGCCGAT
ACCGGGGTTCATACTGTTGGTTGGGTCATTTTGCCGATAAAAACGCGTCAGCGTTTCCGGCGCTTTATACAGA
TGTCCAACGTTGTGCTCCGCCGGGTATTGTGCCCCCCGCGCCTGCAACAGCTCCAGCATTTGCGCTTTCAGCG
CGTGGACGTCGACGCCTTTCTTCACGATATAGTCCTGATGAAAAACGTGGCACATAAAGTGGCCGTAATAGAG
TTTATGTACCAGTTGGCTGTCGATTTCCGGCGGCAGATGCTCGAACCAGTCGGTATCGTTGCGGCGCAGAGCG
ATATCCAGCGCCAGGATATCTTCCACTTCATCAGCGTGTACGGCCTGATAACGAATCGCCGCGCCAGCAGCGG
CGAAGCGGTGCAGGAAGGCTTTGCTGCCTTCTTCCGGCGTACAGGTGAAGAAACCGCCCTCAGCGGATTTAAA
AAATTCATTCAGCCAGCGTTGCGCTTCTGCGACGCCGTCTCCCGCCATTTTTAACAGCAGATGATGCTCATAT
TTATCGCGCCAGTTTTTCATTCGCGGCGGCAGATGGCTGGGAAACAGATGGCCGAATTTCTGCATCGCGCGGT
CGGTGAAATGCGGACGGAAAAATTTTACTTTTTCCAGCATCGCATCGGTTCGGCCTTTTAGCGTGAAAAAGAA
TGGCATCTTGTCGGTGCCGAGTTTATCGATCATCAGAAAGGTATCTTTACCGTACTGCTCCGCAATATCGTAG
ATATCGCGATGCATATATTCTCCCGCCACTGGCAGATTGTCGAAGTTCGCCAGAATATGCCGACGGATTTCAG
TCAGTACCGCGGGCTGATTGGTGCCGATATAAAAAACCTGCTGATTTTTTTCCGCTGCAAAGGTATCAAGGCG
AACGGCAAAAACCGCCAGTTTTCCGGCGCAGCCGGAAGATTCAAACAAACGGTCCGGGTCGGCGTTATAGCGG
GCGGGCGTATCGGCGTTGATATCTCGTACCCGCGTCACGTAGTCGTGATCGTGCGCATGACGACCATCGTGGC
GCACATCCTCATCTTTAATCCGTTCGTCATCCAGCTTGCTCAGGATCTGCTCCGGCGTATGCCCAAGATCGAT
ACCCAGATGATTGACCAGTTGCAGTTTGCCTTGGTCGTCAATACGGGCAAAAAGCGACATCTCGGTATAGGCC
GGGCCGCGCTGCACCAGCGAGCCGCCGGAGTTATTACAGATACCGCCGATGACCGACGCGCCGATGCACGACG
AACCAATCACCGAGTGCGGTTCACGGCCCAGCGGTTTAAGCGCTTTTTCCAGCGCATAGAGCGTCGTACCGGG
CCAGGCCAGAACCTGTTGGCCGTTATCCAGTAAGTGCAGCTTATCCAGTCGCTGGGTGCTGATAATCACAATA
TCGCGATCGTAGTCGTTGCCGTTCGGCGTGGAGCCTTCCGTCAGACCGGTGTTGGCGGCCTGCATCAGAATAA
TTTTGTCAGCGTTAACGCAGGCGTTGAGCACGCGCCACAGTTCCAGTAACGAACCCGGGAAAACAACGGCCAG
CGCGTCGCCCTGACCGGAACGGAAACCCTTGCGATAGCGGGCGGTTTTGGCGGGGTCTGTCAGCAGGTGAGAA
TGGCCTACCAGGCGGGCCAGCTCATTCAGGAACGTTTTATTATCAGTCATTGTTATGGAAGACATTCTCCACT
CCTTGTGGTGGGACAAAATATCAGCGTTAAGCATAGCGCTTGGTATGACAATACGCTTTCTCATTCAAATTGT
CTCTGTGTTGGTGGTATTCATTTGCCGCCTTGATACCATTTGCTGGCGGTTGCGTATTCGACTGTGTGGAAAA

TTAGGAGAATAACCTTCGCTTTCTTCTCCTCTCGCGTTTAGGCTTATGGCAAACTGCGGTTTTTATTCTGTTT
TTACCGCCTCGTCGGGATAAAGATAAAGAGAGACTCAACATGAAATGGTTATGTTCAGTAGGTGTTGCTGTGA
GTCTGGCGATGCAGCCAGCATTGGCGGAAAACCTGTTTGGCAATCACCCGCTGACGCCCGAAGCGCGCGACGC
GTTCGTCACGGATTTACTCAAGAAAATGACTGTCGATGAAAAAATTGGTCAGTTACGTCTGATTAGCGTCGGC
CCTGATAACCCGAAAGAAGCGATTCGCGAGATGATCAAAGACGGGCAGGTAGGGGCGATTTTCAATACCGTCA
CCCGCCAGGATATCCGTCAAATGCAGGATCAGGTGATGGCATTGAGCCGCCTGAAAATTCCTCTCTTTTTTGC
CTATGACGTGGTACACGGCCAGCGAACCGTCTTCCCGATAAGCCTCGGTCTGGCTTCTTCTTTTAATCTGGAT
GCGGTAAGAACCGTAGGACGCGTATCGGCGTATGAAGCGGCGGACGACGGTCTGAATATGACCTGGGCGCCGA
TGGTGGATGTCTCCCGCGATCCGCGTTGGGGACGCGCCTCCGAAGGCTTTGGCGAAGATACATATTTAACCTC
TATCATGGGCGAAACCATGGTGAAAGCGATGCAGGGCAAAAGCCCGGCGGACCGCTATTCGGTGATGACCAGC
GTGAAACATTTTGCCGCCTATGGCGCCGTTGAAGGCGGTAAAGAATACAACACCGTGGATATGAGTTCCCAGC
GTCTGTTTAATGACTACATGCCGCCGTACAAAGCGGGGCTGGATGCGGGGAGCGGCGCGGTGATGGTGGCGCT
GAACTCGCTGAACGGCACGCCAGCGACGTCAGATTCCTGGCTATTGAAAGATGTCCTGCGCGATGAGTGGGGC
TTTAAGGGAATTACCGTTTCCGACCACGGCGCTATTAAAGAACTGATTAAACACGGTACCGCGGCCGACCCGG
AAGATGCGGTGCGCGTGGCGCTAAAGGCAGGGGTGGATATGAGCATGGCGGATGAGTATTACAGCAAATATCT
GCCGGGTCTGATTAAGTCGGGCAAAGTGACGATGGCGGAACTGGACGACGCGACGCGCCATGTGCTCAACGTA
AAATATGACATGGGATTGTTTAACGACCCCTATAGCCATCTGGGACCGAAAGAGAGCGACCCGGTGGACACCA
ATGCCGAAAGCCGCCTGCATCGTAAAGAGGCACGGGAAGTGGCGCGGGAAAGTGTGGTACTGCTGAAAAACCG
GCTGGAAACGCTGCCGCTGAAAAAATCGGGCACCATTGCCGTTGTTGGGCCGCTGGCCGACAGTCAACGTGAT
GTAATGGGAAGCTGGTCTGCTGCTGGGGTCGCGAATCAGTCAGTGACGGTGCTGGCGGGTATTCAAAATGCCG
TCGACGACGGGGCGAAGATCCTCTATGCCAAAGGGGCGAATATCACTAACGATAAAGATATCGTCGATTTCTT
AAACCTGTATGAAGAGGCGGTGAAAATCGATCCGCGCTCACCGCAGGCGATGATTGATGAGGCGGTGCAGGCG
GCGAAACAGGCCGATGTAGTGGTCGCAGTGGTAGGCGAGTCGCAGGGGATGGCGCATGAAGCCTCCAGTCGTA
CCAATATCACCATTCCGCAGAGTCAGCGCGACTTAATCACCGCTCTGAAAGCCACCGGTAAGCCGCTGGTACT
GGTATTAATGAACGGCAGACCGCTGGCGCTGGTAAAAGAAGATCAACAGGCCGATGCGATTCTGGAGACCTGG
TTTGCCGGGACGGAAGGCGGCAACGCCATTGCCGATGTGCTGTTTGGCGATTACAACCCGTCAGGCAAACTGC
CGATCTCCTTCCCGCGTTCGGTGGGACAAATTCCGGTCTATTACAGCCATCTCAATACGGGACGCCCGTATAA
TCCTGAAAAGCCAAACAAATACACCTCACGCTATTTTGACGAAGCGAATGGCCCGCTCTATCCGTTTGGTTAC
GGTCTGAGCTACACCACATTTACGGTGTCTGACGTCACGCTCTCCTCACCGACAATGCAACGCGACGGCAAAG
TCACGGCCAGCGTAGAGGTCACGAACACCGGTAAACGCGAAGGGGCCACGGTCATCCAGATGTACTTACAGGA
TGTCACGGCGTCGATGAGCCGCCCGGTGAAGCAATTAAAAGGCTTTGAAAAAATCACCCTCAAGCCCGGCGAA
CGTAAAACCGTGAGTTTCCCGATTGATATCGAGGCGTTGAAGTTCTGGAATCAGCAGATGAAATATGACGCGG
AACCCGGCAAGTTCAACGTCTTTATCGGCGTGGATTCCGCTCGCGTTAAACAAGGATCGTTCGAGCTACTGTA
AGCCTGCCGTCAACAGGGCGCGATAGCTGCGCCCTGTCTGGCAATGACTTTTTCTGTCCCCCTTTTATTGCCT
GCCACGTCACTTAAACTGCCGGAAAATCCCAGGTTAAACGCAGACTTTTCAACTATGCTTTTTTCTCAAGTCT
CTGAAAAAGGCCGTAAAAGGATGAGGAAAGCATCATGACGATCTCAAAGCTATGGGTAAGTTCGCTGGCGTTG
CTGGCGACGGTGAGTTTACCTCTACAGGCGGCATCGCCCGTGACGGTCGGGTCAAAGATTGATACCGAAGGCG
CACTGCTCGGCAACATGATTTTGCAGGTACTGGAAAGCCACGGCGTTAAAACGGTCAATAAAATTCAGCTCGG
GACGACGCCAGTCGTTCGTGGCGCGATTACCGCCGGAGAGCTGGATATCTATCCGGAATACACCGGTAACGGG
GCGTTCTTTTTTAAAGATGAAAACGATCCGGCATGGAAAAATGCAAAGCAAGGCTTTGAGAAAGTCAAAAAAC
TGGATGCGGAGCAGAATAAGCTGGTCTGGTTGACGCCTGCGCCGGCAAATAACACCTGGACCATCGCGGTACG
CCAGGACATCGCGGAAAAAAATAAACTCTCCTCGCTTGCCGATCTCAGCCGTTATCTGAAAGAGGGCGGCACT
TTTAAACTGGCGGCGTCTGCGGAATTTATCGAACGGGCGGATGCGCTGCCGGCATTTGAAAAAGCCTATGATT
TTACCTTGAATCAGAACCAGCTCTTGTCGCTGGCGGGCGGCGATACGGCGGTCACGATCAAAGCCGCCGCGCA
ACAAACATCCGGCGTTAACGCCGCGATGGCGTATGGCACCGACGGCCCTGTCGCCGCGCTGGGTCTGCAAACC
CTGAGCGACCCGAAAGGCGTCCAGCCTATCTATGCGCCCGCGCCGGTAGTGCGTGAATCGGTGTTACAGGCCT
ATCCGCAGATAGCCGACTGGCTGCAGCCCGTTTTCGCCAGCCTTGATGAGAAAACGCTGCAACAGTTGAACGC
CAGAATCGCCGTAGAAGGGCTGGATGCGAAAAAAGTCGCCGCAGATTACCTCCGACAAAAAGGGTGGGTGAAG
TAATCTGTGAGTGATGCTGCCAAAAAAATCACGGTTAATCGTGTGCTGTTGCTGTTGGTTGTTCTGACCCTGC
TTGCTGTCGCGCTGCCTTTCATTAATTACGCCCCGAATCGTCTGGTGAGCGGCGAGGGGCGCCAGTTATGGGA
GATCTGGCCGGCGACCATCTGGATGCTGACTGGCGCGGGATGCGCGCTGTTTACGTTGTGCTTTGTACCCGGT
AAACGCGGTAGCGTACTGACGCTGATGATGGCGCAGACGCTTTTTATTGTCATGCTGTGGGGAGTCGGCAGGG
TGGCGACCCAATTGGCGCAGGAGGGCAGCCCGCTGGCGCGTACCAGTCTGGGAAGTGGATTGTGGCTGGGCCT
CGGTCTGATGCTGCTGGCCTGTAGCGATGCGATCCGCCGTATTACCACTGGGCCGCTGTGGCGCTGGTTGCTG

CACGCGCAAATTGTCATTGTCCCGCTGGTGCTGCTGTTTTCAGGCACGTTCGACAACCTCTCGCTTCTGAAAG
AGTATACCAACCGCCAGGATGTTTTCGATGCCGCGCTGGTGCAACATTTGATGTTACTGGCGGGCACGGTGCT
GCCGGCGTTGGCGATCGGACTACCGTTGGGCGTCTGGTGTTATTTTTCCGCCTCACGCCAGGGGCCGGTGTTT
ACCGTGCTGAATGTGATCCAGACTATCCCTTCCGTCGCGCTGTTTGGCCTGCTGATTGCGCCGTTGGCCGGGC
TGGTGAAACAGTTTCCGTGGCTTGCCGAATTCGGCGTCGCGGGAACCGGCATGACGCCTGCACTGATAGCGCT
GGTGCTCTATGCGTTATTGCCGCTGGTGCGCGGCGTGGTCGCCGGGCTCAATCAGGTGCCGCGCGATGTGCTG
GAGAGCGCAAGAGCAATGGGAATGAGTAGCGGCCAGCGCTTCAGGCATGTGCAACTGCCGCTGGCGCTGCCGG
TCTTTTTACGTAGCCTGCGGGTAGTAATGGTTCAGACAGTGGGAATGGCCGTCGTCGCCGCGCTGATTGGCGC
GGGAGGTTTCGGCGCGCTGGTGTTTCAGGGGCTATTGAGCAGCGCCATTGATTTAGTCCTGTTGGGCGTCATT
CCGGTGATTGCGCTGGCGGTAGTGATCGACGCGCTGTTTGATTTATGGATCGCGTTTTTGAAGGGAGCAACCG
ATGATTGAATTTAACCATGTCAGTAAAACCTTCGGCGATCAACAGGCTGTTAGCGACCTCAATTTGCACTTTA
GCGAAGGCAGCTTTTCGGTGTTAATTGGCACCTCCGGTTCGGGAAAATCGACCACTCTGAAGATGATTAACCG
GCTGGTAGAGCATGATAGCGGAACGATCCGTTTTGCCGGGGAAGAGATCCGCAGCCTGCCGGTGCTTGAACTG
CGCCGTCGCATGGGCTATGCCATTCAGTCTATCGGTCTTTTTCCCCACTGGACGGTGGCGCAAAATATCGCCA
CCGTACCGCAACTACAAAAGTGGTCGCGTGCGCGGATTAACGATCGTATTGATGAACTGATGGCATTATTGGG
TCTGGAAAGCGCGCTGCGCGATCGTTATCCGCATCAGCTTTCCGGCGGGCAACAGCAGCGGGTCGGCGTTGCG
CGGGCGCTGGCTGCCGATCCGCAGGTATTGCTGATGGACGAGCCTTTCGGCGCGCTTGATCCGGTAACGCGCG
GCGCATTGCAGCAGGAGATGACCCGCATTCATCAGCTACTGGGGCGCACCATCGTACTGGTGACGCACGACAT
CGACGAGGCGCTACGCCTCGCCGACCATCTGGTGCTGATGGACGGGGGCAATGTTATCCAACAGGGATCGCCA
CTTTCTATGCTGACCTCGCCGGAAAATGATTTCGTGCAGGCGTTTTTTGGCCGCAGCGAGCTGGGCGTAAGGC
TGCTTTCGTTACGTAGCGTAGGCGATTATGTACGTCGGCATGAACAGCTCAGCGGCGACGCGCTGGTGGAAGA
GATGACGCTACGCGATGCGCTATCGATGTTTGTCGCCCGTCGGTGCGACGTCCTGCCGGTGGCGAATCAGCAG
GGCGAGCCCTGCGGTACGCTCCATTTCCGCGATCTACTTTCGGAGACGTCCCCCCGTGAAACGACTGTGTGAT
CCGCTTCTCTGGCTTATTGTTCTGTTCTTGCTTCTGCTGTTTGGATTGCCTTATAGCCAGCCGTTCTTCGCCG
CGCTGTTTCCCGATTTACCGCGCCCGGTCTACCAACAGGAGAGTTTTGCCGCCCTCGCGCTCGCCCATTTCTG
GTTGGTGGGCATCTCAAGTCTGTTTGCCGTCGTGGTGGGCGTCGGCGCAGGGATTGCGGTCACGCGAGAAAGT
GGGAAGGAGTTTCGTCCCCTGGTGGAGACTATCGCCGCCGTCGGGCAGACCTTTCCCCCGGTCGCGGTACTGG
CGATCGCGGTACCCGTCATGGGTTTTGGTCAGCAACCAGCCATTATCGCCTTGATCCTGTATGGAGTGTTGCC
CATCCTGCAGGCGACCCTGGCCGGGCTGGGCGCGGTGCCTGCCAGCGTGATGAGCGTTGCCAGCGGTATGGGA
ATGAGCCGTCGCCAACAGTTGTATCAGGTTGAACTGCCGCTGGCCGCGCCGGTGATTCTGGCGGGCATCCGAA
CCTCGGTGATTATCAATATTGGTACGGCGACCATCGCTTCAACAGTGGGGGCCAGTACGTTAGGCACGCCGAT
CATTATCGGGCTTAGCGGCTTTAATACGGCCTATGTTATCCAGGGGGCGCTGCTGGTGGCGCTGGCGGCGATC
ATTATCGATCGCCTGTTTGAAAGGCTGACGCGCGCGCTTACCCGGCACGCAAAATAAAACTGTAACCTGCCAG
CATCACGCCGCCGATACCGCCAATAGCCATCAGCAGGAAAAGGGCGATCACCCCGATTTTCGCTACGCGCATT
ATGTACTCCTTATGTTAATAAAAGGAGTATACATTAAAGCGAATTTGTTAGCTGCTGTTTAAACGCCAAGGGG
ATGAATGTCGCGTCCCTGGGCGCGCCATGCCAGGAGTTGCTGCTGCTGCGCCAGCGTCTGGTTTTCTCCGCAC
CATACCAGTAACGTCTTGCCGTCAAACAGTTCCGGGCGGAACTGGCTAAGCGAATGCGCCAGCACGTCGATTC
GCCATCCCTGTTGGCTGGCGACCCAACCTTCCAGCCACAGGCGGGTGGTATCATGGATATTCCAGCCGATCAC
CAACGCATCTTTTCCCTGTTTCTTACGCGCAGACGCCAGGCAGAGCGCAATATAGTTGATCAGGATACCGTCA
AGAATGCCGAGCAGCGCCTGAAGGGCAGGTTGTTGGCACTGTAATCGTCGCCGCAGCGGGACGAACAGGTTAG
TGGTCAATGTTTGGGCTGGATAATCCTGACAGCGTTCTTTGACCCATAACCGTAAACTGTGCAGATTACTGCT
TTGCAGGTAGTGCAGCAGGATCTCCTGCTGTTCGCGCCAGCCGTTAGGTTGTTCGCTACTGTCGCTACTGAGC
AGCACTTTGACTTTGCTGACCTGGACGCCGTTATTTATCCAGCGCTTGATTTCGCGGATTCTGTCGATATCGG
CATCGTTAAACAGACGATGACCGCCATCCGTTCGCTGTGGTTTTAAAAGTCCATAACGTCTCTGCCACGCGCG
CAACGTGACAGGATTGATATCACAAAGCAAAGCCACTTCACCAATTGTGTAAAGCGCCATCGTTTCACCCTTG
CTCGCGAGGTCCCGGTTTAACTTTAGACGCTCTTTTACGAACCAGGAAGTTTTGCCTGTTTTTTATGCATTAA
AACGCGAAGTAGCGGGTTGCGGCGGGGTGTTTAAGTGATCGTATTCACGAATTCATATTTTTATGCAACAGTT
CAAAGAAAGTTAATCGTACTCAATGTATGTTACGCGCTTTTAATTGAAGTGTGGTTTGCGGGTATGTACGAGT
TTAATCTGGTGTTGCTGCTGCTTCAGCAGATGTGCGTGTTTCTGGTCATTGCGTGGCTAATGAGTAAAACGCG
TCTGTTCATCCCGCTTATGCAGGTCACGGTTCGTCTGCCGCACAAGCTTCTGTGTTACGTCACGTTTTCTATC
TTCTGCATCATGGGCACTTATTTTGGGCTGCATATCGAAGATTCGATTGCCAATACCCGCGCAATTGGCGCGG
TGATGGGCGGCCTACTCGGCGGGCCGGTCGTCGGCGGGCTGGTCGGTCTGACCGGTGGGTTACATCGGTATTC
TATGGGCGGCATGACGGCGCTGAGCTGTATGATTTCCACCATCGTCGAAGGGCTGCTGGGCGGGTTGGTACAC
AGCGTTCTCATACGTCGCGGACGCCCGGACAAAGTGTTTAGCCCGCTGACGGCGGGAGCAATTACGTGTATTG

CCGAACTGGTGCAGATGCTGATCATTTTACTGATAGCCAGGCCGTTTGACGATGCCTTGCATCTGGTCAGTAA
TATTGCCGCGCCGATGATGGTGACGAATACCGTTGGCGCCGCGCTGTTCATGCGTATTTTGCTCGATAAGCGC
GCCATGTTCGAAAAATATACTTCGGCATTTTCTGCTACCGCGCTGAAGGTCGCCCGCGTCAACGGAGGGGATTC
TGCGTCAGGGATTTAACGAAGTGAACAGTATGAAGGTGGCGCAGGTGTTATATCAGGAGCTGGATATTGGCGC
CGTCGCCATCACCGATCGCGAAAAACTGCTGGCTTTTACTGGTATTGGCGACGATCACCATCTACCGGGCAAA
CCCATTTCATCAGGTTATACGCTGAAAGCAATTGAAACCGGAGAGGTGGTTTATGCCGATGGCAACGAAGTGC
CGTATCGCTGTTCGCTACACCCGCAGTGTAAACTCGGCTCGACGCTGGTGATCCCGCTGCGTGGCGAAAATCA
GCGAGTCATGGGCACCATTAAATTGTACGAAGCGAAAAACCGGCTGTTTAGCTCAATTAACCGCACCCTGGGA
GAGGGTATTGCGCAGCTTTTATCCGCGCAGATCCTGGCCGGGCAGTATGAACGGCAGAAGGCGTTGCTGACGC
AGTCAGAGATCAAGCTGTTGCACGCGCAGGTGAACCCGCATTTTCTGTTTAACGCGCTCAATACCATTAAAGC
GGTGATTCGCCGCGACAGCGAACAGGCCAGCCAACTGGTGCAGTACTTGTCGACCTTTTTTCGCAAAAATTTA
AAACGCCCGTCGGAAATCGTCACGCTGGCGGATGAAATTGAACACGTAAACGCTTATCTGCAAATTGAAAAAG
CGCGTTTTCAGTCGCGTCTGCAGGTACAGCTTGATGTTCCATCGACGCTTTCACGTCAGAAATTGCCTGCGTT
TACATTACAGCCGATTGTTGAGAACGCCATTAAACATGGCACGTCGCAACTGCTTGATACCGGCAACGTCGCT
ATTCGCGCCCGGCGCGAAGGGCAGCATTTGATGTTAGATATTGAGGATAATGCGGGACTGTATCAGCCTTCCG
CCGGCAGTAGCGGGCTGGGGATGAGTCTGGTTGATAAACGTCTGCGCGAACACTTTGGCGATGATTATGGTAT
TAGCGTGGCCTGCGAGCCGGACTGTTTTACCCGAATTACATTACGACTTCCACTGGAGGAGGACGCATGATTA
AAGTGCTGATTGTGGATGATGAGCCGTTAGCGCGGGAAAATCTGCGGATTTTGCTCCAGGGGCAGGATGACAT
TGAGATTGTGGGAGAGTGCGCGAACGCGGTAGAAGCGATTGGCGCGGTACATAAGTTGCGACCTGATGTGCTG
TTTCTGGATATTCAGATGCCGCGTATCAGTGGACTGGAGATGGTAGGAATGCTTGATCCGGAACACCGCCCGT
ATATCGTTTTTTTAACCGCGTTTGACGAATACGCCATCAAAGCCTTTGAAGAACACGCTTTTGATTATCTGCT
CAAGCCGATAGAGGAGAAACGGCTGGAAAAAACGTTACATCGTCTGCGTCAGGAGCGCAGTAAACAGGATGTT
TCGTTGTTGCCGGAAAACCAGCAGGCGCTTAAATTCATTCCCTGTACCGGACACAGCCGGATCTATTTGTTGC
AAATGGATGATGTCGCCTTTGTCAGTAGCCGTATGAGCGGCGTTTATGTGACCAGCAGTGAAGGGAAAGAGGG
GTTTACCGAGCTGACGCTGCGCACGCTGGAAAGCCGGACGCCGCTACTGCGTTGTCATCGTCAGTTTCTGGTG
AATATGGCCCATTTGCAGGAAATTCGGCTGGAGGATAATGGGCAGGCAGAGCTGATTTTACGCAACGGCCTGA
CGGTGCCGGTAAGCCGTCGCTATCTGAAAAGTTTAAAAGAGGCGATTGGCCTGTAAAAGACTGTTAGAATATC
GTTTTGCCATAGAAACGACCGAAGGCCTCATGCTGAGTAACGATATTCTGCGTAGCGTGCGCTACATTTTAAA
AGCTAATAATACCGATCTGGCGCGTATCCTGGCGCTGGGTAACGTTGATGCTACGCCGGAGCAGATAGCAATC
TGGTTGCGCAAAGAAGAGGAAGAGGGGTTTCAGCGTTGCCCGGATATCGTGTTGTCCTCATTTCTCAATGGCC
TCATTTATGAAAAACGCGGCAAAGATGAGGCGGCGCCTGCATTGACGGCGGAACGTCGTATCAACAACAATAT
TGTGCTGAAAAAGCTGCGTATTGCCTTTTCGCTAAAAACAGATGATATCCTGGCGATACTTACCGGTCAGTTG
TTTCGTGTCTCAATGTCAGAGATCACCGCGATGATGCGCGCGCCGGACCATAAGAACTTCCGCGAATGCGGCG
ATCAGTTTATGCGTTATTTTCTGCGCGGTCTGGCGGCCCGTGAACACGCGGCGAAGTAATTCTGCGGTATTGT
TCCCGGCAGCGTCCTGTCTGACCGGGAAAACGCATTATTATACTAATTGATTCTATGATACCCGCTCTCTTCC
AACAGTTTCTGCGAGCGAATCATTGACAGATAGTACGCGGAACAGTTGTCAATTGATGATCCTGGCAATTTAC
AGAGGTCGCTTATTTTTGCCTGGGTAAAATCAATATCCACATATTCCGTAGCATAGCTATCATAATAGTCGAT
TCGTTCAGTCAAACCCGGCATACCCTGATAAGCTTCGCCGACTTGACTCAGCATTTTTTGTGCTTCTTCTTTA
TTATTGGCTTTCAGGGTCTTATAAAGTAGTTTATGTTCAGATATTTGACGTAAAACGATATCCCCTTTGTAGT
AATAGGTTAACTTGATTTCAATACCGTTGAGATTACCTACATAGCGTTGTGTTTCCTCTGACTCTTTGCTAGC
GGCTATCTTCTTGATAAACGCTGTCATGTTATTTTGCTTTCCCTGGAGAGTATCGTTTTTCTGATCGCAGCCA
GTTATGCTAACCGATAGAGAGAGCGCGAATAGTGGCAGTGCCATAAGACGTAGAACCTGCATAACAATTCCTT
GTCGTTAAGTATTGGTGTGGCCAGGAATTCAGGGATTATAGGCTTTGGCGAGGGGACTTACAGCGAGGCTGTC
TTTTTTCGGAATTCATAAAGAAAAGACGCTGCCGAAGCAGCGCCCTGAGCGACTTTACCAGTCGATGCAATAC
ATTATGCCTGCCAGTTATTTCGCTTCTTTAAAACCAGCAGCTTCCAGCAGCGTCTGGGTTTGTTTCATGCTGA
TACCTTTGCTGGTATCGCCGGACACCATCGTCCCTGAGATTTGCTGTAACGCTTTAAAGTCCACTTTTTCCAT
ATCCACAGAGACGTTTTCCTGGGCATAGGTATCTTCATAGGTTAATTTTTCTTCCACTCCGGCGATATTTTTA
TATTTCGCGCTCAGCGGATCGAGAATTTTGGCGGCATCTTCTTTCGTTTTAGCGCCTACAGTGGCATAGCTGA
TTTTACTTTCAGACGTCTGCTTAATGATTTTGTCACCTTTATAGGTGTAAGTAATTGAAATTTCTGTCCCCGC
CAGGTTTGCGTTAAAGGTCTTTGATTCTTCTTTATCGCCACAGCCAGCAAGAGAGAACACCAGTACGGAAGCC
AAAGCCGTGGACAATAACTTGCCAGAAATTTTCATCTAAAACTCCATTTTATATAATAATTGGGCTTTTAAAA
TAATTTCAATGAATTAATTTAACCCAGTAATAGCAATGTATCAGGGAGAGATAGAATATGACTTTTAGCCGTT
ATTTAGCAGTCCGGATATGGAGTCTTAGCGCTATTGCTTATTAAGGAAAAAGTTAAAACGTGCGGAGGAGGCG
ATATGCCAGTCAGGATTAAGCGGTTAAAAAAGCCGGAGCATGCTCCGGCTTGTTGCTTATTTCACCTGTTGGC

CAGGCTTCGCGCCGTCATCAGGGCTTAACAGGAAGATATCTTTCCCGCCAGGGCCTGCGGCCATCACCATTCC
CTCGGAGACGCCAAAGCGCATTTTGCGCGGCGCGAGGTTGGCGACCATTACCGTCTGGCGGCCGATCAGCGCC
TGCGGGTCCGGGTAGGCGGAACGAATGCCGGAGAAGACGTTACGCTTCTCGCCGCCCAGATCCAGCGTCAGAC
GCAGCAATTTGTCGGAGCCTTCCACGAACTCAGCGTTTTCAATCAATGCTACGCGCAGGTCAATTTTGGCGAA
ATCGTCAAAGGTGATGGTTTCCTGAATCGGGAAGTCGGCTAACGGGCCGGTAACCGGTGCGGCTGCGGCTTTC
ACCTCTTCTTTAGACGCTTCAACCAGCGCTTCAACTTGCTTCATGTCGATGCGATTGTAGAGCGCCTTAAAGG
TGTTGACCTTGTGACTGAGCAGCGGCTGTTCGATGGCATCCCAGTTCAGTTCGCTGTTCAGGAAGGCTTCAAC
GCGTTCAGAAAGCGTCGGCAGTACCGGTTTCAGATACGTCATCAGCACGCGGAACAGGTTGATGCCCATCGAG
CAAATGGCCTGCAGGTCAGCGTCGCGGCCTTCCTGTTTAGCCACCACCCACGGCGCTTGCTCGTCAACATAAC
GGTTAGCGACGTCGGCCAGCGCCATAATCTCACGGATAGCTTTGCCGAATTCACGGCTTTCCCATGCTTCGCC
AATCACCGCAGCGGCGTCAGTAAAGGTTTTGTACAATTGCGGATCGGCCAGTTCAGCCGCCAGCACGCCGTCG
AAACGCTTATTGATAAAACCGGCGTTACGGGATGCCAGGTTGACTACTTTATTGACGATATCGGCATTGACGC
GCTGGACAAAGTCTTCCAGGTTCAGGTCGATGTCATCAATGCGTGAAGAAAGCTTCGCGGTGTAGTAGTAGCG
CAGGCTGTCGGCGTCAAAGTGTTTCAGCCAGGTGCTGGCCTTAATAAAGGTGCCGCGAGACTTAGACATCTTC
GCGCCGTTCACCGTCACGTAACCGTGAACGAACAGGTTGGTCGGCTTACGGAAGTGGCTGCCTTCCAGCATGG
CAGGCCAGAACAGGCTGTGGAAATAGACGATGTCTTTGCCGATAAAGTGATACAGCTCGGCGTCGGAGTCTTT
TTTCCAGTACTCATCAAAACTGGTCGTGTCACCGCGCTTATCGCACAGATTTTTGAAGGAGCCCATATAGCCA
ATCGGCGCGTCCAGCCAGACGTAGAAATATTTGCCCGGCGCGTTCGGGATTTCGAAACCAAAATACGGCGCGT
CGCGGGAAATGTCCCACTGTTGCAGGCCGGATTCAAACCACTCCTGCATTTTGTTCGCCACCTGCTCCTGCAG
CGCGCCGCTGCGGGTCCACGCCTGCAGCATTTCGCTGAATGACGGCAGATCAAAGAAAAAGTGCTCGGAGTCA
CGCATTACCGGCGTCGCGCCGGACACCACGGATTTCGGCTCGATAAGTTCGGTCGGGCTGTAAGTTGCGCCGC
AGACTTCACAGTTATCGCCGTACTGGTCCGCGGATTTACATTTCGGGCAGGTGCCTTTCACAAATCGGTCCGG
CAGGAACATGCCTTTTTCCGGATCGTAGAGTTGAGAGATAGTGCGGTTCTTAATAAAACCGTTCTCTTTCAGG
CGCGTATAAATCAGCTCGGACAGCTCGCGATTCTCGTCGCTGTGCGTTGAGTGGTAGTTGTCGTAGCTAATAT
TAAAACCGGCGAAATCGGTCTGGTGCTCCTGGCTCATTTCACCGATCATTTGCTCCGGCGTAATACCAAGCTG
CTGCGCTTTCAGCATGATCGGCGTGCCATGAGCGTCATCGGCACAGATGAAGTTAACCTCATGGCCGCGCATT
CGCTGGTAACGGACCCAGACATCAGCCTGGATGTGCTCCAGCATATGGCCGAGGTGGATAGAGCCGTTGGCGT
ACGGCAGCGCGCACGTTACCAGAATTTTCTTCGCGACTTGAGTCATAGTGGGTATTACTTCTTCTGTTGTAAA
AAAGGGCTTTGATAGTACCAAAATGGTAATTACTGCGCCAATCGATAAGGGCATTTATCCGTAAATGAATAAT
TGTTACCGCTGGAGTAAACTACAAAGTTACAATCGTACTTTTCAGAACTACAAAAGGAGTCGGGATGAACGAA
CAATCCCAGGCCAAATCACCAGATACCCTGCGAGCGATGGTCGCCGGGACGCTGGCGAATTTTCAGCACCCAA
CCCTGAAGCATAACCTCACCACGCTTAAGGCGTTGCACCACGTCGCCTGGATGGATGATACGCTGCACGTTGA
GCTGGTCATGCCGTTCGTCTGGAACAGCGCGTTTGAGGTATTAAAAGAGCAATGCAGCGCCGATCTGTTACGT
ATTACGGGCGCAAAAGCGATCGACTGGAAACTGTCGTACAACATTGCGACGCTGAAACGCGTCAAAAATCAGC
CTGGCATTAATGGCGTGAAAAACATCATTGCCGTGAGCTCCGGTAAAGGCGGGGTGGGGGAAATCGTCCACGG
CGGTAAACCTGGCGCTGGCGTTAGCGGCTGAAGGCGCAAAGGTCGGGGTTCTCGATGCCGATATTTACGGTCC
GTCGATCCCAACCATGCTTGGCGCAGAAGATCAGCGCCCGACATCGCCAGACGGTACGCATATGGCGCCGATT
ATGTCTCACGGCCTGGCGACCAACTCGATCGGCTATCTGGTGACTGACGATAACGCGATGGTATGGCGCGGCC
CAATGGCCAGTAAGGCGCTGATGCAGATGTTGCAGGAGACGCTGTGGCCGGATCTGGATTATCTGGTACTGGA
TATGCCGCCGGGAACCGGGGATATTCAGCTAACGCTGGCGCAGAACATTCCTGTTACCGGCGCGGTGGTGGTG
ACTACGCCGCAGGATATCGCGCTGATCGACGCCAAAAAAGGTATCGTGATGTTCGAGAAGGTGGAAGTGCCGG
TGCTCGGCATTGTGGAAAATATGAGTATGCATATTTGTAGCAACTGCGGCCATCACGAACCGATCTTCGGCAC
GGGCGGCGCGCAGAAGCTGGCGGAAAAATACCATACTCAGCTTCTGGGTCAGATGCCGCTGCATATTAGTCTG
CGCGAAGATCTCGATCGTGGTACGCCGACGGTGGTTAGCCGTCCGGAGAGCGAATTTACCGCGATCTATCGCG
AACTGGCCGATCGCGTTGCGGCACAGCTTTACTGGCAGGGTGAAGTGATTCCGGGTGAAATCGCCTTCCGCGC
GGTGTAATCCTCCTGACGTATTCCAAAGAGAATATGACATTCACTGCATTAATATATATTGATGCAGTGAATG
TATTAATAAAATAATAGCCCCCGCATTGTTAATTCCTGCTTAAACAGACAGGTGTTTATCTGAAATAATCGGC
AAAAACATTATTTGAATTGATCATACCTATCCTACTGATTTATTTTTTCAATTGGTTTTAAGGTATCGATACG
TTCATACTGCTCATCAGGACGGCCTGCATACTGTTTGACGTTCACCAGAGGAACAATTACTCACTATGAAAAA
ATATTTGCTGATGGGAATTATAGTTTCGGCTTATGGGATAAGCGTACCCGTTTTTGCCTCCGACACCGCAACA
TTAACCATCAGTGGAAAAGTGACCGCGCCAACGTGCAGTACAGAAGTGGTAAACGCACAGTTGCAGCAGCGTT
GCGGCAACACTATCCACGTTTCAACTCTGCAAACACCCGCCGCTACGCCGATGAGAGGTGTGACAACGCAGCT
ATACACGGTGCCTGGTGATAGCACCCGACAGATTGTTGTTAATCATTACGACTAATTCTTATTAATTCCGCAG
GCCCATATCATTGATATCAATGATATGGGCTTTTTTATTTGTTGTGTTTATGTTTAAGGGCAGTAAATTTCGG

AAATTAATAAGTTTACAAAATAATGATAAGCAATGATTATTTGTTGCAATTATATTGCAATCTGTAAATTGTT
TTAAATCAACAAAACATCGTGTTTACATTGCGATAACTTCCTGTCTATGAGAATTTTCGTTGCAAGGGTTAAT
AACTCTTAACAAATAGAAATTACTTCATTAAGGAAGAGATTATGAAACGTTCACTTATTGCTGCTTCTGTATT
GTCTGCTGTATTTATGAGCGCTGGGGTTTTTGCTGCTGATGAAGATATGGGGGAATTAAAAATAAACGGAGAA
GTGGTGGGAACATCCTGTACTTTCGAAGGTGCAAATAGCGCGACTATTGAATTATCCCAGGTAGGTGTTGATA
GATTGACTGACTTAAACCCTGGCGATATATATACAGGATACACTAGCCCAGAAGCGATTTTAAAAGTAAGATG
TACGAATACAGCTAATCCACGAATTAGTTTTAACCGTTCTCAATTTGTGGATAACATGCAAATCACCAAAAAT
AATGCTACTAATAATGGTGCGGGCTTCGCTGTTTATCTTGATGGTATTCAGGTGAAACCGGATGAGGCGGGGA
ATTACACTCTGAATTCAAGTAAGTTTGAAAATGGTGTATATACCCTGAACTTTTCTGCCCGCTATGCCGCCGT
TGAAAATACTGTAACACCAGGTTCTGTTGAATCTGTACTGACGATGACGGTATTAACTGATTAACAATCGTTT
TTTGGCGGCTAAGCTTTTAGCCGCTTGTATCTCTTTTATAAGGTTATCGGCCATGAAATGGATGATTGCATTA
TGCCTGGCTTGTGCCGCTATGCCTGCGTGGAGCGGCATTTATATATATGGTACACGTATTATTTATCCGGCGC
AGAAAAAAGATATTACCGTACAATTAATGAATGACGGTAAACGAAGTTCACTCATCCAGGCGTGGATTGATAA
CGGTGATACCAGCCTGCCGCCAGAAAAATTACAGGTTCCATTTATTATGACCCCCCTGTAATTAGAGTTGCGG
CAAATTCAGGTCAGCAATTAAAAATAAAGAAGCTCGCTAATAATTTGCCTGGCGATCGTGAAAGTCTTTTCTA
CCTCAATGTATTGGATATTCCACCAAATTCAGACGAGAACAAAGATAAAAATATCATTAAATTTGCGCTACAA
AACCGGATAAAGTTGATCTATCGCCCACCGGGGGTACAGAAAGTCGATAAAGCAACATTTTCCCGCTTGCAGT
TGCTTCGTGCGCCAGGGAGTATTAGCGTGAAAAATAATTCCGCAAACTGGATCACTATTCCAGAAATTAAAGC
AAAAAGTAAAGTAAATAAAGAAACCTTATTACTGGCTCCATGGTCATCGCAGAGTATCACCACAACGGTAGTC
GTAAATAGCTATACCGTGACGCTAATTGATGATTCTGGTAATTATTTAAACGAGACTGTCAAAATAGAAAATT
AAAAGCGGGGAATCCACCTAAGGATAGATAATGGAAAAGTTAAACGCCATCACGCTGTCACTTTTTGCAGCAT
TACTTATTTCACCCGCCTGGGCTGAAGAAGAAACATTCGATACTAACTTTATGTTCGGTGGGTTGAAAGGTGA
AAAGGTGTCTCGTTATCAGATTGATAGCACCAAACCCATGGCTGGCGTTTATGAAATGGACGTTTATGTCAAT
AAAGAATGGCGTGGAACTTATGAGGTTAATATTCAGGATGACCCCGATAGCACCTGTATTTCTCCGGACCTGA
TCGCCAGCCTGGGAATTAAATTTACCCCGCAGAGTACAACAGTAGAAAATGAATGTATTGCGCTGAAAACGGT
GGTTCATGGAGGTAGTGTTTCTTATGACACTGCCGCTTTTAACCTTTATCTGAGCGTCCCTCAGGCTTATGTG
CTGGAATATGAAGCGGGCTATGCGTCTCCAGAGACATGGGACCGTGGAATTAACGCTTTTTATACATCGTATT
ATGCCAGCGAATATTACAGCCATTATAAAAGCGGCGGTAGTGAGAAAAATACTTACGCAAATTTTGTCAGCGG
ATTGAATCTGCTTGGATGGCAATTACATTCTAACGCTAATTTTTCCAAAAGTGAAAATTCAGCTGGCAAATGG
CAGAGCAATACTCTGTATCTGGAACGTGATTTCCCGGCGGTACTGGGGACAATGCGTCTGGGGGAACAGTACA
CCAGCGGGGATATGTTTGATACGGTGCGCTTTCGCGGGGTGCGCTTCTGGCGTGATATGCAAATGCTGCCGCA
CTCTAAGCAAAATTTCGCTCCGGTTGTGCGTGGCGTGGCGCAAAGTAACGCCCTGGTGACGGTTGAACAGAAT
GGCTTCATTGTTTATCAAAAAGAAGTTCCGCCTGGCCCCTTTGTATTTGAAGATCTTCAGCTGGCAGGGGGGA
GCCGATCTGGACGTGAGCGTTAAAGAGGCTGACGGTACTGTTTCACGGTTTATTGTTCCGTATTCTTCCGTTC
CCAATATGGTGCAGCCCGGCGTAGCAAAATATGATTTCGCCGCCGGACGAAGCAGAATTGAAGGAGCCTCTCA
GCAAACTGATTTTCTCCAGGGAACCTATCAGTACGGGGTCAATAACCTGCTAACGTTGTATGGCGGAACGATG
TTAGCCAGCGATTATCGCTCATTTACCCTTGGCACCGGATGGAACACGCTGATTGGCGCGGTATCTGTCGACG
GTACGTTGTCTCATAGCAAGCAGGATAATGGCGATGTTTTTGATGGGGAAAGCTACCAGGTTGCCTGGAATAA
ATATCTCCCGCAATCGGCCACCCATTTTTCTCTGGCAGCGTATCGCTACTCATCACGTGATTATCGCACTTTT
AACGATCATGTCTGGGCTAATAACCGGGATAACTATCGTCGCGATGATGACGATATTTATGATATTGCTGATT
ATTATGAGAATGACTTTGGGCGTAAAAATACTTTCACTCTCAATATTAATCAGACGTTACCCGATGGGTGGGG
ATATTTTACTGCCAGCGCTCTGTGGCGCGACTATTGGGGGCGTAGCGGCACGGGAAAGGATTATCAGTTAAGC
TACAGTAATACCTGGCAGCGGCTCAGTTTTACGCTATCGGCAACTCAGACGTATGACAGCAATAACCGTGAAG
ATAAGCGCTTTAATATCTATTTATCGATTCCCTTAACATGGGGATTAAAGGAGAATGGGGGAAATCGTGATAT
CCATTTATCGAATTCCACCACCTTTGACGATCAAGGTTATGAGGCGAATAACACCAGCCTTAGCGGCACGTTC
GGTAATCGCGATCAGTTTAATTATACGACTAACCTCAGCCAGCAGCGACAGGAACACCAAACGACATTCGGGG
GATCGGTAACCTGGAACGCCCCGCTGGCGACAGTCGGCGGCAGTTACAGCCAGTCCAATAAATACCATCAGGT
GGGCGGAAATATTCAGGGAGGGCTGGTTGCATGGGCCGATGGCGTGCATTTAGCCTCTCGCCTTAATGATACT
ATCGCGATTATTAACGCGCCGTACCTGGAAGGCGCGGCGGTGCAGGGGCGTCCCTATTTACGTACCAATGCAA
AAGGTTATGCCGTATTTGAAGCATTGACGCCATACCGGCAAAATTTTATCTCTCTTGATGTCTCTGAGAGTGA
AAGCGATGTCGCCCTGTTGGGAAATCGAAAAGTTACAGTGCCTTATCGCGGAGCCGTAGTATTGGTTCATTTT
GAAACAGAGACCAGTAAACCTTTTTATTTCCTGGCGCGGCGCGCAGATGGCGAGCCTTTAACATTTGGTTATG
AAGTGGAAGATGACGAGGGAAATAATGTAGGACTGGTAGGTCAGGGGAGCCGGGTATTTATTCGTACTGAAAA
GGTTCCCATTTCGGTAAAAATCGCAACAGATAAACAACAGGGTCTTTTCTGTAAGATCACTTTTGATAAACAG

ATTGATGAAAATAACGTTTATATTTGTCGGTAATCTTTAGGTGACGCCATGAAAAGACTACTATTTTTATTAA
TGATGATGCAGTTTACGCAGTTGGGTTACGCGGCGTGTAATGCGACCTTAACCAACACGAAGGATTATACGAT
TCAATCCGACAGCTTAATGCTGGGGGGGAGGAGTCAGCCATTATTACAAATGGTTTCACAGATGCCAATTGTT
CTAATAGTGATGTTGTGACTAAATTAGAGACATCCGATCATATTATCGGTATGGGACCGAATGACAGTGTTAA
GTTAAAGTTAAAAGTTGAATGGCAGAGCAGTAGTGCGATAAATATGCGCAATCAAAACGGTGTAATTGAAGCC
CCCTATAAGATAACGATTTCAGAAATAAACTCACTGGAGGCGGTGACTGTGTCTGCCAGGGGGGGGTACTCGG
TTACGATAGATAATATAACGGTGATGTCGGGTGCAAAGAATCAATGGAGCAGTAGCGATTGGGTGGTATTTAT
TTTAAGATATATTGGCTGTTGGGGGAATAGAGAGTGTGTTGCAAACGTTATCACTGATCTGAATGGGAAAGGC
GTTTATAAAGCCAGTTTAACGATAAATTACAACCGTAAAGAAACTACCTGCGCCCCCCAGAATCTCACCATCA
CACTGGACCCGGTGCCTATGACTAAACTCCGTGCTAAAGGAGAGGTTAGTGAGTTTTCTAAGCAGGGTGATAT
TATTCTGGATTGCAAAAATCAGGTGCGGAATGCGATGCTGACATCACGGCAAATAGCGGTAAATCTACGTAGT
GATTTAGTCTGGGATGAGAATGAAGCAGTCCTGAAACCGGATAACGATAATGGCGTTGGGTTTATCTTAAGAG
ATTCAAATCGGAGCCTGCTTAAAATAAACAAAGGAGTAGCGATAAATAGTATTTTCAAAACCTATGCCAAAGG
CAGCGCAGTGAATTCGGTGGAAGCTATTCCTGTCTTTGCCACATATTATGTTCTCGATCCTGCAAAAGTTAAA
GCGGGGCCATTACAATCGAAAGCATTAATCGTGGTATCTTATAATTAAATTTATGCGGTACGCACTCTTTATA
GAAAGAGTGCGTATATCATTATCGATGATAAAAAATCTCGCCATCATAGGCTTTGATAATTTTTCCATCGGTA
TCGTTAATCAGTACATACGCGCCGCCCATATAGGTCCAGTGAGTACCGGCATCTGGCGCGGGCAGATTACGTA
ATTTCCACTGTTTAATATTATACTCATCGGTGCGGTACATTTGCGGAACGGTATCGCCGGGCTTGAAGTGGGT
GAAGTCGGCGATAAAGCTTTTTAACTCATATTGATCAATGCCCGTTGTCGCGGGGGCGGCCCATGCAAAACTG
GTTGCAAGCAACAGCGCGCAAGGCAGCAACTTACTTTTCATATTTTTACCCTCCACATATGCGGGTCAGTTAA
CATCGTGTGTATCATTTCTGCTGGTGGCGTATCTGACAAGCGCTATTTTGTTATCTCACGCCCCTTTTCGTAA
AAAGTGTAAACGAAAGCGTCGTGCAGCAACATATCCGCCAGCAAGTTTGCCAGAACCGAATAGGTTTTGAGAA
CTATCTGATAACGCTGAAATTATCGTCCGCGGCTGGCTGTAAATTTACATTGTCTGGCGCAGACAGTATGCTT
AATAGCGAAACCCCGCTTAACACGGTGGGGCTTGACGACTCGGGGTGCCCTTCTTTGTGAAGGCTGAGAAATA
CCCGTACCACCTGATCTGGATAATGCCAGCGTAGGGAAGTCTGACACTGCTCGTGTCCCTTCTTCACGCGGGC
AGGAGCTGAACCATGCAGCCTGACCTGCACTGCCGCACGCTTGCGGCGCATACGTTAAAACACTTTCGCGCGC
TCTCCCCGCTTACGCACTGTATGACGAATGACGTCGTACAAACGTTTACCGCCAATACGTTGCTGGCGCTGGG
CGCTTCACCCGCCATGGTGATTGATCCTGTCGAGGCCAGACCGTTTGCCGCCATCGCCAACGCCTTGCTGATT
AATGTCGGAACATTAACTGCCTCACGCGCTGACGCGATGCGTGCGGCGGTAGAAAGCGCTTATGATGCCAAAA
CGCCGTGGACGCTTGATCCTGTCGCGGTGGGCGCGCTGGAATTTCGTCGGCGATTTTGCCTGGATCTCTTGTC
CCTGCGCCCGGCGGCAATACGCGGCAACGCCTCGGAAATCCTGGCGTTATCCGGCATGGCGCTGGGCGGACGT
GGCGTAGATACCACCGAGGCGGCGTTGGCCGCACTGCCTGCGGCGCAGGCGCTGGCGCGTCAGATAGACTGCA
TCGTTGTGGTTACCGGAGAGATAGATTACGTCACTAATGGTCAGCGCACGCTGAGCATTCCCGGCGGCGATCC
GTTAATGACTCGCATTGTAGGCACCGGCTGCGCGTTGTCGGCGGTCGTCGCCGCCAGTTGCGCGTTACCGGGC
GCCGCGCTGGACAATGTCGCGTCGGCCTGCTGCTGGATGAAACTGGCTGGACAGGCCGCGGCAGAGCGTAGCG
AAGGACCTGGTAGCTTCATCCCGGCTTTTCTTGATGCGCTCTATCATCTGGATGTGGAGGCGGCCAATGCAAC
GAATTAACGCGCTGACGATTGCCGGCACCGATCCCAGCGGCGGGGCCGGTATCCAGGCGGATCTCAAGACCTT
TTCCGCGCTGGGCGCATATGGCTGTTCGGTCATCACCGCGCTGGTGGCGCAAAATACCTGTGGCGTACAGTCG
GTGTACCGGATAGAGCCGGACTTTGTCGCCGCTCAACTTGATTCCGTGTTCAGCGATGTGCGCATTGATACCA
CAAAAATCGGCATGCTGGCGGAAACCGATATTGTCGAGGCCGTGGCGGAGCGACTACAGCGTCATCATGTACG
TAATGTGGTGCTGGATACGGTGATGCTGGCGAAAAGCGGCGATCCGCTGCTCTCGCCCTCTGCGATAGAAACA
TTACGCGTCCGGCTGTTGCCACAGGTATCGCTGATTACGCCTAATTTGCCGGAAGCCGCAGCGTTGCTGGATG
CGCCCCATGCACGTACGGAACAGGAGATGCTGGCGCAGGGGCGGGCGTTACTGGCGATGGGGTGTGAAGCGGT
ATTGATGAAAGGCGGGCATTTAGAGGATGCGCAAAGCCCGGACTGGCTCTTTACCCGTGAGGGCGAGCAACGT
TTTAGCGCGCCGCGTGTGAACACCAAAAATACGCATGGGACGGGCTGTACGCTGTCGGCGGCGCTGGCGGCGT
TACGGCCCCGGCATCGCTGTTGGGGAGAGACGGTAAACGAGGCGAAGGCATGGCTTTCGGCGGCGCTGGCGCA
GGCGGACACGCTGGAAGTGGGGAAGGGCATTGGTCCGGTACATCATTTCCACGCGTGGTGGTAGTATACTGCC
TACAGACCCGTCGTCAGGAAAGATAAATGGAACAAGCGCATACCCGGCTCATCGCGCAACTGAATGAACGAAT
TTCAGCGGCGGATAATACGCCGCTATACATGAAATTCGCCCAGACGGTAAAGGATGCCGTGCGCAGCGGTATT
CTTGAGCACGGTAATATTTTGCCTGGCGAACGCGATCTCAGCCAGCTTACCGGCGTCTCGCGCATCACGGTAC
GAAAAGCGATGCAGGCGCTGGAAGAAGAGGGGGTCGTCACGCGCGCCCGCGGCTACGGTACGCAAATCAATAA
TATCTTTGAATATTCCCTGAAAGAGGCGCGGGGATTTTCTCAGCAGGTTGTCTTGCGCGGCAAAAAACCGGAT
ACGCTATGGGTTAACAAACGCGTGGTAAGTTGTCCGGATGAGGTCGCGCAGCATCTGGCGATCCCCGCAGGCA
GCGACGTCTTTTTACTCAAACGCATTCGTTATGTTGATGAGGACGCGGTATCGATTGAAGAGTCCTGGGTGCC

CGCGCATCTGATTCATGATGTCGACGCGATTGGCATTTCGCTGTATGACTATTTTCGTCGCCAGCACATTTAC
CCTCAGCGTACCCGATCCCGCGTCAGCGCCCGGATGCCGGATGATGAGTTTCAGTCGCATATTCAAATGGATG
GTAAAGTGCCGGTGCTGGTGATTAAGCAGGTGGCGCTCGATCAGCAGCAGCGGCCTATTGAATACAGCATCAG
TTATTGTCGTAGCGACCTATACGTTTTTGTGTGCGAGGAGTAGCGCCTCGCGCGTGGGCGCGCAGTCGCCGCC
GCGATGCCCGACCACCCAGCTCGCCACCGCATTTCCCAGCAGTACGGCATCCGCGAGCGACCAGCCTGACGCC
AGTCCTGCCAGCACGCCGCCAGCATGGCTGTCGCCTGCGCCGATGGTATCCACCACGGTTGCCGGAAACGCTG
GCACAACGCCGGAAGATTGCGCGCTAAAATACCACGCTCCGTCTTTATCGTGGCGCACCACCAGCGGCGCGGA
AAAGCGTTTTACCCATGCTTCGCCGAAAGTGCGGGTATCGGTCGCAAGCCCGGCGCGTTCCGCGGCGATGATG
GCTTCCTGGCGATTGAGGGATACGATGGGCCGGCAGGCCATAATGCGCGCCATTGCATCGTCTGTAATATCAC
CGATTCGCGGGCCAAAATCGATAAAAGGCGTTACATCGTCGACGGTTTCCAGCCACTGGATCAGTGGCTCGCC
ACAGGACGAGGCCAGTTGATAGCCGGACAGGTAGACAAGACTGCCCCGCGGTGTCCTGAGCTGCGACAGCCAG
TCCGCATTCCACTGGTTTTCCACGCCGCTAAAAGACATAAACGTGCGTTCGCCATCCGGTTCGACTAACGCCA
GACACCAGCCGTTATCTCCTTCAACCGCGTCAATCACGCTGGATAATCCCTCTTTTGCCAGACGGCGGCGAAT
CCTCTCCGCCCACACGCCCTGGCCCAGCGGCAGCGCATTGCTGGCGTCAATCCCCAGTCGTTTCAACGCCACG
GCAATATTCAGCGCACAGCCGCCGACATTGACGCTCTGTTGTTGCAGTTCAATATCGCCTCCGCGACGCGGCA
ACGTCCAGGCGTCGGCAATGATATCGATCACTGCCGCGCCGATAACGGTGAGCGGACGCCGGGTATGCAGGGT
CGGTAATAATGTCAGCAACCGTGCGCCGTTCATAGTGCCTCCCGCCGTTGACGATAGCTCGCCAGCGCAACGG
CATAGCGCGCAAAATCCAGCTGATTAACCTTGTCCAGCGTTTGCTTTAACTGGGGGTTAATGGCGCTTACCCC
GTGCAACGCGCCGCAAATGGCGGTAGCCATCGCGCCGATAGTGTCGGTATCGCCGCCGAGGTTAGCGCACAGC
ACGGCACAGCGGTTAGGATCGGTATTCGCCAGCTCCACCATGGCGATAGCGCAGGGAACCGATTCAATGGTGC
TGGTTCCCGCGCCGATGATCTGATAGAGCTGTTCGCTGGCAGATTCGACGCCGTCGGCGTGGCGGACAATGTT
TAGCGCCAGTTCCAGACGTGCCGATAGCGAGGCGCTAAACGTGGTAATACGCGCTGTCTGCGCCTGATGGGCG
ATGGCGGGGAGCGAGTCGGCAATACTTTGCCACGTGTCGCCGTTAACCGCTCGCGAGACGGCCCAGGCGACAA
CCACGGCGCCAGCCACCGCCAGATCGGATTTATGCGTCGGGCTGGAGGCTAACGCTACTTCTGCTATAAAGGC
TGTCAGGTTAGTTGGCGGCAACAGGCAGCCCAACGGCGAAACGCGCATGGCCGCGCCGTTAGTCACGCCGTTA
TTTTCCAGCGCCGCGATGGGTTTGCCGTCGCGAATCGCATTCAGCGCAATTTTTGACGTCGGGCCGAGTACAT
TTTTATTGAACGCATCAAAGCGTTCCGCCCAGGCGAGGATGTTACGCCCAATAATCTCGGGATCAATGTTGCC
TTCGCATTCGAGTAGCGCGTCGGCCAGGCAGAGCGCCATTGCGGTATCATCCGTAAACTCCGCGCGGTTAAAA
TAGCAGGCGGCATTATTCTCTTTTGGCCCGGGCAGAAAGCGGTCAATCCAGCCGAAATGCGCTTTGACGCGCG
TTCTCGGCCACAGCTCAGAGGGCATTCCCATCGCATCCCCTAACGCTTGCCCGTAAAGAGCACCGAGAATACG
TTCTGCTTTCATTTTACTTCCCCTTGTGTCAACGCAATATCGCGATCGTCAATAGCAGTGATCTCTTTGTCCG
ATTCGCGAAAGAAAATCATAAACAGTAGCGCGATGACGGCAATCATCACTGCGCCAAACGTCCACATGCCCGC
CCAGTTGAATGTCAGACCGTTAACCGGTTGCGGATAAGCAAACATTTTTTCCATCATCACGCCGCCGAGGCGA
TACCCCAGCAGGCTGCCGAATCCCTGACAGCAGAGGGTGATAAGCCCTTGCGCAGCGGTACGCATATGAACCG
GCGCTTTTTTATCGACATAAATGTAGGCGGTGACGTAGTAGAAATCGTAACTTACGCCGTGCAGCAAAATACC
GAGGAACAGCAGGGCGTAAGTGAAGTACGTCTCCGCGCCGCCATAGACAAAAAAGCCGTAGCGGATCGCGGCG
GTAATAAGACCAAGTAATAATACCTTTTTAATACCAAAGCGCTTAGTGAAAAAGGGTAGCGCCAGCATAAAGA
AGATTTCAGAGAACTGGCCGAGCGTCATCCAGCCGGTGGCGTTTTTCATTCCCACTTCTGTCAGATAGCCGTT
AGCGAAGATGTAGTAGAAGGCCAGCGGCATCGCAAACAGGAAGGAACAGAAGAAGAAAACGAGAAAGTTTTTA
TCGCGCAGCAGAACCAGCGCATCCAGCCCCAGCATCACTTTAATATCCATCTTGCCGGTGCTTTTTGGCGGCG
TGTCTGGCAGACAGAACGCAAATACGCCCAGCAACGCTGAACTTGCGGCGGTTATGAGCAGTGGAATGTTGGT
GGGGGAAATATCGTTATAGCCTAACATTTGCGGCAAGAATCCACAGGCGAGGCCGGAGGCAATCCACCCAATG
GTTCCCATAACGCGAATGCGTGGAAAATCGCGTTCAACGTCCGGTACGTTAGCGAAGGCAATGCTGTTTGTCA
GCGCGATGGTCGGCATATAGGTCAATGAATAGGCCAGGAGCAGCGGAAAGAATCCGGCAAAGGTGGTTTGCTG
TGCCGCGAAATACATCAGTACGGCACCCGCAAACATCAGTACCGCCAGGACTTTTTGCGCTGAGAAAAAACGG
TCGGTAACGGAGCCGACCAGAATCGGCGACAGGATCGCCGCAATGGCGGTACAGGCATAAGACCAGCCTATTT
CACCGGCGGAAAACCCGCTTTTATTTAGCCATAGCCACAGGGGGACGAACCAGGCGCCCCAGATAAACCACTC
CACGAACATCATGAACGACAGTTTAAAGGTCGTTTTCATTTTTGAATCCTTCATGACAGGTAAGGTACGCCAC
GACAATACCACTTAATAATACCTTTTAAATACCATTGAGCATTAATTTTGACCGATCTAACACTTTTTCGGAC
GTGAATCGAGTCAGCAGATAGCGCCGAAAAAATGCGCTAAAGTTGGCAAAAAATGGTCGGTCAGTACCAGGCTT
ATTGCGACCCGCAATCTGCGGGGCAGGACTATTCACCTTCCGATACCCTATCGGAACTTACGGGAGCATAGCT
ATGACTGATATTGCGCAGTTGCTTGGCAAAGACGCCGACAGCCTTTTACAGCATCGTTGTATGACCATTCCTT
CCGATCAGCTTTATCTACCCGGCAAGGATTACGTTGACCGGGTGATGATTGACAATAATCGTCCACCAGCCGT
TCTGCGTAATATGCAGACGCTCTATAATACTGGACGCCTGGCGGGAACAGGATACCTTTCGATACTGCCCGTC

GATCAGGGAGTGGAGCACTCCGCCGGCGCTTCTTTTGCGGCCAACCCGCGCTACTTTGACCCAAAAAATATTG
TTGAATTGGCGATTGAGGCCGGCTGTAACTGCGTGGCCTCTACCTACGGCGTACTGGCCTCGGTTTCACGGCG
CTATGCGCACCGTATTCCGTTTTTGGTCAAACTGAATCACAACGAAACATTGAGCTATCCGACAGAGTACGAT
CAGACCCTGTACGCCAGCGTTGAGCAGGCGTTCAATATGGGCGCCGTGGCGGTAGGCGCGACGATCTATTTCG
GTTCGGAACAGTCCCGTCGCCAGATTGAAGAGATCTCCGCCGCTTTTGAGCGTGCGCATGAGCTGGGTATGGT
GACCGTATTGTGGGCTTACCTGCGTAATTCTTCCTTTAAAAAGACGGCGTCGATTACCATGTATCCGCTGAC
CTGACGGGGCAAGCCAACCATCTGGCCGCTACGATTGGTGCGGATATTGTGAAACAGAAAATGGCCGAGAATA
ATGGCGGTTATAAAGCGGTCAACTTCGGCTATACCGACGATCGCGTGTACAGCAAACTGACCAGCGATAACCC
GATTGATTTGGTTCGTTACCAGTTGGCGAACTGCTATATGGGACGCGCCGGGCTTATCAACTCCGGCGGCGCG
GACGGCGGTGACACTGATCTCGGCGACGCGGTTCGTACCGCGGTGATCAACAAACGAGCCGGTGGAATGGGAC
TAATTCTGGGACGTAAAGCCTTTAAGAAATCGATGGCTGATGGCGTGAAGTTGATTAATGCCGTACAGGATGT
CTATCTGGATAGCAAAGTCACCATCGCCTGAGTTCAACGAAGAAAAGCCGGATGTGAGCGCATCCGGCCAGCG
ACTGGTGTTATTAACGTAGCAACGGGCAGTCTGGCGGCAGCCGACAGCGTAATGCGCCAGGCAGCACCTCAAT
ATGAAATTCTTGCCCGCTGAGTGGTTCGCCATCCAGATTGAACGTGATTTCATGCGGTGCATGAATATCAAAC
CACGCTGAGGCGCCGTCAATAATATTCGGATTGTCATCTGACTGCGTGAGTGTGGAAAACAGCGCCGGCAGCA
GTTCTTCGCCGGTAAAAATCCGTAGCTGTAACAGTCCGTCGTTAACCAGCGCGGTAGGACATAATTGCTGCCC
GCCGCCGGCCCTGACGACCGTTGCCGATGCCGATCACCAGCGCATCTCCCTGCCAGTGAAAGTTTTCGCCACG
TATTTCGCAGCGGTCAGGGGTCAGCGTATCCATTCTCATCAGACCGTGAATAAGATAGGAGACGCCGCCCAAC
GCGGCTTTAAGTTTTTCCGGCGTTTCGGTGGTAATGCGGGTGCCAAAACCGCCCGTCGCCATATTGATAAAAC
AGGTTTTATCATTGACCCTGGCCATATCAATTTCCATCGCGTTTCCGGCAATCGCCAGTTTCAGCGCTTTGTC
CAGCGCTTCAGGAATACCGGCGCTGGTGGCAAAATCGTTGGCAGTGCCGAGCGGCAAAAGGCCCAGTGCAGGG
GCTACGCCGTCGCGGATCTGGATGAGCGCGGTCGACACTTCATTAATGGTACCGTCTCCGCCGCCGGCAATCA
CCGTTTCAACGCCCAGTCTACGGGCTTCATCAACGTAACGCTGCGCGTCGCCTTTTTCCCAGGTTACCCGAAC
GTGAATCTGTATACCTTCGTCGCGAAGTAGAGTAATGGCCTCACGTAGCGGCTGGTTATCAGCGCTTTTGCCA
TTGAGGATCAGTAAGCTGGCGGGAAAGTTAGCCATCCGTTATGCCTTAATAGGTTGAACAACAATCTCCAGAA
AGTGTATAGCAGAAATGAAAAAGCGAGGTCAGAACAGGATGAAAATTGAAATGTTGCGCACTTATTGCTTTAC
CGTTTTTCTTCCTGCTGTACGCAGCGGTAAGTGAAGCATGGTTTAAGGCAAATGGCGACAAGAGAGATGCCGG
TATGCGCATTGAGTAGCGCTTGTGAGAGTGCAAGGAAATCACAAGGGGTAGTCAGACGCTTTCCGATATAGCG
CCTTTGATAACGAAACGGAGAATTAACAAGACGAACATACGTCAGTTATCAGTATTAATTTCAAACACCATAA
TATCAGATAAAAACCTGATATTTATTACCAAACATACTTTTTAATAGCATAAAATCGAAATGTTCTTTAGACT
CTTTAGCAGAAACACGGCGCACTATTAGAAGCACGCTACTATATATCAAATAAAACCTATTTAGATGATGGCT
TAGCGCGGTGTCCTATAGTCGTACCGTTGCCTGATGTATCGCTTTTCTCATAAACATAAACAGGCATGTGCAT
GAAGAGGTTTATATGAATGTCACTTCAGGCGTGAATGCGCAAACGCCATTGCTCCCTCCTTCTGAACGGGGTA
ATGACGAAAAACCGGTAGCTGAAATCGTTGATTTTAACGCTTATGGCAATAAACCCAGATGTTTAATGTGCCT
GGGAACCACGGCATTGTTTGCAGGTGTTTTCAGTGGGGTATGCTCAGGGGCTGTCGCCAGTGTCAGTAGTGGT
ACTGCATATACTACCGCCCTGACGGTTTTAGGCGCATCGTTTGGCATGGGGGGAATTGGTATGATGGGAATAT
GCGCCGGACTATACCTTAGCGCAAATGGCATCAGAACCCGGCCAGCCTGGCCATAAATGAGCAAACACAGCAA
GTCATAGGGAAAAGACGAATTCCGACGATGACACGGGGTGTTAAGCGTCCAGACCAGAGGCTTATGACTTATA
TAGGAACGCTTTAGATAGAGTATGGGGTGTTATGTATTCAAGAGCCGATCGCTTATTAAGACAGTTTTCACTA
AAATTAAATGCCGATTCAATTGTCTTTGACGAAAACAGATTATGCTCATTTATTATAGATAACCGTTACCGTA
TCTTACTAACAAGCACTAACAGTGAGTATATAATGATATACGGTTTTTGCGGAAGACCTCCGGATAATAATAA
TCTTGCTTTTGAATTTCTCAATGCTAATTTATGGTTTGCAGAGAATAATGGACCACATTTGTGTTATGATAAT
AACTCACAATCATTATTATTGGCTCTCAACTTCTCACTAAATGAAAGTTCTGTAGAGAAACTGGAATGTGAAA
TTGAGGTAGTTATTAGATCAATGGAGAAACTGTATCACATTTTACAGGATAAAGGAATAACTCTGGATACCGA
TTATACGTGACGGTGGGAAGGGGATTTGGTGATCTGGTGGCAATAGAGGATAATCAATCCCAGCAAAACTTAC
ATGGCCTGATGAAGGTGGACTGAGCGTAGGTGCGATGGTTTGAGTATTACTTTGACATGAAATCAAACCTCTA
ATCCGGGAAACATTATTTTAATCCTTGTAAAAGCGGCATTAAAACGTGCCATACCTTTACACGCTTAAATTAT
CAGTTTTTAACCTTAATTTAATAATCTGGAAACTTTTTGTGTAAGTTCTCACAAAGCAGTTATTGATTTAATT
CACTGATGTTAAACGTAAAAGTCAGAATGCTAAGTCCTGAGTCCGACCGGATGCGGGGAGGATTGTGACTTGC
TGGCTGGCCATGAATAGGGGAGGTCTGTAAACCTAAGCGCCCATTTGCGCTTTGGTTAGTGTGGTCAATGTTG
GCTATGGCGGGAAGTACCACTTTTTTATTGATTATACGCAAAAAATAAGCCTGCGTAAGGGAGATTACGCAGG
CCAAGGAGGTGGTTCCTGGTACAGCTAGCATTTATGGGTTATGTTTTTCAGCGCGTTGAATAATACCCGCATT
GAGCGAAGCGGTATGTGATCAATTTCTAAGAATCTTCCCGCCATGAAAAAATAACCGCAATTAACTACTTAGC
ATGGGGGTTACGCGTTGATTCGCCTGAGAAATTACGCATCAATAGTGCGTAATCCAGCGTAACGTCCTGCGGG

ACGGGCATCCAGACGGTATAGCCATCGCCAGGTGCCACCGGCATAGCGTCGCCTTTGGCGTTCTCCATCTGTT
CCAGGGTGAAATTGATATTTCCCTGCGGAGTCATCAGCTCCAGACTATCGCCAACGGAGAATTTATTCTTCAC
CGCCACGGCCGCCAGTTGGCCTTTACGCTCGCCGGTGAACTCGCCGACAAATTGCTGGCGTTCGGAAACGGAG
TACCCGTACTCGTAATTCTGGTAATCGTCGTGCGTATGGCGACGCAGGAAACCTTCGGTATAGCCGCGATGAG
CCAGACCTTCCAGCGTTTCCAGCAGCGTAGGGTCAGAAGGGTTTACCCGCGGCGGCGTCGTCGATGGCCTTAC
GGTAGACCTGTGCGGTACGGGCGCAGTAATAAAAGGATTTGGTGCGGCCTTCGATTTTCAGCGAGTGGACGCC
CATTTTCGTCAGGCGCTCCACGTGGGCGATAGCGCGCAAATCTTTTGAGTTCATGATATAGGTGCCATGCTCG
TCTTCGAACGCGGTCATGTATTCGCCCGGTCTTTGGGCCTCTTCTATCATAAACACTTTATCCGTCGGCGCGC
CGATACCGAGCGTCGGCTCAACGTTCTGTACCGGAATCGGTTCGTGCTTATGCACGATGTTGCCGACAACGTC
TTCTTTTCCTTCCTGCACGTTATATTCCCAACGGCAGGCATTGGTGCAGGTGCCCTGATTCGGATCGCGTTTA
TTGATGTAGCCGGAAAGCAGGCAGCGGCCGGAATAGGCCATGCATAGCGCGCCGTGGACGAAAATTTCTATTT
CCATATCCGGCACCTGCTGGCGAATTTCCTCAATCTCTTCCAGCGACAGTTCGCGGGAGAGAATCACACGGGT
CAGCCCCATCTGCTGCCAGAATTTTACCGTCGCCCAGTTTACGGCGTTAGCCTGTACCGACAGGTGAATCGGC
ATTGCCGGGAAGTGCTCGCGTACCAGCATAATCAACCCTGGATCGGACATGATCAGCGCATCCGGGCCCATCT
CGACGACGGGTTTCAGGTCACGGATAAAGGTTTTGAGCTTGGCGTTATGCGGGGCGATGTTCACCACCACGTA
GAATTTTTTTCCGAGCGCGTGGGCTTCGTTGATGCCAAGCTGCAAATTTTCGTGATTGAATTCGTTATTACGC
ACGCGTAAAGAGTAGCGTGGTTGGCCCGCATAGACGGCATCGGCACCGTAAGCGAAAGCGTAACGCATATTTT
TCAGCGTTCCCGCCGGCGAAAGAAGTTCTGGTTTAAACATCATTTTCTCGTTCTGATGACAGGTCAGATCCGC
CTCACCTGATGAGGCGGCTTGAGGAGAGTCCTCACTTTAAGGGCGGGCATTGTAGCGCCGTGGGAGAGGGGAG
TAAACCACCTTACACCATTGAGTTGACCTGTCCCGGCAGCTCTGAATTCGGGATGCGGACGGTATACCACGCG
AAACGGAAGCGTTTCCAGAAAGAATCGAAATGCAGGTGGTCCAGGCAGTCCATAACGATTTCATTATCCGGGT
AAAGCGTGAAGGCAAAATGCGCCCAGCGTGCGCTTAGCTTCATATTAAACAGCGTTCGATCGTGTTTATTACC
ATGCAGGCGCGATAGGGTCACCATAGCGTGTAGCGTGCCTTTGTTGGCTTCTTGTTGCACCAGTTCCAGATGC
TTATGCGCCAGCGTAATATCCTTCACTTCAAAAATATTCAGCAGGAACAAGCCCCAGGCCTCTTTATGCCCGT
ACTGATGCGCCAGCCAGAGATTATCGAGCAGTTCTTTTTCATAAGCGGAACGTTTTTCGGTATCAGGTTCTTG
TTTACTCAGACGCTGGTTGCAGATGCCAATGCTGAAATGAATATTTTGCAGGTCATTTTCATAGTCGGTATAG
CCTCCGTTATCTATTAATTTGTACGGGGGGCTCTCGCGCAGCGCTAACTGCCGGTGCAAAATCTCCGCCGCGC
GCTGAAAATACCCTAACGCGATGGCAGGATCGTAAGGTAGTAGGCCGTTGTTCTCTTTATTATTGTGTTCAAG
GCCCAGCAGGTTTAGCGCGGCGGGTATATTTCTTTGTGCGCCCAGCTCTGCCAGCGGCACGAAATGTTCCGGG
AGCCCGAACCAGTCGAAATTTTTAATCGCCGCCCGCCAGTTATGGTTATATTGAATGTCGGCGACGCTATCGA
GCCAGGCGAGGCCTTGCGCCTCATCCTTTTCGAAACCCAATAACCCGGCGTACTGAAAGTAGCCACGACGGAT
TTTCGGCACTGCGAATTCCGTTTGCTGCGCATTCTGACAGAGAAAATTATACATCCACCAGGTGTCAGGAAAA
TCGCGCAGGGCGAACTTTATCGTGTCATCAAAGAAGTAATTGTTGAACTTAACTTTTTCCACGATCGAACGCT
GAAGGGTTCGCCAGAGCGCGTCATTATCGTCCAGATCGCTATAGCAGACCCGCAGCCACTTCAGCGTGTTATG
GCGAGATTCCTGGATATGCGCTCGCAGGCTAATCTCTTCGGCTTTGGCGATAATGCGTTCCTGTTCTGCCGGG
GAGTCAACCTCTTTGATGCGCAGATCGCGGTGATCGTCCCACCAGATCAATAGCTCCAGATGTTCACGTTCTG
CGGCAGAAAGGTGATCGCATACGCTTGATTCCAGAAATTCGCGGATTTCCTCGTGAGACCCTCCCCAGCGCGG
CATACGAAACGGGATATATTCATCCAGCACGTAAAATGCCGTCGGGAAAATAGCCAGGCAGACGGTGAGCCAG
TATTTTTGCCCGGCATCGTCATTGCACTCCGGCAGCACGGACGGGCGCGGCAAATTTGGGCAAACCGCGTCGG
CAAACGACAGCCCGGAATGGGCCATCAGCGCGTCCACCTCCTGCGGTGAATGGCGATGATATTCAGCCAGATC
CTCCATTAATGGCTGCCCGGCAACATCCGCGCCCTCGAGGAACTCAACCAGCCAGTCGGGCTGGCCGAAGACT
TTTGAGTTGGTGCTGGTCAGGGCCGCCGCCATCCACTGGCGCGGTTCACAGTCGATAGCATTGAGCACGGCGA
TGACCATCCGTTCGTTGCAGGCGGCGGCGCAGATCCACATCGCTCGGGTGGTTTCTCTCGCCCAGCCATAGCT
GCGATACAGCCACGCACGGTGATTCCAGTATTGCGCCTCGGCAAGCCAGGCGTGAGTGGAGCGGGGGCGGGCG
CGCTGCCAGTTTTTGATCAGCGCTAATCCTTGCGGCCCAGCCTCAACCAGCGTGTCCATATCGTAAAAAGGGT
TGTCCATTTGATTCCAGGCGAAGAAATAGCGCTTTTCCGCTTCGCGGCTGGTAAAGCTCTCCGTCAGCGTCTG
GTTGTAGCGTTCGTCCAGTTCGTCATAGCGTTCTTCGCGCAGCAGGCCGGAAATATCGGCGATATGCCACTCT
CGTTGCCCGTCATTGAGGGGATTTTTGTGGTTGCACATCTCAAACATAGCGTTGAGCAGACGATCATCCTGTT
CCATAAATAGTCCTTTAAATCGGCTCGAAAATTTTGAATTCATTCCACGGCAGTTGTCGCCAGCGGCTGACAG
GGTCTTGCAGATAATGGGTGGCTTCCTGTAAATCCCGTCCGGCAAGCGCTGTTGGCGTGTCACGTAGCGTGAG
CCATACCCGGCGAGCGCGAACGTAGGCGGTGGCGTAATCCTCCCAGGAATCAAAACAGTCCTGAGCGCGCTGG
GCGTTGAGCAATAGCACCAGCCAGGCCTCGTTTTCGTTACACCAGCCGAGCCCGGCGATACAGCGGGTCAGAA
AGGCGGTGCGCATACAGTCGAAGGCCAGCGCGTCGCGGACCTGGCCCTGGGCCGCCAGATGATGATAAAACGT
GCGCGGCGAGCTGCCGATGCCCGCAGGCCAGCGGCGTAAGATATCCTCAACCTTGTGGCGTAACGCCGCGCCG

TCGAGGTTTTCGCTCAAGCCCAGCTCTTTTTTTACCTGCTGCGCCAGATAGCGGCGTTTGTCATCGTCAAGTT
TAAATACCAGCTCAACGCCTTTGATCGGTAGCCCGTTGCGCCGGGCGAGGATATCAGCTAGCGCCAGCGCCCA
TTGCCGCCGTCGGGTATCGCGACGCAGGTCTTTCAGGCGATCTTTTTCTTTCTCCTCGGCGATACGGGCCGCA
ATCAGCGGTTTGTTTTTTTTGTAGTTGCGCCAGCTAAAAAATAGGGCGGCAATAATAAGAACAATCCAGAACA
TTATTGCACCTCCTTGATGACAGGGAGGTAACGCGTGGTTTGTAGCGTCAGGCTTTCAATTTCGAAAGGTTCG
CTACTGGTGTTTTTCCCGGCAAGCGTAGGATGCCATTCATGCTGCGGAAACAGCATTTCCGCAGCCGTCGCCA
CGTTAGGATCAACAACCTGAAGGTAGCGCAGTGCCTCCGGGGCGCTGTGCAGCAGCAGCCACTGTAAAGCCAC
CGGAGGCGTAGGGAAGTGCTCCATTAAATGAAAATTGTCATGCAGCACATTGAGCAGAAAATTGAGGTCATGA
ATATGAGTAATAAATTTCCAACCTGAATATTCCGGCGTCGATTCATAAACTATTCCCATCCCTTCGCAATCAG
CGTGGCGCTCAGGAAGTGGTTGCAGATGGCGTAACAGAGATAAAACATCGTTAGCATATTTCTTAATAGGATG
GCGATTTTGCTTACCTTTAATCTGATCAATGCTCAGGCCGTTATTACCAACCACCAGGCTTATTGTGACGTGG
GGAATATTATTGCCGTCGCGCAGGCTGAACAGGCGAAGTTTTTGTTGTTCCATTTGCCGGGCGTAGTAATCGC
CGTAGCCGCCGGAAAGCGCGCCTTTATTATCAAAATCTCCGTCGCAGTGTTGCATATGCCAGGATTCATACGC
CATCTCACTGCGCAGGCCGGGATTTGTTGCATCAAGTTCAACCAGCACACCGTTATTCACCGTCATCACCGGT
TTTAGCGCCTCGCTGTTGGAGGGATACCAACCTTGTTCACGCAGGCGCTGCATTTTTTGATGCTCCGCTTCGC
GCATTGCCAGCACGGTAAAGCAGTTGATGCGCTGCAGTTTAGTTTCCAGCCGCGTTCCTTCCTGACGCGAAAG
AAATTCCACTAAATCTCTTTCCATTGCCACAAGTTGCGGGTGCTGAACGTTAAGGTAGTAAAAGCGCTCATCG
CCACCCAGCTTTGGCGCCAGCCAGGCGGGGATTTCTGCATCAGCGCTAAAATAAACGGCATAATCGGCGACGG
AACGCACAGGATAAACATGAGGAAAGTCACCGATGATCCAACGATAAAAATGGTTGCTTAACCAGCGCTGGAT
ATTTTCGCTGTCGCCGCGTTGTTGGCTCCGGGAAAAAATAGACGATTTTACATTTGGCGCATTTATTATATTG
CGTGGTTTATAGCGGTGAGATGACGTTTCTGTCATAAAAATCCATGTATAATTAAGCTGGTATTATGTTTATG
TATTTTTAGATTCACTTAATGAATTAATGCGCAATAAGTTTAAAAAACTACTCATAGTATATCGGTAAACTAA
AAAGAGCGGCCAAATAATTTATTGTCCCCGACCGCGTTCGTCGTGGTTTTCGGGATACCGCTCGGCCAAAGGA
AAGAGGACTGTGTGTTAATTCCGCCAGAATAGCGTTGGCGGAAAATATCGTCTTACGACCTTGTTATTGTTAT
GCCAATAATCATTATACCAGGCGACACGCATCCGCTTCCCAGCGATATCCCACCCCGTAGACCGCGCGTATAA
ATGACTGTTCGGCATCCAGCGATTCCAGTTTACGGCGCAAGTTTTTAATGTGGCTGTCGATAGTACGATCGGT
CACCACACGGTAGTCGTCGTAGAGGTGGTTTAACAACTGCTCGCGGGAAAAAACTTTACCCGGTTCAAGCGAT
AAGGTTTTTAGCAGGCGGAATTCAGCGGGGGTTAAATCCAGCGCTTTACCACACCAGGAGGCCTGAAAGCGGC
TTTCATCAATCATTAACGGGCTTTCGGCATCCTGCTGCTGGAGTTCGCGCTGCGGTTTACAGCGGCGCAAAAT
GGTTTTCACGCGGGCCACGACTTCGCGCGGGCTGTAAGGCTTACAGATATAATCATCGGCGCCGATTTCCAGC
CCCAGCAGCCGGTCGATCTCTTCAATTTTGGCTGTGACCATCACAATGGGGATGTCGGAAAAACGGCGAATCT
CCCGGCATAGCGTCAGACCATCGGTACCCGGCAGCATCAGATCCAGCAGAATCAGATCCGGCGGCGTTTGACG
CACATACGGCAGTACCTTGTCGCCGTGATTGATGAGCGTGGGGGCGTAACTCGCCGCGCGTAAGTAGTCAATA
AGCAGCTGACCCAGCTTGGGTTCATCTTCAACAATCAAAATGCGCGGCGTGTTTTCATCAATGGGTAATTCAG
TCATACTTCTCTCTGTAAATCGCGATCCAGCGGTAACTCTACTGTAATGCTAACCCCGCCAAAAGGCGAATGA
GCGGCGTGAAGATGACCGTTATGCGCGTGTACAATATTGACGCATATTGCCAGCCCCAGTCCAGAACCGCCGC
TGGCGCGGTTACGGGACACTTCCGTGCGGTAAAAACGGTCAAAAAGCTTTTGTAACTGTTCATCGCTGACGCC
CGGTGCAGAATCGGCAAAGGTCAGGAATAATGATTTGTCGCGTTGTTCGGCGGAAATATGCAGGCCGCCGCCG
CTGTCGGTGTAGCGCAGACTGTTTTCCAGTAGATTATTAAACAGTTGCATCAGGCGATCCGGGTCGCCGAAGA
CGGTCATACTATCCGGCAACGCGTAATGTAGCGTTAGCCCCCGGCTGGTAAAGCGCTCTCTGAACGCGCCGCC
AGCGACTTCCAACAGGGGAACAAGATCGACGGTCGTTTTTTGGTAAGCCAGCGCGCCCTCGTCAGACAACGAC
AGCTGATGCAGGTCGTCAACCAGTTTGGTAAGCGTACCGACTTCCGCCTGTAGCGAGGTAACCGATTCTGGCG
TGAACTGGCGAACGCCATCCTGAATGGCTTCCAGTTCGCCGCGCAATACCGCCAGCGGCGTGCGCAGTTCGTG
GGAAATATCCGCCATAAAGTCGCGGCGCATCTGCTGGTTTTTTTCCAGCGTACTGGCAAGCTGGTTAAAGTCC
TGCGCCAGTTTTCCCAGCTCATCGGCGCTGGTTGGGGTCACGCGCGTGGTAAAATCCCCCGCCGCCAGTCTGT
GCGTACCCTCGACCAACCGTTTGACCGGGGCGAGCAGGCCACGCGCCAGCAAAAACGTCGCCAGCGCGGCCAG
CAACGTTGATAACGCCACGATCATCCAACTGGCGCGCCGCTGCTGCATATCAAAATTAATATCCGTATTGCGC
GTCAGCCGCTCGACAGGAGAGGCGATAATCGCGCCGACTTCGGCGCCATTGACCAGAATGGGGCGGCGAGTAC
CGTCATGCGGCACCGGACCGCGTGGGCCGACCAGCACCCGGCCATTTTGATCGACGACCCAGAATTGGGTGCG
CCAACCGTGCGGCGGCATACCGGGGCCGGGTTTATCCCGGTCGTTGTCATGCTCAAATGAACGCAAAATCTGG
AAAACGAAGCGATCGTTGTTGCGTAAAAAACGCCAGTTGCCGTGTTGCTGGTACTGGTCGCCCAGCGCATCGC
CCAGCATTTGTAGCCGCTGCTCGTTGCCGTGTTTGATATAGTCAATAAATCCGCGTTCAAAACTGACGCGCAC
GGCCCAATGCATACTGATCAGCAGCACGATGCAGGTGGCGAAAATCGCCAGAAACAGCTTGCCGGTGATTCCG
GGCCGCCAGACTTTCATTACAGGCTCCTTTTACGCCGTGAAATGACCATGTTTTGCTGTGTATCGTTCGGCAC

GCGGGCAAAAATTATCGCCGGTAGCGCGATAATAACCGCCATACATAACCAGGTGTACATAAAGACGTGATGT
GTGGCGCTACTGTCTATCCCGATATGCTGTTGGCCAAACATACCCAGCAGCATTCCGGCAATAGTGACGCCGA
TACTCATCGAGAGCTGCATAATCATCGATAATAAGCTATTGCCGCTGCTGGCCAGCGTATCGGGAAGGTCTTT
TAGCGTTAAGGTATTCATGGAAGAAAAGCGCGCCGAGTTCACCATTCCCTGCAAAAGCAGTACCAGCGGCAGG
AGATAATACCAGCCGAGCAGCGCGACCGACATAAACAACAGGCTAACCAATGCCAGTCCGAGCGTAGTCGCCA
CCAGAACCCGACGATAGCCAAAACGGTTAACTATCTGCACCACAATCCGCTTCATTCCCATGCTGCCTAGCAC
CATGGGGATCATCATCAAACCGGCATGAAACGGTGAAAAACCAAGACCAATTTGTAAAAAGACCGGCGTCATA
AACGGCAGCATACCGCTGCCGATACGCCCGGCAAAACTGCCCAGCAACCCCAGTGAAAACGTGGGCGTGCGGA
ACAATCGCAGGCTGAATAACGCGCCTGAGTTTTTTTGGCATGGAAGAGATAGAGCAGAATGGCTGCCGCGCCG
CCCGCAGCAAGTCCGGCTAATGTCCAGGGCGAAATCCCCATGCTTTTACTACCATCCAGCGCCAGCGTGAGTA
CCGCCATGCCGATAGCTAACAGCAGGAACCCCGGCAGATCAAAGCGCCGGGTCTCTATGGTGTAGTTTGGCAT
CAGCATAAAGGTTGCCATAGCGCCGACGATTCCCACCGGGATATTGATCAGAAAGATCCAGTGCCATGAAGCA
TATTCCACCAGCACCCCGCCCAGCGCGGGGCCGAGTAACGGCCCGATCTGTCCCGGTAGGGTGACGAAGGTCA
TCGCCGCCATATATTGCGCGCGCGGCACGATTTTCATGACCGTCAGGCGTCCGACCGGCACCATCATCGCGCC
GCCTACGCCTTGTAAAACCCGCGCTAATACCAGTTGATTAAGCGTGCCGGAAAGGGCGCAAAACAGCGAACCC
AGCGTGAACAGCACAATGGCGGCAAAGAAGATATTACGCACGCCGATTTTATCTGCCAGCCAGCCGCTGGCGG
GGAGCATTACGGCGACGGTAAGAACATATGACACCACTACCATGTGCATATGCAGCGGGCTTTCCCCCAGGCT
TTTCGCCATGGAGGGCAGCGCAGTGTTGACGATGGTGGTATCCAGCGACTGCATAAAAAAGCCGAATGCCACA
ATCCAGAGTTGCCAGCGGGTATTGTCAGGAAGTTCTGTCATATTTCTACTACCGGTTTGCTATTTTTACGCGA
AAAACGCAGCCGCAGACGATCGAAAAAGAGGTACACCACCGGCGTGGTATAGAGCGTCAGGAGCTGGCTCATG
ACCAAACCGCCGACAATGGTTATCCCCAACGGCTGCCGTAATTCCGAACCGTCTCCGCCAGATAACACCAATG
GCAGTGCGCCGAACAGCGCCGCCAGCGTGGTCATCATTATTGGACGGAAGCGTAACAAGCAGGCCTGGAAAAT
GGCTTGTGCCGGCGTCAGGCCGCCGCTTCGTTGCGCTTCAAGCGCAAAATCGACCATCATAATGGCGTTTTTC
TTCACAATGCCAATTAATAGCATGATCCCTATCAGGGCGATTAGGCTGAAAGGGGCATTGAAGAGTTCCAGCG
CCAGAAGCGCCCCAACGCCCGCCGATGGTAGAGTAGAGAGAATGGTCAGCGGATGGACGTAGCTTTCGTACAG
TATCCCCAGCACAATGTAGACGGTAGCGATCGCCGCCACTATCAAAATAAGCTGTGAATTCATGGTCTGCTGG
AAGACTTGCGCCGTTCCGGAAAAACTGCCGCGTACCGTCGAGGGGACGCCAAGCTGCGTCATGGTGCGATTAA
TGGCCTCTGTCGCCTGCGATAACGATGTGCCGGTCGGCAGGTTAAAGGCAATCGTGGACGCCGCGGAAAGTCC
CTGATGGTTCACCGACAGCGGCGCATTGGCGGGCCGCCATTGGGCGAAATAAGAGAGGGGAATCGCTTTTCCG
TCACGGTTGATAACGAACATTTTCTCCAGCGCGCTGATATCCTGGCTATAGCGCGGATCGACTTCCATCACCA
CTTTATACTGGTTCATCGGCTGATAAATGGTGGAAATTTGCCGCTGGCCGAAAGCATTATTTAACAGACTGTT
TGCGGCCTGAACATCAATACCCAGCCGTGACATGGTGTCGCGGTCGTAGATAAGGTTCATCTCCGCGCCGTTA
TCCTGCTGGTCGGAGTTTACGTCCGCCAGTTGCGGCAGGGCCGAGAGCGCTTTGCGTATTTTCGGCTCCCATT
CGCGCAGCGCCGCCAGAGAGTCAGACAGCAACGTATATTGGTAACTGGCGTTAGCCTGCCGCCCGCCGACGCG
AATATCCTGTACCGCCATCAGAAACAGCCTGGCGCCAGGTTCTTTTGCCAGTTTGACCCGCAGTCGATCAATG
ATTTGCTGCGCCGTCTCTTTGCGTTCGCCACGCGGCTTCAGCGTAATAAACATCATGCCGCTATTCACCCTCG
ATCCGCCGGTAAAACCAGTGACATTATTCACCGCCGGATCGTCGCGAATAATTTTCATAAAATCCTGCAGCTT
GCCGCGCATGGCCTGGAAAGAGATGCTTTGGTCAGCCTGAATACCGCCCATCAACACGCCGGTGTCCTGCTCC
GGAAAGAATGTTTTAGGGATGGCGATATAAAGCCAGATGTTCAGCGCAACGGTGCCAAGAAAAACCACCCCGA
CAAGACGCGTATGGTTAAGCACCCATTTTAACGACGTGCCGTAACCCTGTTGCAAGGCGACCAGCAAACGGCC
AACGCCCCGTTTACGCGGTTGGGTGCGCGGTTTGCTTGATTTAAGCATCCAGCCGCACATCATCGGCGTCAGT
GTGAGCGAGACCACCAGCGAAATGCCAATCGCCACCGAGAGGGTAACGGCGAATTCCCGTAATAATCGTCCTG
GCAGGCCGCCCATTAACAGCAGCGGCAGAAATACCGCCACCAGCGACAGACTCATGGAGATGACCGTAAACCC
GACTTCCCGCGTACCCTGTAATGCCGCCTGCAAAGGTTTCATTCCCGCCTCCAGATGGCGGGCGATATTTTCC
AGCACCACAATGGCATCATCAACGACAAATCCGGTCGCGATAGTCAGCGCCATCAGCGACAGATTGTTGAGGC
TGAAGCCGCACAGATACATGGCGGCGAAGGTGCCGATGAGCGAAACGGGAACGGCGACGGCGGGGATTAGCGT
GGCGCGCCCGGAACGCAGGAATAAAAACACCACCAGGATCACCAGCGCAACAGAGATAGCCAGTGTCTCTTCT
ACCTCTTGCAGCGATGCGCGAATCGTCGGCGAACGATCCTGGGCGATTTGTAAATCGATAGCGGCGGGGATCA
TTGCCCGCAGTTCCGGCAGTTTTGCCCGGATGCCGTCGACCGTCTGAATAATGTTGGCCTCCGGCAGCTTGCG
GATCATCAACAAAATAGCGGGTTTAGCGTTCGTCATCCCGGCGTTACGGACATCCTGCACCGAGTCAGTGACG
CTGGCGACGTCGCCCAGGCGTACCGCCGCGCCGTTGTTATAGTGAATAATCAGCGGCTGATATTCGGCGGCGG
TTTTCAGTTCGTCGTTGGTCTGGATTTGCCAGCGGTGGACGCTATCTTCAATTGCGCCTTGTGGTCGGCGTAC
GTTGGCGCTGTCGATCGCTTCGCGGACCTCATCCAGCGAGACGCCCTGGTTAAAGAGCGCCTGCGGGTTTAAG
CCTACACGTACTGCGGGCAGGGAGCTGCCGCCGACGTCAACATCGCCGACGCCGTCAATTTGCGCGATGGTTT

GCGCCAGTTGGGTAGAGGCGAAATCATACAGTTTGCCCTGTGACCAGCTCTCCGAGGTAAGCGTTAAAATCAT
AATCGGCGCGTCGGACGGGTTGGCCTTGCGATAAGTCGGGCGGCTGGGCATTCCGCCTGGCAACAAGCTTTGC
GCGGCGTTAATGGCGGCCTGCACGTCGCGCGCCGCGCCGTTAATATCACGATCGAAATTAAATTCGAGAATAA
TGCGTGTACTGCCGAGCGAGCTGCTGGAGGTCATTTCATTGACGCCTGCAATGCGTCCCAAAGAACGTTCCAA
CGGCGTCGCCACCGACGAAGCCATGGTTTCCGGCGAGGCGCCCGGCAGCGAGGCGCTAACCATAATCACCGGG
AAATCGACCTGCGGCAGCGGGGCGACCGGCAGCAGACGAAAGCCCAGAATGCCGCACAGCGTAATGGCGGCGG
CAATCAAAATGGTGGCGACCGGGCGGTAGATGAAAAGGGCGAAAAAGCGCATCTACGCCTCCTCTTTATGGCG
CGGAAAGCGACTTTTCACGTACAGCGACAGGCGGTCAAACAGGAGATAAATCACCGGTGTGGTAAACAGAGTT
AATACCTGGCTGACCAGTAGGCCGCCCACCATCGCGATCCCCAACGGGCGACGTAATTCCGCGCCAACGCCGG
TACTCAACATTAATGGCAATGCCCCGAGCAACGCCGCCAGCGTGGTCATCAGAATCGGTCGAAAACGCAGCAG
ACAGGCCTGGAAGATAGCGTCGCGCGGACTCATCCCCTGTTCGCGTTCAGCGGCGAGGGCGAAGTCAATCATC
ATGATGGCGTTTTTCTTCACGATACCGATCAGCAAAATAATGCCGATAATGGCGATAATATCGAGCTCGCTAC
CAGCGATGATCAGCGCCAGCAGCGCGCCGACGCCCGCCGTCGGCAGCGTTGAGAGAATCGTGATCGGATGGAT
AAAACTCTCATACAGCACGCCGAGCACGATATACATCGCCACCACGGCGGCGACAATAAGCCAGACGGTGCTG
CCTAGCGCCGCCTGGAAGGCGAGCGTACTACCCTGAAACTGCGTTGTAATATCTGCTGGCAGGGCGAGCGTTT
TTTCCGTATCGAGAATCGCCTGCACCGCATCGCCGAGCGAATAGCCCTCCGGCACGTTAAACGAAAATGTCGT
AACCGGGAACTGATCTAAATGATTGATGGAGAGCGGAGCGAAGCGCTGCTCAATGCGGGCAATCGCGCTGAGC
GGTACGGTGCCGCCGTCGCGGCTCGTCAGGCGAATCGTCTCCAGCGCCGCCAGGCCCGGCGTGCTGGCGGTAT
TATGTTCCAGCACGACGCGGTACTGGTTCGCCTGGGTATAAATCGTTGAAATCAGGCGTTGTCCGAACGCGTT
GTAGAGCGCGTTATCCACATCCGCCATGCTGATACCCAGACGGCTGGCGCTGTCGCGGTCGACATTCACCCAG
GCCGCTAATCCTCGATCTTGCCAGTCACTGCTTACCTCAGAGAGCTGTGGTAACGACTGTAGCGCGTTCTGCA
GTTTTGGCACCCAGTGGCTGAGCGCATCGAGCGTCGTGGCCTGCAGGGTAAACTGATACTGCGTGCGGCTGAC
CTGCGTGTCGATGGTTAAATCCTGCGTCGGCTGGAGATACAGCGCCACGCCGGGAATCGTCGCCACGGCGGTT
TGCAGCCGGGAGATCACCTGCTGCACGCGGTCATCACGCGCATCCAGCGGCTTGAGGTTAATTTGCAGGCGCG
CGCTATTGAGCGTGGGGTTAGCGCCGTCTACGCCAACAAAAGTCGTCAGGCTTTGCACCGCCGGGTCCTGTAA
TATCCGCTCCGCCACCTGGCGTTGACGTTGCGCCATACTGGCATACGATGACGATTGCGGCGCCTGCAGCGTC
CCCTGGATAATGCCGTTATCCTGTACCGGAAAGAACCCTTTCGGAATGACTATCCACAGCATAACGCTGAGCA
GGAGCGTGGCGAATGCCACGCTCAATGTAAGCCACGGATGGTTGAGCACTTTCGCTAATCCGCGTCCGTAGCT
GGCGATCACGCGGTCAAACATCCGCTCGCAGGCGCGGGAAAAGCGGTTTTGTTTACGCAGAGACTGCTGGCTG
AGCATACGCGCGCACATCATGGGCGTGAGCGTCAAAGAGACGACGGCGGAGATTAAAATCGCTACCGCCAACG
TCACCGCAAATTCGCGGAACAGTCGACCAACAATATCGCCCATAAAGAGCAACGGGATCAGCACCGCAATCAG
TGAAAAGGTGAGGGAAATAATGGTAAAGCCGATTTCGCCCGCGCCTTTGAGCGCCGCCGCCAGCGGTTTTTCT
CCTTTTTCGATGTAGCGCGAGATGTTCTCGATCACCACAATCGCATCGTCCACCACGAAACCCGTGGCGATAG
TGAGCGCCATCAGCGTCAGGTTATTAATGGAAAAATCCAAAAACACCATCACCGCAAAGGTGCCGATAAGCGA
CAGCGGTACGGCGACGCCGGGAATAATTGTGGCGGGAATATTACGTAAAAACAGATAGATAATCATGACGACC
AGCGCGATCGCCAGCATCAGTTCAAACTGGGTATCGCGCACGGAAGCGCGAATATTGGTGGTGCGATCGGACA
GGACCGTGACCTTCACCGATTTCGGCAGGCTTTCGGTAAGCTGGGGCAGCATCTGGCGAATGCTGTCCGCTGT
CGCAATGATATTGGCGCCGGGCTGGCGTTGAACGTTCATCACGATAGCCGGCGCTTGATTCGCCCATGCGCCG
AGCCAGCTATTTTCCGCCCCCTGTTCGACGGTGGCGACATCGCCCAACCGTACCGGCGCGCCGTTTTGATACG
CGATGATAAGCTTGCGGTATTCGTCGGCAGACTGCATCTGATCGTTAGCAGAAAGCGTCACCGCCCGCTCGGG
GCCATCCAGACTGCCTTTCGCCGAGTTGACGTTGGCGCCGGTAATTGCGGTACGGACCGTTTCGCTGGTCAGA
CCGAGCGCGGCGATAGCCTGAGCATTCAGTTTTACGCGTACTGCAGGGCGCTGCCCGCCGGCAAGCGTCACCA
GTCCGACGCCGGAGACCTGCGAGATCTTCTGCGCCACGCGGGTTTCTACCATGTCCTCTACCTGAGTCATTGG
CATCGCGTTTGAGGTGACGGCAAGCGTCATAATCGGCGGGTCCGCCGGATTGACTTTGCTATAAATCGGCGGA
TTCGGCAGGTCGGAAGGCAATAAATTGGTGGCTGCGTTAATCGCCGCCTGTACTTCCTGCTCGGCAACGTCCA
GCGGCAGCGTCAACTGAAACTGTAGCGTTACCACCGACGCGCCGCCGGAGCTTTGCGACGACATCTGCTTCAG
TCCTGACATCTGGCCGAACTGGCGCTCAAGCGGCGCGGTGACGGCGGAGGTCATGACATCCGGGCTGGCGCCA
GGGTAGAGCGTAACAACCTGAATAGTGGGGTAGTCGACCTCCGGCAAAGCGGCGACGGGCAGGAAGCGATAGC
CGATAATCCCGGCGAGTAAAATCGCCGCCATCAGCAGAGTGGTGGCCACGGGGCGCAGAATAAACAGACGCGA
GGGGCCGCCCGTGCTGCCCGGAGGTAATACCTGCATCAGGCGCGCGCTCCTTTTTGACCTTCATGGCGGGAAG
GGGATTTTTCATCCGCCACGGTGGTTTGCGGCTCAACGACCTCGACTTTTGCGCCTTCCGTCAGCCGATCAAT
ACCATCGGTAACGACGCGATCGCCTGCCGACAAGCCAGCGCTGATCACCACGTTTCGGTTATCCTGAATACCG
ATTTTTACCCGCTTCTTGCTGACGTTATTTTCGTCGTTCAGCACCCACACAAAGTGGCCCTCATTGCCCATTT
GCACCGCCGCGGCAGGCACCACAACGGCATTTTGTTCGGTATCGACCAGCATCCGGGCGTTCACAAATTGATT

GGGGAACAGCGTATCGTCCTGATTGGTAAAGCGCGCTTTAATTTTGATCGTTCCCGTTGTGGGATCAATCTGG
TTGTCCAGACTGAGCAACACACCTTCGCTCAATTTGTGCGAGTTAGTCCGATCCCAGGCTTCTACGACCAGCG
TTTTCCCCGCTTTCTGCGCCTGAACCACGGTCGCGATATCGCTTTCCGGCAGAGTAAAAATGAGATCAATCGG
GTGCGTTTGCGTAATGACGACAATACCTGCGGTATCGCTGCTGGAAATCTGGTTGCCGACATCCACCTGTTTC
AGACCCACGCGTCCCGAGACCGGGGCCGTGATACGACTCCAGTCGAGCTGTAACTGCGCGCTGGCGACATTGG
CTTCATCCGCTTTAATGGTTCCCTGGGTTTCGTTGACCAGCGCCTGTTGCGCATCCAGTTCCTGACGGGAAAC
CAGATTGGTTTTTGCCAGTTGCTGATAGCGCGCCAGATCACGACGCGCATTCGCCAGCGTAGCGTTATCTTTC
GCCAACTGTCCCTGAGCCTGCGCCAGGGCGACCTTAAACTGGCTGGGATCGATTTGCGCCAGCAGATCGCCTG
CGTTGACCTGCTGACCTTCCTGAAAGTGCAGGGAGATGAGTTGACCATCCACGCGGCTACGCACCGTAACGGT
ATTCGCGGCGGTCACGGTACCCAGCCCGCTCAGATAGCGCGGTACGGCCTGCGTGGTCGCGGTCGCGGCCTGT
ACCGGCGCCAGAGGGCCGTCGCGCATACCGCGGCGACCTGCGGCGGCGGTATGCGGCGCTTGCGCGGCGACGC
CCGGCGCGGCGGTCGGGCTTTCGCTACGGCTATGCCAGAACCAGAATGCGGCGGCGGCCACTACAACCCCAAT
CGCTATTGCCCAGCGGAAAGTATTACTGCCTTTCATCGGTATAAGTTTCTCATCCTGAAACGTTTTGCGGAAT
GTTGCTTCCTCTATTGTAGTCAGCAGGGAAGGGAGAAAAATGGAGGAATTATGGAACACGGTCGGGATTATTC
TGAATGGCGACTCGCTTTGCGAGGCGCTACGCAACTCTCATCGGTGAAAATCGTTAACGCGCCGGAGTGCGGT
AAAGTGGCGGCACGGCAATGAAGGTAAGCCACCGATTTCAGCAGGACGCTGAAACGGGAAAGCCCCTCCCGAG
GAAGGGGCCAAAATAAGGAAAGGGTTATGATGAAGGTAGTCATCATACTGGTGATACTCTTAGTGATTAGCTT
CCAGGCTTACTAAGACACCAGGGGGAGGAGGAATCCTCCCTAACCCTCACTCCTGACGTTAAGCCACATCAAG
AATAATGTCAATCCATCAGCGCTGGGCTTGCGAGGCGCTTAGCGGAAAACGACCTCTGCCCAGCGAGCCAGTC
CTGCGGTGACGGAGCCGAAATCATCGCCGCCAGCGACGGGTATGCCCGGTAACTGTTCGGAAAGCGCTTTTTT
AATCAATGGCGAACGGGCGCTGCCGCCGGTCAGATAGATGACATCCGGTTTTTCCTGCGCGCTGTCCAGCGCC
AGTTGCACCTGTTCCAGAATACGCGCCAGCGGTTGATCCAGCGCCGCTTCCAGTCCTTGCTGGCTGATAGCGA
CCGCAAGGTCGTCGCTGATAAATGGCAGCCTCGCCGTAACATCCGCCTGGCCGGAAAGGGCGATTTTGCTCTC
TTCCGCGCAGCGTACCAGGCGGTAACTAAGCCGCTGACGCCAGACCTTGAGCAGCAACGCGACCTTGTCTGCT
TCACGCGCGTTGCGAACCAAATCGTTCAACAGACGACCATTCGCGCTACTGTAAAAATCGCTTTGCGCCGGGA
CGTCGTTAATCGCCACGGCGTTCCACCACGGCAGCACCGGTAGGGCGATGCCTTTTTCGGTTTCGCCGCCCAT
GCCCAGCAAAGGCATCAGATTTTTAAAGGCCAGCGCGATATCCAGATCGTTTCCGCCCACGCGGCAACCGCTG
TGCCCCAACAGGCTGTTTTCACGATCGGCGCGCTGACGCCACTGCGGCCCCATCAGCAGCATGGAACAGTCGG
TGGTACCGCCGCCAATATCCACGACCAGCACACGCTTCTCTTCCCGCAGCGTAGCTTCGTAGTCCAGGCCCGC
CGCAACCGGTTCATACTGGAATACCACCTCCTGAAAACCGGCGCGTTTTGCCGCCCGCTCCAGAATACCCTGC
GCCTGGCGGTTCGCGTCATCGCCGCCGAGTCCCTGAAAGTTGATCGGGCGACCGATCACCGCCTGGGTAATGG
CCTCCGGCAATTGGCTGTGCGCCGTATGACGAATATGTACCATCATGGCGCAGACTAAATCTTCAAACAGCGC
GACCTGCTGCGGTTTCAGGCCGCTGGCGCCTAAAAAGGATTTCGGCGATTTGACAAACCAGACCTCCTGGGGA
TCGTCAATATAGTGCGCCAGCGACGCCAGACCAAACTGTACGCTTTGCGCGCCAACCTCAATATCTTCCTCGC
GGTTGTAGCGTATCGCGCGGCGTAGCAGCGCCTGGGTTTCTTCGTCCGTTGCCGGCACATCATGATGGCGATA
CAGCCACTCGCTCACCGCTTCGCGCGTCGGCGCGCACAGCATAGACGGCAGTAATGCGCTGTTATTCTCCATT
GTCAGCAACTGTGGATGCCCGTCGCGCATAATGGCGACCGAGCAGTTTGCCGTACCGTAATCAAATCCGATAA
ACACCTTAAAATCCCCATGCCAGTGAAGAAGGGGCGTGACTTTAGCGAAATGTCGTCGCGGCGACAACTGGAA
TATGAAAGCAAGGCGTTACGTGCGATTAGCGTTTATGCTGAAAGGTCAAATAAAGGAGAAAAGTATGTTTACC
CTGAGCTGGCAGCCGCCTTACGACTGGTCGTGGATGTTGGGCTTTCTTGCCGCGCGTGCGGTGGACGGCGTGG
AAACGGTAGGCGAGGGCTTTTATGCGCGCAGCCTGGTGGTGGGCGAGCATCGCGGACTGATCAGCGTGAGCCC
CCATCTGCCCACCCACACTGTGCAGGTCAGCGTGAGCGCCGGATTACTTCCCGTCGCGCCGGCGTGTCTGGCG
AAGGTTTCGCGTCTGTTTGATCTTGACTGCCAGCCCGCGCAGGTTGCCGCCGTGCTGGGGCCACTGGGCGAGG
ACAGACCGGGGCTGCGTTTACCGGGATCGGTTGATACGTTTGAACAAGGCGTGCGGGCGATACTGGGGCAACT
GGTGAGTGTCGCGATGGCGGCAAGGTTGACGGCGAAAGTGGCGCGCCGCTATGGCGAGGCGTTGCCGGATGCG
CCCGATTATGTCTGTTTTCCCGGTCCGGAAACGCTGGCGTTGGCCGATCCGCTGGCGCTGAAAGCTCTGGGGA
TGCCGCTGCGGCGCGCCGAAGCGCTGATTCATCTGGCGCAGGCGACGCTGGCGGGAAAACTGGCGCTCGCCGC
GCCGCCGGATATTGAGCAGAGCGTTAAAAATTTACAAACGTTTCCGGGAATTGGCCGCTGGACGGCGAACTAT
TTCGCCCTGCGCGGTTGGCAGGCAAAAGATATCTTTTTACCCGATGATTACTTAATTAAGCAGCGCTTCGCCG
GGATGACGCCCGCGCAGATCCGTCGCTACGCGGAGCGCTGGAAACCGTGGCGTTCCTACGCGCTACTGCATAT
CTGGTATACCCACGGCTGGCAACCGTCAATGGATAGCGAAATAGCTGGTATTCAGTAATAAATCCAGCGGTTG
CGCTTCGGCGATAGTGTCGCCATAAATCAGATCCACGCCAATACAGGAGAGCGTATCCAGCACCTGTGGGTTT
TGTACCGGCCCGGCGAGCGTTTTGATGTCCAGATGCTGGGCGTGTCCCTGGATGATCGAGGTCAGCATTTCAT
CCATTAAACTTTCATGAACGTTGGCGATAAGGTCGCTATCCAGCAGTAAATAATCGACGATATGCGGGGGCAG

TAAATTAAACAGCTGTAAATCGCGCCCGACATGGCTGAGAATCACCTGGCAGCCGCGTTGCCGCAGTTTTCGC
AGAGTCGCAGCGGCGGTCTGCGCCTGCTTAACGATAACGTCAGCCGGGATAATCAGATGTAATAACCGTGGCG
GCAGTGGGCTATGCTCCAGTTGTTCCAGCAGCTCATCAATAAGGGTCGCGCTGTATAATCCCGCGGCGGACAG
CGGCAGCGCAACGCTTAATCCCTTGCTGGCGACGGCGGTCGCCGCATGGTGGAAAAACTCATGGAATACCCGT
CGGTCAAGCGCGTGATGCAGCGCGGAGTCGGCTAACCCTGCCCGGAACGCGCGCTCTTCCAACACTTCCCCTT
CGCTGGTCCACAGCCTGAGTGAGACCAGCCAAAAGCTGGTGGCCTCCGGCGTACGCGGCGGCGCGACGTTTCT
GGCAAGCATCAGCAGGTGGTTATTTTTAATCATATGCCACTGTTCTTCCAGCGGCATCATTCCCTTTGAGGAG
GTTAATGCGTGCTGCGGTTCGTACACGGTCAGACGGCCGCGACCGCTATTTTTCGCGGCGTAGCAGGCTATAT
CGGCCTGAGACACGACCTCTGTGAGCTGGCAGTTATGTTCATTTATCATTGTGATCCCGGCGCTGGCGCCGAT
CCGATGCAGGCGTCCTTCCCACATAAAGTGATACTCATTAACGGCGTTGATAAGCCGCGTCACGATAAAGCGT
GCGCTGTCACTATTACAATCCGGCAGCAACAGGCCAAACTCATCGCCACCCAGACGGGCCAGAAGATCGCCGG
ATCGAAGCATACTGAGCATCAGTGACGCGAGTTCGCGCAATAAGGCATCGCCGGCGGCATGTCCCGCGCTGTC
GTTAACCGCTTTAAAACGGTCCAGATCGATAAACACCAGCGCATGATGTTGAGGCGATTCCTGTATTGTTTGC
AGGCGCTGTTGAAGCTGTTTTTCAAAACTAGCGCGGTTGGCAAGATGGGTGAGGGCGTCATGCGTTGCGCTGT
AGCTGAGCTGGCGTAACATTTTTCGTGATTCGGTAACGTCCTGGATAACCAGAACCGAACCAATCTTATCGCC
GTCAAGCGTACTGAGCGGCGTGATACTGTAATGAATATCGTAGCTGCCGCCGTTATGACAATGGAGCACTATC
TCCTGTTCCATGTCCGATCGCGACCTGTCGGCGCGATAAATATCCTCCAGCAGCGGCCCCTTATCACCTGACG
TAATGCGCAGAACCGTTAATAATGGCGCGCCTAAAGCATCCTCTTGCCGCCAGCCGCTCATCTTCTCGGCGAT
CGGGTTCATAAAGGTGATATTCATCGCGACATCAATACACACCACCGCTTCTCCGATAGAGTCAAGCGTGATG
TGCAACCGTTCTTTTTCCTGAAACAGCGCGTCGTTCGCTTGTGTAATACGTTCCATCAACCGCTGGTTTTCCT
GCTCGCTTTGCTTGAGATCGTTAATATCTTCAATCTGAGCAATAAAATAGAGAGGTTTATTGTCTTTATGACG
AACCAGCGAGACGGCCAGTAGCGCCCAGACGACATCGCCGTTGCGGGTGTAGTAGCGTTTTTCCATGGAATAG
CTATTGATGTCGCCGCGCACCAGCATGTTTAGCTGTTCAAGGTCATTATTCAGGTCTTCCGGCCAGGTCAATT
GCTGAAATGTCATCGTGCGTAATTCATCCTGGCTATAGCCAAGAAAATGGCTCAGAGATTTATTGACCTGTAG
CCACTGACCCTCCGTACCGACCAGCGCCATACCAATGGCGGAATACTCCATCGCGTTGCGAAAGCGGGATTCG
CTCTCGGTGATATGTTTGCGCTCCGTGCGAAACGCGTACATCACCATCGTCATCATATTGGCAGGCAGCAGGA
TCATTAAAAAAGGCAGCCACGGCATATTGACCATCACGTCGGTTTTCGGCGTGGCGAGCAGCGTGGGGTCATT
GGCCAGCATGAGCGAGACCACGATAACGGTAGCTAAAAAAATTAAAAACGCCTCCATTCGCGGCAGGCGCACC
GCACTCCACATCAACAGGACGATAACGCACGTAAACGGCCACGGAATATAGCGCATAGCGAGCCAGCTTAGCG
CCAGCGTAATGACCAGCGTCAACAGCGTTTCCAGCAACAGATGCGGGTCGCGATGACGCAATAGATAGTGTGG
TTTAAACAGTAAACCTAACGGCACCAGCGTCAGCGCGCCGATGGCTTCCGATAATACCCAAATCAGAAACGCT
TTCGAGGCGACCGCTTCCGGCGCTATCAGCCAAAATAACAGGCCGCCGAGTAGCGGCGGAATCACGGCGCTGC
CGATAGCCAGACGAAACCAGTCATTCAAATTTTGTAAGGGATTGTAGCTGGGGAGCAGTTTTCGCAGCAGTAT
CGCGCCAGTGGCCGCCTCGATAATATTTATTGCCGTCCAGCTAAAGTTAAGCGAGGCGGCAGGAAATAAAGTC
AGTGAGGCTCCGATGCTGCCGAGCGAGCAGGCGACCGCGATCCCTGGCCACAGACGGCCCGCATGGCGGTAAA
ACGCGACCATCATAATGGAGGTGGGGAACCACAGCGGCGCGAGTTGGGTGCCGACACGCGATAATACGAGCGA
AAAGAGCGTAAAGACAAACGCGACCAGGCCTAAACTGACCAGACGCAATAGCGGGTGGGGGACGGTAACGAAA
ACATGTTGGGATGGTTTACTCATTACCACTCTATCCGAATCGCCGGTAACGTCGCGGAATACCGGACGCGTCA
GTTTACTCTGTGATTATGCTAGTATAAACGCGTAATGATTTATACGCTGGCTGAATATAAATTGTGGCTCCCG
GCGTATTCCTCCTCCAGACGGGGCGATAAATCCGCTGTCCGCGTTAAAACTGAACGCTTTTACGTCGCGACGT
CGAATACGCCGCCAACAAATTCACCGTGGCATAGCGCCGGACTGGTATCGCGCTGCTGAAATCCCTATAATTG
CCGCGTTTGGCGTTTCGGCGCCCCCTTCCTTACATCCAGGTTAATCAGGTCGCTAAATTTATGACTGATCAGT
CTCATCAGTGCGTCATTATCGGTATCGCTGGCGCATCGGCTTCCGGCAAGAGTCTTATTGCCAGTACCCTTTA
CCGCGAATTGCGTGAACAAGTGGGTGACGAACATATTGGCGTTATTCCCGAAGACAGTTATTACAAAGATCAA
AGCCATCTGTCGATGGAAGAACGCGTAAAAACTAACTATGACCATCCGAATGCGATGGATCACAGCCTGCTAT
TTCAGCATCTTCAGGCGCTCAAGCGCGGTTCGGCGATTGAATTACCGGTATACAGTTATGTTGAGCATACCCG
TATGCAGGAAACGGTTCGCGTAGAACCGAAGAAAGTGATTATTCTTGAAGGTATTCTGTTACTGACCGACGCG
CGTTTGCGTGAAGAGATGAACTTCTCTATTTTTGTCGATACGCCGCTGGATATCTGTTTGATGCGCCGTATCA
AACGCGATGTGAACGAGCGTGGTCGCTCAATGGATTCGGTGATGGCGCAATATCAGAAAACGGTGCGTCCGAT
GTTCCTGCAATTTATTGAGCCTTCCAAACAATATGCGGATATTATCGTGCCGCGCGGCGGTAAAAACCGCATC
GCTATTGATATTCTGAAAGCGAAAATCAGCCAGTTCTTTGAATAAATCTGGCGAATTGTGTACCGTTCAGTGA
TAACCTGGTTAGCCCTTGATGTGCTTGATTTAAGGGCTTGATGCGCGAAAGGAGAAAGTGCCATGCGTTTATG
CGACCGAGATATTGAAGCCTGGCTGGATGAAGGCCGTTTGTCTATCACCCCGCGACCTCCCGTAGAGCGCATT
AACGGCGCGACGGTCGATGTACGTCTTGGCAATAAATTTCGAACCTTCCGTGGCCATACGGCGGCGTTTATCG

ATCTGAGCGGGCCGAAAGACGAGGTCAGCGCGGCGCTGGATCGCGTGATGAGCGATGAAATCGTTTTGCCGGA
CGGCGAGGCATTTTATCTGCATCCGGGCGAGTTGGCGCTGGCTGTCACCTTTGAATCCGTTACCCTGCCGCCG
GATTTAGTGGGCTGGCTGGATGGTCGCTCCTCGCTGGCGCGTCTGGGATTGATGGTGCATGTGACGGCGCACC
GTATCGATCCCGGCTGGTCGGGCTGCATTGTGCTTGAGTTTTACAACTCCGGGAAGCTGCCGCTGGCGTTGCG
TCCGGATATGCTCATCGGCGCGCTGAGCTTTGAGCCGCTGTCCGGCCCGGCGGCGCGGCCTTATAACCGCCGT
CAGGATGCCAAATATCGCGATCAGCAGGGCGCTGTCGCCAGCCGGATTGATAAAGACTGAGCCATTCGGCGCA
ATACTGCCGGTCCATTGAGGGTAGCATGAGACGATTTCTGACGACGCTGATGATTCTCCTGGTCGTGCTGGTG
GCCGGATTCTCTGCGTTGGTATTGTTGGTCAATCCGAATGATTTCCGCGCCTATATGGTGCAGCAGGTTGCCG
CGCGTAGTGAATACCAGTTGCAACTGGACGGGCCGCTGCGCTGGCACGTATGGCCGCAACTCAGCATCCTTTC
CGGGCGAATGACGCTAACGGCGCGAGGCGCCAGCGAACCGCTGGTGCGGGCTGACAATATGCGCCTGGATGTC
GCTTTATGGCCGCTGTTGAGTCATCAGCTTCATGTTAAGCAGGTGATGCTGAAAGGCGGGGTGATTCAACTGA
CGCCGCAAACGGAAGCGGTACGCAGTGATGATGCGCCGGTCGCGCCGAAAGATAATACCTTGCCGGACGTAGC
GGAAGATCGCGGCTGGTCGTTTGATATTCGTAGCCTGCGCGTTGCCGACAGCGTGCTGGTTTTCCAGCATGAA
AATGATGATCAGGTCACGGTGCGCGATATTCGTCTGAATATGGAACAAGATGCCGAGCATCGCGGCACGTTTG
ATTTTTCGGGACGCGTGAACCGCGATCAGCGGGATCTTGCTTTATCGTTCAGCGGAACGGTGGATGCTTCCGA
TTATCCGCATAACTTAACCGCGGGTATTGAGCAACTGCGCTGGCAATTGCAGGGCGCGGATTTACCGGCGCAG
GGGATTGAGGGGCAGGGACAGCTGCAGGCGCAGTGGCAGGAGGCGCAAAAACGTCTTTCATTTAACCACTTAA
ATTTAACGGCGAATGACAGTTCTCTGACCGGACACGTTCAGGTAACGCTGGCGGAGCAGCCGGAATGGCAAAT
TGACCTCCAGTCCAGCAAGCTTAATCTGGACAACCTGTTGCCGCATCATAGCGCCGTGGCTCAGACGGGCGGC
GCGGTGTCGCAGGGGCAAAATACGCTCCCCCTGACCAGGCCAGTTATCGCGTCGCGTGTTGGTGCGCCGCCTT
ATCAGGGGCTGCAAAGCTTTACGGCGGAGATTGCTTTACAGGCGGATTCAGTTCGCTGGCGGGGAATGGACTT
TACCCAGGTTTCGACGAAGATGTCTAATCAGGCCGGATTGCTGGATATTACCGAGTTGCAGGGGAAACTGGCC
GACGGAGAGATGTCGCTGCCCGGCACGCTTGACGCCCGCACCGCCAGTCCACGCATCGAGTTCCACCCTCGCC
TCAACCATGTTGAGATTGGGACTATCCTGAAAGCCTTCAATTATCCGATTAGCCTGACCGGTAAGATGTCGCT
GGTTGGCGATTTCTCCGGGGCGGATATTGACGCGGAGGCATTTCGCCACAGCTGGAAAGGAAAAGCGCATGTT
GACATGAGCAATACGCGCCTCGAAGGAATGAATTTCCAGCAACTGGTGCAGCAGGCCGTAGAGCGAAGCGGCG
GCGACGCGCAGCAGTCGCAGGAAAATATGGACAACGCGACCCGACTGGATCGCTTCACGACCGATCTGACGCT
AAATAAGGGCACGCTGACGCTCGACGACATGGTCGGGCAATCTTCTATGCTGGCGTTAACCGGCAGCGGAACG
CTGGACCTGGTTAAGCAGAGCTGTGATACGCAATTTAATCTGCGTGTGCTGGGCGGCTGGAACGGCGATAGCA
ACCTGATCACCTTCCTGAAAGAGACGCCGGTGCCGCTGCGCGTCTATGGCAAGTGGCAGGAGCTGAACTATAC
CCTGCAAGTGGATCAGTTATTGCGTAAGCATTTACAGGATGAGGCGAAGCGTCGGCTCAACGACTGGGCGGAG
CGCAATAAAGATACCCGCAATGGTAAAGATGTGAAGAAACTGCTGGATAAGCTGTAGTTTTTGGCCAAAGGGC
AGAGCCGGATGGCAAGGCTAACGCGCCTCCGGCCTGCCTGGTAAGCAGGCCGGGGCAGGTGAACAGGCGTTAC
ATCTCCTGGTCTGGCTCGCCGTTATGCAGCGGAATTAACTGCACTTTCTGCACTCGATGGCTTTCTACCTGCA
ATGTTTTCAGCAAATAATCACCCACCTGAACCTCTTCACCGGTTTGCGGTATGCGCTGCAAATATTCCATCAA
TAATCCGGCAATCGTATGGTATTCCCGTTTTTCATCCAGCGGTAGCGGCACGTACTGCACCAAATCCTCCAGC
GGCATATGGCCGTTTGCTGTCCAGGAACCGTCCGGGTTCTTCTGAATATCGTGGCGGGCGTCAATTTCCTCGA
CTTCATTCGGCAAATTACCGGCGATAGTCTCCATGACATCGCTTAATGTCACAATGCCTTCAACCGAGCCAAA
CTCATCTACGACAAAGGCAAAATGGGTTCTGGCGTTACGGAACTGCTCCAGCGCAGGCAGCAGCGGCAATGTT
TCCGGGAAAACCAGCGGTTGGCGAATCAGCGCCCGCAGGTTGAGCGGTTCGCCGCGCAGCGATTGTTGCAACA
GATCGATGACGTGGACGACGCCGAGTAAATCTTCCTGTTCATTCTCACCCGTCACGACCAGACGCGTATGCTG
GTTCTTTTCCAGCAGAGCGCGGATTTCCGCTTCCGGCGCGTTGAGATCGATATGCTCGATATCGTGACGCGAC
GTCATGATGCTACTTACGGTGCGCTGGTTGAGATTCAGCACCCGCTTAATCATCCTGCGCTCCTGCGGATTAA
AAATCTGGCTGTCGTCATGGTCTGCCAGCATCGCGGCGGTTTCCGCATCCAGTTCGGCATCCTCTTTTTTACC
GCTAATCAGCCGCATCACGGCCTCCGTTGTGCGCTGGCGCAGCGTATGGTTAGCGGAAAGAAAACGGCGGCGG
TTAAAAATAGCCAGTTGGTTGAGCGCCTCGATCATCACCGAGAAGCCGATAGCCGCGTACAGATACCCTTTCG
GAATGTGGAAACCAAACCCTTCCGCCACCAGGCTAAAGCCGATCATTAGCAGGAAGCTAAGACACAAAATGAC
AATGGTGGGATGGTTGTTCACAAATCGGGTCAGTGACTTGCTGGCGAGCAGCATCAGGCTAATGGCGATTATC
ACGGCCGCCATCATGACCGCCAGGTGATCGACCATCCCCACGGCGGTAATCACGGAGTCGAGAGAAAAGATAG
CGTCCAGCACCACGATTTGCGCGACCACCGCCCAGAATTTCGCCCCCTTACGCTGGGTAGGGTTGGCGCTATC
TTTCCCTTCCAGCCGTTCGTTGAGCTCCATGGTGGCTTTAAAGAGCAGGAAGAAGCCGCCGAACAGCATAATC
AGATCGCGGGCGCTAAAGCTTAACGCCTGTACGCTAAAAAGCGGCTTAGTCAAGGTGACCAGCCACGAGATTG
ACGCCAGCAGCAGCAGGCGCATGATCATCGCCAGTATCAGGCCGGTAATGCGCGCGCGGTCGCGCTGCCCCGG
CGGTAGTTTTTCAGCGAGGATGGCAATAAAGACCAGGTTATCAATGCCGAGAACAAGTTCGATAACGATTAAC

GTTACCAGTCCGGCCCAGATTGACGGATCGGCAATCCATTCCATAGTAAGAGCAATACCTTCTGTAATATGAT
AATTGTCACAATTCGATCATGGATAATACGGCGGAAAAATGCAATGCCGCAGCGCAATTTATCTTTGTTACTC
GGCTATAAAATAACTTTAAAATAGCCGTAATGTTTCTGTCAGAAATTAAATAATGTCAAGAATATATTTAGGA
AATATCGCAGGTGGAGTGGAGTAAATAAATATTGTCAACATCAATAAAATCCTAAAATCATCTGTTAATTCAT
TTAATATTAATCTTAAAAATATAGAATGGCGGGTTTGTTACCTTGCCTGTCATTAGGTTAGCTGCAACAATTC
TTGCAGGATTAAGATAGCGCTCTTAAATATCCTGTCACGACATCGAGAAAACTATTAATACGTAACCTTTATT
TCGCTGAAGATTTTTATCTGAACGAGGATAAAATCTGAGGGGAATGCGAAAATTTAGAATAATCAGTGTATTG
GTAGCTAAGAAGCCAGGGGCGGTAGCGTGCCTGAAAGCCTGAATATAAGCCTTTATTATTCTGTCAATAGCCT
GCGGATGAATTGATTTTTTTCAGAATGATGCCAGTTAATTTTCTCTGCCATTAACTGCAAGGTAAATGTGCAC
GGACGAAGCCGCTATTTAAATTGCACAGGATAATGACTCTGCCAAAGTGATAAATAATCAATGATGAAATCCA
AAATGAAATTGATGCCATTATTGGCGTCGCTCAGCTTGATAAGTGGTTGCACAGTACTTCCGGGCAGCAATAT
GTCTACGATGGGGAAAGATGTGATCAAACAGCAAGACGCTGACTTTGATCTCGACCGGATGGTCAATGTGTAT
CCGCTGACGCCACGGCTGGTTGAGCAATTACGCCCGCGGCCCAATGTCGCGCAACCGAATATGTCGCTGGACC
AGGAGATCGCCAGCTATCAGTATCGCGTCGGGCCTGGCGATGTGCTGAATGTCACCGTCTGGGATCACCCGGA
ATTGACCACGCCAGCGGGCCAGTACCGTAGCTCAAGCGATACCGGCAACTGGGTACAGCCGGACGGCACCATG
TTTTATCCCTACATTGGCAAGGTTAGCGTCGTCGGTAAAACTTTGTCAGAGATTCGCAGCGATATTACCGGGC
GTTTAGCGAAGTACATCGCGGACCCGCAGGTGGATGTCAATATCGCCGCTTTCCGCTCGCAAAAAGCGTATAT
CTCCGGCCAGGTGAATAAATCCGGTCAGCAGGCCATTACTAACGTACCGCTAACCGTCCTGGATGCGATTAAC
GCCGCGGGCGGCCTGACCGATATGGCGGACTGGCGCAACGTCGTGTTGACGCACAACGGCAAAGAACAGCGCA
TTTCGCTACAGGCGCTGATGCAAAATGGCGATCTTAGTCAGAACCGCTTGCTCTACCCTGGCGACATTCTGTA
TGTGCCGCGCAATGACGATCTGAAAGTCTTTGTCATGGGCGAAGTGAAAAAACAGAGCACCCTCAAAATGGAT
TTCAGCGGCATGACGCTCACCGAAGCATTGGGCAATGCGGAAGGCATCGATCTGACCACCTCCAACGCCAGCG
GCATTTTTGTGATTCGTCCGTTGAAAGGCGAGGGGGAACGCGGCGGCAAGATCGCCAATATCTACCAGCTTGA
TATGTCTGACGCCACGTCATTGGTGATGGCGACGGAATTCCGACTTCAGCCTTACGATGTGGTGTACGTCACG
ACCGCGCCGGTTGCTCGCTGGAACCGTCTGATCAATCAGTTGCTGCCAACCATTAGCGGTGTCCGTTATATGA
CGGATACGGCCAGCGACATTCATTCCTGGTAATCGTTATGTTTAACAAAATCTTAGTGGTCTGTGTGGGGAAT
GTTTGCCGTTCCCCGACGGCGGAACGTTTGCTAAAACGCTTTCATCCCTCGCTGACGGTTGCGTCCGCAGGAT
TAGGCGCGCTGGTCGGCAAAGGCGCCGATCCTGCGGCGGCGAGCGTGGCGAGCGCCCACAATCTGTCTTTGGA
AAATCACTGCGCAAGGCAAATTTCCGCGCGTCTGTGCCGGGAATATGACCTGATTCTGACGATGGAGAAACGT
CACATCGCCGCCCTGTGCGACATTGCGCCGGAAATGCGCGGCAAGGTAATGCTGTTTGGTCACTGGGACAGCG
AGCGTGAAATACCGGACCCGTATCGCAAAAGCCGCGATGCGTTTGAGGCGGTGTACACATTACTGGAAAGGTC
TGCCCGCCAGTGGGCGCAGGCACTGAATGCAGAGCAGGGAAAACCATGACAGAAAAAGTAAAACAATCTGCGG
CGGTTACGGGTAGCGATGAAATCGATATCGGGCGCCTGGTCGGAACGGTGATTGAAGCGCGTTGGTGGGTGTT
GGGGACGACGGCCATATTTGCGCTGTGCGCGGTAATTTATACGTTCTTCGCCACGCCCATCTATAGCGCCGAT
GCGCTGGTGCAGATTGAACAAAACGCCGGTAATTCGCTGGTACAGGATATCAACAGTGCATTAGCGAATAAGC
CGCCGGCTTCCGATGCGGAAATTCAGCTCATTCGATCGCGCCTGGTGCTTGGGAAGACCGTTGACGATCTGGA
TCTGGATATTGCCGTCACCAAAAACACCTTTCCGCTGTTCGGCGCCGGGTGGAACGGCTGATGGGGCGTCAC
AATGAAATGGTGAAAGTCACGACATTCACCCGACCAGAGACGATGAGCGGACAGATCTTCACCCTCAAAGTGC
TGGGCGACAAACGTTATCAGTTGGTTAGCGACGGCGGATTTAGCGCGCAGGGCGTTGTGGGCCAGCCACTCAA
TAAAGACGGCGTGACGATGCAGGTAGAGGCGATTGACGCCCGTCCTGATACAGAATTTACGGTGAGTAAATTC
TCAACGCTTGGCATGATTAATAACCTGCAAAATAACCTCACCGTAACGGAAACCGGCAAAGATACCGGCGTTC
TGAGCCTGACGTTCACGGGAGAGGATCGCGACCAGATCCGCGAAATTCTCAACAGTATTACCCGTAACTATCT
GCAACAGGATATTGCGCGGAAATCTGAAGAGGCGGGGAAGAGCCTGGCGTTTCTGGCAAAACAACTGCCGGAA
GTCCGCAGCCGTCTGGATGTGGCGGAAAACAAGCTAAACGCTTTCCGCCAGGATAAAGACTCGGTGGATTTAC
CGCTGGAGGCGAAGGCGGTGCTCGATTCGATGGTTAATATCGACGCCCAGTTGAATGAGTTGACGTTTAAAGA
GGCGGAAATTTCCAAGCTCTTTACCAAAGCGCATCCGGCCTACCGCACCCTGCTGGAAAAACGTAAAGCGCTG
GAAGATGAAAAAGCCAAACTGAACGGGCGGGTGACGGCAATGCCGAAAACCCAGCAGGAGATTGTGCGTTTGA
CCCGCGATGTGGAGTCCGGCCAGCAGGTTTATATGCAACTGCTTAATAAACAGCAGGAGCTGAAAATCACCGA
AGCCAGCACCGTTGGCGATGTGCGCATCGTTGACCCGGCGATTACCCAGCCGGGCGTGCTGAAGCCGAAAAAG
GCGTTGATTATTCTCGGCAGCATTATTCTGGGATTAATGTTATCGATTGTCGGCGTGCTGCTGCGCTCGCTGT
TTAATCGCGGTATCGAAAGCCCGCAGGCGCTGGAGGAGCACGGGATCAGCGTCTATGCCAGTATTCCGCTGTC
GGAATGGCAAAAAGCGCGCGATAGCGTCAAAACCATTAAAGGGATTAAGCGTTACAAGCAGAGCCAACTGCTG
GCGGTGGGCAATCCGACCGATCTGGCGATTGAGGCGATTCGCAGCCTGCGCACCAGCCTCCATTTCGCCATGA
TGCAGGCGCAAAATAATGTACTCATGCTCACAGGCGTCAGCCCCTCTATCGGTAAAACCTTTGTTTGCGCCAA

CCTGGCGGCCGTCATCAGCCAGACGCATAAACGGGTATTACTGATCGACTGCGATATGCGCAAGGGCTACACC
CATGAACTGCTCGGCACCAATAACGTGGACGGCTTATCTGACATTCTGGCGGGCAAAGGCGAGATAGCCTCCT
GCGCGAAACCTACGGCGATCGCTAATTTTGATCTTATTCCGCGCGGTCAGGTGCCGCCTAATCCATCGGAACT
GCTGATGAGCGAGCGCTTCGGCGAGCTGATCGCCTGGGCAAGCAGCCGCTACGACCTGGTATTAATCGACACG
CCGCCGATTCTGGCCGTGACGGACGCCGCGATTGTGGGTCGCCACGTCGGGACGACGCTGATGGTGGCGCGTT
ATGCCGTCAACACCTTAAAAGAGGTGGAAACCAGTCTGAGCCGTTTTGACCAGAATGGCATTCAGGTCAAAGG
CGTCATTCTCAACTCCATTTTTCGCCGGGCGACGGGCTACCAGGATTATGGTTATTACGAGTATGAATACCAG
TCAGATTCCAAATAAAAAATAAGGCGTGCCGGATGGCGCGGCCTGTGACTGTAGGCCGGACAAGCGTAGCGCC
ATGCGGCAAAACTATCTGGGGAATAAGCATGACAACAGACAATCCGCTGATCTCAATTTATATGCCAACCTGG
AACAGGCAGCAGTTGGCTATCCGGGCGATTAAATCAGTACTACGTCAGGACTACCGCCACTGGGAGATGATCA
TCGTGGATGATTGCTCCTCGTCTTATCAGCAGCTACAACAATTTGTTGAGGAGCTGAACGACCCGCGCGTCCG
TTATACCCATAACGCCAGGAATACCGGCGCCTGCGCGGTTCGCAACCAGGCCATTATGCAGGCGCAAGGACAC
TACATTACCGGTATTGATGATGACGATGAATGGACGCCAAACCGTCTGAGCGTGTTTCTGGCCCATAAACACC
AGTTGACTACGCACGCCTTTTTGTATGCCAACGACTATGTGTGCGAGGGCGAGGTCTACTCTCAGCCTGCCAG
CCTGCCGCTGTATCCGAAATCCCCTTATTCGCGCCGCTTGTTCTACAAGCGCAATATTATTGGCAATCAGGTT
TTTACCTGGGCCTGGCGTTTTAAAGCGTGTTTGTTCGATACCGAACTCAAGGCGGCGCAGGATTACGACATTT
TTCTGCGCATGGTGGTGGAGTATGGCGAACCGTGGAAAGTTGAAGAGGCGACGCAAATTTTACATATCAACCA
CGGCGAGATGCAGATTACCTCTTCGCCGAAAAAGTTCTCCGGCTATTTCCATTTTTATCGTAAGCACAAAGAT
AAATTCGATCGCGCCAGCAAAAAATACCAGCTTTTTACGCTGTACCAAATTCGCAATAAGCGAATGACCTGGC
GCACGTTGCTGACGTTGCTCTCCGTGCGTAACAGCAAACGCCTGGCGGCCGGGCTGCGGGGGCGATAACGATG
TTGGAAGACTTACGCGCGAATAGCTGGAGCTTACGCCCCTGTTGCATGGTGCTGGCCTACCGTATCGCTCATT
TTTGTTCCGTCTGGCGCAAAAAGAATGTGCTGAATAATCTGTGGGCCGCGCCGGTGCTGGTGCTGTATCGCGT
GATCACGGAATGCCTGTTCGGGTATGAAATCCAGGCCGCCGCGACCATTGGTCGGCGGTTCACTATCCATCAT
GGTTACGCGGTAGTGATTAACAAACATGTCGTCGCCGGCGACGATTTTACCATTCGCCACGGCGTCACCATTG
GCAATCGCGGCGCGGACAGCCTGGCTTGTCCGGTGATTGGTCACGGCGTTGAGCTGGGGGCGAATGTCATCCT
GCTTGGCGATATTACTATCGGTAATCACGTCACGATTGGCGCGGGTAGCGTGGTACTTGACAGTATTCCGGAC
CATGCGCTGGTGGTGGGAGAAAAAGCACGGGTGAAGGTCAGCACATGAATATTCTGCAATTTAATGTACGGCT
GGCGGAAGGCGGCGCGGCGGGAGTGGCGTTGGATCTCCATCTGCGGGCGCTGCAAAAAGGGCTGACGTCACGT
TTTGTCTATGGTTATGGCAAAGGCGGAAAAAAAGCGTCAGCCACCACCGTTATCCGCAGGTGATAAAACAGAC
GCCGCGCGGCACGGCAATCGCTAATATCGCGCTGTTTCGTTTCCTGAATCGCGATCTGTTTGGCAATCTCGAC
AATCTTTACCGCACGGTTATCCAGACATCCGGCCCGCTGGTGCTGCATTTTCATGTTCTCCACAGTTACTGGC
TAAACCTGGCGGATATCGTGACGTTTTGCGAAAAAGTCAAAGCGCAAAAACCGGACGTCACGCTGGTCTGGAC
GCTGCACGATCACTGGAGCGTCACCGGGCGTTGCGCCTTCACCGACGGTTGCGAGGGCTGGAAAAGCGGCTGC
CAAAAATGTCCGACCCTAAGCAATTATCCGCCGGTCAGGGTGGATCGGGCGCACCAGCTTATTGGCGGCAAAC
GTCAGCGCTTTCGGGACATGCTGCGGCTGGGCTGCCAGTTTATTTCGCCGAGCCAGCATGTGGCCGAGGCCTT
TAACAGCGTTTATGGCGCGGGACTCTGCCGGGTGATTAACAACGGTATCGATCTGGCGACCGAGGCGATTCTC
GCGCAACTATCACCTGTGCCGCTGAATCCGGGCAAACCGCGGATCGCCATTGTGGCGCATGACTTGCGTTACG
ACGGCAAAACTGACCAGCGTCTGGTACACGACATGATGGCGCTGGGCGAAAAAATTGAACTGCATACCTTCGG
TAAATTTTCGCCTTTTACCGGCCAAAACGTTGTTAATCACGGTTTTGAAACCGATAAGCGCAAATTAATGAGC
GCGCTCAATGAGATGGATGCGCTGGTCTTTAGCTCGCGGGTCGATAACTATCCGCTGATCTTGTGTGAAGCGC
TCTCGATCGGCGTACCGGTGCTCGCTACCCACAGCGAGGCGGCGCAGGAGGTGCTGGCGAAATCCGGCGGCCA
GACCTTTGCCGCTACAGATGTACTGCGCCTGGCGCAGCGGCGTAAGCCAGAGATTGCTCAGGCGGTATTTGGC
GCCACGCTGGACGCCTTTCGTATGCGTAGCCGCGTCGCGTACAGCGGTCAACAGATGCTGGAGGAGTATGTCT
CGTTCTATCAGAATCTGTAGCTATCTGCTGCTGCCGCTGATCTACCTACTGGTCAATGTCAAGATTGCCCAAC
TGGGGGAAAGCTTTCCCATTACCATCGTCACTTTTTTACCGCTGTTGCTGCTGTTATTTGTGGAACGCATTAG
CGTAAAAAAATTGATGATCGCCTTAGGCATCGGCGCGGGGCTTACGGCATTTAACTTCCTGTTCGGCCAGTCG
CTGAATGCCGGTAAATATGTCACATCCACGATGCTGTTTGTCTATATTGTGGTCATTATCGGGATGGTCTGGA
GTATCCGATTCAAAACCATTTCCGCGCATAACCACCGAAAGATTTTGCGTTTTTTTATCTGGTGGTGGGTAT
AGTGGTCGCGCTCGCCGCGGTTGAGATGGCGCAAATTATCCTTACCGGCGGCAGCAGTATTATGGAAGGAATT
TCGAAATATCTCATTTACAGTAATAGCTACGTACTGAACTTCATAAAATTTGGCGGTAAGCGTACCACTGCGC
TTTATTTTGAACCGGCATTTTTCGCTTTGGCACTAATCTCAATTTGGCTGAGCATCAAACAGTTTGGTATCAA
AACACCGAAAAGCGATGCTATGATTCTGGCAGGGATAATATTATCAGGATCATTTTCAGGGGTAATGACCTTT
ATCCTGTTTTACCTTCTGGAGTGGGCGTTCCAATATTTGAATAAGGATGCGATAAAGAAAAAACTTCCACTGG
CGCTGGTCTCATTAACCCTGTTTTTGGTTGGGGTAATTATTGCATTTCCTTATATCGCGACACGACTTGGCGA

TTTAGGGACGGAAGGCTCATCTTCTTATTATCGTATTGTGGGCCCCTTAGTCATGGTCGGATATTCCTTGACC
CATATTGATGGTGTAGTCAGATTTGGCTCACTTTATGAATATGTCGCATCATTCGGAATCTTTAACGGTGCGG
ATGTCGGGAAAACCATAGACAATGGATTGTATCTGCTGATTATTTATTTTTCCTGGTTCGCAGTGCTAATGAC
GCTGTGGTATATGGGGAAAGTTTTAAAAATGGCGCTAAATGCGTTTGGCGATAATCGCAATTTTCGGGTACAG
CTCTATCTTTTTACGCCGGTGTCACTGTTTTTTACCGGTTCAATATTTAGCCCGGAATATGCTTTTTTAATCG
TCTGTCCGTTCATTTTGCGCAAGGCGTTAAAAATTTCATAACGGGGTGGTGGCATGTTTTTAAGCGTCGTTAC
CGTCGCCTTTCGCAATTACGAAGGGGTGGTAAAAACCTGGCGCTCGCTGCGCAATCTGGCGCGCGATCCGGGT
CTGTCATTTGAATGGATCGTAGTGGATGGCGGTTCGGAGGACGGCACGCGCGAATTTCTGCAAAAACGTCACG
GTGAGTTTAACCTGCGTTTTATCAGTGAAAAGGATCGCGGTATTTATGATGCCATGAACAAAGGCATTGCGCT
GGCGCAGGGGCGTTATACCCTGTTTCTTAATTCCGGTGATGCGTTCCATGATGATGCCGCGCTGTTTGTCCGC
CAACTGGCGCGGCAAAAGGGCGAGGCGATGTATATTGGCGACGCGCTGCTGGATTTCGGCGGCGGGCATAAAA
TACGCCGGAAGGCTAAACCCGGGTATTATATCTACCATAGTTTGCCAGCCAGCCATCAGGCGATATTTTTCCC
GACGGCAGCGTTAAAAAAGTATCCTTATGATCTGCAATATCATGTTTCTTCAGATTATGCGCTGGCGGCCCGT
CTTTATAAAGCAGGCTATCCTTTTCGGCGAATTAACGGACTGGTATCTGAATTTTCGATGGGCGGCGTGTCAA
CCTCGAATAATCTGGAATTATGTCAGGATGCAAAAAATGTACAGCGAAAAATATTACGTGTGCCAGGATTTTG
GGCGCAATTATCCTATTTATTACGTCTGAAAACGACGGGTAAAACAAAAGCGCGGTATAACAAAGTCTAATCA
CCGGGAGTTATTATGCAGGATCTTAGCGGCTTTACGGTGCCGAAGGGGTTCCGCGGCGCTAATGTGCTAAAAG
TACAATTATGGTGGGCGGTACAGGCGACATTATTCGCCGGGTCGCCGCAAATATTGTATCGCTGGCGAGCCTT
TTTGCTGCGTCTGTTTGGCGCCAAAATTGGAAAGAATGTGGTTATTCGACCGTCAGTAAAAATTACCTATCCG
TGGAAATTAACCGTCGGCGATTATGCCTGGGTAGGCGACGACGCTGTGTTATATACGTTGGGTGAAATTAATA
TTGGCGCACATGCGGTCATTTCACAAAAAGGGTATTTGTGCACCGGTAGCCATGATTATACCAGCGCCCATTT
CGATATTAACGCCGCGCCGATTGTTATTGGCGAAAAATGTTGGCTGGCGACCGATGTTTTTGTCGCGCCCGGC
GTGACGATAGGTCATGGCACCGTCGTCGGCGCGCGCAGCAGCGTATTTAAATCATTACCGGCAAATGCGATTT
GTCGGGGCAATCCCGCAGTGGTAACGCGCCAGCGCGTTCAGAAAGTTACTCCCTAACGGGACTATTTGAGGAA
ATGAAAATGTCAAAAGTCGCTCTCATTACTGGCGTAACCGGACAGGATGGGTCTTACCTGGCAGAATTTCTGC
TGGAAAAAGGGTATGAGGTGCATGGTATCAAGCGCCGCGCGTCATCGTTTAATACCGAGCGCGTGGACCATAT
TTATCAGGACCCGCACAGCTGCAACCCGAAATTTCATCTGCATTATGGCGACCTGACCGACGCCTCCAACCTG
ACCCGCATTTTACAGGAAGTGCAGCCGGATGAGGTCTACAACCTGGGCGCGATGAGCCATGTGGCGGTGTCGT
TTGAGTCGCCGGAATATACCGCCGATGTGGATGCGATGGGCACGCTGCGCCTGCTGGAGGCGATCCGCTTCCT
CGGTCTTGAAAAGAAAACGCGGTTCTACCAGGCCTCCACCTCTGAACTGTACGGGCTGGTGCAGGAGATCCCG
CAGAAAGAGACCACGCCGTTCTACCCGCGTTCCCCCTATGCGGTGGCGAAACTGTACGCCTACTGGATCACCG
TTAACTACCGTGAATCCTACGGTATTTACGCCTGTAACGGCATTCTGTTTAACCACGAGTCCCCGCGTCGCGG
CGAAACCTTCGTCACCCGTAAGATCACCCGCGCCATCGCCAATATCGCCCAGGGACTGGAGTCCTGCCTGTAT
CTCGGCAACATGGACTCGCTGCGCGACTGGGGTCATGCGAAAGATTACGTGCGGATGCAGTGGATGATGTTAC
AGCAGGAGCAGCCGGAAGATTTCGTGATTGCCACCGGCGTGCAGTATTCCGTGCGCCAGTTTGTGGAGCTGGC
GGCGGCGCAACTGGGGATAAAACTGCGCTTTGAAGGCGAAGGCATTAATGAGAAAGGGATTGTGGTATCCGTT
ACCGGACACGATGCGCCGGGCGTGAAACCGGGCGATGTGATTGTGGCCGTTGATCCGCGTTATTTCCGTCCGG
CGGAAGTGGAAACCCTGCTGGGCGACCCGTCCAAAGCGCATGAGAAACTGGGCTGGAAACCGGAAATCACCCT
GTCGGAGATGGTCTCCGAGATGGTGGCGAACGATCTGGAGGCCGCGAAAAAACACTCACTGCTGAAATCTCAC
GGTTACGAGGTGGCCATCGCGCTGGAGTCCTGAGAATGAATAAGCAACGAATTTTTGTGGCGGGCCATCGCGG
GATGGTGGGCTCCGCCATTGTACGGCAGCTTGCGCCGCGCGGCGACGTGGAACTGGTGCTGCGCACCCGCGAT
GAGCTGGATCTGCTCGACGGGCGCGCGGTACAGGCGTTCTTTGCCGGGGCGGGTATCGACCAGGTTTATCTGG
CGGCGGCGAAAGTGGGCGGCATTGTCGCCAACAACACATATCCGGCGGATTTTATCTATGAAAACATGATGAT
AGAGAGCAACATTATTCACGCCGCGCACCTGCACAACGTGAACAAACTGCTGTTTCTCGGTTCGTCCTGTATC
TATCCGAAACTGGCAAGGCAGCCGATGGCGGAAAGCGAGCTGCTGCAGGGGACGCTGGAGCCGACCAACGAGC
CGTATGCCATCGCCAAGATCGCCGGGATTAAATTGTGCGAGTCCTACAACCGGCAGTACGGTCGCGACTACCG
TTCGGTGATGCCAACCAACCTGTACGGCCCGCATGACAATTTCCATCCGGACAATTCACATGTGATCCCGGCG
CTGCTGCGCCGCTTTCATGAGGCCGCGCAGAGCCACGCGCCGGAGGTGGTGGTGTGGGGCAGCGGCACGCCGA
TGCGCGAATTTCTGCACGTTGACGATATGGCGGCGGCCAGCATTCACGTGATGGAGCTGGCGCGCGAAGTGTG
GCAGGAGAACACCGCTCCGATGCTGTCGCACATTAACGTCGGCACTGGCGTGGACTGCACCATCCGCGAGCTG
GCGCAGACTATCGCAAAGGTGGTGGGTTACCAGGGCCGGGTGGTGTTCGACGCCACGAAGCCGGACGGCACGC
CGCGTAAATTGCTCGACGTCACGCGCCTGCATCAGCTTGGCTGGTATCACGAAATTTCACTGGAGGCAGGGCT
TGCCGGTACTTACCAGTGGTTCCTTGAGAATCAGCACCGGTTCCGGGGGTGACAATGTTTTTACGTCAGGAAG
ATTTCGCCGCCGTGGTGCGGACCACGCCCCTCATCTCCCTCGATTTCATCGTGGAAAACGGCCAGGGGAAATT

TTACTGGGCCAGCGTCTCAACCGTCCGGCGCAGGGCTACTGGTTTGTGCCGGGGGGGCGGGTGTGCAAAGACG
AAACGCTGGAGGCCGCCTTTGCACGCCTGACGCAGGCGGAACTGGGCGTGCGTCTGCCGCTGGCGGCAGGGAC
GTTTTATGGCGTCTGGCAGCACTTCTATGACGACAACTTTTCCGGTGAGGATTTTTCAACTCACTACATCGTG
CTCGGCTTTCGTCTGCGCGTGACGGAGAGCGATTTACGCCTGCCTGATGCCCAGCATGGCAGTTACCGCTGGC
TGACGCCGGAACAGCTTCTGGCAAGCGACAATGTTCATGAGAACAGTCGGGCCTACTTCCTTCCTGACGCGCC
GGCGGTGGGCCTATGAAGATCCTGGTCTATGGCATTAACTATTCGCCGGAACTGACCGGTATCGGCAAATATA
CCGGCGAAATGGTGGCGTGGATGGCGCAGGAAGGCCATGAGGTGCGGGTCATTACTGCGCCGCCGTACTACCC
GCAGTGGAAGGTCGGTGAGCGCTATTCCGCCTGGCGCTATCGCCGGGAAGAGGGCGAGGCTACCGTCTGGCGC
TGCCCGCTGTATGTGCCGAAACAGCCCTCCACCTTAAAACGACTGCTTCATCTGGGGAGCTTTGCATTGAGCA
GTTTTTTCCCACTGATGGCGCAGCGTCGCTGGAAGCCGGATCGGATTATCGGCGTTGTACCGACGCTCTTTTG
TACGCCGGGAATGCGCCTGCTGGCGACACTCTCCGGCGCGCGTACCGTTCTGCATATTCAGGATTACGAAGTG
GATGCCATGCTCGGCTTAGGAATGGCGGGAAAAGGCAAGCGCGGCAGCGTAGCGCGGTTAGCGACGGCCTTTG
AACGCAGCGCGCTGCGTAACGTTGATAATGTTTCGACCATCTCTCGCTCCATGATGAATAAAGCGCGGGAAAA
GGGCGTCGCGGCGGAAAAAATCCTCTTTTTTCCGAACTGGTCGGAAGTGGCGCGCTTTCAGGACGTTAATGAC
GCTGACGTGACGGCTTTGCGTCAGCAGCTTGGCTTGCCAGAGGGCAAAAAAATTGTGCTTTATTCCGGCAATA
TCGGCGAAAAGCAAGGGCTGGAAAAGGTGATTGACGCCGCGGAACGATTACGCGATCGACCGTTGATTTTTGC
GATTGTCGGCCAGGGGGGCGGCAAGGCGCGGCTGGAGAACATGGCCCGCGAACGCGGCCTGGCAAATATTAAA
TTCCTCCCGCTGCAGCCTTACGACGCCTTACCCGCGCTGCTGAAAATGGGCGACTGCCATCTGGTGGTGCAAA
AGCGCGGCGCGGCGGATGCGGTACTGCCCTCCAAGCTGACTAACATTCTGGCGGTCGGCGGTAATGCGGTGAT
TACCGCTGAGCCGCACACCGAATTAGGACAACTTTGCGCGCGCTATCCGGGCATCGCCGTTTGCGTGGAACCG
GAGTCGACCGACGCGCTGGTCAACGGGATTAGCCAGGCGCTCGCCATGCCGAAAAACAACACGACGGCACGTG
AATACGCCGAACGCACTCTCAATAAAGAGAACGTGCTACGCCAATTTATTGCAGATATTCGGGGATGATAATG
CGTCAGACAAAACTTTACCCGGTTGTGATGGCCGGTGGCTCCGGTAGCCGATTGTGGCCGCTTTCTCGCGTGC
TTTATCCAAAACAGTTTCTGTGCCTGAAAGGCGATCTCACCATGCTGCAAACCACCATTTGCCGTCTGAACGG
CGTGGAGTGCGAAAGCCCGGTGGTGATCTGTAACGAACAGCACCGTTTTATCGTTGCCGAACAGCTCCGCCAG
CTCAATAAGCTGACGGAGAACATTATTCTTGAGCCCGCCGGGCGCAATACCGCGCCGGCGATTGCGCTGGCGG
CGCTGGCGGCGACGCGCCAGCATACGGACTGCGATCCGCTGATGCTGGTACTGGCGGCGGATCATGCAATCGC
CAATGAAGAGGCCTTCCGCGACGCGGTGCGCGGCGCCATGCCCTATGCCGACGCGGGCAAACTGGTGACCTTC
GGTATCGTGCCGGATCTGCCGGAAACCGGCTATGGCTATATCCGGCGCGGTGATGTCGTGCCGGGCGCGACGG
ATGCGGTGGCTTTTGAGGTGGCGCAGTTCGTTGAGAAGCCGGGGCTGGAAACCGCTCAGGCCTACGTCGCCAG
CGGCGATTACTACTGGAACAGCGGCATGTTTCTGTTCCGCGCCGGACGCTATCTGGAAGAGCTGAAAAAGTTC
CGCCCCGATATTCTGGCCGCCTGTGAACAGGCGATGCGCGGCGTCGATCCGGATCTTGACTTCATTCGTGTGG
ATGAAGAGGCCTTCCTCGCCTGTCCGGAAGAGTCCATTGACTACGCGGTGATGGAGCGAACGGTGGACGCGGT
GGTGATGCCGATGGACGCGGGCTGGAGCGATGTCGGCTCGTGGTCCTCGCTGTGGGAAATCAGCGCCCACACC
CCGGAGGGAAACGTCCACCACGGCGACGTCATCAGCCATAAAACGGAAAACAGCTACGTTTACGCCGAATCCG
GCCTGGTGACCACCGTCGGCGTGAAGGATCTGGTGGTGGTGCAGACCAAAGACGCCGTGCTGATTGCCGATCG
CCACGCGGTGCAGGACGTGAAAAAGGTGGTGGAGAAGATCAAGGCCGACGGACGCCATGAGCACCACATGCAC
CGTGAGGTTTACCGTCCGTGGGGCAAATACGACTCCATCGACGCGGGCGAGCGCTATCAGGTGAAGCGTATTA
CCGTCAAGCCGGGGGAGGGACTGTCGGTGCAGATGCACCATCACCGCGCCGAGCACTGGGTGGTGGTGGCGGG
GACCGCCAGAGTCACCATCAACGGCGAGGTTAAGCTGCTCGGTGAAAACGAGTCCATCTATATTCCGCTGGGG
GCGACTCACTGCCTGGAAAATCCGGGAAAAATACCGCTCGATTTAATTGAAGTGCGTTCCGGCTCGTATCTCG
AAGAGGACGACGTGGTGCGGTTCGAGGACCGTTACGGGCGGGTCTGACGCCTGCAATTATGCCCGGTAAGCGC
AACGCGCCATCCGGCAATGAATAACAATAACGATGCTGGCCATTTACGGTCAGGGCTAACTGTTGCCTGAAGA
AGGGTAAGCACATGACAAAATTAACCTGTTTCAAGGCCTACGACATTCGCGGCAGGCTGGGCGAAGAGCTCAA
TGAAGATATCGCGTGGCGGATTGGCCGCGCCTACGGCGAATATTTAAAACCGAAAACCATCGTGCTGGGCGGC
GACGTGCGTCTGACCAGCGAGGCGCTGAAGCTGGCGCTGGCGAAAGGGTTACAGGACGCGGGCGTTGACGTGC
TGGATATCGGTATGTCCGGCACCGAAGAGATTTACTTCGCCACCTTCCACCTTGGCGTGGACGGCGGTATTGA
GGTGACCGCCAGCCATAACCCGATGGACTACAACGGTATGAAGCTGGTACGCGAGGGCGCCCGCCCGATAAGC
GGCGACACCGGTCTGCGCGACGTTCAGCGTCTGGTGGAAGCGGGCGACTTCCCGCCGGTGAACGAGGCCGCGC
GCGGCAGCTACAGACAAATCAGCCTGCGCGACGCCTACATCGGCCACCTGCTCGGCTATATCAGTGTTAACAA
TCTCACCCCGCTGAAGCTGGTGTTTAACGCCGGCAACGGCGCGGCGGGGCCGGTCATTGACGCCATCGAAGCG
CGCCTGAAGGCGCTGGGCGCGCCGGTGGAGTTCATCAAAATCCACAACACCCCGGACGGCACCTTCCCGAACG
GCATTCCCAACCCGTTGCTGCCGGAGTGTCGCGACGACACCCGCAAGGCCGTTATTGAACACGGGGCGGACAT
GGGCATCGCCTTTGACGGCGACTTCGACCGCTGCTTCCTGTTCGATGAGAAAGGGCAGTTTATCGAGGGGTAC

TACATCGTCGGGCTGCTGGCGGAAGCGTTTCTGGAAAAACACCCGGGGGCGAAGATTATCCACGACCCGCGCC
TGACCTGGAACACGGAGGCAGTGGTGACGGCGGCGGGCGGACGCCGGTGATGTCAAAAACCGGGCACGCCTT
TATCAAGGAGCGGATGCGTACGGAGGATGCCATTTATGGCGGGAAATGAGCGCGCATCACTATTTTCGTGAT
TTTGCCTACTGCGACAGCGGGATGATCCCGTGGCTGCTGGTGGCGGAGCTGGTGTGCCTGAAGAGACAGTCGC
TGGGTGAGCTGGTTCGCGACCGGATGGCGGCGTTCCCGGCGAGCGGGGAAATCAACAGCAGACTGGCGGAGCC
GGCGGCGGCCATTGCGCGGGTGGAAGCGCACTTTGCGGAAGAGGCGCAGGCGGTGGATCGCACGGACGGTCTC
AGCATGTCGTTCGCCGACTGGCGTTTCAACCTGCGCTCGTCGAACACCGAACCGGTGGTGCGCCTGAATGTGG
AATCACGCGGCGATATTCCGCTGATGGAAGCGCGAACACGCACTCTGCTGGCGCTGCTGAATCAGTAACGTCA
GATCTCCCCTTACCGCCTGCGGGTAAGGGGCCAAACACAGGAACAACGATGACAAATCTAAAAAAGCGCGAAC
GAGCCAAAACCAATGCATCGTTAATCTCTATGGTGCAACGCTTTTCAGATATCACCATCATGTTTGGCGGGCT
GTGGTTCATCTGTGAAGTCAGTGGACTGCCATTCCTGTACATGCACCTGTTGGTGGCGCTGATTACGCTGGTT
GTTTTTCAAATGCTGGGCGGGATGACCGATTTTTATCGCTCCTGGCGCGGCGTCAAAATGTCGACAGAGTTAA
CGCTGTTACTGCAAAACTGGACGCTGAGCCTGATCTTCAGCGCCGGTCTGGTGGCGTTTAATGATGACTTTGA
CAACCGCCTCGCCACCTGGCTGGCTTGGTATGCGCTGTCCAGCCTGGGCTTAGTGTTATGCCGTTCATTTATT
CGTTTCGGCGCGGGCTGGCTACGCAATCGTGGTTATAACACTCGCCGGGTCGCCGTCGCCGGAGATTTGCCTG
CGGGTCAGGCGTTGCTGGATAGCTTCCGTAATGAGCCGTGGCTGGGTTTTGAGGTGGTGGGCGTCTATCACGA
TCCTAAACCGGGCGGCGTCTCTGCTGACTGGGCCGGGAACTTCGAGCAGTTAATTGACGATGCGAAAGCCTCG
CGAATTCACAATGTTTATATCGCCATGTCGATGAGCGACGGCGCCATGGTGAAAAAACTGGTGCGCGAGCTGG
CCGACACCACCTGCTCGGTGATCCTTATTCCCGATGTGTTCACCTTTAATATTCTCCACTCGCGCGTGGAGGA
GATGAGCGGCGTTCCGGTCGTTCCGCTGTATGACACGCCGCTATCAGGGATTAACCGTCTGCTTAAACGGGCA
GAAGATATCGTGCTGGCGTCGCTGATTCTGCTGCTCATCTCACCGGTACTGTGCTGCATTGCGCTGGCGGTCA
AATTGAGCTCGCCCGGGCCGGTGATTTTCCGTCAGACGCGCTACGGGATGGACGGCAAGCCGATCAAGGTCTG
GAAGTTCCGCTCAATGAAGGTGATGGAGAACGATAAGGTCGTTACCCAGGCGACGCAAAACGATCCGCGCGTC
ACCCGGGTAGGTAACTTTTTGCGTCGTACCTCGCTGGATGAATTGCCGCAGTTTATCAACGTATTAACTGGCG
GGATGTCCATCGTCGGTCCTCGCCCGCACGCGGTGGCGCATAACGAACAGTATCGTCAGTTGATTGAAGGTTA
CATGCTACGCCACAAGATGAAGCCGGGCATCACCGGCTGGGCGCAAATTAATGGCTGGCGCGGTGAGACCGAT
ACGCTGGAGAAAATGGAAAAACGCGTGGAATTCGACCTGGAATACATCCGCGAATGGAGTATTTGGTTCGATA
TCAAAATCGTTTTTCTGACGATTTTTAAAGGGTTTGTTAATAAAGCGGCGTATTAAGAACCTGACCCACAAGG
CTATTTTACTTGCCATATTGCACCAGGGCAGTGCTCAATCTGGCTGCGCCAATGACGCCTTGTGAACAGGTTC
TGGAGAAAAGAATGAGTTTACGACAAAAAACGATCAGCGGCGCTAAATGGTCGGCTATCGCCACGATAGTGAT
TATCGGTCTGGGGTTAATTCAGATGACGGTGCTGGCGCGGATCATCGACAACCACCAGTTTGGCCTGTTGACC
GTCTCGCTGGTGATTATCGCGCTGGCCGACACGATCTCGGACTTTGGCATCGCGAACTCGATTATCCAGCGTA
AAACGATTGGGCATCTGGAGCTGACCACGCTCTACTGGCTAAACGTTGGGCTTGGGATTGTGGTATTTGCGGT
GGTCTTTTGGCTGAGCGATGCGATTGCCCATGTTTTGCATAACCCGGATCTCGCGCCGTTAATCAAAACGTTG
TCGCTGGCGTTCATCGTGATCCCTCACGGGCAGCAGTTCCGCGCTCTGATGCAAAAAGAGCTGGAGTTCAATA
AGATCGGCATGATCGAAACGACCTCCGTGCTGGCGGGGTTCACCTTTACGGTGATTAGCGCCCATTACTGGCC
GCTGGCGTTAACCGCGATTCTCGGCTATCTGGTGAACAGTGCGGTGCGAACGCTGCTGTTTGGTTACTTTGGC
CGCAAGATTTACCGTCCGGGGCTGCATTTTTCGCTGGCATCGGTGTCCACGAACCTGCGTTTTGGCGCGTGGC
TGACGGCGGACAGTATCGTCAATTACATCAACACGAATTTATCGACGCTGGTACTGGCGAGAATTCTCGGCGC
AAGCGTCGCCGGGGGATATAACCTCGCGTACAACGTGGCGGTTGTGCCGCCGGCGAAGCTTAACCCCATCATT
ACTCGCGTGCTATTTCCGGCATTCGCCAAAATCCAGGACGATACCGAGAAGCTGCGCGTCAACTTCTATAAGT
TGCTTTCCGTGGTGGGAATTATCAATTTTCCCGCGCTGTTGGGCCTGATGGTAGTGGCGAACAATTTTGTGCC
GTTAGTGTTTGGCGAGAAGTGGAACAGTATTATCCCGATCCTGCAATTGCTGTGCGTGGTGGGGCTGTTGCGC
TCGGTCGGCAACCCGATTGGTTCGCTGCTGATGGCGAAAGCGCGCGTGGATATCAGCTTTAAGTTCAACGTCT
TTAAAACGTTTCTGTTTATCCCGGCAATTCTCATTGGCGGCCATCTGGCGGGGGCGATTGGCGTGACGCTGGG
GTTCCTGGTGGTGCAAATCATCAACACCATTCTGAGCTATTTCGTGATGATTAAGCCGGTACTCGGCTCCAGC
TATCGTCAGTATATTCTCAGCCTGTGGCTACCGTTTTATCTCTCATTGCCAACATTTATTACCAGCTACGGCG
CAGGAAAGCTGGCCGACGGCTATTTACCGTTGTCAGGGGTATTCGCACTACAGGTTATGGTCGGTATTTTGAG
TTTTATTTTGATGATTATTTTTTCACGCAATGCGCTGGTGATGGAAATTAAAAATCAGCTTGTTGGCAGTGCA
AAAATGAAAAAGTTATTACGTGTCGGATGACGGGGATATAAGAGGCTATTTATGAAATTATTAATTTTAGGCA
ACCACACATGCGGCAACCGTGGTGATAGCGCCATTATGCGCGGTTTGCTTGATGCCATCCGTCAACAGGCGCC
GGAGGCGGAGATGGATGTGATGAGCCGTTTCCCGGTGAGTTCCGCCTGGTTGCAGGGGCGCCCGATTATTGCC
GACCCGCTGTATCAGTTAAGCCAGAAACAGCAGGCGGCGGCAGGGCTTAACGGTCGGGTGAAAAAAGTATTAC
GCCGTCGTTTTCAGCATAAAATTTTATTGTCTAAAGTCGCCCAGGAAGGATCGCTGCGTAATTTTGCTATCGC

GCCGGAATTTGCCGAATTCGCGCAATTTATTGCGCAGTATGACGCCGTCATTCAGGTGGGCGGTTCAAACTAT
GTCGACTTATACGGTTTGACTCACTTTGAATATCCGCTGTGCGCGTTTATGGCGAACAAACCAATTTATATGG
TGGGACATAGCGTCGGCCCTTTCCAGAATCCTGACTTTAACCAGCTCGCCAATTATGTTTTTGGCCGCACCAG
TGCGCTGATCCTGCGTGAAACCGTCAGCCGCGATATGATGACCGCCGCGCAGATCGATACCCGGAAAGTGGAG
CAGGGGGTGGATACCGCCTGGTTAGTCGAACGCCGTCACGACGAGTTTGTTGCCAGTTATGCTGTGCAGCACT
GGCTGGACGTCACCGCGCGCCGCAAAACCGTCGCGATTACGTTGCGCGATCTGGAGCCGTTTGATAAACGCCT
GGGCACCACTCAGCAGGCCTATGAGCAGGCATTTGCCCAGGTCGTGAATCGCCTGATTGCGGACGGTTATCAG
GTACTGGCGCTGTCAACCTGCACCAGTATCGATCGCTATAACCGCGATGACCGGATGGTTGCGTTACGGATGG
CGAAGTATGTAAACGACTCAGAGAATTTCCATATCGCGATGGACGAACTCAATGACGTGGAAATCGGAAAGAT
CCTCGCCTCCTGCGTACTGACCATCGGGACGCGCCTGCATTCCGCCATTATCTCCATGAATTTTGGCACACCC
GCCATCGCCATTAATTATGAACATAAATCCGCCGGGATTATGCAGCAGCTAGGCCTGGCGGAATTGGCCATCG
ATATCCGTCACTTGCTGAACGGTTCATTACAAAGCGTGGTGGCCGATACGCTCAATCAATTGCCTGCGCTAAA
GGCAAAAGTGGCTAACGCCGTCACCGAGGAGCGGGCGAAAGGGTTCACGATGATCAAATCTGTTCTCGACAGA
ATTCGGGAGGCGCAATGAAAGTCAGCTTTTTTCTGCTGAAATTTCCACTCTCATCGGAAACCTTTGTGCTGAA
TCAGATTACTGCGTTTATTGATATGGGCCATGAGGTGGAGATTGTCGCGTTACAAAAAGGCGATACCCAACAT
ACTCACGCCGCCTGGGAGAAGTATGGCCTGGCGGCGAAAACCCGCTGGTTACAGGATGAGCCCCAGGGACGGC
TGGCGAAACTGCGCTACCGGGCATGTAAAACGCTGCCGGGGCTGCATCGGGCGGCGACCTGGAAAGCGCTCAA
TTTTACCCGCTATGGCGATGAATCACGCAATTTGATCCTTTCCGCGATTTGCGCGCAGGTGAGCCAGCCTTTT
GGGGCGGATGTGTTCATCGCACACTTTGGTCCGGCGGGCGTGACGGCGGCCAAACTACGCGAACTGGGCGTGC
TTCGCGGCAAAATCGCGACTATTTTCCACGGGATTGATATTTCCAGCCGCGAAGTGCTCAGTCATTACACGCC
GGAGTATCAGCAGTTGTTTCGTCGTGGCGATCTGATGCTGCCCATCAGCGAACTGTGGGCCGGTCGCCTGAAA
AGTATGGGCTGTCCGCCGGAAAAGATTGCCGTTTCGCGCATGGGCGTCGACATGACGCGTTTTACCTATCGTC
CGGTGAAAGCGCCAGGGATGCCGCTGGAGATGATTTCCGTCGCGCGCCTGACTGAGAAAAAAGGCCTGCATGT
GGCGATTGAAGCCTGTCGGCAACTGAAAGCGCAGGGCGTGGCGTTTCGCTACCGCATTCTGGGCATTGGCCCG
TGGGAACGTCGGCTGCGCACGCTCATCGAGCAGTATCAGCTAGAGGATGTCATTGAGATGCCGGGGTTTAAAC
CGAGCCATGAAGTGAAGGCGATGCTGGATGACGCCGATGTTTTTTTGCTGCCGTCGATTACCGGTACGGATGG
CGATATGGAAGGTATTCCGGTAGCGCTGATGGAGGCGATGGCGGTAGGGATTCCCGTGGTATCTACCGTGCAT
AGCGGTATTCCGGAACTGGTGGAGGCCGGCAAATCCGGCTGGCTGGTGCCGGAAAACGATGCGCAGGCGCTGG
CGGCCCGACTCGCTGAGTTCAGCCGGATTGACCACGACACGCTGGAGTCGGTGATCACGCGCGCCCGTGAAAA
AGTGGCGCAAGATTTTAATCAGCAGGCGATTAATCGCCAGCTAGCCAGCCTGCTACAAACGATATAAACGAGG
TGGTATGCCCGTGAATAAGTTCTCCCGACGTACCCTCCTGACGGCAGGTTCCGCGCTTGCTGTTCTTCCTTTT
CTGCGCGCCTTGCCGGTACAGGCGCGTGAACCTCGCGAGACCGTCGATATTAAGGATTATCCGGCGGATGACG
GTATCGCCTCGTTCAAACAGGCCTTCGCCGACGGACAGACCGTGGTCTTACCGCCAGGATGGGTGTGTGAAAA
TATCAATGCGGCGATAACGATTCCGGCGGGAAAAACGCTGCGGGTACAGGGCGCGGTGCGTGGGAATGGCCGG
GGACGGTTTATTTTGCAGGACGGGTGTCAGGTGGTGGGGGAGCAGGGCGGCAGTCTGCACAATGTGACGCTGG
ATGTTCGCGGGTCGGACTGTGTGATTAAAGGCGTGACGATGAGCGGCTTTGGCCCCGTCGCGCAAATTTTCAT
CGGCGGTAAGGAACCGCAGGTGATGCGTAATCTCATTATCGATGACATCACCGTTACCCACGCCAACTACGCC
ATTCTCCGCCAGGGATTTCATAACCAAATGGACGGCGCGCGGATTACGCATAGCCGCTTTAGCGATTTGCAGG
GGGACGCCATTGAGTGGAATGTCGCGATTCACGACCGCGACATCCTGATTTCCGATCATGTCATCGAACGCAT
TGATTGTACCAATGGCAAAATCAACTGGGGGATCGGCATCGGGCTGGCGGGTAGCACCTATGACAACAGTTAT
CCTGAAGATCAGGCAGTAAAAAACTTTGTGGTGGCCAATATTACCGGATCTGATTGCCGACAGCTGGTGCACG
TAGAAAATGGCAAACATTTCGTCATTCGCAATGTCAAAGCCAAAAACATCACGCCCGATTTCAGTAAAAATGC
GGGTATTGATAACGCAACGATCGCCATTTATGGCTGTGATAATTTCGTCATTGATAATATTGATATGACGAAT
AGTGCTGGGATGCTCATCGGCTATGGCGTCGTTAAAGGAAAATACCTGTCAATTCCGCAAAACTTTAAATTAA
ACGCTATTCGGTTGGATAATCGCCAGGTTGCTTATAAATTACGCGGCATTCAAATTTCCTCCGGCAACATCCC
CTCTTTTGTCGCCATCACCAATGTACGGATGACGCGTGCTACGCTGGAACTGCATAATCAACCGCAGCACCTC
TTTCTGCGTAATATCAACGTGATGCAAACTTCAGCGATTGGCCCGGCGTTAAAAATGCATTTCGATTTGCGTA
AAGATGTCCGTGGTCAATTTATGGCCCGCCAGGACACGCTGCTTTCCCTCGCTAATGTTCATGCCATCAATGA
AAACGGGCAGAGTTCCGTGGATATCGACAGGATTAATCACCAAACCGTGAATGTCGAAGCAGTGAATTTTTCG
CTGCCGAAGCGGGGAGGGTAAGTACCGCTATTTTTACGAAAATTCCTGGGAAAAAGTTGTTCATACTTAATGT
TATGGTGCCGACTAAGACGTAATGTAAAGCGTGCCATCATTATCCCTGGCAGCAGAGTAATTCATGCTGGCGA
AAACAAGCTAAAGAGCTATAATTCAGCAACCATTTTACAGGTGGAAGAAACAATGATGAATTTGAAAGCAGTT
ATACCGGTAGCGGGTTTGGGTATGCATATGTTGCCTGCCACCAAGGCAATCCCAAAAGAGATGCTACCGATCG
TCGACAAGCCAATGATTCAGTACATTGTCGATGAGATTGTGGCTGCAGGGATCAAAGAAATCGTACTGGTGAC

TCACGCGTCTAAAAACGCCGTTGAGAACCACTTCGACACCTCTTATGAACTTGAATCACTTCTTGAGCAGCGC
GTTAAGCGCCAGCTTTTGGCGGAAGTGCAATCTATCTGCCCACCGGGCGTGACGATTATGAACGTTCGCCAGG
CGCAGCCGTTAGGGCTGGGGCACTCTATTCTGTGCGCGCGTCCGGTCGTGGGCGATAACCCTTTCATTGTGGT
ACTCCCGGATATTATTATCGATGATGCTACCGCCGATCCGCTGCGCTATAACCTTGCGGCGATGGTGGCGCGT
TTCAATGAAACGGGTCGCAGCCAGGTGCTGGCGAAGCGCATGAAAGGTGATTTATCGGAGTATTCCGTTATCC
AGACGAAAGAACCTCTGGATAATGAAGGCAAAGTCAGCCGGATTGTGGAGTTTATCGAAAAACCGGATCAGCC
GCAGACGCTGGATTCCGATTTGATGGCGGTAGGCCGTTATGTGCTTTCAGCCGACATCTGGGCGGAACTGGAA
AGAACCGAACCGGGCGCCTGGGGCCGTATCCAGCTCACCGATGCCATTGCAGAACTGGCGAAAAAACAGTCGG
TTGACGCGATGCTAATGACGGGTGACAGCTATGACTGCGGTAAAAAAATGGGCTACATGCAGGCATTTGTGAA
GTACGGGCTGCGCAACCTGAAAGAAGGAGCGAAGTTCCGTAAGAGCATAGAGCAGCTTTTGCATGAATAAGTA
TTAACAACCGTGATAAATGGTTGGTGATAAACATAATAACGGCAGTGAACATTCGAAGCGGCAAGTTGGCTGA
AGCGAGTGTTGACTGCCGTTTTAGTTTTGTATAAAGGGCTTAAGTAACAAGGGGTTATCTGGAGCATTTTAAT
GCTGATTTTATAAGATTAATCCTTGTTTCCGGATGCAATTAATAAGACAATTAGCGTTTAAGTTTTAGTGAGC
TTTGCCCTGCTGGGCGAGGTTTGTAACAAGTCGATATGTACGCAGTGCACTGGTAGCTGATGAGCCAGGGGCG
GTAGCGTGTGTAACGACTTGAGCAATTAATTTTTATTGGCAAATTAAATACCACATTAAATACGCCTTATGGA
ATAGAAAAGTGAAGATACTTATTACTGGCGGGGCAGGTTTTATTGGATCAGCTGTTGTCCGCCATATTATTAA
GAATACACAGGACACTGTAGTTAATATTGATAAATTAACCTACGCCGGTAATCTTGAATCCCTTTCTGATATT
TCTGAGAGTAATCGCTACAATTTTGAACACGCGGATATTTGTGATTCCGCTGAAATAACGCGTATTTTTGAGC
AGTACCAGCCGGACGCGGTGATGCATTTGGCTGCGGAAAGTCATGTGGACCGTTCGATTACCGGGCCAGCAGC
ATTTATTGAAACCAATATCGTCGGCACCTATGTACTTCTTGAAGTTGCGCGTAAATACTGGTCTGCGCTTGGC
GAAGATAAAAAAATAATTTTCGTTTTCATCATATTTCCACTGATGAAGTTTACGGCGATTTACCGCATCCTGA
TGAAGTTGAAAACAGCGTTACGCTGCCGTTATTTACTGAAACGACGGCATATGCGCCAAGTAGCCCCTATTCT
GCGTCAAAAGCATCCAGCGATCATTTAGTCCGTGCCTGGCGGCGTACCTATGGTCTACCAACGATCGTTACCA
ATTGTTCTAATAACTATGGCCCTTATCACTTCCCTGAAAAACTGATTCCGTTGGTCATTTTGAACGCACTGGA
AGGAAAGCCTTTGCCAATTTATGGCAAAGGGGATCAGATTCGCGATTGGCTATATGTAGAAGATCATGCTCGC
GCGCTTCATATGGTAGTGACTGAAGGCAAGGCGGGGGAGACTTATAACATTGGTGGCCACAATGAGAAGAAAA
ATCTCGATGTGGTATTTACCATCTGTGATCTGCTGGACGAGATTGTACCCAAAGCGACTTCTTATCGTGAACA
AATCACTTATGTCGCGGATCGTCCGGGCCATGATCGTCGTTATGCCATTGATGCAGGTAAAATTAGCCGCGAA
TTAGGCTGGAAACCGCTGGAGACCTTTGAAAGCGGTATTCGTAAAACAGTGGAATGGTACCTTGCAAATACTC
AATGGGTAAACAATGTTAAAAGTGGGGCGTATCAGAGTTGGATAGAACAGAACTATGAAGGACGCCAGTAATG
AATATCTTACTTTTTGGTAAGACAGGGCAAGTAGGCTGGGAGTTGCAACGTTCTCTGGCACCAGTAGGGAATC
TGATTGCCCTGGATGTCCATTCAAAAGAGTTTTGCGGTGATTTTAGTAATCCGAAAGGCGTTGCCGAAACCGT
TCGTAAGCTTCGTCCCGATGTGATTGTTAACGCAGCAGCACATACTGCAGTAGATAAAGCAGAGTCTGAACCA
GAACTGGCGCAGTTACTTAACGCCACCAGTGTGGAAGCCATCGCTAAAGCAGCCAACGAAACTGGCGCATGGG
TAGTGCATTATTCAACCGATTATGTATTTCCTGGTACCGGCGATATCCCATGGCAGGAAACGGACGCTACGTC
GCCGCTGAATGTCTATGGCAAGACCAAACTGGCGGGAGAAAAGGCCCTGCAGGATAACTGCCCTAAGCATCTT
ATCTTCCGCACCAGTTGGGTTTATGCAGGTAAGGGCAATAATTTCGCAAAGACAATGCTTCGTCTGGCGAAAG
AGCGTCAGACACTTTCAGTCATCAACGATCAGTACGGTGCGCCAACCGGTGCAGAATTACTGGCTGACTGCAC
GGCTCATGCGATCCGTGTGGCGTTAAAGAAACCAGAAGTCGCAGGTCTTTACCATCTGGTTGCCGGGGGAACC
ACAACCTGGCATGACTACGCGGCCTTAGTCTTTGACGAGGCGCGCAAAGCAGGGATAACGCTTGCGCTGACTG
AGCTTAATGCTGTGCCGACCAGCGCCTACCCGACGCCGGCGAGCAGACCAGGCAATTCGCGTCTCAATACTGA
AAAGTTTCAGCGTAATTTTGACCTTATTCTGCCGCAATGGGAATTAGGAGTTAAGCGCATGCTGACTGAAATG
TTTACGACGACAACCATCTGATAAATTTAAATGCCCATCAGGGCATTTTCTATGAATGAGAAATGGAAATGAA
AACGCGTAAGGGCATTATTTTAGCGGGGGGCTCCGGCACCCGTCTTTATCCGGTGACCATGGCGGTAAGTAAG
CAATTGCTACCAATTTATGATAAACCGATGATTTACTATCCCCTTTCCACGCTTATGCTGGCAGGCATTCGGG
ATATCCTGATCATCAGTACGCCACAGGACACGCCGCGTTTTCAACAACTGCTGGGAGACGGCAGCCAGTGGGG
GCTGAATCTTCAATATAAAGTACAGCCAAGCCCGGATGGCTTAGCACAGGCGTTTATTATTGGTGAAGAGTTC
ATTGGTAATGATGATTGTGCATTAGTACTGGGTGACAATATCTTCTATGGTCATGATTTACCAAAGTTAATGG
AAGCTGCCGTTAATAAAGAAAGTGGTGCTACCGTCTTTGCTTATCATGTAAACGATCCGGAGCGCTACGGTGT
GGTTGAGTTTGACCAAAGTGGCACAGCCGTTAGTCTGGAGGAAAAACCGTTACAACCGAAGAGTAATTACGCG
GTAACGGGGCTGTATTTTTATGATAATAGCGTGGTGGAGATGGCGAAAAATCTTAAGCCTTCCGCTCGCGGTG
AGTTAGAAATCACGGATATTAACCGTATCTATATGGATCAGGGAAGATTGTCTGTCGCCATGATGGGGCGCGG
TTATGCCTGGCTGGATACAGGGACGCATCAGAGTTTGATAGAGGCCAGTAATTTTATTGCAACCATCGAAGAA
CGCCAGGGGCTAAAAGTGTCCTGCCCGGAAGAGATCGCATTTCGTAAAAATTTTATAAATGCACAACAGGTTA

TAGAACTGGCCGGGCCATTATCAAAAAATGATTATGGCAAATATTTGCTGAAGATGGTGAAAGGTTTATAAGT
GATGATTGTGATTAAAACAGCAATACCAGATGTCTTGATCTTAGAGCCTAAAGTTTTTGGCGATGAGAGGGGA
TTCTTTTTTGAAAGTTATAACCAGCAGACCTTTGAAGAGTTGATTGGACGTAAAGTTACATTTGTTCAAGATA
ATCATTCAAAATCCAAAAAGAACGTACTCAGAGGGCTACATTTTCAGAGAGGAGAAAATGCACAGGGGAAGTT
AGTTCGTTGTGCTGTCGGTGAGGTTTTTGATGTTGCGGTCGATATCCGAAAAGAATCGCCTACTTTTGGTCAA
TGGGTTGGCGTAAATCTATCTGCTGAGAATAAGCGACAGCTTTGGATTCCAGAAGGTTTTGCTCATGGTTTTG
TTACTCTTAGTGAGTATGCAGAGTTTCTGTACAAAGCAACTAATTATTACTCACCTTCATCGGAAGGTAGCAT
TTTATGGAATGATGAGACAATAGGTATTGAATGGCCTTTTTCTCAGCTGCCTGAGCTTTCAGCAAAAGATGCT
GCAGCACCTTTACTGCATCAAGCCTTGTTAACAGAGTAAGCATCGTGTCTCATATTATTAAGATTTTTCCATC
AAATATTGAATTTTCCGGTAGAGAGGATGAATCAATCCTCGATGCTGCGCTATCGGCTGGCATCCATCTTGAA
CATAGCTGCAAAGCGGGTGATTGTGGTATCTGTGAGTCCGATTTGTTGGCGGGAGAAGTTGTTGACTCCAAAG
GTAATATTTTTGGACAGGGTGATAAAATACTAACCTGCTGCTGTAAACCTAAAACCGCCCTTGAGCTAAATGC
GCATTTTTTTCCTGAACTAGCTGGACAGACAAAAAAAATTGTCCCATGCAAGGTAAATAGTGCTGTACTGGTT
TCAGGCGATGTTATGACTTTGAAGTTACGCACACCACCAACAGCAAAAATTGGCTTCCTTCCAGGGCAGTATA
TCAATTTACATTATAAAGGTGTAACTCGCAGTTATTCTATCGCTAATAGTGATGAGTCGAATGGTATTGAGTT
GCATGTAAGGAATGTTCCCAATGGTCAGATGAGCTCTCTCATTTTTGGGGAGTTACAAGAAAATACTCTTATG
CGCATTGAAGGACCTTGCGGAACATTTTTTATTCGTGAAAGTGACAGACCTATAATCTTCCTTGCAGGCGGTA
CTGGATTCGCTCCAGTTAAATCAATGGTTGAGCATCTCATTCAGGGAAAATGTCGTCGTGAGATCTACATCTA
CTGGGGAATGCAAGATAGTAAAGATTTTTACTCTGCATTACCGCAGCAGTGGAGTGAACAGCACGACAACGTT
CATTATATCCCTGTTGTTTCTGGTGATGACGCCGAATGGGGGGAAGAAAGGGATTTGTCCATCATGCTGTGAT
GGATGATTTTGATTCTCTAGAGTTCTTCGATATATATGCATGTGGTTCACCTGTGATGATCGATGCCAGTAAA
AAGGACTTTATGATGAAAAATCTCTCTGTAGAACATTTCTATTCTGATGCATTTACCGCATCTAAATAATATT
GAGGATAATTTATGAAAGCGGTCATCCTGGCTGGTGGACTTGGTACCAGACTAAGTGAAGAAACAATTGTAAA
ACCAAAACCGATGGTAGAAATTGGTGGCAAGCCTATTCTTTGGCACATTATGAAAATGTATTCTGTGCATGGT
ATCAAGGATTTTATTATCTGCTGTGGTTATAAAGGATATGTGATTAAAGAATATTTTGCGAACTACTTCCTTC
ACATGTCAGATGTAACATTCCATATGGCTGAAAATCGTATGGAAGTTCACCATAAACGTGTTGAACCATGGAA
TGTCACATTGGTTGATACGGGTGATTCTTCAATGACTGGTGGTCGTCTGAAACGTGTTGCTGAATACGTAAAA
GATGACGAGGCTTTCCTGTTTACTTATGGTGATGGCGTTGCCGACCTTGATATCAAAGCGACTATCGATTTCC
ATAAGGCTCACGGTAAGAAAGCGACTTTAACAGCTACTTTTCCACCAGGACGTTTTGGCGCATTAGATATCCA
AGCTGGTCAGGTCCGGTCATTCCAGGAAAAACCGAAAGGCGATGGGGCAATGATCAATGGTGGTTTCTTTGTG
TTGAATCCATCGGTTATCGATCTCATCGATAACGATGCAACAACCTGGGAACAAGAGCCATTAATGACATTGG
CACAACAGGGGGAGTTAATGGCTTTTGAACACCCAGGTTTCTGGCAGCCGATGGATACCCTACGTGATAAAGT
TTACCTTGAAGGGCTGTGGGAAAAAGGTAAAGCTCCGTGGAAAACCTGGGAGTAAGTAGATGATTGATAAAAA
TTTTTGGCAAGGTAAACGTGTATTCGTTACCGGCCATACTGGCTTTAAAGGAAGCTGGCTTTCGCTATGGCTG
ACTGAAATGGGTGCAATTGTAAAAGGCTATGCACTTGATGCGCCAACTGTTCCAAGTTTATTTGAGATAGTGC
GTCTTAGTGATCTTATGGAATCTCATATTGGCGACATTCGTGATTTTGAAAAGCTGCGCAATTCTATTGCAGA
ATTTAAGCCAGAAATTGTTTTCCATATGGCAGCCCAGCCTTTAGTGCGCCTATCTTATGAACAGCCAATCGAA
ACATACTCAACAAATGTTATGGGTACTGTCCATTTGCTTGAAGCAGTTAAGCAAGTAGGTAACATAAAGGCAG
TCGTAAATATCACCAGTGATAAGTGCTACGACAATCGTGAGTGGGTGTGGGGCTATCGTGAGAACGAACCCAT
GGGAGGGTACGATCCATACTCTAATAGTAAAGGTTGTGCAGAATTAGTCGCGTCTGCATTCCGGAACTCATTC
TTCAATCCTGCAAATTATGAGCAACATGGCGTTGGTTTGGCGTCTGTGAGGGCTGGTAATGTCATAGGCGGAG
GCGATTGGGCTAAAGACCGTTTAATTCCCGATATTCTGCGCTCATTTGAAAATAACCAGCAGGTTATTATTCG
AAACCCATATTCTATCCGTCCCTGGCAGCATGTACTGGAGCCTCTTTCTGGTTACATTGTGGTGGCGCAACGC
TTATATACAGAAGGTGCTAAGTTTTCTGAAGGATGGAATTTCGGCCCGCGTGATGAAGATGCGAAGACGGTCG
AATTTATTGTTGACAAGATGGTCACGCTTTGGGGTGATGATGCAAGCTGGTTACTGGATGGTGAGAATCATCC
TCATGAGGCACATTACCTGAAACTGGATTGCTCTAAAGCAAATATGCAATTAGGATGGCATCCGCGTTGGGGA
TTGACTGAAACACTTGGTCGCATCGTAAAATGGCATAAAGCATGGATTCGCGGCGAAGATATGTTGATTTGTT
CAAAGCGTGAAATCAGCGACTATATGTCTGCAACTACTCGTTAAGAAAATAAGTTTAAGGAATCAAAGTAATG
ACAGCAAATAACCTGCGTGAGCAAATCTCTCAGCTTGTCGCTCAGTATGCGAATGAGGCATTGAGCCCGAAAC
CTTTTGTTGCAGGTACAAGCGTTGTGCCTCCTTCCGGGAAGGTTATTGGTGCCAAAGAGTTACAATTGATGGT
TGAGGCGTCTCTTGATGGATGGCTAACTACTGGTCGTTTCAATGATGCCTTTGAGAAAAAACTTGGGGAATTT
ATTGGGGTTCCTCATGTTTTAACGACTACATCTGGCTCTTCGGCAAACTTGCTGGCACTGACTGCGCTGACTT
CCCCAAAATTAGGCGAGCGTGCTCTCAAACCTGGTGATGAGGTTATTACTGTCGCTGCTGGCTTCCCGACTAC
AGTTAACCCGGCGATCCAGAATGGTTTAATACCGGTATTCGTGGATGTTGATATCCCGACATATAATATCGAT

GCCTCTCTCATTGAAGCTGCAGTTACTGAGAAATCAAAAGCGATAATGATCGCTCATACACTCGGTAATGCAT
TTAACCTGAGTGAAGTTCGTCGGATTGCCGATAAATATAACTTATGGTTGATTGAAGACTGCTGTGATGCCCT
TGGGACGACTTATGAAGGCCAGATGGTAGGTACCTTTGGTGACATCGGAACCGTTAGTTTTTATCCGGCTCAC
CATATCACAATGGGTGAAGGCGGTGCTGTATTCACCAAGTCAGGTGAACTGAAGAAAATTATTGAGTCGTTCC
GTGACTGGGGCCGGGATTGTTATTGTGCGCCAGGATGCGATAACACCTGCGGTAAACGTTTTGGTCAGCAATT
GGGATCACTTCCTCAAGGCTATGATCACAAATATACTTATTCCCACCTCGGATATAATCTCAAAATCACGGAC
ATGCAGGCAGCATGTGGTCTGGCTCAGTTGGAGCGCGTAGAAGAGTTTGTAGAGCAGCGTAAAGCTAACTTTT
CCTATCTGAAACAGGGCTTGCAATCTTGCACTGAATTCCTCGAATTACCAGAAGCAACAGAGAAATCAGACCC
ATCCTGGTTTGGCTTCCCTATCACCCTGAAAGAAACTAGCGGTGTTAACCGTGTCGAACTGGTGAAATTCCTT
GATGAAGCAAAAATCGGTACACGTTTACTGTTTGCTGGAAATCTGATTCGCCAACCGTATTTTGCTAATGTGA
AATATCGTGTAGTGGGTGAGTTGACAAATACCGACCGTATAATGAATCAAACGTTCTGGATTGGTATTTATCC
TGGCTTGACTACAGAGCATTTAGATTATGTAGTTAGCAAATTTGAAGAGTTTTTTGGTTTAAATTTCTAATTT
TTGAGGGGGGGGATTCCCCTCTATGATTTCATGAAAATTCTAATAATGGGAGCGTTTGGGTTCCTTGGATCA
CGACTTACATCCTACTTCGAAAGTCGACATACTGTGATTGGCTTAGCAAGGAAGAGGAACAATGAAGCTACCA
TAAATAATATTATTTACACGACAGAAAATAATTGGATCGAAAAAATAGTAGAATTTGAACCGAATATTATTAT
TAACACTATTGCTTGCTATGGAAGACATAACGAACCTGCAACAGCTTTAATAGAAAGCAATATTCTTATGCCT
ATCAGAGTATTAGAATCTATCTCATCACTTGATGCAGTATTCATAAATTGTGGAACATCACTGCCACCAAATA
CGAGTTTATATGCATATACTAAACAAAAAGCAAATGAACTCGCCGCCGCCATTATAGATAAAGTTTGTGGTAA
ATATATAGAGTTAAAATTGGAGCATTTCTATGGAGCTTTTGATGGAGACGATAAGTTTACCAGTATGGTTATT
AGACGTTGTTTAAGTAACCAGCCAGTAAAGTTAACATCTGGTTTGCAACAGAGAGATTTCTTGTATATAAAAG
ATCTACTAACAGCGTTCGATTGTATTATAAATAATGTTAATAATTTCCCCAAATTTCATAGTATTGAAGTTGG
TAGTGGAAAGGCTATATCAATTCGTGAATATGTAGAGACTGTTAAAAATATCACAAAAAGCAATTCTATAATT
GAATTTGGTGTGGTCAAAGAAAGAGCCAATGAATTGATGTATAGTTGTGCTGATATAGCAGAACTTGAAAAAA
TAGGATGGAAAAGAGAGTTCTCTCTTGTTGATGCATTAACTGAAATAATTGAAGAGGAAGGGAAATGAAGCTT
TTAATTACCGGTGGATGTGGCTTTCTTGGGAGTAATCTTGCCTCCTTTGCTTTAAGTCAAGGGATTGATTTAA
TTGTATTCGATAATCTATCACGTAAAGGTGCAACAGATAATTTACATTGGTTATCCTCCTTAGGAAACTTTGA
GTTTGTACATGGTGATATTCGCAACAAAAATGATGTTACAAGATTAATAACTAAGTATATGCCTGATAGCTGT
TTTCATCTTGCAGGTCAAGTGGCAATGACTACATCTATTGACAATCCTTGTATGGATTTTGAAATTAATGTAG
GTGGAACTTTAAATTTACTTGAGGCAGTACGGCAGTATAATTCAAATTGTAATATAATTTATTCATCAACAAA
TAAAGTATACGGCGATCTTGAGCAATATAAATACAATGAAACAGAAACTAGATATACTTGTATAGATAAGCCT
AATGGATATGATGAGAGCACACAATTAGATTTCCACTCACCATATGGTTGTTCAAAAGGTGCTGCAGATCAAT
ACATGCTTGATTATGCAAGGATTTTTGGTTTGAATACAGTGGTGTTCAGGCATTCATCAATGTATGGTGGGAG
ACAGTTTGCTACTTATGATCAAGGCTGGGTAGGTTGGTTTTGTCAAAAAGCGGTTGAAATTAAAAATGGTATT
AACAAACCCTTCACTATTTCTGGTAATGGTAAGCAAGTTAGGGATGTTTTGCATGCTGAAGATATGATTTCGT
TATATTTCACTGCCTTGGCAAATGTATCAAAAATTAGGGGGAACGCTTTTAATATTGGTGGTACCATTGTCAA
CAGCCTATCATTACTTGAATTATTCAAATTGCTTGAAGATTATTGCAACATTGATATGAGGTTCACTAATTTA
CCAGTAAGGGAAAGGGATCAGCGTGTTTTTGTTGCAGATATTAAAAAAATCACTAATGCAATTGACTGGAGCC
CGAAAGTCTCGGCAAAAGATGGTGTCCAGAAAATGTATGATTGGACTAGTTCTATATGATGTTTCTGCCCGCG
AAAGCGGGCATAATTAAAGAATGAAATATTTTTTATAATTAAAAGATGAAGCTGACGTGAGGAAACTGAGGTT
GGTTAGAATTCCAAGACATCTTATTATTGCCGCTTCCTCTTGGCTTTCAAAGATAATAATTGCCGGTGTTCAG
TTAGTAAGTGTTAAATTTCTTTTGGAAATTCTTGGCGAAGAATCATACGCTGTATTTACTCTTTTAACTGGAT
TATTGGTCTGGTTTAGCATTGCAGATATTGGGATTGGTAGTAGTCTACAAAATTATATATCTGAGTTGAAAGC
TGATAGAAAATCATATGATGCATATATCAAGGCCGCAATTCATATTCTATTCGCATCCTTAATCATTTTAAGC
TCTACATTATTCTTCTTATCAGATAAATTATCGTCACTATATCTTACTTCATTTAGCGATGAATTGAAAAACA
ACTCAGGAAGTTATTTTTTTATAGCAAGTATATTATTTATATTCATTGGCGTTGGGAGTGTGGTCTATAAAAT
ATTATTTGCGGAACTGTTAGGGTGGAAAGCTAATATAATTAATGCATTATCTTATCTTTTAGGTTTTTTAGAT
GTAGTTGCGATCCATTATTTAATGCCAGATTCGAGTATTACCTTCGCTTTAGTAGCATTGTATGCTCCGGTAG
CAATACTGCCCATTATATATATATCGTTTCGGTACATATATGTTCTTAAAACGAAAGTAAACTTTAACACCTA
TAAATTATTACTATCACGTTCATCAGGGTTTCTGATTTTTTCGTCCTTATCGATAATAGTTTTACAAACTGAT
TATATTGTGATGTCTCAGAAATTATCTGCTGCAGATATTATAAAATATACTGTAACGATGAAAATATTTGGTT
TAATGTTTTTTATTTATACTGCGGTATTACAAGCATTATGGCCAGTATGTGCTGAATTACGAGTGAAAATGCA
GTGGAGAAAGTTGCATAGAATCATTTTCCTAAATATTATTGGTGGGGTATTTTTTGTTGGTCTTGGTACGTTA
TTCATTTATGTTTTAAAGGATTATATCTATAGCATAATTGCTAACGGTATAGATTATAATATTAGTGGGGCTG
TTTTTGTTTTACTGGCTGTGTATTTTAGTATAAGAGTTTGGTGTGATACATTTGCTATGTTACTTCAAAGTAT

GAACCAATTAAAAATTCTTTGGCTCATAGTTCCGTGTCAGGCATTAATTGGTGGTGTGACTCAATGGTATTTT
GCAGAGCATTATGGAATAGTTGGTATTTTATACGGACTAATTTTATCGTTCTCGCTAACTGTTTTTTGGGGAT
TGCCAGTGTATTATATGTATAAGAGTAAAAGGCTAGCATAATATGAAGGTATCATTTTGTATCCCAACGTATA
ATCGAGTAAAATTCATTGAAGACCTTCTTGAAAGTATTAATAATCAATCTTCTCACTCCTTAATTGTAGAAGT
ATGTATTTCAGATAATGCATCAACTGATGGAACGGAAGAGTCAATAAATATCTGGAGAGATAGATTTAATTTT
CCAATTTTGTATCAAAGGCATAACGAAAATATTGGGCCAGATAGGAATTATTTATCTGCAGTTAATATGGGGA
CTGGAGATTATTGTTGGATTTTCGGAAGTGATGATATTCTTACAAAAAATTCCCTTGCATTGATGGAAGATAA
ATTGGCTGCCGGAAGTGACATTTATTTATGTGACAGACGAGAATTAGATATTTCAATGACGAAAATATCTAAT
CCACATCGGCGATGGTTAAATGGTGGCAGTAGATTATTTTCTTTTAGCAATGAAGCTGATTTGATAGAGTACT
TTAGTAAATGTAACTCAGTCGGCGGACTTTTTAGCTACTTAAGCTCTATTATAGTGAAAAGGAATAAATGGTC
TGACGTCATATTTGACGAGTCATACATTGGTACAGCTTATGCGCATGTATATATTTTATTAAGAATAATTAAC
AATATGAATTCGACCCTGCAGTACATATCACTGCCACTGGTTGATTGCAGAGGAGATAACGATACATTCGAAA
GTAATGGGAAGGCTCGTCGAATAAAAATTGATTTCATCGGTTATTTAAAACTGAGGGAGGATTTTTATAATAA
CAATACAAAAATATATATCTCATTTGGACGGGTTCTTACAAAAGAGCGTCCTTGGTTTTATACTTCTTTAGCT
ATGGCATGTTATGGTGATTCCACCGATAGGGCTGAACTTGCATCATTTTATAAGAAGTTGGGATATCCGAAAA
TAGCCACCAATTTAATATTTCGTCTTAAAGGGCTGGCTAGCTACACTAAAAAAATAAAATTAGCAAAAATGGT
AATAAAAAAAATATTTTCATAGTATCAACATCAATACAAATCGAGTTTATAGTATAAAAAATGCTTATGCTAT
TATTGTCGTGACTATTTAGCGTATAGTTTATACGTGTTTTGTAGGATGATTTATTTGCAATTTCCTTAATTAG
AACCTTTAAATACTCTTGCTTTTTCGGATACAGTAACAGATGCTATATATGTATCTATAATATGTATGGACGG
GGTTGTTTTTGACTATTTATCAATTTCAAAATTAAGACTTGTGGCAATTAGAGATTGTCGGAAACCCTAGTTT
TCTTTCTTTTGCTTACTATGCTCTTGCATATCTTAGTGTTTACCATAGAGTGAGTATATTATGGCAGATGCTA
TTTTTTGTGGCATTTTTACTTGGTATAATATTTGGCTTAGTATTATGAACTGATTTTAATGGTGATTTTATAA
TAATTACAAGGCTTGCTTATATTTATTTGGATATGATTCCTTATTCATTTTTAATTATGATCCGTGGTTCATT
TTAATGAAAATGTTTCCTAATAACTATTGGTCGTCCTTGTGATAATCGCTATACAAGCATGAATGGAAAAACG
GAGAGCTATTCAATGATCGTTAACCTATCACGTTTAGGTAAAAGTGGTACGGGAATGTGGCAATACTCGATTA
AATTTTTAACGGCACTGCGAGAAATAGCTGATGTTGACGCAATAATCTGTAGCAAGGTACACGCTGATTATTT
TGAAAAGCTCGGTTATGCAGTAGTTACTGTTCCGAATATTGTTAGCAACACATCAAAAACATCGCGACTTAGA
CCATTAGTATGGTATGTATATAGTTACTGGCTTGCGCTGAGGGTTTTAATTAAGTTTGGTAATAAAAAATTGG
TGTGTACTACACATCACACTATCCCCTTACTGAGAAACCAAACGATAACCGTACATGATATAAGACCTTTTTA
TTATCCAGATAGTTTTATTCAGAAAGTGTATTTTCGCTTTTTATTAAAAATGTCCGTTAAGCGATGTAAGCAT
GTTTTAACGGTATCTTATACCGTTAAAGATAGCATTGCTAAAACTTATAATGTAGATAGTGAGAAAATATCAG
TAATTTATAATAGTGTTAATAAATCTGATTTTATACAAAAAAAAGAAAAAGAGAATTACTTTTTAGCTGTTGG
TGCAAGTTGGCCACATAAAAAATATTCATTCATTCATAAAAAAATAAAAAAGTTTGGTCTGACTCTTATAATTTA
ATTATTGTATGTGGTCGTACTGACTATGCAATGTCTCTCCAACAAATGGTCGTTGATCTGGAACTAAAAGATA
AAGTGACTTTTTTACATGAAGTCTCATTTAATGAATTAAAGATTTTATATTCTAAAGCCTACGCGCTTGTTTA
TCCATCTATTGATGAGGGTTTTGGTATACCTCCTATTGAAGCGATGGCATCAAATACTCCAGTTATAGTGTCC
GATATACCAGTATTTCATGAAGTGTTAACCAATGGTGCATTATATGTGAATCCGGATGATGAAAAAAGCTGGC
AGAGTGCAATTAAAAATATAGAGCAGTTGCCTGATGCAATTTCCCGATTTAACAACTATGTCGCACGGTATGA
CTTTGATAATATGAAGCAGATGGTTGGCAATTGGTTGGCGGAATCAAAATAAATGAAAATAACATTAATTATT
CCCACATATAATGCAGGGTCGCTTTGGCCTAATGTTCTGGATGCGATTAAGCAGCAAACTATATATCCGGATA
AATTGATTGTTATAGACTCAGGTTCTAAAGATGAAACGGTTCCGTTAGCCTCAGACCTGAAAAATATATCAAT
ATTTAATATTGACTCTAAAGATTTTAATCATGGAGGAACCAGAAATTTAGCAGTTGCAAAAACTCTGGACGCT
GATGTTATAATTTTTCTAACGCAAGATGCAATTCTCGCGGATTCGGATGCAATTAAAAATTTGGTTTATTATT
TTTCAGATCCATTGATAGCAGCGGTTTGTGGTAGACAACTTCCTCATAAAGATGCTAATCCCCTTGCAGTGCA
TGCCAGAAATTTTAATTATAGTTCAAAATCTATTGTTAAAAGTAAGGCAGATATAGAAAAATTGGGTATTAAA
ACTGTATTTATGTCCAATTCTTTTGCTGCCTATCGCCGTTCCGTTTTTGAAGAGTTAAGTGGGTTTCCTGAAC
ATACAATTCTTGCCGAGGATATGTTTATGGCGGCTAAGATGATTCAGGCGGGTTATAAGGTCGCCTACTGCGC
TGAAGCGGTGGTAAGACACTCCCATAATTATACCCCGCGAGAAGAGTTTCAACGATATTTTGATACTGGTGTA
TTTCATGCTTGTTCTCCGTGGATTCAGCGTGACTTTGGCGGAGCCGGTGGTGAGGGTTTCCGCTTCGTAAAAT
CAGAGATTCAATTCCTGCTTAAAAATGCACCGTTCTGGATTCCAAGAGCTTTATTAACAACCTTTGCTAAATT
CTTGGGTTACAAATTAGGCAAGCATTGGCAATCTTTACCGTTGTCTACATGTCGCTATTTTAGCATGTACAAG
AGTTATTGGAATAATATCCAATATTCTTCGTCAAAAGAGATAAAATAAATGTCTTTTCTTCCCGTAATTATGG
CTGGCGGCACAGGTAGCCGTTTATGGCCGCTTTCACGCGAATATCATCCGAAGCAGTTTCTAAGCGTTGAAGG
TAAACTATCAATGCTGCAAAATACTATAAAGCGATTAGCTTCACTTTCTACAGAAGAACCCGTTGTCATTTGC

AATGACAGACACCGTTTCTTAGTCGCTGAACAACTCCGTGAAATTGACAAGTTAGCAAATAATATTATTCTCG
AACCGGTAGGCCGTAATACTGCACCAGCGATCGCTCTTGCCGCGTTTTGTGCGCTCCAGAATGCTGATAATGC
TGATCCTCTTTTGTTGGTTCTTGCTGCAGATCATGTGATTCAGGATGAAATAGCTTTTACGAAAGCTGTCAGA
CATGCTGAAGAATACGCTGCAAATGGTAAGCTTGTAACTTTTGGTATTGTTCCAACGCATGCTGAAACGGGTT
ATGGATATATTCGTCGTGGTGAGTTGATAGGAAATGACGCTTATGCAGTGGCTGAATTTGTGGAGAAACCGGA
TATCGATACCGCCGGTGACTATTTCAAATCAGGGAAATATTACTGGAATAGCGGTATGTTTTTATTTCGTGCA
AGCTCTTATTTAAACGAATTAAAGTATTTATCACCTGAAATTTATAAAGCTTGTGAAAAGGCGGTAGGACATA
TAAATCCCGATCTTGATTTTATTCGTATTGATAAAGAAGAGTTTATGTCATGCCCGAGTGATTCTATCGATTA
TGCAGTTATGGAGCACACACAGCATGCGGTGGTGATACCAATGAGCGCTGGCTGGTCGGATGTGGGTTCCTGG
TCCTCACTTTGGGATATATCGAATAAAGATCATCAGAGAAATGTTTTAAAAGGAGATATCTTCGCACATGCTT
GTAATGATAATTACATTTATTCCGAAGATATGTTTATAAGTGCGATTGGTGTAAGCAATCTTGTCATTGTTCA
AACAACAGACGCTTTACTGGTGGCTAATAAAGATACAGTACAAGATGTTAAAAAAATTGTCGATTATTTAAAA
CGGAATGATAGGAACGAATATAAACATCATCAAGAAGTTTTCCGCCCCTGGGGAAAATATAATGTGATTGATA
GCGGCAAAAATTACCTCGTTCGATGTATCACTGTTAAGCCGGGTGAGAAATTTGTGGCGCAGATGCATCACCA
CCGGGCTGAGCATTGGATAGTATTATCCGGGACTGCTCGTGTTACAAAGGGAGAGCAGATTTATATGGTTTCT
GAAAATGAATCAACATTTATTCCTCCGAATACTATTCACGCGCTGGAAAATCCTGGAATGACCCCCCTGAAGT
TAATTGAGATTCAATCAGGTACCTATCTTGGTGAGGATGATATTATTCGTTTAGAACAACGTTCTGGATTTTC
GAAGGAGTGGACTAATGAACGTAGTTAATAATAGCCGTGATGTTATTTATTCATCAGGTATTGTGTTTGGAAC
GAGTGGGGCTCGCGGTCTTGTAAAAGATTTTACACCTCAGGTATGTGCTGCTTTTACGGTTTCATTTGTTGCC
GTTATGCAGGAACATTTTTCCTTTGATACCGTAGCATTGGCAATAGATAATCGTCCAAGTAGTTATGGGATGG
CTCAGGCGTGTGCTGCTGCATTGGCGGATAAAGGCGTTAACTGTATTTTTTATGGAGTGGTACCAACCCCAGC
TTTGGCCTTTCAGTCTATGTCTGACAATATGCCTGCGATAATGGTTACGGGAAGTCATATTCCATTCGAGCGG
AACGGCCTCAAGTTTTATCGTCCTGATGGTGAAATCACGAAACATGATGAGGCTGCGATCCTTAGTGTTGAAG
ATACGTGCAGCCATTTAGAGCTTAAAGAACTCATAGTTTCAGAAATGGCTGCTGTTAATTATATATCTCGTTA
TACATCTTTATTTTCTACCCCCTTCTAAAAAAATAGGGTTTTTGTTTTTGAAAACACACCCGCGGGGGGGGTG
TTTTTTAAACATATTTTTTTTTTTGTTTGGGGGGTGGGGTTGTTTGGTTGGGAGAGAAGGAAATGTTTGTATA
TATAGATAGAGGGTGTAAAAAGAGGAGAGGATCGGGAAAAAGCTCGCTCATGGGCTAAAGAGTTCGATTTAGA
TGCCATATTCTCGACAGATGGGGATGGTGATCGCCCTCTTATTGCTGATGAGGCCGGTGAGTGGCTAAGAGGC
GATATACTAGGTCTATTATGTTCACTTGCATTGGATGCAGAAGCCGTCGCTATTCCTGTTAGTTGTAACAGCA
TAATTTCTTCTGGCCGCTTTTTTAAACATGTTAAGCTTACAAAAATTGGCTCGCCTTATGTTATCGAAGCTTT
TAATGAATTATCGCGGAGTTATAGTCGTATTGTCGGTTTTGAAGCCAATGGCGGTTTTTTATTAGGAAGCGAC
ATCTGTATTAACGAGCAGAATCTTCATGCCTTACCAACTCGTGATGCTGTATTACCAGCAATAATGCTGCTTT
ACAAAAGTAGGAATACCAGCATTAGCGCTTTAGTCAATGAACTCCCAACTCGTTACACCCATTCTGACAGATT
ACAGGGGATTACAACTGATAAAAGTCAATCCTTAATTAGTATGGGCAGAGAAAATCTGAGCAACCTCTTAAGC
TATATTGGTTTGGAGAATGAAGGTGCAATTTCTACAGATATGACAGATGGTATGCGAATTACTTTACGTGATG
GATGTATTGTGCATTTGCGCGCTTCTGGTAATGCACCTGAGTTACGCTGCTATGCAGAAGCTAATTTATTAAA
TAGGGCTCAGGATCTTGTAAATACAACGCTTGCTAATATTAAAAAACGATGCTTGCTGTAAAAAAATTGAATGT
TATTTACTTAATATGCCTATTTTATTTACATTATGCACGGTCAGAGGGTGAGGATTAAATGGATAATATTGAT
AATAAGTATAATCCACAGCTATGTAAAATTTTTTTGGCTATATCGGATTTGATTTTTTTTAATTTAGCCTTAT
GGTTTTCATTAGGATGTGTCTATTTTATTTTTGATCAAGTACAGCGATTTATTCCTCAAGACCAATTAGATAC
AAGAGTTATTACGCATTTTATTTTGTCAGTAGTATGTGTCGGTTGGTTTTGGATTCGTTTGCGACATTATACA
TACCGCAAGCCATTTTGGTATGAGTTAAAAGAAATTTTTCGTACGATCGTTATTTTTGCTATATTTGATTTGG
CTCTGATAGCGTTTACAAAATGGCAGTTTTCACGCTATGTCTGGGTGTTTTGTTGGACTTTTGCTCTAATCCT
GGTGCCTTTTTTTCGCGCACTTACAAAGCATTTATTGAACAAGCTAGGTATCTGGAAGAAAAAAACTATCATC
CTGGGGAGCGGACAGAATGCTCGTGGTGCATATTCTGCGCTGCAAAGTGAGGAGATGATGGGGTTTGATGTTA
TCGCTTTTTTTGATACGGATGCGTCAGATGCTGAAATAAATATGTTGCCGGTGATAAAGGATACTGAGATTAT
TTGGGATTTAAATCGTACAGGTGATGTCCATTATATCCTTGCTTATGAATACACCGAGTTGGAGAAAACACAT
TTTTGGCTACGTGAACTTTCAAAACATCATTGTCGTTCTGTTACTGTAGTCCCCTCGTTTAGAGGATTGCCAT
TATATAATACTGATATGTCTTTTATCTTTAGCCATGAAGTTATGTTATTAAGGATACAAAATAACTTGGCTAA
AAGGTCGTCCCGTTTTCTCAAACGGACATTTGATATTGTTTGTTCAATAATGATTCTTATAATTGCATCACCA
CTTATGATTTATCTGTGGTATAAAGTTACTCGAGATGGTGGTCCGGCTATTTATGGTCACCAGCGAGTAGGTC
GGCATGGAAAACTTTTTCCATGCTACAAATTTCGTTCTATGGTTATGAATTCTCAAGAGGTACTAAAAGAACT
TTTGGCTAACGATCCTATTGCCAGGGCTGAATGGGAGAAAGATTTTAAACTGAAAAATGATCCTCGAATCACA
GCTGTAGGTCGATTTATACGTAAAACTAGCCTTGATGAGTTGCCACAACTTTTTAATGTACTAAAAGGTGATA

TGAGCCTGGTTGGACCACGACCTATCGTTTCGGATGAACTGGAGCGTTATTGTGATGATGTTGATTATTATTT
GATGGCAAAGCCGGGCATGACAGGTCTATGGCAAGTGAGTGGGCGTAATGATGTTGATTATGACACTCGTGTT
TATTTTGATTCCTGGTATGTTAAAAACTGGACGCTTTGGAATGATATTGCCATTCTGTTTAAAACAGCGAAAG
TTGTTTTGCGGCGAGATGGTGCGTATTAAGCTTACCGAGAAGTACTGAATAATAATTGTATAAAATAGCCTGC
GTAAAATCTGAACGCATCAATCGCTACCTTAATATCATACCTTTGAGTTAACATACTATTCACCTTTAACCTG
CCATGACCGTTTGTGGCAGGGTTTCCACACCTGACAGGAGTATGTAATGTCCAAGCAACAGATCGGCGTCGTC
GGTATGGCAGTGATGGGGCGCAACCTCGCGCTCAACATCGAAAGCCGTGGTTATACCGTCTCCGTTTTCAACC
GCTCCCGTGAAAAAGACCGAAGAAGTGATTGCCGAGAATCCCGGCAAAAAGCTGGTGCCTTATTACACGGTGA
AAGAGTTTGTTGAATCCCTCGAAACGCCTCGTCGTATCCTGTTAATGGTGAAAGCGGGTGCAGGTACTGATGC
AGCTATCGATTCGCTGAAACCGTATCTGGAAAAAGGCGATATCATTATTGATGGCGGTAACACCTTCTTCCAG
GACACAATCCGTCGCAATCGCGAGCTGTCTGCGGAAGGTTTTAACTTTATCGGTACCGGTGTTTCCGGTGGTG
AAGAGGGCGCGCTGAAAGGGCCATCTATCATGCCTGGCGGTCAGAAAGATGCCTATGAACTGGTGGCGCCGAT
CCTGACGAAGATTGCTGCTGTGGCGGAAGATGGCGAACCGTGCGTGACCTATATCGGCGCCGATGGTGCTGGT
CACTACGTCAAGATGGTCCACAATGGTATTGAATATGGCGATATGCAGCTTATCGCTGAAGCTTACTCCCTGC
TGAAAGGCGGCCTGAATCTCAGCAATGAAGAGTTGGCGAACACTTTTACTGAGTGGAATAACGGCGAGCTGAG
CAGCTATCTGATCGACATCACTAAAGACATCTTCACTAAAAAAGATGAAGACGGAAACTACCTGGTGGATGTC
ATTCTTGATGAAGCCGCCAACAAAGGGACCGGTAAATGGACCAGCCAGAGCGCGCTGGATCTGGGTGAACCGC
TGTCGCTGATTACCGAATCCGTCTTTGCGCGTTACATCTCTTCGTTGAAAGCGCAACGTGTTGCCGCATCTAA
AGTGCTCTCCGGGCCGAAAGCGCAGCCGGCAGGCGACAAAGCTGAATTTATCGAGAAAGTGCGTCGCGCGCTG
TATCTTGGCAAGATCGTCTCCTATGCGCAGGGCTTCTCGCAGCTACGCGCCGCGTCTGATGAATATCACTGGG
ATCTCAACTACGGCGAGATTGCTAAGATTTTCCGTGCGGGCTGTATCATTCGCGCTCAGTTTCTGCAGAAGAT
CACTGATGCTTACGCAGAAAACGCTGGTATCGCTAACTTGCTGTTAGCGCCTTACTTCAAGAAAATTGCTGAT
GAGTACCAGCAGGCGCTGCGTGATGTTGTGGCTTACGCGGTGCAGAACGGCATTCCGGTGCCGACATTCTCTG
CGGCAGTGGCCTACTATGACAGCTATCGCGCCGCAGTACTGCCAGCTAACTTAATTCAGGCGCAGCGTGACTA
CTTTGGTGCGCACACCTATAAACGTACTGATAAAGAAGGCATTTTCCATACCGAGTGGTTGGAATAATTTCTG
CAAAAATGTTTAAGCCCGGTTTAATACCGGGCTTTTTTTATCTCTATTCTTATTGATTTATCGCTTTTGCTT
AATATTAACTTAATAATCTGTGTTTATCGTAATGAAGATAATCTGAATTGTTTTCGTCTGCGTTGCACTTTAT
ATACTCAGGCGTTAAAACTTTAATATCTTATCAGGATGCGAAATACATCATGATTCATAATTAAGTTAATTCT
GAGAGCGAATAATGAAAATCACGATATCCGGGACGGGTTATGTTGGCTTGTCTAATGGTTTGCTTATTGCACA
ACATCATGATGTGGTGGCGTTAGACATTGTTCCTTCCCGTGTTGAACTGTTAAATGATCGGATATCTCCGATT
GTTGATAAAGAAATTCAGCAATTTTTAAAGGAAGATAACATCAGATTCAGGGCAACGCTGGATAAGTTTGATG
CGTACCAGAATGCAGATTACGTCATTATTGCTACGCCTACGGATTACGATCCCAAAACCAACTATTTTAATAC
GTCCAGTGTGGAATCGGTCATTCAGGATGTGATCAGCATCAACCCCGCAGCCGTGATGATTATTAAGTCTACG
GTGCCGGTTGGCTTCACGGCGGCAATGCGTCAGAAGTTTGCGACAGAAAATATCATTTTCTCTCCGGAGTTTT
TACGTGAAGGTAAAGCCCTTTACGATAACCTTTATCCCTCCCGTATCGTGATTGGGGAACAGTCTGAGCGCGC
CAGAGAATTTGCCGCATTACTCCAGGAAGGCGCTATTAAACAGGAGATTCCAACGCTGTTTACGGACTCCACT
GAGGCGGAAGCTATTAAACTTTTCGCCAATACTTATCTCGCTATGCGAGTCGCTTATTTTAATGAATTAGACA
GCTATGCTGAAACGTTAGGATTAAATACCCGACAAATTATTGAAGGGGTGTGCCTCGACCCGCGTATTGGCAA
TCATTATAATAATCCCTCTTTTGGTTATGGCGGATATTGTTTGCCAAAAGATACCAAACAGTTGCTGGCCAAT
TATCAGTCGGTGCCTAATAATATTATCTCCGCTATCGTTGAAGCAAACCGTACGCGTAAAGATTTTATTGCCG
ACGCTATCCTCGCCAGAAAGCCTAAAGTGGTGGGCATTTATCGCCTGATTATGAAGAGTGGCTCCGATAACTT
CCGCGCCTCATCGATTCAGGGGATTATGAAGCGCATTAAAGCGAAAGGCGTAGAGGTCATCATCTATGAGCCG
GTAATGGAAGAAGATACTTTCTTTAACTCACGTCTTGAACGTGATTTGCACTGCTTTAAACAGCAGGCGGATG
TCATTATTTCCAACAGGATGGCGGCAGAGCTTTTGGATGTTGCTGAAAAAGTGTATACCCGCGATCTTTTCGG
TAGTGATTAATAAGGCAATCGGGGCTGATGAGTCCCGATGATTTATTGACCAAATGGAAATAATGTCTGATTT
TTATCATTAATCCTATGGCATATATTTGCTTTATGACTACACTGTCTCCAGCTTCATCCTTTTTTTAGTTAGG
GTATCTATGACAGTGGATAGTAATACGTCTTCCGGGCGTGGGAACGATCCGGAACAGATTGATTTGATTGAGT
TATTGCTACAGTTATGGCGTGGGAAGATGACCATTATTGTAGCCGTTATTATCGCCATTTTGCTGGCCGTAGG
CTACCTGATGATAGCCAAAGAAAAATGGACATCCACGGCGATTATTACCCAACCTGATGCCGCGCAGGTTGCC
ACCTATACCAATGCGCTCAACGTCTTGTATGGCGGGAATGCCCCCAAAATCTCTGAAGTGCAGGCGAATTTTA
TAAGCCGCTTTAGCTCTGCGTTTTCCGCATTATCGGAAGCGCTGGATAATCAGAAAGAGCGGGAAAAGCTCAC
CATTGAACAGTCGGTAAAGGGGCAGGCGCTGCCACTCTCGGTTTCTTATGTGAGTACTACCGCTGAAGGGGCG
CAGCGTCGGCTGGCGGAATATATCCAACAGGTGGATGAAGAGGTCGCTAAGGAACTGGAAGTTGACCTGAAAG
ATAACATCACGCTGCAAACCAAAACGTTGCAGGAGTCCCTTGAGACGCAGGAAGTTGTGGCGCAGGAGCAAAA

AGATCTGCGTATTAAGCAAATCGAAGAAGCGTTGCGCTATGCGGATGAGGCCAAAATCACGCAGCCGCAGATT
CAGCAAACCCAGGATGTTACCCAGGACACGATGTTCCTGCTGGGGAGCGATGCGCTAAAATCGATGATACAGA
ACGAAGCGACGCGTCCACTGGTCTTTTCTCCGGCCTATTACCAGACGAAGCAGACACTGCTGGACATTAAAAA
TCTGAAAGTGACTGCCGATACGGTGCACGTCTATCGTTATGTGATGAAGCCGACGCTGCCGGTCCGTCGCGAT
AGCCCGAAAAAAGCCATTACCCTTGTGCTGGCTGTATTGCTGGGCGGGATGATCGGTGCCGGGATTGTGCTGG
GGCGCAATGCGCTACGTAGCTATAAGCCAAAAGCCTTGTAAGCTTCTTTGCCGGATGGTGGTCGGCTTCGAAA
GGTAAAATTTCTCATAAAAAACCGGGCGTTGCCCGGTTTTTTATGCTTATTGATGACGCTTACGAAGATTGTC
GATCACGGTGGTGAGGTTGAGATCCTGATCCTGCAACAGCACCAGCAGGTGGTACATCAAATCAGACGCTTCA
TTGGTTAATTCAAAACGATCATTGACGGTGGCCGCCAGCGCGGTTTCTACACCTTCTTCGCCAACTTTTTGCG
CAATACGTTTGGTACCGCTGGCGTACAGTTTCGCCGTGTAAGAGCTGGCCGGATCGGCGGTTTTGCGCTCTGC
CAGTAGCTGCTCCAGTTGATACAGGAATAACCACTGATGGCCGGCGTAGCCAAAGCAGCAGCTGGTACCTTTG
TGGCAGGTCGGCCCGACAGGATTTGCCAGCACCAGCAGTGTATCGTTGTCGCAATCCGGCGCAATGCTGACGA
CATTCAGCACATGGCCTGAGGTTTCGCCTTTGGTCCATAAACGCTGTTTCGTGCGTGAGAAGAACGTGACATG
GCCGGATTCAATGGTTTTGTCCAGCGCTTGTGGGTTCATATAGCCCAGCATCAATACTTCGCCGGATACCGCA
TGTTGCACGATGGCTGGCATCAGGCCATCGGTTTTTTCCCAGTCCAGCTCGCGGCGTTGTTGCTCTGTTAACA
TATCCTGATCTCCACGCCCTGGCCTGCCAGGTACGCTTTTAATTCGCCAATATTGATGATTTGCTTGTGAAAA
ACGGAGGCGGCAAGCGCGCCGTCGACATCGGCATCACGGAATGCCTCAAGAAAGTGTTCCATCGTGCCCGCGC
CGCCGGAGGCGATCAGCGGCACGCGGCAAACGTCACGGACTTTTTTCAACTGCGTCAGATCGTAACCGTTACG
CACGCCGTCCTGGTTCATCATATTCAGGACGATTTCCCCCGCGCCGCGCTGTTGTACCTCTTGCACCCAATCC
AGCGTCTCCCACTGCGTCACGCGGGTACGGTTTTCATCGCCGGTATACTGGTTAACATGATATTTCCCCGTGG
CGTCGTCAAACCAGGTATCAATCCCGACGACAATGCACTGTACGCCAAAACGGTCAGCCAGACGGGTAATCAG
CGTTGGGTCAGCCAGTGCAGGGGAATTGATAGAGATCTTATCCGCCCCGAAAGAAAGAATTTTGGCGGCGTCG
TCAATTGACCGGATGCCGCCCGCTACACAAAACGGAATGTCGATCACCTCGGCAACGCGCGCCACCCAGCTTT
TATCGACTACGCGACCATCGCTGGAGGCGGTAATGTCATAGAACACCAGTTCGTCCGCGCCTTCGTCGGCATA
GCGTTTGGCCAGCGGAACGATATCGCCAATGATCTCATGGTTGCGAAACTGTACGCCTTTCACCACCTGACCA
TCACGAACATCCAGACACGGAATTATACGTTTTGCCAGCATTGGATGGCCTCCTTAACGGTAAATTTCCCTTC
CAGCAGAGCGCGTCCGACAATCACGCCGCGCACGCCGGTGCCGCGCAGGGCGGCAATATCATCGATATCGCCA
ATACCGCCGGAGGATTGAAAGGCGATCTGCGGATATCTGGCGCATATCTCTTCGTACAGTGAAACGTTAGAGC
CCGCCAGCGTGCCGTCGCGAGAAATATCGGTACACAGTACATGTTTCAGGCCGACGGGGAGATAGGTCTCCAC
CAGTTGTTCCAGCGAGACGCCGGAATTTTCCTGCCAGCCGCTAACCGCCACCTGCTTGTTGCCGTGTTCGTCT
ATGCGAACGTCCAGCGCCAGTACCAGCGCCTGCGCGCCAAAACGTTCAAACCAGCCTTTCACCACGTCAGGAG
ATTTCACCGCCGTTGAACCGATGACCACACGGGCAACGCCAGCTTTCAGTAATGCCGCAACGTCTTCTTCGGT
ACGCACGCCGCCGCCGACCTGAACAGGCACGTTCACGCCCGCGACCAGGGTTTTAATCAGCGGTATCTGTCGC
TTAGCCGGATCTTTAGCGCCGGTCAGATCTACCAGATGCAGCACCCCGGCGCCCTGGGCGGCGTAATCCTGCA
AACGGGGCAGGGGATCGTTACCGTAATCCCGCTGCCGGGCGTAGTCGCCCTGGTGGAGACGCACCACGGTGCC
GTCAATTAAATCTAATGCCGGAATAATCATTACATCTCCAGGAAGTTTTTCAGCAACTGTGCGCCCGCCGCGC
CCGAACGTTCCGGATGGAACTGTACGCCGAAGAAATTATCTTTCTGTACCGCCGCGGTAAACGGCTCGCCGTA
ATTGCACTGGGCGATAGTCCACGGGTTGACCGGCATCGCGTAGCTGTGAACAAAGTAAAAATAGGCGCCATCT
TCAATGCCCTGAAACAGCCGGTTGCCCGCCTGCGGATACACGCGATTCCAGCCCATGTGCGGCAGCGGCAGAC
CAAAGTCGGTCATTTTCGGCACGTCTTGTTCGATAATGTTCAGCAGATCCACGCCGCGCGTCTCTTCGCTGCG
ACGACCCAGCAGTTGCATCCCTAAGCAGATACCCAGTACCGGCTGAGTACACGCTTTAATTAAGTCGATCAGT
TCACGTTCGCGCAGTTGATCCATAGCGGCCTGCGCCGTGCCGACGCCGGGCAGAAAGAGTTTGTCAGCGCGTA
ATACGATTTCCGCTTCGCGGCTGACCACCGGGGTGTAACCGTGGCGCGCCACGGCGGATTTACCGAGCTTAA
ATTGGCGCAGCCGGTGTCAAGGATGACGACGTTCATCACAGCACTCCTTTCGAGGACGGTAATGTATCGCCCT
CCACGCGAATAGCCTGGCGTAGCGTCCGACCAAAGGCTTTAAACAAACTTTCGACACGGTGGTGATCGTTTTT
ACCTTTAGTCTTGAGATGCAGAGTGACGCCCATCGTGTAAGAGAGTGAGCGGAAAAAGTGTTCAATCATCTCT
GTGCTCAAATCGCCCACACGCTGGTAGGTAAATTCAGCTTTATATTCCAGATGCGGACGACCGGAAATATCCA
GCGCGCAGCGCGCCAGACATTCATCCATCGGTAGTACAAAGCCAAAACGGCAGATACCGCGCTTGTCGCCGAG
TGCCAGTTTTAATGCCTCACCGAGCGCCAGTCCAGTATCTTCTACCGTGTGGTGATCGTCGATATAGAGATCA
CCCTTAACGGTAATCTCCATGCGAAAGCCGCCGTGGGTGGCGATTTGATCGAGCATATGGTCAAAGAAGCCGA
CGCCGGTATTAATCTTGCTGTTGCCTTCGCGATCCAGCCAGACACTGACATCAATCTGTGTTTCTTTGGTGTT
ACGGATCACATGCGCATAGCGATCGCGTTTCGTTAGCTGTTCGCCAATCATCGTCCAGTTCAGCGTTTCACGG
TGATAGCGTAAACCAGTAATGCCCATGTTATCAGCGAGCTGGATATCGGTCGCACGATCGCCAATCACATAGC
TGTTGGCGCTATCCATCGCTTGTTCCGCAAGATAACGCTCCACCAGCTTCACTTTGGGCTTGCGGCAGTCGCA

GTCGTCTGCGGGCAGGTGAGGGCAGATCAGCACCTCATCAAAGCATACGCCCTGAGAGGTGAAAATCTGCATC
ATCAGGTTGTGCGGTCCGTCGAAGTCCGCCTGCGGGAAGCTTTGCGTGCCAAGTCCATCCTGGTTAGTGATCA
TCACCAGCTTAAAACCGGCTTTTTGCAGCTTCAGCAATACGGGAATCACCTCTGGCTCAAAGGCCAGTTTATC
AAAGCGGTCTACCTGAAAATCGCTCGGCGGTTCGGAAATTAAGGTTCCGTCCCGGTCGATGAAAAGATACTTC
TGACTCATACTGGCTCCGCACGTAAGGCGTCAATGACGCGCTGGTTTTCCTGGCGGGTGCCGACCGTAATCCG
CAGGCAGCCGCTTAAAGAAGGTTGTTTATTCTGATCGCGTAAGATAATGCCCTGATCCCATAAGGATTTAAAC
ACGCTGCTGGAGGCGGTAAACCGCGCCAGAATATAGTTGGTTTCAGAGTCAAAGACGTGTTCTACGCAGGCGG
TCTGTTGCAGGGCATTCACCAGATACTGACGTTCCTGCACTGTCTGCGCCACGCGATCGCGCATTGCGTTTAT
TCCCTGCGGGCTCAGCGCCTGGGCGGCGATATCCGCCACTGGCGTAGAAAGCGGATAAGGGGCGATCACTTTT
AACAGCAGGTTGATCACCTCTTCATTAGCCAGTGTAAAGCCGCAGCGCAGACCCGCCAGCGCAAAAGCTTTCG
ACAATGTGCGCAGGATAACCAGATGAGGATATTCAACCAGCCAGCCTGTCAGCGTGGCCTGCGGGCAAAACTC
AATATAAGCTTCGTCGGCGACGACTATCGCTTTACCGCGTGTCAACTCCAGCAGCGTGCGCAGATCCTGCGGG
TTGATAAGTTGTCCGGTAGGATTATTGGGGCTACAAACGAACACCACTTTTGTGCCGTCAAGGTTGTCGGAAA
TCCCCTGTAGATCCAGCTGCCAGTTTTCAAGCGCGGGAACCGTCCGGCGCTCTACGCCAATGGTTTCGGCGCT
GACGCTGTACATACCGTAAGTGGGCGGACAGTAGAGAATAGCGTCTTTCCCCGGTTCACAGAAGGCGCGGATC
ACCAGCTCGATCCCTTCATCCGCGCCGCGGCTGACCAGCACCTGCTCCGGCTTTACGCCAGCATATTGCGCGT
AGTTTTCAATCACGGCCTTTGGCTGGCATTCCGGGTAGCGGTTAAGCGTTTGTTGGGTGAGCTGAAACTCCAC
CGCTGTCGGGAATTCATTCGCGTTCAGCCAGACATCGCCGTTACCGCCCAGACGGCGGGCAGACTGATACGGT
ACCAGGTTGCGGACATTTTCACGGGCTAAGTCAGCGACGCTGAGAGTGTTTTCAGTGCTCATGCTTGCTCCTT
GAGGGCGTTTACGCGCAGGGTCACGGCATTTTTATGGGCGGTCAGACGTTCTGCCGCCGCCAATGTTTCAATG
GTTGATGCCAGAGCGGAAAAGCCCGCTTTCGACAGTTCCTGAACGGTCATCCGTTTCTGGAAATCCGCTAACC
CAAGGCTGGAACAGGTAGCAGTATAGCCATAGGTCGGTAAAACATGGTTGGTTCCGGAAGCGTAATCACCGGC
GGATTCCGGCGACCAGTCGCCGAGAAATACCGAACCTGCGCTGGTAATCGCATCCACCAAATCGCGCGCATTG
CGCGTCTGGATAATTAAGTGTTCCGGCCCATACTGATTAGAGATGGCGACGCACTGCGCTAAATCTTTGGTCA
CAATCAGACGACTGGCGCTCAGGGCCTGCCTGGCGGTGTCCGCGCGCGGCAGTTCTGCCAGTTGACGTTCTAC
CGCCTCCGCCACCTTGCAGGCAATGTCAGCATCAGGCGTCAGCAGAATCACCTGCGAATCCGGACCGTGCTCA
GCCTGGGAGAGCAGGTCAGAAGCGACGAAATCCGGTGTTGCGCCGCTGTCGGCGATCACCAGTACTTCAGACG
GCCCGGCTGGCATATCGATAGCCGCGCCGTCGAGGCGTTGGCTGACCTGACGTTTGGCTTCGGTTACAAAGGC
GTTGCCGGGGCCAAAAATTTTATCCACTTTCGGTACGGACTCGCTGCCGAAGGCCAGAGCGGCAATCGCCTGC
GCGCCGCCGACGTTAAAGATTTCCTGCACGCCACACAGTTGCGCCGCATAGAGGATTTCATCAGCGATGGGCG
GCGGCGAGCACAGAACCACGTTCTGACATCCCGCAATACGCGCCGGCGTCGCCAGCATCAGCACCGTTGAGAA
GAGCGGAGCCGAGCCGCCGGGAATATACAGACCGACAGACGCGACGGGACGCGTAACCTGCTGGCAACGCACG
CCTGGCTGGGTTTCCACATCTACAGGCGGTAGCGTCTGCGCGGAATGGAACGTTTCAATATTTTTGACGGCAG
CGGCCATCGCCTGTTTTAATTCGTCGCTCAGACGCGCGCCGGCGGCGGCGATCTCTTCAGGGGTGACGCGTAG
CGCTGTCACTTCTGTTTTATCAAATTTAGCGCTGTATTCACGCAGGGCATCGTCACCGCGCGTTTTTACATTA
TCCAGAATATCGCTGACCGTCCGGGTAATACTGTCAGAGGCGGAAATCGCCGGACGCGTCAGCAGCGCACGCT
GCTGTTCAGGGCTACAGCTGTTCCAGTCAATCAGGGTATTGAAGCTCATGGTCTTTACTCCATCACAGGGTTC
CGCCTTATCCGGCCTACAGAACCCAAAATATCAACGCATTACGTAGGCCTGATAAGCGCAGCGCCATCAGGCG
TCAGATCACTCCATCATCTTCTCGATCGGCAGTACCAGAATCGAGCTGGCGCCAAGCGCTTTCAGTTTCTCCA
TGGTTTCCCAGAACAACGTTTCGCTGCTGACCATGTGCATCGCCACGCGCTGTTGCTCGCCTGCCAGCGGCAG
AATTGTCGGCCTTTCGGCGCCTGGCAGCAGGGCGATAACCTCTTCCAGGCGTTCACTTGGCGCGTGCATCATG
ATGTATTTCGATTCGCGCGCCTGAATCACGCCCTGAATACGGGTCAGCAATTTATCGATCAGCTGTTGCTTGC
TCTGTGCCATCTCACCGTCGCGCTGAATCAGACAGGCTTTAGAGCGGTAGATAACTTCGACTTCACGCAGGCC
GTTAGCTTCAAGCGTCGCGCCGGTAGAGACCAAATCGCAGATAGCGTCGGCCAGCCCCGCGCGCGGCGCGACT
TCGACAGAACCATTTAACAGACACGATTTAAAAGAGACGCCTTTCTGGTCGAGGTAGCGTTTGAGGAGGTGCG
GATAAGAGGTAGCGATACGTTTACCGTCCAGCGCGGCCGGGCCGTCCCAGGCTTCGTCAACCGGCGTTGCCAG
CGATAAACGGCAGCCGCCGAAGTCAAGACGGCGCAGGGTAAAATAGCGCGGATCTTCACCCTGCGCGCGGCGG
TTGAGTAGCTCTTCTTCCAGCACGTTCTCGCCGATAATGCCGAGATCGACCACGCCATCCATCACCAGACCCG
GAATGTCATCATCACGCACGCGCAGGATATCAATCGGCATGTTTTCCGCCATCGCAATCAGGCGCTGAGTGTG
TAAATTAATTTTTATGCCGCAGCGGGCCAGCAATTCTCGTGAATCATCGCTTAAACGGCCTGATTTCTGAATA
GCTATGCGTAAGCGGGTGTTGTCTAACATTCTGCGTTCCTCTTTATCCTGTCTGAACCGGTCTGTATCGCGCG
CCAAAAAAAGCCCCCGGAAGATGATCTTCCGGGGGCTTTCTCATGCGTTCATGCACCACTGGAAGATCTGAAT
GTCTTCCAGCACACATCGCCTGAAAGACTAGTCAGGATGATGGTGATGATGGTGGTGTTTAAATTGAACGCGT
GTCATAAAAATTCCGATGAATGCTTATTCATTTGATACCTTTTAACCTAAACCACTTTTACGCCTTAAAGCAA

GCGCTTTTTTCATTTCGCATTTATAGTATGAATTGTCAGTCACGGCGGGCTGGCGTAGCCTTATGTATAAGGC
GTTAAAGTCAGGAGAAAATGGATGAAAAAGGTCGCAATTGTCGGTTTAGGTTGGTTAGGCATGCCGCTGGCAA
TGTCGCTGGCAGCGAGAGGCTGGCAGGTTACCGGCAGCAAAACCACCCTTGACGGGGTGGAAGCGGCCCGTAT
GAGTGGAATTGAAAGCTATCCGCTGCGTCTGGAGCCGGAGCTGGTCTGTGAGGCGGACGATCTTGACGCTCTG
CTGGATGTCGATGCGCTGGTGATAACACTGCCCGCGCGCCGTAGCGGTCCGGGAGAGGATTTTTATCTGCAGG
CGATGCAGGAGCTGGTCGACAGCGCGCTGGCTTATCGTATCCCGCGTATTATTTTTACCAGTTCGACGTCTGT
TTACGGCGATGCGCAAGGCGTGGTGAAAGAGAATACGCCGCGTAATCCGGTCACGGCCAGCGGGCGTACCCTG
AAGGAACTGGAAGATTGGCTGCATAATCTGCCGGGCACGTCAGTAGATATTCTGCGGCTGGCGGGGTTGGTCG
GGCCTGGACGCCATCCCGGTCGTTTCTTTGCCGGAAAAACCGCGCCGGATGGTCAACACGGGGTCAATCTGGT
GCATCTTGAAGACGTGGTTGGCGCAATTACCCTTTTGCTACAGGCGCCAAAAGGGGGGCACATCTATAATATA
TGTGCGCCGGCGCATCCAGCCCGCAACGTGTTTTATCCGCAAATGACGCGCTTGCTGGGAATGGCGCCGCCGC
ATTTTCGTAACGCACCGGACAACGGCAAAGGGAAGATTATCGACGGTAGCCGGATTTGTAATGAGCTGGGATT
TGAATATCAATATCCCGATCCGCTGGTGATGCCGATGGAGTGAGTCGCTGTCCAGCAGGTGGTCGCCATATAG
CGCAAGGGGACGAGTATGAAACCACTGCTGGACGTTCTGATTATTCTTGATGCGTTAGAAAAAGAAGGCAGCT
TCGCGGCGGCGTCGGCAAAATTATTTAAAACGCCGTCCGCCCTCAGTTATACCGTTCATAAGCTGGAAAACGA
TCTTAATATCCAATTGCTCGACCGTAGCGGGCATCGCGCGCGATTTACGCGAACCGGTAAGATGCTACTGGAA
AAAGGCCGTGAAGTGCTGCACACCGTTCGCGAGCTGGAAAAGCAGGCGATTAAACTGCATGAAGGCTGGGAGA
ACGAACTGGTCATCGGTGTGGATGATACCTTTCCCTTTTCTCTTCTGGCCCCGCTTATCGACGCTTTTTATCA
ACGCCACAGCGTGACGCGCCTTAAATTTGTCAATGGCGTTCTCGGCGGTTCATGGGAGACGCTGACGCAGGGA
AGGGCGGATATTATTGTTGGCGCCATGCGCGCGCCGCCGCCTGACACCGGCTTTGGCTTTGCAAGACTGGGCG
ATCTTGAACTGGTGTTTGCTGTCGCTCCGCATCATCCGCTGGTTCATGAAGAGGAACCGCTTAGCCGCCAGAC
GATCAAACGTTTTCGCGCGATTGTGGTTGGCGATAGCGTCCATTCCTCGCGATCAATAGGGTCTGAACTGCTC
GACGCTCAGGAAGCGATTACCGTGTTTGATTTTAAAACCAAGCTGGAGCTGCAAATTAGCGGCCTGGGATGCG
GCTATTTACCGCGTTATCTGGCGCAGCGATTTTTGGAAAGCGGCGCGCTTATAGAGAAGAAAGTTGTGGCGCA
AATCGTCTATGAACCGGTGTGGGTTGGCTGGAATGAACAGACCGCCGGGCTGGCCAGCGGCTGGTGGCGGGAT
GAAATTTTAGCAAATAATGCTATTGCTGGGGTGTATGCAAAATCGACGGTTTAAAAATCAATCGCTTATGAAA
AATAATTTTTTGAACGCGCTCGCATTCTCTTGTCATTTCACTTCAATTTAGGGCAGAATACGCCGCTTGCAAA
AAATGACACTAACCGACGTTTTAATACGCGTCGGTTATTTTTTTGCTTCAAACACATCATTCAATACCAAGAG
GCCGGGCTTCGTACCGGATAGATACTTACTTAAAAATCGACAGTTGTTGTCGCTGAGGAATCCAGAAAATGGG
GCAATTTTTTGCTTACGCGACGGCATTCGCCGTAAAGGAGAATGACCATGTCGCATAACGTTACTCCAAACAC
CTCTCGCGTGGAATTACGTAAAACGCTTACGTTAGTTCCGGTTGTAATGATGGGTCTTGCCTATATGCAGCCG
ATGACGCTGTTCGATACATTTGGTATCGTTTCAGGCCTCACTGACGGTCACGTACCGACGGCGTATGCATTCG
CGTTAATCGCGATTCTGTTTACGGCGTTGAGCTACGGGAAACTGGTGCGTCGCTATCCTTCCGCGGGTTCGGC
GTACACCTATGCCCAGAAATCCATCAGCCCGACCGTTGGCTTTATGGTGGGTTGGTCTTCTCTGCTCGATTAT
CTGTTCGCGCCGATGATCAACATTCTGTTGGCGAAAATTTATTTTGAAGCGCTGGTGCCCTCCATTCCGTCAT
GGGTGTTTGTCGTGGCGCTGGTGGCCTTTATGACGGCCTTTAACCTGCGCAGCCTCAAATCGGTCGCTAACTT
CAACACCGTGATTGTAGTGCTACAGGTGGTGCTGATTGCGGTCATTCTGGGCATGGTGGTTTATGGCGTATTT
GAAGGCGAAGGCGCCGGTACGCTGGCGAGCACCCGTCCGTTCTGGTCCGGCGATGCGCACGTTATCCCGATGA
TTACGGGAGCGACGATCCTGTGCTTCTCCTTTACCGGCTTTGACGGCATCAGTAACCTGTCGGAAGAGACCAA
AGATGCTGAGCGCGTGATCCCGCGTGCGATTTTTCTGACCGCGCTGATTGGCGGCCTGATCTTCATCTTTGCA
ACCTATTTCCTGCAATTGTACTTCCCGGATATCTCTCGCTTTAAAGATCCGGATGCGTCTCAGCCGGAAATCA
TGCTGTACGTGGCGGGTAAAACCTTCCAGGTGGGCGCGCTGATCTTCTCTACTATCACCGTACTGGCTTCCGG
TATGGCGGCGCATGCAGGCGTAGCGCGCCTGATGTACGTAATGGGGCGCGACGGCGTATTCCCGAAAAGCTTC
TTCGGTTACGTTCATCCGAAATGGCGTACGCCAGCGATGAACATCATCCTGGTCGGGGCGATCGCGCTGCTGG
CGATTAATTTTGACCTGGTGATGGCGACGGCGCTGATTAACTTTGGTGCGCTGGTCGCGTTTACCTTTGTTAA
CCTGTCGGTGATTTCGCAGTTCTGGATCCGTGAGAAGCGTAATAAAACGTTGAAAGATCACTTCCAGTATCTG
TTCCTGCCCATGTGCGGCGCGCTCACGGTTGGCGCGTTGTGGGTTAACCTGGAAGAGAGCTCAATGGTACTGG
GCCTGATCTGGGCAGGTATCGGGCTGATTTATCTGGCGTGCGTAACTAAAAGCTTCCGCAACCCGGTTCCACA
GTACGAAGACGTCGCGTAATCGCGCGTATTATCGGCAATAGTAAAACCGGAGGCAGTGCCTCCGGTTTTTTGT
TTGTCATCCAGCACGTTTACCGGATGGCGGCGTCACTCTTTATCCGCTCTGGATTAACAATGCTCGTCAGGCA
TTGGTCAATTACACAATCTCAGTTGCGTACTGCCACAGTGATTTCAGCAACCCCAGCTTCGTTTTATCTTCCG
CATACTGCTGAGAAAGCATCTGCAATTCATTGGCATATTGTTGTAAAAACTCCGGCGTCAGCACCTGACGGCG
ATGCTCTAGCCAGCGCTGCTGCTCTGCGTCATCCAGCGTACCGGGAAAATTGCGTGCGCGGTAATTAAACAGC
AGCTTCTCAATGCGCTTATCGACAAAGGTAATGTCCAGCGCGGGCAGGTTACGTGGCTCGGTTTCGAGTACGA

TTTTCATGGCTGCGCGATCGGCATCGCTGAAAAAACCATCATAGAGCTGGGCATCAACGTTATCCGAGGCGGC
AAAAGGTTCGGCTTCGGCGAAAATCGCCACCACTTTGTCGCGAACCTGCGGGTTTTCACGCAACACTTTCAGG
TTATCCAGGCAATGCTGGCGGTTAATTCCCAGCCGGTCGGCATCCTCCGGGCGCAAGGTATTCGCCTGCGCCA
GTACCGGACATTTATTGATATGCACCAGCTTTACCGGCACTGCGGCGTGATCGCCAAGATCGGCTTTGGCCGT
ATAAAGCCGCTCGCGAAGGGTGTCGCTGTCCAGCTCAAGAAGAGGAGAAATATCGCCTGCTAAATCGACCATG
ATCACCGCGTTACGGTTTTCAGGATGCCACGCCAGCGGCGCGACCCAGCTTGTATTACCGCGCCACGCGCCAA
ACATGCCGGAGACATGCACCAGCGGCTTCATTTGCGGGACGTCAATCAGCGCCGCCAGTTTATGTTTACTGCG
GTGGCTATAAAGATAATCAAACAGCCGCGGCTGGCGTGTTTTCACCAGTTGCGCCATCGCAATAGTGGCGTAG
ACATCCGCCATCGCATCATGCGCGTTGCTGTGTTCGATCCCGTTCGCCTGGGTTAAATGTTCCAGACGAAAGC
TGGGCAGGCCGTCGTCGTTTTCCGGCCAGTTAATTCCCTCCGGGCGCAGCGCATAACAGGCGCGCATGACATC
CAATAGATCCCAACGTGAATTATCATGCTGCCAGCTCCAGGCATAGGGATCGTAAAAGTTGCGATAAAAAATA
TTGCGCGTGACTTCATCATCAAAACGCACATTGTTGTAGCCCACAACGCAGGTTTTAGGAACGGTAAACAGCG
CATGAATGCGTCTGGCGAAAGCGGCTTCGTTTTCTCCTTTCTCACGCGCTTCCTGCGGCGTGATGCCGGTAAT
CAGCACCGCGCCGGGCTGCGGTAGATAATCATCGGCGGGTTTGCAATAAAACACCTCCGGCTCGCCGATAACG
TTGAAGTCGTTATCCGTACGGAGCGCGGCGAATTGCGCAGGTCTGTCGAGGGCCGGATGCGTACCGAAGGTTT
CGTAATCGTGGAAGAGGAATGTGCTCTGTATGCTGTCGTTTTTAACTGTCACTTGATGTACACGCCTGATGTT
ATCTGTATATGAAATATTTTATTCTTTTATTTTGTCGTTGATTGATTGTATTTGTACACCAATTGATATTGTT
GCTAATGTTCGTACATGCGATGGTGAGTACATAAACATTAAAATCATTGAGTACAAAATACTAAAATGACCAT
CAGCGACACTAAACTACGCTCCCTCTATGGTAAACCATATTCCGGTCCATCTGAAATTACTGGTTCTGATTTA
GTACTGTATGGAATTTAGGCCAGGGGCAATCTGGCCGGGATCTCAAAAAATGCATGGACTAAAACGGGTCTAT
GTACAGAGGGACACAACGCTGTGTCCCTTAATTTCATGCGGGTTGAGGCTGGACTAGGCCCAGGCCAGTGGCA
GGATGGATGACAGAACACTCGGGAAATATTTTCTGGCATGACGTTGCATACTATCCGCCACAACGCTGGTACA
GGTAAAGGCGCCATGGAGGCCGTGAAAACGGTCGGACCAGTCGGTAAACTGTTCGCTGGACGGGAAGATCGCC
GGAGACAGCTCCCAGGCTTTCTGCATCAGCGCCTCCGCATAGCCTCTCAGGGCCGGCGGAGAGTCATGCTCAG
TGCCGAAAATGGCGCTGGAGGAATAGCGTGCGAATGCGGTACTGAGACAGAACAGGGTTTCCGCCTGCTTCTG
TGGGGTGGCGTCCGTCAGGTGATAAGCACTGAGGATGTGCTGGTAGTGTGCCGGGCTGAGTTTGCCTTCAGGA
AGCAAGGGGGCAAAAATGGTGGCCAGACGCGACACTGAGGCATCATCAATCAACTTCACGGTGGAATGTTTCT
GGTTCGTGGCGGCAATAAACTGCTGGTTCAGCTCCCCGTGCTCTTCAGTGCTGAGGATGATATCAATCAGCTC
CCCAAAGCGTCGCTGCGTGGCCTGCTGATTAAATGCGGCGAGAAACACCGGAAAGTCATGACGGAATAACTCT
ACCGGAGAGATTTGCGCGGTGGAAACGTTCTCTCCGGCTCGCAGCAGGTAAAAGTTGTCCCATGCAGTGTCAG
GAGTAGGGTTTAGCATTGTTAACAGCGTGTCAGTGGAGAGCATCATCGCCGTGTCTGAATCTTGGGAGGAAAG
CAGCAGGAAATTATCTGCAGCGCGGGTTGTCCAGTCCGGGCTGCCGTCATAATTGCCGAAGAGCCCATTATGG
ATGTGGGGAGACACCGCCGGGTGAGCAAGATACTGTTCATAAAGCGCTGCAGCTTTCTGGCGGGTGCCATCAG
TGCAGTCCACGCCTGTCATACAGTGTAAAATCACCTGATTAAAGAGTCCGCTGTGAGTGGTCATGGCGGCGCT
GTTCCTGTCAAACGAATCCAGCAACACAGGCAGTATGCGCTGCTGATAATATGTGTCAGGAGTCGGCAGGGGA
ACGTCATTATAGGTCTGGCACTGGTGGGCAAAAAAACGTTCCTGAACAGGCCCGAGCCACTCGCGGATAAGCG
CACTTTGCGTATAGGGAGGGTGGGCCAGTACGCTTACCAGCGAACTTTCAACTGGCCGGATACATGCCTCTGT
TAAGCGGCCACCCCGCAATGACTTAACCAGGTCTTCCATTGCCTGGACTTTTTCCTGACTGTATTTATCGGGC
AGGCTGTCAATGGTCATCAGAATACTTTTACCATTATTGTGCTCATGATTTAAAAATCCATCCACAGCGTTCT
CAGTCCAAAGGAGTGGCATTTGCAGCCAGATATCACCTAGACGAATAAAATTTCCGCCCCCTGACACTGACGG
AGGTGATGAATTATCCGGTTCATTTAAAATAACGCCAGAGAGGTTGGCTTTGTGACGTATAAGCGTATTTTTT
AATGTTGTATCAATAAAAGAGGGCTGTTGTTGTGTAGTAATTGAATACTGTAAAAACACTTCATTCATGTCTG
AATTACTAAATTTCGTATTACTGCAGACTACATCACTCAGTGTCGAATAAGTGAATCGGCATTCGTCAAAATG
TAAATGAGATATATCAGAATAAGTAAATTTTGAATTTGAAAAGTTGAACCCCTTAATAATATCGCCGTCTCGC
TCCATGAAGCTGGGGGGCAATATTTTGCTGAATGTAATATTACGGAAATCTGCGTTTGAAAAATCGCGATCTG
CGGCCGTAAGGGTTTCTCCGGCTTTCTGTCGTAAAACTGCATCCGTGAGTTTCTTAGCCCAGTCGGCAGGTGA
TGACGGGGAAGACGTAGCCTGAAGTGAGAGGGAGGGGGATACTGGTGGTGTTCTACCTTCAGAAACCGTGGGC
CTGTTATTGGCTGATACAGAGACTCCACCGGGGAGCGGGGCCGCACCTGTCATTCTGTAATTATAATCACATG
TTTGATTTGCAATATGCTTACGCATGAATGCTGCATTCATTTTTGAGACATCTTCTGCTGAAATATTTTTCTT
GCCTGCATTATTTGTGTCTTTAATATTTTTAACAGTAATCAATGTTTTGTTTAAGGTGTTTTTATCTTTCGGC
TGAAAGAAGTCTTTTATTTTTTGTGGTAACGCGAACTCAATCGATGAGCTCTTTCCTGTTTTAAGTTTTTCAT
TTAAAGAAGCATGCGTGTTTTTAACACTGGTTATTTTTGAGGCGAGCCCGTTTTTCGTATGTTCACGAATAGA
GGTAATTAATTTTTTAACCTGGTTAGGAATAGTAGAAAAATTAATTGCGCCTGATGATATCTTCATTAGAATT
CCTTATCAACATCATTATGGAAAAACGGTCTGGATTTTTAACGCCTTAAAAGTAAGCTTCATATATCGGTATG

GCGATGGAGATCACAATTCACCATGCTATCTGGAAGGTTTCTTTCAGAAAGTGTCCATTCTGTCATCATGTCA
CCGTATTGTGGGACGCTGACGTATCTGCGGAAAAATCCAGGCATAAGAGAGCGGGTCGGGTAACGGAGCCCGT
GTGCACGCAAAAGTAAACCCGCTTCGATGCTCAACCAGTGTTTTTTCGCCGGCATTCGGGATATCGTCGGCGG
ACGGTCCGGCGCGTATGAGAAAGAACTGCGTAAAGCGCGTGAAATCGCTTTTCAGGAAATAATCGTTTTCTTT
TAAATATTATTACGTGTTATTGGAATTGTATCATTTTTATAATTAACAGCGGGTTGTTTGGTAATTCTGCTGA
TTATTCAATGACAGTCCATGTGCGTGTTCAGGAAATGTAAAATATTTATAAAATAAAAGGTTTTATCGTGTTG
GCATGCTCATTTTAACAACTATTTCAAATTGTGATTGAAGCCGACATTTCATAATACAAATTAAATATAATCT
TCAGCTATATCTAATAACAGGAGGTTATATGAGCATTAGTCGTCGTTCTTTTTTGCAGGGGGTAGGCATAGGC
TGCTCTGCCTGCGCGCTGGGCGCTTTTCCGCCCGGGGCGCTGGCGCGCAACCCGATTGCCGGCATTAACGGTA
AAACCACGCTAACGCCAAGTTTGTGCGAGATGTGTTCCTTTCGTTGCCCTATTCAGGCGCAGGTAGTCAATAA
CAAGACCGTTTTTATCCAGGGCAATCCTTCTGCGCCGCAGCAGGGGACGCGCATATGCGCCAGGGGCGGGAGC
GGCGTCAGCCTGGTCAATGACCCGCAACGGATTGTAAAACCTATGAAACGCACCGGGCCACGCGGCGACGGTG
AATGGCAGGTGATTAGCTGGCAACAGGCTTACCAGGAAATCGCGGCGAAAATGAATGCCATCAAAGCGCAGCA
TGGCCCCGAGAGCGTGGCTTTCTCTTCCAAATCGGGCTCGCTCTCCAGCCATCTTTTCCATCTGGCTACGGCC
TTTGGTTCGCCTAATACCTTTACTCACGCCTCAACATGCCCTGCCGGGAAAGCCATTGCGGCAAAAGTGATGA
TGGGCGGCGATCTGGCGATGGATATCGCTAACACGCGCTATCTGGTTTCGTTTGGCCACAATTTGTATGAAGG
GATTGAAGTTGCCGATACCCATGAGTTAATGACCGCGCAGGAGAAGGGGGCCAAAATGGTGAGCTTCGATCCG
CGTTTGTCTATATTTTCCAGCAAGGCGGATGAGTGGCACGCTATTCGTCCCGGGGGGATTTAGCGGTTCTGCT
GGCGATGTGCCACGTCATGATTGATGAACAGCTCTACGATGCGTCTTTTGTTGAGCGTTATACCAGCGGATTT
GAACAGTTAGCACAGGCGGTAAAAGAGACGACGCCGGAATGGGCCGCCGCGCAGGCCGATGTCCCAGCCGACG
TTATTGTCCGGGTGACACGCGAACTGGCTGCCTGCGCGCCTCACGCTATTGTCAGTCCTGGTCATCGCGCGAC
GTTCTCGCAGGAAGAGATCGATATGCGGCGTATGATTTTTACGCTTAATGTGCTGCTCGGTAATATTGAGCGC
GAAGGCGGGCTATATCAGAAAAAAAACGCGTCTGTTTACAATAAACTGGCCGGAGAAAAGGTCGCGCCAACGC
TGGCGAAACTCAACATTAAAAATATGCCGAAACCGACGGCGCAACGCATCGATTTGGTCGCACCGCAGTTTAA
ATATATCGCCGCTGGCGGCGGCGTGGTGCAAAGCATTATTGACTCGGCGTTAACCCAGAAGCCTTACCCGATA
AAGGCGTGGATTATGTCGCGGCATAATCCTTTCCAGACCGTCACCTGTCGTTCGGACCTGGTAAAAACCGTTG
AGCAACTGGATCTGGTGGTCAGCTGCGATGTCTATTTGAGCGAGAGCGCGGCATATGCCGACTATCTGCTGCC
GGAATGCACCTATCTCGAACGGGACGAAGAGGTATCCGATATGTCGGGACTGCACCCGGCTTACGCTCTGCGC
CAACAGGTCGTAGAGCCGATTGGCGAGGCGCGTCCGAGTTGGCAAATCTGGAAAGAACTTGGCGAGCAGTTGG
GATTAGGGCAGTACTATCCGTGGCAGGATATGCAGACGCGCCAACTCTATCAGTTGAACGGCGACCATGCCTT
AGCGAAGGAACTGCGACAGAAAGGGTATCTCGAATGGGGCGTTCCGCTGCTATTACGCGAACCAGAATCCGTT
CGTCAGTTTACGGCGCGTTACCCCGGCGCTATCGCGACGGACAGTGACAACACCTATGGCGAACAGCTTCGCT
TCAAATCGCCCTCCGGCAAAATCGAACTTTATTCCGCAACCCTGGAGGAATTGCTCCCTGGCTACGGCGTTCC
GCGCGTTCGTGACTTTGCGCTGAAAAAAGAGAATGAGCTTTACTTCATTCAGGGCAAGGTGGCCGTGCATACC
AATGGCGCGACGCAGTACGTACCTTTACTCAGCGAGCTAATGTGGGATAACGCGGTCTGGGTTCATCCGCAAA
CGGCGCAAGAAAAAGGCATTAAGACCGGCGATGAGATCTGGCTGGAAAATGCCACGGGTAAAGAGAAAGGTAA
GGCGCTGGTGACGCCCGGTATCCGCCCGGACACGCTTTTTGTCTATATGGGATTTGGCGCTAAAGCTGGGGCC
AAAACGGCGGCGACGACACACGGTATCCACTGTGGCAATTTACTGCCGCACGTGACGAGTCCGGTATCCGGTA
CGGTAGTGCATACCGCAGGCGTGACGCTGAGCCGGGCATGAGGGAGGAGGGAACCATGAATCATTTAACGAAT
CAGTACGTCATGCTGCATGATGAAAAACGTTGTATCGGCTGCCAGGCGTGTACCGTTGCCTGCAAAGTGCTTA
ATGACGTACCGGAGGGATTTAGCCGCGTACAGGTACAAATTCGCGCCCCTGAACAGGCATCCAACGCACTAAT
CCATTTTCAATTTGTCCGCGTCTCCTGTCAGCACTGTGAAAATGCGCCATGTGTCAGCGTTTGTCCTACCGGA
GCGTCATATCGTGATGAAAACGGGATCGTGCAGGTGGATAAATCGCGCTGTATTGGCTGCGATTATTGTGTTG
CCGCGTGTCCTTTCCATGTGCGCTATTTGAATCCGCAAACCGGCGTCGCCGACAAGTGTAACTTCTGTGCCGA
CACGCGGTTGGCGGCTGGCCAGTCTCCGGCGTGCGTATCCGTTTGCCCAACGGACGCGCTGAAATTCGGCAGA
CTGGATGAGAGCGAGATCCAGCGCTGGGTCGGTCAGAAAGAGGTCTATCGCCAGCAGGAAGCGCGTAGCGGCG
CGGTCAGTCTGTACCGTCGTAAAGAAATCCATCAGGAGGGTAAAGCATGAATACTATCTGGGGAGCGGAACTA
CATTATGCGCCAGATTATTGGCCGCTGTGGTTAATTTATGCAGGCGTCGTGGTGCTGCTCATGCTTGTTGGGC
TGGTTATCCATGCGTTATTGCGCCGGATGCTGGCGCCAAAAACGGCGGGCGGCGAAGAGCATCGTGACTATCT
CTACTCGCTGGCGATTCGCCGTTGGCATTGGGGAAATGCGTTACTGTTTGTTTTATTACTGTTAAGCGGTTTA
TTTGGTCATTTTTCTCTCGGCCCTGTAGCGCTAATGGTACAAGTGCATACCTGGTGTGGTTTTGCCTTACTGG
CTTTCTGGGTCGGGTTTGTGCTGATCAACCTCACCACAGGTAACGGGCGTCACTATCGGGTAAATTTTTCCGG
ACTGGTAACGCGCTGCATACGCCAGACGCGTTTTTACCTTTTTGGCATTATGAAAGGGGAAGCGCATCCGTTC
GCGGCAACAGAGCAGAATAAGTTCAATCCACTGCAACAACTGGCATATCTGGCGATTATGTACGCGCTGGTAC

CGCTGTTAATCATCACCGGTTTGCTGTGTCTCTATCCGCAGGTTGCGGGTCTGGGCCCTGTGATGCTGGTGCT
GCATATGGCGCTTGCTATCATCGGCTTACTGTTTATTTGCGCGCATCTCTATCTGTGTACTCTTGGCGACACG
CCGGGACAAATTTTCCGTAGCATGGTTGACGGCTATCATCGTCATCGTACCGCGCCGCGCGGGGATAAGTCCG
CCGTCTGAGCGTATCCATAAAATAATGTCAGCAAGAAAATGTGCCTTGTGTCACATTTTCTTGCAATTTATCC
ATGTTTGTTTAACTGAACTTCCGTAAAAAGAACGGCAAATAGAGACCATCCTGAGGACATGCTGTTGAAACGC
CGTTTGTTTATTGCCGCTTCTTTATTCGCGATGCACCTTTCTCCTGCGCTTGCGGCCGATGCCGTAAGTTTTG
CTCCCCAACCGCCAGCCATTGATGCAGGCGCCTGGGTGTTGATGGATTACACTACGGGGCAGGTTCTTACCGC
CGGTAATGAACACCAGCAACGTAATCCCGCCAGCCTTACCAAGCTAATGACGGGCTACGTCGTCGATCGCGCC
ATCGATAGTCATCGTATCAGCCCTGACGATATCGTCACAGTTGGGCGCGACGCCTGGGCGAAGGATAATCCGG
TGTTTGTCGGCTCCTCGTTGATGTTTTTAAAAGAAGGGGATCGCGTGTCGGTACGCGATTTAAGCCGTGGCTT
AATTGTGGATTCCGGTAACGATGCCTGTGTGGCGCTGGCGGATTATATCGCGGGCGGGCAGCCGCAGTTTGTG
GCGATGATGAACAGCTATGTGAAAAAACTCAATTTGCAGGATACCCATTTTGAAACCGTCCACGGTCTGGATG
CGCCGGGACAACATAGCTCCGCGTATGATCTGGCCGTACTCTCCCGGGCGATTATTCACGGCGAACCTGAATT
TTATCACATGTACAGCGAGAAAAGCCTGACCTGGAACGGCATCACGCAGCAGAACCGCAACGGCTTGTTGTGG
GATAAGACCATGCATATCGATGGGCTTAAAACCGGACACACATCGGGGGCGGGATTTAATCTTATCGCATCGG
CGGTGGACGGGCAGCGTCGACTTATCGCCGTCGTGATGGGGGCGAAAAGTTCGAAAGGCCGTGAGGAACAGGC
GCGAAAACTGTTGCAGTGGGGCCAGCAAAACTTTGCGACGGTGCAGATTTTGCACAGCGGTAAAAAAGTGGGC
AGCGAGCGAATCTGGTATGGAGATAAAGAAAAGATTGCGCTGGGTACGGAACAGGATTTCTGGATGGCGCTGC
CTAAAGCGGAAATTCCGCATATCAAAGCAAAATATGTGCTGGATAGAAAAGAGCTGGAGGCGCCAATCGCCGC
TCATCAACGGGTAGGTGAAATAGAGCTATACGATCGGGATAAACTGATCGCTCAGTGGCCGCTGGTCACTCTG
GAGTCGGTGGGTAAAGGCGGTATGTTCTCTCGTCTGAGCGACTATTTTCAGCATAAAGCGTAAGCCGTTGTCA
CACACCGTTCACCGTCATCTGAGGTGTCAAAAGGGCGGGATTGCGAGGTTGCTCACAAAATGATCACGCTGTC
CTTGTGACCGTAAAAGGCTGTAATTATACTGTACATTCATACAGTATAGTAAAGTGACGGAGGCAGCATGGAT
TACGAAATCAGGCAGGAACAAAAACGCAAGATAGCCGGTTTCCACATGGTCGGTCCATGGGAACACACCGTGA
AGCAGGGTTTTGAACAACTGATGACGTGGGTGGATCGACAGCGGATCGTGCCAGTTGAATGGATTGCCGTCTA
TTACGACAATCCAGATGTCGTACCTGCAGAAAAAACTCCGCTGCGATACCGTGGTGTCCGTGGCAGAGAATTT
CATCCTGCCGGACAATAGCGAAGGCGTAATCGTGACCGCCATTGAAGGCGGCGAATATGCCACTGCTGTCGCG
CGAGTGGAAGACCGTGACTTTGCGAAGCCGTGGGAACGGTTTTTTGATGTCCTGGAGCAGGATAGCGCTTATC
AGATAGCCTCTGCGCCCTGTTTTGAAACCTATCTGAATAACGGCATGGAAGATGGATACTGGGATATTGAAAT
GTATATTCCGGTACAGCGGAAGTGAACGACTGGTTTCCTGGCTGAAAAGAGCGGACGGATAATGTGTTCCGTC
CGCCAGTGCGCTATTTTTGCTATAAAGCAGGTACAATGCATTTTTTGCCATTCCCGTAAGGAGCGCAAGCGTG
CGTGCCGATAAGTCATTAAGCCCTTTTGAAATACGGTTGTACCGTCATTATCGCATCGTGCATGGCATACGTA
TCGCCCTGGCGTTCATTCTTACCTTTCTGCTGGTTCGTCTGTTTTCTATTCCCGAGGGCACATGGCCGCTAAT
CACGTTAGTGGTGATTATGGGGCCCATCTCATTCTGGGGAAATGTAGTGCCTCGTGCTTTTGAGCGCATAGGC
GGTACGATTCTGGGCGCGGCGTTAGGGCTGGTGGCGCTACGCCTGGAGCTCTTTTCCTTACCGCTGATGCTGG
TGTGGTGTGCGATAGCGATGTTTTTGTGCGGCTGGCTGGCCTTAGGGAAAAAACCGTATCAGGCGCTACTGAT
AGGCATAACGCTTGCCGTTGTTGTCGGCGCTCCCGCTGGTGATATGGATACGGCATTATGGCGTGGCGGGGAT
GTGATTTTGGGGTCGCTACTGGCAATGTTGTTTACCGGCATCTGGCCCCAGCGCGCCTTTTTTGCACTGGCGC
ATCCAACTGGCTCATTGCGTCACGGCGTATAATCGGGTGTATCAGGCAGCGCTGTCGCCGAATTTGCTGGAGC
GCCCCCGGTTGGATAAACATTTGCAACGGTTGCTGAACGATGTCGTCAAAATGCGCGGGCTGATAACGCCTGC
CAGTAAAGAAACCCGGATACAAAAATCTATTTCGAGGCCATTCAGACCATCAACCGCAACCTGGTTTGTATG
CTTGAATTACAAATCAATGCCCACTGGGCGACGCGCGCCAGCCATTTTGTGATGCTTAACGCGCACACATTGC
GTGAAACCCAGCAGATGACGCAGCAAACCTTGCTAACGATCGCCCACGCGTTGTTTGAAGGCAACCCGCAGCC
GGTACTGGCAAACACCGGTAAACTGAATGATATTGCTGCCGAGTTGCGGCAGCTAATGAACGAGCAGCAGGGC
GACGCGGTGGCTGAAACCCCCATCCACGGCTATGTTTGGCTAAGTATGGAAACTGCCCGACAGTTGGAACTGC
TTTCACATTTGATCTGCCGGGCTTTGCGCAAATAAAAAGCAGATGGGGCGAATGAATTTGTTGCTGCTTCGAT
TCAGCAAAGATTAAGAGTATGATACTAATATAAGGATAAAATGAGTGCTACTGGCAACGATTTTATCCCCGAA
CCTTCAGGTTTCCCCAGACGGCGACGTAACGGTGTTTCCTCTGGCGGCTTGAAGTATGACGGGGATAAACGTA
ATGTTTATCCACTAGCAGTTGCTTAACGAATCCGAATCTCACATTATCAGGGGTGTAAAAATGGAAACAACCA
AGCCTTCTTTCCAGGACGTGCTGGAGTTTGTTCGTCTGTTCCGTCGCAAAAATAAACTGCAGCGTGAAATTCA
GGACATTGAGAAGAAGATCCGTGACAACCAGAAGCGTGTGCTGCTGCTGGACAACCTGAGCGACTACATCAAG
CCAGGTATGAGCGTTGAGGCGATTCAGGGTATCATCGCCAGTATGAAAAGCGACTATGAAGATCGCGTTGATG
ACTACATCATTAAAAATGCCGAAATCTCCAAAGAGCGTCGCGACATTTCCAAAAAGCTGAAAGCGATGGGCGA
AATGAAACACGCCGATGTAAAAGCCGAATAATGCGCTACGGCAGTGGGAGCGTATATCAGGCTCTCACTGCAG

TTTGACCCCGCCTGTGACCATCTTGAGTAAATGTTGTTTTGGCCAGTGTCGGGTAAGTTTTTTTCTCTGCCAG
CTTACCTTTCAGGCGCGCAATATCATGACGTTTTCGGTCCAGCATCAGACCCACCACGCTACCGCTATGCGCC
ACGTTCAATCCGTATAAATCACACTCTTCGACCAGCGACAGCAGGGCGGTAAAGCCCGGCTTAGGTAACAGCG
TCTGGCTGGCGATAGCGCTAAGCGTAGCCGCCTCACCCAGTCGGAGCGGATTTTGCGTTATACAGGCTTCCTG
CACCAGATTCCAGGCCTGCTGCAAGGTTGCTGAACTTGCTATTAATTTCTGCTGGCGAGGGAGACGGTGGTAA
TCTTGCGTGCGCAGGGTGACCGGGCTTTCCAGTACCAGCAAATCGATTGGCGGCGGTGGCTCGCAGGCGATTT
GCGTGACCGCATTATTATGATCAAACAGCGTTAACTGATGAAAAACGGTGCTATCAGTGGGTTCGATTGAGAC
GCAAAGCTGTGCAAGGGTAGTTTCATCCAGCGAATGGCCAAGATGATGCGCCGTTGCCACCGCCGTAGCGGCG
ATATCTGCGGTGCTGCTGGCCATGCCTTTGGCAACGGGAATTGACGAACGCACATCGACGCGAATCTCGTTAC
TCCAGTGCGCAGGATACTGCCAGTGCGCCAGAACGCGCTCCACCATCGCCCGCGACAATGGACGCTCGTTTAC
CAACGGCGGCGCAGCCGTGACTGCTACGGTGCTGTACCAGTCAACGGGGCAGGAGACCAGTTTCTCACTGCCC
AGAATCCATCCCTGGATAAGTTCCCCGCATGAGGCGGGGCATTGCGCAACAGCCACGTTATCACCTTTCAGGT
ACGAATAGTGTGCGCGCATAGTGTCATGGTAAAAGCATTATTACCATGACTTGCCGCAATGCGTCGTTTAACC
CTCTCAGGCTGGTACACAAAGCCCTTCCGTGGCCGGTAGCGCAATGCGCATAACGTCTTGTGCGATCATTAAC
TCTTCGTTGGTGTTGATGACGGCGACTTTCACCAGGGCATTCTCCGTCTGGATAAATGTCGCATTACGCTGAT
TTTTCTCTTCATCAACGGCTAAGCCCAAAAATTGCAAATTATGGCACACCGCCGATCTGGCGCGCGCTGAGTT
CTCGCCAATGCCCCCAGTGAATACCAGCGCATCCAGTCCTCCCATTTGCATAATATAGCTGCCAATGGTGGCG
CGAATGCGTTCGGCAAACAGAGTGAGAGCGAGTTTAGCCTGCCGGTTACCGGTATTGGCTGCCTGTTCGACAT
CGCGGTAATCAGACGACACGCCAGAAACGCCCAGCAAGCCTGACTCATTATTTAATAACTGATTCAACTGTTG
CGGCGTTTTGCTTTCGCGCTGGGCTATCCACGGCAGAATAGAGGGATCAATATCTCCGCTACGGGTGCCCATC
ATCACGCCTGACTGCGGCGTAAAGCCCATCGACGTATTGACCGAACGCCCGTTCTTGATGGCGCAGATGCTGC
TACCGTTTCCAAGATGACAACAAATCACCCGTAATGCGCTGAGTGGTACGCCCAGCTTTTCCGCCAGAACGCC
GCTGACATACTTATGGCTGGTGCCATGAAAGCCATAACGGCGAATCCCCAGTTCCGCGTAATAATGCCAGGGA
AGCGGGTAAATATAGGCGGGTTCATCCAGCGTCTGGTGAAATGCCGTATCAAACACGGCTACGGAAGGGGCAT
CAGGCAACAGTTGACGAAAAACGTGAATACCCAACGCGTTTACCGGGTTGTGAAGCGGGGCCAGTTCAGCAAG
ACGTTCGATTTGCGCTAACGTTTCATCCGTGACCAGCGTCGAGTCTTTAAAAAACTCACCGCCGTGCGCCACA
CGATGGCCCACCCCGTCAATGTCCCGCAGACTGTTAATGATTTGATAACCCAGCAGCTTTTCCAGAAGCAGCG
TTACCGCATCGCGATGATCGGCAACGGGGACGGTTTCCTGCCATTTTTGGCTGTGCGTCTTAATTGTCACCTG
CGCATCCGCCATACCGATACGCTCAATCAGCCCCTGACAGAGCATGTCGCCCTGCGGCATCTCCAGCAGCTGG
AATTTTAACGATGAGCTGCCGGCGTTAATGGCCATTATTTTGTAAGACATAAAGGTTCCTTCGAATTCAGAAA
ATCCAGCACGGCGTCGAGACCCGAATTATTCAACGCGCTGGTAATAAAAATCTGCCGGGCGCCAGCCTGCGTA
AGCCACTCCGCAACCAGCGAAATACGCTGCGGCTCGGCGAGATCGGCCTTGGTGACCAGGCCAATCGTCGGGC
GGTTCATCGGGGCGGTAAAACCCGGAGAAAAGGGCGACCACTGGGCGTCGGCATTCAGCACCAGGGCGATGAC
GTCGGCCTCGCAGGCGCTGGTCAGCAGGGCGCTGTACAGGCAGCGGTTCTCCAGATATTCGCCCGGCGTATCG
ATGGCCATCGGCGACCATTCAATGGCCTGAGTTTTCTTATAATGGAGCGCTTCGCCGCGCAGGCTTTGGGTCA
GCGAGGTTTTTCCGCACTGGCTGGGGCCGATAAACATCAAACGCTTCATGACTTTACGTCCGGGTGATCGAGC
AAGTGGTGAACTGCATCATTTCGCCCAGCGTGCGCGTTACCTGCTTGAGGGCATACTCGACGGCAGAAACATC
GCCAGTCAGCACCACCGCGCCGGTAAAGCGGTCAAGAAACCCAATCTCCACCGCGCCGGATTTGGTAGCAATA
TCGCAAGCAATAATAGATGCTTCGCTCGGCGTGATGGTAAGGATGCCAATGGCGGACACCGCATCCTGCAGGC
CCAGCTTCTTAAAGAGATCTTTCCCTGGATTAGCAATCAGGTGCGCGAGAGTGACCTGTTTCCCCGGCACGTA
TTCCTGAATCATGCGATCCGTTGTCGGTTGTCTTTCCATTATCCCTCCACCATCTGTCGCCACATGGCTTCAT
GCGGGCGTGGGATCACCGAACGGTAAACCAACAACCCTTTCTCGCCCGCGCTTTCGCTGGCAACCGTCACGGC
GTTATTGACGTCAGAAACGTCGCCCGCCACCACCATGTAGCATTTACCGCCGATACCGAAGGCCATATGCACC
CGCACCAGGGTGACGTTTGAGCCTTTCACCGCCCGGTCTGCGGCGCTAATGCAGGCCGCCACGCTCCAGGTTT
CGACGATACCTACCGCCTGGCGCTTATCGACGCTGTTCAGGCCGCTGATGGCCGGAAGTACGCTGGGATGAAT
ATTCGCCAGCACCAGGCTGTCGACGAGCATTTCGCCGGCCTGCGACGTACCGGTTTCAATGGCCTGCTGGATA
GCGCCGATATCGCCGCCCAGCATCAGTAAAAACTTCCCCGGGCAGATGGTCTTGCTGACCAGTAGATCGACGT
TGGCGCTTTTCAGCATCGCATCACCAAGCTCCATCCCTTTGGCGATGCTGGTGAGTTCTAAAATTCCTATAGC
CTGAGACATGGTTAACCTCTTACAACCGTGATGGCCTGTTCGCTGATGGCCGAAACAATGCCGTCGATGCTGG
CGTGAATAGGTGCGCCGAGCGCGCCAGGCGGGACATCGGCAACGCATTGCCCGCGCGTCACGCGTTCTCCCAC
CGCAACGTTCGCTACCGCGCTGGCACCAATATGCTGGCGCAGCTGCAAAGTGACTTTTTCTACTGACGGCTCT
TCTTCAACCAGCGGCGCTTCCTGGTACCAGGGGCTTAGTCCCAGTTTGGCGATCAGCCGCTTCACCGGCACCA
GGCGATATTTCGCCATTTCGTCGGACGGATTCAGCGGCCCTTCGTAGCGCTGGTTTTGCGCCCGCAGCTCGCG
TTTTAACATGCGGTTGATGCGCATCGGCGAGATCCCCACCGGACAGGCCACGCTTTCGCAAACGTTGCATTCC

GAGCAGGTAAGGGCGCTCAGCAGCAGCTGCGGCGTGGCAGCCTGGTGAAAGTTCACCGCCCGCACCAGCAGGT
GCGGAGAGAGTTCGTGGCCGATCAGGTGTCGCGGACAGAGATCGGTACACAGTCGGCACTGTTCGCAGACGGT
GCGCGCGACGGAAAGCACCGTGCGCTCGTCCTGCATTCTCCGCTGGATAAGCGGATGGCTTTTGGGGAGCACC
AGCAGGCCGCCGGTAGTTTTCGTCACCGGATTATCAAGAGAGGTAATCAGGCCACCCATCATCGGGCCGCCGT
TAATAAAACCAGGGTCGTCGACCGTTGCGCCGCCCGCCAGCGCCAGTACTTCCCGCAGCGACATGCCAATGGG
AACAGTAACGGTCAACGGTCTGGCGACCGCGCCGTTCACGGTCAACGTGCGACGAGTGACCGGAAACTGCTGC
TCGACAGCCCGGGCAATATTCAGCACCGTTTGCACGTTATTGACGACCACGCCGACGCTGGCCGGTAGCGCAG
CCGGGGCGACCCGGCGGCCGGTTGCCATCCAGATAGTCAGCACCTCATCGCCCGCCGGATAAACATCCGGCAG
AATATGCAGGCGGATACCGGCGGGCAGTAGCGGGGTAAGGGCGTCGATGGCCCAGCGGTATTTTTCTTTCAGG
GCAATCACCCCTTCGCGCGCGCCGGTCGCCGTCATGGCGTACTGTACGCCGCGCACAAGGCGCGCAGCTTGCT
GCCACATCAGTTGCTGGTCAACTTTGAGCATCGGTTCACACTCGGCGGCGTTCACCAGAAAAATCTCTACCTG
CGCCTGTAGTTTGACGTGAGCGGGAAAACCTGCGCCGCCTGCGCCGACAACGCCCGCTGCGCGGACGCGCTCA
CGAATTTCATCGGCGCTAAGGGACATTTCAACGCTGTTGATGGCGGTGCTCATAGCAGTTCCTCCAGCAGTTC
ACGAATTTCTTCGGCGCTGGCCGGGCGCGGGTTGGTGCGCAGCGTAACGTCCGCCAGCGCGGCCTGCACCATC
GCCGGAATGCGTGCGGAAAAGTTGGATCTTCCTTCTTTAAGCGCAACGGCCAGTGAGGGAAGGGCGCAGCGTT
GCTTAAGCAGTTCGATTTGCTGAATCAGCGCATTGATCGCCGCGATATCATTGGCTTCTGCCGGGCAAAAACC
GCAGGCTTTGGCCAGCCGCGCGTAGCGTTTGGCGGCGCGCGGAACGCCGGCGTTAAAACGGATAACCGTGGTT
AACAGCAGGGCGTTGGCCAGGCCATGCGGCAGATGAAACTGCCCGCCAAGCTGGTGGGCTATCGCGTGGTTAA
GCCCCAGCCCCGCCTGGCTGAAGGCCATCCCGGCGAGCGTTGAGGCATTGTGCATTTTCCCGCGCGTCGCCAC
GCAGTCGCCTTTTTCCACCGCCGTGGGCAGATACTGGAACACCAGTTTGGCGGCTTTTTCCGCTAGCGCGTCG
GTAAAGTCGCTGGCGCGCGGTGACACCCAGGCCTCCAGGGCGTGGGTCAGCACGTCCATGCCGGTGTTGGCGG
TAATCTGCGGCGGTACGCTGACCACCAGCTCCGGGTCGAGGATCGCCATATCCGGATACAGCGCATTGTTGAA
CAGCGGATACTTGATGCCTTTATCCGGGTCGCTAATTACGCAGGCGCTGGTCACTTCCGAGCCGGTGCCGCTG
GTGGTCGGGATCGCCACGCAGGTTTCGATGTTGATCCCGCTCTGCTGGCTAAACCAGACAATCGCTTTCGCCG
CGTCCATTGCCGAGCCGCCGCCAAAACCGATCACCACCTGCGGTTGCAGAGCCTGCATTTGCGCAATGCCCTG
AACCACTGTGTGGATGGTGGGGTCCGGCGTTATCTCGCTAAAGACGCTGATGCGGTTATCTGCGGGCAGCGCA
TTACGCAGGGTATCCAGCAGCGGAGAGCGCGCCAGAAAGCCATCGCAGATTATCCAGATGTGCTTATTGGTAA
AGCGCTTGAGCACCGCCAGGCTGCCCTGACCGCTGTACAACCGCGTTTGTAGTGAGAAGGTATTCATCGCGAC
CTCAGTTAGCGAATAGAAAAGCCGTTGGTCAGTACGCAGCGCCGGGAACGGGCAAAAGTACGCGCTGACGTGG
TCCCTTCACCGGTTGGCGTGGCGATAGTGAAGGTGGTAAAGCCTTCGCCGCCGACGCCGATCCCGGCATAAGA
GGGGCCGTTTTTGACGAATATCGAGGTTTGCAGGGTGCGGGCCGCGAGGTTCAGGCGTGACACGTTCTGCGAG
TGCATAATGGCGGTATGATGCAGCCCCTCTTCAACCTTCAGGGCCAGCGCCAGTGCGCTATCGAAATCGCTGA
CTTTTACCACCGGCAGCATCGGCATCAGCTGTTCGCTGGTGACCCACGGATCGTCGGCGTTGACAACGGCAAT
CAGCAGACGCGGCGCTTTTGCCGGGACAGCGATCCCGGCGGCTTCCAGCATGGCCGATGGGCTCTTGCCGACC
AGTTTTTTATTCGCCTGGCCTTCAGGCAGGCAGACGGCGCGGAGTTTGTCGGTATCGGTCGGGCTTAGCAGCA
GCGCGCCGAAGGTTTGCATTTGCTGCACCAGACGTTCGGCGACACTCTCCACTACGATCAGGCTCTTCTCGGC
AATGCAGGGCAGGTTGTAATCGAATGACGCGCCGTTGATGATATCTTCCGCCGCTTTCACCAGGTCCGCCGTT
TCATCAACGATGCAGGGCGGGTTACCCGCGCCAGCGCCAATCACCTTCTTACCGCTCTTCATGCCCATTGCCA
CAATGCCGGGGCCGCCGGTAATGGCCAGTACTGCGATTCGCGGGTGGGCCATCATCTGCTGGGTCGCTTCGAA
GGTGGGTTCAGCTACAGTCACCACCAGATTGCGGATGCCGCAGCAGCGGAAGGCAATCTCTTCAATCAGGCTG
ATGAGCTTCAGAGAGACCTTTTTCGCCCCCGGATGCGGGCTAAAGTAGACGCTGTTGCCCGCCGCCAACATGC
TGATGCTGTTGTTGATGATGGTTTCCGTCGGGTTGGTGCTCGGTGCGACCGAACCGATAACGCCAAACGGTGA
GTATTCAAACAGCACCATGCCGCCGTCGCCGGTCAGCGCGGTGGTGGTGAGGTCTTCTACGCCCGGCGTGTTG
TCCAGCGCAGCCTTGTTTTTGAGGAATTTATCTTCTTTGTTGCCCATCCCCGTTTCATTGGCGCTCTCTTCCG
CCAGGGTCGCCAGCAGCGGCGTCAGCTCCTGACGCATCGCGCTGATAATGGCGCTGCGGGTTTTTAGCGGGCA
CTGCTGATAACGTAAGAACGCCTGGTGCGCGGCGTCGATGGCCTCGCTCACGGACTGGAAAATCCCTTTGCCC
TGAGGCTGGACCGGCGTTTGCGCCGGCGTGGTTAATTGCTCGCTAAGAATGGTGCGAATCAGGGTTTCGAGTT
CAGAAGTATTCATTGATGTGTTCCCACGTTAATAGCCGCGATGGCGGTCTGTGCTATGTCCATGTCCTGCTCA
ACGCTGCCGCCGCTGATGCCAAGACCCCCCAATTAATATGCCGTCGCGCCATAGGGCGTAACCGCCGCCAAAG
GTGACCACTTTTCCCTGTAAATGACTTTCCAGGCCATAAAGCGCGGCGCCTGGCTGAACAACATCGCTCAGCT
CATGGGTCGCCGTTTTCATTGCCACTGCGGTCCAGGCCTTTTTCGGCGCCAGTTCGCTGCTGACCAGCAGGGC
GTCCGGCATCCGCCAGGTCACAGTTTCCGTGCCGTGCGCGTCAACGATGCTGACGACTACCGGAACCTGCAGT
TGCTGCGCGCGTTCAACGGCGGCGCGGGTGAGCTGGTGCAGATCGTGGAATGAGAGGGCCACGGGCGTTGTCT
CCTTGCTGCGGGTCGGCTGGCTGGCAGCCAGATAGCGCTTGCTAACTTCGCGGATGATGTTGGCCTGGTGCGC

ATCGCTATCTTCCGCGCGGGCCAGGGCGTACAGGCAGTCCGATAAGCGGTTGATGTAGCGCATCAGCACCTGG
CGTACGTTGACTTCAGTTGCCAGTTCAACCAGACGGCGTTCGGCGCGGCGCGCCAGCGTGCGGGCAAAATGTA
AGCGGCTCGCGGCTTCGCAGCGTCCGGGTAAAATAAAGCTGTGCAGCGGTTCGACGCGGGCCATTGCCCGATC
GATAGCGGCTTCCAGGGCCGAAATCTCTTCGCTGCTGATGTAGCGCTGTTTGGGCGATGGCTGCTCGCTGTCG
CTGGCCAGCTCTGCGCTAAACCAAAAAAGTTGCTGCTGGATGGCCTCGAGTAAGGTGCGGTGATTTTCATCCG
CAGCGGCGCAGGCGCACAGGCTCAGCGCGGCATTCAGCTCATCCAGCGTGCCGTAGGCTTCAACCCGCGGGTG
CGTTTTGCTCACCCGCTGGCCGGTAAACAGTGATGTGGTGCCTGCGTCACCTGTTCGGGTATAAATCGCCATA
TTCGCCCCTTAACGTGAAAGCGTATCGACAATGCCGACAACGGCGAGGTCGATGGCCTCATTTGGCCCGGAAA
AAACGTGCCTGGCGCTGGAGCCGCCGCTGAGCAGAACCAGCTCGCCGATGCCTGCGCCGACGGAATCCACGGC
CACTTCATCGCCGGAGGTTGGCGAGGCGGGGAGTTCGCCATCGGCGCTGACCCGACGCACCAGCAGCAGCTTT
TTCCCAATCAAAGAAGGTGATTTTTGCGTGGAGACAACCGCGCCCGTGACTCGTGCCAGATGCATGATTTACT
CCTGCTTAATTAATTGAATATTCCGCGCATTAGCTGCTTCGCGCGCCAGGGCCGTCACGATGCATTTGCGGTG
GACAACCACACGCCCCCGGCTTAGCAGCGCGACATCGCGATAGCTCAGCACCGAACCCGCGTGCAGAACCAGC
GGCAGTCCGCGCTCATCGGTGAATACCAGCGGCAGACGAGCCAGTTTTTTACCGGGATAACCGGCAGCAGCT
GGCTGTGCAGGCTGAGCTGTACGCGGATGCCAAACGCCAGCGCGTCGTGAATTTTCCGGGCGGCGGTATCGTC
GGTTTCTGCATCCGCAAGCTGACCCAGCAGCGGCAGGTCGACGAGCAGGATGCGCAGCGACGCGTGCTGACAG
AACAGCGCTGGGCAGTCGGCATCGCGAAGCTGCGCAACGCTCAGCGTCACCGTGCTCTGGGCACGGCGCTGCA
GTCGGGAGACAATCTCCTCGACAATGCGCTGCAGAGTTTCGCCGTTCATCGCGGGCCTACCAGCCGGGCAAAG
GCCTGCGGGTTATCGGCACCAGCGGCGTTGGCTTCATCGGTATCAATGTGCATTTCGAGGCGCATGTCCGGTG
AAACGCGAATCGCCACATTGTTGAAAATCAGCCCGCGCTCGTCGCCTTCAATCGCTACCGAAACCCTATCGCC
GTGGGAGACCTTCAGGATCAGCGCATCAAGCGGCGACATATGAATATGCCGCTGCGCGACGATAACCCCGGAA
GGTAGCTCCAGTTCGCCAAAGGGGCTGACCAGGCGGATGCCAGGTGTGCCCTTCAGGTTGCCGGACATTCTCA
GCGGCGCGGCAATCCCCAGCGTTCTCGCATCGGTACGCGAAATTTCCACCTGGCTTACCGAGCGCAGCGGGCC
GAGCAGGCGCACATTGTTGAGCTGTCCTTTCGGCCCTACCAGGGTGACGGTTTGCTCCGCAGCATATTGTCCC
GGCTGCAGTAGCGCTTTTTTTTCGCTGATGGGATGGCCGGGAAACAGCCGCTCATAATCCTGCGCGGACAGGT
GGATATGACGGTTTGAGACGCCCAGCGGAATAGGCCGCTGGCGCATCTCGTCGAGAACTTTACGGACCGTTGA
TTGCAAAAGCTCTTTATCCATTACGCTTCACCTCGCTTGCCGGAATGAATGCAAAGCGTACGAGGTTCGCCCT
TCTGGCGCGGACATTTTGGATCCAGACACAGATTGCAGCTGACCAGTTCTGATTCCTGCGGGGCCTCTTCGGC
TGGCGCTTCAGAAACGACATCAGCTATCTCTGACGCTTCAGGCTCAGGTTCAATGACGGAGGGGGAGGGTGTC
TCTGCGAGCAGGATCCCTTCTCCGGGCCTGGCGATAACCTTGTGGGCCACCAGACCATTCCGCTGTTCGGCAA
ATTGCGCACCGGTGGTGATGGCCGCCTGCACGGAGGCCACATCACCGGTAATTTTAATCACCATCCAGCCTGA
ACCATTGGTCTTTTCGAGGGCGAGCAGCGTGATAGAAGCGGATTTCGCCATGATATCGGCGCAGCTGATAGCC
AGTGCCAGACCACAAACTTCAAGTAATCCCAGTGATTGCTTCACGCGGTGCTCCTTATTCGCCATCGATTAGG
CTGATTTCGGTAAAATGGCCTCAACATCACTGTGCGGACGCGGGATAACGTGGCAGGATTTCACTTCACCCAC
GACGCTTGCCGCCGCGCTGCCGGCATCTACGGCTGCTTTAACCGCGCCGACATCGCCGCGAACCATGACGGTA
ACCAGGCCAGAACCAATTTTTTCGTAGCCCACCAGCTGTACGTTGGCGGATTTAACCATTGCATCGGCGGCTT
CAATGGCGCCGACCAGCCCTTTTGTTTCAACCAGTCCCAGTGCGTTATTCATACTGCCGTTCTCCTGTGATTA
AGCATGGAGATCCCGAAAGGGGATCCCTTTGACCAGCCGTGCCGCGTTATTACCGGTGTTGCGTTGGGCCTGA
CTGTCCTGATGATGCATCAGCGTAAAAAGCGGCGCCGATACGGGTAAATTCTTGTAATGTACGACCAGCGTAT
GTCGATCGCAGGCAATGCCGACCAGCAGCGGCGAGCTGCGCGCCGCCTGCCAGGCGCTGTCAACGACATCTCC
AGCCGGGTGATGCTGAAGCAGGAAAGGGATGCCTTCCTCTTCGATACCCAGCAGCACTTCGTGCCAGAGGCTG
GCGCAGTCGTTGATAACGGTAATGACGATGGCCGGGGCGCTGTGATTACTGTCCATGTGCAAACTCCTTGTGC
CAGGAGAGAATCAGGCCGGTGGCCACCGCGTTTCTTGGGCCTTCGCTGCCGCGAATATTTCCTCGCCCGGCGA
CCAGGCGGTAGTGCGCCAGCGCATCGGTGACCAGCTGCGGAACTTCGAAATCCAGCGACGAGCCGCCGACCAG
CACCACGAACGGAATATCGCGAATGTTGCCGGTTGGACTGACCTGGCGCAGCGCGCGCAGGGCGTTGGTGACA
AAGACGCGTTCTTTAGCGCTGCGGCGAATGGCGCGCACTTTTTCCAGCGCTAAGTCGCCGGGAAGCGGCACCA
GTTCGTCCGGTTTCACCACGCAGACGCGGGCGAACACGGCGGGAGGCAGCGGCGTCGGGAAGAACTGGACGCT
GCCGTCCTCGTGGCGTAAGTGGAACAGGCTTTCGACCTTCGCCAGCGGGTATTTTTTGATCTCTTCCGCCAGA
TAGCGGTCTTCCAGCCCCAGCTCGCGGGCAATAATCATCGTCACCATGTCGCCTGCACCGGCGAGGTGAGTGG
CGATGATTTCGCCTTTAGGGTTGATGATGGAGGCATCGGTGGAGCCCGCGCCGAGGTCAAGGATCGCCAGCGG
TCGGGTGGTACCCGGCGTGGTCAGCGCGCCCAGAATGGCGGCTTCAGCCTCAGCGCCGCCGATCTGCACGTCG
ATATTAAGCTTCTGCTCAATTTCACGGGCAATCATCGCCATCTGCAGACGGTCTGATTTCACCATCGAGGCGA
TGCCGACGGCCTGCTCCAGTGAGAACTCCCCGGCCAGACCGCCGGTGACGCTCACCGGAACCGAGGTGTCAAC
GGCCAGAAGATCCTGAATGAAAATCTCGCTGCTGGGCTTATTGGTTAGCTCGGCCATGGTCTGGCGTACGTGC

TCCAGCATGCCGCCGATATTGGTCCCGGCCTCGCCGGTGACGTTGTCGAGCTTGCCGCAGCCGTCCACCGCTT
TCATGATAGCTTCGGCACCGGCGGCAACATCCACGCGCACGGTACGACCCTGAGCCTGCAGCTCCAGGTTACC
GGCGGGTATTGCGCGCGCTTTGACGTCGCCGGATGGCGTTTTAACCACCACGGCGGAACGGTTGCCAATCAGC
GCGCGCGCCATCGGGACGATGTTTTTCGTCTCATCGGCGTTGAGGTTGAATACGGTGGCGATGCCGTAAGGGT
TGGAGAGGGTCTCGATAACCTTCCCCGGCACGGCGACTTCAATCGCCGCCAGCATCCCCAGCGGAATGCGGTC
GATGTAGAGCACTTCGTCAACGATCGGCAGCGATTTTTCCAGCCGGTTGCTGACCAGCACGCCATCGTCGCGC
TGCAAAATGACGCCGGTAATCTGGTATCCGGCGCGCATTGAGGCGTTGATAACATTGGCGATGTCGGCAAAAT
CGAATGCTGACGATACCACCAGAATATAGGACGAGTCCGCAGGGCGGGTTAACAGCTCCTCCGGCGTAATCGT
GATACCCACACCAAGGCCTGCTCCGCCCGGCGTTTTTGGGTTATGGCCGATCATTGTCGATTCGGTGATGATG
GTTTCGGTAATGGTTTCCATCGCCACATCGCCAATCACCGGCGTGGCTTCGTTAATGCGGATGAGCGAAATAT
CGCTGACATTGATCCCCGCGCGCTTTGCGACGAGGGCGAGCGCTTCCTGAATGCCGAACACGTTACGCAACGT
GCCTTTGATCCCGGTGGTTTCCGCCAGCGCGCTGTGCGTAATCGTCAGTGCGCCAGTCTCATCTTGCCGCGCC
AGTGCGACTTCCGTTGATGAGTTACCGATGTCAATGCCAGCTATATATCGCATACGAAATCCTTAATCGTCGC
CTTTGAGTTTTTTACGCTCAACGTACAGCCCTGCCGCTTCACGAACGAAAGCTGCGCAAATCTTTGCCTGATA
ACGGTTTTCGAGATCGTCGGCGATAGCGAGCAGCTCTTCTTTCGTTGAACGATACGGACGAAGGGCGTTGTAG
ATTTCAAGAATGCGATCGTCCGGTACCGCGGTCAGTTCGGCGGCGCGTTCGAAGTTCATCGCCAGCCGGTCGC
GGCCCGCATCTTTGGCGATAGACGCCTGTAAGCGCAGGGTTTCCGGGGTAATGCGCATATCCTGAGCGGTGAC
TTTATTGCTCAGCACGTTTTCCAGCGTAAAGTCGTCCAGCGTTTTATTGGTGGCGGTTTTCACCCATTCCGGG
TGTTTGTTCGCCAGCGGGTAGTCGCTGACCTTTGCGCTGCGGGACGTGCCGCCTGCCGCAGGAGCCGCTGCTG
GCGCATCGCCCTGCAGGCTGTTCATGCGGCTCAATACGTCCCGAACCATCGATTCAATTGCGTCGGTATTCAT
GGAGTTATCCTTTATCAAAGCGCCACGCGCAGTTCCTGCGGGTTTTTGCCCGTCACGACGTACTTGGTCTCTT
TAATGTGCAAAATGGCCGACTTTGCCTGGTACTTCGGGCGTGCCATCTGGTCATTGAGCGTAGGGACCGGCTG
TGGTGATTCGCGTTTCGCATAACGGGCGGCGTTTTTACCAATCTGACGGTAGGTTTCCAGCGTCAGCAGCGGC
GCCTGTGGGAACAGCTCCAGGTTGGAGAGCGGCGGTAGCCCCTGCTGGTGGATAACCGTAGTGCCTTTCGACT
AGATGCCGATGGAGATGCCGGAGCCGCTCAGGCGGTTACCTTCCACGGCGACGAACGCCACGTCGGAAGATTT
AAAGCAGCGAATCACGCGCGCCCTGATACCTTCTTCTTCAATACCGGCAATGACTTCGCGCAAAATGCTCTTA
TGCGGTAAGCCGACGATATTGACGGTTTGCGCCAGGCCAAATGCCGGGCCGACGGCGATAATGACTTCGTCCT
GCTGAGTGCCCTGGCGCGCTTCGCCCACTTCGGTCAGAAAGCCGTCGCCCGCAGGCGCAGCGGTCTGTGGTGC
TGTGGATACCGCAGGCGCATTAAACGAGACGGGTTTATCGCTGCCCTTCATATCGCGAAGCACGTCTTCAATT
ATCTGGCGCAGCAATTTTTCATTAATTTCCATTTCTCACCCCTTAATCAATCTCGTTGGGATCAAGAGCGCCA
GGGATGTTTTTAATCTCTTCCCAGCGTTCTCCCTGCAGGCGATAACCTGTTGCCGGACCGGCATAGTCATTGA
CGTCATTAACCGCAGAGAGCACTTGTCCGTCGCCGACGATAATGGCGGAGGTGTGCAAATAGTCGCCGGTTAG
CTTGGCTTTCTGGATGTTGAGCATGTCCTGGGCCACGTCGGTAAACCCGCCCTGCGCCAGCGCTTTAACGACT
TCCAGACCGTTGCGGTTTTTATTGATGATTTCTTGGGCGAACTTGATGTCTTCGACGATGTTACGCTCCGGCA
TATCTTTCGAACCGTGGGCATAGGTCGCAGCTTCAACTTCTTCATCGGTAATCGGCGGCAGTCCCATTCCGGC
AAACACGGCCTGCAGCGCGCGGGCGGCTTTGTTACGGATGGCGATAACGTCCTCTTCGCGAACCGGGCGCAGA
CCACCGTCCACTTTCAGGTCACGCTGGATAACGTTGTAGTCGTCAAAGTCTTCCGCATCTTCGTTGGAACCGG
CGAACATGTTGTCGTAGTTCGGCACCGCGGAATAACCAGAGGAGATAAAGTCGGTACCCGGCAGGAACTGCAT
CAGCAGGCGCGCGGTACGACGCATATCGGAGTGGGTGAAGGTCTGGTCGTTACTGGAGGCGCATTCCAGATCC
AGCGAAGAGCAGATCAGGTTTTCCGCCAGCACGGCACGAATGCCTGACGGCACGGCAGACGGTACGCCGATGC
AGCTTACGGAGCCGTTTTGCAGCCCCTGAACGCCCGCGGCTTTGGTGATATAGATGCAGCGCGCTTCCAGATA
CAGCATGGATTTGCCTTCGGCGTAGCCCATCTGCACTTCGGAGCCGGAGCCGGAGGTGAAGCGCATTTTCAGG
CCGCGAGAGGCGTAGGAAGAGGCTAAGAAGCCCTTCGACCATGGGGTATCGTCACCGTCGGTGAAGACCGGCT
CGGTGCCGTAAACGGAGATGGTTTCGGCGTAGCAGGTGTGGCCCAGCATGCCGAGCTTCAGCTCGGTGGCTTC
TTCCAGCGAGCATTGAGTCAGTACCCCCGGACGACCTACCTGAGAACCAACCAGCAGCGCGATGGCGTTGAAC
GGCGCATAGCGCGCTACCGCAACCGTCGTCTCTTGTTCGTCAAACCCGCGCCATGCGCCTTCGGCGGCATCGG
CGGCAATTTGCACCGGGTTGTCTTTAACGTTGGTGACGTGCGCCTGTTGAGATGGAGTACGGCGGGCGCGCAT
TTTCTGCATCGCCATCATCATCTCAACCACGTTCATATGCGAAACCACTTCGACAATTTTCGCTGGGGTCATC
GCGGTGGTTAGCGGAACGATTTCGCTGCGCTTGACGTTCGGATCGCACAGCATGTTAGCCAGCTTGACCGAAT
CCATCGCCATCACTTCTTCGGCGCGGTTCAGGTTGATGCCGTAGCGGGCGATAAAGTGGTCGATCAGGTCGAA
TTCGCTAACCGGTTTTCCGTCCAGCTCGGTTACCGCGCCGTTAACGATTTTTATCGACGGTTTTGGGTCGTTC
GGGCTTTCCATCGCGATAAAGCCTTCTTCGATCCACTCCTTAACAAAGCCGTCCTGATTCACAGGGCGTTTCG
CCAGTGCTTCAAATCTTTTCGATCTCATGAATCAGCCTCGTGGGTATCAGATGTAGGACGGGCGATCGTTTTT
CGGTTCAGAACCGAGGGTCGCAAGGACGGTTTTGCCGATTTCGCGGGCGGAGGTCACCGCCTGACGGACCGCG

CCGGAGTCGCCGGAAATCACCAGAATAGCTTCGTTACTAAAGCTGGTGCCGTGCGCCGGAGAGCTGTACGCCA
CGACTTCAACGTTGGCTGACTTCAGCGCGGTATCGGCCATCAGTACGCCGACGGAAGCCGGAGCGCCGACGAT
AATGCCGCAGGCGCGGCCAATCGGCGCGCCGAACGCTTTTTCCAGCGCATAGCTGGCGCGAGCGGTGTACTGC
AGCTCGATATGTCCGGCTTCGTTGCCGTAAACATCACCGAAGGTGCGGTCCAGCTCTTTCAGCGCGACTTCGA
TACCGCGCTTGACGTCGGATACATCGTTACCGCCTAAAATAATCAGCGAACCGTGGCCCGCTCCGCCTTTGGT
ATCGCGCGGCAGCTCAATGCTGACCACTTCGGTGTTGGTGGCTTTCACCGCTTCGTCCGCCGCCATAATGTGC
GGGCCTGCGCCGGTGCGGGCGCCGAGAATACCGATGGAACGATAGCGCTTCTCAAGCTTCATCGCGTCCAGCA
GGGCGGTATCGACGTTGGCGATAACCAGGCCCAGGGTATCGCCAATGGCGGTCCCGACAAATTCCGTTAAACT
GCAGCTTTTTTCTGCCATAGCCGTCTCTCGTATAGGTTGGGGTTGACCAGGGATGGCCTGTTGTTCCGGCGTT
GCCACACGGGCAATCACCTGCGCCATGATCTGTTCCACCAGCTCATTGCTGCTCATTGGCTAATTCCCTTCGG
TAAGATTTTTTCTACATCGGTGTGAGGGCGTGGGATGACGTGTACGGCTTTCACTTCACCCACGTTGCGTGCG
GCTGCGGCACCTGCATCGGTGGCCGCTTTGACCGCGCCAACATCGCCGCGCACGATGACGGTTACCAGCCCGG
AGCCAATCTTTTCATAGCCCACTAACATCACATTGGCTGACTTAACCATTGCATCAGCGGCCTCTATGGCTGC
GGTTAAGCCTTTGGTTTCTACCATTCCTAGTGCTTCTTGTTGCATAAAGACCTCGCATGGAGTGTTCCGATAG
TTGGGACTATAAGAAGATGCGAGGAAAAAGAATGGCTGACTTGCTGTTGAGCATCAGAAAATAATGAGAAAGC
AATATGACTAAATTGCCTTTTATTTTTATAACTCATTGATAATCAATATTTAATTTATTATTGTCTATATGAT
TATTTTGTTTTTATTATTTCATTTTTACATTTTTGTTAGTGACGAGAGTATTTTTATTTGATATGGCGAAAGA
CGCAAAATGTGATGTTTATTCGAAATTGATAAGTTGGCTTGTGCAATATTTTATTATTTCAACAGGCGTATTT
TATAGCTCAAATTTCCCGTGTGATGGGCTGATAAAAATATCGGAAAAGCAAAATTTTGCTATCGCAACACGAT
TTTTCCGGAAGGCAGGCCCGCAGGTTGCCAACAATTTACAGGCGGCACTTTTTATAGTGAGGTTAGCTGTACT
CTACCGCCAGCCGAAACTTGCGCTGGCCAGGACACGAACTTTGCTCAGAAGGTGTCACATGAATGATTCTCTA
AAGGCGCAATGTGGTGCGGAATTTTTAGGCACCGGACTCTTTTTGTTTTTTGGTATCGGTTGTTTGAGCGCGC
TCAAAGTCGCAGGCGCCAGCCTCGGATTATGGGAGATTTGCATTATTTGGGGGCTTGGAATTTCACTGGCCGT
TTACCTTACTGCCGGAATCTCCGGCGGACATCTTAACCCTGCGGTGACGATTGCGCTGTGGCTGTTTGCCTGC
TTTCCCAAACAGAAAGTATTGCCTTATATTATTGCCCAGTTTGCCGGCGCTTTTGGCGGCGCTCTGCTGGCGT
ATGTGCTTTATAGTAGCCTTTTTACTGAATTTGAGACTGCGCATCATATGGTGCGCGGTAGCGTCGAAAGTTT
GCAATTAGCCAGCATTTTCAGTACCTATCCGGCCGCAGCGCTAAATGTATGGCAGGCTGCGTTGGTGGAAGTG
GTCATTACGTCGATTCTGATGGGCATGATCATGGCATTGACGGATGATGGCAATGGCATTCCTAAAGGGCCGC
TTGCGCCTTTGCTTATTGGGATATTGGTTGCCGTTATCGGCGCGTCTACTGGACCCCTTACCGGTTTTGCAAT
GAACCCGGCGCGTGATTTCGGTCCCAAACTGTTCACATGGTTAGCGGGGTGGGGGAACATGGCGATGAGCGGT
GGGCGAGAGATCCCTTATTTTATCGTGCCGATTGTCGCGCCTGTTATCGGCGCCTGTGCCGGAGCAGCCATAT
ATCGCTATTTTATTGGTAAGAATTTACCTTGTAACCGCTGTGAACTGTAAGAAAATGCGAACCAGATTCACAG
CAATGATGTATCGTAAAAACAGGTGAGATAATTTCGTGATCTGCTTTCACGAACTGATCAAAAGTCTCCCAGC
AATGGGAGACTTTTGATATTGTCATTGTACTTTTTATCAGGGCCAGGATAATGCTAATAAAAAGTGAATGTAA
ATAATGTGTTTTGTTTATAACAATAAATTAACTGAGGGGTTTTATCATGATTTCTGCGAGCGCTCTGAACTCA
GAACTCATTAATAAAATCGCACAGGATTTTGCACAAGCCACCGGTCTGGCGGTTGTGGTTGTCAATATTCATG
GTGATGAAATATCTGAGCTATTCAATTTCACGCCGTTTTGTCAGTTGATGCGGCAGCATCCCCAGCATAGTAC
ACGTTGTCGTATGAGCGATCGCTGCGGGGGACTGGAGGCATCGAAGTCCGATCAACCCTGTATTTATCGTTGT
CATGCCGGTCTAACGGATTTTTCTATTCCGCTGGTAATCGCAGGGCATTTAGTTGGCTTTGTTTTATGCGGGC
AGGTTCGTTTAAGTAATGACGTGGAGCTGGTAAACATTCTTAATGTCGACGATCGCTGGCAGGCTGATCCTGA
GCTCTTGAATGAATTTCGGAATGTGCCAGAGATGGACTATTCACGCGTTATCGCTTCCGCCGATCTTCTTAAA
CTGATAGTTGAGAATTGCCTGAAAAAACAGCTCAATTTCGTGGTTATCAAAGACAATCCGCAGCAATCGGAGG
CAAATAAAACCACTCGTGGTCCCACTCCGCATGACAGTAAAATGAAAAAAGCGCTTCGCTATATTGATGCGCA
TTTGTCCGACGATCTTCGTCTTGAGGATGTCGCCTCACATGTCTATCTCAGCCCCTATTACTTCAGTAAGCTA
TTCAAAAAATATCAGGGAATCGGATTTAATGCGTGGGTAAACCGCCAGAGAATGGTTAGCGCCAGAGAATTGC
TTTGCCATAGTGACTGGAGTATTGCCAGTATTGCGCGTAATTTAGGCTTTTCGCAAACCAGCTATTTTTGCAA
AGTCTTCCGCCAGACCTATCAGGTTACGCCGCAGGCTTATCGCCAGCAGATAAATGAGAATTCTCATCCTCCA
TCGTTATGAATAGCAATATTTTGCTATTGCCGATTTTTGATAGTCTTTTGTATCTTAATCATTTCAGAAAGAA
AATTTATCTGGTGTAACAATAAATTGTCATAGCGCAACAAAATAATAAAATTTAGGGCATAAACTGCATCAGT
TTTTATTTTTGTGATGAAGTACAGTGTCAGGAAAGATAAGTTTTATTACGCTTTGTCGATACGTTTTATCGTC
AATATACCGGTAAGGATGAGTAGATTAACGTCAGATGAGCAACCGCTCAGGCTATTGCTCACAGAAATGTAAA
ACGTAGCACATTATTAATTGCCGTTATGGGTGTGCCGTACAGCCATAACGTAAACCAACAGGTTTGCCACATT
TGTGGTAGGGAAGGGGGTGAAAATCCCCGCAGCCCCCGCTGCTGTGATGCTGACGACCCCGTAAAGACCACTG
ATCGCAAGATTGGGAAGGACGGGCGAGGAGGACGCTAAGCCAGAAGACCTGCCTGTCGGTGATAACCAACAAC

TTCGGTGGGAAGTGGGTTGCGAAGACGCGTACAGTCGAAAGACTGAACATGCGCGTACCTGTATACCCCTACC
ACCCTGAACAGGATCAGGGTAATGGCGGCAAGGCATCACGCATTTATTCTTGCAGGCACAGGAAGCGGTTGCG
GCAAAACCACGGTGACGCTTGGTTTGTTGAGGCTGCTGCAAAAACGCGCGCTGCGCGTACAGCCGTTTAAAGT
TGGGCCAGATTACCTTGATACCGGCTGGCATACCGCTATCTGTGGCGTGGCGTCCCGTAATCTTGACAGTTTC
ATGCTTCCTCCTCCTGTTCTCAACGCCTTATTTTGCGAACAAATGCGGCAGGCGGATATCGCTGTCATTGAAG
GCGTCATGGGGCTGTATGACGGCTATGGCGTCGACCCCAATTACTGCAGCACAGCCGCGATGGCCAAACAGCT
TGGTTGCCCGGTCATCCTACTGGTGGACGGCAAGGCTGTTTCAACGTCGCTTGCGGCTACCGTTATGGGATTT
CAGCACTTCGACCCGACCCTCAACCTCGCGGGCGTGATTGTGAATCGCGTCACTAGCGACGCCCACTATCAGC
TCCTCAAAAATGCCATTGAACATTATTGCTCACTGCCGGTACTGGGGTATGTTCCTCCTTGCGACGGCGTCGC
ATTACCTGAACGCCACCTGGGGTTGATTACCGCCAGAGAATCCCTCGTCAATCAGCAATCATGGCATGATTTT
GCCGCCTCGCTGGAACAAACAGTGGATGTGGATGCGCTACTCTCGCTGAGCGTGTTATCAGCGCTTCCGACGG
GAATGTGGCCTGAGCGCCCCGATAACACAGCGGGGGCAGGGCTGACGCTTGCTCTGGCGGATGATGAGGCCTT
TAACTTTTATTATCCCGATAACATCGATTTTCTGGAGCGTGCCGGCGTTAACATTGTGCGTTTTAGCCCACTG
CATGACCGCGCATTGCCTGATTGTCAGATGATCTGGCTGGGCGGTGGATATCCTGAATTATACGCCGCGGATC
TGGCGGCAAATACGGCGATGTTAAAACATTTGCGGGCGGCGCATCAGCGCGGCGTGGCGATTTACGCGGAGTG
TGGCGGTCTGATGTATCTGGGGAGCACTCTGGAGGATAGCGGTGGGGAAATTCATCAGATGGCGAATATTATC
CCCGGTCACAGCAAAATGGGTAAGCGACTTACCCGCTTTGGCTACTGTGAGGCGCAGGCTATGCAGCCAACGC
TATTGGCGGCGCCTGGCGAGATAGTGCGCGGCCATGAATTTCACTACTCAGATTTTATCCCCGAAACACCCGC
CGTTATGGCCTGTCGTAAGGTTCGCGATGGCCGCGTGTTACAGGAATGGACTGGCGGCTGGCAGACAGGCAAC
ACGTTTGCCAGCTATCTGCACGTCCATTTCGCCCAGCGTCCGGAAATGTTGCAGCACTGGCTGGCGGCGGCGA
GGCGCGTATTATGACGATCCTTGCCTGGTGTATCGCCTGGGTGCTGGATTTTATCATCGGCGACCCTCAACAC
TGGCCCCATCCGGTACGCTGGATAGGCCGATTAATTACGTTTGTGCAGCGTATTGTGCGTCGCTATTGTCCCG
GCGATAAAGCGCTGCGGATTGGCGGCGGCGTAATGTGGGTTGTGGTTGTCGGGGCGACCTGGGGCGTGGCATG
GGGCGTACTGGCGCTGGCGCAGCGGATTCACCCCTGGTTTGGCTGGAGCGTCGAAGTCTGGATGATCTTTACC
ACGCTGGCCGGACGCTCTCTGGCCCGCGCCGCGCAGGAGGTTGAACGACCGTTGCGGGAGAACGACCTTGCGG
AAAGCCGAATAAAACTCTCCTGGATCGTCGGGCGCGATACGTCGCAACTTCAGCCAGCGCAGATCAATCGCGG
CGTGGTGGAAACGGTTGCAGAAAACACCGTTGACGGCATTATCGCGCCGCTCTTTTTCCTCTTTCTCGGCGGC
GCGCCGCTGGCGATGGCCTACAAAGCCGTCAATACCCTGGATTCAATGGTGGGCTACAAACATGAAAAATACC
GGGCGATTGGTATGGTCAGCGCCCGTATGGACGACGTAGCGAACTATCTTCCTGCCCGACTGAGCTGGCTGTT
GCTGGGCATTGCGGCAGGACTGTGTCGCCTGAGTGGTTGGCGTGCGCTGCGTATTGGCTGGCGTGACCGTTAT
AACCACAGTAGCCCAAATTGCGCCTGGTCGGAAGCGTGTGTGGCCGGCGCCTTAGGTATCCAGCTCGGCGGCC
CAAATAACTACTTTGGCGAGCGTGTGGACAAGCCCTGGATAGGCGACGCGCAGCGCGATATTTCCGTGGACGA
TATTTCCCGAACGATTCGATTGATGTGGGTCGCTTCAACCCTGGCGCTGGCGCTGTTTATTGCGGCGCGGTGC
GGGTTATCTGGCGTGGCCTGAGGACGACAGTATGCATTATATCCAGCAGCCCCAAACCATTGAGGCCAACAGT
TTCACCATTATTAGCGACATTATTCGCGAAACGCGTCCTGATTACCGTTTCGCCAGTCCGCTCCATGAGGCCA
TTATCAAGCGGGTTATTCATACCACCGCCGATTTCGACTGGCTGGACATTTTGTGGTTCTCTGCCGATGCGCT
GGAGCAGCTTTGTGATGCGTTACGCCATCCGTGCATTATTTATACCGATACCACGATGGCGCTGTCCGGCATT
AATAAAAGATTACTGGCGACGTTTGGCGGCGAGTGTCGCTGTTATATCAGCGATCCGCGCGTGGTGCGCGCCG
CACAAACGCAGGGGATTACCCGTTCAATGGCCGCCGTCGATATCGCTATCGCCGAGGAGGAGAAAAACAAGCT
CTTTGTTTTCGGTAATGCGCCGACGGCCCTGTTCCGTTTGCTTGAACATAACGTGACGGTGAGCGGCGTGGTG
GGCGTACCGGTAGGCTTTGTCGGCGCGGCGGAGTCCAAAGAGGCACTCACCCATAGCCATTTCCCCGCGGTTG
CCGCGTTAGGGCGTAAAGGCGGCAGTAACGTGGCTGCCGCTATCGTCAATGCGCTGCTCTATCATCTCCGGGA
GGCCTGATGAGCGAGCTTTCTTTTGACGCGCCGGTCTGGCACCACGGTAAAGCGTTACGTAAAGGATATACCA
CCGGTTCCTGCGCGACGGCGGCGGCAAAAGTCGCCGCGCTGATGGTATTACGTCAACATCTGATTCATCAGGT
CTCCATCGTCACGCCGTCGGGCGTCACGCTATGCCTGAACGTGGAGTCGCCGCACATTGAAGGCCAGCAGGCG
ATAGCCGCAATTCGTAAAGATGGCGGCGATGACGTGGATGCCACGCACGGAATGCTGATTTTTGCCCGCGTTA
CGCTCAACGACAGCGGTGAGATTACGCTCACTGGCGGCGAAGGTATTGGTACGGTAACGCGTAAAGGGATTGG
ACTGCCGCTCGGGAGCGCCGCCATCAATCGTACGCCGCGCCATACCATTGAGTCAGCGGTGCGCGAAGCGATA
GGCCCGGCGCGTGGGGCCGATGTGGAGATTTTTGCCCCGGAAGGCGAAGTGCGGGCGCAAAAAACGTATAACT
CGCGGCTTGGCATCCTTGGCGGCATTTCCATTATTGGCACTACCGGCATTGTGACGCCGATGTCGGAAGAAAG
CTGGAAACGCTCACTATCGCTGGAACTGGAGATCAAACGGGCGTCAGGATTAACGCGGGTGATACTCGTGCCG
GGCAACCACGGCGAACGGTTTGTTCGCGAACAAATGGGCGTCGACACACAGGCAGTCGTCACCATGAGCAATT
TTGTCGGCTACATGATTGAAGAGGCGGTACGGCTGGGATTTTGCCAGATAGTGCTGGTGGGCATCCTGGAAA
ACTGATCAAAATCGCCGCCGGGATCTTTCATACCCATAGCCATATTGCCGATGCGCGCATGGAAACGCTGGTC

GCGCATTTAGCTTTACTGGGCGCGCCGCTGGAGTTACTCACCCTGGTCGGCGATTGCGATACCACTGAAGCGG
CAATGGAGCACATTGAAGCATATGGCTTCGGGCATATCTATAACCATCTTGCCAGGCGTATTTGTTTGCGAGT
CATGCAGATGCTGCGTTTTACCAAAACGCCGCCTGTCTGCGACGCCATCCTGTTTTCTTTTGATAACCATATT
CTCGGCAGTAATCGTCCCGTCGACGAGATTGCTAAGGAGCTGCAATGCTAACGGTCGTGGGAATGGGGCCCGC
CGGACGGCATTTGATGACCCCTGCGGCGCTGGAAGCGATAGACCACGCCGATGCGCTGGCAGGCGGTAAACGG
CATTTGGCGCAGTTTCCGGCGTTTGGCGGCGAACGATTTACGCTGGGCGCGGATATTGGCGCGTTGTTGTCCT
GGATTGCCGCCCGCTGGGATAAAGGGATCGTGGTGCTGGCCTCCGGCGATCCGCTGTTTTATGGCATCGGTAC
ACGCCTGGTGGCGCATTTTGGTATCGAACAGGTTCGCATTATCCCTGGCATCAGCGCCGTACAGTATCTGTGC
GCGCAGGCTGGAATTGATATGAATGACATGTGGTTGACCAGTAGCCACGGTCGTTGCGTCAGCTTCGATCAGT
TGGCGAGCCATCGCAAAGTGGCGATGGTCACGGATGCCCGCTGCGGGCCGCGAGAAATCGCGCGGGAACTGGT
CGCGCGGGGCAAAGGGCATCGCTTAATGGTGATTGGTGAAAATCTGGCGATGGAAAATGAGCGGATCCACTGG
CTACCCGTCAGTGCGGTCAACGCCGACTATGAAATGAATACAGTGGTGATCCTGGATGAAAGATGAGCTTTTT
CTGCGCGGCGAGAACGTGCCAATGACCAAAGAGGCGGTACGGGCGCTGGCGCTGTCGAAACTTGAGCTCCACC
GCGCCAGTCACCTGATTGACGTCGGGGCGGGAACGGGAAGCGTCTCAATTGAAGCGGCGCTGCAATTTCCTTC
GTTGCAGGTGACGGCTATCGAGCGTAATCCTGCCGCGCTACGGCTGCTTGATGAGAATCGTCAGCGCTTTGCC
TGCGGCAATATCGATATTCTCCCCGGCGAAGCGCCAATGACCATAACCGGGAAAGCCGATGCGGTGTTTATGG
GCGGCAGCGGCGGACATCTGACGGCGCTGATTGACTGGGCGATGGGCCATCTGCATCCCGGTGGCCGTCTGGT
GATGACCTTTATCTTGCAGGAAAACCTTCATAGCGCGCTGGCGCATCTTGCGCACATCGGCGCATGTCGAATG
GACTGTGTACAGCTTCAGCTTTCATCGTTAACGCCGCTGGGGGCGGGGCACTATTTCAAACCGAACAATCCTG
TTTTTGTTATCGCCTGTCAGAAGGAAGAAAACCATGTCAGAGACATTTGATCCACGCTGTGTATGGTTCGTCG
GCGCCGGGCCGGGCGATCGCGAGCTGATCACGCTTAAAGGCTACCGCCTGCTGCAACAGGCGCAGGTAGTTAT
CTATGCCGGTTCGCTGATCAATACCGAACTGCTGGATTACTGCCCCGCGCAGGCAGAACGCTACGACAGCGCC
GAACTGCATCTTGAGCAGATTATCGAGCTGATGGCGGCGGGCGTGAAAGCGGGAAAAACGGTGGTGCGTTTGC
AAACGGGCGATGTCTCGCTGTACGGCTCCGTACGTGAGCAAGGCGAAGAGTTAACCCGCCGTGGTATCGACTG
GCAGGTAGTGCCTGGCGTCAGCGCGTTTCTCGGCGCTGCGGCGGAGCTGGGTGTGGAATATACCGTACCGGAG
GTATCGCAGAGTCTCATTATTACCCGTCTGGAAGGGCGTACGCCAGTACCGGCGCGCGAGCAACTGGAGGCGT
TCGCCAGTCACCAGACGTCGATGGCGATTTATCTCTCCGTTCAGCGTATTCACCGAGTGGCGGAACGTCTCAT
TGCCGGCGGTTATCCGGCAGCTACGCCTGTCGCGGTCATCTATAAGGCTACCTGGCCGGAAAGCCAGACCGTT
CGCGGCACGTTAGCGGATATTAGCGACAAAGTACGTGATGCTGGCATCCGTAAGACGGCGCTGATTCTGGTCG
GGAATTTTCTGGGTAAGGAGTATCACTACTCAAGACTTTATGCAGCGGACTTTAGCCATGAATACCGTAAAGC
CTGAATCCATTGCGTTGTTTTGCCTGACGCCCGGCGGCGTGGCGCTGGCAAAGAGACTCTCCGCGATGCTGCC
GTTAACCTGCTTTACCAGTGAAAAACTGCGGGAAGAGGGATTTATTCCCTTCGATGGCGGATTCGCTAATACC
GCCCGGCAGGCTTTTACCACTTATACCGCGCTTATTTTTATCGGCGCGACCGGCATTGCCGTTCGTGTCCTGG
CGCCGTTAGTGAACGACAAGTTCAGCGATCCGGCGGTGGTCGTCATTGATGAACGAGGTCAGCATGTCATTAG
CCTGCTTTCCGGTCATGCGGGCGGGGCCAATGCCTTGACGCGCTACCTGGCAGGAATGTTAGGCGCCGATCCG
GTGATTACCACGGCAACGGATGTCAATGAGATGTCCGCGTTGGACACCTTAGCTTTCCAGCTTAACGCCCGCA
TGTCCGATCTTCGCACGGCGGTAAAAACCGTTAACCAGATGTTGGTCAGTCATCAACGTGTGGGGTTATGGTG
GGATGCCGAACTAACGGAAGAGATCGGCCAGTGCGATATTCGCGGTTTTATCCCTGTTGATGATTTGCAGCGG
TTGCCTGAGCTGGATGCGCTTATCTGCGTCTCTTTGCGTAATGACCTCCCTGAGCTTCCCGTACTGCACTGGA
AACTGGTGCCCCAGCGTGTGGTGGCGGGAATTGGCTGTCGCCGCGATACGCCATTTCCCCTGTTAGCGACATT
ACTGGCGCGTCAGCTTGAAGCGCAGAAACTCGATCCGCTGGCGTTAAAAGCGATTGGCAGCGTCACGCTCAAA
AAAGGGGAGCCGGGGCTTATTCAGCTCGCCTCCTGCTGCCGCGTGCCTTTTAAAACCTTTACCGCCGAAGCGT
TGCGTGAATTCGAACACCATTTTCCCGGTTCTGGCTTCGTCAGAAAAACGGTGGGCGTTGGCAGCGTATCCGG
CCCGGCAGCGTGGTTATTAAGCCAGGGACAATTGTTAGGCGAGACCCTGCGAGAACAGGGCGTCACTATTACT
TTGGGAGTTTCACACTGATGTTAAGCGTAATCGGAATTGGCCCTGGCTCACAGTCAATGATGACCATGGAAGC
GATTGAGGCGTTACAGGCGGCGGAAATCGTCGTGGGCTACAAAACCTATACCCATCTGGTTAAAGCATTCACC
GGCGATAAGCAGGTGATCAAGACCGGCATGTGTAAGGAAATTGAACGCTGCCAGGCGGCTATTGAACTGGCGC
AGGCTGGGCATAACGTAGCGCTCATCAGCAGCGGGGATGCGGGAATTTACGGCATGGCGGGTCTGGTGCTGGA
ACTGGTCGGCAAGCAAAAACTGGACGTCGAGGTGCGGCTCATTCCTGGCATGACCGCCAGCATCGCCGCCGCC
TCTTTACTGGGCGCGCCGTTGATGCATGACTTCTGTCATATCAGCCTCAGCGACCTGCTGACCCCGTGGCCGG
TTATTGAAAAACGTATTGTTGCCGCCGGAGAGGCAGACTTTGTTATCTGTTTTTACAACCCGCGCAGCCGCGG
GCGCGAAGGGCATCTGGCGCGCGCGTTTGACCTGCTTGCCGCCAGTAAAAGCGCGCAAACGCCGGTTGGCGTG
GTGAAATCCGCCGGACGTAAGAAAGAGGAAAAATGGCTGACTACGCTCGGCGATATGGATTTTGAACCGGTAG
ATATGACCAGTCTGGTCATCGTTGGTAATAAAACGACCTATGTTCAGGATGGGCTGATGATAACGCCGCGAGG

TTACACGTTGTGAATGAGGGGGAGGTGCTGGTGGTGGGCGGCACCAGCGATGCGCGCGCGTTATGCCGACAAC
TGGATGCGGCGAACGTCGCGTACACCTTATCCGTGGCGACGCCAGCCGGAAAAGCGCTGGCTGGCGACATTAA
AGGACAGGTGCGCTGTGGTCGTCTGGAGTACGGGCAGATGGTCGCCTGGCTAAAAGAAAACCGTACTCGCTGG
GTAATAGATGCATCGCATCCTTATGCAGAAATGGTCAGCCATAACCTCCTTCGCGCCTGTGAAACGGCGGGCG
TGTTGTTAAGTCGTTATCAGCGTCCGGAGCAGCTTAGCAATCTGACGCATCCGCTTCTCTATACGGCGCGCAG
TATTGCCGATGCCTGCGAGATAGCGAGGCGTTTTGGTCCGCGCGTGTTACTGACGACCGGCAGTAAAGATCTG
GCCGTCTGGCGTGCCGGGCTGGCGGAAAAAACGTTGCTGGCGCGCGTACTGCCCGTGGCGGAGGTGATTCAAC
GCTGCAGCGAACTGGGGTTCGGCGTTGGAGAAATTTTCGCACTGTGTGGGCCGTTCAGCGCTGATTTTAACGC
CGCGTTTTACCACCAGTGCCGGGCCGACGTAGTCATTACCAAAGCATCCGGCGCGGAGGGGGGATATCAGGAA
AAAGTTCAGCCCTGTCTGGATGCGGGCATTCCCTGCATCGTTATTGCGCGCCCGACGCCGCTTGTGACGGGTG
ATGAGTTACTGGAAAGCCAGGCCGCCTTTGCGCAGCGTTTATCGCGCTGGCTGGCCGCAGCTAAGGAGTAAAA
ATGAAAAAAGCGCTTCTGGTGGTCAGTTTTGGCACCAGCTATCACGACACCTGTGAAAAAAACATTGTGGCGT
GCGAACGTGATCTGGCGGCGTCCTGTCCCGATCGCGACCTGTTTCGCGCCTTCACTTCCGGAATGATCATTCG
CAAGCTTCGCCAGCGTGACGGTATCGACATCGATACGCCATTACAGGCTTTGCAAAAGCTTGCCGCACAGGGA
TATCAGGACGTGGCGATTCAGTCGTTGCACATTATTAACGGCGACGAATATGAAAAAATTGTCCGTGAAGTAC
AACTCTTGCGTCCGTTATTCACTCGCCTGACGCTGGGCGTGCCGTTGCTGAGCAGCCATAACGATTATGTGCA
GCTAATGCAGGCGTTGCGACAACAGATGCCATCGCTCAGACAAACGGAGAAAGTCGTTTTTATGGGCCACGGC
GCCAGCCATCACGCATTCGCAGCTTATGCCTGTCTCGATCATATGATGACCGCGCAGCGCTTTCCGGCGCGCG
TCGGCGCCGTAGAAAGCTACCCGGAGGTCGACATCCTGATCGACAGCCTGCGTGACGAAGGCGTCACCGGCGT
ACATTTGATGCCATTAATGCTGGTGGCGGGCGATCACGCCATTAATGATATGGCTTCAGACGACGGCGACTCA
TGGAAAATGCGCTTTAACGCGGCGGGCATTCCGGCGACGCCGTGGCTGAGCGGGCTGGGAGAAAACCCGGCTA
TCCGGGCGATGTTTGTCGCGCATTTGCGCCAGGCGCTGAACATGACGGTAGAGGAGGCGGCATGAACGGTAAA
TTATACGCATTAAGTACCGGGCCAGGCGCGCCCGACCTGATAACCGTCCGCGCAGCGCGGATTCTTGGCTCGC
TGGACATTCTGTATGCGCCCGCCGGTCGTAAGGGGGGCGATAGCCTGGCGCTGTCGATTGTGCGCGACTATCT
CGGCGAGCAGACGGAAGTACGTTGTTGCCATTTCCCTATGAGCGCTGACGGCGCCGAGAAAGAAGCGGTCTGG
AACGAGGTTGCCGCCGCGCTTACCACAGAGGTGGAAGCGGGTAAACAGGTCGGTTTTATTACCCTGGGCGATG
CGATGCTATTCAGTACCTGGATTTTTTTACTCCAGAGAATCGGCTGCCCGGAGTGGCTGGAGATTGTCCCCGG
CGTCACGTCCTTCGCCGCGATAGCCGCCCGCGCGAAAATGCCGCTCGCCATAGAACGGCAATCGCTGGCGGTT
ATTTCTTGTACCGCGCCGGAGGCGGAAATAGCCCAGGCGCTACAACAGCATGATAGCCTGGTGTTGATGAAAG
TGTATGGCCGATTCGCCCGTATTAAAGCGTTGCTGGCGCAGGCTGGCCTGCTGGAATGCGCCTTAATGATGTC
CGAGGCCACGCTGCTCGGCGAGCAGTGCTGGCGCCACCTCCACGAGGTCAACGATGATCGAGCGTTGCCCTAT
TTTTCGACCATTCTGGTCAATAAACAGTGGGAGTATGCAGAATGAAACTTGAACAGCAGCTTAGACAGTTGTC
TTTCAGTGGACTGGCTGCAGCGCTATTGCTGATGGTTGTCCCGCAACAGGCGTTTGCGATGCATATCATGGAG
GGCTTTTTACCGCCAGTGTGGGCGCTGGCCTGGTGGTTACTGTTTTTACCCTGCTTGTGGTATGGGCTGGTAC
GCTTACGGCGTATTGTGCAGGAGGATAATCATCAGAAGGTACTGCTGGCGCTGTGCGGCGCGTTTATTTTTGT
CCTGTCCGCGCTCAAAATTCCGTCGGTAACGGGGAGTTGTTCACATCCGACCGGCGTTGGTCTGGCGGTTATC
TTGTTCGGGCCTGGGGTGGTGGCGATTCTCGGCGCGGTTGTGCTGTTGTTTCAGGCGCTATTGCTGGCGCATG
GCGGCCTGACGACGCTTGGCGCAAATGGAATGTCGATGGCGGTGATTGGCCCGGTTGTCGGTTATCTGGTGTG
GAAAATGGCCTGCCGTGCCGGACTGCGCCGCGATGTCGCCGTTTTTCTGTGCGCGATGCTGGCGGATTTGGCG
ACCTATTTTGTGACTTCAGTCCAGCTTGGCGTTGCGTTTCCAGATCCCCATGCCGGAGCGACAGGGTCTGTTG
TGAAGTTTATGGGGATTTTCTGCCTCACACAGATTCCTGTCGCTATTGCTGAAGGTTTATTAACCGTCATGAT
TTATGACCAGTTAACCAAACGGCAAGTGATTACCGTACAAGGACATTAAAATGAAAAAAACGTTGATGTTGTT
AGCGATGGTGGTGGCGTTGGTGATTCTGCCGTTTTTTATTAACCATGGCGGTGAATATGGCGGTTCAGACGGC
GAAGCGGAAAGCCAGATTCAGGCTATTGCGCCGCAGTATAAACCCTGGTTTCAGCCGTTATATGAACCTGCCA
GCGGTGAAATTGAAAGCCTGCTGTTTACCCTTCAGGGCTCTCTTGGCGCGGCGGTGATTTTTTATATCCTGGG
GTATTGCAAAGGTAAACAACGCCGTGATGACCGGGCTTGATCGACTCAGCTATCAAAGCCGCTGGGCACACGT
TGCGCCGCAGCGCAAGTTTTTGCTGTGGCTGGCGATGATAATACTGGCGTTTGTTCTGCCGCCTGTAGGGCAG
GGCATTGAGCTGCTGATCATTGCCGGATTGAGCTGTTGGCTATTGCGCATATCCCTCTGGCGCTGGTGTCGAT
GGATGGCGATACCTTTTGGATTTTTGTTGGTCGGCGTGATAACGATTATCTTCAGCATCAGCCGCGAGCCGCA
AATGCTGCTGGCTGGCATATCTGTTGGGCCGTACTGGATCGGGATTACCCGTGCGGGCGTGGTTACGGCGAAT
GAAACGTTCTGGCGTAGCCTGACGGCGCTGTCCGCGACGTTGTGGCTGGTGATGAACCTACCGTTTCCGCAGT
TAATTAGCTTGCTTAAGCGCGCGCATATTCCACGCCTGCTTACCGAGCAAATCCTGTTAACGTGGCGCTTTCT
CTTTATTCTTCTGGATGAGGCGGCGGCAATTCGTCGCTCGCAAACGCTGCGTTTTGGTTATTGCAGCCTGCCG
AACGGCTATCGATCTCTGGCAATGCTGACTGGACTCCTGTTTACGCGGGTGTTAATGCGTTATCAGCAAATGA

CTACCACGCTGGATATCAAACTGTATCAGGGTGATTTTCACCTGTAAGGACTATTATGCTTGCCACTTCAGAC
CTGTGGTTCCGATATCAGGATGAGCCGGTACTTAAAGGGTTAAATCTGGATTTTTCGCTCTCGCCTGTTACCG
GTTTGGTGGGCGCGAACGGCTGTGGGAAGTCTACGCTTTTTATGAACCTGAGCGGCCTGTTGCGTCCGCAAAA
AGGGGCCGTGTTGTGGCAGGGAAAACCGCTGGATTACAGCAAGCGCGGACTACTGGCGCTGCGCCAGCAAGTT
GCGACGGTTTTTCAGGACCCTGAACAGCAGATTTTTTATACTGATATCGATAGCGACATTGCGTTTAGTTTAC
GTAATTTGGGGGTGCCGGAGGCGGAAATCACGCGCCGCGTCGACGAGGCGCTAACGCTGGTTGACGCCCAACA
TTTTCGCCATCAGCCCATTCAGTGTTTGAGTCATGGGCAAAAAAAACGTGTGGCTATCGCCGGAGCGCTGGTG
CTACAGGCGCGCTATCTATTGCTGGATGAACCCACTGCTGGTCTCGATCCCGCAGGGCGCACTCAGATGCTTG
CTATCATCAGGCGGATTGTGGCGCAGGGCAATCATGTCATTATCTCCAGCCATGATATCGATCTTATTTATGA
AATTAGCGATGCCGTATACGTGTTACGCCAGGGCCAGATCCTGACGCATGGCGCGCCTGGCGAGGTTTTCGCG
TGTACGGAAGCGATGGAACAAGCGGGGCTAACCCAGCCCTGGCTGGTCAAACTTCATACTCAACTGGGACTGC
CGCTGTGTAAAACAGAAACGGAGTTTTTTCATCGAATGCAAAAGTGCGCATTCAGGGAGGCGTCATGACGCAG
GCAGTTATGTTGCAGGGGACGGCATCCGACGTCGGCAAAAGTGTGCTGGCGGCGGGTTTATGCCGCATTTTTT
ATCAGGATGGTCTGCGTACCGCGCCGTTTAAATCGCAAAATATGGCGCTTAATTCTGGTATTACGTCGGATGG
TAAAGAGATGGGGCGGGCGCAGATTTTTCAGGCGGAAGCCGCGGGGATCACGCCAGATGTGCGTATGAACCCG
GTGCTGCTCAAACCGACCAGCGATCGCCAGGCGCAGGTCGTGCTGATGGGGAAAGTGGCGACCAATATGGATG
CGGTGAGTTACCATGACTATAAACCGCGTTTGCGTGAGCAAATCCTTGCGGTCTATAACAGCCTGGCGCAGGA
ATATGACGTTATTGTGCTGGAAGGCGCCGGAAGCCCGGCGGAAATCAATCTGCGCGATCGCGATATCGTCAAT
ATGGGAATGGCGGAAATGGCCCAGTGTCCGGTTATTCTGGTGGCGGATATCGACCGAGGCGGCGTATTCGCCG
CTATTTATGGCACGCTGGCGCTGCTGCATAAACAGGAGCGTGACAGAGTAAAGGGCGTCATTATCAACAAATT
CCGCGGCGACGTGGCGCTACTCTATTCCGGTATCGAACAAATCGAATCGCTTACCGGCGTCCCGGTCCTGGGA
GTGATGCCGTGGCTGGATGTGGATCTGGAAGATGAAGATGGCGTCGCGCTGCAAAACGATAAATATCGCGGTA
ACGCCCCCCGTGATATCACTATTGCCATTGTGCAACTGCCACATATTTCTAACTTTACGGATTTTAACGCTCT
GGCGGCGCAGCCGGATGTGCGCATACGCTATATTCGCCGCCCGGAAGCGTTGACTGACGCTGATCTGGTGATC
CTGCCGGGCAGTAAGAATACGCTGAGCGACCTCGCCTGGCTGCGCGAAAGCGGGATGGCGGATGCGCTATTGC
AGACACATCGGCAGGGCGTGCCGGTGATGGGAATTTGCGGTGGCTACCAGATGCTGGGCGACACTATTGTGGA
TGAGGTGGAGTCGGGGCTGGGTACGCAGCCGGGCCTGGGGCTGCTTAATACGATCACTCGCTTTGCACAGGAT
AAAATCACGACGCAGGTTAACGCGACAATGTCAGGCGAGCTGCCCAGCTGGCTGGCTGCCGCGGCGGGGCTAC
CGGTACGCGGTTATGAAATCCACATGGGGGAAACGGTGCTGCAGGAAGGATGCTGTACGGCCATGACGTTACA
GAAAAATGGATGCTCTGTGGCGGATGGCGCGGTCACCGCCGACGGACTGGCGTTTGGCACTTACCTTCACGGC
CTGTTTGACAGCGATGCGTTTACCCGTGCGGTGGTCAATGGGTTACGGGCACGAAAAGGGCTTGCGCCGTGGG
AAACGACTTTTTGTTATGCGGAGCATAAAGCGCGGCAATTTGATCTGCTGGCGGAAGCGATGCGGCAACATAT
TGATATTGATAAAATTTATACCATCATGCAACAGCATCAGGAGCCGGTATGATGATTCTGGTGACGGGCGGGG
CGCGTAGTGGTAAAAGCCGTCATGCTGAAGCCTTAATTGGCGATGCGCCGCAGGTACTGTATATCGCCACCTC
GCAGATTCTTGATGACGAGATGGCGGCGAGAATTCAGCATCATAAAGATGGCAGGCCGGCACACTGGCGGACC
GCAGAATGCTGGCGGCATCTTGATACGTTGATTACCGCGGATCTTGCCCCTGACGACGCGATTTTGCTGGAAT
GTATTACCACCATGGTGACGAATCTGCTGTTTGCGCTGGGAGGCGAAAACGATCCCGAACAGTGGGATTACGC
GGCGATGGAGCGCGCCATTGACGATGAAATTCAGATTTTAATTGCAGCCTGCCAGCGCTGCCCGGCGAAAGTG
GTACTGGTGACAAATGAGGTGGGAATGGGGATCGTCCCGGAAAACCGTCTGGCGCGCCATTTTCGTGATATTG
CCGGTCGGGTCAACCAACGACTGTCGGCAGCGGCGGATGAGGTCTGGCTGGTGGTCTCAGGTATTGGAGTCAA
AATTAAATGAGTAAGCTGTTTTGGGCCATGCTCGCTTTTATTAGCCGCTTGCCCGTGCCGTCACGCTGGTCGC
AGGGACTGGATTTCGAGCAGTATTCGCGTGGGATCGTGATGTTTCCCTTTATCGGGTTGATTCTGGGAGGGGT
AAGCGGCCTGATTTTCATCCTCCTGCAACCCTGGTGCGGTATTCCGCTAGCGGCGTTGTTCTGTATTCTGGCG
CTGGCGTTGCTGACCGGTGGTTTTCACCTGGATGGGTTGGCCGATACCTGCGATGGTATTTTTTCCGCGCGCC
GCCGTGAGCGAATGCTGGAGATTATGCGTGATAGTCGTCTGGGAACCCATGGCGGGCTGGCGCTTATTTTTGT
GCTACTGGCAAAAATTCTGGTGGTCAGCGAACTGGCGTTACGTGGAACGCCAATGTTGGCGGCGTTGGCGGCG
GCCTGTGCGGCCGGACGCGGCAGCGCCGTTTTATTGATGTACCGTCATCGCTACGCCCGTGAAGAGGGGCTTG
GCAATGTATTTATCGGTAAAGTCAGCGGACGGCAAACCTGCATTACTCTGGGATTGGCGGTAATCGTCGCCAC
GGTATTACTGCCTGGTATGCAAGGGTTGGCCGCAATGGTGGTCACATGCGCGGCGATTTTCATCCTTGGCCAG
TTGCTTAAACGTACGCTTGGCGGTCAAACCGGCGATACGCTGGGCGCGGCGATCGAACTTGGTGAATTGATCT
TTCTGCTGGCTCTGTTATGAGCCGATTATTACGGACGAGACATCTTATGCAGACACTACACGCTTTACTCCGT
GACATTCCTGCGCCGGACGCCGAGGCGATGGCGCGTGCGCAGCAACATATTGACGGCCTGCTCAAACCGCCGG
GCAGCCTGGGCAGACTGGAAACCTTAGCCGTTCAGCTCGCGGGTATGCCGGGTCTTAACGGTACGCCGCAGGT
AGGTGAAAAGGCGGTGCTGGTGATGTGCGCCGACCATGGCGTCTGGGATGAAGGCGTAGCGGTTTCGCCCAAA

ATCGTGACGGCGATTCAGGCGGCGAATATGACGCGGGGAACAACCGGCGTATGCGTGCTTGCCGCTCAGGCCG
GTGCGAAGGTGCATGTCATTGATGTCGGTATTGATGCCGAACCTATTCCTGGCGTAGTTAATATGCGCGTCGC
GCGCGGCTGCGGAAATATTGCCGTTGGCCCGGCGATGAGTCGCTTGCAGGCTGAGGCGCTTTTGCTGGAGGTT
TCCCGCTACACCTGCGACCTGGCGCAACGCGGCGTGACCTTATTTGGCGTAGGGGAGCTGGGAATGGCGAACA
CTACGCCAGCCGCCGCGATGGTCAGCGTTTTTACAGGAAGTGATGCAAAAGAGGTGGTGGGGATTGGCGCGAA
TCTTCCGCCTTCCCGCATCGATAATAAAGTGGACGTCGTGCGGCGGGCGATTGCGATTAATCAGCCCAATCCG
CGCGACGGCATTGATGTGTTGTCGAAGGTGGGTGGTTTTGATCTGGTCGGGATGACCGGCGTGATGCTTGGCG
CGGCAAGGTGTGGCTTACCCGTATTGCTGGATGGCTTTCTTTCCTACTCGGCAGCGCTGGCGGCCTGTCAGAT
TGCGCCTGCGGTGAGACCTTATCTGATCCCGTCGCACTTTTCGGCGGAAAAGGGCGCCCGCATCGCGCTCGCG
CATTTGTCTATGGAACCCTATTTGCATATGGCGATGCGGTTAGGTGAAGGAAGCGGCGCGGCGCTGGCGATGC
CGATCGTGGAAGCCGCGTGCGCCATGTTCCACAACATGGGGGAGTTGGCGGCCAGTAATATTGTCCTGCCGGA
GGGGAACGCAAACGCAACATAACCCGGGCGCACAACACCAGCCATGTGAGAAAAAAGCTATACTTAACGCGCA
CCTGCCGCGACAAAGGAAGGGATATAATGCGTCGTATCACACCTTTTTTTCCGTTTTTCGTCTTGTTGGTAAG
CCATTTCTCGCTGGCTATAAGCTATCCGTTGCCGCCCGAAGGCAGTCGTCTGGTAGGACAGCCTGTCACAATC
GCCGTACCGCAGAACAATACTCAGCCCTTAGAGTCATTTGCCGCCCGCTACGGTCAGGGGCTGAGTAATATGC
TTGAAGCGAATCCCGGCGTGGATGTGTTTTTACCTCAGTCTGGTTCCACACTGGTGGTGCCGCAGCAGTTGAT
TCTGCCTGACACCGTTCGTGAAGGTATTGTGGTAAACGTCGCCGAGATGCGCCTTTATTATTATCCCGCAGGC
ACGAATACGGTTGAGGTGCTACCTATTGGTATTGGTCAGGCTGGCCGTGAAACGCCGCGCAACTGGGTAACTG
CGGTTGAACGCAAACAAGATGGCCCGGTTTGGGTGCCAACGGCTAATACGCGTCGGGAATATGCTAAAGAGGG
AAAAACGCTGCCCGCGATGGTGCCTGCGGGGCCGGATAACCCTATGGGGCTATACGCAATATATATCGGCAGG
TTATATGCCATCCATGGCACTAACGCTAACTTTGGCATTGGCCTTCGCGTCAGTCAGGGCTGTATCCGTTTAC
GCAATGACGATATTAAATATTTATTCGACCATGTTCCTGTAGGAACCCGCGTTCAGATTATCGACAGGCCCGT
TAAATTTTCGGTAGAGCCGGATGGCAGTCGTTGGCTGGAGGTACACGAACCGCTTTCTCGCAACCGTGCTGAA
TTTGAGTCAGACAAAAAAGTTCCATTGCCCGTTACGCCAGTGCTGCGCACGTTTATCAAAGGGGATGACGTTG
ATACTTCGCGGGTTAATGAGGTGCTGGAGCGCCGTTCCGGAATGCCGGTCAACATTAGCGCGGGCAGGCCTGG
CCTTTAAATATCCTGTCGCATTGCTGGTTTTCATGGGCTGGAGTATCTGTATTAGTAGTGAGTGTCATGGCAG
GGGCTTCATTTTCTCAACGAACCGCTAGCCCTCCGATTAAGTCCCCAAAAACTATGGGCGTCAAAAAAACCCGG
CAAACCTTATCAGGTCAACCGGGTAAAGATAAGTTCTTGTTTTTTAAACGTCTCCGGACTCACCAGGATGTCC
CGAAACTAAAATCTGGCTCCTCTGACTGGACTCGAACCAGTGACATACGGATTAACAGTCCGCCGTTCTACCG
ACTGAACTACAGAGGAATCGTGTGGGGTCGTATATTAGCGGCGGAAAAATTTTTGTCAAACCCCATTTAACCT
CTTACAGATTCGATTGCGGGTTATCTCATCACTTTGTTGCATTTGCAGACATTTTTTCTGCAAAAAGCTTTGC
AGCCTTACGATTTCTCGCTCATTATTGAAATGTGGTTTCAACTCTCTTCTTTAAGGTCCATTTTGAACGTCTC
CACTGCCTTACGCCAGGCTGTCGTCCGTACGCCCTGGTATGCCAAACGCAAGAGCTACAAAGTGTTGTTCTGG
CGTGAAATCACCCCACTTGCTATCCCTATTTTTCTGGAAAATACCTGTGTTTTGCTAATGGGCGTGCTCAGTA
CTTTTCTCGTCAGTTGGCTGGGCAAGGAAGCAATGGCGGGCGTGGGGCTTGCCGACAGTTTTAATATGGTAAT
TATGGCTTTTTTTGCGGCTATCGATCTTGGTACTACGGTGGTGGTTGCCTTTAGCCTGGGCAAGCGCGACAGG
CGACGGGCAAGGGCGGCGGCGCGCCAGTCGCTGGTGATTATGACGCTATTTGCCGTTGTGCTGGCAGTGGTCA
TTCATTATTTCGGCAGTGAAATTATTAATATTGTCGCAGGCGAGGCGACGTCAGAAGTAAAGGGGCTGGCGTT
AACGTACCTTGAACTGACAGTGCTGAGTTATCCGGCTGCGGCAATTGCGCTAATCGGTAGCGGCGCGCTGCGT
GGGGCAGGGAATACGAAAATCCCGTTGATGATTAACGGCGGGATGAACATTCTCAATATTATCATCAGCAGCA
TCCTGATTTACGGGGCTTTCTCCTGGCAAGGGCTGGGTTTTGCCGGCGCGGGGCTGGGATTAACCATTTCGCG
CTACATCGGCGCAGTAGCGATTATTTGGGTGCTGATGATTGGTTTTAATCCGGCGCTGCGCATTCCGCTGAAA
AGCTATCTGAAGCCGCTGAATTTCGGCATTATCTGGGAAGTGATGGGTATCGGTATTCCGGCGAGCATTGAAT
CAGTGCTGTTCAACGGTGGCAAGCTACTGACGCAAATGTTTGTCGCCGGAATGGGCACTAACGTTATTGCGGG
TAACTTTATTGCCTTTTCCGTGGCGGCGCTTATCAACCTGCCGGGTAACGCCCTTGGTTCGGCGTCGACTATT
ATCACCGGTAAGCGTCTTGGTACCGGACAAATTGGTCAGGCAGAGCGTCAATTGCGCCATGTATTCTGGATGT
CGACTATCGTACTTACGGCAATCGCCTGGGGGACAGCGCCGTTCGCGGGTCTGTTTGCCTCATTTTATACCCA
GGAGCAGGACGTAAAAGAGGTAGTGAAAGTTCTGCTCTGGCTTAATGCTGTCTTTATGCCAATTTGGGCGGCC
GCGTGGGTGTTGCCGTCGGGTTTTAAAGGCGCGCGCGATGTGCGATTTGCGATGTGGGTATCGATGCTGGGGA
TGTGGGGCTGTCGCGTTGTGGCAGGGTATACGCTTGGTATTGTGCTGGGTATGGGGGTTGTAGGGGTTTGGCT
GGGGATGTTTCTTGACTGGGCCGTGCGCGGCGCGCTGTTTTACTGGCGTCTGGTAAGCGGGCGCTGGCTGTGG
AGATACCCGCGCGTAAAGAGGGAGTAAGCGCTTTTTGCTGTGAAAATCATCAGGAAACGGTGAATTTTAACCG
AAGTGTGGAATAAAGCAGCAAACGAACTTTTTTTGTGAAAACAGGCTTTGACAAGCCAGGGGCACCTCGTTAA
TATTCGCCCCGTTCACACGATTCCTCTGTAGTTCAGTCGGTAGAACGGCGGACTGTTAATCCGTATGTCACTG

GTTCGAGTCCAGTCAGAGGAGCCAGATTTTAGTTTCGGGACATCCTGGTGAGTCCGGAGACGTTTAAAAAACA
ATAAATTATCTTTACCCGGTTGACCTGAGAAGGTTTGCCGGGTTTTTTGACATCCATAGTTTTTAGGGGCTTA
ATCGGGGCCTCGCGGTTCGCTGAGAAAATGGAGCCCCCACCTATGGCATTAACCGATACCGCTATCCGCAAGA
TCAAACCCGTATCAGTATGCTGACATATAGATGTGTTCGGTTCAAACGGAGCGATCACACAAGCTTGCCAGGT
GATGCCCCTGGTGGGAATCGCGACTGTCTCTTTCATCTGATTTCAGATATTTCCCGTGATCTTGATCCGTTCT
TTCCCAAAAGATTGATTTCTTTCATTCGAAACAAGGATGTACAATAAGTAAACCAAAATAGTAACTATAGAGT
TTCTAATGGATACAGTAGAAGAACTAAATGGAACATACTTCTATGCAGGAAAATCCAATCTGACTGCTGCCGA
ACTTCTGTTATGATTTTCTGTGAGAATACCGCAAGCCAGTTTGGGATTGGTGTTGCAGATTTCGGGGCTGTTG
TTGCTTTGGCATCAGGTCGTAATAATCTTTGGACAAGGGCGAAACCCGCTGGAGCGACTAAAGGCACATCATA
CGCGTCCAGGACTGCACGTGCAGTGTTTAAAAAGTCAAAATTTCCCTTTGGAATATCCCTTCCGACATGGTTA
GGCGGTTACACCCCGTGGACAGCCAGACGTGTTATGGTTCGTAACATTGCGCCGTTTGTTGGACGTTTCATTC
CTTTGATCGGGGAAATTATCCTTGCCGCAGATGTGTCACAAATAACATATCGCACTATACGCGACTACAATAC
CATTGCTCGGGGCAATGATAAGTTATGGTAGACAATATTGAACAGCAAGTTTACGAGCTTGTACGGCCTTATG
CTGGCACATATCTGTTCAACATTAAGCAAGTGGAATTAACACCAGAGATTGACCTTGATACTGATCTGAGCAT
TGATGAACTTGAAGCCGAAGATCTCATGAATAAATTCTTCGAGAAACTCAATGTCGAGCGAGGTAACTTCAGA
ATAGAAACCTATTTTCCTAATCACCCTTTTTCATGGCATCCGTTCAAGAAAACGGAACCTGTACCCGTCCCAG
ATTTCACTATCAGCATGCTGATCGAGTCAGCAAAAGCCGGGAAATGGTTATATGACTAATTGTAATGAACTTA
ACAAATGGCTGTACCAGTACCCTTCTTTTTGAGCGATGATGGTGGCGCTAAGATAGTTCTCTTTTCTTTTTAA
AGGGTTCATGTTTACTGTCGCCACCCCTACGCTAACCCCTTTAGTTTCCTCCTTTGCTGTCATACTATCGAAT
GACGTGCCTCACCTGTGAACGCTAAGGAGAATAATATGGAAAATAAAGGCACAAACCTAACGCCCGAGCAGGC
GCTGGATCGCCTGGAGGAGCTATACGAGCAGTCGGTTAACGCGCTCCGTGAAGCCATCGCTGACTATGTTGAT
AACGGTACGCTGCCCGATCCCCACGCCAGGCTTAACGGACTGTTTGTTTATCCTTCGCTGTCTGTAAGCTGGG
ATGGCGCGACACCGAACCCGCCTAAAACACGCGCTTTTGGACGCTTTACTCATCCCGGCTGTTATACCACTAC
CGTTACGCGGCCTGCGCTATTTCGCGCTTATCTTCTGGAACAGCTTAACCTCGTTTACCATGATTATGGCGCG
CATATTGCGGTAGAGGCCTCGCACCATGAGATCCCGTATCCTTATGTGATCGATGGCTCGGCTCTGACGCTGG
ATCGTTCTATGAGCGCCGGGCTAACGCGCCATTTTCCTACCACAGAACTGGCGCAGATTGGCGATGAGACGGC
AGACGGTCTGTTCCACCCCGGCGAATTCTATCCGCTATCGCACTTCGACGCGCGTCGTGTTGACTTCTCGTTG
GCCCGGTTACGGCACTATACCGGTACGCCGGTTGAACATTTTCAGCCCTTCGTTTTGTTTACCAACTACACCC
GCTATGTCGATGAGTTTGTGCGCTGGGGATGCAGCCAAATTCTTGATCCTGACAGTCCCTATATCGCGCTTTC
CTGTGCCGGCGGAATTTGGATCACGGCAGAAACGGAAGCGCCGGAAGAGGCTATTTCCGATCTGGCCTGGAAG
AAGCATCAGATGCCCGCCTGGCACCTGGTAACGGCAGATGGGCAGGGGATTACGCTGGTAAATATCGGTGTCG
GCCCTTCAAATGCTAAAACGATTTGCGACCATTTGGCCGTGTTGCGGCCTGACGTCTGGCTTATGATTGGGCA
CTGTGGAGGGTTACGAGAAAGTCAGGCGATTGGTGATTATGTGCTGGCCCATGCCTACTTACGCGATGATCAT
GTGCTGGATGCCGTTCTGCCGCCTGATATTCCTATTCCAAGCATTGCGGAAGTACAGCGTGCGCTGTACGACG
CTACCAAAGCGGTTAGCGGAATGCCCGGTGAAGAGGTGAAACAGCGACTGCGCACGGGAACGGTCGTCACCAC
GGATGATCGTAACTGGGAGCTTCGTTATTCCGCATCGGCGCTGCGATTCAACCTTAGCCGCGCGGTCGCGATT
GATATGGAGAGCGCGACGATTGCAGCCCAGGGCTATCGCTTCCGCGTGCCTTACGGCACGCTGCTTTGTGTTT
CTGACAAACCTTTACACGGTGAAATTAAACTGCCCGGGCAGGCAAACCGGTTTTATGAGGGGGCTATTTCAGA
ACATTTGCAAATTGGTATTCGTGCTATTGATTTACTACGCGCAGAAGGTGATCGTTTGCACTCACGTAAATTA
CGCACGTTTAATGAGCCACCGTTTCGCTAACGAAATGAGCTGTCGCCTGCTGCTGGACAGGAGTGGAAGAGGC
GTATTTATGTGATATGGAAAGCGGCTGAATGAAGCCTTAGCGGGAGGCGATTCTGCATCCCGCTAAGGCCTTA
TTGTGCCGCGAGCTTCATCACCATCGCCGGATACCTGTCGCCGATAACCGCGTCTATGCCAGGAAACGCGCCG
GAAGGCGAAAACATTGCGCCACACTTCACGCTCCTTGAGCGCCTGACCCACAAGCCCGGATTAACAAGCAGAT
ACCGACCGTGAGAATCGATCAGCTTAAAGGGGTTATCAGAAGGCTTGATACTGAGGATTTTCGCGTCGGACAG
GGATATAGGGTGGTCACGCCATTATCGAACTCATAGCGCCACGAGTGGCGCGGAAAGCCTTCATCATCATAGT
GGCGCACGTCGGTAAAGCGCACCAGCCGGTGCTCGCCGTTCCATTCAAATCGGTGCTCCCGGCACTGCGCCTT
GCCCCAGCGGTCAGCGATGGGATTGCCGAACTCGTCGTACTCATAGTGGTGGTCTGCCCTGTAGCAGCAGGCG
GTCGCCTTTGACTTTCGCCTGATGCTCGCCACCTTCGGGCGGCATGTTCATGCCCAGCAGGTTGTCGTCGTCG
TCAAAGCTGAACTCCTCGTGCAGGGCGTTCCAGCGCGGGTCTTCCGGCAGGCGGTGTTGGGTCAACATTAATA
TCTTGCGGTAATCTCTGTGGGTTTGGATTCGAATTTACGTTAGTGCGTGTGACCGGTAAATCCCGCGACGATC
TGCAGGCAGCGATGGTGCTGATGCGCGGCGGCAATCTGGGCCAGCCGTTCCAGTTCAAAAACTTCCGCGATTG
ATTTTTCCCGCCCGCCGCCTTCCCGCATGTGAGAAAGGTGGCCGGGCGGGTAAATCGATTAAGGCGTGGCTTT
TTTAGGCTCTGCGGCCTTGTTATACAACGCCTGAATCAGCGCCTGCTGTTCCGCTCCCGGTATGATACCCAAT
CTGTGAAAAATATTCCCAATCTGATAAAGCTGCTGATTACCGGTGTCGTCCTGGCGGGTAATCGACGCCTGCG

TGGTTAATTTTTTCAGCGTAAAGGTAATATGTTCCTGACACTCGGGCGTGTTTTCAGAACAAATCTGGCGTAA
CAGCATCAGGTTTTTATTATCGACCCTATCTGCGTTAATCATCAATTCCCTTGCCTGACTCCACTGTTTTGCA
TTCATGTAGTGGTCGATCATCGCCTCCTCTTCGAGAGAGGGAACGTGCTGGTGTAACACCCGTTTGGCATTCA
CCGTGTCGTTGCCATAGCCGTAAAGCTCTGCAAGTCTTTGAAAGGAACGCGACATATCTTCATCAGGCCAGGC
TTTTGCTTGGCTGAGGTAAATCGCCTCGGCGCTGACGGCTAATTTTTGCGCTGCCTCAGGATGGCCAATTTCT
TTGAGTACTTCACCTATTTCATTTGTTGCATCTTCCCATTCGGCGAACGTCAGGGTTTGCGCCTCGGCGGAGA
GGGTTGCGATATGCTTTTCCGCCACAGCATTCTGCTTATGCTGAAAGAGATTCTTCAGCGCTTTGATTTTGTA
CTCGTAGCAATACCTGTCGTCGTAGCAGGTATATTCCGGCGCGCTCATCAGCAAATCGATACGCTTATTCATC
AGCGCAACATCATCAAGCTTGCTTAATAAATCAATCAGCTCCATTTCAATGCGCGGCGAAGGGGCAAAATTGA
ATATTTTATCGACCTGCGTCACATAGGTTGAAAGCGTCTCCTGATAATTCTGTGTCAGCTCGTTTTTCTTATA
AAAAGAGAGCAATCCCGGTAATGAATACTGGAAATAGCTTCCTTTATTGCCGGTTGCTTTGCGCAATCTCGCA
GACAACGCCATCGCCTTCTCTTTATTACCCGTGGCGTAATAGATCTCCATCAGGTCGAGATACTCCTGATAAC
GGTAGGCTTCAGCACTCGGTTGTTGAGCAATAGCCGTATCAAACATTTTCAGCGCGCTATCTGCGTAAACCAA
CGCCTTTGCGGTGTCGCCCTTGGCATGGTACTGCTTCGCAATAATAAGCAGCATTTTTAGCTGAGTATAAGGA
ACAACAATTTTATCAGCGTAATTTTCATTATAAATAGCGGCGGTTTTATCAGGATCTGAGTGCCAGGATTTTA
AAACCGTGGCTAAATAAAAATTCCTCGCGTCGGCATTTTTGCTAAGAAGGGCTAAGGCTTTTTCAGGCTGTTT
GCGATTAACCCAGCCATCAACGTAGCTCTCGTAGGCGGTAATGCTCGACGGTTTATCGAGACCAATTTTGGAC
AGGAAAACCATCGCGGCATCCGGGCTATTATTTTCAGCGATCCACGAGGCCTCATGGTTTAGCGAAAACTTCG
CATCATTCGATTGTGTGTCCAGCTTTTTCAGTAATGCATCCGCACGGCTCATCGGGCCGTACATAAACAGTTC
CCTGTGTAATTCAAACCAGGCTGCCTGCGATTTAAAAGTGGTGTTTTCATTTAAAGGGACGGCATTTTGCAGT
TTCTGTTCGACAACTGACCAGACATCAGGCGATTGCTGACTTTGTGCAACGCTCTGATAAGAGTAAAAAACCG
TGGCGGCTGTGAGTAATGTGGCGAATTTAAATTTCATAGCAACGTGTATCCTTTCAGTTCCGTTTGAATAGCC
TCTTTACAGCAAAGTCATATTTACTTAGTTTGAGGCGTTAATTCCTTTAAGGCTGCCAGTGCAGGTTTGACGC
CGCCTTTTACCGCCGCTGTAAGATGAAGTTTGGCTTTGTTGAGGTCTTTCTCAACGCCATCACCATTCCTGTA
CATGAGTCCAAGGTTATAGTGCGCCTGAGCATTTCCCTGGTCAGCGGATTTTTTAATCCACTTATAGGCTTCC
GGCAGGTTTTTCGGCATTCCTTCGCCGATAAGATTTAAATATCCCACTTCTAACTGCGCATCACTTTCGCCAA
GCTCTGCCGCTTTGAATAAATAGGAGAGATATTTTTTGCTGTCTTGCGTTACGCCATCACCGTCGTTGTACGC
ATAGGCCAAAAAGAGAAGCGCGCCGGGATGATTTTGCTCTGCGGACTGAGTTAACCATTTCATCGCCGCATCG
TAGTTTGTGCATTCAAACTCATACTTACCTAAATGATATTGCGCAGTGCTGTCACCCTGCTTTGCCTTATTGG
TGACGTCAGTCGGCGCATATAGCTCTGAAAGCTCTTCATCGTTGTGGTAACAACGGATGTATTTTATGCCGTC
TTCATCATAGGCTTCTTCTGTACCTTCCTTGTCGCCATCGACATAGGGCAGCGCACTTAAGACCTTACCCGTA
CGATCGTTAAACTCTCTTTTCAGGCCGTTTCTTTCATCATTAGCAAACATAACCTCTTCAACAAGTTTACCGG
TACGTTCGCTCCAGCGGCGATCGGCTCCCTGCTTTTTATCGTCTTTATATTCGGTGGTATAGGTGAGTTTGCC
CTGTTCATTCCAGTGCTTAGCATCCCCATTTAGCATACCGTTTTTATAGGGATAGACTGATTCGCGGTGACCA
TTTTCGTACCACTTTTCGGAATCCCCGTCTAACACATCATGTTTATACATTTTGGATGAGGCAGGCTTTCCGT
TGCTGAACCATTCTTTGACCGCACCATGTTTACGGCCTTCACTATCAAAAGAGGATTCTTCTTTGGGATGCCC
ATTTCCATACCAGCTCTGAGCAGAAAGCTGTTTGCCATTTTTATAAGTCGCTTTAGAAAGGAGTTTGCCCTCT
TCACTGTATTGTTCGAACGTTCCGTCGTTTTCACCTTGGTTATTTCTGTGGTAAACACCACGAATTTTACCGG
TAGAATAATATTCCGTACTGGTGCCGACAATGGTGCCTTTTCTGTATGAATATTTCCCCTTTATTTTCCCATC
AGGGAAATATTCAAATGCCGGGCCTTCCAGTTTCTGATTTAAATAGCTGTGTTTTTGTTTTAAAACGCCGTTA
TCATAAAAATGTGGGCATTCGCCATCTTTAATTTTACCTTGGGCAAAGTAACGTTTACTTTCAACGGCTCCGT
TTTCATGGTATACGATGTATTCCCCTTCGGGTTGACCATCTTTAAACGTCAGCGTTTCCGCTATTTTGTCTTT
TTCATCAAAGTAAGTTTCAGCTTTTCCGTCACGCTTATTTTGCGCATTATAATGATAAATATTTTTGAGATTG
CCGCTTTCATAGAAAGATTTAAATACAGAAACACGTTGTCGGTTAGCGAAGTTTTCTTCGTTGGTATAGCCTT
CAAAGGCTATCTGTTTACTCTGCATCACCTCCCCTGTTTCTTCTGAGGATTCCAGATACCAGCCGGTGACTTT
CCATAATTTTTTCTCTTTGTCATAGGGGAGGGGCATATGGACTACTGTTGTTGCCTGACGTTTAACCGTCGGT
TGCAGTTCATCATCCAGCCACAAGTCAGACGATCTTTTCATGTATTCGGCACCGCACAGCGCTTCGGTACATA
GCTCGATTCCAGTCAGCATGGGCGTGCCGCCAGCATCGCACTGATCGACCGTCAGGTAAAAATAACGGTCATC
GAGAATAGCCGGTCTTAAACTGCGTATTTTATTAATATATTCTTCTGTCGTCAGTCCCATTGATTCTGCGCGC
GCAGAAAGGGTATTAGTATTACAGGGTGTTTTATCTTTAAAATAATAACGGTCGGCGAGTAAAAGTTTCAGGT
CACTGCTAATGGCGGTTTTATTAAAATCGATATTTTCAATTTGGGCGATGAAATTAACGGGTTTGGATTCTGT
ACTGGGGGATTGTTTTGCACATATATTCGCGCAATATAATGAAACTATAATTGTTATGAATATTTTCTTCAAG
ATGTTCTCCTTGTATGAATAATAACTCCCTGAAACTTATTTCATGCTATAGAAACAAAGAGTTAATGTAAAGG
ATGCGAGGAGCATATTCTGGCAGGAGAGAGTTTTCTTCAGTTACCAGTAAGAATAATTTAAGTATTACGAGCG

CGTTGTTTTTAATTTGATAATAACGCGCTGATGATTATTAACGATTATATACCCAATAAACCTCAAGATGCGG
GAGTGAGCACCTGAAAGTGATCTTTAATTCGGGATGTCAGTGAGTCCGCTATATCCGGCAGCCTCTGGTTAAA
AAAGACAGATATTGCGTCCCTGAACTCTCGCGTACTGCTGAAGTAGCGATTATTCCGGGCATGTTCGTTCATT
AATTTCCACATTCGTTCTATCGGATTCAGGTTTGGACTGTAAGGGGGAAGGTAATGCAGCCTGATATTTACCA
CCTCCGCCCAGTCTTTCACTAACTGTGAACGGTGGTAACCTGCTCCATCCGCTGCTCGCTCTTAGCCAGCTGT
CAAATTTGCCCATACACCTGCGCCACATACCGCCCACGCCCGTCCCCATGCCCCAGATCCATTGCACACTCTG
CACCGCTTCCTGTGAACGTAAGCCCATCAGCCTCGCCAGTTCCAGCGCCGCCGCCAGTCCCGGATCTTTCATG
CGGGCCGTCTCCAGCACATGATGATAATGTTCCGGCGTGATCGCCTGTCGCGTCCCGCTGCGCGATGCACCAG
CCAGACCCAGCGATTTATTAGTCAGCCACTCATGCTCTGCCACCTGTTTACGCCCGGCCTGTTGCAGAACAGC
GCGCAGCGAGGCCATTTCATTTTGCAGCGTGCGTTTGCCGATCCCCTGCGCCAACCTTTCATGAATATAGCTT
TCAATGTGGCGCACCTTAATCTGCGCAACCCGCTGCATATGGACGTTCAAAGCCCGTAAGTGATGATCGAAAC
GCTGCACGATATGAATGCAATCGTTCACCGTTTTATGGCTACCGCCACAGTGCTTTGCCAGTGCCTTCATTTC
ACCACCTAACTTGCCCATACACTTCTCCTATGATTTTGAGAACACAAACAGTCTCCGGCGTATGCTCTTTGGC
TGCGCCAGAACGATACCTTTACGCCGGAGTGGCAGTTTGTTGAGGTATAGGGAGTTTCAGACTGTACGCGGTA
CAGGCTGCCTGCGTGAGCAGAAATTCTCCGGGGCATCAGCCCCCCCCTCGGTATCGATCTTTTTCTCTTCTTT
CTTGCTGACAGCGGCCCCAAAAAGGCGTGTCAGATTCGGGAATGAACGCCGGAACCCCGAAAACAGGGGCATG
GCGCGGTGTAGTGGTTGTGCGTCACTTTGTCGCTTTGCGCGACGAAAGTGACGCACCGGTTGAGTGCCGGGCG
CGAAAGATCGCGGATTCTGGCGCAGATTGACGGTCACGCGGCAGCAAAGCCGGACGTCTTTGCAGGCCGCAGC
CCGTAAAGTAAAAGGCGCTGATGTGGTAACAGGTGCCGGGTGGAATCGCTTCCGTCACGGTGCAGCATTGACC
AGAGATCTGGCGCAGGTACCATCGAGATACTCTTTCAGTTCTTTGGTACCGAACGTGTCGATAGCCCGCTGAG
CTGCGCCAAGATTAGACGCCAGCTTGTCACCGCCAATGTCTTTATCGGCTTTAACTGCCGCAGCCCAGTCTTC
TGTTTGCTTCTGCCATGACTCCGCTTGCTGTTGTTGCACTCCCGCCAGCACTTTCGGATAGACGTCCACCAGC
TTCTGCGCCTGCTCCTGCGTCAGGTTCAGTTCACGCGCCACTGGTTCGAACTGCGCCAGCGCGCTGGTATCGA
GTTCCTGACCTTCAGGGGCGGTGAACGCGTATTTCTCCGGCGCGCCGGGCGGCGTTTCTTCTTTCGGTGGAGT
GGGCTTATTGGGATCGGCGGGTGGGTCGTGTGGTTTTTCAGAAGCGGGACCATCTGACGGTGGATCGGCGGGT
GGATTTTCTGACGGTTTTCCTGTACCCGGATCGGTCTGCGTGCCGCCAGCGGTATCGGTTAATGCCATAGGAG
GGGGCTCTATTTACTCAACGAACCGTGAGGCCCCGATTAAGCCCCAAAAACTATGGATGTCAAAAAACCCGGC
AAACCTTATCAGATCAACCGGGTAAAGATAATTTCTTGTTTTTTAAACGTCTCCGGACTCGCCAGGATGTCCC
GAAACTAAAATCTGGCTCCTCTGACTGGACTCGAACTTCATCTGTAGCTCCTGCTGTTAGTGGCTTTCAAAAA
ATGGCGTCTGTTTTTACCATCATATTTACCAGCATTTTTAATGCTGTATTACCATAAAGTCAATTACTATCAA
TTAGTTATGACAATACACTTCACTGAACCTTACTGAACGATCTTCCTGTGCTGCTATAAATTTTATCTCGTTT
ATTCAACAGATTACCGCTATTGCCCGATATGTTTCCTCCCGGAAAGATAAGTAAGGGCAGAAGAATACGTTAT
GACCAATGCTGAGATAGCCAGCCATCTTTATTCCCTTTCTCTATGCTGCTGGATGCGTTAACGTCAATGTAAT
GAACATCAACATTTTTAGGGAAAAGAATGACAAAAAGATTGCCTGTCGCGGAGATAGTGGAAGCATTAAGCAA
ATGGTTTGATGTTAGCCGCTATGATGCGCTGAAAAACCTCACTCTGGAACAAATTTATGCGGAACTGGAAAGG
CGCATGTTTGCCTACAAAGCCAGACAACAATGGGAAACACTGGATGATAAGCATCGTAATGCCGTTATTCACC
ATGATGCGATGATCCACTCTGGCAGGGTCTTGCTGGAGGATAAATGGATCTCTGATAGCCACATGCTGGCTCA
TAGCTATGCCGTCAGGCCAATGACAAGGGATTCACTGTTTAACTATGGACGGGCAATGTATCGACTGGAGAAT
ACACCTCCAGAAGAAAATGTTTCAGTGAGTTCGGATTACATATCTGAATACCTGAAACAGGGAGGGCTGAACC
CTGCTAACAAGATGCTGATTGAAATTGATTTGGAAGAAGCCAGTAGCGACGATCTGGCTGAACACCTGAAAGT
GCTTATCAATCAATGGCAGAAGCACCTGAAAGTGCCTAAACCACCGGAAAAAGATTTTCGTTTTGGTCATAAA
ACATTCCAGAAGATACTCGATTACAAGATTATCCCTCTGATGGACCTCATAGCTTGGGAACAGCTCAACAATC
AGAAAATCAAATACCCTGTATTGGCTGGTATCCTGCACCCTGATATGCGTTATGCCAGAGGTAGCGAGCAAAT
CAAAGACACTGATTATCCGCTTGCCCACGGTTTTTAAACAATGACAATTATTTCAAGTCACTGAATGATTTT
TTCATAAAAAACAATCTGGTGAAAAACTCACCTATTCTTGATGTCATCGCAATGAATGACAAACCAGAAACAA
AAAAGAAAACAAGGGACATCCACTAACCCTGAACTTCCCCTGTATTCATATTATATAAACATCCCCTAAACTC
ACTGGTCCTGAACATCCCTCCGGTTCAGGACTGGTGAACCAACCTAGTTTTTTACAAGCGATCTCCATACAAT
TATCTCCATCGAATAACACAGGAGATAAATATGGTTAAAAGAGAAATAAAAGCAAAGACAACGTTTATCCGTT
TGCCAGAAGTAATAAAACGCACCGGATTTGGTAAAACGTGGATTTACGAACTGATTAAGGCCGGGCGTTTTCC
ATCTCAGGTGAAAACAGGGATAAGAGCAGTGGCATTCATTGAAAGTGAGATTGATGAATGGATTGAAGCGACT
ATTCGAAACTCACGCCAGAACGCAGCATAAAAACAGAAATAGGATCTCGTGTCGAACAGATATCTGTTCGTAC
AGGGCATCTTATTACCCTAAACAATAATGGAGAAAAACATGAAGATAGAACTTATTACAACAAAACAGTTTAT
TGAACAAGCCGAATGTTATTTCAGGAACTATATGGACGGATTGCGGAGGAATGCACCAGATGATTTTTATTAT
TTTCTCAACAATAAATACAATATGAATGACATTATGGAAAGTATCATTAAAAAAACGAGATATTATTTTTATG

ATGACACCGAAGAAGGCAAAAGGAATCGGATTTATGGAGAAGTAAGTCACTGTAAGGTAAAGCAACATCTTCG
TCAGCTTTGGATTATCTATAAGTGTGTATACCGCTAATACTACAGCATTTCATTTTTAATATTAACCAAAAGG
AAAAGCCAATGAATACATTTAAAAATAAAACTACTGAAATCTTTTATGTTGTGTCCTTGCATATCTATGCGGA
GCTTTTTAACTCAAAGGATAAAACAACCAGTAATATGATTATGACGCATGTAATGGATCATGAGTTTGTCTGT
AGGCTAATCGATCTGGCAATGAGGAATGCTGAGAAGCATCTTCTGAAAAAAGCATGGAAAAAAAACGCTGCTG
AAAAACTGTCAGAGGTGGATTTTAAAGGGGTTAAGCAGGCATTAGCCAAAATGCACTACACCGTTCTGGCAGA
GTCAATATGTTGATGTGTTCTGACGTATACCGGCCCCTTCATGGGGCTGATAACCGGACAATTAAGGTGCTGT
TCTGCGGCGAGACTTGTTTTGCTGCTGTTCGAAGGTTGGACTAATGTCTGACAACTGGTCAGGGCACTCTATT
TATAACTATAAATTTGATTGAGATCATTTTTTAATAGAAAATTAGCGTATTACACATTGATAATCTCTTTTAT
GGAAGCATATGTAATTGATGACCTTAACAAGGAAATTACATGAGCACCGCAACTGACTTTAAGACACTCCTCG
ACAATATAAAAATAGATAATGCAGGCCAGATTAGTAAAAGGTATGGTCGTATAACTAAGGCTTTGAACCAATA
CTTTTATAACTTAGATTCTAAGACAGCCAATTCACTACAGGTTGGTTCCTATGGGCGCTTCACAGGGATTCGA
GGGATCTCTGATCTTGATATGCTTTACTTTCTACCTGCAACTGCATGGCCAAGATTCCGAGATCGACAATCGT
ATTTATTACAGGTTGTGAAAACAGAAATCAAGAAAACTTTCAAAAATACAGATATTCGCGGTGATGGGCAAGT
TGTTGTTGTTAAATTTAAGAATCAAGAGGTTGAGGTAGTTCCTGTATTCAGTAATGAAGATGGCACTTTTACA
TACCCGGATACACATGATGGTGGATCGTGGAAGGTATGTAACCCTAGGGCCGAAATGTCGTCTTTTAGGGCAC
TGAATGATGATAGGAAGGGACATCTGAGACGTCTATCTAAAATGATTCGAGCATGGAAAGCTCGTCATGAAGT
TGAGATAAGTGGATTCTTAATTGATACACTGTGTTATAATTTTTTCTCTAATCTAACTGAATATGATGATAAG
AGTTTCAAAAGTTATGATCAACTTTCGCTTGATTTTTTCACTTTCTTAGAGAATGAAGGTGACCGAGTATTTT
ATTATGCTCCCGGTAGTCGCTCAAAAGTGAGCGTAAAAAAATCATTTAATAAAGTAGCAAAATTAACAAAAGA
ATATTGTGAAGAAGCTTTATCTGCTACAAGTGAAAACTCAAGAAACTTAGCTTGGAAAAAAGTTTTTGGCAGG
CCTTTTCCAAATTATACGACAAAAGCACTCAGTAATGTCAATGTATCTGAACAGTTTATTGAAGATCAATATG
AGATGAATTTATATGGTCATGTTTCGATAGAATGTGAGATTAGAAAGAATAATTTACTGGAAGCTCTTCTTTC
AAATCTTCTTGGCGAAGGGCATGATATTAGCACGAATCGCAAGTTAAGATTCTATGTTGATGAGATAAATAAT
ATATCTCACCCATATAAGATTAAATGGAAAATAAAAAATGTAGGTGATGAAGCTGAGCGCCGAGGAAATGTTA
GAGGTGAAATTTTAGACGATGAAGGCGGTTCTGAGCGTTTCGAGACCGCAGATTTCTCAGGACCTCATTTTGT
TGAATGTTATGTTATTTATGGTAATCAAGTTGTAGCAAGAGATAGAATCGACGTACCTATACATAATTAGTCC
CACTTTATGTTAGGTTAGTGTTAAACTAACATTAACCTAACATATGATAATGGTGAGTCGGATGGATAATAAT
ATATTAGAAGGGCAATTAAGAGAATGCTATGGAAGGGTCGTTTACACACATAAAACTCACGAGAAATGTGCAG
ATATCCTTCAAAAAAAACTAGGCTGCATGAAAAATTTACAGTTAGTTCTTTTAGCAATAACTACCGGTAGTTT
TATAACTACAGTGGTGGGAGATGCTAAAACAGGTGCTATAATTGGGTCAGTACTTTCCGCTATCCTCCTATTT
TTAAACTCATATTTAAAGGATTATGATTTAGGCTCTATAGCTCAAAAACACAGACAAGCTGCTGGGGATATGT
GGTTAATCAGGGAAAGATATTTATCTCTTCTCACAGACTTGAAAATGCAAACTAAAAGTATCGAGGAAATTCT
TAAAGAAAGAGACGCCTTGATGATTGAACTTTCTGCAATTTATATCGGAGCACCATCCACGAATTACAAGGCA
TATTCAATGGCTCAAAAGGCACTGAAGGAACTTGAGGATATGACTTTCTCTGATGAGGAAATTGATAAATTTC
TTCCCACTGAATTGAAACGGAAATGAATTTATTTATTCATAGATTAGATAATTCGCATTGGCTCAAATCAGAT
CTGACAGTTATCAGTTATTTATACAGATATCTGTCAGATTCTATTTAGCTTAAATTCTTTTAGCCCGAATGCG
TTTTCCATCAGGTAATATTTTCATTGGTGTCCGGCCACAGCCCATTTTCCCCTGATGAATTTATTCATTATTA
TAGTTAACCAGCAATTCATCAAGATCTGTTTGTAATAATCCAAGAGAGCCTTCTCTTCCTTTGGCAGTCTCTA
TCTTCAGTACTGGCAGTGTCCTGAGACTAATAAAATGGCAATACACCACTTTTCAGTAAATCGGCCGCCGTAA
TCAACGTTTTGATTACGTAGAGCTTACAGCGAACGAAAGTAAAACCGCCAAATTAGATTGACGGTTTTATTGT
GATCAGAGTGCGAAGTCTTCCAGTTTTTTACCTGCTTCGATAGCAGCAGCGATCGCTTTAGGAGTGCGACCCT
GACCTGTCCAAGTCTGCTCGTTGCCGTTTTCATCTGTATATTTGTATTTGGCAGGGCGTGGAGCGCGGGTCGA
TTTGGTTTTAGGTTGGGATGAGCCAACAGCAGCAAGGAGTTCTGCCGGGTCAATACCATCTTCTAACAGTTTC
TGACGGAAAGCTTCCAGCTTAGCCTGACGTTCTTCTTGTTCCTTACGGACGGCACTTTCTTCCTCACGACGAT
CTTCAACGACAGCGGAGAGTTTTTCCAGCATCTCTTCCAGAGTTGCCAGATCTGTTTCACGAGCATGTGCGCG
AAGTGTACGAATATTGTTCAGTGCCTTGAGAGCTTCACTCATAGTATGTTGTCCGGTGTTTTAAAATAGGTGA
TTAATAAGGTTAATGTATATAATGCTGTTGAACAGTCAAAAAATACCAACCAGTAAATAATATAGTAAATATT
ATTGATTAAGCAATCTTAAAACATCCTATTTAACACGGATAACATAGGGCATTTCTTTAAAGAGACATTTTCC
TGCGCTTCTGGCTTGATGGGGCGAACTCTGACGAGATGTGAGAGAGTTGATTCAGGCTTTGTTCTATGGAACG
CTTGAACTCAACAGCGGCTTCTGTACGCTGTTTTATGACTAAGGCAATTATTTCTTCATCGCTTTTTTCTGAA
AGTGCTTTACGGTATAACACGCGTTCGTTAAATAAATCGAGATTACCAGTTGATCTCGCTGATGTGTTAGCTT
TTGAACTGCTAATAGGATTTAACTCGTCAATAAAGTATTCAATAAGAGATTCTCTCTTCTTTTCAAGTCTGCG
AAGCTCAGTATGTGCAGACTTAAATTTTTGAGACTTAACCAATGGAATGTCATCAGAGCGTAGCCTACGATAT

TCATTCCTGTATTCATCTATCGAAATATGATTTTTTAGTTCTTTGACTGTATTTACGTATTCGATTAAATCAC
TGTTGTCTGTTTTTACTTTTCTCATAAACAATACCTCGGAAATAAAATAGTGGATATATTTATATTACGAGAT
ATGGTTCAGGTGTCAAATTGGCATAGGCATAAAAACATTACTTGGTTTTATACCGTTCAAAAATAAGCTCGCC
TGTATCTGTATAAATTTCTATTTTTCCTACTTTCCCATCAGTGCCTTTTATCGAGAAACGAATTAATCTTTCA
TTATGATATTTGTCTAACAGGTCTGGTCGTTCAGTAAAATATCTTGATTTTGTGGAGCGATCTTTCAGATAGT
CGCAGCTTGAACAAACCTGCTTTTTGAAATCCTTCTCAGATAAATCACTTTCTTTTATTTTTGAAGAAATAAA
AGCCTTTAAAGGTCGTTCATACTCCTCTCTTTCTTTAGCTTTGCGTATTTGTTCTTCTTTCTTGCGCTGTTCT
TCAATACGGTAAAGTCTGCCTTGTTCTTCCTGCGCGCGTATTATATCCACTTCAATTATTTTACTAATGATTG
AGGCAGGAACAACACTGATACCTTCAGACCACCCACGATAAGGATTATTGTTTTGTATGTTTCGGACTGTAGC
AGATATTCTGTTTATATCCAGATCTTTATCAAAGGAAACAATACGTTGTCCGTTTTCTTTTGATATGAGTTTA
GCCGTGTTCACAGGGGATTCAAGACCCTTTAATTTGAATGTGGCTACGGCACTGTTGCGACTTCTGTAATATA
ATGCTTTTTTCTTATCTCCAATATAGCATTCAAGATAAGAGAAAATCTTAAACTCCTGAATGCCAAATGTTTC
CTCAAGCATGGTCATCTGCTCAGCTAATATATTTAGTATATCACTCTTGGATAGTTTTTCGTGGAGCTTAATC
TCAAGAGAACCGGGTAGTTTTTTCATTGTTATTTCCTTGTTGTTGTTGTTATTTTATCAGGATACTTTCTGCA
TATGAGAGTCTGCGTCAACAGCGTTTCTTCTTGCTTCCTCATTTTCCTTTGTTCTTTTATATACTTTTCGTGG
TTTATCAACATGTATGCTGTTATTGGGATTAGGAACAGCGTTATTCTTTTTCTTCTTACTGAAAACAAATGCG
TCAGTATAGATTTCTTTTGTTTCAGTTTCTAAGAATAGGGTTCTTCTTTCACTTTTTGACATAGCTGAATAAC
TTTCTGGATACTGTTCTATTTCTGCGCAAAGATTGCGAGCGTTGCTCAACTGTTCTTTACGTTTCTGATCCAG
TTGATGACTCATTCCTTTAGCGAAAATGTTAGAGTTGATATAGAGATGACCATGATGCTCGACGAGTATTCCT
TTCTGTCTGAGTTTTTTAAAAATGTTAGACATATTACCGGAAGCGATATTCAATGCTTTGCACAGCGATGATT
GTGAAAGATTGATCAGATTTCCAAACTCCATATACTCCAAGATATAGGTTAATACACGGAATTCATTCTTGGA
TATATCAAGATCAGAGAAAACACGAAAGTTGGAAGTAAAAGCAAATACATAAGGTTGTGAAAACGAAATAGCT
TTATCTTCCTCAGAAGAAATGACTGTTGTTGATTTTATTTCTTTGTTTTGTAGTAAGCGTCTTGCGATATCAA
TCTTTTGCTCGGTAGTTAATGATTCAAATAAGTGTAATAGATCAGCATCTGGTGTATTCATATTATTCCCTCT
TATTATTTTTGTTATATCTGTTTTAATACTATGCAAAAAAGTGAAAGTCAATAGTAGAACTATAAATAAAATA
GAAAGTATATTTTTATGACAATGTTATATGTTAAAAAAACTATGTTCGAACATAGATCTTGCTATGTTCGAAC
ATAGGACGGATCTATGTTTGAACATAGGAGTTTGCTATGTTCGAACATAGGAAAAAAGTTGTGAAGCCGCGAT
TTTACTGGTCCGGGACTATGCTTATAATTAAATAATTAGTAAGAAGTTAAGAGAGTCCAGTTTTACTTTTGCG
TTATGTTTTAGTGGTTTTACAGAGGGTATTGTATTCCTTAATAACTGCTATCAGCGAAGTGATTGTTTCGAAC
AATCACGAACCGCTGCTTTTATTGTGAGAGCTTAATTAAGATTTCTTTTGTTTTATTAGCGTCTACGCCGCGA
TCTTTTTTTGGATAAATCCATCATTAACAGGGGCAAGCCCTGTCCAGCAAAGTGATTGTTGCCAACAATCACG
AAGTGCTGCTACCAAGGATATAGTTTTCCTATGTGTTTAAGAGCAAATGCTATATGTCAGAGGGAAGATGGTA
TATTCAAGCATAAGATCTGAGTTCTAACTCGGTGGAGGGAATGATCTTTATACTGATCGCTTTATTTTCCATT
GATAAAATATTAATAACTTATAAGTGAACAAGGAAAATAGTTTTATACCAGTATCAGGGGCGTAGCCGTGCGC
CAGCACAATGGATGTATTTTCAAAAAATAATAATTGAATGTATCAGGTAAAAATAAATATCAATAAGCATAAG
TAAGAGTGTTCTATAAACTGTTAATAAACCGCACAGGAAGCTTGGTAAGACCTTGGGAACAGAAATATAACAC
ATATAACCATCTTCACACTTAAAGCTCTTATAACTGATTACAGGCACTAATTTTATATGGAAAAAATTATTAA
TTGAAAAAACATCTTTAACAGGGGCAAGCCCTGTCCAGCAAAGTGATTGTTGGCAACAATCACGAAGTGCTGC
TACTAAAGATATAGTTTTCCTATACATTTAAGAGCAAATGCTATATGTCAGAGGGGGAATGCTATGTTTAAAC
ATAGGATCCGAATTAAAATTTAAAAGGAAAATAGAACTGACTATGATCGAATACATTCGCTTATTACTTCATA
GTAGTAAAATACAAAATGTCAGTTTTAGGGGCGTAGCCGTGCGTAAGCACAGAGATAAAGATCTTTTCAAAAA
GAAGTTTAATAATCAGTAGTAAAATCATAAATTAAATAAAAATATATTTGATTTTTACGCTTACGATAGAGTA
TTAAACAGTAATAAAATAATAAAAGGAAAATACAATGAACAATATCAAAATGATCACACTCTTCCATCCACAT
GACAAAACACCATTTATGATCTGTATTGTAAGCAAAGTAGAAGATACAGAACACGGATTGAAATTAACCCTTG
AAAACGGCAACAACATTTGTGTCAATAATTATAGTCATTATCTTTTATCAGATTCTGTAAGTCGTTGTGATAA
AGACAGGTTGAAAAATATTTATATTCGCTTGGTTTCAGAACTCACACAGATGAGTGAAGAAACTATAAAATCA
CAGATGTTATAAAATATTTATCAAAATATCATAAATATTTTTGATATTACGACTGGTTGCATGGTATTAAATA
AAAACGATAACAGGAAAAATGTCATGCGGAAAATAAAAACAAAGATCTATATACCTACAACTTTAACAGGAAA
CATGGGTAGACACTATATCCCTGTTTTAGAGGATTTAAGAAAAACAATATACAGCGACAGAATATTATCAAGG
CTTGCAGATAGCGGCAATATTGTTATCCACAGTTCAGTTGGGTATCCGGTTGCTAAATATAAAAACACGGGGA
TTAGCATAGGCATAGAACCATTAAATCCAATGATAAGACAAGATCTAACTCTTGGATATATCGTGGTAATCAG
AAACGGAAAAGCATCACAGGAAGTTAATGGTTTACTTAACAGGTCATTGCCAAAAGCGATAAGCACTTTTAAG
GACCATATTAATGAATATGAAGCAGCAAAAAGTAAAATGCTATAACAACCTATGATCTGGTAAAATATTTAAT
CTGATAACTACAGGAGGAGGATGAGCGATGGAAGGTCAGTTAATTTTTTGTTCCGATTCAATACTACGGTTTC

AGTCCGATTACGATGAAACCGCTGCCGTTCCATTGCTATCCATCCAGAACGTCATAGCCGATACCGATCCGTT
CTTTCTTCTCCGTTTCTTCCACCATACCGTTCTGATTGAAGAAGGAACAACGCTGGCAAGTATCTTTCTGGCA
ATAGAACCGTGGAAGGCTTTGTTAGCGGCTTATCTGGACAGAGATGTTGGTGCTTATATAGATGAGGTAAGAA
AACCGTCCGGGCCAACGACATGGGATATTGAATGGATTGGGATTGATCGCCGTAGCATGGTTTACCGCGCTTA
TAAACGTCAGGAGATGCAGGATGGAGAGGATTTTTCTGACTATTTAAACCGGGAACGCGTTCTGACAGATGAA
TTTGAGATTGAAAGTGGCTGTGAGGCTTCTGGCTTCATTAAAGGTGATAAAGAACGATGGAGCATCAGTGGTG
ATGTTCACGAGATAAAGAATCTGCCTGTAATCCTCTATAGCAAACAGACATTGATGACTTCACCGAAAGACGG
TTTGTTGAAGAAAAACATATCGGGTGTGAAGAGTTCGAAGCACAGTTGCTTCATTTATGGCGATACCTCGTTT
TCTTTCAGTGAGGTTATGGAAGCCATCTTCATTAGCGGGCTGTTCTTCTATGCACCAAAAGACGCCGCCAGCA
GTCTTGATGAGCTGAAAGCGAGTCTGGCTGAACTGGAAGAAGAACGCGCTGAAAATCCAAATGCGGAGTCGAC
TGGCAATGAAACAGATGAAGAACCTACCATCGTTGTAGCCGAAGGTGCATTTGATTCGCTCGCTGCTCACATG
GAGTCTGAAAAAGCGGAGTGGCAATCCATTAAGAAGTTATGCCAGCAAGAAGGTGGGCTGCCCATACGCATTG
GCAACATTAAAATGTCTGAGTCTATTCAGATTATACCTTAGTTGATATTTTTACTAAAATGTGAATGCTAAAT
CATAGAGTGGAATTTAATAATGTATAAAGAAGAAAATAAAAATATAGCTCGTAAAAGTGTTTTAAAAGCTGCG
ATTGAGGCGCTTACTTTGTGTAGAAAAGACTCGACTTTAGCTCCTAAAGACTATATTCGAAAAGTTAAAGCGT
TTTATAGGAAAGACGAGAGTGATCCACGTGCGTTTATTGTTGATGAGTTATCGGAGGAAACAATAATTCGGTG
GGAGGAATTCTATGATTCAGTGATTCAGGATAGGACTGCACGTAGTATTAAAGTCGCATATTTATCTGGGCCT
AACCCTGAAAATGATTTAACTGAAATGACTGATATGGGGCTTTTGCCAGAAAATATTTGGGCATTTGAAAGTG
ATGCTAAAATATATAATGAGGCTGTAATTTCAGCACTTTCTTCAAAATTTCCGTTTATAAAATTAATTAAAGC
TAATGTTGGGGATTTTTTTGAGGTGTCACCGCAGAAATTTGATCTTATATATCTTGATTTCTGTGGGCCATTG
CCGAGTAAGAAAGCTGGTCAGAAAACATTAAAGGCAATAACATCAATATTAAAATATCATGCGCTTTCACCTC
TTGGTGTTATGATAACAAATGTAAGTCTACCGTCAAAAGAACAAAACGCGAATGAACATAAAAATATCGTAAA
TTTAGTTGCGAGTTATCTCTATCCAAAATCAACACTGGAGAGTAATAATCCTGAATGGAACTGTACTGATGGT
GCAATTTCTGAAGGTTATAGCCTTGATGAGTGGCATAAAAAAGTTGAATGTGAAATTGAAGATTTCTATGGGC
AGTATATAACGAGATTATTAGTTGATCTGATTTCGGTGATCTCTCCATATGATAATTTCACCTCATCGCATTC
TTTATATAAAAATATGTTTAAAATTTCTAATTACAACGATCTAACTAAAAGCGTAAATGATTTATTTCATTTT
GATAGTAATGGGAATGGTGGGGATATTATAGTTGATTCAGGGCTATTTCCAATCCTTTGGACAATTGCATCAA
TAGATAAAAAAATACAATAATAAAGATAAGAATTACTATCAAGATATTTATTGCGATGATGATTTTAACGATTA
TGCTCAGTCTTTTTTATCGCAAATGAGTGCTAATGGTAATGCTCATGATTTAATAAAAAACATTTCTAATATG
CACTTTCTATTAAATGAAGGGCGTACAGAGAATAATTTCTATAGTGATTCTCTGCGAAATCTCAATAAAATAA
ATTGGTATCAAAAAGTTTATCCATTTTGTGATCTTTTTTTGTTTCATCAAATTAAAGAGGTTTTATTCAGACA
GTTATCCGTACCTTATCATGTAAATATGGAAAAAACACTGAGATGGAAATACAAAGCAAAAGACACCAATATG
TATATGGATATGTTGGTGCTTGATGAATGTCGTTACCTATACGATTGGATGCCTTCACTTGATATGTTTTATA
GTGGGATGATGGATATCGAGAGGCAATTTAGTTTTAGGTTTATTCTTGATGCTGTAGCTAAGCATCGAATGGT
TTATAATAATGAATTCTTTTATGGGACTGCATCTGTTAGTAAATTCGAAACTGATTATGTTGAAAAAGTATTG
AGTGTTAGAAAGAATATAATCTAATCATATAGAGTAAGATCACTTCCTTGGGAAATGATCTTACTCATGAAGT
TAGTTTCATAAAAATAAAATGAAACCACTAATCAATCATACCTCGTGTAACTTTACTGGCTGTCTGACTAAA
ATTATAACGTTCCACAACATCACCTTTAAACCAGATATCCAGTGATTTAATGTGTGCCGTGCTGCTATTGGTC
AATAGGGCAAGGCCGGGTATGTAGTTAGATAATTGGCTTTCTCCCGTCATACTTGAGTAGTTCCACATCTCCT
GTCCATCATTCGTAGCGCGTGTCTGAGGTTCTCCAAAGGCAGCAAGAATTTCTCGTTGGGTAGTTTTACCCTT
GACGATTTTTAATTTAACCGTCTGATGATTTTCATCAGTTAGAGATTTATTACCATGAACGGTACAGCCACCA
AGCGGAAGTATGATAAAAATAGCAAATAATAATTTTTTCATTTTTGTTCTATAAATAGATGAAAGGTAACAGG
TTAGAAAACATACTTTTTAAATACCAGCATCCACCGCTCGCAAAGTTCGTGATTTGAGGTGCTTTTATTTTTC
AGGGGAACATTAACTTTCCACATGGGATTTTTAATATCATCGGTAAAATATTTAGACCAGACCGTTTATTAGC
TGAAAGTTGTTCTCTCCAGTTAGCAATAGCCGCATTATTCATACCTCGTAATGTTCCATTGCCGTAAAACTTA
CCACCCTCTGAAAGCGTATAATTTATTTCCCGGATTTCATCAAAAGTATATGTGCTGTCTTTATCTTTATTGA
AAATACGGACAGTATGTTTTTGAGTATCAATAACAATACCTGTCTGCTGGCATTCATACGAAAGAATGTCGCC
GTCCAGAACTTCCGTAAAGCTGTTGTAGTGCTTTTTCTTCTGTATATCTGCTGCGGTCATGATAATGACGAAG
AGCACCACTAAAAATACGATACCTGTTGCTTCCACTCTTACTTCCTCCATGTAAATGAGTTAAAGAGACTGAA
CCCGCTCACAGAGCTTTGCTTCGTTTTATACGTCCTACAGCACCAAACTCATGGCGTCGGTCTCGGCGTTGCA
AGGCTATCAGAACTTGAAATTATGATCGATATAAAGATCGATAATTAGTTATCGATAGATTATAACGATTGA
AAGTGTGATATCGATCGGTTATTAATTGTTATTATCGATCGAATATCTATTTATGATAGTTAAAAACTATCAT
AAGATTGTTTTTGATCGTTTTAAACGATATGACGAGACGCTAAAAGAATTGAATCCTATCAATTGGTTAAGCG
GTGGTTTTGCCATGTGATATCAGCCGGATCCCGCAACAACTCTAGCCCTGACCGGATTAGGTCAAAAAATATA

CGCAATTTTGATTATCAGGATGGATAAGCAGATGAAGAAGATTTTACTCGTTGCTGGCGCGGCTCTGGCGCTG
GCGGGATGTGGTGAGAAAGGTGAGTTTGAGAAAGCGATCAATGCCAAGATTGGGCAGGACAAATACTGCTTCT
CGCTGGATAACAATAACACCAGCTTCCCGATCCGTCTTGCCAAGCCACGGCTTGATTCGACTGGCACAGGGAC
GAATTCAGTGATCCTCGACGGTTTTATTGAGCAGGGCCTGATGGTTTTTGAACAGGGCTATGACTCGAACGTA
TTAGGGATAACAGAGGAAGGCAAAAAAGCCAAAGTCTGGAGCACAACAGACGGTGCCTGTGTTGGTCGTCGTG
CTGTTGACGAGATTAAGGAATGGACTGAGCCGGGAAACGGTAATCAGAAAGTAGTTCGTGTGTCTTATACGTG
GAAGCTGGTTGACGTTCCGAACTGGATTGACAAAAAGGCATTCGCCAGTGTTAAAGGAATGAATGAACCTGCT
GATGGTGCAATGAATCTCTTTAAGACCAGCAATGGCTGGAAAGCCAATTAAGGAAACCAATATGGAAAACAAT
ACCATCAAATGCCCGTTCTGTTTCAAAGAGAGCCAGCGTGGGGTTCACGTTTGCACAGGTTGCTTAGCGACTG
TGTTATGTGGCACTTATCCCGGCTGGTATGCTGCTGTTGTTTTGTTGTTGACTTTTGGTTTATCGACGTTGAT
CGGTATGGCAGGAGCTACTATCAGCCTTCCGATAATTGCTATCGTAGGGTTTGTTGCTGGCAAAGTTATTTTT
GCTGATAATGTAGTGTTTAGGCGAAGAATGTAATTAAGCATAAAACAAAAACCATATTCTTGATTTATAGGAG
TATGGTTTATATCTATATAAGAGGATTGGATAGAGTGCCCTTAATCGACCTTGAGCTGAGACCTTAATAAATC
AGGTTCGCATCTTACCTGACAAATTGCTCCCTGATATTAGAAACAGATGTGCTGGTAATGAAATTGTGTGGTA
TGATGATTTTAGAAATTGTCTCTTATAAAAGGACTTTGAATAAGATGAATACAATCTATCATTATTGCAGCCC
TGAATCTTTCTTCAGTATTATTCAGAATCAGCGATTATGGCTAAGCTCTATGGATCATATGAACGATTACATG
GAGAAAAAGTGGTTTTACTCTACACTTAAAAAATATTTATATAAAAACTTAGATGCTAACTGCGTTGACCAGT
TTATAGCACATTTGGATGACAATATATCTATTGGAACACCATTTGCTTGTTGTCTATCTAAATCTGGGGATAT
TTTAAGTCAATGGCGTGCGTATGCAAAAGATGGCTTTGGTGTATCCATCGGATTCGACAGAGAAAAGCTCGAT
GTCTATGATGGGATTATAGGTAATAACCTTGATCCTAAACACCGACTAACTCTTAGCGATATATCTTATATGG
ATATTAACGTAATTGAATGCTTAGCTGAAAGGATATTATCAAGATATTCATTCATAAAAAAATACTATATGAA
TGAAATTATATCGACATCAAAATTTAACCGCTACGACAAATGCATCCTTGAATTAATTTCCAATATTATTCAC
TTAAATACCACGACTAAAAACCCAGCATTTAAGGAGGAAAAAGAAGTACGATTAGTTTATCAAACTCTTGATA
CAGGAAGATATGAATACCCAGAATCATCATCAATTAAAGATCTGAAATATAGAATATCCAATAATCAAATTAT
ATCATATTACGAATTAGGTTTCCCAAAAGATGCCGTTTCAGAATTGATATTAGGTCCAAACAATAAATTTAAA
GAAAGCGATATTGTTAACTTTTTGCAGTATAATGGTTTTGAACACAGCATAAAAATATTGAAATCAAAGGCCA
GTTATGGAGCTTAAATAAAAATGTATTTTCTATTTTGCTATTCATTATAATTATTATTACGATATGCCAGTGG
TCTCAACTTTGAAACATGGGACTTTCCTGAATACTCTGAGGGGCTTGATGGTATGAGATCTATGTATTTATCA
ATCTCTGTTTGTACATCTTTCGGACGACTTGGAAAACGAACGGTTTTGTCATGGTCTGATTCATTGCCATAGA
TGCTTGCTTCTGGTGATGTCTGGTCATTGTTAAATTTAGCGCGTTTTTCAAACTCCGAGCGTGGAACATTGAT
ACCCAGCACTTCATCATAGACATAGTTCTCTGCGATACGCTTATCATGGTCAGCATCAGTGGTATCGGGTTTC
CTTCTGGCCCATATAAATTTCTCTCTTATATATCCTACTATCAGTAGCGTGAAACGTTTAAACAAACCATTCT
GGCGGGGCTTGGTCTGTTTCCTACTCCTCATCGGAGCGCGGTTCTTCGCTCTGGTGTTTTGAATAGATGGGGT
TTTCAATTTAGTGGATGCAGTTGGTTCAGGAACAATCAGAACGGGAGATTTCTTCCCTGATACATTTACTTTA
CTGACCTTATTGGGATTACGGTAAGATTTTGTTGCTGGTCTTCTGCTTGTGGCGTGTGTCGTCATAACAGGTT
GCTGTTTAGTTGTAGCCGGGAAGTCAGGGATAACGATTTCTTCGGGTTCAGCAAGATGTGTAATCGTAAAGCT
TCTGACATCAACCACGATCTCCAGTGGTAGCTCACTGAATGTGTTGTAGACCTTCTTCGCTTCCTCTATTTGC
TGATCACGTAAAGCTTGTTCTGTAGCTCGCTGTTCCTGTGACTCGGTGTTATTCCACTTGGCACGATGAACTC
GTTGCATTGCTGGACGGTTTGTTTCGGTTGCTTTGGCAAGCCAGAAGGCTTTTTCTTCAGCGCTGAATGCTTC
TTCTGCTTGGGCAAGGGCATCAGCACATTGAGTTCTGAGAGAACGGTGATCGATACGTTCTTCACGGCCTGCG
GTCTCAAGGTAGCTGTTTGACATCGTGGCCCACGACTCACGCCACTGAACAACCATCTTCTTATCATTCCAGC
TTCTGTCTTTTTTGCCGAAGCCTTCTGGACCAATGGTTTTCAGGGTTAACATGACGTGAGCATGTGGGTTTTT
ACTGTCCAGATCATGAAATGCGATATCAGCAATCATCCCCTTATCGACAAAGTTTTTCTGGCAGTATTCGGCA
ACTAATTTTTTCTTGTCATCATTGCTGAGTTCTACGGGGATAGCGACATCAAAATAACGCGCTGTTTGTCCGT
TGTTCTGGCGTTCCACTCTTTCAACTTCATTCCACAATGCTGAGGAGCTTTCAACAATATGAGCAGGTGCGGA
AACAGGTGCCAATATAATATGACCATGTAGATCTGTTCTGTGGCTGAAATCGTATGTTTCACCGATGCGATCA
TCTGTAATACGTGTACGGGTATGGTAGGCAGCCTTCGCAACGGAAGTCATGCCTTCGCTTCGTTTCACAATTT
TAAAATCCAGATGAAAAATCGCCATTAATAAATTAACCTCAAATATAAATGCTGCCGTTAGTTTTACGTAAGA
AAAACTTCGGGGTTGACGTTGGTTTTGCTTTTAGCCTGCGGCAGGCAACCGTCGGTAGACCCCACACACTGCG
TAGCAGTGTATAGGTGGGCATTGTCTTTAAAAAATAAGCCAATGTAAAAACTCCCCCTTAGTTAATTAATACG
AAATATACTGATAGTTGTAAAATGATGCGTTAAATTAAACGACACGAACTGGATTAAAGGTGTAGGTTTCACG
TCCGAAGGGAAAAAATAAAGAAAAGGCCCGTAATGGCAAAAGAAGTATTAATTGTTCCCGCCGAAAGGCGGGA
TAGCTAAAATTGAAGAAAAAAGCTAATAAAACGGAGAGTGTTATGTGTTGCACAATTCACAGGTTGAGGTTGA
AAAATAGAGTTTAAAGCTGATAGGTGAAAATAAAATAACAAAAGTACAAAAATAAGTAACGCGGAGAGTGCCA

GATGTTTTGTGTTAGTGATTACTATCGCATGTATTGCACTATTTTATTTATGGATGCTCCAGAACTATTCCTT
CTGACAAAACGGTGTAAATTTGACGTTATTTACCTTTGTGATAATTAAATACGTATAAACAATAAGAGGTAAT
AATCATGGATAATGAAACTAAAAGATCAAGAACAGAAAAAACATTAAAACAAAAGGTGGCATTTGCTCAACTT
GAACTTAACCGTTTAAAGTCAATGGAGAAATCAGAACAAAAGAAAGTTGAAACAAGGCTTAAGATTATTCTTG
GGGCAGAAGTAGCCAAGGCTATGAATTGTGGTATCGAACAGGTGGATAAGGAACTTGTTATGGGGATTTTACT
TTCAGCATCTGAGTTGAATGATATTGAAAGAGTCAAATATATAAAAGCAGGGAGATGGTTTCTTGCTCAAATG
GATGGCAGACAAAAATAAAAAATATTAAATAAATTTCATGACAGGGGTTGATATCTAATTAATATCTGTTATC
TATATAAAAGAACAGAGCGCAGGATAATACTTTATTAATACATATTTTTTCCTTTTCAATCAATCATGCGTTC
TGTTCGACCAGTAATCTGTTAATTTTTATCTTTATGTAGTAAACGTCATTATTACTTTTTCATCGGATATGAT
TATATATCTCATGGGAAGGTGATAATGACAAACGCTGTGATAATAAATGTTGTATTTAAATCTCCCAAAAGGC
AAAGAGCGATCTTTGCCTTTTTCAATTCATCCGTCTATCAGATAGAGTTAAATATATCTTACGCCATCTATTG
CCAGAAATGAGAGAAGCGTTATAACAGGAGATATCATTAAAATAAATAAAGGAGATAATAATGGTTTTATTAA
ATAGTAAAAGAAAAAGCAAAAAAGGTTTCTCATTACTCGAATTATTACTTGTTCTGGGGATTATTGCCGCATT
AGTAGTAGCGGCTTTTATTGTGTATCCCAAGGTTCAGGCTTCACAAAGAGCGCAAGCGGAAAGTAATAATATT
GCCACCATACAGGCAGGTGTCAAAGCACTCTATACATCAGCATCCAGCTTTACTGGGCTAACGAATACCGTAG
CTGTTCAGGCAAAAATCTTCCCTGATAATATGTTAAGTGGAACAGGCAATGCAGCTAAACCAATTAACGCATT
CAAAGGGAACGTGACGCTGGCTGCGGCAGCTACAGGTCCGTCATCTGCGGCAGGTTCTTCTTTCACTATCACA
TATGACAACGTGCCAGCAGCAGAATGTGTGAAAATAACCACAGCGGCGGCAGGCAATTTTTACACTGCTAAAG
TGGGTTCTAAAGTAGTAAAAGCTGCAGATGGTACCCTTGATGTAGCAGCTACCGCCGCAGCATGTAATAACGC
TACAAGTAATACATTAGTATTCACGTCTATTTAATTAAAGGGGCACTTTATGCCCCTTTTTGGATCATATTTT
CAGATGGGAGATATAATTATGATAAAGAAAAGAGGATTTACTCTATTAGAGATCACCATCGTTTTAGGTATAG
GTTCTCTTATTGGTTTTATGAAGTTTCAGGACATGAGGAAAGAACAGGAAGCAGTAATGGCACAGGCGGTAGG
CTTTCAAATGAAACAGGTTGGCGAAGCAGTTAACCGTTATATTAGCATTCGCTATAATAAGCTTTCCACACTG
TCATCTTCACGCAGTCAAAGTAGCGATCCGGGACCAAGAACCTGTACGGCTAATGGCTGTGAAATCACTTACC
AGACGCTGGTGAATGAAAGTCTCTTGCCCTCTACATATGCGGGAGTTAATGCCCAAAAGTCTTCTTATAAAAT
CCTGTTAAAACGATCAGGCGTATCACCAAACTATGTTATCAATGGTTTGATTGCTACGACGATACCTTGGGTC
GAAGGTGGAAAAAACGCGATACGACCTGTTAGGAAAATCGATGCAATCAGCAGGTATTGATAGCGGTATGACGA
AATCGCCTGCTCAGGCATCAGGATATAATGGAGTGTGGACAGAAAAGTCTACTGATTACCCGGCAATTAATAA
AGCAGGATTACTGGTTTACCGTGTGGGCTATGATAGTTCTATGTATTCTGTTTACTTGCGTCGCGATGGAACA
TTGCCTATGACTGGAAATCTCAATATGGGCACTAACGATATTAATAATGCCAAGAACATTACCGCATCGGGAA
CGGGGATCTTTGGTGGGGATGTTTCTTCCGCAGGGAAAATAATCGCAGCACAAGAAATAATAGCGCATAATGG
TTATGGTGATGCCATACATTTTGGCGGCGATGCTGTTAATAACGACTATGAAATAACCATGTCTAAGGATAAG
ACCTTATCTATTCATATGGCAAGCAATCGAACAGATCTCACTACGCTGAAAATTAGTGGAGGGCTGTCTACGA
TTGGTAATGGAACGATTAGTGGAACATTAGATACAGGTAAAAGTATCACATCCGGTGGACAATTTAATGGTCA
TAACGGTGGTGGTGATAGTTTCAGTATTGGTGGTGGAGATGCTAACGATTATGAGTTCAGATTAGATACTGTT
AAGCCGCTAACCATCTGGCGAAATGGTGGCACCAGTACTGAAACAAGGCTTCAGGTTTTTGGGAAACAAACCA
ACCAAGGTGATTTTGCTATTACTCCGGGGACAGGAAGCACTGGTAGTATTGCAGCATCTGGAAATATTCAGGG
ACATACTCTACAACCTACATCGACGAATACAACTGGTGGGACATGTCCAAGTATAGGCCTGATATCTAAAGAC
AACATGGGAAATATATTATCCTGCGTTAATGGAAAATGGTCTGTTGTCAGTAACCTGCCAGTTGGTTCACCTG
TTCCTTGGCCTTCAAATACAGCGCCTGTAGGTTGGTTAATATGCAGGGGACAATCTTTTAATACTGCCCTATA
TCCTCAGTTAGCAAAAGCCTATCCAAGAGGAAGACTTCCTGATTTAAGAGGAGTATTTATTCGAGGGCTGGAT
TCGGGAAGAGGACTTGACTCAGGAAGAGTTATTAATTCTTATCAGGATGATCAGATTCAAAATATTACAGGTC
ATATGGCGGCTGATGTATCACAGAGTGGAAACATCGGTAAATATGTGAGTGGCGCTTTCGCGGATAGTGGTGC
GTTAGGTGAAGGTGATGAAGGTCATAAAAGCAATGAAGTAAGGAAATATACGTTTGATGCTTCGAGAGTCGTT
CGGGCAGGTAATGAAACAAGACCTAAGAATGTTGCCATGAATTATATCGTTCAGGCACAATGACTATTTTAAT
GATCAGGCGACTTTAAATGCGAAATGTACGCCTAATGGTCTGGTGGGCCGAGACAGTATGGGTAAGGTTTTAT
CCTGCGTCAGTGGTAAGTGGCAAACAGTAAGTGGTGATAGATTAAAAGGTATTTATATCACGATCACAGACCA
AGTATCGGGCTATAAGTGTGTAATACCAAACAGCGATACAAGGGGATGTTCTTGTCCGGGATCGGCATATAGC
TCAAAATACAGCTATTTATCAGGAACGTTAATTGCTCAATATGATGATCAACGTTGTGGTGGTGGAAAGAATG
ATCACTTTTATAGTAATTACAGAAGATTATATGCATGCCAATAATGTTAACTGTTGAGGAATTAATGTTTGTG
TTTAAACATTATAATAAAATTTAACTTTTAATAAATAACATATTATTTAATAAGTGTCTTTTAAAGATATTCA
CTTTTATAATATATCTGATTGTCTAATATATAGGGAGTAGTTTACAGCTCAATGAGTTGATTCAGTTTTCCCA
AGTGATATGTATTCTCTCCAATGGAGAGTTCCCTTTCGCTTAAATATTTATTTCATTTCAATAATGATTCAAG
CTGTTCAGCAATTTCATCAATGGTATTTATCGATGTATTTAATGCCATTTTATCTACTAGTGTAGGGCTGAAT

TTTAAAATATCAGATTTAGATACTTCGTGCCAGATAGGCAGTATTACTTGGTTTTCACCGCTCATCTCTCTAG
CAGTCAAACCATTTAATTCATACTCTGTCCAGTCCTTTTTGATAAAGGATTTAGATAAAACAACAACTCCAAA
CCGAGAATTTGCTAATCCATAATCGATTGTCTTACGTAGACTTTTACCCCAGCCTAAAGAGAATTCGTCATAC
CATACATTTATCCCTTTTGCTCTTAACAACTCAGCAAGGGGACGGACAAAACTATCTTTATCTTCTGTTGCAT
GAGAGATAAAAACATCATAACTCTCTGAAATATCTTCTGTCTCCTCAAATTTTGTGGTTTGTGGGATATTTTA
TTTGGGGTTTGGGGATATTCCCCTTTTCTGCGATTTCAATTCTCGTGTGGTTTTCTTCTTGTTAACTTATTGT
TCTTTTTCTTCTTTCTTCTTGCGAGAAATTTTTTTTTGCTGGTCTTTTTCTTCTTTTTTAATTGGTAGTAATT
GATGTGGGGGTGTCCATTTTTTTCTTTTTTTTTTTTATTAAATCACATTTTGTGCTTTAGACTTGAAAAATTT
TATATTATAATAGAATGGATATAAAAATTTAGAAAAATAAGGTGTAGAAAATGAAGATGGGGTAGGGACGCGA
TTTTTTTGATTGATTTTTCCTGAAAGTTGGGCCTCTTTACGCTGCTCATCAGAAAGTTGTTTTTGTAGACTAG
CAATGTCTTTTTGTATTCTGTTGATATTTGACATTGTGGTGCTTATAGACATCAGATTAACTCCCTGAGATAT
TTACATATGGTGTATACTACACTATCTATGTACTTGTAGGCGATTGATTTTACCTAATAATATATCGATAAAA
GAGGACTTTCTTATCAGGCCGTTCTAACTCTATACTTCAACACCTGAACATGATCTCCATCATTGAAGTATTT
AGCTCCGCTGGTAAAGGTGCTTAGTCTTTTGGCGTGCCCGGAATGACCACTGTTGCTTGGCGCGTGGACAATA
AATTTACGCGTTTAGTTGGTCATTTTTGCTCCAATATTCTTAATGGATACATTCCCCTTCATTATGAAATTTG
CATTAATATCATTAAAATGAATGTCATTTGATATTTATTATTAGGTATAGAAATGTGTATAATCGATTATTGT
TTTATGTATACGATATGGTGGGAAATGAGTAATACTACAGATTGGATTAATACTATATCTAATGTGATGATCG
CCGGTTCTGCGATTTATGGCGCTTTTAAGGCTAAGAGCTATTTCAAAAGTAAAAATGATGAGGAAGCATATAA
TGATGCAAAAAAATTGATATTTGAACTGTATCCTCAATATAGAATTGATTTGCATAAATTGGTTATCGTTTTG
TTATCAATGACATCCAATAGAATTCCAAATGATATATTGGAAACTCGGTTGGAGAAAATTACCAATAGACTGT
CTAATACACAAATTGAAATTGTAACTATCAGTTCCAATATTATTGAACGCCATAGATGGAAGATCAGAGATGA
TTTTAAAGAGTCTTTTGAAATAATTAAAAATATCTATAGGAATAAATTAAGTTTTCCATTGATTAATGTAGTG
GATGAAGTAAGTCAAAGCACAGAAACTGAAAGGCAAAATATACTTAGAGAATTAATAGGGAAATGTCATGAAG
GTATTGATGCCATTCATACATTCACAGATAATAAATTTAGAGTTGACGAGTATTTCGTTCGTCCCTTTTTAAA
CTAATTTTTCTATAAGAAGATTGATATGAGCAATGAGAAAACATCAGAGGAATCATTTTTCAATAACATAAAA
AGTTCCTTGATCGAGCGTTTCACGACACCATTATATGTATATGTTATTTCAGCCTTCTGTATTGATAATTGGG
ATAAGATATTATTTATTATGTTTGGAAAAGGGAACATTGAGTATAGGACGTCGATAGTTCAAATGCAGGGCAT
TAACTTTTGGCAACCTATTGTTTACGGTATAATCATAACCATAATAATGCCTTTTTTATCAAGAGCGATAGAA
TTTTTCCATCTTAAGTCGGACAGATATTATCTGTACTCTTTCCTCCAAAAGGGACTCTCGTAGGGTAAAAAGT
ATGAACTCTATTAGAGTGATTGAACGTCAACATGAAATAGATATGATTGAAGGTGAAGCAAAACATAAAGCAG
AACTTGAAAGAATAAAATCTGAATCTCAATACAATACGAGCGAGTTAAAAGAAAAACTTGAACGGCTTGATAT
AATTGTCAAAAACCAAGAAGCGCGAAATAATGAACTATTAGAAGAGCGAAAAAAATTAGATGATGATCTCAATA
ATATATTCATTTTTGTAGATGATCTTAGGGAAACTGTATTGAGAGTAAATAATGCTTATAATCGGTCTATGAA
AGAAGATGATGTTGTAAGGAAAAACATGATATTATCCCCTATATATGAGATAATAAAGGAATCAAAATTTAAA
GACATAGAGAAATATCTAGAAAAAGTCGGTGTTCTTGAAAATAGTTTAAAACGTCTCCGAGACTCCGAAAGAA
AAACATTTTCAATGGATGAACTGGAAAATAACGACTATAATGCAATAAATGCCGATTAAGACACGTTATTTAA
AATAATTACCTAAGCATGATAATCATGTTGTAAATATATAATACTGTATTGATAAACAGATTGGATACCACTT
GTGCGATACCCCGTCCAATCATCCATCAATGCCACATGCTTAGCCCATAGCGTCCCATGTTGATACTCTGCTT
TGGCCTTATCTTCTAACTGGTGAGCTAACGCATGTTCAATGATATTCCTCGCGTATTATGAGCACGGCCTCCC
CACTGATTTTCAGAAGGCAACAATTTCAGGATGACAATGAGCTTTTAGCGTATCTTGTTCCTGTTAGACAAAA
TATTTTTATAAATAAAATCAATTAACTACACGTATACTGTGATTTTATACAGCAATGATGTTGTCTTTAGTGG
AATTAAAGCTTATAAAGAATATGGTTATAACTGCATATAGTGATTTTTTTGATGAAGACACTACATAAGTGAT
GTTCATGACTTTTGGGGATGCGTTTAATGAGTAAAAATGTAAAACAGACTTCTGAAAATGTGGCTTCTACTGC
TGCAAAAACGCTGACTGATCCTAATGCCTCAGCCATCCAGAAAAGCCTTGCGGGTTCCGCGCTTTCTCAACGT
GGGACGTCAAACCAGACCAGTGGCAAAATGGAACATAAGGCATCATCGGCCCTTGATAACCCACGCTCTAGCG
AATTAACGAAGCAGCTTGCTGCTTCGGTATTGGCACAATCAAACAAAGGGCGTAAGTGATAAGTAACAGGGAG
AGTAAAACCTCCCTGTATTTTTTGATTTAATGCGATTGTTTGCTTGTAGGAAGGTGATTATTGTTTCTGAGCA
AACTCAAACGGCCTGACCATCTCATACCGATTCTCATCAAGAAAATCAGCCCACCACTGGAGCATCAGGCGGC
GTTGGTCAAGATGCTTGGCCTTATGGGTATAGGCTGCGCGAACGCTGTTACTTTCCTTATGGCTCATTTGAAG
CTCTACAGCATCTTCTGACCATAGACCAGACTCAATTAAGGCACTACACGCCAGTGTGCGGAAACCATGACCG
CAGATGTCCTGTGTGGTGTCATAGCCCATCTTACGTAGCGCCTTGTTAATAGTGTTTTCACTCATGGGTTTGA
ATGAATCATAACAGCCAGTGAAAATTAATTCTGCTTCGTTACCTTCTTCATAGGTAAGCTGGCGGATCTCTTT
CAGTATCTTGAGAGCCTGCCTGCAAAGGGGAACGAAGTGCTGACGTTTCATTTTAGCCCCACGAGTCGAGTGC
TTGACGTTTTCAATCGCTTCCCGCTGTTCGGGGATCACCCATAACTTACTTTTGAAGTCGATTTCCGACCACC

GGGCGAAACGAAGTTCGCTGGAACGAATGAAGATCAGCAGATTGAGTTTAATCGCTAGCGTAGTCAGTCCACG
ACCTTTGTAGGCATCAATACGTTCAAGCAGTAGCGGGATCTCTTCCAGCTCCAGTGCAGGGCGGTGTTCCGTC
TCTGGTTTCTGGACAGCGCCTTCCATATCATAGGCCGGATTATGACGCATAAGCTTTTGCTGGACGGCATGAC
GTAGGATCGCGGTGATGTATTGCTTAATCCGCATGGCAATTTCAAGGTAGCCGAGTGTTTCCGCTTTTTTGAC
CGGGACAAGCAGATCACCCGTATCCAGTTCTGAAACGTTTCTGTCGCCTATATCCGGGAAGACATAGGTTTCA
AGGCGCTTCCAGACAGTATCGGCGTAATCTTCTGACCATTTTGTTTTGGTGGCAAACCAGCTTTTGGCGACGA
CACGGAACGAGCGGGTTTTATCCCGCTTCTCCTGAAGGACTTTTTCATCAGCCTGTTTTTTAGCGTTCGGGTC
AATCCCCTGAGTCAGCAGCCTTTTGGCCTCGTCCCGGCGTTGTCTGGCATCGGCAAGTGAAACCGCAGGGTAA
ACCCCAATGGAAAACACCTTCTGTTTGCCATCAAAGCGATAGCCTAACTGCCAGTATTTTGAACCGTTAGGAT
GCACCAGCAGATAGAGGCCAAACCCGTCAGTGAGCTTGACGGCCTTTTCCGATGGTCTGGTATTTTTTACTTT
GGTATCAGTAAGTGACATGACGGTTCCCTCCGCGTGCTGGTAAAACACAAATCGAACCAGCTTTACCAGCATT
TTTACCAGCAAAAGGGTATGGCTTCGAGTGGTTTTTAGTGAACGAGAGTGAACCTGAAGAAGGGAATAACCAG
TTGATATAAATGCAGAAAGCAGACGTCAGTGAACGTCTGCTTTCCTTAATTTGGCTCCTCTGACTGGACTCGA
ACCAGTGACATACGGATTAACAGTCCGCCGTTCTACCGACTGAACTACAGAGGAATCGTGTGAACGGGGCGCA
TAGTAACGATGTGCGATCGGCTTGTCAAAGGGGGAAATAAGGTTGCGCGTTTGTTTGCTGACAAAAACAACAA
AGCGTTGAAGTTTTGATCTAATTTCTACTTTGCCCCGGCATGGCGCAACTTTGTCTGTAATTGCACAAGTCAA
ATGCTGTGACCTTACCGCAATGGCTATGTGCCGGCGTCTGATGAAACGTGAAAAACTGGCAGGCACTTGGCAA
ATAATTCTGAGACATAACGCCGTAGAGATTAAGGGCAGGGGGCAGAATGAACTTTAGACGTGAAATATTTTGT
AAAAATGGTTGATACAGGCAGTCTGACGCCGGTAGCGGAAATGGCAGATAAATTTCTGGTGCAGGCGAAAAGA
TTTCCGTCAATATCATAGGCAGAATTATGGCGCATCAGCTTTTGGCGACGACACGGGACGAGCGGGTTTTATC
GCGCTTTTCCTGAAGGATTTTTTCATCAGCCTGTTTTTTGCGTTCGGGTCAATCCCCTGAGCCAGCAGCCTTT
TGACCTCGTCACGGCGTTGTCTGGCATCAGTAAGAGAAACCGCAAGGTAAACCCCAATAGAAAACACCTTCTG
TTTGCCATTGAAGCGATAGCCTGTCTGCCAGTATTTTGAATCGATAGAGGCCAAACCCGTCAGTGAGCTTGAC
GGCCTTTTCCGCTGGTCTGGCATTTTTTACTTTAGTATCAGTAAGTGACATGACGGTTCCCCCCGCGTGCTGG
TAAAACGCAAATCGAACCAGCTTTACCAGCAAAAGGGTATGGCTTCGAGTGGTTTTTAGTGAACGAGGGTGAA
CCTGAAGAAGGGCATAACCAGTTGATATAAATGCAGAAAGCAGACGTCACTGAACGTCTGCTTCCCTAAATTT
GGCTCCTCTGACTGGACTCGAACCAGTGACATACGGATTAACAGTCCGCCGTTCTACCGACTGAACTACAGAG
GAATTGTTTCAACGAGGCGCATAATACTGGGCCGGCTATAACGTGTCAACAGTAAAATTAACGCGCCAATTCA
ATTGGTTAATTAACCCACAAAGTGAGGAATTAATGTTCGGATTCCTCGCCGTAGTCGCCTTCGGCAGCGCTTA
CCCGTAAGCGCTGAGAAGGATCCTGGCGATAGAACTGGCAAAAACGCTGCCATAGTGCCGGGAAACGTGGAGC
AAACAGTTCTGGCGCGCTGAAAAAATACTCTGACAACACGGCAAAACATTCTGCAGGGTCGGTGGCGGCATAG
GCATCTATACTGGCAGCGCTTTCGCCAACAAGATCGATTTCATCCTGAATATTATTCATTGCCGCGTGGAGAT
CGTGTTCCCAGCCAGCCACATCGCGTAACGGGATGAAAGGGATGCCGCTGGCGCGATCGCCATTACGCATATC
CAGTTTGTGCGCGACTTCATGAATAATGAGGTTGAAACCCGAAGCATCGAACGAGTCCTGGATATCCAGCCAG
TTCAGAATGATGGGCCCTTGTTGCCAGCTTTGCCCCGACTGTACGACACGCTGGCTATGCACCAGACCTATGT
CATCTTCCCATTCATCATCTACCACAAAGGGCGCGGGATAAATGAGCACTTCATGAAAACCATCAAGCCACTC
AATACCGAGCTCCAGGATCGGTAAGCAAAAAATTAACGCAATACGTGCACTTTTTAACGAGTCGAGCTCAAAT
CCCTGTAGCGCTACCAGTCTTTTCTGCTGCAAAAAACGTTCGGCTAGCGCAATAAGCCGAGCCTGTTCTTGCG
CGGTGAGGTTTACCAGAAGAGGTATAGCCAGCGCATCATCCCACGGCCAGTCTTCGTTCTGGGTTATTTCTTG
TGCTTTCCAGGGCCACTTAATCATCGTTTTGCTCGTAAACTCGTCACTTGAACAAAATTACCCGAATAGGGTC
TGTTAAAATGCCAAATTACCTGGCATCATTGCAATATACGGAGAGATGCCGGAGCGGCTGAACGGACCGGTCT
CGAAAACCGTTGCGGGGGTAACTCCGCCGAGGGTTCGAATCCCTCTCTCTCCGCCACTATTCAAGCACTTACG
TGATTTTCTTATAGTGATGAAAATCACGTTGAGAAAAAAATGAGAAAATTCGGTGAGAAAAAAACGCCAGAATT
TTAACTGGCGCACATCGAAAAGCTCAACGCTTCCTGTCCAGGGTTGGGCTCATTTTCACCTTGCGATCGTAAA
CAAGAACCTGGGATTCGGTTTTGTGGCCACTGTACTTCTGCTTGTCTTTTGCCGTTCCCTCATAGTCTGAAAT
CCCCTTTGCCTTTAGATCGTGGAAAGTGCAGTCAAGAGGACGTCCCAGATCATCCCCCGCAGCCTTTCGCGCC
TTTCTCCACGCCTCGTTAAATCCTTTATAAGAATAACGCTCGCCATACATAGTCCTGATAACAGGGCCTTCCT
CTCCCCATTCACGACATATTTCAACGGCATCACGTAAGCGATCTGTCCAGGATTTAATTTGTTTAACTCCGGT
TTTTCCTTGCTGAATAAAAATTCCTTTCTCCAGTATTTGATTCCAGTTCATTTTCAATACATCAGAAACTCTG
GCAGCACATAAATAAGCTATTTCCATTGCAGCCCTGACGGCTGGCGTTGCGTTATTATATATCGCTCTGTACT
CTTCATCGGTAATATATCGATCGCGTTGAGGCTTAGGAAACTTATCCACACCAACGCAAGGATTACCAGGAAC
ATAACCACGTTGATAACTCCAACGAAATACGCGCGACATAGAGCTGTGTTCATGATTCGCCTGGACACGGCTT
TTTTGCCCACGGGCATCCATATAACGCCGGATATGTTCTGGCTTTATTGCTTTAGCTTCGGCATCACCAAATA
CGGCAAGTATATATTTCTCATGTGCCAGATAATCTTTCTGCGTTCTTGGGGCCAGATCAGCATAATCGGCACT

GGCAAGAAATTTTCGCCATAATTGTGTGAATGTAATTCTGTTTTTTCTACCCTCAACTTTTTTTCGTAAGCCA
CCCAGACCTCAGCTTTAGTTGCATCAGCTGGAGCTATATTTTCTGTTGATCCTCCCGGTTTCCAGTAGTAACC
AGAAGGGCGAAAGAATACACCCTTTGGCATCCACTCATTACCGGGCGCACGTTTGCGACCCATGTTAACTCTC
TATAGCGTCAAAATTCATGCCTGGTACAGGCTGATACCCTGCTGGTGGAAGAAGGCGCGAAACTGGGTGATTT
ATATGATACCAGGTTGTTCTGATTGAACCGTCCCGGCGTTCTATAAAATAAATACCGTTCAGCGTTAATACTT
CTTTCTGGAGTGACTTCTGGCTTGCTCCTGTAGCATCTTCCAGTTCCTCCTCAGTCAGGAAGCGATCGCTCAT
GAGTTGCTTCTCCATCTAACCGGCTGCACCCGGTTTAAATCAGATATTGTTGCTGGTGGGCGGGATCAGTTTC
TGCCAGATAGCTGAAACGTATTTCGCCTGATGCCTGGCATCAGCCAGCGCGTTGTGCATATCGCCTTCGAACG
GCATGTCACGTTTGGGATCGAAGCCGATTGCACGACCCAAAGTAACCATCGTGCGGACGTCATGATCGTTCCG
GTAATTCCACAGGCAGGGGAGGCTGGCACGTTCGAAAGCGCCACGCAGGATAACGTTATCGAAATTGGCACCG
TTACCCCATACCTTCAGGTATTTCAAATCGTCGGCGTGACAGGTGATAAAGTCATTGAACTCAATAAGCGCGG
TCGTAACAGATACTGCATCAGCGCAAATAGCCGCTCGCGCTTCCGGGCTTTGTCTTAACCACCATAGAATGGT
ATCGCCATCAGGAACGGCACCTTGTTCCATTGCGCTTTCAAGGCTAACGGCGGTATAGAACTCAGGTCCAATT
TCACCACTTTGCGGATCGAAGAACACAGCACCGATGGAGACAACAGGCGCGTTAGGTTTTTTACCCATCGTTT
CAAGGTCGACCATTAAGTTGTTCATTCGTTATTTATCTCCGGTTTTGGTGCTGCTGGCACTGGCATCCAGTGT
GTAACAAAGATGTGCTCAATACAGTTCATCTGATTGCCGCCTCGCATGTCAAAAAATAGTCCAGAATGCTCAT
CAAAATATGAAACATAACGGTATCCCAACTTGTTATGAACAATTACTTCTTGCTCGTCTTCTGGCATCCGCTC
ACTACAGCTTATCCAACCATCCGGAATTACCGGAGAGTTGCCAGCATTTGGATGCAGAGCGCGAATGCCTTCG
GCCAATTCTTCCAGGGTGGATGAATATCCGCGTTGGGCATCATTGCCGAACTCGAACGCTCCGGTATCAGGAT
CAGACCATCCATGCTCGCTGTCGTATGCCTCACGCTGCTGATCTATCCATTTGGCGGCGGCTTCAACGCCATC
GCGATAGAAAGTGACTACCGGCGCTGACTGTTCTTTGCCAACCAGACGCGTTATTTCTGATTCCAGAAGTGAA
CCTAAAACTGTACTCGTGCAGTGCTCAGCCCATTCGTTGTTTTCCAACAGGCTGATGATATTTAGCACATCAT
TGTAAACGCCAGTTTCCTGTACTGGCTGGGCGGTGTAAAAATACGGTCTGATAGTCCACTTTTTATTCCAGAA
ATCTCGCGTTTTCTCGGCTTCCTCAAGCGTTGCAACACTACAGCCAACCTTTCCGCACTCTTTGATTACGTGA
TATCCGGCTGGCTCAGCTTCCAGCGATGCCAGCGCACGCTTCAGCACAATAAGAACTTTGGCGTCGTCATCGC
TCAGGCCAAACGGAATATCGTCGCGAGTGTTTTCAAATTCAGCGATAGTTTGCTGTAGCCATTCTTTGGTAAT
AGTGGTCATGGTGTACTCCAGTTATCTTCGATAGCCACACTAAGTCGGTGCAGCCAGTCAGCTAATTTCAGCA
TTGCTTCGCGTTCGCTAAGCCCTTCCGGAAAATCTTCAAGTTCAACGAAAGGCTTAAACCGTCCGAAGGCGTC
GTTCTTAACTGTCAGCTTCTGTTCAAGCGTGGTCTTCTTAACCTTGCTGTGATGCCGTAGAAGGTAAACAGAC
TGAGATTTTTCGGTTTCCGGATCGTATTCGTAGGCAGTTAGAATCATCTGGCTGCCGCCGCGATTTAATCCTC
TCCACATAGTCACTTCCCCTTGCCGATGCCAGCGGCGTTTAATTTCTGCCGTAATTCTACTAATTTGGTTTGT
ACCGCTCTTTGCCTGTGCCATTGCAGGACGAGCATTTCGGACTACCGTTGTGGTCGTAATAACCACTGCCGTT
ACACGCCGTGCAGGGACGCAGCTTCCAGCCAAAAACGAAACGTTGGTAATATTCAGTTCGACGAACTTTGCGT
TCGTGGAAGTTACACATCTCACCCCCTCTCAATACGCAGCGCCTGTTTATCGATGTTGCTCATTGGGCTGTCT
CCTGTGGATAACAAATATCGTCGAAATATTTTTCTGCTACGCGCATGTTGAAGTGATCGAGATTCATCTCCTT
CACCTGGAGTTTTGCCCCAACAATGCCTGTGCATTGATTGACGTAATCCCGGTTTTCTGGGGATTCCGTTACC
CACTCCATAAGGTTTTCGGTGACACTTTTTAAGCAACGTAAAGCGCAGTCCAAATCAGTAAAATGCTGAGCAT
CAGTTATGCAGGAGACGACATAATACGTGGTGACTTTTGGCCCATCAGCGCGTCGTTTAAGCTCTCTTTCGAT
AGCGTTTTTCAGATCAACCAGTTCATGGTCATTGAGTTTGTCGATGTTGCTCATTGGGCAGCCTCCATTAGTT
GCATTACGTGTCGCTTGTGCTCTTCGCTTTGCGGAACACCTGTAAAGTTCACCGCCATGAAATAAGCCAGGCG
ATCCTTGTACGTCACCTCATCCAGCACAACTGCTGGGAGTGGACGACGCCCAAATGCCAATTGCTCCGCACGA
GTCATATCTCGCCAGTAAAGCGGACCATCAGCCAAGATGATTGGAATCTCATTGGTGATGAGTTTTTTCAGGG
TTGTGAGACGCTGCTTGCCGTCAACAACTTCTATGTAAGGAAGTTCACGCGAACACCAGTCAGGTGCCTTTGC
CAGCGCCACTGAGCCGATAGGAAAACCAGAAATAACTGCGTTTAAGAATGCCTGCTGCTCTTCATGCCCCCAG
ACATACCCGCGCTGATAATTGGCATCAAAATCAAGTTCACCACCAATGATCCAGCGAATGTACATATCAACCG
GGTACTCACCGGTGCGCGCGTCGAATACCTGAGCATTGCGAATTCGGTTGCTCATTGAGCTGCTCCTTCAGCT
TTCTTTTCATCAACGCTCCACGCCGTAGCCAGCGCACTTGTTACCTGAAGAAACGAGTGTTTAACCCTCACGG
TGAAAGTTTCTCCGGTGGCTGAAATAGTCTCGATGACTGTTAACTCGCCGCCGCTTTTGTAGTCTGGATAGAA
CTGTGTTACCAGATTGCTATCAACAATCACCGATCCGTCCAGGGTGTACATTTCCAGTTTCATGCTGCACCGC
CTTCAACGCGCTCCCACAAAAGTCTTGATCTGATTGCCTTCACTACAGACTCTTTATCTTTTATGCAGCACAT
TGGCGTAGCTCCATCAGTTCATTAAAGCGGGCCATAAACAGGCCGAAAGCCTGACCGGGGCGAAGTGGGTAGA
TTTCGAATAAATCTGTCGGGGGGATACCTTCCAGTATTACCCAGGGAATACTGTCATCAATATCCAGATCACG
GCGTTCAGTTGCCAGCATGGTCAGATCTGCATACTTCACTACGCTGGCTTCTTCCAGTGGCAAGCCAAACTTA
AAGCGGATCAGTTGATCGGTACGTTTCTCAATCTCGCGATAATCAGGCAGTAACGCTTTTAATGGGGCAGGGA

TATCCTGGCAATACGCTTCGGCTGCGTCGTGCATCAGGGCTTCAAAGGCAAACTCCGGTGATACAAGCTGGCT
GCACAGTACGGAATGCTGCGCCACGCTATAAAATTCAGGAAGATGTCCGGAGAAGCGGCAAATATTGGAAAGC
GCCACGGCGATATCTTCAATATCAATGTCGTCAATAGTTGCGCTGAGATAATCAAATTGTTTACCTGAAAGTG
TTTGAATAAAACTCATCGTTGGTTCTCCTTATAATTTATTTCGCGCTGCACCGCGTGAATTTTGAGTACAGCA
ACCCAACCCACGATGTGGGGTTAATTGCCGCTATGAGTTATCGCTTGGCTTCGCCGCCGAGGGCAGCCGTTAA
ATCAGAAATAAGATTACTGAGTTCGCCTGTCATCAGCGTAATGTCAGCATCAAATCGCTGCACGACATCTTCG
CTGTCGATATCGTCGTTCTGGCTGATTAACTGATCTGCAAATTTTATGCGTTTCAATATACCGTCACAAGAAA
GGGTGAAACTGATACGCTGCTGCCATTCCATTGAAATCTGGGTAACTACCTTCCCAGCTTCGATATGGGTAAG
AATTTCATCGCAGGCAAGATCCTGCTTTTTAAACCGGCCTGTGCCACCATCTTCGAGAATAGCTTTAAGGACC
GCTTCATCGCCGATGGAGAACCCGGAAGGAGCCGCTTCGCTACGAACCCACTCAGTTAGCGTGAGCTCGATAG
GGTTTTCCATCGTCAGCGGCACGACTGGCAAGGAACCTAGGGTTTACGAAGCAGGGCGAGAGAATCTTCTGC
GCGCTTGATGCTGGATGTATCAACAACGATAAACCCGGCTGCAGTGTTTATCCAAATACGAACCAGACTGTTT
TTAGTAAACGCCCTGGGTAACAGGGAATGAAGAACCTCATCACGAATAGAGTCTTTCTCAGTTTTCTTAAGAC
GGCGGCCTTGCTCTCGCTCAAGCGTGGAAACCTTCTTATTAATCTCATCGGCGATCGTTTGTTTAGGTATGAT
TTTTTCTTCACGACGAATAACCAAAAGTAACTGGTTATTGACTGCATGATATAGCACATCTGAATACTGGACT
AATGGTGAAAACCATCCGCTTTTTGCCATATCCTGGCTTCCGCATGGTGAGAAGCGAAACAGCTCAAGTTTCT
TATCAAGAGAGTCTATGTCGATGTTAAAGTCGCGGCTGAAGCGATATATCAGCATATTTTTAAAAAATGGGTT
ATGCATTTTGTTTCCTTAACGCCTCTGCACTGGCGTTTTACGTTGGTTTCTCCACAAAACAGAAAAGAGCACC
TGCTGTAACAGCTTTCCGGGTGGATTGGGTAATGAGCCCGTCGCGCGGAGATGCTCTTTTCTGTTGTGTAAAA
AGGTCGGCGTCACGGCAGAACACTGTCGCCTTCCTCCTGTTGTTGGAAGAGCCGGACGCCGACAAGACTTCAC
ACAGCAATAACGTTGTGGTGGGGCTGTCACTCAGGCGCATGGTCAACCTGACAACCCGGTGTCCTACTGGGTA
CAAATGGAGAAAAACCCGCCATACTTACCGCCGCGCCATTTCGCGGATTACCACAACGAAGAGAGCACTGCCG
GTGTCCGAATTGAACGGACCTTTTCTCTGCCCAACCCTCCTGACTAAACAGGACTGTCTGGAATCGAACCAGC
ACTTATGCCTTGCTCGTCAATGCTCTCATCGTTGTGTGCCTGTCTTTTCACCACATCAGGCTCGGTGGACCTT
GCTATTCCCCAACAGTAAGGATTCGGGTAATCTTTTTAATTCCCCAACAACATAAGGGCTTAACATGTCTCAG
AAGGATGATATTCCTGTCTTTCCCGTAACCGGCTGGCAGGCTGGACCGCTTCCTGGTTACGACGCTCTGGTAG
TGAAATTCCAGTTTCTCTCATCACCGATGCAACCAATTGAGTCTGCTCAGGAAACGCAATTTTTAGTACTTAC
TCCTGAGATGGCTGAGAGCCTGGCTTCAGACTTGCAAAGGCATATTCAGGATTTGCGAAATTCCGACGTTCAC
AGCCCACAAGAAGGCAAGCACTAATAAGGAACACCTGAACTACTTCATTTCCCTTAAAGCGCCGTTGGGTGAT
GGCGCTTTTCTTTGCATTAACCAGCATCATTCCCCCTTCGTGACGTTCAGTTTTACTGGCTTTATCACGGCTG
CGTAGTTGATAAGAATGTTTACGCATGAAACAACGCACTCGGAACAGATAGCTGGCTCGTCCTTACTTCCTTT
AGCGATGAGCTTTTTTGCATCCAGCTCACTGACCCCGCAGAAGGAGCATGTGTAGTAGTTATTCATCTGAACT
CCTGTGTAATGCATCATTGCGAATCATCCGGTCATTCGTATGCCACCGGCGGCTACTTCGTGGGCGTCCTGCC
TGTTCGCTGCTCTATGAGTGCAAATTACATTTAAATTGCACATTGCGCAAGTATAAAATTGCGATATATGCAA
TTTTGAGTCAAAAAAAGCCACTATAATGGTGGCCTTGTCGACGCTTTCTATTAATTGTGTCGTTTGAGTGACT
GCGTCTGGCTTATCAGAACCTTGCCAAAAACACCGAACCTGCACTCGTTGTCTTTGGTAATACTCCATTCCCT
GTAGTTAGTGTTATCAGATATCACCAGTAATTTATCGGGGATCATCTGTAGCCTTTTTACGTATATTTTATCA
TCAAAGCCAAAGACATAGATGCCATCACCATCGAACTGGTTGATGCTTATATCGACAAAAATAAGATCTCCGG
GTTCAATTGTTGGTGCCATGCTGTCACCACGCACGTTAATCACTTTAAGCTCAGCGGCAGGACGCCCGCCGAA
CATAGCTAGTGCTTTGTCCTTGTTATATTCGATAGCATGGATTACATCGATAACATCACCGCCCTGAATGAGT
CCATTACCGGCGCTTGCACTGACATCCAGTATCTCGATACGGAACAAATCCTTCACGTTAGCTGAATCCTTCC
TCATATCACTGTGTTTACATACAGTATTACCTTTTGGGTCTGAGGTAAAGAGTTCTGCTATATCAACACTTAA
GCAGTCAGCCAGCCTAGAAAGTGTTTGTTCGGTAAATTGCTTTTGCTTGCCAGTCTCCAGACGAGAGATGTTT
GCGGCATCCACGCCGATCGCTTCTGCTAGCTCAGCAATTTTCATGTTCTTCGCGCGGCGAAGTTGTCTGACAC
GGTTTCCTATATTCATGCGTTCATTACATTAATTTTTTGCGCATTGTGCAAATCAACTTGCGCAAGTTTGCTG
TATGAAATAACATGCGACATACGCAAAAGAAGGAGGTTTTATGCAATCACCATTGAGAAAATTGCGGAAATCG
CATGGTTATACGTTACAGCACGTCGCTAAAGGGGTTCAGGTTGATCCTGCAACATTAAGCCGGGTTGAAAGAT
GCGAGCAGGCTCCTTCAACAGAGCTTGCTGAGCGCCTGGCTCAATTTTACGCCGGAGAAATTAGCGAGATGCA
AATTTTGTATCCAAACAGATATCAGCTTAGTGATTCGGCGATTTGACCGCCACCACAGCAGAAGGAGTAGATC
CGTGGGACATGAACCTGAATGGAAAGTTGAAAAGCAGCCCCGCTGGCTGGTGGCTGCGATTAAAAAGACGATT
TCCAGTCTGCATGGCGGTTATGAAGAAGCTGCGGAATGGCTGGATGTCACCAAAGATGCTCTGTTTAACCGCC
TGCGTACTGGTGGTGATCAGATCTTCCCGATTGGGTGGGCGCTGGTACTGCAACGTGCCGGAGGAACCTATCA
CCTGGCACATTCAGTAGCCAGGGCATCAGGTGGCGTTTTTGTTCCGCTGGCAGATATGGAAGAAGTGGATAAC
GCAGATATTAATCATCGCCTGCTGGAAGCGATTGAGCAGATCACCAGTTATTCCCAGCAAATCAGGGTGGCTA

TCGAAGATGGCGTTATTGAGCCACATGAAAAAGCCGTGATTGATGAGGAGTTGTATCAGGCGATCGCAAAGCT
GCAACAGCATTCGACACTGGTATACAGAGTTTTTTGCGTGCCAGAAAAGGGTGACGCCCGCGAGTGTGCAGCT
CCGGGCGCCGTGGCGTCAAATTTTATGGAGAAAACCAACGCATGAACAGTTTAACGGTAAATAACCGTTTGTC
GCAACAACCGGGGATGTATGAGTACCGGCCGTTGCGTCATGAATGCAGATTATCAAATAGCCTGGTCGTGCGT
AACCACAGGGAACACAGCCTGACCGTGGGGGATGAATCGTGCAGGAACTTAACCGCTGGTTTCGGGATGGAAG
GGGACTTTATGTCCATGTCATTCGCTGGGAACCAGAAACTGAGCGCGTTATCTATCTGCGCAAGGGCTATCCG
CATGAGTGTTTTAGCCCTTTGTGGAAATTCAGGCGTGATTTTGTTGAGTGTGAAGCGCCAGGAACACATTGAT
TCTGCAATTCCGGGACGTTACACTGTTCAGGCACCTCATAAAGCGGGTGCCGGGCGTGGAAACCCGGAATTCA
ATATAGAGCACAACCGCGCTCATGCGGTTTTTTCTTGTCATGAGCATTGTTACGCCCAAATTATGGTGGGGCG
TGCAGGGCCAGTTTCGGCTGGGCCGGGTTCTATGTTGACCGGTATTTCCACCCCTGTACGTCTCACCACCTAT
AAGGTCGTGGAAAGCCTTGGTGGTGAGTTCATTGAATTCAACATAGAGGCTGCCACTATGGCTACTGTCCCAA
CCCTCGCTCAACCTGAAATTAGAATTATTAACGGCCAAGCCGTTACTTCCTCCCTGGCTGTTGCCGACTACTT
CATCAAGCGTCACGCTGATGTTATCCGTAAAATAGAATCTCTCGAATGTTCCACTCTATTTCGTAAACGCAAT
TTTGCGTTTACATCGATTTCAATAAATCAGCCCAACGGCGGTACTCGCAAACTCCCATGCTATCAAATCACAC
GCGATGGTTTTGCGTTTTTGGCAATGGGTTTCACTGGTAAACGTGCTGCTCAGTTTAAAGAGGCATACATCGA
TGCCTTTAACCAGATGGAGAAACAGCTTTCAACTCCATCGGTGCTGAGCGATGCAGCACATAATGCCAGCGTT
CTTTATTCCTACATTTCATCCATTCATCAGGTTTGGTTACAGCAGCTTTATCCCATGCTGGAAAAAGCGGAAT
CTCCGCTGGCTGTAAGCCTGCACGATCGCATCAATGACGCTGCGGCGCTTGCGAGCCTTATCAATATGACACT
GAACCGTTCAGAGGTAAGGGGGCGCAAATGATCCGGAATATTTTTAAACGGTTCACCAGCCAACGTTTTCATT
GCCCTCGTCCAGGACAGTGGTACAGCACACCAGAAGGGTACGTTCTGCGTATTAGCCTGGTCGATCGCGAATG
TCAGAAGGTTGTCTGTGAGCCTCTTGGGCGTAATTACCGCGTCAACATGCCGCTTATTGCCTTTCGTTCCGGC
AAAAACATGAAGCATCTCGGAGGTGCTGCATGAGTTCCCTTATTCAATTACTCGATCGCCCCATCGCCTACAA
CCCTGCTTTTGCAAAACTGAAAGCCGGGAAGGTAAAAGCTGGCCCGGTTGCGGCAGTATTCCTGTCCCAGCTT
GTTTACTGGCATAACCGGATGGATGGCGGCTGGATGTACAAAACACAGGCTGATATTGCCAGTGAAACGGCGC
TAACCCGCGACGAACAGGAAACAGCACGTAAACGTCTGGTAGCACTTGGTGTACTGGAAGAAGCCCGTCGCGG
TGTACCTGCCACCATGCACTACCGCATCAACACCGCACGGCTTGAAGCGCTGTTGCTGGAAACGGCGAAGCCA
GTGAAAAAGGGCGCTCAGGAGAAAACCAGATTGCGGGACTTCCAGAATGTGGAAACCCCGCAATCTGGATTGG
TGCAACCCCGCAAACCAGATTGCGGTGATGCCGCAAACAAGAATGTGGAAACCCCGCAAACAAGTACGGGGCA
ACCCAACGAACAAGCATGTGGCGATCCCACAATCTTTCCTACAGGAGATTACACAGAGACTACTCAGGAGATT
ACACAGGAGAGTAAAACCCCTTTTTGTCCGGTTGCTGAGCAACCCGACCCCGAAGTGACGCTCACCGATCAGG
CGATTGAGGTTTTAACCCACCTGAACCAGGTAAGTGGCTCCCGGTATCAGAAGTCAAAAACCTCCCTGGAAAA
CATCCGTGCCCGACTGCGTGAGGGGTACAGCGTTGCTGATCTGCAACTGGTTATCGACCTGAAGCATGAGCAC
TGGCACGAGAACGACGAGCAGTACCAGTACATGCGCCCGGAAACGCTGTTCGGTCCGAAGAAATTCGAGAGCT
ATCTGCAAAGCGCTACCCGCTGGGATCAGAAGGGGCGGCCTAAACGCGCTGACTGGGGTGCGAAGAAGCGCGA
TGTGATGGCTTTTGGTCCGGTTGATACAACGATTCCGGAGGGATTCAGAGGATGAGTCTGTTAGCAAAAGTGC
AGGCGTTTATCGAGCTTAATCCGGGGCTGACATCAAATGAGATTGCCGATGCTTTTCCTGAATACGCACGCTT
TGATGTGCAGCGTTCAGCGAGCAAGTTGTATCGGTGTAAGCGTGTTAACCGCCGCCTGGATGGAGATGTATTT
CGCTATTACGCGGGTAAAGACGAGGCAGTGATTTTGACGTTACGACAGAAAAGGTCAGGTCATACAGGTTCGG
GTGATCCGATGGTGATTGCAAAGCTGGTAAGCCGCGCTGAAGAACTGGAATCCAGAGGGTTATTTAATCGTGC
ATCGATAGTGTGGCTGGAGGCATTTAGCGAAAGCCAGTTTATCTACGAACGCGAGGAATTTTTACGCCGCCGT
CAGAAGTGTCTGAACCGCATCAAAAAGAGAATCAGACCCGTAGAGCAGGTTTATCTGGCAGGGCGATTTGTGG
GGAACGTGGAATGACCAGTGAATCCGTTTGTATTGAAAGCAGTGATGTAACGATATCTGTTGATGAATCCGCT
TCGCGCACCTGGCGTCGCCCGTTCCTGAAATGGGCAGGCGGTAAATATTCCATGTTACCCGATCTTTACCAGG
TCATTCCGGCAGGTATGCGCCTGATTGAACCGTTTGTCGGCGGTGGTTCGGTGTTTCTCAACTCAGACAAACA
CGCCTGCTTCCTGCTGGCCGATGTGAATACCGACCTTATCAATCTGTATCAGATGCTGGCTGTTGTACCTGGT
GCGGTGATAAGACATGCTAGGGTAATGTTTGACCGTCTCAATGACGCTGAAAGCTATATGGCGCTACGGGAAG
AGTTCAATGCTCAGGTGATGGACGCTCCGGAACGCGCCGCCGCTTTCCTTTTCCTTAATCGTCACTGCTTCAA
TGGCCTGATCCGGTACAACCGCAACAACCAGTTTAACGTTGGCTGGGGCAAATACCCGTCGCCTTATTTCCCG
GAAGAAGAAATCAGGGCATTTACCGAAATGGCGCACAACTGCGTATTCATGGCGGCAGGATTTCGCCGGACGC
TGGCACTTGCGGGAGAGGGTGACGTTGTGTACTGCGATCCACCCTACGAACCGATGCCCGGCAAGGATGGTTT
TACTCACTACGCCGCTGGTGGCTTTACCTGGGATGATCATATCGCGCTGGCGGAATGTTGTGTTGCTGCTCAT
CAGCGAGGTGCCAGAGTCGTGATCGGCAATTCCACATCTCCGCGTGTTATCGACCTGTACTCGCAGCACGGCT
TTGAAATCCGCTATATCAGCGCCCGCCGCTCAATATCAAGTAAGGGCAGTACCCGCGAGAAAGCGAAAGATCT
CGTGGCGATTCTGTAGGGGGCGGCATGAAACTGACATTGCCATTTCCACCCAGCGTTAACACCTACTGGCGGG

CTCCGAATAAGGGACCGCTTAAAGGTCGTCACATGGTCAGCGCCAGCGGCCGGAAGTATCAGAGCGAGGCGTG
CGCGGCAGTGATTGAGCAGTTACGCCGTCTGCCAAAACCTTCAACAGCCCCGGCAGCGGTGGAAATCACCCTG
TATCCGCCAGACAAGCGGATCAGGGATCTGGACAACTACAACAAGGCGCTGTTTGACGCCCTGACCCACGCGG
GTGTGTGGGAAGACGACAGCCAGGTGAAAAGAATGCTGGTGGAGTGGGGACCAGTTTTCCCGAAGGGGAAGGT
AGAAATCACGATCACGAAATTTGAAACAGGGGCGGGTGCAGCTGCCTGAACATGGAGAAAGAAGCATGAATAA
TTTAATGGTCATTGATGGTATCGAAGTTCGCCGCGACGTTCATGGGCGCTATTGTCTTAACGATTTGCACCGG
GCTGCGGGTGGAGAGCAGAAATACCGTCCGAAGTACTGGCTTGATAATAAGCAAACCCGTGAGCTGATTGAGC
AACTTTTCACCGAGGGCGGAATTCCACCCTCGGAACAAAATCAATCTGTTAGCTTTTTTCAGGGCGGTAGTGA
TACCCGAAGTTTGGCACGTGCTCCAGTAAATACTGTTCGCGGTGGTGCTGAACAAGGTACATACGTATGCAAA
GAACTGGTATTTGCTTATGCAATGTGGATCAGTCCGTCTTTCCATCTCAAGGTGATCCGCACGTTCGATCGGA
TTACCAGTGCGCCACAAATATCTTCTGGTATGGCTGCCGATAAGATGCAGGCGGGGGTGATTCTGCTGGGTTT
TATGCGCAAAGAGTTAAACCTGTCCAATTCATCGGTACTGGGCGCGTGCCAGAAACTCCAGGAGGCAGTGGGA
CTACCTAACCTGGCGCCACAATATGCCATTGATGCTCCGGCTGGCGCGCCGGATGGTTCAAGCCGCCCGACGC
TTGCACTGAGCGCGCTGTTAAAACAGCATGGTATCCGGATGACGGCTAATCAGGCGTATCAGCAGTTAGCAAA
GCTGGGTGTTGTTGAACATCGTGAGCGTTACAGTCGCTCCGCGATTAACGGCATTAAAAAATTTTGGTCGCTG
ACGGCGAAAGGCTGCATGTTCGGCAAAAACATCACCAGCCCGGCAAACCCTCGCGAGACGCAGCCGCATTTCT
TCGAATCCAAATTCCCTGAGCTGCTGAAGCTGCTCGATACCGTTCATTGAGGTGATCGTGAGAGCGTTACTGA
CCCCTGAAATTGCTCCTCGTATGGGCGTTGTATTGTTCAGGCCGGGATCGGAACTGATGCCCCTGTTTATGCA
GGGGCGTGTTCTGCTTGAACCAGAGCCGGAACAATATTCATCTTTCGCCTGCGGCGCGGTCCCGGCGGTATCA
CAGCCGCTGGCGGATGATCCTGCTGTTCGTGATGTGTTCCGTAATGAGTCGGTTATCTATCGTGCTGGTGGTC
TCGATAGTCTGGAAAGCTGGCTACTCCGGGGGAATGGCTGTCAGTGGCCGCATTCAGTCTGGCACAGCGAACA
GATGACAACCATGCGCCACGCACCGGGGGCAATCCGACTGTGCTGGCACTGCGATAACCTGCTGCGCGAACAG
TTTACGGAACGGCTGGAATCAATAGCTGTGGAGAACACGACAAAATGGGTTTTATCGGTTGTTTGTCGTGATC
TGGGTTTTGACGATATGCACGCAGTCACGCTCCCGGAACTGTGCTGGTGGATGGTACGCAATGACCTGGCAGA
AGTCTTACCGGAGAGCGCTGCGAGAAAAGCATTAAGGATGCCGAAGGCAATTGTCCAGTCAGCTACCCGTGAA
AGTGAAATTGTCCCCTCGGTGCCGGCCACCAGCATTGTACAGGATAAGGCGAAAAAGGTACTGGCACTCAGGG
TTGATCCGGAATCGCCGGAAAGCTTCATGTTACGTCCGAAACGCCGTCGATGGATCAATGAAAGATATACCCG
CTGGGTTAAATCCCAGCCGTGCGCGTGCTGCGGGAAGCAGGCGGATGATCCGCACCACCTGACAGGCCACGGT
CAGGGAGGGATGGGAACAAAGGCGCATGACCTCTTTGTGCTGCCGTTGTGCAGAACGCATCACAATGAGTTAC
ATGCGGACACCGTGGCATTCGAAGAGAAATACGGCTCTCAACTGGAGTTGATATTTCGTTTTATCGATCGCGC
GCTGGCGATCGGCGTGCTGGCGTAAATGGAGAACACGCATGAACCTTGAAGCCTTACCAAAATATTACTCACC
AAAATCTCCAAAATTGAGCGATGACGCTCCGGCGACAACCTCCGAATCTTTGACGATTACGGATGTAATGGCG
GCGCAGGGGATGGTGCAATCGAAAGCACCACTGGGGTTTGCTTTATTCCTGGCAAAAGTTGGTATTCAGAATC
CTGACTTCGCGATTGAAGGGCTGATTCATTACGCGGTGGCACTGGATAACCCGACACTGAATAAATTGAGTGA
AGAAACTCGGTTACAGATTGTTCCTTACCTCGTGAATTTTGCATTTGCTGATTATTCCAGATCTGCTGCAAGC
AAGGCTCGCTGTGAGCATTGTGCTGGTACGGGATTTCATCATGTATTACGTGAAGTGGTGAAACACTCCAGAA
ATGGTGAACCCGTCATCAAAGAGGAGTGGGAGAAGGAACTATGTCAGCATTGTCATGGTAAGGGAGAAGTCAG
CACGGTGTGCAGAGGGTGTAAGGGTAAAGGTATTGTCTTGGATGAAAAAAGAACTCGGTTTCATGGCGCGCCT
GTTTATAAGATTTGTGGGCGTTGCAATGGAAACCGGTTTAGTCGTTTACCAACCACACTGGCGCGGCACCATG
TCCAGAAACTGGTACCGGATCTGACGGATTATCAGTGGTACAAAGGATATGCAGACGTCATTGATAAACTGGT
TACAAAATGCTGGCAGGAAGAAGCATATGCAGAAGCGCAATTAAGAAAGGTGACAAGATGAAAGATTTTCAAC
GAAGATAGCGACATGATGCTTGCATATTTCAAAAAATATGGATAAGATTCTCCCAACGATGGGCTTTGTATGT
CTATCGTTGATAAGTCTCAAGAACCCGCCTCCGAGTGGGTTTTTTATTTGTGATCACTTTATTTTTTGTCTTG
CTAAGTTATTGTATGGACAAGAACTAAAATTAAGTGGTGACATTGTGCTCTCTAATAACGAACGTTGGGTTTC
CTTTTTTGACTTTGCTTTTACGCCTACACACGCAGCGGCGCCGAGTATTCCCATTGAAGACATACTCAAGAAA
TTGAAGGTACTGGTGAGCTCAGGGAGTGCTGTAAAGTTATACAATCATAGGTCTAGAGCGCTTAGGATTTCGG
AGATGAAATATTCTATTGGGGATAGCCAGGCGACTCTACTTATCCAGCTTTGTGATAAAAATGGTTCTGACCC
TGTTTTTGGTGAGTTAACAACAGGCAACCTTAGAGTAGAACCTAAGCTTGCCGGTGAAGGTATCGCAGTTTCT
TGTCACATTGTAATATCCACAGATGTTGTCAAAAACACTGCCGATCACCACAAAACTCTCGTTGAATCTGTCC
CCGGTATCAGTAAGTCAGTTCTTGAGCCATTTTTAAATGCTATGCTCAGAGAAGCCTTCGCTGGATGTGAGTT
TAAAAATCCTGCAACTAAAGGTATGTGCCAGCACCGCCCAAAGCTGGAAATCTATTCTCATGGTTCACAAACG
CTGATGGATGCATTAAAAGGTGCAAAGATTCATAACGTTAAACTTGTGAGTACAAGAAGGAAAGGTGGATTGG
ACCAAACGGCGTACACTGAGCTCTCAGAAAGGTCCGTAAAGTATAAAATCATTAGACAGCCGCCATTGAAAGA
TAAAGAAAGGTTGTTAGAGATTTTAAGAAAGAAAGGGCAGCAGTCTGGATATACCAAGGTTTCAATTAGTTAC

TCAAAAGATGGCAAGCAAGCCAGTTTGGATCTTGACCGTAACGAAGATGCTGCCACAAAACTGTTCACTAAAA
GTGAGAGGGTAATATTAGGTAACCTCATCAACCAATGTGAAAGCACAGTACATCTGCAGCTTGAAACAAAAAT
GATAGGGTTGCTCTAACGGGAGTTTCATATGAAACTTTTTTCACCGCTGAGTTATCTCCGCATCAAGCATGAG
GAAAAGGACTGGTATGATTACAAAATACCAGCTGCAGTGTCTCTAATCGTCACTATTGTTTATTATTTTCACG
CTAGCAAAATTTCTTTAATCGAGACTAACGGACTCCTGCTTCAGGTTAATGGGTTACTTCAAGTCTTGATTGG
TTTTTATATCGCAGCACTGGCTGCGGTTTCTACTTTTTCTAGCTCTTCGATCGACGAAGTAATGGCGGGCGTA
CCTCCGACTCTAGTAGAGAAATTCCGAGGGCAGAAGCTTACTGTAGAACTGACGCGCAGGCGCTTTGTTTGTT
ACCTTTTTGGTTATCTAGCTCTTGTGAGCTTTATGTTATTTTGCTTAGGGATGATTTCTATTCTGATTGGGAA
GCCTTTCCATTTGTGGCTGCTCACATTCTGTTCTCCTGATGCAATCTTGTGGCTTAAAACGGTATTTGTTGGC
GTTTATATATTCATCTTAATGAATATCATAACAACAACTTTGCTGGGACTTTACTTCCTTGCAGTTCGGTTCC
ACCAATCATCGCTGTAAAAAATCTAAATACTTTTAGGCTGCCTTCGGGCGGCCTTTTTTATTTCCCCTCATAA
CTGAGAGGACCCACATAACCAGAGGGGGATGAATGTCCGAACCTGTATCCAGTGCGACAGTGTTGGCTGGTGG
ATTAATGGGGGCCAGTGTATTCGGTCTGGCAACCGGAACTGATTATGGTGTGGTATTCGGCGCTTTTGCCGGC
GCGGTGTTTTATGTCGCCACGGCAACCAACATCGGACGCATCAGGCTGGTCGCTTATTTTATTACATCATTTA
TTGTGGGAGTGCTTGGTGCCGGGCTGATAGGTACTAAGCTTGCGGCAATAACGCATTATGAAAAACCACTGGA
TGCACTTGGCGCAGTGATTATTTCTGCAATGTGTATAAAGTTTCTCACTTTTCTCAACAGTCAGGATCTGAAC
ACCCTGTTCAGTATTCTCTCTCGTATCAGGGGAGGGGATCAGATGGTAGCAAATGACCCTTCTGCAGCTCTGA
ATGCCGTAATTTGTGGGGTGATAGTCATCGTTCTGATGTTTTACCGACGCGGTGATGCGACACACCGCCCCCT
GATTTCGTTACTGGCCTATGTCATGGTGCTGGTATATGCCAGCGTCCCTTTCCGGTTTGTTTTTGGTTTATAT
GAATCATCCCACTGGCTGGTGGTGATGGTGAATATCCTTATCTGCGCCGCTGTGCTGTGGGCTCGCGGTAATG
TGGCGCGTCTGGTCGATGCACTGAGGCACTGATGAATCAACAACAATTTCAGCAGGCGGCTGGTATTAGCGCC
GGGCTTTCTGCACGCTGGTTTCCGCACATTGATGCGGCAATGAAAGAGTTTGGTATTACAGCAGTTAATGATC
AGGCCATGTTTATTGCACAAACGGGACATGAATCAGCAGGATTTACTGTTCTGAAGGAAAGCTTCAATTATTC
GGTGGAGGCGCTGAAAAAGACGTTTGGTAAACGCCTGACGCCGTATCAGTGTGAAATGCTGGGGCGTATTGAT
GGTCGCCAGGTTGCCCACCAGCCGCAAATAGCCAATCTGGTTTACGGTGGCCGCATGGGTAACAAAGACGCCG
GAGATGGCTGGAAGTATCGCGGGCGTGGTCTGCTTCAAATCACCGGCCGCGAGAACTACGTCAAATGCGGAGC
TGCGCTGAAGCTTGATCTGATCAGCACACCAGAGTTGCTGGCACAGGAGAAGCATGCAGCCCGTTCTGCTGCA
TGGTTTTTCACATTACGTGGTTGCCTGATGTATTCAGGTGATGTTGTCCGTGTAACGCAGATCATCAACGGTG
GCCAGAATGGACTGGCTGACAGAAATAGTCGTTATAACAAAGCGCGGGCGGCGTTGCTGGTATGACAGCGGTC
TTTGCTTTCGTTAAGGCGCGGTGGAAAACAATCATTGTTTTGCTGATGTTGGCTGGTGCATTTCTTGCCGGGA
TCATCTGGAGTGATCGGGGCTGGCAAAAGAAGTGGGCTGACCGCAATAGCATGGAATCTTCACAGGAAGCGAA
CGCGCAGACTGCCGCACGCTGGATTGAACAAGGGCGCATAATTGCCCGTGATGAGGCTGTAAAAGATGCACAA
GCACAAGCCGCTAAATCTGCTGCCACTGCTGCTGGCCTGTCTGCCACTGTTAGCCAGCTGCGTACCGAAGCAA
CAAAGCTTGCCGCCCGCCTGGACGCCGCAAAGCACACCTCAGATCTTGCCGCTGCCGTCAGAAGCAAAACAGC
CGGAGCCGACGCCGCAGTGCTCGCCGACATGCTCGGACGCCTTGCAGAAGAAGCTCGATATTATGCTGAGCGA
TCTGACGAAAGCTACCGCGCAGGAATGACGTGTGAGCGTATTTACAACTCGGTGAGAGAGTCAACCAACAATC
CCATAGCCCCGCACTAGCGGTGCTTTTTACCGGAGTTTATATGCCACCAAGAACACCTAAATCCTGTCGTGTT
CGCGGCTGTCGCAGTACAACAACAGATCCATCCGGATATTGTGAAAGTCACAGAAGCGAGGGCTGGAAACAAT
ACAAGCCAGGACAATCCCGTCATCAACGCGGTTATGGTTCGAAGTGGGACGTTATCCGTGAGCGCATACTGAA
GCGTGATAAAGGTTTATGCCAGTTATGTCTGCGTGCCGGTGTGGTGCGTGAGGCGAAAACTGTTGACCACATT
ATCCCTAAAGCGCATGGCGGAACTGATGCCGACAGTAATCTGCAGAGTCTGTGCTGGCCCTGCCATAAGGCGA
AGACGGCTCGTGAACGGCTGAAATAAAAACCAGTTTCCACAGCCAGAGGGGAGGGGCGGGGTAAATCCCTGTG
GCCTGACGTCTTCCGGACTGCCCGCCTCATCAAATTTTTACGCGCCAAAAATAAGAAACTTTTTTCCGGAAGG
TTCAACCTATTGAACTGGAGGTTTTGATGGGTGCTGTTGTGAGATCTTCCGGTGGTGGCCGTAAGCGCAATTT
GCCTTCGGGCCAGAAAAGCAAGCTGACCAGGATCGCACCGCCGGAAGAGTTAATGAGTGATATCGCGATCCGC
ATCTGGAAAACGCAGAGCAAAATTTTAATTGAGCGGGGCGTTTTTGATCTTGAAGACGCGCCGCTACTCCTGG
CGTACTGCAATGCGTTTCACTTGATGATTGAGGCCGAAAAAGTCATCGCGGAAGAAGGCCTGACCGTATCAAG
TGAAATGGGTGGTGAGAAAAAACACCCTGCAGTCAATGTCCGTAATGACTCCGTTTCGCAGCTCGCCCGTCTG
GGTTCACTTCTCGGGTTAGACCCGCTCAGCCGCATAAGAATGACCAGCGGAAAAAATGATCCGGACGATGAAG
GGAATGAATTTGATGAGTTTGACTGATGGCTACATATCCGAACGTCAATGCGGCGAACCAGTATGCGCGGGAC
GTCGTGAACGGGAAGATACTGGCCTGCCGGTTAACCATGCTTGCCTGTCAGCGACATCTTGACGACCTGGAAC
GTGCCAAAGATCCGCATTGGCCTTACCGCTTCGATAAAAATAAAGCAGAACGTTTCCTTCGCTTTTCCCAGAA
AATGCCGCACACCTCCGGAGAGTGGGCTCGCCGGAAGTTGCGGATAGAATTTGAACCCTGGCAAAAATTTGCG
CTGGGCGTGCCGTTTGGCTGGGTGCGCAAGGATACCGGTTTTCGCCGCTTCACTGAGATTTACATCGAGGTAC

CGCGTAAAAATGGGAAATCGGCGATTGCGGCCGCCGTCGGTAACTATATGTTCTGTGCAGATGGCGAGTACGC
AGCGGAAGTTTACTGTGGTGCCACAACGGAAAAACAAGCCTGGAAAGTTTTTGCGCCTGCACTGGCGATGGTG
AAAAAGCTGCCGGCGTTGCGTCAGAAGTTCTGTATCAAACCCTGGGCAAAGAAAATGACTCGCCCGGATGGTT
CCCTGTTCGCGCCAATTATCGGTGACCCTGGAGATGGCGACTCACCATCATGTGCGATCATCGATGAGTACCA
CGAGCATGATACTGACGCGCTATACACCACAATGACTACCGGGATGGGGGCGAGGGAGCAGCCCATCACGCTG
ATCATCACCACGGCAGGCTTTGATATTGCCTCGCCTTGCTATGAAAAACGTACTCAGGTGGTCGAGATACTGG
AGCGCATCCGGGAGGGTGGTGAAAACGAGGCAATTTTCGGGATCATCTATACCCTGGATGATGACGATGACTG
GACACAGCCGGAAGCTCTGATCAAAGCCAACCCGAATTACAACATTTCGGTGAAAGAGGGATTCCTCAAGGCT
AAACAGTTGCTGGCGATGTCCACGCCAGGCCAGACCAATAAAATACTCACCAAGCATTTCAACAAATGGGTGA
GTTCTAAAGCAGCTTACTACAACCTGCAGAAGTGGATGACCGCAGCAGACAAAACGCTCAGACTGTCCGATTT
TGCAGGTGAGGAGTGTTATCCCGGCATCGACCTGGCATCAAAACTTGACCTTAATGCAGTGGTGCCGGTATTC
CGCCGTGAAATAGACGGCCTGAGTCATTATTACTGCGTTTCGCCTATGTTCTGGGTACCGGAAGACACCGTCT
ACGCCACGGACCCGGCGTTGAAAACTATTGCAGACCGTTACCAGTCTTTTGTTAATCAGGGCGTGCTGGTTCC
GTCAGACGGTGCAGAAGTGGATTACCGCCTTATCCTGGAAGCGATCCTGAAATTACGGGAAACGGTGAAGATA
GCCGCGAGTCCGATTGACCCCTACGGTGCAACAGGCTTATCTCATATGCTGCAGGATGAAGGGCTTGAACCTG
TCACCATTACCCAGAACTACACGAACATGAGCGACCCGATGCGTGAGATTGAGGCTGCGATCGCTGCTGGCCG
ATTCCATCATGACGGTAATCCCTTGATGACCTGGTGTATTTCGAACGTGGTTGGCAAGTACCTGCCTGGTAGC
GACGATGTTGTTCGCCCGGTGAAAGAAGGCGCAGGCAACAAAATTGATGGTGCAGTTGGCCTGATGATGGGGG
TTGGCCGCGCAATGCTGAACGAGCCGAAAGACTTTCTTTCTAACCTCGATCCTGATGAGGAACTGTTATTCCT
GTGAAATCACTAATTATCGATGTGGCCGGGGTGGCAGGCTTCGGCGCGCTGGTGGGAGGTATTTACCTCAAAT
TTGGCGCGGCGGTTGCTCTTATGGCTGGTGGTAGTGGCCTGCTGCTGTGGGCACTGCTGGCGGCCAGGAGAAT
AAAAACATGCTGATTGATGCCATTTTCAGAAGCAACTCGCTGGAAAACCCAGCTGTTCCGGTCACCGTTGAAG
CGGTCGAAAACGACGGGATCTTTAATGGTGATGTGATTGTTAACCCCCGGACGGCAATGAAACTGGCGGCGGT
GTATGCATGTATCTACGTTATTTCATCCAACGTTGCGCAGATGCCCCTGCACGTCATGCGGCGAACCGGGAAG
AAGGTTGAAACTGCCCGCGACCATCCTGCCTTTTACCTGGTTCATGACGAACCCAATTCCTGGCAGACCAGCT
ATAAATGGCGCGAGCTCAAACAACGTCACATTCTGGGCTGGGGTAACGGATATACCAGAGTTCTCCGTCACCG
CCGAACCGGTGAAGTCACTGGCCTTGAAGCCTGTATGCCGTGGGAAACAACGCTGCTGAACACCGGCGGGCGC
TATACCTACGGCGTGTATAACGAAGAAGGTTCCTTTGCCATTAATCCTGATGACATGATCCACGTCAGGGCGT
TGGGTAACGATCAGAAAATGGGGCTCAGTCCGGTTCTTCAGCACGCCGAAACCATCGGTATGGGTATGAGCGG
GCAGAAATACACGGAAAGTTTTTTCAGCGGTAACGCCAGACCAGCGGGCATAGTTTCAGTAAAAGGAGAATTG
AATGACGGCTCCTGGAAAAGGCTGAAAGAGATGTGGCAAAAAGCCACGGCGATGCTGCGCAGCCAGGAAAACA
GGACAATGTTGCTCCCGGCTGAACTGGATTATAAAGCGCTGACGGTTTCCCCAGTCGATGCCCAGCTCATCGA
CATGATGAAGCTCAACCGTTCCATGATTGCCGGGATTTTCAACGTGCCGGCACACATGATCAACGACCTCGAA
AAAGCCACCTTCTCCAATATTTCCGAACAGGCGATTCAGTTTGTTCGCTACACAATGATGCCGTGGGTGACGA
ACTGGGAGCAGGAGCTTAACCGTCGGTTGTTCACCCGCGCCGAACGGGAAGCCGGGTATTACGTGCGCTTTAA
CCTGGCGGGTTTATTGCGCGGTACTGCCAAAGAGCGCGCGGAGTTCTATCACTTCGCTATCACCGATGGCTGG
ATGAGCCGCAACGAAGCACGCGCGTTTGAGGATATGAATCCGAAAGACGGCCTTGATGAAATGCTGGTCAGCG
TTAACGCCTCCCGGCCAGCCAAATCCACAACCCAGGAGAACACTCAAGATGAGTGAACGTGAAATTCGCTGTT
ACAGCGGCGAGGTGCGCGCAGAAACGCACGACAGCGAGCCCAGCCGGATCATCGGGTATGGTTCGGTCTTTGA
CAGCCGTTCTGAACTGATTTTCGGTTCGTTTCGCGAAATCATCCGGCCCGGTGCGTTTGATGAAGTGCTGAAT
GACGATGTACGGGCGTTATTCAACCATGACCCCAATTTTATCCTGGGTCGCAGAAGTGCGGGCACGCTGGCAC
TGACGGTTGATGAGCGGGGTCTGCGTTATGACATCACCGCGCCAGAAACTCAGACAATCCGTGATCTGGTGCT
GGCACCAATGCAGCGCGGGGATATCAACCAGTCCTCTTTTGCATTTCGCGTCGCCCGCGACGGAGAGGAATGG
TACCAGGACGAGGATGGTGTGGTGATTCGTGAGATTACCCGTTTTTCCCGTCTGCTGGATGTCAGCCCTGTGA
CATATCCGGCGTATCAGGAGGCAGATTCCGCCGTCCGCTCTATGAAAGCCTGGCAGGAGGCGCGCGATAGTAG
CGCACTGCAGAAAGCCATTAACCAACGAATGGCGCGTGAGCGCGTCCTGACCCTTCTTAACGCGTAAGGAAAA
ACCATGAAATTGCATGAACTGAAACAAAAACGTAACACCATCGCGACCGACATGCGCGCGCTGAACGAAAAAA
TCGGCGATAACCCATGGACGGATGAGCAGCGTACCGAATGGAACAAGGCAAAATCTGAACTGGAAGCACTCGA
CGAGCGCATCGCCCGCGAAGAAGAGCTGCGCCGCCAGGACCAGACCTACGTTGATGAAAACGAGGAAGAGCAG
CGCAATAATCAGGATCCTGATAAAGACCCGCAGCAGGACGAAAAACGCGGCCAGATTTTTGATAAATGGATGC
GTCACGGCGCCAGCGAACTGAGTTCCGAAGAGCGCAAAGCCTTACGCGAACTGCGTGCGCAGGGTGTGGCGCC
GGATGAAAAGGGCGGCTATACCGTGCCTGATACCTTCCTGGCGAAAGTGGTCGAACAGATGAAATCCTACGGT
GGTATTGCCAGCGTGGCGCAGATCCTCGCTACATCCGATGGGCGCACTATGGAATGGGCCACTGCTGATGGTA
CCGCTGAAGTGGGTGTGCTGCTGGGTGAAAACGAAGAAGCGGGTGAAGAAGATACCGAATTCGGTATGGATAG

TCTGGGCGCGCTGAAAATGACATCCAAAATTATCCGCGTATCCAACGAGCTGCTACAGGACAGTGCGATCGAC
ATGGAAGCCTATCTCGCCCGCCGTATTGCGGAGCGCATTGGCCGCGGTGAAGCGCGTTACCTTATTCAGGGGA
CCGGCACCGGTACGCCAAAACAGCCTAAGGGTCTGAAAGCATCCGTAACCGGCACTACGCAGACGGCCGCTGC
CGGAGCTGTTAAATGGCAAGAGATTCTGGCGCTGAAACACAGTATTGATCCGGCGTACCGCCGCGGGCCGAAG
TTCCGCCTGGCGTTCAATGACAATACGCTGAAACTCATCAGCGAGATGGAAGACGGTCAGGGCCGTCCACTCT
GGCTGCCTGATATCGTCGGCGTGGCGCCAGCATCAGTGCTGAATGTTCCGTACGTTATTGATCAGGAGATCGA
TGATATTGGCGCGGGCAAAAAATTCATGTTCTGTGGCGACTTCGACCGCTTCATTATCCGCCGTGTTCGCTAC
ATGATCCTGAAGCGCCTGGTGGAGCGTTACGCGGAATTCGACCAGACCGGCTTCCTGGCGTTCCATCGCTTTG
ACTGTATTCTCGAAGATACCTCTGCGATTAAAGCGCTGGTGGGCAAAGGCTCGGCAAGCAGCTAATCCCTCTC
ACCTCTGAACAAACCATGCCGCGTTAAGCGGTTTTTTTGTGCCCGCCACCCGGCGGGCGCAGGAGGATCCTAT
GTTGCTTTCTCCTGAGGAGATCAAGTTGCAGCTCAGGCTGGATGAGGATTACGCCGATGAAGATAAATTTCTT
GAGCTGTTGGGGCGGGCGGTTCAGGCCAGGACAGAAAATTTTCTGAACCGGAGACTTTATACGGCGGAGGCGG
GGGTGCCAGCCGACGATCCGGAGGGGCTTATTCTCTCGGATGACATCAGGATGGGGATGCTGCTTCTGGTGAC
GCACTTCTACGAGAATCGTTCTACCGTCACCGAAGTGGAGAAAGTCGAACTGCCGATGAGCTTTAACTGGCTC
GTCGGTCCATACAGGTACATCCCGCTATGAAACTCAGGCAGGCGCAGGCCAGCGCCACATACCTTTTGCCCGA
CCCAGGCGAACTTGACCAGCGCATTGTTATCCGGCGGCGTGTCGATGTTCCGGCTGATGACTTTGGCGTAACG
CCGACGTACCCGGAGCAGATCCGGGCGTGGGCCAAAAAAGCGCAACCCGGCGCGGCAGCTTATCAGGGGGCTG
TGCAGATAGAAAACAGGGTGACGCACTATTTCACCATCCGTTTTCGCCGCGGTATCACCGCCGATCATGAAGT
GCTCCACGACGATATTTCTTATCGGGTTAAACGGGTCCGTGATCTGAACAGTAAACGCCGCTTTCTGTTGCTC
GAGTGCGAAGAGCTGGGTACCGATAACGGGAGTGACTATGCCGCAGAAAGCATATTTACACGTTGATTTTGAA
CAGCCGGAAACGCTTGTTTTTAACCGGGCGCGTATGCGCCGGGCGTTTGTCAGTATCGGGCAGGTACATATGC
GCGATGCCCGCCGCCTGGTCATGAAGCGGGGGCGTTCCGGACCCGGCGATAATCCTTCATACAGAACGGGAAA
ACTGGCACGCTCCATCGGGTATTACGTTCCGCGGGCATCCAGTCGCCGTCCTGGATTGATGGTGAAAATTGCC
CCTAATCAGAAGAACGGGGAAGGGAACCGCCCGATCTCAGGCGCATTTTACCCTGCCTTTCTGTTCTACGGTG
TTCGCCGTGGGGCGAAGCGTAAGAAAGGCCATCATCGAGGCGCATCAGGCGGCAGCGGCTGGCGTGTGGCACC
ACGTAACAACTACATGACTGAGGTTCTGGATAAACGCCGCAGCTGGACACGTTATGTGCTCTCCCGCGAATTG
CGAAAATCACTCCGTCCTCAGCGAAGGAAGAAAAAATGAAATTAACCCCGATTATTGCGGCACTTCGCAGCCG
TTGCCCTCGGTTTGAAAACCGTGTGGGTGGCGCAGCGCAGTTTAAAGCGATACCGGAGGCCGGAAAGCTCAGG
CTACCAGCCGCGTATGTTGTGCCAGCCGAAGACGTCACGGGTGAGCAGAAATCGCAGACCGACTACTGGCAGG
ATTTGACGGAGGGTTTTTCCGTCATCGTGGTACTCAGCAACGAACGGGATGAAAAAGGGCAGTGGGCTTCTTA
CGACGCAGTTCACGACGTCAGGCAGGAAATCTGGAAGGCGCTGCTGGGGTGGGAACCGGACCCGCAGGCGCAT
GAAATTCAGTATGCGGGTGGGATGCTTCTCGATCTGAACCGCCACGAACTGTATTACCAGTTCGACTTCACGG
TGAAGTATGAAATTACCGAAACAGACACCCGCCAGCAGGATGATCTGGACGGCCTGCCCGACCTTAAAACGCT
CAGTATTGATGTTGATTTTATCGAACCCGGTACCGGGCCAGATGGCGACATCGAGCACCACACCGAAATTACA
TTTCAGGAATAAACCATGTTTGTGAAACCCGCAAAAGGGCGATCGGTTCCCGATCCGGCCCGTGGCGACCTTT
TACCTGAAGGAGGTCGAAATGTTGATGAGAATAACTACTGGCTGCGCCGCGAGGCCGCTGGTGATGTCCGGCG
CACGAATAAAAAGGTGAAAACAAATGGCGATTAGTTTTAATTCCATCCCGTCAGATACGCGGGTTCCGCTGTT
TTATGCCGAGATGGATAACTCGGCGGCAAATACCGCCCGGGACAGCGGGGCATCACTGCTGATTGGTCACGCC
AGCAATGATGCGTCAATTGCCGTCAACAGTCTTGTTCTGGTGTCATCGGTTGATTATGCCCGTCAGATTTGCG
GTGCAGGAAGCCAGCTGGCCCGTATGGTCGGGGCGTACCGTAAGACCGATCCATTTGGCGAACTGTATGTCAT
TGCCGTACCTGAATCCACAGGCGCGGCAGCAACCGTCGCTTTGACGGTAACTGGCGAAGCGACGGAAACCGGA
ACGGTGAATGTCTATACCGGCCGAACCCGCGTTCAGGCTCCCGTGACCAGCGGTGATGACGCTGCGGCGGTGG
CTGTGAGCATTAAGGATGCGGTCAATGCAAACCCTGATCTTCCCTTTACGGCAACATCAGAAGCGGGGGTGGT
GACACTGACTGCGCGCCACAAGGGGTTATATGGAAATGAAATTCCGGTCACTCTCAATTATTACGGCTTTGGC
GGTGGGGAGGTGTTACCGGCAGGTGTGAATATTACGGTTGCCAGCGGCGTGAAGGGGGCTGGTGCGCCAGCTC
TTAACGACGCGGTGGCAGCGATGGGAGATGAGCCGTTCGATTATATCGGCCTTCCGTTTAACGACACGGCATC
GGTGAACACGATGGCAACTGAAATGAATGATTCCAGCGGTCGCTGGAGTTATATCCGGCAGTTGTATGGTCAC
GTTTATACGGCGAAGACGGGGACGCTGTCGGAGCTTGTGGCCGCGGGTGACCAGTTTAACCTGCAGCACATCA
CCCTGGCGGGCTATGAGAAAGACACCCAGACGCCTGCTGATGAACTGGCTGCAAGCCGTACTGCCCGTGCTGC
GGTTTTTATCCGTAACGATCCGGCGCGCCCGACCCAGACCGGGGAACTGGTGGACATGCTGCCGGCACCGAAA
GGCAAACGCTTCACGACGACTGAACAGCAGACGTTACTTTCCCACGGTGTGGCAACGGCGTATGTGGAAAGCG
GCGTGCTGCGTATTCAGCGGGATATCACGACGTACAGGAAAAATGCGTATGGTGTGGCGGATAACAGCTACCT
TGACAGCGAGACGCTGCATACCAGTGCTTATGTGTTGCGCCGTCTGAAATCTGTTATTACCAGTAAATACGGG
CGCCATAAACTTGCTAATGATGGTACGCGTTTCGGGCCTGGTCAGGCCATTGTCACGCCTGCCGTTATCCGTG

GTGAGCTGGGATCAACATATCGCCAGCTGGAGCGGGAAGGCATCGTGGAAAACTTCGATCTGTTCCAGCAACA
TCTGATAGTTGAGCGTAACGCGAACGATTCGAACCGCCTTGATGTGCTGTTTCCGCCTGATTATGTCAATCAG
TTACGTGTGTTTGCGGTGCTTAACCAGTTCCGTCTGCAGTACAGCGAGGAGGCTGCATAATGGGAAAAATTGC
GGGAACAACATATTTCAAAATCGACGGACAGCAACTGTCGGTAACCGGAGGGATTGAAGTCCCCATGAACACC
AAAGTTCGTGACGACGTGATTGGCCTGGATGGTTCCGTTGACTACAAGGAAACCAGCCGGGCACCGTATACGA
AGGTGACCGCCAAAGTGCCGAAAAACTTCCCGGTCGATAAAATTACGTCTTCTGATGTCATGACCATCACATC
AGAGCTGGCAAATGGTCAGGTGTATGTTCTCTCAAACGCCTGGCTGCACGGCGAAGCCAACCATAACCCGGAA
GAGGGCACCGTGGATCTTGAGTTCCACGGTGAGGAGGGATTTTACCAGTGATAAAAGAACTTGTGCTCAAAAA
GCCGATTATGGCGCATAACGAAAAGCTTCATGTGCTGGAGCTGCGCGAACCGTCCTACGATGAAATCGAAGCC
ATTGGTTTTCCGTTCACCGTTTCCGGTGATGGCGGCGTCCGGCTGGACAGTTCGGTTGCTCTGAAATATATCC
CTGTGCTGGCAGGTATTCCACGCTCCTCGGCAGCGCAACTGGCAAAACTGGATATTTTCAAAGCCTGTATGTT
GATCCTCAATTTTTTTACCCGGTCGGAGACGGAGGAGGACTCAGAAAGCGGGTCTACAACACCGCATACTTCT
GGCGAATAAATCCCCTGGAGCTCCGGCGGGCGGCGATATCCGATTTTCTGGAGCTGGAGTCGGAGGCTGTCCG
TATCAATGAGGAAATGAAGCATGGCTGACAGTTTCCAGTTAAAGGCCATTATCACTGCCGTTGACCAGTTATC
GGGTCCGCTGAAAGGGATGCAGCGGGAACTGAAGGGATTTCAGAAAGAAATGGCCGGGCTGGCGATCGGTGCT
GCCGCTGCCGGGACCGCTGTTCTTGGGGCGCTGGCGCTGCCCGTGAATGCTGCGATCGGCTTTGAGTCAAAAA
TGGCTGACATCCGGAAGGTGGTTGACGGCCTGGATGATAAAAAAGCATTCGCGCAGATGAGTGACGATATCCT
GACGCTGTCCACACAGTTACCGATGGCGGCGGAGGGAATTGCAGAGATCGTGGCGGCGGGCGGGCAGGCAGGC
ATTGCCCGCGGCGATTTGATGCAGTTTGCGAACGACGCAGTGAAAATGGGTGTGGCGTTTGATACCACTGCCG
AAGAGTCCGGTCAGATGATGGCGCAGTGGCGGACAGCGTTCAGACTGACGCAGGAAGACGTGGTTGTCCTGGC
CGATAAAATCAACTATCTGGGGAATACCGGCCCGGCAAATGCGAAGAAAATTTCTGATATCGTGACGCGGATT
GGTCCGCTGGGCGGTGTTGCCGGAGTGGCATCCGGCGAAATTGCCGCGATGGGCGCCACCATTGCCGGGATGG
GGGTTGAATCAGAAATTGCCTCCACCGGCATCAAAAACTTCATGCTGTCGTTAACCGCAGGTAATTCGGCAAC
CAAAGCCCAGAAACAGGCTATGGCTTTCCTGAAGCTGAATCCCCGGAAACTCGCTGAGGATATGCAAAAGGAT
TCGCGCGGGGCCATGCTGAAGGTGCTGGACTCGCTCGCGAAAGTGCCAAAAGCTAAACAGGCCGCCGTCATGA
ATGCGCTGTTTGGCAAGGAGTCACTTAGCGCGATTGCCCCGCTGCTGACCAACCTGGATTTGTTACGCACCAA
TTTTGATCGTGTGGCTGATGCCCAGGAATATGGCGGCTCGATGCAGAAGGAATACGCATCCCGCGCGTCCACA
ACAGAAAACCAGCTGGTTCTGCTGAAAAACAGCGTCAATGCGATTTCGGTAACGCTGGGCGATACCTTCCTGC
CCGCCATTAACGAAGCTGCAGAAGCGGTCATGCCTTACCTGGAGCAGCTCCGGACATTCGTTCGCGCGAATCC
TGAACTGGTTCAGTCTGCGGCGAAGTTCGGCGCGGCGCTGCTGGCTGTTGGCGTATCCATTGGCAGCCTGTCC
CGGGCTGTCAAAATCCTGAACAGTGTCATTAACCTCTCTCCGGCGAAAGTCGCCATTGCGGCGCTGGTGGCCG
GCGCTATGCTGATCATTGAGAACTGGGACGATGTTGCTCCGGTGATTAAGGCGGTATGGCAGGAGGTCGATAA
CGTTGCGCAGGAGATGGGCGGATGGGAGACGGTGATTGAAGGGGTTGGTCTGGTTATGGCTGGTTCTTTTACC
GTCAGGACCATTGGTGCCCTGCAGCAGTCCGTCCTGCTGGCCGGACGGCTTTCCGGTCTGCTGGGTAAAATTG
GCCGGATGGGGGCCATGACGCTGACAATTGGCGTGGCGGTGTCACTCTTTAAAGAGCTTAAGGATCTGGAGCA
GGGGGCAAAGGATGCGGGTATGGATGCTGGCGCATTCGCTGTACAGAAGCTGCAAACGAAGGAGCGTGAACGC
GGGTATAACGGTTTTATTCCCAGACTCAAAGAGCTTCTTGGTATGGACACCCCGATTCCGCAGGGGCGTTATC
AACCTTATGTGCCACTGACCCGGCGTTCTGGCGTACTCGGGCGAGCTGTCCCGCCATCAACGCAGCGCAGCGA
ACTCAAAGTGACATTTGAGAATGCACCACAAGGTATGCGTGTGACTGATATACCGAAATCCGGTAATCCATTG
ATGAACATCAGCCATGATGTGGGTTACTCACCCTTTCGTACATCACGATAAACCTGCTCCGGCAGGTTTTCTT
ATGGGGTAAATATGGCTTTTTTCTCCTCAACTGGCTGGCGCGGGCGCCTGCGTGATGCATCATTTCGTGGAGT
GCCTTTCTCCGTTGAAGATGATGAAAGCACGTTTGGACGCCGCGTACAGGTACATGAATATCCGAACAGGGAT
AAGCCCTGGACGGAGGATTTAGGTCGCGCCACGCGCCGCCTGACGATAAATGCTTATCTTGTCGGTGATGATT
ACGCAGACAGGCGGGATCGTCTTATTGGTGCCATTGAAACCGCAGGCCCTGGTACGCTGGTCCATCCGCAGTA
TGGCGAAATGCAGGGCAGCATTGACGGACAGGTCAGGATCACTCACAGCAGTACAGAAGGGCGCATGTGTCGT
GTCTCCTTTCAGTTTGTGGAAAGTGGTGAACTTTCTTTTCCTGTGGCGGGAATGGCAACGGCGAAGCGCCTGG
AAACATCAGGCGGGCTTTTCGACGATGCGATTGACAGTATGTTTTCCACATTCTCGTTGTCAGGTATTTCTGA
TTTTATCCAGAACGATGTCATTGCCGATGCTGCCTCCATGCTGGGCGATGTTGCCGATGCTTTCAGGATGGTT
GACTCCGGCGTGTCTGCCGCAATGCGGCTGTTACAGGGGGATTTGTCTGTCATTCTGATGCCACCGGGCGCCG
CAAGTGATTTCGTTAACGCACTGCAAAAAGCCTGGCGCTCAGGTGACAGGCTCAGAGGCAGTACATCGGATCT
GGTCACGATGATAAAAACGATGTCAGGTATCACCCTTGATCCCGGTCTTTCCCCCCGTGGCACCTGGCCCACT
GACTCCGGATCTGCTGCGAAACAGAAAATGCAACGCAATATGATCGCAGCCGCCATCAGGACAACAGCCATCA
GCACAGCCGTCCACGCCGTGACAACACTGAAGCAGCCGCGTGATGTACCTGATGTCCGGGGCGTAAATCAGCC
TGCAGGAACAGGCCGTGACTCAGACATTATCACTGTCATGCACCCGGCGCTGGATGGTGTACAGACAGTCAGT

AATGGCAGCTTTCCACCGAATTATGAAGATCTGAAAGCTATCCGGACCGCGCTCAATGCTGCGATTGACCAGG
AGCAGTTGCGTATCCGGGATGATGTGCTTTTCCAGCAAATTTCCGTTATGCGGACGGATCTCAATCGCGATAT
TTCTGCACGACTGGCACAGGTTGAACGTACTGCATTGCGAACGCCTGATGATGTTCTGCCTGCACTGGTACTG
GCTGCAGCCTGGTATGACGACGCCGGGCGGGAATCTGATATCCTCACTCGTAATCCCGTTCCCCATCCGGGAT
TTATCCCGGTTGAGCCGCTGAGGGTTCCGGTACGATGAATAATACGGTTTTTTACGCGTCAACGGGCGTGAC
TGGGGAGGATGGACGTCAGTACGGATAAGTGCGGGCATTGACCGTATTGCCCGGGACTTTAATGTCTCGATCA
CCCGGCAGTGGCCTGGTGGAGAAGACGTACCGCCAGTAAAAAATGGTGACGCTGTAGAGGTACTCATTGGCGA
TGATTTAGTTATTACCGGCTGGGTTGAGGCGTTACCGCTACGTTATGATGCGCAGACCATTATGACGGGCATT
GTCGGGCGCAGCAAAACGGCAGATCTTATCGACTGTTCTGCATCGCCTGCACAGCATAACGGGAAAAATTTAT
TCCTGATCGCCAGCGCACTTGCCCGGCCATTCGGTGTGGACGTTGTTGATGCAGGCGCGCCGGCAGCCGCCGT
TATTGAGGCTCAGCCGGAACATGGTGAAACGGTTGTGGACTGTCTGAACAGGCTGCTTGGACAGGCTCAGGCG
CTGGCATATGACGACGAACGGGGACGGCTGGTTCTCGGCAGGCCGGGCAGTATGAAAGCAGCCACGGCACTGG
TACTTGGCGAAAATATTCTTTCCTGTGATACCGAGCGTAGTGTTCGTGAGCGTTTCTCCAGTTATCTGGTTAC
GGGGCAGCGTCCTGGTACGGATGACGATTTCGGCGAGGCAACCATTGCTGCTATCCGGCAGAGTACTGGTGAT
GCAGGCGTCACGCGGTATCGTCCCCACACCATTCAGCAGTCAGGAACTGCCACAACTGACAGCTGCAAATCCC
GCTGTGAATTTGAAGCCCGTCAGCGTGCGGCGAAAACGCTGGAAACCACCTATACCGTACAGGGATGGAGACA
GGGGAATGGCGAATTGTGGAAACCGAATCAGGCCGTGGTGGTGTATGACCCGCTGAACGGTTTTGACAATGAA
ACGCTGGTGATCGCCGAAGTGACGTACAGCCAGGACAATAACGGCACCCTGACCGAAATCCGGGTGGGGCCTG
CGGATGCCTATCTTCCTGAACCATTCAGGCCGAAAGCGAAGAAAAAAGTCAGTGAGGAGGCAGATTTCTGATG
GCTAACCATCCTCTTCAGAACATGATAACGCGCGCAGTCATTACCGCGATTGATACCGTCAGAAAATGCCAGA
CTGCCGGACTGAAACTTATTGCCGGTGAAAAAAAGAGAATGTGGAGCATCTTGAACCTTACGGTTTCACCTCT
GCAGCACAGAATGGCGCAGAAGCGGTGGTATTGTTTCCCGGCGGTGGCCGTTCGCACGGAGTGGCTGTGGTTG
TGGCTGACCGCCGCTTCAGACTGAAAGGGCTGGCGCGCGGGGAAGTCGCGCTATATGACGATCAGGGGCAGTC
GGTCACATTAACCCGAGCCGGAATAGTGGTAAATGGCGGCGGAAAGCCAGTTATTTTCACGAATGCCACTAAA
GCACGTTTTGAAATGCCGATCGAATCCACTGGCGATATCAGGGACAACTGTGACAGCAGTGGAAAAACGATGG
CTGAAATGCGCACGACCTATAACGGTCATACCCATAAAGAAAATGGCGATGGCGGCGGTATAACCGATAAGCC
TGGCCAACCCATGAGCTGACACCATGATCCTTTATGTTAATGGAATCCGTAAGGATGCCACGGCTTCGCTCGA
CTTTCTGACGCGGGCAGTGGTGATTTCTCTTTTTACCTGGCGCCGGGCGGAGCGGGATGACAGGACCCCACAG
CCATACGGCTGGTGGGGGACACCTGGCCTGCTGTTCAGAATGACCGCATCGGTTCCCGCCTCTACCTGCTGA
AACGCCGCAAACTCACCAATAAAACGCCGCAGGATGCCCGCGAATACATGCAGCAGGCGCTGGCGTGGATGAC
AGACGATGGCGTGGCGGCACGTATTGATGTGACATCTGAACGCACAGGAACAGATACCCTGGCAGCTGGCGTG
ACGATATATCAGCGGGACGGGGTAATTCACAATATTACATTCGATGATATATGGAGCAAACTTAATGGCTGAC
AGTCAATTTGCACGTCCTGAACTTCCTCAGTTGATTGCTACCATTCGCAGCGATTTACTGACCCGTTTTCAGC
AGGATGTTGTGTTACGTCGCATGGATGCCGAGGTTTACAGCCGGGTACAGGCTGCTGCCGTACATACGCTGTA
TGGTTATATCGATTATCTGGCCCGGAATATGCTGCCTGATATGTGTGATGAGGACTGGCTTTACCGTCACGCG
AGGATTAAGCGTTGTCCCAGGAAAAATGCCGTATCTGCGAAGGGATTTGCACGCTGGGATGGTATTGCCGGAA
CGCCGGAGATCCCCGCGGGTACACAGATTCAGCGGGATGATCAGGTTACATTCACGACCCTGCAGACGGTGAA
AGCTTCCGGCGGCCTGTTACGTGTGCCGGTTATTGCTGATGTGGCGGGAACTGCCGGTAATACTGACGATGGT
ACGGCGTTACGCCTTGGCACGCCGATTACTGGTATTCCTTCTACAGGTTACGCTGACACTCTGACCGGGGGGG
CTGATACAGAGGAGCCTGAAACGTGGCGCGCGCGCGTCATGGAACGCTATTACTGGATACCACAGGGGGGCGC
TGATCCTGATTACGTCATCTGGGCAAAGGAAATCGCGGGAATAACCCGTGCGTGGACATTCCGCCATTATAAG
GGGACCGGCACCGTTGGTGTGATGGTGGCTACCAGTAACCCGGTGAATCCGGCTCCTGGCGACGATCTCGTTA
AGGCTGTACGTGACCATATTTTGCCGCTGGCACCTGTTGCTGGCGGCGGACTCTTTGTTTTCGCTGCCACTGA
AAAAAGCATTCCGGTAACAGTCGCACTGGCCAAAGATACCCCGGAAATTCGTACTGCCATTATTGCGGAGCTA
AATGCGCTGATGCTGCGTGATGGCGCGCCGTCCGGAAAAATTTATGTTTCGCGAATCAGCGAGGCGATAAGTC
TGGCGACCGGGGAAGTGGCACATCAGCTGCGTGTGCCGGCGGCAGATGTGGTGCTGGGAAAAACTGAACTTCC
TGTCCTGGGGAATATAACCTGGGCCACCTATACCGGGGAGAACGGATAACTATGGCATTACAGGACGAATATA
CGCAGTTACTTTATCACCTTCTGCCGGAAGGGCCTGCCTGGGACGGAGAAAACCCACTGATTGAAGGGCTGGC
GCCGTCGCTGAACCGGGTACATCAGAGAGCGGATGAACTGATGGCTGAAATTGATCCGGCCAGAACCACAGAA
CTGATAGACCGTTATGAACAGCTGTATGGCCTGCCTGATTCCTGTGCACCGGAAGGCGTTCAGACATTACAGC
AGCGCCAGCAACGGCTGGATGCAAAGGCAAATGTTGCTGGCGGTATAAACGAGAGGTTTTATCGGGAACAGCT
TGATGCGTTGGGGTATACCGCTGCCACCATTGAGCAGTTTCAGAATCTCGACAGCACACCCGATCCTGAATGG
GGGGAATTCTGGCGTTACTACTGGCGTGTGAATATTCCGGCTGATGCGAACATCAGCTGGCAGACCTGTACAA
GCACCTGCGACTCTGCGATCAGAACGTGGGGCGATACTGTTGCTGAATGTGTGATTGATAAGCTTTGTCCGTC

ACATACGGTTGTTGTTTTTGCTTATCCGGAAGGAAAAGAGAATGCACAGAATTGATACGCCCACCGCGCAAAA
AGATAAATTTGGTCAGGGAAAAAACGGATTTACGAATGGTGATCCCGCCACGGGCCGCCGCGCAACGGATCTC
AACAGTGATATGTGGGATGCAGTCCAGGAAGAGGTCTGTACTGTTATTGAAGCCGCCGGCATACCACTCAGTA
AAGGCGAACATACGCAGCTTCACGCCGCCATTGGCAGGCTGATCGATGAACAGGTTAAAACCCGTCTTGAAAA
AAATCAGAATGGCGCGGACATCCCGAATAAGCCGCTGTTTCTCCAGAACGTCGGTTTAGGAGAAACGATAAAT
CTCGCTGCAGGGGCCCTGCAAAAATCGCAGAACGGCGGCGATATTCCTGACAAAAAACAATTTGCGAGAACCA
TCGGTGCGGTAACGTCAACCACCATTACACTTGGCGAATCAGGCTGGTTCAAAATCGCCACGGTTGTAATGCC
GCAGGCTACATCAACTGCGGTGATTAAACTGTACGGTGGGGCGGGGTTTAACGCTGGTTCACCTGAACAGGCG
GCAATCAGCGAACTGGTATTGCGTGCCGGTAATGGTTCACCTGTTGGAATAACCGCCACATTATGGAGGCGTT
CACCTTCTGCTGCTAACGAGGTCGCATGGGTTAATACATCAGGCGACACCTACGATATTTATATTAATATCGG
CCAGTATGCGTACTGGTTAATTGCGCAATATGATTACACCGGTAATGCAAATGTCACGCTGCACAGTACGCCT
GAATATTCATCAGTTCAGCCGGGAAACTCAACCAGCGGTCAGACATATACACTGTTTAATAGTCTGATGAAAC
CCACAGCCGGTGACGTTGAGGCACTGTCAGTTAATGGAGGGAGGCTAAACGGTCCGTTAGGCATTGGTACTGA
TAATGCGCTGGGTGGTAATTCGATTGTATTCGGAGATAACGATACAGGGTTTAAGTGGCACAGTGACGGCGTT
CTGGGGATTTATGCCAATAATGCTCTGGTTGGTTATATCGACAATTCCGGGCTGCACATGTCAGTAGATGTTC
TCACTAATGGTGCCGTACGCGCAGGCAACGCAAAAAAACTGTCACTGACGAGCAATAATAATTCGACAATGAC
AGCCACGTTTAATTTATGGGGCGACGCAAACAGGCCAACAGTTATTGAACTGGACGACGATCAGGGATGGCAT
CTGTACAGCCAGCGAAATCCTGACGGTTCGATTGTCTTTACGGTCAATGGAGATATCACCGCTAACACGCTTC
GTGCAAGCGGGGCTATCTATCAGAATAACGGCGACATCTTTGGTTCGCTATGGGGAAATGGCTGGTTAAGTAC
CTGGATTAATAATAATCTCGTCTTAGATGTTCAGTTAGGGGCTGGCACATCAGTGACTACCTGGAACAATGCA
GGTTCCTGGCCTAACACTCCCGGATATGTAGTTACCTCCGTCTGGAAAGATTATCAGGGCGAAAATATTGATG
GTATTAATTATGCGCCTTTGCAAAAACGAGTCGGGAGTCAGTGGTATACCGTACAAGGGGGAACGGTATAATG
AAAAAATATCAGAATATCAAAAATTTCAGACTTATTGACGCGCCCGTAAACAGGGATAAAACTCAGGCTGAAA
TAAATATAGGTGCATATTTTCTGGAGTCGGACGATGGACAGGACTGGTATGAGTGTCAGTCATTATTTTCTGA
TGATACTGCAAAAATAATGTACGACCATGAGGGGGTTATCTGGGGTGTTGTTAATAAGCCAGTCCCGCAACGA
GGAAACACATATTCTGTATCAATGTTGTGGCCGGTTAATATGTCTGTTGCGGAAATAGACGCTGCTGACTGCC
CTGATGATTGCCGTGGTGATGGTACGTGGTTATATCAGGACGGTAAAGTCGTTCAACGGGGTTATTCTCCGGA
AGAGCTGCGTAAAAAGGCGGAGGCTGAAAAAGTTCGCCGCCTTGCTGAGGCTGAATCAGCCATCGCACCACTG
GCACGGGCAGTAAAACTAAAAATTGCCACAGATGAAGAGATTAAACGGCTGGAAGCATGGGAACTTTATAGCG
TAATGGTAAACAGGGTGGATACATCTGCGCCTGACTGGCCGGATATACCACGCTAAATATTCAGGCGGGTTTA
TTACCCGCCTTTTCTTTTTCCTGTCGTTGTGCCATCAACCTGACAGCCGGTACAAATAGCCCCCTCTTGTGTA
CTGACCTGAAAATATACTCACCCCTTAACCACGGAGTTAACCGGATGAGTGATTTTCACCACGGCACGCAGGT
CATCCAATTTAATGGAGGTACGCGCGTCACATCCACGATATCGATCGAAATCGTCAGTATGGTCTTTACGTTT
AGCTTTGCCGAAACATTGCTCGCGTTTATATCATACGTTTGCAAATAATTATCTCCAGGAGCTGATAGTCAAA
CTGCTGGTATCCATAAAAATATGTTCATGCTTAAATTCTATAAAATTACAAAACTCTTCATAATTGTGCCGTA
TAGAACAATTAAAATAATGCCTTAGCCCACCGCAGACACCATCAATGTATGGATCGCCATAAGGTTTACTGTG
CATAATTTCAAGTCCTTTTTTCAATGCCGGATGCTCGCTGCGGTTAACGGCGATAATCCCATTTTCAAGACTC
ATGCTATTACCTTTACGACTTACATGAACAGCAATACCATCAGGTAAATACAAAGTGCCGAGTTTACCTGTAA
GTAACATATCAGCATCAAGATATATGCAGCCACCTCCAGGTTGCAGGTGATGGCAGCCATGTTTGCCCGCCTC
CAGAAAAGCATTACTTCCTTTTAATAAAAAAAGATTTCTGTAAAAATCAAACCGTACATGTCCCAAGCGTTTA
TCATGGGACGAAACAAGGGACTCCTCTGGATTGTTCTTTAAAACTTCATTTAAACTCTTTTTTATCTCACCAA
GCAGATATTCATCTCTGACATTTGCTGGTTGAGCTTCAATTTTAGCGATGTTTTCTAAATAAATATCTGATAG
TTTCTTGTCATACATGCTATAATCCAGGTCGGAATTATAGATAACCTTTATATTTTCATATTGTTTTTCCAGC
TTTGCTAATGCTTTCTTTTGTCCAGCACTAAAATTCCCATCAACTAAAACCCCGATAGTTCTCTCTTTTTCTA
TTATAGCGGCGTTGATAATATTATTGAGATAGGGGTTTTGTTGAGTATTAATAATTGGGATCTGGTTTTCCCC
AAACCTGCTTGGGTTTCGTTCAAACCATTGAAAAAGTAGGGGGTGTGCTGATCTAATGGCAATAAAGGATATT
CAACTCCGGCAAAGTTTGCACTACCTGATGAAGGCAGAGTAATAGCTGGAGTTGCAGTATGAGAATAGTTCTG
GCATGAAAGAAAACCTCTGACTCGAGAAAACATTTTTCAACCCTTACGCTATTAATATAACATACCATGATTT
GAGATTGATAAGATATCGGGTTTTAACCTTAGTTTAATAAATTGGAAGATTTTTGTATGAAGATTTGCTACCG
TATTTTCGTGCCATAGCTATCTGAAACGATAGTTTTTACTTGGTTAGGGGGGGCTTAAAATTACATTTTTGAA
TAAGTATTTTATACTTCTTATACGATATGTTTTTATTGTATTGCAGGGGGACAGGGGAGATTGGCGGGCGAAG
ACCAAAGTTATCACCTGAGCAATGGGCGCAGGCCGGGCGTCTGATTGGGGCAGGAATACCGCGACAGCAGGTA
GCGATTATTTATGATGTGGGGCTGTCGATACACGGGCTGTACTGGATTCTGTTATCGGCTAACCGGAAGGGAG
TTAAAAAACGGGATTACCGCGCTAACTAGAAGCATGAGGGAAGGTTGTGGCATCCGGTGAACCTGTAGGAATC

CCATAACTGATTAAGGACGGAAACCACCAGATGCCACGAAGATGTTATGGCGCATGTTGTTGTGGGAAGTCAA
TAAAGGAGATGGTTTTGTTTAAAAAATAGTCTATCATGGTGAGAAAATGATTTTGATAAGTAGGCTAACTTTC
TGAAAATACTGCGTACAAAAATGCTACTTTTTTCTCATGTTATTAGATAAGTTAATGTTAAATAAGAATATTG
GGACGGTCTCGAAAACCGGAGTAGGGGCAACTCTACCGGGGGTTCAAATCCCCCTCTCTCCGCCAATCATTCA
ACAAAATCAATCACTGACAAAGCGTTTTTGATTTCGTCATACATAAATCCCTGCATTAAAATTTCTTTCACTC
GCTCGATTTTTCTTACTACTGATGCTGTTTTTTACCATTTTGCTGCGCGTCGGGCATCCACTTTTATCTTCTT
TAGCTCACAAGCTCACACTCTTTTGCAACGCCCATCTCACATCTCTGGTGACAAAACAGTTTAACTGCCGTTG
CTTCACACGGATCCTGTGAGGGTATCTTAAAGGAAATGGATTGCTGGAGGATGGAGGAAAAGAGGCGAAATCT
TTTTCAGTGGTGATACCGCCTCCCCTGGACATGAGCTTGCCAGAACCTACGCGGTAAATCCTACGACACGAAG
CCACGCGCAATTTTATGCGAAAACGCTGGCAATAGCCTTGCTGACTGATGCAAGCTATCTAAAATCGCTTGAA
GATGACATCATGCAAAACAATCGCTTAAAAGACTCACCTGTTGATAAGCATGTTGAGGACGAAAAGAAGAAGA
AAAATGCAGCCAGGTAAAAAGGGTTACGTTCGCCGTAAGCGAACGTGATCGCATCCAGCCTTAACGAACCTTT
TTCCGTTCATCTCCCTTCATGTTTTCAAAAAACTCTTTTATGAATGCCCCTAAAACGTCATACGCGAATTCGC
TATTATTGTTCCCGTCGCGACAACATAGGGATTACGTAAAAAAACAGAGGGCAGACATTGAAAATATTGGAAA
TAACGTAAAACGATTTGTCCGTGTTTTTATCTGTCATTTAAACTTACAGAAATGGCTGTACTGCTATCAGTAT
AAAGATAATTTCAGTTGTCCAGATAAATCATTTTTAAAACTATTTGTATTAACAGGGTTTATTCATTTATTGA
ATGGATGCGTGCTCGTTTGGTCTTGTCATCTTGACGCTTTGAGATCACATATTCTATTCTATCATAGACAGCA
GGGAAAATTAGCTGCTGGGATCGATTTTTGATTAATAAAATATTGGATGGAACCACGATGCAATTACCAGAAC
AGGATGAGTTTTCTGATTTTTTTGCTGCCAATGATGATGAACAAGCCTCTTTAAGGCGTAAGTTTTTTTTGGA
GAAACATAAAGAACCGTGTCTGTCTGAGTCTGCATTAGAGGACTACCAGGCGCTGTTTATGAGTATCTACGGA
ATTAATATTGACTGGAAAGAGGGGACTTTTAGCCTGCTTGAGGCACTTTCAGATAATCAGGAGGGAAGCCTGT
CACGGTCAAATTCGATTATGACAGTGAGATTGAAACAGCAACGATAAATTTGGTTGATACGCAGTATGTGTTT
CATCACTACCCAATGGGAAGTGATGGTTTTGATACAGAACTGGTGCGCATTGAGCATATATTGGCTAATAGTG
GATATAGTTTGCGGGTATATCAGAACAGCACTTTTAGTGATACATTATCGTTCTTACTCATTCCGTCAGATGA
GTGGAAACGTGTTGAACAGCATTATAGCCCAGAGCATATTTCTGAATACTTCGTTCCGTATGGAAAACAACTT
GTTATTCCTGAGGTTACTGCTCCAGTCGTAAATTATGTGCCATCCGTTAAACAAGAAGCATCAAATGTCCCAG
CGTTGTTTAATGCTCGGGGTATCCGTATTTGTTTTTTAAGTATAATGCTAATCGCATTTGCAATTTATATTTT
GTGGAATATCCTGACAAAAATAGAGCCTTTATCATCAGGCCAACCTGCTGGCTGTGAAAATCTACAAAATTTA
TACTCAAAATTACGTCCAGAAGTAGCCGAGCCATTAAAAGAAAAAATGCGTAAGAGTTTGGGCTGTAAATGAT
AAATTTTCAGTTTAAGAAAATTACACTAATGGATAGGTAATAGAGATATATTTAATTTGATATTGTGTTATCG
AAATAGTAATAACAGATAATATACATATTCTTGCGGTGGTGGATGAAGCGATTTTTATCTTATTTGTACAGGA
AAAGAAAAAGAGTTTTTTTAGCTACCATGACTCTTATTAATTTTGTTGCCGCAAATCAAAAATACTACGCTTT
TAATTTGTTGTCAGTGTTTATCATGGTGTTCTGGTCAGTTCAGTACCTCCTGCAGCATCTCCTTGTCCAGACT
CAGGTCAGCCACCAGCTTCTTCAGCCGCTGATTCTCATCCTCCAGTTGCCGCAGACGCCGCAGTTCCGTCACG
CCCGGCCCGGCAAATTTTTTCTTCCAGTTAATGGGATGGACTACTTCCTCCCGCTGCGGTTAACTGTACGAAA
TGTGCTCACCAGGAGAATCACCATGAATATCGTATTTCTGGGTATTGATCTGGCTAAAAATGTTTTTCAGCTC
TGCGGGTTAAACCAGGCCGGCAAACCGGTTTATACGAAACGCACTGGCCGAAAAGAATTGCTCCAGACGCTGG
CAAATATTCCTGCATGTCTGATTGGGATCGAAGCGTCCACCGGGGCATTTTACTGGCAGCGTGAGTTTGAGAA
ACTGGGGCACAAAGTAAAGGTCATCAGTCCTCAGTATGTAAGACCCTTTGTCCGCGGGCAAAAAAATGATGGT
AATGATGCACAGGCCATCGCAGTGGCTCTGATGCAACCGACAATGCAGTTCGTGCCGCCAAAAAGCCCCGAAC
AGCAGGATATCCAGGCTTTACACCGGGCAAGGCAGCGTATTGTCAATCACCGCACTGCTACAGTCTGTCAAAT
AAGGGGGCTGTTACTTGACCGGGGGATCCCCATTGGCAGTGCTGTCTCCAGAGCTCGCCGTGCTATTCCTCTT
ATCCTTGAAGATGCAGAAAACGGTCTAAGTTCCCGTATGCGCAGAACAATTGCCGAACTCTATGATCTCTTTA
ACGATCTCGGGCGTCGGATCCATTTTTTTGATAAGGAAATTGAAACAGTATTCAGGCAATCAGAAGCCTGTCC
GCGTATCGCCAAAGTTAAAGGCATTGGTCCTAAAACGGCCACGGCCGTTGTTGCTGCTATTGGCAAAGGAACT
GAATTTAAGAATGGTCGCCACTTTGCTGCATGGCTGGGTCTGGTTCCACGCCAGCATTCGAGTGGCGACAGGC
AGGTGCTGATGAATATGACGAAAAAAGGCGACAAGCATCTGCGGACACTTTTTATTCATGGTGCCCGCGCTGT
CGTCAGGGTTGCCACGAATAACAATGATGGTCATATGAATCAGTGGGTTAACCAGTTAAAGGAACGGCGCGGA
TTTAATAAAACGACCGTGGCGGTCGCTAACAAAAACGCGAGAATAATCTGGTCGATGCTGAGAAATGATACCG
GGTATCAGGTAGTGTGTAATTAATCCCTTGCCAGCAAAAGTTGCAGCGTACTGAGAAATGATGACAGGTTGCA
CCTGCACAATCGGAACCTGATTTTTATACTGGCCTCAGAGGCCGTCCAGTTGTTGAGGCTACCCATTTGGGCA
CAGATTTTCCTGATGCCGGATAGATGTAAGCAACAAGCCAAAACCAGATTCAGTACTTGCAAAACGGAGGTAG
TCCATAGATGTAAAATGTGGCCTCAGAAATTCCCATCTTTCTGCAGACTTCCCCGACGCGGGTGCCGGTTTCA
GCCTGTTTCAGGGCAAATGCAATCTGTTCTTCGGTATAACGGGTCTTTTTCATGACAATGCGGCTCTCTGTGA

GATGGAAGAAAGACTGGAAACTTCAGTTTAGACTGATACTGTTTACAGGGGGAAGGTCAAGCGCGAGGGGCAG
GGGATACTTGATTGTTATCTTTCAAACGCGGTAAGCCTTCTAATAAAGCAACAGCAGCATCAGGCAGAGGAAC
TCGGTGCTTTTTACTACCGAGAGATACTTATGCAAACTTTTCTAAAGCGTTTCAGGTAGGGGAATGGGGCTGT
TTTTTCACAATCTACAGCAACTTTCCCATAGCAGCCAAAGGTCCAAAAATAAACCTTTCATCAGGAAAATCAC
AGCAAAAACAAGAGGTTAAGATGCAAAAAAAGTCCGTTTACACGAGCTTCTTTATGCAGTATTTTATGCAGGA
CAAAACCAATATTACGTTGTAATCGATTGATTTTGTATCAAATATGGCGGTGAGGGGGGATTGACTCGCTGCG
CTCGCCCTTCGGGCAGTCTCCTCGCAAGCTCGTCGTCTGTCCAACTGGCTGCGACGCTACTCAGCCCATCCCT
GGGCCTCGCCCCTTCGGGGCCAGAGCAAGCTCTGTTCAAAAATGCTCCCGGCGTTTTTGTGTCGAACCCCGGT
CGGTGGTTCTCATCCCCCCTTGGTTTGGGGGATACATAATAAGCAAAAAGCCCGTACTTCTGTACAGGCTCTC
AACTTAAATATGGCTGTGAGGGGGGGATTGACTCGCTGCGCTCGCCCTTCGGGCAGTCTCCTCGCAAGCTCGT
CGTCTGTCCAACTGGCTGCGCCAGTTGTCGAACCCCGGTCGGTGGTTCTCATCCCCCCTTGGTTTGGGGGATA
CATATAAGCAAAAAGCCCGTACTTCTGTACAGGCTCTCAACTTAGATATGGCGGTGAGGGGGGGATTCGAACC
CCCGATACGTTGCCGTATACACACTTTCCAGGCGTGCTCCTTCAGCCACTCGGACACCTCACCAAATTGTCGC
TCCAGCATTACTGGAACGGGCGCTAATGTAGGGAAATATCTTTTCTACGTCAATCAACTTTTTTAAAAAAAAG
CGCGTTTATACAAACTTCCACCAATCTGTGGTTTTAATAAGCGAAAACTGCTTTTTTTGCCCGCGCCGGGAAA
TTTGCTATGCTGCACATCCCGTTGAAAACGCTGATAACAGGCGCAATCACATTCCGCACAATACTGCTCAGGA
GATAACATGGAGATAATTTTTTATCACCCGACATTTAACACCGCCTGGTGGGTAAATGCGCTGGAGAAGGCTC
TCCCACATGCGCGCGTTCGTGAATGGAAGGTCGGTGATAACAACCCCGCAGACTATGCGCTTGTATGGCAGCC
CCCGGTTGAAATGCTGGCCGGAAGACGCTTAAAAGCCGTCTTTGCGCTGGGCGCGGGGGTGGATGCAATTCTG
AGTAAATTAAATGCGCATCCGGAAATGCTGGACGCCTCCATTCCTCTATTCCGTCTGGAAGATACCGGAATGG
GCCTGCAAATGCAGGAATATGCCGTAAGTCAGGTATTACACTGGTTCCGTCGTTTCGATGATTATCAGGCGCT
GAAAAATCAGGCGCTATGGAAACCGTTGCCGGAATATACCCGCGAAGAGTTTAGCGTCGGTATCATGGGCGCA
GGGGTACTGGGCGCAAAAGTGGCAGAAAGTCTACAGGCGTGGGGGTTCCCGTTACGTTGCTGGAGTCGTAGCC
GCAAATCCTGGCCTGGCGTGGAAAGTTATGTTGGGCGTGAAGAGCTGCACGCTTTCCTGAACCAGACGCGGGT
GCTGATTAATCTGCTGCCGAATACGGCCCAAACGGTAGGAATTATTAATAGCGAATTGTTGGATCAATTGCCG
GATGGCGCTTACGTGTTGAATCTCGCGCGCGGCGTGCATGTTCAGGAGGCGGATCTGCTGGCTGCGCTTGATA
GCGGTAAGCTAAAAGGCGCGATGTTGGATGTCTTTAGCCAGGAACCGTTACCGCAGGAAAGTCCATTATGGCG
CCATCCGCGAGTCGCCATGACGCCGCACATTGCGGCAGTCACCCGTCCAGCGGAAGCCATCGATTATATTAGC
CGCACCATTACCCAGCTGGAGAAGGGAGAGCCGGTGACGGGGCAGGTGGATCGGGCGAGAGGATATTGACATC
AACCCGGCGCGGGCCGGGTTTCGCTAAAAAACGCTGGCGATACCTGCTATCCTTGTCGGAAATGACTACAGGA
GAGAGCAATGTATCCCGTTGACCTGCATATGCATACCGTCGCCAGCACTCATGCCTACAGTACTCTGAGCGAT
TATATCGCGGAAGCCAAACGCAAAGGCATTAAACTTTTTGCGATTACCGATCATGGTCCGGACATGGAAGATG
CGCCGCATCACTGGCATTTTATTAACATGCGCATCTGGCCGCGTCTGGTTGACGGCGTGGGGATACTGCGTGG
CATTGAGGCGAATATCAAGAACATTAACGGTGAAATTGATTGTTCCGGAAAGATGTTCGACTCGCTGGATCTG
ATTATCGCAGGCTTCCATGAGCCCGTTTTTGCGCCACATGATAAAGAAACCAATACCCAGGCGATGATCGCGA
CCATCGCCAGCGGCAAGGTGCATATAATTAGTCACCCCGGAAATCCAAAGTATCCAGTGGAGGTTAAAGCCAT
CGCGCAGGCGGCGGCGAAACACCATGTAGCGCTGGAAATCAACAACTCTTCTTTTCTGCATTCGCGTAAAGGA
AGCGAAGATAATTGCCGCGCGGTCGCTGCCGCCGTACGCGATGCGGGAGGCTGGCTGGGTAGCGTTAGGCTCT
GATTCCCATACGGCCTTTACGCTTGGCGATTTCACCGAATGCCGGAAAATTCTGGATGCGGTGAATTTTCCGG
AAGATCGAATCCTGAACGTCTCTCCGCAGCGCTTACTGGCCTTTCTCGAATCACGCGGTATGGCGCCTGTACC
GGAATTTGCCGAACTTTAATCGTTATTTACGGGAAGATATCAATGAATGAGTTTTCAATCCTGTGCCGTGTGC
TGGGATCGTTGTTTTACCGCCAACCGCAAGATCCTTTACTGGTTCCGCTGTTTACGTTAATCCGTGAAGGTAA
ACTGGCGGCAAACTGGCCGCTGGAGCAGGATGACATGCTGGCGCGTTTACAGAAAAGCTGCGATATCACGCAG
ATTTCCACTGATTACAATGCGTTATTTGTTGGGGAAGAGTGCGCGGTAGCGCCATACCGCAGTGCGTGGGTCG
AAGGCGCGGAAGAGTCTGAGGTGCGCGCTTTTTTAACGTCGCGAGGGATGCCGCTGGCCGATACGCCTGCCGA
TCACATTGGCACTTTATTGCTCGCGGCCTCCTGGCTGGAAGATCAGTCTGCCGAAGATGAAAGTGAAGCGCTG
GAAACCTTATTTGCCGATTATCTGCTTCCCTGGTGCAATACCTTCCTCGGTAAAGTTGAAGCCCATGCCGTTA
CGCCATTCTGGCGCACTCTGGCGCCGCTAACGCGTGATGCGATAGGGGCCATGTGGGATGAACTTCAGGAAGA
AGATGAAGAATAATGTGATGTAAATCACCCTTAACTGCAACTGGTTTTACATCATTGCATAAAATGTGTGCGT
GATCTCATTAATGCGCCGCTTTTCTGTTATGATGCGCGCCATGAACATACTTCTTTCTATTGCTATCACGACG
GGCATCCTTTCTGGAATATGGGGATGGGTGGCCGTCTCCCTGGGGTTACTAAGCTGGGCCGGTTTTTTAGGTT
GTACGGCTTATTTCGCCTGTCCGCAGGGCGGCTTTAAGGGATTGTTAATTTCCGTCTGTACGCTGTTAAGCGG
TATGGTGTGGGCGCTGGTCATTATTCACGGTAGCGCGTTGGCGCCGCATCTGGAAATTGTCAGTTACGTGTTG
ACGGGGATCGTGGCATTCCTGATGTGTATCCAGGCAAAGCAGCTATTGCTTTCTTTTGTTCCGGGAACATTTA

TCGGCGCCTGCGCGACATTTGCAGGGCAGGGTGACTGGCGGTTGGTATTACCGTCGCTGGCGCTGGGGCTAAT
CTTTGGCTATGCCATGAAAAATAGTGGGCTATGGCTGGCATCACGCCGCGAGCAACATTCAGCGAATACGGCG
GTCACAAAATAAAAAAGCGTGGGGTTCTTCCCCACGCTTTGTCGTATTCATCAGGATTCTGGCGGTACTGACA
GCTCACGGTATTTCACCAGAATATCATTTTGCCTGTCCGCTTTATTCTGCAAATCCCACAGTCCGCGATCGAT
ACCATCATTAATGAGGAAGATAACGCCGGTTTCAATGGCTGACATCAGACACAGCATCACCGGTTCGTTCGAG
GTATAGCCGATTTCGCCTTCCAGTAAGCGCTGGTAATCAATAAAACGGAACACGCCTGCCTGTACTTCATAGG
AAAGGATTGTTTTACTGGTGTTCACCGAAGAAAGGATCTCGCCTGTACTGACGTTAACCACGCGCAGGTTGAC
AGCAATCTGATCCAGCTGATACTGCGTATCGGCGCCAATACCGAAATATCTTGCGCCGACCCCGCCGGATTTG
ACGTTACTTTCATAACCAATAATAGAACCTTCCACCATAATATTTGCCGCCGTCAGCGACTGAAGCGGGATAC
GGTTATTCATCGCCACGGTGCCGTTTTCCTGGGCTGCGCGAATAATTTTCCGTTCATTCAAAAGATTCTGTAA
GCCTTGTCGTTCCAGCGGGATAAACCAGCGCGAATCTTTCAGCGCAGTGACCAACATAGCGGTGGCGCTCTGC
GGCACAGCCGTAGAAAAGTTACTTGCCGGGTAAGGTTTAAATTGGCCCGTTTCATCCTGAATGTTATATACCG
AAACAAAGATCTTACCGGTGGGAGCAGGTAAGTGCGTCAAATCTTTGTAACTTTGTGCGCGGGGCATTAATGT
CGGTTTCGCAGCTTGTTTCGGCGGGGCAGTTAAGCATCCGCTCAACAATAAAACGGCAACCAAAATAAGTAAG
CGCGGCATGATTTATATCCTTTAGTGACTGTAGCTTAAAAATCGGTTGACTGAGTTTGTAAACCTGACACTTC
GATGGTCGAGGTTCTTCCCGTTTTTCTGTCCGTGACGTTGAGCTGGAGCTGTCCGTCGCGATTAGCGATATCG
ATAATAAAATCGTTGGTCACCATACGTCCTGGTTTCCCGGTATTAATATTGGTCAACAAGCCGCCCAGAATTT
GCGATTGAATAGCCTGCGTAAAGTTATCCAACGCTGAGGGGGTTTCGATACCAAAATCGTTATCATAAGCGGG
GTCTTTATATGAATTTTGCGCCTGGGCGCTATTCAATAAAAAGGAACCGTTATTGGGGTTTCCACCAAAGTTA
GGATTACGGAACTGGAACGTCATATTTCCAGCCCAGGTTAATGGCGAAAAAAGCATGAGCAGCACTACTGCAT
GTTTAACACGCATGACAGCCTCCGGATAAAAATCATGTTTTAGAATTCATCACGCGCTAAATCACTCGTACTT
AATAGCGTTTGATCTATTTGTCGGCGATTTAATGCTTCCTCTGTTTGCGCTAATGCGAAGACGACGGTTTTCT
CGAAGTCTCTTTTCATTGGAAATAAAAAGGTCTGGAAAATAACGTCCTGATTTACCGTTATGGTGATCCAGCT
TCCCCAACGCGCACTGGGTCTTTCATTAATGGTCAGATTGCCGGTGTATTCGCTTTCCCATTTGTCGCTGAAT
GCACGATAGAATTCATGTCCTATCGAAGAGACGGTATGGTCGGTTAACAATCCGGGAACCTCGACTTCAACTT
CATTGGCATGCAGGTTTCCGGTAGCGAACAGTAACTCTGCTGCTACAATCCAGGTCAGATAGCGTTTCATGGC
CTTACCGCCTGAGATTATCGTTTGCCCATGAAACTGCCTGGGTGCGATTTTTGACAGCTATCTTTTTGAAAAG
ATTATAAAGATGTGTCTTAACCGTATTCTCGCTGATAAATAGCGACCTCGCGATTTCATTATTAGAGGCACCA
ATACGTAACTTATTGAGGATCTCTTTTTCGCGATGAGTGAGTAATGCGGACTCGGTGCTGTTGTAGCGGTAAT
TTCCTGAGTGCGTAATCAGGTAACTGGCTAATTTTTGTGAAAAATAGCATTCGCCACGCAGAATACCCTGTAA
TCCGCTGACCACGTGTTCCTGGTCTTCAGTGGCGTAAAACACGCCGTTAATATGAGGCCAGTTTTCAATTTCA
CGGTAGGGATAATCGTCAGGGGTATTTAACAATAATGTTTTTATATTATTGTTTTTGCGGCTTAAATTATCCT
GCCAATAGTGGATAAGCTTCTTATCCGCTTCCATCATATCCATTAAAACAATGCAACCGGCTGAGATATCTTC
CAGAGAACGTTGAATATTATGCAGTTTTCCGGTTATGGCCAGCGATTGCTTTAAATGTTGCAATAATGCCGTA
GCTTGCAGAGATGGCTTTGTGATCAACAATAGTGTGTGACCATGACTACTATGGACTTCATTAAACATGATGA
AACTCCACTTTTTTTAATCGCACATCTGACAGCTGCCCCCATAAAATAAAGGCACCAGAAGTACTGACAGATG
TTGCACTGCTGTGGGTTGAAATAGCCCATTATCCAGAAAGAGAAAAATATTTACGAAAATACTTTTAACTGTT
TTCAATCTAGCCATTACAAATCTTAAAGCAAGTGTTAAACTTGTAACAAGATGTAAAAATATATATTAAAATG
TTGTTTTTGGGTTTTTTTGAAGTTTAGATTTGATAGTAAAGTTGTACATTTCGCTGTTATTGCATAGATTTAA
AAAATCATACAATTATAATAATTCATTGATTTTTAATCATTTTAATTATTGTGTGTCATGTTTTGATTTTAT
TTTTTCTTAAAATTTGAGACGAGGCATTAACCTGGACAGCACAAAGACAAAAAAAACGAAGTGTGTCACGTCT
TGTGCGTATTGCCCCCCATAGGAAGCATAAGAACATCCCCATGGCGGCATAACACACACCAACACTTCATTTT
TTGGGTTCGCGATACACTATCTTCTATGGCCAAAAATCAATTATAAAAAATCACATGGCTATCGTTTTATTAG
CACTTTGGTATGAGCTTAAATAACAAAATACCACGCGTGGGTGAGTTATTAAAAATTTTTCCACGGACATACT
CTTCATCGTAACGACGCGTTAACAAAAAACGCATGTCGCTAACAAGGTAATAGATAATTTTCGCTATGTACGA
CCAGGTCCAGGGTGACAGCATGAAAAACAAATTGTTATTTATGATGTTGACAATACTGGGTGCGCCTGGGATT
GCAACCGCGACAAATTATGATCTGGCTCGTTCAGAATATAATTTTGCGGTAAATGAATTAAGCAAGTCTTCAT
TTAATCAGGCGGCCATTATTGGTCAAGTCGGCACGGATAATAGTGCCAGAGTACGCCAGGAAGGATCAAAACT
ATTGTCCGTTATTTCACAAGAAGGAGGAAATAATCGGGCGAAAGTCGACCAGGCAGGGAATTATAACTTTGCG
TATATTGAGCAAACGGGCAATGCCAACGATGCCAGTATATCGCAAAGCGCTTACGGTAATAGTGCAGCTATTA
TCCAGAAAGGTTCTGGAAATAAGGCCAATATTACCCAGTACGGTACGCAGAAAACAGCAGTTGTAGTGCAGAA
ACAGTCGCATATGGCTATTCGCGTCACCCAACGCTAATACCGTTACGACTTTTAAATCAATCCGATGGGGGTT
TTACCATGAAACTTTTAAAAGTGGCAGCATTCGCAGCAATCGTAGTTTCTGGCAGTGCTCTGGCTGGCGTCGT
TCCACAATGGGGCGGCGGCGGTAATCATAACGGCGGCGGCAATAGTTCCGGCCCGGACTCAACGTTGAGCATT

TATCAGTACGGTTCCGCTAACGCTGCGCTTGCTCTGCAAAGCGATGCCCGTAAATCTGAAACGACCATTACCC
AGAGCGGTTATGGTAACGGCGCCGATGTAGGCCAGGGTGCGGATAATAGTACTATTGAACTGACTCAGAATGG
TTTCAGAAATAATGCCACCATCGACCAGTGGAACGCTAAAAACTCCGATATTACTGTCGGCCAATACGGCGGT
AATAACGCCGCGCTGGTTAATCAGACCGCATCTGATTCCAGCGTAATGGTGCGTCAGGTTGGTTTTGGCAACA
ACGCCACGGCTAACCAGTATTAATTTAGCGTCTGCGCTAATAAAAAAACAGGGCATAAGCCCTGTTTTTTTTC
GGGAGGAAATTATGCATACTTTATTGCTCCTTGCCGCACTTTCAAATCAGATTACGTTTACCACGACTCAGCA
AGGCGATATTTACACGGTGATCCCTCAGGTCACATTAAACGAACCCTGCGTCTGTCAGGTGCAAATTCTCTCT
GTGCGCGACGGCGTCGGGGGACAAAGCCATACACAGCAAAAACAAACGCTATCTTTACCTGCTAATCAACCGA
TTGAGTTGTCTCGTCTTAGTGTAAATATATCTTCAGAGGACTCGGTTAAAATTATTGTTACTGTTTCGGACGG
ACAATCACTGCATTTATCACAACAATGGCCGCCTTCTGCACAGTAGTTTTTGATGGTGGCGGAAATGGATTGG
CTGACCTGGGTATAAAGAGGCGATAAAAGCGTCTCATCGTCTCGGCATGTCGCTATAAGGTAACGCCGAACCC
TCGAGGATGACTGATCATTGAGGAGTTAACATGTCCGTAATCAAGAAAAATATCCCTGGCATAGGCCTGTGTA
TCTGCGCTTTTTTTATCCATTCTGCGGTAGGGCAACAAACGGTACAGGGCGGCGTTATCCATTTTCGCGGCGC
GATTGTTGAGCCACTGTGCGATATTTCTACTCATGCCGAAAATATTGATTTAACCTGCCTGCGCGAAGGTAAA
AAGCAAATGCACCGGATTGACCTTCGGCAGGCATCTGGATTACCGCAGGATATTCAGTCCATTGCGACGGTGA
GGCTGCATTATCTCGATGCGCAAAAAAGCCTGGCGGTGATGAATATTGAGTACCGTTAACGGCGGTGTCATAG
ATATAAAAAGAGCGACTCTGTTGAAAGCCCTGCTGTACACTTTGCAGATAAGGTGAGACAAAAGGGGGGGGTT
ATGACATCACGTCTTCAGGTCATACAGGGTGATATCACTCAACTTAGCGTCGATGCGATTGTGAATGCCGCTA
ACGCATCATTAATGGGCGGCGGTGGCGTAGACGGCGCGATTCATCGCGCGGCGGGGCCGGCATTGCTGGACGC
CTGTAAACTCATCCGTCAGCAACAGGGCGAATGTCAGACGGGACATGCGGTTATCACGCCTGCTGGCAAGCTT
TCGGCAAAGGCGGTTATTCACACAGTGGGGCCCGTCTGGCGAGGCGGCGAACACCAGGAAGCTGAGCTACTCG
AAGAGGCATACCGGAATTGTTTGCTGCTTGCCGAGGCGAATCACTTTCGTTCCATCGCTTTTCCGGCAATCAG
TACCGGCGTTTATGGCTATCCACGCGCCCAGGCCGCTGAAGTCGCCGTCAGGACGGTTTCAGATTTTATTACC
CGTTACGCTCTGCCTGAACAGGTATACTTTGTCTGTTATGATGAAGAAACTGCCCGGCTTTACGCAAGATTAC
TTACTCAGCAAGGCGACGACCCTGCCTGATAAAACACGCCTGGAGCGTGCCGTTGAACCGCTATGCGCGCGCC
ATCCCGGAGAGTGCGGCATTCTCGCGCTGGATAACAGTCTGGACGCTTTTGCCGCCCGCTACCGCCTGACCGA
AATGGCGGCGCGGACGCTGGATGTGCAGTATTATATTTGGGAAGACGATATGTCCGGGCGACTGCTCTTTTCG
GTACTGCTGTCGGCGGCGAAGCGCGGCGTTCATGTTCGTCTGCTGCTGGATGATAACAATACGCCTGGTCTGG
ATGATACGTTGCGCTTGCTGGATAGCCATCCTAATATCGAAGTTCGTCTGTTTAATCCTTTCTCTTTTCGTAC
GCTACGCGCGCTGGGATATTTGACGGATTTTGCGCGGCTGAATCGGCGGATGCACAATAAAAGTTACACTGCC
GACGGCGTAGTGACGCTGGTCGGTGGGCGCAACATCGGCGATGCCTATTTCGGCGCTGGCGAGGAGCCGCTAT
TTTCCGATCTGGACGTGATGGCCATTGGCCCGGTGGTCAATGATGTCGCCAATGATTTTGAACGTTACTGGCG
CTGTAGTTCAGTGTCGACATTGCAGCAAGTATTATCCCTTTCTGAGCAGGAACTGACGCAGCGTATCGAACTT
CCCGAATCCTGGTATAACGATGAGATCACCCGCCGTTATCTGCATAAGCTGGAAACCAGCCAGTTTATGGCGG
ATCTCGATCGCGGAACGTTGCCGCTGATTTGGGCAAAAACACGCTTGCTTAGCGATGACCCTTCTAAAGGCGA
GGGGAAGGCGCAGCGCCATTCGCTTCTTCCGCAGCGATTATTTGACGTGATGGGGTCGCCGACGGAGCGTATC
GACATTATTTCCGCTTACTTTGTCCCTACGCGCGCAGGCGTGGCGCAGTTGCTTAATCTGGTCAGGAAAGGCG
TGAAGATCGCCATCTTAACTAACTCTCTGGCGGCCAACGATGTGGCGGTCGTTCACGCAGGGTACGCGCGCTG
GCGCAAGAAATTACTGCGCTATGGCGTGGAGCTCTACGAACTGAAACCGACCCGCGAACATGAAACCGCCGTA
CATGATCGCGGACTCACCGGGAACTCAGGTTCCAGCTTACATGCTAAAACGTTCAGTATTGATGGTAGTAAGG
TGTTTATCGGGTCGCTTAATTTTGATCCCCGTTCAACGCTTTTAAATACCGAAATGGGCTTTGTCATTGAAAG
TGAAACGCTGGCGACGCTTATTCATAAGCGTTTTACGCAGAGCCAACGCGATGCGGCCTGGCAACTGCGGCTG
GATCGCTGGGGACGAATTAACTGGATCGATCGTCAGCAAGAAGAGGAAAAGGTGTTAAAGAAAGAACCCGCTA
CGCGTTTCTGGCAGCGAGTTCTGGTACGGTTGGCGGCAATTTTACCTGTGGAATGGTTGCTGTGAACCCGCGC
GCGGAAAACTACCCGATCGCGGCGCGGCTTTCTTGTTTTACCGGCGGTTTACCTGAAAAGAGAAATTTCAGGA
GCGGGATGCGTAAATGAATTTCATACAGAATTAACGCAATACCCATGACAAATATCAGCCCACACAAGAACCC
GATCAGGTTGGAGGAGATATGCGGCGTAATATACGCGCCAAAGAAAAGCGTTAAGGGATGATGCACCAGATAG
ATAAACAGCGAAGCATTCACGAAATAGGTGACACGCGCGGACTGGAAGTTTAACAAGCGATGCCCCAGTGAGA
ATACCACGTTCACCATCCACAGCCCCATTACCATCGTAATCACGGATTCGGTTTCGTACATCCAGGCGTCGCC
GCTCCCATAACGTTGATTCAGCAGATACGCGATAAAAGCAACGGCAGCGCCTAAAGTGCATCCGCGTGAGGGC
GTGGTGAAGCGCGCTTTCAGATCGGGGTGAATGAAGGCCAACGCACCGAGAATAAAAAACGGCACATAAAATA
GCGTTTGCATCACAATAAAATTGAACATGCCGTCACTGAGGATTGCTGGATATACGATGAAAATAATGCGCCT
GATAGCGGCGTAAGCCATCCCCAGCAGGAAAAAAATAAGCGAAAGCCTGACCAGTGAAATAGCGGCGGGACGA
GGCTTGCTTGTTTCCTGCCTTTTTTGGAACCAGGTAAAAATCCCGATGCTGACGGTGGTTAATATCACCAGCA

CCAGTAAAAACCACAGATGTGAAATGAGTTCCCACGCTAACGTATTATATTTTTCATAGGCAGAGAGTGTAGG
CCAGTTCTCTGTTTTCTCTTTGACATATTGCAACAGGATAAATTGCGGCAAGGTAAGCAAAGGGATTGCGGTA
AGCATGGGAATACCCACACGTTCTACCCGTACTTTCCACCAGCGTTTTAACGGATAACGTAAAAACAACATGT
ACGAAAAATAACCAGAAATAACAAAAAACACCTGCATACGAAAAGCGTGGATAAAATCGTTAAACAGAGTTAG
CCACCACGATGGCGTGGCGCTATTGACATGCCAACTGTGAGTGGAATAGATCAACGAGATATGAAAGGGAATC
CCTAACAACATCAGCCATGCGCGGATAGAGTCAAGAAAATATTCACGCGGCGCGGGTACAGAGCTCATATAAG
GTCACGTATTCTCAGATTTTTCACCTTATCCATAAGGCGAATTATAGTTACATTCGGTAGCAACCCTACACCA
ACTCCGACAACCTGTCTCCAGGATAAGCACGCAAAGTGAAAACAGGCGCGGGAGGTGCTTAATCCATGAGCCA
GCGCGCTGAACAAAGCCTGGATTCAGTTGTCGTAATGCCTGATTATCCATTAAAATGGATCGGATCGATATAA
GCACACAAAGGGGGAAGTGCTTACTTATTATGAAACATAAACGACAAATGATGAAAATGCGTTGGTTGGGCGC
AGCTATTATGTTAACGCTCTACGCATCATCGAGCTGGGCGTTCAGTATTGATGACGTGGCAAAACAAGCTCAA
TCTTTAGCCGGGAAAGGCTATGAGGCGCCTAAAAGCAACTTGCCCTCCGTTTTCCGCGACATGAAATATGCGG
ATTATCAGCAGATCCAGTTTAACAGCGATAAAGCCTACTGGAACAACTTAAAGACCCCTTTTAAGCTCGAATT
TTACCATCAGGGGATGTACTTCGATACGCCGGTCAAGATTAACGAAGTGACGGCGACGACGGTCAAAAGAATC
AAATACAGCCCGGATTACTTCAATTTTGGCAATGTTCAGCACGATAAAGACACGGTAAAAGATTTAGGCTTCG
CCGGGTTCAAAGTTCTGTACCCCATCAACAGTAAAGATAAGAACGACGAAATCGTCAGTATGCTTGGCGCCAG
CTATTTCCGCGTTATCGGCGCAGGCCAGGTATATGGCTTATCTGCGCGCGGCCTGGCGATTGATACCGCCTTA
CCATCTGGTGAAGAGTTTCCCCGCTTTCGCGAGTTCTGGATTGAGCGTCCAAAACCCACCGATAAGCGTTTGA
CCGTCTATGCATTACTGGATTCTCCGCGCGCGACCGGCGCTTACCGTTTTGTGATCATTCCTGGCCGCGATAC
CGTGGTGGACGTGCAGTCAAAAGTCTATCTGCGCGATAAAGTGGGCAAGCTGGGCGTTGCGCCATTAACCAGT
ATGTTCCTGTTTGGGCCAAACCAGCCGTCGCCGACGACCAACTATCGTCCGGAACTGCATGACTCGAACGGCT
TATCCATTCATGCGGGTAATGGCGAGTGGATTTGGCGTCCGCTGAACAATCCAAAACACCTCGCTGTGAGCAG
CTATGCAATGGAAAACCCTCAGGGATTCGGCCTGTTGCAGCGTGGTCGCGAGTTCTCGCGCTTTGAAGATTTA
GACGATCGCTACGACCTGCGTCCAAGCGCCTGGATTACCCCGAAAGGCGACTGGGGCAAAGGTAAGGTTGAAC
TGGTTGAAATTCCGACCAATGATGAAACCAACGATAACATCGTCGCTTACTGGACTCCGGATCAACTGCCGGA
ACCGGGTAAAGAGATGAACTTCAAGTACACTCTGACCTTCAGCCGCGATGAAGATAAACTTCATGCGCCGGAT
AATGCCTGGGTGCTGCAAACACGCCGCTCAACGGGCGACGTTAAACAGTCGAATCTGATTCGCCAGCCCGACG
GCACTATTGCCTTTGTGGTGGATTTCGTTGGCGCCGACATGAAAAAACTGCCGCCGGATACGCCTGTCGCTGC
GCAAACCAGCATTGGCGATAACGGTGAAATCGTTGACAGTAATGTACGCTATAACCCAGTCACTAAAGGCTGG
CGTTTAATGCTGCGCGTGAAAGTCAAAGACGCGAAGAAAACCACGGAAATGCGTGCCGCATTGGTGAATGCCG
ATCAGACGCTAAGTGAAACCTGGAGCTACCAGTTACCTGCCAATGAATAAAACAACTGAGTATATTGACGCAC
TGCTGCTTTCTGAACGTGAGAAAGCGGCATTGCCGAAAACTGACATCCGCGCCGTGCATCAGGCGCTGGATGC
CGAGCATCGGACTTACTCGCGAGAAGACGATTCACCGCAGGGTTCCGTAAAAGCCCGCCTTGAACACGCCTGG
CCGGATTCATTGGCGAAGGGGCAGTTAATTAAAGATGATGAAGGGCGCGATCAGTTGCAGGCTATGCCAAAAG
CGACGCGCTCTTCGATGTTTCCCGATCCCTGGCGAACCAACCCGGTTGGCCGTTTCTGGGATCGTCTGCGTGG
GCGGGATGTAACGCCGCGCTATGTTTCTCGTCTGACAAAAGAAGAGCAGGCGAGCGAGCAAAAATGGCGTACC
GTCGGCACTATACGCCGCTATATTTTGTTAATTTTGACTCTGGCGCAAACCGTCGTCGCGACCTGGTATATGA
AGACCATTCTGCCTTATCAGGGATGGGCGCTCATCAATCCTATGGATATGGTGGGGCAGGATATTTGGGTCTC
TTTTATGCAGCTCCTGCCCTACATGCTGCAAACCGGTATCCTGATTCTGTTTGCCGTGCTGTTCTGCTGGGTG
TCAGCCGGATTCTGGACGGCGCTGATGGGCTTCCTGCAACTGCTTATCGGGCGCGATAAGTACAGCATCTCCG
CGTCTACGGTTGGCGATGAGCCCCTCAATCCGGAACACCGGACGGCGCTGATCATGCCTATCTGTAATGAAGA
CGTTAGTCGCGTTTTCGCCGGTCTGCGCGCGACCTGGGAGTCCGTTAAAGCCACAGGCAACGCCGCGCATTTC
GACGTCTATATCCTTAGCGACAGTTATAACCCGGATATCTGCGTGGCGGAGCAAAAGGCGTGGATGGAGCTTA
TCGCGGAAGTGCAGGGCGAAGGCCAAATTTTTTACCGTCGCCGCCGCCGCCGTATGAAACGCAAAAGCGGCAA
TATTGACGATTTTTGCCGCCGCTGGGGCAATCAGTACAGCTATATGGTGGTGCTGGACGCGGACTCAGTGATG
AGCGGCGAGTGTCTGAGCGGCTTGGTGCGCCTGATGGAAGCGAATCCAAACGCCGGGATTATCCAGTCTTCGC
CGAAAGCGTCGGGGATGGATACTCTGTATGCCCGCTGCCAACAGTTCGCGACCCGCGTCTATGGGCCGCTGTT
TACCGCCGGGCTGCACTTCTGGCAGTTGGGGGAGTCGCACTACTGGGGGCACAATGCCATTATCCGCGTGAAG
CCGTTTATCGAGCACTGCGCTCTGGCGCCGCTGCCGGGAGAAGGTTCGTTCGCCGGATCGATTCTTTCCCACG
ACTTTGTGGAGGCGGCGCTAATGCGTCGGGCAGGGTGGGGCGTCTGGATTGCCTACGATCTCCCCGGCTCCTA
TGAAGAGCTGCCGCCAAACCTGCTGGATGAGCTTAAACGCGACCGCCGCTGGTGTCACGGCAACCTGATGAAC
TTTCGTCTGTTCCTGGTGAAAGGAATGCACCCGGTGCATCGCGCCGTGTTCCTGACGGGGGTAATGTCATATC
TGTCCGCGCCGTTATGGTTTATGTTCCTCGCGCTTTCTACCGCGCTGCAGGTCGTTCATGCGTTAACAGAGCC
GCAATATTTCCTTCAGCCGCGCCAGCTTTTTCCGGTCTGGCCGCAGTGGCGTCCGGAACTGGCAATCGCGCTG

TTTGCATCAACGATGGTGCTGCTGTTCCTGCCGAAGCTGCTCAGTATTATGCTGATCTGGTGTAAAGGCACCA
AAGAGTATGGCGGTTTCTGGCGCGTTACGTTGTCGCTGTTGCTGGAAGTGCTGTTCTCCGTGTTGCTGGCGCC
GGTGCGTATGCTGTTTCATACCGTGTTTGTGGTCAGCGCGTTCCTCGGCTGGGAAGTGGTCTGGAACTCACCG
CAACGCGACGATGATTCTACGCCGTGGGGAGAAGCCTTTATGCGTCACGGCTCTCAACTGCTGCTGGGGCTGG
TCTGGGCGGTGGGTATGGCGTGGCTGGATTTACGCTTTCTGTTCTGGCTGGCGCCGATTGTCTTTTCGCTGAT
TCTGTCGCCATTTGTTTCGGTGATCTCCAGTCGTTCAACGGTAGGATTACGCACCAAACGCTGGAAGCTGTTC
CTGATCCCGGAAGAGTATTCGCCGCCTCAGGTGTTGGTCGATACCGATAAATATCTGGAGATGAATCGCCGCC
GTATTCTGGACGATGGCTTTATGCATGCGGTATTTAACCCGTCGCTTAATGCGCTGGCGACCGCGATGGCCAC
TGCGCGTCACCGCGCCAGTAAGGTGCTGGAAATAGCCCGCGATCGTCATGTGGAGCAGGCGCTAAACGAAACG
CCGGAGAAACTGAACCGCGATCGGCGTCTGGTTTTGCTCAGCGATCCGGTGACAATGGCGCGTTTACACTATC
GGGTCTGGAATGCGCCAGAGAGATACTCTTCCTGGGTAAACCATTATCAGTCTCTCGTCCTGAATCCGCAGGC
GTTGCAGGGACGAACATCGTCAGCGAGATAAGGTCTTCAGGTCTGGAGTGAGGTGAAAAATACCGGCGTGATG
CCGGTATTTTTATAGTGAAATGAGGTATCAGGTGCGTATATTCGCGGTGAGCATAATGGTGATTACCCTGAGC
GGCTGCGGCAGTATTATCAGCAGAACGATCCCCGGACAAGGACACGGCAACCAGTATTACCCTGGCGTGCAGT
GGGATATGCGTGATTCCGCATGGCGCTATATCACTATCCTCGATCTGCCCTTCTCACTGATCTTCGATACACT
GCTACTGCCGCTCGATATTCACCACGGGCCTTATGAGTAATTAACGCTCATCCCATTCGTCTGCCGCAGTACG
GCCTTCCTCGGTATCAAGCGGGGGTTCAAGCTGGAATTCCCCTTCATCCCATTCATGCAAAGTATTCTCTTCC
TGCCATTCCTGGCGGATCTCTATCTCATCATAATCGCCGTCAAACACGCTCTGCGCGGCTTCACCACTCAGCA
TCGGCAGACATTCGCCATCTTCACCTTCATCGGCGAAAAATTCGACCTGCCACATGATGTCCCCATCCTGCAG
TACATATTTCTGAACGTTGAACTGCTGCACATTCGCTTCATCTTGTTCGAGGCCTGGATGGTCAGCCAGAAAT
TCTTCCCGGGCTGCATCGATAGCTTCTTCCAGCGTGGCATACATGGTCATCAGTGTCTCCCTTTGATTTGATG
AGGTATTTAGGGAAAGAATAGCTGATTCTGTAATATTGCAAGTATGAAAGCGCAAAAATTATTCTATTGCCAG
TCTGCGCCGTCGTAAGCTGTTCCATGAATAGATAGCATTGAACAACACCACGCCTGCCGTGACGCAGAATACG
GCGCGGAAGCCATAGCTCGCGGAAATTGCGGCACCCATGAGAGGGCCGGTGACGTTGCCGATATCGCGGAAAG
ATTGGTTGTAACTGAATATGCGCCCGGCTATCTGGTTGGTAGAGTTGTAAACCAGCAGAGTTTGAACGGCTGG
CAGCAGCGCGCCATCCGCCGCGCCAAGCAGAAACCGCAATAGCGCGAGCTGCCACGGCGTTTGCACAAAAGAC
ATTGGAATCAGCAGCAGTACGGAAATAATCAGCGCGACGATAAGGATCTTTTCCGGGCCAATTCTGTCGCCAA
GCTTGCCGAGCCGGGGGGCGCTAAGTAATGCCGCCACGCCGGGAACGGACGCTATCATCCCGCTAATGAACGC
AATATTACTGACATTTCCCGCGAGTTCGCGCACATAGAGCGTCAGGATGGGCGCAATAGACCCCGTCGCCACC
TGAATAATCAGCGTGGTGACAAACAGGCTTAACACCAGGCGGGGATTTTTTAACGAGGCGACCACTTCCCGAA
CGTGAAGCATCTCTTTTTTGCTCACCGGCAGGAAATTCTCGTGAATGAAAAAGAAGGTGAGCAGAAAACAGAT
AAACAAAACGCTGGCGGTAATAAAAAAGACCGGACGAAGGCCGTAATGGTCGGCGAGCAGACCGCCCGCAAGT
GGGCCAAGCAGAGCGCCGCTGACGCCGCCAGTAGAGAGCGTCCCTAACGCCCAGCCGCTTTTGTGGCGCGGCA
CCTGAGTGGCGATGAGCGCATTGGCGTTGGGAATAAATCCGCCCAGCAGGCCTAATAACGCGCGGAGGATCAG
AAATTGCCAGATATTTTGCGCCATGCCCATTAACAGCATCACAATGGCCATGCCGAGAGCGGAACGCAACAGC
ATGATCTTACGCCCTTTACGATCCGCCAGACCGCCCCAAAAAGGGGAAGCGATAGCTGAAAAGAGGAACGTAA
TACTAAAGACCAGCCCGGACCACATGTTGAGCGCGCTATGGCCTGTTACGCCGAGTTGCTCAACGTAGAGAGG
GAGAAAAGGCATGACCAGACTGAATGCTGCGCCGGTTAAAAAACAGCCCAGCCAGGTAACGGTTAGATTGCGT
TTCCAGTTTATGGGGACATCAGAGGGTGACATAGCGTTCCACAGTATGATGCGCGGTTTGCGCTATCATCAAT
TTAATTATGAGCGTACTAATTATAATTATGCGCCGACCTTACCAGCCTCGCAATGTGGGGAGCTTTTAAAGCT
AAAAGAGGGGAAAAATTGCAGCCTGACGGCTGCAATCCTGTCAATAGCGCGACGGTACGCCTTCAGGGCGAGT
TTTAAAGCGGCGATGCAGCCACATATACTGCTCTGGCGCCATCATAATGCACTGCTCAACAATCTTATTCATC
CATGCGGCAGTCGCTTCTGCGCTCTCCAGCGGAGGCGAATATTCCGCAGGAAGAATGATCAGTTCGTAGCCTT
TCCCATCGGGTTTACGGCGAGGCACGAAGGGGATAATACAGGCTTTAGACATGCGCGCGAGCATCCAGGTACC
GGAGGTCGTAGCGGCCTGGTCGACGGCGAATAACGGTACAAAGACGCTGGCGCGCGGGCCGTAATCGTGATCC
GGCGCATACCAGATCAATTCGCCGCTTTTCAATGCCTTAACCATGCCTTTCAGATCTTTGCGATCCAACATCG
ATTTATTGGAGCGTAAGCGTCCCCAGGTCTGTAACCAGTCGAGCAACGGATTATCATTCGGGCGATAAACTCC
GATCCCCGGGTTATGCATACCAAACATGCGGGCGCCAAACTCAAGGGTAAGAAAATGTATTCCTACCAGAATG
AACCCCAGCCCCTGCGCCTTAACTTCACGGATATGCTCCAGACCGCTCGCTTCCATCCAGCGGTTCACTCGCC
GATCGGGCCAGAACCAGGCCATGCCGGTTTCCATCACGCCCATACCGACGGATTCAAAGTTTTTGACCACCAT
AGTGTGGCGTTCCTGCGCGGTCATTTCCGGAAAGCATAATTCAAGGTTGCGATAGGCGATTTTTGCGCGGCGT
TTCATTACGCGTCGCGCCAGGTGGCCCAATGCGCAACCCAATTTATAGATGACCGGGTAGGGGAGTTGCACGA
CCAACCAAAGCGCGCCTATACCCAACCAGGTTAACCAATAGCGCGGGTGCAGTAAGGCCACGGAGAATTTAGG
CAACTTCGTCATTTCTATCCTGTCTTTCAACGAACAATTCCCCGTATTCTCGCATCTTTTTGCGTTGAGCAAA

AATATGTAGCAGGAGAGTGGCGATTAAATCGGCAATTGTTGTTAATTATTTAGTGTAAAGCAGGAAATGTAGC
GCAAAATGTGTGGATGTAAATTGGCGAGACTTGCCTTATCATGCCTGCCCACTTTATTTTTTTGCTGATTGCA
GGATACGTACACCATGCCAGTGTTACACAACCGCATCTCTAATGACGAGCTGAAAGCCAAAATGCTGGCGGAA
AGCGAGCCGCGTACGACAATTTCTTTTTATAAATATTTCACTATCGCCTCGCCGCAGCAGACGCGGGACGCGT
TGTATCAGGTGTTTACGGCGTTGGACGTTTTTGGTCGTGTTTACCTGGCGCATGAAGGCATCAATGCGCAAAT
CAGCGTGCCGCAAAGCAAGGTCGAGACCTTTCGTCAACAGCTTTATACGTTCGACCCCGCGCTGGACGGGCTG
CGTTTAAATATCGCGCTGGAGGATGACGGAAAGTCATTTTGGGTGCTGCGTATGAAAGTTCGCGACCGTATCG
TCGCTGACGGAATTGACGATCCGACTTTTGACGCCAGTAATGTCGGCGATTATCTGAAGGCGGCAGATGTGAA
TGCGATGCTGGACGATCCTGATGCGGTCTTTATTGATATGCGCAACCACTATGAGTATGAAGTCGGCCATTTC
GAAAATGCTCTGGAAATCCCGGCGGATACGTTTCGTGAACAGTTGCCAAAAGCGGTTGAAATGCTGCGGGAAC
ATGCAGATAAAAAGATAGTGATGTACTGTACCGGCGGTATTCGTTGTGAGAAAGCCAGCGCCTGGATGAAACA
CAACGGTTTCAATAAAGTCTGGCATATTGAGGGTGGCATCATTGAGTACGCCCGTCGCGCGCGCGAGCAGGGG
CTTCCCGTTCGCTTTATCGGCAAAAACTTTGTATTTGATGAGCGAATGGGCGAACGAATCTCGGATGAGGTTA
TCGCGCATTGCCATCAGTGCGGCGCGCCCTGCGATAGCCATACCAACTGCAAAAATGACGGTTGCCATCTGCT
GTTTATCCAGTGTCCGCAGTGCGCCAGTAAATTTAACGGCTGCTGTAGTGAACAATGCTGTGAAGAGTTGGCC
TTGCCGGAGGAAGAACAGCGCCGACGTCGCGCGGGTCGCGAGAACGGCAATAAAATTTTTAATAAATCGCGTG
GTCGGCTTAATAGCAAACTGAGCATTCCCGATCCGGCTGAGTAATATTTTGCCGGATAGCAGCGTAAAGGCCG
CTATCCGGCATTTCGCCAGGCGTTACTTCTGCTGAACGCCTTCTACTGAGATGATAAGCTCAACCTCTTGTGA
GGCCGGGCCGAGATCGGTAGTTATATTGAAATCCTTCAGCTTAATTTTTCCTTCGGCCTCAAAGCCCGCGCGC
TTACCGCCCCACGGATCGTCGCCCTGGCCCATCAGCTTCGCTTCCAGCGTCACCGGTTTAGTCACGCCATTGA
GCGTCAGATTGCCGGTAATATCCAGTTCATCGCCCTCTTTTTTCACGCTGGTAGAGGTGAAGGTTGCCTGCGG
GAATTTCGCAACATTAAGAAACTCCGCGCTACGCAGGTGTTTGTCACGTTCGGCATGGTTAGTGTCGACGCTA
TTGGTGTTAATGGTCACGTTCACTTTGTCTGCCGACGGATTTTTTTCGTCAAAAGTGAACGTGCCGTCGAAAT
CTTTAAAGGTGCCGTATAGCCAGCTGTAGCCTAGATGCTGAATGCGGAAATTGACGAACGCATGTTGGCCTTC
TTTATCAATTTTATACTCCGCCGCCACGGCGGAACCGGTCGTGAATAACAAGGATGCGAGGGTGAATCCCAGC
AGGTTTTTTTTCATTTTTGAGCTCCATAGTCAGATGACGACATTCCTGTCATACGTTTCAGTGTGTCGTCTTT
ATCGATGAAATGGTGTTTTAGCGCCATAACCCCATGCGAGAGCGAGATAATGACCAGCGACCAGGCAAACCAC
AGATGCAGCGTTCCGGCGATGTCAGCCTGCGCGCCCGCGTCCGTAAGCGTGGCCGGAATCTCAAACCAGCCAA
AGACGCTAATCGGTTTACCGTCGGCGGTGGAAATCAGGTAGCCGCTAATGATTATCGCAAAGAGCAGGAGATA
CAGGAGGAGATGACCTGCGGCGGCGCCAGCGCGCGTTAAGCGGGAATAGCTGGTCAACGCAACGGGAGGCGGA
GAATAAAGCCGCCAGATAATACGCACGATCAGCGCCATCATCAGTAATATGCCAATACTTTTATGTATTTCCG
GTGCCTGGTGATACCAGCCGTCGTAATAACTGAGCGTGACCATCCATAAACCCAACGCAAACATGCCATAGAC
CACCAGGGCGGTCAGCCAGTGGAGCGCGGCGGAAACCACGCCATAACGCTGTGGAGTATTTTTAAATTGCATA
AACACACCAATGAATATTTCACGAGAGAATGAAAATGGCGTGGAAAAGCGCCGAATGCAACTTATAAATAAGA
ATTTGAATGATATTTATTTTTATTTCAATAATTTTGATGTTGTTTGCGGTTCAGCTTCAGAAGTTTCGAGATA
TTTCACTCCTGTAACCGCGCAACGGCAGGAACTGGCATTATTGTCGTGCTTCAACGTTATGAAATAAAAAGTG
AGGAATTTTCAGGAAGTGTCAGTTAGCGTCAATGAAAAGCAATCAGAAGAAAAGGAGATAAACAATATCCATC
ACCGCTAGCAGTGACCAAAGAATAACGTAAAGCATGAAATGTTCGCGAATATTATTCATCAGAAAATGAAAGA
GACGGCGCATAGGTTACCTTAATAAACAGCCCCTTTACGGGGCCGACAAATTATTGGCTAAAACGGGAAAGCC
GGAACGGCGTCAGATCAAAGGAGGATGTTTTTCCCAACGCAAAATCCGCAGCGATTTCTCCTAACACCGGGGC
GAATTTAAAACCATGTCCGCTGAGTCCAGTGATGACAAGCGTGTTCTCATGGCCAGGCAGCGTATCGATAATA
AAATTCTCGTCCGGCGAATTATCATAGGTACATGCCGCCCCATGTAAACAACCGCCGATACCCGGCAGTACGT
TACGCAGGAAAGGAAATGCTTCCGCGCCATCGCTGGCAACGGCGGCAAAGGGCTTGCGCTCTTCCGGAGCCTG
TATTAGCTGTCCGCCATTGTGTTTGCCGATTTTTAACTCGTCGTTCTCCGCCGGGAAGTCGTAATATTGATCG
CCGTTGGGCATTTCGCCGGTAAAGGCCGGAAAGCGGTTTTTAGTGCTGTAACGTCCATCCGCCTTAAACCAGG
CAAAAACTTTACGTACGGGCTGAACGGGCAGCTCCGGTACCAGCGCTTTGACCCAGGTGCCCGCGCTAATTAG
CGCCTTGCTGGCGTGGTAGCTGCCTTCACTCGTCTCTATCGTCACACCGTTATCATCATGATGAATATGGCTT
ACCTGGCTGTTGAATAGCTGTGCGCAGCCAGCCTCTCGGGCCAGACGAAGCCATGTGGTAATGGCTAATTCGC
TGCGCAGAAAACCGGAGTCAGCTTCAAACAGCCCGATATAATTATCGGGCACGCGAATTTCCGGCCAGCGCGT
CATGAGGGCCGTCGCGTCCAGGCGCTCGACGTTCAATTGCCACTGTTGCGCGCTTCGTGCGACGTTGGCTAAG
AAAGCGGAATCGGCCGGGCCGAGGTTGACGACGCCGGAGCGGACAAAAATAGGCTCTTCATTGTGTGTGGAGA
GTTCATCCCAAAGCGTCTGGGCGCGAAGCATCAGCGGGACATATTTTTCGCCTTCACCATAAGCATGGCGGAT
AAGACGGGTATCGCCGTGGTGGCTGCCCTGTTGATGAGGCGGCATATGCGCATCGGTCATCAGGACCTTTAGC
CCGGCGCGGGTGGCGTAATAACCAGCGGCGGCGCCAACCGAACCGCTGCCGATAATAATAAGGTCGTATTTCA

TCAGCTTCTCTCTGCTATCGCGATGATTTCAGGGTAAATAACTGCGCTGAGATATACAAGCCAGAAATAAGTG
AGGCACCTTACGGTGCCTGAGAGAGGGGGAGCGCGTCACGCTAATGGCGACGATACTCGTTTTCCTGGTAATC
ACCTGATTCTATTTTGGCGATGCCGGCTTCTAATATTGAAATAAATTGCCTGGCTACATCTGTCGTTAACCAG
AGCGTTTGACCAACTTCAGTCCCTTCCGGTTCCGGACGATTTGGGGTCTGGTAGTGCAAACGCAGCATCAGCG
CATCATAGCTATCGACGGTGCTGATGTCCCATCCTACAAGCGGATGGGTCTGAATGACTTCATTATTCTTTTC
CATCATGCCCCCTGGTACGTGTTATAAGACAACGGTTCTCGAGGTTCAATGCGTGTTTTTCTTCTGAAGCAAC
TTCAGTATACCAATTAATAAGGCTATTCACTGCGTTTTTAAAGAGACCGGAGGATAAATTTCTCTTTTTAAGA
ATTATATGAACAATAAAGCGGCAGTTCATTCATATTTTTTTAGGATGTTGTGCAATTATTTTGACGGTCAGGC
GAAATATTCATCAGTTGCGCAAATAAAAAAGCCGGGGCGACCCGGCAAACATACATCACTGCATATCATTTTT
TATTCATTGATGAACCAGTCATCAGCGCTCTCCCAGGTCTCCTGGAGAATCTCGCTAATGCGTTCTTTGTCCT
CTTTTGATGCGCCAATGACGGACAAGTTGTTGGCGGTAGCGTAACGCACGGTGACGTTACCCAAGTTCTCAGG
AAAATGATGGCTAATACGACGGGAGAGTTCGCCCGCCAGCGCATCAATCGCACCGGCAGGCAGAGGTGACGTT
TTGGCTATAGTGACTTCAATACGCATAATGGCCCCCTGTTGAATATACTGGATATATATACAGTTAAATCTAA
TATATAGCAACAGGTAAGCGCATTTTTTATTTTTTTACTGACCAGCGTACTGTTTCACCCGCTAAAAAAGGCA
CCAGCGAATCATCAGCCAGCGCGATATTTTCCGGTACCTGTTGTTCATCGCGAACCAGTTCCACCCACCCCGC
ATTCACCGGCAGGCCATAGAATTGCGGGCCATTCAGTGAACAGAACGCTTCAAAGTGCGCCAGCGCGTTCATT
TCCTCAAACACGGCGGCATAACTGCCAAGGGCGGAGGGAGCGTTGAAACAACCGGCGCAGCCGCAACTAGTCT
CTTTACGATGACGTGAATGCGGCGCTGAATCCGTCCCCAGGAAGGCGCGCGTAAAACCACTGGCAACCAGTTC
GCGTAACGCCTGCTGGTGAATATTGCGTTTCAGAATCGGCAGACAGTACAGGTGAGGACGAATGCCGCCGACC
AGCATATCATTACGGTTAAACATTAAATGTTGAGGCGTAATGGTAGCCGCCAGGTAGTCGTTGCCGTCACGTA
CATACTGCGCGGCATCTTTGGTCGTGATGTGCTCAAAGACCACTTTAAGCGCGGTCAGACGCTGGCGTAGCGG
TTCCATTACGGTGTCGATAAAACGCGCTTCGCGATCGAAGATATCAACCTCCGCATGGGTCACCTCACCGTGG
ACCAGCAATGGCATTCCGAGTTTTTCCATCCGCTCCAGTACCGGCATGATAGCGTCGACTGACGTTACGCCAT
GACTGGAGTTAGTGGTGGCATTGGCCGGGTAAAGCTTAGCCGCAGTAAATACGCCTTCATGGAAACCACGCTC
CAGTTCATCGGCATCGAGCGAATCCGTTAAATAGCAGGTCATTAACGGCGTGAAATCATGCCCGGCGGGCACC
GCATCGAGAATACGCTGGCGGTAGGCGATCGCTGCATCAACGGTCGTGATGGGGGACGCCAGGTTCGGCATCA
CGATAGCGCGACCATAAATTTCGCTGGTATAGGGTACGACCGTTTTTAACATGTCGCCATCGCGAAGGTGAAC
GTGCCAGTCGTCCGGGCGGCGGATCTTTAAAACCTGGGATGGTGCAGTCATTAATAAGCTCCGGCTGAGGAAT
AGTCTTTTTGCCGGAAACAAAGGATAAGCGGAAACGTTTTCGTTTGCACGTAAAAAAAGGCGCGAGCGCGCCC
TCCGAAATCAATTGGTGAAAGGAATAACGATTTCACCAGGTTTAACTTCAATGCCTTTCGCGAGTTTCTTTGC
TAACGCTTCGCCTTTACTGCTGTCCTCACGCAACACGTAAGCGGGCCGCTGGTTAAAGTAGCTACGTAAAGAC
TGATTTAAATAAGGCAGGAGCGTTTGTAGCACCGATTGCATTTTCTCCGGCGTCACGGTGGCGTCTACTACTT
CCATCTCCTGAAGATAGATGGCGCCTTTTTCTTTATCAAAGACCGGCAGGGCTTTTAGCTTGAGTTTCATCGT
CGCTTTTTGACTGCCAAACAGGGAATTCATATCCAGCCTGGCATCGCCAGTAAGGGTGACTTTATTAGGTTCT
TCCCGACCAATCTGGCTGACAAGGTTAGTCAATACGATATGCGCGTCGGCAATGCCGGGCAGACCAATATCTT
TTGAGAAATTATTCCGTTTTTCAAGCGCTTGATTGATTTCTTGTTCGCTAATGGTGTATTGCGTAAGTTGATT
ACAACCCACTAACAGGCCGCTAACTACCAGCGCAGCGGCAAAAAAACTTTTTCATGGCGTTCCTTAGCATGTT
GCCTGTGCCCTAATCTTGACACAAAGCAGCATGTCGCGCCAGCGGACATGGTGCCACTAGAAAAAAGCTGAAA
AAGGCGGCAAGAAAGGGTTGCCGCCTGGCGGGAGTTAGATAGCCATTGAGGAGAGTAAATTAATTTGCGTCTG
CTTCGCCATATTATCGCGATAGTCCGCGACTTTTGTCGGCCAGTGGATACCGGCGACCAGCGTCAGATTACGC
AGAAGCGGAAACAGATGAATATCATCTTCCGATAATTCGCCGTTAACGGCGTTAGGCTGTACGATGAGTTTAT
CCAGCAAACGTAAATCATCGCCGATCTTTTTAATCAGTCCTGCAGAGTGCGCAAGATGGTTGTCAAAACTGCC
AGATGAGGCTTCTTTTTTGCGGATAAAATACTGACGCGCCGCAGGGGTAGAAAATTCATCGAAAGCGGATTTT
GCAAATCGCGGCAGCAGTAGCTGATTAACGTAACCGTTAACTTTGCGCAACCACTCTTCAATGGCTGGATTGC
GTTTCCCGGTTAACAGCGGTTTGCCGTCGAGGTTGTCGACATAATGTACAATATCCATACTTTCAGGAAGGTA
GCGACTATCATCTTTTTGCAGGATGGGCACCATCTTTTGACCAATCATCCGGGTGGGCGTCGCCTCGTCGTCA
TTTTGTAACACGTTAAGTTCAACGGGGATGTTCTTCAGGCCGAAAATCATGCGGGCTTTAACGCAGAAAGGGC
AATGATCGTAAATATAAAGCTTCACGTTTCTCCTCCATTTGACTGTCGGTATCTGACCAGTATGGAGGAGATA
GCGACAGGTATCAAATCAGGCGCCGGGTTCCAGCATCCGGCGCGGCGTGCGTTTATGACTAAATTGCCAGCCT
AAAGCCAAAAAGGTGATAAAGCCGATAATACCGAGCATCATCCACGGTAGTTCAGGCTGCGCAAGCGCTTTCC
CCATATCAAACAACCAGCCGCCGCCGATATAACCAATCGCGCCGCCAATGGCTAATCCCAGACGGCTAAAGCC
CATATAGCTTCCCCGCGCCCTCGCGTCTGCGAGCGACGCGCTGAGCGTTTCGCGCGCCGGTTCGGCGATAACC
GAGCCGATGTAGAAAGCGCAAATAAGCGTAAAAAGCTGCTGTAAATTGCCCACCATCCCGATGGGGAGCATGC
TCAGCGACATGACGAGCAAACCGGCCATCAGCCGATGCTCCAGCCGAAAACGCTTTTCGCTCCAGCGGGCAAT

CGGGTAGAGCAACGTCAGCGAGAGACACGCCTCAATAGCGTACATCCATTTCACGGCAGCAGGCGAACCGGCG
ATATCGTTTACCATAATCGGCAGCATTAACATGACCTGTACCGCCAGCATATAGTAGCCCGCCAGCGTCAGCA
CGTAGGTGACAAACCTTTTATCGCTCATGACGCGGCGCATTCCTTCACGCACCGGCGTTCTGACCGTTGATAG
CTTCCAGGCCGGAAGCAGCCATGCGTTGAAAAGGGCGCATAATATGAACAAAATAGCGCCTGTCGCGCAGACC
AGGCGAAAATCGTATTGTAGCAACCAGCTTCCCAGCAGCGCGCCAATCACCGCGCCCGCGCTGTCCTGCATCA
TCAACAGAGAGAAGAAGCGGCCCCGTTGCTCCGGACGAATTAATTTGACCACCAGCGCTGAACGCGGCGGGTC
AAAAAGCGTACCGCCGAGACCGGAAAGAAAGCAGGAAAACCACAAGAGCCAGGGCTCATGCGCGATACCCATG
GTGGCAAAGCCGGCGGCGCGCATCAGCATACCGGTGACAATCATCGGTTTCGCGCCAAAGCGATCGGCGATGG
CGCCGCCAAAAATGCCCAGACCTTGTTGAATAAACTGGCGCAGGCCGAGCGCGATCCCTACCATTACGGCAGC
CCACCCCATTTGATCGACAAAGCGAATAGAGATGAGCGGGAAGACGACGAAAAAACCCAGCACCACTAACATG
TTATCGATGAGAAGAAAATATTTACCCAGGTTCCTCGCCTGCGAGACGCGCGACATTTCCCCTCCCGGGAAAT
AAAAGATGAGCGTCTTCTATTCTGCGGCGGCGTTTCGTTTTTTCCTACCGTTAGCGGGACAATATTTTTTTAT
CAAAAGTCCTTTTTAATCGAGAGTTTTCATCAAAATGTGGCAGCAATTCAAAAAATGACGATTTGCGCTTTTC
ACAGAGCCTGGTTGCGCAGGTATAGTAATGTTACTGGCGTGCTGAAGACGTTACAGGAAGGAGTAGGTATAGA
ATGTTTGGCTATCGTAGTAACGTGCCAAAAGTGCGCTTAACCACCGATCGTCTGGTGGTACGTTTAGTGCATG
AGCGTGATGCCTGGCGTCTGGCCGATTATTACGCGGAAAATCGTCATTTTTTAAAACCCTGGGAACCGGTCCG
TGATGAAAGTCATTGTTATCCTTCAGGATGGCAGGCGCGTCTGGGAATGATCGGTGAATTTCACAAACAGGGC
TCCGCCTTCTATTTCGCGCTACTTGATCCGGAAGAAAAAGAAATTATCGGCGTGGCGAATTTTTCCAATGTGG
TGCGCGGTTCTTTTCATGCCTGTTATCTGGGCTATTCCATTGCGCAGAAGTGGCAGGGGCAAGGGCTGATGTT
TGAAGCCTTAACCGCTGCGATTCGCTATATGCAGCGCACTCAGCATATCCACCGTATCATGGCGAACTATATG
CCGCACAACAAACGTAGCGGCGCGTTGCTGGCGCGGCTTGGCTTTGAAAAAGAAGGCTATGCGAAAGATTACC
TGTTGATTGATGGACAATGGCGCGACCATGTCCTGACGGCGTTAACCACGCCGTTATGGACGCCGGGGCGTTG
AGCGGCTTACGGAATGAGAGGCAAAGGGAGAAAACGATGAAATATGAATTAACCGCCACTGAAGCGCGAGTGA
TTGGCTGTCTGCTGGAAAAGCAGGTGACAACGCCGGAACAGTATCCGCTTTCCGTCAACGGCGTGGTGACAGC
CTGTAATCAGAAAACCAACCGTGAACCGGTGATGAACCTGACGGAACAAGAGGTACAAGAACAGCTCGATAAC
CTGGTGAAACGCCACTTTTTGCGTACGGTCAGCGGGTTTGGCAATCGCGTCACCAAATATGAACAGCGCTTCT
GTAATTCCGAATTTGGCGATCTGAAACTTAGCGCGGCGGAAGTGGCGCTTGTCACTACGCTGCTGCTGCGCGG
CGCGCAAACGCCCGGCGAGTTGCGTAGCCGGGCGTCGCGGATGCATGAATTCAGCGATATGGCGGAAGTTGAA
TCCACGCTGGAACGGCTTGCCAGTCGTGAGGACGGCCCGTATGTCGTCCGTCTGGCGCGTGAACCGGGTAAGC
GCGAAAGCCGTTATATGCACCTTTTTTGCGGCGACGTCGATGAACTGTCTCTCCAGACGTCTGCGCCGGAAAG
TGCGTCGGGCGATCTTCAGTCGCGCGTCGAAGCGCTGGAAAGCGAAGTGGCGGAGTTAAAGCAGCGGCTGGAT
TCTTTGTTAGCTCACCTGGGAGAGTAATGTGAGAACATTACGGATTGGCATTGTCGGATTAGGCGGTATTGCG
CAGAAGGCCTGGCTGCCGGTATTAACCAACACCGCCGGATGGACGTTACAGGGCGCCTGGTCTCCTTCGCGGG
ATAAAGCCTTACGTATCTGCGAAAGCTGGAGCATACCGTATGTGGATTCGCTGGCGAATTTAGCGTCCGGCTG
CGATGCGGTCTTCGTCCACTCCAGTACCGCAAGCCATTATGCCGTGGTCAGCGAACTTCTCAATGCCGGCGTC
CATGTTTGCGTGGATAAACCGCTGGCGGAAAATCTACGTGATGCCGAACGGCTGGTGGCGCTGGCGGCGCAAA
AAAATTGACGCTGATGGTTGGCTTTAATCGCCGTTTCGCGCCGCTGTACCGCGAACTGAAGACGCGCCTCGGC
ACTGCGGCGTCACTGCGTATGGATAAACATCGTACCGATAGCGTCGGGCCGCACGACTTACGTTTTACTTTGC
TCGATGACTATCTGCATGTCGTGGATACCGCTCTGTGGCTGGCGGGCGGCGAGGCGCGCCTTGCCAGCGGCAC
GTTGCTCACCAGCGAGTCCGGCGAAATGTGCTATGCGGAACATCATTTTTCCGCCGACAAACTACAAATTACC
ACCAGTATGCACCGGCGCGCCGGAAGTCAGCGTGAATCGGTCCAGGCCGTCACCGACGGCGGGCTATATGACG
TGACGGATATGCGTGAATGGCGCGAAGAGCGCGGGCAGGGTATTCTCATCAAACCCATTCCTAGTTGGCAAAC
GACGCTTGAACAGCGTGGTTTTGTCGGATGCGCGCGGCATTTCATTGACTGCGTACAAAATCAGACGGTTCCG
GAAACGGCGGGGGAGCAGGCGATTTTGGCCCAGCGCGTCGTGGAGGCGCTGTGGCGGGACGCCATCAGCGAAT
AACCCTCTGTAACATCTGGCGGTAGTAATTCATCGTAATCCAGGTACTATACCCTCAATAATTCGAGTTGCAG
AAAAGCTAACGCACATGCAGCTCGAAGTATGGCGGGTATATATGCCCACTCTACACAATACCTCTTTTCACAG
TGAATAATGGCAAACCGTGGGGAGTCTGTAAGGCCTGATAAGACGTTTTAACGTCGCCGTCAGGCGCGGTGAT
ACAGCCGGATACGGCAGAAGTTGCGTTATCCGGTCGATGGATCAGCCATGCAGGAGTTTTACGCCAGGGTCTG
GAATACAAAAGAAATGAATTTATTGAAATCGCTGGCTGCCGTCAGCTCGATGACTATGTTTTCACGCGTGTTG
GGCTTTGCCCGTGATGCGATTGTCGCCAGAATTTTTGGCGCAGGGATGGCGACCGACGCCTTTTTTGTGGCGT
TTAAACTCCCCAATCTACTACGCCGGATCTTTGCCGAAGGCGCTTTTTCTCAGGCCTTTGTGCCTATCCTGGC
GGAATATAAGAGCAAGCAGGGTGAAGAAGCGACGCGGATCTTTGTCGCTTACGTTTCCGGCCTGTTGACGCTG
GCATTGGCCGTCGTGACGGTGGCCGGTATGCTGGCCGCCCCGTGGGTGATTATGGTAACCGCGCCGGGTTTTG
CCGATACTGCGGATAAATTCGCGCTGACGACGCAACTGCTGCGGATTACGTTTCCCTATATTCTGCTGATCTC

GCTGGCTTCACTGGTTGGCGCCATTCTCAACACCTGGAATCGCTTCTCTATTCCCGCTTTTGCGCCGACATTT
CTTAATATCAGCATGATCGGTTTTGCATTATTCGCCGCGCCATACTTTAATCCGCCGGTGCTGGCGTTAGCCT
GGGCAGTCACCGTCGGCGGCGTGCTGCAACTGGTGTATCAACTTCCGTATTTGAAAAAGATCGGTATGCTGGT
GCTGCCGCGCATTAACTTTCGCGACACCGGGGCGATGCGGGTGGTCAAACAGATGGGGCCGGCGATTTTGGGC
GTATCCGTCAGTCAGATCTCTCTTATCATCAATACCATTTTTGCCTCGTTTCTGGCCTCCGGCTCGGTCTCAT
GGATGTACTATGCCGATCGGTTGATGGAGTTCCCGTCCGGCGTGCTGGGCGTGGCGTTGGGGACCATCCTGTT
GCCGTCATTGTCGAAAAGCTTTGCCAGCGGCAATCATGATGAGTACTGCCGCCTGATGGACTGGGGGCTGCGT
TTGTGCTTTTTACTGGCGTTGCCGAGCGCGGTAGCGTTAGGCATTCTGGCGAAGCCGCTGACGGTCTCGCTGT
TTCAGTACGGTAAATTCACCGCCTTTGATGCGGCGATGACGCAGCGGGCGTTAATCGCCTATTCGGTGGGGCT
GATTGGCTTGATCGTCGTAAAAGTGTTGGCCCCGGGCTTCTATTCTCGCCAGGATATTAAAACGCCGGTGAAA
ATCGCCATAGTGACGTTAATCATGACTCAGTTAATGAACCTGGCGTTTATTGGACCGCTGAAACACGCCGGGC
TGTCGCTCTCTATTGGTCTGGCGGCATGTCTCAATGCGTCGCTGCTGTACTGGCAACTGCGCAAACAGAATAT
CTTTACGCCACAACCGGGTTGGATGTGGTTCCTGATGCGTCTGATCATTTCCGTACTGGTGATGGCCGCCGTA
TTGTTCGGCGTGTTGCATATTATGCCGGAGTGGTCGCAAGGGTCGATGTTATGGCGTTTGCTACGTTTGATGG
CGGTAGTGATCGCGGGTATTGCGGCCTATTTCGCCGCGCTTGCCGTGCTGGGCTTTAAAGTGAAAGAGTTTGT
TCGCCGGACGGCGTAAATTCAGTGCCTGATAGCGCTGTGCTATCAGGCCTACAAGGCATTCAGGCCGGAAAGG
CGCAACGTCGCCATCCGGCAATGATTAGATAGAAATTTTTTTACCGCCGCGGTGAGAGACGGAAGTCTGACCG
TCAGCCCCGTACAAGGTCGGCTCCTGGTGAGGTTTCAGCACCTCCAGCGCCTGTTGATTACGCTCGATTTGCC
CTTCCAGCAGCCAGCCGTTGTGCTGGTTGAGGTCGCGAAGATGCTGCGTTTTTTCGGTAATCGCCTGCCAGCG
CTCTGCAATGTCATCGTTTGCGCTACGCTGCGCGTTCTGCTCCAGACGGCGCTGTTGTTCCAGATAATCCAGC
GTCGCCAGCAACGAGCTTTTTTCTTCTGTAATACGCTGTAGCTGGCTGCCGTTAATCTGGCCTACGGAAAGCT
GTTGTTGCTCGGCGTCCATCACCGTCTTCAGGTCATTCAGGACGGTGGTCATCTGGTCAAGTATTTCTGACAA
ACGAGTCATACGCTTATTTACTCTGTAAGTAGCTCTGCGCCTCGCGAATGAGCGAGTCTGCTATTTTTCCCGT
ATCCATTTTTAACTCACCGTTACGGATAGCCGTTTTTAATGCTTCGACGCGTTCCATATTAATGTCGCTGACG
CCTGGCTGCATGAGCTTCGCTTGCGCGTCGCTTAACGTTACGCTGGCGCTCGTCGCGGCGGACGTTTTTTCCT
GACGCGTTTTTTGTACCGGCGTGTCGCTGGTTTCGCGCGTCTGGACAGTGCTAACGGGTTTCAAAGGTGAGGT
ACGGTCAATGCTCATTTATTTATCCTCATCGAGGGTTACGTTGTAGCGGCCAGCTACCATCATGGTTGAATAT
CTCATCGGCAGCCGCGACAAAATCTTTACACAATTATAGGTTAATAAGAATATTCCCATCAGAATCGACGGTT
CCACTCACGATTTGACCCGATGTCATGCGCACGCGCGCATTTTGCGCGACGGCGGCATTATTCATCGCCTGAC
CTTCCGCATTGACGCTGAACCCCTCGCCATTGGCGATGACCTGTACTCGTTGCCCCGCTTTGACGCGCCAGGC
CTGACGTATCATCGTAAGCTGTACCGGCTGCCCGGGTGCGAGATCGCGCAAACTGACGGCATCCTGAATCTGA
CGGATATCCAGTACCGTCCGCGGCGGTAACTGATCCAGCCTGCCACGTTTTAGCGTGACGTTGGCCGGCGTCA
ATTTTCCGCCGCGCGCGACGGGCGCGGCAACGGCGACATAATTGCCGGTCGCTTGCACATTCACCTGCAAATA
ACGTTTTTCATTGGCGCAGCGCGCCACCACATTGACGTTGCCCCACAGCTTCGCGCTGCCCGTCATGCTGAAG
GCTGGCTGTTCGCAGCTCGGTAGCAGATTGGGCGATGAACGGAGCGTGACAACCACCTCGTCGCTGAAGCCAG
CCAGACGCTGGGAAAACCACGTGGTCAGCTGGGCGTTGATGTCCTGCGCCATTGTCAGGGGGCTGAACAGCAA
AGCCGCCACGGCGAATCCTCGTTTTAACGTTTGCATGGTACTTCCCCCTGGTTGATGTCATGACAGGATTCTA
CCCGTGTGAAGCAGGCATCAACGCAATAAATAGCGACGCATTTTGCGTTTATTCCGGCGATAACGCGCGCGTG
AAGGCATTTAAGCTGTCGGCTGAATTTTGCCATTTGCGGAGGAGATATGCTCGACAGGCTCGATGCCGCCTTA
CGATTTCAGCAGGAAGCGCTTAATCTGCGCGCGCAACGTCAGGAAATATTAGCGGCGAATATCGCCAATGCCG
ATACGCCGGGGTATCAGGCGCGCGATATTGATTTTGCCAGTGAGTTAAAAAAGGTGATGGTGCGTGGACGGGA
AGAAACCGGCGGCGTCGCGTTAACGTTGACTTCCTCTCACCATATTCCCGCCCAGGCGGTCTCTTCTCCCGCA
GTGGATCTGCTTTACCGCGTACCCGATCAGCCTTCTTTGGATGGTAACACCGTAGATATGGACAGGGAACGTA
CGCAGTTTGCGGATAACAGTCTCAAATATCAGATGGGGCTTACCGTTCTGGGTAGCCAACTCAAAGGCATGAT
GAATGTGCTACAGGGAGGAAACTAATTCGTGGCGCTGTTAAACATTTTTGATATTGCCGGATCGGCGCTTGCC
GCGCAGTCCAAGCGGTTGAACGTTGCGGCCAGTAACCTGGCGAATGCGGATAGCGTCACCGGCCCGGACGGAC
AGCCTTATCGCGCCAAACAGGTGGTTTTTCAGGTGGACGCCGCGCCGGGTCAAGCCACTGGCGGGGTAAAGGT
CGCCAGCGTGATTGAAAGTCAGGCACCGGAAAAGCTGGTTTATGAGCCAGGCAATCCGCTGGCGGACGCTAAT
GGTTACGTCAAAATGCCCAACGTCGATGTGGTCGGCGAAATGGTCAACACGATGTCAGCCTCGCGCAGCTATC
AGGCAAATATCGAAGTCCTGAATACCGTAAAAAGCATGATGCTTAAAACGCTGACATTAGGCCAGTAAAGGAG
GCGCGTATGTCTATTGCCGTAAATATGAATGACCCGACCAACACGGGCGTCAAAACGACGACCGGCAGCGGGT
CGATGACCGGAAGCAACGCTGCCGATCTGCAAAGCAGTTTCCTGACCTTACTGGTCGCGCAATTGAAGAACCA
GGACCCGACTAACCCATTACAAAATAATGAGTTAACGACACAGTTGGCGCAAATCAGTACCGTGAGCGGCATT
GAAAAACTGAATACGACGCTGGGGGCTATTTCCGGGCAAATCGATAATAGTCAGTCCCTACAGGCGACCACGC

TGATTGGACATGGCGTTATGGTGCCTGGCACCACAATTCTGGCGGGTAAAGGCGCGGAAGAAGGGGCCGTGAC
GTCCACGACGCCGTTTGGCGTGGAATTGCAACAGCCTGCGGACAAAGTGACGGCAACCATTACCGATAAAGAT
GGCCGGGTGGTACGGACGCTGGAGATCGGTGAGTTGCGAGCCGGGGTACACACCTTTACCTGGGATGGTAAGC
AAACGGACGGAACAACGGTACCGAATGGTTCTTACAACATTGCGATTACCGCCAGCAATGGCGGAACGCAACT
GGTGGCGCAGCCGCTGCAATTCGCTCTGGTACAGGGCGTGACGAAGGGCAGTAACGGCAACCTGTTGGATCTG
GGTACCTACGGCACCACCACACTCGACGAAGTTCGGCAAATAATCTAAGCCCTTACACTTATCAGGAGTCAGT
CATGTCTTTTTCTCAAGCGGTTAGCGGCCTGAACGCTGCGGCCACCAACCTTGATGTTATCGGTAATAACATC
GCCAACTCCGCCACCTATGGCTTTAAGTCCGGTACGGCATCATTTGCCGATATGTTCGCCGGTTCCAAAGTGG
GGCTGGGCGTAAAAGTGGCGGGGATTACCCAGGATTTTACCGACGGTACGACAACGAACACCGGGCGCGGGCT
GGATGTCGCGATTAGCCAGAACGGTTTTTTCCGCCTGGTAGACAGCAACGGTTCCGTGTTCTATAGCCGCAAC
GGCCAGTTCAAACTGGACGAGAACCGTAACCTGGTCAATATGCAGGGGATGCAGTTGACCGGCTATCCGGCCA
CCGGTACGCCGCCGACCATTCAGCAGGGGGCGAATCCTGCGCCGATCACCATTCCGAACACGCTGATGGCGGC
GAAATCGACCACCACCGCGTCAATGCAGATCAACCTGAACTCAACGGACCCTGTACCGTCTAAAACGCCCTTT
AGCGTGAGTGATGCGGATTCGTATAACAAAAAAGGCACCGTCACCGTTTATGACAGCCAGGGTAATGCCCATG
ACATGAACGTCTATTTTGTGAAAACCAAAGATAATGAATGGGCTGTGTACACCCATGACAGCAGCGATCCTGC
AGCCACTGCGCCAACAACGGCGTCCACTACGCTGAAATTCAATGAAAACGGGATTCTGGAGTCTGGCGGTACG
GTGAACATCACCACCGGTACGATTAATGGCGCGACAGCGGCCACCTTCTCCCTCAGCTTCCTTAACTCCATGC
AGCAGAACACCGGGGCTAATAACATCGTCGCCACCAATCAAAACGGCTATAAGCCGGGCGACCTGGTGAGCTA
CCAGATTAACAACGACGGCACCGTGGTTGGCAACTACTCCAACGAGCAGGAGCAGGTGCTGGGGCAGATTGTG
CTGGCTAACTTCGCCAACAACGAAGGTCTGGCATCCCAGGGCGATAACGTCTGGGCGGCGACGCAGGCTTCCG
GGGTTGCGCTGCTGGGGACTGCCGGTTCCGGCAACTTCGGTAAGCTGACGAACGGCGCGCTGGAAGCCTCTAA
CGTGGATTTGAGTAAAGAGCTGGTGAATATGATCGTCGCGCAGCGTAACTACCAGTCGAATGCGCAGACCATC
AAAACTCAGGACCAGATCCTCAATACGCTGGTTAACCTGCGCTAAGCGCCTGACGGGATAGCTTAATGGATCA
CGCAATTTATACCGCTATGGGGGCGGCCAGCCAGACGCTTAACCAGCAGGCGGTAACGGCCAGCAACCTGGCT
AATGCCTCAACGCCGGGCTTTCGCGCGCAGCTTAACGCGCTACGCGCGGTGCCCGTTGATGGCCTCTCTTTAG
CAACGCGCACGCTGGTTACGGCGTCGACGCCCGGGGCGGATATGACCCCGGGTCAGTTGGACTACACCTCCCG
TCCGCTGGATGTCGCGTTACAGCAGGACGGCTGGCTGGTAGTGCAAGCGGCGGATGGCGCTGAAGGATATACC
CGTAACGGGAATATCCAGGTGGGTCCGACCGGGCAGTTAACCATTCAGGGACATCCGGTTATCGGCGAAGGCG
GCCCGATTACCGTTCCTGAAGGGTCGGAAATCACCATTGCGGCAGACGGCACGATCTCCGCGCTCAATCCCGG
CGACCCGCCAAACACGGTGGCGCCCGTTGGGCGACTGAAGCTGGTCAAAGCGGAAGGCAATGAGGTGCAGCGG
AGCGATGACGGTTTATTCCGCCTTACCGCCGAGGCACAGGCTGAACGCGGGGCGGTACTGGCCGCCGACCCGT
CAATTCGCATTATGTCGGGCGTGCTGGAGGGCAGTAACGTCAAGCCGGTTGAAGCCATGACCGACATGATCGC
CAACGCACGTCGTTTTGAAATGCAGATGAAGGTTATCACCAGCGTAGATGAGAACGAAGGGCGAGCTAACCAA
CTGCTGTCGATGAGTTAATACAGGACATTTTATGATCAGTTCATTATGGATCGCCAAAACCGGTCTGGACGCG
CAGCAAACCAATATGGATGTGATTGCCAATAACCTGGCAAACGTCAGCACCAATGGTTTTAAGCGTCAGCGCG
CGGTATTTGAAGATCTGTTGTATCAGACCATCCGCCAGCCGGGCGCGCAGTCGTCCGAGCAGACGACGCTGCC
TTCAGGGCTGCAAATCGGTACCGGCGTGCGTCCGGTCGCCACGGAGCGTCTGCACAGTCAGGGGAACCTGTCG
CAGACCAACAACAGTAAAGATGTGGCGATTAAAGGGCAGGGCTTTTTCCAGGTCATGCTGCCGGACGGTACGT
CTGCCTATACCCGCGACGGCTCTTTCCAGGTGGATCAGAATGGTCAACTGGTGACGGCGGGCGGTTTTCAGGT
GCAGCCGGCAATCACCATTCCGGCCAACGCGTTAAGCATCACGATTGGCCGCGACGGCGTGGTCAGCGTTACC
CAGCAGGGGCAGGCCGCGCCGGTTCAGGTCGGGCAGCTTAACCTGACCACCTTTATGAACGACACCGGGCTGG
AAAGCATTGGCGAGAACCTCTATATCGAAACGCAATCGTCCGGCGCGCCGAACGAAAGCACGCCGGGGCTCAA
CGGCGCGGGGTTGTTGTATCAAGGGTATGTCGAAACGTCGAACGTTAACGTGGCGGAAGAGCTGGTGAACATG
ATTCAGGTTCAACGCGCCTATGAAATTAACAGTAAAGCAGTATCGACGACCGATCAGATGCTGCAGAAACTGA
CGCAACTCTAAGGGGCCGCCGGTGGGGGATACGCCACCGGCTCCCTGATTTTGAAGATGAAGGTAATGCAAAA
ATACGCGCTTCACGCTTACCCAGTTATGGCCCTGATGGTCGCGACGCTGACAGGATGCGCCTGGATACCCGCT
AAACCGCTCGTGCAGGGGCGACCACGGCGCAGCCGATACCTGGCCCGGTACCGGTGGCGAATGGCTCCATATT
TCAGTCTGCGCAGCCGATTAATTATGGCTATCAGCCGCTTTTTGAAGATCGTCGACCGCGTAATATCGGCGAT
ACGCTCACGATTGTGTTACAGGAAAACGTCAGCGCCAGTAAAAGCTCGTCGGCAAATGCCAGTCGCGACGGCA
AAACCAGTTTTGGTTTTGATACGGTACCGCGTTATCTGCAGGGATTATTCGGTAATTCCCGCGCGGATATGGA
GGCCTCCGGCGGCAACTCTTTTAACGGCAAAGGCGGCGCGAATGCCAGCAATACCTTTAGCGGCACGCTGACC
GTGACCGTCGATCAGGTTCTGGCCAATGGCAATTTACACGTCGTGGGGGAAAAACAGATCGCGATTAATCAGG
GAACGGAATTCATCCGCTTCTCCGGCGTGGTAAATCCACGCACCATCAGCGGCAGCAACTCCGTTCCCTCGAC
ACAGGTGGCGGATGCGCGGATTGAATATGTCGGGAACGGCTATATTAACGAAGCGCAAAATATGGGCTGGCTG

CAACGTTTCTTCCTTAATTTGTCGCCGATGTAAGCGAGGTGTATGTGTTTAAAGCTCTTGCAGGAATCGTTCT
GGCACTGGTTGCCACTCTGGCGCACGCCGAGCGTATCCGGGATCTGACCAGTGTCCAGGGAGTACGGAAAAC
TCGCTGATCGGCTACGGGCTGGTGGTCGGGCTGGACGGTACGGGCGACCAGACGACCCAGACGCCATTTACCA
CCCAGACGCTGAATAACATGCTGTCACAACTGGGGATTACGGTCCCCACCGGCACCAACATGCAGTTGAAAAA
CGTGGCGGCGGTGATGGTGACGGCGTCGTATCCGCCTTTTGCGCGACAGGGACAAACGATCGATGTCGTCGTT
TCCTCAATGGGGAACGCTAAAAGTCTGCGTGGCGGGACGTTATTAATGACGCCGTTAAAAGGGGTGGACAGCC
AGGTGTATGCTCTGGCGCAGGGCAATATTCTGGTCGGCGGCGCGGGCGCTTCCGCAGGCGGCAGTAGCGTGCA
GGTTAACCAGCTTAATGGCGGGCGCATCACTAATGGCGCGATTATCGAACGCGAGTTGCCGACTCAGTTCGGC
GCTGGCAACACCATTAATCTGCAATTGAACGACGAAGATTTTACGATGGCGCAGCAAATTACCGACGCCATCA
ACCGCGCCCGCGGTTACGGCAGCGCCACTGCGCTTGATGCGCGAACGGTACAGGTACGCGTGCCCAGCGGCAA
CAGCTCGCAGGTGCGTTTTCTGGCGGACATTCAAAATATGGAAGTCAACGTGACGCCGCAGGATGCAAAAGTT
GTGATCAACTCGCGTACCGGTTCGGTGGTCATGAATCGGGAAGTCACGCTGGATAGCTGCGCTGTGGCGCAGG
GCAATTTGTCAGTGACAGTCAATCGCCAGCTCAACGTCAACCAGCCGAATACGCCATTTGGCGGCGGGCAGAC
CGTGGTGACGCCACAGACTCAAATAGATTTGCGTCAGAGCGGCGGATCGCTACAGAGCGTGCGTTCCAGCGCC
AATCTGAACAGCGTAGTGCGTGCGCTGAATGCGCTTGGCGCGACGCCGATGGACCTGATGTCGATTTTGCAGT
CCATGCAGAGCGCGGGCTGTCTACGCGCCAAACTGGAAATCATCTGATGATCGGAGACGGTAAATTGCTGGCC
AGCGCGGCCTGGGATGCGCAATCTCTGAACGAACTGAAAGCGAAAGCGGGCCAGGACCCGGCGGCGAATATCC
GTCCTGTGGCCCGTCAGGTGGAAGGGATGTTTGTGCAGATGATGCTGAAAAGTATGCGCGAGGCTTTACCCAA
AGATGGTTTATTCAGCAGCGATCAGACGCGTCTGTATACCAGCATGTATGACCAGCAGATCGCCCAGCAGATG
ACCGCCGGTAAGGGATTGGGGCTGGCGGATATGATGGTTAAACAGATGACGGGCGGGCAGACGATGCCTGCGG
ATGATGCGCCGCAAGTACCGCTTAAATTCTCCCTGGAGACGGTAAACAGCTATCAAAATCAGGCGCTGACCCA
ACTGGTGCGCAAAGCCATACCGAAAACGCCGGACAGCAGCGATGCGCCGCTCTCCGGCGACAGTAAAGACTTT
CTGGCCCGGCTTTCGCTCCCGGCGAGGCTGGCCAGCGAACAAAGCGGGGTGCCGCATCATCTGATTCTGGCGC
AGGCGGCGCTGGAGTCCGGCTGGGGGCAACGGCAAATCCTGCGGGAGAATGGCGAACCCAGCTATAACGTATT
TGGCGTGAAAGCGACCGCCAGTTGGAAAGGGCCGGTGACGGAAATCACCACCACTGAATACGAGAATGGCGAA
GCGAAAAAAGTGAAAGCGAAATTCCGCGTCTATAGCTCGTATCTGGAGGCATTATCGGATTATGTCGCGCTGT
TAGCGCGTAACCCACGCTACGCTGCCGTGACCACTGCCGCCACGGCAGAGCAGGGCGCAGTGGCTCTGCAAAA
CGCCGGATACGCCACTGACCCGAATTACGCGCGTAAATTGACCAGCATGATTCAGCAGTTGAAAGCGATGAGT
GAAAAGGTCAGCAAAACCTACAGCGCGAATCTCGACAATCTCTTTTAAATTGCTCAAGTCCACGTAGTCGCTG
CCGATAACAACGAGTATTGAAGGATTAAAAGGAACCATCATGTCCAGCTTGATTAATCACGCCATGAGCGGAC
TTAACGCCGCGCAGGCCGCGTTAAATACGGTCAGTAATAACATCAACAATTATAACGTTGCGGGTTATACCCG
GCAGACAACTATTCTGGCGCAGGCAAACAGTACGTTAGGGGCTGGCGGCTGGATAGGTAATGGCGTTTACGTT
TCAGGCGTACAGCGCGAATATGATGCGTTTATCACTAATCAGCTACGCGGCGCGCAAAACCAGAGCAGCGGCT
TGACCACGCGCTATGAACAGATGTCGAAAATCGACAACCTGCTGGCCGATAAATCCAGCTCACTGTCTGGTTC
GTTGCAGAGTTTTTTTACCAGCCTGCAAACGTTAGTCAGTAATGCGGAAGATCCTGCGGCGCGTCAGGCGCTG
ATTGGTAAAGCGGAAGGGCTGGTAAACCAGTTCAAAACCACCGATCAGTATCTGCGCGATCAGGATAAACAGG
TCAATATCGCGATTGGCTCCAGCGTGGCGCAAATCAACAATTACGCGAAGCAGATAGCTAACCTGAACGATCA
AATCTCCCGTATGACGGGCGTAGGCGCGGGCGCATCGCCGAACGACCTGCTCGATCAACGTGATCAGTTGGTT
AGCGAGCTTAACAAGATCGTTGGCGTCGAGGTGAGTGTACAGGACGGCGGCACCTATAACCTGACGATGGCCA
ATGGCTATACGCTGGTGCAGGGGTCGACGGCGCGTCAGTTGGCGGCGGTTCCCTCCAGCGCCGACCCGACGCG
AACGACTGTCGCTTATGTCGATGAGGCCGCCGGTAACATCGAAATTCCGGAAAAGTTGCTGAACACCGGTTCG
CTCGGCGGGCTACTGACGTTCCGTTCCCAGGATCTGGATCAGACTCGTAATACGCTGGGCCAGTTGGCGTTGG
CGTTTGCCGATGCGTTTAACGCGCAGCATACCAAAGGTTATGACGCCGACGGCAATAAAGGGAAAGACTTCTT
TAGCATTGGCTCGCCGGTGGTATATAGCAACAGTAATAATGCCGATAAAACGGTATCGCTAACCGCTAAGGTG
GTCGATAGCACGAAGGTTCAGGCGACGGATTATAAGATTGTTTTTGACGGTACAGACTGGCAGGTTACTCGCA
CTGCGGATAACACCACCTTCACGGCGACAAAAGATGCTGACGGAAAACTGGAGATTGACGGTCTGAAAGTGAC
GGTAGGGACCGGCGCACAGAAAAACGACAGTTTTCTTCTCAAGCCGGTTAGCAATGCTATCGTCGACATGAAC
GTTAAAGTGACGAATGAAGCCGAGATTGCGATGGCGTCTGAGTCAAAACTCGATCCTGACGTGGATACCGGCG
ACAGCGATAACCGCAATGGTCAGGCATTGCTGGACTTACAAAACAGCAATGTAGTGGGCGGCAACAAAACTTT
TAACGATGCTTACGCCACGTTGGTCAGCGATGTGGGTAACAAAACGTCAACGCTGAAAACCAGCAGCACCACG
CAGGCGAATGTGGTTAAACAGCTTTATAAACAGCAACAGTCGGTTTCCGGCGTTAACCTCGACGAAGAGTACG
GCAATTTGCAGCGTTATCAGCAGTATTATCTGGCGAATGCGCAAGTATTGCAGACCGCGAATGCGCTGTTTGA
TGCGTTATTGAATATTCGCTAAAGGAGAAGGATGACATGCGTATCAGTACCCAGATGATGTACGAACAAAATA
TGAGCGGCATCACTAATTCTCAGGCCGAATGGATGAAGCTGGGCGAGCAGATGTCTACCGGTAAGCGCGTTAC

CAACCCATCTGACGATCCAATCGCCGCGTCGCAGGCGGTAGTACTCTCTCAGGCGCAGGCGCAGAATAGCCAG
TATGCCCTGGCGCGTACGTTTGCCACCCAAAAAGTGTCGCTGGAAGAAAGCGTACTCAGTCAGGTGACGACGG
CGATTCAAACCGCGCAGGAAAAAATCGTCTATGCCGGAAACGGCACGTTAAGCGACGATGACCGCGCGTCGCT
GGCGACGGATTTACAGGGGATCCGCGATCAGCTGATGAACCTGGCAAACAGCACTGACGGCAATGGTCGCTAT
ATCTTTGCCGGGTATAAAACGGAAGCGGCGCCATTCGACCAGGCGACAGGTGGTTATCATGGCGGCGAGAAAA
GTGTTACCCAGCAGGTGGATTCCGCACGCACGATGGTAATTGGCCATACGGGAGCGCAAATTTTTAATAGCAT
CACCAGCAATGCGGTGCCGGAACCGGATGGCTCAGACTCCGAAAAGAATCTGTTTGTCATGCTCGATACGGCA
ATTGCCGCGCTCAAGACCCCGGTGGAAGGCAATGACGTGGAAAAAGAAAAAGCCGCTGCCGCCATTGATAAAA
CCAATCGCGGCTTAAAAAATTCGCTTAATAACGTCCTGACCGTTCGTGCGGAACTGGGAACGCAACTGAGCGA
ACTCAGTACGCTGGATTCACTGGGAAGCGACCGTGCGCTGGGACAGAAACTGCAGATGAGCAACCTGGTAGAT
GTGGACTGGAACTCGGTCATTTCTTCCTACGTCATGCAACAGGCGGCATTACAGGCGTCCTATAAAACGTTTA
CCGACATGCAGGGAATGTCGCTTTTCCAGTTGAACCGGTAACACCTCTTTTTGAAACATATCACGAAACTGGA
TATGTTTTGTCTGCCCGCGCCATCCACCCCGGCGCGGGCATTTTTTTAGTCGTCATTGAGTTAGTGGTCATAT
TGTCGCGCCCGTAGCGACGCCATCCGTACGTATTATCCCGCCCGATCTCATAGTGACTAAAAGACGCCATGGG
GTGAGGCATCGCCCTGTAAGGGGCATAAAAAAGCCGACCTGGCGGTCGGCTTTGTATCAGCATTTACATCATT
CTACCGGCTGTGGGCGTGTCGCCGGGGCGCTGGCATGACGTGTAGCGCTGTGTCCGCCTGCCGCGCCTTTACC
TTCAAAGTGGAAGGAAGGGCGCTGCCAGTCGCTATGGTGCGGGGTTTCCGGCACATATTCCGGCGCTGGCGCA
CGCGTCATCGGCGCGCTGGCGTGATTATGATCGTCGTCAACGCAGACAACGTCCTGCGGCTTTTCCACCACCT
GGGCCGGTGCGATAACGGTTTCTTCTGCCGTGTGCTCCACAACAGCCGCTTCCGCTTTTGTCTCTTCTTCTGG
CTCGACAACGACAACATCAGCCACGTTTTCAGCCACCGTAATGGACGCGTCGGCGGTTTCTGCTACCGGTTCG
GCGTCAGCAATAACCTCTTGCGCTTCCGGCGTATCGCTTTCGGCAATCAGTTGCGGCTGTTCATCAACCGGCG
CGGCAATCACTTCCGGATGCGTTGTCTCGACCTTGACGACTTCCGGCGCCTGTGGTTCGGCGACGGTCTGCGG
CTCAACGGCAACGTTTTCTACCGCCTGTACCGACGCTTGTGGTTCTAACGCTACGGTCGCAGCGATAACCTGC
GGTTCAGCGACAACCGGTTGTGGCAGCGCCAGATCTGCTTCACGCTGCTCTTCAACAACCTGCGTTTCCTGAG
GACGGACAATCGGGTAGCGGATCCAGACTTTACCGGACGCCATTTCCGGCGACGCGCAGGCGACGGTTAACGG
CATCGGCGATTGTGTCGGGTAGCGCTCGTCACGATAGCGACGGCGACGCTGACCGCTTACGCGCAGGTGGCGC
GGAGAACGGCGGGAGCGACGCGGCATACCGGTATTATCGCGAGGTTCTACGCTATCATCCTGCTCCGGCGCGA
TATCGGCGACGACAGGTAAATCAACTTTCGCCAGCTCGGTACGCGGCGCTGGAACGGGCTGTTCAACGTTTTC
AACCGGGCGTGGTTCGTCGACGACGACCGGCGTGTCAACCGTTTCAACCACTGCGCTGTTGGTAAAGCGGACT
TTCTGATTAAGCTGACGCTGCTTGCGGCGCGGCTGAACCTGCTGAACGCGTTCTTCCTGCTCGGTCTCCTGGA
CAGGCTGTTCTTCGCGGTTAAGCGCTTTTACTTCCTGCTGCGCCTGGCGCTTGTCATCATTGCGGCGGCGGCT
GCGTTCGCGACGCGGCGTCTGTTGCTGTTCATCGCCGGTTTTCACTTTTTCCGCCGTTTCCTGCTGGCGGGTA
TCGCGCGCTTCCGCATTTTGCTGCTGCGTCTGGCGACGGTTACGACGATTATCGTCGCGGCTCTCGCCGCCGT
CACGTCCGGCACGGTTATCACGGGTGTCGCGGCGTTCGTTACGATCGCGACGGTTGTTCTGCCGTGGCTTGCG
ACGATCCTGCTGACGCTCCGCTTTTTCTTCCGCTTTCGGCGCGGCTGTTTCGACGGCTTTCGTTTCTTCACCG
CTGAACAGTTGTTTCAGCGCGTTAAGGAAGCGGCTAAACAGACCCGGCTGGGCAGGCTGCGCGGCGGCAACAT
TATCCTTTTTCGCCGTGGCGACGCTAACCGCCGGTTCGACAGGCGTCGGCGCCGGAGGCACATCTGGCATAGC
GAATGTCGCCAGCGCAGGCTGTTCCGGGCGTTTACGCTCGGCGTACTCTTCTTCAGAAGGCAGCGCCATCGCT
TCTTCGTGCAGCTTCGGCAGCATGTAGCTCAGGGTCGGCGTTTCTTCCCCTTTGCGTACGCGCAGCACGGAAT
AGTGCGGCGTTTCCATCTGATCGTTCGGGACGATGACACAGCGGACGCCATCCTGACGCGTCTCAATAGCGTT
AACTGCCGTCCGTTTTTCGTTAAGCAGATAAGAGGCGATCGGCACCGGAACAATGGCATGGACTTCCTGCGTA
TTCTCTTTCAGCGCTTCTTCTTCAATCAGGCGCAGAATGGACAGCGACAGAGATTCGTTATCGCGCACGGTGC
CGGTACCGCTACAGCGCGGACACACGTGATGGCTGGATTCGCCCAGCGACGGGCTCAGGCGCTGACGCGACAT
TTCGAGCAGGCCGAAGCGGGAAATATGGCTAATCTGAATACGCGCCCGGTCCTGACGCACCGCTTCACGCAGA
CGGTTTTCTACCGCGCGCTGGTGACGTACCGGCGTCATATCGATAAAGTCGATAACGATCAGGCCGCCCAGGT
CGCGCAGACGCAACTGGCGGGCAATCTCATCGGCCGCTTCCAGGTTGGTATTAAACGCCGTTTCTTCGATATC
GCCGCCGCGAGTCGCGCGGGCGGAGTTGATATCGATGGCGGTTAACGCTTCGGTACTGTCAATGACGATGGAG
CCGCCGGAGGGCAGGCGCACTTCACGCTGGAACGCTGACTCGATCTGCGATTCGATCTGGTAATGGCTGAACA
ACGGGATTTCACCGGTGTACAGCTTAATTTTGCTGCTAAAATCCGGGCGGCCCAACGCGGCGATATGCTGACG
CGCCATTTCCATCACTTTTGGATTGTCGATCAGGATTTCACCGATATCCTGACGCAGATAGTCGCGAAACGCG
CGTACGATGACGTTGCTTTCCTGATGGATCAGGAACGGCGCGGGACGGCTTTCAGCGGCTTTCTGAATGGCCT
CCCAGTGTTTCAGGCGGAAGCTTAAATCCCACTGCAACGCTTCGGCGGATTTACCCACGCCCGCGGTACGCAC
GATAAGCCCCATCCCTTCAGGAAGTTCCAGGCTGGCCAACGCTTCTTTTAACTCGGTACGATCGTCGCCTTCA
ATGCGACGGGAAATACCACCCGCGCGCGGGTTATTCGGCATCAGTACCAGGTAGCTGCCCGCCAGACTGATAA

AGGTGGTTAACGCCGCGCCTTTGTTGCCGCGCTCTTCTTTATCGATTTGAACAATGACTTCCTGGCCTTCGCG
TAACACGTCTTTAATGTTCGGGCGACCATGAGCGCTGTAGTTAGCGGGGAAATATTCGCGGGCAATTTCTTTT
AACGGGAGGAAACCGTGGCGTTCTGCGCCGTAATCAACAAAAGCCGCTTCCAGGCTCGGTTCAATACGGGTAA
TTTTGCCTTTATAGATGTTCGCTTTTTTCTGCTCGTGTCCGGGGCTTTCTATATCCAGATCGTACAGACGCTG
CCCATCTACAAGGGCGACGCGCAACTCTTCCTGCTGAGTCGCGTTGATTAACATTCTTTTCATCGTAACTTAC
TCATTATTCTTACGTTGACGACTAAGCTGCGGGCATGGTGACGCCTTACCGGGCATGAACCGATGGCCTCGTG
ACTATTTCGCGTCGCCAACCTCACGGTTATCGTCAGCTCAAAGAGGCGCAGAGTGTCGGTTGCCTGTATTTCA
TACGGAAACACAGCGCAATTATCAGGGAAGCAGCCTGGGCATTACTCTCCAGAGAAGATCCTATCTACCGGTA
AGGACTGCAACCCGCAGCCCGCTAACTGTCTGAAAAATCAATACGTCTTACGCCATTGCTGCGTTGATGATCG
GTCAGACAAAATGGGTTATTCCGCTAAACTTTTTGTTTTAACAAGTTTCGGTGCGGAAACCGCATCATTATTC
CACTGCTAACCTTGTTATAGCAAGATGACTTTTACCATTTATCACCCGCTTACTCACAGTTTTTTCACCAGCG
CGAGCCAATCGCTTTAATAACCAGCAAAACCGCAGTGAAAAATGTTCATCCACTGGCGTAGACGTCTCTATAA
GCATAGAAAAATGTGTGGCGCGAATCTCACAGGCTATTTAGAATCGCCCCCCATGAAAACAGAAACGCCATCC
GTAAAAATTGTTGCTATCGCCGCTGACGAAGCGGGGCAACGCATTGATAACTTTTTGCGCACCCAACTGAAAG
GGGTACCAAAAAGTATGATTTATCGCATCTTGCGTAAAGGCGAGGTGCGGGTGAACAAAAAACGCATTAAACC
GGAATACAAACTGGAAGCGGGCGATGAAGTCCGCATTCCGCCGGTTCGCGTTGCTGAACGCGAAGAAGAGGCC
GTGTCGCCGCATTTGCAAAAAGTGGCGGCGTTGGCGGACGTCATTTTGTATGAAGACGACCATATTCTGGTGC
TGAATAAGCCTTCCGGCACCGCCGTACACGGCGGGAGCGGGTTAAGTTTTGGCGTCATCGAAGGGCTGCGGGC
GTTGCGTCCGGAAGCGCGATTCCTGGAACTGGTGCATCGTCTTGACCGCGATACCTCCGGCGTGCTGTTGGTG
GCGAAAAAACGCTCCGCGCTGCGTTCGCTGCATGAGCAACTGCGCGAAAAGGGAATGCAGAAAGATTATCTGG
CGCTGGTGCGCGGCCAGTGGCAATCGCACGTTAAAACCGTACAGGCGCCATTATTAAAGAACATTCTGCAAAG
CGGCGAACGTATTGTTCGCGTGAGCCAGGAAGGCAAACCGTCAGAAACGCGCTTCAAGGTGGAAGAGCGCTAT
GCCTTCGCCACGCTGGTGCGCTGTAGCCCGGTAACGGGCCGTACGCATCAGATTCGCGTACATACCCAATATG
CCGGTCACCCTATTGCGTTCGACGATCGCTACGGCGACCGTGAGTTTGATCAACAGCTTACCGAAGCGGGAAC
CGGTCTTAAACGTCTCTTTCTACACGCCGCGGCGCTGAAGTTCACGCATCCGGGAACGGGCGAAGTCATGCGC
ATTGAAGCGCCGATGGATAACGCCCTGAAGCGCTGTCTGCAGGTGCTGCGTAACGCAAAATAGTTCCTCTCTC
GGGCAGGTTATCTGCCCGAATCTCACTTTTCTCATAATATTTTTGTTTTGCTGTATCCGACGCATTTTTACCG
TTGTGTACTGTTTATTCTTGCGATCCGGATTTCGGCGTGTTTAAGGACGAACAGATGGCCAATACTAGCTGGA
TGCGCTCATTTACTCAGCGTTGGGGAAAATACGTTATCGTGCCGCTCGTGATCGCGATGCTGACCGGTTGCGA
AGGGAAGGGCGATAGTTCACGACGGGCTTTCGTTGAGTATTTGAATACGCAAATCATTACGTCTCCCGTGGTG
GGCGTCTGTGAGTTGACGCCAGCCCAACGTCAGGCTTTTGGTCATTACGCCGATGACTACGATGTACTATTAG
CTGCCTACGGGCAAATCTTTGCTGTGGTGTATGAGACCGGCCAGGAGGATAAGATCACCGGTGTTATTCAGTC
TGAGCGTGATAATCTGGTTTTCCTGAACGAGCTACGTGTGGCTCGTGACGTAAACAATCGATTTATCCTTCGG
TTGAAAAATGTGAGTAGTGAAAACAGAAAAAAATATTCTGCTTTAAAGCTCCCCTCTGATGTGAAGCCTGTCT
TTGACGCTGCGTGGAATAAAACCGTAACACCGCTGGATGAGACGATGACTCGCTATTATTTCTGCGCGGAAA
GCGGCTCGACCAGCTGGTCGCAATGGGCGAGTTTCTGCAAAAACAGGGAAATAAAGTCCGCTTTGAAACGGAT
GGAAAAGCGGTTTTTGTCGACCTGGCAGCAAAAGAACACTTTCAGGATCAACAATTTAATTTTTTCATTACGC
AGAATGATATAAACCATCTTACTCAGCAGATGGTTGAATTTATAAATAGTCAACAAGATGCGGCGGATGCTGG
CGGCGTTAATTAAAGGAATGTGTCGTGAGGTGAAGCAATGTTAGGGGGGCGTAAACTCATCGCGAGATGGAATT
TAACGAGTCGCAAAGGGAAGGTTTCAGGACAGTAAACGTCCTGAGAGTTGCTCGCGAGAATAAGTTTGTAAGC
CGCTTTTGACGCCGTAGAGCGCGTCAGGCGAGTATTGAGAACATGTGTTTTTACAAAACTGAACTGAGATGGG
GAATTCATTAACGCGCACGTACACCGCTGTTGATACCCTATGGGATAAAACGCACCGTACATCGGTTCGTAAT
ACCAATGCTTTTTTTCTGGTCGGTGAATGGATTGACCAGGAAACGGGCCCGCACGAAGAGAGTGAAGATCGGC
AGGCGCTAAATTGTTCACAACAAGCGCTTTCCAGTTGGTTAGAACTGGAAGCGGTCGAAACTGCTCCCCAATA
AACGGGAGATAACTACCAACACGTGCAGAAACGGCTTTTAGCGGTATTTGAAAAGCCTACAGAATATCCGTTT
ACTGCAGTAGCGGGTTGAAGCCTTCCCGTCGCAGCATCTGGCACAGAGCAATAAGCGGCAAGCCGATCAGCGT
GTTCGGATCGCGGCCCTCCAGGCGCTCAAATAATGCAATGCCTAAACCTTCGCTTTTAAAACTTCCCGCGCAG
TGCAGCGGATGCTCTTTACGGACATAGTCGTCTATTTCCGCTTCGCTTAAATGGCGAAAATGAACGTCAAAAG
GCTCCACTTCGGTTTGTAAGTGCCCGGAGGCGGAGTTATACAGTGCCAGTCCGGTATAAAACGTCACAATATT
TCCGCTGGCTTTGGCTAATTGCTGACGCGCTTTTTCTTCCGTCAGCGGTTTGCCGGTGATTTCGCCGTCCAGA
ACACAAATTTGGTCGGAGCCAATAATCAAATGATTCGGGAAACGCGCGGCGAGCGATTGCGCCTTCGCCTGCG
CCAGCCGCAGCACCAACTGACGCGGCGCTTCGCCCGGCATGGGGGTTTCATCAACATCCGGCGTGGCGCATTC
GAAAGGAATCGTCAGCTTTTCCAGCAGCGCGCGACGCCAGGGAGAGGTAGACGCAAGGATGAGACGGGGCATA
TTTTTTCCCTCAGATATAGCGTAATGATGAGAGCCATCTTAAACTATGCTCTTCGGTCTTCAAGCCTGTCATA

CTGACTCGTCTTGAGCGAAAATAAGTCTGTAACCCGCAATGTGTGCGAATTATTGGCAAAAGGCAACCGCAGG
CTGCCTTTTTCTTTGACTATATGACGTTACGAAGTTAATATGCGCGCCCTATGCAAAAGGTAAAATTACCCCT
GACTCTTGATCCGGTTCGTACCGCTCAAAAACGCCTTGATTACCAGGGTATCTATACCCCCGATCAGGTTGAG
CGCGTTGCCGAATCTGTAGTCAGTGTGGACAGTGATGTGGAATGCTCCATGTCGTTCGCTATCGATAACCAGC
GTCTCGCCGTTTTAACCGGCGATGCGGTCGTTACGGTGTCGCTCGAATGCCAGCGTTGCGGGAAACCGTTTAC
CCATCAGGTTCACACAACGTATTGTTTTAGCCCTGTCCGTTCCGACGAGCAGGCTGAAGCACTGCCGGAAGCG
TATGAGCCGATTGAGGTAAACGAATTCGGCGAAATCGATCTGCTGGCGACGGTTGAAGATGAAATCATCCTCG
CCTTGCCGGTAGTTCCGGTGCATGATTCTGAACACTGTGAAGTGTCCGAGGCGGACATGGTCTTTGGCGAACT
GCCTGATGAAGCGCAAAAGCCAAACCCATTTGCCGTATTAGCCAGCTTAAAGCGTAAGTAATAGGTCTTCCCC
GCGGACGCAGGGATAAACCGTAATTGAGGAGTAAGGTCCATGGCCGTACAACAGAATAAACCAACCCGTTCCA
AACGTGGCATGCGTCGTTCCCATGACGCTCTGACCGCAGTCACCAGCCTGTCTGTAGACAAAACTTCTGGTGA
AAAACACCTGCGTCACCACATCACTGCCGACGGTTACTACCGCGGCCGTAAGGTCATCGCTAAGTAATCGCGC
GATACTCGATGCCTTTTGCATCACAGGCGTGCCTGTGGTGTAAAAGCCGAAGAGTACAAGCGTGATAAAGCTT
AGTGAGGATTTCTCCGCGTAAGCGGAGAACACCGAACCAGGCAGCGACGATACCTTGACACGTCTAACCCTGG
CGTTAGATGTCATGGGGGGCGATTTTGGTCCTTCCGTGACGGTGCCTGCAGCATTGCAGGCACTGAACGCTAA
TTCGCAGCTCACTCTTCTTTTAGTCGGGAATCCCGATATTATCACGCCATTACTTGCCAAAGCTGACTTTGAA
CAACGTTCGCGTCTGCAGATTATCCCTGCACAGTCAGTTATTGCCAGTGATGCCCGGCCTTCGCAGGCGATCC
GCGCCAGTCGCGGGACATCCATGCGTGTGGCCCTGGAGCTTGTGAAAGAAGGCCGAGCCGAAGCTTGCGTTAG
CGCCGGTAATACCGGGGCGCTGATGGGGCTGGCGAAATTATTGCTCAAGCCTCTTGAGGGGATTGAGCGTCCG
GCGTTGGTGACGGTATTACCGCACCAGCAAAAGGGCAAAACGGTAGTGCTTGATTTGGGGGCCAACGTCGACT
GTGACAGTACCATGCTGGTGCAGTTTGCCGTGATGGGCGCCGTTTTGGCGGAAGAGGTCGTCGGGATCAAAAA
TCCCCGCGTGGCGCTACTCAATATCGGGGAAGAAGAAACCAAAGGCCTCGACAGTATTCGCGAAGCCTCTTTA
ATGTTAAAAACAGTCCCAACCATCAATTACATCGGCTACCTTGAGGCGAATGAGTTACTGACGGGAAAAACCG
ATGTACTGGTGTGTGACGGCTTTACAGGCAATGTCACACTTAAAACGATGGAGGGGGTTGTCAGAATGTTCCT
TTCACTGCTGAAATCACAGGGCGAGGGGAAAAAACGGTCGTGGTGGCTGCTGTTATTAAAACGTTGGTTACAA
AAAAGCCTGACGAGGCGATTCAGTCACCTCAACCCCGACCAGTATAATGGCGCCTGTCTGTTAGGATTGCGCG
GCACGGTGATAAAAAGTCATGGTGCGGCCAATCAGCGAGCATTTGCAGTCGCGATTGAACAGGCAGTGCAGGC
GGTGCAGCGACAAGTTCCTCAGCGAATTGCCGCTCGCCTGGAATCTGTATACCCAGCCGGATTTGAACCGCTG
GACGATGGCAAAGGCGTAAACCTGCGGGCGCACAGATAATATCGCTGTCTCCCCGGCGCCGCCAGCGTGCCAG
ACGTTTACAGGTAACGGTATATAACCGAAAAGTGACTGAGCGTACATGTATACGAAGATTATTGGTACTGGCA
GCTATCTGCCCGAACAAGTGCGGACTAACGCCGATCTGGAAAAAATGGTTGAGACCTCTGACGAGTGGATTGT
CACTCGTACAGGTATTCGTGAACGCCATATCGCCGCGCCTAATGAAACTGTCGCGACGATGGGCTTTACCGCT
GCGAATCGCGCGATTGAGATGGCGGGGATCGATAAAGACCAAATTGGCTTGATTGTGGTGGCTACCACATCAG
CAACGCATGCATTTCCAAGCGCGGCATGTCAGATTCAAAGTATGCTCGGTATTAAAGGTTGCCCGGCGTTTGA
TGTCGCGGCAGCGTGCGCAGGTTTCACCTACGCGTTAAGCATCGCCGACCAGTACGTTAAATCCGGCGCGGTT
AAACACGCGCTGGTGGTCGGTTCCGATGTATTAGCCCGCACTTGCGATCCTGGCGATCGCGGTACGATCATTA
TTTTCGGCGATGGCGCAGGCGCGGCCGTACTGAGCGCTTCTGAAGAACCGGGTATTATCTCCACTCATCTTCA
TGCCGATGGCCGTTACGGTGAATTACTGACCCTGCCGAATGCCGATCGCGTAAATCCGGATAACCCGATTTAC
CTGACGATGGCGGGCAATGAAGTCTTTAAAGTGGCGGTCACTGAACTGGCGCATATTGTCGATGAGACGCTGG
CGGCTAATAACCTGGATCGCTCAGAACTCGATTGGCTGGTGCCGCATCAGGCTAACCTGCGTATCATTAGCGC
GACAGCGAAAAAACTCGGCATGTCGATGGACAATGTCGTCGTCACGCTGGACAGGCACGGCAATACCTCCGCG
GCTTCTGTGCCGTGCGCGCTGGATGAAGCCGTGCGTGACGGGCGAATTAAAGCCGGTCAGCTGGTATTGCTTG
AAGCCTTCGGGGGTGGATTCACCTGGGGCTCCGCGCTGATTCGTTTCTAGTATAAGGATTTAAACATGACGCA
ATTTGCATTTGTGTTCCCCGGTCAGGGTTCTCAGAGCGTTGGGATGTTGGCCGAGATGGCGGCAAATTACCCT
ATCGTAGAAGAAACGTTTGCTGAAGCTTCTGCGGCTCTGGGATATGATCTGTGGGCGCTCACCCAGCAAGGTC
CAGCGGAAGAACTGAATAAAACCTGGCAGACGCAGCCGGCGCTATTAACCGCTTCCGTCGCGCTTTGGCGCGT
TTGGCAGCAGCAGGGCGGTAAAATGCCTGCGTTAATGGCAGGTCACAGCCTGGGCGAATATTCCGCGCTGGTT
TGCGCTGGCGTCATCAACTTTGCTGATGCCGTTCGTCTGGTGGAAATGCGCGGTAAATTCATGCAGGAAGCGG
TTCCGGAAGGTACTGGCGGCATGTCTGCGATCATAGGGCTGGATGATGCCTCTATTGCTAAAGCCTGTGAAGA
ATCTGCCGAAGGGCAGGTTGTTTCACCGGTTAACTTTAACTCGCCGGGACAGGTGGTTATCGCCGGGCATAAA
GAGGCGGTAGAACGCGCAGGCGCAGCCTGTAAAGCCGCTGGCGCGAAACGCGCGCTGCCGCTGCCGGTGAGCG
TACCGTCGCACTGCGCGCTGATGAAACCAGCGGCAGATAAGCTGGCGGTTGAATTAGCCAAAATTACCTTTAA
CGTGCCAACGGTGCCGGTAGTAAACAACGTTGACGTGAAATGTGAAACCGATGCCGCCGCTATCCGCGATGCG
CTGGTTCGCCAGTTGTACAATCCGGTACAGTGGACGAAGAGCGTGGAATTTATCGCGGCGCAGGGCGTTGAAC

ATCTTTATGAAGTGGGTCCAGGTAAAGTCCTCACTGGTCTGACGAAACGTATTGTCGACACCCTGACAGCGTC
GGCGCTGAACGAGCCGGCGGCGCTGTCTGCGGCACTTACGCAATAAAAGAGGAAAACCATGAGCTTTGAAGGA
AAGATTGCGCTGGTGACTGGTGCAAGCCGTGGCATAGGCCGCGCAATTGCAGAGACTCTCGTTGCCCGCGGCG
CGAAAGTTATCGGGACTGCGACCAGTGAAAATGGTGCGAAGAACATTAGCGACTATTTAGGTGCTAACGGGAA
AGGTTTGATGCTGAATGTGACCGATCCTGCATCTATTGAATCTGTTCTGGAAAATATTCGCGCAGAATTTGGT
GAAGTGGATATCCTGGTTAATAATGCCGGTATCACTCGTGATAATCTGTTGATGCGAATGAAAGATGATGAGT
GGAACGATATTATCGAAACCAATTTATCATCCGTTTTCCGCCTGTCAAAAGCGGTAATGCGCGCTATGATGAA
AAAGCGTTGTGGTCGCATTATCACTATTGGTTCTGTGGTTGGTACCATGGGAAATGCAGGTCAGGCAAACTAC
GCTGCGGCGAAAGCGGGCCTGATCGGTTTCAGTAAATCACTGGCGCGTGAAGTTGCGTCCCGTGGTATTACTG
TCAATGTTGTGGCTCCGGGTTTTATTGAAACGGACATGACGCGTGCGCTGTCTGACGATCAGCGTGCGGGTAT
CCTGGCGCAGGTGCCTGCGGGTCGCCTCGGCGGCGCTCAGGAAATCGCCAGTGCGGTTGCATTTTTAGCCTCT
GACGAAGCGAGTTACATCACTGGTGAGACTCTGCACGTCAACGGCGGAATGTACATGGTTTAATCACGATTGA
AAAATATTTGCGTTATTAGGGCGAAAGGCCGCAAAATAACGTAAAATCGTGGTAAGACCTGCCGGGATTTAGT
TGCAAATTTTTCAACATTTTATACACTACGAAAACCATCGCGAAAGCGAGTTTTGATAGGAAATTTAAGAGTA
TGAGCACTATCGAAGAACGCGTTAAGAAAATTATCGGCGAACAGCTGGGCGTTAAGCAGGAAGAAGTAACCAA
CAATGCTTCTTTCGTTGAAGACCTGGGCGCAGATTCTCTTGACACCGTTGAGCTGGTAATGGCTCTGGAAGAA
GAGTTTGATACTGAGATTCCGGACGAAGAAGCTGAGAAAATCACCACCGTTCAGGCTGCCATTGATTACATCA
ACGGCCACCAGGCGTAAGTGAACATCTCCAGGCGGTCGTTCGACCGCCTGAGTTTTATCTTTTTGTCCCACTA
GAATCATTTTCCCTCCCTGGAGGACAACCGTGTCTAAGCGTCGTGTAGTTGTGACCGGACTGGGCATGTTGTC
TCCTGTCGGCAATACCGTAGAGTCTACCTGGAAAGCTCTCCTTGCCGGTCAGAGTGGCATCAGCCTGATCGAC
CATTTCGATACTAGCGCCTATGCAACGAAATTTGCTGGCTTAGTAAAGGATTTTAACTGTGATGACATTATCT
CGCGCAAAGAACAGCGCAAGATGGATGCCTTCATTCAATATGGAATTGTCGCTGGCGTACAGGCCATGCAGGA
TTCTGGCCTTGAAGTGACGGAAGAGAACGCAAGCCGTATTGGCGCCGCTATCGGCTCCGGTATCGGTGGTCTC
GGTCTGATCGAAGAAAACCACAGTTCGCTGGTAAAAGGCGGACCGCGTAAGATTAGCCCGTTCTTTGTCCCGT
CGACGATTGTTAACATGGTAGCAGGCCATCTGACTATCATGTATGGCCTGCGTGGCCAAGCATCTCTATTGC
GACTGCCTGTACTTCAGGCGTGCATAACATCGGCCATGCCGCGCGTATCATCGCCTATGGCGATGCGGACGCG
ATGGTGGCAGGTGGCGCAGAAAAAGCCAGTACGCCGCTGGGTGTAGGCGGTTTTGGCGCAGCGCGCGCGCTGT
CGACCCGCAACGATAACCCACAGGCGGCGAGCCGTCCGTGGGATAAAGAACGCGATGGTTTTGTACTGGGCGA
CGGTGCCGGTATGTTGGTGCTTGAAGAGTACGAACATGCGAAAGCGCGTGGTGCGAAAATCTATGCTGAAATC
GTCGGCTTTGGTATGAGCAGCGATGCTTACCATATGACGTCGCCGCCGGAAAATGGCGCAGGCGCTGCGCTGG
CGATGGTTAACGCGTTGCGTGATGCCGCGATTGAGCCTGCTCAGATTGGCTACGTCAACGCGCACGGCACGTC
GACGCCTGCCGGCGACAAAGCTGAGACGCAGGCGGTTAAGTCAGTCTTTGGCGATGCCGCAAGCCGAGTGATG
GTGAGCTCGACCAAATCGATGACCGGCCATCTGTTGGGCGCCGCAGGCGCAGTAGAGTCTATTTTCTCTATTC
TGGCGTTACGCGATCAGGCCATTCCGCCAACCATTAATCTGGATAACCCGGATGAAGGTTGCGATCTGGACTT
CGTGCCGCATGAAGCGCGCCAGGTCAGCGATCTGGAGTACGCTCTGTGTAACTCCTTTGGCTTTGGCGGAACT
AACGGTTCCTTGATTTTTAAAAAGATCTAAGTTAAGCCAGAGACAGACATTAATAAAAAGGTCCGCTTGTCGG
GCCTTTTTTATCAGGCATATCACCTCTTGTTCTGCGTTATTCATCCTGCCACACTAGCAGGCGACGGATAAGG
AGCCACTATGTTTTTAATTAATGGCCATGCGCAGGATCAACTTGCTGTAAGCGACCGGGCGACGCAGTTCGGC
GATGGCAGTTTTACGACCGCACGTATTGTTGATGGCAACATTTGCCATCTGGAAGCGCATCTTCAGCGCTTGC
AGGTTGCCTGTGAAAAATTACGGATCGCTTTTAGCCATTGGGCGACCCTTCGGCAAGAGATGACAATGTTGGC
CACAGGGCATGATTCAGGCGTGTTGAAAGTGATCATTAGCCGTGGTAGCGGTGGCCGGGGATACAGCGCCATG
AATTGTCAGGCAGCTACCCGAATCCTCTCCGTTTCTGCTTATCCCGCTTATTATTCTCAGTGGCGTAAGCAAG
GCATCACTCTTACCCTTAGTCCCATACCGCTTGGGCGCAATCCTTATCTTGCCGGATTAAAACATCTGAACCG
CCTCGAACAGGTGTTGATTCGCTCTCATCTTGAGCAGACGGACGCCGATGAGGCGCTGGTTCTTGACAGCGAG
GGATGGGTTACGGAATGCTGTGCGGCTAATTTGTTCTGGCGTACAGGCGACATTGTTTTTACGCCGCGTCTGG
ATCAGGCCGGGGTGAACGGCATTATGCGACAATTTTGTTTACGCCAACTGGCGCAATCTCCTTTCCAGGTTCT
TGAAGTACAGGCGAGAGAGGAGGCAGTCAGGCAGGCGGATGAGATTATCATTTGCAACGCGCTAATGCCGATT
ATTCCCATACGCGCCTATCACGGGACGTCGTATTCTTCGCGAACACTGTTTCAATTTTTAGCCCCATTTTGTG
AGCATCCGAATTAGTTATGAAAAAATTGTCAGGCGTTTTCCTTCTGCTGTTGGTTGTGCTGGGTATTGCCGCG
GGCGTGGGGATGTGGAAAGTTCGCCATCTGGCGAACAGCACGTTACTTATTAAAAACGAGACTATCTTTACGC
TCAAGGCGGGAACGGGGCGGCTGGCGCTTGGCGACCAGCTTTATGATGAAAAAATCATTAATCGCCCCCGGGT
ATTTCAGTGGCTGCTGCGCGTGGAGCCTGAGTTATCGCACTTTAAAGCGGGAACTTACCGTTTTACGCCGGGG
ATGACCGTACGGGAGATGCTTGAGTTGCTGGAGAGCGGCAAAGAAGCGCAATTCCCGTTGCGGTTTGTGGAAG
GGATGCGCCTTAGCGATTACCTGAAACAGTTACGAGAGGCGCCGTATATTCGCCATACATTGCCGGATGATGA

CTACGCCACTGTCGCTCAGGCATTAAAGCTTGCGCATCCGGAATGGGTAGAAGGGGGGTTCTGGCCCGATACC
TGGATGTATACCGCCAACACCAGCGATGTCGCTATTCTCAAGCGAGCGCACCAAAAGATGGTGAAAGCTGTCG
ATACTGTCTGGAAAGGCCGGGCCGAGGGGCTGCCTTATAAAGATCAGAACCAACTGGTGACAATGGCCTCGAT
TATTGAAAAAGAAACGGCTGTCGCCAGCGAACGCGATCAGGTGGCCTCAGTCTTTATTAATCGCCTGAGAATC
GGTATGCGCCTTCAGACCGATCCCACCGTGATTTACGGAATGGGGACGAGTTATAATGGTAACTTGTCGCGTG
CGGATCTGGAAAAGCCGACGGCTTATAACACGTATACCATAACCGGGCTGCCGCCAGGACCGATTGCATCGCC
CAGCGAAGCGTCATTGCAGGCGGCGGCGCATCCGGCGAAAACGCCGTATCTCTATTTTGTGGCCGACGGTAAA
GGCGGTCACACATTTAACACCAATCTTGCCAGCCATAATCGGTCAGTGCAGGAGTACCTGAAAGTGCTTAAGG
AAAAAAATGGGCAGTAATTATATCGTCATCGAGGGCCTGGAAGGCGCCGGAAAAACCACTGCGCGCGACGTGG
TGGTGGAGACCCTTGAGCAACTGGGTATTCGTAATATGATTTTTACCCGAGAGCCGGGCGGGACGCAACTTGC
CGAAAAACTAAGAAGTCTGGTGCTGGATATCCGATCGGTAGGCGACGAAGTGATTACCGATAAAGCGGAAGTG
CTGATGTTTTATGCCGCTCGCGTACAGCTCGTCGAAACGGTAATCAAACCCGCGCTGGCGCAAGGCATATGGG
TGATCGGCGATCGCCACGATCTCTCGACCCAGGCGTATCAGGGGGAGGGCGCGGCATTGATCAAACCATGCTG
GCGACACTACGCGATGCCGTGCTGGGCGATTTTCGTCCTGACCTGACGCTGTATCTGGATGTCACGCCGGAAG
TCGGGCTAAAACGCGCCAGAGCGCGCGGCGATTTGGATCGCATTGAGCAGGAATCTTTTGATTTCTTTAACCG
GACTCGCGCGCGCTATCTGGAACTGGCGGCGCAAGACTCGCGTATCCGCACCATTGACGCGACCCAGCCGCTG
GATGCCGTCATGCGCGACATTCGCGCCACGGTGACGAAGTGGGTGCAGGAGCAAGCGGCATGAAATGGTATCC
ATGGTTACGGCCCGCCTATGAAAAACTGGTAGAAAGTTACCAGGCGGGACGTGGGCACCATGCGCTACTGATT
CAGGCCTTACCTGGCATGGGCGACGAGGCGCTGTGCTATGCGCTGAGCCGTTATCTGTTATGCCAGCAGCCTG
AGGGCCACAAAAGCTGTGGGCATTGCCGTGGTTGTCAGCTTATGCAGGCTGGCACGCATCCGGACTATTACAC
GCTGACGCCGGATAAAGGCAAAAGCAGCCTCGGCGTAGATGCGGTACGCGAGGTCAGCGAGAAATTGTACGAG
CACTCCCGTCTGGGCGGCGCGAAGGTGGTGTGGATTGCGGATGCGGCGCTATTAACCGACGCCGCCGCCAATG
CGTTGCTCAAAACGCTGGAAGAGCCGCCGGAGCAAACCTGGTTTTTCCTTGCCAGCCCGGAGCCTGCTCGTCT
GTTGGCGACATTACGTAGCCGCTGTCGGCTGCATCATCTGGCGGCGCCGTCCGAATCGTATGCGATGTCATGG
CTGTCGCGAGAAGTGACGGCGTCACAAGAAGCATTGCTCACGGCGCTACGCCTGAATGCGGGGTCGCCCGGCG
CCGCGCTGGCGCTGTTGCAGTCTGAACGTTGGGCGCAGCGTGAGGCGCTGTGTCAGGCGTTGATGGACAGTTT
ACATACGGGCGACTGGTATGCGTTATTAACGGCGCTCAATCATGAGCAAGCCCCGGCGCGCCTGCATTGGCTG
GCGACGCTGCTGGTAGACGCGCTGAAACGTCAGCACGGCGCGTCGTACCTGACAAACGTTGATGCGGACGCGG
TTGTTGCCGCGCTGGCGGGGCCGCTATCCCCGGCGCGTATTCAGGCAATCCTGAACGATGTTTGCCATTGTCG
TGATCAATTACTTCATGTGACCGGATTAAATCGCGAACTGGTATTAACCGATCTCATTCTCCGCATCGAGCAT
TACCTGCAACCAGGCACCTTGTTGCCTGTTCCTCATCTTTGAGAGAGACATTATGTTTTTAGTCGACTCACAC
TGCCATCTTGATGGTCTGGATTATCAATCTTTGCATAAGGACGTGGACGATGTGCTGGCGAAAGCCGCCGCGC
GCGATGTGAAGTTCTGTTTAGCGGTGGCGACGACGCTGCCGGGGTATCGCCATATGCGCGAGCTGGTGGGCAA
GCGCGATAATGTGGTCTTTTCCTGCGGCGTGCATCCGTTAAATCAGGATGAAGCATATGACGTAGAAGAACTG
CGCCTCCTTGCTGCAGAAGACGATGTGGTGGCGATGGGCGAGACCGGGCTTGATTACTTTTACACGCCTGAGA
CCAAAATCCGCCAGCAGGCGTCTTTTATTCATCATATCCAGATTGGCCGCGAATTGCATAAACCGGTGATTGT
CCATACTCGCGACGCGCGTGCCGATACGCTGGCTATCCTGCGCGAAGAAAAAGTGACGGATTGCGGCGGCGTA
CTACACTGTTTTACAGAAGACAGAGAAACGGCCGGCAAATTGCTGGATCTGGGGTTTTATATCTCTTTTTCCG
GCATCGTAACGTTCCGTAACGCTGAACAGTTGCGTGATGCGGCGCGCTATGTGCCGCTGGATCGGTTATTAGT
CGAGACCGATTCTCCTTATCTGGCGCCGGTGCCGCATCGCGGTAAAGAGAACCAGCCTGCGATGGTACGCGAC
GTCGCAGAATATATGGCGGTATTGAAAGGGGTTGCCGTTGAAGAACTGGCGCAGATCACCACCGATAACTTTG
CTCGCCTGTTCCATATTGACGCGTCTCGCCTGTCATCAATCCGTTAAACGAGTTTTTTTAAAGCTCGTAATTA
ATAAGCAAAGCGCGTAAAGTTCACCGCCACAAAAGGGGCGGTGAACGAGCTTGTGGAAACATTCGGAAACTCA
TTTTGGCAGAATGTGATACTTTTTAGGCTATCTGGCGCTGAAACGTGATAGCCGTCAAACAAAATCAGACGTA
TTTATTTTACTCTGTGTAATAAATAAAAGGGCACTTAGATGTCCTGTCCACGGCGGGGTTCTCCCCCCTCGCC
AATGCGTGAGAACGTAGAAAAGCACAAATACTCAGGAGCACTCTCAATTATGTTTAAGAATGCATTTGCTAAC
CTGCAAAAGGTCGGTAAATCGCTGATGCTGCCGGTATCCGTACTCCCTATCGCAGGTATCCTGCTGGGTGTCG
GTTCCGCTAACTTCAGCTGGCTGCCAGCCGTTGTATCGCACGTTATGGCAGAAGCGGGCGGATCTGTTTTTGC
CAATATGCCGCTGATTTTCGCTATCGGCGTGGCGTTGGGCTTCACCAACAACGACGGCGTTTCGGCTCTGGCG
GCGGTAGTAGCGTATGGCATCATGGTGAAAACCATGGCTGTGGTTGCGCCGCTGGTTCTGCATTTACCTGCTG
AAGAGATCGCCGCTAAACACCTTGCTGATACGGGTGTTCTCGGGGGGATTATCTCCGGTGCGATCGCAGCGTA
TATGTTTAACCGCTTCTACCGTATCAAACTGCCTGAGTATCTGGGCTTCTTTGCGGGCAAACGCTTCGTGCCG
ATCATTTCGGGTCTGGCCGCTATTTTTACCGGTGTGGTGCTGTCTTTCGTATGGCCGCCAATCGGTACGGCTA
TCCAGGCGTTCTCGCAGTGGGCGGCTTATCAGAATCCGGTTGTCGCGTTTGGTATTTACGGTTTTATCGAGCG

TTGCCTGGTGCCGTTTGGTCTGCATCATATCTGGAACGTTCCTTTCCAGATGCAGATCGGCGAATACACCAAC
GCAGCGGGTCAGGTATTCCACGGCGACATTCCGCGCTATATGGCGGGCGACCGGACGGCGGGTATGTTGTCTG
GCGGCTTCCTGTTCAAAATGTACGGTCTGCCGGCTGCGGCCATTGCTATCTGGCACTCTGCTAAACCGGAAAA
CCGCGCAAAAGTGGGCGGTATCATGATTTCCGCCGCGCTGACCTCGTTTTTGACGGGTATTACCGAGCCTATC
GAGTTCTCCTTCATGTTCGTGGCGCCGATCCTGTACATCATTCACGCGATTCTGGCAGGTCTGGCATTCCCGA
TCTGTATCCTGCTGGGGATGCGTGACGGTACGTCGTTCTCCCACGGTCTGATCGACTTTATCGTGCTGTCCGG
TAACAGCAGCAAATTGTGGCTGTTCCCGATTGTTGGTGCCGGTTATGCGATTGTGTACTACACCGTGTTCCGT
GTGCTGATCAAAGCGCTGGATCTGAAAACGCCGGGTCGTGAAGACACTACTGATGACGCAAAAGCGGGCGCGA
CCAGCGAAATGGCGCCGGCTTTGGTTGCGGCGTTTGGCGGTAAAGAGAACATCACTAACCTGGATGCCTGTAT
CACGCGTCTGCGCGTGAGCGTAGCCGATGTAGCGAAAGTGGATCAGGCTGGCCTGAAGAAACTGGGTGCGGCA
GGCGTTGTCGTCGCGGGTTCCGGCGTTCAGGCTATTTTCGGTACGAAATCCGATAACCTGAAAACGGAAATGG
ATGAGTACATCCGTAACAGCTAAGCAGTAATGATGTTGGGGAGAAAAAAGGCAGCCATTCGGCTGCCTTTTTT
AATGGCGCTTTACATGCCGCTCTCCGACAGTCTGTCAGAACTGATAATTGGCGGTGAGGCTGACGTTACGCGG
CGCGCCGTAAACAATGGAACCATCAATATTGGTGTCGTAGGTTTTATCAAACAGGTTGTTGATGTTTCCCTGT
ACGGAGAAATTTTTCGTCACCTGATAGCGGGTAAACAGATCGACCAGCGCATAGCTGCCCTGTTCGGCGCGGA
AAGTGCCGTACGGCGTCGTGGTATCTTTGTAAACCCGATTCTGCCAGTTCACGCCGCCGCCGACCGTCAGCTC
CGGTATCGCCGGCAGACGATAGCGGGTGAATAACTTCACGCTGGTGCGCGGGAGATTAGGGTTCACGGCGTTA
CCTTCATTGTCCTCGGCAACATACCGGGTAGCCCCGAAAGTCATCTGCCAGTTATCGGTAATTGCGCCGTTCA
CTTCAAATTCGACGCCTTTGCTCACCGTTCCGTCAGTAGACTTCCAGGCAAACTCCCCATTACTGCCAGGGAT
GGGAATAGTGGTTGCCTGAGCGACGTTATTCTGCTCAATGCGGAACACGGACAACGTGGTGGTGAGGCGACTG
TTCATCCAGTCGGACTTCAGACCCGCTTCATAGTTATTACCGGTAATCGGAGCCAGATATTGACCCGCTTTGT
CGCGTTTATTCTGCGGCTGAAAGATAGAGGTATAACTGGCATAAGCGGACCAGTTGTCGTTGATATCATAAAT
CAGCCCGGCATACGGCGTGGTATGGTTTTTCTCCATGCTGTAGGTCAGTGTATCAACACGCCAGTTGGTATAA
CGAGCGCCGAGAATCAGATGCAGCGGGTCGGCCAGCGAAATGCGCGTAGCGGCATACAGCGATTTCATATGCG
TGGTATCGTCCTGCGCCAGATTTTGTGGCGCCCAGTGGGTTTGGGGGAAATTACCGTTAAAGGCGCTGAAGTT
GCCAATGTCGTCCGGAAAGACGTTCGCCCAGGCGCTAAAATAGCGGTTATTCTGTTTACTATAACTGCCGCCA
AACATCATATTGTGCTGACGGCCAAACAGCTCATAAGCGCCATCGGCAAAGAGATCTATGGCATCTACTTTGC
GCTTGCCGCTGTTCCAGCCCGTACCGCCGACCACGGGGTAGCTTGCGCCATAAGGACTCACCAGCGTACCTGT
CTCTTTATCGACTAACGCATCGATATACATCATTTACTGTCGAATTTGACCTCAGTATGGGTCGCATTCAGG
GTCGCCTGCCAGCTTTCGGCAAAACGCTGTTTAAGCGTGACGAAGAATTTATTTATCTCTTTGTTGTTATAAG
CCCAGTCCGGGGCGGTACTACGCGCGCGGTCATAGCTATTTTTGCTGCCATCGGTGTTCCAGCGCGGCAGACC
GCCCCAGGTTGGGCTGTTGACATCAATTTTTTGATATTCATAACCGGCTGAAAGGTTGGTGGTTGTACCCAGA
TCGGCATCGACAATTCCGGAAAAGAACGCTTTCTCACTGTTATAGCGGTCCAGCCAGGAATCATTATTCTGAT
AACCCGCCACGATGCGCCCACGGACATTGCCGTCCGCGGTGAGCGGACTTTGCAGATCCATGACGTAACGCTG
CTTATTCCAGCTACCGTATTCTGTGGAAACGTTACCTTTGAACTCCCGACTGGTGGCGTGTTTACGGATCATA
TTAATTGAGGCCGAAGGATTACCGGTCCCGGTCATCAGCCCATTCGCGCCGCGAACCACCTCCACGCGCTCAT
ACAGCGCGGTATCCGTTAGCGCATCGCCCAGATTCCAGCGGGACTCAAAATACGTTGGAATACCGTCAACCAT
ATAGTTGTCAATTTCAAACCCGCGCGAGTAATAGCTGATGCGATCGGAGTCAGCCTGGCTTCCGCTGATCCCT
AGCGTATTGGTCATCACTTCGCCCAGGGTTTGCAACTGCTGGTCTTCCATACGCTGCTGGCTGACAATACTGA
CCGACTGCGGGATATCGCGCTGAGTCATCGGCATTTTGGTGCCCGCTGCGGTCGTTTTCACGCTGTAATCCTG
TTCTTCGCGGTTTACGGCGTCAGCGGTCGTCGCGCCTTCGACAATAACGGTATCCTCAGCCGCGAAGGCGGCA
GGCATAAATGCCATCGCCATACCCATAGCTAACAGCGACGGTGCGGCGGTGGACGGAAGGTGTTTGTCCCGGC
GGTATTGAATGAAAGACATCTGAAAATCCTTGCTTGTTATTGGGGTAAGTTCAGGACGTATGACGTTGAACCT
TTTCATTCTTTGCTAGTTAATGTGTATAAAAATGCAAATGAGAAATATACGCATTTGTATTAAATGTGTAAAA
CGGTATAACCAGGGAAACTAGAAATATTTTTTAAGCGTACAAAGGCGTCATGGCAGGGGGATATGGCGGTAAG
TCCAGCATGTAGTCATTTTTCGCTTAATATATCGTGGGTTAAAGTTGAAAGAGCGTCGGTAACTCGCTCATAC
TCTATACTCGCAGTATGGCTCGGCGTAGCATGTGCGCTGCAAGGGCTATTATGAAAAAGGAAAACGTCGTGGC
AGAAGAAACTATATTCAGCAAAATTATTCGTCGTGAAATCCCTTCGGATATCGTTTATCAGGATGAGCTGGTC
ACCGCCTTTCGTGATATTTCACCGCAGGCGCCGACGCATATCTTGATTATTCCCAATATCCTGATCCCAACCG
TCAATGACGTGACGGCGGAACATGAACAGGCGTTAGGTCGGATGATCACCGTGGCGGCGAAAATCGCCGAACA
GGAAGGCATTGCCGAAGATGGGTATCGTTTAATCATGAATACCAACCGTCATGGGGGCCAGGAGGTTTATCAT
ATCCATATGCACCTACTGGGCGGCCGCCCGCTAGGCCCGATGCTGGCGCATAAAGGCTTGTAAGTATGATCAA
AGGATGCATCGCGGTTTCTCTGGGCTTGCTGCTGCTTACCGGCTGCCGGTCACACCCTGAAATTCCGGTAAGC
GACGCGCAGTCGCTGGTGATGGAGTCCACGGTTCTGGCGGCGGGCGTCACCGCAGAGCCGCCTGAACTGACGG

CGTCTGATATCCAGCCATCAGCCTCTTCCCGCGTCTATAACGAAAGGCAAGAGCCCATAACCGTTCATTATCG
GTTTTACTGGTATGACGCGCGGGGGCTGGAGATGCATCCGCTGGAGGCGCCGCGTAGCGTAACCATACCCGCG
CGTTCGTCGGTAACGCTATTTGGCAGCGCCAATTATCTGGGGGCGCATAAGGTCAGGCTTTATCTTTATTTGT
GAGGAGTGAATTTTGATGACAAAAATGCATCGCTACGCCGCCATTGCTGCGCTGGCGATATTTCTCTCCGGGT
GTATGGCGCAACGTCAACCTGCGCCAGTTGAAGAGGTAAAACCAGCGCCAGAACAACCCGCGCAGCCACCGCA
ACCTCCTGTTGTGCCGTCTGTGCCAACCATTCCACAGCAACCGGGGCCGATCGAACATGAAGATCAAACGGGC
CAGCCAGCGCCTAAGGTGCGCCACTATGACTGGAACGGCGCGATGCAACCGCTGGTCAGCAAAATGTTGCAGG
CTGATGGCGTGACGGCCGGAAGCGTATTGCTTGTCGACAGCGTGAATAACCGGACGAATGGGTCGTTAAATGC
GAACGAGGCAACGGAAACGTTACGTAATGCGCTGGCGAACAACGGTAAATTTACGCTGGTCTCCGTACAGCAA
CTCTCGATGGCAAAACAGCAGTTAGGGTTGTCTCCGCAGGATAGTCTGGGAACGCGCAGTAAAGCTATTGGCA
TTGCGCGCAATGTCGGGGCGCAATATGTACTTTACTCCAGCGCATCCGGTAACGTTAACGCGCCTGCATTACA
GATGCAGTTAATGCTGGTGCAAACGGGCGAGATAATCTGGTCAGGTAAAGGTGCGGTCCAACAACAATAATCC
CTTAACGCGCGACGAGATCCTGTCGCGCTATTTTCCCCAGTATCGTCCCGCCGTCGCCGCATCGCAGGGGCTA
AGCGGCGGGAGCTGTATTATTGCCCACGATACTCACCGTATCGTGCTGCGCCGTCATCACGACCCCGACGCTC
CACCAGCCCATTTTTTACGTCATTACCGCGCCTTATCCCAATTGCCGGCCAGTCTTGCGCCGCGAGCGCTTTT
TTATACGCCGGGCTGGATGGCGGTAGAATATCTGCATGGTGTGGTAAATTCCGCTCTGCCAGATGCCGACGAA
CTGGCGGCCTTACTGTATCATTTGCATCAACAGCCGCGTTTTGGCTGGCGTATCGCGCTATCGCCACTATTGG
CGCAGTACTGGTCGTGTTGCGATCCGGCGAGGCGTACGCCGTTTTGGTTGCGGCGGCTCAAACAGTTGCAAAA
AAACGGTGAACCTCGCCCGCTGCGGCTCGCGCCTTTGCATATGGATGTCCATGGCGACAATATAGTATTAACG
TCCGCCGGGTTGAGACTGATTGACTGGGAGTATGCCGGCGACGGCGATATTGCGTTGGAGCTGGCGGCAGTAT
GGGTTGAGGATGAACGCCAGCACCGACAACTGGCAGACGCGTATGCAGCGCGCGCGCGAATCGACGCCCGGCA
GCTATGGCGACAGATACGATTATGGCACCCCTGGGTCATTATGCTAAAAGCAGGGTGGTTCGAATACCGCTGG
CGACAAACCGGCGAGCAACAATTTATCAGGCTGGCCGATGAAACCTGGCGCCAGCTACGTATGAAAGGATAAG
GAGAGCAGTGTGGGCCCAGTAATGTTGAATGTCGAAGGATGTGAGCTGGATGCGGAGGAGCGCGAGATACTGG
CGCATCCGCTGGTTGGCGGGCTTATTCTGTTCACGCGTAATTACCATGACCCGGAACAGCTGCGCGAATTAGT
GCGCCAGATTCGGGCGGCATCGCGTAACCATCTGGTGGTGGCCGTCGATCAGGAAGGCGGGCGCGTACAGCGT
TTCCGTGAAGGGTTTACCCGCCTGCCCGCGGCGCAATCTTTTTTGCGCTGCATGGGCTGGAAGAGGGGGGCGA
CTGGCGCAGGAGGCTGGGTGGCTGATGGCCAGCGAAATGATCGCGATGGATATTGATATCAGTTTTGCGCCAG
TGCTGGACGTCGGGCATATCAGCGCGGCAATTGGCGAGCGTTCTTATCATGCCGATCCCGCAAAAGCGTTGGC
AATGGCAACACGCTTTATTGACGGTATGCATGACGCCGGGATGAAAACGACCGGTAAGCATTTCCCCGGCCAC
GGCGCGGTCACGGCGGACTCCCACAAAGAGACGCCGTGCGATCCACGTCCTGAAACGGACATCCGTGGCAAAG
ATATGTCGGTGTTCCGCACGCTGATTAGCGAGAATAAACTGGATGCGATTATGCCTGCGCACGTGATCTACCG
CGCTATCGATCCGCGTCCGGCAAGCGGTTCGCCGTACTGGCTGAAAACGGTGCTGCGTCAGGAGTTAGGCTTT
GATGGCGTCATTTTCTCCGACGATCTGTCGATGGAGGGCGCGGCGATTATGGGCAGCTATGCTGAGCGCGCTC
AGGCGTCGCTGGATGCGGGTTGCGATATGATTCTGGTCTGCAATAATCGTAAAGGCGCAGTCAGCGTGCTGGA
TAATCTGTCGCCGATCAAAGCAGAACGTGTTACACGTTTGTATCATAAAGGTTCATTTTCACGCCGGGAGCTC
ATGGACTCAGCACGCTGGAAAACAGCCAGCGCCCAGCTCAACCAGTTACATGAACGCTGGCAGGAAGAAAAAG
CAGGTCATTAACTCGAGTTTTCGTGAGGGTGCCAGATGATTATTTATTTACACGGTTTTGATTCAAATAGTCC
GGGCAACCATGAAAAAGTGTTGCAATTACAGTTTATCGACCCGGATGTTCGGCTGGTAAGCTACAGTACGCGG
CATCCTAAGCACGATATGCAGCATCTGCTGAAAGAAGTGGACAAAATGTTGCAGTTGAATGTGGACGAGCGCC
CTTTAATCTGTGGCGTGGGGCTGGGCGGTTACTGGGCGGAGCGGATTGGCTTTTTGTGCGATATCCGTCAGGT
GGTTTTTAACCCGAACCTGTTCCCCTATGAAAACATGGAGGGGAAAATCGATCGCCCGGAAGAATATGCGGAC
ATCGCTACAAAGTGCGTGACCAACTTTCGCGAAAAAAATCGCGATCGTTGTCTGGTGATCCTCTCGCGTCATG
ACGAAGCGCTGGACAGCCAACGCTCAGCGCAAGCGCTTCACCCGTATTATGAAATTGTGTGGGATGAGGAACA
GACTCACAAATTCAAAAATATTTCGCCGCATTTGCAACGTATCAAAGCGTTTAAAACGCTGGGATAACGCCAT
CCGGCAATATGGCGTTATCAGTGACCGGATAACCAGCCTTGCGGCTGGTTTTTTTATGCTCAATTCTCGCAAA
CTGTCTATCTTTTCAGCATGAAAACTTGATGCACATCAATTTTGGTATGACCAATGCACCGTTCATGCTATTC
TCATATCAGAAGCTTATTCTCATTAACGTAACTTGTTGTTAATTAAAGGCTATACTAATAACTTTTAATTAAC
AATTGGTTAATAAATTTAAGGGGGTCACGTTGACTACACCATTAAAAAAGATCGTGATTGTCGGCGGCGGCGC
TGGCGGGCTGGAAATGGCGACGCAGTTAGGCCATAAACTGGGGCGCAAGAAAAAAGCGAAAATCACGCTGGTA
GACAGAAATCACAGCCATCTGTGGAAACCATTGCTGCACGAAGTGGCGACTGGCTCTCTGGACGAAGGCGTGG
ATGCGCTGAGCTATCTGGCTCATGCGCGTAATCATGGTTTCCAGTTCCAGCTGGGGTCGGTGATGGATATCGA
TCGCGAAGCGAAGACCATCACCATTGCCGAGTTGCGTGATGAAAAGGGCGAACTGCTGGTGCCGGAGCGCAAA
ATCGCGTATGACACGCTGGTGATGGCGCTGGGCAGCACCTCTAATGATTTCAACACGCCGGGCGTGAAAGAGC

ACTGTATCTTCCTCGATAACCCGCATCAGGCGCGCCGTTTTCACCAGGAGATGCTGAACCTGTTCCTCAAGTA
TTCGGCTAATCTGGGCGCGAACGGTAAGGTCAATATCGCCATCGTTGGCGGCGGTGCGACGGGGGTTGAGCTG
TCGGCTGAACTGCATAATGCGGTAAAACAGCTGCATAGCTATGGTTATAAGGGGCTGACCAACGACGCGCTGA
ACGTGACGTTGGTTGAGGCTGGCGAGCGTATTCTGCCTGCGTTGCCGCCGCGTATCTCCAGCGCCGCGCATAA
TGAACTGACCAAACTGGGCGTTCGCGTGCTGACGCAAACTATGGTCACCAGCGCCGACGAAGGCGGTCTGCAT
ACGAAAGAAGGTGAATATATTCAGGCCGATCTGATGGTGTGGGCGGCGGGTATCAAAGCGCCAGATTTTATGA
AAGAGATTGGTGGTCTGGAAACGAACCGCATTAACCAACTGGTGGTGGAACCGACGCTGCAAACCACGCGCGA
TCCTGATATTTATGCGATTGGCGACTGCGCTTCCTGCGCACGTCCGGAGGGAGGTTTTGTGCCGCCTCGCGCT
CAGGCGGCGCATCAGATGGCTACCTGCGCGATGAATAACATTCTGGCGCAGATGAATGGCAAACCGCTGAAAG
CTTACCAGTATAAAGATCATGGATCGCTGGTCTCTCTGTCCAACTTCTCTACCGTGGGTAGTCTGATGGGGAA
CCTGACCCGCGGTTCAATGATGATTGAAGGACGTATCGCCCGCTTCGTGTATATCTCGCTATACCGTATGCAC
CAGATAGCGCTGCATGGATACTTTAAAACCGGCCTGATGATGCTGGTGGGCAGTATTAACCGCGTCATTCGTC
CGCGTCTGAAACTGCATTAATTTCTCTGCGCCTCACCGGCTGCTCGCGCCGGTGAGCGCTTCTCCATCTCTTG
CTTCGGAGTTGTCCGAATCCTTCAGCTTTACCGCCTCATCCCGCATTTTTCATCCTCATTCCGATTGGCTTTG
TTCGCATACTGGTTGCAAAATTGTTACCAATAGCAACGAAGGAGGAGCTCCCGTGAATAAATCAATGTTGGCG
GGTATAGGGATTGGCGTAGCGGCTGCGCTGGGTGTGGCGGCAGTGGCCAGTCTCAACGTATTTGATCGTAGTC
CGCAATATGCGCAGGTGATATCGGCTAAACCGATTAAAGAGACGGTAAAAACGCCGCGTCAGGAGTGCCGTAA
TGTCACGGTCACGCATCGCAGACCGGTTCAGAACGAAAACCGTATTGCCGGCTCGGTGCTTGGCGCAGTAGCA
GGCGGCGTGATTGGACATCAATTTGGCGGCGGTCGTGGTAAAGATGTGGCGACGGTCGTCGGGGCGCTGGGCG
GCGGTTATGCTGGCAATCAAATCCAGGGGTCAATGCAGGAAAGTGATACTTACACTACCACGCAGCAACGCTG
TAAGACGGTGTATGACAAATCTGAAAAGATGCTTGGCTACGACGTGACCTACAAAATTGGCGATCAGCAGGGC
AAAATCCGCATGGATAAAGACCCCGGAACGCAAATTCCCCTCGACGGCAATGGCCAACTGGTTCTGAATAACA
AAGCATAAAAAAGCTGTACTCTGCAAGTTAGCCCCTCATTCGCTCAGGCTGAGGGGCTTTTTTTATCCGAAGC
CGCGTCGTTCAGGACTTAAGTAGTTCAGGCCAGATGCGCAGAGTCGTACGCGTAATTTGCATCAGTTGTTCGA
AGGTGGCTCCTTCACGGGCGCTGATCGACATTCCCTGTAAAATACAGTTTATATATTCCGCGAGCGCCTGCGG
GTTACAGTGCGCCGGAATTTCACCGCGCTCCTGACGTTGGCGCAGAAACTGGATGAGCGTCTGTTCCTGCATG
GCGTGGCGTGATTTTACCGTGCGGGCGATATCGCGCGAGGAGGCGGCGAGGGTGGCGGACGTATTAATCATAA
AGCAGCCAGCCGGCGTGTCTGTACTGGTAAAGCAGGCGGCAATTTCTGTGAAGTAATCTTCCAGCGCCGATTC
CACGCTTTTCTCTTCACAAAAGAGCTGGGCTTCGTGCTTTGAGGCAAAGCGAGTGATGTAGCGATCCAGGACA
GCGCGAAATAATCCTTCTTTATTAGTGAATTCCGCATAGAGAGTAGGCGCTTTCGCCCCGGTAGCCTCTACGA
GATCGGCAAGCGAAGTTGCTTCATAGCCGTGCTGCCAGAAGAGAGTCATGGCCTTATCAAGCGCGGCTTCCCT
GTCGAACACTTTTGGTCGGCCACGGCTTTTCTTCGCGCAACTCGTGGTATCCGTTGTCATGATGCCGTTGTAC
CTGTTTAAAGAGCGGTCTCTCTATTATAAAAACAAACCACTTCTGGCTCCAGAGTATTCATTAGAAAAATAAA
TCAATTCTATTCACATGAAAAATAACATCTTTATAAATTATATAACGATCATTATAAAAAGAAGTTGACGTGT
GATGTAGATCACATCTATGATTAATTTATCGATCGTTAAGTTAATGCTTACGACACCCATTCATCTGCTAAAG
GTCATCACTATGAAAAACGTAAAAAACCTCATCGCTGCTGCCGTACTGAGTTCGCTCTCATTCGCCAGCTTCG
CCGCCGTTGAAGTTCAGGCAACGCCAGAAGGTCAACAGAAGTTCGGTACCATTTCCGCGAATGGCGGCACGAA
TCTGGGCTCGCTGGAAGATCAGCTGGCGCAAAAAGCGCAGGAGATGGGGGCGAAGTCTTTCCGTATTACCTCT
GTTACCGGCCCGAATACCCTTCACGGTACGGCGGTAATCTATAAGTAAGCATTAACCCTCATTAATGCCGCTG
CGATTGCCACAATAAAAAAGAAAGCCTCGCGATGCATCGCGAGGCTTTTTTGTCGGTCAGCTATTCACGCTTA
CAGCGACTGCGGTTTCGCCTCTGCTTGACGGGTTACATCCACCGGCATTCCGGAACGCACTTCCATAACATGT
TCCATCACCGCAGCATCGGTCTGCGGCGCGTCCTTAAAGGCCTGCATCGGGCCATTCAGCACAATCGGCAGCA
CCTGCGGATCGTCGCCTATATTTTTAGACAGCGGCTGATGAACTTCCACCAGTCGCACGCCGCCTGGCTCCAC
CGAGGCTTTAATCGGCGTATTGATAATATTCACTTTGGTTCCCGGCGTGACCTGACGGAACAATGTCTCTATA
TCACCGTCGCGCAGACGGATACAGCCGGAACTGACGCGCATACCAATGCCAAAATCGGCATTCGTGCCATGTA
ACAGATAGACGCCGCCGTAGGCCGCTAAACGAATCGCGTGATGCCCCATTGGGTTATCGGGACCGGCAGGCAC
CACGGCGGGGAGATCGATGCCTTGCGCTTTATAACGTGCGCGAATATTGGCGGTCGGCGTCCAGGTTGGATTG
GCGCGTTTATCCGAAATGGTCGTCACCATCGTTGGCGTCAGGGTATCGCCGCCCAGTTGGCCAATCCCGATAG
GGTAGACCGTGACCGTATTTTTCCCCGGTTGATAATAGTAAAGCCGTAATTCCGCGAGGTTAATGACAATCCC
TTCACGTGGCGCGTCCGGTAGCAGCGTTTGCAGCGGAATCGTCAGCACGCTGCCTGCGCGTGGCACGTAAGGA
TCAATGCCGGGGTTGGCCTGTAACAGGGCTAAAAAGCCCACGTTATATTTTTTGGCGATCGCCTCCAGCGAGC
CGCCGTCGTCTTCAACGACATGGAATTTATTTTCTCCCACCAGTCGACTGCCGGGGGGCGGAAGAGGCCACGT
ATTCGCCTGCGCGGGAAGGGCAAGCATGATGGTCGCGGTAAGCGCAAACAACGCCCACCAGCGAGAGAGTCGC
GTATTGTTGAACATTATAATAAGCCCATGATTATTATGTGGTTATTTTTATAAAAGAGGAAATAAGAATTATG

GCGGTTGGCTGTGTCTGGAACTGCAGGGAAGTGCAAAGAATTTGTAAAGATTAGGCCGGACGCAGAGTAATGA
CGCCCGGCCTGACGCTTATGCAATAGCGTTTTCTTCCAGTTGCTGCATAAACTGGCGTACCCAGTCTATACGC
GTTTTACGTTCGCTTAAATCCTGAATAAACTTGAGGCGCGTCGGGCCATCCAGACGGAAATGCTGCGGCTGTT
TTTGCAGCAACCCGATAAGCCAGGCGGGGTCAACGTGGTTTTTCTCGGCGAACTCAATCGTTCCGCCTTTCTC
GTTACCCTCCAGTTTTCGGATGCCGAGCTTTTGCGCCTGTTGGCGCAGGCGGGCGATATCCAGCAGATTACGC
GCCGGATCGGGCAGCAGGCCGAAGCGGTCGATCAATTCCACTTTGATCTCCTCCAGCTCGTTTTCGTTCTTCG
CGCTGGCAATGCGTTTATAGAATGACAGCCGGGTATTAACATCCGGAATAAAATCATCTGGCAGCAACGACGG
CATTCGCAGCTCGACTTCCGTTTGCTGACTGGTGAGATCTTCCAGCGAGGGTTCGCGGCCTGCTTTCAGCGCA
TCGACGGCGTTTTCCAGCAGTTCCATGTAGAGTGAGAAGCCAATGGTTTCCATTGAGCCGCTTTGCTCTTCGC
CAAGCAGTTCGCCCGCGCCGCGGATCTCCAGGTCGTGGGTCGCCAGCGCAAACCCGGCGCCTAAATCCTCCAG
CGACGCAATCGCCTCCAGCCGTTTTTGCGCATCGGTGGTCATGGCCTTAGGATGTGGCGTCAACAGCCAGGCG
TAGGCCTGGTGGTGCGAGCGCCCAACGCGGCCACGCAGCTGGTGCAACTGCGCGAGGCCGAAATGATCCGCGC
GTTCAATGATGATCGTGTTAGCGGTGGGAATGTCGATCCCCGTTTCGATAATGGTAGTACACACCAGCACGTT
AAAACGTTGATGGTGGAAATCGTTCATGACCCGTTCCAGCTCACGTTCACGCATCTGCCCGTGGCCTATAGCG
ATTCGCGCTTCCGGTACCAGTTCCGCCAGCCGCTCTGCGGCTTTCTGAATATTTTCGACGTCATTGTACAAAT
AGTAAACCTGACCGCCGCGCAGGATTTCACGCAGAATCGCTTCCCTTACTACCAGGCTATCATACTCGCGAAC
AAAGGTTTTTACCGCCAGACGACGGGCAGGGGGAGTGGCGATAATGGAGAGATCGCGCATACCGCTCATCGCC
ATATTCAGCGTCCGCGGGATCGGCGTCGCCGTGAGCGTCAGAATATCGACATCGGCGCGCATCGCCTTAATTC
GCTCTTTGTGGCGCACGCCGAAACGGTGCTCTTCATCGACAATCAGCAGGCCCAGGTCGCGCAGCTTCACATC
GCTTTGTAGCAGTTTATGCGTACCGATCAGAATATCGATCTTACCCTCGGCGGCTTCCGCCAGGATTTGTGTC
TGCTCTTTGGCGCTGCGAAAGCGGGACAGCATCTCAATGCGTACCGGCCAGTTGGCGAAACGGTCGCGGAAGT
TATCATAGTGTTGCTGCGCCAGCAGCGTGGTTGGCACCAGTACCGCTACTTGTTTATGGTTTTCCACTGCCAG
GAACGCGGCGCGCATCGCCACTTCGGTTTTGCCGAAGCCGACGTCGCCGCACACCAGCCTGTCCATCGCCAGC
GGTTGGCACATATCGCTCAATACGGCGTTAATCGCCTGTGCCTGATCGGGCGTCGTTTCAAACGGGAAGCTGT
CGCAGAATAACTGATACTGCTCACGATCATGCTTAAAGGCAAACCCCTCTTTTGCCGCCCGTTGCGCATAAAT
ATCCAACAGTTCCGCTGCGACATCGCGCACTTTTTCCGCCGCTTTCTGCCGGGCGCGCGACCAGGCGTCGCCG
CCCAGTTTATGCAGCGGCGCACTTTCTTCCGCGCCGCCGGCATAGCGGCTAATCAGATGCAATGAGGAGACCG
GCACATACAGTTTGGCGTCATTGGCGTAGGTGAGCATCAGATACTCACCCTTGATGCCGCCAGCCTCCAGAGT
GGTCATGCCGGCATAGCGTCCGACGCCGTGCTCCAGATGCACTACCGGCTGACCGACGTGGAGCTCCGCCAGG
TTACGAATCAACGTATCCGGGTTGATCGTGCGGCGGGAATCCAGGCGGCGACGCGCCACCCGCTCACCCAAAA
GATCGCTTTCACAAATCAGCGCCAGATTGCGTTGGGTGTCGATAAAGCCATGTTCTGCGGCGCCAATCATCAG
ATAGCGTCCCGCATCCTGCGCCTCGTCCTGGCGTAATATCCGCTTCGGCGCGATTTTTATGCGAGCCAGGAGC
TCTCCCAGCGCTTCGCGGCGTCCTTCGCTTTCCACAGAAAAAATCACCGGGCCGCTGAAAGATTCGAGGAACT
TACGCAGCGCGTCCAGCGGCGCTTTCTGCTGTGCCTGGATAGCCAAATCCGGTAATTTCTGGAAACCAAGATT
GGTATTGGCGGCTTTTTCCGGCAGATGGTCGGTTTTAAGCTGGAGGCGAGGCCAGCGTTTAAGCTCGGAAAAG
AGCTCATCTACCCGTAGCCAGAGGGCTTCCGGCGGCAAAAGCGGGCGCATAGGATCGACGCCGCGATTTTCAA
AACGCGCCAGCGTATCGGCCTGAAAGCGTTCGGCGCTGGTTTCCAGCGAGCCAGTATTCACCACCAGAGTATT
GGCGGGGAAATAGCTAAATAATGGCGGTAACGGTTCGCTGAAAAAGAGCGGCTGCCAGTATTCGATCCCGGCG
GGCAAGGTGCCTTTGCTGACCTGCTGATAGATGTGTTCGGCGTCGCGTTTTACCTCAAAGGTATCGCGCCACT
GGCTACGGAAAAGCTCAATAGCGGCTTTGTCGGTAGGAAATTCGTGCGCGGGCAACAAATTGATGGCCTCGAC
CTCCTCCAGCGTACGCTGGGTATCCGCGTCAAACAGACGCAGGCTGTCGATTTCATCATCAAAAAAGTCGAGG
CGATAAGGCTGTTCGCTGCCCATCGGAAAGAGATCCAACAGCGCGCCGCGCGTCGCGTATTCGCCATGCTCCA
TCACCTGATCGACATGCCGATAACCGGCGCTGTCAAGCTGCGCGCGCAGAGCGTCGCGGGACAGCCGCTGGCC
TTTTTTCATCACCAGCGCATGACCATGCAGATAGCTATGCGGACAAACGCGCTGCATCAGGGTGTTTACCGGT
ACAATCAAGACGCCGCGCTGCATCGACGGCAACTGGTAGAGCGTGGACAGGCGCGAAGAGATAATTTCCTGAT
GCGGGGAGAAGCTGTCATACGGTAGCGTTTCCCAGTCCGCCAGGTTCATCACCATCTGATCGGTAAACTGGCG
GATTTCATCATGCAATCGCAGAGCATTTTGCATGTCAGGCGCTATGAGTACCACCGGACCGGCATGGCGTTCG
GCTATCTCCGCCACCAGCGTAGCGCAGGCGGCACCGGTAAGTTCGCCCAACTGGCGCTGATCGCCCGCTTTTG
TCGGCAGCGTGTAACGTTGTTTTTCAGGCATAACGGTTGTCAGTATCTCTTTTTGTTTTACGCCGCACTCTAC
GCGTCACCCTTCAGGCTGCTTCATGGTTGGCTGCGCGCCGGGACAGCATGAAGGACTCAGGTATTCAGGGGCA
TATGACTGATAAGCAATGTTCTTTATTATCCGCGATCGTTGTTTATTAGCAAATTGAATCCGCCGGATAACGC
GCCGAACAGGTAAAGTATGAACATGTAAGTGACTTGATTTGTCGGCCTTTTTCAGAATAACGCTGACGAACTC
TTACGGTTCAGACATTTACGCATTCCGCTTTCGCTTATATACTCGGGACTTTGCTAACAGCAACCAGACGGAT
TACATGTATCAACCTGTCGCTCTATTCATCGGCCTGCGTTACATGCGCGGGCGTGCAGCAGATCGCTTCGGTC

GCTTCGTTTCCTGGCTTTCCACTATCGGCATTACCCTGGGGGTGATGGCGCTGGTCACGGTATTGTCGGTGAT
GAACGGTTTTGAGCGCGAACTGCAAAATAACATTCTTGGTTTGATGCCTCAGGCCATCCTCTCTGCTGAACAT
GGTTCTCTTAACCCGAGTCAGATGCCGGAAAAAGCGGTGAATCTGCAAGGCGTTAACCGTATTGCGCCGCTGA
CGACGGGCGATGTCGTACTGCAAAGCGCGCGTAGCGTTGCGGTTGGCGTGATGTTGGGTATCGATCCGGCGCA
AAAAGATCCGTTAACGCCGTATCTGGTCAATGTGAAGCAAAGCGAATTACAGCCGGGTAAGTATAATGTCATC
CTCGGCGAGCAGCTCGCCGGACAGTTAGGGGTCAACCGTGGCGATCAGATTCGGCTAATGGTGCCATCTGCCA
GCCAGTTTACGCCAATGGGACGCCTGCCCAGCCAGCGTCTGTTTACGGTGATTGGCACATTTGCCGCCAATAG
CGAGGTCGATGGCTATGAAATGCTGGTCAATATTCAGGACGCGTCGCGTTTAATGCGCTATCCGGCAGGCAAT
ATTACCGGCTGGCGTCTGTGGCTGGATGAACCGCTGCAAGTCGATACCCTCAGTCAACAGACGCTGCCGCAAG
GCACGAAGTGGCAGGACTGGCGCGAACGCAAAGGCGAGTTGTTCCAGGCTGTGCGCATGGAAAAGAACATGAT
GGGATTGTTGCTGAGTCTCATCGTGGCAGTCGCGGCTTTTAACATTATTACTTCGTTGGGCCTGATGGTCATG
GAGAAGCAGGGCGAAGTGGCGATTTTACAAACTCAGGGGCTGACGCCGCGACAAATTATGATGGTCTTTATGG
TGCAGGGCGCCAGCGCCGGGATTATCGGCGCGCTGTTGGGCGCCGCGCTGGGCGCATTGCTTGCCAGCCAACT
GAATAACCTGATGCCGATCATCGGCGCGTTTCTGGATGGCGCTGCGTTGCCCGTGGCGATAGAGCCGTTACAG
GTGATCGTTATCGCTCTGGTGGCAATGGCCATCGCGCTGCTTTCTACGCTTTATCCTTCCTGGCGCGCTGCCG
CCACTCAACCCGCTGAGGCTTTACGTTATGAATAAGATCCTGTTGCAATGCGACAACCTGTGCAAACGCTATC
AGGAAGGCACCGTACAAACCGACGTGTTGCACGATGTCAGCTTCAGTATCGGCGAAGGCGAAATGATGGCGAT
CGTCGGCAGTTCCGGTTCGGGTAAAAGTACGTTGCTGCATCTGTTAGGCGGGCTGGATACGCCCACCTCCGGC
GATGTGATTTTTAGCGGCCAGCCGATGAGCAAACTCTCTTCTGCGGCAAAAGCGGAATTACGTAACCAGAAAC
TGGGGTTTATCTATCAGTTTCATCATCTCCTGCCGGATTTTACGGCGCTGGAAAACGTGGCGATGCCATTATT
GATTGGCAAAAAGAAACCGGCGGAGATTGACGCTCGGGCGCGAGAAATGCTGCACGCCGTAGGGCTGGAGCAT
CGCGCAACCCATCGACCTTCGGAACTTTCCGGCGGTGAGCGCCAGCGTGTCGCGATAGCGCGCGCGTTGGTGA
ATAACCCGCGTCTGGTACTGGCGGATGAGCCGACCGGTAATCTGGATGCGCGTAATGCCGACAGTATTTTTGA
GCTTCTGGGCGAGTTAAATCGTCTGCAGGGAACCGCGTTTCTGGTGGTGACTCACGATTTGCAACTGGCGAAA
CGTATGAGCCGCCAGCTTGAGATGCGCGATGGCCGCCTGACGGCGGAACTGAGCCTGATGGGGGCGGAGTAAT
GGCGTCGCCTTTATCATTGTTAATTGGCTTGCGCTTCAGCCGTGGGCGGCGGCGCGGCGGTATGGTTTCGCTC
ATTTCCGTCATTTCTACCATCGGTATCGCGCTGGGCGTGGCGGTATTGATTGTCGGTTTAAGCGCGATGAACG
GTTTCGAACGCGAGTTGAATAACCGTATTCTGGCGGTGGTTCCGCACGGGGAGATTGAAGCGGTCAATCAGCC
GTGGACCAACTGGCGTGAAGCGCTGGCAAAAGTGCAGAAGGTGCCGGGCATTGCCGCCGCCGCGCCGTATATC
AATTTTACCGGCCTGGTAGAGAGCGGGGCGAATTTGCGCGCCATTCAGGTTAAAGGCGTCGACCCTAAGCAAG
AGCAGCAGCTTAGCGCGTTACCGTCATTTGTGCAAAATCACGCCTGGGATCATTTCAAAGCGGGCGAACAGCA
GATCATTATCGGCAAAGGCGTCGCCGATGCGCTGAACGTGAAACAGGGCGACTGGGTGTCCATCATGATCCCC
AATGCGAATGCCGACCATAAGCTGCTCCAGCCCAAACGCGTGCGTTTGCATGTCATCGGGATTCTGCAACTGA
GCGGCCAGCTCGATCACAGCTTCGCCATGATCCCGCTTGAGGATGCGCAGCAGTATCTGGATATGGGGTCCAG
CGTTTCCGGTATCGCGCTTAAAGTCCACGACGTGTTTAATGCGAACAAACTGGTGCGTGACGCGGGTGAAGTG
ACTAACAGCTATGTCTACATTAAGAGCTGGATCGGCACCTACGGCTATATGTATCGCGATATCCAGATGATTC
GCGCCATTATGTATCTGGCGATGATTCTGGTGATCGGCGTGGCCTGTTTTAATATCGTTTCCACCTTAGTGAT
GGCGGTAAAAGACAAAAGCGGCGACATTGCGGTATTAAGAACGCTGGGGGCGAAAGATGGTTTGATTCGTGCC
ATTTTCGTCTGGTACGGGCTACTGGCGGGCTTGCTGGGCAGCTTGATTGGCGTGGCGATCGGCGTTGTCGTTT
CGCTTCAGCTTACCGCCATTATTAATGGGATTGAAAAGGAGATCGGGCATCAGTTTTTGTCCGGCGATATCTA
TTTTATCGACTTCCTGCCATCCGAATTACATTGGCTGGACGTTGTTTACGTACTGGTCACGGCGTTGTTGTTA
AGCTTACTGGCGAGCTGGTATCCGGCGCGGCGGGCGAGCAACATTGACCCTGCGCGAGTATTGAGCGGGCAGT
AATCGCGGAATATAAAAACAAGGAGCGGTGATGTATTACGGGTTTGACATTGGCGGAACAAAGATTGCGTTAG
GCGTATTTGACTCAACGCGGCGGCTGCAGTGGGAAAAACGGGTTCCCACGCCCCATACCAGCTATAGCGCCTT
TTTAGACGCCGTATGCGAACTGGTCGCGGAAGCCGACCAGCGATTTGGCGTAAAAGGGTCGGTAGGGATTGGC
ATACCCGGTATGCCGGAAACCGAAGACGGCACGCTGTACGCCGCGAATGTCCCGGCAGCCAGCGGTAAGCCGT
TGCGCGCCGATCTCAGCGCCCGACTGGATCGCGATGTGCGTCTGGACAATGACGCTAACTGTTTTGCCTTGTC
CGAAGCCTGGGATGATGAATTCACGCAATATCCTTTGGTTATGGGGCTTATCCTCGGCACCGGCGTCGGCGGT
GGCCTGGTGCTAAACGGGAAGCCGATTACCGGTCAGAGCTATATCACCGGCGAGTTTGGTCATATGCGTTTGC
CGGTTGACGCGCTAACGTTGATGGGGTTTGATTTTCCTCTCCGCCGTTGTGGATGCGGTCAGATGGGCTGCAT
TGAGAATTATCTGTCCGGGCGCGGGTTTGCGTGGCTATATCAGCATTATTATGATCAATCGCTGCAGGCGCCG
GAGATTATCGCGTTGTGGGAGCAGGGCGATGAGCAGGCGCACGCGCATGTTGAGCGCTATCTGGATTTACTGG
CGGTTTGTCTGGGGAATATACTGACGATTGTCGATCCCGATTTATTGGTGATCGGCGGCGGACTATCGAACTT
TACGGCAATAACAACGCAACTGGCGGAAAGACTGCCGCGCCATCTCCTCCCTGTTGCCCGCGCGCCGCGCATT

GAGCGTGCGCGGCATGGGGATGCAGGTGGGATGCGCGGTGCTGCTTTTTTACATCTTACCGACTAATCAAAAA
AAGAGGTTGTTATGCAGTCGCGTCGGTTTCATCGATTAAGCCGCTTTCGTAAAAATAAACGTCTGTTGCGTGA
ACGTCTGCGCCAGCGGATCTTTTTCAGAGACAGAGTGGTGCCGGAAATGATGGAAAACCCAAGAGTATTAGTC
CTTACAGGTGCAGGAATTTCTGCAGAGTCTGGTATTCGTACGTTTCGCGCGGCGGATGGCCTTTGGGAAGAGC
ATCGGGTTGAAGACGTGGCAACGCCGGAAGGATTCGCCCGTAATCCGGGGCTGGTGCAGACATTTTATAATGC
CCGCCGTCAGCAGCTTCAACAGCCGGAAATACAACCCAATGCGGCGCATCTGGCGTTAGCGAAGCTTGAAGAA
GCGCTTGGCGATCGCTTTTTGCTGGTGACGCAAAACATCGACAATTTGCATGAACGCGCGGGCAATCGCAACA
TCATTCATATGCATGGCGAGCTGTTGAAGGTGCGCTGTTCGCAAAGCGGCCAAATTCTGGAATGGAACGGCGA
TGTGATGCCGGAAGATAAATGTCACTGCTGCCAGTTCCCGGCGCCGCTACGTCCGCACGTGGTGTGGTTTGGC
GAGATGCCGCTTGGCATGGATGAAATTTATATGGCGCTGTCGATGGCGGATATTTTTATCGCCATTGGCACAT
CCGGTCATGTCTATCCGGCTGCGGGGTTTGTGCATGAAGCAAAACTGCACGGCGCACATACGGTGGAACTGAA
TCTTGAGCCAAGCCAGGTCGGCAGCGAGTTTGAAGAGAAGCACTACGGCCCGGCAAGCCAGGTTGTGCCGGAA
TTTGTTGATAAATTCCTGAAAGGGCTGTAGTGCTGTTTGCCCGGTAACGCCTTATCCGGCCTACATTTCACGT
TACGTTCATCTGTAGGCCCGACAGGCGCTTTGGCTGAATTAGCGTCCTGCTTTTAGCTTCTGATAATACTCTT
CATAAATGGCGCTGGCGTCGCCGACATCGTTTTGCCACTCGCCTTTGCTGATGGTTTGCGCATCCGGATAGAG
CGACTTATCATTGGCGACCTCCGGGCTTAACAGCTTACGCGCCGCCAGGTTGGGGGTAGGGTAGCCGATGGTT
TCTGCCACCTCTTTCGCGACGTCAGGACGCAGCAGGAAGTTGATTAGCTTCAGCGCGCCCTCTTTATTTTTCG
CGTTTGCCGGAATAGCCAGGCTATCCATCCAGAAAATACCGCCTTCTTTCGGCCAGACGACCTCCAGCGGCGT
GCCAGCCTGACGAGCAACGAAGGCCGAACCGTTCCACACCATCCCCAGATTGACTTCCCCTTCCATATACGGA
TTCGCCGGATTATCGGAGTTGAAGGCCGCTACGTTCGGCATCAGTTTTTTCAACTCTTCATATGCCGCTTCGA
TCTCTTTCGGATCGGTAGTGTTACCGGAATAACCTAGCTTGCGCAGCGCCATCTGGAAGACTTCTCGCGCATC
GTCGGTTAAAAGCAGGCTGTTTTTATATTCCGGTTTCCACAAGTCAGCCCAGCTGGTAATGGTTTTTGGATCG
ATAGCATCGCTGTTCACGCCAATCGCCGTTGCGCCCCAGATATACGGAACAGAATAATCGTTGTTGGGGTCAA
ACGGTTTGTTGAGCATTTCCGGATCGAGGTTGTGGAAATTGGTTAATTTCGACTTATCGATCTTCTGGATCAT
GCCTTCTTTGCGCATTTTGTCGACATAGTATGTAGAAGGAACAACCAGGTCATACGCGCCATCTTTATAGGTT
TTGAGTTTGGCATACATGGTTTCGTTCGACTCGTAGGTCGAATAAATCACCTTAATGCCTGTCTCTTTAGTGA
ACTGTTCCAGCAGGCCCGGCGGTACATACTCAGTCCAGTTATAGAAATAGAGCGTGTCGTTGTCGGAGGCATG
AGCGGCACTCATACCCAGCGCCAGGGCGCCCGCCGCGAGCAGGTGGCGTGACCATTTTTTCATTTAACGTCCC
CTGAATTACGGGCCTTATCGGCCCTTGGTTTTATCACGAGCAATAAGCTGGCTGGCAATGACCATTACCAGAG
ACAGCACCAATAAAACGGTCGCCAGCGCGTTCACTTCTGGTGAAACGCCGACTTTTACCATGGAATAAATCTT
TAAGGGTAAAATTTCATAGCCCGGCCCGGTGACAAACGACGAGACCACTACATCGTCCATCGACAACGTAAAG
CTCAGCAACCAGCCCGCCGCCACCGCCGGCATCGCCAGCGGCAGAATGATTTTGCGTAAAATCGTTACCTCAC
TGGCCCCCAGGTCTTTTGCGGCTTCCAGCATTCTGACATCAAAGCCTTTCAGGCGCGAATAGACCGTGACGAC
CACAAACGGCAGGCTAAAGGTGATGTGCGAGAACAGCAACGACCAAAAGCCCAGTTGAATGCCGAGTAACATA
AACAGCACCAGCAGGGAGATCGCCATCACGATATCCGGCGACATCATTACTACAAACAGCATCCCGCTGACGA
ATGGCTTCCCGCGAAAGCGGTAGCGATAAAGCGCCACCGCGGTTAACGAGCCAATCAACGTGGCGAAGGTCGC
TGAAAAAATCGCCATCGTCAGTGAATGTTGAGCGGCCTGCAACAGACTGTCGTTATTGACCAGGAGTTCGTAC
CATTTAGTGGTAAAGCCCTGCCAGTTAATGCCGAACCGCGAACTGTTGAAGGAGTTCACGATCAGAATAATGA
TGGGAATATACAGATACGCATAGATGGCAGTCATGAAACCGCCACGGAGCAGCCGACCGATCATTCAAGTTCC
ACCTTCTTATTCAGAGGATGGGGTGCTTTTAGAGTATTGAAAATGATCAATTTTTTCTCTTCTCCAAAATCTC
TCCAAAACGTCTCGCGGTTATCAGCAGCCAGGAAGAAGCAAGCTGCCGCTTGAAGCCGCGAATTTTACAACAA
ATTGCCAGACGAGCGGGAAAAAAAGGCCATTTAAATGACTATTCTCATCCGATCCGGTCATATGCGGCATTAT
TGCACCAGGCAAGAGGTTACTCTGTAGGCAAACATGGAGTACGTGAGTAAACCCTGAACGTGACGTTTGAGAA
AGCGTCGTCTGATTTTATAAAAAACAACATAAACAGCCGCTTTGTTGTTCTGAGCGAACGTGTAGCGTGGTTA
ACTGTTCTTCCGCCATTTTTTGTATAGCCTGGGGGTCTATTCTAAAAATGGCGTGAAAAACCTGATCAGAGAT
GGTTTCGATTATCTCAGCTAACATACTTTGGCTGGTGCTGGGATCCGTTACGAGGAACGCCTGAAACGTCTTA
TCCGCTAGTAAAACCTCTTTTACCATTTTTTCAATAGGTATGTGGGTATGCGGTGGTGGTATTTCAAGCTGTT
TTAATCCACACATCGCCATTGTTTCTAAAAATGCGTTTTGCTGAGTACTTTCGGATATATTCGCATGGTGTAA
AATGCGCGTTGTCTTTTCAATCCCAGACTGAATTTTCGCTGCCAGGTAGGTTGCCTTTTCTTGCGCTTTCCAC
CCATCTAAATGTCCTTTTCTCAATTCAGTATATAAATACCCCTCTATGTTGTCTAATGGAACCGATAATATCG
GCGCGCCAGCAGGATTGAGACATAATGTCGAAGGGAGGGGTAGTCTACGATTTCGCTATATCTTCTCTTAATC
ATGGCCGTCATTTGTGGATGCGATTTTAAAATATCCGGGCGATCTTTCATTAAAAAATAAAGATTCCCCATGA
CTTCACAGCTAAAGGTATCGGTATTTTGAGTGATACGTAACAATTCGTTCTCTTCGTTTTGATCCATGATGCG
AAGAATAATGGTGGCATCATTTTCATGAGGATTATGAACCTGAAATCTATCTCTTTGCGATGCGCAGGCTAAC

TCTTTCAACTCAAAAAAAATCTCTGTAAGCCGCTCTCGTGTGGGGGCGCGTTCGGCAAACAACATCTCATGTA
GACAACGGTCCGCTTTTGCTTTGCCAGTAGAAAAAAGAAGTCTTTAATTTTTTCCCATAAAACTTTCCACCAT
GCTTCTTTCGTATTCCTTGGGGAGTTGGTAAGGATTTCACTTTTTAAAAAACCATTCCCTATAGTAATCGGCA
TATTAATCTCACTTAAACTGGAGTAAAAATGACTACATAATTGATTAAATCGACCCTTTTTATCTCAACTGCG
GGCGCATCGGATGTAATATAATTTTTAAAAGAGACTGACAATCAGTATAAAACCTGAGAGCTTCGCGTATAAA
CGCATTACTGTCTGTGATAGCGTCGCTACAGGTAAAAATAAAAGAAGGCCTGGCGCGGATGATGTTGTAGATT
TGCAATACTGGCGGCAACTGCTTTCATGCGTTTTTTATGCCGGAGGCATGAAGTTTACCCTTGAATAAACTTC
ATGCCTGGATGCGTGTGGATTTGTTAGCGTTGCGCAATTAATCGCTTATATCACTCACCTTCTTATTCAATAA
GCGGGAAGCCCGCCAGTAAATCAATAGCATGAGTCCCATCACAATGGTCAGCGTAATGCTGGTAGCCGCGCCA
AACGGCCAGTCGCGAATATTCAGGAACTGTACCTTAATGACGTTACCAATCAGCAGGTTTTTCGCGCCACCCA
TTAAATCGGAGACGTAGAACAGGCCCATTGCCGGTAGCATCACGAGCAAGCAACCGGCGACAATGCCCGGCAT
GGTTAATGGAATAATGATGCGGATGAACGTTTGCATTTTACTGGCGCCGAGATCGCGTGCGGCCTCCAGTAGT
GGTTTGTCGAGCTTCTCAATGCTGGAGTAGAGCGGCATGACCATAAATGGCAGCAAAATGTAAACCAGGCCGA
TAATAACGGCGCTCGGGGTGAACATGATGCGAATCGGTGTATCAATCACGCCCAGCCACAGCAGGAATTCATT
GAGATAACCTTTAGTGCTCAGGAATATTTTCAGCCCATAGATGCGGATCAGTGAGTTGGTCCAGAAAGGAACA
ATCAGCAAAAACAGCAATAACGGACGTATCTTTTCCGGTAACTTCGCCAGAAACCAGGCAAAAGGATAGCCCA
GCACCAGGCAGGAGAGGGTGGCGATCAGCGCCATATTCAGAGAGTGCAGGAGCACTTCAAAATAGAGCGGATC
GAGCAGGCGCGCGTAATTATCCAGCGTAAAGACCATCTTGACGAAACTGGCGTCGTCACGGGTCAGAAAGCTG
GTGCCAATGATCATCAGGTTGGGCAGAAAAACAAATAACACAAGCCAACCGACGATAGTGACAATCACTACAT
TCTGGAATTTACTTGTGTTCTTCATCAGCCAGTACGACCTCCCAGCTTTCTACCCAACTAATGGCCATTTTTT
GGTCAAGTGAATGGTCGAAGTCAGGATCGTCTTCGTTGAAGAATTCGCTGACCATCACCATTTTGCCATTTTC
CAGTTCAACCACCGACTCCAGCGTCATACCTTTATAATTGCGCTCGCGCACGTAGCCGATAAGCCCTTCGATG
TGGTTATCATCGTTGATCTCTTCGACGCGTAGATCTTCCGGTCGCAGCAGGACGTTCAGTTTTTGGCCCGGTT
CGACAGCGAAATTGACGTATATATTGCACTCCCGACCTTCTACGCTGGCGCGGACGCGCTGTTCATCAAGCCG
TTCAATGACGGTTGCGTCAAAGCGGTTAATTTCGCCGATAAAACCGGCGACAAACAGGTTTTTTGGCTCTTCA
TAGATTTCACGCGGCGTGCCGTCTTGCTCAATCACGCCATTACGCATTACCACGATACGATCGGACATGGTGA
GCGCTTCTTCCTGATCGTGAGTGACAAAAACAAACGTGATACCGAGTTTACGCTGTAATGCTTTAAGCTCGTT
CTGCATTTGTTTGCGCAATTTATAGTCCAGCGCGGAAAGCGATTCATCCAGTAATAACAGGCGAGGTTTATTC
ACCACCGCGCGAGCGATAGCCACACGCTGCTGCTGTCCGCCGGAAAGCTGGTGCGGTTTACGTTGTGCAAACT
CTTCAAGCTGCACCATACGCAGCGCATCGGTCACGCGAGGCGCTATCTCGGCGGCTGGCGTTTTTTGCATTCG
TAAGCCAAACGCGACATTTTCAAACACCGTCATATGCGGAAATAAAGCGTAGCTTTGAAATACGGTATTTACA
TAACGATTTTCGGCGGGAACGTGGGTGATATCCTGGTTATCCAACATGATATGCCCGGCATCAACGGTTTCCA
GGCCGGCAATCAGGCGAAGAACGGTTGTTTTACCGCAGCCGGAAGGGCCAAGCAGCGTGAGGAATTCGCCATT
GTTAATGGTCAAATCCAGTTGCGAAATGACCTCTTTGCCATCGAAACTTTTGCTAATTCCAGACAAAAGCACC
AGCGGTGAAAGCGAACGCGGTTGATTATTCAATTTCTTACTCTGTCCCATGTAAACGCAACGGATGGCTGACC
GCTGCGGGGTTTGTGGTTAACCACCTTAATCACTCTTAATGAGGGCGGTCATTCTACGGCAAACCACTGTGAT
CGCCAATCCTTGTTGCGAATTACTGACTTAGCTTTATAGTCAGAAAGCGTGTCAAAGTGAAATATTCTTGTTT
GCAGGGATAAAAGTGACCTGACGCAATATTTGTCTTTTCTTGCTTATTAATAATGTTGTCACGAAAAGTGAGG
GTGACTGCATGGATAAACTACTTGAGCGTTTTTTACACTACGTATCGCTGGATACCCAATCAAAGTCGGGTGT
TCGGCAGGTTCCCAGCACTGAGGGGCAGTGGAAGTTACTACGTTTGCTCAAACAGCAGCTCGAAGAGATGGGG
CTGGTTAACATTACATTAAGTGAAAAAGGGACGTTGATGGCGACGCTCCCGGCCAATGTTGAGGGGGATATTC
CCGCCATTGGTTTTATCTCCCATGTGGATACCTCTCCGGATTTCAGCGGTAAAAACGTTAACCCGCAGATTGT
CGAGAATTATCGCGGCGGCGATATAGCATTAGGGATTGGCGATGAGGTGTTGTCACCCGTGATGTTCCCGGTA
CTGCATCAATTACTGGGACAGACGCTGATTACTACCGATGGTAAGACATTGCTGGGCGCGGACGATAAAGCCG
GCGTTGCGGAGATCATGACCGCGCTGGCGGTGCTGAAAGGTAATCCTATTCCCCACGGTGAAATTAAAGTGGC
GTTTACGCCTGACGAAGAGGTAGGGAAAGGCGCGAAGCACTTCGATGTTGAGGCCTTTGGCGCGCAGTGGGCC
TATACGGTCGACGGCGGCGGCGTGGGCGAACTGGAGTTTGAAAACTTCAATGCCGCCTCGGTGAATATCAAAA
TCGTCGGCAATAACGTGCATCCCGGTACGGCAAAAGGCGTGATGGTCAATGCGCTGTCGTTGGCGGCGAGGAT
TCACGCGGAAGTGCCGGCGGATGAAACGCCTGAAACCACTGAAGGTTACGAAGGGTTTTATCATCTGGCCAGC
ATGAAAGGCACCGTTGACCGGGCCGAAATGCACTACATCATTCGCGATTTCGACCGTAAGCAGTTTGAAGCGC
GTAAACGCAAAATGATGGAGATTGCCAAAAAAGTCGGTAAGGGGCTGCATCCGGACTGCTATATCGAACTGGT
GATTGAAGACAGTTATTACAATATGCGCGAAAAAGTGGTTGAGCATCCGCATATTCTCGATATCGCCCAGCAG
GCCATGCGTGACTGTCATATTACGCCGGAGATGAAACCAATTCGCGGCGGTACAGACGGGGCGCAACTGTCGT
TTATGGGCCTGCCGTGTCCTAATCTCTTTACCGGCGGATATAACTATCATGGTAAACATGAGTTTGTGACGCT

GGAGGGGATGGAAAAAGCGGTACAGGTGATTGTACGTATCGCGGAGCTGACGGCGAAGCGCGGCCAGTAGCGA
ATGTTACGCATTGTGGTGAAGCGTTTTATTATTTATGGCGGGATATTTTCCGCCATCAATTTTTCTGCCTGGA
GCGCCGAATATACGCCTTCCTGGTCCCAGCGCCAGCAACAATCTGCAGCATGTTTTATGACCGGCGATGAAAC
CTGCATGACGTTTATTGATGATGCGGTGAGGCTGGCCTCGCAGCAATATGGCAAACGCTCAATACAGCTTGTT
CGTAGCCTGCTATTACAAAGCGATATTTATCAATGGCTGGGTAAACCTGAATTAACGCCGCAAATGCTGCTGC
GAGCGCGGGCAATAATGAAAACATTTCCGGCGGGCACTTATCCGGGCGATCGGGCCGATATGTTTGAACACCT
GGCGGCCTTTTATGTTTACAGTGACGATCGCCATATAGAGTATTCATCTACAGAGCAGTGGCGATACGAGATT
AAGGTTGATTACCGTCAGCGAATAGCCTGGCAAGAGCAGGCATTAACGTGGCGTCTTAAAGACAAAAAGGCAT
CGACGGAGGCATTGGTTTATACCTTAAACAGGATGCGCGACGCTTATTCTGATGCGCTGGAAGAGCGGGATGT
CGAATGTGATAGTGCGCGTAAAGCCTATTATTTGGCGAAGATAGACACAACGGAGCGGCAGTGGCTGAGCGTT
ATTTTACGAGATAAAACCTGGGATAATCGTGAGCGCGTCGCCTCTTTTTTGCAGCAGAAAGCGGATATTGCTT
ATAATGCAGGGCATATTAGCGAGGCGATTAATGCGCTAAGTCAGGCGCTAAAAATTGAGCAAACGCTTTATGG
CGCCGAATTCGGCGAAATGACGGTTGACAGCAATAATCTTGCTGGCTTTTATGCACAGGGCCATCATTATAAG
GAAGCGAAAGATCTCTACCTTAAACTTATCGCGTATTACCAGAGCCGTTTAACGCCAATGGCGACGGTAATAT
CACGATTACGCTTTTATTTGCCCGAAAACATCGACCTGGACAGTACCTCCCCCTATCTCCCATTGCTGGAAGA
ATATAAACGTCGGCAGTCCGACGTATCCATGGTGTTGTATGGTATTTCGCTGCTTTATCAGAATAATCAACAG
CTTGAACAAGCAAAAGACTTTGCGGAACGCGCCTTTACGCTTGATGCCGTAGCTTATCCAGCAAAGATGCAGT
ATGAACGATTACAGCGACTGGCTAATATTGCCGAAGGTCTTGGCGATAACGTACTGGCGCGACGTTACCGACA
ATTGTCATTTCGCCATCGAATGGCCCACTCGATTTATCCGGGCGATCCGCAATATAACGATTTCGCCAAACCT
GGCGGCGATCGTTGTGGATAGTTTGCGTCAGCCCGGCGGTCGCCGGGCATTGCGATGTTCTTATCCTTCGAAA
AACCAGTATCCGCTATTCACCAGCGCCGCCAGCATCGCGAGGAAGGACGGGTCTTCCAGCGCATCGCCGAAGT
TTTCAGCGGTCAGTGCAATATGACTGGCGAGCGCCTCCAGAGCGGGGCGATGCGGAGAGTCTATTTTTTCGCC
ATTCGCATAGACCTCATCGCCGATACGCAACACGCGAAGCCCGCCGAGACGTACTAATACCTCGCCTTGCTTT
AAGGCATCGTAAATTTCATCAGGCTGATACGGCGGCTCCGGCGGCGCAATATCCAGTTCGTGGCGTGACTGAG
AGAGAAACTCGCCAAGCCACTGTTGGAAATGCGCGGGTTGATTGATCAAATCGAGCATCATATTACGCAGTTT
ATCCATCTCCTGCGGCAGGATATCCGCCGGATGCTTGCGGGACGGCATATCCGGATCGCTGTAGTATGTATTG
CCCAACTCCCGTTGCAGGACGTAATCGGCAAAACCGCTGATCAGCTCACGGCTATTGGGCGCGCGAAAACCGA
CCGAATAGTTCATGGCGTTTTCCAGCGCATAGCCTTCATGCGGGAATCCTGGCGGAATATACAGAATGTCGCC
CGGCTCCAGCTCTTCATCAATAATGGCCTCGAACGGATCGACTTGTAACAGGTCGGGATGCGGGCAGTGCTGA
CGCATTTGCAGCTTTTCGCCCACGCGCCAGCGACGGCGACCGGTTCCCTGAATAATAAACACATCATACTGAT
CCAGATGCGGGCCAACGCCGCCGCCGGGAACGGAAAAGGAGATCATCAGATCGTCGATACGCCAGTCCGGCAG
AGCGCGGAACGGGCGCATCAGCGCGGCGGTGGGTTCGTGCCAGTGGTTTACCGCCTGTACCAGCAGCGACCAA
TTGCTCTCACCCAGATGATCGTAGCTTTCGAAAGGGCCATGGCTGACCTGCCATTTGCCATCCTGATGGCTAA
CGAGTCGGCTGTCGATTTCGCTTTCCATCGCCAGCCCCGCCAGCTCATCCGGCGAGAGCGGATCGATAAAGTT
GCTAAACCCACGTTTTAAGACCACCGGGCGTTTTTGCCAGTGGCGTTCAAGAAAGTCGGGCCAGTTAAGAGTT
AATTGGTATTCCATGTTAAGTATCCGCAGGCTGGTATCTGACACCGATTATAACGGATGCTTAACGAGATGCG
TGGAAGAACGCACAGAAATGTTTATTCCTCTTTCTGTGTGGGATGCTGTCGGCCAAAAACGACCTCCATACGG
GCACCGCCGAGCAGACTGTCGCTGGCAATGATCTGCCCGGCGTATTGTTCCGTAATCTCGCGCGCGACAGCCA
GCCCCACGCCTTGTCCTGGTCGTAGGGTATCGGCGCGCTGACCGCGATCAAACACCAGGGAACGTTTACTGTG
GGGAATGCCTGGGCCATCATCTTCGACGAAAATATGCAAATGATCGTCGGTCTGGCGAGCCGAAATCTCGACA
AACTCCAGACAATATTTACAAGCGTTGTCCAGTACGTTGCCCATTACTTCGACAAAGTCGTTTTGCTCGCCGA
CAAAACTGATCTCTGGTGAAATATCCATACTGATATTCACCCCTTTACGCTGATAAACTTTATTTAGCGCGGA
GATCAGGTTATCTAACAACGGCGCGACGGGATGCAGTTCGCGGCTTAACAACACGCCGCTACCGCGCATACTG
GCGCGATGCAGATAATAGCCGATCTGCTGGGAAATCCGGCTGATCTGTTCCAGCATCACCGGTTCAGCTTTGC
TGACGCTCATCTTTTCGTTGCGTAAAGAGCGTAATGTACTCTGCAAAACCGCGAGCGGCGTTTTTAAACTGTG
TGTCAGGTCGGTCAGGGTCGTGCGGTATTTGTTATAACGTTCACGCTCGCTTTTGAGCAGTTGATTAAGGTTG
CGCACAAGGCTGGTCAGTTCACGCGTCGTCTCCGGATTGAGCATTTCGCGGTGATGATCTTCAAGCTCGCGGA
CTTCCCGTGCCAGCGCCTCGATAGGGCGTAAGCTCCACCAGGCGGCGATCCACAGTAAAGGAATGACTAACAG
TAAATTGGCGGCCAGCACGTATACGAACCAGCTCCACACCATATAGGAGCGTTTTAGTTCTATCGGAATGGTA
TCGACCACCACGATGGTTAACTGCGGCATCCGCGCCGTGGCAGGATAAATATTTACCGCTACCGAGTGGGTCA
TCTCCGCATCATCGTCATCTTCACGTACTTCTTTGAGTTTTTCCTGCGCGGAATGGTCCTCGCTCAACAGCGT
GCTGGTGGCGTCTACGTTGGTTTCAATTTCATGGAAGCCGTTCGTTTTTAACCATTCCGGTTGAATGCTTTTA
ATCAGCCAGGGGATATTGCGCTGCGTCCATAATAATTTGCCCGTTTCATCGTAAATCAGCGTCATGGTCGGGC
TTTGCATGTCCAGATTTTCAGGCAGCTCAACGCTGATTTTATTATTTTCCCATTTGGCGAGGGTATAAAACAG

GTTGCTTTCGCCGCGCAGCAAACGAAAGGTGGTTTTATCAAAACTTACGCTATAGCCGACCAGCGCCACTATG
CCATATGCCAGAGAAAGCACCAGCACGACGCCGGCTGTCGCCAGCAAAAAACGAACCCGCAGCGACAGCGGCA
GAAAATGGCGAGCAAATTTATTCATTAGCGCAATTCAAAAAGATATCCTTGTCCGCGTACGGTGGTAATGACA
TCGTGCGGATACTGGGCCTGTATTTTTTTCCGCAGACGCCCCATGAGAACATCAATGGTATGACTTTCCCGCA
GTTCCGCATCCGGATACAGCTGAAGCATCAGCGAATCCTTGCTGACCACTTTACCGTTGTTACGGATAAGTGT
TTCCATAATGGTGTATTCGAACGCCGTGAGTTTGATGACCTCTTCATTGACGGATAATTCCCGGCGTGAGAGA
TCCACCTGGAACGGCGGGATGTTGATCACTTGGGAGGCCAGACCGCTATTACGGCGCATTAACGCCTGCATAC
GCGCCATTACCTCTTCGATGTGGAATGGCTTCGTCACGTAGTCATCGGCCCCGGAGCTGAGAACCTCGACTTT
ATCCTGCCAGCCTTCGCGCGCGGTTAACACCAGAACCGGCAGTGAAACATCACTGCTGCGCCAGCGGCGTATC
AAGGAAAGGCCGTCTTCATCCGGCAGACCTAAATCGACAATAGCGATATCCGGAAGGTGTTCATTAAGGTAGT
AATCAGCTTCCCTGGCATCTTCTGCGGCATCGACCTGGTGACCTGAATCCTGGAGCTGAACCTTCAGGTGGTG
GCGTAATAATGCATTATCCTCTACAACCAGTACGCGCATCATCTCTTCTCCCTTGTGTTAACAATAAGAACAG
TCTAGCGTTGATTATGGTGCTTTGGGGATAAACAGTTAATAAACCAGACAAATAGTCACCCTCTTTCTGAAGA
AAAGAGGGTGAGGCAGGCATTATTTAAGTTCGTCGACCAGAGTCACAGCGCGACCGATATAATTTGCCGGCGT
CATGGCTTTAAGGCGCGTTTTTTCTGCTTCCGGCAGGGCCAGACTATCAATAAACTGTTTCATTCCTTCGGCA
TCAACACGCTTGCCACGGGTCAACTCTTTCAGTTTTTCATATGGTTTTTCAATACCATAGCGGCGCATGACGG
TCTGGATCGGTTCGGCTAATACTTCCCAGTTGTGATCCAGTTCGTCAAGCAGATGATCGCGGTTTACTTCCAG
CTTGCTGACGCCCTTCAGGGTGGACTGATAAGCGATAAGCGCATAGCCGATGCCGACACCCAGGTTACGCAGG
ACGGTTGAGTCGGTCAGATCGCGCTGCCAGCGGGAAACCGGCAGTTTGTTTGCCAGATGGTGCAACACTGCGT
TAGACAGACCGAGGTTGCCTTCTGAGTTTTCAAAGTCAATGGGGTTAACTTTATGCGGCATGGTAGAAGAACC
GATCTCCCCGGCGATGGTTTTCTGTTTGAAATGGTTCAACGCAATATAGCCCCAGACATCGCGATCGAAATCG
ATCAGGATGGTGTTAAAGCGCGCGATACAGTCAAACAGTTCCGCAATATAATCATGCGGTTCAATCTGGGTGG
TGTAAGGATTCCACTGGATGCCCAGCGAGGTGACGAACTCTTCGCTGAACTGATGCCAGTCAACTTCCGGATA
GGCGGCGATATGCGCGTTATAGTTGCCTACGGCGCCGTTAATTTTACCGAGGATCTCCACCTGGTTGAGCTGG
CGGAACTGACGCTCCATCCGATACGCCACGTTCGCCATCTCTTTACCCAGAGTGGAAGGCGTTGCCGGCTGGC
CGTGGGTGCGGGAGAGCAGAGGAATGTCGCGATACTGCGTGGCGAGATCTTTAACCGCGTTAATCATCTGACG
CCAGTAAGGCAGGATCACTTCATCGCGCGCGGTTTTGAGCATTAACGCGTGCGACAGGTTGTTAATGTCCTCA
GAAGTGCAGGCAAAGTGGATAAATTCGGAAACGTCATGTAGCGCCGGGATCGCGGCGACTTTTTCTTTCAGAA
AATACTCAACCGCTTTCACATCGTGATTCGTGGTGCGCTCAATGGTTTTAATGCGCGCGGCATCTTCTTCATT
GAAGTTTGCAACAAGCGTATCCAGGTAACCGTTTGCGTCGGCAGCAAAAGCAGGAACTTCCTTGATCGCTGCG
TGCGCGGCTAATTTCTGCAGCCAACGTACTTCGACTTGTACACGAAATTTCAGCAAACCGTATTCGCTAAAAA
TTCCGCGCAGCGCGCTGACTTTATCGCCGTAGCGTCCATCGACAGGGGAAACGGCGGTCAGTGAGGATAATTC
CATAGATCATAACTCCGGGGTTAAATGAGCAAGAATTTGTTTTGCCTGAGTCGTCAGGCGATGACGAGAAAAC
ATAAGCTGTAGGCGACCACCGCCTACCTGATGCCAGAGCACGGCTGCACGGATGCCTGCCAGTAGCGAGGCGC
GGACTTTGGCCTGCACCTGCGGACTTTGCAGAACGGCCGGGGAGCCCGTCACCTGAATGCGCGGGCCCAATGG
GCTAATCACATCCACGTAGATGCCCGCCATGGCGCTCATTAACGTGTCTGACTGTAAATCAAAATGGTCAAGC
TGGCGCTGCAGGCCGTTAATGCGATCGCCTAACGTATTCAACGCGCCTTTGGCGGATGACAGCTTGCGCTCCA
GCACCATCAGGCTAAGCGTATAACGCGTCAGTTCGGCGTTAAGTCCTTGTCGGCTGCTGGCATTCAGCACGCC
GAGCAGTGTTTCCAGACCAAGACGCAGGTTAGCTTCGCTGCCGCCGAACACGCCCAGAGTAGAACTGGGATTC
ATATCAATGACACTGTTGAGTGAAACGTGCAACGCGTCGGCATCACAGTGCCCCTGGTGCGCCAGTTGTTGCA
CCAGGCGTGCCGACTGGCAAATGCCAGACAGCGCGAGGGTGATGTCATAATAATTCTTTGCCACAATCACTGC
TTCCTTGTATCAAATAAAATACACTTCATCTTTTAAGCCGCTTTGAGGCGACTTGAATGGTGTTGTGTATTGA
ATAATCAGACCGTCAGCGGCAGTCGCTGCTCGATAATGCCGCCGCCCAGGCAGACTTCGCCGCTGTAAAAGAC
AGCGGACTGGCCCGGCGTAACGGCGGCAACCGGCTCGTCAAAAATGACCTCGATACGATCGTCATCCAGCGCG
TTAATCGTGCACGGAATATCGGTCTGGCGGTAGCGGGTTTTTACCGTACAGCGCAGGGTGCCGGTGAACGGTT
CGCGATCGACCCAATGTAGCTGCTGGGCGATGAGGCCTACCGACATCAGACGTGGGTGCTCATGCCCCTGGGC
GACAATTAACACATTGTTTTCAACGTCTTTATCCACCACATACCAGGGGTCTTCGCTACCGTCTTTGGTGCCG
CCGATTCCCAGCCCTTTACGCTGACCGAGCGTATGATACATCAGACCCTGATGCTCGCCGATTTCATCGCCGT
CAACGGTAATGATTTTCCCCGGCTGCGCAGGCAGATAGCGCCCTAAAAAGTCGCGGAATTTGCGCTCGCCGAT
AAAGCAGATACCGGTAGAATCTTTTTTCTTGGCGGTTACCAGCCCCAGATCTTCGGCGATTTTACGCACCTGC
GGTTTTTCCAGCTCGCCAACCGGGAACAGACTTTGCGCAATCTGCTCATGGCCAAGCGTATAGAGGAAGTAGC
TCTGATCTTTGTTGCCGTCGAGCCCGCGCAACAACCGGCTTTTGCCGTTTACATCCGCGCGGCGAACGTAGTG
ACCGGTGGCGATATAGTCGGCGCCTAAATCTTCTGCGGCAAATTCAAGGAAGGCTTTAAATTTGATCTCTTTA
TTGCACAGAATATCCGGGTTCGGCGTACGGCCCGCTTTGTACTCTTCAAGGAACAGTTCAAAGACGTTATCCC

AATATTCTGCGGCAAAATTAACGGTATGCAGTTCAATGCCAAGCTTGTCGCACACGGCCTGCGCGTCAGCCAG
ATCGGCTGCCGCGGTACAGTATTCCTCACCATCGTCCTCTTCCCAGTTCTTCATGAACAGGCCTTCTACCTGA
TAACCCTGTTGTTGCAACAGCCAGGCGGAAACGGAGGAGTCGACACCGCCGGACATGCCGACGATCACTTTTT
TTGGGCTTTCTGACATTGGAATACTCGCGACATTGAACTTCAAGGCGGCGTATTCTACCACGCAGCCCTTTAC
TTGACACCCTCTGTAAACGGCCAGTTAAATTCGCCCACTATAGATAAGGGGTAACATTGTCCCGTTTGATAAC
TGCGAATGCTTTCAGCCACCAGCGGAGAACGTAAATTCGGCGCGTTCAGAATCTCGTCGGCGCTCACCCAACG
GCAGCAATCGATGTCATTGTCATGGGGTTCGGTCGCGCAAATATGATCAAGCTTGATAGCGAATAAAAAGCGC
AGGAACGGGGTCTTATCCGGCGCAATCCACTGATGCATACGGATAAAATACTGTGGTTGCGCCGTGATGCCAG
TCTCTTCCCACAGTTCACGCGCGGCGGCCTGCGCCAGCGTTTCATCTGCCTCCAGGTGTCCGGCAGGTTGATT
CCATAACGATTTACCGTTGATGGTCTCTTCCACCACCAGAAATTTATCTTCGGCATGTACGATGCAGGCAACG
GTCACGTGCGGCTTGAACATAGGCATCCTTATTAAAGCGGTTTATCAATGTGGTCATAGATCGCGTCAAGCTG
TTTCAGACGCTCGTTATAAGCTTTTGTCAGGCAGGTGACATCGGCCTTGCACTGCTGACGCTGTTTAAGCCAG
CTCTGCTGGGCGTCCTGCAGCGCGCCGCGACTGCCCATCGCGAACAACCCTTTCAGAAACTGATATTTGGTGT
GCATTTCCACGTTTTTATCATTCAGCGTCAGGTGCGCGCAGATAGCCTTTTCATCCGCAGCCTGCGCTTTCTG
GCAGTCGAAGCTGGCCGCATCGACGGATAACGGTGTTAATAAAGCAAAAGCAATGAGAAGCCGTTTCATCTGT
CTTTCTCCTGTGCGATCTCGCGCCACTGTCCATTATCGAGGCCGTTGAGCGTGTAGTCGCCCATGCTGTAACG
AATTAATCGTAGAGTGGGATGACCGACATGCGCTGTCATGCGTCTTACCTGGCGGTTGCGGCCTTCATATAAG
GTGACTTTTAGCCAACTGGTGGGAATATTCTTCCGTTCGCGAATCGGCGGGGTGCGCGGCCACAGCCAGTCAG
GCTCCGCGACAATCTCAATACCGGCGGGCAGGGTAGGGCCATCGTTGAGCGTGACGCCAGTACGAAGCGCCTG
CAGCGCTGCATTATCCGGGATACCTTCTACCTGAACATAGTAAATCTTGCCGGTGCGTTTGCCCGGTTGCGTG
AGGCGCGCCTGCAGCGCGCCGTCATTAGTCAGCACCAAAAGCCCTTCGCTGTCGCGGTCCAGGCGGCCTGCGG
CATAAACGCCTTGCACAGGAATAAAATCTTTTAACGTGCGGCGACCTGCTTCATCCGTGAACTGCGGCAACAC
ATCGTAAGGTTTATTGAACAAAACCACGCGTTTTGGCTGGTTTTCTTTCCGTGATTTAGATGCTTGTCGTGAG
CTGAATCGCTTAACGTAGTGATTTCTAAAAGAAGTTTTTTGCATGGTGTTTTCAGAACTTATTAATTGCCGCA
TTATAGCGCAATAATGAGTGTCTTTCATGGCGGCAAACAATAGGGTAGTATTGACATGCTCATTACAAAACAT
TAACAAAAAATTGCTCTAACGCAGTCGTGCAGCAGGACGCCATTGCACATGCAGCGCGATGACAGACGAGCAA
ACCAGAAGCGCTCGAAGGAGAGGTGAATGGAAAGCAAAGTAGTTGTTCCGGTGGAAGGTAAGAAGATCACCCT
GCAAAACGGCAAACTCAACGTTCCTGAGAATCCGATTATCCCGTTTATTGAAGGCGATGGTATCGGTGTTGAT
GTCACTCCAGCCATGCTTAAAGTAGTCGATGCCGCCGTCGAGAAAGCCTATAAAGGCGAGCGTAAAATTTCCT
GGATGGAAATTTACACCGGAGAAAAATCCACACAGGTTTACGGCCAGGATGTCTGGCTTCCCGCTGAAACCCT
TGATTTAATTCGTGACTACCGCGTAGCGATTAAAGGTCCATTGACCACGCCGGTTGGCGGCGGTATCCGCTCT
CTGAACGTTGCCCTGCGTCAGGAGCTTGACCTGTACGTTTGTCTGCGTCCCGTTCGTTACTATCAGGGCACGC
CAAGTCCGGTGAAACATCCGGAGCTGACCGATATGGTCATCTTCCGTGAAAACTCTGAAGACATCTACGCCGG
AATTGAATGGAAAGCTGACTCTGCAGACGCTGAGAAAGTGATCAAGTTCCTGCGCGAAGAGATGGGCGTGAAG
AAAATTCGCTTCCCGGAGCACTGCGGTATCGGTATCAAACCGTGTTCTGAAGAAGGCACTAAACGTCTGGTTC
GCGCGGCGATTGAATACGCTATTACCAACGATCGCGATTCCGTCACGCTGGTACATAAAGGCAATATCATGAA
GTTCACCGAAGGCGCGTTTAAAGACTGGGGCTACCAGTTGGCGCGCGACGAGTTCGGCGGTGAGCTTATTGAC
GGCGGTCCGTGGCTGAAAATTAAGAACCCGAACACGGGTAAAGAGATCGTGGTTAAAGACGTTATCGCCGATG
CGTTCCTGCAACAAATCCTGTTGCGTCCGGCGGAATACGACGTTATCGCCTGTATGAACCTGAACGGTGACTA
TATTTCCGATGCCCTGGCGGCTCAGGTTGGCGGTATCGGTATTGCACCGGGTGCGAATATCGGCGACGAATGC
GCGTTGTTTGAAGCGACTCACGGGACTGCGCCGAAATATGCGGGCCAGGATAAAGTCAACCCAGGCTCCATTA
TTCTGTCTGCGGAGATGATGCTGCGCCACATGCAGTGGTTCGAAGCCGCAGACCTGATTGTTAAAGGTATGGA
AGGCGCGATTGCCGCGAAGACCGTGACCTATGACTTTGAACGCCTGATGGAAGGCGCTAAGCTGCTGAAATGT
AGTGAGTTTGGTGACGCGATTATCGCAAATATGTAATAACGATAATTGTTAAAAACTAAAACGGGGACTTAAC
GTCCCCGTTTTTATTATTAGTATTCGAACGGTTATCAAAACTTTATCAAAAAAATTTATCAAAACTCCCTCAA
TTCAGACCGCAATAGTAGTTCATCCTTTACCCCGATCGTCATGATAACCTGAGCCCCCCTGAAAGCTGATAGT
TACCGATTTTGATTGTAGTGGTTTTCTTAATAATGTGTGATTTGGCTATTTTTTGAAAGTGCCTTACCAGATT
TATTGTTATTATCAGGATAATTTTTTAATAAAAAGTGATGGTTTATTGACTACAATAGTGGGTAGGTTAAGTA
TTTTATAAAATTATTAGCATGCTTTATTTGCTTTTCTACAAGGCTGCTAAGAAGTGTTGAAATATCAGCCTGA
TAAAGAATACGTCAGCGAAAGTGAATGCTATATTAGTCATAAAAATTCAGGAGACATATGCTCAAACCTATCT
GCCATAGTGGAAGTATAAAGGTTCCGGAATATCTGGAAACAGATAAGGAAAAAAATGCCGGACGTACTCCACT
GTCTTCAGACATTCAGCAAGTTAGAAATGTTGTTGAAGATGTTCCGCCATTTCCAGAAAGCAGAACGGCGAGA
GGTTCTGTGAGCGCAGCGTACAGGCTTTCTTTTGACGAAGTGTTTTGCGGTCTCAGCAATGAAGAGCGTAAAA
AGGTGTATGGTCGCCTTTTTGGAAAACAAGTACTTGCGCATATTCATTCCAGGTGTCAGCGAGACGCTGACAT

AATAAGAGAAAAAGCGCTCAGGCGGATAAGCCGTGAGTGTGGTACCGAAATAGATTGCACTCTCTTGCTAAAT
AAGATGGTGGATATTTTACAAAATGCCCGGCTGACAATAAATTTTAATGCGGCAAAAATTGACTTTGCCTCTC
TCTTAAAAAATAAAGAATATCTGAACTCTTATGCATTAGGTTGCAGACCAGGAGATTTACCTGCTTATAATGT
CGGACGTGATTCAGTTGAAACTAAAGCATTTGAACTGGAGAGGCTTGCAGATTCACCTTATGCCCCATATGGT
CAGACAGGCGGTTTTTCCGTAGCATATACTCCCAACAGTAGAACTTTTAGCCCTACAAGCAGACCAATTTATG
CTGCACTGGACTTTCTGAACGGTGAAAATGGAGGTGCCAGCGCCTATGGGAAATCATTTTTTGAATTAAATGA
TAATGTAAAAACAAATTGTACATTATCACCTTTTGATATCTACGGCCACAGATTTGGTCTGGATACGAGTAAA
CTATCAACATTTTGGCATATGGAGAACCTGATTGCATCCTGTCAAAATGATTTTTTTGGTTATAATTGCTTTA
AGAGTTTGGTTAAAATGGCTAAGGGCGAAAAATTTTTAGCTCATTCTAATTATGGTAAAGGCTATGAAGGGAA
TTATATAGAAGCTCATATTCATGGTGATGTATGTTTATTCAGAGATATAAAACACGTTTATTTGTCTTTGCAA
GAAAACTCTTACTCGGAAAGTCAACTATATGATTATGCAAAACAAATAAACCTGGTGCTTAATAGAGACTGCA
TAATATTATATTGACAAGCTTATTATCAGGCTCTTTAAAAGAGCATTAAATGATCATGAGCGCATACTTCTTG
AGGTAGGCAGTAGCTGCTATATTTATTAGCTTAGGATCAATCTGGCAGATACCTGGGTGGCGCTCCAGTATGG
CCTAGCAAAATAGAGGAATATGGACCAGTAGAAGCGCCTGCTGGCTACTTTTCACTTAAATCGCTGGACTTCA
CCGGCGCTTCAGATGAAGCGACCTTCACAGTCATCAAGTGGCCTGTATTACCACAGGAGTGATTTACTGGCTA
TCGATATTCCTGCTCATATCAATGACAGTATTTCGCTCTTTCCCGTACATTGCTCTCCCGACCAGCTCACGGT
CCAGTACATGACAGTCCTTTTCCACTTCGCTACGCCTAATTTTTTTTTAGTTGCTCTGTTTTATTGCGCTGAA
TTTGTACCTCAAAACAATAATTTTAAATACTTATTTGAATATTTTTTTTATGATTTTTTCACGAAATGAGTAA
AAAACAAAATGAAGAATGATATAAATAATTAATGCAATAAAAAATGATATCACACCAATTGATGGTCCTGTTT
TGTATACGAATAACATGCATAAAAATATAGCAAATAGATCAAGGGAAATATCCGCGATGAAATCAGTAATTAT
TTTGAAAAAATTCTTCATATTACCTCTTTAAAATATCTATTTTATTCATCATTTTTAGGTCGGGCAGTGCTCG
AAATTCTCACGTACAACGTATACGCTCCGGTTTCTGCGCGCTGCCCGGCTTCACACTGGCTCCAACAAGTACG
CCCGCTGGAATAGATTCCTGGCGTCACATAACTTCACCTGGTTATCCACAAAAGACTCAATAAATAAACATCT
TGAGTACGAAAAATTACAGTGTCAGAGAATGAAATAGCGATTCGCTGTCTTCAAGCAGTGCCTTAGTACCTGG
CACGCGCTCTTTCGCGCTATAATTTTTATCAATGTTTGTATCGCCTGCTATTTTTTAAATGTCTGCTGTATTT
CGTTCATCTTTTCAGCTGCTTTTTTACGCTGACTTTCAATGTCTTTTCTCGATTGTTCAACAAGTTGCTGTGC
TTTTTCGGCTTATTTTTTTGGGTCGAAAGGTTCTTTGATCTCATATCCGGCAATAGAACCATCCTTATTTAAG
GTCATTACCGCACGTGCTGTATTTTTAGCAGTGATGGTAATGTCATAGACGAATTCGCCATTATGAGCTATCT
CTGCTCGTACAACGATATCGCTATCTTCCGTAACGGAAATGATGCCACTGGCGTTAGCGCGTTTAATTGTTTC
CTGAATACGCTCTGGTATAATGCTGAAATTGATCGAGTCGAATTTGAATAAAGCCTCTTTTAGTGCTTCTTGT
TTTTGCTCCTCTGTTATAGTGCCAAAGATGGTTAAGTTGACAGGCGAAGGTCCCCCCAGTAGCCGTTGTTAAA
TTTAAACATCTCTACTTTAGCAGGGGTGTTAACGCTTTGTCGAATGAATGAATACCCGCCCTTGAAGAAACGG
ACTTCAATAATCGCAAGATTGGCTCCACCTTTTGCGTTGATATTGGCAATAGCTTTTTGCATTTCGGCTTTGT
CGTAAAAGAACATTGATTGCTGGTGCTCTTTTTGCTGGATTTTTTGCATGTATTTCTCTTGATTTTTTTCAGC
TTCCGCCATGGCATCTTTATCGTTATCACAGGCGCTAATTAGAGTTGCTGCAATGAAGGTAATCGGAAGTAAG
AGATTTTTATATGTAACATGTATTTTATTCATGTGAAGTATTTCCCTTTTTTATATAAAATAGATAGTCTTGA
CTGGTCGAGACTATCTTGGAAACATAGTAATAATGCGTGTGCATATCTGCAGTAAGTTTTAGTTATGCTGGTT
TTATTTCGGATTTTTACCTGTTACTTTTTGTTCGTAAATAGGTATTTTACGTATTGATGATAAGTTTAGAACC
TATCCCAGTAGGCGTAATCGTTAAAGCCAGTTTGGCGAATAAAAGAGTCTAATGGGATAGGCTCTTAATAAAC
AACAAAACCAAACGTACTTTCAGATTTCAGGCTGGAATGCGCACAGCTGATTGTTGATAAGGGCTACTCATAC
CGACAGGCCAGTGAAGCGATGAATGTCGGTTCCACATACCGGTCTGAGGTATCAACAACAACATGTTCTGAGT
GGTAGCGTTTGCGTTATATCGTCACAGACAGCGCCTCAGGGCGCTGTTTTCGATCGGACATGTCGCTTTCGCA
GGAGGGGACGTTATTCGGCGACCAGCCACATATCAGCCTCTTCAAACATCTCTTCCAGCATACGGTGCAGCCG
TTCTTTCTCGGTTTTTGTACAGTCACTGTTTAATGCGTTCGCCTGCATTGGCTTAACTTTCACTTGCGCATCG
GGAAAAAGTTGATGTACGCGTTTTGTGAGTTCATTGAGGATGATTTCGCGTGCGCCTGGCAAACCTTCAACAT
TTCGCTTGTCATAAACGAGTTCGACGAACATCGCCATTTCCTTTTTACTGGTTGGATGACCAGTATTTAAGCT
GGGTATATAACTGGTGTCAAGGTTTAGCCGCCGTTTTGTCATCCTGACGTTGCGGACGTAAAGTAAATTGATC
GCACGTGGAAGTAAAGGTCGCTAACCTGTTGTCGTGTCAAGAGGTTAGCGGCACTCTCTTCACAGCGGTGTCC
TGCTACCTGATGGTAAATTCCAGGGCGCGACTAAAATCGCAATCGGCCAACACAACAGGGTCGCCTTTTCTGA
CAATAATGCTATTTGTGCCCAGGCATTTTCGGCTCTGTTTCCCCTTAATTTTGTTATCATCAGTGAGCCAGCG
CTGGTTATCATTACCCGTGCATGAATAAAGAATGATTGGTGTGCCTTCTTTTGTACCTTGTCCTGCGGCATCC
AGACATTTCTCACCCTGCGTGATTCTGTCTCGGTGAAATGAGAAAAGCTGAGATTGCACATGCTTACACTCCC
GCAGCGTCAAACGATTCTGTTGATCTGGGCCTGTATCAAGGCACAGACCATCAGTTGTCCGAATTTGTTCCGC
GTCATTTATGTAATAACCACCGGAATTAATCTGGTTTTGTGTAATGACCGGGAACGACAAATTAATACTATAA

GGTGTGGGTAAGCCGTTCGTCGTACATCCACATAAAATAGAGGAGAGGCAGAGAACCAGTGTGGTTTTTCCTG
AAAGCAAAGTCATTCAATTATTACCCTAAAAATAAATAGCGTTACAACTATATTGGCGATTTATACCCACTGA
ATATGACGTAGCATTGAGCGGCAATAGAGATAATTCCGATAGAATCACATAAATACTTCGGTTAAATGCGCGC
AGCCAGCCACTCCTGTGACGATGGGTATATAGCGTTAAATACTTTTGCAATATTAGATGCTATCACAGTGAAA
AACGGCTGTACATTTCTTCACGTTAAACAAAGTGAGAAGGGGGGGCACCCGATATATGATAAAAGAGGTCACA
GGAGTCATTAGCAAAATAAATCTTTTAGATTTTCGCCACTGAAAACATTCTGCAGCCTTTCAGGCAAAGCCTC
ATAGCAGGCTGCGGAGGTAGACGTACTCGCTAAATTTTTCTGAAAAAATGCCGGGTGACGAATTATGAAAGAT
AAACGTTGGCGGCGGTAGGTCCGCGGAGGCCATTAATACGACAAAACTCAACGCGTATACCGGGGATAAGCGC
TTCTGTTTCGTGTTGGCGACATGCTGAAATGTGGACCTGAACATCTTTGCGTCCATCGGAGGGGGTGATGAGA
CCTTTACCGCTCTTACAATCAAAGGTTTTGACAATTCCTGTCATTTTACGAGACAAACAAATTCCTTAATGGG
GATAACGAGGCGCACTATACACGTCAGGAAAAATAATGCCAGCTATATTTGATGGCAATCAAGATTTCCGGAT
GGCTAAAAGTAGTCGTTCGGCACCATTGTAGATAAATAACATTATGTTCTGGAGAAAGTATTCCCTTTCCTGG
CGTCATTGACTGGTGCGGACATCATGGGTTTTGTCGTCGGGCAGGACGGTGAACTAATCTGCCTGGCTTGCTT
TTTATCAGTATTTGTAGGTGGTTGCGGGGTTTTACTACCAAAATCGTAGATAAAAATATCCCATGATGCCTGT
TGTAAACCAGAACAATGGCCTGAACTGGCCAAAACATGGAATGAAAAAATAAGTAATGACCAAAGCATAAAAG
CATGATGTTTCATAGCACCTCCTGTTATATATAATGACTTGTGAAGTTCATCTCCTGAATTATGGAACGTCAT
TGACATTTTTTATTCAACGAAGAGTTAACCACTCTTAATAATAATGGGTTTTATAGCGAAATAGACTTTTTTA
TCGCGTGTTCAATATTTGCGTTAGTTATTATTTTTTTGGAATGTAAATTCTCTCTAAACACAGGTGATATTTA
TGTTGGAATTGTGGTGTTGATTCTATTCTTATAATATAACAAGAAATGTTGTAACTGATAGATATATTAAAAG
ATTAAATCGGAGCGGGAATAAAGCGTGCTAAGCATCATCGTGAATATGATTACAGCGCCTGCGATGGCATATA
ACCGTATTGCGGCTGGAGCGTCACGTGAGGACTGTGAAGCACAATGCGATATGTTCTGGTTATATGGCGAGTT
TGCTTAATGACATGTTTTTAGCCGATCGGTGTCAGGTTTCTTAATGTGGTTGTGAGATTTTCTCTTTAAATAT
CAAAATGTTGCATGGTGGGTTTGTTGTTCTATAGTGGCTAAACACTTTATGGTTTCTGTTAAATATATATGCG
TGAGAAAAATTAGCATTCAAATCTATAAAAGTTAGATGACATTGTAGAACCGGTTACCTAAATGAGCGATAGA
GTGCTTCGGTAGTAAAAATATCTTTCAGGAAGTAAACACATCAGGAGCGATAGCGGTGAATTATTCGTGGTTT
TGTCGATTCGGCATAGTGGCGATAACTGAATGCCGGATCGGTACTGCAGGTGTTTAAACACACCGTAAATAAT
AAGTAGTATTAAGGAGTTGTTATGAAAAATATTATTTTATCCACTTTATTTATTACTACAAGCGTTTTGGTTG
TAAATGTTGCACAGGCCGATACTAACGCCTTTTCCGTGGGGTATGCACAAAGTAAAGTTCAGGATTTCAAAAA
TATCCGAGGGGTAAATGTGAAATACCGTTATGAGGATGACTCTCCGGTAAGTTTTATTTCCTCGCTAAGTTAC
TTATATGGAGACAGACAGGCTTCCGGGTCTGTTGAGCCTGAAGGTATTCATTACCATGACAAGTTTGAGGTGA
AGTACGGTTCTTTAATGGTTGGGCCAGCCTATCGATTGTCTGACAATTTTTCGTTATACGCGCTGGCGGGTGT
CGGCACGGTAAAGGCGACATTTAAAGAACATTCCACTCAGGATGGCGATTCTTTTTCTAACAAAATTTCCTCA
AGGAAAACGGGATTTGCCTGGGGCGCGGGTGTACAGATGAATCCGCTGGAGAATATCGTCGTCGATGTTGGGT
ATGAAGGAAGCAACATCTCCTCTACAAAAATAAACGGCTTCAACGTCGGGGTTGGATACCGTTTCTGAAAAGC
ATAAGCTATGCGGAAGGTTCGCCTTCCGCACCGCCAGTCACAAAACAGGGCTTCTTTACCAGTAACACGTACC
TGCCTGTCTTTTCTCTCTTCGTCATAGTGACGCTGTACATAACATCTCACCAGCATAAGCACAGATAAGGGGT
TGTGGTAAGCAATCAAGGTTGCTCAGGTAGGTGATAAGCAGGAAGGAAAATCTGGTGTAAATAACGCCTGATC
TCACAAGATTCACTCTGAAAAATTTTCCTGGAATTAATCACAATGTCATCAAGATTTTGTGACCGCCTTCGCA
TATTGTACCTGCCGTTGAACGACTACTGAAAAGTAGCAAGGTATGTATTTTATCCAGGAGAGCGCCTTTTTTG
CGCCTGGCAGAAGTCCCCAGCCGCCACTAGCTCAGCTGGATAGAGCATCAACCTCCTAAGTTGATGGTGCGAG
GTTCGAGGCCTCGGTGGCGGTCCAATGTGGTTATCGTATAATGTTATTACCTCAGTGTCAGGCTGATGATGTG
GGTTCGACTCCCACTGACCACTTCAGTTTTGAATAAGTATTGTCTTGCAATCCTGTTACAGAATAATTTCATT
TATTACGTGACAAGATAGTCATTTATAAAAAAATGCACAAAAATGTTATTGTCTTTTATTACTTGTGAGTTGTA
GATTTTTCTTATGCGGTGAATCCCCCTTTGCGGCGGGGCGTCCAGTCAAATAGTTAATGTTCCTCGCGAACCA
TATTGACTGTGGTATGGTTCACCGGGAGGCACCCGGCACCGCAATTTTTTATAAAATGAAATTCACACCCTAT
GGTTCAGAGCGGTGTCTTTTTACATCAGGTGGGCAAGCATAATGCAGGTTAACTTGAAAGATATGATCAATAG
CAGAAACCAGTGATTTCGTTTATGGCCTGGGGATTTAACCGCGCCAGAGCGTATGCAAGACCCTGGCGCGGTT
GGCTGGTGATCGTTCAATAGTGCGAATATGAATGGTTACCAGCCGCTGCGAATTCTACCTGGGTCACTTAATA
AAGCAAAACATTTAAACGCTCAACAACGACAATTCAGACAGTTCGAACTTTTCTTTAAAAATAGGATTAACCA
TGCTTAAAAGTGGTTACTAATTAGCCATTATACTACGGTAGTGATTCACATATTCTTTAACGTTACGTTACAG
GAAGGTTGAAGTGGAAATGCCTGATGAGTCTTCAGGCATTTCTTAAGGTTAGTTAGCTAAGGAACAGTTACTG
AGAACAGGTTTATCATCACCTTCCACCAGTTCAGCCGTTTTGCCTTTGGTATAAAGTTTATAAGTATAATTTT
TGTCTATGGCTTCGTAATTTGCTCCGGAGGCCATTTTCATTAACCGCATCGGGATCATCTCGTTTACCTGACT
GATAATCGCATAGGCGTTTCCAGCCTCGGTGTTGACGTAAATCACCTCCATAACCTGGTTATCATTACAGGTG

TATATAGATTTAGAAATCTCCTGCGAAGCTGCTATAGAGGTGGTACTGGCGCTAAGCGCAGCGAGAAAGAGGG
TAAATGGGATCAATTTACGTTTCATCATTAGTATCCTTTGTGCATAAAATTGTCGTTAATTCCCTGAAGAACG
GGAGATAAAGCGGCATCAATACGATGAATGCTGTAGTATTCCTGTTTACTGACGAAAAGTGCGCCAGAAAAGC
GTATTATAATTTATCCATCACTGACATATGTGATGAGTAGACAAATCTTAAAATCATAAACTATAGGCTGCAA
ATCATTATGGTGCGATAGAAAATGATTGTCACCACTGCTCAATACTATGGCATAAAAACCGAAGAAAACGGTA
CTGTTCACTGTCTCGTTTCATACGGTATCTGTCAGTGAAACTATTTCTTCAAATGATGGTCTTTTTATTATCG
ATCAGATAATGGCATCAACAGGGGTTATTCAGGAGTATATGTGAAAAAGTGGCTTATAGGAGGGATATTGATC
GCAAGTTTTCTGACCGGTTGTCTGATGTGGCACAACATTGATAAATGGTTTAATAAAGATATAGAATTTTTCT
ACGTCGGAGACGATAGCTAAAATTCCTGTGAGTTGGCAACGGGTGTCATATCTTTAGGTATGGCGCCCGGAGC
CGCCGGGCGCAAATTGTAGGTGTATAAATGTCATTTCATTAAAATTTTAGCGACAACATGGACAGTCACTACC
GCGTGCTAACGGCCCTCTGAAGTCACAAGATGAATAAGCCAGTTTTTTGTTTCAGCGGTGACTGAAACATATT
TTGCTCCTGGTGGGCTTACCGCCAGTTGCTGAAAAAGACAACGATGATGTAACTGTTGCAGAGACTGACGAAT
ACGACGACCGTGAATTTTATTTTTGGCGACCATATTTCCTCCCAAAAGGCCGCCCGAAGGCGGCGCTGGGTTT
GATAATGGAGAAGGGCGTTGGTCACTGCCATAACCAACCTGTATACATTAATGCAATTGATAATCATTATCAA
TTGTACTGGTGGAGAAAAAATTGTAAAGATGAAGGGACAGAGGAAATGGCAATGAACGGGATGAGATTTTTCGT
TAACCACTTAAATTCGTGACTAAAAAAGGCTTTTATAGACTTGAAAATGTTTTTCCCGTCCCTATGTAGTCAG
TGGACAAGCAATGCTTGCCTTGATGTTGAACTTTTGAATAGTGATTCAGGAGGTTAATGATGGCACTCAGAAC
CTTGTCAGCACTTCCCGTGTTTGCTGATTCTCTTTTTCTCTGACCGTTTCAACCGTATTGATAGACTTTTCAG
TCAATTAACAGGAGATACGCCAGTCACTGCGACGCCAGCTTACGATCTGCAAAAGCGCGATGCGAATAACTAT
CTGCTTACCGTGAGCGTTCCTGGCTGGAAAGAGGAAGAGCTTGAAATTGAAACGGTTGGCGGCAACCTGAATA
TTACGGGTAAACACACTGAAGAGACGGTAGAGGATCAGACGCACTGGATTTATCGTGGTATTCGTAAGGCGGA
TTTCCAGTTGAGTTTTTCTTTGCCTGAACATGCTAAGGTGAATAATGCGAAACTGGAACAGGGCCTCTTGTTG
GTCGAGATTTACCAGGAGATCCCTGAAAGCGAGAAACCGAAAAAAATTGCCATAGAAAGCAAACCAAAGGCGA
TTGAACACAAATCATAATCGCGTTAATGAAGGCCCGTTTAGACGGGCCTTCATTGCCTGTAGCGAAAAGTATC
CCGTCATGCTTGTAGGCACAGATCGCTAAATATTATAGCCCTGACCTCTATATTATCCCGTGCCACTGACCTC
GTCGCCATTGGACGGCGGTAAGTTGGAATTATCCGCGAGGTCAGTCCAATTTTTGTCCACTCTCGACATTCTG
CCTGTTATGGGCGCGATACCTGAATATAAATTCGTATAGCGGTTGATCGTTTTCACCGCGTTGATGCTATTTC
TGCTAACACTACTTTCATACGTAAAAAAGCTGATGCTTTTTACCTTATTTAATAGAGGCAGGTATGAAATTAG
TGCTCGTCAATCGGCAGGTCATTTTACCGGAAAGCGGAACCGAGTCCTTCCAGTGCCATGCCTCTACGCTTGT
TCGCTTACCGTGCGGTACGCTCGTGGCCGCATGGTTCGCGGGGTTGCGCGAAGGAAGCGAAGATACAGCCATC
TGGCTATCTCGCTATGAACATAATATCTGGACAACACCGCAGCGAGTGGCGGCACGCGAGGGGGAAGCGCACT
GGAATCCGGTGTTATTTTACCCGTCGGATAAATTGTGGTTATTTTATAAAGTAGGCAGCGACGTACACGTGTG
GAAAACCTGGTTTATCACCTCCAGCGATCGAGGATTTACCTGGAGTACGCCAGCTCCGTTGGTTAACGATGAT
ATTTTGCCGCGCGGTCCCGTAAAAAATAAACTCCTGCTGGCATCAAATGGCGCCTGGATTGCGCCAGGGTCGA
TTGAAAGTCCTGAACGGTGGAGGGCATTTGTGGATCGATCCTCTGACGAAGGAAAACACTGGAATATCTCTTT
TGTTCCGCTGGAGCCTGATAACGCTATTTCCGGGACAAACGTAGCGTTGTGGGATGGTGTAAAAAAGGGGATG
TTGTGGGAATGTTGTCTGGAAAACCTCCTGCGCTGGGATGGCGTTATTCAGCCTACGCTGTGGGAGTCTTCTC
CGGGGCATATCCATATGTTGTTACGAAGCACACGCGGGGCAATTTTTCGCAGTGATTCAATCGACTATGGCGC
TACCTGGTCTGTTGCCCGCGCGACATCTTTATCGAATAATAATAGTGGTATCGATCTGGTTAGTATGCAGGAC
GGTACGCTGATTCTTGCGTTAAATCTGGTCAACGGCAACTGGGGAAAGCGCTATCCGCTTTCACTGATTGCTT
CCCAGGATAATGGCGAATCATGGCTACCGCTACTGGACCTGGAAAGTGACCACGGTGAGTATTCTTATCCCGC
TATTATTAGCGAGGGCGGCGTCGTACATATCACCTACACGTGGAACCGGAAAAATATCGTATATTGTCGATTA
CAAACGGTGTAAGGCGCTATCAGTCGAAACCGATAGCGCTGGAATAATAATGCCTTGCCCGGCGTGCTCTCTC
ACGCCGGGATGGCCGAAATAGTCATGATTTACCGGGTAACATCCTTGAAAATGTATCATCTTTTTGTATGTAA
TGGTGAAACAACGCCGCTGCTGCATGCATGGCAATCAGGAAATAGCCGATATTGGCCAGCATTTCATGAATAC
GCTTTATATCCGATTTTAGGGCGCTATCTGGCGTCAAAAATACCGGTACAGTAAAACCAAAAAAGCTCCAGCT
TTTCCCGCCTGATGCCATCATCGCGACGCCCAGCAGCGGTAAGGCGAGAAAAGTGAGATAAAGCGCGATATGC
ATTAATTTAGCGGCTACGTGCTGCCAGTGAGGAGGGGGAGGCGTAATGACCGGATCCGGGTATTTGTGTTTAA
GATATAACCGTAAAAACATCAGCGCCCAGACCAGAACGCCCATATCATAGTGCGTGTCTTTCATAAACAGGTA
AACGCTGGAGCCTTTTGGCGCCCACCCCCGTAACTCAATAGCAGCATATGCGATGCCTGTCAGGAGCAGTGTT
AACCAGTGAAGCGTTATTTGCAGTCTTGAGAAATGTGCCATTATTATTATCCTTTATTAACAACTGCTTGTAG
AGTATGCGTAAAATCTTAACAGAACCTTAAAGCGGGGGCTGAGTCGCAAGGAACAGACAGAGATAGCGGCACG
TAAAATGGGATAAAAAGCCGGCGGCATCGCCGGCTTACTGTTGTTATTGTCCTCTTACGACAACACAGGTTTT
ACCATAAAACGGATATGAACCTGTAGTTTCATAATCAACACTCGACACTAAAGAAATATCGCCCGCTTTTTTC

GCTGAATCAATAGATGCGTCGCCGGTATTTACAATACCCAATACCGTTTGCGCACACGCTTTACCGGTTTTGG
TTGCCGGGAGACTCGTGGCAGTAATTGGCCCTTTAACATCCGTATATAAGCCAATCCCTGTCGGTGATGGTCC
TGTAGCACACCCCGTTAAAAGAGAACCAATAAAGATAATTGAAAAATGTGTTTTTTATTTGTCATGGATTAG
TCCATTATAAATTAATAGAAAACTTACCGTCCGAAAATGTCCGGACAGTCATTAAAAAAGTGGTGTATATTAA
ATATGCGATATTACTCTTTGCACCCTCCTGTTGATGTCGTACCTGACGGACAAATATCCGTTGAAGCCGGATA
TATTCCCCATGCCGTGTTTTTATAACTTGTGCATCCTGTCAGCAATGAGAACATTACGGCTAAAAAAATCATC
ACGAGCCCTGGCGGCGTTAACATTTTTCGGTTGGACTCCATAGCATGTCATTTCATCGGGTTGCCATATGACT
GGCGTTCTGCTGGTAAGTTTCATTAACGCCCGGTTCGTTTAATATTGGTTTAGGTTTAAAGCCAGGAGGCGCT
AATTACGATACCTTCTTACGTTTCAGTACTGACTAATAATGTTGAGAAAATGAACAGCGTCGGGTGGGAAGAC
GCAATACAGAATAGAAAGGAAATAAGAGAATAAGATGGCGTTAATTAACCCGGTATTCCTGGCGTCATTTCCC
TTGCGTTTTATCCCCGTCAAAAGCGGCCTTTAGCTCACGTTGGCGCTCCCGACGTTTGTGCGTATGATGTTTT
TAAAAGATCGTCATACTTTCTGTTCTGCGTCAATTTTTTCATTACAAATGCTCGCCATAATGCCGTCCTCAGG
AGGTCTCCCGACGTTTATGCTGCGCCGGGAGAAACAAAAACAACGCATAATCTGGGACAAATTGATGATTAAA
GGCAAACTGGCGCTCGTGACGTGCGCGCTGACTCTGGTATTCACTCTCGTCGCTTTTCGCCGCGTCGGACACG
GCAGACGGTCGTACCCTGAAGCTGGCCATTGGCCCTGAGCCAACGGAAGGCTTTGATCCTATGCTGGGCTGGA
GCCACGGCAGCTACTTATTGCTTCATGCGCCGTTGCTTAAGCAAAACGCCGACATGAGTTGGGGTAATTTACT
GACGGAAAAAGTGGATACCAGCCCCGTCGGTAAAATCTGGACGCTAACCTTAAAGCCCGGGCTGAAATTCTCC
GATGGCTCGCCATTAACTGCCGAAGATGTCGTTTTTACATACAATAAAGCGGCGAAGAGCGGCGGTAAAATTG
ACATGGGCAATTTTAGCCATGCGCGAGCGCTGGACGCACGCCGAATTGAGATGACGCTGAGCCATCCGCAGAG
CACCTTTGTGAATGTACTCGGGTCGTTAGGGATTGTTCCGGCCAGCCGTTATGATGAAAAAACGTTCGCCCGC
GAACCGATAGGCGCCGGTCCGTACCGACTGGTCAGCTTTCAGCCGGGTCAGCAACTGATCGTTGAAGCTAATC
CCTGGTATGCGGGTAAAAAGAATGACTTTAACCGGCTGGTTTTTGTCTTTCTGGATGAAGATAATGCTTATGC
CGCCGCGCGCAGCGGACAGTTGGGACTGGTGCGCATTGCCCCTTCTATGGCGGTGGCCCCGCAGCAGGATAAT
CTTAAACTCTGGGTACGCGATAGCGTTGAAAACCGGGGCATTGTCTTCCCGATGGTGCCTGCCGGTAAAAAGG
ATGCTAACGGTTATCCTGTCGGCAACGATGTGACCGCTGATGTCGCTATCAGACGCGCAATTAACTATGCCAT
TAATCGTAAGCAACTGGCGGAACAGGTGATGGAAGGCCATGCGATACCCGCCTATAGCGCGGTGCAGGGATTG
CCGTGGCAAAATCCTTCAGTGATATTCAGCGATGGCGATATTGCAAAAGCGCGCGCCATCCTGGAAGAGGCTG
GCTGGAAGATAAACCGTGCGGGCGTGCGTGAAAAAGCGGGTAAAGAAGCGCGTCTGACCTTATGGTATGCCAG
TGGCGACAGCACCCGACGGGATCTGGCCGAGGCGGTGCGCGCCATGTTGCAGCCTTTAGGCATTGTCGTCTCG
TTGCAATCGGGAAGCTGGGAAACTGTAGAGCGCCATATGCACGCTAACCCTACGCTGTTTGGCTGGGGAAGTC
TGGACCCGATGGAACTCTTCCATCACTACAGTGGGAAAGCCGCTGGTGTGGAATATTATAACCCGGGCTATTA
CAGCAACCCTGCGGTAGAAGCACATCTGAAACAGGCTATAGATGCGCCTGACTGGCAAAAGGCGATTCCTTTC
TGGCAGCAGGTTGAGTGGGATGGGAAGCAGGGCGCGGGCGTCCAGGGCGATGCGGCATGGGCATGGCTGCTTA
ATATTCAGCATACCTATCTGGCCAACCCCTGTATTGATCTGGGAAAAGGCGCGCCAGAAATCCACGGTAGCTG
GTCGGTGTTAAACAATCTTGATGACTGGACCTGGACCTGTCGGTGATGAAATTGTTCTTCAGTCATTTTTTGC
GGCTGATTATTCTGTTGGTGCTGGTGGCTGCCGGCACCTTCATTTTGCTTAGCTTTTCGCCCGTTGATCCTAT
CCGTGCTTATATTGGCAACGATCTGCTCCATGTTCCGCCAGAACAATATGCGCGGATTGCGGCGCGCTGGGGG
CTCGACCAGCCGCTGTGGGAGCGTTTCGGTCACTGGTTTTGGCGTCTGCTCCAGGGTGACATGGGGTATTCCA
TGCTGTTCAACATGCCTGTCGCCAGCGTGATCCGGGAACGTTTTGCGACGTCATTCGCGCTGCTGGCGGGCGC
CTGGCTATTGTCTGGTGTGCTGGGCGTGACATTGGGTTTTCTGGCCGGACGTTTTCTCCACCGTTGGCCGGAT
AAGATGATTTGCCGTATCAGCTACTTATTGTCATCGTTGCCCACTTTCTGGATAGCAATGCTGTTGCTGGCGC
TATTTGCCGTCCGCTGGCCGGTATTACCTGTTTGTTGCGCCTGGGACCCCGGAAATAATGCAGGAACAGCTCT
TTTATCGGAACGTTTACGCCATCTTGTGTTGCCGGTATGTGCGTTAAGTCTATTGGGCATGGGGCAAATTGCG
CTGCATACGCGAGAAAAAATAGCCAGCGTAATGAAGAGCGAATTTATTCGTTTTGCGCGCGCTCAGGGGGATA
AAGGATGGTCGCTGTTACGTCACCAGGTGTTACGTCATGCGATTACCCCAGCACTCTGTTTACAGTTTGCTTC
TCTGGGCGAACTGATGGGGGGAGCGTTACTGGCAGAAAAAGTCTTTGCGTATCCGGGGCTGGGACAGGCCACT
ATCGATGCCGGTCTGCGTGGCGATGTCCCGCTACTCATGGGGATCGTTTTATTCTGTACACTATTAGTGTTTG
CCGGAAATACGATTTCAGCCTGGCTTGTGGTGGTGCTTAATCGATCGCTGGAGCGTCCCGATGCTCTATAATC
CTGCCCCGACGTTGTTTCGTCTGATCCTCTCCGTTACCTGTCTGCTGTTTATTGCCGGATACGGTTATGCCAC
GTTGAGCCAGCCGCCAGAGGTTAATCTGCTTGCCCGGCATCTGTCGCCTGATATCCAGCACTGGTTTGGCACC
GATAATCTGGGGCGCGATGTATGGCTTCGTTGCTTTCAGGGGGCCTTCACCAGTTTACAAATTGGCGTTGGCG
CCGCGCTGTGCAGCGGCGTTATCGCGCTAGTGATGGCGGCAGTGGCGCGCATTCATCCCCGGCTGGACCTCCT
GATGCGGTTGATAACCGATGCTATGCTCGCCATGCCGCATTTACTTTTGCTGATCCTTATCTGTTTTACGCTC
GGCGGCGGTAAAAGCGGCGTCATTGCGGCCGTGGCGTTAACGCACTGGCCCCGTCTGGCGCTGATTCTGCGCG

CCGACGCGGAGCGGGTAGCGCAGAGTGACTACCTGACATTGACCTATCGTCTGGGGCATGGACACCTTTATTG
CTGGCGATATCACTATTTTCCGGCGCTATTGCCGCAATGGTTGACGGGAACGTTATTGATGTTTCCTCATGCG
GTATTGCACAGCGCGGCATTAAGTTTTCTGGGGTTTGGGCTGGCGCCGCATGAGCCTTCGCTGGGGCTGTTGC
TGGCGGATGCGTTACGGTTTATTAGCCATGGTAACTGGTGGCTGGTGCTGTTTCCCGGCCTAATGTTATTCAC
GCTGGTGATGCTCTTCGATCAGTTTGCGCGCGCCATTCAGCGGCTGTGGCTAAGGAGTGACGTATGCTAAGTT
TACAACAGGTTACGCTGGAAAGCGCGCGTTATCGCTGGTATGGCACGAGACGCTGGTCGCCGTTATTACAGAA
TGTCTCTTTTGATATCGCACCCGGCGAAATGGTGGCATTGGTTGGCGGCAGCGGGGAGGGAAAAAGTCTGCTG
CTGCAATGCCTGCTCGATCTGCTGCCGGAAAACTTACGCTTTCGGGGGGAGATTACGCTTGATGGCAACCGGC
TGGACAGACATACCATCAGGCAGCTTAGGGGCAATACGTTTAGCTACGTGCCGCAGGGGGTACAGGCGCTTAA
TCCCATGCTGAATATCAGAAAACATTTGAACAGAGCATGTCATCTGACCGGACGCGCCTGGGATGAGACGCAG
ATGGTACAGCTATTACAGCAAAGCGATCTTGAACCGACAGTCCTGGAACGCTTTCCCCGCCAGCTCTCTGGCG
GGATGGCTAAACGAATTCTGGCGTGCCACGCCTCGCTCAGCCAGGCTCGTTATATCCTCGCGGATGAGATCAC
CGCCTGGCTTGATACCGCACTGGCAAACCAGTTGCTTGAGCACTTGCGAGGTCTCTGCGAGCGGGGATGCGGC
GTGCTGTGGGTGACTCATGACCTGCTGCTGGCGGCGCGGTATGCCGATCGCATTGTGGCGCTGCATCAGGGTT
ATATCACGGACAATATCCGCTGCGAGCAGTTACAGCCGGAAGAGATGAGTGAGCCGTTAAAACGGCAATGGCA
AGCATTGCCTGAACTAAACCCGTTATTTATGCCGACCGGAGAGGGAATAGAATGCTGAGCTGCCGCGACCTCG
TTATTCGTCAGGGCGGGAAAGTCTTATGGCAGAACCTGACGTTTAGGATTTCTGCCGGTGAACGGGTAGGAAT
TCATGCGCCAAGCGGAACGGGGAAAACAACGTTGGGGCGCGTCCTGGCCGGATGGCAAAAGCCGACGGCGGGC
GACGTTCTGCTGGACGGCAGCCCGTTACCCCTGCATCAGTATTGTCCGGTACAGCTTGTCCCCCAGCATCCTG
AACTGACGTTTAATCCCTGGCGAAGCGCCGGAGACGCTGTACGTGATGCCTGGCAGCCTGACCCGGAAACGAT
GAGGCGTTTGCATGTGCAGCCCGAGTGGCTGACGCGTCGGCCAATGCAGCTCTCAGGCGGGGAGTTGGCGCGT
ATTGCGATACTGCGCGCGCTTGACCCACGAACGCGTTTTCTGATTGCCGACGAAATGACGGCGCAACTCGATC
CGTCTATTCAAAAAGCGATATGGGTATACGTGCTTGAGGTGTGCCGCAGCCGTTCATTAGGGATGTTGGTGAT
TAGTCATCAGTCTGCGCTACTTGATCAAGTCTGTACCCGCCATTTACAGGTGGAATAGCCGCAAGTCGTTTCT
CCGCATTGCCGTGTCGCCGTTAACGCACCGGGAACCCATGGGTTCCCGGTATTGTCGGCAGAGATTTTACTGC
ATGACGCGTTTATCTGCGTTTTTTGCATCAGCGGTATTACTGTAGTCCCTGGTCTCTTCAACCTGTACAGGGG
CTCTGACAACCAGACTAGGCAGCGCCAGTTCGATATCATTCTCAATGAGTTTTTCAATAATACGGATATTTAT
CTCTTGCTGAATATCCATGTATTTATTGTAATCCGCGGTATTGACGATATGCACCACCTCATAGGTCAGCCGA
TCCTGACCAAAGGCCAGTAGATGGGCGCGGTCGAATTGTGTTTCGCCGACATCGGTAATAATCTTTTTCACCA
TATCGCCGATGAGGCGTAACTTTTCCGGCGGTGTCGCAGTGGCGACGCCAAAAGTAAACACGATACGACGCGT
TTGCATCCGCTTATAGTTGTGTATGGTTTGCTGCAACAATTGCGCGTTCGCACAAACGATCTGTTCGCCGCTG
AGGCTACGGATGCGAGTGGTTTTCAAACCGATATGTTCAATCGTACCGGCTACATCATTAAAGACGACGAAAT
CGCCGATCTCGAAGGGCTTATCGAAACCAATCGACAGCGAAGCGAAGACATCGCTCAGAACGGTTTGTACCGC
CAGGGCAATAGCAATACCGCCCACGCCGAGGCTGGCTACCAGTGCGGTAATATCCACGCCGACGTTCGCCAGG
ATGGAGAGCAACATCATAGACCAGACGAGCACGCGCAAAATCATACCAAGAATGACTAACGTCACCGGGTTTT
TATTGGAGCCTGGGGCATACAGTAAATGACGCATCCATGACTGTACGCCCTGATCAAGCCAAATCGCCATTTG
AATCGCAACCACTAAAAACCAGGCGTGAGAGAGGGTAGAGAATAACCGGTCGGGCAGGGCGACAAAGCGCAGG
CTAAAGAGAAATGCGGCAAAAAAGATTAGCATCTTACTGGTCTTTTTCAGCATCTCGAAAACAATAGGCCGCA
GCCGCCCATGAGCATCATTCTTTTTATCCGGGCGTTGTAGCGCTTTATAGACCACATTAAGTAGCCAGTTGAT
GAGCCAGTAAGTAATGAGGGTGACGAAAAAAACGACCGCCAGATTGATCCAGAACGTCGTTGACATCAGCATC
GCAACGATATTTAGTCGTGCTAAATACTCCATTTTACCTCCGATGAAAAGCAGATGAACATTACAGTATAGAC
GTCACATCACGGAGGCCGATTTCCGGCTTTACTATTGCTGTTTATTCAGCACTTTTGTTCAAAAAGTATGAA
AAAGAGGGGTAGCCGTTTGGCTATCCCGGCGTTTCTGTGCAGACTTATCTACTCCTCTTAATAAAATAAGGAG
TCATTTATGCAACAAGAACATCGTGAGTGTATTGAGCAGTGTTATGAATGCGCGGCGGCGTGTGATATCTGCG
CCTCTTCTTGTCTGCGTGAAGATAACGTCGAAATGATGAAGCACTGCATCCAGTTAGATATGCAATGTGCGGC
CATTTGTCGCCTGGCGGCGCAATTTATGGCATTGGAAAGCGAGTACTCACAAAAATTATGCCGCCTGTGCGCG
GATATCTGCAAAGCCTGCGCCGAAGAGTGTGCAAGGCACGATCACGACCATTGCCAGAATTGCGCGCGGGCAT
GCAGCCAATGCGCAGACGCCTGCCTTAAAATGGCCGCGTAATTTTTCTTCCGCCATTAGCTCAACCGGATAGA
GCATAGAGCTTCTACCTCTAAGGTTCGGGGTTCAATTCCTCGATGGCGGACCAGTTGATATCAAAAAAGGCCA
CCTGCGCGGTGGCCGCTGAGTTTCTGTTGAAATAAGTGCAATGTTATAATATAACAATCATCTTTCTAAGAAA
GATGAGGGTAACGTTTTGGTGATTCATTTAAAAAAACTGACAATGCTTCTGGGAATGCTGTTGGTCAATAGTC
CTGCCTTCGCGCATGGTCATCATGCTCATGGCGCGCCGATGACGGAGGTTGAGCAAAAAGCGGCAGCGGGCGT
GTTTGATGACGCGAATGTACGCGATCGCGCGCTCACCGACTGGGATGGTATGTGGCAATCCGTCTATCCTTAT
CTGGTCAGCGGCGAGCTGGACCCTGTCTTCAGACAAAAGGCGAAGAAAGACCCGGAGAAAACGTTTGAGGATA

TTAAAGCGTATTATCGCAAAGGATACGTGACGAATGTTGAGACGATCGGCATTGAAAATGGCGTTATAGAATT
CCATCGCGACAATAACGTTGCATCCTGTAAATACAATTATGCCGGCTATAAAATTCTGACTTACGCATCGGGT
AAAAAAGGGGTGCGCTACCTGTTCGAATGTAAAGATGCGAATAGCAAAGCGCCTAAATATGTTCAGTTTAGCG
ATCATATCATTGCCCCGCGCAAATCGGCGCACTTCCATATCTTTATGGGAAATACCTCGCAGCAGGCATTGTT
GCAAGAGATGGAAAACTGGCCTACCTATTATCCTTATCAGTTAAAGGCGAATGAGGTCGTTGACGAAATGCTA
CATCATTAAGTCTGATTGCAATGTTAAAAGCTCGCTATGAATAGCGGGCTTTTTTTCTGCCGTTATGCCATAT
TTCTGGCAATCAATATAAAAAATACCTGCGCCCCCGGAGCAACGATGACGCTGTCCATACCGCCCTCGATTCA
ATGCCAAACGGAAGCGGCGTGTCGCCTGATTACTCGTGTCACAGGCGATACGTTGCGTGCTATTCATTTATAT
GGTTCCGCTGTCGCGGGCGGTCTGAAGCCAAACAGCGACATTGATCTACTGGTGACAATATGCCAGCTGCTGA
CGGAGGCCCAACGCGCAACGCTGATGCAGGAATTGCTGGCGCTTTCATCGCCGCCGGGGGCGTCGGCAGAAAA
ACGCGCTCTGGAGGTCACTGTCGTGCTTTATTCGCAGCTGGTTCCCTGGTGTTTTCCACCTGCCCGGGAAATG
CAATTTGGCGAGTGGCTAAGAGAGGACATTTGTCAGGGGATTTATGAACCAGCGCAGCAGGACTGGGACATTG
TGTTACTGATAACCCAAATTCTGGAAACCAGTATACCGCTAAAGGGAGAACGCGCGGAACGGCTGTTCACGCC
AGCCCCTGCGGCGCAGTTGCTGAAGGCCTTGCGTTATCCCCTCGACCTTTGGCAATCCACGGCAGATGTGCAG
GGAGATGAGTATCATATCGTTTTAACCCTGGCGCGTATCTGGTATACCCTTTCTACCGGGAGATTTACCTCTA
AGGATGCGGCGGCTGACTGGCTGTTACCTCAGTTGCCAGAAGATTATGCTGCCACGTTGCGTGCGGCGCAGCG
TGAATATTTAGGTCTGGAGCAACAGGACTGGCATATTTTGCTGCCTGCGGTCGTACGCTTTGTGGATTTTGCC
AAAGCGCACATCCCCACGCAGTTCACATAAGATGCCCCAGGACGTCTGTCAGGTTGCGCAAACGGCGTTCCTC
AACTACTACTTAATAGGTTCTCATCGCTGAAGTAAGCAGATGATCTTATGCGGGCCATCGAATGGATATTCCC
ACATGGCTCTCGTTTTGTTGAGGTGGATATGACTGGTTCCGCATCATCTGTTTTAAAGACAGATACAAGACTA
TTGAGCGAAAACCTGTTTCTTAATTATGGTCTGGAGTCCTTATTTAAGGACGTCGCAATAATAGCTGGCAACG
TAAATTATATTATTTTTGATATCGACGATTACTACACCGTACAGCAGTATATTACTTCCACATTTAAAGCGGT
ACTCATTGGAATTGTTACCCATAATGAATATAACTTTATTGATGAACATCATGCTATATATCGAATAAAGGTC
GATGCCTCTATTGCTGAGTGGCGCGAATTACTAATTTTGGCCGCTGCTGGTCAATTTTTTCCCAGGTCAGAGA
AAGTCAGATTAACGGCGAATGAAAGAAATGTTTTGGCGATGCTTGTTAAAGGAATGGATATCCGACAAATAAG
CTGTGAGCTGAATGTACATTTAAAAACTATTTACTCAGTACGCTACCATGTTCTGACTAAATTAGGTTGTAGA
ACAGTTCTGGACTATCAAATACTGTCCGTTTCTAAAGCCTTTACGCACTGGCTTACAATAAATAATGTTGATA
ATGTAATTTCCCGTGTAAATAGTAAGGTCATTGCTTAATAATGATTTGCTTCTGATGGCGTTTAGGTTCACTA
TGCTTTCTTACATCATCATGAGGCCAGACAGGCATGGCTACTTTTAGTATTAGCGCAATGGCGGCGCAGTGTG
GTGTCACAACAGCAAATATCAGGTCCTGGGAACGCTATGGCCTGCTTCAACCTACCAAAGATGAAGCTGGCAA
TCGTTTTTATTGCATAGAAGATGCTATCAGAATACAGCATATTATCGACGCGTTAAGCAAAGGCTTTTCATTA
AAAGAGATTAAACCTGCCCTCTATGGTGAAGAAACGCATTGCCGCTCGGGATGGTTATTCTACCAGGAAGAGA
TTTTAGCGCAGTGTAGAAAATTCGAACCCGCCAAATTGCGAAAATTACTCTGGCGTTATGGTCGGGAAATTCC
GCCGTGTATCGTAATTGAGTCAATACTGCGTCCTTTACGGCTGTGGTTGTCCGCAGGCGATGATGATGCACGC
CGATTTGAAAAGGCATTACTGGATACTGCGATTATTGAATACGCCACGTTCCAGCTAAGTAGCGTACGAAAGG
CGCCAGCAGGCACAATGCTTATTGCTGCGTTTTCGCTGAACGACCCCATTGAGCTATGGCTGGAAACGATTAA
ATATTGTAGTGAAGGTATGCGAGTTGAAGTCATCCCCTGGCAGGCGCCGATGCCCGATCTTTTATCAACGTCT
TTCGAGCATATTGTTCTGTGGCATGATGAGACATTAACTCCCGCGCAGGAAAATCTTATTCAGGCTTTAAAAG
AATCGGGTAAATTTAGTCTTCATGTGAAAAATGGCTCAGGCTTAACATTACTGCCAGCCGTATACCGGCAGCA
TGAGATGCCCGAAAGCATGCAGCATGTTGCACCGCAAATCCATGCAAACGGCTACGAAAAATCAGGCACCGCA
CGTTGAGGCATCTGCATTATTAACGAACAGGGGAGGAGTAACCCCTCCCTTGTTTATACCCTTCATACTTCAA
GTTGCTTATGTGTTGGCTACGGATTACTCGGCCCATCCATAGGCCTCGCCCTGACGGGCCGTCGCAAGCGACG
TTCAAATCTGCTCCCGGCAGATTTGTCGTTCACCCCAGTCACATAGTTATCTATGCTCCTGGGGACTCACTCC
CTTGCCGCCTTTAAGCAACTCGAATTATTTTGGGTATATATTAGATGTCGTCAGGTGTCTGAAACAAAACGGC
TTCTAAGGCATTACGGTAAATATCAAGCTGCACTACGTCTTTTTCTGTTTCCAGTTTTTCAATAAGTTTGGCG
ATGATATCCTTATTTGAGACATGCTGCTGCGATTGCAATAGTTCGTAAGTAATTTCCTGAATAGCTCGCTGTT
CACATAACATATGTGCAGAAAAAGGATTGCCAGTACTATTTTGTTCGTTGACTAGTGTAATGGTAGTCATATT
TCACCATATTCGATGTAAATTGAAGCCGATCTCCCTGAGCGGGGGAGAGAAATTAGTGACCTATTAAAGTTAG
ACCTATATCATAAAACTGTAATAAAAAATCATAAAAACGTGATATGCATCACATTTGTGTGATTTGTTGTATA
TTTTATACACTTTAAATGAAGGTGAAGATTCCGCAGAATCAACGGCCTGTTCTTTCTATCACTCCAGTTTAAA
CGAATAAGCATTAATACCCATCTGTAATAATTACTTAATGTTATCTTAATAAAGGTAAATTACTGTCAAGCCT
CCGTAAAAGGAGGTTGATTAATGATTCGTCATATCGCCATTTTTCTTTGTTCTTTATTGATGTGCAGCACCAC
TTTTGCCGATTCGGTAACGTCGGTATCGCTTGGCGCGCTCTTAACCGCGCTCAATGAACGCATGTTATTAATG
AAAGATGTGGCTGCTTATAAAATGAAGCACCATCTGCCGATAGAGGATTTCACACGTGAACAAAATGTTTTTG

CCGAGGCTGAAGAAGAAGCGAAAAATAACGGTCTGGACCCGCATTCGATAACCCCTTTTATTCGTTCGCTAAT
GGATGCCAGTAAAGCGATACAGTACCGCTATTTAGCGCAGTGGCGAACCGGCTCGGAACCCTCCTTTCCGATA
CAAACCTTGTCGGTCACCCGGCAACGTATTCGACAACTTGATAATCAAATGTTGATCATTATCAGCCAGCGCT
TGATGGTCGGCGCTTTCAGCCACGACGATATGGTGTGGCTGAGAGCGCAGTTTAATGCGCCGAATCTGAATGA
GAGCGATATCAGTAATGTGTTGGCAGCCTTATCTTTGGTGCGACGCGCGCGTTAACTCCACATGAGGAGAGGT
GGGCCAATACCTGGTGATTGCCTGCGGAGGTAGCGATAAAAGAACGCTTTTAGGTGATTTGGATATAAGCGTG
GCGGAATAGCGGCTGGTGAGACGTGGTTTCGCTTCCCATCTTCCGGTGCATTCGTTAGCATACTGTTTCGCAG
CTAAGCGTAAATGTTTTCAGGAAGAGGTTAATGTGTTCGCGGAGTATGGTGTTCTGAATTATTGGACGTACCT
GGTCGGGGCGATTTTTATCGTTCTGGTACCGGGGCCGAATACGCTTTTTGTGCTTAAAAATAGCGTGGGACGC
GGGGTTAAAGGAGGATATCTTGCCGCATGCGGCGTGTTTATCGGCGATGCGATATTAATGTTTCTGGCGTATG
CGGGCGTGGCGACGCTGATCAAAACAACGCCGGTTCTGTTTAACATTGTTCGCTATCTCGGCGCGTTTTATCT
GCTCTACCTTGGCGCAAAAATCCTTTACGCCACTCTGACCTCAAAAGGGCGTGCGGCGACGGAAACGGTAGTT
CCTTTTGGCGCTATTTTTAAGCGTGCTTTAATTCTTAGCCTGACGAATCCTAAAGCCATACTTTTTTATGTTT
CCTTTTTTGTGCAATTTATTGATGTTACTGCGCCGCATACTGGCGTGTCATTTTTTATTTTGGCCACCACCCT
TGAAATTGTCAGTTTTTGCTATCTGAGCTTTTTAATTCTTTCTGGCGCTTTTGTAACCCACTATATTGGTACC
AAAAAGAAACTGGCAAAAGTGGGCAATTCTTTGATTGGCCTTCTTTTCGTCGGGTTTGCCGCCCGCCTGGCAA
CGCTCCAGTCTTAACCCTCCCGGACCCGTCGTCAACGGCGGGTCATTGCTCTCCTTTCGGTGTTATTGCGTGG
AAAACAGCAAAATAGTAACCAATAAATGGTATTTAAAATGCTGTTTTTGGAGCGTAACCTTTTTACGACAGCG
ATGAGATTATCGCTGAGTAACCTGCGGGAAGAGGGAAGCAAATGCGGCAAATTCCACAAAATCATATCCATAC
ACGTTCCACGCCCTTCTGGAACAAAGAGACGGCGCCTGCCGGAATTTTCGAACGCCACCTTGATAAGGGAACG
CGACCCGGCGTTTATCCTCGTCTCTCGGTGATGCAGGGGGCGGTCAAATATCTCGGATACGCTGATGAACATT
GCTCTGAGCCGGAAGAGATCATGGTGATCAATGCCGGCGAGTTTGGCGTGTTTCCTCCGGAAAAGTGGCACAA
TATTGAGGTCATGACGGATGATACCTATTTTAACATCGACTTTTTTGTCGCGCCGGAAGTACTGATGGAAGGG
GCGACCTCACGAAAAGTCATTCATGCCGGGAGAGAATAATAATGGGAAAGGCCACTTATACCGTCACGGTCAC
GAATAATAGCAACGGCGTCTCTGTGGACTATGAAACGGAAGCACCGATGACGTTGCTGGTACCTGAAGTTGCA
GCAGAAGTTGTGAAGGATTTGGTCAATACCGTGCGTTCCTACGATACCGAAAACGAACACGACGTTTGCGGTT
GGTAAGGATCTTGTAGTATCTATTTGATATAAAGTGATTTTTTAAATCAGAAGCCGCATCGCGCGTCGCCAGC
GTGATGCGGTTTTTGCGTATTGAAAAGCGCTGTTTATTTACATCAGACATGGGTTGAAAAGAAACATTAAGAA
AACATTAAGAATATCGCATTGTTTGCTAATGAGAGCGTATTCATAAAACTTATGATTGGGTATTAAAAACCAG
AAGAGTCTTATAATCATGAAACTCGATACCCGATTAACTTCGTCAGCACTTACTCTTGCGCTTGCCGCTGTGG
TCATCCCGTTTACGGCGGACTGGCAATTGCCTTTGCTTAACGGCGTGGTGGTCCGATGGATTGAAAACGGACA
AGCGTTATGGTTACTGTTTGGGGCGCTATTTACCGCCTGGTATATACGGCCACTTTCCCGTCCCGAAGGGGCA
AAGCAGTTTTGGCTATGGGCTGTTGTCTGGTGGGTGGTGCTATTGGGTAGAAGCACCAGTTGGGGGCGTGACT
ATTTCCCTGACGAGCCGCGTATGCTGTTCAGAACGATTTCAGTGCTATTAATTGCTGCGTTAGTCTTGCCAGT
CCTGTTTTCTGCCGGGCTCCGTAAGGAGATTGTTCGTCGCCTACGGGACGCTCCTCTGCCATTGTGGCTGTTT
ACCGTGACGGCATGTTCATATCTGATTTCCGACACTGTCGAGCATCATCGGTGGTTGTCGCCGATATTCCTGC
ATAACGCTCGTTACACAGATTTGATTGAAGAGCTTTATGAAGTGCCATTTATGATCGGTTTGTTTATGGTGAC
CGTGGTGTTTATGCAGCAGGACAAGCAGGATGAATGTACGGCGCTGGAGATGACCCCGTATCACGCAAAATAG
CGCGGCTGAAAAAGACCATGAATTCGACATGGGATAGATAGCGGTTAGCAAAAAAAGCCCGCACTCGGTGAGG
CGGGCAAATAATACTGGAAGCAATGTGAGCAATGTCGTACCGAACAGGTGGGTTATAAACTCAACTACTCGGT
GTTGATAAAGATAATCGTTATCATTAATGATTGCAACCCTGATATTTATGCGCTTTTATCCACGACGCTAAAA
GGTGATGGATATCACAAACTTCACTTTCCTGACGATGTGCTATTCTTTTTAACGGCAAATAAACCGCTAACGT
TAAGACATAAGTGAGGAAAAAATGGGCATTCTTTCATGGATTATTTTTGGACTTATTGCGGGTATTTTGGCTA
AGTGGATCATGCCAGGTAAAGATGGCGGCGGATTCTTTATGACGATCATTCTGGGGATCGTTGGTGCGGTAGT
CGGAGGATGGATCAGTACGTTCTTTGGCTTTGGCAAAGTGGATGGTTTCAACTTCGGCAGCTTTGCAGTCGCG
GTGATCGGTGCGATTGTCGTGCTGTTAATCTACAGAAAAATCAAAAGCTAAACGCAGAACTTTCAGAGATAAT
AAAGGCTACGACAGGTAGCCTTTATTATTCAAAATGAATTTACCACCAGCCCAGTTCGGTACCCGCCGCCGCA
ATTAGCGCGATGCCCATCAACACCATCGTTGTTTTCCTCATGGTTATGCTCCCGGCGCGGCGTAACCGCCAAT
AAAAAACCATGCCAAAAATAATATTGCCGTAATGAAAATGACGACAGGGAAGAGGATACCCACTCTCATGCTA
TTACCTTCACTGTTCAGGGGGCGCCAGAATAGCAGGGTTTTCTCAGCGACAAAAGGTCGGTTGCTTTGTGACA
GGGCGTGGCTATTACTGACTTAACCCGGGATAAACACCCACTAAAGCGCTGACAGAAAGTATGGAAATAGCGT
ACCCGCCAGGTTATCCTGGACAAAATATCGCTTTAAGGAGTCGTTATGAAATTTACGTGGATTGCGTTGCCAG
GCGTATTGCTGCTGAGCGCTTGTTCATCGTCGCAACCCAACGCCCCCCGGCCGCCGCGCATTGGATGCCTAA
TCCGGCAGCCGTCTACTGTGAGCAACAGGGCGGTACGCTGATGCCGGTGCAGACCCCACAAGGCGTGCGTTCC

GATTGCAAATTGCCCAATGGCGAAATCATTGATGAGTGGGCATTATGGCGCAGAGAGCACCCTGATACGAAAA
AGTGACAACCTGCGGTCCGACGGGATATGATTAAGAACAATCTGATAATGAGTTTCATGCGGCGCAAGAAAAT
ATGTTTCAGTTAAAGCCGGGCGCGTTGGCAATAGTTATTGGCGCTAAGACACCCGCCGGACGGCGGAATATTG
GTAAATCGGTTGAGCTATTTTGCCTGTGCCAACCCGGAGATGAATTCATTAATCCGGTAAATGGACACGTCAC
CTTACTGCCGAAAGAAGCGCCTCGGGCGCTGTGGCTGGTTACGGGCGACGTAGCCTCCGCCGATGGTCAGCAC
GGTTTTGCCTGGGTGCGAGCAGAACATTTGATGCCGTTGTCCCCGGACAGACAACCAGGCCAGGCGGCGGCAC
GACAATCACAGCTCTCGTAGCCACTCTGCCAGCACAATGGCATGGTTCTGGCGAGTATCTTTAGCGGCGTATA
GCAACGTTAGCGGCTGGTGTCGGGCAATATCCGCCAGCCGTTTGCCTTCGTGATGCTGTTGCGCAAGTTCTGC
CCGGTATTGCTGCGCGAAGTGGTTAAAATCAATCAGTTCGCTATGAAAGGCTTTGCGCAATTCGGTGGAGGGC
GTAATGGCCTTATTCCATTCATCATAGACCAGCGCCGATTTTTTAATGCCGCGTGGCCATAGCCTGTCAACCA
ATACCCGATAACCATCGTCTTTTTCCGCCGGGAAATAGACGCGCTTACACTGGATTCGCATTTTTCCCTCCTG
TGATGGTGTCGCTTACGGTACTTTTGTCGGATAGCGGTAATGATTTTAATGGATCAGGCCTGATAAACGAAGC
GTCATCAGGCATTCAAGTGGGTATCAGGTTTATTTATGACGCTATCTCTCGATCGCGTCCGGCGTAGAGGCCT
AAAATCGCCATGATGATAGCCAGAACCGTTACGCCCGATAGCGGCAGATACCAGCTACCGCTGGCATCATGAA
GTTTTCCCATGACGGGCGGCCCACATGCCGCCAGTAAATACCCGACCGACTGCGCCATGCCGGAAAGCGCCGC
CGCCTGATGCGCCGAACTGGCGCGCAGGCCGATAAACGTCAGGCCGAGGATCATCGTCGCGCCTGAACCGAAG
CCGAACAGTAGCGTCCAGATGATCGCCTGGCCCGGCACAAACCAAAGCCCCGCCGCGCCCACTGCGCACAAGA
GTGAGACCAGCGCGGCAATCCAGCGTTGATCGTTAAAGCGAGGCAAAATAAGCGGAATGGCTAACCCAGGCGC
CGCGGTCGCCAGTTGCAGCAGGCCATGTAGCGATCCTGCCTGTGCTTCGCTGTAACCGTGGCTGATGAGTATT
GTTGGTAACCAGCCGATAATCACATAATAAATCAGTGAGTTAAGTCCCAGAAACAACGTAACTTGCCTGGCTA
AAGGCGAACGCCAGATACCGCGTTCATGTAATGCCCGGGAGCTACTCAGGTTAGCTGAGCGGGTAGTGCGCCA
CTGCGGCAACCAGATAAGAAACGCCAACAGCGGAAACAGCATTAGCATTAACAGCGCGCCGCGCCAGCCAAAA
CCGTGCAACGCCAGCGGAACCACCAGCGCGGACCCCAACGCGGCGGCGGCGCCCATCGTCAATGAATACGCGC
CCGTCAGCCTGGCGACATGTTGTGAAAAATCGCGCTTAATCAATCCTGGTAGCAGCACATTACCCAGCGCTAT
CCCGCACCCAATAATGGCAGTTCCGGCAAACAACAATGCAGCGGAGGGCAGGGAGCGGAGAGCAATACCCGCG
CAAATCAGCAGCATTGCGGCAAACAGACTGCGTTCCATCCCAAACCGGCGGGCAATACCGGCCGCCAGCGGCG
AAACCAGTGCAAACGCCAGAAGCGGCAAGGTTGTTAGCAGGCCCGTCTGGGCGGTGGAAAGACCGTAATCCGA
ACGGATTGTCTCCAGCAACGGCGCAGCGCCGGTAAATGTTACGCGCAGCGTAGTGGCTATCATCAAAATACCG
GCAATCAGTAAAGCGCCCTGTTTGCCGCGTGGTGAAAGGGCTGTCGTCATCGTTTTCTCATCTCGTCAGGCGA
GGCGATACCTTAACAGTTTTTACTGTTTGGATGAATCAAGCTAAAATGACAATTTATCGCTAAAATCGGACAA
TATAATGATTGGCCTGAGACTGGACGGTTACGATCCCGACCTTCACCACGATGCCGCTGTGGCCTTTTGTATT
CGCGCCAGGGATGATGAACTGTTTAGCCCTCGCCATCAGCATCGCAAGGGACAGTTGATCCTCGCTCTGCACG
GCGCGATCACCTGTGAGGTGGAAAATGCGATGTGGATGGTTCCGCCCCAGTATGCCGTCTGGCTCCCCGGCTC
GCTGCCGCACAGTAATCATGTGACCGCAGGGGCGGAGCTGTGCTTTTTATTTATTGAACCTGCGGCGGTCGTC
ATGCCGGAGCGTTGCTGCACGCTGAAAATTTCGCCGTTATGCCGGGAGTTGATTTTGTCGCTGGCGCGCAGAA
CCGATCCCGAAAGAGCGCAAATGCCAACGCAGCGCCTTATTCAGGTGTTGTTTGATGAACTGCCGCAGCAGCC
GCAGGAACAATTGCAATTACCGGTATCGGGGCATCCGAAAATCCGCCAAATGGTTGAGACAATGGCGCAGGAG
CCCGCGCGATGGAATACGTTAGGGCAGTGGGCCAGCGTATTCGCGATGAGCGAGCGTAACCTTGCCCGGCTGG
TCGTCAAAGAGACGGGGCTAAGCTTTCGTCGCTGGCGTCATCAGTTGCAATTAATTCTGGCGCTACAAGCGCT
GATCGCGGGACGTAATGTACAGCAGACGGCGCAAATGCTGGGTTATGATTCCACCACGGCATTCATTACCATG
TTTAAAAAAGGCCTGGGGCAGACGCCAGGCCAGTATCTGACGGGCGAGTTACTGCTTCCCCACAATTAAAGAG
ACCAGCCCCGCCGCAATCAGTGGGCCGACCGGCACGCCGCGAAACAGCGCGACGCCAAGTACCGTACCGACCA
GCAGCCCGGCGACGAGCTGCGGTTGGTTTCCCATCAGCGTAATGCCGCGACCGCCCAGCCAGGAGACGAAAAC
GCCGACGGCAATCGCGACCAGCGATTTCCAGTTCACAAAAGAGTGGATCAATGTGGAGGGCGGTAGGGTTCCG
CTGGCGATGGGGGCCATCACGCCGATGGTCAGAATGATAATCCCGACGGTGAGCCCCTGTTTTTCAATCCAGG
GAAAAAAGGTATTCAACGGGGTTACGCGGACAATAATCAGCACCAGAATTGAGACGGCGACAGTGGTGTTGTG
GCTGATAAATCCCAGAGCTGCCAGTCCGAGCAGGATCAGCAGAGTGACATCAAACATTATGGTTTCCTTGCCA
AAAAGTAAGCCTGACTACTTTACGCCCAAACGGGAAAGCGCACAGGCCTTTTTAACGATTGATGCGGATTTCA
GGCAACTGCCTGAAAGACAATTTCTACGTGAAAAACGGCGTTGTCATTAAATTGCCATCAACGCAGCGTACTG
CTAACCGGTGAACCGTCATTGTTGTCTTCCTGAGGATACAATTATGTACGATCCACCGTTTCTTGAAGCGCTG
ATGATAACGGCGTCGTTTTTCGCCATTTTTATCATTATCGTTGTGTCTGTATTGCTCCTTGAAGGGGAAGGGG
ACTAAGCCGTTGGCGATGACTGAAAGCGGCGGAAGACTGCCAGTTCTGGTCGGGCGATGCGCAGATAGTCCTG
TGTATTCATAATGACTGATTTTTCCAGCAGGCCGGCATTAAAGGCAATTTCATCGAAACGCTCAAACAGGAGC
GGATCGGCGACCAGTTTCAGATTGGGGTGAAAACTGAAAGGCGGGATCGCGCCAAAAACGCAGCCGGTCAGCA

TATCGACTTCCGCCGGGCTGGCAAGGGAAGCGCGTAAGCCGCCCAGATGTGAAGCAAGCTGAGAGAGGTCGGC
TTGCTGATCGGCCGCCAGAATAGCAAGAACGTGCTGGTTGACGCCGTTGCCTTTGACTTTACAGACCAGCGCT
TTTGCGCCCTGGCCCAGCGCAGTACCGCGTATTTCAGATACCGCTTCGCATTTCCCTACCGCTTCATGGTTCA
CCACACGATAGCGCGCGTTCTCCTGGGTTAATAAAGCCACTAAACGTTGGTGTGTAGCGACACCCCTTTGCGAC
TTCAGTCATTTCGGCCATTTCTTACTCCTGGTGCAATATGAACATTTACTGCGAAGCACTATGATGAACGATG
TTGTTTTTGCTGTTTGTTCAGATAGAGCTGCGCATCGGAAGCCTGGTAGGCATCATTAATCGTATCATTGGGC
TGCATATTATAAATCCCGGCGGAAAAATGGACTGTCTTATCCGGTGCAATAATTTGCAGGTTACGAATAATAC
GCTCCGGCAGATGGATAGCCAAATCCGCCGCGTAATCAACAAGAATAATACAGAACTCATCGCCGCCGAGGCG
AATGGCGTAATCGCTTTTACGAATCGATGTTTTAATCGCTTTCGCCAACAGGGTAATAATTCGGTCGCCTTCC
TGGTGCCCCTGGGTATCGTTGATCAGTTTTAACCTATCGCAATCAATAGCGACAAATGTCACCGGCGTCCCGG
CATTGACCAGGCGTTGCAGTCGCTGTTCCAGCTCCGGTGTTAATATTTTACGGTTGTAAAGCCCGGTCATGGC
ATCGCTAATATTTTCGCGTGTGACGTTGCGATAAAGCCGAAAGTGCATCCGTACCATATTAAGCAGGAGCGCC
GTTGCCAGTAAGTAAAAGGCCAGCGCTTTCCAGGACGAGACAAGGAAATAGGTTAAATCAAGGGACAACGAAA
CGCGCAGTCCACCCGGGATATCATGGCTATAATTCACATATTGAAACAGATTATTTTTGCTTTGATTAATAAT
AATTTCTTTTCCGGAGTCCATGTCCTTTAGTGTTACGTTAAGATAACGCCAAACCAGCGGACGGTCCTGGGTA
TAAAAAATATTTTTTAAATTATCCTGGTTAACATCCACCATCACGATACCTTTTAACGTTCCCGCCAGATAGA
CTGGCGTTAAAAAGCTCATGACGTTTTCCTGGGTAATATCATCCATGTAAATACTTGAAATAACCGTACGGCC
GGTAAAAAGTTTATCTATATCTTTTCTGTTAATACCCATTGTGCCTTTTTGTAAAAAAGTCCAACGATGCATG
GCGAAGCGCCGTGAGTTGATTAAATCATAGAAATGGACATAATGTTTATTTAAATCAACGTAATAACGGAATT
TAAGCTCCAGTGTGCCCAGCCCGTGACTGTGGTCGTCCTGCGAACGGTTGATGCTTACCGCCTGATCAAATGC
AGGCAGTAAAAGGGCGTCATCAAGATTATCGGTACACTGTGTAGCGGCGGTTTGCAGGGTGCCATGTAGCCCC
GGATAATTATGCCGGGTCAGGTTGACGCCGTGCGTACCGTTAATAGCCTCAAAGGCGTCGCAAAACGCGCGGC
GTTTTTCTGCAGTGACGGGGTCTCCCGGCGCTTCAAAAGTTCGCATCAAATGTGCGGCGATGCTCTGATTCTG
GTACTTATCGTACAAAAACGACGAATCGGCTCTCTCCATGATATAACGCATATAGGCATTCATTGCCCTGTCG
CTGGCAATAAGTTCGTAAATCAGGAAGGACGATGTCAAAACGATGACGCTTGCCGAGATAAAGTGCCTGAGCG
CTTTATGATGCAAATTCATGATGAACTACAGCCCTGAAAGTCGGATGCGTTAGTTTACCATAGCGATGAGAAT
TCCTCTAATAGACTGTTTAAAAATAAAACGTGTTGATGTTATGAAATAAAAATCATTCTCGATTTAATAAACC
AATTAATACGCTGTGTTTATATTTTCATGTGTGACTATTGTGATAAAAACTAAATTTTTAACCAACAATACTG
ATCTATTTGTGACAAAAAGAAAATAACCTTTATTATGATGATGAAAAGAGTGTGAAGTGGTAAGAATAATAAT
TTACTTCCGTTCTGAGGCTCTATGAAACGATTTAAAGAGATGGCTGTGATTTTATTTTTTGCATTACTGGCAG
CAGGTTTTACGTCGTCGGCGATGGCGGATGATCCTTTTTCACATGTTTTGGATCATGTTTTTGATAAAGTCCC
TGCCTGTCACATTGTTCCTCCTGGAGTGGGGCAACCAGGACATTTGGGGCCTTGGTCTTGGCCTTGGTGTTAT
TAACATGATGGAATTCTATTAATGGGTTAAATTACCCGATATGCCGGAATACCTTCATTCAGACTATTCGCGG
GTAATTGCTTCAGTCATAAAAACCGAACACCAGGTTTATTAAAATCCGCCCGACATATGAACGGGCGGATTTA
TTTATACGCTTTGATTGGCGCTCTGTTTCTGAAACAATTCGCGGAATACCGGATAAATATCCTCCTGATCGCG
AATATGCTGCATGGCGAAGTTATCGAACGTCGCCTGCAGATGTTCATACTCGCGCCATAAGGTCTGGTGGGCG
CGGCGGGTAATCTCGATATAGCTGTAATAGCGCACTACCGGCAGCAGCTTTTTCGCCAGAATCTCATGACACA
GCGGTGAATCGTCGGCCCAGTTATCACCGTCTGACGCTTGCGCCGCATAGATGTTCCACTGCCCCGGGTCGTA
GCGCTCTTTAACCACTTCATCCATGAGTTTAAGCGCGCTGGAGACAATCGTCCCCCCGGTCTCTTGCGAATAA
AAGAACTCATGTTCGTCCACTTCCTTCGCCTGGGTATGGTGGCGGATATAAACCACTTCTACGTTCTTATATG
TTCGGCTCAAAAACAGATAGAGCAGAATGTAAAAACGCTTGGCCATATCTTTGGTTGCCTGGTCCATCGAACC
CGAGACGTCCATCAGACAGAACATCACCGCCTGGCTGGAGGGCTCAGGCCGTTTTTCATAATTTTTATAGCGT
AAATCAAAGGTGTCGATAAACGGCACTCGCTCGATTTTAGCCCGTAGTTCGGCAATTTCCCGACGTAACCGCT
CCTCTTCAAGCAGTTGCGCTGGTTCGCTATGGCTGATGGTCTCCAGTTCCGTTTCCAGCGCGTGCAGTTCGCG
GCGTTTTCCTGCCGTCATTGCTGTACGGCGCGCCAGAGAGTTTTGTAGCGAACGTACCACGCTGATATTGGCC
GGTACGCCGTTTGAGGTGAAACCGGCGCGGTGAGTTTTATACTCGTTAAGCTGGCGATGCTGGTTTTTCTTCA
GATTAGGCAGCGCTAAATCTTCAAAGAGCAGATCCAGATATTCATCTTTTGAAATCTGAAAAACAAACTCATC
CTGGCCTTCGCCGTCCTGGCTGGCCTGACCTTGACCGCTGCCGGAACCGCCGCCGCCACCGCCTTGCGGACGC
TCAATGCGATCATTCTGGATAAAGTGATCGTTACCCGGATGGACGCGATGGCGCAGACCGCCGCGCCCCTGAT
GAAACATCGGTTCGCTAATATCATCGGTTGGAATAGAGACGGACTCTCCGCTGTCGACATCGGTCACAGAGCG
TTTATTAATCGCTTCGGAAATTGACTGCTTAATTTGTGCTTTATAACGGCGCAAAAAGCGCTGGCGATTCACC
GTGCTTTTATTTTTGCCGTTAAGACGTCGGTCTATGAACCAGGTCATATGCCCCCCGTACTGCCAACTGAGCG
CCTGTTCTGTCAGTGCCGGATAGCGCTAATGCTTAGCCAGTCTACAGAACATTACTTTGTAGGCCCGGTAAGC
GCAAGCGCCACCGGGCATTAACATCACGACGATTTACGCACGCGCAGGTACCACTCGCACAGCAAACGAACCT

GCTTACGGGTATATCCTTTCTCCATCATCCGATCGACAAAGTCGTCATGCTTTTTCTGCTCGTCAGTCGACGT
TTTGGCGTTAAAAGAAATGACCGGCAACAGCTCTTCAGTATTAGAGAACATTTTTTTCTCAATCACCGTGCGC
AGCTTCTCGTAACTGGTCCAGTTTGGATTACGGCCGCTATTGTTGGCTCTGGCGCGCAGGACAAAGTTTACTA
TCTCATTACGGAAATCTTTCGGGTTACTGATACCCGCTGGTTTTTCAATTTTTTCCAGTTCGGCGTTCAGCGA
CTCGCGATCGAACAGTTGACCGGTATCCGGGTCGCGATACTCCTGATCCTGAATCCAGAAATCCGCATAAGTC
ACGTAACGATCAAAAATGTTCTGCCCGTACTCAGAATAAGATTCGAGATAGGCCGTCTGGATCTCCTTGCCGA
TAAATTCGGCGTATTTCGGGATCAGGTAGCCTTTCAGGAACTCCAGATAACGCTCAGCTTGCTCCTGCGGGAA
CTGTTCACGCTCGATTTGTTGTTCCAGAACGTAGAACAGATGTACCGGGTTTGCCGCCACTTCCGCATGGTCG
AAGTTGAATACGCGGGAGAGGATCTTAAAGGCGAATCGGGTGGAGAGACCGTTCATCCCTTCGTCCACGCCCG
CATAGTCGCGATACTCCTGGTACGATTTAGCTTTAGGGTCGGTATCTTTCAGGCTTTCACCGTCATAAACGCG
CATTTTCGAGTAAATACTGGAATTTTCCGGCTCTTTCAGGCGGGAAAGAATCGAGAAGCGCGAAAGCGTTTCC
AACGTGCCCGGCGCGCAGGGCGCATGAGCCAGCTCGCTATGATTAAGCAGTTTCTCGTAGATTTTGATCTCTT
CGGAGATTCGCAGGCAATAAGGCACCTTCACGATATAGACACGGTCGAGGAACGCTTCGTTATTTTTATTGTT
ACGGAAAGTCACCCATTCAGATTCGTTTGAGTGGGCAAGTATGATGCCATTAAAGGGCAGGGCGGAAATACCT
TCCGTACCATTGTAGTTACCTTCCTGAGTTGCGGTCAGCAAGGGGTGCAGCACCTTAATAGGCGCTTTAAACA
TCTCGACGAACTCCATGATCCCCTGGTTAGCGCGGCACAGCGCGCCAGAGTAACCATACGCATCCGGATCGTT
CTGGGCGTGATGTTCGAGTTTACGAATATCCACCTTGCCGACCAGCGCCGAAATATCCTGGTTGTTTTCATCG
CCGGGTTCCGTTTTGGCGATGGCAATCTGCTCCAGAATGGAGGGCCAGACTTTCACCACGCGGAATTTAGTAA
TATCGCCGCCAAACTCATGCAGGCGTTTGGCCGCCCACGGGGACATAATCGTACCGAGATAGCGGCGCGGAAT
ACCGTACTCTTTTTCCAGAATCTGCGCGTCTTCCTGCGGATTAAACAGGCATAACGGATGATCGTTGACCGGA
CTACGCTCGCCGTTGGCGCTTAAAACATAAATTGGCACGCGCTGCATTAGCGATTTCAGCCGTTCAGCGAGCG
ATGATTTGCCACCCCCCACAGGTCCCAGCAAATAGAGAATTTGCTTCTTCTCTTCCAGCCCTTGCGCCGCATG
TTTCAGATAAGAGACAATCTGTTCAATCGCGTCTTCCATGCCGTAAAACTCTTCAAACGCAGGATAGCGAGCA
ATAACCCGGTTGGAGAAGAGTCGGGAAAGTCGGGGTTCCTGGGCAGTGTCGACCATATTAGGCTCGCCGATAG
CCATTAATAGCCGCTCTGCCGCGTTGGCATAAGCACTGCGATCTTGCCGACAAATGGTAAGAAAATCCTGCAG
TGTGAACTCTTCGTCCTTGGCAGCTTCGTAACGCTGGCGATAGTGATCGAATATATTCATGATATGCCATCCT
TTCGTTTTTTCGCACATGTTAAGAGCCGTTCATATGAATAGTAGAGGCTCCCGGAAGAGGTAGACTGAACGCC
GTCTCTGCTAAGGAAAAAAAAGCAACTCTCATGCCATTTTATTGGTCAGAAGATCAACGTGTCCGGTGATACA
CCTTCTTAACATTAAGCGTAGATGGCATTTGCAAAACTTGCCTGTACGCGTCAGTGGATTTCGATGACATATC
AATAACTCATCAGGTAGAAAAGGCTGGTGGATATCAGAAAAAGCGTTCCCGGTCACATAACGATCATCAGCCG
ATCATCTGAAAATAATCGGTGATGGTGATATTAAACGGTTACATTGTGTAAAATAGTTGGGCTTACAGGTATT
GGCGGTTACACTTTTGCCGCAGAACATTTGAATAAGGAATTATGGATTGTGACCAAACTTAAACTTCTGGCTC
TTGGCGTGTTTATTGCAACATCCGCCAGCGTCGCGCACGCTGAAAGCAACCTGACGTTGGGCGCAGGCGTTGG
TGTGGTTGAACACCCTTATAAAGATTACGATAGCGATGTTTATCCGATACCGGTCATCGCTTATGAAAGCGAA
AACTTCTGGTTCCGTGGTCTGGGCGGCGGGTATTACTTGTGGAATGACAATGCCGATAAGTTGTCAATCATGG
CATACTGGTCCCCGATGTACTTCAAACCGGGCGACAGCGATGACCATCAGTTGCGTCGTCTGGACAGGCGTAA
AAGTACTATGATGGCGGGCGTTTCTTACGCGCATCACACCCAGTATGGATTCCTGCGTACCTCACTGGCAGGG
GATACGTTGGATAACAGTAACGGTATTGTCTGGGATCTGGCCTGGCTGTATCGGTATACCAATGGCGGGCTGA
CCTTAACGCCAGGTATCGGGGTTGAATGGAATAGCGAGAACCAGAACGATTACTATTACGGCGTATCACGTAA
AGAATCTTCGCGTAGCGGCCTGCGCGGCTATAACCCGAACGATAGCTGGAACCCGTATCTTGAACTTAGCGCT
AACTATAATTTCGCCGGTAACTGGAGCGTATATGGTACTGCGCGCTACACCCGCCTGTCGGATGAAATTACCG
ACAGCCCGATGGTGGATAAATCCTGGACCGGTATCCTTTCTACTGGCGTGACCTATAGATTCTGATTGATTGA
ATTTGCCGGAAAAACCATGACAAAGCCTGCGTTATGCAGGCTTTGTGCTAACTGAAACGCGAACATGGCGGCT
TGCATAAATGCGCTGTTGACCGGGTATGGTACAGCCTTACGTGATTTATACTTTGTTACATCAATTTAAAAAA
CTAGCCTTTTATATGAAAAATCCAGGTGATTACAACTGCGCTAACAATTAGCGTAATAATTGTTGCAATGCTG
GCTGATATCGCCCTGTCACGTCCCGCTTATGCTATACCCATTCTTCACAGTGAGTAAGACGAATGAGCATAGT
ATTCAATACCGTAGCCAAGCCCAGCGGCAGTCTGTGTAACTTATCCTGCAAGTACTGTTTCTATCTTGATAAA
CCCCGGGGGCAGCGCGTCATTTCTGACGATGTGCTGGAGACGTATATCCGCCGGGTAATTGATGATACGCCAT
CCTCAGAGGTCTCGTTTTGCTGGCAGGGGGGAGAGCCGACGCTATGCGGTCTTTCTTTTTACCAAAAAGTGGT
GCGCTTGCAGCAACGCTATGCCAACGGCAAAACTATCTACAACAGTCTGCAAACCAATGGCGTATTAATCAAT
GAAGAGTGGGCGGCTTTCTTTGCGCAGCACCAGTTCCTGATTGGTATATCGATTGATGGGCCGCAAGTCGTTC
ATGATAATTACCGGAAAACGCCGTCAGGGCGGGCGTCTTTTTCCCGAGTCGTTAATGCTATCCGCCTTCTGCA
GGCAAATGATGTCGAGTTCAACACGCTCACTGTCGTGAATGATGCGTCATGCCGTCATGGCAACGCTATTTAT
CATTTTTTGACGCAGGAACTGGAAAGTAAACACCTGCAATTTATTCCCATTGTTGAGCCGCTCGCGCAAAAAG

CGCAGCGTTCTTTGACGTTATCTGACAATGAGGATTCGCCTTCGCTGATGCCCTTTTCCGTCACGCCTGAAGG
GTGGGGCGCCTTTATGTGCGATGTTTTTGATCAATGGATACGTCACGATGTCGGACGCATATTCGTACAGCTT
TTTGATAACTTACTTGGCGTCTGGATGGGGGAGCCCGCCACGCTTTGTACGATGCAGTCGACCTGCGGGCAAA
GTTTGCTGGTGGAGCAGAATGGCGACGTGTTTAGCTGTGACCATTTTGTTTTTCCCGCCTATAAACTGGGCAA
TCTGCAGCAACACTCTTTAGAAGAAATGGCGGCCTCTCCTTTTCAGCAGCAGTTTGGCGCGGCTAAAGCAAAC
CTTTCCTCACGCTGCCAGAACTGTACGTGGCGCTTTGCCTGTCACGGCGGTTGTCCGAAACATCGAATTTGTA
TGGACGGCGGCGAACGGCAAAATTATCTCTGTAAAGGATATCTGGAGTTCTTTCAACATGTGACGCCCTATAT
GAATGTGATGCGTCAATTATTACTAAATCAGCGACCCGCCGCGCATATTACCCGCATCGTCGACATGATTGCG
GATGACGTTCGTCAGTGACAAGAAAATCGCCCCCTTGCGCGGTATGATGCCGGACATTACCGTAGGGTCTAAG
AGTCCTGTATTGTTTTACCGTCGAGTGACCTTGAAAAGGAAATAAAATCAGTGAAAAACGTAACGTTTTGCGG
GCAGGTGACGCTTCCTGCCATCGGACAGGGAACATGGTATATGGGCGAGCGCGCGGATCAACGTCAGCGCGAG
GTTTCGGCGCTGCGCGCGGGGCTGGATTTGGGGTTACGCCTTATTGATACGGCGGAAATGTATGCCGACGGCG
GGGCGGAAAAGGTCGTGGGCGAGGCGCTGGCAGGGCGACGCGATGAGGCATTTCTGGTGTCAAAAGTGTACCC
GTGGAACGCTGGCGGCCAGAAGATAGTTGCCGCCTGTGAGGAAAGCCTGCGTCGTCTGAAAACAGATTATCTC
GATCTCTATCTATTGCACTGGGCCGGAAGCTTTTCATTCGAAGAGACCGTTGAAGGTATGGAGCGGCTAATCG
CGCAGGGGAAAATTCGCCGTTGGGGAGTCTCTAATCTTGATTATGACGATATGCAGGCGCTGTGGCGCATCCC
TGGGGGACAGCAATGCACGACAAATCAGGTGCTCTATCATCTGGCGTCACGCGGTATTGAGTATGATTTATTG
CCGTGGTGTCAACAACAGCGAATGCCAGTGATGGCCTATAGCCCGCTGGCGCAGGCTGGCCGGTTACGTAGCG
GTTTACTGAATCATCCGGTGGTGAATGAAATTGCGCAAGCGCATAACGCAACGGCGGCGCAAATCTTACTGAC
GTGGGTGATTGCACATCCGGGCGTGATGGCGATCCCGAAAGCCGGAAGTACAGAACACGTGAAGCAAAATGCG
GCGGCGCTTGAGATCGCCTTGTCAGCAAACGAACTTGCCTTACTGGACAACGCCTGGCCTGCGCCAAAAGGGA
AAACCGCGTTGGATATGGTGTGACGCCGGATAAAATGTGTAAGCGCCGCCATCCGGCAGGTCATGCCTGATGG
CGGCTGACGCCTTTCAGATCCTATAGCAACGCCGTTATCCATTCACTTTTAGCGTTTAGCGACGCAGATAGTC
TGCGCCAGACGAGAGGGTTTTTCTTCTGTGGCTTGTTGCGGCGCAGTAGCGTAAACCGTTTCAACGCAGACAA
ACGTTTTATAGCCGTCATCCGGCATGTCGCCCATGCTGACGGACAACGCCGGGCCAGGGTTCCAGCCTACGAC
GTTAAGGTGATGGTGGTGAACTACATCAATCGTGCGGTTTAATGTCGCATCGTGGATCACGCTACATGCTTCA
GGATTGAGGTAAACGCGATCGGTACGGTCCGGAAAGGTTTGTATACCGCCGGCCAGGACACCTTCTTTAGCGT
CGTTAACTTTATCAATAAACCGATCGCCGAGTCCGCTGACCTTAACGTTGGCGATGTCGCCAACGTTAAAATA
GCTGTGGAGCGCGGATGTGGTTGCAAACTCGCCATGCGCTTCCAGTTCAATTTCACAGGTTTTGCCCACTTTA
AAACGTGCCAGCAGCGTGAAATCGTGCGGCCAGTATTTCCGGGTGGCCTCACTGCTTTGCAGCTCAAATGTCA
GCATTACGCCATTATCATCTTCATTATGCGCTTTTAGCGCCCACGGTAGATTACGCGCAAAGCCGTGTGACGG
TAGACCCTGTTGAGCCGCAGGTCCAAACCAGGGCCAGCAGATCGGTACACCGCCGCGTAGCGCCACGCCGGTT
TTGAAAGGCGTATTATTACTGAGCCACAATACCTCTTCCTCGCCAACGGGTTTCCAGGAGAGAAGGTGCGCGC
CCTGTAATGCAAAAGAGGCTTTAACCTGCGGATGGTCAACGACGATCAGATCCAGGTCATCAAGCTGACGACG
GGAAAGGACAGGGGTAAGCTGTTCGATAACCGGGAGGGCAAAAATTTTATTAATCATTACGCAATCCTCTTAA
CCCTAAATAATTCGAGTTGCATGACAAGGCGCTTGTGCCTTGACCAGTGCTTGCGCTGGCCCGTAGAGGCGAG
GCCGCAGGCCGAGTAACGCGGCAAGTGAACGACAAATTCATCGGGAACGAATTTGAACAACCGTAGGTAACCT
CCGGCCAGGGACATCCCTGATTCATCCCGATAAGTTCGACTTCCGTCAGTGATTCGGGTGAGAAAGCATAGCC
AACACACCTGCAACTTGAAGTATGACGGGTATATCTTTAAGCAAAAAAAGAGCGACCTGAGTCGCTCTTACCA
ATGACTGTGTCATCTCAACTTATTTGGAGATGTGAGCAATCAGGTCCAGTACTTTGTTGGAGTAACCGGTTTC
GTTATCGTACCAGGAGACCAGTTTCACGAAGTTGTCGTTCAGCGCGATGCCTGCTTTAGCATCGAACACGGAG
GTGCATACTTCGCCGTTGAAATCGGTAGATACAACGTCGTCTTCGGTGTAACCCAGAACGCCTTTCATTTCGC
CTTCGGCAGCAGCTTTAACAGCAGCTTTGATCTGCTCGTAGGTAGCCGCTTTTTCCAGACGAACGGTCAGGTC
AACAACGGATACGTTCGGAGTCGGAACGCGGAACGCCATACCAGTCAGTTTGCCATTCAGTTCTGGCAGTACT
TTACCTACCGCTTTAGCAGCGCCAGTAGAGGACGGGATGATGTTCTGAGATGCGCCGCGGCCGCCGCGCCAGT
CTTTGTGAGACGGGCCGTCAACGGTTTTCTGGGTTGCGGTAGTCGCGTGAACAGTAGTCATCAGACCTTCGAT
GATGCCGAAGTTGTCGTTGATAACTTTAGCCAGCGGCGCCAGGCAGTTGGTGGTGCAGGAAGCGTTGGAAACG
ATGTCCTGGCCTTCGTATTTGTCAAAGTTAGCGCCTTTAACAAACATTGGGGTGTTGTCTTTAGATGGACCCG
TCAGAACCACTTTTTTCGCGCCAGCGGTGATGTGTTTACGCGCGGTTTCGTCAGTCAGGAAGATACCGGTAGC
TTCAGCCACTACGTCAACACCAACTTCGTCCCATTTCAGGTTAGCCGGATCGCGTTCAGCGGTAACACGGATT
TTTTTACCGTTAACGATCAGATGACCGTCTTTCACTTCAACAGTACCGTCGAAACGGCCGTGAGTGGAGTCAT
ATTTCAGCATGTATGCCATGTACTCTGCGTCTAACAGGTCGTTGATTGCAACGATCTCGATGTCAGAACGTTT
CTGAGCAGCACGGAAAACAATGCGACCGATACGGCCAAAACCGTTGATACCTACTTTGATAGTCATATATTCC
ACCAGCTATTTGTTAGTGAATAAAAGGTTGCCTGTAAAATTACAAAAACCTTACGCAGCGTCAAGCGGAATCG

TGTCAATCATTGCGACAAATCAATCCTCTGCACAACCTTTGCGCGGCTGATTCGCCTCACTCTTCCTTTCGGC
TTGAAACCACATGGGGGCGCCGCCCCGAATTTTAAAGGGCAACCGAGATAATAATGTGACATCAGTCACGATT
CGTGAGCCGCCATTTCGCAATGATCAAGTTTAGAACAAAAGTTGGTACTGAGGTGTTAATGTTTTGTTAGAAT
CGGTCAGGCAATGTGAGCACGTTTAAAGTGAGATGTGAGCAAATGGCTAACCAACCTTCAGCAGAAGAACTCA
AGAAAAAATTGTCCGAAATGCAATTCTACGTGACGCAGGATCGTGGCACAGAACCGCCATTTACCGGACGCCT
GCTACATAATAAACGCGATGGGGTTTATCACTGCCTGGTCTGTGATACGCCGCTTTTTCACTCCCACACCAAA
TACGATTCAGGCTGCGGCTGGCCTAGCTTTTACCAGCCTGTCAGCGAAGAGGCGATTCGTTACATAGACGATT
TTTCTCATGGCATGCAGCGTGTCGAAATTCGTTGCGGTAACTGCGATGCGCATCTCGGACACGTTTTCCCGGA
CGGGCCGCAGCCAACCGGTGAGCGCTATTGCGTTAACTCGGCCTCGCTGGCCTTTTCTGACGAGAAAAACGGC
GATCAACTGAAAGGCTGAAAAAACGATTCAGCAAATTATTCCACAGGAGCGGACACATCATGAATCTTGATGA
GATCATTAACAGCATGACGCCGGAAGTGTATCAACGTTTATCCACCGCCGTTGAGCTGGGTAAATGGCCGGAT
GGCGTGGCGTTGACGCCGGAACAGAAAGAAAACAGCCTGCAACTGGTGATGCTATGGCAGGCGCGGCACAATA
CCGAAGCGCAACATATGACGATCGATACTCACGGTCAGATGGTGATGAAAAGCAAGCAGCAGCTCAAAGCGGA
TTTCGGTATTACGTCAAAGCCGATTGCGACATTGAAAATGCAGTAACGGCAGCCGGATAAGAGGTAGCCGCCA
TCCGGCGGTTTCATTGCCAATGCGTTGTGCTTACCGGGCCTGCTCATTGCTGTCAGGCACAGGCCCGCCATAT
TTTTACCCATTAACGCTTAATGTCCAGCGAGTCCGCCAACTGTTTCGCTAACTGATTATTGTCATCGGCGGCC
TATTCCCAGAACATTGCGCCGGCCAGTCCGCGCTTTTTAATCTGGTCCGCGTTGATCGATCGCCACCGAGCGC
GGATTTTCATAAGAGAGGGCAAGCAGTGCCTTCCTCCGGCAGACTTCACTGACAGCAGCAGGTCAGGCGTTTG
AGTCATACGTATTGGCGTTGAAATACTGTGTGCCATAGGGCCATGTCGTAAGTCATCAGATTGATAAAATCGA
GCTACGGGGCTATAGCTTTCACGTCCAGCCAGCTTTTCGGACTTTCCGCATTCGCGCCGACGACGAGGGTGAC
CCATTGGCCCATTAGCTGACGAAGCGCTTTTAAATGCGACGTTAAACGAATATTCCCAGTCAAGATCGATCCC
ATCGAGACCATATTTGTCGACAATCTTCTGCGCCGGTTGAATAAACACCGCACAGGCTTCTTTAACGATCCTC
TTCGACATCTAAAACAGAAACACGCCACAGAATGGAGTGGCGAAAAAATAACCGCAGCAGATTAGCATCTTCA
TCCATGGAAACCTGCGCAGGCGTTCACGCCGTCTTTCGCGATACGGCTTCCATCTTTTTGGTTTTATCGCCGT
TTGTTTCGTTGAGTTTGTGATCTTGGTTGGTAAATACAGATCATTATCTGTGTTTTAAAGATTTATCGTTGAC
CTTAAAGATTTTATGGTTAAGTATGATGAGCATTGATGCACACCAAACACAAAGGAAGATGCAATGAAAGCAC
TGGCGAGATTTGGCAAGGCCTTTGGCGGCTACAAAATGATTGATGTCCCGGAGCCGGTATGCGGTCCGGAAGA
CGTGATTATCGAAATCAAAGCGGCGGCGATCTGCGGCGCGGATATGAAGCACTATAACGTTGACAGCGGGTCC
GACGAATTTAACTCCGTTCGCGGTCATGAATTCGCCGGACGTATCGCGCGGGTGGGCGAAAAAGTGAAAGACT
GGAAAGTCGGCCAGCGCGTGGTTTCTGACAACAGCGGACACGTTTGCGGCGTTTGCCCTGCCTGTGAACAAGG
CGATTTTCTGTGTTGTACAGAAAAAGTGAATCTGGGTCTGGATAACAACACCTGGGGCGGTGGATTTTCCAAA
TACTGCCTGGTTCCGGGAGAAATCCTGAAAATCCATCGCCATGCCCTGTGGGAGATCCCGGAAGGCGTGGATT
ACGAGGAAGCCGCAGTACTGGACCCTATCTGCAACGCCTATAAATCTATCGCCCAGCAATCCAAATTTTTACC
CGGCCAGGACGTGGTAGTCATCGGCACCGGTCCGTTAGGTCTGTTTTCGGTCCAAATGGCCCGCATTATGGGG
GCGGTAAATATCGTGGTGGTTGGCCTGGATGAGGATGTTCCTGTCCGTTTCCCTGTGGCCAAAGAACTCGGCG
CGACGGCGTTTGTTAACGGCTCCACGGAAGATGTCGTCGCACGCTGCCGGGAGATTTGCGGCAAAGACAACCT
CGGTCTGGTGATCGAATGCTCCGGTGCCAACATTGCGCTTAAACAGGCCATCGACATGCTGCGGCCAAATGGT
GAAGTGGTTCGCGTCGGTATGGGGTTTAAGCCGCTCGATTTCTCCATTAACGACATTACTTCCTGGAACAAAA
GCATCATTGGGCATATGGCCTATGACTCCACATCCTGGCGCAATGCGATTCGCCTGCTTGCCAGCGGCGCTAT
CAAAGTAAAACCGATGATCACCCATCGTATCGGTCTTTCTCAGTGGCGCGAAGGGTTTGACGCAATGGTCGAT
AAAACAGCCATTAAAGTGATCATGACCTACGACTTTGATGAATAACCCTTCTGCTAAAGGAACGTTATCGCAT
GGAACAGATAACAAAACCGCACTGCGGCGCCCGGCTGGATCGCCTGCCGGATTGCCGGTGGCATTCATCAATG
TTTGCCGTCGTCGCCTTTGGTCTGCTTGTTTGCTGGAGTAATGCCGTCGGCGGATTAATTCTTGCCCAGCTCA
AGGAGTTAGGCTGGACCGATAATTCCACGACAGCCACCTTCTCCGCCATTACCACGGCGGGGATGTTTCTCGG
CGCGCTGGTGGGCGGCATTATCGGCGATAAAACCGGACGCAGGAACGCCTTTATTCTTTATGAAGCGATTCAC
ATTGCCTCGATGGTCGTGGGCGCATTTTCACCGAATATGATGTTCCTGATTGCCTGTCGTTTTGTGATGGGCG
TGGGATTAGGGGCACTACTGGTCACGCTGTTCGCCGGATTTACCGAGTACATGCCCGGCAGAAACCGGGGAAC
CTGGTCGAGTCGCGTTTCGTTTATTGGCAACTGGTCATATCCATTGTGTTCCCTGATTGCAATGGGGCTGACG
CCGCTAATTAGCGCGGAATGGAACTGGCGCGTGCAGTTACTGATCCCGGCTGTGCTGTCGCTGATTGCTACCG
CGCTTGCCTGGCGATACTTCCCTGAATCTCCCCGCTGGCTGGAATCGCGCGGGCGTTATCAGGAAGCCGAAAA
GGTTATGCAGGCCATTGAAGCAGGCATCGAACGTCAGACAGGAAAACCGCTGCCGCCGGTTGTTATTGAAAAC
AGCGGCAAGCCGCCGCGTTCCGTACCGTATTCAGCGTTGCTCACTGGCGTATTACTGAAACGCGTTATTTTGG
GTTCCTGCGTGCTGATTGCCATGAACGTTGTGCAATACACGTTAATTAACTGGCTGCCCACCATCTTTATGAC
ACAGGGCATCAACCTCAAAGATTCGATCGTTTTAAATACCATGAGTATGTTCGGCGCACCGTTTGGCATATTT

ATCGCTATGCTGGTAATGGATAAAATTCCGCGCAAAACCATGGGTGTCGGTTTATTAATTCTGATTGCCGTGC
TGGGATATATTTATTCGCTGCAAACCAGCATGTTGCTTATCACCTTAATTGGCTTTTTCCTGATTACCTTTGT
CTATATGTATGTCTGTTATGCCTCGGCGGTCTATGTGCCGGAGATCTGGCCAACAGAAGCGAAACTTCGCGGT
TCCGGCCTGGCGAACGCTGTCGGCAGAATCAGCGGTATCGCTGCCCCCTATGCGGTGGCGGTATTACTCAACG
GTTATGGCGTGACGGGCGTTTTCGTCCTGCTGGGCGCTGTCTCGATTATCGTCGCCGTCGCAATAGCGACTAT
CGGAATCGAGACCAAGGGTGTTTCGGTTGAAAGTCTGAGCATTGATAAGGTCACGAGTAAATAAAGGAATAAA
AATGAAAAATTCAAAAGCGATACTAAAAACGCCGGGCACCATGACAATTATAGCGGCTGATATTCCAGTACCA
AAAGAAAACGAAGTATTGATCAAAGTGGAATATGTCGGTATTTGCGGTTCAGATGTTCACGGTTTTGAATCCG
GGCCATTCATTCCGCCGAAGGATCCAAATCAGGAAATTGGTCTCGGTCATGAGTGTGCTGGTACGGTCGTTGC
GGTCGGCAATCGGGTAAGCAAATTTAAGCCAGGCGATCGGGTTAATATCGAGCCGGGCGTGCCGTGCGGCCAC
TGCCGCTATTGTCTGGAAGGAAAATACAATATTTGTCCGGATGTTGATTTTATGGCGACGCAGCCGAATTATC
GCGGGGCCTTAACGCACTATCTGTGCCATCCGGAAAGTTTTACGTACAAGCTTCCGGACAATATGGACACTAT
GGAAGGTGCGCTGGTGGAACCTGCTGCTGTTGGAATGCACGCGGCAATGCTGGCGGATGTTAAACCGGGTAAG
AAAATCGTCATTCTCGGCGCGGGCTGCATTGGTTTAATGACCCTGCAAGCGTGTAAGTGTCTGGGGGCGACCA
ATATCGCGGTAGTGGATGTGCTGGAAAAACGGCTGGCAATGGCTGAACGACTGGGCGCGACAACCGTTATCAA
TGGGGCGAAAGAAGATACTGTCGCGCTCTGCCAGCAGTTCACCGACGATATGGGCGCCGATATTGTGTTTGAA
ACCGCCGGTTCCGCCGTCACAACTCAGCAAGCGCCGTATCTGGTCATGCGCGGCGGGAAGATCATGATTGTTG
GCACTGTCGCAGGAGATTCAGCGATTAATTTCCTCAAAATTAACCGTGAAGTCTCCATCCAGACGGTATTCCG
CTATGCCAACCGCTATCCGGTGACTATTGATGCCATCTCCTCCGGGCGTTTCGATGTGAAATCAATGGTGACG
CATATTTACGATTACAAAGACGTACAACGTGCATTTGAAGAGTCGGTGAATAACAAACGCGACATTATTAAAG
GCGTTATTAAAGTTTGCGATTAATGAAAAACGGGAGAAACAGCGATGTTGGCAGATATAAAATATTGGGAAAA
TGATGCACAAAATAAACATTACGCGATTGCGCACTTTAATGTGTGGAATGCGGAAATGTTGATGGGAGTGATT
GATGCGGCGGAAGAGGCAAATTCGCCGGTTATTATCTCCTTTGGTACCGGATTTGTGGGCAATACTTCATTTG
AAGACTTCTCTCATATGATGGTTTCCATGGCTAAAAAGCCACTGTGCCGGTAATTACCCACTGGGATCATGG
ACGTAGCATGGACATTATCCATAATGCCTGGACGCACGGCATGAACTCGCTGATGCGTGACGCTTCTTCCTTT
GATTTTGAAGAAAATATTCGCCTGACCAAAGAAGCCGTGGATTTCTTCCATCCGCTGGGTATTCCGGTAGAGG
CGGAGTTAGGACACGTAGGGAACGAAACGGTGTATGAAGAGGCGTTGGCGGGGTATCACTATACCGACCCGGA
TCAGGCGGCGGAATTTGTAGCGCGCACCGGTTGTGACTCGCTGGCGGTCGCCATCGGCAACCAGCATGGCGTG
TATACCTCTAAACCAAAACTGAATTTTGAGGTCGTGGAGCGCGTGCGCCAGGCAGTTTCCGTACCGCTAGTGT
TACACGGCGCTTCCGGTATTAGCGACGCGGATATCAAAAAAGCCATTTCGCTGGGCATCTCGAAAATCAATAT
CCACACCGAACTGTGTCAGGCCGCGATGGTAGCGGTAAAAGAGAATCAGGATCAGCCGTTCCTGCATCTGGAA
CGCGAAGTACGCAAAGCGGTAAAAGCCCGTGCGCTGGAAAAAATCAAACTGTTTGGTTCAGATGGTAAAGCGG
AATGATGATATGGACGAAATTGAAGTTCTTTGCATAGGGGCGGCCATTGTCGACATTCCTTTGCAACCGGTTA
GTAAAAATATCTTTGATGTCGATTCGTATCCATTAGAACGAATTGCCATGACCACAGGGGGAGATGCGATTAA
TGAAGCCACGATTATTTCGCGTCTCGGGCATCGCACTGCGCTAATGAGTCGCGTTGGCGACGATGCGGCAGGA
TATTTTATTCTCGACCACTGTCGGAAGGAGAATATCGATATTCAGAGCCTGAAGCAGGATGCGGATATTGATA
CCTCAATTAATGTTGGGCTGGTGACTGATGATGGTGAGCGCACCTTTGTCACTAACCGTAACGGCAGCCTGTG
GAAGCTAAATATTAACGATGTCGATTTTGACCGTTTTTCTCAGGTCAGGCTGTTATCACTGGCAAGTATCTTT
AACAGCCCGCTGCTGGATGGCAAAGCATTAACGGCTATTTTTACGCAGGCGAAAGCGCATCAACTGGTTATCT
GCGCTGACATGATCAAACCGCGGCTGAATGAAACTCTGGACGATATTCGCGAAGCGCTAAGTTTTGTCGATTA
TCTGTTTCCTAATATTGATGAAGCCAAAAAGCTGACCGGGAAAGAGACGCTGGATGATATTGCCGATTGTTTT
TTGCAATGTGGCGTAAAAACAGTGGTGATTAAAACTGGCAAGAAAGGCTGCTTTATCAAAAGCGCCGATATAA
AAATGGAGGTGCCTGCGGTGTCAGGCATTACCGCCATCGACACTATTGGCGCTGGTGATAATTTTGCATCGGG
ATTTATCGCCGCGCTACTGGAGGGAAAAACGCTGCGTGAATGCGCACGGTTCGCCAATGCGACGGCCGCGATA
TCGGTACTCAGCGTGGGCGCCACAACGGGCGTGAAAAACAGAATGCTAGTGGAACAACTCCTGAATGAATACG
AAGGGTAATAGCGGCAGATGAAAATGATACCTTTAGGTAATACAGATATAGCGCTTTCCCGTATGGGACTCGG
CACCTGGGCTATCGGCGGCGGTCCGGCATGGAATGGCAACCTCGATATGCAGGTCTGCATCGATACTATCGTT
GAGGCGCACCGTTGTGGTATTAACCTGATTGATACGGCGCCGGGCTATAACTTTGGCAATAGCGAAGTGATTG
TCGGCCAGGCGCTGAAAAAAATTACCGCGTAACGAGATGGTGGTGGAGACCAAATGCGGGATCGTCTGGGAGC
GGGAAGGGAGTCTGTTCAATAAAGTCGGCGATCGCCAGCTCTATAAAAATCTTTCTCCGGACTCTATTCGTAA
AGAAGTCGATGCCAGTCTCCAGCGACTAGGGATTGATTATATCGATATCTATATGACCCACTGGCAGTCGGTA
CCGCCGTTTTTTACGCCGATTGCGGAAACCGTTGGCGTGCTCAATGCTCTGAAAGCAGAGGGGAAAATCCGTT
CTATCGGGGCGGCGAATGTAGATGTCAGCCATATCCGCGAATACCTTAAACATGGCGAGCTGGACATCATACA
GGCGAAATACAGCATTCTTGACCGGGCGCTGGAAACGGAGCTGCTGCCGCTATGCCGCGAGCACGGCATCGTG

GTACAGGTTTATTCGCCACTGGAGCAGGGATTGTTAACCGGCACCATTACCCGTGACTATGTTCCCGGCGGGG
CGCGCGCGAATAAACTCTGGTTCCAGCGTGAGAATATGTTGCGGGTTATTGATATGCTGGAGCAGTGGCAGCC
ATTGTGCGACAAATACCATTGCGCCGTCCCGACGCTGGCGCTGGCGTGGATATTGCGACAAAGCGAGCTGATT
TCGATACTGAGCGGCGCCACGTCGCCGGAGCAGGTACGAGAGAATATCGCTGCGCTGTCCATCACCTTAACGG
ATGACGATGCGTTATTGATGAGACAAATGGCGGAAGCGCTGGACAATCCATAAGCAATACATTACACCTCTTT
TTTGGCGAAATGCAGATAAAATCTGTTATTCAAACGGCTTATTTTGAAAGTAAACATAGTCTGTGAGATACTC
AACGTTTATCATGACGTCGAAAACAGAGGTGTAATCGTGGCGGCGAAAGACAGGATTCAGGCGATCAAACAAA
TGGTCGCTAATGATAAAAAAGTAACAGTCTCTAACTTGAGTTCAATTTTTCAGGTAACCGAAGAGACAATTCG
ACGCGATCTGGAAAAACTCGAAGATGAAGGCTTTTTGACTCGAACTTATGGCGGTGCGGTATTAAATTCGGCG
GTGCTGGCTGACAATATCCATTTTTATAAGCGTGCAAAATCGTTCTATGAAGAGAAGCAAATTATAGCCCGTA
ATACGCTTCCGTTTATTAAAAACAAAACAACGATGGCCGCCGACTCCAGCAGTACGGTAATGGAATTGCTAAA
ACTCCTGAAAGACCGAAACGATCTTACTTTGTTAACTAACTCAGCGGAAGCTTTCCATGAACTGGCGCAATCG
GACATTAATCTTGTTTCTACGGGCGGTGAGTTAAATAAAAATACGCTATCGCTGCAGGGGCGTATCACTAAAG
AAATAATCAGCCGTTATCATGTTGATATTATGGTAATGAGTTGTAAAGGGCTGGATATGGCGAGCGGGGCGCT
TGATTCTAACGAGGCGGAAGCTGAAATCAAAAAAACGATGATTCGCCAGGCCACAGAGGTGGCGTTATTGGTG
GACCACTCAAAATTTGATCGTAAAGCGTTCGTGCGGTTGGTCGATTTTAGTCATATCCATTACCTGATAACGA
ATAAAACGCCAGGTGATGAATGGATCGCATTTTGCGATAAAAATAATATCCAGCTTGTCTATTAATCGATAGA
TTATCCTTGCCTGGATTGTTTTCGCCTGGATTATGCCAAAAAAAACGGCAGATGATAGCAAGTGCGGCAAATA
CCCCTTTGCCGAGAGACAATAATTTATTATTTTGCTGCGTTAGAGGCCATCCAGATGGAAGACAATGATCATA
TCGGCGCCAGACTAGACCGCTTACCGTTATCCCGGTTTCATTTTCGTATTTTTGGAATAATTAGTTTTGGCCT
GCTATTAACGGGGTTTTTGAGTTATTCAGGAAATGTGGTTCTGGCCAAATTGATAAACAATGGCTGGTCGAAT
AATTATCTTAATGCCGCGTTCACATCGGCACTGATGCTTGGCTATTTTATTGGTTCGCTTACCGGCGGTTTTA
TTGGGGATTACCTGGGGCGTCGAAAAGCGTTTCGCATTAATTTGTTGCTTGTGGGCATCTCCGCAACGGCTGC
CGCATTTGTTCCCAATATGTACTGGCTGATTTTCTTTCGTTGTCTGATGGGGACAGGCATGGGGGCATTGATT
ATGGTTGGATATGCTTCTTTTACCGAGTTTATCCCTCCCATTGTACGAGGAAAATGGTCCGCGCGTCTCTCTT
TTGTAGGGAACTGGTCACCGATGCTGTCTGCGGGAATTGGCGTAGTGGTCATTGCGTTTTTAAGCTGGCGGAT
GATGTTTCTTCTTGGTGGGATAGGGATGCTGCTGGCGTGGTCTCTTTCGGGGAAGTACTTTATCGAATCGCCC
AGGTGGTTAGCAGGTAAAGGGCGGCAACGCGAGGCTGAACAGCAGCTTCTCATGATTGAATCGCAAATAGAAA
AAGAAAAAAGTATTGTTTTGCCGCCGCATCAGGAAAGCAATAAATCCACGGAATCGCGCTTTGAAAACGGTAC
ATTCTGGCTCTTATTCAAAGGACAAATGTTGCGGCGCACGTTAGTGGCGATCACTGTATTGATTGCGATGAAC
ATTTCGCTTTATACCATTACGGTATGGATCCCGACAATTTTTGTTAATTCAGGAATTGATGTTACAAAATCCA
TTTTCATGACCGCCATCATTATGATTGGCGCGCCGGTGGGCATATTTATCGCCGCGCTCATTATTGATCATTT
CCCCAGACGGTTATTTGGTTCGCTGTTGCTTATCGTTATTGCGGTAGTAGGATATTTTTACTCAATACAGACT
GAGGAATGGGCCATTCTGGTTTATGGACTAGTCATGATTTCCTTTCTGTATATGTACGTCTGCTTCGCCTCGG
CGGTTTACGTTCCCGAATTATGGCCTACGCACCTTAGACTACGTGGATCGGGTTTTGTGAATGCGGTAGGGCG
GATCGTCGCTGTCTTTACCCCCTACGGCGTGGCGGTTTTACTGACCCGGTATGGATCTGTCACCGTTTTTGTT
GTGCTGGGTATGCTGCTGGCGGCGTGCGCGCTGATATTATTTGTTTTGGCATTGAAACGAGAAGAGTTTCGT
TAGAAGCGCTCTCCAGCCTGCGTTGAGCTTTGCCTGGTAGCGGCGCTGACGTGACGTATCCGGCCCAAAGATC
TATGCAATGGTCTCACCGTTGCGTGATGTATTGACCGGATACGATACCTGCTCCGCTACCTGGCGACAGCCTG
GCCCTGCGTCTCTTCCCAGTCTGCCAGCGTATACAGCGTCGCGCCTGCCGACGCCATCTCCATAAACGCGTGT
GCGCTATCCTGCGGGTGAATATTCACGCCGCGACAGCCATCGGTGATAACGTTAACGGCATAGCCCAACTCTA
GCGCATCCAGCACCGTAAATTTTACGCAGTAATCGGTCGCCAACCCCATCACGATAAGCTCGGTAACGTCATG
CTCGCGAAGCCAGGTATCAAGGGTAGTTTTCTGCCGATGTTCGTTGTCAAAAAACGCGCTGTAGCTATCAATC
AGCGGGTTCTCACCCTTATAGATGCAGGCGTCAATCGCATGTTGGTTAAGCAGGGGATGTAACGCCGCGCCGT
CGGTATGTTGAACGCAGTGATCCGGCCAGAGCGTTTGCGGCAGCCCGTCCAGCTCTCCCTGACTATAGGGTTC
CGCCTGATGCTGGCTGGCGAAGCTGCCATGCTGCGCGGGATGCCAGTCCTGGCTGGCCAGCACCGGAATCTGC
CGGGGCTGGCACCAGTCAATAAGAGCGTTGGCGATGTCGATGGTACTGTCGCCTTCCGCTACAGCCAGCGCGC
CGCCGGCGCAAAAGTCATTTTGCAAGTCGACCAGTAATAAGGCCCGGTTTGTCATTATCTTCTCCTTGTCGTC
GGGATTTTTTAATCATCCGGCGTCAATTCACCACGCAAGTTTTGCGCCATCGCGCTACGAATCGCCTGAGTGT
CCAGACCCTGGCTTAACAAATAATGCAGTTTGGTTAAGGTAGCTTCGACGGTCATATCGGCACCGCCAATCAC
GCCAGCGTGCGCCAGCGCATTACCGGTAGCGTACCCGCCCATATTGACTCTCCCTGACATGCATTGCGTCAGG
TTAACCACCACGATGCCGCGCGAACTGGCCTCTTTCAGCTCCTGTAAAAACGCTTTATTTGCGGCGCGTTGC
CTACGCCATAAGAGCGCAGAATCAGCGCTTTTACCGGCTGACGCAGAAAGTTACGTACTACCTCGGCGGAAAT
CCCCGGATAAATCGTCACTACGCCTATCGGCTGCGGCGTAATAGGGTGAACGATTAATTCACCAGAACCGTAG

GGCGCGGGAGGCGTATTTAAACGGCGAATGTGAATTCCTGCTTCCAGCAAGGGCGGCAGATTAGGAGAGGCAA
AAGCGTCAAAACCATCCGCATGTGCTTTGGTCGTGCGATTGCCGCGGAACAGACGATTATTAAAAAACAGCGT
TACTTCATTAACAGGGTAGTTGGCGGCGACATAAAGCGCATTCAGCAAATTAATTTGCCCGTCGGAGCGCAGC
TCAGCCAGCGGAATTTGTGACCCTGTCACAATAACCGGTTTACCCAGATTTTCCAGCATGAAAGAGAGCGCCG
ATGCGGTATACGCCATCGTGTCGGTGCCGTGCAGAATAACAAAACCGTCATACTCATCGTAGTGCGCTTTGAT
ATCTGCAGCGATATGCTGCCAGTCTTCCGGCGTCATATCTGATGAATCCATCAGCGGGGCATATTCATGGATG
GTAAAATCCGGCATCTCCGGGCGGTGGAATTCCGGCATCAGCGCCAGCTGGCGCTGAAGGTGCCCGGACACCG
GGATATACCCTTTATCTGAGCGCTGCATACCGATAGTACCGCCGGTATAGGCAACGTAAATTGATTTCTTTTG
CATGATAATGTTCAGTCGTGTCGCGAAGGACGTATTAATGATTATCGGATGGCGGCTATGAGCAGACGCCGCC
ATCCGGTATGGCTATTGATGAAGCATTAGCGAACGTTCGCGCAAGTCAAACAGAACGCATAACGGTTTTGGGG
ATCGTTAAATGCCGCCAGTTTATCCCCCTCGGCCTTCACCGTGTTGGCGGCGGAAACGAGCGGCGCCGGGAGC
ATAGCCTGAATGGCCTCCGGCAGCATGGCGCGTACTGATCCGGTCATACTGTCCATGACCATATCAAGGACCG
TCGGTTCGTCCTGATAGTAATCAAGATGCCACTGTTTCAGTTTCGCCAGCTCCGCCGCTTTGGCGACGGCGTC
GTCAAAGTCGCCGAGACTGTCCACCAGACCATTGGCTTTCGCGTCTTCTCCGGTCCAGACATGGCCTTGTGCG
ATTTTATCAATTTGCTCCGGCGTACGCTTACGCGCGTCTGCCACCAGCGTGATAAAGCGTTTGTAGCCGTACT
CAATACTGAGTTGCATCATCTGCTGCACTTCCGGTGACAGTGCTTTGGTCATCGAAATATCCGCCAGCGGCGA
GGTGGAAACGCCGTCGCTGTGTACGCCAATCGACGACAGGCTGTTTTCTACCGTATTGATGACGCCGAAGATG
CCAATTGAACCCGTCAGCGTGCTGGGGCTGGCCACGATATAGTTTGCCGGCGTAGAGATCCAGTAACCGCCGG
AGGCCGCCATGCCGCCCATTGAGACCACCACCGGTTTGCCAGCCGCTCTTGCTGCCGCCAGTTCGGCGCGGAT
AACTTCGGAGGCGTTGACGCTCCCGCCTGGGCTATTGACGCGCAGCACAATCGCTTTCACTTTAGGATCAAGG
CGTGCGTCGCGGATCTGCGATGCCGTAGTGTCGCCGCCGACATTCCCTGGCGTTTCTTCGCCATCCATAATCG
CGCCATTGGCGAAAATAACCGCAATAGTACCGCCGGTATCCGCAGGCGTTTTCAGCGAATAATCGTAATAGCT
GATCGCGCGATAGTTATTTTCGGTTTTGCTCCAGCCAAACTGCTTCGTCAGCGCTTTTTCAACATCTGCGCTG
GAGGCGAGGGCGTCCACCAGTTTATGGTCGAGCGCATATTTGGCGGTGTCGCCGCCCACGCTGGTTAACCCGT
CGATAATAGCCTGCGCGCCGGGGAACAGTTGTTGCGGCGAAATCTGGCGATTGGCAGAAACGGTATGCAGGTA
GTTCTGCCACAGTTCGCCTATCCAGCGGCTGTCGGCCTCGCGGGCGGCGGGCGACATATCGTCGCGGATAAAC
GGCTCGACGGCGGATTTATAGGTGCCGACCCGGAAAACGTGGGTAGAGACTTTCAGTTTATCCAGCAGCGTTT
TGTAGTACAGGCCATTCGTAGCGAAGCCGTGAAGATCTACCTGACCCTGTGGGGAAAGCCAAATTTTATTGGC
GAAACTGGCGAGGTAATACTGACCCTGGCTGTAGTTTTCACCCACGGCGAAAACCGGTTTGCCGCTATCGCGG
AATTCGCGCAGCGCTTTACCGATATAGCGCATTGACGGCTGATCGGCACCGGTGAAGTTTTTCAGATCCAGAA
CGATACCCGTGATGTTACGGTCATCTTTCGCCTGACGAATAGCGTTGACGATGTCAAACAGAGAGTTTTCCTG
CAGACGGTCGGAGCTGGCGCCAAATAACTGGCGACCCAGCGCGCCCAGACGATGATTGGTGGAGGGTTTATCC
ACAATGACGCCGGAAATATCCAGCAGCAGAGCGCCGCGCGCCGTTTGTTCACTGTTGCTGCCGTTACCGATCT
GCATCCAGATCCCAACGCCCACCAGGACCAGAAAAATAAAGAACAGGTTGAGTACCATTTCACGGACGAAGTT
CAACACTCGCCACGTCCATTTAAAAAATCCGGCAATAAATCGCCACAGGGTTCGCATGTATTCTCCCTATCTG
AGTAACAACGCTTATACCTTGGTGAGGATAAAGCATTAAGATGTGGCTATCGTAATGACCCATCAGGCAGTTG
TCAGCAGGAATCACTGCCTGCGCTGTAACAAAATCCGCTCCCGTGTTAATTTTGTGAATAAATATCATTGCTG
GAGTTAATCAAATGGATGCACTAGAACTGCTGGTCAACCGTCGTAGCGCCTCTCGTCTCGCAGAACCGGCGCC
TGTCGGAGAGCAATTACAGAACATTCTGCGGGCGGGAATGCGTGTTCCCGATCATAAATCTCTACAGCCGTGG
CGTTTTTTTGTGATTGAAGGAGAGGGGCGCGACCGCTTCAGCGCCGTGCTTGAACGAGGCGCTGTCGCTGCCG
GAGGCGATGAGAAAGCGATCGAAAAAGCGCGTAATTCGCCGTTTCGCGCGCCGCTGATCATTACCGTGGTGGC
GAAATGCGAAGAAAACCATAAGGTTCCCGTGTGGGAACAGGAGATGTCTGCCGGATGCGCGGTGATGGCGATG
CAAATGGCGGCGATTGCGCAAGGGTTTAATGGTATCTGGCGTAGCGGCGCATTAACGGAGAGCGCTATCGTGC
GGGAAGCCTTTGAGTGCCGCCCGCAGGATAAAATTGTTGGTTTTCTCTATCTCGGCACGCCGCAGCTCAAAGC
GTCAACGACGATCAGTACCCCCGACCCCACGCCGTTTGTGCGTTATTTCTGACGCTGAGGGATAAAACTGTCT
GGATTCTGAGCAAAGGCGCGGTAATTCAGACAGTGATACTTACCACATCAGGGAATGAGCGCTACCATAGCGC
GAATGCAATGACAGGAGATGTCCATGAGCGAGCAAGCCATTCGTTTAACGCAATACAGCCACGGCGCTGGTTG
CGGTTGTAAAATTTCCCCTAAAGTGCTGGAGACTATCCTGCATAGCGAGCAGGCGAAGTTCGTCGACCCGAAC
CTGCTGGTGGGTAATGAAACCCGCGATGATGCGGCGGTTTACGATCTGGGTAATGGCACCAGTATTATCAGCA
CCACCGACTTCTTTATGCCGATAGTCGACAACCCGTTTGATTTTGGCCGCATTGCGGCAACAAACGCCATCAG
CGATATTTTTGCGATGGGCGGCAAACCGATTATGGCGATCGCGATCCTTGGCTGGCCGATTAATACCCTGTCG
CCCGATATTGCGCGTGAAGTGACCGAGGGGGGGGCGTTTTGCCTGCCGTCAGGCCGGTATCGCGCTGGCGGGC
GGACACTCTATTGACGCCCCGGAGCCGATCTTTGGTCTCGCGGTCACAGGCGTAGTGCCGACCGAACGGGTGA
AGAAAAACAGTACCGCGCAGGCGGGATGCAAACTCTTTCTGACCAAACCGTTGGGGATTGGCGTGTTGACCAC

CGCCGAGAAAAAAATCGCTGCTTAAACCTGAACATCAGGGGCTGGCGACGGAAGTCATGTGTCGGATGAACGTT
GCTGGCGCGGCGTTTGCCAATATCGACGGCGTAAAAGCCATGACTGACGTTACCGGTTTTGGCCTGCTGGGGC
ACCTGAGCGAGATGTGCCAGGGCGCAGGCGTGCAGGCGCTGCTTTGCTATCAGGACATCCCTAAACTGCCGGG
CGTGGAAGAGTATATTGCTCTGGGCGCCGTACCGGGCGGCACAGAGCGCAACTTCGCCAGCTATGGTCATCTG
ATGGGCGACATGTCGCGTGAAGTTCGTAGCCTGCTGTGCGATCCGCAAACGTCAGGCGGTCTGCTGTTGGCGG
TAACGCCGGACGCCGAAGACGATGTTAAAGCCACCGCGGCGGAATTTGGTATCGATCTGACCGCGATTGGCGA
ACTGGTCGAGGCCCGCGGCGGTCGCGCTATGGTTGAGATTCGTTAACCTGATGCGGTTGTTTATTGCCGAAAA
ACCGAGTCTGGGGCGCGCCATTGCGGATGTGCTGCCAAAACCGCACCGTAAAGGCGATGGTTTTATTGAGTGC
GGAAACGGGCAGGTCGTCACCTGGTGTATTGGTCATTTGCTGGAACAGGCGCAGCCGGATGCGTATGACAGCC
GTTATGCGCGCTGGAATCTGGCTGACCTGCCTATCGTGCCGGAAAAAATGGCAGCTTCAGCCTCGTCCTTCCGT
CACCAAACAGCTCAATGTGATTAAGCGCTTTTTGCATCAAGCCGGTGAAATTATTCACGCCGGCGACCCGGAT
CGCGAAGGACAGCTTCTGGTTGATGAAGTGCTGGATTATCTCCAGCTTCCTGCCGAAAAGCGCCAGCAGGTGC
GGCGTTGTCTGATAAACGACCTTAACCCGCAGGCGGTCGAGCGTGCTATTGACAGGCTGCGGGCGAACAGCGA
CTTCGTGCCGCTGTGCGTCTCCGCTCTGGCGCGAGCGCGAGCGGACTGGCTGTATGGCATTAATATGACCCGT
GCTTACACGATCCTGGGCCGTAATGCCGGCTATCAGGGCGTGTTATCCGTGGGACGCGTGCAGACGCCTGTAC
TGGGGCTGGTGGTGCGGCGAGACGAAGAGATTGAGAACTTCGTCGCCAAAGACTTCTTTGAAGTAAAGGCGCA
CATCGTTACGCCTGCCGACGAGCGTTTTACCGCTATCTGGCAGCCGAGCGAGGCGTGCGAACCTTATCAGGAT
GAAGAGGGGCGCTTGCTTCATCGTCCGCTGGCGGAGCATGTAGTGAACCGAATCAACGGTCAGCCCGCGCTGG
TAACCAGTTATAATGATAAACGGGAATCAGAATCCGCGCCGCTGCCGTTTTCGCTCTCGACGCTACAGATTGA
AGCCGCCAAACGCTTTGGCCTGAGCGCGCAAAACGTGCTTGATATTTGTCAGAAGCTATATGAAACCCACAAA
CTGATTACCTATCCGCGTTCCGACTGCCGTTATCTGCCGGAAGAACACTTTGCCGGACGGCAGGCGGTCATGA
ACGCGATTAGCGTCCACGCCCCGGATTTACTGCCGCAGTCTGTGGTTAATCCTGACACGCGCAATCGCTGCTG
GGATGACAAAAAAGTGGATGCGCACCACGCGATTATCCCGACGGCGCGCAGTTCTTCTGTCCATTTGACGGAA
AACGAAGCGAAAGTGTACACCCTGATTGCGCGTCAGTATCTGATGCAGTTCTGCCCGGACGCGGTGTTTCGTA
AATGCGTTATTGAACTGGAAATCGCCAAAGGAAAATTTGTCGCCAAAGCGCGTTTTCTGGCGGAGGCCGGTTG
GCGGACGTTACTGGGCAGTAAAGAGCGCGACGAGGAAAACGACGGTACGCCGCTGCCGGTTGTCGCCAAAGGT
GATGAGTTGCTGTGTGAAAAGGGGGAAGTGGTCGAGCGCCAAACCCAGCCGCCGCGTCATTTCACCGATGCGA
CATTGCTTTCCGCGATGACCGGAATTGCCCGCTTCGTGCAGGATAAAGATCTGAAAAAGATCCTGCGCGCGAC
CGATGGGCTGGGGACGGAAGCCACGCGCGCCGGGATTATCGAGCTGCTGTTCAAACGTAGCTTTCTGACCAAA
AAAGGGCGCTACATTCATTCTACCGATGCTGGCAAAGCGTTAATACATTCGCTGCCGGAAATGGCGGCCCGTC
CAGATATGACCGCGCACTGGGAATCTGTTTTGACGCAAATCAGCGAAAAGCAGTGCCGTTACCAGGATTTTAT
GCAACCGCTGGTCGGCACGTTATATCAGCTGATCGATCAGGCTAAGCGCACGCCGGTGAAGCGTTTCAGAGGG
ATAGTCGCGCCAGGCGGTGGAGAAAAGAAAAAGAGCGCGCCGCGTAAGCGAGCGGGCAAAAAAAGCCCGCCTG
CTGAGGAGACAGGCCGTCAGACCGAATAAGCGCAAAGCTATCTGGCCTGACGACGGGATTAAATGACGCCCTG
CGCCAGCATGGCGTCGGCGACTTTCACGAAACCGGCGATGTTGGCGCCCTGAACGTAGTTGGTATGCTTATTA
TCGCCGCCGTACTCGACACAGGCGTGGTGGATATCCAGCATAATGTGATGCAGACGGGCGTCCACTTTCTCGG
CTTTCCAGCTCAACCGCGCGGCGTTCTGCGCCATTTCCAGCCCTGATGTCGCCACGCCGCCCGCGTTGGCGGC
TTTACCCGGCGCAAACAGTACGCCCGCTTCAAGGAAGAGATCGGTCGCTTCAATCGTGGTTGGCATATTTGCC
CCTTCCGCCACGGCTTTCACGCCATTGGCAATCAGTACGCGCGCGGCGTCAACGTCCAGTTCGTTCTGCGTCG
CGCATGGCAGGGCGATATCCACCGGTACGCTCCAGGGCTGCTGGCCTTCCAGATAGGTTAAGCCAAACTCACG
GGCGTAGTCAGCGACGCGACCGTCACGGCTGGCTTTAATTTCACACAGACGCGCCAGCTTCTCTGGCGTGAAG
CCACTTTCATCGACCACCGTCCCGCTCGAATCCGACGCGGTAACCACGCGCGCGCCAAAGGCCATCGCTTTCT
CAATGGCGTATTGCGCTACGTTGCCGGAGCCGGACACGGCGACGCGCATCCCTTCAAAACCGAGACCATGACG
TTTAAGCATCGCTTCGGTGAAGTAGACCAGGCCATAGCCCGTCGCCTCCGGACGAATCAGGCTGCCGCCGAAG
GAAAGCCCTTTGCCGGTGAAGACGCAGGCGCTGTTATTGGAAAGCTTGCGCATCATTCCCGCCATAAAGCCCA
CTTCGCGCCCGCCCACGCCGATATCCCCGGCAGGCACGTCGGTATCCGGCCCTAAATGGCGATACAGTTCAGT
CATCAGCGCCTGGCAGAAACGCATCACTTCGCCTTCGCTTTTCCCTTTCGGATCGAAATCGCTGCCGCCTTTA
CCGCCGCCCATCGGCAACGTGGTGAGGGCATTTTTAAAGGTTTGCTCAAAGCCGAGGAACTTAAGGATGGAAA
GGTTTACCGAAGGATGGAAGCGCATACCGCCTTTATAGGGGCCGATAGCAGAGTTGAACTGCACGCGCCAGGC
GCGATTTACCTGCACCTGGTTTTTGTCGTCCAGCCACACTACGCGAAACTGAATTACGCGCTCTGGTTCAACC
AAACGTTCCAGTAAAGACATGTGGCGGTAACGGGGGTTTTGTTCAAGAAACGGCCACAGGGTGGTCATGACTT
CACGGACGGCTTGCGCGAACTCGGTTTGATGCGGGTCGCGCTTTTGTACATGGTTGAGGAACGATTCCAGAGA
ACATGTCTGATCCATAGATATAAAAACCTCTTATATATTAATGTGGTTGTATTTGCGTTTTATCAGAATATGT
ATTTGCAGTTTTCGACTATACCATCAGCAATGTGACGGTAAGCAAGAAAAAAATGACGCTGCTAAGTAAATTC

ATAACGGGGCGAGGGTCATAACTAAAAGCGATGACAAAAGAGGATTAAAGTTTTCACGGCGGCTTACCGTTAT
ACTGAGAATATACTTAGAAAAATAAGGAACTCCTATGTGCCGAAGCCTCGTGGTCGCCGTGATGGCGATAATG
CCAATGGTCGGGCTGGCAAACCAGAACAGTCGTCCTGATATTCAGGTGAATGTCCCGCCGGAAGTTTTCAGCA
CGCGCGGTCAGTCGTCCCAGCCCTGCATTCAGTGCTGCGTTTACCAGGATCAGAATTACTCCGAAGGCGCGGT
AATAAAAGTGGAAGGCGTCGTGCTCCAGTGCCAGCGTGACGAAAAAACCATCAGCACGAATCCGTTAGTCTGG
CGTCGCGTAAAGCCATAAACGCCTGCAATAGCGGAATATCCGCCGGGGCGAGGGGGTATTCCAGCGCCTCTTC
CGGCGTACACCAGGCTAATGCCTGATGTTCCAGCGCACGTATCAGACCGTTAAACGCAGGCACATGCCAGGCG
TGCAAATGAATCCGTCGGCCAGACACGTCGCGTTGATGGCTGGCGATATAGACGCCGGGCGTGGCGTCAATCC
CTAACTCTTCGCGTAGCTCGCGGATAAGCGCCTGCGGCTGGGTTTCGCCGGGTTCGACTTTACCGCCGGCAAA
CTCCCATAAACCGGCTTGATCCGCATGAGCTGGGCGCTGGGCCAGCAGAATTTTGCCGTCGCATTCAATGATG
GCGGCAACAACATCGAGTTTTTTATCATTGTCGTTAAGGTCGTTATTCCTGCCGGGAAGCGTCGCGCTATCC
CGGCCTTACGGTTAATCAGATAGCGGAATCAAACACGAAACGTCGCCCAGACCGGCGCATGGTCGGAGGGTTT
TTCCATACTGCGAATGTCGTAGTCTATACCGGTTTCTGCGCAACGTTCCGCCAGCGGAGCGCTTGCCAGCAAG
AGGTCAATGCGCAGTCCGCGATTATCCACGAAACCTTTTGAGCGGTAATCAAACCACGAAAATTTATCCATCG
TTTGCGGATTCGCCTGACGGAACGTGTCCACTAATCCCCACTTCAGTAACCGTGACATCCATTCACGCTCCTC
CGGCAGGAAGGAGCATTTACCGGTACGCAGCCAGCGTTTGCGGTTCTCTTCGCCGATGCCAATATCCAGATCG
GTCGGGCTGATATTCATGTCGCCCATAATTAATACCGGATTATCGCACTTGAGTTCCGTTTCAAGATAGTTTT
GCAAGTTCTGGTAAAACTGCGCTTTCGCGGGGAACTTCAACGGATGATCCCGGTTTTCCCCCTGCGGGAAATA
GCCGTTAATGACGGTGATATTACCCAGCGGCGAGGGGATTTCCGCCATGATAATGCGGCGTTGCGCCTCTTCG
CCATCATCCGGGAAACCGCGTCTGACGGAAATGGGCGTGGCTTTTGTTAGCAGCGCGACGCCGTAATGGCCCT
TTTGTCCATGATAGAAAACGTTATAGCCCAGCTTCGCGACCTCTTCGAGCGGGAACATTTCGTCGTGGACTTT
TGTCTCCTGTAGACCGATCACATCGGGCTGGTGTTTTTCGACAATCGCTTCAAGCTGATGGGGACGGGCGCGC
AGGCCGTTGATATTAAAAGAGACAAATTTCATAGTCACTGCCACTTTGCAAGGTGAATCGTGTTGGGATGGTA
ACAGAAATTTTACCGGGTGACTGCCACTCATCGTCAGATTCGGCCAATGGAAGCGTATGGTGTTTTCTATAGC
GCGAAAATGGCACGGTTTGCGTGCAGATGCGCCAGAACGGGGCGGTAATTTCCAGTAAATTTCGTATAAGATC
ACAATTCCAGAATTATATTTGGCAACTGAATAATATTTCGCTTTTTCCTCTTTTTAGCCACCGATTATGCGGA
AATATTCACTTTTCATGCAGTAAAAGTGAATATCGCTACGCTGTAACCCATTGATTTACCGTTTATCACTTCT
GTTTATGCAATGTTAACGCTGGCTGGCACGAATGCTGCAATCTACATTTACAGCGCAAACACTCATATTAATT
AACATATAAATAACTTTATTTTTACCAAAAGAGGTCGCTATGTCTCTGTCAGTTACGCGCGAAAACTTTGATG
AATGGATGGTGCCGGTATATGTTCCCGCGCCTTTTATTCCTGTACGCGGAGAAGGCTCACGCCTGTGGGATCA
GCAGGGGAAAGAGTACATTGATTTTGCTGGCGGGATCGCGGTCAACGCATTGGGACATGCGCATCCGGCGCTG
CGCGAGGCGTTAAATGAACAGGCGAATCGCTTCTGGCATACCGGGAACGGTTACACCAATGAACCGGCGCTGC
GACTGGCGAAAAAACTGATCGACGCGACCTTCGCCGAACGCGTTTTCTTTTGTAATTCAGGCGCTGAAGCAAA
TGAAGCAGCATTAAAACTGGCGCGCAAATATGCCCACGATCGTGTCGGCAATCATAAAAGCGGCATTGTAGCC
TTTAAAAACGCGTTCCATGGTCGCACGCTCTTTACCGTCAGCGCTGGCGGTCAACCCACCTATTCACAGGACT
TTGCGCCGCTGCCGCCGGATATTCGTCATGCAGCCTATAACGATCTGAACTCCGCCAGCGCGCTCATTGATGA
CAATACCTGCGCCGTAATCGTAGAGCCTGTGCAGGGCGAGGGCGGCGTGATCCCCGCCACGAAAGCTTTTTTG
CAGGGGCTGCGTGAATTGTGCGACCGGCATCAGGCGTTACTGATTTTTGATGAGGTGCAGACGGGCGTCGGAC
GTACCGGCGAACTGTATGCATATATGCATTATGGCGTTACGCCCGATATCCTGACGACTGCTAAAGCGTTGGG
TGGGGGCTTCCCGATCGGCGCGATGCTGACCACGCAAGACTATGCCAGCGTTATGACGCCAGGCACGCATGGC
ACCACCTACGGCGGTAATCCGCTGGCGACGGCAGTTGCCGGAAAAGTACTGGATATCATCAATACGCCGGAGA
TGCAAAACGGCGTCAGACAGCGCCACGATGCGTTTATTGAGCGCCTAAATACCATTAATGCACGTTTTGGCAT
GTTCAGTGAAATTCGCGGTCTGGGGCTACTGCTTGGTTGTGTGCTACAGACGGAGTTTGCCGGAAAAGCCAAA
CTCATCGCCCAGGAAGCGGCAAAAGCAGGCGTGATGGTGCTCATCGCCGGCGGCGACGTGGTGCGTTTTGCGC
CAGCGTTAAATGTTAGCGACGAGGAGATAGCCACCGGGCTTGATCGTTTTGCCCTGGCGTGCGAACGCCTTCA
GACGGGAGGCGCATCATGCGGGTGATTCGTCCTGTCGAACATGCGGATATCGCCGCGCTGATGCAGCTTGCAG
GCAAAACGGGCGGCGGCTTAACCTCGCTACCGGCAAATGAAGCGACGCTGGCGGCCCGCATTGAACGCGCGCT
AAAAACCTGGTCCGGCGAGCTGCCAAAAGGCGAACAAGGATATGTATTCGTTCTCGAGGATAGTGAAACGGGT
GAGGTCGGCGGGATCTGCGCCATTGAGGTCGCGGTCGGCCTTAACGACCCCTGGTATAACTATCGTGTCGGTA
CGCTGGTTCATGCCTCCAAAGAGCTGAATGTATATAACGCGTTGCCAACATTATTCCTGAGTAATGATCATAC
CGGCAGCAGTGAGCTTTGCACGCTGTTTCTCGATCCCGAATGGCGTAAAGAGGGTAACGGCTACCTGTTGTCA
AAATCGCGCTTTATGTTTATGGCCGCGTTTCGCGATAAATTCAATGAGAAAGTGGTGGCGGAAATGCGCGGCG
TCATTGACGAGCACGGTTATTCTCCCTTCTGGCAAAGTCTCGGCAAACGCTTTTTTTCTATGGATTTCAGCCG
GGCGGATTTTTTATGCGGTACCGGGCAAAAAGCGTTTATCGCCGAGCTGATGCCCAAACATCCTATTTATACC

CACTTCTTATCTGAAGAGGCACAGGCCGTGATTGGCGAAGTCCACCCGCAAACGGCGCCAGCCCGCGCGGTGC
TGGAAAAGGAAGGTTTTCGCTATCGCCACTATATCGACATCTTCGACGGTGGGCCGACGCTGGAGTGTGATAT
TGACCGCGTGCGGGCCATTCGTAAAAGCCGACTGGTGGAAGTCGCGGAAGGGCAACCCGCGCCTGGCGACTAT
CCGGCATGTCTGGTCGCCAATGAAAACTATCACCACTTTCGGGCCGCGCTGGTGCGTGCCGATCCGCAGACTT
CACGGCTTGTACTTACCGCCGCGCAGTTGGATGCGCTGAAATGTCGCGCGGGCGACCACGTTCGGCTGGTACG
CCTTTGCGCTGAGGAGAAAACCGTATGACGCTATGGATAAACGGCGACTGGATAACCGGTCAGGGCGAACGCC
GCCGCAAAACGAACCCGGTGAGTGCGGAGATACTTTGGCAGGGGAATGACGCTAATGCGGCACAGGTCGCCGA
GGCCTGCCAGGTGGCGCGCGCGGCGTTTCCTCGTTGGGCCAGACAGCCTTTTGCGGCACGACAGGCTATCGTA
GAGAAATTTGCCGCCCTGCTGGAGGCGCATAAAGCCGAGCTCACGGAGGTCATCGCGCGTGAAACCGGTAAAC
CGCGCTGGGAGGCGGCAACGGAAGTGACGGCGATGATCAATAAGATTGCCATCTCGATTAAGGCTTACCATGC
CAGAACAGGCGAACAAAAAAGCGAACTTGTCGATGGCGCCGCGACGTTGCGCCATCGTCCTCACGGTGTGCTG
GCGGTATTCGGCCCTTATAACTTTCCCGGCCATTTACCGAATGGCCATATTGTGCCCGCGTTGCTGGCAGGCA
ATACGCTGATCTTCAAACCTAGCGAGCTAACGCCATGGACCGGGGAAACGGTAATAAAACTCTGGGAACGGGC
GGGGCTACCGGCAGGCGTTCTTAATCTGGTGCAGGGCGGCCGGGAGACCGGACAAGCGCTAAGTTCGCTCGAC
GATCTCGACGGACTGCTGTTTACCGGCAGCGCCAGTACCGGATATCAGCTTCATCGCCAGCTATCCGGCCAGC
CGGAAAAAATACTGGCCCTTGAAATGGGCGGAAACAATCCGCTCATTATTGAGGATGTGGCAAATACAGATGC
GGCGGTACATCTGACGCTGCAATCGGCGTTTATTACCGCCGGACAGCGCTGTACCTGCGCGCGACGCCTTCTG
GTAAAACAGGGTGCGCAGGGAGATGCATTTCTGGCGCGGCTGGTTGACGTCGCCGGACGTCTGCAGCCCGGCA
GATGGGACGACGATCCGCAGCCGTTTATCGGGGGACTGATTTCAGCGCAGGCGGCACAGCATGTGATGGAGGC
CTGGCGTCAACGAGAGGCATTAGGCGGTCGCACGCTACTGGCGCCGCGGAAGGTCAAAGAGGGAACCTCTCTG
CTGACGCCTGGCATCATTGAGCTGACGGGCGTCGCGGATGTGCCGGATGAAGAGGTGTTTGGTCCGCTGCTGA
ACGTCTGGCGTTATGCGCATTTCGATGAGGCGATTCGTCTGGCGAATAATACCCGTTTTGGTCTGTCGTGTGG
GTTGGTGTCGACGGATCGCGCGCAGTTCGAACAGCTCTTGCTGGAGGCGCGGGCAGGGATCGTTAACTGGAAT
AAACCGCTCACCGGGGCAGCGAGTACCGCGCCGTTTGGTGGTATCGGCGCGTCTGGCAACCATCGGCCCAGCG
CCTGGTATGCCGCCGATTATTGCGCCTGGCCGATGGCCAGTCTGGAATCTCCCGAACTGACGTTGCCTGCGAC
ATTAAGCCCCGGCCTCGACTTTTCTCGCAGGGAGGCGGTATGACGGCGCATGAAGTTAATTTTGACGGGCTGG
TGGGGCTTACGCACCATTATGCCGGGCTATCCTTCGGCAATGAGGCATCGACCCGCCACCGTTTTCAGGTGTC
GAATCCTCGTCTGGCGGTAAAGCAGGGGCTGCTAAAGATGAAAGCTCTGGCGGATGCCGGCTTTCTCCAGGCG
GTGATCCCGCCGCATGAGCGGCCTTTTATTCCGGCGTTGCGTCAGCTCGGCTTCACGGGTAGCGATGAGCAGA
TTCTGGATAAGGTTGCGCGTCAGGCGCCACGCTGGCTTTCCAGCGTGAGTTCCGCGTCGCCAATGTGGGTTGC
GAATGCGGCGACGGTTTGCCCATCGGCAGACGCGCTGGACGGGAAAGTCCACCTGACGGTGGCGAATTTAAAC
AATAAATTTCATCGCGCTCTTGAGGCGCCTGTGACCGAAGCGCTGCTACGCGCCATATTTCGCGATGAAAACC
AGTTTTCAGTGCATAGCGCGTTACCGCAGGTCGCATTATTGGGAGATGAAGGCGCGGCGAATCATAACCGTCT
TGGCGGCGAGTATGGTTCGGCAGGCGTGCAGCTTTTTGTCTATGGGCGCGAAGAGGAGAATGAAATACGACCC
GCTCGTTATCCGGCGCGCCAGAGCCGCGAAGCCAGCGAGGCCGTGGCGCGTCTTAACCAGGTGAATCCGCAAC
AGGTTATCTTCGCTCAGCAGAACCCGGAGGTCATCGATCAAGGCGTATTCCATAATGATGTCATCGCCGTTTC
GAATCGACAGGTATTGTTTTGTCACGAAGCGGCGTTTGCCCGGCAGAAAGTGCTCATTAATCAGTTGCGTACG
CGCGTTGACGGTTTTATGGCGATAGAGGTGCCCGCCGAAGAGGTTTCTGTATCAGATGCTGTGGCGACCTACC
TGTTTAATAGTCAGTTGCTAAGCCGTGACGACGGCTCAATGCTGTTAGTGTTGCCGCGGGAATGTCAGGACCA
TGCCGGCGTCTGGCGCTATCTGAATAAGCTGGTGGCGGAGGATAACCCCATCAGCGCGATACAGGTGTTTGAT
TTGCGAGAAAGTATGGCCAACGGCGGCGGCCCGGCCTGTCTGCGATTACGCGTGGTGTTAACAGAAGAAGAAC
GACGGGCGGTGAATCCAGCGGTAATGATGAATGACGCTCTGTTTACGGCCCTTAACGCGTGGGCGGATCGTTA
TTATCGCGATCGCCTGACCGCCGCCGATCTGGCCGATCCGTTATTATTGCGAGAAGGCCGGGAGGCGCTGGAT
GTGTTAACGCGTCTGCTGGATTTGGGGTCGGTTTATCCTTTCCAGCAAACGGGGGCGGCTGATGGATAACTTT
CTGGCGTTGACGCTAAGCGGCACGACACCACGGGTGAGGCAGGGGAAGGGCGCGGGTTTTCGCTGGCGCTGGT
TAAGCCATGGTCTGCTTGAACTCACGCCCGATGCGCCGGTCGACCGCGCGTTAATTCTTTCTGCGGGGATACA
CGGCAATGAAACCGCGCCGGTCGAGATGCTGGATAAGCTGCTGTCGGCGCTGTATTCAGGCAGCTTGACGTTA
ACGTGGCGAGTGCTGGTGGTACTCGGCAATCCGCAGGCGCTGGCGGCGGGAATACGCTATTGTCACAGCGATA
TGAACCGTATGTTTGGCGGGCGTTGGCAGTCCTTTGCCGAAAGCGATGAAACGCGGCGGGCGCGTGAGCTGGA
GCTAAGTCTGGATACCTTCTTTTCATCTGGCCAGGCGCGGGTACGCTGGCATCTGGATCTGCATACCGCCATT
CGTGGCTCGCATCATTTGCGTTTTGGCGTATTGCCGCAGCGCGACCGCCCGTGGGAGACAGATTTTCTGGCGT
GGCTGGGCGCGGCAGGACTAGAGGCGTTGGTATTTCATCAGGCGCCCGGCGGTACGTTTACGCACTTTAGCTC
TGAACATTTCGGCGCGCTTTCCTGTACGCTGGAGTTGGGAAAGGCGTTGCCGTTTAGGCAAAACGATCTGACG
CAGTTCAACGTAACCTCGCAGGCGTTGTCGGCGTTGCTGAGCGGTGTCGAAACGTCAACCTCGTTTTCGCCGC

CGCTACGCTATCGGGTGGTGTCGCAAATCACGCGTCACAGCGACAAGTTCGCGCTTTATATGGATGCGCAAAC
GCTGAATTTTACTGCCTTTGCGAAGGGAACGTTGCTGGCCGAGGAGGGGGATAAGCGCGTGACGGTGACGCAT
GACGTTGAATATGTTCTCTTTCCTAATCCCTCTGTCGCCTGCGGATTGCGGGCTGGATTAATGCTGGAAAGAC
TGCCCTGATAAATGTCCCTGTAAGCTCACGTTAGGATTACAGGGATGATTTGGGACACAGTTTCATGCTATTA
CGGATTATTCCTGGAGAATTGCTTTATTAATAAGCGTTGCTGCGTTATAGTTCTGCATAATTTCTGCTATATC
ATGCTGTTGTATATTTCTCTTTCAACTCTGCGCAATTTATTCTTTTTTTCTTCACAATTGGCGAAAAAGTGTT
TTTTATACTTTCATTGTTTTACCGTTGTTATGACTAATTGACGCTAAGACGGTTAAAGTTAATCACGACAACA
CGGCGCTGTTTTAAATATGCCGTAAAAATAACTGAAAGGAAGGATGGAAATATGCGTAAACTGACTGCTCTAT
TTGTTGCCTCTACCCTGGCGATGGGGGCTGCGAATCTGGCTCATGCCGCTGAGACTACCACCGCCGCGCCAGC
GGATGCGAAGCCAATGATGCAGCATAAAGGTAAATTTGGCCCACATCACGATATGATGTTTAAAAACCTGAAC
CTGACAGACGCGCAGAAGCAACAAATTCGCGACATTATAAAAGCGCAGCGCGAGCAAATGAAACGTCCGCTGC
TGGAAGAGCGTCGCGCCATGCATGATATTATTGCCAGCGATACCTTCGATAAAGCCAAAGCCGAAGCGCAAAT
TACGAAAATGGAAGCGCAGCGCAAGGCCAATATGCTGGCGCATATGGAAACCCAGAATAAAATTTATAACGTT
CTGACGCCGGAGCAGAAAAAGCAATATAACGCTAATTTTGAGAAGCGTCTGACAGAACGCCCGGCGCAAGAAG
GTAAAATGCCTGCTGCGGCAGAATAACTTTCCGCATAATTTAAGACCGCCGGTCTTGTCGACAACACAATCGT
TGAGGTGGACAGGATCGGCGGTTTTCTTTATGACTGGCGACAGTCGGTGTGCAGTTCAATGATCTCATATTGA
CCTGACATTAACGGTTTACAGAGAATCTTATAGCCGTCCTGATCAAAGCCCGCAGGGGTGCGCACCAGCGTGG
CGGCCTCATCCAGCGAGGGGACCGCCCCCAGCCATAACCAGTTGTTGATAATGTGGAACTGGGTCATTTGTGG
ACGGCTCTCTTTTAACGCGATAGCGCCTTTCCAGGGCCAGCAAACCACACGTAACCGTTCCAGCGAGGCCTGC
AGACGAAGAAAATGCGCCTGCGGGGTTTCTTGCCCACAACACGCGCCAGCGCAGCGCTTCAGAGCGAAACGAA
AACAGGCGCGCCCACGGCTTACCGGTTCAAGCCCCAGCAGGCCATAACACAATTTTTGTTCATCGGCGAGGTT
CTGTAAGCTTTGCAGCGCCGCCCGACGGTTGGCGAAGAGGCCAAACAGGTTTGGCTCATGGGAAAAATCGACG
CTGCGGGCGGAAACGACCTCAATTTTTTGTTCGCTCAACTGTAGCGAGCAAAGCTGGCGATTACGACGTAACC
GCTTGTTAAACAGCGGCTGTTGTTCTTTGATCAGCCGTGCCTCCAGCAGCAACGCGCCCATTTCCCCAGCGGT
GCAGATCCAGCTTATCCGCCGCGCCTGTCGCAGCATAGCGGCCTCGTCAGGCGTTCGCAGATGTGAGAGCACC
CGGCTGCGAATATTCACGCTTTTACCGATATAAAGCGGCAGCGTATCGCTTTCGCTATGAAATACATAGACGC
CCGGTAAGGCTGGCGCTTCGCTGAGGAAAGGACGTAAATGTTCTGGATATTCGTAAATGGCCGCCGCTTCAAA
CTCAAGGCGGGGGGCTGATTGACGCCGTACCACAGTGATCTCCGTTAACTGGTTATTCATCCAGTATAACAGC
GGATGGGTGATTAAAAAAGAGGCTGGGAAGAGAGGAGGCCGGATAGACGAGCGATATCCGGCACAACAGTAGA
AGTACGCTTACCTTTTCCAAAAGTCGTCGAATACCGTAATCGGTAAACGGCGCTTATGTTCAGTTTTTACATA
CCAGCCTTCGATAGTTTTAGCGATGGCGGGGTCCAGCGTTTTGCCTTCCAGGTAATCGTCAATATTGTCATAC
GTTACGCCCAGCGCGGCTTCATCAGGCAGTGAAGGGCGGTCGTCTTCCAAATCGGCGGTGGGGACTTTTTTAT
ACAGATGCTCAGGACAGCCCAGCGCTGCGAGAAGCTGTTTGCCCTGGCGTTTGTTAAGGCGATGAAGCGGGTT
AATATCGGTACCGCCGTCGCCATATTTGGTGAAGAACCCAGTTATCGCTTCCGCCGCATGGTCGGTGCCCACC
ACGACGCCGTGAGTCATACCGGCGATGCTATATTGCGCTTTCATTCGCTCACGCGCTTTTTCATTGCCGCGAA
CAAAATCGCTCAGCTCAATTCCCGCTTCGCGCAGCGCCTGTTCGCTGGCGAGGACGGCGCCTTTAATATTGAC
GGTGAGTACTCTGTCCGGCTGAATAAAAGCAATGGCGTCCTGGCAATCCTGTTCATCGGCCTGTACGCCATAA
GGCAGACGAACGGCGATAAACTGCAGCGCATTATCACCGGTCTCTTCTCGCAATTCGGCGATAGCCATCTGGC
TGAGTTTCCCGGCCAGCGTCGAATCCTGCCCGCCGCTGATGCCTAACACCAGCGATTTCAAAAAGGGATAGGT
TTCCAGGTACGCTTTAAGAAAATCCACGCTGCGGCGAATTTCTTCTTCAGGGTTGATATGCGGTTTCGCGCCA
AGCGCCTGGATTATCTCTTGCTGCAGAGTCATTTAACCCCCTTCGACATTAAAAAAATGAATCTTTACCCTGT
TAAAGCTAACCCTTCGCCGCTGAAACAACAAGGTTTGGCGGGCTAAGCGGTGTTTAAAACAACATGTCGCTGC
GTTAGAGAATTTTCCGAAAGACTTTTTGTGGCGGGTCGCGGCTTGAAAAATATTCTGATGTATTCCAGGCTTA
CATAACACGCTGATAAACAAGAGGACGGAAAATGAACAAGAACGTAGCAGGAATTTTAAGCGCAGCGGCGGTA
ATGACTATGCTGGCAGGCTGTACGGCGTATGATCGTACAAAAGACCAATTTGTTGAGCCTGTCGTTAAAGATG
TTAAAAAAGGAATGAGCAGGGCGCAAGTTGCGCAGATTGCGGGCAAACCGTCATCTGAAGTGAGTATGATTCA
TGCGCGCGGTACCTGTCAGACCTACATCCTGGGTCAACGTGATGGTAAAGCGGAAACCTACTTTGTTGCGCTG
GATGATACCGGGCACGTGATTAACTCCGGCTACCAGACCTGTGCGGAGTACGACACTGACCCACAGGCGCCGA
AGCAGTAACAACTGTACATTGCCTGAACATTCAAGGAAACCGGCCTGCGAGCCGGTTTTTTTGTGCCTGCCAT
AACCTTATTTATTATCGCGAATTATTTGCCCGAAATGTGAGGGGGGTCATAACGCCAGGTCAATGAGAGACAA
TTTAGTGGGTCAAGGAAATACCATCCGGCGGTCCGATCCCGTATACTCATTTCAGCCACCTAAAAAAGTAAAT
CCGGTTACGCAAGTCGCCCAGAGTATGGATGCAGGTTGAGGCTTGCGGCGTATCTGGTTGACAGATAATCGCA
CATGAGGAAGGTTTTTATGGAAAAGAAACACATTTATCTGTTTTGTTCTGCGGGCATGTCAACATCGTTACTG
GTATCAAAAATGCGCGCTCAGGCTGAAAAATATGAAGTGCCGGTGATTATTGAAGCATTCCCTGAAACCCTGG

CCGGTGAAAAAGGCCCGGCAGCGGACGTCGTGTTATTAGGCCCGCAAATTGCTTATATGCTCCCCGAAATTCA
GCGTTTATTACCCGGTAAACCGGTAGAAGTCATTGATTCAATGTTGTACGGCAAGGTAGATGGCTTAGGCGTG
CTTAAAGCTGCGGTAGCGGCAATTAAAAAAGCCGCCGCGAATTAATTATTTTTTAATTTTTCCCGTCAAAGAG
TTATTTCATACACTAACCGCAATATTTTATTGCGGTATTTAAGGGTATTTTTCTATGAGTAACGTCATTGCTT
CACTTGAAAAGGTACTCCTTCCTTTTGCTGTAAAAATAGGAAAGCAGCCACACGTTAATGCAATCAAGAACGG
CTTTATTCGTTTAATGCCATTAACCCTTGCGGGGGCGATGTTTGTATTAATTAATAACGTTTTCCTGAGCTTT
GGGGAGGGGTCGTTTTTTTATTCCATGGGCATTCGGCTTGATGCCTCAACCATTGAAACACTTAATGGATTGA
AAGGCATCGGCGGGAACGTCTATAACGGCACATTGGGCATTATGTCGCTCATGGCGCCATTTTTTATCGGCAT
GGCGCTGGCGGAAGAGCGCAAGGTTGATGCGCTGGCCGCCGGATTGCTTTCCGTGGCGGCATTTATGACCGTA
ACGCCGTATAGTGTCGGCGAGGCCTATGCCGTAGGCGCTAACTGGCTGGGCGGCGCGAATATTATCTCCGGTA
TTATTATTGGCCTGGTGGTCGCGGAGATGTTTACATTTATTGTCCGGCGCAACTGGGTGATTAAACTACCGGA
CAGCGTACCGGCTTCGGTGTCTCGTTCATTCTCAGCATTAATTCCCGGCTTTATTATTCTCTCTATTATGGGG
ATTATTGCCTGGGCGCTGTCTAATTACGGTTCTAACTTCCATCAGATTATTATGGACACTATCTCTACGCCGC
TGGCATCGCTGGGTAGCGTGGTAGGGTGGGCATATGTCATTTTTGTACCGCTGCTGTGGTTCTTTGGTATTCA
TGGTTCGCTGGCGCTGACCGCGCTGGACAGCGGCATCATGACGCCCTGGGCGCTGGAAAACATCTCTATTTAC
CAGCAGTATGGCTCCGTCGATGCGGCACTGGAAGCCGGTAAAACGTTCCATATCTGGGCGAAACCGATGCTGG
ATTCTTATATCTTCCTCGGTGGTAGCGGCGCAACGCTGGGTCTGATCATCGCTATCTTCCTGGCATCCCGTCG
CGCGGACTATCGCCAGGTGGCAAAACTGGCGCTGCCGTCAGGTATCTTCCAGATTAACGAACCCATCCTGTTT
GGCCTGCCGATTATTATGAACCCGGTGATGTTTATCCCCTTCATTCTGGTTCAGCCGATTCTGGCGGCGATTA
CGCTGGTGGCTTACTATTTGGGTATTATTCCGCCGATTACCAATATTGCGCCGTGGACCATGCCAACCGGGTT
GGGGGCGTTCTTTAACACCAACGGCAGTGTCGCCGCGTTGCTGGTTGCGCTATTTAACCTGGCGGTCGCTACC
CTGATTTATCTCCCCTTCGTGGTGGTGGCTAACAAAGCGCAGAACGCCATCGAGCAGGAAGAAAGCGAAGAAG
ATATCGCTAACGCACTGAAATTCTAAACTCCGCGCCGGCATGGGATGCCATGCCGGGCTGAATCAGAGGAAAG
ACTATGTTTGATCTTGAAAATGTTGTAGATAACCAGACAGCGGCTGAGGAACTCGAAGAAGTCGTTATGGGGC
TTATCATCAATTCTGGTCAGGCGCGCAGCCTGGCATATGGCGCGCTCAGAAAAGCGAAAGAAGGCGATTTTGA
GGCGGCGAAAGCGATGATGGACCAGTCGCGCATAGCGCTCAATGAAGCGCATCTTGTGCAGACAAAACTCATC
GAAGGCGATCAGGGCGAAGGCAAAATGAAAGTCAGTCTGGTCCTGGTTCACGCTCAGGATCATTTGATGACCT
CAATGCTGGCTCGTGAACTGATCGCCGAGTTGATTGAGCTCCATGAGAAACTGAAATAACAGGGGGCGGATGA
TGCAGCTACAGGTTAACGCAACGGAAATCAAAACGGTTTATGAGCAGCAGCTCTTCAATGGCAAAAATTTCCA
TGTGTTTATCTATAACAAGACGGAAAGCGTCACCGGGCTGCATCAGCACGACTACTATGAATTTACCCTGGTA
TTAACCGGACGTTATTACCAGGAGATTAACGGGAAGCGTGTGCTGCTGGAACGTGGAGATTTTGTTTTTATCC
CGGTGGGGTCGAATCACCAAAGTTTCTATGAGTTTGGCGCAACGCGTATTCTGAACGTAGGAATTAGTAAACG
TTTTTTTGAACAGCATTATCTTCCGTTACTGCCGTTTTGTTTTGTGGCATCGCAGGTATACAGGGCGAATAGC
ACCTTCCTTACCTATATTGAAACGGTGATTGCGTCGTTGAATTTTCGCGGCAATGGGCTCGACGAATTTATCG
AAGTGGTGACATTTTATATTATAAACCGCTTACGCCATTACCGCGAAGAGCAGGTTTATGATGATATTCCACA
ATGGCTGAAAGCAACCGTGGAGATAATGCATGATAAAACGCAGTTTGGCGAACATGCGCTGGAAAATATGGTG
CGCCTGTCGGCAAAATCCCAGGAGTATCTGACGCGCGCCACCCGACGTTATTACAGCAAAACGCCGATGCAAA
TCATTAATGAGATTCGCATTAATTTTGCCAAGAAACAGTTGGAAATGACCAACTATTCGGTTACGGATATTGC
TTATGAAGCCGGGTATAGCAGTCCAAGTCTGTTTATTAAAACGTTTAAGAAGATGACCTCATTTACGCCGAAT
AGTTATCGGAAGCGATTGACGGAAATTAATGAGTAACGGTTTGCATTGCTCGATTAATTACATTTCACGCTTG
CCCCAATAGCGCGGGGAACGTCACGCTATTTCTTACGAGCGGGCATAGCGTAATACATATCAGTGTCATACAC
TGAAGGGAGATATTATGAGCCAGAAATTAAAAGTCGTCACTATTGGTGGCGGGAGCAGCTACACCCCTGAATT
ACTTGAAGGCTTTATTAAGCGCTACCATGAATTACCTGTTACCGAATTATGGCTGGTTGACGTTGAAGACGGG
AAAGAGAAGCTGGGCATTATTTATGATCTCTGCCAGCGAATGATTGATAAAGCAGGCGTTCCGCTAAAATTGT
ATAAAACGCTGGATCGCCGGGAAGCGCTGAAAGACGCTAATTTTGTCACTACCCAGCTACGCGTTGGTCAACT
CAAAGCCCGTGAACTGGACGAGCGTATCCCGCTTAGCCACGGCTATCTGGGGCAAGAAACCAACGGCGCTGGC
GGTTTATTTAAAGGGTTGCGTACCATTCCGGTTATTTTTGACATCATTAAGGATGTTGAAGAATTATGTCCGA
ATGCGTGGGTCATTAACTTTACTAACCCGGCGGGGATGGTGACGGAAGCGGTTTATCGTCATACCAACTTTAA
AAAGTTCATTGGCGTATGTAATATTCCTGTCGGCATGAAAATGTTTATTCATGACGTGCTGGCGCTGAATGAG
ACTGACGATCTTTCCATTGACCTGTTTGGTCTAAACCATATGGTCTTTATTAAAGATGTGCTGGTGAATGGCA
CCTCACGGTTCGCAGAATTACTGGATGGCGTGGCGTCCGGTCAGTTGAAAGCGTCAACCGTAAAAAATATCTT
TGATCTGCCGTTTAGTGAAGGATTGATTCGCTCGCTGAACATGCTGCCGTGCTCTTATTTGTTGTATTACTTC
AAGCAAAAAGAGATGTTGGCGATTGAAATGGGCGAATATTACAAAGGCGGCGCGCGCGCTCAGGTCGTACAAA
AAGTGGAGAAACAACTCTTCGACTTGTACAAAAATCCTGAGCTAAACGTGAAGCCGAAAGAGCTTGAGCAACG

CGGCGGCGCTTATTATTCCGATGCCGCTTGTGAAGTCATTAACGCTATTTATAATGACAAGCAGACTGAGCAT
TACGTTAATATTCCACATCATGGGCATGTCGAGAATATCCCGGCGGACTGGGCGGTGGAAATGACCTGCATTC
TGGGACGCAATGGCGCGACGCCGCACCCGCGTATCACCCGTTTTGACGAAAAAGTGCTGGGGCTTATCCACAC
TATTAAAGGATTTGAGGTCGCGGCCAGCAATGCGGCGCTGAGCGGAAACTTTAATGATGTGCTGCTGGCGCTT
AACCTGAGTCCGCTGGTGCATTCCGACCGCGACGCAGAAGTCCTGGCGCGTGAGCTCATTCTGGCGCATGAAA
AATGGCTGCCTAATTTTGCCGCTTGCATCGAAGCGCTTAAAGGTAAGCACCACTGACAGAGGTAGGCTATGGA
ACGCGTGTTGATTGTGAATGCCGATGATTTTGGCCTGAGTAAAGGGCAAAATTACGGCATTGTTGAAGCATAT
CGCAACGGCGTTGTGACCTCCACGACGGCGCTGGTCAATGGCGAGGCTATTGACCATGCGGCGCAGCTTAGCC
GCGAGCTGCCTGCGCTTGGGGTGGGAATGCATTTTGTTTTGACGTTAGGCAAACCGGTGTCAGAGATGCCGGG
CCTGACGCGAGACGGCCTGTTGGGGAAATGGATCTGGCAAATGGCGGAGGAGGATACGCTTCCGCTGGATGAA
ATTGCGCATGAACTGGCGTGTCAGTATCAGCGTTTCATCGACGTATTTGGCCGCGAGCCAACGCATCTCGACA
GTCACCATCATGTGCATATGTTCCCGCAAATTTTCCCCATCGTCGCCCACTTTGCGGCGCAGCGCGGGATTGC
GTTACGTATTGACCGCCAGACGGTGTTGAACGCCGACGATCTGCCGTCGGATTTGCGCAGCACTCAAGGATTT
AGTAGTGAATTTTACGGCGAGGAGATCACCGAAGCGTGCTTTTTACGCATTCTGGATGCCTCGGCGCATCGAG
GAGAAGCCTCTCTGGAGGTGATGTGTCATCCGGCGTTTGTGGATAACATTATTCGCCAGAGCGCCTATTGTTA
CCCCCGCTTAACTGAACTGGAGGTGTTGACGTCTGCGTCACTCAAGGCAGCGATTGCGGAACGTGGATATCGT
CCTGGCTGTTTTCTTGATATTTAGCTCCTGAGTTTGCGGCGCAAGAATGCGCCGCATTTTTTGCCGTTTATGC
AGGAATCGCGTTAATTTTCCCCGCACGCGACCAGACACGGTGTGCCGCCATCAGCGTCAGCAGAGTGTCCATA
AAGTGGTGGTCAACGTTATCTGCCTCAACAAGTCCTTCTTCCCCCTGGCTATCAATGTTTAAAAGCGCCTTAA
ACCGTCGGGCGTCGCCTGCCAGTGCGATGGGCTTCAGATGTTTGTAGGCTTCCAGAAGGTAGTAACGAGCATC
GCCGCAACTCTCGATATCGGCAATATTGCCGCACGGTACTATTACCGCATCGACCGTTAGCGACGGCGCGCCG
GCAAAGGTTGCCGCAATAGTTAATGTTGAACCGTCATCAGCCGTTACTTCACCCATCCGTGAATAGAGCAGTT
TTGCGTGGACGCCTTTGGCTTTTAAAGCCTGCAAAATGGTCAACAGTTCGGCGGCGTTAACCTTGTCGTTCAG
CAGAATAGCGACCACTCGGCCTTTAACATCGCCGTCCGGCACCGCATACAGGCTGAGCGCCGGATCTTTTTTC
AGACCGTTGACATCAGGAGGCGGGGCAATTTGCGTTTGCTCATGGGTGAGTGCAAAACCGAGATTATGCGCGA
CGCCCTGAGCCAGCGTGACATCAATATGCGCTAACTGATCGACCACCCGCTCCCGAATATACGCGCGGGCGAC
TTTACCCAGTTCAAAGCTGAACGCGTCAATAATATGCTGCTGTTCTATCGGCGTCTGACTGAGCCAGAACAGG
CGAGGATGCGAGTAATATTCACCGAAAGAGGGGCTGCGTTCGCGTATTTTATTGCCATCCACGCGTTCCTGGT
ATGATTCGAAGCCGCCGCGTTTTGGCGCTGGCGGCGTTTCGCGCGGCCAGTTGTCATTAATGGAGTTCGGTTC
ATAGTTCGCCGGATTGGTGTCGATATCCATCCGGTGCATACCGTCACGCTGGAAGTTATGGTAGGGGCAGGTA
GGGCGGTTAATCGGAATTTCGTGGAAGTTTGGCCCGCCCAGACGACTGATTTGCGTGTCGGTATAAGAGAACA
GACGCCCCTGTAACAGCGGATCGTTGGTAAAATCAATGCCGGGAACAATATGCCCTGGATGAAATGCCGCCTG
TTCGTTTTCAGCGAAAAAGTTATCCGGATTACGGTTTAACACCATTTTGCCGACGCGTTGTACTGGCACCAAC
TCTTCCGGAATCAGTTTGGTCGGATCGAGCAGATCAAAATCGAATTTAAATTCATCCTCTTCGGCAATGAGTT
GTAGGCCCAGCTCGTATTCCGGGAAATCGCCCGCTTCGATGGCCTCCCACAGATCCCGGCGGTGAAAATCCGG
ATCGCGACCGGTCAGTTTTTGTGACTCATCCCATACCAGCGAGGCTTTACCCGCCAACGGTTTCCAGTGGAAA
CGGACAAAGGTCGCTTTCCCTTGCGCATTGATCAGACGGAAGGTGTGAATGCCGAACCCTTCCATCGTCCGAT
AACTGCGTGGAATGCCGCGATCCGACATTGCCCACATGACGTTGTGCAGCGTCTCAGGCTGTAAGGAAACATA
GTCCCAGAAGGTGTCGTGCGCGCTCTGTCCTTGCGGAATGGCCCAATGTGGTTCGGGCTTAACGGCGTGCACA
AAATCCGGAAACTTATGCGCATCCTGGATAAAAAAGATCGGCGTGTTATTACCGACGAGATCGAAAATTCCCT
CTTCGGTATAAAATTTGGTCGCAAAACCGCGGATATCACGTACGGTATCAGCAGACCCCGCGCCGCCCTGTAC
TGTCGAAAAGCGAACAAAGACCGGGGTAATTTTCTGTGGATCGCACAGAAACGCGGCTTTCGTGATGTCGCTC
AGGTCTTTATATGACTGAAAATAGCCGTGCGCTGCTGATCCGCGGGCGTGAACGATCCTCTCCGGGATACGCT
CATGGTCAAAGTGGGTAATTTTTTCGCGCAGGATAAAATCTTCCAACAGCGTAGGGCCACGGCTGCCGGCGCG
AAGAGAATTTTGATCGTCGGCGATGCGCACGCCCTGATTGGTGGTCAGCGCATAATTTTCGCTGCCTTTACGA
AAGGCGTCGAGGGCGGTGAGCTTATCATTCGCGGTCTCGGGCGCTTTTAAGCTGCCGGGGGCCGTTGGTTGCG
CGCCCGGCGGCGTCGGTTCTGGCGTTGGGCGATGTGAACCGTCTGACGGCGCCAGGGAGTCCAGGCCGGGCTG
TGATTCACGGGTATCATGCACGGGGGACTGATGGGGGGATTTCTCATTATGCGACATTGAACTCGTCTCCTGC
TTTCATCACTGAAATGGGCGTGGTAGTAGTCAAAATCACTCTAACTATAGAACATTTCAGCAATTATGCCGGG
GGAGAGCGGAACGGCAGACAAGGCTCAATAGGGAAATCGCACGCGCGGAACAGGACGTCGGGGGCGAAGATCG
GCTAAAATAGTGTTTTTCTGATTTTTAGCCTTAGAGCCTATCCCAGTAGGCGTTATTGGCGCAGCCAGTTTGG
ACACGGACAGCGCGCAAAAACCGGAGCGTACACGTAGTACGTGAGGATTTTGAGCACTGCCCAGGACCAAAAT
GGCAAGTAAAATAGCCCTAATGGGATAGGCTCTTAGCCTCTTTTAGTGAAGCATTAATGATGAAACCTCTTCG
CCAGCAAAATCGCCAGATTATTAGCTATATTCCGCGTGTTGAACCCGCGCCGCCGGAACATGCGATAAAAATG

GATACGTTTCGCGACGTCTGGATACTGCGCGGTAAATATGTTGCCTTTGTTCTGACGGGGGAGTCATTTCAAC
GGTCGCCCGCCTTTAGCGTGCCAGAATCGGCGCAACGTTGGGCGAATCAGGTTCGCCAGGAAAACGAAATCGC
TGACTAAGAAAAAAACAAAACGCCGGTACTGGAGACCGGCGTTTTTAGTGACAGACATTTTGAGTTGCCGATC
GTCGTGTCTCAGACGATGTGAACTCCCGATAATGTATTATCGATGCGCCAGTTCAGCGTCTTCTTCGCTATCG
AGAATGGTTTTATCGGTCTGCTTCAGCCACTGGCTGGTCAGCGTACCCGCGGTCATTGAGCCGCTCACGTTCA
GTGCGGTACGGCCCATATCAATCAACGGTTCGACGGAGATTAACAGCGCGACCAGCGTGACCGGTAAGCCCAT
GGCAGGCAGCACAATCAGCGCGGCGAAGGTCGCGCCGCCGCCAACGCCGGCAACACCCGCCGAACTTACGGTA
ACGATACCCACCAGCGTGGCTATCCATACGGGGTCTAAGGGGTTGATGCCGACCGTCGGCGCAACCATTACCG
CTAACATTGCAGGATAAAGACCGGCACAACCGTTTTGGCCGATGGTCGCGCCAAAAGAGGCAGCAAAGCTGGC
GATGGACTCCGGCACGCCCAGGCGGCGGGTTTGCGCTTCGACATTAAGTGGAATGGAGGCGGCGCTGGAGCGG
CTGGTAAAGGCAAATGTCAGCACCGGCCACACCTTGCGGAAATATTTCAGCGGGCTAATCCCGTTCACGCCCA
ATAGTATGCCGTGAACCACAAACATTATCGCCAGACCAAGATATGACGCTACCACGAAGCTTCCAAGCTTAAT
GATGTCCTGCAGATTAGAGCCGGCAACCACTTTCGTCATCAGCGCCAGTACGCCGTAAGGCGTAAGTTGCATT
ACCAGACGCACCAGCTTCATCACCCAGCTTTGCAGGGTATCGATAGCAACAAGCACACGCTCGCCTTTTGGCG
CATCATCTTTCAGCAGTTTAAGCGCGGCCACGCCCAGGAATGCGGCGAAAATCACCACGCTGATAATAGACGT
AGGATTTGCGCCAGTCAGATCCGCAAACGGATTTTTCGGTACAAAAGACAGCACCAGTTGCGGCACGCTGAGA
TCGGCCACTTTGCCGACATAGCTCGTCTCAATGGCATTCAGACGCGCGGTTTCCGCGCCGCCCTGAACCAGAC
CTTCCGCCGTCAGTCCGAACAGATTGGTGACCAGCACGCCCACCAGCGCTGCGATAAGGGTAGTAAACAGCAG
CGTGCCGATAGTCAGAAAACTGATTTTCCCCAATTGAGAGGCGTTATGCAGTCGTGCCACGGCGCTTAAAATA
GAGGCGAATACCAGCGGCATCACAATCATTTGTAGCAATTGCACATAGCCGTTGCCGACAATATTGAACCACT
GAATGGAGTCTTTTAACACCTGACTGTCAGCGCCATAAATGGTGTGCAGGGCCAGACCAAAGACCACGCCCAT
CACAAGGCCGACAAGCACTTTTTTAGCCAGACTCCACTGTTTATGACGGGCCTGCGCCAGTACAAACAGCAGA
ATGACGAACACCGCAATGTTCGCAATTAATGGAAAATTCATCCCCGTTCTCCTGATTTTTTTATTATGAAGCG
CTGTGTTTTAGCGATTCTGTTCGCGCAAGATTAGCAGAACTGTGAGCTGGCTCTTATATTCAAATGGAATGGT
TTATACCAAAAATGATTTTGGCGTTGATTTACTAATCAATCTGGTGATTTATAAAACGATTGAACTGCATTTG
CAGGATAGTAATCATCTGCGAAGACCAGCGTATGACGTTGTTTTCCGGCATGGACTGTGGCATCCACACCGCC
CAGGCAAACAACGCCATGCATAAAAATCGCTCCAGTTGATTGCCTTTGCGCGGCGCCAGAATAGGCAGGCGGA
AACGCCAGCGGCACGGCCACAGTAAGGGAACGCCTGCGGGGGTGAGCATATCCGCGACGATATGGCTTAAATA
GCCAAGTACCATGCCTTGCAGCGCATCCGCCGGGATAATCCACGTATCCGGTACCTTGAGATAAAAGGTCGCC
AGTAGGGCAAAGACCGCCAGCAGACTATGGGTAAACCCCCGGTGACCGAACGCGCGGGCGATCGGTTTCGATA
CCCATTTCAGGCGCTGACCAAGAAAAGATTTGGGGTGGTCGATATCCGGCAGCAGACAGGTCAAAATAGCCGA
AGGAATAATATGCCACCAGTCGCCGTGCGCAAGGACGGGCGTTAACTCGGCATTCTTGGCGAAAACCGCGCTG
GCTATAGAAAAAAGAAGATGTCCTTCCGCCGTCATGATAAAACCCATTAAACTGTCAATTCATACAGTATAGG
GTTTTTATACAGTAGGCGGAAGTGGTGACGTTGTAACTCTTTGTCTCTGTCAAAGCCGCTTGCCGTCTGGCTC
AGCGGCTAATTGCCCGTTGACTTTGTTTCGGAAACATCTTAACCGAGTAGATGCGCCGCCGTCAGCTCTGTCA
GTGAGTTAAGTTTGACATTCGCCAGCGCAAAGCGCGGATCAGGCTGGTTTTCTTCTGCTGGCACCACAATGGC
GCGCATTCGCGCCGCCTTTGCCGCGATTAGACCATTCACCGAATCCTCCAGCGCCACGCAGGTTAATGGATCA
ACACCTAATTTGGCCGCACAATCCAGATAGACCTGCGGATGCGGTTTGCTGTAGGGCAATTTTTCCGCAGAGG
CCAGCGCGTCAAAACTGTCGCGCAGTTCAAACATGGTGAGCACTTTTTCCAGCATATGCAGCGGCGATGCCGA
GGCAAGCCCCACTAATAGCCCCTGCGCTTTACACAGCGCCACGGCCTCGCGCACACCCGGTAAAAGAGGGCGC
GTCTCTTCGATAAGCGTAATCGCGCGGGCAATAACACGGTTTGTCACTTCCTGGCGATCGGGGCCGTTCCACG
GTTGCTGCGCAAACCAGAGATCGACAACCATATCAATGCGTAGCCCAAGCGTGTCTGGCAGTTCATGGCGCCG
AGTAATATCTACGCCTAAACTTGCCATCACGTCCAGTTCAGCGCGATCCCAGAGCGGTTCGGAATCGATCAGT
AGTCCATCCATATCGAAAATTGCAGCAAGAATTTGACGTGGGGTTGACATCACAACCTCTCCTGTTGAGATTA
AAAAATAACGAATTAAGCGTTCATTATGACCATGTTCTTAGTATATCGCCCTTAAAGATCAGGCGAAAACGGG
AATCAACAGGTAGACTTAGGCCAAAACGACGCGTCTTTATCAAGGGGAACTCATGACGTATCAACAAGCTGGA
CGCATTGCAATCCTAAAAAGAGTTGTTGGGTGGGTTATTTTTATTCCGGCACTGCTTTCCACGCTCATCTCAG
TCCTTAAATTTATGTATGCGCACAGTGAAAAGCAGGAGGGGATAAATGCGGTATTGCTGGATTTTACCCACGT
AATGATAGACATGATGCGCGTGAATACGCCGTTTCTGAATGTTTTTTGGTACAACTCGCCGACGCCGAATTTT
CAGGGAAGCCTGAATATCGGTTTCTGGCTCATTTTTATTCTGATTTTTGTCGGGTTGGCGATGCAGGATTCCG
GCGCCAGAATGAGCCGCCAGTCACGTTTTTTGCGTGAAGGCGTGGAAGATCAGTTGATCCTGGAAAAAGCAAA
AGGCGCGGAAGGGCTAACGCGTGAGCAGATAGAGTCACGTATCGTGGTGCCGCACCATACCATCTTTTTACAG
TTTTTCCCGCTCTATATTTTGCCGGTCATTATTATCGTGCTCGGCTACTTTTTCTTTTCCTTGTTAGGCTTCA
TGTAAACTGAAAAATGCCGGATAGCCCGTATCCGGCATGGGCTGTACCTATACCGCAAGCAGCCGATCCATCG

CTTTTTGCGCAGTGGCCAGGTGCTGACCGCCAAACAGCCTTGCCCGATTAAGCAGGGTGTAGAGCTGGTAAAT
CGGCTGCCTGTCGAGAAAATCCAGCGGTAGCGGCGAAACGGACTGATAGCCGTCATAAATCTGCGGTGGCTGA
TCGGTATGCAGCGGCAGCATCGCCAGATCGCATTCTCTGTCGCCCCAATAGCAGGCTGGATCGAAGATATAGG
GGCCGTCAGGGCCAAGCGCGCAGTTCGCCGACCATAGATCGCCATGCAAAAGAGAAGGCTGCGGTTGATGCGA
CGCCAGACGTTGCTGAACATGTTCAACAATGGCGTCAATATTACCAAAAGTAATGCCTTTTTCCGCGGCGAGT
TCAAGTTGCCAGCCAATGCGCTGTTCGGCGAAAAAAGTCGACCAACGGCGTTGCCAGGTATTCGGCTGCGGCG
TGGTAGAGAGCGCGTTGTCAAAATCGAGGCCGAATTGAGGTTGGTCGCTCCATTGGTGCAGGCGCGCCAGCTG
TTGCCCAAGAATAAACGCATTATGGGCATCCAGCGGGCGGGGCGAGAGGTAGTCCATGACTAAAAAGCTGTAA
TCCCGATCCGAACCTACCGCCCATACCTTAGGCACAACAACGGTTTTACTGCGGGAGAGCAGCTCCAGCTGAT
CGGCTTCAGCGGTAAAACCGCGAAGCATTTCTCGTTCATCGCACTTTACGAAAAAATCATGGCCTGAATAACG
TAAATGCCATGCGGCATGAACTTCTCCGCCAGGCAGTTCATTACGCAGTTCGATTTCGCCTTCACCGACTTGC
TCGCTTAAAAGACGACTGATAGCTTGCCACATAATGTCTCTCCCCACGTTGTCTGCCAGATCATAAGGTTAGC
GCAAAAATGCGGTTAAAAAATACGAACTAACGCACATCGCGGACGCGCCGCGGCGGGAATGGCACTTATTGTG
CGTTTTTACTCCACGCCTCCCAGGTCGCCACGGTAATGTCGACATCCTTATCTAATGCGCTTTTCGCAAGTCC
GGCGACAAGCGCTTTGATTTCATCAGCGCTCTGAGCGCTAACAAAGCCAAACGTATTGGTGCCGAGCTCATGC
ACATTCCCTTCATCGTCCGTTAATGTCAGCAAAAAACCGCTGCGCGTCAGATGATTATTGAGTTCGTTAATCT
CGGTCAGGGAATCTTCATGGAAGGTTACGGTAATAACATAACGGGTAATATCGCCACTGGCCATCGTTCACCT
CGTTGGGAGCAACTGTGTTTTTAACAGCGGCGGATGCGCCGCCCGTTACCGATTTTCCGGTATCCCCCTCGCC
GAGGGGGATACGATTATTGCGCGGAAAGATAGGCGTATATTTTTTGGGTATCGTCACGGGAACACAGACGCGT
ACCTTGATTGAGCGTAGCGGCGCTGCCTGCCGCCACACCAAAGCGCACCATCTCTTCCAGCGACGCGTCTTGC
GCCAGTTTTAACGTCATCGCGCCCACCATACTGTCGCCTGCGCCGACAGTACTTTGACTTTTCACCGGGGGAG
GAACAACCTGGATGCAATTCTCACTGTCTATGCCCAGCGCCCCTTGCGGCCCCAGAGAGACCACTACCCGGCG
AGCCTTGCCGCTTTGCACTAATTCCTGGGCCGCTTTACGCACATCATCAGGCTGCGTCAGATCGCGATTAACC
AACGCGCTCAATTCTTTCAGGTTAGGTTTCACCAGTTCGATATCGCCAAGCGCCAGCGCGGCGGTGAGCGCGT
CGCCAGAGCTATCAATAATGCAGCGAATGCCTTGCTTCTGTGCGGCGGAAATCAGTTGCGTGAGTTTCTCGAC
CTTAACGCCGGGCGGAAGGCTGCCGCTGATAACCAGAATTGCCCCGGATTCAATTTCCAGTACCTGTTCTTCG
AGCTGGCGAAATTCGTCATCGTCCAGCGTGGCGCCGGGCATAACAAAACGATACTGTTCGCCGCTGGACTCGA
CGTGGACGTGCAGGTTCTGACGCGTCCAGTCTTTCGCATCGACGGTAGACACGGGGACATTTTCGTCAGCCAG
TAGTGCGACCAGATGTTCGCCGGTAGCGCCGCCAGCCGGGAAAATTGCGGTCGCGGTGCCGCCAAGATGCGCA
ATGGCGCGAGCGACATTAATACCCCCGCCGCCGGGTTCGAATACCGGCGCGGAGCACCGCAGTTTACCTTCGG
GATAAATTTGCGGCGTGATTGTTGCGCTATCGAGAGAGGGCGCAAGCGTCAACGTATAGATACGTACCATCGT
TACCTCCTGTTAGGCTGATTTCAGTCTGGCACTTAATCCCCGGAAAGTGAAGGAATTAACAATATGATTTTCC
GGATCTTTATTTAAAGAAGTACGATGTAAAGTTATATAAATAAATGAATGCTTCGGGAGGGTTCTTATTCAAA
AAATGAAATAGTAATTGATTGCTATGTTATTCGGGGCTTCTTATAATTCGCTACCTTTCCAGGGGCGTTTGTC
ATTGATCCCTATGAAAGTTTCCGGGACCGTCGTGGTCTCTTTTTTAGTTGTACGGACTCTATATAAATGAAGC
TTTTGAAGGCAGTACCCGCTGTAGTTATGCTGGCGGGTGGCGTGTTCGCGTCTCTGTATGCCGCCGCCGATGA
TTCCGTTTTTACTGTCATGGATGATCCCTCCACCGCGAAAAAACCTTTTGAAGGTAATCTGAATGCGGGCTAC
CTTGCTCAGTCAGGCAATACTAAAAGTTCTTCCTTAACGGCTGACACGGCGATGACCTGGTATGGACAACGTA
CTGCCTGGTCATTATGGGGCAATGCCAGCAATACCTCCTCTAATGATGAACGCTCTTCAGAAAAAATATGCGGT
AGGCGCGCGTAACCGTTTTAATATGACGGATTATGATTATACCTTTGGCCAGGCCAGTTGGCTGACAGACCGT
TTCAACGGTTATCGTCAGCGCGACGTACTGACCGCCGGTTACGGGCGGCAGTTCCTTAATGGTCCAGTGCACA
GCTTCCGTTTTGAATTTGGTCCAGGGGTTCGCTATGACGAACATACGGACGATACCACTGAAACGCAGCCGTT
GGGTTATGCTTCCGGCAGCTATGCCTGGCAGTTAACTGACAATGCTAAGTTTACACAGGGGGTGTCAGTATTT
GGCGCAGAAGACACCACACTAAATTCGGAAACAGCCCTCAATGTCGCCATTAACGAACATTTTGGTCTAAAGG
TGGGGTATAATTTAACGTGGAACTCGCAGCCGCCTGAATCAGCGCCTGAACATACCGATCGTCGCACTACGGT
AACGCTGGGCTATAAGATGTAATATTGTACCGGGTTACGTTACGTAACCCGGTTATTCCGTGGTGACGCCTCA
GAAGAAGAAGGTGATGTCTCCGTTGATATAAGAATAGTCGTCTCCGGTGCGAAATTCCGACGTGACTTTGTTT
AAAGAAAGAGAAAATGCGACGGGAGAATAGCCGATGGTGGCACCGACGCGATATTCATCCACGGTTTTATTAA
TATCCACCGTTTCCATTTTTGTTTGCAGCGTTTTCCCCTGTAACGTGTAGTTTTTATCAACTTCCCTGCGCTC
AACGCCAGCAAATACTTTATAGATCAATCCCTCTTTTTGAGTGTGTATTAACCCTCGGCGGGACAATAACCCA
AACCCATTATCAGAGTTGAAGGTCTTATCATTTCCCAGCGCAACGGTTGCGCCGTACCCCAAATACTGGAAGA
GATTGCCGCCGACAGCAGAGACTTCTGGATAGAAGCCCACATTGACACCCAGAATATCAATAGCTGGCGTAAG
GGAGACCATTCCTTTTGCCGTGTAACCATAACGGTTTTCAATTTGATCCTGCCAGGCAGGGTATTTTTCTGCG
CCTATTATTCGATGCGCCCGATTCTGAACCTCCTGACCGCCTGCGTCAGGACCAATAACGCCGATATCGGTAC

CTAAACGATAACGTAACCAATTGGTGGCTAATGAATTCCACTCCAGTCCGGTATGAAGAAAGGCGCTGAACGC
GCGGTCGCCTTTGACGGCTTCAGGCTTTCTCTTATTGGCGCCTGATGGAGAATAAATATCTTGCGCGATATGA
AAAGAGAGTTGACTGGCGTCAGTGAGATCGTGGCTGTAGCCTAAAAAGAGCCCGGCAGTATAATCACCCCGGT
CTGCAGCCGGGTGCCCATAAAGGGCATTTAAGGATGGTTGAAATATACCTGCATCATCATTCGCCACTGAAAT
AGCAAGGCTACTGGCATTGGCCATTGTGGTCGTACTGAGTATGGCGGCAATCATCGTTGCGCAATAGCTGTAT
TTGTTCACTTTTTACCCCTGAATATGAAAGTGAATACTCTTATTTTTACAAAGTAATAAGCACAGCCGCATGA
TGCGCAGTGCTTATTGAATCTTTAAATATAACTAAACTCCTGCCAGCAGCGAGTCATTGAGAGGATACGTTGC
CTTAATGTTGAAAAATGGTGTTAAAAAACACGCGCCAGATATTATTGAATATCCATTTTTCATTCGCTATCTG
AGTGCGAGAAATTATTGGCTTCACGATTATGCATATAATACGATGTTTTTTGGTATCAATATGAATATCACGT
TGTATTCTTTTGAGCTCATTTTCTATGATGTCTTCGATGTTTATCTGTTATTAATTTTTACCGTGATAGTGTT
GTCTTTAATGATGAGCGCATCTAACGGCTGGTAGGGTAATATAACAAATTATTGCCATCTGAATTATTAGGGT
AGTTATTATTAAGGAAGAAAAAGTGAATAAAACCATTAATCTGCTAAAATTACTGCCCGTAGTATTATTAAGC
GCATGTACTACATCGTATCCTCCCCAGGATATAATATCGGCACCCGAGTTACCCCATCGTAACGTACTCGTTC
AGCAACCTGATAACTGTAGCGTTGGCTGTCCTCAAGGAGGAAGTCAACAAACAATCTATCGCCATGTCTATAC
GCTCAATAATAATAGCGCCACGAAATTTGCCAACTGGGTTGCCTATAGCGTGACAAAGACCAGCCAGGCAAGC
GGTCGCCCGCGGAACTGGGCGCAGGACCCCGATTTACCGCCCTCGGATACGTTGGCCCCTTCGGCCTATAAAA
ATGCCCATACGCTATTAAAAGTCGACAGGGGACACCAGGCGCCGTTGGCAGGATTGGGCGGCGTTTCGGACTG
GCCGTCGTTAAATTATTTATCGAATATTACGCCGCAGAAATCCGCCCTGAACCAGGGAGCATGGGCTGCACTG
GAAAACCGGGTGCGCGAACTTGCCAAACAGGCTGATGTATCTGTAGTGCACGTAGTGACCGGCCCCCTTTTTG
AGCGGCATATCGCCACATTGCCAGAAGATGCGACGGTAGAAATTCCCAGCGGGTACTGGAAGGTTTTATTCAC
CGGAACGGCGCCGTCAAAAAGTGAAGGAAATTACGCTGCGTTTATTATGGATCAGAATACGCCCCGTTCGGCG
AATTTTTGCGACTATCAGGTTACCGTGGAGGCTATCGAACATAAAACGAAGCCAGTGCTGACGCTGTGGTCTG
CCTTGCCTGAAGCGGTAGCCAGCGAGGTGAAAACGACAAAGGGGAGTCTGGTGCAGAAGTTAGGTTGTCGATG
AGAAGCGCTATACGGCGCGTAGAAAGATAATGGAGAAACCCTGTCAAGGGTCTTGATTTGCTACAGAGTGATG
CAATCTCCCTTTTTTTAGTGTTACCATCGTCATGCCGGACGAAAATAGCGATTTTCGTCTGTGTCGAAGGTTG
TGCGCCAATTTAGCAATGGTTGGCTAGATGGATACACAACTCACTGTCAATAAATTCATTTTCTCTTTGTATG
TGATCTTGCGTATAAGTACTATCCTTCATTCACATCCATTCACGTTCGTTTAAACCTGTTTCACCAGTTCCGC
GTTATTACTAGTAATAACGCATATATATGTTTCATACCGTTTCACATTGATCCCTTTCGTGCCGTAAGATGTG
TGTACCTAATCTAACTTAAGCAGGGAACTGTCATTCATAACACAGCGTTTATTGATATCAATGGTAGATTATA
TTACGGTGATAATCTCGGGATGATCCGCATATGAGTAGATGTGGATTAGGGCAGAGTAGCGGCAGGACTAATA
ATAACCGGCGGCGAGAAATATATAATTGAGGGGCTATTCATAAATCATTATTAGGAAACTCAAGCACTATCAT
GAATGCATCAAAAGTAATTTACGTTGGTAATGCTGTTCAGCAAACCCAACAAGATTTACGCTCCAAAACTAAA
TAAAACGGCGGCATTGCCGCCGTATAAATTATTTATCGCTTCTTAGTAAAACGAATCTTATATGCGCTGAAAA
AACAAAGGAAGATGCAATGTATACTATAAAGGCAATCATCAAATGCTCAGGGAACAACCATCCCATGCTAACA
GGAATCACACTTATTCCAATGCAAAATAGGAACATTATGAAATAATGCGCTGACTTTGTTTCCTGAGTTTTAT
TGGAGAAATACTTAGCCAGGACGCCTTTAAATACTCCAGATATAACTAACCAAACTGGTGTAAAAAGCCCGAA
ATCTGCGTATAGTTCACCATACCCGAAAGCAGGAGCGCCCTGATTCCTGTAGAATGCATCAGGGAAAAATACT
TTTGCCAGATACAATGCGCCAAAATCTTCAGGCTTATCAGGCCAAATAGCCCTCGGAATTCTCGAGTAAACCT
CGCTCTCCATTAGTAATTTTCCGTACATAAAGTCAAAGTTTGAGGAAGCAACAAGAACAGCATTACGGGTATA
ATCCGAGTAATTCGCCATATTTTCTATCGGATTCCCATCAGTAAACGTATATGCAAAGAATGCCGTTACAATG
ACTGCTATAACAGCAAACGATTTCACCAGGAACATAAATTTAATTTTTTTATTTTCAATATATGAAAGATAAA
GGATGAATGCTATAAATATACTAAATATTGGTCCTTTGTTACCATGCAAGAAAATAAGTATACAGTTAAATAA
AACTATGGAAATACAAAATAACTTTGATTTTTTGTATGTAAAGAACGCGCAAATAGAAGCGACAAGAGAAAAC
ATTAATGAAGGATAGAAGTATATACCATACCCTGTTCTGGTTAATTCGTATATTCTTCTTGGGCTAAGTATGT
ACTCTCTGAACTCCATCAACACTGGAAGGTAAATGATAAAAGCAAGGAAGGTGAATAACAATGAAGTAAACAC
ATACCTTGGTTTATTTTTTGTGTTTGATTTGTTGTATATGGGTTTTCTTTGTGTGTAAAGATAAGAAATAACA
AATGATGCTATGTATGTTATATAGCAAGTATAGAAAAACAAATATTCCACTGTATACTTCCGCACCTCTGAGA
GGTATACAAATATACCTTCAAACACGTACAGTGTAGGCACTGTTATTATCAGGTATGGGACCATTACATTTAT
TATTTTTCCCTCAATTCTTACGGAAAGAGTATAGAGATAAACTATAAATAATAAGCATAATACAATGTATGAA
ATTATAAGCATATAGAGCCTTTAGAAAAAATGCTTAGTATTAAAAATATTAATAATATTGCCATCAGGCAAAC
AAGGTGTGGTACGGAAAATCTTCTGGCAAGATATTCTGAATCTGGTAGTCTATAATCATTACTCATCACTATC
TTCCCGATATCAAAACCGTGTTATTGACATATTAACACTCATTAACACTCCTATCTACTGCGCCTCCGATAGC
ATGAAGCTATCAAGCGTTTGCGGCACAAACATCAAGCAATGTAAATGCTTTTGATAATTACGCATGATTGTTA
CTGCGTTTATATACAGTGGTATTCGAAGGTTCATCATGAGGTTCCCGAGCCCTGTAAAAGACTATACAGAGAA

AGGGCTATCACTCGACGAGTAACTCATCTCACATCGTGCCGCGACATACTTCAAGCAACACAGACGAATTACA
AGTACGGCATCATAAGGGAGACGTTGCTAGTTATAGAAGCTTCATAGCAGCATGTAATGGCTCTTTGCTGGTA
TGTGCTTTGGAAAGTGGGTTTCGTATTGTGCGTTATCGCATGCTGCCAGGTCCCCATCTGGAGAATATAGATA
GCGGTCGTCGAAAGCTATCTGATGTGAATGACTCATACGGTGGCAGTAAGTCGAGACTCAGAGTGACCATGTA
CATCATTAACGATACGCGTGCTGGGTAGTGTGTTGGGATTCAGGTTCTGTAGAACAGAAAGATGCAATCTCCC
TTTTTTTAGTGTTACCATCGTCATGCCGGACGAAAATAGCGATTTTCGTCTGTGTCGAAGGTTGTGCGCCAAT
TTAGCAATGGTTGGCTAGATGGATACACAACTCACTGTCAATAAATTCATTTTCTCTTTGTATGTGATCTTGC
GTATGGGTCACCACTGCAAATAAGGATATTACATGCCTGTTATTACTCTTCCTGATGGCAGCCAACGCCATTA
TGACCACCCTGTAAGCCCGATGGATGTTGCTCTGGACATTGGTCCTGGCCTGGCGAAAGCCACCATTGCGGGC
CGTGTGAATGGCGAGCTGGTTGATGCTTCCGATCTGATTGAAAATGATGCGACGCTTGCCATCATCACCGCAA
AAGATGAAGAGGGTCTGGAGATCATTCGTCACTCTTGCGCGCATCTGTTAGGTCACGCAATCAAGCAACTTTG
GCCGCACACGAAAATGGCGATCGGCCCGGTTGTCGACAACGGTTTTTACTATGACGTTGATCTTGACCGCACG
CTAACTCAGGAAGATGTCGAAGCGCTCGAAAAGCGGATGCATGAGCTCGCCGAGAAAAATTACGACGTTATCA
AGAAAAAGGTGAGCTGGCATGACGCGCGCGAAACCTTCGTGAAGCGCGGCGAGACTTACAAAGTCGCTATTCT
TGATGAAAATATCGCCCATGATGATAAGCCAGGCTTGTACCATCATGAAGAATATGTCGACATGTGTCGTGGT
CCGCACGTGCCGAATATGCGTTTCTGCCATCACTTTAAACTGATGAAAACTGCTGGCGCATACTGGCGCGGTG
ACAGCAATAATAAGATGCTGCAGCGTATTTACGGTACGGCATGGGCAGATAAAAAAGCCCTGAACGCTTATCT
GCAGCGCCTGGAAGAGGCCGCAAAACGCGACCACCGTAAAATTGGTAAGCAGCTCGACCTGTATCATATGCAG
GAAGAAGCGCCGGGCATGGTGTTCTGGCATAACGACGGCTGGACTATCTTCCGCGAGCTGGAAGTCTTTGTTC
GTTCTAAACTCAAAGAGTACCAGTATCAAGAAGTTAAAGGCCCGTTCATGATGGACCGTGTGCTGTGGGAAAA
AACCGGGCACTGGGACAACTATAAAGATGCGATGTTCACCACATCCTCAGAAAACCGCGAATATTGCATCAAG
CCGATGAACTGCCCGGGCCACGTTCAGATCTTTAACCAGGGTCTGAAATCCTATCGTGATTTGCCGCTGCGTA
TGGCGGAATTCGGTAGCTGCCACCGTAACGAGCCATCAGGCGCGCTGCATGGTCTGATGCGCGTACGCGGCTT
TACGCAGGATGATGCGCATATCTTCTGCACCGAAGAGCAGATTCGCGATGAAGTTAACGCTTGTATTCGTATG
GTCTACGATATGTACAGCACCTTTGGCTTCGAGAAGATCGTCGTCAAGCTTTCCACTCGTCCTGACAAGCGTA
TCGGCAGCGATGAGATGTGGGATCGTGCTGAGGCGGATCTGGCGGTTGCGCTGGAAGAAAATAATATCCCGTT
TGAGTATCAACTGGGTGAAGGCGCATTCTACGGTCCGAAAATTGAATTTACCTTATATGACTGCCTCGATCGT
GCATGGCAGTGCGGTACAGTACAGCTGGACTTCTCCTTACCGTCTCGTCTGAGCGCCTCCTATGTAGGCGAAG
ACAACGAGCGCAAGGTGCCGGTAATGATTCACCGTGCGATTCTTGGGTCGATGGAACGCTTCATCGGTATCCT
GACCGAAGAGTTCGCTGGTTTCTTCCCGACATGGCTCGCGCCTGTTCAGGTAGTCGTGATGAATATTACCGAT
TCGCAGTCTGAATACGTTAACGAATTGACGCAGAAACTACAAAATGCGGGCATTCGTGTAAAAGCAGACTTGA
GAAATGAGAAGATTGGCTTTAAAATCCGCGAGCACACTTTACGTCGTGTCCCTTATATGTTGGTCTGTGGTGA
TAAAGAGGTGGAAGCAGGCAAAGTTGCCGTTCGCACCCGCCGCGGTAAAGACCTGGGCAGTCTGGACGTAAAT
GACGTGATTGAGAAGCTGCAACAAGAGATTCGCAGCCGCAGTCTTCAACAACTGGAGGAATAAAGTATTAAAG
GCGGAAAACGAGTTCAAACGGCGCGTCCGAATCGTATTAATAGCGAAATTCGCGCGCAAGAAGTTCGCTTAAC
AGGTCTGGAAGGCGAGCAGCTTGGTATTGTGAGTCTGAGAGAAGCTCTGGAAAAAGCTGAAGAAGCCGGAGTA
GACTTAGTCGAAATCAGCCCTAACGCCGAGCCGCCGGTTTGTCGTATAATGGATTACGGCAAATTCCTCTATG
AAAAGAGTAAGTCTTCTAAGGAACAGAAGAAAAAGCAAAAAGTTATCCAGGTGAAGGAAATTAAATTCCGTCC
TGGGACAGATGAAGGCGACTATCAGGTAAAACTCCGCAGCCTGATTCGCTTTCTCGAAGAGGGCGATAAGGCC
AAAATCACGCTGCGTTTCCGCGGTCGTGAAATGGCCCACCAGCAAATCGGTATGGAAGTGCTTAATCGCGTGA
AAGACGATTTGCAAGAACTGGCAGTGGTCGAATCCTTCCCAACGAAGATCGAAGGCCGTCAGATGATCATGGT
GCTTGCTCCTAAGAAGAAACAGTAAGGCCTTCAAGTAACAATTCTGTGAAGCCTTCGGGCTTCACAGGTTTTG
TTCGCCTATGTTTCGTTTATTAACAATGCGAAGTGGAAGTTATTAAAATGCCAAAAATTAAGACCGTACGCGG
TGCTGCTAAGCGCTTCAAAAAAACCGGTAAAGGTGGTTTTAAGCACAAGCACGCTAACCTGCGTCACATTCTG
ACCAAAAAAGCGACTAAACGTAAACGTCACCTGCGTCCGAAAGCCATGGTTTCTAAAGGCGATCTGGGCCTGG
TTATCGCGTGCCTGCCGTACGCATAAGCCGTTAACGTTTTTAACTTTTTAATTAGAATAGATACAGGAGAGCA
CATATGGCTCGCGTAAAACGTGGTGTAATTGCACGCGCACGTCACAAGAAAATTTTGAAACAAGCCAAAGGCT
ACTACGGTGCGCGTTCTCGCGTATACCGCGTTGCCTTCCAGGCAGTTATCAAAGCTGGTCAGTATGCTTACCG
TGACCGTCGTCAACGTAAGCGTCAGTTCCGTCAACTGTGGATTGCGCGTATCAACGCAGCAGCACGTCAGAAC
GGTATTTCTTACAGCAAATTCATCAACGGCCTGAAAAAAGCCTCTGTTGAAATCGACCGTAAGATCCTGGCTG
ATATCGCCGTATTCGACAAAGTCGCGTTCACCGCGCTGGTTGAAAAAGCGAAAGCAGCTCTGGCATAAGCCAG
TGAAAAGAGGGAGCCTGGCTCCCTCTCTTTTATTTACTGTTCAGTGAGTTGACAATTGACCTGTAACCCTTTT
CAATAAAGGATATTCGGTTTTACCACATAAGGTAACGCAAGCATGAACGCTGCTATTTTCCGTTTCTTTTTTT
ACTTTAGCACCTGATCCCAGGAGGCTAGCGCGTGAAAAACGAAACGAAAAACAGCGCAAAAAAGCCTCCTGAT

GGAGGCTTTTTTTGTATCTGAAATCGAGAGATTACGTCCAATGAACCGGTGTCTTCACCGACACAATGAGGAA
AACCATGTCACATCTCGCAGAGCTGGTTGCCAATGCAGCGGCCGCCATTAACCAGGCGTCAGATGTTGCCGCG
TTAGACAATGTACGCGTCGAATATTTGGGCAAGAAAGGGCATTTAACCCTTCAGATGACGACCCTACGCGATC
TGCCGCCGGAAGAGCGTCCGGCAGCCGGGGCAGTCATTAACGCCGCGAAAGAGCAGGTACAACAGGCGCTTAA
CGCGCGTAAAGCCGAGCTGGAAAGCGCAGCGCTTAACGCCCGTCTGGCCGCTGAAACCATCGATATCTCCCTG
CCGGGGCGCCGTATTGAGAACGGCGGTCTGCATCCGGTCACGCGTACCATTGACCGTATTGAAAGTTTCTTCG
GTGAGCTTGGTTTTACCGTGGCGACTGGCCCGGAAATCGAAGATGACTACCATAACTTCGATGCGTTAAACAT
TCCGGGTCATCATCCGGCCCGCGCCGATCATGATACCTTCTGGTTTGACGCGACCCGTCTGCTGCGGACCCAG
ACGTCTGGCGTCCAGATCCGGACGATGAAAGCGCAGCAACCGCCGATCCGTATTATTGCGCCGGGCCGTGTAT
ACCGTAATGACTACGACCAGACGCATACTCCGATGTTCCATCAGATGGAAGGTCTGATTGTTGATACCAACAT
CAGCTTCACCAACCTCAAAGGTACGCTGCACGACTTCCTGCGTAATTTCTTTGAGGAGGATCTGCAGATTCGC
TTCCGTCCTTCTTATTTTCCGTTTACCGAACCTTCTGCAGAAGTGGATGTGATGGGGAAAAACGGCAAATGGC
TGGAAGTGCTGGGCTGCGGGATGGTGCATCCGAATGTGCTGCGCAATGTCGGTATCGATCCGGAAATCTATTC
TGGCTTCGCTTTTGGTATGGGGATGGAACGTCTGACCATGTTGCGTTATGGCGTCACCGACCTGCGTTCATTC
TTCGAAAACGATCTGCGTTTCCTCAAACAGTTTAAATAAAGGCAGGATAAAACAATGAAATTCAGCGAACTGT
GGTTACGCGAGTGGGTTAACCCGGCAATTGATAGCGATGCGTTGGCTAACCAGATTACGATGGCTGGCCTGGA
AGTAGACGGCGTTGAGCCGGTCGCGGGAAGCTTCAATGGCGTTGTGGTTGGGGAAGTGGTTGAGTGCGCCCAG
CATCCGAACGCTGACAAATTGCGTGTAACGAAAGTCAACGTCGGCGGAGAACGCCTGTTGGATATCGTCTGCG
GCGCGCCAAACTGTCGTCAGGGGTTGAAAGTAGCGGTGGCCACCATTGGCGCTATTCTGCCGGGCGATTTCAA
AATTAAAGCGGCAAAACTGCGCGGCGAACCGTCTGAAGGGATGCTGTGCTCCTTCTCCGAACTGGGGATTTCC
GACGATCACAGCGGCATTATCGAACTGCCGGCGGATGCGCCGCTGGGTACCGATATCCGTGAATACCTGAAGC
TTGACGACAATACCATCGAAATTAGCGTCACGCCGAACCGTGCGGATTGCTTAGGTATCATCGGCGTTGCGCG
TGATGTGGCGGTACTGAACAAAGCGCCGCTCCAGGAGCCTGAAATGGTGCCGGTTACGGCAACCATCAGCGAT
ACGCTGCCGATTACGGTAGAGGCCGCTGATGCGTGTCCTCGCTATCTTGGCCGGGTGGTAAAAGGCATCAATG
TTAACGCGCCGACGCCGCTGTGGATGAAAGAGAAACTGCGTCGCTGCGGTATTCGCTCCATCGATGCGGTGGT
TGACGTCACGAACTATGTGCTGCTCGAACTGGGTCAGCCAATGCATGCTTTCGATAAAGATCGCATTGATGGC
GGTATTGTCGTGCGGATGGCAAAAGAGGGTGAAACCGTCGTCCTGCTGGACGGTAGCGAAGCGACGTTAAATG
CGGACACGCTGGTGATCGCCGATCACCATAAGGCGCTGGGGATAGCCGGTATTTTTGGCGGCGAACATTCCGG
CGTGAACGGCGAAACGCAAAATGTGCTGCTGGAGTGTGCGTACTTTAATCCGCTGTCTATCACCGGTCGCGCT
CGCCGCCACGGTCTGCATACCGATGCGTCTCACCGCTACGAGCGCGGGGTGGACCCAGCGCTGCAATATAAAG
CGATAGAGCGCGCTACTCGTCTGCTTCTTGATATCTGTGGCGGTGATGCGGGTCCGATAATTGATGTGAGCAA
CGAAGCAACGTTGCCGAAACGCGCCACCATTACGCTGCGTCGCAGTAAACTGGATCGCCTGATCGGGCATCAT
ATTGCGGATGAGCAGGTCAGTGATATTCTGCGTCGTCTGGGCTGTGAAGTGACCGAAGGGCAGGACGAGTGGA
AAGCCGTAGCGCCGACCTGGCGTTTCGATATGGAGATTGAAGAAGATCTGGTGGAAGAGGTGGCCCGCGTTTA
CGGCTATAACAATATTCCGGACGAGCCAATTCAGGCCGGTTTAATCATGGGGACGCACCGTGAAGCCGATCTG
TCGTTGAAGCGGGTTAAAACCATGCTGAACGACAAAGGCTATCAGGAAGTGATTACCTATAGCTTTGTCGATC
CTAAAGTACAACAGCTCATCCATCCAGGCGCAGAAGCGCTGCTGCTGCCAAACCCTATCTCCGTTGAGATGTC
GGCGATGCGTCTGTCTCTGTGGAGCGGATTGTTGGCGACGGTGGTCTATAACCAAAACCGTCAGCAGAATCGC
GTGCGTATTTTTGAAACCGGTTTACGTTTCGTTCCGGATACACAAGCCAATCTGGGTATTCGACAGGATCTGA
TGCTGGCTGGCGTGATTTGCGGTAACCGCTATGATGAGCACTGGAACCTGGCAAAAGAGACCGTTGATTTCTA
TGATTTGAAAGGCGATCTGGAAGCAGTATTGGATTTAACCGGCAAACTGGGTGATATCCAGTTTAAGGCGGAG
ATGAATCCGGCTCTGCATCCGGGACAGTCTGCGGCGATTTATCTGAAAGATGAACGTATTGGTTTTATTGGGG
TTGTTCACCCTGAACTGGAACGTAAACTGGATCTGAATGGTCGTACGCTGGTGTTTGAACTGGAATGGAATAA
GCTCGCAGACCGTATCGTGCCGCAGGCGCGGGAGATTTCACGCTTCCCGGCCAACCGTCGCGATATTGCGGTT
GTTGTTGCAGAAAACGTTCCCGCAGCGGATATTTTATCCGAATGTAAGAAAGTTGGCGTAAATCAGGTAGTTG
GCGTAAACTTATTTGACGTGTACCGCGGTAAGGGTGTTGCGGAGGGTTATAAGAGCCTCGCTATCAGCCTGAT
CCTTCAGGATACCAACCGTACACTCGAAGAAGAGGAGATTGCCGCTACCGTCGCCAAATGTGTAGAGGCATTA
AAAGAGCGATTCCAGGCATCATTGAGGGATTGAACCTATGGCGCTTACAAAAGCTGAAATGTCAGAATATCTG
TTTGATAAGCTTGGGCTTAGCAAGCGGGATGCCAAAGAACTGGTTGAACTGTTTTTCGAAGAGATCCGTCGTG
CTCTGGAAAACGGTGAGCAGGTGAAACTCTCTGGTTTTGGTAACTTCGATCTGCGTGATAAAAATCAACGTCC
GGGGCGTAACCCGAAAACGGGTGAAGATATTCCTATTACAGCACGGCGCGTGGTGACCTTCAGACCCGGGCAG
AAGTTAAAGAGCCGGGTCGAAAACGCTTCGCCCAAAGAAGAGTAATCAGATCCAGGCATAACATCCATCAGGG
TGTATTGCGGCAGATACGCTGCCATCCCGAAAGTAGATCAGCGGGATGACAGCGTATCTGTCAGCCTGAAAGA
TGACCCGTATAACTAAAAAGGCCGCATTGCGGCCTTTTTCTTTCAAGTAAAGGATACCCACCGTAAAATCACG

CTACCTCTTTTCAGCTGAATGGATAACATGCTGACTTTTGCCCGCCAACAACAGCGACGAAACGTTCGCTGGC
TTCTGAGCCTGTCACTGCTGGTGCTACTGGCTACACTTCTGAGCTTATGCGCAGGCGAACAGTGGATTGCCCC
CGGTGACTGGTTAAGCGCCCGGGGGGAACTGTTTGTCTGGCAAATTCGCCTTCCCCGCACGCTTGCGGTATTG
CTGGTTGGCGCTGCGCTGGCGCTATCTGGCGCCGTGATGCAGGCGCTGTTTGAAAACCCACTTGCTGAACCGG
GTCTGCTCGGCGTTTCGAATGGGGCCGGTGTTGGGCTTATTGCCGCCGTCTTACTGGGGCAGGGGCAACTGCC
AGGATGGGCGCTGGGACTGTGCGCTATAGCCGGCGCGCTCATTATTACGTTAATCCTGCTGCGTTTTGCGCGT
CGCCATCTCTCTACCAGCCGCTTGTTGTTGGCGGGCGTCGCGCTGGGCATTATCTGTAGCGCGCTGATGACGT
GGGCTATCTATTTTTCCACCTCTTTCGATCTGCGGCAATTAATGTACTGGATGATGGGAGGATTTGGCGGCGT
TGACTGGCAGCAGAGCTGGCTAATGATTGCGCTCATCCCGGTACTGATCTGGATATGTTGCCAGTCGCAACCG
CTGAATATGCTGGCGCTAGGGGAAACCTCGGCGCGGCAGCTTGGCCTGCCGCTGTGGTTCTGGCGCAATTTGT
TGGTCATCGCCACCGGCTGGATGGTGGGCGTCAGCGTGGCGATGGCGGGGGCGATTGGTTTTATCGGTCTGGT
TATTCCGCACATCCTGCGCTTATGTGGTTTAACCGATCACCGGGTTTTACTTCCCGGCTGCGCGCTGGCCGGG
GCTATCGCCCTGCTATTGGCTGATGTGGTCGCCCGACTGGCGCTGGCGTCGGCTGAACTGCCTATCGGGGTGG
TCACCGCCACATTGGGGGCACCAGTGTTTATCTGGCTGCTACTCAAATCCGCGCGTTAGCGCATCGGTAAACA
ACTGCTTTCATCATTCGCAGTCTATGATTAAAGGCTAACGATGAAACCCGCTATTCTACCGACATCAGGGAGA
CACCATGCAAAACTCGCTTCTTAATACTCACGTTACGACGATTGATGGAGAGGTCACTACACTTGAAAAATAC
GCCGGAAAAGTGCTGCTCATTGTAAACGTCGCCTCCAGATGTGGCCTGACGCCGCAATATGAACAGTTGGAAA
ATATCCAGAAGGCGTGGGCAGATCAGGGGTTTGTCGTGTTGGGTTTTCCCTGCAACCAGTTTATGGGGCAGGA
GCCAGGCAGCGAAGAGGAGATTAAAACCTATTGTGCCAGCACCTGGGGCGTCACGTTCCCGATGTTCAGTAAA
ATTGATGTGAATGGCGACGCGCGCCATCCGCTGTATCAAAAACTGATAGCCGCTGCGCCAACGGCAGTCGCGC
CGGAGAAGAGCGGCTTTTACGAACGGATGGTAAGCAAAGGGCGTACGCCACTCTATCCGGATGATATTCTGTG
GAATTTTGAAAAATTTTTGGTAGGCCGGGATGGTCAGGTGGTACAGCGTTTTTCGCCGGATATGACGCCTGAA
GATCCTATTGTGATGGAAAGCATAAAAATCACGTTGGCAAAATAATGTCTCAATTGATGCAATTAAAGGATGT
CGCCGAGAGCACCCGCCTGGGGCCGCTTTCCGGCGAAGTAAGCGCAGGAGAGATCCTTCATCTTGTCGGGCCG
AACGGTGCCGGGAAAAGCACGCTACTGGCGCGTATGGCGGGGTTAACCTCCGGCGAGGGGAGCATCAGGTTTG
GCGGAGCGCCGCTGGAGGCATGGGCGACGGCAACGCTTGCCCAACACCGCGCTTATCTTGCGCAACAGCAAAA
TCCGCCGTTCGCCATGCCGGTCTGGCATTACCTGACATTGCATCAACCTGATAAAACGCGAACCGGGCAGCTT
AACGAGGTGGCCGATATGCTTGGATTAGGCGACAAACTGGGGCGAAGCGTGAATCAGCTTTCCGGTGGCGAGT
GGCAGCGTGTTCGCCTTGCTGCCGTTGTACTACAAATTCATCCCGACGCTAATCCGGTCGGTCAATTATTGCT
GCTTGATGAGCCAATGAACAGTCTCGATGTTGCGCAACAAAACGCTCTGGATCGGGTATTACATCATTTATGT
CAGGCAGGAATTGCGATTGTGATGAGTAGCCACGATCTGAACCACACGCTGCGCCATGCGCATAAAGCCTGGT
TACTAAAGCGCGGTAAACTCATTGCCTGCGGCCGCCGGGAAGAGGTGCTCACCCCCTCTTATCTGGCGCAAGC
CTACGGTCTGCGCTTTCGACGACTCGACGTCGAGGGGCATCCAACGCTCATTTCGGCCACTTAAGTAAAATCG
TTTCTTGAAAAGCAGGTAATTTCGACGCTAAATTAATACCAAAATAAAAACAGAGGATTGTTGCGGCATGCGT
TTTTGGCTTCTTGTTATCACAGCATTGTTCCTGGCAGGATGCAGTACTCATCGGGCACCGGCGCCAAATGCCA
GATTATCAGACTCCATTACCGTCATTGCGGGTCTGAACGATCAGTTGCAGTCCTGGCGCGGTACGCCATACCG
TTACGGCGGCATGAGTCGCAGAGGCGTGGACTGTTCCGGCTTTGTGGTGGTCACTATGCGCGACAAGTTTGAT
TTGCAGTTGCCTCGTGATACGCGTGAACAATCAAAAATCGGCACCAGAATCGATAAAGATGAATTGCTGCCCG
GAGATTTGGTTTTCTTTAAAACCGGTTCGGGCGAAAGTGGTTTACATGTTGGTATTTACGATACAAATAATGA
GTTTATTCATGCCTCTACCAGTCGTGGCGTGATGCGTTCATCGCTGGATAATGTGTACTGGCGAAAAAACTTC
TGGCAGGCACGGCGAATTTAACCTCGCAGACGATATAAATTTTATAGTCTATTTTTAACATATCGCCGGTTGA
ACTTATGACATCTGTTGTCATCATATATAATGATATAACCGTAGCATTTACAAACGTACTTTTATTAAAAACG
GGCGTGTTCACGCCCTGTGAAAAGTACAACATTGGATATTGGTTTATAATCAGAGTAAGTTAAAAAAAGGAAC
CGGTTCACCGGTAAACAAGAAAAATGATGACGCTTCAATTAGTATCTGAAGTTACGTCTGTTTCCGTCCGTAG
TGACTGGATGGCGAATAGCGCCCTAACCATGGGACTGGCGTAATGATTGCTTCACTTGATGAGCTTTACCATT
CCGAACTCTTTTTCCTTCCCGTCATGGATGAAAATGCTCGGCTTGTTGGTTTAGAGATTATTGCAACGTTTGC
CGCTGAAGACGGCGCCGTGCGGATGCCGACTGAGTTGGTAGCGCCTCGTCTTTCGGTGGAGGAACAATACTGT
CTGTTTGTTGAAAAACTGGCGTTGTTAGAAACCTGTCAGCACTTTTTTATTCAGCATAAACTTATTGCATGGC
TTAATTTACCGCCCGCAATTAGTGACTTATTATTATTAGATAGCGAACTTTTTTCCCGGGCAGCGCGCTTTCC
GTTTCTTGAATTAGCTATAAATGAGAACTATCCTGGGTTAAACCAGGGCAAAAACAATGAAACGTTAGCTAAT
CTGGCAATGCATTTCCCATTAATGCTGGCAAATTTTGGGGCAGGCGAGGCGTCGACTAAAGCTATTTTCGATG
GGCTTTTCAAACGAGTCATGTTGGATAAAAATTTTATTCAGCAGCGAGCTGAAATGATCTCATTCGAACCATT
CATGCACGCTATTGTTGCCCAGATCTCCTCCAGCTGCGAGTCGCTGATGATTGCCGGTATTGATAATGAGGCG
ATGTTTTCGCGCGCAGCGCCATTGGGATTTAGCGCATTCCAGGGCGGACTATGGCCTCCGGTGCCGGTAAGCC

AGCTCATTAAACTCGTTCAGCGATAACCTTTCTGTTTGCCGGTGTTTAACCGGTGATTAACCGTCTACACTGA
AGACAGGAGGCCCTATGACCCTGTCTTTTACTGCCCGTTGGCGCGATGAACTGCCAGCAACGTATACCGCGCT
TTTACCCACACCGTTAAAAAATGCGCGACTGATTTGGTATAACGACAAACTGGCGCAGCAGTTAGCAATTCCC
GCGTCGTTATTTGACGCTACCAATGGCGCTGGCGTTTGGGGCGGCGAAACCTTATTACCCGGTATGTCGCCTG
TCGCGCAGGTATATAGCGGGCATCAGTTCGGCGTCTGGGCGGGACAACTCGGCGACGGACGCGGTATTTTGCT
GGGCGAACAGCTCCTGGCGTATGGCTCGACCCTCGACTGGCATCTGAAAGGCGCCGGATTAACACCCTATTCG
CGAATGGGGGATGGACGCGCCGTCCTGCGTTCAACCATACGAGAAAGCCTTGCCAGCGAAGCCATGCATTATC
TGGGTATTCCCACTACGCGCGCGTTATCTATCGTAGCCAGCGATACGCCGGTTCAGCGAGAGACGCAGGAAAC
GGGCGCGATGTTAATGCGTCTGGCGCAAAGTCATATGCGTTTTGGTCATTTCGAACATTTCTACTACCGCCGT
GAGCCGGAAAAAGTTCAGCAACTGGCTGATTTCGCTATTCGTCATTACTGGCCGCAATGGCAGGATGTCCCGG
AAAAATATGCTTTGTGGTTTGAAGAGGTGGCGGCGCGTACCGGAAGACTCATCGCCGAGTGGCAAACCGTCGG
TTTTTCGCACGGCGTTATGAATACGGATAACATGTCGATTCTGGGGCTGACCATCGACTATGGCCCCTTTGGT
TTTCTCGACGACTACGATCCCGGCTTCATTGGTAACCATTCCGATCATCAGGGACGTTACCGCTTCGATAATC
AACCGTTGGTGGCGCTGTGGAATTTACAACGTCTGGCGCAGACGTTAACCCCTTTTATTGAGATCGACGCGTT
AAACAGAGCGCTGGATCGTTACCAGGACGCGTTGTTAACGCACTATGGGCAGCGAATGCGCCAGAAGCTGGGT
TTCTTTACCGAGCAAAAAGACGATAACGCTTTACTGAATGAATTGTTTAGTCTGATGGCGCGCGAAGGGAGTG
ACTATACCCGTACATTTCGGATGTTAAGCCATACGGAGCAACAGAGCGCTTCCTCGCCGTTGCGGGATACGTT
TATCGATCGCGCGGCGTTCGATGCCTGGTTCGATCGCTACCGGGCGCGATTGCGCACAGAGGCGGTGGATGAC
GCGCTGCGCCAGCAGCAGATGCAACGCGTGAATCCTGCGGTTGTGTTGCGCAACTGGCTGGCGCAACGCGCGA
TAGATGCGGCTGAACAAGGAGACATGGCGGAACTGCATCGGTTACATGAAGTTTTACGTCAACCTTTTACCGA
TCGAGATGATGACTATGCCAGCCGACCGCCGGAGTGGGGAAAGCGGCTGGAGGTCAGTTGTTCAAGTTAGCCT
GACGCCAGCAGCATCGTCTGCTGGCGGAGCGTTATTTGGTTAACAGTAGCTTACCAGCCTGAGTTTTGCGCAA
CAGATACTCCTGACCGTTGTGATCGATGATGACTTTGCCGTCCGGACCTAATAGCGCCTGACTGTTGACGCGC
CGTTCAGCGGCAGGGAGAAGCGTTTCATTGTCTATTTCCTTAGGGTGAGGCAGCTCGGTGTTATCCATACGCG
ACATGTTATTTATTAATAATCAAACTCACAATGATTATTATTATCAATAATTAATGCGATAACAGCAAGTGCT
AATTCTGTGGAGTGATATACGCGCGGGGAGAGAAAAGGGCGGGTAACGCATCGAAGAAACGCCCATCCCGGCA
GGATGGGCGAGCTATTAAAAGCGGCTGTCTACCGCTGCGGCAAGTTTTTCCAACAAAACTTCCGTATCTTCCC
AGTTCAGGCAGGGATCGGTAATGGACTGACCATAGATTAATGGTTGACCGCTGATAATTTTTTGCGTGCCTTC
CCGTAAAAAACTCTCGGCCATAATCCCGGCAATTGCCGTGGAGCCATTACGAATCTGCTGGCAAATATCATCA
CATACCTCCAGCTGGCGGCGATGCTGTTTTTGACAGTTGCCGTGGCTGAAGTCGACGACCAGATGTTCCGGCA
GATCAAATTCATGCAACGTATCGCAGGCGGCGGCAATATCTTCAGCGTGGTAGTTCGGTTTTTTACCGCCGCG
CATGATGATGTGCCCATACGGGTTGCCACTCGTCTGGTAGATGGTCATCTGTCCGTCTTTATCCGGCGAGAGA
AACATATGGCTGGCGCGGGAGGCGCGAATAGCGTCAACGGCAATGCGAGTATTGCCATCCGTACCATTTTTAA
AGCCGACCGGACAAGAGAGCGCAGAAGCCATTTCCCGATGGATTTGGCTTTCGGTGGTACGCGCGCCAATCGC
TCCCCAACTGATCAGATCGGCAATAAACTGGCCAGTGACCATATCAAGAAATTCTGTGGCGGTCGGTACTCCC
AGTTCGTTCACCTGCAATAGCAAGCGACGCGCCAGTTCAAGCCCATAATTGACGCGGTAACTTCCATTCAAGT
CAGGATCGGAAATCAGGCCTTTCCATCCCACAACGGTGCGCGGTTTTTCAAAATAGGTGCGCATGACGATTTC
CAGACGCGCCTGATGCTTTTGGCGTTGTGCCTGGAGCTGCGTCGCGTATTCCATGGCAGCATTCAGATCGTGA
ATCGAACAGGGGCCAATGACGACCAGTAGCCGTGGATCTTCACCATTTAAAATCTTTTCTATCCGGCGTCGGG
AGTCGGTAACGTGACTGGCGACGGAGGACGATACAGGATACCGCTGCGCGAGTTCGGTCGGTGTTACCAGGCT
GTCGATACGCGCAGTACGGAGTTCGTCGGTTCTGTTCATTACAAGTCTCAGAAATTTGTTGCTTCAACGGCAG
GACTACCGGGAAGTGATGGGCATCACGATAAACCAATCCCTGCTGATTTCAAGTGCGAGGCGTGTCACACCTC
GCAGGCAGAACGGCATGATAACAAAAACCAACCCATTAAACTAGTTTGCTAGTACATCCTGCGGTTAAGCCCC
ATAATATCGAGAATCTTGGTGGCTATCTCTTCAACGGAATAATTAGTGCTGTTCAGGCAGGGGATCTGATTCT
TACGATACAGCGCTTCGACCTCCGCCACTTCCATGCGGCACTGACGCAACGAGGCATAGCGACTGTTTTCACG
GCGTTCTTCGCGAATTGCCGCCAGGCGTTCAGGGTCAATTGTCAGGCCGAACAGTTTATGTTGTAGCGGTTTT
AACGATGTGGGCAGCGTCAAATTATCCATATCATCGGCGATGAAAGGGTAGTTAGCCGCACGAATACCGAATT
GCATCGCCAGATAGAGGCTGGTGGGCGTTTTTCCACAGCGCGAAACGCCAAGTAAAATGACCTGAGCCTGATC
GAGGTTGCGCAATGAAATGCCATCATCATGCGCCAGGGTATAATCGATAGCGGCAATACGGGCGTCATACTTG
TTCAGGTTACCCGGATTAAGCCCATGAGTACGATGAGCGATCGGCGTCGGATCCAGTTTCATCTCCTGCTGAA
GCGGCGCCACCAGCGCCTGGACGATATCCTGGCAGAAACCTTCGCTCTGTAAGATGATGGCGCGAATTTCAGG
TAAAACAATCGAGTAAAACACCAGCGGGCGAACGCCGGTTTGCTGATAAATGGCGTCAATTTGGTCTTTGACG
GCGCGGGCGCGACTTTCGTTTTCCACGAATGGCAGCGTGATGCTGCTGATCGTCACCGGAAACTGCGACATAA
CCGCGTGACCCAGGACCTCGGCGGTGATGGCGGTACCATCAGAAATATAGAAAACATGGCGATCAACAACATT

ATCCATTCTGTTCATCCTGAAGAGTAAACATAATTATCTGAAAGCATAAATTAAAAGTAACGGGAAACGTATA
ACTTGTCGCTTATTTTATAAAATGACAGGCGCTTTTTATAATTAATGAAAAGTGTGTTTCATTTCCTGGAAAC
GCAGTTTATACCGGGGGTTATTCAGGGAATGCCGTGCTATGCGCGGGTTTGCGCAACTACGTAAACAATCTGA
AAAAATGAAAAAATAAATTTGCTTGAACGATTCACCGTTTTTTTCGCGGGTTTAAGTATGCCAGGATAAATAC
GCAACTATGTGTTTCTTAAACTCGTTCATTTATCACAAAAGGATTGTTCGATGTCCAACAATGGCTCGTCACC
GCTGGTGCTTTGGTATAACCAACTCGGCATGAATGATGTAGACAGAGTTGGGGGCAAAAATGCCTCCCTGGGT
GAAATGATTACTAACCTTTCCGGCATGGGCGTATCCGTTCCGAATGGTTTTGCAACCACTGCCGATGCGTTTA
ACCAGTTTTTGGATCAAAGCGGCGTAAACCAGCGCATTTATGAGCTGCTGGATAAGACAGATATTGATGATGT
CTCCCAGCTGGCGAAAGCGGGCGCGCAGATCCGCCAGTGGATTATCGACACTCCTTTCCAGCCGGCGCTGGAA
AACGCCATCCGCGATGCCTATGCGCAGCTTTCGTCGGATGATGAAAATGCCTCTTTTGCCGTGCGTTCCTCCG
CCACGGCGGAAGATATGCCAGATGCTTCGTTTGCGGGTCAACAGGAAACGTTTCTTAACGTTCAGGGCTTCGA
CGCGGTGCTGGTGGCAGTGAAACACGTATTTGCTTCTCTGTTTAACGATCGCGCCATCTCTTATCGCGTTCAC
CAGGGATATGATCATCGCGGCGTAGCGCTTTCCGCTGGCGTGCAGCGGATGGTTCGCTCCGATCTCGCCTCGT
CAGGGGTGATGTTCTCTATCGATACCGAATCCGGCTTTGACCAGGTGGTCTTTATCACCTCGGCATGGGGACT
GGGTGAAATGGTGGTGCAGGGCGCGGTTAACCCGGATGAATTTTATGTCCATAAGCCAACCCTGGCGGCAGGC
CGTCCGGCCATCGTACGCCGCACCATGGGGTCGAAAAAAATCCGCATGGTTTACGCCGCCACCCAGGAGCACG
GCAAGCAAGTGAAGATTGAGGATGTGCCGCAGCAACAGCGCGACATTTTCTCGCTCACCAACGAAGAAGTGCA
GGAACTGGCGAAACAAGCGGTGCAGATTGAGCAGCACTATGGTCGTCCGATGGATATCGAATGGGCGAAAGAC
GGTCATACCGGTAAGCTCTTTATTGTGCAGGCACGTCCGGAAACCGTGCGCTCCCGTGGCCAGGTGATGGAGC
GTTATACGCTACATGCGCAGGGGAAAATCATTGCCGAAGGTCGCGCCATTGGTCATCGTATCGGCGCGGGTAC
GGTAAAAGTTATTCATGATATCGGCGAAATGAACCGTATTGAGCCTGGCGATGTGCTTGTCACCGATATGACC
GACCCGGACTGGGAACCGATCATGAAAAAAGCGGCGGCGATTGTCACTAACCGTGGCGGGCGCACCTGTCACG
CGGCGATTATCGCCCGCGAATTGGGCATTCCGGCGGTTGTCGGCTGCGGCGACGCCACCGAACGCATGAAAGA
TGGCGAAAAAGTAACCGTCTCCTGTGCGGAAGGTGATACCGGTTATGTGTATGCCGACATGCTGGATTTCAGC
GTAAAAAGCTCCAGCGTAGATACCATGCCAGAGCTACCGCTGAAGGTCATGATGAACGTCGGCAACCCGGACA
GGGCATTTGACTTTGCCTGCCTGCCGAATGAAGGCGTAGGTCTGGCGCGACTGGAATTCATCATTAACCGTAT
GATTGGCGTTCACCCACGCGCGCTGCTGGAGTTTGACGACCAGGATGCCGCGCTGCAAAACGACATTCGTGAG
ATGATGAAAGGGTTTGACTCGCCGCGTGAATTTTACGTTGGTCGACTGACGGAAGGGATCGCGACGTTAGGCG
CCGCCTTCTACCCGAAACGCGTCATCGTTCGCCTGTCTGACTTTAAGTCCAACGAATACGCCAACCTGGTCGG
CGGCGAACGTTATGAGCCGCATGAAGAAAACCCGATGCTGGGCTTCCGCGGCGCGGGCCGTTACGTCGCAGAA
AGTTTCCGCGATTGTTTTGCGCTGGAATGCGAAGCGGTGAAACGCGTGCGCAACGATATGGGCCTTACCAACG
TCGAAATCATGATCCCGTTCGTGCGGACGGTCGACCAGGCGAAAGCGGTCATCGAAGAGCTGGCGCGCCAGGG
GCTTAAACGCGGCGAGAACGGGCTGAAGATCATCATGATGTGCGAGATCCCGTCCAATGCCTTGCTGGCTGAA
CAGTTCCTCGAGTATTTTGATGGCTTCTCGATTGGTTCTAACGACATGACGCAGCTGGCGTTGGGGCTGGATC
GCGATTCCGGCGTGGTGTCTGAACTGTTTGACGAGCGTAACGACGCGGTGAAAGCGCTGCTGTCGATGGCGAT
CCGCGCGGCGAAAAAACAGGGCAAATATGTTGGAATTTGCGGTCAGGGTCCGTCCGACCATGAAGACTTTGCC
GCCTGGCTGATGGAAGAGGGGATCGACAGCCTGTCGCTGAACCCGGACACCGTGGTTCAGACCTGGCTGAGTC
TGGCGGAACTGAAGAAGTAGTGTAATACCGGATGGCGGCGTTAACGTCGCCATCCGGCAATTTATACCGCAGT
GTGCTCCTGGCGCAGCCGCTCAATAATATCCTGCCGCAACAGAAACTTCTGCACTTTGCCAGAGGCGGTTCTG
GGCAGTTTTTCTACGATGACGATCCGTTCCGGATATTTATACTTCGCTACCCGTTTTCGACTGAAAAAAGCGA
TCACCTCTTCCAGCGTCAGCGAAAGGTGCGGCGGTTTTAAGACTACATACGCGCATGAACGTTCGCCTAAGCG
TTCATCAGGCATAGCAACGACGCAAGCATCGTGTATGCGGGGGTGTTGCAATAAAATATCCTCCACCTCGCGA
CTGCTGATATTTTCACCTCCACGTATAATAATATCCTTTTTGCGCCCGGTAATTTTGATATAACCGTCTTCAT
CCATGCGGCAGAGGTCGCCGCTGTAATACCAGCCCTCGTTATCCAGTGCGCGGGCGGTAAGTTCCGGCTCGTC
CAGGTATCCCATAAAAACGTTCGGCCCGCGCGAGGCTTCTTCGCCTTCGTGGCCTGCTGGAAGCGTATTGCGA
TCTTCATCCACGATTTTAATTTCTACACCGGTGGCGGCATAACCATCGGTATTCATCATGCGTGAAGTCGAAT
CACCCAGATTAACCATCGAGTGTGGAGAACTTTCTGTAGAACCGTAAATACTCAATAATTTGATACCGCGCTG
CTGGCAGTCGCGGGCAACCTTTTTGGGAATAATCGTGCCGCCGCATAAGAAGAATCGCAGTGAGGAGAGGTCG
GCAGGCTGTTGCTCAACGGCACAAAGCAGATCGTAAATAAACGGCGTCGCGCCTGACATACAGGTACAGCGTT
GCTGCGCTAATAAGGTAAGGCAGGCTTCTGGGGTAAAGATGTCCAGCAATACGCTACGCGCCCCGATTAAAAA
GGGCGCGGTGACGCCGTGTAAAAATCCGGTGGCGTGGCCCAGTGGCGCAGGCATCAGGAACACATCTTGCCAG
GTTAAATTCAACCGCGCGCAATACGCCCGTTCACTGGCAAGAATATTATTGTGGGTCAACATCACCCCCTTTCG
GCATTCCTTCTGTGCCGGAGGTAAACAGCACTGCCGCCAGTTCATCACCGTGAATGTTAATGTCTGACTGCAA
CGGTTCGCTGCGGTCGATGATTTGACTAAGCGCAAGCGCTGTGGTGGCAGGCGCCAGTTTATCCACGCCGACA

ATATGCGTCAGATGGCGCAGTTGATTTTGTAGCGGAAGGATAAGATCGACCGGACGATTCTGTTTGAATACGG
TGGGGGCGAAGAATATTTTAGCCTGACATTTGTTCAGCACCCAAACCAGCTCTGCTTCTCGCCAGGCGGGAAG
TAGCGGGACAGATACCGCGCCCGTTTTCAGGCAGGCCAGATAAATGAGGGTAAACTCACACCAGCCCGGAAGC
TGAAAGGCCACGCGATCGCCCGGTTGAATCCCCTGAGACAGTAACCAGTTTGCCAGGCGGCTTGCCGCGTAGT
CGAGCGCGGCGTAGGTCCAGGACGCGCCATGATTATCGACCACGGCGATTTTATCGGGTACGGCGCGTGCGGT
TTGCCGCCAGTAGTCGCCCAGTGAAGCATCGCCCCAGTAGCCGGACTCGCGATAGGTTTTCCGCCTTGCGGCG
TCAAACGTTAATGTAACACTCATTGACTTGTTCTCTATTAATACAAATGACGTAATCTCCGGGGGCAAGCCGA
ATAAGAGCCTATCCCATTAGGGCTATTTTACTTGCCATTTTAGACCTGGGCAGTGCTCAAAATCCTCACGTAC
TACGTGTACGCTCCGGTTTTTGCGCGCTGTCCGTGTCCAAACTGGCTGCGCCAATAACGCCTACTGGGATAGG
CTCTAAGACGCCGTAGCGTCGTTATCCGGTGTTTCAGAGCGTCAGCCGTAGCGAAACTCAACGCCGAACGTTC
CCGCCGGGTATTCCCATTTTTCGAGAAGAGTATTGCCGCAGAGAACCCTGCAGGTCCCGCATTCCAGGCAGCC
CGCGGAGTCAAAATGAATATTTCCCGCATCGTCCAGCTTATAGAGTCCGGCAGGGCAGGCTTTTATTAATTTA
TTAAACTCCTGCATGTCAGGCGAGGAGCGCAAAATGATATGTGGGTGTCCTTCATCTACGTAAAATTTATTTA
CGCCCAATTTGACGTCAACATTCACCTTATTGTCCGAACTCATAATGCCGTAGCCCCTTTTATGCCGTCTTTT
AATAGATTCACCAGTCCAATTTTTTTCGCATGTGACAGGATAGTTTTACGCAACGGTGGGACTGGACCACCAT
CAACGGTGAACAGATCGCCCACAATGTTTGCCGCCATTCGCGGGTACTCGGTAAAAAGCCGTGGGTTTTCCAT
TAATCCGGGGAGTTTGCGGAAATGCCGCATATCGCGTAACACGCCGTTTTTCTCCAGCGCGCGTTTGTAACCG
GCAAGACTCGCGGCGGAAAAATCTTGCCGCGCTTTAGCGTCAATGGCGGTTTGAGCGGCCGCTTGCGCGGACG
CGATGGCTAAATCCATGCCGCGGACGGTATAGCCGAGATTCAGGCAAAGACCGGCGGCGTCGCCGACAATCAT
CACGCCGTCATTTGCCAGCTCCGGCATCATTGCTATGCCGCCTTCGGGCACCATATGAGCGGAGTATTCGAGC
AGCGTTCCCCCTGAATCAAAGGGCGTATCGCCGGATGCTGTTTAAAATCTTCCAGCATTTGCGGTACGCTTTT
GGTCGCGTGAGCAATGTCGCCCAGGCCACATACCAGCCCCAGAGAAACCGAATCGCGGTTGGTATAAAGAAAC
CCGCCGCCCATGAGCCCGTTGGAGGGCGCGCCAGCGAATAACCAGGCGGCGCCTTCATGGGACGCCAGATTAA
AACGCTCTTCAATAAGTGCCGGAGAGAGCCCAATCAACTCTTTTACGCCGACGGCATAGTGATGCGCAGACGA
GGCAGGCACCATGTCCAGTGAACGGCCCAGCATGGAATTCACGCCGTCCGCGAGAATCACAATATTCGCATCA
AGAATATCATCCCCGGCCTGAACGCCAGTGACGCGATTGCCTTCACGTACCAGAGCGTCGACCCGTACACCTG
GGATAAACTGCGCGCCAGCCTGCTCCGCCTGCGCCATTAACCACGGGTCCAGTTTGTTGCGCAGTACGGTATA
TGACGTAAGGGGCGGAGTAGGGGGCGCGCGGTGAAAATCAAGTGTGACGGCGCTTTCTTCGGTTAAAAAGGAG
ATTTTTTCACGGGTAACGACGCGTTCGATGGGGGCGGACGTTGCGAATCCCGGCATAATACTTTCGATACTGT
GAGCGTAGAGCCGGCCACCGGTCATATTTTTGCTGCCCGCGCTGTTTCCGCGTTCAATAACCAGTACATCCAG
TCCCGCTTTTGCCATCACATACGCGGCGACCGTTCCTGCTACGCCTGCGCCCACCACAATAGCGTCAAATTTA
TCATCCGACATGCTTTATGCTCCTTTCCCGCCGTATCGACGACGGGAGATGTGCGTTGAAAAAGGAATTAGCG
GCTTAACTGGTCGATGAGGGCAGGAACAACCTTATAGATATCGCCCACCAGGCCATAATCGGCATAATTGAAA
ATGGGCGCGTTTTTATCTTTGTTAATTGCGACAATAATTTTTGCGCCGTTTCCACCCACCATATGTTGAATTT
GGCCGGAAATGCCCAGCGTCAGATAGAGATCGGATTTCAACAGAACGCCGGATACGCCAATATAACGCTCACG
TTCCATCCAGTTTTCACCTTCCGCAATGGGGCGGGAACAGCCCACTTCTGCGCCGAGAACGGCCGCCAGTTTA
TGCACCATATCCAGATCGCTTTGCGCCGCCAGGCCTCGCCCTACGCCGATGACCCGTTTGGCTTTGCTCAAAT
CGACGCTGCTTAGCGATTTCGCGCGCCGCTGCTGGCACAATACCTCGTGGCGTGGGGCAATATATGCCAGCGT
GGTCTGCGGGCAAGTATGACCGGGGAGAGGGGCGGCGGGTTCCAGCACGCCGGGTGCCAGCGTAATAATCGCC
TGCGGACTGTTTAATTTCTCTTTGCCGAAAGCAAGTCCGCCGTACATGCGATGCTCTGCGTGTAGCGTCCCGT
CGAGGAGGTCAATAGACGTCGCGTCGTTCACCATCATGGCGTCAAGCTGGATGCTCAGCCGGGCGCCCAGCGC
TTTACAGCGCTTGGTCGCCGCCATGAGCAGCAGCCCATTTTGGTCGCCTAATAATGAGGCCAGCGTTTCGGCA
TAGTTTTCCACGCGCTGCAAATCCGTATGCGATTCAAGAACGACTATCTCATCAGCGCCAAGCGCTTTCACGT
AGTCGATCTCCGTGTTTCCTTGCACGATGGCGTAAACTTTTTCTCCCCACTGCCGCGCGCCGGTCATTAATTC
GGCATAACGCTCTACGTTATCGCTAAATACCCATACGTTAGCTAAATGATTCATGATAATTTTTCTCCGTTGG
GTTTAGTTCAGGGATTTTTTCAAATGGTCGGCCAGTGCGGTAATGGCTTCCGGCGAATCGTTATCGAGAATGA
TATGCTTGCGTTCTGTCTGCGGCGGTACCGAGATGGCGACCAGTTCCGCCAGTGGCGTGGTTGGGGTCCAGTG
GATATCGCTCGCCAGCCACGACGTAACGGCTTTTTTCCCGGCGCCGAGAATGGCTTTCATTGACGGGATGCGT
GGAACGTTAATATCTGAGGTCACGCAAAGCACCGCGGGCAGCGTGAGCTCGATAATTTCAACGTCTTCTTCCA
GTGTGCGTTCAACGATGATTTTGTCGCCGTCGCGTTGAATATGGCTTACTGCGTTGATGACGGGGAGTTTCAG
GAGTTCGCCAGTCAGCAGGCCCACCTGTTGGGCATAAATATCGCCGGAGCCCTCCCCGAAGAGCAGCAGGTCA
AAGCCAATTTTCTCCGCCGCGGATGCTAGCGCCAGCGCGGTTTCTTTGGGCAGGGCGTGCTCCAGTTGCGCAT
CCTGAACCATGAAAAGCGCATCCGGCCCGCGGGACTTTTGAATTTTGTAACAGAGAGCCGCCGACGGTGAGCG
CGGCGATCTCGTCGCCACCGCCAGCTAACTGCGCGGCGGCCTCAATTGCATTTAAATCAAACTGGCTGATTTT

GGCCTCGGCACGATCAAAATTGAGCGAATGCTCCGGGGTCACGGTAATGTCCTGTTCTTCAGGCACCAACTTA
AAGCAGGTTATTATTTTCATTGCATCTCCAGTGAGTCGCGTGCGGATAAGTAACGTAAATCTGACTGAAAATA
GTCTGCGGCAGTCTGAAGAGGTTTACGATGGGTCAATCTGTTTTCCTTGCCAACAATCTTGCACAAAAAATCT
GCGAACAGAGAATCGTTAGCAACCATCTGAAAAGGGCGTCATAAAAATATAAATACGGTAGTGAGAATAATAA
GTGCGAAGCATATCATCATTTTTTAACATGAAACGTGCGATGTTGTTTCTGTGAGGTTAAAAGAAAACATCAA
AGATATGTCGGCTATGTATTTTTGTCTGATAATACGCCATCGACAGGTAATCTGAGCTGTCTGCAAAAGAACA
TAATATCTTGCATTCGGAACGAATAATCTTGTTTTGCTGGTAATCTCCACGGTTGTACAACACTGACGCTCGC
TTTATTTTATCCTGGTTATCTTTTTAAACCTGACAGAGGTTATTATGTATCAGCGCTGTTTTGACTCCGCCGC
GGAGACGATTTTTGAACAGGACAAGACCCCTCGATTTTCACGTTTTGTCATTAGCGATGATCCAAACTGGGAG
TCTGGCCATCATATGCATGATAATGAAACAGAGCTTATTTACGTCAAAAAAGGGATAGCCAGGTTAATTATTG
ACTCTTCACTCTACGTCGCGCATGCGGACGATATTGTCGTCGTTGAGCGGGGGCGGCTACACGCTGTAGCATC
CGACAGCAATTCGCCGGCTACCACCTATACCTGTGCGCTGTATGGGTTTTGTTTTCCAGGTTGGGAAGAAAAC
CAACTATTGCAGAGTCATTCCTGTCCGGTGGTTTCCGTGGTTCAGGGGAAAGAGGTCATTAAAAGTATCTTTA
ATGAATTAAGCGTGTTACTTCCGCAGGGGAAAAACGTATTGGCTTCCTCTGTATACGATGCATTTGCTTACAC
TCTGACCGCACTTTACTATGAAAATTTTAAAAATGCCTATCGTTCTGAACAAGGCTACATTAAAAAAGATGTA
TTAATTAAGGATGTGCTTGTTTATCTCAATAATAATTATCGTGAAAAAATCACGCTTGACCAGCTATCGAAAA
AATTTCGTGCCAGCGTCAGTTATATATGCCATGAATTTGCCAAAGAATATCATATATCCCCTATCAACTATGT
TATTCAACGACGTATGACCGAGGCCAAGTTTGCGCTGACAAACACTGAGTCGCCACTGGCAGAAATTTCATGG
CGGGTAGGATATGAAAATGTGGACCATTTTGCCAAATTGTTTATGCGGCATGTAGGATGTTCTCCCAACGATT
ATCGTAAGCAATTTAAAAATAGTCTGGTTGAGCAGGCGTATTTGCTGCCAAATACCTGACGGCGAAATATATC
TGATTTATTCGGCCTGATGCCGTAAAGCAGTCAGGCCGAATAAGACGTTCCGGTACGATCCTTACTTGTTCTG
ATAGTCTTTCAGAATCTGGCGTCCTGCCACATAGATCATAATTTCATCCGTGCCGCCACCGATACGTTCGCAG
CGAACATCGCGCCAGAAGCGGGAGACACGGGCTTCGTCAGTATAACCAAGCCCACCCATAATCTGAATCGCAT
CGTCAATCACTTCCATCGCTGTGCGTGCGCAATACAGCTTAGCCAGCGCGGCGCTGGTACGCAGTGACTCTTC
CCGATCGGCCTGCCAGGCGACTTTCAACACCATATTGCGCATGTTTTCGATTTTGATGGCCATTAACGCCAGC
TTTTCCTGGATCATCTGGTTATGGCCGATGGGTTTACCAAAGGCGATACGCTGGTTGGCGTAACGGGCGGCAT
CTTCAAATGCGCATTCGGCGAAACCTGTACTGCGCGCCGCATTAATCAAACGCTCCATCTCAAAATTGTACAT
GACATTGAGGAAACCCATGCCTTCTTCACCGACCCGGTCGCTTTCGTCCACCTCGACATTATCGAGATAAACT
TCGCATGTACTCAACATGTGCCAGCCAATTTTGTGCAGCGGATTAATCTTTATGCCCGGTTTATTTGAGTCCA
CCCACCAAAGCGTGAACGCTTTTTTAGGATCTTTTGGCTCCGTATCGCGCGCCAGAACCAGCATGTAAGGATA
TTCTTTTGCACCGGTGATAAATGTTTTTTGGCCGTTAAGATAAACCTTGCCGTTTTTACGCGTCCAGGTTGTT
GTGGCGCTATTATTATCAGAGCCCGCGCCAGGTTCTGTTAAGGCTAGCGCATATGCTGGATCGCCGGTGGCCA
GAGTACTTTCTGCTGTTTTACGCAGTTGTTCTGCGGAACCAAAACGGCGCATGCTGTGAATGCATTGGCCGTT
GGTAATCAAAAAGGCCGGAGCGCCGCATTTTGAGATTTCCATCAGCGCCAGCATTTGCGTTACATAGTCAGCC
GGAATGCCGCCGAATTCTTCCGGGACGCCCAGCATCGAAATACCGTTTTCCGCCAGCGCCTGCATAAATTCTT
TGGGATAGGTGCCCGTTTGATCGCAGGTACGGAAATACTCTTCCGGAAAATTGCTGGTGATTAACTCGCGAAT
ACTGGCCAGTAATAGTTCTTGTTCTTCAGTTAATGAAAAATCCATATTTGTACTCCTTTTAATGTGTTGCGTC
AGGAAGCGTGAATCCCATAGTGCTATCGGTAAATAATCTTGTGTCCATCAGTTTTAAATCTGGTGAGATAACC
GGTGAAAAATCCATCTTGTCAAGAATATCCCGTTGCAGATCGACGCCAGGCGCAATTTCAATTAAATGCAGGC
CATCTTGTTTGAGAGTGAAAACCGCACGTTCAGTGATGTAACGCACGTCTAACCCGCGCTCAAGCGCAATTTT
TCCACTGAACGTAATTTCCGGCAGTTCGCTGACGAATTTTTTTCACGCGACCTTCCTGCAGAATATTTAATTT
GCCATCGGTAATTTCAGTTTTTAAACTTCCGGCCGTCAGAGTTCCGCAGAAAATAATTTTTTGCGACGTTGCG
CTAATATCGATAAACCCGCCGGTGCCCATAATTTTGCCGTTGAATTTATGTACACCGACATTACCGTGTTGAT
CCACTTCGGCAAAACTTAAATAGCAGACATCGAGCCCACCGCCGTGATAAAAATCAAATTGTGAGGTCATATC
GAGGATCGCGCGGGTATTGACATTGGCGCCGAAGGCCACACCCTGAGAGGTAATACCGCCCACCGGGCCAGTT
TCTACGGTCAGCACGAAGTCATCGGCGCAGCCTTCCTCGCGAGCGACCAGACCAATGCCGTCGGCAATGCCTA
CGCCCACATTGCCTACCGCGCCTTTACGCATTTCAAACAGCGCGCGTCGGGCTACCAGCTTGCGCTGATTAAG
GGGCAGGGTAAGCTGGGTACTATCATCAAGAGTAAAATCCCCGGAGATAAATCGGTTCACCGGCGCGCCGCCA
TAGAGTTGCGTCTGGTCGGCGTCGACAACCACAATATCCACCAGATAGCCGGGAATACGCACGGATTTTGGAT
GCAGCGTGGCTTTTTTTACCATTTTCTGCACCTGCATCATTACAATACCGCCATTATTGTGAACGGCCTGGGC
GATCACCAGTGCATCAAGATACATCACCTCATCTTCAAATGATGCATAGCCTTCGCTGTCGCAGGTGGTGGCG
CGGATAAAAGCCACATTAGGCGCTATCGCTTTATAATAGAGATATTCTTTATTATCGATCTCTACCAGCTTAA
TGAGATCTTCTTTAGTCACGTCGTTTAATTTTCCGCCCTGTTGGCGAGGGTCGACAAAGGTACCAATACCGAT
ATCGCTCAGAATACCCGGCTGGTGTGCGGCGCTGGCGCGCAGCGTCTGGGTTAATACGCCTTGTGGGTAGTTA

TAGGCGGCGATTTTATTTTGTTCAGCCAGTTCAGAAATACGGGGAGATTGCCCCCAATGTCCGCATAGCGCCC
ATTTTACCAATCCCTCCTGCGCCAGCGGGCTGATGCCGCGATCGGCGCGGTCACCTAATCCGGTAGGGCTAAT
AATAGATAAATCTCGTGGTGACTGGGTTGTTTGATATTTATCTGCCAGTGCGGTAATTAATGTTGTCGCTTCG
AGAATACCGCCGCCAGCGCCCAGAATACATAAAGTGGCCTCATCTGGAATATAATTTACTGCTTCTTGTGCAG
AGAGCACGGGAACGCGGCCATTAATGCGTGATGGCTTATTGATTTTCATATTTACTCCACTATTATCCCTGAA
AGCAATATCGATATAACCGTGTTAAGGTAATAAAGACAGCGGCTTTCAATGAATATGGAACATATTCATATCA
GATAACTATTTATTTTATTGTGGCAGAAAGAGAATATTCCGCCACACGATAAAGTATTACGCCTGGTGCAATA
TAGTTAATACGGTACGCAGATCGGCTACCGATATTTGTCCCGGCGCAGATGCTTTTTTCACCGCGCCAAACGT
TGCTGCAGAACCGAACACTTCGCCGGCAAGACGAGATATTACCCCGGTTTTCGACATCGACATGGTAATAATC
GGACGATCCGCATAGCGCTCCTGCATTTCTACAGTGGCGGTAAGTAAGGTCAGGACATCGGCTTTAGTCTGTG
GCATGACGGCGATCTTCGGAATATCGGCGCCCAGTTCCTGCATTTTACGCAGACGCTGAACAATCTCTTCCGC
TGCGGGCGTTTTATGAAAATCATGGTTAGACATGATCACCGCAACATTGTGTTGATGAGCATAGCCGACGGTG
GCTTTCACCTCGTCGTCGCCGGTAAAAAGCTCAAGATCGATCATATCGACCAGACCGCTGTCAACCGCTGCAC
GATTCAGAGCGATATACTGTCCGGTAGTTAGCGCCTGTTCGCCGCCTTCTTTCGCGCTGCGGAAGGTAAATAG
CAAGGGTTTATCGGTAATAATCTCCCGGATGGCGCCGGCGGCCTCAAGTACGCTTTCCGCCGTTGTCACGTTG
GCAAAATGGTCAACGCGCCACTCCAGAATATCGAAATCCGCTTCACGGTAGGCGAGTGCTTCCGATTTCACAT
CGGTAATGGTTTTTCCCATTAGCGACACAATGATCTTTGGCGCGCCTTCGCCAACCACGAGATCTCTTACAGT
TACGGTTTTCATTTGGTACCCTTCATTGTCATTCGTCGCAATTATAATTTTACCGGTCAGGCTGTGAATCCCA
TCACCTGCTTCACATAGTCCAATGGAAATGCTTTTCCTGTCCACAGTTCAAACTGTTCGGCTCCTTGCCATAA
CAACATGCCGTAACCATCAATTGTTTTGCAACCAGCTTGTTGCGCCTGCTGCAATAACTTTGTCATATGCGGA
TTATAAACACATTCGGTGACCAGCAATTCAGGGCGGAGCAGGGAAACATCGCCGATCAGGGATTCGTTCTCCA
ATGGTTTCATGCCAACTTTGGTGCCGTTCGTCAGAATATCTGCGCTGGCAAGCGCTTCAGTAAAAGCGTGCTG
ATCGGCAAGATCGGTGACGGTAACGACGCAATCGGTGTTTTCATTCACCCGCTTCGCAAAGGCAACCGCTTTT
TCGAAAAAGTCATCTTTACGGTTAAACAGTTTAATTTCCTTAATGCCTTCAATGGCTGCCTGGGCGCCAATGG
CGGTAGCCGCGCCTCCCGCGCCGAGCAGCACCATGGTTTTTCCCCGAATATCGAAACCGCTTTCTTTAATTGC
CCGAATATGGCCCGTACCATCCGTGTTGTAGCCGCGTAGATAGCCGTCATCGTTAACAATGGTGTTGATAGCG
CCAACCAGTTTTGCTGCTGGCGTTAATTCATCGACATACTCACACGCGAGCTGTTTGTTTGGCATGGAGACGC
CCGTACCGCGCATTTTTAATGCTTTCAGTCCTTCAATGGCGCTGGCAAACGTCGTGTTATCCACTTCGAAAGC
CATATAGGTATACGGTAGCCCCGCTTTTTCTAACGCTTTGTTTTGCATTTCGGGCGATAAGCTATGACGGATA
GGATACGCCATAAGACCAATTAATTCATATTTTGCGGTAACATCCATTTTATACTCCTTAATAAGTGGACAGT
GCAGAGCAGGGCGCATGCCTGTTGCGCATCGTTAACATTTTTACTGGAAATAACGCTCGCCAAATCGGACATC
GTTTTGCGGGATCTTAAATACGCGATAATAGCGAATAAATACAATAATGGCGGTGATAAATGTCAGTAGTGCA
AAGGCAAAATCTAACAAAATGATATATTGCAGGCCAATAGTAGAGAGATAACCGGTGATCAGTGGAATAATAA
AGTTAGCTACGCCCCCCATCATCATATATATACTGGTGACTTTAGCTTTACTCTTAGGGAAAAATTCCGACAT
TACCGATACACCTAATTGTAAAATACCTCCAGCAGCGGAAAAACCAATCACGAAGGCGCCAGCATTACAGATC
AGTGGAGAGGGATACAGGTAAATTGCCGCAGCGGTGAGTGTCGCCAGCCCGGCATTGAAAACATTGGCCCAGA
TAGGGCGAACCATTTTTTTCAGCAATGCGGCAAAAATAAACACGCAAACCAACGATCCCATACTGTAATAAGA
GATGGTTTTCAGCGCGTCGGATTCCGCCATTCCGGCGAAGGCCATCGCATATTTAGGCATCCAGACCACAATC
ACATAGAAGGTTGAGAACGCGGCGACGCCAAATAAAACGGAAGCTACGCCTTCCAGCCAGACGAGGGGAGTAC
TGTTCATCTGGGGTAATTCTTTCGCAATACTGGCATCGACAAGTTGGCTGGGAAAACGGCTTTTCAACAGCAT
CAACGTGATGAGGACGAAAAGGATACCGGGGATTACCACCGCGTAGCCGTACCAGATATGGTTGACTAACAAG
GCGCTGACAATAAGGGGATAAATCATTTGCCCAAAAGAGACCATCGCTTTAACCAGAATAACTGCCGAGCCCG
ATGCTTTGGGAAAACACTCCATTAATGCAGGGTATCCGCCGGTATCCAGCGCGGAGTTCGCCACGCCGACACA
CACCGCTAATATGAAAGCGATCATCAGGTTAGGACTGGCGGGAATACCGAAGAAAAATAGCATATATAGTACG
GCGCCAAGCAGTATTATTGCCCGACGGCCAAATTTATCGGACAGTACGCCAAAGAATAAAATACTGACCAGAC
GACCAAGACCAATGCCAGAGATTAAATAGGCGATACCGGCACTATCCGTGGAGAATTTCTGTGCAAGGGAGGT
CATGTTCTGGGCTAGTGTAATAACACTCATGCCGTGGAGAAAGTAACTAAAATAAATGCAGATAACAGCCAGC
AAATATGGCATGTTGCGAGCCTTGTTCTGAGACATTTGTTCACAGCTCCTTGCTGATGGTTTGATTCAATGTC
CTGCTTATTACTGCTTTACTACATGCAGCGATTGTTCAGTAACACTATAAGTCGGTACTGTCGTTATATGTAC
ATAACATGGCGTTTAAAATGCCAGGTTTATTGCATTGATAGCATTTCATTTGTTAGCCATGAAATATAAAAAT
TGCTGCGAATTTGTTGTTTAATACATATAACTCGTGATGCTCATCGCAATTTTTCTGATAAGTGTGAAGATAA
TGAATAATAATTAACACGAAAATTACATTTTTTGTTTCCCGGTGATAATGGCTAACGTTTTATTTTGCATAGC
AAGGCAATAATATTGCAACTGGCACGCTAACATTTATTGCGCGGTTGACGCTGCTTCAGCGTGATGTTGTGAT
TCAGCCCGACTTCGGTAACCGATGAACAGTGCGAGGAGAAATCCCACTGCGGCGATACCGGTATCAAACCACA

TAATATGTGCAATGCTCATTTCTGAAATCCGCGCTGTGATCAACGGAATAGTGAACGTCGCGATACTGCCGGC
GCTGTAATAAATGCCGGTCGCTTTACCTTTCTCCTGTGGAAAACGGGCCGCCATCAGCGTCAGGCCGATTTGT
ACTACGCCACCGGCGGAAGAGAATCCAATGACGAATGCGAAGATCATAACAACATAGGCCTGAGGATGCAGGC
AGACCGTCAGAAGCGCAATAAAAGAGATAAAGGTATAAAACATCAATAATGTGGTAGAGCGAATTGTTTTTCT
GACCAGCGGGGCGGTTATAAAAACGCAGAGTAGCGATCCGCAGGTATAAATACTTAACAGTTTGATGGATTGG
GTATACGACATTCCTGCGACGAACTGTCCATACTGCGCCAGCCACTGGCTGATAAGATAAAAGGTTGCCATCG
AGATATAGCCATAAAGGGTGTAGCTGATAAGATCGATGAGGGAGCAATGATGTACGCCTGCGACAGGCGCTTG
CGAGATTTTGGGCTTCAATATCCGACCAGGATAGGGTGGAAAAGGACAGCGTAATAAAAACAGAGCATTAATA
AGCATGATCGCCCCGGCAAGCAGGAAGGACCAGCCAAACCACATATTAGCCCAGACTAATAAACTGATAATAA
TGGGCAACAAGAATTGCCCGCCAGAGACAAATGCTTTGATCAGAATATTTGCCGTACTGGGGGAGCGAGGGAA
GGCTTCCATCAAACTGGGATAGGTACCTGCATCCAAAAAACTGTTGGCCATACCCGCTAAAAAGCCACAGGCA
TAAGCGACAAAAATCGTCTGAGCATAAAGAATGCCAATGAAAAAAATCAGGTAACAGGCGGTCCCCAGAATGA
TAAAAGGACGGCGACCAAAGCGATCGGAAAGCATACCGGCGATTAACAAAACGCTTAGCCGGCCAATACCAAG
CGAAGAGATCACAATAGATACGCCTGCGGCACTGGTATGCCATTGCTGTTCAAGCGATGACATGTTCAGGCTC
ATCAGGATAACCCCCATGCCATGTACAAAGTAATTAAAATACAACCCTACAGCGGTAGGGAAATAACGATTTT
TCATATTATCTGACTCAGAGATGCCGCCTGAAAGTTACAGTGGTATACACAGGCAGGAAGCATATTGGATAGG
GTGACAAGTCAATGTCGTGATTGTGTGGTTTTCTATCCGGTATCAATACGATGCGGCGATAAAGTTTACGTCT
AACACAGCCGCTCCAGATCATGTGCGAAAAGTTCGGCAGCAACAGATTGATAAATTTTCCACTCGATAAAATC
ACCCTCTGTATAAATGGACTGAAAAGAAGATAAATCAGGGAAGTAACGCATTGTATTATTGCCGATGCGGTTA
TTTATTTTGTCAACAATGGCGTTAATTCGTCGCTGTCTCCTGGCTTTCATTTCCTTCAGCCTGGCAATGATTT
CTGGAGAGATGGGTGTGTCGCCAGCTTCCCATCGTTGCCAGGTTGTCGAATTATTATCCTGGGTGATATAGAT
TGTGCATTCTTCAATCGTCATATCAAAAATACGCCGTAGTGCTTGTAGTTCTAACGCGTTCATCATTTTTTAG
GCTTTTATATGAAAAAAGTGATGCTATAAATAGCACCACCGACACCGTTTCGACTTGAACAGAATCACACTAT
TTTGTGCAATATTATTTGTTCGTTGCAGATATCGCGGTGTTGAAGGGAAACGGGAGCTGGCTTATGTAACAAT
GCAGCGAAAAAAGTTAAAAAAAAAGCCCATCGTAGGAGACGGGCAAAGACTACACACAGCAATTCGTTGTTTCA
CTCAGGGGATTTCCATGCTTATAAATCAATGTGTTGATTTATAACCGTGTGCTAATAGTAGGCATGAGCACTT
TTTGCGTCGATCAGATTCGTCTCAATAGTTTAAAAACTGTAACGGATTTGCGACAGAAATGAGATGCGGCGGG
GAACAGACGGGCGAATCAAAATGGGGTGTGCGTTGAATTGATAAACGTCGCTAAAGCATCGGGCGGCGATGCC
GCCCGACGGGATTATGACTGCTTGTTGGCTTCCTCGATGTCATCCAGCTCTTCAAGGATCTCCTCTGGTTCAT
TCGTCGGCGCCGGAACTTCATGTACCCATGCGGAGAACAAACGCCAGGTCACGGCCAGAAGCACCGGTCCAAT
AAACAGACCAATCATACCGAATGCAATCAGGCCGCCGATTACCCCGGAAAGGATCAGAAGCAGCGGTAAATCG
GCACCCATGCGGATGAGTACCGGGCGAATCACGTTATCCAGAGTGCCGACGACGGCGCTCCAAACCAGCAGCA
CGGTGCCCCAGGTGGTGTCGCCTGTCCAGTAAAGCCAGATAATGGCTGGAATCAATACCGGCAACGGCCCCAA
CTGAACCAGACACGAAAGGATCATCACTACCGTCAACAGGGTAGCGTAAGGCACGCCGGAAATCGCCAGCCCG
ACGCCGCCAAGTACCGCCTGTACCAGCGCGGTAACCACCACGCCAAGCGCCACGGCGCGGATCGCCTGGGCAG
CCAGCAGAACCGCCGCATCGCCCGCGTTTAGCCGCGAGACGACAGGCAAAATGGCGTATACCCATCGCCACTTG
TTCGCCGCGCCAGTAAAGCAGGGCGCTAAACAACAGCATCAGGGCACAGTGCATCATAAAGCGTCCGATATGC
GCCGCCTGACCGACAAACCAGGTCGTGGTTGTGCCGATATAAGGCCGCACTTTCGCCATAATAGCGCTACCGC
CCATATCCAGCAGGCTATGCCATCCGGCATACAGTTTGGCGCCAACCAACGGAATATTATTTAACCATGCCAG
ATCGGGTAGCGTCATATCGCCGCCGGTCACTGCATGGATGAGCGGGCCGCTACCATCGACAATGCTGTTCACC
AGCAAGGCGACGGGAATAACGAAGAGCAGCACTAACAGCAGCGTCATGACCAATACGGCAAGCGAGCGGCGGC
CCCACAATATTTTTTGTAATTTCAGCAAAACGGGCCAGGTCGCGATAACAATCGTACCTGCCCATGCAAACCC
CAAAATAAAAGGCTGTACTATCCACAAACAGGCCACAATCATGATGGCTAAAAACAGCACCGATAGCAGCACT
TGCGCAATATCCCTGGGCTGACGAACATTTACCATAAATACTTTTCACCTTTGTCTTTGCGCCAGAACGTTGG
CGCGACGTGAACATGCAAACCCACACCCTATAATGATGAGCAATTTCAGCGGTTTTTAACAGGCCGATTCTGCA
TGTAATTCTGTTGGGCGCACAGGAAAAAAATGTGATACAACAAATAACGCAACACGCAAACGATTAAGCATCC
CTTCCTGTGCGTAGACCGCTCGCGCTCGCCGGGTTGGGCGCCACCGTTCACAACATAGACCGCAGGAAAAGGT
CAATATAATGATTCCACAAATTTCTCAGGCACCCGGCGTCGTTCAGCTGGTGCTCAATTTTTTGCAGGCGCTG
GAGCAGCAAGGGTTTACGGGCGATACGGCAACCAGCTATGCCGACCGTCTGACAATGTCGACCGATAACAGTA
TCTACCAGCTTCTTCCCGATGCTGTTGTGTTTCCCCGTTCTACGGCGGATGTCGCGCTTCTTGCCCGTCTGGC
GGCGGAGCCGCGCTTTACGTCGCTGATATTTACCCCTCGAGGCGGCGGTACTGGCACGAATGGCCAGGCGCTA
AACCAGGGCATTATTGTTGATATGTCCCGCTATATGAACCGCATTATTGAAATTAATCCTGAAGAGGGATGGG
TGCGGGTAGAGGCGGGAGTGATAAAAGATCAGCTTAATCAAGCGTTAAAGCCGTATGGCTATTTTTTTGCGCC
GGAACTTTCCACCAGTAATCGCGCCACGCTTGGCGGTATGATTAATACCGATGCCTCCGGTCAGGGTTCGCTG

GTCTATGGAAAAACGTCAGATCACGTCTTAGGCATACGGGCCGTATTGTTGGGGGGCGATATCCTTGACACGC
AATCGATGCCCGTAGAGCTTGCCCGGACGCTGGGTGAAAACACGACGACGCCAGGCCGCATCTATCAGACCGT
CTATCAGTCCTGCCTGGAAAACCGCCAACTTATCCTCGATAGCTTTCCAAAGCTCAATCGCTTCCTGACAGGC
TACGATTTACGCCACGTCTTTAACGATGACATGACCCGCTTCGATCTCACCCGCATCTTAACGGGGTCGGAAG
GGACGCTGGCGTTTATTACTGAAGCCCGGCTGGATATTACCCCGATACCCAAAGTCCGCCGTCTGGTGAATGT
AAAATACGACTCCTTTGATTCCGCGCTGCGTAACGCCCCTTTTATGGTTGATGCGCGCGCCTTATCCGTCGAG
ACGGTCGATTCCAAAGTCCTTAACCTCGCGCGGGAAGATATTGTCTGGCACTCCGTTAGCGAATTGATTACCG
ATGTGCCGGATAAAGAGATGCTTGGCCTGAATATCGTGGAATTCGCGGGTGACGATGAAGCGCTTATTGACGG
ACAGGTCGAGGCGCTGTGCCACCGGCTGGATGGCCTGATGGCGCGAGCGGAAGCGGGCGTGATCGGCTGGCAA
CTTTGTACCGATTTGACGGGTATTGAGCGTATTTACGCGATGCGTAAAAAAGCGGTAGGGCTGCTGGGTAATG
CGAAAGGCGCTGCCAAACCGATTCCTTTCGCGGAAGATACCTGCGTGCCGCCTGAACATCTGGCCGATTACAT
CGCCGAATTTCGCGCTCTGCTGGATGGTCATGGCTTAAGTTACGGCATGTTCGGCCACGTGGATGCCGGGGTG
CTGCATGTGCGTCCGGCGCTGGATATGTGCGATCCGCAGCAGGAACTGTTGATGAAACAGATCTCCGATGAGG
TCGTCGCGCTGACCGCCAGATACGGTGGGCTGTTGTGGGGCGAGCATGGAAAAGGTTTTCGTGCGGAATACAG
CCCGGCCTTTTTCGGCGAAGTTCTGTATGGCGAGCTGCGCAAAATAAAAGCGGCTTTTGATCCGCATAATCGA
CTTAACCCGGGTAAAATCTGTCCGCCGCAGGGGATAGAGGCGCCGATGATGAAGGTGGATGCGGTAAAACGCG
GCACCTGGGATCGGCAAATTCCGCTGGCGGTACGGCAGACCTGGCGCGGCGCTATGGAGTGTAATGGTAACGG
CCTGTGCTTTAATTTCGATGCGAAAAGCCCGATGTGCCCGTCGATGAAAATTAGCCTTAACCGGATCCACTCG
CCGAAAGGGCGGGCGACGCTGGTGCGGGAATGGCTCCGTCTATTGGCCGATCGCGGCGTCGATCCGTTGAAGC
TGGAAAAGGAGTTGCCAGAAAAACGCGCCAGCTTACGCACGCTGATTGCCCGCACCCGGAATAGTTGGCACAA
ACGCAAAGGCGAGTATGACTTTTCCCATGAAGTTAAAGAGGCCATGTCCGGCTGTCTGGCGTGTAAAGCCTGT
ACGACCCAGTGTCCGATTAAAATCGATGTTCCGGAGTTTCGTTCACGTTTCTTACAGCTTTACCATACGCGTT
ATCTGCGCCCGGTACGCGATCATCTGGTGGCGACGGTCGAGACCTACGCGCCGCTGATGGCGCGTGCGCCAAA
GACCTTTAACTTTTTTATTAACCAACCGGTGGTTCGTAATCTGGCGAAAAAACATATCGGTATGGTGGATTTA
CCTCTGCTTTCTGCCCCTTCATTGCAACAGCAACTGGTGGGCCATCCGTCTGCCAATATGACGCTTGAGCAAC
TGGAACGCATGAGCGCCGAGCAAAAAGCGAAAACGGTACTGGTGGTTCAGGATCCGTTTACCAGCTACTACGA
CGCCCGGGTCGTGGCGGACTTTATCCGCCTGGCGGAAAAACTGGGCAGGAGGCCGGTATTGTTACCGTTCTCG
CCGAATGGTAAGGCGCAGCATATTAAAGGTTTTCTTAATCGCTTTGCGAAAACGGCGAAAAAAACCTCTGAGT
TCCTTAATCGGATCGCCGCGTTGGGGATGCCGATGGTGGGCGTCGATCCGGCGCTGGTTCTGTGTTATCGCGA
CGAATACAAACTGGCGCTGGGTGAACAGCGCGGCGATTTTCATGTGTTGCTGGTGAATGAATGGCTGGCGCAG
GAAATAAATGCAAGACTGTCCGTCGAGGTGAGCGGCGAACCGTGGTACTTTTTTGGTCATTGTACGGAAGTCA
CGGCTTTACCCGGCGCGCCTGCGCAGTGGGCCGCCATCTTTGCCCATTTTGGCGCGAAGCTGGAAAATGTCAG
CGTAGGCTGCTGCGGTATGGCGGGCACTTATGGACATGAACTCACCAATCATAAAAATTCGCTGGGAATTTAT
GAGCTATCCTGGCATCAGGCGATGCAACGTCTACCGCGAAACCGCTGCCTGGCAACCGGCTATTCCTGCCGAA
GCCAGGTTAAACGCATTGAAGGTACGGGCGTGCGTCACCCGTTGCAGGCATTACTGGAGATTATAGGTTGATC
TGGAAACGTGAAGTGACCCTCGATGCGCTTAATGCGATGGGGGAAGGAAATATGGTTGGCCTGCTTGATATCC
GCTTTGAGCGTATTGGGGACGATACGCTGGAAGCGACAATGCCCGTCGATCATCGCACCAAACAGCCGTTCGG
CTTGCTGCATGGCGGAGCGTCTGTGGTGCTGGCGGAAAGTATCGGCTCGGTCGCAGGTTATCTGTGTACGCAG
GGGGAGCAAAAGGTGGTGGGGCTGGAGGTGAATGCCAACCATGTACGGTCGGCGCGTCAGGGGCGCGTACGAG
GCGTCTGTAAAGCGTTGCATACCGGGGCTCGCCATCAGGTCTGGCAGATAGAGATCTTTGATGAACAGGGACG
TCTGTGCTGTTCTTCGCGTCTTACTACGGCGATTGTGTGACGTTGTCACCATAAAAGTGTGATGCATATTGCT
TTTGGTCAAAATGTGCGCAGAAAAAGGTGATATACTGTGCACGTTTACACATAAGCGAGGATGATATGTCTAC
TGATTTAGACCCGACCCAACTGGCGATTGAATTTTTACGCCGTGATAAAACCGAACTTTCTCCCGCGCAGTAT
TTGAAGCGTTTAAAACAGCTGGAGTTAGAGTTTGCCGATCTCCTCACCCTCTCTGCAACCGAACTGAAAGAAG
AGATCTATTTCGCCTGGCGGTTGGGCGTGCATTAATAAAGTCTGTGTAGGCCGGATAAGGCGTTAGCCGCCAT
CCGGCTATGTTACCAGGCAATCTCACCAGACACTGTCCCCAGGTAATAGATAACCCCCTTGATTTGTTTAAA
AAAAATACAGATCGCCATTTGTGCTAAAAAGGCGCGCGTTCTTTATGAGATTATTCTTATGCCCCTTCAAGAG
CTAAGCCATTAAGAGTGCCGGAGATAAGCGCCGGATGGGGCCGGAACCCTTAAGCCTGTATTGAACAGACTTA
AGGGTTTTTCTTTTCCGGGCGCGTAAAAAACTAAGGTCTGTCAATAACCGCTGGCATGTCGTCAGCCGTATGA
TTATTCAAAATACGGTTAGCCAGCACACCTGACATCATCGATCCGTTAACGTTCAGAGCCGTGCGGGCCATAT
CAACGATAGGTTCGATGGCAATAAAAATACCGACCAGCGCAACGGGAAAATTCAACGTCGACAGGACAATCAG
CGCCGCGAACGTACCGCCGCCGCCGACGCCGGCTACGCCAATAGACCCTAGCGCAATGGCAGGCAACATCGCG
GCCAGAAAATGCAGCGAGAGCGGATCAATGCCGATGGTGGGCGCAATCATCGCTACCATAATAGCCGGATAAA
TCCCGGCGCAGCCATTTTGCCCCATACTGGAGCCAAACGAGGCGGAAATATTGGCAATAGTGCTTTGTACGCC

AAATTTTTCCTGGGCGGAAATGGCCAGCGGGATAGAGGCTGCGCTGCTGCGGGAAACAAACGCAAAGGTTAAG
ACGGGCCAGACCATACTGAAATAACGCGCTGGATTATTACCGCTGAGGATCAGCAAGATGGCATGCACGATAA
ACATCATGAAAATCGCGATATAACAGGCGCCGATAAAACCAAGTAAGCTGGCGAATTGTTCAAAGTGGTATGA
TGAAAATACGGTAGTCATTAATGCCATGACGCCATAGGGCGTTAATGCAATAACGATACGTACCATCTTCATG
ACCCAGATCTGGATAGCGTTAATGCCTGCGCTTAATTTCTGACCTTCTTCCGGCGCCTCTTTTTTGACCTTTA
ACAGAGCGATCCCCGCTATTAGCGTGAAGATAACGATGCCGATAACGGAAACACTTCTGGCTCCCGTAAGATC
AAGAAAAATATTAGTCGGGATCAGCGATGTCACTAACTGGGGAATCGACACCGCGGCAGTCTTATTGACTTCT
TCTGTAGTTAACATTGACGGCATGTGCACAAAAGCGCTGGCGTCAAGCCCCAGCACATGAGCGGTCAGTAATC
CGATAAAACCGGCAACCATCACCAGGCATAACATGCATCCGACGATCGTCAGCGACATTTTTCCGATGCCGGC
GCTATTTTCCAGTTTATTGATGGCGGAAAGAATAGAAATAAAAATTAGCGGTATAGCGACCAGTTTTAACAGG
TGGACGTAACCATTGCCGACGATGCTGTACCATTCGATAATAGTTTTAGTGTGATTTGCTGAAATAAAATGCA
GGGATAAACCAATAACAGCCCCTAAAAAGATAGCGATAAAAACGCTTTTCGACAGGGAATCGCTTTTTTGATT
AATTCTCAAGATGACCCACGAGAAAACCAAAAAAAGTGCGATATACGCGAGCGTAACAACCATATTTTAAGCC
TCTTGCCTCATTCAGCACCTGAAATGCCGGAAAGAGTTTACATAAACCTATAGCTCAAACTGAGTTATAGAAC
CGCAGCGGATTATAAAGAGCGCAACGCCAGGTATCCATACAAAAAATGGGGTTCTGACCTCGCCGCCCGGCAA
TGTCGACAGCCTATTAATTAAATAGTCATTTTCTATACATCTTTTCGTTTTTGACCTGCCAGAACGGTTAATA
TCTTATAAATCATTACTTATCAAAAAGTTAAGTTGTTTTTTGTCTGTTGTATGACCTGGCGGACAGGGTCTAT
GCTTAATAAAAGGCGCTCAATATGACCATTTGTTGGAAAGCCCCTGCGGTTAAGGGGTTGAAGTGATAATCAT
TATCACTAACATGTTGTTATATCCTGGTGATTTAGAACGCGAGGTAACTCTATGGAATTGCATTCAGACACGT
TTAACCCGGAGGACTTCCCCTGGCAAGGGCTGACGCTAACGCCAGCGGCGGCAGCGCACATACGTGAACTGGC
AGAAAAGCAGCCGGGCATGTTGGGCGTGCGGTTAAGCGTAAAACAGACGGGATGTGCCGGATTTGGCTATGTT
CTGGATACCGTTCGGGAGCCGGATAAAGACGATCTGGTTTTTGAAGCCGAAGGCGCAAAGCTTTTCGCACCGT
TACAGGCGATGCCTTTTATTGACGGAACAGAAGTGGATTACGTACAGGAAGGGCTGAACCAGTTATTTAAATT
TCATAACCCCAAAGCCCAGAATGAATGCGGCTGCGGCGAAAGTTTTGGGGTATAGGCGGTACTATGTCTCGTA
ACACTGAAGCAACGGAAGAGGTCGGCATCTGGACAGGCGGACGCCTTAATTACAAAGAAGGATTTTTCACGCA
ACTACCCACTGATGAACTGGCAAAAGGCATCAGCGAAGAGGTGGTGCGCGCGATTTCCGCAAAGCGTAACGAA
CCTGAGTGGATGCTGGAATTTCGTCTCAATGCCTATAGAGCCTGGCTGGACATGGATGAACCGCACTGGCTGA
AGGCGCACTACGATAAACTGAACTATCAGGACTACAGCTATTACTCTGCGCCATCATGCGGAAACTGCGACGA
TAGCTGCGTTTCCCAGCCGGGAGCCGTACAGCAAACGGGCGCGAATACCTTTTTGAGTAAAGAGGTTGAAGAC
GCCTTCGAGCAACTTGGCGTTCCGGTTCGCGAAGGGAAAGAGGTCGCCGTCGATGCGATTTTTGATTCGGTCT
CCGTAGCGACCACATACCGTGAAAAACTGGCGGAACAGGGCATTATTTTCTGTTCTTTTGGCGAAGCGATACA
CGATCACCCTGACCTGGTCAGGAAATATCTTGGCACCGTCGTGCCGGGGAACGATAACTTTTTTGCCGCGCTG
AATGCGGCAGTCGCTTCCGATGGGACATTTATCTATGTTCCCAAAGGCGTGCGTTGCCCAATGGAGTTATCCA
CCTATTTTCGCATTAACGCCGAGAAAACCGGTCAGTTTGAGCGCACGATTCTGGTGGCGGATGAAGGCAGCTA
TGTGAGTTATATCGAAGGCTGCTCCGCGCCCGTACGCGACAGTTATCAGCTCCACGCCGCGGTAGTGGAAGTC
ATCATCCATAAAGATGCTGAAGTAAAATATTCCACCGTACAAAACTGGTTCCCGGGCGACAACAACACGGGCG
GCATTCTGAACTTTGTGACCAAGCGGGCGTTGTGCGAAGGGGAAAACAGCAAGATGTCGTGGACCCAGTCGGA
AACCGGTTCGGCGATTACCTGGAAATACCCGAGCTGTATCCTGCGCGGTGATAATTCTATCGGCGAGTTCTAT
TCCGTTGCGCTGACCAGTGGACATCAGCAGGCTGACACCGGCACCAAAATGATCCATATCGGTAAAAACACGC
GCTCAACCATTATCTCAAAAGGCATTTCTGCCGGCCACAGCCAGAACAGCTACCGTGGGCTGGTAAAAATTAT
GCCGACGGCAACCAACGCCCGAAATTTCACCCAATGCGACTCCATGTTGATTGGAGCGGACTGCGGCGCGCAT
ACGTTCCCGTATGTTGAATGCCGCAATAACAGCGCGCAGCTGGAGCACGAGGCGACAACATCACGCATCGGTG
AAGATCAGCTTTTCTATTGTCTGCAACGCGGCATCAGCGAAGAAGACGCTATTTCGATGATCGTCAACGGTTT
CTGCAAAGACGTTTTCTCGGAACTACCGCTGGAGTTCGCCGTTGAAGCGCAAAAACTGCTGACGATTAGCCTT
GAGCACAGCGTCGGTTAATGATGAAGGAAAACCCATGTTAAGCATTAAAGATTTACAGGTCAGTGTGGAAGAA
AAAGCGATTTTGCGTGGCCTGAATCTGGAGATTCGCCCCGGCGAAGTTCACGCCATTATGGGGCCGAACGGTT
CCGGGAAAAGCACGCTCTCCGCCACCCTGGCGGGACGTGAAGATTATGAGGTCACCGGCGGCTCGGTTACGTT
CAAGGGTAAAGATCTGCTTGAACTGTCGCCGGAGGAACGGGCGGGCGAAGGGATTTTTATGGCCTTCCAGTAC
CCGGTAGAAATCCCCGGCGTCAGTAACCAGTTTTTCCTGCAAACCGCGCTGAATGCCGTGCGCGCCTACCGCG
GGCAGACGTCGTTGGATCGCTTTGATTTTCAGGATCTGATGGAAGAAAAGATCGCATTGCTGAAAATGCCGGA
AGATTTACTGACCCGTTCGGTAAACGTCGGTTTTTCCGGCGGGGAGAAAAAGCGCAATGATATTTTGCAAATG
GCGGTGCTGGAGCCGGAACTGTGCATCCTTGATGAGTCGGACTCCGGACTGGATATCGATGCGTTGAAAATTG
TGGCGGAAGGCGTCAACGCGTTACGCGACGATAAACGCGCTTTTATTATCGTGACCCACTATCAGCGCATACT
GGACTATATCAAACCCGATTACGTCCATGTCCTCTATCAGGGGCGAATTGTCCGATCCGGCGATTTTACTCTG

GTCAAACAACTGGAGGAGCAGGGTTATGGCTGGCTTACCGAACAGCAGTAAAGCGCTACAGCAATGGCAGCAT
CTCTTTGAGGAAAAAAGAGAAAGTCGAACTGAGCAGGCAAGACAACATTTACAACAGATGTTACGTCTGGGAT
TGCCGACGCGTAAACATGAAGACTGGAAATACACGCCGCTGGAGGGCTTAACCCATAGCCAGTTTATCCAGCA
GTGTGCGACTATCAGCGCCGCACAGCGTGATGCGTTGGCGCTGCAAATCGATGCCGTGCGGCTGGTGTTTGTC
GATGGTCGGTTTATGCCAGAGCTCAGTGATAGCACGCAAAACAGCGGATTTGACGTTAGCGTCAGGGATGAGC
GCCAGACGCTTGCCGCGCCGGTACAGCCTGAGGTTTTTCTGCATCTGACGGAAAGCCTGGCGCAATGTGTCAC
GTATATACAGGTACGGCGAAATCAGCGTCCGACCAGGCCGCTACTGTTAATGCATATCACGCAGGGCGTGGAT
GGCGACGAGCTGAACACCGCGCATTATCGCCACCATCTGGCGCTGGCGGAAGGCGCGGAAGCGACGGTTATTG
AGCATTATGTCAGCCTTACAGCGGCAAAACATTTTACCGGCGCGCGTCTGACGATGAACGTTGCAGACAATGC
GCAACTGCATCATATCAAACTGGCGTTTGAAAATGCGTCCAGCTATCACTTTGCGCATAACGATCTTCTGCTG
GCGACAGACGCCTCGGCGTTTAGCCACAGTTTTCTGCTGGGCGCCGCAGTACTACGTCACCACAGCAGCTCAC
AACTGAATGGTGAAAACGCGACGCTTCGACTGAATAGTCTGGCGATGCCGGTGAAAAATGAAGTTTGCGATAC
GCGGACATGGCTGGAACATAACAAAGGCTACTGTAACAGCCGCCAGTCGCACAAGACCATCGTCAGCGACAAA
GGACGCGCGGTCTTTAATGGCCTGATTAACGTGGCGCAACACGCCATTAAAACCGACGGCCAGATGACCAATA
ATAACCTGTTGTTGGGGAAACTGGCTGAAGTCGATACCAAACCGCAATTAGAGATCTATGCCGATGACGTGAA
GTGCAGCCACGGCGCGACGATCGGGCGTATTGACGATGAGCAGATGTTTTATCTGCAATCACGCGGCATCAGA
CAGCAGGAGGCGCGGCACATGATTCTTTACGCGTTTGCCGCAGAGTTAACGGAAGCTATCCATGATAGCGCCC
TTAAACAGCAGGTGCTGGCGCGAATCGGCCAGCGACTGCCCGGAGGCCTGGTATGACATTTCCTGTAGAAAAA
GTACGGGCGGATTTTCCCATACTGCAGCGTGAAGTTAACGGCCTGCCGCTGGCTTACCTGGACAGCGCAGCCA
GCGCTCAAAAACCTAATCAGGTGATTGATGCTGAATCTGCCTTCTACCGTCACGGCTATGCTGCGGTACATCG
AGGTATCCATACGTTAAGCGCGCAGGCGACCGAAAGCATGGAGAATGTGCGTAAGCAGGCGTCGCGGTTTATT
AACGCCCGCTCCGCAGAAGAACTGGTGTTCGTGCGCGGTACGACGGAGGGCATTAACCTTGTCGCCAACAGTT
GGGGAACGGAAAATATTCGCGCCGGGGATAACATTATCATCAGCGAGATGGAGCATCACGCCAACATCGTTCC
CTGGCAGATGCTGTGCGAGCGCAAAGGCGCTGAACTGCGCGTGATCCCGTTGCATCCTGACGGTACGCTGCGG
CTGGAGACCTTAGCTGCGCTGTTCGATGACCGGACCCGACTGCTGGCCATTACCCATGTTTCCAATGTGCTGG
GGACGGAAAACCCACTGCCGGACATGATTGCGCTGGCGCGCCAGCATGGGGCGAAAGTGCTGGTGGATGGCGC
CCAGGCCGTGATGCACCATGCTGTTGACGTCCAGGCGCTGGACTGCGATTTTTACGTTTTCTCCGGCCATAAA
CTTTACGGGCCGACCGGCATCGGCATTCTGTATGTTAAAGAGGCGTTGCTGCAAGAAATGCCGCCGTGGGAAG
GGGGCGGGTCGATGATCTCGACCGTCAGCCTGACGCAGGGAACGACATGGGCGAAAGCGCCCTGGCGTTTTGA
GGCGGGAACGCCGAATACTGGCGGCATCATCGGTCTCGGCGCGGCGATTGACTATGTGACGTCGCTGGGACTG
GATAAGATTGGCGATTATGAGCAGATGCTGATGCGCTATGCGCTGGAGCAACTGGCGCAGGTGCCTGATATCA
CGCTGTATGGTCCGGCGCAGCGATTGGGCGTCATCGCGTTTAATCTGGGTAAACACCATGCTTACGACGTCGG
CAGCTTTCTTGATAATTACGGTATCGCGGTACGAACGGGACATCACTGCGCAATGCCGCTCATGGCCTGGTAT
GGCGTGCCGGCAATGTGCCGGGCTTCGCTGGCGATGTATAACACCCATGAAGAAGTGGACCGACTGGTGGCAG
GATTAACGCGTATCCACCGCTTATTGGGATAACAGTGAGGTTTTATGGCTGCGCTACCGGATAAAGAAAAACT
GCTGCGTAACTTTACGCGTTGCGCTAATTGGGAAGAGAAATATTTATATATTATTGAGTTGGGGCAGCGCCTG
GCGGAGTTAAATCCGCAGGATCGTAATCCGCAAAATACGATTCATGGCTGTCAGAGCCAGGTGTGGATCGTGA
TGCGGCGAAACGCCAACGGCATTATCGAATTGCAGGGCGACAGCGACGCGGCGATCGTTAAGGGCCTTATGGC
GGTGGTATTTATTTTGTACCACCAAATGACGGCGCAGGATATTGTGCATTTTGATGTCCGGCCATGGTTCGAA
AAAATGGCGCTTGCGCAGCACCTCACACCTTCTCGTTCTCAGGGGCTGGAAGCAATGATTCGCGCAATTCGCG
CTAAGGCCGCCACTCTTAGCTAAACTGTACAGACAGCGTTCTACCTGATGTCAAATCCTTTGTCGGACGGCGA
CGTCGCTTTATCCAGAGTGGACAGAGCGTATATTTTGTTGTTCTGATAAGCGAAGCGCTACCAGTCGTCGCAG
AGGGCATCACCAGGAATACAGGAGGTTTGGTATGAAACGTGCGTCTTTCATTACGCTAACTATTATCGGCGCG
TATAGCGCGTTACAGGCAGCCTGGGCGGTTGATTATCCATTACCGCCCGAAGGCAGCCGTCTTATTGGTCAGA
ATCAAACCTATACCGTACAGGAAGGTGATAAAAACCTGCAGGCTATCGCCCGGCGTTTTGATACGGCGGCGAT
GCTCATTCTTGAAGCGAACAATACGATTGCGCCGGTGCCTAAGCCCGGTACGCTAATTACCATTCCCTCGCAG
ATGCTATTGCCGGATGCGCCCAGGGAAGGCGTTATCGTCAACCTCGCGGAGCTACGGCTGTATTATTACCCGC
CGGGAGAAAACCGCGTTCAGGTGTATCCCATCGGCATCGGTTTGCAGGGACTGGAAACTCCCGTCATGGACAC
CCGGATAGGGCAAAAGATCCCCAACCCGACGTGGACGCCGACGGCGGGCATACGCCAACGTTCGCTTGAGCGG
GGGATCACGCTGCCGCCGGTGATCCCTGCCGGGCCAAATAACCCGCTGGGACGCTATGCGCTGCGTCTGGCGC
ACGGTAATGGGGAATATCTCATTCATGGCACCAGCGCGCCGGATAGCGTAGGTCTGCGCGTGAGTTCCGGTTG
CATTCGCATGAACGCGCCGGACATCAAAGCGTTATTTGCGCAGGTCAGAACGGGGACGCCGGTAAAAGTGATT
AACCAGCCGGTGAAATTCTCTGTCGAGCCGAATGGCATTCGTTATGTAGAGGTGCACAGGCCGCTATCGCCGG
AAGAAGAGCAAAACGTGCAGACGATGCCCTACGTGTTGCCTGCGGAATTTACCGCGTTCAGAAACGCGCAAGG

GGTGGATAGTCGCCTGGTCGATAAGGCGCTATACCGGCGAGCCGGGTATCCAGTCAGCGTGAGCGCCGGGCAG
ACGCCAGCGGTAAATACGACCGCAGTCGAATCCGCTCAGAACGGTTTTGTCGGGGAAGAGGGGCAAACGCGCG
CGACGCAGTAGCCTTCGCGCAAACGACGGACAAAAAAAATGGCGCACGATGTGCGCCATTTTATATCGTGCGT
CAAATTACTTACGGACGTGGGATACTACTTTGTTGTCCAGACGCTGGTTCTCGCGAGCTGCGTCGTCTTTAGC
AGCCTGAACGTCGGAACGCATTGCATTTACGTCATTACTCAGTTGCTCAACTTTAGCGCTCAGCGTCTGTACA
TCGGAAGACAACTGATCGATTTTAGCGTTGTTTGAACAACCGGCCAGTAACGTGGAACCCAGAACTACTGCAC
CTAAGATCAGCTGGTTAGTACGGTTCATAACAATAACATTATACTTTGCGTGGTTATATGGGTAAAAAAAATG
GCGCACGATGTGCGCCATTTTTATTACGCAGGTACTATTACTTACGGTATTTAGTAGCCTGGTTGTCCAGACG
CTGGTTAGCGCGAGCTGCGTCGTCTTTAGCAGCCTGAACGTCGGAACGCATTGCGTTCACGTCGTTGCTCAGC
TGGTCAACTTTAGCGTTCAGAGTCTGAACGTCAGAAGACAGCTGATCGATTTTAGCGTTGCTGGAGCAACCAG
CCAGCAGAGTAGAACCCAGGATTACCGCGCCCAGTACCAGTTTAGTACGATTCATTATTAATACCCTCTAGAT
TGAGTTAATCTCCATGTAGCGTTACAAGTATTACACAAACTTTTTTATGTTGAGAATATTTTTTTGATGGGAA
TGCACTTATTTTTGATCGTTCGCTCAAAGAAGCATCGAAATGCATGAAAGTCCCTAAAAAACCGAAAGAAAAC
AGGGGGCTTCCATCGGATTCTTCTTAGATAATCCGCAATTAGATAGTAAAATCCGATTTGATTTTTTTATTAA
TAGTGGCTATCGATTAAATAAAAAAAGCGCCGGAAGGGCGCTTTTTTAAACAAATTAATTCACACAACAATTA
TTACAGTACGTGAACAGAGGCGGTGTTAGTGGTGCCGCTTGGAACCAGCGCGCCGGAGACCATCACTACGACG
TCGCCTTTCTGGGCGAGGCCGCTTTGCAGGGCGACATCTTTGCCCAGACGGTAGAAATCATCAGTAGAATTGA
TTTCTTTCACCAGCTGCGATACCACGCCTTTGCTCAGTACTAACTGACGGGCGGTCACTTCATTGGTGGTCAA
TGCCAGGATGGTGGCGTCCGGGAAGTATTTGCGCACGGCGCGAGCGGATTTACCGCCCTGGGTCGCTACCACG
ATCAGCGGCGCTTCCAGTTTTTCGGCGGTTTCTACCGCGCCGCGGCATACTGCTTCCGTAATGCGCAGTTTAC
GGCTGTCGTTGTTGTAGTCCAGACGGCTGTTCATGACGCGGTCGGTACGTTCGCAGATGGTCGCCATGATAGA
AACGGCTTCCAGCGGGTATTTCCCTTTGGCGGATTCGCCGGACAGCATAACCGCATCCGTACCGTCGAGGATG
GCGTTCGCTACGTCGCCTGCTTCTGCGCGGGTCGGACGCGGGTTTTTGATCATAGAATCCAGCATCTGAGTCG
CGGTGATCACGACTTTACGTGCGCGGATACATTTTTCGATCATCATTTTCTGCGCAAAAATCACTTCTTCAAC
CGGGATTTCTACGCCCAGGTCACCACGCGCAACCATGATGCCGTCGGAAGCTTCGAGAATTTCATCGAAGTTG
TTCAGGCCTTCCTGGTTTTCGATCTTAGAGATAATCTGGATGTTTTCGCCGCCGTGGGCTTTCAGATGCTCGC
GAATTTCGACAACGTCAGAACGTTTACGGATAAAGGACGCAGCAACAAAGTCAACGCCCTGTTCGCAACCGAA
GATCAGGTCCTGTTTGTCTTTTTCAGCCAGCGCTGGCAGAGCAATAGAAACGCCCGGCAGGTTAACGCCTTTG
TTCTCGCCCAGATCGCCGTTGTTCAGCACCTTACAAATGACTTTATTGCCTTCGATGGCGGTCACTTCCATAC
CGATCAGACCATCGTCAACCAGGACGGTGTTGCCAACGCTCAGGTCGCTGGTGAAGCCTTCATAGGTGACGGC
AACGATTTCATTATTACCGACCACGGATTTGTCGGTGGTAAAAGTGAAGGTTTGACCCGCTTTCAGCGAAACG
TCGTTACCGCCTTCCAGTTTAATGGTGCGGATTTCTGGTCCTTTAGTATCAAGCAGAATAGCGGCTTTTTTCC
CGGTTTTGCTCATCACGTTGCGCAAGTTCTGGATACGCTGACCGTGTTCTGCATAATCGCCATGAGAGAAGTT
CAGACGCATCACGTTCATGCCCGCGTCCAGCATTTTGCTTAACATCTCTTCGGATTCGGTTTTCGGACCGATA
GTACAAACAATTTTCGTCTTTTTCATGACAGTCTTAGTCTTTAAGTTGAGGAGGATATGGAAATCTGGCTTCG
GGCGCGCACGGCCCGGAAGCTAAGCCTGTGTTGCTAAAGTGATGATGCCTCGCTCTAAGGATAGGTGACATCG
AAAGAGCGTGCAGAGGAATGTGTGCTCGCGTTTCAGCCCAACGGGAAGGGGATAATTTTAGTCGTTGAGTTTT
ACAGTCCAATGCGCTGAAACCATTCAAGAGACAATTGGCGCGCATTATACGCTGAAATTTACAAAAAAGGAAA
ATGAAAACCCTGTTTCAAAAGACATCTGTGCAGATCCACAACGGTTTGTTATTACGCTGTTTACGATGTCGCT
GGCGATAACGCCGCCCGAACAGGGGCGGCGTTGCGCAACAGTGGGTGTTGTTGCGCATAATGTCAATCGAGCA
ACTTTTTGCCTTCCAGGTCGATGGCGATGTTTTGGTTATGTGCTTTTCGATAGCACAGAATCGCTATCCCGAT
CGCCGCCCAGCTAATTCCGAGTATCAGAGCGTGAATGTTCATGTTTAAACAGACGTAGCCGATGACGATCATC
CCCATGATCGGCGAAATGACATGGGCGAAAATGCGCCGCTGGGCTTTCATCCGGAAATTGAACAGGATAAACA
CACACAGGTTCACGACAAAAAAAGCGAAAAGCGCGCCAAAGTTCACCAGCGTGGAGATAGACTCAATCTTGCC
GGAAAACAGGTAGCTGAGCAGCAGGGTGACGCCGCCGGTAAACAGAATGGCATAATCCGGCGTTTTACGCGTG
GTATGGACGTGAGCGAGGAAGGCGGGCAACATTCGGTCGCGCCCCATTGAAAATAGCACTCTGGACACCGCCG
TTTGCGCGGTAATAATGTTGCCGACGGCGCACACAAACGCCACAATGATGGAAAATGCCACTTTGAGCCAGAT
ACCGCCAACGTTGCCGGCAATGGTGAAAAATGCTTCATTCGTCGCATTGCCTTCCGCGAAGCGGTCGACATTA
CCGGTGGCGAACGCCGCAAACGCCACAACGATAATAAACAGTACCGTCGCCAAAACTAACGCCAACAGCGTTG
CTTTACTGACGGCGCGTCCCCCGCCTTCGCTTTCTTCACTTAATGTACTGATAGCGTCAAAGCCGAGAAAGTT
CAGCGCAGCCACCGAAATCGCCGTCGCGATGAGCCCTGGGGTGAACCACTGTGAATCGAAAAAAGGCCGGAAG
GAAAGCGTTATCTGACTACTGTCCAGCAGCATCAGACGGACAATGAGTAGCACGAAAATCGCCAGAATACCGA
GTTGAATAAAGACCAGTAACTTATCGAATTTCGCCGTTTGCTGTATCCCGAGATAGTTGACTCCGGTGCCGAT
AGCCACATAAATCAACGGCCAGACCCAGACCGGGATTTGCGGTAAAATAGCGTGGACCGCGACGCCGCCCAGC

ACTGCCACGAGCGTAGGGAACAAAAGATAATCCAGCAGTAATATCCATCCGGTCAGAAACCCTACGGCGGAT
TAATGCATACGCCAGCATACGCATAGGCCGAACCCGACGAGGAGATATGCTGCGATAAGGTAGAGTAGCTGTA
CGCGGTAAAAAACATCGCGATAGCGCCAATAATATAAACCAGCGCAACCATACCGTGCGAAAGATTATAAACG
ACGCCATAAAGAGAGACTGGCGCGATAGGCACCATAAACACCAGACCATAAAGCACTAAATCGGTAAGAGACA
ATTTTTTGACTTTTTTCATGATAAATACCTTAATGGCTTTTTATGCCAGATCGCGTAAAAACGCGCTTACCGC
TGCGAGGCAGGGCGCGATATTTTCTTCATGCGGGTTATGGCTGCTGCCAGGAATAGTGACCGCGCGGCAGTCT
GAAATGTGAGAGAGAAGCGGAGAGACGACGCGCTCGGTTGCCTGATCGTTTTCGCCGCGCAGTACCAGAACAG
GACAGCGTATCTGGCACAAATGGGGCGTAATATCCCAGTCGGCAAGTTTGCCATTTGCCGCCAGTTCATTGGT
TCCCCATAAAATGTGATAACCCGTAGGGTCGGCGGCGAACTGCGCATTAGAGCGCTGAACATGAACGGCTTCT
TCCGCGAGGGTATAAACGTGTCTGGCATAATAAGCGACCAGCTGTTCCGGTGGGTTTTGATAAATGACGGCGG
GCATAATGACGTTTTTGATATCGTCATCGGACATTGGCGTCAGAGCGTTAAAGAGCCGTATGGCTTCCTGTTG
CCACAGGGCGATACTGGCCGGCGAACTGGCCAGTATCGTCCCGCGAAGCCCGGCAGGCTGTAAGCAGGCGTGC
TCTGCGGCAAGCATTCCGCCCCAGGAATGTCCCAGTATCGAATACCCGTGTCCAATACCCAGGTGATGAATTA
ATTGGGTTAATTGACTGAGATAACGCGCAATAGTCCAGAAAGACGCGTCAGCATGAGGAAAATGCGTTGAACG
ACCGCAGCCATATTGATCGTAAAATATTACCATGCGCCCGTCATCAGCCAGACGGCGATAATTTAATAAATAA
TCAAAGCCGCCAGAAGGGCCGCCATGAAGGATAATGAGCGGCGTGAGTAATGATTTTCTGTCTCCATAAATCC
GATAGCTTGTCAATATGTTTTCAGAAAACATTTCGCCATAGAGATAAGTATCATTCATATTCTTAATGACTCA
GTGTGTGGCAGGGCAGGCGAAATTTATATTCGCCTGGGTAATATTATTTTTTTATTGTGTGTAAAACATTACA
CCAGAAGGTGGCGTAATGTTCCCATTGCAGAAACTGCGGGCTTCCCCAGTGCGGCGAACAATCACTGGCAAAG
CAACATACTTTCCCTTTATGGTAAGTACCCATAACTAACAGTGGATCGCCATTAATTTCAGCAAGGACTTGAC
TATTTTCTTTGGCGATAAGCTTGTTATAGCCCAACAGCGGCGGCCATTCGCTAAATGGCTGGGTAATAACATG
CTCTACCGCAGTATTTACTGCTTTACATCCTTGCGGTAGTTCTACGCGGTCATCTACATCCAGCATATCAACC
GGCAATACCTCCGCCAGAACCGTATTTTTATAATTGGCTTTTGCTTCTATTCCCGTAAAGGATAAATACCCGC
CGATCATCAACAGACCGCCGCCCTCAGCAACATAATCGGCGATAAGTTGTAATGCATCAGGAATAATGTCCAT
ATTATAAAACGTTCTGTTTTGTAGCAGGAACGTATTGCTACCGATATCACTGATGACGATCGCATCATAGCAG
GCTAATGCTTCGGCGGTATGTGGAAAACGAGTTTGCACAATATGGGCAGGCATGTAGTCCACATCGATATTAC
CCTGGCGTAAGCAGGATAATAAATAGTCTGCGCCTTCTTCATATTTGCTCGATGTAAAGCTATCAAAACCTTT
AGAGTGAATCATATGAATGTGCCAGGATTCGCCAATAAATAAAATTTTCATTATGTCACCTGTTAATTAATGT
GAATGACTTTATTAGATTGGGTACGCATTCGATGTAAAACCTGCAAGTGCTGAGGCATACTCGACGCACCGAC
TTGTTCCACCGCCAGAGAAGCGAAAGCGGAGGCATATTGAATGGCTGCGACCATGTCTGCTTGTCGCGCCAGC
GCGGCGGCTAAGGCGCCATTAAAAGCGTCACCGGCCCCGGCGGCATCTTTTACCACGGCGCTCCACGCAGGAA
TATACAACGTGCGGGCGTGCTCACAGAGCAGAGATCCACGGGCGCCAAGGGTAATGATGACTTTTTTCACCCC
GGATTGCAGAATAATATTTCCGGCTTTTTTCGCATCATTAATATCAGTAATGGTTACGCCGGATAAAAAAGAG
GCTTCAGTTTCATTCGGCGTGATGATGTCAATATCAGATAATAAATGCGTCACCTGTTTCGTATAGGGGGCAG
GATTCAGCATGATCATTTTATTTTCTTGTTTGCCCAGACGAATAAATTCAGTCAACGCTTCAATGTTCGTTTC
GAGCTGCATTAACATAACGTCAGACTCGGCGATATAACGGTGTTGCTCGTTAATCTCTTGCGTGGTGAGCATC
ATATTGGCGCCAGGGTAGATGGCGATAATATTATCGCCATCCACGGCGGAGACATAGATCAATGCGCTACCGG
TGGGCGCTTTATCTGTTTGATACGCGCTGTATGAGGCGATACCTGATTGAATAATATGATTCATAGCAAATGC
GCTGAACTGATCTTTGCCCACTTTAAAAATAAAATGTGCCGCAGCGCCGGCATTGTTCGCCGCTAAAGCCTGG
TTGGCTCCTTTACCGCCAGGATAGAATCCAAATTGACTGGCCAGAATGGATTCTCCGCTTTGTGGAAACCACG
GCACGGTTGCTGAAACATCAATATTGAATGAGCCTAAAACACACACCTTGCCTGCTTTTTTTGGGTTTTCCCG
GCGCAGTTTTGCATCCTGATGGATTGGCGTGGCGATAGTGCTGGAGATATCAATCTTATTTTCAGTGATAAGC
TTACTCAGCGCGTCACGGTTTAAAATCCCTCCGCCATAGCAACGCTGAAGCATACCTGATTTCTGGAAATAGC
GTAGATCGGAACGAATAGTCTCCTGGCTAACCTTATAAAGGTCTGAAAGTTTACTGACGCTAACACTATTATC
CTTTATCAGTAAATTAATGATGGCATGACGTCTTTCTTCTTTAAACATATTGCCTCCGGGTAGTGAGTTGAAT
TGTATTTATGGCAATGTTGTCATGCGGTGAATTCAATCACAGATTATGCGGTCAACCGGAAGTAACCCCAAAT
GAATGTCAATAATCAGAAGCGCAGCTAATGTGTTAAATATTAATTGCTTACAGAAGGGGAAAATAGTTTTGTG
AGTGTTGTCATATATTTTCTATGTAATATGACGGGGTGAACGTAAAATAGTTGGGCTTGCACGATTCAGTGAG
TATATGCAGCCGGACAGCGTTCGCTTATCAGTCGCTACCCTCGACGAAATAAGACGATATCGCTCTCCGGCCC
GGGGGTTATATACGCTCGATTTTTGCCGGCAGCCCCTGACGTACTGCCGCGCCAGAAACCCAGTCGAGCCAGG
TGTTGGTAATGGTTCTTGTCGGATCGGCAAAGCCCAGATCGTTAAGATTTATGCCTGCCCTGATTTGTGGATC
ATACGGCATAGGCACGCCATCCAGAGAGTGCTGCGTTGCGCCCATCTCGCGGTGGCCATATCCGTGTTCGATG
GCGATGACGCCTGGCATCACGCCATTTAACAAACTGATTTGCGCCACGACCTGACCGCCCGGCGTAATGATCC
GTACCCGATCGCCATGTTGCAGTCCATAACGCTCGCCGTCTTGCGGATTCAGCGCCACCAGGTTTGCTGGCTT

CACATGGTGTAAGCGCGGGATGACGGCTGTTGAGCTGGACATGGTATTTGATTTAAATGAAATCAGTTTCAGC
GGCCATTGCCCAATGGGAAACTGGTCGTCAATCGCACGACCATCTGACAAACGCGCCGGATACCAGACCGGGC
AACCGCTGAAGCGCTCCCCGGTGATGGCGTGACGGTGGGCGGCGACATCTGCATTCCAGATCTGTAAGGGTTT
TTTCCACGCGTTACCTAACCGTTGCTCCGTATAGCCGCTATCCTCGGGCGCAAACCGGCCGCCACGCGAGTAG
ATAAACGCCACGCGACCGACCTCATCAGCTTTAAGCGTGTGCTGAATTGCTGGCAGAATGCGGCTGACGCCGG
TAAGCGAAATATCTTCCTGATTTGCCAGCGCGACCGGCGTCTTGCCCATAAAGGCGATATTAGCGGCTACGCG
CAGATAGAAGTCTTCCGCCCGGTTCAGTGGAAAAGTATTGCCCTGCGGATCGGTTATCGCCCGGTCGCCGAAG
CCGGGCAGATGGAGCCGTTTTGCTACCGCAATACAAAATGCTTCCATTGAGACAGGTTGCCCGTCCACCGTTC
GGCGAGTGGCGGGGGCGACAACCGGCCAGCGGGCGGTAGTGGCTTTACTGGCTACGCCGCCCCAGGGCGCCGT
AAAGCCCCAGCTCTCAAAATTGTGCGTATCCGGCACAATGTAATCCGCCAGCGCCGTCGTTTCATTCATAAAG
GCGTCAATCGCGATAAAGAGCGGCAGTCGGCGAGGGTCTTTTAGCTTTTCTTCCGCCACGGCGCGTAGACCGG
GAACACCGTAAAACGGGTTGCTCATATTGGAAATCCAGGCTTTAAGCGGATAAGGATAGCCTTCGAGCGCGGA
GGTCAACAGTTCGGTAAGCTGGCCTGCCACAAAGGGATACCACGGCGCTTTGGCTGGATAAGGGGATTGCCCG
CCGGCAATTTTGTCGCGGTATTCTTCCGATGCTTCATAAGCGGTTTTGCTACGGGCAATACTTAACCCGGACG
GTTTCACTTTTCCGGCAAAACTGTTCATGTTGTAGCGGGGGCCGTCGCTAACGCCGTTGAATTTGCCGCCGCC
GACAAAGACGCCGCCGGACAAGCTGAGGTTGCCGATCAGCGCGTTAAGCATCATGACCGACCAGGCGTTATAA
AACCCATTGCCGGCCATCATGCCGCCGTGACTGATGACCGCGGCTTTACGTCCGTGACTGGTAAAGGTTTCCG
CCAGCGCGATAATTTGCGCTTCCGGTACGCCGCACTGTTCGCTGTATTGCGCCAACGAGAGCTTTTCTGCCGC
CTCTTTCAGGCGTTGCAACCCGCTCTTCACCGTGACCCGTTGGCCGTCGGCGAGCGTAACGTACTGCGTCACG
AAAAGCCGTGCCTGTCGGCAAGTGGACGCATCGACCAACTCGCCGTCGGTATTCAGCACGACAGGGGTCTCTT
CGCCATCGGGCGTAAGATGGCGCAGCGTCAGGTGTTGTCCGGCAAGCGTCGGCAGTTCATCCGCAATGACCAG
GTGCGTGGCGTTGGTCCAACTTTGCTCGCCGGCCTGCTGCATAGCCTGTACGCCGGGAATCGCCAGATAATCA
GCATTATAACGTTCATTATCCATGATCCAGCGGATCATCCCCATTGCCAGCGCCGAATCGCTGCCGGGCATGA
CCGGTTGCCAGCGACCGCGAGGATCGGCGAGCACCGTTGATAAGGGGAGGGCGGGGGCAACCACGACGTATTG
AAAATTCTCACGCAGTCGGGCGCTCGCCAACTGACGTGCCTGGCGTTTAAACGGATTGCCGGACTGTGCCGGG
GAGGTGCCCATAAAGAGCGCAAACTCCACGTTTTCCCAGTCGGGTTTGACATGCGGGTTTTTATCCAGATCGC
CCATCAATGCCCCGGAGCCGGCCCGGTAAGCCAGTCCACAGTAGGCGCCATGCGCGCCGAAATTCTTGCTGCC
GAAGCTATTTAGCGCAAAACGACGCAGAAACGCATCGCGGCCTTCGTCGCTGGTATTCGTGACCAGTAACTGA
TTGGTTTTGGGCCCGAAACTGGGGTGCTTTGCGTCAATTGGCGTATCCGGCGCATGAATAGCGCGCAGTCCGT
CCACATGACCTTCGCCAAACAGATCGCCGCCTTCCACGGCTTCTTCAATAAGTTGCTCAAAGCTGATGCGCTG
CCATTTCCCTTCGCCGCGTTTACCCACGCGTTTCATCGGTTCAAGCAGTCGTAGCGGACTGTACAGGCTTTCC
AGCAGCGTGGCGCCGCGCGCGCAGGCGGTTGAGCGGGCGTCAAGACCGCTTTCTCCCGCCAGTTGCTCCATGG
CTTCGCTAAAAGGGACGGACGAGTCAATCGGGTGTTCCTGCGACAAGGGGTGATAGGGATTGCCGGCGATGCG
TATCACTTTGCCATCGGCATTAACCCGGGCGCGGATACCGCACTGTGTCCAACAGCCGAAGCATTGCGTCATG
GCGATGGTTTGTTGTGGATTTTGCTGCCAGTGCGTTTGCGCCTGCGCCTCCGGAATTAACGCATTGCCAAAGA
TGCGGTCGCGCGTTACCTTGCCGGATGTCCCGTTTAACAGGCCATCAATTGCGCGTTTCGCCACATCACGGTA
GCTCAGACCAAAAGTGACCATCCCACCGACGGCGAGACCGACTTTTAGCCACTGACGACGGGTTAAATTAGCC
ATGTTGTAATCTCCTGGTGAGTCCGTTCAGCGTTTCACGAATAATAATCAGTAGCGCTATCCACAGGCCGAAG
GTGCCGAGAATAGCCAGCCAGCCATCCGTTCCGCCTGGTAACGAGTAAGGGTTAAATTGCGCGTTGAACTTGG
GGACGGTTTGTACCTGAATCAACAATGTCCAGCGCATCAGCCAACATAGCGCCAGCGCGCTGAGAACCAGCAG
GACGCGTCTTAGCTGCGATAACGGATGACGTAGCGTCAGGCTACAGAACAGCAGCGTGAATACCCACAGCGCT
AGCCAGCCGACAGCGTAATATTTGGCTGACAGGGCGACGGTAATCCACTGACGGATTGCCGTGCCGGAAAGCG
TATCGCCGCTAACCCACATGGCCACAACCAGCCCCAGCGCCGCCAGCGTCCAGATTTGTCCCCACAATATTTT
TGGCAGCCTTACCGAGTCGCGTCGGGCGGCGACAATCATCAGCGCGAAGAACGCCTGTAAGGCGCTAAGGAAC
ATCGCCACGGGGAAGGCGTAGCTAAACCAGATTGGGCGCGCCAGCACAACGGAGACTTCGCGGCCGGTATAAA
TCAACAGGCCCACCGCGCAAAGCGCGCTGGCTAACGCCAACCATTTAGTGACGTTGTAACTTTTATTGAATAA
TCGTTTAATCTGCTGCGCCAGGAACCACAGAGCGAGAAATCCGGTAAACAGCGGCAGGAACAACGCTCCCCAG
GGCATCCACGACCAGGGCGTCGGCCAGGCATAGAAATGCCAGACGCGGGCAGTCTGGTGCAGATCCGCCGTCA
GCGCCAGCGGTGCGGTAATCGCACAGGTAATGGCAATCAGTAATGCCCGATTTTCTTCTGTTGCGGCGTCTTT
TTTCCGCCAGTGAAGATAACAGGCAAACAGTGCGGCGCAGGCGGCAATGCCAATAAAAAAGAAATATTGTACC
GCCCACGGCAGCCAACTAATGTCCTGCGGGTGAGCCAGCACTTCTTCAATGATGAGTGAATGCGTCATTCAGA
CCTCCTGCCAAAGCGCGGGCTGCGCACGGCCCATTAATGGGGTGACAAAGGCGTCGTCCAGACCCAGGTAGAA
AACATGGGGCGACGTGCCGTTTTCCGGCTTTAATACCTTGATAGCGTCGCGATGCTGATGAAGCATGGTGGCG
ATGCGGCTATGGGGATCTTTGATATCGCCAATAATACGCGCGCCGCCGACGCAGGACTCTACGCAAGCGGGTA

ACAGCCCGGCTTCCAGACGATGGACGCAAAACGTGCATTTATCGGCAGTTTGCGTTTCATGATTGATAAATCG
GGCGTCGTAAGGACACGCCTGGACACAATAGGCGCAGCCGACGCAGCGTTTGTTATCCACCACCACAATGCCA
TCTTCCCGCTGAAAGGTGGCTTGTACCGGGCAGACCGGCACACAGGGGGGGTTATCGCAATGGTTGCACAGAC
GCGGCAATAGCACATTCGTGACTTCCTGACTACCTTCACGCTGGACCTGGTATTGGTTCACCGTCGTACGAAA
CGCGCCCTGCGGCGTTTGGTTTTCAATAGTGCAACTTACGGTACAGGACTGACAGCCGATACAACGCCGCAGG
TCGATAAGCATGGCGTAACGGTGTCGGGGAGAGCCTTCATGCCGCTCCGGCGAAAAAGGAAATTTCGCTTCAG
CCAGCGGAACCAGCGAGGCGCCAGCGGTCAGGACGCCGAGCTGCTGGAGAAATTGCCGTTTACTGCTGTCCAT
ATTGACTCCCGTCCACATTGCCAACAATGAAACATTTGTCACGATGTTGGTTCATGTGTAATGAATAAAAAAT
GTTGATGATTTACAGTGTAAAAATCGAGACGGGGTGTGCTCTATTGTGGTTAACCACATACCCGGCTTGTTGT
TGATCTAAAACAACATAATTGACAGGGATATTGGGGTGAGAGGTAAAACCGTAAGGCGCCTGGCGGTGTTGGC
GGCAGTAGGGCTACTTTGTCATGGCGCGTGGGCAGGGACGTGGAATATCGGTATTTTGGCCATGCGCGGCGAG
GCGTCTACGCGTAGCCACTGGCAACCGTTGGCAAAGACATTAAGCCAACAGCTTCCAGGCGAAACCTTTCATA
TCCAGCCGCTGGATCTGCATCAAATGCAGGAAGCCGTTAACCAGGGAACCGTGCAGTTTGTGATAACCAACCC
GGCGCAATTTGTCCAACTGAACAGCCATGCGCCGCTGCGCTGGTTAGCTTCCCTGCGTTCCACGCGCGATGGG
AAAGCGGTGAGTAATGTTATTGGCAGCGTGATTTTGACCCGGCGCGATAGCGGCATCACCACGGCGCATGATC
TCATCGGTAAGACAGTCGGCGCGATTGATGCTCAGGCGTTTGGCGGCTATTTATTAGGCTATAAAGCGCTCAG
CGACGCGGGCTTACGCCCGGAGCGCGATTTTCATCTTCGTTTTACCGGATTTCCTGGCGATGCCTTAGTCTAT
ATGCTGCGCGAAAAAGCGGTGCAGGCGGCAATTGTGCCAGTGTGCCTGTTAGAAAATATGGATCAGGAAGGAT
TGATTAATAAAAAGGACTTTATCGCGCTGCTTTCCCGACCGACGCCACTGCCTTGCTTAACCAGTACGCCGTT
ATATCCTGACTGGTCGTTCGCGGCGCTACCTGCGGTAAGCGATGCGCTGGCGGATCGCGTAACGCGAGCGCTA
TTCAACGCGCCCGCCGCCGCGTCATTTCACTGGGGCGCGCCAGCGTCGACCAGTCAGGTGGAAGCTTTGCTGC
GTGATGTTCGTCAGCACCCTCAGCAGCGCCGACTGTGGCTGGATGTCAAAAGTTGGTTAATCCAGCACCAGCT
AATGGTCGGTGGCGTGATTCTGGCGTTCCTGTTGCTCACGCTCAATTATATTTGGGTCATGCTGCTGGTGCGT
CGACGCGGAAAACAACTGGAACGTAATAATGTAGTCCTTCATCAGCATGAGCGGGCGCTGGAAACCGCCCGGC
AAATGAGCGTGTTGGGTGAAATGACCTCCGGGTTTGCCCATGAGCTTAATCAGCCGCTTTCCGCGATTCGACA
TTATGCCCAGGGGGGCCTGATTCGACTGCGCGCTGCAGATGAACAGCATCCCTTGCTGCCGGCGCTGGAGCAG
ATTGACCAGCAGGCGCAACGCGGTGCGGATACTCTGCGTAACCTGCGTCACTGGGTCAGCCAGGCGCAGGGCA
ACCCGGTGCTAACCGAAGCGTGGAAGGCCATAGCCATTCGCGAGGCGATTGATCATGTCTGGCAATTGTTGCG
TATGGCGCAACAGTTTCCGACAGTGACTCTGCATACCGAGGTTAGCGCTGCGCTGCGCGTAACGCTGCCGTCA
GTGCTGCTGGAACAGGTGCTGGCGAATATCATTCTTAATGCGGCTCAGGCGGGCGCCACCCATTTATGGGTCG
TTGCTGAACGCACTGAACACGGCATCAGTATCGCTTTACAGGATAACGCCGGGGGAATCGATGAGGCGCTATT
ACGTCAGGCGTTTCAGCCGTTTATGACCACCCGCAAAGAGGGGATGGGCTTAGGGCTGGCGATTTGCCAGCGG
CTGGTGCGGTATGGGCGGGGCGATATCAGTATCAGGAACCAGACCGCGCCGGACGGTCTGTCGGGAACGGTGG
TCACGATACATTTCTTACATGAAAATGGGGGCAGGGATGGCGACAATTCATCTACTGGATGATGATACGGCGG
TCACTAACGCGTGCGCGTTTTTACTGGAAAGTCTGGGATATGACGTAAAATGCTGGACGCAGGGGGCGGATTT
TTTGGCGCAGGCCAGTCTGTATCAGGCCGGGGTCGTATTACTGGATATGCGAATGCCGGTACTGGATGGGCAG
GGCGTTCATGATGCGTTGCGCCAGTGCGGAAGTACCCTGGCGGTTGTTTTTCTTACCGGGCATGGCGATGTAC
CGATGGCCGTGGAGCAGATGAAACGCGGCGCCGTCGATTTTCTGCAAAAACCGGTATCGGTAAAACCGCTACA
GGCGGCGCTGGAGCGTGCGCTGACGGTTTCATCGGCAGCGGTGGCGCGTCGTGAGATTATACTGTGTTACCAG
CAGTTGACGCCGAAAGAGCGTGAGCTGGCCAGCCTGGTGGCAAAAGGATTTATGAACCGTGAAATTGCGGAAG
CGATGAATATCGCGGTGCGTACCGTAGAGGTGCACCGCGCCAGAGTCATGGAAAAAATGCAGGCCGGTAGCCT
GGCGGAACTGATTAGGCGTTTCGAAAAAATGGCCTCGCCAGAGACCAGAATACGAACAACGTATGAGCCATGA
ATAATAAACCGCGTTATTTCTTTGATTTTTATCAAATTTTGTTTAATTGAGAAGACTGTTATATTCTGTTGGT
TTGAGCTGGCGACGAGTAAAGCGTATGACAAAACCGACAAAAGATGACGAACTGTACCGCGAGATGTGCCGGG
TGGTGGGCAAGGTCGTGCTGGAAATGCGCGATCTGGGGCAGGAGCCTAAATATATTGTTATTGCGGGCGTGTT
AAGAACCGCGCTGGCGAATCAACGCATCCAACGTAGCGCGTTAGAAAAACAGGCTATGGAAACCGTGATTAAC
GCTCTGGCGCGGTCATAGCCGGTATCATGGTGGGAACGGCCTGTTGGCAATGACGAAATGCTAGTGGGCATCG
CCTTTGAATCGTCGCGATTGTCACCATGGCGGGCTGGTTGCCCGCCGTTAGCGATGCGCAGGGTTATCAGGTG
ATGGCTAAGCGCAGCCCTAACGTATCAGGGTACGGCTCAAAATAATTCTGGCTGCGTAGATAGTCATCTGGAT
GTTCCGCCAGATAGTGCTTCAGCAGCGTTAACGGTGCCAGGATAGGTAGTTGGCCCGCCCGGTAGCCGAGAAT
CACCTCGCGTAATTCCGCGCGTTGCCTGCTGTTCAACGCGCGGTGAAAATAGCCCTGGATATGCATCAGCACA
TTGGTGTGATTTTTACGTGAAGCGGGGTGGCGCAGGATAGCCATCAGTTTTTCTCTGTATCGGACAAAGAAGG
CATCCAGATCGCCCCACTCATGCAGACGCGCGACGAAAGGGCCAATTTCGCGGTAGCCGGCCTGGTGGTGGGC
GAGAAGCTGAAGTTTGTATCGGCTATGAAACGCGAGCAAACTGTGACGACTCAAGCCCTGCGCGCGTAGCGCA

TTTAATTCATGTAGCGCGAAGATACGTGCGATGAAATTCTCGCGTAGTACCGGATCGTGCAGGCGACCATCTT
CCCCTATCGGCAGCCAGGGATATTTGTCCATCATCGCGGCGGTAAACAGCCCCGTGCCAGCTTTGCGGCCCCT
GTTACCCTTTTCATCATACAGGCGAACGCGCTCCATTCCGCAACTGGGCGATTTTGCACACACAATAAACCCC
GCCAGTTCGCCTATTGTTGGCAGAAACCGATCGGCAAAGGCATTCATTCGCTCGGTGACGTCATCGTGCGGGG
CATGGCTATAGCGCATCCGAATGTCGCCACAGGTCGTCTGGATAAGACGCAGCGCAGGGCGAGGCACGGGCAG
GCCGATCGCCACCTCCGGGCAGATCGGTTTGTAGTCCACCCACGGCGCCAGAGAGTTCATGACAAAATCCATA
CGCTTATGTCCGCCATCAAAGCGTACCGTGGCGCCAGTCAGACATCCGCTAATGCCGACTACGGGTTTGTTAT
TCATGATTCCCCCTTATTGAAAGTACGACGACTGACGCCAATGGCGCAAAATGTTATCTCACGCTTATTTAAA
ACTTACACAACTTTGTTTTTTTGTCTAAGTTTTCGCGGAGATTTTTTTTGCGTAGTTAAATATCAATAAGATA
GAATGAAGGGAAGAAATCTATTTCAGCGCTTATAGCGTGATAACCTCCAGCGAAGCGACCACGTTGCGCCACT
GGGCAAGCTGTTTTTTCTGCCCGGCAGTGAGCTTTCCGGTGGTCACCAGGAACCATTTTCTCTGGGCGAACAG
CTCGGGCGCCAGCGCGGCTGGTGGAGAGGGAAGCACATTGAGCCGCAGCTCTTGTCCGGTACGAGTCAACGCT
TCCAGCCAGATTTCACAGTGATCCGAGAGATGCCATCCGCTAATAAAGGCATTGTTGCCAGGCGCTTTTCTGT
CGCCTTCAAGGCAAAAAGAGGTGTAAGCAATAATGATGCCATCCAGTATTTCGCGCAGCGTCATCACGGCGGG
AAGGTGCGCGGACACTTTGCTGCGCAACGGACGCAATAAATGGGTCACCAGTTCTGCGCGAGGATATTCCCGG
CCACAGTCATAAATCAACAGCCGCAGCGCGTCAATTCGCCCTTCGTGGAGATGTTGGAGCATCGTCTCCTGGA
TCGTTATCCAGTTATCGCCCAGGCGAATGACCGGACGCGACAGTAAGGGTTTGACCTGGCTTATCGGTACGCC
TTTTCTCACCCAGTCGAGGATGCTAAGCGCTTGTCGAATATCGTCATCGCTGTATAAGCGATGCCCGCCATCA
GTACGCTGCGGTTTCAATAAACCATAGCGTCGCTGCCATGCCCTTAGCGTGGCGGCATTGATACCGCATAGTC
TGGCAAATTCGCCTATAGAATACGACATGGTAAAGCCCGTTAAATAAGGGGATTCTACTATATCATGATCAAA
TGAAGACTAAGGTAGTGATAAATGGTGCGGCATACCAGGGCATCCGTATGGATTTTGTCGATGATGAGCAATA
CTGCGTGGCGTAAGGCTCATCAAAATATGACCAATGCTTAATACCATCGGACGCCCCTGGTTAATACTCTATT
AACCTCATTCTTCGGGCACAGTTAAGTAACTCTGTCACTTTATGAACCTGTAGCTTTCTCATCATATTCATCC
GGTGTGTTTCGACGGTTTTTATACTGATATGTAGCTTTTCGCTGATCCCATGATTGGTATACCCCTCGTCAAT
AAGTTTAAGAACCTGACGCTCGCGCAAAGTAAGCAGTTGATGATTGGTCGTGTCAGCGTTTAATTCAGCCAGG
ATAGCTTCCCGATTCAACGTTGGGTCAATGTAACGCTTGTTTACTGCTACTGTTTGCAATGCCGCTAACAGAA
CTTGCTGACTACTGCTTTTTAAAACATAGCCATTAGCACCTGCGGCTAAAGTTTTAATGGTCATATACTCTTG
TTGGTATGCTGTGTAAACCAGAATATTCATTGCTGGCCAACGCTGATGTAATTGAGGAATGATATCCAGGCCA
TTGATGCCAGGTAAGCTAAGATCAAGGATAAGTATGTCAGGCTCGTATGCGCAACAGGCATTATAAACCTCAA
GACCATTTTTAACATGCTCTACAATTTTAAAATGAGGCCAGGGTAATAAGGCATTCATAATGCCGTTAATGAT
GATTTCATGATCGTCTACTAATAAGATCTTATATTCTTTCATTTTGCTGCCCTCGCGAAAATTAAGATAATAT
TAAGTAATGGTGTAGTTTTTGAAGATCATACGTATTTTCTGGCGTAAGTCGGTTAGTTCCTCCAGCGCGATGA
TTTTCCCCATTTTTACGCGATTCTCAATGTCTATGACATAGCATACCAATTCAGTCTGCCCTATTTGACCTAA
ACAGCCTTTTAATGTGTGAATTAACTGATCGATTTTTTGTCCAGCCGATACAGCATTTTCAATATCAGCCAGC
AAGAGGTCCAGTGATTGGAAAATCTTGCTATTAATGACCATATCATCTGTCGCCAGTAACGCTGAGCAGCGAC
TCGGATCCTGCTCCTGTAGCTCTATATTTCGCAAAAGTTGATATTCTGCGGCAATACTGATATAGCGAGCTAA
GGTAGCCAATGTCACTGGTTTGGTAATGTAATGATGAATCCCATTTTTTTACAACGATGAATATCTTCTGTCG
CTACGCTAGCGGATAGCGCCACAAACATGCAGTCAGGATCTAAATTATTCGGCTCATCGTGCCATAATTGTAC
ACATTCAATACCATCTATTTCTGGCATTCTAATGTCAATCAGTACTAAATCGAATCGCTGCTGTTGTGATAAA
GTCAGAGCCTCGTTACTACTGGCGGCAACAGTGACGTGTTGTCCCAGGCTGACAAGCATTTTGCCGATGATAT
CCCGATTAATATCGGCATCATCAACCAAAAGAATCTGCAACTGCCAGGGGGTAACAAATTATTTATTACTGGT
ATATTTGGTGTACACAATATTAACTGTTGCGCCAGGTCGTAGAGTTTTCCGGGGAAATACAAAAGCTCTGCGT
TGAGAAGCGCATTTTGCTGGTGGGGTGGTTCGCCGCGTATTCCCCAGCAAGCCAGTTGGCGATGCAGGCAGAA
CGGCGCTGATAGTGTCCCTTTAATTGGTTGAGGCGGCTGGTATTCTTGTAAGGGTAATACTAGCGAGACACAG
GTTCCAACCCCGGGGACACTTTTTAGTGTCAGATTACCGCCCATCATTTTAGCCAGGCTTGACGCAATAGTCA
GTCCAATTCCTGTACCTTGCGAATTTGTGTCTGCTTGATAAAAAGCAGTAAAGATTTGAGACTGCTGCTGTAT
TTCAATCCCTTTACCGCTATCGCTAACCAGAAATATTAATTGTTCCTCATGACGCTTGACCGTCAGACGTATC
CCTCCGGTTTCGGTAAATTTTACCGCATTCCCGAGTAAATTAACCAAAATTTGCCGTAAACGGATACTGTCGG
TATGAAAATAGAGAGGGACATGTTGACCGACAAAAGTACGTAATGACAGTTTTTTGCTTTGCGCTGGCCCCTG
GATGGTTTGCATTGCCTGGTCTAGTAACGGCAGTAACGCTGTTTCTTCCATATGTAATGTGAAATGACCAGAC
TCGATGCGTGAAAAATCCAGCAGATTATTAATAATAGCTAACAAAGACAGTGTACAATTTCTGGCGGTATCAG
CTAATCCTTGCTGCTCTATGTTTAAAGGGGTGGTTTGTAATAATTCAATCGCACCGAGTACGCCATTCATCGG
AGTACGTAACTCATGACTTATTACCGTAAGATGAATGCTTTTACGTTTGTTAGCTCGCTCAGCGCGTTTTTTT
GCTTCATTTAGCGCCTGGGTGCGCTCTGCGACTTTGTTTTCCAGATTGTCGTATTGGACTTGTAGAGTATCAA

GCAGTTGGTTAAAAGCACCGGCAATACTATCTAATTCATCCAGTCGTTGTGCTGGTAAACGTGTGCTCAGCGG
TGCAGTTGCGGTTTTATTAATGACATCGACAAAACGCCATAACGGTTTGGCCAGTGAGCGATGTAGTAACCAG
CAAAAAGCCGACGTCATCAACACCAATGCTGTTAATGTAAAGGGGATTTGTTGAAGGATAATTTTTAAGATGC
GATTATGTAGATTACCGTATGGGTACAGCGTAACCAGACTCCATCCGGGGCCATGCAAGGTTGTGCGTAATAT
CAGAAATCCGGGAATTTGCTGCCATCCATCATGCAGCGTTACATTTTCTAACTGTGTACGTATTTTTTGCGGG
ATGTATGAAAACGGCAATAAGTGGTTGTTTTGATCCAGCCATACTCGAATACTATCATCTAATGGCAGGTGGC
TCTTAGTAATGAGATCGGGAAGTTTAACCGTCACCCCAAAAAATACGCCTTGCTGATCGGCAACCGCAACGGA
AGCGTGCCATCCTTTGCCGTTTATGTATTCTGGTTCACTCCAGTAAAACCCGGCATGGGTTGGGTATAAAGGA
AAGCTTTTTCGCGTTAAAGGCTGTAGAGTTGAATAATCTGAAGGGTTATCAGTAGATAATAACGAAATCTCAT
TTTTATGATTAAGAATAAAACTATCGCGACGAAAGCTATTTTCATCGATATCAGAGGACTGCAGAAAGAGACG
GTGCTTCTCTCCGTTTAGCGTCGGCGTGCAATTTGAAGGACCGACAGATAGATGCCGGCTCACCTCAGGGAAA
ATATCGTTATGATGAATCTCAGTCGCTAATGAGCATTGATACATTAAATTTTTAGCGTCACGTTCAGCTTCTT
CAAACCGTTGATTGCTTAGTACAATATTCATCTCGGATAGAACGGATAAATCCTCTATTATATGCTGCCGTTT
CTGAACCATTGATATATAAGCTGCGGTAAGCACAGATAGCAGCCAAATAATTATTGTTGTTAATAAAAATAAA
AAAGTTAGCCTGATTACTAAAGATGTTTGCAGCGTATTCTTGAGATTGAGCAAATTCATAATGCTTCCCTCCA
GTTGCCTGTTGCAAAATCTTTGGCACTTGATCACTATCGCAGTACATATAGTTTCATCAGAAGATTAATCGAT
GGTGTTATCATTAGGAAGATAAATTTCTTCATATATAACCCAGTCGATGACTACAATTACTTTTTAATAAGAT
GGCGATGTAAAAACATCGTAACAGTTTATTTAATAAATAATTTTTCAAATTGTAAGTTTTTATGTCAGTGCTG
AAAATGTAATTGTGAATTTATCGGAAAATCCGAATGATAGAGTCGCCTGTGACAAGGTATATGTAGACAGCAT
CCTGATATTGTACAAGAAAAGTATAGTCGAAATAAATGTGAATCAGGCTTTTTACGGATGTGGTTGTGAGCGA
ATTTGATAGAAACTCCCATTTATGTCTGAGGAGGGATTCATGCTGGCAGTTTTAAAAGGCATTGCATTAATTC
AGGATATCAAGGCCGAAGGTAATAGCCGATCCTGGATAATGACTATTGATGGGCATCCTGCCAGAGGAGAAAT
TTTCTCAGAAGCATTTTCTATTTCTTTGTTCTTAAATGACCTGGAAAGCTTACCTAAGCCTTGTCTTGCCTAT
GTGACACTACTGCTTGCAGCACACCCGGACGTCCATGATTATGCTATACAGCTCACAGCGGATGGGGGATGGT
TAAACGGTTATTATACCACAAGTAGTAGCTCTGAGCTTATTGCTATTGAGATAGAAAAACACCTGGCTTTAAC
TTGCATTTTAAAAAAATGTAATACGCAATCACCATAAACTTTATTCGGGTGGGGTATAAAATGGTAGTAAATAA
ACGTTTAATCTTAATTTTACTATTTATACTCAATACAGCAAAGAGTGATGAGTTATCATGGAAAGGTAATGAC
TTCACCCTTTATGCCAGACAAATGCCATTAGCAGAGGTTTTACATCTGCTCTCAGAGAACTATGATACGGCTA
TTACTATTAGCCCATTGATAACAGCTACATTTAGTGGAAAAAATTCCGCCTGGACCACCGGTCGATATTTTGAA
TAACCTGGCAGCACAATATGATTTGCTTACCTGGTTTGATGGCAGCATGTTATATGTATATCCTGCATCGTTA
TTAAAACATCAGGTTATCACTTTCAATATTTTATCTACTGGACGGTTCATTCATTACTTACGCAGCCAGAATA
TCCTTTCATCACCGGGATGCGAGGTTAAAGAAATTACCGGTACCAAAGCTGTGGAGGTGAGCGGTGTTCCCAG
CTGCCTGACTCGTATTAGTCAATTAGCTTCAGTGCTGGATAATGCGTTAATCAAACGAAAAGACAGTGCGGTG
AGTGTAAGTATATACACGCTTAAGTATGCCACTGCGATGGATACCCAATACCAATATCGCGATCAGTCCGTCG
TGGTTCCAGGGGTCGTGAGTGTATTGCGTGAGATGAGTAAAACCAGCGTACCGGCGTCATCGATGAACAATGG
TTCACCCGCTACACAGGCATTGCCCATGTTTGCTGCCGACCCACGCCAGAATGCAGTGATCGTTCGTGATTAT
GCGGCCAATATGGCCGGGTATCGGAAACTTATCACAGAATTAGATCAACGCCAGCAGATGATAGAGATTTCGG
TGAAAATTATCGATGTTAATGCTGGAGATATTAACCAGTTAGGCATCGACTGGGGAACGGCAGTGTCGCTGGG
TGGCAAGAAAATTGCGTTCAATACAGGTTTGAATGACGGTGGTGCTAGCGGTTTTTCAACGGTAATCAGCGAT
ACCTCAAACTTTATGGTGCGTTTGAATGCCCTAGAAAAAAGCTCTCAGGCTTATGTACTTTCCCAGCCATCTG
TGGTGACTTTAAATAATATCCAGGCTGTGCTGGATAAAAATATTACTTTCTATACCAAACTGCAGGGAGAAAA
AGTGGCTAAACTTGAATCCATCACTACGGGTTCTTTGTTGCGCGTTACACCTCGCTTGTTAAATGATAATGGC
ACGCAAAAAATAATGCTTAATCTTAATATTCAGGATGGACAACAAAGTGATACGCAAAGCGAAACAGACCCGC
TGCCCGAAGTGCAAAATTCTGAAATTGCTTCGCAGGCCACATTATTGGCCGGGCAAAGTCTATTGCTGGGAGG
ATTTAAACAAGGTAAACAAATCCACTCGCAAAATAAAATTCCTTTATTGGGCGATATTCCTGTCGTAGGCCAT
TTGTTTCGCAATGATACGACTCAAGTACATAGTGTAATCAGGCTTTTTTTGATTAAAGCCTCAGTAGTAAATA
ATGGCATATCTCATGGTTAATCCAAAGAGTTCCTGGAAAATACGTTTTTGGTCACGTTTTTCAAAGGCGGGAG
TGTTGGCCGAAGGAAGGAAGCCTGCCGCGGGGGGGGAAAGGATGGGATATTTGTATTCCCCTTATTATAAATA
AAAAAAATATTCTGAGAGAACAAGAGAATAATTATTTTTTGTTGATGCCGGGAAAGCCAGAGTTAGAGTTAAT
GGCCGCAGATTTAATCCAAATAAGCCGCTACCATCCAGTGGGGTTTTGCAGGTTGCGGGAGTGGCTATCGCGT
TTGGTAAACAGGATTGTGAACTTGCTGATTATCAAATACCCGTTTCCAGATCAGGGTACTGGTGGTTGGCTGG
CGTATTCTTGATTTTCATCGGTGGAATGGGTGTCCTGTTAAGTATTAGTGGTCAGCCTGAAACGGTAAATGAC
TTACCTTTGCGGGTTAAGTTTTTATTAGACAAAAGCAATATTCATTATGTGCGGGCGCAATGGAAAGAAGATG
GCAGCCTGCAGTTGTCCGGTTATTGCTCGTCAAGCGAACAGATGCAAAAGGTGAGAGCGACTCTCGAATCATG

GGGGGTCATGTATCGGGATGGTGTAATCTGTGATGACTTATTGGTACGAGAAGTGCAGGATGTTTTGATAAAA
ATGGGTTACCCGCATGCTGAAGTATCCAGCGAAGGGCCGGGGAGCGTGTTAATTCATGATGATATACAAATGG
ATCAGCAATGGCGTAAGGTTCAACCATTACTTGCAGATATTCCCGGGTTATTGCACTGGCAGATTAGTCACTC
TCATCAGTCTCAGGGAGATGATATTATTTCTGCGATAATAGAGAACGGTTTAGTGGGGCTTGTCAATGTTACG
CCAATGCGGCGCTCTTTTGTTATCAGTGGTGTACTGGATGAATCTCATCAACGCATTTTGCAAGAAACGTTAG
CAGCATTAAAGAAAAAGGATCCCGCTCTTTCTTTAATTTATCAGGATATTGCGCCTTCCCATGATGAAAGCAA
GTATCTGCCTGCGCCAGTGGCTGGCTTTGTACAGAGTCGCCATGGTAATTACTTATTACTGACGAATAAAGAG
CGTTTACGTGTAGGGGCATTGTTACCCAATGGGGGAGAAATTGTCCATCTGAGTGCCGATGTGGTAACGATTA
AACATTATGATACTTTGATTAACTATCCATTAGATTTTAAGTGAGTGGAAAATGACAACTTTGACCCGGTTAG
AAGATTTGCTGCTTCATTCGCGTGAAGAGGCCAAAGGCATAATTTTACAATTAAGGGCTGCCCGGAAACAGTT
AGAAGAGAACAACGGCAAGTTACAGGATCCGCAGCAATATCAGCAAAACACCTTATTGCTTGAAGCGATCGAG
CAGGCCGAAAATATCATCAACATTATTTATTATCGTTACCATAACAGCGCACTTGTAGTGAGTGAGCAAGAGT
AAAGTAAAAATATCTTAGAGCCTATCCCACCAGGCGTTAATTGGCGCAGCCAGTTTGGACACGGATAGCGCGC
AAAAACCGGAGCGTACACGTAGTACGTGAGGATTTTGAGCACTGCCCAGGTTCAAAATGGCAAGTAAAATAGC
CCTAATGGGATAGGCTCTTAGTTAGCACGTTAATTATCTATCGTGTATATGGAGGGGAATGATGATAAAGAAA
AAGGCTGCGTTTAGTGAATATCGTGATTTAGAGCAAAGTTACATGCAGCTAAATCACTGTCTTAAAAAATTTC
ACCAAATCCGGGCTAAGGTGAGTCAACAGCTTGCTGAAAGGGCAGAGAGCCCCAAAAAGAGCAGAGAGACAGA
GAGTATTCTTCATAACCTATTTCCACAAGGCGTTGCCGGGGTTAACCAGGAGGCCGAGAAGGATTTAAAGAAA
ATAGTAAGTTTGTTTAAACAACTTGAAGTACGACTGAAACAACTTAATGCTCAAGCCCCGGTGGAGATACCGT
CAGGAAAAACAAAAAGGTAAAGCATAATGTCTTCAGGAAACATCTTATGGGGAAGTCAAAACCCTATTGTGTT
TAAAAATAGCTTCGGCGTCAGCAACGCTGATACCGGGAGCCAGGATGACTTATCCCAGCAAAATCCGTTTGCC
GAAGGGTATGGTGTTTTGCTTATTCTCCTTATGGTTATTCAGGCTATCGCAAATAATAAATTTATTGAAGTCC
AGAAGAACGCTGAACGTGCCAGAAATACCCAGGAAAAGTCAAATGAGATGGATGAGGTGATTGCTAAAGCAGC
CAAAGGGGATGCTAAAACCAAAGAGGAGGTGCCTGAGGATGTAATTAAATACATGCGTGATAATGGTATTCTC
ATCGATGGTATGACCATTGATGATTATATGGCTAAATATGGCGATCATGGGAAGCTGGATAAAGGTGGCCTAC
AGGCGATCAAAGCGGCTTTGGATAATGACGCCAACCGGAATACCGATCTTATGAGTCAGGGGCAGATAACAAT
TCAAAAAATGTCTCAGGAGCTTAACGCTGTCCTTACCCAACTGACAGGGCTTATCAGTAAGTGGGGGGAAATT
TCCAGTATGATAGCGCAGAAAACGTACTCATGAAAAAAGACCCGACCCTACAACAGGCACATGACACGATGCG
GTTTTTCCGGCGTGGCGGCTCGCTGCGTATGTTGTTGGATGACGATGTTACACAGCCGCTTAATACTCTGTAT
CGCTATGCCATGCAGCTTATGGAGGTAAAAGAGTTCGCCGGCGCAGCGCGACTTTTTCAATTGTTGACGATAT
ATGATGCCTGGTCATTTGACTACTGGTTTAGGTTAGGGGAATGCTGCCAGGCTCAAAAACATTGGGGGGAAGC
GATATACGCTTATGGACGCGCGGCACAAATTAAGATTGATGCGCCGCAGGCGCCATGGGCCGCAGCGGAATGC
TATCTCGCGTGTGATAACGTCTGTTATGCAATCAAAGCGTTAAAGGCCGTGGTGCGTATTTGCGGCGAGGTCA
GTGAACATCAAATTCTCCGACAGCGTGCAGAAAAGATGTTACAGCAACTTTCTGACAGGAGCTAAAAATGAAT
CGAATTCACAGTAATAGCGACAGCGCCGCAGGAGTAACCGCCTTAACACATCATCACTTAAGCAATGTCAGTT
GCGTTTCCTCGGGTTCGCTGGGAAAGCGCCAGCATCGTGTGAATTCTACTTTTGGCGATGGCAACGCCGCGTG
CCTGTTATCAGGGAAAATTAGTCTTCAGGAGGCAAGCAATGCGTTGAAGCAACTGCTTGATGCCGTACCCGGA
AATCATAAGCGTCCATCATTGCCTGACTTTTTGCAGACCAATCCCGCGGTTTTATCAATGTTGATGACGACAT
TAATACTCAGCGTTTTTGGTAATAACGCTCAATCGTTATGCCAACAGCTTGAGCGGGCAACTGAGGTGCAAAA
TGCATTACGTGATAAGCAGGTAAAGGAGTATCAGGAGCAGATCCAGAAAGCGATAGAGCAGGAGGATAAAGCG
CGTAAAGCGGGCATTTTTGGCGCTATTTTTGACTGGATTACCGGCATATTTGAAACTGTGATTGGCGCCTTAA
AAGTTGTGGAGGGTTTTCTGTCCGGTAATCCCGCAGAAATGGCTAGCGGCGTAGCTTATATAGCCGCAGGTTG
TGCAGGAATGGTTAAAGCCGGAGCCGAAACGGCAATGATGTGCGGTGCTGACCACGATACCTGTCAGGCAATT
ATTGACGTGACAAGTAAGATTCAATTTGGTTGTGAAGCCGTCGCGCTGGCACTGGATGTTTTCCAGATTGGCC
GTGCTTTTATGGCGACGAGAGGTTTATCTGGCGCAGCTGCAAAAGTGCTTGACTCCGGTTTTGGCGAGGAAGT
GGTTGAGCGTATGGTAGGTGCAGGGGAAGCAGAAATAGAGGAGTTGGCTGAAAAGTTTGGCGAAGAAGTGAGC
GAAAGTTTTTCCAAACAATTTGAGCCGCTTGAACGTGAAATGGCTATGGCGAATGAGATGGCAGAGGAGGCTG
CCGAGTTTTCTCGTAACGTAGAAAATAATATGACGCGAAGCGCGGGAAAAAGCTTTACGAAAGAGGGGTGAAA
GCCATGGCAAAAGAAGCGGCAAAAGAAGCCCTGGAAAAATGTGTGCAAGAAGGTGGAAAGTTCCTGTTAAAAA
AATTCCGTAATAAAGTTCTCTTCAATATGTTCAAAAAAATCCTGTATGCCTTACTGAGGGATTGTTCATTTAA
AGGCTTACAGGCTATCAGATGTGCAACCGAGGGCGCCAGTCAGATGAATACTGGCATGGTTAACACAGAAAAA
GCGAAGATCGAAAAGAAAATAGAGCAATTAATAACTCAGCAACGGTTTCTGGATTTCATAATGCAACAAACAG
AAAACCAGAAAAAGATAGAACAAAAACGCTTAGAGGAGCTTTATAAGGGGAGCGGTGCCGCGCTTAGAGATGT
ATTAGATACCATTGATCACTATAGTAGCGTTCAGGCGAGAATAGCTGGCTATCGCGCTTAATCTGAGGATAAA

AATATGGAAGCGAGTAACGTAGCACTGGTATTACCAGCGCCTTCCTTGTTAACACCTTCTTCCACTCCATCTC
CCTCCGGGGAGGGAATGGGTACTGAATCAATGCTTCTGTTATTTGATGATATCTGGATGAAGCTAATGGAGCT
TGCCAAAAAGCTGCGCGATATCATGCGCAGCTATAACGTAGAAAAACAACGGCTGGCCTGGGAACTGCAAGTC
AATGTTTTACAGACGCAAATGAAAACAATTGATGAAGCGTTTAGAGCATCAATGATTACTGCGGGTGGCGCAA
TGTTGTCGGGTGTACTGACGATAGGATTAGGGGCCGTAGGCGGGGAAACCGGTCTTATAGCGGGTCAAGCCGT
AGGCCACACAGCTGGGGGCGTCATGGGCCTGGGGGCTGGTGTAGCGCAACGTCAAAGTGATCAAGATAAAGCG
ATTGCCGACCTGCAACAAAATGGGGCCCAATCTTATAATAAATCCCTGACGGAAATTATGGAGAAAGCAACTG
AAATTATGCAGCAAATCATCGGCGTGGGGTCGTCACTGGTCACGGTTCTTGCTGAAATACTCCGGGCATTAAC
GAGGTAAACATGGTGCAAGAAATAGAGCAATGGTTGCGTCGGCATCAGGTGTTTACTGAGCCTGCATATTTAG
GGGAGACCGCCATATTACTTGGGCAGCAGTTTATATTATCGCCTTACCTGGTGATCTATCGTATTGAGGCAAA
AGAAATGATTATTTGTGAGTTCAGGCGCCTGACGCCCGGGCAACCTCGACCACAGCAATTGTTTCACTTACTG
GGACTTTTACGCGGGATATTTGTGCATCACCCGCAGTTAACATGTTTAAAGATGTTGATAATCACCGACGTTC
TGGATGAAAAAAAGCCATGCTACGCAGGAAATTATTGCGCATCCTGACAGTAATGGGAGCGACCTTTACACAG
CTTGATGGCGATAACTGGACAGTTTTATCCGCCGAGCATCTTATCCAGCGACGTTTTTAAATGACCTTCCTGA
CGTAAATCATTATCACGTGAAAATAACAATCAATAGGTATGATGATGAAAGAAGATCAGAAAAATAAAATACC
CGAAGACATTCTGAAACAGCTATTATCCGTTGATCCGGAAACCGTTTATGCCAGTGGTTACGCCTCATGGCAG
GAGGGGGATTATTCGCGCGCCGTAATCGATTTTAGTTGGCTGGTGATGGCCCAGCCATGGAGTTGGCGTGCCC
ATATTGCATTGGCTGGCACCTGGATGATGCTTAAAGAATACACGACGGCCATTAATTTCTATGGACATGCCTT
GATGCTGGATGCCAGCCATCCAGAACCGGTTTACCAAACGGGCGTCTGTCTCAAAATGATGGGGGAACCCGGG
TTGGCGAGAGAGGCTTTTCAAACCGCAATCAAGATGAGTTATGCGGATGCCTCATGGAGTGAGATTCGCCAGA
ATGCGCAAATAATGGTTGATACTCTTATTGCTTAAATAACAGAACGAAATATGAAAATTCATATTCCGTCAGC
GGCAAGTAATATAGTCGATGGTAATAGTCCTCCTTCCGATATACAAGCGAAGGAGGTATCGTTTCCTCCCCCT
GAAATTCCAGCGCCTGGTACCCCCGCAGCCCCTGTGCTGCTTACGCCTGAACAAATAAGGCAGCAGAGGGATT
ATGCTATACATTTTATGCAATACACTATTCGTGCGCTGGGTGCGACAGTCGTGTTTGGGTTATCGGTTGCTGC
AGCGGTAATTTCTGGCGGGGCAGGATTACCCATTGCTATTCTTGCGGGGGCGGCGCTCGTGATTGCTATTGGG
GATGCTTGCTGTGCGTATCATAATTATCAATCGATATGTCAGCAAAAGGAGCCATTACAAACCGCCAGTGATA
GCGTTGCTCTTGTGGTCAGTGCGCTGGCCTTAAAATGTGGGGCAAGTCTTAACTGCGCTAACACCCTTGCTAA
TTGTCTTTCTTTATTAATACGTTCAGGAATCGCTATTTCTATGTTGGTTTTACCCCTACAGTTTCCACTGCCC
GCGGCTGAAAATATTGCGGCCTCTTTGGACATGGGGAGTGTAATTACCTCCGTTAGCCTGACGGCGATAGGTG
CGGTACTGGATTATTGCCTTGCCCGCCCCTCTGGCGACGATCAGGAAAATTCTGTTGATGAACTTTATGCCGA
TCCCAGTGCGTTATTGGCGGAACAAATGGCAGCGCTCTGTCAATCTGCTACTGCACCTGCATTAATGGACAGT
TCTGGTCATACATCTCGGGGAGAACCATGAAACCTGTTAGCCCAAATGCTCAGGTAGGAGGGCAACGTCCTGT
TAACGCGCCTGAGGAATCACCTCCATGTCCCTCATTGCCACATCCGGAAACCAATATGGAGAGTGGTAGAATA
GGTCCTCAACAAGGAAAAGAGCGGTTATTGGCCGGACTTGCGAAACGAGTGATAGAGTGTTTTCCAAAAGAAA
TTTTTAGTTGGCAAACGGTTATTTTGGGCGGACAGATTTTATGCTGTTCCGCTGGAATAGCATTAACAGTGCT
AAGTGGTGGAGGCGCGCCGCTCGTAGCCCTGGCAGGGATTGGCCTTGCTATTGCCATCGCGGATGTCGCCTGT
CTTATCTACCATCATAAACATCATTTGCCTATGGCTCACGACAGTATAGGCAATGCCGTTTTTTATATTGCTA
ATTGTTTCGCCAATCAACGCAAAAGTATGGCGATTGCTAAAGCCGTCTCCCTGGGCGGTAGATTAGCCTTAAC
CGCGACGGTAATGACGCATTCATACTGGAGTGGTAGTTTGGGACTACAGCCTCATTTATTAGAGCGTCTTAAT
GATATTACCTATGGACTAATGAGTTTTACTCGCTTCGGTATGGATGGGATGGCAATGACCGGTATGCAGGTCA
GCAGCCCATTATATCGTTTGCTGGCTCAGGTAACGCCAGAACAACGTGCGCCGGAGTAATCGTTTTCAGGTAT
ATACCGGATGTTCATTGCTTTCTAAATTTTGCTATGTTGCCAGTATCCTTACGATGTATTTATTTTAAGGAAA
AGCATTATGGATATTGCACAATTAGTGGATATGCTCTCCCACATGGCGCATCAGGCAGGCCAGGCCATTAATG
ACAAAATGAATGGTAATGATTTGCTCAACCCAGAATCGATGATTAAAGCGCAATTTGCCTTACAGCAGTATTC
TACATTTATTAATTACGAAAGTTCACTGATCAAAATGATCAAGGATATGCTTAGTGGAATCATTGCTAAAATC
TGAAGTTATTAGCGACGATGTTCGACGGTTGCTGCTGGAAATCATGTTTGCGGGCGTTAACCATAGCCTGATT
TCCCAGGTACATGCGATGTTACCAGCGCTAACGGTTATTGTTCCGGATAAAAAATTACAGTTGGTATGTCTGG
CATTATTGTTGGCGGGTTTAAATGAGCCGCTAAAAGCCGCGAAAATTTTATCGGATATAGATTTGCCAGAGGC
TATGGCGCTGCGTCTGTTATTTCCTGCACCAAATGAGGGGTTTGAAAATTGAATATTTCTGATATGAGCGTAG
TGCCTGTAAGCACTCAATCTTATGTAAAGTCCTCTGCAGAACCGAGCCAGGAGCAAATTAATTTTTTTGAACA
ATTGCTGAAAGATGAAGCATCCACCAGTAACGCCAGTGCTTTATTACCGCAGGTTATGTTGACCAGACAAATG
GATTATATGCAGTTAACGGTAGGCGTCGATTATCTTGCCAGAATATCAGGCGCAGCATCGCAAGCGCTTAATA
AGCTGGATAACATGGCATGAAGGTTCATCGTATAGTATTTCTTACTGTCCTTACGTTCTTTCTTACGGCATGT
GATGTGGATCTTTATCGCTCATTGCCAGAGGATGAGGCGAATCAAATGCTGGCATTACTTATGCAGCATCATA

TTGATGCGGAAAAAAAAACAGGAAGAGGACGGTGTTACCTTACGTGTCGAGCAGTCGCAGTTTATTAATGCGGT
TGAGCTACTTAGACTTAACGGTTATCCGCATCGTCAGTTTACAACGGCGGATAAGATGTTTCCGGCTAATCAG
TTAGTGGTATCACCCAGGAAGAACAGCAGAAGATTAATTTTTAAAAGAACAAAGAATTGAAGGAATGCTGAG
TCAGATGGAGGGCGTGATTAATGCAAAAGTGACCATTGCGCTACCGACTTATGATGAGGGAAGTAACGCTTCT
CCGAGCTCAGTTGCCGTATTTATAAAATATTCACCACAGGTCAATATGGAGGCCTTTCGGGTAAAAATTAAGG
ATTTAATAGAGATGTCAATCCCTGGGTTGCAATACAGTAAGATTAGTATCTTGATGCAGCCTGCTGAATTCAG
AATGGTAGCTGACGTACCCGCGAGACAAACATTCTGGATTATGGACGTTATCAACGCCAATAAAGGGAAGGTG
GTGAAGTGGTTGATGAAATACCCTTATCAGTTGATGTTATCGTTGACAGGACTGTTATTAGGAGTGGGCATCC
TGATCGGCTATTTTTGCCTGAGACGCCGTTTTTGAGCCGACCTGATCCCGAGGTGTTGCAACTTTATCGTTAT
TTCTGGCAACCTGCTCGTTACGCTGTACCGGAATGGCTGGATAAGCTGGGCTTTCATCTTTCAAACTGCTGGC
GTTATGGCGATCGGCCCGAGTTGGATCGTCTTCTTGACAGAGCGTTAAATAGACTAAGAGGAAGCTCTGTTAT
TCCAGCCTGTTTAAATGACAGGCAAAAACGGCAGGTTCGTCTTGCGCCGCGTATATCGGCATTTGCCTTTGGG
CTGGGATTATTCAAACTCAGGTGTAGTGACTATTTTATGCTACCAGAGTATCGGCAATTGCTTCTACAGTGGT
TTAGCGAGGATGAGATCTGGCAGCTATATGGTTGGTTGGGGCAAAGAGATGGCAAATTACTTCCTCCGCAAGT
GATGCAACAAACTGCATTGCAGATCGGTACCGCCATTCTTAATCGGGAAGCGCATGACGATGCGGTTTTACAT
GCGCTATTAGTATTATTACCCCCTCCGCAGCGTATACTTTGGCCGAAGACTTCTCTTACCGAGATTATCTTCA
TGGAGCATTTGCTATGAGTTTTACTTCACTTCCTCTGACGGAAATTAACCATAAGCTACCCGCTCGAAATATT
ATTGAGTCACAGTGGATAACATTACAATTAACTTTATTTGCGCAAGAGCAACAAGCTAAGAGAGTTTCACATG
CTATTGTGAGCTCCGCTTACCGTAAGGCTGAAAAAATCATCCGAGACGCCTATCGTTATCAGCGTGAACAGAA
AGTTGAGCAGCAACAAGAACTAGCGTGCTTGCGTAAAAATACGCTGGAAAAAATGGAAGTGGAATGGCTGGAA
CAGCATGTAAAACATTTACAAGAAGATGAAAATCAATTTCGTTCATTGGTCGATCACGCAGCGCATCATATTA
AAAATAGTATAGAACAGGTTCTGTTGGCCTGGTTCGACCAACAGTCGGTAGACAGTGTTATGTGCCATCGTCT
GGCACGCCAGGCCACGGCTATGGCGGAAGAGGGAGCGCTTTATTTGCGTATTCATCCTGAAAAAGAGGCATTG
ATGCGTGAAACTTTTGGCAAGCGGTTTACGTTGATTATCGAGCCTGGTTTCTCTCCCGATCAGGCTGAACTTT
CCTCAACACGATATGCCGTTGAGTTTTCACTTTCTCGTCATTTCAACGCGTTACTGAAATGGTTACGTAATGG
TGAAGATAAAAGAGGTAGCGATGAATATTAAAATTAATGAGATAAAAATGACGCCCCCTACAGCATTTACCCC
TGGCCAGGTTATAGAGGAACAAGAGGTTATTTCGCCTTCAATGTTAGCTCTCCAGGAGTTACAGGAAACGACG
GGGGCAGCGCTCTATGAGACGATGGAAGAAATAGGAATGGCGCTGAGTGGTAAACTGCGCGAAAGTAATAAAT
TCACTGATGCTGAGAAACTGGAGCGCAGGCAGCAGGCTTTGCTGCGTTTGATAAAACAAATACAGGAGGATAA
TGGGGCAGCGTTGCGTCCGCTTACCGAAGAGAATAGTGATCCTGATTTACAGAATGCGTATCAAATTATCGCT
CTTGCAATGGCGCTTACTGCCGGCGGGTTGTCAAAAAAGAAAAAACGCGATTTGCAATCGCAACTGGATACGC
TTACAGCGGAGGAGGGATGGGAACTTGCCGTTTTTAGTTTACTGGAACTTGGCGAAGTGGATACCGCTACGCT
GTCCTCGCTGAAGCGTTTTATGCAACAGGCGATAGACAACGATGAAATGCCCTTATCGCAGTGGTTCAGACGC
GTGGCAGACTGGCCGGATCGCTGTGAACGGGTCCGTATTTTGCTAAGAGCAATAGCCTTTGAACTTAGCATAT
GCATCGAACCCTCGGAGCAAAGTCGTTTGGCCGCAGCATTAGTACGTTTGCGTCGTTTGCTGTTATTCCTTGG
CCTTGAAAAAGAGTGCCAGCGTGAGGAGTGGATTTGCCAGTTGCCGCCTAATACATTACTGCCGCTACTACTC
GATATTATTTGTGAGCGCTGGCTTTTCAGTGATTGGTTGCTTGATAGACTTACCGCTATAGTTTCTTCATCGA
AGATGTTCAATCGGTTACTCCAACAACTTGATGCGCAGTTTATGCTGATACCCGATAACTGTTTTAACGACGA
AGATCAACGTGAACAAATTCTCGAAACGCTTCGTGAAGTAAAGATAAATCAGGTTTTATTCTGATACCTGGCT
TTCAATATTTAGGTAAATTGGCTTTCTGGCTCATCATGAGGCGTCAGGATGGATTGGGATCTCATTACTGAAC
GTAATATTCAGCTTTTTATTCAATTAGCAGGATTAGCTGAACGGCCTTTAGCGACCAATATGTTCTGGCGGCA
AGGACAATATGAAACCTATCTAAACTATCATAACGGTCGTATTCACTTATGTCAGATACTCAAGCAAACCTTC
TTAGACGAAGAACTGCTTTTTAAAGCGTTGGCTAACTGGAAACCCGCAGCGTTCCAGGGTATTCCTCAACGAT
TATTTTTGTTGCGCGATGGGCTTGCAATGAGTTGTTCTCCACCTCTTTCCAGCTCCGCCGAGCTCTGGTTACG
ATTACATCATCGACAAATAAAATTTCTGGAGTCGCAATGCGTTCATGGTTAGGTGAGGGAGTCAGGGCGCAAC
AGTGGCTCAGTGTATGCGCGGGTCGGCAGGATATGGTTCTGGCGACGGTGTTATTAATCGCTATTGTGATGAT
GCTGTTACCTTTGCCGACCTGGATGGTTGATATCCTGATTACTATCAACCTTATGTTTTCAGTGATCCTGCTC
TTAATTGCTATTTATCTTAGTGACCCTCTCGATTTATCGGTATTTCCGTCTTTATTACTTATTACTACATTAT
ATCGTTTGTCACTCACAATCAGCACATCACGGCTGGTACTGTTACAACATAATGCCGGTAATATTGTGGATGC
TTTCGGTAAGTTTGTCGTAGGAGGAAATCTCACCGTTGGGTTGGTCGTATTTACCATCATTACTATCGTGCAA
TTTATTGTCATTACAAAAGGTATCGAGAGGGTGGCGGAAGTTAGCGCACGTTTCTCGCTTGATGGGATGCCAG
GCAAACAAATGAGTATCGATGGCGATTTGCGTGCCGGAGTTATCGATGCAGACCATGCCCGTACATTAAGACA
GCATGTCCAGCAGGAAAGCCGCTTTCTCGGTGCGATGGACGGTGCGATGAAATTTGTTAAAGGCGATACGATT
GCCGGTATTATTGTTGTTCTGGTGAACATTATCGGCGGTATCATTATCGCTATCGTACAATATGATATGTCGA

TGAGTGAGGCTGTTCACACTTATAGCGTACTGTCAATCGGAGATGGTTTATGTGGGCAAATTCCATCGCTGCT
GATTTCCCTTAGCGCGGGAATTATTGTCACCCGTGTCCCGGGTGAGAAACGCCAGAACCTGGCGACAGAGTTG
AGTTCTCAAATTGCCAGACAACCTCAGTCGCTCATATTAACCGCTGTGGTTTTAATGCTCCTCGCTTTAATTC
CTGGCTTTCCTTTTATCACTCTCGCTTTCTTTTCAGCGTTGTTAGCATTGCCAATTATCCTCATTCGCCGCAA
AAAGTCTGTGGTTTCCGCAAATGGCGTCGAAGCACCGGAAAAAGATAGTATGGTTCCCGGCGCATGTCCTCTA
ATCTTACGTCTTAGCCCGACGTTACATTCTGCCGACCTGATTCGTGATATTGACGCCATGAGATGGTTTTTAT
TTGAGGATACCGGCGTCCCTCTCCCTGAGGTGAATATTGAGGTTTTGCCTGAACCCACCGAAAAATTGACGGT
ACTGCTATATCAGGAACCCGTATTTAGTTTATCTATTCCCGCTCAGGCGGATTATTTATTGATAGGCGCGGAC
GCTAGTGTGGTGGGTGACAGCCAGACGTTACCGAACGGATGGGGCAGATCTGTTGGCTTACAAAAGACATGG
CCCATAAGGCGCAAGGTTTTGGACTGGACGTTTTCGCGGGCAGCCAACGTATCTCTGCCTTATTAAAATGTGT
CCTGCTTCGGCATATGGGAGAGTTTATTGGTGTTCAGGAAACGCGTTATCTAATGAATGCGATGGAAAAAAAC
TACTCTGAGCTGGTGAAAGAGCTTCAACGCCAGTTACCCATTAATAAAATCGCTGAAACTTTGCAACGGCTTG
TATCAGAACGGGTTTCTATTAGAGATTTACGTCTTATTTTCGGCACCTTAATTGACTGGGCGCCACGTGAAAA
AGATGTCCTGATGTTGACAGAATATGTCCGTATCGCGCTTCGCCGTCATATTCTGCGTCGTCTTAATCCGGAA
GGAAAACCGCTGCCGATTTTGCGGATCGGCGAAGGTATTGAGAACCTCGTGCGTGAATCCATTCGCCAGACGG
CAATGGGGACCTATACTGCGCTGTCGTCTCGTCATAAGACGCAGATCCTGCAACTTATCGAGCAGGCGCTGAA
GCAGTCAGCCAAATTATTCATTGTCACTTCTGTCGACACCCGACGTTTCTTGCGAAAAATTACAGAAGCCACC
TTGTTCGACGTACCGATTTTGTCATGGCAGGAATTAGGAGAGGAGAGCCTTATACAAGTGGTAGAAAGTATTG
ACCTTAGCGAAGAGGAGTTGGCGGACAATGAAGAATGAATTGATGCAACGTCTGAGGCTGAAATATCCGCCCC
CCGATGGTTATTGTCGATGGGGCCGAATTCAGGATGTCAGCGCAACGTTGTTAAATGCGTGGTTGCCTGGGGT
ATTTATGGGTGAGTTGTGCTGTATAAAGCCTGGAGAAGAACTTGCTGAAGTCGTGGGGATTAATGGCAGCAAA
GCTTTGCTATCTCCTTTTACGAGTACAATCGGACTTCACTGCGGGCAGCAAGTGATGGCCTTAAGGCGACGCC
ATCAGGTTCCCGTGGGCGAAGCGTTATTAGGGCGAGTCATTGATGGTTTTGGTCGTCCCCTTGATGGCCGCGA
ACTGCCCGACGTCTGCTGGAAAGACTATGATGCAATGCCTCCTCCCGCAATGGTTCGACAGCCTATCACTCAA
CCATTAATGACGGGGATTCGCGCTATTGATAGCGTTGCAACCTGTGGCGAAGGGCAACGAGTGGGTATTTTTT
CTGCTCCTGGCGTGGGGAAAAGCACGCTTCTGGCGATGCTGTGTAATGCGCCAGACGCAGACAGCAATGTTCT
GGTGTTAATTGGTGAACGTGGACGAGAAGTCCGCGAATTCATCGATTTTACACTGTCTGAAGAGACCCGAAAA
CGCTGTGTCATTGTTGTCGCAACCTCTGACAGACCCGCCTTAGAGCGCGTGAGGGCGCTGTTTGTGGCTACCA
CGATAGCAGAATTTTTTCGCGATAATGGAAAGCGAGTCGTCTTGCTTGCCGACTCACTGACGCGTTATGCCAG
GGCCGCACGAGAAATCGCTCTGGCCGCCGGAGAGACCGCAGTTTCTGGAGAATATCCGCCAGGCGTATTTAGT
GCATTGCCACGACTTTTAGAACGTACAGGAATGGGGGAAAAAGGCAGTATTACCGCATTTTATACGGTACTGG
TGGAAGGCGATGATATGAATGAGCCGTTGGCGGATGAAGTCCGTTCACTACTTGATGGACATATTGTGCTATC
CCGGCGGCTTGCAGAGAGGGGGCATTATCCTGCCATTGACGTGCTGGCAACGCTCAGCCGCGTTTTTCCAGTC
GTTACCAGCCATGAGCATCGTCAACTGGCGGCGATATTGCGGCGGCGCCTGGCGCTTTACCAGGAGGTTGAAC
TGTTAATACGCATTGGGGAATACCAGCGAGGAGTTGATACTGATACTGACAAAGCCATTGATACCTATCCGGA
TATTTGCACATTTTTGCGACAAAGTAAGGATGAAGTATGCGGACCCGAGCTACTTATAGAAAAATTACACCAA
ATACTCACCGAGTGATCATGGAAACTTTGCTGGAGATAATCGCGCGGCGTGAAAAGCAATTACGCGGCAAGCT
TACCGTACTTGATCAGCAGCAACAGACGATTATTACGGAACAGCAGATTTGCCAGACGCGCGCTTTAGCAGTG
TCTACCAGACTGAAAGAATTAATGGGCTGGCAAGGTACGTTATCTTGTCATTTATTGTTGGATAAGAAACAAC
AAATGGCCGGGTTATTCACTCAGGCGCAGAGCTTTTTGACGCAACGGCAGCAGTTAGAGAATCAGTATCAGCA
GCTTGTCTCCCGGCGAAGCGAATTACAGAAGAATTTTAATGCGCTTATGAAAAAGAAAGAAAAAATTACTATG
GTATTAAGCGATGCGTATTACCAAAGTTGAGGGAAGTCTTGGGTTGCCATGCCAGTCTTATCAGGATGATAAC
GAGGCGGAGGCGGAACGTATGGACTTTGAACAACTCATGCACCAGGCATTACCCATTGGTGAGAATAATCCTC
CTGCAGCATTGAATAAGAACGTGGTTTTCACGCAACGTTATCGTGTTAGTGGCGGTTATCTTGACGGTGTAGA
GTGTGAAGTCTGTGAATCAGGGGGGCTAATTCAGTTAAGAATCAATGTCCCTCATCATGAAATTTACCGTTCG
ATGAAAGCGCTAAAGCAGTGGCTGGAGTCTCAGTTGCTGCATATGGGGTATATAATTTCCCTGGAGATATTCT
ATGTTAAGAATAGCGAATGAAGAGCGTCCGTGGATGGAGATGCTTCCAACGCAAGGCGCTACCATTGGTGAGC
TGACATTGAGTATGCAACAATATCCAGTACAGCAAGGGACATTATTTACCATAAATTATCATAATGAGCTGGG
TAGGGTGTGGATTGCAGAAGAATGCTGGCAGCGCTGGTGTGAAGGGTTAATTGGCACCGCTAATCGATCGGCT
ATCGATCCTGAATTGCTATATGGAATAGCTGAATGGGGGCTGGCGCCGTTATTGCAAGCCAGTGATGCAACCC
TCTGTCAGAACGAACCGCCAACATCCTGCAGTAATCTACCACATCAGCTAGCGTTGCATATTAAATGGACAGT
TGAAGAACATGAGTTCCATAGCATTATTTTTACATGGCCAACGGGTTTTTTGCGCAATATAGTCGGAGAGCTT
TCTGCTGCGCGACAACAGATTTATCCTGCCCCTCCTGTAGTAGTCCCTGTATATTTAGGCTGGTGCCAGCTTA
CGTTAATTGAACTTGAGTCTATCGAAATCGGCATGGGCGTTCGGATTCATTGCTTTGGCGACATCAGACTCGG

TTTTTTTGCTATTCAACTACCTGGGGGGATCTACGCGAGGGTGTTGCTGACAGAGGATAACACGATGAAATTT
GATGAATTAGTCCAGGATATCGAAACGCTACTTGCGTCAGGGAGTCCAATGTCAAAGAGTGACGGAACATCTT
CAGTAGAACTTGAGCAGATACCACAACAGGTGCTCTTTGAGATCGGACGTGCGAGTCTGGAAATTGGACAATT
ACGACAACTTAAAACGGGGGACGTTTTGCCTGTAGGTGGATGTTTTGCGCCAGAGGTGACGATAAGAGTAAAT
GACCGTATTATTGGGCAAGGTGAGTTGATTGCCTGTGGCAATGAATTTATGGTGCGTATTACACGTTGGTATC
TTTGCAAAAATACAGCGTAAACCTGATAAGAAAAATAATATGCGAACAATATAATAGCGTTCCAGGTCGTGTC
ATGAGAGATACAGTATGTCTTTACCCGATTCGCCTTTGCAACTGATTGGTATATTGTTTCTGCTTTCAATACT
GCCTCTCATTATCGTCATGGGAACCTCTTTCCTTAAACTGGCGGTGGTATTTTCGATTTTACGAAATGCTCTG
GGTATTCAACAAGTTCCCCCAAATATCGCACTGTATGGCCTCGCGCTTGTACTTTCCTTATTCATTATGGGGC
CGACGCTATTAGCTGTAAAAGAGCGCTGGCATCCGGTTCAGGTCGCTGGCGCTCCTTTCTGGACGTCTGAGTG
GGACAGTAAAGCATTAGCGCCTTATCGACAGTTTTTGCAAAAAAACTCTGAAGAGAAGGAAGCCAATTATTTT
CGGAGTTTGATAAAACGAACCTGGCCTGAAGACATAAAAAGAAAGATAAAACCTGATTCTTTGCTCATATTAA
TTCCGGCATTTACGGTGAGTCAGTTGACGCAGGCATTTCGGATTGGATTACTTATTTATCTTCCCTTTCTGGC
TATTGACCTGCTTATTTCAAATATACTGCTGGCTATGGGGATGATGATGGTATCGCCAATGACCATTTCATTA
CCGTTTAAGCTGCTAATATTTTTACTGGCAGGCGGTTGGGATCTGACACTGGCGCAATTGGTACAGAGCTTTT
CATGAATGATTCTGAATTGACGCAATTTGTAACGCAACTTTTATGGATCGTCCTTTTTACGTCTATGCCGGTG
GTGTTGGTGGCATCGGTAGTTGGTGTCATCGTAAGCCTTGTTCAGGCCTTGACTCAAATACAGGACCAAACGC
TACAGTTCATGATTAAATTATTGGCAATTGCAATAACCTTAATGGTCAGCTACCCATGGCTTAGCGGTATCCT
GTTGAATTATACCCGGCAGATAATGTTACGAATTGGAGAGCATGGTTGAATGGCACAACAGGTAAATGAGTGG
CTTATTGCATTGGCTGTGGCTTTTATTCGACCATTGAGCCTCTCTTTATTACTTCCCCTATTAAAAAGTGGCA
GTTTAGGGTCCGCGATTTTACGTAATGGCGTGCTTATGTCACTTACCTTTCCCATATTACCAATCATTTACCA
GCAGAAGATTATGATGCATATTGGTAAAGATTACAGCTGGTTAGGGTTAGTCACCGGAGAGGTGATTATTGGT
TTTTTAATTGGGTTTTGTGCGGCGGTTCCCTTTTGGGCCGTTGATATGGCGGGGTTTCTGCTTGATACTTTAC
GTGGCGCGACAATGGGTACGATATTCAATTCTACAATAGAAGCTGAAACCTCACTTTTTGGCTTGCTTTTCAG
CCAGTTCTTGTGTGTTATTTTCTTTATAAGCGGCGGCATGGAGTTTATATTAAACATTCTGTATGAGTCATAT
CAATATTTACCACCAGGGCGTACTTTATTATTTGACCAGCAATTTTTAAAATATATCCAGGCAGAGTGGAGAA
CGCTTTATCAATTATGTATCAGCTTCTCTCTTCCTGCCATAATATGTATGGTATTAGCCGATCTGGCTTTAGG
TCTTTTAAATCGGTCGGCACAACAATTGAATGTGTTTTTCTTCTCAATGCCGCTCAAAAGTATATTGGTTCTA
CTGACGCTCCTGATCTCATTCCCTTATGCTCTTCATCACTATTTGGTTGAAAGCGATAAATTTTATATTTATC
TAAAAGACTGGTTTCCATCTGTATGAGCGAGAAAACAGAACAGCCTACAGAAAAGAAATTACGTGATGGCCGT
AAGGAAGGGCAGGTTGTCAAAAGTATTGAAATAACATCATTATTTCAGCTGATTGCGCTTTATTTGTATTTTC
ATTTCTTTACTGAAAAGATGATTTTGATACTGATTGAGTCAATAACTTTCACATTACAATTAGTAAATAAACC
ATTTTCTTATGCATTAACGCAATTGAGTCATGCTTTAATAGAGTCACTGACTTCTGCACTGCTGTTTCTGGGC
GCTGGGGTAATAGTTGCTACTGTGGGTAGCGTGTTTCTTCAGGTGGGGGTGGTTATTGCCAGCAAAGCCATAG
GTTTTAAAAGCGAGCATATAAATCCGGTAAGTAATTTTAAGCAGATATTCTCTTTACATAGCGTAGTAGAATT
ATGTAAATCCAGCCTGAAAGTTATCATGCTATCTCTTATCTTTGCTTTTTTCTTTTATTATTATGCCAGTACT
TTTCGGGCGCTACCGTACTGTGGGTTAGCCTGTGGCCTGCTTGTGGTTTCTTCTTTAATAAAATGGTTATGGG
TTGGGGTGATGGCTTTTTATATCGTCGTTGGCATACTGGACTATTCTTTTCAATATTATAAGATTAGAAAAGA
TCTAAAAATGAGTAAAGATGACGTAAAACAGGAGCATAAAGATCTGGAGGGCGATCCTCAAATGAAGACACGG
CGTCGGGAAATGCAGAGTGAAATACAAAGTGGGAGTTTAGCTCAATCTGTTAAACAATCTGTTGCGGTAGTGC
GTAATCCAACGCATATTGCGGTTTGTCTTGGCTATCATCCCACCGATATGCCAATACCACGCGTCCTGGAAAA
AGGCAGTGATGCTCAAGCTAACTATATTGTTAACATCGCTGAACGCAACTGCATCCCCGTTGTTGAAAATGTT
GAGCTGGCCCGCTCATTATTTTTTGAAGTGGAACGCGGAGATAAAATTCCTGAAACGTTATTTGAACCCGTTG
CAGCCTTGTTACGTATGGTGATGAAGATAGATTATGCGCATTCTACCGAAACACCATAAATGCTTTTGGTATG
CTTCTTCAGGCCACTGCGAAGGTTAAGAGGGTAATAGCGTATAGAGCAGTGCTTGACGATAAAGGTGAGAGAC
TGAAAATAATCGCTTTTAGCCTGGCACAAGCACCAGATAGCGTATTTTAAAATTAAACAAGATAATGGATTGG
TGCGTCTGAATGGACTCGAACCATCGACCCCCACCATGTCAAGGTGGTGCTCTAACCAACTGAGCTACAGACG
CATTAAAATGATGGTGCGTTCAATTGGACTCGAACCAACGACCCCCACCATGTCAAGGTGGTGCTCTAACCAA
CTGAGCTATGAACGCAACGTTGTAGGTGACAACGGGGACGAATATTAGCGTCACAACCGCAATGAGGCAAGAG
GGAAATCGCAATTTTCTTCCTGAAATCACCTGATTGCGGTGGAAATATGCAACATGTCGAGAAAATAGCCGCC
ATGCGACGGCTATCGTCGTATTATCGCGCAGCGCGCTGCAAAATGATGGCGGACGGCTGACGTTGTAGATAGC
GCATCCGTAGCATCATTAACACCGCCGCCGAGGTCAGGCCGATGATGAACCCCATCCAGAAGCCTGCCGGTCC
CATACGATCCACCACCAAATCCGTTAACGCCAGGATATAACCGCTGGGTAAACCTAACACCCAGTAGGCGGTA
AAGGTGATAAAAAAGATGGAACGCGTATCTTTATAACCGCGCAGAATACCGCTGCCGATAACCTGTATAGAGT

CGGAAATCTGGTAAACCGCAGCGAGCAGCATTAATTGCGCGGCAAGCGCCACGACCTCAGGGTTGTCATTGTA
GAGCAAAGCAATATGCTTACGCAGAGTAACGGTAAAAATAGCGGTAACCACAGCCATACAAATGCCGACGCCT
AAACCGGTACGCGCTGCGGTTTGCGCATCCAGCGTTGAGCCCTGGCCCAGACGATAACCCACTCGAATCGTTA
CCGCCGCAGCCAGCGACATCGGCAGTACGAACATCAGCGAGCTAAAGTTAAGCGCAATCTGATGACCGGCGAC
ATCCACAATACCTAATGGCGAAACCAGCAGCGCAACGACCGCAAATAACGTCACTTCAAAGAACAGCGCCAGC
GCAATCGGCAACCCCAGTTGAATCAGGCGCTTCATGACGATGCTATCAGGTTTGCCAAAGCCTTTTTCATTAC
GAATATCACGCATTGAACGCGCGTGTTTAATGTAAGAAAGCATGGCGATAAACATCACCCAATAGACCGCCGC
AGTCGCAACGCCGCAGCCGATACCGCCGAGTTCCGGCATACCAAAATGGCCATAGATAAAAATATAGTTCACC
GGAATATTCACCAGCAGGCCCAAAAATCCCATCACCATACCCGGTTTGGTTTTGGCCAGACCTTCGCACTGGT
TTCGCGCTACCTGAAAGAAAAGGTATCCTGGCGCGCCCCACAGCAGCGCGCGAAGATAACCCACGGCTTTATC
GGCCAGCGCCGGATCAATATTATGCATAGAGCGGATAATGTATCCGGCATTCCACAGGACGATCATCACCAGC
ACGGAGACAAAGCCCGCCAGCCAGAACCCTTGTCGAACCTGATGCGCGATACGCTCACGACGGCCGGAGCCAT
TGAGTTGCGCAATCACAGGCGTCAAGGCCAGCAGTAAGCCGTGACCAAACAAAATGGCGGGAAGCCAGATAGA
GGTGCCGATAGCGACGGCAGCCATGTCCGTAGCGCTATAGCCTCCCGCCATGACGGTATCGACGAATCCCATT
GCGGTCTGTGCCACTTGCGCAAGGATCACCGGTATCGCTAACGCTAATAACTGACGCGCTTCACTGGTATACT
TCTGCACGTATTCACCTTTTATTTTGTTGTTATATGAAAGACTAAAAAGCCGCCGTAGTGGCAGCCAAAAGAA
ATAGCAGGGGAAATTTCAGTCTATTGTAGCGGGGTATTACTATTTCTCCAGTGAAAAAACAGTTGTTAACGGC
GCATTGCTGGCAAGCTGTTTTTCCACCTGCTATTGTGCTGAACAGTTCTGCTTTTATTTATTTCAGGAGTTGA
AGATATGTTTACGGGGATCGTACAGGGTACCGCGAAACTGGTATCGATTGATGAAAAACCCAACTTTCGCACC
CATGTGGTGACGTTACCGGACTACATGCTGGAGGGGCTGGAGACGGGGGCGTCGGTAGCGCATAACGGATGCT
GTTTGACGGTGACTGAAATAAACGGCAACCAGATAAGTTTTGATCTGATGAAAGAAACGTTGCGCATCACCAA
TCTGGGCACGTTGAGGGTAGGTGATGAGGTCAACGTAGAGCGTGCCGCTAAATTCAGCGATGAAATCGGTGGT
CATCTGATGTCGGGGCATATTATTACGACGGCGGAGATCGCGAAAATCCTGACCTCCGAAAATAACCATCAGG
TGTGGTTTAAAGTGCAGGATGCGTCCCTGATGAAGTATATCCTGTACAAAGGCTTTATCGGCGTCGACGGCAT
CAGTTTGACCGTCGGCGAGGTAACGCCAACGCGCTTCTGCGTGCATCTCATTCCGGAAACGCTGGAACGTACT
ACGCTTGGCAGAAAAAAACTGGGTGAGCGTGTGAATATCGAGATCGATCCGCAAACGCAGGCGGTTGTCGATA
CCGTAGAACGCGTACTGGCTGCGCGAGAAAATGCGGTCAGAAATCAGGCCGACATTGGCTAACGGAAAATAAG
ATTCCCCCGCATGAAATGCGGGGGAGATGATTAGCGAGGAACGCGCAGTCCGTTTTCAACGCCGCGCGTAAAT
ACCACCTGCCAAAGCTGGATATCACGCGCGCGAAACGCACCCGCGCAGGCGTTGAGATAATAGCTGAACATAC
GTTTAAAGCGCTCGTTATAATTGCCCGCAATCTCTGGCCAGGCATTGATAAAGCGCTCATGCCACGCCATCAG
AGTCGTGTCATAGTCGGCGCCAAAGTTATGCCAGTCTTCCATTACAAAGTGTGATTCGCTGGCCTCGGCGATT
TGACGAACAGACGGCAAGCAACCGTTGGGGAAAATATACTTATTGATCCACGGATCGACATTATGATCGGTTT
TTTTAGAACCAATAGTATGGAGCAGGAAAAGACCATCCGGTTTTAAATTCCTGTCAACAACCTCAAAATAGGT
GTTGTAGTTTTTTGGCCCGACATGTTCGAACATGCCGACGGAGACGATTCGGTCAAACTGATCGTTAAGGTCG
CGATAATCTTCCAGGAGAATGGAGACATCCAGACCTTCGCAACGCGTCTGCGCCATTTTTTGTTGTTCAGCGG
AGATCGTTACGCCCACCACGCTAACGCCATAATGGGTCGCCATATATTGCGACAGACCGCCCCAGCCGCAGCC
GATATCCAGCACACGCATCCCCGGTTGTAGCTGTAGCTTTTCGCAAATTAATTTCAGCTTGGCTTGCTGTGCC
GCTTCCAGCGTATCGGCATCTTTCCAGTAGGCGCAAGAATATTGCATATAGGGATCGAGCATACGGGAAAAAA
GGTCATTACCAAGATCGTAATGTTCCTTGCCGACAATCCAGGCGCGTTTTTTACTTTGTAGATTAATCAGACG
CGCGCCGAGGATACGGAGCGTATCTTTAACATGGTGGGGGAGTTGATTTTCAAGACCGGCACGTAAGACTTTG
CTGAAGAAAATATCCAGACGCTCGCATTCCCACCAGCCATCCATGTAACTCTCACCTAACCCCAACGATCCTT
CCTGAAGGACGCGTTTAAAAAAATCGGGATTCTTAACACGAATGTCAGACGGGGCGGAACCATTAATTGTAAT
ATCCGCGCGACTTAACAATTCGTTGGCGATTCGGTACCAGTTATCATCCGGTACGCTGACTTCTTCTATACAC
GATGAACTCATAGTTTCTCCATCACTGGACGGTGATCAGAACCTTAAAACAGCGTAGACGCTTTTTTGGCTTT
GTGAGAAATCCCACGGACAATTCCGTGAGCCAGTTATCCGACGTAGAACAGAGGAAGGGAGGAACCCCTTGCCG
AAAAGGCCATCCATGGTGAATCGGGAACGCTCCGGTTCCCGTTAATGCCTAATAATTATCGTAATATAAACAA
CCGGGAATCAGTATAGGCCGCAATTTTTGATGATTCAACCGAAAATTGTTAGCGTGCTAATTACCAGCGGTTA
ACAAGTCTTTAGTGAGACTTGTCGTGGGCGACATTGGCATGGGGAAATCCGGTCTCAGCGCAATCTGCATGAG
ACTGCATTTTGTAGCCTAACGCCGCCAGAACAACGGTAGAGAGCATTACGCTGGTGGTGGTCAGCAGCGGCGT
GCTGATGAGCCATGAAACCACCAGGCTTGCCAGGAAACATAACCCCAGTTGCAGCGTATTTTGTAGCGCCGCA
GCGCGCCCCGTGGCTTGAGGGAAAGGGCGTAACGCTTGCGCGACGACGATAGGGTAGATGGCGCCATTAGCAA
TCGCCATTACGCAGAAAGGAATCAGGATCTCCACCAGTGAGGCATTATTCAGAAGGCCCGCTCCCCAGGTGGC
GATAACGCTCAGCGCGTACAGCCCTAACAGCCACGGAAGCAATTGGTAGCCCTGCCATTTTTGTAGCGCCGCG
CGGCAACCATATCCGCCAATCAGAAAAGCGATGGTTTGCGGAACATAGCTCAGGCCAATCACTGCCGGGCTGT

AACCCATCGCGCTGAGAATAAACGGGGACCCTGTTAGCCACGCAAAAAAGCTGGCGGAACAGGCGGCATAAAT
CAGCACATTACCGCGATAGGTTTTAGAGCGTAACAACGTCGCGAAAGTGAGTTTATCCTGTCCTTCGGTACGC
GCTTTTACCGATGGTTTTAAACGCAGCGCCGGCAACATCAGCAACAACGTAATCACGAACAGAGTGGCGAAAA
TCGCCTGCCATGAGAAATGCGTCAGTATCCAGCTTCCCAGTAAGGGCGCAAGCGCCGGGGATAATCCCACCAG
TGGCATGATCGTGGCGAAGATACGGTTAATTTTTTGCGAAGGGTAATAATCGGTCACCAGCGCTTGCCAGATT
ACCGTTGCCGCGCAAACGCCGACGGCCTGCACGAAGCGCAGCGTCAGCAGTGCTGCCGCGCTTTCTACCCACA
GCATCCCCAGACTTCCGAGTGCAAAGATAGATAACCCTAGCAGTAAAATCGGTTTACGGCCATAACGATCCGA
TAGCGGTCCCCAGAGGAGTTGCGCGACGGCAAAGCCAGCTAAAAACAGGCTTAGGCTGGCGCTGACCGCGGCG
GCAGGCGTTTGTAAATCCGCCTGTATGGCGGCAAACGCGGGCAGGTACATGTCCGTAGCCAGAAATCCCAGCA
CGCTAAGACCGGCTAACCAGACTAAAAAACCTTTTCCAGGTTGCATATTTGTTTCTCTTTCTTATCAGACAGA
CATTGTCGCAGAGTGTAGGGAGTGAAATTACGGTTGTGAAACGTTAATATTTGTCCAGTGAATTCAAAATTTT
TGTAGGCAAATTATGTGGTCGGAATACTCGCTTGAAGTGGTTGACGCCGTAGCGCGCAATGGCAGTTTTAGCG
CGGCGGCGCAGGAACTGCACCGTGTGCCTTCAGCAGTCAGTTACACCGTTCGTCAGTTAGAGGAGTGGCTGGC
CGTACCGCTTTTTGTACGACGTCACCGTGATGTTGAACTGACGCCTGCTGGCGTCTGGTTTTTAAAAGAAGGG
CGTTCTGTTATCAAAAAAATGCAGATCACTCGCCAACAGTGTCAGCAAATTGCTAATGGTTGGCGTGGGCAGT
TAGCTATTGCGGTGGACAATATCGTCAGACCAGAACGTACCCGGCAGATGATCGTCGATTTCTATCGCCATTT
TGACGATGTGGAACTGCTGGTTTTTCAGGAGGTGTTTAACGGCGTCTGGGACGCGCTCTCCGACGGGCGTGTC
GAACTGGCGATTGGGGCGACGCAGGCGATTCCGGTAGGGGGGCGTTACGCTTTTCGGGATATGGGGACCCTGA
GCTGGAGCTGCGTAGTGGCAAGCGATCACCCGCTGGCGTCGATGCCTGGGCCGTTAAGCGATGATACCCTGCG
CAACTGGCCGTCGCTGGTCAGGGAAGACACTTCGCGAACCTTACCGAAACGGATTACCTGGCTGCTGGATAAC
CAAAAAAGGGTCGTCGTACCGGACTGGGAGTCATCGGCAACCTGCCTGTCGGCAGGACTATGCGTGGGAATGG
TGCCGACGCATTTTGCCCGACAGTGGATAGACAGCGGAAAATGGGTGGCGCTGACGTTAGAGAATCCGTTTCC
CGATGCGGCCTGTTGCGTGACGTGGCAGCAAAACGAAGCCTCGCCCGCGCTGGCATGGCTGCTGGACTATTTG
GGCGATAGCGAAACGTTGAATCGGGAGTGGCTGCGGGAGCCAGAAGAGGCTCCCGACAGCGGGGATTAACGCC
GATAGTCGCGGAACGGGCCGTCAGCGACCGAGCGACGCTCAACCAGGCGTGGATGAACTTCAATAGACTGTGA
CTCTTCGCGCTTATTGACGATGCGATCCAATAGCATATTAAATGCGGTTTCGCCTAAAGAGTCTTTGGGCTGG
TGAATCGTCGTCAGCGCCGGGGTAAAGTAACGGGCGTTACGCACATTGTCATAACCGATCACCGAAACGTCCT
GCGGTACGCGAAGCCCCATTTCGTCAGCCGCGCAAAGCGCGCCCATCGCCATAATATCGCCGCCGCAGAAAAC
GGCGGTAGGGCGATGTGACTGCGATAAAATTTGCTGCATCGCGTGGTAACCGGATTCCGGCTCGAAGTCGCCC
TGAACAATCCAGTTGTCCGGCACGTTGATCAGCGCCTCCTCCATGGCTTTCATAAAGCCTGCCAGCCGGCCCG
CGCCGGTGTTGCGCTCCAGCGGGCCGGGAATAACGCCGATATCCCGGTGGCCGCGCTCAACCAGATAACGACC
CGCCATATAGCCGCCTGCAAAGGCGTTATCAATCACCGTGTCGGTAAAATCGGCCTTCGCTTCACCCCAGTCC
ATCACCACCATCGGAATATGGCGATACTCTTCCAGCATGGAAAGCAGAGGTTCTGGATACTCAGAACACATCA
CCAGCAGGCCATCCACGCGCTTTTGCGCCATCATGGACAGGTAGGCGCGCTGTTTTTCCAGGTTATTCCAGGC
GTTGCCTAAAATCAGCGTATAGCCTTTCTGGAAACAGTTTTTTTCAACCGCCTCGATAATTTCGGCAAAATAG
GCCGCTTCGCTGCTGGTCGCCAGTAAGCCTATCGACTTGGTATGGTTAACCTTCAGGCTACGCGCGACGGCGC
TGGGAGAGTAGTGCAGCTCTTTAATTGCCGCCCAGACCGCGTTACGCGTTTCTTCAGCGACAAAACGCGTTTT
GTTGATTACGTGTGATACAGTTGTAGTGGAAACGTTTGCTCGTTTCGCTACATCTTTAATTGTTGCCATTACA
TTTCACTCCAGACCCTCGCTAAGCTCCTGAGAAACGCCAGGTAAACGTTTGCCTACACACACCCTAATCGCAA
CATCAGGGTTGCGGCACGCCGGAAAAACGACACGGAACGTCAGGAAGGGGTCAATGGCCGTTACGCTAATTCA
GTAAGCCTGGAATTTTGTCTGATCTTGAGGAAAAGGGGAAGCGTTAAAAACAGTTATCAATATAAAACATGAG
AGATTTTTGTGCGGGAATGTGCAAAAATGCGCTATATCACTTTTTGTGTGGGAATAAAAAGGAGAAAACTTGA
TGAGTACCGACCTGAAATTTTCGTTGATTACTACGCTGATTGTTCTGGGCGTGATTGTCGCAGGTGGATTAAC
GGCCGCTCTGCACTGATTTCCTCTCTTCTGAGGGTGGGCGTCTCCTCTATTCTATCCCTCTCTAAAACGCAGA
AGGCGTACCTTCTGCGTACTCAGACTTATGCGGCGAGATTTTTCGCAACAAATTCCCAGTTAACCAGCGCCCA
GAAATGCTCCAGATAGTTTGGACGGGCGTTGCGGTAGTCGATGTAATACGCGTGTTCCCACACATCAACCGTC
AGCAACGGCGTTGCATCCGTAGTCAGCGGCGTACCGGCATTTGAGGTAGAAACGATAGCCAGTTTGCCATCAG
CGCTTTTTACCAGCCATGTCCATCCGGAACCAAAGTTTTTGATAGCGGCATCGGTGAACTGCGCTTTAAATTC
CGCGAAGCTGCCAAAAGATGCCGCGATAGCATCAGCCAGTTTCCCGGTCGGTTCGCCGCCAGCGTTTGGCGCC
AGGCAGTTCCAGTAGAAGGTGTGGTTCCACACCTGAGCGGCGTTATTAAAGATGCCGCCTTCGGAAGTGCGCA
CGATCTCTTCCAGCGATTTGCCTTCAAACGCCGTGCCTTTGATCAAATTGTTAAGATTGGTCACGTAAGTCTG
ATGATGTTTGCCATAGTGATATTCCAGCGTCTCAGCAGAAATATGCGGCGCCAGAGCATCTTTGGCATACGGT
AATGCAGGTAATTCGAACGACATTGCTTCTCTCCTTATTATTAATATTGCGCACGCACAATACCCCTGGTGCG
CGTAAGGATAGGGTAGCAAATTGAGCGGTGGCGCAAAAGAAGAAGTTATCCTGTCGTTAAACGACAGGATAAA

ATATTAGCGAATTGTTTTAGGCGTCATCACCCGGCGGGCGCCGACATAATGACGTTGCCAGTATTCTTCGTTC
AGTGAGGTGATCTTAATTTCCTGACCGCTGCGCGGAGACTGGATAAACTTACCGTTGCCGACATAGACGCCAA
CGTGATCGGCGGTGCCGCGCCCCTGCGTACGGAAGAACACCAGATCGCCGTTTTTCAACTCGCTACGCTCAAT
GGGCGCCGCGTCACGCAGGTGATACATCTCATTCGCCGTTCGGGGGATACGGATCTTCACTAAATCTTTATAG
GCATAATAAACCAGACCGCTGCAATCAAAACCGGTACGCGGCGACGCGCCTCCCCAATGATACGGTTTACCAA
TCTGGTTCATGAGTTTGGACATCGCGGTTTTCGTCGCTTTTTGTACTTTTGCACGATGCGCATCGGCAATAGA
GACCGGCAATGTTCCTTTCCCCTTCACGCAGTGCGGCTTACGGCCTTTACGCGTCGTGCATTTTTCCGTTACG
TCGAGGGCGGCGATTGACGCGGGCGCGGTTCTGTTACGACGATGCGTCGTATGACGAGATTTCGTGGCGGCGG
TGGTACGTTTTGGCGTAGAAGCGGAAGAAGAACGGCTGGTTTTTGAGGTTTTTTTACGTTTTTTTTGCTGGT
CTTACTGATCTTTTTCGTTGTGCTTTTCTTTTTATCTGCCGTTTTCGTGACATGAGAAACAGCAGGCGTCGCT
TTGGCTTGCCTGGAAGCATGGGCCATTGGCGTAAAAGGGAGAGTAGTAAAAAGTAAAGCACAGAGCGTGATCG
AGATTTTGTTTATCCGCGCCACTGAGCAATCCTCTGTTAAAGGCAGGCGATTCATGCCTGCGTATGATTTACA
AAACCTGATAATTCTAATCTACAAAAGCACAGATTGGTAATTGCTTTTGGTTTCTGAAGCTGTGTTTCGTTAA
CAGATGTAAAAAAAATAGCAATTAATGCTAAACACGAACGCGTTATCGACGAATCACTGCGTGGCAACCCGCT
TAAAAGATATTTTGTATACAACTTATTCCCGCAGACGGTAAAATAATAAGGCATGATGAAAATGTTATTTTCC
GTTGCCAGCCTGACCCGTTACAATGACAGGCTTCAGTCGTAAAAGTAGTTTAAGGAAGCGAGACAATGAGCAC
CACTATCGAAAAAATTCAGCGCCAGATCGCTGAAAACCCGATTCTCCTGTATATGAAAGGTTCACCGAAACTG
CCAAGCTGTGGTTTTTCTGCGCAGGCCGTTCAGGCGCTTTCAGCCTGCGGGGAACGTTTTGCCTATGTTGATA
TCCTGCAAAACCCGGATATTCGCGCTGAATTACCGAAATACGCCAACTGGCCGACATTCCCACAACTGTGGGT
GGACGGCGAGCTGGTCGGCGGGTGTGATATTGTGATTGAGATGTATCAGCGTGGTGAGTTGCAGCAGCTTATT
AAAGAAACTGCGGCAAAATATAAAACTCAGGAGCCGGACGCAGAGTAATCTGTGAAAGGGGAAAGCGACCTGT
AAAGGTCGCTTTTTTACCGCCATCGTGAGCAAAAGCGGCGTCGTTAGCCACGTTATTTATCCGTCGGCAAAGG
TAACGGCCAGCCGCCCAGGCGCTTCCAGCGATTGACGATTTCACAAAACAGCACCGCTGTCCGCTCGGTATCA
TACAAAGCGGAATGGGCCTTTTCGCCGTCAAACTCCATACCTGCCGCCAGGCACGCTTTTGATAACACCGTTT
GCCCCAGCGCCAGTCCGCTCAATGCCGCGGTATCAAAGGTGACGAACGGATGAAACGGGTTACGTTTCAGCGA
GGCGCGCTCTGCGGCGGCCATCATAAAACTGTGATCAAAAGTGGCGTTATGCGCGACCATAATGGCGCGGCTA
CAACCGCTTTCTTTGATACCTTTACGCACCATTTTGAAAATAGCGTGTAGCGCCTCATATTCACTCACCGCGC
CGCGTAGCGGGTTAGAGGGATCGATACCGTTAAACGCAAGCGCTTCCGGCTGTAAGTTGGCTCCAGCGAACGG
CTCCACATGGAAATGCAGCGTCATGTCCGGCATCAGCCAGCCTTGTTCATCCATTTCAGCGTGATGGCGGCG
ATTTCGAGCAGCGCATCGGTTTTAGCGTTAAATCCTGCAGTTTCAACGTCGATGACGACAGGATAAAAACCAC
GAAAACGGTCGCACAGACCGGAAAGTTGAGCGTTATCGGACATCTGAGTCTCTTAATACGAAAAAATGCAGCG
CGTATTATGGCAAATTTTACGCCGGGATGCAGTAAAACAACGGGCGCAGTGACCTGCGCCCGGCGGGGGATCA
GTTACCCAGACCGCGGCCTGCGTCTTTAGCTTCAATCAATTCGATTTTATAGCCATCCGGATCTTCAACGAAG
GCGATGATAGTCGAACCGCCTTTTACCGGCCCCGCTTCACGGGTCACGTTGCCGCCGTTCTGACGAATGCGTT
CACAGGCTTCAGCGGCATTGTCAACGCTCAGCGCGATATGCCCGTACGCATTGCCCATGTCATAGCTCTCAAC
GCCCCAGTTATAGGTTAGTTCGATGACCGCTTCTTCCGTTTCCGGACCATATCCCACAAAGGCCAGCGAGTAT
TTGTATTCCGGATTCTCACTGGTACGCAGCAATTTCATGCCCAGAACGTTGGTGTAAAAAGCGATGGAACGCT
GCAAATCGCCGACGCGCAGCATAGTATGCAATAAACGCATTTTCGTTGTCCTTAAAATAGTCAAGATATTCAA
AGTATAGCGGCGATGTGCGGCCGCTATCAATGAAATTACAGAGTAGGGTAGTCAGTGTAGCCTTCCGCGCCGC
CGCCATAAAAACTTTCGGGACGCTGTGGATTAAGTTCCGCTTTACGTTGCAGACGCGCCACCAGATCCGGGTT
AGCGATGTAATCGCGACCAAACGCCACGGCGTCAATCAGCCCTTTTTCGATCAGATCTTCCGCTTTTTCTGGC
GTATAGGCGCCAGCGCCGATGATGGGGCCGTGGAAACACGCGCGCACTTTTTCACGGAATGCGTCGCTGTACG
GTTCTCCGCCCGCCCAGTCTGGTTCAGACATATGCAGATAAGCAATGCCGCGTTTGCCTAACGCTTCAATCAG
ATAAAGGGCGTCAGCTTCTTCATTCGGGCCGTTATCGACATTCTGGAAAGTGCCGACAGGAGAAAGGCGGATG
CCGATACGGTCGGCGCCCCATTCCTTAATGCCGGCATCAACCACCTCCAGCACCAGACGGGCGCGATTCTCTA
CGCTGCCGCCGTACCGATCGGTACGGTGATTAGAAGACGGAGAGAGGAACTGGTGCAGCAGATAACCGTGCGC
GGAGTGCAGCTCCACCAGGTCGAAGCCTGCCTCTCGGGCATTGGCGATAGCCTGGCGGAAATCGTTGACGATC
CCGGGGATCTCGTGAGTATCCAGCGCGCGCGGCGTGGAAGTTTCTACGCGGATGGCCTGGCCGTTTTCGTCAC
GCAAAGAAGTGCGAGTTCCCGCATTGATAGCGGATGGCGCAACGGGCGCTTGTCCGCCTGGTTGCAGGCTGGC
GTGTGAAATACGGCCCGTATGCCACAGTTGCACCGCCATATGGCCGCCCTGAGCATGAACTCCCTGAGTGATT
TTTTTCCAGGCGGCGATCTGCTCATCGCTGTGTAATCCCGGTGCGCCGGCATAGCCTTTCGCCTGTGCGGAGA
TTTGTGTGGCTTCGCTGATGATCAATCCGGCGCTGGCGCGTTGACGGTAATATTCCGCCATTAACGGCGTAGG
AATGTCGCCCGGTTCAATACTCCGCAAACGCGTCAAAGGCGCCATAAATACGCGATTTGTCGCCGTGATGGCA
CCCACCTTGAGGGGGTAAACAGTTTTGCTGATGACATAGTGGCTCCTGATATAAGTAGACCGGTCGTCTTGTA

ATAAAGGAAATAAAAACGCCTGTTACTGCTCAGGCGTTGCAATGATGGTCTTAACATGCGCCAACGCATTTTC
CAGCGGCGCGGCGCTACGGGATATTTTGGCCTGCAAATTGGCGCCCAGCCACAGGGCATAAAGTACCTGCGCC
TGTGGCAACGGCTGGCCGGTGAAATGTAAACAGTGGCTATTACGGCCATCTTCCAGCGCGCGTGCAAGCAGTG
CCATGATCTCCTGCGCCCCTTTATCCATCGTAGAGCGCATATCCTCCGACAGATCGCAGACCTCCGCTGACAG
TTTCACCGTCAGGCAACCGCTGATGGCGCCTTGCTGGCAAAACTGCTTTAACGTTTCCTGATACCAGGCGAGA
AGGCGATCGCGATGATTACCGGGACCGGAGGTGAAGTGCGCCGTAAGACGCTGATGATAGCAGGCGTAATGTC
GCTCAAGCATGGCGACGCCGAAGGCTTCTTTCGAGCGAAAGTAGTGATAAAACGACCCTTTAGGCACCTGGGC
GGTTTTGAGCAATTCGCTAAGCCCCATGCCGGTAAAACCCCGTTGCATACAAAGCTGCTCGCCGGTGGCCAGC
AGATGTTCGCGGGTATCATGTTCGGTTTGCTTATTCATAGCAGGCAGTATAGTAGACCAGTCGGTCTACTGCA
AGCAGAGTTGCCATAATGTCAGTTAGCGTCTTCAATAGTCATAAGCGCCAAACGTTGAGGAGGGGATGTGGCC
GAGCAGTTGGAGTTTTTTCCTGTAGCAAGCCCATGTCGCGGTATCTGCCAGTCTGATGAGCGTGGCTTTTGTC
GGGGCTGTATGCGCAGCCGCGACGAACGGTTTAACTGGCAAAAAATGAGCGATGCCGAGAAACAAAATGTACT
GCGGCTTTGTCGCCAGCGTTTCTTGCGTAAAATACGCGCAAACAAGCCTCTCCCATCGGAAGATCCTCAACAA
CCTTCACTCTTTTAAACGCGGAATTGCGTATACTCAACCTCATTATTCTCTATGAGGAATCTGTTATGGTTCA
GCGTGTCACCATCGCTCCACAAGGCCCGGAGTTTTCGCGTTTTGTCATGGGTTACTGGCGTTTGATGGACTGG
AATATGTCCGCGCGTCAGTTAGTGAGTTTTATCGAAGAACATCTGGATTTGGGCGTCACTACCGTTGACCATG
CGGACATTTATGGCGGTTATCAGTGCGAAGCGGCATTTGGCGAGGCGCTAACGCTGGCGCCGCATTTGCGGGA
AAGACTGCAAATCGTCACCAAATGCGGTATTGCCACCACTGCCAGAGCGGAAAATAAGCTCGGTCATTATATT
ACCGATCGCCGCCATATTATTCTGAGCGCCGAACAGTCATTAAAAAATCTGGCGACAGATTATCTGGATATGT
TGTTAATTCACCGTCCCGATCCGTTAATGGATGCGGATGACGTCGCCGAGGCGTTTCAACATCTGCACCAAAG
CGGCAAAGTGCGCCATTTTGGCGTTTCTAATTTTACGCCAGCGCAATTTACGTTGCTGCAATCGCGCCTGCCT
TTCACCCTGGCGACCAACCAGGTGGAGATCTCGCCGGTTCACCAGCCTTTGTTGCTGGACGGCACATTAGATC
AATTACAGCAACTGCGTATTCGTCCGATGGCCTGGTCATGTCTTGGCGGCGGTCGGCTGTTTAATGATGAGGC
TTATCAGCCGTTGCGCCAGGAACTTTCCGTTATCGCGCAAGAACTCAACGCCAGTTCTATTGAACAGGTGGTC
TATGCCTGGATTTTACGTTTGCCGTCGCAGCCGCTGCCGATTATTGGCTCGGGTAAAATCGAACGCGTACGTG
CCGCACTAGAAGCGGAAACGCTGTCATTGTCGCGTCAACAGTGGTTCCGAATCCGCAAGGCGGCACTGGGTTA
TGACGTGCCGTAATCGCCAACTCGTTGACTTTCCGTACATGTTTTCGCCAGTGGTTTACACTTAACAGGCGAC
CACATGTAACGGAGGTTTTATGAAGCGATTAAGTTTAGCGATGGTGACGCTGCTGGCCTGTGCGGGTGCGCAG
GCTGCCAGCGAGAAAGTAGAGATGAATCTGGTGACGGCGCAAGGCGTAGGACAGTCTATCGGCACCGTCGTCA
TCGATGAAACCGAAGGCGGCTTAAAATTTACCCCACACCTTAAAGCGTTGCCGCCGGGCGAGCATGGTTTTCA
CATTCATGCCAACGGTAGCTGCCAGCCAGCGATTAAAGACGGCCAAGCGGTTGCCGCAGAAGCCGCTGGTGGT
CATCTGGACCCACAAAATACCGGCAAGCATGAAGGACCGGAAGGCCAGGGGCATCTGGGCGACCTCCCGGTGT
TAGTCGTTAATAATGATGGTATCGCCACCGAACCGGTTACTGCGCCGCGTCTGAAGTCTCTTGATGAAGTGAA
AGATAAAGCGCTCATGATCCATGTGGGCGGCGATAACATGTCCGATCAGCCGAAACCGCTCGGCGGCGGCGGA
ACGCGTTACGCCTGCGGCGTCATTAAATAACCCGGAGACAGTGTTCCCTGCGCTGGAGCCTGTTCCAGCTGCG
ACAGCGAACAGTGTAAACGCCAGATAATCGCCGCCAGTTCATGCGCGGCGGGTTGATGATGATGTGCGAGCGT
GTCGCAGATACGCTGTAGTTCCTGTAGCGTAACGGCAAGCGGGCGTTGCTGTACGCCGCGCTCGCTCATCACA
TCGCGCAGCAGGGATATACAAATATCCCGCACCCGGGAAAGCGGATCGGAACGCGACTCCCAGGCGCGCAGCT
GCCACACCCACATGCGAGCAGTTCAGCAGTACCACTCCCCAACGTAATAACCACCGGCGCGCCAGCGCATCCTG
GCTATTGCTGAGCTGACTGACGTGATGATAGGTCAGCGATTCAAACTCGCTTTCGTTATGGGAAGGGTGACGG
CTAAGCTGATCGACGAAATCGCGGCGTAGCGCGCGGATATGGCGGCGGCTTTTTACGGCATCGGAACCGGGGC
GCAAAATCGCAAACGCCAGCCAGGAAATCGCGACGCCGACGATCTTGGCGGTATTATCGTTAAGGAAGTCGGC
GAAGTCGTACACCGGTGGATTGGTCACGGCGATGAATGAGCCCATAAAAACGATAAGCTGCCCCCACAGTCCC
GCCAGCTTAGGCATCTGCAGTTTAAGCAGTTGCATCGTGACGAACAGCGGAAAAAGGAAAAGCAAGAATTGCC
ACAGATCGGTGATTTGCACCATCAGGCCAAATTTCACCACGAAGCTGAATAAGGACAGCAGCACTAAAGTACG
CATCAATAAGGAAAGCGACTTAAACGGCGTGGCGACGATGGAGTAGAGGACGCAACTGATCGCCGCCAGGGTT
AATGCGCCGGGCCCGGATTCCCATTGCGCGCCAATACTCCAGGCGCCAATTACCGTGAGCGTGCAAAAGGTAC
GCACGCCGCTCCAGATGGCCTCTGCGTTATCCGTATGCCGCGCAAGGCCGGGAGTGCGGCGGATATGGATTTG
ATCTACCGGCGCGCCGCTTTCGATCAGATATAACTGACGGCTGCTGCGCAGATATAGCTGGCAAAAATAGCGT
AATCGCTGCCAGAACGCGAGGTGACGATAATCCTGTTCATCCTGCGGGCGTAGCGGCGCAATAATCCGCGCCA
CGGTGTAGCTGTCCGCGCGCGGTTTTGCCAACTCAGCTAAGAGTTGTTCAATGACTTCGCGGCTGTTTTCCGG
CGGCGTCGGCCAATTTAAAAGCATTCTACGCAGACTGGAGATCACGCTGGTTAGCCGTAATTGCTGGTGTAAC
AATGCATTTAGCAATGCATTCTGGCGGCGAAAACGGTAGTGACTCCAGAAGGCCTGAATACGCAGCAGGTTCA
TGGTCAGGATTTGCCCAATGACCCCCTCATGCGCGGAGCGGATAGCGTCGGTAGTCTCCGGCTGCCACAGTAG

ACTGGCATGTTCCAGCAAGCGCGCATGCATGTTTTTCAACGCGGTGAGCAGGGCGGTACCATCCGATGTGCTG
GGTAGGATCATCATCATCATGCCGCCACAGAGGATGCCGACGATAACTTCACATACACGTGCCTGGGCGATAT
CCCACAACTGCGTGATTTCTACAATATTAACCATCGGGAACGCGATGATCGCCGCCGTGTAGCCGGACAACTG
GAAAGCATAAGCGGCATTATTGGTAAAATGCGCGCAGGCCCAGGTACAAAAACCGATCCAGGCGGCCATGCTA
AATAAGAACAACCAGGGTTCATTCAGCGTATGGCCGGCAATAATCAACGCGGCGGTCGCGCCGAGTAAACTCC
CGGCAATACGCCCCAGACTTTTACTGATTACGCCGCCTACGGTCGGAAAGCTCACCACTGCGGCCGAAGTCAT
CGCCCAGTAGGGCTCATCCAGATTGAGATAATAAGCGAATGTCAGCGCGAGACACATGGCAATGGTATTGCGC
AACGCGTAACGCCACTGGCCGGAGGTCGCTTTAAACCAGGGCGTATTTCGCAGGGCTGGCAGGGAGAGTTTCA
TTACCGCTGCCCAATGGCTACGCTGCAGGTAGTGCCGGAGACGAGCGTGATATCCTGCGGCAAAGCGTCAAAT
TCGATACGTACCGGTACGCGTTGCGCCAGACGTACCCACGGTACGTTGGGTTTAATATCGGGCACTAAACCGG
AGTCACTTTCCACGCTTTGGTCGTAAATAGCGCGCCCGATACTGCCAACGTGACCCTGTAACTTAACATTGCC
ACTATACAGTGTTATTAATGCAGGCTCACCTTCGCGGATATGGCGTAGTTTGGTTTCTTCGAAATATCCCATT
ACATAAAATGAATGGCTATCTACCAGGGCGAAAAGTGGCTTACCGGTACTGGCATAATCACCGGTACGCGTGG
AAAGGTTGGTCACCCAGCCGGATACCGGCGCTTTTACTTCCGTCTGGCTAAGCTGCCACTGCGCCTGTTTTAA
CGTCGCCAGTGCGACATCTACGCTGGCCTGCATCGCTTTAACATTCAGATTGGCGCTATCCAGATCTTCAGCA
GAGATGTAGTTACGCGATAAATGTCGACGTCGATCGGCTTCATTATTCGCTTTTGCCAGATCGGACTGCGCTT
TGGCCAACTGCGCCTGCGCGTTAAGTTCTGCAATATGGAAGGGCGTTTTATCAATGACAAAAAGAACGTCCCC
GGCGTTGACGAACTGATTATCTTTGATATTGAGTTGGGTAATGCTGCCAGAAACCTGCGGCGTTACGCTGACC
TGTTCCGCGCGGATTTTACCATCGCGCGTCCACGGTGACTGCATATAGTAATTCCATAACCACCATGCGGCCA
GAACGGCGACGACGGCGACAATGAGAGTAGAAAAATATTTTATTGTTTTTAACGACATATTTACCACGCGATC
AGTAAAACGAGGCCCAGACATACGCAAAGCGTAAAGAGGGAGAGATCCATTAACAGGGGATGCCAGATTTCAT
CAGAGTATATCCGGTCACGCAGGAGTCGGTGAATAAACAACCAGATAACGAACCCAAGCGCAAAGGCTTTAAA
GAATGGCGGAAAGTAAACAGATGCTCCCAACATGAGATCTTGTAAGGGCAATCCTGTGGCGTTGAGTATACAC
TTCACAAGCAATGTTCCTTTGCGTCAGACATCGATGCTTTAGTTTTAGCCAAAACTGAAGCTACAGGTGCCAG
GTGCGCACTATGTCTGAAAAAATGTCTATTGGTAAGCAAATTTAGCAATACATTTGTTTTGAGAATACAAATA
CTGCACACTATTCTAAAATCAGCATAATAACTTAGCAAGCTAATTATAAGGAGATGAAATTGGAATCGCCACT
AGGTTCTGATCTGGCACGGTTGGTGCGCATTTGGCGTGCTCTGATTGACCATCGCCTCAAGCCTCTGGAATTG
ACGCAGACACATTGGGTCACGTTGCACAATATTCATCAATTGCCGCCTGACCAGTCGCAGATTCAATTGGCTA
AAGCGATAGGCATTGAGCAGCCATCGCTGGTACGCACGTTGGATCAACTTGAAGATAAGGGGCTAATTTCGCG
GCAAACCTGCGCCAGCGATCGTCGCGCTAAGCGCATTAAACTGACCGAGAAAGCGGAGCCGCTGATCGCTGAG
ATGGAAGCGGTCATTCATAAAACGCGCGGTGAAATTTTGGCTGGGATTTCTTCAGAGGAGATTGAGCTTCTGA
TTAAACTTATCGCCAAACTTGAACACAATATTATGGAATTGCACTCTCACGATTGAGGTGCAGGGGCATACGT
GTGGCCATGTGACCACACGTAAAGCCTGGTTTAGCGTGGAGAGACGGTAACCTGGCTGCCGTTGCTGGCCAGC
ACGACACGCTGACCTGCCGAGAAACGCGTATTTCCTTGTTTCTGCACAACCATAATGGTGTTGCCATCGTCTT
TACGAATTTCCAGTTCCACACCCTGGGTTTTATTCATTGCGCTCTGGACGCCCTGGCCCGCTACGCCGCCAGC
CACCGCGCCTGCCGCGGTCGCCAGTGAACGACCCGTACCGCCGCCGATAGTGTTGCCGAGGAATCCGCCCAAC
ACCGCGCCGCCGATAGCGCCAATCACGTTAGAATCATCACCGCCCTGAATTTGAACCGGACGAACGTTAACGA
TAGTACCGTACGTTACATTCTGAACTTGTTTAGCCTCGGATGCGGTATAAACATCACCCGAAAGGCTATCATT
GTTAACACACCCAGCTAAGGATAACCCCATCAGTGAAACGGCCAGTACACGTTTAATCATTTACCCATCTCCT
GTTCTTCACGAAACGCTATGTCAGCATCCCTATAGCTAAATTATATGGCATTTATGCAGTTAAGGTCATATCT
TCTGCTGAAAGAATGTAAAAATCATAATCAGAAGTTAATTAACCAATCTTAAACGAAGTCGGAAGCAAGAAAA
GATAGGTCATTGATATCCGTGGACGACACGGCAAAAAAACGTGACGTGGGCATAGCATTACAAAATACGTTAT
GCCGAGTGTTAAGGCTTAACCCAGCTTAAAAGGAAGAGGTATGAAATCGGGCCGTTTTATTGGCGTTATGTCC
GGAACCAGCCTTGATGGCGTGGATGTTGTACTGGCGGCAATTGATGAAACTATGGTTGCGCAACAGGCAAGTC
TCACCTGGCCTATTCCCGTTCATCTGAAAAAAGGCATTCTCGATATTTGCCAGGGACAGCCGCTTACGCTCTC
GCAACTCGGCCAGCTGGATACCCAGTTAGGCCGGCTTTTTGCACAGGCGGTTAATGCGCTGTTAGCGCGACAA
CGTTTGCAGCCGCGGGATATCGTCGCCATTGGTTGTCATGGGCAAACGGTCTGGCATGAACCGACCGGCGAGG
CCCCGCATACGCTGCAAATTGGCGATAATAATCACATCGTCGCCCATACGGGGATTACAGTGGTCGGCGATTT
TCGGCGGCGGGATATTGCGCTGGGCGGACAAGGCGCGCCGTTGGTGCCTGCTTTCCATCACGCGCTGTTGGGA
CATCCGACGGAAAAACGTATGGTCCTGAATATCGGCGGCATTGCCAATTTATCTCTGCTTTTCCCCGGACAGG
CGGTGCGCGGGTACGACACGGGGCCGGGTAATATGTTGATGGATGCGTGGATTTGGCGACAATGCGCGCAGCC
CTACGACAAAGACGCGGCATGGGCGAAAGAAGGTCAGGTTATTCTTCCATTGCTGCAAAAGATGTTGCGCGAT
CCGTACTTTGCTGCGTCGGCGCCCAAAAGTACAGGACGCGAATATTTCAACTATGGCTGGTTAGAGCGTCATC
TTACGGCTTTCCCTGGCGCAGACGCGCGCGATGTACAGGCCACGCTGGCGGAGCTGACGGCGGTCTCCATCGC

CCAACAGGTGCTGCTCAATGGCGGCTGCGAGCGGCTGATGGTCTGTGGCGGCGGAAGCCGGAATCCGTTAGTG
ATGGCCCGTCTGGCGGCGCTGTTGCCCGGTATCGAGGTTTCGACCACCGATAAGGCGGGGATCAGCGGCGATG
ATATGGAAGCGCTGGCCTTTGCATGGCTTGCCTGGCGAACGCTCGCGGGCCTGCCGGGAAATTTACCTTCCGT
GACGGGGGCTACAGAAGCCAGTGTTTTAGGCGCTATTTACCCGGCTAACCCAATAACTCAGAGTTAACTGAAT
TTTTCCCGGACCCTATATCGGTAAACTAGCAAGGTCAGGAAGGGGCGTTTGCCCTTCCAGAAGCAGGAGAGCT
TTCGGGATATGCCTATGAAAAAGATCGCAATAATGTGTTTACCTGTGCTGCTGACCGGCTGTAGCGTTTATCA
ACAATTTGTTGAACGTATGCAAACGGATACGCTGGAATACCAGTGTGATGAGAAGCCGCTTACCGTTAAAGTC
AATAACCCGCGTGAAGAGGTCAGTTTTGTTTATGACAATAAGCTGCTGACCCTGAAGCAAGGAATCTCGGCCT
CGGGCGCGCGTTATACCGACGGGATTTATGTTTTCTGGTCGCAAGGCGAGAGCGCCACGGTCTATAAACGAGA
CAGAATTGTGCTTAATAACTGTCAGTTACAAAATCCGAAGCGTTGAGATTTTCAGTATGGGGGCGCACAATAG
CGCCACCCACTGATTATTTCTGATCAACTCCATGTCTGATAACGACCAATTGCAGCAGATCGCGCATCTGCGC
CGTGAATACACGAAAGGCGGCTTGCGTCGCCGCGATTTACCCGCTGAACCGTTAACCTTATTTGAGCGCTGGC
TTGGGCAAGCGTGCGACGCCAGACTGGCCGATCCGACGGCAATGGTCGTGGCGACCGTAGATGACAAGGGGCA
ACCGTACCAGCGTATTGTATTGCTCAAGCATTATGATGAAAAAGGCCTGGTCTTTTATACCAACCTGGGAAGC
CGCAAGGCGCATCAAATTGAGCACAATCCGCGTATCAGCCTGTTATTTCCCTGGCATATGCTTGAACGCCAGG
TGATGGTGACAGGCAAAGCCGAGCGTCTCTCCACACTTGAGGTGGTGAGGTATTTCCACAGCCGTCCGCGCGA
CAGCCAGATCGGCGCATGGGTTTCTAAACAATCCAGCCGCATTTCCGCGCGCGGTATCCTCGAAAGCAAATTC
CTGGAGCTCAAGCAGAAGTTTCAACAGGGCGAAGTGCCGTTACCCAGCTTTTGGGGTGGTTTTCGCGTGAGTA
TCGAACAGATGGAGTTCTGGCAGGGCGGCGAACATCGTCTGCACGATCGCTTTTTATACCAACGCGACGACGG
CGCATGGAAAATCGACCGTCTTGCGCCCTAAAGATGCAAAAATCTTGTTTTAAGCACTGGTGCGGATGAATCC
GCCGTTTTATGCTATGTCTCTTTCGCGTCTGGCGAAAAGTCGTGTACCGGCAAAGGTGCAGTCGTTTTATACA
TGGAGATTTTGATGGCAAGCAGTAACTTGATTAAACAATTGCAAGAGCGGGGACTGGTAGCCCAGGTGACGGA
CGAAGACGCGTTGGCAGAGCGACTGGCGCAAGGCCCGGTCGCGCTCTATTGCGGCTTCGATCCTACCGCTGAC
AGCTTGCATTTGGGGCATCTGGTTCCATTGTTATGCCTGAAACGCTTCCAGCAGGCAGGGCACAAACCTGTTG
CGCTGGTAGGCGGCGCGACCGGTCTGATTGGCGACCCGAGCTTTAAAGCCGCCGAGCGTAAACTGAACACTGA
AGAGACCGTTCAGGAGTGGGTGGCGAAAATTCGTAAACAGGTTGCCCCATTCCTGGACTTTGATTGCGGCGAA
AACTCCGCCATCGCGGCCAATAACTATGACTGGTTCGGCAGCATGAATGTGCTGACTTTCCTGCGCGATATCG
GTAAACATTTTTCCGTTAATCAGATGATCAACAAAGAAGCGGTGAAGCAGCGTCTGAACCGTGACGATCAGGG
TATCTCTTTCACGGAGTTCTCTTATAACCTGCTACAGGGGTACGACTTCGCTTGTCTGAATAAACTGCACGGC
GTTGCCTTACAGATTGGCGGTTCTGACCAGTGGGGGAACATCACTTCCGGTATCGACCTGACCCGTCGCCTGC
ACCAGAATCAGGTATTTGGTTTGACCGTTCCGCTGATCACCAAAGCTGACGGCACGAAATTCGGTAAAACCGA
AGGCGGCGCTGTCTGGCTGGATCCGAAGAAAACCAGTCCGTACAAATTCTACCAGTTCTGGATTAACACCGCA
GATGCCGATGTTTATCGCTTCCTGAAATTCTTCACTTTTATGGACATTGAGGAGATCAATGCCCTGGAAGAAG
AAGATAAAAACAGCGGTAAAGCGCCGCGCGCGCAGTATGTACTGGCCGAGCAGGTGACGCGCCTGGTTCATGG
TGAAGAGGGGCTGGTCGCGGCGAAACGGATCACTGAATGCCTGTTCAGCGGTTCACTGAGCGCGCTGAGCGAA
GCTGACTTTGAGCAGTTGGCGCAGGACGGCGTACCGATGGTTGAAATGGAAAAAGGCGCCGACCTGATGCAG
GCGCTGGTAGACGCCGAACTCCAGCCGTCGCGTGGTCAGGCGCGTAAGACTATCGCCTCCAACGCTGTCACTA
TCAATGGTGAAAAACAATCCGATCCTGAATACATCTTTAACGATGAGGACCGTCTGTTTGGCCGCTATACCTT
ATTACGTCGCGGCAAAAAGAACTATTGTCTGATTTGCTGGAAATAAGGCATTAAGTAACAGGGAGTGAGAAAT
CACTCCCTTATTTTTGATGTTCAGGTAAAAAAATGAAGAATATCCTCGCTATCCAGTCCCATGTTGTTTTCGG
ACATGCGGGCAACAGCGCCGCCGAATTTCCCATGCGCCGTCTTGGCGCGAATGTTTGGCCGCTGAATACCGTT
CAGTTTTCTAACCACACGCAATATGGTAAATGGACCGGCTGTGTCATGCCGCCCAGCCATCTGACTGAGATTG
TCCAGGGGATTGCGGACATTGGCCAGTTAGCGCACTGCGACGCCGTACTTAGCGGTTACCTGGGATCTGCCGA
GCAGGGAGAACACATTCTCGGTATTGTGCGCCAGGTTAAAGCAGCAAATCCGCAGGCAAAATATTTCTGCGAT
CCCGTGATGGGGCATCCGGAAAAGGGTTGTATCGTGGCGCCTGGCGTCGCAGAGTTCCATGTGCGTTATGCGC
TACCCGCCAGCGATATTATCGCGCCTAACCTGATTGAACTGGAAATTCTCAGCAAACATAGCGTGAATAATGT
GAATGACGCCGTGCAGGCGGCGCGAGAACTGATTGCTCAGGGGCCAGAAATTGTACTGGTAAAACATCTCGCC
CGCGCCGGTTACAGTTCAGAACGCTTTGAAATGCTGCTGGTGACAGCGCAGGAAGCCTGGCATATTAGCCGTC
CGCTGGTGGATTTCGGTTCACGTCAGCCGGTTGGCGTTGGCGATGTCACCAGTGGTTTATTGCTGGTCAAACT
TTTGCAGGGCGCGACGTTGCAGCAGGCGCTGGAGCACGTTACGGCGGCAGTCTATGAGATTATGATCGCCACC
AAAACGATGCAGGAGTACGAACTGCAGGTCGTGGCGGCCCAGGACAGGATCGCTAACCCTGAACATTACTTCA
GCGCAACGCGGCTGTAATTATTGCTGCTAAAAAGAGGCCGGATGGCGGTACAGCGAGCTATCCGGCAACATGA
CATTAATTCAGTCCTTCCGCTTTAAGCGCTGCCGCAACGGTCGGTCTTTTTGCGACACGCTGCATATAACTTT
CAATATGCGTTAACCCGTCCATATTCAGTTTTACGCCATAGGCCCAACGCAATACGGTAAACAGATAAGCGTC

TGCGATGGTAAAACGCTGGCCGCAAATCCACTGGTCGTCTGAAAGCGATTCATCCACATACTGAAATTTCTTT
TCCAGAGCGGCGCGAACGGCGGGTTTAAGAGTTTCCGGCGTGTCAGGGCGGAACAGCGGGGTAAAGCCTTTAT
GCAGTTCCGTTGCAATATAGTTGAGCCATTCCAGCGTATGATAGCGAGCAAGACTACTTACCGGCGCCAGAAG
CTGGCGGTCGGGAACGCTGTCGGCGAGGTACTGCATAATGGCGACGCCTTCAGTCAACAGTGTGCCGTCATCC
AGCAGCAGAGCCGGAACCTGTCCTTTAGGGTTGACTGCCAGGTAATCATCACCGTTTTCCAGGCGTTTTTTTG
CGAGATCGACGCCATCAAGCGTAAAGTCTTTGCCAATTTCGCGCAGGGTAATGTGGGAAGCAAGAGAGCAGGC
ACCCGGTTTGTAGAACAGTTTCATCGGTAACTCCTTTTTGCTGAGGTTTAAAGTATGGTAGTGCGGGGACGAG
CAAAAAAAAGCCGCTAACGCACAGTTAGCGGCCTGAATGTTGTTTCCTGGAAAATTAAACTGTAGCGGCTTTA
GAGCCTTTTTCAGCGGTATCATCGTCCTGGGTCATACGGTTCAGCTTAGGCGCTGTCAGCAGCATCAGGACGG
CGATAACGGCGGTTGCTATGCCAATCTGCATAAATACGCGACCATAGACTTCCAGGGACATCAGTGGGTCAGT
CACGTCGCTCGGCACAGCCATCAGGTTGGCGACATAACCGCCGATGATGTTAGCGCCTGCCGTTGTCAGGAAC
CAGCTACCCATGATGAAACCCATCAGACGCTGCGGAACCAACTGTGCGACCATTGCCAGTCCCAGACCGGAGA
TCATCAGCTCACCGATACTTTGCAGGCCGTAGCTTGCGATCAGCCAGTTTACGGATACGATGCCGGCATCGTT
CGCAAATTTGGCGCCCAGCGGCAGAATCAGGAACGCGCCGGAACACAGCACCATACCAATGGCGAATTTCATC
GGCATCGGCAGCGTATCGCCCATCCTGTTATAGATAGCGGCCAGAATCGGGCTGCCGATAATGATCCAGAATG
GGTTCAGCGCCTGATACTGTTCCGGTTCGAACGCGATACCGAGAATAGAGTGCTCGACGTTACGAATGGCAAA
GAAGTTCAGCGACGTCGGCATCTGGCTGTACAGTACGAAGAAGATAATCGCCTGCAGCATCAAAATAAAGGCG
ACGATCATTTTACGGCGAGCCGCGCCGTGCATGGAAAACGCTTCTTTACCGAAGATAATGACGATGCCAAGAG
CGATTACGCCCAGCACCATACGCGCGATATCCTGGTTGTGCAGCAGCCAGGTGGCGACCGCAATCAGAACGAC
AATACCCACGATTGTCAGCAGCAGATTACGGAAGTTGATCGGTTCAAAGTCGGGTTTGGAACCGTAGCTTTTC
ACCCAGCGCTGGCAGAAAGCAAAGTTAACGACGGTTATCAGCATACCGACGACGCTAAGCGCAAACGCGGTGC
TCCAGCCATAACGCGCCGCCAGCCACGGCGTCGCCAGCATAGAGAAGAAGGAGCCGATGTTGACGGACATATA
ATACATCGTAAATGCGCCGTCGAGACGCGGATCATCCTTCGCGTAGCAGGTAGACAGCAGGGAGGACGGGTTA
GCTTTAAACAGACCGTTACCCACAGCGATAGCCGCCATGCCCATATAGACGATGCCAGCGTCATGACCAGACC
ATGCGACCAGCGCATAACCGATAGCCAGCACCACGGCACCCAACATAATAACGCGTTTGGTGCCCAGAATTTT
ATCACCTAACCAACCGCCAATGGCGACCAGGCCGTACACCAGCGCACTGAAGGAAGAGAAAAGGGTGATGGAA
TCCGCTTCTGACATACCCAGTTGTTTTACCAGGTAGACGGCCATAATGCCTTGCAGGCCGTAATAACCAAAAC
GCTCCCATAACTCTATCGAGAAGATGAGATAGAACGCCTTCGGTTGTTTGAAAGCGTTCAAACTGACGCTTTC
AGTTGGTTTTTTGTTTGCAGTAGACACTTTTACCTCTTTTTTACGCCCCGTATTAACAGGGATTGTCTGGCGC
GTGATGAGTCAGACTCATCCGTCCGATTGTTATATTGGAGGGGAAACGGCAGGTAATGTTCACTATCTTTCCT
CTCCAGACAAGAGCTTTGTAATAATCTGTTACATATAACTCTTCTGGTTGAATCGTCCGATGTCAGAAATGTT
ATCCAGCGTTAAATTTCTTCATTTGAAGAATGGCAGATTTTTACACTGCAAAAAGGCCATTAGTTTGGGTGAA
AGGCGATATCTTCTGCTATTTAAGGTAAAAAGCAGTGATATAGGCCATTATCTGAAAAAATGGCAGAGTAATG
TTTTGTAGTGAAAATGGGGCGTCAGTCTATCCGGTTGTAAATGTAACCTTAGATGTCATTAAATTTCACATGA
AGTTATCAAAGTGATCCTGATCACGGATAGATAGAAATTTTTGCGATTAAAAAATGATTAACCTGACGTGGAG
GGAAAACGAAAGCACATTACATCAGAAAAAGGACAGAGACTGGAATGATTGATGGCTAAAATAGATCCAGGCC
GAAGGTGGGAGCGCGCTGGTGTGACGGACGCACTCCGACCATATCAAATATCGACTTTTTCTTTGTATTCGCA
AAGGTCTTCAATGAGGCAGGAGCCGCAGCGGGGTTTTCGGGCGATACAGGTATAGCGGCCATGCAAAATTAAC
CAGTGATGGCAGTCCACTTTGAACTCATTCGGCACCACTTTCAGCAACTTCTCTTCTACCTGTTCGACATTTT
TACCCGGCGCGAACTGTGTTCGGTTGCATACACGAAATATATGCGTATCAACGGCGATGGTCGGCCAGCCGAA
GGCGGTATTTAGTACTACGTTAGCGGTTTTTCGCCCCACGCCGGGAAGCGCTTCCAGCGCCGCCCGATCCTCC
GGCACTTCGCCATTATGCTTGTCCAGTAAAATACGGCAGGTCTTAATCACGTTTTCCGCTTTACTGTTAAATA
AACCGATGGTTTTGATATAGGATTTCACGCCTTCTACGCCCAGTTCCAGCATCGCGGCTGGCGTATTGGCGAC
CGGGTAAAGTTTGGCGGTAGCCTTGTTCACGCTGACGTCGGTTGCCTGCGCGGAAAGCAACACGGCAATCAGC
AACTCAAACGGCGACGTGAAATTGAGTTCCGTTGTGGGGTGCGGATTGTTGTCGCGCAGGCGCGTCAGAATTT
CCAGCCTTTTTGCTTTGTTCATCAGACTTTCCCTGTTTCACCTGCCGGAACAGCGGAAGGCGCGGTTTCGGCG
CGGCGTTTCTTCATTTTTTCATCAATAAGATATTTACCGCCAGCATGAGCCCCAGGCCGATAAACGCGCCTG
GCGGCAGCATCGCCAGCAGGAAAGGCGAGTCGGTGTGAAATATCTCGACCCGTAATACTTTTGCCCAGCCTCC
CAGCAGGCTATCCGCGCCATCGAAAAGCGTACCATTCCCGAGGATCTCGCGCAGCGAACCCAGCACGAACATC
GCGCCTGTCGCGCCCATGCCGATAGAAAAACCGTCCAACGCGGAAAGCCACGGTCCTTTTTTCGCGGCAAACG
CTTCGGCGCGACCAACAACAATGCAGTTCGTTACAATCAGCGGAATAAAAATCCCTAAAGACTGATACAAACC
GAACGCATACGCATTGATCAGCATCTGTACTGCGCTTACCACCGAGGCAATGATCATAACGTAAATCGGGATA
CGGATTTCTGCCGGCGTCCAGCGACGTAGTGCGGACACCGTAAGGTTGGTCAGCGTTAATACCAGCGTTGTGG
CCAGACCCAGCCCCAGAGCGTTGGTTGCCGTAGACGTCACTGCCAGTAAAGGGCACAGGCCAAGTAGCTGCAC

CAGAGCAGAGTTATTTTTCCATAACCCCTGAACAACAATGTCTTTTATTTCGCTCATGGCTATTCTCCACAGG
CGGTTAAATGGGGGAGCTGAGCGGGCAGTGATTCGGCGTACAGCCCGGCGCGTTTAACCGCATTCACTACGGC
ACGCGGCGTTATTGTCGCCCCGGTAAATTGATCAAAATCACCGCCATCTTTTTTCACCGCCCAGTGCGTATCA
TTTTCACCGCTGATGGTTTTACCGCTAAAGTGGGTAATCCAGTCAGACAAACGGCGCTCAATTTTATCACCTA
ACCCTGGCGTCTCATGATGTTCCGTCACACGCGTCCCCAGGACGGTACCGTTGAAATCTGCGCCAACAATAAG
CTGGATAGCGCCGGAATAGCCATCTGGCGCGGTGGCCTCAATGATGGCCGCTACGGGTTTATCATCCTTGCGA
GCGATAAAAACACGGTGTGTGCCTTTGCCGAGGGCGGGCGCATCGACCAGATAACAACTCTCTTGCAGATTAT
TATTGTAACGATCTGGCGGCAATACCTGATCGAACAGGGCATGTTGTTGTTGCAGCGCTTGTTCGTGGATGGT
TGATTTTGTCATCTGATTAATCACCGCGGTCAATCCCGTAGAGCCTGCGGCAAAAAGCGCCAGCGTAATACCG
TGTTTACGAATCGTTTTCAGCATGGTTTATCCTTTGCGATGTCCATAGACGCGGGGGCGCGTGTAGTAGTCGA
TCAGAGGAACCGTGATATTCGCCAACAGGACAGCAAAAGCAACGCCGTCCGGATAGCCGCCGAAGCTACGAAT
CAGCCACACTAATACCCCAGCCAGCGCGCCGAAAATTAAACGTCCATGGTTGGTCGTGGAGGCGGTAACCGGA
TCCGTTAAGATAAAAAATGCACCCAGCATGGTGGCGCCTGACAGCAGATGAAGCTGCGGCGAGGCCAGCGTAG
CGGGCGAGAAAAGCCAACCTAACGCGGCGCATAGCGCCAGCGTCAGCAGAAAACTGACCGGGATATGCCAGCG
GATAGCTTTTTGCCACAGCAGGAATACGCCGCCGACAAGCCAGGCGAGGTTAACCCACTGCCAGCCCACGCCG
GCCAGCGCGCCGCTGTATATCGGGTACTGCATAATTTGCTCAACGGAATGTCCGGCGCGCAAGGACGTTTTAA
ACGTATCCAGCGGCGTCGCCTGGCTGATACCGTCAATACCGATACGTAGCAGCGTCATATCGCCGCCGCTGGC
GGTATGCCCGCTAAAAATCATCCGTAACGTGTCCAGCATATCGGGCGTTGTTGCGGCTATTTCATAAGGCGGC
AACCAGCTTGTCATTTGTACCGGAAAGGAAATTAATAGCACCACGTAACCGATCATTGCCGGATTAAAGGGGT
TTTGTCCCAGCCCGCCATATAACTGTTTAGCGATAATAATGGCGAATCCGGTTCCCAGCACGACCATCCACCA
GGGCGCAAGCGGAGGGATGCTGACCGCCAGCAAAAGCCCCGTCAATAACGCGGAATAATCCTGCAGGTGACTG
GCGACCGATTGTTTGCGCAGCCTGAGGACAATGGCTTCTGCGACGAGCGCCGTGATAGCAGCCAGAACGATCT
GGAAAAGCGTTCCCCAACCAAAAAACCACGTCTGCGCGGCAATCCCCGGCAGAGCGGCGATTACTACCAGCAA
CATGATTCGCGATGTCTGGCGCTGGTTATGGGTATAAGGCGAGCTTGCGATTCTGAATACCATTTAATCCTCG
TTAACAACCTGCTGCTGTGCTGCTTTTTTTGCCTGAACGCGGGCGATAGCCGCAGCAACCGCGGCTTTACGCG
GGTCGGCCGGTTCGGCAGGTTCGCTTCCGGCCTGCTGCTCCTGCTTACGGGCTTTGGCGCGGGCGATGGCCGC
TTCCACCGCGGCTTTACGCGGATCGACCGGTTCGGCAGGTTCGCCTCCGGCCTGCTGCTCCTGCTTGCGGGCT
TTGGCGCGGGCGATAGCCGCTTCCACCGCGGCTTTGCGCGGGTCGACCGGTTCGGCAGGTTCGCTTCCGGCCT
GCTGCTCCTGTTTGCGGGCTTTGGCGCGGGCGATGGCCGCCTCCACCGCGGCTTTGCGCGGATCGACCGGTTC
GGCAGGTTCGCTTCCGGCCTGCTGCTCCTGTTTGCGGGCTTTGGCGCGAGCGATAGCCGCCTCCACTGCGGCT
TTACGCGGGTCGCCGCCGGATATCGCGCTGTCAGCCACCGGATGAGCGGCTTGTTTAGCGCGCGCTTGCGCTT
TACGCGCTTCACGGGCCGCGATGACGGCGCTATTGTCTGGCAGACTACCCGCCTGAATAACCACCGGTTGCGT
GGCCTGCGCCTGTTTCTCTTTTACCCGCGCCAGCGCAGCGGCAATGGCATCCTGATCTTTTGCCGCTGGTTGT
ACCGCTGCGCTCTTATGACGTGCAAGGCGGGCGGCTTTTTCGCGCTCCAGTCTTGCCTGACGAGCCTCAAAAC
GCGCTTTGGCTTCGGCGGCGCGTTTTTCTTCAAGCCTGATCGCATTAATCTCCGCTTTTTCCTGACGGAAATA
TTGGACGAGCGGAATATTACTGGGGCAGACCCAGGCGCAGGCGCCGCATTCTATACAATCAGCAATATGGTGA
GCGGTAGCCTTGTCATGCTGCTGCCCTTTGCTGAACCAATAAAGCTGCTGCGGTAAAAGGTCGGCAGGGCAAG
CGTCAGCGCAAGCGCTGCAACGAATACAGCTTTTCTCTTCCTGAGGCGCTCCCATTTCAGTCACGGATGGCGC
CAGCAGGCAGTTGGTGATTTTTACGACCGGTACGTCAAGCCAGGGAAGGGGGAATCCCATCAACGGGCCGCCC
ATGATGACCATCTGGTCAGCAGAAGGGCAGAATCCGGCATCGTTAAGCAAATGACGAACGGGAGTGCCGAGCC
GCGCCCAGACGTTACCCGGACGGCTTACCGCTTCACCCGTTAAGGTGACGACGCGTTCTGTGATCGGTTCGCC
GTCAACCACGGCGCGTTTAACGGCATAAGCCGTACCGACGTTTTGCATCAGCACGCCAATATCAGAGGAGCGC
CCGCCGTGGGGAACCTGTTTTCCCGTCAGTATTTGGGTGAGTTGTTTTGCGCCGCCGGAAGGGTATTTGGTTG
GGATCACACGTAACGAAATGTCGTGAGCGTCAGCCAGTACGGCGCGCAGCATGGAAATAGCCTGCGGTTTGTT
ATCTTCAATGCCGATTAATACCTCACGCGGTTGCAGAATATGCGCCAGGATGCGAATACCTTCCACAATCTGC
GCGGCGCAGTCCTGCATCAGACGATCGTCCGCCGTAATGTAAGGTTCGCACTCTGCGGCATTGATAATAAGCG
TGGTGATTTTATCGCCGCCGCCCTGCAATTTAACGCCGGTGGGAAACCCTGCGCCGCCAAGCCCGGCAACGCC
ATACTGATGAATGCGTTCAATGAGCGCTTCACGGCTGTGGGCGCGGTAATCAGACCAGCCCTCGCGTTCAATC
CAGCGGTCCTCGCCGTCGGCATCAATGATGACGCTTAACTCCGCCAGCGCCGAAGGATGTGCCGTCGAGTGGG
GGGCAATAGCGATAACCGTCCCGGAGGTGGGCGCGTGTACCGGCAGCATTCTCCCACGTCCACGGGTCAGCGC
CTGGCCGCGCAGCACGCGATCGCCGACGCTAACGCACAACTCGCCCTCAGCGCCAATGTGCTGTTTTAAGGGG
ATAACGAAGCGCGGCGCTAAAGGCACCTGACGCAGCGGCGTGCCGTTTGACTGGGTCTTCATTTCGGGCGGAT
GAATACCACCGTCGAAATCCCAGATTTTATCTTTTCTGAAAGCGGAAAATAGTTTAAGCATGTTGTTCCACGG
GAATGATGCGAACGGGAATGGTGTTCAAATCCCATTTCCAACTGTCGGGCGTCTCATTCACCGGACGTAATTC

GATGCAGTGCGTCGGACACGGATCGACGCACAGATTACAGCCAGTACAGAGATCGCTCATGACCGTGTGCATG
GCGCGCGTTGCGCCAACAATGGCGTCAACCGGGCAGGCCTGAATACATTTTGTGCAGCCAATGCAGTTATTTT
CATCAATAACTGCCAGCATACGTACTGGCGCGGCCTGCTGTTCTTCACCATCGCATGGCTGCGGCTCTACGTT
TAGCAGCTCTGCCATTTTCAGCATGACGGCTTCGCCGCCGGGCGCGCAACGATTGATTTTCTCGCCCTGGAGA
CCCACGGCTTCAGCGTAGGGGCGACAGCCGGGATAGCCGCACTGACCACACTGGCTCTGCGGCAAAATCGCAT
CGATTTTTTCAACAACCGGATCATCTTCAACCGCGAATCGGCGGGAGGCATAACCCAGAATGGCGCCGAACAC
CAGGCCCAGAAGGGTCAGGGCGCCAACGGCAATCCAGATAGTATTCATTACAACTTCACCAAACCACTAAAGC
CCATAAAGGCCAAAGACATTAATCCTGCGGTAATCAATGCTATCGCGTTACCACGAAAAGGGGCGGGCACATC
CGCCACGGCAAGGCGTTCGCGGATGGCGGCAAACAGAACCATCACTAATGAAAAACCGACGGCGGCGGAAAAT
CCGTACAGCGCCGACTGAAGAAAGTGATGACCGAGATTAATATTCAGTAACGCTACGCCCAGAACCGCACAGT
TAGTGGTAATCAGTGGTAAGAAGATGCCTAACAAGCGGTATAACGCCGGGCTGGTTTTCCGTACAACCATTTC
GGTAAATTGCACGACCACAGCAATGACGAGGATAAAGGCCAGCGTCCGCAGATAAATCAGATCCAGCGGAATA
AGTATCCAGGTGTCGATAAGCCAGGCGCATATCGACGCCAGCGTCATGACAAAGGTCGTCGCGAGGCCCATCC
CCATGGCGGTTTCCAGTTTTTTGGATACGCCCATAAAGGGACAAAGGCCCAGGAATTTGACCAGTACAAAGTT
ATTGACCAGTACAGTTCCGACAAAGAGCAGCAGGTAATCAGTCATGTTTCGGCCTGAAATAAAAAAGCCGCCT
ATTATCTGTCAAACTACCACAGGCGACAACAGGTTAACTGTAGGGTTATTACGGATTAACAAACGTTGTTTTA
ACGCGCGCTGAACGTTTGAGATAAGGCACAATGAGCGCCGTCGCCAACAGTGGGAAGAGTAACTGACGCACGG
CAAAGGCATCTGAGACGGGCGAAAAGGCAAATGCTTTAACCGCCAGTAACACCGACACCAGCAGCCAGATAAT
GTAATGCTTCGGCACGCAGCGTCTGCGCTTGAAGAATGCGATCGTCAACCACAGGGTATAGTACCACATGGCG
ATAGCGGTAATAAATGAAATGCCCCATAGCAGTAAATTACCGGTTTCCTGTCCGCTAAGCGTCTTGAACGTTT
GCGGTGTGGCAAGCGCCATAACGTAGAGTAATAACGCCAGCGAGGCGCTCAACAACGCGACTAACAGCCAGGC
TAACGGGCCAAGAAGCCAGCCTCCAATTCGTTGTGGCGGTGTTGTAGTCATATCTTCTCCGGGGTTACGCGCA
TTAACGGTCATGCCGCTCATTTTGGGGCAAATATTATAAGGTTTTTGATGTTAAACGCTACTTTCTTTATTGC
ACATAGCGCCAGACGGATTGCGGAACCTGACCTACATCAAACAGGCGTCCTCCGGAAACCAGTTCGGCACGGC
GGTGATCTGCAGCGCGATACATATTAACAATGTCCATGTCATCAGTAAGGGTATAGTTAAGGTGGTCGAACAG
TTTTTCCAGACTTTCGAGCGAACTAATTTTGCGGAATTTTAATAGATAATCCTGAACAGTCATATAAAGTTGA
TCCATATTAAATATAATGCCAACGGAGTAGTCACTGATAAAAAATAAACAAAACCGCACCAGTGAGAGGTCAG
GGGTGCAGGTAAAAAGGGAATATTCTTACCGGTAAAGAGTCAGGCGCCACATGGCGCCTGGGGCGAAAGGTTA
ACGCGCTTTCCTACAACAGGCAATAAACGTCAGACCAGATAGGACATACTTTCGTTTTTTTGCCGGTTTTTG
ACTTTTCTTTGCCCCCCGGGGCGCCGCTTACCGTGCCACGCGAAAATGCCTTTACATTATCTGACGCTTACTT
CTTCTGAACGGAAGCCGTGGGCTCTGGTGGTTGGTAATTGTCAATATGGTTGGCAATACCTAAAAGAATGACT
GAGATAACGAGAACTATCCACCCAGTTAATTCAATAAGACGATTCATTATTGACGACATTTGCTTTGATTTAT
CCATAAACACAGGGGGTAAATGTTACTTTCCTGGCGAACGGTCGGCAAGAGGGAGGGAAACGTTTTCAGCCTC
TGTTCTGTAACAAGTTAATCATTTGATGCTACGTTGATGGTCAGGACGCTATTGCGGCTAATCGCACGATGGT
GTGGAATAGCGATATCTAAAACCGGAGGCAGTGAGATGAATGACAAAATCCGCGTTGGGTTAATCGGTTATGG
CTACGCCAGTAAAACCTTTCATGCGCCCTTAATTATGGGAACGCCAGGGCTAGAGCTTGCGGCGGTATCCAGC
AGCGATGAAACAAAAGTGAAAGCAGATTGGCCGGCGGTTTCCGTGGTCTCTGAGCCCCGACATTTATTTAACG
ATCCGCATATCGACCTTATCGTGATCCCGACGCCCAACGATACCCATTTCCCGCTGGCGAAAGCTGCGCTGGA
AGCCGGTAAACATGTGGTGGTTGATAAACCCTTTACCGTGACACTGTCACAGGCGCGAGAATTGGATGCGCTT
GCGCGGAGCACTGGCCGGGTACTTTCCGTCTTCCACAACCGACGCTGGGACAGCGATTTTTTGACGCTGAAAG
CCTTGCTGGCGGATGGCGTGCTCGGCGAGGTGTCCTTGTTTGAATCCCACTTTGATCGCTTCCGTCCCCAGGT
GCGCGACCGCTGGCGCGAGCAAGGCGGGCCGGGAAGTGGTATCTGGTACGATCTGGCGCCGCACTTGTTGGAT
CAGGCCATTCAGCTTTTTGGCCTGCCGGTCAGCATGACGGTGGATTTAGCGCAACTGCGTCCGGGCGCGCAAT
CTACAGATTATTTCCACGCGATTTTATCCTACCCCCAGCGCCGCGTGATTTTGCACGGTACGATGCTGGCCGC
GGCGGAATCCGCGCGATACATCGTTCATGGTTCGCGCGGTAGCTATGTTAAATATGGCCTCGATCCCCAGGAA
GAACGGTTAAAAAACGGCGAGCGTTTACCGCAGGAAGACTGGGGATACGACATGCGTGACGGCGTATTGACCC
TGGTTGAGGGAGAGACGCGCAAAGAAGAGAACTGGCTTACGTTGCCGGGTAACTACCCGGCATATTATGCCGC
CATCCGCGATGCGTTAAACGGTAACGGTGAAAATCCCGTGCCGGCAAGCCAGGCGATTCAAATTATGGAGTTG
ATTGAACTGGGCATGGAATCGGCAAAACATCGCGCGACGCTGTGTCTTGCCTGAGATGTCGCATTTCTCCGCC
TGGGATAGCATTAAATCGCAGGCGGAGAGAGGGCGTTAGCATCAATAAAGCAATCTTACGCCGCGGCCACTTT
TTCGCGCAGCGCACGTTTTTCGCCATCGCTCAAAAAGGCGATTTCCAGACCGTTAATCTGCGCCTGGCGAATC
TGTTCGCGGCTCAATCCTGCCGCTGGCGCGGCAACGTGATATTCATGGATAATATCTACGCCCTGAACAGCCG
GATCGTCCGTATTAAGACTGGCGAGAACGCCATGCTCAAGGAAGGTTTTGAGCGGATGATCTGCCAGCGATGC
GACGGTACTGGTCTGAATATTCGAGGTCAGACAGGATTCAATACCGATACGCTGTTGTACAAGAAAATCCATT

AACGCGCGATCTTCGACAGCTTTTACGCCATGTCCGATACGCTCGGCCCCCAGTTCGCGAATCGCCTGCCAGA
TACTTTCCGGCCCTGCGGCTTCGCCAGCGTGAACGGTAATATGCCAGCCCGCATCGCGCGCCCGATTAAAGTG
CGACAAAAACAGACTGCCGGGAAAGCCCAGCTCATCACCGGCTAAATCCAGCGCGGTGATGTTCTCTCTGTGC
GCCAGCAGCGCATCAAGCTCCTGTAAACATGCCGCCTCGCCGAAGGTACGGCTCATAATGCCAATGAGTCGCG
CCTCGACGCCAAACGTGTTACAGCCGTCGCGTACGCCGTCGATAACGGCTTCAACCACGCCTGCTATCGGTAA
CTGATGCGCCATCGCCATATAGCCGGGCGAAAAGCGAAGCTCGACGTAATGCAGGCCGTTACGTGCGGCATCT
TCAATATTTTCAAAGGCCACACGGCGGCAGGCGTCAAGCGATGCCAGGACTTTTACCCCCAATCGAGCTTCGT
GAGAAAGCTCACTAAATCGGGTTCGGTTGAGGTGACCTGGACATGCGGAATCAGCGTTTCCAGCGTTTGGGCG
GGAAGGGCGATGTTGAACTGCCGACCAAGATCCAGAATTGTTTGGGCGCGAATGTTGCCATCAAGGTGGCGGT
GGATGTCAGTCAAAGGAAGCGTAATATCAATCATGGGCGCACTCAATTTTTAATAAAAGTGCGCACCATTATA
CTACAGATTGATAATGCTCTGGAAATTTTGCAAAAACGGAGTCATTACGTTGCAACTTCGCGAGAGCGCGGGA
GAAATTTTGTATCATTCTCTTTAACGCGCCCCCAGTCAGCTCACGGGGGCCGTCTCTGTTATCGCCTCTCAGG
ATAAAGGGTCAACCCCCCGCTTGTAGACAGTATCAGCGAACGGTGCGGTGGCAAAATCCATATCCGAGATCGT
AAAGGGGAGCAGATCGGCTTCGCCAAAACGATCGGCGACATAATCCCACTACGTACACCATGTCCCGCGTCGG
TCAACTTCCTGTGAAAAATCGAACATATCCCTTCTTTATGCCTGAACGGTATTCATCAGGCCAACCATTTCAT
CTTTTACCGACTGAACCTGCGGGCCGATAATTACCTGCAAATTATGCTGATTGAGCTGTACGATGCCTGTCGC
GTGATTGTCCTTCAATGCCTGGACATCGACAAACGACATATCTTTCACTGACAGGCGTAGGGGGGATGCAGTT
GTCCAGACTCACGATGTTTTCCGTGCCGCCTAATGCTCACGACGAGCACCATGTGCGGAGTCAAACGATAGCC
GCTCGCCTGGTAACGCAAAAGTAGAGGCTGCGCGTCGTTCGGCGCAGACCGGGTGCAGCGTCGCGACACCCGG
CAAGGCGATTATTGTTGAGGCAGCGCCGGAACGTCCGGTACGGAAAGCGCCGGCATCCCAAACAGGCCGACAA
AGTCTTCCAACGGCATTTTTTGTCCGTTGAGCGTGATCTGGCCATTGGCGTACTGCAAACTGGAGGCGATAGT
ATTATCCTTCAGCGTTGTCAGTCGGAACATCTGACCCATTGCGGACAGCCCCTGCACCTGCTGTTTTGCCAGT
TTTTCCGCATCATCCTGCTGATAGCCTTCCAGCTTTGCTATCTGCGTCATAAACTCGACCGCCATGTCCATCG
GAATTGCCAGTTTCGCATCCAGCGATTTCACGGAGCGGTCGACTTCCTGCGCCAGCGTTTGCGGTTGTGCCGT
TGTGGTTGCCGGATCTTTGAGGAACAGCGACAGATTCAGCGTGGTTTCCCCTTTGGCGTTTTTCCAGCTAAGC
GGCGCCAGGGTCAGTACCGGATCGCCTTTTAACAGCACGGGGAGAGCGCTAAAGAACGCTTCAGTGACTTTTT
GCTGATAAAGCTCAGGATTCTGCGCCACGTCCGGCTGATTCAGCAGCGCCTGCGTTTGTGCATGATATTGCTG
ACTAAACTGGTGCCAGGCTTCGCCGTCGATTTGACCGACTTTCAGCGTTAATTTACCGCTGCCCAAATCCTGA
TTCTGCACTTTCAGGCTGTTAAGCGAGTAATCCAACTGGCTGTTAATGGTTTTTCCGTCGTTGACGAGATCGG
ACTTGCCGGCAATTTCCATCCCTTCCAGCACCGCCATCTCTTTACCCTCAATGGCGATGGACAGTTTATCCAG
CGTTAACTTTTGGTCGCCCACACGTTCGCCAAAGCTTGCCAGTTTACTGGTACCGTCGGTTTTCAAATTATTA
AAGGTGAGTTGGACCTTCTGGTTATATTCGTTAACGGCATCGACCAGGCCGCTCTGCGCTTCGCCCGACAGCG
AAACCACATTACCGTCTTTATCGGCGTTAAGCTGGAACTCGCCGCCGCTAAAGGCGACCTTTTCACCGGCATT
CTCGTAGTTTAACGGCTTGAGAGAGATATCGGAGCGGGTATCGCCGCCATAACCAATGCGGGTATTGATAACA
AAGGGCGTGTCGCCTTTCGCGATATCAAAGAGGGGTTTTGCCGCATCGTTATTCACCAGCGTGGTTTTTACCG
ACGCCATAGAAGGGATCAGATTGAGCGTTTTGAGCTGGGCGAGCGGGAAGGGGCCATGTGAAACGGACTCGTC
CAGCACAATGCTTTGCCCGGGCTTCAACCAGGTAGTGTCTGCGCCGGCAACCGGTTTGACAACCAGTTGCAGA
TGGCTACTGAACACGCCACGCTGGTAGTTTTGATAACTTAATTCGACACCGGCCTCCGGCGCAGTACGCTTGA
GCTGTTCATTGGCCTGAGTCACCATTTCTGAAAGATGGTTCTCCAGCTTTTTCCCCGTATACCAGGCGCCGCC
TGTCCAGACGATGCCAAGTGCAATCACTACACCTGCAGCTACCAGTGTTTTTTTCATATCAATTGTCCATAAA
AATGAAACCAGGCGGATTAAACCGCCTGGAGGAGTTTTACGTCTGTTATAAGCTTAGCAAGAGTTGTTAAAAA
ATTCAGTACGTTGCTACAGCTTGTTATAAACACGCGCTAAACGGCCTGTGCCGCTGGCGTTAACCGGAGACTC
ATCCGCGCCGATAAAGGCCGATTCACCCGGCTTCAGTACCAGACGCTGTTCATCTTTACGTAACACCGCCTCA
CCCTCAACGCAGAACAGAATCGCGGCGCTGTGTTGGCCGATGCTCGTCTCCTGAAGCGCCAGGTCGTGCAGTG
AAAAAGCAAAATCGTCAACCGGAATTGGGAAGTCCAGCTCCGCGCCGCTTTTCACCGGGGCAGTCAGCAACTC
GCCGGCAGGTTTAGGTTCGAACTTCACGTTCGCGACCAGCTCAGGGATGTCGATATATTTGGCGTAAGGCCA
GCGCGCAGAACGTTATCGGAGTTCGCCATGACTTCCAGCGCAACGCCCTGCAGATAAGCATGAGGCGTTTCGG
CAAACAGGAACATCGCCTCGCCGGGATTCAGTTTGACCACATTCAGCAACAAAGGAGAGAAAAGCCCGCTGTC
GTCAGGATAATACTCTGAAATCACGCGGATCGTTTGCCACGGTTCGCCTTGCTGGCTGTTAAGCGCCGCTTTG
AGTACGGCTAACGCGCGGGATTTTTCTTCGCCTTGCATATTCAACAGGCTGGCGAAAAGCTGGCTCAGACGTT
CAGCATTCGGCGCCTGCAAAAAGTGGGCGATAGCGGAATGCGCGCCGGCGACAGGTTGCAGTAAAGAGACAAT
GTCAGAAAATTCGCGGAACGCGTTCATCGCCAGGAAAGGCGTCAGGGCAAAAACCAGCTCTGGTTTATGGTTA
GGATCTTTATAGTTCCGCTCTGCGGCATCCATGGGGATACCCGCCGCATTTTCTTTCGCGAAACCGATTTCGG
AATTGCGCTTATTCGGGTGCACTTGAATAGAGAGCGGCTGCGCGGCGCACAGTACTTTAAACAGAAACGGCAG

TTCGCCGAAACGGTTGGCTACCGCTTCGCCCAGCATGGCGGTTTTATTCTTTTCGATGGCGTCACGCAGGGAG
ACGGTTTCGCCGTTGGCGGTGGTGATTCGCGAGCTGCTTTTGGGATGCGCGCCCATCCAGAGTTCAGCCATTG
GCTGCTGCTGCGGATTGGCGATGCCATAAAGTTCCGTTAACGCAGTTTTACTTCCCCAGGCATAGTTTTGCAC
TGAGTTAATGAGTTTTTGCATTATCAAGCCCTGTTTCAATGTGGAGATCAATGGGAGGTATTAAACCAAGAAA
CCCGCATGAAGTAACCTCCGGGCGTAAAAAGTCGTACTTGTCTCAGTTTTTGTTAAAAAATTGTGTAGGATAG
TGTTACTCGCTTTGAGTCGGGCAATGGAACACCTGCCCAGAGAATAACCATACCGAGCGGTAAGTGAGAGCAC
AATGTCAAACAAACCCTTCTTTTATCAAGACCCTTTTCCCCTCAAAAAGGACGATACCGAGTACTACTTGTTG
ACCAGCGAACATGTTTCCGTCGCCGAATTCGAAGGACAAGAGATCCTGAAAGTCGCGCCGGAAGCGCTAACGC
TGTTGGCGCGTCAGGCCTTCCACGATGCGTCTTTTATGCTACGTCCCGCGCATCAGCAGCAGGTGGCGGATAT
TCTGCGCGATCCGCAGGCCAGTGAAAACGACAAATATGTCGCGCTGCAATTCCTGCGAAACTCCGATATCGCG
GCCAAGGGCGTGTTGCCCACCTGCCAGGATACCGGTACGGCCATTATTGTCGGCAAAAAAGGCCAACGCGTCT
GGACTGGTGGAGGTGATGAAGCGGCGCTGGCTCGCGGCGTATATAACACCTATATCGAAGATAACCTGCGCTA
TTCGCAGAACGCGGCGCTGGATATGTACAAAGAGGTCAATACCGGTACTAACCTGCCGGCGCAGATCGATCTC
TACAGCGTCGATGGCGATGAATATAAATTCCTTTGTATTGCTAAAGGCGGCGGTTCGGCGAATAAAACCTACC
TCTATCAGGAGACAAAAGCGCTTCTGACGCCGGGTAAGCTCAAAAATTATCTCGTCGATAAGATGCGTACGCT
GGGCACCGCCGCCTGTCCGCCGTATCACATTGCGTTTGTCATTGGCGGCACCTCGGCGGAAGCGAACCTGAAG
ACCGTTAAGCTGGCCTCGGCAAAATACTATGATGCGCTACCAACCGAAGGCAATGAACACGGGCAGGCCTTCC
GCGATATTGAGCTGGAAAAAGAATTGTTGCTTGAGGCGCAAAATCTTGGCTTAGGCGCGCAGTTCGGCGGCAA
ATATTTCGCTCACGATATTCGTGTCATCCGATTGCCGCGTCACGGGGCATCGTGTCCGGTAGGTATGGGCGTG
TCCTGCTCCGCGGATCGAAACATTAAAGCGAAGATCAACCGGGAAGGGATCTGGATCGAGAAGCTGGAGCGCA
ATCCCGGTAAATATATTCCTGAGGCGCTGCGCCAGGCGGGAGAGGGCGAGGCGGTGCGCGTTGATCTTAACCG
TCCCATGAGCGAGATACTGCAACAATTGTCGCAGTATCCGGTATCAACGCGCCTGTCGCTGAACGGCACAATT
ATTGTGGGCCGCGACATCGCGCACGCTAAACTGAAAGAACGGATGGACAGCGGCGAAGGTTTGCCGCAGTACA
TCAAAGATCATCCTATCTATTATGCGGGGCCGGCAAAAACGCCGGAAGGGTACGCCTCCGGGTCGCTTGGGCC
GACTACCGCAGGACGGATGGACTCTTATGTTGATCAGCTCCAGTCGCAGGGCGGCAGTATGATCATGTTGGCG
AAAGGCAACCGCAGCCAGCAGGTGACCGATGCCTGTAAGAAACATGGCGGCTTCTACCTGGGCAGTATCGGCG
GCCCGGCAGCCGTTCTGGCGCAGGGCAGTATCAAGCGCCTGGAGTGCGTGGAATATCCTGAACTGGGTATGGA
GGCTATCTGGAAAATTGAAGTGGAGGATTTTCCGGCCTTTATTCTGGTGGATGATAAAGGCAACGATTTCTTC
CAGCAGATTCAGTCATCGCAGTGCGCACGCTGCGTTAAGTAATGTCGCCGCCCTGCGGGGCGGTAACATGATT
CGCTATTTTGCCGGAGAACGGCGCAAGCGCCTTATCCGGTCTACACCGTTGTGTTGTTATCGTTAATACTCAT
AAGACCGCAAACATGTGAGCAATGTCGACACATGATCTCATCAACTTGAGTAAGGAGCAGGTAATGGTAACGG
TACGCCGCGAGAAAGATTCAATGGGCGCGATTGAAGTCCCGGCAGATAAACTGTGGGGGGCGCAGACTCAACG
TTCGCTGGAGCATTTTCGGATTTCCACGGAGAAAATGCCCGTCTCGCTCATTCACGCTCTGGCGTTGACCAAG
CGCGCCGCTGCGAAGGTCAACCAGGACTTAGGGTTGTTGGCGGCGGAAAAAGCCAGCGCGATTATCCAGGCGG
CTGATGAAGTGCTGGCGGGAAAACATGCTGATGAGTTTCCGCTGGCGATTTGGCAGACCGGGTCCGGTACGCA
AAGCAACATGAATATGAATGAGGTGTTAGCGAACCGCGCCAGTGAAATTTTGGGCGGCGTTCGCGGTATGGAA
CGCAAGGTGCATCCCAATGATGACGTCAATAAGAGTCAAAGCTCAAACGATGTGTTCCCAACCGCCATGCACG
TGGCGGCGTTACTGGCGTTACGCGAACACCTTATCCCGCAATTATCCGCATTAACGGATACGCTTCGCGATAA
ATCCCACGCTTTCGCCGATATTGTCAAAATTGGCCGTACTCACCTGCAGGACGCGACGCCGCTCACCTTAGGC
CAGGAGATTTCCGGTTGGGTAGCCATGCTGGAACATAACCTCAGGCATATTGAGCACAGTTTACCGCACGTCG
CGGAACTGGCGCTCGGCGGAACCGCAGTAGGGACAGGGCTTAATACCCACCCGGAATATGCCCGGCGCGTGGC
CGAGGAACTGGCGACGATTACCGCGGCGCCGTTTGTTACCGCCCCGAATAAATTCGAAGCGCTGGCGACCTGT
GACGCGTTGGTACAGGCGCATGGCGCATTAAAAGGACTGGCGGCCTCGCTGATGAAAATCGCCAACGATGTTC
GCTGGCTGGCGTCCGGCCCGCGCTGCGGCATTGGCGAGATTGCTATCCCGGAGAATGAGCCAGGCAGTTCCAT
TATGCCTGGTAAAGTGAATCCGACCCAGTGTGAAGCGGTAACGATGCTATGTTGCCAGGTGATGGGTAACGAT
GTGGCCATCAATATGGGGGGCGCATCGGGCAACTTTGAGCTCAACGTCTATCGTCCGATGGTTATTCATAATT
TTCTGCAAACGGTGCGCCTGCTGGCCGATGGCATGGCGAGTTTCAATCAACACTGTGCGTCAGGAATCGAGCC
AAACCGCGAGCGTATTACGCAGTTGCTGAATGAGTCGCTGATGCTGGTCACTGCGCTCAATACGCACATCGGT
TACGATAAAGCGGCGGAGATTGCGAAGAAGGCGCATAAAGAAGGGCTGACGCTCAAAGCCTCAGCCGTGGCAT
TAGGATACCTTAGCGACGAGGAGTTCGACGCCTGGGTACGTCCGGAGTTGATGGTTGGCAGTATGACGCCGGG
ACGTTAATCCGCCACATACAGGTGCAGTCGCGGAATAATCAGTCGCAGCGGCTGCGCCTGTGGTTTAAAACGG
TGCTGAATATTGTCGGCGTCATAGTTTTCCAGCCCGCCAATATCCGGCACGCCAGCGCCGTTGTCCGTGTTGA
TTAATGCGATAATCGGCGTGGGGCAGGCGTGTTGCACCTGTTTTTGCTGGCCTTTATACCAGATGCGGGCGAT
CGGCTGTACTTTAACCGGACGCTTAATCTTGAGTCGGGCCTGCTGCGGTAAGGCAGCGATATCCTGATATTCC

CGTTCCAGCTTGAATTGCCATTGTTCGCGCGTCCACGGCGGGACGCTGCGCGGCGACGCGAGGCTTTTTTTCA
ACTGGCTCAGAACCTCATCCCGGCTTAAATTTTTAATAATATGTTTATTTGCCCAACCGAAACGTATGGTGGC
GGGGTTATTGATAAGCGTCAGCGTGCGGTAAGCGTTCAGCGTAATTAAGCCGGGTAGATGCCGATGCACCCAC
TCAAAACGTGCCGCTGAGGGAAGACCGGACTCTACCGTAACCATCTGCTCGAAGGTGGTTTTCAACTGGTTAA
TGCGCTGTACCTGATTTTCCAGATCTAGCTGTGTCGCATTATCCACTTGATAGCATAAAACGCCAGGCAGGCG
GACGGCGGCTTTACTGCTGCGGTTTTCGGACTGCTGTTGAATAAACAAATGGCGATAGTGACGTAGCGCCAGC
GTTTCGGCCTCTTTCCCCAGATGTTGCGTTACCTCGATAGTATCCAGCGGCGCATGTTCCGCCTCTTTGGTCA
CCTGCGGCAGTGAAAAGACACGGGCGATAAGTAGCGAATGCTGCTGTAGATTATCCGTCAGCGCCGCCAGGTG
TTGTTCTATCTGGCGAAAAGTGCCATTCAGGCGTTCAACGAGATCGTAGCGGGACATGGTATCACTCACTTTA
GTTACAACATACTATCTATATAGCACAGTTCCCGCCGCTCAACCAGCGTGCAAAATGCGTCAGGCAGCGGTCA
TGTCGGGCATCGCGTGCCAGACCGGCCAGCGAAAACTAAATTTTGCGCCGCCCAGTTCGCTCTCGTCACAGAC
GACGCTACCGCCCATCGCCAGGGCGATAGAGTGTACGATGGCGAGTCCAAGCCCGCAGCCGCCTGTCGCCCGG
TCTCTACTGGGGTCGAGTCTGACAAAAGGTTCAAATATGCGCTCACGCGCATCCGCTTCAATGCCGGGGCCAT
CATCTTCGACTATCAACGTTGCCTGACTGCCGTCGAGTAACAGGCTGACCTGAACGGTGGTACGACTGTAGCG
CAACGCATTATTCAGCAGGTTATCCAGCACTCGCGACATTAACCGCATATCAAGCGCGCCATAATCGCCGATT
ACACATGTGAGAAGGTTTACGGCACGTTCCGGGGTCACGCTCTGCACATCCTGCAAATGCGCGAGCAACCATG
CGGGGAGGTCGGGTTCGGTCAACATCAGTTCATTTTGTGGTCGGTCAAGGCGCGCATAGGTGAGCAGTTCTTC
AATCAGCGCTTCCAGTTGACCAATATCGCGATTGAGCGCCTGTGATTCCGGCGGTGTCAGATTTTCACTCATT
TCAAGCCGATAGCGTAAACGTACCAGCGGCGTGCGAAGCTCATGGGCGATACCGTCAATCAGCTGTTTTTTAC
TGGCGATCAGGGCATTAATGTTATCCGCCATCTGGTTGAACGCGACGCCCAGACGTTCGAAACTGGAGCCATT
ATCGAAGTGCAGGCGTTCAGTCAAATGACCCTCGCCAAAGCGCTGCGCGGCGGATTCGAGCCTGAGCATTTCT
TGCCAGTGCGGGCGCATCCAGATAAACACAGGAAAGGCGAGAGAAAAGGCAATGAGCGCCATTAAGGCGATAT
CCAGCAGTCGCATTTGATGCAGGAAATAGAGATAGGGAACCGGGCCAACCGCCAGGACGTAATGGCTGCGCGG
GATACGCTGAATAAAGGTGTATTGATCGTCAAGCGCGACAATATCGCCTTCACGCAGTCGCTGCGTGGTGGCG
GCATCAAGCTTATAGTGATTTAACGGTTCAACGCGTAAATCGAAAGAGAGGTTCAGGTCCATCTCTTTTAACG
TTTTCCCCCATTCACGCGGCGGAATTTCCCGCAGTTCGCTGCGCATCAGATAGAGCGAACTTTTCATCAGATC
GTCGAGCGATTGCCTGCCCGCGCGTTCGGCGGTGAATTTGTACACCAGTCCGACCAATAAGGTCATGACCAGA
AAACAGACGAACAGCAAAAGGTAAAACTGCACAAACAGCTTTTTCATCAACGCCCACCGGATCTCAATACATT
AGCTCTATCGCGCGCTGACGAAAAAGATTTCCCGTCAGCGCCGCTGGTGGGTTATAAAAGCAGGCTTTGCGTC
AACTGACAACCTCATTGTCCGGCGTTAAGGTCCGGACTTTCGTGAACAATGCGTCCATCGACAAGCGTCAAAA
CAGCGCGAATGGTATCAATTCTGTCTTCCGGCATCGTCATAAATGGCTGATTGAGTACCGCCAGATCGGCCTG
TTTTCCGACGCGAAGCTGGCCCCGGTGTTGTTCGGCAAAGGCGAGCCAGGCGGCATGACGTGTATATAACGCT
AGCGCTTCCAGACGCGTGAGCCGCTCGCTGGCGGGTTTACGCACGGAACCGCCTGACGCTATACCGGTGATAT
GATATTCGATAGCGTGCCAGACACCGGCAATGCCAATACGCGTCGAATCGGTACCGCCAGCTACGACAAGCCC
TTTATCCAGCGCCAGTCGAACCGGCGGCGAATTGTCCGTCGCGCCGGGTGGATTGGCGTCACGGATGGCAAGC
CCCTCAAAGTAAGGCCCCATTTGCACAGTGTATGCCAGACCCAGCTTACGCATTCGCTCAAGCGTCTGTGGCG
AACCGGTATTCAGATGCGCAATAGACCAGCGGAGAGGGCGCAGATCGTACTGCTGCGCTACCTGCTCAAAAAT
CGTCAATATCGCGTCGGCGCTGTCATCGGTATAGGCGTGGATCTCAAGCGGTATTCCCCGTTTTGCCGCAAAT
GTCGCGACCTGGCGAAGCGCGGTTTTGTCCTGCTCTGAGGAAGAAAACCCTGGGGCCATCCGCACGCCGTCAT
TCATTCCGGCCACCAGGCTTTCCCCCAGGCCAAGAAAAGCCAGTTGCCCGTCATCGGCACGCGCCGGGCGAAA
GGCCATCAGGTTGCTGAACCACTGCGCTTCATGACCTTTCGCTTCTGGCTGTACCGGAATGCGATACCCCACG
CGCAGCGGTAAATCCCCCCTGGTTTCGCATTGCAAATAAAGGCTCATAAGCGGCGGCAGGCCCGGCAGAGGGG
TCAATGATGCCGGTCACGCCGCGAGCGTTCATATCAGCGAAAAATTGTCGCAGACCGCCCTCGCGATCGGCGT
TACTACTTATGCTGGCAAAAAGCTGGTTAAACGCGGCGATGTCACCAAATAATTTCCCCGTGGCGCTACCTTT
TGCGTCGCGCTCTACGCGGATTCCCGCTAAATCAGGAGGAGGGGTGTCGTTAAGGCCAAGTACGTCTATACCG
CGCTGATTCACTAACGCATAGTCGTAAAGATACTGAATATAAGCCGGATGATCGGGAAGGGCGTGGCTCAATT
CGGCTACCGTCGGCGCCCGGTTTTCTGCAAATTGCGCCGGTATCCACGATCCCACTACGGCTACCCATTGATC
GTGGGGACGACGGTTAGCGTCGGCGCGTAATTTATCCAGCGCGTCTTTAAGCGAAGGGCTGTCGTACCAGTAG
GTTTCGAATGTCCAGGTTTGTCCGCCGCGAATGGCGTGGATGTGGGTATCGGTCAGGCCGGGTATCACGGTTT
TACCCTGTAAATCGATGGTACGGGTATGGTCGCCGCGCCATTCATCTGTCGCCGTATCATCGCCAATCGCCAC
AATCCGCGAGCCGGAAATCACCAGCGCGCTGGCCTGCGGCTGGGCATCATTCAGAGTGATGATATTACCGTTA
TGCAAAATAATATCCGCTGCCGGGCGGGCGGCGGTGGCTGCGCCTGTGGCCGTGAGCAGAAAGAGAAGCGCGG
TTCTGGAAAGCAGAGGCGGGACGAGACTGATCATTAGCATGTTCCTTTTGTCGGGCGATAAGCAAACCATAGA
GAGCGAACAAGAATTGTTCAATCTGTGCCCAGGGAACAGTGCGTTCTGTTTTTAGAACGGTGGTGATAAAAAA

AGAAAAGCCCGCCGAAACGGCGGGCTTGAGAAGAATTAATGAATAATTAGAACTGGTAAACCATACCCAGCGC
GACGATATCATCAGTGCTGATACCGGCATCTTTATAGAACTGGTCGTCATCATCCAGCAGGTTGATTTTATAA
TCAACATAGGTGGAGAAGTTTTTGTTGAAATAGTAGGTAGCACCTACATCAGCATATTTAACCAGGTCTTTAT
CATCGCCATTAACGTTGTTATAGGTCAGATCTTTACCTTTAGACATCAGGAAAGAAACCGCCGGGCGCAGACC
GAAATCAAATTGATATTGGGCTGTAACTTCGAAGTTTTGCGTTTTGTTTGCTACGCCGCCAGCATAACCCGCA
TCCGTTTTACCATAAGGCGTCATATTACGGGTTTCAGAATACATAGTCGCTAAATAGATGTTGTTGGCATCAT
ATTTCAAGCCAGCTGTCCATGCATCCGCTTTGTCGCCGCCAGCAATCGTGCCGCCAGCATTGACTTGCTCATT
AGTACGATCGGAAGTGGTATACGCAGCGCCTGCGCTGAAGCCCATGCCAATATCATAGGTAGTAGAAATACCG
AAGCCATCACCGTTATCGTAACGAATATCGTCGCCATTATTACGGTTATTGGTACCGATATTTACATCGTCAG
CGCTTTGGCTTTCATTTTTACCCTGATATTGCAACGCGAAGTTCAGACCGTCTACCAGACCGAAGAAATCAGT
GTTACGGTAGGTGGCCACGCCGTTAGCACGACCGGTCATGTAGTTGTCAGCGTAAGTATAGGAGTCGCCGCCG
AACTCAGGCAGCATATCGGTCCAGCCTTCTACGTCGTACAGGACGCCGTAGTTACGACCATAGTCGAATGAGC
CGTAGTCGCCGAATTTCAGGCCGGCGAATGCCAGACGAGTCCAGGAATTAGCGCCTTCGCCTTCGGTAGTGTT
AGCCTGAACGTTATATTCCCACTGGCCGTAACCGGTCAGTTGGTCGTTAATCTGCGTTTCGCCTTTAAAGCCG
ACACGCATATAGGTCTGATCGCCATCTTTAGAAGAGTCATCAGAGAAATAGTGCAGGCCGTCTACTTTCCCGT
AAAGGTCCAGTTTGTTGCCGTCTTTGTTATAAATTTCAGCGGCGTGTGCTGCGCCAGCAGCCAGCAGAGCCGG
GATGACAAGTGCCAATACTTTTCTTTTCATTTTTTTATCCTTAAAAACTTAACTTATTTGCAAAAGATTGAAC
TTCTACAGATTCGTTGAATCAAAGGCATCCTAATCTGAATAATATTATTTCAGCAAATGCTAACTTTTCTTAT
ATGTTATTAATTAATACAAAAGTTACTATCGGAAACGCATATATTTATGGTGAATATATTTGTTATTATGTAT
TCATGTATGTGATTTGTTTTATTTCACAATTTGCGAAAAAAGATGGCAATATAGCGGCGGGCAGACATTCTGT
TATCAATTTTGTGAACTGGCTTCGAGTTTTTTTAATTTAACTTTCTTGAGCGAAAAAGACGTAATCATGAACC
ACAACGCAGAGAAAACGGTAATAAAGGGGCCGTTTCGACTAATAAAAATAGCATTCATGTATACAATGTCTGT
TTTTTGAACAGTGGCGGTGATGAGCATCGCCACTATGACTTGATAAAGGCAGGTAAAATTTGTCGGCTAACTT
ATCCCGTCGTTTCGTCCCAGGCATGAGGGGCAAAAAGATAGCCTTTATTGCGAATGGTCTTTATACGATAGGG
CTCAGCAGCGCTATCCAGCAGTTTCTTACGCAGGCGGGAAATCGCCACATCCACGCTGCGATCCAGACCATCA
TAATTGACGCCGCGCAGCGTTTTTAGCAACGCGTCGCGATCCATAATTTGTCCGGCGTGTGTCGCCAGTTCCC
AGAGCAATTCAAAATCGGCGGTGGAGAGCGATATGAAATCGCCATTGAGCTGGACCGCCCGGTTAAGGGGATC
GATGGTTAACGCGCCAAAACGCAGCGCTTTATGCGGCGTAAGCGCGCTCTCCTGAAGACTTTTTGCCTGTTGC
GTTTGTTCGCTTTGCCGCAGGTGTAAGCGCAGACGCGCCAGCAAAACGGCCGGAGGCGTGGTTTTGAGGATGT
AGTCGCAGGCGCCCATCTCCAGCGCCAGAATATGATTCATATCACTGTCCAGCGAGGTGAGCAGGACAATCGG
GCCTTGCCAGCGGTGGCGTAAATCGCGACATATCGTCATGCCATCTTTACCTGGAAGCATAATATCCAGTAAA
ACCAGATCGGGCTGAGTGGTGAGGATAAGATCCTCCGCCCGGTCACCACGCGGCTCAACAATGACATCAATAT
CATGTTTCGCCAGATAAGCGGCAATGAGAGAACCCACTTCAGCGTCGTCTTCAACAAATACAATGCGGTTCAT
ATTATAAATGGAGAATAGAAAACGCCAACATACACCGCCTCTGTTTTCCCTTCCATAAATCTTTTCTAAACGA
GAGCGGTTCCGTTATGCTACACGCTGTTGTTATTAGCGTGTTAAGGCAAGGTAATGGGACTCGTGATTAAAGC
TGCCCTGGGGGCGCTGGTCGTCGTATTGATTGGTCTGCTGTCAAAAACGAAAAATTATTATATCGCCGGATTA
ATTCCTCTGTTTCCAACGTTTGCGCTTATCGCGCACTATATCGTGGCCAGCGAGCGGGGCATCGACGCGATGC
GTACCACCATCGTCTTTAGCATGTGGTCGATCATTCCTTATTTCATTTATCTTGCAACGCTATGGTATTTCAG
CGGCGTGATGCGCCTGCCCGTCGCGCTTGGCGGTGCGGTGGTCTGCTGGGGGCTAAGCGCGTGGCTGCTGATT
TTTTGCTGGATTAAATGGCATTAATATTGGCAAAGACCGTGGTTTCCCCATCGTAATACAGACGGATATTACG
ATGGGGAGGTTGTCAACCCACCAGCATCCACGTAGCCGGAACCGCCGCGACCAGCAATAAACCAATCAGCACG
ACTTCCTGACGATTTAACACGTTGTCATGCTTGTGCGTCCGACGCGCATACAGGAAAACCAGTAATCCAGGCG
CGTACAGTACGACGGACAGCAGCAGGTGCATGGGACCCGACGCGTATAACAACCATAAGCCATAAATACAGGC
TCCGATACCGACCGCCCGATGGAGGGGACGTGTTGCAATCTTCAGCAGAAATGCGCCGACCAGAAAGTAGGGC
ACCAGAATCATTTCTGACGCAATCGTCAATAGCGTGTTGTAGTCCGAACCGGTCAGCCAGATCAGCACCAGAC
AGATTTGCACACAGATATTGGTGAGCCATAACGATGCAGAAGGCGCGCCTTGCGCATTTTGTCGCGCGAAGAT
GCCCGGGAAGGATTTATAGGCTGCCGCCAGAAACGGCACCTCTGCCGCCATGATAGTCCAGCTTAGATAAGCC
CCGCAGACAGACACAATCAGTCCCGCTGCAATAATAATTTCTCCCCACGGCCCCATCATTTTTACCATCAGTC
CGGCCATCGACGGGTTACGCATCTCCGCCAGTTCCGGACGCGCAAGAACGCCAAGAGAAAGCAGCGTGACCAG
GAGATATATTCCCAGCGCCGCGAGAACCGCCAGTAGCGTGGCGCGGCCTACATCGCGTTTATTTTTCGCTCGG
GCCGAGACGACGACGGCGCCTTCAACGCCAATAAATACCCACAGCGTAATAAGCATGGTGTTCTTGACCTGTT
CCCATACGGGAATACCGAGTTCAACACCGGTGAAGTCCAGTGTGAAGGTATCCAGTTTGAACATCATGATAGC
CAGCACAATAAACGCGCCGAGCGGCAACAGTTTCGCCAGAGTCGCGACCAGATTGATGCTGGCTGCCGTTTGT
ACGCCGCGCAGGATGAGGAAATGGACAATCCAGAGCAGGACTGAAGCGCCGACAATAGCCTGCCAGGTATTAC

CGTCGCCAAACAGACGCAGCGCAGGCGTATCGGTAAAGAAACTCAGCGCTGAAAAAACGATAACCAAATAGGA
TACGTTGGCGATGACCGCGCACAGCCAATAGCCCCAGGCAGAACAAAAGCCAATCAGTTCGCCGAAGCCTTCG
CGGGCGTAAGTAAAGATGCCGCCGTCAAGCTCGGAGCGAATGCGCGTAAGGATTAACATGGCGAATGCCAGTA
AGAGTATCCCCACGCCGGTAATGGCCCAGCCAATTAACAACGCAGCCGGACTGGCAACGGCGGCCATATTCTG
GGGCAGACTGAAAACACCTGCGCCAAGCATTGAGCTTAAAACCAGCGCGGTCAGCGCGCTCAGTCCCAGTTTT
TTTTCCATTGGTATCCAGCTATAAAAATGAAATTGGAAGAATAATTATTATGGCAAAACGCATAAAAATGGTG
AAAGCCACTAAGGCGCGGGATTTTACGGAGAGAAGGGACCGCATGCAACGGCCTGGATAAGATTTATCGCCAA
AAAAGCGAAAAGTGAGATTCATCGCGATATTTGACGGAACTGGCATCCCGCGAGAAAGTGCCGGATGGTGGCT
TATCAGGCCTGCCGTTGAACGATCGTGTAGGCCTGATAAGGCAGGTATGCCGTCATCCGGCGAGAAAGCCTGA
TGGCGGCGCGCGCCTTATTTATAGAGGTCGGCGCTGATGGTGATGTTATTGCCGCGCTCCTGCCACTGACGCG
TAATATGATAATACTTAGCGCCTTTCTTCGCCGCGCGTTTGGCGACCTGATAGGAGATTTCTGTCATATTGCC
GTAGTTGCCGGTGAACTTAACGCTGTCGAAAGGCACCATCATCGCAGCCGTCGCTTTATTCAGCTCCTCAACT
TTTGTGCCATCCGGAAGTGTGACAGTGTAACGTCCGCCTTTGGAGGATTGCGTTTCGAAGAAACGGCCTACTT
CGGCGCTCGGCGAGGCTGACGTGGCGACGCCTGGAATTTCGACTTTCTTCGCAGCTTCGCCGCCCTGAGCCAG
CGCGGCGCGGCCAGCTTCAGAGTCTGCCGGAATAGCGTCCGGGCTCTGGACGATGCGTTTTTTCGCATCTTGT
TTATAGATAAATGCAGTGATGCGCTGGTTTCCGCCCTGATTCGCATCTATCTGACGAACAATATAGAAAGCGT
ATGCGCCTTTTTGTTTCGCCGCTTTCGTAATGGCATCGTTAACTTCCGGCTGGCTACGGTAGAAGCCCTGAAC
GGTGACGGTATCATACGGTTCTAATTGTACGGCCTGGTCTTTCGGTAATTCCACGATGCCATTAATGACACGA
TTTTTGGGCGCGTCGGCTTTCGGCGCGTCGGCTTTATACACGTCCGCCACAACACGCCAGTTACCACTGTTGC
CGAACTCCGAGGTGTCGACAACATAGAAGGAGGCTGCGCCTTCTTTATCTGCGCGGCGGGACACGGCGGAGAC
CGCATCGCCAATAGCATTAAAACGACCGGTAATAACGATGCGGTCATAAGGTTTTAGTGCAGCCGCTTGCTCC
GGCGTCAGTTCTGTTGCTGCATGGACAGAAAAAGCAGCCGCAGAAAGCAGAGCCGACGCCAGGAGAGTGTTCT
TAAGCTTCATAAAAATAATCCTTCGCCTTGCGCAAACCAGGTACTGGTATTGTTATTGACTAGAAACGTGGCT
GATTATTGCATTTAAACCATATGGATGTCTGCGGGATTTTTCACGCATCGTGTCATGTATCCTATCTTTGATG
ATAACATTTGCCGATATGTCGAAAAAAACGGCGTCCGGGCGGCTAAGCTAATGAAGCGCATAAATTTAATAAG
TTAATTTAATGTTAATTTCTTGATCGCGCCAGGGGTTTAATGATGTTTTGCTGTCAAATTTGAGCGAATTATC
AGCCCTCGCAGGCCATTTTCAAGGCATTATGGCTGCTTAGCGGCTGAATCGTTCATGTTTTGCAAATTTGATA
AAAAATTCTGTTTTTTGGCGCTAGATCACAGGCGTTATTTTCAGTAGGTTATAGAGAGTTTGTAACGGTTTTA
TTCTGCTGGGGTAACATTTACCTCAAACGCGGGCAGGCGTACGCTTCTCGCGCTTACTGACAAACCATCATCA
AAAATATCGATGGAAGGGAACATTTATGCGAATTGGCATACCAAAAGAGCGGTTACCCAATGAAACCCGTGTC
GCTGCGACGCCGAAAACGGTAGAGCAGCTACTGAAGCTGGGGTTTAGCGTCGCTATCGAAAGCGGGGCGGGTC
AACTGGCAAGTTTTGACGACAAAGCGTTTGCGCAGGCTGGCGCGGATATTGTAGACGGCAATGCTATCTGGCA
GTCGGAAATTATTCTCAAGGTTAATGCGCCTGAAGAAGAGGAAATCGCTTTACTGAACCCCGGGACTACGCTG
GTCAGTTTTATCTGGCCTGCGCAAAATCCTGGGTTAATGGAGAAACTTGCCGAACGTAAGGTTACGGTGATGG
CGATGGACTCCGTGCCGCGTATTTCCCGCGCGCAATCGCTCGACGCGTTAAGCTCAATGGCGAATATCGCGGG
TTATCGCGCCATTGTAGAGGCCGCGCATGAATTTGGGCGCTTCTTTACCGGCCAAATCACTGCCGCCGGGAAA
GTGCCGCCGGCGAAGGTGATGGTGATTGGCGCGGGCGTTGCCGGTCTTGCGGCTATCGGCGCGGCAAACAGCC
TGGGGGCGATTGTCCGCGCGTTTGACACTCGTCCTGAAGTCAAAGAGCAGGTCCAGAGTATGGGGGCTGAGTT
CCTCGAACTGGATTTTAAAGAGGAAGCCGGTAGCGGCGATGGTTACGCCAAAGTGATGTCCGAGGCGTTTATC
AAAGCGGAAATGGCGCTCTTTGCCGCCCAGGCCAAAGAGGTCGATATCATTGTTACCACCGCGTTGATTCCGG
GCAAACCTGCGCCGAAGCTGATTACCCGCGATATGGTCGATTCTATGAAGGCCGGCAGCGTGATTGTCGACCT
GGCCGCGCAGAACGGCGGTAACTGCGAATATACCGTCGCTAATCAGGTTGTGACGACGGACAATGGCGTGAAA
GTGATTGGCTACACCGATCTACCGGGGCGTTTGCCGACGCAGTCATCCCAACTTTATGGCACCAACCTGGTTA
ACCTGCTGAAGCTGCTGTGCAAAGAGAAAGACGGCAATATTGACGTGGATTTTGACGATGTCGTGATCCGTGG
CGTGACGGTGATTCGTGACGGCGACATTACCTGGCCTGCGCCGCCAATCCAGGTTTCCGCGCAGCCACAGGCC
GCGCCTAAAGCAGCACCTGCGCCAAAAGAGCCGGAAAAGCCCGCTTCACCCTGGCGGAAGTATGCGTTAATGG
CGCTGGCGATCATTCTTTTTGGCTGGCTGGCCGATGTCGCGCCAAAAGAGTTCCTCGGCCATTTTACCGTGTT
TGCGCTCGCCTGCGTGGTTGGTTATTACGTGGTGTGGAACGTGTCTCATGCGCTTCATACGCCATTAATGTCG
GTGACCAACGCAATTTCAGGGATTATTGTTGTCGGGGCATTATTGCAAATTGGTCAGGGCGGCTGGGTCAGCT
TCCTGAGCTTTATCGCGGTGCTTATCGCCAGCATTAATATTTTCGGTGGCTTCACCGTGACTCAGCGTATGCT
GAAAATGTTCCGGAAAAACTAAGGGGTAACACATGTCTGGAGGATTAGTTACAGCTGCATACATTGTTGCCGC
GATCCTGTTTATTTTCAGTCTTGCCGGGCTTTCAAAGCATGAAACGTCCCGCCAGGGGAATAACTTCGGTATT
GCCGGGATGGCGATCGCGTTGCTGGCGACCATTTTCGGGCCGGATACCGGCAACGTGGCATGGATTCTGGTCG
CCATGATTATTGGCGGCGCGATTGGCATCCGCATGGCGAAAAAAGTTGAGATGACCGAGATGCCGGAGCTGGT

CGCGATTCTGCACAGTTTCGTGGGCCTGGCGGCGGTACTGGTTGGCTTTAACAGCTATCTGTACCATGACGCC
AGTCTGGCGCCGGTGCTGGTGAATATTCATCTGACCGAAGTCTTCCTCGGTATCTTTATTGGCGCAGTCACTT
TCACTGGATCGATCGCAGCGTTTGGTAAACTGCGCGGCAAAATCTCTTCTAAGCCGTTGATGTTGCCTAACCG
TCACAAAATGAACCTGGCGGCGCTGGTTGTTTCTTTCCTGTTGCTGCTGGTTTTTGTCCGCACCGAAAGCGTG
GGTCTACAGGTTCTGGCGCTGCTGCTGATGACCATTATCGCGCTGGCGTTTGGTTGGCATCTGGTCGCCTCTA
TCGGCGGGGCGGACATGCCGGTGGTTGTTTCAATGCTGAACTCCTATTCCGGTTGGGCTGCCGCGGCGGCTGG
CTTCATGTTGAGCAACGATTTGCTGATTGTGACCGGCGCGCTGGTCGGTTCGTCAGGCGCGATCCTGTCTTAC
ATTATGTGTAAAGCGATGAACCGCTCTTTCATCAGCGTTATTGCTGGTGGGTTCGGTACTGACGGCTCCTCAA
CGGGCGACGATCAGGAAGCTGGCGAACATCGCGAAATCACGGCGGAAGAGACCGCTGAGATGCTGAAAAACTC
CCACTCCGTCATTATTACGCCGGGGTACGGGATGGCGGTGGCGCAGGCGCAGTATCCGGTGGCGGAAATCACC
GAAAAACTGCGTGCGCGCGGTATCAATGTGCGTTTCGGCATTCATCCGGTGGCGGGTCGCCTGCCGGGGCATA
TGAACGTGCTGTTGGCAGAGGCGAAAGTGCCTTATGACATCGTGCTGGAAATGGACGAAATCAATGATGATTT
CGCCGATACCGATACCGTGCTGGTGATTGGCGCGAACGATACGGTGAACCCGGCGGCGCAAGACGATCCCCGC
AGCCCGATCGCCGGTATGCCGGTTCTCGAAGTGTGGAAAGCACAGAATGTCGTGGTCTTTAAGCGTTCAATGA
ATACTGGTTATGCTGGTGTGCAGAACCCGCTGTTCTTTAAAGAGAATACGCAAATGCTGTTTGGCGATGCCAA
AGCCAGCGTGGATGCAATTCTGAAAGCGCTTTAATCTTTTAAACGTGCGGCGGCCTCTTCGGAGGCCGTTTTT
ATACACTTCATCCTTTACGCTGCCTCTTTGTTGGCTGCGCTCAGAAACCCCGGTCACATCGTTATCTATGCTC
CCGGGGATTTCTTCCCTTGCCGCCGCGAGGCATCTTGAATGATTTTGTGTATGTATTGCATTTTATCTTTTGT
TTAAATCGCTAAGCAGAACGGCGATACTCTGCCCGCCGGTGGTCTGTTCGAGGGCGATTTTGACAATGATGGT
TAACGGAACGGAGAGCAGCATGCCCACCGGACCTAATAGCCAGCCCCAGAAAATTAACGACAAAAATACCACT
AAGGTTGAAAGGCCTAAACCACGTCCCATGATTCGTGGCTCAAGGATATTGCCGAAGACCAGATTAATCAGCA
GATAGCCTGCCAGCACCAGCAGCGCATCATAAAACCCGTTAAATACCAGCACCTGAATAATGGGCGGCAGGGC
AGCGAGCACCGATCCAATATTAGGAATATAGTTCAGCGCGAAGGCCAGTAGTCCCCAGACAAAGGCGAAGCGG
ACATCCAGCGCGGCCAGCATTCCCCATGCGACCAGTCCCGTCACGATACTGATCGCTGTTTTTAGCACCAGAT
AATGGGAAACGCTATCAAGCGCGCGTTGAATTGCCGCCATGCCCTCCACCGGACGGCTCATCATCTGCTGCAG
TTTTTCAGGCAATTGTGGAACCTCCAGCAGCATAAATACCACGGTCAATAACAATAAAAAGATGGACGACATG
GCATTGGACAGCTGGGTGAGCAGGTTCGTTACCAGAGTCATTGCGGCGTTTGGATCGATATATTTCGCCAGCT
CATCAACCGACACGCCAATGCCTGCTCGCTGCAACCACGGTTCGATACGTTGCAACGGTATCACCAATGAGGA
GCGATACTGCGGTAGGGTACGGGCCAGTTCATTTAATGATGTCCCCAAATAGGCCAGTAGCAGTACCATTAGC
ATGACAATAATCACAATGAGCAATGACACGGCGATAACGCGCGGAATTCGACATCGGGTCATCCGTTGCACAA
CGGGATTGAGAACGACCGCAATAAATAACGCCAAAATAAAGGGAACAATGATATCTGCGGCGAAGCGGATACC
GCTCAGAATAACCACCAGCATTCCCAGCATAATGACGATTTTTAGACCATTGAGCGTGATGATCGGTTTTGCC
ATATCAGTCCCTGTTTTCTGATGCCGACACGAATAATAATGTGATGTCGGTCGACCTGTTCTGGTTAAAATCA
AATACTTCAGGTAAAGAAGTGAAAATATTTTGAGTTAATTCCTGGCTTATGATACAAATCAGGCGTGTTCAAC
TACCGAGGACAATTATCATCCGCGATGACGAGAAGCAACACTGCGGATAATTGTAATATTATGGACAATATGT
TCAGCGCTTTTTTCTCCACGCAAACGCATCTTCACTCTACCTCCTTTTTCCTGCTTATCGGCGAGCAGCACTG
GCGTAACGCACTTTAGTCTCTCACTTCTGCCTGAGCTGGCGCAATGCGCTGTGCTAACTGATTTTTTTTTTTA
GTTTGCTGCGTGCAGCAGACTCGAAAGAGTATATTCTCATGCAGGAGAAGTGTCATGTTTTACTGGATTTTAT
TAGCTCTGGCTATCGCGACTGAAATTACCGGCACGCTCTCAATGAAATGGGCTAGCGTGGGTAATGGCAATGC
GGGTTTTATTTTAATGCTGGTGATGATCACGCTGTCATATATATTTCTTTCTTTTGCGGTAAAAAAGATTGCC
CTGGGTGTAGCGTATGCCTTATGGGAAGGGATCGGCATTTTATTTATTACCATTTTTAGCGTGCTGCTATTTG
ATGAAGCATTATCAACCATGAAGATTGCCGGCTTGCTGACGCTGGTCGCCGGCATAGTATTGATCAAATCAGG
GACGCGTAAACCCGGCAAACCGGTGAAGGAGGCGACTCGTGCAACAATTTGAATGGATACACGGCGCCTGGCT
TGGGCTGGCAATAATGCTGGAAATCGCGGCTAACGTACTGTTGAAATTTTCGGACGGCTTTCGCCGTAAATGC
TATGGCATCTTATCGCTTGCCGCTGTTCTGGCCGCCTTCAGCGCGCTTTCCCAGGCGGTAAAAGGGATAGACC
TGTCGGTGGCGTATGCGTTATGGGGCGGTTTTGGTATTGCCGCCACGCTGGCGGCGGGCTGGGTGCTCTTCGG
CCAGCGCTTAAACCCCAAAGGCTGGGTTGGCGTGATATTGCTGCTCGCCGGAATGGTCATGATCAAATTTGCC
TGAGACGATCGCTGCCTGTCGGTTCAGGCAGCGATTCCCGCATTAATCCTGCGCCAGAGCTTTCAGCTTATCG
CGAAAGCCGGTGACGGATATAGCGCGGTTATCGGCGCGCCAGCGATCTTTAGCCGCAGGGGCGGAGCTTTGTA
CGCCAATGAGCTGCCATCCGCTATCCGTGTGCAATAAGAGCGGCGAGCCGCTGTCGCCAGGTAACGTGTCGCA
CTGGTGTGAGAGTACGGCATTCTGCGCCCAGCCGGTCACGACGCAATCCTGATGGGAATAGAGCGCATTCAGA
TGATCTTCCGGATAACCGGACTGCGTGACTTTTCGGTCTGCGGCTTTCAACGCCGCAGTGAGCGCCGCTTTAT
CGCCGGTAAATAAAGGCACTGGCGTGATACCGGAAGGTGGGTAGCGCAATATTACCAGACCAAAATCCCAGCT
TGCCGCGGCAGGCGGCACAATCCAGCCATCGCCATCCGCTTTCAGGCGTCGGCCCAGCGACGGTTCAACGCGG

CCTTCAATGCCGTGGATTTCATAGCGCCATACTCCTTTTCGCGCCACAAAACGCAGCGCAACCGCCTTGTCTG
GTTTTCCTTTCGGTGGCGTTAGCAGGCAGTGACCTGCTGTCAGGGCGATATGCGGGGAGATAAGCGTTGCCGT
ACACAAATTACCGCTGGCGGTCTCTAGTTGCCCGATGGCATCCCACGGCGATTGGGTAGGATCGGCGACGGGC
ACCCGATCATCATGGTTAAAAAAAAGCGTTTTGACGTCGCTGGCATCCGTGGCGTCTGTTTCAGCCCCCTGGC
TTTCGCCATAACCGGTAAAAAACACACTATGCCCAGTAATACAGCAATGGTTTTATGCATATCACACTCTGGT
GGGTAATTATGATTATTAACAACAAACCCTCAGTACTACTATAGACGTGACGGTAAGAAAGTGGGAGTAAAAT
CAGATAACTACACTCAGGAAAGATAGAAATGCGTGGCAATAGCGGCGCATAAAATAAGCACGATAAGAATGAG
CTCAAAGCGGTAGCGAGACACTTTTTTGCCCTCCCGGAAGGCGGCGCTAAACCAGAACAATTTAGCGCCGTAA
AAACGGGCGCGGGGGCGCGCCGCAGCATGTTACGACTTACGCAGCCGGTTGAGTAGTTGGTTTTGACGGGGTT
TTGGCTGGCGCTTTACCACCTTTTTTCTGGTGCTTTTTAGCCGCCTGCGCTTTTTGCTCAACGGTTTTTTTAT
GCTGTTTTTTGTGGTGCTGGGTGTTCTGGGTTGTTTTGGCAGGCGCTGCGGTTTTCGCAGGCGTTGCAGTCTC
AGCCGCGAATGCTGCGGAAGAGAGACCCATTGCAGCGGCAACAACCAGAGCTAATACTTTTTTCATTTTGATA
CCCTCGATTTGGTTTTCATTCAACCCCACTGCGGGGCCGGTGAAATGACTATATCGCTGTTATCAAAAGGCTT
CCGTGAGTGATTGGTTTCGGCATGTAACGCTATGTACAATCCCCCGGTAACGCGATGTCAGCCCCCACCATAA
TAGGTAGCTTTACGTTACCGGCCGTTATTTTTGGCCGCAGAGTCGGTAATATCCGCCCTCCAGAATAACAGGC
ACACCGTATATGAATCCGATAATCTGGGTGGTATTTACGCTTCTGGCGCTGGATGCAATCAGAGAAGTTATGG
GCGTATCAAGCCTGACAGGTCTATGGTGAACAGTGAGTCGTGGCGTGAAACCAGCCCGGCGATAGCGCACGTT
GCTATCGCCGGAATATTTCTTTTCAGGCATGAAGACGGTGATGTAACCGCGTCCCGACCAGGATGGCCAGCAC
CAGCATTAGCGCGATAAAACCACCTACGCCGTTCCAGCCGTAACTATGCCAGAATACACCGCCTAGCGTCCCG
GCAATACTGGACCCCAGATAATAGCTGAAAAGATAAAGTGACGAGGCCTGTCCCTTCGCCCGACGCGCACGCG
GGCCAATCCAGCTACTGGCCACGGAGTGCGCGGCGAAAAAGCCGGCGGAAAAAAGCAGCATCCCAGCAAAAAT
CAGCCAAAGCGAGGTAAAAAGCGTCAGTAAAAGCCCACCCAGCATTACTGCGGTAGAGAACAGCATCACCGGC
CCCCGGCCATAGCGTGAGGTCATGGCGCCAGCTTTCGGCGAGCTCCATGTGCCGGTGAGATAGGCTACGGATA
ACAGTCCGACCACGGCCTGGCTTAATTCCCAGGGGGAGAGCATCAGGCGATAACCAATGTAGTTAAACAGGGT
GACGAACGCGCCCATCAGTAAAAAACCTTCTGCAAACAGCAGAGGTAACCCCCGATCGCGCCAGTGGAGACGG
AAATTAATAAATAGCGTTTTAGGCCGTAGCGACGTGGGACGGAAATGTTGCGAGGCGGGTAAAATCTTCCAGA
ACATCAGCGCGGACGCCAGCGCAAAGCATCCGATGGCCGCCAGCGCGATGCGCCAGTTAAAAAAGTCGGTCAT
GACGCCGCTTAATAAACGCCCGCTCATCCCGCCGATGGAATTACCGCTAATATAAAGCCCCATAGAGAAAGCG
ACAAAGCTGGGATGGATTTCTTCGCTCAGGTAGGTCATTCCCACCGCGGCGACGCCGCTTAACGACAGACCGA
TCAGCGCGCGCATAATCAGAATACCATGCCAGCTGGTCATCATGGTTGATAACAATGTGCAGCAGGAGGCTAA
TAACAAGGCGGTGACCATCACCGGTTTACGGCCTATCGCATCAGAAAGCGGGCCGGTAAAAAGTAAGCCGATA
GCCAGCATGGCGGTAGAAATAGAAAGTGAAACGCTGCTGCTGGCGGGGGAGACGCCAAACTCCTGGGAAAGTA
CCGGTAGTATAGGCTGAACGCAATAAAGGAGCGCGAAAGTCGCCAGGCCCGCGGAAAAAAGGGCCAGCGTGAC
GCGCATGAAAGGCGCTGTACCGCGTTTAATAAATTGAACCGGCTGAGAATGGCGTTGTTCATCGATATCGCTC
GCCGTGGCGTCATCGAGAATAGTTGTACGACTCACGTTAAATCCTTGCTTTACATCTCCGTCAACAGGAGACG
GGTATAACCGACATGTAAAGCATAGAAAAAGCAAAATATTCTGTCTAATATATTAATTGTCTCAAATAAGACG
TTAAAAATATGAATATCGAATTACGTCATTTACGTTACTTTGTGGCGGTAGCAGAAGAGCTGCATTTTGGTCG
CGCCGCCGCGCGCCTCAATATCTCGCAGCCGCCGCTGAGTCAGCAGATACAAATTCTGGAGCAGCAGGTCGGC
GCGCGATTACTGGCGCGGACTAACCGCAGCGTAGCGTTAACCGCCGCCGGGCGACAGTTTCTGGTGGATAGCC
GACACATCCTGAGTCTGGTAAACGACGCCGCCGCTCGCGCCGAGCGGTTGCATCAGGGCGAAGCGGGTGAAAT
CCGTATTGGCTTTACGTCTTCAGCGCCTTTTATTCGGGCGGTATCGCATACCTTTTCGCTGTTCCGCCAGTCC
TATCCGGGGGTGCATATGCAAACGCGTGAAATGAATACGCGTGAGCAAATCGCGCCGCTCAACGAAGGCGTAC
TGGATATTGGCTTACTGCGGAATACGCCGTTGCCCGACACACTTAATCGCGAGGTGATTTTGCATGAGCCGCT
TATGGCCATGATCCCGCGCGAACATCGGCTGGCGCAAAAGCCGGTCGTCAGTCTGACAGAACTGGCGGAAGAA
CCTTTCGTCTTTTTCGATCCGCATGTCGGGACGGGCCTCTACGACGATATCCTCGGCCTGATGCGGCGCTATA
ACCTGTCGCCGACCATCACGCAGGAGGTTGGCGAGGCAATGACCATTATCGGTCTGGTAGCGGCGGGACTGGG
TGTCTCGATTCTGCCGGCATCCTTTAAGCGTGTACAGATGAGCGAGATGCGCTGGGTTCCCCTGGCGGAAGAG
GACGCCGTATCTGAAATGTGGCTGGTATGGCCAAAGCATCATGAACAGAGTCATGCCGTACAGCGGTTTTGCC
AACTGCTATTACTGGCTGCCCGACGGGATTAATTACGCTTAAAAAGGGTGAAAATGTGCAGTTAATCACACGG
CTAAGCAAAAAGTTGACGGCGAGTATTGAAGTGTTTCACCATAGCACACAGATTATTTCGGAGCGCGAAAATA
AAGGGAGTGAGCGGTGGTTGCTGATAGTCAGCCTGGGCATATCGATCAAATTAAGCAGACCAATGCTGGCGCA
GTGTATCGCCTGATTGATCAGCTCGGACCGGTATCGCGAATTGACCTGTCTCGTCTGGCGCAGTTGGCGCCTG
CCAGTATTACGAAAATTGTTCGCGAAATGCTGGAAGCGCATTTGGTTCAGGAACTTGAAATTAAAGAGGCGGG
CAGTCGCGGACGTCCCGCCGTAGGGCTGATGGTGGAAACGGAAGCCTGGCACTATTTATCTATTCGTATTAGC

CGTGGCGAAATTTTCCTTGCACTGCGCGATCTTAGCAGCAAACTGGTGGTAGAAGAGTGTCTGGCGCTGCCGT
TAAACGAAGCTACGCCGTTGCTTGAGCGAATTATTACGCACGTTGATCGGTTTTTTACCCGCCATCAGCAGAA
ACTGGAGCGTCTGACCTCCATTGCCATTACGTTACCGGGCATTATCGATACCGAAAACGGCGTTGTGCACCGG
ATGCCGTATTACGAAGATGTCAAAGAGATGCCTTTGGGAGATGCGCTGGAGCGGCACACCGGCGTACCGGTTT
ACATTCAGCATGATATTAGCGCCTGGACGATGGCAGAGGCGCTTTTTGGCGCCTCACGCGGCGCGCGCGACGT
TATCCAGGTGGTGATTGATCATAATGTGGGGGCGGGCGTTATCACCGACGGTCATTTGCTTCATGCGGGTAGT
AGCAGTCTGGTAGAGATTGGGCATACCCAGGTCGATCCTTATGGTAAGCGCTGTTATTGCGGTAATCATGGCT
GTCTGGAGACCATCGCCAGCGTCGATAGCGTGCTGGAACTTACGCAGCTTCGGCTTAATCAGTCGATGAGTTC
AATGTTGCACGGCCAGCCGTTAACGGTAGATTCACTGTGTCAGGCGGCGATGCAGGGAGATCTATTAGCAAAA
GATATTATTAGCGGCGTTGGCGCGCATGTCGGACGCATTCTGGCTATCATGGTGAATTTATTTAATCCGCAAA
AAATTCTTATTGGTTCGCCGCTAAGTAAAGCGGCTGATATCCTTTTTCCAGCCATTGCTGACAGTATCCGTCA
ACAGGCGCTGCCCGCCTACAGCAGGAATACGGTTGTGGAAAGCACGCAGTTTACCAACCAGGGTACGATGGCC
GGGGCGGCGTTGGTAAAAGACGCGATGTATAACGGCTCTTTGTTGATTCGTCTATTACAGGGTTAACATTTTT
TAACTGTTGTACAAAAATTTGCGCTAACTCAAGCTGATTGCCCTTGCCATATCTTAGACTTTCTCCACTGTAT
TATTTTCCTGGCTTATATTTTCGAAGCATAACGGTGGAGTTAGTGATGCTGAAGCGTTTCTTTATTACAGGTA
CAGACACTTCTGTTGGGAAGACGGTGGTTTCCCGCGCATTACTACAAGCGTTAGCGTCGAGTGGTAAGAGCGT
TGCAGGGTATAAACCTGTCGCTAAAGGGAGCAAAGAGACTGCAGAAGGGATGCGCAACAAAGATGCGCTGGTA
TTGCAAAGCGTCTCTTCGCTGGAGTTGCCGTATGAGGCGATTAATCCGATCGCGTTAAGCGAAGAAGAAAGCA
GCGTCGCGCATAGCTGTCCTATAAATTACACCTTGCTGTCCAACGGTCTGGCGAGCCTGAGCGATAAAGTGGA
CCACGTTGTTGTGGAGGGCACTGGTGGCTGGCGTAGTTTGATGAATGATTTACGTCCATTGTCTGAATGGGTG
GTACAGGAACAGTTACCGGTATTGATGGTGGTAGGGATTCAGGAGGGGTGCATTAATCATGCGCTACTGACTG
CGCAAGCTGTCGCTAATGATGGACTGCCCTTAATTGGCTGGGTGGCGAATCGTATCAATCCTGGCCTGGCGCA
TTATGCTGAAATTATTGATGTGCTTGGGAAAAAACTGCCAGCGCCGCTGATTGGCGAGCTACCGTACCTGCCG
CGCGCCGAGCAGCGTGAGCTGGGGCAGTATATTCGTTTGTCAATGCTCGGCAGCGTGCTGGCGGTAGATAGAA
TCATGGCGTAACGTTCGCGAGAGCACTGACGCGACTACACAGGCAATCAGCAGACCGGGGAGTAACTGATACT
CGCCGGTCATTTCACAAATCATCAGGGTGGACATTATCGGCGCATGGGTTGTCGCCGCCAGTAGCGTCGCCAT
ACCCGCCAGCCCCAATAAAATCGCTATTTCATCCGAGCCTGGCAGCCAGAATCCCCATATCCGACCCAGAAAC
ATTCCAATTGATAATCCGACAAATAATGTTGGCGTAAAGACGCCGCCCGGCGCGCCAGACCCGCTGCTGGCGA
GCACCGCCAGGATTTTGCACGCAAAAATTCCGCCAATCAGCGAGAATAGCGGCGGAGAGAGCAAAAATGATTG
TACGACGCTATAGCCGTTTCCCCATACCGTGGGCGTGAGTAACGACAGTAATCCGACAATAAGCCCGCCCAAC
GCCAGTTGCCAGGGCGGGGACAGTTTTAAGCGTAAGAAACTATTATGGCTGGCCGTCATCAGCCACATCAGTA
ATGGACCGCACAGACCGGCGACCAGGCCGGTACTGACTATCATGACGTACTCCCGCGCGTGGAGATCAACGGT
CAGATGTACGGTATAGAGTAGTGAATCGCTACCGTTTAATAGATGGGTGGTTAGCAGGGCGACGACGGCGGAA
ACGACAACCGGGCCGAGGGAAGCCAGCATTAAGGTACCGAACAGGATTTCCGCGATAAACAGGCTGCCCGCCA
GCGGCGCATGGTAAGCGCCCGCCATGCCCGCAGCGGCGCCGCTGGCGATCCATAATTTCCATTCCTCACGCGG
CGTGAAGCGTCGGGCAAAGCTGGATGCCGCCAGCGCGGCGAGGAGGATCATTGCGCCTTCGCGACCAATTGCG
CTGCCGCTGACCACGACCAGCAGCGAGGCCAGCGATTTCACCAGACTGGCGCCAACGTCAAATTGCCCGTCGG
TTTGCAATGCTTCCATATAGTCAGTGGGCGCATGGGGGCGCTGTTGGTTCATTTTTTGCCACCCCCACAGCAA
CAGACCAGCGGCCAGGCCGCCAAGCGCTGGCGTAATCAGCCGTCGCCAGGGCGAAAGCCCCTCAGCCGCATTC
ACCAGACTGCCGGTGTCATTACTCAGGAAAATCCACTCCAGCAATTGCATCGCATGACGGAAGGCGGCGACAG
CCAGCGCCGCGAGAATACCGATGAGCGTAGCGATCAGCAGGCGACGAAACATCGCGCGCAGATCCGGGTAAGC
GTGTAAACGATGCATGAGCGGGTAAATAGGGAGTTTCAGGATAGCCTATTCTGCGGGGAAATGGCGTCAGACG
CAAAAGCACTATGTGCGATATAATATTGCGTACAATAATGATACTGCCGGATAGCGAGAGGCCATCCGGCGGC
AACGTTAGCTGCTTTCGTGAATATTTAGCGCCCGACGCGTGCGGCCTGAGCTGAGATAATCGGCGATGTAATC
CTGAGAGATTTCACCGTTATAGCGTCCGTCTTCATCAACGATCGGCATCCAGCTAGTATTACTTTCGTACAAT
CTGGACAGCACAATACGCAGATTATCTTCCGCTTTGCCCGTAATACGGAAAGGATGGGTGATATCGGCGCAGA
TTCCGCTGGCGTTCCGCGCTTCCCGGCGCTTCACAAATCCCAGTGGCTTACCGTCGTTGTCAATGACGGTAAT
GGCGCGAATATCGTGATCATCCATAATGCCGAATGCCTCGCTTAACGGCATGGAAGGCCGCGCGGTAATGGTT
GGCTGTTGGTCGGTAACATCGCCCGCGGAGACCAGTAGCAAACGCTTTAACGTTCTGTCCTGACCGACAAACG
ACCCGACAAACTCGTTAGCCGGTTTGGCCAGCAGTTCATCAGGACTGGCGCACTGTACAATTCGCCCCTGACG
GAACACGGCGATACGGTCGCCGAGCTTTAGCGCTTCGTCGATATCATGGCTGACCAGCATCACGGTCTTTTTT
AGCTTACGCTGCATATCCAGAAACTGGTTCTGGATCACTTCGCGGTTAATGGGGTCCACCGCGCCGAAGGGCT
CATCCATCAGCAGTACCGGCGGATCGGCGGCCAGAGCGCGGATCACGCCAATACGCTGTTGCTGTCCCCCGGA
CATCTCCTTTGGATAGCGATGCAGAAATTTTCTGGCATCCAGCGCCACCATGTCCATCAACTCTTCGGCGCGT

TGCTTACAGCGCGCTTTGTCCCAACCCAACATGCGCGGCACAACGGTAATATTTTCTTCAATCGTCATATTAG
GAAACAGGCCGATCTGCTGGATAACATACCCAATATTGCGGCGTAGCGTTACCGTATCCATATCGTTGGTATT
CTCGCCGTTGATAAGAATATTACCGCTGCTGGGCGCGATCAGCCGGTTGATCATCTTTAACGTGGTGGTTTTG
CCGCAGCCGGAGGGACCGAGTAAGACGCACATTTCGCCTTCCGGCACGTTCAGGTTGACGTTGTCGACGGCTT
TTAGCGGCTGACCCTTTTTCTGTACAAATTGTTTGGTGAGATTTTCCAGTTTTATCATTATCGTATCCCCTTA
GGTGTCAGTACGACTTGCAGACGATGCAACAGCCAGTCAAGTACAATTGCCAGTAGACAGATCATTAGCGCGC
CGGCAATTAACATGCGAATATCGCTGCCGCTAATGCCGTTTAACAGTAGCAGCCCCAGACCACCAGCGCCGAT
CACCGCGGCAATCGCCATCACGCCGATATTCATCACTACCGCGGTGCGGATACCCCCGAAAATAACCGGTAGG
GCCATTGGGATTTCGACCCAGCGCAGCCGTTGCCAGAAAGTCATACCGATGCCGCGTCCCGCTTCACGCAGAC
CGGGCGGCAAACTATCAAGCGCAGTATGGGTATTACGGACAATCGGCAGTAAAGAGTAGAGAAAAACGGCCGT
CACAGCGGGCAGAACGCCAATACCATGCCCGATCAGCGAAAAGAGCGGGATCATTAATCCAAACAGCGCGATA
GACGGAATGGTAAGAAGTAGCGTGGCGGCGCCCAATACCGGCATTGCCAGCCATTTGTGGCGTACAATCAAAA
CGCCTAACGGTACGCCGATGATAATCGCCAGACCGACGGCCAGCGCGACCAGCCAGAGATGCTGAAAGGTAAG
GCTGGCGAGATAGCCTGCATTATCCAGCATATAATGTATCGTATCCATAGGCGCTCCTTACAGCAGACCTTTG
TCCTGCAAAAATTGATGGGCTACCTGTTGCGGCGTGCGATGCTCGATATCGACCTGGGCGTTTAGGGTCGATA
TCACATCGTTATTGAGCAGGCCGGAAAGGGTGTTTAAGGCGTCATCAAGGCCAGGATTGGCTTCCAGCACCTC
TTTACGCACCACGGGCGTGACAGCGTAACTTGGAAAGAAGCCTTTATCATCTTCCAGCACTTTCAGATCAAAC
CCTTTCACCCGTCCGTCGGTGGTATAGACCAGCCCGGCGTCAACCAGCCCATCCCGAACGGCGTTATAGACCA
GCCCTGGGTCCATCTGTCGTATTTGCGGGCGATCAAGCTGCATTTGGTAGGCTTGCTGAAGGGGCTTCATCCC
ATCGCTGCGCCCGGCAAATTCCAGATCGAGACCGAGCATCCAGTTGTGGTCAGGATCGTTCTGCCGGACCTGT
TCGATTTTTGCCACCATTTGCGAGATGGTGGTGATATTTTCCGACTCGGCGCGTTTGCGCTGCATCGCGAACG
CGTAAGTATTGTTCATGTCAGCCGGTTTGAGCCATACCAGGCCCAGCTTCGCATCCAGGCGTTTTACCGTGTC
GTAGGTTTCCTGTGGACTCATGCGCTTATCGATACGGTTGAAAATAATCAGCGATGTACCGGTGTATTCCCAG
GTAATATCAATTTGTTTATTCACCATCGCATTACGTGAAATCACCGCGGCGATATTGGTTTGCGGCTGAACCT
GAAAGCCTTTCTTCTGCAAATACTGAACGGTCATAGCGGAAAGAATATGCTGCTCGGTAAAGCTTTTGGTGGC
GAGAACCAGCGGCGCGGCCTGCGTCTGGCTACTGAACAACAGCGTTGCGGCAAGCCATCCCAACAGGTGTTTT
TTGAATCTCATTTAACGCTCCTTATTGTTATTTATGCGTTCAGACGGTATGAGGGCTAAGGCGTCGCCCAAAC
CAGGCAAGCAGAGTGTCAAGAATCAGCGCGAATAACGCCGTGGCCGTTGCGCCGAGGATCAACGTCGGAAAAT
CGTTGAGATAAATTCCCGGGAAAATCAGCTCGCCGTAGCTGCTGGCGCCGATCAGAAAGGCCAGCGGCGCGGT
GCCGACATTGATGGCGGTCGCGATGCGAATACCGGACAGCATCACTGGCCACGCATTAGGTAGCTCTACCTGA
CGAAGCCGCTGCCATTTTGTCATCCCAATCCCGTTCGCCGCTTCAATCAGTGAGGCGGGAACGGAACAGAGGC
CCGCATAGGTATTGCGGACGATAGGCAGAAGGGAGGCCAGAAATAGCGCGACAATGGCGGGCGTATCGCCGAT
CCCGATAATCACCATCGCTAAGGCCAGAACGGCCAGCGGCGGCAAGGTATTACCAACATTAAAGATTTGCATG
ACATATTCAGCAAATCCTTTTGCCGCAGGTCGGCTGAGTAAAATCCCGCTCGGGATACCGACCAGTAAAGCAA
AAAACATGGACGTAAAGACCAGCATCAAATGCCGTTGCCCGAGATACAGCAGATCGACCTGACGCGCTTTCAG
CGTCTCCAGACCGATTCCCCAGATAAACAGCGCCAGTAACAGCAAAATGACGATAGTGAACCCAAGTACACGT
TTTAGGGTGAGGGTGTGCATTGCGGTGTGTCTCCCTGTTTGCATGTGTTATGGCGACGTTAAGAGTCGCGTGT
TGTTATGCCATGTTCCGGCAGGGTACTTTGAGCTATAGCAACAGGTTGGGAAGGATTCCAGCGAAGCGTGATG
TTTAACACTATTAAACATCTATTAACAATTATGCTTTGCCCTTATGCGGCTTAGCGTAAACATTCACGATAAA
GAATTATCTTAAAAACAGAAATGTAGTGTGACGTTGTCACTTTTTGTAGATAAAAATAGGCCGCAATGGGAGA
AAAGGCAGGTAAAATCCTGCCTTTTGTGGGGATTAACGGAATAACGGTTTTACAGCGACGGGAATAATAAGTT
GAGCTTGCCATTGCGCCAGAGTCAGACGCGCCAGTTGCCCCAACGCCTGATAAAACGGATGCCCGGCATGATC
GATAAATACGTCCAGAAAGCGGGACGACCACGGAAACAGATGCCAGGCCAGGAGTTGTTCGCATTCATGATGA
CGATCATTCTCGGCAAGCCAGGCCGCCAGCAGCAACAGCGACCCAAAATGATCTTCTGGCTCGTTTTGTTGCA
TTTCAAACTGAATTCCGTTTTCGCGCATCCACTGACGTAGCGCCAGCGTAGAATCGCCAAATAATACGCTCTC
ACGATCCAGCCAGACGGAACCCCACGGCGGAGACGGCAGAGCGTAAGGGCCGATAAACAGACGCTGCCAGGCC
TGTGGCAACGACTCTTCGCTGTGGGTCTTAAACATAGCCGCCACAGGCGCAAGCGCCTCGGCATCAAGCGGCC
ACTGCGCCTGCCAGTCGTCGGTTAATAGCGCCTGAACCAGAGGAGCGGTTTCGTGGCTTTCCGGCGAATAATA
AAACAACGCGCCAAGAACACGCGCCGTTACTGAAAAATCATCACGTTGTAAAAAAGTGGTCATTCAAACAATC
CCGAAAGGTGCGGGTCGCCCCGCACGCATAAATTAACCTGCGATGGCCATACCTACGGTCATATGCAGGCCAT
AAAAAAGTCCGCGGCCAATAATTTCGCCCGCCAGCACCAGCACGACACCGAGCAGCAAACCAACGGTATGCGG
TTCGCGGCGACGGATTAGCGGACATAGCCAGCAGCCTAATCCGGCGGCAAGCAAGACAATGCGCCAGACCTGT
AGCATACCGTAATCCGGCGCGAGATGGCTGGCCTGTTGCACGGAACTGTGAATGGTTGAGAGAGACAGTCCTT
GTAGTACGATGACCGCCACGCACACCACCAATGCTAGGCCGCTAATACTGGCAAACGTCACGCTGCAAAATGG

GACGCGCGCGATGCGTAGCAGTAGCGCCGCAAACACCGGGCCGCACAGGAATGCCGTGAGGAAAAAGGCCAGC
GTGGTGTAGCCATTATACCAGGTTGGCACGGTATCTATCTGATAAACGAGCGTCATTGCCCAGATGAATGCAA
CGCCGAGCGCCATACTGACCAGCAGCCACACTTTACCCAGCGCCGGGGGCATTTTACCGAGTACCGCCACCAG
CCACCAGATGCCGCCTACGGCAAAGAACACCGATCCTGCGGCGATTTCATTACTCAGTGCGGAAGCGCCTACG
CGGTTAAGCGAGTTAAACGCGCGCATCGGCGAGCCGAGATGCATAATCGACGCGAGGAATCCTAGTCCCATCA
CCAGCCACAGAAAAAACATGCTACGCACGATGCGCTGACGGGCGATGGTATCGTCTTTTGCCGTCAGCCAGCC
CAGTCCGCTAACGATTAACGCGCCGACCACGCACTGACCAAAGACCGTGAAGATCATCAACGGCCATTCATGC
CATCCACTTCCCATCTTATACCTCCTTCGGATTGGCCAGATAACCGGTAGTGTCCCCGGTCGGGCGGCTGTTG
GCGTTCGGTTTAATCACAATATTCGGCTTCGTGAAATGCGCGCGCGGCAACGGCGCGACGGCAGCCAGCTCTC
CGTGTTTTTTACGCAGTTCGTCTATCGGGCCAAAGTCCAGCGCCCGCAGCGGGCAGGACTCCACGCAAATCGG
CTTTTTGCCGTCGGCGACGCGATCATGACAGCCGTCGCACTTAGTCATATGACCTTTGGCGGCGTTGTACTGC
GGCGCGCCGTACGGGCAGGCCATGTGGCAGTAGCGGCAGCCGATACAGACCTCTTCGTTCACCACCACAAAAC
CGTCATCGCGCTTGTGCATCGCGCCGCTCGGGCAGACTTTGGTACAGGCCGGGTCTTCGCAGTGGTTGCATGA
AATGGAGAGATAATAGGCAAAGACGTTCTGGTGCCAGACGCCGTTATCTTCCTGCCAGTCGCCACCTGCGTAT
TCATATATCCGCCGGAAACTGACGTCGGGAGTGAGATCTTTGTAGTCCTTGCAGGCCAGTTCGCAGGTTTTGC
AACCGGTGCAACGACTGGAATCAATAAAAAATCCATACTGGGTTGTCATCGGTTACTCCTTACGCCTTTTCAA
CCTGAACAAGATTACTGTGTGACGGATTACCTTTCGCCAGTGGGGAAGGCCGGTGCGACGTCAGAATGTTAAT
GGAGCCGCCGCGATCGACTTTGTCGCCAAACATGTCGGCATTTAACCAGGCGCCCTGGCCGATAGCGGTGACG
CCAGGCAATATGCGCGGCGTGACTTTTGCCAGAATGAGCATCTCTCCATTCTGGTTAAAGACCCGCACGGTGT
CGCCATGCTGAATGCCGCGCGCTTGCGCATCAATCGGGTTGATCCAGATCTCCTGCGGACACGCCTGTTGCAG
GATGTCGATATTGCCGTAGCTGGAGTGGGTACGCGCTTTATAGTGGAAGCCGGTTAGCTGTAGCGGGTAGCGC
TGACGAAGCGGATCGTCCCAACCGTCAAAGCCTGGCGTATAGGCGGGTAAAGGATGGATAATCTCATCTTTCT
TCAGCTCCCACGTGTTGGCGAGGGTTGCCAGACGCTCGGAATAGATTTCGATTTTACCGGACGGCGTTTTCAG
TGGGTTGGCGGCAGGGTCTTCGCGGAATGAGCGGAAAGCGACGTAGTGCTCCTCCGGGCATTTTTTCTTGAAA
ATCCCTGTCTGTTTCATCTCTTCGTAATCCGGCATTTCCGGGTTACGCGCTTTGGTTTTTGCGTGCAGGTATT
TGACCCACTCATGCTGCGTGCGGCCTTCTGTGAAGGTCTGGTATACGTCCGGACCAAGCCGTTTAGCCACTTC
ACTGAGCGTCCAGTAAATGGGCTTTCTTTCAAATTTCGGCGAGGTGGCGGGCTGACCAAGAATGACATAGCCC
ATATTGCCTGCGGATTCGTGGGAAATCAGATCTTCCTGCTCGGTCGGCATTAAATCAGGCAGCAGAATGTCGC
AATATTTTGCCGACGCGGTCATAAAGTGCTCGATGCCGACGATCATTTCACATTTACTGTCGTCCTGAAGCAC
CTCGTGGGTGTGGGCGATGTCGCCATGCTGATTGATCAGCGTATTACTGGCGTAGCACCACAAAAATTTGATC
GGCACGTCGAGCTTGTCTTTTCCCCGCACGCCGTCGCGTGTGGCGGTCATTTCCGCGCCATGATCGATAGCGT
CGGTCCAGGTAAAGACCGAAATCTGGGTTTTTACCGGGTTCTCCAGCATAGAAAACCATTCGACGCCCAGATC
CCAGGTGCCTTCGCGCACGCCGGAATTACCGCCATTTATGCCGACGTTACCGGTGAGTACAGACAGCATGGCG
ATTGCGCGTGCGGTCTGCTCCCCGTTAGAGTGGCGTTGCGGTCCCCAGCCCTGGCAAATGTAAGCCGGTTTCG
CCGAGCCGATTTCCCGCGCCAGTTGAATGATTTTTTCCGCCGGAATACTGGTAATCTGCGCGGCCCATTGCGG
TGTTTTAGCAATACCGTCGTCGCCCTGACCCAAAATATAGGCTTTATAGTGCGCGTTACGCGGGGCCGAGGCG
GGCAGAGTGGTTTCGTCGTAGCCGATGCAGTATTTATCCAGAAACGGTTTATCAATAAGATCTTCGGTAATCA
GCACCCAGGCGATTGCCGCCGCCAGCGCGCCATCGGTACCAGGGCGAATCGGCAGCCACTCGTCTTCGCGGCC
GGCGGCAGTGTCGTTATAACGCGGGTCAATCACGATCATCCGGGCGTTGGAGCGCTCGCGCGCTTGTTCGACA
TAGTATGTCACGCCGCCGCCGCTCATACGTGTTTCCGCGGGGTTATTGCCAAACATCACCACCAGTTTTGTAT
TGGCGATATCATCCGGACTGTTGCCGTCGTTGCCGCCGAACATATAGCTCATGGCCGCGCTGATTTGGGCGGT
GCTGTAGCTACCGTAACGGCTGAGATAGCCGCCGCAGGCGTTCATCAGGCGATACGGTACGTTAGAATTGGTA
ATATTGCCGCCGTCCACGCCGGTACCGTAAAGGACATGTACCGCTTCGTTGCCGTAGTCCTTGAGGATACGTT
TCAGGTTATCGCCGATGGTATCCAACGCTTCATCCCAACTGATACGTTCGAATTTGCCTTCTCCTCGTTTACC
AACGCGTTTCATCGGATATTTCAGGCGATCGGGATGATTCATCCGGCGACGAATAGAACGGCCGCGCAGGCAG
GCGCGTACCTGATGGTTGCCATATTCATCGTTGCCCGTGGTATCTGATTCAACCCAATATACGGTGTCATCTT
TTACGTGTAGGCGCAGCAGGCAGCGGCTGCCGCAGTTAACGGTACATGAACTCCAGACCGCTTTTTCTGCCAC
ATTGCCGGATGCGAGGTCTGCTGCGGCGTGTGCGATGCGGGAGAACGGAAGGGTGAAAGCGCTACTGGCAAGC
GCAAGGCTACCGATAGCGGAGGTTTTCACCAGGCTTCGTCGGCTGATACTGGCCGCCATTAATGCCTCAGGGT
TGGTGATTTTCATAACAGCTCACTATGCTCATCCAAAAAAGAGTTTTAAATGTCGTTGTGTTCGCAAAACGGG
GAGCTCCGGGCGCGTAAAAACGTCCCGGAGCAGGCGGCTTAGATTTTTTCGATTTCCACCAGATTTGTATGTT
GAGGATTCCCTTTTGCCAGGGGGAAGGACGCAGCGTCGTGAGCGTATTGATGCAGGCGCCGTGGTCAATTTTA
TCGCCAGCCATATCCGCATTATGCCATGCGCCCTGACCCATCGCGCTGACGCCCGGCAGAATACGCGGCGTGA
CTTTGGCGGGTAGACGCACCTCTCCACGATCGTTAAAGACGCGAACCTTATCGCCATTCGCGATACCACGTTT

TTGCGCATCGAGCGGATTTATCCACACCTCCTGGCGACAGGCGGCCTGCAGAACATCAATATTGCCGTAGCTT
GAATGGGTACGGGATTTATAGTGAAAGCCAAACAGCTGTAGCGGGAAGGTGCTCCGCTTCGGATCGTCCCATC
CTTCAAAAGTAGGGGTATAAATCGGCAGTGGGCTTATCACGTCGCCTTCCGCCAGCTCCCAGGTACTGGCGAT
GTGAGCGAGTTTGCTTGAGTAGATTTCGATTTTACCGGACGGCGTTTTTAACGGATTGGCCTGCGGATCTTCA
CGAAATTTTTTATAGGCGACAAAGTGGCCGTTGGGATCTTTGCGTTTATAGATGCCCATTTTTTTCAGATCGT
CATACGCGGGCAATGCCGGATCTCTTGCCTGCATTTTGGCGTACAGATATTGCAGCCATTGCGCCTGTGTACG
GCCTTCGGTAAACCGCTGATACACATCGTCGCCGAGGCGTCTGGCGATTTCGCTGAGTACCCAGTAGATGGGC
TTGCGTTCAAACTTAGGCGTCGTCGCCGGTTGGATAAAAATCAGATATCCCATATTGCCGGCGTAATCATTGG
GGATGATATCTTCCTGTTCGACGGTCATGAGATCCGGCAGTAAAATATCGGCGTACTTCGCGGAAGAGGTCAT
AAAATTGTCGATGACCACAATCATTTCGCATTTCGTTTCATCCTGAAGAATTTCATGGGTTTTATTAATATCC
GAATGCTGATTAATCAGGGTGTTGCCAGCGTAGTTCCATAGAAATTTGATCGGGACATCCAGCTTATCTTTGC
CGCGTACGCCGTCGCGCAGGGCGGTCATTTCCGGGCCGCGCGCAATGGCGTCCGTCCAGCTAAAGCAGGAGAT
GGTGGTTTTAACGGGATTCTCCAGGACCGGCAGACGTTCAATGGTAATGGTGTAAGTGGATTCGCGTGCGCCG
CTATTACCGCCATGAATGCCGACGTTGCCGGTCAGAATCGGTAACATGGCGATGGCGCGAGCGGTTTGTTCGC
CGTTCGCCTGACGCTGCGGGCCCCAACCCTGACAGATGTAGGCCGGCTTGACGCTGCCTATCTCGCGCGCCAG
TTTGATAATCCGATCGGCGGGGATGCCGGTAATATGCGATGCCCACTGCGGCGTTTTGGCGATACCGTCCTCG
CCCTGGCCCAAAATATAAGCCTTATAATGGCCATTGGGGGGCGCGTCAGCCGGAAGGGTCTTTTCATCGTAGC
CGATGCAATAGTTATCGAGAAAAGGCTGGTCAACGAGATTTTCGTTAATCAATACCCAGGCAATTCCCGCCAC
CAGCGCGGCATCGGTGCCGGGGCGAATCGGTATCCACTCGTCTTCACGTCCGGCAGCGGTATCGGTATAGCGC
GGATCTATTACGATCATGCGCGCATTTGAGCGTTCACGCGCTTGCTCCAGAAACCAGGTAATGCCGCCGCCGC
TCATGCGTGTTTCAGCCGGATTATTGCCGAACATGACCACCAGTTTACTGTTTTCGATATCAGATGTGCTGTT
GCCGTCGTTGGAACCATAGGTGTAGGGCATGGCGCAAGAAATTTGCGCGGTACTGTATGAGCCGTATTGATTC
AACGAACCGCCGTAGCAGTTCATCAAACGCTTCACCGGCGAGGCGGCAGGCGACGAGCGGGTTATGTTGCCGC
CTACGATGCCGGATGAATAGTGGATATAAACGGCTTCATTGCCGTAGGTCTCTACGGTCTTTTTCAGGCTGGC
GCTAATGGTGTCCAGTGCTTCCTGCCAGCTAATGCGTTCAAATTTGCCTTCGCCGCGGCGGCCAACGCGTTTC
ATGGGATAGTTAAGGCGATCCGGATGGTTAATACGCCGACGGATTGAACGGCCCCGCAGGCAGGCGCGAACCT
GATGATTGCCGTAGACATCGTCGCCAGTGTTATCAGTTTCAACCCACCAGACTTCATTATCTTTTACATGCAG
ACGCAGGGCGCAGCGACTGCCGCAGTTGACCGAACAGGCGCCCCAGACGATTTTATCTTCTTCGTTGCGCATA
GCCTGCTGCACCGCCGCGGCCGCGGTACGCATCCCAAACGGTAAAGATACGCCTCCGGCAGCCAGCGCCAGTG
AGCCAAGCGCCGCTGATTTCACTAACGTCCTGCGGCTAACGCCTGTTTGTTGCTTTTCTCCGGGCATCACTCA
CTCCATTGTCGTTATTATTTCGCTATATATTGATAACTATAACGAAATTATTAACATGCTAATGATGTAATGA
GTTTACCCCTTTGGGAGTAAGGATTATTAATCCATGTCAAAGAGCGTTACCCTCCGGACCCGGAGGGCAGGAG
GAGGTTATTGCGGTTCGGCGGATGAACCGTCCTGAGCGGGAGAGGTATTGGCTGGCGCATTGCCGCTGCTGGT
GCGGGTATAAAGAATTTTATGCGTGTCGTTCGCGCAATGGCCCACCACCTGTGAGTCCGGCTGATCGGCCTGG
TCGTTAGGAACGATAGTGAGCGTAAAATTATCCGCCGGCACGCCGTTATTGATGATACGTTGTTCAATATCGC
TTTTTACTCTCTCGCACGATCCCGGTGCCGCCATCACCAGCGAGGGGGCGCTAACTAACGCCAGAGCGGCTAT
TGCAGTTGCGATTTTCATCATGAACTCCTGTGATTAATGACGATGTTCTAAGCTTAGCATTTTTCGTGTAAAT
AACTGTATTTGCTAATATGATGATAAATATCTTTTCATCCTGGTGATACGTGTGAAGAAACCCCTTTTACTGA
CGTTATTGTGCATGATACTGGCAGGTTGTGATAACCCGAAATCCCTTGAGTCTTTTACGCCGGAAATGGCCAG
TTTTTCGAACGAATTTGATTTTGATCCATTACGTGGTCCGGTAAAGGATTTCAGCCAGACATTGATGAGTGAG
AATGGCGAAGTGGCGAAGCAGGTCACCGGTACGCTTTCACAGGAAGGCTGTTTTGATACGCTTGAGTTACACG
ATCTGGAGAATAATACGGGGCTGGCACTCGTGCTGGATGCCAATTATTACCGGGATGCTCAGACGCTGGAAAA
AAAGGTGCAGTTACAGGGGAAGTGCCAGCTGGCGGCGTTGCCTTCGGCGGGCGTGACCTGGGAAACGGACGAC
AACGGTTTTGTGGTATCGGCGACGGGCAAAGAGATGAAGGTCGAGTACCGCTATGATAGCGAAGGCTATCCGT
TAGGTAAGACAACCATAAACTCGCAGAATACGCTCTCTGTGACGGCAAAACCCTCCGCGGACCCGCGTAAAAA
GCTGGATTATACGGCAGTCAGCCGGGTGGATGATCGACAGGTCGGCAACGTCACACAGAGCTGCGAATATGAT
GCGTACGCGAACCCTGTTGACTGCCGGCTTGTTATCGTCGATGAGAGCGTTAAGCCGGCAGTGTTCCACCATT
ACACCATTAAAAATCGTATCGATTACTACTAAACCGGGCGCGGTGCGACTCTCCGTGACTACCGCGCCGCGTT
TACTGCGCGGTGGGTTTCAGGAGCGTAGAACCGGACGTTTTATGTTCATCCAGATACTGCTGCTGGAAGATAC
ACATGCGGATGGTGTTACGGTATTCGCCGTTAATGAAGAACTCATGAATCAGCTCGCCTTCTACTCTAAACCC
GAGTTTACGGTAGATGTGAATCGCTTTTTCATTCTCTTTATCGACGATCAAATACAATTTATAGAGATTGAGT
ACGGTAAATCCGTAATCCATCGCCAGTTTGGCCGCGCGTGACGCCAGGCCTTTTCCCTGGTACTCCGGCGAGA
TAATAATCTGAAATTCCGCCCGCCGATGCACATGGTTAATTTCCACCAGTTCTACCAGCCCGGCGTTTTCACC
GTCGCATTCGACGACAAAGCGCCGTTCGCTTTGGTCGTGGATGTGTTTGTCGTAGAGATCCGACAGTTCGACA

AAAGCCTCATACGGCTCTTCAAACCAGTAACGCATAACGCTGGCATTATTATCAAGCTGGTGGACAAATCGTA
AATCTTCACGCTCAAGCGGGCGCAGTCTGACCTTGTAGTCACTTGTCATACTGTATCCTTACACTTCCTTTA
GTTATCCTGCATTAGGGCGTAATAACGCGACCTGTACGACGATCCAGACACCGCAGCGTATTGGGCTCCCAAT
AGGCGTTGATGTTGGCGCTTTGCTCGCATTTATCGCGGTTATCAAAGGCGGCATCGGCTTTGTCCCACTCTTT
TTCTGCGCGGGTATTCACTTTCTGGCGCAGACTGCGAGTGTCGTTCCATTGTTCCTTTTCCATAGCCGCTTCC
TGACGACTTTGTGCGCTATCTCCCGATTCAATCACCAGTTTACTGGTTTCAGCGAATGCGGAAGTAGTATATA
CAACGGCCGCCAACGTAAGCATTGCCGTCAGGCACAGGCGTTTGCTCAGCGTATTGTTCATCACAACATCCTT
TCATTAGAGGGGAAAAGCGGTAAACCACCTGTAAATTCTACACCAATCCTTATGCCTGGCATATACACAAGAA
TGGCCGGATAACCGTAACGATATTCTGTTGTATTATCACTTATCATTTCGTAACCAAATGAATCTCATGCTAA
AGACGACTCTGCTTTTCTTTGTCACCGCCCTCTGTGAAATCATTGGATGTTTTCTTCCCTGGCTATGGCTTAA
GCGCGGCGCCAGCGTGTGGTGGCTTCTGCCCGCCGCTGCGTCACTTGCGTTATTCGTCTGGCTTTTGACCTTG
CATCCGGCGGCGAGTGGGCGGGTTTATGCGGCTTACGGTGGGGTATATGTCTGTACTGCGCTCCTGTGGTTAC
GTGTTGTCGATGGCGTCCGGCTCACGGTTTATGACTGGTGCGGCGCGCTGATCGCGCTGTGTGGAATGCTGAT
TATCGTCGTCGGATGGGGACGTACGTAACATTGCCGCGCGACCAGAACATTTTGTGATCTGGCAGCGCTTTTT
ACATCAATATACTTGTATGGTAGTAGCTCAGTTGGGTAAATTTCCTGCATCCCATAATGCGATGTAAGGAATA
AAAATGAAGATTGTAGGGGCTGAAGTTTTTGTTACGTGTCCAGGGCGTAACTTCGTCACCTTAAAGATAACCA
CGGAAGACGGGATCACCGGTCTGGGGGATGCCACCCTGAACGGACGCGAGCTCCCGGTGGCCTCGTACCTTAC
CGATCACCTGTGCCCACAGCTCATCGGGCGGGATGCTCGCCGCATCGAAGATATTTGGCAGTTCTTCTACAAA
GGCGCGTACTGGCGCCGTGGCCCTGTCACCATGTCCGCCATTTCCGCGGTTGATATGGCGCTATGGGATATTA
AAGCCAAAGCCGCCAATATGCCGTTGTATCAGTTACTCGGCGGCGCTTCGCGTGAAGGCGTCATGGTGTATTG
CCATACCACAGGCCACACCATCGACGATGTGCTGGAAGACTACGCGCGTCATAAAGAGCAAGGGTTCAAAGCA
ATCCGCGTGCAGTGCGGCGTTCCGGGGATGAAAACCACCTACGGCATGGCGAAAGGCAAGGGGCTGGCCTATG
AGCCCGCGACAAAAGGCCAGTGGCCGGAAGAACAATTGTGGTCAACGGAAAAGTATCTCGATTTTACGCCAAA
GCTGTTTGACGCCGTGCGTAATACGTTCGGGTTTAATGAACATCTTCTGCACGATATGCATCACCGACTGACG
CCTATTGAGGCTGCCCGCTTTGGCAAAGGTATTGAAGACTATCGTTTGTTCTGGATGGAAGATCCGACGCCGG
CTGAAAATCAGGCATGCTTCCGCCTCATCCGCCAGCATACGGTCACGCCGATTGCGGTAGGCGAAGTCTTTAA
CAGTATCTGGGATTGCAAACAGCTCATTGAAGAGCAATTGATTGACTATATCCGCACCACGCTTACCCATGCC
GGTGGTATTACCGGGATGCGCCGCATTGCCGATTTTGCCTCGCTCTACCAGGTGCGTACCGGTTCGCATGGGC
CGTCCGATCTGTCGCCGGTTTGTATGGCCGCTGCATTACATTTCGACCTGTGGGTGCCTAACTTTGGCGTGCA
GGAGTTTATGGGCTACTCCGAACAGATGCTGGAGGTTTTCCCGCATAACTGGACGTTCGAGGGCGGCTATATG
CATCCGGGCGACAAGCCAGGACTGGGCATTGAGTTCGATGAAAAACTGGCGGCGAAATATCCGTACGATCCAG
CCTATTTGCCGGTGGCCCGCCTGGAAGACGGCACACTGTGGAACTGGTAAGGAGCGAAAAATGAAAAGCATCG
TAATCGAAAAACCGAATACATTAACTATCGAAACGCGCGCGCTTCCGCAGCCTGCGCCGGGCGAAGTTCGCAT
TAAAGTGAAACTGGCGGGTATCTGCGGTTCCGATAGCCATATTTATCGGGGACATAATCCCTTTGCAAAATAT
CCCCGTGTCATAGGCCATGAATTTTTCGGTGTCATCGACGCGGTAGGCGACAACATCAATCGCGACCGGATAG
GAGAACGGGTATCCGTCGATCCGGTGATCAGTTGCGGGCATTGTTACCCTTGTTCAGTCGGTAAACCCAATGT
GTGTACCTCGCTGGTGGTGCTGGGCGTGCATCGTGACGGCGGTTTCAGCGAGTATGCGGTGGCGCCGGCCCGT
AATGCTCATCGCATACCGGACAACATCGCAGATCATCATGCCGTCATGGTTGAGCCGTTTACCATTGCGGCAA
ATGTGACGGGACAAGTGAATCCGACCGAGCAGGACGTGGCGCTTATCTATGGCGCAGGCCCGATGGGACTGAC
GACCGTACAGGCGCTGAAGGGGGTTTATCAGGTTAAAACGGTCATAGTCGTTGATCGCATTGAAGAGCGTTTA
GCGATGGCGAAGCAGAGCGGCGCTGACTGGACCCTCAATAATGGTCAACATTCACTGGCGGAATTTCTACAGC
AGAAGGCGCTAAAGCCGACCTTGATTGTCGATGCCGCCTGTCATCCCGCTATCCTGCAAGAGGCGATTACACT
TGCATCGTCTGCGGCACGCATTGTCCTGATGGGATTTTCCAGCGACCCGTGTGAGATTGTACAGCAGGGTATT
ACCGGAAAAGAGCTGGCTATTTATTCATCGCGTCTTAATGCCAATAAATTCCCGGTCGTGATTGACTGGCTGA
ATAAAGGGCTTATTGATCCGGATAAGCTGATAACGCATACATTTGATTATCAACATGTCACCGACGCGATTGA
GCTTTTCGAGAAAGACCAGCGTCAATGCTGCAAAGTCTTACTCACGTTTGCGAAATAATATAAATAATTGCGA
GTAAGTGGTACGCATCTTACCTCTTAGAGATTGCCTATATGACACAAATAAAGCATGAACGATCGACTCAGGA
TTTAGTGCGCGCTGCCGTATCTGGATGGTTAGGCACCGCGCTTGAATTTATGGATTTTCAGTTATATTCACTA
GGCGCGGCTCTGGTCTTCCATGAGATATTTTTTCCGGAACAGTCTGCCGCGATGGCGCTTATTCTGGCAATGG
GCACCTATGGTGCAGGTTATATAGCCAGAATTATCGGCGCCTTTGTTTTTGGTAAAATGGGCGACCGAATCGG
ACGAAAAAAAGTATTGTTTATTACCATCACCATGATGGGGATCTGCACCACGCTGATTGGCGTACTGCCGACG
TATGCGCAAATCGGCATTTTTGCGCCAGTATTACTGGTGACATTGCGAATTATACAGGGGCTGGGCGCCGGGG
CGGAAATTTCCGGCGCGGGTACTATGCTGGCCGAGTATGCGCCGAAAGGAAAGCGTGGTATCATTTCCTCGCT
GGTCGCGATGGGCACCAACTGCGGTACGTTGAGCGCGACGGCGATCTGGGCGGTGATGTTCTTTGCGCTTGAA

CGCGAACAACTGATCGCCTGGGGATGGCGTATTCCTTTCCTGGCGAGCGTCGTTGTTATGATATTCGCGATCT
GGCTGCGAATGAATCTGAAAGAAAGTCCGGTATTCGAAAAAGTAAGCGAAGGCGAAAAATCGCCAGCGTTAAC
GCCTGCGTCAGAAAATACATTGGGCGCGATGTTTACCAGCAAATCTTTCTGGCTGGCTACCGGTTTACGTTTT
GGGCAGGCGGGCAATTCCGGGCTTATTCAAACCTTCCTTGCCGGATATTTAGTTCAGACGTTATTATTTAACA
AATCTATCCCAACCGATGCGTTAATGATAAGTTCCGTAATTGGTTTTATCACTATCCCACTCTTGGGGTGGTT
GTCCGATAAATACGGTCGTCGGCTGCCGTATATAATATTGAATATCTCCGCGATTATTCTCGCCTGGCCGATG
CTGTCTATTGTGGTAGATAAAACGTATAGCCCTGGCGTAATTATGGCGGCGCTCATTGTTATTCATAACTTTG
CCGTGCTGGGATTATTTGCGCTGGAAAACATCACCATGGCGGAAATTTTCGGATCGCGTAATCGCTTCACCCG
GATGGCGATTTCAAAAGAGGCAGGGGGCTGGTGGCGGTCGGTTTTGGCCCCGTCCTCGCAGGGATCTTTTGTA
ACATGACCGATTCCTGGCTGCCCATCTTAATTATGCTGGTGCTCTACTCGTGTATTGGTCTGATTTCTGCGTT
GTTGATGCCGGAGGTCAGAGATCGCGATCTGAGCTTGCCTGAGGATGCGGCGGAAGCGACAGCGGCAGAGAAG
TTACGCCACTCCGCAACCCAGACGAGTTAAATCGTGTCGACGGTATGGCCGGAACGCTAATGCGGTTTCGGCC
ATGCTGCCCGGTCACTTTTGTTTAATTAGCGTAAATCCAAATGTACCAGGGCAGGCGTTACTCTGCGACTGTT
CCGCTTTCAGAATATAGAGGTTATAAAATGCAAAACAGGCTTTTATCGGCAAAGGCGACGCTTCCTGATTACG
ATCGTGCGGCGCTGGTGGCGCGGATGGTCCATCTCGGTTTTGGCGCGTTTCATCGCGCGCATCAGGGGGTTTA
TACAGATATTCTGGCTGCGGAGCAGCATAGCGACTGGGGCTATTATGAGGTTAATCTTATTGGCGGCGAACAG
CAGATTGCCGATCTAAAGCAGCAGGATAACCTCTATACCGTGGCGGAGATGTCGGCGGAAGCCTGGACGGCGC
GTGTGGTGGGCGTCGTCAAAGCGGCGTTGCATGTTCAGGTTGATGGTCTGGAACGTGTGCTGGCGGCGATGTG
CGAACCGCAAATAGCGATTGTGTCTCTGACCATTACCGAGAAAGGGTATTGTCATTCGCCAGCCACGGGCCAA
CTTCTGCTGGAACATCCCATGATTGCCGCCGACCTGCAAAATCCGCATCAACCGCTAACGGCACCGGGTATTA
TTGTCGAAGCGTTAGCGCGTCGTAAAGCCGCAGGCTTACCGGCATTTACCGTGATGTCCTGCGACAACATGCC
GGAAAATGGGCATGTGACGCGTCAGGTTGTGACGGCTTACGCCCGGGAGGTGGATGCGGAACTGGCGATATGG
ATCGAGCAAAACGTGACATTTCCGTCAACCATGGTTGATCGGATTGTCCCTGCGGTCACGCCGGAGACGCTGG
ATAAAATCGAACAACTGACCGGCGTGCGCGATCCGGCGGGCGTTGCCTGTGAACCGTTTCGCCAGTGGGTGAT
AGAGGATACGTTTGTGGCGGGTCGTCCGCTGTGGGAAAACGCGGGCGCGACGTTGGTCGCCGACGTGGTTCCG
TTTGAAGAGATGAAACTGCGAATGCTTAACGGCAGTCATTCATTCCTGGCTTATCTGGGGTATCTTGCCGGCT
ATCAGCATATTAATGACTGCATGGCTGACGACAACTATCGTCTGACCGCGCAGGCGTTAATGTTACGCGAGCA
GGCGCCCACGCTGAAAGTCCAGGGGGTAGATTTACAGCGTTATGCCGATCAGTTGATTGCGCGTTACCGTAAT
CCGGCGCTGCGTCATCGTACCTGGCAGATTGCGATGGATGGCAGCCAGAAACTTCCGCAGCGTATGCTGGACT
CCGTTCGCTGGCATCTTGCTAATCATAGCGATTTTGATTTGCTGGCGCTGGGCGTGGCGGGGTGGATGCGTTA
CGTTGGCGGCGTGGATGAGCAGGGTAAGGCGATCGACGTCAGCGATCCGCTATTGCCGGTTATTCAGCGCGCC
GTGGCAAACAGTGAAGAAGGCGCCAGTCGTGTTAAGGCGCTGCTGGGAATGGCTGAAATTTTCGGCAACGATT
TGCCGCAGGCGGAGCGGTTTACGCAAAAAGTGCAAGAAGCCTATGACAGTTTACTGACATATGGCGCGAAAGC
GAGCGTGGCGAAATATGCCGAGCGCTTGAAATAGCGTAACAGAGACCCGGTAGCATTATGCTACCGGGAAGGC
CGGAACGGTTCCTGGCGGCCTGTGGTATCAGTTCATGCCAAGACGAATCAATTCAACCGGCGCGAATTTACCT
TCACAACCTTCGACAATCACCGTTTTGCCGCGGCGAATTTGACCTGCATCCAGACCTTCGCTCTCAATGGCTT
TGATAATCCCGGTATGACCCGTGCCGCTTATCATGACCCGGCTACCGGTAGTGATTGCGTTACGGTTACGATC
GTATGTCATCATCGTGTTTTCTCCTTTCAAACTTACTGCCAAAGCCCGCAGGTTTCCCCGCATGACGGGCGAC
ATATAAATACGCTTATCGTTGCGGATTTTTTTTGTTTTTGATCAAGCTCACGGCTTTTTAGGCGGGAATGTGC
AGGGTGAGAAAGCGGAGAAGGCGTCTCACCCCGACGTTAAAGGGGGTTAGTCTTCGCTAAACCAGCCGCTGTT
TTCCTGACGGATAAGCTGGACGGATTCGCTAATTTCCTGCAAATGCTGGGTCATTGCGCTCTCAACCGCATTG
CCGTCACGATTTTCCAGCGCGGAGAAAATATCATGGTGCTGGCGCAACAACATCTCCGGCGGAGAAACATGAT
CCAGACTCATATAGCGAACCCGGTCGATTGTCGCTTTGATATTTTCAATGGTATCCCACGCTAACTGGCAATC
GGCGATCTGCGCCAGCTTTTGGTGGAAATCGTCATCAAGCTGGAAAAAATCATCAATCTGTTTGCGCTCGATA
GCAATATGTTGCAGGTGCAGGTTTTGCGCCAGTTGATAGCACTGGTTGTCGGTGATCATCGCCGCCGCACGGC
GGACCACCGCGCATTCGATCGCCTGCCGAACAAAGCAGCCATTCTGTACCTGAGAAAGCGAAATCTTATTCAC
ATAACTACCGCGCTGAGGGCGGATCTGAATCAACCCATTTTCCGCCAGCTTTATAAAAGCCTCGCGCACCGGT
TGACGCGAAACGTTAAAACGGGTTGAGACCTCTTTTTCTGATAGCGGCGTGCCAGGCGGGATAAGACAATGCA
CGATATCACGCCGAAGAATACGGTAAATTTGCTGGTTGACAGGCTGTGTAGGGTTAAGCTGCGTTTCGACGGT
CATTCATTCTTACTTTTTAGGCGGTTGACCCGGATACTCTACCACTTTTCCGTGGCGGTCTATACCGATCATG
TTTCTGGTTGCAGATACGTTTGCCAGGGATTACCCGGCCCGCAGACCGGGTATAACCTCTTAGCTACGGTGGA
CGCTGAGCCCTGCGAAAGATTGGGTAACAGGCATCATTTCAACGGTATTAATATTCACATGCGCGGGGAGTGT
GGCCACCCACCATACCGCTTCGGTAATGTCTTCTGGCGTGAGCGCGGTGGTATTTTCGTAGGTTTTACCCGCT
TTCTCATCGTCGCCTTTGAAACGAACGCTGGAAAACTCGGTACCGCCAACCAGGCCCGGTTCAATGTCGGTCA

CGCGAACCGCCGTGCCGTGTAAATCCGTGCGCAAATTCAGGCTAAACTGGCGCACAAAGGCTTTCGTCGCGCC
ATAGACGTTGCCACCAGCATAAGGCCAACTGCCGGCGGTTGAACCGATGTTAATGATATGACCGCGATTACGT
TCGACCATTCCTGGCAGCACGGCGCGGGTCATATAAATTAAGCCTTTATTATTAGTATCGATCATAGTTTCCC
AGTCTTCTACGCTGGCTTTATGCGCCGGTTCAAGACCCAGCGCCAGACCGGCGTTATTGACCAGCACATCAAT
ATCGCGCCACTGCGCAGGCAGAGAGGCCATCATCTCTTCGATGGCCGCGCGGTTGCGCACGTCCAGTTGGGCG
GTCAATACGTTCTCGCCAAGCTCATCTTTTAACGCTTGCAGGCGTTCATGACGACGTCCCGTGGCAATGACTT
TATGTCCATTCTCAACAAAACGGCGTGCAATGCACTCGCCAAACCCTGCGGTTGCTCCAGTTACTAAAACGAT
CATCACACAGTTCCTCAACGCTTTTTGTGTTGTATTACCATAGCACGCGGCGTTTATGAAAGGGTAATCCTGG
TTAACACGGTTGCGGTCAGCATAGGGGGAAGCTTACTCTTATGATCAACCTATTTTCAGGAGTGAAAAATGTC
GACGAATCCTTTGTTAGACCAGAGTATGTTGCCCTATCAGGCACCGCGTTTTGATCGGATCAAAGATTGCCAT
TATCGTCCTGCTTTTGATGAGGGCGTTCGGCAAAAACGCGTGGAAATCGAGGCCATCGTCAATCATCCGGCGG
CCCCTGACTTTACGAATACGCTTCTGGCGCTGGAGCAAAGCGGGGCGCTTCTGTCACGCGTCACCAGCATTTT
TTTCGCGATGACGGCCGCGCACACTAACGATGAACTCCAGCGGTTGGATGAGGCCTTTTCTGCCGAGCTGGCG
GCGCTCTCCAACGATATTTATCTGAATAGCGCGTTATTCGCTCGCGTGGATGCCGTCTGGCAACAGCGTCACT
CACTGGGGCTGGATGATGAGTCGCTACGGTTGGTCGATGTGATCCATCAGCGTTTTGTGTTGGCAGGCGCGCA
GCTTGCCGAAGAGGATAAAGCGCAACTGAAGGTATTGAATACTGAATCCGCGACCTTAATGAGTCAGTTTAAT
CAGCGTCTGCTGGCGGCAAGTAAAGCGGGCGGGCTGGCGGTCGATGACGCGCATTGCCTGGCAGGATTAAGCC
CGGAAGAAATGACCGTCGCTGCTGAAGCGGCGCGTGAAAAAGGCCTGGAGGAGCGCTGGTTCATTCCGCTCCT
TAATACGACGCAACAGCCTGCGCTTGCTACGCTGCGCGATCGCCAGACTCGCGAAAATTTATTCGCAGCGTCA
TGGACTCGGGCGGAAAAAGGAGATGCCCACGATACCCGCGCTATCGTTCAGCGTCTGGTAGAGATTCGTCGCT
GTCAGGCAAAACTGCTGGGTTTCCCCAATTATGCCGCATGGAAAATGGCCGATCAGATGGCGAAAACGCCGCA
AGCCGCACTGAGCTTTATGCGTGGCATTGTGCCGCCAGCGCGTCAGCGTGTACTCAATGAACAGGCGGAAATT
CAGAACGTCATTGATGGTGAGCAGGGCGGCTACACCGTTCAGGCCTGGGACTGGATGTTCTATGCCGAACAGG
TACGGCGGGAAAAATATGCGTTAGATGAAGCGCAACTGAAGCCCTATTTTGCCTTAAATACGGTGTTGCAAGA
GGGGGTTTTCTGGACCGCCAACCAGCTATTCGGCATCACCTTCGTCGAGCGTTTTGACATTCCGGTGTATCAC
CCTGATGTTCGGGTGTGGGAGATTTTCGATTCCGATGGTGTCGGCATGGCGTTATTTTATGGCGACTTCTTCG
CGCGGGACTCGAAAAGCGGCGGCGCGTGGATGGGGAATTTTGTCGAGCAATCCACACTCAATGAAACCCGGCC
CGTTATATACAATGTATGTAACTATCAGAAACCGGTTGATGGGCAGCCTGCATTACTGCTTTGGGACGACGTT
ATTACGCTCTTCCATGAGTTTGGTCATACGTTACATGGTTTGTTTGCCGTTCAGCGCTATGCCACGCTTTCAG
GTACCAATACGCCCCGCGACTTTGTCGAGTTTCCCTCCCAGATTAATGAGCATTGGGCGAGCCATCCACGCGT
GTTCGAACGCTACGCGCGTCATGTCGACAGCGGTGAAAAAATGCCCGCTGATTTACAGGAAAAAATGCGCAAG
GCGAGTTTATTTAATAAAGGTTACGATATGACTGAATTGCTCGGCGCCGCATTGCTGGATATGCGCTGGCATA
TGCTGGAGGAGAGCGTAGCAGAGCAGTCTGTCGCTGAGTTCGAGCAGCAGGCGCTGGCCGCCGAGCATCTTGA
TTTACCGGCAGTGCCGCCCCGCTATCGCAGCAGTTATTTTGCCCATATCTTCGGCGGCGGCTACGCAGCGGGA
TACTACGCCTACCTGTGGACGCAAATGTTAGCGGATGACGGTTATCAATGGTTTGTAGAGCAGGGCGGCCTGA
CGCGTGAAAACGGGCAGCGTTTTCGTGATGCTATCCTTTCCCGGGGGAATAGTACTGATTTAGAAACGCTTTA
TTCAGCCTGGCGTGGACATGAACCGCATATTGACCCCATGTTGCAATATCGTGGGCTCGATCGCTAAGCTGAT
ACGGTTGTTCGGCACTGTCGAACAACCGCCTGAAAATGAATCCTGCACTTCTGATGACAGCCAGTTAAATTTA
TGGGGTTAAATTGTCTGAATATAAAGAGGGCGTGCATTCGGATTTTTCTACTTATTTTCCCGTGGTGGCTTGA
TTGCCAAAGATTTATTACCGCTTCGCTGCTGATGTTTTAAAAAATATATTTTCCGCCATGTGATGGTATTATG
TAAATAATGCAGAAATGAATTTGACACTGCGCACAGGGCGACTAGATTTAGAACTGTATCACATGATATGAGA
AGACATATCATATTTAAAACGCAACATCATCATGAGGATTATATTATGGCAGAACATCGCGGCGGTTCCGGTA
ATTTTGCAGAAGATAAAGAGAAAGCCTCAGAAGCAGGTCGTAAAGGCGGCCAGCATAGCGGTGGGAACTTTAA
AAACGACCCACAACGTGCATCTGAAGCGGGTAAAAAAGGTGGCCAGAATAACCACAGTGGCGGACGGAAATCC
GGCAATTAATTTTACTCCTGATATTTCGTAAATATATTCTGAAATATTGCACATACGATGCGGGCCATAGGCT
CGCATTTTTTTATTCTGACCGGGAGTTGTTCTGACCGGTCAGATGAAATTATTGTTTATGCTTTCTTCGTTAA
CAGAAAAAGCAATTCAGCGAGGGCGAAGTGTACGCATTTTTCTAATACTTCCTGACAATCTCCGTTGAAATGT
TGTCGGCTTGTAAAAGTTGTATTGTTAATATTCCAGGCAAACCAAACGGTGCCCGCCGGCGTGCCATCTTCGC
CGCCTTCCGGACCGCCATAGCCGCTGATCGCTATGCTGATATTGACCTCAGCCTGATCCTTAGCGCCCTGTGC
CATTTCAGTAACGACCGCTTCGCTGACGGCGGTATGTTCGGCAAGGCTGTGGCGTTGTACGCGTAAGATTTTT
GCTTTGGCTTCATCAGTAAAGGTGACATACCCCACACCGTAAAATGAGGGCGTATCTTCTGCTGCGCACAATG
CTGAAGCCAGTTTCCCGCCAGTGCAGGATTCTGCAGTGGTCAGACGGAGTTTCTGGTTAACCAGTAAATCACC
AAGTTTTTTTGTCAGGCTATCGACGGATTCGGCTTCATCAAGGGTGACAATATCGTGCTGTTCTAATCTCATC
GTTTATTTTTCCGTTACCAGAGACAAAAGAAATCTGTTCGTTCTTCTCAAGTATGGCATATTTATTTTATGAC

ATTGTCAGTGAATGATTATTTTATCCAGTTACCAACAAACCCGCTGTTGCATAACTTGCTTACTTTCTTACGG
CGATGACTGTCATTGATTATGCGTAAGGCCATTATCCTCAAAAAATAAAGCCGATTAATCTGAATTGCTATCA
AAACGTTGATGCATATGACTTCAGCTATGTGGCAAGTTCTCTATTACGCCGTTACGCTTTCGCGCTCACCGGC
GCTGATGTCGGTTGCATATTAATCCGGATGGGTTGATTTTATTGCTTAATATTCTGCGGGTAAATTCATGTTT
GTAAAAACTAAAAATAGCGGCCAGACTTAATACGTCAAATAGACGTTATTAAGGAGAGAGCATGAAACTACAG
GTGGCTGCATTTTTAAGTTTTCTGATAATGCCTTACGCGCTAGCGGAAGATCAGGGGGATTAAAAAAAGACGT
GGCACCGCCGCCGCCTCATGCTATTGAGGATGGTTATCGCGGTACGGATGATGCAGAAAAAATGACCATCGAA
CAGGCAAAAACCCTGCATGATGGCGCGACCGTTTCGCTGCGCGGCAACCTTATTGATCATAAAGGTGACGATC
GCTATGTATTTCGTGATAAATCCGGCGAGATTAACGTGATAATTCCTTCCGCCGTATTTGATGGGCGGGAAGT
TCAGCCGGATCAAATGATCAACATTAACGGTAGTCTGGACAAGAAAGCGAAGGAACCGCTTGTCCGCGTGAAC
CGACTGCAAAAATAACGCGCGCTAACCGGCAATCTCCAGGTCAGCCATACGGGGCACGCGGCTGGCCTGATAC
CATGGGTCTTGTAAAAGCGCCTGGGGGACAAAAGGTCCCATAATGTCTTATTCAGGTCTGGTTGCGTATCGTT
TAAGCTTCATTGATATTGACGGTGCTACAGTCGTTAGCGATCCGTTATCCTCGCAAGAATTTGCCCGGTAGCG
TATAAGCAAAACACAAATCTATCCATGCAAGCATTTACCGCTGGTGAACCGGCGGTTTTTTTTTGCCTGCCAT
CCAGACAAAAGCGGCCCCTTGCGCAACGATGCAGCAAGGGGCCGGCTCTGCGAGGGATTATCTCCGTTCATTA
ATCATCCCATTTATGACTGAACCAGGCGGCCAGAAATCCGGAAAATAAAATAATTCCTAATACAACGATAAAA
AGGTTAATACTGCCTAACATCTCGTTTCTCCTTATTTTAGCGTGCATCCTCTTTTTTAAAAAACCTCGTACAG
ACTATAGTACTAATTTATACTTAAGCAAAGTCTGAATTGCGTATTTATTTTACTGGAAGGGTAAAGCATTAGA
ATATTTTCGTCAGCGCCCAGGAATATTCCTGAATTACCGCAGTGACAGCAGATAATTAGCCTGCAATATATAG
GGTTAACCCGGTGCGCCAGAAACGGCGCACATGACTGGCTGCAGATAATTAACAGGTGATAGCCTGTTGCGTA
GGGGTAATCCGCATTCCTTTTAACATCAGTGCCCATGCGAACACAATGGCGATTATCAGGACAACGAAAAGCG
ACCAGGCGGGAAGCGTCGTCAGGATGACGCCGCTGACCAGCGGGTTTACCGCCGCACCCAGCCAGCCCAGCGA
TTGCGCTGAAAAATAGCTGGCTTTCATCCCGGCAGGCGCAATGTTGTCAATTAACATATATTCGCCGGGGGCA
TAAATAATTTCGCCAATCGTGAATACTGCCGCTGAAAGCCCCCAAAAAAACAGATTGTCGCCTGAAAAAATAA
ATCCGATGAGCCCGGCGATAAAAAAGACGGTGCCGGTCGTCATCATTGGTCGAATATTGACGGCGGTAAGTCG
ACGGCCCACGGCGTATTGCAAACTGACCACAATAACCGCATTAACCGGCAGGACGACGGCTACCACTTTTTCC
GCGAAGCCGCTATCGGCAACGACCATAACGTACTGTGAAATACAGGACGCAAAGGCCCCCCACAAAGGAGGCC
AGAAATGCCGAAAGCGTAAACCACAATAATGCTTTGTCGCGCAGCAGAACGGAGGGAGACCAGATAGCGGCAT
TTTTCCCCTCGCTGGCGGCAACCGATCGTGTCACCCATACCTGAATAAAGACCAGCGGGAATGCGGAACAGAT
GGCGGCAAGCCAGAAGGGTAAATTGATGCTTTGCATCACCAGCAGCGTGCCGAGCGGCGGGCCAACCGTCCAG
CCGATATTGAGTACGGTATAATTGAGCGAAAAAATCCGTGTTTTTGTTGTCGCGGTGAGGTTATCGGCGAACC
AGGCCTTCAGGACGGTAGAAAAAACCGAATAGGCGCAGTTAATAAGCGCAAAAAGCAGGACAACCAGTACGAC
ATTATGCACCATTGGGATGGCGATAAATCCGCAGGCGAAGGCGATAATCGCGAGTAACATGTAGCGCTTTTTA
TCAAACTTGTCCGCCAGAATCCCAAAGCCAAGACTAAAGATGACGCCGATCGTTAACGCGCTGGTCAGCGCGT
AGCCAATCAGATCCACGCCTAAACCGTACTGACGGTTAAGATAGATGGTCATAAAGGGGAGCGTGGCACCGCG
TCCAATTGTCAATAACAGCGATGAGGCGAGTAGAGCCAACGTCGAACGTCTCAATCCTGGATTCATTTTTCCT
GCCTGAAATGGAGGTTACGTATCGGGACGTTTTAATCTACCGTGCCCGCAAAAGCATGATGTCTATACGCTAT
AGCGTAGTCTTAACATACCATAACGCAGGATTTTGCCCATCGTGTGTTGTTCAATGCCATGCTCACGGTAAAA
AGAAAAGTCCTGTTATGGCGCACTGATTTCCGATAACTTACCTTTTTACAAAATTTTAATAATGACAGGGGCC
GATGATGTCGCGTAAAGACGGTTTTTTGGCGTTACTGGTGGTTGTCGTATGGGGGCTCAATTTTGTTGTCATC
AAAGTAGGCCTGCACCATATGCCGCCATTATTGCTGGCAGGATTACGATTTTTACTGGTGGCGTTTCCGGCAA
TTTTCTTTGTCGCCCGGCCTAAAGTCCCGCTTACGCTGTTGCTGGGCTATGGGCTGACGATAAGCTTTGGGCA
GTTCGCCTTTCTCTTTTGCGCGATAAAGTTTGGTATGCCCGCAGGACTGGCGTCGTTGGTATTACAGGCGCAG
GCCTTTTTTACCATGGCTTTGGGGGCGTTTGTTTTTAGTGAGCGGTTACAACGTAAGCAGTTAGCGGGGATCG
CCCTGGCGATTATAGGTGTACTGGTATTGATTGAAGCCAGTCTCAACGGACAGCATATAGCGATGTCGGGATT
TATGCTGACTCTGGCGGCGGCATTTAGCTGGGCCTGCGGCAATATCTTTAATAAAAAAATTATGCAACATAGT
CCGCGCCCGGCGGTGATGTCGCTGGTGGTCTGGAGCGCGCTGATCCCGATCCTGCCGTTCCTGCTGTCATCGC
TGCTACTGGAGGGGGCGGATCACATTACGCAAAGCCTTATTACCATCGATATGACGACGATATTGTCGCTGCT
TTATCTGGCTTTTGTCGCCACTATACTTGGCTACGGCATCTGGGGCGCGTTACTGGGGCGCTATGAGACCTGG
CGCGTGGCGCCGTTATCATTGCTGGTGCCGGTAGTCGGGCTGGCGAGCGCCGCGGTGTTACTCGGGGAAACGT
TGACCGGGATGCAGCTTGCGGGCGCAGTACTGATTATGGCTGGACTCTATATCAATGTGTTTGGATTTCGTGT
GCGCCGTACTGCCCGCGTCAGGGGATGAGGCATTATGTTACTAAGAAGACAAACGCTAATTTCAGACCATGCC
GCGAGGTAATAGCGGCGGACTTGTCATCGCCAGACGGTACTAAAAAAATGCCCCGCAAAACGGGGCAAAGAGG
TTAAAGGTGTTGGTCGTTATAATAGGGCACGCCTAATTCATCTGACTTATCGCTGTTCACGCTCACGGGGTGA

AGGTCATTTGGCGGATCGCCTACACAGGGCAGCAGCATCACATCGCGGCTATTTTGCGCCACAGGTAACAAAG
TTTGTTCTGCAATGCCATATCCGGAGGCGATAAGTAACAGACCCGGCAGGGCGGGAAACAGCATTTTCATAGT
GCTCTTCGCGTGGCGCATAAACAAACTAGTAGTTGCCATGGTTCAGCGGCAGCATATACCGTGATTCGCCATG
CATATTGGTGATCCGGTATTTGTGTGGCGGCACATCAAAGTAGTTTTTGAACGTCCGGGTCAATGTTTGCTGT
GACTCAAAGCCATAGCGTTCCGCCAGATAGAGAATGGGCTCGTTGCTCTCTTTTAATTTTTGCGCGATTTCCG
TCATTTTACGGCTGCGGATGTATTGGCCTAATGAATGACCGGTCTCTTTTTTAAACATCCGTTGCAGGTGCCA
TTTGGAATATCCTGAACGCTCAGACACTTTTTCCAGTGAGAGCGGCGACTCCAGGTTATCCTCGATCCAGTCC
AAAATGCTATGAATAGTAATAGCGTCAGTGTTGCGTCTGGACATCGTCATACCTCTTTTTTGTCTACGGCAGA
ATTTTCTTGAGCAAATACTCAAGCGTTGCCACTTCGTCCGCCGTTAAGTTTTTTGTTAATTCCTGATGCAGGT
CTTGCCCTGGTCGTTGATGACATTGCTCACAAATTGCCGCGCCGTCCGGCGTTAGCTTCACCAGTACGCCGCG
TTTGTCATTAGGATTCGGCAGTCTTTCGATCCAGCCTTTGCACAGCAGGCGGTCGAGCATTCGCGTCAATGCG
CCGAGATCGACAGACAGCACTTTTTTAAGTTCAACCGGGGTAATACATCCCGCGCAGCGTATCGAGCAAAGCA
CTTTAAACTGTGTTGCGGTGATATCCAGCGGGGATAAATAGTTATTTAACAGGCGATCTTTTTTTGATTTACC
ATGTAGATCAAGCGACCCAGCGGAATGATTTCATTGAACAGATCACTGGTGCTTTTCAAAATGGTTGCCCCGG
CAAGTAATTAATTTCGTCAGATACTATTGCCCAGGCAAGTATAAGTCAACTAAATGAATTGGCCGATGCCACG
ATTTGCTAAAAGGCGTCCGGGGTATTGTGCTGTGATAACTTGTAACTAATTGATAATTACAGGTTATAGGTTG
TAAAGAATATTGACTTGCACACTGGCGAAATGGCGCGCCTGGGCAATATCACTTTATACTTCCGGTTCATGAA
ACGCTGATGGGTAAGAGATAGTATTATGATGGATTTGTTTAAAGCGATTGGATTGGGGCTGGTCGTACTGCTC
CCGTTAGCCAATCCGCTAACCACCGTGGCGCTGTTTCTTGGCCTTGCGGGCAATATGAATAGTGCGGAACGCA
ACCGGCAGTCCTATATGGCTTCGGTTTATGTCTTCGCTATTATGATGGTGGCGTACTACGCCGGGCAGTTAGT
TATGAACACCTTCGGTATTTCGATTCCAGGGCTACGGATCGCCGGGGGGTTAATCGTGGCGTTTATCGGCTTC
AGAATGCTTTTCCCGCAGCAGAAGGCGCATGAGTCGCCGGAAGCGAAAAGCAAATCGGAGGAGCTGGCAGACG
AACCGACGGCTAATATTGCATTTGTTCCACTGGCTATGCCAAGCACCGCAGGACCGGGGACCATCGCAATGAT
CATCAGTTCCGCTTCCACGGTGCGTCATGGCGGCGAGTTTCCCGACTGGGTCATTATGGTCGCGCCGCCGATT
ATTTTTCTTGCCGTGGCGGTGATCCTGTGGGGATGCTTACGCAGTTCTGGCGCTATTATGCGGCTGGTGGGTA
AAGGGGGGATTGAAGCGATCTCCCGCTTAATGGGATTCCTGCTGGTGTGTATGGGCGTGCAATTTATTATCAA
CGGCGTGTTGGAAATTATTAAAACGTACCACTAAGGCGCATTTTCGTCTGCCGGAAACCTGTTCCCGGCAGAC
GAATGTCTTAATGCGGTTGTTCTTCCAGCGTCACTGGCCATTTGCGGAATATGAGCACAGCCCATACCAGCGC
GGCACAGGCGGGTATGGCGCCAATATAGCCGATGGCCGACATTGACCAGTGCAAACTCACCTGATTGCCTACC
AGCGCGCCAGCGCCAATACCAATATTAAAGATGCCTGAAAATAGCGCCATTGCGACATCGGTCGCGTCCGGGG
CCAGCGCCAGTACTTTCACCTGCATACCGAGCCCTATCACCATAATCGCGATGCCCCAGAAAATGCTCAGTAT
GGCCAGATGCGCTTCGCTATCCGCCGCAGGCAGCAGCAGAAGCAGACAGACAACCAGCAGGGCAATAGCGATA
CTGACGAGCGACGAGGCATGGCGATTACCCAGTTTACCAAACACGAGGCTGCCGATGATCCCGGCGCCGCCGA
GGATCAGTAACAGGACGGTAGCAAAATTCGCACTGAGCCCGGCGACATTCTGAACGAACGGTTCAATATAGCT
ATAAGCTGTGTAATGCGCAGTCACCACAACGACGGTCAGCACATAGAGGCTCATCAGCGCCGGGCGCCGGAAT
AGCAAAGGTAAACTTTTTAGTGAGCCGGAATGCTCGCTCGGCAATTTCGGTAGAAGCTTAATCAGACATAGCA
GCGTAATTAATGCGCCCATGCCGATGGCAAAAAAGGTGGTGCGCCAGCCAAAATACTGACCAACAACGCGACC
TATCGGCAGCCCAAGCACCATGGCCAACGCGGTGCCGGTCGCTATCAGACTTAATGCCTGCGCGCGTTTCCCG
GCAGGGGCCAGGCGGATCGCCAGCGAGGCGGTTATCGACCAGAAAATAGCGTGGGCGAAAGCGATACCAATAC
GGCTAATGACCAGCACGGTAAAGTTCCATGCCAGAAAGGAGAGCACATGGCTGGCAATAAACAAAACAAACAG
ACAAATTAGTAGCTTACGGCGCTCCATTTGACTGGTAAGAAGCATAAAAGGCAGCGACATGACGGCAACTACC
CAGGCGTAAATGGTTAGCATGATGCCGACCTGCGCGGTTTGCATATGAAAGCTTTCAGCAATGTCTGATAGCA
AACCAACCGGCACAAATTCTGTGGTATTGAAAATAAAAGCGGCAATAGCAAGCGTAACTACCCGTAGCCACGC
GACCTTGCGGGAAACTGGATTGATTGTCATAAAAATATTTCGGGTTAGTTGCGAGAAGGTGAGGCCGCGATCT
TAAAACGTTTACTGGCGAAAAGACAATCATTTTGTGACCTGCTTCTCATTTACTTCGCAGGCCGTTTCGTCTT
CAGTCGACGCTCGCCAGTTGGGCGTTAAGTAACTCGATAAAGGCCTCAAGCTGACGCGTCATCGCCCCGCGCC
GCCACATTAGCCATGTGTTAAGCCAGCGCCACTTCTCCGCCAGCGGCCAGGCTTCCACCTGATGATGTCCTGG
CATACTGTTGAGCATACTTGCTGGCATCAGCGCAATACCTGCGCCGGCAATTACGCACGCCAACATTCCATGA
TATGACTCCATTTCGTGAATCGTACCGGGGGTCGCGCCATCGGCATGAAACCAGCTTTCGAAATGCCGTCGGT
AAGAACAGTTGGCGCGAAAAGCATAGATGTTATAACCATTCACCTCGCTGGCCCGCGAAACGACGGAATGGCC
GTGTGGCGCGACGATCATCATCTCTTCCTGGTAAGCCGGTATACCTTCCAGCCCCGGATGCATTAGCGGGCCA
TCAATAAAGGCGGCGTTCAGCTTTCCCTCGAGCACGCCATCCAGCATGGCGCCGGAAGGACCGGTCGTTAACG
CGAACTGAATTTTAGGGTAACGCTGGTTATAACCCGCCAGCAGCGCGGGGATACGTACGGCGGCAGTACTTTC
TAACGCGCCGAGTGAAAACAGTCCTTGCGGTTCCTCTCCGGCAACCACCATGCGGGCTTCATCGACCAGCGCC

AGGATCTGCTGGCTGTAACGTAAAAAGTTATGCCCGGCTGGTGACAGCCTGAGTCGCTGGTTTTCGCGAATAA
ACAGAGAGACGCCCAGGTCGGCTTCAAGCTGGCGGATACGCGTGGTCAGGTTGGACGGAACGCGGTGTACTTT
TGCCGCCGCCTGCGTAATGCTGCCCGTCAGAGCGACAGCGTTAAACATTTCCAGTTGCGTCAGGTCCATGGTT
TTCTTAATTCGTGAATAGTCTGGTTAATATTATTCATTTTTCAGAAATGGTAAAGGGCGTCATGATGGTACAA
CTCCCCTGATAACGAGAGAAAGAACATGACCATGATGACTGCAACACAGGCGTTATCCGTTAATCCTGCGACC
GGCCAGACGCTGGCGGCAATGCCCTGGGCTAACGCGCAAGAGATTGAACATGCGCTGAGTCTGGCGGCGAGCG
GTTTCAAAAAGTGGAAAATGACATCAGTAGCGCAACGGGCGCAAACATTACGCGATATTGGTCAGGCGCTTCG
CGCTCATGCAGAAGAGATGGCGCAATGTATTACGCGTGAAATGGGTAAACCCATAAAGCAGGCGCGTGCGGAA
GTGACGAAATCCGCCGCGTTATGCGACTGGTATGCGGAACATGGCCCGGCAATGCTGAATCCTGAGCCTACGC
TGGTAGAAAATCAGCAGGCGGTGATTGAATACCGTCCGCTGGGCGTCATTCTGGCGATAATGCCGTGGAATTT
TCCTTTATGGCAGGTGTTACGCGGGGCAGTGCCGATACTGTTGGCGGGGAATAGCTACTTGTTAAAACATGCG
CCCAATGTCACGGGTTGCGCGCAGATGATAGCCCGAATCTTGACGGAAGCCGGTACTCCCGCTGGCGTCTATG
GATGGGTGAACGCCAATAATGAGGGCGTGAGTCAGATGATTAACGATCCGCGAATCGCGGCGGTGACGGTGAC
CGGTAGCGTGCGCGCCGGCGCGGCGATTGGCGCTCAGGCTGGGGCGGCGCTGAAAAAGTGCGTACTGGAACTG
GGGGGCTCGGATCCGTTTATTGTACTCAATGATGCCGATCTCGAGCTGGCGGTAAAAGCTGCGGTTGCAGGAC
GCTATCAAAACACCGGACAAGTCTGCGCCGCCGCCAAACGTTTTATTGTCGAAGAGGGCATTGCGCAGGCGTT
TACCGATCGTTTTGTGGCGGCTGCCGCAGCATTAAAGATAGGTGATCCTCTGGTTGAAGAGAATGACCTGGGG
CCGATGGCGCGCTTTGATTTGCGTGATGAACTCCATCAACAGGTGCAGGCTTCCGTCGCCGAAGGCGCACGGC
TACTGTTGGGCGGGGAGAAAATCGCCGGGGAGGGCAACTATTATGCCGCGACCGTTCTGGCGGATGTTACACC
TGACATGACTGCATTTCGTCAGGAACTTTTTGGGCCTGTTGCCGCCATTACCGTCGCAAAAGATGCGGCGCAC
GCCCTCGCGCTGGCGAATGACAGTGAGTTTGGCCTGTCGGCGACGATCTTTACCGCCGACGATACGCTGGCCG
CGGAGATGGCGGCGCGTCTTGAGTGCGGCGGGGTATTCATTAATGGGTACAGCGCCAGCGATGCGCGTGTGGC
ATTCGGCGGGGTGAAGAAAAGTGGCTTCGGTCGCGAGCTTTCGCACTTTGGTTTACACGAATTTTGTAATGTG
CAGACCGTCTGGAAGAACCGCGTCTAATTGCCCTGACGTCGCGTGGCGCCCTCTGCTATACTCGCAGGCCTTT
TCAGACCTGCGAGCAAGGAGCATGTTGTGGCCTGGGCCATGGATAATGCAATTTTAGAGACGATTTTACAGCG
CGTTCGCCCCCTCATTGGCCAGGGCAAAGTCGCTGACTATATTCCGGCATTAGCGTCCGTCGAGGGTTCAAAG
CTGGGGATAGCGATTTGTACCGTGGACGGGCAGCATTATCAGGCGGGCGATGCGCACGAACGCTTCTCTATTC
AGTCTATTTCGAAAGTGCTCAGTCTGGTGGTGGCGATGCGTCATTATCCGGAAGAGGAGATCTGGCAGCGCGT
CGGAAAAGATCCTTCCGGCTCGCCGTTCAATTCGTTGGTTCAGTTAGAGATGGAACAAGGCATCCCGCGTAAT
CCATTTATTAATGCGGGCGCGCTGGTGGTATGCGACATGTTGCAAGGCCGGCTTAGCGCGCCGCGTCAGCGGA
TGCTGGAAGTTGTGCGCGCGCTTTGCGGCGTATCGGATATTACGTATGACGCTACGGTCGCGCGCTCTGAATT
TGAGCATTCCGCCCGTAACGCCGCTATTGCTTGGCTGATGAAATCATTCGGTAATTTTCATCATGATGTGCCT
ACCGTGTTGCAAAATTACTTCCACTACTGTGCGTTGAAAATGAGCTGCATGGAACTGGCGAGGACGTTTGTCT
TTCTGGCGAATCAGGGCGAGGCGTTTCATCTCGACGAGCCGGTAGTGACCCCCATGCAGGCCCGGCAAATTAA
TGCGCTAATGGCGACCAGCGGGATGTATCAGAATGCCGGAGAGTTTGCCTGGCGGGTAGGATTGCCCGCTAAA
TCCGGCGTGGGCGGTGGTATTGTCGCCATTGTGCCGCATGAAATGGCGATTGCCGTCTGGAGTCCGGAGCTGG
ATCCTGCGGGAAACTCTCTGGCAGGTATCGCCGCGCTTGAACAATTAACACAAACGTTAGGACGTTCGGTATA
CTGATGAATGCGCTTGAACCGCTCTTTGCCCGCCTGGCGCGATCGACATTTCGTTCGCGTTTTCGTCTCGGGA
TAAAAGAAAGACAATATTGCTGGGATAAAGGCGCGGAGGTGATTGATAAACACGCCGCCGATTTTATCGCCCA
GCGACTGGCGCCAGCTCACCCGGCGAATGATGGTAAGCAAACGCCGATGCGGGGCCATCCGGTATTTATCGCG
CAACATGCGACGGCAACCTGCTGTCGCGGGTGCCTGGCGAAGTGGCACCAGATTCCTCAGGGAGAACCGCTGA
GCGAAGCGCAGCAACAGTATATTGTCAGCGTTATCCATTACTGGCTGGTCATACAAATGAATCAGCGGTAGAG
CAGCGCGTCGTTGCTGACGCTTTCCCCGATAAATCACCCTATACCACTGATAGCAGCCGTTGACCGTTATATA
TCTCATGTGCTGAAGTGTCTTTTTAGTTACAACATACGTTTGGTTGTTTTTTTTCAATGCCTGTCAGCACTGT
GCAAAAAATGTTCTACAGGGCAGGGGATCCGTGTATCACCAGAAGCTTGCAACCTTGATCTGGATCACGTTTA
ATGACAGTGCTTTTTTACCTGCTGAAGATAATCATGATCGTTAGACCACAACAGCACTGGATTCGCCTGATAT
TTGTCTGGCATGGTTCCGTGTTATCTAAAATCTTTTCACGACTGCTCCTTAACTTTCTTCTTTCTATCGCCAT
TATTATCATGCTGCCCTGGTACACAATGCTGGGTATTAAGTTTACCCTTGCGCCGTTTAGCATACTGGGCGTC
GCTATCGCCATTTTTCTGGGGTTTCGCAATAATGCCTGTTATGCGCGTTACGTCGAAGCGCGACATTTGTGGG
GGCAGTTGATGATCGCTTCACGCTCAATACTGCGTGAGGTGAAAACAACCTTGCCTGATGAACGCGGTATCGA
GGATTTTGTTCGGTTACAAATCGCTTTTGCCCACTGTTTGCGTATGACATTGCGTCGACAGCCTCAAACGCAA
GTTCTGGGCAACTATCTTGACCAGGAGGCGTTACAGAAAGTTGTCGCTTCCCATTCCCCTGCTAACCGTATCT
TATTGCTGATGGGAGAGTGGCTGGCGATTCGCCGCCGCAGCGGAAAGCTTTCCGATATTTTATTCATAGCCT
CAACAACCGTTTAAATGATATGTCGTCAGTGTTGGCCGGCTGTGAACGCATCGCCAACACGCCGGTGCCTTTT

GCCTATACGTTAATTTTGCATCGTACGGTTTATCTTTTCTGCATTATGTTGCCATTTGCACTGGTGGTTGATC
TGCACTATATGACGCCGTTTATTTCGGTGCTTATTTCTTATACGTTTATCGCGTTGGATGCGTTAGCGGAAGA
GTTGGAAGATCCGTTCGGTACTGAAAATAATGATTTACCGCTGGATGCGATATGCAATGCGATAGAAATCGAT
TTACTGCAAATGAATGATGAAAGGGATATTCCGGAAAAACGGATACCGGATAAGCGCTATCAACTGACCTGAG
TTTGTCTTAACAGGCGCAGAATGTTCATCTGCGCCATAACCTGAAGAGCAAAGCTTAATAGCCTTCTCCAACG
CCGGTTTTGGCTGGACCGGAGTAGTGGACATGAACGATTCGCCATCCTGTGTTTGGTATCTTCGCCCACACCT
GGCTTTCGCGGCCTGTCGTGTGTTGAGTCTGCCCATTATCACGGCGTACCGCGGTAAAATGCCAGTCGAACTC
TGCAACGGCGGCATTGCCATACACATGAATAGCAGGGGGAGCGTCCAGTTTTAATGTGCGTTTACTGAAAGTT
TTACCCATAGTGGTGCCATAAAAATTTTCAGCGATCTGCGACCATCCGCGTTCGTGACCGCGTGGATGAATAA
AACTGGTTTCCGGTGAAACATAAAAGAGCTGCTTGCCTAACGTTGGGTCTGCCTTATCTATTGAATAAAGGTA
ACGGTTAATGCCTTCTGTAATTTCTTGTTTAACGGTCGACGTAGCTTCATTTTGTGCGGCGGTTGGCGTTGCC
GCTGATGCCGATGACACCATCAGAGTTAACGTTATCGCGGCGGCCGAAAACAATAGTGACGTTTTTTGCATAA
TAGTTATACCCATATGGTCTGTTTTTGAGACGCTTTATCTTTCCTGTTACGTTCACTTTTATACTAAAGGAGC
ATGGCTCACGAACAATAAAAAACATCCTTCTATAAAACTACCCCGAAAAGGGAGTAGTGCAATTAAGAATATC
GTATTTGTTTGTAACGACATGACTCAAAATATCAACATTTAGAGTTTCAGTATTGTTGTTGTCCATTGACTTA
CTTTTATGTTTATTACCGCTGGTAAATAGTTAATCTTATGAATAGTGATATGCAAATGACCGTTTGCGCTATG
TGATAATTAACCATAGTAAAAATACACGAAGCGAAGAAGTGCTATTTCAGTAGTACTGATATTTTCATAACGC
TAATTTAGAAATAAATGTAAACGTAACAAATTATACACAAAAATAAGAAGGACTGTGGCCTCAACTGACTTGA
TTATGATTCCGTCTTAACGAATGTCAGCCGAGTATTCAGTGCCATTCTCGCCCTGGCATCCCCGACCGTAAGC
CAGTTCTCTACTGGTAACCCCCTTGTTATTACAGCAGAAAACAGGGCATATCATTGATGTAGTCATGCTTAAC
TTGCATTTTAACATCACTAATACATAGCAAGCTAATATTACACTTTGAAACTTAACGCACGGTAATTGTTAGT
GATATTGTCCTGGACAGTTCCGCGAGCAGGTTTTGCGGAAAGTCCAAAAATAAGACAAATAAGGCATATAAAA
TGAAAAAAGTAGTGGTGTTATCTGCGGTAGCCGCAGCCGTAATGATGGCTGGAGCCGCCAACGCAGCAGAAAT
CTATAACAAGGATGGCAATAAACTGGATTTGTATGGCAAGGTCGACGGCCTGCATTATTTTTCCAGTAATCAT
AGTACGGATGGCGATCAATCTTATATCCGTATGGGTATTAAAGGCGAGACTCAAATTACCGATCAACTAACCG
GTTTTGGACAGTGGGAGTATCAGGTCAACGCCAATCGTCCGGAAGATGGTGACTCCAGCGGTTCCCCGCAAAG
CTGGACGCGTCTCGGTTTTGCTGGTTTGGCATTTGCCGATATGGGGTCTGTTGATTATGGTCGTAACTATGGC
GTGTTATACGACATTGGTTCATGGACTGACGTACTGCCTGAATTTGGTAATGATTCGTATGAAGCTTCCGATA
ATTTTATGACCGGACGCGCGAATGGCGTTCTGACTTATCGCAATAATGATTTCTTTGGTCTGGTTGATGGTCT
GAATATTGCGTTGCAGTATCAGGGTAAAAATGACGGCCTGTCGAAAGAAGGCGATCCTTTAAGTAATAATGCC
AGAAAAAGTATCGCGTACCAGAATGGCGATGGTTTCGGGGCATCAGCGACATATGATTTGGGCATGGGGGTGA
GCCTGGGGGCAGCCTATACCAGTTCCAAACGCACGCTTGATCAGATGACGCAAGATAAATACGACAATGGCGA
CCGCGCTGAAGCCTGGACTGGCGGCGTGAAGTATGATGCCAATAATATTTACCTGGCGGCAAACTATACCCGT
ACTTATGATATGACCTATATGGGCGATACATTGGGCGGCTTTGCACATAAAACCGATAACTGGGAAATGGTAG
GGCAATATCAATTTGATAATGGCTTACGCCCGTCACTGGCATTCCTGCAATCCAGAGCCAATGATGTTGATGG
ACTGGGTTCGTTCGACCTTGTGAAATATATTGATGTGGGTTCCTATTACTACTTCAATAAAAATATGTCCGCG
TATGTTGACTACAAAATCAATCTGCTAAAAGATGGCAACCCATCGAATCCTAATACAGACAACACCGTGGCGC
TCGGCTTAGTCTACGAGTTTTGATAAAACTTGCTGATTAAGACAGTTTAGGGATACCCTCCTTTTCCCAAAAC
TGGGTCACTGCTTAACCTTTATTATTGTTAATTTATTCTTTTATTATTTCAGGACGCTTCTGGCGTCCTTTTT
TATTGCCTATTCGTTGACTATTCGACTTCGATCTCGCGAATGCGCATTCTGCTACTGTCATCTATCTGCACTT
TGCCTGAATGACAAAAGGGACAACAGAGTGTGGATGTTTGAGAGGTGAAGGGTTGATTACAGGACAGACACCA
ACCTTGCGCCGGTGCGATAGTAATGTGGAAACGCGCATTGTCGGCAAGGGTATGACGAGCGGCGACGTCCAGG
CCAAAATGCAGCGCCGGCACCTCAACACAGGCCATAACGCCAATCTCCAGCCAGACTTGCTTTACCTTGCTGA
ACTGGTGATTTACTGCCTGTTCCTCCAGCAATTCGATAATATTTTGCGCCAGGGCTAATTCATGCATGGGCCA
GCTCCTTCATTCAACAATATTTACGGCGACACAAGGATCAATTACCTGTAACTGTTGCGACCACAATGAGAGT
GGAATAGTGGCCAGTTCGCGAGCAATGACGCCGTCAGGTTGGGCGTGACGTGTGGTAGGCGAGATAATACGGT
AATCAAGAATATTGCCTGACGTCGTCAGTCTGGCCTGATGAACTAACCATCCTCGCGCGGTTTCCACTACCGC
TATTTTCCATTCGCCATCATCAAAACGATTCCAGCGCAGGGTGGGAAGGGCGCTCAAACTTCGGGCGAGCGTA
ATGCAGCGGGCCAGCAGACGTAGCGCAGTCGTTTTACCGCTAAGACAACAGGCTGACAGCAGCGGATATTCGA
TAGCCAGAGGCGAGACATCCAGGGGACCATGGGTTATTAACGTATGGTCCGGGGACGATGCCATCGGTAGTGT
AGGTGTAATGATATTCTGTACTGCCGCCAGCCAGCGGGCGGTTTCAGAACGCTCTTGCAAAAACCAGGCTTCC
CAGCCCTTTACGCCAGCGGCAAGCCAGGTCTGAGGATCATCGTCAAAAAAGATTGTGCTTGCCAGCGGGGATA
ACTCTCCGCGACGTAACAACGCCCATTCTTCAGCGGTTGCCTGTCGGTGGCTAAACAGGGGGAGCCAGTCTGT
CAACGCGCGACGGAGCGTTTCTCGCGCCAGCTCTTGTTGTGCGGCCTGCATATCTGGCTTCTTTTCAGGAAAC

AACAGTAAAGCCGCCGCCGCGCTGTGCGCCTGAGCGCACAACGTAAACAAACGTGAAATAAGCGTTTTTGCGT
ATACCGGCGTTGCGCCCTGTAACAAACGACCGATCGGCGCCTCCTGCCAGATAATCCGTAGCGGGTGAGAATA
GACGGTCAATCTTTGCACGAACTATTACCTTCATCGATCACCATAAACCCGGACTTACTGGTTTCGCAAACCG
GACAGCACCAGTAATCAGGTAACTGGCTGAAGGGCACACCGGGTGGAATTTGCCATACGTCATCGCCCAGCGC
AGGGTCGTAAACCCACCAACATACTTTGCACTCCATGCGCACCACTGGCTCATTTTCTGTAACCGGGTATTCT
TTCAACCATTCCAACGTATTTTTTAGCCCCGCGTAGGCATCTTCGATATCATCGATGCCTGCAACGGCGATAT
CAGGAATGTCCGCTATCTCAATCATATCTTGCAGTAATGTGTTCATATCATTGAAATAATTGACCCGCCATAC
GCCGGGAAAATGCGTGCTTACAATCCGGCATTTACCATATCCGCGTGAAAAAATAGCGCTACAACCTTCGCCT
AAAAAATGATCCAGATACTCGCGATCGGCATCGTTTACTGGCAACAGCGTTAAGTTAATGATATGCGGCTCTT
TTACCGGCTGTTTTACGCGCTCGGCTATTTCATGCGCGATAAAGGCACCATTCATCAATCCCGCCGGTTGTGG
CGGCAATTCTGGCGTAATCGGCGGCTGCGGATGCGTCTGCCAGATGCTCTCTGGGATCGGGCCAATGATAATT
TCATCGGCAACCCGTTGCTGGTCAGCGTTATATTCACGCACACGCCAGACGCCCGTAAAAACCGTCTCCTGAA
TACGCTGTTCTTTTCCATCGGATAATAGTACCCGGGCGGAGATTTCACCTTCGCCGAGCAGTTGATCCAATAA
ACCGCTATTCTGTTGCTCAGTGGCATTCAGCTTATAGCTCAGGGGAAAGGGGAGCGTTGCCACTGGGTTATCC
ATATCGCGAATAAGCGTAGTCAGGATGTGTCTGGCAACACGGAGCTCAGCGGGCAGCGCCTGTTCCGGTAAAA
CCGGCGTCAGCGGGAGATTAATGCCGGCGGGGATAGGAAGAAAGTTAAAGTCTTCCTCTTCTGGCTGCGAGCC
TGGTCCGGCAATATTAACGACTGGAATAGTTCTGGAATAATTCACGTGTGTTACCTTTCATTCTATTGCGTGA
TAACGGGAATATCCTGACGATAGCCCGGTGTGGTCAGCAATGCGGCCACCCGCTGAGAGTATTCTTGCCAGCT
ATATAATCCCGCCAGACTGCCGACAAAACGGTGCTGGCGAAAGAAAAGCAGCGCCGGATATTTCAGAATGCGA
TACTCCCGGGCGATCAGTTCACTCTGTTCGCTATCCGCAAATGCCGCGGTATAAGACGTCGCCGGAAATTGCT
TGAGGATCTCCGGGATGATGACCCAACTGTCGGCGACTTCCGGATGTTTGTTGGGGTCGCTTTGCAGCAAAAC
CAGCACAGTCGGATGCGCATTAATCAACGACGGGAGCGTTTGCGCATCGGTAACCAGAAAGCCTTTATCCGCC
AGTGGACAAACAGGATTTTGGGGTGAGATAGTCATTTTTTTAAATGCTCCGGTAATTCAGGCGTGCGAGACAA
TAAATCGGCAAAACCGGTCAGCGGATCGCTTTGCGTGAGCATGGCCTGTTCCAGTGAGTCCAGCGCCGCCAGT
ACCTCAGCGGCTTCCGCTTCGTCCATTTCTCGTCGTGCTGCGCCGCCAAATGTCAGCAGCCATGCGCCTGCTA
AGGGGGAGCCACAAGCCGAATATCAACGTCGATAAGCTCGCCGTGTCGGTCGCGGCATTTGGCGAACCACTGA
CCGGGTGAAATAACCTGCACAGGAACGCCGATACACATTATGATTTCTCTCTCTGTAGCACCCGAATATCGCC
GACCCGGCAAGCGCTTTGCGCATCTGGGCGTTCATCTTCGTAGCGTTCCATACACAGGCTGTAATGATTGAGT
CTTTCAGTGGGCAAGGCCGCTGAAGAGGCGGTGATACCCCATTGCGCCAGCACTTCCTGCGCGAGATAGACCG
CAGGCATCAGTTGCGCCTTTACCTGCGGCGTAAGACTCCCGCCATAGTCATCCAGACATTCCGGCTGCACGCC
AATCAGCACAATCTCCGATAACTGGCCGCCGGTTAATTGCAGCAATGCCAGCACTTCAGAAAAACTGGTCTGA
TGTAAACTGAGTTTTTTCGCCGTCAGATAGGCGGGAACCTGTTCATCACGAAACATCGCCAGCGAACCTGGCG
CCATACCAAAATCGATAGCATCCATGATTAACAGTTTTTCGGTCTGTTGCAGCCAGGGCAGCAGCGCCAGCCC
CTGCGTACCGCCATCAACAACGTCAACTTTCTCGTTATCGGCATACTGTTCAAACAGCTTTTCCGCCGCTCTG
ACGCCGAACCCTTCATCTGCCCAAAGCAGGTTGCCAAGCCCTAAGATTGTTACTTCTGCCATTGTTATCACCT
GAACCAGCGCCAGCCGCTTATCATGACGGAAATCACGCTTTGTCGGCTCATAATGTCTTCGCGGATAGCGGTA
TAAACATGCGCAATGACGAAGGCGATAATGAACCACATGCCCAGCCGGTGCCAGAAATGCAGCGCGAGGCTAT
CGTTGCCAGTCAGGCGAATCATCCAGCCAAACCATTGATACGCCCAACTGTCTGTTCCAAGTCCTTCGCCATA
GAGCGCAAAGCCGCTACACACCATCAGTACGGACATCCAGAAATAGAACATTACCGCCAGTCCCGCTACCGGA
TTATGTCCATAGTAACGATGGGCCTCTTTTTCGAGGAAAAAATACCAGCGGATTTCGCTGATAACGCCTTTGC
GCCAGGCGCGGCGCCAGAACGGAACCAGAAACATCTCCCTGGCGTACTCATTGCCGACACATGCCCAGTAAAT
ACGAAACAGCAGCGCGACAGTAAAAATATACGCCGTGGTAAAATGGATCAGTCGTATCCAGCCCATCCAAAAC
ATAAAGGTCGCTTCACCTTGAATCGATGGCAGGGGGCGGCCAATGAAATATCCTGTAACAGCCAGGACGACAA
TGGATAACGCCGTGATCCAGTGCCATAAACGGACCGGAGCTTCATAGATATAGTACCCCTGGCTGGTTATTTC
TTTGAGATGCATTGAGACCTCCGAATCGTCGTCATCGTACCTGAACCCTGACCAGCTCCCCTCCATGATTATC
AATAACATGGGTGGAGCAGGCCAGGCAGGGATCGAAGCTGTGCAGAGTACGTAAAATCTCCAATGGTTGATCG
GGAACGGCGAGTTTAGTCCCCATTAACGCCGCTTCGTACGCGCCAATTTGACCTTTATCGTCACGTGGCCCGG
CATTCCAGGTTGTCGGTACCACACACTGATAGCTGTCGATTTTCTGATTTTCAATTTTTATCCAGTGCCCCAG
CGCGCCACGCGGCGCCTCGCTGAAACCAACGCCTTTTGCCGATGTAGGCCAGGTATCGGGTTCCCAGAGCGTG
ACGTTTGCCGTCGCCGTATCGCCGGACTTGAGGTTACGCATCAGACGATCGAAGAAATATTGCAGCGTATTGC
CTGCCCATACGGATTCCAGCGCACGCGCGGCAGTACGTCCTAATGTAGAAAAGAGCGCTTCTTTAGGCAGCTT
TAATACACTCAGCAGTTGATCCACGGGCTCTTTAAATTCCGGTTTATTTTGATGATATCCAATCAGATAACGC
GCCAGCGGACCTACCTCCATCGCATGTCCCTTCCAGCGCGGCGATTTAATCCATGAATATTTAGCGGACTCAT
CCACCTGTTCGATAAAAGTTTTTGTGCCTTTATAGTGTTCGCCCATGACCAGTTGCGGTTCGGTCAGACCATC

CCAGGGATGCAGGCCCTTATCGTTATTGCCGTTACCATAGGTATACCAGGAGTGAGTGACAAACTCCTGAATC
TCATCGGGGGCGGTTAAATCGACAGGATGAATTTCGTCGAAACGACCGTTAATGATAGCCCCACGCGGAAGCA
GAAGATTACTGGCGGAATAATCGTTAGGGTTATCCGGAAACTCGCCATAAGCCAGCAGGTTCATGCTCGATAA
CCCGCCGCCGATTTTTGCCCAGTCTTTGTAATAACTGGCAATCAGCAAGACGTCCGGAAGATAAACCTGTTCG
CAGAACTGGCGAGCTTTTTGGATGATGGAACTGACAAGGTTCAGGCGTTCCATATTCACTGCGCCGACCGCGC
CCGTTTCGTCAAGGTTGATGGCGCAAGGAACGCCGCCCACCAGCCAGTTAGGATGCGGGTTTTTACCACCGAA
AACGGTGTGAATTTTTACGATCTCTTTTTGAAAATCGAGCGCTTCAAGATAGTGGGCGACCGCCAGCAAATTG
GCTTCCGGCGGAAGTTTCATCGCCGGATGTCCCCAGTAGCCATTACGGAAAGGGCCAAGTTGACCGGACTCGA
TAAACCGCTTCAGTCGATTTTGCAAATCGCGGAAATATCCCGGCGAGGATAGCGGCCATGCGGAAAGGCTTTG
CGCGATGGCGGAGGTCTGATGCGGGTCGGCTTTCAGGGCGGCAACCACATCAACCCAGTCCAGCGCGTGCAGA
TGATAAAAATGCACCAGGTGATCGTGAACGTGTAGCGTAGCCTGCATCATATTACGGATACAGTTTGCGTTGT
CCGGAATCGCGATTCCCAGCGCATTTTCTACGGCACGGATTGAGGTCAGAGCGTGTGTACCGGTGCAGACGCC
GCAAATGCGTTCAACAAATGCCCAGGCGTCTCGCGGATCGCGACCTTTCAGGATAACCTCCAGGCCGCGCCAC
ATGGTGCCGGTCGACACGGCGTTAGTAATGACATTATTACTGTCGATATTCACTTCGCAACGCATATGCCCTT
CGATGCGAGTAACCGGATCGACCACAATTCGCCGTCCGCTATTGTCCAGCGTAAAACCCTGAGTCTGATAAGG
ATATGCCATAATTATTTTTTCTCTTCCGGAGCGTTTTCTGATGACGTGTTGTTTTTATTGCGGGCGTGTTTTA
TGGCGCTCACCGTGGCGTGAGCGATGGCGGCAGCGCCCGCGACGCCGGCAACGCCCAGTCCGATTTTATCGGC
GGTAGCTTCAATGCCGGTTTGCGGAATGCCCGTTGCCCGGCTGTAAAAAGAACCGTAATCCCAGAATCCATCT
TCCGAACATCCGAGGCATCCATGACCCGACTGGATAGGAAAAGACACGCCATCATTCCAGCGAACGGTTGAAC
AGGCGTTATAGGTGGTCGGGCCTTTACAGCCCATCTTGTACAGGCAATACCCTTTTCTGGCGCCTTCGTCATC
CCACGCTTCCACAAACTGACCGGCATCGAAATGCGCGCGTCGGTAACATTTGTCATGAATTCGCTGGCCATAA
AACATTTTTGGTCGTCCAAGACGATCAAGGGGAGGGATGCGATCAAATGCCAGCATATAGGTGATAACGGCGG
ACATCACTTCAGGTATAGGCGGGCAGCCTGGCACTTTAATAATGGGTTTATCGGTAATTAATTTGTGAACCGG
CGTGGCTTTAGTTGGGTTGGGACGGGCTGCCTGCACGCAGCCCCATGATGCGCAGGCCCCCCAGGCGATAATC
GCTTTAGCATCCGCGCTGACGCGTTTTAACTTCTCCAGGAACGGCTCGCCAGCCAGTATGCAAAACATCCCAT
CTTCATTCAGCGGCGCGTTACCTTCCACGGCAACGATATAATTTCCTTTGTACTCACGCATCACATCCGCCAA
CGCCTGCTCAGCTTGTTGCCCTGCGGCGGCCATAATGGTGTCGTCATAATCCAGGGAAATGAGCGACAAAATG
GCATCTTTTGCCAACGGGTGCGCAGAGCGAATGAAAGATTCGGTACAGCAGGTACATTCAAGACCATGCAGCC
ATATCACTGGCGTACGCGGTTTATTCTCCAGGGCATAAGCTATCTGAGGAATCATCGAACTGCTCAGGCCTAA
AGACGTTGCGGTAAGGCTGCAAAACTTGAGGAAGCTACGCCTTGTCACGCCGTGTCTACGCATAACTTGATAA
AATGTATCTTGTGTTTGCATAACGTTTATCCCAAACCACTATAGTTAGTGTGGGCATCATATGTTGCATTTCT
TGTCTTTCTCAACGTGTTTTAGTAGCAATTATGTTTCTAATTCGTAACAACTGATTACATGTGTGCTACTCAC
ATTACTCAAAAACATAAATTAATTTAATGTTATTGGTGATGTGGAGTAAATATAGCGTTGTGAAGATATCTCT
TAATGTTGATAAGAAACCGTGTTTTTTATTTATAACATGATGGGATTTTTTTAAATGGTCACACGGATAACGC
GTTTCATGTTAGTTCGCTGTGTTTAACTCTCCTTTAAACCCGTCAGTTAAATTTATTAGCTTGTAAAATAAGA
TCACAATTAATTACGTGCTGCATAACCATTGATAGGTTTAATTTAATATATTCATCCAATTCATTTTACAAAC
AAGGAGAGCATGATGAAAAAAAGTAAAGCAATTTTATTTATCGCTCCGGCGCTGTTTTCATACGCCATGGCTG
CACAGGCCTGCACAACCCTTGCTATTCAGGATAAACAAGGCGATATTTTTCATGGGCGTACGCTGGAATATAT
GCAGGATTTACCCTCATGGTTAACGTATTACCCGGCGGGAACACAGTTTGATAAAAAAACGCCGGATGGCAGC
CAGGGGATAAGCTATCAGGCGAAGTATCCGATACTGGCCATTACTTCCACCATTACTGATGGCGATAGCAGAG
ACATACTTGAAGGCATGAATAGCGCGGGATTGTCATTTAGTGAAAACATGATCATGAATGCGCAGTTGCCGCC
ATTGCCCGCGAGTGAATATAAACAGGCAATTCCTGTCACCTCGTTGGGAGAATGGGCGCTGGCCCGTTTTGCT
ACTGTCGGCGAGGTAAAACAGGCAATAAAAGAGGGCAAATTCTGGTCACCGGAACTGCATCGGTTTGGCGATC
TGAAATCGCCATTTCATTACGCGTTTTACGACAAGAAAGGAGGAAGCATTGTCGTTGAAGTGGAAAATGGCAA
ATTCCATGTCTATGACAATCCCACTCGCGTGATGACAAATGGTCCCGCATTCCCCTGGCATCTGACAAATCTG
AATAATTATACGCAATTAACCAATGTGGACCGCTCTTCAGGCACATTGGGCGGGATAAAAGTGATGCAGCCTG
ATAGCGGGATTGCTATTGCAGACTTGCCATCGTCCGATACGTCGGTTTCCCGTTTTATACGCGGCGTTTATTA
CACCACATATGCGCCGCAAGCGACATCCGCCCACGACGCAATGAATACGCTTGCACATATTATGAGCCGTTTC
GATCGTCCGAAAAATATTACGGTTGATTATATGGGGAGTGAAGGGGAAGGCAACGCTACCCGCAAACCGGTCT
CAGAATATACGGTCTGGACAACGCTGTCTGACTTAACGCACGGTGAGATGATGGTGCGCGGCTACAATGATAT
CAACTATAAAACCTGGTCTCTTTCTCAGTTTAAGAACGCGACAGCGCCCGTGTTCGAGAAAATCAACGTCAAA
GGATAAATAGCTCATCTCGGTTTAGTTTCCCCGGTAGTCTCGGCGAAGGTAATGGAGGCTACCGGCGGTTGCT
TCTCTTTATCATGCACGGTAATTAATTATCACGATCACCAGCGCCAGGATGGTATGCTAAATCCATTCAAAAT
CCCCTGGATATGGATTGGAGGGCGCATGGTTATACCTGAGGTCAGGCAGGAGAGGCTCTATCAGAAGATAGCC

AACCTCATCATTAAGCTGATCAATGATAATATTTTTCCCCCGGGAACTTTTTTACCGCCAGAACGCGAACTGG
CAAAACAGCTTGGCGTGAGCCGCGCCTCCTTGCGAGAAGCCCTTATTGTACTGGAGATCTCCGGATGGATCGT
GATTCAGTCCGGTAATGGGGTTATCGTTTCAGATAAAAAACACCTTGCCAGCGACTATACCATCGAAGAAATT
CTCTCTACTCGCGAGCTGGTTGATAGCCATTGTGCGCGCTTAGCCGCACAAAACGATAACGTTGACGTCATCA
ACCAAATAGAGGCGATTTATCATCGTATGGATCAAGCCATTAACGATAATAACGTCCACGGCTTTTACTCGCT
GGATAAAGAGTTTCACCTTGCGATCTCTGAGGCTAGCCGTAACCGGGTGTTGTTCGATATGTCCCGTATGTTA
TGGGAACAACGGATTAATATCCCTTATGCCGGGCTTGATGAGCAGTCTGGAGACAGAAATGTTTTACTTAATC
TCAACAAACAACATAAAGCGATAGTGGATGCAATGCGCCAGTCTGATGCTGACAGCGCTTATGAGGGATCGCT
GGAACATTTACGCTATGTGAGAAAGATTGTCGGCGGCTAAGTTTGTCGGTAAATAACACCGCATGGCGGATAT
GCCATGCGGTAGAACTCGCCGTAAATCAATGGAAAACAGCATTAATCTTTAAGTAGCGCTCCGGATAGCGATC
AAGTTCTTTAACTGCATCAGGGATCTCATCAAGATTGACGACCTTACTGACAACATCTTGCGGATTAATCTCC
AGCGTTGACAGCATGCGTAATGCCTCATCAAATTCACCGGCACTGGTGCGGGAACCGCGTAAATCCAGTTCTT
TATAGGTGATCAGGTTCGTCGGCAGCGACGTTTCCTGTTTTGGCCATCCGGTAAACGAAATACGACCAGCAAA
CGATGCTAAATCAAGCATATTTCGAATAGCGCTATTTGCGCCGGAAGCTTCTATCACGACCTGCGCCATGGTG
CCGTGGGTGATATTTTTAATAGCGTCGATAACCTGATCGTCAGCGGCGTTCAGCACATAAGGAACGCCAAGCT
TTTGCGCATAGCGTAGCCTTTCTTCCACAATATCGATCAGAATAGGCGTTGCCTTGTAATGACGCGCGCTTAA
TGCCGCCATAAGACCAATGGCCCCCGCGCCGATGATGGCAACATATTCCCCAGCCTTAACGTTTGCCCGATGC
AGCGCGTGCAGAGCGATGGTCAGCGGTTCTGCCAGCGGCGCCATTTCAGAAGGCACATTATCTGGTATTTTAT
GAATTAAATGTGCGGGATGAGTAACCACCTCCTGCATTGCGCCATCGACATGCACGCCAATGACGTTTAAATT
TTCACAGCAATTAGTTCTGCCGACAGAGCAAGGGTAGCAGTGACCACAATAAATATAGGGGTCAACAATTACG
CGGTCACCTTCTTTAATATTGTCCGGCATTCCGGCGCCTTCCTGAAGAACAATTCCGGTCACTTCATGCCCAA
TAATTCTTGGGTAAGTAACCAGTGGGTTCGTTCCACGAAAAGCCCCGATATCCGATCCGCAAATGCCTGCAGC
ATCAACCTTAATTAATACTTCATTTTCTTTCTTATGGGGATAATCCACCCAGCGGGTTTCAACTTCACCAGGC
TTATTAATGAACACAGCTTTGACTTTCATTGGCTTTGAATCCTTTACTTTTTACAAACACGAAGACCAACAGG
CTTGAAACGATAGCGATAGCACCAGCCAGATAGAACGCATAGTCATATTTACCGCTAAATTGAACGATAAAAC
CGGTGACAAGCGGACCGATAATACCGGATATATTAGCCAGGCCATGCATGGCTCCTCCGACGCTCCCTACCTT
ATCTTTATGAACAACATCCTGAATAACCGCCCAATAAATTGGGCCTGTTAAATAGAGCAGGAAGAGTGAAACT
GACATGAGCGTAATTGCGCTACCAATAGTGCTAACGGTACCGGAAACGGCAACACATACCGCCGCGCCTGCCA
GACATACGCCCAGAATAAGTCGACGTGAGAGCAGAGCATTACCGGTAATCCGATAAATTACATCAGAACAGAC
GCCGCCGAGTACCATACCAATGGCGCCGATGACCCAGGGAATTACCGTGGCGATACTAATTTCTTTAATATCG
AGATGTAACGAATGGTTCAGGTAACTTGGGAACCAGGTCAGGAAGAAAAAAGAATATAGTTGTAGCTGAAAAA
AGCCAGAGTTGTCGCCCATACCATTGGTTGTTTCATGTAATAGCCGAGTGAAGGCGTAGCCCGGCCATCATCA
GATAAAATAACGTCCTCATGATTTTCAAAATCAATGCGTTCTTCCGGCGCCAGGCGTTTACTCATTGTTGGCT
TATCGCTAACGATAAAGTACCACAGTAGTACCCAGACCAGGCCAAATAAGAAAATAATGCCAAATGCGGGGCG
CCAGCCTAAGGATAACGCCAGCAGGCCCACTATCGGCCCGGACACCGCGCCGCCGAGCGGGGAACCGGCGCTA
AAAATTCCGATCGCCGTGGCAGACTCCTTTCGGGAAATCCAGTTATTGATGATTTTATTTCCGGCAGCGCTGA
CCGGTCCTTCAGCCATACCGAAAAGAACGCGCACGATAAGCATTGTCCACAATCCGGTGACCAAACCAGTTAA
GCCACAAAATACAGACCATAGTAACGCTGCGATGAGAAAAACCGTTTTTGGCCCAACTTTATCGCTGGCCCAG
CCACCAATAAAATTAAACAGGGCGTATCCGATAAAAAACGCGCTGAAGATCATCCCCATCTCCGCGGCGTTAA
ATCCTAACTCCTTTTCGATCAAGGGGGCAGTTATCGACAATGCTGAACGATCCATATAGTTGAGCATGTAAAC
TATAAATAGAAGAAAAACGATAAGCCATCCCATTGTTTTTCTTTTCATGATTAATCCTCTTATTGTGGTCTAG
CGGCCTGACCACAATATTTTAAGGTTATTTTCAAATCACCTCTCTTGTGGTTAGAACGTGATCAATATAACAA
AAATTTATCGCAGAGGATTTTTATCCATCGCAAGCGTGATGGATAAAATTATTTAAACTCAGATAATTACTTG
AAAATTCAATCGGGGCTATCGTTTTCCCCTAATCCACGCCCCGGTAACGTTTGACATGCGGCGTTAACGGAAG
GTGGTGCTGTCTGTGGGTATTTTTCATCGGGCGTAATATAAACCTGACTCGTGGCGACATAGCCATCATTAAA
AGTGGCTTCGATATAGGTAGCTTCCCAACCTGTCTTCGGTTCATTTAACGTAATACTGATCTTATTGTTGGCG
GGAATATCTATTGTAAGCGGCTTATAGCGGATGCCACAGGCATAACGAAAATCACGCGCATTTGGATTGTTAG
CTGTCCAGCGAACGACTTTTACCGGCGCTTCTGAGAAATAGACCGTAAGAAGATGGTGGTGAATAAGGCCGAT
CAACTGTGGTAATGTTTTTTTACTTTGAAACCTATTAATAAAAGGAACGAGGCTTTCTTCAGCGAACTGGTTA
ATTGAGTAGTGGTTCATATTAGGCACTATACGTAATGATTTTACGCCAGGAAGTTTGCTATAATAAAAGCGAG
TGTTGTCTGGTACGAAAAAATCATCGCCACTTGCATTAATAATATATTTCGGTATTGCAAGTCGGGGTTGATA
AATAGTATTGAGATATCTTAACGGATCTATTATTTGCCTTAGTTGGGTAAATGTGGGGGATTTTATTTTCTCA
TCTATGCCTTGCTGGTAGTAGGGGTAGAATGTAATGGGCCAATTTCCACCGTATGATTGATAAATATGCTCAA
GTGAAGCATCAATATCAAGTAGATCAATGGCGAAGGGAACAATTGCTTCAACATCAGGATCGGCGATAGCTGA

CAACCAGGTGGTCCACCCACGCTTTGAAATACCTGTAATGATAAAGGAATTTATATTCCATTGCGTTAACTCC
TTTTTTGCTAATCTCATCGCTTGCGATAGGGCAGTGACCATAGGAATATTTAAAGGCATTAACTCACGTTTCT
CCGGCGCTTCCATAAATAATGCCCAGCTTCGGGAAACGCTCTCGTCCTCTTTCAGTGGTTTTTTATCATCCTG
AAAGGTTAAATATTGGTTAGGTATATCACTCACTGATATTACGATGGTATTCGTATCACGGGAGATGCTTGCC
AGTGTTTCCTGGGGGAAATCACCGGGTTTACCAGTGATTTGTACGCCTTTATCATAATTAATTCCGTTGTTTA
CAACCACCAAAGCATGGTGCTCTTTTGCCGTTTCAGGGATATAAATATCAACGTTATGTCGCCATTGCGCAGG
GGTTACCATGTCATCTGGCGACCAGTGTTGCGATAATAATTCATAGTGTCTGACTACCACCTGTGCTAATGGA
GCTGTATTAATCAAGGTATATTTTAGCGGTGTGCCTGAAATCTGCTCCCGGTAGTCGGGGAGAACATGAGACA
AATCACACTCCGGGCTGTTTCGACAAAAGAAGATGTTGTTATGAAGTAGTGTATAAACTTTTGTAGAAATGAA
AAACAGTACGATAACAACGAGATATGTTTTTTTCATGCTTTCCCCGCGCTTTACAAGGGCAAATGCCAGCCAG
CATAATTTCCATAAATACCTCTTAAACATTCAATATAAGTACAATACTTTTGATAAATAAGGGAAGAAGGGAA
CAATAATGTTTGTGACGTTGTCATATTATTCTGTTTCTGGTTTCACTATTAGCACTGAACTTTTTGAAAGGTA
AATCATTGCCGTCACCAGACCAATAATGGCGACAGAGATAGCTATACCGATCCAGACGCCATACAGAGCATAA
CCCAGGACAAATCCGCCCAATACGGGGGATATAAAATCAGCGATTGCGATTAATGCCTGCGCCGTTCCGATGA
CGATACCTTGTCGGGCAGGCGATACATGGACGGACAAGGCGGCAAGATAAGTGGGTTTGGCTAGCGCATCAGC
GATGCTAATACAAACGATAGCAAAAACAAGCACAGGTATGGTCGTGGCGATACCCGCAGTGAGAAATCCAGTA
CAAAGAAGGGCGAAGATGAGGATAATTAGCTTTCGCTCCGAAAAATATTGGCTCACCCAACCTAACAGAAATA
GCTGAACGAAAATATTAATAACCCCGTCCGCCATTAACAGATAGCTTAACGCTTTTGGCCCAAAGGGAAGCCC
ATTCCAGATGAAGGTGTCAGAAAGAAAAACAGGTAATTGTGAAGAGTACATGCCATAGGCGAAGAAATGACAA
AGCATTACGATGATTAAGACGCGAAGGACAGGCATCTTTAAAATAGCGCGGGCAGAGAACGCCGCTATTTTAT
CTGTGGTACGTGATGTTGTGGAAGGGTCTTTTAACCCCCAAATCGCGACCAGGGCAGACCCAAGGACAAGTAT
AAAGGCGGCGTAGATCGGCGCGCTCAGACTGATACGCGATAACCACCCGGAAACGCCTGCCCCGACAATACCG
CCTAAACCAATGCAGCCTGTGAGGATACCGATTGCCTGACGCCGATTTCTGACGTGCGTACAATCGGCAATAT
AGGCTGCGGCGGCTGACAAATTCCCGGCGCTAATGCCAAAAAGCGTGCGGGCGAGCAGGATAAACAGGATACA
TTGCGCGTTGGCGAGTAATAGTAAACTTATCGCCGCAATAGCCAGCGTGACAATCAGTATTCGCTTGCGGCCA
ACGCGATCGGAAAGGTGGCCAATCAGGGGCGCCGCACAAAATTGGCTAAAAGCTTCAGTGGCGATGATGATTC
CAATGATAAGCGGCGAACCGCCCATCTCGCGGATATAAAAAGGCAGTACCGACATTACAGCCGCTGTACTGGC
GGCGTAAAGGCCGACAAGCAGGAAAACAGGCAAAATACGCCGCTTGTTGAGCGGGGTGATAGTTTCGCTGTCG
GTGTTCTCATAGACATTAGTATTCATCTTTAATCCCTATGGCAAAACGAGAGAGATAATTTTCAATATCGTTG
CTCTGTTTGCCGTGAAAGAATGCGCCGACATAGCCATCGGGTCGAATCACCACGCATTGACCCGGCGCCAGAT
GATAGGATTCCCGGAAGTGCCCCAGGGTATCGATGAGCTCGTCCTGCTCGCCAATGTGATGAATACGCAGTCC
TCGCCGCGCATCGATGACCTTACCGTGGGTTTCGTAGGCCAACAGATTCCAGTCCGGACCTTGTAGTAACTGA
AATAATCGTAATGATTGGCCGCCGGCGCCCAAAAGAGGAGCATCCGGAGCTCTTTCTCCCGCTTGTAACCCAT
GCTGACGTTCGGGCAACGTGTGGGCTAAGGGGGAGTCGGTATACTGGATGTCAAGTTGTTGAACGTCGCGTTC
TCGTTTGATGCCACCCTGTTTTTGAGAGTCAAGTAATCGGGTCGAAAGGTGGAGCAGTGATTCGGCTACCGGG
CGGCGTTCCTGTTCATAGCTATCGAGAAGCTCTTCTCCTGCGCCCCGCAGCGAGGCTGCCATTTTCCACCCCA
GGTTATAGGCATCCTGGATGCTGGTATTTAATCCTTGCCCACCCGTCGGCGGATGCACATGAGCGGCATCACC
GGCTAAAAATACCTTACCCACACGGTAATGTTCCGCTATGCGCGCATTCATCTGGTACTTCGATACCCAGGGA
ATCGAATGGATGCGAACATCCGTCCGCCCGATTCGTTCAGTAAGAAAAGCCGTGAGCACGTCGGCTGAGAAAT
TCTGCGAGTCGTCCGGGGCCAACAACGCCTGGATTTGAAAGAGCTGCGTGCCGGCGAGAGGGCAAATCGTTAT
CATCCGCGTCATATCGCCATCGTTAAAATGGTGCCATACATCCCGGTTTAGACCGGAAAGTGAAGCGTCAGCC
ACAAGCGCATGGATTCCCAACGTACGACCGGGGAAGCTGACGCCTAATTTTTTTCTGACAAAGCTTCCGCCGC
CATCCGCACCTATCAAATAATGCGCGATGATGACCTCTTCTCCAGCCGGCCCGGCAACATACGCGGTGACTGT
GCGTGGGGTTTGCGCAAAATGTCGTAACTCGCAGCCGAACTCCACCCGATGTCCCCATGCTTTTAATTGCTCT
CGCATAATCGTTTCTGTCACGTTCTGTGGAACCATCAATGGCAGATGGTAGGGTTCGGCGTGGGTCGGTTTTG
TATGATGCGCAATATCTGAATCTACATAGCTTCCGTCGTGACGGTAGGTTCGTAGTCGGGGATAGAGCCCGCC
GGCGGCGACCACTTTATTGAGTATTCCAAGATCCTCGAAGATTTCTTGCGTTCGCGGCTGAATACCTTTACCG
CGGGAGCCGATAAAAGGCGTCGTTCTTTTCTCAATCAGGCGAAATGAGACCCCGTGACGCGCCAGTTCGATGG
CTAAAGTAAGCCCGGTAACGCCAGCGCCGCAGATCAGTACATCTGTAGTGGTGTATCTTGTCATCGTCTCTCC
GATATGTGTATTATGCACATAAGAGGAGACATGCTATGTCAGAAAATAAAAATGTGCAAGATACACATATTTC
CGAGCAAATGAAGGCGCTTCACGGTGCGTTGATTCGCGTGGTGAGCGCGCTTAACCGGCCTCGTAATGATGAG
AAATTAATTGCAGAGGCGGGGATACAACTCGATCGTGCGCTATTTTCGATCCTTATTAGTATCGAACGTTTAG
GGCCGATTGGCGTTGTTGAGTTAGCCGAACGTGCCGGTCGTGATTATACGACGGTAAGCCGACAGGTCGCCAA
ACTGGAGAAGTTGGGATTGATAATACGGCAACATAATGCCATCGATCGTCGTATACGCGAGGCCGTTATCTCC

CGTACAGGAAAGGCGATGACGGAGCGTATAGACGCCGCTCGAGAGCAAATGGGCAAAGTTGTTTTTAAAGACT
GGCCTCAGGATGAACTTGATGTCTTTGTTCGGCTAATGCAAAAGTTTGCCGATGCAATGGATAGCGATGCTTC
AATGCCGGAATAAGCAAAAGAAAAGGGCGGTCAAATCCGATCGTCCAGGCTAATATCGGTATCTGACATTGAT
TAAATAGCCAGCAAAGTAAATATAACAACAACATTTATCGAGTTAATTTTCACCCTTTGAAAGGCCAACGTAT
ACTGCAGAATACGGCTATATAACAATCGCCGTATCAATGGTTTTTCAAAGGGTAAATATATGCTTACCGAAAA
ATATGATTTTCGCATTACCGATCAGATGACTATCCCACTCAGACCTCATTGGATAGCCAATGATTCATACAGG
GAAAAGTGTAAAATGCTTGTCCTCAACCGGAGTAAAGGGGAAATTCATAAGGTAGACTTTAGTAAATTAACAG
ATTATATCAAAGAAGGTGATGTAATCTGTTTTAACGACAGTACTATAATAAACCATATGTTTATCTGTAAAAC
AAGACAGAATAGGCTAATTAAAATTGTTTTAGAGGGATTTTTACCAAATAATAGAGTAATTATTTCCGGACTT
TTAAAGGAAAGGCTTAACGCGAATGATGTTTTCTATTTGGTTGATAACCCTGAAATTTCCATTAAGATAGAGC
AAAGGTTTAGCGAGGAGAGCCAGTACCGGGCCGTCGTGGAAAATCATGAGGCCTTAATTTGCTATTTAGCCAG
TCATGGTGAGCGTCTTGATGAGTATGTGGACTCCAGCTTGTTTTATAAATATCCAGATGCTTACCGTTCCGTT
TTTTCTAAAAAGTATGGTTCTTTGGAAATACCTTCTGCCGGCATTCATTTTACCTGGGATCTTATCCAAAAAA
TCAAAGATAAAGGTGGGTTAATATCCTTCATCACTTTACATGTTGCATCAACAGAAATGTTGTCCAACAGAAA
AATACAAACAAAATGTGTAGAGGAAGTGACTATCAACGAAGAATACTACGAGGTTCCGCAAGCAACAGCGGAT
ATCATTAATACTGCGAAGCAAAATGGCGGACGTATTTTTGCCGTAGGAACTACCGTTACACGATGCCTGGAGT
CCGCGTATTCGAGGGAGCACAATTGCCTCAAAGCAAGTTCTGGCTGGACTGCGCTTTATATACATCCAGGCTA
TCAACTGAAAGTGGTTGACTGCCTGCTAACGAACTTACATCAGCCAAAAACGACGCATATGGTGTTAACTGGT
CAGTTTGCTGGGGTTGATCTACTCATGAAGGCCTATGCCTCAGAAGATATCCAGTCATGTCAGTTCGATATGT
TTGGTGATTGCATGTTAATCATACAGGATGAAGGGTAGGGATGAATAAAATAAAACCGGTACACATAATGCTA
CCGGTTTTACCGAGAATAAGCATCAGGGGATTCGGGCAATAACCTTTATTTCGAAATCAAAGCCGGCCAGCCA
GGTAACGCCAACAGCGGTCCAGTTAGGATAAGGGGGATAGGCAAATATCTCCTGTTTCACCGCCATCACGTCG
TTAAGTTGTTGCTCTGGATCGGTATGGAAGGTGGTGACATCCACTATGTCATCGAATGTGCATCCTGCTGCAG
TCAGTGTTGCTTTCAGGTTTTCAAAAGCAAGCCTCACTTGTTTTTGAAAATCAGGCTCCGGCGTTCCGTCTGT
GTGACTGCCTACCGTTTGACGGGAAAGAGAGTATTTCCTGAGTGATTAGCTTCTGTGTCGTGTCATTTTGTAG
ACGGCATCATTTTCACGCTTTCCAACACCAATGATAGTTACCGTGATAATTTCATCTTCGACGCTGTAGACCA
GTCTGTATCCTGCACCACACAGCTTAATTTTGTACTAGTCAGGGATTACAAACGCATTGCCAGAGGAAGCGAT
AAAATATGGTAAGTGATACTCTTGAACAGCGGATTTATGAACTGGTAAGAAGCCATGATGGAATTTACCTCTT
TAAGAAAAAAGAACTGACACCGTCAACAGATTTGGATTCAGATCTACGGCTGGAAGATGATGAAGCACTGGCA
TTGATGGATGATTTTTTTACGACATTTAATGTAGATAGAGGGAGTTTCTCGATCACAACATACTATCCGCCAG
AACCACCGCTAAAGCATTTGCTTAATCCGTTTCGCAAAAACGATATTCCTCAGGTGGCGGATTTCACTATAGG
TATGCTCATTGCGTCTGCCAGGGCTGGTCGCTGGTTGTATGACTGATTCACGCTGGGGGGCTGCTTTTCGCCT
TCCGCCATTATGGTGCGCATAATGTATATTATGTTAAATTCACCAGAACATTTAAAAGACATGTTAAAGCGGC
TGGATTCGTGTATGTCAAAAAACTTCTGGCCCATAAGTTTGATCCCTTCCCTGCCGTGACGCTGCGCGAATTC
TGTTGATTTGCACCCGCTTATCCTGCACACGATTTCACGCAGCACACGCCACGCAGGCATAAAAAAACCGGCC
ACTGTGACCGGTTCGTCGCGCAGGTTTTTGGCTCAGTGGGTTTTACCCTGGTCCGCTGCTTTTTCTGACTGAT
GTTCTGCAGCTCTGACAACGGCTATAAACGCTTTGATACCCGGATCCACATCCGGCGACAACATGCGCATTGT
TGATTTGGCATTCCTGCCGAACCTCGTCAAAAATCGCAGGATAAGCGAACGTGACTTTAATCAGCGTGCGCAG
CATCCTTTTGGCGACTTCCATTTCACCCGCCAGAATTAATTCGTGTATCTCCTGGATAATCTGAACCCGCGGC
CCGATGTCCTTTTCGATGTCATACGCGGCCATGTTTTTTACTCCTATAATCAGCACGATATTTTTCGACGGCA
CCCAGGTCGTTATGATACTGACAATACACTGGAAAATCGGTAGCTACTAAAATTTGCAGTTACCAGGATTTAA
GATGATTTTAAAAATGTTTATAAATTCAGGCTGCGCCGCAGTGATTGCACTGATTTCCACGTAACATGATAAT
CAATGGCTTTGATACCCTTTAAACGAACAAATATGAGCTTAACATGGCGACAATAAAGGATGTTGCATCCATG
GCCGGTGTATCAACCGCGACTGTATCACGCGTCATTAACCAGACCGCGTGGGTTGAACCCGTCACGCGCGAAC
GTGTAGAGAAAGCCATGCGCGATCTCAATTATCGCCGGAATGCCGCCGCTATCGCGCTGGCGAAACGTAGCGG
CGATATGCTCGGATTGTTAACCGGCAATCTGGCCGATCCTTTTTTTGCCCGCCTTGCTCGCGGCGTAGAAGAT
GTTTCTCGTAAGCAGCAGTATCGCTTGATGGTATGTAGCGGCGGCCACGACGAAGAAATGGAAAAAGCGGGAC
TGGATTTTCTGGTTAATCAAGGCTGTGAGGCTATTGTTGTACACGCCAGCCGATTACCGGATAAAGAGCTGCT
GCGCTACGCAGCCCACTTTCCAGCGCTGGTTGTTGTTAATCGTTATATCGCCGGTATGGCCAACCGGTGTATC
TGGCTGGAAAATCGCAGCGCCGCCAGGGAAGCGACCCGATATCTCCTGGCGAACGGGCATCGGCGCATCGCCT
GTGTGACATCTGACTTGCCAATTATCGATCGTCAGGAACGCCTTGATGGTTACCGTCAGGCGCTGGAAGAATA
TGGTATTTCTCCAGACCCTCGCTGGGTGATTAGCGTTCCGTTTAACGAAGAAGGCGGCGAGCGGGCCGCGCAT
CAACTTATTAATAGCGGGCTTCCCCTTACGGCAGCGGTCACGTTTAACGATGTGATGGCCGCTGGCATTATGC
GTATTCTTCATCAACGCGGCGTTCAGCTTCCGCAACAGCTCTCTATTGTCGGCTTTGATGATGTGGTGCTGGC

CCGGTATCTTTATCCGGCGTTAACAACAATGCATTATCCGGTCGAACAAATGGCGCGTTGTGCCGCGCAACTG
GCGATACAGTTATACCAGGGCATTACGCCGCCGCCCAGCAGCAATCATTTCAATGCCGAACTGGTGATCCGCG
ATTCTGTCGCGCCCTATTTTTCACGATGAATGCGTCGTCAACAGTAAAAACTGTGATGACTATCAAATTAACC
CTTCTCATGTAATCGGTTACATGGACGAAAGTAATGGATCACAGTAGTGAGAAGCGTATCCCCCTACCCTTGA
GCTACCTTACTGACCGGAGCTCCATTATGAGTGCATCACAAAACAAGAAGAAAACGCTGTCATTGGGTTTAGC
GCTAATCCCAGTCATCTCCATGTTGTTATTGTTGATTATCGGTTACGGCATCATGGGGCTACGTATCGAACCG
CTCCTTCTGTGCTCTGCGGCGGTAGCGGCAGGCATTGCATGGTGGCAAGGATACTGTTGGGAAGACATCATCA
ACTCAGTGGTGGATAAGCTGGCTAAAGCAATGCCGGTTATCATGATCCTGATCTGCGTGGGCGGTTTGATTGG
CACCTGGATGTTTAGTGGCACCATTCCCTACATGGTGTACTGGGGCTAAAACTCATAAGCCCGGAATATATTT
TGATTGCAGCCTTCTTTTTAACCAGCGTGGTCTCCGTCTGTACCGGTACATCATGGGGATCCGCCGGGACGGT
TGGCGTTGCGTTAATGGGGGTTGCCGCCGGGCTGGACGTGTCTCTTGCAGCAGCAGCTGGCGCCGTTGTTTCT
GGCGCTTATTTCGGCGACAAAATCTCACCGTTGTCCGACTCGACTAACTTTGCCGCGATTGTCGCAGACACTA
CTCTGTTTGAGCATATTCAGCATTTACTTTGGACGACCCTGCCAAGTTTCCTGCTTGCCGCCGTGGTTTATCT
TATCGCCGGACACAGTAATATGCTGGGCGAGGTGGCTACGCCGCAGCGCGTTACGGACATCATCCACTCGCTG
GAGTCGCTATACCATTTTAATATTATTCTTATTTTGCCGCCGGTGATCGTACTGTGGGGCGCAATTCGCAAAA
AAGCCTGTCATCCCATTAATGTTAAGCGCCTGCGTACTTGCCTTGTTCCTCGGCGTCATCATGCAGGGACTGA
GCATCAAACAGGGACTGGACGCTTTCATTGATGGATTTGATATCGCCATGTTCCCACAAGGCGCAGAAGGTGT
CGTGGCCGATGTTCCGCGACTGCTAAACCGGGGCGGAATGTTCTCGATGATGGGAACGATACTGTTAGTTTTC
TGCGCTTTTTCATTTGCCGGAGCGCTAACGTTAACCGGCGCGTTAACGATCATCATTAACCGACTGCTGACCA
TTATTCATAGCGTTGGTCAACTCATTGCCGCCACTATCGGCACCACAATTTTGGTCACAGGAGCGACCAGCGA
CGGTAAACTGGCATTGTTAGTTCCCGCCGAACTGTTTAAAGATGCCTATCGTCGTATGGGGCTGGATACTAAA
AACCTTTCACGGACGATAGAAGATGCCGGTACCGTTATCGAACCTCTCCTGCCGTGGACCTCGGCTGGCGTCT
ACATGGCAACCACGCTGGGCGTTAGCACGCTTGATCTTCTGCCCTGGGCTATCCAGTGCTATGCGGCGATCTT
CTTCGCATTAATTTATGGTTTTAGCGGAGTTGGTATTGCCAGAACTGCCTCCGCTTCAGAAAAGTCGCCGCAA
ACGTCAGTGACGAAATAATAAGGAAACAAGATGGATTTTAACCAGATTATCAATCGCAACAATACTGGTTCGG
TAAAGTGGGATTTTATTGAGCGTCACTTTGGCGACGGCGCTGGGAAACTGCTGCCGATGTGGGTTTCTGATTT
CGATTTTGCCTGCCCGCCTGAGGTTCAGGCGGCATTGCATCAGCGAATCGAGCACGGCGTATTTGGCTATAGC
GAACGTGATGAGGCCTATTTTAACGCCCTGCTTCACTGGTTTTCGTCACGCCACCAGCTTACGTTAAAACAGG
AATGGGTATGCAGCGTGGAAGGCGTCGTACCAGGGTTGGCGCTCTTAGTGCAAATGTTGACGCATCCAGGCGA
CGGCGTAGTGGTACAGGGACCATACTATGGCTCCTTCGCCAAAATAATCACGCTTAATGGTAGAAAACTGCTG
GAGAATCCCCTTAGCGAGTCGCCGGACGACGGCTACCAGATGGATTTTTGCCAACTGGAAAGGCTGTTTCGCC
ATGAGCGCCCTCCGCTGATGATTTTGTGTAATCCACATAATCCAACCGGGCGTTGCTGGTCGGCCAACGAGCT
GGAGCAACTGCTTTTATTATGTGAAGCTTATGATGTCACTCTCATCTCGGATGAAATATGGGCCGATCTATTG
CTGCCCGGCGAAACCTTTACCTCGGTACTGCATTTAGGGGAGCGCTGGCATAAACGGGTCATCTCCGCCACTG
CGGCCAGCAAAACGTTTGGCTTATCGTCGTTGCGAATTTCTAATTTTCTCATTCCCGATCCTACGCTTCGCCA
GCGATTCCTGTCTCGTCTGGACGCTCATGGGCTGGACGTCTTTAACGCCCTCTCCGTTCAGGCGGCGACTACC
GCATGGAATGAAAGCAGACAGTGGCTGGATTCGCTACTGGATTACCTGGCGGAAAACCGCCGCTGGTTTGTTG
AACAGGCGACAGAGCACCTTCCCTGGGCAAGAATTGTCCCGGCGCAGGGAACCTATTTATTATGGATGGATTG
TAAAAAACTGGGACTGGATGACGAACAGCTAAAATGGGTAATGGCGGATGTCGCGGGTATTGCTCCTTCAATG
GGCAGCGGCTTTGGCCCGGCGGGCAGCGGATTTATCCGTTTAAATCTGGGATGTCCACGTACCTACCTTGAAA
TGGCAATAGACGGCCTGAAGCGAATATCGGTGAAGACAATCAAAGGAATACCAACAGGCGGATGAATTTTTCG
GGCGGTAAACTGACACCGGTGTTATTTCTGCGGCATGATATCGCCTGAGCGCCAGTAAAAAACCGGCCATCAA
GGCCGGTTGGGTCCGGAGCGCGGTTTCACTATCAGGGGCGTCTGAATAACACGATACTGCGCCCGGCCAGATC
GAAATCCATTTCTTCGGTAATCCGTATTCCCTGCTGCGCGTTATCGGCAGTCGTCAGTTCAAGTACCCAGCCG
CCTTCTCCCCACATCGGAATACGGAACGGTACGCTGCCTTCAAACGGATTGAAAAGCAGTAAGGCGTCATGCC
AGATCCCCGCTTCCGGCTGAAGATCGGGACGACTAATGCATACGCCTATGGTCGATCCCTCATCCCATTGTTC
GGATTGCTGCGCCCCTCCGCCGGCGTTAAACCAGCGGATCTCCAGACCGTCACGCCAGCTTTCCCGGCGCAGC
AGCGGTTGCGTGGCGCGCAGTTGAATCAGATGACGGGTAAACTCCCGTAGCGCATTGGCGGTTTCCGGCAAAT
TATCCCAGTGGACCCACGATATTTCACTGTCCTGACAGTAGCCATTATTATTGCCCATCTGACTGCGGCCAAA
CTCATCGCCCGCTAATAACATCGGCGTGCCGTGCGAGAAAAGCAAGGTGGTCAGGAAATTACGTTTCTGTCGC
TCGCGTATCGCATTGATGCCCTCGTCGTCGGTTGGCCCTTCTGCGCCATAGTTACAGGAGCGGTTATCATTAT
GCCCGTCATTATTATCTTCGCCATTGGCTTCATTATGTTTGTCGTTATAAGACACCAGATCGTTCAGCGTGAA
GCCGTCATGGGCAGTAATAAAGTTAACGCTTGACCAGGGGCGACGGCCGCGCAAATCATAGAGATCGCCGGAA
CCCAGCAGGCGTGCGGCGAAATCATTTGTGACGTTGTCACCTTTCCAATATTCGCGTACCGTATCGCGGTATT

TATCATTCCATTCGCCCCACCCTGGTGGAAAGCCGCCAACCTGATAGCCGCCCGGTCCGATATCCCAGGGTTC
GCCAATCAGTTTCAGTTTTGCCAGCACCGGGTCTTGCGTTACCGCATCAAAAAAACCGCCTCTCTGATCAAAG
CCTTCCGGCTCACGACCAAGAATGGTGCCCAAATCAAAACGAAAGCCGTCGACATGCATTGACTCCGCCCAAT
AGCGAAGGGAATCCATCACCATTTGCAGTACCCGTGGATGAGAGGTATTGACGGTATTACCTGTCCCTGTATC
GTTAATGTAATAGCGATGCTGATCGGGCATGGTGCGATAGTAAGAGAAGTTATCGATACCTTTAAATGACAGC
GTGGGGCCAAGCTCATTCCCTTCTGCCGTATGGTTATAGACCACGTCCAGTATCACTTCGACGCCGGCATCAT
GGAAAGCGCGCACCATATCGCGGAATCCCTGAATACCTTTTGGACCATAGTAGCGCGTCGCCGGCGCAAAAAG
CCCAGCGAGTTGTATCCCCAGAAATTTTTCAGTCCTTTATCGAGCAGATGCTGATCGTCCGGGAACCAGTGAA
CGGGTAAAAGCTCGACGGAGGTAATACCCAGACTTTTAATGTAATCCACGCTGGCTTTATGTCCCATGCCCTC
AAACGTACCGCGTAGAGGCTCAGGGATCGCGGAATTAAGCTGGGTAAAGCCTTTTACATGCGTTTCATAAATA
ATGGCGTTCGACCACGGTATGGAAGGCCGTTGTCTATCCTCCCAATTCGCCTCAGCCGGATCAATCACCCGGC
ATTTAGGCGTAAATGGCGCGCTATCCTGGTCATCAAAGGTCAAATCTTTATCGTCATGAAGTAATTGATACGC
GAAATGCGCATCGTTCCACTGAATATCGCCCACCAGCTCACGGGCATAAGGGTCAATAAGCAGCTTGTTGGGG
TTAAAACGATGACCATTTTCCGGGTCATAAGGACCGTAAACGCGGTACCCATACAGCGCGCCGGGCTGCAACT
TCGGGACATAGCCGTGCCATATTTCGTCGGTATATTCCGGTAACTCCAGACGAGCGATTTCATGCTCCCCGCT
CGGATCGTAAAGACAAAGCTCGACGCGTTCCGCATGGGCGGAAAAAATAGCGAAATTCACGCCTTCGCCGTCA
TAATTTGCGCCAAGCGTATGGCTGTATCCTGATCTGATTTCAAAAGCCCTGTTTGTTGTCATTCTCTTTCTCC
ACCCACGGTAAACGTTGTCCTGTCACGGCACATCAACACATAAACTCAGTCGCTCATTAAAACCAGTACGCCG
CCCTGGTGTGAGGCCAACGACAGGCGTTCACCAGGCATTAACCTCTCCTGGGTAAGCACATTGCGATAACGAT
GCTGATTAAGCTGTCCGGGTATAATAATGTCGGTTCCTGACCAGAAACCGCTATGCGATTGTGACAATAAGCC
GTCACATTCGGCGAACACCAGTCGGGGCGCAACAACAATCAGCGCATCATCATGATTGACTCTGGCGTAAGCG
ATGACCTTGTCGGCACGCTGACCAACAGCCCGCAGCGGTATATAGTCGCCAAAACGGAAGAGCTCATGATTCT
GCTGACGCAAACGCAGTAAGGCGGCGGTGACATACTGATTCACCTGCCCGTTCAGCCAACTCTCTTCGCGCGA
GAAAACTCCCGGCGTTAGTTGGTCTAACTGCTGCGCCAGCGTGGCGAAATCCGGTTCGCGGCGGTTGTCGGGA
TCGACAAGACTAAAATTCAACGCCTCGCTTCCCTGATAGATATCAGGTACTCCCGGCGCGGTTAACTTGATAA
CGGTCTGCGTCAGGCTGTTAACCAGCCCTGCGCGGATGAAGGGTTGCAAGGAACGATAAAAATCCTGCAAAAA
TGTCTGATTATCCGGCGCCAGTAAATAACGGGCGTAATCAAGCATTGCCGTTTCGTAAGCCTCATTACTGTCC
ACCCAATCGGTACGTAGCTTCGCTTCGCGTAGCGCTTTCTCCACAAAGGCTTCAAAGCGTATTTTCAGCGCGT
TTAAACCTGTTTCGTCCTGTGGTTGTAGCATCGGGGGCCAGACGCCGGTCAGCGCCTGATATAACATCCACGT
ATCCGCCGATTTTGGCGCAGTGCCATCATTCAAAAATTTGACATGGGTCTGGTTCATCTGCCGCCAGCGGGCA
AGGCATTCGCTCCACTGTTCCGGCGCTTCGGTAAGCGTATACAGGCGTGCTCGCGCATCCTCGCCTCGCTTGG
TATCGTGGGTCGACGTACCGGATAACGCATCAGGCTGTCGCGCCTGTCGGGTTTTCATCTCATGGTGAAAGCG
TTCGATGGAGAAGTGATGCGTGACGGGCTCGGCGCCGACTTCATTTAACGCCAGTCCCATGTTTTGACGAAAG
AAAAGCGTATCCTCTACCGATTTCGCCATCAGCGGACCGGTGAGTTGCTGGAACCGAACGCGAAACTGTGTCG
CCTCCTCCTGGCTGGAGGCGGGAACATCGCCAGTAAGAAGACGGCTCAGAAATGTAAGCGCTTCTGGCTGCGG
AGGATTTTTCAAGCGTCTTTACCCGTTCCACAATACGGTGTAACAGGCAAATATCGGTCGGCGGTAGCCCTTC
TGCCGTGCCGTAAGTGCGATATACGGGAAAGGCAATTAATAACTCGCGTAACGCCTGCCGAACGATGCTCTCT
TCCTGTGCGATCTGTAGCTCGCTGGCAATCGACAGCGCAAGCGCGAGCAGCCGGGTAAATTCCCCCTCGAAAT
TGCGATCGACCATCAGCAATTTAGCCGCCCGTAATTCCGCCCGCATATCCACTGGCGCGCCTTTAACGGTCTC
GTAAGCCTGGCGCAGATTGTCTATCTGCTCATCGTCAACCAGCACCTCCGCCAGCGAGGCGATAAATTCATAG
CCTGTGGTGCCGGAAACCGGCCAGTCATCAGGAAGTTGCTCGCCTTTAGCGAGAATTTTTTCCACGGTGATAT
AGCACGCCGGCCCCACTTCCTGACGTAAACGCGCAAGATAGGCTTTCGGATCGGCAAGTCCGTCGATATGATC
GATACGTAAACCGTCAACCGCCCCCGTTCTGACCAGCTCCAGAATCAGCCGATGTGTATCGTCGAAGACGGTT
TTATCTTCCACCCGCATTCCTACCAGCCCTGTGACCTCAAAGAAACGCCGATATGAAAGCGAGCGAGCGGCAT
CGCGCCAGGACATTAATTTCCACGGCTGGCGCTCATGCAAATCAGCAATAGCGGCTTTATCGGTAAGCGCCAG
TATTTCGGCCTCGCGCCCCTGCCACGTCGCCGGCGCAAGCGGATAATAGTTATCATAATAGGCGAACGCGGGT
TTACCCGTAGCGGGATCGGGCTTGATCGCAATTTCTCCGTTTTCCAGCACGGCGTCAAACGTGTCGCCTAAAA
AAGGCAGCGTCAGTCGGCGCGACCAGTCAATATCGAAATGGCGCGCGTAACGGCTCTCCTTGCCATATTCAAG
CACGTCGCGCCACCAGGCGTTTTCCAGCGAGCTGGCCATGTGATTAGGCACAATATCGATGATCAATCCCAGA
CCTTGCGCTTTAAGTTCGGCCACCAGTCTTTCAAATCCTTCGCGCCCGCCAATAGAAGGCTCAATCTCATTCG
CATCGGTGACATCATAACCATGCGTTGACGCTTTGGTGGCGGTAAAGATGGGTGAGGCATAAAGATGGCTGAT
ACCCAGATTTTTAAGGTAAGGCACCAGCGCCGCAGCGCGATCGAAGGTCATGCCATTACGAAACTGAAGACGA
TACGTTGCAGTTGGGATCATGATGCAGACTCTCCCTGGGCTAAGCGGACAATAAGAGAATGTTGGGAAAGCGA
CCCGGTGGATTCATTCGGCCAGGCGAAGAGGGTCTTACCCGGTAAATCGGGCAGCAATACCGTCGTGGCGCTA

ATATTCAGCGCCAGTGACAGCGTTCCTCCCGGAAAACGCCAGCTAACGGCAATAAACCCGGGCGCGGTTTGCA
ATACCGTTCCTGAGCTCTCACGGGCAGCGGACAACAGCGGCACGATATGCTTCTGGCGCAAAAGCAGTAGTTC
GCGGGTAAATGCCAGCCACGCTTTACCCTCTTCACTGTGCTGTTGCTTCCAGTTAAGTTTTGAGCGTTGAAAG
GTCTCTGGCGCATTCGGGTCCGGAACATTTTCCCCTGCATGATCGGCAAACTCTTTTGCGCGACCTTCACGAA
CGGCGCGGGCTAAATCCCCATGGAAATCGGTAAAAAAAGAAAAGGACGGCTTTCGCCATACTCTTCGCCCATA
AAAAGCAGCGGAATATGCGGTGAAAGCAGCAACGTGGCGAGCAATACTTTTGTTCGTTCAGCGCCCGCCAGGG
TTATCAGTCTGTCGCCCTGGGCGCGGTTACCGACCTGATCGTGGTTCTGAATAAAATCCACAAAGGCGACCGG
AGGTTGTCCGGTACTTTTTACGCCGCGAGGTTCGCCGGTTTGGGGGGAAATTTCTCCCTGATAAGCGAATCCT
TCGGCCAGCGCTCTCGCGAGGTGTTTTTCCGGGGTATCAGCAAAATCGTTGTAGTAGGCCTGGGTCTCTCCGG
TCGCAAAAACGTGGACGGCATTATGAAAATCGTCATTCCATTCGGCGGTAAACAGCGGCGCATTGCCATCCTG
ATCACGGGGATGCAGAGAAATAATATTGCGGCTATCTTCGGTAGTCAGATGAATGGGTCTGTCGGTAATGTCT
TCCCGAATACGTTGTGCGATTTCAACCAGCACATGCCTGGCGCTACTGTCCTCAATCTGATCGATAGCGTCAA
AGCGTAAGCCGTCGAGATGGTATTCTGTCAGCCAGTATAACGGCGCCTCGATGATATAGCGCCGCACGGCGTC
GACATCATAGGCGATACCATTTCCCCACGGCGTCATGCGCTCTTTGTGGAAAAACGCCGGCGCCAATAGCGGT
AAATAATTTCCCTCCGGGCCGAAATGGTTCAGCACAATATCCAGGACGACGGAAAGACCATACCCATGCGCGG
CGTCAATAAACGCCTTGAAATCATCCGGCGTCCCATAGGCAGAATGCGGCGCGTAAAGCAGTACGCCGTCATA
GCCCCAGCCACGCTCGCCGCCAAATTGCGCAACGGGCATCACTTCAATAACGGTAACGCCGAGTTCAGCGAGA
TAAGGCAGCTTCGCTATTGCGGCGCGGAAGGTGCCTTCCGGAGTGAACGTGCCTGTATGCATCTCATACACCA
CGGCCTGCTCCCAACGGCTACCTTTCCACCCGGTGTTGCGCCACGTGTAGCTGCCTGGATCAACCACATATGA
CGGACCGTTGACGTCAGTTTTTGGGCGCGGGAAGCCGGATCGGGGACCACCATGCCATCGTTGAGTACAAAG
TTATACTCCGTACCTGGCGTCACCCCGGAGACGTCCAGCGTAAACCAGCCGTCACCGCTCGCCTGCATTTCCT
GGTCTTTACCCGCAAGCCTGAGCATAACCTTTTGCTGACCGGTGGCCCAAAGACGAAAGCGGACAACATCAGC
GGCGATATATTCAGCCCCCCAGCTTTTGCAAAAAATTTTTGAACTCATTTGTCGCTCCGTTTAACCCATAACC
TCTACAACATCCGAAGAACAAGCATAGACTATGGGGCTAATCTTTACGTGACACTGTCACTTAACAGGCATTC
TTTTTCCTATCTGCGTGTAAAACCGTGGAATTATTGTCGGGATTGCTCAGATGTACCGAAGTGCGCGGTATCG
CCTCGCCCGTGTTCGCCAGAGCCCTGAACACGCTGTAGCTGGATAACCTCCCCCTGCTCCCAGGCGTTAGCCC
CCTTAACAACGGGATGTTCAGGCAGATCCTGCCGCTCGCGTTTAATGGCCTGGGTATCAATTCCCAGACGGCG
ATCCCGATCGGCCAGCAGCGCGGGGTCAAGCTGCCCGTCGCCAGTAAAGCCCATCATCAGCGCAGGGATACCC
ATTGGCAGGGTTTTATCCCGGTCGGTATGCCAGGTGTGCCAGGTCTTGCCATAAGTATTAACAATCTTACTCA
TCAACGCTTTATCAGCGACCTGCGGTAAGCCAGGCGCCACCAGGCTACCGGATTTCACCTCGTACTGGTGGCT
GTGCCACAGTTTTTTCTCCTCGGGAGGAAGCGTTTTAAATAAACGTTCGCTGATAATGTACTCCACCCCCATC
AGGCGCGCGTTTTTCGTATTGCCGTCGTAAATCACCGCCTGCATGACATCTTCGTTCAGGACGGTAACGTAAT
GGTGCGCTTCCATCTGTCCGTTTTTATCACCGCTATAAAAATGGAACCCGTCAAGGTAGGTACTGATGGCATC
GACAGGTGGTCTGGATTGAATAGCCGCCGCGCCTGTTTCCAGGGTACGTAATTTGGCAGAGGTTTTTTCCCCG
GGAATAGGCGTTTGCGGCGGCGTATTATTGGCGCCGCATCCGGCCAGAAAAGGAATGATAAAGAGTGCCAGCG
GTAAAATTTTCATTAGGTCTCCATATAGTGAAAGGGCTCTAATGAAAATAGTGCAATACCACGTGATTGGTAG
AAGAAATGGAAGATGTTATTTGTGACGGTGTCACAAATAGTAAAACCCGACACACAGGCCGGGTTTTGATATC
AGTTTACTCTTATTTTGAGAGTTCTTTCTTGATTTCGTCTTTTATTTTGCTTAATTCACTCTGCGGGTTTTTC
TTACAAAGCTCAACGGCTAGCGGCACTGCTGTCGTCTCAGTTTCCTGGAAATCTACGTAGTCCCCGCCTTTAA
AATCTTCATCTTCATTCAGCACCCAGAAAGCGACTGGCGCCATCGTCTGCGGATTGAGGTCAACAAACTCCTG
GCAGCTCATACCTTTAGGCGTAACGTTTGTTTTAGTAGTATCTGTTGCCGCATTCACGCTAACACTGGTTACC
AGCGCTGCCACGATAATACCTGCTGTAGCAAGGGAGAATTTATTCATTGTTGTTGCTCCAATAATTATATAAA
TAACCTCTACATCTGCGCGTTAACCCGTTAATGTAGAACACAGAAAATAATACCCCTCCTCTCTCAGGAATAA
AGATCACCCTCCGTGATATGGCTATTATTGATATTTACGTTGACTGATTTTGATGTTTGATGAATAAAGCAAC
ATGGCGAGCAGATTTTAAGGCGGAAAAGTAGGCCACGACAATAGCCGCAGACGTGGTCTACGGCGCGTTGAGG
TGATAGAAATACGGCAGGATCTCAGATAGACCGGGAGCAAGCCCTCCGTCTGAAAGTATATTCATCAGCCCGA
TCAACCCATCGTTACCGGGCTGGAGAGGTTATCAGGCGTTAAGCTGGTAGTCGAGGGTGATTTCCGCATTCAG
AACCTGCGATACCGGGCACCCGGCTTTTGCTTTCTGGATGATTTGATCGAAAGTTGAAGCATCAATATCTGCT
ACCGCGACTTTGCTTTGTAGCGCAATTTTGGTAATGGCGAAGCCCGCCTCCACCTTATCAAGCGATACGTCAG
CGGTGGTGTCAATGGATGTCGGCGTAAATCCAGCCTCTCCCAGCATGAGTGATAGCGCCATGGAGAAACAGGC
CGCATGTGCGGCGCCAATTAACTCTTCCGGGTTGGTTCCCTGCGCGCCTTCGAACCGGGTATTGAATCCATAC
GGTTGTTGATTAAGCACGCCGCTTTCTGTTGATACAGTACCTTTACCCCGTTTGATGTCGCCTTCCCAGTGTG
CCTGACCTTTCTTATGGATTGTCATTTTTGCTCTCCTGTTGGCTCAAAAAATAAAGTATAGACCATGCTGCTA
ATCTGCGTTGTCCATGCCGATGGAGGTAGCATGTTGTCGATTACCCGCACTTCAGATATAGAAAATAGTTGTA

TGCTTTTTCAGTGATGAATATTAAACCAACAGGGCTCCCTTTCTTTAAAGAATATATCCCTCCTTTTGTCCTT
ACTTTTTCTTCTGGAGGCGTATTCCATTATGTATATTTTATATGTATATTGAGATTTATCCTATCACATTGTA
ATAAAGGATAAAAATCACAATAAGTAGCCTTTTAAATGTATATTAATTGACCACTATTTGTATGAGGATATGT
AATTGGCAAACGTAATATATTTTCTTCAGGGGTAAACAATTAGAGTTCCGCCTTGCGTTAAAATAACTCCAGG
ACTAATCTTAACCACAGCCGCAAAAATATTTGCCATGCTGAATACGAATTAATTTTCTGCGAGCCGTACTAAA
TAAATACATGTTCACGGAGGCATTATATGTTTACTTATCACTCAGCAAATACCTCAGCAGCACAACCGGCCTT
GGTCAACGCGATTGAACAAGGATTACGCGCGGAGTTAGGCGTTGTCACTGAAGATGATATTTTAATGGAGCTG
ACCAAATGGGTTGAGGCTTCCGACAATGACATCCTCAGTGACATCTACCAGCAGACCATCAACTATGTGGTAA
GCGGGCAGCACCCGACTCTGTAAAACCTGCTATGCTGTATCGGCAACCTTAGGGGTTACGTTTCTTTCGCGTA
AGCGAATTTGTCCTTAAAACTAGCAACAGGATTGAGGAGTTAACATGAAATCGAACCGTCAGGCTCGTCATAT
ACTTGGACTGGATTACAGAATTTCCAACCAGCGTAAGGTTGTTACTGAAGGCGACACCAGCAGCGTGGTGAAT
AACCCAACCGGCAGAAAGCGCCGCGCCGACAGCCAAAAGTAATCGTACAGTCCTGAAATGACGTTAAACACCA
TTGCATCTGCAATGGTGTTTTTGTTTATAGGAATGACCTTAACGTCTCACAATGCTTTATACTCAGGGTCGGT
TCCCTGGTGGAAGGCAATATATGATGATAAACGACGGTGCCAGGGACAGTTATTCGAAAAAGAGTGAGTAACA
TGGAAACTATAACCAAGAAAGCACGTTCCCTTTATATCCCTTACGCTGGCCCTGTATTGCTGGAATTCCCCCT
GTTAAACAAAGGTAGCGCTTTTAGCGTAGAAGAGCGCCGCAACTTCAATTTGTCAGGGCTGCTGCCAGAGGTC
GTCGAGTCGATTGAAGAACAGGCCGAACGCGCCTGGCTCCAGTATCAGGGGTTTAAAACAGAAATCGATAAGC
ATATTTACCTGCGTAACATCCAGGATACCAACGAAACGCTCTTTTATCGACTGGTGCAAAACCATCTTGAAGA
GATGATGCCGGTTATCTATACCCCGACCGTGGGCGCGGCCTGTGAGCGCTTCTCTGAGATTTATCGCCGGGCG
CGCGGCGTATTTATCTCTTATCCGAACCGTCACAATATGGACGACATTTTGCAGAATGTGCCTAACCACAACA
TTAAAGTTATTGTGGTGACGGATGGCGAGCGGATTCTGGGGCTTGGCGATCAGGGCATCGGCGGGATGGGGAT
CCCTATCGGTAAGCTGTCATTATACACCGCCTGCGGCGGCATTAGTCCGGCCTATACCCTTCCCGTTGTGCTG
GATGTCGGCACCAACAATCAACAGTTACTCAACGATCCGCTTTACATGGGCTGGCGTCATCCGCGCATTACCG
ACGATGAGTATTATGCGTTTGTCGATGAGTTTATTCAGGCCGTTAAGCAGCGCTGGCCAGATATCCTGCTACA
GTTTGAAGATTTCGCGCAGAAAAACGCAATGCCGCTGTTGACGCGCTATCGTGATGAGATTTGCTCCTTTAAC
GATGATATTCAGGGCACCGCAGCCGTGACCGTCGGTACGTTGATCGCCGCCAGCCGCGCTGCCGGCAGTCAGC
TTAGCGAGCAGAAAATCGTTTTCCTCGGCGCGGGTTCCGCCGGATGTGGGATTGCTGAACAAATCATCGCGCA
GACTCAGCGTGAAGGTTTAAGCGAAGATGCCGCTCGACAGAACGTTTTTATGGTCGATCGCTTCGGCCTGTTG
ACGGACCGGATGCCTAATTTATTGCCGTTCCAGGCAAAACTGGTGCAGAAATGCGACAATCTCCAGCACTGGG
ATACCGAAAATGACGTGCTGTCGCTGCTGGATGTGGTGCGTAACGTGAAGCCGGATATCCTGATCGGGGTTTC
CGGCCAGACCGGGCTCTTTACTGAAGAGATCATCCGCGAGATGCATAAGCATTGCCCTCGCCCGATCGTCATG
CCGCTCTCTAATCCCACCTCTCGCGTGGAGGCCACGCCACAGGACATTATCGCCTGGACGGAAGGCAACGCGC
TGGTGGCAACCGGCAGTCCATTCTCGCCGGTCATCTGGAAAGATAAGATTTATCCGATCGCACAATGTAATAA
CGCCTATATTTTCCCCGGCATCGGTCTGGGGGTTATCGCTTCCGGCGCATCGCGCATTACTGATGAAATGCTG
ATGTCAGCCAGTGAGACGCTGGCGAAACATTCTCCGCTGGTCAATAACGGCGAAGGATTAGTGTTACCAGCGC
TTAAAGACATCCAGGTCGTGTCGCGCGCTATCGCTTTTGCCGTAGGTAAAATGGCGCAGCAACAAGGCGTAGC
GGTGAAAACCTCTGCGGAAGCGCTGCAACAGGCGATTGACGATAACTTCTGGAAACCGGAATACCGCGACTAC
CGTCGGACTTCCATTTAATACGTACCCGGCGGTGAATGCCGCCGGATATCATTTTCTTCTTTAGTTTTAGTGA
GTCTGTCGACACACCTGAATTCCTTTCCCTGCTTATTTGTCAAGCTACTAATATTAGCCACCTTTATGTATTG
AACGGGGATTTATCAGGATAATCCTGGCTAAACTATTACATTGTATTTCTGCCGCCTTGCATAACTCATCAGA
AATATTAATCCTAAAGAGGTAATAGCACCTTACCTGCTATTACCTGCTATGCAGTTCACTATTAATAAAAGGA
GAAGGTTATGAAAGCTGCTGTTGTCACACAAGATCATCAGGTTGATGTTACTGAGAAAACGCTGCGTCCCCTT
CGCCACGGTGAAGCTTTGCTAAAAATGGAGTGCTGCGGCGTATGCCATACCGATCTACACGTGAAAAACGGCG
ATTTCGGTGACAAAACCGGCGTCATTCTGGGACACGAAGGCATTGGCGTCGTGGCGGAAGTCGGTCCTGGTGT
CACCTCGCTGAAACCCGGCGATCGCGCGAGTGTCGCGTGGTTTTATGAAGGATGCGGACACTGTGAGTATTGC
AATACGGGCAATGAAACCTTATGCCGCAACGTGAAAAATGCCGGTTACACGGTAGATGGCGGCATGGCCGAAG
AGTGTATTGTCGTCGCGGATTATGCGGTGAAAGTACCGGAAGGTCTGGACTCTGCGGCGGCAAGTAGTATTAC
CTGCGCGGGCGTTACCACGTATAAAGCGGTGAAGATTTCTCATATTAAACCTGGCCAGTGGATTGCCATCTAC
GGTCTGGGCGGTCTGGGTAATCTGGCCTTGCAGTACGCGAAAAACGTTTTCAATGCGAAAGTGATCGCCATTG
ATGTCAATGATGGACAACTGAAACTGGCCGAGGAAATGGGCGCAGATCTGACCATTAATTCACGTACGGAAGA
TGCGGCTAAAATTGTGCAGGAAAAAACCGGTGGCGCGCACGCAGCGGTGGTTACCGCGGTCGCTAAGGCGGCG
TTTAATTCCGCAGTAGATGCCGTTCGCGCCGGAGGTCGCGTGGTGGCAGTCGGGCTGCCGCCAGAAGCCATGA
ACCTTGATATCCCTCGCCTGGTGCTGGATGGCATTCAGGTTGTCGGTTCGTTGGTGGGGACACGCCAGGATCT
GACCGAAGCCTTCCAGTTCGCGGCGGAAGGAAAAGTCGTGCCAAAAGTCGCTTTACGTCCGCTTGAAGATATC

AACGTTATTTTTAAAGAGATGGAACAAGGGCAAATTCGTGGCCGTATGGTCATCGATTTCCGTCGTTAAACGA
TTTGCTGTGGTCGTCTGAGTATACAGACAGCAGATGAAAACAAGGCGCTACCAGGATAGCGCCTTGTTTATCA
GATTGCGAAAGAATTACTGAATACCTTCTTCGCTCTCTTTTTTTGCTTCCGCCTTCTCGATTTCACGATACCA
GCGTGGATGATGTTTCTTCGCCCAGCGACGGCTGACCTTCCCTTCGATCATGCCTTTAATCGAGCCTTTCACC
CAAAATGCCATATACATATGGATGAGGATCGCGTGCATTAAAATAATGCCCGCCGCCGCATGAATTAACAGGC
TATAACGCACCACCTGCATGGGGAAATACTGCGCGAAATACGGACGCCAGATAATCACGCCAGTCACCAGCAG
CACAAAAATCATGCTCATAATGGACCAGAACATCATCTTCTGTCCGGCATTGTATTTGCCGACATCCGCCACC
TTATGCTCATTGCCTTTCAATACTTCGACAATATTTTTCAGCCACGGAATATCTTTTTTATCCGGGATGTTGT
GGTGGACGAAACGCACGAACATAAACATCAGCGCGATGAAGATGGCAATACCGAAGAACGGGTGCAAAATACG
TCCCATCTGCGGCGTGCCGAAAGTTTGCGTCAACCATTGTAGCGTCGGGAAGAAAAACGAAATTCCCGACAGC
GCTACCAGGAAGAAGCAGATCACCACCGTCCAGTGACAGGCGCGGTCGACAAATTTCGTGCGCACAATCATTT
TCGACTTACTCATGATGATCCTCCTCATCGTCGTCCGTTTCTTTATTCGGGCCAATACCGATGTAGTGGTAAA
TCAGACCGGCGAAAGTGGCGATAAAGCCCGCTGCGGCCAGCGGTTTTAGCGCCCCTTTCCACAGATTGATTGA
GGTATCGATCTGCGGATCTTTCGGCAAGCCGTGGTACAACTCCGGCTGATTGGCATGGTGCAGCACATACATG
ACGTGCGTGCCGCCGACGCCCTGCGGGTTGTAGATACCGGCATGTTCAAAGCCACGCGCTTTTAACTTCTCCA
CGCGTTGCTCGCCCAGTTCCAGCATCTCTTTTTTGGTGCCGAAGTGAATAGCCCCGGTCGGACAAGTTTTGAC
GCAGGCCGGTTCCTGGCCCACGCTGACGCGATCGACGCAGAGCGTACATTTATAAACGCGGTTGTCCTCTTTG
TTGAGGCGCGGAATATTAAACGGACACCCGGCGATACAGTAACCACAGCCGATACAGTGTTCAGATTGAAAGT
CAACAATCCCGTTCGCGTACTGGATGATAGCCCCGGCAGATGGACAGGCTTTAAGACAGCCTGGATCTTCACA
GTGCATACAACCATCTTTACGAATCAGCCACTCCAGCTTGCCGTTCTGCTCGGTTTCGGTAAAGCGCATCACC
GTCCAGGACTTGGCGCTGAGATCGGCAGGGTTATCGTACACCCCGACACAATGACCAACGTTATCGCGAATAT
CGTTCCATTCAGAACAGGCTACCTGGCAGGCTTTACAGCCGACACAGGAGGAGACGTCGATCAGTTTCGCGAC
TTCGGCTTTGTAATCACGCGCCTGCGGAGGCGGCGTGATGGCGTTTGTTGCGGACCGTTTAATAATGTCCTGC
GTTTCCATAGACATATGTTCGCCTCCCTTACGCCTTCTCGATGTTAACTAAAAACGCTTTGTATTCCGGCGTT
TGCGAGTTGGCATCACCGACATTTGGCGTCAGAGTATTGGCGATATAGCCTTTGCGCGCGACACCCTCAAAGC
CCCAGTGAATCGGGATCCCCACCGTTTCAACCTGCTGACCGTTGACATGCAGGGTGCGAAGACGACGAGTTAC
CACCGCGACCGCGCGAATAAACCCGCGTTTACTGCTCACCTTCACATAGTCGCCGTTAGCAATACCTTTCGCT
GCCGCCAGCGTCTCACTGATCTCCACAAACTGTTCCGGCTGAGCGATAGCGTTAAGTAGCGCATGTTTGGTCC
AGGTATGGAAATGCTCTGTGAGGCGGTAAGTCGTGCCCACGTACGGAAACTGCTCCTTCTTACCCATGCGCAG
CGCATCTTCTTCATACAGACGCGCAGCCGGGTTCGAGACCACATTCGGGTGCAGCGGGTTAGTGCCCAGCGGC
GTTTCCATCGGCTCGTAATGCTCAGGGAACGGCCCTTCCGCCATTTTATCAATAGCAAACAGACGTCCCAGCC
CTTCCGGCTGCATGATAAACGGATTGGTCCCGCTCCCCGGCGCCGCGTTGTTAAAGTCCGGAATATCGTTCCC
TGTCCACTTCGCGCCGTTCCACTGGATCAGCATCCGTTTTGGATCCCACGGTTTACCCTGCGGATCCGCCGAA
GCGCGGTTATACAATACGCGACGGTTTAGCGGCCACGCCCACGCCCAACCCAGTGTATTGCCAAGCCCTGACG
GGTCGGCGTTATCACGTCTGGACATCTGGTTGCCCTGCTCCGTCCAGCTACCGGTATAAATCCAGCAGGAGGA
GGATGTCGTACCGTCATCGCGCAGTAATGCAAAACTACTCAACAACTGGCCTTTCCTGGCAAGCAGTGTACCG
TTGGCGTCATAGAGATCTTCCAGCGCATAGCCGTTGTTCTCTTTCGCCACTTCTTCCGAATGCGGTTCATCCG
GCTGCTTGTAATTCCAGCTCATCTTCAATAGTGGTTCGGCGCCTTTACCGCCTTCTGCGCGATACATTTCACG
CAGACGATGGTAAATACCGGCCAGAATCTCGCCGTCGTTACGCGCTTCGCCCGGCGCGTCCTGACCTTTCCAG
TGCCACTGTAACCAGCGACCGGAGTTAGCGATAGATCCATCCTCTTCCGCAAAACAGGTAGACGGCAGACGGA
AGACTTCGGTCTGAATCGTTGTCGGATCGACATCATTAGATTCGCCGTGGTTCTGCCAGAACGTTGACGTTTC
AGTCACCAGCGGATCGATAACGACCAGATACTTCAGTTTGCTTAAGCTTTGCACCACTTTGTTTTTGTCCGGG
AAGGACGCCACCGGGTTAAAGCCCTGACAGAAATAGCCGGTGACTTTCCCGCTATCCATCATGTTGAAATATT
TGATCACGTCATAGGACTGATCCCACTTAGGCAGCCAGGCAAAGCCCCAGTCATTCTCTTTCTGCGCCGCATC
GCCATAGAAAGATTTCATCAGGCTGACGAAGAACTTAGGATAATTACCCCAGTAGTTGACCTGATCGGCCAGC
GTCGCTTTGGGGGTATTCGCTGCCAGATAAGTTTGCAGATCGGCCTGTTTTTCTGACGGTAGCGTCAGGTAAC
CCGGCAGGCTGGTGGAGAGCAGTCCTAAATCCGTTAAACCCTGAATATTAGAGTGGCCGCGCAAGGCGTTCAC
ACCGCCGCCAGCCATCCCCATATTGCCGAGCAGCAACTGGATCATCGCCATAGTACGGATGTTCTGCGCCCCG
ACGGTATGTTGCGTCCAGCCCAGCGCATACAGGAAGGTGGTAGTGCGATCCGGCGCGCTGGTAGAGGCCAACA
CCTCACACACCTTCAGGAAATCCGCTTTCGGCGTACCGCAGATGTTTTCCACGACGTCCGGCGTGTAGCGCGA
AACGTGCTGCTTCAGCAGATTCCACACACAGCGTGGGTGCGTCAACGTTTCATCACGTTTGGCATAGCCGTTT
TCATCGAACTGGTAGTTCCAGGAGGATTTATCGTACTGACGCTTTTGGGCGTCATAGCCGCTGAAAAGACCGT
CATCAAAGGCAAAATCATCCCGCACCAGCAGGCTGGCGTTGGTGTAATGCTTAACGTATTCAGCGTTGATTTT
GTTATTTTCGATCAGGTACAGCAAAACGCCGGACAGAAAGGTAATGTCTGTACCGGAACGTATTGGCGCATAG

ATATCCGCCACCGACGCCGTACGCGTAAAGCGTGGATCAACGACAATCAGCGTCGCATCGTTATTATTTTTCG
CTTCCATCGCCCAGCGGAAACCCACCGGATGCGCTTCTGCGGCATTGCCGCCCATCACCATCACGACGTTGGC
GTTTTTAATATCCACCCAGTGGTTGGTCATCGCACCGCGACCAAATGTTGGAGCAAGACTTGCTACCGTTGGT
CCGTGTCAGACGCGCGCCTGGTTATCTACCGCCAGCATACCGAGAGAACGCGCAAACTTCTGCGTCAGCATAC
CCGTTTCATTGCTGGCCGCCGAGGCGCACAGCATCCCGGTAGAGAGCCAGCGGTTAACCGTGACGCCCTGCTC
ATTCTTTTCAATAAAGTTGGCGTCGCGGTCGGCTTTCATTAATTTAGCAATACGAGTGAAAGCGTCATCCCAG
CTAATGCGCTGCCACTTGTCGGAACCCGGCGCGCGGTATTCCGGATAGCGTAGGCGATCTTCACTGTGAACAT
AGTCCAGCAAACCGGCCCCTTTCGGGCACAGCGCGCCACGGCTTACCGGATGATCCGGGTCGCCTTCAATATG
GTAAATCGCTTCTTTCGCGTTTTTTGCCCCATCTCCCAGGCTATACATTAATAGCCCGCAACCTACGGAACAG
TATGTGCAGGAGTTACGGATCTCTTTTGCGCGCAACAGCTTATAGTTTCGCGCCTGAGCCAGGGCCATTTTAG
GGGTAAAACCCAGTGCGGCCACCGTTGTTCCCGCCATACCGCCCGCGCAGATTTTAAAAAATTGTCTGCGGCT
GACGTCCATTGCTTTCCTCTTTTTTCCACGGATGCACGCCTCCTTCGAACTGCAGCAAAACCTCAGCCCAGGA
TGCGTGTATAACTTCGCGAGGAAACTAACAGCAAAATCGATAACTTTTTTGCGTCAAATCAATATTGAAACGG
TTCCGCTATTAAATAGCCCTATTTTTAAACCGCAATAACTCCAGGGAGGTACTAAAAAATGACACTGCATGGC
ATCGCCCTCGGAAAAAGTGCTATAAGTGGCAGTTTATTTTGCGCGGTATTCAGGACAGTCACAAAGCATGACA
TCACAAAAAGCAACGCTAATAGGGCTTGTTGCCATCGTTCTGTGGAGCACGATGGTTGGGTTAATTCGCGGCG
TCAGCGAAGGTCTCGGACCGGTAGGCGGCGCAGCCATGATCTATTCATTAAGCGGTCTGCTGCTGATTTTTAC
CGTCGGATTACCTGATATCCGCCGTTTCCCGGGGCGATACCTCATCGCAGGCAGCGTACTGTTTGTCAGTTAT
GAAATATGCCTGGCGCTTTCTCTCGGTTATGCCGCCACTCGCCATCAGGCTATCGAAGTCGGCATGGTTAACT
ATTTATGGCCCAGCCTGACCATTCTGTTTGCCATTCTTTTTAATGGGCAAAAAACTAACTGGCTTATTGTTCC
TGGATTGCTTATTGCACTTACTGGCGTTTGCTGGGTTCTGGGGGGCGAGAATGGTCTTAATCCAGGAGAAATT
ATCAGCAATGTCGCCACCAGTCCGCTAAGTTATTTACTGGCATTTCTTGGGGCGTTTATATGGGCGACGTACT
GCACCGTCACAAATAAATATGCGCGTGGTTTTAACGGTATCACAGTCTTTGTGTTACTCACCGCCGTGGCGCT
GTGGTTCCACTATTTTCTTACGCCGCAACCTGCCATGATATTTAGCCTACCGGTGATTGCTAAACTTTTTACC
GCGGCATTAACGTTAGGGTTTGCCTACGCCGCATGGAATGTGGGCATACTTCATGGCAATGTGACTATCATGG
CCGTAGGGTCTTATTTTACCCCCGTCATGTCCTCTGCTCTGGCCGCGCTGTTACTCAGCTCACCACTGTCATT
CTCCTTCTGGCAAGGCGCAGTCATGGTTTGCGTAGGTTCGCTACTTTGTTGGCTGGCAACGCGGCGGCGCTAA
GCGACGGGCGGGCCGATATTGATATTAAATCGACCCGCTGTACCTTGACACTCTTCATAACACAACTACGCTT
TTATCTATTGCCTGTGCGTATTTGACGATTTTCCATTTTCACGTGCTCCTCCTGCGCCATACCAGGATTGCGC
TGGTAAAAATGAATTTTATAAAATCGTTAAACATGTTAATGCAATGATATAATTATATTAACAAATATATAAT
TAACGTTAGCTCGCAACTGTTATTAGTGCATATAATAATCAGTAGTTACTACCAAAAATAAAACCATAAACAG
CCCAATAGTCCCCTCCGAAAATATATCTTATTAGACAGTCCCTATTTGAATTAATACTCTCAAATGTATTAAG
GAGATCTCGATCACACAAATTAAAATAATTTGTAATCTTATGAAACTTATTATTGAACTTATGCCACTCCGTC
ATTTAAAAATAGTCTGCCATTGACAAACGCCTCGTTTAACAATGGTTGAGGAAACACGCTAAGAAAATTATAA
GGATTATTAAAATGAAACTTAAGTTAGTGGCAGTGGCAGTGACTTCCCTGTTGGCAGCAGGCGTTGTAAATGC
AGCCGAGGTATATAACAAAGACGGCAATAAACTGGATCTGTACGGTAAAGTTCACGCTCAGCATTATTTCTCT
GATGATAACGGAAGTGATGGTGACAAAACTTACGCGCGTCTGGGCTTTAAAGGCGAAACTCAGATCAACGATC
AGTTAACCGGTTTTGGCCAGTGGGAATATGAATTCAAAGGCAACCGTACTGAAAGCCAGGGCGCTGACAAAGA
CAAAACCCGTCTGGCGTTTGCTGGTCTGAAATTCGCTGACTACGGTTCTTTCGACTACGGTCGTAACTACGGC
GTTGCTTACGACATCGGCGCATGGACCGACGTTCTGCCAGAGTTCGGTGGCGATACCTGGACTCAGACTGACG
TCTTCATGACTGGCCGTACTACTGGTGTTGCCACCTACCGTAACACTGACTTCTTTGGCCTGGTTGAAGGTCT
GAACTTTGCCGCGCAGTACCAGGGCAAAAACGACCGTGACGGCGCGTACGAGTCTAACGGCGACGGTTTCGGT
CTGTCCGCAACGTATGAGTACGAAGGTTTTGGCGTAGGTGCGGCCTATGCGAAGTCTGACCGTACTAACAACC
AGGTGAAAGCAGCGAGCAACCTGAATGCTGCGGGTAAAAACGCTGAAGTCTGGGCTGCTGGTCTGAAATATGA
TGCGAACAACATCTACCTGGCGACCACCTACTCCGAAACGCTGAACATGACCACCTTTGGTGAAGATTCAGCG
GGTGATGCGTTTATCGCGAATAAAACCCAGAACTTTGAAGCAGTTGCTCAGTATCAGTTCGACTTCGGTCTGC
GTCCGTCCATCGCTTACCTGAAATCCAAAGGTAAAAACCTGGGTACTTACGGCGACCAGGATCTGGTTGAGTA
CATCGATGTCGGTGCTACCTACTACTTCAACAAAAACATGTCCACCTTCGTTGATTACAAAATCAACCTGCTG
GACGACAGCGACTTCACCAAAGCGGCTAAAGTGTCTACCGACAACATCGTTGCTGTTGGTCTGAACTACCAGT
TCTAATTTTGTTGTAGTCTGAATACAAAGCCAGTCCTTTCAGGGCTGGCTTTTTCTCTTGATGTAGACTCGGT
CATAAGGAGTATGCCGTGCAGACGTTCACTGGTCGTTGTCTTTGCGGACAGAGCCATTTTACCGTTGACGTCG
AAATGCTGGATGTCTATGCCTGCCATTGTACGCTGTGTCAAAAATGGTCGGGTGGAATAGCCATGTATCTGGA
AGCCTGCGGGCAACCACAGCTAGCCCCCTTTCCCCGGAACCGCCATACTTTTCATCCTCTTCGCGCGGCGAAC
GCTACTTCTGTTCCGGCTGCGGGTGTCCGCTGTGGTTTCGGCTTACCGATACCGATCGCTATTTTATTCCCTG

GACCTTACTGGAACTGAACGAAGTTGATCGCCGTCGGCTGATTCTGGCTGCGGAAATTTATACCGAAACCCAG
CCTGCCTTCTGGCGACTGACGGGGCAATACGCACGCCTCAGCGGTAAAGAGGTTGAGGAAATGGACTCCCCTG
CCAGCTAGCGCATTAAGCGCTTATCTCACCAGGCGTTATGTTATCGGCGTTGGGCTTTTGCCAGGCTGAACCA
CATCCCTACCGCCAGCAAAAGAAGCATACTCCCGGCGCTGCTTAACGCCACGCTATGCGACCAGATAAAGGCA
TGTCTGGCGGCGTCCAGTATTGCCTGCCCCTGCGTGGGCGGTAGGCTATTCGCCAACTGCACGGCTTCTCCCA
TTGAAGATGACGCTCTGGCAATCTCTTGCGCCTCAAGCCCGGCAGGCAGACGGATAGACGCGGAGAAGCTACG
GCTTAACAACAGACCGAAAATGGCAATGCCCAGTCCCGCGCCCAGTTCATAAGCCATCGTTTCTATCGCGCCG
GCCGCCGCCGCTTTTTCTGCCGGCGCAGCGGCCATAATTGCCGACGTGGAAGCCAGCAATGCGCTGGCGGCGC
TAAATCCCAGCAGCGCCATCAGCCCCCAGGCCTGCCATTGTTGGGTGCTGAAATCCGTCATCGCCAGGCCATA
AAAACTCAGCGCGCTTAACGCCATGCCGCCCGTCGCGACCAGCCGTAACCCCAGACGCGAGACCAGCGCGCCC
GCAATCGGCCCGCTGAATCCACTGGCGACCATCACCGGCAGCATAAATACCCCGGCCTCATAAGGCGATAGCC
CGTGAACAAACTGTAGCTCTTGCGCCATCAGCAGCTCAAAACCCACCAGAGTGATCATCGCGGTCATCGCCAT
CACGACGCCGCTCAGGATAATGCGATGGGTAAATAGTCGCATATCAATCATCGGACGCGATGTCGCAAGCTGT
GTGCGGAGAAAGAGTCCCAGGAGCAGCGTGCCGGTAAGCAGGGTAAGCGAGATGGCCCACAGCGACAAATTCC
CTTTCAGCGCGGTTTTAGCGCTATAGACCAACAGCAAAATGGCAATAATCAGCATCACCGCATGGCCAAGATT
GAGCGGTTGATCGCGACGGCCCGCCTGGCGAGGAACATAGCGGGCGGTTAAGCCCATGACGACCAGCACAATC
GGCACGTTGATCAGGAAAACCGATCCCCAGTAAAAATGCTCTAATAATATGCCGCCGATGAGCGGCCCAAACG
CCGCTCCGCCCGAACCTACCGCTGCCCAGACGCCCAGCGCCATGTTGCGATGCTTCTCTTCACAAAAGGTAGC
GCGTATTCCGGCCAGCGTCGCCGGTACAATCATCGCCGCGCCAATAGCCAGTAATACCCTGGTGGCGATAAGC
CAACTGGCGGTATGCGAAAACGCTGCCGCCAATGACGCCAGGCCAAAGAGCGTCCCGCCCAGCATCAGCAGGC
GTTTAAAACCGATACGATCGCCAAGCGCGCCCATCGGCAACACCATGCCAGCCATGACCAAAGAATAAATATC
AATGATCCACAGCAGCTCGTTGCCGCTGGCCCCCAGTGTCATACTCAGTGTCGGGGCGGCGACATGCAAGACC
GTGGCATCAATGGCGACAGGAATATAGACCAGCACAATAATGACTAACGTTAACCACTGACGAAACATAACTC
TCCCTTCAAAAACTCAAATTTGGACGCTTGTCCAGCTTCAGGATCCTACGGTAAGTTGAACGAATGTCCAGCT
TTTTGTTACACTGCGCGCATTGGCTAATGTGGGGAAAATATGAGTTACCTTAACCGGGATGAACGCCGCGAAG
TGATCCTGCAAGCGGCGATGCGCGTGGCGCTGGCAGACGGGTTTGCCGCCATGACGGTGCGACGAATTGCATC
AGAAGCCGATGTCGCCGCCGGCCAGGTGCATCACCACTTCAGCTCGGCGGGCGAGCTCAAAGCGCTGGCCTTT
GTCCATTTGATCCGCACCCTGCTTGATGCCGGGCAAGTTCCGCCGCCCGCCACCTGGCGTGCACGGCTTCACG
CTATGTTGGGTAGCGAAGACGGCGGCTTTGAGCCTTACATAAAATTGTGGCGAGAGGCGCAAATTCTGGCCGA
CCGGGATCCGCACATCAGAGATGCTTATCTCCTCACGATGCAGATGTGGCATGAGGAAACGGTCACAATTATC
GAGCAGGGCAAACAGGCAGGAGAATTTACGTTTACTGCTAACGCCACGGATATCGCCTGGCGGCTTATCGCAC
TGGTGTGCGGGCTGGACGGTATGTATGTACTGGGGATTCCGGAAATGGCCGATCCCGCGTTTAAATTCCATCT
GGATCGTATGATTACGCTTGAACTCTTTGCGTAAAGTTACAAATAATTTAATAAAAATCGCTTTTAATTTACA
AATAGTAACAATTAAGAAACATCATATCTCCTCATCGCGCCGCGCTGGCCTGGTTTTTTATCTATTCTTTCAC
TCAATATTCTAAAAAAACTTCAATAAGTTCTCAAAAGAGCGCGTGACGCTCTGTCTCTTTTTTGTGTTTTTCT
TCAAATGATATGCGCATGTGAGGGGTAAAATGACACGACAAAACGAGAATTATAACCGTTATCTCTTGAGTGA
CTGGCGACCCGAAAATCCAGCCTTCTGGGAGAATAAAGGGAAAGGCATCGCCAGAAGAAACTTATTGATTTCA
GTAAGTTGTTTGCTCCTTGCATTTTGCGTCTGGATGTTGTTTAGCGCTGTTGCCGTCAACCTCAATAAAATTG
GATTTAATTTTACCACCGACCAACTCTTTTTATTAACCGCACTACCTTCTCTTTCCGGCGCAATATTACGCGT
TCCCTACTCCTTTATGGTACCACTGTTTGGTGGCCGTAAATGGACCGTATTAAGTACCGTAATATTGATTATT
CCCTGCACCTGGCTGGGATTCGCCGTGCAAAATCCTGCCACCCCTTTCGGGGTATTTATGCTCATCGCTCTGT
TGTGCGGTTTTGCGGGGGCAAACTTCGCGTCCAGTATGGGCAACATCAGTTTTTTCTTTCCAAAAGCCAGACA
AGGTAGCGCGTTGGGTATTAACGGCGGACTGGGAAATCTTGGCGTCAGTGTGATGCAGTTGATTGCGCCGCTG
GTTATTTTTCTGCCGATATTTACTTTTTTAGGCGTCCGGGGCGTGCCGCAACCTGATGGCTCCCTGCTCGCCC
TCACTAACGCAGCCTGGATCTGGGTGCCGTTACTGGCTGTCGCGACGCTTGCCGCCTGGTTTGGTATGAACGA
TATCGGCAGTTCAAAAGCGTCTGTCGCCTCGCAATTGCCGGTGCTAAAACGTCTGCACCTGTGGCTGCTTAGC
CTGTTGTACCTGGCGACATTCGGGTCATTCATCGGCTTTTCCGCAGGTTTCGCCATGCTCGCCAAAACGCAAT
TTCCCGACGTAAACATTCTGCAACTGGCGTTCTTTGGCCCCTTTATCGGCGCGCTGGCGCGATCGGCAGGCGG
CGTGATCTCCGATAAATTCGGCGGCGTTCGCGTCACGCTCATTAATTTTATCTTTATGGCGCTGTTTACCGCC
TTGCTATTCCTCACACTGCCAGGCTCCGGCGCAGGCAGCTTTAGCGCGTTCTATCTTGTTTTTATGGGCCTGT
TTTTGACAGCGGGTCTCGGCAGCGGTTCTACCTTCCAGATGATTGCGGTGATCTTCCGTCAGATCACGCTCTA
CAACGTCAAACTGCGCGGCGGGAGCGATGAGCAGGCGCAGCGCGAAGCGGTGACGGATACCGCCGCCGCGCTC
GGTTTTATCTCCGCTATTGGCGCTGTGGGCGGCTTCTTTATTCCAAAAGCGTTTGGCACCTCTTTGGCCTTGA
CCGGCTCGCCGGTGGGCGCCATGAAAATCTTTTTGCTGTTTTACATCGCTTGCGTCCTGCTGACCTGGCTGGT

CTACGGGCGTCGTAAGCCTAAACAACAATAACAACATACGCACCTGTAGGACGGCCATCCGGTAATGGTTTAA
CGCCAAATCGACAGGATGGCGGAAAATTTATCGAAGCAGGAGAAATGTCATGAGTAAACTGTTGGATCGCTTT
CGTTACTTTAAACAAAAAGGCGATACCTTTGCCGACGGCCATGCTCAGGTGATGCATACCAACCGCGACTGGG
AAGACAGTTATCGCCAGCGCTGGCAGTTTGATAAGATCGTGCGCTCAACCCACGGCGTCAACTGCACGGGCTC
CTGTAGTTGGAAAATTTATGTCAAAAATGGTCTGGTGACGTGGGAAACCCAGCAAACCGACTACCCGCGCACG
CGTCCCGATTTACCGAACCATGAACCGCGCGGCTGCCCGCGCGGCGCCAGCTACTCATGGTATCTCTATAGCG
CCAATCGTTTGAAATACCCTCTGGTGCGTAAGCGGCTGATAGAACTGTGGCGCGAGGCCCTTTCCCGGCACAG
CGATCCGGTACTGGCATGGGAATCTATAATGAACGACCCGCAAAAATGCCAAAGTTACAAACAGGTGCGCGGA
CATGGCGGCTTTATTCGTTCGAACTGGAAAGAGCTGAATCAGCTCATTGCCGCTGCTAACGTCTGGACAATCA
AAACCTATGGTCCGGATCGTGTGGCGGGGTTTTCACCTATTCCGGCAATGTCGATGGTTTCTTACGCCGCCGG
AACACGTTATCTGTCATTGCTTGGCGGTACTTGTCTGAGCTTCTATGACTGGTATTGCGATCTTCCACCCGCG
TCGCCAATGACCTGGGGCGAGCAGACTGACGTGCCGGAGTCCGCCGACTGGTATAACTCAGCCTACATTATTG
CCTGGGGCTCTAACGTGCCGCAGACGCGTACCCCGGACGCCCACTTCTTTACCGAAGTGCGCTACAAAGGCAC
CAAGACGATCGCCATCACCCCGGACTATTCCGAAGTCGCTAAACTGTGCGATCAGTGGCTGGCGCCAAAGCAA
GGTACCGACAGCGCGCTGGCGATGGCCATGGGTCACGTTATTTTAAAAGAGTTTCATCTCGATAATCCCAGCG
ACTATTTTCTTAACTATTGCCGCCGTTATACCGACATGCCAATGCTGGTTCTGTTGGATGAGCGCGCAGACGG
CAGTTATGTGCCGGGGCGTATGATGCGCGCGTCGGATCTGGTGGATGGACTGGGAGAAGCCAATAATCCGGAA
TGGAAAACCGTCGCGCTCAACAGTACGGGCGAACTGGTCGCGCCCAATGGTTCTATCGGTTTTCGCTGGGGCG
AAAAGGGAAAATGGAACCTGGAACCGGTGGCGGCGGGCGTCGAAACTGAACTGTCGCTCTCTTTACTCGGTCA
GCATGATGACGTGGCGGGTGTCGCCTTCCCCTATTTTGGCGGTAACGAAAACCCGCATTTTCGCAGCGTGCGG
CAGGAACCGGTGCTGGTACGTCAGTTACCGGTGAAACGCCTGGCTCTGGCGGACGGTAGCGAGCGGATGGTCG
TCAGCGTTTACGATCTGGTGCTGGCTAATTATGGGCTGGATCGTGGTCTGGATGACGGCCATAGCGCCAATAA
TTATAACGACGTAAAAGCCTACACGCCGGCCTGGGGCGAACAGATTACCGGCGTGCCGCGTCGACATATCGAA
ACTATTGCACGTGAATTTGCCGAGACCGCCCATAAAACTCACGGTCGTTCGATGATCATCCTCGGCGCGGGAG
TGAATCACTGGTATCACATGGATATGAATTACCGTGGGATGATTAACATGCTGGTGTTCTGCGGCTGTGTTGG
ACAAACCGGCGGCGGCTGGGCGCACTATGTCGGCCAGGAGAAGCTGCGGCCGCAAACCGGCTGGCTGCCGCTG
GCCTTCGCGCTGGACTGGAATCGCCCGCCACGTCAGATGAACAGTACCTCGTTTTTCTACAACCATGCCAGCC
AGTGGCGCTATGAAAAACTGACTGCGCAAGAGTTGCTGTCGCCGCTGGCCGATCCGGCTAAATTTTCCGGACA
CCTGATCGATTTCAACGTGCGCGCGGAACGTATGGGCTGGCTACCCTCGGCGCCGCAGCTTAATCTTAACCCG
CTCAGCGTGAAAGCGAGCGCCGATAAAGCCGGGCTGTCTGCGGCGGATTATACCGTGCAGGCGTTGAAATCAG
GCGCTATCCGTTTTGCCTGCGAACAGCCTGACAGTGGTCATAATCACCCGCGAAATTTATTTGTCTGGCGCTC
TAACCTGCTCGGCTCCTCCGGGAAAGGTCATGAGTACATGCTCAAATATCTGCTGGGCACCGACAGCGGTATT
CAGGGCGAAGCGCTGGGTTCCAGCGAAGGAATTAAGCCTGAAGAGGTGGAATGGCAGTCCGCCGCGATTGAGG
GCAAGCTCGATCTGTTGGTCACGCTTGATTTCCGTATGTCCAGTACCTGTCTGTTTTCCGATATCGTTTTGCC
GACCGCCACCTGGTATGAAAAAGACGATATGAATACCTCGGATATGCATCCGTTTATCCACCCCCTTTCCGCC
GCGGTCGATCCTGCCTGGGAATCCAAAAGCGACTGGGAGATCTACAAAGGTATCGCCAGCGTCTTTTCTGAAG
TGTGTGTCGGACATCTCGGACAGGAAACCGATGTGGTGTTACACCCGCTCCAGCATGATTCCCCGGCGGAACT
GGCGCAGCCATTTGATATTCTCGACTGGCGTAAAGGCGAGTGCGAACTTATTCCGGGCAAAACCGCCCCCAAT
ATCGTCGTGGTTGAACGCGATTATCCGGCCACCTATGAACGTTTTACCTCGCTGGGTCCCCTGCTGGATAAAT
TAGGCAACGGCGGTAAGGGTATTGCCTGGAATACGCAAGATGAAGTCGATTTCCTCGGTAAGCTCAATTACAC
CAAACACGATGGCCCGGCGAAAGGACGTCCGCGGATCGACACCGCGCTGGATGCTTCGGAAGTGATTCTTGCC
TTAGCGCCGGAAACTAACGGCCAGGTGGCGGTGAAAGCCTGGCAGGCGTTGGGCGAGATGACCGGGCGCGAGC
ACACCCATCTGGCCATCAATAAAGAAGATGAGAAAATTCGCTTTCGTGATATCCAGGCGCAACCGCGCAAAAT
TATCTCCAGCCCGACCTGGTCAGGGCTGGAAAGCGAGCATGTCTCTTATAACGCCGGCTATACCAACGTACAT
GAACTGATCCCGTGGCGCACGTTGTCGGGGCGTCAACAACTCTATCAGGATCATGCGTGGATGCGCGCTTTTG
GCGAAAGCCTGGTGGCATATCGTCCGCCGATTGATACCCGCAGCGTCAGCGAAATGCGAGAAATTCCGCCCAA
CGGTTTCCCGGAAAAAGCGCTTAACTTCCTGACGCCGCACCAGAAATGGGGAATTCACTCCACCTACAGCGAA
AACCTGCTGATGCTGACCTTGTCGCGCGGCGGGCCGATTGTCTGGATCAGCGAAGCCGATGCCCGTGAATTGG
GTATTGAAGATAACGACTGGATTGAAGCCTTCAACGCTAATGGCGCCCTTACCGCTCGTGCGGTGGTCAGCCA
GCGCGTCCCGCCGGGTATGACCATGATGTACCACGCCCAGGAGCGGATTATGAACATTCCGGGTTCGGAAGTG
ACCGGGATGCGCGGCGGGATCCATAACTCCGTGACCCGGGTCTGTCCAAAACCGACGCATATGATCGGCGGCT
ATGCGCAACTGGCTTACGGCTTCAACTATTACGGAACCGTGGGTTCGAATCGCGATGAATTCATCATGATCAG
GAAGATGAAAAACATTAACTGGCTGGATGATGAGGGCCGGGATCAGGTACAGGAGGCGAAAAAATGAAAATAC
GCTCACAGGTTGGGATGGTACTGAATCTTGATAAATGTATTGGCTGTCACACCTGCTCCGTCACCTGTAAAAA

CGTCTGGACCGGACGTGAAGGGATGGAATACGCATGGTTCAATAACGTCGAAACTAAACCGGGTATCGGCTAT
CCCAAAAACTGGGAAGACCAGCAGGAGTGGCAAGGCGGTTGGGTTCGTGATGTTAATGGCAAGATAAGGCCCC
GTCTGGGCGGCAAAATGGGGGTAATTAGCAAAATTTTCGCCAACCCGGTGATTCCGCAAATCGATGATTACTA
TGAACCCTTTACCTTCGATTATCAGCATTTACATAATGCGCCGGAGAGTAAGCATCAGCCCACTGCCCGTCCT
CGTTCACTGATCGACGGAAAACGGATGGACAAGGTGATCTGGGGGCCAAACTGGGAAGAGCTGCTCGGCGGCG
AGTTTGAAAAACGCGCCCGCGACCGCAACTTCGACAACATACAAAAGGAGATGTACGGTCAGTTCGAAAACAC
CTTCATGATGTACCTGCCGCGCCTGTGCGAACACTGCCTCAACCCCAGTTGTGTCGCCACCTGCCCAAGTGGC
GCTATTTATAAGCGTGAAGAAGACGGCATCGTGCTAATCGACCAGGATAAATGCCGGGGCTGGCGTTTGTGTA
TCAGCGGCTGCCCGTACAAAAAAATCTACTTTAACTGGAAGAGCGGCAAGTCTGAGAAATGCATATTTTGCTA
TCCGCGTATTGAATCCGGCCAGCCGACCGTTTGCTCGGAAACCTGCGTCGGGCGCATCCGTTATCTTGGCGTT
CTGCTCTACGACGCCGACAGGATTGAAGAAGCGGCCAGTACCGAACACGAGACGGATTTGTATGAACGTCAGT
GTGATGTGTTTCTCAATCCGCACGATCCGGCGGTCATCGAAGAGGCGCTTAAACAGGGCATCCCGCAAAACGT
CATCGACGCTGCCCAACGGTCGCCGGTGTACAAAATGGCGATGGACTGGAAGCTGGCGCTTCCGCTGCACCCG
GAATACCGTACCCTGCCGATGGTCTGGTACGTACCGCCGCTGTCGCCCATCCAGTCATACGCCGACGCGGGCG
GGTTACCGCACAATGGCAATATTCTGCCGGCGGTGGAGACGCTGCGTATTCCGGTCCAGTACCTGGCCAATAT
GCTCAGCGCGGGCGATACCGGTCCGGTCATTCGGGCTCTAAAGCGGATGATGGCGATGCGCCACTACATGCGC
TCGCAGACGGTTGAAGGCGTGACGGATACCCGCGCGATTGACGAAGTCGGTCTGAGCGTACAACAGGTTGAAG
AGATGTATCGCTACCTGGCGATTGCCAATTATGAAGATCGTTTTGTCATTCCCACCAGCCACCGGGAAATGGC
GCGCGATGCCTTTCCGGAGCGCAACGGCTGCGGCTTTACTTTTGGCGACGGTTGCCACGGGTCGGATACCAAA
TTCAACCTGTTCAACAGTAGCCGTATTGATGCCATCAATATCACCGAAGTACGTGATAAAGCGGAGGGGGAAT
AATGCAAATCCTCAAAGCGATTGGCCTGTTGATGGAGTATCCGGACGACGAGTTGTGGGAATGTCGGATGAA
GCGCTTGCCCTTATCCAACACGATGCGCCAATGCTGGCCGACTTCACTCGCGAGCTGCTTTATGCGCCGTTAC
TGGATAAACAAGCAGAATGGTGTGAAGTGTTTGACCGCGGGCGCGCCACTTCATTGCTGTTATTCGAGCATGT
TCATGCGGAGTCCCGCGATCGCGGCCAGGCGATGGTCGATTTGTTAAGCCAGTATGAAACCGTGGGCCTGCAG
CTCAACTGCCGCGAACTGCCGGATCACCTGCCGCTCTATCTGGAATACTTAAGCGTGTTGCCGGAAGCGGAAG
CGCGCGAGGGATTACAGAATATTGCCCCTATTCTGGCGCTACTGGGCGGTCGTCTGAAACAGCGAGGAGCGCC
GTGGTATCAACTGTTTGACGCCCTGCTGACGCTGGCGGGCAGTACGCTTACAAGTGACAGTGTCACCAAACAG
ATTGTTCAGGAATCGCGGGATGATACCCGTCAGGCACTGGATGCCGTCTGGGAAGAAGAACAGGTGAAGTTTA
TCGAAGATAACGCCACGACCTGTGACAGTTCACCGTTACACCATTATCAACGACGCTTTAGCCAGGACGCGGC
GCCGCAGTACGTCGACGTCAGCGCGGGAGGCCCGAAATGATACAGTACCTGAACGTCTTTTTTTACGATATCT
ACCCCTATATTTGCGCCACGGTCTTCTTCCTCGGCAGTTGGCTGCGCTATGACTACGGACAATATACCTGGCG
CGCGTCGTCCAGCCAGATGCTGGATAAGCGTGGGATGGTGATATGGTCGAATCTGTTCCATATTGGCATTCTG
GGGATCTTTTTTGGCCATCTGTTTGGAATGCTGACGCCACACTGGATGTATGCCTGGTTCCTGCCGATTGCGG
TAAAACAGCAGATGGCGATGATCCTCGGCGGCGTGTGCGGCGTATTAACGTTGATCGGCGGCGCGGGATTACT
GTGGCGTCGTCTGACCAATCAGCGTGTGCGGGCGACATCCACTACACCAGATATTATCATCATGAGCATTTTG
CTGATTCAGTGCCTGCTTGGCCTGAGCACTATTCCGTTTTCGGCGCAATATCCGGATGGCAGCGAAATGATGA
AGTTGGTGGGCTGGGCGCAAAGTATTGTCACCTTCCGCGGCGGGTCTTCGGAAATGCTGAGCGGTGTGGCGTT
TGTTTTCCGCGTTCATCTGGTGCTGGGAATGACTATTTTCCTGCTCTTCCCGTTCACCCGTCTGGTTCATGTC
TGGAGCGCGCCGTTTGAGTATTTCACCCGTCGCTACCAGATTGTTCGTACCCGCCGCTAATTCTCTTTGCCCG
GGGGGACGCTTACCGGGCAAAGAAAAACGTCTGGGCGTCGTCATTCGGCAACGCACAGGCTATGATGATTCTT
CAGTCAAAGGAGAACATCAATGAAACCGCAGATTTACCATGTTGACGCCTTCACCAGCGAACCCTTTCGCGGC
AACTCTGCTGGCGTTGTCTTACATGCGGATACGCTAAGCGACGCGCAGATGCAGCTGATTGCCCGTGAACTTC
GTCACTCGGAAACCGCCTTTTTGCTAAAAAGCGAAGAGAGTGATGTGCGCATACGCTACTTTACACCGACAGT
AGAAGTCCCCATCTGTGGTCATGCCACCGTGGCCGCGCATTATGTTCGGGCAACGGTACTGGGGTTGGGAAAC
ACCACCGTCTGGCAAACCTCGTTAGCGGGCCGTCATCGCGTTGAAATTCATGCCGAACACAACGACTATCGCA
TCACGCTGGAACAAGGTCAGCCCTCTTTTGAACCACCGTTAGTCGGAGAGATTCGGGCTGCCATTATCACCGC
ACTGAACCTGACTGAAGATGATATCGTGCCGGGAGCGCCAATTCAGGTGGCTTCAACAGGACATGCAAAAGTG
ATGATCCTGCTCAAACCAGACGTTGATATTGATGCCCTTTCTCCGAATCTGGCAGCCCTGACGGCAATCAGCC
AGCAAATTGGCTGTAACGGCTTTTTTCCTTTCCAGATTCGTCCGGGAAAAAACGAAACCGATGGGCGAATGTT
TTCCCCGGCGATAGGAATTGTGGAAGATCCGGTAACGGGAAACGCCAATGGCCCGATGGGGGCATGGCTCGTC
CATCATGGCCTGATGGCGCATGACGGTAAAACGCTGCAGATTCAAGGCCATCAGGGGCGCGCTTTAGGCAAAG
AGGGTACTGTTGATGTTACCGTCACCATTCGCGATAACCAGCCGGAAAACGTCACCATCTCCGGCCAGGCGGT
CATTCTGTTTCATGCCGAATGGGCCATTACTTTTTAATCTTTGTCAGGCCCGCAAATCCTCGCGGCAAAAAAT
TATTTTCCCGCCTCCGGATGGGTATCAAATGCAGCCATTACCGCGGCCAGCTCCGCCTCGGTAAAACCGTGCT

TTACATCATTTACGCCGAGACCAAACTGCTGCTGAAGTAACTGATACAACGACGGTACATCCGGTACATTGAC
CTGTTCAACGGCATGTCCCTGGTGGTAACGCGTGAAGTGGAAATTCGTTAATGTCAGCTTGCCGCCATCAGGC
AAATGGCGGCACATCAACAAATGATGACGAAAATGGGATTGCGGCCAGTGAGCCGACCAGAAATTGCCCATCA
CATGATCGCTCTGCTGCTGCACCCCCAGATCAAAACAGTACATCGACTGCCAGTGCTCATGGTGGCGGAATTG
TAAAATCCATGTGCTTCCCTCCTGCATCAGGCGGTATTCGCCATGCGGCGTTTGCTGGGCAATTTCAGCCTGC
AGACGAAGCGGCGCGGTTAGCGTTTGGCCGCCAAACCCCACGTCAGCGATCCACTGTTCATCCTCAACATCAA
CCAGCAGCAAACGGTGCGTGCGCGGCGGTAAACTGGCGGGATGAGACAGAATGACCCGCCCCAATAAGCTTCG
GACGTTAAAACCGATGTCGCGTAACGCACGTTCAAACAGACCATTCAGTTCAAAACAGTATCCACCGCGTCGG
GCATAAAGCAGTTTCTCCTCTAATGCCGTCTCATCGAGCTGTATTTCACGAGGCAGTAGCACATCCAGATTTT
CAAAAGGAATCGCGCAGTTGTGCGCTAAGTGAAGCGTTCTGAGCGCTTCAACCGTAGGAACCCCCAACGGCTG
ACAATGTAATCGCGTAAAATAAGCATGTAAAAAAGAGGTCATCTGAGGCTTCCATTAACATTAATGCTTGCGT
TCGGCACACAGGCTGAGGCGGATATCGCTGCGCAACAGGATACCCTATCCGTGGAATAGCGTTGGATACTTTT
GAAAAAATAAGACAGGCCGGCTGTCGCGCCGGAGGTACGTGGAATTTTGGCGTACAAACGGAAATCGGGTTCT
GAGAATCTCTTTGCGACACGCCGCCAAAATAGCGGCGTGTTAAAATTTTTAACGCCGGCTCAGCGAGTCTGAT
TTCTAACAAAACTGGCTAAACATAAACGCTTTTTAATAATTGTCCAAATAGTTATGGTAGCGAGCTTTTATGT
CGGCCGCCCATTGCGCTTTCGCCTCCTGCATGTTGCTGGCCCGAGCGATATAATATTCTAAACACGGAATGGC
GCCTAATGCCTGAGGTAAGGCATTATATGCTGCCATTCGCTCTCTGACGGTTTTATATTTTTCATCAATATCA
TAATCATAGTACGCTGGCGCTTCAAAAAGCCCTCTTCCAGTCTCATACAAATCTCTTCAAAGGACCCCTTAAC
CTCAACGGTATGTTCGCCATCCGATATCCTGACGATTGGCTCACGTCCAGCCATCAGCGAGAGTTGTTCGAAT
GTTAACCTCCGACCATCAGAAAGGTCGACATCTATAGTATACAGCCCATCACACACAGCATCAGCAAGGTGAT
TATGGATATTCCCTGCATTGGCACAGAACTCATCAACTTTAGGCGTAACATTGCAATAATGTTCAATTACTCG
CGTAAATCCGTAGGCCAGTCCTGCGGTAAGCACATACAAGATGCCTTTCATGATTTTTGTTGATAATTTATTT
TCATAATCTCCTGCAGCAACATGAGGTAGCTTATTTCCTGATAAAGCTCTGGCATAGGTAGAAACTGATGTAT
ATGGCATATCCTGCTCCTTCAAATTTTGCTCAATAGCTTTATATGTCCTACTCCTCTCTCATTATGACGATAT
GTCAATCAACAAAATTGCTCAAAGGCATACATTTTCAGGAGGAAATGAGAATAACAGGCGCAACGGCCTGATC
TTATGCTGCTTCAATATCGTCAGGTGGTTTATCGTTCGAGGTCATATCTATGACCATAAATCCCTGCAGGGAT
TAACCCTGATTAATTATTGCGCAACGCAATCACTGCGGATTTGTGCTGAAAGCTTATCTGGCTGAATCGTGAC
CACACCGCACCACATCTGACCGCACAAATAAACACGACATCCTCATTTTCTTCTTTCCGTATGTTTTGCGTCA
TTTAACAATTGCAGGTAGGATGCTCCGCCGGCGGTTTTATCCCTGTCACAGAATCAGGGAAAAGCGGCATTCG
CCATTGCAGGCAATCGATTGGTGAGCGCCCGTCGCGGCATAAGGAATTTTTCTGTCAGGACAGGCTATAACAG
GTAACGTAATGAAAACACAGACTACGCACGCCGCAGAACAACATGCGGCGAAACGACGCTGGCTGAACGCCCA
TGAAGAGGGGTATCACAAAGCGATGGGGAATCGTCAGGTTCAGATGATTGCTATCGGCGGCGCGATTGGCACC
GGTTTATTTTTAGGTGCAGGCGCCCGATTGCAAATGGCCGGACCGGCACTGGCGCTGGTCTATCTGATATGCG
GCATTTTTTCTTTTTTCATTTTACGCGCGCTGGGCGAACTGGTATTGCATCGCCCTTCCAGCGGGAGCTTCGT
CTCCTACGCCCGTGAATTTTTGGGTGAAAAAGCGGCCTACGTCGCAGGCTGGATGTACTTTATTAACTGGGCG
ATGACCGGGATCGTCGATATCACCGCAGTGGCGTTATACATGCACTACTGGGGCGCATTTGGCGACGTTCCGC
AATGGGTGTTCGCGCTCGGCGCGTTAACCATTGTCGGTACCATGAACATGATCGGCGTAAAATGGTTTGCCGA
AATGGAGTTCTGGTTCGCGCTGATAAAAGTACTCGCCATTGTTATCTTCCTGGTGGTAGGCACGATTTTTCTC
GGTACCGGTCAACCGCTTGAGGGTAATGCCACCGGTTTTCACCTGATTACAGATAATGGCGGCTTTTTCCCGC
ACGGCCTGTTGCCCGCGCTGGTACTGATTCAGGGCGTAGTGTTCGCTTTTGCGTCGATTGAACTGGTCGGTAC
CGCCGCTGGGGAGTGCAAAGATCCACAGACGATGGTGCCAAAAGCCATCAATAGCGTGATCTGGCGTATTGGT
CTTTTCTATGTAGGATCGGTCGTATTGCTGGTTTTGCTGCTGCCGTGGAACGCCTACCAGGCGGGGCAAAGTC
CGTTTGTCACCTTCTTTTCTAAACTTGGCGTGCCTTATATCGGCAGTATTATGAATATCGTGGTGTTAACCGC
CGCGCTTTCCAGCCTGAACTCCGGGCTTTACTGTACGGGGAGAATTTTACGTTCAATGTCGATGGGGGGTTCC
GCGCCGAAATTCATGGCGAAAATGAGCCGTCAGCATGTGCCCTATGCGGGTATACTGGCGACTCTGGTGGTTT
ACGTGGTCGGCGTATTCCTGAATTACCTGGTCCCCTCCCGCGTGTTTGAGATCGTTCTTAATTTTGCCTCGCT
CGGCATTATCGCGTCGTGGGCGTTTATCATGGTGTGCCAGATGCGTTTACGCCAGGCGATAAAAGAAGGCAAA
GCCGCAGACGTCAGTTTTAAACTGCCCGGCGCGCCTTTTACCTCCTGGCTAACGCTGCTGTTTTTACTAAGCG
TGCTGGTACTCATGGCGTTTGACTACCCGAATGGAACTTACACTATCGCCTCGTTGCCGCTGATCGCCATTCT
GCTGGTCGCCGGCTGGTTTGGCGTGCGACGTCGCGTGGCGGAGATCCATCGCACTGCGCCAGTGACGGCGGAT
TCGACAGAGAGTGTCGTATTGAAAGAAGAAGCAGCGACATAACATTCCCCTTCCCCGGCAGCCCCAGGCCTGC
CGGGTTTAATGCGCGTTGCCGCCAACACCGCATCGCGCCCAATGCTTAGTCAGCCTTAATCAACTGCGCCACA
TCATTGCTGTTAATTTCCCGCTGGTTGCCTTCCTGATCGGTATAACTGGTCATCCCCGTCTCTTTATCTAACT
GCGGCTTACCCTGAGTTACGATCGTTTGCCCATTCTTCGTCGTCATCACATAGTTGGTTGTACAACCCGCAAG

CATAAAAAACATCATGCCCAAACCTGAGCATATAAATAACTTTTTCATATTTTCCTCGTGAATATAATGAGTT
AACTACAGCAAAAAGGCAGTGTAATTCTCACAGAATCATCAGGCTATCGGCGGGGTGTAACGAGGCGTATCGG
TGCGTTTAAAGAGATGACTGGAGGGGTTTCCCCCTCCATACCCTATTTACAGAGCAATGCGAATAACATCATC
CGGCTGCGTCGCTTCCTGTTCACGTGTCGATTTCTGCTTCACGCTGACGTAGAGCGTTTTACCGTCTGCCGAT
AGCGCCAGGCTATTCGGGTACGTCGGCGTATCGAACGTTTTAACAACGTTATAGGTCTTCGCATCGATCACGC
TGACCTGACCTGCCTGACGATGTGTCACATACGCCTCGTTACGTGTCGGGTTAAACAGGACGGCCAAAGAAGC
AGGCGCCGCGATTTTGGCAAGAATATTGCCATTACGGGTATCGACAACCAGAACCTCAGTCGCCTTCGAGTCG
GTGATAAACGCGCGATGACCTGCGGTATCGAGACTCAGGTTAATAAAGAAGTGTTCTTTACCGTCATCCAGCA
ACTTCTTACGACTGAGAATTTTATTGCTGGCGGTATCGATGGTGATAAATTCGCCATCCGCATTGGTGGTGTA
CAGGCGTTGCGCTTTACTGTCGAGCGCCAGACCCGTACTCATTTTGCCGGTATTTTCGATCGTCGTTTTCAGT
TTGATGGTTTCGCCATCCACTACCCAAATAGCACTCTCTTTACCAACACCGCTAATGTAGATCGTGTTGGTCG
CCGCATCGGCAACCAGCTCACGGGGCTGTAACGGACGAACCTCTTCAGTACGTTTGCGCGCATCAAGTACCAG
ACGACCTTTTACATCACCCGTTTTGGCATCAATCGCGGTAACAGCGCTGTTAATGGTATTGCCAAACCACAGC
GTTTGGGTCGCGGCATTGATGGTGGCGCCGAACGGCTTGAGATCGTTATGAATCGCTTGCGTGATTTCCAGCG
TCACCGGGTCGAGACGATAAACTACGCCGCCTTTATCCAGTTTACGGCTTTGCGATGTAGCCAGCCAGAGCGC
GTTTTCTTGCTGACTCCAGGCCATCTCATAAGCGCCTTTGCCTACTGCTTTACGCAGCATGTCTTCCGCCGCC
AGCGTGCTAAATGAGGATGCCACGAGGAGCGAACCTAACAATAAAGAACCACGCAGGCGCGGCGAAAAAAGAT
GACGTAAGTGCATGACGACTCCCTTTGATAAAAACGTGTATAGCTGCTTCACACTACTTCGCTGCGCGGGATC
TTCGTTCCGCACTTATTATAAGTTGAGAATAATAATCATTATTTATCAGAAAGTGGAGCTTTTCCTCGCCTGC
GAATGTCATGGCTCTTAAATACCGCACATTTTCTTAACTTAATGTGCGATTAAAATTTCAAAACTTTACATAT
TGACTACCCTTAATGTCACGTTTTCCGTTGATACGCTTACATTTATCCGGCATTTTCATGAAAATTATTTCCG
TTAGACAGCGACTTTATCCCGCCCTCTTATTGCCTCTGACCTTCTCCCCGGTCCTTCAGGCCGCCAGCGCGTC
CAATGAACAAACCATGATAGTCACCGCAACGCCGCAAACCGTATCCGAACTGGATACACCTGCCGCCGTTAGC
GTCATTGAGGGAGAAGACATGCGTCTGGCGACGCCGCGCGTTAATTTATCTGAGACTTTAACCAGCGTCCCGG
GATTGCAGGTGCAGAACCGGCAGAACTACGCGCAGGATCTGCAAATCTCTATCCGTGGATTTGGCTCGCGTTC
CGCCTTTGGCGTGCGCGGCATTCGTCTGTATGTTGATGGCATTCCCGCCACCATGCCGGATGGACAAGGCCAG
ATTTCGAATATTGATATTAACAGCATACAAGACGTTGAAGTGTTACGCGGCCCCTTCTCAGCGCTATACGGCA
ATGCTTCCGGCGGCGTAATAAATGTCACCACCGAAACCGGGATTGAGGCCAGCAGCTACTACGGCAGTTATGG
AAGCTGGCGCTATGGGCTAAAAGCCACGGGCGCGATGGGTGACGGCACACAGCCTGGCGACGTGGATTACACG
GTATCAACTACCCGTTTCACCACCCACGGCTACCGCGATCACAGCGGCGCGCGAAAAAATCTGGCTAATGCCA
AACTGGGCGTGCGCCTTGACGATGTCAGTAAACTTTCGCTGATTTTTAATAGTGTGGACATTAAGGCGGACGA
TCCCGGCGGACTTACCGAATCTGAATGGAAAGCCGATCCGCAACAGGCGCCGCGCGCTGAACAGTACAATACG
CGTAAAACCATCAAACAAACTCAGGCAGGATTGCGTTATGAACGTCAGCTCAGCGCGCAGGATGATATCAGCG
TGATGGCCTACGCCGGAGAGCGGGAAACCACGCAATACCAGTCTATTCCCCTGGTGGCACAGCTAAAACCAGC
CCAGGCTGGCGGCGTAATTACACTGCAACGCCACTATCAGGGAATTGATTCCCGCTGGACTCACCGGGGAGAA
TTAGGTGTGCCGGTCACCTTCACTGGCGGGTTAAACTATGAAAACATGAGCGAAAACCGCAAGGGTTACAATA
ACTTCCGTCTCAACAACGGCCCCCCTGAGTTCGGGCATAAAGGCGATTTACGGCGGGATGAGCGTAACCTGAT
GTGGAATGTCGATCCTTATCTGCAAACCCAGTGGCAGCTTACGCAAAAACTCTCGCTGGATGCTGGCGTGCGC
TACAGTTCCGTGTGGTTCGATTCTAACGATCACTATATCGCGCCCGGCAATGGCGATGACAGCGGCGACGCCA
GCTATCACCACTGGCTACCCGCCGGATCGCTAAAATATGCGTTAACTGACGCCTGGAATCTCTATCTTGCTGC
CGGTCGCGGATTTGAAACTCCCACTATCAATGAACTTTCTTATCGCGCTGACGGCCAAAGCGGGTTCAACTTT
GACCTCAAACCGTCGACCAATGATACGGTGGAAGTCGGCAGTAAAACGCGGATTGGCAATGGCTTGTTGACCG
CCGCGTTATTTCAAACCGATACCGATGATGAAATTGTCGTCGCCAGTAGTATGGGAGGACGCACTACCTACAA
AAACGCGGGCAAAACGCGCCGCCAGGGCGCAGAGCTCGCGTTGGACCAGCGCTTCGCCGGCGACTGGCGGGTG
AAAGCATCATGGACCTGGCTGGATGCGACCTATCGCAGTAACGTTTGTCAGGGGCAAAACTGTGATGGAAACC
GGATGCCCGGCATCGCCCGTAATATGGGATTCGCCTCATTAGGTTTTATCCCGGATGAGGGGTGGTACGCCGG
AACAGACGTTCGGTATATGGGCGATATCATGGCCAATGATGAAAATACCGCCAAAGCGCCTTCGTATACCGTT
GTTGGACTAAATACAGGGTATAAATTTAATTACAGTCAGCTTACCGTCGATATTTTTGGGCGGGTAGATAATC
TATTTGATAAAGAATATATCGGTTCGGTGATCGTTAATGAATCTAATGGCCGATATTATGAGCCAGCGCCAGG
GCGTAACTATGGCGTGGGGATTAATCTGGCATGGCAATTTGAATAAATATCCGGCAATAGCGGGGCATCCCCG
CTATTGATTAGTCATAGTTTCATGAAAGTTACATGAAGTACTGATTTTTAATCGTCGCTACGTTTTGTTGCAA
AACGTTCTGTAGGCAGTGGCGACTGGCTTCTTTATCACCAGTCACTAACGCATTAAGCAATTTTCTGTACAGA
TTAACGCCGTTTTGATAATCATCAAGTTTAAAATTATCATATAAAAAATGCAGACTTGGCCCCAGGCGTACCC
ATAATTGCTCGATCATGGCATACAGCGTTGGCATATCCGCGTAGTGGTAGATCTCAAAGCGGAATGCGCGGTT

GGCCAAAAGAATTTTCTCCATAACGCCACTTTCCAGCGCCTGTTGAAATTCATCTAATAGCGCATTCAGCGTC
TGCTCGCGATCCGGGGTAATCTTACCCGCAGCGGCGACCACCGCCATCTCCTCCAGGGCAGTACGAATCGTAT
TAATTTCCAGCAAACTTTCCAGCGAGATCTCCGGCACCATAAACGCCTGTGCTGGCGCCACCGCCAAAGCACT
GGAAGAGACCAGCCTTAACAGGGCCTCACGCACTGGCGTAATACTGACGCCTAACTCTTGAGCGATATTTTTA
GTGATAAGCCTTGCGCCTGGTTTTAATGCGCCAATAGACAATTGATGCTTTAACCGATTTTCAACCTGTGTGG
TCAGGCTTATATGTTGTGTTTTTTCCGTACCCGGCATTTTCCTTCCTTCACCTCTTGGCAGAACAACATCGCA
ATCGAGTCCCGAAGAAGTTTCGGAACTTAATTGTAAAAAAGATAATCCAGGCCTGAACGGCCAAATAAATAAA
AGTGATAATTTACTTGCGTGATATTCTCATCTTTTACAACAATACAGGTTTCTTTATGGCAACCGTTTTATCT
CCGTCATTCCTTCATGTATCGAGATTTTTGACCGGTTCAGGCCGCTGAGCGAGATAAGCTGCCCCACCGCGAT
CTGAATGATGAATATAAGTAAAGCCGGAATTTTAAAATTTGCACATTTTTATGGCGACATAATGCCGCCATTT
TTTCTTTACGCATCGTCCGCTAAACGTATCACGACTTTGCCAAAGTTCTTCCCCGCCAGCAGCCCCATAAACG
CTTCTGGCGCATTTTCCAGCCCATCAGTAATCTGCTCCCGGTAATGTATTTTCCCTTCTTTGATCCAACGCCC
CATTTCTTGCTGAAACTCATGTATCCGGTGACCATAGTCCTGGTTGATGATAAAACCTTGCAGACGAATACGC
TTTTTAAGTAATGTCGCCATCAGCAGAGGCAGTCTGTCTGGACCGTCCGGCAACGCTGTCGCATTGTAGCCGC
TGACCAGACCGCAGAGCGGAATCCGCGCCGACGTATTCAGTAGCGGTAAGACGGCGTCAAAGACTTTACCACC
GACATTCTCATAATAGATATCGATACCCTGCGGGCAGGCCTGCGCAAGCTGTTGTGGGAAATTATCAGCGTGA
TGATCAAGGCACAGATCAAATCCCAAAACGTCTGTCGCATGACGGCATTTTTCCGCGCCGCCAGCAATACCCA
CGACCCGGCAGCCCTTCAGCTTACCAATTTGCCCAACCGTTGCGCCTACGGGGCCGGTGGCGGCGGCGACAAC
CAGCGTTTCGCCCTCTTTCGGCTGGCCGATATCCAGTAACCCCATATAGGCGGTAAACCCCGGCATTCCTAAA
ATACCCAATGACCAGGAAGGATGTTGCGGATTATCACCCAGCTTTACTAACCCTTCGCCATCTGAAATGTCAT
AATCCTGCCAGCCATTGTAACTCAACACCCATTCGTCTGGTCGGTAATCAGGATGGTTTGACGCGACAACGCG
ACTGACCGTACCGCCAACCATAACGCAGCCAGGTTCAACAGGCGGCGCATATGAGGGTTCATCGCTCATGCGG
CCACGCATATAAGGGTCGAGTGATAAAAAAACGGTACGCAATAGCACCTGCCCTTCACCGGGCGTCGCGACGT
CATCTTCTTCGAGGCGGAAGTTATCCATCTGCGGCGCGCCGTGGGGACGCGAGGCCAAAACCCAACGACGATT
TCGGTTGGTCTGTTGCTTCATTCTATTCTCCTCATGCCTGGCATAATCCATCAATAATAGTCGAACATTAGCT
TTCTGAGGGAGTTAAACACTCTCAGATATCAGGCTGTGCGCTTTTATGAACGACTTTCTTTCCCCTCTCGCTA
AATATGAATTCTTGCGCTAATTCGTTGATTTATGCCGCCTTCAATAGTATTTCTAACACTATCGTAAACATTC
CCTGGGGTTCCCTATGACGATTCGCTTTGCCGATAAAGCAGACTGCGCCGCGATTACCGAAATCTATAACCAT
GCGGTATTACATACTGCGGCCATCTGGAACGATCGGACGGTCGATACGGATAATCGCCTTGCCTGGTATGAGG
CCCGTCAATTACTGGGCTATCCGGTGCTGGTAAGCGAAGAAAATGGCGTGGTGACTGGCTATGCATCATTTGG
CGATTGGCGCAGTTTCGACGGTTTTCGCTATACCGTCGAGCACTCGGTTTATGTTCATCCGGCGCACCAGGGG
AAAGGACTGGGGCGAAAGCTGCTCAGCAGACTGATCGACGAAGCCCGACGCTGCGGCAAGCATGTGATGGTGG
CCGGTATTGAGTCACAGAATGCCGCCTCGATACGCCTGCATCATTCGCTGGGATTTACCGTTACCGCGCAAAT
GCCGCAGGTCGGCGTAAAATTTGGCCGTTGGTTAGATCTTACGTTTATGCAGCTCCAGCTTGATGAACACGCC
GCGCCGGACGCCTGCTGATGAACCAGTCTTTGACGCTTATCTTTTTGATCGCCGCCGGCGTCGGGCTGGTGGT
ACAAAACAGTATTATGGTGCGTATTACGCAGACATCATCGACGATTCTGATCGCTATGCTGTTGAACTCGCTG
GTAGGGATCGTTTTATTTGTGACGATATTGTGGTTTAAGCAAGGCGCTGCGGGTTTCGGCGAGCTGGTCGCCA
GCGTTCGCTGGTGGACATTGATCCCCGGTCTGCTGGGGTCATTTTTTGTATTTGCCAGCATTAGCGGGTATCA
GAACGTTGGCGCCGCGACGACTATCGCGGTACTGGTCGCCAGCCAGTTAATCGGCGGACTGGCGCTGGATATC
GCGCGTAGCCACGGCGTCACGTTACGCGCAATGGTCGGGCCGGCGTTTGGCGCGCTGTTGCTGGTCATTGGCG
CATGGCTGATTGCGAAGCGCCAGTTTTAATGGGTTACAGGATCGTGCCGCCTTTGGTGAGCTGCTCGCGGCGC
GCGTCCCGATCTTCTTGATATTGCCTGCCGTGATGCGCGATAGCCGTGCGTAAACGCTGCTGCTGCACATAAC
GATCTTCGCGACGTAATTCAGCGTCATCGCTCAAGGCAATCAGCAATTCATTCATATGGGCAATTACGCTCTC
CGCGATGGCGGCATCCACGCGGGCGCTCACTTCCTCCAGGTGCGACATAAGCTCTCCTACTCTTTATTCTGTA
ACAGCATGATTCGCCTATTATACGCGTTATTGTCGCCCGATGCGGTAATTCGCCGCTGCCCTCCTGATTTAAC
TTACGTCAAGTGAAAACACGGGGCTTAATAGACCGAGAATTCGCGCCTTTTTGCCTTTCTTCCGCCGTTTGCT
TGCGTATAGACCCCGTAAAGTAGACGCGTGAAGAAGTCTAAGGGATAACAACGTGGCAAAATCTCTCTTGCGC
AGCGGCAATCTGGATGATTATCAGGCCGTGGGCGGCGGCGGTCAGGCCGTATTTGAATCAGCATTGCAAATTC
GCGAAACGCTTCGTCTGCGTAAACAGCAGGCGATGGTTGACTGTCTGGCGATTCCGCAACTTAACGATAATGG
CGATCGGGTTGACTGGTATTCGCCGATTGAAGGTCAGGCGATAGCGTGGAAAGCGGCTGACGAAGAGACACGT
TCTCGGGCTTTACGTTATTTAGCCAGCACTTTTGAAAGCGCGGCTGCACTCAGTCGCAAAAGTCTGCAATCCG
GTAAAACAGCGCTCCAGCTATTTGGGTCGCTACTTGAGAAAGCGACGCAATTTCCCGGCGAAAATCATGTTTT
CCTGGTTAACGGCAAACCGGTTATCACCTTCTGGGGATTCGTTAATCTCAACGAAAATACACGCGATGATGTG
CTGGACTGCCTGCGGGTAACCGAGGCAATCCCCGACATCCCTCTGGTCGAGCCGGAGCCGGAGCCCGAAGAAA

AGCCGCTGGTAGAGGCGGCATTTAGTCAGGCGGATGAACCTTTGTTAACGTCTGTCATTGAGCCGCCGAAAAT
GCCGGAGGAACCTGTTGCGCCGCCCGTTATCGTTAGCGAGCCGAAGCCGGCCACACCCATCCCGGTTGCTGAG
GCGAAAAGAGCTCGCCGTCTACCGCTGTGGAGCTTACCTGTCGCTGCCGTGGTTATCGCCGCCGTCGCCACGC
CTTTTTTCTGGCCATCTTCATCCGTTGACGGGTCCTCTCCTGTTCGCGCCGCGCCTGTCGCTACCGCGAAAAC
TGACGTTACCCCATTGCCTGAACTCACCGCGCATTTGCCGCTGCATCGCGCTGAGGTGACGCCCGCCCCCAAA
GCGGCGCCGCTGCCGGAAGCGCCTGTTATCATTGCCGCTATTCCCAAAGACGCGCTGGTGATGGACAGTACGC
AGATGAAGCTTGGAACCACGCGCTTTTTAAACGGTAGCTGGCGGGTCAGCGTGGATGTCAAAGATCCGATTAC
CGGCAAGCCGCCCTCTCTGCGTTATCAGATCCAAAATAACAAAGGTATCGCGCGCGTAGTACATGGCGATAAC
GTGGTTTGTCGGGCGGAAATTTTCTCTGGTCTGCATCAAACGGGCGAGCTGATGATTAAAAGTCGCGGCAATG
CGCGCTGCACCGACGGTTCACGTTATCCGATGCCGGAAATTACCTGTAAAGCAGGCGTCAATGACGTCGCAAC
CTGTACCGCCCGTTATGGCGACCACGCGGCAATTCCGTTGACGTTTAAAAAGATAGGTGCCTGATTTTATGTT
GGTCAATCTGTGTGATTACAAACAGAGCGTCACGCTCATTGCAAACAGTGGCGTCCAGTTTCTTGATTTTGGC
TTAACGCCGCAAGAGTCGGCCCACTACGGACGTTTTGTGCGCAAAACGGCAAACGGCCCGCTACTGCGCCTCG
ATTTTGATCTGACCAGCGGGCGCTACACGTTGCCTGGACGTGCTGGCGGCCAACCCGAAGTCGTGAAACCGGA
AAGCACTCAGACGCTGCACTATTCTCTGGATGTACTCGACGGAATCTGGCTTCCCCTGCCGTTCCTGCGTTTT
AATCCGCCGCGTACCTTTATTGACGGGCCGGATAACTGGGCGCGCATTCAGGTACGTAAACTCAGCGAGCCAG
ATAGCGCCGGAAACACACACCGGATCACCCTCGCCTTCGACAGTCAACTGGCAAAAAATATGCCTGCGGCGTT
GGCGCCTTGCGAAAACGATCTGCTCAATGGCACACGTTTTGCGCTCGCCTGGCAGGATGAAGAAGTTGCCGAT
TTTCTTGACCAGACATGGATTGACGGCTGGCTGCGCGAGTCGTTTCTTCAGTATGCCTCACAGGTGGAAAACC
GTTCGGAACAGGCCATACAACAAGCGCTGCGTAGCTTTGAGTATCAGGCGCACTGGTTGAATTTACTGACGCT
GTTAGGCGAACAGCTTACGGTGCCGGAAGTAAAGTTTGTGACACATACGTTAAGCACGCCCGCAATCCCGGTC
GATCTGATTCTGGATGTCGGCAATACGCATACCTGCGGCGTCTTGATCGAGGATCATGGCGATGCGAATGACG
GGTTACGCCAGACGGCAGAGCTTCAGGTGCGTTCTTTAAGCGAGCCGCAGTATCTGAACGATCCTTTATTTAC
CAGCCGGGTGGAATTCTCGGAAGCACGCTTTGGCAAGCAACACTTTTCTGTCGAAAGCGGACGAGACGACGCC
TTTGTCTGGCCTTCCATTGTGCGTGTGGGCGACGAGGCTCGCGCCCTTGCTATGCAGCGTGTGGGCACCGAAG
GCAGCAGCGGCATCTCCAGCCCGCGTCGCTACCTGTGGGATGAAACCCCCGCCTTGCAGGACTGGCGTTTTAG
CCAGATCCACGGTAAAACGCAGCGCGAAGCGCTTGCCACCGCGTTTCCGCTGATGAATCTGATGAACGATGAC
GGACAGCCGTTATTCCGCCTGCCGCATGAAGAAAGGCTACCGGTTTTTTCGCCGCAATACAGCCGTAGCACCT
TGATGACGCATATGTTGTGCGAAATTCTGGCTCAGGCGCTGGGGCAAATTAACAGCGTCGCCACCCGGCTGCG
GCTCGGCTTTCCCGCCTCGCCGCGCCAGCTACGCACCCTGATTTTAACGCTGCCTTCCGCCATGCCGAAACAG
GAGCGAGAAATTTTCCGCCAGCGAATGTTTGAAGCGCTGGCGCTGGTCTGGAAAGCCATGGGCTGGCATCCGC
AGGATGAGGATTTCACCACGCCTAAGCAGCGTGAAAAAAGCGTGGTGCCGGTACCAGAAATACAGATGGAATG
GGACGAAGCAAGTTGCGGCCAACTGGTATGGCTTTATAACGAAGCCATTTCGCATTACGCCGGGCGTACCGAA
TCCTTTTTTAATGCGCTCGCCAGACCGGATCGCCAGCCTGAACCCGGCGTCGTCCCCGGACGCGCGTTGCGCG
TGGCATCGATTGATATCGGCGGCGGCACGACGGATATGGCGATCGTTCACTATCAGTTAGATGACGGCGTGGG
GGCCAATGTAAAAATTACGCCTCATCTCCTGTTTCGCGAAGGTTTTAAAGTCGCCGGGGACGATCTGCTATTG
GATATCATTCAGCGCTGTGTACTCCCTTCCCTGCAAACGGCGTTGCAACGGGCAGGCGTTACGGATGCCGCCG
CCCTGCTGGCGACCTTATTTGGCGATTCGGGACGAATTGATACTCAGGCCATTTTGCGTCAGCAAACGGCGCT
GCAATTATTTATGCCGCTGGGCCATGCGGTACTTTCCGCCTGGGAGCAAAGCGACATTAACGATCCGTTTGCC
GGTTTACACGCGACATTTGGCGATCTGCTGATCCGCCGCCCAACGAGCAACGTGATGAACTATATCCAGCAGG
CGATCGATCATGCCTTACCGTCTGGTTCGCCGACATTTGATATATTCAATGTGCCGTTGCAAATCCAGTTTAG
CCAGCTTCAGGAGGCTCTGCTGGCCGGTCAGTTTACGCTAACAACGCCGCTTCATGCGGTCTGCGAAGCGATC
TCCCACTATCACTGCGATATCTTGCTGGTGACGGGGAGACCGACCTGCTTACCCGGCGTACAGGCGCTCATCC
GGCACTTACAACCGGTGCCGGTTAATCGCATTGTGTGGATGGATAAGTATCAGGTACATGAATGGTATCCCTT
CAGTCAGCAAGGGCGTATCGGTAACCCAAAATCCACTGCCGCGGTGGGCGCGATGTTATGCAGCCTGGCGCTG
GACTTACGTCTGCCGCGTTTCAATTTTAAAGCGGCTGATATCGGCGCGTATTCGACTGTACGTTATCTCGGCG
TACTGGATAATACCGTTAATACCCTTCGCGATGAAAACATCTGGTATCACGAAATCGATTTGGATAAACCCGG
CGCAACGCTGGACGCTCGCTTGCATTTTCCGCTACGCGGTAACGTGACGTTGGGCTTTCGTCAACTGGCGAAC
AGTCGCTGGCCGGCAACACCACTTTATTGCCTGAGCATTAATTCCGCCGAACTGGCGAAAACCATCGCTGGCG
ACGGCGTACTGAATGTGCGACTGAAATTACGCGGCAGTAGCAAAGATAGCGCCCCGGAATCGTTCATATTAAG
TGATGCCTGGCTCCAGGACGGGACGCCGGTCGCCGCCGATGCGCTGACATTAAAATTGAATACGCTGGCTGAC
CGCCGCCACAGCGGCAGCCACTACTGGATAGATAGCGGGAGTGTATACCTGAAATGACAAAACCGTTAAACGC
CACCCAGGCTGTTATCGAGTGGGTTAACAACACGCGCCGTTACGCAACGCGACTGGATGACGAAGCCGACGCT
CTGCTGGCGCAACTTACGCTTGCCGCGGCGGATGAGTCAGCGCTCAACGCCGCCTGCGCGTCACATGGCTGCG

TTGGTCTGTACGGGTATGCGCAATCCGCAAAAGCGCATCTGTTAACGACGCTATGCGGTAACGAGAATGGCAA
GCTGGAAATTATCACGCCGGACCGTGACTATGATTATTTCAGCCACATCAATCCGGGTCATGCGCCAGCGAAT
ATGGCTATTCGCTTTACGCGCGACATTTTTTCAAACGAGAGCGGCTGGCCGTTGCGCCTGCGCTTGATCAGTG
AAGCCGAACTGGTGCAGATCTTTATTGCCTGGACCAGCGCGTCCCCCATTTGTCGCCAGGTCGAAAAAATCCAT
TATCACATCACGCCTGGAGAAATGGCAATCGCTTCGCCAACCGCAGCCCGTCCCCGGCGTTACCGCTGAAGAG
GTAGCGACCATCGCGAGTTTCTGGCGCTCTTGCCTGCCGTCCGCCAGACAACATATTGATGATGCGACATGGC
AACACTTTGCCTCGCTCTTGCCTGCGCTGGATCTCACGACACGGGCGCACGCCTGGGCATTACTGTGGGGAGA
GCAACCGGAGATCACCCAGCAATGGCTGGCGCTGGCGCATATGTTGCAACAAACCGGCCATGCCGGGGAACTG
GCGGCGCCGCTCAGCCTGCTGGTCGATCATTTTGGCTTACCGGCGGAAAACTTCCTGACGCAAATGGCGCTAA
CCGCCAACGATACGCAAATCGATGTGGTGGTGCATCCGGTTAAGGAAGGCCGACTGCTCAATGCGGTCAGCCT
TTCGCTTGATTCTCTGGCGCTGCTTACCCGCGAACTGGTGCTAACCGTTGAAAACAACGTACTGGATAACGTA
GATTTACTGGATATTCCGGTCGCGCCAGACAGTCATCCTCATCCGTTATGGCGGGCGAAATTAGGCTGGATGC
TGGCCCATTACCGCCAACAGGTACAGCCCGATGTTCTGGTGATTTGCAATGCTCTGGCATCTCGCTCGCAAAC
GTCCACTGCCGCCCGTCACTTACTGGAGTGGGTTAACGCCACGCAGCCGCAGCATGAATCAGCGTTACCCGGC
GTCGTCTGGGCTATTACTCCACAGGATGCCCGTTTTGCCACCCAGCAAAATCTGGATGAAGCCGTCCAGCAGT
TGATGGGTAAACCTGGCGTGCATTGGGGAACGTTGCAGGCGCTGGATAAACACAGTATGCAGCGTTTGGTGGA
ATGGTTATCGCAGGCGACATCGGCACCACAGCGACAGGCGCGCTTACAGGCGTTACGCGAACAACTGCGAGGC
CGCGTGCGCGATCTGTTACCGATGTTTGATGACGCGCGGCTACCGGTTGAAACCGTGATCCGGCGTCTTCAGG
CCCAGGCCGCCAGACATGGCGACCTGCTGGCCGGGCTGTTACCGCCAGTACAGAATTTCGAAGCGTTGCTGCG
CACCCGACAGTCACGAGAAGAACAGGTCTGCGGTTTATTTAATGACGCGATCGATCTCTTTGCCGACGAACCA
ACGCGCGCTTCAGCGTCAGAAGGTCACGAAACAGGCTACCAGGCGCACAAAATGTGGATCAATCATCTGCGCC
AGTGGGCCCACTGCCGGGATAATGCGCAGCGTCTGGGACTGGAACCGCAGATGCTCAACGCCGTGGCGGAGAT
TTTAATTACCGCCAGCTATCGCCTCGGTTTACCTCAGCAGTTACAGAAAACCATGCAGCGCGAGGAGGTGAGC
GGCGCGCAGCTTCATGCCATCATTGGTAACTTCATCGCCTGGCTCGGCTACGCCAATATCGAGGAGGCCCAAC
GCCCGGCCAGCCGCGTTCAGAAAGGCGCAGCCATTTTCGCCGCAACACCGCGTTCGACAATGTTGCGTCTAAC
AAAGCTTGACGAACAGCCCGTTCACGCCGCCAGCCGCTATGTCTATGACTGGCTTGTTGCGCTGTATACCCTG
GCCAATGAGAACGCAGGATATCGGCATCCGCAGGACGTTACCGACGTAGACAGAGCGCAATTAATTGCCCTGA
TAGCCTGACCTGGCGGAAAAACCATGCCCGGTAACCTGTATCGGGCTTGAGGTCAGAAAAGCGAAAACAGCAA
TGCGACGGGGAAACTTATTGGCCATGTCGCGCCAACCAAAAAGGCGCTCAGGAAACGAATTCGTTTTTTGTCA
TGAGTCAAAAACCAGGTGATCAGGGATGATATCACCGCCATGACGGCGTAAAAAATTAATATCTTTTGAAAGA
GGGTCATATCAGGCATCCGCGCCAACCAGAGGAAAAAACGCGCGCAATATGCTCTTTTTATTTGACGCACATC
AAGGTTTATCAGCCTTAACAGCAAAACGTTCTCAATGCACGGCTCCGCTCACAGAATATCCTGTTTACGTATT
ATTGCGGATCTCGCCAACCCGTACTATACCCATAGGGGTTATGATGAAAGGTGGCGAAAATGAATGTTTCCAG
TAAAACCGTCGTGCTGATTAATGTCTTTGCTGCTGCAGGACTTTTGGGTCTGATCTCGTTGAGATTCGGCTGG
TTTGCTTGAGGTGTTCAACCTTTGAGTACTTGCTGCGCATGTTGGCGGGGAGGTCCCCCTCTCCGCCGACACC
CGCCTATTTTTTCACGATTTTTCTGAACAGTAAATGATCCTTAATGCTTAACCATGATGTGACGGACAAGTGT
GTAGTCTTCCAGACCATACAATGACATATCTTTGCCGTATCCCGACTGTTTTTGCCCTCCATGCGGCATCTCG
CTAACCAACATGAAATGGGTATTAATCCAGGTACAGCCATACTGCAATCTGGCGCTAAGCCGGTGCGCCCGCC
CAACGTCCTGAGTCCAGACGGAGGATGCCAGACCATAGCGAGAATCGTTGGCCCACCTCAGAACCTGATCTTC
ATCATCAAAGACGGTTATACTGACCACCGGGCCGAAGACCTCGCGTTGCACGATGGCATCTTCCTGTTTTGCG
TCTGCCAGCAACGTCGGAGCGAAATAGTAACCTTTACCTTCCGTTTGACTTCCGCCAGTAATCACACGGATAT
GGCTGAGGGCCTTCGCCTCCTCTACAGCCGCTGTGACGCGCTTCAGGTGCGCCAGCGAACTTAACGGCCCCAG
CTCTGTCGACTCATCCTCCGGCGCGCCCATTTTCAGGCTGCTCACCGCGTTCCCTAGCTTTTCGACCAGCGCA
TCATAGATACCGCGCTGCGCGTATATACGGCACGCCGCAGTACAATCCTGTCCGGCATTATAGAAACCAAAAG
TCCGGACGCCCTGCGCCACCGCATCCAGATCGGCGTCATCAAAAACAATCACCGGGGCTTTACCACCGAGTTC
CATATGCGTACGTTTTATCGCTGGCGCAGTATGGCGCAGAATATGCTCCCCGGTCGCGATAGAACCGGTTAAA
GAGACCATGCGTACTTTTTCATGACCGGTCAATACATCGCCAACAGTCTGCCCTCGTCCGAACAGGACATTGA
GCCCCCCCGGCGGAAAGATATCCTTCGCCAGCGCGGCAAGTTTAAGCGCCGTCAGCGGCGTGATTTCAGAAGG
CTTAATCACCACGCAGTTACCTGCCGCCAGCGCTGGCGCCAGCTTCCAGGCCGCCATCATGAGCGGATAATTC
CACGGCGCGATAGAGGCAACCACGCCAATCGGATCCCGGCGGATCATCGACGTATGTCCTTCCAGATATTCGC
CCGCCGCCAGCCCACTCAAACAACGGGCCGCGCCGGCAAAAAAGCGAAAGACATCCACGATTGCCGGAATTTC
ATCATTAATTACGCAGTGCAAAGGCTTACCGCAGTTTTGCGATTCAAGCCGGGCGAACTCAAGGGCGTTTTGC
TCGATGCTGTCCGCCAGTTTTAACAGACATTCCGCCCGTGCTTTGGGTGTGGTCTGCCCCCATTCGGCAAACG
CGCTATCGGCGGCCAGCACTGCGGCGTCAACCTGCGCGGGCGACGCTTCCGCAATCTCAAGTATGACCTCGCC

TGTCGCCGGGTTATAAACAGACTGCCTTTCTCCCTCACCGTCCACCAGCACGCCATTAATCAGTAATTGATAT
TGCATAGCATTTTCCCATATCGTCATTAATCGATTTCCGGCGAACGGGCCGCCGCTTTCCTAAAAATAGACGG
CGCGCCAGATGCTCACCCGGCACACTCGGGAAATTTAGTCAGGTTGTGAGCTGACACGCATTCCACCTCACAC
AAGGCTTTTAGCGCTCAGCATCTCGCGAATCAGGCTGCCAAGCTGTTTTACGGCCTGTTCTTCCCGTTCTCCC
CAATGCCATGCGGTGTTAAAACGAAAGAAACGCGTCCAGGCGCCGGACGTTGAAAACATTTTCCCCGGCGCGA
TACTGATAAGGTGTTCAAGCGCTTTGGCGCTAAGTTCACCGGCATCAAGCTGCTCAGGTAGCTCCAGCCAAAG
AAAATAACCGCTATCGTTATGGTGAATTTTTACTTCCGCCGGGAGATGGCGCAAAAGGGTTTGCCAGGCTTGC
TGTTTACGTTCGGCAAGCTGCCTTCGCAATCGTCGCAGATGCGCGTCATATCGACGTGTCGAGAGATAATCCA
CTAACGCCAATTGCATGGGGGAACTGGTGGATAATGTGCTCATTAACTGCAAACGCTGAATTTGTCTGGCATG
GCTTCCTCCCGCAACCCAGCCAATGCGAAAACCGGGAACCAGACATTTGGAAAAAGACGAACAATGCAACACG
GTATCGTCGCGATCCCATGCTTTGGCAGGCAGAGGTTTCTCGCGGCCAAAGTAGAGTTCGCTATAAACATCGT
CTTCAATTAGCGTCACGTTATGGTGCGTGAGTAACGCAACCAGCCGCGCTTTTTTCTCAGCACTCAGCGTGAA
CCCTAACGGATTCTGGCTGTTGGTCATTAACCAGCACGCTTTCACCGGATATTCCTGTAACGCCTGTTCCAGC
GCCATAAGATCGATACCTTCTTTAACATCGGTAGGTATCGACAACGCTTTCAGGCGCAATCGCTCCAGGGCCT
GTAGCGCACCGTAGAAACAGGGGTTCTCCACCACCACCCAGTCTCCCGGCGCCGTCACCGCCTGTAAACTAAG
ATTCAACGCTTCCAGCGCGCCGGCGGTAATGACAATCTCGTCAGGAGAAACGGTAATCCCCTGCTGCGCGTAA
CGGCGTGCTATCGCATAGCGCAATTCTGCATTACCTGGCGGCAAGTTTTCGATGACGCTCATTGCCGTCGCGG
TTTTACTGACCTGCGCCAGAGAGCGGTTCAATTGCGGCAAGGGAAACAGACGCGGATCGGGAAACGCTGAGGC
AAAGGGCACCACAGAGGTATCACGGCTCGCCTGCAGCATCTCAAAAATATAGGTATTGATATCTACGGCTTCA
TCCCGAATAACGTGCGCTGCCGTCGCGACTGACCGACAAACCGGATGCGGCGCGACATAATAACCAGATTGCG
GACGGGCGATAATCCGTCCCTGACTTTCCAGCAACTGGTACGCATGACCGACAGTCATAAAACTCATGCCGCT
ACTGGCGACCTGCTCCCTCAGCGAGGGTAATCGATCGCCGGGTTGCCAAACGCCAGAGGCGATTTGTTCTCTA
ATTTGCTCCGCCAGACGTTGATATTTTTCATTATTCGATCTCATTGAGCGCTGCTTGCAGGTGGCAACCGCA
GTGTATATCAGTTGTTAAAATTAACAATGATTTGCTAACTGTTATGGTGCTTTCCAACAGGCGTAATGATTAG
ATTTCGCAACAAAACGCCGTTTCAGGGTCTCTGCTTAACGGGAAAGTCAAACCAAGTGTTCGGGTATGGTTCA
TGAGTAAGAGAGAAGTGCCACCATTCAGTATCCAGTGGCGTAAAACCCGCATCACGCATCAATGTTTGTAATA
ACAACCTATTTGCGCGTTGCTGAGCGGTAAGCATGACATTATCGGGATGGGATAACGGACTAAAACAGTCAAA
GCCGGTACCAAAATCCAGACTATTATCTGGCGAGCGTTGCGCCGCGGACGCAGTACAATTCACCAGTGGTCGT
CCGGGATCGTATATTGGTATTTTACTGTCAAGTGGAACAATCGTTAGATCAAGCGTACTTCCCCGACTGTGGC
CGGATCTGGCGGCAAGATAACCTTCCTCAAACAGACGATTTTTCTCTACCTGCGGATAAAATTCGTTTTTCAT
TTGGTTTTGAGAAGGATCTTTGGCCCACGCGATAAAGTCGTTCACTGCGCTTTGCGGACGGTAGCAATCATAT
ATTTTTAAGGTAAGTCCGAAGGGGCGTAAACGATCGGCGACCTGCTTCACTGCGTTCGCGGCTGGTCGTGTTA
ACAGGCAAACAGGCGCGTTATAGCCTTTAATGGGGCGACCAATAAAGTTATGGGAACTGAAGTATCGCATGTC
TACCTGTACGTCGGGGGCGACCGTAGTAATATCGACAAAGTCTTTTGGCTGGTGGAGATCAATATGGTTTTCA
GCTGACATTGCCTGAACGGACAACAGGCTGAACAGGAGGAAAGAAGATACGCATTTTACCATATGTTACACCT
CGCGAGAGTCACAGGGGTGATTATGTATTTGAGCAGGTAAAGATTAGCGGACACCGCTTAAACAGATGGTTAA
AATAAACATCATTCCCCCTGTAATCTGTGCCTTATCACAAACGGAGAGTTTATATTTTGCTTATACGACCACT
GCGCCTCCTGTTCTATACCCAAAATAATTCGAGTTGCGTAAAGGCGGCAAGGGAGTGAGTCTCAGGAGCACAG
ATAACGATGTGTTTTCACAAAGGCGAGTCTACCTGACCAGAAAGGTCATTTGATAATCAGTAGTACAGTTGTT
GTCGCCTTCCATAACTAAATAACATTGTTTCGCCAAAGATAAGACCACTATATAGTCAGGAAGGCAATTAGTG
AGGAACACGTCGATTGTCGGCGATAATGCACTGAAATTACGATTCACTGCAGTTGTGCAAACACATAGAATCC
CCCTCGAACCGAGATAATTCGGTCTGCTGGTCATAGCCATTATTCAGTAAGACCGCAGGTTGCAGCGGCGGAT
CACGGGCGTGAAGAAAAATCCTGTGCCCCAAATTTTTTGGACAAGCGCTGAACCTGCCGTATACTTTTACTTA
ACGTACACTCCGCCATAAAAATAAACCCTGCGATGATACCGTAATGCCATAGGCATAACTTAAGGATATGCCA
TAAATCAAGCGAAGCATGATTTAACTTCTCAGTGATTGCGATAGTCTGTACAAATATTATGGGATGAAATAAT
AAGACGCCTAACAAAAATATCCCTGCCACGATCATGGCAAGTAATTGAAAAAAAGCCCATCCGATAACGAAAC
GGTAAAGTAACAGCAATAATAGCCGTCCACATTTTCTTAGCCATTCTGACTGCAAAATGCCCCAGGATGCTGT
CTTTTCGTGAATTTCACCATCTGATTTCTTCATTTTGAGCCTCCTCGCAGGTTTTTATAATTTTATCGCCCAA
CTGGAAATAAAGCCGTCAGCTAATCGTTACAACAAATATAATTAAGACAAAAACTAAAGAGTAAGGTATTTAT
ATCATAAGCACTATCAGTATTGGCCTTCTGCCCTACCGCTAAACATCTCATTGTTGTTAGCCTAATAATACTT
TTAGTTTAACTTCTTATAAGACAATTTCTACACGGTTGAGCAACTATTTACTTTCTCTAAAAATAATATAGTG
CGTAATTAATCATTACTCATAGTACATGATGATGTGAGAATTAAGAAAACCGTTTTACTTTCATTCGTTTTAT
CTGACATATTTCATGGCCAGGAGGCGTGGGCATGACTAAAGCTACGGGTCGATTTGAACAATTGAACAATAAT
GTTGACGGTTCAGGACAAAGCAAAAATCAGGTGTTTCACCGATAGGCAAACCGATGGGCAACATGGGATAATA

TTTCGAATACCACCTATTCCAGTAATGAAGTATCATATAATCACTTGTGGTCTACATTATGCCAATTACTATC
GGGAGAGGATTTTTAAAGAGTGAGATGTTTTCTCAAAGTGCTATCTCTCAACGCTCCTTCTTTACGTTACTAT
GGGAAAGGATTAAAGATTTTTTTGTGACACGCAAAGATCCACTGCCGATCAATATATAAAGGAATTATGTGAT
GTTGCCTCTCCGCCTGACGCGCAACGTTTATTTGATTTATTTTGCGCGTTATATGAATTATCATCTCCGTCAT
GCAGAGGAAATTTCCATTTTCAGCACTATAAAGATGCTGAGTGTCAATATACAAATCTCTTTATTAAAGATGG
CGAAGATATTCCGTTATGCATTGTGATACGTCAGGATCACTACTATTATGATATAATGAATAGAACAGTGCTC
TGTGTTGATACTCAGCCTGCCCACCTGAAAAGATATTCTGATATTACTATAAAAGCTAGCACTTATGTTTGTG
AAGAGCTATGTTGCTTGTTCCCTGAGCGGTTACTACTCTCGCTATCCGGCGGTATAACATTTCCCGTTGACCT
AAAAAATATAAAAGAGACGCTTATCGCCATGGCAGAAAAGGGTAATTTATGTGACTGGAAGGAACAGGAAAGA
AAAGCCGCCATCTCCTCCAGGATTAATCTGGGCATTGCCCAGGCAGATGTACCACCTATTGACGACGCTATCA
AAAACAAAATAGCCGCAAAGGTTATTGAGAATACTAACTTAAAGAATGCAGCGTTTGAACCAAATTATGCACA
AAGCTCAGTTACTCAAATTGTTTATTCATGTTTGTTTAAGAATGAGATACTAATGAATATGCTGGAAGAAAGT
TCCTTTCATGGCCTGCTATGTTTAAACGAGCTTACTGAATATGTAGCGCTACAAGTTCATAACAGCCTTTTTT
CTGAAGATTTATCTTCACTCGTTGAAACAACAAAAAATGAGGCTCATCACCAGAGTTGATCACACCATAAATA
GTATGGATTTACAATCCCCTGGCAATACCAAAACCATCTAATACAATAACTTTAAATATATACTCCCAGGGAA
TAATGGATTTACTATGGAACGAATTGGCTCGTTATTTTTATAATTGATATACTGAGTATAAGTATATTTTCTC
ATACAACTTATCGCACTTATTGACAAACCGTTTTCGCTAATTTGAAGTGTGGAATAATAACTCAAAGAAATTA
TTGGCATCATAGCCTCACGACGTAACTGGAAGGTGGCATGATGCTTACAAAAACGTTATCAGTAGTTTTACTG
ACCTGTGCGCTGTTTTCAGGCCAGTTACTGGCGAAACAGCAAGACCACGCGTTCGTATGGTTTGCGACGGGAG
GGCACCAGCTTAGACACGAAGCCGACAGCGATGAATTACGGGCGGCAGCGGAAGAATCCGCGGAAGGCTTGCG
CGAGCACCACAACTGGCAGAAATCGCGCAAACCAGAAAGCTATTTTCGTTAATTAAACGCCCCTGCGCTTCGG
CAGCGTTTTCATCAGCGCTTCAGGGCTCACTGAAGCGACAACGCTGCCGGGCTGGGGCATTTTTAAGATGTGG
TTTTTAATTTTGCCAATGACATGCATTTCGCAAGGGCGGCAGTCGAATTTTAGCGTCAGTACCTCATCGCCAT
GCACCAACTGCATCGGCGTGGCTTTCCAGCTCTTAATATTTCCTTTCGCCTGCTTTGGACAAAGGTTGAACGC
AAAACGCAGGCAGTGTTTGGTGATCATGACCGGTACCTCGCCCTTCTCCTGATGCGCCTCATAGGCCGCATCA
ATCAATTGTACGCCGTAACGGTGATAAAATTCCCGTGCTTTGTGGTTGTAGACATTAGCGAGAAAGCTGAGAT
GCGTTTGCGGATAGACCGGCGCAGGCTGCGCCACGGGTTTACGACGACCTCGTTGATAATGGGCCAGCCGCGC
CGCGTCAAGCATATCAATCGCCTCCCGACGGAACTGATTGAGCAGGCTATTTGGCACGAACAACGCCGCCGGT
AATGTCACCTGCATATCACGCGCGTAGTAAGGCGTCTGTCCCAGCTTCGCCAGTCCGGCTTTTAGGTTATTCA
ACGCTTTTTCAGAGTTGTTGGCTTCCTCAAATACCCCGTCAAGCGTATGCGTGATGCAGACACCGTCTTCACT
GGTCAGCGTCAGAATAAGCTGTTCCTGCCAGCCGCCCAGCATGATATCAACCGCCACACGGCGCTCACTGGAG
GTTTTTGTCAGCGCTTGCTGCCAGTTATGATCCAGATTACGATTCAACGGATGATGCGGACGGACTTTATGCA
GGTCGGCAGGCATATCATTTGGCCAAACGCGGTAGCGGTTATGACCGGTTTTCTCCACCGTATTGGCGCGAAA
ACCTACCACTTCACGCTTAATCAACACGTTCAGACCATCGCCATTCGCCAACGGCTCCGTAGCTTCTACGTCG
AGATAATCCTTCGCCACATTCAGCACCTCACCTACCGGCAAGCCAATAAATTTTGGTGAATCAAATGCGCCAA
TATCACCTTTACGCGCGTTAACAAAATAGTCGGTGCTGCCGCGATGGAAAGTTTTCTCCGTGGAGGGAACAAA
AAAGTGTTCGGTCCGACCAACCGACGCACGCGCCAGATCGCCACGTTGCTCGATAATCGCGTCCAGCATCTGA
CGATAATGCGCGGTGATGTTTTTGACATAGCTCATGTCTTTATAGCGCCCTTCAATCTTGAAGGAACGTACGC
CTGCATCGATCAACGCGCCGAGGTTAGCCGTTTGGTCGTTATCTTTCATCGATAGCAAATGTTTTTCGTAAGA
GACCACCCGCCCCTGATCGTCTTTTAACGTATACGGTAAGCGACAGGCTTGCGAACAGTCGCCCCGATTGGCG
CTGCGCCCGGTTTGCGCATGAGAGATATAACACTGCCCAGAATAAGCGACACACAGCGCGCCATGAATGAAGA
ACTCAATCGTGGCGTCAGTAGCCTGATGAATAGCCGCTATCTGACTCAAATTAAGCTCGCGCGCCAGTACAAT
CTGTGAAAAACCGACATCGGCAAGAAACTTCGCTTTTTCCACGCTGCGAATATCACACTGTGTACTGGCGTGA
AGCTCAATCGGCGGGATATCCAGTTCCAGAATGCCCATATCCTGCACAATCAGCGCATCCACCCCGGTGTCGT
ACAAATCGGTGATTAACCGCTGCGCGGGCTCCAGCTCATCATCATGCAGGATAGTATTCAGCGTGACAAAAAT
CCTGGCGCCATAACGGTGAGCAAACGGAACCAGATCGGCGATATCGCGCAGACTGTTACTGGCGTTATGACGT
GCGCCAAAACCGGGTCCGCCGATGTAGACGGCATCTGCGCCATGCAAAATCGCTTCGCGAGCAATTGCGGCGT
CACGGGCCGGACTTAATAATTCGAGATAGTGAGGTTGCTGGCGCATACAGTCTTTCTTATCCAATAGCAGCGA
AATGGCGGCTATTGTAGTCAGAAGCGCAAACTAACGAAATAGTTTTCCCGCTTAACCACGCGGGTAATGGATC
AGCGAGTGAAAATGAACCGTCTGGTCACTGCTATTGCGATAGGTATGCGCCATATCACCGGCAAAACGCAATC
CTTTACCGGCGTCTATGGTTTGCCATACCCCACCAGTACACATGTCCAGCCGACCCTGAATCACCACCACATG
TTCGACAACGCCGGCTTTATGCGGCGTTGATTCGCTGACCGCGCCAGGCGCCAGCAATAGGGAAAAATAGTCA
AAACAGAGTTCAGGATCCCATGGAAACAACGGTGTGATTACCATCGCTTGCTGCTGGGGATCGAAGGTCGGAG
GAAATTCGGCCTGCGGCGGCGAAATAAACACTGAGAATGGAACGTTCAGGCCCGTCGCAATTTTCCACAGCGT

TGCGACTGTCGGGCTGGATTCATTACGCTCTATCTGCCCCAACATGGCTTTTGAGACGCCGGAGATCTCCGCC
AGACGCGATAAACTCCAGCCTCGCTGATGGCGTAACATTCTTAACGTGGTCGCCAGATAGTGTGTCAGGTTAT
CCATTTTCCCCCCCAGTAAGGCCATAGCGTATCACTTGTACGCTATAACGCACAATGCTACATTGACTGACCG
TTATAACGCACAAGAGGCGATCATGCGTCTGTTATCACTGCCGTTACCTACTGTATTATCCGGTCTGGTCGCC
GTTCTGGTCGGCTATGCAAGTTCCGCCGCCATTATCTGGCAGGCGGCGCTTGCCGCTGGCGCAACACCCGCAG
AGATCGCCGGCTGGATGACCGCCCTCGGTATAGCCATGGGCATAAGCACACTAACGTTAACCTTGTGGTATCG
TGCGCCGGTTTTAACCGCCTGGTCAACGCCTGGCGCCGCTCTGTTGGTAACAGGGCTTCAGGGATTATCGTTG
CCAGACGCGGTGGGCATATTCATTGTCGCCAATGCCCTGATTGTGCTGTGCGGCGTCACGGGATTGTTTGCGC
GTTTGATGCGGATAATCCCCCATTCTCTTGCCGCTGCAATGCTGGCGGGAATACTGCTACGCTTCGGCCTGCA
GGCGTTCGGGACGCTCAACGGAGAGTTTGTCATGTGCGGCGGTATGCTATTGGCGTGGCTACTCTTTAAAGTA
TTCGCGCCCCGCTATGCCGTTATTGCTGCCATGGTGATGGGAATAACGGTGGCGCTCATCCAGGGTAAGGTGG
CGATGAGCGGTATTCATTTCGCGCCGGTGTGGCCCACCTTCGTTCCCCCCCACTTTTCATTCGCTCAAAGCCT
GAGCGTCGCGGTACCGCTCTTCCTGGTGACGATGGCATCGCAAAACGCCCCCGGCGTCGCCACAATGAAGGCG
TCTGGCTATCAGCTTCCTGTCTCTCCGTTAATGATTTTTACCGGATTGCTGGCGCTGCTGCTTTCGCCATTTG
GCGTTTATTCTATCTGCATTGCCGCCATTACAGCCGCCATTTGTCAAAGCCCGGACGCGCACCCCGATCCAAC
GCGTCGCTGGTTAGCGGCCGCCGCCGCAGGCGTTTTTTATTTACTTGCCGGGTGGTTTGGCGGTTCGATAACC
GGGCTGATGGTCGCCCTGCCGGTTAGCTGGGTGCAAATGCTGGCTGGTCTGGCACTGTTAAGTACGATTAGCG
GTAGCCTGTATCAGGCGCTTACTCACGAAAGTGAGCGTGACGCGGCAGTTATCGCATTTCTCGTCACCGCCAG
CGGGCTAACGCTGATGGGCATCGGATCGGCATTCTGGGGGTTGATCGCTGGCGGGATAGGCTATGCAGTATTA
ACGAGAACGCGCCGCCCATCGTTGTCTGGCTGATGGTGACCGCTAATCTGCCGGGGTTTGCGCCATCTTGCGA
TGGCGCCGCAGAATTATTTTTTGTTAACGTCAAACATAGAGGTATCCGTCGCCATATCATCCACGACCTGTTT
CAACGTCTCAAACGCCATTGGCGTACTGGAGTTACTCAGGTCTTTACCTTCACCCTGGCGTACTACCTTGACG
ACCGGCTTGTTGGTCGCGGCATCTATCAGCTCACCTTCAAAATAGAGGTGGGTATCCATGGTCCGGTGGCCCG
TCGCCATTTGCGTGCCCGCCACAATCAGCGCTACAGGGATCACCTCATAGAATTGCAGCCCTTCTTTGCTGGT
ATCCACGCCCGTAATCGCCCCGCGGAAAATCAAACTGCGCGGCCCTGGCGTCGTGACCAGCGGTTTGCGCTGT
TCAATTGCTGATTTTACTTTAGTGTTCGTATAAGCCAGCAGTTTATCAAGAACTTGCTGCCCGACCTGGGTCG
TTGGTTTGGGGACGGGATAGTATGTTATCGGATTGTAAACAATACTGTCATAGTTAGAGTCATTGAAATGTGG
ATCTACCCAGCGCAACACCGGTTTCCCCGTGGCGGAGGTAGTTTCCTGCAGGTCCGAATAATTTTTTAAGAAT
CCAGAATACTTATCCGGCTGTGTGACTTTAGACGCACAGCCGGATAACGCCAGCAAGCCAGTAAGCACTGCAA
CTTTAAATAAAGTATGAGTACGCATGAAACGATCCCTTTAGTCTTCGCAATGATGCAAAGGAAATAATAGCAA
AACGAAATCATTCTGGATGCACTAAAAATGGTAACGACAGCACAATTTGAACGCAGCGCCCGACAAAAAAATG
CCGAACGCAGCGAGACTTACAGAACTTGCATCGCTCAGGCCGTTTTTCTCGCCAGCATCGTAGCAAAGCGCAG
TTTGATACGGTTGCCGTTTTCGTCCGTGCGGTGAAGCTCGCCGACATCTTCGTTGTATTTCAGCATGTCCCAT
CCTTCGTAATAGCGGCGTAATTCGCCTTCTTTAAAAGCAAACGGGAATCCCACCGTACAGGGAAAGTCCGGCG
TATCCATTGCCGCAACAATCAAATTATACCCGCCGGGTTTTGTGCAGCGTTGCATATTGGCAATTAAACTGGG
AATGGTTTGCGCCTCAAGAAACATCATAACCACCGTCGAGAGAATAAAGTCGTACTCACCGTCAAAGGTCAAT
GTATTCAGATCGACAAGGTCGGTTTGCAGATTATCCAGCCCTTCTGCCGCTTTAATACGCTCAAGATTAGCCA
TACTGGCCGGATTTTTATCCCACGCGGTAACGTCATAACCGTTCGCGGCCAGATACAGACTATTACGGCCATT
GCCACATCCCAAATCCAGCGTTCGGCCTGGCGCGACAACCTTTGCCGCCGCCAGCACATCCGAATGCGTGCGG
GTTAAACCGTATTTCTCAGTAAAGTAATTTTCGTCACGAACGGTCATTTTTTTCCTCCGTTTTCAACAGTGCA
GCGCGCTCAGTGCGAATAAGCAACGTCCCCTGCACCAGCAATAGAAATGTACGAACCAGCAATAAAGCGATAA
TAGCGTTAGTGAAAATAAACAGCGGGACGGCCAGAATATGGAAAAGCCCTGATTCACTACCGTGACCAAGATG
TAACCCCGTGGTCGCCAGCGCGGAAACGCCGAACGAAAAACTCCAGAAGGAAGCATTAAACGGTTGTGATAAA
TACCAGGGCATTAATCGCAGCATAAATAAAAGCTGTAACAGACCGTAGCCGAATAGCATTTTCGCCAGCGTGT
CGCCCTCCCCGCCATTGACGCTCAGCCAGGCGCTACAGGCGACCAGCGCGGGCGCCAGTTGGATACCCAGCGA
CGTACGCAGCACCGCCGGTAATTCGCCGCAGCTACGCAGTCGCTGCAAAATAACCGGCTCCAGACTTAACCAC
GAAAAGACGCCTGCGCCTAAAAACACCAGTCCGGCATCATTGTAACCGAGCGCTCCACAGGCCATAGCGCTGA
TAAAATTATTCGCTACCGTGGGCAGATAAAGCCCCGGGGTTGTCGCTTCTTCCGGATGCGCTCCGCGCCAAAG
TCCCGCCGTCTGCCAGGCGGCATACGCCAGTTGTATGACGACCCCCACGCTGAATAGCGCAACGGCCAACGGG
CGATACCAGGGAACGAAACCGATCGCGACCAGCATCGTGGTGGCGGGAAACAGGCTGACAAAACTGCTCATTA
CCGGATGTCGCACTTCAGCCATCACGCTATGGGGAAAGCGCACCAGACGGCTAAGGAACGCCAGGGTCAAGAG
TGCCCAAATAATCATCGCCAGGATCACCATGATGTCGCCGGGCCAGTGGCTTATTCCCCATATCTGGCTGGCA
TAACGCCAGGCGAATCCCATACCGATAGTGCCAAGGACGATGCCAAAATAGCCCGCAGGCAGATTGAGCACGC
GATCGCTCTGCTTGTGATTACGCATTTTGTTTAATTTATAAAGTATATTTGAAATGCATTTTATGGAATTTCT

GGTTGTTCCGCCAGTACCTTTGCTACGGTTATCTTAATCGTTCACACGGAATTTCCCTATGATCAAATATCGA
CTGAGTGAAGAACCTCGTGCTTTTACTTATCAGGTAGACGGTGAGAAGAAAAGCGTCTTATTACGACAAGTTA
TTGCCGTAACGGATTTTAACGATGTTAAAGCCGGAACCTCCGGCGGCTGGGTTGATGCCGATAATGTGCTGTC
ACAACAGGGGGATTGCTGGATTTATGATGAAAACGCGATGGCCTTTGCGGGCACGGAAATTACGGGTAACGCG
CGTATCACGCTGCCGTGCACGCTCTACAATAACGTGCGAATAGGCGATAACGTCTGGGTTGATCGCGCTGATA
TCAGCGATGGCGCACGGATAAGTGACAACGTCACCATTCAGTCCTCTTCCGTGCGCGGAGAGTGTGCGATTTA
TGGCGATGCCCGCGTGTTAAACCAGTCAGAAATTCTCGCCGTTCAGGGACTAACGCATGAACATGCGCAGATT
TTACAAATTTACGATCGCGCCACCGTGAACCATTCGCGGATTGTCCATCAGGTACAGCTTTATGGCGACGCCA
CTATCACACACGCTTTTATTGAGCACCGGGCGGAGGTCTTTGATTTCGCGCTCATCGAAGGCAACAAAGACAA
TAATGTCTGGATCTGTGACTGCGCCAAAGTCTATGGCCATGCGCGGGTTATCGCCGGTACAGAGGAAGATGCG
ATCCCTACCCTTCGCTACAGCTCGCAGGTAGCGGAACATGCGTTAATCGAAGGCAACTGTGTGTTAAAACATC
ATGTGCTGGTAGGCGGACATGCTGAGGTACGCGGAGGCCCGATTTTACTGGACGACCGCGTGCTGATTGAAGG
CCATGCCTGTATACAGGGCGAAATTCTGATTGAGCACCAGGTCGAGATTAGCGGACGGGCGGCGGTGATAGCC
TTTGACGGCAATACTATTCACCTGCGCGGCCCAAAAGTGATCAATGGCGAGGATCGTATTACGCGTACCCCCT
TAGTCGGTTCCCTTTAATCCGCATCAATAATGCGGGCATACATATTAACGTCGTGATAGTCGCCGTTAAGATA
CTCTGCCTGTTTCATACAGCCTTCCAGCGTAAAATGGTTGCGCCGTGCGACCGCGTTACTGGCCTGATTATCA
ACGCGGCATTTAATGACGAAGCGCCGAATATCGCCGCGCCGGGCATAATGTGTCATCAGCGCCTGTAATGATT
GCGACATTATCCCTTGTCCCTGGAGGGACTCATCCAGCCAGTAACCGATATAGGCGGCTTTGTTTACCGGTTC
AATGGCATTAAATGAGAGTACGCCCGCCATCTCATTCTGGCAGAAAATCAGGTACATTTTGGCGTAACCACGC
TGGTGCAACAGTATATTACCCTGCACATGCTTACGCGTTTCCTCCTGCGACGTGACATATTGCGGCCAGTCGA
GCGACTGTTGTAGCCACGCTTTGTTTTTGAGTACCAGTTCGTGCAGCGCCGGAACATGACTTTCATCTGCCGC
TCGCAGTTCCAGCGTCGTGCTAACAGGAATAATTTCCACCATCGCGTTGCCCTTCTTCGTTGTTGGTCGTGAT
CACATAAATAGCCTGGTTTACCGATAACCCGCAGGTGATATGAGCGTTTTTATTGTATCGCCATCACATTATT
TTTTATTGGCCATATTTAGCGTATTTCCGCGTGACACATTTCACACTTATCCAACCATTCTCCGCCTATAAAA
CGCTCAACGACTGATTGTTTTATCACCACGGAGACGCCAATGACCTTTTCTGTGCAGGAAACGCTTTTTTCTT
TACTGCGGCTAAACGGGATTTCAGGACATGAAAGCAGTATTGCAAACGTTATGCAGCACGCGTTTGAACAGCA
GGCCAAAGACGTCTGGCGGGATCGCTTAGGCAATGTCGTCGCCCGTTATGGCAGCGACAAATCCGACGCGCTT
CGCCTGATGATTTTTGCGCATATGGATGAAGTCGGTTTTATGGTACGCAAGATCGAACCCTCCGGCTTTTTAC
GTTTTGAACGCGTGGGCGGCCCGGCGCAAATTACTATGCCCGGTTCGGTCGTGACGCTTGCCGGACGTTCAGG
CGATATCATGGGCTGTATCGGTATTAAAGCATATCACTTCGCGAAGGGTGACGAGCGCACCCAGCCTCCCGCG
CTCGATAAACTCTGGATTGATATCGGCGCAAAAGATAAAGCGGATGCCGAACGAATGGGTATTCAGGTGGGGA
CGCCAGTAACCCTTTACAACCCGCCGCACTGTCTGGGCAACGACCTGGTGTGCAGTAAGGCGCTGGATGACAG
GCTGGGGTGTACGGCGCTACTGGGCGTCGCCGAGGCCCTCGCCTCCACACCGCTCGATATCGCGGTATTCCTG
GTCGCGTCGGTACAGGAAGAGTTCAATATTCGCGGTATTGTTCCCGTTTTACGACGCGTGCGCCCCGACCTGG
CGATTGGTATTGATATCACCCCCTCCTGCGACACGCCTGACCTGCAGGATTACTCTGATGTGCGGGTCAACCA
CGGCGTCGGCATCACCTGTCTGAACTATCACGGACGCGGTACGTTGGCGGGACTGATTACGCCGCCGCGTTTG
CTGCGGATGCTGGAGACCACCGCGCACGAAAATAATATTCCCGTACAGCGAGAAGTCGCGCCAGGCGTCATCA
CCGAAACCGGCTACATTCAGGTTGAACTGGACGGTATTCCCTGCGCCAGTCTTTCTATTCCCTGCCGCTATAC
CCACTCGCCAGCCGAAGTCGCCAGCCTGCGCGACCTGGCTGATTGTATCCGTTTACTGACTGCGCTGGCTAAC
ATGTCGCCAGAACAGTTTCCCATTGAGCCTGAAACAGGCGCTACACAAGAGGCACGACCATGATGAAAAAGAT
CCTTGTGGCATGTGGTACCGGCATGTCGACATCAACCATGATTGCACAAAAACTGCAGGACTTTCTTGCGGAG
CAAGGCATTGCGGCAACCACGGCGCAATGTTGTCTGAATGAGATCCCGTTGAATTGTAACGGTATGGATCTCA
TCGTAACGTCCATGCGTACGCACAGTGACTATGGCATCCCGACGCTCAACGGCGCCGCCCTGCTGACAGGCAT
CAACGACGACGCATTAAAACAAGAAATCAAGGCGCTGTTAACACAATAAAGGGGGCATTATGTTTGATTACAT
CCTGTCTCTCGGCGGCACCGTTTTTGTGCCCATCATTATGATTATCATCGGCTTAATCTTTCGTATTCCCTGG
CTGCAGGCGGTGAAAGCCGGGGTGACGGTCGGCATCGGTTTTGTCGGCATGGGGCTGGTGATCGTCATGGCGA
TCGACAGTCTCAGTCCGCCGATTAAAGTGATGATTGAGCGTTTTGGCCTGACGCTGCATGTGTTTGACGTCGG
CGCCGGGCCCGCATCCGGCGTCGGCTATGCGACGGCCATCGGCGCGATGATCATTCCCGTTATCTTTCTGCTC
AACGTCGGTATGCTGGTGACTCGTCTGACCAAAACCATGAACGTTGATATTTATAACTACTGGCACTACGCCA
TTACCGGAACGGTCGTCCAGTTGATGACCGGCAGTCTGATTTATGGCGTACTGGGCGCGATTTGCCACGCCGC
CTTGTCGTTGAAAATGGCCGACTGGACGGCGAAACGAGTACAGAACATTGTGGGTCTGGAAGGGATTTCTATT
CCGCAAGGGTATGGCTCCAGTTCCGTGCCGTTGTTCGTATTGCTGGATGCGATATACGAAAAAATACCGTTTA
TGAAAGGACGCAATATTGATGCTCAGGAGATCCAAAAACGGTATGGGATGGTTGGCGACCCGGTGATCATTGG
CGTTGTTCTCGGGCTGATCTTCGGACTTGCCGCAGGCGAAGGCTTTAAAGGGTGCGCAACCTTAATGATTACC

GTGGCGGCGATTATGGTGCTGTTTCCGCGCATGATTCGCCTTATCGTTGAGGGATTAATGCCCATTTCCGACG
GCGCGCGTAAGTTCTTCCAGAAACACTTTAAAGGGCGCGAAGTCTTTATCGGTCTGGATACCGCAGTGACGTT
AGGCCACCCGACCACCATCGCGGTTGGACTGCTGTTAATTCCCATCATGTTAATTCTGGCGAGCATACTGCCG
GGCAATAAAGTCCTGCCGCTGGCTGATTTACCGGTTGCGCCGTTTTTTATCTGCATGGCCACGGTTATTCATC
GCGGCGATCTGATTCGTACTCTGCTCAGCGGCATCATTGTCATGATTACCGTTTTGCTGATCGCCACCCAGTT
CGCGCCGTACTTTACCGATATGGCGCTCAAAGGCGGCTTTAGCTTCGCCGCTGAAAACGCGCAAATTACCGCG
CTGTCGGTGGGAAATATGTTTGGCTGGTCAATATCTGAATTGATGTCGCTGGGCATGATTGGTGTGGTTATTG
TGGTCGGCATTGTCGCCAGCATCATACTTGTCTTACGTAAACGTGAACTACCGGAATAAGGAGCAACCCAGAA
TGAGTTGGCTAAAAGAGGTAATAGGAACGGAAAAAGCCGTTATCGCCATGTGCCATTTACGGGCGCTGCCGGG
CGATCCCGGGTTCGATACCAGGAAAGGCATGAATTGGGTAATTGACCGCGCCCATGACGATTTAATGGCGCTA
CAAAATGGCGGCGTGGATGCGGTGATGTTCTCCAACGAATTTAGTCTGCCTTATTTAACGAAAGTCAGACCGG
AAACGACCGCCGCGATGGCAAGAATCATCGGTCAGTTAATGAGTGAAATCCGCGTACCGTTTGGGGTAAACGT
GTTGTGGGACCCTGTGGCCTCGTTTGATTTGGCGATGGCGACCGACGCAAAATTTATTCGCGAAATTTTCACC
GGCGCTTACGCCAGCGATTTTGGCGTCTGGGATACGAATGTCGGCGAAACCATTCGCCATCAGCATCGCATCG
GCGCAGGCCACGTGAAAACGCTGTTTAACATCGTGCCGGAAGCGGCGGTGTATCTGGGAAACCGGGATGTCTG
TTCAATTGCGAAATCGACCGTATTTAACAATAACCCCGATGCCCTGTGCGTCTCGGGGCTGACGGCGGGCGCT
CGCACGGATAGCGCCATACTGAAGCGGGTAAAAGAGACCGTCCCCGACACGGTCGTGCTGGCGAATACCGGGG
TGTGTCTGGAAAACGTCGAGGAGCAGCTCTGCATTGCCGACGGATGCGTAACGGCAACCACGTTTAAAAAAGA
CGGCGTCTTCGCCAACTTTGTCGACCAGGCGCGCGTGGCGAAGTTTATGGAGAAAGTACGCCATATCCGACAA
TAAAATGGGGATAAGCAAAACGCTTATCCCCTGCAGAGAGCGATAACTCTTCTGCCGATCTCCGTCGCCTTCG
CCAAAAGACACGCCGGGGATCGGTTTCAGAGTAACCCGAACCGGCAAGGAGTCAACGATCGATGATTAATGAT
GTTAAGTGGGTTCAGGCGCAACGCCATGCCAGCGACTGGCGACACGCGCTGGATATCGCCGTTCGTCCGCTGA
TTACCTTCGGCGCGGCGGCCCCCTGCTACCTGGACGGTATTATTGAGAACACCCTTAAATGGGGGCCATATTA
TCTTATCGCGCCGGGGATTGCGTTGCCCCATGCCAGACCAGAGCAAGGCGCCAACCATAATCAAATCAGCGTC
ACTACGTTAGCAACGCCGGTCGCTTTCGGCAATGCGGATTGCGATCCCATCTGGCTGTTGCTTTGCGCCAGCG
CCACGGATGCCGAAGCACATATTCGCACCATTCAACGCATCAGCCAGTGGCTAGACTCGCCGGAAAGCGTCGC
GATGCTGCAAGATGCGCCAAATGACGCCGCGCTTTTCGCGCAATTAACCGCATTGCGCTGATGGAGGTTTCGA
TGATCCTGCACCCTTCACTGGCCTCCGCCGATCCGCTGCGCTATGCCGAGGCGTTGACCGCTTTACACGATGC
GCCCCTCGGCTCGTTGCATCTGGATATCGAAGATACCAGTTTCATCAACAATATTACCTTCGGTATGAAAACC
ATTCAGGCAGTCGCACAACACACCCGCCATCCGCTCTCATTTCACCTGATGGTCTCATCACCGCAACGCTGGC
TACCCTGGCTTGCAGCTATCCGCCCCGGCTGGATTTTCATTCATGCCGAAAGCGTGCAAAACCCCCCGGAGAT
TCTGGCGGATATTCGCGCTATCGGGGCGAAAGCAGGACTGGCGCTTAACCCGGCAACGCCGCTATTGCCTTAT
CGTTATCTGGCATTGCAACTGGATGCGCTGATGATCATGACCAGCGAACCCGACGGACGCGGACAGCAATTTA
TCGCCGCGATGTGTGAAAAAGTGAGCCAGGGCAGAGAGCATTTCCCTGCTGCGGAATGCTGGGCGGACGGCGG
TATTACACTTCGCGCCGCACGGCTGCTTGCCGCCGCAGGCGCACAGCATCTGGTGATTGGCCGGGCGTTATTC
ACGACGGCGAACTATGATGTGACGTTGTCACAATTCACCGCGCTATAAAGGAGTCATATGCTGATGAGCCAAC
AGCGCCCCGACCGTATCAAACAGATGTTGCATTACCTTTGGCAACACAGGCACCTCTCAACACAGCAAGCTAT
TGAGCTTTTTGGCTATGCAGAGGCAACCGTGCGCCGGGATTTTCACTATATTGCCAGCCGTTATCCCGGTATG
GTTCGCGGCCATGGTTGTATTGACTTCGACGACAGTACGGAAGATAAAGAATATGTTTTCGACGTTAAGCGGA
CGTTACAAAGCGAGGCGAAACGCGAAATCGCTGCCCTCGCCCGCACGTTCATCAAGGATGGAGACTGCTTTTT
TCTCGACTCCGGCTCCACCTGTCTGGAACTGGCAAAATGTCTGGTCGACGCCAAAGTAAAAGTCATTTGCAAT
GACATCAAAATCGCTAATGAACTGGGTGGCTTCCCACACGTAGAGAGTTACATTATCGGCGGACTGATTCGCC
CCGGTTACTTTTCGGTGGGCGAGAGTCTGGCGCTGGAGATGATTAACGCCTTTGCCGTTGAACGCGCCTTTAT
CTCCTGCGATGCGCTGTCGATTGAAACGGGCATCACCAACGCTACCATGTTTGAAGTGGGCGTTAAAACGCGC
ATTATTCAGCGCTCACGCGAGGTCATATTGATGGCCGATCACTCAAAGTTTGATACTGTAGAACCCCATGCGG
TGGCGACGCTGTCCTGTATTACTACCATTCTTAGCGACAGCGCGCTCCCGTCTGCTATCGCCCGACGTTATCA
GCAGGCGGGGTGCCGTCTCATCATGCCAGACCCCTCATCAGGAGTGCGTTAATGGACATCAGGCAAATGAACA
AAACCCATCTGGAGCACTGGCGCGGATTGCGAAAACAGCTCTGGCCCGGTCACCCGGATGACGCCCATCTGGC
AGACGGCGAAGAAATCCTGCAAGCCGATCATTTGGCATCATTTGTTGCGATGGCAGACGGGGTGGCGATTGGC
TTTGCGGATGCCTCAATCCGCCACGATTATGTCAATGGCTGTGACAGTTCGCCCGTGGCTTTTCTTGAAGGTA
TTTTTGTTCTCCCCTCGTTCCGTCAACGCGGCGTAGCGAAACAATTGATTGCAGCGGTGCAACGATGGGGAAC
GAATAAAGGGTGTCGGGAAATGGCCTCTGATACCACGCCGGAAAATACAATTTCCCAGAAAGTTCACCTGGCA
TTAGGATTTGAGGAAACAGAGCGCGTCATTTTCTACCGGAAGCGTTGTTGAGGGAAATATTCAGGCTCCTTAT
AGAGAAGTGGTCACTGATTAAAAAGCAGCCTCAATATACAATAATTCCTGTTTCTAAAACGGAGTCCATCAGG

ACGTCGGGAATAAGACCCGGCAGATGCAAGCCGGGCCTTCATTGCTTACGGTCTTGAGGCCGTACCGTCTGGT
GTGCGCGCCATTGTCCAGGATGGGATTCCCCCTCGCACGGATATTTCGCCGCTTTGGCTTCATCAATGTCAAT
ACCCAGACCCGGCTTATCGTTCAGATAGGCATAACCGTGGTCAATCTCAGGGCAGCCCGGGAAGACATCACGC
AGCGCGTCGTTCATTGGCGTGTATTCCTGAATGCCAAAGTTCGGTGAACTCAAATCCAAATGCATATTGGCGC
AAACCCCAACTGGAGAAATGTCGCCCGGCCCGTGCCATGCGGTGCGCACACCGTTCAGTTCGCTGTAGACAGC
AAGTTTCCTCGCCGGCGTAATGCCGCCGATGGTACTCACGTGGCAGCGAATGTAGTCGATGAGCCTGTTGTCG
ATGAGCGGCTTCCACTCATTCACATTCACAAACAGCTCACCCATGGATATCGGAGTGGAAGACTGCTGGCGCA
GCATTTTCAGCCAGTCGATGTTTTCCGGTGCCACCGGATCCTCAAGATAAAATAGTTGATACTGTTCAAGCGT
TTTGGCCAGATTAATGGCAGTCACCGGGGTCACGCGCTCATGTACATCATGAATAAATTCGATGCCAAAACCG
AGCTTGTTACGCAGGTGGTCGAACAGACGCGGAACGCTTTTGGCGTAGGCGTCCGGGTCAAAGTAGATGCCGG
GCGTTTTGCTGCGCGGTGAACGTTTCGGCTGAATGTTCTTCGCACGTGCCAGCTGCGTGGCGATCAGTTTAAG
GTCATCGGTCCCCGCACCGCCATACATTCCCATCTGGCAGCGAACGTACTGGTAACCCTCTTCCATTCTGGCT
CGGATGTTATCTTCCACTTCAACTTCATCACCACCATCGGTGTGGCAGTACAGTGGGATCCCATCACGACATT
TGCCGCCCAGCAGATCATAAACTGGCATCCCCGCTAGCTTACCTTTGATATCCCACAGTGCCATGTCCACGCC
GGACAATGCATTGTTCATGATCGGGCCATTGCGCCAGTAGCCGCTCACCACGCCTGACTGCCAGATGTCCTCA
ATACGGGTCGGATCTTTGCCAACCAGAAAAGGCGCCATGTACTCATCGATCGCGCTTTTTACCGCGAAGATAC
GCTGGGTAAATGTCGCACATCCCAGCCCATACAGCCCTGGCTCGTTGGTTTCTATCTTAACGACTGCCAAATC
AATGCCGCCCGGCGCCGTCAGAATCGTTTTCACGTTGGTAATTTTCAGGTTACTCACTTTCACTCCTTAACTC
AGCGAATTTACCGCATGTCATACCGTTGTGGATGATACAACAGATATACGTAAGTTGTCCTACAACTTTATGC
TGTTATATCACGCTTCTTTTACAGCGGATCGCTGCGAGTGCCATTCTGTGACAGCGATCAAACTGCGTTAAAT
TTAAACCAAATATATACATATAATTCAATTAATTAATATTAATTACGCGGTCGGCATGTGGAGCTGTTTGTGA
TCCTTGTTGCAAATTCATGGGTGAACTGTTGATTTTATCAGGGCGAAAATTCAACCTAAAAAAGGATAGTCGT
CATACAAATTAAACAAGTGAGGAAACAACATGACCGCTTTATTTGATTTAACTGGGAAAACGGCGCTGGTAAC
GGGTTCTGCACGAGGACTGGGCTTTGCCTACGCAGAGGGTCTTGCCGCTGCGGGTGCACGGGTTATCCTGAAT
GATATTCGCGCCACGCTGTTGGCCGAATCAGTGGATACGCTGACCAGAAAAGGCTACGACGCGCATGGCGTGG
CTTTTGACGTCACCGATGAACTGGCGATTGAGGCAGCTTTTAGCAAACTTGATGCAGAAGGGATCCATGTTGA
TATCCTGATCAATAACGCCGGTATTCAGTACCGCAAACCAATGGTCGAGCTGGAGCTGGAAAACTGGCAGAAG
GTGATCGACACTAACCTGACCAGCGCATTTTTAGTCTCCCGCTCGGCGGCAAAACGGATGATCGCCCGCAACA
GCGGCGGCAAAATTATTAATATCGGCTCGCTTACCAGCCAGGCGGCCCGCCCGACTGTTGCCCCGTACACGGC
AGCAAAAGGCGGCATCAAAATGCTCACCTGCTCGATGGCTGCTGAATGGGCGCAGTTTAATATCCAGACTAAC
GCCATTGGACCTGGCTACATTCTGACCGACATGAACACCGCGCTTATTGAAGACAAGCAGTTCGATAGCTGGG
TGAAAAGCAGCACCCCTTCTCAACGTTGGGGTCGTCCGGAAGAGCTCATCGGCACCGCCATCTTCTTATCATC
CAAAGCATCAGATTATATCAACGGGCAGATTATTTACGTCGATGGTGGCTGGCTCGCAGTTTTATAAGTCGCC
CGCACTCTACCTACTCGCCTTTAAAATTGATGGCATGTGTTTACCCCGATGCATGCCGATATAACACAGGTAT
TATTATGAAGGCTTCAAGACAACGACTCTTTATTCTCACATTGCTGTTTATTGTGACTGCAATTAATTATATG
GATCGCGCCAATCTTGCCGTCGCTGGTTCGAATATCCAGAATGATTTCAGTCTGACGCCAACACAACTGGGTT
TGCTTTTCTCCATGTTTACCTGGGCCTACGCTGCCAGTCAAATTCCTGTCGGCTATGTTCTTGATCGCATTGG
CTCACGTATTCTTTATGGTGGTGCGATTATTCTGTGGAGTATTTTTACCTTTATGATGGGGTTCGCCTCACAC
CATTTATTCGCGACGGCAACCGCTTCATTTGCAATGCTTCTGGCCTGCCGCGCATTAATTGGTGTTGCCGAAG
CGCCATCCTTCCCGTCTAACACAAAAATAATCGCCACCTGGTTCCCGGACCATGAACGTGCCCGTGCGACCGC
GATTTATTCCAGTGCACAATATATCGGTCTCGCGCTGCTGACGCCTGCCCTCGCCTTTATTGTGGCTAACTAC
GGTTGGGAAATGTCGTTTTATCTGTCCGGTGGCGCCGGTATTCTGTTTGGCATCTACTGGCTTATGTATTACC
GCGATCCACAGCACAGCACTGCTGTGAACCAGGCCGAACTGGATTATATCAAAGCAGGCGGCGGCTATGGCTC
GGAGAACCAATCCTCCGTGAGTGCAAAAATCAGCTGGCAAAATATTAAATTCTTCCTCAGCAAAAAAACGATT
TGGGGTTTGTTCATCACCCAGTTTGCCTGCTCGTCTACGCTCTATTTTTTCCTGACCTGGTTTATTGTTTACC
TGGAAAAAGGACTGCATCTCTCTATTTCCAAAGCCGGGATAGGCGCCATGCTGCCCTATATTATGGCGATGCT
TGGGGTGCTCTGTGGCGGTACGCTAAGCGACATGCTGCTGAAAAAAGGAAAATCCCGAACGCTGGCACGAAAA
TTACCCGTTATGGCTGGCCTGTGCGTCACCATGATTATTGGCCTGGTCAATTTCTTTGAAAACCAGCCAGTTA
TTGCGATTGTCATTCTGTCTGTTGCGTTCTTTGCCAACGCCTTTTCAAACCTGGGCTGGGTGGTCTGGAGCGA
TGTAATTCCCCGTAATTTTCTCGGGACTATGGGTGGCTTTTTAAATATTTGCGGCAACCTTTCGGGGATCGTT
AGCCCAATTGTTATTGGGGTTATTCTCCAGCGCACGCAAAACTTCCAGTATGCCATGTGGTATATCGCAGGCG
TCGCCGGACTGGGCTTATTAGCCTACATATTCCTGGTGGGCAAAATTGAAGTGATCCTGCCTGGAAAAAAGAA
TGCCGACACTGTGGATAAGAATGCCATTAACCCGGCAACTGCCAACAAATAATGAGGTCATTATCAAAATGGA
AAAAATAACCTGTAACGCTTGTCTGGCCCATGCGGAAAAAGACGTACGCTTTGAATCACGTGAAATCGAGCAC

AGTGAACATGACGTCGTCGTGAAGGTCGCCTGCGGCGGCATCTGTGGCTCTGATATTCACTACTATCAGCATG
GCCGCGCGGGGATGTCTGTCCTCAAGCATCCGATGGTGATTGGCCATGAGTTTGTTGGCGTGATCAGCAAAGT
GCCAGCAGGCAGCGACCTGAAAGTGGGCCAGACGGTTGCGGTGAACCCGTCCAGCCCATGCAATCAGTGCGAG
ATGTGCCTTTCCGGCCATCAAAATCTGTGCGGCTCCATGCGCTTTATGGGCAGCGCGCAGTTCAATCCGCATG
TGAATGGCGGTTTTTCTGAATACGTGGTGGTAAAACCGGAGCAATGCATTCCTTACGACAGGCGCGTGCCCGC
AAACGTGATGGCCTTTTCGGAGCCGCTGGCGGTCGCCATTCATGCAGTAAAAAAGGCAGGCCAGTTGACCGGC
AAACGCGTACTGGTGATTGGCGCAGGCCCCATTGGTTGTCTGATCCTCGCGGCGGCGCGCAGTGCCGGAGCAT
CTGAACTGGTAGCATCCGATCTCAGCCCACGCTGTCTGGAACTGGCACGCCAAATGGGGGCCACAGCGGTGAT
GGATCCGCGCGATGAAGAACAGGTTGCCCATTATCAGCAGCATAAAGGCTACTTTGATGTCGTGTTTGAGGCC
TCCGGCGCGCCGATTGCCGTGGCATCCACCGTCGACTTCACCCGCCCTGCCGGCACCATCGTTCAGGTGGGGA
TGGGCGCCAGCCCGGTAAGCTGGCCGGTGTCAACGATGCTGGTTAAAGAACTCAACTGGGTCGGCTCATTCCG
TTTTATCGGTGAGTTCATCACCGCGGTACGCTGGCTGGAAGATGGGCGCGTCGATCCTCGCCCGCTTATCAGC
GCCGAGTTCCCGCCCCAGCAAATTGAAGACGCGCTGATTACCGCCACAGACAAAAATGTCTCTGCTAAGGTAC
TCATTCGTTTCGATTAACGGTGAAAAGCGCCCGGCCGGGCGCTTTGTTCTTAAAAGAGAATTGTTATATAATA
AAGCACTTCAGCGACATCTTAACGGATACCCATCTTGAGCATAAAATCCATTCAAAAACAGAATGTTGTAAAT
GAAATTTATGATCAGATAAGTAGCAAACTGCTGGACGGCAGTTGGGCGCCGGGTAGCCGTTTGCCCTCAGAAG
TGGAACTGACCGCCTCATTTAACGTCAGCCGGGTCAGCGTTCGCAGCGCAGTACAGCGTTTTCGTGACCTGGG
GATTGTGGTGACGCGTCAGGGCAGCGGCAGCTACGTGAGCGAAAACTTCACCCCGCAGATGTTGAGTAACGAT
CCCCGCCCAATCATGCACCTTAGCCGCGAAGAGTTTCACGATATGATGATTTTTCGTCAGACCGTGGAGTTCA
AATGCGTGGAGCTCGCCGTCACACACGCCACCGATGATGACATTCGCCAGCTCGAGGAAGCATTGAACAACAT
GCTGATCCACAAAGGTGATTATAAAAAATACTCGGAAGCGGACTACGAGTTCCATCTGGCGATTGTCAGGGCA
TCGCACAACAGCGTGTTCTACAACGTGATGAGCTCGATTAAAGACATCTATTACTACTATCTTGAAGAGCTTA
ACCGTGCGCTGGGTATTACCCTTGAAAGTGTGGAAGCCCATATCAAGGTCTACATGTCGATAAAGAATCGCGA
TGCCAGCACGGCCGTCGAAGTGCTCAATGAAGCGATGTCAGGCAATATTATTGCGATCGAAAAAATCAAATCT
ACAGAGACATCAGGGACAAAATAACCGTTGGTTACAAGCTCAAGTAGTAGAGCAATTTAACATATCTGAATCC
GAAATAGTTGCCATCAACTATTTAGTGACATAGTCCCACTTTAAAATCGTGGCAGTGCGGCCTGAAATCAGCG
AAGCTTCAAATAGCCAAGCCCCCTTAACATACAATAATTTTCATCATCATTCCCTTTATTGCTTAACCGCTTC
TGCCTGAATTTTTATATCGACAACTTTCGTCATCCCCATATCGACCAGATAATCTACCCCCCACTGGGTGCGA
TCTATTTGCGTGACAAAATCACCACCGCAAACCTGCGCCTTGAATATCGGGCTCATATAACAGCCAAACTTGG
TGGTGGTTAAGGTGACGGGCTTCGTTACTCCCTTCATTGTTAGCAAACCGTCAATAGAAACCGGTTTATTATC
TTCAAAATGCCATTTTGTTGATTTAAATACCATTTCTGGATATTTTTCCGCATCCAGAATATCGCTACTGCGA
ATATGGTTATCAAACGCATCCACCCCGGTATTTAAGGCGCTCACGGGAATCGTCACGCGCATCTCTGCATCTT
GCTTTTCCGGCTGATATTTCAGTTCGCCGCTAATAGCATAAAACCCGCCATGATTGGTTGAGGTCTGGAAATG
ATCGATATTAAATACCGCATTGGTATGCGTATTATCAAATTTATATACATCTGCATGAGCAGCACTTAATAAA
CCCAGAGACAGGACAACACCAAGAAAGCTCTTTTTCATTATATTCACTTTATATCTTAAATAAGGTGAAGCTA
TCTTAATGTTCCCTGTCCGCAGGATCGAGAGGGATAAACAGGAAGGACTGTTGCAAAAATAGAACGATGGAAA
GGCCGTGTCACAAAAACAGCGGCTCACGAATGAGTTTATGTCAGGGCTATTTCCGGAACCTGTACCGCCTTTG
TGGCGGTACAGAAGGCAAACTGACTAACGCATGATACGATCGTCGACATAACGGCGCTTATCCGGCGCGGGCG
GGAAATACTGATACAGCCAGGTTTCGCTGATAGCTTCCCCCTGGCAGCGCAGGAACATACGCATATCCACCGG
TGCCGTAGAATCCGAGGTCGGATACCAGTCAAACTGGATACGATAACCGTCGAAAGGCTCGACGTAGAGGATC
TCAATCTGCTTCGCCTCGCCACTGGAAAGCGTAATCACCGGCTCAATGCCTTTTGGCGCGGCCGCTTTCAGAT
CGCCGCCGACAAAATCAATAGCAAAACGGCGCGCCCATTTATCCGGGTAATGTTCGCCCGGCGCCCAACCTTC
GGGAAAGCCGCCCATCCCTGTACGGGTCGCCATGACCCGCGCAAGCGGAGATTGTACCGGCGGCTGCGCGCTC
CAGTACAAACGATAATTAAACGCCAGCGTATCCCCGGCTTTGATCGCTTTTTCCGGCTGCCAGAAACAGACCA
CATTATCCAGAGTTTCGCCAGTGGTTGGGATCTCCATCAGGCTAACTGCGCCTTTCCCCCACTTACTACGCGG
CTCCACCCACAGGCTCGGACGTTTGTTGTACCAGCCCATCACATCCTGATAATGCGAGAAATCGCGATCGAGT
TGCAGCAGGCCGAACCCTTTTGGGTTATCGTCCATATATGCATTGAACTGCAATTTCTGCGGATTATTCAGCG
GGCGGCAAATCCACTCGCCGTTACCCCGCCACATCGCCAGCCGATCGGAGTCGTGAATTTGCGGGTGAATGGT
GTCGCATACCCGACGTTCATTATTCCCACAGCTAAACATACTGGTCATCGGCGCAATGCCAAGTTGCTTTATA
TCTTTACGGGCGTAGAGATGGTTTTCTACATCCATGATCACCTGCGATTTTTCGCAATGAATCACAAATTTAT
ACGCGCCCGTCACGCTAGCGCTGTCCAGCAGAGCGTAAACGGTAAACGTAGTATCGCCCGGCTTCGCGGTGTC
AAACCAGAATGCGGTGAAGTCAGGGAACTCTTCCTGACCGTCAGTATAGGTATCTATCGCCAGCCCGCGAGCC
GACAGGCCATACTGATAAGTATCATCTACCGCCCGGAAATAACTGGCGCCCAGGAAGGAGACGACATCGCGCC
GCGCCAGTTCCGGCGCTTTAAAGACACGGAAACCGGCGAAACCGAGATCGCTCTGCCCTTCCAGTTGCGTCGT

ATCGACGCCGGCATCGTTGTATTTAAACAGTTCCGGGCGAAAATGAATCTCGCGAGCAAGATGCGTCGTCGTA
TCAACGGAAAACATGCGTACGCGACGACGAAAGCCCATCCCTACGTGGAAAAACTGAATATCGAGCTGCCGGT
TGGCAACACCGTTCCAGAGTGAATGCGCCGCGTCATACTGAATGCTGTTATAGGCCTGCGGCGTCAGATTGGC
GAGCGTATCCGGCAAGGGTCCCGGCGCGCCGCCCCAGGGCTTTTTCGCTAACGCCTGGGCCATTGATTGCAGA
CCAGCAAAATCAAAACGGACAATTTTACCATCCGCAATATCGGATTCCGCGGCAAACGCCGCCTGAGAAAAGA
GTGAAGCAATACCGCTGGAACCGCACACGGCGGCCATTGCCATTGAACCTTTAATAAATCGTCTACGATTCAT
ACCTGAAAATGAGTCCTTCTGGTCTTGTGAATGGTCCCGCGCTGGTTTTTTCAGCAGCAAGAATGTGCCCGTT
TCTGACCGCTCACTCTAGACAAAATTCATTAATAATCCAATTGTTGGTCGCCTGGAAACTGTGCAAAAGCGTA
AATATTTTATGTCGATAAGAACCGACCGAATCGGATGTAAACCTCACGCAGCATTCTTCAGGTTATGAAAAGT
TTATTCCAGACTAACGTGATGTTTCATGACTCGTTTAAACAGCGCCAGACGCGCACGCATAAAGCGGTTTTCC
ACTTTAAAACCGGCAAGTTTGTTCAGCCCAATCCGCAACAGACGATCTCGCCCCCGAATGCTGGCGGGCCAGC
GAACCGGGCCGTGCAGCACGTCGCGCGTGCGGCTGGCATGACGAGCGAAATTCACCCAGTAATCCGCCACCTG
CGAGGCAAAGGCCAGGTCATTCTCATTCACATAATGGCATGTGGGCTCAGCCCGCGTTAACGTATCGAATACA
TACGGTATTTCATTGCCATGACAGGCGCCATTCGCGTAGGTGTTATGCTCCGCCTCGGCGACATAATCAAACC
AGTAACGCCAGCACGGTTCGCCGATCCGCTGCTGCGCTTGCATGACGACATAACCCAACGTGGTAAATACCAT
ATCCCGACAGACCTGCCGCCCTAGCGCTTCGTCGCCTTTCACTCCAGGATAAAGCAGCCGGATTAACCCCAGG
CCAACACGCCGCTCACGGCGCATTTTTTGAATTTGACCGGCAATATCGACGCCAAAAACGGCCAATACGCTGG
CTTCATCGCTATTCGAGCCGATCATCACCGGTATTGGATGCTGTTTCGCCGCAAAGAAGGTTTCCAGCATCGG
ATGCGGCAGCACGACGTCGCCGGAAATCGGCGTCGGCGCAATTTTAAAAGGCGCGTCCAGCGGCCAAAACGTC
TCTGCCGGTAAAGCGCGTAGCTGTTCGGCAGTGGCATGGGCTAAACCAAGATGTTCCGCCAGCGCAACGCCTT
TTTTTAGCGCCACCTCGCGGGGCGTATCCGGCAGCGTATAACCACTCTGAATAATCGCTTTATGAAAAAGCCC
CTTCGCCAACGGCGAAGCCATCAACGACAGCACGCTGCGGGCGCCGGCGGATTCGCCAAATAACGTCACATTC
TGCGTATCGCCGCCAAATGCGGCAATATTGTCCTGCACCCAACGCAGCGCAGCGATTTGATCGAGTAAAGCGA
AATTATGAATACACTCCGCCCCTTCGCCTTCCAGCGCCGGATGCGCGAAAAAGCCCAGATGGCCAAGCCGATA
ATTGACCGTCACCACGATTGCGCCGCGCTTCGCCAGCGCTTGACCGTCATAGGGCGGCAAACTCCCCGCGCCA
ATGGTGTAACCGCCGCCGTGTAGCCAGACCATGACCGGCAACGGTTCATGACGCACGGCGGGAGCCCAGACGT
TCAGATAGAGACAGTCTTCGGAAAAATTTCCCGGATCGCCGCCGCCGAGTTCGCGACACCAGGTAATATCCTG
CCAACTGGCGCAGGAGAAACAATCCGCCTGGCGCACATCCTGCCAGGGCGTAACAGGCTGCGGCGCACGCCAG
CGTAGCTCCCCTGTTGGCGGCGCGGCATAAGGTATACCGCGCCAGATATGGGTTTCTTCCTGTACGACGCCCA
CGATTTTACCGTGACGCGTCGCCACCAGCGGGATGGAGGGATTCAACATAACGCCTTCCTTTTACAGTATCCT
CACAGCCTGTGAAGCTTACCGCTTTTAAAGCAGACCGCAACGGCGTTTACTGCGTTCTTCCGCTTCCTGATAT
AACGAAAATTCATCCCGTACAGCACATCCCAGCGCCTGTTCAAAGGCGTCGCGGCTGGCATTGCCGTGGCTTC
TGGCCAGCGTTTCATTTCCCAAATTCGACTGAAAAATGCCGGCTGCGCTGACCGGAAGAAAATCTTCATAGGT
AATCGGCTGCGCGATTACCCAACCGCGCTCAATAAGCGGCTGCGGATCGTCGCCAGGATGGATCGCCTGACGA
TGCGCCTCACCGGAAGGCGTTAAGCGATAGCGGAACCAGGCAAGCCCCTGCTGGCGAAGCAGAAATTCGCTAT
CCGGAAAGGCGTTAAAGACCTCCCGCAAATGGAGTTGATGCGTGAAGTTATCCTTACCCGTTCCCGCCTTGTG
CAACAGCTCGTCATATAATCGACGACCTTTTGGCGTCAGCGCGACGCCACGTTGCTCAATTTCGCCGAAGCGC
GCCGTGTGCGTACCCTGCTTTTCATCAACGAAGAGCACCTGCTCTTCCAGGGCTTTGAAACTGGTTTGCCGCA
ACAGAATAGGCACTTCGCGGCGAGGCGGACCTTCGATAAGAGTTTTAGGCGTAATGCCGCACTCCGGCATCAT
GGCCTGTACCCGGTCGATATCCAGAGTACGCGGCGTCAGATGGTTAATGTGACATCCCGGAAAACAGACCACG
TCGGCAATTAAACGATGCTCACGATGCAGGCTGCGGTACGTTTCTTCATCAACGGTAGCCTGACGATGCCAGC
GAAAAGTCTCCAGCGTTTCACGGACAAATTCTTCGGCCTGAGCAGCACTAAAACCGCCCTGCTCGTCGTACTC
ATCCAGCAATTGTCGGCAGCGCGATGTAAAAATATCCCGCTGCGATAAGATTTCCGCCGCCCGCTGGCGTAAT
GCCGCGTTTTCAATGAGCTCCAGGCGTAATAGCGAAGTGAACATGCGAAAGGGATTACGCGAGAGCGAAGCCT
CGTCAATGGGACGAAATGCCGTGGAGTGCACAGGCACGCCCGCCTGAGAGAGATCGTAATAACTTACTGGGTA
CATCCCCATGATGGCGAATATCCGACGCAAGGTAGAAAGTTCTTCTGCGGTGCCAACACGAATGGCGCCATGC
CGTTCCACGTTCAATCGCGCCAGTTCATCGGCATTAGCCAACTGCTCATGCAGTTTGGGATTATTTTCCAGCA
CCGCCAGATTCACGTCAGCCACAAGTTCCAGTAACGTACCGTACTGCGGAACTTCCTGCTGGTACATTGCCGA
CATTGCCTGCGAAAAGTGTTCCCGAATCTCATCTGCCGTGATGGTGTTCGCCATAATGTCATGCCTCCAGTGA
ATGTTACCTGGAGTGTAGAAAACCTCATCCATTCCGGGGAGAAGAATTTATAAAGTGTGATCCGACCTGAATT
CATTATGCAGAACGGATTGACATGAGCTGGCATATCCCCGACCAGATCGCGGCTTAACACGCTTAACGGCAGT
TGAATCATCAGTAAAATAAAACAGTTGTAACTTGAAATGGTAAAATGTTAATAATATTTTGCTATCAGGTTAT
CAAAAACAAACAACTTTACTTGTCACCCGCACGGCTCTGTTTTTAACATCGCTTATGGAAAAAAATGGTCTGT
TTAGTCAGCGCATTCGGTTGCGCCACCTTCATACATTTGTAGCCGTTGCGCAACAGGGAACTCTGGGGCGCGC

GGCTGAAACTCTTAACCTGAGCCAGCCAGCGCTTTCTAAAACGCTGAACGAACTGGAACAACTCACCGGAACC
CGTCTGTTTGAACGCGGGCGACTGGGCGCGCAATTAACGGTTCCTGGCGAACAATTTTTGACTCATGCGGTAA
AAGTATTAGATGCATTGAATACCGCCGGGCAGGCGCTGAATCGCAAAGAGGACGCCAGCGCCGATGTGGTGCG
TGTTGGCGCGCTCCCCACCGCTGCGCTGGGTATACTCCCGGCGGCTATTGGGCGGTTCCATCAACAGCAAAAA
TCGACCTCGTTACAGGTCGCGACCATGAACAATACGATGTTGCTTGCGGGTCTGAAATCAGGCGAGATCGATC
TGGGTATTGGCCGCATGTCAGACCCGGAATTAATGGGCGGTTTAAATTACGAACTGCTGTTTTTAGAATCGCT
TAAATTAGTGGTACGCCCCGGTCATCCCCTGTTACAGGAGACGATCACGCTCAGCCGGGTTATGGAATGGCCG
GTAGTCGTCTCGCCAAAAGGCACGGTACCGCGCCAGAATGCGGAAGCCCTGTTGCAAAGCCAGGGGTGTAAAA
TGCCTGCCGGATGTATCGAAACGCTATCCGCATCGTTATCACGACAACTGACCGTAGATTACGATTACGTCTG
GTTCGTCCCCTCTGGCGCGGTTAAAGAAGATCTTCGCCAGGCGACGCTGGTCTCATTACCCGTGCCGACCCAG
AGCGCGGGCGAGCCGATAGGTATTCTTACGCGAGTTGATATCCCGCTCTCAACCGGGGCGCAAATGCTGATTG
CGGCGATCCGTAAATCGATGCCGCTGTGATAGCGGCTGTCGGAGAATAAACGCGCCCGCGGCTAAAATAGCCC
GCTGGCGCGACGCTTACTAAAGGAAGGATGATCGCATCGTCGCGCCAGTATCCTGTAAATGAAACGCATCAAC
GGCTTGCGTCAAGCGGGCGGCCTGTTCTTCAAGCGAAGCCGCCGCCGCGGACGCCTCTTCGACCAGCGACGCG
TTTTGTTGCGTAACCTTGTCCATCTCAGAAATCGCCTGACTGACCTGAACAATACCGCGACTTTGCTCATCAG
AGGCTGCCGCGATATCGAGCATAATATCGGTGACGCGTTTGACGGCATCCACAATCTCATTCATGGTGCTCCC
CGCAGCGATAACCTCTTCAGATCCTTGTTCAATCAGGCTGACCGATGCGCCGATCAAGCCTTCAATCTCTTTT
GCCGCCTGCGCGCTGCGACTGGCGAGGGTACGAACCTCACTGGCGACCACCGCAAAACCGCGTCCTTGTTCCC
CGGCGCGTGCCGCCTCGACCGCCGCGTTCAGCGCCAGGATGTTGGTCTGGAAAGCGATACTGTTAATGACGGC
GGTAATTTCCGAGATTTTTTTCGAGCTGGTGGAAATATTCCCCATCGTTTGTACGACCCCGGAAACCATCTGG
CCTCCGCGGCTGGCTTTACCGGAAGCATCTTCCGCCAGTTTGCTGGCGTGATGCGCATTATCCGCATTCTGCT
TAACGGTTGCCGTCAACTGTTCCATACTGGCCGCGGTTTGTTCAATGGCGGCAGCCTGCTGTTCAGTACGAGA
AGATAAATCCGTATTACCAGCGGTAATTTCGCTGGTGCCTCGATAAATTTCCTCCGCCCCCTGACGCACCGCC
CCTACCGTTTGTTGCAACGCATGCTGCATCTGCTGAAGATGATGACTCAGGCGACCAATTTCACTGCGTCCTG
TGGGCTCGTCAGCCATGGTCAGATCGCCGGCCGCAATGCGTTCGATACGTGACGCAGACTGCTGCAAGGGTTG
TATCACCGTGCGACGCAGCACCATGAAGGTCATTAACGTCAATACCAGCGCCAACGTAAATGCGCCAATCATA
AACATCATACCGAGTCGGGTACGTTGGTAAGCCTGCTCGCTAAGTAGTCTTGCCCGCTCGGTGCGCAGTTCAA
TCGCTTTCAACAGGACATGGTTATATGCTGCATCCAGTTGTTTTGCCTGCTCGTTTTCATGATTAATGATCGC
TTCGAACATGCCATTTTTGGCAAATTTCAGCATCGGTTGCAGGCCGTTGATATAGGCGGTGTAGCGGGCGTTT
AACTCGTTATCTAACGCGTCATCAGCGGGTGTTTTCACCGCTCTGGACATATAAGCGTTAAAGCCGTCCTGAG
ACTGCTTGATCCGCGTTTCCGCAGCGGCAATATTGGCTTTCATTTCATCCATTTCAGCAATCCGGCTCGCCGC
GCCAGCGTGGATCATATTGATACGTGCGGTACGTAAGTGGTTAGCGCTGTTGGATAATCCCATCCGTACCTGA
ATCTCGTCCGTGACATCACGCTGATCGCGATCGGCCTGTAACAGAAAATAGCCTGCCAGCCCGGCGCTCAAAG
CAAAAAGAAGAAGGATGCCACCGAGAATGGAGGAAAACAGCGGAACCAGCCGAATATGATGCAGGAAGCCCAG
TTTATGCGAGGCTTGCATCGAGAATGTGTTGCCCATGACCGTCGACTCTCTTGTAGGTTTTATGCGTAAAACA
CGCCCGTTAGATAGTCATCGGCACTTCGTGACGTTTGCTTATCGCGAAAAGCATCGCCTTCGTCACATTTTCA
CAACATTCATTCCAGAAAATTCAGAAAAGCGCCAGCGGAAAATCCGCTGGCGGATGAATCAGTTATCGCCAAA
GTGGATAACAGTACGGATGGATTTACCCTGATGCATCAGTTCAAAGGCATCGTTAATCTGCTCCAGCGGCAGG
CGGTGAGTAATAAAAGGATCTAAACGGATTTTACCGTTCATCGCATCTTCGACCATCCCTGGCAGCTGCGTGC
GCCCTTTCACGCCGCCAAAGGCGGAGCCACGCCAGACGCGACCGGTCACTAGCTGGAAGGGACGGGTTTTGAT
TTCCTGCCCTGCCCCCGCTACGCCAATAATAATGCTTTCGCCCCAGCCTTTATGACAACATTCCAGCGCTGCT
CGCATCACGTTAACGTTGCCGATACATTCAAAACTGAAATCTACGCCGCCATCAGTAAGCTCAACAATAACGT
CCTGGATCGGTTTGTCGTAATCATTCGGATTAATAAAGTCCGTGGCCCCCATTTCGCCCGCCAGCGTAAATTT
TTCCGGATTCGTGTCGACAGCTAAAATACGCCCGGCTTTAGCCTGAACCGCGCCCTGAATAACCGCCAGCCCA
ATACCGCCTAAACCAAAAACGGCAACGGTGTCGCCCGCTTTCACTTTAGCGGTGTTATGGACGGCGCCAATAC
CAGTCGTCACGCCGCAGCCCAACAGACACACTTTATCCAGCGGCGCCTGCGGGTTCACTTTCGCCAGAGAAAT
TTCCGCGCAAACGGTATATTCGCTGAACGTGCTGGTTCCCATATAGTGATAAACGGGTTCGCCGTTATAAGAA
AAGCGGGTAGTGCCATCCGGCATCAGCCCTTTTCCCTGAGTGGCGCGTACGGCCTGACAAAGGTTAGTTTTAC
CGGACTTACAGAACTTACACTCGCCGCATTCCGCCGTATACAGTGGAATAACATGATCGCCGGGTTTCAGGCT
GGTGACCCCCTCGCCGACCTCGACTACCACGCCGCCGCCTTCGTGACCCAGGACTGCCGGGAATACGCCTTCC
GGATCATCGCCGGAAAGGGTAAAAGCATCGGTATGGCAAACGCCGGTATGAGTGATTTTGACCAGAACTTCGC
CTTTTTTCGGCGGCGCAACGTCAATCTCGACAATTTTTAACGGCTGACCGGGACCAAAAGCAACTGCTGCACG
TGATTTCATATATCTCTTCCTGTTACGGTGAGGGAAATTGTTATTTTAGATAAGCGCGAAGAAGATGGCCGAT
TTCCGCCATCCGAACCGCGCGCTGATCTGGCGTAGTCTCCCCGCTGACCAGCTCATCTTTCAGATGGATTTCA

ACCATTTCACTCATCAGGCCATTAGACGCGCCGCGCACGGCGGCAATCTGTTGCAGAATCGCCAGACAAGGTT
CGCCAGACTCAAGCGCCCGCTCCAGCGCTTCGACCTGCCCGCGTATGCGACGAACACGGGTAAGGATACGTTT
TTTATCTTCAGGTGAATGCGGCATTCGACCTCCATATACTATAGGGGGGTATACTATCAGAATTTTATGTTTA
TGTAATTGTAAAATTACACACAATATAAAAATCAATAGCTTATAGATTTCCGGCATAGACCATCAAGAATCAG
TTCACATTCAGCATATTCAGCTCATGGGAACTTTTTCAGGCCACAGACAATAAACACCAAAGCTTTCTTTCTA
AGCTGCAATGTTCTGCCCGACTGGGGCACAGAGGTAATGCCGGTAAATCCAGTCCCATTACCCAGAAGTAAAT
ATTTGCTTAATAGTTTTTATTTACTCACCAGGTGGTAATGAATAAAGGTTATGATATGCAGATGTCAGTCTTG
TAAGAACGTAACATCACAACTGCGATACACCGCCGACGGGTAGCGAAAATATCTTAACCCTGTGTCTTTCCAG
GCTTAGTCAATGTGGACAAAAAATCAAATGAGCGTTATTTGAAAGATTTTTAATACCCAAAAGCGTCATCATT
ATTGTTAATTTGAAATCAATCTCAGGTAATAATCCATGCCTATTTCGATTTGTAAACATGGTGCTCCTTTTGT
TGTTCAACATGAAAATCGGTACGGTTCCGGTGCAAGTCAGTCATCATCACTTTCAAAATCAATCCGCCATATT
AGCAACTCTCATGAGGAAATAAAATTTATAAGTTGCTATAGTGCAAATGGAGCCTGTTTTTCCAATGCACAGA
TGCTAGCCAATGCCTCAGGGAGGCCTGTTATAGGTTACTATGGGAAAATAAATAAGTTAACAGCCAGTCTGGA
TAATAGCGGTCGTATATTCCGCCCACAGCATAAGCTTGCGGCCAATATTTGTTATGTAGGTAATCGGCTTCTG
AGCGCTCCAGTTCAACTTGGGTTTGGCCTGAAACATCTGCTAACCTGTCACTCAAATGGTAATGTCAGATAAA
GTGTCATGCCATGTCCTTCCCGGCATTGCTATTCCACATCCTACAGATATGGGCGCTCAGTTCTTTCCTCAAT
CGTTGCTATGGGAAGTTCTGCCGTCATTTCACGCTCTTGCCAACCAGCATTTTATTAAAGCCTGACTTTTACC
AGAGTAATTTCATAAGAGTGCCCATCTTCTGGTCTAAAAATAATAAATCTATTGTTATTTTTAGTAAAGGATT
TCGACAAAATAAAACTATCATGATATCTGGCCAGGACGTAGTACCAGCCTCCATCATACAATATCATTTCGTA
TTCTTTCTTTAATTGTGGTTTATAGATCCCAACAAAAAAAGAAAATATAAAAAAATATATAATAAAACCAAGC
ATAACTATAGTGAAACAATGCCTTCGCACCCAACTGAGTTGCGTAAAACAAATCACCGGCATCAATTTACTAT
[truncated: 478,076 more chars]
